# *PTBP2* – a gene with relevance for both Anorexia nervosa and body weight regulation

**DOI:** 10.1038/s41398-022-02018-5

**Published:** 2022-06-09

**Authors:** Yiran Zheng, Luisa Sophie Rajcsanyi, Beate Herpertz-Dahlmann, Jochen Seitz, Martina de Zwaan, Wolfgang Herzog, Stefan Ehrlich, Stephan Zipfel, Katrin Giel, Karin Egberts, Roland Burghardt, Manuel Föcker, Saad Al-Lahham, Triinu Peters, Lars Libuda, Jochen Antel, Johannes Hebebrand, Anke Hinney

**Affiliations:** 1 Department of Child and Adolescent Psychiatry, Psychosomatics and Psychotherapy, University Hospital Essen, University of Duisburg-Essen, Essen, Germany; 2 Center for Translational Neuro- and Behavioral Sciences, University Hospital Essen, University of Duisburg-Essen, Essen, Germany; 3 Department of Child and Adolescent Psychiatry and Psychotherapy, University Hospital of the RWTH Aachen, Aachen, Germany; 4 Department of Psychosomatic Medicine and Psychotherapy, Hannover Medical School, Hannover, Germany; 5 Department of Internal Medicine II, General Internal and Psychosomatic Medicine, University of Heidelberg, Heidelberg, Germany; 6 Translational Developmental Neuroscience Section, Department of Child and Adolescent Psychiatry, TU-Dresden, University Hospital Carl Gustav Carus, Dresden University of Technology, Dresden, Germany; 7 Department of Psychosomatic Medicine and Psychotherapy, Medical University Hospital, Tübingen, Germany; 8 Centre of Excellence for Eating Disorders, University of Tübingen, Tübingen, Germany; 9 Department of Child and Adolescent Psychiatry, Psychosomatics and Psychotherapy, University of Würzburg, Würzburg, Germany; 10 Department of Child and Adolescent Psychiatry, Psychosomatics and Psychotherapy, University Hospital Frankfurt, Frankfurt(Oder), Germany; 11 Department of Child and Adolescent Psychiatry, University of Münster, Münster, Germany; 12 Department of Biomedical Sciences, Faculty of Medicine and Health Sciences, An-Najah National University, P.O. Box 7, Nablus, Palestine; 13 Institute of Nutrition, Consumption and Health Faculty of Natural Sciences, University Paderborn, Paderborn, Germany

**Keywords:** Clinical genetics, Genetics

## Abstract

Genetic factors are relevant for both eating disorders and body weight regulation. A recent genome-wide association study (GWAS) for anorexia nervosa (AN) detected eight genome-wide significant chromosomal loci. One of these loci, rs10747478, was also genome-wide and significantly associated with body mass index (BMI). The nearest coding gene is the Polypyrimidine Tract Binding Protein 2 gene (*PTBP2*). To detect mutations in *PTBP2*, Sanger sequencing of the coding region was performed in 192 female patients with AN (acute or recovered) and 191 children or adolescents with (extreme) obesity. Twenty-five variants were identified. Twenty-three of these were predicted to be pathogenic or functionally relevant in at least one in silico tool. Two novel synonymous variants (p.Ala77Ala and p.Asp195Asp), one intronic SNP (rs188987764), and the intronic deletion (rs561340981) located in the highly conserved region of *PTBP2* may have functional consequences. Ten of 20 genes interacting with *PTBP2* were studied for their impact on body weight regulation based on either previous functional studies or GWAS hits for body weight or BMI. In a GWAS for BMI (Pulit et al. 2018), the number of genome-wide significant associations at the *PTBP2* locus was different between males (60 variants) and females (two variants, one of these also significant in males). More than 65% of these 61 variants showed differences in the effect size pertaining to BMI between sexes (absolute value of *Z*-score >2, two-sided *p* < 0.05). One LD block overlapping 5′UTR and all coding regions of *PTBP2* comprises 56 significant variants in males. The analysis based on sex-stratified BMI GWAS summary statistics implies that *PTBP2* may have a more pronounced effect on body weight regulation in males than in females.

## Introduction

Anorexia nervosa (AN) is a life-threatening psychiatric disorder defined by disordered eating and extremely low body weight [1, 2]. Obesity, defined by a body mass index (BMI) at or above 30 kg/m² [3], is a major hazard to human health. For both AN and variance in BMI environmental and genetic factors are relevant. According to twin and other family studies, AN and BMI variation are both highly heritable [4–7]. Moreover, BMI-associated loci in part also have an effect on AN and vice versa [8]. Monogenic and/or polygenic genetic mechanisms can be relevant for both AN and BMI variation. Thus far, variants in 16 genes, which play a role in the leptin-melanocortin signaling pathway, were identified in monogenic forms of obesity [4]. No monogenic form has been described for AN. For both AN and obesity, genome-wide association studies derived chromosomal loci with a genome-wide significance (*p value* < 5 × 10^−8^) for the analyzed trait [9].

The recent GWAS meta-analysis for AN (Watson et al. 2019) included 33 datasets comprising 16,992 cases and 55,525 controls of European ancestry from 17 countries [10]. A total of eight chromosomal loci are associated with AN. We found that one of these is also associated with BMI [11]. The Polypyrimidine Tract Binding Protein 2 gene (*PTBP2*) is the nearest gene. The PTBP2 protein is a splicing regulator that can be recruited to the S region DNA and interacts with other chromatin-associated factors. A role in controlling a genetic program that is essential for neuronal maturation [12] and differentiation of male germ cells [13] has been described. The higher level of *PTBP2* expression in patients with obesity compared to individuals without obesity suggested a role in obesity development [14].

AN is nine times more prevalent in females than males [15], making the female sex a robust and reproducible risk factor for AN [16]. Studies on the sex specificity of obesity are often based on hormone level differences [17, 18] or on inflammation markers [19]. A genome-wide association study based on the waist-to-hip ratio (WHR) showed a narrow-sense heritability estimate difference between sexes (~50% in females and ~20% in males) [20]. Pulit et al. found that WHR variant effects were generally stronger in females than males [11].

In general, GWAS hits may point to genes harboring mutations with large effect sizes (e.g. the melanocortin 4 receptor gene (*MC4R*) locus) on the analyzed phenotype [21]. We analyzed AN GWAS [10] data and BMI GWAS data [11] to identify a genetic region relevant for both AN and body weight regulation. We aimed to discover mutations in the *PTBP2* gene with a major gene effect on AN or body weight regulation. Thus, we performed a mutation screen of the *PTBP2* gene in 192 females with AN (acute or recovered) and 191 children or adolescents with (extreme) obesity. In addition, a sex-specific analysis of the chromosomal region of the *PTBP2* gene were performed on sex-stratified BMI GWAS data [11].

## Methods

### Study groups

We, Sanger, sequenced the coding region of the *PTBP2* gene in 383 German individuals including 192 females with AN (acute or recovered, diagnosed according to DSM-IV criteria [1]) and 191 children and adolescents with (extreme) obesity (BMI percentile ≥90th, 93.7% were extremely obese with BMI percentile ≥97th [22]). The ascertainment strategy was previously described in detail [23]. Briefly, the 192 independent female AN patients included 148 individuals with acute AN and 44 individuals with a history of AN. The acute patients had a mean age of 19.52 ± 9.08 years and a mean BMI of 15.71 ± 1.81 kg/m^2^. The recovered individuals had a mean age of 33.09 ± 9.52 years and a mean BMI of 19.91 ± 2.41 kg/m^2^. The phenotypes of the study groups are shown in Table 1. Written informed consent was given by all participants and in the case of minors by their parents. The study was approved by the Ethics Committees of the Universities of Aachen, Dresden, Essen, Frankfurt, Hannover, Heidelberg, Marburg, Tübingen, and Würzburg, and was performed in accordance with the Declaration of Helsinki.

**Table 1:** Description of the study group.

Study group	Sex	Individuals	Age		BMI (kg/m^2^)	
		N^a^	Mean ± SD^b^	[Min, Max]	Mean ± SD	[Min, Max]
**AN**	Female	192	22.63 ± 10.80	[11.93, 67.41]	16.67 ± 8.17	[9.03, 25.21]
**Extreme obesity**	All	191	13.88 ± 2.48	[6.50, 24.42]	32.34 ± 6.02	[21.27, 53.96]
	Female	106	13.87 ± 2.71	[6.50, 24.42]	32.07 ± 6.45	[21.27, 53.96]
	Male	85	14.07 ± 2.18	[8.05, 19.73]	32.67 ± 5.47	[23.17, 48.52]

^a^*N* the number of individuals, ^b^*SD* standard deviation.

### Look-up of “AN GWAS SNPs” in BMI GWAS

Our initial analysis was a look-up of eight genome-wide significant loci for AN [10] in the large-scale GWAS for BMI on up to 806, 834 individuals of European ancestry (434, 794 female and 374, 756 male) [11].

### Sanger sequencing

The *PTBP2* gene (14 coding exons) is located on chr1: 96,721,665–96,823,738 (GRCh38/hg38). The genomic sequences of the *PTBP2* gene were extracted from the Archive Ensembl Database (http://www.ensembl.org/index.html). For the cDNA and protein sequence transcript variant 1 of the *PTBP2* gene (PTBP2-201, ENST00000370197.5) was used. Primer pairs were designed using the online software PRIMER3 and Primer Premier 6. Primers (sequences can be obtained upon request) were analyzed using the BLAST and in silico PCR functions of UCSC Genome Browser to verify the designed primer’s specificity. Polymerase chain reaction (PCR) amplified DNA samples were bi-directionally sequenced by Microsynth Seqlab GmbH (Göttingen, Germany).

All sequences were analyzed using the software SeqMan Pro software by DNAStar, Inc. (version: 10.1.0) and evaluated by two experienced scientists. Samples with variant patterns were confirmed with bidirectional sequencing. Hardy–Weinberg equilibrium (HWE, R package “genetics” [24], RStudio Desktop 1.4) was fulfilled for all analyzed variants.

### In silico mutation analyses

#### Conservation analysis

The conversation analysis of human *PTBP2* gDNA was compared to 35 other species (ten primates, five rodents and related species, ten laurasiatherian, five sauropsids, and five fishes) in the software MegAlign by DNAStar, Inc. (version 10.1.0) using the cluster W method.

#### Functional relevance of detected variants

In silico analyses were performed for all detected variants and the AN GWAS SNP rs10747478 to predict the putative effect of variants. All detected variants were looked up their *p* value in both AN GWAS [10] and BMI GWAS [11]. The in silico analyses tools to explore the effects of variants on the function or structure of mRNA and protein ensued. An overall prediction of all detected variants was first analyzed by MutationTaster2 (http://www.mutationtaster.org/). Then the deleteriousness of SNPs was evaluated by the integrated PredicSNP2 (https://loschmidt.chemi.muni.cz/predictsnp2/) [25], which includes the prediction results from combined annotation dependent depletion (CADD) [26], deleterious annotation of genetic variants using neural networks (DANN) [27], functional analysis through Hidden Markov models (FATHMM) [28], FunSeq2 [29], and genome-wide annotation of variants (GWAVA) [30]

Synonymous variants may alter mRNA stability or splicing pattern. The putative alterations were analyzed by ESEfinder3.0 (http://krainer01.cshl.edu/cgi-bin/tools/ESE3/esefinder.cgi) to quantify the activation level of splicing enhancers. To explore the putative changed alternative splicing, Spliceman (http://fairbrother.biomed.brown.edu/spliceman/), and Spliceman2 (http://fairbrother.biomed.brown.edu/spliceman2/upload) [31] were applied.

DNA methylation has a remarkable impact on various human development progresses throughout life [32]. The enrichment of large-scale genomic and multi-omic studies, for example, genome-wide association studies (GWASs) and expression quantitative trait locus analyses (eQTLs), provided increasing evidence that genetic variants play a role in DNA methylation [33, 34]. Thus, a brain eQTL database server (xQTL, http://mostafavilab.stat.ubc.ca/xQTLServe/) was recruited to examine the detected variants in our study. The brain xQTL composes three data sources: gene expression (RNA sequence), DNA methylation (mQTL, cis methylation sites), and histone modification (haQTL, H3K9Ac) [35].

MicroRNAs, ~22 nucleotides base length, non-coding RNA molecules, act as endogenous translational repressors of protein-coding genes in humans by binding to target sites in the 3′ UTRs of mRNAs. If variants are located in microRNAs or their binding sites the function will be disrupted [36, 37]. An integrated web-based database, PolymiRTS Database 3.0 (http://compbio.uthsc.edu/miRSNP), was used to look up the microRNAs and their binding sites in *PTBP2*.

The putative alteration of post-modification and transcriptional factor binding were analyzed with Regulation-Spotter (https://www.regulationspotter.org/) and FABIAN (https://www.genecascade.org/fabian/), respectively [38, 39]. The effects of variants on alteration of transcription factor binding sites were filtered with known binding sites derived from three sources (ENCODE 3, Ensembl Regulation 102, and FANTOM5) [38].

The utilized nine in silico tools were summarized in the Supplementary Table S4-2 and classified into four catalogs with different prediction aspects: overall prediction, deleteriousness of single nucleotide alteration, mRNA splicing alteration, and downstream modification. When the evaluated variant was predicted as pathogenic in at least one software for all catalogs, it was denoted as “may trigger functional consequence”. Otherwise, the putative functional impacts of the variant remained unclear.

The PTBP2 protein function prediction in our study was also examined in GeneMAMIA (https://genemania.org/), a website to generate hypotheses about gene function [40]. The proteins, which interact with PTBP2, were analyzed for previous hints for association with either body weight regulation or AN. The GWAS catalog (https://www.ebi.ac.uk/gwas/) was then recruited to explore the BMI or AN GWAS hits in or within close proximity of the interacting genes [41].

#### Linkage disequilibrium (LD) analyses

Linkage disequilibrium (LD) is defined as an association of alleles of two SNPs at different closely located loci [42]. Two calculations, squared correlation coefficient (*r*^2^) and disequilibrium coefficient (D′) are widely used to quantify LD between two loci. The values range varies from 0 (no LD) to 1 (strongest LD) for each *r*^2^ and D′. Here threshold values for *r*^2^ and D′ were set to 0.3 and 0.8, respectively.

The software HaploView 4.2 (Download: https://www.broadinstitute.org/haploview/haploview) was used to analyze LD structures or haplotypes between detected SNPs. LDlink, a web server (https://ldlink.nci.nih.gov/?tab=home), includes ten online analysis applications which can easily and efficiently investigate the LD in selectable population groups (1000 Genome Project) [43]. Here, three applications were utilized to interrogate the LD in a larger genomic region in the European population. The AN-related SNP rs10747478 and identified known variants were first summarized and analyzed in LDmatrix (https://ldlink.nci.nih.gov/?tab=ldmatrix). LDtrait (https://ldlink.nci.nih.gov/?tab=ldtrait) and LDexpress (https://ldlink.nci.nih.gov/?tab=ldexpress) were then used to find all possible variants with strong LD with detected variants (threshold values for the parameters: distance ±500 kb; LD: *r*^2^ ≥ 0.3, D′ ≥ 0.8).

#### Assessment of heterogeneity of effect sizes at the *PTBP2* locus based on sex-stratified GWAS

We calculated *Z*-scores of the differences in effect sizes of each SNP for BMI between sexes according to ref. [44]. If the absolute value of the *Z*-score for one variant was larger than 2, this was considered a different effect size at the significance level of 0.05. The variants located in the 1000 kb upstream and downstream regions of rs10747478 including the genomic region of the *PTBP2* gene (variants in these regions) of the sex-separated summary statistics by ref. [11]. were used as a data source for the corresponding effect sizes of the associations between the SNPs and BMI (Supplementary Tables S9–S11). Figures were plotted in GraphPad Prism V9.3.0.

## Results

### Look-up of AN GWAS hits in BMI GWAS

Eight genome-wide significant AN risk SNPs (alleles), identified in the recent GWAS meta-analysis for AN [10], were looked up in a large GWAS meta-analysis for BMI [11] (Supplementary Table S1). One of the eight AN-SNPs (rs10747478) was genome-wide significantly associated with BMI (Supplementary Table S1). SNP rs10747478 (chr1: 96,435,899, GRCh38.p13) is significantly associated with both AN (effect allele = T, beta = 0.076, *p* value = 3.13 × 10^−8^) and BMI (effect allele = T; females: beta = −0.016, *p* value = 9.03 × 10^−10^; males: beta = −0.02, *p* value = 4.83 × 10^−12^; combined sexes: beta = −0.018, *p* value = 9.26 × 10^−20^). The T-allele is associated with decreased body weight in both sexes and increased risk for AN.

Nearest to this SNP are two processed pseudogenes, (ubiquitin-conjugating enzyme E2 W pseudogene 1) *UBE2WP1* and (eukaryotic translation elongation factor 1 alpha 1 pseudogene 11) *EEF1A1P11*. The Polypyrimidine Tract Binding Protein 2 gene (*PTBP2*) is the nearest coding gene, 285.8 kb downstream of rs10747478, located on chromosome 1. The *PTBP2* locus is genome-wide associated with BMI [45]. The PTBP2 protein is expressed at high levels in the adult brain, muscle, and testis [46, 47]. In a recent study, the enriched expression level of the PTBP2 protein was found in patients with obesity compared to healthy individuals with normal body weight [14].

### Mutation screen-detected 25 variants in *PTBP2*

**Fig. 1:**
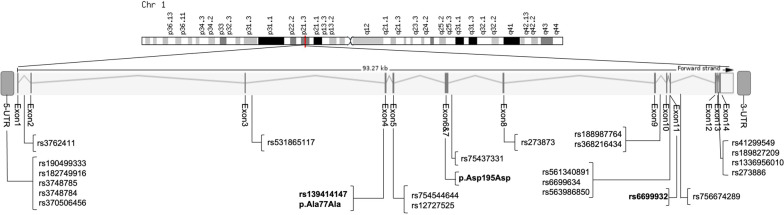
Genomic structure of the *PTBP2* gene (derived from Ensembl, GRCh38.p13) including the location of the 25 detected variants. Bold: Variants located in the coding region.

We performed a mutation screen by Sanger sequencing in the *PTBP2* gene in 192 female patients with (acute or recovered) AN and 191 children or adolescents with (extreme) obesity. All 14 coding exons and the 5′ UTR of the *PTBP2* gene, as well as a part of flanking intronic regions, were sequenced. Twenty-five variants were identified, including four synonymous variants, and 21 intronic variants including one deletion (Fig. 1). Four intronic SNPs were only detected in the patients with (extreme) obesity and six variants including two novel synonymous were only found in female patients with AN. The frequencies of alleles of all detected variants in *PTBP2* are shown in Supplementary Table S2. Among all sequenced individuals, frequencies of all detected variants were in Hardy–Weinberg equilibrium (*p* value >0.05).

The known variant rs139414147 (p.Gly49Gly) and a novel (not described in Gnomad: https://gnomad.broadinstitute.org/ and NCBI: https://www.ncbi.nlm.nih.gov/) variant (position 1:96769818, p.Ala77Ala) are located in exon 4, another novel variant (position 1:96777737 and p.Asp195Asp) is located in exon 6. In exon 11 the only frequent SNP (rs6699932, p.Leu376Leu, MAF for AN = 0.169, MAF for obesity = 0.178) was detected. The rare synonymous variants rs139414147 (p.Gly49Gly) and rs6699932 (p.Leu376Leu) were identified in both study groups. The two novel SNPs (p.Ala77Ala and p.Asp195Asp) were each only detected once respectively in two patients with AN.

Of the remaining 23 variants 15 were found in both phenotypes, four variants (rs531865117, rs754544644, rs368216434, and rs6699634) were only detected in patients with AN and four variants (rs188987764, rs563986850, rs756674289, and rs1336956010) were exclusively identified in patients with (extreme) obesity.

All detected variants with dbSNP ID were looked up in two recent GWAS sources for AN [10] and BMI [11]. The *p* values and effect alleles of detected variants are shown in Table 2 (*p* values were plotted against the chromosomal location of the corresponding variants; Supplementary Table S3). Two intronic SNPs (rs12727525 and rs273873) were significantly associated with BMI in males and in both sexes combined. One intronic SNP (rs3762411) was only significantly associated with BMI in males. No genome-wide significant hit for AN or BMI was identified in females.

**Table 2:** Variants in *PTBP2* looked up in GWAS datasets for BMI and AN.

dbSNP_ID	MA^a^	BMI^b^							AN^c^	
		Combined sexes			Female		Male			
		Freq.^d^	beta^e^	*p* value^f^	beta	*p* value	beta	*p* value	beta	*p* value
**rs190499333**	A	0.007	0.015	3.04 × 10^−1^	0.026	1.98 × 10^−1^	0.0088	6.89 × 10^−1^	NA	
**rs182749916**	A	NA^g^							−0.121	2.82 × 10^−6^
**rs3748785**	A	0.306	−0.010	1.05 × 10^−6^	−0.007	9.81 × 10^−3^	−0.014	3.66 × 10^−6^	−0.009	5.62 × 10^−1^
**rs3748784**	A	0.486	−0.009	3.09 × 10^−6^	−0.005	5.84 × 10^−2^	−0.013	4.03 × 10^−6^	0.012	3.81 × 10^−1^
**rs370506456**	A	0.006	0.037	1.18 × 10^−2^	0.051	1.22 × 10^−2^	0.038	8.59 × 10^−2^	NA	
**rs3762411**	G	0.485	−0.010	**2.90** × **10**^−**8**^	−0.007	3.86 × 10^−3^	−0.013	3.84 × 10^−7^	0.013	3.41 × 10^−1^
**rs531865117**	C	0.010	−0.014	1.38 × 10^−1^	−0.021	1.11 × 10^−1^	0.0004	9.76 × 10^−1^	NA	
**rs139414147**	G	0.002	−0.006	8.19 × 10^−1^	−0.027	4.31 × 10^−1^	0.026	4.74 × 10^−1^	NA	
**rs12727525**	C	0.332	0.014	**2.33** × **10**^−**12**^	0.011	1.37 × 10^−4^	0.019	**1.64** × **10**^−**10**^	0.060	4.22 × 10^−5^
**rs75437331**	G	0.019	0.006	4.21 × 10^−1^	0.004	7.02 × 10^−1^	−0.002	8.90 × 10^−1^	0.048	4.69 × 10^−1^
**rs273873**	T	0.327	0.015	**4.69** × **10**^−**17**^	0.012	8.00 × 10^−7^	0.020	**4.53** × **10**^−**14**^	NA	
**rs188987764**	G	0.002	−0.009	7.46 × 10^−1^	0.015	6.97 × 10^−1^	−0.06	1.29 × 10^−1^	NA	
**rs368216434**	G	0.0005	−0.051	2.35 × 10^−1^	−0.075	2.00 × 10^−1^	0	9.96 × 10^−1^	NA	
**rs561340891**	D^h^	NA							0.006	9.36 × 10^−1^
**rs6699634**	C	0.007	0.003	8.06 × 10^−1^	0.007	6.63 × 10^−1^	0.005	7.76 × 10^−1^	NA	
**rs563986850**	C	0.0003	−0.011	8.87 × 10^−1^	−0.045	6.43 × 10^−1^	−0.034	7.77 × 10^−1^	NA	
**rs6699932**	G	0.191	−0.006	5.58 × 10^−3^	−0.006	4.12 × 10^−2^	−0.008	1.84 × 10^−2^	−0.066	1.37 × 10^−4^
**rs41299549**	C	0.020	0.004	5.77 × 10^−1^	0.002	8.76 × 10^−1^	−0.002	8.19 × 10^−1^	0.048	4.61 × 10^−1^
**rs189827209**	C	0.003	−0.036	6.57 × 10^−2^	−0.023	4.02 × 10^−1^	−0.046	1.16 × 10^−1^	NA	
**rs273886**	G	0.481	−0.009	3.31 × 10^−6^	−0.005	4.09 × 10^−2^	−0.013	7.44 × 10^−6^	0.014	3.04 × 10^−1^

^a^MA: minor allele (minor allele).

^b^BMI: data extracted from BMI GWAS summary statistics [11].

^c^AN: data extracted from AN GWAS summary statistics [10].

^d^Freq.: frequency of minor allele in BMI GWAS (combined sexes).

^e^beta: effect size of minor allele.

^f^*p* value: *p* value extracted from corresponding database, the *p* values in bold were genome-wide significant.

^g^NA: not available.

^h^D: deletion.

### In silico analyses

#### Conservation analysis of the detected variants in *PTBP2*

Human *PTBP2* gDNA was compared to orthologous gDNA of 35 other species in five superorders (ten primates, five rodents and related species, ten laurasiatherian, five sauropsids, and five fishes) by MegAlign DNAstar, Inc. (version: 10.1.0) with the cluster W Method. The overall alignment comparisons are shown in the supplement (Supplementary documents SD1–5).

Conservation analysis of all detected mutated nucleotide positions is shown as a percentage (Supplementary Table S4-1). The genomic positions of the two novel variants (p.Ala77Ala and p.Asp195Asp), one intronic SNP (rs6699634), and one intronic deletion (rs561340891) are 100% conserved in the 25 analyzed primates, sauropsids, and laurasiatherians. Among 36 species, the two novel synonymous variants, three intronic SNPs, and one non-coding region deletion are highly conserved (percentage of conservation larger than 85%).

#### Functional effects of the detected variants

Although all detected variants are synonymous and do not alter the amino acid sequence of encoded proteins, there is still potential to change the accuracy and efficiency of mRNA’s splicing or folding process and stability [48]. MutationTaster2 first analyzed the deleteriousness of detected variants to generate an overall prediction, and then an integrated predictor PredictSNP2 evaluated the pathogenesis of single nucleotide exchanges. The potentially varied mRNA splicing pattern was estimated in three in silico prediction tools. The advances in high-throughput molecular techniques show growing evidence that genetic variants have an impact on the establishment of DNA methylation, histone acetylation, and microRNAs which are involved in multiple important human developmental processes, such as transcriptional regulation, genomic stability, and metabolism [32, 36, 49–51]. Thus, detected variants were looked up in the brain xQTL and PolymiRTS Database 3.0 databases to understand the functional consequences of the variants in gene expression, methylation, histone modification, and putative microRNA function alteration [35]. However, no detected variants were significantly associated in xQTL and none of them were located in microRNA-relevant regions (results generated by xQTL in Supplementary Tables S4-7–9).

FABIAN and RegulationSpotter showed the putative alteration of gain- or loss-probability of transcription factor binding sites and the potential histone modification [38, 39]. The two predictors are available only for single nucleotide variants and all 24 SNPs were predicted to affect at least one known TFBS in FABIAN and alter histone modification in RegulationSpotter (Supplementary Table S4-11~12).

Predictions of the in silico analyses were illustrated in Supplementary Tables S4-3 (detailed results of separated predictors were in Supplementary Tables S4-4~12). The two novel variants (p.Ala77Ala and p.Asp195Asp), one known synonymous variant (rs139414147, p.Gly49Gly) and five non-coding region variants (four SNPs and one intronic deletion) were predicted as “may trigger functional consequence” based on the predictions of all available analyses.

The AN hit rs10747478 is located in ~285.8 kb upstream of the coding region of *PTBP2* and thus not included in the sequenced region of our study. The potential functional consequences due to this SNP were estimated by PredictSNP2, ESEfinder3.0, RegulationSpotter, FABIAN, and looked up in xQTL (Supplementary Table S1–3). This SNP may alter splicing enhancers and a putative methylation feature (cg13557213, beta = 0.307, *p* value = 2.53 × 10^−13^) located in the ~247 kb upstream region of *PTBP2*. However, the interaction between this SNP and our candidate gene *PTBP2* was not significant (*p* value = 0.85) and FABIAN did not imply an effect on a known transcription factor binding site.

The PTBP2 protein function prediction in our study was also examined in GeneMAMIA and 20 genes were explored to be interacting with *PTBP2*. Half of the associated genes were either demonstrated to associate with body weight regulation in previous functional studies or to harbor genome-wide significant BMI or weight GWAS hits (Supplementary Tables S4–13).

#### Linkage disequilibrium analyses for detected variants

LD analysis for detected variants (except the intronic deletion) was performed in Haploview. Two different LD blocks (Supplementary Table S5-1) were revealed in the study groups with female patients with AN or obesity. The LD blocks of female and male individuals with obesity were slightly different.

The detected variants and the AN GWAS hit rs10747478 were analyzed via LDmatrix (Supplementary Table S5-2) based on the genotypes of the 1000 G project (European). Seven SNPs with dbSNP ID and the two novel variants cannot be analyzed in this tool. However, none of the identified variants had an LD structure exceeding the predetermined threshold value (*r*^2^ > 0.3 and D′ > 0.8). The strongest LD between detected variants and rs10747478 is for rs6699932 (*r*^2^ = 0.142, D′ = 0.614; for all LD data see Supplementary Table S5-3).

Whether these detected variants were strongly linked to the known AN or BMI GWAS SNPs was analyzed by two tools (LDexpress and LDtrait) in LDlink. The genome-wide significant trait-associated variants which exceeded the threshold (overlapping ± 500 kb, *r*^2^ ≥ 0.3, D ≥ 0.8, *p* value < 5 × 10^−8^) are summarized in Supplementary Tables S5-4 and S5-5. Four intronic variants (rs3762411, rs3748785, rs3748784, and rs273886) were in strong LD to the variants that were significantly expressed in the subcutaneous adipose tissue (Supplementary Tables S5-4). These four SNPs and the other two detected intronic SNPs (rs12727525 and rs182749916) are highly linked to the BMI GWAS hits via LDtrait (Supplementary Tables S5-5). Moreover, the intronic SNP rs182749916 and one frequent synonymous SNP rs6699932 are in the strong LD with SNP rs720090, a CC-GWAS (Case to Case Genome-wide Association Study) hit for both AN and MDD (major depressive disorder) [52].

#### Sex-specific analyses on sex-stratified BMI GWAS

**Fig. 2:**
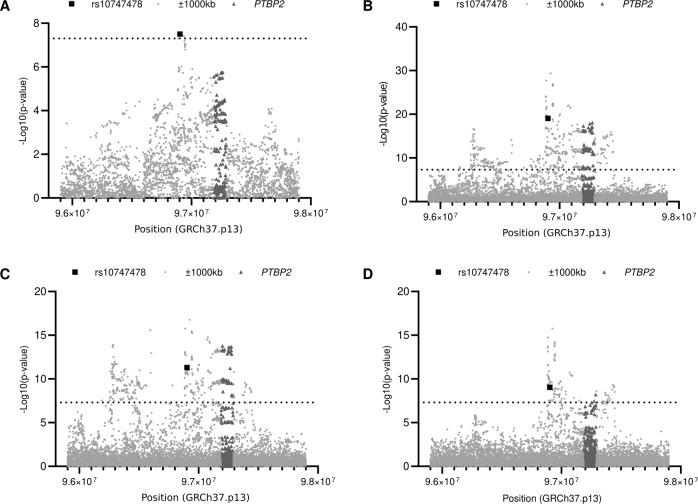
Plots of the chromosomal location and *p* values of variants located near rs10747478 (±1000 kb) on BMI GWAS and AN GWAS data. Variants were extracted from Pulit et al. BMI GWAS [11] and Watson et al. AN GWAS [10]. **A**: AN, **B**: BMI (combined sexes), **C**: BMI males, **D**: BMI females. The −log10(*p* value) for the association are shown on the y-axis and the chromosomal locations are ordered on the x-axis. The variants which surpassed the dashed line are genome-wide significant. Light gray dots indicate ±1000 kb genomic region of rs10747478, black square pointed to SNP rs10747478, and variants located in the *PTBP2* genomic region plotted with dark gray triangles.

A look-up was performed in the AN GWAS summary statistics [10] (https://www.med.unc.edu/pgc/download-results/) and the BMI GWAS summary statistics [11] (https://zenodo.org/record/1251813#.YT8QxJ0zZEZ) in the region flanking rs10747478 ± 1000 kb (Supplementary Tables S7, 8). The variants were plotted with respective *p* values (Fig. 2). The distribution of genome-wide significant variants for BMI located in the *PTBP2* gene was significantly different between females and males.

For the *PTBP2* genomic region (rs10747478 ± 1000 kb), 72 SNPs were genome-wide associated with BMI in both sexes combined, 60 of these were significant in males and two variants (one of the two variants is significant in both sexes) in females (significant variants in both sexes summarized in Supplementary Table S6-1). In the region, ~1200 kb upstream of *PTBP2* including the SNP rs10747478, 99 (in females) and 377 (in males) variants were identified as genome-wide significant and the downstream region of *PTBP2* gene only 20 (in females) and 17 (in males) significant variants were identified (Supplementary Table S6-2).

A *Z*-score was utilized to quantitatively compare the difference in effect size between sexes [44]. The Z-scores of more than 65% of genome-wide significant variants located within the genomic region of *PTBP2* have a sex-specific effect size on body weight regulation (Table 3). This percentage is twofold higher than within the nearby genomic region (size around 2000 kb).

**Table 3:** Assessment of *Z*-scores from sex-stratified BMI GWAS.

Regions	annotation	|*Z*-score|^b^ ≥ 2	|*Z*-score| < 2	The percentage of variants with a|*Z*-score| ≥ 2 (%)^c^
*PTBP2* genomic region	all variants	80	810	8.99
	significant variants^a^	40	21	**65.57**
*PTBP2* ± ~1000 kb genomic region	all variants	1064	20415	4.95
	significant variants	256	438	36.89
*PTBP2*− ~1000 kb upstream genomic region	all variants	720	10977	6.16
	significant variants	216	392	35.53
*PTBP2* + ~1000 kb downstream genomic region	all variants	264	8628	2.97
	significant variants	0	25	0.00

^a^significant variants: the variants with *p* value smaller than 5 × 10^−8^.

^b^|*Z*-score|: absolute value of *Z*-score.

^c^The percentage of variants with a |*Z*-score| ≥ 2: the percentage of variants with a *Z*-score (absolute value) larger than 2.

**Fig. 3:**
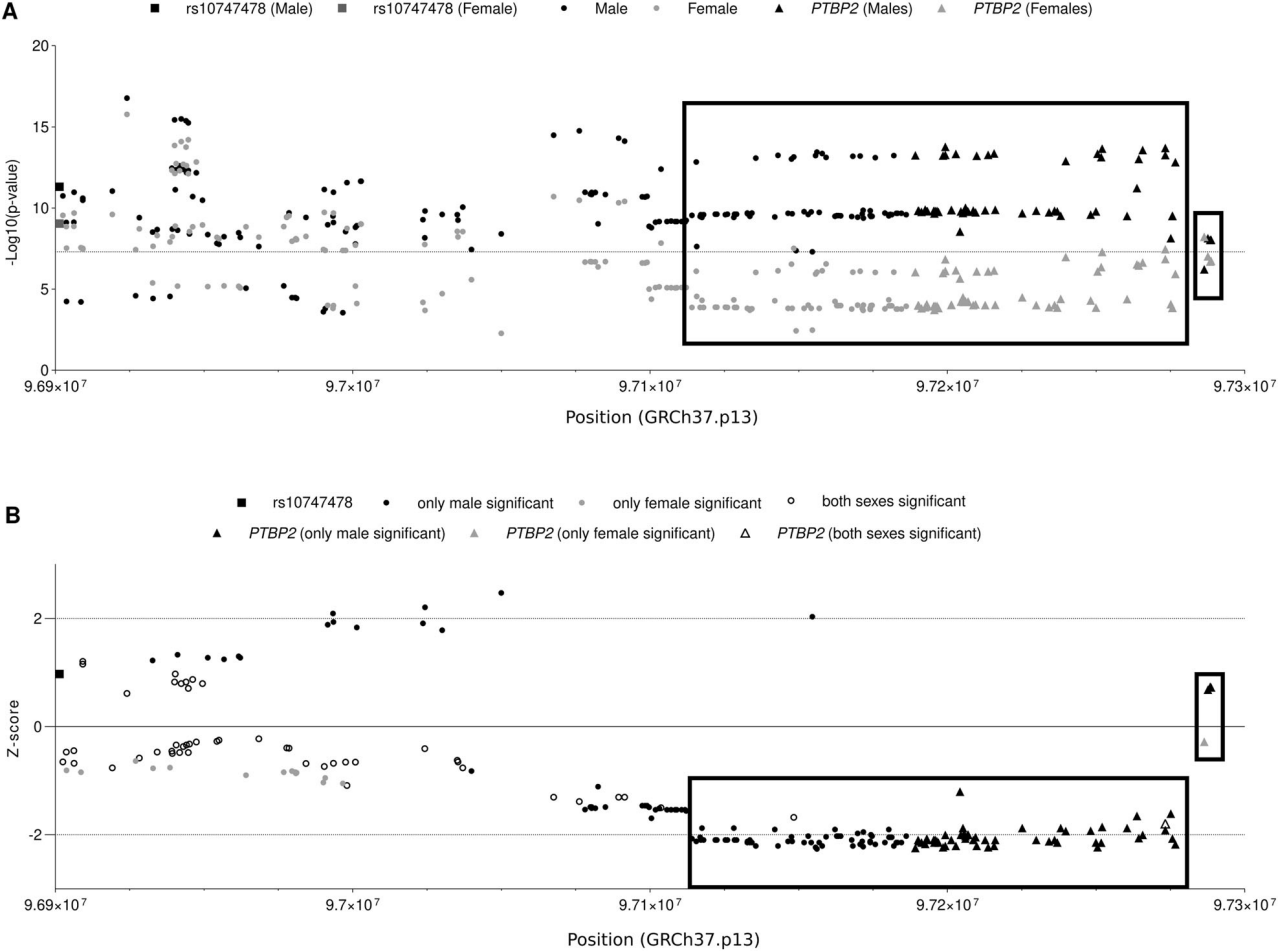
Differences between effect sizes and *p* values in females and males. Variants located in the genomic region from rs10747478 to the *PTBP2* gene were extracted from sex-stratified Pulit BMI GWAS [11] with a significant *p* value (*p value* < 5 × 10^−8^) in at least in one sex. **A** Plots of position and *p* values (for males and females), **B** Plots of *Z*-score of variants. A negative *Z*-score shows a larger effect for males than females (effect difference = beta_female_ − beta_male_). The variants with MAF larger than 0.42 were excluded. Dots indicate the variants located between rs10747478 and the *PTBP2* gene, triangles point to the variants located in the *PTBP2* gene and the SNP rs10747478 was plotted with a square. Colors are corresponding to the significance of variants: gray = only significant in females; black = only significant in males; white = significant in both sexes. Boxes indicate two putative clusters in strong LD.

To explore if the SNP rs10747478 or the *PTBP2* gene has a sex-specific effect on body weight regulation, the variants located in the genomic region (from rs10747478 to *PTBP2*) whose *p* value is at least in one sex significant (*p* value < 5 × 10^−8^), were plotted with *p* values and *Z*-scores in Fig. 3. For the variants located in the region from rs10747478 to ~100 kb upstream of the *PTBP2* gene, the *Z*-scores were distributed randomly, and most variants had similar *p* value in both sexes. No clear pattern could be described for this region. The significant variants located in the genomic region of *PTBP2* and its ~70 kb upstream region can be classified into two clusters (shown in Fig. 3). The SNP rs12563540 with the lowest *p* value in males (females *p* value = 1.439 × 10^−7^, males *p* value = 1.69 × 10^−14^, *Z*-score = −2.002) and SNP rs12060538 (females *p* value = 6.089 × 10^−9^, males *p* value = 6.066 × 10^−7^, *Z*-score = −0.279) with the lowest *p* value in females were included in the two clusters separately.

The LD analysis was used to identify, if the clusters are caused by different LD block structures in the *PTBP2* gene and its ~70 kb upstream region (shown in Supplementary Table S6–3). The variants rs12563540 and the other 117 variants (115 variants only significant in males and two variants significant in both sexes, the variants with a frequency of minor allele larger than 0.42 were excluded) are in one LD block structure. The effect sizes are larger in males than females with a *Z*-score of approximately −2 and the minor alleles of these variants have positive values of beta. Another LD block structure included four variants at the 3′UTR of the *PTBP2* gene and was led by variant rs12060538. The *Z*-scores of variants in the second LD block were nearly 0 and the beta values of minor alleles were negative.

## Discussion

Recent GWAS showed that SNP rs10747478 is associated with both, AN and BMI variance [10, 11]. The *PTBP2* gene is the nearest coding gene to rs10747478. We sequenced all coding exons of the *PTBP2* gene and identified 25 variants, including four synonymous SNPs, 20 intronic SNPs, and one intronic deletion variant. The *p* values for all analyzed variants located in the *PTBP2* gene were derived from the recent GWAS for BMI [11] or AN [10]. Three and two SNPs reached genome-wide significance for BMI in the combined sexes and male datasets, respectively. However, no detected variant was significant in females in the BMI GWAS nor in the AN GWAS.

The synonymous variants p.Ala77Ala and p.Asp195Asp, which were each detected once in patients with AN, were evaluated as “may trigger functional consequence” relied on the simulated results of available in silico prediction methods. The molecular evolutionary analyses showed that the mutated nucleic acid positions of the two SNPs are highly conserved (88.89 and 97.22% conserved percentage among 36 species in five superorders, respectively). Additionally, they were located in well-known functional domains of the PTBP2 protein. The PTBP2 protein has four RNA-recognition motifs (RRMs), which can recruit mRNAs and regulate the process of metabolism [53]. The variants p.Ala77Ala and p.Asp195Asp are located in RRM2 and RRM3, respectively. Thus, the two novel variants may have an impact on the stability of mature mRNA and protein. However, their effects on body weight regulation and AN development remain unknown.

Intronic SNPs were habitually neglected for decades. One intronic SNP (rs188987764) and the non-coding region deletion (rs561340981) were predicted as “may trigger functional consequence” in in silico analyses and were highly conserved among 36 species (conservation percentile larger than 85%). Thus, they may impact on the structure or stability of protein or mRNA. However, no previous studies nor GWAS data revealed an association between the two non-coding variants and BMI or AN. In the BMI GWAS [11] three intronic SNPs (rs3762411, rs12727525, and rs273873) were genome-wide significantly associated with BMI. Except for rs3762411, three intronic SNPs (rs3748785, rs3748784, and rs273886) may also express specifically in subcutaneous adipose tissue and the four SNPs were in strong LD with BMI GWAS hits [54, 55]. The intronic SNP rs12727525 was highly linked to multiple BMI GWAS hits [11, 56, 57]. A recent case (AN) to case (major depressive disorder: MDD) GWAS detected rs720090 which is located in intron 9 of the *PTBP2* gene [52] (*p* value = 2 × 10^−8^). The AN GWAS [10] was used for the Case to Case - GWAS approach. Intron 9 of the *PTBP2* gene was not included in our sequencing approach. However, it is in strong LD with the frequent synonymous SNP rs6699932 (p.Leu376Leu; *r*^2^ = 0.348, D′ = 1) and in perfect LD with one detected intronic SNP rs182749916 (*r*^2^ = 1, D′ = 1) analyzed via LDtrait. Additionally, SNP rs182749916 was also in strong LD with another BMI GWAS hit rs12066944 (*p* value = 2 × 10^−13^) [54]. Thus, the *PTBP2* locus is associated with BMI, AN, and MDD.

PTBP2 controls a genetic program essential for neuronal maturation. Thus, Ptbp2 null mice display cyanosis and die immediately after birth due to respiratory failure [12]. More specific knock-out models for Ptbp2 are currently not available. GeneMANIA reported 20 genes which are interacting with *PTBP2* and half of them were shown to be relevant in body weight regulation.

The sex-stratified BMI GWAS [11] showed that genome-wide significant variants for BMI were more frequent in males than in females in the *PTBP2* genomic region. LD analyses and comparison of the effect size differences (measured in *Z*-scores) between sexes identified two clusters in *PTBP2* and its 70 kb upstream region. One cluster in strong LD is composed of 60 significant variants located in 70 kb upstream of *PTBP2* and 57 significant variants in *PTBP2* and displays a larger effect size on BMI in males (*Z*-score of approximately −2) and is associated with increasing BMI. Lead SNP rs12563540 displays the lowest *p* value in males. Another LD block (four variants located in the 3′ UTR of *PTBP2*) is driven by one (only significant in females) variant rs12060538 with ambiguous effects (*Z*-score of ~0) on both sexes and may promote reduced body weight. Thus, variants in the LD block (5′UTR and the coding region of the *PTBP2* gene and its ~70 kb upstream region) with larger effect sizes in males are associated with increased body weight.

Recently, a dual-luciferase assay on the *PTBP2* putative promoter showed that one intronic SNP (rs12409479, 386 kb upstream of *PTBP2*) may regulate the expression level of the PTBP2 protein. By analogy, the ~70 kb upstream region of *PTBP2* containing the putative promoter may regulate the expression of the PTBP2 protein and/or mRNA.

Whereas the effects size of variants located in the *PTBP2* gene and its upstream region on BMI shows sex-specific differences between males and females, it is unclear what functional effects the variants in the *PTBP2* gene have. Two processed pseudogenes, *UBE2WP1* and *EEF1A1P11*, are located closest to the SNP. However, their effect on AN or body weight is not very likely.

## Conclusion

SNP rs10747478 near the *PTBP2* gene is genome-wide and significantly associated with AN [10] and body weight regulation [11].

A mutation screen of *PTBP2* in 192 females with (acute or recovered) AN and 191 children or adolescents with (extreme) obesity revealed 25 variants. These include one intronic deletion and four synonymous variants (two of them are novel). The two novel synonymous SNPs (p.Ala77Ala and p.Asp195Asp), one intronic SNP (rs188987764), and the intronic deletion (rs561340981) located in highly conserved and functional RRMs may have an effect on structure and stability of mature mRNA or protein. The in silico analyses on detected variants implied that the *PTBP2* gene may be relevant for BMI, AN, and MDD.

Previously Pulit et al. demonstrated that heritability and variants effects on WHR were larger in females than in males. However, the sex-stratified BMI GWAS summary statistics [11] showed that the *PTBP2* gene and it is ~70 kb upstream region may have a larger effect on body weight regulation in males than in females.

## Websites

### Database

https://zenodo.org/record/1251813#.YT8QxJ0zZEZ

https://www.med.unc.edu/pgc/download-results/

### Software

http://www.mutationtaster.org/

http://krainer01.cshl.edu/cgi-bin/tools/ESE3/esefinder.cgi

http://fairbrother.biomed.brown.edu/spliceman/

http://fairbrother.biomed.brown.edu/spliceman2/upload

https://sourceforge.net/projects/spicev2-1/

https://www.medcalc.org/calc/odds_ratio.php

https://www.socscistatistics.com/tests/fisher/default2.aspx

https://www.broadinstitute.org/haploview/haploview

https://ldlink.nci.nih.gov/?tab=home

https://ldlink.nci.nih.gov/?tab=ldmatrix

https://ldlink.nci.nih.gov/?tab=ldtrait

https://ldlink.nci.nih.gov/?tab=ldexpress

https://ldlink.nci.nih.gov/?tab=ldhap

## Supplementary information

gDNA alignment report (Primate)

gDNA alignment report (Sauropsidas)

gDNA alignment report (Laurasiatheria)

gDNA alignment report (Rodent)

gDNA alignment report (Fish)

Supplementary Table
