# Supplementary material for: PTBP2 – a gene with relevance for both Anorexia nervosa and body weight regulation
Source: Transl Psychiatry. 2022 Jun 9;12:241. doi: 10.1038/s41398-022-02018-5 (PMC9184595; doi:10.1038/s41398-022-02018-5)
Supplement: Supplementary file 2 — gDNA alignment report (Sauropsidas) [file 41398_2022_2018_MOESM2_ESM.pdf]

```
Majority                 --------------------------------------------------------------------------------
                                  10        20        30        40        50        60        70        80
Human                    TGCCTCCAAATGTTAAACTCCAACTATTGTAGCATTTTAACACATGGCTCCATTTGTGTCTATTTCCACCTAGCGTATTG 80
Kakapo                   -------------------------------------------------------------------------------- 0
GoldenEagle              -------------------------------------------------------------------------------- 0
JapaneseQuail            -------------------------------------------------------------------------------- 0
MediumGroundFinch        -------------------------------------------------------------------------------- 0
GoodesThornscrubTortoise-------------------------------------------------------------------------------- 0

Majority                 --------------------------------------------------------------------------------
                                  90       100       110       120       130       140       150       160
Human                    TGAATATTTTCTACAGGCGCACACTAATCACCAACTGAAGTGAGAATCACATTTTCCTCCACTTGGCTTGGTACTACAGG 160
Kakapo                   -------------------------------------------------------------------------------- 0
GoldenEagle              -------------------------------------------------------------------------------- 0
JapaneseQuail            -------------------------------------------------------------------------------- 0
MediumGroundFinch        -------------------------------------------------------------------------------- 0
GoodesThornscrubTortoise-------------------------------------------------------------------------------- 0

Majority                 --------------------------------------------------------------------------------
                                 170       180       190       200       210       220       230       240
Human                    TCTTGAAGCAAAAGCCTGTATTTCAGTCTGAGGAGTTCTACTTGCTTGAGAAAGTACTGTTGTTACCAAGTGGTCTCGTT 240
Kakapo                   -------------------------------------------------------------------------------- 0
GoldenEagle              -------------------------------------------------------------------------------- 0
JapaneseQuail            -------------------------------------------------------------------------------- 0
MediumGroundFinch        -------------------------------------------------------------------------------- 0
GoodesThornscrubTortoise-------------------------------------------------------------------------------- 0

Majority                 --------------------------------------------------------------------------------
                                 250       260       270       280       290       300       310       320
Human                    CAGTGGGGTAAAGATTTTTGTTTTAGAGGCTGGAGCCGGTCTGCAAATAAAAGATGGCTTAGGAGAAAAGCTATCCAATG 320
Kakapo                   -------------------------------------------------------------------------------- 0
GoldenEagle              -------------------------------------------------------------------------------- 0
JapaneseQuail            -------------------------------------------------------------------------------- 0
MediumGroundFinch        -------------------------------------------------------------------------------- 0
GoodesThornscrubTortoise-------------------------------------------------------------------------------- 0
```

```
Majority                   --------------------------------------------------------------------------------
                                     330       340       350       360       370       380       390       400
Human                      TTTTTTCCTTGGCCCGCATAGGTTGACGGTAGCCAAGCTGGTACTTGGCTTGGAGTGAAAAGTGGAGGCTGCCTGGGGGC 400
Kakapo                     -------------------------------------------------------------------------------- 0
GoldenEagle                -------------------------------------------------------------------------------- 0
JapaneseQuail              -------------------------------------------------------------------------------- 0
MediumGroundFinch          -------------------------------------------------------------------------------- 0
GoodesThornscrubTortoise   -------------------------------------------------------------------------------- 0

Majority                   --------------------------------------------------------------------------------
                                     410       420       430       440       450       460       470       480
Human                      GTCCTGCAGTCAGGGCAGAGAGAGTGTGGAGAGCTGTTGGGAGGTGTCCCACCCACGCCCCCACAAGGAGGGGAAACCAG 480
Kakapo                     -------------------------------------------------------------------------------- 0
GoldenEagle                -------------------------------------------------------------------------------- 0
JapaneseQuail              -------------------------------------------------------------------------------- 0
MediumGroundFinch          -------------------------------------------------------------------------------- 0
GoodesThornscrubTortoise   -------------------------------------------------------------------------------- 0

Majority                   --------------------------------------------------------------------------------
                                     490       500       510       520       530       540       550       560
Human                      GTCGCCGCGGTCTGCAGCAGAACGCCAGGTTCCAGAGGACGGCAGGCGGAGGGCGGAGGGAGAAAGGCGCGCGAAGGCGC 560
Kakapo                     -------------------------------------------------------------------------------- 0
GoldenEagle                -------------------------------------------------------------------------------- 0
JapaneseQuail              -------------------------------------------------------------------------------- 0
MediumGroundFinch          -------------------------------------------------------------------------------- 0
GoodesThornscrubTortoise   -------------------------------------------------------------------------------- 0

Majority                   --------------------------------------------------------------------------------
                                     570       580       590       600       610       620       630       640
Human                      GGAGGAGGCGTCGGCTGCGGCGCCCACTCCCCCTAGTCCCAGCGCCCTGCCGCTCGCCTCCCGCTGCCGGGCGCCGGCGC 640
Kakapo                     -------------------------------------------------------------------------------- 0
GoldenEagle                -------------------------------------------------------------------------------- 0
JapaneseQuail              -------------------------------------------------------------------------------- 0
MediumGroundFinch          -------------------------------------------------------------------------------- 0
GoodesThornscrubTortoise   -------------------------------------------------------------------------------- 0
```

```
Majority                 --------------------------------------------------------------------------------

                                 650       660       670       680       690       700       710       720

Human                    GCTCGGCTCTTTCCGCCGCCGCCGCTGCCGCGCGGTGGCCCGTGCGCCTCGGCACCTTCGGCAATTTCCGTCGGGCCCCA 720
Kakapo                   -------------------------------------------------------------------------------- 0
GoldenEagle              -------------------------------------------------------------------------------- 0
JapaneseQuail            -------------------------------------------------------------------------------- 0
MediumGroundFinch        -------------------------------------------------------------------------------- 0
GoodesThornscrubTortoise-------------------------------------------------------------------------------- 0

Majority                 --------------------------------------------------------------------------------

                                 730       740       750       760       770       780       790       800

Human                    GCCGCCATTTTCTCGCCGCTTGTGTGGCTCGCTGGCTGCGTGGCTCGGTTCTTGTGAGCGAAGCTTTGTCCGGTTCGGCA 800
Kakapo                   -------------------------------------------------------------------------------- 0
GoldenEagle              -------------------------------------------------------------------------------- 0
JapaneseQuail            -------------------------------------------------------------------------------- 0
MediumGroundFinch        -------------------------------------------------------------------------------- 0
GoodesThornscrubTortoise-------------------------------------------------------------------------------- 0

Majority                 --------------------------------------------------------------------------------

                                 810       820       830       840       850       860       870       880

Human                    ATGGACGGGTATGTAATCGGGCCGGCGAGAAGGTGTGTGTGAGAGAGGAGTTGGACCGTCCTTCGGCCCGGTCCCGGGCC 880
Kakapo                   -------------------------------------------------------------------------------- 0
GoldenEagle              -------------------------------------------------------------------------------- 0
JapaneseQuail            -------------------------------------------------------------------------------- 0
MediumGroundFinch        -------------------------------------------------------------------------------- 0
GoodesThornscrubTortoise-------------------------------------------------------------------------------- 0

Majority                 --------------------------------------------------------------------------------

                                 890       900       910       920       930       940       950       960

Human                    GGGGAGAAACCCTCCCGCGGCCCCTCCCAGGGCTGGGCTGCCGTTACCCAACCCCCGCCCCATCGCACACACCCCTCCCT 960
Kakapo                   -------------------------------------------------------------------------------- 0
GoldenEagle              -------------------------------------------------------------------------------- 0
JapaneseQuail            -------------------------------------------------------------------------------- 0
MediumGroundFinch        -------------------------------------------------------------------------------- 0
GoodesThornscrubTortoise-------------------------------------------------------------------------------- 0
```

```
Majority                  --------------------------------------------------------------------------------

                                   970       980       990      1000      1010      1020      1030      1040

Human                     TTGCTTCCCCCGGCGGGCTTTGGTCGAGAAAATGAGAAGAAAGCGGGCTTGGAGGCTGGGGAGGCATAGGGGCGATGGCG 1040
Kakapo                    -------------------------------------------------------------------------------- 0
GoldenEagle               -------------------------------------------------------------------------------- 0
JapaneseQuail             -------------------------------------------------------------------------------- 0
MediumGroundFinch         -------------------------------------------------------------------------------- 0
GoodesThornscrubTortoise  -------------------------------------------------------------------------------- 0

Majority                  --------------------------------------------------------------------------------

                                  1050      1060      1070      1080      1090      1100      1110      1120

Human                     GGGGTGAGGATCCCGGAGTGGGAGCGGGCACCGGCTTGGCGGCGGGGGATGGGGTGGGAACCCCATAACGTCTCCCCGGT 1120
Kakapo                    -------------------------------------------------------------------------------- 0
GoldenEagle               -------------------------------------------------------------------------------- 0
JapaneseQuail             -------------------------------------------------------------------------------- 0
MediumGroundFinch         -------------------------------------------------------------------------------- 0
GoodesThornscrubTortoise  -------------------------------------------------------------------------------- 0

Majority                  --------------------------------------------------------------------------------

                                  1130      1140      1150      1160      1170      1180      1190      1200

Human                     CTGTCCCTGCCCCCTCTAGGAGCCATTTCGATCCGTACCTTGGGACCCGACCCTTGGGTTAGCGGTGCCTGTGAGAGCGA 1200
Kakapo                    -------------------------------------------------------------------------------- 0
GoldenEagle               -------------------------------------------------------------------------------- 0
JapaneseQuail             -------------------------------------------------------------------------------- 0
MediumGroundFinch         -------------------------------------------------------------------------------- 0
GoodesThornscrubTortoise  -------------------------------------------------------------------------------- 0

Majority                  --------------------------------------------------------------------------------

                                  1210      1220      1230      1240      1250      1260      1270      1280

Human                     GTGGGATGGGCAGAGACAGGCCTTTGGATTGGGGGAGCCTCTAGGGGAAGAGGAGAGAGGCACCCCATGTGGACCCCAGC 1280
Kakapo                    -------------------------------------------------------------------------------- 0
GoldenEagle               -------------------------------------------------------------------------------- 0
JapaneseQuail             -------------------------------------------------------------------------------- 0
MediumGroundFinch         -------------------------------------------------------------------------------- 0
GoodesThornscrubTortoise  -------------------------------------------------------------------------------- 0
```

```
Majority                 --------------------------------------------------------------------------------

                                  1290      1300      1310      1320      1330      1340      1350      1360

Human                    CATGAGCAACCTCTCCGGCCTCGCCCGGCCCTCCGTGGTCGGGAGAGATGCCGGTGGCGGGAGCTCCGGGGAAAGCCTAG 1360
Kakapo                   -------------------------------------------------------------------------------- 0
GoldenEagle              -------------------------------------------------------------------------------- 0
JapaneseQuail            -------------------------------------------------------------------------------- 0
MediumGroundFinch        -------------------------------------------------------------------------------- 0
GoodesThornscrubTortoise-------------------------------------------------------------------------------- 0

Majority                 --------------------------------------------------------------------------------

                                  1370      1380      1390      1400      1410      1420      1430      1440

Human                    TGGGAGCCGCTGGGGAAGGGGGGAGGGCGTGCGGCGGCGGGAGGAAGGGGGAGGGCAGATGTCATACTCCTTTGTTTTCA 1440
Kakapo                   -------------------------------------------------------------------------------- 0
GoldenEagle              -------------------------------------------------------------------------------- 0
JapaneseQuail            -------------------------------------------------------------------------------- 0
MediumGroundFinch        -------------------------------------------------------------------------------- 0
GoodesThornscrubTortoise-------------------------------------------------------------------------------- 0

Majority                 --------------------------------------------------------------------------------

                                  1450      1460      1470      1480      1490      1500      1510      1520

Human                    TTTGAGTCTGGTAGTGGGGGCGGGAGGGAGGAAAAAATGCCTTTTTGTGGGGACTGAAAACATTCAAGGCTTGGCAAAAG 1520
Kakapo                   -------------------------------------------------------------------------------- 0
GoldenEagle              -------------------------------------------------------------------------------- 0
JapaneseQuail            -------------------------------------------------------------------------------- 0
MediumGroundFinch        -------------------------------------------------------------------------------- 0
GoodesThornscrubTortoise-------------------------------------------------------------------------------- 0

Majority                 --------------------------------------------------------------------------------

                                  1530      1540      1550      1560      1570      1580      1590      1600

Human                    GGCCCAGAAATTAAATCATCTAAAAAACGAAGTGTTTGAAGCCGACATCAGTCACATGGGCAAAGGCCAACAGCAATGGC 1600
Kakapo                   -------------------------------------------------------------------------------- 0
GoldenEagle              -------------------------------------------------------------------------------- 0
JapaneseQuail            -------------------------------------------------------------------------------- 0
MediumGroundFinch        -------------------------------------------------------------------------------- 0
GoodesThornscrubTortoise-------------------------------------------------------------------------------- 0
```

```
Majority                   --------------------------------------------------------------------------------
                                   1610      1620      1630      1640      1650      1660      1670      1680
Human                      AGAAACAAGAGCAGATAGTCAACAAAGGGCTATCTCTTTAGCTACTTTGGTCAGTTTCTGGAAAAACGACTATAAAAACC 1680
Kakapo                     -------------------------------------------------------------------------------- 0
GoldenEagle                -------------------------------------------------------------------------------- 0
JapaneseQuail              -------------------------------------------------------------------------------- 0
MediumGroundFinch          -------------------------------------------------------------------------------- 0
GoodesThornscrubTortoise   -------------------------------------------------------------------------------- 0

Majority                   --------------------------------------------------------------------------------
                                   1690      1700      1710      1720      1730      1740      1750      1760
Human                      CAGAACCATGCTGCATCTTTGCGAACCTGAGTCAAGACGGAATGCTGAGTGAGGTGAGAGGCAAGACTGTGAAATGGTAG 1760
Kakapo                     -------------------------------------------------------------------------------- 0
GoldenEagle                -------------------------------------------------------------------------------- 0
JapaneseQuail              -------------------------------------------------------------------------------- 0
MediumGroundFinch          -------------------------------------------------------------------------------- 0
GoodesThornscrubTortoise   -------------------------------------------------------------------------------- 0

Majority                   --------------------------------------------------------------------------------
                                   1770      1780      1790      1800      1810      1820      1830      1840
Human                      GGACCGATGTCTCGCTCATATGCTTCACACCTGAAAAGACTCAGTAATTTAACTCAATGTTAGAAAGGGGGCACGCTTAA 1840
Kakapo                     -------------------------------------------------------------------------------- 0
GoldenEagle                -------------------------------------------------------------------------------- 0
JapaneseQuail              -------------------------------------------------------------------------------- 0
MediumGroundFinch          -------------------------------------------------------------------------------- 0
GoodesThornscrubTortoise   -------------------------------------------------------------------------------- 0

Majority                   --------------------------------------------------------------------------------
                                   1850      1860      1870      1880      1890      1900      1910      1920
Human                      GGTCAAAAATACAGCAGTATGCAGCTCTTTTGGGAGTGTAAACATAGTATCATTTAACAAGTACATTTATTAAAATCAGT 1920
Kakapo                     -------------------------------------------------------------------------------- 0
GoldenEagle                -------------------------------------------------------------------------------- 0
JapaneseQuail              -------------------------------------------------------------------------------- 0
MediumGroundFinch          -------------------------------------------------------------------------------- 0
GoodesThornscrubTortoise   -------------------------------------------------------------------------------- 0
```

```
Majority                 --------------------------------------------------------------------------------
                                  1930      1940      1950      1960      1970      1980      1990      2000
Human                    GAGTATTTGAAGAATCAACAATTTGGTTAAGCGCCTAGAGTTTGGTGTGTTTTAAAAGGTACTAAAAACAAGATTTTATA 2000
Kakapo                   -------------------------------------------------------------------------------- 0
GoldenEagle              -------------------------------------------------------------------------------- 0
JapaneseQuail            -------------------------------------------------------------------------------- 0
MediumGroundFinch        -------------------------------------------------------------------------------- 0
GoodesThornscrubTortoise-------------------------------------------------------------------------------- 0

Majority                 --------------------------------------------------------------------------------
                                  2010      2020      2030      2040      2050      2060      2070      2080
Human                    AAAGTTAACACAGTCTCACAGAATTCAAATCATCCTAGATGTTACCTAAAGATTATTTTCCAGATGAAACAAATCCAGAG 2080
Kakapo                   -------------------------------------------------------------------------------- 0
GoldenEagle              -------------------------------------------------------------------------------- 0
JapaneseQuail            -------------------------------------------------------------------------------- 0
MediumGroundFinch        -------------------------------------------------------------------------------- 0
GoodesThornscrubTortoise-------------------------------------------------------------------------------- 0

Majority                 --------------------------------------------------------------------------------
                                  2090      2100      2110      2120      2130      2140      2150      2160
Human                    AAGTTCAGTATTCCCCCCAGATCAACACGGAAATTGATGTCAAAACTTGCACTAGAATTCATGTTTCCTGATTCCTGGTT 2160
Kakapo                   -------------------------------------------------------------------------------- 0
GoldenEagle              -------------------------------------------------------------------------------- 0
JapaneseQuail            -------------------------------------------------------------------------------- 0
MediumGroundFinch        -------------------------------------------------------------------------------- 0
GoodesThornscrubTortoise-------------------------------------------------------------------------------- 0

Majority                 --------------------------------------------------------------------------------
                                  2170      2180      2190      2200      2210      2220      2230      2240
Human                    CCTGTGGAGACTACTAAAGAGTTTAAGCGTTAAATTATTAATCTCAGTATACTGGCATTGTAGAAATGGCATAGTGTTTT 2240
Kakapo                   -------------------------------------------------------------------------------- 0
GoldenEagle              -------------------------------------------------------------------------------- 0
JapaneseQuail            -------------------------------------------------------------------------------- 0
MediumGroundFinch        -------------------------------------------------------------------------------- 0
GoodesThornscrubTortoise-------------------------------------------------------------------------------- 0
```

```
Majority                  --------------------------------------------------------------------------------

                                   2250      2260      2270      2280      2290      2300      2310      2320

Human                     GGGTTTTGTTCTTTTTGGCTATTGAATGTGTTGTTAAAAAATACCACAAGTTGTCTCACATTTGAAAGTTACTTTTAGAA 2320
Kakapo                    -------------------------------------------------------------------------------- 0
GoldenEagle               -------------------------------------------------------------------------------- 0
JapaneseQuail             -------------------------------------------------------------------------------- 0
MediumGroundFinch         -------------------------------------------------------------------------------- 0
GoodesThornscrubTortoise  -------------------------------------------------------------------------------- 0

Majority                  --------------------------------------------------------------------------------

                                   2330      2340      2350      2360      2370      2380      2390      2400

Human                     AACCAGGTTGGTAACAGTACAGGTAAGTCACGGATCATCTGTGGTATTTTTTATCCCCATCTGTGTGTGAGTGGCTGGAA 2400
Kakapo                    -------------------------------------------------------------------------------- 0
GoldenEagle               -------------------------------------------------------------------------------- 0
JapaneseQuail             -------------------------------------------------------------------------------- 0
MediumGroundFinch         -------------------------------------------------------------------------------- 0
GoodesThornscrubTortoise  -------------------------------------------------------------------------------- 0

Majority                  --------------------------------------------------------------------------------

                                   2410      2420      2430      2440      2450      2460      2470      2480

Human                     GATTTATGAATGATGGACTATCCTAAAAAGTTTTAGAGCCAAAGAGTGAGTGATTAGAAGAATAACAGGAGGTTGAAACT 2480
Kakapo                    -------------------------------------------------------------------------------- 0
GoldenEagle               -------------------------------------------------------------------------------- 0
JapaneseQuail             -------------------------------------------------------------------------------- 0
MediumGroundFinch         -------------------------------------------------------------------------------- 0
GoodesThornscrubTortoise  -------------------------------------------------------------------------------- 0

Majority                  --------------------------------------------------------------------------------

                                   2490      2500      2510      2520      2530      2540      2550      2560

Human                     ACTTATTTTTTCTTTGCAGAATCGTCACTGAGGTTGCAGTTGGCGTGAAGGTAGGAAAATACTATGTTTGAAACTGGGAT 2560
Kakapo                    -------------------------------------------------------------------------------- 0
GoldenEagle               -------------------------------------------------------------------------------- 0
JapaneseQuail             -------------------------------------------------------------------------------- 0
MediumGroundFinch         -------------------------------------------------------------------------------- 0
GoodesThornscrubTortoise  -------------------------------------------------------------------------------- 0
```

```
Majority                 --------------------------------------------------------------------------------
                                  2570      2580      2590      2600      2610      2620      2630      2640
Human                    TGTTGGATCTATTATCATAATTACTGGAAAATTTTAGCTTTTGCTTTTGAAAACTATAACGTAGCTAACAAAACCCAAGT 2640
Kakapo                   -------------------------------------------------------------------------------- 0
GoldenEagle              -------------------------------------------------------------------------------- 0
JapaneseQuail            -------------------------------------------------------------------------------- 0
MediumGroundFinch        -------------------------------------------------------------------------------- 0
GoodesThornscrubTortoise -------------------------------------------------------------------------------- 0

Majority                 --------------------------------------------------------------------------------
                                  2650      2660      2670      2680      2690      2700      2710      2720
Human                    GTAAAATGTTTCCTTTCATTTGTAAAGTTGGACCATATAAACAGTTTGTAATCACCATAAGTTTTTCTAGAACTGTCAGT 2720
Kakapo                   -------------------------------------------------------------------------------- 0
GoldenEagle              -------------------------------------------------------------------------------- 0
JapaneseQuail            -------------------------------------------------------------------------------- 0
MediumGroundFinch        -------------------------------------------------------------------------------- 0
GoodesThornscrubTortoise -------------------------------------------------------------------------------- 0

Majority                 --------------------------------------------------------------------------------
                                  2730      2740      2750      2760      2770      2780      2790      2800
Human                    CTGTAAGAATAACCTTTTAAAGTAAGTATGAATGATCAAGAAATTGAAAATGCTTTCACACATGGCTTCTTGTAATATAA 2800
Kakapo                   -------------------------------------------------------------------------------- 0
GoldenEagle              -------------------------------------------------------------------------------- 0
JapaneseQuail            -------------------------------------------------------------------------------- 0
MediumGroundFinch        -------------------------------------------------------------------------------- 0
GoodesThornscrubTortoise -------------------------------------------------------------------------------- 0

Majority                 --------------------------------------------------------------------------------
                                  2810      2820      2830      2840      2850      2860      2870      2880
Human                    AACGATACCCCAATTTTCTGAGGGGAAATAATGATTTTAAATGGATGGTAAGTGGTTTGACCTATATCTTATAATTTTGT 2880
Kakapo                   -------------------------------------------------------------------------------- 0
GoldenEagle              -------------------------------------------------------------------------------- 0
JapaneseQuail            -------------------------------------------------------------------------------- 0
MediumGroundFinch        -------------------------------------------------------------------------------- 0
GoodesThornscrubTortoise -------------------------------------------------------------------------------- 0
```

```
Majority                --------------------------------------------------------------------------------
                                 2890      2900      2910      2920      2930      2940      2950      2960
Human                   TAATCTAAGTTTTTTGTTTTTTATTACTTTTTGTCATGAAAATGAATATATTTTGGTTAATAATAAAGCACATTAGAAAT 2960
Kakapo                  -------------------------------------------------------------------------------- 0
GoldenEagle             -------------------------------------------------------------------------------- 0
JapaneseQuail           -------------------------------------------------------------------------------- 0
MediumGroundFinch       -------------------------------------------------------------------------------- 0
GoodesThornscrubTortoise-------------------------------------------------------------------------------- 0

Majority                --------------------------------------------------------------------------------
                                 2970      2980      2990      3000      3010      3020      3030      3040
Human                   GAATTACACTGATCCTTAAAAAGGAAGTTGTTGAGGCAGTTAGCTTCAAATTTGAATTAGAAGTTAAGAGCTGGTTTGTC 3040
Kakapo                  -------------------------------------------------------------------------------- 0
GoldenEagle             -------------------------------------------------------------------------------- 0
JapaneseQuail           -------------------------------------------------------------------------------- 0
MediumGroundFinch       -------------------------------------------------------------------------------- 0
GoodesThornscrubTortoise-------------------------------------------------------------------------------- 0

Majority                --------------------------------------------------------------------------------
                                 3050      3060      3070      3080      3090      3100      3110      3120
Human                   TATTAAAGAAAGTCTTTATGGTAGAGATCCTTTGGAATCCTTCCTTTTTGGCTAAGAAAACTACTCATCGTTTAAACTTT 3120
Kakapo                  -------------------------------------------------------------------------------- 0
GoldenEagle             -------------------------------------------------------------------------------- 0
JapaneseQuail           -------------------------------------------------------------------------------- 0
MediumGroundFinch       -------------------------------------------------------------------------------- 0
GoodesThornscrubTortoise-------------------------------------------------------------------------------- 0

Majority                --------------------------------------------------------------------------------
                                 3130      3140      3150      3160      3170      3180      3190      3200
Human                   AGTTCTAATGCTTAACATTTTCTTGAGTACTTGCAACTTTTAATGTCATGATGTATTGTAATTTTTTTTTGTTTTTTGTT 3200
Kakapo                  -------------------------------------------------------------------------------- 0
GoldenEagle             -------------------------------------------------------------------------------- 0
JapaneseQuail           -------------------------------------------------------------------------------- 0
MediumGroundFinch       -------------------------------------------------------------------------------- 0
GoodesThornscrubTortoise-------------------------------------------------------------------------------- 0
```

```
Majority                 --------------------------------------------------------------------------------

                                  3210      3220      3230      3240      3250      3260      3270      3280

Human                    TTTTTGAGACGGAGCCTCACTCTGTTGCCCAGGCTGGATGCAGTGGCACGATCTTGGCCCACTGCAACCTCTGCCTCCTG 3280
Kakapo                   -------------------------------------------------------------------------------- 0
GoldenEagle              -------------------------------------------------------------------------------- 0
JapaneseQuail            -------------------------------------------------------------------------------- 0
MediumGroundFinch        -------------------------------------------------------------------------------- 0
GoodesThornscrubTortoise -------------------------------------------------------------------------------- 0

Majority                 --------------------------------------------------------------------------------

                                  3290      3300      3310      3320      3330      3340      3350      3360

Human                    GGTTCAAGCAGTTCTCCTGCCTCAGCCTCCTGAGTAGCTGGGATTACAGGTGCGCACCACCACGCCCAGCTAATTTGTAT 3360
Kakapo                   -------------------------------------------------------------------------------- 0
GoldenEagle              -------------------------------------------------------------------------------- 0
JapaneseQuail            -------------------------------------------------------------------------------- 0
MediumGroundFinch        -------------------------------------------------------------------------------- 0
GoodesThornscrubTortoise -------------------------------------------------------------------------------- 0

Majority                 --------------------------------------------------------------------------------

                                  3370      3380      3390      3400      3410      3420      3430      3440

Human                    TTTTAGTAGAAATGGCATTTCACCCTGTTGGTCAGGCTGGTCTCAAACTCCTGACCTCATGATCTGCCTGCCTCTTATGA 3440
Kakapo                   -------------------------------------------------------------------------------- 0
GoldenEagle              -------------------------------------------------------------------------------- 0
JapaneseQuail            -------------------------------------------------------------------------------- 0
MediumGroundFinch        -------------------------------------------------------------------------------- 0
GoodesThornscrubTortoise -------------------------------------------------------------------------------- 0

Majority                 --------------------------------------------------------------------------------

                                  3450      3460      3470      3480      3490      3500      3510      3520

Human                    TCTGCCTGCCTTGACCTCCCAAAGTGCTGGGATTACAAGTGTGAGCCACCGTGCCCGGCCTGTATTGTCATTTTTCATTT 3520
Kakapo                   -------------------------------------------------------------------------------- 0
GoldenEagle              -------------------------------------------------------------------------------- 0
JapaneseQuail            -------------------------------------------------------------------------------- 0
MediumGroundFinch        -------------------------------------------------------------------------------- 0
GoodesThornscrubTortoise --------------------------------------GACAAATTTGAATGTAGACACATGCATCAGTAGGTTGATGCA 42
```

```
Majority                 --------------------------------------------------------------------------------

                                   3530      3540      3550      3560      3570      3580      3590      3600

Human                    GCAAGCCTCTCTGTCCTGAGAGGCTTTCTGCTTAGTCCAGTGATGCTGACTGTTCAAACATTTTTTCGAATTCTTAAAAT 3600
Kakapo                   -------------------------------------------------------------------------------- 0
GoldenEagle              -------------------------------------------------------------------------------- 0
JapaneseQuail            -------------------------------------------------------------------------------- 0
MediumGroundFinch        -------------------------------------------------------------------------------- 0
GoodesThornscrubTortoise AGGCAATTTTTGTGAACGTAACTTTGTAGTGTAGACCAAGGGTGGGCAACTTGTGGGCCGCGAGACTGTTTATTTACATT 122

Majority                 --------------------------------------------------------------------------------

                                   3610      3620      3630      3640      3650      3660      3670      3680

Human                    TGTATCCATAATGGGGGGAGGGGGAAGGGATAGCATTGGGAGATATAGGGGGGAGGGGGGAGGGATAGCTTTAGGAGATA 3680
Kakapo                   -------------------------------------------------------------------------------- 0
GoldenEagle              -------------------------------------------------------------------------------- 0
JapaneseQuail            -------------------------------------------------------------------------------- 0
MediumGroundFinch        -------------------------------------------------------------------------------- 0
GoodesThornscrubTortoise GACTGGCTGCAGCTCCCAGTGTCCGTGGTTTGCCATTCCCGGACAATGGGAGCTGAAGGAAGTGGTGACCAGCATGTCTC 202

Majority                 --------------------------------------------------------------------------------

                                   3690      3700      3710      3720      3730      3740      3750      3760

Human                    TACCTAATGCTAAATGACAAGTTAATGGGTGCAGCACACCAACATGGCACATGTATACATATGTAACAAACCTGCACGTT 3760
Kakapo                   -------------------------------------------------------------------------------- 0
GoldenEagle              -------------------------------------------------------------------------------- 0
JapaneseQuail            -------------------------------------------------------------------------------- 0
MediumGroundFinch        -------------------------------------------------------------------------------- 0
GoodesThornscrubTortoise TGCCAAAACCATGAGGTGGGACTCAGGAAATCATGGGATTGGTTTAAAAATCATGAGACTATTTTTTTTAAACAAAATGT 282

Majority                 --------------------------------------------------------------------------------

                                   3770      3780      3790      3800      3810      3820      3830      3840

Human                    GTACTCATGTACCCTAAAACTTAAAGTATAATAATGATAATAATAATTGTATCCATAATGATGTGGTGTTCTTTCAAATA 3840
Kakapo                   -------------------------------------------------------------------------------- 0
GoldenEagle              -------------------------------------------------------------------------------- 0
JapaneseQuail            -------------------------------------------------------------------------------- 0
MediumGroundFinch        -------------------------------------------------------------------------------- 0
GoodesThornscrubTortoise ATGTTGAGATCTCTTTATTTTCCTGTTGGTTTTTGAGCCCTTATATTACCTTTGGTTTACATTTTTTAAGCTTTTCTCCA 362
```

```
Majority                ---------------------------------------------------------------------------------
                               3850      3860      3870      3880      3890      3900      3910      3920
Human                   CTTTTGGGGATGGTACTGTGTTTTCTTGCTTATTTTCTGTCCTCTTTCCTATAGTGAAATAGATATTTGCCCATTCTTTA 3920
Kakapo                  -------------------------------------------------------------------------------- 0
GoldenEagle             -------------------------------------------------------------------------------- 0
JapaneseQuail           -------------------------------------------------------------------------------- 0
MediumGroundFinch       -------------------------------------------------------------------------------- 0
GoodesThornscrubTortoiseTAAACACAAAGGTTAGAAACTTTTTTTTTTTCAAAAAAATGAAAGCTGAGATTTTCCCATAATCAGCCATCTCCAGGAGA 442

Majority                --------------------------------------------------------------------------------
                               3930      3940      3950      3960      3970      3980      3990      4000
Human                   GGCTTGTTTCTTGAGTTCATTTTGTACAGACTAAAGGAGGAATAATACGCTCTTTTTGGTGATAGTATTTTCCAAGTTGA 4000
Kakapo                  -------------------------------------------------------------------------------- 0
GoldenEagle             -------------------------------------------------------------------------------- 0
JapaneseQuail           -------------------------------------------------------------------------------- 0
MediumGroundFinch       -------------------------------------------------------------------------------- 0
GoodesThornscrubTortoiseCTTGGATATAAGTAAAATATCAAATATTGCAAGATTCACCCTAAAACAGAGAGAGTTTGCAATACTGTGAAACCAAACAT 522

Majority                --------------------------------------------------------------------------------
                               4010      4020      4030      4040      4050      4060      4070      4080
Human                   GTTAGTCATGGGATTTAAAATCAGGAATAGCTAAAGATTGCAAAATGGTAGATGTTGTAGGTTCCATCCAAACAGATTTC 4080
Kakapo                  -------------------------------------------------------------------------------- 0
GoldenEagle             -------------------------------------------------------------------------------- 0
JapaneseQuail           -------------------------------------------------------------------------------- 0
MediumGroundFinch       -------------------------------------------------------------------------------- 0
GoodesThornscrubTortoiseGCTATTAAATGGCCTTGTAGTGTTAATATACCTAGGTTAGATTTAGGCCCAAATCCTGCAATCTACAACATTTCGGGACT 602

Majority                --------------------------------------------------------------------------------
                               4090      4100      4110      4120      4130      4140      4150      4160
Human                   TGTAAATTTTGTAAAAGAATAAATGATACAAGTGTGAGTTGTCGACTGTTAGTGGAAAAGGAGAGGAGTTTGTAGACAAG 4160
Kakapo                  -------------------------------------------------------------------------------- 0
GoldenEagle             -------------------------------------------------------------------------------- 0
JapaneseQuail           -------------------------------------------------------------------------------- 0
MediumGroundFinch       -------------------------------------------------------------------------------- 0
GoodesThornscrubTortoiseCTTGTAGCCATGAGGAGTCCCAATGACTTCAAAAGGATCATTGACCAATAAGGCTAATGACTCTTACATATTGCGCAACA 682
```

```
Majority                 --------------------------------------------------------------------------------
                                  4170      4180      4190      4200      4210      4220      4230      4240
Human                    GCTCAGAATGAGTAGACAGGAAAAAACTGAGGAGACTTGGAATAGGATGTCCAGGTGTAATGTATTGGTTACACCAGTTT 4240
Kakapo                   -------------------------------------------------------------------------------- 0
GoldenEagle              -------------------------------------------------------------------------------- 0
JapaneseQuail            -------------------------------------------------------------------------------- 0
MediumGroundFinch        -------------------------------------------------------------------------------- 0
GoodesThornscrubTortoise TGGACTAATCCACCTGCATATTGTACAATACAGGATTTAGGTCTTAATTTATATGTTCATCTTAATACCAACTAAAATAT 762

Majority                 --------------------------------------------------------------------------------
                                  4250      4260      4270      4280      4290      4300      4310      4320
Human                    TTTTTTTTTTTTTTTGAGACAGAGTCTCACTCTTCGCCCAGGTGGAGTGCAGTGGCACGATCCTGGCTCACTACAAGCTC 4320
Kakapo                   -------------------------------------------------------------------------------- 0
GoldenEagle              -------------------------------------------------------------------------------- 0
JapaneseQuail            -------------------------------------------------------------------------------- 0
MediumGroundFinch        -------------------------------------------------------------------------------- 0
GoodesThornscrubTortoise ATTTGTTATCAGCTTTGAAGGGGAA---ACCCTAACCCCAACAGGAGTGTGAGCCATTGTGTGCAATCTTTAATTGCTTG 839

Majority                 --------------------------------------------------------------------------------
                                  4330      4340      4350      4360      4370      4380      4390      4400
Human                    CGCCTTCCGGGTTCACTCCATTCTTCTGCCTCAGCCTCCCGAGTAGCTGGGACTACAGGCGCCCACCACCACGCCCGGCT 4400
Kakapo                   -------------------------------------------------------------------------------- 0
GoldenEagle              -------------------------------------------------------------------------------- 0
JapaneseQuail            -------------------------------------------------------------------------------- 0
MediumGroundFinch        -------------------------------------------------------------------------------- 0
GoodesThornscrubTortoise CAGAGAGGGGGAGAACATACTATAGCAGGGGTAGGCAACCTATGGCACACGTGCAGAAGGTGGCACACAAGCTGATTTTC 919

Majority                 --------------------------------------------------------------------------------
                                  4410      4420      4430      4440      4450      4460      4470      4480
Human                    AATTTTTTGTATTTTTAGTAGAGACGGGGTTTCACCGTGTTAGCCAGGATGGTCTTGATCTCCTGACCTTGTGGTTACAC 4480
Kakapo                   -------------------------------------------------------------------------------- 0
GoldenEagle              -------------------------------------------------------------------------------- 0
JapaneseQuail            -------------------------------------------------------------------------------- 0
MediumGroundFinch        -------------------------------------------------------------------------------- 0
GoodesThornscrubTortoise AGTGGCACTCACGCTGCCCAGATCCTGGCCACCGGTCTGAGGGGCTCTGCATTTTAATTTAATTTTAAATGAAGCTTCTT 999
```

```
Majority                    --------------------------------------------------------------------------------

                                     4490      4500      4510      4520      4530      4540      4550      4560

Human                       CAGTTTTTTGTTAGTTGTTTGAGGAGAACACTTTAGAAGATGATAAAAAAAAATTGCAAATCGTTTTTAAAAATCCATTG 4560
Kakapo                      -------------------------------------------------------------------------------- 0
GoldenEagle                 -------------------------------------------------------------------------------- 0
JapaneseQuail               -------------------------------------------------------------------------------- 0
MediumGroundFinch           -------------------------------------------------------------------------------- 0
GoodesThornscrubTortoiseAAACATTTTAAAAACCTTATTTACTTTACATACAACAATGGTTTAGTTATATATCATAGACTTATAGAAAGAGACCTTCT 1079

Majority                    --------------------------------------------------------------------------------

                                     4570      4580      4590      4600      4610      4620      4630      4640

Human                       TTTAATATAAAAGTCTGAATAATTAAAGTTAATTTAATTTTCTGGAATAATGGTTAAGAATTTTTGCTTTGGTGTCAGGC 4640
Kakapo                      -------------------------------------------------------------------------------- 0
GoldenEagle                 -------------------------------------------------------------------------------- 0
JapaneseQuail               -------------------------------------------------------------------------------- 0
MediumGroundFinch           -------------------------------------------------------------------------------- 0
GoodesThornscrubTortoiseAAAAATGTTGAAATGTATTACCGGCACGCAAAACCTTAAATTAAAGTGAATAAATGAAGACTCGGCACACCATTTCTGAA 1159

Majority                    --------------------------------------------------------------------------------

                                     4650      4660      4670      4680      4690      4700      4710      4720

Human                       CTGTGTACAAGTAATTAAGTGTTCTTTAAAACTTAAAAC--ATTTGTGAATTTGCTTTAAAGACACTGATGCATGTGCAT 4718
Kakapo                      -------------------------------------------------------------------------------- 0
GoldenEagle                 -------------------------------------------------------------------------------- 0
JapaneseQuail               -------------------------------------------------------------------------------- 0
MediumGroundFinch           -------------------------------------------------------------------------------- 0
GoodesThornscrubTortoiseAGGTTGCTGACCCCTGTACTATAGGATGCTTGATGGTTTTGGGTTTTTTTTGTTCATCAGCAGTCTTAAGAGTGGTTGAT 1239

Majority                    --------------------------------------------------------------------------------

                                     4730      4740      4750      4760      4770      4780      4790      4800

Human                       ATTTAAAAAAATTCAGGCCGGGCGCGGTGGGTCACACCTGTAATCCCAGCACTTTGGGAGGCCGAGGCGGGCGGATCACG 4798
Kakapo                      -------------------------------------------------------------------------------- 0
GoldenEagle                 -------------------------------------------------------------------------------- 0
JapaneseQuail               -------------------------------------------------------------------------------- 0
MediumGroundFinch           -------------------------------------------------------------------------------- 0
GoodesThornscrubTortoiseATCTAGTACTAACTGCACTAGCTGTGATTATTATATCGTGTGCAATAACATGTCTACCTTTTCATTGCTCATGTTTCAGG 1319
```

```
Majority               --------------------------------------------------------------------------------

                                4810      4820      4830      4840      4850      4860      4870      4880

Human                  AGTTCAGGAGAGACCATCCTGGCTAACACGGTGAAACCCTGTCTTTACTAAAAATACAAAAAATTAGCCGGGTGTGGTGA 4878
Kakapo                 -------------------------------------------------------------------------------- 0
GoldenEagle            -------------------------------------------------------------------------------- 0
JapaneseQuail          -------------------------------------------------------------------------------- 0
MediumGroundFinch      -------------------------------------------------------------------------------- 0
GoodesThornscrubTortoiseCCATAAGCTAATACTTCTCAATTTAAAACAGGAAGAATTTCTTTCCTCTCCTTTGGAATACTGTTGCACAATTAGGTGCA 1399

Majority               --------------------------------------------------------------------------------

                                4890      4900      4910      4920      4930      4940      4950      4960

Human                  CGGGTGCCTGTAGTCCCAGCTACTCGGGAGGCTGAGGCAGGAGAATGGCATGAACCTGCGAGGCAGAGGTTGCAGTAAGC 4958
Kakapo                 -------------------------------------------------------------------------------- 0
GoldenEagle            -------------------------------------------------------------------------------- 0
JapaneseQuail          -------------------------------------------------------------------------------- 0
MediumGroundFinch      -------------------------------------------------------------------------------- 0
GoodesThornscrubTortoiseTTATGAGAGGTAATAATTTCCTTTGAAGGACCCTGCACTGGCCAATTGGAGAGGCAGGATACTACAGAAGATGGACTATT 1479

Majority               --------------------------------------------------------------------------------

                                4970      4980      4990      5000      5010      5020      5030      5040

Human                  GGAGATTGCACCACTGCACTCCAGCCTGGGCGACAGAGACAGACTCTATCTCAAAAAAAAAAAAAAAAAAAAAAAAAAAA 5038
Kakapo                 -------------------------------------------------------------------------------- 0
GoldenEagle            -------------------------------------------------------------------------------- 0
JapaneseQuail          -------------------------------------------------------------------------------- 0
MediumGroundFinch      -------------------------------------------------------------------------------- 0
GoodesThornscrubTortoiseCATGGCAATTCCTCTCCATAATTTTCCATTACAAGAGGAGAAAGGCTTTAACTACTCCCTCTCTGTCCCCAGAAATGTGG 1559

Majority               --------------------------------------------------------------------------------

                                5050      5060      5070      5080      5090      5100      5110      5120

Human                  AAAAAAAAATTCAAACTGCTTCACAGCTTTTTTTGCTATTTCTTAGGTGTAGGTAATTGTGTTAAAATTTTGTGCCTTTT 5118
Kakapo                 -------------------------------------------------------------------------------- 0
GoldenEagle            -------------------------------------------------------------------------------- 0
JapaneseQuail          -------------------------------------------------------------------------------- 0
MediumGroundFinch      -------------------------------------------------------------------------------- 0
GoodesThornscrubTortoiseAGATGTGGCACCAAGTT--TGGACAGTTTCTTCCTGCAACAAGCGAAGCCTTGCACCAGGGCTGGAGCCTGGGGAATCCA 1637
```

```
Majority             --------------------------------------------------------------------------------
                              5130      5140      5150      5160      5170      5180      5190      5200
Human                TTCTTGAAATTATTAATTTATATATTAGCACACATATAAACATCTTTATTTACGTGATTGAATTCAAACTATTGTTTTGG 5198
Kakapo               -------------------------------------------------------------------------------- 0
GoldenEagle          -------------------------------------------------------------------------------- 0
JapaneseQuail        -------------------------------------------------------------------------------- 0
MediumGroundFinch    -------------------------------------------------------------------------------- 0
GoodesThornscrubTortoiseATCCAAGTCTCCCTGCATCATATCTGGCAAGTTTCTCTTCTCAAGCTCAGCCTGGCAGTAGGGCTGAGGAGTCTTTCTCC 1717

Majority             --------------------------------------------------------------------------------
                              5210      5220      5230      5240      5250      5260      5270      5280
Human                CCCACATCTTGCCTTTTTTTTTTTTTTTTGAGTCGGAGTCTCCCTCTGTCATCAGGCTGGAGTGCAGTGGCATGATCTCG 5278
Kakapo               -------------------------------------------------------------------------------- 0
GoldenEagle          -------------------------------------------------------------------------------- 0
JapaneseQuail        -------------------------------------------------------------------------------- 0
MediumGroundFinch    -------------------------------------------------------------------------------- 0
GoodesThornscrubTortoiseAGCTAAAGGAGCCAGACTTTGAGGGAAACAGTCAAAAGAATTTCCCTCCAGAAAGTGGGCCTCCAGTCAGCAGGGCTGGG 1797

Majority             --------------------------------------------------------------------------------
                              5290      5300      5310      5320      5330      5340      5350      5360
Human                GCTTACTGCAACCTCTGCCTCCTGGGTTCAGGCAATTCTCCTGCCTCAGCCTCCCTAGTAGCTGGGACTACAGGCACACG 5358
Kakapo               -------------------------------------------------------------------------------- 0
GoldenEagle          -------------------------------------------------------------------------------- 0
JapaneseQuail        -------------------------------------------------------------------------------- 0
MediumGroundFinch    -------------------------------------------------------------------------------- 0
GoodesThornscrubTortoiseAGATGAATGTGACGTCGGAGATGGGCCTAGGAGTGGGCCTCGTGGGCAGCAAGGCTCGGAAACCGCCCTGCTTGTCTTGC 1877

Majority             --------------------------------------------------------------------------------
                              5370      5380      5390      5400      5410      5420      5430      5440
Human                CCGCCATGCCA--GACTAATTTTTTTTTTTTTTTCGAGACGGAGTCTCGCCCGGTCTCCCAGGCTGGAGTGCAGTGTCGC 5436
Kakapo               -------------------------------------------------------------------------------- 0
GoldenEagle          -------------------------------------------------------------------------------- 0
JapaneseQuail        -------------------------------------------------------------------------------- 0
MediumGroundFinch    -------------------------------------------------------------------------------- 0
GoodesThornscrubTortoiseCTGCTTTGCCCCCCGCGGGGGGAATAGTTTGCCTCAGACAGGCAGGTGACGAGGAGCATCGCAGAAGCGCCAGTAGCTGC 1957
```

```
Majority                   --------------------------------------------------------------------------------

                                   5450      5460      5470      5480      5490      5500      5510      5520

Human                      GATCTCTGCTCACTGCAACCTCTGCCTCCTGGGTTCACGCCATTCTCCTGTCTCAGCCTCCCAAGTAGCTGGGACTACAG 5516
Kakapo                     -------------------------------------------------------------------------------- 0
GoldenEagle                -------------------------------------------------------------------------------- 0
JapaneseQuail              -------------------------------------------------------------------------------- 0
MediumGroundFinch          -------------------------------------------------------------------------------- 0
GoodesThornscrubTortoise   TGCTACTGAGCATGCGCACAGCGCACGGGCATGCCCAGTGACAGCTGAGGGAGCCCAATCTGATGGGGAGCAGAGCCTGG 2037

Majority                   --------------------------------------------------------------------------------

                                   5530      5540      5550      5560      5570      5580      5590      5600

Human                      GCGCCTGCCACCACGCCTGGCTTCTTTTTTGTATATTCAGTAGAGACAGAGTTTCACCATGTTAGCCAGGATAGTCTCTA 5596
Kakapo                     -------------------------------------------------------------------------------- 0
GoldenEagle                -------------------------------------------------------------------------------- 0
JapaneseQuail              -------------------------------------------------------------------------------- 0
MediumGroundFinch          -------------------------------------------------------------------------------- 0
GoodesThornscrubTortoise   CACTCGGCAACCTTCGGCGGTTTCCGGGGGGGCCTGGCCGCCATTTTCTCGTTGCCTCTCTCCGTTCGCTGCCTGCGCGG 2117

Majority                   --------------------------------------------------------------------------------

                                   5610      5620      5630      5640      5650      5660      5670      5680

Human                      TCTCCTGACCTCGTGATCTGCCTGCCTCGGCCTCCCAAAGTGCTGGGATTACAAGCGTGAGCCACCGCGCCTAGCCAAAA 5676
Kakapo                     -------------------------------------------------------------------------------- 0
GoldenEagle                -------------------------------------------------------------------------------- 0
JapaneseQuail              -------------------------------------------------------------------------------- 0
MediumGroundFinch          -------------------------------------------------------------------------------- 0
GoodesThornscrubTortoise   CTCCCTGCCCTGCTCGCCAGCGCTTTGTCTTGTTCGGCGATGGACGGGTGAGTACAGGGGCGGTGGGGGAGCGGGGGGCG 2197

Majority                   --------------------------------------------------------------------------------

                                   5690      5700      5710      5720      5730      5740      5750      5760

Human                      TGCCAGACTAATTTTTGTATTTTTAGTAGAGACAGGGTTTCACCATTTTGGCCAGGATGGTCTTGATCTCTTGACTCCAT 5756
Kakapo                     -------------------------------------------------------------------------------- 0
GoldenEagle                -------------------------------------------------------------------------------- 0
JapaneseQuail              -------------------------------------------------------------------------------- 0
MediumGroundFinch          -------------------------------------------------------------------------------- 0
GoodesThornscrubTortoise   CTGCCGCGCCCGCCGGGTTATTGTTGCTTTCCTGTCGGGGCTGGGCAGAGCGGGGCTCGCTCTCCCCCCCGTGGGTGTCT 2277
```

```
Majority                 --------------------------------------------------------------------------------

                                  5770      5780      5790      5800      5810      5820      5830      5840

Human                    CATCCGCCCGCCTTGACTTCCCAAAGTGTTGGGATTACAGGCTTGAGCCACCGCGTCCGGCCCATATCTTGCCTTTAAAA 5836
Kakapo                   -------------------------------------------------------------------------------- 0
GoldenEagle              -------------------------------------------------------------------------------- 0
JapaneseQuail            -------------------------------------------------------------------------------- 0
MediumGroundFinch        -------------------------------------------------------------------------------- 0
GoodesThornscrubTortoise GGGAGGAGCAGGGTGCCCAGCACACCCCACTGAATAGCGGGGATTAGCTCCTGCAGACTCCTTGTGGGGCAGCTGGAGAC 2357

Majority                 --------------------------------------------------------------------------------

                                  5850      5860      5870      5880      5890      5900      5910      5920

Human                    AAAATAGCTTTATTGAGATATAATTTGACATAAAATAAACTACCCATATTAAATATACAATGTTTTATCTCATATATACC 5916
Kakapo                   -------------------------------------------------------------------------------- 0
GoldenEagle              -------------------------------------------------------------------------------- 0
JapaneseQuail            -------------------------------------------------------------------------------- 0
MediumGroundFinch        -------------------------------------------------------------------------------- 0
GoodesThornscrubTortoise CGACTGGGGCCAGTGAGGATGAGGGCGGTGGGGGGAGCTTCTGCATAACGCGGACTCAGATGCGGGGCTGGGGCGGGTCC 2437

Majority                 --------------------------------------------------------------------------------

                                  5930      5940      5950      5960      5970      5980      5990      6000

Human                    CTTGTGAAACCACT----GCAATCAAGATAATGAACATCTATCACTCCCTACAGTATCATCTTGCGCCTTTATAGTCCCT 5992
Kakapo                   -------------------------------------------------------------------------------- 0
GoldenEagle              -------------------------------------------------------------------------------- 0
JapaneseQuail            -------------------------------------------------------------------------------- 0
MediumGroundFinch        -------------------------------------------------------------------------------- 0
GoodesThornscrubTortoise CTTACTGACTCACCTAGGGCTGCGTGAGTCCTTAGCGCCCTGTTCATTCTGTGGCCTTCTCAGTGGTGGGTGTCTGTGTG 2517

Majority                 --------------------------------------------------------------------------------

                                  6010      6020      6030      6040      6050      6060      6070      6080

Human                    CCTTTCTGCTTTTCTTTCCCCAAGGAACCACTGATCTGCTTTCTGTCACTATAGATTAGTTCACACTTTTTAGAATTTTA 6072
Kakapo                   -------------------------------------------------------------------------------- 0
GoldenEagle              -------------------------------------------------------------------------------- 0
JapaneseQuail            -------------------------------------------------------------------------------- 0
MediumGroundFinch        -------------------------------------------------------------------------------- 0
GoodesThornscrubTortoise TGTGTGTGGGGGGTGCCCCCATTCCCGCCCTTTTATCCCCTCCCCCTCATTCGTGGTGTTAGGGGAAACGCTGTGAGCAA 2597
```

```
Majority                 --------------------------------------------------------------------------------

                                  6090      6100      6110      6120      6130      6140      6150      6160

Human                    GATAAATGTAATCATATGGTATGTACATTTTTGGTCTGGGTTAGTCCACTCAGAATAATTATTTTGAAATTCACCCATGT 6152
Kakapo                   -------------------------------------------------------------------------------- 0
GoldenEagle              -------------------------------------------------------------------------------- 0
JapaneseQuail            -------------------------------------------------------------------------------- 0
MediumGroundFinch        -------------------------------------------------------------------------------- 0
GoodesThornscrubTortoise CACCCCTGCTTGTTTTGGTGCTGGCCAGAGGAGGAGGAGGAGCTGGCAGATGGCAAGCTCTTTTGTTTTCAATAGCTGTT 2677

Majority                 --------------------------------------------------------------------------------

                                  6170      6180      6190      6200      6210      6220      6230      6240

Human                    TGTCTTTCAATAGTCCATTCTGTTTTATTGCTTAGTAATAGTACACTTTATGGCTATACAGCAATTTGTTAATCCATTCA 6232
Kakapo                   -------------------------------------------------------------------------------- 0
GoldenEagle              -------------------------------------------------------------------------------- 0
JapaneseQuail            -------------------------------------------------------------------------------- 0
MediumGroundFinch        -------------------------------------------------------------------------------- 0
GoodesThornscrubTortoise TAATAGCCCCCTGGCTTTTTTTTTTTTTATTTAAGGTAGAAAGATGCCTTTGTGTATACTGGAGACAGGCAGTTAAAGCC 2757

Majority                 --------------------------------------------------------------------------------

                                  6250      6260      6270      6280      6290      6300      6310      6320

Human                    AGTCTTGAGGGACATTTGTATTGTTTTCCGTTTTGAGCTAATAAAAATATAGTTGTGAACATTACTGTACAAGTCTTTGT 6312
Kakapo                   -------------------------------------------------------------------------------- 0
GoldenEagle              -------------------------------------------------------------------------------- 0
JapaneseQuail            -------------------------------------------------------------------------------- 0
MediumGroundFinch        -------------------------------------------------------------------------------- 0
GoodesThornscrubTortoise CAAGTAGGGAAGGGTTTGCAGGCATCAACCATATGGGACAGCAAGGGCCTTGATTCCTGGCAGGGAGCTTGGGTGGGTAG 2837

Majority                 --------------------------------------------------------------------------------

                                  6330      6340      6350      6360      6370      6380      6390      6400

Human                    ATGGACATATGCTTTCATTTCTCTTGGTGGGGCAGGCTATGATTGGAAAGTCTGGATCATGTGATAGGCATATGTTTAAC 6392
Kakapo                   -------------------------------------------------------------------------------- 0
GoldenEagle              -------------------------------------------------------------------------------- 0
JapaneseQuail            -------------------------------------------------------------------------------- 0
MediumGroundFinch        -------------------------------------------------------------------------------- 0
GoodesThornscrubTortoise TTATACACAAGGCCCTCTTTTCATTGGGGCTGGGAGCTGCTGCTGGTGTTTATAGCTACTGTCAGCATAAACACTTGTGT 2917
```

```
Majority                --------------------------------------------------------------------------------
                                  6410      6420      6430      6440      6450      6460      6470      6480
Human                   TTTTTAAGGAACTGCAAACTTTTTAAAAGTAGTTGTACCATCTTACATTGCCATCAGCATGGTATGAAATTTCCAGTCCT 6472
Kakapo                  -------------------------------------------------------------------------------- 0
GoldenEagle             -------------------------------------------------------------------------------- 0
JapaneseQuail           -------------------------------------------------------------------------------- 0
MediumGroundFinch       -------------------------------------------------------------------------------- 0
GoodesThornscrubTortoiseGTGTGCAGCATCTTACTGCCCCCATTTACTTGCCTCATCCTTTTGAGACAGATTAAACAACCTCTTGTGTTTGTGTGATA 2997

Majority                --------------------------------------------------------------------------------
                                  6490      6500      6510      6520      6530      6540      6550      6560
Human                   TCCACATCCTCTTTAATGCCTGTTATCTTCTGTTTTTGATTATAGATATTGTAGTGTATATGAAGTGATATTTCACTGTG 6552
Kakapo                  -------------------------------------------------------------------------------- 0
GoldenEagle             -------------------------------------------------------------------------------- 0
JapaneseQuail           -------------------------------------------------------------------------------- 0
MediumGroundFinch       -------------------------------------------------------------------------------- 0
GoodesThornscrubTortoiseAATGTATAATCTATTGGTAATGTGATTTTAAAGTCTTT-TTATGGTTGCTTGTCAGCCTAATGTTGGTGTTACAAACCCT 3076

Majority                --------------------------------------------------------------------------------
                                  6570      6580      6590      6600      6610      6620      6630      6640
Human                   GATTTAATTTGCATTCCTCTAATATCTATGATGTTGGACATCTTTTCGTGTGCTTTTTGTCAGCTGTTATCTTTGGTGAA 6632
Kakapo                  -------------------------------------------------------------------------------- 0
GoldenEagle             -------------------------------------------------------------------------------- 0
JapaneseQuail           -------------------------------------------------------------------------------- 0
MediumGroundFinch       -------------------------------------------------------------------------------- 0
GoodesThornscrubTortoiseGACTAAAAAGTCAATCCTTTGTGCTGCTCACAAATTGATACCCATGGAAGTGGCATGCTTTACTTATCACATTTTGTTTG 3156

Majority                --------------------------------------------------------------------------------
                                  6650      6660      6670      6680      6690      6700      6710      6720
Human                   GTGTCTGTTTATATCTTTAGCCCAATTTTTTATTTGGTTATTTATTTTAAATTGAGTTTTGAGAGGTCTTCATTGTGGTT 6712
Kakapo                  -------------------------------------------------------------------------------- 0
GoldenEagle             -------------------------------------------------------------------------------- 0
JapaneseQuail           -------------------------------------------------------------------------------- 0
MediumGroundFinch       -------------------------------------------------------------------------------- 0
GoodesThornscrubTortoiseTAATATTTTATGATATTTGTGTTAATTAGGGTAGCCTGGCTATATTCTGTTTTGGGTTGTTATATTCTGCCTACCCAGTG 3236
```

```
Majority                  --------------------------------------------------------------------------------
                                   6730      6740      6750      6760      6770      6780      6790      6800
Human                     ACAAGTCCTTTATCAGCCATAAGGCCTGCAAATATTTTCTGCCACTCATCTTCTTATTCTCGCATTGTCTTTTGAAGAGC 6792
Kakapo                    -------------------------------------------------------------------------------- 0
GoldenEagle               -------------------------------------------------------------------------------- 0
JapaneseQuail             -------------------------------------------------------------------------------- 0
MediumGroundFinch         -------------------------------------------------------------------------------- 0
GoodesThornscrubTortoise  CTAGATCCTGCAAATAGATCTACATAAGAGCATGTCTGTGCTGTTGTTCCACATGGGTGTAGAGTGCCATTTGTAGGATC 3316

Majority                  --------------------------------------------------------------------------------
                                   6810      6820      6830      6840      6850      6860      6870      6880
Human                     AACAGTTATTAAATCTGATGGACTCCAGTTTACCAATTTTTTCTTTTATGGATTGTAGTTTTGGGGTCTTGTCTGAGCCA 6872
Kakapo                    -------------------------------------------------------------------------------- 0
GoldenEagle               -------------------------------------------------------------------------------- 0
JapaneseQuail             -------------------------------------------------------------------------------- 0
MediumGroundFinch         -------------------------------------------------------------------------------- 0
GoodesThornscrubTortoise  TAGCATTCAATTTGGTCATCTTTTTCAATTCAGAAATAGTATTAATCACTTTCTGCCCTGAACTGTTCTGTAAGGATCAC 3396

Majority                  --------------------------------------------------------------------------------
                                   6890      6900      6910      6920      6930      6940      6950      6960
Human                     TTTTTGCCTGACTGTAAGCCATGAAGATTTTTTTCCTTTTGTTTGTTAATAATAATAATAATTTTTTTTTAGAGACAGGA 6952
Kakapo                    -------------------------------------------------------------------------------- 0
GoldenEagle               -------------------------------------------------------------------------------- 0
JapaneseQuail             -------------------------------------------------------------------------------- 0
MediumGroundFinch         -------------------------------------------------------------------------------- 0
GoodesThornscrubTortoise  TGTTAAAGAATGAACAGAAAGATTATCTTATGTACTACATCTGTCCTTAAATTCTCCTATTCTCACCCTATCCATTATCC 3476

Majority                  --------------------------------------------------------------------------------
                                   6970      6980      6990      7000      7010      7020      7030      7040
Human                     CCTGACTCTGTCACCCCAGCTGGAGTGCAGTGGTGCAATCGTAGCTCATGATAACCTTGAACTCCTGGACACAAGGGCTC 7032
Kakapo                    -------------------------------------------------------------------------------- 0
GoldenEagle               -------------------------------------------------------------------------------- 0
JapaneseQuail             -------------------------------------------------------------------------------- 0
MediumGroundFinch         -------------------------------------------------------------------------------- 0
GoodesThornscrubTortoise  CACAAAGTCCAGAGACTTCCTCTTCAGACTTACTGGCATTGGCCAAAATGGATATCTATCAGACTCTGGAAGGAGGAGGC 3556
```

```
Majority                 --------------------------------------------------------------------------------
                                   7050      7060      7070      7080      7090      7100      7110      7120
Human                    CTGCTTCAGCCTCCCAAGTAGCTAGGACTATAGGCACACAGCACCATGCCCAGCTAACTTTTAAATTTTTTATAGACCCG 7112
Kakapo                   -------------------------------------------------------------------------------- 0
GoldenEagle              -------------------------------------------------------------------------------- 0
JapaneseQuail            -------------------------------------------------------------------------------- 0
MediumGroundFinch        -------------------------------------------------------------------------------- 0
GoodesThornscrubTortoise TGGATGGGGTGGGTTCTCTGACTGTGGGACCTAATTCCTCTCACTTCTTAAGTCCCATGTCCAGGCAGAGCTTCTTTGGA 3636

Majority                 --------------------------------------------------------------------------------
                                   7130      7140      7150      7160      7170      7180      7190      7200
Human                    GGGTCTCTCTGTTTTGCCCAGGCTGGTGTTGAACTCCTGGCCTCAAGCGATCTTTCTGCCTCAGCCTTCCAAAGTGTTGG 7192
Kakapo                   -------------------------------------------------------------------------------- 0
GoldenEagle              -------------------------------------------------------------------------------- 0
JapaneseQuail            -------------------------------------------------------------------------------- 0
MediumGroundFinch        -------------------------------------------------------------------------------- 0
GoodesThornscrubTortoise AAGTGTTCACTGACATCATGGACACCTTTTGTAGCTGTGTGTACTCTCAGGGATTCTTTGTTCAGTGTGCTGCCTGGATG 3716

Majority                 --------------------------------------------------------------------------------
                                   7210      7220      7230      7240      7250      7260      7270      7280
Human                    GATTAAAGGCATGAGCCACTACACTTGGCCACCTTTGTTTTCTAATAGAAGTTTTATACTTTGGTTTAACATTTGGGTCT 7272
Kakapo                   -------------------------------------------------------------------------------- 0
GoldenEagle              -------------------------------------------------------------------------------- 0
JapaneseQuail            -------------------------------------------------------------------------------- 0
MediumGroundFinch        -------------------------------------------------------------------------------- 0
GoodesThornscrubTortoise TCTTCTATTGAACCTTTGAGCCCGTTCCCATTCCTGTTTTGTTTCTTTTTGACTCCTCTCCTCTTTTGAGGGGGTGGTCT 3796

Majority                 --------------------------------------------------------------------------------
                                   7290      7300      7310      7320      7330      7340      7350      7360
Human                    CTTTCATTTTGACTTAATATTTTGAATATGGTACAAAGTGTGGGTCAGAAGTATTTGGTTTTGCATGGGGATATCCAGTG 7352
Kakapo                   -------------------------------------------------------------------------------- 0
GoldenEagle              -------------------------------------------------------------------------------- 0
JapaneseQuail            -------------------------------------------------------------------------------- 0
MediumGroundFinch        -------------------------------------------------------------------------------- 0
GoodesThornscrubTortoise TTGCTATTTTAATTTCTAAATACTAAAGTTAATTAAAATCTAT-TAAGAGTTCAGTTTTATGGGGAAATCATATTTATTT 3875
```

```
Majority                   --------------------------------------------------------------------------------

                                    7370      7380      7390      7400      7410      7420      7430      7440

Human                      TTTACAGCATCATTTGTTGAAAAGACTATCCTTTCTCCATTCACTTGGCTTTGCAGCTTCATGAAAAATCACTTATGTCT 7432
Kakapo                     -------------------------------------------------------------------------------- 0
GoldenEagle                -------------------------------------------------------------------------------- 0
JapaneseQuail              -------------------------------------------------------------------------------- 0
MediumGroundFinch          -------------------------------------------------------------------------------- 0
GoodesThornscrubTortoise   TGAGTCAATTTGTGGATTGAAAACTCAGACATGTTTCTTACTATTAAGTCAGGTTTCTTGTTTTCTTTACAGCATCGTCA 3955

Majority                   --------------------------------------------------------------------------------

                                    7450      7460      7470      7480      7490      7500      7510      7520

Human                      GTATGTGTGATTTTATGTCTGTACTCTTTTTTGTATTGACCCATTTTCCTTTATCTAGATGCTGGAAACATACTGTCTTA 7512
Kakapo                     -------------------------------------------------------------------------------- 0
GoldenEagle                -------------------------------------------------------------------------------- 0
JapaneseQuail              -------------------------------------------------------------------------------- 0
MediumGroundFinch          -------------------------------------------------------------------------------- 0
GoodesThornscrubTortoise   CTGATGTTGCAGTTGGCGTGAAGGTATGATGTGAAGCTAGCTGATGATAAACTGGCAACCTGTTAAAATGATATATGTTA 4035

Majority                   --------------------------------------------------------------------------------

                                    7530      7540      7550      7560      7570      7580      7590      7600

Human                      ATCATTGTAGCTTTATATTGAGTCTTGAAACCACTTACTTAGTCTTGGCCCTCCAACTCTGTTCTTTTTCAAAGTTGTTT 7592
Kakapo                     -------------------------------------------------------------------------------- 0
GoldenEagle                -------------------------------------------------------------------------------- 0
JapaneseQuail              -------------------------------------------------------------------------------- 0
MediumGroundFinch          -------------------------------------------------------------------------------- 0
GoodesThornscrubTortoise   ATTTGTGGAGTTGAAGTGAGCTGTAAGATTTTATTAAGTGATTGTCAGCTGAC-------ATTTTGTCATCGAGATATGC 4108

Majority                   --------------------------------------------------------------------------------

                                    7610      7620      7630      7640      7650      7660      7670      7680

Human                      TTAACTCTTCTGAGTCCTTTGTATTTTCATATGAGTCAGTTCTTAGAAAAACTGCTTGGATTTTACTGGATTTGACTTGA 7672
Kakapo                     -------------------------------------------------------------------------------- 0
GoldenEagle                -------------------------------------------------------------------------------- 0
JapaneseQuail              -------------------------------------------------------------------------------- 0
MediumGroundFinch          -------------------------------------------------------------------------------- 0
GoodesThornscrubTortoise   TTTAATAAACAGAAAGCTTTAAGTTGGAAACTGACCAGAAATGGGAGAATCTGTTATCAAAACCAGTCACTTGGATTGCA 4188
```

```
Majority                   --------------------------------------------------------------------------------
                                    7690      7700      7710      7720      7730      7740      7750      7760
Human                      ATCTGTAGAGCAAATTTGGGGAAAATTGGCATCATGACTACAATTGATGTCTCTATTAGGTCTTTAATTTCTTTAAGCAT 7752
Kakapo                     -------------------------------------------------------------------------------- 0
GoldenEagle                -------------------------------------------------------------------------------- 0
JapaneseQuail              -------------------------------------------------------------------------------- 0
MediumGroundFinch          -------------------------------------------------------------------------------- 0
GoodesThornscrubTortoiseCATGATATAAACTTATTCTAAAGATTAGTCTCTCAGAAATAACGTTTGGGCAACATGCTGTTCTCACTGCTGCATGATGG 4268

Majority                   --------------------------------------------------------------------------------
                                    7770      7780      7790      7800      7810      7820      7830      7840
Human                      TTTTTTTTTTTTTTTCAGTGCACACGTTTTGTGCATTGTTTGATTAGATTTACCCATAAGTATTTTATGTATTTTGAAGT 7832
Kakapo                     -------------------------------------------------------------------------------- 0
GoldenEagle                -------------------------------------------------------------------------------- 0
JapaneseQuail              -------------------------------------------------------------------------------- 0
MediumGroundFinch          -------------------------------------------------------------------------------- 0
GoodesThornscrubTortoiseCCTTAAAAGCTGATTTTTTTCCCCCCCTCT---CCCTACCTGCAAAAAACTCCAAATAGGTATTCAGTTCTTGTTGACCT 4345

Majority                   --------------------------------------------------------------------------------
                                    7850      7860      7870      7880      7890      7900      7910      7920
Human                      ACTGTAAATGATATTTTAAAAACTTAATTTCCAATTGTTTGCTAGTGTATAGAAATGATTATGGCATATTGCTCATATGT 7912
Kakapo                     -------------------------------------------------------------------------------- 0
GoldenEagle                -------------------------------------------------------------------------------- 0
JapaneseQuail              -------------------------------------------------------------------------------- 0
MediumGroundFinch          -------------------------------------------------------------------------------- 0
GoodesThornscrubTortoiseCATAATTGGAGCAGAATATGAGAATGAAGTTTTGACCTGCAGATCTGAGAGCAGAGGTTTGCTGTTTTTTTGTTTTTTGT 4425

Majority                   --------------------------------------------------------------------------------
                                    7930      7940      7950      7960      7970      7980      7990      8000
Human                      TCTGAACCTTTGCTAAACTCATTTAGTAGATCTAATAGTTTTTTTTATATATTTCATCAGATTTGCTTGAGAGGGAGTCT 7992
Kakapo                     -------------------------------------------------------------------------------- 0
GoldenEagle                -------------------------------------------------------------------------------- 0
JapaneseQuail              -------------------------------------------------------------------------------- 0
MediumGroundFinch          -------------------------------------------------------------------------------- 0
GoodesThornscrubTortoiseTCCTCCCCCTCAACTCACATTTCTTCAATGAAAGTTCTCCAGTTATAAGAATTGTACCATATAGTTAATTTCAGTTAGCC 4505
```

```
Majority                 --------------------------------------------------------------------------------

                                  8010      8020      8030      8040      8050      8060      8070      8080

Human                    CAGTCACCCAGGCTGGAGTGCAGTGACGTGATCTTGGCTCACTGCAACCTGCACCTCCTGGGTCAAGCAATTCTCCTGCC 8072
Kakapo                   -------------------------------------------------------------------------------- 0
GoldenEagle              -------------------------------------------------------------------------------- 0
JapaneseQuail            -------------------------------------------------------------------------------- 0
MediumGroundFinch        -------------------------------------------------------------------------------- 0
GoodesThornscrubTortoise CTCTGATTCTTTCACTCTTGAAATGAATGTTGCATTTGAGAATCAGATCAATACTTCATAGGAGCTGAATGCGACAAAAA 4585

Majority                 --------------------------------------------------------------------------------

                                  8090      8100      8110      8120      8130      8140      8150      8160

Human                    TCAGCCTCATAAGTAGCTGGGACTGCAGGTGCACACCACCACGCCCAGCTAATTTTTGCATTTTCAGTTGAGGCAGGGTT 8152
Kakapo                   -------------------------------------------------------------------------------- 0
GoldenEagle              -------------------------------------------------------------------------------- 0
JapaneseQuail            -------------------------------------------------------------------------------- 0
MediumGroundFinch        -------------------------------------------------------------------------------- 0
GoodesThornscrubTortoise TATAGTTAGCATGAAGGGAACAATGGCACAGGGCTGGCCTTGAACTATACAGACAAATATGTTGCCACAGGTGCACAAGT 4665

Majority                 --------------------------------------------------------------------------------

                                  8170      8180      8190      8200      8210      8220      8230      8240

Human                    TCACCATGTTGGCCAGGCTGGTCTGGAACTCCTGACCTCAAGTGATACACTTACCTAGGCCTCCCAAAGTGTTAAGATTA 8232
Kakapo                   -------------------------------------------------------------------------------- 0
GoldenEagle              -------------------------------------------------------------------------------- 0
JapaneseQuail            -------------------------------------------------------------------------------- 0
MediumGroundFinch        -------------------------------------------------------------------------------- 0
GoodesThornscrubTortoise TATGAACAAATGAGAGTTTTCTAATATTTGTATATTACTTGTTGAAGAGCATATTTGGGATTATGGAAGTGAACACAGCA 4745

Majority                 --------------------------------------------------------------------------------

                                  8250      8260      8270      8280      8290      8300      8310      8320

Human                    CAGGCGTGAGCCACCACCCCCAGCCTATGCCATTAAATTTTCTACATAGATAATTTATACCCAATTGTTTTTAAATAGTT 8312
Kakapo                   -------------------------------------------------------------------------------- 0
GoldenEagle              -------------------------------------------------------------------------------- 0
JapaneseQuail            -------------------------------------------------------------------------------- 0
MediumGroundFinch        -------------------------------------------------------------------------------- 0
GoodesThornscrubTortoise AAATTCTTTTATTATGAAATAGACTCTTGAAAGTAGCACCCCATAAAATAAAGTTCCAGCTGTACTAGAAACAACTAGAT 4825
```

```
Majority                 --------------------------------------------------------------------------------
                                   8330      8340      8350      8360      8370      8380      8390      8400
Human                    TTCTTTCTTTTCTATCTGGATGCCATTTTTTGTCTCCTTTTTTTGTCTGTGGTATCCAGTACAATGTTTAATAGAAGTGT 8392
Kakapo                   -------------------------------------------------------------------------------- 0
GoldenEagle              -------------------------------------------------------------------------------- 0
JapaneseQuail            -------------------------------------------------------------------------------- 0
MediumGroundFinch        -------------------------------------------------------------------------------- 0
GoodesThornscrubTortoise TGCACAATTTCAGCACTTTAACCCCTGTCAGTATCAGTCTCTTAGGGAATGCCCTGAATTGTGTGGACAAGGGTGAACCA 4905

Majority                 --------------------------------------------------------------------------------
                                   8410      8420      8430      8440      8450      8460      8470      8480
Human                    TGAGAGAATATCTTTTCCTTGCTCCTGATATTAGGGTGAGAACTCTAAAATTTCTTCCTTAACTGACACAGTTCACTAGT 8472
Kakapo                   -------------------------------------------------------------------------------- 0
GoldenEagle              -------------------------------------------------------------------------------- 0
JapaneseQuail            -------------------------------------------------------------------------------- 0
MediumGroundFinch        -------------------------------------------------------------------------------- 0
GoodesThornscrubTortoise CTGGGATAGTGTGCTTGGACTTTCAGAAAGTCTTTATCAAGGTCTCTCACTAAAGGCTCTTAAACAAGCTAAGTAGTCAT 4985

Majority                 --------------------------------------------------------------------------------
                                   8490      8500      8510      8520      8530      8540      8550      8560
Human                    GAAGCCATGTGGGGCCAGATGTTTGTTTTGTCGGGAGGTTTTAAACTACATATTCAACTTATTTAATAGATATAGGGCTA 8552
Kakapo                   -------------------------------------------------------------------------------- 0
GoldenEagle              -------------------------------------------------------------------------------- 0
JapaneseQuail            -------------------------------------------------------------------------------- 0
MediumGroundFinch        -------------------------------------------------------------------------------- 0
GoodesThornscrubTortoise TGGATAAGAGGGAAGGTCCTCTCATGGATAAGTAACTGGCTGAAAGATGGGAAACAAGGGAGGAATAAATGGTCAGTTTT 5065

Majority                 --------------------------------------------------------------------------------
                                   8570      8580      8590      8600      8610      8620      8630      8640
Human                    TTCAGTTTGTTTAGTTCTTCTTGAGTGAACTTTGGTAATTTGTATCTTCTGTAGAACTAGTCCATTTCCTTTAAGTTGCT 8632
Kakapo                   -------------------------------------------------------------------------------- 0
GoldenEagle              -------------------------------------------------------------------------------- 0
JapaneseQuail            -------------------------------------------------------------------------------- 0
MediumGroundFinch        -------------------------------------------------------------------------------- 0
GoodesThornscrubTortoise CAGAATGGAGAGAGGTGAATATTGGTGTCCCTGAGAGATCTGTACTGGGACCAGTACTGGTCAAGGTATTTGTAAATGAT 5145
```

```
Majority                  --------------------------------------------------------------------------------

                                  8650      8660      8670      8680      8690      8700      8710      8720

Human                     GAATGTATTTGTGTAGAGTTGTTCATGATACTACTATTTTTTTTTTTTTTTTTTTTTTGAGACGGAGCCTTGCTCTGTCG 8712
Kakapo                    -------------------------------------------------------------------------------- 0
GoldenEagle               -------------------------------------------------------------------------------- 0
JapaneseQuail             -------------------------------------------------------------------------------- 0
MediumGroundFinch         -------------------------------------------------------------------------------- 0
GoodesThornscrubTortoiseCTGGAAAAAGGGGTAAACAGGGAGGTGGAAAAGTTTGCAGATGATATAAAATTACTTTAAAATAGTTAAATCCAAAACAG 5225

Majority                  --------------------------------------------------------------------------------

                                  8730      8740      8750      8760      8770      8780      8790      8800

Human                     CCCGGGCTGGAGTGCAGTGGCACCATCTCGGCTCAATGCAACCTCCACGTCCCGGGTTCACGCGGTTCTCCTGCCTCAGC 8792
Kakapo                    -------------------------------------------------------------------------------- 0
GoldenEagle               -------------------------------------------------------------------------------- 0
JapaneseQuail             -------------------------------------------------------------------------------- 0
MediumGroundFinch         -------------------------------------------------------------------------------- 0
GoodesThornscrubTortoiseATTGCGAAGAGTTACAAAGGGATCTCACAAAACTGGGAGATTGGGCAACAAAATGGCCAATGAAATTCAGTGTTGATAAA 5305

Majority                  --------------------------------------------------------------------------------

                                  8810      8820      8830      8840      8850      8860      8870      8880

Human                     ATCCCAAGTAGCTGGGATCACAGGCACACACCACCACACCTGGCTAATTTTTTGTATTTTTAGTAGAGACGGGGTTTCAC 8872
Kakapo                    -------------------------------------------------------------------------------- 0
GoldenEagle               -------------------------------------------------------------------------------- 0
JapaneseQuail             -------------------------------------------------------------------------------- 0
MediumGroundFinch         -------------------------------------------------------------------------------- 0
GoodesThornscrubTortoiseTGCAAAGTAATACATATTGGAAAATATAATCCCAACTATACATACAAAATGATGGGGTCTATAATTAGCTGTCACCATTC 5385

Majority                  --------------------------------------------------------------------------------

                                  8890      8900      8910      8920      8930      8940      8950      8960

Human                     TATGTTGGCCAGGTTGGTCTCGAACTTGTGATCTGCCCGCCCTGCCTCACAAAGTGCTGGGATTACAGGCGTGAGCCACT 8952
Kakapo                    -------------------------------------------------------------------------------- 0
GoldenEagle               -------------------------------------------------------------------------------- 0
JapaneseQuail             -------------------------------------------------------------------------------- 0
MediumGroundFinch         -------------------------------------------------------------------------------- 0
GoodesThornscrubTortoiseAA----GAAAGAGCTCTTGGAGTCATTGTGATTTTTTTTTCCCCTGAAAACATCAGCTGAATGTCCAATGGCAGTCAAAA 5461
```

```
Majority                  --------------------------------------------------------------------------------

                                   8970      8980      8990      9000      9010      9020      9030      9040

Human                     GCGCCCAGCCATGATACTACATTTTTACACTTTTGGTATCTGTTGAATCCTTAGTGATGTCAGCTTTCTCATTCCCAGTA 9032
Kakapo                    -------------------------------------------------------------------------------- 0
GoldenEagle               -------------------------------------------------------------------------------- 0
JapaneseQuail             -------------------------------------------------------------------------------- 0
MediumGroundFinch         -------------------------------------------------------------------------------- 0
GoodesThornscrubTortoise  AGTTAAAAACTTTAGAAAACAGTATGAAAGGGATAGATAATAAGACAGTAAATATAATGCCA-TTGTATAAATCCATGGA 5540

Majority                  --------------------------------------------------------------------------------

                                   9050      9060      9070      9080      9090      9100      9110      9120

Human                     GGGATAATTTGTGTCATCTTTTTTCCTTGTCTGTCTGGATTGAAGTTTATTGATTTTATTAATCTCCAAAATACCAGCTT 9112
Kakapo                    -------------------------------------------------------------------------------- 0
GoldenEagle               -------------------------------------------------------------------------------- 0
JapaneseQuail             -------------------------------------------------------------------------------- 0
MediumGroundFinch         -------------------------------------------------------------------------------- 0
GoodesThornscrubTortoise  CTGCCCATAGCTATTGTGTACAGTTCTGGTTGCCCCATCTTGAAAA-----AGGTATCTTAGAAATGGAAAAGACGGAGA 5615

Majority                  --------------------------------------------------------------------------------

                                   9130      9140      9150      9160      9170      9180      9190      9200

Human                     TTGGTTTGAGTTTTCTCTGCTGTTTTTCTGTTTTCTATTTTATTGATTTCTGCTATTTTTATTTTGTTTTGCTTTGTGGT 9192
Kakapo                    -------------------------------------------------------------------------------- 0
GoldenEagle               -------------------------------------------------------------------------------- 0
JapaneseQuail             -------------------------------------------------------------------------------- 0
MediumGroundFinch         -------------------------------------------------------------------------------- 0
GoodesThornscrubTortoise  AGGGCAATAAAAATGATTGGGGTATGGAACAACTTCCTTATGAGAAGAGATTAAGATTGCACGGTTAGTCTGAAAAAAGA 5695

Majority                  --------------------------------------------------------------------------------

                                   9210      9220      9230      9240      9250      9260      9270      9280

Human                     TAGTTTGATCTTCATTTTCTACTTAGTTAACATTGAAGTTAATGTCATTAACTTAGTACTTTTACTTATATAGGGATTTA 9272
Kakapo                    -------------------------------------------------------------------------------- 0
GoldenEagle               -------------------------------------------------------------------------------- 0
JapaneseQuail             -------------------------------------------------------------------------------- 0
MediumGroundFinch         -------------------------------------------------------------------------------- 0
GoodesThornscrubTortoise  CGCAACGAAGGTCTATAAAATCATGAATGGGGTAGAGGAAGTGAATAAGGAAGTGTTGTTTATCCCTTCACATAACACAA 5775
```

```
Majority                   --------------------------------------------------------------------------------
                                    9290      9300      9310      9320      9330      9340      9350      9360
Human                      TTGCTGTATGTTTCCCTTTTAAGTACCGCTTTCACGGCATTCCTCAAATTTTGACACGTATATTTTTATTCACTTCAAAA 9352
Kakapo                     -------------------------------------------------------------------------------- 0
GoldenEagle                -------------------------------------------------------------------------------- 0
JapaneseQuail              -------------------------------------------------------------------------------- 0
MediumGroundFinch          -------------------------------------------------------------------------------- 0
GoodesThornscrubTortoise   GAACCAGGGGTCACCCAATTAAATTAATAGGCAGCAGGTTTAAAACAAACAACACACAGTCAGCCTTTGGAATTTGTTGC 5855

Majority                   --------------------------------------------------------------------------------
                                    9370      9380      9390      9400      9410      9420      9430      9440
Human                      TACTTTTTTAAATATTTCTTTGACCCATGGGTTATTGGAAAGTATGTTATTTATGTTATTTAATTTCCAAATATTTGGAG 9432
Kakapo                     -------------------------------------------------------------------------------- 0
GoldenEagle                -------------------------------------------------------------------------------- 0
JapaneseQuail              -------------------------------------------------------------------------------- 0
MediumGroundFinch          -------------------------------------------------------------------------------- 0
GoodesThornscrubTortoise   TAGGGGATGTTGTGAAGGCCAGAGTATAACTGGCTTAAAAAAAGAATTAGTTAAGTTCATGGAGGATGGGGCCAAAATGA 5935

Majority                   --------------------------------------------------------------------------------
                                    9450      9460      9470      9480      9490      9500      9510      9520
Human                      TTTTCCATATATCTTTCTGTTATGTATTCATAATTTTAGTTTTATTCTGGCCAGAGAACATATTTTCTAAGACTTGGATC 9512
Kakapo                     -------------------------------------------------------------------------------- 0
GoldenEagle                -------------------------------------------------------------------------------- 0
JapaneseQuail              -------------------------------------------------------------------------------- 0
MediumGroundFinch          -------------------------------------------------------------------------------- 0
GoodesThornscrubTortoise   GTCGCTTGGGATGACTTGGAGCATGGATTAACGTCCCAGTTTGCAACTGTCTCCATCCCATAATTGTATTTGTATCCCAT 6015

Majority                   --------------------------------------------------------------------------------
                                    9530      9540      9550      9560      9570      9580      9590      9600
Human                      TTTGTAAACATGGAGACTTGTTTTGTGGTCCAAATATATATGTTGTTAAGTATTCACTGAATACTCTGATGATTCTCTGC 9592
Kakapo                     -------------------------------------------------------------------------------- 0
GoldenEagle                -------------------------------------------------------------------------------- 0
JapaneseQuail              -------------------------------------------------------------------------------- 0
MediumGroundFinch          -------------------------------------------------------------------------------- 0
GoodesThornscrubTortoise   AACTTTTGTGTCTTTTTTTTCAAAATCCCACAAACCCACATGTCCCTCCTTGCTCTCCACTATCTCAGACAGAAGCATGA 6095
```

```
Majority                 --------------------------------------------------------------------------------

                                   9610      9620      9630      9640      9650      9660      9670      9680

Human                    AGATTTGGAGAAATCTCTGTGCAGTTCTTTTCTCTGGTACTTTGACCTGTATGTAAACTCTAGTTACTTTGGTCTTCTCA 9672
Kakapo                   -------------------------------------------------------------------------------- 0
GoldenEagle              -------------------------------------------------------------------------------- 0
JapaneseQuail            -------------------------------------------------------------------------------- 0
MediumGroundFinch        -------------------------------------------------------------------------------- 0
GoodesThornscrubTortoise AGCCTGCACAGTTCTGCACTATTGTCAGGAGCATTGCAAGCACAGGGTTCACGATCCTGTAGTAGTTGCAGAGCTGCAAG 6175

Majority                 --------------------------------------------------------------------------------

                                   9690      9700      9710      9720      9730      9740      9750      9760

Human                    GGCTCTTGGCTCTTTCACAATTAAAGTAGTCTTTGAGGCTCAGCCTGCTTTCCTCATAGCTATGCTATGGCCTGGACACT 9752
Kakapo                   -------------------------------------------------------------------------------- 0
GoldenEagle              -------------------------------------------------------------------------------- 0
JapaneseQuail            -------------------------------------------------------------------------------- 0
MediumGroundFinch        -------------------------------------------------------------------------------- 0
GoodesThornscrubTortoise AAGACCTGAATGGGTGGGTAGTGGGGGCATAATGATTCCTTGGAGGACAGATTGTCAAGGGACATAGCAAGAATCAATTG 6255

Majority                 --------------------------------------------------------------------------------

                                   9770      9780      9790      9800      9810      9820      9830      9840

Human                    CAAGGGAGTATAAGCTGAGGCAAACATGGACTCATTTGTTTTCTAACTTTCAGGGATTATTGTCCATCATTGCCTGATGT 9832
Kakapo                   -------------------------------------------------------------------------------- 0
GoldenEagle              -------------------------------------------------------------------------------- 0
JapaneseQuail            -------------------------------------------------------------------------------- 0
MediumGroundFinch        -------------------------------------------------------------------------------- 0
GoodesThornscrubTortoise AATGTTGTTGGTGTGCATGGAACAGATGGAGATGGTGGAGCACCA-CTTCTGGACCTGAGAAACAAGCAATGACTGACTG 6334

Majority                 --------------------------------------------------------------------------------

                                   9850      9860      9870      9880      9890      9900      9910      9920

Human                    CCAGTGTCTTGAAAAGCAATTATTCTGTATAGTTGCTTGATTGTTTGGTGTTGTTTCGGCGAGGCAAATCTGGGTCATGT 9912
Kakapo                   -------------------------------------------------------------------------------- 0
GoldenEagle              -------------------------------------------------------------------------------- 0
JapaneseQuail            -------------------------------------------------------------------------------- 0
MediumGroundFinch        -------------------------------------------------------------------------------- 0
GoodesThornscrubTortoise CTGAAATCGCATCGTAATGTAAGTTTGGGAAGATGACCAGTGGCTGAGGAACTTTCAGATGTATAAGGCCATGTGCCTGG 6414
```

```
Majority                --------------------------------------------------------------------------------
                              9930      9940      9950      9960      9970      9980      9990     10000
Human                   TATTCTGTCTTGACTGGAAGTAGAAGTCCACTGTTTTTTTTTAATTACATAATATGGTCTCAGCAGTTGTAGATCTACCT 9992
Kakapo                  -------------------------------------------------------------------------------- 0
GoldenEagle             -------------------------------------------------------------------------------- 0
JapaneseQuail           -------------------------------------------------------------------------------- 0
MediumGroundFinch       -------------------------------------------------------------------------------- 0
GoodesThornscrubTortoise ATCTGTGTAGGGAGCTTTCTCCAGCCCTTCCGCCCAAGATACCAAAATGAGAGCTGCGCTGACAGTGGAGAAGTGAGTGG 6494
```

```
Majority                --------------------------------------------------------------------------------
                             10010     10020     10030     10040     10050     10060     10070     10080
Human                   CATTCTTTGTTAATGACAATGGAGGATACTCGTGTGGCTGCACCACAGTGTAGTTACTGGACATACCACAATTTAGTTTC 10072
Kakapo                  -------------------------------------------------------------------------------- 0
GoldenEagle             -------------------------------------------------------------------------------- 0
JapaneseQuail           -------------------------------------------------------------------------------- 0
MediumGroundFinch       -------------------------------------------------------------------------------- 0
GoodesThornscrubTortoise CGCTCACAAGTGGAACACCTGATTGTTACCTGTCTGAAACCAATTTGGGGTCAGGAAATCCATGGCTGTTTTCATGTGAG 6574
```

```
Majority                --------------------------------------------------------------------------------
                             10090     10100     10110     10120     10130     10140     10150     10160
Human                   CTACATCGATGGACATTTAGGTTTGATTCCAGTTTGTTGTTATAACATGTGGACATATTTAGAATAGAAGTAAAAATGCT 10152
Kakapo                  -------------------------------------------------------------------------------- 0
GoldenEagle             -------------------------------------------------------------------------------- 0
JapaneseQuail           -------------------------------------------------------------------------------- 0
MediumGroundFinch       -------------------------------------------------------------------------------- 0
GoodesThornscrubTortoise TGTACAGAGCCTTTAAATGCCTCATGCTATGCAGGATTGTGACTGCAATGTGCAGGACATACTGGATGGCTTTGGAGCTG 6654
```

```
Majority                --------------------------------------------------------------------------------
                             10170     10180     10190     10200     10210     10220     10230     10240
Human                   GTGTCAAATGAAATTTATACTTAAAGAGTTACTGAAAACCACAATGCCAGAACACTCATTTGTTTTTCACAAGTGTGTAT 10232
Kakapo                  -------------------------------------------------------------------------------- 0
GoldenEagle             -------------------------------------------------------------------------------- 0
JapaneseQuail           -------------------------------------------------------------------------------- 0
MediumGroundFinch       -------------------------------------------------------------------------------- 0
GoodesThornscrubTortoise TGGGGTTCCTGAACTGTGGTGGAGCAACAGATGGCATGCATATCCCTATTTTGGCACCAGAACATGTTGCCACTGTATAC 6734
```

```
Majority                --------------------------------------------------------------------------------

                                10250     10260     10270     10280     10290     10300     10310     10320

Human                   GGGAGTGCCTATTTTTCCATACCCTTGGCAATTCTAGGTTTTATCAAAATTTAAGAATACTAGCTTATTTTACACTAAAA 10312
Kakapo                  -------------------------------------------------------------------------------- 0
GoldenEagle             -------------------------------------------------------------------------------- 0
JapaneseQuail           -------------------------------------------------------------------------------- 0
MediumGroundFinch       -------------------------------------------------------------------------------- 0
GoodesThornscrubTortoiseGTTGGTAGAAAGGGCTCTTTTCTATGATTATGCAAGCATAGATGGATCACTGGGGGATGCTTCATCGACATCAATGTCGG 6814

Majority                --------------------------------------------------------------------------------

                                10330     10340     10350     10360     10370     10380     10390     10400

Human                   ATTATTTTGTAATTTATATTTTGAAATGGGTGAGGTTTTTCAGATTTGGTTATTTATATTTCATTTTCTGTGAACTGCCT 10392
Kakapo                  -------------------------------------------------------------------------------- 0
GoldenEagle             -------------------------------------------------------------------------------- 0
JapaneseQuail           -------------------------------------------------------------------------------- 0
MediumGroundFinch       -------------------------------------------------------------------------------- 0
GoodesThornscrubTortoiseGTCCAGGAAGGTACATGTCACTCACATCTTTAAGAACA--CAGGACTGTTCAGAAAGCTACAAGCATGGACTCTTTGCCT 6892

Majority                --------------------------------------------------------------------------------

                                10410     10420     10430     10440     10450     10460     10470     10480

Human                   GTTTGTGTACTGGACCTAGTTAATTCAAATATATATATTTGAATTAAGTGATTTTATTGTTTAAAGTCCATTTGATGTGT 10472
Kakapo                  -------------------------------------------------------------------------------- 0
GoldenEagle             -------------------------------------------------------------------------------- 0
JapaneseQuail           -------------------------------------------------------------------------------- 0
MediumGroundFinch       -------------------------------------------------------------------------------- 0
GoodesThornscrubTortoiseACTGGTGGATTGCCGCTGTCAATATTGAAATGCCAATCCTTTGCTCCCCTTTCTCATGAAGCCATAGATTGGTCACCTTG 6972

Majority                --------------------------------------------------------------------------------

                                10490     10500     10510     10520     10530     10540     10550     10560

Human                   AAACATTTTTAGCCTCAGCTTCTTCACCTTTTAAATTAGGATTTTAATAGTTCCACAAACTAGGTCTAATGTTAATGTAT 10552
Kakapo                  -------------------------------------------------------------------------------- 0
GoldenEagle             -------------------------------------------------------------------------------- 0
JapaneseQuail           -------------------------------------------------------------------------------- 0
MediumGroundFinch       -------------------------------------------------------------------------------- 0
GoodesThornscrubTortoiseACAGCACCAAGGAATGATTCAGTACTGGATCAGCAGGTATTGAGTGACAGTTGAATGTGCATTTGGTCATTTGAAGGGTC 7052
```

```
Majority                  --------------------------------------------------------------------------------

                                   10570     10580     10590     10600     10610     10620     10630     10640

Human                     TTACATTTAGAATATCCAAGTTTTTATGGCTTTAGGATACAAATGAAATCTCATTTTCCATTATTTTATAAATTTGCTGT 10632
Kakapo                    -------------------------------------------------------------------------------- 0
GoldenEagle               -------------------------------------------------------------------------------- 0
JapaneseQuail             -------------------------------------------------------------------------------- 0
MediumGroundFinch         -------------------------------------------------------------------------------- 0
GoodesThornscrubTortoise  CCTGGCGTTGCCTACTCACAAGATTGGACCTCAGGGAGAAAAATATCCCACAGCTGACTGCTGTGTCCTGCCTAATATCT 7132

Majority                  --------------------------------------------------------------------------------

                                   10650     10660     10670     10680     10690     10700     10710     10720

Human                     ATTCAGCTTTTCTTGATTTTAGACTTTTTTTTGTTAGCTCAGTGAGACTATTAAGAAAGAATATTTGTGAGTTTGTAATA 10712
Kakapo                    -------------------------------------------------------------------------------- 0
GoldenEagle               -------------------------------------------------------------------------------- 0
JapaneseQuail             -------------------------------------------------------------------------------- 0
MediumGroundFinch         -------------------------------------------------------------------------------- 0
GoodesThornscrubTortoise  TTAAAGCAAAAGGGGAAAAGTTTCTGCTGGGATGGAGGGCAGAGGTGGAGTGGCTGCCTGCTGAATTTGAATGGGGCTAT 7212

Majority                  --------------------------------------------------------------------------------

                                   10730     10740     10750     10760     10770     10780     10790     10800

Human                     GGAGATTTGTTGCATTTCTTTAAAATGGCTTTTTATATTCTTCGTAATTTGTAATATCAGGGGTGTCATTTCTGTACTCT 10792
Kakapo                    -------------------------------------------------------------------------------- 0
GoldenEagle               -------------------------------------------------------------------------------- 0
JapaneseQuail             -------------------------------------------------------------------------------- 0
MediumGroundFinch         -------------------------------------------------------------------------------- 0
GoodesThornscrubTortoise  TAGAAGAGATGAACATGGAGCTGTACAGCTCAGGGAGGCTTTGAAAGGCAATTTTAACAGTGAGTGGAGTAATGTGTGGT 7292

Majority                  --------------------------------------------------------------------------------

                                   10810     10820     10830     10840     10850     10860     10870     10880

Human                     TAACCATTTTGCCAATGTGAAATCCTTGCAAAGTAATCGGCCTATTACTTGGGAAGACAGAATTGTATGAAATGACTGTA 10872
Kakapo                    -------------------------------------------------------------------------------- 0
GoldenEagle               -------------------------------------------------------------------------------- 0
JapaneseQuail             -------------------------------------------------------------------------------- 0
MediumGroundFinch         -------------------------------------------------------------------------------- 0
GoodesThornscrubTortoise  CATGTAGTATGCTCTATCTGGCCCTGCTCTTTTGTGGCCTGGTAGGAATTGTGTGGTGATTACTGTAC-ATGTAGGAATA 7371
```

```
Majority                 --------------------------------------------------------------------------------

                                  10890     10900     10910     10920     10930     10940     10950     10960

Human                    GAATAATTCAGGATGGTCCCCAATACGGTAAAATGTTAACTTTTTAGATGTAGATTTTAAGTATGGTGAAATTTGTAGCA 10952
Kakapo                   -------------------------------------------------------------------------------- 0
GoldenEagle              -------------------------------------------------------------------------------- 0
JapaneseQuail            -------------------------------------------------------------------------------- 0
MediumGroundFinch        -------------------------------------------------------------------------------- 0
GoodesThornscrubTortoise GAACATTGTCAACTCACCTTCGCCATTGGGAGGTGAACTATCTCAAATTCAACAGGTTAATTATTAATACTTAGAGGATT 7451

Majority                 --------------------------------------------------------------------------------

                                  10970     10980     10990     11000     11010     11020     11030     11040

Human                    GTGTTCTTTAGTAAGTTAACTATTAATATATCAAGACCTATAACTGGACAACTAATAAATAACCTGTTTCTTAAAATGTT 11032
Kakapo                   -------------------------------------------------------------------------------- 0
GoldenEagle              -------------------------------------------------------------------------------- 0
JapaneseQuail            -------------------------------------------------------------------------------- 0
MediumGroundFinch        -------------------------------------------------------------------------------- 0
GoodesThornscrubTortoise TTCTTGCCATGCTCTATTATGAGGAGAACATCACCAGACATTTAAATTGTTTTATTTAACTAAAACAACAAGCAGCATAA 7531

Majority                 --------------------------------------------------------------------------------

                                  11050     11060     11070     11080     11090     11100     11110     11120

Human                    AGCTTTTTCTAAATATTCTGAAAGTACTGGTTGTAAATTGCTGAACTCACAATTTAAATTATGATATGACTATTGAATTC 11112
Kakapo                   -------------------------------------------------------------------------------- 0
GoldenEagle              -------------------------------------------------------------------------------- 0
JapaneseQuail            -------------------------------------------------------------------------------- 0
MediumGroundFinch        -------------------------------------------------------------------------------- 0
GoodesThornscrubTortoise TATTTTAACAAAATAATCATATGCATTTTTGAATTTAGTTAAACATTCACGTTTTTTTAAATCAGGTTTGTTTTTGTTAA 7611

Majority                 --------------------------------------------------------------------------------

                                  11130     11140     11150     11160     11170     11180     11190     11200

Human                    ATCCTCATTGGCTTGCATAGAAAATTACAATCAAATAATGCTAAATGTAACTTTTTTGGTAATAGTTGTGTGTAACTGGT 11192
Kakapo                   -------------------------------------------------------------------------------- 0
GoldenEagle              -------------------------------------------------------------------------------- 0
JapaneseQuail            -------------------------------------------------------------------------------- 0
MediumGroundFinch        -------------------------------------------------------------------------------- 0
GoodesThornscrubTortoise AATTGTTTTAACTAAAGTAGTTAAATGAATTTAAAAAATTAAATAGACTATGTCAGCCAGGTCAATATGAGAAACTTAAA 7691
```

```
Majority                 --------------------------------------------------------------------------------

                                  11210     11220     11230     11240     11250     11260     11270     11280

Human                    TGTTCATTATAGATGTGTTATTCAAAACTTTGGGTTTTTAGGTTTTGACTTTTCTTAAAATACTTTTCTTAAATGCTACT 11272
Kakapo                   -------------------------------------------------------------------------------- 0
GoldenEagle              -------------------------------------------------------------------------------- 0
JapaneseQuail            -------------------------------------------------------------------------------- 0
MediumGroundFinch        -------------------------------------------------------------------------------- 0
GoodesThornscrubTortoise ATATTGGCTTCTGCAGCTAACTCAGTCGTCTTCACCTTAATTTTCCTGTTTGTTCATAATCTGGAAAAGAAAAACAAGCT 7771

Majority                 --------------------------------------------------------------------------------

                                  11290     11300     11310     11320     11330     11340     11350     11360

Human                    TAATGCTTTCTCTAGGGTATTTTTATTAGAATTTCAATTAACAGATATTTTGATGTCTGCGAAATGACAGTACTATGTTA 11352
Kakapo                   -------------------------------------------------------------------------------- 0
GoldenEagle              -------------------------------------------------------------------------------- 0
JapaneseQuail            -------------------------------------------------------------------------------- 0
MediumGroundFinch        -------------------------------------------------------------------------------- 0
GoodesThornscrubTortoise ATCCTGCTTTTTCAAATCCAAAACAATTTCTCAATTTGGAATGAATTAGTCCAAAGAGAGAAAATATTCTTTCTACACTG 7851

Majority                 --------------------------------------------------------------------------------

                                  11370     11380     11390     11400     11410     11420     11430     11440

Human                    GAAATTGATGTATTGGCTCCTTGAGGGCAGTGACCCTATTTGTTATGTTTGTTCCTCAATCATTAATAGTACACATATTA 11432
Kakapo                   -------------------------------------------------------------------------------- 0
GoldenEagle              -------------------------------------------------------------------------------- 0
JapaneseQuail            -------------------------------------------------------------------------------- 0
MediumGroundFinch        -------------------------------------------------------------------------------- 0
GoodesThornscrubTortoise ACAGAAGAAGCTACTGCTGTTTAAAGTGAGATTATCACTTCAACAGTCTCTGAATCAAAATGCTTAAGTGACTTCTGCTG 7931

Majority                 --------------------------------------------------------------------------------

                                  11450     11460     11470     11480     11490     11500     11510     11520

Human                    AATAGTACATATACACTTAATATTTAATAACTCTTAGAAGTGTCATAGGCTTTAATATGCTCATACTCTAATGAGCCATT 11512
Kakapo                   -------------------------------------------------------------------------------- 0
GoldenEagle              -------------------------------------------------------------------------------- 0
JapaneseQuail            -------------------------------------------------------------------------------- 0
MediumGroundFinch        -------------------------------------------------------------------------------- 0
GoodesThornscrubTortoise CTGAAAGTGACTTTCTTTAAAACATCATCAGCAAATAAATATTTCTTGAATGGTTCACCCTTAGCTCTGAAGTTTATTAG 8011
```

```
Majority                --------------------------------------------------------------------------------

                                 11530     11540     11550     11560     11570     11580     11590     11600

Human                   TATCAATAAAAAGATAAATAACAAGACTATGTCTTTTAGGACTTTAGTTAGAGAAATAAGGACTCAAGGTTTTAATTAAG 11592
Kakapo                  -------------------------------------------------------------------------------- 0
GoldenEagle             -------------------------------------------------------------------------------- 0
JapaneseQuail           -------------------------------------------------------------------------------- 0
MediumGroundFinch       -------------------------------------------------------------------------------- 0
GoodesThornscrubTortoise TTGGTATTATGGAGGGATGATTGCTGGATGTCCATGTGATAGCTAACTCCTCTTCTTCAGCAGTTAAGGGTTGACCCTGG 8091

Majority                --------------------------------------------------------------------------------

                                 11610     11620     11630     11640     11650     11660     11670     11680

Human                   TGCTATATGGCTACAGTAGGAAAGATTGGTTTGGGTTGGTAGGCTCAGAATAGTGGTCTGTGACCTAGCTAGATTGGGAG 11672
Kakapo                  -------------------------------------------------------------------------------- 0
GoldenEagle             -------------------------------------------------------------------------------- 0
JapaneseQuail           -------------------------------------------------------------------------------- 0
MediumGroundFinch       -------------------------------------------------------------------------------- 0
GoodesThornscrubTortoise TACGGAGTATTGAGAATATTTGCAAGAAAATGAGCTGGAGATAGTGCTTGTCCCATTTTGTTTTTTTTAATGTTTGTGAT 8171

Majority                --------------------------------------------------------------------------------

                                 11690     11700     11710     11720     11730     11740     11750     11760

Human                   ATAGGAGAAAGAGTGGAGAGCAACAGGCGTTGAGAGAAGGACGTAACCAAAGTTGTGTCTATAGGTT--GGCATAAATTT 11750
Kakapo                  -------------------------------------------------------------------------------- 0
GoldenEagle             -------------------------------------------------------------------------------- 0
JapaneseQuail           -------------------------------------------------------------------------------- 0
MediumGroundFinch       -------------------------------------------------------------------------------- 0
GoodesThornscrubTortoise TTAACTCTGTCATTCCATATTTCTCTCTTTAAGATCTCACTCAGTTCCTTCCAAATTTCAACAGCATCAGCAATAAAACA 8251

Majority                --------------------------------------------------------------------------------

                                 11770     11780     11790     11800     11810     11820     11830     11840

Human                   ACAGCCATTGATCAGTGAGTTGAATAGTTTGGCCTTTATGGCTAACAGCTTGTATATGGATATGAGAGAAAAGATAGAAT 11830
Kakapo                  -------------------------------------------------------------------------------- 0
GoldenEagle             -------------------------------------------------------------------------------- 0
JapaneseQuail           -------------------------------------------------------------------------------- 0
MediumGroundFinch       -------------------------------------------------------------------------------- 0
GoodesThornscrubTortoise CCTATTTCCATGCATTTTGTTCAAGGCTACAGAAATAGGCTTCAGAGTACTCAGCATGTGTTCAACATTGGGCTTAAGAG 8331
```

```
Majority                    --------------------------------------------------------------------------------
                                     11850     11860     11870     11880     11890     11900     11910     11920
Human                       GGCAGATAGGTTATATAGAAGACCTCAATTACAGGTAGTGAAATTTGTACTTCATCTGGTAGGCATTATTCTATATACCT 11910
Kakapo                      -------------------------------------------------------------------------------- 0
GoldenEagle                 -------------------------------------------------------------------------------- 0
JapaneseQuail               -------------------------------------------------------------------------------- 0
MediumGroundFinch           -------------------------------------------------------------------------------- 0
GoodesThornscrubTortoise    AAATGTTGAGAACTGTGACTGTGATCAAAATCGTGAAAAAAGTAGATGGCACTAACTGTCATCAGATTAGGCCAGTTCTT 8411

Majority                    --------------------------------------------------------------------------------
                                     11930     11940     11950     11960     11970     11980     11990     12000
Human                       GTAATTTTCCACATAAAAGTAGCTCATTACTCTGCTTTCTTTCTTTCTTTTTTTTTTTTTTTGCTATAGCAAATTTATTG 11990
Kakapo                      -------------------------------------------------------------------------------- 0
GoldenEagle                 -------------------------------------------------------------------------------- 0
JapaneseQuail               -------------------------------------------------------------------------------- 0
MediumGroundFinch           -------------------------------------------------------------------------------- 0
GoodesThornscrubTortoise    GATATAGTGCTCAAAACAGTCCACTACTGAGTTCCATCACACATCTTGTGGGAGAGTTAGTGTGGTTCCTCCCACTTTTT 8491

Majority                    --------------------------------------------------------------------------------
                                     12010     12020     12030     12040     12050     12060     12070     12080
Human                       TAACAACACAAAGTATTATCTTATAGTTTTGTAGGTGAGAAATCTGACACAGGTGGGCTAAAATCAAGGTGTTAGCAGGG 12070
Kakapo                      -------------------------------------------------------------------------------- 0
GoldenEagle                 -------------------------------------------------------------------------------- 0
JapaneseQuail               -------------------------------------------------------------------------------- 0
MediumGroundFinch           -------------------------------------------------------------------------------- 0
GoodesThornscrubTortoise    TCAGAGCGGCTGCTGCAAAGTGGTTGTTACGGAAGTATTTTGCAGTTTCAACAACATTAACCTTTATTTCTGCAACACTG 8571

Majority                    --------------------------------------------------------------------------------
                                     12090     12100     12110     12120     12130     12140     12150     12160
Human                       CTGTGTTCCTTTTTGCAGGATCTGGGGGGAATTTGTTTCCTTGACATAACTCGGCTGTTAGCTTCATCTTCAAAGAAGGA 12150
Kakapo                      -------------------------------------------------------------------------------- 0
GoldenEagle                 -------------------------------------------------------------------------------- 0
JapaneseQuail               -------------------------------------------------------------------------------- 0
MediumGroundFinch           -------------------------------------------------------------------------------- 0
GoodesThornscrubTortoise    AAGTCTTTGGCTAGGAGGTGCATCAAATGAGCACTGCAACGGTATGTTATTAGCTTGGGACTCTCTTTTAAATAATTTCT 8651
```

```
Majority                   --------------------------------------------------------------------------------

                                   12170     12180     12190     12200     12210     12220     12230     12240

Human                      ATGGCAAAGAAGACTCTGTCTCACATCACATCACTCCGACACATTCTTGTGCCTCCGTTTCCCACTTACAGGGACCTTGT 12230
Kakapo                     -------------------------------------------------------------------------------- 0
GoldenEagle                -------------------------------------------------------------------------------- 0
JapaneseQuail              -------------------------------------------------------------------------------- 0
MediumGroundFinch          -------------------------------------------------------------------------------- 0
GoodesThornscrubTortoise   TCTCATCTTGGCTGCATTTTCAGCATTGTCTGTGACCAAGCTGCATACTAGACATTTGAATTTTTTTCCAAGTTCGTTAT 8731

Majority                   --------------------------------------------------------------------------------

                                   12250     12260     12270     12280     12290     12300     12310     12320

Human                      GATTACAATGGGCCCAACTACATAATAGAGGATAAATGT-TTAAATTACAAATTAATGAAGTGGCAACTGAAACAAGTTT 12309
Kakapo                     -------------------------------------------------------------------------------- 0
GoldenEagle                -------------------------------------------------------------------------------- 0
JapaneseQuail              -------------------------------------------------------------------------------- 0
MediumGroundFinch          -------------------------------------------------------------------------------- 0
GoodesThornscrubTortoise   AGCTTTTACTGCTACTTGTAAGTATTCTGCTCTATGTGCATTTCCTGATGTATCAATTGTTTCTACAAGGAAACATTCCC 8811

Majority                   --------------------------------------------------------------------------------

                                   12330     12340     12350     12360     12370     12380     12390     12400

Human                      TAACCTATTCTCAAGAAAGGGCTTTGCTGGAATGGTGGCTCACACCTGTAATCCCAGAACTTAGGGAGGCTGAGGCAGGC 12389
Kakapo                     -------------------------------------------------------------------------------- 0
GoldenEagle                -------------------------------------------------------------------------------- 0
JapaneseQuail              -------------------------------------------------------------------------------- 0
MediumGroundFinch          -------------------------------------------------------------------------------- 0
GoodesThornscrubTortoise   TTCTTCCATTATCACACAAGCACATACAACAGGATCATTGTGGACGTTGCTCCACCCATCAAGACTCGGGTTAACAGTTT 8891

Majority                   --------------------------------------------------------------------------------

                                   12410     12420     12430     12440     12450     12460     12470     12480

Human                      AGATCACTTGAGCCCAGGAGTTTGAGACCAGCCTGGTCCTCATAGCAAGACCCCATCTCTACAAAAACTTTTAA---AAA 12466
Kakapo                     -------------------------------------------------------------------------------- 0
GoldenEagle                -------------------------------------------------------------------------------- 0
JapaneseQuail              -------------------------------------------------------------------------------- 0
MediumGroundFinch          -------------------------------------------------------------------------------- 0
GoodesThornscrubTortoise   TACTCTCTCTGGCCGTGGTTCACTGCTCCAGGCCAATGGGAGCTGCTGGAAGCGGTGGCCAGTACGTCCCTCAGTCCGTG 8971
```

```
Majority                 --------------------------------------------------------------------------------
                              12490     12500     12510     12520     12530     12540     12550     12560
Human                    TTAGATAGATCTGGTGGCATGGGCCTGTAGTCCCAGCTACTTGGGAGGCTGAGACAAGAGGATCACCTGAGCCCAGGCGG 12546
Kakapo                   -------------------------------------------------------------------------------- 0
GoldenEagle              -------------------------------------------------------------------------------- 0
JapaneseQuail            -------------------------------------------------------------------------------- 0
MediumGroundFinch        -------------------------------------------------------------------------------- 0
GoodesThornscrubTortoise CCGCAGCTAGTGGGAGCCGCGATAAGCTGAACCTACGGACGCGGCAGGTAAACAAACCGGCCCTAGCTACCAGGGGCTTT 9051

Majority                 --------------------------------------------------------------------------------
                              12570     12580     12590     12600     12610     12620     12630     12640
Human                    CAGTGAGCTATGATTGTATCACTACACTCCAGCCTGGGCGAGAGGAGATCCAGTCTCAAAAAAAAAGGAAAAGGGCTTTC 12626
Kakapo                   -------------------------------------------------------------------------------- 0
GoldenEagle              -------------------------------------------------------------------------------- 0
JapaneseQuail            -------------------------------------------------------------------------------- 0
MediumGroundFinch        -------------------------------------------------------------------------------- 0
GoodesThornscrubTortoise CCCTACACGAGCGGCGTCCCAATTTTGGGAAACACTGCCATAGAATAACAACTATATATTTTTCTCAAACATGAGAATTC 9131

Majority                 --------------------------------------------------------------------------------
                              12650     12660     12670     12680     12690     12700     12710     12720
Human                    CTGAGGGTCAAATGAAGTACAGTTGACCCTTGAACAACATGATTTTGAATTACACAGGTCCACTTATGCATAGATCCACT 12706
Kakapo                   -------------------------------------------------------------------------------- 0
GoldenEagle              -------------------------------------------------------------------------------- 0
JapaneseQuail            -------------------------------------------------------------------------------- 0
MediumGroundFinch        -------------------------------------------------------------------------------- 0
GoodesThornscrubTortoise AAGAATAGTCCAGAAGGAAGACAAGCAGTCCTTAAGAAAGAACTATGAAATAAAAAAATTTACCAACCTGCAGATCCTGT 9211

Majority                 --------------------------------------------------------------------------------
                              12730     12740     12750     12760     12770     12780     12790     12800
Human                    TATACAGAATTTTCTTCTCCCTCTTCCACCCAAGACAGAAAGACCAACTCGTCCTCTTCCTCCTTAGCCTACTCAGCGTG 12786
Kakapo                   -------------------------------------------------------------------------------- 0
GoldenEagle              -------------------------------------------------------------------------------- 0
JapaneseQuail            -------------------------------------------------------------------------------- 0
MediumGroundFinch        -------------------------------------------------------------------------------- 0
GoodesThornscrubTortoise CTGTTCAGACATGTTCCTTTCATCATCTTTAACGCAACTTTCTCCTGAGAAGGATCATTTCTCATGATGTTGTTTCATTT 9291
```

```
Majority                 --------------------------------------------------------------------------------

                                  12810     12820     12830     12840     12850     12860     12870     12880

Human                    AAGACAGTCAGGATGAAGACCTTTATTATGACTGATCTCCAAAAAGATTGGCTTCAGTATTTCATGGAGTGCTATGAATT 12866
Kakapo                   -------------------------------------------------------------------------------- 0
GoldenEagle              -------------------------------------------------------------------------------- 0
JapaneseQuail            -------------------------------------------------------------------------------- 0
MediumGroundFinch        -------------------------------------------------------------------------------- 0
GoodesThornscrubTortoise CAGTAATCAGGCCTTGCATTTCTTTGTTGCCCTGTTTGCATTTTGCACGCATGCGCGTCTTACTCACAGGAAGAGGAACT 9371

Majority                 --------------------------------------------------------------------------------

                                  12890     12900     12910     12920     12930     12940     12950     12960

Human                    AGAATCCTGAGTCTCTATATTGTTAATTATTTTAGTCTCTTGGTC--TACGTGTGTTCCTAAACTGTAGTAGCTTTCTTA 12944
Kakapo                   -------------------------------------------------------------------------------- 0
GoldenEagle              -------------------------------------------------------------------------------- 0
JapaneseQuail            -------------------------------------------------------------------------------- 0
MediumGroundFinch        -------------------------------------------------------------------------------- 0
GoodesThornscrubTortoise TCATTAAAATATTTCCAAACTGGGTTTCTTTTACGGCCTGCTGCCATTATAGGTTTTCTTTTCTAGTGAGAGAATGGTAT 9451

Majority                 --------------------------------------------------------------------------------

                                  12970     12980     12990     13000     13010     13020     13030     13040

Human                    ATTTAGTTCTAGGCATACTACATTAGGGGATAAGGTGGTTAAGTGATATTTTCACTTTAAAAGTAATAAGGTGTCACAGA 13024
Kakapo                   -------------------------------------------------------------------------------- 0
GoldenEagle              -------------------------------------------------------------------------------- 0
JapaneseQuail            -------------------------------------------------------------------------------- 0
MediumGroundFinch        -------------------------------------------------------------------------------- 0
GoodesThornscrubTortoise AGTAGATCACAGATCAATGAAGGCTACACTCAGAAAGACCTCAAAACTTCTGGAATATGCTGCTCAGACAGTTTCGCCTT 9531

Majority                 --------------------------------------------------------------------------------

                                  13050     13060     13070     13080     13090     13100     13110     13120

Human                    AAGTTATCACGAGATTTTTTTGAGCAGTACTTCTTTTTTAAAACTATTATAATGAATTTTAGACACAATAGTATCATGAA 13104
Kakapo                   -------------------------------------------------------------------------------- 0
GoldenEagle              -------------------------------------------------------------------------------- 0
JapaneseQuail            -------------------------------------------------------------------------------- 0
MediumGroundFinch        -------------------------------------------------------------------------------- 0
GoodesThornscrubTortoise TGTTTCTACTGCCTGTCCCTCACTTCTCATATTTATCTCCAGGCTTCTTCCCCTTGTCCAGATGAATGTTGGGGGCAGAA 9611
```

```
Majority                 --------------------------------------------------------------------------------

                                  13130     13140     13150     13160     13170     13180     13190     13200

Human                    CCATTGTGTATCTATCATTCAGTCCCCAAAACCATTAGTAACCCATAGCTAAAATGATGATATCCACACTCTGCTTTTCC 13184
Kakapo                   -------------------------------------------------------------------------------- 0
GoldenEagle              -------------------------------------------------------------------------------- 0
JapaneseQuail            -------------------------------------------------------------------------------- 0
MediumGroundFinch        -------------------------------------------------------------------------------- 0
GoodesThornscrubTortoise TAGAACTTTTTGAAACTTTGCACATTTAGAGAGAGGTCAGAGATTGACTCTGTGTACACAAGTTTGCAGAGGGACAGTAG 9691

Majority                 --------------------------------------------------------------------------------

                                  13210     13220     13230     13240     13250     13260     13270     13280

Human                    ACTTCTGTTATTTTGAGATAAATTTCAGATATTCATATTGTTTTATCTGTAAAGGCTTCAGTGTATATCTTTAAAGAATA 13264
Kakapo                   -------------------------------------------------------------------------------- 0
GoldenEagle              -------------------------------------------------------------------------------- 0
JapaneseQuail            -------------------------------------------------------------------------------- 0
MediumGroundFinch        -------------------------------------------------------------------------------- 0
GoodesThornscrubTortoise CGTTGAGGTCTGTTATTTCTCACCTCTATATATGATTTATTTATTAAAAAAATATTTGTTGTTAACAAGTGTGTTATCTC 9771

Majority                 --------------------------------------------------------------------------------

                                  13290     13300     13310     13320     13330     13340     13350     13360

Human                    GGGACTCTCCTCTCCTTTTTAAAATATAATCAAAAGACTGTTAGGACACACACACAAAGTTACCTCATATTATAAAATGT 13344
Kakapo                   -------------------------------------------------------------------------------- 0
GoldenEagle              -------------------------------------------------------------------------------- 0
JapaneseQuail            -------------------------------------------------------------------------------- 0
MediumGroundFinch        -------------------------------------------------------------------------------- 0
GoodesThornscrubTortoise TGGAAACACAAATCCAGTTTGAGAACTGCAAAGCTAAGCATCTTTGATGGTGTTTTCTAGACCAGTGCTTCTCAAAGTGG 9851

Majority                 --------------------------------------------------------------------------------

                                  13370     13380     13390     13400     13410     13420     13430     13440

Human                    CAAATTTGTATTAAAATTTCTAATTGTCTCATAAAAGTCATAGTATTTTTATAGGGTTTTTTTTTTAGTGGGGGAAGGA 13424
Kakapo                   -------------------------------------------------------------------------------- 0
GoldenEagle              -------------------------------------------------------------------------------- 0
JapaneseQuail            -------------------------------------------------------------------------------- 0
MediumGroundFinch        -------------------------------------------------------------------------------- 0
GoodesThornscrubTortoise TGGTCTG----CAGACTGGTGCCGGTCCACGAGCCATCAGCTGCCAGTCCGCAACGAGTTTCCTCATAAGAGCGTCAAAT 9927
```

```
Majority                  --------------------------------------------------------------------------------

                                   13450     13460     13470     13480     13490     13500     13510     13520

Human                     GTCACGATAAAATAAGATCAGCAAATTTAACAGGTCGTTATTACCTCTTAAATATCTTTTAATGTACAAGCTCTCCGTTC 13504
Kakapo                    -------------------------------------------------------------------------------- 0
GoldenEagle               -------------------------------------------------------------------------------- 0
JapaneseQuail             -------------------------------------------------------------------------------- 0
MediumGroundFinch         -------------------------------------------------------------------------------- 0
GoodesThornscrubTortoise  AGGATAATAACATGGCACCTGTCCCTGACACATTGGGGAAAAAAAAATTGCTGGTCCCCCACATCAGATAGTGCTCAGTC 10007

Majority                  --------------------------------------------------------------------------------

                                   13530     13540     13550     13560     13570     13580     13590     13600

Human                     CTGTTTTTTGTTTTTTTTTTAAGTTGCATTTCATTTGTTGAAGAAAACTGGTTATTGGTCTTACACAGTTGCCTATAGAC 13584
Kakapo                    -------------------------------------------------------------------------------- 0
GoldenEagle               -------------------------------------------------------------------------------- 0
JapaneseQuail             -------------------------------------------------------------------------------- 0
MediumGroundFinch         -------------------------------------------------------------------------------- 0
GoodesThornscrubTortoise  TAGAACAGTGCTTCTCAAGCTAAGTCCCATTGGGTAGATAGAAAGATGAACCTAAATAATCTATACAGAAGCCCCTGGAA 10087

Majority                  --------------------------------------------------------------------------------

                                   13610     13620     13630     13640     13650     13660     13670     13680

Human                     TACATTTACTGATTGCGTTATGATTTTGTAATTTAGTATGTTTTCCATATTTCCAGTAAATTGGTAGTTAGATCTAGAGA 13664
Kakapo                    -------------------------------------------------------------------------------- 0
GoldenEagle               -------------------------------------------------------------------------------- 0
JapaneseQuail             -------------------------------------------------------------------------------- 0
MediumGroundFinch         -------------------------------------------------------------------------------- 0
GoodesThornscrubTortoise  CTCCATAAAATTGGGTCCCTAATCCATGCACTGTTGGAACTCCTTAACGAAACTTTTCTTAAACATTACATGAATATATT 10167

Majority                  --------------------------------------------------------------------------------

                                   13690     13700     13710     13720     13730     13740     13750     13760

Human                     CTTGCTCAGGGTCTCTATTTTTTTGGTAATATTTTTAAGTGGTGGGTTTTTATTTCTGTTAGACATGGTTTCTGACTTTT 13744
Kakapo                    -------------------------------------------------------------------------------- 0
GoldenEagle               -------------------------------------------------------------------------------- 0
JapaneseQuail             -------------------------------------------------------------------------------- 0
MediumGroundFinch         -------------------------------------------------------------------------------- 0
GoodesThornscrubTortoise  CTCTCATACTATAGAATTAGAATTTATAATCCCTATTCCATGAGGAGATACATTATAGTTCAAAGGTATCTTTATTAAAA 10247
```

```
Majority                   --------------------------------------------------------------------------------

                                    13770     13780     13790     13800     13810     13820     13830     13840

Human                      CGTGTATGTCATGTTTTTCCCAGGAACATTTCTTATACTTTACCTTGTTTGTGCTAGGCATAGTCTGTGCTCAATAATTG 13824
Kakapo                     -------------------------------------------------------------------------------- 0
GoldenEagle                -------------------------------------------------------------------------------- 0
JapaneseQuail              -------------------------------------------------------------------------------- 0
MediumGroundFinch          -------------------------------------------------------------------------------- 0
GoodesThornscrubTortoise   CTGTCTTTAGATAAGATGCTTTTTGAGGGGGAAAAGCATATCAAAATCCAATTTTAAATTTAAAAAAATCCATTTTTTTT 10327

Majority                   --------------------------------------------------------------------------------

                                    13850     13860     13870     13880     13890     13900     13910     13920

Human                      TCTCCTTTTTTTTTCTTTTTTTTTTTTTTTTTTTTCCTGCTTTTTGAGAGACAAGAGTCTTGCCCTGTTGCCCAGGCTGGA 13904
Kakapo                     -------------------------------------------------------------------------------- 0
GoldenEagle                -------------------------------------------------------------------------------- 0
JapaneseQuail              -------------------------------------------------------------------------------- 0
MediumGroundFinch          -------------------------------------------------------------------------------- 0
GoodesThornscrubTortoise   TTAAAATAATTGATTTTTATCCACCCTGGTATTTACGGTTCCCAGAAGCCTGAAAGGCAAAAACTCTGTGGGCTCTCCCC 10407

Majority                   --------------------------------------------------------------------------------

                                    13930     13940     13950     13960     13970     13980     13990     14000

Human                      GTGCAGTGGCCCAATCTCAGCTCAGTGCAACCTCTGCCTCCCGGATTCAAGCGATTCTCCTGCCTCAGCTTCCCAAGTAG 13984
Kakapo                     -------------------------------------------------------------------------------- 0
GoldenEagle                -------------------------------------------------------------------------------- 0
JapaneseQuail              -------------------------------------------------------------------------------- 0
MediumGroundFinch          -------------------------------------------------------------------------------- 0
GoodesThornscrubTortoise   CTAGGTGAACTCCTAGGCAAACTCCTCTGTTTGCTTCTCCTCTGTTCACTGCTCACTAGGTTGGCAGCTGGCTGCCTTTT 10487

Majority                   --------------------------------------------------------------------------------

                                    14010     14020     14030     14040     14050     14060     14070     14080

Human                      CTGGAACTACAGGCGCCTGCCACTACACCTGGCTAATTTTTGTGTTTTTAGTAGGGATGGGGTTTCACCACGTTGGCCAG 14064
Kakapo                     -------------------------------------------------------------------------------- 0
GoldenEagle                -------------------------------------------------------------------------------- 0
JapaneseQuail              -------------------------------------------------------------------------------- 0
MediumGroundFinch          -------------------------------------------------------------------------------- 0
GoodesThornscrubTortoise   ATAAGGCTGCTGGCTCAAAACAAGTTACCCAGGTTTGAAGCCTCACCTGGTTCACACTTCAGTCAACCACTCAGGACTCA 10567
```

```
Majority                   --------------------------------------------------------------------------------
                                    14090     14100     14110     14120     14130     14140     14150     14160
Human                      GCTGACTTGGAACTCCTGACCTCAAGTGATCTGCCCACCTTGGCTTCCCAAAGTGCTGGGATTATAGGCATGAGCCACTG 14144
Kakapo                     -------------------------------------------------------------------------------- 0
GoldenEagle                -------------------------------------------------------------------------------- 0
JapaneseQuail              -------------------------------------------------------------------------------- 0
MediumGroundFinch          -------------------------------------------------------------------------------- 0
GoodesThornscrubTortoise   CCAGGAACAAAGTTCTCCAAGTCACACTTTTCAAACAGGCATGCAGTCAAGCAGTCCCTGCAGTTAGCAGCAGTCAAACA 10647

Majority                   --------------------------------------------------------------------------------
                                    14170     14180     14190     14200     14210     14220     14230     14240
Human                      TGCCTGGCCAATAAGTGTCTCTTAATGAGTATTCTTAAAGGATATCTAAAAAGAAATGTAACTTAAAATTCACATAATAG 14224
Kakapo                     -------------------------------------------------------------------------------- 0
GoldenEagle                -------------------------------------------------------------------------------- 0
JapaneseQuail              -------------------------------------------------------------------------------- 0
MediumGroundFinch          -------------------------------------------------------------------------------- 0
GoodesThornscrubTortoise   TAGCCAAACAAATGGTCACAGCAGACAGACGCTCAAATCTTCACCCCACCAGCTCACAACACAGCCCCTCTAATGCAGCA 10727

Majority                   --------------------------------------------------------------------------------
                                    14250     14260     14270     14280     14290     14300     14310     14320
Human                      TAATACTATACAGTATTAACATTTAATGTAAGTTGGAACTATTGATGGTGGACAAGAATGTTTTCTTGTCAAGAACATAC 14304
Kakapo                     -------------------------------------------------------------------------------- 0
GoldenEagle                -------------------------------------------------------------------------------- 0
JapaneseQuail              -------------------------------------------------------------------------------- 0
MediumGroundFinch          -------------------------------------------------------------------------------- 0
GoodesThornscrubTortoise   TCTACCTCAAAAGCACTGACCATAGTTTCTCTTGGATGGTTCCCAGACAATTTCCCTGTTTGCTGCTCATCCTGAGTCCT 10807

Majority                   --------------------------------------------------------------------------------
                                    14330     14340     14350     14360     14370     14380     14390     14400
Human                      GATTTAGAATGAAAAGATGAGGGTTTGACTTTTATAGCATTGAACAGGTTAACCTCTGAGCTTCAGTTTTATACATTTTT 14384
Kakapo                     -------------------------------------------------------------------------------- 0
GoldenEagle                -------------------------------------------------------------------------------- 0
JapaneseQuail              -------------------------------------------------------------------------------- 0
MediumGroundFinch          -------------------------------------------------------------------------------- 0
GoodesThornscrubTortoise   CTTCTGTCTCCTGCTTCTCTGGCTCAGATGCTCCATGGGTGTGGAGGAGGAGACCCTGAAGGCTGCAGTGGCAGCAGCTG 10887
```

```
Majority              --------------------------------------------------------------------------------
                           14410     14420     14430     14440     14450     14460     14470     14480
Human                 AAATGGTTTCAGTTGTACCTACTTCCAAAATTATTTTGAATGTTAGATGAGATGAGGAAAAGACTTATGCTGAAAGGATG 14464
Kakapo                -------------------------------------------------------------------------------- 0
GoldenEagle           -------------------------------------------------------------------------------- 0
JapaneseQuail         -------------------------------------------------------------------------------- 0
MediumGroundFinch     -------------------------------------------------------------------------------- 0
GoodesThornscrubTortoise CAGATAAAACATATGGATGTACTATTGTCTGTGTAGTCACGATGGAAAGTGACATTTAATATGCAGAACTCACTCTCTTT 10967

Majority              --------------------------------------------------------------------------------
                           14490     14500     14510     14520     14530     14540     14550     14560
Human                 AGAATGGTTGTTGTTGTAGTTCTTTAATCTGTAAATCTACATTTGGAAGGAACACTTTTATGGAAATCAAATACAGTAAA 14544
Kakapo                -------------------------------------------------------------------------------- 0
GoldenEagle           -------------------------------------------------------------------------------- 0
JapaneseQuail         -------------------------------------------------------------------------------- 0
MediumGroundFinch     -------------------------------------------------------------------------------- 0
GoodesThornscrubTortoise CCTCCCCTAAAGTTTTAGGCACTATATTCTCAGTTCTGCTTGGGGTATTTGTGCACCATGCCAGTCACAGGACCAGTCGT 11047

Majority              --------------------------------------------------------------------------------
                           14570     14580     14590     14600     14610     14620     14630     14640
Human                 TTAATTTCTAAGAGGTCCTGGAGTTCAGGTTAAAAATAGCCTCAGACAGCACTAAATATCTTTATTAGTGAACAAAAAAA 14624
Kakapo                -------------------------------------------------------------------------------- 0
GoldenEagle           -------------------------------------------------------------------------------- 0
JapaneseQuail         -------------------------------------------------------------------------------- 0
MediumGroundFinch     -------------------------------------------------------------------------------- 0
GoodesThornscrubTortoise CGTGAGTAGTGCCCCCACCCCACGCTAGAGTTTGGGGAAATGAGGAAAGAATTATTCGGTTGCATGAATCTAAAAGTATA 11127

Majority              --------------------------------------------------------------------------------
                           14650     14660     14670     14680     14690     14700     14710     14720
Human                 TAAAAATAACTTAGAATTTAGCACAGAGAAGCAAAGAGATGAAAATAGGAGAAGCGTTAAAAATGTGTTGGAAAGGAAGA 14704
Kakapo                -------------------------------------------------------------------------------- 0
GoldenEagle           -------------------------------------------------------------------------------- 0
JapaneseQuail         -------------------------------------------------------------------------------- 0
MediumGroundFinch     -------------------------------------------------------------------------------- 0
GoodesThornscrubTortoise CTGTAATGACACTGAATTCTGGTTCTGTTTTCCACAGGCAGGAGTGGCTTTAGATCAGGAGTGAGCAAACATTTTGGCCT 11207
```

```
Majority                --------------------------------------------------------------------------------

                                 14730     14740     14750     14760     14770     14780     14790     14800

Human                   GAAGTTCTAACAAACATCTAATTAGCGTTCCAGAAATTAAAGATTGCAGAAAGGCAAAATTTGAAATGCTAAAGGCTGAT 14784
Kakapo                  -------------------------------------------------------------------------------- 0
GoldenEagle             -------------------------------------------------------------------------------- 0
JapaneseQuail           -------------------------------------------------------------------------------- 0
MediumGroundFinch       -------------------------------------------------------------------------------- 0
GoodesThornscrubTortoiseCAGGGCCACATCTGGGTATGGAAATTGTATGGTGGGCCATGAATGCTCACAAAACGGGGGTGGGGATGAGGACTCCAGCT 11287

Majority                --------------------------------------------------------------------------------

                                 14810     14820     14830     14840     14850     14860     14870     14880

Human                   AATTTTTCAGAATTGGTGAGCTATATGAGTTCTTAGATTTAGGAGGCACAAGAAAACCTGGACACATAACAGTAAAAGAG 14864
Kakapo                  -------------------------------------------------------------------------------- 0
GoldenEagle             -------------------------------------------------------------------------------- 0
JapaneseQuail           -------------------------------------------------------------------------------- 0
MediumGroundFinch       -------------------------------------------------------------------------------- 0
GoodesThornscrubTortoiseCGGGATGCAGACTCTGGGGATGAGGGGGTTGGGGTGCAGGAGGGGGATCAGGGGTGCAGGCTCCGGGCGGCACTTACCTC 11367

Majority                --------------------------------------------------------------------------------

                                 14890     14900     14910     14920     14930     14940     14950     14960

Human                   CAGAGCACCAAAGACATAGAGATCTTTAAAGCAGCCAGAGAAAATCAGATTCCCTAAAGAATAAGAACTAGAAAGT---C 14941
Kakapo                  -------------------------------------------------------------------------------- 0
GoldenEagle             -------------------------------------------------------------------------------- 0
JapaneseQuail           -------------------------------------------------------------------------------- 0
MediumGroundFinch       -------------------------------------------------------------------------------- 0
GoodesThornscrubTortoiseAAGCTGCTCCCGTAAGTAGCGACATGTCCTCCTTCCGGCTCCTATGTGAAGGCATAGCCAGGCAGCTCTGCACACTGCCC 11447

Majority                --------------------------------------------------------------------------------

                                 14970     14980     14990     15000     15010     15020     15030     15040

Human                   CTAATATGCTTCATCAGCATGGGCATCCAGAAGATAGTGGAATAATATTTTCAATGTGTTGAGTGAAAATAGCTGCCCAC 15021
Kakapo                  -------------------------------------------------------------------------------- 0
GoldenEagle             -------------------------------------------------------------------------------- 0
JapaneseQuail           -------------------------------------------------------------------------------- 0
MediumGroundFinch       -------------------------------------------------------------------------------- 0
GoodesThornscrubTortoiseCATCCACAGGTGCCCTCCCTGCAGTTCCCACTGGCCGTGGTTCCAGCCAATGGGAGCTGTGGGGGCGGCATTTGGGGCGG 11527
```

```
Majority                  --------------------------------------------------------------------------------

                                   15050     15060     15070     15080     15090     15100     15110     15120

Human                     CTGTTATTTTATAGCCAGCCAAATTATCATATAAGGAGAAGAACAAAATAAAGACACTTTTTGAAACACGTCATTCTGCC 15101
Kakapo                    -------------------------------------------------------------------------------- 0
GoldenEagle               -------------------------------------------------------------------------------- 0
JapaneseQuail             -------------------------------------------------------------------------------- 0
MediumGroundFinch         -------------------------------------------------------------------------------- 0
GoodesThornscrubTortoise  CGGCAGTGCAGAGCCCCCTGGCTGCCCGTACATGTAGGAGCTGGAGGGAGGACATGCTGTTGCTTCCAGGAACTGCGCAG 11607

Majority                  --------------------------------------------------------------------------------

                                   15130     15140     15150     15160     15170     15180     15190     15200

Human                     TCCAAAAAAGCAATTTAGGAAATGTATCAACAGACCCACTGAAGGAATTTCTGGAGTTTGTACTTCAGGAAGAAAGCCAA 15181
Kakapo                    -------------------------------------------------------------------------------- 0
GoldenEagle               -------------------------------------------------------------------------------- 0
JapaneseQuail             -------------------------------------------------------------------------------- 0
MediumGroundFinch         -------------------------------------------------------------------------------- 0
GoodesThornscrubTortoise  AGCCACAACATGAGCGGAGCAGGGCAAGCCCCAGACCCCGCTTGCCGATGGGAGCTTGAGGGCCAGATTAAAATAGTGGA 11687

Majority                  --------------------------------------------------------------------------------

                                   15210     15220     15230     15240     15250     15260     15270     15280

Human                     TGACACCAGAAAGGAAGTCTGATATAGAAGTGAGGCTAATCAAGAAATTGGTAGGTCTGTGAGTAAATCTAATTACTGAC 15261
Kakapo                    -------------------------------------------------------------------------------- 0
GoldenEagle               -------------------------------------------------------------------------------- 0
JapaneseQuail             -------------------------------------------------------------------------------- 0
MediumGroundFinch         -------------------------------------------------------------------------------- 0
GoodesThornscrubTortoise  CAGGCTGTAGTTTGCACACCCCTGTTTTAGCTGATATCTTACTCCTGCAGGTAGATCAATCAAATATTTTAATTTTAGCT 11767

Majority                  --------------------------------------------------------------------------------

                                   15290     15300     15310     15320     15330     15340     15350     15360

Human                     CATATAAGAATGTAATAGCAGTGATTGATAAGGGGTTTAAAAATAGAATTAAAAATATTAAATCATAACAATATGTGAGA 15341
Kakapo                    -------------------------------------------------------------------------------- 0
GoldenEagle               -------------------------------------------------------------------------------- 0
JapaneseQuail             -------------------------------------------------------------------------------- 0
MediumGroundFinch         -------------------------------------------------------------------------------- 0
GoodesThornscrubTortoise  CCTTGAAGAAATTCTTAAGTCTATATAAATTGTATATTTATGTATAACCTATGCATCTAATTCCTCAAGTATAAGTATTT 11847
```

```
Majority                 --------------------------------------------------------------------------------
                                15370     15380     15390     15400     15410     15420     15430     15440
Human                    GAGTGGTAGAGACACTGGAGTTTGAGTTCTGATGATCTAGTATTGATTGGAGCTGTGTTGTCAGTTACAGTAGCTACTAG 15421
Kakapo                   -------------------------------------------------------------------------------- 0
GoldenEagle              -------------------------------------------------------------------------------- 0
JapaneseQuail            -------------------------------------------------------------------------------- 0
MediumGroundFinch        -------------------------------------------------------------------------------- 0
GoodesThornscrubTortoise TAAAAATGGATAAATCAGAAATTTGTATTTCACCTGGCTTCTTTATAACCAAAATTCAGATGGCTTAACTACTTTATATT 11927

Majority                 --------------------------------------------------------------------------------
                                15450     15460     15470     15480     15490     15500     15510     15520
Human                    TCATTTGTTACTGAGCACTTGACATGTATGTAGTTTAAGTTGATCTTCTACTTAATTGAAAAATACACATTTCTGAGCCT 15501
Kakapo                   -------------------------------------------------------------------------------- 0
GoldenEagle              -------------------------------------------------------------------------------- 0
JapaneseQuail            -------------------------------------------------------------------------------- 0
MediumGroundFinch        -------------------------------------------------------------------------------- 0
GoodesThornscrubTortoise TCATACCTGAGAAAATACTGCAATCTTCTCATTACAAACTTTCCATTTCTCTCAACTGTAAAATCTTGGCTAATCAAATA 12007

Majority                 --------------------------------------------------------------------------------
                                15530     15540     15550     15560     15570     15580     15590     15600
Human                    TAGTCTGAAAGAAGAATGTAAAATATCCCAAGTTTTCATATTGGTTACATGTACAGCGGATAATATTTTGGTATTTTGGG 15581
Kakapo                   -------------------------------------------------------------------------------- 0
GoldenEagle              -------------------------------------------------------------------------------- 0
JapaneseQuail            -------------------------------------------------------------------------------- 0
MediumGroundFinch        -------------------------------------------------------------------------------- 0
GoodesThornscrubTortoise TA-TAAAAATTCAGCCTGCCCCAAACCATGGATTTAAGGTATTGTCTTCTGTACACAAATGATGATTTTTACCTACCTGA 12086

Majority                 --------------------------------------------------------------------------------
                                15610     15620     15630     15640     15650     15660     15670     15680
Human                    TTAAATGAAATACATTATGTGTGTTGCCTTTTCCTCTTTTTTCCTTTCCTTTTTTTAAGATAGGGTCTTGCTCTGTCCAC 15661
Kakapo                   -------------------------------------------------------------------------------- 0
GoldenEagle              -------------------------------------------------------------------------------- 0
JapaneseQuail            -------------------------------------------------------------------------------- 0
MediumGroundFinch        -------------------------------------------------------------------------------- 0
GoodesThornscrubTortoise CCAGCAGTGAGTGCT-----GGGTGAGAGAGTTAGGGCCCTAACTGAACTAATTAAAGGATAGTCTGACAACCTCTGTGC 12161
```

```
Majority                  --------------------------------------------------------------------------------

                                15690     15700     15710     15720     15730     15740     15750     15760

Human                     CAGGCTGGAGTGCCGTGGCATGATCATGGCAGCCTCAACCTCCTGGGCTCCAGTGACCCTCCCACCTCAGCCTCCCAAGT 15741
Kakapo                    -------------------------------------------------------------------------------- 0
GoldenEagle               -------------------------------------------------------------------------------- 0
JapaneseQuail             -------------------------------------------------------------------------------- 0
MediumGroundFinch         -------------------------------------------------------------------------------- 0
GoodesThornscrubTortoise  TGTTAAGGACAATGGAAGTCTTGGGGAAAACATCAAGCTGGAGCGGAGGGAAATGAGCCCATCATTGGGACTCTACTTGT 12241

Majority                  --------------------------------------------------------------------------------

                                15770     15780     15790     15800     15810     15820     15830     15840

Human                     AGCTGGGACTACAGTTGTGTGCCACCACACCTGGCTAATTTTTGAGTTTTTTTTTGTAGAGACAGCGTCTCGCTGTATCG 15821
Kakapo                    -------------------------------------------------------------------------------- 0
GoldenEagle               -------------------------------------------------------------------------------- 0
JapaneseQuail             -------------------------------------------------------------------------------- 0
MediumGroundFinch         -------------------------------------------------------------------------------- 0
GoodesThornscrubTortoise  AGATGATGATGATGTATTCGCTCACACTGAGGATTCCATTCTGGGGGAGGGAACCCCAGTTATTAGGAAGAGACAGGTAA 12321

Majority                  --------------------------------------------------------------------------------

                                15850     15860     15870     15880     15890     15900     15910     15920

Human                     CTTAGGCTGGTCTCAAACTCCTGGGCTCAAGTGATCCTCCTGCTTTAGCCTTGCAAAGTGCTGGGATTACAGGACTGAGT 15901
Kakapo                    -------------------------------------------------------------------------------- 0
GoldenEagle               -------------------------------------------------------------------------------- 0
JapaneseQuail             -------------------------------------------------------------------------------- 0
MediumGroundFinch         -------------------------------------------------------------------------------- 0
GoodesThornscrubTortoise  TAGTAGTAGGAGATCTGATTATTAGAAATATAGATAGTTGGGTTTGTGATGATTGGGAATACAAATGGTGGATTGTCTGC 12401

Majority                  --------------------------------------------------------------------------------

                                15930     15940     15950     15960     15970     15980     15990     16000

Human                     CTGGCCACCTTTTTCTTTCTTTCTTTTTTTTTTAGACGGAGTCTCCCTTTGTTGCCCAGGCTGGAGTACAGTGGCTCTAT 15981
Kakapo                    -------------------------------------------------------------------------------- 0
GoldenEagle               -------------------------------------------------------------------------------- 0
JapaneseQuail             -------------------------------------------------------------------------------- 0
MediumGroundFinch         -------------------------------------------------------------------------------- 0
GoodesThornscrubTortoise  AGAGAGAAGGTTGTGGACGTCATGAGTCAATTTAGGTGAATTTTTGTGATGTGCTGGGGAAGAACCAGTGATCATGGTAC 12481
```

```
Majority                   --------------------------------------------------------------------------------

                                     16010     16020     16030     16040     16050     16060     16070     16080

Human                      CTCTGCTTCCTGCAAGCTCTGCCTCCTGGGTTCACGCCATTCTCCTGCCTCAGCCTCCCGAGTAGCTGGGACTACAGGCG 16061
Kakapo                     -------------------------------------------------------------------------------- 0
GoldenEagle                -------------------------------------------------------------------------------- 0
JapaneseQuail              -------------------------------------------------------------------------------- 0
MediumGroundFinch          -------------------------------------------------------------------------------- 0
GoodesThornscrubTortoise   ATGCAAGTACCAGTGACATAGGAAAAAGTAGGAAAGAGGTCCTGGAAGCCAAATTTGGGCTACTTGGTAAGAGATTAAAG 12561

Majority                   --------------------------------------------------------------------------------

                                     16090     16100     16110     16120     16130     16140     16150     16160

Human                      CCTGCCACCACACCCGGCTAATTTTTTGTATTTTTAGTAGAGATGGGTTTTACCATGGTCTCGATCTCCTGACCTCCTGA 16141
Kakapo                     -------------------------------------------------------------------------------- 0
GoldenEagle                -------------------------------------------------------------------------------- 0
JapaneseQuail              -------------------------------------------------------------------------------- 0
MediumGroundFinch          -------------------------------------------------------------------------------- 0
GoodesThornscrubTortoise   TACAAGACCTCCTTGGTGGCATTTTCTGATATGCTTCCAGCTCCATGTGCAGGACTAGTTAGACAGGCAGAACTGCAGGA 12641

Majority                   --------------------------------------------------------------------------------

                                     16170     16180     16190     16200     16210     16220     16230     16240

Human                      TCTGCCCACCCTGGCCTCCCAAAGTGCTGGGATTACAGGCGTGAGCCACTGCGACCGGCCCACTTTTTCTTTTTACTTTT 16221
Kakapo                     -------------------------------------------------------------------------------- 0
GoldenEagle                -------------------------------------------------------------------------------- 0
JapaneseQuail              -------------------------------------------------------------------------------- 0
MediumGroundFinch          -------------------------------------------------------------------------------- 0
GoodesThornscrubTortoise   TCTCAGTGTGTGGATGAAATGTTGGTGTTTGAAGGAGGGATTTAGGTTTATTAGGAACTGGGGAACTTTTTGCGAGAGGA 12721

Majority                   --------------------------------------------------------------------------------

                                     16250     16260     16270     16280     16290     16300     16310     16320

Human                      AAAAATGTGGCTAATAGAAATTTATGAGATTATATTTATGGTTCATACTACGTTTCTTTTGGACAGTGCCAGAGTGAATC 16301
Kakapo                     -------------------------------------------------------------------------------- 0
GoldenEagle                -------------------------------------------------------------------------------- 0
JapaneseQuail              -------------------------------------------------------------------------------- 0
MediumGroundFinch          -------------------------------------------------------------------------------- 0
GoodesThornscrubTortoise   CGAGCCTATCCTGGCAGAATGGGCTTCTCATAACCCAAAAGGGAACCAGATTGCTGGCATGTAAAATTAAAAAGGTCGTA 12801
```

```
Majority                --------------------------------------------------------------------------------

                                16330     16340     16350     16360     16370     16380     16390     16400

Human                   AGATAAGCTTGCATTTTAAAATCCTAAGGGTAAATGCAATAGAGATAGAACGCAAATAATTGGGGAGGGGGGTTGACTGA 16381
Kakapo                  -------------------------------------------------------------------------------- 0
GoldenEagle             -------------------------------------------------------------------------------- 0
JapaneseQuail           -------------------------------------------------------------------------------- 0
MediumGroundFinch       -------------------------------------------------------------------------------- 0
GoodesThornscrubTortoiseGAGGAGGTTAGAAACTAAGAG--CTGAGGGAAAGCTGACGGGTGTATAGAAGCACACAGTTTAGAAAGAGACATTCCATA 12879

Majority                --------------------------------------------------------------------------------

                                16410     16420     16430     16440     16450     16460     16470     16480

Human                   AATTAAAGATGTATAATCCAAAAGAAGGCAAAAAAAAAGGAAAGACACAAAGTGAGCTTATATGTTAATAGCGTGGAAAG 16461
Kakapo                  -------------------------------------------------------------------------------- 0
GoldenEagle             -------------------------------------------------------------------------------- 0
JapaneseQuail           -------------------------------------------------------------------------------- 0
MediumGroundFinch       -------------------------------------------------------------------------------- 0
GoodesThornscrubTortoiseGAGGGAGTATTTATTAAATGGGGTATTCTATATCCTAGTAAAGAGTAGGGGGTAGCAGCTGATGATAAAGTACAGGTAGG 12959

Majority                --------------------------------------------------------------------------------

                                16490     16500     16510     16520     16530     16540     16550     16560

Human                   GTTCTATGAAGGGTTGTTTAGAGCCTTTAAGGGCAAAATTTTCTTGTCTGGTTGCAGGAAAGAGTGGCATTGTTTTTGTT 16541
Kakapo                  -------------------------------------------------------------------------------- 0
GoldenEagle             -------------------------------------------------------------------------------- 0
JapaneseQuail           -------------------------------------------------------------------------------- 0
MediumGroundFinch       -------------------------------------------------------------------------------- 0
GoodesThornscrubTortoiseAACTGAAGAGAAACAGTCAAATGAAAAAGAGTTCCCTTTAGTTACATCACATGAAAGCAGACATCTAAATATTAGCAAAT 13039

Majority                --------------------------------------------------------------------------------

                                16570     16580     16590     16600     16610     16620     16630     16640

Human                   CACTTTTAAAGCTACAAAAACAAGTAATGATGAGGAAAATTGGGACATGTTGAGGAAGTAAGTAATTTTTTGGAATGTAG 16621
Kakapo                  -------------------------------------------------------------------------------- 0
GoldenEagle             -------------------------------------------------------------------------------- 0
JapaneseQuail           -------------------------------------------------------------------------------- 0
MediumGroundFinch       -------------------------------------------------------------------------------- 0
GoodesThornscrubTortoiseTTTATAAGTGCTTGTTTACAAATGCTAGAAATCTAAATACTTAAATGCGTGAACTTGAGTGCCTGGTGTTAAATGAAGAT 13119
```

```
Majority                --------------------------------------------------------------------------------

                                16650     16660     16670     16680     16690     16700     16710     16720

Human                   GTTGAGTTAGAGTAGTAGAAGACAAAATTTAAAAAGTAGTTTTTGACAGTAATATGGAGAGCTTTGAGTGCAAAGGATTT 16701
Kakapo                  -------------------------------------------------------------------------------- 0
GoldenEagle             -------------------------------------------------------------------------------- 0
JapaneseQuail           -------------------------------------------------------------------------------- 0
MediumGroundFinch       -------------------------------------------------------------------------------- 0
GoodesThornscrubTortoise ACTGGTATAACA-AGAATCACAGAAATTTCGTGAGATGATGATAAACAATGGCACAGAGTAATACCAGGGTACAAAATAT 13198

Majority                --------------------------------------------------------------------------------

                                16730     16740     16750     16760     16770     16780     16790     16800

Human                   GCAGTGAGGACCTTTGAAAATTGAACCTGCTTTCTCCTATCAGTATTTATGTTTAAATAAACTTGATTCTTTGTGAGTAT 16781
Kakapo                  -------------------------------------------------------------------------------- 0
GoldenEagle             -------------------------------------------------------------------------------- 0
JapaneseQuail           -------------------------------------------------------------------------------- 0
MediumGroundFinch       -------------------------------------------------------------------------------- 0
GoodesThornscrubTortoise ATAGGAATAACAGAGTAGGTTGCACTGGGTGGGGGGGGGGGGAGAAGAGGAGGAGAGAAGTGGTACTGTATGTGAAAGA 13278

Majority                --------------------------------------------------------------------------------

                                16810     16820     16830     16840     16850     16860     16870     16880

Human                   TGTTATATATGTTTTCATAACTACTGTTGTAGTAACGTTTCTTTTTTTTTACTAGCTTGCTTTCATGCCACGCTGTTGAT 16861
Kakapo                  -------------------------------------------------------------------------------- 0
GoldenEagle             -------------------------------------------------------------------------------- 0
JapaneseQuail           -------------------------------------------------------------------------------- 0
MediumGroundFinch       -------------------------------------------------------------------------------- 0
GoodesThornscrubTortoise CAGCATAGAATCGAATAAAGTAAAAGTTTCATTCATTCAAGACAGTACCATAGAATATGTATGAGTAGAATTTCCATTCT 13358

Majority                --------------------------------------------------------------------------------

                                16890     16900     16910     16920     16930     16940     16950     16960

Human                   TAAATATTTATGGGCCATTTTAAGGCTTCTAATCTTCATTTGTGATGAACTTTTGAGGAAGAGTTAGGTTTTGTAAGTGT 16941
Kakapo                  -------------------------------------------------------------------------------- 0
GoldenEagle             -------------------------------------------------------------------------------- 0
JapaneseQuail           -------------------------------------------------------------------------------- 0
MediumGroundFinch       -------------------------------------------------------------------------------- 0
GoodesThornscrubTortoise TAAATAACAGTATAGCAATAGTAATTGCTTGATCAGGATGGTGATGGTGATTGTGAAATGCTCAGGGAGATTAGCAGTTA 13438
```

```
Majority                   --------------------------------------------------------------------------------
                                    16970     16980     16990     17000     17010     17020     17030     17040
Human                      TGAACTCCAATAAAC-----ACAGTTCCACTTTCCTTAACTTCTCAGAAAGATACTTTGCTATTTGATACAGATAGTTGT 17016
Kakapo                     -------------------------------------------------------------------------------- 0
GoldenEagle                -------------------------------------------------------------------------------- 0
JapaneseQuail              -------------------------------------------------------------------------------- 0
MediumGroundFinch          -------------------------------------------------------------------------------- 0
GoodesThornscrubTortoise   CAAAAACAGAAAACCCAGTAATAATGGAGGATTTCAGCTATGCTCATACTGACTGGGTAAATGTCACCTCAGGATGGAAT 13518

Majority                   --------------------------------------------------------------------------------
                                    17050     17060     17070     17080     17090     17100     17110     17120
Human                      CTAGGCAAGCTATTAGCAGGGTTTCAGGTAAGACATATGTGTTTGCTATTTGTTTTAAGCACTCTTCCCTCATTTATTCA 17096
Kakapo                     -------------------------------------------------------------------------------- 0
GoldenEagle                -------------------------------------------------------------------------------- 0
JapaneseQuail              -------------------------------------------------------------------------------- 0
MediumGroundFinch          -------------------------------------------------------------------------------- 0
GoodesThornscrubTortoise   CCAGAGAAATTTTTGGTCCCCATTCATGACTGTTTCTTGAGGCAACTACTTCTGGGAACCCACAGGGGGAGAGGCAGTTT 13598

Majority                   --------------------------------------------------------------------------------
                                    17130     17140     17150     17160     17170     17180     17190     17200
Human                      AATTGTAGCAAACATCTACTACACCCATTTCCAGTTCTTTGTCTTAATAGTTCAACTATTATAAAAAGGAGTGGTATATA 17176
Kakapo                     -------------------------------------------------------------------------------- 0
GoldenEagle                -------------------------------------------------------------------------------- 0
JapaneseQuail              -------------------------------------------------------------------------------- 0
MediumGroundFinch          -------------------------------------------------------------------------------- 0
GoodesThornscrubTortoise   CTGATTTAGTCCTAAATGGAACACAGGATCTGGTCCAACGAGTCAATATAGCCAAACTGCTCAGTAATAGCTACCATAAT 13678

Majority                   --------------------------------------------------------------------------------
                                    17210     17220     17230     17240     17250     17260     17270     17280
Human                      TATATATAAACTCTTGCTTGGCTTTGGTTTGATTGAATTAATAAGGTGAGTTTTTTTTGTTTGTTTGTTTTGAGATGGAG 17256
Kakapo                     -------------------------------------------------------------------------------- 0
GoldenEagle                -------------------------------------------------------------------------------- 0
JapaneseQuail              -------------------------------------------------------------------------------- 0
MediumGroundFinch          -------------------------------------------------------------------------------- 0
GoodesThornscrubTortoise   ATAATTAAATTTCACATCCTTGTAGGGGGGAAAATACCAAAGAAACCCACCACAGTAGCAGTTTAAGTTAAAAAAGGGGA 13758
```

```
Majority              --------------------------------------------------------------------------------
                                17290     17300     17310     17320     17330     17340     17350     17360
Human                 TCTCGCTCTGTTGCCCAGGCTGGAGTGCAGTGGTGGGATCTCGGCTCACTGCAGGCCATTCTCCTGCCTC-AGCCTCCCG 17335
Kakapo                -------------------------------------------------------------------------------- 0
GoldenEagle           -------------------------------------------------------------------------------- 0
JapaneseQuail         -------------------------------------------------------------------------------- 0
MediumGroundFinch     -------------------------------------------------------------------------------- 0
GoodesThornscrubTortoiseACTACACAAAAATGTGAGAGCTAGTTAAATTGAAATTAAAAGGAACAGTTATGAATGAAATGCTTGCATGGAAACTATTT 13838

Majority              --------------------------------------------------------------------------------
                                17370     17380     17390     17400     17410     17420     17430     17440
Human                 AGTAGCTGGGACTGCAGGTGCCTGCCACCACACCTGGCTAATTTTTTGTATTTTTAGTAGAGATGGGGTTTCACCGTGTT 17415
Kakapo                -------------------------------------------------------------------------------- 0
GoldenEagle           -------------------------------------------------------------------------------- 0
JapaneseQuail         -------------------------------------------------------------------------------- 0
MediumGroundFinch     -------------------------------------------------------------------------------- 0
GoodesThornscrubTortoiseAAAACACCATAGCAGAAGCCCAGACTAAATGTATATCCCAAATTTAAAAAAAATAAAAAAAAAATACAGTAAGAGGACCA 13918

Majority              --------------------------------------------------------------------------------
                                17450     17460     17470     17480     17490     17500     17510     17520
Human                 AGCCAGGATGGTCTAGATCTCCTGACCTTGTGATCCGCCCGCCTCGGAAATACTTTGCATTGTTGGATTGATTGGGTTTG 17495
Kakapo                -------------------------------------------------------------------------------- 0
GoldenEagle           -------------------------------------------------------------------------------- 0
JapaneseQuail         -------------------------------------------------------------------------------- 0
MediumGroundFinch     -------------------------------------------------------------------------------- 0
GoodesThornscrubTortoiseAGGCTAAACAATGAAGAGCCGGTTAGATGCAAAAAGACACACTTAAAAAAATTCATAATAAAATCCTACTGAGGATAACA 13998

Majority              --------------------------------------------------------------------------------
                                17530     17540     17550     17560     17570     17580     17590     17600
Human                 AGCCCCTGTTGTACTTAAGTGATTATGAACAAGTCTTTTATAATCTGAATTTCCCTTTCCTAGTTTGTAATATTGAGGAT 17575
Kakapo                -------------------------------------------------------------------------------- 0
GoldenEagle           -------------------------------------------------------------------------------- 0
JapaneseQuail         -------------------------------------------------------------------------------- 0
MediumGroundFinch     -------------------------------------------------------------------------------- 0
GoodesThornscrubTortoiseTAAACTAGCAAGTCAAGTGTAAATGTAGAATGAGGCAGGACAAAAAAGAATTTAAAGAGCAATTAGCAGAAAACACATCA 14078
```

```
Majority                   --------------------------------------------------------------------------------

                                   17610     17620     17630     17640     17650     17660     17670     17680

Human                      AATAATTTCTAACTTGCATGATTGTTGTGAAGATTAAATAAGACAATGATATTGGTAGGAGCTCTTTATAATCTTTCTCT 17655
Kakapo                     -------------------------------------------------------------------------------- 0
GoldenEagle                -------------------------------------------------------------------------------- 0
JapaneseQuail              -------------------------------------------------------------------------------- 0
MediumGroundFinch          -------------------------------------------------------------------------------- 0
GoodesThornscrubTortoise   CCTTCGTATCAGTGCACATAACAAAATTCATGTGGGGTGGAGCCAAGGGGTTCGGAGTGTGGGAAGGGGCTCAGGCTGGG 14158

Majority                   --------------------------------------------------------------------------------

                                   17690     17700     17710     17720     17730     17740     17750     17760

Human                      CTTATGTACAATTCAAATATATGTCGGTTTAAATACCAAAATACCACTTTAAAAAATCTGGGCTGAAATGGACACTTCTA 17735
Kakapo                     -------------------------------------------------------------------------------- 0
GoldenEagle                -------------------------------------------------------------------------------- 0
JapaneseQuail              -------------------------------------------------------------------------------- 0
MediumGroundFinch          -------------------------------------------------------------------------------- 0
GoodesThornscrubTortoise   ACAGGGTGCAGGGCTTGGGTGCAGGGCTCTGAGGGCTCCAGCTGTGGGTTCAGGCTCTGGCGTGGGTCTGGAGATGGGCT 14238

Majority                   --------------------------------------------------------------------------------

                                   17770     17780     17790     17800     17810     17820     17830     17840

Human                      ATGTAGGAGTAGTGCTTTAGAAAATAATGCTTTGTTTCGTATTCTTTTCCTAGTATTTTATGTCTAGTAACTCATAAGGA 17815
Kakapo                     -------------------------------------------------------------------------------- 0
GoldenEagle                -------------------------------------------------------------------------------- 0
JapaneseQuail              -------------------------------------------------------------------------------- 0
MediumGroundFinch          -------------------------------------------------------------------------------- 0
GoodesThornscrubTortoise   CAGGGCTGGGGCAGAGGGTTGTGGTGCAGGGGTGTGAGGGCTCCGGCTGGAGGATGTGCTCTGGGATGAGGTCAGGGATG 14318

Majority                   --------------------------------------------------------------------------------

                                   17850     17860     17870     17880     17890     17900     17910     17920

Human                      AAAAATGATCAGATAAGTATATTTTTTAGATGCTGATTACAGCATTATTTAAATAGTAGAAATAACAACTGTTCAGTGAT 17895
Kakapo                     -------------------------------------------------------------------------------- 0
GoldenEagle                -------------------------------------------------------------------------------- 0
JapaneseQuail              -------------------------------------------------------------------------------- 0
MediumGroundFinch          -------------------------------------------------------------------------------- 0
GoodesThornscrubTortoise   AGGGGCTCAGGGCTAGAGAAAGGATTGGGGTACAGGAGTTGAGGGCTCAGAGGTGGGACTGGAGATGAGGGGTTTGAGGT 14398
```

```
Majority                 --------------------------------------------------------------------------------

                                  17930     17940     17950     17960     17970     17980     17990     18000

Human                    AAGACAATGAATAAATTCTGTTCATAAACATTACAAATGTTTTTAAATATTTTTCATATGTGTGAAAAGTAGGATTAAA 17975
Kakapo                   -------------------------------------------------------------------------------- 0
GoldenEagle              -------------------------------------------------------------------------------- 0
JapaneseQuail            -------------------------------------------------------------------------------- 0
MediumGroundFinch        -------------------------------------------------------------------------------- 0
GoodesThornscrubTortoise CCAGGAGCGTGCTTAGGGGCTACAGCTGGGAGAGAGGACCCCACCCAACTCTCTCTCCCCGCAGCAGCAGCAGCACCAGC 14478

Majority                 --------------------------------------------------------------------------------

                                  18010     18020     18030     18040     18050     18060     18070     18080

Human                    AATAACTTTGACAAATGCATACAATTTATATGTGTCCCCAAAAGCTGGAGGGCAGTGTACCAAAATAGAGTAGTTTTCTC 18055
Kakapo                   -------------------------------------------------------------------------------- 0
GoldenEagle              -------------------------------------------------------------------------------- 0
JapaneseQuail            -------------------------------------------------------------------------------- 0
MediumGroundFinch        -------------------------------------------------------------------------------- 0
GoodesThornscrubTortoise AGCACTGGGTTGGCGGGGGAGATGCCTCTCCCTGCTACAGCAGCTCTGGGGTTGGGGCTGCAGGATAGGCTCCCCTTCCC 14558

Majority                 --------------------------------------------------------------------------------

                                  18090     18100     18110     18120     18130     18140     18150     18160

Human                    CAGGTTGA--TGGGATTAGTTTTTTGTAATTCTTTATATATTTTCTCTATATTCCACATTTTAAGTATTTTATGATAAAG 18133
Kakapo                   -------------------------------------------------------------------------------- 0
GoldenEagle              -------------------------------------------------------------------------------- 0
JapaneseQuail            -------------------------------------------------------------------------------- 0
MediumGroundFinch        -------------------------------------------------------------------------------- 0
GoodesThornscrubTortoise TATTTGGGCTTCCCAGCCATGGCTGGGTCTGGGCCACTCTGGTCATGCTTGAGGCTAGCCCGGGCTGTGCCACAGCAAGA 14638

Majority                 --------------------------------------------------------------------------------

                                  18170     18180     18190     18200     18210     18220     18230     18240

Human                    TTATTAATATTGATAAAGTTATTAATGTTGCTTAATTGATTAAACCCTGATATTTTCTTGTTTCTTTAAAAAACGTTGCA 18213
Kakapo                   -------------------------------------------------------------------------------- 0
GoldenEagle              -------------------------------------------------------------------------------- 0
JapaneseQuail            -------------------------------------------------------------------------------- 0
MediumGroundFinch        -------------------------------------------------------------------------------- 0
GoodesThornscrubTortoise CTTGGGGCCAGAGGAGGGGCGCCCTGCACCCTTGCTGCACCAGATTGTGGGCCAGGCTAGGTGGGGGCCGGGGAAGGGAT 14718
```

```
Majority                 --------------------------------------------------------------------------------

                                 18250     18260     18270     18280     18290     18300     18310     18320

Human                    TAACTTGATATAAATCTTTTTAATTGTACAAAAAAACACATAAAATTTACTATCTTAACCATTTTTAAGTGTACACTTAA 18293
Kakapo                   -------------------------------------------------------------------------------- 0
GoldenEagle              -------------------------------------------------------------------------------- 0
JapaneseQuail            -------------------------------------------------------------------------------- 0
MediumGroundFinch        -------------------------------------------------------------------------------- 0
GoodesThornscrubTortoise CCCCCTCCACCGGCCCCGGCCGGTCCCCAAACAGGTTCCTTAAGCACCTGCGCAGTACTAAACAGGCTGCTGCGTGGCCA 14798

Majority                 --------------------------------------------------------------------------------

                                 18330     18340     18350     18360     18370     18380     18390     18400

Human                    ATTGTTTAATTATTCTTATACGTACTTAATAATATTACATTGCAAAACCCATCTCCAGAACTTTTTGGAGAACTGAAACT 18373
Kakapo                   -------------------------------------------------------------------------------- 0
GoldenEagle              -------------------------------------------------------------------------------- 0
JapaneseQuail            -------------------------------------------------------------------------------- 0
MediumGroundFinch        -------------------------------------------------------------------------------- 0
GoodesThornscrubTortoise TGCAGCTTCCAGGAAACTTAGATGAGAAGTGAGGAAGAGGGAGAGGACTTATAGGAGAAATGAGGACACGTATGCATGTA 14878

Majority                 --------------------------------------------------------------------------------

                                 18410     18420     18430     18440     18450     18460     18470     18480

Human                    CTATACTGATTTTAAAACTCCCTCTTTTGTCCTTCTGCTGGTAACCAGCATTCTATTTTCTGTGTCTATGAATTTGACTA 18453
Kakapo                   -------------------------------------------------------------------------------- 0
GoldenEagle              -------------------------------------------------------------------------------- 0
JapaneseQuail            -------------------------------------------------------------------------------- 0
MediumGroundFinch        -------------------------------------------------------------------------------- 0
GoodesThornscrubTortoise ATGTCTAATTAGTTAGAGAAAGACAAGTGAACTTAAAACAAATTACAGCAATGAGTTACCACTTGTTGGAATACAGAGTT 14958

Majority                 --------------------------------------------------------------------------------

                                 18490     18500     18510     18520     18530     18540     18550     18560

Human                    CTTTAAATACCTCACATAAGTGGAATTATACAGTATAGGTTAAGCATCCCTAATCTGAAAATCCAAAGTCTGAATGCTAC 18533
Kakapo                   -------------------------------------------------------------------------------- 0
GoldenEagle              -------------------------------------------------------------------------------- 0
JapaneseQuail            -------------------------------------------------------------------------------- 0
MediumGroundFinch        -------------------------------------------------------------------------------- 0
GoodesThornscrubTortoise CTTGGGCTGGTCTACGCACAGTTCTTGTCCTGATTTAACTAAATTGTATTTAAATCAGTAAAAAACTGTTTAGACAAGTC 15038
```

```
Majority                   --------------------------------------------------------------------------------

                                    18570     18580     18590     18600     18610     18620     18630     18640

Human                      AAAATCTGAAACTGGTGTGTGTTTTTTTTTTTTTTTTTTTTTTTTTTGAGACGGAGTCTTACTCTGTCGCCCAGGCTGGA 18613
Kakapo                     -------------------------------------------------------------------------------- 0
GoldenEagle                -------------------------------------------------------------------------------- 0
JapaneseQuail              -------------------------------------------------------------------------------- 0
MediumGroundFinch          -------------------------------------------------------------------------------- 0
GoodesThornscrubTortoise   TTTTGTCTGTATGGTGATGTGGCAGGGATGAAACTGGGGAGTTTTGTAGCACGCCAGTGAACCTGCATGGAATTTCTTTT 15118

Majority                   --------------------------------------------------------------------------------

                                    18650     18660     18670     18680     18690     18700     18710     18720

Human                      GTGCAGTGGCGCGATCTCTGCTCACTGCAAGCTCCACCACCCGGGTTCACGCCATCTTCCTGAGTAGCTGGGACTACAGG 18693
Kakapo                     -------------------------------------------------------------------------------- 0
GoldenEagle                -------------------------------------------------------------------------------- 0
JapaneseQuail              -------------------------------------------------------------------------------- 0
MediumGroundFinch          -------------------------------------------------------------------------------- 0
GoodesThornscrubTortoise   CGACTATAGGTAGGATGAGAATGTGTATGGATGTAACTCATATTCTTGGGGCCCTGGCCTTGGGGAGTCTAGTGATACTA 15198

Majority                   --------------------------------------------------------------------------------

                                    18730     18740     18750     18760     18770     18780     18790     18800

Human                      CGCCCGCCACCACGCCCGGCTAATTTTTTGTATATATATATATATTTTTTAGTAGAGACGGGGTTTCACTGTAGCCAGGA 18773
Kakapo                     -------------------------------------------------------------------------------- 0
GoldenEagle                -------------------------------------------------------------------------------- 0
JapaneseQuail              -------------------------------------------------------------------------------- 0
MediumGroundFinch          -------------------------------------------------------------------------------- 0
GoodesThornscrubTortoise   TGTTGCTATACAAAGTGAGTTGGTTTCTGTTAGTTAT---------TTTTGGAGCAGATTGGGGATATTGCTGTTTGAGG 15269

Majority                   --------------------------------------------------------------------------------

                                    18810     18820     18830     18840     18850     18860     18870     18880

Human                      TGGACTCTTATCTCCCGACCTCGTGATCCGCCTGCCTCGGCCTCCCAAAGTGCTGGGATTACAGGCCTGAGCCACCGCGC 18853
Kakapo                     -------------------------------------------------------------------------------- 0
GoldenEagle                -------------------------------------------------------------------------------- 0
JapaneseQuail              -------------------------------------------------------------------------------- 0
MediumGroundFinch          -------------------------------------------------------------------------------- 0
GoodesThornscrubTortoise   TCAGTGTGTAAGGTAAGATGTCTATACATAAATGCACGTCCTCTTCACTAAATAAGCATAAACCAAATAATATGCATGCT 15349
```

```
Majority                --------------------------------------------------------------------------------

                                 18890     18900     18910     18920     18930     18940     18950     18960

Human                   CTGGCCAATCTGAAACTTTTTGAGCACTTACTTGATGCTCAAAGGAAGTGCTTAGTGGAGCATTTTGGATTTTAGATTTT 18933
Kakapo                  -------------------------------------------------------------------------------- 0
GoldenEagle             -------------------------------------------------------------------------------- 0
JapaneseQuail           -------------------------------------------------------------------------------- 0
MediumGroundFinch       -------------------------------------------------------------------------------- 0
GoodesThornscrubTortoiseCTATTTCACTAAAAATTCATCATGTGACAACAGAATATT--AATTAAATTCATATCTATCTATCATGCAAATTGAAATCT 15427

Majority                --------------------------------------------------------------------------------

                                 18970     18980     18990     19000     19010     19020     19030     19040

Human                   CAGATTAGGAATGCTCAGGTTGTGTTTGTCCTTTTGTGACTGGCTTATATTCACTTAATATAATGTCCTCAGGGTCCATC 19013
Kakapo                  -------------------------------------------------------------------------------- 0
GoldenEagle             -------------------------------------------------------------------------------- 0
JapaneseQuail           -------------------------------------------------------------------------------- 0
MediumGroundFinch       -------------------------------------------------------------------------------- 0
GoodesThornscrubTortoiseCTAAATGAGTGCTTATTGTTCTGTGCCCTCTCCTTCCCCCCAATGTACTACCTCAAACTCTTAGTATTTTCATCCCTGGA 15507

Majority                --------------------------------------------------------------------------------

                                 19050     19060     19070     19080     19090     19100     19110     19120

Human                   CATTTTGTAGCATGTGACAAGATTTCCTCCCTTTTTAAGGCTACATAGCATTCCATTGTGGTACACAAAAGTTCATTTGA 19093
Kakapo                  -------------------------------------------------------------------------------- 0
GoldenEagle             -------------------------------------------------------------------------------- 0
JapaneseQuail           -------------------------------------------------------------------------------- 0
MediumGroundFinch       -------------------------------------------------------------------------------- 0
GoodesThornscrubTortoiseCACAATGTGTTTTTTTAAATCCTGAAGACACTTTATATTTTGGATTTGCAAGAAATTTACACTGAAGAAGAATGACTCAA 15587

Majority                --------------------------------------------------------------------------------

                                 19130     19140     19150     19160     19170     19180     19190     19200

Human                   AGCCCCCTGTGTCATAACTCAGACTTCCATAACAAAATACCATAGACTGAGTGACTTAAAAAACAGAGATTCGTTTTCTC 19173
Kakapo                  -------------------------------------------------------------------------------- 0
GoldenEagle             -------------------------------------------------------------------------------- 0
JapaneseQuail           -------------------------------------------------------------------------------- 0
MediumGroundFinch       -------------------------------------------------------------------------------- 0
GoodesThornscrubTortoiseCCTACTGGAATGGTAGTCACTGATTATTTTGAACACTTTTTTTTGAATGCTGCTACTCCTCCATCATACTGCTAGTTTTC 15667
```

```
Majority              --------------------------------------------------------------------------------

                               19210     19220     19230     19240     19250     19260     19270     19280

Human                 ACAGTTCTGGAAACTGGGAAGTCCAAGATCAAGGTACCAGCAAGGCAACTTTCCTTCTGAGGCCACTTCTCTTGAAGTGA 19253
Kakapo                -------------------------------------------------------------------------------- 0
GoldenEagle           -------------------------------------------------------------------------------- 0
JapaneseQuail         -------------------------------------------------------------------------------- 0
MediumGroundFinch     -------------------------------------------------------------------------------- 0
GoodesThornscrubTortoiseAGTTGCCTGCAGATGCTGTTAGAAGCTAAGGGTACATCTACACTTCAATAAAAAACCTGTGGCACCAAGTCTCAGAGCCT 15747

Majority              --------------------------------------------------------------------------------

                               19290     19300     19310     19320     19330     19340     19350     19360

Human                 GGCCACCATTTTACTTTGTGTGCCTCACATGACCTTTTCTTTGTGAGCACACTTTGGGGGAGTGGGTGGCAGAAGCTCTC 19333
Kakapo                -------------------------------------------------------------------------------- 0
GoldenEagle           -------------------------------------------------------------------------------- 0
JapaneseQuail         -------------------------------------------------------------------------------- 0
MediumGroundFinch     -------------------------------------------------------------------------------- 0
GoodesThornscrubTortoiseCGTCAA---TTGATTCAGGCCAGCTGTGGCTCTGCTGTTTGCAATTGTAAACATACCTTGAGTGTTTCACAGTGATGTTA 15824

Majority              --------------------------------------------------------------------------------

                               19370     19380     19390     19400     19410     19420     19430     19440

Human                 TGGTATTTCTTCTCATAAAGGCATTAATCCCATCATGAAGGCCTTACTCTCATGATCTCATGAAATCCTCCAGTTAACTC 19413
Kakapo                -------------------------------------------------------------------------------- 0
GoldenEagle           -------------------------------------------------------------------------------- 0
JapaneseQuail         -------------------------------------------------------------------------------- 0
MediumGroundFinch     -------------------------------------------------------------------------------- 0
GoodesThornscrubTortoiseTGCTCATGGTTTGACATGTGTCCCTAGGCTGAGACTAACTGTGGTAAATATACAATGTAACTAAAACCACCATTTAACAT 15904

Majority              --------------------------------------------------------------------------------

                               19450     19460     19470     19480     19490     19500     19510     19520

Human                 CTAAAGGCCCTGTTTCAAAATTGCATCACATTTGGGGTTAGGACTTCAGCATGTGATTTTTGGGGAAACATAAGCATTGA 19493
Kakapo                -------------------------------------------------------------------------------- 0
GoldenEagle           -------------------------------------------------------------------------------- 0
JapaneseQuail         -------------------------------------------------------------------------------- 0
MediumGroundFinch     -------------------------------------------------------------------------------- 0
GoodesThornscrubTortoiseTTTTTCTGTTTTCTTTCATCACAGAAAAAGTGTCCTTCCTATTCTCTACCCCGATATAACGCTGTCCTTGGGAGCCAAAA 15984
```

```
Majority              --------------------------------------------------------------------------------

                              19530     19540     19550     19560     19570     19580     19590     19600

Human                 GTTGATAATACCCTGCATATAGTTGACCCATACTTCTCCACTCCAGGTAATTACTATACTAAATTTGATGTTTACCATTC 19573
Kakapo                -------------------------------------------------------------------------------- 0
GoldenEagle           -------------------------------------------------------------------------------- 0
JapaneseQuail         -------------------------------------------------------------------------------- 0
MediumGroundFinch     -------------------------------------------------------------------------------- 0
GoodesThornscrubTortoiseAATCTTAACAAATTATA---AGTGAAACTGTGTGAAATCGAACTTGTTTTGATCCACCGGAGCGCTGCTTTACCGTGTTA 16061

Majority              --------------------------------------------------------------------------------

                              19610     19620     19630     19640     19650     19660     19670     19680

Human                 CCATAAATTTCTCATTTACTACATGTTTATGTATTTTTATCTTAAGCAATATATGGTTTTGTTTTGCATGTTTAAACTAT 19653
Kakapo                -------------------------------------------------------------------------------- 0
GoldenEagle           -------------------------------------------------------------------------------- 0
JapaneseQuail         -------------------------------------------------------------------------------- 0
MediumGroundFinch     -------------------------------------------------------------------------------- 0
GoodesThornscrubTortoiseTATCTGAATTCATGTTATATCGGGGCAGAGGTATACGTATCATCGGTGAATCAGAATCTCTCCCAAGAGTTAACAGCATT 16141

Majority              --------------------------------------------------------------------------------

                              19690     19700     19710     19720     19730     19740     19750     19760

Human                 AGATGTACTATCATGCTGTATGTATTCTGTAATTTGCTTTTCTTGTTCAGTGTTATTTTTGTTAAGTTTATATATATATA 19733
Kakapo                -------------------------------------------------------------------------------- 0
GoldenEagle           -------------------------------------------------------------------------------- 0
JapaneseQuail         -------------------------------------------------------------------------------- 0
MediumGroundFinch     -------------------------------------------------------------------------------- 0
GoodesThornscrubTortoiseTCTTTTTTAATTAT--TATATTCTCTCCACCAGTTGTAATTAAAAACTGTTTAGATATTGTATCACAGTAGAGTAGATGA 16219

Majority              --------------------------------------------------------------------------------

                              19770     19780     19790     19800     19810     19820     19830     19840

Human                 TATTTTCATTGCCATATAGTATATGATATATGAATATATCATCTTGACAAATGAGTTAATATTTGACGAGATTGATTTGT 19813
Kakapo                -------------------------------------------------------------------------------- 0
GoldenEagle           -------------------------------------------------------------------------------- 0
JapaneseQuail         -------------------------------------------------------------------------------- 0
MediumGroundFinch     -------------------------------------------------------------------------------- 0
GoodesThornscrubTortoiseTATTGCTGGCTCTTACTACTATGTTTGAAGTAGAGGGATAGTTTTGTTCATTTGTTGACTATTTTTAGTAATATCATTCA 16299
```

```
Majority                   --------------------------------------------------------------------------------

                                   19850     19860     19870     19880     19890     19900     19910     19920

Human                      CAAATATTTTAAATTTTTTTCCATTACAAACTGCTACTGTAAGCATTAATATGCATATTCTAACGTGTTAATATTAGAGT 19893
Kakapo                     -------------------------------------------------------------------------------- 0
GoldenEagle                -------------------------------------------------------------------------------- 0
JapaneseQuail              -------------------------------------------------------------------------------- 0
MediumGroundFinch          -------------------------------------------------------------------------------- 0
GoodesThornscrubTortoise   CTTCGTGTTTTGTTTATTTCCATTGTCAGAGGAGACTACAAATTTGAGTGATGCTTTTAATAAGCCTTCTTTAACCCAAG 16379

Majority                   --------------------------------------------------------------------------------

                                   19930     19940     19950     19960     19970     19980     19990     20000

Human                      TTCTCATAGATTGTGTCTTTTCAAATCGATTGAATATTAACATATTGCTTTCATAGGTAATTATATTAATTATACTGTTA 19973
Kakapo                     -------------------------------------------------------------------------------- 0
GoldenEagle                -------------------------------------------------------------------------------- 0
JapaneseQuail              -------------------------------------------------------------------------------- 0
MediumGroundFinch          -------------------------------------------------------------------------------- 0
GoodesThornscrubTortoise   CAGATCTGTTACGTTAGGTACAAGAATCAACTTCCGAAAAGTTTTTCTCTACTTAGGAGTTAATAAAATATTGGGCAGAC 16459

Majority                   --------------------------------------------------------------------------------

                                   20010     20020     20030     20040     20050     20060     20070     20080

Human                      GCGCTGCATGTAGATTCTTGTTTCTATACATTCTTTGAAAATCGGCATTGTTGGACTTTTTAATTTTGCTGATCAAATGT 20053
Kakapo                     -------------------------------------------------------------------------------- 0
GoldenEagle                -------------------------------------------------------------------------------- 0
JapaneseQuail              -------------------------------------------------------------------------------- 0
MediumGroundFinch          -------------------------------------------------------------------------------- 0
GoodesThornscrubTortoise   AACTTTTGGATATATATATATTTTTAGACTTTTAGAAATTTTTTTGACAGGTCCTCACGAGGCAGTTAAACTTGGTAGCCGC 16539

Majority                   --------------------------------------------------------------------------------

                                   20090     20100     20110     20120     20130     20140     20150     20160

Human                      ACATGGGAAGGATATCTTGTGGTTTTAATTTGCATTCCCTATTAATGCAAAAAAAAAAAAACATTGATTCCTGGGAGTTT 20133
Kakapo                     -------------------------------------------------------------------------------- 0
GoldenEagle                -------------------------------------------------------------------------------- 0
JapaneseQuail              -------------------------------------------------------------------------------- 0
MediumGroundFinch          -------------------------------------------------------------------------------- 0
GoodesThornscrubTortoise   CAAGTTTTCAATCCAGTAATAGATTTTAAAAATGTTTGCGAATTGGCTGCTTATTTCTACTCTTTGCTTCTTAAATGTGG 16619
```

```
Majority                --------------------------------------------------------------------------------

                                 20170     20180     20190     20200     20210     20220     20230     20240

Human                   TTATATACTGTGCAGACTAATTGTTTTGTTATTTTTTTTGGTTTGTTTGTTTTGTTTTGTTTCATTTTTGTTTTTGTTTT 20213
Kakapo                  -------------------------------------------------------------------------------- 0
GoldenEagle             -------------------------------------------------------------------------------- 0
JapaneseQuail           -------------------------------------------------------------------------------- 0
MediumGroundFinch       -------------------------------------------------------------------------------- 0
GoodesThornscrubTortoiseAAAGAGATTTCTACTAGAGTTAAAATATATTATTCTGGAGATGTGGGAAAAATAGTTGACAGTAGATTTTTTGCCATCCT 16699

Majority                --------------------------------------------------------------------------------

                                 20250     20260     20270     20280     20290     20300     20310     20320

Human                   GAGAGACAGAGTCTCACTGTGTCATCCAGGCTGGAGTGCAGTGGCTCGGTCACAGTCACTGCAGTCTTGACATCCCAGGC 20293
Kakapo                  -------------------------------------------------------------------------------- 0
GoldenEagle             -------------------------------------------------------------------------------- 0
JapaneseQuail           -------------------------------------------------------------------------------- 0
MediumGroundFinch       -------------------------------------------------------------------------------- 0
GoodesThornscrubTortoiseGAATGGTTTCATGGTTAGACCTTAGGGGATTTTGATGACTTTAATGGCCTGATAAAACTGGGAGAGTGTGTAACATAACC 16779

Majority                --------------------------------------------------------------------------------

                                 20330     20340     20350     20360     20370     20380     20390     20400

Human                   TCTAGCAATTCTCTCACCTTTGTTTCCTGAGTAGCTGGGACTACAGGAATACACCACTATGCCCAGCTAATTTTTCAATT 20373
Kakapo                  -------------------------------------------------------------------------------- 0
GoldenEagle             -------------------------------------------------------------------------------- 0
JapaneseQuail           -------------------------------------------------------------------------------- 0
MediumGroundFinch       -------------------------------------------------------------------------------- 0
GoodesThornscrubTortoiseTGTGGCATTCAGATTAGACACATTTTCAAAGTAATGCGTGTTGGGACAAACCATTTTGAAACTAATTATAATATGGATAC 16859

Majority                --------------------------------------------------------------------------------

                                 20410     20420     20430     20440     20450     20460     20470     20480

Human                   TTTTTGTAGAGATGGAGTCTCACTGTATTACCCAGGTTTGTTGCGAACTCCCAGGCTCAAGCAGTTCTCCCGCATTGGCC 20453
Kakapo                  -------------------------------------------------------------------------------- 0
GoldenEagle             -------------------------------------------------------------------------------- 0
JapaneseQuail           -------------------------------------------------------------------------------- 0
MediumGroundFinch       -------------------------------------------------------------------------------- 0
GoodesThornscrubTortoiseTAAATTAGTTGCAACATTTAAAGCAAAGATCTAAACACTGTGGCTAGTCATTGCACTTTCTTCCTCCTTTTCTATAGGTT 16939
```

```
Majority                 --------------------------------------------------------------------------------
                                  20490     20500     20510     20520     20530     20540     20550     20560
Human                    TCCTAAAGTGCTGAGATTACAGGCGTGAGTCGCTGTGCCTGGCCTGTTTTGTTAGTTACATGTCCTACAGATAACTCTCT 20533
Kakapo                   -------------------------------------------------------------------------------- 0
GoldenEagle              -------------------------------------------------------------------------------- 0
JapaneseQuail            -------------------------------------------------------------------------------- 0
MediumGroundFinch        -------------------------------------------------------------------------------- 0
GoodesThornscrubTortoise T-----ATCGTATAATTTTTGAAAATTAGAAGATTTGATTGTATTTGTTGTGCAATGGTATTATACACAGTTAAACATTC 17014

Majority                 --------------------------------------------------------------------------------
                                  20570     20580     20590     20600     20610     20620     20630     20640
Human                    CCTCTCTCTGACCTGTTTTTTTTACACTGATGAATAGTTGTAAATTTTAATGGGACATTTATGTTTTTCTTTTAAAAAAA 20613
Kakapo                   -------------------------------------------------------------------------------- 0
GoldenEagle              -------------------------------------------------------------------------------- 0
JapaneseQuail            -------------------------------------------------------------------------------- 0
MediumGroundFinch        -------------------------------------------------------------------------------- 0
GoodesThornscrubTortoise CCTACATTCCACTTCTTTTAATTTTTATATTTCAGATTTACAGTACATGAAGCAATTTATGCTTCTGAAGTTTGTAAATT 17094

Majority                 --------------------------------------------------------------------------------
                                  20650     20660     20670     20680     20690     20700     20710     20720
Human                    CATTTAACCTAAGGCCTTACGTATAGGCTTGTGTATTTTATTTTGAAAGTTTTAAAATTTTGATTTTCACATTTATGGCG 20693
Kakapo                   -------------------------------------------------------------------------------- 0
GoldenEagle              -------------------------------------------------------------------------------- 0
JapaneseQuail            -------------------------------------------------------------------------------- 0
MediumGroundFinch        -------------------------------------------------------------------------------- 0
GoodesThornscrubTortoise CCTTTTGGGAGTTGCATATTGAAGGCTTAACTTGGCAGTCAAATCTTTTTTTGAAAATTTGGTCCTGTGTGAGGAAACTC 17174

Majority                 --------------------------------------------------------------------------------
                                  20730     20740     20750     20760     20770     20780     20790     20800
Human                    TTAATCTACCTGGAATTTATTCTTTGTGTAGCATATGTTACAGGTTTAATTATTTTTTCT---ATATGGATAGCAAGCTT 20770
Kakapo                   -------------------------------------------------------------------------------- 0
GoldenEagle              -------------------------------------------------------------------------------- 0
JapaneseQuail            -------------------------------------------------------------------------------- 0
MediumGroundFinch        -------------------------------------------------------------------------------- 0
GoodesThornscrubTortoise ACAGTGTCACTTTTACACATCTGCTTTTCTGGATAGAACCACACTTCAAATATTTTTCTTCACACTTGTGGTGAAAGGCT 17254
```

```
Majority                   --------------------------------------------------------------------------------

                                    20810     20820     20830     20840     20850     20860     20870     20880

Human                      AGTATGTACTTGATTGAATAGCTCAGCTTTTCTCCAATGATCTGCTTTCTCAGCTCTTCAGGTTTCCGTATATTCACAGA 20850
Kakapo                     -------------------------------------------------------------------------------- 0
GoldenEagle                -------------------------------------------------------------------------------- 0
JapaneseQuail              -------------------------------------------------------------------------------- 0
MediumGroundFinch          -------------------------------------------------------------------------------- 0
GoodesThornscrubTortoise   GCATTGAGGGGGACAGGATGAGATAGGAAGGACAAAACAGAATTTGTCGGCATGAGCGAGGATTATTTAAAAGAGGCTGG 17334

Majority                   --------------------------------------------------------------------------------

                                    20890     20900     20910     20920     20930     20940     20950     20960

Human                      ACCATTTCTGTGCTATGTCTTGTAGCAGTACAACAGTGTCTTAATTACTTATGGCATCAGCATTAAGTTTTGGAATCTTG 20930
Kakapo                     -------------------------------------------------------------------------------- 0
GoldenEagle                -------------------------------------------------------------------------------- 0
JapaneseQuail              -------------------------------------------------------------------------------- 0
MediumGroundFinch          -------------------------------------------------------------------------------- 0
GoodesThornscrubTortoise   GTCTAATATCCTCCATGTTTAAGTTGTAAATTAGGAGTTCATCAAGTAGTTTGTTTGTTTAGTACAGAGTCTTCCCTTCG 17414

Majority                   --------------------------------------------------------------------------------

                                    20970     20980     20990     21000     21010     21020     21030     21040

Human                      TACAGTGAGTGTCAAAACTTTGTTTTTCAAAATTGTCTTGGTGTTTTCTTCACTGCATTTATTTTAAGATCATTTGGCTA 21010
Kakapo                     -------------------------------------------------------------------------------- 0
GoldenEagle                -------------------------------------------------------------------------------- 0
JapaneseQuail              -------------------------------------------------------------------------------- 0
MediumGroundFinch          -------------------------------------------------------------------------------- 0
GoodesThornscrubTortoise   TAGGGTAAATACATAGGGTTAGTGGTCTGGCTGTTACTTAAGACAGATCAGGATTCCATGATTTTAAGAGTTGTTGCTTT 17494

Majority                   --------------------------------------------------------------------------------

                                    21050     21060     21070     21080     21090     21100     21110     21120

Human                      AGTTCTGTGAAGGCTCTTTGGGTCTTATTTATACTTGCCTTAAATTTAGTAGTTAATTGGGAGAACATTATAGCTGCATT 21090
Kakapo                     -------------------------------------------------------------------------------- 0
GoldenEagle                -------------------------------------------------------------------------------- 0
JapaneseQuail              -------------------------------------------------------------------------------- 0
MediumGroundFinch          -------------------------------------------------------------------------------- 0
GoodesThornscrubTortoise   GCCAAGACTGAAATAGCATCCATCTTTCCAAAACAAAAAGAAAAAATTATGAGACTCTTGGGACTTGAAAGCCTTCTAGA 17574
```

```
Majority                --------------------------------------------------------------------------------

                              21130     21140     21150     21160     21170     21180     21190     21200

Human                   ATCAAAATAAAATGGCAATTTTTTTTGAGCTGAAAGGCAGAGAACTATAACATTTCTTTGAAGAATTTTCCACTTTCTTC 21170
Kakapo                  -------------------------------------------------------------------------------- 0
GoldenEagle             -------------------------------------------------------------------------------- 0
JapaneseQuail           -------------------------------------------------------------------------------- 0
MediumGroundFinch       -------------------------------------------------------------------------------- 0
GoodesThornscrubTortoise CTCAAAAATGCATGTGCCTAGATCCGGCACTGGTAGAGATGTGGCCTGTGTGTCATCTATGTTCAGGCCTGAGACTAAGC 17654

Majority                --------------------------------------------------------------------------------

                              21210     21220     21230     21240     21250     21260     21270     21280

Human                   TTCATGGTATTTTCCCTAAGAGACATGCTTAATGCAGAAAATATATTAAAAGGATTTTAAACAGCTTATATGTAAGGGAT 21250
Kakapo                  -------------------------------------------------------------------------------- 0
GoldenEagle             -------------------------------------------------------------------------------- 0
JapaneseQuail           -------------------------------------------------------------------------------- 0
MediumGroundFinch       -------------------------------------------------------------------------------- 0
GoodesThornscrubTortoise AGGTAATTGCCCTCAAATGAAGCAATGAAAACAGAATCTGGATATATCCAGGTCAGTACATGGAGCAGCATGGGAATTCC 17734

Majority                --------------------------------------------------------------------------------

                              21290     21300     21310     21320     21330     21340     21350     21360

Human                   GAGGGAACAGGGATACTTGGACTAATATTTCAATATTTCTAAAATTATTGGTTTATTGAGTACGTTCCTAGAAAACTGTT 21330
Kakapo                  -------------------------------------------------------------------------------- 0
GoldenEagle             -------------------------------------------------------------------------------- 0
JapaneseQuail           -------------------------------------------------------------------------------- 0
MediumGroundFinch       -------------------------------------------------------------------------------- 0
GoodesThornscrubTortoise CTTTGGATGTAGGGGAAGGGCTGTGTCTGACAAGCAAACTGAAGTCTTAAATATGCAGTAATATTATATTACACGGGTTT 17814

Majority                --------------------------------------------------------------------------------

                              21370     21380     21390     21400     21410     21420     21430     21440

Human                   TTATTGAAAGCTTCAAATTTCTCAGAATTGTATAGCATTTTTGAATTCTCTCTTTTATTACTAATATCAAGCACCTATGT 21410
Kakapo                  -------------------------------------------------------------------------------- 0
GoldenEagle             -------------------------------------------------------------------------------- 0
JapaneseQuail           -------------------------------------------------------------------------------- 0
MediumGroundFinch       -------------------------------------------------------------------------------- 0
GoodesThornscrubTortoise TTTTGAGAAACTACCATAATTTTTTTTTTTAAACAACCATGTATAATCTGTTTAATTGGTAGTAAAAATAACTGAGGTAT 17894
```

```
Majority                   --------------------------------------------------------------------------------

                                    21450     21460     21470     21480     21490     21500     21510     21520

Human                      TTTCTCCTATTTCTTGATTTGTCTTGTCACAGAATTTATTTTACTGGTCTTTTCAAAGAACCAACCTTTTTACTTGTTTT 21490
Kakapo                     -------------------------------------------------------------------------------- 0
GoldenEagle                -------------------------------------------------------------------------------- 0
JapaneseQuail              -------------------------------------------------------------------------------- 0
MediumGroundFinch          -------------------------------------------------------------------------------- 0
GoodesThornscrubTortoise   CTTTTGTTTTTTTTTTTTTTTTTTTTAATGATAGCTGCCATCACTAGTTATTTTTGTCCATTGCAAACAGAGGTAAAATA 17974

Majority                   --------------------------------------------------------------------------------

                                    21530     21540     21550     21560     21570     21580     21590     21600

Human                      CTTTTTTGTTGGGGGCAGTGGAGGGATTCACTTTAATTTGATCCTGAACTCCTTTATTTTCTTTTAAAATGCATTCAAGG 21570
Kakapo                     -------------------------------------------------------------------------------- 0
GoldenEagle                -------------------------------------------------------------------------------- 0
JapaneseQuail              -------------------------------------------------------------------------------- 0
MediumGroundFinch          -------------------------------------------------------------------------------- 0
GoodesThornscrubTortoise   TCCTTCTCTGGAAATGTATTGGTGGGCTGACTGCACAGAAGTGCAGGTATTTTTTGAATTCAACCATCTTGTCTTCCATG 18054

Majority                   --------------------------------------------------------------------------------

                                    21610     21620     21630     21640     21650     21660     21670     21680

Human                      TTAAAGGTAATCAGCTGATAGCTTTGGCTTTTTTTTTTTTTTTTTACTTTTAGAGTTCTCTTTTTATTGTAAACTTCATT 21650
Kakapo                     -------------------------------------------------------------------------------- 0
GoldenEagle                -------------------------------------------------------------------------------- 0
JapaneseQuail              -------------------------------------------------------------------------------- 0
MediumGroundFinch          -------------------------------------------------------------------------------- 0
GoodesThornscrubTortoise   ATTTTACTTCCATAATTTTGATGTCAAACTATAATATCCTCTCCTTAGCAAATATTGGGGTAGTAATTTAGATTTTCAAA 18134

Majority                   --------------------------------------------------------------------------------

                                    21690     21700     21710     21720     21730     21740     21750     21760

Human                      TAAAGGTTTTAAGCTACACAAAAGTAGAGGGAATGGTATATAATACACTTTTTTCTGTTGGATAATATTAATGCAAATCT 21730
Kakapo                     -------------------------------------------------------------------------------- 0
GoldenEagle                -------------------------------------------------------------------------------- 0
JapaneseQuail              -------------------------------------------------------------------------------- 0
MediumGroundFinch          -------------------------------------------------------------------------------- 0
GoodesThornscrubTortoise   CAGAAAGAGAGAAC-ATTTCAATGAGTTCCATCTGCCACATTTCAGTCATCTTTGTACTTGAGAACAGTCATATAGAGT- 18212
```

```
Majority                 --------------------------------------------------------------------------------

                                  21770     21780     21790     21800     21810     21820     21830     21840

Human                    CAGATCATTTCATATAATCTGCAGATATATCAGAATGTGTGTCTCTCTATTAAATGTGACATTGGTACCATTATAAAGTT 21810
Kakapo                   -------------------------------------------------------------------------------- 0
GoldenEagle              -------------------------------------------------------------------------------- 0
JapaneseQuail            -------------------------------------------------------------------------------- 0
MediumGroundFinch        -------------------------------------------------------------------------------- 0
GoodesThornscrubTortoise --GAATAATGGAAAGGTTCCATAGCTTCTCTGAATTGGCTTTCAGTTTTGCCTGTTTTAAGTTATTCTTTTGATCTAATT 18290

Majority                 --------------------------------------------------------------------------------

                                  21850     21860     21870     21880     21890     21900     21910     21920

Human                    TAAAATAATTTCTTAATGTCGTCTAATCTCCAGTATTCAGAATACTTTCTTTTCATTATTTATTTTTTCTTGCCTTTTCT 21890
Kakapo                   -------------------------------------------------------------------------------- 0
GoldenEagle              -------------------------------------------------------------------------------- 0
JapaneseQuail            -------------------------------------------------------------------------------- 0
MediumGroundFinch        -------------------------------------------------------------------------------- 0
GoodesThornscrubTortoise AAAAATGGTTTCCTCTGACAGTGACAAGACTTATTGCTGTTTCATTTTATTAAATTTAAAGTCAAATCATCTTGCCAATT 18370

Majority                 --------------------------------------------------------------------------------

                                  21930     21940     21950     21960     21970     21980     21990     22000

Human                    TTTTTGTTGTTGTTAGATAAAGTGAGTCATCTGTACAATTTCCACTTTCTGGCTGGGCGTGGTGGCTCACGCCTGTAATC 21970
Kakapo                   -------------------------------------------------------------------------------- 0
GoldenEagle              -------------------------------------------------------------------------------- 0
JapaneseQuail            -------------------------------------------------------------------------------- 0
MediumGroundFinch        -------------------------------------------------------------------------------- 0
GoodesThornscrubTortoise TGAACCCCAAAATGTGTAGAGCAGAATCTAGAATCAACAAGAGTGTGAGGCAAACTAAAATTATTTAGAAGTAGTCTGTT 18450

Majority                 --------------------------------------------------------------------------------

                                  22010     22020     22030     22040     22050     22060     22070     22080

Human                    CCAGCACTTCGGGAGGCTGAGGCGGGCAGATCACGAGGTCAGGAGATCAAGACCATTTTGGCTAACACAGTGAAAACCTG 22050
Kakapo                   -------------------------------------------------------------------------------- 0
GoldenEagle              -------------------------------------------------------------------------------- 0
JapaneseQuail            -------------------------------------------------------------------------------- 0
MediumGroundFinch        -------------------------------------------------------------------------------- 0
GoodesThornscrubTortoise TAAATAACTTTGAGAACTAGGGCTCAAAACTGAATAAGCTTAGTGACTGTAATAGCTTGGTGTGTTGTCTAGTATGTTGT 18530
```

```
Majority                    --------------------------------------------------------------------------------

                                      22090     22100     22110     22120     22130     22140     22150     22160

Human                       TCTCTACTAAAAATACGAAAAAATTAGCCAGGCGTGGTGGGGGGCACCTGTAGTCCCAGCTACTCGGGAGGCTGAGGCAG 22130
Kakapo                      -------------------------------------------------------------------------------- 0
GoldenEagle                 -------------------------------------------------------------------------------- 0
JapaneseQuail               -------------------------------------------------------------------------------- 0
MediumGroundFinch           -------------------------------------------------------------------------------- 0
GoodesThornscrubTortoise    GAATTTTAGATATGGATACTTCAAAATGTGTTATATATTCTAATATTCCTCTTCATATTCCTATGTGTGTGATTATATG 18610

Majority                    --------------------------------------------------------------------------------

                                      22170     22180     22190     22200     22210     22220     22230     22240

Human                       GAGAATGGTGTGAACCCGGGAGGCGGAGCTTGCAGTGGCGCCACTGGACTCCAGCCTGGGTGACAGAGCAAGACTCCGTC 22210
Kakapo                      -------------------------------------------------------------------------------- 0
GoldenEagle                 -------------------------------------------------------------------------------- 0
JapaneseQuail               -------------------------------------------------------------------------------- 0
MediumGroundFinch           -------------------------------------------------------------------------------- 0
GoodesThornscrubTortoise    CTTGATTATATGAATCCTGGTATAAGCATACTACTTAGTATTGTGATATCCTCAGATTAGTTTAGTATGTATTTAGAATG 18690

Majority                    --------------------------------------------------------------------------------

                                      22250     22260     22270     22280     22290     22300     22310     22320

Human                       TTAAAAAAAAAAAAAAAATTCCACTTTCTGGATTCTGCTGATTGTACCGCTGTGTTGTCATTTCATATGTTCTTCATGTA 22290
Kakapo                      -------------------------------------------------------------------------------- 0
GoldenEagle                 -------------------------------------------------------------------------------- 0
JapaneseQuail               -------------------------------------------------------------------------------- 0
MediumGroundFinch           -------------------------------------------------------------------------------- 0
GoodesThornscrubTortoise    AAGAACTATCCAAAATATACAGGATGCTTTTCAAACAGAGGTAGATGATATCTGCCTCAAGGACAGCACAGTCTGAAACA 18770

Majority                    --------------------------------------------------------------------------------

                                      22330     22340     22350     22360     22370     22380     22390     22400

Human                       AACAAGTTTACCTGAAATACAGGGGTTTGATCAGACTCTGGTGTAATTTTTACCAAGAATATTTTGCAGATGTACTCTAT 22370
Kakapo                      -------------------------------------------------------------------------------- 0
GoldenEagle                 -------------------------------------------------------------------------------- 0
JapaneseQuail               -------------------------------------------------------------------------------- 0
MediumGroundFinch           -------------------------------------------------------------------------------- 0
GoodesThornscrubTortoise    TCTTGATTATTATAAAGATTTCATAAGTAAAGCAGTTTGTGGTCAGTACTCAGAATGGAAACAGACTCCATGCAAAGTAA 18850
```

```
Majority                  --------------------------------------------------------------------------------

                                   22410     22420     22430     22440     22450     22460     22470     22480

Human                     GTAATTCCTGTTGTATGTCAGCTTGCTCCATCTATTACAGAGCGCATCATCCTTTCACCTAATAGTTTTTAGTAGCTAAT 22450
Kakapo                    -------------------------------------------------------------------------------- 0
GoldenEagle               -------------------------------------------------------------------------------- 0
JapaneseQuail             -------------------------------------------------------------------------------- 0
MediumGroundFinch         -------------------------------------------------------------------------------- 0
GoodesThornscrubTortoise  TATTTGGTGATTCAGTAACTGGCACTCACCCTTTTTAGTAATGACCCAATGGCCTGGGTTAGTGCTGAGGGCATTGGTGC 18930

Majority                  --------------------------------------------------------------------------------

                                   22490     22500     22510     22520     22530     22540     22550     22560

Human                     GATTGCATACTTTTATTAGGTATTGCA-AAAGTGGCGCTATTCTTTTTTTCTGTGTATATTAGAATTCTTAAGAATTATA 22529
Kakapo                    -------------------------------------------------------------------------------- 0
GoldenEagle               -------------------------------------------------------------------------------- 0
JapaneseQuail             -------------------------------------------------------------------------------- 0
MediumGroundFinch         -------------------------------------------------------------------------------- 0
GoodesThornscrubTortoise  TAATGGAGATACCTTTGAATTAAGACACAAAACGGAGGTCCTAACCTCTTTTGGATATAAAAAATCTAGTAGCCTATTAA 19010

Majority                  --------------------------------------------------------------------------------

                                   22570     22580     22590     22600     22610     22620     22630     22640

Human                     TCCATCAACTGTTTGGTCATACGTAAGTACCTTTTTATAAGAAAAGCAGGAGAGAATTTGACTTCTTAAGTTTTCTGATG 22609
Kakapo                    -------------------------------------------------------------------------------- 0
GoldenEagle               -------------------------------------------------------------------------------- 0
JapaneseQuail             -------------------------------------------------------------------------------- 0
MediumGroundFinch         -------------------------------------------------------------------------------- 0
GoodesThornscrubTortoise  TGATGCCATGCAAAGGAAGGGAGGTTTATGCCCTTTTTAATTCATTTTGACTTATGAGGAAGGCCTGAAAGATTTAAAAA 19090

Majority                  --------------------------------------------------------------------------------

                                   22650     22660     22670     22680     22690     22700     22710     22720

Human                     AGTTGTTACCCTAAAACCTCCAGTATAATCAATATGAGTTCCTTTTTGTTGCTGTTAGTATCGTCGCAACAGCAAAGAGT 22689
Kakapo                    -------------------------------------------------------------------------------- 0
GoldenEagle               -------------------------------------------------------------------------------- 0
JapaneseQuail             -------------------------------------------------------------------------------- 0
MediumGroundFinch         -------------------------------------------------------------------------------- 0
GoodesThornscrubTortoise  AACAAACAAACAAAAAATCCTACTTGTTTTACATTAGGCCTCTGAGTGTTTTTTCTATCCTGGAAGGAAATGAAACCTGA 19170
```

```
Majority                   --------------------------------------------------------------------------------

                                    22730     22740     22750     22760     22770     22780     22790     22800

Human                      TTAATAACATTTATTTTCTAGTGTATTGCAGTAATCATTCTTCTTTTTTTTAAATTTCTAAGCTGTTTTATTAAATGAAA 22769
Kakapo                     -------------------------------------------------------------------------------- 0
GoldenEagle                -------------------------------------------------------------------------------- 0
JapaneseQuail              -------------------------------------------------------------------------------- 0
MediumGroundFinch          -------------------------------------------------------------------------------- 0
GoodesThornscrubTortoise   TCACTGAGAGGGGAAGTATATTTTTTTTAAGTACACCTCTACCTCAATATAACAC-----------------GAATTCGG 19233

Majority                   --------------------------------------------------------------------------------

                                    22810     22820     22830     22840     22850     22860     22870     22880

Human                      AGAGAACAATGCTAAGCAGCTTGTATGGTGTGTGTGTTGTGTGGGTTTTTATTTTGTATGAATGTTAAAAACACGGTGGC 22849
Kakapo                     -------------------------------------------------------------------------------- 0
GoldenEagle                -------------------------------------------------------------------------------- 0
JapaneseQuail              -------------------------------------------------------------------------------- 0
MediumGroundFinch          -------------------------------------------------------------------------------- 0
GoodesThornscrubTortoise   ATACAACACGGTAAAGCACTGCTCCGGGGGGGTGGGGCTGTGCACTCCTGTGGATCAAAGCAAGTAAGATGTAACGCAGT 19313

Majority                   --------------------------------------------------------------------------------

                                    22890     22900     22910     22920     22930     22940     22950     22960

Human                      TAAAGCCTGTAATCCCAGCATTTTGGGAGGCCAAGGTGGTTGGATCACAAGGTCAGGAGCTTGAGACCAGCCTGGCTCTA 22929
Kakapo                     -------------------------------------------------------------------------------- 0
GoldenEagle                -------------------------------------------------------------------------------- 0
JapaneseQuail              -------------------------------------------------------------------------------- 0
MediumGroundFinch          -------------------------------------------------------------------------------- 0
GoodesThornscrubTortoise   TTCACCTATAATGCATTAAGATTTTTTGGCTCCTGAGGACTGTGTTATATTGAGGTAGAAGTGTATTTAGACTGCTTCAG 19393

Majority                   --------------------------------------------------------------------------------

                                    22970     22980     22990     23000     23010     23020     23030     23040

Human                      CTAAAAATAAAATGTGGTGAAACCCCGTCTCTACTAAAAATACAAAAATTAGCCCTTTGTTGTTGCACGCGCCTATAGTC 23009
Kakapo                     -------------------------------------------------------------------------------- 0
GoldenEagle                -------------------------------------------------------------------------------- 0
JapaneseQuail              -------------------------------------------------------------------------------- 0
MediumGroundFinch          -------------------------------------------------------------------------------- 0
GoodesThornscrubTortoise   ACTAATTTTTAACACCTATTTAACTCAAATGCTTGGGGGGAAAGGATCCCTCTTTAAGGATTCAACACCAAATCTCCTCT 19473
```

```
Majority                   --------------------------------------------------------------------------------

                                   23050     23060     23070     23080     23090     23100     23110     23120

Human                      CCAGCTACTCGGGAGGCTGAGGCAGAAAAATTGCTTGAACCTGGGGAGGCTGAGGCAGAAAAATTACTTGAACCCGGGAG 23089
Kakapo                     -------------------------------------------------------------------------------- 0
GoldenEagle                -------------------------------------------------------------------------------- 0
JapaneseQuail              -------------------------------------------------------------------------------- 0
MediumGroundFinch          -------------------------------------------------------------------------------- 0
GoodesThornscrubTortoiseCGTATCTGCATTCCACATTGAAATCATCCAAAAAGAAGACTAAGAAAGAACAGACTTGATGTTCTTCATATTTTTATGAA 19553

Majority                   --------------------------------------------------------------------------------

                                   23130     23140     23150     23160     23170     23180     23190     23200

Human                      ACAGAGGTTGCAGCGAGCCGAGATTGCGCCACTGCACTCCAGCCTGGGCAACAGACTGAGATTCCATCTAAAAACAAAAC 23169
Kakapo                     -------------------------------------------------------------------------------- 0
GoldenEagle                -------------------------------------------------------------------------------- 0
JapaneseQuail              -------------------------------------------------------------------------------- 0
MediumGroundFinch          -------------------------------------------------------------------------------- 0
GoodesThornscrubTortoiseCCTTAAACCAAAAAGAAATCCATCCTCCAACAAACAAACAATTGTTTTCAGCGTTCCTTTATATATTATAAAGAAGTGC 19633

Majority                   --------------------------------------------------------------------------------

                                   23210     23220     23230     23240     23250     23260     23270     23280

Human                      CATGTATATATGTAAGTTTGAGGAAAAGAGTTGTATGAAAATTCAGGATAATTAATAAAAGTAAACGAGAACTTCTTTAC 23249
Kakapo                     -------------------------------------------------------------------------------- 0
GoldenEagle                -------------------------------------------------------------------------------- 0
JapaneseQuail              -------------------------------------------------------------------------------- 0
MediumGroundFinch          -------------------------------------------------------------------------------- 0
GoodesThornscrubTortoiseAAAACACAACAGTCTGCTTTGAACTTTTTCTAATAGGAGTGCTAGCCCTAGCACTTTGGTCAAATTCTAATTTCAGTATT 19713

Majority                   --------------------------------------------------------------------------------

                                   23290     23300     23310     23320     23330     23340     23350     23360

Human                      ATATATATATATTTTTTAATTTTCTCAGTTAATGTTTGAATTTTTTTAACGTAGAAAATGTCAATGGGGTTATCTGGGGA 23329
Kakapo                     -------------------------------------------------------------------------------- 0
GoldenEagle                -------------------------------------------------------------------------------- 0
JapaneseQuail              -------------------------------------------------------------------------------- 0
MediumGroundFinch          -------------------------------------------------------------------------------- 0
GoodesThornscrubTortoiseTGCCTGAATTCCCCATTTAGTTTCAGTGGATATTAACTTCCTATTTTAAAATATTTTGTTAAGTTGTGCACTGTTGGACA 19793
```

```
Majority                    --------------------------------------------------------------------------------

                                      23370     23380     23390     23400     23410     23420     23430     23440

Human                       GAAAGATTATAGTCCATGAGTCCCCCTGTTGTATATATACCTGTATTAAAATATGCAATAAATGCTATTTTAAAAAATGT 23409
Kakapo                      -------------------------------------------------------------------------------- 0
GoldenEagle                 -------------------------------------------------------------------------------- 0
JapaneseQuail               -------------------------------------------------------------------------------- 0
MediumGroundFinch           -------------------------------------------------------------------------------- 0
GoodesThornscrubTortoise    CCTCTTGTGGTCCCTGGAGATTGCATTTCAGATGTGGATGAAGTGATTTTGTGTATGGAATTATTTGACTTGAGGGATGC 19873

Majority                    --------------------------------------------------------------------------------

                                      23450     23460     23470     23480     23490     23500     23510     23520

Human                       CTTATTACATAGAACAGAAGACCAGAATACTAGCTCTCCACGTCTTATGTCTTGATCAGTGAAGATTAGCTAGCAAAACA 23489
Kakapo                      -------------------------------------------------------------------------------- 0
GoldenEagle                 -------------------------------------------------------------------------------- 0
JapaneseQuail               -------------------------------------------------------------------------------- 0
MediumGroundFinch           -------------------------------------------------------------------------------- 0
GoodesThornscrubTortoise    TATGTAAATATACTGTATTGTTTAGATTTTAATTTTTTAATTTACTGTCACTTTCTGAAGAGGGAGAATCTGCCAAATCT 19953

Majority                    --------------------------------------------------------------------------------

                                      23530     23540     23550     23560     23570     23580     23590     23600

Human                       ACCATAACTTAGGGATAGCTTTATTTGTGGGAAGTGTCATTTAGTATGTCATGTATTAAAAATCATTTGTAGTCTTTCAG 23569
Kakapo                      -------------------------------------------------------------------------------- 0
GoldenEagle                 -------------------------------------------------------------------------------- 0
JapaneseQuail               -------------------------------------------------------------------------------- 0
MediumGroundFinch           -------------------------------------------------------------------------------- 0
GoodesThornscrubTortoise    TATGTAATGTTCATTTAAAGCTCTCTGAAGGGATTTGCCCTGTCAAACTGATAGTTTTAATATCAACATTAAAGATGACA 20033

Majority                    --------------------------------------------------------------------------------

                                      23610     23620     23630     23640     23650     23660     23670     23680

Human                       AGAGCATCTTATCTTTCTTGTAGGTGATAGTATTGGCTATCTATGTAGTGTCTTAAGTTCTTTAAATTATCCTTATTCTT 23649
Kakapo                      -------------------------------------------------------------------------------- 0
GoldenEagle                 -------------------------------------------------------------------------------- 0
JapaneseQuail               -------------------------------------------------------------------------------- 0
MediumGroundFinch           -------------------------------------------------------------------------------- 0
GoodesThornscrubTortoise    AAAATAGAAGGGAAAAAAAACTCTTACTGTCTTTTTCTTTTTAGAGAGGATCTGACGAACTTCTTTCAGGCAGTGTTCTC 20113
```

```
Majority                   --------------------------------------------------------------------------------
                                    23690     23700     23710     23720     23730     23740     23750     23760
Human                      ACTTTTTGTGTACATTGTTTATAAAATAATGTGATCATCTTAATCTACCATTTTGATTATGGTTTGATCATTCTTTTACT 23729
Kakapo                     -------------------------------------------------------------------------------- 0
GoldenEagle                -------------------------------------------------------------------------------- 0
JapaneseQuail              -------------------------------------------------------------------------------- 0
MediumGroundFinch          -------------------------------------------------------------------------------- 0
GoodesThornscrubTortoise   AATAGTCCGAACTCTAACATGAGCAGCATGGTAGTTACTGGTAAGTGTTCCAGAGGAGAAATCTAACATTATATCTCAGA 20193

Majority                   --------------------------------------------------------------------------------
                                    23770     23780     23790     23800     23810     23820     23830     23840
Human                      ATTTCCACTTGTTAGCAAAGCCTTTATACTTACTAAATTATGATAAAAATGATTATTATAACATGCCCGGTTTTACTTAA 23809
Kakapo                     -------------------------------------------------------------------------------- 0
GoldenEagle                -------------------------------------------------------------------------------- 0
JapaneseQuail              -------------------------------------------------------------------------------- 0
MediumGroundFinch          -------------------------------------------------------------------------------- 0
GoodesThornscrubTortoise   ATCTTTTCCTGTTTACAAGGGGAAAATAGTAAAATTAAAATAGTAAAAGTAGATTTCAT-------CCTGTTGTACAAGA 20266

Majority                   --------------------------------------------------------------------------------
                                    23850     23860     23870     23880     23890     23900     23910     23920
Human                      ATGTCTTATTTAAACGTTCTGATTGTTATTCTTTCTTATTCAGGTTTCTTATTGTCATGTTTTATGTCTGTTTTCAGGTG 23889
Kakapo                     -------------------------------------------------------------------------------- 0
GoldenEagle                -------------------------------------------------------------------------------- 0
JapaneseQuail              -------------------------------------------------------------------------------- 0
MediumGroundFinch          -------------------------------------------------------------------------------- 0
GoodesThornscrubTortoise   ATAAATTGTTGGCATGCACCAGACTTTAACAATCCTGTATCTATATTGAAACACTCAAAAAATATTTGAACGAGTAAACC 20346

Majority                   --------------------------------------------------------------------------------
                                    23930     23940     23950     23960     23970     23980     23990     24000
Human                      TAATTGTCCTCTCACAAATGTATCCCATTTTGGGTGACAAAATACCTGATCACCTTATCCTTATAGTTTTCTCCCTAGAA 23969
Kakapo                     -------------------------------------------------------------------------------- 0
GoldenEagle                -------------------------------------------------------------------------------- 0
JapaneseQuail              -------------------------------------------------------------------------------- 0
MediumGroundFinch          -------------------------------------------------------------------------------- 0
GoodesThornscrubTortoise   AATTAAATTAATGGAAATTTACAGATGATGAAACAATTCTTAGGGCTGGGGAACTGGATGGGACATGAAAGAAATTAAAC 20426
```

```
Majority                 --------------------------------------------------------------------------------
                                  24010     24020     24030     24040     24050     24060     24070     24080
Human                    TCTTTTTTGTTATATTAATCAAAGTTTATTTGTGAGAATGTTATGAAATATAAGAAAATGAACATCATGAACCTGTCACT 24049
Kakapo                   -------------------------------------------------------------------------------- 0
GoldenEagle              -------------------------------------------------------------------------------- 0
JapaneseQuail            -------------------------------------------------------------------------------- 0
MediumGroundFinch        -------------------------------------------------------------------------------- 0
GoodesThornscrubTortoise TTTTTAATATGGAATGAGTTATACAATTCTCTTCAAACTAAGGAGTGATACAGAGCAAACAAAGGCTAACAGGAACTGCT 20506

Majority                 --------------------------------------------------------------------------------
                                  24090     24100     24110     24120     24130     24140     24150     24160
Human                    CAAATAAAAAAACTTGAATGT-TACCAATACTATTGTATATTCCATATGCAGCAATATTGCTTTTTTTTTTTTAGAGTCT 24128
Kakapo                   -------------------------------------------------------------------------------- 0
GoldenEagle              -------------------------------------------------------------------------------- 0
JapaneseQuail            -------------------------------------------------------------------------------- 0
MediumGroundFinch        -------------------------------------------------------------------------------- 0
GoodesThornscrubTortoise ATAGAAATTTAACTTTGTCATGTGTAGGCATCCACATGTCTGGGCATCCCTGCTGGATTAGCCTGTTCATTTCACACTAG 20586

Majority                 --------------------------------------------------------------------------------
                                  24170     24180     24190     24200     24210     24220     24230     24240
Human                    TGCTCTGATACCCACGCTGGAGTGCAGTGGTGTCATCTCGGCTCACAGCAAGCTTCGCCTCCCTGGTTCACCCCATTCTC 24208
Kakapo                   -------------------------------------------------------------------------------- 0
GoldenEagle              -------------------------------------------------------------------------------- 0
JapaneseQuail            -------------------------------------------------------------------------------- 0
MediumGroundFinch        -------------------------------------------------------------------------------- 0
GoodesThornscrubTortoise TGGAGTAACTTTATGAATAATTAAAATTCTAATAACTCCTCCGCTAGACTGCCACAGCACTAGACTCTGATCCTAGTTTC 20666

Majority                 --------------------------------------------------------------------------------
                                  24250     24260     24270     24280     24290     24300     24310     24320
Human                    CTGCCTCAGCCTCCCGAGTAGCTGGGACTTCAGGC--ACCCGCCACCACACCCGGCTAATTTTTTGAATTTTTAGTAGAG 24286
Kakapo                   -------------------------------------------------------------------------------- 0
GoldenEagle              -------------------------------------------------------------------------------- 0
JapaneseQuail            -------------------------------------------------------------------------------- 0
MediumGroundFinch        -------------------------------------------------------------------------------- 0
GoodesThornscrubTortoise ACTGCCTTAGGATGGAAGTAGCTTTAGGCTCAGAGCAGCACTTCAGTGCAGCACGTGTTACTGTTCCTTTGTCAAACTGC 20746
```

```
Majority                  --------------------------------------------------------------------------------
                                  24330     24340     24350     24360     24370     24380     24390     24400
Human                     ACGGGGTTTCACCCTGTTAGCCAGGATGGTTTTGATCTCATGACCTCGTGATCCGTCTGCCTCGGCCTCCCAAAGTGCTG 24366
Kakapo                    -------------------------------------------------------------------------------- 0
GoldenEagle               -------------------------------------------------------------------------------- 0
JapaneseQuail             -------------------------------------------------------------------------------- 0
MediumGroundFinch         -------------------------------------------------------------------------------- 0
GoodesThornscrubTortoise  TGGGGACTCCAAATAAATTGGCTTTTCAGTAATTGAAACCTGTTCCTCTTTCTTTTATAACTTCCTTACAGCTATTTCAG 20826

Majority                  --------------------------------------------------------------------------------
                                  24410     24420     24430     24440     24450     24460     24470     24480
Human                     GGATTATAGGTGTGAGCTACTGCGCCCAGCCCAATATTGCTTTTTAATCCCCCTTCTTACCCTGCCATGGGTAGGTGGCT 24446
Kakapo                    -------------------------------------------------------------------------------- 0
GoldenEagle               -------------------------------------------------------------------------------- 0
JapaneseQuail             -------------------------------------------------------------------------------- 0
MediumGroundFinch         -------------------------------------------------------------------------------- 0
GoodesThornscrubTortoise  AGTGTATCTGTCAAAGGAGCTTTGCTCAGCCTGCTGTTTTGGCACACCTACCACTTAGGTCTTTCATTCTGGTGGCAGAG 20906

Majority                  --------------------------------------------------------------------------------
                                  24490     24500     24510     24520     24530     24540     24550     24560
Human                     GCCACAACTTTTGCCTTATTTTTTCAATATGGATTTTTCATGTAAATGACAAAAATATATTTAGTTTTTTTTTAGTTTAT 24526
Kakapo                    -------------------------------------------------------------------------------- 0
GoldenEagle               -------------------------------------------------------------------------------- 0
JapaneseQuail             -------------------------------------------------------------------------------- 0
MediumGroundFinch         -------------------------------------------------------------------------------- 0
GoodesThornscrubTortoise  TGGTTTAGTTCATACCTCTAGTCTGTTAGTGGGTGATCTAAATAATTGTAATCAGAAGACTTAGTTCGCCACTTACTGTG 20986

Majority                  -----------------------------------------------C---------T--A--A-AT---C-----T--T
                                  24570     24580     24590     24600     24610     24620     24630     24640
Human                     GGAAACGGTATGCCTATATAATGTTTTCCATGTCTTGTGTTTTAATTCAATAATGATTCTAATAAATAGCCGTATTTTTT 24606
Kakapo                    -------------------------------------------------------------------------------- 0
GoldenEagle               -------------------------------------------------------------------------------- 0
JapaneseQuail             -------------------------------------------TTAACGGTATCTGTTAGAAAATATTTTCCTTCTTTTT 37
MediumGroundFinch         -------------------------------------------------------------------------------- 0
GoodesThornscrubTortoise  CTAGGTGAATTCTCCACAGCAGTGCAGCAAGGTGGTCTAGTA-ACTCCTGCTACCAGTGGACCATATCAACAGTAATCCT 21065
```

```
Majority                       G-A--------T--T-CT-TCTGAGTTCTTATAATAGTG--GCATAGGTTCAGAGCCTGTATTGTGTTACTCTCAAGGGT

                                        24650     24660     24670     24680     24690     24700     24710     24720

Human                          GGATAGTTTTATAATACTCATTGCTGTGATATACTCTTTT-GTGAAAATACCCATTTTATCTTTTATTGGACATTTGGGT 24685
Kakapo                         ---------------------------------------------AGGTCCTGCCCCTGGGTCGGGGCAATCCCAGGCAC 35
GoldenEagle                    ------------------AACAGGGATATTACAAAAGAGAAACACAGGTTCGAAGATGGCATTACTATAAAGGCAAAGGA 62
JapaneseQuail                  GAAAATTCATATGTTCCTGTAGGAGTCCATAAAATAATCTCAAATAAGCCCAGGAACCTAATACTGTTTCTATGTAAGGT 117
MediumGroundFinch              -------------------------------------------------------------------------------- 0
GoodesThornscrubTortoiseCCAGTAAAAGGTGGTGCTTTCTGAGTGCTAATGACTGTG--GCATAGCTTTAAAGCCACTACAGAGATCCTATAAATTAT 21143

Majority                       TGTTAGAGGTTTGGGAGA---TTAGATA--TTTACTTTAGCCTGGGGTGATGGCTTTGGTATTGTTTGTTAACAATAATC

                                        24730     24740     24750     24760     24770     24780     24790     24800

Human                          TGATGGACATTTGAATAT---TTAAAGATTTTGACTTAGGCCGGGCGTGGTGGCTCAAGCTTGTAATCCCAGCACTTTGG 24762
Kakapo                         AGCTACAGGCTGGGCAGA---GAAGAGA--TTCAGAGCAGCCTGAGGAGAAGGACTTGGGAGTGTTGGTTGACAAGAAAC 110
GoldenEagle                    GGTAAGCAAGACAGGCAG---TGAGATA--GATACATCAGA-TGGAAAGACAGATTTTCTACTAACTGCTAAGAAGTAGA 136
JapaneseQuail                  GCAAAATGGTTTGGGAGAACCTGTGATA--GATGGGATGTTATGAGAGCACAGCTCTGAAAAAGTTCTGCAAAACTAATT 195
MediumGroundFinch              -------------------------------------------------------------------------------- 0
GoodesThornscrubTortoiseTTTTATATTAAAGGAAGATTTCTGGATACCAGTGCTTTACCCAGCTCTCCTGGTTTCCTTTTTGCTTCTGGACAATATTC 21223

Majority                       AGATTTTGTGCCTCTACATTTTAAGGTCGAGTGATGAT---ATGCTTGGCTTACTTAGTGTCATCAACATCACCGT----

                                        24810     24820     24830     24840     24850     24860     24870     24880

Human                          GAGGCCAGGGCGGGCAGATCACAAGGTCAGGAGTTTGAGACCAGCCTGGCCAACATAGTGAAACCCCCATCTCTACTA-- 24840
Kakapo                         TGAACATGAACCGCTTCAGTGTGTGCTTGAGCCCAGAA---ACCCCCCGTGTGCTGGGCTGCATCAACAGGGGCGT---- 183
GoldenEagle                    AGGATTAGTACTTCTACCAATCCAAAACCAGACAAGAT---GTGTTTAGATTTATAAGGGGGAAAAACGACAAAAA---- 209
JapaneseQuail                  ACATTTTCTGACATCAAAGTGTAAAGTGGAATGGTGACTTCATATATCACTTACGTGACCTTCTAAAAATGACCGT---- 271
MediumGroundFinch              -------------------------------------------------------------------------------- 0
GoodesThornscrubTortoiseCTATTTCATTTCTAGGTATCTTTGGACACAATAATAGT---ATGCTTGAGTTAGAAAGTGTCCTCCTTGCCATCTTTTCT 21300

Majority                       --AAGCTCCATTTCAGGGCAGGGGATCCTGATTGTTTCCTTTGCTCCGGATGGCTCCCTATTTAAGCACCGTGTGAACTT

                                        24890     24900     24910     24920     24930     24940     24950     24960

Human                          --AAAATACCATAATTAGCGGGGTATGGTGGTGGGTGCCTGTAGTCCCAGCTACTCAGGAGGCTGAGACAGGAGAATCGC 24918
Kakapo                         --GAGCAGCAGGTCGAGGGAGGGGATCCTGCCCCTCCGCTCTGCTCTGGTGAGGCCCCACTTGCAGCACTGTGTGCAGTT 261
GoldenEagle                    --AATCCCCAATACAGTACAAGAGAAACCAACTGTAACCCTAACCTCCAATGGTGCAAAGTCTACTAACCATGCTAGAAT 287
JapaneseQuail                  --GAGACACATTTTAGGCCTAATTGTCAAAATCTGATCTTTTGATCTGTATGGCTGCTTAGTAAAGAGCAGTATGAACTG 349
MediumGroundFinch              -------------------------------------------------------------------------------- 0
GoodesThornscrubTortoiseTTATAGTCTTTTCCAGCTTCTGGGAAAAACTATGATTCCTTCTCCCCTGCTCCCAAGCTTTCTGTGCATCAGGACTAAAT 21380
```

```
Majority                    CTATGTTATTGAGTCGTAGATT--GAGTAAAGAAGTAACACTGCTTTCCATTCCTACAGATGCTTTAGACTGAGTCTCAT

                                      24970     24980     24990     25000     25010     25020     25030     25040

Human                       TTGAACCCAGGAGGCGGAGGTTG-CAGTGAGCTGAGACCGCGCCATTACACTCCAGCCTGTGTGACAGACTGAGACTCCA 24997
Kakapo                      CAACGTAAGAAGGACATGGAAC--TGTTGGAGAAGTCCAGAGG-----AGGCCACGAGGATGATCGGGGCTGGAGCACCC 334
GoldenEagle                 CTACAGGAATGATATCTATATG--GAGTAAATATATAAAAATGCACTGGATTTATAGAGATGGTTATAAGCAATTCTAAA 365
JapaneseQuail               TTGTGTTTTGGTATTTAAGACT--GTAGAGAGCAGTAACACTCCTTGCCCTTCCAAAATATGCTGTCTTCGAGGAGAAAT 427
MediumGroundFinch           -------------------------------------------------------------------------------- 0
GoodesThornscrubTortoise    CCATGTTATTTAATCGAAGATTCTAAACAAAAAAGCTATATTACTGTCAAGTTTTACTGCTACATTACAGTTAATGTGAT 21460

Majority                    TCTCTAAATAAGCCAATGTAAAATACCAGGTTTGAACTGCTTTAACA-ATACAAGCTGTGTTGAGGACTACAACCATATA

                                      25050     25060     25070     25080     25090     25100     25110     25120

Human                       TCTCAAAAACAAACAAAAAAAAACAACAACAAAAAAAGATTTTGACT-TTAAGAACAATACTGCTGTGAACTTCTTTGTA 25076
Kakapo                      CCCATACGGACACAGGTTGAGAACATTGGGGCTGTTCAGCCTGGAGA-AGAGAAGCTGCGTGGAGGCCTTAGAGCAGCTT 413
GoldenEagle                 TCTCTAAAGTCCTCAATGTTACTTTCAAGGATAGTACTTCTTTAACACATGCAATCCGTAAAGAATACTACAACCAGATA 445
JapaneseQuail               ACTACAAATAAGATACTGGAAAATACCCTGTGGGACTTGGACCAACA-AGACAAGTTCTGATCTGAACTCGAAAAAAATA 506
MediumGroundFinch           -------------------------------------------------------------------------------- 0
GoodesThornscrubTortoise    TTTCAAGTGAGCCAATATTAAAACACAAATTTGAGAGAAATAAATTAGTTTAAATTGACTGCAAAATGGAAATGTTTCT 21540

Majority                    CTTTTTTCTGATTGGAGCTATGTCAGTGTTTCTTG-AGAGGATATTAGCATGTG--GGAATATATTCATTGTACACAGAC

                                      25130     25140     25150     25160     25170     25180     25190     25200

Human                       CTTGTTTCCTGGTGCATTTATGCAAGCGTTTTTGAGAGGGTATATTTAAAGGTATAGGTATATATTACTAGTGTGTAAAA 25156
Kakapo                      CCAGTGTCTGAAGGGGGCTACAAGGATGCTGGAG----AGGGACTCTGCATCAG--GGACTGTAGTGACAGGACAAGGGG 487
GoldenEagle                 CTTTTTTAAAAAAAAAAACATAACAATGTTGAAAGCAAAAAAGAATAGCATTTG--GCATGATGAACTTTGTATACAATC 523
JapaneseQuail               CTTTTACTTGGTACAAGGCTTGTAAGAATTACTTGAGTAACTTCAAAACTTCAAT-AAAAGAAAGTAAATCAACACAGAC 585
MediumGroundFinch           -------------------------------------------------------------------------------- 0
GoodesThornscrubTortoise    TTCTTCTCAAATTGGTCCTTCTTCCCAGGCTCTTATAGGGGCCACCAGCTGGTTG-TGAAAGACTTCATTGTACTCAGAT 21619

Majority                    TGAA-TGAATGAAATGTTAATGAAGGATTTTGTAAGATAGAGTTCATCCATATGTTCTGCCCTGTAGTGATTTTTGGTC-

                                      25210     25220     25230     25240     25250     25260     25270     25280

Human                       TAAACTTTATGAGACAGTACTAGATTATTTTCCAGTGTATTGTTCCTATTTATGTTCTCACCAGTAGTGCGTAGGAGTTC 25236
Kakapo                      TGA--TGGGTTCAAACTGAACCAGGGGAAGTCCAAGTTAGATCTCAGGCAGAAGTTCTTCCCTGTGAGGGTGCTGAGGC- 564
GoldenEagle                 TAAG-TAAATGAAATGTTTAAGAGGGATTTTATAAGAATGAGAATAACAAATCATTCAGTCTAGTGGTCAGCAATGGGT- 601
JapaneseQuail               TGTT-GGAAAGAAATGTTGATTAAGGACATTGTTTGCAGGAGTACAAAAAGATGAATAGCTACTAATAAATTTTAGGAC- 663
MediumGroundFinch           -------------------------------------------------------------------------------- 0
GoodesThornscrubTortoise    CGAA-CAAATGCA---CTAAAGAATTTTGTTGAAAAATATTGATTTTCCATACTATTGGGCCTTAAAACAACTATGGTC- 21694
```

```
Majority                    --GCTGTTTCAAGTCGCCAAGAGAATTTGT---GGTTTCTGATTTC-TAATAGTGTTCAATTTTAGGTGGAATA----TT
                                    25290     25300     25310     25320     25330     25340     25350     25360
Human                       TTGCTGTTTCAGATCCCCACCAGCACTTGGCTAGGTATTTAATTTCTTAATTTTTAACAATTTGGTGTGTAGTATATTCT 25316
Kakapo                      --GCTGGCACAGGGCGCCCAGAGAAGCTGT---GGCTGCCCCATCCCTGGCAGTGTTCAAGGCCAGGTTGGACA----CA 635
GoldenEagle                 --GCTGAAGGCAGTTGCTGAAAGAGAAGGT---AGTTGCAGATATCCAAATGATATTGACATCTAGGAGAAGCAA---TA 673
JapaneseQuail               --TTTGGTCAAATGCATCTAGGCGATTTAT---GAATTATGAAGT--TGATAAGAACTGATTTAAGATAGAATA----AT 732
MediumGroundFinch           -------------------------------------------------------------------------------- 0
GoodesThornscrubTortoise    -AAGATTTTAAAATGTGATTAGAGTATCC---AACTTAAAACACC-TAATAGGGCTCAGTTTTCAGAGAAATGGGGTTT 21768

Majority                    TTGGT-----TTTGTGCAACCTAGACTATTGAATGGTGTCTACAA-------G-CTTTACCATTATAGGCATATGAAAAT
                                    25370     25380     25390     25400     25410     25420     25430     25440
Human                       CTGGTCTTCCTTTGTCTTACCCTGATTATTAATGAGGTCTTACATCTTTTTTCCCCCCACATTTATGGCCATGGATGTTT 25396
Kakapo                      GGGGC-----TTGGAGCAACCTGCTCTAGTGGAAGGTGTCC-------------CTGCCTGTGGCAGGGGGCTGGAACT 696
GoldenEagle                 TCAGT-----TATTGTCCACCTAGTAAATTGATTGCTGCACAGAATCAGAGTGACTTTTCCAGAACAGGACAACAAAAAG 748
JapaneseQuail               TTGGT-----TGAATGCAACAAAGACTGGAAAATGAAATCATCAA-------G-CTTTACAATTATATCCAGAGGAACAA 799
MediumGroundFinch           -------------------------------------------------------------------------------- 0
GoodesThornscrubTortoise    CGTAC-----TTTGAGAAAATCAGAGCCTTTCAAGGTGTCTCAAGGTAGGTCTTCAGAATCATTAGTCACTTTTGAAAAA 21843

Majority                    TCATATTGTGTAATTTTATTTTCTAGTTAAACCTGTTTGTTGTTTTAATAAACTGGATGCTTTTAATTTCAG---TTATG
                                    25450     25460     25470     25480     25490     25500     25510     25520
Human                       CTCTTTTGTGAAATTCTGTTAAATGCCTATTTGTGTTTTTTGC-TTGATAAAATGTGAGCTTATTATTTAACTGATTTTG 25475
Kakapo                      GGATGAGCTTTAAGGTTCTTTCCAACTGGGACCAGTCTGGTCTTACACTAACCTGAAGAAATTAAATTACAA---TCAAG 773
GoldenEagle                 TCACAGGGTAGAATGAGAATTTGCAGGTACTCCTAATTGTTAAGTTAGTTACTGGGCTGCTCATGGGGTCAG---TTAGA 825
JapaneseQuail               ACGTATTAAGCAACACCATTCACTGTTAAAATATAAGGAAAGCAAAGAGATAATGGAGGCATCTAGTTTTGG---TCATC 876
MediumGroundFinch           -------------------------------------------------------------------------------- 0
GoodesThornscrubTortoise    TCTTAGCCTATACTTTTATTTTACAGTCAAACTAGCCACAAGTTTCAATGAACTGAATAATCTTAACCGGAACTCTTCTA 21923

Majority                    TAATGTGCAGATAG-GCTGGGGTTGTTAAATTTAGTCTTTAATCTCGGATTATGGAGATGTTGATAGACTAGAATTTGTT
                                    25530     25540     25550     25560     25570     25580     25590     25600
Human                       TAGTTTTAAAAAATTGCTGGGGGCGGTGGCTTGCGTCTGTAATCTCAGCTTTTTGGGAGGCTGAAGGAGGAGGATTGCTT 25555
Kakapo                      TATTGTGGTAATGG-GCTACCTCCCCGAAATGTAGTTTTTAAACCCGTATT-TCCAGATCAAACTATACTAAAAACAGAA 851
GoldenEagle                 GGAGAGGCAGGCAG-ACAGGGGTAGTAAAGCCTAGTGAAAATTTTCTGATTATGGAGATATGCATAGCCTTTTGGCTCTT 904
JapaneseQuail               CAAACAAATGAAAA-GCTGTTACTGTACAAGTATATCCAGCAGCAAAAGAAATAAAATTCTTGACAGGAGTCAGTATGTT 955
MediumGroundFinch           -------------------------------------------------------------------------------- 0
GoodesThornscrubTortoise    TAATGCCCAGATCC-GTTGCCCTTTCTAATGGTAATAATAAATATTGGAAAAAGAGGAGGGAACTCTAAAAGAAATATAA 22002
```

```
Majority                    GATGTTTTAAGAAGGTT-----TGACTGCAGTATTTTGTTTGAGATTTTTTA-AGGGTGTTTTGGTAACCTGCTCAGATT

                                    25610     25620     25630     25640     25650     25660     25670     25680

Human                       GTGGGGCCCAGGAGTTTG----AGGCTGCAGTGAGCTATGATCGTTCCATTGCACTCTAGCTTGGTGACAGAGCAAGACT 25631
Kakapo                      GATGTTTTGAATAATTT-----TTAATATCTTTCTAAGTTTGGGATTTTTTA-GGAACGCTTGGATCACCTTTTCAAATT 925
GoldenEagle                 AAATTCTTTATCAGGCT-----CTATTGCAATATTTTCAGTGACAAACTCCA-AGGGTCTTGCAGTAACCTGAAAAAATT 978
JapaneseQuail               TGTGTTTTAGGATTGAT-----TGACAACACGATATCGTCTCAAAAAAAAAA-AAAAAGTTGAAGTTCTCTGCCCAGATC 1029
MediumGroundFinch           -------------------------------------------------------------------------------- 0
GoodesThornscrubTortoiseGAATCCCTAACAAAGATGTTCCTGAATGCAGTAATATAATTGTCATTTTCAACGGGGAGTCTTGAAAATATCCTTGGATA 22082

Majority                    TAATTTGAATGAATTCAT-AAAACATTAGCTG-TTATACATTCTATGATTGTTCTTTGACATA--TCTTTTTGCAGAAAT

                                    25690     25700     25710     25720     25730     25740     25750     25760

Human                       TTGTCTGAAAAAAAAAAAAAAAAAAGTGTGTG-TTTTACAGTTACTGATTGTCTTTTGGTTAT--TTGTGTTGCTGGTGT 25708
Kakapo                      ---------TTACTTCAT-ATGACTTCAGGTTTGAATGCATTCCAGGATCGTTCCCGTACACA--GCTCTTTGCAGAAAC 993
GoldenEagle                 AAAGTGCAATCAAGTAAT-GGGCTTCCTCCTCAAAATCTATTTTAAGAACCAGCATTGCCAGA--TCAAAATACAGAAAA 1055
JapaneseQuail               TCTTGAGACTGAGGTGAT-ACAAGATTAGC---AGGTACATTCTTTGATATATGAATAACTTA--TTATTTATCAATTAT 1103
MediumGroundFinch           -------------------------------------------------------------------------------- 0
GoodesThornscrubTortoiseAAACTTTCAGGAATTCATCAAAACATAACCTGTTTCCTCAGTCCAGCTCTCTTATGAGACATAAGCCTCTGAACAGAAAA 22162

Majority                    TATTTCTGAATTACTGTATGTTTTTTTATGAAATTTTGAGGCTGGGTTGTCTATGTGTCTATTGAAGCGGGA----ATTT

                                    25770     25780     25790     25800     25810     25820     25830     25840

Human                       CTTTTCCCAGTT--TGTAGCTTGCCTTGTCACTTCTTTATGCTGTCTTGTCTGTCTGTCTGTCTGTCTCCCTCCCTCCCT 25786
Kakapo                      TGTCTCTCACACAGTGTAAGATTTGTGATGAAATACTGAGACCTGGAT------GTGAATAATGAAGGGGAA----AAGT 1063
GoldenEagle                 TACAGAAGATGTTTTGAATATTTTTTAATACACTTCCGGGTTTGGGATCTTCTAGGAACATTTGGATCACAT----TTTC 1131
JapaneseQuail               TAATAGTGGATTACTACAAGATTTTATATAAAGGTACAAGAAAAAATAGTCTATATTTTTACTGAAGAGGGAGGTCACTT 1183
MediumGroundFinch           -------------------------------------------------------------------------------- 0
GoodesThornscrubTortoiseTATTGACTAACTCCCACATCTTGTCTGGTGAGATCTAATGGACAATCCTTCCAGCTGTATCCAGAGCCAGGAAAT-ATTT 22241

Majority                    GTATTTCCCTGCATGCGTGTGTACTGTTCAGTTTGTTCTTTTACGAC--GTCGTATATAGGGTGCTTCTTGGAATGGTTC

                                    25850     25860     25870     25880     25890     25900     25910     25920

Human                       CCCCCTCCCTCCGTCCGTCTGTCCCTCCCTCCCTCCCTCCCTCCCTCCCTCCCTCCCTCCCTCCCTTCCTTCCTTCCTTC 25866
Kakapo                      GTATTTCCAAGCAGCCACGTGGAGCGAATGGTTTGTACCCTGACGTG--GTAGTTTACAGAGAGCTTCTGTGACCGGTTT 1141
GoldenEagle                 AAATTTTCCTTCATAGGTATAAAATCCTTAGTTTGACTTTTTGTTAA--ATCCAATCTAGGATTGTCCCAGGAACAGCTC 1209
JapaneseQuail               GTAGCATACCACAGGGACCTGTGCTGTTCAGCATATTCTTAAATGAT--GTGGAAAAAGGAGTGATTATTGAGGTGACCA 1261
MediumGroundFinch           -------------------------------------------------------------------------------- 0
GoodesThornscrubTortoiseTTCCAGCTTTGCAAGCGAGAGGTCAGTACAGATTCTTCTTCAACCACTCATAATGTAAATGATCAACTAAGGTATAATTA 22321
```

```
Majority                     AGTCTG-----GAAGCTAAATTATAGATTATTG--TAGTTCTGATGGTTTACTACGGAGACTTATGGTATAAAGTCTCGA

                                     25930     25940     25950     25960     25970     25980     25990     26000

Human                        CTTCCTTCCTTCCTACTTTACTTTAGATTCAGGGTTACATGTGCAGGTTTGTTACATGGGTATATGGCATGAAGCTGAGG 25946
Kakapo                       GGGTTG-----GAAGGGACATTAAAGATCATC---CAGTTCCAAGCCCCTGCCACAGTATCTCAGTGTTTAACATCCCAA 1213
GoldenEagle                  TTTCTA-----GAAACTCGGTTATCAAACATTG--TAGGATTTGTGATGAAATACTGAGACCCGGCATAAAAATACTGGG 1282
JapaneseQuail                AGTTTGCTGATGATGCTGAATTATTCATGGTAA--TAAAAACAAAAGCTGACTGTGAAGAATTGTGGAAAGCTCTCATGA 1339
MediumGroundFinch            -------------------------------------------------------------------------------- 0
GoodesThornscrubTortoiseAGCCTA-----AAAGGAAAACCAAGCATTTTTC--TTATTCAGATTCTTGACCAAGACTACTTAAGGCTTATAGGTTCGA 22394

Majority                     TAT----TACAAG----CCTGTCTGCTAA--TGTCAGCTTAATACGTACCGTTTGGATTTATATTCGTGTCTCTCTGCGA

                                     26010     26020     26030     26040     26050     26060     26070     26080

Human                        TTTGCAATACAAGTGATCCTGTCAGCCAGAGAGTGAGCTCAGTACCCAACAGTTAGATTTACAATCCTTTCTCCCTCCCC 26026
Kakapo                       CAT----TAGAAG----CTTCAGGCTTAA--TGTCTCCCTTAGACAAACTGTAGGGGCTCATTCGCGTGCTTATCAGTGT 1283
GoldenEagle                  GGG----AAAAAG----CCTATTTCCTAA--TGGCCATGTAGAGCGAACAGTTTGTACCCTGATACG-GTAGTTTAGAGA 1351
JapaneseQuail                TAC----TCCATG----ACTGACTGATAAAATGGCAAGTTAAATTCTGTCCTGAAAAATGGAAGTAATATGTAAAGGAGA 1411
MediumGroundFinch            -------------------------------------------------------------------------------- 0
GoodesThornscrubTortoiseAATGTCATTTAAA----AATGTATTATAGGTCAACAGACTAATAGGTTCATCTCTAATCTATCTTCTGGCCTCTCTGCCA 22470

Majority                     GCACCATTAGTTCACCAGTTTATA-TGTCGAGGAGTCGTACTTATTCTGATTTATTAGTTTTAAGGTGACTTTAAATGTT

                                     26090     26100     26110     26120     26130     26140     26150     26160

Human                        CCACCACTAGTTC-CCAGTGCATATTGTTGCGGATTAATACAGATTCTTAATGTATAGTTTTAATGTGGCCACAAATTCC 26105
Kakapo                       GCTTGATAAGTTGATCTCTTTATA-ACTCAGGAGGTCGTATTTATTCTGATTTATGTGGGAAAAACAGACTTTCAAAGAG 1362
GoldenEagle                  GAACCATGGCTACACCAGTTTCTG-TGAAGAACAGCCTAACACACTGCAATAGTTAATTTTTATGTTCATCTGAATTATT 1430
JapaneseQuail                AAAAAAAAACAAAGCCAAGCTATACACACAAAATGCTGGAATCTAAATGAATTACTACCATGCAGGAAAGAGCTTAAGTT 1491
MediumGroundFinch            -------------------------------------------------------------------------------- 0
GoodesThornscrubTortoiseTTGAATTTATATATATGTGATAGTATTGACATGAGTCAGAGTTCTGCTATCACACTCAAAGCATCATGACTATGAAGCCT 22550

Majority                     CTTTGAAGCTAAGATATTATTCTTTTTCA----GCTTCCAAAGGTTGTATACT-AAGAA------TGGCTCTTCTATAAA

                                     26170     26180     26190     26200     26210     26220     26230     26240

Human                        CTTTGCATCTTAGATAAAAATAATTTATA---TGATCCCATTCTTTACACATTCCAAATTTTCCTTTGCTCTTCTGGACA 26182
Kakapo                       CCGTAAAGCCCCACGGATACTTTCCCTCA----GCATCCAAAACGGGTGCAC--AAGA-------TCCCTCTGCTATGTA 1429
GoldenEagle                  TTTTGAAATAGTATTAGTGGTCAGCTATA----TAGTCAACAGGTTGTTTCCTTAAGAA------TGACTGTGTAGCCGA 1500
JapaneseQuail                CTGTGAAACTAACAGCTCATTGTTTAGCA----GCACTCAAATGTGGAAATC---AGAC------TGGCTTTATCAGAAA 1558
MediumGroundFinch            -------------------------------------------------------------------------------- 0
GoodesThornscrubTortoiseTTATAAAGAGAAGATATAATCCTTGATCACTCTGTTTGTAAAGGAAGCATGATTTAGAACAG---TAGCTACTCCATAAC 22627
```

```
Majority                  ACTCA----TTGAGTGATGGATCTAGTGCACGCTTTTACAATTTGTAGTTTTAAAGCATTTGGCTTTTT--GCTTTGTGC

                                 26250     26260     26270     26280     26290     26300     26310     26320

Human                     TCTTCCATGTTGATTGCTGGAAGTCTTGAAGGCAATCCCAAATAACAGTTTTTAGCTTTTCAACTGTTGCTGTGATACGG 26262
Kakapo                    CTTCA----CTGGATGTTTCATCAAGTAAATGCTTGTTTTAAATGAAAGCTTAAAACATCGTAATTCTT--CCT--GTGC 1501
GoldenEagle               AGGCA----CTGAGTGATGAGGACAGGGCTCCCTGTCAGGGTTGGGTCCTCTGAGGGGACGGGGTGCCCGAGCCTGGCGC 1576
JapaneseQuail             AATAA----TAGAAAAACAGAAATAAAACAAGAAGAAAAAATAAATGATATAAAAGCATTTGGTTTGTAACGAATAATGA 1634
MediumGroundFinch         -------------------------------------------------------------------------------- 0
GoodesThornscrubTortoise  ACTC-----TCGAGAAATGGCTCTGCTTCCCTCTTTTGCACCGTGTAGTTCTCAAGAACTTCTCTTTCT--GATTTGTCC 22700

Majority                  TGCCTTTCA-AGTCTTTTATAGTGACGTTAAGAGGGGTTTTTATGAAGCCTGGCTGTTTTGGCGTCAAATTCGAAAAGTC

                                 26330     26340     26350     26360     26370     26380     26390     26400

Human                     TTTCTTATAGAGATTTTTGTAGTAGAATTTATCAGTATTTTCTTTCATGTTCAGTTTTCTTGTGTCTTAATTTAAAAATC 26342
Kakapo                    TGCCTATGA-AGGATATAACAGTGACTTAAAGTTGGTATCTTATGAAGCCTGGCGGCTGTGCCGACCCTTCCAAAACAGC 1580
GoldenEagle               GGCCTCTCT-GAACGTTTTCCATGACGAGAAGGGGCAGGCTCCCGGCGCCGGGAAGTTTTGGCACCGAGCACGCAAAGTC 1655
JapaneseQuail             GGATCGTCACAGTCTTCTAGAATAAAATTAGGAAAGGTGTGGAGTAAGGCTAATTGAATTACTGG-AAATATGGAACGAC 1713
MediumGroundFinch         -------------------------------------------------------------------------------- 0
GoodesThornscrubTortoise  TGGTCTTCATTTTCTACTATTCCTGGGAAACAAGACGTATTTATGAC-CTTGTCACTTCTGTCATCAGATTCCATAATAC 22779

Majority                  TTTCCATACTGCAATCGCTAAATTGTA-TATAGATTCTGATTGAAGTTTAAGGAGCTGT-CTGCATGCATTTACCCCATT

                                 26410     26420     26430     26440     26450     26460     26470     26480

Human                     CTTACATACTCAGAGTTCATAAAGATACTATATTTCTTCTGAAATTTTAAAAAATTTTGCTTTTTATATTTATCAGAAA 26422
Kakapo                    CTAACCCACTGCAATAGCTAATTTGTA-CGTTCATCTTAATTACTGTTTGAGGTACTAT-TAAGAGGCCAGAAGGCTGTT 1658
GoldenEagle               TCTCCGCGCAACAAGCGCGGTCCGGCGGTCCAGACACCGCCCGAGGACTGAGGAGCCCC-CGGAGCGGGCCTGCCCCCAC 1734
JapaneseQuail             TTCCAATAGAGGAGAAGTTAAATTTACAAAGAGTCTCTAAATTAAATTGAAGGAGGTGT-ATGCATTCATGGACGGCATG 1792
MediumGroundFinch         -------------------------------------------------------------------------------- 0
GoodesThornscrubTortoise  TGTCTAAGCTGTTGTCTTCATAGAATA-AAAGAAGGAAGAGGGCAATTCATATTTCTGT-AACCAGGATATGACCTATTT 22857

Majority                  GTTATACGTAGGTCTCGAGTTCCCTAACCATCTGGTTGCGTGAATTGGCGGTTTCCGAGG-GGTGTTTCTCCTTCGCACA

                                 26490     26500     26510     26520     26530     26540     26550     26560

Human                     ATTATTTTTGTGTATTGTATTTAGTAGGGATCTGGTTTCATTTTTTTGCCCTTATTAAGATAGTCAATTAAATTGAAATC 26502
Kakapo                    TCCTTACAAACGGGCCGCGTACCCGAAGCACCGGGGGGCTTGCCCTGGGGGTGCCCGGCCTGGCGCGGCCCCTCTGCACA 1738
GoldenEagle               CGCCCCCGGCGGCCTCGGGCGCTCTCCCCGGGCAGGACCGTGAGTCGCGCTCCTCCGAGGCGACCTCTCTCCCTCGCGCA 1814
JapaneseQuail             GTTAGGAGAAGACAGAAAGTTACCTATCTATTTTGTTAAGACAACTAACGGATTATAAAG-GATATTATTAGGCAGCAAG 1871
MediumGroundFinch         -------------------------------------------------------------------------------- 0
GoodesThornscrubTortoise  CGTGAAAATACATCTGAAAACAACTTACCTTCACACAGCCCAAAGAGTCTACCTACTACT-AGTGCTACCCCAACAGTGA 22936
```

```
Majority                      TTTGTAATAAGTACTTAGACGTTCGTTTGTCTCTGTGTTAAGGGTTCT--CTCCCGACTCGTTGTTCTGTGATGCTGTGG
                                       26570     26580     26590     26600     26610     26620     26630     26640
Human                         TCTGTAATGACTTTTTCATATTACAGGTATATATATCGTAAGGCAAAGGCTGAAAAAACTATCATAACAAAATGCAGAAT 26582
Kakapo                        ATTCCATGAGGAGACGGGACATGCTCCCGGCGCCGGGAATTGTGTCCCGGCTCCTGAGGCGGGAAGCGGTTACGCCGGGG 1818
GoldenEagle                   CGCACAAACGCTCCCGAAGCGGTGCGCTGGGGCGGGAGGAGCGGGAAC--CCCCCGCCTCGCTGCGCGGCGGGTCCCGGG 1892
JapaneseQuail                 CTTGAAACAAGCAGTAAGAAATACGTTTAACACAGTGTTTAACTATGT--TGTGCAACTTGCTGTTTTGCAATGCTGTGA 1949
MediumGroundFinch             -------------------------------------------------------------------------------- 0
GoodesThornscrubTortoiseTTCATAATAAAGACTTT--TGTTTGTTTGTTTTTTTTTAAAGCCTTCT--CTCTCCTTTCTTCCCTCTCTGGTCCTATAC 23012

Majority                      CTGGTAAGGGAGATAAAATCGTTTTTGTGCGC-CTTTCCCTCTTCCCGCCCCAGGCGGGGCAGGATCGGGGCATCTT-CG
                                       26650     26660     26670     26680     26690     26700     26710     26720
Human                         ATGGTGGCATAAATAAAAGAGTTTTTTTGTTT-CTTTTTCTGTCATAGCAGAAGTCCTGTTGGTATAAGAAAGTGGTATC 26661
Kakapo                        CAGGAATGGGTGTCGTTGTCCCCCCTCCCCGCCGCCGCCAGCTTCCCGCCCTCGGCGGGCCTCGATCGCGCCACCTCCGG 1898
GoldenEagle                   CGCGGGCCGGCGAGGAACCCGCTGCCGCGCCCGCCTTCCCGCTTCCCGCCCCAGGCGGGGCAGGCTGGCGGCGCCTCGCG 1972
JapaneseQuail                 ATGCCAAGAGGGAGAGGATAAACCTGGTCCG---TTTAATACCCCCCGCCCCAGGCCGGGCAGTGTCCGGGCAGCT---- 2022
MediumGroundFinch             -------------------------------------------------------------------------------- 0
GoodesThornscrubTortoiseCTTGTAAAGCACTTAAAAACATTTTTGTAAAAATAAAAAATAATTTCACGACAAGAAGCAAAAGATCAGATTATTTTTCA 23092

Majority                      C-TCTGCT--ATGGCGGCAGCTTCAGGGTCGGGTA--TTTCACATGCTCGGACGGCTGGTGCCTGCTCGGTTGGCGTTTG
                                       26730     26740     26750     26760     26770     26780     26790     26800
Human                         AGTCTGCTCCATAAGGTTATTCACTGGGCCAAGTTTCTTTCATATTGTTGCTTCACTGATCCCTAGGCTTTTGTTCTTGG 26741
Kakapo                        CACCCGCCTCATGGCAACGGCTTCAGCGCCGCCCG--GGGCGCATGCTCGGGCGGGCGGCGCATGCTCGGTTAGCGGCGG 1976
GoldenEagle                   ACTCTGCTCGGCCCCGGCAGCT--GATGGCAGAAA--CTTCAGCGGCGCCGACGGCGCATGCCCGCGCGACTGGCGCATG 2048
JapaneseQuail                 -----G----ATGGCAGAAGCTTCATGGTCTCGTA--CTGCGCCCGCTCGGAACTCTGGCGCATGCTCCG-T-------- 2082
MediumGroundFinch             -------------------------------------------------------------------------------- 0
GoodesThornscrubTortoiseCTTACACT---TGTCTAAGATTTTAAAGTGGGAT---TTTCAAAGGAACCCAAGGGAATTAGGCATCCAGCTCCCATTTA 23166

Majority                      CTCGGGAGGGGGTTGAC-GGGGAGCGGACTCCGGCCCTCGGTAACGTTCGGCGTTTTCCGTGCAGGCCAGGCCGCCATTT
                                       26810     26820     26830     26840     26850     26860     26870     26880
Human                         CTATGTGGTCTGTTGATGTGTGATTGTGCTCTTCTCCTTGTTTAAAAAAAAAGTTTTCCCTGTATTTGTGTATTTCATTG 26821
Kakapo                        AGCGGGAGGGGGGCCCC-GGGGAGCGGAGCGCGGCGCTCGGTAACGCTCGGCGGTTTCCGTGCAGGCCAGGCCGCCATTT 2055
GoldenEagle                   CTCCGTAGCGGCGAGG--GACGAGCGGAATCCGGCACTCGGCAACGTTCGGCGGTTTTCGTGCAGGCCGGGCAGCCATTT 2126
JapaneseQuail                 ---GTGAGGCGAAGGAC-GAGGAGCGGAATCCGGCCCTCGGCAACGTTCGGCAATTTCCGTGCAGGCCCCGCCGCCATTT 2158
MediumGroundFinch             -------------------------------------------------------------------------------- 0
GoodesThornscrubTortoiseATTCAGTGAGACTTGAGTACCAAATTTCCCTAGTCTCTCTTGAAAATTC--CATTCACAATTCTCTTCAAATAGACGGTG 23244
```

```
Majority                  TCTGACAGCGTTCCCGTCCGCGTTTGCTGCGCGGCTGTCTGTCCCGCTTGTCAGCGCTTTGTCC-TGCTCGGCGAT---G
                                   26890     26900     26910     26920     26930     26940     26950     26960
Human                     TCTTTTTTTTTTAAAGTCAGCTTTTGATTTTACTGATCCTCTTCATTGTACCATTGTTTTCTCTATTTTTATTGGT--TG 26899
Kakapo                    TCTGACCGCGTTCCCGTCCGCGTTGCCCGCGCGGCTGCCCGTCCCGCTTGCCCGCGCTCTGTCC-TGCTCGGCGAT---G 2131
GoldenEagle               TCTCACAGCCTCCTCGTACGCGTCGCCTGCGCGGCTGTCTGTCCCGCTTGTCCGCGCTTTGTCC-TGCTCGGCGAT---G 2202
JapaneseQuail             TCTCGCAGACTCTCCGTGCTCGCGTGTTGCGCGGCTCTCCGTCCCGCTGGTCAGCGCTTTGTCC-TGCTCGGCGAT---G 2234
MediumGroundFinch         -------------------------------------------------------------------------------- 0
GoodesThornscrubTortoiseAATGATACGGCAGATAGACACCTTCTCTTTCCTTCAAGGATTTCTGTACGTTAACCTTTTCTCC-TATTTTTTCATATTG 23323

Majority                  GACGGGTGAGT-GTGAT-CGAGGCGGTG------A---G--A-T-----G-CTTCGTCGCCGG-TCGGTGGGGGCGACCT
                                   26970     26980     26990     27000     27010     27020     27030     27040
Human                     CGTGAGTCAGGGCTTTTGCAAGTCTTTAATAGGTAGGTGGTATTCAGTCCACTTCTTCATCATCTTGGTGGCACCTCTGT 26979
Kakapo                    GACGGGTGAGTCGCGAT-CGCGGCGGTG--------------------G-CACCGCCGCCGG-TCGGTGGCGTCGACCT 2187
GoldenEagle               GACGGGTGAGTAGTGAG-CGGGGCGGTGTGGGCGAA--GGTACTGCCGCGGTTGTGCCGCCGG-TCGGCGGTGG-GACCC 2277
JapaneseQuail             GACGGGTGAG-----------AGCGACG------------------------GCCGACGCTGG-TTGGGGGGGGTGGGAT 2278
MediumGroundFinch         -------------------------------------------------------------------------------- 0
GoodesThornscrubTortoiseTCTGGGTGACTTGCAAAATAAAACGAGGTAAAGAATCTGTAATTCTTATTGCATCATTATTGG-CCTCACAGGCCATCAT 23402

Majority                  GTCCCGCGGCCAGGG---CGC-------------------------------CGTCTCGGTGGGTGCGGGGAGC-GGCCG
                                   27050     27060     27070     27080     27090     27100     27110     27120
Human                     TTAAGGAGACCAGGAATACTCACCTGGGTACGTACCTTTCTTCCTCTCTGATAATCTATTTTTCCTCAGGAACTTAATAT 27059
Kakapo                    GTCCCGCTGCCAGGG----GC-------------------------------GTCCCCGAGGCTGCGGGGAGC-GGCCG 2230
GoldenEagle               GTCCCAGCGCCGGGGGCTCGCTCCCCGCGGCGGCTGGCTGTCGGCGGGCGGGCGTCTCCGCGGGTGTGGGGAGC-GGCCG 2356
JapaneseQuail             GAGGCCCGGCCGTGC---CGC-------------------------------CCGCTCGCTCGGTGGTGGCACC-GCTCC 2323
MediumGroundFinch         -------------------------------------------------------------------------------- 0
GoodesThornscrubTortoiseCATCCACGATCATGAAGACATATT---T----GTAGGAAAACTTCAACATTTCATTCTGACCTTGGCAGGTTGA-AACTA 23474

Majority                  CTGCGCCGGAGGCGGTGGGGACGGTGCGGTGTTCCCC-CG----GCCGCTGCGGGGCAGTACGTGGTCGGAAGGGTCTCA
                                   27130     27140     27150     27160     27170     27180     27190     27200
Human                     TTGTGTCCTTGCCCGGTGTCTTTCTGTCATGGTTCCCTAGGCTGCCATCTCCACAGGAGAAAATGTTTGGTTAGTTCTCA 27139
Kakapo                    CTGCCCCGGAGGCGGTGGGGACGAGGCGGGCTTCTC-----------GCCGCGGGACGGCGGGTGGGCAGAAGGGGCTCA 2299
GoldenEagle               CTGCCCCGGCAGTGGTGGGGACGGGCCGGCTTCCCGC-CGCCGCGCAGCTGAGGCCCAGTACGAGGGGGGAAGGGGCTCA 2435
JapaneseQuail             GCACGCCGGGTCCCGTGGCGGCTTTGGGGTGTGGGAG-CG----GCCGCTGCCCGGCGGCACGGGGACGGCCGGGTACTG 2398
MediumGroundFinch         -------------------------------------------------------------------------------- 0
GoodesThornscrubTortoiseATAGTTTAGAGTCTAAGAGAACGGATCTGGAAACAACTCTGTTAAGCATTGATAAACATTCTGCAGTCAGAACATACATA 23554
```

```
Majority                     GCCGCTTGGTA-ACTCGGGCCCCTCG---GTGGG-GCCCTCCGCTTAAAGTGCTGGGGGCTCTGGGCCGCC-CCCTACTT

                                     27210     27220     27230     27240     27250     27260     27270     27280

Human                        GTCCCAAACCATAGCAGAGCCCAGAGACTCTTGTCACCCTCAGCCTTCAGTCCTTGGTTGCCTTCTTCGCAGACCTGGCA 27219
Kakapo                       GCCGCTCCGTA-ACGCGGGCCCCTCG---GCGGG-GGCTGCCGTTTAAGCTGCCGAGGGCTCTGGGCCGCC-TTGTACTT 2373
GoldenEagle                  GCCTCTTCGTA-GCGCGGGCCCTTCG---GTAGG-GTCTTCCGTTTAAATTGCTGGGGGCTTTGGGCCGCC-CCCTACTT 2509
JapaneseQuail                GCTGCGGGAGG-ACTGGAGT---------GCGGG-GCCCTGCGCGGGATGGGAAGGGGGAACTCGGCCTCC-CCGCAACG 2466
MediumGroundFinch            -------------------------------------------------------------------------------- 0
GoodesThornscrubTortoise     AGGATTTGGAGTACTTGGCTTCCAAA---GTACACAACCTTTACCAAAACAGACTTCAAATCAAACACTCT-AACTTATG 23630

Majority                     CTTGTTGCACCTGGGATTTTTGCTTTGGATGTCCAGAGGGCCCA--CTGTT-TGCTCCCCGCCGTCTCTTGTATCGATGC

                                     27290     27300     27310     27320     27330     27340     27350     27360

Human                        GCTCTTCCACCTGAAAGTGATACATGGTATTAATAGTTGGACTAGACTATTATGATAAACAGAGTATCTAATAACCATGA 27299
Kakapo                       CGCGTAGAGGCGCCGCGTCTAACGAGGGAGCGCGATCTGCCCCA--TCTGT-CCCCTCCAGCCCCGAGTGCTGTCGTTCT 2450
GoldenEagle                  AGTATAGTACCCGAGATGCTGGATCTGGCTGTAAAGAGGGAGCG--CTGTC-TACCCCCCTCCGTCCCCTCCAGAACCTC 2586
JapaneseQuail                CTGGCCCCTCTTGGGGTCTTGGCTTTACACAGCCGAGGGCTTCGGGCCGTG-GGATCCCGCCCCCCTGCTGAGGCGGCGG 2545
MediumGroundFinch            -------------------------------------------------------------------------------- 0
GoodesThornscrubTortoise     TTCCTTTCAGAAAGGAATTCTTCCCCAGTTGTCCAGAGGTCCCAATCTCTC-TATTCCATGTTATTTCTGGTATCAATAC 23709

Majority                     GTATAGTTTCCTGCTACCCCTCGCGCTTGCCTCGACTGTGGGTTGGCGTGTCGAGCCCCGTAGGGTTTGGGTTTGTTTTG

                                     27370     27380     27390     27400     27410     27420     27430     27440

Human                        GAATAAAAAACAAGAAATTTGTAACATTGAAATGAGTGCTAAATGGCCTTTAATCTGTACTTGCTTTTGCACATTTTTTT 27379
Kakapo                       GTCCCGCCTTTGCCTACCCCCAGCGCCGGGTTCCCCCCTCCTCAGCCGTGGGGAGCCTCTTCGGGCTCGGGTTTGTTTTG 2530
GoldenEagle                  TTGTTCTTTCCTTCTCCCCCCCCCCCGCGCCCCGACCGTGGGGTGAGGAGGAGCGCTCTGTAGGGTTCGGGTTTGTTTTG 2666
JapaneseQuail                GTACAGTGTGCTGCTACCCCT-GCGCTGCCCTCGGCTGCCCCTCGGCCCGTCCGTTCCCCCAGGCGCTGGGGGTGTTTTG 2624
MediumGroundFinch            -------------------------------------------------------------------------------- 0
GoodesThornscrubTortoise     TTTTACATCCTTGCATTTTCTCAAAGCTATGTGGTGTTAGGGTGCAGTCGTCAACCCCAGAAGTGTTTTGTATTTCACTT 23789

Majority                     GAGCTGGCTGGAGTGGAGGAGCGA-TA-CCTGCAGGAGCC--ATTTCGAGCTCTTTTGTTTTCAGAAGCTGCTTAATAAG

                                     27450     27460     27470     27480     27490     27500     27510     27520

Human                        TTAAGTGGTTTCTTTCTCTGACGTGTAGAGTGTATCTTCCTATTTCTGAATTTTCTTGTTTCTGTGGGATGTTAGTTTCT 27459
Kakapo                       GAGCCGGCTGGAGGGGAGGAGCGA-TA-CCAGCAGGAGCCAGATGTCGAGCTCTTTTGTTTTCAGAAACTGCTTAATAAG 2608
GoldenEagle                  GAGCTGGCTGGAGGGGAGGAGCAAATA-CCAGCAGGAGCCAGATGTCGAGCTCTTTTGTTTTCAGGAGCTGCCTAATAAG 2745
JapaneseQuail                GAGCAGGATGGGGAGGAGGAGCGA-TA-CCGGCGGGAG----A--T------CTTTTGTTTTCAGAAACTACCCGCTT-G 2689
MediumGroundFinch            -------------------------------------------------------------------------------- 0
GoodesThornscrubTortoise     AAGCTATATATATTTATATCAAAATTAGTTTTTACGAGTC--ATTTGTGACTCCTTTGCGTG-AAAAGTCCATAAATAAC 23866
```

```
Majority                    CCCTCTGACTTCCTCCCCATC-C-CAGGTAGAAAGATGCCTTTGTGCGTGTTGGAGACTGGTTGTAGATGCCATA-----

                                    27530     27540     27550     27560     27570     27580     27590     27600

Human                       CTTGAAATGGTCATTGATATTACTCAGGAAGAAAAGTTTCTGTGGTCAGATTTAATAAGAGATATCTATGCTAGTC--T- 27536
Kakapo                      CCCTCTGGCTTCTCCCCCCTC-CTCAGGTAGGAAGATGCCTTTGTGCGTGCCGGAGACTCGCTGTGAAGGCCATA----- 2682
GoldenEagle                 CCCTCTGCCTTCCTCCCCGGC-C--AGGTAGAAAGATGCCTTTGTGCGTGCTGGCGACTGGCTGTAGAAGCCAAA----- 2817
JapaneseQuail               CCCTCTGACTTACTCCCCTCC-C-CAGGTAGAAAGGTGCCTTTGTGTGTGTAGGAGACGGGTTGTAGGAGCCAGA----- 2762
MediumGroundFinch           -------------------------------------------------------------------------------- 0
GoodesThornscrubTortoiseATTTGAGAAGAAAATCCTAGA-T--GCTTGAATAGATTATTTTAAAGGTGAAGACAAGAGCTAACAACTGCTATGTATTC 23943

Majority                    -GAAGGGAAGTTT-----TTGGAGGAGTCTACCATT--TAGGACAGCGAGGTCCTTCGGTCCTGGTTGGTAGCGGGGGTG

                                    27610     27620     27630     27640     27650     27660     27670     27680

Human                       TAACATTTAATTT--CTTTTAGTTGAATCTAAAATTGATATCATTCTTAGATTAATTAAAAATTTTTTAAAGAGCATGTA 27614
Kakapo                      -GAAGGGAAGGTT-----TAGAAGGAGTCAGCCATT--CAGGACAGAGAGGGCCCTCGGTCCTGGC------CAGGGCTG 2748
GoldenEagle                 -GAAGGGAAGGTT-----TTGAAGGAGTCGGCCATA--TGGGACAGCGAGGGCCCTCGGTCCTGGCTGGGAGCTGGGGTG 2889
JapaneseQuail               -GAAGGGAAG--------TCGGAGGAGTCGA--------GAGGCAACGAGGCCCTCCG--TCTGGAGGGTAGCGCGGGTG 2823
MediumGroundFinch           -------------------------------------------------------------------------------- 0
GoodesThornscrubTortoiseTGGAAAGCAGTTTTGTCTTTGCTTAAGATTAGGGTT--CAGGGCTCAGAGTTTTTCTAGCAATAGTTGATGACATCATTT 24021

Majority                    GG--CAGTCACTGTGCGCT-GGCCTTCTTTATTTGGACCTGGACAATGAG-GTTGATGTTG----GTCGTCT-TCATTA

                                    27690     27700     27710     27720     27730     27740     27750     27760

Human                       TTCTTTAGTATTTTGATTCTAAGTACCACTAATTTGATTTGTTTCAACAGTCATTAATGTGGGGCAGTCATCAATCACTA 27694
Kakapo                      GG--CAGTCACTATGCACA-GGCCTTCTTTTTGTTCGACCTGG--------------TGGTC----GTAGCCT-TCCTCA 2806
GoldenEagle                 GG--CAGTCAGTGTACACAAGGCCTTCTTTCTGTTGGACCTGGAAAATGAG-GCTGGTGGCT----GTAGCCT-TCGTCA 2961
JapaneseQuail               GG--CAGGCAAGA-GCGCTCAGGGCCCGAAGTTTTGGACCCGAAGAGGGAG-GGAGCTGCTCTGT-GCTGTCT-TCATGA 2897
MediumGroundFinch           -------------------------------------------------------------------------------- 0
GoodesThornscrubTortoiseACACCTACAGCTGAGAGCTGCACCCTCAAAAGCACAGATTAAAACTATTAGAGTAAATGTTGTCC-TTCATCA-TTTCTG 24099

Majority                    GTGTAAATACAACATCTCCCAGCTGCTATATGTCCTAT--GAGTTGTTTGCTCTGGTTCAAACAGAGAAGCAATCTCT-T

                                    27770     27780     27790     27800     27810     27820     27830     27840

Human                       CCATTGATCATTTATTTCATATACATTGTAAGTTAAAT--GACCAGGCTGAAATAATTCAGATGAAATTATTCTGTCAGT 27772
Kakapo                      GTGTAAATACAACGTCTCCCAGCTCCTAAATGCCCTATT-GAGTTGTTTACTCTGCTTCAAACACAGAAGCAATCTCT-T 2884
GoldenEagle                 GTGTAAATACAACATCTTCCAGCTTCCAAATTTCCTATT-AAGTTGTTTACTCTGCTTCAAACAGAGAAGCAATCTCT-T 3039
JapaneseQuail               GTATAAACACAGAACCTGCCTGCTGCCAGATGCCCAAC--AAGTTGTCTGTTCTTGCTCAAACAGAGAAACGATCCCT-T 2974
MediumGroundFinch           -------------------------------------------------------------------------------- 0
GoodesThornscrubTortoiseAAGCTGATGTAAAATTCCATTCTTGTTTTCTATTTTAAACTAGTTAGGGTCAGAAGTCTTTGGAGAGCCTGAGCCTCC-T 24178
```

```
Majority                 TGGTATGTTT----AAGCATTTATCCTTTCAACTG--TGAGAGTTTTAAAGGCTTTTTATGGTTAGTTGTGATTATAATG
                                 27850     27860     27870     27880     27890     27900     27910     27920
Human                    TAATGTGGTAATGTTAACATTTATCAGTAGTGCTGAATCACTTCTTTTGGGCATAATAGTGGTAAGCTTTTTGCTCAATG 27852
Kakapo                   TGGTATGTTT---CAAGCACGCAGCCTTTCAGCT----GAGAGTTCTAAAGGCTTTTTATGGTTAGTTGTGATTATAATG 2957
GoldenEagle              TGGTATGCTT---TAAGCATATAACCTTTCAACTG--TGAAAGTTTTAAAGGCTTTTTATGGTTAGTTGTGATTATAATG 3114
JapaneseQuail            TGGCATGT--------------AACCTTTCAACTG--TGAGAGTTTGGAAGGCTTATTATGGTTACTTCTGACTGTAATG 3038
MediumGroundFinch        -------------------------------------------------------------------------------- 0
GoodesThornscrubTortoise TCCAGTGTTT----ATGAAATCATCCTTTTTATAT--TGATGAATGGCAGATATTGACTCACTGAAAAGTATTCCAAGCA 24252

Majority                 TTGGG--AGTTACAATTCCTTTCT--GAGGAG-CTTGTCATTAATGAGT-------ATTACACTGTGGAATGCTTTTACA
                                 27930     27940     27950     27960     27970     27980     27990     28000
Human                    CAAAGACAGGCATAATTGCTTTTTTCAAATAAAAATGTAATTTGTAAAAATACTCCAGTAATTTTTGTAAAATCTCAAAA 27932
Kakapo                   GTAGG--AGTTGCAAAGCCCTGCT--GAGGAGGCTTGTCATTAATGAGT-------ATTACACTGTGGAATGCTTTCCCG 3026
GoldenEagle              TTCG---AGTTACAAATCCTGTCT--GAGAAG-----TCATTAATGAGT-------ATTACATTGTGGAAAGCTTTCACG 3177
JapaneseQuail            TTGGG--AGTACCAAGTCCAGACG--AAGGAGTCTTGGCACTCACGAGTGT----TATTATGCTGTAGAATGTTTTTACA 3110
MediumGroundFinch        -------------------------------------------------------------------------------- 0
GoodesThornscrubTortoise AAGAG--AGATTAAATGGGACTCT--GATACTCTATGAAATGGATCAGAA------TGGAAACAGGGAAATACACCTCTA 24322

Majority                 T-GTTCATATTTTGCTGTTAT-TTTTTTTTGGTGGGGTTTTAGGTTTCTTTTGGAATTGT-GTT---ATATACT-TGAG
                                 28010     28020     28030     28040     28050     28060     28070     28080
Human                    TTGCAAATAATTTGACGTTATTCTCAGTAATGAAGGCTGTTAAATCTCCTCTCTTAACGGCAGGTATGAAATCATTTGAG 28012
Kakapo                   T-ATCCTTGTTTTGCC------TTTTCTGTTGGGGTGGGCTTAGGTTTCTTTTGGGATTGT-GTT---ATACACT--GAG 3093
GoldenEagle              T-ATTCATGTTTTGCTTTCAT-TTTTTTTTGGTGGGGACTTAGATTTCTTTTGGGATTGC-GTT---ACATGCTGTGAG 3251
JapaneseQuail            C-GTTCATATTTTGCAGTTTTCTTTTTTTCTGTTGGTGTTTTCTGCTACCTTTGGAGTTTT-ATT---GCATAGT----- 3180
MediumGroundFinch        -------------------------------------------------------------------------------- 0
GoodesThornscrubTortoise C-CTCAATATAATGCTGTCCTTGGGAGCCAAAAAATCTTAACGCATTATAGGTGAAACTGT-GTTTATATTGAACTTGCT 24400

Majority                 TT--------GTTCTGTCACACTGTCTAAGAAAAAGATGGACCTTGGTGTACCTGTTGGAGAGTTTCTTGTAGGTGT--G
                                 28090     28100     28110     28120     28130     28140     28150     28160
Human                    GAAGAAAAAAGTTCATAAATACAAACTTAAAAAAAAATGGAAAGACTTTTCCATGCAAAAAGATGCATTGCAGGAAGGAG 28092
Kakapo                   TT--------ACCATGTCACAACCTCCAAGAAAAAGATGGGCCTTGATGTACCTGTTGCAGAGTTTCATATAGGTGT--A 3163
GoldenEagle              TT--------GTGTTGTCACAACCTCTCAGAAAAAGATTTACCTTGACATACCTGTTGGAGAGTTTCGTATAGGTGT--A 3321
JapaneseQuail            ----------GTTAAGTTACACTGTCTATGAGGATGGCGTACCTTGGTGTACCTGTTGCGGAGTTCCATGTAGATA---G 3247
MediumGroundFinch        -------------------------------------------------------------------------------- 0
GoodesThornscrubTortoise TT--------GATCCACCAGAGTGCGCAGTGCAAAAGAGGGAGGTGGAGCCATTTCTTGAGTGTGTTTTGGAGTAGC--T 24470
```

```
Majority                   GATGATTAAATTTTATATTAGTGTACCTGGCAATATATTCTTTTTG-TTGTGTCTTATTTTAAAACCTGTATTCGTCACT
                                   28170     28180     28190     28200     28210     28220     28230     28240
Human                      GAAGAGTGTCCTTCATAGCACACCACAAGTCAGTATTAGAGGCTTGCTTTTCTACTTGAGTAGACCCAAGATTCCCCACC 28172
Kakapo                     CATGGTTAAACTTCATATAGGTGTACCTGGTTAAAT-TTCATATAG-GTGTACCTGGTTAAACTTCATATAGATGT-ACC 3240
GoldenEagle                CCTGATTAAATTTTATACAGGTGTACCTGCCAATGCATTCTTTTTT-TTCAAGCTAATTTCAAAAAATGTGTTCGTTGCT 3400
JapaneseQuail              GATACCTATCTACTATTTTAGT--ACCAGCCAATACATTCTTTTT---CCAGGCTCATTTCAAAACCTGTGTTTATCACT 3322
MediumGroundFinch          -------------------------------------------------------------------------------- 0
GoodesThornscrubTortoise   ACTGTTCCAAATTTGTGTTATATCAGGTGGCGTTATATCAAGGTAG-TGGTGTATTAGAATTTGACCTCAACTCTGGGTA 24549

Majority                   TTCTGTTGTGCGCAGTTCTCTGGGCTAGGCTATAA-TACTG-ATTAACTGTTTTTTTACTATGGGTTTGTGGGATAAGTT
                                   28250     28260     28270     28280     28290     28300     28310     28320
Human                      TATCGTCTTCCAAAGTTACTTGTGATCCACTGCAATCACAAGTTTTACAGTGTGTATACTCTAGGTGTATGGAAGGATTT 28252
Kakapo                     TGCCAATGTGTTCTTTTTTACAGGCTAATTTAAAA-CAATGTGTTCATTGCTTTCTACTGTTAGATCTGTAGGGTAGGCT 3319
GoldenEagle                TTCTGTTGTAAGAAGATCTATAGGGTAGGCTATGT-TGCTGAACTAGCTGTCTAGTTAATAGGGGTGCGTGAGAAAAGTT 3479
JapaneseQuail              TTCTTCTGTAAGGAGATCTCTGGGTTGAACTGGA--------------TGCCTGGTTAACA-GGGTTTGTGAGAAAAGTT 3387
MediumGroundFinch          -------------------------------------------------------------------------------- 0
GoodesThornscrubTortoise   TTCTGCAGGGCACCAGTGTC--GTCTAGGTCATAAGTCCTGCACTAACTGATCTTAAACTGGTGTCTTTAACAATATGCT 24627

Majority                   ---CTGTACTTATTTTTAGATATCTATTTAATCA----GTGTGTGTTCTCATGTTATGTAGTTACCTATGTTTTCTAAAT
                                   28330     28340     28350     28360     28370     28380     28390     28400
Human                      GA-ATTCACAAATAGTCAAACATCAGAAGAATAAGCTGATAATAATACATTTGAAAAGAGGTCAAGTATATTTTTGAAAA 28331
Kakapo                     ---GTGTAGCTGAACT-AGCTGCCTAGTTAATAG----GAGTGTGTGGAAAAGTTCTGTACTTACGAATTCTTTCACTCT 3391
GoldenEagle                ---ATGTACTTACTTTTAAATTCTTTTATTTTCA----GCCTATGATCTCATGTAGTCCAGAAACCTGAGTTTCCTAAAT 3552
JapaneseQuail              ---CTGTGTTTACTTTAAGTTCTTCCTTTTTTCACCCTGTTTGTGATCTCACGTCATCCAGAAACCCATGTTTCCTAAAA 3464
MediumGroundFinch          -------------------------------------------------------------------------------- 0
GoodesThornscrubTortoise   TCCCTATGCTTTTCACTAGGCAACTAGGAAAGCAGATGTTGCATGCCCCATAAATACTTTCTTAACTATGTACTTTGCAT 24707

Majority                   GTAGTGACAGATAGTGT--TTTTTTGAAGTAATATTTATATGAGACATTGA----ATTTTTCTGGA-----GAAGCTTAT
                                   28410     28420     28430     28440     28450     28460     28470     28480
Human                      CTAG-GAGAGTTAAAGTGTTTTTGTGAACTAAGGAACAAATAAGGCATTGACAATATTTTACTTGATTAGGAATTTTGTT 28410
Kakapo                     GTGATCACAGAAACCCG--AATTTCCGCTGAATAAAT-TTCCTGGCAGTGC----AGCTTTGGAGT-----CATGTTTAC 3459
GoldenEagle                AAATTTCCTGACAGTAC--TGCTTTAAAGTAATATTTACTTGAAACTTTGG----GCTCTTTTGGA-----GAAGCCTAT 3621
JapaneseQuail              GTACTGCCTGGTAGTGTGTTGCATTGGAGTAATGTTTACATGAAACTTTGA----ACTCCGCTGTA-----GAAGCCAAT 3535
MediumGroundFinch          -------------------------------------------------------------------------------- 0
GoodesThornscrubTortoise   ATGGTGAAAAAAAGTGCTCTTATTCTAAGGTTTAGCTATAAAACACATTTA----TTTTCTATAGAACAAGGAAACATGC 24783
```

```
Majority                  ATGGTGGGTGAGAC-T---GTGGCTTTTGTATGTTTCTTTTGTTT-TTACTCTTAGTTCTTCTGTTTTCTTTGGCTGTAC

                                   28490     28500     28510     28520     28530     28540     28550     28560

Human                     CAGGTGAGTAAAACTTTAAGCATCACTTAAGTTTTGTTTTTGTTTCTTACTTTTAGAATATCTGATCTGGACTTCTGTGG 28490
Kakapo                    TTGAAACTTTGGAC-T---CTGGCTTTTGAAAGTTGCTTTTGTAC-TTACCCTCAGCCCTGCTGTGTTCTTTGAACCTAT 3534
GoldenEagle               AAGCTGGGGAAGTC-A---GTGGCTTTTCTAAGTTTCTTTTGTAC-TTCCTCCAACTCCTGCTATGTTCTTTGAATATAC 3696
JapaneseQuail             GGAGCAGGTG-G---------AGCTTTTGTATGTTTCTGTATTCTGTCACTTTTTTTTTTTTTTTTCCTTTGGCCACAC 3605
MediumGroundFinch         -------------------------------------------------------------------------------- 0
GoodesThornscrubTortoise ATCTCCTCTGTGGTGT---GCTATTTCTGTGCCAAATTTAGTCTTCTGAAACTTTATTCCTTTCAACTGTAGGGCTGCTC 24860

Majority                  TTTCTTGATTGGGGAAGT--TGTTG-TCTATTGTGATTCCTAATTGTTTTGTCATTGCA-GTA------AACTTGAACAG

                                   28570     28580     28590     28600     28610     28620     28630     28640

Human                     ATTTATGGAAGAGCTGGCTTTATTTCTCTATCCTGTGGGAGGGGCAAACTTTGATTTTGTATGTGGAGTAACTTGACCTT 28570
Kakapo                    TTTCTTGATTGGGGTAAT--CGTTG-GCTATTGCAATT-------------GCACTGC-------------TTTGAAAAG 3585
GoldenEagle               TTTCTTGATTGGGGAAGT--TGTTG-GCTGTTTTGATTCCTCATTATCTTGGCATTGCA-ATA--------CTTGAACAG 3764
JapaneseQuail             TTTCTGGGTTGGGAAAGT--TGTTA-ACTAATGTGATCCCTAATCGCTCTGATGTTGCA-GAACAAACCAACAAAAGCCC 3681
MediumGroundFinch         -------------------------------------------------------------------------------- 0
GoodesThornscrubTortoise AATAAAAATTGCAGAAATATTTTTA-TGTATGGAAAAGTATATCTGTTTTCTAGTTCCTTGTACATGGAAACAGACAAAC 24939

Majority                  TTGGTTTTATG-----------AGGACAAATTGAT-TG----ACTTCTTGTTGTTGTTTCTTTTTTTTTTTACAGCATTG

                                   28650     28660     28670     28680     28690     28700     28710     28720

Human                     CAGTTTTTTTGGTCTGAGAACAAAGGCCAAAAAATATGTAACAGTTAATAACGTAATTCACTTACTTAGTCAATAAATAT 28650
Kakapo                    TTGGCTTTATA-----------AGGATAAATTGGT-TG----ACCTCTTGTTGTTTTCTCTTTTTTCTTTTACAGCATTG 3649
GoldenEagle               TTGGTTTTATG-----------AGGATAAATTGGT-TG----ACTTGT-GTTCTTGTTTTTTTCTTTTTTTACAGCATTG 3827
JapaneseQuail             TTGGTTTTATG-----------AGAACAAATTGAT-TG----ACTTAT-------GTTTCTTTTTTTTCTTACAGCATTG 3738
MediumGroundFinch         -------------------------------------------------------------------------------- 0
GoodesThornscrubTortoise TGTGTTTCCTG-----------AAATCTGCCAAATCTT----ACCTCTTTTCTCACTCTCTCTTCTCCCCCACGCAAAAA 25004

Majority                  TCACTGATGTTGCAGTTGGTG-TGAAGGTAGGATGTGTACTTAGCAAATAAGGGGGCAAACTGTTGATACAAAATGAACT

                                   28730     28740     28750     28760     28770     28780     28790     28800

Human                     GTGGGGACATTGTACTTGGTTGACACTTGGGAATATTGTAGTGAGCACTACTGATGTGAGCCAGGCACTTTGCCTGGTAA 28730
Kakapo                    TCACTGATGTTGCAGTTGGTG-TGAAGGTAGGATGGCACTTGGCAAATAAGGGGGCAAATTGTTGATACAAAGTGAACC 3728
GoldenEagle               TCACTGATGTTGCAGTTGGTG-TGAAGGTAGGATGTATACTCAGCAAATAAGGGGGCAAATTGTTGATACAAAATGAACT 3906
JapaneseQuail             TCACTGATGTTGCAGTTGGTG-TGAAGGTAGGATGTGCACTTAACAAATAAGCGGGTTCACTGCTGATACAAAACGAGCT 3817
MediumGroundFinch         -------------------------------------------------------------------------------- 0
GoodesThornscrubTortoise TAAAAGAAAATAAAATAAAAAATCATCAGTGGGCAGATACACAGAATGGAAAATTTCAGCCTGAGGGGATTAAATTTCCT 25084
```

```
Majority                      GCTTTGTGCGTGTGTAATTTCAAGTTGTAAGATTTTAAGCAGT----CATTGTCAGCTGATATTTAGTCAGCTGTATG--

                                       28810     28820     28830     28840     28850     28860     28870     28880

Human                         TCACTGTTCGTGTATAATTTAATCCTAAGGGCTGTGATGTAGATGT-TATCCCTGTTTTACAAATAAAGAACTTCAGGCT 28809
Kakapo                        ACTTTCCGCGTCGGTAATCTCAAGTTGTAAGGTTTTAACCAGT----CATTGTCAGCTGATATTTAGTCAGCAGTATA-- 3802
GoldenEagle                   ACTTTGAGCATCAGTAATTGCAAGTTGTAAGATTTTAACTAGT----CATTGTCAGCTGGTATTTAGTCAGCTGTATA-- 3980
JapaneseQuail                 GCTTTGAGCATGTGTCATTGCAAATTTTAAGACTTTAAGCAGT----CATTGTCAGCTGATATTTAGTCAGCAGTACG-- 3891
MediumGroundFinch             -------------------------------------------------------------------------------- 0
GoodesThornscrubTortoise      CAAAGTTGAGTGACTGGAAACAAGGGATTGGAATGGAAACAGTTACCCAACGTTAACTGTAGCAGTGGCTGCTGCTGC-- 25162

Majority                      TGCTTTA-TTAACTAAAAATCCCTAGAGGTAAATTGGAGGCTCTACACAAAA--GGCTTTTCTAGGCACTGAACTGTAGA

                                       28890     28900     28910     28920     28930     28940     28950     28960

Human                         TAGAATGATTAAGTACTGTTACCTAACTGATAAGTGGAAGCTGAGCTCCAGAGCACCCTTTCTTAATACAGTATTTTCTA 28889
Kakapo                        TGCTTTA-TTAACCAAAAATCCCTAGAGATAAATTACAGCCTCTACACAAAA--GGCTTTGCTAAGCACTGAACTGTAAA 3879
GoldenEagle                   TGCTTTA-TTAACCAAAAATCCCTAGAGGTAAATTGGAGGCTCTACACAAAA--GACTTTTCTAGGCACTGAACTGCAGA 4057
JapaneseQuail                 TGCTTTA-TTAACAAAAAATCCCTAGAGGTAAATTGGAGGCCCTACACA-----GACTTTTCTAGGCAGTAGACTGTAAA 3965
MediumGroundFinch             -------------------------------------------------------------------------------- 0
GoodesThornscrubTortoise      TCCTATAATCCAGTAGAAAACTCCATCAGTGCAGAACATCTGAAAAAATGTAT-TGATATTAAACCCACTAAAATTTGGG 25241

Majority                      CAGT--AGTACTAGATTAATGTAG-TAGTGTCAGCTTCTTATTTT-AAAGC---TTGATTTTGCAGATTA-T--TTTTTT

                                       28970     28980     28990     29000     29010     29020     29030     29040

Human                         CTTGTCATGAAAACATGAAGAAAGCTAGGAAACATTTTCTGTGTTGATAATGAGCCAATAATGTGTATCAGTGGTTCTTA 28969
Kakapo                        CAGT--GGCACTAGATTAACGTAG-TAGTGTCAGCGTCTTATTTTTGAAGC---TTGATTTTACATATTAGTC-TTTTTT 3952
GoldenEagle                   CAGT--GGTTCTAGATTAATGTAG-TGGTGTCAGCTTCTTATTTT-AAAGC---TTAATTTTGCAGATT------TTTTT 4124
JapaneseQuail                 CAGG--AGTTCTAAATTAATGTAG-TGCCATCAGATTCCTATTTT-AAAGC---TTTATTT-GCAGATTA-----TTTTT 4032
MediumGroundFinch             -------------------------------------------------------------------------------- 0
GoodesThornscrubTortoise      CAATTAACAATCCAGTGTCTGTGG-TAACTTTTAACTGTCTCATTAGAAAT---ATGCTTTGATAGATATTTAATTGCAT 25317

Majority                      CAAAACAACACTTTCTCGT-G---TTGCATAGTAATTGTAATAGACATGTTTTCTTCTTCTTCCTCTCCCCTCATCTTTT

                                       29050     29060     29070     29080     29090     29100     29110     29120

Human                         AACTTTAGCATGCATCAGAAACACTTGGGGATTTTTTTTTTTAAACCTGGGGTTTTTAGATCCCACCCTCAGAGTTTCTT 29049
Kakapo                        CAAAACAGCACTTTCTCATTG---TTGCATAGTAACTGTAAAAGACATGTTTTCTTCTCCCTCCCCTCCCCCCATCCTTT 4029
GoldenEagle                   CAAAACAACACTTTCTCGTTG---TTGCATGGTGAATGTAAAAGACATGTTTTCTTCTTCCCCCTCCCCCCATCTCTTTT 4201
JapaneseQuail                 CTAAGCAATGCTTTCTTGT-------------------G--------TGCTTTGTTGTTT-TCTTCTCCCCTCATCCTCT 4084
MediumGroundFinch             -------------------------------------------------------------------------------- 0
GoodesThornscrubTortoise      TCACAAAACACTTAACAAT-G---TGGCATACAAGTGATCATGTATATCTTAAAATATGGTTTAACAATGCTAATTTGAC 25393
```

```
Majority                 TTTC--T---AAAACAGTT--ACCACTGAAGTTAGA-----AAAAA--T-------CTGTTTTTA---TTAGG----ACA

                                 29130     29140     29150     29160     29170     29180     29190     29200

Human                    ATTCAGTAGGTCTGGGATTGGACCTGAGAACATATACTGTCAGTAAGCTTCCAGGTCTAATCTTAGGATTAGGGACCACA 29129
Kakapo                   TTCC------AAAACAGTT--ACTACTGAAGTTAAA-----AAAGA--------------------------------CA 4064
GoldenEagle              TTTTTTTTTTGAAACAGTT--ACCACTGAAGTTAAA-----AAAGAAGTGA-----CTGTCTGTA---ATAGG----ACA 4262
JapaneseQuail            TT------------CAGTT--AACACTGAAATTAGA-----AAAAG-----------TTGTTTTA---TCAGT----ACA 4127
MediumGroundFinch        -------------------------------------------------------------------------------- 0
GoodesThornscrubTortoiseATCCCTTACAAGAAGAGGTGCATTGTTATACATTGA-----ATGAATTTATATGTCCTGTTTTTA---TAAAG----CCA 25461

Majority                 CAACAATATGGGTGGTAG----------AATTT--------ATGTAGACAGAGAAGTGAGTGTTTTCTTTGCT--TTACT

                                 29210     29220     29230     29240     29250     29260     29270     29280

Human                    CTTTGAGAATGATTAGAGTATTCAATTAGATCTTAGGGACTACACTGTGAGAATCAGTAATGTATACAATTCTGCTCATC 29209
Kakapo                   CACAAATATGGGAGGTAG----------AATTT--------ATGTAGACAGAGAAGGGGGCGTTCGCTCTGCT--TTACT 4124
GoldenEagle              CAAAAATATGGGAGGTAG----------AATTTGTT-----ATGTAGACAGAGAAGTGGGTGTTTGCTTTGCT--TTACT 4325
JapaneseQuail            AAACAAAATGAGTGCTAG----------AATTT--------ATGTAGACAG--AAGTAAACATTTTCTTTGCT--TGACT 4185
MediumGroundFinch        -------------------------------------------------------------------------------- 0
GoodesThornscrubTortoiseTAGCTAAACTGGCAGTGGTTAACAATAAAATGTTATAA---ATACAAATACTTGAGTAACTAAGTTCACAACTAGTAATT 25538

Majority                 TTTTATTTTTCAAGTTC---AGTTATGAATTTT-ATCATATTTATTATAATAAGGTGATTGCTTTCGACTTGTTGTTAAT

                                 29290     29300     29310     29320     29330     29340     29350     29360

Human                    ATGTTCTTATAAAACTATGCCATGCCAGATCTTCCATATCTTGATTAGAAATTGGTTAAGAAATTAGGCTTGGTGTAAAT 29289
Kakapo                   TCTTGTTTCTCAAGTT-----GTTATGAATTT--ATCATATTTATTAAAACAAGGGAAGAGCTTCCAAATTGTCGTTAAT 4197
GoldenEagle              TTTTATTTTTCAAGTTC---AGTTATGAATTT--ATCGTATTTGTTATAACAAGGGAAGAGCTTCCAAATGGTTGTTAAT 4400
JapaneseQuail            TTTTCTCTC-CAAGTTC---AGTTATGAATTTTTATCATATTTATTTTAATAAGGAGTTTCCTGTAGGTTATTAATTATT 4261
MediumGroundFinch        -------------------------------------------------------------------------------- 0
GoodesThornscrubTortoiseTTTAATAGTATCTGCTGGA-AGTTTTAAAAAATTACTACCCATCCTAAGTTTTGGTGATTTATATTGACCAGTTTATCAT 25617

Majority                 TCAGTATT-TTTTTCATTGTTGTT--TTTGATTTTTTT---GTTGGAATTGGATCTGTACTTGATAGAAGCAGGCTGAGA

                                 29370     29380     29390     29400     29410     29420     29430     29440

Human                    ACAGTATCAATGTGGCTTGAAACTGGTTGTATTTTTCTGTTTTTTTTTTTCTTTGTGCATTTGATGCTAATAAGTAGAAA 29369
Kakapo                   TTACTGTT-TACTTCACTTCTGTG--TCCAAATCTGTT---GTGGGAATCAGATCAGTACTTTGTAGAAGCAGACTGAGA 4271
GoldenEagle              TT-------TGTTTCACTTTTGTA--TTCAAATCTGTT---GCAGGAATTAGATCGGTACTTCATAGAGGCAGACTGAGA 4468
JapaneseQuail            TCACTTCTGTATCCAGTTGTTGTT--TTTGTTTTTTTTTTTACAAGAACTGGATCAGTACTTGATAAAAACAGTTCGAGA 4339
MediumGroundFinch        -------------------------------------------------------------------------------- 0
GoodesThornscrubTortoiseTCTGTAGTAATGTAAATTGACATT--TATGATTATCATACCAGTGGGCTTGTGCATATATGTGCATACATTAAGCTCACA 25695
```

```
Majority                 AATAATGAGA-ATGTAAAGATAA-GTTGTATGCGTGAACTTTAGGTT-TGTTAATGTATGTATGAAAGCAGTTCTATT-T
                                29450     29460     29470     29480     29490     29500     29510     29520
Human                    GCTCTTAAAATATTTAAGGGAGACAACCTATGGATATCTGTTTACTC-TTCTCATATTTGTTGTAATACAGTTTATTCAT 29448
Kakapo                   AATAATGAGA-ATGCAAAAATAATGTAGGGCAAGGGAACTTGAGGTTATATTAATGAATGCACGCAAGCAATTCTGTT-T 4349
GoldenEagle              AATAATGAGA-ATGCAAAGATA-TATGGGACAAGGGAACTTCAGGTTATGTTAATGGATGCATGCAAGCAGTTCTATT-T 4545
JapaneseQuail            AATAACGAGA-ATGAAAAGACC----T--GTGCGAGAACTTTAAATT--GTTAATGCATGTAAGAAAAAAATCCTATT-T 4409
MediumGroundFinch        -------------------------------------------------------------------------------- 0
GoodesThornscrubTortoiseAGCAAGAACA-GTTTAAAAATAA-GCTTTATGCTTGAAAGTGTGTTTTCAGTGGAATCTAGCTTAAACATCAAGAATT-G 25772

Majority                 CAGTGGTGTAAATGTAA-TTTGTATA--AGC-AATGGTTCATATTTGTCCAGGGACTTTTTAGCTATACTATGTAACTGC
                                29530     29540     29550     29560     29570     29580     29590     29600
Human                    TATGAGTTTAAATACAAATTAGCGTAA-AGCTTATGGTTAATTTGGATTTTTTGGACTTTCTGAAATGCTTTGATAATTC 29527
Kakapo                   CAGTAGAAACAGAATTCTAGTATATA-CAGTTAAGTCTTCACATTTGTCCAGGGGCTTTTTAGCTATACTGCATCACTGC 4428
GoldenEagle              CAGTGGTAGAAATGT------------------AGACTCCACATTTGTCCAGGGACTTTGTAGCTATACTATGTAGCTGC 4607
JapaneseQuail            CAGGGGTGTGCATGGAAGTTTTCTTA-TGAA-AATGGTCTCTGCTTGTCTGGGGAGTTT------GTACTACGTAGCTGC 4481
MediumGroundFinch        -------------------------------------------------------------------------------- 0
GoodesThornscrubTortoiseTAACTTTGTTTATGAAACCTTGTATAACTGCAGAAGACAAAAACCTAAACAGTAAATCTGGAAATGAA-TAGTTAAGAGA 25851

Majority                 CTTACCACCTTATGCTGCTTTTTGTTAACTGCTTGGTGAACC-AGGTTTTGTGTAGTAAAGCTCTTTGGCTCAGATTCAG
                                29610     29620     29630     29640     29650     29660     29670     29680
Human                    ATTGCCTCCTTTGCACATTTTTTTCATTTTGATGGTTGCATTTAACTTCTTTGTACTGA--TTTTTTAAATCAG--CCAG 29603
Kakapo                   CTTACCACCTAACACTGCTCTGTGTTAACTGCTTGGTTAGCC-AGGTTTTGTCTAGTAAAGCTCTTTGGCTCAGATTCAG 4507
GoldenEagle              CTTTTCACCTGAAGCTGATCTATGTTAACTGCTTGGTAAGCC-AGGTTTTGTCTAGTAAAACTCTTTGGCTGAGATTCAG 4686
JapaneseQuail            CTTA------AATGCTAGTTTATGTGAACTGCTTGGTGAACC-AGGTTTTGTGTAGTAAAGCTGCGTGGCTCAGGTTCAG 4554
MediumGroundFinch        -------------------------------------------------------------------------------- 0
GoodesThornscrubTortoiseCTTACTGCTTTATAATGCTACTTAACTCCTCTTTATCCCACTTAACTTATATTGCACACTCAACCTTAATTTTGATCCAT 25931

Majority                 TAT-TGGTTCTACTGGATTTGGACT----AGTTCTTATAT-AATACAACTAGCTTTTAGATAGCAGAGGCTATTTTGTCA
                                29690     29700     29710     29720     29730     29740     29750     29760
Human                    TAT-TAATTTAACCTATTTTCAATACTAATATCCATGTATGTAAATATCCATAACTAAGGCAAAACAGTTGATTTTAGCT 29682
Kakapo                   CAT-TGGTCCTGCTGGATTTGGACT----AGTTCTTATGT-AATACAACTAGCTTGTAGATAACAGCAGCTATTCTGTCA 4581
GoldenEagle              TAT-TGGTCCTA---GATTTGGACT----AGTTCTTATAC-AATACAGCTAGCCAGTAGATA-CAGCAGCTGTTTTGTCA 4756
JapaneseQuail            TAG-TGGTTCTGCTGGATTTGGGCT----AGATCTTAGAT-AATACAATTAGTCTTTAGATAGCAGAGGCTACTGTGTCA 4628
MediumGroundFinch        -------------------------------------------------------------------------------- 0
GoodesThornscrubTortoiseAACATTGTAAAAATATATTTAAAGTTCAAAATTCCTATATTAATAGTACTAAATCATATTTAGAGGATCCATTTCCGATT 26011
```

```
Majority                 GATTT-AAC------CCTGTATTATGTTTCTAAGTAAGCTTGCTTCTAGGAGGAGCTGTAATAA-ACTGGT-------TA

                                  29770     29780     29790     29800     29810     29820     29830     29840

Human                    AGTTTAAATATTAGTCCATTCATATTTTTGTAAGTACATAACATTTTCAAAATAGCTAAAATGTCACTGGCTGGTTTGAA 29762
Kakapo                   GAATGTAGA------CCTGTAGTATGTTTCTGAGCAAGCTTGCTTCTAGGAGAAGCTGTAAGAA-GTCAGT-------TA 4647
GoldenEagle              GATGT--AC------CTCGTATTACGTTTCTAAGCAAGCTTGCTTCTAGGAGGAGCTGTAAGAA-GCGAGT-------TA 4820
JapaneseQuail            AATGA--A-------CCTGCGTTATGTTT--GAGTAAGCTCTCTTCTAGGAGGAGTTGTAATAA-ACTGTG-------TG 4689
MediumGroundFinch        -------------------------------------------------------------------------------- 0
GoodesThornscrubTortoiseCACTATAACATA---CCCACTGTTACTCACTACCAAAATCCCTACCCACTTGGTACTACACAAA-ATAGAGATAAAAATA 26087

Majority                 CA--GATTCTGGTCCCATCTGGCTTCATGTGTTCGCTCAGT-ATTGAAG-CT-AGCTTAAAATCACTCTCTGAATCAAGA

                                  29850     29860     29870     29880     29890     29900     29910     29920

Human                    CAACTGTGCAGTTACCGAAGAGATGAATGTATAACCTTTTCTGTAAAGAATTTTAAGACATGACCTTATTTTTTTGCAGA 29842
Kakapo                   CA--GATTCTGGACCCATCTGGCCCCACAGGTTCAGGCAGT-ACTGAAGACA-AGCTTAAAATCACTCTCTGAATCAAGA 4723
GoldenEagle              CATAGATTCTGGACCCATCTGGCTTCACATGTTCGCGCAGT-ATCGGAG-CA-AGCTTAAAATCACTCTCTGAATCAAGA 4897
JapaneseQuail            ------TTCTGGGCCTACCTTGGTCCATGTGCGTGTTCAG--ATAACAG--------ATAAATCACTCTTGGAGTCAAAA 4753
MediumGroundFinch        -------------------------------------------------------------------------------- 0
GoodesThornscrubTortoiseTACACTTGCAGCTTTTATGTCACTTTGTGTGTAAAATCTGT-GTTCTTGGCTTAACTGGTAGACATTTACCTTCTTGAGC 26166

Majority                 GAAAAGTGTAGTTATTCCAGTAATTCTGTGGGAGCT-----AATCCCAATCA--GAAATATAAACTGGTATAATT-TTTC

                                  29930     29940     29950     29960     29970     29980     29990     30000

Human                    AAGAAATCTCTTCATTCCAAAGATTTTGCCTTTGCTTTCGGTGTTCTAGCCACTGAAGTATATACCTGTTTAATTTTAAT 29922
Kakapo                   GGAAAGTGTAGCTCCAGTA----CTGGGTGGGAGCT-----AATCCCAATCA--GAAACACAAACTGGCATGATTATTTC 4792
GoldenEagle              GGAAAGTGTAATTATTAAAGTACTTCTGTCAGAGCT-----AATCCCAATCA--GAAATATGAACT-------------- 4956
JapaneseQuail            GAAAAGTACAGTTATTAAAGTAGTTCTGTGGGAGCTC----AATGCCATTCA--GAAATATAGACCAGTAAAATTGTTTC 4827
MediumGroundFinch        -------------------------------------------------------------------------------- 0
GoodesThornscrubTortoiseCAGGAATTCTGATACACCCCACATTCCACTCCTGCCT----AAT---AAACA--ATTGTATAAATTGCTGGCCATCTATG 26237

Majority                 TCTGTTGAAGGGAGGGATA-C-----CA-----TTTAAATTGTATTTAATAGTTGGGAGGAATTTC-CGGGTAAATTCAA

                                  30010     30020     30030     30040     30050     30060     30070     30080

Human                    AATGATGAAGATATTGATATCTAACTCACCAGAATGAAGTTCTAATTTTCTAAAGACCTGAATTATCTGGATGAATAAAG 30002
Kakapo                   TCTGTTGATGGGAGGAAAA-AAGCGTCAACTGCTTAAAACTGTATTTAATAGTTTGGAGGAATACC-TGAGGAAGTTCAA 4870
GoldenEagle              ---------------------------------TTAGGAATGTATTTAATAGTTTGGAGAAATACC-CAGGGAAGTTCAA 5002
JapaneseQuail            CCCATTACCTGGGAGGACA-G------------TGTCAACTGCCTACAAGTGTAGGAGTGTATTTGACAGTTAAATTCAA 4894
MediumGroundFinch        -------------------------------------------------------------------------------- 0
GoodesThornscrubTortoiseTCTTCTAGAAGCTGGTCTA-CAAACCCACTGCCAGTTATTTCTTTAGAGCAATTGGTAGAAACCTATCCTATGAATCTGA 26316
```

```
Majority                 GTCTTTCAGAATTAAACTGCCTCA--TGAACGGTTTTG-GGCAAAACAATTTCTAA--A-TTGCTCTCATTCTTTTCTAA

                                30090     30100     30110     30120     30130     30140     30150     30160

Human                    GACCATTGGGATGGAGGATTTATA---GAACGTTTCTGAGAAAGTCTAGCTGTTGTGGCTTTGGTTTGTTTGTAAACAAT 30079
Kakapo                   GTCTTTCAGAAGTAAACTGCCTCC-----ATGGTTTTGCGGCAAAACGATTTATAA--A-TTGCCCTCCATCACGTCTAA 4942
GoldenEagle              GTCTTTCAGAAGTAAACTGCCTCATGTGAACAGTTTTGGGGCAAAACAATTTCTAA--A-TTGCCCTCAATCCTGTCTAA 5079
JapaneseQuail            GTCTTTCAGAAATAAACTGCACCACATGAAGAACTTTGTGGCAAAGCAATTCATAA--AATCACTCTCAGTCATTTCTAA 4972
MediumGroundFinch        -------------------------------------------------------------------------------- 0
GoodesThornscrubTortoise AGGTGT-GGCGTCAAACCCCGTTT-ATGAGACATGCTG-GAAAAAATAGTCTCTAATTATTTGATTGCATACTATCATAA 26393

Majority                 TGAAGAAGCAATTTCAGGCA---GACTATGTATTGTTT--GAACCTTAAATTCTGACATTCTCAGTTTTGGAGAAGTTAA

                                30170     30180     30190     30200     30210     30220     30230     30240

Human                    TGAGATAGCTTTCCAAAGCTTTTGCTTATAGAATCTAATCACTTTTTCTATTTATTGTCTTTTGGGCTTAGAGTAAAGAA 30159
Kakapo                   TGAAGAAACAATTTCAGGTA---GACCATGTATTGCTT--AAGCCTTAAATTCTGACATTCTCACTTCAGGAGAAGTAAA 5017
GoldenEagle              TGAAGAAGCAATTTCAGGTA---GACTGTGTATTGCCT--GAACCTTAAATTCTGACATTCTCACTTTGGGAGAAGTAAA 5154
JapaneseQuail            TGAAGAAGCAGTTTCAGACA---GTCCATGTATTATTT--GAACGTAAAATTCTGACACTCTTAGTTCAGGAGAAGTT-- 5045
MediumGroundFinch        -------------------------------------------------------------------------------- 0
GoodesThornscrubTortoise TGCATATTCTCAAGAGAGCA---AAATTGAGATTATATCGGTAACTTTAATTTGGGCATTTCCCATCTTTGAGTGCCT-- 26468

Majority                 -GGATTTAGTATC--TT-CATTTTTTAGTATGAGTGACTTAGA-T-TTAGGAGAATTTGAGGGTTCATGAAATATTTTGT

                                30250     30260     30270     30280     30290     30300     30310     30320

Human                    GGGAAGGGAACACTGTTGCCTTTGTGAGAATGGGAGAGGGATAGA--TGGGAGAGTAGAGGGATGAAGTTAATTTTTTAT 30237
Kakapo                   TGGATATAGTATCCCTTTCATTCGTTGCTGTGAGTGACTTAG--------GAGAATTTGAGTGTTCATGAAGTATGTTGT 5089
GoldenEagle              TGGATTCAGTATCCCTTTCATTCTTAACTATGAGTGACTTAGAATCTTAGGGGAATTTGAACGTTCATGAAATACGTTGT 5234
JapaneseQuail            --GGTTCAGCATC------AATTCTTAGTATGAGTGACTTAGACTCTTAGAAAAATTTGAGCATTCCTGAAATACTTTGT 5117
MediumGroundFinch        -------------------------------------------------------------------------------- 0
GoodesThornscrubTortoise -GACTTTGTTAAG------AATATTAAGATTGCATATTTTGTGTTTTGAAATATATCCAGGGGATCCTCTC-TGATTAGC 26540

Majority                 G---ATGTAGTACGATTTTGGAACCATATTTTCAGTTACTAGCCAGACGATGAAGTGAAGGTACTTCAGATGACATAATT

                                30330     30340     30350     30360     30370     30380     30390     30400

Human                    TA-TAGGGATTAACATTTTTAACTATTCCCAATAGTGTAACATTTGACATTTAAGTGAAAGGCTTTTAGATTA-ATATTT 30315
Kakapo                   G----TGTAGGACGATATTGGAACCATATTTTCAGATACTAGACA--CGATGAAATGAAGATACTTCAGATGACATAAGT 5163
GoldenEagle              AAGCATGTAGTGTGATTTTGGAACCATATTTTCAGGTACTAGCCAGACAATGAAGTAAAGACACTTCAGATGACATAACT 5314
JapaneseQuail            G----TGTAGAGCAACTCTGGATCCATATTTTCAGGTACTAGCCTGGCGATGAA-TGAAGGTACATCAAATGACAT---T 5189
MediumGroundFinch        -------------------------------------------------------------------------------- 0
GoodesThornscrubTortoise ATTTATTTGGAAACA-GTAGAAGGGATAGGTTCTGTGCATCACAACTAGGTGGATTATCTGTGCCTTAGATTTCTCTGTT 26619
```

```
Majority                   GCTTCT-TTTTGTTGTCTGTAGAAGTTGTGTTTTTAATGTAATA-----AAGAAAGTGAGACAAGTGTATCTGAGGTAGT

                                  30410     30420     30430     30440     30450     30460     30470     30480

Human                      TTAAATTTAAAGATGTCTTATGCTAATTTGTTTTTGTTTTCATA-----GAGAGGATCTGACGAACTACTCTCAGGCAGT 30390
Kakapo                     GCTTCT-TTTTGTTGATTGTAGAAGTTGTATTATGAATGTAATATAAAAAAGAAAGCGAGCCAGGTGTATCTGAGGTACT 5242
GoldenEagle                GCTTCT-TTCTGTTGATTGTAGAAGTTATATTATGAATATAATA-----AAGAAGGTAAGACAGGTGTATCAGAGGTAGT 5388
JapaneseQuail              ACTGCT-TTCTGCTGCCTGTAGAAACTGAGGTTGTAATGCAAGA-----AAGAAAGCGAGGGAAGTGTATCAGAA--AAT 5261
MediumGroundFinch          -------------------------------------------------------------------------------- 0
GoodesThornscrubTortoiseCTTGTTATTTTGACATCTGGTAAATTGCTCAATCAGACACAATTTTCCTATCATACATAGTTTATTAATTGTGAGTATTC 26699

Majority                   GTTTAAATTCAATACAAAG----AAATGAACAGAAGTGTCAGGAAGATGTGATCTTTGCCTTGTAAGAAAGGTCTTGTTT

                                  30490     30500     30510     30520     30530     30540     30550     30560

Human                      GTTCTCAGT-AGTCCGAACTCTAATATGAGCAGCATGGT-AGTTACAGGTAAGTGTTACTCTCTTAGCAGTCTGTCATTT 30468
Kakapo                     GCTTAAAATCCATACAAAG----AAAGGCACAGAAGTCTCAAAAAGGTGTGCTCTTTGCCTTGTAAAAAAGGGCTTCCTC 5318
GoldenEagle                GATTAAAATCAATACAAGG----AGAGGAACAGAAGCGTCAAAAAGGTGTGGTCTTTGCCTTGTAAGAATGGCCTTATTC 5464
JapaneseQuail              GATTAAGTTAAATACAGGG----AAATGCAAGCAAGTAT-ATGAAGACATGATCTTTGTCATGTGAAAACGGTATTGTT- 5335
MediumGroundFinch          -------------------------------------------------------------------------------- 0
GoodesThornscrubTortoiseATTTTAATTCATTATGTAATATTAAATAAATCTAATTATCAGGTTAAT-TAAACAATACCAAATAGGATAACAATTGCTT 26778

Majority                   TC-GGATTTTAAA---AGGAAACAATCAATTTGTGTATTGAAGTATTGGGTATAAATAAAAAGGATGATTGT---TTTCT

                                  30570     30580     30590     30600     30610     30620     30630     30640

Human                      GC--CATTTTAGGG--GGCAGAATGTTAAAACTCAAATTTAACCCTGTTATACCAGGAAAGCCTTTCTTTTTAAATGCGT 30544
Kakapo                     TCTGGACTTTAAA---AGGAAACAATCCATTTGTGCATGGAAGTAGTGGGAATAAATAGAAAGGGTGATTGT---TTTCT 5392
GoldenEagle                TCTGGATTTTAAA---AGGAAACAATCAATTTGTGCATGGAAGTAGTGGGAATACATAAAAAGGATGATGGT---TTTCT 5538
JapaneseQuail              -----GATGTAGA---AGAAAACAATC----TGTATGCAGGAGCATAGGGTATAA--AATAAAGACAATGGT---TTTCT 5398
MediumGroundFinch          -------------------------------------------------------------------------------- 0
GoodesThornscrubTortoiseGTAGCTCTTTTAATCTGGTAAAATGGCAGTCTTTGTGTTTAGCTGTCAGTCACAAAATAGGAGGACATTTCC---ACTGT 26855

Majority                   GAAGAAAGCTGTTAGATTTGAGTGTAGTAAAGTGGATTGTATCC---TGGAGCTTCTGTAAC--TCTCAAGATACTAATA

                                  30650     30660     30670     30680     30690     30700     30710     30720

Human                      GTTGAAACATTTTAGAGTAAATTTTACTAAAGTATATATGAGCCGAGTGGAAAAAAAAAAAAAACCTGAAGCTGGTAACT 30624
Kakapo                     GAAGAAAGCTGTTAACTGTGGGAGAAGTAAACTGGATTGTATTC---CTGAGCTTCTGTAATTATCTCTAGATACTAATA 5469
GoldenEagle                GAAGAAAAGTGTTAGATACGGGAGCAGTAAACTGGAATGTATTC---TGGAGCTTTTGTG----TCTCAAGATACTAATA 5611
JapaneseQuail              GAAGGAAGCTGTTAAATATGAGTGGGATAAAGTGCACTGTCTCC---TTGAGCTTCTGTAACTGGCTCAGGACACCAAGA 5475
MediumGroundFinch          -------------------------------------------------------------------------------- 0
GoodesThornscrubTortoiseCTCCCAATTTATATGATTTTATGTTTGTCAAAAGACTCACACCG---TGAAGATCAGCTCAC--TTCCATGTTAAATATA 26930
```

```
Majority                    AGAATGTAAAATGGAATTTCTCAGTAATTTATGTTTGCAGTTTGTGTA-GCAGTTTGATGCTAGCTTCAGTTATGTTTTG
                                    30730     30740     30750     30760     30770     30780     30790     30800
Human                       TTGGTGAACAAGTTACTCTTAAATTTGATTGCTTTTCATTTTTGAACCACAAATGTAATTCTGTCTCACATTATAGTCCC 30704
Kakapo                      GGAATGGAAAATGGAATT-CTGGCTAATTGATGTATGCAGTTTGGGTA-GCAGCCTGATGCGAGCTTCAGCAGTGTTTCA 5547
GoldenEagle                 AGAATGGAAAATGGAATTTCTCGCTAATTGATACATGCAGTTTGGGTA-GCAGTCTTATGCTAGCTTCAGTCATGTTTTG 5690
JapaneseQuail               AGAATGTGACATGGAATTTCTTAGCAATTTATATTCATAGTTTGTGTAAGCAGTTTGATGTTGGCTCTAGTCATATTTTC 5555
MediumGroundFinch           -------------------------------------------------------------------------------- 0
GoodesThornscrubTortoiseAAACAGTTAAAGTAAAGAGAACAGTTCATCAGGATTTAAGCACATAAT-GTTAATCCATAAAATTTACATGTGAGATTAG 27009

Majority                    TTGGAGGCCTTGTGAAGCA--TTTGTTTTTTTTTT-TTTCTGGTGGCTGTTCGT--TGCTGTTGATGAA-CTTATGTTTT
                                    30810     30820     30830     30840     30850     30860     30870     30880
Human                       TTCATCTGCCCCTCTGGTATTTTTATTTTGTTTTTATTTTTTGTGTTTGTGTGTCATATAATATTTTTACTTTAAGTATT 30784
Kakapo                      TGGAAGGCCATGTGAAGCA--TTTGCCTTTTATGT-TCTCTGGTGACTGTTCAT--AGCTCTTGATGAA-CTGATGTTTT 5621
GoldenEagle                 TGGTAGGCCATGTGAAACA--TTTGTCCTTTATGT-TCTCTGGTGACTGTTCGC--TGCTCTTGATGAA-CTGATGTTTT 5764
JapaneseQuail               TAGGAAGCATTGTGAAACACCTCTGTGTTTCTTTT-TTTCTGGTGGATGTTCAT--TGCTGTTAATAAAGCTAACGCTCT 5632
MediumGroundFinch           -------------------------------------------------------------------------------- 0
GoodesThornscrubTortoiseTTGGGTTTTTTTTAAAGGTACTTAATTTAATTTTT-GTTTCTGTGGCTGACTTT--AGGAGTTGTCCCTATGTGGGTTTG 27086

Majority                    AAAGTTGAGGAATGCTTTACCTATAGGTTGTGTATAAACCACAGGGAACATTCACTTGGCCTTTTCTTAGCTTTTTTCTT
                                    30890     30900     30910     30920     30930     30940     30950     30960
Human                       TATGCCACAAACCCTAAAACCCAAATTTTAAGTAAAAACCGAAGAATTCAGCTACATAGTTGTTTAAAACCATTGCAACC 30864
Kakapo                      ACAGTTGAGGAATGCTTTATCTATAGGTGGTTTATAAACCACCAGGAACATTCATTCGGCCTTTTCTTGGCTGTTATCTT 5701
GoldenEagle                 AAAGTTGAGGAATGCTTTACCTATAGGTGGTATATAAACAACTGGGAACATTCATCTGGCCTTTCCTTGGCCATTTTCTT 5844
JapaneseQuail               AAAGCTGAGGGATGCTTTACCTAATAGTTG-GTATTTATGACAGGGAAAATTCACGTGGCTTTAACTTAGCTGTCATCTG 5711
MediumGroundFinch           -------------------------------------------------------------------------------- 0
GoodesThornscrubTortoiseCAAATAATCACACACACTTACTCTTAATTAACTGTGCCTAATAAGGAGAGAACACTAGACCTTCTAGGAGTGTTGTCCTG 27166

Majority                    TTCTTTGGGGTGCTTTGGG--AATGTGTA--GGGAGCAGGTTTTGAGT-CCAAGGTGTTGCATCA--GCAGTTTTAATGC
                                    30970     30980     30990     31000     31010     31020     31030     31040
Human                       ATAGTTGCTATGGTTTTAACTAATTTTTTGCTGAAACAGTATTTTAATTCTGAGTTGATCCTTTTATGTTGTCTACCTGC 30944
Kakapo                      TTCTTTGGGGTGCTTTGGG--AACGTGCA--GGGAGCAAGTTTTGAGT-CCAAGGTGTTGCATCA--GCAGTGTTAATGC 5774
GoldenEagle                 TTCTTTGGGGTGCTTTGGG--AATGGG----AGGAGCAAGTTTTGAGT-CCAAGGAGTTGCATCA--GCAGTTTTAATGC 5915
JapaneseQuail               TTCTTAGGGGTATATTGGG--AGTGGGTA--GAGAGCAGCTTTTGAGTGCCAAGGTTTTGCATCA--GCAGATTTAATGC 5785
MediumGroundFinch           -------------------------------------------------------------------------------- 0
GoodesThornscrubTortoiseCTGGAAAAAATAAAATCGA--AACACATGATACTTATAGAAGATGATTTACATTGTTATGGTTTCATGCATCTTCAGGAA 27244
```

```
Majority                 TCTTTTAA--TACT--GCTTTTATCGTGATTGG-ATAGCTATTGGAAGTTGGCAGGGATTTACCTGTGT------TAGAT
                                  31050     31060     31070     31080     31090     31100     31110     31120
Human                    ATATTTAAGATGATTTGTTTTAATCTTCAAAGTTAGAGTTAAAAAAAATTGTATATGTCTGAAATAATGTTG--TTTGTA 31022
Kakapo                   TCTTTTGG--CACT--GCTTTTACTGTGACTGG-ATAGCTCTTGGAAGTGGATTGGGATTTACCTGTGT------GAAAT 5843
GoldenEagle              TCTTTTGA--CACT--GCTTTTATTGTGACTGG-ATAGCTGTTGGAAGTGGGCAGGGATTTACCTGTGT------GAAAT 5984
JapaneseQuail            TCTTTTAG--TACT--GCTTTTATCATGATTGG-ACAGCCATTAGAAGCAGACAAGGATTTACCTGTACTTGCACATGAT 5860
MediumGroundFinch        -------------------------------------------------------------------------------- 0
GoodesThornscrubTortoise CCTAATAA--TGATTAGCCTGTAACATTTTCAACACAGCTTGTTAATATTAGAAGCTGATTCCTTGTGG-----CTATCA 27317

Majority                 CAACTTATCCAGGATATTTGTTTGACCTGATGCTTTCTTGAGGTTTTCTGCCTACTGTATG-TATGTTTATTAGTC--TT
                                  31130     31140     31150     31160     31170     31180     31190     31200
Human                    GCATCTGTCAGTAAAACAATATAGACCAAATTTTAGACCTTAGTTTTATGGCTGATGGGCCAAATTTTTTTTTGTATTTT 31102
Kakapo                   CAACTTACCCAGGACATTTGTTTGACCTGATGCTTCCCTGAGGTTTTCTACCTACTGTAT--TATGCTGATCAGTTAGTT 5921
GoldenEagle              CGACTTATCCAGAATATTTGTTTGACCTGATGCTTTCTTGAGATTTTGCGCCTACTGTATG-TATATATGCTGATCAGTT 6063
JapaneseQuail            CAACTTACCGAGGGTATTTGTGTGACCTTACACTTTCTTGAGGTTTTTTGCCTGCTGTATG-TATGCTGAGCAGC----T 5935
MediumGroundFinch        -------------------------------------------------------------------------------- 0
GoodesThornscrubTortoise CATCTTAGATTTTACAAGTTATTGGACTCCTGCCCCTTGGTTGGTTGCCAGGCAGGGAATG-GAGAAAACTTAGGC--CT 27394

Majority                 ATTTGGTATGTTTGTGATGTATTCATTTATATTTGAA-GAAAGAGATTGTGAT-TT---TGACCCTTCTGTTTGAAAAAT
                                  31210     31220     31230     31240     31250     31260     31270     31280
Human                    GTTTAGTTTTGCTTGAATTGACGTGTTCTCATCTGAATGGGCCAAATTTTGAATTTGATTATTATTATTATCAAATGAAT 31182
Kakapo                   AGACGGCATGTTTGTGATATATTCAGTTGTATTTGGA-GAAAGGGATTGTGACACA---GGACACTTCTGTTTGGAAAAT 5997
GoldenEagle              ATACAGTATGTTTGTGATGTACTCAATTATATTTGCA-GAAAGGGATTGTGATATA---TGACCCTTCTGTTTGGAAAAT 6139
JapaneseQuail            -----------CTATGGCATATCTGGTTATACTTGAA-GAAAAAGACTGTGA----------CCCTTCTCTTAGAAAATA 5993
MediumGroundFinch        -------------------------------------------------------------------------------- 0
GoodesThornscrubTortoise TCTTGGCCAGCATGGACTGGCATCATATA-GCCAGAA-GCAATATAGTCTCCT-TT---TCCTCCAGGTCTTGAAGATAA 27468

Majority                 TTGCTTTGCTT-TTCATCATCAGTCATGTCGATACAAATTTGAAAGTTCTATTA-ACTATTGTCTGGCTG--GTTTCTCT
                                  31290     31300     31310     31320     31330     31340     31350     31360
Human                    TTACCTCACAACTCCACATTTTACATTATTTACCTGAAACCAAAGTTCCTTTTTCTGTATTTTAACTTTGA-ATTATTTG 31261
Kakapo                   TGGCTTTATTT---CATCATCAATCATGTCGACACAAATTTCATAGTTCCATTA-ACTATTGGCTGGTT--------TCT 6065
GoldenEagle              TTGCTTTGCTTTATCATCATCAGTCATGTCGATACAAATTTGAAAATTACATTA-ACTATTGTCTGGCTG--GTTTCTCT 6216
JapaneseQuail            CAGTTTATCAAAATCATCATTAGTGTTGTTGATACAACTATGGAAATTGTATTA-ACTATTGTCTGGCAG--GCTTCTCT 6070
MediumGroundFinch        -------------------------------------------------------------------------------- 0
GoodesThornscrubTortoise CAGCCTGGCTGGTTCCTCCTCACAAAAACCTATCTTCTTTGGCAAGTCAGAATGTGGCACTGTCACTCTGAAGTCACTGA 27548
```

```
Majority                    CTTAAACAGCAAAG--TGAAT--GTTAGTTATATTTTTATACCCATTTGTAAGAGAGTAATAGTAGAAGTTAACTACTGT

                                     31370     31380     31390     31400     31410     31420     31430     31440

Human                       CTTGAAGAACAAAT--TATATTTGGCAATAAAAGGATCATGAAAGACTAGAAGACATTTTGATTTCCCTTAGGTAGCTGT 31339
Kakapo                      CTTAAACAGCAAAG--TGAAC--GT--GTTACGTTTTTACACCCATTTGTAAAAGAGTAATAGTAGAAGTTAACTACTGT 6139
GoldenEagle                 CTTAAACAGAAAAG--TGAAT--GTTAGTTATATTTTTAAACCCATTCGTAAGAGAGTAATAGCAGAAGTTAACTACTGT 6292
JapaneseQuail               CGTAAGCATAAAAG--TGAGCTTGTTAGCTATGTTTT-AAACCTGTTTGCTA-AGGCTAGCAGTGGAAGTTAACAAGTAT 6146
MediumGroundFinch           -------------------------------------------------------------------------------- 0
GoodesThornscrubTortoise    CATCACCAGTGGGGATTGATTGGGTCAATTTTATGCAAATGAAGAACTTTTGGAAAGTCCCACAATTAGTTT-TTATTTT 27627

Majority                    AGTCTGGGAATT-TTT--GAATTAGAAA------AAGGCTTTTCAGCTTTTGGGACTGCATGTAGGCAAGGTAAGTATGA

                                     31450     31460     31470     31480     31490     31500     31510     31520

Human                       GCTTAATGTATACATACATCTTGACACACACAAACACAAACACACATCTCTGACAATTCAGTTAACTAAAAATTATAAGG 31419
Kakapo                      AGTCTGGGAATTTTCT--GAATTAAAAA------AAGGCTTTTGAGCTTTGGGGACTGCATGTGGGCAAGGCAAGTATAA 6211
GoldenEagle                 AGTCTGGGAATTGTCTTTGAATTAAAAA------AGGGCTTTTCAGCTTTAGGGACTGCATGTGGGCAAGGCAAGCGTGA 6366
JapaneseQuail               AGACAGGA-----TTT--GGATTGGAAA------AAGGGGTTTCAGCTTTAGGAACTGCATGTAGGCAAGGTGAGTATAA 6213
MediumGroundFinch           -------------------------------------------------------------------------------- 0
GoodesThornscrubTortoise    ATTTTGGAAATAAATGGGCAGTCAGTCATTCTACAGAACAGAAAACTATTTTGTGATGCATGCAGGTAGAGT--CCATCA 27705

Majority                    A-TAAAGGGAAAACTTGCTTATAATGTTTTGTTAACAGTTTTACAGT--CTTAGTGTTATTTAT----TAATGAGGCTGC

                                     31530     31540     31550     31560     31570     31580     31590     31600

Human                       ACTTAACAGATAA--TAGTCATGATGTGAAGATA--AATTTGTCAACAGGTCATTATCATTGATTTTGCTATGTAATGAT 31495
Kakapo                      AGTAAAAGGAATACTTGCTTATAATGTTTTAGTAACAGTTTTACAGT--CTTGGGCTTAC---------AATGAGGCTGC 6280
GoldenEagle                 AGTAAAATGAATACTTGCTTATAATGTTTTATTAACAGTTTTATAGT--CTCAGAGTTACCTGC----TAATGAGGCTGC 6440
JapaneseQuail               A-TAAAGGGAAAGCCTGCTTGTAATATTCT-----CAGTTAGATAGT--CTAACTGCAAATTTC----TAATGAGGTTGT 6281
MediumGroundFinch           -------------------------------------------------------------------------------- 0
GoodesThornscrubTortoise    A-CTTGGGGAAAGTTGACTTTAGATTCTCTGTCAACAAGATCACCATAATTTAAAATTATTCATAC-ATACTGGACCTTC 27783

Majority                    T-AAATTTAAAGCTATTACCATTTGTTTGTGCTGTTC-A-GTGCTTTGATTTAAAACATTTTT-GTTCATCAAAATTTTG

                                     31610     31620     31630     31640     31650     31660     31670     31680

Human                       TACAAAGCAAAATCACTGGGCCTTGATTTTGATAGTCAAAGTACCATGTACACATAATTGAACAAATAGGCAGAAGTGTG 31575
Kakapo                      T-AAATTTGAAGCTATTACCATTTATTTGTGCTGTTC---GTGATGTGATTTAAACCATTTTT-GTTCATCGGAGTGTTG 6355
GoldenEagle                 T-AAATTTGAAGCTATTA-----TTTTTGTGCTGT-----ATGCTTTGATTTGAAACATTTTT-GTTCATCAAATTGTTG 6508
JapaneseQuail               TTAAATTTAAAGCTATTACCATTTATTTGTGCTAAGCAAGTTGGCTTGATTTAAAGCATTTTTTGTTCAACAAACTCATG 6361
MediumGroundFinch           -------------------------------------------------------------------------------- 0
GoodesThornscrubTortoise    CTTATTTCACAGTTGCTGCTGTCAGTTTAACTTTAGACAGCATTTTTTATTCAAAAATCTGACTGAAAGGTAAAACTATC 27863
```

```
Majority                 AAT-----GTTCAAT--TTTGTTTTTATTTTTGTGCTGAGTCACCAGAAATTAATACCAAAAGCTGAGTAAAGAATTTAT

                                  31690     31700     31710     31720     31730     31740     31750     31760

Human                    TAACTCTGGTTTGTCGGTTTCTCTTTTTGTTTTTACAATGCATACTGTGATCGATAATGGGAAATTAATTTGGAATTAAT 31655
Kakapo                   AAT-----GTTCAAT--CTTGGGTTTATTTATGTGTTGAGTCACCAGAAATTAATGCCAAAAGGCGAGGAAAATATA-AA 6427
GoldenEagle              AAT-----GTTCAAT--CTTGATTTTATTTTCATGTTGAGTCACCAGAAATTAACACCAAAAGCTGAGTAAAAAAAAATAA 6581
JapaneseQuail            AGT-----GTT--------T----T-ATTTGTGTGCTGAGTCAGAAGAAATTAATACCAAAAGCTGAGGAAAGCATTCAG 6423
MediumGroundFinch        -------------------------------------------------------------------------------- 0
GoodesThornscrubTortoiseACT-----GTAAAAAAGTCTTTTTTTTTTTTAAAACGAAATTCCCAGTTGTTGGTACAGTAATTATATAGGCTGTGTTGT 27938

Majority                 AACTAAAAAT--GGAGGTGGAGAAAA--TGACTAATACTATTTGAAAAC------TTGGTTTACATCGAGTAAATGTGGG

                                  31770     31780     31790     31800     31810     31820     31830     31840

Human                    TTCCAAATATTTAGGGCTAGTGACAGAGTAACTAGTAGCAGCCTTGGCCCCTTTTTCAGTCTCCCCCCAAAACCAAAAAG 31735
Kakapo                   ACTCAAAAAT--GGAGGTGGAAAAAA--TGACACATACTCTTTGAAAAC------TTGGTTCACACAGAGTAAATGTGGG 6497
GoldenEagle              AACTTAAAAT--GGAGGTGGAAAAAA--TGACATATACTATTTGGAAAC------TTGGTTTACATAGAGTAAATGTGGG 6651
JapaneseQuail            CGCAGAATGA--AGGAGTGGAGAAA---TGATGAATGCTATTTGAAAGC------TTGGTTTACGTGGTGTAAATGTGGT 6492
MediumGroundFinch        -------------------------------------------------------------------------------- 0
GoodesThornscrubTortoiseGATTACAAGT--GGATGCCTTGACATA-TTGCTAATGGTCTGTGAAGATCACCAGTTTGACTCCAGCTTGGCTCTGTAGG 28015

Majority                 GATTTG----TACAACATAAAATATGTGGTGGGAGCATGC-TTCCAAGTTAAAATCTGTCAGAGGAGCAGTGTGCTT-AA

                                  31850     31860     31870     31880     31890     31900     31910     31920

Human                    TATGTA-----ACTGAATAAAATACGTGGAACAACTGTTGTTTGGAAGTGATGAGCAGACGGTATAAGATCATCCTT-GA 31809
Kakapo                   GATTTG----CACAACTCAAAATATGTGGTGGGAGCATGC-TTCCAGGCTAAAACCTGTCAGAGGAGCAGTGTGCTTTAA 6572
GoldenEagle              GATTTG----CACAACTCAAAATATGCAGTGGGAGCACGC-TTCCCAGTTAAA-TCTGTCAGAGGAGCAGAGTGCTTTAA 6725
JapaneseQuail            GTTCTG----TCTAAAATCAAATGCGTAATGAGAACATAC-TTCCCAGCTAACATCTGTCAGAGGAGCAGTGTGCTT-AT 6566
MediumGroundFinch        -------------------------------------------------------------------------------- 0
GoodesThornscrubTortoiseAACTTAAAATTACCGTATAATGAATGTTGTGTGACTCTGTGTTCCAACTTGTAAATCATAAACTTATCATTGTGCTT--T 28093

Majority                 TTTCTGAAAGAGTTAGAACATGTCAAAGCTGAATACTCTTGTGTGTACATGTGAAGTAATTCATA--TTTGATTTGGTCT

                                  31930     31940     31950     31960     31970     31980     31990     32000

Human                    GAGAAGGGGTACACGTGAGGTAAGTATCATGAACACTCTGGCTTTCTGTCTTGGACTATTTTACTGCTCTAAACCACAGA 31889
Kakapo                   TTTCTGAAGGCGTTAGAACATGTCAAAACCCAGTAATCTTCTGAGCACGTGTGAAATAGTTCATA--TTTGATTTGGTCT 6650
GoldenEagle              TTTCTCAAAGCGTTAGAACATGTCAAAACCCAATCATCTTGTGAGTACATATGAAATAATTCATA--TTTGATTTGGTCT 6803
JapaneseQuail            TTTCTGGAAGAATTAGAATATATCAATGCAGATGACTTGTGGCCGTA-ATATAAAGTAATTCATA--CTTGAGTTGATAT 6643
MediumGroundFinch        -------------------------------------------------------------------------------- 0
GoodesThornscrubTortoiseTTACTGACAGATTCAGATAAGCCAGGAGCTGTATGGGCAGCTATTTACCTGTAAAGAAAGTAGCT--TCAGATCAGAT-T 28170
```

```
Majority                     GTAGTGTGTTGTTTTGCTTACCTAGAATTCATGGAGGC-A-GA-A-CGTGACTCTGCACAAAGTTTACCCCTTCCGTTTC
                                      32010     32020     32030     32040     32050     32060     32070     32080
Human                        GAAGTGGAGAGCCCAGAGTATACAGGTCTCACTGAGCTGAAGAGACCAAGAGTGGGTGTCAGGGCTGCCCAATCATTTGG 31969
Kakapo                       TTAGTGTCTTGTTTTGCTTACCTAGAATTCATGGAGGCAAGGAAA-CAGCACTCTGCAC--AGTTTACCCCTTCCGTTCC 6727
GoldenEagle                  GTAATGTGTTGGTTTGCTTACCTGGAATTCATGGAGGCAAGGAAA-TGTCACTCTGCACACAGTTCACCCCTTATGTTAC 6882
JapaneseQuail                GTAGCGTCTTGTTTGGCTGTCTTAGAATTCATGG------------CGTGCCTTTGCACAAAGTACACCCCTTACAGTTC 6711
MediumGroundFinch            -------------------------------------------------------------------------------- 0
GoodesThornscrubTortoiseCAGGCATGTTAGCAGGGCAATGTATATGAGAAGCTTAC--------CCTGCCACTGTGCTATAGATAAGCAGAGGACTTC 28242

Majority                     AATTTACAGAGTAGTACGAGAATAATGAGCTTTAGCTTTGTGTCTGATGTTACTTTGTGAATTCCCGTTGTATATAAGCA
                                      32090     32100     32110     32120     32130     32140     32150     32160
Human                        AATTTATGGAGAAGTCCTGGAGAAAAGAGAGCTGTCTAGGGGTTTAGGAATAGAGCAGGGGGCAGAATTTAGTATGGTCA 32049
Kakapo                       AATTTCCAAAGTAACAGGAAAAGAATGAGCTTTAGCTCTGGGTCTGATGTTACTTTGGGAATTCCCCTTGTATAGAAGCA 6807
GoldenEagle                  AGTTTACAAAATAATACAAGAAGAATGAGCTTTCGCTCTGTGTCTGATGTTACTTTGTGAACTCCTGTTGTATATAAGCA 6962
JapaneseQuail                AATTTAGATAGCAGTATGAAAATACTGAGATTTAACTTCGTGTCAAATGTTCCTGTGTGAACTCCCACTATATATAAACA 6791
MediumGroundFinch            -------------------------------------------------------------------------------- 0
GoodesThornscrubTortoiseATTTACCAGGGCTGGAAACTTGTAGTAGTGCTAAATCTATATACATTTTTTAAGATGAAGTTTATAGTTATATGCACAAT 28322

Majority                     GTTTGTGTAAA--GTGTTTTTG--AAAAGAACAGAGTTAAAGGTTCCAGTTATGATACTAT-ATTACGTGTTTCAGACAT
                                      32170     32180     32190     32200     32210     32220     32230     32240
Human                        TTTCACTTCATTCACAGTTTTGG-ATAATGACTTGGTTACATGTGTGCAGGTTGAGGCTTT-GCCAGGTCTGTCAGAAAT 32127
Kakapo                       GGTTGTACAAA--GTGGTTTTG--AATAGAACAGAGCTAAAGATTCCAGTTACGATAATAT-ATTACATAGTTCAGTCTG 6882
GoldenEagle                  GTTTGTGTGA---GTATTTTTG--AATAGAACAGAGCTAAGGATTCCAGTAATGATAATAT-ATTACATAGTTCATAGAT 7036
JapaneseQuail                GTTTGTGTAAA--GTGTTTTGAGTAAAACAACAGATTTAAGGCTTCCAGTAATGACAGTA--ATACCGTG--TCGATTGG 6865
MediumGroundFinch            -------------------------------------------------------------------------------- 0
GoodesThornscrubTortoiseATTGGTAAATA--TACACATTATTTAATGGACTGATTTACATGAAGTGTTTGTGTGACCATGACTAAGTGTCCCTAACAT 28400

Majority                     TTTCTCTTCAGTTTTTAATT------TCTTGATGTAAT-C-GTACATCTAAAATTTGTAG-TTTGTACATTCATAATTTT
                                      32250     32260     32270     32280     32290     32300     32310     32320
Human                        TGTCTGCTCTGGGGCTAAATGGTGATTCCATAGGTTGCACGGTACTCTGAAACATTGGAGTTCTGAGAAGTCAGAATGCA 32207
Kakapo                       TTTGAAATGACCTGCTAGTT------ACCTACTAA-----------TCTCAAACTTCT-------------CAT------ 6926
GoldenEagle                  TTTTTTTTTAATTTTCAGTT------TCTTGAAGAAATGCTATACATTTTAAATTTATAGATTTCTTCATGCATAGTTTT 7110
JapaneseQuail                TACCTCTTCAGGTTTTCATT------TCTTGAAGGAAT-CTGTACATCTGAAGTTTATAGATTTCTATGTGTACGCTTTT 6938
MediumGroundFinch            -------------------------------------------------------------------------------- 0
GoodesThornscrubTortoiseTTTCCCCCAAATCTGGCAAC------CCGAAATGTCAA---GTGACTCTAAAAAGTGT----TTGTACATTTAAAAAGGT 28467
```

```
Majority                     GACAATTGTCATGTTGTATAAAATGTTT----T----TTT--AAAGTGTGCGTCTTGGTTGCTTTGTTTTTATTATATGT

                                     32330     32340     32350     32360     32370     32380     32390     32400

Human                        GACATATTGCAGGGTACTTCAAACACTCCATTTAGACTACAGAAAGCCTGGACCTGAATAGAAAGGATCACACCCTATAG 32287
Kakapo                       ------------------------------------------------TGCTCACTGGCTGTGTTGGTTTGAGTATCAGA 6958
GoldenEagle                  AAAAATTGCCATATTGTATAAAATGTTTGCTTTTATGTTTTAAAAAGGTGCATCTTTGTTGCTCTGTTTTTATTACATGA 7190
JapaneseQuail                GACAATTGTCATGTTGTATAAAATGTTT----------TTTCCAAGTGTATGTTTTAGTTGCTCTGTTTTTATTACGTGT 7008
MediumGroundFinch            -------------------------------------------------------------------------------- 0
GoodesThornscrubTortoise     GACTGTAGTGACTCCATA-ATGAAGTCTGCAATGAGATTT--GAAATGGTAGGGGAGATTAAATCAATTTTCTTATATGT 28544

Majority                     G--CCTTAGTCACTAAATCTTGAATTGCGTGCTAGTATCCCTTCTGTG----AGTTAAGGTTGCAT--GTTAA--TTG-C

                                     32410     32420     32430     32440     32450     32460     32470     32480

Human                        GAAGTGCCATCTATAAGACTGAGATAAAAACCAGAAACCTAGCCCGATAGGAAGTGAAGGATTCACCAGTTAATCTAACC 32367
Kakapo                       A--TCT-AATAACTGTAGCTTGAATTGCTGGCTTGTGTTCCTTTTG-----------C-------------------- 7002
GoldenEagle                  GGTCCTTAGTCACTAAATATGAAATAGCCTGCTAGTTAGCTTCTAGTCTCAAACTTCTCATTGCTC--ATTGGCTATGTA 7268
JapaneseQuail                GGTCCATAGTTA-TATAGTTTCAGGGACGAGTTAGCATCTCATCTCTGT---AGTCAGTGAGGAAT--GTCAATTTTGGC 7082
MediumGroundFinch            -------------------------------------------------------------------------------- 0
GoodesThornscrubTortoise     T-----CGGCATTTCACTACTGAGCTTGGTGTCAACACCCCTTCCTAG----ACTTAAGATTGAAT--TTTCA--GGAAA 28611

Majority                     GGTTTGAGTATCTGGGTTTAATAGC--TGTAGTT----TGAATTGCTGTCTTATGTTCTCTTTACAGTTATGATGCTTTA

                                     32490     32500     32510     32520     32530     32540     32550     32560

Human                        AGTAACTGATGAAGGATTAATCAGTAATTTAGTTGCCTTCCAAAACAAAACAACACCCCCAAGAAAGACATGATATAGTT 32447
Kakapo                       ----------------------------------------AGCTA-----TGATGTTCTCTTTACAGTTATGATACTTTA 7037
GoldenEagle                  GGTTTGAGTATCAGAATCTAATAAC--TGTAGTT----TGAATTGCTGTCTTGTGTTCTCTTTACAGTTATGATGCTTTA 7342
JapaneseQuail                GCTTTGA-CATCTGGGTCTGACAGC--TGTAGTT----AGAGTTGCTGTCTTGTGTTCTCCTTATAGTTAGGAGGCTTTA 7155
MediumGroundFinch            -------------------------------------------------------------------------------- 0
GoodesThornscrubTortoise     CTTGAAAGTAAATCTGTTCACTAGC--TCAAGTT----AACAATCAAAAGTTATTTTCTTTTTGAAATT-TAAACTTAAA 28684

Majority                     ATCTGGC----------TAAGTAAACTGATTTGGCACTTGGAGTGATAGAGGCTTTTTCGACTGTTGAAGTGAACGTATG

                                     32570     32580     32590     32600     32610     32620     32630     32640

Human                        TTCCTGCAACTATCTTCCATAATATCTAGTATACCATCAAAAATTACAAGTGATGTGCAGAAGCAGGAAAGTAACCCATG 32527
Kakapo                       ATCTCGA----------TAAGTAAACTGATTTGGCACTTGAAGTGATAGAGCCTTTTTCAACTGTTAAAGTGAACGTATG 7107
GoldenEagle                  ATCTGGA----------TAAGTAAACTGATTTGACACTTGGAGTGATAAAGCCTTTTTCGATTGTTGAAGTGAATGTATG 7412
JapaneseQuail                ATCTGGC----------TAAGTAAAC----TTGGTACTTGGAGTGGTGGAGGCCTTTTGA------------AATGTAGG 7209
MediumGroundFinch            -------------------------------------------------------------------------------- 0
GoodesThornscrubTortoise     CTCTCAG----------CTCCTTTTCCCTTTCCTCTAAGTCAGTGGTTCCCAGTCTTTTCTCTGCCATGCCACACCTTGA 28754
```

```
Majority                     AAGTATATAATGTATCTTTTCTCTGG--AGTAGTTAAAAGAATCTTTAG--AGAGGTAT-AAAATGTGATGTAAAGAATG
                                       32650     32660     32670     32680     32690     32700     32710     32720
Human                        AAATATAAAGAACACATTTAATAGGAACAGATTCCAAAATGACCTAGAGTTAGAATTAGCAGACAAGGACGATAAGTATT 32607
Kakapo                       AAGTATATAATGTAGCTTTTCTCTGG--AGTAATTAAAAGAATCTTTGG--AGGGGTAT-AAAATGTGATGTAAAGAAGG 7182
GoldenEagle                  AAGTATATAATGTATCTTTTCTCTGG--AGTAGTTAAAAGAATCCGTAG--AGGGATAT-AAAATGTGATGTAAAGAATA 7487
JapaneseQuail                AAGTATATAATCTGTCTTTTCTCAGG--AGTAATGACAAAAATCTTTAG--AGACACAG-AAGCTGTGATGGAAAGAATG 7284
MediumGroundFinch            -------------------------------------------------------------------------------- 0
GoodesThornscrubTortoise     GCTCCACGGGCTCATCCTCATTCTGGCTGTCAGTGGATAGGGGACGGGC--AGAGGTG--ACGACCTGTTGCTCACACAG 28830

Majority                     TAGATCACAGAGGTGCAAGGAATAACTTTGGTGTGATTTTGGTGCGTGTTGAAGGTGATAGTTCTCTTTTACTGTATT-C
                                       32730     32740     32750     32760     32770     32780     32790     32800
Human                        TTAAGCTGTAAAGGAAAAGATTGAAATAATGAGTGATGGAGGAGTGTGTTGAAGA-GATAAAAAACTATAAAGAAAGCA- 32685
Kakapo                       TAGATCACACAGATGCA-GGAATAACTTTGGTGAAATTTTGGTGCGTATTGAAGGTGATATTTCTCTTCTACTGTATTGC 7261
GoldenEagle                  TACACTACACAGGTGCATGGAATAACTTAGGTGAACTTTTGGTGCGTATTGAAGATTATAGTTCTCCTTTACTGTATTTC 7567
JapaneseQuail                TACCTCACAGAGATGCAGGGAATAACTATATGGTGA----GCTGCTAGTTGGTATTGATAGTTCTCTT----TGTA---- 7352
MediumGroundFinch            -------------------------------------------------------------------------------- 0
GoodesThornscrubTortoise     GAGGGGAGGGGGGGAAAAGAGGGAAGTTGAGCTCTAATTGCTCTCCTCCTGGAGGAGGTGGTGCAGTGAGGCAGCTTCCC 28910

Majority                     --AGTGTGTGCCACTTCAGTCTAATATATCGTCTTTCTCCTGGTTGGCATGGATAGCTGTGCTGGAAATGGGGGCTGA--
                                       32810     32820     32830     32840     32850     32860     32870     32880
Human                        --TGTAGAAATATTCTAATTGAAAAATACAGTTTTTGAAATAGTTGGGACTGATTCACTGGTTGGAACTAGTAGCAGATT 32763
Kakapo                       --AGTGGGTGCCACTTCAGTCGGATGTATCATCTTTCTCCTGCATGGCCTGCATAGTTGTGCTGGAAACGGGGACTGA-- 7337
GoldenEagle                  --AGTGTGTGCCACTTCAGTCTAATCTATTATCTTTCTCCTGGATGGCACATGTAGCTGTGCAGTAAATGGGGCCCGA-- 7643
JapaneseQuail                --AGCGTGCACCACTTCAGCTTAACATATCCTCTTTCTCCTCATTGGCATGGGTAGCTGTGCAGTGAATGTGGCCTGA-- 7428
MediumGroundFinch            -------------------------------------------------------------------------------- 0
GoodesThornscrubTortoise     CTTTCCCCTCCCCCCTTATTCTCACTTGCAGTTTTTCAAAAAGTTCCACAGCACAGCTGT-TTGGACATGATGGCACA-- 28987

Majority                     ----ACAATTGAGTGACTCATTTTG-ATATGTGCATTGCGCATTTCATG-----TGCAATGGGTCAGG----TGTACTTC
                                       32890     32900     32910     32920     32930     32940     32950     32960
Human                        GGAGACAATAGAAGAAATGTTAGTGAACTTGAATACTGTTGAATACAGTG---ATTAAATCTGTCAGAGAGGAGAAAGTA 32840
Kakapo                       ----ACAATTGAGTGACTCATTTAGTGTATGTGCATTGCGCATTTCATG-----TGCAGTGGCTGAGG----TGTACTTT 7404
GoldenEagle                  ----ACAATTGACTGACTCATTTTGTATACATGCATTGCACATCT---------TGGAATGGCTGAGG----TGTACTTC 7706
JapaneseQuail                ----GCAATTGAGTGACTCATTTGG-GGATGAGCATTGCACGTTTCAGGCTGTCTGCAGTCAGTCTGGCT-ATGACACTC 7502
MediumGroundFinch            -------------------------------------------------------------------------------- 0
GoodesThornscrubTortoise     ----CCAGTTGGGAAACATTGCTCT-AAATATGATAAGTGTCTTGGATGTGACACTTAAAACTTCAGGTCCATATATCTC 29062
```

```
Majority                 TATCTGATG-------A-----------------TGGAGCGAAGAAGCAGTCGTTCCTTCATGGGTGTTTT---------

                                  32970     32980     32990     33000     33010     33020     33030     33040

Human                    TATCTAATGGACAATGAAGCAGTCTGACA-----TGTAAGTTGAATTTCAGAGTGAGAATGTGGGGGAATAAAACATCTA 32915
Kakapo                   TGCCTGCTG--------------------------GGAGAAAAGAAGCAGTCATTCCTTCATGAATGTGTG--------- 7449
GoldenEagle              TACCTGACG--------------------------GGAGAAAAGCAGCAGTCATTCCTTCATGGGTGCATT--------- 7751
JapaneseQuail            TAACTGATGAGCAGAAAAAGGTGGGGGGGGCGGGTGGAGCGGAGGGGAAGGTTTTCTTTCTTTGTTCTTTTTTCTAATAC 7582
MediumGroundFinch        -------------------------------------------------------------------------------- 0
GoodesThornscrubTortoise TTTTAGATAT----AAACTAAT------------TATGACGAGATCCCCGAGGTGCACCCAGGGACCGTGGGACTGCTGT 29126

Majority                 --T-ATTTGGTACATAGCATTTGGGAATTTGTAACATT-GTCTACACTGCTTTGGGCTACA------GCTGGCCAAGATC

                                  33050     33060     33070     33080     33090     33100     33110     33120

Human                    TCTGTTTGAGATAGTAATGACTGAGAACTTCTAAAATTTGAATAAAATATATCAACTTACATTCCTAAGAAGCTCAGGTA 32995
Kakapo                   ----ATTTGGTATATAGCAGTCGGGAATTCGCAACGTT-GTCAACACTGCTTTGGGCTGT-------GCTGGCCAAAAAC 7517
GoldenEagle              ----ATTTGGTACATAGCATTTGGGAATTTGTGACGTT-GTTGACAGTGCTTTGGGCTAC-------ACTGGCCAAAACC 7819
JapaneseQuail            ATTTACTTGGTACATAGCATTTGGGAATTTGTAACACT-GTAGA-ACTGCTTTGGGCTACA-----CGCTGGCCAAGAGT 7655
MediumGroundFinch        -------------------------------------------------------------------------------- 0
GoodesThornscrubTortoise CCTGCCATAACTCTTTCCAGTCTGGGCTGTCTCTCAAT-GCCTTGCTAGTGACAAGCAGCA-----AGCACCTCCAGGTG 29200

Majority                 TGATCTTCAGCT-GC-CAAGTGTGGACAGCCCTAGTTA--TACCTAGGAGGGAAAAC-TCATATACTC----CTGTAAAC

                                  33130     33140     33150     33160     33170     33180     33190     33200

Human                    AACTCCTCATGTAGGCTAAATAAAAAGAACCATACTTAGACACATAGTAGTCAAAATATTGAAAACTAGAAATGAAGAGC 33075
Kakapo                   TGAACGTCAGCTTGC-CAAGTGTGGACAGCCCCAGCTG--TACCTAGGAGGGAAAACCTCATATATTC----CTGTAAAC 7590
GoldenEagle              TGAGCTTCAGCT-GC-CAAGTGTGGACAGCCCCAGTTA--TACCTAGGAGGGAAAAC-TCATGTACTC----CTGTAAAC 7890
JapaneseQuail            GGAGCTTCAGCT-GC-CAAATGTGAACAGCCCAGGTTA--TGTATAGGGGAAGAAACTTCATATGCTA----TTGTAAAT 7727
MediumGroundFinch        -------------------------------------------------------------------------------- 0
GoodesThornscrubTortoise CTGTGATCACTCAGCACAACTGCATGTAGAGCTCCCGC--AGCCCAGCTAGGTTGCA--TGAATGTTC----CCTGAGCC 29272

Majority                 ATAGTTTTGTAATTTTGAAAGA---GGG-AATAGCCTTTTTCCCCCAAGAGTCAGGGCATCAGTCATTGCAGTATGCTTA

                                  33210     33220     33230     33240     33250     33260     33270     33280

Human                    AAATCTAAACAGCTGCGAAAAAAACGTCTAACAGGAGACCAACACCTCGTATTAAAGGGGGAAAAAAAGCGAATTTCTTT 33155
Kakapo                   ATAGTTTTGTAATGTCTAAAGATGTGGG-AATGGCCTTTTTCCCCCCAAAGGCAGGGCATCAGCCTTTGCATGCT--TAA 7667
GoldenEagle              ATAGTTTTGTAATGTTTAAAGATGTGGG-AATGGCCTTTT-CCCCCAAAAGTCAGGGCATCAGCCATTGCAGTATGCTTA 7968
JapaneseQuail            GCAGGTTTGTTGATCTGGGAGA-------AGGATCTTTTTTCCCCCAAGAATCAGAGCATC--TCTTTTTAGTATGCCTA 7798
MediumGroundFinch        -------------------------------------------------------------------------------- 0
GoodesThornscrubTortoise ACTCATGAATCACACAGAGAAA---GGC-ACCAGCCGAATCCCCCCAGCTCCCAGCAC-TGACTCAGGGATATATCATCT 29347
```

```
Majority                     AGGAAGGATGGAATTGTTAGTGTGTA-TTAGTTGGTTATGTTAGGATTGGGTAG----AAGATGTTAAGAAGTATT----
                                     33290     33300     33310     33320     33330     33340     33350     33360
Human                        AGACTGAATGGCATAACTTGGCTCTA--CAGGGGATAAGAGGAGTACTGGGAATGGTTAATATGTGTATAAATTTTAAAA 33233
Kakapo                       AGAGAAGACGCAATTCTTGGTGTGTGTGTAGTTGGTTACGTTATGGTTGGGTAG----AAGATGCCAAGAAGTGCT---- 7739
GoldenEagle                  AGGAAAGAGGGAATTCTTAGTGTGTATTTAGTTGGTTATGTTAGGATTGGGTAG----AAGATGCTAAGAAGCACT---- 8040
JapaneseQuail                GGGAAGGAGGAAATTGTTAGTGGTT--TTAGTTGGTAATGTTAGGGTTGGGCAG----GCGGTGTTAGAACATAGC---- 7868
MediumGroundFinch            -------------------------------------------------------------------------------- 0
GoodesThornscrubTortoiseTGCATTGCTCAAGTTGAGCGGTACAAATTTATTAATTGGTTTACCAGTGGAAAG----TGGATATACACCAGCCTTTGCA 29423

Majority                     -GTCTTAGTAC-TTTCTGTTAGTTTTCCAAAAAGTTATGTGTCACAAAGTCCCTCTTTTTCATGGTTGCTGAAAT-TTAA
                                     33370     33380     33390     33400     33410     33420     33430     33440
Human                        GGGCTCTCTCTTTTCCTATCAGTTTCTTTGAAAGCCTTTATGTAAACAACAACTTTGTATTATGGGGTTTATTATGTAAA 33313
Kakapo                       -CTCCTGGTAC-TTTCTGGTAGGTTTCCAAACAGTAATGTGTCCCAAAGTCCCCCTTTTTCAAGGTTGCTGAAAT-TTAA 7816
GoldenEagle                  -GTCCTAGTAC-TTTCTAATAGTTTTCCAAACAGTAATGTGTCACAAAGTCCCCCTTTTTCCTCGTTGCTGAAAT-TTAA 8117
JapaneseQuail                -ATATGAGTAC-TTTCTGTTGGCTTTCCAACAAGTT---CATCACAAAGGCCCTCTTTTTCCTGGTTACTGAAAT-TTAA 7942
MediumGroundFinch            -------------------------------------------------------------------------------- 0
GoodesThornscrubTortoiseAACCTGAGCACATTTGCCACACACTTCAGGCAGATTCACTGGTAAAGATAAACAGTAAAATAAGTTTGACTACAA-AAGA 29502

Majority                     CAGATGTTAATATAATAGTATTGAGGAAGGGCT------GAAACTAAAAGAAATATGAGGTGATAGAGTACA-TAAGAGT
                                     33450     33460     33470     33480     33490     33500     33510     33520
Human                        TGGATGTTTATATGACAACAGTTAGTACAAAGTG-----AAGATAAATGGAATTCTTCAGTGGTAAGGTTCTTATATTTT 33388
Kakapo                       CACAGGGGAATATAATAGTATTGGGGAAGGG--------GAACCCATGAGAGACACGAGGTGATAGAG------AAGAGG 7882
GoldenEagle                  CATGGGGGAATATAGTAGTATGGGGGAAGGGCAGTAGCTAAAAGCAAAAGAGATAGGAGCTGATGGAGAACAGGAAGAGA 8197
JapaneseQuail                CAC-------------TGTA--GAGGAAGGGCA------GTCGCTAAAACCAGCACAAGTTGAAGGAGAGAAATAAGAGA 8001
MediumGroundFinch            -------------------------------------------------------------------------------- 0
GoodesThornscrubTortoiseTAGATTTTAAGTGATAGGCAAAAAGTCAGAGTTAGTTACCAAAATAAAATAAATATAAGCAAGCAGGTTAAACTCTCAAT 29582

Majority                     GCCTGAAGTAG--CAGTGTT---TGTA-AATAC-A------------------A-A-----A-T-TC--TGTAAGCACTT
                                     33530     33540     33550     33560     33570     33580     33590     33600
Human                        ACCTGAAGTGATACAGTGTTAACTCTAACATAAAAAGATTCTGTTAAGGTTGCATATCAAAATTCTAAGTGTAATCTCAT 33468
Kakapo                       GCATGAAGTAG--AGGGGT--------------------------------------------------TGC-------- 7902
GoldenEagle                  GCACAAAGTAG--CAGTGTC---TGGAGAAAAGGAAGAG---------AGCACAAAATAGCAGTGTC--TGTAAGTACTC 8261
JapaneseQuail                GCCCTGAGTAG--CAGTGTT---TGTA-AGTAC--------------------------------TC--CCTGAGCAATA 8041
MediumGroundFinch            -------------------------------------------------------------------------------- 0
GoodesThornscrubTortoiseTCTTAGATTGGG-CAACATC---TAGATTAAGCAATTTTTCTCACCCTACTGGATATTGCAATTCGT--TGTACACAGAT 29656
```

```
Majority                   TTCA-GTT-TATCCAGTTCTTTT-TTCTTTTTTTTTAGACTCAAGTTCTGAGGGTTAATCTCTTTTCTTCCAAAATTATT
                                    33610     33620     33630     33640     33650     33660     33670     33680
Human                      TTAAAAAGATAAAAAGAAGTATAACGAAAAGCCAATAGAGGAAAAATTGGAGCATTGACTACCAGTTTTCAAGAATTACT 33548
Kakapo                     -TA-------------------------CT-TCATTTAACTCGAGTTCTGAGGAGTAATCTCTTTTCTTACAAAATTATA 7955
GoldenEagle                TTGAAGCGTTGTCCACTTCTTTT-TTTTTTTTTTTTGCCTGAAGTTCTGAGGGTTAATCTCTTCTCTTACAAAGTTACA 8340
JapaneseQuail              CTCA-GTTCTTTTTTTTTCTTTT-TTCTTTTTTTTTAAACTCAAATTCTGAGATCT--TCTTTTCTCTTCGGAGATTATT 8117
MediumGroundFinch          -------------------------------------------------------------------------------- 0
GoodesThornscrubTortoise   TTCACGTTGAAACCGGGGTCAGT-CTCCTCTGTTGGAGTCTTAAGTCTTCTCAGTGT-CCTTGTTGCTTGCAGCATAGGT 29734
```

```
Majority                   G-AACGCTGGCCCTTTTTTGCTTCTAATAACTGAGAGTTTC-GTTATGGTGGTAATACATATTGTGGATATATTGCGACT
                                    33690     33700     33710     33720     33730     33740     33750     33760
Human                      GCAAAGCTACGGTAATTAAAAATAGCATCATAATAACATAAAGATAAATAGGTCAGTGAGACAGAGCAGAGAGTTCAGAA 33628
Kakapo                     G-AGCCCTGGCCCTTTTTTGCTTCTAATAACTGAGAGTTTC-CTCATGGTGGGCATA---------GATACATTGCGACT 8024
GoldenEagle                G-AACACTGACCCTTTTTTGCTACTAATAACTGAGAGTTTC-CTTGTGGTGGAAACACATATTGTGGATATATTGTGACT 8418
JapaneseQuail              A-AAGACTGACCAGTTTTTACTCTCAATAATGGAGAGCTTC-GTTATGGTGGAAACAAATATTTTGGTCATATTGGGTCT 8195
MediumGroundFinch          -------------------------------------------------------------------------------- 0
GoodesThornscrubTortoise   G-GGAGCAGGGGAAGCCAAGCATGTGGCCACTGTCTTCTGT-TTTATACCCTTAATCCATGTGCT-TGTAAAACACAAGT 29811
```

```
Majority                   TTTTATTTGAGCTGTACAGTGTA----------------GG---A----CA---CTCCTGGGAAG-TGTCAAC----AGA
                                    33770     33780     33790     33800     33810     33820     33830     33840
Human                      GTTAACTCACACAATTATGGATAACTGATTTTTGACAAAGGTGCAAAGTCAATTCAGTGGAGAAAAAGTAGACTTCCAAC 33708
Kakapo                     TCTTATTTGAACTGTACAGTGTA-------------------------------ATTCTAGCAAGCTGTCAGC----AGA 8069
GoldenEagle                TTTTATTTGAGCTGTACACTGTATAATGAAAATATGTGTGGAAAAAAGTCAGAAATCCTAGGAAGTTGTCAAA----AGA 8494
JapaneseQuail              TTTTATTTAAGCTGT----------------------------------------T-----------T------------ 8212
MediumGroundFinch          -------------------------------------------------------------------------------- 0
GoodesThornscrubTortoise   ACAGGCATGTCTCATGGACATTGCTGAGTCTCCAGGCAAGGCTGAG---CAATTCCCCTGGTGTGGCCTCATG----CAG 29884
```

```
Majority                   GAAGTTTACTCTAAACTTTAGAAATGGGAGAATAGATGTAAATAGTTTTTCTTTTCAGTTCTTCTTCTT-TTTCTGTT
                                    33850     33860     33870     33880     33890     33900     33910     33920
Human                      AAAGGGTGCTACAGCAGTTGGAGCTATCCGTATGCAAAAAAATGATCTTGGATCTGTATCTCACACTGCATATGAAAATT 33788
Kakapo                     GAAGTTGAGACTAAACTTTAGAAATGAGAAAATACATGTAAATAGCTTCTTCCTTTCAGTTTCTTCTTCTT-TTTCTGAA 8148
GoldenEagle                TAAGTTTACACTAAACTTAAGAAATGAGAAAATGTATTTAAATAGCTTTTTCCTTTCAGTTTTTTCTTCTT-TTTCTGAA 8573
JapaneseQuail              --A--TAA---TACACTTTAAAAAAGGAAGCATAGGAATA---A----------------------------T------- 8247
MediumGroundFinch          -------------------------------------------------------------------------------- 0
GoodesThornscrubTortoise   CTGAGTCATTGCATTGTCATTCCCTTGCTGGACAGTGGCCGGTGATGTCTGTTCAACAGCACCCACCTTGC-TGTTAGTT 29963
```

```
Majority                    ACCTCTTT-GCCTTCA----CTGTTGTAATTCCAAATACTTAAAAAAA---A--GA-TGGTAGGATAAAACTCCTT----

                                    33930     33940     33950     33960     33970     33980     33990     34000

Human                       AACTCCAAATGGATCATGGCCTAATGTAAAACCAAAAATTATAAAATCTCTAGAAAAAAATACAGAAAATCTTCGTGACT 33868
Kakapo                      ACCTGTTT-ACCTTCA----CTGTTGTAATTCTAGATACTTAAAAAAACTCATGGAGTGGTAGGATAAAAAACCTG---G 8220
GoldenEagle                 ATCTCTTTTGCCTTCA----CTGTCATCATTCTAGATACTTAAATAAA---AGGGAGTGGTAGGATAAAAAAAGT----- 8641
JapaneseQuail               --CTGTTA-----------------GAAATTCCAATAAATTACCAAAA----------GGTAAGTTTAGCCTCCCT---- 8294
MediumGroundFinch           -------------------------------------------------------------------------------- 0
GoodesThornscrubTortoiseACCTCCCTTGTCAAT-----CTGGGTGCCTCCCAAACCCTCAGCATATTTTA--G--TGAAAACCATGCACCATTT---T 30031

Majority                    TTGAATTATTCATACAGACAAATGCCATG----TGAAGGTCTGTTTTATTGTATTTAAACTATTAGAATGATACTTTTGA

                                    34010     34020     34030     34040     34050     34060     34070     34080

Human                       TTGGATTAAGCAGGAATGTATTTGATATGACCCTAAAAGCACAATCTATTAAAAAATGGAATTTGACATCAGAATTAAAA 33948
Kakapo                      TTGACTCATTCATACAGACAAATGCCATT----TGAAAGCCTGTTTTATTGTGTTCAAACTATTAGAATGATACATTTGA 8296
GoldenEagle                 TTGAATCATTCATACAGACAAATGGTATG----TGATGGTCTGTTTTACTGTATTTAAAATATTAGAATGATAGTTTTGA 8717
JapaneseQuail               TTAGGCTAAACTTACAGACAAATGCCATC----TGATGATTTGTTTTATTTTTGGTAAACTA------------------ 8352
MediumGroundFinch           -------------------------------------------------------------------------------- 0
GoodesThornscrubTortoiseTATAATTTCAAATGCATTGACGATACACA----TACACCTCTACCCCGATATAAGGCGGGTTTGCATACAAGGCAGTAAA 30107

Majority                    GCTATCTT-----------TGCTTGTCTGATATA--ATTACTAGTAGATTGCTAGTAG---ATTA-GACAAAGCCTC-AG

                                    34090     34100     34110     34120     34130     34140     34150     34160

Human                       TCTTCTGCCATTCAAAAGAAACTGCTAGGGAATGAAAAGACAAGCCATAAACTGGAAGAACATCATAACAAAGCATATAG 34028
Kakapo                      GCTATCTT-----------TGCATGTCTGATATA--ATTACTAGTAAATTCCTAGTAG---ATTA-GACAAAAAGTC-AG 8358
GoldenEagle                 GCTATCTT-----------TGCATGTCTAATATA--ATTACTAGTAGATTGCTCATAG---ATTA-GACAAAGACT---G 8777
JapaneseQuail               --------------------GTGTTTCTCATACT--AGT--TGG---GCTGCTAGTAG---ATTA-GACAAAGTTTC-TG 8400
MediumGroundFinch           -------------------------------------------------------------------------------- 0
GoodesThornscrubTortoiseGCTCTGAC----------ACACTGCTCTGACACACTGCTCTGAGCAGCGTGTTAAGGGT--GCTG-GACCAGGCCCG-GG 30173

Majority                    CT----AAGGGCATCGATATGAG--AAAAATTGTTTGATTGTGAATTGGATGAACTGTT-TAGTTTTG-------TGTTA

                                    34170     34180     34190     34200     34210     34220     34230     34240

Human                       CTGATAAAGGACTTCTATCCAGAATATTAAAAGATTCAGTATATGTAAAATCAGCAGTC-AAGCAACAAACTACTCAATG 34107
Kakapo                      CT----AAGGGCAGTGATATGAG--AAAAATGGGTTGATTGTGAATTAGATGAACTGTTTTAGTTTTG-------TGTTA 8425
GoldenEagle                 CT----AAGGGCAGCAATATGAG--AAAAATGACTTGATGGTGAATTGGAAGAACTATTTTAATTTTA-------TGTTA 8844
JapaneseQuail               CT----AAGGCCATTGGTACGA-----AAGCTGTTTGCCTGTGAATTGGAGGAACTGT------------------GTTA 8453
MediumGroundFinch           -------------------------------------------------------------------------------- 0
GoodesThornscrubTortoiseCT----GAGGGGTTCGATAAGGGGCAGAAGGTCTTGGGGGGTGGTCAGGGGCTACCCCCCAGGGTCTGGGGCGGGCAGGA 30249
```

```
Majority                  GCTGGGATAATGTT-TTAAATCGACTTTTGTGTTCAATAATATTTAGTTGTAGCAACTGTAGACAGTAAAAAATTTGCAG
                                   34250     34260     34270     34280     34290     34300     34310     34320
Human                     AATTGGCCAAAATATTTGAACAGGTAGTACAGCACAA-AAGATACACAGATGGTAAGTA-AGCACGTGAAAAAGCTGGTC 34185
Kakapo                    GCCAGGATAACGTTCTTAAATCAAATTTGGTGTTCGGTAATACTTAGTTGTAGCAAGAATAGACAGCAAAAAATGTGCAG 8505
GoldenEagle               GCTAGGAGAATGTT-TTATATCAACTTTTGTGTTCAATAATACTTAGTTGTAGCAACAGTAGACAGCAAAAAATGCGCAG 8923
JapaneseQuail             GCTGGGATGATGTT-TACAGTTGAACTTTATATTCAACAATATTTTGTTGCAGCAGCTCTTGTAAGTAGAAA-TTTTCAG 8531
MediumGroundFinch         -------------------------------------------------------------------------------- 0
GoodesThornscrubTortoise GCTGTGGGAGGGCACTTTTGGGGGCCCCGCAGTGCCAGAGTGGCCTGGGGGATTAGCGGGGGGCTGGGAGCAGCTCGCTC 30329

Majority                  AACATTC-TAGCAGTGA----AGTGGGAAT--GAATTGTGATTAT----GTTTAGAAGTTTAGAACTGGCGTCTGGATTT
                                   34330     34340     34350     34360     34370     34380     34390     34400
Human                     AGCATCATTAGTTATTAGGGAAATGAAAATTAAAACTACAGTGAGATAATATTACAAACCTATTAATAGTGACTAAAATT 34265
Kakapo                    GACATTC-TAGCAGTGA----AGTGGGAAT--GAAGCGTGACCAC----GTTCAGAAGTTCAGAACTGGCATCTGGATTT 8574
GoldenEagle               AACATTC-TAACAGTGA----AGTAAGAAT--GAATTGTGACTAT----CTTCAGAAGTTTAGAACCACCGGCTGGATTT 8992
JapaneseQuail             AACGTTC-TAACAGTGA----AGTGGGAGT--GATTTGTGATTGTA---GTTTGTAAGTCTGGAACTGGCATCTGGATTT 8601
MediumGroundFinch         -------------------------------------------------------------------------------- 0
GoodesThornscrubTortoise TGCTTCCCTCGCCCCAGCCCCAGCTGTGTT--GCTTGAGGGAGGGG---ACTTGGGGAAAGGATCCCCTCCCCACACTC 30404

Majority                  -------GTCTCTTCAGCGGTAGTGTG-----GT---------G---------------CA-----C--C----------
                                   34410     34420     34430     34440     34450     34460     34470     34480
Human                     AAAAAGAGTGCCATCTTGGAAAGCATGAGTTGGGAAGAATGTGGAAGAATGTTAATATTCACAATACTGCTGATGGGAAT 34345
Kakapo                    -------GTCTTTTCAGCAGTGG--------------------------------------------------------- 8590
GoldenEagle               -------CTCTCTCCAGTGGTGGTGTGTTGTGGTTTAACCCCAGCCAGCAACTAAGCACCACACAGCTGCTCACTTACTC 9065
JapaneseQuail             -------GTTTCTTCAGCAGGAGTGTG------T---------------------------------------------- 8622
MediumGroundFinch         -------------------------------------------------------------------------------- 0
GoodesThornscrubTortoise -------AGCGCAGCTGCGGAAGCGGA--GCAGCCCTGCCGCAGCCCGCTCCACTCTGCCAGCTCTCCACCGCAGCACTC 30475

Majority                  ------------------G-A---------------------------C-------------------C--TT--C----
                                   34490     34500     34510     34520     34530     34540     34550     34560
Human                     GTAAAATCGTAGTACTTTGGAAAACAATCTGGTAGTTTCTCCATGATCCAGGTTATTTGTCTCCTAAGCATTTACCCAAG 34425
Kakapo                    -------------------------------------------------------------------------------- 8590
GoldenEagle               CCCTCACCCAGTGGGATGGGAGCAAGAATCAGAAGGAAAAAGGTAAAACCTGTGGGTTGAGATAAGAACAGTTTAATAGA 9145
JapaneseQuail             ---------------------------------------------------------------------------A---- 8623
MediumGroundFinch         -------------------------------------------------------------------------------- 0
GoodesThornscrubTortoise CGCTTCCGTCGCTGGTGAGTATAGAGGGGCGTCCTTTCTCCAACCTCTCCACACTCACCAGCCCGCTCCGCTTTCCTTGC 30555
```

```
Majority                       --A-----------------AATATGTACTTGATGGCAAAAGTTA-----TC-----A--TGAAATACCTAAAGTG-GA-
                                      34570     34580     34590     34600     34610     34620     34630     34640
Human                          AGAAAAGAAGGCATCTATTCATTAAAAAACTCTCATGTTAATGTTCATAGCCATTTTATTTGTAATCACCAAAATGTGAA 34505
Kakapo                         --------------------AACATGTCCTTGGTGACAAAAATC------CC--------CCAACTTCTTACAGTGAAAC 8636
GoldenEagle                    ACAGAAAGGAAGAAACTAATAATAATAACATGACAACAATAATTAAAGGATTGGAATATACAAAACAAGTGACGCACAAT 9225
JapaneseQuail                  --A-----------------AGCATGTCCTCAATGGCAAAAGCAA----------------------------------- 8649
MediumGroundFinch              -------------------------------------------------------------------------------- 0
GoodesThornscrubTortoise       CCTGGTCCCAGCCGTGTG--GCTGGGGAGCGGCTGGGGAAAGGTCCTGCCTGGTGGGAAGTGGATCACCACAGCTGGGAG 30633

Majority                       ATAATTGCTCACCACT-GCTG---T---------AATTTCTGTGC-GCCATCCTGTGCAGG-CGATTC-G--C---ATAT
                                      34650     34660     34670     34680     34690     34700     34710     34720
Human                          ACAAACATTCATCAACAGATGAACTA-ATAAGTAGATTCCTGTTTTGGCATCATATGCTTTTCCATACAGTGGAATACAA 34584
Kakapo                         ATATCTGCTCTCTTTTTAACA---G---------AATTTCTGTAAATTTACCCTGTTGAAAATTACTCTC--C----TAT 8698
GoldenEagle                    GCAATTGCTCACCACTCACCAACCGATGTCCAGTAGTTCCCGAGCGGCGATCCCCTCCAGGCCGACTCCCCCCAGTATAT 9305
JapaneseQuail                  -------------------------------------------------------------------------------- 8649
MediumGroundFinch              -------------------------------------------------------------------------------- 0
GoodesThornscrubTortoise       CTGGTGGAGTGGAACTGGCTGGGGTTGGGCTGTTCCGCTTCCCGCCACCGGTGAGTGCGGGGAGGTTGGGAGTGTGAGTT 30713

Majority                       -TG------A---------A----------TA-CCCCT-GAACGGTTTG--AT-CAGTTCT--TCT-----TG-------
                                      34730     34740     34750     34760     34770     34780     34790     34800
Human                          CAGCCATGTAGAATGAGCTATTGATAGACACAACAGCAAGAATGAATCTCCAAACAATTATGTTATGTAAAAGATGGGAG 34664
Kakapo                         TTA---------------------------T--TCCCT-GAACAGTTTA--CTCCAATTCT--TTT-----T-------- 8731
GoldenEagle                    ATGCTGGGCATGACGTCGCATGGTATGGAATACCCCTTTGGCCAGTTTG--GGTCAGTTGT--CCTGGCTGTGTCCCTGC 9381
JapaneseQuail                  -------------------------------------------------------------------------------- 8649
MediumGroundFinch              -------------------------------------------------------------------------------- 0
GoodesThornscrubTortoise       GGGGAGGATACCCCCCCACACACACTCACCTATGGCAGGAAGCGGAGCG--ATGCAGCCCCAGCCCACTGCAGTCTGCCG 30791

Majority                       C----TAGTTGTGACCCTCTAGTTTTCTTTTTAA-TGAGA-TCTGGGGCTGGGCAATCTTTGTC-AG-CTATAAGCAGTC
                                      34810     34820     34830     34840     34850     34860     34870     34880
Human                          AAAAAAAGACTACATCCTGTATGATTCCATTTATATAAAACTCTGGGAAATGCAAACTAATCTAAAGCAAATTCATGGTT 34744
Kakapo                         T----TCTTTGTCACTTTTTTGTTTTCTTCTTAATGCTTATTCTGCCACTGTGTAATGTATGTGGAA-AAATAAGGAGAG 8806
GoldenEagle                    CCAGCTTCTTGTGCCCCTCCAGCCTTCTTGCTGGCTGGGCATGAGAAGCTGAACAATCCTTGACTTG-GTATAAACACTA 9460
JapaneseQuail                  --------------------------------------------------------------------CTGAAACAATAC 8661
MediumGroundFinch              -------------------------------------------------------------------------------- 0
GoodesThornscrubTortoise       CATCCCAGCTGTGGCGCTCCACTTCCCGATGCCAGTGAGTGCAGGGGGGAGGGCATTCTTTCCCCAACCTCCCCGCACTC 30871
```

```
Majority                   CTTAGCAGT-ATTAAAGACATATGTGGCTGTCAGGATGCTTTGTATA--G--GCAAGGATGGACCGGTGTAG-------

                                     34890     34900     34910     34920     34930     34940     34950     34960

Human                      CTTAGGAGT--TTAAGAGTGGGAAAGGACAGGCAGGAGGGGTGGCAAAGGGACACAGGAAAACTCGGGAGTGATGGACAT 34822
Kakapo                     CAAAATGGAGAGGAAAGAAAAATGTTGAGAATTAGTGTGCTCTGTA-------GCAAGCATGCATCAGTGTAG------- 8872
GoldenEagle                CTTAGCAGCAACTGAAAACATCAGTGTGTTATCAACATTCTTCTCATACTGAATCCAGGACATAACACTATACCAGCTAC 9540
JapaneseQuail              C---------ATGAAAGACACTTGC------------TGCCTTTGAT------GATAAAATGGACCCTGTTAG------- 8707
MediumGroundFinch          -------------------------------------------------------------------------------- 0
GoodesThornscrubTortoiseCCCAGCAGTGGGAAGTGGAGTACCAAGGCTGGGAGGTGGCGGAGTGGAGCGGGCTGGGGCTGGGCTGCTCTGCT---TCC 30948

Majority                   -GGAAGGCTTGTTAAGTTTGTTTCAG--G-----G-----TTGTGTGTATGTCATGTTTTTTGTTGTCAGAA---A----

                                     34970     34980     34990     35000     35010     35020     35030     35040

Human                      ATAAATTATTGTGATGGTGGTTTTATGGATGTATGCATATGTCAAAACTTGTCAAATTTTATAATTTAAATACATATACA 34902
Kakapo                     -GGAAGGCTTAGTGTACCTGTTTGAT--------------TTGTTTTGATGTCTTGTCACTAGTTG---GAA-------- 8926
GoldenEagle                TAGAAAGAAAATTAACTCTATCCCAGCCGAAACCAGGACATGGTATGGGGAACATGTCTTTCATTGTAAGAAGTTATTTT 9620
JapaneseQuail              -AGGTGGCTTTCAAATTTGTTCTCAG--A-----A-----AAGTGTATAATTCCTTCTTTTTGTTTTCGGAA-------- 8766
MediumGroundFinch          -------------------------------------------------------------------------------- 0
GoodesThornscrubTortoiseCGCCGCTGCTGGTAAGTGCCTGTCAGGGGGCGGGGCGGGGTGGGGGTAGATAGGGATTGGAGCAGTCAGGGGCCAGAGG 31028

Majority                   -----TCGGAGGACA---CTTGTTTTCCTTTTGATAGCAGGGTGTTTGGATGGA--------GA-A-GA-GTTCCACTTA

                                     35050     35060     35070     35080     35090     35100     35110     35120

Human                      CATCCTAGAATGGCCGAACTTCAGAACATTGATAACACCAAATGCTGGCAAGGATGTGGAGCAACAGGAACTCTCACTTA 34982
Kakapo                     -----ACAGAGCACT---TATGTTTGCCTCTTAATTGAAGGGGTTGTAGAAAGA--------GA-AATGTGATCACCTTC 8989
GoldenEagle                TTGTCACCAAGGACA---TCTGCTCTCTTTTTAACAGTAACTTCGGTAAATTGACCCTGTTTGAAATGACACTCATATTT 9697
JapaneseQuail              ------------------CTTATTTTTCTTACGGTGACAGGATGGTTGGGTACT----------------ATGTC----- 8807
MediumGroundFinch          -------------------------------------------------------------------------------- 0
GoodesThornscrubTortoiseTTGGGTAGGAGGTTACGTCCTGGGGGTGATTGGGGATGGGGGTCTCTGGAGGGGGGCGGTCAGGGAACAAGGAACCAAAAA 31108

Majority                   ATGCTCAGAAAAGT-TGAT-TAATAC-----C----G-----AACTTTGAGATTTTTTTGCCTACTGCACAGCCTTTCAC

                                     35130     35140     35150     35160     35170     35180     35190     35200

Human                      CTGCTCAGTGGAATGCAGAGTGGTACAGCCACTTTGGAAGACAATTTGGCAATTTCTTAGAAAACTATACGGCCGGGCAC 35062
Kakapo                     ACCATCTGAAAAATAGGAGCCAATACC----------------AGCATGAAAATACCTTTCCCATTTCACAGACTCACAC 9053
GoldenEagle                ATTCCCAGAACAGCCTACTTCAATTCTTTTTCCTTTG----TAACTTTGGGGTTTTCTTCCTTAATGCTCATTCTTCCAC 9773
JapaneseQuail              ------------------------------------------------------------A--A-----------TT--- 8811
MediumGroundFinch          -------------------------------------------------------------------------------- 0
GoodesThornscrubTortoiseCGGCCAAAGCAAGTTTGATATAATGCGGTCTCACCTG----TAACACTGAGATTTTTTTGTCTCCCGAGAACCGCGTTAT 31184
```

```
Majority                   AGTGGTTTAGGTGTGAAATCAGATAGGGCTGAAA--TGGAGACAAAGTAATAACCTTGGAACTGA-ATTTCACACTGTAG

                                   35210     35220     35230     35240     35250     35260     35270     35280

Human                      AGTGGCTTACGCCTGTAATCCCAGCGCTTTGGGAAGCCAAGGCAGGCGGATCACCTGAGGTCAGAAGTTCAAAACCAGCC 35142
Kakapo                     ACTGGTTTGGGTTGGAAAGGACCTTAAGCTCATC--CAGCTCCAACCCCCTGCCACGGGCAGGGACATCTCACACTAGAG 9131
GoldenEagle                TGTAATATATGTGGAAAAACAAGGAGAGCTGAAAA-TGGAGAGAAAGGAAAAATGTTGAAAATTAGTTTGTACTCTGTAG 9852
JapaneseQuail              -------TAAAAAAAAAAAAAGAAAAGGCTGAAAG-TGGAGAGAAAATAAAAGTATTGAAGATTA------ATGCTGTGA 8877
MediumGroundFinch          -------------------------------------------------------------------------------- 0
GoodesThornscrubTortoise   ATCAGGGTAGAGGTGTATTTAGATGTGATCTGCT--GAAAGACACTGTTCTAACCTTTCTCCTGATATCTCACATGGCAT 31262

Majority                   CAGGTA------A---TACAGAAGTCTTCTGATTCCTACTTG----GTTTTGAAGTCTGTCGCAAATTGGAAATCCC--G

                                   35290     35300     35310     35320     35330     35340     35350     35360

Human                      TGGCCAACATGGAGAAACCCGATCTCTACTAAAAATACAAAATTAGCTAGGCGTGGTGGTGCATGCCTGTAATCCC-AG 35221
Kakapo                     CAGGT-----------TGCTCCAGGCCCCTGTGTCCAACTTG----GCCTTAAACACTGCCAGGGATGGGGCAGCCACAG 9196
GoldenEagle                CAAGTATGCATCAATGTAAGGAAGGCTTAGTATTCCTATTTGATATGTTTTGATGTCTGTCACTAATTGGAAATCACGTA 9932
JapaneseQuail              ATGCT-----------TACAGGAAATTTCTG--------TTG-------------TCTGTTGCAAATTGGAAGTC----A 8921
MediumGroundFinch          -------------------------------------------------------------------------------- 0
GoodesThornscrubTortoise   CCTTTATATGCAATATCACAGTTATATACAGATGAGGAATATGGGAGTTAACAAGATGCACCCCCAAAGTATAGCAA--G 31340

Majority                   CTTCTCTTTTGTCACAGAACCCCTAGATTTGTGTATTCATTGGGTGGAGTTTGTGATGAGC-AATGTGATCTTTATCTCC

                                   35370     35380     35390     35400     35410     35420     35430     35440

Human                      CCACTCAGGAGGCTGAAGCAGGAGAATTACTTGAACCCAGGAGGTGGAGGTTGCAGTGAGCCAGGATCACGCCCATTTCA 35301
Kakapo                     CTCCTCTGGGCACCCTGTGCCAGCGCCTCAGCACCCTCACAGGGAAGAGCTTCTGCCTTA--GATCTAACCTGAACTTCC 9274
GoldenEagle                TTTTTCATTTGTAACAGAACACCTATATTTGCATCTTAATTGAGGGGGTTGTAGGAGGAGC-AATGAGATCACTTGCACC 10011
JapaneseQuail              CTTTTCCTTTGTAAAAGAACCCCTAGACTCGTGTATTAATTGAGAGGACTTTGGGAAGAGA-AATGTGATCATTTGCCCA 9000
MediumGroundFinch          -------------------------------------------------------------------------------- 0
GoodesThornscrubTortoise   TCACACTAATTTTATCTAACTTTTAGATATTTGCATTGTTTAGTTTAAACTTGTAATAAAT-GTTATGGTATTAATCATG 31419

Majority                   GTCTGATCAGTT-----TGAACTA-ATATCAACTTTAATAT-C-TTCCTTGTTTCTTAGAGCTT-TGGGCT-----GTGT

                                   35450     35460     35470     35480     35490     35500     35510     35520

Human                      CTCCAACCTGGGCAACAAGAGCGAGACTCCATCTCAAATATACTTTCATTGTATGATCCAGCAGTTGTGCTCCCTGGTAT 35381
Kakapo                     CCTGGTTCAGTC-----TGAACCC-ATCACCCCTTGAGTAT---TT-CTCATTTCTGAGAGCTT-TAGGCC-----ATGT 9338
GoldenEagle                ATCTGAAAAATA-----TGTACTG-ATACCAACATGAAAATACCTTTCCTGTTTCTTAAAGCTT-TGGGCC-----ATGT 10079
JapaneseQuail              GTCTGAAGAGCT-----TG-ATGA-ATATCAACATTCATATGCCTTCCTAGTTTCTTTGAGCTC-TGAGAT-----CTGT 9067
MediumGroundFinch          -------------------------------------------------------------------------------- 0
GoodesThornscrubTortoise   CTCAGTTATTTTAGCAATGAACTA-ATATCAGCTGTTATAT---TTGCAAGAGAATTATAGTTGGTAAATT-----GTAA 31490
```

```
Majority                   TTTCACAAGCAAATCAA--GCTGCATTCCAGTTTTCTCTGTCTCTGTGATACTTATGGCACAAATGCTTGGA--TAGCC-
                                    35530     35540     35550     35560     35570     35580     35590     35600
Human                      TTGCCTAAAGGAGTTGAAAACTTAGTCTGCGCAGAAAGTAGCACATGGATGTTTATAGCACCTTTCTTTATAG-TTGCCA 35460
Kakapo                     TTTCACAAGCAGATCAA--ACTGTGTTCCAGTTTTCTCTGTTCCTGTAATGCTTGAGCACCAAATGTTTGGG--TAGCC- 9413
GoldenEagle                TTTCACAAGCAGATCAA--GCTGCATTCCAGTTTTCTCTGTCACTGTAATACTTGTGTAACAAATGCTTGGG--TAGCC- 10154
JapaneseQuail              TTTCACAGGCAAACGAA--GCTGCATTCCACTTTTCCCTGCCTCTGTGACACCTAAGGCACAAATGCTTGAA--TAACC- 9142
MediumGroundFinch          -------------------------------------------------------------------------------- 0
GoodesThornscrubTortoiseCTCCTCAAGAGAAACAATAGGTGAAATTAAAGTTTTTTTTTTAATAGATAGGTATTCTTCCAGGGCATAAACCTTGCCC 31570

Majority                   --ACTTCACAG--------ACGTCTCTTGGGAACT---TAACCAGGTTAAAGCTGA-----GTTGACACAACAGTGAGTT
                                    35610     35620     35630     35640     35650     35660     35670     35680
Human                      GAACTTCATAGCAACCAAGAAGTCCTTTAGGAGGTGAATGGCCAAATAAACTGTGGTGTATATTCAGACAGTATAATGTT 35540
Kakapo                     --ACTTCACAA--------GCTTCTCTTGAGAACT---TAACCAGGATAAAGCTGA-----GCTGACACAACAGTGAGTT 9475
GoldenEagle                --ACTTCACAA--------ACCTCTCTTGGGAACT---TAACCAGGTTAAAGCTAA-----GTTGACACAACAGTGCATT 10216
JapaneseQuail              --ACTTCAAAC--------ACCTTTCTTGAGAACT---TAACCAGGTTGAAGCTGA-----GCTGACACAGTAATGAGTT 9204
MediumGroundFinch          -------------------------------------------------------------------------------- 0
GoodesThornscrubTortoiseAAACTACACAG--------AAGTTGCTGTGGACCTAGGTAATGGAGGCTGAGCAAATATG-GGTAAGGGAACTGTAGGAC 31641

Majority                   ATTTCCTTTTAGTGGGATATG---TACCAAAGTGTGTGGTTGCATAAGT-GCTTGTAGCAATATAGACTTGG------T-
                                    35690     35700     35710     35720     35730     35740     35750     35760
Human                      AATCAGCACTAAATGGAAATGAGCTATCAAGCCATGGAGAACCTTCAGT-GAACATTACTAAATAAAAGAAGCCACTCTG 35619
Kakapo                     ACATCCTTTTAGTCGGATTTG---TGCCAAAGTTTGTGGTTGCATAAAT-GCTTGTAGCG-CATAGAATTGG-------- 9542
GoldenEagle                ACATCTTTTTAGTCAGATTTG---TACCAAAGCGTGTGGTTACATAAAT-GCTTGTAGCAATATAGACTTGG-------- 10284
JapaneseQuail              ATGTCATCTTTGGGGCAAATC---TGACA-----T--GGTTGCATAAGC-TCTTGTAACAATATAGACTTTATATTGGTT 9273
MediumGroundFinch          -------------------------------------------------------------------------------- 0
GoodesThornscrubTortoiseATTCACTGTAGGAGGAAGAAA---GAGGAAAATGTTTGTTACAGTGGGTCATTTGTATCACAGCATAGCAAGGAGATGTC 31718

Majority                   GGAGTTCTGAATAGGAACAAGTTGCT-AGGGCTCGGGAGACGGTAGGACTGTTGCTTTTGGTGGTAGTTGG--CAA-TGA
                                    35770     35780     35790     35800     35810     35820     35830     35840
Human                      AAAATTCTGCATACCATATGATTCCA-ACTATATGACATTCTGGAAAAAGCAAAATTATGGAGACAGTAAAAATCAGTGG 35698
Kakapo                     GGAGGACAGAACAAGAGCAAGTTGCT-GGGGCCAGGGAGAAGGTAGGGCAGTTGCTTTTGGTGGTAGCTGGTTCAA-TGA 9620
GoldenEagle                GGAGGACAGAATAGGAACAAGTTGTT-AGGGCCCGGGAGACAGCAGGACTGTTGCTTTTGGTGGTAGTTGGTTCAAATGA 10363
JapaneseQuail              GGGAAGGGCAATAGAAACAAACTGCTTAGGACTAAGGAGAAGGTAGGACTGATGCTTTTGGTTGTGG------------- 9340
MediumGroundFinch          -------------------------------------------------------------------------------- 0
GoodesThornscrubTortoiseTAATTTTTAAATTTAAATTTTCAATTACATCTCAGTTTTTCACAGTAACGTTAAATTAAGTTGCCATAAG--AAAGTTT 31796
```

```
Majority                     TGTTGTTGTTTTACAAGTTGTTAATGGACTGTGGTGTCTGATTTAGGGGATTGGG----G--TTT--ATAGCTGCAAGT

                                     35850     35860     35870     35880     35890     35900     35910     35920

Human                        TTGCTAGGGTTTAGGGGTGGAGGGAGGGATGAACTGGTGGAGTACAGAGGATTTTAGGGTACTGAAAATATTCTGCATGA 35778
Kakapo                       GGTTCTAGTTTTGCAAGCTATTAATGGACTGTGATGTCTGATTTAGGGGTTTGGG----G--TTT--ATAGCTGCAAGT 9692
GoldenEagle                  GGTTGTTGTTTCACAAGCTGTTAATGGACTGTGATGTTTGATTTAGGGGGTTGGGTGGGGTTTTTTTATAGCTGGAAGT 10443
JapaneseQuail                ---TGTTGTGTTTTAATTTAAT-----TTCTGTGGC-----ATGT---G--CTGGG-------CTT--ATAGTTGAAAGA 9393
MediumGroundFinch            -------------------------------------------------------------------------------- 0
GoodesThornscrubTortoise     TACTGATGGATTACATTTTTGTCTAACATAGATTGC-TCTGACCTCAAACACTTAAAAAATTGCATTTTTTAATGCTGCC 31875

Majority                     ACATACA---ATTGTTA-CTTTTCATAAAATTTAGTTTTTTCATA-AATGTGTAACATCAGGA-TGAATTTTGG----AC

                                     35930     35940     35950     35960     35970     35980     35990     36000

Human                        TACTATAATGATAGATA-CATGTCATACATTTTTCCAAACCCATAGAATGTAAGACACTTGGAGTGAACCTGGAGGTAAC 35857
Kakapo                       ACATACG----TTCTTAGCTTTTCATAAAA-TCAGGTTTTTTATG-AATGTGTAACATCAGTATTGAATTATAG----AC 9762
GoldenEagle                  ACATACAGCCATTTTTAGCTTTTCATAAAAATCAGTTTTTCTATG-AATGTGTAACATGAGGATTGAGTTATAG----AC 10518
JapaneseQuail                AAAAAAA---------------CC---AAACTTAGTTTTCTCCTA-GATGTG-----------T--------------- 9427
MediumGroundFinch            -------------------------------------------------------------------------------- 0
GoodesThornscrubTortoise     ACAAAAAAGAAGTGACAATTCATGCTAGTATTTTTTTTTTTCAAA-TTTAACTATTTCCAACA-TGAAGTTGGC----CC 31949

Majority                     ATAATAGCTTTAGTG-TTTTAATTTATTATTGGAGAATAGCAGAGTT-TAAGTAGAAAATATCTTCTTT------T-G-T

                                     36010     36020     36030     36040     36050     36060     36070     36080

Human                        TACAGACTTTGAGTGATTATAATGTGTCAGTGTAGGTTCATC-AGTTGTAACAATGTGCCATCTGGTGGGAGATGTTGAT 35936
Kakapo                       AAAATAGCCCTAAATCTTCTAATTTATTATTGCAAAATAGCAGAGAAACAAGTAGAAAATACCTTCTTTTCATATTGGAT 9842
GoldenEagle                  ATAATAGCACTAGCGTTTTTAATTCATTATTGCAAAATAGCAGAGAATCAAGCAGAAAATACCTTCCTT----------- 10587
JapaneseQuail                ----------------CCTTACTTTAATATTTGTTAATA----------------------------------------- 9450
MediumGroundFinch            -------------------------------------------------------------------------------- 0
GoodesThornscrubTortoise     ATTGTTCATTGGTTGGTATTATTCTGGGCTTTGGGATTAGCTGTTTTCTTTCTAAAGCAGCTCTGACTTCTGAGTTGGCT 32029

Majority                     ---------GTGGATCTTGTTTTGAGAATTAGGAATTGC-------T-CTGGAGGATCAGATGTGTACTTGTTGG-TTCC

                                     36090     36100     36110     36120     36130     36140     36150     36160

Human                        AAAGGGGAGGCTATTTGGGGCAGGAGGCAGGGAAATCTCTCTGCTTTCCTCTCCATTTTGCTGTGTACCTAAACTTACTC 36016
Kakapo                       CTTTTCATAGTGGATCTTATTTTGAGAGGTAGGAATTGC-------TACTGGAGGACCAAATGGGTACATCTCAG-CTCC 9914
GoldenEagle                  ---------ATGGATCTTATTTTGAGATGTAGAAACTGC-------TGCTGAAGGACCAAATATGTACATCTAGG-TGCC 10650
JapaneseQuail                -----------------T--TTTAACAATTAATAATTGT-------T-----A-----A------TACTCGTTAC-TTCA 9487
MediumGroundFinch            -------------------------------------------------------------------------------- 0
GoodesThornscrubTortoise     CCAGG-ATGGTCTCTGCAGCCTGTATGATTGAGAGTCACA----TGTTCCAGATGATCTGTCGTCTATTGGTTGCATTTA 32104
```

```
Majority                  ATGAATCTTTATTCAGAAAGTTAATAATACATCTGGGTGGTATCTCTAGCGAATATTTCT--CAT--CTGTTCTAACTTA
                                   36170     36180     36190     36200     36210     36220     36230     36240
Human                     TGAAAAATGTCTTTGAAAATTTACTCATGCGCTTGCATTGCATTTCTTTTGGATATTACTGGTCTAAGTAGTCCAGTTAA 36096
Kakapo                    ATGACTCTCTAACCAGAAAGCAAAGAATACATCCAGGTGGTAGCTCTAGCGAGTAGTTCT--CAC--CTGTTGTAACTTA 9990
GoldenEagle               ATGATTCTCTATCCAGAAAGCTAAGAACACATCCAGGTGGTACCTCTAGCAAGTTGTTCT--CAC--CTATTCTAACTTA 10726
JapaneseQuail             GTTAATTGTTA------------------------GGAGATGTCCCCAGCCAACATTAC--------------------- 9522
MediumGroundFinch         -------------------------------------------------------------------------------- 0
GoodesThornscrubTortoise  ACATGGCTTTATTCAGGATATGTATGATTTTTCTGTTCTTTGGATCTGATCCATGACCCT--CTTGGTTGCTTAAAAGAA 32182

Majority                  CTACCAGTTATTTTCAGCTGCTTTAGAGGAACA----CTTACTTTCCTGGTAA-----AGGTGGAATAGAAA-CTTGAAA
                                   36250     36260     36270     36280     36290     36300     36310     36320
Human                     AAGACAAAGATTGTCAGACTTGATTAAAAAGCAAGGCCTAACTACACTGTCAATACGAGGGTCAGGTAGAAATATCAAGA 36176
Kakapo                    CTACCGGTTATGTGTAGCTGCTTCAGAGGAACA----CTTTCTTTCCTGGTAA-----AAGTGGAGTAGAAAGCTTGAAA 10061
GoldenEagle               CTACCAGTTATGTACAGCTGCTTCAGAGTAACA----CTTACTTTCCTGGTAA-----AAGTGGAATAGAAAGTCTGAAA 10797
JapaneseQuail             ------ATTGTTTACAGC-----------------------------------------ATGGAAAAGGA---TTGAAG 9551
MediumGroundFinch         -------------------------------------------------------------------------------- 0
GoodesThornscrubTortoise  AGCTAAGTGGTTTTTTTGCCCTTTATAGGAATT----ATCAATACCTTGCTAA-----AGGAGTGAAAGATC-CTGACTA 32252

Majority                  TGC----TGGAATAAA--ATTTGTGTAATTCCTTTTGG---AAGTAGGAAGCATCAGTTAGT--ATTGGACTGGATTATT
                                   36330     36340     36350     36360     36370     36380     36390     36400
Human                     TGCAGGTTGAAAGTAAAAAGATGGAAAATGATATTCCGTGCAAACAGTAAGCACAAGAAAGTTGATATGATTATATTACT 36256
Kakapo                    TGC----TGGAATAAA--ATCTGTGTGTTTCCTCCTGG---AAGTTGGAAGCATCAGTTGGT--ATTAAACATGGCTCTT 10130
GoldenEagle               TGC----TGGAATAAATTGTTTGTGTACTTCCTCCTGG---ATGTTTGAAGCGTCAACTGGT--ATTGGACTAGAATCTT 10868
JapaneseQuail             CAG----GGGGAAAAA------------------------AAGTACAGAGGATAAAGTATG--GAAAGGCAGACATATT 9600
MediumGroundFinch         -------------------------------------------------------------------------------- 0
GoodesThornscrubTortoise  TTC----TTAAACAGGCAACTTCAAGAACTCAGTTAATGCTAAATAAATAGTCTTTCTAATC--TTTGGGAGGGTTTAAG 32326

Majority                  GCCTACAGTCTTTTATGACA----ATAGGAAATTTAAAG-TTT---CCAAATAAGGTTGAGGGGTCAGTTT--------G
                                   36410     36420     36430     36440     36450     36460     36470     36480
Human                     AGACAGAAAAAATTTAAGACAAATATTACCAGATATAAAAAAGCATATTTCACAATGTTGAAGGGTCAGTTTATCAGAAAA 36336
Kakapo                    GCCTACAGTCTTTCATGACA----ATTGAAAGTTTGAAGCTTT---CCAAATGAGGTTTAAGTGCAGATTT--------G 10195
GoldenEagle               GCCTGCAGTCTTTCATTACA----ATTGGAAGTTTGAAGGTTT---CCAAATAAGGCTGGGTGGCATGGTTTAAGTGGAG 10941
JapaneseQuail             CTAACCAGTTACTT-C--------ATAGGGAATTTAAGG-------CCAAGTGA----------------T--------- 9639
MediumGroundFinch         -------------------------------------------------------------------------------- 0
GoodesThornscrubTortoise  CCCCAAGGAATCTTATGCAT----CTAATAGACTTAAAATAGTAGCATATGTATAGCACAGCCATCAATCTTTTAAATTG 32402
```

```
Majority                ---------ATTTGTTTTTTTACTCTTCAGC-T-CTGGTTTAATAGT----CCATCTTGATCTTGAGATGCTTTG-AGAA

                                 36490     36500     36510     36520     36530     36540     36550     36560

Human                   CATTTCTTAATTTGTATGCCAATAATAACAAATCAAAGTATATTAAGAAAAACATCACAGAACTAAGTAGAGAAACAAAA 36416
Kakapo                  ---------ATTCTTTTTTTTACTCTTCAGCCTGCTGGTTTAATAGT----CCATCTTGATCTTCAGATGCTTTGGAGAA 10262
GoldenEagle             ---------ATTTGATTCTTTACTCTTCAGCTTTCTGGTTTAATAGTAGT-CTGTCTTGATCTTGAGATGCTTTG-AGAA 11010
JapaneseQuail           ---------------------------G----------T--AATAGG------------A-------------------- 9648
MediumGroundFinch       -------------------------------------------------------------------------------- 0
GoodesThornscrubTortoiseTTGCTGGTTATTCACTATCTTGGAGTTCTGTGTATAGATTTGGAAAACTA-TGACAGTACTTTTGAGAATGGGTGTATGT 32481

Majority                ATTTG-CTCTTTTCTACATTGTATTT-----TGAGAACTTGGAAGTTTTTAGGAGAGATTGATCCTTGTCTCCTTTTAGC

                                 36570     36580     36590     36600     36610     36620     36630     36640

Human                   ATCCA-CAATCATAGCCTGGTTTTTTAAAACATAATTTATGGCAATTGATAGGATAGTACAGTCCTAGCCCCCAAATAAA 36495
Kakapo                  ATTTGGCTCTTTTCCACATTGTATTT-----TGAGAACCTGGAAACTTTTCCAAGAAACTGAGCCTTGTCTCCTTTCAGC 10337
GoldenEagle             ACTTGGCTCTTTTCTACAATGTATTT-----TGAGAACCTGGAGGCTTTTAAGAGGAATTGAGACTTGTCTCCTTTCAGT 11085
JapaneseQuail           ------------------T--T--CT-----TAAGCACTTG----------------------------------TGA-- 9665
MediumGroundFinch       -------------------------------------------------------------------------------- 0
GoodesThornscrubTortoiseCTGTGTTTATTTTCTTCAGTGAAATTCCAAGTGATAATTTTGTTGTCTATTGTAGAGATTACTTTTAACTAACATTTTAC 32561

Majority                TTAAAGAATTCTTTGCTA--TCCTTTCATGCTGTCTTTGTTT-T------------TTTTCA-GAATTGGGTACATGGCA

                                 36650     36660     36670     36680     36690     36700     36710     36720

Human                   AAGATACATAATTTGAAAAGTGCTATCATGTTGGCTTAACTCACATTTATAAAACTATACCCAGTAACTGTAAAATAACT 36575
Kakapo                  TTAAAGACTTCTCAGCTT--CCCTTGATTGCGCTCTCAATTTCTGCCAACTTTGTGTTTTCACAAAGAAGGTACATGGCA 10415
GoldenEagle             CTTAAAGACTGCTTGCTC--TCAATTTCTACAGACTTTGTGTGT------------TTTTCATGAATTGGGTACATGGCA 11151
JapaneseQuail           -------------------------------------------------------------A--GA------------CA 9670
MediumGroundFinch       -------------------------------------------------------------------------------- 0
GoodesThornscrubTortoiseTCAGAGAGAGCTTTGGGA--TTTTTCCATCATGTAGATGTCTTTTGAAGGTGGGATTTGTTTGGAATTGGCAACAGGGAT 32639

Majority                TATACCATACATATAGTT------AAAAAGGA-----TAATAGCTT-----ATTTTTCTCTAAGATTC---TTCTTTACT

                                 36730     36740     36750     36760     36770     36780     36790     36800

Human                   TCAAGTGTACATAGAACATTCACAATGATAGACCTTATATTGGATTATAAAATAACTCAATAAATTTCAAATGATTGACA 36655
Kakapo                  TATACCATGCATATAGTT------AAAAAGGA-----TAAAAGCTT-----ATTTTTCTCTGAGATTC---TTCCTTACT 10476
GoldenEagle             TATACCATGCATATAGTTGCAAGAAAAAAGAA-----TAAAAGCTT-----AATTTTCTCCTACATCC---TTCCTTACT 11218
JapaneseQuail           TACTCCATATATGTAGGG-------------------------------------TTCT--------------------- 9692
MediumGroundFinch       -------------------------------------------------------------------------------- 0
GoodesThornscrubTortoiseAATACTAAACAAATAATTCAGTTTAGTAACAA-----TGATCATTTGGATGATCCTTTTGGATGATGC---TTTTCCACA 32711
```

```
Majority                 GATAAAG-ATGAGTGTAAAAATGGTGAA-----ATTAGTTGTTAAGAT-TATA-A-A-----TG-TGGGCTGAGGATTGC
                                 36810     36820     36830     36840     36850     36860     36870     36880
Human                    GCTAATGTATCTGACTGACTATAGTGGAATTAAATTAGAAATCAAAA---ATAGACATCTAGGAAAGTACTAAATATTAG 36732
Kakapo                   GAAAAAA-ATGAGTGTAAAAATGGTCAA-----ATTAGTTGTTAAGATATATC---------TGCTGGGCTGAGGACAGC 10541
GoldenEagle              GAAAAAATATGAGTGTAAAAATGGTGAA-----GTTAGTTGTTAGGATATATAAACAATACATGGTTGGCTGAGGACAGC 11293
JapaneseQuail            GATGAAG------------------------------------------A-----------------------GA---- 9702
MediumGroundFinch        -------------------------------------------------------------------------------- 0
GoodesThornscrubTortoiseCCGAGTGGCCGATATTTTTCCTTGTGAATGAACATTAATTTTTATCTTCTCCAGAAAATAGCAGTGGGGGAGGAGCTTAA 32791

Majority                 ATGAAAAAGAATTGTAGTG---GGGAAAAATGAGAATGCA-GATAATAAGGAT-TATAGGTGGTCGGAT---TA--TT-C
                                 36890     36900     36910     36920     36930     36940     36950     36960
Human                    AAATTACACAATCGTGAACAATACATGGATTATAGAAGTGTTACCACAAGGACATATTTTTGACTGAATGAAAATATATC 36812
Kakapo                   ATGAAAAAGCACTGAAGTG---GGGAAAAAAGAGAATACA-GATAATAAGCATTGAAAGGTGGTCATAT---TC--TACC 10612
GoldenEagle              ATGAAAAAGAATTGAAGTGAGGGGGAAAAAAGAGAATGCA-GAGAATAAATATGGAAAGGTGGTCGGTCATATT--GTAC 11370
JapaneseQuail            -------------------------------------------------------------------------------- 9702
MediumGroundFinch        -------------------------------------------------------------------------------- 0
GoodesThornscrubTortoiseACTGAATAGATTTTTCAGATCTGGTAAAAGTTAAATTTCATGATAATACAGGCCTGTAGGCAATCAGCTGTTTAAAGTGC 32871

Majority                 CAGTTAATTTGATTCTGAATTAAG-----GTGATGTTATAA----------------C-A-----------------T--
                                 36970     36980     36990     37000     37010     37020     37030     37040
Human                    AAAATTTGTTGGATACTACCCAAGGGTGTGTATGGTGAAAAGTATGTCTTCAAATACCTATATTACAAAAAAAGGTTTAA 36892
Kakapo                   CAGTTAATGGGATACTGCATTAAA-----GTGATGTTATA---------------------------------------- 10647
GoldenEagle              CAGTTAACTTCATACTGAATTAAAAGCAGGTGATGTTGTAAGTGAGTTACTAAGCACCCCACAGAGTTCATGGCCTTATCT 11450
JapaneseQuail            -------------------------------------TAA---------------------------------------- 9705
MediumGroundFinch        -------------------------------------------------------------------------------- 0
GoodesThornscrubTortoiseCAAATACTGTAATTGTAATTTTGGCAGAATTGAAACACTGACATTTGTCCTGGCTTTCCACTATTTACTACTTAGCCTGT 32951

Majority                 --------A----A--C-------G-----CAG--TT---T----A---------T----G-----A----C---T--A-
                                 37050     37060     37070     37080     37090     37100     37110     37120
Human                    AACTCCCAATTTAAGACTGACCAAGGAAAAAGACATAAATTACCAATACTGGAAATCAAGGTCGAGATAATCACTTTCAG 36972
Kakapo                   ------------------------------CAT--GT------------------------------------------- 10652
GoldenEagle              GATAAG--ATTTCATGCTAATATAGTTAAATAG--TATAGTCTTAAAAAATAAAAATCAAGTAGACATACTC-CTTTTAT 11525
JapaneseQuail            -------------------------------------------------------T------------------------ 9706
MediumGroundFinch        -------------------------------------------------------------------------------- 0
GoodesThornscrubTortoiseTGCATC--AGGGAAGACCTTTTAGGCTTGTCTG--TTGTTTCCTTAATCTATCTGTGATTGAGATGAACCAC-AGTACAT 33026
```

```
Majority                    ------T-------TAGTGAGAATAAGCTTATAGTTTAAATTATAT-GATTTGTAAAATACTTTTTAACCAGCATTGA-A

                                   37130     37140     37150     37160     37170     37180     37190     37200

Human                       AGAAAGTGGATATATGCAGAGTATAATAGTATAACATAAATAATATAAAGTGGAGATAGCACTTTCAATAAAAGCAGAGA 37052
Kakapo                      --------------AAGTGGGAACAAGCTTATAGGTTAAACTATAT-GATTTGTAAAATACTTATTAACCTGCATGGA-A 10716
GoldenEagle                 GTAAGGTTCTGGTGAAGTGATAACAAGGTTATAGTTTAAATTGTAT-GCTTTGTAAAATACCTATTAACCTGCATGGA-A 11603
JapaneseQuail               ------------------------AAGCTTATAGTTGAAATTATGT-GCTTTATAAAGTAC-TGTGAAGCAGCAGTGA-A 9759
MediumGroundFinch           -------------------------------------------------------------------------------- 0
GoodesThornscrubTortoiseAATTGCTACAGCCATTCAGTGGTTGATTACGCAGGTATACCATTGTTAAATGACTGAACATTTTCTAACAATAATTGA-G 33105

Majority                    TTTCTTTTGGATTGTATGTTTGGACTTGGTTTGTCCTGTTCTGAATGA-G-----ATCTTCTGTGATTTTGTT-------

                                   37210     37220     37230     37240     37250     37260     37270     37280

Human                       TATATAGAATATAATAGGAAAATAAGTCAATATTAGTAATGTTGTTAAGGCTAACAAATTCAGCAACTTAGATGAAAAGT 37132
Kakapo                      TTTCTTTTGGATTGTCTGTTTGGACTTGGCTTGCCCTGTCCTGAATGA-------GTCTTCTGTGAT------------- 10776
GoldenEagle                 TTTCTTCTGGATTGTATGTTTGGACCTGGCTTGTCCTGTTCTGAATGA-------GTCTTCTGTGATCCAGTA------- 11669
JapaneseQuail               TTTCTTTTGGGTTTTATGTATCGACTTGGTTTGGCCTGTCCTCATTGATGAGGACATTGTCTGTGATCCTATA------- 9832
MediumGroundFinch           -------------------------------------------------------------------------------- 0
GoodesThornscrubTortoiseTTCACCTTCCAGTGGCTTTGAGCTTCTGTTTTTTCATGTATTAAACTGGG-----ATCATCAGTCCCTTTTCT------- 33173

Majority                    TGTGTGAGAGGTGTAACAGTCTATT-A--ATGTAGTTTAG--ATTATGTGTTTTGATAGTAGTGCGTGTA-GTCAGAGAG

                                   37290     37300     37310     37320     37330     37340     37350     37360

Human                       TTCTTGACAAATGCAAAACTGACTTAAGTATATAGCTTAACAGCTATATACTACTGTATTATACAAATTGGAATTTATAA 37212
Kakapo                      -GTGCGAGAGGTGTCAGGGCCTATTCACCATCTAATTTGG--ATTAGGTGTTTTGACAGTAGTGCGTGCAAGTCAGAGCG 10853
GoldenEagle                 TGTATGAGAGGTGTAACAGTCTATTAATTAGGTAATTTAG--ATTATGTGTTTTGACAGTAGTCCGTATA-GTCAGAGCA 11746
JapaneseQuail               TGTGTAAAATGTGTAGCAGTCTGTT----AGGTAGTTCGG--TGTATGTGCTTTGATAGCAGTGTGTGAA-GTCAGGGAG 9905
MediumGroundFinch           -------------------------------------------------------------------------------- 0
GoodesThornscrubTortoiseCCCACATATATCATTAAACTTTATC----TCCTGTTGAAA--AGTATCATTTAAAATGGTAAGTGAGAGACCACACAGAG 33247

Majority                    TAAGGCAGAT------AGAGTATTTCCTCTTCAC--A-GTGTTTGTGATAGTGTATTT-ATGCAGTGTTTCATTAAAAAT

                                   37370     37380     37390     37400     37410     37420     37430     37440

Human                       TTAAAAAAATCTTTCCACAAAGATTACTCTAGTCATAGATGGTTTCACTATTGAATTTTATGAAATTTTAAAGGAATACT 37292
Kakapo                      TAAGGCAGAT------CCAGTATTTCTTCTTCACTAAAGAGTATGTGAGGGTGTATTT-ATACAGTAGTTCATCAAAAAT 10926
GoldenEagle                 TAAGGCAGAT------AAAGTATTTCTTCTTCACTA--GAGTATGTGAGAGTGTATTT-ATGCAGTGTTTCATTAAAAAT 11817
JapaneseQuail               TAAGGCAGGT------CGGCTATTTCCTCTT------------------------------------------------- 9930
MediumGroundFinch           -------------------------------------------------------------------------------- 0
GoodesThornscrubTortoiseTAAGGTGGAG------AGCTTTCTTTCAGTTCC---AAATGCTTCTAATAATAGTTTT-GAAAACTGAATGAATTTCATA 33317
```

```
Majority                   GTTTCATGTTAAAAATGAAGCATTCATTAAATTCT---CCATTGTATGTAACTTGTATAAATTT-AGAAATGCT-TGTTT
                                     37450     37460     37470     37480     37490     37500     37510     37520
Human                      AGCTGTCTTTGAAAATGGAGGAGGGAATACTACCCTGTTCACTTTATGAAACCACTATAACTGACAGCAAAACC-TGACA 37371
Kakapo                     GTATCCTGTCAAGAATGAAGCGTTCATCAAATTCTGAGCCATTGCGTGTAAACTGAATGAAGTTGAGAAATGCTACATTT 11006
GoldenEagle                GTTTCATGTTAAAAATGAAGCATTCATTAAATTCTGATCCATTGCATGTAAATTGAACAAAGTTTATAAATGCTGTGGTT 11897
JapaneseQuail              -------------------------------------------------------------------------------- 9930
MediumGroundFinch          -------------------------------------------------------------------------------- 0
GoodesThornscrubTortoiseTTTTCAAACTCAATATTTT-TATTTTGAAAGTTCC---CTTTAATATGTACCTTGTAAAAATGTACAAAGGGCAGTGTTA 33393

Majority                   AGGGGTTTATAGTGTGTTTTCC------T--------C------CCAAGGATAAATCAGACACTGAATATTGATGCAT--
                                     37530     37540     37550     37560     37570     37580     37590     37600
Human                      AGGTAATATATCAAGATTTCAGACTAGTATTTCTCATTAACCTAAATGCATATATTCTTAAGAAAATGTTGGTAAATCA 37451
Kakapo                     AGAGGTTTGAAGTGTCCTGTCC---------------C------CCAAGTTAAAATCAGACATTGAACATTGAAGCAG-- 11063
GoldenEagle                GGGGGTTTAAAGTGTGTTGTCCTGTTGTTTCCAACATCCATTCCCCAAAGCTAATTCACACACTGAATATTGAAGCAG-- 11975
JapaneseQuail              -------TGGAGTG--------------------------------------------------GGATGTTG-------- 9945
MediumGroundFinch          -------------------------------------------------------------------------------- 0
GoodesThornscrubTortoiseTTCAATCCATGACTCATATAAAACACATTGATTTCCGCTGGCTGGCAAAGAGAGGGAAGAATCAAGACATCACTGCTT-- 33471

Majority                   --TGTGTTTGCAAGTGTG--TATCTTTTAGGGGGGAATGT---ATGTCTGGGTTTGAATTCTCATTTGATTTTATTCCTG
                                     37610     37620     37630     37640     37650     37660     37670     37680
Human                      CCTCCGGTAGAATATGAGAGACAATTCTGAGACTTATCGCAGGATTGTAAGGTTTGTTTAACATTTGAAAATGAACTCAG 37531
Kakapo                     --TGTGTTTACAAGTGTGAGTATCTATTAGAGGGGAATGTTTAATGTCTGCGTTGGAATTCTCATATGCTTTTATTCCTC 11141
GoldenEagle                --TGTGTTTACAAGTGTG--TATCTGTTAGAGGGGAATGTTTAATGTCTGGGTTGGAATTCTCATATGCCTTTATTCCTC 12051
JapaneseQuail              -------------------------------------------CTGTCTAGGTTAGAATTCTCATTGCAGTCTCCCATTG 9982
MediumGroundFinch          -------------------------------------------------------------------------------- 0
GoodesThornscrubTortoise-TCTGTTAGCAGGTGACTCTAGAATTTTTGAGAAACTGC---ATGTTTTATTATCTGTTGGCACTCCATCCTTTTCCAT 33546

Majority                   TGC------ATTAAATTTCTGGCTTTTGCTTT-TAAATTGTTCTCAGATGTTGTTAGAGATGAAGTG-TTTTACAATGCT
                                     37690     37700     37710     37720     37730     37740     37750     37760
Human                      TGTAATTTGTCACAATGATAGAATGAAGAAAGAAGAATTTCTCTCAGAAAACTAGGAAGAGAAGGAGCTTTTACAATGTA 37611
Kakapo                     TCCCATACCATCAAGTTTCTGGCTTTTCCTTT-TAAACTGTTCCCAGATGTTGTAAGAGATGAAGTG-TTTTACAATGCT 11219
GoldenEagle                TCC------GTTAGGTTTCTGGCTTTTTCTTT-TAAATTGTTCCCAGATGTTGTTAGAAATGAAGTG-TTTTACAATGCT 12123
JapaneseQuail              TGC--------CAGATTTCTGGGTCTTGCTTT-TAAATTGTTCTCAGATGTTGTTAGAAATGAAATA-TTTTACAGCACT 10052
MediumGroundFinch          -------------------------------------------------------------------------------- 0
GoodesThornscrubTortoiseCAGATGTAGAGTAAACTGCTTTCTGTTGTTACATGCAACACTCTCATCCATTTGATGAGCTGAGATG-GAGACAGCCATT 33625
```

```
Majority                  GTTAGATTTCTTTGAATCATGAGAT-----CTATCTCCTTGCTTGTC-ATAGCCCTTGGGAAATTCGTGCTTGTAGGC-G
                                   37770     37780     37790     37800     37810     37820     37830     37840
Human                     AATAAGGGGTATCTACTAAGAACCTACAACAAATTTCATACTTAATGGAGAAATATTGAATGTTTTCTTCTTAAAAACAG 37691
Kakapo                    GTTAGATTTCTTGAAATCATGAGAT-----CTGTCTCCTGGCTTGTC-ATAGCCCTTGGGAAATTCGTGCCTGTAGGCTG 11293
GoldenEagle               GTTAGATTTCTTTAAATCACGAGAT-----CCATCTCCTGGCTTGTC-ATACCCCTTGGGAAATTCATGCCTGTAGGCTG 12197
JapaneseQuail             GTTAGAATTCTTTG--TCATGTG-------CTATCTCCCTACTTGCC-ATGGCTGTTGGGAAGTTCCTGC---------- 10112
MediumGroundFinch         -------------------------------------------------------------------------------- 0
GoodesThornscrubTortoiseCATTTCTCACTCTGAGCCATAAGTTAAAGGGAATCTCTGTTTTCATT-TTAAACAATCAGCAACTTGGAATTTCTCAC-A 33703

Majority                  AGAATGACTGTGGTAAA---ATAGTGCAGCTGATGTTCTC--CTTTTTTCTTTCATTTAGGGGAGTA---GGGGAGAGAG
                                   37850     37860     37870     37880     37890     37900     37910     37920
Human                     AAATGAAGCAAGGATGTCTGTTTTCTCAATTTTTCCCCTTAGCTTTCTGCTTACATTAAGGCAGGTGAAATAAAAGACAT 37771
Kakapo                    AGAATGACTGTGGTAAATGTGCAGTGCAGCTGAAGTTCTC--CTTCTTCTTTTCATCTAGGGGAGGAGTGGGGGAGAGAG 11371
GoldenEagle               AGAATGACTGTGGTAAATGCACAGTGCAGCTGAAGTTCTC--CTTTTTTCTTTCATCTGGGGGAGGAAGCAGGGAGAGAG 12275
JapaneseQuail             -------------------------ACAGCTCAGGTTCTC--CTTTGCTCCCTCACACAGAGG---------GGAGGGAT 10156
MediumGroundFinch         -------------------------------------------------------------------------------- 0
GoodesThornscrubTortoiseAGAGTATCTATGCAAAG---ATAAAATGACCATTACAACA--GTTCATTTTTATACTTAAGACTCTA---GATATAACTG 33775

Majority                  GGAGGTG--GGGGG-----GGAAAAGCATCCTGTTCTTGAT---AGAGGTTTGTAATAGCAGCAAATTTGAA---TACTA
                                   37930     37940     37950     37960     37970     37980     37990     38000
Human                     AAAGATTGTAAAGGAAGAAAGAAAACTGTCTTATTCACCATTCAACATGCTTTTAAAGTAGAAGACCTGAAGGACTCTA 37851
Kakapo                    GGAAGTG--GGGTG-----GGAAAAGCATCCTGTTCTTGAG---AGAGGTTTGTAATAGAAGCAAATTTGGG---TGCCA 11438
GoldenEagle               AGAAGTGGTGGGGG-----GCAAAAGCATCCTGTTCTTGAA---AGAGGCTTGTAATATCAGCAAATTTGAA---TACCA 12344
JapaneseQuail             GGGGGTC------------GGGAAATCATTCTTTCATTGTT---AGAGGTTTGGAACGTCCGCATATTTGA----TACTA 10217
MediumGroundFinch         -------------------------------------------------------------------------------- 0
GoodesThornscrubTortoiseTAATATCAAGCAAC-----TTCAAAGTGTGGGGAGAGCAAT---CAATCATTTGAAAGGCTAAAAGTTTGAA---TATTG 33844

Majority                  CGAATGAGATG-T-TTATCAGAAAATGAATTTTTCATTATTTTTGGGTGTATTCT--ATGTTTTCTAATGGGATTTTAAG
                                   38010     38020     38030     38040     38050     38060     38070     38080
Human                     CAACAGATTTGGTAGAACTAATAAGTGAATTTAGAATAATTATAGGATACAAGTTCTATGTACAGAAATAGTCTCCTTAT 37931
Kakapo                    TGAATGAAAGAAT-TTATCAGAAAATGTATTTTTTGATATCTTTGAGTATATTCT--GTCTTTTCTAGTGGGAATTAAAG 11515
GoldenEagle               CAAATGAGAGGAT-TTATCAGAAAATGTATTTTTCAATATCTTTGAGTGTATTCTTTATCTTTTCTAGTGGGAGTTTAAG 12423
JapaneseQuail             CGAATCAGAAG---TTATTGGAGAACTATTTTTTCAGTATTTTT---TGTAT-----ATGTTTTCTAATGGGATGTCAAG 10286
MediumGroundFinch         -------------------------------------------------------------------------------- 0
GoodesThornscrubTortoiseCGTTTGAAATC---TAATCTTAAATTTACCCTTCCATTTCTACAAGATCAATAAAAAAATAATTAAAATATGCACAAAAT 33921
```

```
Majority                    AAATTACTTTAGATGATTCTGATTGATTTTTTAATAAAGTGCAGTTATGTCATTTGTTTACTGTTTTTTGTATTATTGGT
                                      38090     38100     38110     38120     38130     38140     38150     38160
Human                       ACTTGGCAGCAAATTCTTAGAAAATGAAATTTTAAAAAATCTGTTTATAAAAGTATCAGACAAAATATCAGGAAAAAAAT 38011
Kakapo                      AAATTACTTCAGCTGATGCTG-TTGACTTTTTAAGAAAGTGCAGTTTTGTCACTTGTTTACTGGTTTCAGTAGTGTTGGT 11594
GoldenEagle                 AATTTATTTTAGATGATGCTG-TTGACTTTTTAACAAAGTGCAGTTTTGTCATTTGTTTACTGTTTCTTGTAGTATTGGT 12502
JapaneseQuail               AAATTACTGTAGCTGATTCTGATTGATGACATTTTAAAATGCAGTTAGTTTGTTTGGTTATTCTTGCTAGTGTTATCAGT 10366
MediumGroundFinch           -------------------------------------------------------------------------------- 0
GoodesThornscrubTortoiseTAATGTTAAAATAAAATTATAATATTTCCTTTAATACAGTGTTTCAAAGTGTTTTGCATGTAGCCTTGTAGATTTGTGAA 34001

Majority                    TTAGTGTTT---ATGTAGTACA-----------------CGGATCTAACCTTGGTTCTAATAAGCCTTTTATGAAACA-T
                                      38170     38180     38190     38200     38210     38220     38230     38240
Human                       TTGACAAAAGATATGTAAGACATCTACACCAATATGAAACACTGCTAAGAGAAGTTTTAAAAAGACTTACATGAAGACGT 38091
Kakapo                      TTAGTGTTC---ATATA--A-------------------CGTATCTAAACATGCTTCTAGTAAGCCTTTAGTAAAAAA-- 11648
GoldenEagle                 TTAGTGTTC---TTGTAGTATAGTT----------GTAGTGCATCTACACGTGCTTCTAATAAGCCTTCTGTGATACACT 12569
JapaneseQuail               TCAGTGTCT---CTGTAGTGC------------------TAGAAGGGGCCTGCATGTTCACACGCTTCTGATAAGTCA-- 10423
MediumGroundFinch           -------------------------------------------------------------------------------- 0
GoodesThornscrubTortoiseCTACTAGTTGG-AGATAAGGGAGTA----------TGGACTGACCTCTCATTTGTACTCCTGAGTCCCATATTTCATTTT 34070

Majority                    -GAT----TTCTTATGTTATGTTCAGCCATAACTTCTGATGAGAGTCT--TC---------ATAGCCTTGAATTATTGGG
                                      38250     38260     38270     38280     38290     38300     38310     38320
Human                       AGACCATGTTCATGAATTAGAAGGCTCAATATGTTAACATGCCAGTTCTCCCTAAATTGGGATGGCATTGAATCTATAAA 38171
Kakapo                      -GAC----TCAGTTTGTTATGTTTAGCCAGAACTTCTGCTAAGAGTCTCTTC---------ATAGGCTGGAATTATTGGG 11714
GoldenEagle                 CAAT----TTGTTATGTTGGGTTCAGCCATAACTTCTGATCAAAGCCT--TC---------ATAGCCCTGAATTATTGGG 12634
JapaneseQuail               --------------CGTTATGTGCAGCTGTAACTTCTTCCTGGAGCCT--TC---------GTAGCCTCAGATTATTGGA 10478
MediumGroundFinch           -------------------------------------------------------------------------------- 0
GoodesThornscrubTortoiseCAGT----CCCTGATTTCGTATTCCTTAAGAACAAATAAAGTTAGTTT--CT---------GGCTGTTCTGGTTGTTGAG 34135

Majority                    AAAAGAGCTGATAATATTTCTCTTTAA-AAACAGTTGT-GAAATATGACTGAGCTTGGAAAA---AACCTCTTTGGTAAC
                                      38330     38340     38350     38360     38370     38380     38390     38400
Human                       GACTTATAAATTGAGAGTACATTCTA--AAATTTATGTGGAAATAAAAAGGAGCTAGGACAATTAAAACCATTTTGAAAA 38249
Kakapo                      AAAAGAGCTGATAATGCTTCCCTTCCATAAACAGTTGTCAAAAAAGGACTTTGCTTAAAAGACTAAACCTCTTTGATAAC 11794
GoldenEagle                 AAAAGAGCTGATAATGTTTCTCT---------------------------------------------CTCATTGGTAAC 12669
JapaneseQuail               AAAAGAGCTGATAATATTTCTCTTCTATAAACAGTTGTTAAAAATTCTCTGATGCTTGAAAAACTAAACTTCTCGG-GGT 10557
MediumGroundFinch           -------------------------------------------------------------------------------- 0
GoodesThornscrubTortoiseAGCCTTCAAAATGAGACGTTTTTGTAACAAAAGAAGG--GATCTCTGCCTGAGTTTGCAAAG---AGCCTGAAATGAAAC 34210
```

```
Majority                   TCTGAA----GCCAGAGTTGT----------TATTCTTTTGTGTTTTTTTACCTCA--T--------G------TAGTG
                                   38410     38420     38430     38440     38450     38460     38470     38480

Human                      AGTAAATTTTGCCACAGTTCTCAAGACTCACTACAAACCTGTAGTTAATCCAAAACAGTTTACTACTAGCATAGTTAGTG 38329
Kakapo                     TCTGAA----GCCAGAGTTGT----------TATTCTTGAGGGTTTTTTTGCCT----------------------TG 11837
GoldenEagle                TCTCAA----GCCAAAGTTGT----------TACTGTTT-GTGTTTTTTTTACTT----------------------TG 12711
JapaneseQuail              TCTCAG----GCCAAAATTGAGAAGTTTG-GTGTTTTTTTGTGGTTTGTTAGCCTCATTTTA--AACGGAGCTAGTAGTG 10630
MediumGroundFinch          -------------------------------------------------------------------------------- 0
GoodesThornscrubTortoiseTTTGAG----GTGTGAACTGA-------C-CTGTTCTTTTTTATCAGTGCTAACTCAGGT----GCCAGTAATAGTAGTT 34274

Majority                   G-ATGTAATAACATTTGGGGAATGTTTGAAATATTTAAA--GCCCTATCTGCACTGTGAGGAAAA--TA----GTTGGCA
                                   38490     38500     38510     38520     38530     38540     38550     38560

Human                      ACACAGAATAGCGTTCAGGGAATAACTCATGCATTAAAGTTGCCTTGATCACTCAGTGAAGAAAGAGAAGGCCTTTCAAC 38409
Kakapo                     G-ATGTAATGAAATCTGGGGAATGACTGAAATATTTCAA--GCCCTATCTGAAGCGTGAGGAGAAGATA----GCTGGCA 11910
GoldenEagle                G-ATGTAATAAAATCTGGGGAATGTTTGAAATTTTTAAA--GCCCTATCTGGAATGTGAGGAAAA--TA----GTTGGCA 12782
JapaneseQuail              G-CTGTAGTAAGGTTTGGGGGAT-TTTGAAATACT-GAA--GTCCCATCAGCCCTGTGAGGAAAA--TC----ATTGGTA 10699
MediumGroundFinch          -------------------------------------------------------------------------------- 0
GoodesThornscrubTortoiseT-AAATGTTAGCATTGTTAATAATGTTACTGCTTATTAA--AACATGTAAGAAAGGAGAAAACTATGTATGAAAATCTGA 34351

Majority                   AGGACTTCTA-AATAGTATACTTTTTATT--AGCCTGGAAAGATAATTTGACTGTAACAACTTCCTG-ATATTACATCGC
                                   38570     38580     38590     38600     38610     38620     38630     38640

Human                      AGATGTGCTAGAACAGCTGAATATACATACAAAAAAGCAAAAGAAACTTAAACCCTACATCATTCTGTATAAAACAATGA 38489
Kakapo                     AGGACTTCTA-AATAGTATAGTTTTGTTT--AGCCTGGAAGGATAGCGTGACTGTAATGACTTCCTG-ACATTCCATAGC 11986
GoldenEagle                AAGACTTCTA-AATAGTATATTTTTGATA--AGCCTGGAGGGATAATATGACTGTAATGACTTCCTG-AAATTACATAGC 12858
JapaneseQuail              AGGACTTGTA-AATAGTG-GCTTTTTATT--AGCCTGGAAAGATAGTATGACTGTGACAACTTCCTG-GAATGGCATCGG 10774
MediumGroundFinch          -------------------------------------------------------------------------------- 0
GoodesThornscrubTortoiseCTGCTTGCTA-AACAACAAGCTCTTTCAT--CCTACTGCTAAATAATTTAAAGGT-ACACCTCTACC-CTGATATAACGC 34426

Majority                   TACTTAATATA---------ACCTGTGA------TAGCATGAAGCTTCATAGTTGTCTGC---TTTGGGGGAAAGGGGGG
                                   38650     38660     38670     38680     38690     38700     38710     38720

Human                      GATTAATTATATAAAAACTAATATATAGAGTTTTTAGGAAAAAGCATAGGATATACTTGCAACTTTGGGATAAATGAAGA 38569
Kakapo                     TGCTTAATATA---------ACCTGCGA------TAGCATGAGGCTTCATAGTTGTCTGT---TTTGGGGGAAGGGGGGG 12048
GoldenEagle                TACTTAATATA---------ACCTGTGA------TAGCATGAAGCTTTATAGTAGTCTGT---TATGGGGGGAAAA---- 12916
JapaneseQuail              TACTTAATATA---------ACTTGTGA------TAGCATGAAGTCTCA-AGTAATCTGC---TGTGGGGGAGTGGAGGG 10835
MediumGroundFinch          -------------------------------------------------------------------------------- 0
GoodesThornscrubTortoiseAACCCGATATA---------ACACGAAT------TCAGATATAACGCCGTAAAGCAGTGC---TCCAGGGGGCCGGGGCA 34488
```

```
Majority                 ------GGAATCTGAAATCCT------GCTACATAAATTTAGCTC-GTGTAGTGAGGG-------ATTCTGAGTTAACAG
                                  38730     38740     38750     38760     38770     38780     38790     38800
Human                    TTTCTTAAAATAGAAAAATCTTAAAGGAATATATAAACTTGACTCCATCAAAATGGGA--AATTTCTGCTTCATCAAAAG 38647
Kakapo                   ------GGAATCCAAAATCCT------GCTACCTAAATCCAGCTT-GTGTAGTGAAGA-------ATTCTGAGTTAGCTA 12108
GoldenEagle              --------AATCTGAAATCCT------CCTACATAAATCCAGCTT-GTGTAGTGAGGG-------ATTCTGAGTTAGCTA 12974
JapaneseQuail            ATT---GGAATCTGGAGTCCA------GCTACATAAATTTAATTC-CAGTAGTGAGGG-------ATTCTGAGTTAACAG 10898
MediumGroundFinch        -------------------------------------------------------------------------------- 0
GoodesThornscrubTortoise-----GGGCTGTGCACTCCG------GCGAATCAAAGTAAGTTCAGTATAATGTGGTTTCACCTATAATGCGGTAAGAT 34556

Majority                 TTACTGTTA---AAGCAAGGAGCTAGAAGATTGATTTGGTGAGCTTGGTATTTTT-----------TGCTTTTCTTTGTA
                                  38810     38820     38830     38840     38850     38860     38870     38880
Human                    ACATTGTTAAGACATTGAAAAGACAAGGCACAGACTTGGAGAATATAATATTTGTGTATCAATACATGCATATGACTGTA 38727
Kakapo                   TTACTGTTA---AAGCAAGGAGCTAGAAGAT----------------C---------------------T--CCTTTATG 12146
GoldenEagle              TGACTGTTA---AAGCAAGGAGCTAGGAGATTAATTTGATGAGCTAGGGGCTTTT-----------TCTTTTTCTTTGTA 13040
JapaneseQuail            TGACTGCTA---AAGCAGGGAGCTTGAAGGTTCATTTGCTAAGCTAGTTACTTTT-----------TACTTCTCTTAGTA 10964
MediumGroundFinch        -------------------------------------------------------------------------------- 0
GoodesThornscrubTortoiseTTTTGGCACCCGAGGACAGTGTTATATAGGGGTAGAGGTGTACATTGGATTGTT-----------TGATGTAAAGAAAA 34625

Majority                 TAATTCCTCTTTTAGATTGCAGAAATAAAAGTATCAACAGT----------ATTTATGAAAA-------TTTCATGAATT
                                  38890     38900     38910     38920     38930     38940     38950     38960
Human                    TTAGTCCATTTTCATGCTGCTGATAAAGACGTACCCGAGACTGGGCGGGCAATTTATGAAAGAAACCAGTTTAATGAATT 38807
Kakapo                   TAATTCCCCTGGTTGATTCC---A--------------------------------------------TACATGAATG 12177
GoldenEagle              TAACTGATTTTGTAAATTGTAGAAGAGAGAGTATATACAAT----------AACTGTGTTCA------CTTACAGAAATT 13104
JapaneseQuail            TAATTGTTCATTTGAAGAGCCAGAATATTTGCAACAACTGT----------ATTTAATAAAA--GATTTCTTTACGAATT 11032
MediumGroundFinch        -------------------------------------------------------------------------------- 0
GoodesThornscrubTortoiseAAATTCCACTTAGAGGTAACAAATATAAAACAATCACTAGT----------AAGTAAAACTATTGGAAACTTGAAAATCT 34695

Majority                 TACTAAACTATAGAAATGGTATATTCTATTAAAAGTATTTAAAGGCATCTTTATATGTATA---TTTAAGCTTCTTTGAA
                                  38970     38980     38990     39000     39010     39020     39030     39040
Human                    TACAATTCTATGTGACTGGGGAAGCCTCATAATCATGGTGGAAGGCAAGGAGGAGCAAGTCACATCTTACGTAGATGGCA 38887
Kakapo                   TGCT---------------------------------------------------------------------------- 12181
GoldenEagle              AACTAAAATATCGAAGTGGTATCTTCAATTAACAGCATATAAAATAATTTTTATACGTATAATTTTCAAGTTTCTCTGAA 13184
JapaneseQuail            AAGTGAAATATAGAAATGCTATTTTCAATTTAAGGTACTGAAAGATTTCTTTATATGTATA---TTTAAGCTCCTTTGAA 11109
MediumGroundFinch        -------------------------------------------------------------------------------- 0
GoodesThornscrubTortoiseTCTTCCACTTTAAAAAATAAATAATTTTATGCAAACATTTCTTAGCATCCCATATTGAATATACTCTATTCTTTAATTAA 34775
```

```
Majority                  ATACGTA---AAAAT----CTAGT---GGATATCTTTTATCTTTTTCTGAAAATGA-CCT-TAGGTCATTTTTA------

                                39050     39060     39070     39080     39090     39100     39110     39120

Human                     ACAGGCAAAGAAAAGAGAGCTTGTGCGGGGAAACTCTCCCCTTTTTAAAACCATGAGACT-TACTTCACTGTCACGAGAA 38966
Kakapo                    -------------------------------------------------------------------------------- 12181
GoldenEagle               ATACATATTAAAAGT----CTCATTTGGTATATTTTTCATCTTTTTCTGAAAGTGATCCCACATGCCAGTTTTATGTAGT 13260
JapaneseQuail             ATACATATTCAGTCT----TGAGT---GGACAACTTTTTTCTTTTTCTGAAAATGTCCAATTTGCACATTTTTA------ 11176
MediumGroundFinch         -------------------------------------------------------------------------------- 0
GoodesThornscrubTortoise  AAATGTA---AAGAT----GTATTATAAGATGTAGCTTAATTTAATAAAAAAATTAAATTATGGGTATTTTTTAACACAT 34848

Majority                  -----------A---CATACATTAACATGCTGTT--TTCACA------CCGTA--GTTAGAAATGCTC--TCATGAGGGC

                                39130     39140     39150     39160     39170     39180     39190     39200

Human                     CAGCATTGGAAAGACCTGCCCCCATGATTCAGTTAACTCCCACCAGGTCCCTCCCACAACACATGGAATTCAAGAGGAG 39046
Kakapo                    -----------------------G-CATGCTGGT--CTTACA------CCACA--GATGGAAATGCTC--TCATGAGGGC 12225
GoldenEagle               TCCATTGGTTGAAGCCATAAATTAACATGCTGTT--TTCACA------CTGCA--GATGGAAATGGTC--TCGTGGGGGC 13328
JapaneseQuail             ---------------CATAGATTAACATGCTATT--GCCACA------CCGTGCTGTCAGAAGTACTCAGTGACAGGGGC 11233
MediumGroundFinch         -------------------------------------------------------------------------------- 0
GoodesThornscrubTortoise  CGTAGGACTGAAAGGAGCACATTAGTTTTTATTT--TCCTGAAGTTTTCAGAAGTTTGACAAATGCTGTGTATTAATATA 34926

Majority                  AAGGGTGTGATAACATGGGCTTCCTTGA--AAGGACAAT--CTTGAT-CTGCAACAAATTTGAAGTTCACTCA---CTGC

                                39210     39220     39230     39240     39250     39260     39270     39280

Human                     ATGTGGGTGGGGACACAGCCAAACCATATCAATGACAAAGGACTTGTATCCCAAAGGACTTGTAAAAAAAAAACTCCTCC 39126
Kakapo                    AAGGATGTGATAACGTGGGCTTCCCTGG--AGAGACA-T--CCTGA--CTGCAACAAAATCAAGGTTCCCTCA---CTGC 12295
GoldenEagle               AAGG--GTGATAACATGGGCTTCATTGA--AAGGACAAT--ATTGACTCTGCAACAAATTTAAAGTTCACCCA---GTGC 13399
JapaneseQuail             TGGGGTATGAGAGCATTGCATTCCTTGA--GAGGGCT-----TTGATTCTGCAAGAAATCTGAAGTTCTCTGA---CTGT 11303
MediumGroundFinch         -------------------------------------------------------------------------------- 0
GoodesThornscrubTortoise  GATCAACAGTTATCATATTTATGCAGAA--ATATGCAAA--CTTGGC-ACATAACACATATGAAGTCAAATAATG-CTCC 35000

Majority                  AAGAAGTTAGTTTG---GCAATTCCCTTGTTT--T-TGTGTAAAAGCTTTTTGGC----AGATACTGGTTTCATGAAGAT

                                39290     39300     39310     39320     39330     39340     39350     39360

Human                     AATCAATAAATATG--AGCAGCCCAATTTTTTTTTAAGTGGCAAAAGAAGTGACATTATAAGGATGGGTAACAAGCACAT 39204
Kakapo                    AGGAAGTTAGTTTG---GCAATTCCCTTGTTTATTCTGTGTAACAGTGTTTTGGC----AGATGCTGGTTTCATGAAGAA 12368
GoldenEagle               AAGAAGTTAGTTTG---GTGATTCCCTTGTTT----TGTGTAATAGCATTTTGGC----AGATGCTGGTTTTGTGAAGAA 13468
JapaneseQuail             AAGAAGTTGGTTTG---GCCATTCCCTGGTTT------------ACCTTTTTGGC----AGACATTGAATCCAGAGCATT 11364
MediumGroundFinch         -------------------------------------------------------------------------------- 0
GoodesThornscrubTortoise  CTGGAATTACTGTGCGTGAAGTGATACTGAATGCTTTCCTTAAAAGATTTTGGGCCCTTAGAAACCCACATTACAACAAG 35080
```

```
Majority                  TTTTTGTGGATGTCTCTGATCTGGATAGT----GTTTTAGC--TTTTAGGGAGGATTAATGTATATTCATGTT-CTTGA-

                                   39370     39380     39390     39400     39410     39420     39430     39440

Human                     GAAAAGATAATGTTAGTCATCAGGTGATCACAAGTCAAAACCATTAGAGATAGAATTTACACCAACTAGAATGGCTAAAA 39284
Kakapo                    GTGTTCTAGATAGCTCTGATCTGAATAGG----AGTTTATC--TTTTAGGGAAGATTAATATATATTCATGTT-CTTGA- 12440
GoldenEagle               ATGTTTTGGATATCTCTGATCAGAATAGTTACTGGTTTAGC--ATTTATGGAAGATTAATGTAGATTAATGTT-CTTGA- 13544
JapaneseQuail             TGTCTGTGAATGTCTATGGTATAGTTAAT----GTTTTAGG--TTGTATACAGTACTGCTTGATGTTTGT----CTTGA- 11433
MediumGroundFinch         -------------------------------------------------------------------------------- 0
GoodesThornscrubTortoise  TGTAGCAGCAACTCCCTGCTGTTCAGACTGAGAGAGCCTCT--TTATGGGCTGGCATAGAGTCCACCCATGATGCATAAA 35158

Majority                  ---CATTGGTAGGAATAAAAATATGGCGTTGAAAACTTG-TGCTGTTGAGGACTAGTTGTCAGTAGATGACC-----TAT

                                   39450     39460     39470     39480     39490     39500     39510     39520

Human                     TTAAATAGATTGGCAATACCGAATGTTGATGAAGATGTGGAACTGTCTTTAATTACTGCTGAGTATATAACA----TAGT 39360
Kakapo                    ---CATTGGTAGGAATGAAAATATGGCGTAGAAAACT----GCTGTTGAGGAGTAGCTGTCAGGCGATGACC-----TAT 12508
GoldenEagle               ---CATTGGTAGGAATAGAAATATGGCATTGAAAACTTG-TGCTGTTGAGGACTAGGTGTCAGAAGATGACC-----TAT 13615
JapaneseQuail             ---TGTTGGTAGGAATACAGATGTGGCATTGAAAACTCC-TGCTCTTGAGGGCAAGCTCTCAGAAGATGACC-----TTC 11504
MediumGroundFinch         -------------------------------------------------------------------------------- 0
GoodesThornscrubTortoise  CCCCATCTGTCAATGTATCAGAATAGCTTTTGTTAACAC-AGTCAGGAAACTAGACTCAATAGTACATAGTCATAATCAG 35237

Majority                  TAATAGTAT--AATAGTAGTTTGTACTTTTTATTTTGTCCTTTATAATCTCTTTCCCTCTTACTAAAAT-GAATATTCAG

                                   39530     39540     39550     39560     39570     39580     39590     39600

Human                     TGGGAAAATAGTTTGCTTTTTTTTTTTTTTTTTTTTTTTTGAGACAGAGTCTTGCTCTGTCACCAGGCTGGAATGCAGTG 39440
Kakapo                    TAATAGTAT--AATAGCACTTTGTACTTCTTATTTTGTCCTTTATAATCTGCTTGCCTCATGCTAAAAT-GGATATTCAG 12585
GoldenEagle               TAATAGTAT--AATAGTAGTTTGTACTTTTTATTTTGTCCTTTATAATCTCCTTCCCTCATCGTAAAAT-GAATATTCAA 13692
JapaneseQuail             TAATAGTCTGCAATAGCAGTTTGTACTTCTT---TTGTCCTTTGCAACCTCTTTCCCGTGTTCTAAAAT-GAATACTTAA 11580
MediumGroundFinch         -------------------------------------------------------------------------------- 0
GoodesThornscrubTortoise  TCATAATGAAACAATGTAACTTGATTTTTTTGCATTTTATTAGAAAATGTCTTTTACGCTAATTGTCATAGAGTAATCAT 35317

Majority                  GTAGTTTCTTACA-TGTTGCTA-TTGTAGGAACTTT-CCTTTGCAGTTTTTGTT--------------GGAAATAAAATT

                                   39610     39620     39630     39640     39650     39660     39670     39680

Human                     GCATGATCTCAGCTTACTGCAACCTCCGCCTCCTGGGTTCAAGCGATTCTCCTGCCTCAGCCTCCTGAGTAGCTGGAACT 39520
Kakapo                    TTAGTCTCTATCA-----------TGTAGGAACTAT-CCTTTCCAGTCTATGCC---------------AAAATAAAATC 12638
GoldenEagle               TTAGTCTCTTACCATGTTGCTA-TTATAGGAACTTT-CCTTTGCAGTCTATGGT---------------GAAATAAAATC 13755
JapaneseQuail             GTAGCTTCTATCA-TGTTGCTA-TTGTAGGAAATTT-TCTTTGCAGCTTGCGATT-------------GGAAGGGAAGTT 11644
MediumGroundFinch         -------------------------------------------------------------------------------- 0
GoodesThornscrubTortoise  GCATTTTTTTAAAACTTGCTTAATTTTAGGAGTTTTGCCAGTTTAATTTTTATTCATTGGA-----GGGGAAAAAAAACT 35392
```

```
Majority                  ACAGTGGTTGTTTTTG----TTTTTTTGTTTTTTTACTTTTATTTTAGAATTCTT--------TTTGCTTGTGATCTTC
                                   39690     39700     39710     39720     39730     39740     39750     39760
Human                     ACAGGTGCACGCCACCACACCCGGCTAATTTTTTGTATTTTTAGTAGAGACTGGGT---------TTCGCCATGTTGGCC 39591
Kakapo                    ACAGTGGTTGGTTTT-----TGTTTGTGTTATTTTACCTTAGATTTTAGATTTCTT--------TGTGTTTGTGATCTTC 12705
GoldenEagle               ATTGTGGTGGTTTTTG----TTCATTTGTTACTTTAACTTATATTTGGACATTCCTGTCACCTACGTGCTTGTGATCTTC 13831
JapaneseQuail             GATGTGGCAGCTTCAG----TTTGTTTGGCTTATTTATTTTTGTTTTTAAACTTAAATTTGGACTTTTCTGTCGATCTGT 11720
MediumGroundFinch         -------------------------------------------------------------------------------- 0
GoodesThornscrubTortoiseACAAATATTTTTCTTAGCTCTGGTATTACTGCTTGTCCTATTAATGAATAATTCTCTGTATAGACACCAGAATAGCTCCT 35472

Majority                  AGTCTTGTTTTACGGTCTCAAACTTGCTT-GA---T-CGGCACACAGCAGTTTGCAAAGATGGGGAAAAGAGTTAAGTGA
                                   39770     39780     39790     39800     39810     39820     39830     39840
Human                     AGGATGGTCTTGCTCTCTTGACGTTGTGA------T-CTGCCCACGTCGGCCTCCCAGAGTGCTGGGATTACAGGCCTGA 39664
Kakapo                    AGTCTTGTTAAACAAACTCAGACTTGCTTTGATTCTTCAGAACACAGCAGTTTGCACAGATGGGGAAAAGAGTTAAGTGA 12785
GoldenEagle               AGTCTTGTTATACAATCTCAAACTTGCTTTGATTCTTTAGAACACAGCAATTTGCATGGATGGGGAAAAGAGTTAAGTGA 13911
JapaneseQuail             GTTTGTGATCTTTGGTCTCGAACTTGCTT---------GACACACAGCAGCTTGCAAAGATGGGGAAAAGAGCTAAGTGA 11791
MediumGroundFinch         -------------------------------------------------------------------------------- 0
GoodesThornscrubTortoiseGGCCCCAGTTGAAGGGTGGAAAGATTCCA-GAGCTCGGGCGACAACCCAAGCT-CAGAAGTCTCCACAGCAGTGAAACAG 35550

Majority                  ACATTGCTCTTGGATCTTGCATTCTTTATTGAATCTGATGGACTATGTTCTTCAGAGATCTGAGTTTAGTTTCCTTATCT
                                   39850     39860     39870     39880     39890     39900     39910     39920
Human                     GCCACTGCGTCTGACCTAGTTTACTATTTT----CTTACGGAGTTAAACATTAACCTATTTTATGGTCCATAACTTCTAC 39740
Kakapo                    ACATTGCTCTTGGATCTTGCATTCTTCCCTGAATCTGATGTGCTATGTTCTTCAGAGATCCGAGTTTAGTTTCCTTACCT 12865
GoldenEagle               ACATTGCTCTTGGATCTTCCATTCTTCATTGAATCTGATTCACTGTGCTCTTCAGAGATCTGAGTTTAGTTTCCTTATCT 13991
JapaneseQuail             AAATTGCTCTTGAATCTTCCATTCTTTATTGAATTTGATTCACCACGTTCTTCAAAGATGTGAGCTTGTTTTCCTTGTTT 11871
MediumGroundFinch         -------------------------------------------------------------------------------- 0
GoodesThornscrubTortoiseCCCCACAGCCCAAGCCCTGTGAGCCCGAGTCGACTGCTAGGGGCCAGGCATGAGATTTTCTTTGCTGTGTAGACATACCC 35630

Majority                  CTGAAGCTCTCGATAGAATTTACCTGAATTGGTTGAAAGGTTTCTTTGCGTTGCAT-AATTTGACTAGCAGAACTGGTAT
                                   39930     39940     39950     39960     39970     39980     39990     40000
Human                     TTTTAGTTTTTTACCCAGTAAGAACATGTTTGCTGTAAAACTTGTATGTGTATCAAAAACTTTACTGATAATGGTCCCAA 39820
Kakapo                    CTGAAACACTCGAAAGAATTTACCCGAATAGGTGGAAAGGTTTCTTTGCTTTGCAC-GATTTGGCTAGCAGAACTGGTGG 12944
GoldenEagle               CTGAAACACTCAATAGAATTTACCTAAATTAGTTTAAAGGTTTCTTTGCATTGCAT-AATTTGACTAGCAGAACTGGCAT 14070
JapaneseQuail             CTGAACCGCTCGATAGAATTGACCTGAACT----TAACGGTTTCTTCGTGTTGGAT-GATTTGACTATTGGAAGTGGTGT 11946
MediumGroundFinch         -------------------------------------------------------------------------------- 0
GoodesThornscrubTortoiseTATAAGGTCAGGGGCAAAGCATCTGCTACTGTGTGGATTCTTCCTTCCCACTCCGTCAGTGGGGGTGCCACTACTGGAAT 35710
```

```
Majority                    GCTTGA---GAATAAGACTAGTGTATGTT--CAAGATGCT-TGGGAAATGGGTTATAATTCTGAAATTTATGTGTGAGAG

                                     40010     40020     40030     40040     40050     40060     40070     40080

Human                       ACTTGA----AATAACCCAAATGTTTATCAACAGGAAAATGGATAAATTGTGGTATAATCATATAGTATATTACTACT-- 39894
Kakapo                      GCTTGA---GAATAAGACTAGTGAATGTT--CAAGGTGCT-TGGGGAATGGTTTATCATTCTGAAATTCATGTGTGAGAG 13018
GoldenEagle                 GCTTGA---GAATAAGACAAGCAAATGTT--CAAGATGCT-TGGGAAATGGGTTATAATTGTGAAATTTGTGTGTGAGAG 14144
JapaneseQuail               GCTTGA---GAATAAGACTACTGTGTGCC--CAAGATACT-TGGGAAGTGAGCTGTAATTCTGAAAT-CATGTA------ 12013
MediumGroundFinch           -------------------------------------------------------------------------------- 0
GoodesThornscrubTortoiseACTGCATCCAGTTCTGCTGTTTACATTTT--TAAAAGGATGTTGAAAATTGAAGAGGGCGCAGAGAGGAGAGAGAAACAG 35788

Majority                    TGCTGGG--TGATGAAGAGCTTTGCCCTTGGAG-CAAAATGCCAG-GCCTTTTTGGCTTAGCACCCTCTAAAAGAAAATG

                                     40090     40100     40110     40120     40130     40140     40150     40160

Human                       TACTAATAACAAAAGAATGGTTAGTAGTACATGCGAAAACATGGGTGAATAGTAAGCATTGTATGAAGCAAAAGAAACCA 39974
Kakapo                      AGCTGGG--TGATGAAGAGCTTTGCCCTTGGAAACAAGATGCCAC-TCCTTTTTGGCTTAGCACCCTCTAAAAGAAGATG 13095
GoldenEagle                 AGTGGAG--TGATGAAGAGCTTTGCCCTCGGAG-CAAGATGCCAG-GCCTTTTTGTCTTAGCACCCTCTAAAAGAAGATG 14220
JapaneseQuail               --C-----------AAGAACGTTGCCCTTGAAG-TAGAGTGCAAG-GCCTTTTTGGCCTATCTCCCTCTAAAAGAAAATA 12078
MediumGroundFinch           -------------------------------------------------------------------------------- 0
GoodesThornscrubTortoiseTTTGAGGCCTGGAAAAATGCCTTACTGTGAGAGACTTAA-GCTCA-ATCTGGTTAATTTATCATAAAGAAAATGAGAGTG 35866

Majority                    CACATAAAACCAAAGC---CAAGCGCC----CCAAACCTCAAATTGT----ATAA-------AGTCTTTTCTTTG----C

                                     40170     40180     40190     40200     40210     40220     40230     40240

Human                       GACAGACCTGAAAGAGTACATAGTGTGATTCCATTTCTTGAAAGTTTAAGAGCAGATCATTAATTTTTTGTTTAGAAACC 40054
Kakapo                      CACATAAACCCAAAGC---CAAGCACC----CCAAACCGCTAATTGC----ATAA-------AGTCTTCTCTCTG----C 13153
GoldenEagle                 CACATAAAACCAGAGC---CAAGCACC----CCAAAACACAAATTGA----ATAA-------AGTCTTATCTCTG----C 14278
JapaneseQuail               CACATAAAAACTGGTC---CAAACGC------------ACAAGTTG-----ATAA-------AGTCTTTCCTTTG----C 12127
MediumGroundFinch           -------------------------------------------------------------------------------- 0
GoodesThornscrubTortoise-ATATGAGGCGTAAGTGGATAAGTGTTTGTGCCGGAAGTCAGATTCTGATCATGATCATGATGGTCCGTTCTGAC----C 35941

Majority                    TGAATGTATGTGATTAAAG---AAGTATTTATG--TCTGTGCTGTAATTCAGTAGGTGCTTCTGTCAT-GTAGTAGTGTC

                                     40250     40260     40270     40280     40290     40300     40310     40320

Human                       AGAATGTTGGAGATTAACA-GAAAGGGAATATGGTGGCATTTTCTGGTGTGATGGAAACGTCCTATGT--TAATAAACTA 40131
Kakapo                      TGAATAAATCTAAGTAAAG---AAGTATTTATG--TCTGTGCTGTATTTCAGGAGGTGCTTGTGCCAC-GTAGTAGTGCC 13227
GoldenEagle                 TGAATAAATGTAAGTAA-------GTATTTATG--TCTGTGCTGTAATTCAGGAGGTGCTTCTGTCAC-ATAGTAGTGCC 14348
JapaneseQuail               TGAATGGATGTTTTAAAAA---AAATAAAGTTGG-CCTCTGTTGTAATTTCGTAGGTGCTTATGTCATTATAGCAGTGTC 12203
MediumGroundFinch           -------------------------------------------------------------------------------- 0
GoodesThornscrubTortoiseTTAAAGTCTATGATTAGTGTAGAAGTATTTTCACAGGGAGACTATAAAGCACTACAGGGTTCTTTAAT-GTAGCAGAGAA 36020
```

```
Majority                  AGTTT-AAAGAGAA-CGCAT--TTGCTTACTTGAAATGCTTATATTAAATTGCTTT-ATTAGACAGCTGTCATCTTAAGA
                                    40330     40340     40350     40360     40370     40380     40390     40400
Human                     AGTTGCAAAACAAATCCAAAAGTTGTTTGAAGCAGTAGATTATCTCTGAGAACTCTGAATTTCCAGTAGTTGTGTCTTGC 40211
Kakapo                    AGTCTGAAAGAGAA-CGCAT--TTGCTTACTTGAAATGCTTATATTAAACAGCTTT-ACCAGATAACTGTCATCTTAAGA 13303
GoldenEagle               AGTCT--AAGACTA-CGCAT--TGGCTTACTTGAAATGCTTATATTGAATTGCTTT-ACTAGACAACTGTCATCCTGAGA 14422
JapaneseQuail             AGTTG-------------------ACTTACTTGAAATGCTTATATTAAATTGCTTT-GTAAAACAGCTGTCATCTTATGA 12263
MediumGroundFinch         -------------------------------------------------------------------------------- 0
GoodesThornscrubTortoise  AGGTATAACAAGAA-CGAATGGCTGGAAGTTAAAGCTAAACAAATACAAATTAGAA-ATAAGATACAGATTTTTAACAGT 36098

Majority                  TAA-CCTTTTTGAAGTAAA-TGGCAATTACCAGGCAG------TTA-CTGCTTTTGATTT-TGTAATTTAACTGGTCTGT
                                    40410     40420     40430     40440     40450     40460     40470     40480
Human                     TGTAATGAGAAATATGAGATTGGGAATGACTAGGTGAGTCCATGTAGCAGGGGCCTAGATGTGGGATTTGATGTGTGTGT 40291
Kakapo                    TAGCCCTTTTTGAAGTAAA-AGACAATTACCAGGCAG------TTA-CTGCTTTTGATTT-TGTAATTTAACTGCTCTGT 13374
GoldenEagle               TAATCCTTTT-GAAGTAAA-TGACAATTACCAGGCAG------TTA-CTGCTTTTGATTT-TTTAATTTAACTGCTCTGT 14492
JapaneseQuail             GA--CCTTTTTGAAGTAAA-TAGCAATTAACAGCCAG------CTAACTGCTTTTGATTTCTGTAATTACTCTGTTTTCA 12334
MediumGroundFinch         -------------------------------------------------------------------------------- 0
GoodesThornscrubTortoise  CGAGGTTCAATGAGACAAACTACCAAATAAAGAGTAGG---ATTCTCCGGCTTTTGATGTTTTTAAAT---CGAGACTGG 36172

Majority                  GTGCGAGTGTGTTTTGTTTCTTTGCGTG--TTTTTAGGTTACATATTGAATGAAGTGCA------GAGGTTTCTATGCTG
                                    40490     40500     40510     40520     40530     40540     40550     40560
Human                     GTGTGTGTGTG-TGTGTGTGTGTGTGTGTGTGTGTAGCTTGGACTTTAACAGATTTACATTTTAGAAAGAATTTTTGTTC 40370
Kakapo                    GCCGAAGTACGTCATGTGTCTTAA----------AGTTTTACATACTGAATGAAGTGCA------GAGCTATCTAGGCTG 13438
GoldenEagle               GTCCAAGTGTCTTTTGCAGCATAGCTGG--TTTTAAGGTTACATACTGAATGAAGTGCA------GAGGTGTCTAGGCTG 14564
JapaneseQuail             AGTAGACTGTGACTTCTTTTGCTGCATGGCTGTTTAAGTTACATATTTAATGAGGTACA------GGGGTTTCTATGCTG 12408
MediumGroundFinch         -------------------------------------------------------------------------------- 0
GoodesThornscrubTortoise  ATGCCTTTATGCTTTAATCAAATACGAG--TTATTGGGCTCAGTATGGGGATAAATGAG------TGAAATTCTATGGCC 36244

Majority                  CTTATGAACTT-ATAGGGAAAAATAATCATGTATATTTGGTAG-GT-ATTTTTCATACA-GTATATGATACCTTTGTTTT
                                    40570     40580     40590     40600     40610     40620     40630     40640
Human                     CATTTGTATTTCACAGGTAGGATCAGTATTGTATAGAGGGGAGAAT-AGTTTTAAAACATTACGGTAGTTCATTCCAAAG 40449
Kakapo                    CTTAAGAACTG-ATAAGGAAAAAGAACCAGGTATATTT---A-----ATTGCTCACATG-GTAAGAGATATCTTTGTCTT 13508
GoldenEagle               CTTAAGAACTG-ATAAGGAAGAATAGCCATGTATATCTAGTAAGGTCATTGGTCGCACA-GTACATGATACCTTTGTCTT 14642
JapaneseQuail             CTTACAAACCT-ATGGGGAAATGTGATGATGTATATAAGACAGTGT-GCTCTGCTTACA-TTATAAGGTATGTTTGTTTT 12485
MediumGroundFinch         -------------------------------------------------------------------------------- 0
GoodesThornscrubTortoise  ATTTTATAC---ATGGGGGCAGACTAGAAAGCCTAATGATTCCCTCTGACTTTAATATATGAATATAATGCTGTAGTTAT 36321
```

```
Majority                  TTCC--AAAAGCTTGGCAAGTATGTCTGGGAGGGAGTTTGCAGAAGTTTGCTATACGCTTGTGATTTCATGGTGAAC---

                                 40650     40660     40670     40680     40690     40700     40710     40720

Human                     ATACCAAGGTCCTGAATTTTAGGGTCTTGGAAGGAGAATGCAGGAAATGATAAAACG--AGTTATCTGGAAGAAGACAAG 40527
Kakapo                    TTCC-CAAAAGCTTGGCAAATACATGTGGGAGCGAGTTAGCAGAGGTTTGCTCTACGGTTGTGATTTCATGGTGAACTCG 13587
GoldenEagle               TTTAGTAAAAGCTTGGCAAGTATATATGGGAGTGAGCTAGCAGAGGTTTGCTATACGTTTGTGATTTCATGGTGAGC--- 14719
JapaneseQuail             ACCA---AAAGCTTAGCAAGCATGAATGGGA----GTTTGCAGAAATCTGTCACATGCTGTTGGTTTCATGGTGAAC--- 12555
MediumGroundFinch         -------------------------------------------------------------------------------- 0
GoodesThornscrubTortoiseCACTG-AGAGGCTACCCTCACTGCTCTGAAGAGTTGAAGATGGTAGACTTCTCCATTCTCTTCCCTTCATGGAATTCTCC 36400

Majority                  ------AT-CCAT-ACAGAGATCTGTACA---TTTGTTACTTAGGTTCGTGAACATTGATTTATGACAGTAAAATAAATG

                                 40730     40740     40750     40760     40770     40780     40790     40800

Human                     TTTGGAATAATATTAAAGAGTTTTGTCTTAGAATCACTGGGTTGGTAGAAGATGATGCCATTAACAAAGGGGGAAAAGTA 40607
Kakapo                    AAGAACATGCCAT-ACACAAACCTGGACA---TCTGTGAATCAGGCTCGTGAACACTGATTTGTGACAATAAAACGAATG 13663
GoldenEagle               ------ATTCCAC-ATACAAACCTGTACA---TCTGTGACTCAGACTTGTGAACACTGATTTATGAGAACAAAACAAATG 14789
JapaneseQuail             --------------------------------------------------C--CA--GGTTTACAACAGTAAAATAAATG 12581
MediumGroundFinch         -------------------------------------------------------------------------------- 0
GoodesThornscrubTortoiseCATAACAACTCAG-GCTGGGCTCTGAGAGGATTTTTTACATAAGTGCCTGCCTATTGATTTATTCTATGGACTTGTTTA 36479

Majority                  TAGAATTTATGATAGATGCTGGTGTAAGGAATTCACATT-TGCT--TGTTCTTCTGAAGCAAAACTGTTATAACTTTTTC

                                 40810     40820     40830     40840     40850     40860     40870     40880

Human                     GGAAAAGGAGACTCGTTTTTGGTGGGAAAAAAGAATAATCCGCTACTGTTCTGTTTTTTTTTGTTTGTTTTTTGTTTTTT 40687
Kakapo                    TAGGATTTATGATAGATGCCACTATAAGGAATTCACATT-TGCT--TGTTCTTCTGAAACAAAACTGTTAAAACTTTTCC 13740
GoldenEagle               TAGAATTTATGATACATGCCAATATAAGGAATTCACGTT-TGCT--TGTTCTTCTGAAGCAAAACTGTTAAAACTCTTTC 14866
JapaneseQuail             CTG--TTTATAATATGTGCTGGTGTAAGAAACTCCCGT---------GTTCTTCTGAAGAAAAAC--TTGTAATTTTTTC 12648
MediumGroundFinch         -------------------------------------------------------------------------------- 0
GoodesThornscrubTortoiseTCAA--TTAC-ATAGATTTGTCTCTGGTCCTCTGGCACCATATT--TGTTCTCCCCAAACTTTGTC-ATATGACCTCTGA 36553

Majority                  AAGTTTAATGACTAAATCTTGTGATTGTCTCTG---AATGTATTCGTGAGATGTTAAATG---TGTTTCAAAATGAAGAG

                                 40890     40900     40910     40920     40930     40940     40950     40960

Human                     ATTTTTTATTGATCATTCTTGGGTGTTTCTCGCAGAGGGGGATTTGGCAGG-GTCATAGGA--CA----ATAGTGGAGGG 40760
Kakapo                    AAGTGTAATGACTAAATCTTGTGATTGACTCTG---AATGTATTCATGACATTCAAAATG--ATGTTTCAAAATGAAGAG 13815
GoldenEagle               AAGTGGAATGACAAAATCTGGTGATCGACTCTG---AATGTATTCATGAAATGCAAAATGGTATGTTTCAAAATGAAGAG 14943
JapaneseQuail             AAGTATAATGACAAAATTTTATAACTATCTACATC-AGCGTTATCGTGAGATGTTAAATT---CATTTCAAAATGGAAAG 12724
MediumGroundFinch         -------------------------------------------------------------------------------- 0
GoodesThornscrubTortoiseCAGTTTCACAGTTTCATCTGCTTGTTAGTTCCTTTAGAAATTGTAGGTAAATGTTAAATGGC-TTTGCTGACATGAATGA 36632
```

```
Majority                   AAAACTCTTTA------ATTGTTCATTAAACACAAA--TACAG-TAACTGAGT--ATCCACTCTGTTGAGTA-ATTTGTG

                                   40970     40980     40990     41000     41010     41020     41030     41040

Human                      AAGGTCAGCAG------ATAAACAAGTGAACAAAGGTCTCTGGTTTTCCTAGGCAGAGGACCCTGCGGACTTCCGCAGTG 40834
Kakapo                     AAAACTCTTC-------GCTGTTCATGAAACACAAAAATACAG-TAACTTAAT--ATCCACTCTGTTGAGTA-ATTTGTG 13884
GoldenEagle                AAAACTCTTTTA-----ACTGTTCACAAAACACAAA--TACAG-TAACTGAAT--ATCCACTCTATTGAGTA-ATTTGTG 15012
JapaneseQuail              AGAACTTTTAAA-----ATTGCTCTTAAAACA------------TAACTGAGT--AGCCACTCTATTTGGC---TTGGTG 12782
MediumGroundFinch          -----------------ACTGTTCATGAAACACAAA--TACAA-GAACTGAAT--ATCTACTCTGTTTAGTA-ATTTGTG 57
GoodesThornscrubTortoise   AAGGGTCCATAGCTCACATGGTGACTTGAATATTTAGCATCAA-GTACAAAGTGCATCTTATTTGAAAAGTTTGCTTATG 36711

Majority                   T---TCCTTTTGGATTTTT----CTGCTGTG-GATTAAGAAATTAA-C----AGGTTTTCTTAAAGC--CTGTTTTACTA

                                   41050     41060     41070     41080     41090     41100     41110     41120

Human                      TTTGTGTCCCTGGGTCCTTGAGATTAGGGAGTGGTGATGACTCTTAACGAGCATGCTGCCTTCAAGCATCTGTTTAACAA 40914
Kakapo                     C---TCCTTTTGGATTTTT----CTGCTGTG-GGTTAAGAAAT---------GGCTTTTT-GAAGGC--CTCGTTTACTG 13944
GoldenEagle                C---TCCTTTTGGATTTTT----CTGCTGTG-GATTAAGAAATTAA-C----AGCTTTTC-AAAAGC--CTGATTTACTA 15076
JapaneseQuail              T---TGATTTTGGATTTTTTT--CTGCTGTG-GTTTAAGAGACGTATC----AGGCTTTC---ATCG--TGGGTTTCTTA 12847
MediumGroundFinch          C---TCCTTTTGGATTTTT----CTGCTGTG-GATTAAGAAATTAA-C----AGTTTTTTTGAAAGC--CGGATTTACTA 122
GoodesThornscrubTortoise   TAGATATTTTCTAAAATATTA--CTGACCTGTAAATATGAAATGAAGTTTA-AAAATGTCTTTCAGC--CAATGGTAATG 36786

Majority                   GGAACAA---GAAGTTCAT--------GTAA------------TCATTCTTGTGTTCTGCTAGTCAG-CAGTTGCTGGTT

                                   41130     41140     41150     41160     41170     41180     41190     41200

Human                      AGCACATCTTGCACCGCCCTTAATCCATTTAACCCTGAGTGGACACAGCACATGTTTCAGAGAGCACAGGGTTGGGGGT- 40993
Kakapo                     GGAGCAA---GAAGTTCAT--------GTAA------------TCACTCTAGTGTTCTGTTAGTTAG-CAGCTGCTG--- 13997
GoldenEagle                GGAGCCA---GAAGTTTAT--------GTAA------------TCACTCTTGTGTTCTGCTAGTCAG-CAGGTGCTGATG 15132
JapaneseQuail              GGA---------G--------------GCA-----------------TTTTACATTCTGCCAGTTAG-AAATTGCTCATT 12886
MediumGroundFinch          GGAACCA---GAAGTTTAT--------ATAA------------TCATTCTTGCACTCTTTCATTCAG-CAGTTGCTGTTG 178
GoodesThornscrubTortoise   ACAACAA---GAAATTCAAAGCAGAGGATAAAATGGATGGGGCTCCTTCTCGTGTTCTTCATATCAGGAAATTGCCTGGT 36863

Majority                   TAAGTAACTTGTTGAAGCTGATGGCAATTTAA-TTTGAGATATTTCAGATTTGTAAAGTTCCTGCAGGTTTTGGGAAGA-

                                   41210     41220     41230     41240     41250     41260     41270     41280

Human                      AAGGTCACA-GATCAACAGGATAAGAATTTTT-CTTAGTACAGAACAAAATGAAAAGTCTCCCATGTCTACCTCTTTCTA 41071
Kakapo                     -AAGTAACTTGTTGAAGCTGAAGGCAACTAAAATTTGAGATATTTCAGAGTTGTAAAGTTCTTGCAGGTTTGGGGAAGA- 14075
GoldenEagle                GAAGTAACTTTTTGAAGCTGAAGACAGCTCAA-TTTGAGATATTTCAGAATTGTAAAGTTCCTGCAGGTTTTGGAAAGA- 15210
JapaneseQuail              TAGGCAACTTCTTGAAGACAGTTGGAATCAGT-TGT-AGG--TTTTAGATTTGTGGAGTTCCTGTAGGGTTTGGGAAGG- 12961
MediumGroundFinch          TAAGTAACTTTCTGAAGCTGGTGGCAATTGAAATCTGAGATATTTCAGATTTGTGAAGTTCCTACAGGTTTTGGGAAGA- 257
GoodesThornscrubTortoise   GAAGTGACA-GAAACAGAAGTTATTGCTTTAGGCTTACCTTTTGGTAAGGTAACCAACATCCTGATGCTAAAAGGAAAAA 36942
```

```
Majority                   -TCATTCATTT-G---GTACTGGGGTTGCTTCTGTTGTTTACATAGTAATTGCTGTACTGTTGCAAATTAATGCCTG---

                                   41290     41300     41310     41320     41330     41340     41350     41360

Human                      CACAGACACG--GCAACCATCCGATTTCTCAATCTTTTCCCCACCTTTCCCCCCTTTCTATTCCACAAAACCGCCAT--- 41146
Kakapo                     -GCATTCATT------GTACTGGGGATACAAT--------ACATAGTAATTGCTGTACTGTTGGAAATGAATGCCTG--- 14137
GoldenEagle                -CCATTCATTT-G---GTACTGGGGATGCTTCGGGTGTTTACATAGTAAATGCTGTACTGTTCCAAATTAATGCCTG--- 15282
JapaneseQuail              -TCATTCATTC-ATCAGTACTGGGGTTGTTTCAGCTCTTTGTGTAGCATTTGCTGTACTGTTGCAAACTGATGACTG--- 13036
MediumGroundFinch          -GCATTCATTT-G---GTACTGGGGTTACTTCAGCTGTTAATGTAGTAATTGCTGTACTGTTGCAAATTTATACCTG--- 329
GoodesThornscrubTortoise   ATCAGGTATTCTGCTTGTTTCAGAGTTCCATTTATAGTACAATGATTAAGTATTTTCTAATTCCAAATATACTGGTAAAA 37022

Majority                   GCTTATTAAGGGA-A--CCAAATATGCTGCTGAATTATCTTAATATGCTTGACTATGAACTGATTCAGTGTCTGCTGCTA

                                   41370     41380     41390     41400     41410     41420     41430     41440

Human                      TGTCATCATGGCCCGTTCTCAATGAGCTGTTGGGTACACCTCCCAGACGGGGTGGTGGCCGGGCAGAGGGGCTCCTCACT 41226
Kakapo                     GCATGTTAAGGGA-A--GAAAAAATGCTGCTGAGTTATCTTAATATGCCTGACTATGAACTGATTTAGTCTCTGCTGCTG 14214
GoldenEagle                GCGTATAAAGGA-----AAAAAAATGCTGCTGAATTATCTTAATATGCCTGACTATGAACTGATTCAGAGTCTGCTGCTA 15357
JapaneseQuail              GCCTATTAGGGAA-AA-CCAAATTTACCACTGAATTTATTTAATTTGCTTACCTCCGAATGGATTCTGTCTCTGCTG--- 13111
MediumGroundFinch          TCGTATTAAGGG-----GGAAAAATGCTGGTGAATGATCTTGATCAGCTGGACTATGAGCAGATTCAGTGTCTGCTGCTA 404
GoodesThornscrubTortoise   GCTTACTAAGATT-AATCCAGATGTAATGGTAAATAA--TTAAAATGAAAAGTCTTGCATTCTTTCAGTTTGCATTCCT- 37098

Majority                   CTTCTGCCTGTTCAGTTGAGCT-------GTCCAGTCTACAGACGAGGGCTTCCAAGCAAGGCTA-------------GG

                                   41450     41460     41470     41480     41490     41500     41510     41520

Human                      TCCCAGTAGGGGCGGCCGGGCAGAGGCGCCCCTCACTCCCCAGTAGGGGCAGCCGGGCAGAGGCGCCCCTCACCTCCCGG 41306
Kakapo                     CTTCTGCCTATCCAGTTGAGCT-------GTCCAGGCTACAGACAAGGGCTTCCAAACAAGG------------------ 14269
GoldenEagle                CTTCTGCATATTCAGTTGAGCT-------GTCCAGTCTACAGACCAGGCCTTCCAAGCAAGGCTA-------------GG 15417
JapaneseQuail              CCTCTGACTATTGAGTTGAGAT-------TTCCAGACTAGAGATGAGGACTTTCGAGCAAGGCTG-------------GA 13171
MediumGroundFinch          CTTCGGCCTGTTCAGCTGGGCT-------GGCCTGGCTGCAGACCGGGGCTCCCAAGCAAAGCTA-------------GA 464
GoodesThornscrubTortoise   CTTTCATGTGCTCCATACTATTT---CTAGTCTATTTTATATTAGAAAGCAGTGAAGGGCTAATA-------------GT 37162

Majority                   T--GGGTAGCTG--CAAGCAGAAGGA--ACTGCTGTT---ATCA-CTGAGTGAGCTAATA--CTGTTAGTAGTCTTGAAC

                                   41530     41540     41550     41560     41570     41580     41590     41600

Human                      ATGGGGCGGCTGGCCGGGCGGGGGGCTGACCCCCCCACCTCCCTCCCGGATGGGGCAGCTGGCCGGGCGGGGGGCTGACC 41386
Kakapo                     ---------------------AAGGA--ACTGCTATT---ATCA-CCGAGTGAGCTAATA--CTGGTAGTAATCTTGAAC 14320
GoldenEagle                T--GGATAGCTG--CAAGCAGAAAGA--ACTGCAGTT---CTCAACTGAATGAGCTAATA--CTGCTAGTAATCATGAAC 15486
JapaneseQuail              T--GAGTAGCTG--TGAGCAGAAATA--ACAGTTGT----ATTA-CTGAGTGAGC-AGCA--GTGTTGACAGTCTTGACC 13237
MediumGroundFinch          T--GGGTA-TTG--CAATCAGAAGGA--ACTGCACTT---ACCA-CTGAGTAACCTAATG--CTGCTAGTAAACTTCAAC 531
GoodesThornscrubTortoise   TCAGAGCAGATGA-CACTCAAAAAAAT-ATGGCTTTCAG-GCTAACCATTTAGGCTAAC---CTCTTCAAAGGTATCATT 37236
```

```
Majority                    TTTGCAGTATTCTACCCA----TGTAAATATTAT-GTTAGA---AAAA-TAGGACATTGAAG----AGAAAGAGTGAATG

                                    41610     41620     41630     41640     41650     41660     41670     41680

Human                       CCCCCACCTCCCTCCCGGACGGGGCGGCTGGCCGGGCTGGGGGCTGACCCCCGCCACCTCCCTCCCGGACGGGGCGGCTG 41466
Kakapo                      TTTGCAGTATTCTACCCA----TGTAAATATTAT-ATTAGA---AAAC-TAGAACCT---------------AGTG---- 14372
GoldenEagle                 TTTGCAATATTCTACCCA----TGTAAATATTAT-GTTAGA---AAAA-TACAACATTGAAG----AGAAAGAAGGAATA 15553
JapaneseQuail               TTTAGGGTACTCTGCCTG----TGT----------GTTAGA---GGAA-TGGGCCATTAGAG----AGGAAGAAGAAATG 13295
MediumGroundFinch           TTTGCAGCATTCTACCCT----TGTCAATATTAT-GTTAGA---AAAAGTAGAACGTGAAAG----GGAAAGAATG---- 595
GoodesThornscrubTortoise    TTATGTGCACAAACCCCA----GTTTGCTAAACTGGGCAGACTTAAAATGGTGTCTTTGAAT----ATATGAGGTTTATT 37308

Majority                    -TTTATGTTTCGACTTATACCGAGT---T---TTAGTGTATTATA-TTTGCAGTACAAAGCAG-TGTAGTTTGAATAATA

                                    41690     41700     41710     41720     41730     41740     41750     41760

Human                       GCCGGGCTGGGGGCTGACCCCCCGACCTCCTTCCCGGACGGGGCGGCTGGCCGGGCAGAGGGGCTCCTCACTTCCCAGTA 41546
Kakapo                      -------------CCTGGGTGG-------------GTGTATTATA-TTTGAAGTAGAAAACAG-GGTAGTCTAAATAATG 14424
GoldenEagle                 TTTTATGTTTCAACTTATACTGAGTGCCTGGGTTAGTGTATTGTA-TTTACAGTACAAAGCAA-TGTAGTTTGAATAATG 15631
JapaneseQuail               -TTTGTGTAGCCATTTGTACTGATT---T---TTAGTGTATGATA-CTAACAGTACAGAGCAA-TCTAGGCTGACTAATA 13366
MediumGroundFinch           -TTTATGTTTCAACTTACACCT-------------GCATGTTATG-TCAGTAGTAGAAAGCAA-TGTAGTTAGAAAAACA 659
GoodesThornscrubTortoise    TGCCAACTACGGACCCATTTCAGAGAGCTTGGTTTTGGTTTTTTT-GTTTTTGTTTGTTTGGG-GTTTTTTGGGGTAAAA 37386

Majority                    GA-GAGTTTCCATA-ACTT-CCTCTAACTTGTTTCTCAGTTTTAT----CCAGCTAAATTTTTCCTTAGTTATATCTGAT

                                    41770     41780     41790     41800     41810     41820     41830     41840

Human                       GGGGCGGCCGGGCAGAGGCGCCCCTCACCTCCCGGAAGGGGCGGCTGGCCGGGCGGGGGTCTGACCCCCCCACCTCCCTC 41626
Kakapo                      TA-GAGTTTCCATA-ACTT--CTCTAATTTGTTTCTCAGTTTCAC----CCAGCTAAATTTTTCCTTAGTTACATCTGAT 14496
GoldenEagle                 GAAGAGTTTCCATA-ACTT--CTCTAACTTGTTTCTCAGTTTTGT----CCAGCTAAGTTTTTCCTTAGTTATATCTGAT 15704
JapaneseQuail               AA-GAGTTCCCATA-ACTT--TTCTGACATATGTCTTAGTTCCAT----CCAGTTACATATTTCCTTAGTTCTATCTGAT 13438
MediumGroundFinch           GA-GAATTTCTATA-ACTTGCCTATAAATTGCTTCTCAGTTCTAT----CTAGCTAAATTTTTC-TTAGTTATATCTGAT 732
GoodesThornscrubTortoise    CATGAAATCTTATC-AAATCCCTTTATTATGTGAATGTGTTTTATT---CTAGCAATACAAAGTGTAAATAGTGTGTAAA 37462

Majority                    TAGAAATGTGTCCTTACA-TTGAGAAGGATAACACATGGTCC-----TTCTCATGGTCCTGGCTAATCTTGGTATTAGGT

                                    41850     41860     41870     41880     41890     41900     41910     41920

Human                       CTGGATGGGGCGGCTGGCCTGGCGGGGGCTGACCCCCACCT------CCCTCCCAGACGGGGTGACTGCCGGGCGGAGAC 41700
Kakapo                      TAGAGATCTGTACTTACA-CGGAGAAGGACAACACATGGTCC-----TTCTCATGGTCCTACTTAGTCTTGGTATCAGGC 14570
GoldenEagle                 TAGAGATCTGTCATTATA-TTGAGAAGGACAAGACATGGTCC-----TTCTCATGGTCCTACCTAATCTTGGTATTAGGT 15778
JapaneseQuail               TGGATATATA-CTTTGCA-CTGAGAACAAGAAGAC------------TTCTCA------------------GTATTAGCT 13486
MediumGroundFinch           TAGAAATGCATCCTTACA-TGGAAAAGAAAAACACATAGTCC-----CTCTCATGGTTCTGTCTGATCTTGGTATTAGGA 806
GoodesThornscrubTortoise    TACTATATTATTATTATAATTATTATTTTAATTCTTGCTTTATAGGCTTTTTTGGAGCTGGC-AACAGAAGAAGCAGCT 37541
```

```
Majority              GTTACTCAGTTTTAAAGGAAATCTGTTGT-------TGATTTGATAGGCA--CA-GCTTTGTTGGTTGCTATGTACCCTT
                               41930     41940     41950     41960     41970     41980     41990     42000
Human                 GCTCCTCACTTCCCAGACGGCGTGGCTGCCGGGCGGAGGGGCTCCTCACTTCTCAGATGGGGCGGCTGCT--GGG--CGG 41776
Kakapo                ATTACTCAGTTGTAAAGGAAATGTGCTGT-------TGATTTGATAGGC----A-GCTTTGTTGGCTGCTGTGTACCCTT 14638
GoldenEagle           GTTACTCAATTTTAAAGGAAATCTGTTGT-------TGATTTGGTAGGCA--CA-GCTTTGTTGATTGCTATGTAGCCTT 15848
JapaneseQuail         GTCATTTATTTTCAGAGGAAGTCAGTTGG-------CAATGTGATGGGCA--CATGTTTTGTTGGTTG----------TT 13547
MediumGroundFinch     ATGATGCAATCTTAAGGGAG--CTCTTGC-------TCATTTGATAGGCG--CA-GCTTTGTTGCTTGCTATGTACCCTT 874
GoodesThornscrubTortoiseATCACTATGGTTAATTACTATTCCGCTGTGACACCTCATCTTCGTAACCAACCCATCTATATTCAGTATTCTA-ATCATA 37620

Majority              AAGAGCTTAGAGTATTCAATAAACTGTA-TCAAGATTCTTCAGAGCTGATGTTGAAAACTGAGTAAA----CTTTAATGG
                               42010     42020     42030     42040     42050     42060     42070     42080
Human                 AGGGACTCCTCGCTTCTCAGATGGGGCGGCCGGGCCGAGACGCTCCTCACCTCCCAGACGGGGTCGCGGCCGGGTAGAGG 41856
Kakapo                AAGAGCTTAGAGTACTCAGTAA-----------GGTTCTTCAGAGGTGATGTTAAAAACTGAGTAAA----CTTTAATGG 14703
GoldenEagle           AACAGCTCAGAGTATTCAATAAAGTGTA-TCAAGATTCTTCAGAGCTAATGTTGAAAACTAAGTAAA----CTTGAATGG 15923
JapaneseQuail         AAGAGTGAAGAATCTTCCATAAGCTGCA-TGAAGATACTACAGAGCTGATGCTAGAAATTGCAT-------CTTTAATG- 13618
MediumGroundFinch     ACAAGCTTAGAGCATTCAGTAAACTGTA-TAGAGATTCTTCAGAGCTGATGTTGAAAACCAAGTAAA----CTTTAATGG 949
GoodesThornscrubTortoiseAAGAGCTAAAGACTGATAATACACTAAA-CCAGGTATATTTACTGTT-ATGGAGTATAACAAATGC-----ATACAATAG 37693

Majority              CTCTGTGTGTGTA-TACACA-TACGTACTACTCTTAAGAGCT-----AT-TCAG-TG----T-ATTTAAACTGTAACATA
                               42090     42100     42110     42120     42130     42140     42150     42160
Human                 CGCTCCTCACATCCCAGACGGGGCGGCGGGGCAGCGGCGCTCCCCACATCTCAGACGATGGGCGGCCGGGCAGAGACGCT 41936
Kakapo                CTCTGTGTGTGCAACACACA-TACGTACTACTCTTAAGAGCTTCTGTATTTCAGGTG----TGAATTAAACTGT--CATA 14776
GoldenEagle           CTCTATGCGTACA-TACACAGTATATACTATTTTTAAGAGCTTGTGGATTTCAGGTGTGGATTAAACAAACTTTAAGATA 16002
JapaneseQuail         -TCTGTGTATGCA-TACGTA-------CTGCTCTTAAGAAC-----------------------CTTTGGATGTCAGGTG 13666
MediumGroundFinch     CTCTATATGTGTG-TACACA---------------------------------------T-ATATAAACTTTAACATA 986
GoodesThornscrubTortoiseCAAATTAAGCTTAGCATACATTCAACTATACACTATGTAGCC-----------A-TGAATATATTTTTATATAAACCAAA 37761

Majority              CTGTTCTT--TTTATGGACACACATACAGTGCACTATGTTAAAATGGTAAAGGTTTGATTTT-ATGCGTCTTGCAACTGT
                               42170     42180     42190     42200     42210     42220     42230     42240
Human                 CCTCACTTCCTAGATGGGATGGCGGCCGGGAAGAGGCGCTCCTCACTTCCTAGATGGGATGGCGGCCGGGCAGAGACCTC 42016
Kakapo                ACGTTCTA--TGTATGAACAC--ATACAGTGCACTAGGATTAAATGGGAAAGGTTTGATTTT-ATGCATCTTGCAACCGT 14851
GoldenEagle           CTGTTCTG--ATTATGAGCACACATACAGTACACTATGGTAAAATGGTAAAGGTTTGATTTT-ATGGATCTTGCAACTGT 16079
JapaneseQuail         CCATTTT---TGTGTATATATACACACAGAACATTGTGTTCCAATGCTAAAGGTTTGATTTT----------A----TGC 13729
MediumGroundFinch     CTGTTCTC--TTTGTGGACACAAATCTAGTGCAGTATGAGAAAAAGGTAAGGGTTTGATTTTTGTAAGTCTTTCAACTGT 1064
GoodesThornscrubTortoiseCTTTATTT--CTGATAGCTGCA-GTTTATTGTAATATATAAAAGACATAGAGTCTGGAATTGTACATGTTGCAATTACAT 37838
```

```
Majority                 GCT-GTCTCTAAAGGTGGTATTCT--CAGGTTAATT---TAGTATGCAG-TATTGAGGTGCCTTGTAAAA-CATAT-TTA
                                  42250     42260     42270     42280     42290     42300     42310     42320
Human                    CTTTCTTTCCAGACTGGGCAGCCAGGCAGAGGGGCTCCTCACGTTCCAGACGATGGGCGGCCAGGCAGAGACGCTCCTCA 42096
Kakapo                   GCT-GACTAGAAAGGTGAGATCCT--CCTGTTATTT---TAGTATGCAG-TATTGAGGTGCCGTGTAAAC-CACAT-TTG 14922
GoldenEagle              GCT-GCCTAGAAAGGTGATATCCT--CAGGTTAATT---TAGTATGCAG-TATTGAGGTGCCCTGTAAAA-CATAT-TCA 16150
JapaneseQuail            GC----------AGACAGTATTCT--TAGGTTAATT---TAGAATGTGG-TTTTGAGGTACTTTGCAGGA-CAT---TTT 13789
MediumGroundFinch        GCT-GTCTCTGAAGGTAATAGTCT--CAGATGAATT---TATTATGAAG-TATTGAAGTGCCTTGTAAAA-CATAG-TCA 1135
GoodesThornscrubTortoise CTTAGAATTTTATTAATGTATTTC--ATGGTTAATTGTTTCTAATTAAA-TTTTGAATATTTTCATAAAATCAGTC-TTA 37914

Majority                 CTAACTATTCTTGAGG-TTCTATTGTAGTGTCCACAACAGAATGAAATTCGTTTATTTTTGTAAGGAG-TGGGTAGTA--
                                  42330     42340     42350     42360     42370     42380     42390     42400
Human                    CTTCCCAAACGGGGTGGCGGCTGGGCAGAGGCTGCAATCTCGGCACTTTGGGAGGCCAAGGCAGGCGGCTGGGAGGTG-- 42174
Kakapo                   CTGACTGTTCTTGAGG-TTCTCCTGTAATGTTCACAACAGGATGAAGACCATT--------------------------- 14974
GoldenEagle              CTAACTATTCTTGAGA-TTCTATTGTAGTGTCCACAACAGAATGAAAAATGTTTATTTTTGTAAAGA--TAGGTAGTA-- 16225
JapaneseQuail            CTGCCTAGCATGGAGA-TTTTATTATAGTGCCCATAACTAAATGAGATGCTTTCACTTCTAAAAGGAGGTGAGCTGCA-- 13866
MediumGroundFinch        TTAACTATTCTTGA---TTCTCTTGTAGTGTCCACAACAGAATTAAACCCATTTATTTTAATAAGGA--TGGATAGAA-- 1208
GoodesThornscrubTortoise ATATTGACTCTTGGTT-TAGAAGTTACATGACTAAAACTTCAAGCTTCTTGGTTCTGGCATTTGAGAGTTCAGTACCATA 37993

Majority                 ---ACTTGTTTCTTTCCTCATTT----CCATTTTAGCAGTGACCTGGAT--TAGTGTTAAGGGTGTTGTGGAACTACTTT
                                  42410     42420     42430     42440     42450     42460     42470     42480
Human                    -GAGGTTGTAACGAGCCGAGATCAC-GCCACTGCA-CTCCAGCCTGGGC---ACCATTGAGCACTGAGTGAACCAGACTC 42248
Kakapo                   -------------------------------------------------------------------------------- 14974
GoldenEagle              ---ACTCTTTTCTTTCCTCATTT----CCGTTTTAGCAGTGACCTGGAT--TAGTGTTAAGGGTGCTGGGGAAATACTTT 16296
JapaneseQuail            ---ATTGGTTTCTTTACTCTTTT----CCATTGTAGTGGTGACGTGGAT--TGGTGTTAAGGCTGTGGAGGAAGTACTTT 13937
MediumGroundFinch        ---ACT-TTTTTTTCCCTCATTG----CCATTTTAGCAGTGACCTGGAT--TAGTCTTAAGGGTGCTGTAGAAATACTTC 1278
GoodesThornscrubTortoise TGGAATTGTGCCATCACTAATGCAGAGTCCTACTCTCAGTGTTTTGAATCGTAAATTCGATAGTATTGATAACCTCAGTT 38073

Majority                 TGAAGG---T-C---------GGGGAAATAACTGATAAGAAATTCA--ATAATGTTCAACGGATGTCAAGTTTATTC--C
                                  42490     42500     42510     42520     42530     42540     42550     42560
Human                    CGTCTGCAATTC-CGGCACCTCGGGAGGCCGAGGCTGGCGGATCACTCGCGGTTAGGAGCTGCAGACCAGCCCGGCCAAC 42327
Kakapo                   -GGGG----T------------------------------------------T--T------------------------ 14981
GoldenEagle              TGAAGGAAATACTTTTGAAGTGGGGAAAGAACCAATAAGAAATTAAAGATAATCTGCAAAGGATGTCAGGTTTATTC--C 16374
JapaneseQuail            TGAAG----T-----------GGGAAAATATGTAATAAAAA----------ATGTTTAAAGGTTGTCAAGTTTATTC--C 13990
MediumGroundFinch        TGAAG----T------------GGGAAAGAACCTGTAAGAAATTCAAGATAATATTCAAGGGATG---G-TTTA------ 1332
GoodesThornscrubTortoise TGTTTGGTCTGCTCTTCTCACTGGACAGTTCCTGAAGAAAAATTCA--TTAATGATCACCTTGGGAAAAATGTATTC--A 38149
```

```
Majority               ATTTTAAAATTTTGTTTTTTTTAAGATCACATGAATATTTTGTGGGCTTTTCGA-GCGAGGAAGTTTTGGGAG-TTGACG

                              42570     42580     42590     42600     42610     42620     42630     42640

Human                  ACAGCGAAACCCCGTCTCCACCAAAAAAATACGAAAACCAGTCAGGCGTGGCGGCGCGTGCCTGCAATAGCAGGCACTCG 42407
Kakapo                 ----------TTTGTTT-------------------------GGGTTTTTC----------AGTTTTGG---------- 15005
GoldenEagle            CCTTTAAAATTTTTTTTAATGAAGGATCACCTAAATATTTTGGGGGCTTTTTGA-GAGAGGAAGTCTTGAGGGATTGAGA 16453
JapaneseQuail          ATTTTAATAATTTCTTTTTTTTTAAGTGG----AAGATAATGTGTGTGTGTGAA-ATAAAGACCTACTGGGGT-TGGACA 14064
MediumGroundFinch      -----AATATTTTGTTTATTGAAAGATCACATAAAGATTTTGTAGGCTTTTTGA-GGGAGGAAGTCTTGAGAA--TGAAG 1404
GoodesThornscrubTortoiseCTAACTGAGAATTTTGTTGTTTGATCTTGGATGTGTATAGAACCGGCTGTTCTT-TCAAAACAGTTGTAATACATTTTT- 38227

Majority               AGGGGTTGAGGGATAAAAGAAAAAAGGGAAGGATAAAA--AGCAGGGAA---AGAAGCAAAGGCCTCTTTAGCTTTGTCA

                              42650     42660     42670     42680     42690     42700     42710     42720

Human                  GCAGGCTGAGGCAGGAGAATCAGGCAGGGAGGTTGCAGTGAGTCGAGATGGCAGCAGTACAGTCCAGCTTCGGCTCGTCA 42487
Kakapo                 -----------------------------------------------------------------T----T-- 15007
GoldenEagle            AGGGGTTGGGGGTTAAAAGAAAAAAGGGAAAAACAAAAAAGGCATGAAA---AGAAGCAAAAGCCTCTTCAGCTATGTCA 16530
JapaneseQuail          AGAAGTAAAAAAAAAAAGAAAAAAGGGGAGGGGGGAGAGCAGCAGGGGAGACAGAAGCAAATGACTCTTTAGCTTTTTCA 14144
MediumGroundFinch      AGGG-TTGGGGCATAAAAGAAAAAAAGAAACAACAA----GGTGTGAAA---AGAAGCA----CCTCTTCAGCTGTGTCA 1472
GoodesThornscrubTortoise----TTAATAGAACACGGAACTGTTTCAACGATAACA-TAGCAGGG------GCAGTAAAGGTCTTCGTAGTATTTGAG 38295

Majority               --AGTGCC-TATCTTACAAATAGTTTG--AGGGAAGGATTGTCTTGT-ACGTTTTTAATATGGATCTTTTTATGATTATT

                              42730     42740     42750     42760     42770     42780     42790     42800

Human                  TCAGAGGGAGACTGTGGAAAGAGAGGGAGAGGGAGACCGTGGGGAGAGGAGGGAGGGGGAGGGGGAGGGGGAGGGGGAGG 42567
Kakapo                 -----------T------------------GGGTGGGCTTGTTTTGT--------------------------------- 15025
GoldenEagle            --AATGCC-CATCTCACAAGTAATTTGATAGGAAAATATTATCTTGT-ACATTTTCAATATAAACCCTCCTCTGATTTTT 16606
JapaneseQuail          --ACTATC-TGTCTTGTAACTAGTTT----GAGGAAAAGTTTTCCATAGAAATCCTGCTCTGGTTGTTTTGTGGCCTACT 14217
MediumGroundFinch      --AGTGCC-CATCTTACCATT--TGT----GGAAAGGGTTATCTTGTTATGTTTTTAATATATACCCTCCT--GATCTTT 1541
GoodesThornscrubTortoise-GTTGCTGTATCCTACAAAGAATAGGGGAAAGAAGCATTGACTCCTCACTGTTGAAATGCTAATATCTTCATGTTCATT 38373

Majority               GCT---GTTCATTTTTAAGT-CATTTTAGTGATGATT----GT--AATT-ATATTTAAGGTGT---TTTAAT--TATTGT

                              42810     42820     42830     42840     42850     42860     42870     42880

Human                  GCTGTTCTGTTTTTTTAACTATGCTTTAGGGATGATATATAATGTACTTCATGACCAAGGAATCAATCTAATCTTTTTGT 42647
Kakapo                 -------------------------------------------------------------------------------- 15025
GoldenEagle            GCA---GCCCATTTTCGAGT-CATTTTTGAAAAGACT----GTTTAATTGATATTTACCATGT---TTTAAG--AATTAT 16673
JapaneseQuail          ACT---ATTCAAAGC--GAT-CATTTACGTGATAATT----GT----C--ATATTTTAGGAGT---TATAGT--TGTTGC 14276
MediumGroundFinch      GCA---GCTCAGTTTTAAGT-CATTC--------AT------T--AATTTATATTTGTCATGT---TTTAAG--AACTGC 1596
GoodesThornscrubTortoiseTCTTGTGTTCCCTTTTCTAT-CAGACTAATGCTGTCT----GTTGTGACAATCTACAGTTTCT---ATTAGTC-TCTTGT 38444
```

```
Majority                CATGAAGATGTAATTTTCTGCCCTAAGTTGTGTTTTC---CAGGAAA--AATGA-----ATGTCCCCAAAATGAGAATTG
                                 42890     42900     42910     42920     42930     42940     42950     42960
Human                   AATGATGGCATAAAATTC--CTTTAAGCCTTGTTTTAATACAAGAAGTCAGTTTATTTAACCTCCATGTACAGAGGAAAT 42725
Kakapo                  --------------------------TTGGTTTTTT----------------------------------AT----A--- 15038
GoldenEagle             GATGAAGAAGCAGTTCCCTGCCATAAATTGTGTTTTC---CAGGACA--AATGA-----ATGCCCCCAAAATGAAAATTG 16743
JapaneseQuail           C-TGTAGATGCTACCTTCTACCCTAAGTTCTGTTTTC---CAGGACA--AATGA-----CTGTCCCCAGATTGAGCAACG 14345
MediumGroundFinch       AATGAAGATGTAATTCCCTGCCCTAAGTTGTGTTTTG---CAGAAAA--AATGA-----ATGTCCCCAAAATGAAAACTG 1666
GoodesThornscrubTortoiseCATACTTAAGTCATTT-CTAGTATA-ATAGTGTGTTA---ATGGCAA--ATTTTTTTTTATGTCTTTAAGAGAAGTTTTC 38517

Majority                AC-GTTTCCT--G-----------T-TTTCTGATAATCATTATTTATTTGTTTTTTTTTTTTTTT-TTC-AATAGGGGT
                                 42970     42980     42990     43000     43010     43020     43030     43040
Human                   ACTGAGTTTTAAGATTTTTCTATTTTTTTTCTGACAAAAATGAAA-GTCTGCATATATTCTAAGAGACACCAATTTGAAT 42804
Kakapo                  -----------------------------------------------------------------------AATAGGGGT 15047
GoldenEagle             ACCATTTCCTATGAAGGAAGATGAGTCTTTCTTTTAATCTTTTTGTTTTTGTTTTTGTTTTTTTTTTTTTTAATAGGGGT 16823
JapaneseQuail           ATAGCATTCT--------------------CTGAAGACGAGTTTTTATTTTCTTTTTTTTTTTTTCCCCCAAATAGGAAT 14405
MediumGroundFinch       A---TTTCAT------------------TTCCTGTGATCATTAAATATTTGTTTTTA--------------AATAGGGGT 1711
GoodesThornscrubTortoiseTGGAAACCCTTGGTTGTTGTTTTTTTCTTTCTGCTAAAATTAAGTTCAGTATTAATTTCCCTACCTGTTAGTACAAACAT 38597

Majority                GTTAGAA-TTGAGCTGAACTCCGTATTTCTATTTGTAT---AGATTTCCCTTTTAGT-TTCAGCTAGATATAATAATTTA
                                 43050     43060     43070     43080     43090     43100     43110     43120
Human                   ACTGTTG-CTTATAAGAGCAAATAGCATAAAAGACCTTT--AAAAATTGGTGATTATATTTGCCTGGAAA-AATACTCCA 42880
Kakapo                  GTTAGAA-CTGAGTTAAACACCATATTTCTATTTGTAT---AAATTTCCCTCTTAGT-TTCAGCTAGA----ATCATGTA 15118
GoldenEagle             GTTAGAA-TTGAGCTAAACTCCGTATTTCTATTTGTAT---AGATTTCCCTTTTAGT-TTCAGCTAGATATAGTAATTTA 16898
JapaneseQuail           GCTAGAA-TTGAGCTGAACTCACCGTTTATATTTGTGT---AGATTTCCCTTGATGT-TTCATCTGGATGTAATAATTTA 14480
MediumGroundFinch       GTTAAAA-TTGAGCTAAACTCCATATTTCTCTTTGCAT---AGATTTCCCTTGTAGT-TTCAGCTAGATATAATAATTTA 1786
GoodesThornscrubTortoiseACTAGATCTTTTGGAGCGCTGAGTATTCTCCTATCCATTAAAGAAATACTTTAGATTGTCCATTTTTAAAGTGCCAGCTG 38677

Majority                TTATATATGTATATTTAAATATTAAAACAAT-AAGTTAATTAAAATGTTATTGAAATAGACTA--AATATATTTAATTAT
                                 43130     43140     43150     43160     43170     43180     43190     43200
Human                   TGTAATTCAGATGACTCAAAAGGACGTTGGTAGACAATGTTACAAGGACACTTTTATGGTTATCAGATATAGTACAGGAT 42960
Kakapo                  GTATGTACATATATTCCACTATTAAAACAAT-AAGTGAATTTAAATGTAACTGAAATAGCCTA--AATATATTTAATTCT 15195
GoldenEagle             ATATAT--GTATATTAAAATATTAAAACAAT-AAGTTAATTTAGATGTCATTGAAATAGACTA--AATATATTTAATTAT 16973
JapaneseQuail           --ACAT-----------------AA-ACA-----------------------------------------TTTAATTA- 14497
MediumGroundFinch       ATATATATGTGTATTAAAATATTAAAACT-T-AAATTAATTAAAATGTTATTGAAGTAGAATA--AATTTATTTAATTA- 1861
GoodesThornscrubTortoiseTAAGAAATGGATACTTAAAGGTAAATAGAATGAAATTTGTAAAGGGAATGATAAAACATGATGCCAGCATGGGCAATAAG 38757
```

```
Majority                 TATAATATTT-----TAATTTGAATGCTGGACAAAATTATTGTT-CAGCAGTTCTCATTGT------GTTTTGAGGTGG

                                  43210     43220     43230     43240     43250     43260     43270     43280

Human                    TTCATTTTTATGTTCCAGCTTCCAGCTTTATCTAAAATATTTCTTGTAATAGATTTTACTGTAGAATTGTTTCCTAAGGC 43040
Kakapo                   AATAATACTT-----TAACTGGAAATGCTGGACACAATTATTGTT-CA---GTTCTCATTGT------GTTTGGAGGTGG 15260
GoldenEagle              CATAATATTT-----TAATTTGAAATGCTGGACAAAATTACTGTT-CAGCAGTTCTCATTGT------GTCTGGAGGTGG 17041
JapaneseQuail            -----TGTTA-----CAATTTGAAGTGCTGATGAAAATTATTGTT-CAGCACTTCTTGTTGT------GTCTTAAGGTGG 14560
MediumGroundFinch        -----TATTT-----TAATTTGAAATGCTGGACAAAATTACTGTT-CAGCAGTTCTCATTGT------GTCCAGAGATGG 1924
GoodesThornscrubTortoiseTGGTGGATTCATGTACAACTTTAAACAATAGGAACACTATCAGTC-ACGTATTTGCCTTTCA------TTTTTTTTGGGG 38830

Majority                 CTTTGTTTCAGTGG---------TGGGTGAAACAACTGTGCTTAAGCTGCTCTCATTTCAAAG-T--TTGCCATGTA--A

                                  43290     43300     43310     43320     43330     43340     43350     43360

Human                    ATCTGTCTCAACAGCCTTTCCCTTATCTGTGCAGACTTTTCTTTCTCTGTACTTGTTGAATAGGTGCTTTTTGTCAAAGA 43120
Kakapo                   CTTCATTTCAGTGG---------TGGGTGAAACAACTGTGCTTAAGCTGCTCTCATTTCAAAG-C--TTGCCATGTA--A 15326
GoldenEagle              CTTTGTTTCAGTGG---------TGGGTGAAACAACTGTGCTTAAGCTGCTCTCATTTCAAAG-C--TTGCCATGTA--A 17107
JapaneseQuail            CTGCATTTTGGTGG---------TGGGTAAAACAACTGTGCTTGAGCTGCTCTCGTTGCAAAG-T--TTGCCTTGTA--A 14626
MediumGroundFinch        CTATATTTCAGTGG---------TGGGTTAAACAACTATGCTTAAGCTGCTCTCATTTCAGA-----TTGCCATGTA--A 1988
GoodesThornscrubTortoiseTCCTGTGGCAGTGTCC-------TGCCTGCTTCAGTTAACCATCTTCTGCTCTGATGTGATGC-TGTCAGCCTTTTGGCA 38902

Majority                 ATATACT-GCTTTACT-----TAGA-TTTCAAGTTGTTGCA--CTTATCTGA---AAAGAGGAAGTCTGCCAAATCTGAA

                                  43370     43380     43390     43400     43410     43420     43430     43440

Human                    AAAAAATAATTTTACAGGTGAAAAAATTTCATGTTATTAGAAACTTAATCTCAGGTAAATAAATGTTTAATAAATTGATA 43200
Kakapo                   ATAGACC-GCTTTACT-----TGGA-TTTCAAGTTGTTGCA--CTTAACTGA---AAAGAGGAAATCTGCCAAATCGGAA 15394
GoldenEagle              ATATACT-GCTTTACT-----TAGA-TTTCAAGTTATTGCA--CTTATCTGA---AAAGAGGAAGTCTGCCAAATCTGAA 17175
JapaneseQuail            ATATACT-GCTTTATG-----TAGA-CTTCAAGTTGTTGCA--CATATCTGA---AATGAGAAAGTCTGCCAAATCTGAA 14694
MediumGroundFinch        ATATTCC-GCTTTACT-----TAGA-TTTCAAATTGTTGCA--CTTATCTGA---AAAGAGGAAGT-------------- 2042
GoodesThornscrubTortoiseATATCCTTGCTTTCAT-----AACACTTTTTTGTTTTAGCA--CTAAGTTGGCTCAGTCTTGCAAGCACCCTTATTTTAT 38975

Majority                 TAGGTTTGGTTATGCTTGCTTAA---GTTCTTTTAAAGGTTTTCCCTGTCAAACTGACAT--TTTTAATATCATCTTTAA

                                  43450     43460     43470     43480     43490     43500     43510     43520

Human                    CAGTAAGGACATTTTCTAATGAATTGGTTCCTTCAAGAGATATAAAAGTGAAA-TATTTCAAAAATACAACTGTTCCTAA 43279
Kakapo                   GAGGTTTGGTTGTGCTTGCTTAA---GTTCTTCTGAAAGTTTTCCCTGTCAAACTGACAT--TTTTAATATCATCATTAA 15469
GoldenEagle              TAGGTTTGGTTATGCTTGCTTAC---GTTCTTCTAAAGGTTTTCCCTGTCAAACTGACAT--TTTTAATATCATCTTTAA 17250
JapaneseQuail            TAGGTTTGGTTATGCTTGCTTAA---GTTCT---AAAGGTTTTCCCTGTCAAACTGACAT--TTTTAATATCATCTTTAA 14766
MediumGroundFinch        -----------------------------------A---------------------------T-----CATCTTTAA 2053
GoodesThornscrubTortoiseCC--TTTTTTTATGTATAAT-AA---ATTAATTGACAGTACAGTGCTATAAATATTCTGTGACTTCACCAGCATGATGGA 39049
```

```
Majority                   AAA-T-CACAAAATAGAGGAAAAAAAA-TCTCTT--------ACTGTCTCTTTCTTTTTAGAGAGGATCTGA--CGAACT

                                  43530     43540     43550     43560     43570     43580     43590     43600

Human                      GTAGCATTAGAATTTGATTTTGTTAAA-TTTGTAACTATTTAGCAGTGACTATGAGGCTTGACAATTCTAAGGTACAGTC 43358
Kakapo                     AAACT-CACAAAATAGAGGAAAAAAAA-TCTCTT--------ACTGTCTCTTTCTTTTTAGAGAGGATCTGA--CGAACT 15537
GoldenEagle                AAA-T-CACAAAATAGAGGAAAAAAAC-TCTCTT--------ACTGTCTCTTTCTTTTTAGAGAGGATCTGA--CGAACT 17317
JapaneseQuail              AAA-T-CACAAAATAGAGGAAAAAAAAATCTCTT--------ACTGTCTCTTTCTTTTTAGAGAGGATCTGA--CGAACT 14834
MediumGroundFinch          AAA-T-CACAAAATAGAGGAAAAAAAA-TCTCTT--------ACTGTCTCTTTCTTTTTAGAGAGGATCTGA--CGAACT 2120
GoodesThornscrubTortoiseGAAATGTACAGAGCAGGAGCCAGTTGGCTGTGATGGGTGTGGACAGTGTAGTAACCTGCTGGGAAGACTAAAAATGAGTT 39129

Majority                   TCTTTCAGGC---AGTG--TACTCAATAGC--CCGAACTCTAACAT------GAGCA------GCATGGTA--GTTACTG

                                  43610     43620     43630     43640     43650     43660     43670     43680

Human                      TCTTTAATTTTCTAGTGATTTCTTTGTAGCTACGGAACTGGAATATCCCTTTAGGTAT-----ACATATTTCTGCCAGTG 43433
Kakapo                     TCTTTCAGGC---AGTG--TACTCAATAGC--CCGAACTCTAACAT------GAGCA------GCATGGTA--GTTACTG 15596
GoldenEagle                TCTTTCAGGC---AGTG--TACTCAATAGC--CCGAACTCTAACAT------GAGCA------GCATGGTA--GTTACTG 17376
JapaneseQuail              TCTTTCAGGC---AGTG--TACTCAATAGC--CCGAACTCTAACAT------GAGCA------GCATGGTA--GTTACTG 14893
MediumGroundFinch          TCTTTCAGGC---AGTG--TACTCAGTAGC--CCGAACTCTAACAT------GAGCA------GCATGGTA--GTTACTG 2179
GoodesThornscrubTortoiseGTTGGTAGGT---CATG--TACAGAATATC--CCATTGTCTGGTCTAG--TGGGGCATTCATCACATAATTTTGTTACCA 39200

Majority                   GTAAGTGA---TGCCCTTTCAAAG--GAGAAATCTATCATTTATACTTGGAC-TCATTTCCTGTTTACAAGGGGAGAACA

                                  43690     43700     43710     43720     43730     43740     43750     43760

Human                      GTGATTGT---TACTCTGTAATAGCAGGATTTCTGAAAATTATTGCTAAACATGACATTTTGCT--CTACCTGAAAGCA 43508
Kakapo                     GTAAGTGA---CGCCCTTTCAAAG--GAGAAATCTATCCTTTATACTTGGAC-TCATTTCCTATTTACAAGGGGAAAACA 15670
GoldenEagle                GTAAGTGA---TGCCCTTTCAAAG--GAGAAATCTATCATTTATACTTGGAC-TAATTTCCTGTTTACAAGGGGAGAACA 17450
JapaneseQuail              GTAAGTGA---TGCCCTTTCAAAG--GAGAAATCTATCATTTATTCTTGGAC-TCATTTCCTGTTTGCAAGGGGAGAACA 14967
MediumGroundFinch          GTAAGTGA---TGCCCTTTCAAAG--GAGAAATCTATAATTTATACTTGGAC-TCATTTCCTGTTTACAAGGGGAGAACA 2253
GoodesThornscrubTortoiseAAATTTGAAAGTTACATTTGAAAGCTAGGCAGTTTCATTTTTAAAATGGTCC-TTTCCTTACATTTGAGACAGAGAAATG 39279

Majority                   CTGAAA---CGAGAATCCGTTGTGCTATAG-AGGGATAAACTGTTGGCATGCATTAGAATGTAGTGACTTTTAA-TTTAC

                                  43770     43780     43790     43800     43810     43820     43830     43840

Human                      GTTAAA-----CCATTCCATAGAGTCT-----GGAACATTTTACTTAGAACTCTTTTAAGATTTTCATTTTAACTTTTAC 43578
Kakapo                     CTGAAA---CCAGAATCCGTTGTGCTATAG-AGGGATAAACTGTTGTCATGC-------TGTAGTGATCTTGTG-TTTAC 15738
GoldenEagle                CTGAAA---CAAGAATCCGTTGTGCTATAG-AGAGATAAACTGTTGGCATGCATTAGACTGTAGTGACCTCTA--TATAC 17524
JapaneseQuail              CTGAAA---TGAGAACCCATTGTGCTATAG-AGGGATAAACTGTTGGCATGCATTAGACTGTAGTGACTTTACA-TATAG 15042
MediumGroundFinch          CCAAAA---CAAGAATCCGTTGTGCTATAG-AGGGATAAACTGTTGGCTTGTATTAGAATATAGTGACTTTGT--ATTTC 2327
GoodesThornscrubTortoiseCAAAGAAAGTGAGGGTCAGTAGATTCATTGCAAAGATGAAATATGAAAAAGG--TGAAAAGTACAAACGGTTAA-GCTAA 39356
```

```
Majority                    TGACAAACCTTGGGTATC-TGAGAAGAGTAATCCCATAATACAATAGGCAACTTTCAGATGTCTGTACATTAAATTGTTG

                                   43850     43860     43870     43880     43890     43900     43910     43920

Human                       AGACATATTTTTGAGGTGATGTCATTCTTTTTGCTATAATGTTTTGAAAGTATTCCAGTAGATTATTTCATAGTTTTACT 43658
Kakapo                      TCACAAACCTTGGGTATC-TGAGAAGGCGAATCCCATAAAACAAGAGGCAGCTTTCAGATGTCTGTACGCTAAATTGTTG 15817
GoldenEagle                 TCACAAACCTTGGGTATC-TGAGAAGAATAATCCCATAAAACAAGAGGCAACTTTCAGATGTCTGTACACTAAATTGTTG 17603
JapaneseQuail               GGTAAATCCTCAGGAATC-TGAGAAGAGTGATCCCATGAAACAAAGAGCAAGTTTCAGTTGTCTGTACAGAACACTGATG 15121
MediumGroundFinch           CTACAAACCTTGGGTGTC-TGAGAAGAATAATCCCACAAT-CAACAGGCAACTTTCAGATGTCTGCACATGAAATTGTTG 2405
GoodesThornscrubTortoise    AGGGATGAGAGAGGGATC-TGTAATTAGAAAAGATGGGGTACATTAAACAGC---CAAGTTTTTGAAC-TCAAAGTAAAA 39431

Majority                    GTGGGTGATGGGATGAAAAATTACACTTCTCTTGTACAGCTGCCTTCAAAGTAAGGAATCACA-----ACTAC-------

                                   43930     43940     43950     43960     43970     43980     43990     44000

Human                       ATTTATAAAGGTTTTAACAACTCATCTTCTTGCATATAAAGGTAGTGAGAGCCTTATTTCATAGTGTTACTATTTATGAG 43738
Kakapo                      GTGGGTGATGGGATGAAAAATTACACTTATCTTGCACAGCTGCCTTCAAAGTAAGGAATTGCA-----ACTGC------- 15885
GoldenEagle                 GTGGGTGATGGGATGAAAAATTACACTTGCCTTGTACAGCTGCCTTCAAAGTAAGGAATCACA-----ACTAC------- 17671
JapaneseQuail               GTGGATGACAGGATGAAAAATTACACTTGTCTTATTCTGCTGCTTTCAAAGTAAGGAATCAGA-----ACTAC------- 15189
MediumGroundFinch           GTGGGTGATGGGATGAAAAATCACAGTTCTCTTGTACAGCTGCCTTTAAAGTAAGGACTCTCA-----GCTGCAAAGGTA 2480
GoodesThornscrubTortoise    ACAAGTTGC---ATTTATGACTGAATTTCCCATGATTGGTTGTCAGCAACATGGTTATTTATG-----AAAAAGTCTAAT 39503

Majority                    --------------A----CAAGCAAAGGTAAACAGCTT-AACAATTCCTGTATGACTTCAGCTTTTTTACACATGGTCA

                                   44010     44020     44030     44040     44050     44060     44070     44080

Human                       GGTTTTAACAACTTATCTTCTTGTATATAAAGGTAGTGAGAGCCTTCTCTAGGTGCCTTCTGTGTTTTTCCAGCTCATGC 43818
Kakapo                      -------------------CAAGCAAAGGTAAAGAGCTT-AACAACTCTGGGATGACATC--CTTTTTCACACATGGACA 15943
GoldenEagle                 -------------------CAAGCAAAGGTAAACAGCTT-AACAACTCCTGTATGACATCAGCTTTTTCACACATAGACA 17731
JapaneseQuail               -------------------CAGTTAAAGGTAAACAGCA--AACAACTCAC-TATGACTTCTGTTTTTTCACATGTAG-CT 15246
MediumGroundFinch           AACAGCCC-AGTAAAATCTCAGGCAAAGGTAAACAGCT----CAGTTCCTGTGTGACATCAACTTCTTTACTCATGGTCA 2555
GoodesThornscrubTortoise    CACA-TTA-GGTTTATACCTGACCAAAAAAAAAAGATGT-AAAAGTCTTTTGAATGTTTCATGTCATTTTAATAATTTTG 39580

Majority                    TCTGTGAGTCTGGGCGTTACAGGGAAGAACACTGTGC--CAGTTTTATAGT--ATAGTTTGGATTCAGGAAAA-------

                                   44090     44100     44110     44120     44130     44140     44150     44160

Human                       CTAATATGCTTTTGACAACTACACCACACAACTACTTGGAAATACAAGAGTTAATAGTTACCTTTTACGGAAACCTGGTG 43898
Kakapo                      TCTGTGAGTCTGGGCATTGCAGGGAAGAACACTG--C--CAGTTTTATAGC--ATAGTATGGATTCAGAAGAG------- 16010
GoldenEagle                 TCTGTGAATCTGGGCATTGCAGGGAAGAACCCTGTGC--GAGTGTCATAAT--ATAATATGGATTCAGGAAAA------- 17800
JapaneseQuail               TCTGTGAGCCTGGGTGTTAT-GGGAAGAACGCTG--------CCCTATAG---------TGGACTCAAAAAAA------- 15301
MediumGroundFinch           TCCACAAGTCTGGGTGTTACAGGGAAGACCACTGTGC--CAGTTTGATAAT--ACAGTGTGGATTCAGGAAGA------- 2624
GoodesThornscrubTortoise    TAAAGATATTTTGTCCTAAAAGACATGTAAACAGAACAACAAAATAACATT--AAAGTTCGGATTCAAAACAA------- 39651
```

```
Majority                 AAGATTTTTGCTTTGT-TCT-GTTTAGACT--GCTAA------AGCAGGAA----ACTT-----CACTT---TG-CTTCT

                                 44170     44180     44190     44200     44210     44220     44230     44240

Human                    AGGCTTTTTGCGTGGTGGCTGTTGCAGAAACAGCCAACAGAATAGCAGAAGTACCACCTATCTGCACCTACCTGCTTTTT 43978
Kakapo                   CAGAGTTTTT---TGC-T---GTTTG--CT--GCTAA------AGCAGGAA----ACTTTT---CACTT---TG-CTCCT 16062
GoldenEagle              CAGAGTTTTGCTCTGT-TCTAGTTTAGACT--GCTAA------AGCAGGAA----ACTT-----CACTT---TG-CTGCT 17858
JapaneseQuail            AGAGAATTTGGTCTGT-TCTCATTTAGACT--GCCAA------AGCAGTAA----ACTA-----CACTT---TG-CTTGT 15359
MediumGroundFinch        GAGACTTCTG----G------GTTT---CT--GCTCA------AGCAGGAA----GCTT-----CACTT---TG-CTCCT 2670
GoodesThornscrubTortoise ACATTTTTACCTTGTGTGCTGCAGTACCTAGGCACA------AGTAGGTG----AGTTAAGAACAGAA---TGACTGCT 39718

Majority                 TCAGTTTGAGATCGC----AGTGGTAGAATCTCAGAATGTCAA--TGCAAAGTGGGTATGGTTGTTCTTTAAGCTTA---

                                 44250     44260     44270     44280     44290     44300     44310     44320

Human                    TTCTCTTTAACTCTTCTTTGGAATTTGTTTCTCATACAGGAAAGAGTAGCAGCATTCATTTTTTTTTCTTAGCTTAGAG 44058
Kakapo                   TCAGCCTGAGATTGC----AGCGGTAGAATCTCAGAATGTCAG--TGCAAACTGGGCATGGTTGTTCTTTAGGCACA--- 16133
GoldenEagle              TCAGTCTGAGACTGC----AGTGGTAGAATCTCAGAATGTCAA--TGCAAAGTGAGTGTG-TTGTTCTTGAGTCAAA--- 17928
JapaneseQuail            TCAGTCCGAGGTGGC----A---CTAAAATCTCAAT-----------------GG-TAGG-TTGTTCTTTAACCA----- 15408
MediumGroundFinch        TCAGTTTGAGATCAC----AGTGGTAGAATATCAGA----------GCAAAGAGACTGTGGCTGTTCTTGAATCTGT--- 2733
GoodesThornscrubTortoise CTAACTGTGCATCTCCTTGATTGTCAGTTTGTCAGAATGAAATGTTTTAATATAGACTTTATATTTTTATAAGCTTA--- 39795

Majority                 -----------T---TTTTGGTGTTGCTAGGTAGAAAGGCTTTTT-----------CATTAA-ATTGGCACACACTAAAA

                                 44330     44340     44350     44360     44370     44380     44390     44400

Human                    TCTTTTTATTTTGTTTTTTGTTTTTCTAAAGCTAGAAAACACTGCTTTGCCTTGAAGTCTAAAACTGCTTAGAATTAAAA 44138
Kakapo                   -----------T---TGCTGGTGATGCTGGGTAGAAAGACTTCATGAATTGGCACACACTAAAATTGGCACACACTAAAA 16199
GoldenEagle              -----------T---TTTGGGTGGTGCTAGATGGAAAGGCTTTT------------CATGAA--TTGGCACATACTGAAA 17980
JapaneseQuail            ---------------CTATGGTGTTACTGGATAGAAAGGCTTTT------------AAGGAA--TTAGCGCGTAATAAAA 15459
MediumGroundFinch        -----------T---TTCTGGTGATGCTAGATAGAAAGGTTTTTT-----------CATTAA--TTGTCACACACTAAAA 2786
GoodesThornscrubTortoise -----------TGTATTATGATGTTCTTAGGATCAAAGGTGAAATAGTCAAATGTGGTAGAATATTATGAAACATTAAAA 39864

Majority                 TGCCTT--CTATTGCAGTTAATGCTTCT--------GTAAGTTGAACGTGCA-----TTGAGTTCC-------CTTGGAG

                                 44410     44420     44430     44440     44450     44460     44470     44480

Human                    CTTCTTTGCCTCTCAGTTTCATATTTGATAAAGAGAGTTAGGAAAACTTTTATAAATGTGTGTCTCTGAAAGACTTGGAG 44218
Kakapo                   TGTCTT--CTATTGCAGTTAATACTTCT--------GTACGGTGAACGTGCA-----TTGAGTTCC-------CTTGGAA 16257
GoldenEagle              TGCCTT--CTATTGCAGTTAATGCTTCT--------GTATGTTGAACGTGCA-----TTGAGTTCC-------CTTGGAG 18038
JapaneseQuail            AGCCTT--TCATTGCAGTGTATGGTCCT--------GACATTTGAACTTGCT-----TTGATTTCC-------TTGTGAG 15517
MediumGroundFinch        TGCCTT--CTTTTGCACTTGATGCTTCT--------GTATGTTGAATGTACA-----TTAAGTTCC-------CTTGGAA 2844
GoodesThornscrubTortoise -GAAAT--CAATTTTATGTAATACTTC---------ATAAGTTAAATATGCAG----CTGATTTTTACCTA--TTCCAAC 39926
```

```
Majority                   AGCTCACTT---CAGAGGTCTTGCTT-------TTTCTCTTTTATTATT-TACAGTACTCTTAATT-T-------TTTT-

                                     44490     44500     44510     44520     44530     44540     44550     44560

Human                      AATGAAGATGTACCTAAGTTTTGTCACT---GATTTTCATTGGACAGAAGTATAAGGTTTTGCCGTGTCGTATTTTATAT 44295
Kakapo                     GGCTCACTT---CAGAGGTCTT-----------TTTCTCTT----C-------------CC------------------- 16287
GoldenEagle                AGCTCACTT---CAGAGGTCTTGCTT-------TTTCTCTTTTTTCATTTCCCCCCCCCCCCC------------CTTT- 18095
JapaneseQuail              AGCTCACCT---CACAG-TCTTGCTT-------TTTCTCTCTG----------AGAACGTCTAAC----------TTCT- 15565
MediumGroundFinch          AGCTCATTT---CTGAGGTCTTGCTT-------TTTCTTTTTTCTTTTTCTACTGTACTATTAATTTTGTGTGGGTTTT- 2913
GoodesThornscrubTortoiseATTACATTTTCTCAGACACCTTCAAAGGTAGGGTTTCTGTACAACTATTAAAAATTAAACTGAATTTTTTTCCTCCTCCA 40006

Majority                   ---TTCTCTTTTTTTCCTTT---TTTTAG-AGATGTGGAGGAATTAGTTCA--TGGTA--TCAGAGAGT--TGCTGGGCT

                                     44570     44580     44590     44600     44610     44620     44630     44640

Human                      TTGTACACAGTTGAAAGTATAACGGTGAATGAGTAGGACTAAAATAATTTACAAATTATATGAGAAAGT-AGGCCAGGTG 44374
Kakapo                     -----CCCCTTTCTCCCT------------AGATGTGGAGGAATTGGTTCA--TGGCA--TCAGAGAGTGGTGCTGGGCT 16346
GoldenEagle                ---TTTTTTTTTTTTCCTAC---CCTCAG-AGATGTGAAGGAATTGGTTCA--TGGCA--GCAGAGTGT--TGCTGAGCT 18162
JapaneseQuail              ---TTTTCTTTTTTTCTTTT---TTTTAG-AAATAGAGAGGAATTAGTTCA--TGGTA--TCAGTGTTG---CTTGGGCT 15631
MediumGroundFinch          ---TTCTCTTTTTTTCCCTCTTGCCTTAG-AGATGTGGAGGAGTTAATTCA--TGACA--TCAGAGGGT--TGCTGCACT 2983
GoodesThornscrubTortoiseAAACAATCCATTGTTCTTGTA-ATTTCTA-AGCTGTAGACCCTACACTGCAAATGCTTATTCCAACAATCATGCTTGCTT 40084

Majority                   TTCCAAGTAGTTGCCTGGTTCTCTGT----TTTAGTGTGCTTGATCAG---ATTTTTTTGTATTTATGTGCTCAGC-AGT

                                     44650     44660     44670     44680     44690     44700     44710     44720

Human                      CTGTGGCTCAT-GCCTGTATTTCAGC--ACTTTGGGAGGCTGAGGCAGGTGGATCACTTGAGGCCAGGAGTTCGAG-ACC 44450
Kakapo                     TTCCAAGGAATTGCCTGCTTTGCTGC----TTTAATCTGCCTGATCA----A--TTTTTCTGTCTTTGTGCTGAGC-AGT 16415
GoldenEagle                TTCCAAATAGTTGCCTGGTTCACTGC----TTTAATGTGCTTGATCA----ATTTTACCGTATTTCTCTGCTGAGC-ATT 18233
JapaneseQuail              TCCCAAATAGTTGCTTGGTTCACAGT----TTTAGTGTGCAATATATGT--ATATTCTCACCTGTATATACTAGTTGAGC 15705
MediumGroundFinch          TTCCAGTCACTTGCCTGGTTCGCTGT----TCTACTGTGCCTGAT---------TTTCTGTATTCATGGGGTCAGC-AGT 3049
GoodesThornscrubTortoiseTGCTATGCAGTCCCTTTGACCTTAGTGGAATTAAGTGTAAGGAATAAGCACACACTGAAGAAAGTGTGTTTTCAGGCTTT 40164

Majority                   AGAGTGGCTGATATTA-GTAGCAGACTCTTTCCTT--TT-AAATTA--TTAGTAAGAGGTTGGGATTCAGGGTGCAATTT

                                     44730     44740     44750     44760     44770     44780     44790     44800

Human                      AGCCTGGCCAACATGGTGAAACCCCATCTCTACTGAAAATAGAAAAA-TTAGCTGGGTGTGGTGGTGCACACCTGAATTC 44529
Kakapo                     AGAGTAGCTGATATTA-GTAGCAGACTCTTTCCTT--TT-AATCTA--GTAGTAAAAGACTGGGATCTAGGGTGCAATTT 16489
GoldenEagle                ATAGTTGCTGATACTA-GTAGCAGACTCTTTCCTT--TT--AATC----TAGTAA-AGATTGGAATTCAGGGTGCAATTT 18303
JapaneseQuail              AGTATGTACAGTGTTA-GTACCAGATTTACTACTAGATTAAAAGCAAAGTAAACAAAGGTTAAGATTCTGGATACAGCTT 15784
MediumGroundFinch          ATAATCACTGATATTA-ATAGCAGACTATTTCCAT--TTTAAATTG--TTAGTAAGAGGTTGGGATTCAGGGTGCAATTT 3124
GoodesThornscrubTortoiseCATGTAGAAAGCCAGA-TCAGACAAATCCTTACTTACTT-GAATAGTCCTGATTG-AGCTTACTAGTATGAGTAAGAGTT 40241
```

```
Majority                   CATGTTACA--TTTAGGGTGC--TGTGGTGGCAGCTGTTGCCAGGATGGGTGGTTCTGT--GCT-TTCTCATCCTACATC

                                     44810     44820     44830     44840     44850     44860     44870     44880

Human                      CCAGCTACA-TGGGAGGCTGTGGCACGAGAATCACTTAAACCTGGGAAGTGGAGGTTGTAGTCAACCAAGATCATGCCAC 44608
Kakapo                     CATGTTACA--TTTAGGGT----TGTGATGGCAGCTGTTGCCAGGATGGGTAGTTCTGT--GCT-TTCTCGTCCTACATC 16560
GoldenEagle                CATGTTATG--TTTGGGGTGC--TGTGGTGGCAGCTGTTGCCAGAATGGGTACTTCTAT--GCT-TTCTCATCCTACCTC 18376
JapaneseQuail              CTCTATGTA--TTTAACATGCTGTGTAATGGCAGCTGTTGCCAGAGCGGGTG-------------TTCTCTTCCTGCATT 15849
MediumGroundFinch          CATGTTACT--ATTAGGGTGC--TGTGGTGACAGCTCTTGCTAGAATGGGTGGCTCTGT--GCT-TTCTCATCCTACATC 3197
GoodesThornscrubTortoise   TGCTGTACAACCTAAGGACCAGAACTTGCAAAAGCTTCTTCTCAGACATACATTTTAATTGACA-CATTACTACTATTTG 40320

Majority                   CTTAGCACAGGCAGGAAAACC------TCCAACTCCATAT--GTTTCTGCTCCAT---TCTTTATGTTGGAGTGCTCATA

                                     44890     44900     44910     44920     44930     44940     44950     44960

Human                      TGCACTCCAGCCTGGCAATAC------AGCAAGACTCTGTCTTAGATAGATAGATAGATAGATAGATAGATAGATAGATA 44682
Kakapo                     CTTAGCTCAGGCAGAAACCCTCC----CCCAACTCCACAT--GTTTCTGCTCCAT---TCTCTATGTTGGAGTGCTCATA 16631
GoldenEagle                CTTAGCACAGGCAGAACCCCCCCA---CCCAACGCCACAT--GTTTCTGCTCCAC---TCTCTGTGTTGGAGTGCTCATA 18448
JapaneseQuail              CTTAGTACAAACAAGAAAAAA------AGCAACTGTTTGT--GTTTCTGCCCCGC---TCAAT--GCTGGAGTGTTCATA 15916
MediumGroundFinch          TTTAGCACAAGCAGAAAAACC------TCCAAATCCAC----GTTTCTACTACAC---TCTTC-TGTTGGAGTGCTCATA 3263
GoodesThornscrubTortoise   CTTAAAAATTAGAAGAAGACCAAATGTTTGAGTTGCTTAAAAATGTGTGTTTTATGTGTTTTTAAAAATAAATTAACATA 40400

Majority                   GACTTGCAGAA---T-CGTTAATGCAGCATTAAACTGTACT-GCCAAAAAGA-GTAGTTTTTCCTTG---GAGCAGC---

                                     44970     44980     44990     45000     45010     45020     45030     45040

Human                      GATACTGTGAAA--CCCAGTGCTAGAGTGTTTATATTTAATAAGTAAAGATAGGTAGAGTATCGGAACAAAAACAGCATG 44760
Kakapo                     CACTTGC------------TAATCCAGCAGTAAACTGTACT-GCCAAAAAGT--TTAGTTTCCCTTG---GAGCAAC--- 16690
GoldenEagle                GACTTGCAGAA---TGAGCTAATCCAGCATTAAACTGTACT-GCCAAAAAGT-GTAGTTTTTCCTTG---GAGCAAC--- 18517
JapaneseQuail              CTCTTCGCAA----------GATACAGCACTAAAGTGTACT-GCTAAGAAGA-GCTGCTTTCTGTTG---GAGCAGC--- 15978
MediumGroundFinch          CACTTGCAGAG---TACGTTAATGCAGCATTAAACTAGGCT-AACAAAATGAAGTCGTTTTCCCTTG---TAGCAGC--- 3333
GoodesThornscrubTortoise   CTCTTCAAAATACTTTTGTCAGAGTAGCATTAACTAATGCTTGACATGATACGGTAAACTTTCTGTCCC-AAGTAGTGT- 40478

Majority                   -TTA-CTGATCAGTCATG----TGACTGCACAGTTGCTAGATTCAGTGC-AGTGTGGGGTGGA------GGAGTGTGGCA

                                     45050     45060     45070     45080     45090     45100     45110     45120

Human                      GGAAACTAACAATTTTGGGGAATGAATGAGTAATCA--GGATTCAAAGCAAGTATCAAATGAGTC----GGGAAATTAGA 44834
Kakapo                     -TTA-GTCATCAGTCCCG----TGGCTGCACAGTTGCTAGATTC------ACTGTGGGGTGGA------GGAGTGTGGTG 16752
GoldenEagle                -TTAACTGATAAGTCATG----TGGCTGCACAGTTGCTAGATTCAGTGC-ATTGTGGAGTGGA------GGAGCATGGCA 18585
JapaneseQuail              -TTA-CAGGTTGGTCATA----TGACCATGCAATTTGTAGATCATGAAC-ATTGTAGGGAAAA------TGAGTCAGGTG 16045
MediumGroundFinch          -TTT-CTGATCAGTCATG----TGATTGCACAGTTG-TAGACACAGTGT-AGTGTTGAGTGGA------GGAGTGTGGCA 3399
GoodesThornscrubTortoise   -TCACCAGGTCTTTTTTTCT--TACTTTCCTTATTCCTTAATTCGTTTC-AGTGTGAGGAATATCATGTAAGTTGTGGCA 40554
```

```
Majority                 TCAGAAGAGA------------GGAGAACCACTTCCATTGGCAGTTCTGTAACCAAA----GGTGAAGAGTT--TTTTTC

                                45130     45140     45150     45160     45170     45180     45190     45200

Human                    AAAGTAGAAATTGAAGTTTTTAGGGGTGCTATT----AGAGAAATTCAGTGAGGAAGAGAGGGTTCACAGTTCCTTGTTG 44910
Kakapo                   CAAGAAGAGA------------GGAGAACCACTTCTGTTGGCAGTTCTATAACCAAA----GGTGAAGAGAT---GATAC 16813
GoldenEagle              GCAGAAGAGA------------GGAGAACCACTTCCATTGGCAGTTCTGTAACCAAA----GGTGAAGAGATGATACTTC 18649
JapaneseQuail            TCAGGAGA---------------------------------------------------------GAAAGC--------- 16059
MediumGroundFinch        GCAGAAGA-----------------AAACCACTTCCACTGGCAGTTCTGTAACCAAA----GGTGACGAGGT--TATTCC 3456
GoodesThornscrubTortoise TCTGCAGTTGTGTATTGTCTCTGGTATACCTTTTAGAATATTCAGTATGCATTCATATGTTGGTGATTGATTTGATTTTT 40634

Majority                 TTTGGAATAGG--------TCAGAAATGTGGTGTAGT-----GC-ATTCATAGCCATTTTAG---AACTCCTGAACATAT

                                45210     45220     45230     45240     45250     45260     45270     45280

Human                    ACTGATGGATGTGGTGATTTAACAAATTACCCATGATCCTTGCCGACACCCATTTATGTAAAAATTGCAGGTAAAAGCTA 44990
Kakapo                   TTTGGAATAGC--------TCTGAAGTGGGATGTAGT-----GC-ATTCATAGCCATTTTAG---AACTC---------- 16866
GoldenEagle              TTTGGAATAGA--------TCAAAAATGTGGTGCAGT-----AC-ATTCATAGCCAGTTTAG---AACTCCAGAACATCT 18712
JapaneseQuail            ---------------------------------CAAT-----GT-ATTTGTGGTCATTTTAG---AGCCCCTGGA---AC 16094
MediumGroundFinch        TTTGGAATAAG--------TCAGAAATGCAGTGCAGTTTAGTGC-ATTCATAGCCATTTTGG---AACCTCAGAACATAT 3524
GoodesThornscrubTortoise CCTGGCATGCACACTTTTGTCTTTTCTATTGTGTATTGTTTTACTACTTAAAGGTACTGTAATAAAACATATGAAATGAA 40714

Majority                 GTTTATCTCTTCTGCTTTTCT---------------TACTTCCTTCTGGTTCTGA--------------AAG--------

                                45290     45300     45310     45320     45330     45340     45350     45360

Human                    GTGGAGAACAAAGGTCAGTCAGCAGGAGAAAAGTGATGTACTGATCAGAGGTGAAGATGCTGGCTTTAGAATTGAGGTGA 45070
Kakapo                   GTTTATCTCCCCTGCTTTTCT---------------TACTTCCTTCTGCTTCTGA--------------AAG-------- 16909
GoldenEagle              GTTTATCTCCTCTGCTTTTCT---------------TTCTTCCTTCTGGTTCTGA--------------AAG-------- 18755
JapaneseQuail            ACTTATCTCTTCTGCTTTTTG---------------TGCTTTCTTCTATGTCTGA--------------AAC-------- 16137
MediumGroundFinch        GTCTCTCTTTTCTGTTTTTCT---------------TACTTCCTTCTGGTTCTGA--------------AAG-------- 3567
GoodesThornscrubTortoise AACCCTCTTTTCTGCATTTTTCGTCAGAAAATCACCTAGCCCTTTTAAAATTTGATCAATTGGCAATAGAAGTAGTTATG 40794

Majority                 -AGGTCCCT--T---CTTGTTTTCATTTTCTTGATGTCCTCTGCATTCCTGGA---TATTT--AGTGCTTCAGCAATTT-

                                45370     45380     45390     45400     45410     45420     45430     45440

Human                    AAAACTGGTTTTGAGTTTGGAAGAGAAGTATTCAAGTAGAAACCATTTTAAAATTTTATTTGCTTTGAATCCGTGATTAC 45150
Kakapo                   -AGGTCCCT-------T-GTTTTCATTTTCCTGATGTCCTCTGCATTCCTGGA---CATT---AGTGCTTCAGCACTTT- 16973
GoldenEagle              -AGGTCCCTGTT---CTTATTTTCATTTTCTTGATGTCCTCTGCAGTGCTGGA---TATT---AGTGCTTCAGCAATTT- 18824
JapaneseQuail            -AGGTACAT------CT--TTTCCATTTTCTTGACATCCTCAGCAGTCCAAGA---TGATAATACTGCTTCAGCAATTT- 16204
MediumGroundFinch        -AGGTCCCT------CTTTTGTTCATTTTCTTGATGTCCTCTTCAGTCCTGGA---TATTT--AGTGCTTCATCAGTTT- 3634
GoodesThornscrubTortoise TAGATGTGTGCT---CTTATTGTTACTATGTGATTATCCTAAGAATTTTTAAAAC-TGTACTTATTGCTTTAAGAAACTC 40870
```

```
Majority                   ------GCTTTACTTAAATGAGAAACTTGAATGAAAAGGAGT----TTGG----GTTGTGTGTGT-TGAGGGTTTGGATT

                                   45450     45460     45470     45480     45490     45500     45510     45520

Human                      ATGAACATCTTCCCTAAGCAAGAGGCTTTGAAAGAGTTGAATAATGTATGGAAAATGGTGTGCTTAAAGATTTTTTACAT 45230
Kakapo                     ------GCTTTACCTAAATGAGAAACTAGAATGAAAAGGAGT----TTGG----GTTGTGTGTGTGTGAAGGTTTGGATT 17039
GoldenEagle                ------GCTTCACTTAAATGAGAAACTAGAGTGAAAAGGAGC----TTGG----GTTGTGTGTG--TGAGGGTTTGGATT 18888
JapaneseQuail              ------GCTTTTCTTAAATGAGAAAACGCAATTAAAGGGAGT----TAAGA---GTTGTGTGGG---GGGCATTTGGATT 16268
MediumGroundFinch          ------GCTTTACTTAAACAAGAAACAGCTATGCAAAGGAG-------------TTGTGTGC---CAAGGGTTTGGGTT 3691
GoodesThornscrubTortoise   TTGG-TATTTTTGTTTTTTAAAGAGGTTGGATAATACAGACT----GCAAGTAGTTTGCTTATCTGTGACCACTTCATTC 40945

Majority                   TGAGCAGTGGGT--GGGAGTGTGAGTTCATTGTTAACAGGACAAAGT------TCCTGAACGTTACCAAA-TTTTCCATG

                                   45530     45540     45550     45560     45570     45580     45590     45600

Human                      TCATATACAATGAATAAAATTTATATGCATAAATAAAAGCATTACCTCATGCCTTCTTAACTGAACATAAACATTTGAAT 45310
Kakapo                     TGGGCAGTGGGA--GGG------AGAACACTGTTAACAGGACTAAAT-------CCTGAATGTTACCAAA-TTTTCCATG 17103
GoldenEagle                TGGGCAGTGGGA--GGGGGAGGGAGAGCACTGTTATCAGGACAAAGT-------CCTGAATGTTACCAAA-TTTTCCATG 18958
JapaneseQuail              TGAGCATCGAGTATGGGAGTGGAATGTCATTGTTTACAGGACAAACTAGTTTTTCCTGAACATTCTGAAAATCTTCCATG 16348
MediumGroundFinch          TCAGCAGTGGA----------------CATTAAGA-CAGGACCAAGT-------CCTAAACATTACCAAA-TTTTCTATA 3746
GoodesThornscrubTortoise   TGACTTTTTGTTCAGTTACTGTGTATTGTTTCATTAAAACACAAATTGGAATATTTTTAAAGTACCATACTTGCATTATC 41025

Majority                   TATTTTTAGAGAAACTTTTAGTTCAGGAA--------------AGCTAAATGTAA-GTTAATTTATGAAAGG--AAAAAA

                                   45610     45620     45630     45640     45650     45660     45670     45680

Human                      TATTACTGGTTTTACACGCCCAAACAAAAGTTCTGGGTTTACTGGAGGAATGGAAGGGAAAGATCTCTTTCATTCCCCCA 45390
Kakapo                     GATTTTCAGAGAAACTTTTAGTTCAGGAA--------------AACAAAATGCAA-GTGAATTTATCAAAGG--GAAAA- 17165
GoldenEagle                TATTTTGAGAGAAACTTTTAGTTCAGGAA--------------AACTAAATGTAA-GTTATCAAAGGAAAAA--AAAAAA 19021
JapaneseQuail              TGTTTGTAGAGGAACTTGAAAATTGGGAA--------------AGCTAAATGGGT-GTTTATTTATCCAAAG--GAAAA- 16410
MediumGroundFinch          TATTTTTAGATAAACTTTTAGTTCCAGGA--------------AATTAAATGCAA-ATTAATTTATGAAAGG--AAAAAG 3809
GoodesThornscrubTortoise   TTTTCCTATCAGAACTTTTTGCTTAATTTTTCACTATTGTTTTGGTTAACTCTAATATGAATATTGGAAATATTAAAAAA 41105

Majority                   GAAAGC--AAGGGATTC--AAAATTGAAATATGAATTAGTAGAATTTTAGGTTTTTATGACAAGAAAT-ACTGAAGCTTG

                                   45690     45700     45710     45720     45730     45740     45750     45760

Human                      GTAAACCTTTCCCATTC--AGGATCATGGGAAGATGCTGAAGAATTGTAGAACTGTAACACAGGCAGA-CCTAATGCTTC 45467
Kakapo                     --------------------AACCTGAAATATAAATGAATGTCACTATGCGTTTTCAGGCCAAAAAAT-ACAGAAGCTTG 17224
GoldenEagle                AAAAGCTAAAGGGAAAACAAAAATTGATATATGAATTATTAAAATTTTGAGTTTTCAGCAGAAAAAAT-ACAGAAGCTTG 19100
JapaneseQuail              -------------A------GAGTTAAAAGCTAAAGGAGT--AAGAAAAGAACTATATGTTTGGGTTT-TCTGAAGCTTT 16468
MediumGroundFinch          GAGGG---AAAGGAAAA--AAAATTGAAATAAGAATTAT---AATTTTAAGGTTTCGTAACAAGAAAT-ACGAAAGCTTG 3880
GoodesThornscrubTortoise   AAAAACTTCAGTTATTCAGTAAATCACATTATGATTCTGTAGATTTGTTTTTTGTTGTTTTTACTCCTCATTGTAACCTG 41185
```

```
Majority                    TCTAAGAAGCCTTAA-ACTT-TAAATACAAATTCTTTTTTGCTCAAAAAGTTCTTTA------AATGG--AAAAGAGTGC
                                     45770     45780     45790     45800     45810     45820     45830     45840
Human                       ATAAGCTAATGTTTCCATTTTTGGACCCTTTTTCGGTGTTGCTGGAGTGTGTTTATATTTGAAAATAGGCTTTGTTATAT 45547
Kakapo                      TCTAAGAAGCCTCAA-ACTT-TAAATAGAAATCCTTTTCTCCTTAAAAAGCTCTTAA------AATG---AAAAGAGTGC 17293
GoldenEagle                 TCTAAGAAGCCTCAA-ACTT-TAAATAAAAATTCTTTTCTCCTCAAAAAGTTCTTGA------AATGG--AAAAGAGTGC 19170
JapaneseQuail               TCTAAGAAGCCTTAATGCTT-GTAATGTGAGTTTTTCTCATTT--GGGAGTTATT----------TGG--AATAAATGGC 16533
MediumGroundFinch           TCTAAGCAGCCCC--------TACATACAAATTCTTCCTT-----AAAAATTCTGAG------AATGG--AAAAGCATGT 3939
GoodesThornscrubTortoise    TGGAAGTAGTCTTCTGAAAT-CTCCCAAAAGCTCACTTTTGTCCTACTAGCACTATACTTGCTGCTAG--AAAGGCCAGA 41262

Majority                    T-TT----GATTCCATT-ATGTGGATTTAC------GGAGTTTGATGTATTTT-AACATATACATTCTT-CTGCTTTCCA
                                     45850     45860     45870     45880     45890     45900     45910     45920
Human                       TATTAAAGGAGCCCCCATTCCCAGTTTCACATTCTCATAGTATCATTTATTTAAAATGAAGACTTTTATAATATTTGGCA 45627
Kakapo                      T-TT----GATTCTCTG-GGCACCCTGTGCC-----AGCGCCTCA-GCACCCT-CACAGGGAAGAACTT-CTGCCTTAGA 17359
GoldenEagle                 T-TT----GATTCCATT-ATGTGGACTTG-------GGAGTTTGATGTATTTT-AACAAATACGGA-TT-CTCCTCCCCA 19234
JapaneseQuail               T-TT----GATTCCATA-ATGTGGATGGAT-------GAGTTT---GTGTTTA-ATGATGCAAATTCTC-CTACCTTCCA 16595
MediumGroundFinch           T-TT----GATCCTGTT-AGGTTGATGTATGGGATAGAAGTTTGATGTATTT--AACAGATACA------TTGCTTCCCA 4005
GoodesThornscrubTortoise    TATT----GACTTCATTTGAAGCCACTTCCTGTTGTTCAGTAAGAAACAAATC-ATTATTTTCAGCATTACTGCCTTCTC 41337

Majority                    TCTTGTTTTTATTTCCC-----T-----CA--CTGGTTCAGGATTTTGGTTTTGTCATGTTTTGTGGAATTTTTTTGTTT
                                     45930     45940     45950     45960     45970     45980     45990     46000
Human                       CTAAGATATGAATACTTAGTAGTCACCTCATGGAAGCTATTAACTGAGGGAGTGTCAGGTAGTGTAAATGTTGATAGTTT 45707
Kakapo                      TCTAACCTGAACTTCCC---------------CTGGTTCAG---TTTGAACCCATCACCCCTTGTCCTATCGCTCCAGTC 17421
GoldenEagle                 CCTTGTTTTTGTTTCCCCCCTTTT--TAAG--CTGATTTAGGGTTTGGGGTTTGTGTTGGTTTGTGGGATTTTTTTCTTG 19310
JapaneseQuail               TCTTGGTTTTAGGAGAC------------A--GTTTCCAAACAAACTGATTCTGT-----TTTATACAAGCCAAACCTCT 16656
MediumGroundFinch           TTTTGTTTTTATTTCCC-----------AG--CTGACTGAGGGTTTGGGTTTTAT-----TTTTGGGAGTTTCTTTGTTG 4067
GoodesThornscrubTortoise    CCCTGGTGGTAGATTATGGTGGTT--TCCATTATACTTCCCTAATTTGGCATTGCACTTTATTGTCTAGATCTAGTAGAT 41415

Majority                    -----A-------------TCTGTTGGAGAGTT----A----ATTT-----T----T-T-TGTGT-GGT---TAGGTTTC
                                     46010     46020     46030     46040     46050     46060     46070     46080
Human                       ACTACAGTAACTGGCTTGGTCTTTGGGAGAAGAGGACAGATCTTTTTTAGGCAACCTGTATGTAATGTAATGTTTGTTTT 45787
Kakapo                      -------------------CCTGATGCAGAGTC---------CCTC----------------------T---CCAGCATC 17448
GoldenEagle                 GTACCAAAGAAAAAGCA--TTTGTAAGAGAGTTTTCAAATGAATTTATTCTTTACATTT-TATACAGGT---CAGACCTC 19384
JapaneseQuail               ---------------------TGGT---AGGT-----------TTC-------------------------TAGGCATC 16675
MediumGroundFinch           -------------------CGTGT--GGGAGT----------ATTC-----T----T---GGTGTGGGT---GGGGTTTC 4101
GoodesThornscrubTortoise    AGTTTAGTTTTGCTGCCATGCCACTGAAGGAATATCTAAGATGTTTAGCATTCTATTGT-TACGTCAGTAGGTATATTTT 41494
```

```
Majority                   TTAGTATGTGCTTTTTAGTCGTTTTTAAGGATAACTGA-T----AGTAATGATCTCATTATGAA-GCAGCTGTGGTCTGA

                                   46090     46100     46110     46120     46130     46140     46150     46160

Human                      TGAGATCCTACTATAGTGTAGTTGTTGAAGAGCACAGACTATGGAATCATGCTCCCTGAGTTTAATCCTTTCTCTGCTAC 45867
Kakapo                     CTTGTA-GCCCCCTTCAGACA-CTGGAAGCTGCTCTGA------------GGTCTCCACG------CAGCTTCTCTTC-- 17506
GoldenEagle                TTGATATG---TCTCTAGGCATACAGAAGCATAAATGATTTCTCAGAAATGATCTCATTAACAA-GCAGCAGTGCTCTGA 19460
JapaneseQuail              CAAAACTG------TAAGTGGTTTCCAAGGAAAAAAAA---------AATAATCTCATTATGAG-CCGGCAATGGTCTGA 16739
MediumGroundFinch          TCTGTTTGGGTTTTTTTGGCATCCATAACCATAACTCA------AGAAACAATCTCATTA-----GCAGCAGTGGTTAGA 4170
GoodesThornscrubTortoiseTGAAGAGGTGAATTTTCATTG-GGTCAAAGGTAACTGAATATGTATTGGTAGTAACAATATGAATATATCTATAAGCT-- 41571

Majority                   GTCGTG-TCTAGTTCCTGTAGTATCTGTTTCCTGTTATTAAATTTTTTATTTTTATTGTGTAATTTGTAAAGGATATTCA

                                   46170     46180     46190     46200     46210     46220     46230     46240

Human                      TTCCTG-TCTATACCACTTAGTAACTATCTAATCTTGGGCATTTAACTAATTGTTACCTGTAAATGGGGTTGATAGGTCA 45946
Kakapo                     -------TCCAGGCTGAACAGCCCCAATGTTCTGGTTTTGACCTTGCTGTTTTCAACATCTGATCCCC---TGATACTTT 17576
GoldenEagle                GTAGTGGTCTGGTTTCTGTAGTATCAGTTGCCTGTTATAAAAGTTTTCATATTAGTTGTGCAGTATATAAAGGAGGTTGG 19540
JapaneseQuail              G------ATAAGTTCCTGTAGTATCTGTAACAAATTTTGATATTAACTAGGTATAAAGGAGTATTCTGGAAAAGTACCCA 16813
MediumGroundFinch          GTTGTGATCTGGCTCCTGTAGTATCAGATGCCTGTCATTAAAAGTTTCATATGAGTTCAGTAATATGTAAAGAAGATTGG 4250
GoodesThornscrubTortoise-TCTGAACCTTTTTCTTTAAATGCTTTGTTTATAATACATATTTTTTTCTTTTTATTTAGCGGGCTCAAGCTGTTCTTCA 41650

Majority                   TGG-AAAAACAAT--CCCAGACAAACAAAACTCC-----------CCATTGTTAGTG-CAGT---GTTTTAATTCAAGTC

                                   46250     46260     46270     46280     46290     46300     46310     46320

Human                      TAATGATAGCAAT--CTGAGAGTTGTAAATTGCCCGGCATGTAGTTAATTAGCAGTGAATATTAAATATTGATGCAAAAT 46024
Kakapo                     TCC-AAACACATA--CCTAGATGAACAGAAGAA------------AGAGTGTTTGT--CTTT---GTAATTCTCTGAGCC 17636
GoldenEagle                TGG-AAAAATAGT--CCCAAACAAACAAAAAACC-----------CCAATCTGAATG-CAGT---ATTATAATTCAAGTC 19602
JapaneseQuail              GGA-GAAAACAAC--AACAAACAAAAAAAACTCC-----------CCATCACAAATG-CAAT---ATTTTAATTCAAGTC 16875
MediumGroundFinch          TGG-AAAAATAAT--TCCAAACAAACAAAAACCC-----------ACAATCTGAATG-CAGT---GCTGTAATTCAAGTC 4312
GoodesThornscrubTortoiseGGC-AGTAACAGCAGTACAGACAACAAATACTCCCATTAGTGGAACTACTGTTAGTGAAAGT---GCCGTGACTCCAGCT 41726

Majority                   A--------TTCTTTATAGAGGTTTGTGGAAAGGGG-ACTAG-TTGT--TTTTGGCTGTTCTGTTTGATTTTAAGCAGTT

                                   46330     46340     46350     46360     46370     46380     46390     46400

Human                      AAAATTACATCTTTTCTATATTTTTTCCAAGAAG---------TCAAGATCTTTGTCTTATCCTTTGATTCTCAGAAGTT 46095
Kakapo                     ---------TTATTGGTAAAGGC--ACAGACAAAAA-CCCAG-TTGT--CACTAGCAGTGCCCCTGAATTTAGGGCAAAA 17701
GoldenEagle                A--------TTCTCTATAGAGGTCAGTGGAAAGGGG-GACATTTTGG--TTTGGGCTTTTTTGTTTGTTTTTAAGCACTT 19671
JapaneseQuail              C--------TTCCCTATAGAGGTATGTGGGAAGG----------T----T-----------TGTT----TTCATGCAGTT 16918
MediumGroundFinch          A--------TTCTCTACAGCAGTCTGTGAAAAGGGG-ATTAGCTTG---TTTGGG-TGTTCTATT---TTTTAAACATTT 4376
GoodesThornscrubTortoiseCAGAGTCCAGTACTTAGAATAATTATTGATAATATGTACTACCCTGTAACCCTGGATGTTCTTCACCAAGTAAGCCAGTT 41806
```

```
Majority                 TCTTGG----TAT---TGTTTTCAAGCTTCTTTT-T-----TCTGTCTACATTTTTTAAATAGCATT-AA-AAATATCCT
                                  46410     46420     46430     46440     46450     46460     46470     46480
Human                    TCCTACGTTCTTTCTGTGTTATTCAGCTATTTTACTTCTGCTTAATATCCACTATCCAGAAAACATCCAACACAAATCCA 46175
Kakapo                   TCTTG-----------TGGTTCACAGCCTCATTT--------CTTACTAAATACCTACTGTAGAACT-----ATTACCCT 17757
GoldenEagle              TCTTGG----GAT---TGCCTTCAAATTTCTCTTCTGCCTGTCTGCCTGCCTTTGTAAAATAGCATTTAA-AAAAACCCC 19743
JapaneseQuail            TTTTGG----CAT---TATGTTCAAGCTTCTATT--------CTGCCTACTTTGGTTAAATAGCAT-----GCAGATTCT 16978
MediumGroundFinch        TCTTGG----TAT---TGTATTCAAATTTCA-TT--------CTGTTTGTCTTTTTGAAGTAGCAC--AA-AAACCCCCT 4437
GoodesThornscrubTortoise CTCTATGTAACTT---TAGTTTATATGTTTTGTAATAAATTTCTATACATACAATCTTGTTATTTTTGAC-AGATATTCT 41882

Majority                 GTAGAGAAGGTTAAGTCTTCATTTCAGTGTTATTT--CTGAATATATCCTCTCATATCTTTTTTTT----TTTATTGTGA
                                  46490     46500     46510     46520     46530     46540     46550     46560
Human                    AAATAAATTATTTAATTTCCTTAATAATAGGTTTAGGCAGGCTTTTGCCTTTTGGGAATTATTTTTGGAAAATCATTTGA 46255
Kakapo                   AAACCAAGTTTTAGGGGTGAAACTGGGAGATGTTT--TTCATTGTG----CTCATCTTGTC-TTTT----ATCACAGTGA 17826
GoldenEagle              GTAGAGTAGGCTAAGTCCGCAGTTCAGTGTTATTT--CTTAATATATCCTCCCATCTCTCTCTTTT----TTTAGTGTGA 19817
JapaneseQuail            GTAGAGCAGGTTAAATCAACATTTCGGTGTTATTC--CAGTATGTGTATGTTCTTATATCTGTCTTG---TTTATTAGGA 17053
MediumGroundFinch        GTAGAGAAGGATAAATCTTCTGTTCAGC-----TT--CTGAATATATCCTCTCATAGCTCT-TTTT----TTTACTGTGA 4505
GoodesThornscrubTortoise CTAAATTTGGTGCTGTGTTGAAGATAATCACATTCA-CAAAGAATAACCAATTTCAAGCTTTACTGC---AATATGGTGA 41958

Majority                 ACCTGGAAG--TACTTTTTCCAGACTTGCTCAG--------------TTTACAA-------TTTCAGATTGT-ATACTC-
                                  46570     46580     46590     46600     46610     46620     46630     46640
Human                    AAGGGGAAGCCTTTTTTCTCCTTATGGGTACAGTGATAGGTAGTATCTTTATTGGTCTTTTTTTTCCTTGTCTTTCTCC 46335
Kakapo                   CAGTTCCAG--TAGGGTTTTTTG--TTCCTCAG--------------CTGCCA---------TGCAGTTAGA-ATACT-- 17876
GoldenEagle              ATCTGGAAG--TACTTTTTCCAGACTTGCACAG--------------TTTATAA-------GTTCAGATTGT-AGACTC- 19872
JapaneseQuail            ACCTGGAAGG-TACGTTTTCTAGACTTGCTTGGGT--------TTCTTTTACAA-------TTTCAGATTAC-AGATGC- 17115
MediumGroundFinch        ACCAGGAAG--TACTTTTTCCAGACTTACACAG--------------TTTACAA-------GTTCAGATTGT-AAACTC- 4560
GoodesThornscrubTortoise TCCAGTAAATGCACAGCAAGCAAAATTGGTAAGTA--------CAAAATTACT--------TTTGGGCTAGTGATATCC- 42021

Majority                 -AGAAGTAATTGGATG------------TGAAATGGAAACTTG-TGTGTTAATTTTTTTG-TACCCATT-----------
                                  46650     46660     46670     46680     46690     46700     46710     46720
Human                    CAGAAGGAAAAGGACAGACCAAATTAGGAGAAACTGAAATTTGCTTTTTTGAGTATCCTGATAC-CATAACTAT---AAC 46411
Kakapo                   --GTAATACTTTAATGC-----------TTTTATGAATACTCT-TGCTCTAATTCTCTT------CCTT----------- 17925
GoldenEagle              -AGAAGTAATTAGATC------------TGAAATGGAAACCCA-TGTGTTAATTTTTCTG-TACCCATT----------- 19926
JapaneseQuail            -AGAAATGATTGGGTC------------TGAAATGGAAACCTG-TACATTAATTTTTTTTGTACCTGTC----------- 17170
MediumGroundFinch        -AGAAGTAGTTAGATG------------TGAAATTGAAACC---TCTGTTAATTTTTTTG-TAGCCATT----------- 4612
GoodesThornscrubTortoise TGTGGTGATGGAATTGTT---ATTATTTGTGCAGGGTATTAC-TATGACACTGATTTAACCACACATTCTTTTATTAAA 42096
```

```
Majority              TCATGAAGCC--AAATGGGTTT-----AAGTATTCTTTC--TGTCCAAA-----TTAATGTC--------TTCCTTATGC

                               46730     46740     46750     46760     46770     46780     46790     46800

Human                 ACATGGGGCTTTAGGTTGGATTGTGAAGTATTTGTTCTCGTTATTCAAACAAACTAATGAACAAGAACAAATTATCAAGT 46491
Kakapo                AGAGAAAGGC--ATGAAAGGT------GCCTGCTTTTGC--TGCCCTGT-----AGGGCGTC--------TTACATATG- 17981
GoldenEagle           TCATGAAGCC--AAATGAGTTC-----AAGTATTCTTTA--TGTCCAAA-----TTCATGTC--------CTCCTTTTGC 19984
JapaneseQuail         TCCTGAAGCC--AGATGGGTTG-----AAACATTCTTTA--TGTCCAAA-----CTAATGCC--------TTCCTTTTAC 17228
MediumGroundFinch     TCCTGAAGCC--AAATGAGTTC-----AAGTATTCTTTC--TATCTGAA-----TTGATGTC--------TTCCTTATGC 4670
GoodesThornscrubTortoiseTCTGAAGGCC--AAATGGTATTTATACAATTGCTCTAAACTTGCCTAAAAAGCATAAATAATAAG--CAGTTTACTACAT 42172

Majority              TTCAGTTCTCTTATTACTTTT--------TCTTGAAAAGAATTTAAAACTTTAGTGAACT---AATTTTTTTTTTTTATC

                               46810     46820     46830     46840     46850     46860     46870     46880

Human                 TTCTGTACTCAGACTGCTTTG--------TCAAAAAAAGGTCTTAGTCCTGTATCAGAATAATATGTGTTCCTTGTTGAC 46563
Kakapo                ----AATCCCTCCCTATTTTT---------GGTGAAAGAAACTTGCAGAATTGTTGGAC----AACATTAAGTTTGCATT 18044
GoldenEagle           TTCAGTTCTGTTGTTACTTTT-------CTCTTTAGAAGAATTTAAAACTTTTGTGAAC----AGTTTTTTTGATGTAGC 20053
JapaneseQuail         TTCAGTTCTCTTATATTTTTT--------TCAGCAAATGAATTTAAAACATCAGTGAAAA--GGATTTTTCAGCTATAGC 17298
MediumGroundFinch     TTCAGTTCTGTTATTACTTTTT------CTCTTCACAAGAATTTAAAACTTTAGTCAATT---AAACTTTTTTTTTTTTT 4741
GoodesThornscrubTortoiseCCAAACAGTAACAAAACATTTATAAGCATTTGAGGAAGAAATTTACAAATGGGCTAAACCCCAGGATACTTACTTCCATA 42252

Majority              TGAGTTGTAGT----------AGTATTGAATCTTTTCCGGATT-----T-TTTATGGC-TTCAGGGAATCTGTAA--TCA

                               46890     46900     46910     46920     46930     46940     46950     46960

Human                 TGATGCCGATTGTTGTGTGTGCCCATTATGTGTTTTAAAGTATATGTGTATTTTTTGGATTCTGTGCTTTAGCTT--ACA 46641
Kakapo                CGTATTG--------------ATTATTGAACTACTGGCAATTTGA---T-CATATGGA-ATTAAGGAAGATGCTA--GCA 18103
GoldenEagle           CAAGGCATGGT----------AGTGATAAATCTTCTTCGGATT-----T-TGTAGGGC-TTAAGAGAATCTGTAA--TCA 20114
JapaneseQuail         TGAGCCA------------------TCGTAGCTTTCCAGGGCC---------TA-----TTGAGAGACTCTGTAG--TCA 17344
MediumGroundFinch     TTTGCTGTAGCCAA-------GGTGATGAATCTTTCCCAGATT-----T-TCTAGGGT-TTCAGGGACCCTGTGA--TCA 4805
GoodesThornscrubTortoiseTGAGTTCCAACATGGTTATTTAGCAATGAACCCTTAAAGAATTTATTTT-TTTATACCGTTCAACAAACGTGTAACATCA 42331

Majority              TTTC---TTAAGTTCTTTGTAAATGAGACATGTTGTAGAAACTTGCCTATA-T-C-A---T-AATCC--------TTATA

                               46970     46980     46990     47000     47010     47020     47030     47040

Human                 TTTTCAATTAACGGGCCTAGAGATGATTT-TGTTAGATAAGATACTACAAAATGCAAAGTTCAGTCTCTTCCTTTTGACA 46720
Kakapo                CGT----TTGACTGCTGTCCAAAAGACAT-TGCTCAAAGTACAAGGCAATA---C--------------------TTGAG 18155
GoldenEagle           TTGA---TTAAGTTCTTTGTAAAGGAGACATGTTGTAGAAATCTGCCTAAATTGTAATTCTTAAAAC--------TAATG 20183
JapaneseQuail         TTGC---TTAAGGTCTTGGTAAAGGAGGCATTTCATAGAAACTGACCTACT-----------------------CTGTA 17397
MediumGroundFinch     TTTA---TTAAGTCCCTTGTAAATGAGACATGTTGTAGATACTTGCCTATG---T-------AAAAC--------TAATA 4864
GoodesThornscrubTortoiseTTGCCAATTACTAACTTCAGATAAAAAACAGTTTGAGACAATTCATCCTTAGTTCTAGTCTTAATCC--------ACATA 42403
```

```
Majority                 CTTTTTCTTGCCT-TTTCGTTCTTAGAGTTTG-ATGTGTCTTGATGTTATTG---AGAGATTTGTTCATTGTTTTCTGGA

                                47050     47060     47070     47080     47090     47100     47110     47120

Human                    AATTCAGGTGCCCTGTGCGCACACAAATCTTT-GTCAGTAGTGAAAGGACATGTGATAGCTTCCTGTAGTTTTTTCATAT 46799
Kakapo                   CATTTTCTAACCAGCTTTGTCATCAGTATCC--AT-CCTACAGACTATATAG---ATGAAATTGG-CAGTGTGTTC---- 18224
GoldenEagle              CTTGTTTTTGGCT-TTTCATTCTTAGAGTTTGGAGGTGTCATGATGTTAT------GAGACCTGGTCATTGTTTTCTGGA 20256
JapaneseQuail            ACTCTT-TAATCTAATGCCTGCTTGGAGTTTG-GTGTCATTTTATGAGACC----TGTCCATTGTTGGTTTTCTTTTGCA 17471
MediumGroundFinch        CTTGTTCTTGCCC-TTTCATTCTTAGAGTTGGGAAGTGTCATGGTGTTATTA---TGAGACTTGGCCATTGTTTCCCAGA 4940
GoodesThornscrubTortoise AGATTTACAACCTCCTTTCTTCCCAAATCCTTTCCTCCCCTCCTCCTTGCTGACAAGCAATTTTTCCATTGCATCTTGGT 42483

Majority                 TTATGTAGAAACCTATTCTGGTTTT-----------TATGTTGATATTTTCAATATCTGATTCC-TTATATTTTTCTAAA

                                47130     47140     47150     47160     47170     47180     47190     47200

Human                    TTCTGGAGTGGTGAAGAGCTACCCAGCCAAGATTTTCAGGGCAATATATTCAGAAGCCAGAGCT--TACTCTTCTGTACA 46877
Kakapo                   ---TGCTGACACTCACTACAAGTC-------------AAGTGAGTGTCTTAAGTTCCTCGTGTG----TCTTTTGCTAAA 18284
GoldenEagle              GTATGTAGAAACCTATTCTGGTTTT-----------TACCTTGATATTTTCAACATCTGATCAC---ATATTTTTCCAAA 20322
JapaneseQuail            ATACATAGAAACC---TCTGGTTTA-----------GTTATTGATGTTTTCAACATAAGATTCCATTATACTTT-CTGAA 17536
MediumGroundFinch        GAATATAGAAACCTATTCTGGTTTT-----------GACTGTGATATTTCCAATATCTGACTCTGTGATATTTTTCTAAG 5009
GoodesThornscrubTortoise TCATGTAGGCTTTAACCCCAGTATGCAGGGAAGGCTTATGCTGGCTTCTTTTAAGACAGATTCC-TTTTGTCTTATTTAA 42562

Majority                 CAGATACCTTGGTGAGCAGATGAA-AGTAAAGGTTTGCC-TT------TATAATCCTCTGTGCCTTTTATTGGTTAAAGT

                                47210     47220     47230     47240     47250     47260     47270     47280

Human                    AAGACAACCTAAGGGGATTTTTAAATGCATTGGCTTGCCGTTCAAGTATATAAACCTAAGTGAAACTCCTAGAGTAAAAT 46957
Kakapo                   GA-ATAGCCT------TAGAAGAA-AGTTCAAGTTGGA------------TAATTGAGGATGACTTCCAT--GTGGGTTC 18342
GoldenEagle              CAGATACCTAGATGAACAGAAGAA-AGACACAGTTTGT--TT------TGTAATCCTCTGAGCCTT--ATTGGTAAAAGC 20391
JapaneseQuail            CAGATGCTGTGCTGTGAACTTCACTGGTAAAAGTATGCAATC------CAGAAAACAGCATTGCTCTAACAGGCCTAAAT 17610
MediumGroundFinch        TAGATAGCTAGGAGAACAGAAGAA-AGAGATGGTTTGTC-TT------TGTAATCCTCTGGGCCTTTTATTGGTGAAAGT 5081
GoodesThornscrubTortoise AACCATCTGCAGTCCCCCCCTAAACATCAGAGGCCTTCTTTT------TAGAAACTTCACCTTATCTTATTAGCTATTCT 42636

Majority                 TTACACCATCAATAG------TCCAGTTGTTATTACTT--------CTGCTTTAGTAATCCTATACTTTGAATA------

                                47290     47300     47310     47320     47330     47340     47350     47360

Human                    ATGACTTAAGTATAGCTTGAGTCCATATATTATCACTTAGAGATACCTTCTCTGATCATCCAATAAAGTCATTACTACTA 47037
Kakapo                   TTTTACCAC--ATAG------TTCTGGTCTTATATCTC--------CTGCTTTA-----CACATAGTTTGATTA------ 18395
GoldenEagle              ATACACAATCAAAAA------ACCAGTTGTCACTAGCA--------GTGCCCTAGCAGGCCTAAACTTAGAGCA------ 20451
JapaneseQuail            TTAGAAGAAAAACTT------GTGAATTGTTCCCTCCT--------TTCTTTTG--AAATCCACACTGCAAAGAT----- 17669
MediumGroundFinch        GTACACAGTCAAAAA------AGCAGTTATCAATAGCA--------GTGCCTTAGCAGGCCTAAACTTTGAACA------ 5141
GoodesThornscrubTortoise TTGAACCAGACATCC------TCTCTACTTCATTCCTT--------CTGGCCTTATAATTTAGTATTTTCAAGGCCTGCC 42702
```

```
Majority                    -AAATCTTGCTTGTC---ACAGCCTTTTTTTTCTGTGAATCTCTGCTTTAG----AAACATTTACCT--AAACCAATCTT
                                    47370     47380     47390     47400     47410     47420     47430     47440
Human                       TCAATCTTCCAGTCCTAGACAAAGTAACTTTTAAGCACATCACTGTTTTACCTCACAGCATATACCGTAGAAAGTATCTG 47117
Kakapo                      -CTGTCCTTAGTGTT---GTCGCATTTCGTTTCAGTTAAGCATTTCTATTT----AGACATTCA-----AGATTATTCTT 18462
GoldenEagle                 -AAAACTTGTGAATC---ACAGCCTCTTTTCTTTCTGAATCTCTGCTATAG----AAATATTAACCT--AAACCAAATTG 20521
JapaneseQuail               -ATATCAAACTTCTT---ATAAAACAATTTGCCTCAGACCTGTTGCTGCT-----AAGCAGTGGTTT--TAAAAACTCTT 17738
MediumGroundFinch           -AAATCTTGTCAATC---ACAACCTCATTTCTTTC------TCTGCTGTAG----AAATATTAACCT--AAACCAAAATG 5205
GoodesThornscrubTortoiseTTCAACTTGCTTGTC---AGGGCCTTTCTTTACAGCACGTGTATTCCTTAA----CCAAACTTAAGC--TAACCCTGATT 42773

Majority                    CTTTAGTTTCTAAATAATCTAGCTTTTCA-TGGAAGTTATTGGTTTTGGTTGAATTGAGT---------T-GTTGTGTCT
                                    47450     47460     47470     47480     47490     47500     47510     47520
Human                       GTTCAGGTACTAGTTAGTCCAGCTCCCTACTATAAGGTATCAGGAATCATATAATC--ATCTTGTTCACTCTCTGTACCC 47195
Kakapo                      CTTT--TTCCTGAATGGGTTTGAGCAGTA-TGGCAGAACATCAGTCTGAGGGAAC--AA------------TCACATCT 18524
GoldenEagle                 CTTAAGTTGTCAAAAAAATGGCCTTATCA-TGGAAGTGGGTGGTTTTGGTAGAACTGGAG-----------GCTGTGTCT 20589
JapaneseQuail               TTTTTTTTTCTCCAAACAAGACCTGTTCA-CAGAAGTTGTCACTTTCTGTTGAATTGAGCTGTCTTTGATGGTTCTGTGG 17817
MediumGroundFinch           CCTAAGTTGTCAAAAAACCTACCTTTTCG-TGGAAGGAAGGGGTTTTGGTGGAACTGGGG-----------GCGGTGTCG 5273
GoodesThornscrubTortoiseCTTTAATTTCATATTTATCCTGTGTGGCT-TACAAGAGATATTTGAAAAATGTGTT-AGTGTAAGACATTGCTAATTTTA 42851

Majority                    TTAGGG-GTAAAACTGGATGTGAGGTTTTTAAAATGATCATCCTATTCTTTTATCGTAGTAATAGTTCCATTAG-----
                                    47530     47540     47550     47560     47570     47580     47590     47600
Human                       TCAGAACGTAAAACATTGCTAGACACATCCCTGAATACCCAGTAAATG--TTTAAGGAATTAATGAGTGAATTAATTTTG 47273
Kakapo                      ACTAGC-TCACAGTTGGTTGCAAG------GGAGGCCAGCTTCTCATG-TTCCAGGTCAGCATTAGATGTTTTAA----- 18591
GoldenEagle                 TTAGGG-GTGAAACTGGAGGTGAGGTTTTTAAAAATGATCATCCTACCCTTTTATCGTGGTAACAGTTCCATTAG----- 20663
JapaneseQuail               TTAGAG-GTAAAGTTCGACATGAGGTTTCTAAAAATGATTATCTTATTCTTTTATCATAGTAATAGTCCCATGAG----- 17891
MediumGroundFinch           TTATGG-GTGAG-CTGGAGATGAT-TTTTTTAAAATGAACATCCTACCCTTTTATCATAGGAAGAGTTCCTTTAGTGAGT 5350
GoodesThornscrubTortoiseCTAATG-TCCTAAACTCTTCCACTGCTTTTTAAAGAGAGTAATCTTTT-TTATACAGAGATAATGTGTACCCTAA----- 42924

Majority                    ----------------AATGTTTTTTTGAT----T-----------T---GATTCAGCTGACATT-CAGTTAGAATACTG
                                    47610     47620     47630     47640     47650     47660     47670     47680
Human                       AAGATACGTTGGAGACCAAGATAATCTGACAGTCTTATAGATGTTGTTGAACATACACAAACACATTATATAGTTTATAG 47353
Kakapo                      -------------------GTTTCTGACAT--------------------GGATAAGTTGAAGTTGCATTGTCAAAACCA 18632
GoldenEagle                 -------------------GTTTTTTTGGT---------------------ATTCAGCTGCTGTT-CAGTTAGAATACTG 20702
JapaneseQuail               ----------------AATTTTTTTTTAAT---------------------ATTCAACTCTCTTT-CAGATAAAGTACTG 17933
MediumGroundFinch           TGTGTTGGGGTTTTTTGGGGTTTTTTTGTTTGGTTGTTTTTTTTTTTTTTAATTCAGCTGCCATT-CAGTGAGAATACTG 5429
GoodesThornscrubTortoise--------------TAATTGTTGTTTTATGTGTTGTGGTAGCACTTATAGGGCCCAATAAGATATCAG-GACCTCACTG 42988
```

```
Majority                 TAGTACTTTAATGCTTTTATAAATACTCTTGCT---CTAATTTTTTATCTTAGT---AG-----------------AAAG

                                  47690     47700     47710     47720     47730     47740     47750     47760

Human                    GTTGTGTTGAATGTTTTAGGAAATACCATAAATTACCTAAAGTTTTATACAAAATGGAGTATACATGTATATTTCTGAAT 47433
Kakapo                   CAGACCTGC--TGCTTCCTGAACTACTCCTGGT---GTTTTCCTTCACCTTTTC----T-----------------GAAA 18686
GoldenEagle              TAGTACTTTAATGCTTTTATGAATACTCTTGCT---CTAATTCTTTTTCTTAGTCTGAG-----------------AAAG 20762
JapaneseQuail            TAG---TTTAATGCTCCTAAAAGGATTCTCATG---CTTATTTTTCTT----------------------------AAAG 17979
MediumGroundFinch        TAGTACTTCAATGCTTTTATGAATACTCTTGCT---CTAATCTTCTCTCCTAGT----G-----------------AAAG 5485
GoodesThornscrubTortoiseTGTAAGTAAAATGAATGAGTAGAGTTTTTAGCG---TTAAAGTGTTAGATTAAAAAAAA-----------------AAAA 43048

Majority                 GCATGAAAGATGT--ATTTTTTCTATGTCCTATAGGGATTCTTG-----CAGATGAATTGTTTCCTATTTTTTTGGGAAA

                                  47770     47780     47790     47800     47810     47820     47830     47840

Human                    GTGTCAAGATCCTTCATTTTAGCTAAGTCTCAGAAATATCCTTGAGATCCTAAAAGACTAAGAACCATTGATCTGGCATA 47513
Kakapo                   ACATTGTCAAAGT--ATA--TGCTATTTTCTATCAGAATC------------ATGCTTGGTTTGACCTCTTTGGAGAGCA 18750
GoldenEagle              GCATGAAAGATGC--ATGCTTTCTATGCCCTATAGGGAGTCTTA-----CATATGAATCCCTCCCTATTTTTTTGTGAAA 20835
JapaneseQuail            GCATGAAAGATGT--GTTCCTTCTTTCTCCTTTGCAGGAT------------------------CATTTAAAAAGAAAA 18032
MediumGroundFinch        GCATGAAAGAGGA--ATGGTTTCTCTGCCCTATAGGGAGTCTTG-----CAGATGAATCCTTTCCTATTTTTTTGTGAAA 5558
GoodesThornscrubTortoiseTCCTTTGAAAAGCCAAAATCTGTCTTGAGCAACACAGACTGTGC-----CAGCTAAATTGAAAATTGACTGGAAAGGAGA 43123

Majority                 GAAAATT----------CTTGCAAA-ATTT----TTGAATAA-AC--ATTTGATTTGCATTCTTAGTAA-TAATTTGAAC

                                  47850     47860     47870     47880     47890     47900     47910     47920

Human                    GTAGATATACTGCAAGTCCTTCAAACAACTGTATTTAAATATTACCTATTTTATAGGCATTTTGAATAA-TATTTTTATT 47592
Kakapo                   CAACGCT----------CATGCAAAGATCT----GTGAATGA-ACTGGTTTTGTTTTGATGCCTAGCAA-TGACTGGAAA 18814
GoldenEagle              GAGAATT----------TTTGCAAA-ATTG----TTGGACAA-AC--ATTTGGTTTGCTTTCTTATTGA-TTGTTTGAAC 20896
JapaneseQuail            AAGC-------------CTTGCAAA-ATTG----TTGGATAC-GC--ATTTGTTTTGCATTGGTG-----TAATTGGTGC 18086
MediumGroundFinch        GAAA-------------CTTGTGAA-ATTG----GTGAACAA-AC--GTTTGATTTGCATTCTTACTGA-TAATTTGAAC 5616
GoodesThornscrubTortoiseCAAAAGAGAA-------CAAGAAATGACTTGCCCTTATTTAA-ATACATTTGAAGTACATTAGAAGTAAGAAGACTGTGT 43195

Majority                 CACTGGTAATTTTTTAAA----A-----T--A----AATTC------AAGCATGTTGGACTA-TGT-------A-ATTAC

                                  47930     47940     47950     47960     47970     47980     47990     48000

Human                    TTTTGGTGAAGTTTTGGAATGAAGACAGTTAACTAATTTGTTGATTGAATTATGTTACTTTACTTTAAATCTCACGGTAA 47672
Kakapo                   ----GGCAGTTTAGGCA-------------------AACTA------AAACATGATCTAGTAACGG---------GATTC 18856
GoldenEagle              CACTGGTAATTTGATAAACAATATGGAATTAAGGAAAATTC------TAGCATGTTGGACTGTTGTCCAGAGGACATTGC 20970
JapaneseQuail            -ATTAACATTTATTTGA-----------------------------TAATATGAGCCACTG-------------ATAAT 18122
MediumGroundFinch        CACTAGTAATTCTCTAA-----A--------A----AAGGC------CAGCATGTTTGAC---------------ATGGC 5658
GoodesThornscrubTortoiseCACTAGGCCTTCTAAAAAACTAGTGGGCTGCCTGGGGGTCAGTTGAAAAGCATGATGAGATAGTGAAGTTGTTAAATGAT 43275
```

```
Majority                    TGAAAAAATAATGCTTAATCCTTGAGCATTTTTGAA-ACATATTTGTCACCTGTATCCATCTTCAAGG-TGTATAGGTGA
                                     48010     48020     48030     48040     48050     48060     48070     48080
Human                       CAGAATAAGGTAATTAAGTTCATGAGTGATTTAGAGTATATTGATGTTAATAGTCACAAAATATTAGGTAGTTTAGACGG 47752
Kakapo                      TGAGAAAGCAGAGCTGAAGCC---AGCATATTTCAA-ACATATTT-TTGCCTCTGCATGTTTTTAAAG--ACATTAGTTA 18929
GoldenEagle                 TCAAAATACAAGGGCTGGTCCTTGAGCATTTTCTAA-ACAGCTTTGTCATCAGTATCCATCTTCAAGGGTGTACAGATGA 21049
JapaneseQuail               TGGATAAATGGTATGAAATTA---AGGAAAGCTGAT-GCATATTA---GACTGT-----TGTTCAAAG--AAATTGCTGA 18188
MediumGroundFinch           TCAAAATGTAAGGCCTGATCCTTGGGCCTTTTCTAA-ACAGTTTTGTCACCTGTATCCATCCTCCAGACTGTGTCGGTGA 5737
GoodesThornscrubTortoiseTTTTTTCATCATTCTTACTATAGAAGATAATGGGAA-ATTAATTTTCCAGTGGCACACTTCAAAGGAAATGAGAATGTGA 43354

Majority                    ----A-----ATAGCAGGTT--------ATGTTAATATTCACTACAAGTGAATTTACTGTTTTAGGTACTTTGTAAGTCT
                                     48090     48100     48110     48120     48130     48140     48150     48160
Human                       CAATAGTAATATATCAGGAGAGGCT-CATTGTAGATAAGCCAT-AATATGGATTGACTGCATTATCATTTTTATAAACTT 47830
Kakapo                      --------------CATACT--------A-ATTAAT-GTT--TATAAG--GACCTGCAGTGACAGG----ACAAGAGGGT 18977
GoldenEagle                 AAGGAAATTGACAGTATGTT--------GTATTAATATTCACTACAAGTCAAGTTACTGTCTTAAGTACTTTGTGTGTCT 21121
JapaneseQuail               --------------GAGTTT--------AAGTTATGATTC--T-CAAAC--AATTTCTG-AACAGC----TTGGTCATCG 18236
MediumGroundFinch           -----------CAGCAGGTT--------ACATTAGCAGTCACTACAGGTGAAGTTACTGTTTTAGGTACTTTCTATGTCT 5798
GoodesThornscrubTortoiseCCTTACAGAAATTGAAGACTTAGTAATAAAGTTAAAGAGCAATTTGAAAAACTCAAGTGATGTAAGTAC-TTGGAAGAGA 43433

Majority                    TT-GTA---TGAGCTCAGCCTTAAGAA-A-TTTAGAGAAGTATAAATT--TATATTTGCTTTGAAATTTCTTTTCTTTCT
                                     48170     48180     48190     48200     48210     48220     48230     48240
Human                       TACATTCTTTGATCCCACCTTCAAGTACATTTTTCAAATCAAACAATTTATA-ATTTCTCAGGTAAAATTATAATCTTCC 47909
Kakapo                      TCAGGA---TGGGTTCAAACTGAACCA------GGGGAAGTTCAGGTT---AGATCTAAGGCAGAAGTTCTTCCCTGTGA 19045
GoldenEagle                 T--GTA---AAAGAACAGCCTTAAAAGAAATTTAGACTTGAATAATTGAGGATGACTTCTTTGTGAGTTCTTTTACCACT 21196
JapaneseQuail               TT---------A-CCCATCTTCAAGAC------TGTGTAGGTAATATA-----ACATACTTCCAACTTCCCTTTATGGTT 18295
MediumGroundFinch           TTTGTA---AAGGAGCAGTCTTAAGAC---TGTAAAGGAGCAGAATTC--TACACTTGGATTGAGATGACTTTTGTCTCT 5870
GoodesThornscrubTortoiseTGGTAT---TTAGCTGAGTGTTGTGAAGAAAATAGTGAGATATTGACAAGGGTGTGTGCTAAGAAGTCTCTGGCCCTATG 43510

Majority                    TAATTCTGATCCCTT--GT----T-TTTTGTGCAAAATTTGAT----TATTGTT--TATTGTTGTTGAATTTCAT--TTC
                                     48250     48260     48270     48280     48290     48300     48310     48320
Human                       TAATTTTGGTCAAACCAGTTTTTTCACTTTTTAAAAATGTTCATATTAGTTCATTATAAGATGATTAAATTACAAGATAC 47989
Kakapo                      GGGTGCTGAGGCGCT--G-------------GCACAGGGTGCC----CAGAGGA---GCTGTGGCTG--CCCCAT--CCC 19099
GoldenEagle                 TAATTCTGAACCCTTATATCTCCTCTTTTGGACATAATTTGAT----TATTGTCCTTATTGTTGTGGAATATCAT--TTC 21270
JapaneseQuail               TACTGCAAGTTACT---------------------AACTTAAG----TACT-----------TGGTG-----TAT--CTT 18332
MediumGroundFinch           TAATACTGAACCCT---G-------TTTTGGACA-AATTTGAT----TATTGTC---ATTGTTGAGGAATTTCAT--TTC 5930
GoodesThornscrubTortoiseCAACTTCACTCATTCAGATAGCATAAATAGTGAAAAATGCATTCCTCTACCACTTGTTTTCCAGGAAAATGTCAT--TTC 43588
```

```
Majority                   AGTTAAGAAT-TTATATCTAGATATGCAG-----ATTCTTGTTC-------CTGGATCAGTTTGTGA------CAATATA

                                    48330     48340     48350     48360     48370     48380     48390     48400

Human                      AGTCATAAATCTTGGGGATAGTGATGAGGATACTCTCGGAGAAATGCATTGTTAGGTAATTTTGTCACTGCATAAATGTC 48069
Kakapo                     TGGCA-GTGT-TCAAGGCCAGGTTGGACA--------CAGGGGC-------TTGGAGCAACCTGC-----------TCTA 19151
GoldenEagle                AGTTAAGCAT-TTATATTTAAATATACAA-----ATTATTTTTC-------CTGAATTAGTTTATAA-----GCCATATG 21332
JapaneseQuail              CAGTA-AA-----AGAGCTACCTCAAAAG-----------------------GAAGGCAGCCTTTG-----------ATA 18372
MediumGroundFinch          AGTTAAGTGT-TTATATTTAAATATACAAGATTTATTCTTGTTC-------CTGAACCAGGTTGGA-------CAGTATG 5995
GoodesThornscrubTortoiseCTCTTGGAAT-A-GATTCTGGACATGCTGATCCATGTTTTTATCACCACCTCTGGATTGGATCATGA-----TATCTGCA 43661

Majority                   GCAGAATATGATTCTGTAAACATTGAGGGA--AGAATCCTATCTGCTATA-------TTATGATTCTTTGCAAGGGAGGC

                                    48410     48420     48430     48440     48450     48460     48470     48480

Human                      ATAGAGTACATTTGCACAAAC-CTGCTACACATGTAGGCTATATGGTATATGTAGCCTTTTGCTCCTAGGCAACAAATTT 48148
Kakapo                     GTGGAAGGTGTCCCTGCCCATGGCAGGGGGTTGGAACTGGATGAGCT----------TTAAGGTCCCTTCCAACACAAAC 19221
GoldenEagle                GCAGAAAATCATTCTGTAAACATTGAGGGA--AGAATCCCATCTACTAGA-------TCAGAATTTGTTGCAAGGGAGGA 21403
JapaneseQuail              AATGAGCATGACTTTGT---------GAGA----TTTTCTTACTGCTTAC-------TCTTGACTCCTTGCATCTG---- 18428
MediumGroundFinch          GCAAAATATTATTCTGTAAATATTAAGGGA--AGGATCCCATCCACTAGA-------TTAGAATTTGTAGCAAGGGAGAA 6066
GoodesThornscrubTortoiseACTGAAGTGAAGTGAAAAAACACT--GAGACAAGACTCTGGTGTACTCTAAGCTA--TCATGTCTCTTTTAGAGGAAGGC 43737

Majority                   --CAGCATGTTACTGTTCTTGAT-CTGCAAATATTTATACTT----AATA-AAATCT-TTTTTTATGTTAGATGTGTAAG

                                    48490     48500     48510     48520     48530     48540     48550     48560

Human                      GACAGCATGTTACTGTTCTGAATACTGGAGGCAGTTGCAACACAGTAATAAGTATTGATGTATCTAAACAGAAAAGGTAC 48228
Kakapo                     --CAGTCTGTGACTGTCTGTACA-TTGTAAATGATTGTACAT---------AAACCT-----TTCAGTGAAAC---TATG 19281
GoldenEagle                --CAGCTTGTAAG-GTTCCAGGT-CAGCAAATATCTAAACTTTTCTAATA-AGTTCTCTTTTTTACGTTAGATGTTTAAT 21478
JapaneseQuail              --CGGTTATTTACTGTTCTTAC--CTGTGAGGAAATTCATTT---------AAACAT-----GTCTATTATAA------- 18483
MediumGroundFinch          --CAGCATGTAAA-GTACAAGAT-CAGCAAATATCTAAGCTT----AAGA-AGTTCT-TTTTTTATGTTACATGTGTAAG 6136
GoodesThornscrubTortoiseTTCTGGACCTTGCAGATGCTGAC-ATGCACAAAGTGATACTTTTCAGTCACAAACAT-TGTCTTAGGGGACATCTACGTG 43815

Majority                   TTTCTGAATTTTTTAACATCATCTGATATTAG-TATGTTGAAGTTGTGTTGTCAGAACTACAGGCCT--TTTATTACTAG

                                    48570     48580     48590     48600     48610     48620     48630     48640

Human                      AATAAAAATATGGTATTATAATCTTATGGGACCACCATTGTATATGTGGCCTGTCAATGACCAAAAT--GTAGTTATGTG 48306
Kakapo                     TTCAGGAAATACATAAC-TTATTTCTTATAAG-CAACTT--AGTTGC-TTGTC----CTGCAG--CT--TTTAAATCTAA 19348
GoldenEagle                TTTCTGAGCTTTTTAGCACCAGCTGACATTGA-TATGTTGAAGTTGTATTGTCAGAACCACAGGCCT--TCTACTACTGC 21555
JapaneseQuail              ----AGCATATTTTATCGTCAGCTGGCATTGA-TATGTTGAATTTGC------AGAATCACAGGCCT--TGCATTGTTAG 18550
MediumGroundFinch          TTTCTGATGTTTTTAACACCAGCTGACATGAG-TATATTAAAGTTGTGTTCTCAGAACTACAGGCCT--TTTACTACTGC 6213
GoodesThornscrubTortoiseCA-GTTTGACACCCAAGGCTGTCCCATGCTAGCTGACTCAGGCTCATGGGACTTGGGCTAAGGGGCTGCTTAATTGCAAT 43894
```

```
Majority                   GTAAATGACTG---TCAGT--TTTCTTTTAGCTTTTGA-GAATGTTTTGTCTGTTTTGATTTTCATTTTATTATCTTGCT
                                   48650     48660     48670     48680     48690     48700     48710     48720
Human                      GCACATGACTG---TGATTGTATTCTTTAGTCATTGGACAGTTTCTTTATGAGATTACATTTTAATGTTGTTG-CTTATT 48382
Kakapo                     GAAAGTCCCT----TC--T---TTTTAAGAACTTAAGA-AAAAGTGTTTGCTGGGTTGCTTTTAGGCTCAACTTCTTGTG 19418
GoldenEagle                TTGAACCACTA---CCAGTGTTTTCCTTCAGCTTTTCT-GAAGATATTGTCTGTTCTCATTTTC-TATTATAATCTTGCT 21630
JapaneseQuail              ---------TG---TT------TTCTTTTAGCTTTTCA---GTGCTGAGTCCATTTTGACTTTC-TATCATAATCATGCT 18608
MediumGroundFinch          TTAAACTACTG---CTAGT--TTTCCTTTAGCTTTTTTTGAAAATATTGTCGAAGTCTATTCTTATTCCATTATCTTGCT 6288
GoodesThornscrubTortoise   CTAGATGTTTGGGCTCAGGCAGTAGCCTTGGCTCTGGGACCTTGTG-AGAGGGTGGGGACCCAGAGTTTGGGCTCCAGCC 43973

Majority                   AGGCTCTGATCTCTTTGGA-GAGCATAA---ACCTTGTTATGAACATCTTTGAATTGTTTATTTCTGTGTTGATGCAC--
                                   48730     48740     48750     48760     48770     48780     48790     48800
Human                      AATAATTACTCTTTATAA--TACTGTTAAGAAAC--ATTAACAGCAATCTTAAATAGTTGATAATTTCATTTAATAAT-- 48456
Kakapo                     AGGCCTTTTTCTTTTCAG--GTGAAGGA----CTGCCATATGGAAGTTTTAGAAGCAGTTATATATATGAAGATGCTC-- 19490
GoldenEagle                TGGTTCCGATCTCTTTGGA-GAGCATAA----CCTTGTCATAAACATCTGTGAATTCATTTTTT-TGTCTTGGTGCAC-- 21702
JapaneseQuail              CATCTCTCATATCCTAGAA-GAGCGTAAGTAACCTTCTTGTGAGAACCTTGAATTTGTTTCTTCATGAACACAGCAGC-- 18685
MediumGroundFinch          CGACTCGGATTTCTTTAGA-GAGCATAA----TC---TCATGAAGATCCATGAGTTAATTTTTTCTGTGTTGGTGCAC-- 6358
GoodesThornscrubTortoise   AAGCCCAAATGTCTTAGCCTGAGCACGAGTCAGCTGGCACAGGCCAGCTGTGGGTATCTGATTGCAGTGTAGATATACCC 44053

Majority                   -AA-A---TGACTTGAATG-GCAGTTTAGGTAAACTGACATTTGGTTTGGTAACAGGATTTTGAGAATCCAGAGCTGAAG
                                   48810     48820     48830     48840     48850     48860     48870     48880
Human                      -AATAGGTGGATATGATTTTATTCCTTTGAAAGCCTGGCATTTGAATTTTCAAGAATTATTTAAGTATTCTCAGCTATGT 48535
Kakapo                     -AT------GCCATGAATT-GCA--TTGACGAAACCC-----CCGCACACTCACAAAAATTAAGGGATGTAGATACGAAG 19555
GoldenEagle                -AGCAGACTGACTAGAAAG-GCAGTTTAGGTAAACTG-----TGGTTTGGTAACAGGATTCTGAGAAACCAGAGCTGAAG 21775
JapaneseQuail              -AA-----TGACTTGAAGG-ACAGTTTAGGTAAACTGAAGTACAGTTTGATAGCAGGATTCTGAGAAACAAGAGCTGAAG 18758
MediumGroundFinch          -AA-----TGACTAGAAAG-ACTCCTTAAGTAAACTAAAATATGATTTGGTAACAAGATTCTGAGAAACCAGAGCTGAAA 6431
GoodesThornscrubTortoise   TGAGAATTATAGAGGAAGA-GCCAGTTCTCTGTAGTGACATTGGGACTTGTATGAGTGATTGAGTATTTTAATGATAGTA 44132

Majority                   CTTGGATCTTTTAATGA-------TGTTTTTGATACTGC-AATGTTTTAGAAGACA--TTAGTT-ATGGATATTGTAAA-
                                   48890     48900     48910     48920     48930     48940     48950     48960
Human                      TTTTAAGTTCTTTTTGGAAATTTGTAGTTTTTTTAC-ACAAATAGGAAAATTCATACATTCTTTTATTGTTGATATATAA 48614
Kakapo                     ----------TGAAAGA----------CGTTGATGCAA-----GTTTTACAAAACGG-CTTGTC-AAAGACATGG----- 19603
GoldenEagle                CCAGGATATTTAGAAAA-------TATTTTTGACTCTG--GGTGTTTTTCAAGACA--TTAGTT-ATGCACATTGTAAA- 21842
JapaneseQuail              -----------------------------------------------------------CC-------------------- 18760
MediumGroundFinch          CCAGGATCTTTAAATAAATATTGCTGTTTCTGACCTTGCTAATGGTTTTGAAGACA--TTAGTA-AGGCAGATTGCAAA- 6507
GoodesThornscrubTortoise   CTTGGGGCCTATCTGGAGCCAGGGTGGAGTACATATGTCTCCCTTTCCAGATGATGGGCTAATTCAAGGATACTGCCATT 44212
```

```
Majority                   TGGTTGTATATAAATAA-TTTTTTCACGTTTTAATGCTGT----AAGTTCAATGAGTA------TGTAGTTGCT--T-AT
                                   48970     48980     48990     49000     49010     49020     49030     49040
Human                      GGACTGTTTTTTAATGTCTTTCAGCCAATGGTAATGATAGTAAAAAATTTAAAGGAGAAGATAAAATGGATGGTGCTCCT 48694
Kakapo                     -GGTTGAAGCAGAAGAA---TTGGCAGGGTTTTTTACTAT----AGGTGTCACCAATT--------TCATTGCT--C-AC 19664
GoldenEagle                TGATTGTATATAAACAACTGTTTTCACCTTTCAGTGCTGT----GTGTTCAGTAAATACATAACTGCAGTTGCT--T-AT 21915
JapaneseQuail              TGG--ATATATAAGGAA-TATCTTATTGACACTATTCTTC----AACTTCTAAGAGAA------TCAAGTC--------- 18818
MediumGroundFinch          TGATTGTACATAAATAA-TGTTTTTACCTTTCAGTGCTGT----ATGTTCAGTAAGTA------TGTAGCTGTT--T-AT 6573
GoodesThornscrubTortoiseTAGGAGCCCCCAAAATAGTTTGTCAACACCATGATTCTGG----CATCCCAATCTGAATT---CTTAAGTTCCA--GGAT 44283

Majority                   -TCTGTGTGTTTTAAGTTCAAGAAAT---TCCCTTATTTATAAAAACTGGAAAAGTT-------GTTTACTTTGTGATGG
                                   49050     49060     49070     49080     49090     49100     49110     49120
Human                      TCTCGTGTACTTCATATTCGAAAATTACCTGGGGAAGTAACAGAAACTGAAGTTATTGCTTTAGGCTTACCTTTTGGTAA 48774
Kakapo                     ATTTGTGTGTTTTAAGTTCAAGCAG-------------TA-AAAAACATGTCAAA---------ACTTACTTTGCAACTG 19721
GoldenEagle                -TCTGCAGCTTTTAAGTCTAAGAAAG---TCCCTTCTCTTTAAGAACTAGAAAAGGC-------ATTTCCTCTGTGGTGG 21984
JapaneseQuail              --CCT--TTTT-------CAG--AA--------------G----AACTACAAAGGTT-------ATTTCCTCTATGAGGG 18860
MediumGroundFinch          -TCTGCAGATTTTAAATGTAAGAAAAAAATCCCTTATCTTTAAAAACTGGAAAAGTT-------GTTTTCTCTGTAAAGG 6645
GoodesThornscrubTortoiseGTCCCTCAGAGACCATTTGAGGACATCATACTTCTAATAAGAAGATTTGTCACAACT-------GGTCATTTTGAACAAT 44356

Majority                   AGT-A---------T-A-G------GTTACTTTTTGCTGAACTTCTTGTGAGGCTTTTG-CTTTTCAGGTGGAGGACTT
                                   49130     49140     49150     49160     49170     49180     49190     49200
Human                      GGTGACCAACATCCTTATGCTGAAAGGAAAAAATCAGGTACACTTCTTTCAGGGTTTATGAAATGTTAAACCCCAAACTA 48854
Kakapo                     AGC----------------------TCCCCAAATTCCTGGAGCTTTAAATGATACTATTG-ATCTT------GAACTTGT 19772
GoldenEagle                -------------------------GTTACTTTTATGCTGAACTTCTTGTGAGGCTTTTTTCTTTTCAGGTGGAGGACTG 22039
JapaneseQuail              -------------------------GTTACTTTTATGCCAAACTTCTTGTGTGACTTCTT-TCTTTCAGGTGGAGGGCTA 18914
MediumGroundFinch          AGTAACCTCCTCTGTAAAGGG----GTTACTTTTTCACTGAACTTGGTGTGAGGCTTTGG-CTGTTCA---GGAGGATTG 6717
GoodesThornscrubTortoiseACTTA--TGAGTGTTAAGGTT----GTCGCTAACTAGATACATAGTACATGTGACATATCTGTATTTGGAT-AACCTCAT 44429

Majority                   TTTAATGG-----AATTTGTAGG--AAGAGTTAT--GTATGAAGTTGCTCATGCCAT--GAACTGTATTGCTG----AAA
                                   49210     49220     49230     49240     49250     49260     49270     49280
Human                      TCCAGCAGGTGTGAATTAATTGTAAAAGGGAGAA-AATAGTAGGTTATTTTTCTTAAA-GAACAGAAGTCTTATTCTCAC 48932
Kakapo                     TTTTACGT-----CATTAGCAGG--CAGTGACTTTGGTAGGTAATTCATTGTAACAG--AATAC---------------- 19827
GoldenEagle                CTTAATGG-----AAGTTTTAGG--AACAGTTAT--ATATGAAGATGCTCATGCCAT--GAACTATATTGCTG----AAA 22104
JapaneseQuail              CTCAATGG-----AAGTTCTAG---------TGT--GTGTGAAGGTGCTGGTGCCAT--GAACTGTGTTACTG----AAA 18972
MediumGroundFinch          CTTAATGG-----AATTTGTAGG--AAGAGTTATCTGTGTGGAGATGCTCATGCCAT--GAACT-CATTGCTG----AAA 6783
GoodesThornscrubTortoiseTAAAACCT-----AAATCATAGTGGAAGCACGTTCCAGAGGGACTTTCCCATCCCATCTGAATGATAACTTTGGGGCAAC 44504
```

```
Majority                  ---TCCACAC-GATA----AAGGATAATATTTTAAAGTGGTTTAGATAAA--AATTGAAA--GATGTTAATGAAAGTTTT
                                   49290     49300     49310     49320     49330     49340     49350     49360
Human                     TTTTTCGAGTAGATGTCTGTGGGAAACTAATGCTAGGTAGCCAAAGTGAGTGAATCACTATAGTTTTTAAACTTTGTCTT 49012
Kakapo                    ---TTGGCAC---CG----AGGCATGTAATCATAGAATGGTTTGGGTTGG--AA----AG--GACCTTAGAGCTCATCCA 19889
GoldenEagle               ---TCCACAC-CAAA----AAAAAAAAAATTTTAAAGGGATGTAGATAAA--AAGTGAAA--GATGTTGATCAAAGTTTT 22172
JapaneseQuail             ---TCTACAA---------AAGAAAAATGTTCTAAAGGGGTATAGATAAA--AA----GT--GAATTTTAGGAGAGTTTT 19032
MediumGroundFinch         ---TCCACATACAAA----AAAGGTAAAATTTTTAAGTGATATAGATAAA--AAGTGAAA--GATGTTGATGAAAGTTTT 6852
GoodesThornscrubTortoise  ---TCTACCCTGATAGGCTATGGATGATCTTCTGGTAGGACTAGATTTCC--AGTTTTAACTAATGTAATTTTATAATGT 44579

Majority                  ATGAAAGTTGT-CTTGCCAAAGACAGGAG---AG-TTTGAAGATTTGCAGATTTTTTAAG-GTGGCTGTTTTCAATTTGG
                                   49370     49380     49390     49400     49410     49420     49430     49440
Human                     TTGAATTGACTTTAGGGCTCTAATTGAATAATTGTTATGAAATTGTGGAAAATACAAATGAAGTATTTCTTTTACTGTGG 49092
Kakapo                    GCTCCAACCC--CCTGCCACGGGCAGGGA---CACCTTCCACTAGAGCAGGTTGCTCCAA-GCCCCTGTGTCCAACATGG 19963
GoldenEagle               ATGGAACTTGG-CTTGTCAAAGACAAGGGATGAAGCAGAAAGATTGGCAGATTTTTTAACTATGGCTGTCACCAATTTCA 22251
JapaneseQuail             ATGAAAGTTA------CCAAAGACAAAAG---A----TGAACAAAAGTGTATTTTTCTTA--TATCTACTATCACTTTTA 19097
MediumGroundFinch         ATGAAAATTGG-CTTGTCAAAGACAAG-----------AAGAATTGGCAGGTTTTCTTGTTGCAGCTGCCATCAATTTCA 6920
GoodesThornscrubTortoise  CTAAGAGATGTAGTCACCCTGGAATGGACTAAGGATTTTCAGACATTCAGAATCCTAAAG-GTGATCTTTTTTAAATAGG 44658

Majority                  TTGTTTACATTTGTGT-----GTTTGAGTTGGATCAG-TTGCAACATTTCTAAATCATTTTGAGGCTGAGCA---TCACC
                                   49450     49460     49470     49480     49490     49500     49510     49520
Human                     CTCTGTTCTTTGGTATCAGAAATCAGCAGGGGTACTAGTCGCATCTTTCCTTAAGCTTTATGAGAATATGAAATTTTATT 49172
Kakapo                    CCTTGAACACT----------GCCAGGGATGGGGCAG-CCACAGCTTCTCTGGG-CACCCTGTGCCAGCGCC---TCAGC 20028
GoldenEagle               TTGTTCACATTTGTGT-----GTTGAAGTTGAATCAG-TGAAAACATCTCAAAATTGTTTTGTAGCTGAGC----TCACC 22321
JapaneseQuail             TTATTTATGTTT---------GCGTGTTTTAACTCTA-CTGCACCATTACCAACATAGCAAAAGAGGAGGCA---TTA-- 19162
MediumGroundFinch         TTGTTCACATTTGTGT-----GTTTAAAGTAGACCAG-TAGAAACATTCCAAAATCATTTTGCAGCTGAGC----TCACC 6990
GoodesThornscrubTortoise  CTATTTAAAAAAATATAT--AATTGGAGATATACCAATCTCCTAGAACTGGAAGGGACCTCGAAGGTCATCG---AGTCC 44733

Majority                  ---AAACTCCTGG----AGCATT-AGATGCTGCTTTTGATCTTGATCC-TGTTTTTACTTAATTAGGATGGAAC-GTTAA
                                   49530     49540     49550     49560     49570     49580     49590     49600
Human                     TAAAAATGTTAAATGATACCATTTAGAAGAATTTATGAAATTTAACACTCCCTTTTATATACTTGAGGTTAATC---TGA 49249
Kakapo                    ---ACCCTCACAG----GGAAG--AGCTTCTGCCTTAGATCTAACCTG-AACTTCCTCTGGTTCAGTTTGAACCCATCAC 20098
GoldenEagle               ---AAACTCCTGG----AGCTTT-AAACGATACTATTAATCTTGATCT-TGTTTTTACTATATTAGCAGGGAAT-GTTAA 22391
JapaneseQuail             ------CTTTTGG----AGT----AGACTCAGGTGGACATTTG--------TGTGTGGACAACCGGAATGATAA-TGCAC 19219
MediumGroundFinch         ---AAACTCCTGG----AGCTTT-GAATGGTACTGTTTATCTT-------GTTTTTACTTAATTAGCAGGGAAT-GTTAG 7054
GoodesThornscrubTortoise  ---AGCCCCCTGGCTTCACTAGC-AGAACCAGTTTTTGCCCCAGATCCCTAAGTGGCCCCCTCAAGGACTGAACTCACAA 44809
```

```
Majority                 CAGTTGTTTTGGCAGTGTATTTCTTTG-----TATAATCAGACTCTTTAGCAT---G-----CAATGTAACAATGT--TT
                                 49610     49620     49630     49640     49650     49660     49670     49680
Human                    CATTTAATTTGTAATTTTGTTCTTTGTCTCTATATAATTATACTGCTTTTAAAATGACTTGGAAATCTTACATTCT-CTA 49328
Kakapo                   CCCTTGTCCTGTCACTACAGTCCCTGA-----TGAAGAGTCCCTCTCCAGCAT---------CCTTGTAGCCCCGT--TC 20162
GoldenEagle              CAGTGACTTTGGTAG-GTAATTCATTG-----TATTAACAGAATATTTAGCAC----------AAGCTAATAATAT--TT 22453
JapaneseQuail            AGAATGTTTGAACACTATATCTTTCTG-----TAAATTAAGAAACTTTAGGAG---G------TAGATAGCAAAG----- 19280
MediumGroundFinch        GAGTGACTTTGGTAG-GTAGTTCATTG-----TATTAACAGAATAATTAGCACTGAGGCATACAAAGTAATAATAT--TT 7126
GoodesThornscrubTortoise CCCTGGGTTTAGCAGGCCAATGCTCAAACCACTGAGCTATCCCTCCCCCTTATTATTTCTGTAGAAGCAAGCAGGGACCT 44889

Majority                 CGAGTGCAGTATTTAACTTGGGGAGTAAACGTAGAT----------TCTGTTTCTTTTGAT--AGCACATT------GAG
                                 49690     49700     49710     49720     49730     49740     49750     49760
Human                    GGGTAGAAGTGGTAAAATTCTGATGCTAG--AAAATGTTTACATTTTCTCTAGCATTTGTTGCAAGAATAGATTTGTGGG 49406
Kakapo                   AGACACTGGAAGCTGCTCTGAGGTCTCCACGCAGCT----------TCTCTTCTCCAGGCTG-AACACATCG-----AAG 20226
GoldenEagle              CAAGTGCTGTATTTAACTTGGGGAATAAACATAGAT-----------GTGCTGGTTTTGAT--GGCACATT------AAG 22514
JapaneseQuail            -AAAATAATAAACCACCACCGGCAATCAGCTAAAAC------------CACATCTCTGGAT--GGGAGA--------GAG 19337
MediumGroundFinch        CAAGTGCTGTATTTAACTTGGGCAGTAAACATAGATATGTCATAGATGTGCCATTTTTGCT--AGCACATT------AAG 7198
GoodesThornscrubTortoise CGAGTGCAGCTCTTGTGGCAGGAAGCATA-GTAGATGCAAAAACAGGCTTTGTCTTTGGATTTCCCATTTTCCTGCAGAA 44968

Majority                 TAAAATTAGTTATTAGTTAAC-----ATTAATGATGTCTAAACTT---------AGGTAATT-TACTTCTGAAGTT---T
                                 49770     49780     49790     49800     49810     49820     49830     49840
Human                    TGTGAAAAATCATAGGTGGAAGGCAGAAGCAGCGTGTACTCGGTTTTCTTCTGAGGTTAGCTTTAACTGTATACCTAAAC 49486
Kakapo                   TAAAATTGGTTATTAGTTAAC-----ATTAATGATGCCTAAACAT---------AGGTAATT-TACTTCTGAAGCT---T 20288
GoldenEagle              TAAAATTGGTTATTAGTTAAC-----ATTAATGATGTCTAAACAT---------AGGTAATC-TACTTCTGAAGTT---T 22576
JapaneseQuail            AGAATACAGCTATTAG-------------AATGAATTCCTTTTTT---------TTTCTATC-TGCATCACTATGC---T 19391
MediumGroundFinch        TAAAATTACTTATTAGTTAAA-----ATTAATGATGCCTAAACTT---------AGGTAATT-TACTTCTGAAGTT---T 7260
GoodesThornscrubTortoise CAACATATGCTACAAGTGGTCTCTGGATCAAGAATTTCTCCATATGATACCAGTAGATGACCATATTTGTTAGGAT---C 45045

Majority                 TCAAAT---TGATTACAATTAATAGTAATTGGAAAGATT---TGAAGCAGTACGGTTCATGTGATTGGTAAGTGTAATAA
                                 49850     49860     49870     49880     49890     49900     49910     49920
Human                    TTCAACAAGAGATTTCTAATTCTGCTTATCAGAACATTTAAAAGTAACCTGAAGGATTATATTCTAGATAATTGATTAAA 49566
Kakapo                   GCAAAT---TGATTACAATTAATAGTAATTGGAAAGATC---TGAATTAAGACAGTACATGTGATTGGAAAGAATAAGAA 20362
GoldenEagle              GCAAAT---TGATTACAATTAATAGTAATTGGAAAGATC---TGAAGAAGTACAGTACATGCGATTGGAAAGTATAATAA 22650
JapaneseQuail            GTAATT---TGCTTTTATTTCATTTCAGTTGCACTGCAC---TCTAGCCATAGTATTTTTATTTTTCTTAAGGGCAGGGG 19465
MediumGroundFinch        TCAAAT---TGATTACAATTAATAATAATTTGAAAGATT---TTAAGAAGTAGGTTACATGTGACTGGAAGGTATTGTAA 7334
GoodesThornscrubTortoise TCATGTCCATACTGCCAATGTGGAGTGTCCTGAATGCTTA--TCCCTCAGTACCTCTGCTCCCACCAGTACTTGCAAAGA 45123
```

```
Majority                  TTT-----AAT----AATAGGGTATATTTAAATTTTTCTGAAGATA--T---G-CATGTGCATGTGAGTGAATTAAATT

                                   49930     49940     49950     49960     49970     49980     49990     50000

Human                     ATCATCTGAAT---AGATTGCTTAGATATTAATTTATTTGAATTTA--TA--G-TATTAAAAATTGTGCTACTTAAATT 49638
Kakapo                    TTT-----AAT----AGTAGGCTATAATTTAAATTTTTCTAAAGATATATGTAGACATGTGCATGTGAATGAACTAAATT 20433
GoldenEagle               TTT-----AAT----AATAGACTGTAATTTACATTTTTCGGCAGATAACTT--GGCATGTGCATGTGGATGAACTAAATT 22719
JapaneseQuail             TG-------------ATTGACGTTTGTTTTGGATGGTCC--TG------------------------------------- 19493
MediumGroundFinch         TTT-----AAT----AATACTGCATACTTTACTTATTTCTACAGATA--------CA-----------TG--------- 7376
GoodesThornscrubTortoise  TTCTGGCCAATCTCAGAAGAGATAAGGTTTCAGTATTCTTGAAGGTGCCC-----CACACTGTCCCAAGCAGTTACAGTT 45198

Majority                  -----------TTGATGCTCTTAACGG-T----------T---T--GG-TAAT-ATT-TTTTCCTGAGATTTAGAAATTT

                                   50010     50020     50030     50040     50050     50060     50070     50080

Human                     TTCAGGCATTTTTGGAACTAGCAACCGAGGAAGCAGCTATTACTATGGTTAATTACTATTCTGCTGTGACACCTCATCTT 49718
Kakapo                    AATGGTTGGACTCGATGATCTTAAAGGTCTTTTCCAACCTAGTTGAGTCTATG-ATTCTCTTCGATGAGTCTATAAATGT 20512
GoldenEagle               -----------TTAATACTCTGAATGCCT-----------------GAACAAT-ATATCTTTTTGCAAATTAAGAAACTT 22770
JapaneseQuail             -----------------------------------------------------------------------TGCAACTTC 19502
MediumGroundFinch         -------------------------------------------------------------------GATTTAGAAACTT 7389
GoodesThornscrubTortoise  TTCATGTCTGCTGCAAGAGGTTATCTTGTTCTCTTTGCCTTACTCCTGGCAAG-AAGGAACTCCTGAGGTTCAGTGTTCT 45277

Majority                  T--------------------------------T-AGAAA-----AAAC-CAAATAATCA--CAAAACCCCCAAACAT-TA

                                   50090     50100     50110     50120     50130     50140     50150     50160

Human                     CGTAACCAACCAATATATATCCAGTACTCGAATCACAAAGAACTAAAGACAGATAATACATTAAACCAAGTAAGTATGTG 49798
Kakapo                    TAATGCTTTGAATGCCTGAACAATGTATCTTCTTAGAAATTCAGAAACTTTAAAAGTCAGGCAAAACCCCCAAACCTTCA 20592
GoldenEagle               T----------------------------------AAAAA-----AAACCCAAACAAACACACAAAACCCCCAAACTCCTC 22812
JapaneseQuail             C----------------------------------AACAG----------C-----------CAATA-------------- 19514
MediumGroundFinch         -----------------------------------AGAAA-----AAA--T-----------AAAA-------------- 7402
GoodesThornscrubTortoise  TGAGTTAGGTGCTTCAAATTGTTCTTACGCTTTTAGGCGGTTACAGAAGGAATTACAGTTCTAAATTGAAAATACAAACA 45357

Majority                  ---AACCTTAGGATGTGAGAAATAAAATGTGGCTATT----A------TTATCAATTTGGAT--AATTTCTGTCTGCATC

                                   50170     50180     50190     50200     50210     50220     50230     50240

Human                     TAGGTACATAAATAAAATGGCCTAGAACATA-TTATGAAT-CTCATAAACATTAATAGGAAGGAAATGTTTACCTGTTCA 49876
Kakapo                    GAAAGCTTCAGGATCAGAGAAATAAATAGTGGCTATT----ACTGTTATTATCAATTTAGGT--AACTTCTGTCTGCAGC 20666
GoldenEagle               ---AACCTCAGGATGGGAGAAATAAAATGTGGCTATT----A------TTATTAATTTAGTT--CATTTCTGTCTGTATC 22877
JapaneseQuail             --------TACCACATTAGAACTGCATTATAGTTAGA----A--------AACAATCAGTAG--ATTTTGAAACAGCAGC 19572
MediumGroundFinch         ---AATCTCCGGATGGGAGAAATAAAATGTGGCCATT----A------TTATCAGTTTGGGT--AATTTCTGTCTGCATT 7467
GoodesThornscrubTortoise  GGCACATATGGATGCTTTTAAATGCCCTCCATACATGCCTGACTACCTTCGTCTTTCTGCATT-AAAGTCCAATTGCATT 45436
```

```
Majority                   GCTATGCT--ATGGTAATTCATT----TTTGTTCTACTTCAGTTGCATTTCA-----CTCTAGC-----TGTAGTAT---

                                   50250     50260     50270     50280     50290     50300     50310     50320

Human                      GATGTTCCTTATAGGCATTTTCTT--GTTATTTTTAAGGTATTTTCATGCAGAATGTTTCTAAGTATTAAATACTATGTC 49954
Kakapo                     ACTATGCT--ATGGTGATTAATA----TTTTTACCACTTCAGTTGCATTTCA-----CTCTATC-----TATAGTAT--- 20727
GoldenEagle                GCTATGCC--ATGGTAATTCATT----TTTGTTCCACTTCAGTTGCATTTCA-----CTCTAGC-----TGTAGTGT--- 22938
JapaneseQuail              AGACTGA---ATGTGGATGCA------CTTGTTCTA----GGTCTTAATTAA-----CATCGAT---------ATAG--- 19622
MediumGroundFinch          GCTATGCT--GTGGTAACTC-------CTTGTTCTACTTCAGTTGGATTTCA-----CTCTAGCC----TGTGGTAT--- 7526
GoodesThornscrubTortoise   TCTCTGAAT-TTGACAACTCTTTCTGGCCCATTGTAAAGCAGTTGAAGGTAA-----ATCTACCTGCAGCCCATTGT--- 45507

Majority                   -TTTTACATTTTTTTAGGGCCTAAGTGAAGTG-CATTTGTTTTAT-CTG---------CAACATTGAGTAGCCACTATAC

                                   50330     50340     50350     50360     50370     50380     50390     50400

Human                      ATTTTGAATTCCTTTAATCCCCTGGTCAAATGTTCTTTTTCTTGTTGCAAGTTAAA---ATTTTTGTGTAGAAACT-TAT 50030
Kakapo                     -TTG-GCAT-TTTGAAGGGCCAAAGGGAAGTG-CATTAGTGT-----------------AACATTGAACAGCCACTATAC 20786
GoldenEagle                -TTTTACATATTTTAAGGGCCAAAGGAAAGTG-CATTAGTTTTAGACTGTG-------CAACATTGAACAGCCACTATAC 23009
JapaneseQuail              -ATA---ATCTTTTATCTGCCTGACTGTGGGT-TATTTATTT-----------------GAACTCACGTTGCTGATGGGC 19680
MediumGroundFinch          -TTTTACATATTTTTAGGGACTAAATTAGGTG-CATTCATGTTAGGCTG---------CAGCATTGAACAGCCACTATAC 7595
GoodesThornscrubTortoise   -CGTCAGATTAATATGAGGCTTCACACATTTGACACTCCTTCCCTCCCCCCCGGCCCCCAATTTTGTGTCAATGTTACTG 45586

Majority                   CATATTGTATGTGT-GTTAGGATAGTTG--TTTATACAACT----TAGTTAG-GCTCA---------GT---GGATTTTT

                                   50410     50420     50430     50440     50450     50460     50470     50480

Human                      CAGGTAGGATTTATTATTAGCATAAACTG-TTTATACAACTCTCCTGCTTAGAATTTAAGGTAAGAAGTTCAGTGCCTTT 50109
Kakapo                     CATAGTCCATGTGT-GTTAGAGTAGTTG--CGTAGAAAACT----TACTCAGTGCTCT---------GT---GGGTTTTG 20847
GoldenEagle                CATATAGTAAGTGT-GCTATGGTAGATG--CTTAGAAAACT----TAGTGCT-GCTCA---------GT---GCATTTTG 23069
JapaneseQuail              AAAATTCTTTGTAG-TTTAGGATTAGTT--TCAGTACTATT----TGGTCA---------------------GGATTTTC 19732
MediumGroundFinch          CATACTGTAAATGT-ATTAGCATAGTTG--CTTCAAAAACT----CAGTGC----TCA---------GT---GTATTTTA 7652
GoodesThornscrubTortoise   TGTTTTGGACATAG-CTAATGATAGCTGATTTTGAGCTATT----TCTCTGCCTGTGA---------GTTCAAGATTTTT 45652

Majority                   AATT---AGATGTGCAGCCTATGTGCAGATGTCCCAGTTAAAAGCTGTTTATGTTGATGTTTTGTTTGCTTTACTATGTT

                                   50490     50500     50510     50520     50530     50540     50550     50560

Human                      GGTTATTTGATTTTTTTTATAGGTCTTAATTTCTTACGGAAAATTTTTTTTTGAAGATGTGTTAATGGTTTTCCTGGAAA 50189
Kakapo                     AAA----AGAGGTGCAGCCTCTGTGCAGATGTCCCAGTTAAAAGCTGTATAAGTTAATTTTTTGCTTGCTTGACCATGTT 20923
GoldenEagle                AAAC---AGAGGGGCAGCCTATGTGCAGGTTTGCCAGTTAGAAGCTGTGTA-GTTAATTTTTTAATTGCTTGACCATGTT 23145
JapaneseQuail              CTT----TGTCTTGTGGCTTGTATCTGGAC---TAAGGCTCCAGCTGTTATTACTGATGGGATGCTTCATACAAGGAATA 19805
MediumGroundFinch          AAAC---AG--GCACAGCCTATGTGCAGATGTGCCAATTAAAAGCTGTAT----TAAAGTTTCTTTGTTTCGCTATGTT 7723
GoodesThornscrubTortoise   ATTTTTTATGTGGAAAGTATCTTTCCTGGTGGCAGTGGCATTAGCTTGCTAAGTGGGTAAGCTTTCAGCTGTAATAACTG 45732
```

```
Majority                 TGTTTATTTAAG-ATGAAATTCCTGTGAAGTGGTTGGTTTTG---TATGGTAAGATA-ATCTTATTA----------TTA
                                  50570     50580     50590     50600     50610     50620     50630     50640
Human                    AGAAATCTTAAGGATGAAGAATATGTGAATGTATTGATGTTTTAAAAATGTAATATAGAAGATATTGT--TTAAAATTAA 50267
Kakapo                   TGTTTATTTCA---TAGAATCCCAGCC---TGGTTTGGGTTG---GAAGGGACATCA-AGCTCAT------------CCA 20981
GoldenEagle              TACTTATTTAAG-TTCAAATTCCTGAGAGGTGGTATTCTTTG---TACGATAAGATA-GTTTTATTA----------CTG 23210
JapaneseQuail            TGATTAGTGCAG--TGCAGTACTGGCC---TGACAGGGTAAG---GATAATAAGGTATAGCATACTT----------TTA 19867
MediumGroundFinch        TACTTATTTAAG-CTCAAATTCCTCTTAAGCAATAGTCTTTG---TAGCGTAAGATA-GTTTTAATA----------TTG 7788
GoodesThornscrubTortoise GATCTATCTTAT-ACAAATATCAAGAGATACGGTTGGTATTGCTATTTCATCAAATATTTCTTCCAAAGATGGTAACTGA 45811

Majority                 ATTGGTCAATATT----TTACCATGCTCGTGGCCCTTTTCCATTATACAAAT-------T---T-------------TA
                                  50650     50660     50670     50680     50690     50700     50710     50720
Human                    TGTATTCAAAAATAGCATTAGAATGTTTTCCGACCTACATTATAAGACTGCTGTAATGATCTAAAACTTTAGCTGTTTTA 50347
Kakapo                   GCT--CCAACCCC----CTGCCACAGGCAGGGACCCCTTCCACTAGAGCAG------------G-------------TT 21029
GoldenEagle              ATTGGTCAAGATT----TTCCTTTGCACGTGGCCCATGTCCATGGTAAAAATCCACCTATTGCTACTGGTTAATGCTTTA 23286
JapaneseQuail            AGT---CAATACT----CAACCATACTCCTGTCTCCCCAGAGATCCTCAAA----------------------------- 19911
MediumGroundFinch        ATTGGTCAAGATT----TTTGTATGCACATGGCTCATGTCTGTGATAAACA----------------------------- 7835
GoodesThornscrubTortoise ATTCCTCCCTGTTGAGCCAATCATTCTCGTGGCACTTTTTCTGTATTTGCATGTTTCTTTTAGTGAAGTCTTTCTCCACA 45891

Majority                 ----A-GTTATAAAACTT----TATGT---------CTAACAAAGTATGGATCA---------------------ATGCA
                                  50730     50740     50750     50760     50770     50780     50790     50800
Human                    ATATA-GTTTTAAAACTAATGATATCTTTCTCTG--TCAGTAAAATACAAACTTT------TT-----------CTTAAT 50407
Kakapo                   ------GCTCCAAGCCCC----TGTGT---------CCAACCTGGCCTTGAACA---------------------CTGCC 21069
GoldenEagle              GTTCAGGTGGTAAAACTTCCA-CATGTAGTCGTGGCCTAGCAAGGCAAGGATAATGGG---------------GTATATA 23350
JapaneseQuail            ------ACAACAGAAGC-------C------------TGACAGAGT------------------------------AAGCA 19937
MediumGroundFinch        ------GCAAAAAAACC-----CAT------------TAACAAAA------------------------------ATGTA 7862
GoodesThornscrubTortoise AGCCACATTAGAAGGCTTTTGGGATTCTACGTGTAGCAGACAGAATTTGTATCACAGGTACCATATCTAAATGGCTGGCA 45971

Majority                 AAGAATGTTGAAGC-TACAGTTTCCCAGAGCAC-TTCTGCCCCCCCCT---C--------C------------------
                                  50810     50820     50830     50840     50850     50860     50870     50880
Human                    AAAAATGTAATGGAAAACTGTTCCTCATAGAT--TTTTGTCACTTTACAAAG---TG--ACAAAATCATTTTGTTAGTTT 50480
Kakapo                   AGGGATGGGGCAGC-CACAGCTTCCCTGGGCACCCTGTGCCAGCGCCT---C---------------------------- 21117
GoldenEagle              AAGTATGTTGAAGTACAAAGTCTTGCAGAACACACTCCGCCCCCCCCC---CCCCCCCCCCCCCCCCCGCCAAGCCCCCA 23427
JapaneseQuail            TAGAATGTGTATCC-TGCAATTTT--AGAATA------------------------------------------------ 19966
MediumGroundFinch        ATTAACATTGA-----------CC--AATGTA------ATCT-------------------------------------- 7885
GoodesThornscrubTortoise GACTGTATTTCCTATTGCTATTACCTGTCACACCTGCAGCCCACCCTTGCACAGTTTGTACAGTGTGAGGCAAGGCAGTT 46051
```

```
Majority                   ----AAAGCA--AT-------CTTTAAGGTTAACAGATGCTGA----CTTCATACTATTTCATATTTTTCATGT--TTTT
                                    50890     50900     50910     50920     50930     50940     50950     50960
Human                      ATGGAAAATAAGCTTGTAAAACTTTTTACCTAAAAGATAGGACTGAAATTTCAGCTTTTTTAATTTGATGATGAGTTTTT 50560
Kakapo                     ------AGC---AC-------CCTCACAGGGAAGAGCTTCTGC----CTTAGATCTAACTTGAATCTC--------CCCT 21169
GoldenEagle                AACAACAGCAAAACCCTTTAATTTTAAGGTTAACAAATGCAGA----CTTCAGAGTACTTCACATTCTTCATGT--TTTC 23501
JapaneseQuail              ----AAA-----AT-------ATGCAAGCTTTACAGAACTTAA----TCTCATAATTTTTCTTTTTTTC--TTT--TTTT 20022
MediumGroundFinch          ------A-----AC-------CTTCAAGGTTAACAAATGTGGA----CTTCAGACAACTTCATATTCTATGTGT--TTTT 7941
GoodesThornscrubTortoiseTCTGATACCAGCCTGAATCTTTTTTTAATTTGACATATGCAAAGCAGCCACATGGCAGCAACCATCATACAAAGCGTTGT 46131

Majority                   ATTTTATTTTTAAATATAATATCTTCTTTTTCTGTTCTTTCAAGCGTTTG--TG---------AATTCC---ACATTTTA
                                    50970     50980     50990     51000     51010     51020     51030     51040
Human                      AATTTCTTTTGAAAAAGAATGTATGCTTCTAATAATTTATCAAGAGGAAGAATACCAAAAGAAAATATCTGCTCTTCTTT 50640
Kakapo                     CTGGCAGGTT--AAAGCCATTCCCCCTTGTACTGTCCTTACAGGCCCTTG--TC---------AAAAGT---CCTTCTCT 21233
GoldenEagle                ATCTTATTTTGAATGTAATATCTTCTTTT-TTGCTCTTTCAAACATTT---TA---------AATTCC---AGATTTTA 23565
JapaneseQuail              TTTTTTTTCATATGCATTATAGACTCCATTTCCTTCATTTCAAGCGTTC---TG---------AATTCC---AGATTTTA 20087
MediumGroundFinch          ATAATATTTT-GAGTATAGTATCTCCTTTTCTTGTTCTTTCAAACATTT---TG---------AATTCC---AAATTTTA 8005
GoodesThornscrubTortoiseTGCCTAAATTCAACTACAGTGGATGACGTTTCCGTCCCTCAGTGCTGTTGAGCC---------AATGCCCACACACTCTC 46202

Majority                   TCTTTAGCTTGGATTTTTG---ATGTTTCACGTATTAAAAATAAGAATCTG---TTTTTTTTTTTTTAGGATCTTGTTTA
                                    51050     51060     51070     51080     51090     51100     51110     51120
Human                      CTTTTTACTTAGATTTTTTTGCATTTTTAACTTATTTTTAACAAATAATTGTTTATACTTATGGTGTACAATGTGATGTT 50720
Kakapo                     AATGAAGCTTAAATTCCTG---ATAGGTG--ATATTCTTTGTACTATTCTA---TTATTGATTGGTCAAGATCTTCTTT- 21304
GoldenEagle                TCTTTAGCTTGAACTTTTG---ATGTCT---GTACTAAAACAAAGGTCCTG---TTTTTCTTGTTTTGGGAACCTATTGA 23636
JapaneseQuail              TCTTTAGCTTGGA-TTCTG---AGGTATC-TGTATGAAAAACAAGAATCTGA--TTTTCCGTTTTTTAGGAATCTGTTGA 20160
MediumGroundFinch          TCTATAGGTTGAACTTTTG---AAGATTTACATGCTAAAGTAAAGGGCCTG---TGTATTTGCTTTTGGGAGCCTG---- 8075
GoodesThornscrubTortoiseACTCTCTGTTAGTTTTGTT---TCTCTTCAGGCATCTCACATTTGAATTGGCCGGTTTTTGAAAGTTAGAATATTGGTCA 46279

Majority                   TTCTTGTGGATATGTTT----GTCACTTGATCAGACTA---AAAATCTTCTCTTCCTACGTTCTATCACTTTCATCAGGG
                                    51130     51140     51150     51160     51170     51180     51190     51200
Human                      TTAATACATATATATTATGGAATGATCAAATCTGGCTGGTTAACATATTCATCTCTTCAGTACTTACAATTTCTTTATGG 50800
Kakapo                     TGCACATGGCCATGT---------ACATGGTAAAA--------ATCCACCTATTACTACTGGTTAACGCTTTAGTTCAAG 21367
GoldenEagle                CACTTGTGGAGAAGTTT----GTCACTGGATCAGACCA---AAAGTCTCCTCTTCCTACGTTTTATCTGTGACATCAGGC 23709
JapaneseQuail              CTCTTGCAGC-AAGTCT----CTCATTTGATCCTTCCA---AAGATCCTCTCTTTCTATCTTCTGTCATCCGTATCAGGC 20232
MediumGroundFinch          -------GGAGAGGT--------CACTGG-TCAG-------------CTCCCTTCCTACATTCTATCACTTACATCAGGG 8126
GoodesThornscrubTortoiseTTTGTCTGTTTTTGTGT----GGTGCCTA-TTATAGTA---TTAA--TTCTTTTATTTTGAGACATCCATTGAGTCAGAG 46349
```

```
Majority                 T-AGGTACATATAGGA-TA-T--TTTTTAAGGAAAGAACATAGCAGAAATAA--AA--CTGTATTGTTTTCTGTTATAC
                                 51210     51220     51230     51240     51250     51260     51270     51280
Human                    T-AAGAACATTTAAAATTCCTTTAGCTATTTTGGAAGTATACAGTGGAATGTGGAAAAACTGTTTTTCCTCTGCTCACAC 50879
Kakapo                   T--GGTAAGACTTCCACGA----TGTAGTAATGGTGTAGCAAGGCAAGGATAATGGG--GTATAAAGTACTTGGAAGTAC 21439
GoldenEagle              T-GGGTATATATA-------------TTTAAGGAAAGAACCT--CAGAAACAA--AA--CCACAATGTTCTCTGTTATAC 23769
JapaneseQuail            C-AGGTGCATACA-------------CTTAGGAA---AACCT--CAGAAATAG------CTGTGGTGGTTTCT-TTGTGT 20286
MediumGroundFinch        TAGGGTACGTATAGGAAAACT--TTCTTTAAGAAAAGAACTTTCCAGAAATGA--AA--CCACAGTGATCTCTATTATAC 8200
GoodesThornscrubTortoise TGAGAATCCTATAGGGGTAATGCCTTTCTAAAGAAGAGACAGAGTGAGAGGGAGAATGTGTGTGTCTAATTGGGTTTTTT 46429

Majority                 T--------CAAAGTATTTTTGTTT-GCGGTTTTCT--GCTGGTTGTTTTATACAGATGATTGTTTTCAGTAGCCAGTGT
                                 51290     51300     51310     51320     51330     51340     51350     51360
Human                    CACAACAATCAACACAGAAGGCTTCTGTGGCCTCAAATGTGGGGGAATTTTTTTCCACGCCAAG--CAAGCAGTCAGGTC 50957
Kakapo                   ----------CAAGTATTGCAGAAC-ACACTTTTCT--GCTCCCCAAAACAAGAAGAAAAAAAT-CCCACCAACCAAAGA 21505
GoldenEagle              T----CTCCCAATTTGTGGTTGTCT-GCGGTTTCCTTAGCTGGTTGTTTTATGCAAATGATTGTGTTTAGTAGCCACTGC 23844
JapaneseQuail            T---------CCAGCCTTTCTTTTG-GTGGTTTTCT---TTCATTGCTTTATACAGGTGGTTGTTTTCAGTAACCACTGT 20353
MediumGroundFinch        ----------AATTTGCGATTGTCT-GCAGTTTGTT--GCTGGTTGGTTGAGACAGATGATTGTGTTCAGTAGCCAGTGT 8267
GoodesThornscrubTortoise T----TTCCCAAAGAATTTTTTTTTAGCATTTTTTTGTGGTTAATTTTTTACACCTGTGATTAGTTTGAGAGGTCTTGAT 46505

Majority                 A---ATATTTGATCTTTCTGTTTGGT-ATAA--CATTATTTTAAACTAGATATCTTCA--ATTCCATTGCCTTT-CTCTT
                                 51370     51380     51390     51400     51410     51420     51430     51440
Human                    TGCAGTGTTCTCCAACTTAGCTCAGTTTCAA--CACTGTCTGCCGGGAGATAGCATCA-GATCCCACAGGTTGAGGGCTC 51034
Kakapo                   A---GCCTAAG----------T-----A--------------AAACTGAACATGT-----AACCTTTTAATTTT-AACAT 21547
GoldenEagle              A---ATATTTAATCTTTCTCTCTGGTCATAATCCACAACTGTAAACTAGATATCTTCATAATTCCATTACCTTT-CTCTT 23920
JapaneseQuail            G---ATACTTGATCTTTCTGTCTGGGGAGAATCCATGATTTTAAACTAAATATTTTCATAATTCCATTGCCTT--CTTTT 20428
MediumGroundFinch        A---ACATTTG-----TCTAAT--------------------AAACTAGATATCTTCA--GTTGCATTGCCTTT-CTGTT 8316
GoodesThornscrubTortoise TT--ATCCTCCACAATTAGAGAGATGACTAAAAGATTCTAGGAAATGAGGTAATGGC---AGTTTGTTGGACC--ATGAT 46578

Majority                 AGTCAAATATGTCCTGTGTGGATA--TGTGCCTTATGT-TCTACATGTTGTTAACA-TTAAATTTCTTATATTTAGGTAA
                                 51450     51460     51470     51480     51490     51500     51510     51520
Human                    AGTCCCACAAGATCTCCTGAGACACCAGCAAGTCAGGCCTCTGGAACTTCTGA----TTGACCAGCTTCAAGTTGGGGTT 51110
Kakapo                   TAACAAATGCAGCCCTCGGAGA-------ACTTTGTGT-TCTTCATGTT--------TT---TCTCTTATTTTTGAGTGT 21608
GoldenEagle              GGTCAGATATTTCCTGTGTGGATA--TGTGTCCTATGT-TCTACTTGTTGATAACG-TTAAATTTCATATATTTAGCTAA 23996
JapaneseQuail            AGTCAAGTATGTTCTATATGGATA--TATACCTGATG---CTACTTCTT---AACA-TTGAGTTTTGTGTATTCAACATA 20499
MediumGroundFinch        AGTCACTTATTTCCTGTCTGGATA--TGTGTCTGATGT-TCTACA-GCTGATAACA-TTAAATCACATATATTTAGCCAA 8391
GoodesThornscrubTortoise CAGTGAGAATGGCTTAGGTGGATAATCCTGCTGTTAGT-ACTGCAGTCTTTGAGCACTCAAATGCTTTGAACTTTGGGAA 46657
```

```
Majority                   G--------------GTGATACAAGTAGTATTTTCTATATTAGCA-ACAGCACTGAATAATACTGTTAGTAAGTATCTTG

                                   51530     51540     51550     51560     51570     51580     51590     51600

Human                      CCCACAACCC-CCTTTTTGGATTTCATTAATTTGCTAGAGCAGCACACAGAACTCAAGGAAACATTTACTGAGATTTACT 51189
Kakapo                     A--------------GTTTCTCCTTTTTTATGTTCTTTCTCCCAA-ACACTTCAAAAGTTCAAGCTAAAGAGAAAAGCTG 21673
GoldenEagle                T--------------GTGACACAAGAAGTACTTTCTACATTAGCA-ACAACAATGAATAATACTGTTAGTAAGCTTCTTG 24061
JapaneseQuail              G--------------GTGATACA---AATATTTTTGACATTAGCA-GCAACTATGAATATATCTGTTAGGAAGTATCCTG 20561
MediumGroundFinch          G--------------GTAAAACAAGTAGTGCTTTCTATGT--AGA-ACAGCACTGAATACTACTGCTATTAGGTGTCTTG 8454
GoodesThornscrubTortoiseATTCTGCCTTGTCTTGTGCTGGGAGCAGAAGACCCAAGGGTAAGA-ATTCTGTGAAATAATAGTTTCAAAAAAGAATTCC 46736

Majority                   TCTTTAGTATATAG--A-TGATTGTACTGAAAGATATAATAAGT-TTCTAAGAATGC--AA--A-TTA-CTTTTTCTTGT

                                   51610     51620     51630     51640     51650     51660     51670     51680

Human                      AGTTTATTATATAG--------GATATTAAAAGGATAATAAGC-CTCTAATCATGC--------CTTGATCTTTCAGGT 51252
Kakapo                     GAATTTAAAGACAG-------CCATACTAAAACAA--------------AG-------------------TTCCTGTTCT 21713
GoldenEagle                TCTTTAATGTATAA--ACTGATTGTATTGGAGGATGAAATAAGTGTTTCAGGAAAGCCTAAGTATTTACCTTTTTTTTGT 24139
JapaneseQuail              GCTTTT-TATATGA--AGTGATTGTACTGGAAGATAAAATAACTGTTCCAAGAAAGCACAAATACTTAAATTTCTCTTGT 20638
MediumGroundFinch          TCTTTAGTATTTA---------CAAACTGAAAGGT-------------TA--------------------CTTTTTTTTT 8493
GoodesThornscrubTortoiseAGGTGAGTAATTTTTCATTGATAGCACAATGGATATTAGCAATCTAAATGAGAATTTGAAATGATCTCTTCTTTGCTTTT 46816

Majority                   AGTTGGTTGTTGGTGTGTAGTGGTATTTGT--GAAACAGA-AG-TGACTCGTGCAGAAAGTATGTTTATTTATAAATT-A

                                   51690     51700     51710     51720     51730     51740     51750     51760

Human                      GACCAGTCCCCATCTTAAAGTGGGGCTGCCAGCCATCAGTCAACTCATTAGCATACAAAATATATCATTTTGGAGATTTA 51332
Kakapo                     GGGAACCTGTTGGTACTTGTGGAAATGTTT--GTCAC-------TGGCTCAGACCAAAGGTCTCT-TCTTCCTATATT-C 21782
GoldenEagle                AGTTGGTTGGTGGTGTGTACTGACAGTTGT--GAAACAGATAT-TGACTCCTGCAGAAAGGATATTTATTTATCAATT-A 24215
JapaneseQuail              AGTTGGTTATTGGGGTGTAGTGATGATTGT--GATCCAGA--G-TAACTTGTGCAGAAATCAGGT----TTATCAATT-A 20708
MediumGroundFinch          AATTGGTTGTTGATGTGTACTGGCAGTTGT--GAACCAGA--G-TGATTTCTGCAGGAAGTATGTGTATTTGTACATT-A 8567
GoodesThornscrubTortoiseAGGAAAATGTCAATGCATAATTGTTTTAACTAGTTATAAAAAA-TGAAACTTGCCAAGAATTTCCTAAAAAAAAAATA-A 46894

Majority                   TTTC-ACTAG---TACAGATATGAGGAAATT----TATCG--TCAGTGTCTTGTGATCAATTTTTCAATATTTTTTTCC-

                                   51770     51780     51790     51800     51810     51820     51830     51840

Human                      TTAGGAATTTAGAGTTGTATGCCAGGAAATGGGGTTGAGGACCAAATGTCTATTTTACAATGTCACAATACAGTATTATG 51412
Kakapo                     TGTC-TGTGA---CATCAAGCCGGGTATATA----TATT---TAAGAACCTCAGAAGCAAAACCACAGTAGTTCTTCCCA 21851
GoldenEagle                TTTC-GCTA----CACTGATATGCAGGGATT----TATCA--TCAGCATCTTGTGATGAATTTTTAAAAATTTTTTCCC- 24283
JapaneseQuail              TCTCTGCTCG---TGCAGGTATAAGAAGATC----TACAG--TTAACCTTTC--------CCCC----CACCCCCCTCC- 20766
MediumGroundFinch          TTTC-ACTAG---TGCAGATATGCAGGAATT----TATAG--CCAGTGTCTTGTGGTCATTTTT---ACTTTTTTTTCC- 8633
GoodesThornscrubTortoiseTGGTAATTAAGGAAACAGGCATCAGTCAACTGGACTATCA--TGTGACATTTCAGCACAGTATTTGAATGTGGCCTTCTG 46972
```

```
Majority                  --CT----TATTTTCTGTTATTAATGGGTG------AGATCAAATTTTTATTGTATGCAACTTT---TTTT-----GCCT
                                   51850     51860     51870     51880     51890     51900     51910     51920
Human                     CATTACAGTAGTTGCCATGCTGTGCGGTAGATCACCAGAACCTATTCTTCCTGTCTGAAACTTTGTACTTTGACCATCAT 51492
Kakapo                    AGCT----GGTTGTTTTATGCAGATGATTG------TGTTGAAGCCACTGCAATATTTAATCTT---TCT------GTCT 21912
GoldenEagle               --CT----TCTTTCCAGGAAATAATAGGTG------AAAGCAACTTTTCATTGTATGCAAAATC---TTT------GCCC 24342
JapaneseQuail             ---------ACTCCCTCATGTCAAAGAAAG------AAGTTAAACCT-TATTGTATGCCAGATC---TTTG-----CCCT 20822
MediumGroundFinch         --CT----AATTTTCAGGAACTAATGGGTG------AAAGCAACTTTATATTGTATCCAACTTT---TTTT-----GCCT 8693
GoodesThornscrubTortoiseTATT----TGTATTTGGTGAGTAGTGAGGACCTCATAGAAGAAATGATTGTAGGGGGCAACCTTA-GTTTGAGTGATCAT 47047

Majority                  GTACCCATATAGTTCCATTTCTATATTAGGAGA---TTTTGTGAATATGAGAGAAGGATATTTTATAGTAAAATTATACA
                                   51930     51940     51950     51960     51970     51980     51990     52000
Human                     CACCCCTTCCCTGTTCACTCCTCCCTCCCCCAGC-CTCTGGTGACTATTTTTCTACTCTCTACTAATGTGAGTTCAGCTT 51571
Kakapo                    G--GTCATA-ATCTCCATTTATAAACTAGAAGGGGGCTACAAGGATGCTGGAGAGGGACTCTTCATCA-GGGACTGTAGC 21988
GoldenEagle               ATAGCCACATAGTTACATTTCTATATTAGGAGA---TTGCGTGAATATGAAAGAAGATTATTGTAAAGGAAAATTACACA 24419
JapaneseQuail             G----AGCA-------ATTTCTGTACAGGGAGA---AATTGTGAATGTGAGAGGAGGA-ACCCTAGAGGAGAAT------ 20881
MediumGroundFinch         GTACCCACATAGTTACATTTCTGTATTAGGAGA---TTGTGTGCTTAT-AAAAAAGTTTATGATACAG-AAAATTATGCA 8768
GoodesThornscrubTortoiseCGCTAATTCAGTTCAAACTAAATGGAAGGATAAACAAAAATAGATCTGTGACTAGGATTTTTTATTTTAAAAGGGCTAA 47127

Majority                  TTT----AGAATAAGGGT-T-A---A----------T--------C-------T-----T-----CAGAACA-TTTTAG-
                                   52010     52020     52030     52040     52050     52060     52070     52080
Human                     TTTT---AGATTACATATGTAAGTGAGATAATGTGTTATTTGTCTTTCTTTGTTTGACTTATTCACTTAGCACACACAGT 51648
Kakapo                    GAT----AGGACAAGGGG---------------------------------------------T-GATGG-GTTCAA- 22014
GoldenEagle               TCT----AATTTAAAGGCTTCATATAT---------TTTCAAGGTCAATTATGTATTTTTGGCCTCAGAACA-CTTTAG- 24484
JapaneseQuail             --------GAATGAGGAA-------------------------------------------------GAATA-TTTTGCT 20903
MediumGroundFinch         TCT----AAAATAAAGGT--------------------------C-------------T-----CAGAAC--TCTTAG- 8796
GoodesThornscrubTortoiseCTTTAAAAAAGTAAGGAAATTAGTTAGGGAAGTGGATTGGACTGAAGAACTTGTGGATCTAAAGGCAGAGGAGGCCTGGA 47207

Majority                  GTCATATTTA--TGAATTTCTAGA----A--TTAAAT------------A--GTGTAGGGAAAAGCTCTTCTAGGATGAA
                                   52090     52100     52110     52120     52130     52140     52150     52160
Human                     GTCCTCTTAAAATGACATTTAATATCCCATTTCTAGTTCGTTATTATTTAAGTAATTTGCAAGTACATTCTCATTTTAAA 51728
Kakapo                    ACTGAAACAG--GGGAAGTTCAGG-------TTAGAT----------------CTAAGGCAGAAGCTCTTCCCTGTGAGG 22069
GoldenEagle               GTCATATTTA--TGTATTTCTCGAAAA-A--TTAAAAAAAAAAAAA--AAGTGTGCAGAGAAAAACTCCTCTAGGATGAA 24557
JapaneseQuail             GCAGTAGCTC--TTAATTTCTAGA-------GTAAAT-----------------ATTCATTACAGTTCTTGTCATAGAAA 20957
MediumGroundFinch         GTCATATTTA--CATATTTCTTTT-------TTAAAA---------------GTGCAGAGAAAAGCTCCTCTAGGATGAA 8852
GoodesThornscrubTortoiseATTACTTCAAGTTAAAGTTGCAGAAACTA--TCAGAAGCCTGCATCCCAAGAAAGGGGGGAAAAAAAATCATAGGCAGGA 47285
```

```
Majority                    ATTTTA-----A------------------T-GAAATGGTTTTTAAGATCAT--G---ATACTATTAA----G-----T-
                                     52170     52180     52190     52200     52210     52220     52230     52240
Human                       AGATTAAACGAA--AGGAACATGTAAAGAATAAAGCTGGGGGCTGGGCTTGGTGGCTTATACTGGTAATCCCAGCACTTT 51806
Kakapo                      GTGCTG----------------------------AGGCGCTGGCA----CA---G-----ACTTTTC------------- 22096
GoldenEagle                 ATCTCATTAAGATCATAGAATCATTTAGGTTGGAAAAGACCTTCAAGATCATCGAGTCCAACCATCAACCATGCCCACTA 24637
JapaneseQuail               ATTACAC--------------------------ACACAAAACATAGGACTGT------GTATCTTCAA----G------- 20994
MediumGroundFinch           ATTTTA------T------------------GAAATTGTTTTCAAAATCA---------GCTATTA------------- 8885
GoodesThornscrubTortoiseCTTGTAGACCAAGCTGGATGAGCAAGCATCTCAGAAAGGTGATTAAGAAAAAGCAGAAAGCTTACAAAGAGTAGGAAGTG 47365

Majority                    ------GTCAG-AGAGTATGTTATTT----TCTCGATTTTAGGACTCTTC-----AAGGTGA----A-------------
                                     52250     52260     52270     52280     52290     52300     52310     52320
Human                       GGGAGGCTGAGGCGGGTGGATTACCTGAGGTCAGGAGTTCAAGACCCGCCTGACCAACATGGTGAAACCCTGAGTCTACT 51886
Kakapo                      -------CCAG-AGAAGCTGTGGCTG----CCCCATCCCTGGCAGTGTTC-----AAGGCCA------------------ 22141
GoldenEagle                 AACCATGTCCT-GAAGTACCCCGTCC----ACTCGCTTTTTGAATACCTCCAGGGATGGTGACTCAACCACTTCCCTGGG 24712
JapaneseQuail               ------GCAAA-ACAGTGTGTATATT----TTCCAACTCTTGTA--TTT----------TGG------------------ 21033
MediumGroundFinch           -------TCAG-ATAGTAAGCTATTC----ATTCTTTGCTAGGA--CTT------AGGGTGA------------------ 8927
GoodesThornscrubTortoiseGGAGGGATCAGCAAGGAAAACTATCT----TATTGATGTCAGAACTTTCC-----AAGACCAATGCAGGTTCGAGTAAGG 47436

Majority                    ------ACTTGAAAAC---A--AGTATCTCAGTAAAAA--CT--TTTCTA-GTTCCTAGCT---TGAATGTTCA--TAGC
                                     52330     52340     52350     52360     52370     52380     52390     52400
Human                       AAAAATACAAAAAAAAAAAAAAATTAGCCAGGCATGGTGCCAGGTGCCTGTAATCCCAGCTACTTGGAGGCTGAGGCAAG 51966
Kakapo                      ------GGTTGGACAC------AGGGGCTTGGAGCAAC--CT--GCTCTA-GTGGAAGGTG---TCCCTGCCCA--TGGC 22199
GoldenEagle                 CAGCCCATTCCAATGTTTGACAACCCTCTCAGTAAAAAAATT--TTCCTA-ATATCTAACT---TAAATCTCCC--TTGC 24784
JapaneseQuail               -------CTTCAGAAC---------ATTTCAGGTGGTA------TTTCT--G------------TGCCTTTTTG--TAGG 21075
MediumGroundFinch           ------ACTTAAAAAC------AGTTTTTCTGCAAAAA---C--TGCTTG-GT---TGCAT---TGAATATTCA--TAGC 8981
GoodesThornscrubTortoise------ATTTGATTGAGAGAGGAAAATCAAAATGAAAAC-CA--ATTGTA-ATTCAAAACCAAGTGATTGTTTA--TTAA 47504

Majority                    TTGGGATTGAG-------------G-------TTGAGTGATCTGA--------A---CA--ACT--C-----TAC-AATT
                                     52410     52420     52430     52440     52450     52460     52470     52480
Human                       AGAGAATTGCTTGAACCCGGGGGGGTGGAGCTTGCGGTGATCCCAGATGCGTCACTGCACTCCAGCCTGGGTGACAGAGC 52046
Kakapo                      AGGGGGTTGGAA--------------------CTGGATGATCTTG-------------------------------AGGT 22228
GoldenEagle                 CTCAACTTGAGGCCATTTCCTCTCGTCCTATCTCCAGCCACCTGACAGAAG--AGACCAGCACCCACCTCACTAC-AACC 24861
JapaneseQuail               GGAGG---------------------------AAGTGTGCAAAGA---------------------------A--AAAT 21098
MediumGroundFinch           TTGAA--TGA----------------------TTGAATGACTTGG----------------ACT--------TAA-AATA 9012
GoodesThornscrubTortoiseTTCGGAATGAG-T--TGCAACTTGGACTGGGGAGGAGCACTTTTACCAACT--AA--CAATACTATCAGGACAAG-AATA 47576
```

```
Majority                 TCCTTTCTGTTTGAAACA--G-------A----TT--CTTTTAGTTTAGTCTTCTATA-----T-A--------AGTTCC
                                  52490     52500     52510     52520     52530     52540     52550     52560
Human                    GAGACTCTATCTCAAAAAAAAAAAAAAAAAAAAAAAGAGTAAAGCTGAGGCCTACACATGGATGGATGTGGAGGAGGGGC 52126
Kakapo                   CCTTTCCAACCCAAACCA---------------TT--CTATGAGTCTATGATTCTATA-----------------ATTCC 22274
GoldenEagle              CCCCTTCAGGTAGTTGTAGAGAGCGATAAGGTCTC--CCCTCAGCCTCCTCTTTTCTAG--ACTGAACAGCCCCAGTTCC 24937
JapaneseQuail            TCCTTAGTGTTTCCAATA----------------------TTAGTTCT-----CAGCA----------------GGAAAC 21135
MediumGroundFinch        TGCCTTGTGTTTGCTGCA--G------------TT--CTTTTTGTTTC-TTTTTTATA-----T-A--------GGTTCC 9061
GoodesThornscrubTortoise TACACACAACTTGAAACAATCTATTTACATCCAACGACAAGAAACTAAGCAGTCTATAA--TCAACTGCTCACTAAAAAC 47654

Majority                 ------TATTACTC-----A-TTGT--T-------C-TTAGTTAATTG---GTTCTTCTATGAAAATGCCCTAGGACCTC
                                  52570     52580     52590     52600     52610     52620     52630     52640
Human                    ACTTAGGATGACTAGTGGAAGCCCCATTGAGAGGGGATATTTGAATAATGAACTTCAAAGTGGTTAGGGAATAAGCCACT 52206
Kakapo                   -------ATTGCTT-------TTCT----------C-TTGGTCAGATA---TTTCCTGTATGGATATGTCTTACGTTCCC 22326
GoldenEagle              CTCAGCTGCTCCTCATGAGACTTGTGCTCCAGGCCCCTCACCAACTTGGTTGCCCTCCTCTGAACACGCTCCAGCACCTC 25017
JapaneseQuail            ----------------------------------------GTTACTTG----TTCTTGTATAAGAGTTGCGTTGAACTTT 21171
MediumGroundFinch        ------TATTTCT--------TTGT------------TTAATAAAATAGGAGTTTCTCACTGAAAATGC--CAGGACCTG 9113
GoodesThornscrubTortoise CAGAGCAGATACACAAACCAAATAAACTCTA----CACGCATTAATTG-----AATACTATAAAAATACACCAGCTAGCC 47725

Majority                 AG--TC-----T------A---------------------TGTTGAGAATTTGGC--C----------A-----------
                                  52650     52660     52670     52680     52690     52700     52710     52720
Human                    TAT-TCCAGACAGAAGGAATAATCAATGCAAAGTTCCTTCTGTTGAAAAAAATGCTTTTATAAT-TTTATTTTTCATCTT 52284
Kakapo                   -----C-----T----------------------------TGTTGGTAACATCAC------------------------- 22343
GoldenEagle              AGCGTCTTTCCTGTAGTGAGGGGCCCAAAACTGAACACAGTACTTGAGGTGCGGCCTCACCAGTGCCGAGTACAGGGGGA 25097
JapaneseQuail            ---------------------------------------------AGAATTTGTT------------------------- 21181
MediumGroundFinch        AG---------T----------------------------TGCTCAGATTGTGGC--C---------------------- 9132
GoodesThornscrubTortoise ACAATAGCAATTAATACAACAATTTGACTCTCATATACTATATACACAACTTGGTACCAAGTAAGGGAAGGGAAGAAGTT 47805

Majority                 -------C------------------A---TATTTCT---ATAGGC-A-G---------------TTAGCTACCTGTGAA
                                  52730     52740     52750     52760     52770     52780     52790     52800
Human                    CTAAAGCCCTTCAGTCTCCTTTGGTCATCTTTTTTTTGTAGTTGGTTATGCCTTA---A----CAACAGTACTTTGAGGA 52357
Kakapo                   -------------------------------ATTTC----ATATAT-------------------TTAGCTAATGTGAAA 22369
GoldenEagle              CAGTCACCTCCCTGCTCCTGCTGGCCACACTATTTCTGATAGAGGCCAGGATGCGATTGGCCTTCTTGGCCACCTGGGCA 25177
JapaneseQuail            --------------------------------TTTCT---GTAGAA----------------------ACTACCTGTGAA 21204
MediumGroundFinch        -------------------------------ATTT-----AAAGGC-----------T-------TTGTCAACCTGTAAA 9158
GoodesThornscrubTortoise AAGCAAGCAGAGTATCTACAGAAATTAAAATGCACATACCACACTCCAGGCGCAGCTGACCAGCAGTACAGACACGAGGA 47885
```

```
Majority                   CATT-----C------------------CA----AACCTCACCAGTTTTTTCTATGCTAGC-T---T----A--------

                                   52810     52820     52830     52840     52850     52860     52870     52880

Human                      TACTAGTTACAGTATTTAAAAAATACGTCACTATATGCTGAGTTATTTTTTCAGTGTTAGCATTTATGTTTATCTTGTTC 52437
Kakapo                     CAAG----------------------------A--------AGTACTTTCTACATTAGC-C----------------- 22393
GoldenEagle                CACTGCTGGCTCATATCCAGCCGGCTGTCAA-CCAGCACCCCGAGGTCTTTCTCTGCCGGG-CAGCTTTCCAGCCACTCT 25255
JapaneseQuail              CATT----------------------------------------GAATCTTTAAAGCTTG-------------------- 21224
MediumGroundFinch          CATT-----C----------------------AATTTCACCAGGTTGGTCTATGCTGA-------------------- 9189
GoodesThornscrubTortoise   CATGGCGAATTGGGGAGGGGAAGTGATCCAA-AGAACCAAAACGACACGACATGAGCCAGC-TAAATCCGGAGGAGACCT 47963

Majority                   -----------------------------------ACTGAAG---G--------T--------------GACTTTATTCG

                                   52890     52900     52910     52920     52930     52940     52950     52960

Human                      TCATTCTCTTCATCCCCCCTTTGTTGTCTTTTCGTGTTTCAGAATGGACAATTAGATTTTGTTAAAGTTGTATGCTTTCC 52517
Kakapo                     -----------------------------------ACAACAA------------T--------------GACTA-ATACT 22411
GoldenEagle                GCCCCAAGCCTGCAGCGCTGCATGGGGTTGCCGTGACCGAAGTGCGGGACCCGGCACTTGGCCTTGTTGAACTTCATACG 25335
JapaneseQuail              --------------------------------------AAT---------------------------GATTGAATGCC 21238
MediumGroundFinch          -----------------------------------ATTGAAG---G-----------------------AACTCTATTCA 9208
GoodesThornscrubTortoise   TAGCTGGAAGGGTGATCAGGCCTTCCAGCTAACACACGGATACTCCTG-CAGGTTTTGGCATGAGATACAGTTTTATTTG 48042

Majority                   ACTGG--T-A-------GATTTAGCTTAT---G------CTGTCTTTCCTGCTTAATTTCAAGCAG--------T-----

                                   52970     52980     52990     53000     53010     53020     53030     53040

Human                      TCTAGGATAAAGTAATGAAGAGCGTTTACTAAGCTGTTACCCCCATTCCCCCCAAATACCAAAGAGGCTCTTATTCTGGG 52597
Kakapo                     ACTAG---------------TAAGCTTCT-----------TGTCTTTAATGTATAAACTGAAATG--------------- 22450
GoldenEagle                ATTGGCCTCAGCCCATCGATCCAGCCTGTCCAGATCCCTCTGTAGAGCCTTCTTACCCTCAAGCAGATCGACCCTGCCTC 25415
JapaneseQuail              ATGGA--------------CTTAACTTGT------------GTACCTTATGCTTCATTTT--GCA--------------- 21275
MediumGroundFinch          GCAGG------------GATGTGGCTTAT---G-------TGTCAATTATATTTAAATTGAAGTA------------T-- 9252
GoodesThornscrubTortoise   CACACTCTTACTCGTGTCA-GCGTCTGATTGGTCCCTAGCTCCTTCACCAACCAGGTTCCGAGCTGGTACCCTCTACATC 48121

Majority                   CCAACTTGG----ATGTGCAAATT--------------C--AATGTTTTGACTCACAGCTTTAATAAAAT---T------

                                   53050     53060     53070     53080     53090     53100     53110     53120

Human                      TCAACAAAACTCCCTTTAGAATTT---CTGGTACCAGGCTGTGCGTGGTGGCTCACACTTGTAATCCCAGCACTTTGGGA 52674
Kakapo                     --AA---------AGGATGAAATA-----------------AATGTTTTGGAA--A-GCTTTAAGTATTT---------- 22489
GoldenEagle                CCAACTTGGTGTCATCTGCAAACTTGCTGACGGTGCA-CTCAATCCCCTCATCCAAATCATTGATAAAGTTATTAAACAG 25494
JapaneseQuail              ---------------GCTCATAT-------------------AGGTTTTCAGTC-CTGTTTTAATAAAA----------- 21309
MediumGroundFinch          CCTATTTGG----ATGTGAGAAT------------------AATGCTTTGAAT--A-TCATTAGTAAAG----------- 9296
GoodesThornscrubTortoise   AATAGGAGCTGCACCCGGCAGACTACTTTTGCACACAGCCCTATGCTTTTACACACAGCACCAACTTGACCTTTTGACCG 48201
```

```
Majority                 -A----G-T--------------G-----------------------GACATC--TGGTTTT-A--C-A--------A--

                                 53130     53140     53150     53160     53170     53180     53190     53200

Human                    GGCTGAGGTGGGCGGATCACGAGGTCAGGAGATCGAGACCATGGTGAAACCCCGTCTGTACTAAAAATACGAAAATTAGC 52754
Kakapo                   ------------------------------------------------ACATT--TGAGTTT----C------------- 22502
GoldenEagle              AACGGGGCCCAACACCGAGCCCTGGGGAACACCACTTGTGACCCGCCGCCAAC--TGGTTTTCACCCCATTCACCACAAC 25572
JapaneseQuail            -------------------------------------------------------------------------------- 21309
MediumGroundFinch        -A------T--------------------------------------GACATC--TTGTTT------------------- 9310
GoodesThornscrubTortoiseACAGAAGGTTCGAGAACAGGCACGCTGGTATTTTTGCACATGGCACTGGC--C--TGACCCTTAGGTGACCAGGGAAAAG 48277

Majority                 -----G-----G-----------A--------------G-A--------------------TAAGATGTTGTTGGCTCA-

                                 53210     53220     53230     53240     53250     53260     53270     53280

Human                    TGGGCGTGGTGGCGGGCGCCTGTAGTCCCAGCTACTCGGGAGGCTGAGGCAGGAGAATGGTGCAGACCCAGGAGGCGGAG 52834
Kakapo                   -------------------------------------------------------------TAGTTGGTTGTTGGTCTG- 22520
GoldenEagle              CCTCTGGGCTCGGCCATCCAGCCAGTTTTTCACCGAGTGAAGAGTGCACTTATCCGGGACACAAGATGCCAGTTTCTCAA 25652
JapaneseQuail            --------------------------------------------------------------GAGAAGTTTCTAACT--- 21324
MediumGroundFinch        --------------------------------------------------------------AAGATATGGTTGTCTCA- 9327
GoodesThornscrubTortoiseAATGGGAATAGGCACACGGCACTATGGTGTAGCACACAGTACCAGTGTGACCTTT------TGACCGACAATTGGCCAAA 48351

Majority                 -GAGTATCACATG-------------------T-----------A----T--A-A----G---------GAAAGTGAGTT

                                 53290     53300     53310     53320     53330     53340     53350     53360

Human                    CTTGCAGTGAGCCGAGATCGCGCCACTGCACTTCAGCCTGGGCGACAGAGCGAGACTCTGTCTCAAAAAAAAAAAAAAAA 52914
Kakapo                   -GACTGACACTTG------------------------------------T-------------------GAAACAGAG-- 22542
GoldenEagle              GGAGTATGCCATGACAGTGGCAAAGGCCTTGCTGAAGTCAAGATATGTTTTCAAAAATAGTTATCAGCAGGAAGTTAGTT 25732
JapaneseQuail            ------ACAAATG------------------------------------------------------------------- 21331
MediumGroundFinch        ----TATCACATG--------------------------------------------------------GAAACTGAATT 9347
GoodesThornscrubTortoiseCAGACGCCATGTTTGAGCATAGGCTTTAGGGCTGCGACAAGAACATGTAGGGATAAAGTGAGAAAGGTCAAAAGCCATGT 48431

Majority                 ATTGATTG-TCTTGCAA--AGGGCATGCTGAGTACTTAGATCAGTT-----------A--A---------A----A----

                                 53370     53380     53390     53400     53410     53420     53430     53440

Human                    AAGAATTCCTGGTACCAGCAGGGCACTTTGGCTCATGCCTGTAATCCCAGCACTTTGAGAAGCTGAGGTAAATTCAGGGC 52994
Kakapo                   ATTGACT---CCTGCAA--AATGCATGTTTAGTAGTCA-ATGATT----------------------------------- 22581
GoldenEagle              ATTCACTG-TTTGTTAG--GAGTTATGCTGAACTTTAAGATCAGTTTTTCTGCAAAAACTACTTGGGAGCATTGAATCTT 25809
JapaneseQuail            -----TC------------AGGGAATGCAGATTGCTTAGATAATGC---------------------------------- 21360
MediumGroundFinch        CTTCATCT-CCTTGGTG--GAGAGATGCAGAGCACTTAGATCAGT----------------------------------- 9389
GoodesThornscrubTortoiseAGAGTTGGACCTTGCAA--AGGGAATTAAAACCAATAGTAAAAGGTTCTATAGTCATATAAATAAGAAGAAAACAAAGAA 48509
```

```
Majority                  ---AG---------------GCCATGGA--------A---------------ACA-----TT------------------

                                   53450     53460     53470     53480     53490     53500     53510     53520

Human                     AGCAGTGAACTGGTCACACCACTGCACTCCAGCCTCAGCAACAAAGCAAGACACAGTCTCTTAAAAAAAAAAAAAAGGAAG 53074
Kakapo                    ---------------------TCACTAA---------------------------------------------------- 22588
GoldenEagle               CATAGCTTTAACAATTGAATGCCATGGACTTACAGTATTCCTTGTGTACATTACACTTCATTCTGTTGTGTATAGGTTTC 25889
JapaneseQuail             ---------------------CCATTC----------------------------------------------------- 21366
MediumGroundFinch         --------------------GCTATGG----------------------------------------------------- 9396
GoodesThornscrubTortoise  AGAAGAAGTGGGACTGCTAAACACTGAGGTTAAGGTATGGCCCAATATCTAAACAAATACTTTGCCTCAGTCTTTAATGA 48589

Majority                  -----TTG--T--TTAATGGAACAGGGGTTTTTAAT---------CA--G-----GGTATTATGTGTTC---------A-

                                   53530     53540     53550     53560     53570     53580     53590     53600

Human                     AAATTCTGGTTCCTTTAAGGGAGATTTGTTGTTGTTTTCTTCTTTTAATGGTGAGGGTACAAGTTGGTCACAGGAGGGAG 53154
Kakapo                    ------TG------CAGAGAAACAGGGGCTTAGAAT---------C---------AGCACCTTGTGATG----------- 22627
GoldenEagle               TATTTCTG--TCTTTAATAAAATAGAAGTTTCTAACTAAAAATGCCAGGGACCCAAGTTGTTTAGATAATGGCCATTTAT 25967
JapaneseQuail             --------------------A-TAGGCTTTGTCA---------------------GATCTTATGCCTTC----------- 21393
MediumGroundFinch         -----TTG-----TTAACAGAACGTGGGTTTTTA-----------C---------AATAGTCTGAATTG----------- 9435
GoodesThornscrubTortoise  GGAGCTTA--TGGATAATGGTACGATGACAAATGGG-AATGAGGATATGGAGGTAGATATTACCACATCTGAGGTAGAAA 48666

Majority                  -----------AATTTT---------TATCACTTTTTCCATTTGTGCTTAATTGA---A----T-T-------G-ATAT

                                   53610     53620     53630     53640     53650     53660     53670     53680

Human                     AAGAGAAATTTAGCTCTTTTGTTAATGAACTAGACTTTCTGTTCGTCTGTTTTTCACCCACTCCCTCCTCCTGTGAACCT 53234
Kakapo                    -----------AATTTT---------TAACATATTTTCCCTTTATTTCCAGGAAA----------------------T 22663
GoldenEagle               AGGCTTTGTCAACTGTTAAGCATTCAATTTCAACAACTTCATTTATGCTAAATTGAAGGAACTCTGTGCAACAGGGATGT 26047
JapaneseQuail             -----------AATTTC---------CATGACTTGGTTT-TCTGTGCAGTATGGA---------------------TAT 21430
MediumGroundFinch         -----------AATATT---------CCTGAACCATTCC---TGTGCTCAGTGGA--------TCT-----------T 9471
GoodesThornscrubTortoise  CAAAACTTGAACAGCTTAATGGGACTAAATCGCTGGGCCCAGATAATCTTCATCCAAGAATCCTAAAGGAACTGGCACAT 48746

Majority                  GGCT----------C---G-----GG----TGT-AGTATC-----------AATTTTGTTCTGGATGC-----G----G-

                                   53690     53700     53710     53720     53730     53740     53750     53760

Human                     GCCAATTTCTGATTTGGTTCCTGTGGCTGCTACCAGTAT-----A----ACACCTGTTTGCTGTGTGTTCACTGTGTCTC 53305
Kakapo                    AGT---------------A-----GG----TG--AAAAC------------AACTTTTTACTGGATGC------------ 22693
GoldenEagle               GGCTTACATATCAACTATGTTTAGGG----TGT-AGTATCCTATTTAGGAGAATAATGCTTTGGATGCCATTTGTAGAGA 26122
JapaneseQuail             GGCT--------------------------TA--TGTGTC-----------AATTATGTTTAGACTGC------------ 21459
MediumGroundFinch         GGC-----------C---------G-----TGT-AGGAT-----------TAATGGT----TGGGCTC----------G- 9499
GoodesThornscrubTortoise  CAAATTGCAAGCTCATTAGCAAGAAATTTTTAA-AGAATCCTGTAAACTCAGGGGTTGTACTGTATGACTGGAGAATTGA 48825
```

```
Majority                   -A--AGATTTT-T--------A--AAG-----GTCCTATGTAATCTGGATTTTTCTA-TC--TGATGGTGAGCCA-----
                                   53770     53780     53790     53800     53810     53820     53830     53840
Human                      CAGTCCATTTGGTTCCATTCATTTTCTGTGTTCCAGGAATTTGATAAAATTATTCTCAGC--TAATGATAACCTTCCCTC 53383
Kakapo                     -A--AAATCTT----------------------TGCA--CGTATCTGCATCGTTATATTA--CTATATTAAGACG----- 22739
GoldenEagle                TG--AGATTTTGTTTAGAAATAGACAGCTGATGTCATACGGAAACTGGATTTTTCTTCTCCTTGGTGGAGAGCCAAAGAG 26200
JapaneseQuail              ----AGGATTC------------------------------AGTTTAGATTGGTGAA-----TAATGC------------ 21488
MediumGroundFinch          ------ATCTT----------A--AAG-----GTCCTCTCCAACCCAAATGTTTCTA-----TGATTCAGGGCTA----- 9546
GoodesThornscrubTortoiseTAGCATAGTTCCTATTTTTAAGAAAGGGAAAAAAAAAATGTGATCCGGGTAATCACAGGCC-TGTCAGTTTGACATCTGT 48904

Majority                   ------------------T----------------GG----TTTTTG---G----------AATA---TGAAT----A--
                                   53850     53860     53870     53880     53890     53900     53910     53920
Human                      CCTTCAGTGTTTGTTTATTTTATATTCATTCACTTCTAGGCATTTTAATAGAGTTTCAT--AATAGAAGACATGCAAATG 53461
Kakapo                     -----------------------------------------TTTTTG--------------AACA---TGAA-------- 22753
GoldenEagle                GGCTTA-CATCAGTGCAATGGCTGTTACAGGAGAGGGGGGTTTTTTGGTAGTCTTGAGTTGAATATAATGAATATTCACG 26279
JapaneseQuail              ------------------------------------------TTTTG--------------AAT---------------- 21496
MediumGroundFinch          -----------------------------------GG----TTTCT---------------AAT----TT---------- 9558
GoodesThornscrubTortoiseAGTATG-C---AAGGTCTTGGGAAAAAATTTGAAGGAGAAAGTAGTGAAGGAC--------ATTGAGGTCAATGGTAATT 48972

Majority                   -----TTTATGGTTTATGTTTTTTTAAAATTCTGCT--TTTTAAAGCTATAACTTGAT--TTCTAAATGAACTAATATTT
                                   53930     53940     53950     53960     53970     53980     53990     54000
Human                      -AGTATGCATTAACCATGAATTCTTTCACATCCGTTCATGAAATCACCAGGTCCTATTAGTTCACACTTTTTAATTATCT 53540
Kakapo                     -----AGA--AGGTTATTGTAAAGAAAAATCCCACA--TCTAAAAGGCATTATATAGT--TTCAAGGTTAA-TGATGTAT 22821
GoldenEagle                TTAGATTTCTGATTTATTTTTTTTTAAATTTTTTTAAACTATAAGGCTATAACTTGAT--TAGTAAATGAACTAATACTA 26357
JapaneseQuail              ----------GCCTTATGTAGAGGCAAGATTCTGCT-------TAG----AATTTG-----------CCAACTGATGTTG 21544
MediumGroundFinch          -----TTTTTGGTTTACATTTTTTTTAAACTATGCA---TTTGCAGCTATAACTTGAT--TTGTAAATGAGCTAACACTG 9628
GoodesThornscrubTortoiseCAAACAAAATACAACATGGTTTTACAAAAGGTGGATCGTGCTAAACC---AACCTGAT--CTCCTT-TGAGAAAGTAACA 49046

Majority                   ----TGTTTTAGTCATAGTACTCTCTTTGTGATTTTTTTAACTTTTTTTC---CGTAATGCATATATTGTAGC-CTGCTC
                                   54010     54020     54030     54040     54050     54060     54070     54080
Human                      GCCTGGTTTTCTCCATCTTTCCTGCTACTTGTATTGCTCAAACACACTCACCTGTTGGCTATATTACTACGACAGTTCTC 53620
Kakapo                     ------TTTTGGCCACAGAACACTTTCGAGGATTTTTTATACTATTTTTC--AAGAAATACATAAATTGTG----TG--- 22886
GoldenEagle                ACACTGTTTTAGTCATGCTATTCTCGTGGTGATTTTTTTATTTTTTTTTTTTCCCCAATGTAGATATTGTATCATTATGC 26437
JapaneseQuail              -------CATGGAAAC-----------TG-GATTTTTCTTTCTCTTTTT--------GTTGACAAACT------------ 21585
MediumGroundFinch          ----TGTTTTAGTCAT-----TCTCCCAGTGCTTCTTAAAACTTTTTCT----CCCAATGCAAATATTGTAGC-CTGCTC 9694
GoodesThornscrubTortoiseCA--TTTTTTAGACAAAGGAAACACGTTGGATCTAATTTACCTCAATTTC---AGTAAGGCATTTGATACAGTTCCACAT 49121
```

```
Majority                  TTTTCAAAGCGTATTTATTTTTATTACAGTTCTTGCATTAAGATTTTTGTATTGGATGAAGCTTT--TCAAT-TCTAGTG
                                   54090     54100     54110     54120     54130     54140     54150     54160
Human                     TTTATAAACTACTTTCTTTTTCTTTTCCCTTCTGGAATTTTTTTCTCGCTAGTACAGCCAGTACGTATCTTTCTCAAATG 53700
Kakapo                    ----CAAAGAGAAACTCTTTTTGGGTGAAATCTTGCTTCAGCGTGTTT---TTGAAAGTAGTTA----------CCAGCA 22949
GoldenEagle               TCTTCAGTGCATGTTTATTTTTATTA--GTTACAGCAGCAAGAGTTTTGAATTGGATTAGGCTTT--TCAAG-TCTGGTG 26512
JapaneseQuail             -----AAAGAGGACTTTGCATCAGTGCAGTGGCTATTACAGAATT---------------GCT----------------- 21628
MediumGroundFinch         TTTTCAGTGCATATTTATGTTTGTTAAAGTTTTAGCAGTAAAATGTTTGAATTGGATCAAGATTT--TCAAG-TCCAGTG 9771
GoodesThornscrubTortoiseCGAAAAAATGGGGATCAATATGAAAATTGAAAGGTGGATAAGGAACTGGTTAAAGAGGAGGCTACAGTGGGT-TGTACTG 49200

Majority                  AA---AGCTAAATTACTTGGT---GAAGTAGCTACTTGCCAA--------TAAGCTTCCATTTTAAGATCAGTATTTTTG
                                   54170     54180     54190     54200     54210     54220     54230     54240
Human                     CACATATCTAAGTTACTC--CCATGCAGTAGGTCCTGTTCATGCTCAGGCTAAAGGCCCTTTCTGAGCTTACTACTTTTT 53778
Kakapo                    GG---AAGTAAGTTACTC--------ACTGTTTGCCAGGAGT--------TAGGCTGAACTTTTAAGATCAGAGTTTCTG 23010
GoldenEagle               AA---AGCCAAACTACTTGGT---GAAATACCTACTTGCCAA--------TAAGCTTCCAATTTAATAACAG--TTCCT- 26575
JapaneseQuail             -------CTTATTCATTTGGT---GAATTAGCTTCTTGCCAA--------TAAGCTTCCACTTCAATAGCACTATCTTGG 21690
MediumGroundFinch         AA---AGCCAAACTACCTCAT---GAAATAGCTACTTGCCAG--------TAAGTCTCCAGTTTAACAGCAG--TTTCAG 9835
GoodesThornscrubTortoiseAA---GGGTGAACTGTCAGGCTGGAAGGAGGTTACTAGTGGAGTTCT--TCAGGGATCGGTTTTGGGACCAATCTTATTT 49275

Majority                  CAGTAGTTTTTAGTTGGCTTGTGAGAT---TGCTTGCAGCATGCTTTTGCCTCTTCTCGTTTTTCTGCAATTTTGTCTTA
                                   54250     54260     54270     54280     54290     54300     54310     54320
Human                     GTCTTGTCTTATGACTACTCCCACCCTATTTGCTTATGCTTCTGTCCCAAAAGTAATGGTGCATCCTGTAACCTGGCATG 53858
Kakapo                    CATTGAATCTTCAT-AGCTTGAACAAT---TGAGTGCCGCAGACTTAGAGTATGCCTTGTGTACATTCCACTTCAGTTTA 23086
GoldenEagle               CAGCAGTATTTAGTTGGCTTGTGAGGTGTCCCCCTCCAACATGCCCTTGCCCCCTCCCAGTTTTCTGCAGTTTTGTCTTT 26655
JapaneseQuail             TAGTAGTTATTAGCTGGCTTTTGAGAT--------------------TGTCCCTTCCCAGTTTTCTGCAGTTTTGTCTTT 21750
MediumGroundFinch         CAGTAGTTATTAGTCAGCTTGTGAGGT---CTCCTCCAACATCCCTTGGCCTCTCCCCAAGTTTCAGCAATTTTGTTTTA 9912
GoodesThornscrubTortoiseAATCTTTTTATTACTGACCT-TGGCACAAAAAGTGGGAGCGTGCTAATAAATTTTGTGGATGACACAAAACTGGAAGGTA 49354

Majority                  TT---GCTGTATAGGGAAGGATCTTTGAGGTTA------T-----TTAT---GATAGATTTTGTTTCTACCAGAAGACAA
                                   54330     54340     54350     54360     54370     54380     54390     54400
Human                     TTTTCTCTTGATGTCAGTCGTTTGCTGCCATTATGTCACTGCTCTCATGCATGGAATATTGTGGGTTGGCCTGCCTTTTG 53938
Kakapo                    TT---GCT-TATTTGGATAGGTTTCTGTGTTTA------------ATAA---AATAGGAGTTTCTAACAAAAAAAGCCAA 23147
GoldenEagle               CTCCTGCTGTATAGGGAGGGATCTTTTGGGTTACTTGTTTATCTCTTATCTCCACAGACTCCATTTCTACTGCAAGACAA 26735
JapaneseQuail             TT---CCT-------GAAGCATCTTTCACATTG----------------------------T-CTTGTGTCAGAAAAC-A 21790
MediumGroundFinch         TG--TGCTGTATAGGGAAGGATCTTTCAGGTTG-----TTGCTGCTTAG--GCATAGACTCTATTTCTACCAAAAGACCA 9983
GoodesThornscrubTortoiseTTA--CCAATGCAGAGAAGGACC----AGGATA------------TCATACAGGAAGATCTGGATGACCTTGTAAACTGG 49416
```

```
Majority                 GGTTATACTTTTAGTACCTTACTCTTCTTGATTTGAGTTGTTAACTTTTATGGATCCTGATTTTGCTCATAAATTT-TGT

                                  54410     54420     54430     54440     54450     54460     54470     54480

Human                    CACCACCCTTCCTCCACCTCACACTTCACATGGCCAGTGTTAAATATCTTGAGGTCTCCCTTTAGCTATTT--CTTCTCC 54016
Kakapo                   GGACCAAAGCTGTGTAGATAATGGCCATTTATAGGCTTTGTCAACTCCTAAGCATTCTACTTTCAAACCTTGATTTCTGC 23227
GoldenEagle              TATTATTCTTTTAGTACCTTGCTGTTCTTGACTTAACCTATTAACCTTTATGGACACTGATTATGCTGGTACAATT-TGT 26814
JapaneseQuail            GACTATATAAATAATG--TTCTTCTCAGTGCTTGGGGTT--TGTTTTTTTTTGTTCTTTTTTT------TA--TTT--GC 21856
MediumGroundFinch        GGTTATTCCTCTAGTACCTTGCTCCCCTTGACTTGGCTT-TTAACCTTTATGGACCCTGATTCTGCTAATAAAATT-TGT 10061
GoodesThornscrubTortoise AGTAAAAGTAATAGGA--TGAAATTTAATAGTGAAAAATGCAAGGTCATGCATTTAGGGATTAATAACAAGAATTTTTGT 49494

Majority                 TAAAAGATTGGAGCTGTTGTTTTAACAGTA----AGTTTACCTTTCAGTGGCTCTGA--ACAAGTTGTGCTTCT-ATGTT

                                  54490     54500     54510     54520     54530     54540     54550     54560

Human                    TAAAAGCTATCCCCTGATAAGCAAAGAGTAGATTAGGTTTGGATTGAGAGTGTTAAC--TCCGAATGAGATACTGCTATG 54094
Kakapo                   TAAATTGAAGGAACT-CTGTGC-AGCAGGG-------------AT--GTGGCTTAGAT-ATCAGTTATGCTTA----GAG 23285
GoldenEagle              TAAGAGAATGAAGCTGTTGTTTTAACAGTAGTTGAATTCACCTTTCAGTGTCTCTGA--ACAAGTTGTGTTCCC-ATGTT 26891
JapaneseQuail            TAAA-GAATGAAGTTGGTGCTTTAACATCA-----GTTTACCTTTCAGTGGCTCTGA--ACAAATTGTGTTTCC-ATGTT 21927
MediumGroundFinch        TAGAGGATGAGATTTGTTGTTTTAACAGTA----AATTCACCTTTCAATGGCTCTGA--ACAAGTTGTGCTTCT-GTGTC 10134
GoodesThornscrubTortoise TATAAGCTGGGGACACATCAGTTGGAAGTAACAGAGGAGG---AGAAAGACCTCAGAGTATTGGTTGATCACAGGATGAC 49571

Majority                 TTAAATGTT--GGTT-TTCTATTCCCAAGTGTAGTTAA-ATGATGCTTCTTTAGCTCAGAAGTGCCATTTAAAGTTAGAA

                                  54570     54580     54590     54600     54610     54620     54630     54640

Human                    CACATAACCAGAGTGGCTAAAATTAAAAGGATAGGTAATAAAATACTGGTGACAACCATTTTTGAAAACTCGAAATATCT 54174
Kakapo                   TGGAATAT----CCT-ATTTAGGTAGGAGAATAATGCT-TTGA-ACATCATTAGTACAGAGGAG--ATTTT--GTTAGAA 23354
GoldenEagle              TTAAATGTTGCAGTT-TTCTATTCCCAAGTGCAATTTA-ATGATGTCTCTCCAGCTGAGAAGTACCATTTAAAATGGCAA 26969
JapaneseQuail            TTAAAAACT--GGTT-TTCTGTACC-AAGTGTAGTTAA-ATAA--------------GAGAGTG---TATAAAGTTAGGG 21985
MediumGroundFinch        ATAAATGT---GGTT-TTCTATTCCCAAGTGCAATTTA-ATGATGCGTCTTCAGCTGAGAAGTACCATTTATAATGACAA 10209
GoodesThornscrubTortoise TGAGTCGCCAATGTG-ATATGGCCATGAAAAAAGCCAATGTGGTCTTGGGATGCATCAGGCGAGGTATTTCCAGTAGAGA 49650

Majority                 ATAGGAGATTA-AGTT--G-TGTA-GGTGTCATGTAGTGCCAGGATCTCTCGATAACTGTCCTGAGAGCTAATTTTAATT

                                  54650     54660     54670     54680     54690     54700     54710     54720

Human                    ACTAAAGTTGAGCATATAGCTATT--CTTTGATGTAGTAAACTCGCTCCTAGTTAACATACCTGAGAACATGTACATAGG 54252
Kakapo                   ATAGACAACT---------------GATGTCCTGTGGAACCTGGATTTTTC-----TTCTCTTTGGTGCAGAGCCAAAGA 23414
GoldenEagle              ATAAGAGACTAAAGTTGGGGAGAAAGGTTTCATACAGTGCCAAAAACTCTTAATAACTGTTCTGAAAGCTAAATTTCACT 27049
JapaneseQuail            AGAGGTGCTT--------------------CATGTGGTGCAAGGAACTTTAC-TATTTGTCCTAAAAGATAATTCCA--T 22042
MediumGroundFinch        ATAAGAGACTACAGTT----AGAGAGGTGTCATACAGTGCCAGAATCTCTCAATAACTCTCCTAAAAGCTAAATTTAATT 10285
GoodesThornscrubTortoise TCAGGAGGTTTTAGTACTGTTATAGAAAGGCACG-AGTGAGACCTCATCTGGAATACTATGTGCAGTTCTGGTCTCC-TA 49728
```

```
Majority                   TGT-TTAACAA--ATGTTTTAGAAAATTCACTTGTGTATGTACATGTTTGTTTAGTATATGATACACTTTT--------T

                                     54730     54740     54750     54760     54770     54780     54790     54800

Human                      TATGTTAAAAGATATGTACAAGAAAGTTCATGGAACACTGTATGTAATAGCCCAAAAAACTATAAATCATC--------- 54323
Kakapo                     GGG-TT--------TACCTCAGTGCAGTGGCTGTTACA-GAACAGGTTTGGTTTCGTTTTGGGGTTCTTTG--------- 23475
GoldenEagle                TGT-TC-ACAA--ATGTTTTGAAAAATTCACTTGTGTATGTGCATGCATATTTAGTATATGACACACACACACGCTTTAT 27125
JapaneseQuail              TGT-TTGACAG--ATGTTTT-GAAAATTCACGTCTGTG--TACACAAGTGTTTAGTATATGACAGAGTTTG--------T 22108
MediumGroundFinch          TGT-TCCACGA--ATGTCTT-AAAAGTTCACTTGTGTATGTTCATGCATATTTAGTGTATGATACACACTT--------- 10352
GoodesThornscrubTortoise   TGT-TTAAGAAGGATGAATTCAAACTGGAACAGGTACAGAGAAGGGTTGATCCGAGGAATGGAAAACCTGT------CAT 49801

Majority                   TACTGCCATTACTGAAATAG-GAAGTTTGTGTTAGGCTATACTGGATGGATAATTAAAGGTAGTAA--AAGAACATGTTC

                                     54810     54820     54830     54840     54850     54860     54870     54880

Human                      CAAATGTTCAACAGCAGCAGAATATGTTGTGTTGTATTCATTTAGTGGAATATTGTATAGTAATAAAAATGAACAAGCTA 54403
Kakapo                     -----CCAGT-CTGAACTGGCTATAATCATCCCTGGAGGTGTTTAAAAGATGTGTAAGTGTGGTGCTTGGGGACATGGTT 23549
GoldenEagle                TACTGCCATTATTGAAATAG-TAAGTTAGTGTTAGGCTATGCTGGCTGGATAATCAAAGGTAGAAA--CAGTACATGTTC 27202
JapaneseQuail              TACTGCCCTTATTGAAGTAG-GAAGTTTGAGTTAAGCTATACTGGCTGGATAATTGAAGATTACAA--AAAAGCTTCTTT 22185
MediumGroundFinch          ------CATTACTGGAACAG-GAAATTACTGTTAGGTGATACTGAATGGATAACCAAAAGTAGTAA--AAGTACATATTC 10423
GoodesThornscrubTortoise   ATGAAAGGAGACTCAAAGAGCGTGGCT--TGTTTAGCCTAACCAAAAGAAGGCTGAGGGGGAGATA---TGATTGCTCTC 49876

Majority                   TCTTTGTGTTCAAGAAA--AGCAGTAATCACCG--GTTAGGTTAATTTTTTCTGTCTATCTAGAATCTGGTCTTAA--AT

                                     54890     54900     54910     54920     54930     54940     54950     54960

Human                      CTGCTGCCTGCAAAACATGGGTAGTGTACATCATAATTATTGGAAGAAGCCAAACCAAAGTAGAA--TACATCTGAT-AT 54480
Kakapo                     TAGTGGTGGACTTG------GCAGT----------GTTAGGTTTATGGTTGGACTCGATCTTAAA---GGTCTT------ 23604
GoldenEagle                TCTTGGCTTTCAAGAAA--AGCAGTAATAACCGATGTTGTGTTAATTTCTTCTCTTTTCTGAGAATCCAGTCTTAA--AT 27278
JapaneseQuail              TCT--ATGTTCAAAAAA--AGCAATAGTGACTA--ATTTGTTGTTTTTTTTTTTCCATCTTCTATCTGATATTAA--AC 22257
MediumGroundFinch          TCTTTGCATTCA--------GCAGC---------TGACACGTTCATTTCTTCTGTCTTCCAAAAG-CCATTGTTAA--AT 10483
GoodesThornscrubTortoise   TATAAATATGTCAGAGG--AATAAATACCAGGGA-GGAAGAGGAATTATTTAAGCTAAGTACCAGTGTGGACACAAGAAC 49953

Majority                   GTTTTGGTGTAAATTT-ACTTTATGAAGGT-GGGCTTCATTTTT--ATGAATTTTATTAGTTCAGCTTTTA-TTCTCTGA

                                     54970     54980     54990     55000     55010     55020     55030     55040

Human                      GATTCCATTTATGTAA-CTTGGAGGAAGCGTGAGTATGGCTTCT---AGAGTGTTGATAATATACTGTTTT-TTATCTGG 54555
Kakapo                     -TTCTGACCTAAATG--ATTCTGTGATTCA-GGGTTAGATTTCT-----GATTTTATTATTTTAATTTTTAACTCTATAT 23675
GoldenEagle                GTTTTGGGGTAAATTTTACTTTAAGAAGGT-GGGCTTCATTTTT--ATGAATGTTACTAGTTCAGCTTTCA-CTCTGCAA 27354
JapaneseQuail              ATTTTGGGGTAAATT----CTTGTG--------GCTTCATTTTG---TGAATTCTATCAGTTCAGCTTTCA-GTATCTGA 22321
MediumGroundFinch          CTTTTGGCATAAATTTTACTTTAAGAAGGT-GGGCTTCATTTTTTAATGAATGGTACCAGTTCAGCTTTCA-TTGTACAA 10561
GoodesThornscrubTortoise   AAATGGATATAAACTGGCCATCAGGAAGTTTAGACTAAATTAGAGGAAGTATTCTAACCATCAGTGGAGTGTTGTTCTGG 50033
```

```
Majority                   GTCATGCTTTTGCGGATA-G-GTAATAAG---CTTTT-TATTGGTTTTTTGCCCTACGACTGATAACTTTTTTTGGGCAA
                                    55050     55060     55070     55080     55090     55100     55110     55120
Human                      GTAATTATTTCACAGAGATGTCCAATTTGTGGTATTT-TATGGAGCTTTATACTTAGGTTTTGTGTCCTTTTGTGTGTGT 54634
Kakapo                     TTAAGGCTGTAACT--------TAATAA-----------A---------TGAACTAAGACTAACACTGTGTTTCGGTCA- 23726
GoldenEagle                GGCATGCTTCTGCCAATA-A-GTAATAA----CTTTTGTCATGGTTGCTTGCCCTACCACTGAAAACTTTCTTTGGGCA- 27427
JapaneseQuail              AGTATGCTCCTGCGAATA-G-GAAATCAG-------------------TGGCTCTGTCAGACTTACTTGCATTTATGTAG 22380
MediumGroundFinch          GAGAGGCTTTTGCCCATA-A-GTAATAAACAGCTTTTGTCATGTTTACCTGCCCTACTGCTAAAAACATTTTTTGGGCAA 10639
GoodesThornscrubTortoiseAACAGCCTTCCAAGGGGA-GCGGTGGAGGAAAAAGACATATCTGGCTTTTAAACTAAGCTTGATAAGTTTGTGGAGGGTA 50112

Majority                   ---TTCTATTGTGATGAGAATTCTTGTTG-CAGTCTCCCT--------TCCTGTTCCCCCAA--------------CC-C
                                    55130     55140     55150     55160     55170     55180     55190     55200
Human                      GTGTAATATTTTGATAAGAAGTTTTATTA-AAATCTAGGTTTGCAAGCCAACACTGCTCCATGAATTATAAGAAAAAATA 54713
Kakapo                     ---TGCTGTTCTCACAGGGATTTTTATT-----------------------TATTCCCCTAA------------------ 23762
GoldenEagle                ---TTACATAGCATTGAGAATGCTTGTTGCCAGTCCCCCCCCATCCTGTCCTGTCCCCCCCATCCTGTCCTGCCCCCCCC 27504
JapaneseQuail              ---CACTATTGC--CAAAGGTCCTTGAG-----------------------TGGCTGCCCA------------------- 22413
MediumGroundFinch          ---TTCCATAGCACTGAGAATGCTTGTTGCCAGTCCCCCC-------ATCCTGTCCCTCCCA--------------CC-C 10694
GoodesThornscrubTortoise--TGGTATGATG--GGGTAGCCTAATTTTGGCAATTAATTGATCTTTGACTATTAGCGGTAAGTATGCCCAATGGCCTG 50187

Majority                   T--CTGGTTTTTGATTGCCATAT-GTTCTTTAGTGCTTAATTCTGTATTTT-TTTGTGT-TATTG-TATTAATATTTATC
                                    55210     55220     55230     55240     55250     55260     55270     55280
Human                      TATGAAGTCATTAAAAATGAAATCATAAAGAAATGTATGAAGTAATTTGTTACTGATGTGAGAGAACATGATTGTTAAGC 54793
Kakapo                     T--ATGGATATTGTAAGCATTAT-GTTCTTCAGTCCTT--CTTTGTATTTG-TTAAAA------G-TGTTAACAGCAAGA 23829
GoldenEagle                C--CTTTTTTCTGATTGCCATAC-ATTCTTTAGTGCGTATTTCTGTATCTT-TTTGTGTATATTG-TTTTAATATATATA 27579
JapaneseQuail              --------TGTT--TTGTCTTGT-GTTCTTTAGTGTGTTACCCTGTATATT-TTCGTGT-----G-TATTAATTTCCATA 22475
MediumGroundFinch          C--CTGTTTTCTGATTGCCATAT-GTTCTTTTGTGCCTAATTCTGTATTTT-TTTGTAT-TACTG-TAATA-TATTTATC 10767
GoodesThornscrubTortoiseTGATGGGATGTTAAATGGGGTGG-GATCTGAGTTACTACAGAGAATTCTTT-CCTATGTGTCTGGCTGATGAGTCTTACC 50265

Majority                   TATTTTTTCACGGAAATG--TT-TTAGCCTTTTCAGTTCTCAATATGGCTATTTTTTCCAGTAAGTATAAAAAGTTTCAA
                                    55290     55300     55310     55320     55330     55340     55350     55360
Human                      CATAAAAGGAAGATAGAGAACAATGAATAGTATTAAATAAAAGTATGAATAGTATTGAATAAAAATATGAATAGTATTGA 54873
Kakapo                     GGTTT-------GAATTG--GA-TCAGGCTTTTCAGGTCTGGTGGAAGCCAGATTTACCTGATGGAATAGCTCCTTGCCA 23899
GoldenEagle                TACTTTTTCACTTAAACA--TT-TTAGCATAGGCAGTTCTCTATATAGCTCTTTTTCACAGTAAG--AAAAAAGTTTAAA 27654
JapaneseQuail              TATTT----------A-------TCTACTTTTTATCTTCTGAAACTGGATGTTTATTCCCCGAATCTCCACATGTGTGAA 22538
MediumGroundFinch          TGCTTTTTTACAAAAATG--TT-TTTGCCCAGGCACTTGTCTACATAGCT-TTTTTTACAGTAAGTAAAAAAAGTTACAA 10843
GoodesThornscrubTortoiseCACATGCTCAGGGTTTAG--CTGATAGCCATATTTGGGGTCAGGAAGGAATTTTCCTCCAGGGCAGATTGGCAGAGGCCC 50343
```

```
Majority                 ATAAAGT-GTAGTTTAGGT---TAGCATTTTTTAAAAAGTAAAATTGTGTTAGAGGCTTGTCTATGTGAA-------CTA

                                55370     55380     55390     55400     55410     55420     55430     55440

Human                    AAAAAAT-ACAGACTAAGT----ATAATATTTATATATATATGCTTGTAGATGTAGCTAGTATCTCTGGAAGGATATCGA 54948
Kakapo                   ATAATCTTACAGTTTAGG-----AGCAATTCCTCAGTAGTAGTAGT-TCTTACAGAATCAGCTACGT-----------TG 23962
GoldenEagle              TAATAGCAGAAGCTTAGTT---TAAGATAGTTTAAAAAATAAAATTGTGTGAGAGACTTGTATACGTGAG-------TTA 27724
JapaneseQuail            ACCAAGCCATAGTCTTGATATGTGGCAGCAGAGAAAAAGAAAATGTGTCTTACTGCATTCATCCTCT----------CCT 22608
MediumGroundFinch        GTACAGT-GTAGTTGAGGT---TAAGACTGTGTAAAAAATAAAATTGTGTGAGAGGCTTGTGTATGTGAA-------CTA 10912
GoodesThornscrubTortoise TGGGGGCGGGGGGTTGCCTTCCTCGCAGCATGGGGCATGGGTCACT-TGCTGGAGGATTCTCTGCCCCTTGAAGTCTTTA 50422

Majority                 AGCTAAAGCTGTTAGATGCTGGTTAGGCA---AAGACTTATATATTTA-----A--GTGGATTAAAGTTTTTCTTTTCAC

                                55450     55460     55470     55480     55490     55500     55510     55520

Human                    AGATAATACTGTGAGGGGTTGGTAGGGGAAAATAGACTGGGTGACAGAGTTGCAGGGGAATTTTTAATCTTTAATACCAC 55028
Kakapo                   GGAAAGACCTTTAAGATCAT--CAAGTCC---AACCTTTAC--CC-CA----------GGACTGAGGTTTCCCCCTTCAT 24024
GoldenEagle              AGGTAAAGCTGTTAACTGCT--CTAGGAA---AAGACATATATATATATAAAAATAGTACAACAAAATATTTCTCATAAA 27799
JapaneseQuail            AGCAGTATCTGTTGGATACTTACTACCCCCTCAATTCTGATCAATTTA-----A--AAGGTGTAAGGTTTTTGTTGT--- 22678
MediumGroundFinch        AGTTAAAGCTCTTAGTTGCTGTTTAGGAA---AAGATACACATATTTA--------GTACATCAAAATGCTTCTTTTAAG 10981
GoodesThornscrubTortoise AACTATGATTTGAGGACTTCAGTGGCTCAGACATGGGTTAGGGGTTTGTTATGGGAGTGGGTGGATGAGATTATTTG-GC 50501

Majority                 CT--TTCCTTAAACTTATTGCAGCAAAAGTTGAAAA--------ACTGTGT--GAAGTGTA-GATATTA-TTTGCAATGT

                                55530     55540     55550     55560     55570     55580     55590     55600

Human                    TTTTATACC-ATGATCATGGATTCCTATTTTAGAAAACAT-TTTATTATTT--GAAGCAGATGTTAGTGTTTTTCTAAGT 55104
Kakapo                   C---ATCCC----CTCGAGGCAATGAAAGTTCAAGA--------ACTGTGT--GAAATGCA-AATGATA----------- 24075
GoldenEagle              TTTGTTCTTTAAACTTACTGCAGCAAAAGTTGAAAA--------ACTGT----AAAATGTA-GATATTACATAGCAATGT 27866
JapaneseQuail            ----TTTCTAGAACAAATAGAAAAAAGTCTTAGAAA--------ATCTTCC--GTGGTATA-AGAACCT---------G- 22733
MediumGroundFinch        C---TTCTTTAAACTTACTGCAGCAAAAGTTGAAAA--------ACTGTG---AAAATGCC-AGTATTA-GTAGCAATGC 11045
GoodesThornscrubTortoise CTGCATTGTGCAGGAGGTTGGACTAGACGTTCATAATGGTCTCTGCTGTCCTTAAAGTCTATGATTCTATTATATGTATT 50581

Majority                 TA--ATAGATTCA-AAATCAATT--ATATTGATGTAATACAATAATTATTGTTT-T-AAAGAAACATTA----A-CTTTG

                                55610     55620     55630     55640     55650     55660     55670     55680

Human                    TGTGGTAGTCTATGCTTGTTATTATAGATAAATACAGTAAGAACATGTTCACTAATGAAGAAGATAATATTGCAATGAGC 55184
Kakapo                   --------------AAACCAGTT----ATTCAGGTAATACAATAATTATT-------AAAGGAACATTT------CTTTG 24124
GoldenEagle              TA--ATAGATGGA-AAACCAATT---TATTCAGGTAATACAATAATTATT-------AAAGGAACATTT------CTTTG 27927
JapaneseQuail            ------------G-AAAGCAGAA--ACACTGAACTAGAACAGGAAGTTTAGCTG-T-TAGGAAAAGATG----------- 22785
MediumGroundFinch        TA--ATAGATGCA-AAATCAATTGGGTAATACAGTAATTCATAAATTATTGTGT-T-AAATAATTATTAGAGAAACATTT 11120
GoodesThornscrubTortoise TA--ATAGAATCACTTATTAATCATATATTGGTTTAATATGAGCACAATTTTTT-CCAAACAAACAATAAA--ACTTAAC 50656
```

```
Majority                   CAAATACA-TATATATGAGTAATTATATA-CTGGCTAGAGATTCTTTTATTAG--------TAACA--CTGGGATTACCT
                                   55690     55700     55710     55720     55730     55740     55750     55760
Human                      TACTTAAAATATTTAAATACATTTAAACATTTGAATAGATACTGCTCTATAAAACTAATTTTACCAATTTACAATTATTT 55264
Kakapo                     CAAATACA-TATATTGGAGTGATTCTAT--CTGGCTAGGGATGCTTTTATTAG--------TAACA--CTGGGATTACCT 24191
GoldenEagle                CAAATACA-TATATGTGAGTGATTGTATA-CTGGCCAGTGATACTTTTACTAG--------TAACA--TAGGGATTACCT 27995
JapaneseQuail              TAAAAGAG-TG-GTAGTAGTAATT-TGCG-ATGGACAGATCTTTTCTTTTTCC--------TTTC---CTATCACTTCCA 22850
MediumGroundFinch          AATATACA-TATATGTGAGTAATTATATA-CTGGCCAGTGATACCTTTACTAG--------AAATG--CTGGGATTACCT 11188
GoodesThornscrubTortoiseCAGTTGTG-AAAACATCCACTATTACTCT-CCTGTTTTAAGCTCTTTAATTAGAGCTTATTTAGTC--ATGAGATTTGTT 50732

Majority                   GAAGCCTAATCCAAATAAAATCTTTAGTTTAATAAAAACT----CAAATTCTTGGACATTATGCTGTGGAA---AAGTTA
                                   55770     55780     55790     55800     55810     55820     55830     55840
Human                      TTTCAATAATAGTATCAAGATGTCTACTAAAATAATCTAT----CACATTTCAAAATGTTGAGTTTTAATTAGTAAGTCA 55340
Kakapo                     AAAGCCTAATCCAAATACAAGCTGTAGTTGAACAAAACCCCCCTCAAATTCCTGGACCTTATGCTGTGGAA---AAGTTA 24268
GoldenEagle                AAGGCCTAATCCAAATACAAGCTTTAGTTTAAAAAAAA----------TTCTTGGACATTATGCTGTGGAA---AAATTA 28062
JapaneseQuail              GTAGTGAAAACACAGGAAATTACTGTGTTTACTAAAAATGGG---AACTATTTGTAGCTTTGGCCAGAGAACTGGAGTTT 22927
MediumGroundFinch          GAAGCCTAATTTAAATAAGAACTTTAGTGTAACAAAAAGT-----AAATTCCTGGACATTGTACTGTGGAA---ACGTTA 11260
GoodesThornscrubTortoiseCCTGGGAAGTACAGAAAAATAGTTCTTATTAAGAACCACTGA--CATTCAATGAAAAATAACACTGATGTG---GCTTTC 50807

Majority                   ATTTGTCAAA---AT-TATATGTTGAAAAGCATACGAAAGATTAAACT-GTTCTGTAAATATAGATCTGAGC-ATCATGG
                                   55850     55860     55870     55880     55890     55900     55910     55920
Human                      TTTTGACTAATACATAAATTTATAGATAAAATTAAGTAGCATTTAAATGATTGTTTAGATATATATCTGGACTCTCAAGC 55420
Kakapo                     ATCTGTCAAA---AT-GATATGTTGAAAACCACACAAAAGATAATACA-GCTCCGTAA-----GAACTGATC--TCATGG 24336
GoldenEagle                ATAGGTCAAA---AT-TGTATGTTGAAAACCACACAAAAGTCAAAACA-GCTCTGTAA-----GATCTGAGCATTCGTGG 28132
JapaneseQuail              GCCTGTTTGG---ATAGAAATGTTGGGATGTTTGTGAAATATGTTACTTGGTCTGGCTGTTTGGATCCAGAA--TTGAAA 23002
MediumGroundFinch          ATATGTTAAA---AT-TGTTTGTTGAAAAGCACACAAAAGACGAAACA-GTTCTGTCCATACTGATCTGAGCCATCATGG 11335
GoodesThornscrubTortoiseTTTAAACACA---AATAAAATGTGAAAATTCATATGTAGATGTACAGT-GTTTTAGAAACCTAAAAGGGCCTGATACATG 50883

Majority                   TATATACTTTTCTGGTGATGTAATA------AAAACAGAATTTTCTTATCTGTTTCAGAGAATTTTTTAGTTTAAATGCT
                                   55930     55940     55950     55960     55970     55980     55990     56000
Human                      CGTGTGATCTG-TGGTTATATAATATTTTAGTAAAATGACTTTTCTTGTTTATTCTCCTGAGTGTCCTAATTCTTTTGTA 55499
Kakapo                     TACATACTTTTCTGGTGACGTAGTA------AAAACAGAGGTTTCTTACCAGTTTCAGAGAATCTTTTAGTTTAAAGCCT 24410
GoldenEagle                TATATACTTTTCTGGTGATGTAATA------AAAACAGAAGTTTCTTTCCAGTTTCAGAAAATCTTTTAGTTTAAAAGCT 28206
JapaneseQuail              CATGGATATTCTTGGCT-TCTAAAA------ATGAATTGATTCCATTCTCTGTTTTTAACATTGACTGAGTTGTCATGGT 23075
MediumGroundFinch          TGAATATTTTTCTGGTGATGTAA--------AAAAAAGAGGCTTCTT---AATTTCAGAAAATTCTTTAGTTTGAAAACT 11404
GoodesThornscrubTortoiseCATCAACTCCATTTGGAATTAGAAA------AATACGTAATTCTTTGAGTTGATACAGCCATGTATTCCACTTAAATGTG 50957
```

```
Majority                 T----TTTCTCTTTCTCATTTCTGATTGTTAATTTTTCATATTTAAA-T--TTGAAGTTTGTC----ATGAATTTAAATT
                                  56010     56020     56030     56040     56050     56060     56070     56080
Human                    T----TCTCTTTCATTAACCTGTTGATATAGATTTTTCAAACTGTAA--A-CCTATGTATAGGTATTTGTGGTATTTGTC 55572
Kakapo                   TGATTTTTCACTTTCTCATTTCTCATTGTTAACGTTTCATATTTAGCTT--TTGAAACCTGT-----ATGAATTCAGGTT 24483
GoldenEagle              TTAT-TTTTGCTTTCTCATTTCTCATTGTTAATGTTTCATATTTAATTT--TTGAAATTTGTA----ATGAATTCAAATT 28279
JapaneseQuail            G----TA-GGCAAACTGAGTTAAGAGCTTAAATTCTTAATTTTAA------CTTAGGTATACC----TTTAGTATAACTT 23140
MediumGroundFinch        T----TTTCTCGTTTTCATTTCTGATTGGTAATGTTTCATATTT----T--TTGAAACTTT------ATGAATTGAAATT 11468
GoodesThornscrubTortoise TG---CACCCTTAGCACATCTGAGCTGGAGAATTTTGCCTAGCCGTACTGGTAGAGGAGCGGCGCTCGTGTCTTGTGATC 51034

Majority                 ATTGATAAAGCT----TAAGTATGATA-GGCATTGGAT---ATCTTGTTTCAGTATCACTATAATTCTTTTATTCT---T
                                  56090     56100     56110     56120     56130     56140     56150     56160
Human                    ATTGTTATATTT----T-AGAACTATTTGCCTTTGTATGTTTTTTGACCACAGATAAGGTAGAAAGAAATTATTTCCTTA 55647
Kakapo                   ATTTAGAAAGCT----GAAGTATGACA-GGCATTGGAA---ATCTAGTTCCAGTATCACTGTAACTCTTTTGTTCT---T 24552
GoldenEagle              ATTTAGAAAGCT----TAAGTATGAAA-GGCATTGGAA---ATCTGGTTTCAGGATCACTGTAATACTTCCATTCT---T 28348
JapaneseQuail            ACTGTTCTCAAT----TTAGTCACATA-TGAGTGAAAT---ATTTTTATGCAA-GTGATCAGATTAGGTCTAAATA---A 23208
MediumGroundFinch        ATTCAGAAAACT----GAAGTATGAAG-GGCATTGGAC---ATGTAGTCTCAGTATCGCCATAATTCTTTTATCCT---T 11537
GoodesThornscrubTortoise ATAGCTGCTGCTCCTGGCTGTATGAGGCGGCATCACCCC--ATCCTCCCTCAGTTCCTTCTTACTGCTCATGGCCTGAGT 51112

Majority                 TGCAAGTTTGTTTG-----TAATGTGATATCTGTAATAC--ATTGTTGAAGCCTGTT---GTACTTTACAATGTAT---T
                                  56170     56180     56190     56200     56210     56220     56230     56240
Human                    TCATAGACTGTCATCATAATAACAGGAAAGGGATAATAGTAATAAAGGCAGTCTTTTGGAGCATTTAACATAATATTTGA 55727
Kakapo                   TGCAAGTTTGTTTG-----TAATGTGTTGCCTCTAATAC--ATTGTTAA-GCCTGAC---AAACTTTGCAATGTAT---- 24617
GoldenEagle              TGCAAGTTTGTTTG-----AAATGTGCTATTTGTAATAC--ATTTTTGAAGCCTGTC---ATACTTTACAATGTTT---- 28414
JapaneseQuail            TTTACCTGGACATA-----AAATCTG--AGCTGTATTCT--GCAGTTACAGCTGAGTG--ATAATTGAAAATTTCT---T 23274
MediumGroundFinch        TGCAAGTTTGTTTT-----TAACATG---TTTGTAATGC--ATTGTCCA-GCCTATT---GTACTTTGCCTTGTTTCTTC 11603
GoodesThornscrubTortoise TGTCGCTGTGTGTGGTTCCTAACTTCACAACCTCTCTCT--TTCTTAGTGACCTCTCTA-GGAGTATATAGTTAATTAGT 51189

Majority                 TCTTCTGGTTTTTT-TTAATTTTGTAGAGATTTAATTTGAGAACTAGGTAA----GGCTGTT-GTGTGATGAGCTTCA--
                                  56250     56260     56270     56280     56290     56300     56310     56320
Human                    TTGACAGATTTCCTACAAATACTGTGTTTATTCAATTTTATGGGTATATTTTTTCGCATTTTAAAATCTCAGAAATTTGA 55807
Kakapo                   TCTTCTAGTTTTTT---G-----GCAGAGATTTAAATTGAGAACTAGGGAA----GACTGTT-GTGTGATGAGCCTCA-- 24682
GoldenEagle              TCTTCTAGTGTTTT---------GTAGAGATTTGAATTGAGATCTAGGGAA----GACTATT-GTGTAATGAGCTTCA-- 28478
JapaneseQuail            TATTCCC-TTCTCTCTGGAAATCACAGCCATTGGCTTTCCTATGAAGGTGA----GGCTTTT-GGAGGATTTAATACA-- 23346
MediumGroundFinch        TCTTCGGGTTTTTTTTTATTTTTGTAGAGATTTAAATTGAGAACTAGAGAA----GGCTGTT-GTGTGATGAGCTTCA-- 11676
GoodesThornscrubTortoise ACCCTTGGTATAGTGTTAGTTTTAGTTAGCTTTAGTTTAAGTACTGTATAT----ACTTGTTCATAAGCTGGATTTTTTT 51265
```

```
Majority                 GGTGAAAAATAAAAGTATTTTTTTC-TATATCGTTTGGCTATCTGTCACTTTGAGGATAAAGTGGTAT----------TA
                                   56330     56340     56350     56360     56370     56380     56390     56400
Human                    TGTGTCCTGCAATAGTCTATCTTCCATAGTTCAATTGGCAACATCTTATTAGTCATACATAAAACAATAG------TGCA 55881
Kakapo                   GGTGAAAAAGAAAAGGATGTTTT-------------------CTGT--CTTTGAGGATTAAGTGCTC-----------TA 24730
GoldenEagle              GGTGAAAAATAAAAGTACTTTTT---CATAAACTTTGGCTGTCTGTCACTTTGAGGATAAAGTGGTA-----------TA 28544
JapaneseQuail            AATTCTCACTATTGATTTTTTATTTTTTT----TTTATTTTTTTTCCCTCTTCCTCTTATTTCTTCT----------GA 23412
MediumGroundFinch        GGTGAAAAGTGAAAGTACTTTTTTCCCACAAAGTTTGGCTCTCTGTCTCTTTGAG-ATAGTGTTATAA----------TA 11745
GoodesThornscrubTortoise AGTAAAAAAGGAAAGCATCAGAGAAGGGGGTCGGCTTGTGAACGGCTATAGAGAGGGAGAAGTGGGACACAGCCCCTCTG 51345

Majority                 TTTTGTAATTT----TGAATAGGTGAAGTCTGGCTGCTTGGAAGCTCA--GTCTCACCATGTTTTCCAAACTGG-TACGG
                                   56410     56420     56430     56440     56450     56460     56470     56480
Human                    TTTTATAATTAATGGTGTATTTGATACCATAAAATGTATGGGAAATATTCTTCATATTATATCTTGAAGTTTACCTGAGA 55961
Kakapo                   TTGTGTAATTT----TG----------------CTACTTGGAAGCCCACAGTCTCACCATGTTTACCAAAGGGA-TAGAG 24789
GoldenEagle              TTATGCAACTT----TGAACAGGTGAAATCTGGTTACCTGGAAGCCCA--GTCTCGCCACGTTTTCCAAAATGA-TACAG 28617
JapaneseQuail            TTTTTTAGTTAG---TAAATAAAGTTAGTC---CTGTTAATAAATTTA---T-TTTTAATCCTCATCAACC----CATGA 23478
MediumGroundFinch        ATGTATAATTT----TGAGCAAGTGAAACCTGGCTGCCTGGAAGCTCA--ATCTCACCATGTTTGCCAAAATGG-TACGG 11818
GoodesThornscrubTortoise CTCCCCAATAGGAG-CAAGGAGAGGCAGCACAGCCAGAAGGGAAGCAGCGAGGCCAGAATCTCTCCCCTTCTGGCCACGC 51424

Majority                 -GTTAGAGCCCTGTGTATACGTTACAG-TGGACTTCAGATCCTAG---TTCAGTGTGTGGTTTTGAATTTTTAGTCTTCT
                                   56490     56500     56510     56520     56530     56540     56550     56560
Human                    ATTTTGTGGTTTTGGACTAGACTACACGTTGGTTTCTATTCTAAAAATTTTGGTGTCAGAAGTTAAGTGTAGTATGAAAA 56041
Kakapo                   -GTTAGAGCCTTGGGTAGAC-TGACAG-TGGACTTCAGATCCTAC---TTCACACTGTGGTTTTTGACATTT--TCTTCT 24861
GoldenEagle              -GTTAGAGCCCTGTGTAGACGTAAGGA-TGGACTTCAGATACTAG---TTCAGTGTGTATTTTTGAAGTTTTAGTCATCT 28692
JapaneseQuail            -ATTTGAACCCTAATAATAAGATATAT-ATTTCTTCTAGTCACAG---GACTGTTTTTAGTTTT----TTTTAGAAGAGT 23549
MediumGroundFinch        -GTTAGAGTCCTGTGTAGAC-------------TTCAGATCCTAG---GTCAGTGTGTATTTC--AAATTTTACTCTTCT 11879
GoodesThornscrubTortoise TGCTCTTCCCCTGAGTGTCTGAAGCAGCTGCAGCTCCGGGGCTGG---CTTAGGCTGTGGCCACGTCTCCTGGCCTGGCC 51501

Majority                 GTGTTCCACCAGAATTTGTCTTTCTGTGCAGCAGACCAAAATAAAATACGTACTATTGCATGGATTT--CCACTTCTAGC
                                   56570     56580     56590     56600     56610     56620     56630     56640
Human                    ATGTATTTTCTGAATGTTCCTATTTTAAGATTGTATTAGAA-ATAATAAGCTTTATGGGAGTTCTTTTGGATAATTCAGT 56120
Kakapo                   GTGTTCCATCACAATTTGTCCTTCTGTGCAGCAGACCAACATAAAATACATACTCTTGCATGGATTTGACCCCTTCTAGC 24941
GoldenEagle              GTGTTCCACCAAAATTTGTCCTTCTGTGCAGCAGACCAAAATAAAATACATACTATTGCATAGATTCAACCACTTCTAGC 28772
JapaneseQuail            AAGTAGTTGTTGAGTGTATTTTCCAAAGATGAAATGAAAGATTGAATA--TATCACTGCATATTTT-----ATGGCCAGA 23622
MediumGroundFinch        GTGTTCCACCAAAATTTGTCTTTCTCTGCAGCAGACCAAAATAAAACACCTACTATTGCATGGAT---------TCTAGC 11950
GoodesThornscrubTortoise CGGCTCAGCCCGGCTGTGCTGCCCAGCCTGCCAGAGCAGCTCCAAGCA-GGCCAGAGACATCCTTCCC-TGGCCCATCCC 51579
```

```
Majority                   ATTGTCA--GTTGGGAGTTACTGCTAGCTGGATTTTGACCGGTCCTGATGTGTTTAGGTTTTTGTCGTT--TTGTCTGCT
                                  56650     56660     56670     56680     56690     56700     56710     56720
Human                      AACCCCCTTGTTGAGATTTACAACGTACCTGTTTTATGGAGTGATTAA--TGTTAAAGTTGTATTCTCTAAATTTCTGTT 56198
Kakapo                     ATTGGCA--GTTGGAGGTAACTGCTAGCTGGATTTTGACCCTTTCCCATGTAGTTAGGTTTGGGTTGTT--TTGGCTGCC 25017
GoldenEagle                ATTGTCA--GTTGG-AGATGCTGCTAGCTGAATTTTGACCAGTTCTGATGTCATTAGGTTTTTGTTGTT--TTGTCTGCT 28847
JapaneseQuail              AATACAA--AAAAGGAAGTCTTAAT-GCTTGTTTTCGTCATATCCAAATGTGTTCTGATTTTCAACATTAATTCATTAGT 23699
MediumGroundFinch          ATTGTCA--GTTGGGAGATACTGCTAGCTGAATTTTAACCAGTCTTGA-ATGTTGAGGTTTTTATAGTT--TTGCCTGCT 12025
GoodesThornscrubTortoiseAGCTAAG--GTGGGAAGGAATGGGATGGGGAGAATGTGGGGGTCCTGG---GCTAGGGGTGGGGTCATT--TTGGGGGGT 51652

Majority                   GTAGTAAGTTGGTAAAAGACTTAGAATGTGTTCTTCAGTGTAAGGACTTTTTAAAAAG----T---T-TCTCAGTCC--T
                                  56730     56740     56750     56760     56770     56780     56790     56800
Human                      TAAAACATTTTCTGATGGTACATGTATTTATAAAACTACATCTTGTTCTTTTGAGAATAAATTACATATCATACCTTTGC 56278
Kakapo                     ATTGGAAGTTGG-AAAAGACTTAGAAAGTGTTCTGCAGTGCAAGGACTTTTTAAAAAG-----------CTCAGTTC--T 25083
GoldenEagle                GTAGTAAGTTGG-AAAAGACTTACAAAGTGTTCTGCAGTGTAAGAACTCTTTAAAAAG---CTCAGTCCTTCAGTCC--T 28921
JapaneseQuail              GTCCTTGTCTCTTAAGACAGT-----TCTGTGCTTTAGCTTTCAGTCCTTTATGTTA-----------CTTCAGT----- 23758
MediumGroundFinch          GTAGTAAGGTGG-AAAACACT----GTGTGTTCTTCAGTGCAAGAACTTTTTAAAAG------------CTTAGTCA--T 12086
GoodesThornscrubTortoiseCGTCACAGGGCTTACTCCCCTGACCCCCAGCTCCCCCCCCCCCAAACTTTCCCACCAG---TT-GCTGTCCCAGCCCATC 51728

Majority                   GGATTTATTTTTT--TTGGGGGGGTGG-TAGTTTTTTTGCATGACCTGCCTTTAAGGCATACTGTT-TTT-AGCTAAGAG
                                  56810     56820     56830     56840     56850     56860     56870     56880
Human                      ATGTTCAACATTGATCCCAAAGCCTGGATAATTTTTTTGAATGAAATACTAGTAGAATATTTGATTTATTAAACAGGTAG 56358
Kakapo                     GGAATCAATTTTT--TTGAGGGGGGAG-AGGAGTGAAGGGATGACCTGCCTCTAAGGCATACTGGT-TTT-AACTAAGAG 25158
GoldenEagle                GGATTTTTTTATTACTTGGGGGGCAGG-TAGAGTTAAGGCATGACCTGCCTTTAAGGCATACTTTT-TTTTAGCGAAGAG 28999
JapaneseQuail              -GACATATTTA-----TGGGCA----------------------------------A--------------------- 23775
MediumGroundFinch          GGAAATTTTT-----TTGGAGG---------------------------------------------------------- 12103
GoodesThornscrubTortoiseAGGGTAAGCTGCTGGTGGGCTGGGTGA-CACTTTGTTTACTTAGGTTTACCTCCATGCCTGCGGACACTTGAGGTAAACA 51807

Majority                   AA--TAGCTTGAGTTT-ACGTTTAGCTATATCTATTGCTGGATAGCTGAAGTAGA-GGTAGAACCATTCAAATTAGA---
                                  56890     56900     56910     56920     56930     56940     56950     56960
Human                      TACCTGGCTCATGGCAGGTGCTTAAAAATATCTGTTGAGGGGCCATTTAATTTT--GTTTAATCTGTGTAAGTTCTAGGA 56436
Kakapo                     AA--TAGCTTGAATTT-ACATTTAGCTAAATCTAA----AGATAGCTGAAGCAGAAGGTAGAAACATTCAAATTAGA--- 25228
GoldenEagle                AA--TATCTTGAATTTTAGGTTTAGCTAAATCTAAAGATGGATAGCTGAAGTGGAAGGTAGAAACATTCAAATTAGAATG 29077
JapaneseQuail              --------------T-----------AGTTTTATTACCATATAGCTGAAGTA---AGT---------------A----- 23806
MediumGroundFinch          ----------------------------------AG-GGTTGATGGTAGTGGTACAGGGT-------------------- 12128
GoodesThornscrubTortoiseAA--CTGGCTCGGCCTGCCAGAGGCTTATCCTGATGGCCCAGGAGCCAAAGTT---TGCCAACCCCTGAATTATAGGGTC 51882
```

```
Majority                ----GGTTTAGCTGTTATAAAAAGAT-TACGTGATTTATGGAAATATTT--TATGATGTTTAGCT--ATCTTTTTTCCAT
                                56970     56980     56990     57000     57010     57020     57030     57040
Human                   ACAAAGTGAACTAGTAGTGTAACAGGTTGCAAAGTATGTGACAATAATGGGTATGTTTCTAAACTACATAATCTTAATTT 56516
Kakapo                  ----GGTTTAGCTGTTAGAAAAAGCTAGAAGGGAGTGGTGGAAACAGTT--CCTAATGTATAGATGTATCCTTCTCCCAT 25302
GoldenEagle             AAGAGGTTTAGCTGTTAGAAAAAGATATACGTGAGTGATGGAAACAGTT--CCTGATGTGTAGATTTATCCTTTTTCCAT 29155
JapaneseQuail           ------------------------------AGTATTTCTTAACATATTT--TTCAAAGCAGAACT---TCTTCCATCCAG 23851
MediumGroundFinch       ------------------------------GTGGGT-ATGGAGTTACTA--CATGATGT--A------------------ 12155
GoodesThornscrubTortoise CGCTTATGAATAGGTCATAAAAAAAT-TTCAATTTTTACTTATCCATCT--TAGGGGGTTCAGCT-----TATAAACAAA 51954

Majority                CCGTCCCTTTAATGGATATATTCAGGATGTCTTTGTTTTTGAAAAAGT-AAAACTT------GTGTTC-CA------G--
                                57050     57060     57070     57080     57090     57100     57110     57120
Human                   CCAGCGTGCTCAGGCAGTTCTTCAAGCTGTGACAGCTGTCCAGACAGCAAATACTCCTCTTAGTGGCACCACAGTTAGCG 56596
Kakapo                  TAATTCCTTTAATGGAAGTATGGAGAATGACTTTATTCTTGAAAAAGT-AAGACAT------ATGTTCACAGGTTTAGTC 25375
GoldenEagle             CACTTCCTTTAATGGAAATATGGAGAATGACCTTATTCTTGAAAAAGT-AAAAAGT------GGGTTC------------ 29216
JapaneseQuail           CTGTCTCTTTATTTCATATGTTCTGGCCTGAGTTCTTTTTT----TGT-GAATTTT------A----------------- 23903
MediumGroundFinch       -----CCTCTAA--GACATAC------------TGTTTTT---------AA----------------------------- 12178
GoodesThornscrubTortoise CCGGCTTATGATTGAGTACATACGGCATTTCTTTGGTGATGCCTTCGCCAAAACAC------TGTCCATCATGTGAGGGG 52028

Majority                A-A---C---------------TTGCTAATTGCTTTGAATAGTTAGTTAAATGTCATACCT-GGTTTGGCTTTTGATGTT
                                57130     57140     57150     57160     57170     57180     57190     57200
Human                   AGAGTGCAGTGACTCCAGCCCAGAGTCCAGTACTTAGAATAATTATTGACAACATGTACTACCCTGTAACACTTGATGTT 56676
Kakapo                  AGAGACCCCGAGCTTGCCAATCTTCCTAATTGCCTTGAAAAGGTACTTAAATGTCATACCT-GGTTTGGCTTTTCATATC 25454
GoldenEagle             ----------------------TTCCTAATAGCATTGAAAAGGTAGTTAAATGTCATACCT-GGTTTGGCTTTTCAAGTC 29273
JapaneseQuail           -----------------------TTGGAATTCTGTGCAAGGAATTCTGGAA------A-----------------ACATT 23937
MediumGroundFinch       -------------------------CTAAT----------AG-------------------------------------- 12185
GoodesThornscrubTortoise ACAATTCCCAACAACAGTCCCCATGTACAGTACTTGCTGTGCTTAGGTGAAGCCCACGTCAAGGATCGGTGTTTGATTTT 52108

Majority                CAAATC---TGTAGTGTGGATGTTCTTGA---CATGTAAAATTG--AGTCTATTTCATTGTGTGTTTTTAAT--TTTGAT
                                57210     57220     57230     57240     57250     57260     57270     57280
Human                   CTTCAC-CAAGTAAGTTTAATCTGCATAATTACCTATAAATTAGAGAAATAATAATATAGTAAATAAGTAATGTTTTGAT 56755
Kakapo                  CACATC---TGGAGTGTGGATGTTCTTGA---CTTGTAAAAATG--AGTCCATTTCGTTGTGTGTTTTTAAT--CTAGAG 25524
GoldenEagle             CAAATC---TGGAGTGTGGATGTTCTTGA---CATGTAAAAAAACCAGTCCATTTCATTCTGTGTTTTTAAT--CTTGAG 29345
JapaneseQuail           CAAAAC---TGTCTCGTGGAGCTTTGTAC---GATATGA----------TTTTTT-----TATATTATTATT--TTTA-- 23992
MediumGroundFinch       ------------------GA------T-----------------------------------ATT--------------- 12191
GoodesThornscrubTortoise CAAATCATTCACAAGGTGGACTCAGGTGA---CATGGGAGCTTT---GTCTAAAGCAGCACCTGCTCAAACA--GGCAGT 52180
```

```
Majority                 TAAGTTGGCTTGGCTTATACTAACTGAGTTA--AACTTGAATTC----T-A-----T-----T------A--------TT
                                57290     57300     57310     57320     57330     57340     57350     57360
Human                    TTTAATGTTTTAACAGATATTTTCTAAGTTTGGTGCT-GTATTGAAGATAATCACATT----TACAAAAAATAACCAGTT 56830
Kakapo                   TAAGTTGGGTTGGCTTAGACAAACGGAGTTAAGAACTTGAATTC----T-----------------------------TT 25571
GoldenEagle              TAAGTTGGCTTGGTTTAGGCAAACTGAGTTAAAAACTTGAATTCTGGATTATGACTTAGGCATACCCTTAGCAAAACTTT 29425
JapaneseQuail            ---------TTTCCTTGTTCTAGTAGAAATA----CTTG---------------------------------------TT 24020
MediumGroundFinch        -----------------------TTGAAT-----------------------------------------------------T 12198
GoodesThornscrubTortoiseCCAGCTTGCTTCGGAACCAATGACTGTGTTA--GATCAGAGGTTGCTCTCAGGTTCTGAGAGTTTTATCACTTCCTGGTC 52258

Majority                 TTGTGCTTAAGTCCGTC---ATAAATGAATGCATTCTTTTTA--TGCATGTAGCGAGA--TTAGGTTGAGATGGT-TTTT
                                57370     57380     57390     57400     57410     57420     57430     57440
Human                    TCAAGCTTTGCTCCAGTATGGTGATCCAGTAAATGCTCAACAAGCAAAACTAGTAAGTCTTTCTTTTGAGATGGTGATTT 56910
Kakapo                   CTATACTCAAGTCAGTC---AGAAATGAATGCAGTATTTTTA--CACAGGGAACGAGA--TTAGGTAGAAATGAC-TTTT 25643
GoldenEagle              CTGTGCTCAAGTCCGTC---ATAAATGAATGCAATGTTTTTA--TGCAGGGAATCAGA--TTAGGTGTAAATGG--TTTT 29496
JapaneseQuail            TTGCTCTTCCCTCCCTG---TTAACTGCATG-GTCTTTCTTA--TGGATAAGCCCA-----TGGTTCATCATGGT-TCTT 24088
MediumGroundFinch        TTATATT----------------------------------------------------------T--AGAT-------- 12210
GoodesThornscrubTortoiseTGGAGCTGGTACCTTTCCAAAGAGATGGAGGAGTCCCTCCCCATTGAGTGTGGCGAGG--AAAAGGTCACATGAG-ACTG 52335

Majority                 CTTGGCATTAATTCAG----AGTGGAATTCTGTATTTACATCTGAGT-ATAATTGAGGACTTCTTGATTCTCTTCACTCT
                                57450     57460     57470     57480     57490     57500     57510     57520
Human                    TTTTTTATTGAAAT-GTATATGTAGAAAAATATATATATATGTATGT-ATACTTAATGATATCTTGTAGCATTACAGATA 56988
Kakapo                   CCTGGCACCAATTCAG----AACGACATTCTGTAGTTATAGCTGAGTGAGAACTGAAGACTTCTTCATTCACTTCTCTCT 25719
GoldenEagle              CCTGGCACAAATTCAG----AATTATATTCTGTGGTTACAGCTGAGTGATAATTGAGGACTTATTTATTCTCTTCCCTGT 29572
JapaneseQuail            CTTAGCATTAGT------------GACTTCTGTATTTGCATC--------AATAAA-----------TTTTCTT------ 24131
MediumGroundFinch        ---------A--------------------------------------AAT-------------------------- 12214
GoodesThornscrubTortoiseATTAAGGTTGCTCCAGGTTGAGTGGGGTCTTGTCTTTCCAAA-GAGACGCAGCCTGGTACAGTGTGAATCCTGGCATGCA 52414

Majority                 GGAAA---CAGCCATTAGCTTTCTTAT-A----A-C--T----G--GACTTAACACCTGTACTT-CTTCCTATTGATTCC
                                57530     57540     57550     57560     57570     57580     57590     57600
Human                    GTAGA----AATTGATGGCATTAATATTAAGA-AACCTTAATATTTGTCATAAAAGAAATATTTATTTTTAGGAAAATCA 57063
Kakapo                   GCAAATTGCAGCCATCAACTTTCTTACAA--------------G--GATTTAACACCCGTAAATGCTTCCTATTGATTTC 25783
GoldenEagle              GGAAATCACAGCCATCAGCTTTCTTATGAAGACAACGCTTTTGGAGGACTAAACATACAGAAATGCTTCCTATTGATTCC 29652
JapaneseQuail            --------------TTAACTTTTTTA---------------------CTTTGTGCCTG-GCG--CT----AGTAGTAT- 24167
MediumGroundFinch        ---------------------------------------------CTAA---------------------------- 12218
GoodesThornscrubTortoiseGGAAAGTGCAGGATCTGTCAACCACACCCTAGTACCATTCAGGGAGGATGGAGATCCTGTACTG--TTGACCCTGGTTCC 52492
```

```
Majority                    TCCCCCTCCTTC---C---------TTT-T-TGTTTCTACTTTTTTATTTAGTATATAATAGTAGTCCTG--TTC-GTAG
                                     57610     57620     57630     57640     57650     57660     57670     57680
Human                       TAGTTGTAGTTAACCATGATGTGGGTTTCTATTATTTACTTTTTTTTTTTTTTTTTAATTTAAGGACAAAAGTTTGTAC 57143
Kakapo                      TCCCCTTCCCCC---C---------TTTGT-TGTTTCTGCTTTTTTAGTGAGTAAATCAAAGTAGTCCTG--TACTATTT 25848
GoldenEagle                 TCCCCCCCCCCCCCCCCCCCCCCCCTTTAT-TGTTTCTACTTTTTTAGTGAGTAAACAAAGTAGTCTTG--TTC-ATAA 29728
JapaneseQuail               -----------------------------T-TGTAGTTACTTTATT---------------------------CC-GTTG 24189
MediumGroundFinch           --------------------------------------ACTTTTTT---------------------------------- 12226
GoodesThornscrubTortoise    CACCCCTGCTTCTTGATCGCAGACGT---T-CCACTCTACATCTTGCTCTAAGATGAAATCACTGGCCCA--GGCACCAG 52566

Majority                    TTTTCTTTTTTTATTAATCCTCTTTGGTATAAGCCCTCAAGCCCAGGATTTGAACTCTGATAAT---------TTTTCTT
                                     57690     57700     57710     57720     57730     57740     57750     57760
Human                       TCTTAAGAAAAAAGCAGGACTATATTGTGAATGTGCACCAGCATAAGATTTGAAGT--GGCTTTTGCTGGGAATTTCAAA 57221
Kakapo                      CTTTCTTTCTTTATTAATCCTCTTTGGTAGAAGCTCTCAACCCCAGAATTTGAACCCTAATAATGACAAATCTTTTTCTC 25928
GoldenEagle                 TTTTTTTTTTTTTTAAATCCTCTTTGGTATAAGCCTTCAACCCATGGATTTGCACTCTAATAAT---------TTTTCTA 29799
JapaneseQuail               CTGTCTGTTTCCATT-------CAAGATAAAAAC----AAAGCAAAAAACAGAACTA-G--------------TTTACCT 24243
MediumGroundFinch           ----------------------TTTGGTAGAAGA---------------------------------------------- 12238
GoodesThornscrubTortoise    TGAACCTTTCCCTTGAAGAGCATCCAAAGTTGTCCCATCAGTCCTGGAGTAGCA-TCTGTCACAGTTAACCATTTTATCT 52645

Majority                    CTAGTCACAGTTGGTGTG--TTTTTTTGGAA-T--TATACAGTTGTTGTTCTATTGTTCTAAGATGTAATCAGATGTTTT
                                     57770     57780     57790     57800     57810     57820     57830     57840
Human                       GTAATTAGTTTTCAAAAGAGTTTGGCTGAAA----TATACAGATGTGGCTCAAATGCTATGATAGGCTCTCAGTTTATTT 57297
Kakapo                      CTAGTCACAGTTGGTGTGGGTTGTTTTGGAAATA-TATGGAGCTATTTGAGTGCTCTTCTAACCTGTAATCAGAGGTTGA 26007
GoldenEagle                 CTAGTCACAATTTGTGTG--TTTTTTTGGAAATA-CAGATAGCTGTTGAACTGTTCTTCTAACATGAAATCAGAAGTTGA 29876
JapaneseQuail               CTAACTAC---------------------------TTTACAGTTCTTATGCAATCGTTCTGAGTCTTAATTAG----TA- 24291
MediumGroundFinch           -------------------------------------------------------------------------------- 12238
GoodesThornscrubTortoise    TCATCCCCAGATGATTCCATTGTGCCTGGCTCTTCCACTCCTTTGGTATTTGAGTATTTCAAGGCATACCAGGATCTTTT 52725

Majority                    GTAGCTCATACACTGCCTTTCATGTTTTTTAGCCAGAAGTGGTATAAA-GGAAGTCTTATCACTTGTTTCAC---TTATA
                                     57850     57860     57870     57880     57890     57900     57910     57920
Human                       AAAACT-ATATATTCTCTTTAATGTCTTTTTTTTAAAGGTTTTATGAATAGGTTTCTAGTCACTTTTTATTCA--TATCA 57374
Kakapo                      GTAGCTCATGCACTGGCTTACATGTTTTATAGCCAGAAGTGGAAAAAG-GGAAGTCTTAACACTTGCTTCTC---TCATA 26083
GoldenEagle                 ATAGCTCACACACTGACTTGCATGTTTTATAGCCAGAAGTAGAACAAA-GGAAGTCTTAACACTTGCTTCGC---TTGTA 29952
JapaneseQuail               ----TTCTTACACAGCTTTACATGATACGTCACAGAAATTGAGGTGGA-AAAAAATTGACTGTGCTTTTCATCT-TGATG 24365
MediumGroundFinch           -----------A----------------------------AG-------GGAAGTCTTATCACTTGTTTCAC---TAATG 12269
GoodesThornscrubTortoise    CCAGTTCATG-GCTGCTTCTCTGGGTATTCAAGCAGAGTTCCTGCAGG-AGAATACCCATAAATTGTTGGATATGCTGCA 52803
```

```
Majority                 TCTAATTGCGTTCTGATTTCCAGC----ATTGATTAA---TTCATGAATATT---ATATCTTTCAACAGTGTTGTTCTTT
                                57930     57940     57950     57960     57970     57980     57990     58000
Human                    TTTATCTTTTTTGTCATTGTTACATATGGAGGATGTAAGGTCATTGAGAGATGTAAAATTTCCCATCAAGGCTGCCACTG 57454
Kakapo                   TGTAATTGTGTTCTGATTTCCAGC----ATTGATTAAA--TTCATTAATATCCATCTATCTTGGGACAGTGTTGTTCTTT 26157
GoldenEagle              TCTAAATGCATTCTGATTCCCAGC----ATTGATTCA---TTAATATCCAT----AGAACTTATAACAGTGTTGTTCTTT 30021
JapaneseQuail            AGGAAATGAGTTATCATAAACAGCT---GCTGGGGTA---TCCTTGAACAGT---CCATCTTTCAA--GTATTGTAG--- 24431
MediumGroundFinch        TCTAAGTGCATACTGATTTCCAGC----ACTGATTAA---TTCATTAATATT---ATATCTTATGACAGTGTTGTTCTTT 12339
GoodesThornscrubTortoise CCTGTTTGCCCCGTTAGTCCCCCTCCCTATTAGCAATGA-CCTCTGAGTGCCTGCTAAGTTCTCTGAAGCAATCCAGGTC 52882

Majority                 AGCTCTTGACTCTG-ATGTTTAC-TCCACTGATATATTTTTATTCAG-TGT--TA---------------TGTACTTTTT
                                58010     58020     58030     58040     58050     58060     58070     58080
Human                    AAAACTGCGCACCACATCTTTGCTTCCA--AGTACCTTAATCTTCTGTTTGTATATCTTAAATCTACTTTTGCTCTGTCT 57532
Kakapo                   AGCTCTTGACTCTA-ATGGTTAT-TCCACTGACATATTTTTAGGCAG-------------------------TGGTTGTA 26210
GoldenEagle              AGCTCTTGACTCTG-ATGTTTAC-TCTACTGACATATTTGTGGGCAGCAGTTGTATTACCTTTTAACCAAAGTAATTTTT 30099
JapaneseQuail            AATCATTAAGATTG-GAAAAGA--TCTA--GATGTAGTC-CAACCAT-------------------------CACCACCA 24480
MediumGroundFinch        AGCTCTTGACTCTG-ATGTTTAC-TCCACTGACATGTTTTTAACCAA-AGT--T----------------AATAGTTTTT 12398
GoodesThornscrubTortoise AGTACTGCTTATTG-CGAAGCACATGGAGAAATGCTACTTCATTCCAGTGCAGCAATTTGAGGGTTTTTATTTACATCCA 52961

Majority                 TTTCTGGCCTTCTTTCCAAAGCAAGACTTCTTCCATCCAACTGGCTAGTTC-----T-GCTTATTC---A-AAAAAGGAA
                                58090     58100     58110     58120     58130     58140     58150     58160
Human                    CTTTAACTTTTTGTGCCATAGTATCTCATTATTATTACTATTCCATTATTAAGCCATTAAAAATTC-----ATTATTAAG 57607
Kakapo                   TTACTGGCCTTCTTTCCAAAGCAAGACTTCTTCCATCCAACTGGCTAG-----------------------------GAA 26261
GoldenEagle              TTTCTGGCCTTCATTCCAAAGCAAGACTTCTTCCATCCAGCTGGCTAGCTCTTTTATTGCATATTCTCAAGAAAAAGGAA 30179
JapaneseQuail            TGCCCGGTAAGCCATCTGTGCCG---CTTCTGTGCCACATCTGCCTTTTTC-----------------------AT-GAA 24533
MediumGroundFinch        TT-CTGGCCTTCTTTCAAAAGCAAGATTTCTTCCATCCAACTGGTTAGCT--------GCTTATTC---AGCAAAAGGAA 12466
GoodesThornscrubTortoise CCCCCAGATTTCCTGGTTGTAACCGCTGTGAACGACAGAGCCCGACAGAGCAGATGTAAGTCAACTCCCAAGGATAAGGA 53041

Majority                 CTCCAGTTGGAATTTTGCTGTATTTCTTTACAAGAATTCTGGACAGTATTCTTAA---TA---ATACCTTTCTGGTGTTT
                                58170     58180     58190     58200     58210     58220     58230     58240
Human                    CCATTATTATGAAACCATTTAATAAGCTTACATTTATCTTAACTGGTTTTCTTCCTTTCATTCATTCTTTTACTTTCAGA 57687
Kakapo                   CTCCAGCTGGAATTTTG-TC----------CAAGAATTCTGGACAGCATTCCTAA---TA---ATACCTCTAAGGTGCTT 26324
GoldenEagle              CTCAAGCTGGAATTTTGCTGGAATTCTGCACAGGAATTCTGGACAGTATTCTGAA---TA---GTACCTTTCTGGTGGTT 30253
JapaneseQuail            CGCCTCTAGGGCTGCTGACT---------ACCTTCTCCCTGGGCACCCTTTTC-----CA---ATACCTCACCACTCTTT 24596
MediumGroundFinch        TTCCAGTTGGAATTTTGCTGTAATTCTTTGCAAGAATTCTGGAGAGTATTCTGAA---TA---GT--CTTTCTGGTGTTT 12538
GoodesThornscrubTortoise CTCTAAATGTATGGACCCGGTGTATCCCCTGAAGCCCCAACGGAGGAATTTCAAG---CATTTATCACCAAAGACTGGTT 53118
```

```
Majority                 TGTAC-AATGATGTTAATGCTTTCCTGCTTCTTCTAGGAAAATCTTGTCTGG-T----CTCTTA-------TT----TTG

                                  58250     58260     58270     58280     58290     58300     58310     58320

Human                    TTAATTATTGTCTCTAATACTTAAATGCTTCCTCCAGATCAAGATTACTTCTGTGGAGCCCAGACCATTTTTTACAATTG 57767
Kakapo                   TGTAC-AATGATGTTAATGCTTCCCTGCTTCTGCTAGGAAAATCTGGTCTGG------CTCTTC-------CC----ACA 26386
GoldenEagle              TGTAA-AATGATGTTAGTGCTTCCCTGCTTCTGCTAGGAAAATCTTGTCTGGTTCTTTGTTTTG-------TT----TTG 30321
JapaneseQuail            TGGA--ACAGAAATT-----TTTCCTGATATCCAACCTAAACCCTCACCT----------CTCA-------TC----GTG 24648
MediumGroundFinch        TGTAC-ATTGATGCTAATGCTTGCCTGATTCTTTTAGGGAAATGT-ATCTGG------ATCTTA-------CC----CCA 12599
GoodesThornscrubTortoise CGTGGCTAAGACTTTGTTGCAGTCCATGTTGCATCGCAGAGTGATGGCCTCAGTGGCGACCATGAGAAGGGCT----TCC 53194

Majority                 TTCCCTTGTCA-TGTGTTGGCTGCATCATATTT----------ATTAGTTG----GTTTTTCCTTTTCTATGGGTGAGCT

                                  58330     58340     58350     58360     58370     58380     58390     58400

Human                    TTTCATTGCATTTCCATCCACACAACCTCATTTTGGGAGCAAAATTAGTTGTAG-GTAGCAGAAAGGCTATACAAGTAAT 57846
Kakapo                   CCCCCTTCCCC-TGTGTTGGCTGCCTCATGTTT----------ATTAGTTG----GGTTTT----TTCTGTCAGAGAGCT 26447
GoldenEagle              TTCCCCTATC--CATATTGGCTGCATCATATTT----------ATTAGTTG----GTTTTTCATTTTCTATTGGTGAGCT 30385
JapaneseQuail            TT-GCTTGTTA-TTTGGGAAAAGAGGCCTACTT----------ACACCTTG----CTACCACC--TCTTTTCTGGCAGCT 24710
MediumGroundFinch        T--CCCTGTC--TGTGTTGGCTGCATCATGTT-----------ATTAGTTG----GTTTTTCCTTTTTTATGAGTGAGCT 12660
GoodesThornscrubTortoise TGGCTGTGGAA-TCTGTGTATTGCTCCCCATGTGC-------AACAGGCCATAGAGGATTTGCCCTTCAATGGCTGAGTC 53266

Majority                 CGTAGT----TCATAATGGACTTTTTTGTGC-GGGTCCTTAA--ATTCTGAG-GCT---CTAGATT-TCATCAGATTTT-

                                  58410     58420     58430     58440     58450     58460     58470     58480

Human                    AATAGCATAATTGTAATAGTTTACCAAGTGCCGTAGTCTCATTTTTTCTGACCACTACTCTATATTGTCAACAAAACAGG 57926
Kakapo                   TGTAGT----TCATAATGGATAGTTTTGCTCTTGGTCCTTAA--ATTCCGAG-GCT---CAGGATT-TCATCAGATGTT- 26515
GoldenEagle              TGTAGT----TCATAATGGACATTTTTGTACTGGGTCCTTAA--ATTCTGAG-GTG---GTGGATT-TCATCAGACTTC- 30453
JapaneseQuail            CTTAGT----GAGCAGTGAGGTCTCCTCTGG----CCCTCAT--GTT-------TT---CTAGACA-AAACAAACCCGT- 24768
MediumGroundFinch        CGTGGT----TCAGAAGGGACGTTTTCCTAC-----C-TTAA--ATT------------GTTGGTT-TCATCAGCTTTT- 12714
GoodesThornscrubTortoise CTTTTT----TCCAAACAGACGAAACTCTGC-ACTCCTTCAAGGACTCTATG-GCTACCCTACAGT-CCTTGGGGGTGTA 53339

Majority                 TGTGATGTCAGTCCTTTG-GGTCATCCTCTTTCTC--CGGTGT-GATCCTTCTCAG-------CATTAATAAT---GCTT

                                  58490     58500     58510     58520     58530     58540     58550     58560

Human                    GTTTTCCCCTGCGTTACAAGAGGATGCTAAGATTTAAAGAGGTGAATTACTTACAGTGAACACCACCTATAAGGTAGACA 58006
Kakapo                   TGCAATATCAGTCCTCTG-GGTCATCCCCTTTCTC--CGGTGT-GATCCTTCTCAG-------CATGAATAAC---GCTT 26581
GoldenEagle              TGTGATGTCAGTCCTTTG-GGTCATCCCCTTTCTC--CAGTGT-GATCCTTCTCAG-------CATTAATAAT---GCTT 30519
JapaneseQuail            TCCCTCAGCTGTTCCT---AGTAAGACTTGTGTTC--CAG----ACCCCTCACCAG-------CTTTGTTGA----ACAT 24828
MediumGroundFinch        TGTGATGTCAGTCCTTTG-GGTCATCCCCTTTCTC--CGCTCT-GATCTTTCCCAG-------CATTAATGAT---GCTT 12780
GoodesThornscrubTortoise TTTGCTGGCAGTGCAGAGATGTCACTGTGGTAGTCAGCAGCGG-CATCATCTGTAG-------CCCACATCTGG--GCAG 53409
```

```
Majority                   CCTTTCTTGCTTTGATACTTTTCTACT--TTTTGTGTCTGGT-------TCCTTAGTGCAAACAGTTCTTAAAATTTTCC
                                   58570     58580     58590     58600     58610     58620     58630     58640
Human                      TTTTTTCAGTCTTAGCCTTTCTCTCCA--GTATGCTGCTTCTCACTGGTTCTCGGGTATAATCTAATCTTACTGTGCTTT 58084
Kakapo                     CCTGTCTTGCTTTGATACTTCTCTACT--TTCTGTGTCTGGT-------TCCTTAGTGCAAACAATCCTTAAAACTTCCC 26652
GoldenEagle                CCTTTCTTGCTTTGATATTTTTCTACT--TTTCGTGTCTGGT-------TCCTTAGTGCAAACAATCCTTAAAACTTTCC 30590
JapaneseQuail              GCTCCAGGGCTTCAGTGATTTTCT-----TTTAGTGAGGAGT-------CTGAAACTGAACACAGTACTTAATAGTG--- 24893
MediumGroundFinch          CCTGTCTTGCTTTGTTACTTTTCTACT--TTTCGTGTCCAGT-------TCCTTAGTGCAAGCAGTTCTTAAAAAAA--C 12849
GoodesThornscrubTortoise   CCTTTCCCTCCCTGAGACAGCAGGACTACTTCAGAAAGGGGTATAGGTCCCGGAGGAGGAGGCGGCATTCTGGTTTTGCC 53489

Majority                   TTTCGGAGCTC-TTTATTAATTATTCACCAC-----TATTTTTTTCCTTTGAAACAAAATTCTGGTTA-GCC---TCTAA
                                   58650     58660     58670     58680     58690     58700     58710     58720
Human                      CCGCTTCAGGCATAGGTTAAT-AATTAGAAT-TCTTTTTTTTTGTCCAAAAAATGATTATCCTCCTTTCACATGCCCTGG 58162
Kakapo                     TTTAGAAGCTCTTCTAGTAATTATTCACCAC-----CATTTTTGTCCAGAAAAACAAAATTCTGGGTA-ATC---TCTAA 26723
GoldenEagle                TTTAGGAGCTC-TTTAGTAACTATTCACCAC-----CATTTTTCTCCTTTCAAA-AAAATTCTGGGTA-ACC---TTTAA 30659
JapaneseQuail              ------------T-----AAT--CTCACCAG-----TGCTGCATACAGAGGGACAGTTACCCCCCTTGTGCT---GCTGA 24946
MediumGroundFinch          TCTGGGA-----TCTATTAATGGTTCACCAC-----TATTTTATTTCTTTCAAA-AAATCTCTGGGTA-GCC---ACTAA 12914
GoodesThornscrubTortoise   AGTCGACATACTTTTCCCAAGAGCCTTCCATGTACTCATTTTCACTCCTTGGACCAGAGCTGTGTTCTGGTCATGGCTAC 53569

Majority                   CTGTTT---T--GTTTTTTTTATGT------AATTTGGAGTTCTTATGCAAGCCCT----TCTGGGTCATCATTAATAT-
                                   58730     58740     58750     58760     58770     58780     58790     58800
Human                      CTACCAAAATCAATGTGGTATGAGGTTTTATGAATAGGAGACAATCTAGAAGTCATGGTTTCTGTGACATTTTTCACAA- 58241
Kakapo                     TTGTTT---TGGGTTTCTTTAATAG------AATTTAGAGTTCTTATGCAAGCCTT----TCTGAGTCATCATTAATAC- 26789
GoldenEagle                TTGTTT---T---TTTTTTTTATGT------ACTTTAGAGTTCTTATGCGAGCCCG----TCTGAGTCATAATTAATAT- 30722
JapaneseQuail              CTGCAC---T--GCTTCTGGTACTG------AAGCCAGGATGCCATTGG----CCT----GCTTGGCCAC--TTGGCGC- 25004
MediumGroundFinch          CTGTTT---T---TACTTTA-ATGT------GTGTTGGAGTTCTTATGTGAGCCCT----TGTTGGACATGATTAATATC 12977
GoodesThornscrubTortoise   CTTCTC---CCTGCCTGCTCCATTTGGGAACAGACTGGCTTGCTTTCACAGTGCTTGGA-TCTCGATCACCATTGACATT 53645

Majority                   ----TTTTACATGTTATTATATGAGA-TACTTCACTGAGAGT----TGTAAAGGATTTGCTGTGGTCTTTTTCT--T---
                                   58810     58820     58830     58840     58850     58860     58870     58880
Human                      ----CTTCAAACGTTTAAATATA-GTCTACATTTTTCTTGGT-----GTGGAATATTTTCCTTGCTCTTCTGAC--CCCA 58309
Kakapo                     ----TCTTGCATGGTATTACATGAGA-TACTTCACTGAGAGC----TGAAAAGGATTTG----AGT---T---------- 26843
GoldenEagle                ----TCTTACATGGTATTATATGAGA-TGCTTCACTGAAAGTGAGATGAAGAGGATTTGCTGTGGTTTTTGTCTTGTTTA 30797
JapaneseQuail              ----T--CAGCTGGC-TCATGCT----CATCTCACTGTCAGC-------CAATGCTGTC---AGATCCTTTTCT------ 25057
MediumGroundFinch          ATGTTATTCCATGTTATTATATGAGA-TGCTTCACTGAAAGTGAGGTGTAAAGGGTTTGCTGAGGTCTCTGT----T--- 13049
GoodesThornscrubTortoise   TGGATTGCAAGCATTGTCAAGTGGGGCTATGCCATCCAGTTCC---TTTGCATCCTCCTCCCCTCTCCTTCCCC-ATCCC 53721
```

```
Majority                 ----------------AGATGAG------------------G---G--TTA---AGTCTTTGACA---CATCTTTTATG-

                                  58890     58900     58910     58920     58930     58940     58950     58960

Human                    AAGAAAAGTATTTGTTAGAGGACCAATTAAATGAAATTTGAGTGGGGATTATACTGTGTTTGAGCTATAATTCTTAAGGG 58389
Kakapo                   -------------------------------------------------------AGTCTTGAACA---CACCTTTTCTG- 26865
GoldenEagle              GAAAATGAGTTATCACAGAGGGCTGTTGAGTTAGTGTAAGAGACTGGGTCAGTTAGTCATTAACAGTCCAACTTTCATG- 30876
JapaneseQuail            -------------------TGTG-------------------------------GATGTGTGACA----ATTTCTTCAT- 25082
MediumGroundFinch        ----------------AGATGA--------------------------CGA---AGTCTTGAACAATCTACCTTCTATG- 13083
GoodesThornscrubTortoiseTCTTCAGGGACACCTCTCATGAGATGCTGCTCCTCGAGCAGGATTGCTCTCTGCTGGCTTTGGGA----GTAGTGGAAG- 53796

Majority                 --AAACTAT--A-ACATCTGTCTCTTCAGAATTTGTTCTGAA--GCCTTGAATTTTATTTAGATCCAACTAAGAGGTGAC

                                  58970     58980     58990     59000     59010     59020     59030     59040

Human                    GAAAATTGTTTATATGTGTATCTTGTCTGAAGTAATACATAGTTACATTAAATCTCATAAAAGTACCTCAATTGTGTCAA 58469
Kakapo                   --AAGCTAT--A-ACATCTGTCTCTTCAGAATTTATTCTGAA--GCCTTGCCTTTTATTTAGACCCAACTAAAAGGTGGC 26938
GoldenEagle              --AAACTATGTA-ACATTTGTCTCTTCAGAAGTTGTTCTGAA--ACCTCAAATTTTATTTAGATCAAACTAAGAGATGGC 30951
JapaneseQuail            --GTTCTT-----CTAGCTGCCCCTTCAGAATCTGTTCTAAAC-ACTCCCATTTTCCTTTAGACAGGACCAGAAGGCGAT 25154
MediumGroundFinch        --AAACT----A-GCATTTGTCTCTGCAGAATTTGTTCTGAA--GCCTTGAATTTGACCTAAACCAAAT---GAGATTAC 13151
GoodesThornscrubTortoise-GGATTCCGCT-GGAACACCAGGGTCAGAACTTCTATTTCTG-GTACTTTCTGGTGCCCAAATCCAAGGGAGGGCTAAG 53872

Majority                 TTACTTAAAATTTATG-C-T------GTTTATAA-----TTAAAAGCATTTTTTAATATAGTATTAATAGTATCAGTAAA

                                  59050     59060     59070     59080     59090     59100     59110     59120

Human                    CTGTTTGACATTTTTG---TTTAATCTTCTGTGAACAGATACTAAGCATCCA--ACTATGATATTGATGTTTGTGCACAG 58544
Kakapo                   TTACTTCAAAGTGA-----T------ATTTATAA-----TGCAAACCATTTT-------------------ATCAGTCAA 26983
GoldenEagle              TTACTTAAAAATTATCTCAT------GTTTATAA-----TTAAAACCATTTTTTAATAAAGCACTAATAATATCAGTAAA 31020
JapaneseQuail            TCTTCTAAAACTTA-----T------GTTTATAA-----TAAAAAGCATTTTTAAATGGAATGCTAACAGTGTCAGTAAA 25218
MediumGroundFinch        TCACTTAAAATTAATTTCAT------GTTTATAA-----TTAAAAGCATTTTTTAATAAAGCATTAAAAGTATCAGTAAA 13220
GoodesThornscrubTortoiseATTCTTGACCTTCATGGCCTCAACAGATTTATCAG-ATATGTGACATAGCCTCCCCCGTTGTCTTGGAACGACTGGTTGG 53951

Majority                 CTTTTCAAATAGAGTCTGAATATGTCTTTCAGCAAATGGTAATGACAACAAGAAATTCAAAGGTGACGATAA--AATGGA

                                  59130     59140     59150     59160     59170     59180     59190     59200

Human                    GTATGCACTCAGGTCTTCTTGACAAAGATCTA-AAGTTATAATTCTAAGAGAGTAGATAATTGTTTTACTAAGTACTTAT 58623
Kakapo                   CTTTTCAAATCTAGTCTGACTCTGTCTTTCAGCAAATGGTAATGACACCAAGAAATTCAAAGGGGACGATAA--AATGGA 27061
GoldenEagle              CTTTTCAAATAGAGTCTGAATATGTCTTTCAGCCAATGGTAATGACAACAAGAAATTCAAAGGTGACGATAA--AATGGA 31098
JapaneseQuail            GTCTTCAAAT--AGTCTTAATGTGTCTTTCAGCTAATGGTAATGACAACAAGAAATTCAAAAGTGAAGATAA--AATGGA 25294
MediumGroundFinch        CTTTTCAAATAGAGTCTGAATATGTCTTTCAGCCAATGGTAATGACAACAAGAAATTCAAAGGTGATGATAA--AATGGA 13298
GoodesThornscrubTortoiseCTACTC---TGGACCTTCAGGATGCCTATTTTCATGTGGCAATTTTGCCAAGCCA--CAGAACATTCCTTTG--ATTTGT 54024
```

```
Majority                    TGGGGCTCCTT--CTCGTGTTCTTCATATCAGGAAACTGCCTGGGGAAGTGACAG--AAACAGAAGTT-----ATTGCTT
                                     59210     59220     59230     59240     59250     59260     59270     59280
Human                       TCTGGTATCAGAACTCACACTTCACTTATTACAGAAGGAAAAAGAAATTTGTTTCCTAAACTTGCTTTGCCCCATTACCT 58703
Kakapo                      TGGGGCTCCTT--CTCGTGTTCTTCATATCAGGAAACTGCCTGGGGAAGTGACAG--AAACAGAAGTT-----ATTGCTT 27132
GoldenEagle                 TGGGGTTCCTT--CTCGTGTTCTTCATATCAGGAAATTGCCTGGGGAAGTGACAG--AAACAGAAGTT-----ATTGCTT 31169
JapaneseQuail               TGGGGCTCCTT--CTCGTGTTCTTCATATCAGAAAACTGCCTGGGGAAGTGACGG--AAACAGAAGTT-----ATTGCTT 25365
MediumGroundFinch           TGGGGCTCCTT--CTCGTGTTCTTCATATCAGGAAATTGCCTGGGGAAGTGACAG--AAACAGAAGTT-----ATTGCTT 13369
GoodesThornscrubTortoiseCATAGCACCAT--TTCCAGTATGTGGTGCTTCCCTTCAGCCTGTTGGGTTTTTACC-AAATGCATGGT-----CATGGTG 54096

Majority                    TAGGTTT---ACCTTTTGGTA-AGGTAACCAACATCCTGATGCTGAAAGGGAAAAATCAGGTAATTTGTTGCTTTCC---
                                     59290     59300     59310     59320     59330     59340     59350     59360
Human                       TGTAATCTAGACCCTTTGGTA---A-AATTTGCATTTTTGATAACTACAGTAATGTTCATTTTACTTGCCTGCATCTGGG 58779
Kakapo                      TAGGTTT---ACCTTTTGGTA-AGGTGACCAACATCCTGATGCTGAAAGGGAAAAATCAGGTAACTTGTTGCTTTCC--- 27205
GoldenEagle                 TAGGTTT---ACCTTTCGGTA-AGGTAACCAACATCCTCATGCTGAAAGGGAAAAATCAGGTAATTTGTTGCTTTCC--- 31242
JapaneseQuail               TAGGCTT---ACCTTTTGGTA-AGGTAACCAACATCCTGATGCTGAAAGGGAAAAATCAGGTAATTTGTTG--------- 25432
MediumGroundFinch           TAGGTTT---ACCTTTTGGTA-AAGTAACCAACATCCTGATGCTGAAAGGGAAAAACCAGGTAATTTGATGCTTTTTG-- 13443
GoodesThornscrubTortoiseATGGCAT---ACCTCAGGAAGGAAGGAATCCATAACTTCCCATACATAGATGACTGTTTACTGAGGGGCAG-TATCCA-- 54170

Majority                    -TTTTTTTAGTATGCTTTCTGTCTCATTTCAAAATATGCT-GTTAAAATGCTTTTGAAATAAGTCATA-CAACAGTAAGT
                                     59370     59380     59390     59400     59410     59420     59430     59440
Human                       CTCTACTTAGAGAATATCACATAATATTACATAATCTGACAATTGGTACCAGTTTAAATCCATCAACGTCAACTACAAGT 58859
Kakapo                      -ATTTTTTAGTATGCTTTCTGTGTAATGTCAAAATAGGCT-GTTAAAATGCTCTTGAGATAAGTCATA-GAACAGTAAGT 27282
GoldenEagle                 -TTTTTTTAGTATGCTTACTGTCTAATTTCAAAATATGCT-GTTAAAATGCTATTGAAATAAGTCATG-TAACAGTAAGT 31319
JapaneseQuail               --TTGCTT--T------TCTTTCTCATTTCAAAATATGCT-GTTAAAATATTATTGAAATAAGTCACA-CAACAGTAAGT 25500
MediumGroundFinch           -TTTTTTTCATATGCTTGCTGTCTGATTTCAAAATGTATT-GTTAAAATGCTGTTGAAGTAAGTCATA-AAGCAGTAAGT 13520
GoodesThornscrubTortoise-GAGAGGAAGTCC-TTTTCCATGTCAACACTACATGCACTTGCTCAAACATCTGGGACTCATCCTAAA-CATCGGAAAGT 54247

Majority                    ATTCAATAGA----A--AAAAAAGCCTTTGTTCCTTTCTTTT-TT-AATGAATTTATAGTGTCTTTTA--GTAAATTTT-
                                     59450     59460     59470     59480     59490     59500     59510     59520
Human                       AAGCCCTCTACTCTACTTTAAAAATCTCTTCCCATTTCTTCA-GTCAACAGTTTTTCTGTTTTTCACAATGCCTATGTCA 58938
Kakapo                      ATTAAAACCA---------AAAAGCCTTAGTTTTTTCTTTTT-----ATGAATTTAGAGTGTCTTTTA--GTAAATTAT- 27345
GoldenEagle                 ATTAAAAAAAAAAAAAAAAAAAGAGCCTTTGTTCCTTTAATTTTTTTAATGAACTTATAGTGTCTTTTA--GTAGATTTGA 31397
JapaneseQuail               CGTAATGAGG-------AAAGAAACAGTTGTTTCTTTCTTTA----AATCAGTTTGTGCTGTCTT-----GTAAATTCT- 25563
MediumGroundFinch           ATTCTAGAAA-------AAAAGAGCTTTTGTTCCTTTCTTTTTTTAAATGAATTTATGCCATCTTTTA--CTAAATTTT- 13590
GoodesThornscrubTortoiseCAACTTTGGTTCCCAT-TCAGAAAATTGAGTTAATTATCGCCCGT--ATAGACTCTGAGAGCATTTTT--GTCAACT--- 54319
```

```
Majority                    GGGGGGGAACAGGGATTGTTTGGCAGCTACCTAAAGCAAAAAG--TTAG------ACTGTAAGATAAATATTT----AGT

                                      59530     59540     59550     59560     59570     59580     59590     59600

Human                       GATAGAAATTTTCCCTTCTACCATATCTCTCCTCACTCTCACTTGCTTGAAGAGGAAAATCACATGGAAGCTTCATCAGC 59018
Kakapo                      GGGGGGGAACAGGG--TATGGTGGAACTGCATACAGCAAAAAG--TTAA-------CTGTAAGAGAAATA-------GTC 27407
GoldenEagle                 GGGGGGGAACAGAGGATGGTTGGCAGCTACGTAAAGCAAAAAG--TTAG-------CTCTAAGATAAATATTT----AGT 31464
JapaneseQuail               GTTTGGGAATGTGGATTGGTTAACGGCTAACTAAATTAGGAAG--TAGGATAAATACTGTAAGTTAAATACGC----AGT 25637
MediumGroundFinch           GGGGGGAAACAAA----ATTTGGCAGCTACATAAAGCAAAAAG--TTAG-------TTGGAAGATAAATATTT----ATT 13653
GoodesThornscrubTortoiseCACAGTTTTCAGGCAATTCACCACCTTTGTCTCAGTCCGCAGTC-TCAGCCTTTCACAAGAATCCAGATGTGCCTGAAGC 54398

Majority                    TCTGCATCTTCACAGGTG---AATTTTTGGAGATGCATTAGTTTATCAAATAAAGTTGGTATGTGTAGAATGACATTT--

                                      59610     59620     59630     59640     59650     59660     59670     59680

Human                       TTTTCTTGCA-AAATCTG-CTAATACTCACACTTGTCTTTCTCTTTTAA-TAAAAGGCATAGATGTTTAGTTATCTAAGA 59095
Kakapo                      CCTTCATCTTCAGAGGTG---AATTTTTGGAGATG----A-------A--TAAACTTGGTATGTAACAAATGACAT---- 27467
GoldenEagle                 CCTGCATCTCCACAGATG---AATTTTTGGAGATGCATTAGCTTAGCAAATGAAGTTGGTATGTAGAGAATGACATCT-- 31539
JapaneseQuail               TCTGCATTCCTTCAGATG---ATGTTTTGAAGATGAAGTGGTTTAGCAAATAAAGTTGGTAATTGGAGGATGACATCT-- 25712
MediumGroundFinch           CTTGCATCTTCACTGGTG---AATTTTTGGAGATGAAGTAGCTTATCAAATAACATTGGTATGTGTAGAATGACATTT-- 13728
GoodesThornscrubTortoiseTCTGTTTGCACACAGTGGTCCAGTTTGCAAGGTTGCATCTTTGTCTCCTTCAGATGTGTCTCAGGACAGTTTACTGTC-- 54476

Majority                    T--CTGAAGACTGATAT--ATTTGTTAAAATGTGTTATCAGACC-CTTTAAGTGTTTTTT----GTATGCTTCTAA----

                                      59690     59700     59710     59720     59730     59740     59750     59760

Human                       CCAATAAACACCAAAGCCTATCGTCTTCTGCCTGTCAAGAGACTGCACTATCTGTCATCC-------CCTTTCTCATTTA 59168
Kakapo                      ---CTGAAGACTGGTAT----TTGTTACAATGTGTTGTCAAATG-CTTTAAGTGTTTTAT----GTATGCTGCTAA---- 27531
GoldenEagle                 T--CTGAAGACTGATAT----TTGTTAAAATGTGTTATCAAACC-CTTTAAGTGTTTTTT----ATATGCTACTAA---- 31604
JapaneseQuail               T--CTAAGGATTTATGT----TTGGGAAAATGTGTTCTTAGACC-TCTTA--TGTCTTC--------TGCTTCTAA---- 25771
MediumGroundFinch           T--CTGAAGGCTGATATCAATTTGTTAAAATATGTTATCAAACC-CTTT---TTTTTTA----ATGTACGTCTAA---- 13794
GoodesThornscrubTortoiseCAACTCTTCACTTGGACAGATTGGTCCATCTTCCTCCACTGGTC-TTGGTCATATCGTAGTGATGGACACTTCCGGAGGA 54555

Majority                    ----TATCAATATTATCTGCTTTGTA--AATACTGAAAATT---AGTTGTTTT-ATTACAGGCATTTTTGGAACTTGCTA

                                      59770     59780     59790     59800     59810     59820     59830     59840

Human                       TTTCTCTCTATATTGGCCACTTTTCACCAGTTTCAAGCATTCTCAGCTTCTTTCATTTTAAATGAATTTACCTTCAACTT 59248
Kakapo                      ----TATGACTGTTATCTGCCTTGTA--AATACTGAAAATT---AACTGTTTG-ATCACAGGCATTTTTGGAACTTGCCA 27601
GoldenEagle                 ----TATCAAGATTATCTGCTTTGTA--AATACTGAAAATT---AACTGTTTA-ATTACAGGCATTTTTAGAACTTGCTA 31674
JapaneseQuail               ----TACAAATTTTATCTACTTGAAA--TTTACCTGT--TT--AACTTGATT---TTACAGGCTTTTTTGGAACTTGCAA 25838
MediumGroundFinch           ----TATGAAGATTTCCTGCTTTGTA--AATACTGAAAATTTTAATTCTTTTA-ATTACAGGCATTTTTGGAACTTGCCA 13867
GoodesThornscrubTortoiseTGTTTGTCAGGGTGTTCCCTTTGTCC--GGCCCTTACCAACC--AGTTCTCTT-GTCACCGATGCCTCCCTGATGGGCTG 54630
```

```
Majority                  CAGAAG-AAGCAGCTATCACTATGGTTAATTACTACTCTGCTGTG-ACGCCTCATCTT------CGTAACCAACCTATTT
                                  59850     59860     59870     59880     59890     59900     59910     59920
Human                     TCAATCCATCCATCTATAACTCTACGTACTTTCTATCACCCTGCCTCCCCTCTCCCTTTTCACTCATAACCAGACTTCTT 59328
Kakapo                    CAGAAG-AAGCAGCTATCACTATGGTGAATTACTACTCTGCTGTG-ACGCCTCATCTT------CGGAACCAACTTATCT 27673
GoldenEagle               CAGAAG-AAGCAGCTATCACTATGGTTAACTACTACTCTGCTGTG-ACGCCTCATCTT------CGTAACCAACCTATCT 31746
JapaneseQuail             CAGAAG-AAGCAGCTATCACAATGGTTAATTACTATACAGCTGTG-ACGCCTCATCTT------CGTAACCAACCTATCT 25910
MediumGroundFinch         CAGAAG-AAGCAGCTATCACTATGGTTAATTACTACTCTGCTGTG-ACACCTCATCTT------CGTAACCAACCCATTT 13939
GoodesThornscrubTortoiseCGGAG---CGCACATGGCGCTGCTGAAAAGTCAAGGTCAGAGATGCAGGCCTCA-CTG------CATAAC-AGTGTGCTG 54699

Majority                  A--TA-TCCAG------TATTC-CAATCATAAAGAACTAAAGACTGATAATACGCTTAAC------CAGGTATATTTACT
                                  59930     59940     59950     59960     59970     59980     59990     60000
Human                     AAATAGTCTATCTTTGTTGTTTATTATCTCATTTCTCTACTTACTATTATTTGGTTTTAGCACTGTTGACCCAAATTGCT 59408
Kakapo                    A--TA-TCCAG------TATTC-CAATCATAAAGAACTGAAGACTGATAACACACTTAAC------CAGGTATATTTACT 27737
GoldenEagle               A--TA-TTCAG------TATTC-CAATCATAAAGAACTAAAGACTGATAACACGCTTAAC------CAGGTATATTTACT 31810
JapaneseQuail             A--TA-TCCAG------TATTC-CAATCATAAAGAACTGAAGACTGATAATACACTGAAC------CAGGTATATTTGCT 25974
MediumGroundFinch         A--TA-TCCAA------TATTC-CAATCATAAAGAACTAAAGACTGATAACACACTTAAC------CAGGTATATTTACT 14003
GoodesThornscrubTortoiseG--CACTTCAGGC----CATCT-ACAAGGTCATGCTTTTCTGGACCATATTGGGCTCAGTGGTTCACATACCTACAGACA 54772

Majority                  GTTCCGTAGTATTACAAATG-TAGACAGTGGAAGATGAGGCATGGCATGTGTTCTTGGCTCATGTGGC--ACATGTTTTG
                                  60010     60020     60030     60040     60050     60060     60070     60080
Human                     CTTCCTCAGCGTCACCAGCGATTAGTAGAGTAGCTAAATGCTAGTGATATGATTTAGTCCTTGTCTTACTTAATACCTAG 59488
Kakapo                    GTTCTGTACCATTGCAAATG-CAGGCAG---AAGATGAGGCTTAGCATGTATTCTTGGCTTATGTGGC--ATATGTTCTG 27811
GoldenEagle               GTTATGTAGTATTACAGATG-CAGACAGTAGAAAATGAGGCATGGCATGTATTCTTGGCTCATGTGGG--ATATGTTTAA 31887
JapaneseQuail             ATTACATGGTATTAGAAATG-TAGACAGTGGGGTGTGTGACA-AATATAC-CGCATCAGTCAAGAA-T--ACCTGTTATT 26048
MediumGroundFinch         GTTCAGTAGTATCACAAATG-CAGACAGTAGAAGATGAGGCATGGCATG--TTCTTGGCTCATGTG----CCATGTTTGA 14076
GoodesThornscrubTortoiseGTACCACCACAATGTATATG-TGAACAA-GCAAGGGGGAGCACACTCCAGGATGCTGTGCCAGGAGGCAATCAGATTGTG 54850

Majority                  ATAAT-T-TGGTTTTGT---CCACATTCTAATACTACTGTA---GTCTTCTCTGTGATCATTCTATCTTGTGTCATTGAA
                                  60090     60100     60110     60120     60130     60140     60150     60160
Human                     ATAATATTTGGCACTGTGCACCATTGTCTTAAAATTCTCTTTCTTCCTGGTCTCTGATACTTCTCACCTGGATTTATCCT 59568
Kakapo                    GTCAG-T-TAGTTTTGT---CCACATTCTAATGCTACTGTA---GTCTTCTCTATGATCATTCTGTCTTGTGACATTGAA 27883
GoldenEagle               ATAAT-T-TGGTTTTGT---CCACATTCTGATACTACTGTA---GTCTCACCTGTGATCATTCCATTTTGTGACATTAGA 31959
JapaneseQuail             TAAGT-C-TACTTTTGC---CCACATTCAGATACTACTGTA---GTTTTTCCAGTGGT----------TTTGGTAATGAA 26110
MediumGroundFinch         ATCGT-T-TGGTTTTGT---CCACATTCTAACACTCCTGTG---GTCTTATCTATGATTGTTCCATTTTGTGGCACTGAA 14148
GoodesThornscrubTortoiseGTAAT-TCTGCACTTAC---CTAGGATCCAGAATCATCTTACGGACCATCTCAGCAGGAATTTTTCCTGAGTCA-TGAA 54925
```

```
Majority                 GGGTCTTTGCT---------TTCTGTG-TGATCTTTCCATTAGGATGAA-TC--------T----------TT-------

                                 60170     60180     60190     60200     60210     60220     60230     60240

Human                    ACCTCTCTATTTCTTCCTTGTCTTGTAACTCCCCTTCTACCTGGGAGGCCTCACCTGCTGTCACAGCTCCATTAAACCTC 59648
Kakapo                   GGGGATTGGCT---------TTCTGTG-TGATCTTTCCATTAGGATGAAATC--------TGGAGAGGGACTTTCGACAA 27945
GoldenEagle              GGGA-TTTGCT---------TTCTGTG-TGATCTTTCCATTACAATGAG------------------------------- 31997
JapaneseQuail            -GA--TTTGCT---------TTCAATG-TGATCCTTCAATTAGGATGAA------------------------------- 26146
MediumGroundFinch        AGGCCTTTGCT---------TTCTGTG-CCGTCTTTCCGTTACAATAAA------------------------------- 14187
GoodesThornscrubTortoise TGGTCTTTGTAGACAAGT-GTCCTCTG-TTATCTTCTCAGCTTTGAGCATTC--------TGGCAATTGACTTATTCTAA 54995

Majority                 ----------------G----------------T---------------------A----------------G-----C-

                                 60250     60260     60270     60280     60290     60300     60310     60320

Human                    TTATGATTCAGACAGAGTTCACATCACATTTTCTAGCTCAGACCTCTCCTAAGAGACCCACACTAAGAACCTGGCGGACT 59728
Kakapo                   GGGCCTGTAGGGACAGGCCAAAGGGAATGGCTTTAACCTGCCAGAGGGGAGATTGAGATGAGCTCTGAGGCAGAAGCTCT 28025
GoldenEagle              -------------------------------------------------------------------------------- 31997
JapaneseQuail            -------------------------------------------------------------------------------- 26146
MediumGroundFinch        -------------------------------------------------------------------------------- 14187
GoodesThornscrubTortoise TCTTGAGGGTACCTTAGTCTGGAATCCTTATCCTGTGCTTTCCATCTCAGGTGGCAGTTGGATCTCCTGTAAGCTTTTCC 55075

Majority                 ------T----------------T-------A------------------------------------C-----------

                                 60330     60340     60350     60360     60370     60380     60390     60400

Human                    AATGAATATCTTCAAACAGTGTGTTCAATCTAGCTCCTGTTTTTGGTGTCCGCTGTGTTAGGAAAGTGCAAGATACCAAC 59808
Kakapo                   TCCCTGTGAGGGTGCTGAGGCGCTGGCACAGACTTTTCCCAGAGAAGCTGTGGCTGCCCCATCCCTGGCAGTGTTCAAGG 28105
GoldenEagle              -------------------------------------------------------------------------------- 31997
JapaneseQuail            -------------------------------------------------------------------------------- 26146
MediumGroundFinch        -------------------------------------------------------------------------------- 14187
GoodesThornscrubTortoise CCCAGTTCTGATCATCCCACAAGTCATGCTCAAGCTGAGGTTGGATCGGGCCACACTCATACTTATTACCTCGGTATGGC 55155

Majority                 ----G-------------C--------------T-T-----------T-C----------C------T----A-------

                                 60410     60420     60430     60440     60450     60460     60470     60480

Human                    TTCAGTCTCATTAACAAGCCAGTAAACATGGTATTTTTCCCTTTCCTTCCAACCTGTCATCAGATATTATTGATTATACC 59888
Kakapo                   CCAGGTTGGACACAGGGGCTTGGAGCAACCTGCTCTAGTGGAAGGTGTCCCTGCCCGTGGCAGGGGGTTGGAACTGGATG 28185
GoldenEagle              -------------------------------------------------------------------------------- 31997
JapaneseQuail            -------------------------------------------------------------------------------- 26146
MediumGroundFinch        -------------------------------------------------------------------------------- 14187
GoodesThornscrubTortoise TGAGGCAGTTCTGGTTCTCTGACCTTGTCGCTCTGTCAGTTCAGCCTTTCATATCCCTTTCTTTCCATCCTGACCTACTC 55235
```

```
Majority                    ------A----T--T---------------------A---T----ATTTTCTTAATGTG--TCCTTGATGTCAAAAGGAA

                                      60490     60500     60510     60520     60530     60540     60550     60560

Human                       TCCTAAATTTTTGTTGAATCTGGCCTTTTTTCTCCAACTCTTGCTACCATTGCAGTCTAAGCCAGTGTTATTCAAGATGC 59968
Kakapo                      ATCTTAAGGTCTTTTCCAACCCAAACCAGCCTGGG-ATCGTGTGAATTTGCTTAATGGC--TCATGAATGTAAAAAGAAA 28262
GoldenEagle                 ----------------------------------------------TTTTCTTAATGT---TCCTTAATGTAAAAAGGAA 32028
JapaneseQuail               ----------------------------------------------TTTACTTAGCGT---------------AAAAGAA 26165
MediumGroundFinch           ----------------------------------------------TTTTCTTAATGTG--TCCTTGATGTCAGAAGGCA 14219
GoodesThornscrubTortoiseACACAGAAACATGGTTGCCTCTTATTTCCCATGCTCAGCTTCCCGAATCTCATGATGTGGTTCCGTCATGACTAAATGAG 55315

Majority                    AAGATTCT-------GTAGTGTGTTTTAA-------TTTTAATATTTTTCATAACA-T--A---AGTTTTCATCTTAGTT

                                      60570     60580     60590     60600     60610     60620     60630     60640

Human                       TGGTCTCTCAAGGTAGTAGTGTGTGTCAAACTGAAATCAGCATACTTCTAGCAACAGTATGCTTAATGTCAATATTGGCA 60048
Kakapo                      AAGATTCC-------AAAGTGTCTTTTCA-------CTTTAAAATTTTCCTTAAGA-C--A---AGTTTTCATCCTGATT 28322
GoldenEagle                 AAGATTCT-------GTAGTGTCTTTTAA-------CTTTAATTTTTTTCATAAAA-T--A---AGTTTTCATCTTAGTT 32088
JapaneseQuail               AAGATTGT-------GAAG-ATGTTTTAA-------CTTGAATATTTTTCACAG-----------ATTTACATCTCAGTT 26219
MediumGroundFinch           AAGGTTCT-------GAAGTGTGTTTTTAATAGTTATTTAAAAGTTCTAAATAACT-TCAA---AGTTTTTGTCTTAGTT 14288
GoodesThornscrubTortoiseCAGGAAGT-------GTGATGTTTGGTGGCTGT---TTAACAAGTCCTACTCAGCAATAGA---AAACCCTCTACCAGAA 55382

Majority                    TAGA-ACTGTAGGATGAAA---AAATTAACTTTCTAATT---CTG-ACACCTGAGATTTTAATGCCAATTTGAATTTCC-

                                      60650     60660     60670     60680     60690     60700     60710     60720

Human                       TAA--ATTTTGACACAGACTTCTTATATTCATGCAGAGTAGCCTGGGCTTCTGGACCGTCAATCATAATTTAAGTAGCA- 60125
Kakapo                      TAG--GCTATAGGATGAAA---AAATGAACTTTCTAATT---CTG-ACACCTGAGATTCTAATTCCAAGTTGAATTTCC- 28392
GoldenEagle                 TAGAGACTGTAGGATAAAA---AAATTAACTTTCTAATT---CTG-ACACCTGAGATTCTAATGCCAGTTTGAATTTCC- 32160
JapaneseQuail               TAGAAAGTGTAGAATGAGA---AAATTAACTTTTTAATT---CTG-ACACCTGAGATGTTAATCTC--TTTAAATTTCC- 26289
MediumGroundFinch           TAGAGACTGTAGGATGGTA---CAATTAACTTCCTTGTTA-ATTGTACACCTGAGATTCCAATGCCAATTTGAATTCCC- 14363
GoodesThornscrubTortoiseTGG--CCTGTCAGGCAAAATT-GAAGCAGTTTTCATTGTG-TTCGCTGGCCTGTGGAGTTCAAGATAACCTGAATAGAAG 55458

Majority                    CTAGTGGTAGTGACTAATGCCAATGCCTATATTTCAGTGCTTTGGATGATCACTCTGGTAGCAC------TGGTAACCTC

                                      60730     60740     60750     60760     60770     60780     60790     60800

Human                       -TTGATGTAGTAATAATTCT---CTTTCATATCTCCACTTCCTTGGCCTTCTGACTGAACTCACCATTT-TGACATCTTC 60200
Kakapo                      CT---GGTGATGCCTAATGCCAATGCCTATGTTTTGGTGCTTTGAACGATTGCTCTGGTAGCAC------TGGTAACCTC 28463
GoldenEagle                 CTAGGGATAATGACTAATGCCAATGCCTACATTTCAGTGCTTTGAATGATCACT--GGTAGCAC------TGGTAACCTC 32232
JapaneseQuail               CTGGTGATAATGAGTAATGTGAATGCCTACATTTCAGTGCTTTGAATGATTACTCTGGTAGCAC------TGGTGGGCTC 26363
MediumGroundFinch           CTAGTGGTAGTGGCTAATGCCAATGCCTAAATTTCAGTGCTTTGGATGATCACTCCAGTAGCAC------TGGTAACCTC 14437
GoodesThornscrubTortoiseCCAGCTGAAGTACTTGCTGC--ACCTTCAGCTGTTGGGCCTTGTGCTTAGCTCATTGAGAGTATATCTGGCAGTGATATC 55536
```

```
Majority                  AGCTTCTGTGATCTGCTGTTCCTGGAGAACAATACCTTAATGATCACTGTAGAGACAAAT-GTTCTTAATAACAAAAAAA

                                   60810     60820     60830     60840     60850     60860     60870     60880

Human                     CCCTACCTCCATTATTTTTCCACAGAGTACTAGGAATCATCTC--AACGTCAAGTCATATTGCTTCTAATCACAATACCC 60278
Kakapo                    AGCTTATGTCATCTGCTGTTCCTGGAGAACAATAACTTGATGA---CTGGAGAGACAAAT-ATGCATAATAAAAGCAAAA 28539
GoldenEagle               AGCTTCTGTGATCTGCTGTTCCTGGAGAAGAATACCTTAATGATCACTGTAGAGACAAAT-ATACTTAATAACAAAAAAA 32311
JapaneseQuail             TTCTTCTGTGATCTGCTGTTCCCAAAGAACA---CATTAATGCTCAGTGCAGAGATAAAT-GTGCATGTTAGCAGAAAGA 26439
MediumGroundFinch         AGCTT--GTGATCTGCTTTTTCTGGAGAATAATACCCTAATGATCACTTTAGAGACAAAT-GTACTTAATAACAAAACAT 14514
GoodesThornscrubTortoise  AGCAT-TTCATCCTCCTATTCATTGAAGATCAGTCTTTTCCAAT-GCCAGGGTAGCAAG--GTTCTTAAAAGGACTTTGT 55612

Majority                  TTTTTTG-------TTTTGCTCTTGCCTC----TGCAGAACCTATTTCACAATAAATATAGCTATATATGTG--------

                                   60890     60900     60910     60920     60930     60940     60950     60960

Human                     TTTAGTGACT---TTTTTGTCCTTGCAATAAAGTCCACAGTCCTTATCATGGCTTATAAGACTCTAAATCAGTCCTCAGC 60355
Kakapo                    TGTTTTC------------CTCTTGCTTC----TGCAGAACCTATTTCACAATAAAGATCATTACATATGGGGGGGTTAG 28603
GoldenEagle               TGTTTTG------------CTCTTGCCTC----TGCAGAACCTATTTCACAATAAATATAGATGCATATGTA-------- 32367
JapaneseQuail             AAAAATA-------TTTAGATTTTGCCTC----TGCAGAACCTATTTTCAAAGAAAGGTATGTATGTATAC--------- 26499
MediumGroundFinch         CATTTTGATCCCCATTTTGCTCTTGCCTC----TCGAGGACCTATTTCACAACAAATACAGATACATATGT--------- 14581
GoodesThornscrubTortoise  CTTCATCCTCCAGTCTAAGAGTTTCTTCC----TGTGGAATCT-TAGCACAGTTCTAGCAGCTTTTAATGAGGCTTCCAT 55687

Majority                  -------------------TC-----------------T-------T---------T-------CTGTTTTTAGTAGA

                                   60970     60980     60990     61000     61010     61020     61030     61040

Human                     TTGCCTCTCCATTCTCCTCTCATGCCTTTTTAGCTATATTGGACTTCTTTAAATAGCTTGCATATACTGTATTTTCTCTG 60435
Kakapo                    ACTAAGTGATCTCCGGAGGTCCCTTCCAACCGTGATGATTCTGGGAGTCTGTGATAAGT-------CTGTTTTTAATAGA 28676
GoldenEagle               -----------------------------------------------------------T-------CTGTTTTCAGTAGA 32382
JapaneseQuail             ------------------------------------------------------------------CTGTTTCTAGT--- 26510
MediumGroundFinch         ------------------------------------------------------------------CTATTTTTAGTAGA 14595
GoodesThornscrubTortoise  TTGACCCCTTGGCATCTTGTCTGTTTCTGCT--------TTTGTG--TCA--GAAGACT-------TTGTTCCTGATAGT 55748

Majority                  ATAGGACAATTAGCAAAAGT---AAC----------GTGGTGACCTTTTTATACAAATTATTGGGAGAAAAAAG-GACT-

                                   61050     61060     61070     61080     61090     61100     61110     61120

Human                     ATGTCTCGATCTTACAGATTGCTGTTCCATTTGCTAATACTATTTTTTCTCTTCATCTCTTTATGCCCAACTTCTGCTTC 60515
Kakapo                    ACAGGAAGATTAGCAAAAACCTAAACCA------CGGCAGTGACCATTTCATACAAATTATTGGGGGAGAAAAGAGACA- 28749
GoldenEagle               ACAGGAAAATTAGCAAAAGT---AAC----------GTGGTGACCATTTTGTACAAATTATAGGGGGAGAAAAA-GGCA- 32447
JapaneseQuail             A------------CAGAAAT-----G--------------TTTTGTTTTTTTTCCAAT----AGGAAAA----------- 26544
MediumGroundFinch         ATTGAAGAATTGGCACAAGT---AAC----------ATGGTGACCAATTAATGCAAACAATAGAGAGATAAGA--GACT- 14659
GoodesThornscrubTortoise  C-ATAACATCCATAAGGAGGGTTGCGGG------CACTGATGACCTCCTTATACACAATTCTCTAAGGACAAAGTGATT- 55820
```

```
Majority                 --TCAACTTACCACAAGTCAGATGA------TTGTTTTAAGGTTAATTTTTT---ATGTTTCCTTTTTGTTG--------

                                   61130     61140     61150     61160     61170     61180     61190     61200

Human                    TCTTTGTCTTTCAAGTATCAGTTTAGAAGCCTCTTCCTGAGATAAGTCTTCTGTAACATTTCAACTAGATTAGATCCCTT 60595
Kakapo                   --TCATCTTACCACAAGTCAGATGA------TTAATTTAAGGTTAAT--------ATGTTTTCTTTAAGATG-------- 28805
GoldenEagle              --TCAATTTACTACAAGTCAGATGA------TTGTTTTAAGGTTAATTTCTT---ACGTTTTCTTTTTGGTG-------- 32508
JapaneseQuail            -------TTAGCAAAAAT--AGTGA------T-GTTGCAAAATAAAT-----------TTTCCT---------------- 26581
MediumGroundFinch        --CAAACCTACCAGAAGTCTGATGG------TTATTTTAAGATTAATTTCTT---ATGTTGCATTTTTGATG-------- 14720
GoodesThornscrubTortoise -T---CCTACCACACCTGAAATTT------TTG-TGTAAAGTGGTTTCTC-----AGTTTCATTTGAATCG-------- 55875

Majority                 ----TTACAAAAGACAGTGT-AAGTACCAATTT---A-TTTCTAATACTTGTATTTTTTTTTATG-A--GTAGGTGCCTA

                                   61210     61220     61230     61240     61250     61260     61270     61280

Human                    CCTTTCTTAGTTCTCAGTATCACACATAAAATTTATAATTTGTTAAATGTGTATATTTTCTGTCGGACTGTAAGTTCTGA 60675
Kakapo                   ----TTAAAAAAGACAGTGT-AAGTGTCCTGTC---T-TGGGTAAGGCACGTAGATAGTGAAATGAAGAGAAGCTGCGTG 28876
GoldenEagle              ----TTACAAAAGACAGCGT-AAGTACCAAGTTA-CAGTTCCTGTTGCTTGTGTCCTGTCTTAGGTAAGGTACATAACTA 32582
JapaneseQuail            ---------------AATGT-GATGACCTTTTT------------------TATTTTATTTCAAG--------------A 26613
MediumGroundFinch        ----ATACAAAAAACAGTGT-AAGTGCCAAATT---AATTTCTATTAATTGTGTCCTCTATTAGG----ACATGTAAATA 14788
GoodesThornscrubTortoise ---GT--------CAGTAT-ATTTACCTATGTT----CTTCTCAAAGCTGCATTCATCTCAATACAT--TAGATGTCAG 55936

Majority                 GTG--ATGTCATAGCTGTTATC-G--G-TTTGA--AAGCAATTTCAGGGTATTCTGTTGAGAGAC-----ATTTCGGTTC

                                   61290     61300     61310     61320     61330     61340     61350     61360

Human                    GTACCATGTCTGCTTTGCTTTCTGCTGTATTTATTAAACATTGACATTCAGGGTGAAAATCAGAAGTCATAGTAACAATC 60755
Kakapo                   GAG--ACCTCAGAGCAGCTTCCAG--GGTCTGA--AGGGGGCTACAAGG-ATGCTGGAGAGGGACTCTGCATCAGGGACT 28949
GoldenEagle              GTA--AAATCATAGCCGTTACC------TTTAA--AAACAATTTCAGGGTAATCTGTTGAAAGGC-----ATCTCGGTTC 32647
JapaneseQuail            TTG--TTTTCATT-CAATCATC------TTTGA--AAG----TG--------TCT--------------------GTTT 26649
MediumGroundFinch        GTA--AAGTTATAGCTGTTGCC------TTTGA--AAGCAATTTCAGGGTAATCTATTGAAAGAC-----ATTTTGGTTC 14853
GoodesThornscrubTortoise GTG--ATGTCTAGCCTTCTATCTG--GACAGGACTAAACCATTTCT---TCTCCTCACCTGTTTCTGTCATGTGCAGATC 56009

Majority                 ATATCAA---GTAAAGTGT----TGATT--TTCCTATTGATCTTTCTAAA---CATTTTAG---TTCTTA--TAGAATCT

                                   61370     61380     61390     61400     61410     61420     61430     61440

Human                    TTAATTATCAATAAAAAGTAT-TTTATGCGATCCTCATATTTATTAAAGAAAACACCTTAATACCTTTCA--GAAAATCT 60832
Kakapo                   GGAGCGACAGGACAAGGGG----TGATGGGTTCAAACTGAACCAGGGGAAGTTCAGGTTAG---ATCTAAGGCAGAAGCT 29022
GoldenEagle              AGATCAA---GTGTAGTGT----TGATT--TTCCTATTGATCTTTCTAAA---CATTCCAG---TTCCT---TAGAATCT 32709
JapaneseQuail            ATGTAAA------------------------TCC--T----------AAA-----TG-TAG---TTTTT---TAGAA-C- 26679
MediumGroundFinch        AGATCAA---GTATAGTGT----TGATT--TTTCTGTTGGTCTTTCTAAA---TAGTCTCG---TTCCT---TAGAATAC 14915
GoodesThornscrubTortoise ATATGAAGGGACAAATGGTCTCTTCACAGACAATCTCTAAATGGATAACATCCCATATCAAGACTGCTTATGAAAAAACT 56089
```

```
Majority                    TTTGCTTTT-TTGGTGCTAAGGGT--TTGTCAAAGGTTGAGCGTCGATTCTCTTTCTATCCCATCCCT-ATAGTGTTCA-
                                     61450     61460     61470     61480     61490     61500     61510     61520
Human                       TGTTTATTTATTGGAAAGTAGTGTATTAGGCAAAGATGCGTCTTCTCTTGGCTTTCTAATTCAGCTTTTATAATGTTGAG 60912
Kakapo                      CTTCCCTGTGAGGGTGCTGAGGC--GCTGGCACAGGGTGCCCAGAGAAGCTGTGGCTGCCCCATCCCTGGCAGTGTTCAA 29100
GoldenEagle                 TTTGATAGTTTGTCTGCTAAAGGTACTTTTTAAAAATTGAGGGTTGGTTTTCTTTTTTTCCCCCACAAAATGTCAGTTAT 32789
JapaneseQuail               -------------------------------------------------------------------------------- 26679
MediumGroundFinch           TTTGAT-------------------------------------------------------------------------- 14921
GoodesThornscrubTortoise    TTGGCTATGCCTCCTCAGAGGGTTCAAGCTCATTTGATGAGAGCCCAAGCAAAGTCAATAGCGTTCCTCAGTGCTGTCTC 56169

Majority                    GAA-ATGAT-AATTGAAGGGCTT---TATCTACTT-TTCAAATGTAGAGTAA-GCTT-ACATGTGTCAGC-TTT-CACT-
                                     61530     61540     61550     61560     61570     61580     61590     61600
Human                       GAATATAATTACTTGATGCATAAAAATATCAATCTTTTTAATAGTATAATAAGACATATAATGTAATCTCCTTTTCACCT 60992
Kakapo                      GGCCAGGTTGGACACAGGGGCTTG--GAGCAACCTGCTCTAGTGGAAGGTGTCCCTGCCCATGGCAGGGGGTTGGAACT- 29177
GoldenEagle                 AAAGAAAAGAAAAATAAATACCTT--CATTTACTTCTGAAAAAATAGAGTAGAGGTTGAATTCTGTCAGCACATACAGT- 32866
JapaneseQuail               -------------------------------------------------------------------------------- 26679
MediumGroundFinch           -------------------------------------------------------------------------------- 14921
GoodesThornscrubTortoise    AATATTGGACATTTGCAGGGCTGC--TATATGGTCATTCATATACCGATTTATGAACTACACCTGTCACATTGTCTGAA- 56246

Majority                    -GGATGTACATACTGTTTTTTCCTATCTCTTACATTGT-GTACTCT-ACGTTGGCTG-CGTCTTTAGTGG-ATTGTTTTT
                                     61610     61620     61630     61640     61650     61660     61670     61680
Human                       GGGATTTAAAAATTGTTTTTACATAGTCCTTACATTATAACACACATAAGTAGGAAATGGTTCTTTTTGGTTTTTGTTAT 61072
Kakapo                      -GGATGATCTTAAGGTTCTGTCCAACCCAAACCAGTCTGGAATTCT-ATGATAGCTGCTGCCTTTAGAAACAATAGTAAT 29255
GoldenEagle                 -GGATGGACATACTCCCTTAAGCTTTCTCTTACATTGTCTTTCTCTTACATTCTCTGGCTTCTGTGGTGGTACTGTTTTT 32945
JapaneseQuail               -------------------------------------------------------------------------------- 26679
MediumGroundFinch           -------------------------------------------------------------------------------- 14921
GoodesThornscrubTortoise    -GTGGCTCACCACTGCAAATTACTATCTCAGGGCTGGCTGTCAGAAAACGAGGGCAGACACCCCAAACGG-GTTGTACTA 56324

Majority                    TTG-TTCA-GT-TTCA----TTTAACTT-ATGTGAAGTATTGATTTTTCAG-----TTTATGCATCGAGTCGTAT-AATG
                                     61690     61700     61710     61720     61730     61740     61750     61760
Human                       TAGAAACAGCTTTTCAGAAACATGTCTTTATTAGGAATATCTTTAGTTGACACTGGACAGTATATTGTGTCTTCTGGAAG 61152
Kakapo                      GTG-TTCAAGGACGTCTTGGTTCAGATT-AAGTATAGTATTGATTTTCCTG-----TTGATGCTTCTAAACGTTTCAGTT 29328
GoldenEagle                 GTACTTCACGTGTTCACCTTTTCAACAT-ATAGAATGTACTGACTTTTCAGCAGTTTTTATGCCACCATACGCATTAATA 33024
JapaneseQuail               -------------------------------------------------------------------------------- 26679
MediumGroundFinch           -------------------------------------------------------------------------------- 14921
GoodesThornscrubTortoise    TAATTAGATTACACCAAGCCAGTAACAA-ATGTGAGCTTCTAGCTCACTATTCCAGTGTTACCATGGAGTCACAGACAAG 56403
```

```
Majority                TCCTGTTTTCATGT-CT-CTTATCTTGCCAAACA-ACTTTTTGTTAATTGTGATTTATTTTTTTTAAAACCTTACATGTC
                                 61770     61780     61790     61800     61810     61820     61830     61840
Human                   TCCTGTTTATATGATCATTTTATTTTAACATATAAGTGGGTTGTTTCTTATTCTTTATTTCTTCCAGGAGATTCCATTTC 61232
Kakapo                  CCTCAGAATCTTGTGATAGTTTGGCTGCTAAAGGTACTTTTTAAAAACTGAGATTTTTTTTTTTTGTTCCCCAAAATGTC 29408
GoldenEagle             CCCAATTTTAACATATTCCCAGTCTTTCAGGTCTCTATTTTCTTTAATTTAGAACGAAATAATATAAATAGGTACTTGGC 33104
JapaneseQuail           -------------------------------------------------------------------------------- 26679
MediumGroundFinch       -------------------------------------------------------------------------------- 14921
GoodesThornscrubTortoise TCCTGTTACCCTCTCCAGCCTATCTTGCCAACCAGACAAATTGAACTTTGTGATTAAAGTTTATTTAAACCTAAAATCAC 56483

Majority                AGTAC-ACAGGATAATAAATATCT-TATGAGACCTA-TATTAACCGCAGAACGGTTGGATCTTCT-TTTTTTTCTCAGGA
                                 61850     61860     61870     61880     61890     61900     61910     61920
Human                   ATCACTAACTGATAATAAAAATAAATATGAGATAAAACATTTGCAGCAGTAAGATTATGTCATTCATTTCCTTCTCACAG 61312
Kakapo                  AGTGATAAAGAAAAACAAATACCT-TCATTTACCTAGAAAAAAGAGAATAGAGGATGAATTCTGTCTGCACATCTATGGA 29487
GoldenEagle             AGTAA-GCACTCTAACTAGTATCT-TATGAGGCATTCTGTAAAACACAAGACAGTTGGATATTCTTTTTTTCTGTAAGGA 33182
JapaneseQuail           -------------------------------------------------------------------------------- 26679
MediumGroundFinch       ---------------------------------------------------------------------T---------- 14922
GoodesThornscrubTortoise CCCACATCAGGTTTCTCCCAGTCC-CAAGAGACCAGTTACTCACCCTAGATCAACTGGTACTCCAGACCTTTCACCAAAC 56562

Majority                AGAATA-AGTCCTTTAAGAAG-TTCTT-TAGACTTCTA-TTTTATTTTTTATGCTAAATACTTAGGTATTGATTGTCTT
                                 61930     61940     61950     61960     61970     61980     61990     62000
Human                   AGTACACATTGCTTTATGCAGATTCTTTGAGAGACCTAATGGAATTATCCTTTGCTAATTTCTTGGGCAAAGATTCTCCT 61392
Kakapo                  TGGACATAGTCCTTTAAGAAGCTTTCCCTTGCATTGTCCTCTGGCTTCTGGGCTAAAACTCTTTTTGTACTCCATGTGTC 29567
GoldenEagle             AAAATAAAGGTGGTGCAGATATTGCTTGTAGACTGCCTTTTTTATTTTTTTATGCTCAAAACTTAGGTTTTTTTTTTTTT 33262
JapaneseQuail           -------------------------------------------------------------------------------- 26679
MediumGroundFinch       ------------------------------------------TATTTTTATA---------------------------- 14932
GoodesThornscrubTortoise ACAATGGTCACAGTCAATTCTGTAGTAAACTGCCTATAGATTTATTTGCTAAGAAAAAAGAATGAGTTATTGAGAGGCTA 56642

Majority                TGACATGTAATTG-TAAGCATTGTCTTTCTTTTCAATTCCTTTTGATAGTGACTTTTT--CGAAATCAGTA--TATAAAG
                                 62010     62020     62030     62040     62050     62060     62070     62080
Human                   TGACATCTGCTTAATTTACAATCTGTATATTTTACATTCATATTAATGGAGAATTTATGATGAAATCAGTGGTTTTAGGA 61472
Kakapo                  CACCTTCTCAATGTAGAGGATGGGCTCACTTTTCAACAGCTTTTAATACACATTAATA--CCCAATTTGAACATATTCCC 29645
GoldenEagle             TAATCTGTATTTG-AAAGCTTTCTCATCCAGCCAAATTCCTTTTGAAATTGTCTTTTT--CTGGATGATCAAGCAGGAAG 33339
JapaneseQuail           -------------------------------------------------------------------------------- 26679
MediumGroundFinch       ---------------------------------------------------------------------------GC--- 14934
GoodesThornscrubTortoise CGACAGGTAAAAAATAACAAGTGCAGTTTGTAACTCCAAATGGTGGCAGTGACGTAAT--AGACACCAGTTTCCCAAAAG 56720
```

```
Majority                 TGCTTTAGGGGTCTCCAT-TTAATACTTG-GAGTTGAGTTATATGAT-----TAAGTA-TTGG-C-AAGGTATTAT-TTA
                                  62090     62100     62110     62120     62130     62140     62150     62160
Human                    TGCTTTAGGAAAATCCTGCATAATCTACCAGGGTGGGGTTATTTGATATTGGTAAATAATTAGTCTGAAGTATTACATTA 61552
Kakapo                   AGCATTCCAGGTCTCTATTTCTTTAATTTCAAGCTAAATAATAGAAA-----TAGGTACTTGGCAGTAAGTATTCTGTTA 29720
GoldenEagle              ATGTGAAGAAGTCAGGATGTTAGTACATGTAACTGGAAAGGTAGGAT-----AAACAAGTTGGAGAAAGGTGCAAATATC 33414
JapaneseQuail            -------------------------------------------------------------------------------- 26679
MediumGroundFinch        ----------------------------------------GAT-----TA------------------------------ 14939
GoodesThornscrubTortoiseTCTTTCAGGGCTACCCAGAATAACTCTGGGGAGCTCCGTTTTCTCAT-----TCAGTTTTTCTGCCATGTTAGAATCCAA 56795

Majority                 -TAGTATAAACATAG------ATTGAT-A-AATG-TGTTT-TCTATG-TTT-CTATATAC-GACAAGTC-ATATAAATAG
                                  62170     62180     62190     62200     62210     62220     62230     62240
Human                    GTATTATAAGCTTAATTTTGTGTTGTTGATTTTGATAATTTTTCATGACTTGCCAAATATTAAAAAGTAGATATTTAAAG 61632
Kakapo                   CTAGTATCTCCAAGGCATTCTATACAACACAAGG-TGGTTGGCTATTCTTTTTTCTGTAAGGAAAAATAAAGGTGATGCA 29799
GoldenEagle              TTAGCTGTAAAGAAG------ATAGAAAAAAATG-TGTTTAACCAAGGGGTACTTTAAACAGCCAGGCCTTTAGAAATAA 33487
JapaneseQuail            -------------------------------------------------------------------------------- 26679
MediumGroundFinch        -------------------------------------------------------------------------------- 14939
GoodesThornscrubTortoiseACAGTCTAGAGATACAGGAGCCTTTCTTTGAATT-CATATCTATATCTTCTCACAGAAGCCCACCCTCCCACAGAGCTGG 56874

Majority                 GATCTGGAAGGTAAGCTTCATTTAGATATGCGATTGTG-TTATCTCCGGCAGTGTAGCTAGGAAGAT-AATT-TAGTTGG
                                  62250     62260     62270     62280     62290     62300     62310     62320
Human                    AATTTGGAAGTTAAGGTTTCTCTCAGTGTGTGTTAAATCTGATCCCCAACAATCTCTTCAAAAAAGTAAGTTATCAATAG 61712
Kakapo                   GATGTTGCTTGTAGTCTTCTTTTGGATTTGAAATTGTCTTTGTTTCTGGATGTTCAAGCAGGAAGATCTAAAGAAGTTGG 29879
GoldenEagle              TGACAGTAGGGAAAGTTCCATTAAGCTATGCTATTCTGAATTTCTCAGAAATAGTAGCTTCCAAGATTATTTTTAGTTAT 33567
JapaneseQuail            -------------------------------------------------------------------------------- 26679
MediumGroundFinch        ----------------CCCA------------------------------------------------------------ 14943
GoodesThornscrubTortoiseGAGCGCAGACGGTAGCGGGGCCCACACAAACGCTGGCGGCCAAGACCTGCAGCGGAGCTGGGGGCTGGGACCCCTGGCGC 56954

Majority                 GTT-TCAT--GTGGAGTTGAAA-AGTTTTGTTTAGGCTTATGATGTGCCCTTTC--TTTTCA---T-TTATT-TGCTGCT
                                  62330     62340     62350     62360     62370     62380     62390     62400
Human                    TAAACGATGAGAAAAATGGAAATAGTTTTATTTTGGCTTTTCATGTTTTCAGTCCATATGCAGTGTTTCATTGTGCTGCT 61792
Kakapo                   GATTTAGTCCATGGAACTGGAA-AGATAAGATAAAAAATATGAAGAGGAGTGCAAGTGTCTT---AGTTATAAAGAAGAT 29955
GoldenEagle              GTACTCAG--AATGAGTTGACT-CTGAATAAATAGATTCAAAAGGGACCATTTAAATTTTCA---TCTCAGCTTTATAAT 33641
JapaneseQuail            -------------------------------------------------------------------------------- 26679
MediumGroundFinch        -------------------------------------------------------------------------------- 14943
GoodesThornscrubTortoiseATTGCCAGGAGTGGAGCTGTGG-GGTCTGGTCCGGGCCCCAGCTCTGCCCCTTC--CTCTAA---CATTACTCCGCACCC 57028
```

```
Majority                  TCTATGAAGCTTTTTATCCTGATTGACGTT-GCA-ATTTTGATTA-GGTGTTTTA-TT-TAAATTTAAAATTTATATCAA
                                    62410     62420     62430     62440     62450     62460     62470     62480
Human                     TTAATTATGCTTTCTGTGCCTAATATCTTTCCTGTGTTTTGATTTCCATGTTTTAGCCATAAACTTTAAATTTACATGAA 61872
Kakapo                    TAAAAAAAACTTTTAAACATAATGAAAGTAGGCAAAGTTCCATTAAGCTGTTTTATTC-TGAATTCCTCAATAATAGCAG 30034
GoldenEagle               TCTATGAAAATCCTTTTCCTGTGAGACATCAGAACACTGTGAGAGGAGCATTTAAACT-CAGATTTAAAACAAACATCTC 33720
JapaneseQuail             -------------------------------------------------------------------------------- 26679
MediumGroundFinch         -------------------------------------------------------------------------------- 14943
GoodesThornscrubTortoiseCCTGGGGGGCACACCACACAGATTGAAGATTTCTGATATACATAATGGCCACTTGCTT-GAAATTAACTTTTTCTGTTAA 57107

Majority                  ATCCCTTGTTTCTTTTTGTTTCTGTTCTCAGGATGA-TTTGCTCTGTGAGTAAGT-TGTATGGGTAATTTTCA-TTAATT
                                    62490     62500     62510     62520     62530     62540     62550     62560
Human                     AACTTTTTTTTCTGATTGTTTTTGCTTACTGTCTAAACCTTCACTGTGAGTAGGCACATTTGGAAGGTCTTTCTGCAAAT 61952
Kakapo                    CTTCCAGGACTGTTTTTAGTTCTGTGCTCGGGATGAGTTGGCTCTGAAGAAAACT-TAAATGGTCCATTTTGAATCTGTT 30113
GoldenEagle               GTACCTTGTTTCATACAGTATCTAGACTCAGAAGGATTGAGAACAGAGTGACCTC-TGTAACTGTAATTCTCT-TTAATC 33798
JapaneseQuail             -------------------------------------------------------------------------------- 26679
MediumGroundFinch         ----------------------------C--------------------------------------CCT---------- 14947
GoodesThornscrubTortoiseAGCCCTTAAGTCCTTCATTAGCATTTCACAAGATTTCTTTGATGTGTAATTTAGT-TGCAAGAGTATTTGCCATCTAAAT 57186

Majority                  TTTT-CCAGTTCTTAATGCCAGGTATAT-T-TTTCAACACAGTCATCGCAACATTGAG-AATATTTAAA-G--TT--AAT
                                    62570     62580     62590     62600     62610     62620     62630     62640
Human                     TATCTTGAGCTTTTAATGTCAGAAATCTTTGCTCCAACAAGATCACCACATTCCTGATTGCTGAACCACAGGACT--GGT 62030
Kakapo                    TTCTCTCAGCTCTTAATTCCATGTAAATCCTTTTCCTGTGAGACATCAAAACACAGAGGAGCATTTAAACTCCTTTAAAC 30193
GoldenEagle               ATCAGCCTGTCATAAATGCATGGTTTTAGTCTTTCTGCACAGACATGGCAACATTTAGAAATAAATGTATGTTATACTTT 33878
JapaneseQuail             -------------------------------------------------------------------------------- 26679
MediumGroundFinch         -----C-------------------------------------------------------------------------- 14948
GoodesThornscrubTortoiseCTTTACTATTACGTTGTGACAGGTATAGATAAGTGAAAACAGTACAAGTAACTTTCCACTAGTTTCAATGAAGTTTAAAC 57266

Majority                  CTGTGTGTTCCTGCTATTTTTTGTGAATTTACTT--AT-TGGCCTAGAAGTGAGAATG-CTTTATTTATTCACTTTAATT
                                    62650     62660     62670     62680     62690     62700     62710     62720
Human                     TTGTGTGCTACTGCTATTTCTTGGGATTTTGCAAATACATGAGCTTGAAGTGAGCTTGCATATATTTTTTAAATTTGATT 62110
Kakapo                    CAACCATTTCTTACCTTGTTTTGTCTGGTATCTGGACTGAGGAATCGAGATGACAGTGACCTCTGTAATTCTCTTTAATC 30273
GoldenEagle               CTGAATGTTGCAGCTGATCTTTTTAAATTAATTTCCATTTTGCAAAAATAAGAAAAAAGCTTTACATACTGCTCAAATGT 33958
JapaneseQuail             -------------------------------------------------------------------------------- 26679
MediumGroundFinch         -------------------------------------------------------------------------------- 14948
GoodesThornscrubTortoiseATCTGAATACATTCTATACATTCAGCAATTACTTTGATCTATCCTAATAGAAAGTAAATTGGCCTGAATACACTCTTACA 57346
```

```
Majority                  TTTAACAATTGCTTATTTGTGTATG---ATGCTAACTAT-TATTTTGAATTTATGGATTAATATACTCATA-GCCATAAG
                                   62730     62740     62750     62760     62770     62780     62790     62800
Human                     TTACTCCCTTTCTTATTTGTGGTCACCTCCTTTAACCAACTATTTAAAATACCTGGATTTTTGCTCTCATACACCCTCAG 62190
Kakapo                    ATTACGGGTTTAGTCTTTCTGCAC----AGACACTGCAA-CATTTGGAAAGGAAGAAATACTACATTTAGA-AAGAAAGA 30347
GoldenEagle               CTTAAAAAATGGCTATTTATATATG---ATACGAAATGT-TATTTTGAACTTGTTTGACAAAATACTCACT-GCCATAAT 34033
JapaneseQuail             -------------TTTCTTGAAAAA---ATGCTCACTGT-TGTATTCGTTCTATAGATTACCCTTTTT--------TGAG 26734
MediumGroundFinch         CC--AAA--TGACTACTTAGGTATG---ATGCTAGATAT-TATTTTGAGGTTGTTTGACAAAATACTC-T------TAAG 15013
GoodesThornscrubTortoise  TCTAACAATCGCTTTTGGGTACTGC---TTGTGAGTCACCTAAATGGAATACATGGCTGCATGTACTCAAG-GAAGAAGA 57422

Majority                  AATTT--TATAGAT----TATATCCTTATATGCT-----------TCCTGTATTCCTTC---------GTGTATTCACAT
                                   62810     62820     62830     62840     62850     62860     62870     62880
Human                     CTCCTATTTCTTTCCTCTTTTGCCAACAGATGATTAATTTACATCTTGTTTGCTTATTTCTCTAGCTCTTGTACCTTAGG 62270
Kakapo                    AATATCATATAGAT----TTTATCCTTACATGGTGA---TGTT-GTCATGTATTCCTTC---------GTGTATTCACAT 30410
GoldenEagle               AATTT--TACAGAT----TATATCCTTATTTGCC------------AATGTATTCCTTC---------AAGTATTCACAT 34086
JapaneseQuail             AATGT----TAAGT----CATGTACAT-T--CCT---------------TCACTCCATC---------GTGTGTTTACAC 26779
MediumGroundFinch         AATTT--TAATATT----TACCATTTTTTTAGT------------CCTGTATTCCTTC---------ATGTACTCACAT 15066
GoodesThornscrubTortoise  AATTTT-TAAAGATGTGATGCAGACGTGTATTCCAC---GACCCATCCTACGTCCCCTCTGCATTGGAGTTTAGTCTCTG 57498

Majority                  TA-ACTTTTGTGGAATGAAAT---ATGATGTATAAGCATATATGGATAGTGCTGACAAGGGTCATATGTTAAAATTG-AC
                                   62890     62900     62910     62920     62930     62940     62950     62960
Human                     TATACTTCTGCATCTTCCACCTACATAAAGTTTTTGTTTTTGTTTTTAATGTTCAGAGGGA--AAATTTCAAAGGGT-CC 62347
Kakapo                    TA-ACTTTAGTGGAATGAAAT---ATGATGTATAAGCATAAATGGGTAGTGCTAACAAGGGTCAGATGTTAAAACTG-AC 30485
GoldenEagle               TA-ACTTTTGTGGAATGAAAT---ATGATCTATAAGCATAGACGGATAGTGCTAACAAGGGTCATATGTTAAAATTG-AC 34161
JapaneseQuail             TA-ACTTCAGTGCAGTGAAGT---ATGATGTGTAATCATATGTGAATAGTGCTTTCAAGGGTTGTATGTTAAAAGTA-AC 26854
MediumGroundFinch         TA-ATTTTAATGGAATGAAAT---ATGATGTATAAGTATGTATAGACAGTGCTGACAAGGGTCATACGTTAAAATTG-AC 15141
GoodesThornscrubTortoise  CA-TGTTCTTTGCAAAGGAACTGAGGGAAGTTGAAGCAACACCATCTCATATAGCCAGGAG--AGGCATTATAGCCACAG 57575

Majority                  TTCACAAGTTTTGGTTCTTTGGGTTA--TACAGTTTAA-ACTTTTCCAGTC--ACTTTAAGGAGTG-------AGTTTTA
                                   62970     62980     62990     63000     63010     63020     63030     63040
Human                     TGGAGGGCTTTCTAACAATTGGCATATTCATTGTTAGATAATTTGGCAGTCGGACCTCAGTATTGCTGGGAAAGAATTCA 62427
Kakapo                    TTCACAGATGTTGGTTCTTTTGATTA--TACAGTCTAG-CCCTTTCCAGTC--ACTTGAAGGAGTG-------AGTTTTA 30553
GoldenEagle               TTCACAAGTTTTGGTTCTTTGGGTTA--TACAGTCTAA-ACTTTTCCAGTC--ACTTTAAGGAGTG-------AGTTTTA 34229
JapaneseQuail             GCCACAAGTTTCGGTTCTGTAGGTTG--CACGATTCAG-CATTCTCCAATC--ACTTAATGACATG-------AATGTTA 26922
MediumGroundFinch         TTCACAAGTTGTAGTTCATTGTGTTA--TACAGTTTGA-ACTTTTCCAGTC--ACTGTAAGTATTG-------ATATTTA 15209
GoodesThornscrubTortoise  CGCGCGAGCACCATCCCTTTTGAGTAC-TGCTAGGCAA-AATTCTCCAGCT--CCGGTGTGGTGGGCATGCACACACCTA 57651
```

```
Majority                       ATTGCTATG-TGCTTCTTC-----TGTGCTTACAGAAAGAGTAGGAAATAATT----------------------ATGCA

                                       63050     63060     63070     63080     63090     63100     63110     63120

Human                          ACTCCTAAAAAACTTAGTG-----TGTGTTTAGTAATTTTGTATCAGCACTTTTAGCATTAACCTAGATACTGTGACTCA 62502
Kakapo                         ATTCCCATG-TGCTTCTCC-----TGTGCTTACAGAAGGAGTAGGAAATAATT----------------------ATGCA 30605
GoldenEagle                    ATTGATATG-TGCTTCTTC-----TGTGCTTACAGAAAGAGTAGGAAGTAGTT----------------------ATGCA 34281
JapaneseQuail                  ATTG--GTG-TGCTGCTTC-----TGTGCTTGCAGATAGAGTAGGAAATAATT----------------------ATACA 26972
MediumGroundFinch              ATTGATATG-AACTTG--------T--------ATAAAGAGTAGGAAATAATT----------------------ATGCA 15250
GoodesThornscrubTortoise       AGTGGAATACTGCTGCATCACATCTCGAACCACAGTAACAGTAAGAAATTGTTTGTTTTAAAAAATTGTCTTAAAATAGA 57731

Majority                       ACTCGGCCAAATATCTGACCAGCTTGAAAGTTGTGTTTTTCATACATTAGGGAAAAAA---AGAACCTGATTGTTATCAA

                                       63130     63140     63150     63160     63170     63180     63190     63200

Human                          AACCCAGCTCTTTTCTCATAAGGTATCATTTTCTCT-TTGATAACATTGAAGTAGTCACCTAAGAGTGAATTTTGCTACT 62581
Kakapo                         ACTTGGCCCAATATCCGGCCAGCTTGAAAGTTACATATTTCATACATTAGGGAGAAAA---AGAACATGGTTGTTACCAA 30682
GoldenEagle                    ACTCGGCCAAATATCTGACCAGCTTGAAAGTTGTGTATTTTATACATTAGGGAAAAAA---AGAACCTG-TTGGTATCAA 34357
JapaneseQuail                  ACTCAGCCAAAGGTCTGAACAGCCTGAAATTTCTGTTTTTCGTACGCTGGGGGAGGAA---AAAAAC--ATCTTTATCAA 27047
MediumGroundFinch              ACTCAACCAAGTATCTGGCTATCTTGAAAGTTGTG--TTTCATGCATTAGGGAAAAGA---AG--------TGTAATTGA 15317
GoodesThornscrubTortoise       TCTTTTTCATCTGTCTTTTAAATGTTGATGCTGCTTTTGTTTAACAGTAGAACAAAAA---ACATTCTACTAGAACACTG 57808

Majority                       AAGATATTTTTATAG--TTACAGTAATT-AATGTTAAATGTAGAGGTAAA---TTTTGTTTTCCCAACTGGTGTTCACCA

                                       63210     63220     63230     63240     63250     63260     63270     63280

Human                          TATCTTTTGATATTTATCTATTGTGTAGAAACATCAGTTCTATTACTTAT--CTCACATTTTCCCTATAGGAATGCTACA 62659
Kakapo                         AAGCTGTTTTTACAG--TT---GCAGT--ACTGTTAAATGTGGAGGTAAA---TCTTGTTTTCCCAACTAGTGTTCACCA 30752
GoldenEagle                    AAGATGTTTTTACAG--TTACAGTAATT-AATGTTAAACATAGAGGTAAA---TTTTGTTTTCCCAACTAGTGTTCACGA 34431
JapaneseQuail                  AAGGCATTTTTATGGAATTACAGTAATT-ATTGTTAAGTGCAGAGTTAAA---TTTTGTTTTCTCAGCTAGAGTTCATCA 27123
MediumGroundFinch              AAGAGATTTCAAT---------GTAATT-AATGTTAAATGTAGAGGTAAA---TTTTGTTTTCCCAACTGGTGTTCACCA 15384
GoodesThornscrubTortoise       CAAATACTGTGATCC--TTGACATAACCTCTTTTTACATATTATTTTAATGCCTTCTGTTTATTCTTCTGTCTCTCTCTA 57886

Majority                       --GGTCTTT-----------T----TTCTTTCCCCCCCTTT-ACTTTCCTTATTCA-TTGAT--TTGTTTCAGTATGAGG

                                       63290     63300     63310     63320     63330     63340     63350     63360

Human                          TTAGTAAATGAATACCTATCTTCAGTGCTTTCTTCACCTTTACACCAGGGTATTCAATTAATAGTTATGTTATTCTGGCT 62739
Kakapo                         --GGTCTTTGTGTTTTTTGTTTTGTTCCCCCCCCCCCCCCCACTTTCCTTACTCA-TTGAT--TCCTTTCAGTATGAGG 30827
GoldenEagle                    --GGTCTTT----------------TTCCCCCCCCCCCTTCGACTTTTCTTACTCA-TTGAT--TCGTTTCAGTATGAGG 34490
JapaneseQuail                  --AGTCTTT------------------------AATC-------GTTCCTTATCAAATTGAT--TCATTTCAGTTTGAGG 27168
MediumGroundFinch              --GGTCTTT----------------TTTTTTTCCCCTC-----CTTTCCTTATTCA-TTGAT--TTGATTCAGTATGAGG 15438
GoodesThornscrubTortoise       -ATGCCTCTTTACAAAGAATGCTTTATTGTGGTTTTAACTTTTGGCAACTTTACAGTATAT--CTGCATTAAACCTGAG 57962
```

```
Majority                  AATATCAT-GTAAGTTGTGAGATATGAAATTTTGTAGTAGCTCTGATACCTT---TAAAATAA-TGCAGT--ATGTATTA
                                    63370     63380     63390     63400     63410     63420     63430     63440
Human                     GATAAAGTTGATTGATACATGGCTTCCACTTTTATGTGATGATAGGTACCTTGCCTAACCCAAATGTAATTCAAATCCTG 62819
Kakapo                    ATTATCAT-GTAAGTTGTAAGCTATGAAATCGTGTCGTAGCTCTAATACCTT---TAAAATA--CGCGGT--ATGTGTTC 30899
GoldenEagle               AAGATCAT-GTAAGTTGTGAGATATAAAATTTTGTAGTAGCTCTAATACCTT---TAAAATA--TGCAGT--ATGCGTTA 34562
JapaneseQuail             AGCATTGTTGTAGGTTGTGGGATGTGAAAC--TGAGGTAACTCTGATACCTC---TGAAATAA-TGCATTG-ACTTACTC 27241
MediumGroundFinch         AATATCAT-GTAAGTTGTGAGACATGGAATGGTATAGTATCTCTAATACCTT---TAAAATA--TGCAGT--ATGCATTA 15510
GoodesThornscrubTortoise TTAAATACCAAGCAGCATGGAAAACAGGTTGATTCAGTAGATCTGACAAACT---GGGTCCCA-AGTTTTA-CTGCACTA 58037

Majority                  ATATGGTGGTTATTATTTTTA-----CCTGGCA-TGCTCACTTGTCTCTGTT-GTTTTGTATTGTTTTGCTTCCTAAGGC
                                    63450     63460     63470     63480     63490     63500     63510     63520
Human                     ATGTGTATGAACTCTCTTTCAGTT--TTTACCAGTGCTGTATTGAAGTGGATGAATCTGTGTTGAATTATTTTCTAGGTT 62897
Kakapo                    ATATGGCGGTTATTACTTTTA-----CCTGGCA-TGCTCACCTGTCTCTCT--GTTCTGTATCATTTTGCTTCGTAAGGC 30971
GoldenEagle               ACATGGTGGTTATTATTTTTA-----CCTTGCA-TGCTCACTTGTCTCT----GTTTCGTATTGGTTTGCTTCCTAAGGC 34632
JapaneseQuail             GAATGGTCGTTACTCGTTTTG-----CCTGGTG-TGCTCACTT-T--------GTTTTGTATTGTTTTGTTTCATGCGGC 27306
MediumGroundFinch         ATATGATGGTTATTATTTTTA-----CCTGGCA-TGCTCACTTGTCTCTCTT-GTTTTGTATTGTTTTGCTTCATAAGGG 15583
GoodesThornscrubTortoise AAATGCTACTATAAATCTCCAGGTATTTTGATA-TGCAAAATTTTTCTAGGT-AAGGGAATTTCAAAAGCTTCCAGGAAT 58115

Majority                  TACTGTAA--TGA---AAGTATTAAATGGAACCCT--TT--TTTTTA--CTGCAC-TTTTTTTTTCTCAGGAA--AAGAC
                                    63530     63540     63550     63560     63570     63580     63590     63600
Human                     AGTTTTTAGGTAGGTAGATTAGTCCATAAAAAAATTCGTA-TATTAAGAGAAAAGGTTTTATATTCCCAGAGGTGAGAAT 62976
Kakapo                    TCCTGCAA--TGA---AAGTATTAAATGGAACCCC--TT--TTTC----CTGCAC-TTTTTCTTTCTCAGGAA--AAGAC 31035
GoldenEagle               TACTGTAA--TGA---AAGTATTAAATGGAAACCC--TT--TTTT----CTGCAC-TTTTTCTTTCTCAGAAA--AAGAC 34696
JapaneseQuail             TGTTCTAA--TGG---AAGTAGTAACTGGAACCCT--TT--TTTTCACTCAGTACGTTTCTTTTTCTCAGGAA--GAAAG 27375
MediumGroundFinch         GACTGTAA--TGA---AAGTATTAAATGGAACCTC--TT--TTTTT---CTGCCC-TTTT----TCTCAGAAA--AAGAC 15644
GoodesThornscrubTortoise GACCTTTA--TGAGCTACATAATATGTTTAATCTTAATTGGTTTTTAAACTAAATAACTTTAGTTTAAATGGA--CAAAA 58191

Majority                  ATCTGATC------TTTCTGATACTTT--CAGTTTTGGC--------AGTGGACGTAGTTACATAGATTTGTGTTTTT--
                                    63610     63620     63630     63640     63650     63660     63670     63680
Human                     ATTTGAATTAAAAGCTTTATATACTTAAGCAACTGTAGA-GGTTTACTTTTTCCATAACACGACATGTTTATTCTTCT-- 63053
Kakapo                    ATCT-----------TTCTGATCCTGT--CAGTTTGGGC--------AGTGGAAGTAGTGACATAGCTGTGTGGTTTT-- 31092
GoldenEagle               ATCTAATC------TTTCTGATACTGT--CAGTTTTGGC--------AGTGGACGTAGTTACATAGATGTGTGTTCTT-- 34758
JapaneseQuail             TTAT---C------TTTCTGATACTTC--CAGTTTTGGC--------AGC------AGCTATGTAGACACATATTTTT-- 27428
MediumGroundFinch         ATCTAATC------TTTCTGATACTGT--CAGTTTTGGC--------AGTGGAAGTAGTTACATACATTTGTGTTTTT-- 15706
GoodesThornscrubTortoise CTGTGCTCACT---TTTATCAGACTTCAGCAGTTATGACCAAGCTATATTGTATATTATTAAATACATTTTTCCTCTTTC 58268
```

```
Majority                   ----GTGATGTCATCATGTGGTT---------------ACCTTTTGAATT--------TCTAAACT------CCTGCTTG

                                    63690     63700     63710     63720     63730     63740     63750     63760

Human                      -AATGAAAAGTTAACAGTATGTTCACCTGTTCTCTAGTTTGTTTCCAGCTTATATCCAGCTGAACTAGAAATGGTGATTA 63132
Kakapo                     ----GTGCTGTCATCATGTGGTT---------------ACCTTTTGGATT--------TCTAAACC------CCTGCTTG 31139
GoldenEagle                ----GTGATGTCATCATGTGGTT---------------ACCTTT-GAATT--------TCTAAAAC------CCTGCTTG 34804
JapaneseQuail              ----GTGATGTCATC-----ATA---------------AACCTTTGAATT--------TCTAAAA-------CCTGTTTC 27469
MediumGroundFinch          ----GTGGTATCAT-----GATT---------------ACCTTT-GAATT--------TCTAAAAT------CCTACTTC 15747
GoodesThornscrubTortoise   AGGCATTAGATGGTCAAAATATTTATAATGCTTGCTGTACCCTACGGATTGATTTT--TCCAAACTGGTGAATTTAAATG 58346

Majority                   TGTT--A-TAAAAACAATAAAATCC----ACCATACATGGA-------------T--T--T----GAGGATTTT--GTTT

                                    63770     63780     63790     63800     63810     63820     63830     63840

Human                      TATAGGAGCAGAAAAAGTACAAGATA---ATCATAGCGGTAACATAGTCC---TTTATAATTCATGATGTATTTTCACAT 63206
Kakapo                     TGTT----TAAAGACCCCAAAATCC-----CCACACATGAA------------------------GAGGATTTT--GTTT 31184
GoldenEagle                TGTT----TAAAAACCAAAAAATCC-----CCACACATGGA------------------------GAGGATTTT--GTTT 34849
JapaneseQuail              TGTTACA-TAAAAATAATAAAACCAAATAACAACAGAAGGAAACAGAAGTG--CTTGTTGTGGAAGAGGATTTTCTATTT 27546
MediumGroundFinch          TGTT----TAAAAACAAAAAAAAAC-----CCATACATGGA------------------------GAGGATTTT--GTTT 15792
GoodesThornscrubTortoise   TGAAGTA-CAACAATGATAAAAGTAGG-GACTATACTCGACCTGATCTTCCATCTGGTGATGGGCAACCAGCATTGGACC 58424

Majority                   TTCTTTTTATTAAAGAGGGTGGATAACAGACACCAGTTT---GTTT-ACCTC---TCTGTGGATACTAC--TTTCTGAT-

                                    63850     63860     63870     63880     63890     63900     63910     63920

Human                      ATGTTGTCTTTAATTTACTCCTCTTAATAACCCTTGAAG-AAGCATGACAGCCATTGTCATCATTCCTGTTTTCCCGATT 63285
Kakapo                     TTTCT-----GAAAGAGGGTGGATAACAAACACCAGTTT---GTTTTACCTC---TCTGGGGATACTAC--TTCCTGAC- 31250
GoldenEagle                TTCTTTTCATGAAAGAGGGTGGATAACAGACACCAGTTT---GTTT-ACTTC---TCTGTGGATACTAC--TTTCTGAC- 34919
JapaneseQuail              CTCTTTTTAGTAAGAAGGGTAAAAAGCAGGCACCGGGTT---GTTT-ACCTT---TCTGTGAATACTAC--TTTCTGAT- 27616
MediumGroundFinch          TTCTTTTTACAAAAGAGGGTGGATAACAGACACTAGTTT---GTTT-ACCTC---TCTG--GATACTAC--TTTCTGAC- 15860
GoodesThornscrubTortoise   CAGCTATTGCTGCAGCATTTGCAAAGGAGACGTCACTTCTAGGTATGATTTTAAATTTTCAAACACTTCCCTCCCCATT- 58503

Majority                   ----ACTTGTCAGTCTTGTGTATTATTTCATTGTGTTAAA---ACTGAGAAGTTTGGATGTGT-TAGTTTAGCATTTTCC

                                    63930     63940     63950     63960     63970     63980     63990     64000

Human                      AAGAAATTGAGATTCAGAGAGGTCAAATAACTTGCTTAAGATGACCGAAAAGAGTCTAGGCTTTTAATTAATCCTAGACC 63365
Kakapo                     ----ATTTGTCAGCCTTGTGTATGATTCCACTGTGTTAAA---GCTGAGAAGTTTGGAAGCGT-TAGCTTAGCGTTAGCC 31322
GoldenEagle                ----ACTTGTCAGTCTTGTGTATTATTTCATTGTGTTAAA---ACTGAGAAGTGTGGAAGTGT-TAGTTTAACATTATCT 34991
JapaneseQuail              ----GCT-GTCAATCTTCTTCATTATTTCTTTGTGTTACA---TCAGAGAAATTTGGCTGTAT-T--C--AGCATTTTCC 27683
MediumGroundFinch          ----ATTTGTTAGTCTTTTGTATTATTTCATTGTGTTAAA---ACTGAGAAGTTTGGACATGT-TAGTTTAGAATTTTCC 15932
GoodesThornscrubTortoise   ----CCCAATTAATCAAATGCAGTCTTTTTTCTTATTAATC--ACTGTAATTATTTAATGTTTATATTATGGTAGAGCCC 58577
```

```
Majority                    C----TTCCTGTTAGAGTTTTCTACA----TAAT-TAAAATGGCAGTGGAA-----------------TGTTAACT-GTT

                                    64010     64020     64030     64040     64050     64060     64070     64080

Human                       T--GGCACTCGTCAG--T-TTCAGAAC---TTTTCGGGAATGACATTGGATTTGCTAATTTC-ATCAAGATAATCTGATT 63436
Kakapo                      C----TTCCTATCAG--CGTTCTA-------------AAATGGCAGGGAAA-----------------TGTGAAGT-GTC 31365
GoldenEagle                 C----TTCCTATTAGAGCCTTCTACA----TAAT-TAAAGTGGCAGTGAAA-----------------TGTTAACT-GTA 35044
JapaneseQuail               C----TTTGTCACAGAGTTTTCCACA----TAAT-TAAAGTGGCAATGGAA-----------------TATTAACT-GTA 27736
MediumGroundFinch           C----TTCCCGTTAGAACCTTCTACA----TAAT-TAAAATGACAGTGGAA-----------------TGTTAGCT-GAG 15985
GoodesThornscrubTortoise    AAAGATATATGTTAGGGTTGTTTTCAGTTGCAGTGTACAATTGTAGTAAGACAGACAGTTTCTGCCACTGCAAACTTACT 58657

Majority                    ATACAATCTCTTGAAA-AATAAGATAAGTACTT-CTGGTTTCTGTA--GTCTC---GGTTTTTTGTTGGTTGGT----TT

                                    64090     64100     64110     64120     64130     64140     64150     64160

Human                       GGGGGTGAACATTTCAGGTTAAGGAAAATACTAGTTTTTTTCTTTTTTGCCTC----ATTTTATGGGAATTGCTATTATG 63512
Kakapo                      ATAGAATCTCCTGGAA-AACAGGTTCAATACTT-TTGGTTTCTATA----------GGTTTTTTGGTGGGTT-------- 31425
GoldenEagle                 ATACAATCTCTTGAAA-AATAGGATAAGTACTT-CTGGTTTCTGTATAGTCTTGG-GTTTTTTTGTTGGTTGATTGGTTT 35121
JapaneseQuail               ATACAGACCGTTGAAATAACAAGATTAATAATTACTGGCTTCTGCA-----------CTTTTTTCCCCTTTAG------- 27798
MediumGroundFinch           ATACAACCTTTTGAAC-A-TAAGACAAGTACCT-CTGCTTTCTGTA--GCATCAG-CGTTTTTCCTTGGTTGGT-----T 16054
GoodesThornscrubTortoise    CTATAAATAGATGAGAGAGAGAAACTAGAATAGGACAGAATATACACATTATCAAAAGTTTTTGCCTAGTTACTTACATT 58737

Majority                    TGTTGGTTTTGTTTGGTTTTATTTTCTTTTATATAAAGAGTCTTGAAACCTACAAAATCCTCACTTACTTTA-------T

                                    64170     64180     64190     64200     64210     64220     64230     64240

Human                       TGTTAGGATAATTTGGTTTGAAGCAACTTTGTACAATGTGGCATCAAATAGCAGTTACCTTCTAAAACTGTACTGA---A 63589
Kakapo                      -------TTGGTTTGCTCGAAAGGAAAGAACAATAAAGAATCTTGAAACCTACAAAATCCTCACTTACTTCA-------T 31491
GoldenEagle                 TGTGGGTTTTGTTTGGTTTTTTTCTCTTCTAT--AAAGAGTCTTGAAACCTACAAAATCCTCACTTTCTTTA-------T 35192
JapaneseQuail               --------AAGTTTG-T-----------------AAGGAGTTTTGACATCCACAAAATCCTA--TTTTTTTA-------T 27843
MediumGroundFinch           TGTGGGTTA-GTTTAGCTTTATTTCCTGTAAAGGAAAGAATCTTGAAACCTACAAAATCCTC------TTTA-------T 16120
GoodesThornscrubTortoise    TTTTAAGTTTCCCTAATCCATCCTTCCTTTTGTTCTGGTGTCAGTCAACTTCCTCCACCGTTGGTGACCTAAAGTATCTT 58817

Majority                    CCTTACTAGTAGA-ACAATAAGCTTTCTAC-TAGGAAGATTGATATTGGTGCTATTT----GAAGCCCCTCTTTACTGTG

                                    64250     64260     64270     64280     64290     64300     64310     64320

Human                       TCATATAAGTACTGCCATCCAGCAATAAATATATGTATAATAAAATTAACCTTATTGAG-CAGAGTAGTGTGGTACTACT 63668
Kakapo                      CCTTACTAGTAGA-A--GTAGGATT------------GATTGACACTGGTGCCATTT----GAAGCCCCTCTTTACTGTG 31552
GoldenEagle                 CCTTACTAGTAGA-ACAATAAGCTTGCTAC-TAGAAAGATGGATATTGGTGCCGTTT----GAAGCCCCTCCTTGCTGTG 35266
JapaneseQuail               TGTTAATAGTAGA-ACAATAAGCTTGTTAC-TGGGAAGATTGATATTTGTGCTATTT----GAAGGCT----------TA 27907
MediumGroundFinch           CCTTACTAGTAGA-ACAACAAGCTTTCTAC-TAGAAAGATTGATGTTGGTGCCTTTTTT-TGAAGCCCCTCCTTAATGTG 16197
GoodesThornscrubTortoise    CCTTTCTTCTGGCCACCTCATGGCGAAATC-TTTGGGACTTGATATTGGTCTTGTTAAAATCAAGTAAGCTTCTCTATTG 58896
```

```
Majority                    ACATAAGGATTAATTGA--T--TTGTTTTTATT-GT-TTCTT----CTCACAGAGTTGGACTGCATTGCACTTA-CGTTG

                                     64330     64340     64350     64360     64370     64380     64390     64400

Human                       TTGGTAGGACTTCCTGT--TTGTGATCCTTATTAGTTTTTTTAAAACTAACCTATTTGCAATTTTTAAAAATTAAAAGTA 63746
Kakapo                      ACACAAGGGTTAATTGA------TGTTCTCATTTGT-CTCTT----CTCACAGAGTCGGACTGCATTGCACTTA-CATTG 31620
GoldenEagle                 ACATAAGGATTAATTGACATTTTCATTTTTATT-GT-TTATT----CTCACAGAGTGGGACTGCATTGCACTTA-CGTTG 35339
JapaneseQuail               ATTTATGAATT--T----------ATTTTTATT-------TT----CTCATAGA-TTGGAGTGCATTACATTTA------ 27957
MediumGroundFinch           ACATAAGGATTAATTG------CTGTTTTTGTT-GG-CTCTT----CTCACAGAGTCAGACTGCATTGCACTTAACGTTG 16265
GoodesThornscrubTortoiseACTTCAGCAGGAACAGG-ATCTGTGTCCTTATTGGCATTGTA----TGCAGGTGATGACATTTTGGCTTGCTTTCCTATA 58971

Majority                    -TGTTATGTCTG--TAGCT--CTGAGTTTTAAATAAGTGGACTATTTTCTGTTTCTGGAGTTAACTATATGAATATTACT

                                     64410     64420     64430     64440     64450     64460     64470     64480

Human                       TTAGCATGGATGCTTAGCTGGTTGTATTTTAGGTTGAAGTAGCATTTTCTATTTAAGGAAGATAATATTTGGGGGAAAGT 63826
Kakapo                      -TGTTCTGTCTG--TAGCT--CTGAGGTTTAAATCAGTGGCCAGTTTTCTGTTTCGGGAGTTAACGATACGAATATCGCT 31695
GoldenEagle                 -TGTTATGTCAG--TAGCT--CTGAGTTTTAAATAAGTGGACTATTTTATGTTTCTGAAGTTAACTATATTAGTATTGCT 35414
JapaneseQuail               -TATTCTGCC-----------------------T--------CATTGT---TTTCTGAA--TAATCATGTGAC--TTACT 27998
MediumGroundFinch           -TGTTATGTCTG--TAGCT--CTGAGTTTTAAATAAGTGGACTATTTTGTGTTTCTGGAGTTAACTATATTAATATTTCT 16340
GoodesThornscrubTortoise-TGCAGTGTCCA--TT-TT--CTTCAACTAAGAAAGATGCTTTGCTTTTTCTATTAAAAA--AAAAATACCAAAGAAATA 59043

Majority                    AATTAATTTCTAGCTATTATCTTGAAATAT---TAA-GAAATTTG---TCTTTTGTCTTTTTTTTTT-ATT-----T--T

                                     64490     64500     64510     64520     64530     64540     64550     64560

Human                       AATACATGGACAGCTGGAACAGCAAAATTTGATATATGAAATTTGAATCCTTTTCCTGGTAGCTTTTAAGTTCGAGTTTT 63906
Kakapo                      AATTAATTTCTAGCTCTCATCTTGCAATAT---TAACCAAGCTGT---CTTGTTGTCTTGTCTCTTTTGTT--------T 31761
GoldenEagle                 AATTAATTTCCAGCTACCATCTTGAAATAT---TAA-AAAGTCTG---TCTTCTGTCATCTTTTTTTGTTTGTTTGTT-T 35486
JapaneseQuail               AATTAATTTCTAGCTATTGTCTTGAGATAG---TAA---AATCT--------TTGTTATTTTCTTTT------------- 28051
MediumGroundFinch           AACTAATTTCAAGCTGTTATCTTGAAATAT---TAAGAAAATCTC---TCTTCTGTCTTATTTTTT---T---------- 16401
GoodesThornscrubTortoiseAAAGAACAGATGACAAAAATGAGACCATGTATACAAAGAAATTCC---ATCTTAGCATTTTATTTTTCATAATTACTGAT 59120

Majority                    T--T-T----TAAGCGGGCCCAAGCTGTTCT-TCAGGCAGTGA---CAGCTGTGCAGG--CAACAA-ATGCTCCCATTAG

                                     64570     64580     64590     64600     64610     64620     64630     64640

Human                       TGTTGTTGAGTTTTATTACTAAAATTATACTTTCACTAATTTTTATTTTTTGTTTAAGATTGCTGATATGCTTTATTCAC 63986
Kakapo                      ---T------GAAGCGGGCCCAAGCTGTTCT-TCAGGCAGTGA---CAGCTGTGCAGG--CAACAA-ATGCTCCCATTAG 31825
GoldenEagle                 TTGTTTTTGTTAAGCGGGCCCAAGCTGTTCT-TCAGGCAGTGA---CGGCTGTGCAGG--CAACAA-ATGCTCCCATTAG 35559
JapaneseQuail               ---C-------AAGCGGGCCCAAGCTGTTCT-TCAGGCAGTGA---CAGCTGTGCAGG--CAACAA-ATGCTCCCATAAG 28114
MediumGroundFinch           ---T------AAAGCGGGCCCAGGCTGTTCT-TCAGGCAGTGA---CAGCTGTGCAGG--CATCAA-ACGCTCCCATTAG 16465
GoodesThornscrubTortoiseTTTTTCCTTTATATAGATCATCACTCTTATCTCTAGCGCC-----CTGTTATGCAAGTTCAAGAA-CTGATAAGAT-AT 59193
```

```
Majority                   TGGGACCACTGTTAGTGAGAGTGCAGTGACTCCAGCTCAGAGTCCAGTACTTAGAATAATTATTGACAACATGTA--CTA

                                     64650     64660     64670     64680     64690     64700     64710     64720

Human                      TTTTACAGGCCCTAG-ATGGTCAGAATATTTATAATGCCTGCTGTACCCTAAGGATTGATTTTTCCAAACTTGTGAATTT 64065
Kakapo                     TGGGACCACTGTTAGTGAGAGTGCAGTGACTCCAGCTCAGAGTCCAGTACTTAGAATAATCATTGACAATATGTA--CTA 31903
GoldenEagle                CGGGACCACTGTTAGTGAGAGTGCAGTGACTCCAGCTCAGAGTCCAGTACTTAGAATAATTATTGACAATATGTA--CTA 35637
JapaneseQuail              TGGGACCACTGTTAGTGAGAGTGCAGTTACTCCAGCTCAAAGTCCGGTGCTTAGAATAATTATTGACAACATGTA--CTA 28192
MediumGroundFinch          TGGGACCACTGTTAGTGAGAGTGCAGTGACTCCAGCTCAGAGTCCAGTACTTAGAATAATTATTGACAACATGTA--CTA 16543
GoodesThornscrubTortoiseTAGAACC--CATCAG-AAGAATCAACTGAGATTCCCTGAG----CAGTGCATAGA-TAAACATTCATAGCATCTAA-CCA 59264

Majority                   TCCTGTAACCC-TGGATGTTCTTCAT-CAGG--TAA--GTCAAATTTCATTTTAACTTG----AGCTATGATTTGGGAGG

                                     64730     64740     64750     64760     64770     64780     64790     64800

Human                      GAATGTAAAATACAACAATGATAAAAGTAGGGATTATACTCGACCTGATCTTCCATCTG-----GGATGGACAACCTGC 64140
Kakapo                     TCCAGTAACCC-TGGATGTTCTTCAT-CAGG--TAA--GCCCAATTTCTTTTGAACTCG----AGCTCTCATTTGGGAGG 31973
GoldenEagle                TCCGGTAACCT-TGGATGTTCTTCAT-CAGG--TAA--GCCAATTTTCATTTTAACTTG----AGCTATGATT-GGGAGG 35706
JapaneseQuail              TCCTGTAACCC-TGGATGTTCTTCAT-CAGG--TAA--GAAAAATTTCATTTTAATTTG----ACCTTGTAATTAGCAGG 28262
MediumGroundFinch          TCCAGTAACCC-TGGATGTTCTTCAT-CAGG--TAA--GTCAGATTTCACTTTAGCTTG----AGCTATGATT-GGGAGA 16612
GoodesThornscrubTortoiseTCTTGTTGTTGGTAGAGGCAGTGCATTCAGGACTTA--CTGTACTTATGCTTTGAGAGAGAGAAAATTTTAATACACTAT 59342

Majority                   AACTTGTGTGTAAAATTTTACAGTGTTTTTT-GGCAGATATTCTC--TAAATTTGGTGCTGTATTGAAGATAATCACATT

                                     64810     64820     64830     64840     64850     64860     64870     64880

Human                      ATTGGACCCAGCTATTGCTGCAGCATTTGCCAAGGAGACATCCCTCTTAGGTATGATTTT-TATTGTC-TTAACCACTTT 64218
Kakapo                     AATTTGTGTGTAAAATTTTACCGTGTTTTTT-GGCAGATATTCTC--TAAATTTGGTGCTGTATTGAAGATAATCACATT 32050
GoldenEagle                AACTTCTGTGTAAAACTTTACAATGTTTTT--GGCAGATATTCTC--TAAATTTGGTGCTGTATTGAAGATAATCACATT 35782
JapaneseQuail              AACATGTGTATAAAACTTT-----GTTTTT--GACAGATATTCTC--TAAATTTGGTGCTGTATTGAAGATAATCACATT 28333
MediumGroundFinch          AACAGATGTGTAAAACTGTACAATGTTTTTT-GGCAGATATTCTC--TAAATTTGGTGCTGTATTGAAGATAATCACATT 16689
GoodesThornscrubTortoiseATCTTTTTTTTTGTTTTATCATTTTAATTT-GGTATCGACCCTG--TAAATTTCATGAT-TATATAAACTAGTAAGCTT 59418

Majority                   CACAAAGAATAACCAGTTTCAA-GCTTTACTGCAGTATGGTGA-TCCAGTAAATGCACAGCAAGCAAAGCT--AGTAAG-

                                     64890     64900     64910     64920     64930     64940     64950     64960

Human                      TCTCCCATTTTGCCAAATGGAAAAGTACCAGTAAGTATAATGAATCCCCCCATTTTGGACCTTACCAAATTGTGTTAGGT 64298
Kakapo                     CACAAAGAATAACCAGTTTCAA-GCTTTACTGCAGTATGGTGA-TCCAGTAAATGCACAGCAAGCAAAACT--AGTAAG- 32125
GoldenEagle                CACAAAGAATAACCAGTTTCAA-GCTTTACTGCAGTATGGTGA-TCCGGTAAACGCACAGCAAGCAAAGCT--AGTAAG- 35857
JapaneseQuail              CACAAAGAATAACCAGTTTCAA-GCTTTACTCCAGTACGGTGA-TCCAGTGAATGCACAGCAAGCAAAACT--AGTAAG- 28408
MediumGroundFinch          CACAAAGAATAACCAGTTTCAA-GCTTTACTGCAGTTTGGTGA-TCCAGTAAATGCACAGCAAGCAAAGCA--AGTAAG- 16764
GoodesThornscrubTortoiseTACCCAG--TTCCTGTTCTGGATGTCCTTTTATATTTTGAAGT-TTTAGGGTTCATTTTGTCAGCAAAGTTCAGGTTGGA 59495
```

```
Majority                   --TAT-AGTTTTCTTTTAAG--------------GCTGGATATCTTCCTGTA---CTCTAAATATTTGT-----------

                                    64970     64980     64990     65000     65010     65020     65030     65040

Human                      TTCGTGAGTTTTCTTTTTCTCAAGTGAGAAGGCATATGAATACTGTTAAGAAAAACCCAAAGTATTCTTT---------- 64368
Kakapo                     --TAG-AGTTTTCTTTTAAG--------------GCTGGAGACCTTCCTGTA---CTCTAAATGTTCGT----------- 32174
GoldenEagle                --TAT-AGTTCTCTTGTAAG--------------GCTGGAGACCTTCCTGTA---CTCTAAACATTTGT----------- 35906
JapaneseQuail              --TAT-AGTTTTCTTTTAAG--------------GGTAGGTATGTTTCTGTG---CTCTAGACATTTGT----------- 28457
MediumGroundFinch          --TAT-AGTTCTCTTCTAAA--------------GCTAGAGATCTTCCTGTA---CTCTCAGTATTTGT----------- 16813
GoodesThornscrubTortoise   TTTAG-AGATTTTTACAAGACCCAATGAACGGTGTTGTTTATTTTCTTACC---CACTTATTGTTTGTTTCCTTAGGAG 59571

Majority                   --AAAATGTTAATATAACGGATTACTGACTCTTATAATGTGTCATACTC--CTCTACTATTTG-TGTGAA-TTTTTTGAG

                                    65050     65060     65070     65080     65090     65100     65110     65120

Human                      --ATAAGTCTAATTAAAATTATATGTTAGTTTTGTTCCTTTTAATACCTTCAGCTTCTCTTCCATGCCACTCTCCCAGAA 64446
Kakapo                     --AAAATGCTAATGTAAC------CTT-CCCTGATGA--------------CTTTAGTACTT--TGTGAA-TTCTTTGAG 32228
GoldenEagle                --AAAATGTTAATATAACAGAGTACTGACTGTAATAATGTGTCATACTC--CTCTACCACTTG-TGTGAA-TTTTTTGAG 35980
JapaneseQuail              --ACAATGTTAACAGAATG-----CTGACTACTATAG--------------TTGTAATATTC----C-----TCCTCCAG 28507
MediumGroundFinch          --AAAATGTTGATGTAACAGAGTACTG-CCATAACAATGTGTCATACTC--CTCCACCATGTG-TGTGAACTTTTTTGAG 16887
GoodesThornscrubTortoise   TTAGGAAGCTAGAAGGAAGGATCAGCCATTCATGTAAAGTGCTGCATTCTGTTTTGACAGTCAGTAAATAGCTTTTTGAA 59651

Majority                   --ATTAGAA-A-A---ATAATTGGGAGTTACTA---AA-TGTTAATAT--TGTGTAAAATCTGTGTTTGAAGATACTATG

                                    65130     65140     65150     65160     65170     65180     65190     65200

Human                      CAAAAGAAGAAAAGCTCTAATGAAGAAATACTGTTGAATTCTTAATGCCATATTTACACTTTAAACTTGATGTCACTATG 64526
Kakapo                     --ATGAAAA---A---ATAGTTGGCAGGTATGA------TATTAATAT--TGCATAAAATCAGTGTTTGAAGATACTATG 32292
GoldenEagle                --ATCAGAA---A---ATAACTGGGAGTTAATATATAA-TGTTC-TAT--TGCGTGAAATCGGTGTTTAAAGATGCTATG 36048
JapaneseQuail              --TTTTGAG---------------------------------------------AAATC--T--TTG---A------- 28524
MediumGroundFinch          --ATTAAAACAAA---ATAAGTAGAAGTTAGT-------TGTTA-TAC--TATGTGAAATCAGTGTTTAATGATAGTATG 16952
GoodesThornscrubTortoise   CTATTTGATTATAGGCAGAGCTGACAG-CACCACCTAA-TACTGAGGT--TGTCCAACACTTCTGATTTCAGTTGCTCTG 59727

Majority                   TTTAAGGATTCTATGTCTAAAGAATTT---TATACATCTATGATTATGACCAAATATTTTAATA----AGACTGCACAGA

                                    65210     65220     65230     65240     65250     65260     65270     65280

Human                      CCTGAG---TCTAGGCCTAAAGTTCTTGTGTGTGTTTTTCAGTGCTGATCTGATTGTTTTAATTT--AGCATTGCATGGA 64601
Kakapo                     TTTAAGGATTCTATGTCTAAAGATTTG---TATACATCTATAATTATGACCAAATATTTTAATA----AGACTTCACATA 32365
GoldenEagle                TTTAAGGATACTATGTCTAAACAATTG---TATACATCTATAATTATGAACAAATATTTTAGTA----AGACTGCACAGA 36121
JapaneseQuail              ------------------------------------------------AAATTTTTTA------------------- 28534
MediumGroundFinch          TTTATGGATACTGTGTCTAAAATATTT---TAGATATTTG----TATGAACAAATATTGTAGTA----AGACTGCACAGA 17021
GoodesThornscrubTortoise   AC-ACTGTTGTCAATTGTACATAACTTAGCCAAACTTCAATGATTTGGGCTGATAATTTCCATGCCAGATATCTGCCTAA 59806
```

```
Majority                GATGGAATAATATGCAAGACTGCAATAGTGAAAATG--TCAACCAGAAGTAAGAAT-----GTAGGA--A-TGTAATGG-
                                 65290     65300     65310     65320     65330     65340     65350     65360
Human                   AACAGTGAAATGTTAAGAGACAAAACATACATAATGGGTGGA-GGCATATGGGATTTTTTTTGAAGCATATTGTAGTGGC 64680
Kakapo                  ----------TATGCCAGAGTGCCTTGGTGCAGATG--TCAACAAGCAGTAGAAATCTG-TGTAGAAGTACTGTGATGT- 32431
GoldenEagle             GGTGAAATAATATGCAAGACTGCAATAGTGAAAATG--TCAACCAGAAGTAAAAAT-----GTAGGA----TGTAATGT- 36189
JapaneseQuail           ------A------------------------AAA---------------------------------------------- 28538
MediumGroundFinch       GATGAAATAATATGCAAGACTGCAATAGTAAAAATG--TCTACCAGAAGTAAGAAT-----GTAGGA----TGTCAGGG- 17089
GoodesThornscrubTortoiseTACTGAATGTTTTTTTTAAATAT-TCAGCCAAAATGGCTCAGCCATTTCTGAGAACAAGGCATGGGGAAAATACATTTG- 59884

Majority                -TGTTGCATAATTAATTCACTGTAAGCGGCATTTTCTGTAA-A-----T-------T-----------------------
                                 65370     65380     65390     65400     65410     65420     65430     65440
Human                   CTTTTAAAAAAACAATTTAAACTAGAAGAAATTTTCTGTAAAATTAATTTTGCTATTTCTTTGCATTTTGTCTTAATCAT 64760
Kakapo                  -TGGTGCAGAATTAATTCACTCTAAGCAGGAAATTCTGTAAGATAGAATCACAGAATGGTTTGG--GTTGGAAAGGACCT 32508
GoldenEagle             -TGATGCATAATTAATTCACTGTAAACAGCAGATTTAGTAA--------------------------------------- 36229
JapaneseQuail           -----------------------AAGGGGGAGTTTATATAA--------------------------------------- 28556
MediumGroundFinch       -TGATGCATAATTAATTCACTGTAAGCAGCAAATTTAGTAA--------------------------------------- 17129
GoodesThornscrubTortoise-TTTTCCCCTTGTTAAAAAATTCAAGTGACCTTTCCTTTGAGAAAGTTTTTAGTGCTCCACACC--CCCACTTCAGAGCA 59961

Majority                -----T-------T---------------T----------A----------G-------------AA-------------
                                 65450     65460     65470     65480     65490     65500     65510     65520
Human                   TTATATTTTACTTTGTACCACAATGGACTTGAGCCACCTAATAAAAATACAGTGCAAACATATGCAACTTATAGTTGATA 64840
Kakapo                  TAAGATCATCCAGTTCCAACCCCCTGCCATGGGCAGGGACACCTCACACTAGAGCAGGTTGCTCCAAGCCCCTGTGTCCA 32588
GoldenEagle             -------------------------------------------------------------------------------- 36229
JapaneseQuail           -------------------------------------------------------------------------------- 28556
MediumGroundFinch       -------------------------------------------------------------------------------- 17129
GoodesThornscrubTortoiseGGGACTTGAAATTTGACAGGGGATGATTTTTGTGTCAGGGATGTGCCTTTTGCCATCACGGTGAAAATCCTCCCAAATTT 60041

Majority                --------------------A--------G------C------T----G------G--C---------------------
                                 65530     65540     65550     65560     65570     65580     65590     65600
Human                   GCGTGGAATCAAGATGAAGAAAAAAATAAGGAAAATCTTACTTTGTAAGTAAAATGTGCAAAGTGATTTATAAGTTAAGA 64920
Kakapo                  ACCTGGCCTTGAACACTGCCAGGGATGGGGCAGCCACAGCTTCTCTGGGCACCCTGTGCCAGCGCCTCAGCACCCTCACA 32668
GoldenEagle             -------------------------------------------------------------------------------- 36229
JapaneseQuail           -------------------------------------------------------------------------------- 28556
MediumGroundFinch       -------------------------------------------------------------------------------- 17129
GoodesThornscrubTortoiseGGCCAAGTCGTGAGCCTTAAAAAATCACAGTTCACACATAGACTCATAGACTCTAGGACTGGAAGGGACCTCGAGAGGTC 60121
```

```
Majority                   ----A----------------T-------G-A----CCCTTTT--AG-----------------TT--------------
                                    65610     65620     65630     65640     65650     65660     65670     65680
Human                      TGGAATTTTAGAGTTTCCTTTTATTTGATGTATTTCTTGATTTCTAGTATTGGTAAGATTATCTTTAAGTCATGATTTTT 65000
Kakapo                     GGGGAGAGCTTCTGCCTTAGATCCAACCTGAACTTCCCCTGTTTCAGTTTGAACCCATCACCCCTTGTCCTGTCGCTCCA 32748
GoldenEagle                ------------------------------------GCCCATT------------------------------------- 36236
JapaneseQuail              -------------------------------------------------------------------------------- 28556
MediumGroundFinch          ------------------------------------CCCTTTT------------------------------------- 17136
GoodesThornscrubTortoise   ATCGAGTCCAGTCCCCTGCCCTCATGGCAGGACCAAATACTGTCTAGACCATCCCTAATAGATATTTATCTAAC---CTA 60198

Majority                   -T----A---------GAATTGTT-GTTGGCAGATCCTTTCTAAGCA-TCAATACAAATGTGTGATTTGTGGATTGGATA
                                    65690     65700     65710     65720     65730     65740     65750     65760
Human                      ATATATATGGAACTCAAAATTGATAGTTGACAAACTAGCTTTTACAATTTAGTTTATGTCTGTCATTTCAAAAGAAAAAA 65080
Kakapo                     GTCCCTAATGTAGAGCGAATTATT-GTTGGTAGATCCTTTCTTACCA-TCAAAACAAAGATGTGATTTGTGGATTGGATA 32826
GoldenEagle                ----------------GGATTACT-GTTGGCAGATACTTTCTAAGCA-TCAAAACAAAGGTGTGATTTGTGGATTGGATA 36298
JapaneseQuail              ------------------------------------------------------------------------ATTGGACG 28564
MediumGroundFinch          ----------------GAGTTGTT-GTTGGCAGGTACTTT-TAAGCA-TCCATACCAAAATGTGATTTGTGGATTAGTTA 17197
GoodesThornscrubTortoise   CTCTTAAATATCTCCAGAGATGGA-GATTCCACAACTTCCCTAGGCAATCTATTCCAGTGTTTAACTACCCTGACAGTTA 60277

Majority                   TAGATTTGTTA-AAGTATTTAATTATAGTATCCT----A--AAATATAA-AATCTAGACTTC--TTTGTAACTATAAATG
                                    65770     65780     65790     65800     65810     65820     65830     65840
Human                      TAGTATAATT--AAGTAGTTATTGGAAATATTTACTTCCCAATATATAACAGTCATGCCATCAGTTTGTGAATATGAAAT 65158
Kakapo                     TAGATTTGTAA-AAGTATTTAATTATAGTATCCT----A--AAAGAAAT-TACCTAGACTTC--ATTATAACCATAAATG 32896
GoldenEagle                TAGGTCTGCAA-AAGTATTTAATTATAGTATCCT-------AAAAAAAA-AATCTAGACTTC--ATTATAACTATAAATG 36367
JapaneseQuail              AAGATC------A-----------ATAGCATGTC----------------------ATCA-------------------- 28585
MediumGroundFinch          CAGATTTGTGA-AAGTACTTAGTTATAGTATCCTTAAAAAGAAAAAGAA-AATCTAAACTAA--TATGTAACCATAAATG 17273
GoodesThornscrubTortoise   CGAACTTTTTCCTAATGTCCAACCTCAATCTCCCTTGCTGCAGTTTTAA-GTCCATTGCTTCTTGTTCTATCATTGGAGG 60356

Majority                   CCCAGAAGAGAGAGTGTA----GTCTTACTCATGTGACCAGACTGTGAAGAAGCTTTG-CAGTAGTGACAGGTGAGT---
                                    65850     65860     65870     65880     65890     65900     65910     65920
Human                      CTGTAAGTGGTATATATGTAGAGAGAGATACTCTAAGGCAGTTGGTATTTGATTCTGGTAACTAATAAGAATTAACTACC 65238
Kakapo                     CCCAGAACAGAGAGTGTATGGGATCAGACTCATGGGATCAGACTATGAAGCAGCTTTG-GATGAGTGACAGGTGAGTGTT 32975
GoldenEagle                CCCAGAAGAGAGAGTGCA----GTCTTACTCATGGGACCAGACTATGAAGAAGCTTTG-CAGCAGTGACAGATGAGA--- 36439
JapaneseQuail              ---------------A-------------------GACCAGAC-----------------A------------------- 28595
MediumGroundFinch          CCCAGAAGAAAGAGTGCA----AT-TTACTCATGTGACCAGACTGTGAAGAAGCTTTG-CAGCAGTGATGGGTGA----- 17342
GoodesThornscrubTortoise   CTAAGGTGAACAAGTTTTCT---TCCTCTTCCTGTTGACACCCTTTTAGATACCTGCA-AACTGCTATCATGTCCCCTCT 60432
```

```
Majority                   ----------------T---T-T--CA---------AA--GCATGATCTTA-----CTTTT------------GTTT---

                                  65930     65940     65950     65960     65970     65980     65990     66000

Human                      -------------TGTTAATTCTGGCAGCTGCAAACAAATCTGACCCTTTATTCGAATTTTC----------TGGTGTAT 65295
Kakapo                     TTAATGGAGGCATCCCTCCCTGTGGCACGGGGTTGGAACTGGATGATCTTAAGGTTCTTTCCAACCCAAACCAGTCTGTG 33055
GoldenEagle                ----------------------------------------GCAGGATCTTA-----CTTTTT-----------GTTT--- 36460
JapaneseQuail              ---------------------------------------------------------TTTT------------------- 28599
MediumGroundFinch          ----------------------------------------GCAGGATCTTG-----CTTTT------------GTTT--- 17362
GoodesThornscrubTortoise---------CAGTCTTCTCTTTTCCAAACTAAACAAACCCAATTCCTTTAGCCTTCCTTC--A------T-AGGTCATG 60493

Majority                   --TATAGGAAATTCTCCTATCCTGGATAGGAGTGGCAAGAACATGTGGCTGGTTTTATTCATTATAGCAGGAGAGAGCCT

                                  66010     66020     66030     66040     66050     66060     66070     66080

Human                      CCTAGCAGAAGTCATAGTGTTTATTATGACTGTAGTAGATGCTTTAGGTCACTTACACTCTTTCCTGACAGAAT------ 65369
Kakapo                     ATTCTATGAAACTCTCCCATCCTGGGTAAGAAAGGCAAGAACAGGTAACTGGTTTTATTCATTATAACAGGAGAGAGCCT 33135
GoldenEagle                --TATTGGAAACTCTCCTATCCTGGTTAGGAGAGGCAAAAACAGGTAACGGGTTTTATTCATTATAGCAGGAGAGAGCCT 36538
JapaneseQuail              --------GAATT------------A----AATGATAATGATGTGTG------TACATCCATGACAACAAGA-------- 28641
MediumGroundFinch          --TATAGGAAACTCTCCTATCCTGGATAGGAGAGGCAAGAACAGGTAGCTGGTTTTATTCATTATAGCAGGAGAGAGCCA 17440
GoodesThornscrubTortoiseTTCTTAAGATCTTTAATCATTCTTGTTGCTCTTCTCTGGACCCTCTG-CAATTTTTCCACATCTTTCTTGAAATG---CG 60569

Majority                   ATGGATAAATTATGACAAAGT-CAACAGTATGTCATCTAGACCAGATGTTTTGAATTAATTGGATGGAATA--TGTTCGC

                                  66090     66100     66110     66120     66130     66140     66150     66160

Human                      ---C-TCTACTACCTCTGTGT------GTGTCTAGCTTAATTCG--TAATTCAAGTTAATAAGATTGAATATTTCACAGT 65437
Kakapo                     ATGGATAAATTATGACAGAG--CAACAGCACATAATCTACACCAGTTGTTTGGAATTAGTTGGATGAAATAATTGAATGA 33213
GoldenEagle                ATGGATTAATTATGACAAAGATCAACAGTATGTCATCTAGACCAGATGTTTTGAATTAATTGAATGGGATA--TATTCAC 36616
JapaneseQuail              ----------------AAAG-----------------TAAACCAAAGGCGACGACTAGATTGTGTG-TGTG--TGTATAC 28685
MediumGroundFinch          ATGGATAAATTATGGCAAAGATCAACAGCATGTCATCTAGACCAGATGGTTTGAATTAATTGAATGGCATA--TGTTCAC 17518
GoodesThornscrubTortoiseGTGCCCAGAACTGGACACAGTACTCCAGT-TGAGGCCTA-ACCA---GCGCAGAGTAAAGCGGAAG-AATGACTTCTCGT 60643

Majority                   ATCTG--TGGCAATAGAAAAGTTGAACCAAAGGGGAAGGCTAG-T--GTTTTTTTTGTTATTATGATTATATTT-TTCAA

                                  66170     66180     66190     66200     66210     66220     66230     66240

Human                      GTGCAGTTTTTAA-AGGCAAAGTCAGGAATGAAAGAGTGAAACATGGTTTTTGCTTATAATTATTACTATAGATTATTAA 65516
Kakapo                     ATCTG--TGGCAACAGAAAAGTTGAACCAAAGGGGGAGGCTGG----ATTTTTTTTGTTATATTGATT-------TTCAA 33280
GoldenEagle                ATCTG--TGGCAATAGGAAAGCTGAACCAAAGGGGAGGGCTAGTTTGGTTTTTTGGGTTTTTTTGTTTGTTTTTCTTCAA 36694
JapaneseQuail              ACT----T--------AT--------------------CTAA------TTTT----TTATTCTGATT-------TTTAA 28715
MediumGroundFinch          ATTTG--TGGCAGTAGAAAAGGTGAACCAAAGGGGAAGGTGAG----ATTTTATTATTATTATTATTATAATT-TTCAA 17591
GoodesThornscrubTortoiseCTCTTGTTTACAACACACCTGTTAATGCATCCCAGAA--TCACGTTTGCTTTTTTTGCAACAATATCACACTGTTGACTC 60721
```

```
Majority                    GGGTTAAA--TGGAATCAATTTTTATTTTCAAACCTTCATGTGCAGTCTGGGTTTGTGGA-AGGCTGATTCAAATTGGTA

                                     66250     66260     66270     66280     66290     66300     66310     66320

Human                       ACTCTTTACTGATAGACATTGTTTTAGAAAGAATCAAAATTTATTGAGTAGCTTCTAGGTCATATTCATTCAGCTAGGCA 65596
Kakapo                      GAGCTAAAA-TGGAATCAGTTTATGTTTTCCAGCCTTCCCGTAGAGGCCGGATTTGTGGG-AGGCTGATTCAAGTTGGTA 33358
GoldenEagle                 GGGATAAA--TGGAATGAATTTATATTTTCAAACCTTCACATGCAGTCTGGGTTTGTGGA-AGGCTGATTAAAATTGATG 36771
JapaneseQuail               GGGTTAAA---TGAATTACTTTGTATTCTTTAACATTCATGTGCTGTCTGTTTTTTTGGA-AGGTTGGTTAAAACTGATG 28791
MediumGroundFinch           GAGATAAA--TGGAATTAATTTGTATTTTCAAACCTTCACATGCAGTCTGAGTTTGAGGA-AGGCTGATTAAAATTGATA 17668
GoodesThornscrubTortoise    ATATTAAGCTTGTGGTCTACTATGACCCCTAGATCTCTTTCTGCCATACTCCTTCCTAGACAGTCTCTTCCCATTCTGTA 60801

Majority                    TGCTGGTTAAGTGTTGCTTGGCAGCATAGAAAGTTCTTATTG----CCTATAGATGACAGAATAG---CTGAGAACTTCA

                                     66330     66340     66350     66360     66370     66380     66390     66400

Human                       CTTGGCAGATACATTACTTTGTTAAAATAGATGGTCTCATCAAGATTTTACATATAGTTTCTAAGAAGTTGAAAAATTCA 65676
Kakapo                      TGCTGGTTCGGTGTTGCTTGGCAGCACAGAAAGTTCTTACTG----CCCATAGATGACAGAATAG---CTGAGAACTTGA 33431
GoldenEagle                 TGCTGGTTAAGTGTTGCTTGGCAGCATAGAAAGTACTTGTTG----CCCATAGATAACAGAATAG---CTGAGAACTTCA 36844
JapaneseQuail               TACTGGTTAAATGTTGCTTGGCAGCATAGAAAGAGTGTATTA----CCCACAAGTGACAGAATAG---CTGAGAATATCA 28864
MediumGroundFinch           TGCTGGTTAAGTGTTGCTTGGCAGCATAGAAAGTGCTTATTG----CCTGTAGATGACAGACTAG---CTGAGAACTTC- 17740
GoodesThornscrubTortoise    TGTGTGAAACTGATTGTTCCTTCCTAAGTGGAACACTTTGCA----TTTATGTTTATTGAACTTCATCCTGTTTACCTCA 60877

Majority                    GG-GCAAATTTGAAAAGCTCGCACCTCAGACTAAGTTGGCAATGAGTATCTCTTAGTAGAGGAAAGA-AAAAATAGGCTT

                                     66410     66420     66430     66440     66450     66460     66470     66480

Human                       CATGCAACTCATCAAAGAAACAATTTAAAGATAAAATACCAA---GAAACTCCAGCTAAAGATAAAATACCAAGAAACTC 65753
Kakapo                      A--GCAAAAGTGAAAAGCTTGCACCTCAGACTAAGTTGGCAATGAGTTTTTCTTAGTAGAGGAAAGA-AAAAACAGGCTT 33508
GoldenEagle                 GG-AAAAAGTGAAAAAGCTCGCACCTCAGACTAAGTTGGCAATGAGTATCTCTTAGTAGAGGAAAGA-AAAAATAGGCTT 36922
JapaneseQuail               TG-TGAAAGTGGAAAAATTCACGTCTCTGATGAAGTTGGTGAAGAGTATCTGGTAGTAGAGGAGAGAAAAAGCAGGCTTT 28943
MediumGroundFinch           ---ACAAAATTGAAAAGCTCACACCTCAGGCTAAGTTGGCAA-ATTTTCCTCTTGGTAGAGGAAAGACAAAAATGGTCTT 17816
GoodesThornscrubTortoise    GA-CCATTTTTCCAATTTGTCCAGATCATTTTGAATTTTGACCCTGTCCTCCAAAGCAGTTGCAATC--CCTCCCACTTT 60954

Majority                    AGTTTTCTGTTGT---T---AAAGAATAATGACTATATTGAA----ATAAAGACCTTT--ATTAAATA-GTGGAGGATGG

                                     66490     66500     66510     66520     66530     66540     66550     66560

Human                       CAGCTAGTATTAACAATGGACTACAATAATCACTTCTTTGGACCCTACTGAGGTGTGTGTATTTGCCCATCTGGATATAC 65833
Kakapo                      AGTTTTCTGTTCT-------TGTAAGTA--GATTATATCGAA----ATCAAGACCTT-----C--ACA-GTGGAGGATGG 33567
GoldenEagle                 AGTTTTCTGTTGT-------AAAGAATGATGACTATATTGAA----ATAAAGACCTTC--ATTAAATG-GTGGAGGATGG 36988
JapaneseQuail               GGGTTTCTACTGTGCTTCTTAAAGAATTATGACACTATTGAG----ATAAAGACCGTC--ATTAAAAA-GTGGAGGATTG 29016
MediumGroundFinch           AATCTTCTGTTGT-------AAAGAATGAGGACTATATTGTA----ATAAAGACTTT---GTT--ATG-GTGGAGGGTGG 17879
GoodesThornscrubTortoise    CGTATCGTCCACAAACTTAATAAGCATACTTTCTATGCCAAC----ATCTAAATCGTT--GATGAAGATATTGAACAGAG 61028
```

```
Majority                 TCATTTTTATCCTTATCACCAAATGAAATC-ACTGGTTATTGATTAAAAGAA---CTGTGA---GGTGGTCAAGACAG--

                                  66570     66580     66590     66600     66610     66620     66630     66640

Human                    TTAGATGTGCTTGGATCCTGGGTGGAGGC--TTGGTTAGAAGTCACGTGGATTCTTCTAGGGAATTTATCACCATTTTT 65911
Kakapo                   TCATTTTTATCCTCATCACCAAATGAAATC-ACTGGTTATTGATTAAAAAAAATACTTTAAAAAGGTGGTCAAGACA--- 33643
GoldenEagle              TCATTTTTATCCTCGTCACCAAATGAAAT--GCTGGGTATTGATTAAAAAA----CTGTTA---GGTGGTCGAGAC---- 37055
JapaneseQuail            TTATTTTCATTCTTATCACCCTATAAAGT--AATGAACATTGATTAAAAGT-------TGA---GGCAGCCAAGACAG-- 29082
MediumGroundFinch        TCATTTTTGTCCCTATCACCAAATGAAAT--ACTCAGCATTGAGTCAAGAA----CTGTGA---CATGGTCAAGACAGG- 17949
GoodesThornscrubTortoise CCGGTCCCAAAACAGACCCCTGCGGAACCCCACTTGTTATCCCTTTCCAGCAGGATTGGGAGCCATTAATAACTACTCT- 61107

Majority                 ---GGGATCACAAAAAAATAAG--AGATGTTTTCAAAAAGCAGAGTGTAAGTTTTCATG-CTGT-TGATAAAAGTAAAGTT

                                  66650     66660     66670     66680     66690     66700     66710     66720

Human                    CTTGTGATCTTATGACCTTGCTGAAACTTTTAAAACAATAATAAAGTCATTTTTTTTCTCTTTGAGATGGAATCTCACTC 65991
Kakapo                   ---GGAATCACAAAAGATAAA---GAGGTTTTCAAAAACCAGAGAGGAAGTTTTCGTG-CTGT-TGAGAAGAGTAAAGTC 33715
GoldenEagle              ---GGAATCACAAAAAGTAAGAGACATGTTTTCAGAAAGCAGAGTGTAAATGTTCATG-CTAT-TGACAAAAATAAAGTT 37130
JapaneseQuail            ---GGAATGGCAAAAAAATAAG--AGATCTTTTCAAAAAGCAGAATGTAAGTGTTGA-----G-----AAAAAGTAAAGCT 29147
MediumGroundFinch        ---GGGATCACAAAGAATTGA--ACATGTTTTCAAAAAGCAGAGTGTAAGTTTTCATG-CTGT-TGAGGGAAACAAG--- 18019
GoodesThornscrubTortoise ---CTGAGTACGGTTATCCAGCCAGTTATGCACCCACCGTATAGTAGCCCCATCTAAA-TTGTACTTTCCTAGTTTATCT 61183

Majority                 AGCCA-------------AGAGTAAAGAAGATTTG--TAACT-TGATTTATGATGATCTACTTTAAGATAATCTACTCCA

                                  66730     66740     66750     66760     66770     66780     66790     66800

Human                    TGTCGCCCGTGTCAGAGTGTGGTGGCGCAGTCTCGGCTCACTGCAACTTCCGCCTCCCGGGTTCAAGCGATTCTCCCGCC 66071
Kakapo                   AGCCAGAATAAAGTTACAGGAATAAAGATGATTTA--CAACT-TGATTTACAATGATCTAATTTAAGATAATCTACTCCT 33792
GoldenEagle              AGCC--------------AGAGACAAGAAGATTTG--TAACT-TGATTTATGATGATCTATTTTAAGATAATCTAGTCCT 37193
JapaneseQuail            AGCTT-------------AGGACAAGCAGAATTTT--CAACT-TGGTTTCTGATTATGTGCTTTAAAGTAGTCAGCCTCA 29211
MediumGroundFinch        ------------------AGAGAAGAGATGATTTG--CAGCT-TGATTTATGGTGGCCCACTTTAAGATAATCTACTCCA 18078
GoodesThornscrubTortoise A--TAAGAATATCATGAGAGACCGTATCAAATGCC--TTACT-AAAGTCTAGGTATATCACATCCACTGCTTCTCCCTTA 61258

Majority                 GCAAAGC----AGTCTGGAAA--TACTTGTGGAT-TTGGAATACAATTTAAATTAGGGATATTCTTTTCAGAGATGAA-T

                                  66810     66820     66830     66840     66850     66860     66870     66880

Human                    TCAGCCTCCTGAGCATCTGGGACTACAGGCGTGTACCACCACCCCTGGCTAACTTTTGTCTTTTTTCGTAGAGATGAGAT 66151
Kakapo                   GCAAAGC----AGTGCGGAAA--TACTTGTGTAT-TTGGAGTATAATTTAAATTGGGGATATTCTTTTCAGAGATGAA-T 33864
GoldenEagle              GAAAAGC----AGTCTGGAAA--TAGTTGTAGAT-TTGGAGTACGATTTAAATTAGGGATATCCTTTTCACAGATGAA-T 37265
JapaneseQuail            GGAAGGT----AGTCTGGAAA--TATTTGTAGACCTTGGAATCCACTTTAAATTAGTGATATTGTTTACTGACATGTA-T 29284
MediumGroundFinch        GAAAAGC----AGTCTGGAAA--TACTTGTAGAT-CTGGAACACAATTTAAATTAGGAATGTCATTTTCAGAGTTGAAAT 18151
GoodesThornscrubTortoise TCCACAA----GGCTCGTTATCCTATCAAAGAATGTTATCAGATTAGTTTGACACGATTTGTTCTTTACAAACCCATGCT 61334
```

```
Majority                    AG-TATCAAAT--ATTAGATTGTGT--TTCCGAGTGGAGGCAGATG-----------TAAGTATTTCATTGTGAT-----

                                    66890     66900     66910     66920     66930     66940     66950     66960

Human                       TT-CACCATGTTGGCCAGGCTGGTCTGGAACTCCTGATCTCAGGTGATCTGCCCGCCTCAGCCTCCCAAAGTGCTGAG-A 66229
Kakapo                      AG-TGTCAAAT--ATTAGATGGTGT--TT--GAGTAGAGGAAGATG-----------CAAGGATGTCGCTGTGAG----- 33921
GoldenEagle                 AG-TATTATGT--ACTAGATCATAT--GTCAGAGTAGAGGGAAATG-----------CAAGTATTTCATTGTAAT----- 37324
JapaneseQuail               AA-TATAAAAT--ATTAGGTGGGA--------GGCGTGTGCAAATG-----------TATTACTGAAACTAGTAA----- 29337
MediumGroundFinch           AA-TATCAAAT--ATTAGATTGTGC--CTCAGAGTGGAGAGAAATG-----------CAGATACATCAATGTACT----- 18210
GoodesThornscrubTortoiseGGCTATTCCCT--GTCACCTTACCACCTTCCAAGTGTTTGCAGATGATTTCTTTAATTACTTGCTCCATTATCTTCCCTG 61412

Majority                    ATACAAGAATTTGTCTGGATGGGATGCAGCTTTCAGG--TTT-TAAATAGGTTTAAAATAGTAG-ATAGAAACTAGATCT

                                    66970     66980     66990     67000     67010     67020     67030     67040

Human                       TTACAGGTGTGAGCCACCATGCCTGGCCGCTTTTGTTCATTTTTTGAGCGTTGTACATTTTTACATTAAAGGCGTGTAAA 66309
Kakapo                      ATACAAGG-----------TGGGATACGCCTTTCAGG--TTT-GAACTAGGTTTAAAATAGTG---CAGAAA--AGATCT 33982
GoldenEagle                 ATGTAAAAATTTGCTTGGATGGGATACACCTTTCAGG--TTT-TAAGTAAGTTTAAAATAGTAACACAGAAACTAGATCT 37401
JapaneseQuail               AAAGAAAAAAATTGTGTGGATGGAATGCAGCTGTCAAG--TTTATAAATAGACTTAGAATGACA---TAAAAGGTTGATCA 29412
MediumGroundFinch           ACATAAGAATTGGTATGGTTGGAATGGACCTTGCAG--------------GTTTAAAATAGTGGTACAGAAACTACATCA 18276
GoodesThornscrubTortoiseGCACAGAAGTTAAACTAACTGGTCTGTAGTTTCCTGG--GTTGTTTTTATTTCCCTTTTTATAG-ATGGGCACTATATTT 61489

Majority                    TTAACCTACAATTGCTGC--AGAAATTGACTG-TAGTCATTTAGATTTATAT-TTGAAATACTTGATA-AACGGT-GTAT

                                    67050     67060     67070     67080     67090     67100     67110     67120

Human                       TTAAGTTGCTCGCTTTGCCAAAAAATTGTCTAGTGTCTTTTTCCACTTCTGTGTTGAATTTTTTAGTATAACGCATTTTT 66389
Kakapo                      TTAACCTACAATTGCTGC--AGAAAGCAACTG-AAGTCATTGAGGTTCCTAT-TTGAAATACTTTATA-AACGGT----T 34053
GoldenEagle                 TTAAACTACAATTGCTGC--AGAAATCGACTG-TAGTCATTTAGATTCATAT-TTGAAATACTTGATA-AACAG----AT 37472
JapaneseQuail               TTAACCTGAAATTGCTGT--GGGAACTGACTGCAAACCATTTGAATTCACCT-CTGAGATACATTTTG-ATCCCA-GTGT 29487
MediumGroundFinch           T-AACCTACAAGTTCTGC--AGAAATCAATTG-TAGACATTTAGATTTATAT-TTGAAATATTTGATA-AATAGTTGGAT 18350
GoodesThornscrubTortoiseGCCGTTTTCCAGTCTTCT--GGAATCTCCCCC---GTCTCCCATGATT-TCC-CAAAGATAATAGCTA-GAGGCTCAGAT 61561

Majority                    GCTTCA-TGTATTTGTTGCA-GAA-TGTTGTAATTCAGGTTTGATTTGTGTTTGGGAAGTTATTTTT-TAGTAA---TAG

                                    67130     67140     67150     67160     67170     67180     67190     67200

Human                       GTTTCTTCATCTCTGCCTCATAAGAAAAAGGAAAAAGTGTTTAGCCTTTCCTAAGTGACTTCTTTTTTTTATCTGTTTTA 66469
Kakapo                      GCTCA--TGTGTTTGTTGCA-GAA-TGTTGTAATTCAGGCTTGATTTGTGCTTGGGAAGTTATTTTGTCAGTAA---TA- 34125
GoldenEagle                 GCTA---TGTGTTTGTTGCA-GAA-TGTTGTAATTTAGTCTTGATTCGTGTTTGGGAAGTTATTTTT-CAATAA---TAG 37543
JapaneseQuail               TCTTCA-GGTATTTGTTGCG-GA--TGTTGTAATTCAGTTTTGATCAACGTCTGGGAAGTTGTTTT--T--T-------- 29551
MediumGroundFinch           GCTAAA-TGTATTTGTTACA-GAA-GGTTGTAATTCAGGCTTCACTTGTGTTTCGAAAGCCATTTT--CAGTAG---TAG 18422
GoodesThornscrubTortoiseACCTCC-TCTATTAACTCCTTGAG-TATTCTAGGATGCATTTCATCAGGCCCTGGTGACTTGCAGGCATC-TAA---CTT 61635
```

```
Majority                    ATGTTAATGC-TGTTTTTATAGTTCTTTTTTTATGCAAAATATGA-A---CGTGATAAATCTTTTTACTTCA-GTTTTTT

                                      67210     67220     67230     67240     67250     67260     67270     67280

Human                       ATGTTTTTGCATCTTGTTATAACTGGGCTTTTTAACTATACTCAAAAATAGGTTAAAAGTAGGTGAGTTAAAGGTTTTAA 66549
Kakapo                      -TGTTAATGC-TGCTTTTAAAGTGCCTTTCTAAAGCAAAATAGGACATTAAGTGATACATTTCTTTACCTT---TTTTGA 34200
GoldenEagle                 ATGTTAATGC-TGCTTTTAAAGTGCGTTTTTGAAGCAAAATATGACAT--CGTGATAAATCTTTTGACTTCA-GTTTTTT 37619
JapaneseQuail               -----------TTTTTTAATAGTTATGTTTTACTTCACA---------------------TTTTTTACTTCA-TTTTTGA 29598
MediumGroundFinch           ATGTTGCTGC-TGCTTTTAAAGTGCTTTTCTGATGCAAAGTATGA-----CGTGTTAAAACTTCTGACCTC--GTTTTTT 18494
GoodesThornscrubTortoiseTTCTAAGTGA-TTTTTTACTTGCTCTTTTTTTATTTTATCTTCTA-----ATCCTACCCTCTTCCCATAAGC-ATTCACT 61708

Majority                    GTGTTATATATATTATCAGAAGTTT--TAATGGA-AAGATGGCGTCTGTGAAATGGACTTTAGTGATTTGAAAATTA---

                                      67290     67300     67310     67320     67330     67340     67350     67360

Human                       AAATCAGATAATGGACCCTGGTTTTGATAATTCACAAGAGATAACCATTTATTTTCATATTTTTTATGTAAATACATGTT 66629
Kakapo                      GTCTTAAATGTATTATCAGAAGATT--TAATGGA-AAGATGACGTCTGTACAATGGCCTGTAGTGATCTGAGAATGAA-- 34275
GoldenEagle                 GAGTTAT--ATATTATCAGAAGATT--TAATGGA-AAGAC---GTCTGTGAAATCGACTGTAGTGATTTGAAAATTA--- 37688
JapaneseQuail               ATCTTAAATATGTTGTCAGAAGATT--TAATGGA-AAGATGGCATCTGTGAAATAAACTTTAGCAATCTGAAAATTA--- 29672
MediumGroundFinch           GAGTCATGTATATTATCAGAAATTT--TAATGGA-AAAATTATGTCTGTGAAATGGACTGTAGTGATATGAAAATTA--- 18568
GoodesThornscrubTortoiseATACTAGACATTCCTTCAGACTTCT--CAGTG---AAGACGGAAACAAAGAAGTCA--TTAAGCATCTCTGCCATTTC-- 61779

Majority                    TCAGTGTAATTCTAGGAATATATTAT-AAAGAAGTTACAAAATAAC-AAAGGATGAGGTATGTAAATGTATGAAAGGAAT

                                      67370     67380     67390     67400     67410     67420     67430     67440

Human                       TCTGTACAAAATTAAAATTGCTCTGTATTTGCAGATTTGCATTATCTTTTATTGTAATTGACATTTTACTTAGAATTTGT 66709
Kakapo                      TCAGTGTAATTCTAGGAATATATTAT-AAGGAAGTAACAGAATAAC-AAAGGATGAGGTATGAGAAAGTATGGAAGGAAC 34353
GoldenEagle                 TCAGTGTAATTCTAAGAATATATTAT-AAAGAAGTGACAAAATAACAAAAGGATGAGGTATGTAAATGTATGAAAGGAAC 37767
JapaneseQuail               CCAGTATAATTTTAGAACTGTATTATTGAAGAAGCGACAAAAAAAC-ACAGGATGAGGTATGTAAAAGTATGAAAGGAAA 29751
MediumGroundFinch           TCAGTGTAATTCTAGGAATATATTAT-AAAGAAGTTACAA----ATGAAAGGATGAGGTATGTAAAAGTGTGAAAGGAAT 18643
GoodesThornscrubTortoiseCAAGTCTCGTTACTGTTTCCCCCTCCTCACTGAGCAGTGGGCCTACCCTGTCCTTGGTCTTCCTCTTGCTTCTAATGTAT 61859

Majority                    T--TAAGAATCTC--TTGCTGTTGTTGACTTCT---GATAGATTGATACTAGCTTGTGGGAT-GCT-TATTGGTCTTACT

                                      67450     67460     67470     67480     67490     67500     67510     67520

Human                       TT-TAAAAGATAGCTTTAATACCTGCGTAGCATGTTCATGGGTATGCCATACCTTTGAAAGTGGACATGTAGGCTATTCC 66788
Kakapo                      C--TAAGGATCTCCCTTGCTGTTGTTGACTTCT---GACAAACTGATACTAGGTCATGGGT------TACTG----T--- 34415
GoldenEagle                 A--TAGGAATCTC--TTGATGTTGTTGACTTCT---GACAGACTGATACTAGCTTGTGGGAT-ACTGTATTGGTCGTAAT 37839
JapaneseQuail               T--AAAGAACCT-----GCTGTCTCTGACTTCT---GATAAGTTGCTACCAGCTTGTGGGAT-GC--TATTGGCCATAGT 29818
MediumGroundFinch           C--TAAGAATCTC--TTGCTGTTGTTGACTTCA---GACAAACTGATACTAGCTTGTGGGA---CTCTATTGGTCTTAA- 18712
GoodesThornscrubTortoiseTGATAAAAAGTCTTCTTGTTTCCCTTTATTCCCATAGCTAGTTTAAGCTCATTTTGTGCCTTTGCCTTTCTAATCTTGCC 61939
```

```
Majority                 TTT----T--TAAAAAAACAGCATGTATTTGTTTCT----------CCAGTTTTGCATGTTTTGATTAGGGTAGCTGTTT

                                67530     67540     67550     67560     67570     67580     67590     67600

Human                    ATACTTTTTATTAAAACACC-CTTGTACTTTTTTGCCT--------CTCAAATTATTTTCTTAGACTAGATTCCTAGAAT 66859
Kakapo                   -----------A-----------TGTATTTGTTGCT----------CCAGTTCTTCATGTTTTGATTAGGGTAGCTGTTT 34463
GoldenEagle              TTT----TTTTAAAAAAAAAAGCATGTATTTGTTTCC---------CCAGTTTTGCATGTTTTGATTAGGGTAGCTGTTC 37905
JapaneseQuail            ATT----T---GAAACAACAACACATCTTTTTTGCT----------CTGGTTTTGCATGT---GACTAGGGTAGCTGT-- 29876
MediumGroundFinch        -----------AAAAACCCAGTATGTATTTGTTTCT----------CCAGTTTTGCATGTTTTCATTAGGGTACCTGTTC 18771
GoodesThornscrubTortoise TCTGCATTCCTGTGTTATTTGCCTATATTCGTCCTTTGTGATCTGACCAAGTTTCCATTTTTTATATGACGCCTTTTTAT 62019

Majority                 ACTAGTGGTAGCGCATGTGTGAATGACTAAACTTCATTGATTAAT-AGTGAC-TCCTGTATGTACAGTCATTGTCAGAGT

                                67610     67620     67630     67640     67650     67660     67670     67680

Human                    TAGAATTATTGGGTCAAAGAAAATGTTAAAGCCTGTTTACTTTATTTGCAAATTTCTTCATCTAAAGGTGATATTAACTT 66939
Kakapo                   ACTACTGGTAGCTCATGGGTGAATGACTAAACT-CATTGAGTA---AGTGAC-TCCTGCAGGTACAGTCATTGTCAGAGT 34538
GoldenEagle              ACTAGTGGTACCTCATGTGTGAATGACTAAACTTCATTGAGTAAC-AGTGAC-TGCTGCATGTACAGTCATTGTCAGAGT 37983
JapaneseQuail            GTCACTAATGGCACTCGTGTGAATGACTAAACTTAATTGAATAGC-AGAAAT-ACCTGTGTGTGCTGTTATTGTCTGAGT 29954
MediumGroundFinch        ACTTGTAGTAACGTGTGTGTGAATGACAAAACTTCATTGAATA---AGTGAC-TCATGTATGTGCAGTCATTGTCAGAGT 18847
GoodesThornscrubTortoise TTTGTAGGTCACGCAAGATCTCGTGGTTAAGCCAAGGTGGTCTTT-TGCCACATTTTCTATCTTTCCTAACCATCGGAAT 62098

Majority                 AATCT----TGGAGCCTAT------CTTAGTGACAAATTGAAATAGTCTTAGGTTTATAGTTTCATATTTTAACCAATTT

                                67690     67700     67710     67720     67730     67740     67750     67760

Human                    AAACTAACATTAATTATATATGAGGCTGGGTGTCCTACTTTTTCCCCTGCCAGCATACTATTTTTTAATTTCACAAGATT 67019
Kakapo                   GATCT----TGGAGCCCAT------CTTGGTGACAAATTGAAATGGTCTTCAGTTTATAGTTTCATATTTTAACCAATCT 34608
GoldenEagle              GATCT----TGGAGCC----------TTAGTGACAAATTGAAATAGTCTTAGGTTTATAGTTTCATATTTTAACCAATCT 38049
JapaneseQuail            TATCT----CGAAGCCTAT------CTTAGTGACAAATTGAAACGGGAATAGGCTTATAGCGTCTTATTTTAACCAATTT 30024
MediumGroundFinch        AATGT----TGGGGCC----------TTATTGA--AATTGAAATAACCTTAGATTTATGGTTTCACGTTTTAACCAATCT 18911
GoodesThornscrubTortoise AGCTTGCTTTTGGGCCCTT------AATAGCGTCCCTTTGAAAAACTGCCAACTCTCCTCAGTTGTTTTTCCCCTCAGTC 62172

Majority                 ATGCATAAACAGGCAAAATGCTAAGTGTAATACCTATTGCAATT--ATTAAAGAGTGCTAAATTTGAATGAA--GGTTTT

                                67770     67780     67790     67800     67810     67820     67830     67840

Human                    AAT--TTGATAGG-CAAATGCCTATTTTAATTTCTGTTAAGATTTAGTTTACAATCTTTAAATGTTTATTTGATAGTCAC 67096
Kakapo                   ATACATAAATAGGCAAAATGCTAAGGGTAATACCCATTGCAAGT--ATTGAAGAATGCCAAACTAGAGGGAA--GGTTTA 34684
GoldenEagle              GTGCATAAACAGACAAAGTGCTAAGAGTAATACCAACTGCAAGT--ATTGAAGAGTGCTGTATTTGAAGGAA--GGTTTG 38125
JapaneseQuail            CTG-------AG--AAAATGCTT-----------TAATG----------AAAGAAGGCTTGATTTTTATTT----TTTCT 30070
MediumGroundFinch        ATGCATAAACATACAAAATGTTAAGAGTAGTACCAAGTGCAGTT--ACTAAAGGGTGCTAAATTGGAAGGAA--GGTTTG 18987
GoodesThornscrubTortoise TTG-ATTCCCAT--GAGACCTTACCTATCA-GCTCTCTGAGCTT--ACCAAAATCCGCCTTCCTGAAATCCATTGTCTCT 62246
```

```
Majority                     ATTTTCCTACTATCCTCCCAAGC--TAAATAGAGTAAAATAAGTA-CAGTTCTTATGGTTAAGTTACTGTGGTAAAACAT
                                     67850     67860     67870     67880     67890     67900     67910     67920
Human                        AATTTGCTAAATTGCTTGCCTGCATTCTTTCTGTTAGGATGTACGGTAATTCTTATAAAATGCATAGTAAGGATATTAAT 67176
Kakapo                       ATTTTCCTCCTATCCTCCCAAGC--TAA-TAGAATAAAATAAGTA-CAGTTCTTAGGGTTGAGCTACTATGGTCGAACAC 34760
GoldenEagle                  ATTTCTGCACTACCCTCCCAAGC--TAAATAGAGTAAAATAAGTA-CAGCTCTGATGGTTGCGCTACAGTGGTAAAACAT 38202
JapaneseQuail                CCTTCCTCTTTTCTCTCCCAG-----AGATAGAGTAAAATTAATA-CTGTTGTTACAATTAAGGTAATGTGATAAAACAT 30144
MediumGroundFinch            ATTTCCCCACTACTCTCACAAAC--TAAATAGAGTAAAATA-----CAGTTCTTACAGTTAAGTCACTGTGATAAAACAT 19060
GoodesThornscrubTortoiseATTTTGCTGTACTCCCTTCTACCCTTCCTTAGAACTGCAAACTCT-ATGATTTCATGATCACTTTCACCCAAGCTTCCTT 62325

Majority                     GAAGTGCCACCAGATCAA-AAATACCTAAAGGCATGAAACAGTCA-GT-T------GATT--ATGGGAAAAACCTTTTCA
                                     67930     67940     67950     67960     67970     67980     67990     68000
Human                        ATGCATATTTTGGATGAGCCAAGCCTTCAG--CTTTATTCACTTATGT-TAGGTCAGTTT--TTGGAAAAGTATTTTTCA 67251
Kakapo                       AAAGTGCCACCAGAAGAA-AAGTACTTAAA------AAAGAGTCA-----------GATG--ATAGGAAAAACCCTTCTG 34820
GoldenEagle                  GAAGTGCCACCAAAACAA-AAATACCTAAAGGCAAGAAACAGTCA-----------GATG--ATGGGAAGAAACTCTCCA 38268
JapaneseQuail                GAAATGTTACCAGATCAA-AGCTGACAAAAGGAA--AGAAAGTCA-------------C---ATAAGGAAAAAGCCTACA 30205
MediumGroundFinch            GAAGTGCCGCCAGAACAA-AAATACCTGAAGGAAAGAAGCAGTCAAGTATTCAAGTGATA--AGGAAGAAAACCTCTTCA 19137
GoodesThornscrubTortoiseCTACTTTCAAATTCTCAACAAGTTCCTCCCTATTTGTTAAAATCAAGTCTAGAACAGCTTCCCCCCCAGTAGCTTTTTCA 62405

Majority                     GTTTAAATACATCTGGAAATA-GTGTA--GAAGCCAGAGGATGTGGTGGCTACACTTT-AGTTGCATGGAGTTCAAAATC
                                     68010     68020     68030     68040     68050     68060     68070     68080
Human                        ATAATCTCTCTTTTTAAATAATTGATCTCAATCCCAAAGTATTCATTTGTAGGACATGAAGACACA--ATTTTCTTCTTC 67329
Kakapo                       GTTTAAATATATCTGGAAATA-GAGTA--GAAACAAGAGGATGTGGTGGCCACAATTTCAGTTGCA-GGAGTTCAAAATG 34896
GoldenEagle                  GTTTAAATACAGCTGGAAATA-CAGTA--GAAGCAAGAGGATGTGGTGGCTACACTTA-AGTTGCATGGAGTTCAAAATT 38344
JapaneseQuail                GTTTAAATTCATCTGGAAATA-GTGTT--AGAAGCAAAGGGGATGGTGGCAACGTTTG-AGTTACGTGGCATTCAAAATT 30281
MediumGroundFinch            CCTTAAATACATCTGG--GT----GTA--GAAGCAAGATGATGTGGTGGCTGCAATTA-AGTTGCATGGAGTTCAAGATC 19208
GoodesThornscrubTortoiseACTTTCTGAAATAAAAAGTTGTCTGTAATGCAGTCCAGGAACTTATTGAATACTCTGT--GCCCCGCGGTGTTATTTTCC 62483

Majority                     AAACGCGTGGTAGAAGAGTAAAGAAGTTTTCAATTTGTTTTCCATCAGTTGTTACTATAGAA--GATAATGGGGAAATTT
                                     68090     68100     68110     68120     68130     68140     68150     68160
Human                        ATACGTTTGGT-GAATATTTTGA----TCTCAGTTTCCACTTCTCTTTTTGCTTTTTAAACATTGTTTATGGTGATATCC 67404
Kakapo                       AAAAGCATACTAAAAGAGGAAAGAAGTTTTCAATTTCTTTTCCATCAGTTGTTACTATAGAA--GATAATGGGGAAAT-- 34972
GoldenEagle                  AAAAGCGTGGTAGAAGAGTAAAGAAGTTTTCAATTTGTTTTCCGTCAGTTGTTACTATAGAA--GATAATGGGGAAATTT 38422
JapaneseQuail                AAAAGCGTGGTAAAAGAGTAAAGAAATTTTCAATTTGTTTTCCGTCAGTTGTTACTATAGAA--GATAATGGGGAAATTT 30359
MediumGroundFinch            AAACACGTGGTAAAAGAGTAAAG---TTTTCAATCTGTTTTCCAT-ACAAGTTACTGTAGAA--GATAATAGGGAAATTT 19282
GoodesThornscrubTortoiseCAACATATATCTGGATAGTTGAA--GTCCCCCATCACCACCAAATCTTGGGCT-TTGGAGGA--TTTTGTTAGTTGTTCG 62558
```

```
Majority                   AATTTTCCATTGGCCCTCTTCACAGGAAATCAGAATGTGGCCT---TAATAGAAATTGAAGACTT-ATTAAGGAAGTTAA

                                    68170     68180     68190     68200     68210     68220     68230     68240

Human                      AATATTGCATGAGTGAGGTCAT--GCCTTTTAGAATAATAACTGAATCTTTTCTTCTAATTATTTAATCAGATGACTTCA 67482
Kakapo                     ------------------------------CAGAATGTGGCCT---TAATGGAAATTGAAGACTT-ATTAGGGAAGTTGA 35018
GoldenEagle                AATTTTCCACTGGCACTCTTCACAGGAAATCAGAATGTGGCCT---TAATAGAAATTGAAGACTT-ATTAAGGAAGTTAA 38498
JapaneseQuail              AATTTTCCACTGGCACTCTTCACAGGAAATCAGAATGTGGTCT---TAATAGAAATTGAAGACTTTATTAAGGAAGTTAA 30436
MediumGroundFinch          AATTTTCCGGTGGCCCTCTTCACAGGAAATCAGAATATGGCCT---TAATAGAAATTGAAGACTT-ATTAAGGAAGTTAA 19358
GoodesThornscrubTortoiseAAAAAAGCCTCATCCACCTCTTCC--ACCTGATTAGGTGGCCTG--TAGTAGACTCCCAGCACGACATCACCCGTGTTTT 62634

Majority                   --AGAGCAATTTGGAA-AACTCAAGTGCTGTAAGTACCAAGGAGAGATGCTATTCATTTGAAG--GTTCTGAGAAACTAG

                                    68250     68260     68270     68280     68290     68300     68310     68320

Human                      GCAAACTAATATTGGTGGGATTAACTTCCTGCTCAGTCTTCCAGTAACAGTACTTGTTCAAAATGAAATAATTGATAGAG 67562
Kakapo                     --AGAGCAATTTGGA--------AGGGCTGGAAGTACCAAGGAGAGATGCTATTCATTTGAAG--GTTCTCAGAAGCCAG 35086
GoldenEagle                --AGAGCAATTTGAAA-AACTCAAGTGATGTAAGTACCAAGGAGAGATGCTATTCATTTGAAG--GTTCTGAGAAAATAG 38573
JapaneseQuail              --AGAGCAATTTGAAA-AACTCAAGTGATGTAAGTACCAAGGAGAGATGTTATTCATTTGAAG--GTTCTGAGAAACTAG 30511
MediumGroundFinch          --AGAGCAATTTGAAA-AACTCAAGTGATGTAAGTACCAAGGAGAGATGCGATGCATTTGAAG--GTTCTGAGAAAACAG 19433
GoodesThornscrubTortoise-TACCCCTTTTAGCCTAACCCAGAGACTCTCAACACTTCCATCTCCTA-TGTCCATCTCCA------CCGCAGTCCAAG 62705

Majority                   TGACACTGACAAGAATGTACAATATGAAGCCTCTAGCCCTGTGGGACTACTTTTGCTATTTGAATGAGCAA--GTAGTGA

                                    68330     68340     68350     68360     68370     68380     68390     68400

Human                      TATTTATTTCAAAAATTTTTAAGAACACAAATATAAGG-TGATACTCTTTTGCTAAAATTTGTGGGGGGCTTCACTTTAC 67641
Kakapo                     TGACACTGACAGGAATGTACAATACCAAGCCTC------TGTGGGACCACTTTTGCTATCTGAATGAGCAA--ACAGTGG 35158
GoldenEagle                TGACACTGACAAGAATGTACAATATGAAGCCTCTAGCCCTATGGGACCACTTTTGCTGTTTGAATGAGCAA--GTAGTGA 38651
JapaneseQuail              TGACACTGACAAGAATGTACAATATGAAGCTTCTAGCCCTGTGGGACTACTTCTGCTATCTGAATGAGCAA--GTAGTGA 30589
MediumGroundFinch          TGACACTGACAAGAATGTGCAATATGAAACCTCTAGCCCTGTGGGATCTCTTTTGCAATTTGAATGAGCAG--CTAGTGA 19511
GoodesThornscrubTortoiseTGTGTACATTTTTAATATAAAAGGCAACACCTCCTCCCTTTTTCCCCTGTCTATCCTTCCTGAGCAAACTATACCCATCC 62785

Majority                   AAAATTGCATTCCTGCCCCA---CTTGTTTTGT-GAGAAACTCATTTCCTGTAAAAGTAG-ATTATGGCTGC--ACTGAT

                                    68410     68420     68430     68440     68450     68460     68470     68480

Human                      CTTGTATGATTTTTATATTAGATCTAGCTACTTGACTGAATTTAATTACTTTAGAATCAGTACTATAAATTTTAATTGAT 67721
Kakapo                     AGAATTGCACTGCTGCCTCA---CTTGCTGTATTGAGAAGCTCATTTCCTGTTGAGGTAG-ATGATGGCTGC--ACTAAT 35232
GoldenEagle                AAAATTGCATTCCTGCTCCA---CTTGCTTTGT-GAGAAACTCATTTCCTGTAAAAGTAG-ATTATGGCTAC--ACTGAT 38724
JapaneseQuail              AAAATTGCGTTCCTGCTCCA---CTTGTTGTGT-GAGAAACTCATTTCCTGTAAAAGTAG-ACTATGGCTAC--ACTGAT 30662
MediumGroundFinch          AAAACCTCATTCCTGACCCG---CTTGTTTTGT-AAGAAACTCATTTCCTGTAAAAGTAG-ATAGTGGCTCC--ACCGAT 19584
GoodesThornscrubTortoiseACACCAACATTCCAGTCGTG---TGTATTATCCCACCAAGTTTCAGTAGTGCCAACA-AT-GTCATAGTTGT--ATTTAT 62858
```

```
Majority                   TTAT-GTTTCATCACCACCTTCAGATTGGATCATTATAGTTC-TTATATTTGTAT-TAA-GGTGAAGGTGACAGGCTTCA
                                   68490     68500     68510     68520     68530     68540     68550     68560
Human                      TTAT-TTTACACAGTAATTTTTTAAGACTAACATTTATGTCTGGCTTTTCTGTTT-----CCTTTAA-TGCTAGGTATCA 67794
Kakapo                     TTGT-GTTTCATCAGCACCCTCAGATTGGATTAGTACAGTA----------------AA-GGTGAAGGTGACAAAGCTCA 35294
GoldenEagle                TCAT-GTTTCATCACCACCGTCAGATTGGATCATTATAGTTCGTTATACTTGCAAGTAAAGGTGAAGATGACAAACTTCG 38803
JapaneseQuail              TCAT-GTTTCATCACCACCTTCAGATTGGATCATTATAGTTCATTATATCTGTAACTAAAGGTGAAG---ACAGACTTGG 30738
MediumGroundFinch          ACTT-GTTTCATCACGGCCCTCAGGCTGGATCGTTATAGGT-----TCCTTG--------GGTGAAGACTGGCAGCTTCA 19650
GoodesThornscrubTortoise   TTATTAGCACTTCCAGTTCTTCCTGCTTATTACCCATAATTC-TTGCATTTGTATATAGGCATCTAAGATACTGGTTTGA 62937

Majority                   GGTTCTGTGCAATGCAGCAGTCTAGCCATGGGTTGGAGA--AAGTA-CTGAGAATGTGCAGCA--------GTAGAGCAC
                                   68570     68580     68590     68600     68610     68620     68630     68640
Human                      CAGTGTCTCAGAAAATGTAATTTGTTCAAAGAAAGGAGACTGAGTGCTTTGTAATGAGTTTAAAATCTGACATCTAAAAT 67874
Kakapo                     GGTTCTGTGCAATGCAGCAGCCTAGCCATGGGTTGGAGA--AGGTA-CTGAGTATGTGCAGTA--------GTAGAGCAC 35363
GoldenEagle                GGTTCTGTGCAATGCAGCAGTCTAGCCATGGGTTGGAGA--AAGTA-CTGAGGCTGTGCAGCA--------GTAGAGCAC 38872
JapaneseQuail              GGTTACTTTCAGTGCAGCGGTCTAGCCATGGTTTGGAGA--AAGTA-CTGAGAATGTGCAGCA--------CTAAAGCAT 30807
MediumGroundFinch          GGATCTGTATAAAGCAGCAGTCTAGCCA--GGGTGGAGA--ATGTAACTGAGAATGGGCAAGA--------GTAGAGCAC 19718
GoodesThornscrubTortoise   TCTTGCCTCCCA-GCTTTGCCCTGACCCTCCTTTCTCTC--TGCCATTATAGCCCGTGCTCCCT------CCTATTTCCA 63008

Majority                   ATTGAACTACT--GTGCTTCTGCTAATG---C-GAAGGTGATGTTC---TGTAGATGTCTGTATTCATATGGAGATATTC
                                   68650     68660     68670     68680     68690     68700     68710     68720
Human                      ACATAATCACAA-ATGTGGCCGACTATTAGTC--ATTCACATATTC-TGTTTCAAAGAACACAGTGATCAAAAATCCTTC 67950
Kakapo                     TTTGAACTACT--GTACTTCTAATGAAG---CAAAAGGTGATGTCC---TGTAGATGTCTGTATGCATGAGGAGATACTC 35435
GoldenEagle                ATTGAACTACT--GTGCTGCTCCTAATGAAGCAGAAGGTGATGTCC---TGGAGATGTCTGTATGCATAAGGAGATATT- 38946
JapaneseQuail              GCTGAACTATT--GTACTTCTGGTAAAA-----CAAAGGGATATTC---TGGAGGTGTCCGTATTCATATGGAAATTTT- 30876
MediumGroundFinch          ATTGAACTGCT--GTGCTTCTGCTAATG------AAGGTTTTGTC----TGGAGATGTCTGTATGCATATGGAGATAC-- 19784
GoodesThornscrubTortoise   ACCCATCTCCCAGGTCTTGTTCCCCACTTACCTGTGGGCTTTGCTCACCTGTCCCCGTCGAACCTAGTTTAAAGCCCTCC 63088

Majority                   TTA-----------AGCAT-----CAAAAATAGCTCATTTT--CATAGTAAATGGTG-AGTACCCTCAGCATA-AGCAG-
                                   68730     68740     68750     68760     68770     68780     68790     68800
Human                      AGATTATAAATTGAGACAT---CATTATAATGGATCATTTTATCATGTTAAGAGGTGTAGTTAATTTATTAAGGGGTAGA 68027
Kakapo                     TTA-----------AGCAT-----CAAAAATAGCTCATTTT--CGTAGTAAATGGTC-AATACCTTCAGCATA-ACCAG- 35494
GoldenEagle                CTA-----------AGCAT-----CAAAAATAGCTCATTTT--CATAGTAAATGATC-AGTACCCTCAGCATA-AGCAG- 39005
JapaneseQuail              TTG-----------TGCAT-----CAGAGCCAGCACATTTT--CACAGTAAATGAT--AGTGCCCTCAACAGA-AGCAG- 30934
MediumGroundFinch          CTA-----------AGCAT-----TAAAAATAGCCCATTTT--CATTGTAAATGATG-AGTACTCTTAGCATA-AGCAG- 19843
GoodesThornscrubTortoise   TTACTAGGTTAGCCAGTCTGTGCGCAAATAAGGCCTTTCCC--CTTCTCGAAAGGTG-AACGCCATCTGTGCCTAGCAGT 63165
```

```
Majority                      -CAAACTTAAATA--TTTCTATGC------CAGT--AATCTGGTCTACCATATTCAGATTAGAGTGCAAGTGTTTCTGTC

                                        68810     68820     68830     68840     68850     68860     68870     68880

Human                         TCACTTTTAGAAAAATTGCTGGAAGTAATTTTTCATGATCATGTTATCTACATTCTAAAAATTAGGAGAGAGACTGTGTA 68107
Kakapo                        -CAAACTTGAATA--TTT---------------------------------------------------------TTAAGTT 35516
GoldenEagle                   -CAAACTTAAATA--TTTGAATGC------CAGT--AATCTGGTATACCATAGAAAGATTAGAGTGCCAGTGTTTCTGTC 39074
JapaneseQuail                 -AAAGCGTACTTAGGTTGCTATGA------AAGT--AATGCCTTCTATGTATTTCCATGGAAACAACAACAAATACAAAG 31005
MediumGroundFinch             -CAGACTTAAATG--TTTGAACAC------CAGT--ACTTTGGTATACCATG---AGATGAGAGTGCTGGTGTTTCTGTC 19909
GoodesThornscrubTortoise      CCTTCCTCGAATAGCATCCCGTGGTCGAGGAAGCCAAAGCCCTCCTGGCGACACCATCTTCGCAGCCAGGCATTCACCTC 63245

Majority                      TGAACAGCAGGTTTATTTGG---AGCCTTGATCTTCAAGTAGAAGAACCGGAAATATTATTGCCAAATCTTGAATATTGA

                                        68890     68900     68910     68920     68930     68940     68950     68960

Human                         CAAAGAGTGTTTATTTTAGAGCTTTCCTTGTATTTCAAATTGAATAACAGGCATTCTCATCATAAAGTTTTTAAAAGAAA 68187
Kakapo                        TGAATAGCAGG--AATCTGG---AATCCTG-----------------TAGAAAAA--------------T----ATTGA 35555
GoldenEagle                   TGAACAGCTGGCTAGTCCAG---AGTGTTGATCTTTGTGTAAATGCACCAAAAGTACTCTTGCCAAATCTTGGATATTGA 39151
JapaneseQuail                 AGCACAGTAACAGTATTTGGT--AGAGCAAATCCTCAACTACAAAACTCAGCTACAAAACTGT-GACTTTTCAACAGTCA 31082
MediumGroundFinch             TGAGAAGCTGGCTAATTCAG---AGCATTG-CCTTCATGCAGATGTACTGGAAGTATTCTTGCCAAATCGTGGCTATTAA 19985
GoodesThornscrubTortoise      CACGATGCATCTGTCTCTGCCTGGGCCCCTACCTTCGACAGGAAGAATCGAAGAGAATACCACCTGTGCTCCAAACTCCT 63325

Majority                      -------CAGTCATTAA-TGCCTCATCTTTGGAGTATTAACTGAGACGCTTA-TGTAGTGCATGACAGTCAT-CTTGACC

                                        68970     68980     68990     69000     69010     69020     69030     69040

Human                         GGCAAAGCAGACTTTCTGTAGGAAATCATTGACGTTAAAATAGTTATAATTGTGAACAGATACAACATTTATTCATGAAG 68267
Kakapo                        -------CAGTTACTAA-TGCCTCATCTCTGGAGGATTAACTGAGACGTGTA-TGTAGTGCATGGCAGTCAT-CTTGGCC 35625
GoldenEagle                   -------CAGTTATTAA-TGCCTCGTCTATGGAGGATTAACTGAGACTCATA-TGTAGCACGTGACAGTCAT-CTTGACC 39221
JapaneseQuail                 -------CCACCATTAGCTGTGTTTTTTTGCCAGCAGTGAACAAGA-GCCTG-TGTGTTGCACTCAAACAAA-TCTGTGC 31152
MediumGroundFinch             -------CAGTCATTAA-TGACTCATCTACAGACCATCAACTGAGGCTCATA-TGTAGTGCATGCAGGTCAT-CTTGACC 20055
GoodesThornscrubTortoise      -------TAACCCATAC-TCCCAGAGCCCTGTAGTCACTCTTGATCTGCTCAGTGTCACACCTCACAGTATCATTTGTGC 63397

Majority                      CAACATGGGTTAAAAATATGACTAGT---------AGGTTCTGTTATA-GTTG--GCCAGTTGGTCCTTACTCGGCCTGT

                                        69050     69060     69070     69080     69090     69100     69110     69120

Human                         GTAAACATGTAGGTCTTATAGAATATTGTTTCTCAAAATTTTGCTGCACGTTGAAGTCACCAGATTCTACCCCAGGCTGA 68347
Kakapo                        CAACAGAGTTCGAAA---TG--GAG----------AGTTTCTGTTCTA-GTTG--GCCTATAGGTGAT---TCAGAGTAC 35684
GoldenEagle                   CAACATGGCTTAAAAATATCACAAGTCTAATTGAGAGTTTCTGTTATA-GTTG--GCCAGTTGGTCCTCACTTGGCCCAT 39298
JapaneseQuail                 CAGCAGATGTGACCCAC-TGTTTCA----------TGG--CTGTTGTG-ATGG--CATTGTTGCTAGGAACATGTTGCCT 31216
MediumGroundFinch             CAGCATGGGTTAAAAATATCACTAG----------GGGTTCTGTTACA-GTTG--GCCAGTTGATCCTTCCTCGGCCTGC 20122
GoodesThornscrubTortoise      CCACATGGATGAGTAGCATG--GGGTAGTAGTCAGAAGGCCGGATAAT-CCTC--GACAATGCCTCTGTA-ACATCTCGG 63471
```

```
Majority                   ATGTAGTTCAA------GTTACAGAGATTCTGGTGAGGTTTGACTCTACA---GAGGTGTCTTCTTTCCAGT---TGTTG
                                    69130     69140     69150     69160     69170     69180     69190     69200
Human                      GAATAGTTAAAGAGTGGGTCACAGGCATTCCTATGTTTTTAGGATCTCAA---GATGGTTTTACTATGTAGCAAGAGTTG 68424
Kakapo                     CTCTA---CA-------------GAGATTCTGCTGAGGTTTGACTCTACATTCAGGTTGTCTTCTTTCCTGT---TGATG 35745
GoldenEagle                AGGTAAATCAGAGTACCTTTACAGAGATTCTGTTGAGGTTTGACTCTACAGTCAAGATGTCTTGTTTCCAGT---TGTTG 39375
JapaneseQuail              GTGAAGTCCAA------CTTTCATTGGTCCTGATAAGAAGGAATTCAGAA-----GGTGCCAAATCTGAAGTGTATGGTG 31285
MediumGroundFinch          ACGTGATTCA-------------AAGATTCCAGTGAAGTTTGACTGCACA-------TGTCATCTTTCCAGT---TGTTT 20179
GoodesThornscrubTortoise   ATACGGGCCCCTGGCAGGCAGCATACCTCCCGGGATGAACGGTCAGGGCAACAGATGGGTGTCTCCGTCCGCCTCAGCAG 63551

Majority                   AGAGTGACAGCTTCAGGGTAGCTCTGCGCTGGTAGCAGTCTGCAA----AAGATCTCTGCTAG-CTACATTGCCCTAAGC
                                    69210     69220     69230     69240     69250     69260     69270     69280
Human                      AGAATTACTGCTACAGAAA-----AGAGCTTGTAACTTAATGTCAT--TTAAATCTATTTTGG--TACAGTTCTCCTGGC 68495
Kakapo                     TGAGTGACAGCTTCAGGGT-GCTCTGCACCGGTAGCAGTCTACAA----AAGATCTCTGCTAGACTACATTGCCCTAACA 35820
GoldenEagle                AGAGTGACAGCTTCAGAGTGGCTCTGCCCTGGTAGCAGTCTGCAA----AAGACCTCTGCTAGGCTACATTGCCCTAAAG 39451
JapaneseQuail              GGTGTGGTAAGA-CAGGCCAGC-CAAAATTGGCAGTGGGCTACGT----GGTCTTCAGACTGG--TATGGGGCCTGAATC 31357
MediumGroundFinch          TGCCTCACAACTTCAGGGTAGCTCTGTGCTGGTAGCAGTCTGCAA----AAGATCTCTGCCAATCTGCATTGCCCTAAAA 20255
GoodesThornscrubTortoise   AGAGTCTCCAAC-CACCACTACCCTACGTTTCTTATTATCAATGGTGGCAGCAGACCTCCCAGCCTTAGGGGTACGAGGC 63630

Majority                   TCTGTATTAGG-AAC-GTTCAGG-TAGTTTTTGCTGTAGTGGAAAGTAAGGACATCTCCAGTTAAGTTATTCAGCTTCAG
                                    69290     69300     69310     69320     69330     69340     69350     69360
Human                      ACAATTTTAATTAGCTGTTAAACTGAATTTATAATTTTCTATATGTTTGTGATACATAAAATTAACTGGAAAACTTTGAT 68575
Kakapo                     TCTGTAGTAGG-AAC--TTCAGG-TAGTCGTTGCTGGAGTGGAAAGCAAGGACACCTCCAGGTAAGATATTCAGCTTCAG 35896
GoldenEagle                TCTGTAATAGG-AAC--TTCAGG-TAGTCATTGCCAGAATGGAAAGCAAGGACATCTCCAGATAAGATATTTAGCTTCAG 39527
JapaneseQuail              TGTGCACTTAC-AAGAGTGCAGT-TATCTTCTTCTCTAGCTGGCTCTAAAAGTTTGAGCCTTTAGCTTAGTCAGTGTCGT 31435
MediumGroundFinch          TATGTAGTAGG-AAC--TTCAGG-TAGTTATTGT-GGAATGGAA-GAAAGAACATCTTCAGATAGGATACTGAGCTGCAG 20329
GoodesThornscrubTortoise   TTCGCCTCCTT-AACTGTTGGGGATGATTCCTTCTCTCCTGTATCAAGAAGAGCATAACGGTTATCTATTACCACAGCAG 63709

Majority                   GGCGATTATCCGTGA---TTGTATTGGGGATT--TTCAGGCATCTTGAATTCTAAAGAGC-ACAATTTTTGCTCAGAAGT
                                    69370     69380     69390     69400     69410     69420     69430     69440
Human                      TTTCTTTCTTTGTTACATTTGTATGGGTTATTCATTTGGTAATATTTATTAATTTATTATTAGAGATTTCAGCTATTTGC 68655
Kakapo                     GGCAGTTATCCGTGATTATTGCATTCAGGCTT--TTCAGGCATCTTGAATCCTTAAGAGC-CCAATTTGTGCTAGGAAAT 35973
GoldenEagle                GGCAATTATCCATGATTATTGCATTAAGGATT--TTCAGGCATCTTGAATCCTAAAGAGC-ACAATTTGTGCTCAGAAGT 39604
JapaneseQuail              GTTGGTTAAA----------GTTGGTGGTTTG--TCCAGGCTCCAGGAAATCCAGAAAG--ATAACCCTTTTCC------ 31495
MediumGroundFinch          GGCTATTATC----------GTATTAGGGTTT--TTTTGACATCTTTAGTTCTAAGGAGC-ACAATTCATGCTC------ 20390
GoodesThornscrubTortoise   CAGGATTTGCAGCAG-----GGGTGGAGCACT--GCCTGCTGCCAGAAGTAACCAGCTGCCAGTGTCCACCCTGAGCCAT 63782
```

```
Majority                 CATAA----TGTTCATATACACAGCAGG-TT--G--------C------------------------------------G

                                 69450     69460     69470     69480     69490     69500     69510     69520

Human                    TAATATAATGAATCCCATACACCTGTCATCTTGGTTCAATAGCGCTGACACTTAGTTTTATCTATTCAGTTTTTCTTTTG 68735
Kakapo                   GGTAA-----GTTTATATCC-CAGCATGATG------------------------------------------------- 35998
GoldenEagle              CATAAGCAGTGTTCAGGTAAACAGAAGAGTGCTGGATTGTATCCATTGTATCAGGAAGCGCAGGAAGTGCAGCAACAGTG 39684
JapaneseQuail            ---------TGTCTCAAAACACAGCACG---------------------------------------------------- 31514
MediumGroundFinch        ---------CGTTCACATGCACAGA------------------------------------------------------- 20406
GoodesThornscrubTortoise CTCCTCCTCCACTGGTGTGT-CAGTAGTCCTGTGAACTGGGACAGCTACGTCAGCTGTCTCCACATGGATACTGTCCAGG 63861

Majority                 --T---------A--------TTTTGTAGCATGGCCTC------------------T---T--------------T----

                                 69530     69540     69550     69560     69570     69580     69590     69600

Human                    TGTTCTAAAGCAAATCCTTGACATTGTGACATGTCTTCTCAAATATTACCATAGTATCTCTAAAATAATTACATTTTCTT 68815
Kakapo                   ---------------------TGCTGTAGTATGGAATA------------------------------------------ 36015
GoldenEagle              TTTGTCTCCTGGATATTCCAATTTTGTAGAAAAGAATAAGATATGCAACTGGTCCTTCAGTCAAGGAACTGCACATAAAA 39764
JapaneseQuail            ------------------------CATCGGTTTACCTG------------------------------------------ 31528
MediumGroundFinch        -------------------------------------------------------------------------------- 20406
GoodesThornscrubTortoise AATTGCTCATGGATACGGATGTTCCTCAGCCTAGCCACCTCCTCCTGTAGCTCTCCTACTTGCTGCCTGAGAGATTCCAC 63941

Majority                 --------A---------T---A----------A---T----AT-A-----T---------AA----C-----T------

                                 69610     69620     69630     69640     69650     69660     69670     69680

Human                    ACCAAACCATAATATGATTATTACACTTACCAGA-ATTGACAATGATTTCTTAATGTCCTCAAATACCCAGCTTACATTA 68894
Kakapo                   -------------------------------------------------------------------------------- 36015
GoldenEagle              AAAAGAAAAAAAAATTTATCTTATTTTTGGAGAATGTTTTACATAAACATTTTAGCCACAGAAACTTCTCATATCCAAGA 39844
JapaneseQuail            -------------------------------------------------------------------------------- 31528
MediumGroundFinch        -------------------------------------------------------------------------------- 20406
GoodesThornscrubTortoise CAGTAGGCACCTTTCACATTGGATGCCACCCCCAGCCTGGATATCAGTAAGTGGAAATTGCAAGTTACAGTCTTTTCAAG 64021

Majority                 ------TC--AA------T-AA------T--A--------CTGTGGGATGCATTTGGGACATATTTCTCCGAAGAGGTGC

                                 69690     69700     69710     69720     69730     69740     69750     69760

Human                    AAATTTCCCCAATTATTTTGAAAATGCTTTTA--------AAGTTTGATTTGTTCAGAACAAGGTCCTATGGAGGGCCGC 68966
Kakapo                   ------TC--------------------------------CCTTTAGCTGC-TTTGGGTCGGGTGTCTC---GGCTGTGC 36053
GoldenEagle              CACTTGTCTCAAAAGAAATCAATTTTATTCAAATGCATATCTGTAGGATGCACTTGGGACACATCTCTCCCAAGAGATAT 39924
JapaneseQuail            ----------------------------------------CAGAGGGCTGCATCTTGATCTTTTTCTTC--AATGAGGAA 31566
MediumGroundFinch        ------T---------------------------------CTGTGGGATGCACTTGGGACATATCTCTCCCAAGAGATAC 20447
GoodesThornscrubTortoise CCCACACCAGAATCTGGGTAAA--AGCATCCA-T-----GCTTTGGTGCTCTGTCTGGCTACAGGCGCAGGTGGAGGAGA 64093
```

```
Majority                   TTATCGTGTTGCTTTCAGATGTTTTGCTGTTCTTACTCGAGCTGGAGTGTGTTTTACTATCATTCTTTTCTT--T---T-

                                  69770     69780     69790     69800     69810     69820     69830     69840

Human                      ACATAGTATTTGGTTGTTACATCTCATTAGTCTGTTTCAGTCTGTAATAGTCCTCTCCCTCTCTCTACTCCCAATCTCTC 69046
Kakapo                     TTCTTGTGCCCCTTCTCACTGGTAGAGCATGAGAGACTGAAAACACGAGTGTGTTACCAACATTATTCTCAG-------- 36125
GoldenEagle                TTACCATGTGGCCTTCAGTTACTTTGAGGTACTTATTCGAGCTGGAGTGTGTTTTGTAAGGATTACTTCTTTTTTTTTTA 40004
JapaneseQuail              TTCACATGTCAGTGCTCCATGGACTGCTGTTTTGACTCCAGCTCATAGTGGTGATACTGTGTCTCATCTCTG-------- 31638
MediumGroundFinch          TTACCATATGACCTTCAGTTACCTTGAAGTACTTACTTGAGCTGGAGTATATTTTATAAGTGTTGTTTCCTT-------- 20519
GoodesThornscrubTortoiseCAGAAGCAGTGCTGCCACAGGTGTTGCGGGTCCTCCTCACCATCGTAAGCCTCCCTCTGTCAAACTCTCTCAAATTCCTG 64173

Majority                   -T-----------T---GTT----------TTTT-TT-------T--------T-G--T---------------------

                                  69850     69860     69870     69880     69890     69900     69910     69920

Human                      ATTCATTTACTTATGAAGTGATGTT-----TCCTTTTAGAAAGTTTCACAGATTAGACTAGTCTGGCTGTTTCTTTAGAG 69121
Kakapo                     -----------------ACT----------ATT----------------------------------------------- 36131
GoldenEagle                ATGCCTTAAACTGTTATGCCCTTCTGAAGATCTTGTTTCTGGTTTCTCCTGCTTTGTGTGCTTTGGGAAGAGAGACAGTG 40084
JapaneseQuail              -------------------------------------------------------------------------------- 31638
MediumGroundFinch          -------------------------------------------------------------------------------- 20519
GoodesThornscrubTortoiseTTCAGAGACTTCTTAGATTTTAGCAGTTAAATTTTTTTGAGTGTTCCTACCCTGGGCTTGCTCCAGCCTGGGGCTGAGC 64253

Majority                   -GGAATAA---------------T-TGT-T--------AT-TAGGGTAGATGTCAG----------T--C------CTAG

                                  69930     69940     69950     69960     69970     69980     69990     70000

Human                      TGTCATGTTAACTTGTTCCTCTATCTGCTGTGTTTCTTATATAAACTTAAAGTTAGTTCCAAAGGCTTGATAAGATTTAG 69201
Kakapo                     -GGTATAA-----------------C------------AT-TAGGTTAG----CAG--------------------CTAA 36156
GoldenEagle                GGGATTAAATTTCTCTCTCTTCTTTTTTCTCCTCATGAGT-TTGGAGAGAAGGCAGCAGTTTTGTGTTGCCCCTCTCTGC 40163
JapaneseQuail              -GTAACA-------------------CTGT-------GAT-CCAGGAAACTATCA--------------C------CTGC 31670
MediumGroundFinch          -GG----------------------------------GT-TTGGGGAG----------------------------T-- 20532
GoodesThornscrubTortoiseAGGACTTCCACTGCAATTGCAGCTCTGGACTGCTGCGCAC-CATGCCAGGTCCCTGTCTGGACCCCTGAGCTGAAGGTAG 64332

Majority                   AGCTTTT-----TTTT-T------T---T-T--------------G-C---G---AATGCTTTTGTGCT----T-----

                                  70010     70020     70030     70040     70050     70060     70070     70080

Human                      AGCCTTT----TTTTTTTTGAAGTGTTTCTTGTGCTTCCTATTGTGTCTCAGTAGGAGACATCTAATATCTGGTTGCCC 69277
Kakapo                     AGTTGTT-----TTTC--------T------------------------------GTTAATTTTGTCA----------- 36181
GoldenEagle                CACTTTT-----TTCTCTCCACTCTTAATTTGATTTTTCTTTATCAGACCTTGGTAAATGCTTTCAAGCTATCCTCATCC 40238
JapaneseQuail              AG---CT-----CTGT---------------------------------------ATTGGTTCTG-------------- 31688
MediumGroundFinch          -----TT-----TTTT-----------------------------------------ATGCTTT---------------- 20545
GoodesThornscrubTortoiseCAAGCCTGTCGCTTCTGTGCTGTCAATGTCTCCCTTCGTGGGTCCAGGCAGTGTGGAGTGAGATGCTGCTTGATTCAGAT 64412
```

```
Majority                 --C------------T-------TG----------G-------------------A------------------------
                                  70090     70100     70110     70120     70130     70140     70150     70160

Human                    CACTTTTAGT--GATTTTAATGATGCTTTAAGACTGATTGTGGATTTGGGTGTTGACATTCTGATCCTTTCGTTACTTTT 69355
Kakapo                   -------------------------------------------------------------------------------- 36181
GoldenEagle              TGCAAGAGATCCATTTCCTGTCTTGGATCATCATAGGTGTCTACAGTGCTTGACAAAGAGAGGGGCACATGCACTCTTTG 40318
JapaneseQuail            -------------------------------------------------------------------------------- 31688
MediumGroundFinch        -------------------------------------------------------------------------------- 20545
GoodesThornscrubTortoise CCATGGAGAAAAGATCTGGACCTGTGCAGGGTGGGAGTAACATAGACTGGAACGAGGAACCTGCGGGCGAGAGTGCAGA 64492

Majority                 --------T---G------G--------------T-CTAGCT--A-------------------TG--AA----G-----
                                  70170     70180     70190     70200     70210     70220     70230     70240

Human                    TTTTCTACTAATGATTTTAGTATCCAATAATGGTTGCTGCCTTAAT-TAATTTCACTAGTGGGTTGCAAATATTGCTTTT 69434
Kakapo                   ------------------------------------CTAATT--A----------------------------------- 36188
GoldenEagle              TTGTGGCTTGAAGCCATGTGTCTCCAGAAATATCTGCTAGCTAAATCTCTGGGCTCTTCTGAACTGGGAAATGAGAATAT 40398
JapaneseQuail            -------------------------------------TAGAT-------------------------------------- 31693
MediumGroundFinch        -------------------------------------TAGCT-------------------------------------- 20550
GoodesThornscrubTortoise AGACTGAATTTTGACAAGAGCCGTTGGAGGGAGATTGGGACTGGGAACCAGTAGGGGTGGAGAATGTGAATGGGGGAGCG 64572

Majority                 ---------A------------------A-------T---------T-------G-----TA----------CTGAATG-
                                  70250     70260     70270     70280     70290     70300     70310     70320

Human                    TCTCCCCTAATTCACATATTCCTTCTCTACATTAATTAGCTGAAAATCTTTATAAAGTAGAATTTTCCTTCATCAACTAG 69514
Kakapo                   ------------------------------------------------------------TA----------TTGAAGG- 36197
GoldenEagle              GAGAATGTCAGATAATTTTCTGTCTCCCATACTGTGTTCTTGTCTGTTTGTTAGGTACTGTATGTGAATTAGCTAAATA- 40477
JapaneseQuail            ------------------------------------------------------C-----------------CTG----- 31697
MediumGroundFinch        ------------------------------------------------------------------------CT------ 20552
GoodesThornscrubTortoise AGAGGAGAGAGATTGGATGAGGAGAGGCAGGGAAC-TTGGAATGACTGGGGAAGGAGACTGAGAACAAAGAGCTGAGGGT 64651

Majority                 -GTA-TTTAAGTAG------TATCTTTCTGGGT------G----A---------------G--A---------T-G----
                                  70330     70340     70350     70360     70370     70380     70390     70400

Human                    AATTATCAACCTTGTAATTCAATTTGTGCAGGACAATTAGTTAAATTCTTGCATTTGTTTGCTAATTTTAAGTTGGTGCC 69594
Kakapo                   -GGA---GAAGGAA-------AACATGCAGGA------------------------------------------------ 36218
GoldenEagle              -GTATTTTAAGTAGCATATCTATCTTCCTGAGTTTTAGAGGGCTACCTTGACTATCTCCTGAGAGGGGTTTCATTGCACA 40556
JapaneseQuail            ------TCAGGC--------TTGCGTACCGTGT----------------------------------------------- 31716
MediumGroundFinch        -------TAAG----------ACCCTTCTGAA------------------------------------------------ 20567
GoodesThornscrubTortoise TGAAGTGGGAGAAGTGGGAGTGGCTGTGTGGGGACACTGGGACCAAGGCAGACTGGAATGGTGACACAAGGACTTGCTGG 64731
```

```
Majority                     ---------------------------------------------T-C--------T----C-------------CT---
                                      70410     70420     70430     70440     70450     70460     70470     70480
Human                        CAGCTTTTGTTTTCAGGTAAGTACTTAAACCAGCTTATGAACAGGTACAGTATAGAT---TCTTCCCACTGTCTGCTTTA 69671
Kakapo                       -------------------------------------------------------------------------------- 36218
GoldenEagle                  GATTCATGGTCTATACAGGCCTTAGAAGCTCATCAAAAACATAGTTCCTATGAATATAGCACTTTTCAAGGGAGTCTTCC 40636
JapaneseQuail                -------------------------------------------------------------------------------- 31716
MediumGroundFinch            -------------------------------------------------------------------------------- 20567
GoodesThornscrubTortoise     GCAACAAGACTAGGGGTAGGAGAGTAGTTCTCAGCCTGCAGCCTGTGCACCACTTGTGTCCCAATCAGCACACAGCTGCA 64811

Majority                     --C----G---------G-----T--A-----T--------------------------G----T--------------T
                                      70490     70500     70510     70520     70530     70540     70550     70560
Human                        AACTTTTGAAACTGTCAGTTGTTTGCAGGTTCTGGTAGCCCAGAGTGGTTTGAAATGGTGACCTTAGCCCAGAAGTATGT 69751
Kakapo                       -------------------------------------------------------------------------------- 36218
GoldenEagle                  TTCACAAGGATTTTCAGGCATCTTGAACATCTTGAATCTTGCAAGTAATAAAAAGAGCAGTGAATTCTTCTATCCATTAT 40716
JapaneseQuail                -------------------------------------------------------------------------------- 31716
MediumGroundFinch            -------------------------------------------------------------------------------- 20567
GoodesThornscrubTortoise     GCCATATGACATCCACAGGACCATATAGTAGGTAGTATTGGATGTCACCAGGTGGTATTGGATGTGGTCATATAACACAT 64891

Majority                     -----------------T--------------------G-------T---------------------------------
                                      70570     70580     70590     70600     70610     70620     70630     70640
Human                        AATTATTTAATTTGGCCTTGACCAGTGGCCCAAAAAAGGAAGGGGGTGGGGTGGATAAAGGACTAAACAAAATTAATTAA 69831
Kakapo                       -------------------------------------------------------------------------------- 36218
GoldenEagle                  ATCAGAAAGTCCTTCATTCAGGTGAGGAAAGCCTTAGAGAAGAAATTATTCTAGGAATGTTCTTCCTCCGCTTCAGAGCC 40796
JapaneseQuail                -------------------------------------------------------------------------------- 31716
MediumGroundFinch            -------------------------------------------------------------------------------- 20567
GoodesThornscrubTortoise     TGTGGGCTGTGTAATGGTAAATAGGTTGAGAACCACAGGGCTAGAATGGGAAGCTTGATAAGTGGAGACGGACTGGTTAC 64971

Majority                     ---------AT----CT---A----GA----------------------------A---------A--------G-----
                                      70650     70660     70670     70680     70690     70700     70710     70720
Human                        AATTTAGAAATTTTTCTCTCAGAAGGAATTC-ATGCCCATAAAACAAAGACCAGGATACATTCGTATTTTATCGGTAAAT 69910
Kakapo                       -------------------------------------------------------------------------------- 36218
GoldenEagle                  TACCCATAGATCGGACTCTTAATCAGAGAACTGCATCAGTCATTCAGGATGTTACAACATTTTGTAAATTTGTAGAGTGC 40876
JapaneseQuail                -------------------------------------------------------------------------------- 31716
MediumGroundFinch            -------------------------------------------------------------------------------- 20567
GoodesThornscrubTortoise     CTGAGGAGAATGGGACTGGGATGGGGAGCTAGGTTAAGGAAGAGACAGGACTCGGACAAGGCCAGATTAGGGAGGATTGA 65051
```

```
Majority                    ----------T---A----------T---------A-T------A--------------A-------A-------T----
                                     70730     70740     70750     70760     70770     70780     70790     70800
Human                       ACCAATAGATTATAAATCACTTTTCTCGTATACATAATTGTGAAAAAAAAAAACCATCCAGGTATGCATGTTATTTATTC 69990
Kakapo                      -------------------------------------------------------------------------------- 36218
GoldenEagle                 TTTTGCCATCTGAGATCACCTTTGCTGCTGTTACCACTTCTCTCAACCTCCACAATCACATTGGGAAATGAGAGATCCTA 40956
JapaneseQuail               -------------------------------------------------------------------------------- 31716
MediumGroundFinch           -------------------------------------------------------------------------------- 20567
GoodesThornscrubTortoise    CCAGAAGGATTCTCACTTCAGGGAATGGGCAGAAGACTCTACTCACTAGTGCACAGTCTATTCCAGAACCTCTAATGGAA 65131

Majority                    -------------A------------ATTT--T-T-----------TGTTGTTAATCTTGCGTGT-------T-------
                                     70810     70820     70830     70840     70850     70860     70870     70880
Human                       ATTGAGAGGATATACATCTCTCAGGTGTTTTTTGTCGTATGTGTACTTTTGTGTAGTCTGCCTGTTTATTCTT-TTTGAA 70069
Kakapo                      --------------------------ACAA----------------TGTAAGTATTCTTGCCTAT--------------- 36241
GoldenEagle                 GTGTCATGAGTCCATGTTGACTAGATAGAAATTCTTACTGTCCTTTTGCAGAAAACCTTGAGTGTGGTTGATTGCATCAG 41036
JapaneseQuail               ----------------------------------------------TGTTTCTGTTCTTGTGTGGG-------------- 31736
MediumGroundFinch           --------------------------A-------------------------A--TCTTG-------------------- 20574
GoodesThornscrubTortoise    CCTCAGACTGCTGAATCTCACCGTTCGTTTGCTGTTAGCAAATATCTGTGATTAACCACGAATAA---CCACTGTGCATC 65208

Majority                    ---------------------A---A---TTTT-----------TT---GT-GGTGG-------C---------------
                                     70890     70900     70910     70920     70930     70940     70950     70960
Human                       TAGGATGATATGTACAATTAAAAAAAATCTTTTGTGAAATGTGGTTTTCTTAAATACATTATTGCATTTCATACACAGTG 70149
Kakapo                      -------------------------------CA-------------------GGTGG----------------------- 36248
GoldenEagle                 CAGCAGGAGAAAATACTGTTTATTGAGCTTTTCTTTCTTTCTTTTTGTGGTGGGTTGACCCTGACTGGATGCCAGGTGCC 41116
JapaneseQuail               --------------------------------C----------------ATTTGTGG----------------------- 31745
MediumGroundFinch           ----------------------------------------------------GGT------------------------- 20577
GoodesThornscrubTortoise    CGAAGAAGTGAGTTGTAGCCCACGGAAGCTTATGCTGAAATAAATTTGAGTCTCTAAGGTGCCACAAGTACTCCTGTCCT 65288

Majority                    -------G----------A--C-------------------------A--A-----------------T-----AA-GT-
                                     70970     70980     70990     71000     71010     71020     71030     71040
Human                       ATGAAGGGAAAAACTACAAAGCTAGACAGATTGCAAATTAGTATTGCAGTAGTGAGGTAATAGAGCAATACCCAAATGTT 70229
Kakapo                      -------------------------------------------------------------------------------- 36248
GoldenEagle                 CACCAAAGCCGCTCTATCACTCCTCCTCTTCAGCTGGACAGGGGAGAAAAAATAAAACAAAAGGCTTATGGGTCAAGGTA 41196
JapaneseQuail               -------------------------------------------------------------------------------- 31745
MediumGroundFinch           -------------------------------------------------------------------------------- 20577
GoodesThornscrubTortoise    TTTTGCAGATACAGACTAACACTGCTGCTACTCTGAAACCTGTCACTAGTAGCGTGTGTGTCTCGCTCTTCTCTAATGTT 65368
```

```
Majority                    -----A-A--------A----A--------T------------------T-----T-------A---A------T-----

                                     71050     71060     71070     71080     71090     71100     71110     71120

Human                       TTCTTAAAATATATGTAGGTGATGCCTGTGTTCTTTACTTTTGTGTGTGTGTATGTGTTTTTAAACAAGATTCATTGTAG 70309
Kakapo                      -------------------------------------------------------------------------------- 36248
GoldenEagle                 AGGACAGAGAGAGATCACTCAACCAATTACTGTCATCGGCAAAGCAGACTCAACTTAGGGAAAATTAACTTATTTATTGC 41276
JapaneseQuail               -------------------------------------------------------------------------------- 31745
MediumGroundFinch           -------------------------------------------------------------------------------- 20577
GoodesThornscrubTortoise    AATCCACATATCATATAGAGGAGGACAGCCTACTACTGCTATAAATTACTCTTTTTGCTCAAGAGGCAAAGGTCTGCCCA 65448

Majority                    ---T--------A------T--------------C----T-------------------------------T--------

                                     71130     71140     71150     71160     71170     71180     71190     71200

Human                       TCTTTTAGATATAATTTAATAAATTTAGGCTGTTCAGGGTTTCAGTATCCTACAAAGAAGAACGCTGCTTCTAAGTTTTT 70389
Kakapo                      -------------------------------------------------------------------------------- 36248
GoldenEagle                 CAATCAAACCAGAGTAGGGTAAGGAGAAATGAAGCCAAATCTTAAAACACCTTCCCCCCATCCCTCCCTTTTTCCCAGGC 41356
JapaneseQuail               -------------------------------------------------------------------------------- 31745
MediumGroundFinch           -------------------------------------------------------------------------------- 20577
GoodesThornscrubTortoise    CCTTTGCTGATAACCCGTTTGGAGGTCATGTGATCAAATTTCTGTGTCTTCAGTTTGTACATTTTAAAAACTAGCAAATT 65528

Majority                    -----------------------A---A-C----T----G------A-----------------G---------A-----

                                     71210     71220     71230     71240     71250     71260     71270     71280

Human                       GGGTTTCTTGAACCAGAGCTTAGAAAGAACAAAAATATGGCATGGTATTTTCATGGTTTAGTAAGGCTACTTTTAGTTAA 70469
Kakapo                      ----------------------------------T--------------------------------------------- 36249
GoldenEagle                 ACAGCTTCACTCCCGGATTCTCTACCTACCCCGCTGCCAGCGGCGCAGGGGGACAGGGAATGGGGGTTACGATCAGTTCA 41436
JapaneseQuail               -------------------------------------------------------------------------------- 31745
MediumGroundFinch           -------------------------------------------------------------------------------- 20577
GoodesThornscrubTortoise    ACACACACATGCAAACTCTGCTAAAAAAACATTACTAGGGTTGCAAAGTCAAGTCAGAGGTTAGGAAATGCAGAATGAAG 65608

Majority                    ---------TGTCTCTCCTGATGTTTACTTCT------C--G-------------------------A--------T-C-

                                     71290     71300     71310     71320     71330     71340     71350     71360

Human                       TTATTGTGATGTAGGCCATTAAGTAGACTTTTCTAAAACGAAACCCCAGAGGAGAGGGGTTTTGAAGATACATATATACT 70549
Kakapo                      ---------TGTCTAACATGGTATTTAAGTCT------------------------------------------------ 36272
GoldenEagle                 TCACA-CGTTGTCTCTGCTGCTCCTTCCTCCTCAGGGGCAGGACTCCTCACACTCTTCCCCTGCTCCAGTGTGGGGTCCT 41515
JapaneseQuail               ----------GATTCACCTGATG---AAAACT---------G-------------------------------------- 31765
MediumGroundFinch           -------------------------------T------------------------------------------------ 20578
GoodesThornscrubTortoise    CTTAT-CTGTG-CAATCTTAATTTGGCCTTCTTGTGAACATGCATATGATAGTCTTTACATGATCACATACCATTTTTCC 65686
```

```
Majority                   -----------A----T------GGATTTTTTG-----G------------GG--------TC---------T-------

                                     71370     71380     71390     71400     71410     71420     71430     71440

Human                      GGTTTCTAAGAACTAATCTTTTTGGATCATTGGTAT-TGGTAGAAGGGATTCGCTATGTTTTCTGCCACCATTGGATCTC 70628
Kakapo                     -----------------------AGATTTTTTA------------------GG--------------------------- 36284
GoldenEagle                TCCCACGGGAGACAGTTCTCCACGAACTTTTTGAGCGTGGGTCCTTCCCACGGGCTGCAGTTCTTCATGAACTGCTCCAG 41595
JapaneseQuail              -----------------------TGATATTCC-------------------AG--------------------------- 31776
MediumGroundFinch          -----------------------GAATTTTTT----G--------------GG--------------------------- 20590
GoodesThornscrubTortoise   ---------ATAGGGCTTCTGTCTTATTCTGTGTACAAGATGGACCTGCTCTGGGGAGGAATCAGGGTTGTGTAATAAAA 65757

Majority                   ----T------T-------------------------------C---------G------------G-------------

                                     71450     71460     71470     71480     71490     71500     71510     71520

Human                      TGTATAAAGTTTGGCTCCACTATCAAAATGCTTAGATAGATGCCTTTGCAATAGTTGCCACCCTAGGAATAGCTCAAAAA 70708
Kakapo                     -------------------------------------------------------------------------------- 36284
GoldenEagle                CGTGGGTCCCTTCCACGGGGTGCAGTCCTTCAGGAGCACACTGCTCCAGCGTGGGTCCCCCAAGGGGTTACAAGTCCTGC 41675
JapaneseQuail              -------------------------------------------------------------------------------- 31776
MediumGroundFinch          ----T--------------------------------------------------------------------------- 20591
GoodesThornscrubTortoise   CAGATGGAAGGTGTGTAATGAATAAGAGAGGGGATTGCAGGACCAAAGAGGATGAATGGATTTTGTGGTTAAAGCAGTTG 65837

Majority                   -------C-T-----------------------C-C-G---C---G-----------------------T------T---

                                     71530     71540     71550     71560     71570     71580     71590     71600

Human                      TTATTAACTTAATACTGGTTACTTAAATTCCACCTCTGTTGCTTGGAGTTGTGCTTTTTTTTTTTTTTTTTTTTTTTGAG 70788
Kakapo                     -------------------------------------------------------------------------------- 36284
GoldenEagle                CAGAAAACCTGCTCCAAAGTGGGCTTCTCTCTGCACAGGTCCACAGGTCCTGCCAGGAGCCTGCTCCAGTGCGGGCTGGG 41755
JapaneseQuail              -------------------------------------------------------------------------------- 31776
MediumGroundFinch          -------------------------------------------------------------------------------- 20591
GoodesThornscrubTortoise   AATGCTGCCTTGAGAATTGGTTTATATCCCTTCCCCTGCCACGGAGTTCCTAGGTGGTGCTGGGCAAGTTGATTTGTATA 65917

Majority                   ------------T--------C-----G---------------C-----------------------G-----------C

                                     71610     71620     71630     71640     71650     71660     71670     71680

Human                      ATAGAGTCTGGCTCTGTCACCCAGGCTGGAGTGCAGTGGCATGCAATCTCGGCTCACTGCAAGCTCCGCCTCCTGGGTTC 70868
Kakapo                     -------------------------------------------------------------------------------- 36284
GoldenEagle                CTTCCCATGGGATCACAGCCTCCTTCAGGCACATCCACCTGCTCCAGCGTGGGGTCCTCCCCAGGCTGCAGGGGGACAGC 41835
JapaneseQuail              -------------------------------------------------------------------------------- 31776
MediumGroundFinch          -------------------------------------------------------------------------------- 20591
GoodesThornscrubTortoise   CGTTCTGAGCACTGACACCTGCAATTAGTCATTGGGAGCTGTACTTTGAAAAATATAAAGTGATATAGAATGCTAAATTC 65997
```

```
Majority                   -------------G--T-----------------G-A--------------------------------T-----T---
                                    71690     71700     71710     71720     71730     71740     71750     71760
Human                      ACGCCATTCTCCTGCCTCAGCCTCCCAAGTAGCTGGGACTACAGGCGCACGCCACCACGCCCAGCTAATTTCTTCAT--A 70946
Kakapo                     -------------------------------------------------------------------------------- 36284
GoldenEagle                CTGCCTCACCATGGTCTTCCCCACGGGCTGCAGGGGAATCTGTTCTGGTGCCTGAAGCACCTCCTCCCCCTCCTTCTTCA 41915
JapaneseQuail              -------------------------------------------------------------------------------- 31776
MediumGroundFinch          -------------------------------------------------------------------------------- 20591
GoodesThornscrubTortoiseTCTGAAAAAATCCGGTTCAAGGAGTCTCAAATTGAGCACCCAAAATTAGTGAACGCTTTTTTAATTTCTCTATGCTTCAG 66077

Majority                   -T-----T-G--------GGG-T-----------------------T------------T----------C-G---C--
                                    71770     71780     71790     71800     71810     71820     71830     71840
Human                      TTTTTTTTAGTAGAGACGGGGTTTTACTGTGTTAGCCAGGATGGTCTTGATCTCCTGACCTCCTGATCCTCCTGCCTCGG 71026
Kakapo                     -------------------------------------------------------------------------------- 36284
GoldenEagle                CTGACCTTGGAGTCTGCAGGGTTGTTTTTCACATGTTCTCACTCCTCTCTCCAGCTGCAGTTTCTGTGCCACAGCAACTT 41995
JapaneseQuail              -------------------------------------------------------------------------------- 31776
MediumGroundFinch          -------------------------------------------------------------------------------- 20591
GoodesThornscrubTortoiseTTCCCCCTAGGTAATATGGGGATAATAACCCCTCATTTCACAGTCTGTAATGAAAATGAATGTGAAGCGCTCAGATACTG 66157

Majority                   ----------T--------------C---------------------G------G-TT-----------A----------
                                    71850     71860     71870     71880     71890     71900     71910     71920
Human                      CCTCCCAAAGTGCTGGGATTACGGGCGTGAGCCACCGCGCCTAGCCTGCTTAGAGTTTTTTATGTAATTATTTTGTGACA 71106
Kakapo                     -------------------------------------------------------------------------------- 36284
GoldenEagle                TTTTTTCCCTTCTTAAATCTGTTATCACAGAG-GCGCTACCACCGTCGCTGATGGGCTTGGCCTCGGCCAGCGGCAGGTC 42074
JapaneseQuail              --------------------------------------------------------TT---------------------- 31778
MediumGroundFinch          -------------------------------------------------------------------------------- 20591
GoodesThornscrubTortoiseTAGTAATGAGTGCCATAGAAAAGCCCATGAGG-AAATTGATAATTCTGTCTTTAGAGCAGGGTTTAATTAGTGTACAGTA 66236

Majority                   ----------------------T-----------------------------------T----GAA-------------
                                    71930     71940     71950     71960     71970     71980     71990     72000
Human                      CAAACATTTAAAATACTGTAGCTTAGAATGAAATTGTCTCAATCCTAGGAGCCAAAAGTTTGATGAATATGAATACTAAT 71186
Kakapo                     -------------------------------------------------------------------------------- 36284
GoldenEagle                CGTCTTGCAGCTGGCTGGCAGTTGCTCTGTTGGACATTGAGGAAACTTCTGGCATCTTCTCACAGAAGCCACCCCTGTAG 42154
JapaneseQuail              -------------------------------------------------------------------------------- 31778
MediumGroundFinch          -------------------------------------------------------------------------------- 20591
GoodesThornscrubTortoiseAAGAAAACATGCGGGCACACACTGAACAATGAGGAGAAAAGAAAATATTGAAACTACTCTTTGTGAACACTGTCCATTCT 66316
```

```
Majority                   ----------------------------------------C--T-------T----------A--A-T-T--------A-

                                   72010     72020     72030     72040     72050     72060     72070     72080

Human                      ATTAACCATTTTTGTGTTCTGTGTCAGGCACTGTTCTTAGCCCTTTACAAGTAAGTTAATACATCA-TCTCTAAATGTAG 71265
Kakapo                     -------------------------------------------------------------------------------- 36284
GoldenEagle                CCCGCTGGCTACCAAAACCTTGCCACGCAGACCTAATACACTTTTTTTTTTTCCCCATTGGAACAGTCTCTAGTCTCAT 42234
JapaneseQuail              -------------------------------------------------------------------------------- 31778
MediumGroundFinch          -------------------------------------------------------------------------------- 20591
GoodesThornscrubTortoise   CTGCACTGAATAAGTCATTGATGTAAAAAGCGGCAGAGTCCTGTGGCACCTTATAGAGTAACAGAAGTGTTGGAGCATAA 66396

Majority                   -------T-----------T-T------------------------------------------C---AA--------

                                   72090     72100     72110     72120     72130     72140     72150     72160

Human                      TTACCATTATCGATCCCATTTTATAGATTTGGAAAGTAGGGTACAAAGAACAGTTTGCCCAGATTCCTTTAACTTTTGAG 71345
Kakapo                     -------------------------------------------------------------------------------- 36284
GoldenEagle                CTCTTAATCTGTGGGATCTTTTCTCAGTCAGCGGTTTTCATCTCTTCTTCCTCCTGGCTGCTATGACCAAAAGAGAGGGT 42314
JapaneseQuail              -------------------------------------------------------------------------------- 31778
MediumGroundFinch          -------------------------------------------------------------------------------- 20591
GoodesThornscrubTortoise   CCGTTCATGAACAGCATTATCTCCTGTAAGTGTAAACAAACTTGTTTGTCTTAGCGATTGGCTGAACAAGAAGTAGGACT 66476

Majority                   -----GA-------------AA-------A-T-T--T----------G------T---T---A---A-----------T-

                                   72170     72180     72190     72200     72210     72220     72230     72240

Human                      TGGCCGAGCCCTAGCACTGAAAGCCAGGTAGTCTGGTTCCAAAGTCCGTACTCCTGACTAATAAATAGTGGAATAAAATT 71425
Kakapo                     -------------------------------------------------------------------------------- 36284
GoldenEagle                GGTTTGACATTATTTTTAAGAAATGAGAGAGTATTGTGTTTTGTGGTGCAGACCTCCGTTTCATCGATATTCCCTGTATG 42394
JapaneseQuail              -------------------------------------------------------------------------------- 31778
MediumGroundFinch          -------------------------------------------------------------------------------- 20591
GoodesThornscrubTortoise   CAGTGGACATGTAGGCCTTAAAGTTTTATATTTTTTTTTGTTTTTGAGTGCAGTTATGTAACAAAAAAAAATCTACATTT 66556

Majority                   ---------------------------T-------C----------------------T----------T----------

                                   72250     72260     72270     72280     72290     72300     72310     72320

Human                      AGATTAGCTTGACATTTTAGCCTGTCGTCTTCTCACCTGTAGAATAGTTATCAAGAATAGTAATATACATATAGTAAAAA 71505
Kakapo                     -------------------------------------------------------------------------------- 36284
GoldenEagle                GTCATTTTTAATGTCTGTGACAGACAGTCATAGCTCTCTGTTCTGGATCCTTACGACTTCCTGGGCTATTTCTGCTAGTT 42474
JapaneseQuail              -------------------------------------------------------------------------------- 31778
MediumGroundFinch          ----------------------------------------------------------T--------------------- 20592
GoodesThornscrubTortoise   CTAAGCTGCACTTTCACGATAAAAAGATTGCACTACAGTACTTTTATGAGATGAATTGAAAAATACTATTTCTTTTGTTT 66636
```

```
Majority                  ------T--------------TTGCTTAT---T----T-------------T-------------------T--T-----
                                   72330     72340     72350     72360     72370     72380     72390     72400
Human                     TTTAAGTCCCATCATTTTAGCTTGCTTTTTTTTTACATACTAAGATATTTTTTGTTACCAAGTCCATTAAGTATTTAGCC 71585
Kakapo                    -----------------------GTTTTT---T----------------------------------------------- 36291
GoldenEagle               CCTGTGTTGTTAG-GCCTTGCTTGCTTATTCTTGCCTTCACTGAAAGGTGCTTCCATGAGACATGAGCAGATTTTGGATT 42553
JapaneseQuail             ---------------------TTGCCACT--------------------------------------------------- 31786
MediumGroundFinch         ---------------------T-GCTGAT--------------------------------------------------- 20599
GoodesThornscrubTortoiseATCATTTTTACAGTGCAAATATTTGTAATATAAATAATATAAAGTGAGCATTGTACACTTTGTAGTCTGTGTTGTAATTG 66716

Majority                  ---T-----T-TT-----A--T---AA-A----------------------TT---A------T-----------A----
                                   72410     72420     72430     72440     72450     72460     72470     72480
Human                     TGATTATCTTATTGATTAAAATACCAAAAATGTGTATTGTTCATGCATTGATTCAAAATTGGGTACAAAACGGGTAGATA 71665
Kakapo                    -------------------------------------------------------------------------------- 36291
GoldenEagle               CTTTGTGAATGTTGCCCCAGTTGTTAAGACCCTTTCTAAATATCTGGAATTTTTCTACTGTACTAGTTGTCTAGGAATAC 42633
JapaneseQuail             -------------------------------------------------------------------------------- 31786
MediumGroundFinch         -------------------------------------------------------------------------------- 20599
GoodesThornscrubTortoiseAAATCAATATATTTAAAAAAGTAGAAAAACATCCAAAAATATTTAAATACTTTTCAATGGGTATTCTACTGTTTAACAGT 66796

Majority                  ----------------------G---TT-------T-T---T---A-----------------------------T---
                                   72490     72500     72510     72520     72530     72540     72550     72560
Human                     TGTAACATGCAAATAATAAATTGAACTTATATTTTATCTTTTTGACAAACAACACGTTAAAATTTATGTCAATCTTATT 71745
Kakapo                    -------------------------------------------------------------------------------- 36291
GoldenEagle               TAGCTTCTTTCCAACCAACTCCGCTATTTTGGAGCTTTCACTAGTATTGGGGATTGTGATTTTCTTTATTTCTGGTTTGC 42713
JapaneseQuail             -------------------------------------------------------------------------------- 31786
MediumGroundFinch         -------------------------------------------------------------------------------- 20599
GoodesThornscrubTortoiseCTGATTAAAACTGCGATTAATTGTGATTAATTATTTTAATTATGATTTTTTTTTTAGTTAATTGCATGAGTTAACTGAT 66876

Majority                  T------------T--------T-----A------------------------A--A------TCT------CT-----
                                   72570     72580     72590     72600     72610     72620     72630     72640
Human                     TTTTCAAAATCCTTTTCTATATTTTATTAAGACTAATTATAGCAAATAATAGCCATAAAATTAAACCAAAAAACTCATCT 71825
Kakapo                    ----------------------------------------------------------------TCC------------- 36294
GoldenEagle               TCCCTTTCTTAGCTGCCATAGCTGATGTAGAACCAAGCAGCATATTTTGTTATTAGGAGTAATCTTTTTGTTTCTTCTGT 42793
JapaneseQuail             -------------------------------------------------------------------------------- 31786
MediumGroundFinch         -------------------------------------------------------------------------------- 20599
GoodesThornscrubTortoiseTAATCAATAGCCCTATTACAAATAAGCAAGGTGCTTTATAGAATATACAAATACAGGATCCCCACCTGAGGAGCTTACAG 66956
```

```
Majority                 -------------------------------------------T---------T---T-------CT-------------

                                 72650     72660     72670     72680     72690     72700     72710     72720

Human                    ACTGCAGACATATTTGAAAGCTGTCCCCCAAGAAAGACCAAAGTAATCACACATACCTAAACATGCTAGTATTTCCCAGA 71905
Kakapo                   -------------------------------------------------------------------------------- 36294
GoldenEagle              GTGGTGTTTTGGTTTTTTGTTGTTGTTGTTTTCTTTTTTTTTTTCGGTGGTGGTGGTTTTCTCCCCTCCCAAAAACAGTC 42873
JapaneseQuail            -------------------------------------------------------------------------------- 31786
MediumGroundFinch        -------------------------------------------------------------------------------- 20599
GoodesThornscrubTortoise TATAAGTATTGTGACAGGGTCAGGCCAGGTGGCCACAAGAGAGTGGTCGAAGGTAGATACATTAGCTCCAGGTTAAACAG 67036

Majority                 --------------------------G----T----------------------------A---A-----G-A-------

                                 72730     72740     72750     72760     72770     72780     72790     72800

Human                    AATATTAAAAATACATAGATTTTGAAGCACATTGCTGAATGCCATAGTAACACCAAACTAAAAAATACATGTAAATTA-A 71984
Kakapo                   -------------------------------------------------------------------------------- 36294
GoldenEagle              TAAGTGGCCATAGAGAAACTTGTAGGGATGATGGTGTGTTTGCACTGTGGAGTTGAGGTCATGCACAGGAGTATGCTGTG 42953
JapaneseQuail            -------------------------------------------------------------------------------- 31786
MediumGroundFinch        -------------------------------------------------------------------------------- 20599
GoodesThornscrubTortoise CTCCCTTTTCGCTGGGTAAGATAACAGTGACTATTCCAGAACACTCAGGAACTTTCTAGAACTAATTAAGGCAGACAGGC 67116

Majority                 -A-T----A------T--C--CTTAGGTA-----T-C--------------------------------AA--G------

                                 72810     72820     72830     72840     72850     72860     72870     72880

Human                    AAATTAATATTTTATTTTCAACATAAATCAGAAATACTTGGTTAGAGGTCACATGATAATACCTGTCCTAACTGTCCCAT 72064
Kakapo                   ---------------------CTCAGGTC--------------------------------------------------- 36302
GoldenEagle              GAGTGGAGATTGTTGTGTCAGCACAGGCACATGCTTCTCTCCTTGGGTTATGTGTCAGTCTGAATTGAGAAAGGGATTTT 43033
JapaneseQuail            ----------------------TTAGTTT-------C------------------------------------------- 31794
MediumGroundFinch        ----------------------ACAGGCA--------------------------------------------------- 20606
GoodesThornscrubTortoise TAATTAGGACACCTGTAGCCAATTGGGAAGTTACTAGAATTAATTAAGGCTAATCAGGACACCTGGTAAAAAAGGCTCTC 67196

Majority                 --CT------T---C------------T--------------G--A---A-----------T-C-T------------A-

                                 72890     72900     72910     72920     72930     72940     72950     72960

Human                    AACTTCGGCTTTCTCATTTCATGTGTTTTAATATCTAAG-AAGTAAAAAATAGTTGGAGCATGCCTCTTTATATGTGCAA 72143
Kakapo                   -------------------------------------------------------------------------------- 36302
GoldenEagle              TGTTTGTTCTTTTACTACTGTTTTCTATTGGTCTTAGTTCTTACTAGCCATCTTGTTTGTGTGCTTCTAAATACTACAAT 43113
JapaneseQuail            --C---------------------------------------A------------------------------------- 31796
MediumGroundFinch        -------------------------------------------------------------------------------- 20606
GoodesThornscrubTortoise TACTGTGGAATAGACAGGAGCCACACATCTCGAAGAGTGCAAGTTACGGAACAGGTAATTATCCTTTACTGATTTTACAA 67276
```

```
Majority                  -------T----------------------------G-----AGGTTTGCTACT--------------------------

                                   72970     72980     72990     73000     73010     73020     73030     73040

Human                     TTTTAAATTATGTTGACTTCTTTGGATTATTGAACTGTCCTCTGCTTTGCTATA-GCTGAATTTTTAAAATGTTAATTTT 72222
Kakapo                    ------------------------------------------AGCTTTCCATCT-------------------------- 36314
GoldenEagle               GTGTTTTTTTAGGAATCTGTCTGCTGACAAGCTCTTGGCAGGAGGTGAGCCACTCATCAGAATCATTTTCCCCCTCGGGA 43193
JapaneseQuail             ------------------------------------------ATGCAATT------------------------------ 31804
MediumGroundFinch         ------------------------------------------AGGTGA-------------------------------- 20612
GoodesThornscrubTortoise  GAGAAGTTGTAATTCGTAACCACATGTTGAATATAAGGTTTGAGTCCTAATAGACACTTCTTCAACAATTAAATATAGTT 67356

Majority                  ---------AGAAGAGTATT-------A------G--A-------A----A------TG---A-----------AT---

                                   73050     73060     73070     73080     73090     73100     73110     73120

Human                     GAAGTCTTAAAATGTGTCTTTTGGAAAAAACATTGGAAGAGCTAAAGTAGATAGAGTTGTTCATTTAAATTGTTTATTTT 72302
Kakapo                    ---------AAAAGAGCACT------------------------------------------------------------ 36325
GoldenEagle               AAGTCCAAGAGAAGGGTACTTTTTAGTAACAAGAGGGAGGGAGGGAAGGCAGGGGCCTGGAAACAATTCAGTATCATGAC 43273
JapaneseQuail             ---------AAGCCAGTGTT------------------------------------------------------------ 31815
MediumGroundFinch         ---------AGAATGGTGCT------------------------------------------------------------ 20623
GoodesThornscrubTortoise  TTAGCAGTCTGACTAGCATTAAAGTCTACGAGTAGAGAATCTAAAAATATATGTAAGTGGAAAACCATTGTATCTATCAC 67436

Majority                  ------T-TCGTATT--------------T------T-T-TTGTTTAGGCT--------------------C----CA--

                                   73130     73140     73150     73160     73170     73180     73190     73200

Human                     TTATTTTATTTTATTTTTTTTAGTTGGAGTACTGGCTCTGTTGCCTGGGCTGGAGTGCAGTGGCGGGATCTCTGCTCACT 72382
Kakapo                    ---------C--GTG--------------T-----------CATTTTTGCT----------------------------- 36340
GoldenEagle               ACTTGATATCACATGATGAC-ATAAGTTTTGATACTTCTGTTGTTGAAGCTTAATAAT-GGGTTTGCTGGCCCATGCAAG 43351
JapaneseQuail             ---------CAACT--------------------------CCATACACA------------------------------- 31829
MediumGroundFinch         ---------CGCAT---------------T-----------TGTAGAAG------------------------------- 20637
GoodesThornscrubTortoise  TCTACATGTGGTTTTACTAG-GACATTGACAAGCTCTTTTTTATTTGGGAATGTGTTT-TTATATTGTTTGCATTACACT 67514

Majority                  -------------GTTTCATT-----------GGTACTTTATCTTCTTGGTTTGGCACATATGTG-TGTTTTACTGTATC

                                   73210     73220     73230     73240     73250     73260     73270     73280

Human                     GCAAGCTCCGCCTCCTGGGTTCACGCCATTCTGCCGCCTCAGCCTCCTGAGTAGCTGGGGCTACA--GGTGCCCTCCACC 72460
Kakapo                    --------------TTTCCTTTT-----------TCCTTTGAGCAATTTATTTGGCAT-TATGTC-TGTATTAAAGTTAC 36393
GoldenEagle               ATAACGAACAGTAGTCACATTTTGTAGAGCTTGGTACTTGATCTCCTTGGTTTGGCACATGTGTGGTGTGGTACTGTGTC 43431
JapaneseQuail             -------------GTTCCCT--------------GATCATATTCTGCCAGTTTG-CCTGGATGAGCTGATTGAGACCCTC 31881
MediumGroundFinch         -------------GTCTCAT------------GATTCTTGATCTCCTTGGTTTGGCCCATGTGTGATGTGGTGTGGTATC 20692
GoodesThornscrubTortoise  AATGCCTAGAGGACTGAGATT----------GAGTATCCACTGTGCTAGGTATAATACAAATACA---TATTAAGACATA 67581
```

```
Majority                   ACACGTGGA---GTGCATAGGTAAATGTAGTTGTATGGAAAAGTTTCTTCTTTTTAGCTAGACTGCTTT-GAGATGTTGA

                                   73290     73300     73310     73320     73330     73340     73350     73360

Human                      ACACCTGGCTAATTTTTTTTGTAGTTTTAGT--AGAGACAGGCTTTCACCGTGTTAGCCAGGATGGTCTCAATCTCCTGA 72538
Kakapo                     AC-CGTGGA---ATGCA--GGTGAATGAACTTATATTGAACAGACTTAACTTTTCAGTTGGACTGCATT-GCCACTTTGG 36466
GoldenEagle                ACATGTAAATGTGTGCATAGGTAAATGCTCAAAGAAAGCAAAGTTTCTTAGCAGTAACTAGAGTTCTGT-GAGATGATGC 43510
JapaneseQuail              TCATTTGGTGATGTG-ATGGGGGTACGTGGCTGTCTGGAAATGTGACTTGTTTTTCACTTCACTGTTGT-GTC-TGTTGA 31958
MediumGroundFinch          ACACGTGAA---AT--------AAATACT---GGAAAGAAAAGTTTCTTCACAGTAGCTAGAGTTCTTG-GAGATAATGT 20757
GoodesThornscrubTortoise   ATCCTTGTC---CCAAACAGGTAAATCTATAAGTATGCAAGGCAGACCAAACTCATGGGAAACTGAAACAGAGTTATTGA 67658

Majority                   TTGTGTGTACATTCACTAACGGTGTATGGTTAAAGTCTTGGAATTTTTTTCAGGATGTCTGT--TCTAGGCGAAT---A-

                                   73370     73380     73390     73400     73410     73420     73430     73440

Human                      CCTCGTGA---TCCGCCCACCTTGGTCTCCCAAAGTGCTGGGATTACAGTCATGAGCCACCGTGCCTGGCCTCATTTAAA 72615
Kakapo                     T-GCATGAACAGCTTCTAGAGAAAAGAAGGAAACATC-----AGGCTTCCTGGAAT-CATGT--TAAGAGAGGTT---A- 36533
GoldenEagle                CCGTATATACATTCACTAACAGT--ATGGCTACTGTCTTCTCATTTTTTTCAGGATGTCTGT--TTGAGAGGAAC---A- 43582
JapaneseQuail              A-GTGCAC-CATTCACTGCCTCTCTATGCTAACACTCACTAACTGGTCTCCATAAA-TATTC------AGCAAAC---A- 32025
MediumGroundFinch          TTGTATGTGCGTTCACTAGCAGTGAATGGTTGGTGTC-----ATGTTTTTCAGGATGTCTG------AGAGGAAC---A- 20822
GoodesThornscrubTortoise   CTTTGCAGAAGGTCACTAAGGAAGTCCGTGGAAAGTCTGGGACCTGAACCCAGAACTTCTGAGTCCTAGTCTAGT---AT 67735

Majority                   GTTTTTGAGTGTTTGGTTGAACTTTTTTTTTT-ATT-GCCATATATGAAGGAGTTCAGTATTCTTGTTTG-AGTTGTAAC

                                   73450     73460     73470     73480     73490     73500     73510     73520

Human                      ATTTTTTAACACTGGGTAAAATTTTTATTTTTGAGTCACAAATTAATTTGAAGCTGTGTCTTTTAGAGAGCTTATTCAAC 72695
Kakapo                     ATGAATACGTTTTAAGCTGAACTGGTTTTCAG-ATG-GATGTATAT----GTATACACTGACCTTGTTTA-AAACATAA- 36605
GoldenEagle                GGTTTTGAGGGTTT-GTTGCACCTTTTTTTTT-ATT-GCCATATAAGAAGGAGCTCAGGATACACATTC--AGTTGTGAC 43657
JapaneseQuail              GTGTTGGTATGTTGGTGTGATTATTTTTTTTT-TCT-GC-----ATGGAGGAATTCAGCAGCACTCTTT-----TGCTT- 32092
MediumGroundFinch          GTGTTTGAGGATTT-GTTGCACATTCTTTT-------GC----TA--A--GAGCTCAGGACACATATTCGCAATTGTGAC 20886
GoodesThornscrubTortoise   CTTAACACAAGCCAAGCGCTACTTTTATGTTT-TTTAGCCTAATGTAAACAGTTTCTTCTCTATTTTATAAGGTTTTAAT 67814

Majority                   TTTATATGTGATC--AATTAATCTTG-AA-AAATGTTTTAGTTGTCTTGAAT-CTTTATTGCAT-GGTATAGGTGTATTA

                                   73530     73540     73550     73560     73570     73580     73590     73600

Human                      TCTTAATTCCATTATAAATAATATTTGAATGAATGTGTTTTACATCTGGAATGATTTTTATCATCTGAGTAAAGGAAATA 72775
Kakapo                     ---ATGTGCAACC--AACCTGTATTG-TG-TCTTATTTTAAGTAACTTGACT-CCCTTTTCCA--GGCCTTAGATGGTCA 36675
GoldenEagle                TTTACATATGAAC--AGTTAATCTTG-AA-AAAACC-CTAGTTGCTGGTAAT-CTTTAGTGCATTGGTATTGGAGCTGTC 43731
JapaneseQuail              ----CTTGTGCTC--T-TCCATGTTA-GA-CTGTCCTTCTGTTGTCATG----TCACACAGCA----ACTACACGTAATG 32155
MediumGroundFinch          TTTAGATGTGAAC--AATTAATCTTG-AA-AAAAATGCTGGTTGGCGAGAAT-CTTTCTTGCATTATTAGAGGTGTATGA 20961
GoodesThornscrubTortoise   TTGTTTAATGATTCTAAGCTCTCTGATAA-AAATGTTACAGTTTACAAATAT-GCTCACGATAT-AACATATATGTACAC 67891
```

```
Majority                 TGAT-GTTGGTAGTGAT-TGCTGGGCCGA---TTTTGTTTTGTTCTGGCACAAGAATCTTTTTCCAGAGTTTGAC-TACA

                                73610     73620     73630     73640     73650     73660     73670     73680

Human                    TGTTTGATGGTAGTTGGGAACTTTGAAGGTATTACGGGCTACAGATGAGAAAAAAATGTTACTACGTCATTTCATGTTTA 72855
Kakapo                   AAAT-ATTTATAATGCT-TGCTGTACCCTACGGATTGATTTTTCCAAACTGGTGAATTTGAATGTCAAGTACAAC-AACG 36752
GoldenEagle              TGCT-GCTGGTAGAGGT-GGGTGGGGTGA---TTTGGTTTTGATCTGTTGCAAGAAGCCTTTCCCAGAGTGTGAC-TACA 43805
JapaneseQuail            AAAT-ATTGGTGGGGA--AGTTCAACCGA--------TACTGCCATACCACTGACATCCATCTCCAGTGTGGACC-AGCA 32223
MediumGroundFinch        TGTT-GCTGGTAAAGAT-GGTTGGGGTGAGGGTTTTTTTTTGTTCTGGTGTTTGAATCCTTTCCTAGAGTTTGAC-TACA 21038
GoodesThornscrubTortoise TTAA-AAAAACATTGATGTGCTGGGCCAA-------GGTATACTTTAGCAAAAAGAGCTGATAAGATTGCATCTCTAACA 67963

Majority                 -GTACTAATTAGGAGTGTCC-TCTTCTTCCTTTTTTATTTAG-GATGTTCTATCAGCGT-TCAATGCTTCTATTGC--AA

                                73690     73700     73710     73720     73730     73740     73750     73760

Human                    CATACTGACCAGCATTTTCAATGAAATAAGCTTATTACTATAGGACTATAAAATGTTTT-TCAAAGGTTTAACTGTCCAG 72934
Kakapo                   -ATAAAAGCAGGGACTACAC-TCGTCCTGATCTTCCATCTGGTGATGGACAGCCAGCGC-TGGACCCAGCTATTGC--TG 36827
GoldenEagle              -GTGCTTATTAAGAGTGTG--TCTCCTTCCTTTCTGGTTCAG-GATGTTCTTTCAGCGTGTCTGTGTCCCAACTGC--AA 43879
JapaneseQuail            -TAATCAAATAGGAGGCATT-ACTTTTGGAGCATCTATTTACAAACATTGGAATGCCAC-TAATGTCTTCTGTTGG--TT 32298
MediumGroundFinch        -GTGCTTATTAAGAGTGCA--TCTCCTTCCTTTCTCATTCAG-GATGTTCTTTCAGCCCATCTATCCCCCAGCTAC--AA 21112
GoodesThornscrubTortoise -GCAAAAAACAAAAACAGCCCTCATTCCCCAAAATGTGTTAGCTGTAATCTAGAAAGAT-TAAATACAGCTATTG----A 68037

Majority                 GGGAATTCTCTAGTGCAGTGTC-TTTTCTAT-ATGTAATTTTGGCCTCGTTTTATTTGATTGAATGAGTTATTTTTACTG

                                73770     73780     73790     73800     73810     73820     73830     73840

Human                    GGCAGATACTTAAGACTATCTGATCATCCATTAAAAACTTTTCACATAGTCTTGCTTAAATGGATCCATTATGTTTACCC 73014
Kakapo                   CAGCATTTGCAAAGGAGACCTC-TCTTCTAG-GTATGATTCTTGCCTTGTTCTTTTGGATTGCAA-AGTCACTTGATGGA 36904
GoldenEagle              GGGATTTCTCTTGTGCAGTATT-CTTT-TGT-ATGTGCTTTTGGTCACTTTGAATTTGTCAGAAGGAGGAATTTTTGCTG 43956
JapaneseQuail            TGATACTGG---GAGTAAAGCT-GTGCATGA-AAGTCATTTTGACTCAGCATGAGTTA--CAAATGGCATATATTCACTG 32371
MediumGroundFinch        GGGATTTCTCTTGTGCAGTACC-TTTTATAT-ATGTATTTTTGGTCGCTTTGATTTTG---GAA---GGAGTTTTTGCTG 21184
GoodesThornscrubTortoise CCAAATTCATGAATGCAGGGTCACTGTGTTG-AAATAATTTAAGATTCGCTCCATATCATGTATACTATTCTGTTGATTA 68116

Majority                 ATT--CAGTGTTGGTTTTTGTTATTTGAACAAGTTTTTTTAAACTTTGTTTATTCTTGAA-ACTCTGAATA--------T

                                73850     73860     73870     73880     73890     73900     73910     73920

Human                    ATTA-TATTGTTCTCAGCTGTAGTTTTAAGAAATTTATTTCA--TTTGTTAATACTTACTTTCCATTACCTTCCCCTTTT 73091
Kakapo                   AAT--GGCTGTTGGTTCTTCTTCTGACAATTAGAGATCTTAGGCTGTAGTAGTGTCCAAA-GGCCCATTCA--------G 36973
GoldenEagle              TTT--CAGTGTAAGTTTTAGTTACTTATGCAGCTCCCTTTAAACATCGACTACTCTGGAA-ACTATGAATG--------C 44025
JapaneseQuail            AGT--TTCTGTTGCGCTTGGTCATTTGAACCTAAGATGTCA---TGTAGGTGTTCTAGAGTACTGAGAATT--------T 32438
MediumGroundFinch        TTT--CAGTAGAAGTTTTAGTTAATTACATAACTCCCTTCAAATACTGACTACTCTAGAA-ACTCCAAACA--------C 21253
GoodesThornscrubTortoise ATTACCAGTGCATCATTCTACAAATTGATCATGGTTTTTATCAAAATATTTTTACCTGATAATTCAGAATAGCCAGCTGT 68196
```

```
Majority                 GGTTATCTATTGGTTTTCTGTTTCTTAATTCTAAAGTATTTATATTATCTTT-CAGCTTTCTTGCAGACACAAGTCAGTG

                                  73930     73940     73950     73960     73970     73980     73990     74000

Human                    CTGTGACAATCCGTTGATACTTGAAGACCTCTCTTGTATTCATCTTAGGGTTAATATTTTTAAGGCCAAACAGCCTAAAT 73171
Kakapo                   TGTTCACTCTCTATTTTGAGCTGAATTGTAGTAACACAGTTACATTGTCTT--TAGACATCTTGCAGAAATGAATGAGAG 37051
GoldenEagle              GGGTATATTTTGGTTTGCTGCTTTTTAAGACTAAAAGATTTACATCAACTTCACAGCCTTCTGCCAGCCATAAATCAGTG 44105
JapaneseQuail            GTTTCTCTACAGAATTTCTTTTACTCCACACTGGAG-----ATGCCCTCTT--CCAGTTTCTTTGAGTGACTGCTTCATG 32511
MediumGroundFinch        AGATATGTCTTGGTTTGCTACTTCTTAAGTCTAAAATATTTCCATCAACTTAGCAGCTTTCTGCAAGCCACAAGTCAGTA 21333
GoodesThornscrubTortoise AAATAGCTACAGAATAAGGATCACTTTTTTTCTGTGGATGTGTAATTTCTCT-GAACCTTCTAACAGATG-AAGTCTCTG 68274

Majority                 TTTGTACTGATCACCTAGAGATCT--ACAATGTGAATCTCTA-TTGTGG-TT------------T---------GGTTC

                                  74010     74020     74030     74040     74050     74060     74070     74080

Human                    TCTATGGTAATCAACTCCAGCCTTGTGTAATGAAATCTTCTGCATAAAGATAGGTTTAAATCAAATCAGATTGCAGATTT 73251
Kakapo                   AATGCAGGGGAAAAGGAGAAATCC--ACAAAGTGGATGGAAA-ACTCAAGTT-----------------------AGTTC 37105
GoldenEagle              TGTCTACAGACTCCATACAGAGGT--ATAACATGAATCTTGA-TTGTGTTTTAAAGACTTAGAGCTCTGTGTCATGGTCC 44182
JapaneseQuail            GTTGCTCTGTTCTGCTAGCAGTCT--GCAGAGAAGACCTCTG-CCA-----------------------------GGCCT 32559
MediumGroundFinch        TTTCTACAGACTCCACACAGAGGT--TAAGTATGAATCTTG--TTG-----------------------------GGTCC 21380
GoodesThornscrubTortoise TGCTATATAATCACTTAGAAACCTG-ACATCCAAAAAGACAA-TCCTGGCCCTGACAGATGATTATTCATTCATATACTG 68352

Majority                 TGTTATTTGGTCTAAGCTAATG-GTGCTTCCTTTTGT-TCTTATGTATTCAAA--AGTTATCTTCATTATGTGC-TTGTG

                                  74090     74100     74110     74120     74130     74140     74150     74160

Human                    TATTGAAGAAATTGTGTTTTTAAGAGTTGACAAATATATGTTGTATGGCTAAAACAAAGAAAATACTTCTGTTGCTTCTG 73331
Kakapo                   TACT-TTTGTTCCCAAATATG---TCCTCCCCTC----TCTCATATATTCCTG----TCAGCCTC-TCCTGGCC-ACGTT 37171
GoldenEagle              TGTGATTTGGACAAAGCTAAAGGATACTGCCTTTTGG-TCTCATTTGTACATA---GTTTTCTTCATTATGTGA-ATGGG 44257
JapaneseQuail            TGTT--GCCGTAAAATCTAATGTGAGCTTCCTGTGGT-CATTTTCTAAACAAA--AAGCAACAACATTTCCAGT-TAAGA 32633
MediumGroundFinch        TGTGATTCAGACTAAGCTAAA---TACATCCTTTCTG-TCTCAAGAATTCACACCTGTTTGCTTCATCATGGGA-TTGGG 21455
GoodesThornscrubTortoise CATTCTCTGCTGTGAAAGAACA-AGGTGATCAGAAGT-CCTTGAGATTGTAAAATGGATGTCTTTTTAATGAGCCTTTTA 68430

Majority                 TATTTCGTTGCAGGGCAATTACAT-TGTTTGTGAC-AAAAAATAAATTCTGGCTGTTTCATTTGTACCAATGTTTATTCC

                                  74170     74180     74190     74200     74210     74220     74230     74240

Human                    CATTTAGTAGAAGAAAAACTATATATGTTTGTGACCAAAGTATAAAATATGATTCTTTCCAGGGAGGTAAAGGTTATGC- 73410
Kakapo                   GCCCACCTTCCCACCCAA----GT-T---TGTAAG-AGACAACATGATTTGCATGTTTCATTCATGCTAATGCTTATTTG 37242
GoldenEagle              TATTTCCTTGATGG-CAATGGCAT-TGTCTTTGAT-AAGAAGTAAGCTCTGGCTGTGTAAACTGGACCTACCTTTAATCC 44334
JapaneseQuail            TATTTAGCTTCAGGACAGTTACCT-TGATTATTCC-GTTAAGGACATTCATGCCACCTGAATCCTAAAGAAGACTATC-- 32709
MediumGroundFinch        TGTTCCCTTGCTGGGCAGTGACAT-TGTATCTGAC-AGTAAAAAAACTCTGGCTGGATAATTTGCACCTTCCTCTAGTCC 21533
GoodesThornscrubTortoise TATTTTGAAAGAAGGAAAACTCAT-TAATGGACAG-AAAGGAGTGATTTCAGGTGAATTGTCAAAAGCAATAGTTCCTTT 68508
```

```
Majority                 --A-TATCATAGTATGATTAGAAAG---GTATGAAGTTTTCTTAAAGTGATGG-AGATTATCAGT-TATGCTTTTATA-A
                                  74250     74260     74270     74280     74290     74300     74310     74320
Human                    -----AGAAGATTTTCACTAGCAGC--TCTAAAAGGCTACCCTCAATTAATTG-CCATGAACATTTCATAGCCCTAGAAG 73482
Kakapo                   TAG-TATCATATTATGAAAAGAACA---TTTTGTTGGTTTCCTTCAATGAGGGAAGATTAT-GCTCTCTGCTTAAAA--A 37315
GoldenEagle              --A-TATTTTAGTCGATTTTAAAAG---GTGAGAAATCTTCTGAAAGTGATGACTCATTCTCGGTGTATGCTTTTTTT-A 44407
JapaneseQuail            -----GTGCTGGAATGATAGGCAGT---GCTTGGATCAACCATAGTGTGCTGG-ATTTTATCAGT-TATGTCAGAAAG-C 32778
MediumGroundFinch        --A-TATCTTAGTGGTTTTTAAAAG---GTAGGAAGTCTTCTAAAAGTGATGGTAGATTCTCAATATAAACTTTTTC--A 21605
GoodesThornscrubTortoise CGAGTGGCATAATATGAAAAAAAAACACATAGGGAAAATCCTCTATATAGGGA-AGATCACTAGC-TGTGTTTTGATATG 68586

Majority                 GATGCAAGTAT-TCTGAGTGTTATT---TTTTTTCCTATATTCTAATAT-----TTTGAGTTTTGGATTATGCTT--TGT
                                  74330     74340     74350     74360     74370     74380     74390     74400
Human                    GATGTTGGCTCATTTCAGTGTCATCCTGGTTTATTCTTTATTGTATTATTCAGCAGTCATTTTAACACTATGCTAGACAC 73562
Kakapo                   AAGAAAAGGAA-TC----TGTAGTT---TCACTTCTGGTGCTGTGGTAC------ATGAGTACTAATGTCCTGTC--AGG 37379
GoldenEagle              GATGTAAGTGT-TCCGAGTGAGATT---TTTTTTCCCAAATACCAACAT-----TAGGAGTCTTGGATTCTGCTT--TGC 44476
JapaneseQuail            CCAGCAAGTAC-AGTAATAGTGTTTG--CTGCTTCCTGGACGCTGCAGC-----TTTGTAGAGAAGAATATGATG--CGT 32848
MediumGroundFinch        GGTGCAAGTGT-TC-AAGTGATATT---CTTTT-CCCAAATACCAACA--------GGAGTCTTGGATTGTGCTT--TGT 21669
GoodesThornscrubTortoise TAAGCAGTTATATCTGATCAATATCA--GTATAATCTATATTCCAAGATACAAACTGGAGTTAAGGCTTATTGTA--TAT 68662

Majority                 TTCTGAGTTTAAAAGAGCTAGCAGGTTTTGTGTTCT-GTGATGGTGATTCTTGACAGTACTCTAATGATTTAGATTTAAT
                                  74410     74420     74430     74440     74450     74460     74470     74480
Human                    TTTAGAGATTCAGAAGAGTAACAGGGTTTCTGTTCTCATGAAGCTTATCAGAGACAGAAAACATATGAGTTAGATCTAAT 73642
Kakapo                   TTTCGAGTTCATCAGAAGTAGTAGGTGAAAGGCTCT-GAGAAAGAGAGAGATAAATGTATTTT--TGACTCTTAGCAAAC 37456
GoldenEagle              CACTGTGTGTAAAAGAGCTAGCAAACTCTGTACTCT-GTAATGGTGACACCCGCCATCACCCCAGCAATACAGAATTAAT 44555
JapaneseQuail            AACTGATCTTTTAATG--AAG-GAACTGCGTGCAAA-AAAACCTTGGTTTTGGATAATGGTCT-GTGATTTACA-TACGT 32922
MediumGroundFinch        CACCATGTATTAAAGAGCTAGCAAATGGTGTATGCT-GTAATGGTGGTGCTCGCCACCACCCCAACAATACAGAATTAAT 21748
GoodesThornscrubTortoise ATCAGGGGTCAGCAAC-CTTTCAGGAGTGGTGTGCT-GAGTCTTCATTTATTCACTCTAATTTAAGGTTCTGTGTGCCAG 68740

Majority                 TAAT--AGCTTGTAATCTTTGTATAAGGACTGTATTTTTTATTTTTGAATTATTGTATAATCCATTGTTT----------
                                  74490     74500     74510     74520     74530     74540     74550     74560
Human                    TGGA-GGCAAACTGAAATATATAGTGGAGTTAGTGTGGTTATCAGCACATAAATGAGTGATCCATCAACAAAAGGAGAAA 73721
Kakapo                   TAAT---GAATGCATGCTTTGGGAGAGAGTTGTAATTTGTGGTTTTAATTTAT--CACAAGTTGTTGCCT---------- 37521
GoldenEagle              CCTC--AGCTTGTATTCTATGAATCAGGACAGTAGATTTTATTTTGCCAGTATTGTATAAAGCATTGTTT---------- 44623
JapaneseQuail            TGTTTAGCCACAGAAACTTCTTGTATCCAAGGCATTTGTCTCAAAAGAAACAAACCTCATTCAAATGTAT---------- 32992
MediumGroundFinch        TAATGAAGCTTGTGCTCTATGAATAAGGACAGTAAATTTTGCTTTTGAAATATTGTATAAGGCATCGTT----------- 21817
GoodesThornscrubTortoise TAAT--ACATTTTAACATTTTTAGAAGGTCTCTTTCTATAAGTCTGTAATAGATAACTAAACTATTGTTGTATGTAAAGT 68818
```

```
Majority                     AAGTTAGGTTTCAAAGG-TGCAAAGAAGCTTCTTTTTAATTGATA--TGTTTGTTGGTATAATCTCTAGACTGTTGGGTT
                                     74570     74580     74590     74600     74610     74620     74630     74640
Human                        TTGGGAGGGTTTTATGGGCCAAAAACAGCATGATTAAATGTGATA---GAGTATATGTCATGTTTTAGGTGTGATGAACA 73798
Kakapo                       AAGTTTCACTCCTACTTCTGTCAAGATGCTATTCTTCAAAATGGAATAGTACTCGGGCATAATCTGCTGACTTTTAG--- 37598
GoldenEagle                  AAGCTGAGTTTCAA-----GCACAGGAGTTTCTCTTTAATTGCTAAGTATTTGTTGAGACAATGTCTAGACTGTCAGGTT 44698
JapaneseQuail                ACAAAAGGATCCAAAGGATACAATAAGGATACATCTCTTGAGAGA-----TTTACAGTATAACCTCTAAGCTACCTTAGC 33067
MediumGroundFinch            AAGCTGAATCTCAA-----GCATAGGAGTTTCTCTTTAATTGCTG--T--TTGTTGATATAATGCTTAGATTTTCCAGTT 21888
GoodesThornscrubTortoise     AAATAAGGTTTTAAAAATGTTTAAGAAGCTTCATTTAAAATTAAATTAAAATGCAGAGCTCCCCCCCGGACCGGTGG-CC 68897

Majority                     TGGAGACATGTTTTGTTATTTTGATATTTTTTGAACTTCCCATT-ATAGTAATTTC-GATTATATTGTCTTACT--TATT
                                     74650     74660     74670     74680     74690     74700     74710     74720
Human                        TTCAGTTATGTGTGACGAATAGGATAATTGAAAAAATATGAAAG-GCTATGATGCCAGAAAGTATTATGGACA--AGAT 73875
Kakapo                       --AAAACCTGATTTATTCTGTT-GTGATCATTGTTCCTCCCACT--TAGTCTTTTC---TCATATTTTCTTCTTT-CATC 37669
GoldenEagle                  TCTATACTTTTTTTTTTTTTTTGGTATTATTTTATTTTCCCATTACTTGGAAGTTCGAACAATACTGACTGACT--TATT 44776
JapaneseQuail                TGGAG-TATGTTTTGTGAGGATCAATTTTTAAAAATTTTTTATT--TATTTACTT-----TGTATTCTTTTATTT-TCCT 33138
MediumGroundFinch            --AAG---TTTTTTGTTCTAT--ATGTTTTTAGAACTTCCAAGCTATGGCAAAAT--AATTACATTGACTTACA--CATT 21957
GoodesThornscrubTortoise     AGGACCCGGGCAGTGTGAGTGCCATAAGTTGTCTACCTGTGATATATAGTAATGTAAGAGTAAAGTATTATAATGATGTT 68977

Majority                     GTTATACGTTGATTTATACTGGTGGTA-------------ATTTAA-GAGCGTTGGAGATGTTAGCTGTGTTAATA--TA
                                     74730     74740     74750     74760     74770     74780     74790     74800
Human                        CTTAAAACCAGTGTTACCCAGGGAGTATGA----------ATTTAA----TATGGGAATTCTTAAACTCCTTTATGACTG 73941
Kakapo                       AATTCCCATT--TTTCTTCTAAAGAAA-------------ACTTCA-GAGCAACATAGATTTCA--TGCCTTGGTT--TT 37729
GoldenEagle                  CTTGTACACTGATTCATAATGGTGGTA-------------ATTTCACGAGCCCTGGAGATGTGAGCTGTGGTAATA--CA 44841
JapaneseQuail                GGTACTTGTC-ATTTTTAATAGTACTA-------------ACTTAT---GTGTTCTTAATATTAGCTGTGATAATA--CA 33199
MediumGroundFinch            GTTTTACGTTGACTGCCAGTGGTGGTAGTTCTTTTAAGTGATTTTGTGAGCCCTGGAGATGTCAGGTGTGATAATG--T- 22034
GoodesThornscrubTortoise     ATTATTATCC--TCCATATAGGTATTACAAACTCTGGAT-ATTCAGTTAAGGTTAAAATTAAAAGAAATTCAGACA--CA 69052

Majority                     -TTTGTGGGAAT--GGACAGCCTTGCTGACCCAAGCCTGGCATAGTGGTGTCTGTTGGATGTTCAGTTTT--CAGATGCA
                                     74810     74820     74830     74840     74850     74860     74870     74880
Human                        GAAGATGAGCATCAGAGTGTCTGCGACCATTTGATGATATTAT-GTACCAAGTTTTAGATGTTTGGCTTTTTTCAGGTTA 74020
Kakapo                       -GGTACAGGATG--GGTCAGCTT----GAAGTAAGTCTGAGCTAATGCAGCTCTTTGTGTTTAGAGAAGT--TAGAAG-- 37798
GoldenEagle                  -TTTGTGGGAAG--GGACAGCCTTGCTGGCCCAGACCTAGCAGAAGGGTGTCTGCAGTATGTTCAGTTTC--CAGATGCA 44916
JapaneseQuail                GCTTGTAGGAAA--GGACGGACTTGCTAACCCAGACATAGC---GAGGTGTCTGTGGGTCACTCAGTTCC--CGGATTCA 33272
MediumGroundFinch            -TTTGTGGG------GACAGCCTTTCTGGCCCAAGCCTGGCAGAGGGGTGTCTGTAAGATGTTCAGTTTC--CAGATGCA 22105
GoodesThornscrubTortoise     CTGTAAAGGCATCCAGGCAACCTT---AACTCCACCCTGTAATGATGTTGTGCAATAACCATACTGTTATAACTAATGCA 69129
```

```
Majority                 TGAC----TGTGTGAAAACAGCT-TG----TTTCATG--TAGAGGGCTCATTAGTTTTGT-----GCAGTGCT-TAGGTC
                                  74890     74900     74910     74920     74930     74940     74950     74960
Human                    TGAA----AGCGGGGGATGAGTTAAGAACCACTGCTG--TGAAGGATTCATCAACTATTTT--TAG--GCAGT-TGGGTA 74089
Kakapo                   GCAC----TTTGTGAATGCTGT--------------------GAGCCCAGGAAAAGGGT-----G-AATATT-GTAGTT 37846
GoldenEagle              TGAC----TGTGTGAAAATAGCTATGTTGCTTCCATACCCAGCAGGCACACTAGGTTTGT-----GCAGTGCT-TGGCTC 44986
JapaneseQuail            TTGC----TGTATGAAAACAGC--T-----TTCCATGGCCAGAAGGCCTAATAGTTCCAT-----GCAGCACT-TAATTC 33335
MediumGroundFinch        TCAC----TGTATGAAAATAGC--------------------AGGCACACAAGGTTTGT-----GCAGTGCT-GAGCTC 22154
GoodesThornscrubTortoise TAATAGCATTTGTGGTCTCAGGTTAGTTAAACTTATTTTTAAAGACATTATATATTTTGTTACTAGTATCGCTACAGGGC 69209

Majority                 AAAGTGG--TAAGTCCTTTTA-GAACTTTAATGTG-TTGGGATGTACATTGCAG---GTAAAATCAAGC-TAGCTGT--C
                                  74970     74980     74990     75000     75010     75020     75030     75040
Human                    AAAATGACCAATTTAGTTTTAAGAAACTGACTGTGGCTCCAGAGTATGTTGGAGAAGTGAAAATGGAGACTAGGAATAAC 74169
Kakapo                   AAGG-----------CTACT--GAAC---GCTGTG-CTA-TATCTGCCCTTTT----CTAAAATCAGAT-CACTTG---A 37900
GoldenEagle              AAAATGA--CAAGTCCTACCA-GAACTTTAATGTG-TTGGGAAGTACACTGCAG---CTGTAACCAAGC-TAGCTGTG-C 45057
JapaneseQuail            AAAATGG--TACATCTTTTCA-GAACTTAAATGTG-TTGGAATGTGCATTGCTG---GTAAAGCTAAGC-TAGCTT---- 33403
MediumGroundFinch        AAAGTAG--TAAGCCCTACCA-GAACCTGGATGTG-TTAGCAAGTACACTGCAG---ATGTAACCCTGC-TAACTG---C 22223
GoodesThornscrubTortoise AAAGTTCCCTAACTGATTTTC-ATACTTTAACTTT-TTGGGATTTCTTTTAAGG---GTAACCTTAACTGTAAATATC-C 69283

Majority                 AGGAGCT--ATTTCTGGTCCAG--AAGTTGTACATTTCAGTCCAGTATTGCACTCTAAACAAATG------T-GGAATAT
                                  75050     75060     75070     75080     75090     75100     75110     75120
Human                    AGGTGGGAGACTATTAGTCTAATTAAGATGTAATTATAAATCTAAGCTAGGAACGTAAAATGAGAA-----T-GCAAAGT 74243
Kakapo                   AGGATAC--ATTTCCTAT--GG--A-GTTGTAAATTCCA---CATACTCCCACCTCCCACAAATT------TTGGAATGC 37964
GoldenEagle              AAGAGCT--AGCTCAGGTTCAG--AAGTGGTACATTTCAGTCCAGCATT--AATCAAGAGAAACA---------AAAGAT 45122
JapaneseQuail            ----------TCTTTGGTCCAG--AGGTGATGCGTTTCTGTCCAGTATT--ACTCAAGAGAAACG---------AAAGAT 33460
MediumGroundFinch        AAGAGCT--AGCTCAGGTCCAG--AAGTGGTACATTTCAGTCTAACATTTCAGTCCAAACAGATGTGTGTATTTGAAAAT 22299
GoodesThornscrubTortoise TGGGTTTGTAATGCTTGTTTGG--GGGCTAATCTGAACATCCCAGTAATGTGTTTTCAACCATTGAAT---TACTGATAT 69358

Majority                 TTGATTT--TTGTATTTTCCAGTGTA-ATTTATGTATTACTGTATGATTTCTATTTGATGTAG-TTTATATTTAGGATAA
                                  75130     75140     75150     75160     75170     75180     75190     75200
Human                    AAGAAACAAATATGGGGAAAATTATATGTAAAAGTAATAGGACTTGGCATCTTACTGATGTGA-TTGATTATGAGAAAAA 74322
Kakapo                   TCGACTT---CACATCCTCCAGTCTG-ACTTGCAAAGCACTGTATCACTTCTAGCTACAGCAG---GA-GTTTGGGGAAG 38036
GoldenEagle              CTGATTT--TTGTATTTGGAGGTGTG-ATTTGTGTTTTTCACCAT-GTGTATATATGAGGTAGTTTTATATTTAATATAA 45198
JapaneseQuail            CTCGCTT--TTGCATTTGAAAGTCCAAATGTTTCTA---CTGAATGATCTGTGTTTCTTGGAA----ACATT-----AAA 33526
MediumGroundFinch        TTGCTTTATTTGATTTTTTCAGTGCATATGTATATATATATATAT-ATATATATATATGGTAGCTTTATATTTAGCATAA 22378
GoodesThornscrubTortoise CTACTCTCAACTTTCACTCCAGCTTA-ACATAGTTCTGATTTGGGAATTTCCTTTTTATTTAATTTAAATGTTCAGGTAT 69437
```

```
Majority                 TGACTTGATTGAATGAT---TCATTAATTTTTAGAATGTGCTGTTAATCTTTATTTCCTAGTATTTGTTATTAATC-TCA
                                 75210     75220     75230     75240     75250     75260     75270     75280
Human                    TGAAGCATGTGGAGGAGTCCACTGGACAGTAGGAAATTCAGCCTAAGACTTGGGTAAGAGTTCTGTGGAGTTGTGAATTC 74402
Kakapo                   TAAAAATACTGTAAGAA---TCCAAAGTCTGAAGAACCCACTGCTGAAGTTTATTTCCTTGTTTTCTCTTTTAACT-TCC 38112
GoldenEagle              TGACTTGATTGAATGG------ATTAATTTTTAGGATGAGCTGT--ACCTTTACTTCCTAGCATTTCTGAAGAATA-TAA 45269
JapaneseQuail            TAACTTGCTTTAAAAAG---T--TCTATTTCTTCTGTGTAGCATTAAGAGCTA-TACATACTATATGAAAGCATGC-TCA 33599
MediumGroundFinch        TGAATTGATTGAATGCTAATTTGCTAATTTTTAAGATGAGCTGC--ATCATCACTTCCTAGCACTCCCTAAGAAT--TAA 22454
GoodesThornscrubTortoise GAACACTAAACAAGAATGCCAAAGAGAATGTTACAATGTGATATTTCTAATGATTTGAAGATGTATATTATAAACCTTCA 69517

Majority                 AGTTGCTT-AATTATTAAAATTT---CTGTGTTTTAAAATC--TATGATATTTAAATG-TTAGTGATTAGAGTGTAATGA
                                 75290     75300     75310     75320     75330     75340     75350     75360
Human                    AGAGGCCAGAGATGTGATATTTAAAATTTTGGTTCAAGATTTCCCAGGTATAAGAAAGCAAGAGGATTAAAGCATTGTAA 74482
Kakapo                   TATTGTTT-AAGTATTGAAATCT---CTCTTTTCACGGGTTGATAGGGCAGGTGGATGAGTATTTGTAAGTGTGACACCA 38188
GoldenEagle              ACCAACTT-AGTTACAAAAATGT---CCAT-TTTTTAAATC--TCTCATATTTAGCTG-TTAGTGCTTCAACTGAAAGAA 45341
JapaneseQuail            AAATGCTA-ATGGAATTAAATTA---GTCTGATATAGAATG---AGAATAGTCAAAAGTCAAATAATTAGGTCTTAATGG 33672
MediumGroundFinch        AGTGACTT-TATTACAACAGTTT---CTGTATTTTTAAGTC--TGCCATATTTAACTG-GTAGTGCTTAGAGTGAAAGGA 22527
GoodesThornscrubTortoise CTTTATTCCAACTTTTAGGACCA---TTGTCCCCTAACACCTATATTTCCCTTCCTTAGTCACACACCACTTCCTCCTCA 69594

Majority                 TAGGTGTGCCCACAATGTATATGGAC----TCAAGTTAAGTTATACTTATTTGCCTTGTTGTTAA-TAGGTATAATGTAG
                                 75370     75380     75390     75400     75410     75420     75430     75440
Human                    TTAAACTTTAAGCAGTGCATATTTATG--TTATAGATAAGATAAA-CAAGAAATCTAGGGATCAAATAGGATTAAAATTA 74559
Kakapo                   TAGGGATGAGTGCTATAGAATTGCCC----TGGAGGCAGTTAGTAATTCTTTGGTCAGCACGTAA-TAAGTAGAATTCAG 38263
GoldenEagle              -AGGTGTGCCCAAAATGTAAATGGAA----TAAAATCCAGGTATATCAATTTGCCTGGTATATAA-TGAAGATGGACAAA 45415
JapaneseQuail            AAAGCGTTTCCAC-ATAGATGTGGTA----CCATGGTAAG-AGTCCATGTGAATTCTGTTGTTAG-TAGGCATCCCCTAA 33745
MediumGroundFinch        -GGATATGCCCAAAATGTAAATGGAC----TCAAATCCAGGTAT--TGACTTGCCTGGTGGATAA-GGGAGATAACGGAG 22599
GoodesThornscrubTortoise CTGTTGTCCTTCACATATCTCTGTTCACATCCACCCTGAGTTCCCCTTCCTAACCTTCCTGTCAAGTCAATATGGTGCAG 69674

Majority                 ACAGTGATAATTAGGTATATTT-TTGTTTATTTTT-TATACTTTTTTATAGCATTATTTTAACAGGGAATGTGAGTTTCA
                                 75450     75460     75470     75480     75490     75500     75510     75520
Human                    GTAGTGATCATTCAGTACAGTAGTTACGTACTGTTATTCACAAGAGTATATAAATCAAATTACAAGGAATTAAGGATATA 74639
Kakapo                   ACATGGGGCTACAGTCATATTA---GCTTAAATGC--AC-CTCTTTAAATGCACTGTTTTTCCAAGCAGCAGGAAAATCA 38337
GoldenEagle              ACATACATAATTAGGTCTTTTTAGAGAGTGTTTTCATAGAGTTCTAAGTAGTGTCATGGTAAGAGTTCACGTGAATTTCA 45495
JapaneseQuail            GAAGTAAGAATTGGGAAAATAG---GATTTTCTTT---TATTAAGGTGTAGCATTAGACTAGCAGGTAATGGGTCTTTTG 33819
MediumGroundFinch        ACACACATATATATATATATGT-GTGTGTATATGT-TGGATTTTTTTACAAAAGTGTTTTCATAGAGTAC-TAGTTGTCA 22676
GoodesThornscrubTortoise AGAGTAGTTATTGAAAATAATTTTCATTTTTGCTGTCATTCTGCATAACTTGGGTACTTCAACAAGGACT-AAAGAATAA 69753
```

```
Majority                 T-T-TTTGTAGGCAATTTTTCATAAGTTTCACTTAATATG-TTGTCATAGTATTTTTTAAAGGATAGT--GTTAGAAACA
                                  75530     75540     75550     75560     75570     75580     75590     75600
Human                    AAC-GTGATAAGAAAGTATGCACTGTACTCTTTGAGGAAGTTTGCCATAGAAAGGAAGAAGAAATAGGATGGTA-GATCA 74717
Kakapo                   ----AAGGTAGACGA-CATGAGTAAGTTGCAGTGGAG-T--CAGTCAT-GTTTATTTTGATGAGTAGT--GC-AGATACT 38405
GoldenEagle              C-TGTTAATAGGCAACCTTTCAGAAGTACTAATTAAT-TG-TGAAAATAGGATTTTTTTAAAAAAAGT--GTTAGTAACA 45570
JapaneseQuail            TGTGTGTGTATGTGTGTTTTGTTTTGTTTTTCTTAAAATT-TCGGCACAGATTACTTCTAAGGGGAGA--GT---AAA-A 33892
MediumGroundFinch        --TATTAATACACATTCTTTTAGAAGTACCAATTAAT-TG-TGAAAAT-GGGTTTTCTAAAAGAGAGT--ATTAGGAACA 22749
GoodesThornscrubTortoise TGT-TCTGCGGATAATTCCACAAAGAAGGGGCTGGATAGG-GTGTCATCGTATTCCGCAAAGGCAGTTTGGCTTCATGTA 69831

Majority                 TTAGGTTAGCA-GTTAGAGTTGGTTGTCTTTCT--ATTTAGGCACTATTTAT-----CTTAAA--AGGGAGAAGGGGAGC
                                  75610     75620     75630     75640     75650     75660     75670     75680
Human                    GAAGTAAAGCAGGACCCAGTGGGGGGAGTGTTTGCAGTGAGGCAGTATGTATAATCATTTAAAACATGGGTTTGGAGTCC 74797
Kakapo                   ATAATATGGTT-GCTACATTACTTTCTCCCTCC--ATAAAAGCATTCATTGT-----TTGGAA--AGAGACTTAAGGGAG 38475
GoldenEagle              TTAGGTTAGCA-GGTAGAGTTGCTTTTCTTTCA--ATTTTGGCACTAATTAC-----CTTGAA--GGGGAGAAGGAAAAC 45640
JapaneseQuail            GCAGGCAGG---AACAAATTTGGTGGTTTAGC---TTGGAGCCATCTACTAT-----ATGATG--TGTAAGCCTTGGGGT 33959
MediumGroundFinch        TCAGGTTAGCA-GTTAAAGTTGGTTTTCTCTCA--TTTTTGGCACTATCTAC-----CTTTAA--GAGGAGAAGGGAAGC 22819
GoodesThornscrubTortoise CATTGTTCCCA-ATAGGAGTTGGTGGCCATTTTGAAAGAGGGAACTGTTCGC-----CTCATTCCATTGAGAAAAATAGT 69905

Majority                 ATGCAGGAATTATGTTTATATTGTGCTTTATCAGATGGTTGTCTGTTGTGTTTTTGAA-GTTTAGATTTTTCTT-----C
                                  75690     75700     75710     75720     75730     75740     75750     75760
Human                    TCTCAGGTTCCATGTTTGTAATGGACATAAT--GATAATAATCCCTTTCATTTAAGGCTGTTGTGAGGATTAAATGTGTT 74875
Kakapo                   CTGGATCCATTTTCTA-ACGCTGCTCATGGCCAGTTGGGTGAGCTTTGAGTTGTCAAA-ATGGGAATGGTGCTT------ 38547
GoldenEagle              ATGCAGGAACAATGTAAATATTCAGCTTTAACAGATAGCTGTCTAACATGATATTTAA-GTCTAGATTTCTGCCCCGCCC 45719
JapaneseQuail            TTTTTTGGGTTTTTTT--GGTTTTTTTTTTT----TGTTTGTTTGTT-TGTTTTTTGA-GATC------TTCTT------ 34019
MediumGroundFinch        ATGCAGGAATAATGTATATATTATGCTTTAACAGATGGCTGTCTAACATGGTAGTGAA-GTCTAGATTTTTTTT-----C 22893
GoodesThornscrubTortoise AGATAGTAACCATTTTTAAAGAGAGCATTTTTGTGTAATTATCGGTCGTAGATTAGCA-GTACCCTCAACCCCC-----C 69979

Majority                 TCAGTTCAATTTTCCATGTAAAA-------TCT-TGGTTTTTGGTTTGGT----TTTTCTTTTTTTTTTTTACTTTGAGA
                                  75770     75780     75790     75800     75810     75820     75830     75840
Human                    AATGTGCAAATAACTTTACACAGTGCCTGGTATATAATAAAT-GCTTGCTACCTATTAACTAGTATTTGTTTCTAAGGCT 74954
Kakapo                   ---GTTTGAAT----ATGGAAAA--------CA-TGGTTCTCAGTCTGG-----GCTCCTTACTGTGCAAAAAGGCACAA 38606
GoldenEagle              TCAGGTCAGCTTTCCATATAAAAGAGTACTTGTGTGGTTTTTGGTTTTGTGGTGTGTTGTTTTTTTTTTTTCCTTTGAGA 45799
JapaneseQuail            --AAGTCAGCTTTT--TGTAGAC---------T---ATTTCTGGTCTGAT----TTTTATTTATTTATTTTACTTTTTGA 34079
MediumGroundFinch        TCAG-TTGATTTTCCATGTAAAA------TTCT-TGGCTTTTTGTTTT------TGTTCTTTTTTTTTTTTTTTTTGAGA 22959
GoodesThornscrubTortoise TCCCCTCAATTAATCCTAAAAAA-AA---------GTTTTATGATATAGC----CAATTTCAATGAGTTTTACATTGAAG 70045
```

```
Majority                 AATTTA---TTTGGCATT--ATGTCTGAATTAAAGCTGTACTTTAGAATACAGGTT------CATTATCTTTATTAGACA

                                  75850     75860     75870     75880     75890     75900     75910     75920

Human                    AATTTAAGTCCTAGAATTGATTGCAAGGATTAGATCAGGAGTATAGTGGACATGTTGGGATTTAAATATTTAAATATAGA 75034
Kakapo                   AATAGA---AGTGACAT---AAATAAGAA---AGGGCAAAGGATAGAAGAAACCCT------CAAAAGCTTCAATGGACA 38671
GoldenEagle              AATTTA---TTTGGCATT--ATGTCTGAATTAAAGCTGTACTGTCGAATACAGGTT------AGTTATCAATATTCAACA 45868
JapaneseQuail            GAGATG---TTTGGCATT--TTGCCTGCATTAAAGCTATACTGTGGAATACAGATT------CATTATATTCAGCAGAAA 34148
MediumGroundFinch        AATCTC---TTTGGCATT--ATGTCTGATTTTAAGCTGTGCCTTGGAATGCAGGTT------AATGAGCTGTATTCGACA 23028
GoodesThornscrubTortoiseGATCTA---CCGTATTTT--GTTGTTGAGATCATTGGGGACTTCAAGAAAAACCTATTAGAGCGCTAAATTTCTTAAAGA 70120

Majority                 GTTGATTTTTT-AACTTTTGAC-TGCGTTAAA-ATTTTTCTTGCCACTTTGGTGCAGAAA-------TGTAGGTACTAAG

                                  75930     75940     75950     75960     75970     75980     75990     76000

Human                    GATGCTTTTTAGGACCATTGTTAGAACCAGAAGAGATTTTTTACCAAGTTCACACAGAAA-------TGTAGGTGCATTG 75107
Kakapo                   CTTGCGCCTGC-AATTTATAGC-TGCGTT-----TTTATTCAGAGACTCAAAAGGAGAAGG-----TTGAGAACACTCAG 38739
GoldenEagle              GCCTAACTTTT-AAGTT-TGAC-TGCATTAAA-ATGTATCTTGCCACTTTGGTGCATA---------TCTAGGTAAGAGA 45935
JapaneseQuail            TTTGATTGCACCAACTTCTTTG-CACCTTGCT-GCATGAACCGTCTCTTGGTAAGAGAA---------ATAAATGTCAAG 34217
MediumGroundFinch        GACAAAATTT--AAATT-TGAC-AGTGTGAAA-ATGTCCCTTGCCACTTGGGTGCATAAAAACAGTTTCTAGGTAAGAAG 23103
GoodesThornscrubTortoiseTTCCATTATTTTAAATTTTAATGCAAATTAATTGATTTACTTGTAAATTCTCTTAAGTCCTTCTGTATTTAAAGAGTGAG 70200

Majority                 TGTTAACATGGAGGTTCCTG---------GCATCATGTTGAG--AGAGGTTAATGCATTTTAATTTCAGTTGGTTTTAAG

                                  76010     76020     76030     76040     76050     76060     76070     76080

Human                    GCTGGGCATGGTGGCTCACACCTGCAATCCCAGCACTTGGG----AAGGCTGAGGCAGAAGAACTGCTTGAGGCCAACAT 75183
Kakapo                   TTATAATATGGAGGTTGGCA---------A-ACTGCGATGTG--TGAAAT-ATGGCAGAATGATAGCAG-TGACCTTCCT 38805
GoldenEagle              AGTAAACATCAAGGTTCCTG---------GAATCGTTTTAAA--AGAGGTTAATGCATTTTAATCTTAATTGGTTTTAAG 46004
JapaneseQuail            TA--AACATCGAGCATCCGG---------GAGTCATGTTAAG--AGAGGTTAATACATTGTAATCTTAATTAGTTTTAAG 34284
MediumGroundFinch        GG--AGCATGGAGCTTCCTG---------GCATCATGTTGA----GAGGTTAATGCATTTTAAT------TGGTTTTAAG 23162
GoodesThornscrubTortoiseTGCTGTGATGGCGGTGCCTGCTGGTGGAGGCAGCACATGGAGCTAGGAGCTGAGGGAGAGGAGCTCCTGTCACCTATTCA 70280

Majority                 CTAGACAGTAAGATGGATAA-ATATCTATACGCTGACCTTCTTTAAAAATACAACCACTATAAT-CCAACAACGATGCTT

                                  76090     76100     76110     76120     76130     76140     76150     76160

Human                    TTTGAGACCAGCCTGGGCAACATATTAAGACCCCGTCTCCACCAAAAAAAAAAAAAAAAGAAGTAGGTGCAGAGCTGGAA 75263
Kakapo                   CCTGCTTCTTGCTTTGAATA-TTTTATTTCCTTTTATTTGCTTTAGAATTGCGAGCTCTGTAATGTCACTAACGATACCT 38884
GoldenEagle              CTAGACAGTAAGATGGATAA-ATATCTATACGCTGACCTTATTTAAATAAACAACAACCACCAC-CAAACAAAAATAATT 46082
JapaneseQuail            CTAGACAGTAAGAGGGATAA-ATAATTACATACTGATCCAGTGCAAC----CAACCTATAT------------------T 34341
MediumGroundFinch        CTAGACTGTAAGACAGATAA-ATATCTATACACTGACCTTCTTTAAA-ATAAAACAACCAA-----AAACCCCAGCC-TT 23234
GoodesThornscrubTortoiseTGGAGCCCCTCCCCCAGTAA-GCACCTCCTCGCACACCAGTCCCCAGCCCTGAGTCCCTCCAAACCCAATCTTC-TGCTG 70358
```

```
Majority                 GTGGAACCAACTTGTTCTGTGTTTGTTTTAAATTAATTGT-GCTCCTTTTTCCAGGCCTTAGATGGTCAAAATATTTATA
                                  76170     76180     76190     76200     76210     76220     76230     76240
Human                    GCAGAACCGAAATCATCAGTGTTACAGTCATTATTCTTTCCTGTCACCATTATATGTCTTTATGAAGCAAGGGAGAAAGA 75343
Kakapo                   TCAGAGGGTACTCAGTCAGTGTCTGCTCTGCCCTATTCACAGATGGCTCCCAAAGGGCATGTTTCTCCATGAAAGAGTGG 38964
GoldenEagle              ATGTAACCAACCTGTATTGTGTATTTTTTAAATAAATTG--ACTCCCTTTTCCAGGCCTTAGATGGTCAAAATATTTATA 46160
JapaneseQuail            GTGTA------T-------TATTTATTTTAAATTAATTGT--TTCCTTTTTGCAGGCCCTAGATGGTCAGAATATTTATA 34406
MediumGroundFinch        GTGCAACCAACTGTTATTGTG--TCTTTTAAATAAACTGT-GATCTTTTTTTCAGGCCTTAGACGGTCAAAATATTTATA 23311
GoodesThornscrubTortoise CTGGGGGCAAGGGGGGCCTGAGACTGCCCCAG--CAGCTG---CTGCTGCGACTGGCCCATGGGCTGCCTGAGCTGCTCAG 70433

Majority                 ATGCTTGCTGTACCCTACGGATTGATTTTTCCAAGTTGGTGAATTTGAATGTCAAGTACAACAAAGATAAAAGC--AGTG
                                  76250     76260     76270     76280     76290     76300     76310     76320
Human                    AGAACAGATGAAAGAAGTGAGGATTTTGAAGTTGGTTGAAAGATTTGATTGAATTCTGATCTAAAAATTATAAGGCACTT 75423
Kakapo                   AGCTCCGTTTTGAGCTTCGTGTGATGCTCTTCAGGTTCTTTTGCATGCTCAAAAAGAATACCCTAAATGGAACC--TGTG 39042
GoldenEagle              ATGCTTGCTGTACCCTACGGATTGATTTTTCCAAATTGGTGAATTTGAATGTCAAGTACAACAACGATAAAAGC--AGGG 46238
JapaneseQuail            ATGCTTGCTGTACTCTACGGATTGATTTTTCCAAATTGGTGAATCTAAATGTCAAGTACAACAATGATAAAAGC--AGGG 34484
MediumGroundFinch        ATGCTTGCTGTACCCTACGAATTGATTTTTCCAAATTGGTGAATTTGAATGTCAAGTACAACAACGATAAGAGC--AGGG 23389
GoodesThornscrubTortoise GCAGCTCCCAGGCCAACCACAGGGGCCACGGAAAGTTGCGGCATCCG--TGACCTGCATGACAAACATGGAGCCTTACTG 70511

Majority                 ACTACACTCGTCCTGATCTTCCATCTGG---TGATGGTCAGCTAGCTCTGGACCCAG--TCATTGCTGGAGCATTTGCTA
                                  76330     76340     76350     76360     76370     76380     76390     76400
Human                    GTTTAACAAGTTGAAAGTAGGAAAGTAGACATAAGACTCTACTAGATTTGGGGAAACTCTCAAAAATGGACTGGAAATTC 75503
Kakapo                   GC-ATTCTTGTTAAAATCCTTGCTGTAC---CTGTGCTCTTATATGTATATAATGAGA-TCATTAACAGAAAATGTGGTG 39117
GoldenEagle              ACTACACTCGTCCTGATCTGCCATCTGG---TGATGGACAGCCAGCGCTGGACCCAG--CCATTGCTGCGGCATTTGCAA 46313
JapaneseQuail            ACTACACTCGTCCTGATCTTCCATCTGG---TGATGGACAGCCAGCGCTGGACCCAG--CTATTGCTGCAGCATTTGCAA 34559
MediumGroundFinch        ACTACACTCGTCCTGACCTTCCATCTGG---CGATGGACAACCAACTCTGGACCCAG--CTATTGCTGCAGCATTTGCCA 23464
GoodesThornscrubTortoise ATGAGACTGCTAAAAATGATTTGATTGT---TGTTGCTTAGTT--TATTTGAAAAAGC-TGTCCAGTAGGGTTTTCACTG 70585

Majority                 AGGAG-ACTTCTCTTCTAGGTATGATTTTGACTTTGTTGTTATGTGTTGCATAGTTATTTCATGAAAATATTTATTAATC
                                  76410     76420     76430     76440     76450     76460     76470     76480
Human                    AGCTAAAAGTGGATAACAAAATATTTCTAGAATTAGCATTTGTGGGGTGTGTGTGT-TTTCACTCTAGTATTTGTCAAGC 75582
Kakapo                   AGGA---C-TCAGTTCTGGCTTTTGCTTATGTTATGCTATTATATGCTGGATAGTTGTACATTAGATGCTTTTTTTTGTT 39193
GoldenEagle              AGGAG-ACATCTCTTCTAGGTATGATTCTGACGTTGTTCTTTTGTATTGCAAAGTCACTTGATGAAAATGTTTATTAATC 46392
JapaneseQuail            AGGAG-ACTTCTCTTTTAGGTATGACTCTGACTTTATTGTTATGTGTTTCAGAGTCACTTGATGAAAATACTTATTAGTC 34638
MediumGroundFinch        AGGAA-ACCTCTCTTCTAGGTATGATATTTACATTCTTCTTCTGTATTGCAAAGCCACTTCATGAAAATATTCATTAATC 23543
GoodesThornscrubTortoise ACAACTGCTTACCTACCCTGGCCAGATTTGAACCAGGGGCAATAGGGTGCATTTGTATTCCATTATCAATCCCCCACATC 70665
```

```
Majority                 CTTC----TATCA-ATTCTAAATCTTAAATTGCAATAGT----GTCCTAAGTCCTGTT---CAGGGTTCAGTCTATTTTG

                                76490     76500     76510     76520     76530     76540     76550     76560

Human                    CCAGATGAAAGCATAGACAGAATGTAAGACTGGATTTATCTAAGTCTGGAATTGTGTAACATTAAAGGAATAGTAGCAAA 75662
Kakapo                   GGCT----TTTTA-ACTCTCTGCTGTAATCTGTA----T----GTCAGAAGTGAGGTT---TAGGTTTAATACGATTTTG 39257
GoldenEagle              CTTC----TAACA-GTTAAAGATCTTAAATTACGATACT----GTCCAAAGGCCCATT---CAGGGTTCAGTCTATATTG 46460
JapaneseQuail            --------T----------------TAAATTGCAATAGT----GTCCTGAGGCCTA----------TTCAGTTTATGTTG 34680
MediumGroundFinch        CTTC----TAACA-A--------CTTAAATTACAATAGT----GTCCAAAGACCCAGG---CACGGTTCAGTCTAGATTG 23603
GoodesThornscrubTortoise ATCCAGATTCTAA-ATACTAAATATTTATTTCCCTTTCT----TCTCTAGACTGTGGC---CTGGAGAGGGATAGCTCAG 70737

Majority                 TGGTCTGAGTTTTAG--TAA--CATAAT-TGTGTTGTCTTTTGAAGCCCTGCATAAG-TGAGT---GAGAGAATGCATGA

                                76570     76580     76590     76600     76610     76620     76630     76640

Human                    TGAGCAGAGTGTTGGCTCAAGCCTAAGCTTGAGCCTAAGCTTGACTCTATGGTAAAGTCAAGTCAAGGGAGAATAGAAAG 75742
Kakapo                   CAAGCTGAGTTTTAG--TGA--CAGAATGTGGGTCTTGGTGTTTAAATCTGCATTAA--TACT---AAACCATTACATAT 39328
GoldenEagle              TGATCTGCATTGTAG--TAA--CACAAT-TATATTGTCTTTAGAAGTCCTGCATAAA-TGAGT---GAGAGAATGCATGA 46531
JapaneseQuail            CAGTGTGAATTCTAG--CAA--TATAGT-TGCATTATCCTTAGGAGCCCTGGAGAAG-TGAAT---GAAAGAATGCATGA 34751
MediumGroundFinch        TGGTCTGAATTGTAG--TAA--CATAAT-TATATTGCCTTTAGAAGTACTGCAAAAAATGAGT---GAAACT-TGCATGA 23674
GoodesThornscrubTortoise TGGTTTGAGCATTGGCCTGC--TAAACCCAGGGTTGTGAGTTCAATCCTTGAGGGGGCCATTT---GGGGATTTAGTTGG 70812

Majority                 CAAT--ATGATAAA-TGCACAAAGTATAAGAAAAGGTCAAGTTAGTTCAGCTTG-TGTTCCAAAGTCTGA--TCCTTCCA

                                76650     76660     76670     76680     76690     76700     76710     76720

Human                    GGGGTCACCATAAA-GGTCAAAAGTGGGTTTAGTGGTTGTGTGGGAATAGGCAGATCAAGAAAAGAATGAAGTTAGGAAA 75821
Kakapo                   CTCT--GTTTCCTTCTGGATGGAATTAACCCTTTGCTTCAGGAAATGGAACTTTATGATGCAGTGTAGGAA-CACTTACA 39405
GoldenEagle              CAAA--ATGAAAAA-TGCACAAAGTATAAGGAAAAGTCAAGTTAGTCCTACTTT-TATTTCCAAATCTG---TCCTCCCG 46604
JapaneseQuail            CAA---ATGAGAAA-TGCACAAACTATAA-ATAAGCTCTCGCTCTGAATGCGTG-TCTTCGATTGTACCG--TTGTCCCA 34823
MediumGroundFinch        CAAA--ATGACAAA-TGCACAAAGTACTAGAAAAAGTCAAGTTAGTTCAGTTTG-TATCCCAAAATCTG---TCCTTCCC 23747
GoodesThornscrubTortoise CGATTGGCCCTGCTTTGAGCAGGGGGTTGGACTAGATCAGTGCTACTCAAAGTGGTGGTCCATGGACCCATGTCGGGCTG 70892

Majority                 TCTGTCAT-GAGTGTCAGTCGGCTGCTAGTAAC---GACCTTGCTAACTTTACCAGCCATGTTTT---------AAAAAA

                                76730     76740     76750     76760     76770     76780     76790     76800

Human                    GGAGATAT-AAGTGTTGAATGACCATTACAAAAAGAGACAGAGGAAAGAAAAATGAAGATGTATCAAAAGAAGTTGCTAA 75900
Kakapo                   GCTTCCAA-AACTGTTACGCAGGGACTTGAAAA---GAACTTACTATCTCCTCCAAACCCATTT---------------A 39466
GoldenEagle              TCTTTCAT-GTGTTCCTGTCAGCTGCTACTACC---CACCTTGCTCACACTCCTACCCAAGTT-T---------GTAACA 46670
JapaneseQuail            AATGACAG-AAAAATAAAAGGGATGT----------GACACAAGTAACTTAAACAGCATTTCTTG---------AAAGTG 34883
MediumGroundFinch        TCTATCAT-GTGTTCCTGTCTGTGGC----------CACCTTGCTCACCTTCCCAGCTAATTTGT---------AAGAGA 23807
GoodesThornscrubTortoise TGAGTCATCGGCAGCCAGTCCGCAGCGAGTTTCCTAGAATGTAAGTACCAGAAGGGTCATGCTTGGCAAAAAAAAAAAAA 70972
```

```
Majority                   GACTGAGTGTGATGTGCATGTTTTCATCATGTT-AATGCTTA----TTTG-TGATTTCAGATTATGAAGCTACCATTTTG

                                   76810     76820     76830     76840     76850     76860     76870     76880

Human                      TATGGATGGCAAAGTAGATGTTTTTAAGAAATCATGAGACCAGAGTCTTGGAAAAGTCATAGGATGATGCAGGGAATGGA 75980
Kakapo                     GGCTCAGCATAAGAAGCAGGCCATTAACAGGTACAAAAA--G----CTGG-TGGTTCCTTGACATAAAGCCACAAAAAAG 39539
GoldenEagle                GACTTAATGTGATTTGCATGTTTCATTCATGCT-AATGCTTA----TTTG-TAATATCAGATTATGAAAATAACATTTTG 46744
JapaneseQuail              TCCTGAGTGTCA-GTGAATGCTTTCATAATCTT-------TA----TCT--TGATTATTA---ATGGAGTTTTTATATTT 34946
MediumGroundFinch          GACTTAACATGATGTGCATGATTTCTTCATGCTGAATGGTTA----TTTG-TAATATCAGATTATGAAACTAACATTTTG 23882
GoodesThornscrubTortoiseAAGGGAGGAAGGGGAGAAAAATAACAATTTAAATAATGCGGA----CTGAATGGGGCCAGTGGCTGGTATCCCAGACTGG 71048

Majority                   CTGGATTCCTAGAATGGGGAAAGATTATGTTTTTTGGTTATAGTAAGCTAATTCTGGGTTGTG-ATTTGTAATTTTGGTG

                                   76890     76900     76910     76920     76930     76940     76950     76960

Human                      GAAGAGGGAAATAAAGCCAGGTGCTGAAGTCTTTATGTAATGGGAGGAGATGTTCCAGTAATCCAATGGCTATTTTGATG 76060
Kakapo                     CTTCATACCAAGTCTAGGGATTAAATCCAGTTCTCAGTCACAGTTATGCACTCCTGCATTAAG-ATGACTACTCCTGGTG 39618
GoldenEagle                TTGGTTTCCTTCAATGAGGAAAGATTATGCTCTTTGCTTAAAAAAAAAGAAATAAAGGTTCTA-GTCTGGGATTTCTGTA 46823
JapaneseQuail              GAAGAAAGAGAGAGGGAGGTAACTTTTCTGCTTTTGCTAATGACCAGTTGCATGAGCTTTGTG-TATCTTAACTTTGCTT 35025
MediumGroundFinch          CTGGTTTCCTTCAAAGGGGAAAGATTATGTTCTTTGGTTAAAATACCCAAATCCTTTGGTGCT-ACCTGTAGTTTCTGT- 23960
GoodesThornscrubTortoiseCAGCAAGCTGAGCGGGGCTGGCAGCTAGGACCCAGGCTGGCAGTAGGCTGAGCAGAGCTGGCA-ACTGGGACCCCAGTTG 71127

Majority                   GCAATGGGTAACATAAAAAAGTTTATCGTT--------------GC----ATTTCTAGTGTTGT--------------GT

                                   76970     76980     76990     77000     77010     77020     77030     77040

Human                      GGAAAGAGTGTGGTATGATTGGGTGGCATTGAC-ATCGGAAGCCATCCTCATTGATGGTGGTGGAACAGCAGTTTGAAAG 76139
Kakapo                     CCTATGGTTTTCACACAGAAGTTCATCATA--------------G-----ATTTTTAGTCTAGC--------------TT 39665
GoldenEagle                GAAAAAGGAAAAAAAAAAAAAATCTGTAGTT--------------GC----ACTTCTAGTGCTAT--------------GG 46871
JapaneseQuail              CATCTGGCAAATAGAAATACTCTGATCTTT--------------GCATTAGTTTCCTCTTTCAT--------------TT 35077
MediumGroundFinch          --G--------AATAAAAAAAATCTATAGCT--------------GC----ACTTCTAGTGATAT--------------GT 23998
GoodesThornscrubTortoiseGCAGGAACCAACGGATGGAACCTCTTAGCCCGCTGCCAGCCGGGGTCCCAGCCACCAGCCCCGCCCAGCCCTCTGCCAGC 71207

Majority                   TAACTGAGTTCTGT---TTATCCTGTCA------TAGCTTTTGTGTTCATCAGAAGCTGTAGGTGGAGATGCTCTGAAAA

                                   77050     77060     77070     77080     77090     77100     77110     77120

Human                      TAACATTGTGCGGTGAGGTAGAGTGGCACATGATGCATCCTTATTCTTACCTTTGGAGAAAAGTTGAGGGAGACCAAAAA 76219
Kakapo                     TAATTTGCCTCTGA---TTAAACTGTCA------TTACTAATGTGCATTTCAGAAGTCATGCAAGAAGATGCACCAAATG 39736
GoldenEagle                TACATGAGTTCTA----ATATCCTGTC--------AGCTTTTGAGTTCATCAGAAGAAGTAGGTGGA-ATGCTCTGAGAA 46938
JapaneseQuail              TGACTGCCTCCTGC---TTGCCTTATT-------TTCTTATCACAGACTTCAGGCTCTACTGCTGAACAACTTCAGAAAA 35147
MediumGroundFinch          TATAGGAGTTCTAT---ATATCCTGTCT------CAGCCTTTGAGTTCATCAGAA---GTTGGTGAA-ATGCTCTGAAAA 24065
GoodesThornscrubTortoiseTGAGTGAATGCAGCC--CCAAGCCGGCAG-----CAGGTTCAGTGGCGGCCAGGACCTGCAGCCTGCCATTAAAAAAAAA 71280
```

```
Majority                   AGAC----TACAGATAATTTTATC-TTCTGACTATTAGCAAATTAATT-----AATAAATGCTT--------TAGGAGG-

                                  77130     77140     77150     77160     77170     77180     77190     77200

Human                      TGACT-TTTTGAGGGAATTGTAGAAGTTTCATTAGAAGAAAAGTAAGTTTTTAATTAAAAAGTTAATCTGAGGAACAGGT 76298
Kakapo                     GGA------ATAAATAATGTGACA-TGCTGTGTTTAAGCTTCCTTGAG-----GAAAAAGACCT--------CAGAAAG- 39795
GoldenEagle                AGAA----TACAGATAAATATATC-CTCTGACTATTAGCAAAATAATT-----AATGCATGCTT--------TGGGAGAA 47000
JapaneseQuail              TGGG----TTCTGTTGGTATTAT--TGCTGCCT--TAGCCAATCAAGT-----AGTTGTCATAT--------TTGGA--- 35203
MediumGroundFinch          AGG-----TACAGATATGTCTGTC-TTCTGACTATTACCAAAATAATC-----AATGCATGCT----------------- 24117
GoodesThornscrubTortoiseAATCAGCTCGCCTGCCACCTTATGGTACCAGTTCCTGGCACATTGGAA-----AAAAATTGCCAGTCCGCCACATCAGAT 71355

Majority                   A-----A-----TT-----T---AGTA-ATGAGAATTCAT--CA----G----ATTATTATTCTTGTTAAGTTTTTATTC

                                  77210     77220     77230     77240     77250     77260     77270     77280

Human                      AGAATAAAAGTGTAGTTGTTAGTGGTAGAAGAGAATGGATTCCATAGGGCAAAATAAGAACTCAAGGGAAGG-TTGGTGG 76377
Kakapo                     ------------CT---------AGGAAAAAGATGTACA--------------ATAGTTGTATGTTTTCA-TTAATACCC 39839
GoldenEagle                ATTTGTAATTTGTTTTTTTTT-TAATTTATCACAATTCATTGCATAA-GTTTCACTCCTACTTCTGTCAAGTTGCTGTTC 47078
JapaneseQuail              --------------------------A------------------------------TTATACTTGTTTG---AATATGG 35224
MediumGroundFinch          -------------------------------------C-------------------CTACTCCTGTCAAATTGTTTTTT 24141
GoodesThornscrubTortoiseAGTTTTAGAAGCACTGAACTA-GATGACCTGAGGTCTCTTCCCACCCTGTTATTCTATGATTCTTGTGCCAGTTTTACCC 71434

Majority                   AGGAAGAGCACCTTTCA--TTTTTCTATATTTAATAGTACTC--AGACATTAAATAAT--AACTTTTATACAAGCTGATT

                                  77290     77300     77310     77320     77330     77340     77350     77360

Human                      AAGAGGAAGAGGATTGAATTGTTTCAAGAAAGAATAGCAGTTGTCATCCTTATGAAAAGTAAAATTTTTATTTTCAAATC 76457
Kakapo                     AGAAAAAGCACA----A--CCATGTTATCCGTAATTGT--TC--AAAGCCAAGATAA---AGCTACTGACCAAGCT-ATG 39905
GoldenEagle                TTTGAGAACACCTTTCA--CTCTTCAAAATTTAATAGTACTC--AGGCATAATCTCAT--GACTTTCATAAAACCTGATT 47152
JapaneseQuail              AAGTACAAGGCTC------TCGCTCTATGCAAAAAAGG------AGAGAAAAAAAAAA--AGGCACAAAATACATTATAT 35290
MediumGroundFinch          GGGGAGAGCACCTTTCA--CTCTTAAAAATGTAATAGCACTC--AGATACTGAATCAT--AACTTTTATACAAGCTGATT 24215
GoodesThornscrubTortoiseAGTAAAAGCAAAAATAAGGTGTTACTTTTATCGATTGGTCTA--AATCATTAATAGGTGCAAATTTCAAGCATATC-ATT 71511

Majority                   TATTATGTTATGATGATTGTT-CAACCCACTTAATGTTTTATAT-A----TT--TTTTATTTGGTGCTTT--CCCATTTT

                                  77370     77380     77390     77400     77410     77420     77430     77440

Human                      AGGAAATGTAAAATGTGCCTTCCAGACCCCTTGGTGGTATACATGGGAGATTGGTTCTAGGACACACACAGTCCCATCCC 76537
Kakapo                     TGATACCATTTGATCACATTCACAAAGAAGCTAACGTGGTAAAGTA----GT--TTCAAATAAGTCCTCAAATCTAGTTG 39979
GoldenEagle                TATTATGCTGCAATCATTGTT-CTTCCCACTTAATGTATTTTCTCA----CTTTTTTTCTTTCATCATTT--CCCATTTT 47225
JapaneseQuail              GAATATGAAAGGAGGA-------AACAAAAGGAACTTGAAAG------------TCTGATTGGGTGGTTAT-GCAGTTTA 35350
MediumGroundFinch          TATTATGTTGTGATCATTGTT-CTTCCCGCTTAGTCTTTTTT---------T--CTTTCTTGGATAATTT--CCCATTTT 24281
GoodesThornscrubTortoiseCTTTGT-TTATAAAGATCATTAAAAGAGAGTCACCTTTTTAGAAAAAAACTTGTTTTTATTTGGAGTGTTCTCCTATAGA 71590
```

```
Majority                   TGTTCTTATTAAT---ACTCACGAACAGAGTAGTTTTAGTATTTTATTTTTGAGTACTTTGGGCCTACTTGAATTAAGTT

                                    77450     77460     77470     77480     77490     77500     77510     77520

Human                      CCACCCTCTGACCCC-ATACACCCCCTGGATACTCAAATCCACTGATGCTCAAGTTCCTTGCATAAAATGGTATAGTGTT 76616
Kakapo                     CGTGCTAACTAGT---AATCATAAACCAAGTTGTTCTTGTATTTATTTATTAACTATTTTTGACTTGCTTAAATCAGAAT 40056
GoldenEagle                TCTTCTAAAGAAA---ACTTAAGAACAACATAGATTTCATGCCTTGTTTTTGTACAAGATGGGCCAACTTGGAGTAAGTC 47302
JapaneseQuail              TGGCTGTATTTGT---ATTCAGATACAGAGAAAGGTTAGAACAGC---TTTAAGTTCAGAGAGCTG--TAAGATGAAGGT 35422
MediumGroundFinch          TGTTCTGAAGAAA---ACATAAGAACAACATAGATTTAATGGTTT---TTTGTACAGTCTGGGCCGAACTGAAGTAAGTT 24355
GoodesThornscrubTortoise   TTTATTCATTTATCCTACTCCCTGTTTGAGTTGTAATTCTATTTTATTCCCGAGAAACCTAGATCTTTTTAAATTGAAAA 71670

Majority                   TGAGTATCTCTGATAAAGAGG----GGCGTGTGCTCTCTGT---TTATGAAATGTATGAGTTACTTAGTGAATGATGT--

                                    77530     77540     77550     77560     77570     77580     77590     77600

Human                      TGCATGTGACCTATACACAACCTCTTATGTGTACTTTAAATCATCTCTAGATTACTTATATTACCCAGTACAATATAAAT 76696
Kakapo                     GGCAAATCAGAGAACAAGAAC----GGAGTTTGCTGTAGGT---TTGTAATTTGTCTGACCTTTCCAAGAAATGAGGTCT 40129
GoldenEagle                AGAGTTAATCTAGTAAAGAGG----GGCCTCTGCTCTCTGTGTTTTACAGAATTTAGAAGGCACTTAGTGAATGATGT-- 47376
JapaneseQuail              TGGGAATCTATGA-----AGT----GAAGTATGGCAG--A------ATGATAGCAGTGACCTTCC-------TCCTGT-- 35476
MediumGroundFinch          TGAGTTACTCTACGAAAGAGG----GGCCTCTGCTCTCTGT----TATGAAAGGTAGAAGGCACTTAGTGAATGATGT-- 24425
GoodesThornscrubTortoise   AAAGAATTAATCATTGAGAGGCT--ATGATGTGCTATGCATGTCCCCTGAGACACATGCTTTAATTGTTTCCATCTGAGG 71748

Majority                   --GTTCTAAAGTATCGGTTAAATATTTTATTTAT-GGCTACTGAATG-ATGCAT-TGTCTC-AC-----------A----

                                    77610     77620     77630     77640     77650     77660     77670     77680

Human                      GTTATGTAAATGGTTGTTATAGTGTATTGTTTAGGGAATAATGACAAGA-ACAACTTTCTATACATTTGCAGTACACCAT 76775
Kakapo                     CTGTGCTATAGGATCAGTTAGAAACGTTATCCAT-GGTCCTAGCCCACATGCAT-CGTTAC------------------- 40188
GoldenEagle                --GAATCCAGGAAAAGGGTGAATATTGTAGTTAA-GGCTACTGAATG-ATGCGC-TATATCTACGCTTTTCCAAAATCAG 47451
JapaneseQuail              ---TGCTTATTTATCTT-CATATTTTATATTTTT-ATTTGCTTTAGA-----AT-TGTC--------------------- 35524
MediumGroundFinch          --GAACCCAGGAAGAGG-TAAATATTGTAGCTAG-GGCTACCGAATG-TTGCAC-TGTA--------------------- 24478
GoodesThornscrubTortoise   C-GTTCTAAACTTTGTAATATAAGCTTTGCTTGTCAGCTCTTGAAGGTGCATATGTGAGGACACACGACTTAAGAATGTG 71827

Majority                   -----TT-----A-A--TT--------A----T---T------------------CA---------------------

                                    77690     77700     77710     77720     77730     77740     77750     77760

Human                      TGTTTTACCCCCAAATATTTTTGATCCAAGGTTGGTTGAATCGGAACCCAGAGATACAGAGGGCTGACTATACTTTAAGA 76855
Kakapo                     -------------------------------------------------------------------------------- 40188
GoldenEagle                ATTACTTGAAGGATACATTTCATATCTAGCTGTAAATTCCACACATGCCCACCTCCCACAAATTTTGGAGTGCTTGACTG 47531
JapaneseQuail              -------------------------------------------------------------------------------- 35524
MediumGroundFinch          ------T------------------------------------------------------------------------- 24479
GoodesThornscrubTortoise   AACCTTTAGAGCAGAGGTTCTCAGGACAAATTTTTTTGGTGGCTTCAGAGTGCGGCCACCAACTCTTGCTGATGGCCACT 71907
```

```
Majority                 ---A---T------T-----G--------------A-----A---AC--------TT------------A----------
                                   77770     77780     77790     77800     77810     77820     77830     77840
Human                    ATTAGAATTAGCTGGGTGTGGTGGTGGGTGCCTGTAGTCCCAGCTACTCGGGAGGCTGACGCAGGAGAAAGGCGTGAACC 76935
Kakapo                   -------------------------------------------------------T------------------------ 40189
GoldenEagle              CACAACCTCGAGTATGACTTGCAAAATGGTGTATCACTTGTAGCTACTGCAGACGTTTTGGAAAGTAAAAAATACCGTAA 47611
JapaneseQuail            -------------------------------------------------------------------------------- 35524
MediumGroundFinch        --------------T----------------------------------------------------------------- 24480
GoodesThornscrubTortoise CAGACAATTTTTCCTAAAATGCTTTAGAAAAACAAATAAATATGCACATATACATGTCCAAATCAGTGTAATTTATTCAT 71987

Majority                 ---------G--------G-------------T---------C--T----T---------------A---T---------
                                   77850     77860     77870     77880     77890     77900     77910     77920
Human                    CGGGAGGTGGAGCTTGCAGTGAGCCGAGATCGTGCCACTGCACTCCAGCCTGGGCGACAGAGCGAGACTCTGTCTCTAAA 77015
Kakapo                   -------------------------------------------------------------------------------- 40189
GoldenEagle              GAATCTAAAGTCTCTGAAGAACCTGTGGCTAATGTTTGTTTTCTTTTTTTTCTCTTTGAACTTCCCATCATTTAAAAAAT 47691
JapaneseQuail            -------------------------------------------------------------------------------- 35524
MediumGroundFinch        ---------------------------------------------T---------------------------------- 24481
GoodesThornscrubTortoise TGCTAGCTAGTTAAGTCTGTTGTGAAAAGCCATATTAACAAACATACAAGTATCACTTTTCACAGCAGCCTTACTTAGCC 72067

Majority                 -------------------A--------------G---------------A-----------------------------
                                   77930     77940     77950     77960     77970     77980     77990     78000
Human                    AAAAAAAAAAAAAAAATTAGAAGGGGCTGTCAGGCGTGGTGTCTCATGCCTATAATCCCAGTCATGCCTGTAATCCCAGCA 77095
Kakapo                   -------------------------------------------------------------------------------- 40189
GoldenEagle              GAAACCTCTCATTTCATGGATTGATAGGGAAGGTGGATGAGTGTTTGTGAAGAAGTTTGACACCATAGAGATGAGTACTG 47771
JapaneseQuail            -------------------------------------------------------------------------------- 35524
MediumGroundFinch        -------------------------------------------------------------------------------- 24481
GoodesThornscrubTortoise CTGTCAAGCCTGGGGAGAAATTAAACTCTGGACTGGGGGTGAGGGGAATGAGGGAGAATTGATGGGGGCAGGAGAAGTAG 72147

Majority                 -------------------G------A-----------G---------------------A-------A--C--------
                                   78010     78020     78030     78040     78050     78060     78070     78080
Human                    CTTTGGGAGGCTAAGGTGGGCATATCACTTGAGATCATGAGTTCAAAACCAGCCTGGCCAACATGGTGAAACACTGTCTC 77175
Kakapo                   -------------------------------------------------------------------------------- 40189
GoldenEagle              TAGAAATGCCCTTGAGGCAGTTAGTAATTCTTTTGTCAGCACGTAATGACTAGAACATAGACATGGGGATGCCCTATGAG 47851
JapaneseQuail            -------------------------------------------------------------------------------- 35524
MediumGroundFinch        -------------------------------------------------------------------------------- 24481
GoodesThornscrubTortoise CAGGGGGCTGGAGCCTGTAGCCCCGCACCCAGAGCTCAGTGGCTGGTGGATGGAGCCTAAAGCCCTGCAGCCAGAGCCTG 72227
```

```
Majority                 ---T-------C-----------A------------------------C--------T--G-------------G-----

                                78090     78100     78110     78120     78130     78140     78150     78160

Human                    TACTAAAAATACAAATATTAGCCAGGTGTGGTGGTGGGCGCCTGTAATCTTAGCTACTCAGGAGGCTGAGTCAGGAGAAT 77255
Kakapo                   -------------------------------------------------------------------------------- 40189
GoldenEagle              TAGTAAGGAGGCAAAAACAAAGTAAAGAAGCTGCTTATTAATAAAAGACCTATCATATTAGTTGTTGTAAGGCAGGGATT 47931
JapaneseQuail            -------------------------------------------------------------------------------- 35524
MediumGroundFinch        -------------------------------------------------------------------------------- 24481
GoodesThornscrubTortoise CTGTCCCAGTACCCCAGGGCTGAAGCCAATGCCTGAGCCATGCAACCCCTGGGAAGGTGGGGAATTCACCTACTGCCTGC 72307

Majority                 T-----A------------------------A--------------------------------------------GACA

                                78170     78180     78190     78200     78210     78220     78230     78240

Human                    TGCTTGAACCCAGGAGGTGGAGGTTGCAGTGAGCTGAGATCATGCCATTGCACTCCAGCCTGTGTGACAGAGTGAAACTC 77335
Kakapo                   ------------------------------------------------------------------------------CA 40191
GoldenEagle              TAGAAGAGAGTGAGTTGAAGATTCATGATTTATAAAACGGTGCCAAATGCAGGATTACTTATGGAACTCTAATTCTGACA 48011
JapaneseQuail            -------------------------------------------------------------------------------- 35524
MediumGroundFinch        ----------------------------------------------------------------------------GACA 24485
GoodesThornscrubTortoise TCCTCCAGTGTTGTGCCCCAGCAGTCTTCAGAGGGTGGAGCAGGGCCCTGCTGGCAGCCCCAGCAACCAACACCAAGGCA 72387

Majority                 TTTTTCTGAAAC---------------------T---G---AC-A---C--------------------------A-G--

                                78250     78260     78270     78280     78290     78300     78310     78320

Human                    CATCTCAAAAATACAAAAAGAATTAGAAAGGGCTTTAGAGTCCCACCACTAATGCTCTTCTGTTCAGTAGTACACAAGAC 77415
Kakapo                   TTCGTCTACTAC-----------------------------A-------------------------------------- 40204
GoldenEagle              TTTTCCTGAATCTTGAGAAGTTGCTTTCCAGTCTCAGGCTTACTAATGCATTTCTTTGTATATAATGCTTATAGTATGCA 48091
JapaneseQuail            -TTCTCTAAT---------------------------------------------------------------------- 35533
MediumGroundFinch        ATTTTCTGAA-------------------------------A-------------------------------------- 24496
GoodesThornscrubTortoise TTCTGGAGCAACAGGGAGGAGCAGAGGCTGCTATTTTGTCCCCCATCCCATTACAGCCCAGGAGGCTGTGGCTGCAAGAA 72467

Majority                 ------------G-T----TTGTC---------------------AGG-------------T-C------T--------T

                                78330     78340     78350     78360     78370     78380     78390     78400

Human                    TAATAACTACGAGATGTTATGAACAGGGAAAAGAAGTAAGACCAAAAGAAATCATAA--ATTACTTTGGATGGAACTTTT 77493
Kakapo                   -------------------TCCTC---------------------AGC-------------------------------- 40212
GoldenEagle              TCAAATTAACAGGTTTAATTTGTCTGGCATAGTGGAATTACACTGAGGATGCTTGGATCATTGCATCACTTTTGATATGT 48171
JapaneseQuail            ---------------------GTC-------------------------------------------------------- 35536
MediumGroundFinch        -------------------TTGTC---------------------AGG-------------------------------- 24504
GoodesThornscrubTortoise AAGCCCCTGGTGGCTGCATGTGGCCCCAGTGGACACATTTGAGAAACGGTGCTTTAGAGGATACAGCCTTTGGAGAACTT 72547
```

```
Majority                 --A-T----G--------A----T-------T-TTC----T-A------------T--A----A------A---------
                                  78410     78420     78430     78440     78450     78460     78470     78480
Human                    AGAATGGCAGATGGAGGGATAAATTTGACATTGTTCAAAATCATAGAGTAAACTCTCAAAAGTAAGGGGAACCTAAGGAG 77573
Kakapo                   -------------------------------------------------------------------------------- 40212
GoldenEagle              TTAATAATTGGGGTTACTATACTTGTTATAGTTTTCCAAGTGACTTTAGCTTTAGTGGACTTTATGTTCTAGTATTTTAG 48251
JapaneseQuail            -------------------------------------------------------------------------------- 35536
MediumGroundFinch        -------------------------------------------------------------------------------- 24504
GoodesThornscrubTortoise CCACTTCAGGTCCTGGTAAGCTGTGCTTTTGTATTCTGCTTTACTATGCTAATTGTTAAGAACATTATAAAAATGCAGTC 72627

Majority                 ----G----T---------------TA------------A------A--------G-----T-A------G--------
                                  78490     78500     78510     78520     78530     78540     78550     78560
Human                    ACAAGATGATTAAATGTAATAGGAGATACTGGAACAACAAAAAAGGACATTAGATGCAATTTGA-----GGAAATCTGA 77648
Kakapo                   -------------------------------------------------------------------------------- 40212
GoldenEagle              GCCTGAAAGTCACTTCGACGCTTTTGTAATTTTTGTCTACAA----AACTACGTAAGTTTTCTAACCTTTTGATTTTAAT 48327
JapaneseQuail            -------------------------------------------------------------------------------- 35536
MediumGroundFinch        -------------------------------------------------------------------------------- 24504
GoodesThornscrubTortoise TTCTGTTGTTTTCAATATAGTAGTGGTACATAGAAGCCCCAGTCAGGATTAAACCTGTATATTTAAGGTTGGTTAAAGCT 72707

Majority                 ---------T-------------A--T----T------T-----------------G--A-A-----------A---
                                  78570     78580     78590     78600     78610     78620     78630     78640
Human                    TAATGGACTTTTGTTGATTTATCGATATTGGTTGATTAATTGCAAGAACTAATAAATTGTAATACCATACTAATGTATGA 77728
Kakapo                   -------------------------------------------------------------------------------- 40212
GoldenEagle              CAGGCTCATTCTTTTGTGTATATTAATTTTTTTGCTAGTAAAATTATGAAGATGACAGTCATATTAGCTGATTAAATAT 48407
JapaneseQuail            -------------------------------------------------------------------------------- 35536
MediumGroundFinch        -------------------------------------------------------------------------------- 24504
GoodesThornscrubTortoise TTGTGGAAATACAGAAACATTTCTAATTAGAATCATGGTTATATATCTGTTCAAAGAGGGAAAAAGGCTCTTAAGTAGTG 72787

Majority                 ---------T-----------G-----------ATATATTGA--TT---A------------T-AAAGGTACAT----GA
                                  78650     78660     78670     78680     78690     78700     78710     78720
Human                    TGCTAATAATGGGGGAACTGCGGTTTCCAGGTTATATAAGAACTCTGTAA--------TGTCTTAAATTTGTTT---GTA 77797
Kakapo                   ----------------------------------TGTGATAA----------------------AATAGTGTCA----GA 40232
GoldenEagle              TTTAATTCTTACTCTCTGGGTGGACCTCTTTAAATGCAGTGTTTTTCCAAGCAGCAGGAAAATAAAAGGTACAT----GA 48483
JapaneseQuail            ----------------------------------AATAGTGA--T-------------------AAAGGTAG-------- 35553
MediumGroundFinch        ---------------------------------ATACATTTG----------------------AAGGATAC-------- 24521
GoodesThornscrubTortoise CTGAGGGATTTTACTTAGTAAGAATATATAGATGAATATTGGTAGTTTCAATTTGCTTCTGTTTATTCTTCCATTGAAGG 72867
```

```
Majority                   AAT----A--TT-------A-CT----A----A---A-A-C----------------------A----------T-----

                                    78730     78740     78750     78760     78770     78780     78790     78800

Human                      AATCTAAACCTTTTAAAATAGCTAAAAATTATAGATACAATTTCTAAATGATTTAATTAACAAAACCAGTTTAATTCTTC 77877
Kakapo                     AAT----------------------------C------------------------------------------------ 40236
GoldenEagle                CATGAGTAACTTTCATTGCAGCTGTGGAACGTTTTCAGAGCATCAGTGAAGTCAGTCATGTTTATTTTGATGAGTAGTAG 48563
JapaneseQuail              ----------------------------------------C--------------------------------------- 35554
MediumGroundFinch          -AT----------------------------------------------------------------------------- 24523
GoodesThornscrubTortoiseTACTGATATTTTAAGTATTACCTCAGCAACAAATTGATAACACAACACAGCATTTTGACATGAAGCGATTGATATGCAGT 72947

Majority                   A----------------T-----------TG--------T----T----------A-A--------------T--AT-

                                    78810     78820     78830     78840     78850     78860     78870     78880

Human                      AGTTGAAATGAGCAAGGTTAGGGATACCTGTGTGTTTCAGTGTAATATCTGAAAGGAAAAGAATAGATACATAATTTATA 77957
Kakapo                     -------------------------------------------------------------------------------- 40236
GoldenEagle                AGATTATGTATCATGGTTGTTTTTTTCTGCTGTAAAAGCATTCATTGTTTTGAAAGAGATTTAAGGGAGCTGGGTTCATC 48643
JapaneseQuail              -------------------------------------------------------------------------------- 35554
MediumGroundFinch          -------------------------------------------------------------------------------- 24523
GoodesThornscrubTortoiseAATAGGTGATACAAAACTAGAAAAGATAAATGCAGCTACATATTATTGTATATGTTATAAATTGCATACATGGGTGTATA 73027

Majority                   ---------TTCGGGGTTAGT-------------T-----ATTGGG--T---TATCTTTTG---AATGA----------A

                                    78890     78900     78910     78920     78930     78940     78950     78960

Human                      TCACTTCTCTCTGGACTTAGGGTCTCTTGTCATATACATGACTGGGCATCCATCAGCTTCAGTGGAGGAAAGGTTAGGAA 78037
Kakapo                     ---------TTCGAGACTGAG-------------A-----ATTGGA------CATCTTTTC---AATG------------ 40268
GoldenEagle                TTCTAACTGCTCATGGCCAGTT---GGGTGAGCTTGGTATATTGTAACTCTGCATCATCTGTCAAATGAGGATGATGACA 48720
JapaneseQuail              ---------TTCAGAGTGTGC--------------------TTAG----------------------------------- 35570
MediumGroundFinch          ---------TTCATGTCTAGT-------------------GTTGT------------------AA-------------- 24542
GoodesThornscrubTortoiseCGAATATGTTTTGGTATGTGTTAAAGAGTTTCCAGCATAAATTAGGAATGGCTGCCTTGTG-CAAATGACAAATCTATAA 73106

Majority                   ------T--------T--------------------T--------AGTC----T-------T-----------------

                                    78970     78980     78990     79000     79010     79020     79030     79040

Human                      TAATAGTGTCAGAACTAACATGAGAATTTTAGGAGATTACTGAGTCACTCAAAATGTCTGGGTGACCCAGCTGATGAGGG 78117
Kakapo                     ---------------------------------------------AGTC------------------------------ 40272
GoldenEagle                CAGTGATTTTTTTGTTGTTGTTGTTTGGGTTTTGGGGTTTTTTACTAGTTGCCTTTTCTGTTTGTTTTTACAGAATTTGA 48800
JapaneseQuail              ----------------------------------------------GTA------------------------------ 35573
MediumGroundFinch          ---------------------------------------------AGTG------------------------------ 24546
GoodesThornscrubTortoiseCGTGAGTTATGAAGTTCATCCCAGAAGAAAGACGTTGTGTCGTCTGCACAGGGGTACAAAAATAATATTTTAAGACTGTT 73186
```

```
Majority                 -------A--T-A-------T---------C----------T----------T-------T----AG-T-T---------

                                  79050     79060     79070     79080     79090     79100     79110     79120

Human                    CCATTTCACATGAAAAAGATTCCTGCAACACCTT---CTTTTCTTTAGCACATGATCGTTTATGAAGCTCTTTTACATTG 78194
Kakapo                   -------------------------------------------------------------------------------- 40272
GoldenEagle              GCTCTACAGCTGAAAAACTTTATAAAAAGGCTCCATAGTACTGTTGGTGCCTTAGCCATTTGGATAGTTGTCAAAATGGG 48880
JapaneseQuail            -------------------------------------------------------------------------------- 35573
MediumGroundFinch        -------------------------------------------------------------------------------- 24546
GoodesThornscrubTortoise TTAAAAAATATTATTGGGACTTTTTTTTATCCCTTTACCTGTAAAATATCAGTTACTACGTTTCTAGCTGTAGAGGGCTC 73266

Majority                 ---------T-T---AA--------A-------------T------T---T-CT----A-----ACTTAAAATATC-GA-

                                  79130     79140     79150     79160     79170     79180     79190     79200

Human                    TTTTATCTTTATAGCAATCCTCAGAATAGATCAAGCTTTTTTTTTTTTTTTTTTTGAGATGGAGTCTTGCTCTGTCGCCC 78274
Kakapo                   ----------------------------------------------------------------TTTTAAAA-----GA- 40282
GoldenEagle              AATGGTGCTTGTTTGAATATTCAAGAACAGGGTTCTCATTCTGAGCTGCTTACTGTGCAAAAACACACAAAATATGTGAC 48960
JapaneseQuail            ---------------------------------------------------------------------GATTATC---- 35580
MediumGroundFinch        ----------------------------------------------------C-----------ACACAAA--------- 24554
GoodesThornscrubTortoise AGAGGGAGCTATTAAAAAACCTCTAAAAATGGCTTGAAATAGCTTTTCCATGCTTAAAAATGTTACTTCTAGTCCTCAAA 73346

Majority                 -------A-------------A-----------------CT-------------TG-----A---T---G---C----T-

                                  79210     79220     79230     79240     79250     79260     79270     79280

Human                    AGGCTGGAGTGCAATGGTGCGATCTCGGCTCACTGCAAACTCGGCCTCCCGGGTTCGTGCCATTCTTCTGCCTCAGCCTC 78354
Kakapo                   -------------------------------------------------------------------------------- 40282
GoldenEagle              ATAAATAAGAAAGGACAAAGGATAGAAAACCTCAAAAGCCTAAATGGATGCTTGTGCATGCAATTTATAGCTGCATTTTA 49040
JapaneseQuail            -------------------------------------------------------------------------------- 35580
MediumGroundFinch        -------------------------------------------------------G------------------------ 24555
GoodesThornscrubTortoise CGTCCTGAAATAGTAAGTGTTAAGTCTGATTGGGAAGCTCT----GCTTCCCAGTGGTACAAAGCTGCTGTTTCTCGCTC 73422

Majority                 -----------C-------------T-----CA-----A----------------G----G-------------------

                                  79290     79300     79310     79320     79330     79340     79350     79360

Human                    CCGAGTAGCTGCTACTACAGGTGCCTGCCACCACGCCCAGCTAATTTTTTTGTATGTTTAGTAGAGATGGGGTTTCATCG 78434
Kakapo                   -------------------------------------------------------G------------------------ 40283
GoldenEagle              ATTCAGATACTCAAAAGGAGAAGGATGAGAACACTTAAAAGTTCTGAGAGTCATAAGGTCGAGTTTGGCAAACTATGATG 49120
JapaneseQuail            -------------------------------------------------------------------------------- 35580
MediumGroundFinch        -------------------------------------------------------------------------------- 24555
GoodesThornscrubTortoise CTCCAGTGATTCTTTAGTGTCATGGTATTTTCATAGATAAAAATGGCCTACGTTTAAACTGTGCTAGGCTTCACGTGGAA 73502
```

```
Majority                   --------------G------------G--C----------T------T-T------------T----------A----

                                   79370     79380     79390     79400     79410     79420     79430     79440

Human                      TGTTAGCCAGGATGGTCTCGATCTCCTGACCTTGTGATAGATCAAGTATTTTTATCCCTCTCATCAACATGTGTAAACAG 78514
Kakapo                     -------------------------------------------------------------------------------- 40283
GoldenEagle                TGTGAAATATGGCAGAATGATAGGAGCGACCTTCCTTCTGTTTGTTTCTTTCATCTTAATATTTTATTTCCTTTTATTTG 49200
JapaneseQuail              -------------------------------------------------------------------------------- 35580
MediumGroundFinch          -------------------------------------------------------------------------------- 24555
GoodesThornscrubTortoise   AAAGTTGTGCAGTGGGCACATGGTATTGTGCCCCTTTGT-CTTGTGCATCTTTCCTGAATTTTTACTATGCAGCTAATGT 73581

Majority                   -T-T-----------T---------T---T---GA--------------------A------GCTATGCCCTGTTCAGGT

                                   79450     79460     79470     79480     79490     79500     79510     79520

Human                      TTTTTTCCTCACATATCTGCTGCCTTAGCTAAAGATGCAAATCATTAAAGTTAAAATTATAATTTATGTAGCTAAAAGTA 78594
Kakapo                     --------------------------------------------------------------GGAGAAACTAGAACGGGC 40301
GoldenEagle                CTTTAGAATTCTTATTTCTGTAATGTCGATAAAGATGTCTTCAGAGTATACTCAGAGTGTCTGCCATGCCCTGTTCACAA 49280
JapaneseQuail              ---T----------------------------------------------------------GCCATTCGCTGTTCATGG 35599
MediumGroundFinch          --------------------------------------------------------------GCTGTGCCTTATTCACGT 24573
GoodesThornscrubTortoise   ATGCATATGTGACCTTCTTTAACTATACTTTTTGAGCTTACCGAAAACAGACACGAAAAATGGGTATGTACAATATAAAT 73661

Majority                   ATGGTTTCCAGAAGGTTTGTTTCTCC---ATGAAATAGT----AAAACTTTATTTAGAG---CTTAA----TGTGTTGTT

                                   79530     79540     79550     79560     79570     79580     79590     79600

Human                      ATCTGTTAGAGCCAGTGTATACAATATGTGTAATATGCTTTCTATAACTTTCTCAAAGGGCCCTTGGTAAGTTTATTGTT 78674
Kakapo                     -TGGTTTCCAAAAAGTTAGGAAATC-----TGACAGAAA----AAACCTGTGAATAAAC---AGCAAC---TGTATTATG 40365
GoldenEagle                ATGGTTCCCAAAGGGCATGATCCTCC---ATGAAAGAGT----CAAGCTCCATTTTGAG---CTTAA----TGTGATGCT 49346
JapaneseQuail              ATGGCTCCCAAAAGGTGTGCTTCACC---ATGAAATAGT----GGAGCTTCATTTTGAG---CTTGA----TGTGATGCT 35665
MediumGroundFinch          GTGATTCTCAGAGGGCATATTTCCCC---ATGAAAGAGT----GGAACTCCATTTGGAG---TTTAG----TGTGCTGTT 24639
GoodesThornscrubTortoise   ATCTATTTCTGCATATTTTTTTGTCTGG-ATTAAATTTTTTC-AAAATGTTTCCTAGGG---AACAAGTTTTATACATTG 73736

Majority                   CTTCAAGTTCTGTTGCGTGCA-AAAAAGAATACCA-----TAAGATGGACCTGTGGTATTCTTGTTAAAACTCTTGCTAT

                                   79610     79620     79630     79640     79650     79660     79670     79680

Human                      TTCTCA-TTTAGCTGGGAGTT-AAGAACAGAACTATTTTCTAGAATGTATTAATTATATAAATAGTGCATCATGAAATAT 78752
Kakapo                     CTGAA----CTGCAACAAACA-AAAAAGATTATCG-----TAAGAATGGCTCAAGGTTTAACTACTAATATGTAATAAT 40435
GoldenEagle                CTTCAAGTTCTTTTGCGTGCACAAAAAGAATACCA-----TAAGTTGAACCTGTGGCATTCTTGTTAAAATTCTTGCTGT 49421
JapaneseQuail              CTTCAAGTTCTGTTACGTTTG---CAGCGATGCTA-----T--GTTGCACCTCTGGCATTCCTGTTAAAACTCCTGCTTT 35735
MediumGroundFinch          CTTCAGGTTCTTTTGCATGCTCAGAAAGAATACCA-----TGAGATGGACATGTGACATTGCTGTAAAAATTCTTGCTGT 24714
GoodesThornscrubTortoise   CTCTATTTCCTCTTATTTACATCACAAAAATCTGAAATACTAATATAATTCAGAAGTATACTTAAATCAACTACTGCTAT 73816
```

```
Majority                   A------GCTATTTTTTATATGTATATCATGAGATTGCTAACA-GAAAATGTGTTAAGG-ACTCAGTTCTGACATTGGC
                                    79690     79700     79710     79720     79730     79740     79750     79760
Human                      TT---TGTAGGTTTTATATTTTTATTGCAGCAGACAGC-ATTTAGATTAAACCTAAAAATACCTGATTGACAAAGTAGT 78828
Kakapo                     A------CTTAGATATCTAAATTGGAGTTA-GATATTAC-AGCT-GGAAATAGGTTGCTGTGGTTAGCGCTCCCATTGGT 40506
GoldenEagle                A------GCTGTTCTCTTATATGTATATAATGAGATCACCAACA-GAAAATGTGGTAAGG-ACTCGGTTCTGACTTTCGC 49493
JapaneseQuail              A------GCTATTTTAGTATAT---------GAGATCTCTAACA-GAAAATGTGGTAAGA-CTTCAGTTTTGATCTTGTC 35798
MediumGroundFinch          A------GCAAT-CTTTTATACGTATATCATGAGATTGCCAACA-GAAAATGCACTAAGG-ACTCACTTCTTCCTTTTGC 24785
GoodesThornscrubTortoise   ATAGTTAGCAAGGAAAGTAAATGTAT-CATTTTTATGGCTGTCACAAAAATGTTCTGGCTCTCAGAATACAGGGAGTGGG 73895

Majority                   TTATGTTATACTGTTATATGCTGGATAGTTGTATAA--TTTGGGTGC--CTTTTTGTTAGTGGCTTTTTAGGAGTATACT
                                    79770     79780     79790     79800     79810     79820     79830     79840
Human                      TTTTCAGACAAAAGTATATTGAGGGAAATTGGAGAATCTTCAGAGGCTTTAAAAGTCCAGGAAAATCTTAGACTACTCCA 78908
Kakapo                     TTATCCCTTGCTGTCAT-TGCTG---GCTTGCTT----TCTGGGTGC---TTTCCACTAATGCCCCTTCTTAAG---ATT 40572
GoldenEagle                TTCTGTTATGCTGTTATATGCTGGATAGTTGTACAA--CTTGGGTGC--CTTTTTGTTGTTGGCTTTTTAACAGTATACT 49569
JapaneseQuail              TTCTGTTACATTGTGCT--GTTATATAGTGGGTTG---TTTTGTAGC----TT-TGGTACTTTTTCCTAAAGTGTATGAA 35868
MediumGroundFinch          TTATGTTACACTTTTATATGCTGCGAACTTGTACAAAACTTGGGTGCAACGTTTTGTTGCTGGCTTTCTAGGAGTATACT 24865
GoodesThornscrubTortoise   TAAAAACATTTAGAAACAAATTACATATTTGAAGC---CCCTAGTTCTCCTCCCTATCAGCCCCTCCACTCCTTGGAACT 73972

Majority                   GTAATTCATATGTCAGAAGTGA-----TATTTAGGTTTATCATAATTTTGT---------GAGTTGAGTTTTAG------
                                    79850     79860     79870     79880     79890     79900     79910     79920
Human                      GTGATTTAGAACGGTGTAAAGATAGCCTAAGTAGTTTTTCT--AAGTTATCAGCCGCGGGGAGGAAATAATGAAAGAGAA 78986
Kakapo                     CTTATTAAATTATC-------------TATTTCAAAGAATAACAATTGTGT---------GAATGGTGTTGAAT------ 40624
GoldenEagle                GTAATTTACATGTCAGAAGTGA-----CATTTAGGTCTAACATAATTTTGT---------GAGTTGAGTTTTAG------ 49629
JapaneseQuail              GTAATTCATATGTCAGAAGTGA-----GGCTTAGGTTGATT---AGTTTAT---------GAGTAGAGGTTTAG------ 35925
MediumGroundFinch          GTAATCCATATGTCAAAAGTGA-----GATTTAGGTTCAACACAATTTTGT---------GGCTTGTGTTTTAG------ 24925
GoodesThornscrubTortoise   ATATTACAAATATCAAATTTTATGACCAACTTACTTCAGTCTTGAATTAGTTGTACAATAGAATGCACATTTAAATTGCC 74052

Majority                   TGACAGGATGTGAGT-TTTGTTATTCTTGGTTTTTAAATGTGCCT-TAATTGCATACCAAT-------------------
                                    79930     79940     79950     79960     79970     79980     79990     80000
Human                      ATACATACGAGTGGCATTAAGGACAGGTGGTGGCTTCTTTTACCTATTAAAGCATTCTTACACCATACTGTGAACTGTGT 79066
Kakapo                     TAATCTT-TGTCAGC-TTTCTTCTGCTCAGCTGTGGAAAGAGCCA-TAAAGACTTCACTAG------------------- 40682
GoldenEagle                TGACAAGATGTGAGT-CTTGTTAGTCTTGGTGTTTAAATCTGCCT-TAATACCAAACCATT------------------- 49688
JapaneseQuail              TGACAGGGTGAGACT-CTTAGTCTTTCTATATCATATATATATATAAACACACACACACACACACACACACACACACATA 36004
MediumGroundFinch          TGACAGGATGTAAAT-CTTGTTAGTCTTGGTTCTTAAATCTGCAC-TAATTCCAAACCAA-------------------- 24983
GoodesThornscrubTortoise   CAATAGGATAAATGTATTTTGTTCTCTAAATGTTGATGTGCATCTGTTGTTGTTTGCCAATTATGAAGTAAGGGATTGGA 74132
```

```
Majority                   ----ACATATCTCAGTTTACTTC-TGGATATAGTTAA----TGTTCCATTT-CAGTAAATGTAAATTTATGATTTAGTGT
                                   80010     80020     80030     80040     80050     80060     80070     80080
Human                      AAAAGGAAGGATCATTGGACATAATAGATTATCTGACAGGGTGGTCCGGAA-TGGTGGAAGTAAATCGACAGGTGAGGAC 79145
Kakapo                     ----TCATCTTCAGATTCATTTA-TTCCTATAAATGC----TGGACTGGCT-GAAAGCTTCTAAAGTTAGGGAAAGGTGC 40752
GoldenEagle                ----ACATATCTCAGTTTCCTTC-TGGATGGAATTAA----CCCTTCTCTT-CAGTAAATGTAACTTTATGATGTAGTGT 49758
JapaneseQuail              T---ATATATATATATATATATA-TATATATATAAAA----TGTTATATTT-CAGTTTCCTTCTGCTTGAAATTAACCCT 36075
MediumGroundFinch          ----ACGTATCTCAGTTTCCTTC-TGGGTGAAGTGAA----TGCTCCACTT-CAGTAAATGTAACTTGATGATGTA---- 25049
GoodesThornscrubTortoiseCAGGGAATGTCTCAGAGGAGACC-AGGATAGTGTAAAAACGTGTGTAATTTGCAGAAAAGACCAAATTAGTCATTTCT-T 74210

Majority                   ATTCACTCA--ATTGTAACTTTGTAAGC----TGTTGTGCAGTGCTTTTAAATGT-AAACTTATA---CCTCCCTCAAAG
                                   80090     80100     80110     80120     80130     80140     80150     80160
Human                      ACTGAATTAGCATTCAGACAGTGGGGACCTCACACCTTGTATTCCCTTTTTTGGTAAGATTTTTATTACCTCCTTAGAAA 79225
Kakapo                     ATACACACA---CTGGAAGATTGGAAG-----TGCTCTGCTATTTTGTCATTT-----TCTAATA---CCTAAATGATTG 40816
GoldenEagle                AGTAACAC----TTCCAGCTTCATAAGC----TGTTATGCAGAGATTTTAAAAG--AACCTGCTA---TCTCCCCCAAAC 49825
JapaneseQuail              CTTCATTAGTGACTGTTACTTTATGATG----TGGTGTAGTAAAACCTAAAATGTGAAACTTATAG-GCCCTCCTAAACG 36150
MediumGroundFinch          ----ACTC----CTGTAGCTTTGTAAGC----TGTTAAGCAGAGGTGTTAAG-------CTGCTG---TCTCCCTCAAAG 25107
GoodesThornscrubTortoiseCCTTGTAAAACATGTAAATAATGAACAC----AGGTGAAAGCTGCTTTGAGAAATTTAACTCATG---CATCTATCACTG 74283

Majority                   CATTCAGACTAAGTATAAGAGGAGC-CTAGTATTCAGTTCAAGTAGATAG-TGGTTAC-TTGACATAAAGTCACAAAAAT
                                   80170     80180     80190     80200     80210     80220     80230     80240
Human                      GAGGTAAATTCATC-TATTTCGAGCCCAAATTTTTCTGTCTAGAAGATTGGTAGTCTTATTTTTACTAAAGTACAAGATT 79304
Kakapo                     C-TGTTGACCTGGTGTAAAATAAGC--TGTT--TCCCTTCAGGTGGACAT-CTGTCAC-TC--CTTACTGTCA------- 40880
GoldenEagle                CATTCAGGCTAAGTATAAGAAGCATGCCAGTAACAAGTACAAAAAGATGG-TGGTTAC-TTGACATAAAGTTACAAAAAA 49903
JapaneseQuail              CTTTCAGGCTAAGTATAAGAGAGCA--AAGTAATGAGTGCCAGGAGGTAG-TGCTTAC-TTGACATAAAGTCACAAAGAA 36226
MediumGroundFinch          C---CATAGGAAGCATGGCAGGAAC--TAG---TCAGTACAAGTAGATGG-TGATTAC-TTGACATAGAGCCATGAAAAG 25177
GoodesThornscrubTortoiseCCCCCAGATTATAC-TAGAGGAATTACCATACTTAGTTTTTGATTAACAGATGGTCTC-TCCTAACACTTCATCCAACTT 74361

Majority                   GTTTCGTAACAGGT----CTATGTACTTAATCCATTTCTCAGTTATAGTTTTACATACCTG---CATTAAGATGATTATT
                                   80250     80260     80270     80280     80290     80300     80310     80320
Human                      ATTTAGTAAAAGGTAGCATTCTCTGGCTTTTTTTTTTTTTTTTTTTAATGTTTCAGATCTGTCACACTAAAAAGGGCTT- 79383
Kakapo                     --TTCTTCACATTT----CTGTCTGCGTGACC---TCCACAGG---AGCTGAGTGTAACTGA-ACGTGACAGTCAGCATA 40947
GoldenEagle                GGTTCGTAACAGGA----CTATGGACTAAATCCAGTTCTCAGTTGCAGTTGTGCATACCTG---CATTAAGATGACCACT 49976
JapaneseQuail              G-TTTGTAGCCCGA----CCAGGAAT-GAATCCAATTC--AGTCATAGTCTTACATACCTA---CATTAAGATGATTATT 36295
MediumGroundFinch          GGTTCATAACAGGT----CTAT-----AAACCCAGTTCTTGGT--CAGTTTTACTTACCTG---CATTAAGATGATTAC- 25242
GoodesThornscrubTortoiseATACAACAACTGAT----CTTAACAATCAGTCTTTTCAACATTAAAAACATAACATTTTATT-GTATTTAAACTTTTTTC 74436
```

```
Majority                  TATACTGCGTATTGTT-TTCACACATAAGTTCATAAT-ATTTTTTTAGTGTAGCTTTAA--TTTGCCTATTATTAAAATG
                                   80330     80340     80350     80360     80370     80380     80390     80400
Human                     TAGACCAGGTGTTGTGGCTCACACCTGTAATCCCAGCACTTTGACAGGTCAAGGTGGGAGGATTGCTGGAGGCCAGGAGT 79463
Kakapo                    TACAATACAAAGCACT-CAAAAGGACATTTCCAAAACGATTTTTTTAGAGCAGAACTGG--ATTGTTATAAAATTGTTTG 41024
GoldenEagle               CTTACTGCCTATGGTT-TTCACACATAAGTTCATTAT-ATATTTTTAGTATAGCTTTAA--TTTGCCTTTTACTAAAATG 50052
JapaneseQuail             TCTACTGTGTATTGCT-TCCACACACAATTTCACATT-TTATTTATAGCGTCACTTTAA--TTTGTCCATTAATAAAATA 36371
MediumGroundFinch         ---AGTGCCTGTGGTT-TTCATGGA--AGTTGATAAT-ATATTTTTAGTGTAGCTTCAA--TCTGCCTACTATGAAAATG 25313
GoodesThornscrubTortoiseTGAAATAATATTTATT-TAAAAATT--GGCTGTGAGTACTTTTTAAAAAAAAAAATACAG--TTTTGATACTGTTTTTGAG 74511

Majority                  GTGTTAGTAATTTGAATTTCAGAGGT--CTCTCAAAAAGATATTCTGAT--AGGGAATAAA----TAATT-GTGACATG-
                                   80410     80420     80430     80440     80450     80460     80470     80480
Human                     TTGAGACCAGCCTGAGCAACATAGTGAGATCTCAGTCTCTACTAAAAATAAAAAAAATTAGC-CATGCCTGGTGGCATGT 79542
Kakapo                    AGGGCTTTAATTTGACATTCACAGGTTTCCCTGTGTATTACAATCTGATTTGGGGAATAAA----CTTCCAGTTCAGTG- 41099
GoldenEagle               GTGTTACTAATTTGAATTTCAGAAGT--CGTGCAAAAAGATAAACTGAG--AGGGAATAAA----TAAT--GTGACATG- 50121
JapaneseQuail             TCATTAATAACTTGAACTTAAGAAGT--CACACAAAAA-ATATGCT------GGGAGTAAG----CAATGCATAACATG- 36437
MediumGroundFinch         GCGTTAGCAATTTGCATTTCGGAAGT--CACAGAAAAGGATAAGCTGAG--AGGGAATTAA----TAAT--GTGATATG- 25382
GoodesThornscrubTortoiseGGGACAGCATTATGGGATTCAGTGTGACTTCCAGAAGACATTTTCTCATCTTGCAAGCAAAGATGTTTTTTTCTGTCTA- 74590

Majority                  --CTGTATTTCAACTT--------GGTTGAGG-GAAA-GATCTCCATGGTCTAGAA----GAAAGGTGTGTAATTTA--A
                                   80490     80500     80510     80520     80530     80540     80550     80560
Human                     GCCTGTAGTCCCAGCTACTTAGTAGGCTGAGGTGGGAGGATTGCCTGAGCCCAGAAGATAGAAACAGCAGTGAGCCATGA 79622
Kakapo                    --TTCAAGGCCAGATT--------GGATGGGG-CATT-GAGCAACCTGGTCTAGTA----CAGGGGTACTTTATTTA--A 41161
GoldenEagle               --CTGTGTTTAAACTT--------CATTGAGG-AAAAAGACCTCAAAAGGCTAGGA----AAAAGGTGTATAATAT---- 50182
JapaneseQuail             --CTGT------------------GTTGAGG-GTAA-AGGCTCCATGGCTGGGAA----GAAAGATCTGTAATGT---- 36486
MediumGroundFinch         --TTGTATTTTAACTT--------CATTGAGG-AAAA-GGCCTCAAAAGGCTAGAA----AGAAAGTATGTAATAT---- 25442
GoodesThornscrubTortoise-CTCTACAAACTTTT--------TTATGAAA-GTTATGTTCTTTCTGGTTTGTAGCTTTGAGGGATGAGGGGTTCAT-A 74658

Majority                  TTGTATGTTTTCATTAATATTTTGAAGAAAACGAAATCCTG--TTATCTTTAATTTTTGGAAGCTAAAATAAAGCTTGTG
                                   80570     80580     80590     80600     80610     80620     80630     80640
Human                     TCATGCCACTGCACTCTAGCCTGGGTGACAGAGCAAACCTTG-TCTCAAAAAATAAAGGGTGGGGAAGCTTTAGCCTAGG 79701
Kakapo                    CTGCATCCTTTAGTAATTGTTTTTTTGTAAAAGTAACGCTA--TATTCTTCAGCTTTAGCC--CAAGAGCAAAACATGCA 41237
GoldenEagle               TTGTATGTTTTCATTAATATCCAGAAAAAG-CAAAAACCTG--TTACTTGTAATTGTTGGAAGCTAAAATAAAACTAGTG 50259
JapaneseQuail             TTATGTGTTTTCATTAATATCCGGAAGAAG-TAAAGTCCTG--TTATCTATGATTGT------------------T--A 36542
MediumGroundFinch         TTGTATGTTTCCATTAATATTTTGAAAAAAACAAAAGCCTG--TTATCTGTAGTTATAGGAAGCTAAAATAAAGCTAGTG 25520
GoodesThornscrubTortoiseTTATCTTCTTCCATGGGATATGTGAAAAATCGGAAGTATTGAATAGTGTTCAATATTTTGGATATATTTTCATTTTTTCG 74738
```

```
Majority                    CCAA-----AGCTTTCTAATACCA----G--TTTATCTTGTTTATAAAGTAGCTAAT--ATTTCTAA----ATAGTTTCA

                                    80650     80660     80670     80680     80690     80700     80710     80720

Human                       CTTTT----AATTCTCAATTACCATTATAAATTTATTTTAATTATTAACTAATTACTTTAATTCTCAATTACTATTTTTC 79777
Kakapo                      TCAT-----ATTTATGTTATATT--------TTTATGTTGCTATTAATATACGTTAA--GTATATAGTATTACACTTTGA 41302
GoldenEagle                 ACAA-----AGCTTTCTAATACCA----G--TTGATCATGTTGTCAAAGTAGCTAAC--ATTTCTAA----GTAGTTTCA 50322
JapaneseQuail               ---------AGCTTTCCAATACA-----G--GACATCTTCTTGATAAAGTAGCAACA--GTTTCTGA----ATAGCTGCA 36600
MediumGroundFinch           ACAA-----AGATTTCTAATACTA----G--TTGATCATGTTTACAACATAGCTAA---ATTTCTAA----ATAGTTTCA 25582
GoodesThornscrubTortoiseCTTATCTCAGCCTCTTTAATGGCT----GGTTCTGTTGCTCTAATACAGTGCCTAAT--ATCTGTGCA--TTTCTTCTGT 74810

Majority                    GAT---AAGGCATCATATCTTAAACAGTAATGTGTAAGTTAGTTAATTATA----AAAACCCATGTTGT----------T

                                    80730     80740     80750     80760     80770     80780     80790     80800

Human                       CATGAAAAGGCTTGATGTTCTAAACAGTATTATACCACCAAGTTATCTCTACTGGAAAACCCAGATTTCAAGAAAAAAAA 79857
Kakapo                      CAC---CAAGTACATTTTGAGAAACAAAACTGCGTAACAGTGGTCCATCTA----ATTTCATATCCTGTC---------T 41366
GoldenEagle                 GAT---GAGTCCTCAAATCTTAAACAGTGTGTTGCATGATAACTAATAAAC----ATAATACAAGTTGT----------T 50385
JapaneseQuail               GGT---AAGTCAT------T-AAACAGTCAACAATGAGTTGAGTGATGATC----AATAGCCATGATATAA-------AT 36659
MediumGroundFinch           TAC---AAGGTCT--------AAACAGTAACATGGTAACTTTTTCATAATA----GAAACTCTTCTTAT----------T 25637
GoodesThornscrubTortoiseGAT---ACAACAGCAGAACATAGCTAGTTAAGGGGAAATCAGTTAATTGCA----GAATGCCTCTCTGC----------C 74873

Majority                    CTTATTTATTAAAAACATTTTTGTCTCTTCTATTAGAATGGCAAATCAAAATGCTGTGGA--------GAATTACTTA-A

                                    80810     80820     80830     80840     80850     80860     80870     80880

Human                       AATGTATTTTGAAAACAATTATGTCTTAAATATTTAAAATTCTTTTAAACATGTTTTTAACCATCTTTAAATTTTTTTGG 79937
Kakapo                      CTTGACTCTTAGCATTACTTCTGCCTTCTCTGCTGGAGGACCTAGCACAGCCCTCGTAGT--------GGA-TGCTCA-C 41436
GoldenEagle                 CTTATTTATTAAAAATTTTTTACTTGCTTAAATCAGAATGGCAAATCAGGGTGCAGTGGA--------GACCTACAGA-A 50456
JapaneseQuail               TTTATTCATGAAACAAGATTTTG--ACTTCTGTT-GAGTGGCAAATGTTAAAGACCTACA--------AAATAAACCC-A 36727
MediumGroundFinch           TTTATTTATTAAAAACCTTTTCATTGCTTTAATCAGAATGGCAAATCAAAGTACAGTGGA--------GAC-TGCAGA-C 25707
GoodesThornscrubTortoiseCTTTACTATTTGAAACATTCAGGGCACAACTTTAATGATGACCTTTTGGAAAACTTCGGC--------ACATCATTTTTA 74945

Majority                    CAAGTAATGTACTTT--A----T-T-TTGTGTCTTTGTA-ATTTGTC-TGAGCATTTCAAGAAATGAGGTCTGTGTGTGC

                                    80890     80900     80910     80920     80930     80940     80950     80960

Human                       TAAGTAATTTTCATTAGATTGTTAAAATGTATAGTGGCACGGTAGGG--TTGTATCTCAAGATATGAAGCAGTTTTTCAT 80015
Kakapo                      ACAGTAGTATACCTGGGAGGAGTGC-TTTCCTCCCCAGA-GGGTGCT-CAGGCATTGGAACAGGCTGCCCAGGGCAGTGC 41513
GoldenEagle                 CAAGAACTGTGTTTT-----------CTCTGGGTTTGTA-ATTTGTC-TGACCTTTCCAAGAAACAAGGTCTATGTGTGC 50523
JapaneseQuail               TAAAAAATGCAATTC-------------------CTGTT-ATATGTCCTGACCCTTCTAGGAAATGAGGGCTGT--GTGC 36785
MediumGroundFinch           CAAGAATTCTGTTCCACAGCTGTTTGCTGTGGCTTTGTA-ATTTGTC-TGACCTTTTCAAAAAATGAGATCTATGTATGC 25785
GoodesThornscrubTortoiseCATCTAACGTACTTT-------TCTCTTATTTATTGGTACATTTGTATATAGCAGTATAAATTTGCATGGCCGTCAACTG 75018
```

```
Majority                  TA-AGTAATCAGTTAAAAATGTATTG-GATGAATAGATGATCCTGGCCTGCAT--GCATTATTACTTATTCATCTA----
                                   80970     80980     80990     81000     81010     81020     81030     81040
Human                     AAAAATAAAGTATTAACCTTAGGCTGGTATGTTTAGATCAATACTGTATTTAAATAGATTGATAATTTTTTATATATTTT 80095
Kakapo                    TGGAGTCACCGGCCCTGCAAGCATTC-ACACACTGTGTAGCCGAGGCCCTCA---GTGCCATGGGTTAGTGGT------- 41582
GoldenEagle               TA-TGTAATCGGTTAGAAACGTATCA-ATCAAATAGATGATCCTAGCCTGCAT--GCATCATTACTCATTCATCTA---- 50595
JapaneseQuail             TA-TATAAGCAAGTAGAAAATTGTTG-CATGTGTAGATTATCCTGTCTCACAT--GCGTTGTTACTTGTTCATATC---- 36857
MediumGroundFinch         TA-AGTAATCAGTTAAAAACACATCA-GTCAAATAGATGGTC-TAGCCTGCAT--GCATCATCACTCATTCATCTA---- 25856
GoodesThornscrubTortoiseTGGAAAAGAAAGTGAAAAGTGGATTTTGAGGAATATAGGAAT-TGCCATGCA---GAACTACCAGTGGTCTATCTAATC- 75093

Majority                  ------CTACATCCTTGGCTGTGATAAAATAAGGTTT-CAGAAATCTTTG-AGACTGTAAGTTGGACATC-----TTTTC
                                   81050     81060     81070     81080     81090     81100     81110     81120
Human                     ATGAATTTATACTTTTTCCTGAGATTTCAGGAGGGAAAAATGAGTAGGAGATGACTGAGAGCTTAAAGTTTGGGAGTGTC 80175
Kakapo                    --------G--GCCTTGGCAGTGGTGGGCAATGGTT----GGACTGGATG-AGCTTTAAAGGTCTTTCA------AACCG 41641
GoldenEagle               ------CTACATCCTCAGCTGTGATGAAATAAGGTGT-CAGAAATCTTTG-AGACTGTAAATTGGACATC-----TTTTC 50662
JapaneseQuail             ------CTACATCCTCGGCTATGATAAAACTGAGTTGTCAGAAATCTTAG-AGACTGTAAATTGGACATA-----TTCTC 36925
MediumGroundFinch         ------CTACATCCTCACCTGTGATAAAATAAGGTTT-CAGAAATCCTTG-AGTCTGTAAACTGGACAA------TATTC 25922
GoodesThornscrubTortoise------CTGTATACTT-TCTCTGACAGCAACCAGTAC-TATATTTCAATGCAAGATGATAGACAACCCTC-----ATTAT 75160

Majority                  AGTGAGTTTTGCTATTTTTTGAAAGAAGTGGAT----AATTAC-------T-AATGGATAG-AGTGGGCTGGT-------
                                   81130     81140     81150     81160     81170     81180     81190     81200
Human                     AATTAACTCAGCATTCTTTTAAAAAACATGTGTCATATATTACAGCATTTTCTTTTATTTGAAGTGAGTAAATGTATCTT 80255
Kakapo                    AGTGGATTTTGTGATGCAGATTATATGTTACTT----TATTGC-------CCATTTAATAT-AACTGGCTGTTG------ 41703
GoldenEagle               AATGAGTCTTGTTATCTTTTGAAAGAACTAGAA----AATCAC-------T-AATGGACAG-AATGGGCTAGT------- 50722
JapaneseQuail             AGTGAGTCCTGCTATTTTTTAGAATAAAGGGGAA----AATTTC-------T-AATGAACCA-GGTGGATTGCT------- 36985
MediumGroundFinch         AATGAGTGTTAATGTTTTTTGAAAGAAGGAGAA----AATTAC-------T-AGTGGACTA-AATGAGCTGGT------- 25982
GoodesThornscrubTortoiseCGACAATTATACAATATTATGTTGGGGGTGGGTCCT-AAACCC-------CAGATAGTTAGTGGTTGGCTTATA------ 75226

Majority                  TTCAAA----TGAATG-ATCATGTGCATTT-ATTTG-TTATTTTGTTTTGTTTTTTTTTCATATTATATATGAAGAGATA
                                   81210     81220     81230     81240     81250     81260     81270     81280
Human                     TTTAAATTCCTTAGTAATTTTTGAGCACTCCATATGTATAAAGCATGTGAATATTTGGTAGCATTTTACA--AATGTCCA 80333
Kakapo                    TTTACA----TAGACC-ATCTAATACGGTC--T----CAAATCTGCTGAGTTTTTATAGGATGGAAGGGAGCAAGAGAAG 41772
GoldenEagle               TCCAAA----TGAAAT-ATCAGAAGCAGATGGTTTGTTTTTTTTTCTTTCCTTTTTTTTCCACATCCTATATGAAGAGAGA 50797
JapaneseQuail             TTCAAG----TGAGT--ATCATGGCCATCATATCA--GAATTAGGCTTTTTTTTTCCCCCTTTTTATGTGTGAAGCAGAG 37057
MediumGroundFinch         TTCAAA----TGAATG-ATCAGAGGCAATT-------TTTTTTTCTTCTGTTTTTACCTCACATATGAAAC-AATAGATA 26049
GoodesThornscrubTortoiseTCCTGAAGCTTGATTG-TCCATGTACATGTACTTAGCCTCTCTAATATAACTGGATGTTCTTGTTATCCATAAAAATGTC 75305
```

```
Majority                   TAAACTTTTTTTTGTTC---CCTTCAAAATATATAGCCTATGTTACAGAATAAAACC-TTTATATTAATAAT-AAGTGTT

                                   81290     81300     81310     81320     81330     81340     81350     81360

Human                      GAGATTTGTGAGAGTTCCTGAGATCTTCATAGGGGGCCCACAAAGTTTAGTATTACTTTTCACGGTAATACTAAAGTGTA 80413
Kakapo                     ATAATGGACTTTTGTTC---CTTTTAGGTATTCCAGTCAGTCCCACA-AATACCACA-TCTAATGCAATAAA-AGATGGT 41846
GoldenEagle                TACACATTCTCCCCCCC--CCCCCCAAAAGTTACAGGAGATCTTACAGAAAAAAACC-TTTGAATAAACAGC-AACTGTG 50873
JapaneseQuail              AAAAGTTATGGGAGATT----TTTCAGGATAAAAAGCCTTTTGTGTA-AACAGCAGC-TATATGGTAATG---AACCGCT 37128
MediumGroundFinch          TAGTCTCCCTCCTCTTCAACCCCCCTAAAAAAGTTACAGATGTCACAGAAAAAAGT--TTTGGATAAACAAC-AGGTGTA 26126
GoodesThornscrubTortoise   TAATCCCTTGTTGAATC---CCACTAAACTCTATGCATTGTGCTAAAAAATAAAGCA-TTCTTATTGTCTTT-AAATGTG 75380

Majority                   TTATACTGAATTTTAATTAATAAAATATT--C------T--TTA-----AATAAGAACTACTTGGGATTGCAAGTACTGA

                                   81370     81380     81390     81400     81410     81420     81430     81440

Human                      TTTTGCCTCTTTTTACTTTTTCTCTTAATAGCATACAGTGGTAACTGAAGGCTAATAGTATGTGTGTTTATGTGCTTTAA 80493
Kakapo                     GTTTGTTGCCTTTGATTCAGGGAGATTTTCCCTTTCTCTTGTCG-----CGTGAGAGATGAGTGGGATTGTCATTACTGA 41921
GoldenEagle                TTATACTGAACTGCAACAAACAAAAGATT------------TTA-----AATAAGAACTACTTATAATTGCAAGTACTAA 50936
JapaneseQuail              AAATAAAAACTAAATGCTAATAAAATGCT--------------A-----AACAAAATTTACTTGGGATTGCAA-----GA 37184
MediumGroundFinch          TTATACTGAACCACAACATACAAAATATT------------TTA-----TATAAGAACTACTCAAAATAGCAAATACTGA 26189
GoodesThornscrubTortoise   TTGCCCTTTAATTTTATTAGATACCTCCTTCCCCTTTTTCTTGAGTTACAAGAGGGACTAAATAGGATTGCCTAGTTAAC 75460

Majority                   TA-TTTGTTATTC-TTAGTTTAGCTCCCTGTTTGATATCACTATTGCAAGTTCTTTGCTATACTTGGAAATATGCTGT--

                                   81450     81460     81470     81480     81490     81500     81510     81520

Human                      AAAGTTCGTGGTT-TTGGCCAGGCGCAGTGGCTCAGACCTGTAATCCCAGCACTTTGGGAGGCTGAGGCAGGTGGATCAC 80572
Kakapo                     TAACTTTCTATTCCTCTGGTTCGACCCCTGCATACTAACAATTTTGAATCTTCTAAACTTTATTAGAAGACATTCTGTAT 42001
GoldenEagle                TA-TGTAATA----ATACTTTAACTCCAATTTAGATATTT---------GTTTAATGCTATAGCTGGAAATATGTTGT-- 51000
JapaneseQuail              TA-TATCTTA-------GTTTAACTTCAATTTAGCTG-------------TTCATTACTACAGCTAGAAATATACTGT-- 37241
MediumGroundFinch          TA-TTCGTTACTC-ACAATTTAGGTATTTGTGTAATATTACAGCTGCAAATATACTGTTGTGCTGGAAAATTCACTGT-- 26265
GoodesThornscrubTortoise   CTCAGTGGCATTCGTTATTTTTTATACCTGTGTTATGACACTATAGAGCAGTGTTTCCCAAACTTGGGATGCCGCT-TGT 75539

Majority                   -TGTGGTTAGGCCTCCCAGAGCTTTATC--CCTTGCTGTCTTTGCTGGCGTTCTTTGTTAG-AACTTTTCACTAATTCC-

                                   81530     81540     81550     81560     81570     81580     81590     81600

Human                      CTGAGGTCAGGAGTTCAAGACCAAAACCAGCCTGGCCAACATGGTGAAACCCCATCTCTACTAAAAATACAAAAATTAGC 80652
Kakapo                     CTTTAATTTCCCTTCTTAGAACACTTAG--TAGTGGGGTTTTTTTGTATGTGCCTTGACAG-GAAATAACCACAAAATA- 42077
GoldenEagle                -TGTGGTTAGGTCTCCCACAGCTTTATC--CCTTGCCGTCGTTGCTGGCTTGCTTTATTAG-CACTTTCCACTAATGCG- 51075
JapaneseQuail              ----TATTATGACTCCCACAGTATTATT--TCTTGCTGTCTTTCCTAGGCTTTTTATTAT-AACTTTTCACTAATTTCT 37314
MediumGroundFinch          -TGTGCTTAGGGCTCACACAGCTTTATC--CATTGCTGCCATTGCTGGCGTTCTTTGTTAG-CACTTTCCACTAGTGCC- 26340
GoodesThornscrubTortoise   TTAGGGAAAGGCCCTGGTGGGCTGGGCT--GATTTGTGTACCTGCC-GCATCCACAGGTTC-AGCCAATCATGGCTCCCA 75615
```

```
Majority                   CTTTC-TTATGGTTGTTGCT--AAAT-TAATCTGTTTTAGAGGATAATAATTGAAAA--TATTTCTGTGTGAATGGTG--

                                  81610     81620     81630     81640     81650     81660     81670     81680

Human                      CAGGCATGGTAGTGGGTGCCTGTAGTCTCAGCTACTCGAGATGCTGAGGCAGGAGAAT-CACTTGAAGCTGGGAGGTGGA 80731
Kakapo                     CTTTC-CAATGCTTGATCAG--TAAG-TAAGCACTATTACAGAGTAGTGATGGATGTCCTGTTCCCTTTGAGGGGGCA-- 42151
GoldenEagle                TTGTC-TTATGGTGGTTCTT--AAAT-TAATCTATTTCAAAGGATAATAATTGAAAA--TATTTTCATGTGAATGCTG-- 51147
JapaneseQuail              CCTTT-TTAAAAGTCTTATT--AAAT-GAATCTGTTTCAGAGGATAACAATTGAAAA--CATTTCTGGATGAACGCTG-- 37386
MediumGroundFinch          CCTTT-TTGAGATTGTTGCT--GA-------CAGTTTTGAAGGATAATAATTGAAAA--TGCTTTTGTAGGAATGGTG-- 26406
GoodesThornscrubTortoiseCTGGC-CGTAGTTCGCTGCTCCAGGC-CAATGGGGGCTGCAGGA-AGCCACAGCCAGCACATCCCTCGGCCCACCCTGC- 75691

Majority                   --TTTAATTAATCCTTTTT-----------TTTAACTTCT-TCAGTGGGACCAAAGAAAGTCTTGACGAGAGTTTGATGC

                                  81690     81700     81710     81720     81730     81740     81750     81760

Human                      GGTTGCAGTGAGCCAAAATCTCGC----CATTACACTCCAGCCTGGGGGACAAGAGCAAGACTCCATCTCAAAAAAAAAA 80807
Kakapo                     --TTTGTGCAACCCTTTTC-----------ACGAACAGAT-TGGACTGA--CGATGGAAATCTTGCCTAAATTGTAACCT 42215
GoldenEagle                --TTGAATTAATCTTTGTT-----------TTTAACTTCT-TTAGTGGGACCAAAGAATGTCCTGTAGAGAGTCTGATGC 51213
JapaneseQuail              --TTTAATTAATCTTTTCT-----------TTTTACTCCA-TCAGTAGGA-CCAAGTGAGTCCTATAGAAAGTTTAATGC 37451
MediumGroundFinch          --TGTAATAAATCATTGTT-----------TATAACTTCT-GTGGTGGGGCCAAAGAATGTCCTTAGGAGAGTCTGATGC 26472
GoodesThornscrubTortoise-TTCCAGCAGTTCCCATTGGCCTGGAGCAATGAACCGCTGCCAGTGGGAGCTGCAATCGGCTTGACCTGTGGTCGCGGC 75769

Majority                   TGTGAAGAGTTTTTGCCTTTTAA----TGAGTTACTTGTGTTTGTTGTGCCCA-ATTATGGAAAGAGCGATAA-ACACTT

                                  81770     81780     81790     81800     81810     81820     81830     81840

Human                      AAAAAAAAGTTATTGGTTTTAATTTTGGAAATGACAACCATTAATAGAACCCACATTAGGCCGGGCGCGGTGGCTCATGC 80887
Kakapo                     TGCTCACTGCTCC--CCTTCT---------GC-GCCCGTGGATGTTCTG--AG-AATATGATTGCACTAAAAG-ACAGTA 42279
GoldenEagle                TATGAAGAATTTT-GCCTTTTAAAGTATGAATTACTCGGCTATGCTCTGCCCA-ACTATGGAAAGAGCAATAA-AAACTT 51290
JapaneseQuail              TATGAAGAGTTTTGGCTTTTAAA-----GCATCTTTTGTGT-CATTACATTCA-ATTATGGAAAGAGCCTTAA-TAACTT 37523
MediumGroundFinch          TGTGAAGAATTTTTGCCTTTT------TGAGTTACTTGGCTTTGCTGTGCCCA-GCTATGGAAAGAACCATAA-ACACTG 26544
GoodesThornscrubTortoiseAGGTAAACAAACCAGCCCAGCAG----CCCGCCAGGAGCTTTCACTACACAAGCAGTGTCCGAAGTTTGGGAA-ACACTG 75844

Majority                   CACTAGTCA------TCT-------TTAGATTCTTTTATT------------------TATTAATTT--GTTGGACTGGT

                                  81850     81860     81870     81880     81890     81900     81910     81920

Human                      CTGTAATCCTAGCACTTTGGGAGGCCGAGACGGGTGGATCACGAGGTCAGGAGATCAAGACCATCTT-GGCTAACACGGT 80966
Kakapo                     AAATACTCA------ACT-------TAGATTTCTTCTTTT------------------ACATATTTT--GTTAAGCCAGT 42326
GoldenEagle                CACTAGTCA------TCT-------TTAGGTTCTTTTATT------------------TGTAAAAAT--GCTGGACCGGC 51337
JapaneseQuail              TGTTAGGCA------TCT-------TCAAATTCCCTTCTTGTATTTTTGTATTGTATGTATTGATGT--ATTGGAATAGC 37588
MediumGroundFinch          CACTGGTCT------TCT-------TTAGATTCCTTTACT------------------CCTGAAAAT--GCTGGACTGGC 26591
GoodesThornscrubTortoiseCTATAGACTAATATCTTT-------TAAAACTCTTCTATTTTGAAAATCCTTTCACATCATTAATTTTAATTGCCCTTCT 75917
```

```
Majority                  TTAAAGTTTCTTATATTAGGGAG-----AAATG-TCTAGACATATTTGAAGAT--TTGAAATGCTATAATATTTTGACGC

                                   81930     81940     81950     81960     81970     81980     81990     82000

Human                     GAAACCCCGTTTCTACTAAAAATACAAAAAATTAGCCGGGCGTGTTGGCGGGCGCCTGTAGTCCCAGCTACTTGGGAGGC 81046
Kakapo                    GTCTTTTTTACTGAACTCTGTA------AAATG-TGCAAATAAAAT-GTGGAT--C--AAATACTATAAAAGCT------ 42388
GoldenEagle               TTAAAGCTTCTAAGGTTAGGGAG-----AAGTG-CCTAGACACACTTGAAGAT--TTGAAATGCTTTAGTATCTTGGCAT 51409
JapaneseQuail             TTAGAGTTTCTAAAACTATGGAG-----AGGTGCTTTAGGCACATTAAAAGAC--TTGAAGCACACTAATATTTTGACAC 37661
MediumGroundFinch         TTAAAGTTTCTAAGGTTAGGGAG-----AAATG-TCTAGACATACTAGAAGAC--TGGCTATGCTCTAG----------- 26652
GoodesThornscrubTortoise  TTCAACTCTTCTGTATCTGTTTTT---GACATGGAATTACCAGAACTGAACATGTTTTCGAAGGGAGAATATAACTTTGA 75994

Majority                  TT---CATAAAACTAGCA----------TGGCCCCGTATAAAGTAAGTTGTT---C------TG--C-------------

                                   82010     82020     82030     82040     82050     82060     82070     82080

Human                     TGAGGCAGGAGAATGGCATGAACCTGGGAGGCGGAGCTTGCAGTGAGCCGAGATCACGCCACTGCACTCCAACCTGAGAG 81126
Kakapo                    -----------------------------GTTCCTGTATAAACTA----------------------------------- 42404
GoldenEagle               TT---CCTAATACCTAAATGATTGCTATTGACCTCGTATAAAATAAGTTGTTTCCCTTCAGGTGGACATCTGTCACACC- 51485
JapaneseQuail             TT---CTCATACCTAAA-----------TGGTCAC-TGCTAAGTAGGTTTTT---C------------------------ 37699
MediumGroundFinch         -------------------------------------------TA----------------------------------- 26654
GoodesThornscrubTortoise  TT---TATAAAAGCAGCAAAGAATCCTGTGGCACCTTATAGACTAACAGACGTTTTGGAGCATGAGCTTTCGTGGGTGA- 76070

Majority                  --AC--------T------T---------A----------------------T--A---A---------T-A---T----

                                   82090     82100     82110     82120     82130     82140     82150     82160

Human                     ACACAGCGAGACTCCGTCTCGAAAAAAAAAAAAAAAAAAAAAAAGAACCCACATTAACAAAAACTCCTTGTGATTCTCCAT 81206
Kakapo                    -------------------T------------------------------------------------------------ 42405
GoldenEagle               TTACTGTCATTCTTCACATTTCTGTCTCTATGACCTCTGCCGTACATGAGTGTAAATAAACATGACAT-TCAGCATATGA 51564
JapaneseQuail             -------------------------------------------------------------------------------- 37699
MediumGroundFinch         -------------------------------------------------------------------------------- 26654
GoodesThornscrubTortoise  ATACCCACTTTGTCGGATGCATGTAGTGGAAATTTCCAGGGGCAGGTGTATATATATGCAGGCAAGAA-TCAGTCTAGAG 76149

Majority                  A-----A-G-------A-----------A--A--A---TTTAGAA---TAGAACTGTAGGTTTCAAAGTTGA--GAGGAA

                                   82170     82180     82190     82200     82210     82220     82230     82240

Human                     AGTTTTAAGACTATAAAGGGGAGCTGAGACCAAAAAGTTTGAGAACTTTTTCACTGTGGGATACAAAGTTGAA-GACAAT 81285
Kakapo                    ----------------------------------------AGAT---GAGGCATATAGGTGACAA----G--------- 42428
GoldenEagle               AATACAAAGCACTCAAAAGTAAATTTCCAAAACAATTTTTTAGAA---CAGAACTGGATTTTTTAAAGTTGTTTGAGAAA 51641
JapaneseQuail             -------------------------------------TTGAGAG---TAAAGCCATATTCTTCAGTTTTACCCCAAGAA 37738
MediumGroundFinch         -------------------------------------------------AGACCTGG---T------------------ 26663
GoodesThornscrubTortoise  ATAATGAGGTTAGTTCAATCAGGG----AGGATGAAGCCCTGTTC---TAGCAGTTGAGGTGTGAAAACCAAGGGAGGAG 76222
```

```
Majority                 ---AATTTGATATTCACG---TTCAGCGTGTAGTACAGTC--AT--------T---------------------------
                                 82250     82260     82270     82280     82290     82300     82310     82320
Human                    TATAGTTAAATAAGCAGGAGATACAGAGTATTTTAATGAACAGTA-GATGGTTATAAGAGTCAAGAGATGTACAGAGAAA 81364
Kakapo                   ---AACCTTCCACTGA-----TCCTAGGAGTAGCACAGTC---------------------------------------- 42460
GoldenEagle              TTTAATTTGATATTCACAGGTTTCCCTGTGTAGTACAGTCTGATTTGGGGAGTAAACTTCTTCTTCCGTAGGGGTACTTT 51721
JapaneseQuail            CA-AAACCAATACTTGTG---TTAGGCATATAGGACTGT---AT------------------------------------ 37775
MediumGroundFinch        ---AGCTTGACATT------------------------------------------------------------------ 26674
GoodesThornscrubTortoise ---AAATTGGTTTTCACA--CCTCAACTGCTAGAACAGGGCTTCATCCTCCCTGGTTGAACTAACCTTGTTATCTCTAGC 76297

Majority                 -TGTTACTGCTTCG--TATATCAATTATTTTACTTGGAAAAA-CAGTTATATTCCT----T--A----------------
                                 82330     82340     82350     82360     82370     82380     82390     82400
Human                    ATGCTAGTGATTAGAGTTTTTAGATTATTTTAATTTTGGTAACTAATTATAATTCCTCAGTTAAGATTTTTTGGCTTAGT 81444
Kakapo                   --------GCCTAG--TACCCCAGTGACTTGACCAGGCAAAA-CCGTCAGCTCCCT------------------------ 42505
GoldenEagle              CTGTAACTGCATCC--TTTATTAATTTTTTTTCTTGGAAGTA-AAGCTATATTCTTCAGCTTTAACCAAAGAGCAAAACA 51798
JapaneseQuail            ---TTACACTTTCC--TATATCACGTGATTGGTTTTAAGAAA-CAGTAA--TC--------------------------- 37820
MediumGroundFinch        ----------T--------------------------------------------------------------------- 26675
GoodesThornscrubTortoise TTGCTTCTTGCTTG--CATATATATACCTGCCCCTGGAAATTTCCACTACATGCATCCGATGAAGTGGGTATTCACGCAT 76375

Majority                 -----C-----------------T--T-----------T-----A-GATAT-----------------T----------A
                                 82410     82420     82430     82440     82450     82460     82470     82480
Human                    GGGCCCTTAGAATTACTTCTTCCTGTTAGAATTAAAAGTGAAACCAGAAATTAAAGTGATTTAATTTTTTTATTGTGGCA 81524
Kakapo                   --------------------------------------------ACACAAT----------------------------- 42512
GoldenEagle              TGCGTCATATTTATGTTACATTATTATCTTGCTATTAATATATGTTAGTATATAGTATTACACCTTCCTTTCACACAAAA 51878
JapaneseQuail            -------------------------------------------------------------------------------- 37820
MediumGroundFinch        -------------------------------------------------------------------------------- 26675
GoodesThornscrubTortoise CAAAGCTCTTGCTCCAAAATGTCTGTTAGTCTGTAAGGTGCCACAGGATTCTTTGCTGCTTTTACAGATCCAGACTAACA 76455

Majority                 ----T-----------AACTTGGTATGTTTATTCTAGCAATAGACTTTCTAATTTCAT----ATCCTTTCTCTTGCTGCT
                                 82490     82500     82510     82520     82530     82540     82550     82560
Human                    AAGATCTAGTCCAAATATCTTTG-ATGGACTTTTTGAAAACAGACTTTATTGTTAAAAAGGGAATTTGAGGCTTGGTACT 81603
Kakapo                   ----------------------CCATGTTT--CCTTGGAAGAGGGAAACTAAAAGCAC----A-----------GC---- 42549
GoldenEagle              TGTATTTTGAGAAACGAAATTGCTATGCTTGGCCTAGCAGTGGTCCATCTAATTTCAT----ATCCCTTCTCTGACAGCT 51954
JapaneseQuail            -----------------ACTACGCGTTTGTATGCCAGCAGTGG----TCTAATTTGGT----ATCCTTTCTCTGACAGAT 37875
MediumGroundFinch        -------------------------T----------------------CTAA---------------------------- 26680
GoodesThornscrubTortoise CGGCTACCCCTCTGATACTTTGATTTATGTAATGCCATAAAATATTTTCAGAATTAATGTCTATCCCTAACATTGTTTTT 76535
```

```
Majority                    G-TGT-ACTTCTTTGTTCT--CTTATAGAAGACATTGG-TAGCT-TC-TAATGGGTGACCATAGGTTAGCATGCCTGAGA

                                     82570     82580     82590     82600     82610     82620     82630     82640

Human                       GA----ACTTCTTCTGCATGCCATATTGAAGTTGGTGGTTTTTTGTT-TGTTTGCCATCAGTGGGATCTCATACAGACTA 81678
Kakapo                      -----------T--G-----------------------------------AAGGTTTAGCCTAAGGGTAACGTGCTT---- 42578
GoldenEagle                 AGTGTTACTTCTGCGTTCT--CTGATGGAAGACCTAGCGCAGCCCTCGTAATGGATGATCATACAGTAGCATACCTGGGG 52032
JapaneseQuail               GCTGTTACTTCTATGTTCTTGCATATAGCAGACATAGGACAGCCTTAATAATGGGTGATCACTTGTTAGCATGCCTGAGA 37955
MediumGroundFinch           -------------------------------------------------------------------------------- 26680
GoodesThornscrubTortoiseCTTGTGGTTTTTTTTTTACCATTACTACATAACAGTTACTGGCTATCCCACTCAGTCACAATAAATTAATATTACATAAT 76615

Majority                    TGGGTACTTTTCTAGTCCTGAAGTAGCTAGTGTT--TGACTTGTGATTA---ATATTACATATAATACAATGGTCTATTT

                                     82650     82660     82670     82680     82690     82700     82710     82720

Human                       TGGAATTTAATAGAATTTTTAAGATACTGTTTTTCCTAAAAGGTAGCTAACTAAAGGCAGGAAAATAGAATGATGTA-AC 81757
Kakapo                      ---------------CT-TCAGGGAATTGG-----------------------ATTACAGA-------AG---CCACAA 42608
GoldenEagle                 GGAGTGCTTTCCTAACCCCCAGGTAGCTAGTGCT--TGACTTGTGATGAAGCATATTATATGTCACTTTATTGCCTATTT 52110
JapaneseQuail               GAAGTACTTTCCTAGTCCCGAAGTTGCTAGTGCT--TGGCTTATGTGC-----TGAAGCACATTACACATTGCTTTATTT 38028
MediumGroundFinch           -------------------------------------------------------------------------------- 26680
GoodesThornscrubTortoiseTAGCTAGTCCTTTTGTCCAGAACAATAAAATCTC--AATGTGTTAATTATTTATCTCTCATGAATTCCCCTGGTAAAAGA 76693

Majority                    AATATAATTGGT-----TGGTGTTTCCACTGTTTACCTAGTATATTATAAACTATTTTAGTTGCTTAT-TTGT-------

                                     82730     82740     82750     82760     82770     82780     82790     82800

Human                       AATATAATGACTACATCTGGAGTTTCTGCTACTAATAAATAATTGTATTGTCAACTTTAGTCAAATATTCTGTGCATTCA 81837
Kakapo                      AGTATAATCAG------TGGTGCT----------GCCTAGAATC------------------------------------ 42636
GoldenEagle                 AATGTAACTGGC-----TGTTGTTTCCATAGACCATCTAATACAGTCTCAAATCTATTAAGTGTTTTTGCAGGA------ 52179
JapaneseQuail               AATATAATTGGC-----TGCTGTTTCCACTGTTTCCATAGTCTATCAAATACTGTTTGAAGTGCTGATATAAGCATAGCT 38103
MediumGroundFinch           AATGTAAGTGAT-----TGCTATT----G-----ACCTAGTATA------------------------------------ 26710
GoodesThornscrubTortoiseAACATAATTAATG-AGGTGGAGCACGGAAATTTTACTTACTTTATGTGAAAGAAACTTTGTAACCACACTTATTTTACAC 76772

Majority                    -TCGCCGCTAT--AAACAAGATAAGGTTTTTTTTTTTGTTATGT--TG-AGATAATTTCTCAAAT-TGACAAGTAATGTA

                                     82810     82820     82830     82840     82850     82860     82870     82880

Human                       GTCAGAGCTATCTGAAAATACTGACGAATAGTATTACAATATG---GGGAGGTCACTTCTCAAGACTGCAAGAAAAAAAA 81914
Kakapo                      -------------------------------------------------------------------------------- 42636
GoldenEagle                 -TGGAGGGGAGAAAGACAAGATAGGGTTTTTCTTTTTTTTATGTATTATAGTCAATCCCACAAATATCACATATAATGCA 52258
JapaneseQuail               TTTGCCTCAATGGAAGAAAGACAAGATTTTTCTTTCTGTTCTGTATTCCAGACAGTTCCACAAATGTCACAAGTAATGTG 38183
MediumGroundFinch           -------------------------------------------------------------------------------- 26710
GoodesThornscrubTortoiseCTCTCCATTTTTCTTCCTGCTGCAATAACTATTTTAGTAGTGTTGGGAATATTTTATTTTATTATTGACGAGTACGGTC 76852
```

```
Majority                 ATAAT-AG-TT-TGTATGTTT-CTTTTGATTTA----AGTAGGTTTTCTCTTCATCTCGTG-A-TTATATTT--AGACTT
                                 82890     82900     82910     82920     82930     82940     82950     82960
Human                    TTGTTGTGTGTAGGAATGTTTGCCTAGGAATTA----GGAATGTATACTTGACAACAAGTGAACGTATCCATTGAATGTT 81990
Kakapo                   -----------------------T--TGAATTG----AGAACGTTGCCTCCTCACCTCA--------------------- 42666
GoldenEagle              ATAAA-AGATCATATTTGTTGCCTTTTGATTCA----AATAGGTTTTCCCTTTATCTTGTGGCATGAGAGAT---GACTG 52330
JapaneseQuail            ATAAA-AAGAT-TGTATTTATTGCCTTTATTTA----AGTAAATTTTCCCTTTATCTCATCCAGGGAGATTACTAGAATT 38257
MediumGroundFinch        ----------------------------A--------AATAAGTT----------------------------------- 26719
GoodesThornscrubTortoise ACAATTAGCTTCTCAGTATTTACTCACTATCTGAGACAGGAGAATTCTTCTGCCTTTCCCTTATTTTTATTTTTAAACAA 76932

Majority                 AATTAA-TGTTACTTAGAAGTTTGTATATCTCTGGTTTGGCCTCTGAATACTAACA-TTTG-A---TTATAGATTAATCG
                                 82970     82980     82990     83000     83010     83020     83030     83040
Human                    AAATGAGAACAACTTATGGGGAGGATAGTGAGAGGTATAGTATTTAACATGGAAAAGTTAGTAATATAGTAGAATATTTG 82070
Kakapo                   --------GTTATGCAGAGGTT-------------------C-------------------------------------- 42681
GoldenEagle              GAATTGTTGTTACTCAGAACTTTCTATTCCTCTGGTTTGACCCCTGAATACGAACATTTTTAATCTTTTAAACTTTATCA 52410
JapaneseQuail            TTTTAAATATTACTTAGTACTTTATGTATCTCTGATTTGGCCTGTGAATACTAACACTATG-----CTATTGATTCATCA 38332
MediumGroundFinch        -------------------------------------------------------------------------------- 26719
GoodesThornscrubTortoise AATTAACTCCTCAGGGAAAGAAGGGAGAAAACTCAGTTCTCATCTGCAAACTAAAGATTTCTATTTAGATGGGTTAGTGG 77012

Majority                 GAAGGCATTCTCTATGTTGAAT---CTTTATTAGAATACTTATTT----GTATTTTCTTCTGGGTATTATTAGACAAGAG
                                 83050     83060     83070     83080     83090     83100     83110     83120
Human                    GTTGAAAAGATCCACATTGG------TGTATTCAAAGACACACTGAAGACTATTGTGCATTAGTAAGTATTAAACATGAG 82144
Kakapo                   --------------------------T----GA-GAGACATGTAG----GCCTTTTCATAGG------------------ 42708
GoldenEagle              GAAAGCATTCCATGTCTTTAATTTCCTTTCTTAGAACACTTAACT----GTTTTTTCTTCTGTATGTGCTTTGACACGAA 52486
JapaneseQuail            GAAGGCATTCTGTATGTTGAATTTCCTTTTCTAGAATGTGTGTTT----TTATTTTTGTGTGGGTTTTGGCAGGAAATGA 38408
MediumGroundFinch        -------------------------------------------------GTTTTTCCTTCTG------------------ 26732
GoodesThornscrubTortoise GCCCAACTGGTCAAGGTTACATGGTCATGAGTTAAATATTCATTTATCATCATCATCCTCCAGGCATCAGAAGACCACAG 77092

Majority                 AATACT--ATTTTCCAGTTGGAGTGTGA---CAGAGATTTGCTTTAAGTCTAATTGAGTATTGAAGGAT-----CATTTG
                                 83130     83140     83150     83160     83170     83180     83190     83200
Human                    AATTCTTAATTTTAAAATTGGACCCTAATTTCTGAGATTTACTTTAAGAATAAAAGGGGATGAAACGAGATTGGCCATGA 82224
Kakapo                   ------------ACCAGCTCCAGTGTTA---CAGA------------------TTGAGT-TTGAAGCAT-----CA---- 42745
GoldenEagle              ATGACC--AGAAAATATTTGCACTATGG---CAAAGTAGTGATGGATGTCCTGTTCCCT-TTGAGGGGT-----CATTTG 52555
JapaneseQuail            CAAAAT--ATTTTTCAGCGTGTTTGTGA-ATCATGACTGATCAGTAATACTCCTTAAGTACTTCAGAAA-----AATAGG 38480
MediumGroundFinch        -------------------------------------------------------------------------------- 26732
GoodesThornscrubTortoise AGTGAA--ATCTGCAAGGCCTAGTGCAGTAGTAGAAAATAGCATACAATAGAATAAAGTAATAAATAATTCAAACAATTA 77170
```

```
Majority                  T-CTTTT-TTGTTAAACTT-TGTGTTTATTTCTTTGAG-CCCA-TATAAT-TCCACATTTATCT--TTTAATTTTTATAA

                                  83210     83220     83230     83240     83250     83260     83270     83280

Human                     GTTGATCATTGTTAAAGTTGGGTGATGGCTACTTAGAGTTGCATTATTTAGTCACTTTTACTTTTGTATATGTTGGAAA 82304
Kakapo                    -------------------------------------------------------------------------------- 42745
GoldenEagle               TCCAGCCCCCGCCAAATCTCTCTCTCTCTCTCTCTCCCCCCCTCTCAGTGCTGCCCACCTCCCCCGTATTTGTTTTTC 52635
JapaneseQuail             GACTTATGTCCTGTTCCCTTTGAGGAAACATTTATCCAGCCTGGGAAAATCTCCTTATTTTTATGGTTTGGTTTTTATCA 38560
MediumGroundFinch         -------------------------------------------------------------------------------- 26732
GoodesThornscrubTortoiseTGATTTTTGTGCTAGTCTTATCATTTTATTTGTTGGAGACTAAATAAAATGTACAAATATATCTAATTTAACTGTAAAAG 77250

Majority                  ACTT-TTTGGCAAAA-GTA-ATACAGAATT-----GGA-----GTGAGGA-AGATGTCTTTCCTAGGCAATTAGTT----

                                  83290     83300     83310     83320     83330     83340     83350     83360

Human                     TCTTCCAAAACAAAATGTATAGCCATAAAAAAAAGGGAGCAAAGTAAGAAAAAAAGGAGAGCCTAAAAAAAAAGGTACAG 82384
Kakapo                    --------------------------AGTT-----GAA-----GTGAGGT---GTCCCTGCCCATGGCAGGGGGTT---- 42782
GoldenEagle               ACAAATTTGTGTAACAAAACATAAAGAATT-----GGA-----ATGAAGATAGAAATCTTCCCTAGGCAATTAGAA---- 52701
JapaneseQuail             ACTTGTTTAGCAAAAGGTAACTAAAGAGTG-----GAA-----ATAAATATAGATGTATTTTCTAGATAATTCAT----- 38625
MediumGroundFinch         -------------------------------------------GTGGGGA------TCTGTC------------------ 26745
GoodesThornscrubTortoiseCTAGTGCAGGCATAGCACAGGGGCACAATTTCGCAGGAT---GGTGAGGA-AGAGGTGTATCCTAGGGGGGCAGTATGCT 77326

Majority                  -----AA--TACTAAAATAATCTCATTGCGTACACCCTTGTTAAATTCTTACTGTCCGTTC-CGTTTCC-TTCTCATTCT

                                  83370     83380     83390     83400     83410     83420     83430     83440

Human                     GCAAAAAGGTGTGAAAAGAAACTCATCAAGAGCAAGAGTATTAAATGAGAAACATATGCCACTGACAGAGTTCTCAGAAG 82464
Kakapo                    -----GG--AACTGGA----------------------TGA-----TCTTAAGGTCCTTTC-C----------------- 42810
GoldenEagle               -----AA--AAAAAAAATTGTAACCATGCTTACTCCCTGCCTTCTCTCTCCCTGTGTGTTC-CCTTTTGATACTAAATCT 52773
JapaneseQuail             -----AA--TTCTATAATAATCTCTTTCCATCCACAATGGTAAAGACCTTCCTCTCCCTCCATGTTTCCCTTCTGATACT 38698
MediumGroundFinch         -----------------------------------------------------ATGC----------------------- 26749
GoodesThornscrubTortoiseCACTAAA--TACCCAAGGGATTCTACTGGGAAAACCCATCATAAATTAATATTGTCCCTGCACAATGCCTTAGCCTTTTT 77404

Majority                  GGAT-AGGGGTGTTTTTAAATAAGTGG-TGA-TGTATTTACTTTT-TCTGCTTA-TATTTTGGGATTGACTGAAACTGAA

                                  83450     83460     83470     83480     83490     83500     83510     83520

Human                     AGATAAAGGCTGAATTAAAAAGAGATGGAGGGGAAAGTTTTATAAGTCAAAAACTTAGGTTAAGGTTTGTGAATATTAAA 82544
Kakapo                    -------------------------------------------------------------------------------- 42810
GoldenEagle               GGGATTATTCTTTTTTTAAATTAATGGTTGAATGTATTTACTATTATCTGCTTAGTACTTGGGCATTGACTGAAACTGAA 52853
JapaneseQuail             AAATCAGGGGTATTTTTCAATATGCAGATGACTATATTTACTGTTTCCTGCTTAATATTCGGTCATTGACTGAAACTGAA 38778
MediumGroundFinch         -------------------------------------------------------------------------------- 26749
GoodesThornscrubTortoiseGGAGGGGAGGAGCTGAAGTGTAGGTGGCCATTGGCAGCTGAGTTCCTGGATTAACTTTGTTAGGAGGCAGAGAGACTAGG 77484
```

```
Majority                     GAGCTTTTCTTTTTTCTG---ATGTCTTTTAATA-AATACTTTAAGGTCAAAAGGGCGAAAACTT---T--A-AGTGTAA
                                     83530     83540     83550     83560     83570     83580     83590     83600
Human                        GAGCGCTTCAGATTGCCAATGAAAGGTCATAGTA-GTTTCTATAAAGTCAAAAGATCAAAAACTTGACTTAAAAGAGTAA 82623
Kakapo                       --------------------------------A-----ACCCTAA----------------------------------- 42818
GoldenEagle                  GAATTTATTTTTTTTATG---ACTTCCTTTAAAATAAAACTTAATGTGGGAAGGGAAGTAGTCTTAGGTGCATAGTGAAA 52930
JapaneseQuail                GAACTTTGCTTTTTCCTG---TTGTCTTCTTCTGAAATAAATCAGGGGAAAAAAAGCGCTAGAT-------AGTGTAAAA 38848
MediumGroundFinch            -------------------------------------------------------------------------------- 26749
GoodesThornscrubTortoise     CAGGATTACCACTATGGAGCCATGTCTCTGGACTGAATATTCCTATGTCTTCATGGCTAGAACTTTTTTTCCCCCACTGA 77564

Majority                     GATTATTTTTA--CTGTCTACCTAGGATACTGGAA--GACTTGTCCAGAGATGTGGTA-C-TAGCATGATGATATAAGCT
                                     83610     83620     83630     83640     83650     83660     83670     83680
Human                        GATCTCTAAAACCAAAGGTAAC-AGCAGATTCTTATGAAATGCACCAGAAAGCTGGAA----AGAAAGAAGAAATAAAAT 82698
Kakapo                       ----------------CCAGTCTAGGATTCTATGA--GGAATGCC----------------------------------- 42845
GoldenEagle                  AGTTATATTTAGACTGTCTACCTGGCATACTGGAA--TATTAGTCAAAAGTTGTATTAGCTTAGCGTGATCATATAAGCA 53008
JapaneseQuail                AATTATATTTAATCTGTCTACCTGGTGTATTGGAA--GACTGGTCAAAAGCTTTGCTAGCTTAACATCATCATACAAACA 38926
MediumGroundFinch            ---------------------C----ATACTG------------------------------------------------ 26756
GoodesThornscrubTortoise     CCCTATTACTATGGGTAATACGTAGGCTACTCGATTTGCCTTGCTCTGCAAGGTTGTAACCTAGCCTAAAGCATCAAGTT 77644

Majority                     TTAGAAATTTTGCCAA--AGTGCTGAAAGCT--------------A--------AAGTTAA---GCATTTAAGTTATGT-
                                     83690     83700     83710     83720     83730     83740     83750     83760
Human                        GTGAACTTATGGAACAAGAGTGCTAAGGGCCTGTTCTACCCAGGTATGTTATGAAATCAAA--TTCATTTAGTTTGGGTG 82776
Kakapo                       --------TTTCAAAG--GGTGATGGAAAAT----------------------------AA---GGATG----TAA---- 42876
GoldenEagle                  TTAGAAGTTTTGCCAA--AATACTGAAAGCTGAGAAGCCAGTTGCAGTAAAAGAAAATAAA---ACGCTGAAGTTATAT- 53082
JapaneseQuail                TCTGGAATTTTGCCAA--ACTGTAGAGAAA--------------------------GTTAA---ACACTTAAGTTCTGT- 38974
MediumGroundFinch            --------------------TCCTGGAA-------------------------------AA---GTACT----------- 26771
GoodesThornscrubTortoise     CAAGTGAAGAAAGTAACTAATACCAAAGCGTAATATAAAGGAAATATAATGGTGAAGATAACTTGCATTAATATTTAGT- 77723

Majority                     TTCTTT----TTTTTTGCACATTTT----GCTACATTGGTTTCCTTTT---ACTGAACT------------CTATAATAT
                                     83770     83780     83790     83800     83810     83820     83830     83840
Human                        TTTGTTCCTTTATGTTGGGAAGATAAAAAAGAACATATAATTCGTTTCTTGAGTGAACCAGTATTTATGAGAAATAAGCT 82856
Kakapo                       ------------TGATGCAGAGAGC----ACAATTAGCGTTTACTCTT---ACTGATTTGT----------CAATATTTA 42927
GoldenEagle                  TTCTTTATTGTTTTTTACACATTTT----GCTATGTCGGTGTCCTTTT---ACTGAACT------------CTGTGAAAT 53143
JapaneseQuail                TTCTGTT---CTTTTTACACGTTTT----GCTACATAAATGTCCTTTT---AATGGACT------------CTGTAAAAT 39032
MediumGroundFinch            ------------T--T-------------GATACAATGGTGCTCATTT---ACTGAAC-------------CTACATGAT 26808
GoodesThornscrubTortoise     TTCACTGACTTGACTGGTATATTCCAGCAACAAGATTAATTAAGGTCC---ATCTAGCCCA----------GTATCCTGT 77790
```

```
Majority                   GTGCAAATAATAATTTTAAT----CAAATATTTTAAAGCTATT----CCTGTATAGGCTATGTACAGTGCATATGTATTA
                                   83850     83860     83870     83880     83890     83900     83910     83920
Human                      GGGTGTGTAAGAATTTAAGGGATGAAAATATTCCAAGTTGATTGGTAATTGAAAAGTAGATGAACGGTGCTAACACAAAA 82936
Kakapo                     CTTTAGACCATTGTGATAAC----TAGAAACTTTAAGCATTTT----AGAGTACAGGCTGAATACATTTTAGA-GTATGA 42998
GoldenEagle                GTACAAATAA-AATTTTGAT----CAAATATGATAAAGCTGTT----CCTGTATCAACTATGTATAGTGCATATGTATCA 53214
JapaneseQuail              GTGCAAATAAAAATTTGATT----AAAATATGATATTGATATT----CCTGTATGAGCTGTCTGCAATGTATATATGTTG 39104
MediumGroundFinch          GTGCAAATGGCAATGTGGAT----CAAATACATTAAAGCTGTT----CCTATATAAACCATGTATAGTGCAAGTGTATCA 26880
GoodesThornscrubTortoiseCTTCCAACAGTGGTCAATGC----CAGGTGCCTCAGAGGGAACG--AACAGAACAGGTAATCATCAAGTGATCCATTCTC 77864

Majority                   CGAGAGTCTTTCCCGGATCC-TGGGAGTAGCACGCTTGGCAGA-------TACCCCAGTGACGTGACCAG--ATTAAACC
                                   83930     83940     83950     83960     83970     83980     83990     84000
Human                      GGGAATTCTGTCTAGAATAAATGAAAATATGGTGACTTAAAGAT----AGTAGGACAGCAATAAGACTGGAAATTAGAAC 83012
Kakapo                     AGACAGGCT---GAGAACAT-TGGGGCTGTTCAGCTTGGAGAA-------GAGA--AGCTGCGTGGAGAC--CTCAGAGC 43063
GoldenEagle                CAAGAACCTTTCACAGATCC-TGGGAGTAGCACACTTGCCTAG-------TACCCCAGTGACTTGACCAG--GCAAAACC 53284
JapaneseQuail              CAGAGATCTTTCACAGCTCC-TGGAAGTAGCACAGTTGTCAGG-------TGCAGCAGTGACATCACCAC--ATGCAACA 39174
MediumGroundFinch          CAAGAGCCTTTCCCGAATCC-TGGGAGTAGCACACTCAACTCA-------TACCCCAGTGATGTGAGCAG--ACAAAACC 26950
GoodesThornscrubTortoiseTGTCGCTTATTCCAAGCCTC-TGGCAAGCAGAGGCTAGGGACACCATCGCTGCCCATCCTGCCTAATAACT-ATTGATGG 77942

Majority                   AGCATTTCTCTATAGG---ATCTGAGTTTCTTTGGA---TGATGGAAAGTA--GAAGCTCAACTGATGCTGTAGC-TGAT
                                   84010     84020     84030     84040     84050     84060     84070     84080
Human                      AATAAAATAAGAAGGAAATGATAACATAGATTGGGAAAGGATAGGAAAATACTGAAACAAAATCTGTGTTGTATAGTGAT 83092
Kakapo                     AGC-TTCCAGTGTCTG---AAGGGGGCTACAA-GGA---TGCTGGAGAGGG--ACTCTTCATCAGAGACTGTAG--TGAT 43131
GoldenEagle                AGCAGGGCTCTACAGG---ATGTGAATTTCCTTGGA---AGAAGGAAAAAA--AAAGCACAGCTGAAGGTTTAGC-TAAT 53355
JapaneseQuail              ACCAGATTTCTGTAGG---ATCTGAGTTACTTTGGA---AGAGTGG------------TCAAGTGTTTTTCTAG------ 39230
MediumGroundFinch          AGCATTTCTGTACAGG---GTCTGAGTTTCCTTGAA---GAAGAAAACGTA--GAGGCACAACTGAAGCTATAGC-TGAG 27021
GoodesThornscrubTortoiseACCTATCCTCCATGAATTTATCTAGGTTTTTTTGAACTCTGTTATAGTCTT--GGCCTTCACAACATACTCTGGC-AAAG 78019

Majority                   GGTAACATGTTTTTTCAGGGCGTTGGATTTCAAAAGCCACAGAGTATG-T---T---------TTGAGTCTTTAATTTAG
                                   84090     84100     84110     84120     84130     84140     84150     84160
Human                      GGTTTCTTGTTCTTTCAACCGGGAGAGTGTTGTGTTGAAGGAACTTGTATTCATTTTAATTACTTGAGTGTATAATTAAG 83172
Kakapo                     AG-GACA-AG-------GGGTGATGG-TTTCAACTGAACCAGGGGAAG---------------TTCAGG-TTAGATCTAA 43185
GoldenEagle                GGTAACATGTGTCTTCAGGGCATTGGATTACAAAAGCCACAGAGCATAATCAGTGGTGCTACCTTCAGTCTTGAATTTAC 53435
JapaneseQuail              GGAATCATAT---------------TATAT--AAAACCATAGAATAAC-----------------CAGT--TTGATGCAG 39274
MediumGroundFinch          GGTAACATGGTTCTTCAGAGAACTGGATTACTAAAGCCACAGAGCATG---------------TTGAATCTTGAATTTAG 27086
GoodesThornscrubTortoiseAGTACCACAGGTTGGCTGTGCGTTTTGTGA--AGAAATACTTCCTTTGTTTGTTTTAAACCTGCTGCCTTCTAATTTCAT 78097
```

```
Majority                ATGGTTGCCTC-TTCACCTTG--CATGCTAAGGTTCTGAGA---GATGCATAGTCCTTTTCACATGGCCAGCTCTCTAGT

                                 84170     84180     84190     84200     84210     84220     84230     84240

Human                   AAGGCAAGCACATTTTGCTAAGATTTTCTTTATTTGCATGA---ATTAAATAATGATGTTCAGAGTGATTTGTCTCTAGG 83249
Kakapo                  GGCAGAAGCTC-TTCCCTGTGAGGGTGCTGAGGCGCTGGCACAGGGTGCCCAGAGAAGCTGTGGCTGCCCCATCCCTGGC 43264
GoldenEagle             AACGTTGCCTC-CTCACCTAGTTCATGCTAAGGCTCTGAGA---GATACATAGGCCTTTTCACATGACCAACTCGCATGT 53511
JapaneseQuail           CTGTGTGCCTC-CTCACCT----CAT-TTATGGTG-TGAGA---GATGCATAGGCCTTTTCACTTGACCAGCTCTGCAGT 39344
MediumGroundFinch       AGGGTTGCCTC-CCCACCT----CATGCTAAGGTTCTGAGA---GATGTGTAAACCTCTTCACATGGACAAATGCACAGT 27158
GoodesThornscrubTortoiseTTGATGACCTC-TTGTTCTTG--TGTTATGAGGAGTAAATAACACTTCCTTATTTACTTTCTCCACACCAGTCATGATTT 78174

Majority                TGTGGACATAGTCTGAAGTTTGAAGTTGAAGTGTGGAGTACCT---CTCAAA-G-TGGTGTGAAATAAAGATGTCATGAT

                                 84250     84260     84270     84280     84290     84300     84310     84320

Human                   AGCTGAAAGACTACTACCTTTGTTTCATAAGCTGTAATTATTTGATTTCAAGTGTGAGTTTATGAAGAAGATAGCAC-TT 83328
Kakapo                  AGTGTTCAAGGCC--AGGTTGGACACAGGGGCTTGGAGCAAC----CTGCTC---TAGTGTGAGGTGTCCCTGCC-TGTG 43334
GoldenEagle             TGTATACTTAGTCTGAAGTATCAAGTTGAAGTGAGGAGTACCT---CTCAAAGGGTGATGGAAAATAAGGATGTAATGAT 53588
JapaneseQuail           -----ATATAGTATGAAGCATGAAGTTGAAGTGAGCAGTACCT---TTCAAA-------GGGCAATACAGATACA----- 39404
MediumGroundFinch       TGGGGATTTAGTC--AAGCACCAAGTTGAAGTGTGGAAAGTCT---CTCAAA---TGATGAGAAACAAGGATGTAATGAT 27230
GoodesThornscrubTortoiseTATAGACCTCAGTCATATCTCCCTTTAGTCGTCTCTTTTCCAAG--CTGAAAAGTCCCAGTCTTATTAATCTCTCCTCAT 78252

Majority                GCGGAGAGCACAATTAGTGTTTATTTTTACTGATTTTATCAACATTTACT-T---CC---------AACTAGACA-----

                                 84330     84340     84350     84360     84370     84380     84390     84400

Human                   GCGGAAAAAA--GTGAATTACCATTATGTCGAAAGTGATACTTTTTTACATTTGGCCTTTTACTGTAAATCGTGGAAGTG 83406
Kakapo                  GCAGGGGGTTGGAACTG-GATGATCTTTAAGGTCCTTTCCAAC------------------------CCAAACC----- 43383
GoldenEagle             GCAGAGAGCACAATTAGTGTTTATTTTTACTGATTTGATCAACATTTACT----------------AACTGGACA----- 53647
JapaneseQuail           GTGGGGAG------------------------------------------------------------------------ 39412
MediumGroundFinch       GCAGAGAGCACAATTAGTGTTTATTCTTACTGATTTAATCAACATTTACTTTAGACCATCTTGATAAACTAGACA----- 27305
GoodesThornscrubTortoiseATGAAAGTGATTATATACCCTAATCATTTTTGCCCTTTTCTGAACTTTTTCTAAACCTGTAGATATTATTAGGGAGGATT 78332

Majority                -TTTCTGTCATTTTGGAGTACAGTAGAAACAGTTTTGGTGCACAAGAAT---GTTTATTTGAAAAGAA-----AGTACTT

                                 84410     84420     84430     84440     84450     84460     84470     84480

Human                   AATTTTCCAAGCCTGTGACTTGG-AATTATATTTTTCATGCTTTG-TTTGCTTTTCCAGAGGAAATAGAGTATTATATAT 83484
Kakapo                  -ATTCTGTGATTCTA---TACAGGAGAAACAGTTCTGGTACATAG-AAT---GTTTGTTTGAAAAGAA-----AGTACTT 43450
GoldenEagle             -TTTTAAGCATTTTGGAGTACACTGGAAACACCTTTGGTGCACAAGAAT---GTTTATTTGAAAAGAA-----AGTACTT 53718
JapaneseQuail           --------------------CG---------GTTCTGGTGCACAAGAAT---ACTTATTTGAAAAGAGGAGAGAGTAATT 39460
MediumGroundFinch       -CTTCAAGCATTTTGGAGTGCAGTGCAAACAGTTTTGCTGCACAATAAT---GGTTATTTGAATAGAA-----AGTACTT 27376
GoodesThornscrubTortoiseTTTGCTGTAATCTAAGAAAAAAAGAGCAATATGTGTGCTTCACAAGAACTACATTTGATTGAAAACAA-----AATGGTC 78407
```

```
Majority                    TTTATACCT---CATCAGTTCTTTGAAGACTTGATTTGAAT-AA--AA-------AATATTTTTCATTTTTCTTCTTTCT
                                 84490     84500     84510     84520     84530     84540     84550     84560
Human                       TTGATATTTTAACATAATACAGGTATGTACTTTAGTTCAAATAATTAATGGTTTTGTTGAAATCAATTATTTTTCTTTAC 83564
Kakapo                      TTTATACCT---CCTCAATTCTTTGAAGACTTGATCTGAAT-AA--GA-------AAGAATTTTCACTTTGCTTCTTGCT 43517
GoldenEagle                 TTTACACCT---CATCACTTCTTTGAAGACTTGATCTGAATCAA--AAAAA----ATAATTCCCCATTTCTCTTCCTTCT 53789
JapaneseQuail               TTTACACCT---CGTCTCTT---TGAAGCCTTGATTTGAGT--------------AATATTTAATATTTCTATCCCTCAT 39520
MediumGroundFinch           TTTATACCT---CATAAGTC---TGCAGACTTGATCTGAA--AA--A--------AAAATTCTACGTTTCTCTTGCCT-- 27436
GoodesThornscrubTortoise    TTTAGAGAA---GTGAAGTAGCGTATAAAGT-GCTTTGAAGCAA--GAAAATAGCGAGAACTTTGGTTTTTCTCCTTA-T 78480

Majority                    GTTAATTTAGCAACCCATTTCACAACCCTTTGGATAACTTTAACAGTT-GAATGGTTTGCTTTGTAGAGTGTTGTATGCA
                                 84570     84580     84590     84600     84610     84620     84630     84640
Human                       TACACATCAGCAAACAATTTTTCTAAACACTATAAAAATTAACTTTCAGGAACCATTTGGAATGGTCAGCCGTAAGCCAT 83644
Kakapo                      GTGAATTTAGCAACCCCTTCCTCAAGCCTTTGGAGGACGTTGACAGC--GAATGGTCTGCTGAGTAGAGTGTTGTCTGCA 43595
GoldenEagle                 GTTAATTTAACAACCCATTTCACAACCCTTTGGATAACATTAACAGTG-GAATGGTCTGCTCTGTAGAGCATTGTATGCA 53868
JapaneseQuail               GTTCACTTA---ATTCATTTTACAACCCTCTGGACATCTACAGCAGTTAGAATGGTCAACTTTGTAGACT--CATATTCA 39595
MediumGroundFinch           --TAATTTACCAGCCTATTTCATAACCTTTTGGATGACTTTA-CAGTT-GTATTGTTTGCTGTGTAGAGTGGTGTATACA 27512
GoodesThornscrubTortoise    ATTTA-TTACTGATCCATTTCACTAAGCATTGGAAAATACGAATTGTG-GAAGGAATTTTTTTATAGTGT---AAATATA 78555

Majority                    GCACTTGTACTGATAAAGGTGCTTTTTT-T--TAAATGACTTGTGTGTGTTTCGTAAAATATGCTTTATTTTAAAATTAG
                                 84650     84660     84670     84680     84690     84700     84710     84720
Human                       GCAGCCATCGTGCTTAAATTGGTTTTTG----TAAACGACTTGTGTGTGTTTCGTAAAATATGCTTTATTTTAAAATTAG 83720
Kakapo                      GCACGTGTGCTGATAGAGGGACTTTTCTATACAAAATGACTTGTGTGTGTTTCGTAAAATATGCTTTATTTTAAAATTAG 43675
GoldenEagle                 ACACTTGTGCTGATAAAGGTACTTTTTT-TTTTAAATGACTTGTGTGTGTTTCGTAAAATATGCTTTATTTTAAAATTAG 53947
JapaneseQuail               ACACTTGTACTGATGTAGGTGCTGTTCT----TAAGCGACTTGTGTGTGTTTCGTAAAATATGCTTTATTTTAAAATTAG 39671
MediumGroundFinch           GCCCTAGTACTGATATAGGAACTTTTTT-TAGAAAATGACTTGTGTGTGTTTCGTAAAATATGCTTTATTTTAAAATTAG 27591
GoodesThornscrubTortoise    GTATTTGAACTTCTAATTTAGATATTTT----TAAATGACTTGTTTGTATTTCGTAAAATATGCTTTATTTTAAAATTAG 78631

Majority                    GGCTTCCTGTTGCAGCTGTTCCAGGAGCTCTGAGTCCTTTGGCTATTCCAAATGCTGCTGCTGCAGCTGCAGCTGCTGCT
                                 84730     84740     84750     84760     84770     84780     84790     84800
Human                       GGCTTCCTGTTGCAGCTGTTCCAGGAGCTCTGAGTCCTTTGGCCATTCCAAATGCTGCTGCAGCAGCTGCTGCAGCTGCT 83800
Kakapo                      GGCTTCCTGTTGCAGCTGTTCCAGGAGCTCTGAGTCCTTTGGCTATTCCCAATGCTGCTGCTGCAGCTGCAGCTGCTGCT 43755
GoldenEagle                 GGCTTCCTGTTGCAGCTGTTCCAGGAGCTCTGAGTCCTTTGGCTATTCCAAATGCTGCTGCTGCAGCTGCAGCTGCTGCT 54027
JapaneseQuail               GGCTTCCTGTTGCAGCTGTTCCAGGAGCTCTGAGTCCTCTGGCTATTCCAAATGCTGCTGCTGCAGCTGCAGCTGCTGCT 39751
MediumGroundFinch           GGCTTCCTGTTGCAGCTGTTCCAGGAGCTCTGAGTCCTTTGGCTATTCCAAATGCTGCTGCTGCAGCTGCAGCTGCTGCT 27671
GoodesThornscrubTortoise    GGCTTCCTGTTGCAGCTGTTCCAGGAGCGCTGAGTCCTTTGGCTATTCCGAATGCTGCTGCTGCTGCAGCTGCTGCTGCT 78711
```

```
Majority                 GCTGGCCGTGTGGGAATGCCTGGAGTTTCAGCTGGTGGCAATACAGTCCTCCTGGTTAGCAATTTAAATGAAGAGGTCAG

                                  84810     84820     84830     84840     84850     84860     84870     84880

Human                    GCTGGCCGAGTGGGTATGCCTGGAGTCTCAGCTGGTGGCAATACAGTCCTGTTGGTTAGCAATTTAAATGAAGAGGTTAG 83880
Kakapo                   GCCGGCCGGGTGGGAATGCCTGGAGTTTCCGCTGGTGGCAATACAGTTCTCCTGGTTAGCAATTTAAATGAAGAGGTCAG 43835
GoldenEagle              GCTGGCCGTGTGGGAATGCCTGGAGTTTCAGCTGGTGGCAATACAGTCCTCCTGGTTAGCAATTTAAATGAAGAGGTCAG 54107
JapaneseQuail            GCTGGCCGTGTGGGAATGCCTGGAGTCTCAGCTGGTGGCAATACAGTTCTCTTGGTTAGCAATTTAAATGAAGAGGTCAG 39831
MediumGroundFinch        GCTGGCCGTGTGGGAATGCCTGGAGTTTCAGCTGGTGGCAATACAGTCCTCCTGGTTAGCAATTTAAATGAAGAGGTAAG 27751
GoodesThornscrubTortoiseGCTGGCCGAGTGGGTATGCCTGGAGTTTCAGCTGGTGGCAATACAGTCCTCCTGGTTAGCAATTTAAATGAAGAGGTCAG 78791

Majority                 TGAAACAATTTTA---TCTTTTTTTTTTTTCCTCTTTAGTCACACTTCATTTGTCAGTGTTTTAT-ATTTTTTTGCATTT

                                  84890     84900     84910     84920     84930     84940     84950     84960

Human                    TAAAATAATCTCTAATGTTTATTCTTTAACTCCATTTCATT-TGTGAAAGTTTTTCATGTTTATT--TCATTTTGCACTT 83957
Kakapo                   TGAAACCACTTTA----CTACTTCTTTTGCCCTCTTTAGTCACCCTTCATTTGTCCCTGTTTTATGATTTCTTTGCATTT 43911
GoldenEagle              TGAAACAATTGTAATTTTTTTTTTTTTTTTCCTCTTTAGTCACACTTCATTTGTCAGTATTTTATAATTTCTTTGCATTT 54187
JapaneseQuail            TGAAATAATTTGA--CTCTTTTATTTTTTTCCTTTTTACCCATATGTCATTGGTCATCATTTTAT--TTCTTTGTTTTTT 39907
MediumGroundFinch        TGAAACAATTTTA----CTTTGTTTTCCCCCCTCTTTAGTCACACTTCATTTGTCAGTATTTTATAATTTCTTTGCATTT 27827
GoodesThornscrubTortoiseTGAAACTATTAAC---TCTTCTTTTCCTTTATTTTTAATCACACTTCATTTGTAAATGTTTTTC-ATTTTTTTGCACTT 78867

Majority                 GGCCTC--TTATATTTGGAT--TTTAAGCTCAAA-GTATTTTTGCTGTT---TTTTTTTA--ATAATCCCTACCCAAAGT

                                  84970     84980     84990     85000     85010     85020     85030     85040

Human                    GCCTTTCTTTTTATGTACAT--TCATTAGTCAAA-GTATTTTCTGTATGTTTCTACTTCTGAATAAAATCTACATATTGT 84034
Kakapo                   GGCCTC--TTACATTTGGGT--TTTAAGCTCAAAAGTATTTTTGCTGGT---TTTCTTTA--GTAATCCCCTCCCCAAGT 43982
GoldenEagle              GGCCTC--TTACATTTGGAT--TTTAAGCTCAAA-GTATTTTTGCTG-----TTTTTTAA--TAATCCCCCA----AAGT 54251
JapaneseQuail            GGCCTC--TTATATTTGGAT--TTTAAGCTCATA-GTATGTTTGCTGTTACATTTTATTACCTTACTACTCCCCCAAAGT 39982
MediumGroundFinch        GGCCTC--TTATATTTGGAT--TTTAAGCTCAAA-GTATTTTTGCTG-----TTTTTTTA--ATAATCCCTA----AAGT 27891
GoodesThornscrubTortoiseGCCTCTATTTTCACTTGCATACTTTATGATCAAA-GTATTTTTTCTCGTTTTTTGTATGTCTAAATTAATTTGGAAACTA 78946

Majority                 TAATTTTAAAGTTATTTTTGTTAAATACTTTTGTTTTCCTAGGTAGCTAACAACTTAAAATGTCCTTTGATAGTTGGCTA

                                  85050     85060     85070     85080     85090     85100     85110     85120

Human                    TTAGGTAATACTTTATTTCCTTAAACAGTCATGTCTCTTTATATCTATATCTATATATG-TATATTTTTTAACCTA---A 84110
Kakapo                   TAATTTGAAAGCCC-------TT----CTTTTGTTTTTCCAGGTAGCTAACAACTTAAAATGTCCTTTGATAGTCGGCTA 44051
GoldenEagle              TAATTTTAAAGCCATTATTGTTAAATACTTTTGTTTTCCCAGGTAGCTAACAACTTAAAATGTCCTTTGATAGTCAGCTA 54331
JapaneseQuail            TAATTTTGAAGTTATCCTTGTTAAATACTTTTGTTTTCCAAGGTAGCTAATGACTTAAAATGTACTTTCATAGTTGGCTA 40062
MediumGroundFinch        TTATTTTAAAGCCA---TTGTTAAATACATTTTTGTTCCTAGGTAGCTAACAACTTAAAATATCCTTTGACAGTCAGCTA 27968
GoodesThornscrubTortoiseTAAACTTTATATTTTTTCTATTA--TAATTTTGTTTTCCTAGGTAGCTAACAACTTAAAATAGCCTTTGATATTTGGCTG 79024
```

```
Majority                   CCTATTTTTTCT--AAGAAAAATATGGT-AACTACTGCATGTTTGACTTTTGAATTGAAAGTTCTA--TTT------AAA

                                  85130     85140     85150     85160     85170     85180     85190     85200

Human                      CCTATTTTTCCT--AAGAAAAATATGGTGAAGTACTGTA-GTTTGGCTTATCTTTTTGTTGTTCTC------------AA 84175
Kakapo                     CCTATTTTTTCT--AAGAACAATGTGGT-AACTACTGCATGTTTGACCTTGGAATTGAAAGCTCTTCTTTT--------- 44119
GoldenEagle                CCTATTTTTTGT--AAGAAAAATGTGGT-AACTACTGCATGTTTGACATTTGAATTGAAAACTTTATTTTTACA---AAA 54405
JapaneseQuail              CCTGTATTTTCT------TAAGTATGGT-AACTATTGCATGTTTGACATTTGAATTGAAA-------------------- 40115
MediumGroundFinch          CCTATTTTTTTTCTAAGAAAAATTTGGT-AACTTCTTCATGTTTGA----------------------------A-- 28014
GoodesThornscrubTortoiseCCTATTTTTCCT--AAGAAAAATATGGT-AAATACTGCATGTTTGGCTTTCGATTTGTAACTTAAATATTTTCACAAAAA 79101

Majority                   TAGCAACTGTGCTTTCTTTTGCA-ATTATAATC-ATGTAAACTAGAATTT-CTGTTTTGTTTCTTCTAATTGTTTGCTGT

                                  85210     85220     85230     85240     85250     85260     85270     85280

Human                      AGGCAAATGTGATCTAATTTGCAAATT-TAACT---GTAAAACAATTTTG--CTCTTTGCCTTTTCTAATTATTTGCTTG 84249
Kakapo                     TTACACCTCTGCTTTCTTTTGCA-ATTATAATC-CCCTAAACTCGATTTTTCTGTTTCGTTTCTCTTAATGGTTTGCTGT 44197
GoldenEagle                TAGCAACTATGCTTTCTTTTGCA-ATTATAATC-ATGTAAACTAGAATTT-CTGTTTTGTTTCTTCTAATTGTTTGCTGT 54482
JapaneseQuail              -TTTTGTTTTGTTTTATTTTACA-ATTATGATC-ATTTAAACTTAGAATTCCTGTTTTGTTTCTTCTAATTGTTTGCTGC 40192
MediumGroundFinch          TACCAACTATTCTTTCTTTTGTA-ACTGTAATC-ATGTAAACTAGAATTT-CTGTTTTGTTTCTT---ATTGTTTGCTGT 28088
GoodesThornscrubTortoiseCAGCAAATGTATTCTAATTTGTAGATTATATTTTATGTAAAGTAGAATTG--TGTTTTTCTTTTC-TAATTATTTGTTGC 79178

Majority                   TTCATTTCATGCTTGTATCTCACA------TTTTAAGGTCTT-AATTTATGTGAACTACTCTAACACGTTTTTCTTTGAA

                                  85290     85300     85310     85320     85330     85340     85350     85360

Human                      TTCATTTCATGCTTATATGTCATTGCATTTTTTTTAAATCTT-ATTTTATGTGAACTACTCTAATACATTTTTCTTTGAA 84328
Kakapo                     TCCATTCCATGCTTGGAGCTCACC------TTTGAAGGTCTTTAATTTATGTGAACTACTCTAACACGTTTTTCTTTGAA 44271
GoldenEagle                TTCATTTCATGCTTGTATCTCACA------TTTTAAGGTCTT-AATTTATGTGAACTACTCTAACACATTTTTCTTTGAA 54555
JapaneseQuail              TTCATTCCATGCTTGTATCTCACA------TTGTAAGGTCTT-AATTTACGTGAACTACTCTAACACGTTTTTCTTTAAA 40265
MediumGroundFinch          TTCATTTCATGCTTGTATCTCACA------TTTTTGAGTCTT-AATTTATGTGAACTACTCTAACACATTTTTCTTTGAA 28161
GoodesThornscrubTortoiseTTCATTTCATGCTTACATGTCACTGCAT--TTTTAAAATCTT-AATTTATATGAACTGCTCTAACACGTTTTTCTTTGAA 79255

Majority                   GGTTCTCCCAAAGATCTTGACGAGGCACTCTTCCAGTCTCTCTTAGTAATTTTTTTTCTTTGCAGTTACTCATTTGTCT

                                  85370     85380     85390     85400     85410     85420     85430     85440

Human                      GGTTCTTCCAAAGATCTTGACGAGGCACTCTTCCCGTCTTTCTTAGTAATTTTTTCTTTGCAGTTATTAGTCATTGTCTT 84408
Kakapo                     GGTTCTCCCAAAGATCTTGACGGGCCACTCTTCCAGTCTCTCTTAGTAATTGTTTC--CTTTGCAGTAACTCGTTTGTCT 44349
GoldenEagle                GGTTCTCCCAAAGATCTTGACGAGGCACTCTTCCAGTCTCTCTTAGTAATTTTTTT--CTTTGCAGTTACTCATTTGTCT 54633
JapaneseQuail              GGTTCTCCCAAAGATCTTGACGAAGCACTCTTCCAGTCTCTCTTAGTAATTTTTTT--CATTGCAGTTTCTCATTTGTCT 40343
MediumGroundFinch          GGTTCTCCCAAAGATCTTGACGAGGCAGTCTTCCAGTCTCTCTTAGTAATTTTTTTTTCTTTGCAGTTACTCATTTGTCT 28241
GoodesThornscrubTortoiseGGTTCTCCCAAAGATCTTGACGAGGCACTCTTCCAGTCTCTCTTAGTAATTTTTTCTTTGCAGTTATTATTCATTTGTCT 79335
```

```
Majority                      TTAAAAAA--------A----TAAAAAAATGGTGGCAAAGCATTTTCACCTTAA--CTGT-ATCTTTCTTGCTAACTCTG
                                       85450     85460     85470     85480     85490     85500     85510     85520
Human                         CTAAAAAT--------A-TTTTTAACTTTATTCCCCACCCCTTCCTTCCTTACCACCCCCTCCACCCAGTTAAATAAA 84479
Kakapo                        TTAAAAAAATAACAATAAGACGAAAAAACTGGTGGCAAAGCATTTTCACCTTAA--CTGT-ATCTTTCTTGCTAACTCTG 44426
GoldenEagle                   TTAAAAAA-------TAATAATAAAAAAATGGTGGCAAAGCATTTTCACCTTAA--CTGT-ATCTTTCTTGCTAACTCTG 54703
JapaneseQuail                 AAAAGTAA-------------TAAGGAAAAATTGACAAAGCACTTTCACCTTAA--CTGT-ATCTTTCTCGCTAACTTTG 40407
MediumGroundFinch             TAAAAA---------------TAAAAAAGTGGTGGCAAA-CATTTTCACCTTAA--CTGT-ATCTTTCTTGCTAACTCTG 28302
GoodesThornscrubTortoiseCAAAA------------------------------ATA--TTTTTCACCTTAATACTGTTATCTTTCTTGCTAACTCTA 79383

Majority                      A---AATCTAAAGGGGTTT-ATTTAGTTTTGCATTTTTACTGTCCTTT-ACATTTATTTTGATGGCTGTGAAAGCTGGTA
                                       85530     85540     85550     85560     85570     85580     85590     85600
Human                         ATTTATTGCTAAGTTATTTTCTTTAGTTGTACATTTTA--TGTCTGGTATATTTTATTTTGAATGCTATGAAAGCTGGTA 84557
Kakapo                        A---AATCTCCAGGGGTTTGATTTAGTTTTGCATTTTGACTGTCCTTTTACATTTCTTTTGATGGCTGTGAAAGCTGGTA 44503
GoldenEagle                   A---AATCTAAAAGGGTTT-GTTTAGTTTTGCATTTTTACTGTCCTTT-ACATTTATTTTGATGGCTGTGAAAGCTGGTA 54778
JapaneseQuail                 A---AATCTAAAGGAGTTT-GTTTAGTTATGCATCTTTACCGTCCTTT-ACTTTTATTTTGATGGCTGTGAAAGCTGGTA 40482
MediumGroundFinch             A---AATCTAAAGGGATTA-ATTTAGTTTTGCATTTTTACTGTCCTTT-ACATTTATTTTGATGGCTGTGAAAGCTGGTA 28377
GoodesThornscrubTortoiseA---AATCTAAGGGGTTTT-ATTTAGTT-TGCATTTTTACTGTTTTGTACAATTTTTTTTAATTGATGTGAAAGCTGGTA 79458

Majority                      TGAAATGTGGGAAGTTTG---TATCAGTCTAG-----CT-------------GTTCCATTTTTAACTGGCCTCTGTCACT
                                       85610     85620     85630     85640     85650     85660     85670     85680
Human                         TGAAATGTGGGAAGCTCAGTGTATCAGTTTATGATGTCTAATTTGAATATTTGTTTCATTTTTAATTGGCCTCTGTTAAT 84637
Kakapo                        TGAAATGTGGGAAGTTTG---TATCAGTCTAG-----CT-------------GTTCCATTTTTAACTGGCCTCTGTCACT 44562
GoldenEagle                   TGAAATGTGGGAAGTTTG---TATCAGTCTAG-----CT-------------GTTCCATTTTTAACTGGCCTCTGTCACT 54837
JapaneseQuail                 TGAAATGTGGGAAGTTTG---TTTCAGTCTAG-----CT-------------GTTCCATTTTTAACTGGCCTCTGTCACT 40541
MediumGroundFinch             TGAAATGTGGGAAGTTTG---TATCAGTCTAG-----CT-------------GTTCCATTTTTAACTGGCCTCTGTCACT 28436
GoodesThornscrubTortoiseTGAAATGTGGGAAGTTTGATGTATCAGTTTGT-----CTAGTTGGAATACTTGTTTCATTTTTAACTGGCCTCTGTTAAT 79533

Majority                      GTAAATCATTAAGCTTTTTTTATCATCTGGTACTATTTTGTCATCCACAGTCTTGCATGTTAAAATGTTTTAGATCAGAG
                                       85690     85700     85710     85720     85730     85740     85750     85760
Human                         ATGAA-CATTAGCTTATTTTATCCATTTAATACTATT--ATCATTCACAGTTCTGCATGCTAAAATGTTT-GAATCAGAG 84713
Kakapo                        GTAAATCATGAAGTTTTTTTTATCATCTGGTGCTATTTTGTCATCCA--GTCTTGCATGTGAAGATGTTTTTGGTCAGAG 44640
GoldenEagle                   GTAAATCATTAAGCTTTTTTTGTCATCTGGTACTATTTTGTCATCCACAGTCTTGCATGTTAAAATGTTTTAGATCAGAG 54917
JapaneseQuail                 ATAAATCATTAAGCTTATTTTATCATCTGGTACTATTTTGTCATCCACAGTCTTGCATGCTAAAATGTTTTAGGTCAGAG 40621
MediumGroundFinch             GTAAATCATTAAGCTTTTTTTATCATCTAGTACTATTTTGTCATCCACAGTCTTGCATGTTAAAATGTTTCAGATCAGAG 28516
GoodesThornscrubTortoiseATGATTCATTAAGCTTATTTATCATTTTGTTACTATTTTCTCATTCACAGTCTTGCATGTTAAAATGTTT-GGATCAGAG 79612
```

```
Majority                 TGCCATT-------TTGAAAGATTTTTTGCCTGCATTTCATAACCAGCCATGCTTATGCAGTTAAAGTTCAAATTTTAAA
                                   85770     85780     85790     85800     85810     85820     85830     85840
Human                    TGCCTTTGTTTTTATTTTAAGAC-TTTTGCCTGCATTTCATAACCAGCCATGCTTATGCAGTTAAAGTTCAAAGTTTAAA 84792
Kakapo                   TGCCGTT-------TGGAAAGAGGTTTTGCCTGCATTTCATAGCCAGCCATGCTTACGCAGTTCAAGTTCCAAGTTGAAA 44713
GoldenEagle              TGCCATT-------TTGAAAGATTTTTTGCCTGCATTTCATAACCAGCCATGCTTATGCAGTTAAAGTTCAAATTTTAAA 54990
JapaneseQuail            TGCCATT-------TGGAAAGGTTTTGTGCCTGCATTTCATAACCAGCCATGCTTACGCAGTTGAAGTTTAAATTTCAAA 40694
MediumGroundFinch        TGCCATT-------TTGAAAGATATTTTGCCTGCATTTCATAACCAGCCATGCTTACGCAGTTAAAGTTTAAATTTTAAA 28589
GoodesThornscrubTortoiseTGCCATT-------TTGAAAGAT-TTTTGCCTGCATTTCATACCCAGCCATGCTTATGCACTTAAAGTTCAAAGTTTAAA 79684

Majority                 ATTTCTATTGCATGCTTTTTTTA-TTTATTTTCATGTTGAGATGAAATGCTGTAGTTTACTCTT-GATATGAATGTACTT
                                   85850     85860     85870     85880     85890     85900     85910     85920
Human                    ATTCCTAT-GCATGCTT--T-----CCTTCCCTATGTTGAGATGAAATGCTGTAATTTACTCTTTGATATAGATGTACTT 84864
Kakapo                   AGTTCTATTGCATGCTCTTTTTA-TTTATTTTCATGTTGAGATGAAATGCTGTAGTTGACTCTT-GATATGAATGTACTT 44791
GoldenEagle              ATTTCTATTGCATGCTTTTTTTA-TTTATCTTCATGTTGAGATGAAATGCTGTAGTTTACTCTT-GATATGAATGTACTT 55068
JapaneseQuail            ATTTCTATTGCATGCT-TTTTTA-TTTATTTTCGTGTTGAGATGAAATGCTGTAGTTTACTCTC-GATATGAATGTACTT 40771
MediumGroundFinch        ATTTCTATTGCATGCTTTTTTTTAATTTATTTTCATGTTGAGATGAAATGCTGTAGTTTACTCTT-GATATGAATGTACTT 28668
GoodesThornscrubTortoiseATTTCTATTGCATGCT---------TTTTTTTCATGTTGAGATGAAATGCTGTAATTTATTCTTTGATATGGATGTACTT 79755

Majority                 TACCCATATTTGTCTTGGGTGACTACATTTTACTCAGTTTTT--CTTGTACAGTACATAAACCAACCATTTTCTGACCAA
                                   85930     85940     85950     85960     85970     85980     85990     86000
Human                    TACCCATATTTGTCTTGGATGACTACATTTTACTCAGTTTTT--CTTGTACAGTACATAAACCAACCATTTTCTGACCAA 84942
Kakapo                   TACCCATATTTGTCTTGGGTGACTACATTTTACTCAGTTTTT--CTTGTACAGTACATAAACCAACCATTTTCTGACCAA 44869
GoldenEagle              TACCCATATTTGTCTTGGGTGACTACATTTTACTCAGTTTTT--CTTGTACAGTACATAAACCAACCATTTTCTGACCAA 55146
JapaneseQuail            TACCCATATTTGTCTTGGGTGACTACATTTTACTCAGTTTTT--CTTGTACAGTACATAAACCAACCATTTTCTGACCAA 40849
MediumGroundFinch        TACCCATATTTGTCTTGGGTGACTACATTTTACTCAGTTTTT--CTCGTACAGTACATAAACCAACCATTTTCTGACCAA 28746
GoodesThornscrubTortoiseTACCCATATTTGTCTTGGGTGACTACATTTTACTCAGTTTTTTTCTTGTACAGTACATAAACCAACCATTTTCTGACCAA 79835

Majority                 ATTCCTGCATTTCCTATGTACTGACCTATATTTTATTTTTTTTTGTTCCCCACTTCCTTATTTTTTTTTCTTCTGCAT
                                   86010     86020     86030     86040     86050     86060     86070     86080
Human                    ATTCCTGCATTTCCTATGTACTGACCTATATTTTATTTTGTTTTTGTTCCCCAATTCC---TTATTTTTTTCTTCTGCAT 85019
Kakapo                   ATTCCTGCATTTCCTATGTACTGACCTATATTTTATTTTGTTTTTGTTCCCCACTTCCTTATTTTTTTTC--TTCTGCAT 44947
GoldenEagle              ATTCCTGCATTTCCTATGTACTGACCTATATTTTATTTTTTTT--GTTCCCCACTTCCTTATTTTTTTTTTCTTCTGCAT 55224
JapaneseQuail            ATTCCTGCATTTCCTATGTACTGACCTATATTTTATTTTTTTT--GTTCCCCACTT----ATTTTTTTTTCTTCTGCAT 40923
MediumGroundFinch        ATTCCTACATTTCCTATGTACTGACCTATATTTTATTTTTTTTTTGTTCCCCACTTCCTTATTTTTTTTT-CTTCTGCAT 28825
GoodesThornscrubTortoiseATTCCTGCATTTCCTATGTACTGACCTATATTTTATTTTTTTTT-GTTCCCCACTT-----C--TTTTTC--TTCTTCAT 79905
```

```
Majority                   TGCTGTATTCCCTTCCCCATTTCATCCTTTTCCCTTTGTGTTCAACTTCCCTTT-CCTTGTCTTTACCCAAATTCCCATG

                                    86090     86100     86110     86120     86130     86140     86150     86160

Human                      TGCTGT-TTCCCTTCCCCATTTCATCCTTTTCCCTGTGTGTTCACCTTCCCTTT-CCTTGTCTTTTCCCAAATGCCCATT 85097
Kakapo                     TGCTGTATCCCCTTCCCCATTTCATCCTTTTCCCTTTGTGTTCAACTTCCCTTTTCCTTGTCTTTCCCCCAACTCCCATC 45027
GoldenEagle                TGCTGTATTCCCCTCCCCATTTCATCCTTTTCCCTTTGTGTTCAACTTCCCTCT-CCTTGTCTTTACCCAAATTCCCATG 55303
JapaneseQuail              TGCTGTTTCCCCCTCCCCATTTCATCTTTTTCCCTTTGTGTTCAACTTCCCTTT-CCTTGTCTTGACCCAAATTCCCGTG 41002
MediumGroundFinch          TGCTGTATTCCCTTCCCCATTTCATCCTTTTCCCTGTGTGTTCAACTTCCCTCT-CCTTGTCTTTACCCAAATTCCCGTG 28904
GoodesThornscrubTortoise   TGCTGT-TTCCCTTCCCCATTTCATCCTTCTCCTTTTGTGTTCACCTTCCCTTT-CCTTGTCTTTCCTCAAATTCCCATG 79983

Majority                   CCCTTCCCTGTCTTACCCTTT--TCTCCTTGTCTTT-GTCTGCGTTCCCTGTCTTCATTCCCTATGTTCATGCTTCTGTG

                                    86170     86180     86190     86200     86210     86220     86230     86240

Human                      CCCTTCCCTGTCTTATCCTTTATTTTCCTTGTCCTT-GTCTTCATTCCCTGTCTCCATTCCCTATGTTCATGCTTCTGTG 85176
Kakapo                     CCCTTCCCTGTCTTACCCTT----CTCCTCGTCTTTTGTCTCCATTCCCTGTCTTCATTCCCTATGTTCATGCTTCTGTG 45103
GoldenEagle                CCCTTCCCTGTCTTACCCTT----CTCCTCGTCTTT-GTCTGTGTTCCCTGTCTTCATTCCCTATGTTCATGCTTCTGTG 55378
JapaneseQuail              CCCCTTCCCGTTTTACCCTTT--TCTCCTCGTCTTT-GTCTGTGTCCCCTGTCTTCATTCCCTATGTTCATGCTTCTGTG 41079
MediumGroundFinch          CCCTTCCCTGTCTTACCCTT----CTCCTTGTCTTT-GTCTACGTTCCCTGTCTCCATTCCCGATGTTCATGCTTCTGTG 28979
GoodesThornscrubTortoise   CCCTCCCTTGTCTTATCCTTTATTTTCCTTATCCTT-GTCTACGTTCCCTGTCTCCATTCCCTGTGTTCATGCTTCTGTG 80062

Majority                   CTTGAACAAAATGT--TCCTCGGACCAACTTGCCCCAATTAACCGCCTTGAAACCATGATCCATGACCACCTCACCATTC

                                    86250     86260     86270     86280     86290     86300     86310     86320

Human                      CTTGAACAAA-TGT--TCCTCGGACCAACTTGCCCCAATTAACCGCCTTGAA-CCATGATCCATGACCACCTCACCATTC 85252
Kakapo                     CTTGAACAAAATGT--TCCTCGGACCAACTTGCCCCAATTAACCGCCTTGAAACCATGATCCATGACCACCTCACCATTC 45181
GoldenEagle                CTTGAACAAAATGT--TCCTCGGACCAACTTGCCCCAATTAACCGCCTTGAAACCATGATCCATGACCACCTCACCATTC 55456
JapaneseQuail              CTTGAACAAAATGT--TCCTCGGACCAACTTGCCCCAATTAACCGCCTTGAAACCATGATCCATGACCACCTCACCATTC 41157
MediumGroundFinch          CTTGAACAAAATGT--TCCTCGGACCAACTTGCCCCAATTAACCGCCTTGAAACCATGATCCATGACCACCTCACCATTC 29057
GoodesThornscrubTortoise   CTTGAACAAAATGTGTTCCTCGGACCAACTTGCCCCAATTAACCGCCTTGAA-CCATGATCCATGACCACCTCACCATTC 80141

Majority                   TGCGGGAACCACCCTTCGTTATGGATGATCTGTTCATCTCCGCTCTTCCTCGACTCTTCTCTCTTCTTGTCTTACGCTGC

                                    86330     86340     86350     86360     86370     86380     86390     86400

Human                      TGCGGGAACCACCCTTCGTTATGGATGATCTGTTCATCTCCGCTCTTCCTCGACTCTTCTCTCTTCTTGTCTTACGCTGC 85332
Kakapo                     TGCGGGAACCACCCTTCGTTATGGATGATCTGTTCATCTCCGCTCTTCCTCGACTCTTCTCTCTTCTTGTCTTACGCTGC 45261
GoldenEagle                TGCGGGAACCACCCTTCGTTATGGATGATCTGTTCATCTCCGCTCTTCCTCGACTCTTCTCTCTTCTTGTCTTACGCTGC 55536
JapaneseQuail              TGCGGGAACCACCCTTCGTTATGGATGATCTGTTCATCTCCGCTCTTCCTCGACTCTTCTCTCTTCTTGTCTTACGCTGC 41237
MediumGroundFinch          TGCGGGAACCACCCTTCGTTATGGATGATCTGTTCATCTCCGCTCTTCCTCGACTCTTCTCTCTTCTTGTCTTACGCTGC 29137
GoodesThornscrubTortoise   TGCGGGAACCACCCTTCGTTATGGATGATCTGTTCATCTCCGCTCTTCCTCGACTCTTCTCTCTTCTTGTCTTATGCTGC 80221
```

```
Majority                   TTGCTCTTCTCTCCTTCTAAAGATGGTTACGCCCCAAAGTCTGTTTACCCTCTTCGGTATGTTATTGTTAGCACTATACT
                                   86410     86420     86430     86440     86450     86460     86470     86480
Human                      TTGCTCTTCTCTCCTTCTAAAGATGGTTACGCCCCAAAGTCTGTTTACCCTCTTCGGTATGTTATTGTTAGCACTATACT 85412
Kakapo                     TTGCTCTTCTCTCCTTCTAAAGATGGTTACGCCCCAAAGTCTGTTTACCCTCTTCGGTATGTTATTGTTAGCACTATACT 45341
GoldenEagle                TTGCTCTTCTCTCCTTCTAAAGATGGTTACGCCCCAAAGTCTGTTTACCCTCTTCGGTATGTTATTGTTAGCACTATACT 55616
JapaneseQuail              TTGCTCTTCTCTCCTTCTAAAGATGGTTACGCCCCAAAGTCTGTTTACCCTCTTCGGTATGTTATTGTTAGCACTATACT 41317
MediumGroundFinch          TTGCTCTTCTCTCCTTCTAAAGATGGTTACGCCCCAAAGTCTGTTTACCCTCTTCGGTATGTTATTGTTAGCACTATACT 29217
GoodesThornscrubTortoise   TTGCTCTTCTCTCCTTCTAAAGATGGTTACGCCCCAAAGTCTGTTTACCCTCTTCGGTATGTTATTGTTAGCACTATACT 80301

Majority                   TTTATTATTGATTTGATTTTTTTTTT-T--TTTTGTTTGTTTTTT-TTTTT--TTTGTTTCACCTTAATTCTTATTTGTA
                                   86490     86500     86510     86520     86530     86540     86550     86560
Human                      TTTATTATTGATTTGATTTT-----------------------------------TGTTTCACCTTAATTCTTATTTGTA 85457
Kakapo                     TTTATTATTGGTTTGATTTTTGGTTTCTTGTTTGGTTGGTTTTCTATTTTTTTTTTGTTTCACCTTAATTCTTATTTGTA 45421
GoldenEagle                TTTATTATTGATTTGATTTTGTTTTATTTTTTTTTTTTGTTTTTT-TTTTTTGTTTGTTTCACATTAATTCTTATTTGTA 55695
JapaneseQuail              TTTATTATTGATTTGATTTTTGTTCTGT--TTTGGTTTTTTTGTTGGTTTTGTTTTGTTTCACCTTAATTTTTATTTGTA 41395
MediumGroundFinch          TTTATTATTGATTTGGTTTTGTTTTTATGGTTTTGTTTGGTTGGT-TTTTT---TTGTTTCACCTTAATTCTTATTTGTA 29293
GoodesThornscrubTortoise   TTTATTATTGATTTAATTTT-----------------------------------TGTTTCACCTTAATTCTTATTTGTA 80346

Majority                   GCTAGCACTTT-GGCTTAAAGTTGAATAGTAAATCTTTTG-CTATTTTTCTTTTGCTGTTTAAAA-CTCTCCATAGA-CA
                                   86570     86580     86590     86600     86610     86620     86630     86640
Human                      GCTAGCACTTT-GGCTTAAAGTTGAATAGTAAATCTTTTG-CTATTTTTCTTT-GCTATTTAAAA-CTCTCCATAGA-CA 85532
Kakapo                     GCTAGCACTTT-GGCTTAAAGTTGAATAGTAAATCTTTTGGCTATTTTTCTTTTGCTGTTGAAAA-CTCTCCATAGAACA 45499
GoldenEagle                GCTAGCACTTC-AGCTTAAAGTTGAATAGTAAATCTTTTG-CTATTTTTCTTTTGCTGTTTAAAA-CTCTCCATAGA-CA 55771
JapaneseQuail              GCTAGCACTTT-GGCTTAAAATTGAGTAGTAAATCTTTTG-CTGTTTTTCTTTTGCTGTTTAAAA-CTCTCCATAGA-CA 41471
MediumGroundFinch          GCTAGCACTTT-GGCTTAAAGTTGAATAGTAAATCTTTTG-CTATTTTTCTTTTGCTGTTTAAAT-CTCTCCATAGA-CA 29369
GoodesThornscrubTortoise   GCTAGCACTTTTGGCTTAAAGTTGAATAGTAAATCTTTTG-CTATTTTTCTTTTGCTGTTTAAAAACTCTCCATAGA-CA 80424

Majority                   CAGATT----------------TTCTTTTAATGCATGCTAATATATTTTGCATGGTCTTTAATTTAATAT-CATTCACAT
                                   86650     86660     86670     86680     86690     86700     86710     86720
Human                      CAAAAT----------------TTGTTTTAATGCATGCTAATTTATTTTGCATGGTCTTTAATTTAATAT-CATTCACAT 85595
Kakapo                     CAGATTGATTGATTTATTTATTTCTTTTAATGCATGCTAATATATTTTGCATGGTCTTTAATTTAATAT-CATTCGCAT 45578
GoldenEagle                CAGATT----------------TTCTTTTAATGCATGCTAATATATTTTGCATGGTCTTTAATTTAATATTCATTCACAT 55835
JapaneseQuail              CAGATT----------------TTCTTTTAATGCATGCTAATATATTTTGCATGGTCTTTAATTTAATAT-CATTCGCAT 41534
MediumGroundFinch          CAGATT----------------TTCTTTTAATGCATGCTAATATATTTTGCATGGTCTTTAATTTTATAT-CATTCACAT 29432
GoodesThornscrubTortoise   CAAGAT------T---------TTCTTTTAATGCATGCTAATATATTTTGCATGGTCTTTAATTTAATAT-CATTCACAT 80488
```

```
Majority                    AGCTTT-GAGGGTTTATCAGAAATT-ATTCTTTTCAAAATTCACTATTCA-AAATCTTTATCTCCTTTATTCATTTGTGA

                                     86730     86740     86750     86760     86770     86780     86790     86800

Human                       AGCTTT-GAGGGTTTATCAAAAATT-ATTCTTTTCAAAATTCACTGTTCA-AAATCTTGATCTTCTTTATTCATTTGTGA 85672
Kakapo                      AGCTTT-GAGGGTTTATCAGAAATG-ATTCTTTTCAAAGTCACTATTCAGAAATCTTTATCTCCTTTATTCATTTGTGA 45656
GoldenEagle                 AGCTTT-GAGGGTTTATCAGAAATT-ATTCTTTTCAAAATTCACTATTCA-AAATCTTTATCTCCTTTATTCATTTGTGA 55912
JapaneseQuail               AGCTTT-GAGGGATTATCAGAA----ATTCTTTTCAAA-CTCACTATTCA-AAATCTTTATCTCCTTTATTCATTTGTGA 41607
MediumGroundFinch           AGCTTT-GAGGGTTTATCAGAAATT-ATTCTTTTCAAAATTCACTATTCA-AAATCTTTATCTCCTTTATTCATTTGTGA 29509
GoodesThornscrubTortoiseAGCTTTTGAGGGTTTATCAAAAATTTATTCTTTTCAAAATTCACTATTCA-AAATCTTGATCTTCTTTATTCATTTGTGT 80567

Majority                    GAGTGTTGAGTTTGAGTGAGTGATCTTGTTGCTGTCTGAATGTTCCATTGATATGTCA-AAATGTATTACTTAGGTGACT

                                     86810     86820     86830     86840     86850     86860     86870     86880

Human                       GAATGATGAGATTGAG----TGATCATGTTGATGTCTGAATGTTTCATTGATATGTCAGAAAGATAATCTTTAGGTGACT 85748
Kakapo                      GAGCGTTGAGTTTGAGCGAGTGATCTCGTTGCTGTCTGAATGTTCCATTGATATGTCA-AAATGTATTACTTAGGTGACT 45735
GoldenEagle                 GAATGTTGAGTTTGAGTGCGTGATCTTGTTGCTGTCTGAATGTTCCATTGATATGTCA-AAATGTATTACTTAGGTGACT 55991
JapaneseQuail               GAGTGTTAAGTTGGAGCGAGTGATCTTGTTGCTGTCTGAGTGTTCCATTGGTATGTGA-AAATATGTAACTTAGCTGACT 41686
MediumGroundFinch           GTGTGTTGAGTTTGAGTGAGTGATCTTGTTGCTGTCTGAATGTTCCATTGATATGTCA-AAATGTATTACTTAGGTGGCT 29588
GoodesThornscrubTortoiseGAATGTTGAGTTTGAGTGGGTGATCTTGTTGATGTCTGAATGTTTCATTGATATGTCA-AGATGTGCTGTTTAGTTAATT 80646

Majority                    GGC-TTTAAAATGAACTTTTTCTTTTGGGTTACCTTT----GACTTTGAAAA-GGTGTTTATGGTGATGTGCAGCGTGTG

                                     86890     86900     86910     86920     86930     86940     86950     86960

Human                       TCCACATAAAATTAATTCCA-TTTTTGGATTACCTCTCTGTGGCTCCAAAAAAGGTGTTTATGGAGATGTGCAGCGTGTG 85827
Kakapo                      GGC-TTTAAAATGAACTTTTTCTTTTGGGTTACCTTT----GACTTTGAAAA-GGTGTTTATGGTGATGTGCAGCGTGTG 45809
GoldenEagle                 GGC-TTTAAAATGAACTTTTTCTTTTGGGTTACTTT-----GAATTTGAAAA-GGTGTTTATGGCGATGTGCAGCGTGTG 56064
JapaneseQuail               GGC-TTTAAAATAAACTTTTTCTTTTGGGTTACCTTT----GACTTTGAAAA-GGTGTTTATGGTGATGTGCAGAGTGTG 41760
MediumGroundFinch           GGC-TTTAAAATGAACTTTT-CTTTTGGGTTACTTTT----GACTTTGAAAA-GGTGTTTATGGTGATGTGCAGCGTGTG 29661
GoodesThornscrubTortoiseAGA-CTTAAAATTAATTTTT-CTTTTGGATTACCTCTCT--GACTCCAAAAAAGGTGTTTATGGGGATGTGCAGCGTGTG 80722

Majority                    AAGATTTTATACAATAAGAAAGACAGTGCTCTTATACAGATGGCTGATGGAAACCAGTCACAGCTGGGTAAGATTAGTTT

                                     86970     86980     86990     87000     87010     87020     87030     87040

Human                       AAGATTTTATACAATAAGAAAGACAGCGCTCTAATACAGATGGCTGATGGAAACCAATCACAACTTGGTAAGATTAAACT 85907
Kakapo                      AAGATTTTATACAATAAGAAAGATAGTGCTCTTATACAGATGGCTGATGGAAACCAGTCACAGCTGGGTAAGATTAGTTT 45889
GoldenEagle                 AAGATTTTATACAATAAGAAAGACAGTGCTCTTATACAGATGGCTGATGGAAACCAGTCACAGCTGGGTAAGATTACTTT 56144
JapaneseQuail               AAGATTTTATACAATAAGAAAGACAGTGCTCTTATACAGATGGCTGATGGAAACCAGTCACAGCTGGGTAAGATCAGATT 41840
MediumGroundFinch           AAGATTTTATACAATAAGAAAGATAGTGCTCTTATACAGATGGCTGATGGAAACCAGTCACAGCTGGGTAAGATGAGTTT 29741
GoodesThornscrubTortoiseAAGATTTTATACAATAAGAAAGACAGTGCTCTAATACAGATGGCAGATGGGAACCAGTCACAGCTGGGTAAGATTAGATT 80802
```

```
Majority                 TCTTTTTAT------ATTTCCATTAGT---CT-AAGTACTTGAAAATTATGC-TGTGTAAAA----------TCAGGTGA
                                87050     87060     87070     87080     87090     87100     87110     87120
Human                    ATGTTTTATCTATACATCTTCACTTCTGCTTTCAAATGCATAATGTGAATGTGCGAATAAAAATAAACTCCTTTACATCA 85987
Kakapo                   TCCATTTCT------ATTCCCGTTAGT---CTTAAGTGCTTGAAATGAACAC-TGTGTAAAA----------TGAGGTGA 45949
GoldenEagle              TCTGTTTGT------ATTCCCATTAGT---CT-AAGTACTTG-AAATCATGC-TGTGTAAAA----------TCAGGTGA 56202
JapaneseQuail            TCTATTTAT------ATTTGCA-------------------AAAAATTAAGC-TGTGTAAAA----------TTAGGTGA 41884
MediumGroundFinch        TCTGCTTAT------ATTCCTATTAGT---CT-AAGTACTTGGAAATTAGGC-TGTGTAAGA----------TCAGGTGA 29800
GoodesThornscrubTortoise CATTTATGCCT---CATTTAAATCTGTAAATTTAAGTATTTG-AAATCATGT-TGTGGAAAT----------TCAAATGA 80867

Majority                 AC-TGAACATGTTT--------ACATTTGTT--AGTTTGTCTTGTATCCTGG-TTATCCACAAGATCCAAAATCTTATTG
                                87130     87140     87150     87160     87170     87180     87190     87200
Human                    GTAAGAAATTATTTTAATGGCCAGGGCTCAAAATGTCATTTTAATGTGAATAGTTGTATGTGACGAACATATTTGTATTT 86067
Kakapo                   AC-TGAGCATGTTC--------ACATTTGTC--AGTTTGTCTTGTATCCTGG-TTATCCCCAAAATCCAGAGTCTTACTG 46017
GoldenEagle              AC-TAAGCATGTTT--------ACATTTGTC--AGTTTGTCTTGTATCCTGG-TTACCCTCAAGATCCAAAATCTTACTG 56270
JapaneseQuail            AC-TAAAGATGTTT--------ACATTTGAA--AGT---TCTTGTGTGCTAG-TTACCAACAAGATCTGAAATCATATTT 41949
MediumGroundFinch        AC-TAAATATGTTT--------ATATTTGTT--AGTCTGTCTCTTATCTTGG-TTATCCACCAGATCCAAAATTTT-TTG 29867
GoodesThornscrubTortoise ACATGTTCATATTTGTCAGTCTACTGTTATTCTAGTTTTTCTCAGATCCCAGACTGTCCATGAGACATGAAAACTTATTA 80947

Majority                 C------ATGCACTT---GTATTCCAGAAGGTCTGCAC-----------------------------AGTAGTCTAAA-
                                87210     87220     87230     87240     87250     87260     87270     87280
Human                    TTCTGCTATCCTCCTACTGTTTTTCA-AATGCACTCATAATAGGAAGAGATTTAGCTTCAAAATTGTAATAATAGTTTC 86146
Kakapo                   C------ATGCACTT---GTATTCCAGAAGGTCTGCAC-----------------------------AATAGCCTAAA- 46057
GoldenEagle              C------ATGTACTT---GTATTCCAGAAGGTCTGCAC-----------------------------AGTATCCTAAA- 56310
JapaneseQuail            C------ATCCACAT---GTATTCCA-AAAGTCTG----------------------------------GCGGTGTAGG- 41984
MediumGroundFinch        C------ATGCACTT---GTGTTCCATAAGGTCTGCACTTAAATG----------------------AACACCCTAAA- 29914
GoodesThornscrubTortoise ATCA---ACCCATTTT--GTATTCTAAAAAGAATGTGTGTATGTGCTGCTGTATGTGAACAGAATAATAGTGGTGTTGGA 81022

Majority                 --------------------------TGAATTCA--ACAGTTG-----------AAT-------GATGGTCTCTAATTG-
                                87290     87300     87310     87320     87330     87340     87350     87360
Human                    ACATGAGAATTATATCATTTATATTGAAGATTCATAGTGATTTTTATTCTAGGTACTCCTTGTTAATAGAGGCTAACTGT 86226
Kakapo                   --------------------------TGAATTCA--ACAGCTT-----------AATCGCATGTGATGGTCTTTAAGTA- 46097
GoldenEagle              --------------------------TGAATTCA--ACAGCTG-----------AAT-------CACA---TGTGATGG- 56340
JapaneseQuail            --------------------------ATAACTCA--GAAGTTG-----------AAA---------------CTAACT-- 42008
MediumGroundFinch        --------------------------TGAATTCA--ACAGCTG-----------AAT-------GATGATCCCTGAATA- 29947
GoodesThornscrubTortoise CGAGAAAGAGGTGTTAATTTATTTTTTGAACTAA--ACATTTGCTCCAAATTCTAATTTAACCCTATGGACATCAATTTG 81100
```

```
Majority                   --------------------ATATTA---------------AATACTCATGAAG-------ATTCTGCA---------GA

                                  87370     87380     87390     87400     87410     87420     87430     87440

Human                      TCATACAGTCCATAGAATCCTTCTTAATGCCATGTTATTTTAATTCTTCTAAAGTCTTGCGATACCAGGTTCTTAGAAAA 86306
Kakapo                     --------------------AAATGA---------------AATATTCATGCAG-------ATTCTGCA---------GA 46126
GoldenEagle                --------------------TCTTTA---------------AATACTCATGCAG-------ATTCTGCA---------GA 56369
JapaneseQuail              ---------------------TTCTA---------------AATACTCACGT---------ATTCTGCA---------GA 42034
MediumGroundFinch          --------------------AAATTA---------------AATATTGACAAAG-------AGTCTGCA---------GA 29976
GoodesThornscrubTortoiseCTA------CTACACTGGCCATATCAGCACAAT--------AGCACACACAAAA-------ATACCACAATTAAATCAGA 81159

Majority                   CCTAGTTCTTGATTC-------C-----------------------------AGTCTTTTAGCACGCT------------

                                  87450     87460     87470     87480     87490     87500     87510     87520

Human                      TGAAAAGCTTGTGCTAAAAACACTGTTGTTAATCCCTTTGGGAATCTTAAAAGGCACTTTAGTCAGGTTACACTTTATAT 86386
Kakapo                     GCTGGAA-TCGATTC-------C-----------------------------TGCCTTTTAGCTCATT------------ 46157
GoldenEagle                CCTAGTTCTTGATTC-------C-----------------------------AGTCTTTTAGCACGTT------------ 56401
JapaneseQuail              CCTAGTTTTTGCTT--------------------------------------AGTCTTCTAGCACACC------------ 42064
MediumGroundFinch          CCTAATTCTTGATTC-------T-----------------------------AGTCCTTTAGCACACT------------ 30008
GoodesThornscrubTortoiseAAAAAAGTTTGATCA-------TAATACTATGTAGTGGTGTATATGTTGGTTAGTGTCATGGTGTGCTTTTTCACCTGGA 81232

Majority                   ------------GACCCTTTGATT--------------------TGTTTGGGTGTTTGTCTTGTTTG---CATGT-----

                                  87530     87540     87550     87560     87570     87580     87590     87600

Human                      CATTACTTAAATAAGTAATTGATTATTTTAATTAAACTT-ATGTCGTTTGACTTCATACATCCTCGAATACATAAATCTA 86465
Kakapo                     ------------GACCCTTTGATT--------------------TGCTTGGGTGGTGGTCTCGGTTG---CTTGG----- 46197
GoldenEagle                ------------GACCCTTTGATT--------------------TGCTTGGGTGTTTTTCTTGTTCG---CTTGT----- 56441
JapaneseQuail              ------------AACTATTCGATT--------------------TTTCT---TGTTTGAAGGGCT------GTGT----- 42098
MediumGroundFinch          ------------GACCCATTGATT--------------------TGCTTGAGTGCTTTTCTTCTTTG---CATGT----- 30048
GoodesThornscrubTortoiseATAAATTATTTGGACCATTCAAATAACAGAATAAAATAGGATTGTGTTTGCATGTTTGTTTTACTGAATACATGACTAAA 81312

Majority                   ----GCGA-TGTA------------TCACAAGCTG---------------------------------------------

                                  87610     87620     87630     87640     87650     87660     87670     87680

Human                      GAACACACCTGCACATTCACACCAGTGATTGACTGTAAATGCCAGGATGTTCTTACATTTCTGTTCATTGAGATTCAGGG 86545
Kakapo                     ----GCGA-GGTA------------CCACAAGCTG--------------------------------------------- 46215
GoldenEagle                ----GTGA-TGTA------------TCACAAGCTA--------------------------------------------- 56459
JapaneseQuail              ----ACAG-TGTA------------TCACATGCTG--------------------------------------------- 42116
MediumGroundFinch          ----GTGA-TGTA------------CCACAAGCTA--------------------------------------------- 30066
GoodesThornscrubTortoiseTATAGCAG-TGCAGTTTTGAAAAATTTACAAACTAACTTTGAAATGGAATTATTTTTCGCAATGTTGGCTAAAATCAGTG 81391
```

```
Majority                 ------------------------------------TTGAAAAGT----AATACTGAATTGAGTAAAC-T-TTGTCT

                               87690     87700     87710     87720     87730     87740     87750     87760

Human                    ATAATGGTGCCTCAGACTGATTGTATTCACATTGTTAATTTGTAAATTTGCCATTTTTGATGTAAGGACGAGTGTATTTT 86625
Kakapo                   ------------------------------------TTGTAAAGT----CACACTGAACTGAGTAAAC---CTGTCT 46249
GoldenEagle              ------------------------------------TTGAAAATT----AACACTGAATTGAGTAAACTTCCTGTCT 56496
JapaneseQuail            ------------------------------------CTGAAAAGT----AATACTACATTGAG-AAACCTGTTGTCT 42152
MediumGroundFinch        ------------------------------------TTGAAAAGT----AGCACTCAATTGAGTAAAC---CTGTCT 30100
GoodesThornscrubTortoiseATTTAATGAGAAAGTATATGCCATCCTTGCAATGTT---TTGAAATGT----AGTATTGAATTTAATAAAC---TTGTCT 81461

Majority                 TTTTCAGCCATGAGCCATCT----TAATGGACAAAAAATGTATGGAAAGATTATTCGGGTTACCCT---TTCTAAACATC

                               87770     87780     87790     87800     87810     87820     87830     87840

Human                    TGCTCAGAGATGAACAACTTGCTATATTAGACCAATTTTGGCCTTTGACATCACATAGAAAATTTTGAATTTCAAACACA 86705
Kakapo                   TTTTCAGCCATGAGCCATCT----TAATGGACAAAAAATGTACGGCAAGATTATTCGGGTTACCCT---TTCTAAACATC 46322
GoldenEagle              TTTTCAGCCATGAGCCATCT----TAATGGACAAAAAATGTATGGAAAGATTATTCGGGTTACCCT---TTCTAAACATC 56569
JapaneseQuail            CTTTCAGCTATGAGCCATCT----TAATGGACAGAAAATGTATGGGAAGATAATCCGTGTTACCCT---TTCTAAACATC 42225
MediumGroundFinch        TTTTCAGCCATGAGCCATCT----TAATGGACAAAAAATGTATGGAAAGATCATTCGGGTTACCCT---TTCTAAACATC 30173
GoodesThornscrubTortoiseTTTTCAGCCATGAGTCATCT----TAATGGACAAAAAATGTATGGAAAGATTATTCGAGTTACCCT---TTCCAAGCATC 81534

Majority                 AGACAG----------TACAACTACCTCGAGAGGGACTTGATGACCAAGGGCTGACTAAAGATT------TTGGTAATTC

                               87850     87860     87870     87880     87890     87900     87910     87920

Human                    TAATAGGTTTGAATTTTAGTGTTTCTTGGCAATAGTCTTAGCAACTCAGATGAGGTATAAAACTATCCATCTGGCTGTCC 86785
Kakapo                   AGACAG----------TACAACTACCTCGAGAGGGACTTGATGACCAAGGGCTGACAAAGGATT------TTGGTAATTC 46386
GoldenEagle              AAACAG----------TACAACTACCTCGAGAGGGACTTGATGACCAAGGGCTGACTAAGGATT------TTGGCAATTC 56633
JapaneseQuail            AGACAG----------TTCAACTACCTCGAGAGGGTCTCGATGATCAAGGGCTGACTAAAGATT------TTGGTAATTC 42289
MediumGroundFinch        AGACAG----------TACAACTACCTCGAGAGGGACTGGATGACCAAGGGCTGACTAAAGATT------TTGGTAATTC 30237
GoodesThornscrubTortoiseAGACAG----------TACAGCTACCTCGAGAAGGACTTGATGACCAAGGGCTCACTAAAGATT------TTGGTAATTC 81598

Majority                 ACCTTTGCATCG-----------CTTCAAGAAAC---------CTGGTT----CAAAAAACTTCCAAAATATATTCC---

                               87930     87940     87950     87960     87970     87980     87990     88000

Human                    ATTTTTAAAATGGTGGTATGTGTTTTAAAGAGATACGAAAGTGCTGATTATGTCAAGATACTTAGTACATATTTTCCTCT 86865
Kakapo                   ACCTCTGCATCG-----------CTTCAAGAAAC---------CTGGTT----CAAAAAACTTCCAAAATATATTCC--- 46439
GoldenEagle              ACCTTTGCATCG-----------CTTCAAGAAAC---------CTGGTT----CAAAAAACTTTCAAAATATATTCC--- 56686
JapaneseQuail            ACCTCTGCATCG-----------TTTCAAGAAAC---------CTGGCT----CCAAAAACTTCCAGAATATCTTCC--- 42342
MediumGroundFinch        ACCTTTGCACCG-----------CTTCAAGAAAC---------CTGGTT----CCAAAAACTTCCAAAATATATTTC--- 30290
GoodesThornscrubTortoiseACCCTTGCATCG-----------CTTCAAGAAAC---------CTGGCT----CAAAAAATTTTCAGAATATATTCC--- 81651
```

```
Majority                   --CTCCTTCTGCAACACT-----------------TCATC--------TGTCCAATATTCCGTAAGTGTTGTCTGTCAG
                                   88010     88020     88030     88040     88050     88060     88070     88080
Human                      TTCTCTGTGTGCACTTCTGTACTCTGGTTGGAATAGTCATTGCTGTTGTTTTCATATATGTTGTAAGTAGCACTTAACAG 86945
Kakapo                     --CTCCTTCTGCAACACT-----------------TCATC--------TGTCCAATATTCCGTAAGTGTTGGCTCTCAG 46491
GoldenEagle                --CTCCTTCTGCAACACT-----------------TCATC--------TATCCAATATTCCGTAAGTGTTGGCTGTCAG 56738
JapaneseQuail              --CTCCATCTGCAACGCT-----------------TCATC--------TATCCAATATTCCGTAAGTCTGGTCTATTAG 42394
MediumGroundFinch          --CTCCTTCTGCAACCCT-----------------TCATC--------TGTCCAATATTCCGTAAGTGTTCATTGTCAG 30342
GoodesThornscrubTortoise   --CTCCATCTGCAACACT-----------------TCATT--------TGTCCAATATTCCGTAAGTATTGTATGACAG 81703

Majority                   TAAGCCA-----GTAGTATTTAT--GTGAGTCATAACACACTG-AATTGACTAATAATTTCTTG--CTTATGCTTTTTTT
                                   88090     88100     88110     88120     88130     88140     88150     88160
Human                      TACTTCATAATAGGAGTATTCTCAAATATGTCATTTTTTTCCCTAATTGTTTGAGGTATGGGTGGCATTGTGTCATTTAA 87025
Kakapo                     TCAGCCA-----GTTGTGTTAAT--GTGAACCACAACACACCG-AACTGACTAAGAATCTCTTC--CTTATGCTCGTTTC 46561
GoldenEagle                TAAGCCA-----GTAATGTTAAC--GTGAGTCACAACACACTG-AATTGACTAATAATTTCTTG--CTTATGCTTATTTT 56808
JapaneseQuail              GAAGCCT-----GTAGTATTTTTT-GATACTCATAATACAGTG-AATTAGTTTG---TTTTCTA--ATTATGCTTGTC-- 42460
MediumGroundFinch          CAATCTG-----ATAATATTTAT--GTGAATCACAGCACACTG-AACTGACTAATGATTTCCTG--CTTATGCTGATTTT 30412
GoodesThornscrubTortoise   TACGCCT-----GCGGAATTCAC----AAGCCATAAAATACTA-GATCCACTAATAATTAATT---CTTATGTTTTTTGT 81770

Majority                   ----------------CTTTCTGTCTGTAGACCATCAGTAGCAGAAG------AGGATCTACGCACATTGTTTGCTAACA
                                   88170     88180     88190     88200     88210     88220     88230     88240
Human                      ATAAGGGATGCATAGTCTTTATGGTTGGTAATTACCTGTGGTAGAATTACCTCAGATCTTCCAGATAGTTTTTGTTTTAT 87105
Kakapo                     ----------------CTTTCTGTCTGTAGACCCTCGGTAGCAGAAG------AGGATCTACGCACATTGTTTGCTAACA 46619
GoldenEagle                ----------------CTTTCTGTCTGTAGACCATCAGTAGCAGAAG------AGGATCTACGCACATTGTTTGCTAACA 56866
JapaneseQuail              ------------------TTTTGTCTGTAGACCATCAGTAGCAGAAG------AGGATTTACGCACACTGTTTGCTAACA 42516
MediumGroundFinch          ----------------CTTCCTGTCTGTAGACCATCAGTAGCAGAAG------AGGATCTACGCACATTGTTTGCTAACA 30470
GoodesThornscrubTortoise   A---------------TTTTGTTTTTCTAGACCATCAGTGGCAGAAG------ATGATCTGCGCACACTGTTTGCTAACA 81829

Majority                   CTGGAGGCACTGTGAA--AGCATTTAAATTTTTT-------CAGTAAGTAACTT----TTCTGTA----------CATAG
                                   88250     88260     88270     88280     88290     88300     88310     88320
Human                      GTAAAGACTGTTTTATGTAATATTTTAATTTTTTGCCATGCTCGTAGTCTGCTTGGGCTGCTGTAACCAAAATACCATAG 87185
Kakapo                     CTGGAGGCACTGTGAA--AGCGTTTAAATTCTTT-------CAGTAAGTAACTT----TCCTGTA----------CGTAG 46676
GoldenEagle                CTGGAGGCACTGTGAA--AGCATTTAAATTTTTT-------CAGTAAGTATCTT----TTCTGTA----------CATAG 56923
JapaneseQuail              CTGGAGGCACTGTGAA--AGCATTTAAATTTTTT-------CAGTAAGTAATTT----TTCTGTG----------CGTAG 42573
MediumGroundFinch          CTGGAGGCACTGTGAA--AGCATTTAAATTTTTT-------CAGTAAGTACCTT----TTCTGCA----------CATAG 30527
GoodesThornscrubTortoise   CTGGAGGCACTGTGAA--AGCATTTAAATTTTTT-------CAGTAAGCAAGTT----TTCCTTGA----ATCCTTATGT 81892
```

```
Majority                      CTTTAACAAA--AGACAGCCTAAGTGTGTTT-TTGCAGCGTGTCTTAAGAAAAAGTTCTT------AGAA-----AATAG
                                        88330     88340     88350     88360     88370     88380     88390     88400
Human                         ACTGAGTAGATGAAACAACAAAAATGTATTT-CTTCAAAGTTTTGAGAAATAAAACTTTGGGGTCTAGAAGTCCCAGATG 87264
Kakapo                        CTCTACCAAA--AGACCATCTAAGCGTGTTC-TTGCAGCGTGCCTTAAGAAAAAG------------------------- 46728
GoldenEagle                   CTCTAACAAA--AGACTGCCTAAGTGCGTTT-TTGCAGCATGTCTTAAGGAAAAGTGCCT------AGAG-----GATAG 56989
JapaneseQuail                 TTTTAACAAA--AGACAGGATAATTGCATTTTTTTCAGTGTGTCTTAAGGAAAGGTGCTT------AGAG-----TATAG 42640
MediumGroundFinch             CTCTAAAAAA--AAAGAG--T-------T---TTGCAGCTCATCTTAAGAAAAAGTATAT------ATCA-----AATTG 30582
GoodesThornscrubTortoiseTTTTAACAAA--AGATTGACTTCATATGTTT-TAACAGAATGTCTTGAAAAAATACTCTTCTGTAGAGAA-----AAGAG 81964

Majority                      CAAATATTAGGAG-CAACTCCTGTAGTGTTCGAGTTCCTAATGCCGTTATTTTGTTTCAGAAGAGATCACAAGATGGCAC
                                        88410     88420     88430     88440     88450     88460     88470     88480
Human                         AAACTGCTAGCAGATTGTTTCTGGTGAGGTCTCTCTTGGCTTGCAGCCAACCATCTTCCCCCTGTGTCCTCATGTGGCCT 87344
Kakapo                        CAAACATTAGGAG-AAGCTCCTGTTGTGTTCGAGTTCCTAATGCCGTTATTTTGTTTCAGAAGAGATCACAAGATGGCAC 46807
GoldenEagle                   AAAACATTAGGAG-CAACCCCTGTAGTGTTCGAGTTCCTAATGCCATTATTTTGTTTCAGAAGAGATCACAAGATGGCAC 57068
JapaneseQuail                 CAAATATTAGGAG-CAACTCTTGTAGTGTTCAAGTTCCTAATGCCATTATTTTGTTTCAGAAGAGATCACAAGATGGCAC 42719
MediumGroundFinch             CAAACATTAGGAG-CAACCC-TGTAGTGTTCAAGTTCCTAATTCCATTATTTTGTTTCAGAAGAGATCACAAGATGGCAC 30660
GoodesThornscrubTortoiseAAGTTTTTAGGCC-CTTATTTAGTATTGGTTGAGTTCCTAATGTTGTTGTTTTGTTTCAGAAGAGATCACAAGATGGCAC 82043

Majority                      TTCTTCAGATGTCAACAGTAGAAGAAGCTATTCAGGCTTTGA------TTGATCTTCACAATTACAATCTTGGAGAAAAT
                                        88490     88500     88510     88520     88530     88540     88550     88560
Human                         TTCCACTG-TGCAAGCACGTTAAGAGTCTCTTCCCCTTGTAAGGATCCTAGTCCTGTTGAATCAGGGCTTTACCCTTAAT 87423
Kakapo                        TTCTTCAGATGTCAACAGTAGAAGAAGCTATTCAAGCTTTGA------TTGATCTTCACAATTACAATCTGGGAGAAAAT 46881
GoldenEagle                   TTCTTCAGATGTCAACAGTAGAAGAAGCTATTCAGGCTTTGA------TTGATCTTCACAATTACAATCTGGGAGAAAAT 57142
JapaneseQuail                 TTCTTCAGATGTCAACAGTAGAAGAAGCTATTCAGGCTTTGA------TTGATCTTCACAATTACAATCTTGGAGAAAAT 42793
MediumGroundFinch             TTCTTCAGATGTCAACAGTAGAAGAAGCTATTCAGGCTTTGA------TTGATCTTCACAATTACAATCTGGGAGAAAGT 30734
GoodesThornscrubTortoiseTTCTTCAGATGTTAACAGTGGAAGAAGCTATTCAGGCTTTGA------TTGATCTGCATAATTACAATCTTGGAGAAAAT 82117

Majority                      CACCA--TCTGAG---AGTTTCTTTCTCTAAG-TCAACTATTTAA--AAAGGCAGATTG-AAAATGAGGGTGTATTGCAT
                                        88570     88580     88590     88600     88610     88620     88630     88640
Human                         GACCACATTTAACCTTAATTACCTCCTTAAAGGTCCTGTTTTTAAGTACAGTTACATTGGGGGTTAGGACTTCAACATAT 87503
Kakapo                        CACCA--TCTGAG---AGTTTCCTTCTCGAAG-TCAACCATTTAA--AAAGGCAGATTGGAAAAGGAGGGTGTCTTGCAT 46953
GoldenEagle                   CACCA--TCTGAG---AGTTTCTTTCTCGAAG-TCAACTATTTAA--AAAGGCAGATTG-AAAATGAGGGTGTATTGCAT 57213
JapaneseQuail                 CACCA--TCTGAG---AGTTTCTTTCTCTAAG-TCAACTATTTAA--AAAGGCAGATTG-AAAATGAGGGTGTATTGCAT 42864
MediumGroundFinch             CACCA--TCTGAG---AGTTTCTTTCTCTAAG-TCAACTATTTAA--AAAGGCAGATAG-AGAATGAAGGTGTATTGCAT 30805
GoodesThornscrubTortoiseCACCA--TCTGAG---AGTTTCTTTCTCCAAA-TCAACAATTTAA--AATGGGAGATAA-AAAATGAAGGTGTATCGCAT 82188
```

```
Majority                   TG-TTTGGTGTTGTCAC----CTATTGACTGTTCAGGAAAGTGGGGACCAGAGTTTGTCTTCTGTTTTTTTA-----TTT

                                    88650     88660     88670     88680     88690     88700     88710     88720

Human                      AAATTTGGGGAGGACACAATTCAGTTAATAGTACTTAAATTTGTAAATCA---TTTATCCCAAATTTTATAAGATATTGT 87580
Kakapo                     TG-TTTGGTGTTGTCAC----CTATTGACTGTTCAGGAAAGTGGGGACCAGAGTTTGTCTTCTCTTGTTTTTAGGGGTTT 47028
GoldenEagle                TG-TTTGGTGTTGTCAC----CTATTGACTGTTCAGGAAAGTGGGGACCAGAGTTTGTCTTCTGTTGTCTTT-----TTT 57283
JapaneseQuail              TG-TTTGGTGTTGTCAC----CTATTGACTGTTCCGGAGAGTGGGGACCAGAGTTTGTCTTCTATTTTTGGA-----TTT 42934
MediumGroundFinch          TG-TTTGGTGTTGTCAC----CTTTTGACTGTTCAAGAAAGTGGGGACCAGAGTTTGTCTTCTGTTTTTTTA-----TTT 30875
GoodesThornscrubTortoiseTG-TTCAGTGTTGTCAC----CTATTGACTGTTCAGGAGAGTGGGGACCAGAGTTTGACTTCTGTCTTT----------C 82253

Majority                   TTTTT-----TTTCCTTCCATGCTG---TTATCATTCCTTGGTTGTTTTAAAAAATAAAAAA--------TTTAAAAGAA

                                    88730     88740     88750     88760     88770     88780     88790     88800

Human                      CACTAAAGTTTTGACTTGCTAGTAGGGCTTATAATTTTTGGACTATCTGTAGTGGTAAATAATTCATCATTTCTCATTGC 87660
Kakapo                     TTTTAATTATTTTCTTCTCATGCTG---TTATCATTCCTTGATTGTTTTAAAAACTTAAACC--------CTTCAAA--- 47094
GoldenEagle                TTTTTTTT--TTTCCCCCCATGCTG---TTATCATTCCTTGGTTGTTTTAAAAAATAAAAAA--------TAAAAAAAAA 57350
JapaneseQuail              TCTTT-----CTGTTTTCCATGCTG---TTATCATTCCTTGGTTGTTTAAAAAATAAGAAAA--------AGTTTAAGA- 42997
MediumGroundFinch          TTTT----------C----ATGCTG---TTACCATTCCTTGATTGTTTTAAAAAA---ATAA--------TTTAAAAAAA 30927
GoodesThornscrubTortoise------------------ATGCTG---TTATCATTCCTTGGTTGTTTTAAAAAAAAAAAAAAAAGAACGTTCATAAGAA 82311

Majority                   T------GCAGTGATATT-AACTGCTGTT----TGTTCAACTGT-----TGTTTAGATAAACCATCATTTTGTTTAAAAG

                                    88810     88820     88830     88840     88850     88860     88870     88880

Human                      T-----GCTAGGTAGATTTTGCT-TTCTTGAACTGTGTGACCATAAAAGTATGTACTGACACCATCATTTCCTTTAAAAC 87734
Kakapo                     -------GCAGTGATACT-AACTGCTGGT-----GTTCAGCTGT-----TGTT--GATAAACCATCTTTTTGTTGAAAAG 47154
GoldenEagle                TTAAAAAGCAGTGATACT-AACTGCTGTT-----GTTCAACTGT-----TGTTTGGATAAACCATCATTTTGTTTAAAAG 57419
JapaneseQuail              -------GCAGTGATACT-AACTGCTGTT-----GTCCAACTGT-----TGTTTAGATAAACCATCACTTTGTTTAAAAG 43059
MediumGroundFinch          T------GCAGTGATGTT-AACTGCTTTTCAACTGTTCAACTGT-----TGTTTAGATAAACCATTGTTTTGTTTAAAAG 30995
GoodesThornscrubTortoiseAATAAAAGCAGTGATATTTAACTGTTGGT----TTTTCATCTGT-----TGTTTAGGGAAAC-ATCATTTTGTTTAAAAG 82381

Majority                   A-TTTCAAGTTAATAC-CACAGTTTTTCAACTTAGTTGACGTACGTGCCTTAAAAA-GGAA-AACTAGTAC----TGCTG

                                    88890     88900     88910     88920     88930     88940     88950     88960

Human                      ACACACACACAAACACACACACACACACGCATACACTCCCACACAAAACTCTATGGTGTACTATTTAGTATGGTGTACTA 87814
Kakapo                     A-TTTCAAGTTAATAC-CACAGGTTTTCAACTTAGTTGACGTACGTGCCTTAAAAAAGGAG-AACTAGTAC----TGCTG 47227
GoldenEagle                A-CTTCAAGTTAATAC-CACAGTTTTTCAACTTAGTTGACGTACGTGCCTTAAAAA-GGAA-AACTAGTAC----TGCTG 57491
JapaneseQuail              A-TTTCAAGTTAATAC-CACAGTTTTTCAACTTAGTTGACATACGTGCCTTATAAA-GGAA-AGCTAGTAC----TGCTG 43131
MediumGroundFinch          A-TTTCAAGTTAATAC-AACATTTTTTCAACTTAGTTGACGTACGTGCCTTAAAAA-GGAA-AATTAGTAC----TGCTG 31067
GoodesThornscrubTortoiseCGTTTCAAGTTAAGCC-CACAATTTTTCAAGTTAGTTGACATATGTGCCTTAAAAA-GGAA-AACTAGTGT----TGCT- 82453
```

```
Majority                   GAA---TTGCATAACTAG----T-AAAAAGCAAATTTGGT----TGTCTGGGGCACAT-TGTTACATGATAATTTAAATA
                                   88970     88980     88990     89000     89010     89020     89030     89040
Human                      AAAAGATTATCAAACTTGGTATTTAAAAAGTTAGCTATATACTTTTTACAAGAGACATGTAAAGCAGAAGAATATGAAAG 87894
Kakapo                     GAA---TTGCATAACTAG----TGAAAAAGCAAATTTGGT----TGTCTGGGGCACAT-TGTTACATGACGATTTTAATA 47295
GoldenEagle                GAA---TTGCATAACTAG----T-AAAAAGCAAATTTGGT----TGTCTGGGGCACAT-TGTTACATGATAATTTAAATA 57558
JapaneseQuail              GAA---TTGCGTAACTAG----T--GTAAACGAATTTGGT----TGTGTGGGGCACAT-TGTTACATGATAATTTGAATA 43197
MediumGroundFinch          GAA---TTGCATAACTAG----T-AAAAAGCAAATTTGGT----TGTCTGGGGCACAT-TGTTACATGATAATTTAAATA 31134
GoodesThornscrubTortoise   -A--------GT-------------AAAATGAATTTGGT----TGGCTGGGGCACAG-TGTTATATGAGAATTAAAATG 82505

Majority                   TGTTTAGGCAGGGGTGTGTAAAAAGGTTAAGCTTTTGTTTTCTCCTGCTTG--ATAGATTTCTTT-ATCTGCTGGCTTGT
                                   89050     89060     89070     89080     89090     89100     89110     89120
Human                      ATTGAAAGGAAAAGGATGTAAAGAGAT---ACTAATATACTGGACAGATAG---TAAGCAAGACAAAACTTAAATACCTT 87968
Kakapo                     TGTTTTGGCAGGGGTGTGTAAAAAGGTTAAGCTTTTGTTT-CTCCTGCTTG--ATAGATTTCTTTTATCTGCTGGCTTGT 47372
GoldenEagle                TGTTTAGGCAGGGGTGTGTAAAAAGGTTAAGCTTTTGTTT-CTCCTGCTTG--ATAGATTTCTTT-ATCTTCTGGCTTTT 57634
JapaneseQuail              TGTTTAGGCAGGGGTGTGTAAAAAGGTTAAGCTTTTGTTT-CTCCTGCTTGGAATTAATTTCTCT-ATCTGCTAGCTCGT 43275
MediumGroundFinch          TGTTTAGGCAGGGGTGTGTAAAAAGGTTAAGTTTTTATTTTCTCCTGCTTG--ATAGATTTCTTTTATCTGCTGGCTTGT 31212
GoodesThornscrubTortoise   TATTTAGGCAGGGGTGTGTAAAAAGGTTAAGGTTTTGTTTCTCCTGCTTGGAATT-ATTTTATT-ACTTATTGGGTTGT 82583

Majority                   CACTTACTTTTCTTTTTAATTGGATAAGATGCATTACGCTGAAAGAGTAAAACG-ATTTTATTTGATTTTTGCTACTTTT
                                   89130     89140     89150     89160     89170     89180     89190     89200
Human                      TATTAACTTAAGACAGAATTGATAAAGGATTTAGTTCACTAGAAAGATATAACAGTTTATGTATATGTGTGCCTAGTGAA 88048
Kakapo                     CACTTAGTTTTCTTTTTAATCGGATAAGATGCATTAGGCTGAAAGAGTAAAAAGGACGTTATTTGATT--TGCTACTTTT 47450
GoldenEagle                CACTTACTTTTCTTTTTAATTGGATAAGATGTATTATGCTGAAAGAGTAAAATG-ACATTATTTGATTTCTGTTACTTTT 57713
JapaneseQuail              CACTTAATTTTCTTTTTATTTGG-TAAGATACATTGTACTGAAAGGGTAAAACA-TTGCTATTTGATTT-TGCTACTTTT 43352
MediumGroundFinch          CACTTA-TTTTCTTTTTAATTGGATAAGATGCATTACGCTGAAAGAGTAAAATG-ATTTTATTTGATTTTTGCTACTTTT 31290
GoodesThornscrubTortoise   CACGTACCTTTCTTTTTAATCAGATAAGATACATTACTTTGAAAGATTAAAACACAACATATTTTATTTCTGCTACTTTA 82663

Majority                   T--GCTTTGTGCTCTTTT--TT----------------ACAAAAGGCCTTTTGTA----TTTCATGGTTCTGG-TCTAGA
                                   89210     89220     89230     89240     89250     89260     89270     89280
Human                      ATAGCCTCAAAATATATAAATAATAAGTAGATAGAACTACAGGTAGACTTGAGCAAATCTATTATAGTGGTGGATTTCAA 88128
Kakapo                     TT-GCTTTGTGCTCATTTGTTT----------------GGAAAAGGCCTTTAGTA----TTTCATCGTTCTGG-TCTAGA 47508
GoldenEagle                T--GCTTTGTGCTCTTTT--TT----------------TCAAAAGGCCTTTTGTA----TTTCATGGTTCTGG-TCTAGA 57768
JapaneseQuail              T--GCTTTATGCTTTTTTTTTTTTTT-TAAAGGGAAAAAAAAAAGGCCTTTTGTA----TTTTATGATTCTGG-TCTAGA 43424
MediumGroundFinch          T--GCTATGTGTTCTTTT--TT----------------AAAGAAAGCCTTTTGTA----TTTCATTGTTCTGG-TCTAGA 31345
GoodesThornscrubTortoise   A----------CTTTTTT---T----------------GTAAATGGCCTTCTATA----TTTCATGATTCTGG-TCTAGA 82709
```

```
Majority                   TTCAGTTATGAATGTAGGCATTAGTTAAAATTAACAAGGTACAGAATATTAATTTCTTCAAAGGACAAAAAGTGACTTCT

                                   89290     89300     89310     89320     89330     89340     89350     89360

Human                      TTCACTTTTCTGAATTACATGTCAAACTGACCAAAAAAAGACAAAATCAACAAAGATACAAATGATACAATACAATTACC 88208
Kakapo                     TTCAGTTCTGAATGTAGGCATTAGTTAAAATTAACAAGGTACGGAATATTAATTTCTTCAAAGGACAAAAAGTGACTTCT 47588
GoldenEagle                TTCAGTTATGAATGTAGGCATTAGTTAAAATTAACAAGGTACAGAATATTAATTTCTT-AAAGGACAAAAAGTGACTTCT 57847
JapaneseQuail              TTCAGTTA                                                                         43432
MediumGroundFinch          TTCAGTTATGAATGTAGGCATTAGTTAAAATTAACAAGGTACAGATTATTAATTTCTT-AAAGGACAAAAAGTGACTTCT 31424
GoodesThornscrubTortoise   TTCAGTTATGAATGTAGGCATTAGAGAAAATTAACAAGGTACAGAATATTAATTTCTTAAAGGAGAAAAAAGTGATTTCT 82789

Majority                   GTGACTTTGAGCCCTACGTGAAAAGCAT---TGTGG----AATCTTAACCTTTTTGTACACACTCTTGTGGGATGTATCA

                                   89370     89380     89390     89400     89410     89420     89430     89440

Human                      TTAAC--AGAATTATGTGTCTAAGTTATGCATCCAACACAGTTTTTAAACGCATATGGAACATTTTTGAAAAATCAGTCA 88286
Kakapo                     GTGACTTTGAGCCCTGCGTGAAAAGCAT---TGTGG----GATCTTAACCTTTTTGTACACACTCTTGTGGGATGTATCA 47661
GoldenEagle                GTGACTTTGAGCCCTACGTGAAAAGCAT---TGTGG----AATCTTAACCTTTTTGTACACACTCTTGTGGGATGTATCA 57920
JapaneseQuail                                                                                               43432
MediumGroundFinch          GTGACTTTGAGCCCTACGTGAAAAGCAT---TGTGG----AATCTTAACCTTTTTGTACACACTCTTGTGGGATGTATCA 31497
GoodesThornscrubTortoise   GTGTTTTTGAGCCTCATGTGGAAAGCAT---TGTGGGTAGAATCTTAACCTTTTTGTACACACTCTTGTGGGACGTATCA 82866

Majority                   TATAAATGTCAGCACTAAGTAATGTCTTGTTTGTGGCTGAATATTTTTXGTAGATGTTTTTXGAAGTTG-ACATGACTTA

                                   89450     89460     89470     89480     89490     89500     89510     89520

Human                      CATACTAAAC--CATAAAGCAAGCTTCAGCTAATTTCAAAGAGTATCATACAGACCTCATTCTCTATTGCACAAGTAAGT 88364
Kakapo                     TATAAATGTCAGCACTAAGTAATGTCTTGTTTGTGGCTGAATATTTTTCGTAGATGTTTTTTGAAGTTG-ACATGACTTA 47740
GoldenEagle                TATAAATGTCAGCACTAAGTAATGTCTTGTTTGTGGCTGA                                         57960
JapaneseQuail                                                                                               43432
MediumGroundFinch          TATAAATGTCAGCACTAAGTAATGTCTTGTTTGTGGCTGAATATTTTTCGTAGATGTTTTT-GAAGTTG-ACATGACTTA 31575
GoodesThornscrubTortoise   TATAAATGTCAGCACTAAGTAATGTCTTGTTTGTGGTTGAATATTTTTTGTAGATGTTTTT-GAAGTTG-ACATGACTTA 82944

Majority                   XGXXXXXXXXXXXXXXXXXXXXXXXXXXXXXXXXXXXXXXXXXXXXXXXXXXXXXXXXXXXXXXXXXXXXXXXXXXXXXX

                                   89530     89540     89550     89560     89570     89580     89590     89600

Human                      TAGAATTAATAACAAAAAGATGCATATAAAATCTGTATCCTGAGATTTAAAAATAAGTCTCAGTATTGCATGAGTCAGAA 88444
Kakapo                     CGTGCATTTCAATATATATTGCCATCTTTAGTTTGTAATTAAGATATGGAATATGGTTGTGGATTTCTGAGCATGTACAG 47820
GoldenEagle                                                                                                 57960
JapaneseQuail                                                                                               43432
MediumGroundFinch          CGTGCATTTCAATATATATTGCCATCTTTAGTTTGTAATTAAGATATGGAATATGGTTGTGGATTTCTGAGCATGTACAG 31655
GoodesThornscrubTortoise   TG                                                                               82946
```

```
Majority                  XXXXXXXXXXXXXXXXXXXXXXXXXXXXXXXXXXXXXXXXXXXXXXXXXXXXXXXXXXXXXXXXXXXXXXXXXXXXXXXX

                                   89610     89620     89630     89640     89650     89660     89670     89680

Human                     GTTTGTTTGTTTGTTTGTTTTTGAGCCAGTGTCTTGCTCTGTCCCCCAGGGTGGAGTGCAGTGGTGCTATTGTGGCTCAC 88524
Kakapo                    ACTGGTCAAGCTAGTTCAGGAAATGGTGCATGTATTTTTTCAATGAAGAAGAAAGTTTGCTGCAGAAGCTGGCAGGAAAT 47900
GoldenEagle                                                                                               57960
JapaneseQuail                                                                                             43432
MediumGroundFinch         ACCGGTCAAGCTAGTTCAGGAAATGGTGCATGTATTTTT-CAATGATGAAGAAAGTTTGCTGCAGATGCTTGCAGGAAAT 31734
GoodesThornscrubTortoise                                                                                  82946

Majority                  XXXXXXXXXXXXXXXXXXXXXXXXXXXXXXXXXXXXXXXXXXXXXXXXXXXXXXXXXXXXXXXXXXXXXXXXXXXXXXXX

                                   89690     89700     89710     89720     89730     89740     89750     89760

Human                     TGCAGCCTCGACCTCCCAGGCTTAAGCAGTCCTTGCACTTAAGCCTCCAAAGTAGCTAGGACTAAAAGCATGCACCACCT 88604
Kakapo                    TTTGGGGCAGTTTCCTCAAACTGACAAACCAGGTGGGACCAAAGTTTATGTGCCTTTAGTCTTAATTTACCTTGCATTGT 47980
GoldenEagle                                                                                               57960
JapaneseQuail                                                                                             43432
MediumGroundFinch         TTTGTGGCAGTTTTCTAAAACTGACAA-CCAGGTGGGACCAAAGTTTATGTGCCTTTAGTCTTAATTTACCTTGCATTGT 31813
GoodesThornscrubTortoise                                                                                  82946

Majority                  XXXXXXXXXXXXXXXXXXXXXXXXXXXXXXXXXXXXXXXXXXXXXXXXXXXXXXXXXXXXXXXXXXXXXXXXXXXXXXXX

                                   89770     89780     89790     89800     89810     89820     89830     89840

Human                     GCCCGGCTAATTTATTA---TTATTATTATTTTTTGTAGAGATGAGTTCTCACTGTGTTGCCCAGACAGAAGTTATTTTA 88681
Kakapo                    AATATTCAGTTTTAATAAATCTCTTCAAACTATTTTGTATTTAGAAATTGATCTGACTTTACTATAAACATGGCTCAGAA 48060
GoldenEagle                                                                                               57960
JapaneseQuail                                                                                             43432
MediumGroundFinch         AATATTCAGTTTTAATAAATCTCTTCAAAATATTTTGTATTTAGAATTTGATCTGACTTTACTATAAACATGGCTCAGAA 31893
GoodesThornscrubTortoise                                                                                  82946

Majority                  XXXXXXXXXXXXXXXXXXXXXXXXXXXXXXXXXXXXXXXXXXXXXXXXXXXXXXXXXXXXXXXXXXXXXXXXXXXXXXXX

                                   89850     89860     89870     89880     89890     89900     89910     89920

Human                     ATGGGAATATAAAATACTTAAAATTAAGCGATAAAGTACTATATACAAATTAGATATATGTGGTACAGTGAAAGAGAAGT 88761
Kakapo                    TCTACAGATCCAGTTCATTTGAAAGCAGTTCTCTGTCAGGCTGAACTGTTACCTTGATTCTGTTTCAATGACCAATGCTT 48140
GoldenEagle                                                                                               57960
JapaneseQuail                                                                                             43432
MediumGroundFinch         TCTGCAGATCCAGTTAATTTGAAAGCAGTTCACTGACAGTCTGAACTGTTACCTTGATTCTGTTTAAATGACCAATACTT 31973
GoodesThornscrubTortoise                                                                                  82946
```

```
Majority                  XXXXXXXXXXXXXXXXXXXXXXXXXXXXXXXXXXXXXXXXXXXXXXXXXXXXXXXXXXXXXXXXXXXXXXXXXXXXXXXX

                                   89930     89940     89950     89960     89970     89980     89990     90000

Human                     CTTGACGGGAAGTTTTAACTTTAAGAATAACAGTGTTACCCCCAGGGAAGGAGATATTAAAGACAAGACTGGAAATCAAT 88841
Kakapo                    TTTGAAATTGATGTACTTAGTTTCAAGATCCATAGATTCTGTTATCTATGTAGAGAGAGAAAAAAAAAAACAAATGGTCA 48220
GoldenEagle                                                                                                57960
JapaneseQuail                                                                                              43432
MediumGroundFinch         TTTGAAATTGATGTACTTAGTTTCAAGATTCATAGATTCTGTTATCTATGTAGAAAAAAAA--------------GGTCA 32039
GoodesThornscrubTortoise                                                                                   82946

Majority                  XXXXXXXXXXXXXXXXXXXXXXXXXXXXXXXXXXXXXXXXXXXXXXXXXXXXXXXXXXXXXXXXXXXXXXXXXXXXXXXX

                                   90010     90020     90030     90040     90050     90060     90070     90080

Human                     GGTATAAAAGACAGTTGAGAGAAAAAAACATGCCACAAGTTGGATTCTTTGAAAAGACTAAAAAAATAGACAAACATCTA 88921
Kakapo                    TGTATATTTTCTATTAGTTGAGTTTTTACATCTTTAGAATTGTAAAATTCAGTCTAGTTTGAAAGTGGCACAATTAAAAA 48300
GoldenEagle                                                                                                57960
JapaneseQuail                                                                                              43432
MediumGroundFinch         TGTATATTTTCTATTAGTTGAGTTTTTACATCTTTAGAATTGTAAAATTCAGTATAGTTTGGAAGTGGCACAATTAAAAA 32119
GoodesThornscrubTortoise                                                                                   82946

Majority                  XXXXXXXXXXXXXXXXXXXXXXXXXXXXXXXXXXXXXXXXXXXXXXXXXXXXXXXXXXXXXXXXXXXXXXXXXXXXXXXX

                                   90090     90100     90110     90120     90130     90140     90150     90160

Human                     AAGATTGATTAAAAAATGAGAGTGGTAACACAAAAAAATTTTTTTGAATTATAAAGGGGAGAAGCATTGATAAAGCTCCA 89001
Kakapo                    TTAATTTTCTAACAAAGTTGGGAGGTTTGACGGTGGTTTAATTTCATTTTGTGTGTACTCTGCTTACCTCTGTAGCATGC 48380
GoldenEagle                                                                                                57960
JapaneseQuail                                                                                              43432
MediumGroundFinch         TTAATTTTCTAACAAAGTTGGGAGGTTTGATGGTTGTTTAATTTCATTTTGTGTGTACTCTGCTTACCTCTGTAGCATGC 32199
GoodesThornscrubTortoise                                                                                   82946

Majority                  XXXXXXXXXXXXXXXXXXXXXXXXXXXXXXXXXXXXXXXXXXXXXXXXXXXXXXXXXXXXXXXXXXXXXXXXXXXXXXXX

                                   90170     90180     90190     90200     90210     90220     90230     90240

Human                     GTTTCCACATCATATAAACACCACTATGTCTGTAAATTTCTTGAGGTCTTTTAAAATTTTTATTATTCGTTAATAATCTC 89081
Kakapo                    TCAATAAACACTTCTGTAGCTCTGTATTCACCTTTTCTGTCTTTCTCTGCTGCTTTCTCTCTCTCCTCTTCTTTGTTTTT 48460
GoldenEagle                                                                                                57960
JapaneseQuail                                                                                              43432
MediumGroundFinch         TCAATAAACACTTCTGTAGCTCTGTATTCACCTTTTCTGTCTTTCTCTGCTGCTTTCTATCTTTTCTCTTCTTTGTTTTC 32279
GoodesThornscrubTortoise                                                                                   82946
```

```
Majority                 XXXXXXXXXXXXXXXXXXXXXXXXXXXXXXXXXXXXXXXXXXXXXXXXXXXXXXXXXXXXXXXXXXXXXXXXXXXXXXXX

                                 90250     90260     90270     90280     90290     90300     90310     90320

Human                    TTTCATAGATGGATTAAGGGAAATGTAAA-AAATTACATTAAATCAAAAATGTCATTTCAATTGTGAAACATTGACTTTT 89160
Kakapo                   CACTTCTACTGTGCTTCTAAATTCATGTTTATTCTCTGCCAGGGTGGGAAAAGCATAATACTCCAAAATACAATTTACGG 48540
GoldenEagle                                                                                               57960
JapaneseQuail                                                                                             43432
MediumGroundFinch        CACTTCTACTGTGCTTCTAAATTCATGTT-ATTCTCTGCCAGGGTGGGAAAAGCATAATATTAAAAACTGCTATTTATGG 32358
GoodesThornscrubTortoise                                                                                  82946

Majority                 XXXXXXXXXXXXXXXXXXXXXXXXXXXXXXXXXXXXXXXXXXXXXXXXXXXXXXXXXXXXXXXXXXXXXXXXXXXXXXXX

                                 90330     90340     90350     90360     90370     90380     90390     90400

Human                    TCCTTTATTATTGCCTTGTCCATTTATGCCATATTTCTTGGAATAGGAGAGTTCAAGTGTAAGATTCACATTGTTTAAAA 89240
Kakapo                   CATTTTACCA-AAATCTGATGCTGTGAAAAATACCAGCTCTTTATTTTCACAAAGATAGTGACCCCTTTGCAGGCATATG 48619
GoldenEagle                                                                                               57960
JapaneseQuail                                                                                             43432
MediumGroundFinch        CTTTTTACCATAAATCTGATGCTGTGAAAAATACCAACTCTTTATGTTCA----GATAGTGACCCCTTTGCAGGCATATG 32434
GoodesThornscrubTortoise                                                                                  82946

Majority                 XXXXXXXXXXXXXXXXXXXXXXXXXXXXXXXXXXXXXXXXXXXXXXXXXXXXXXXXXXXXXXXXXXXXXXXXXXXXXXXX

                                 90410     90420     90430     90440     90450     90460     90470     90480

Human                    TTTTATTCATTTAAAATTAGTAATTCAGGTAATTTCCTTCTGCTCTGTTTACTCCTTGTGTACTTTCAGTTTTTGACAAA 89320
Kakapo                   TGCATATGAAACATTTCCCACCCTGTTGTAACTTACTGCTTTAAAGACATAACTTTTATTGTAGTTCGTTAGCTCTCTCT 48699
GoldenEagle                                                                                               57960
JapaneseQuail                                                                                             43432
MediumGroundFinch        TGCAAATTAAACATTTCCCACCCTGTTA                                                     32462
GoodesThornscrubTortoise                                                                                  82946

Majority                 XXXXXXXXXXXXXXXXXXXXXXXXXXXXXXXXXXXXXXXXXXXXXXXXXXXXXXXXXXXXXXXXXXXXXXXXXXXXXXXX

                                 90490     90500     90510     90520     90530     90540     90550     90560

Human                    GCTTTTGTTTTCTTTTTTACTTAAAAATTTTGGTATTCTAATTATCATCCCCCTTCCCCTTTAGATCACTTCAGATCCTA 89400
Kakapo                   TTCTATCTTTTTAGTTTTGAAGCAGATTTATGCACTAATAGATCTCTCAGGTTTGCCATGCTCTCTTGCTGCAGTTTCAT 48779
GoldenEagle                                                                                               57960
JapaneseQuail                                                                                             43432
MediumGroundFinch                                                                                         32462
GoodesThornscrubTortoise                                                                                  82946
```

```
Majority                   XXXXXXXXXXXXXXXXXXXXXXXXXXXXXXXXXXXXXXXXXXXXXXXXXXXXXXXXXXXXXXXXXXXXXXXXXXXXXXXX

                                    90570     90580     90590     90600     90610     90620     90630     90640

Human                      TTGTCAGCAATCAGCTGGCTAGAACTCTCTTGTTACCTGGCTACTTCCTGAGGTAGCTGTTTGGTTATTTAGCTTTATTT 89480
Kakapo                     CTCTCATTTTTTTTGTGTCTGCTAAAGGTTTTCTACTAATCATACAATATACTCATGGGCTACACTTACAAAGTTGGGCT 48859
GoldenEagle                                                                                                57960
JapaneseQuail                                                                                              43432
MediumGroundFinch                                                                                          32462
GoodesThornscrubTortoise                                                                                   82946

Majority                   XXXXXXXXXXXXXXXXXXXXXXXXXXXXXXXXXXXXXXXXXXXXXXXXXXXXXXXXXXXXXXXXXXXXXXXXXXXXXXXX

                                    90650     90660     90670     90680     90690     90700     90710     90720

Human                      TAATCCCATTCCTCTATCACTCATCTGTTCTGTATGCAGTTACTATGCCTGGGACTTATTTTCTTATTCTTTCCCTCCAT 89560
Kakapo                     TTTTTTTTTTCATGCTAGATTAGTGGACCATACATATTTCAGACCAGGATATTTCACCTCATCGGAGACATGGGTTCAAA 48939
GoldenEagle                                                                                                57960
JapaneseQuail                                                                                              43432
MediumGroundFinch                                                                                          32462
GoodesThornscrubTortoise                                                                                   82946

Majority                   XXXXXXXXXXXXXXXXXXXXXXXXXXXXXXXXXXXXXXXXXXXXXXXXXXXXXXXXXXXXXXXXXXXXXXXXXXXXXXXX

                                    90730     90740     90750     90760     90770     90780     90790     90800

Human                      TATCTCAAAGCTCTGCTGTTTGTGCTGTCTTTTTATTTAGACAGCTTTTTTTCTGACAACGTTAAGTCTACTTATTTATA 89640
Kakapo                     TTGAATGTAAAAACAGTTGGGATACTTTAATAGTGTTTCTTCATATTACTTTGAACTGATTGTTGTGGGTTTTTTTCCC 49019
GoldenEagle                                                                                                57960
JapaneseQuail                                                                                              43432
MediumGroundFinch                                                                                          32462
GoodesThornscrubTortoise                                                                                   82946

Majority                   XXXXXXXXXXXXXXXXXXXXXXXXXXXXXXXXXXXXXXXXXXXXXXXXXXXXXXXXXXXXXXXXXXXXXXXXXXXXXXXX

                                    90810     90820     90830     90840     90850     90860     90870     90880

Human                      TCACTTTATTTCTCAAACTCACACTTAATAACACATTTTATCTGAGGTAGTTTATCTAAACAAAAGTTTGTTCACTTAAA 89720
Kakapo                     CCCTCGATTAGCAATAGGATGAAAACTTTTACAGAGTCTTCTTTTAAGAGTAACTGTATGATCCCAAAGCATTACATTGG 49099
GoldenEagle                                                                                                57960
JapaneseQuail                                                                                              43432
MediumGroundFinch                                                                                          32462
GoodesThornscrubTortoise                                                                                   82946
```

```
Majority                    XXXXXXXXXXXXXXXXXXXXXXXXXXXXXXXXXXXXXXXXXXXXXXXXXXXXXXXXXXXXXXXXXXXXXXXXXXXXXXXX

                                     90890     90900     90910     90920     90930     90940     90950     90960

Human                       ATAGTTATAGAACTTTAAAATTTTATAATTCAGTGGGTTTTATTGTAAAAGGATTTTATGTACTGTCACTGTAGATAATT 89800
Kakapo                      TAAAAGGTGTCAGTGTTCAATTCAAAGCATATTGCTGTCACTTCTCCAGTGCGACTACTGTAGATTTATTGATGTCAGAT 49179
GoldenEagle                                                                                                  57960
JapaneseQuail                                                                                                43432
MediumGroundFinch                                                                                            32462
GoodesThornscrubTortoise                                                                                     82946

Majority                    XXXXXXXXXXXXXXXXXXXXXXXXXXXXXXXXXXXXXXXXXXXXXXXXXXXXXXXXXXXXXXXXXXXXXXXXXXXXXXXX

                                     90970     90980     90990     91000     91010     91020     91030     91040

Human                       GGTTTTACAGGTGTAAGCGTAAATTTGCAAGTATATGAAATTAAGTATGTGAAATGCAGTGTGAGAAACAAATGAGAAAA 89880
Kakapo                      TTCTTTGTGACCTAATGCCTGTTCTTTGGGTTGGCTTTCAAATCAATAGCTATCAGCTTTTTAAAGGTCACGGTACAACA 49259
GoldenEagle                                                                                                  57960
JapaneseQuail                                                                                                43432
MediumGroundFinch                                                                                            32462
GoodesThornscrubTortoise                                                                                     82946

Majority                    XXXXXXXXXXXXXXXXXXXXXXXXXXXXXXXXXXXXXXXXXXXXXXXXXXXXXXXXXXXXXXXXXXXXXXXXXXXXXXXX

                                     91050     91060     91070     91080     91090     91100     91110     91120

Human                       TTCAGACTTGTGAGAAGATTGTTTTGTTTTTTTTCTGTGCTTAGCCTTGGCGAAAAATCTGAGAAAACTTAAAATATATA 89960
Kakapo                      TAGGGGGCTTGCATTAGTCTTCACCACATAAAATAAAATACATTTATCAGAAATACACCAAAGATACGACTTAAGGTATA 49339
GoldenEagle                                                                                                  57960
JapaneseQuail                                                                                                43432
MediumGroundFinch                                                                                            32462
GoodesThornscrubTortoise                                                                                     82946

Majority                    XXXXXXXXXXXXXXXXXXXXXXXXXXXXXXXXXXXXXXXXXXXXXXXXXXXXXXXXXXXXXXXXXXXXXXXXXXXXXXXX

                                     91130     91140     91150     91160     91170     91180     91190     91200

Human                       ACACTGGAGTGACAATCAGGAAGAGTGGACTCTTTTTTTTTTTAATTTTTTTAAACAAATTAGGGAAGTAAGTTCAATGG 90040
Kakapo                      TTGCAGTGATCACCCAGTCTGAAATGCCAGTAGAATCAGATTCCATCCATACCTGAAACATACGAATCGACATCTTTTTA 49419
GoldenEagle                                                                                                  57960
JapaneseQuail                                                                                                43432
MediumGroundFinch                                                                                            32462
GoodesThornscrubTortoise                                                                                     82946
```

```
Majority                  XXXXXXXXXXXXXXXXXXXXXXXXXXXXXXXXXXXXXXXXXXXXXXXXXXXXXXXXXXXXXXXXXXXXXXXXXXXXXXXX

                                   91210     91220     91230     91240     91250     91260     91270     91280

Human                     CTAGTGGTTATCATAATAAGAAAAATTTAAGGAAGTTATAGGTAAAATAAGAATTTTACTTTTGTTTCTTTTCTGAATGG 90120
Kakapo                    CAACTCATGCACCCAAACACGTTGATGTGATTATCTCCCAGTTGACTCGATCAGTAGCTAAGGGTCTGATAGAGCTGATC 49499
GoldenEagle                                                                                                57960
JapaneseQuail                                                                                              43432
MediumGroundFinch                                                                                          32462
GoodesThornscrubTortoise                                                                                   82946

Majority                  XXXXXXXXXXXXXXXXXXXXXXXXXXXXXXXXXXXXXXXXXXXXXXXXXXXXXXXXXXXXXXXXXXXXXXXXXXXXXXXX

                                   91290     91300     91310     91320     91330     91340     91350     91360

Human                     TACCTCATATTTTGACAGCATTATCCTTTTCAGATCTGTTTTATATTTAATTGGTTTATTATACATGTAAGTATGATTAG 90200
Kakapo                    TTTTCAATTTTCCATCTATACGATATGATTTCCCATGTTTTTAAAAGTAAAAATGCGTCTCACTTCCTTTGGCATATCAG 49579
GoldenEagle                                                                                                57960
JapaneseQuail                                                                                              43432
MediumGroundFinch                                                                                          32462
GoodesThornscrubTortoise                                                                                   82946

Majority                  XXXXXXXXXXXXXXXXXXXXXXXXXXXXXXXXXXXXXXXXXXXXXXXXXXXXXXXXXXXXXXXXXXXXXXXXXXXXXXXX

                                   91370     91380     91390     91400     91410     91420     91430     91440

Human                     ATTCATGGATTTAACCAGTTCTTTGTATCTTCTGTGTCAAAATGTATACTGTAGTATACTGTTTATATTAGAAACTCTTT 90280
Kakapo                    AACGTGCCTGCATGTTTCCACTAGAAATGTGAATCTCTTGCCAGGCTTATTTCCTTCAGAAGAAGTTTCTATACTTCTCT 49659
GoldenEagle                                                                                                57960
JapaneseQuail                                                                                              43432
MediumGroundFinch                                                                                          32462
GoodesThornscrubTortoise                                                                                   82946

Majority                  XXXXXXXXXXXXXXXXXXXXXXXXXXXXXXXXXXXXXXXXXXXXXXXXXXXXXXXXXXXXXXXXXXXXXXXXXXXXXXXX

                                   91450     91460     91470     91480     91490     91500     91510     91520

Human                     AAAAGTGGATGTTCTATTCTTAATTTCCTGATCTTCCACATTGTCTTTATCTGTAACTCTACTGTTTTTTGTTGGTTTTT 90360
Kakapo                    CATTTTGAAGAGTTCTGTGGCAAAAACCTCAAGGGAAAGAATATATATTGAATTTGATTTTTATTTTTTTTAATCAATTC 49739
GoldenEagle                                                                                                57960
JapaneseQuail                                                                                              43432
MediumGroundFinch                                                                                          32462
GoodesThornscrubTortoise                                                                                   82946
```

```
Majority                  XXXXXXXXXXXXXXXXXXXXXXXXXXXXXXXXXXXXXXXXXXXXXXXXXXXXXXXXXXXXXXXXXXXXXXXXXXXXXXXX

                                   91530     91540     91550     91560     91570     91580     91590     91600

Human                     GTTTTTGTTTTGAGACAGAGTCTCGCTCTGTCACCCAGGCTGGAGTGCACTGTGGCACCATCTCAGCTCACTGTAACCTC 90440
Kakapo                    AGGAACTTTTATTAGGAGGTTATATTTGTATCAGCCGAAATTAAAAGCAATGATAGTCATAATTGTCATGAAAGCATTTT 49819
GoldenEagle                                                                                                57960
JapaneseQuail                                                                                              43432
MediumGroundFinch                                                                                          32462
GoodesThornscrubTortoise                                                                                   82946

Majority                  XXXXXXXXXXXXXXXXXXXXXXXXXXXXXXXXXXXXXXXXXXXXXXXXXXXXXXXXXXXXXXXXXXXXXXXXXXXXXXXX

                                   91610     91620     91630     91640     91650     91660     91670     91680

Human                     CGCCTCCTGGGTACAAGCAATTCTGCCTCAGCCTCCTGAGTAACTGGGACCACAGGCACGAGCCACCACGCCCAGCTAAT 90520
Kakapo                    TCATTAACTCGGTGAAGAAAAATCACTGCTGCTGTATTGATACATAGTCTTTAAAGTGGGATCTCTTCAGAATACACAGG 49899
GoldenEagle                                                                                                57960
JapaneseQuail                                                                                              43432
MediumGroundFinch                                                                                          32462
GoodesThornscrubTortoise                                                                                   82946

Majority                  XXXXXXXXXXXXXXXXXXXXXXXXXXXXXXXXXXXXXXXXXXXXXXXXXXXXXXXXXXXXXXXXXXXXXXXXXXXXXXXX

                                   91690     91700     91710     91720     91730     91740     91750     91760

Human                     TTTTGTATTTTTAGTAGAGATGGGGTGTTACCATATTGGCCAGGCTGGTCTCGAACTCCTAGACCTTGTGATCCGCCCAC 90600
Kakapo                    TTTCATATCCTAAACTCGAACTTTTCCAAACCTTGTTTTTCTTGAGACGGGACTGCATTTTGTATAGTAATTTGTACCTT 49979
GoldenEagle                                                                                                57960
JapaneseQuail                                                                                              43432
MediumGroundFinch                                                                                          32462
GoodesThornscrubTortoise                                                                                   82946

Majority                  XXXXXXXXXXXXXXXXXXXXXXXXXXXXXXXXXXXXXXXXXXXXXXXXXXXXXXXXXXXXXXXXXXXXXXXXXXXXXXXX

                                   91770     91780     91790     91800     91810     91820     91830     91840

Human                     CTCAGCCTCCCAAAGTGTTGGGATTACAGGCGTGAGCCAACGCGCCCGGCATCTACTGTTAATACTTTATTATCTAGTTG 90680
Kakapo                    TTTGTTGTTATCAAATGTAAATAAGTTTCTGTATTTTTAGCTCTTGGGAATGTGAAAATGCAAAGCATCATTTTTATTAA 50059
GoldenEagle                                                                                                57960
JapaneseQuail                                                                                              43432
MediumGroundFinch                                                                                          32462
GoodesThornscrubTortoise                                                                                   82946
```

```
Majority                 XXXXXXXXXXXXXXXXXXXXXXXXXXXXXXXXXXXXXXXXXXXXXXXXXXXXXXXXXXXXXXXXXXXXXXXXXXXXXXXX
                                  91850     91860     91870     91880     91890     91900     91910     91920
Human                    ATTATAGTCATTTAGACCTTCAGTTTAGCCAAAGTGCATGACATATTAGAAATGGTCTTTTTTAGTTATTTTGACTGGTG 90760
Kakapo                   AATTTTACTACAAATAAGGTTCCTTAGACTAGATTTTTCTCTGCTGGAGCAATGACATCACTTGGGAAAGTCCAAAAGTG 50139
GoldenEagle                                                                                               57960
JapaneseQuail                                                                                             43432
MediumGroundFinch                                                                                         32462
GoodesThornscrubTortoise                                                                                  82946

Majority                 XXXXXXXXXXXXXXXXXXXXXXXXXXXXXXXXXXXXXXXXXXXXXXXXXXXXXXXXXXXXXXXXXXXXXXXXXXXXXXXX
                                  91930     91940     91950     91960     91970     91980     91990     92000
Human                    AAT---AAAACA--TATAACTTTCTAGCATCAGACCAGTTCATTTAGTTTAAAAATGATCCAGCAGCTATGTCAAGAAAT 90835
Kakapo                   ATTCTTCAATCAGGTAACGTCCACTGGCAAATACAATATAGAACTGAAGTAAACCAGAGCCATTCTGACTAAAAATAGGG 50219
GoldenEagle                                                                                               57960
JapaneseQuail                                                                                             43432
MediumGroundFinch                                                                                         32462
GoodesThornscrubTortoise                                                                                  82946

Majority                 XXXXXXXXXXXXXXXXXXXXXXXXXXXXXXXXXXXXXXXXXXXXXXXXXXXXXXXXXXXXXXXXXXXXXXXXXXXXXXXX
                                  92010     92020     92030     92040     92050     92060     92070     92080
Human                    AGTCATGTAATGTTCTGTTTTCACACACTGCACTATGTTCTGGGGTAGAGACAAAGGTTTGGATTTTGGAATATTTGCAT 90915
Kakapo                   GCAAAACTTCAAATTTAAGTGAATGCACTTGAAAAGATTTCTTGTGTGTTGTTATTTCACGTGTTACTAGAAGACACCTT 50299
GoldenEagle                                                                                               57960
JapaneseQuail                                                                                             43432
MediumGroundFinch                                                                                         32462
GoodesThornscrubTortoise                                                                                  82946

Majority                 XXXXXXXXXXXXXXXXXXXXXXXXXXXXXXXXXXXXXXXXXXXXXXXXXXXXXXXXXXXXXXXXXXXXXXXXXXXXXXXX
                                  92090     92100     92110     92120     92130     92140     92150     92160
Human                    TATACCAATTGAGCTTCCCAAATCTGAAATGTTCCAATGAGCATTTCCCTTGAGCATCATGTTGGCCCTCAGTTTTGGAT 90995
Kakapo                   TGCCCTGCAGGAATAACAGCTCCTGTGACTAGTTGTGAATTGCCATTTTGGATGTTTTACATTAATTTTCAGTTCTGTAA 50379
GoldenEagle                                                                                               57960
JapaneseQuail                                                                                             43432
MediumGroundFinch                                                                                         32462
GoodesThornscrubTortoise                                                                                  82946
```

```
Majority                    XXXXXXXXXXXXXXXXXXXXXXXXXXXXXXXXXXXXXXXXXXXXXXXXXXXXXXXXXXXXXXXXXXXXXXXXXXXXXXXX

                                     92170     92180     92190     92200     92210     92220     92230     92240

Human                       TTTGGAACATTTCAGATTTCAGATTTCTGCATTAAGGATACTCAACCTGTACAGACTGGGAAAGGTATAAGCCATGTTAA 91075
Kakapo                      CAAGCCATCTTACGAAAGAAAATCCCTTTGATTCATTGTGAGTGTCCTTGTGCTTATTGTAGTGTTTTACAGCATGACAG 50459
GoldenEagle                                                                                                  57960
JapaneseQuail                                                                                                43432
MediumGroundFinch                                                                                            32462
GoodesThornscrubTortoise                                                                                     82946

Majority                    XXXXXXXXXXXXXXXXXXXXXXXXXXXXXXXXXXXXXXXXXXXXXXXXXXXXXXXXXXXXXXXXXXXXXXXXXXXXXXXX

                                     92250     92260     92270     92280     92290     92300     92310     92320

Human                       AGAATGAGTTTCAACTTTGTCCTTAAGGGTTTTAAGCAGGTATTGACGTGATCAGATTTGTAATTTATGAAGGTCATCAA 91155
Kakapo                      CACCCATCAGGCATTTATCTGCACTATTTAAGGCCTTAAATTTCATACCCTGGTTTACTCTAGCTAATAGATCTAATTTT 50539
GoldenEagle                                                                                                  57960
JapaneseQuail                                                                                                43432
MediumGroundFinch                                                                                            32462
GoodesThornscrubTortoise                                                                                     82946

Majority                    XXXXXXXXXXXXXXXXXXXXXXXXXXXXXXXXXXXXXXXXXXXXXXXXXXXXXXXXXXXXXXXXXXXXXXXXXXXXXXXX

                                     92330     92340     92350     92360     92370     92380     92390     92400

Human                       GCTGCTGTATAGAAAATGGATTGGAAAAATTATCCAACAGGAAGACAAATATGAGACTGAGTTATCCAGATAAAAAATAA 91235
Kakapo                      ATGCTTAGATAGTAAGTAGTTCTGTTGAAACACTAGAAAAAGGGTGAGAATTGTGTCTTTCTCTGCAAACAGGTATATAA 50619
GoldenEagle                                                                                                  57960
JapaneseQuail                                                                                                43432
MediumGroundFinch                                                                                            32462
GoodesThornscrubTortoise                                                                                     82946

Majority                    XXXXXXXXXXXXXXXXXXXXXXXXXXXXXXXXXXXXXXXXXXXXXXXXXXXXXXXXXXXXXXXXXXXXXXXXXXXXXXXX

                                     92410     92420     92430     92440     92450     92460     92470     92480

Human                       TGTCAGCGAGAAGTAGAGAAACAGATCATGAATTTGAGGGACAATTAGGAGGAAGGCTATCACAATATTTAGCAATTAGG 91315
Kakapo                      ATCCACTCGTCCACCTCACGAAGTAGTGTTCCCACTATGAAATGTTTTACAGTGAGC                        50676
GoldenEagle                                                                                                  57960
JapaneseQuail                                                                                                43432
MediumGroundFinch                                                                                            32462
GoodesThornscrubTortoise                                                                                     82946
```

```
Majority                 XXXXXXXXXXXXXXXXXXXXXXXXXXXXXXXXXXXXXXXXXXXXXXXXXXXXXXXXXXXXXXXXXXXXXXXXXXXXXXXX

                                  92490     92500     92510     92520     92530     92540     92550     92560

Human                    ATGTGTAACAGAAGGGAGAAAAAGAGCTAGGGATGCTTATTAAGTTTCTTTCTTGTGTAGCTCACTATACGATTTGTTAG 91395
Kakapo                                                                                                    50676
GoldenEagle                                                                                               57960
JapaneseQuail                                                                                             43432
MediumGroundFinch                                                                                         32462
GoodesThornscrubTortoise                                                                                  82946

Majority                 XXXXXXXXXXXXXXXXXXXXXXXXXXXXXXXXXXXXXXXXXXXXXXXXXXXXXXXXXXXXXXXXXXXXXXXXXXXXXXXX

                                  92570     92580     92590     92600     92610     92620     92630     92640

Human                    CAGGATTGTTGAGGAAGATAGATATTCATAATCTGGATTTTCATTCTATGAAAGTAGCTGGCTTTTTTGCCAGAATGCCT 91475
Kakapo                                                                                                    50676
GoldenEagle                                                                                               57960
JapaneseQuail                                                                                             43432
MediumGroundFinch                                                                                         32462
GoodesThornscrubTortoise                                                                                  82946

Majority                 XXXXXXXXXXXXXXXXXXXXXXXXXXXXXXXXXXXXXXXXXXXXXXXXXXXXXXXXXXXXXXXXXXXXXXXXXXXXXXXX

                                  92650     92660     92670     92680     92690     92700     92710     92720

Human                    TGTCTAGTTCAAATGAAAATGTACTTAAGTCTTATCACCAGTGGCTATGGTAAATTGGTGATTTATTTTAAAATCATAAG 91555
Kakapo                                                                                                    50676
GoldenEagle                                                                                               57960
JapaneseQuail                                                                                             43432
MediumGroundFinch                                                                                         32462
GoodesThornscrubTortoise                                                                                  82946

Majority                 XXXXXXXXXXXXXXXXXXXXXXXXXXXXXXXXXXXXXXXXXXXXXXXXXXXXXXXXXXXXXXXXXXXXXXXXXXXXXXXX

                                  92730     92740     92750     92760     92770     92780     92790     92800

Human                    AAAATTCAAATTTTTAAACTGTTACGTTAAAAGAATTATGTTTGTTTTGAGCTTTGATATTGTTTGTTAATAGACATGCT 91635
Kakapo                                                                                                    50676
GoldenEagle                                                                                               57960
JapaneseQuail                                                                                             43432
MediumGroundFinch                                                                                         32462
GoodesThornscrubTortoise                                                                                  82946
```

```
Majority                    XXXXXXXXXXXXXXXXXXXXXXXXXXXXXXXXXXXXXXXXXXXXXXXXXXXXXXXXXXXXXXXXXXXXXXXXXXXXXXXX

                                     92810     92820     92830     92840     92850     92860     92870     92880

Human                       TTCTTTTTATAGCCATGAATCATCTTAATGGACAGAAAATGTATGGAAAAATTATTCGTGTTACTCTGTCTAAACATCAG 91715
Kakapo                                                                                                       50676
GoldenEagle                                                                                                  57960
JapaneseQuail                                                                                                43432
MediumGroundFinch                                                                                            32462
GoodesThornscrubTortoise                                                                                     82946

Majority                    XXXXXXXXXXXXXXXXXXXXXXXXXXXXXXXXXXXXXXXXXXXXXXXXXXXXXXXXXXXXXXXXXXXXXXXXXXXXXXXX

                                     92890     92900     92910     92920     92930     92940     92950     92960

Human                       ACTGTACAGCTACCTCGAGAGGGACTTGATGATCAAGGGCTAACAAAAGATTTTGGTAATTCCCCATTGCATCGTTTTAA 91795
Kakapo                                                                                                       50676
GoldenEagle                                                                                                  57960
JapaneseQuail                                                                                                43432
MediumGroundFinch                                                                                            32462
GoodesThornscrubTortoise                                                                                     82946

Majority                    XXXXXXXXXXXXXXXXXXXXXXXXXXXXXXXXXXXXXXXXXXXXXXXXXXXXXXXXXXXXXXXXXXXXXXXXXXXXXXXX

                                     92970     92980     92990     93000     93010     93020     93030     93040

Human                       GAAACCTGGATCCAAAAATTTTCAAAACATTTTTCCTCCTTCTGCCACCCTTCACCTATCTAATATCCCGTAAGTATATA 91875
Kakapo                                                                                                       50676
GoldenEagle                                                                                                  57960
JapaneseQuail                                                                                                43432
MediumGroundFinch                                                                                            32462
GoodesThornscrubTortoise                                                                                     82946

Majority                    XXXXXXXXXXXXXXXXXXXXXXXXXXXXXXXXXXXXXXXXXXXXXXXXXXXXXXXXXXXXXXXXXXXXXXXXXXXXXXXX

                                     93050     93060     93070     93080     93090     93100     93110     93120

Human                       AGCTAGAGTGTATTGAGATACATTCTATTTTGATAAAATATGAAATTTATTCTTAATCTTCACTTTTTCTTCCCATTCAA 91955
Kakapo                                                                                                       50676
GoldenEagle                                                                                                  57960
JapaneseQuail                                                                                                43432
MediumGroundFinch                                                                                            32462
GoodesThornscrubTortoise                                                                                     82946
```

```
Majority                    XXXXXXXXXXXXXXXXXXXXXXXXXXXXXXXXXXXXXXXXXXXXXXXXXXXXXXXXXXXXXXXXXXXXXXXXXXXXXXXX

                                     93130     93140     93150     93160     93170     93180     93190     93200

Human                       TTTTCCTAGTCCATCAGTAGCAGAAGAGGATCTACGAACACTGTTCGCTAACACTGGGGGCACTGTGAAAGCATTTAAGT 92035
Kakapo                                                                                                       50676
GoldenEagle                                                                                                  57960
JapaneseQuail                                                                                                43432
MediumGroundFinch                                                                                            32462
GoodesThornscrubTortoise                                                                                     82946

Majority                    XXXXXXXXXXXXXXXXXXXXXXXXXXXXXXXXXXXXXXXXXXXXXXXXXXXXXXXXXXXXXXXXXXXXXXXXXXXXXXXX

                                     93210     93220     93230     93240     93250     93260     93270     93280

Human                       TTTTTCAGTAAGCAAGCTTCCTTATCTTTAAATTAGTGACCTGATAAAATTTTTAAGTAGTTTTTGTTCTTTTCGTTTAT 92115
Kakapo                                                                                                       50676
GoldenEagle                                                                                                  57960
JapaneseQuail                                                                                                43432
MediumGroundFinch                                                                                            32462
GoodesThornscrubTortoise                                                                                     82946

Majority                    XXXXXXXXXXXXXXXXXXXXXXXXXXXXXXXXXXXXXXXXXXXXXXXXXXXXXXXXXXXXXXXXXXXXXXXXXXXXXXXX

                                     93290     93300     93310     93320     93330     93340     93350     93360

Human                       AGAAATTTTTGATTCCGGAATGGCCAAAATTCAAGTATTTAATAAGCCCTTTCTAATTTGTATGAAGTGTCTAATTTTAT 92195
Kakapo                                                                                                       50676
GoldenEagle                                                                                                  57960
JapaneseQuail                                                                                                43432
MediumGroundFinch                                                                                            32462
GoodesThornscrubTortoise                                                                                     82946

Majority                    XXXXXXXXXXXXXXXXXXXXXXXXXXXXXXXXXXXXXXXXXXXXXXXXXXXXXXXXXXXXXXXXXXXXXXXXXXXXXXXX

                                     93370     93380     93390     93400     93410     93420     93430     93440

Human                       AATTTTGTTTCAGAAGAGATCACAAAATGGCTCTTCTTCAGATGGCAACAGTGGAAGAAGCTATTCAGGCCTTGATTGAT 92275
Kakapo                                                                                                       50676
GoldenEagle                                                                                                  57960
JapaneseQuail                                                                                                43432
MediumGroundFinch                                                                                            32462
GoodesThornscrubTortoise                                                                                     82946
```

```
Majority                      XXXXXXXXXXXXXXXXXXXXXXXXXXXXXXXXXXXXXXXXXXXXXXXXXXXXXXXXXXXXXXXXXXXXXXXXXXXXXXXX

                                       93450     93460     93470     93480     93490     93500     93510     93520

Human                         CTTCATAATTATAACCTTGGAGAAAACCATCATCTGAGAGTGTCTTTCTCCAAGTCAACAATTTAAAAATGGAAGATGA 92355
Kakapo                                                                                                         50676
GoldenEagle                                                                                                    57960
JapaneseQuail                                                                                                  43432
MediumGroundFinch                                                                                              32462
GoodesThornscrubTortoise                                                                                       82946

Majority                      XXXXXXXXXXXXXXXXXXXXXXXXXXXXXXXXXXXXXXXXXXXXXXXXXXXXXXXXXXXXXXXXXXXXXXXXXXXXXXXX

                                       93530     93540     93550     93560     93570     93580     93590     93600

Human                         AGATTGGGGGTGAATCACATTGTTCAATGTCATCACCTATTTGACTGTTCAGAAAAGTGGGGACCAGAGTTTGATTTTTT 92435
Kakapo                                                                                                         50676
GoldenEagle                                                                                                    57960
JapaneseQuail                                                                                                  43432
MediumGroundFinch                                                                                              32462
GoodesThornscrubTortoise                                                                                       82946

Majority                      XXXXXXXXXXXXXXXXXXXXXXXXXXXXXXXXXXXXXXXXXXXXXXXXXXXXXXXXXXXXXXXXXXXXXXXXXXXXXXXX

                                       93610     93620     93630     93640     93650     93660     93670     93680

Human                         TTGTTTTTGTTTTTTTGGGGTTTCTTTTTTTTTTCCATGCTGTTATCATTCCTTGGTTATAAAATGAAATGGCATATGTA 92515
Kakapo                                                                                                         50676
GoldenEagle                                                                                                    57960
JapaneseQuail                                                                                                  43432
MediumGroundFinch                                                                                              32462
GoodesThornscrubTortoise                                                                                       82946

Majority                      XXXXXXXXXXXXXXXXXXXXXXXXXXXXXXXXXXXXXXXXXXXXXXXXXXXXXXXXXXXXXXXXXXXXXXXXXXXXXXXX

                                       93690     93700     93710     93720     93730     93740     93750     93760

Human                         AAGGCAGAGTTGTTAACTGCTATATTTCATCTGTTCTATAGGGAAGCCATTTTGTCTGTTTAAAATTTCAGTTTAATTTT 92595
Kakapo                                                                                                         50676
GoldenEagle                                                                                                    57960
JapaneseQuail                                                                                                  43432
MediumGroundFinch                                                                                              32462
GoodesThornscrubTortoise                                                                                       82946
```

```
Majority                  XXXXXXXXXXXXXXXXXXXXXXXXXXXXXXXXXXXXXXXXXXXXXXXXXXXXXXXXXXXXXXXXXXXXXXXXXXXXXXXX

                                   93770     93780     93790     93800     93810     93820     93830     93840

Human                     GCTTTTTTTTTTTTTTTTTTTTTCCTTTCAACTTAGTTGACATACGTGCCTTAAAAAGGAAAACTAGTGTTGCTATTGTG 92675
Kakapo                                                                                                     50676
GoldenEagle                                                                                                57960
JapaneseQuail                                                                                              43432
MediumGroundFinch                                                                                          32462
GoodesThornscrubTortoise                                                                                   82946

Majority                  XXXXXXXXXXXXXXXXXXXXXXXXXXXXXXXXXXXXXXXXXXXXXXXXXXXXXXXXXXXXXXXXXXXXXXXXXXXXXXXX

                                   93850     93860     93870     93880     93890     93900     93910     93920

Human                     CATTTACTAGAAAAAAGGAATTGGTTGTTTAGGGCACACTGTTATATGGGAATTAAAATATGTTTAGGCAGGGGTGTGTA 92755
Kakapo                                                                                                     50676
GoldenEagle                                                                                                57960
JapaneseQuail                                                                                              43432
MediumGroundFinch                                                                                          32462
GoodesThornscrubTortoise                                                                                   82946

Majority                  XXXXXXXXXXXXXXXXXXXXXXXXXXXXXXXXXXXXXXXXXXXXXXXXXXXXXXXXXXXXXXXXXXXXXXXXXXXXXXXX

                                   93930     93940     93950     93960     93970     93980     93990     94000

Human                     AAAAGGTTAAGTTTTTGTTTCTCCTGCTTGGAACTTATTTTGAATTACTGGCTTGTCACCTTTTTTTCTATTTAATCAAA 92835
Kakapo                                                                                                     50676
GoldenEagle                                                                                                57960
JapaneseQuail                                                                                              43432
MediumGroundFinch                                                                                          32462
GoodesThornscrubTortoise                                                                                   82946

Majority                  XXXXXXXXXXXXXXXXXXXXXXXXXXXXXXXXXXXXXXXXXXXXXXXXXXXXXXXXXXXXXXXXXXXXXXXXXXXXXXXX

                                   94010     94020     94030     94040     94050     94060     94070     94080

Human                     TAAGATACATGATATTGAAAGAATAAAGCAGCATTTTTAGTTTTTACTACCTTAGGCTTTATTGCTTTGAAAACAACATT 92915
Kakapo                                                                                                     50676
GoldenEagle                                                                                                57960
JapaneseQuail                                                                                              43432
MediumGroundFinch                                                                                          32462
GoodesThornscrubTortoise                                                                                   82946
```

```
Majority                  XXXXXXXXXXXXXXXXXXXXXXXXXXXXXXXXXXXXXXXXXXXXXXXXXXXXXXXXXXXXXXXXXXXXXXXXXXXXXXXX

                                   94090     94100     94110     94120     94130     94140     94150     94160

Human                     GGCCTTTTGTATCTCACAATTCTGGTCTAGATTCAGTTATGAATGTAGGCATTAGTTAAAATTAACAAGATGCAGAGTAT 92995
Kakapo                                                                                                     50676
GoldenEagle                                                                                                57960
JapaneseQuail                                                                                              43432
MediumGroundFinch                                                                                          32462
GoodesThornscrubTortoise                                                                                   82946

Majority                  XXXXXXXXXXXXXXXXXXXXXXXXXXXXXXXXXXXXXXXXXXXXXXXXXXXXXXXXXXXXXXXXXXXXXXXXXXXXXXXX

                                   94170     94180     94190     94200     94210     94220     94230     94240

Human                     TAATTTCTTAAGACAACAAAGTGATTTCTGTAAGTTTGAGCCCTATGTGGAAAGCATTGTGGAATCTTAACCTTTTCGTA 93075
Kakapo                                                                                                     50676
GoldenEagle                                                                                                57960
JapaneseQuail                                                                                              43432
MediumGroundFinch                                                                                          32462
GoodesThornscrubTortoise                                                                                   82946

Majority                  XXXXXXXXXXXXXXXXXXXXXXXXXXXXXXXXXXXXXXXXXXXXXXXXXXXXXXXXXXXXXXXXXXXXXXXXXXXXXXXX

                                   94250     94260     94270     94280     94290     94300     94310     94320

Human                     CACACTCTTGTGGGACGTATCATATAAATGTCAGCACTAAGTAATGTCTTGTTTGTGGCTGAATATTTTTCGTAGATGTT 93155
Kakapo                                                                                                     50676
GoldenEagle                                                                                                57960
JapaneseQuail                                                                                              43432
MediumGroundFinch                                                                                          32462
GoodesThornscrubTortoise                                                                                   82946

Majority                  XXXXXXXXXXXXXXXXXXXXXXXXXXXXXXXXXXXXXXXXXXXXXXXXXXXXXXXXXXXXXXXXXXXXXXXXXXXXXXXX

                                   94330     94340     94350     94360     94370     94380     94390     94400

Human                     TTTGAAGTTGACATGACTTACGTGCATTTAAATATATATTGCCATCCTTAGTTTGTAATTAAGATTTGGAATATGGTTGT 93235
Kakapo                                                                                                     50676
GoldenEagle                                                                                                57960
JapaneseQuail                                                                                              43432
MediumGroundFinch                                                                                          32462
GoodesThornscrubTortoise                                                                                   82946
```

```
Majority                  XXXXXXXXXXXXXXXXXXXXXXXXXXXXXXXXXXXXXXXXXXXXXXXXXXXXXXXXXXXXXXXXXXXXXXXXXXXXXXXX

                                   94410     94420     94430     94440     94450     94460     94470     94480

Human                     GGATTTCTGAGCATGTGCAGACTGGTCTAGCTAGTTCAGGAACTGGTGCATGTATTTTTCAAAGATAAAGAAAGTGTACT 93315
Kakapo                                                                                                     50676
GoldenEagle                                                                                                57960
JapaneseQuail                                                                                              43432
MediumGroundFinch                                                                                          32462
GoodesThornscrubTortoise                                                                                   82946

Majority                  XXXXXXXXXXXXXXXXXXXXXXXXXXXXXXXXXXXXXXXXXXXXXXXXXXXXXXXXXXXXXXXXXXXXXXXXXXXXXXXX

                                   94490     94500     94510     94520     94530     94540     94550     94560

Human                     GCGAAAATATGCAGGAAGATTAATTTTGTGGCAGTTTTCTAAAACTGACAACCAGGTGGGACCAAAGTTTATGTGCCTTT 93395
Kakapo                                                                                                     50676
GoldenEagle                                                                                                57960
JapaneseQuail                                                                                              43432
MediumGroundFinch                                                                                          32462
GoodesThornscrubTortoise                                                                                   82946

Majority                  XXXXXXXXXXXXXXXXXXXXXXXXXXXXXXXXXXXXXXXXXXXXXXXXXXXXXXXXXXXXXXXXXXXXXXXXXXXXXXXX

                                   94570     94580     94590     94600     94610     94620     94630     94640

Human                     AGTCTTAATTTACCTTGCATTGTAATATTCAGTTTTAATAAATCTTCAAAATATTTTGTATTTAGGAATAGATCTGACTT 93475
Kakapo                                                                                                     50676
GoldenEagle                                                                                                57960
JapaneseQuail                                                                                              43432
MediumGroundFinch                                                                                          32462
GoodesThornscrubTortoise                                                                                   82946

Majority                  XXXXXXXXXXXXXXXXXXXXXXXXXXXXXXXXXXXXXXXXXXXXXXXXXXXXXXXXXXXXXXXXXXXXXXXXXXXXXXXX

                                   94650     94660     94670     94680     94690     94700     94710     94720

Human                     TAATAAAAACATGGCTCAGAATCTACAGGTCAAATTAATTTGAACAGTTCTTGTCAATCCGAATTGTTGATTCTGTTTAA 93555
Kakapo                                                                                                     50676
GoldenEagle                                                                                                57960
JapaneseQuail                                                                                              43432
MediumGroundFinch                                                                                          32462
GoodesThornscrubTortoise                                                                                   82946
```

```
Majority                    XXXXXXXXXXXXXXXXXXXXXXXXXXXXXXXXXXXXXXXXXXXXXXXXXXXXXXXXXXXXXXXXXXXXXXXXXXXXXXXX

                                     94730     94740     94750     94760     94770     94780     94790     94800

Human                       ATGACCAATACTTTTTGAAATTGATGTACTTAGTTTCAAGATTCATAGATTCTGTTATCTATGTAGACAGAATGGTCATG 93635
Kakapo                                                                                                       50676
GoldenEagle                                                                                                  57960
JapaneseQuail                                                                                                43432
MediumGroundFinch                                                                                            32462
GoodesThornscrubTortoise                                                                                     82946

Majority                    XXXXXXXXXXXXXXXXXXXXXXXXXXXXXXXXXXXXXXXXXXXXXXXXXXXXXXXXXXXXXXXXXXXXXXXXXXXXXXXX

                                     94810     94820     94830     94840     94850     94860     94870     94880

Human                       TATATTTTCTATTAGTTGAGTTTTTACATCTTTAGAAATGTAAAATTCAGTATAGTTTGAAAGCGGCACAATTAAAAATT 93715
Kakapo                                                                                                       50676
GoldenEagle                                                                                                  57960
JapaneseQuail                                                                                                43432
MediumGroundFinch                                                                                            32462
GoodesThornscrubTortoise                                                                                     82946

Majority                    XXXXXXXXXXXXXXXXXXXXXXXXXXXXXXXXXXXXXXXXXXXXXXXXXXXXXXXXXXXXXXXXXXXXXXXXXXXXXXXX

                                     94890     94900     94910     94920     94930     94940     94950     94960

Human                       AATTTTCTAACAAAGTTGGGAGGTTTGATGGTTGTTTAATTTCATTTTGTGTGTACTCTGCTTACCCCTGTAGCATGCTC 93795
Kakapo                                                                                                       50676
GoldenEagle                                                                                                  57960
JapaneseQuail                                                                                                43432
MediumGroundFinch                                                                                            32462
GoodesThornscrubTortoise                                                                                     82946

Majority                    XXXXXXXXXXXXXXXXXXXXXXXXXXXXXXXXXXXXXXXXXXXXXXXXXXXXXXXXXXXXXXXXXXXXXXXXXXXXXXXX

                                     94970     94980     94990     95000     95010     95020     95030     95040

Human                       AATAAACACTTCTGTAGCTCTATATTCACCTTTTCTGTCTTTCTCTGCTGCCTTTTCTCTCTCCTCTTCTTTGTTTTCAC 93875
Kakapo                                                                                                       50676
GoldenEagle                                                                                                  57960
JapaneseQuail                                                                                                43432
MediumGroundFinch                                                                                            32462
GoodesThornscrubTortoise                                                                                     82946
```

```
Majority                   XXXXXXXXXXXXXXXXXXXXXXXXXXXXXXXXXXXXXXXXXXXXXXXXXXXXXXXXXXXXXXXXXXXXXXXXXXXXXXXX

                                    95050     95060     95070     95080     95090     95100     95110     95120

Human                      TCCACTGTGCTTCTGAATTCATGTTTATTCTCTGCCAGGGTGGGAAAGGAGTAATAATATTACAATTCTATGGCTTTATA 93955
Kakapo                                                                                                      50676
GoldenEagle                                                                                                 57960
JapaneseQuail                                                                                               43432
MediumGroundFinch                                                                                           32462
GoodesThornscrubTortoise                                                                                    82946

Majority                   XXXXXXXXXXXXXXXXXXXXXXXXXXXXXXXXXXXXXXXXXXXXXXXXXXXXXXXXXXXXXXXXXXXXXXXXXXXXXXXX

                                    95130     95140     95150     95160     95170     95180     95190     95200

Human                      CCATAAATAAATCTAGATGCTGTGAAAATATACCAGCTGGTTTTTTTTAATTTAAAAGATGGTAACTGCTTTTCAGGAGG 94035
Kakapo                                                                                                      50676
GoldenEagle                                                                                                 57960
JapaneseQuail                                                                                               43432
MediumGroundFinch                                                                                           32462
GoodesThornscrubTortoise                                                                                    82946

Majority                   XXXXXXXXXXXXXXXXXXXXXXXXXXXXXXXXXXXXXXXXXXXXXXXXXXXXXXXXXXXXXXXXXXXXXXXXXXXXXXXX

                                    95210     95220     95230     95240     95250     95260     95270     95280

Human                      ACACATATTAAACATTTCCCACCCTGTTATAATCTACTGCTTTAAAGACATAACTTTTATTGTAGCTTGTTAATTCTATC 94115
Kakapo                                                                                                      50676
GoldenEagle                                                                                                 57960
JapaneseQuail                                                                                               43432
MediumGroundFinch                                                                                           32462
GoodesThornscrubTortoise                                                                                    82946

Majority                   XXXXXXXXXXXXXXXXXXXXXXXXXXXXXXXXXXXXXXXXXXXXXXXXXXXXXXXXXXXXXXXXXXXXXXXXXXXXXXXX

                                    95290     95300     95310     95320     95330     95340     95350     95360

Human                      TCTTTTGTTCTTGTTGTTTTTTTTTTTTTTCCAGTAGATTTATGCACTAATAGATCTTTTGGATTTGCCATGCTCTCTTG 94195
Kakapo                                                                                                      50676
GoldenEagle                                                                                                 57960
JapaneseQuail                                                                                               43432
MediumGroundFinch                                                                                           32462
GoodesThornscrubTortoise                                                                                    82946
```

```
Majority                    XXXXXXXXXXXXXXXXXXXXXXXXXXXXXXXXXXXXXXXXXXXXXXXXXXXXXXXXXXXXXXXXXXXXXXXXXXXXXXXX

                                     95370     95380     95390     95400     95410     95420     95430     95440

Human                       CTGCAGTTTCATCTTTCATCTTTTGTGTCTGCTAAAGATTTCTTACTAATCTTAGACTACCTTGTGAGTTAACAAAAAGA 94275
Kakapo                                                                                                       50676
GoldenEagle                                                                                                  57960
JapaneseQuail                                                                                                43432
MediumGroundFinch                                                                                            32462
GoodesThornscrubTortoise                                                                                     82946

Majority                    XXXXXXXXXXXXXXXXXXXXXXXXXXXXXXXXXXXXXXXXXXXXXXXXXXXXXXXXXXXXXXXXXXXXXXXXXXXXXXXX

                                     95450     95460     95470     95480     95490     95500     95510     95520

Human                       ACTTGATATTACTGGGAAGAAAGAGCAGCATATCTGCATATCAGCATATCTGCTTTTGCCTTGGGTGGAAGAATGATAT 94355
Kakapo                                                                                                       50676
GoldenEagle                                                                                                  57960
JapaneseQuail                                                                                                43432
MediumGroundFinch                                                                                            32462
GoodesThornscrubTortoise                                                                                     82946

Majority                    XXXXXXXXXXXXXXXXXXXXXXXXXXXXXXXXXXXXXXXXXXXXXXXXXXXXXXXXXXXXXXXXXXXXXXXXXXXXXXXX

                                     95530     95540     95550     95560     95570     95580     95590     95600

Human                       ACATTCAAGTATTTAAAAACTTAGAGTAATTTGCATTTAACAACTGAGGATGTTACTACTGTAACTTGTTAAGCAGTTAC 94435
Kakapo                                                                                                       50676
GoldenEagle                                                                                                  57960
JapaneseQuail                                                                                                43432
MediumGroundFinch                                                                                            32462
GoodesThornscrubTortoise                                                                                     82946

Majority                    XXXXXXXXXXXXXXXXXXXXXXXXXXXXXXXXXXXXXXXXXXXXXXXXXXXXXXXXXXXXXXXXXXXXXXXXXXXXXXXX

                                     95610     95620     95630     95640     95650     95660     95670     95680

Human                       AATTAGGGTGCTATTTATATTCAAAATATTTCGGTCAGTTTTTCTAAACAGATATATTGGTATCAAATAACTTTGGAGTT 94515
Kakapo                                                                                                       50676
GoldenEagle                                                                                                  57960
JapaneseQuail                                                                                                43432
MediumGroundFinch                                                                                            32462
GoodesThornscrubTortoise                                                                                     82946
```

```
Majority                   XXXXXXXXXXXXXXXXXXXXXXXXXXXXXXXXXXXXXXXXXXXXXXXXXXXXXXXXXXXXXXXXXXXXXXXXXXXXXXXX
                                    95690     95700     95710     95720     95730     95740     95750     95760
Human                      ACACATGTTTCTTAGGTTTGTTCTGTTATAAAATATCCCACACTTATGCAAAACTTACACATAGGATAATAAAAAGTAAA 94595
Kakapo                                                                                                     50676
GoldenEagle                                                                                                57960
JapaneseQuail                                                                                              43432
MediumGroundFinch                                                                                          32462
GoodesThornscrubTortoise                                                                                   82946

Majority                   XXXXXXXXXXXXXXXXXXXXXXXXXXXXXXXXXXXXXXXXXXXXXXXXXXXXXXXXXXXXXXXXXXXXXXXXXXXXXXXX
                                    95770     95780     95790     95800     95810     95820     95830     95840
Human                      TGAAGGAACATAAAATATGTTTTACTATACTTAATGTAGTAATTCAGTTACCTCCAAGAAAGAAGAATATAATAAGTAAA 94675
Kakapo                                                                                                     50676
GoldenEagle                                                                                                57960
JapaneseQuail                                                                                              43432
MediumGroundFinch                                                                                          32462
GoodesThornscrubTortoise                                                                                   82946

Majority                   XXXXXXXXXXXXXXXXXXXXXXXXXXXXXXXXXXXXXXXXXXXXXXXXXXXXXXXXXXXXXXXXXXXXXXXXXXXXXXXX
                                    95850     95860     95870     95880     95890     95900     95910     95920
Human                      ATAATTTTGTTTACACTAGTGTCACTGTTGTCATCTGAAGAAGAACCATTAGAGTCAACAGTCTGGGATGATTTGGAAGC 94755
Kakapo                                                                                                     50676
GoldenEagle                                                                                                57960
JapaneseQuail                                                                                              43432
MediumGroundFinch                                                                                          32462
GoodesThornscrubTortoise                                                                                   82946

Majority                   XXXXXXXXXXXXXXXXXXXXXXXXXXXXXXXXXXXXXXXXXXXXXXXXXXXXXXXXXXXXXXXXXXXXXXXXXXXXXXXX
                                    95930     95940     95950     95960     95970     95980     95990     96000
Human                      TAGGTGATCTGTGTTTTTTTCTTAAAATAACATCGTAGCACAGAGGAACTCTACCAGCATCAGATAAGTCTAAAGTCTAA 94835
Kakapo                                                                                                     50676
GoldenEagle                                                                                                57960
JapaneseQuail                                                                                              43432
MediumGroundFinch                                                                                          32462
GoodesThornscrubTortoise                                                                                   82946
```

```
Majority                   XXXXXXXXXXXXXXXXXXXXXXXXXXXXXXXXXXXXXXXXXXXXXXXXXXXXXXXXXXXXXXXXXXXXXXXXXXXXXXXX

                                    96010     96020     96030     96040     96050     96060     96070     96080

Human                      AAAACCAATACCAGGGTTAGTGAATAGTAATTGAACAAAGCTTCTCAAGGTGAAGTTTTTCTCAGTAATTAGTGGTGGCT 94915
Kakapo                                                                                                      50676
GoldenEagle                                                                                                 57960
JapaneseQuail                                                                                               43432
MediumGroundFinch                                                                                           32462
GoodesThornscrubTortoise                                                                                    82946

Majority                   XXXXXXXXXXXXXXXXXXXXXXXXXXXXXXXXXXXXXXXXXXXXXXXXXXXXXXXXXXXXXXXXXXXXXXXXXXXXXXXX

                                    96090     96100     96110     96120     96130     96140     96150     96160

Human                      ATAATATTTGAAAGTAGGTTCTGTCACCTTTTCAGGTGCTCATTGTGGGTGGATTGCCTTGGGAAGGAGAAACTAAGTC 94995
Kakapo                                                                                                      50676
GoldenEagle                                                                                                 57960
JapaneseQuail                                                                                               43432
MediumGroundFinch                                                                                           32462
GoodesThornscrubTortoise                                                                                    82946

Majority                   XXXXXXXXXXXXXXXXXXXXXXXXXXXXXXXXXXXXXXXXXXXXXXXXXXXXXXXXXXXXXXXXXXXXXXXXXXXXXXXX

                                    96170     96180     96190     96200     96210     96220     96230     96240

Human                      CTGAGCTGTGTGTGTGGACTTAGTTTGTGCCAGTTGCTAAGCTGGTGGGAGTTCCCAGTCTCCCTAGTGGGAGATAACCA 95075
Kakapo                                                                                                      50676
GoldenEagle                                                                                                 57960
JapaneseQuail                                                                                               43432
MediumGroundFinch                                                                                           32462
GoodesThornscrubTortoise                                                                                    82946

Majority                   XXXXXXXXXXXXXXXXXXXXXXXXXXXXXXXXXXXXXXXXXXXXXXXXXXXXXXXXXXXXXXXXXXXXXXXXXXXXXXXX

                                    96250     96260     96270     96280     96290     96300     96310     96320

Human                      GAAACCAGCTCTTCCTGCCTAAGGACTACAGCAGATTCTATACTGTCTTACAGTATGCCCTAATACGGCCTTCCTTCCAA 95155
Kakapo                                                                                                      50676
GoldenEagle                                                                                                 57960
JapaneseQuail                                                                                               43432
MediumGroundFinch                                                                                           32462
GoodesThornscrubTortoise                                                                                    82946
```

```
Majority                 XXXXXXXXXXXXXXXXXXXXXXXXXXXXXXXXXXXXXXXXXXXXXXXXXXXXXXXXXXXXXXXXXXXXXXXXXXXXXXXX

                                  96330     96340     96350     96360     96370     96380     96390     96400

Human                    AGCACCAAGTCACTAAAGGGGGATTTTGACATTCTAGAATATTTTTAATTCAGTACATCAAATTAACCCAGGCCTTGGCA 95235
Kakapo                                                                                                    50676
GoldenEagle                                                                                               57960
JapaneseQuail                                                                                             43432
MediumGroundFinch                                                                                         32462
GoodesThornscrubTortoise                                                                                  82946

Majority                 XXXXXXXXXXXXXXXXXXXXXXXXXXXXXXXXXXXXXXXXXXXXXXXXXXXXXXXXXXXXXXXXXXXXXXXXXXXXXXXX

                                  96410     96420     96430     96440     96450     96460     96470     96480

Human                    TATAGTGAATTGCTTATTAAAAGTTAGGTAAAAATAAAAACAGCTTTGTCGTATATTAGTACTCTATGACTCTTAGAGTG 95315
Kakapo                                                                                                    50676
GoldenEagle                                                                                               57960
JapaneseQuail                                                                                             43432
MediumGroundFinch                                                                                         32462
GoodesThornscrubTortoise                                                                                  82946

Majority                 XXXXXXXXXXXXXXXXXXXXXXXXXXXXXXXXXXXXXXXXXXXXXXXXXXXXXXXXXXXXXXXXXXXXXXXXXXXXXXXX

                                  96490     96500     96510     96520     96530     96540     96550     96560

Human                    AACAAGCTAACAAGTATTCTTAGGACTTAGCTGCTTTGTTAATAGAGGTCTTAACTTGAATATTTAGAAATAATCAGAAA 95395
Kakapo                                                                                                    50676
GoldenEagle                                                                                               57960
JapaneseQuail                                                                                             43432
MediumGroundFinch                                                                                         32462
GoodesThornscrubTortoise                                                                                  82946

Majority                 XXXXXXXXXXXXXXXXXXXXXXXXXXXXXXXXXXXXXXXXXXXXXXXXXXXXXXXXXXXXXXXXXXXXXXXXXXXXXXXX

                                  96570     96580     96590     96600     96610     96620     96630     96640

Human                    AATTATTTTTTGGTATTGAAGATGTATTTTGGAAATGGGTTTTAAAGTACCTACTTAAGGAAGAGATATCTACAGTTTAT 95475
Kakapo                                                                                                    50676
GoldenEagle                                                                                               57960
JapaneseQuail                                                                                             43432
MediumGroundFinch                                                                                         32462
GoodesThornscrubTortoise                                                                                  82946
```

```
Majority                  XXXXXXXXXXXXXXXXXXXXXXXXXXXXXXXXXXXXXXXXXXXXXXXXXXXXXXXXXXXXXXXXXXXXXXXXXXXXXXXX

                                   96650     96660     96670     96680     96690     96700     96710     96720

Human                     ATTTAACGTTAGCATTTGAAAGCAATGAAGAAAATTTTTCCTTAACCAGGAAGTGTAATGAATCATTATATTAGAGATTA 95555
Kakapo                                                                                                     50676
GoldenEagle                                                                                                57960
JapaneseQuail                                                                                              43432
MediumGroundFinch                                                                                          32462
GoodesThornscrubTortoise                                                                                   82946

Majority                  XXXXXXXXXXXXXXXXXXXXXXXXXXXXXXXXXXXXXXXXXXXXXXXXXXXXXXXXXXXXXXXXXXXXXXXXXXXXXXXX

                                   96730     96740     96750     96760     96770     96780     96790     96800

Human                     AATTAACACTTTGGAAGCATTTTAGCTCCTAGAGTTAAGGTTAGTATAGTGAAATTATGGTGATTAGCTTAATTCATTCA 95635
Kakapo                                                                                                     50676
GoldenEagle                                                                                                57960
JapaneseQuail                                                                                              43432
MediumGroundFinch                                                                                          32462
GoodesThornscrubTortoise                                                                                   82946

Majority                  XXXXXXXXXXXXXXXXXXXXXXXXXXXXXXXXXXXXXXXXXXXXXXXXXXXXXXXXXXXXXXXXXXXXXXXXXXXXXXXX

                                   96810     96820     96830     96840     96850     96860     96870     96880

Human                     TGTACATCTATTTGTATCACCATATAATTACTAGGGAAAGGACAAATCACTTTTCAAAGCCTCAATGTTATTTGTAAAAT 95715
Kakapo                                                                                                     50676
GoldenEagle                                                                                                57960
JapaneseQuail                                                                                              43432
MediumGroundFinch                                                                                          32462
GoodesThornscrubTortoise                                                                                   82946

Majority                  XXXXXXXXXXXXXXXXXXXXXXXXXXXXXXXXXXXXXXXXXXXXXXXXXXXXXXXXXXXXXXXXXXXXXXXXXXXXXXXX

                                   96890     96900     96910     96920     96930     96940     96950     96960

Human                     GGAGGCAATAAGTTCTGCTCTGCTCCCCTCACAGGATTATTACAGAGGCTCAAATAGGATAAGTGAAGGAATGCTATGCA 95795
Kakapo                                                                                                     50676
GoldenEagle                                                                                                57960
JapaneseQuail                                                                                              43432
MediumGroundFinch                                                                                          32462
GoodesThornscrubTortoise                                                                                   82946
```

```
Majority                  XXXXXXXXXXXXXXXXXXXXXXXXXXXXXXXXXXXXXXXXXXXXXXXXXXXXXXXXXXXXXXXXXXXXXXXXXXXXXXXX

                                   96970     96980     96990     97000     97010     97020     97030     97040

Human                     ATTAGTTAACTAATGCCAGTTCCCAGACTGCCAGATTTGCCCACAGTAAACTTCATTATCCACAATACATGTTATCCTAA 95875
Kakapo                                                                                                     50676
GoldenEagle                                                                                                57960
JapaneseQuail                                                                                              43432
MediumGroundFinch                                                                                          32462
GoodesThornscrubTortoise                                                                                   82946

Majority                  XXXXXXXXXXXXXXXXXXXXXXXXXXXXXXXXXXXXXXXXXXXXXXXXXXXXXXXXXXXXXXXXXXXXXXXXXXXXXXXX

                                   97050     97060     97070     97080     97090     97100     97110     97120

Human                     TATGTCTTTTTGCTACAAAGAAATACTAATGTTTTAAAATTATTTACTAGTTTCATAAAGCAGAAAGATGTAAAATGCAG 95955
Kakapo                                                                                                     50676
GoldenEagle                                                                                                57960
JapaneseQuail                                                                                              43432
MediumGroundFinch                                                                                          32462
GoodesThornscrubTortoise                                                                                   82946

Majority                  XXXXXXXXXXXXXXXXXXXXXXXXXXXXXXXXXXXXXXXXXXXXXXXXXXXXXXXXXXXXXXXXXXXXXXXXXXXXXXXX

                                   97130     97140     97150     97160     97170     97180     97190     97200

Human                     TTATAGAGAGCATCCCAAGCCAGATGAAAAGAAACCATGGATTTAGCTTTATATTTTCTGTTTGAACTTGATAGCCCAAT 96035
Kakapo                                                                                                     50676
GoldenEagle                                                                                                57960
JapaneseQuail                                                                                              43432
MediumGroundFinch                                                                                          32462
GoodesThornscrubTortoise                                                                                   82946

Majority                  XXXXXXXXXXXXXXXXXXXXXXXXXXXXXXXXXXXXXXXXXXXXXXXXXXXXXXXXXXXXXXXXXXXXXXXXXXXXXXXX

                                   97210     97220     97230     97240     97250     97260     97270     97280

Human                     CATAAAATGCACATATTTAATTATAGACTGCACTTAAGATTTCATATGTATAAAAAAGTTGCTGTGAAACAGGTAATTTT 96115
Kakapo                                                                                                     50676
GoldenEagle                                                                                                57960
JapaneseQuail                                                                                              43432
MediumGroundFinch                                                                                          32462
GoodesThornscrubTortoise                                                                                   82946
```

```
Majority                   XXXXXXXXXXXXXXXXXXXXXXXXXXXXXXXXXXXXXXXXXXXXXXXXXXXXXXXXXXXXXXXXXXXXXXXXXXXXXXXX

                                   97290     97300     97310     97320     97330     97340     97350     97360

Human                      AACACTCAGGAATTTCAAGGCTAAGGAAGAAAGACATACTCTCATGAGCAGCTCTGAACTTCAAACAGGATTTTCTGGCT 96195
Kakapo                                                                                                      50676
GoldenEagle                                                                                                 57960
JapaneseQuail                                                                                               43432
MediumGroundFinch                                                                                           32462
GoodesThornscrubTortoise                                                                                    82946

Majority                   XXXXXXXXXXXXXXXXXXXXXXXXXXXXXXXXXXXXXXXXXXXXXXXXXXXXXXXXXXXXXXXXXXXXXXXXXXXXXXXX

                                   97370     97380     97390     97400     97410     97420     97430     97440

Human                      GCCTTAATATATTAAAATGGAGCCTAAATCTGAAAACGTTTTTTTCATTCAGAAGAAAGTACAGAATTCAGTGAAATTGA 96275
Kakapo                                                                                                      50676
GoldenEagle                                                                                                 57960
JapaneseQuail                                                                                               43432
MediumGroundFinch                                                                                           32462
GoodesThornscrubTortoise                                                                                    82946

Majority                   XXXXXXXXXXXXXXXXXXXXXXXXXXXXXXXXXXXXXXXXXXXXXXXXXXXXXXXXXXXXXXXXXXXXXXXXXXXXXXXX

                                   97450     97460     97470     97480     97490     97500     97510     97520

Human                      GTATACTAGGAAATCTGATGGCAAGAATTCAGCAGAAAGTCTTAATTTTAAACCGTAATATTCATTGATTATGCAAAACC 96355
Kakapo                                                                                                      50676
GoldenEagle                                                                                                 57960
JapaneseQuail                                                                                               43432
MediumGroundFinch                                                                                           32462
GoodesThornscrubTortoise                                                                                    82946

Majority                   XXXXXXXXXXXXXXXXXXXXXXXXXXXXXXXXXXXXXXXXXXXXXXXXXXXXXXXXXXXXXXXXXXXXXXXXXXXXXXXX

                                   97530     97540     97550     97560     97570     97580     97590     97600

Human                      TTAGTTTTATAGTTATTCTTTGTTATTTTGAATAGCTTCCCTTTAAATTTTCTAGTTTCAGATTTTGCAAATAAAAGTGT 96435
Kakapo                                                                                                      50676
GoldenEagle                                                                                                 57960
JapaneseQuail                                                                                               43432
MediumGroundFinch                                                                                           32462
GoodesThornscrubTortoise                                                                                    82946
```

```
Majority                    XXXXXXXXXXXXXXXXXXXXXXXXXXXXXXXXXXXXXXXXXXXXXXXXXXXXXXXXXXXXXXXXXXXXXXXXXXXXXXXX

                                     97610     97620     97630     97640     97650     97660     97670     97680

Human                       ACATGTTAAAGTTATAGAGTATTAGATTATTCACATTAAGAACTGTTCAAATACGCGGAAAAATAAAACCGTAATGAACA 96515
Kakapo                                                                                                       50676
GoldenEagle                                                                                                  57960
JapaneseQuail                                                                                                43432
MediumGroundFinch                                                                                            32462
GoodesThornscrubTortoise                                                                                     82946

Majority                    XXXXXXXXXXXXXXXXXXXXXXXXXXXXXXXXXXXXXXXXXXXXXXXXXXXXXXXXXXXXXXXXXXXXXXXXXXXXXXXX

                                     97690     97700     97710     97720     97730     97740     97750     97760

Human                       CCTATGCACTTCACTCAACTATATCACTACCTAACATATTCCATATTTTAAACGTAATTATATATTAGTTGAAAGAAAAC 96595
Kakapo                                                                                                       50676
GoldenEagle                                                                                                  57960
JapaneseQuail                                                                                                43432
MediumGroundFinch                                                                                            32462
GoodesThornscrubTortoise                                                                                     82946

Majority                    XXXXXXXXXXXXXXXXXXXXXXXXXXXXXXXXXXXXXXXXXXXXXXXXXXXXXXXXXXXXXXXXXXXXXXXXXXXXXXXX

                                     97770     97780     97790     97800     97810     97820     97830     97840

Human                       GTTACAGAATGAACTGAAATCCCATATGAACCTCTTTCTGATCTTAGTTCAAACCTTATTCTTTCCCAAGAGGTAATTGT 96675
Kakapo                                                                                                       50676
GoldenEagle                                                                                                  57960
JapaneseQuail                                                                                                43432
MediumGroundFinch                                                                                            32462
GoodesThornscrubTortoise                                                                                     82946

Majority                    XXXXXXXXXXXXXXXXXXXXXXXXXXXXXXXXXXXXXXXXXXXXXXXXXXXXXXXXXXXXXXXXXXXXXXXXXXXXXXXX

                                     97850     97860     97870     97880     97890     97900     97910     97920

Human                       TACCTTCAGTTTAGTATTTTATTATTCAGTGGTTGCTTTTCAAGAGGAATTTGTCCTATATTCTGTAGGCTAAATTTCTT 96755
Kakapo                                                                                                       50676
GoldenEagle                                                                                                  57960
JapaneseQuail                                                                                                43432
MediumGroundFinch                                                                                            32462
GoodesThornscrubTortoise                                                                                     82946
```

```
Majority                    XXXXXXXXXXXXXXXXXXXXXXXXXXXXXXXXXXXXXXXXXXXXXXXXXXXXXXXXXXXXXXXXXXXXXXXXXXXXXXXX

                                     97930     97940     97950     97960     97970     97980     97990     98000

Human                       GTTTTGCCTAGTTATAAAAATGGCAAATAATATGATATTGGGTGTTTAAATAGTACAATATTGTTCAAAACACACTTTAT 96835
Kakapo                                                                                                       50676
GoldenEagle                                                                                                  57960
JapaneseQuail                                                                                                43432
MediumGroundFinch                                                                                            32462
GoodesThornscrubTortoise                                                                                     82946

Majority                    XXXXXXXXXXXXXXXXXXXXXXXXXXXXXXXXXXXXXXXXXXXXXXXXXXXXXXXXXXXXXXXXXXXXXXXXXXXXXXXX

                                     98010     98020     98030     98040     98050     98060     98070     98080

Human                       AATTTTTAACAGACTGTTTGGACATTGATAATTCCCCTATCAAATCTCATGACATTTAACCAATTTTTAACAAGGATTTT 96915
Kakapo                                                                                                       50676
GoldenEagle                                                                                                  57960
JapaneseQuail                                                                                                43432
MediumGroundFinch                                                                                            32462
GoodesThornscrubTortoise                                                                                     82946

Majority                    XXXXXXXXXXXXXXXXXXXXXXXXXXXXXXXXXXXXXXXXXXXXXXXXXXXXXXXXXXXXXXXXXXXXXXXXXXXXXXXX

                                     98090     98100     98110     98120     98130     98140     98150     98160

Human                       AAATTGTGTTTAAGAAATAAGTCATTTGGGCCTCAGGTTTCTCTTAAGGATGTAAACAAGGAGCTATATCATTGGTATTT 96995
Kakapo                                                                                                       50676
GoldenEagle                                                                                                  57960
JapaneseQuail                                                                                                43432
MediumGroundFinch                                                                                            32462
GoodesThornscrubTortoise                                                                                     82946

Majority                    XXXXXXXXXXXXXXXXXXXXXXXXXXXXXXXXXXXXXXXXXXXXXXXXXXXXXXXXXXXXXXXXXXXXXXXXXXXXXXXX

                                     98170     98180     98190     98200     98210     98220     98230     98240

Human                       TAGACGCTGAATTATGTGTAAGGTCCTTAAATACAAACATACCCTTTGTAGCTTCACAGAGCCACCCGTTTTGCACTTCT 97075
Kakapo                                                                                                       50676
GoldenEagle                                                                                                  57960
JapaneseQuail                                                                                                43432
MediumGroundFinch                                                                                            32462
GoodesThornscrubTortoise                                                                                     82946
```

```
Majority                     XXXXXXXXXXXXXXXXXXXXXXXXXXXXXXXXXXXXXXXXXXXXXXXXXXXXXXXXXXXXXXXXXXXXXXXXXXXXXXXX

                                      98250     98260     98270     98280     98290     98300     98310     98320

Human                        CTCGCTTTTCTTCCTCCCTCTTTGGCTCCCATTTCAATGGACAGAAAGACTATGTGGCATCTCTATTTTCATTCACTTGG 97155
Kakapo                                                                                                        50676
GoldenEagle                                                                                                   57960
JapaneseQuail                                                                                                 43432
MediumGroundFinch                                                                                             32462
GoodesThornscrubTortoise                                                                                      82946

Majority                     XXXXXXXXXXXXXXXXXXXXXXXXXXXXXXXXXXXXXXXXXXXXXXXXXXXXXXXXXXXXXXXXXXXXXXXXXXXXXXXX

                                      98330     98340     98350     98360     98370     98380     98390     98400

Human                        TTTATTGTCTTGGTATGTTGTATAGGTTTTTGAATCAGAAAATTTTAACATTTTTAGATATTTCAAACATACAAAAAATA 97235
Kakapo                                                                                                        50676
GoldenEagle                                                                                                   57960
JapaneseQuail                                                                                                 43432
MediumGroundFinch                                                                                             32462
GoodesThornscrubTortoise                                                                                      82946

Majority                     XXXXXXXXXXXXXXXXXXXXXXXXXXXXXXXXXXXXXXXXXXXXXXXXXXXXXXXXXXXXXXXXXXXXXXXXXXXXXXXX

                                      98410     98420     98430     98440     98450     98460     98470     98480

Human                        GAAGAATATTTAACAAACCCTTGTCAGCTTCAGTTAGCAACTTAGATCTTCCTCGTTTCCTCCACCCAATTCTCATCTCC 97315
Kakapo                                                                                                        50676
GoldenEagle                                                                                                   57960
JapaneseQuail                                                                                                 43432
MediumGroundFinch                                                                                             32462
GoodesThornscrubTortoise                                                                                      82946

Majority                     XXXXXXXXXXXXXXXXXXXXXXXXXXXXXXXXXXXXXXXXXXXXXXXXXXXXXXXXXXXXXXXXXXXXXXXXXXXXXXXX

                                      98490     98500     98510     98520     98530     98540     98550     98560

Human                        TTTATTAATGCAGATCCCAGATATATTTTATCAAGAAATTTTGTAGCATATATTTGTGTTGTTTTATTCGTGGTCATAGT 97395
Kakapo                                                                                                        50676
GoldenEagle                                                                                                   57960
JapaneseQuail                                                                                                 43432
MediumGroundFinch                                                                                             32462
GoodesThornscrubTortoise                                                                                      82946
```

```
Majority                   XXXXXXXXXXXXXXXXXXXXXXXXXXXXXXXXXXXXXXXXXXXXXXXXXXXXXXXXXXXXXXXXXXXXXXXXXXXXXXXX

                                    98570     98580     98590     98600     98610     98620     98630     98640

Human                      TGCTTTCTCTAATATCTAGACTAGCCTAGCTGGTCAATACTACTTTTCTTCAAATCCTAGAACCTCCCTATTAAAAATAT 97475
Kakapo                                                                                                      50676
GoldenEagle                                                                                                 57960
JapaneseQuail                                                                                               43432
MediumGroundFinch                                                                                           32462
GoodesThornscrubTortoise                                                                                    82946

Majority                   XXXXXXXXXXXXXXXXXXXXXXXXXXXXXXXXXXXXXXXXXXXXXXXXXXXXXXXXXXXXXXXXXXXXXXXXXXXXXXXX

                                    98650     98660     98670     98680     98690     98700     98710     98720

Human                      AGTCACCAGACCAGTGGCAAATTAGTATCACCTAGGAGTTTATTAGAAACTCAGTCTATGAGCCTACTGAATCTACATTT 97555
Kakapo                                                                                                      50676
GoldenEagle                                                                                                 57960
JapaneseQuail                                                                                               43432
MediumGroundFinch                                                                                           32462
GoodesThornscrubTortoise                                                                                    82946

Majority                   XXXXXXXXXXXXXXXXXXXXXXXXXXXXXXXXXXXXXXXXXXXXXXXXXXXXXXXXXXXXXXXXXXXXXXXXXXXXXXXX

                                    98730     98740     98750     98760     98770     98780     98790     98800

Human                      TGACAAGATTGGCAGGTGATGCATTTGGACATAAGTTGGAGAAGCACTCAATGCATTTTTTCAGTTCTATAAAAATCAGC 97635
Kakapo                                                                                                      50676
GoldenEagle                                                                                                 57960
JapaneseQuail                                                                                               43432
MediumGroundFinch                                                                                           32462
GoodesThornscrubTortoise                                                                                    82946

Majority                   XXXXXXXXXXXXXXXXXXXXXXXXXXXXXXXXXXXXXXXXXXXXXXXXXXXXXXXXXXXXXXXXXXXXXXXXXXXXXXXX

                                    98810     98820     98830     98840     98850     98860     98870     98880

Human                      TTCAAAGTTTTTTCCTGAGTGTGGAAATTATTTCATTAGTAATTTTAAGTTATGCAGATATAGTTAATACCAAGAAATAA 97715
Kakapo                                                                                                      50676
GoldenEagle                                                                                                 57960
JapaneseQuail                                                                                               43432
MediumGroundFinch                                                                                           32462
GoodesThornscrubTortoise                                                                                    82946
```

```
Majority                   XXXXXXXXXXXXXXXXXXXXXXXXXXXXXXXXXXXXXXXXXXXXXXXXXXXXXXXXXXXXXXXXXXXXXXXXXXXXXXXX

                                   98890     98900     98910     98920     98930     98940     98950     98960

Human                      TTGAACTGAAAAGGAACCCGGATATCTAGTTTTAACCAGCTGTTTTAAAGATAAACAGTTCTAGAGATCAAATGAATAGT 97795
Kakapo                                                                                                      50676
GoldenEagle                                                                                                 57960
JapaneseQuail                                                                                               43432
MediumGroundFinch                                                                                           32462
GoodesThornscrubTortoise                                                                                    82946

Majority                   XXXXXXXXXXXXXXXXXXXXXXXXXXXXXXXXXXXXXXXXXXXXXXXXXXXXXXXXXXXXXXXXXXXXXXXXXXXXXXXX

                                   98970     98980     98990     99000     99010     99020     99030     99040

Human                      TTAGCAACATATTTATTGTAACTTACCTGTAGTTAGAATCCAAGTCCCTGGCTTTCCAATCCAGTCTTCAATGTATAATA 97875
Kakapo                                                                                                      50676
GoldenEagle                                                                                                 57960
JapaneseQuail                                                                                               43432
MediumGroundFinch                                                                                           32462
GoodesThornscrubTortoise                                                                                    82946

Majority                   XXXXXXXXXXXXXXXXXXXXXXXXXXXXXXXXXXXXXXXXXXXXXXXXXXXXXXXXXXXXXXXXXXXXXXXXXXXXXXXX

                                   99050     99060     99070     99080     99090     99100     99110     99120

Human                      CTGATCAAAAGAAATACAGGCCTGCTATTATTCATAAATTTAAACTATTATGTACAAGTTGATAGCTTATAATAGAAATC 97955
Kakapo                                                                                                      50676
GoldenEagle                                                                                                 57960
JapaneseQuail                                                                                               43432
MediumGroundFinch                                                                                           32462
GoodesThornscrubTortoise                                                                                    82946

Majority                   XXXXXXXXXXXXXXXXXXXXXXXXXXXXXXXXXXXXXXXXXXXXXXXXXXXXXXXXXXXXXXXXXXXXXXXXXXXXXXXX

                                   99130     99140     99150     99160     99170     99180     99190     99200

Human                      AGTATGATTAGTGTTAAAAGTCAAATTTAGTCTGACAGGGAAGGAATGTCCATGTGAACTGATTTTTTTTAATAGAATGC 98035
Kakapo                                                                                                      50676
GoldenEagle                                                                                                 57960
JapaneseQuail                                                                                               43432
MediumGroundFinch                                                                                           32462
GoodesThornscrubTortoise                                                                                    82946
```

```
Majority                   XXXXXXXXXXXXXXXXXXXXXXXXXXXXXXXXXXXXXXXXXXXXXXXXXXXXXXXXXXXXXXXXXXXXXXXXXXXXXXXX

                                    99210     99220     99230     99240     99250     99260     99270     99280

Human                      AAATTAATACTCAATTCCTGATTTGTTTATTACATTTTGTGGTTTAATGAAATGATACTCAGGTCATTTTCATAAATAGC 98115
Kakapo                                                                                                      50676
GoldenEagle                                                                                                 57960
JapaneseQuail                                                                                               43432
MediumGroundFinch                                                                                           32462
GoodesThornscrubTortoise                                                                                    82946

Majority                   XXXXXXXXXXXXXXXXXXXXXXXXXXXXXXXXXXXXXXXXXXXXXXXXXXXXXXXXXXXXXXXXXXXXXXXXXXXXXXXX

                                    99290     99300     99310     99320     99330     99340     99350     99360

Human                      TTTTCTATTAATGCTTCCTCAGTCACATTCTCTATTCCTATTGCATAGCACAGTCTAACAGTTGAAATGCCTAAGCCTTA 98195
Kakapo                                                                                                      50676
GoldenEagle                                                                                                 57960
JapaneseQuail                                                                                               43432
MediumGroundFinch                                                                                           32462
GoodesThornscrubTortoise                                                                                    82946

Majority                   XXXXXXXXXXXXXXXXXXXXXXXXXXXXXXXXXXXXXXXXXXXXXXXXXXXXXXXXXXXXXXXXXXXXXXXXXXXXXXXX

                                    99370     99380     99390     99400     99410     99420     99430     99440

Human                      CTGAATGATGACTGATATTCTCTGTGAGAGTTAGAGCTGTACTATAAATATACATTACCTGTGCAAAGTATGCCTTCAGG 98275
Kakapo                                                                                                      50676
GoldenEagle                                                                                                 57960
JapaneseQuail                                                                                               43432
MediumGroundFinch                                                                                           32462
GoodesThornscrubTortoise                                                                                    82946

Majority                   XXXXXXXXXXXXXXXXXXXXXXXXXXXXXXXXXXXXXXXXXXXXXXXXXXXXXXXXXXXXXXXXXXXXXXXXXXXXXXXX

                                    99450     99460     99470     99480     99490     99500     99510     99520

Human                      GGCTATTCCCCATAACCTTAGCAAAATTTTAAAATATTCTTATGGCTAGCAATCTTATCTATTGTCATGTCTTACTATTT 98355
Kakapo                                                                                                      50676
GoldenEagle                                                                                                 57960
JapaneseQuail                                                                                               43432
MediumGroundFinch                                                                                           32462
GoodesThornscrubTortoise                                                                                    82946
```

```
Majority                  XXXXXXXXXXXXXXXXXXXXXXXXXXXXXXXXXXXXXXXXXXXXXXXXXXXXXXXXXXXXXXXXXXXXXXXXXXXXXXXX

                                   99530     99540     99550     99560     99570     99580     99590     99600

Human                     CCTGCTTACCACCCCCATTTCTCTCTCAACCTTGTTGCTGCATTAGCTGTCCATCACTTAACACTGTTTATTTCTCTGTT 98435
Kakapo                                                                                                     50676
GoldenEagle                                                                                                57960
JapaneseQuail                                                                                              43432
MediumGroundFinch                                                                                          32462
GoodesThornscrubTortoise                                                                                   82946

Majority                  XXXXXXXXXXXXXXXXXXXXXXXXXXXXXXXXXXXXXXXXXXXXXXXXXXXXXXXXXXXXXXXXXXXXXXXXXXXXXXXX

                                   99610     99620     99630     99640     99650     99660     99670     99680

Human                     TTTCTTCCATCATTCTCTTCTTTGCAACTAAATGATTTAGACTTTTTATAACCATAATCAACCAGTTAGATTCTTTTTAT 98515
Kakapo                                                                                                     50676
GoldenEagle                                                                                                57960
JapaneseQuail                                                                                              43432
MediumGroundFinch                                                                                          32462
GoodesThornscrubTortoise                                                                                   82946

Majority                  XXXXXXXXXXXXXXXXXXXXXXXXXXXXXXXXXXXXXXXXXXXXXXXXXXXXXXXXXXXXXXXXXXXXXXXXXXXXXXXX

                                   99690     99700     99710     99720     99730     99740     99750     99760

Human                     TTGTGGTTATTGAGTTGATAAATGATAGTGCATTTTATGTAATATTCAGGTGTTATGCTATTTTTATCTGGTTCACTTTA 98595
Kakapo                                                                                                     50676
GoldenEagle                                                                                                57960
JapaneseQuail                                                                                              43432
MediumGroundFinch                                                                                          32462
GoodesThornscrubTortoise                                                                                   82946

Majority                  XXXXXXXXXXXXXXXXXXXXXXXXXXXXXXXXXXXXXXXXXXXXXXXXXXXXXXXXXXXXXXXXXXXXXXXXXXXXXXXX

                                   99770     99780     99790     99800     99810     99820     99830     99840

Human                     AAACCTCCCTCCCCCCACTCCCTTTTTTTGTGGGTTTTTTTTTTATTATTATTATTTTCCAGTTTTGTGTTGTTTTGCTT 98675
Kakapo                                                                                                     50676
GoldenEagle                                                                                                57960
JapaneseQuail                                                                                              43432
MediumGroundFinch                                                                                          32462
GoodesThornscrubTortoise                                                                                   82946
```

```
Majority                   XXXXXXXXXXXXXXXXXXXXXXXXXXXXXXXXXXXXXXXXXXXXXXXXXXXXXXXXXXXXXXXXXXXXXXXXXXXXXXXX

                                    99850     99860     99870     99880     99890     99900     99910     99920

Human                      TTTAACTTCTGAGATTGCAGTGGTTCCAGATGGTTGAAATCACTAGTAGTGATTTACATGAAGCATTCATGTATTTATTA 98755
Kakapo                                                                                                      50676
GoldenEagle                                                                                                 57960
JapaneseQuail                                                                                               43432
MediumGroundFinch                                                                                           32462
GoodesThornscrubTortoise                                                                                    82946

Majority                   XXXXXXXXXXXXXXXXXXXXXXXXXXXXXXXXXXXXXXXXXXXXXXXXXXXXXXXXXXXXXXXXXXXXXXXXXXXXXXXX

                                    99930     99940     99950     99960     99970     99980     99990    100000

Human                      CTGGTATATTCAACTATGGTGGAGGGAAATTGGTCGAAGAATTACGGTTAAAGAGCTCCTTTAAAAAGGAGATTTGTAGA 98835
Kakapo                                                                                                      50676
GoldenEagle                                                                                                 57960
JapaneseQuail                                                                                               43432
MediumGroundFinch                                                                                           32462
GoodesThornscrubTortoise                                                                                    82946

Majority                   XXXXXXXXXXXXXXXXXXXXXXXXXXXXXXXXXXXXXXXXXXXXXXXXXXXXXXXXXXXXXXXXXXXXXXXXXXXXXXXX

                                   100010    100020    100030    100040    100050    100060    100070    100080

Human                      ATTCTAAAATGTGATTCTGATTCATATAGTCATGATTCTTAGAAATTAGCTACAGGAAGAGATAGAAACATTGCTATAGT 98915
Kakapo                                                                                                      50676
GoldenEagle                                                                                                 57960
JapaneseQuail                                                                                               43432
MediumGroundFinch                                                                                           32462
GoodesThornscrubTortoise                                                                                    82946

Majority                   XXXXXXXXXXXXXXXXXXXXXXXXXXXXXXXXXXXXXXXXXXXXXXXXXXXXXXXXXXXXXXXXXXXXXXXXXXXXXXXX

                                   100090    100100    100110    100120    100130    100140    100150    100160

Human                      TGTCTTTGCCCTAAGTTTGGAGTTTTATAGTGTAATATATATATAATAGCTCTAATTATAAATACACTTTTACTCTGGAA 98995
Kakapo                                                                                                      50676
GoldenEagle                                                                                                 57960
JapaneseQuail                                                                                               43432
MediumGroundFinch                                                                                           32462
GoodesThornscrubTortoise                                                                                    82946
```

```
Majority                   XXXXXXXXXXXXXXXXXXXXXXXXXXXXXXXXXXXXXXXXXXXXXXXXXXXXXXXXXXXXXXXXXXXXXXXXXXXXXXXX

                                   100170    100180    100190    100200    100210    100220    100230    100240

Human                      AAAGCATAGACATTGACATAAGAGACATTTAAAAACCATTATTTTCTCATCTGTAAAATGGAGAGAATTACTTCATATGT 99075
Kakapo                                                                                                     50676
GoldenEagle                                                                                                57960
JapaneseQuail                                                                                              43432
MediumGroundFinch                                                                                          32462
GoodesThornscrubTortoise                                                                                   82946

Majority                   XXXXXXXXXXXXXXXXXXXXXXXXXXXXXXXXXXXXXXXXXXXXXXXXXXXXXXXXXXXXXXXXXXXXXXXXXXXXXXXX

                                   100250    100260    100270    100280    100290    100300    100310    100320

Human                      TCACTTATAAGGATTATGTGAAATACTGTTCCATTCCATTAGTAGTTTTTCTTTTCCTCTAATTGGGGGTGGGGGCAGTT 99155
Kakapo                                                                                                     50676
GoldenEagle                                                                                                57960
JapaneseQuail                                                                                              43432
MediumGroundFinch                                                                                          32462
GoodesThornscrubTortoise                                                                                   82946

Majority                   XXXXXXXXXXXXXXXXXXXXXXXXXXXXXXXXXXXXXXXXXXXXXXXXXXXXXXXXXXXXXXXXXXXXXXXXXXXXXXXX

                                   100330    100340    100350    100360    100370    100380    100390    100400

Human                      TTTGGCATTCTATTTATGGAAGGTGTTTTTTTCCCTAAGATCATTTTGATGTCTCAGAAGACATTTGCTATGTACGTTGG 99235
Kakapo                                                                                                     50676
GoldenEagle                                                                                                57960
JapaneseQuail                                                                                              43432
MediumGroundFinch                                                                                          32462
GoodesThornscrubTortoise                                                                                   82946

Majority                   XXXXXXXXXXXXXXXXXXXXXXXXXXXXXXXXXXXXXXXXXXXXXXXXXXXXXXXXXXXXXXXXXXXXXXXXXXXXXXXX

                                   100410    100420    100430    100440    100450    100460    100470    100480

Human                      ACATTTGCCATGTACATTGTAGAGAATACAAAGATAAACAGAACCACAGTACAGCTGGTTCTATTAGACATTGAAAATGC 99315
Kakapo                                                                                                     50676
GoldenEagle                                                                                                57960
JapaneseQuail                                                                                              43432
MediumGroundFinch                                                                                          32462
GoodesThornscrubTortoise                                                                                   82946
```

```
Majority                  XXXXXXXXXXXXXXXXXXXXXXXXXXXXXXXXXXXXXXXXXXXXXXXXXXXXXXXXXXXXXXXXXXXXXXXXXXXXXXXX

                                   100490    100500    100510    100520    100530    100540    100550    100560

Human                     ATATTCTAGCATATTTAATATATTAGGGAACAATTTAAGCATAATGTGAATTTCATGTTTACTGAAGATTTCATCCTGAG 99395
Kakapo                                                                                                     50676
GoldenEagle                                                                                                57960
JapaneseQuail                                                                                              43432
MediumGroundFinch                                                                                          32462
GoodesThornscrubTortoise                                                                                   82946

Majority                  XXXXXXXXXXXXXXXXXXXXXXXXXXXXXXXXXXXXXXXXXXXXXXXXXXXXXXXXXXXXXXXXXXXXXXXXXXXXXXXX

                                   100570    100580    100590    100600    100610    100620    100630    100640

Human                     AAACAAATGAAAGCAGAAAATTACAACCAACTGAACCAGCTGTGTAAGAAATATATATATGGTGTGCACACATGTACATA 99475
Kakapo                                                                                                     50676
GoldenEagle                                                                                                57960
JapaneseQuail                                                                                              43432
MediumGroundFinch                                                                                          32462
GoodesThornscrubTortoise                                                                                   82946

Majority                  XXXXXXXXXXXXXXXXXXXXXXXXXXXXXXXXXXXXXXXXXXXXXXXXXXXXXXXXXXXXXXXXXXXXXXXXXXXXXXXX

                                   100650    100660    100670    100680    100690    100700    100710    100720

Human                     CCTCAGACATCTATTAGCATTCTCAGTTTTCCACAAGTTAAAAGCCACGCTCATCCATATCTGGTGCTAATAACTTCCTA 99555
Kakapo                                                                                                     50676
GoldenEagle                                                                                                57960
JapaneseQuail                                                                                              43432
MediumGroundFinch                                                                                          32462
GoodesThornscrubTortoise                                                                                   82946

Majority                  XXXXXXXXXXXXXXXXXXXXXXXXXXXXXXXXXXXXXXXXXXXXXXXXXXXXXXXXXXXXXXXXXXXXXXXXXXXXXXXX

                                   100730    100740    100750    100760    100770    100780    100790    100800

Human                     TCCAATTTCAGATAGCTGTTTTCAACACATCTGACACCTTCTTTTTTTCAAGCAAACTTCAGGTCTTTTTCAAGATAAAG 99635
Kakapo                                                                                                     50676
GoldenEagle                                                                                                57960
JapaneseQuail                                                                                              43432
MediumGroundFinch                                                                                          32462
GoodesThornscrubTortoise                                                                                   82946
```

```
Majority                   XXXXXXXXXXXXXXXXXXXXXXXXXXXXXXXXXXXXXXXXXXXXXXXXXXXXXXXXXXXXXXXXXXXXXXXXXXXXXXXX

                                    100810    100820    100830    100840    100850    100860    100870    100880

Human                      TCATTTATAGTAGTGTCTATATATTTCTTAACCATTTAACATGTAAACTGTGCTGCCTTTTAAGAAATTAATTCTATCCT 99715
Kakapo                                                                                                      50676
GoldenEagle                                                                                                 57960
JapaneseQuail                                                                                               43432
MediumGroundFinch                                                                                           32462
GoodesThornscrubTortoise                                                                                    82946

Majority                   XXXXXXXXXXXXXXXXXXXXXXXXXXXXXXXXXXXXXXXXXXXXXXXXXXXXXXXXXXXXXXXXXXXXXXXXXXXXXXXX

                                    100890    100900    100910    100920    100930    100940    100950    100960

Human                      TTTTAGTGTCAGAGTTGAAGATTTTGAGTGTTGTGCCCTAACCTTATCATTTTCCTCATAAGTCCTGTGGTTTTTATTGT 99795
Kakapo                                                                                                      50676
GoldenEagle                                                                                                 57960
JapaneseQuail                                                                                               43432
MediumGroundFinch                                                                                           32462
GoodesThornscrubTortoise                                                                                    82946

Majority                   XXXXXXXXXXXXXXXXXXXXXXXXXXXXXXXXXXXXXXXXXXXXXXXXXXXXXXXXXXXXXXXXXXXXXXXXXXXXXXXX

                                    100970    100980    100990    101000    101010    101020    101030    101040

Human                      ATGATTTTGCATAGTGTGGCAATTCTTGGAAATGCTTATGTTGTGTTATAGTATAACTGACTTACTACTTTCAAATAGCT 99875
Kakapo                                                                                                      50676
GoldenEagle                                                                                                 57960
JapaneseQuail                                                                                               43432
MediumGroundFinch                                                                                           32462
GoodesThornscrubTortoise                                                                                    82946

Majority                   XXXXXXXXXXXXXXXXXXXXXXXXXXXXXXXXXXXXXXXXXXXXXXXXXXXXXXXXXXXXXXXXXXXXXXXXXXXXXXXX

                                    101050    101060    101070    101080    101090    101100    101110    101120

Human                      TCTAGTCTGATTCATGTTATGACAGTATACAGTATAGCAAAATAAGGACATCAGGGAACAGGAGGCTGAGCATATCACCA 99955
Kakapo                                                                                                      50676
GoldenEagle                                                                                                 57960
JapaneseQuail                                                                                               43432
MediumGroundFinch                                                                                           32462
GoodesThornscrubTortoise                                                                                    82946
```

```
Majority                   XXXXXXXXXXXXXXXXXXXXXXXXXXXXXXXXXXXXXXXXXXXXXXXXXXXXXXXXXXXXXXXXXXXXXXXXXXXXXXXX

                                  101130    101140    101150    101160    101170    101180    101190    101200

Human                      TTTACCAATGGTAGCTTGAGCAAGGCACTTCATTTATCGAAGTCCATTTTCTCATCTGTACAATGGAGGTGATGATAGTA 100035
Kakapo                                                                                                      50676
GoldenEagle                                                                                                 57960
JapaneseQuail                                                                                               43432
MediumGroundFinch                                                                                           32462
GoodesThornscrubTortoise                                                                                    82946

Majority                   XXXXXXXXXXXXXXXXXXXXXXXXXXXXXXXXXXXXXXXXXXXXXXXXXXXXXXXXXXXXXXXXXXXXXXXXXXXXXXXX

                                  101210    101220    101230    101240    101250    101260    101270    101280

Human                      CATGTCTTTTCTGTCTTGTGTTATTTGTAGTTTCAAGTAAGATATGAAATATGCTGTAGAAATACAGGGTTGGGAATATC 100115
Kakapo                                                                                                      50676
GoldenEagle                                                                                                 57960
JapaneseQuail                                                                                               43432
MediumGroundFinch                                                                                           32462
GoodesThornscrubTortoise                                                                                    82946

Majority                   XXXXXXXXXXXXXXXXXXXXXXXXXXXXXXXXXXXXXXXXXXXXXXXXXXXXXXXXXXXXXXXXXXXXXXXXXXXXXXXX

                                  101290    101300    101310    101320    101330    101340    101350    101360

Human                      TAGTGGTACAAAAATCAACTTTTGGCCCAATTTTAAGGTGAGATAATTATGAGACATTTTTCGACCTCAAAGTCTTTGTT 100195
Kakapo                                                                                                      50676
GoldenEagle                                                                                                 57960
JapaneseQuail                                                                                               43432
MediumGroundFinch                                                                                           32462
GoodesThornscrubTortoise                                                                                    82946

Majority                   XXXXXXXXXXXXXXXXXXXXXXXXXXXXXXXXXXXXXXXXXXXXXXXXXXXXXXXXXXXXXXXXXXXXXXXXXXXXXXXX

                                  101370    101380    101390    101400    101410    101420    101430    101440

Human                      CTTTTCTCTCTACACCATTGTGAATAACACAGTGTATATATGAGATTAATTTATTACCTCGACTTTCCATTTTTGTTTTG 100275
Kakapo                                                                                                      50676
GoldenEagle                                                                                                 57960
JapaneseQuail                                                                                               43432
MediumGroundFinch                                                                                           32462
GoodesThornscrubTortoise                                                                                    82946
```

```
Majority                   XXXXXXXXXXXXXXXXXXXXXXXXXXXXXXXXXXXXXXXXXXXXXXXXXXXXXXXXXXXXXXXXXXXXXXXXXXXXXXXX

                                    101450    101460    101470    101480    101490    101500    101510    101520

Human                      TAAAGTAGTTTAATTTTTTATATCTATTTATAAAGTTAACTGCAGGAGTGTACCTGCAGTTAACTTTATACAGTTAAGAA 100355
Kakapo                                                                                                      50676
GoldenEagle                                                                                                 57960
JapaneseQuail                                                                                               43432
MediumGroundFinch                                                                                           32462
GoodesThornscrubTortoise                                                                                    82946

Majority                   XXXXXXXXXXXXXXXXXXXXXXXXXXXXXXXXXXXXXXXXXXXXXXXXXXXXXXXXXXXXXXXXXXXXXXXXXXXXXXXX

                                    101530    101540    101550    101560    101570    101580    101590    101600

Human                      GAGGTTGGTTTCAGTTTTAAAGATTGTATGTCAGTGTAAAACATCCATTATTATGCTTTCAAGTAAAATAGCTCCTAAAA 100435
Kakapo                                                                                                      50676
GoldenEagle                                                                                                 57960
JapaneseQuail                                                                                               43432
MediumGroundFinch                                                                                           32462
GoodesThornscrubTortoise                                                                                    82946

Majority                   XXXXXXXXXXXXXXXXXXXXXXXXXXXXXXXXXXXXXXXXXXXXXXXXXXXXXXXXXXXXXXXXXXXXXXXXXXXXXXXX

                                    101610    101620    101630    101640    101650    101660    101670    101680

Human                      ATATGGATTACTATCAACTTTCAAGGAAAATAGCTCCTAAAAATATGGATTACTACCAACTTTCAAGGAAAATAGCTCCT 100515
Kakapo                                                                                                      50676
GoldenEagle                                                                                                 57960
JapaneseQuail                                                                                               43432
MediumGroundFinch                                                                                           32462
GoodesThornscrubTortoise                                                                                    82946

Majority                   XXXXXXXXXXXXXXXXXXXXXXXXXXXXXXXXXXXXXXXXXXXXXXXXXXXXXXXXXXXXXXXXXXXXXXXXXXXXXXXX

                                    101690    101700    101710    101720    101730    101740    101750    101760

Human                      AGAAATATGGATTACTAGCCACATAAATTGCTAACATTTGCTTTTATTATTATTATTATTATTATTTTAGACACAGTCTT 100595
Kakapo                                                                                                      50676
GoldenEagle                                                                                                 57960
JapaneseQuail                                                                                               43432
MediumGroundFinch                                                                                           32462
GoodesThornscrubTortoise                                                                                    82946
```

```
Majority                   XXXXXXXXXXXXXXXXXXXXXXXXXXXXXXXXXXXXXXXXXXXXXXXXXXXXXXXXXXXXXXXXXXXXXXXXXXXXXXXX

                                     101770    101780    101790    101800    101810    101820    101830    101840

Human                      GCTCTGTCACCCAGGCTGGAGTGCGGTGGTATTATCTCGGCTCACTGCAACCTCCACCTCTCGGGTTCAAGCAATTCTCC 100675
Kakapo                                                                                                      50676
GoldenEagle                                                                                                 57960
JapaneseQuail                                                                                               43432
MediumGroundFinch                                                                                           32462
GoodesThornscrubTortoise                                                                                    82946

Majority                   XXXXXXXXXXXXXXXXXXXXXXXXXXXXXXXXXXXXXXXXXXXXXXXXXXXXXXXXXXXXXXXXXXXXXXXXXXXXXXXX

                                     101850    101860    101870    101880    101890    101900    101910    101920

Human                      TGCCTCAGCCTTCCGAGTAGCTGGAATTACAGGCACCCACCACCACACCCAGCTAATTTTTGTATTTTTAGTAGAGACAG 100755
Kakapo                                                                                                      50676
GoldenEagle                                                                                                 57960
JapaneseQuail                                                                                               43432
MediumGroundFinch                                                                                           32462
GoodesThornscrubTortoise                                                                                    82946

Majority                   XXXXXXXXXXXXXXXXXXXXXXXXXXXXXXXXXXXXXXXXXXXXXXXXXXXXXXXXXXXXXXXXXXXXXXXXXXXXXXXX

                                     101930    101940    101950    101960    101970    101980    101990    102000

Human                      GGTTTTACCATGTGACCAGGCTGGTTTCGAACTCCTGACCTCAAATGATCCACCTGCCTCAGCCTCCCAAACTGCTGGGA 100835
Kakapo                                                                                                      50676
GoldenEagle                                                                                                 57960
JapaneseQuail                                                                                               43432
MediumGroundFinch                                                                                           32462
GoodesThornscrubTortoise                                                                                    82946

Majority                   XXXXXXXXXXXXXXXXXXXXXXXXXXXXXXXXXXXXXXXXXXXXXXXXXXXXXXXXXXXXXXXXXXXXXXXXXXXXXXXX

                                     102010    102020    102030    102040    102050    102060    102070    102080

Human                      TTACAGGTGTGAGCCACCGCTCCCAGCCTATTTGCTAATATTTAACCTCTTGAGAGTCTTTAATTCTTTTTTTCAACAAG 100915
Kakapo                                                                                                      50676
GoldenEagle                                                                                                 57960
JapaneseQuail                                                                                               43432
MediumGroundFinch                                                                                           32462
GoodesThornscrubTortoise                                                                                    82946
```

```
Majority                      XXXXXXXXXXXXXXXXXXXXXXXXXXXXXXXXXXXXXXXXXXXXXXXXXXXXXXXXXXXXXXXXXXXXXXXXXXXXXXXX

                                       102090    102100    102110    102120    102130    102140    102150    102160

Human                         TGTTCATTACCTGCTATGTGCCAGCTTAGATGCTTTGGATGAAATACAGGTTGGTGCGAAAGTAATTGCGATTTTTGCCG 100995
Kakapo                                                                                                         50676
GoldenEagle                                                                                                    57960
JapaneseQuail                                                                                                  43432
MediumGroundFinch                                                                                              32462
GoodesThornscrubTortoise                                                                                       82946

Majority                      XXXXXXXXXXXXXXXXXXXXXXXXXXXXXXXXXXXXXXXXXXXXXXXXXXXXXXXXXXXXXXXXXXXXXXXXXXXXXXXX

                                       102170    102180    102190    102200    102210    102220    102230    102240

Human                         TTAAAATTAGAAAAACCACAATTACTTTTGCACCAACCTAAATAACTAAAACACAATTTCTTCCTATCATGGGGCTTTCA 101075
Kakapo                                                                                                         50676
GoldenEagle                                                                                                    57960
JapaneseQuail                                                                                                  43432
MediumGroundFinch                                                                                              32462
GoodesThornscrubTortoise                                                                                       82946

Majority                      XXXXXXXXXXXXXXXXXXXXXXXXXXXXXXXXXXXXXXXXXXXXXXXXXXXXXXXXXXXXXXXXXXXXXXXXXXXXXXXX

                                       102250    102260    102270    102280    102290    102300    102310    102320

Human                         GTTCAGTATGAGATAAGTAAAGAATTATATGGGTTGATAAATTTTCTGGAGGAACTATTGGGTGTTATTACAGTGTGCTC 101155
Kakapo                                                                                                         50676
GoldenEagle                                                                                                    57960
JapaneseQuail                                                                                                  43432
MediumGroundFinch                                                                                              32462
GoodesThornscrubTortoise                                                                                       82946

Majority                      XXXXXXXXXXXXXXXXXXXXXXXXXXXXXXXXXXXXXXXXXXXXXXXXXXXXXXXXXXXXXXXXXXXXXXXXXXXXXXXX

                                       102330    102340    102350    102360    102370    102380    102390    102400

Human                         AATCTTTAGCAGTTAGGAAAGGATTCTTGAAGGTAGTTGTATTACCTGGGTTGGAGGTAAGTGTTGGGGACACAAGTGTT 101235
Kakapo                                                                                                         50676
GoldenEagle                                                                                                    57960
JapaneseQuail                                                                                                  43432
MediumGroundFinch                                                                                              32462
GoodesThornscrubTortoise                                                                                       82946
```

```
Majority                  XXXXXXXXXXXXXXXXXXXXXXXXXXXXXXXXXXXXXXXXXXXXXXXXXXXXXXXXXXXXXXXXXXXXXXXXXXXXXXXX
                                   102410    102420    102430    102440    102450    102460    102470    102480
Human                     AGAAACAGAACAGTTTGTTTAAAAGTTGGGGACAAAAAAGGACCAGAATCAAGATTACGGCACCCTGGAGAAAGTTAAGC 101315
Kakapo                                                                                                     50676
GoldenEagle                                                                                                57960
JapaneseQuail                                                                                              43432
MediumGroundFinch                                                                                          32462
GoodesThornscrubTortoise                                                                                   82946

Majority                  XXXXXXXXXXXXXXXXXXXXXXXXXXXXXXXXXXXXXXXXXXXXXXXXXXXXXXXXXXXXXXXXXXXXXXXXXXXXXXXX
                                   102490    102500    102510    102520    102530    102540    102550    102560
Human                     ATGGCTCGGTCATAAATTAGGAGACAAGGAACAAATAGAGAAATACGTTTGGATAGAATAGTCAGATTATAGGCTGTGCT 101395
Kakapo                                                                                                     50676
GoldenEagle                                                                                                57960
JapaneseQuail                                                                                              43432
MediumGroundFinch                                                                                          32462
GoodesThornscrubTortoise                                                                                   82946

Majority                  XXXXXXXXXXXXXXXXXXXXXXXXXXXXXXXXXXXXXXXXXXXXXXXXXXXXXXXXXXXXXXXXXXXXXXXXXXXXXXXX
                                   102570    102580    102590    102600    102610    102620    102630    102640
Human                     AAGTTGTTTGGGCTCCACTAGGGTTTCTAAATGCAGTTATTGGTAAACTGGACTTAGGAGAAGGGACAGGGTTGACATGA 101475
Kakapo                                                                                                     50676
GoldenEagle                                                                                                57960
JapaneseQuail                                                                                              43432
MediumGroundFinch                                                                                          32462
GoodesThornscrubTortoise                                                                                   82946

Majority                  XXXXXXXXXXXXXXXXXXXXXXXXXXXXXXXXXXXXXXXXXXXXXXXXXXXXXXXXXXXXXXXXXXXXXXXXXXXXXXXX
                                   102650    102660    102670    102680    102690    102700    102710    102720
Human                     CAAATCTAGTGAATTTATGTTCATTAAAATATCCCTTTTGAATTGATTTAGTTTTTGTATGTTTCTTTTTTATAATTCCA 101555
Kakapo                                                                                                     50676
GoldenEagle                                                                                                57960
JapaneseQuail                                                                                              43432
MediumGroundFinch                                                                                          32462
GoodesThornscrubTortoise                                                                                   82946
```

```
Majority                     XXXXXXXXXXXXXXXXXXXXXXXXXXXXXXXXXXXXXXXXXXXXXXXXXXXXXXXXXXXXXXXXXXXXXXXXXXXXXXXX

                                      102730    102740    102750    102760    102770    102780    102790    102800

Human                        AAAAATCATACAAATAAGTTAAACTTATCCATCTAAAAGCTGCACACAAAACTTGATGTTAGATTCTTTTAAAATTACAA 101635
Kakapo                                                                                                        50676
GoldenEagle                                                                                                   57960
JapaneseQuail                                                                                                 43432
MediumGroundFinch                                                                                             32462
GoodesThornscrubTortoise                                                                                      82946

Majority                     XXXXXXXXXXXXXXXXXXXXXXXXXXXXXXXXXXXXXXXXXXXXXXXXXXXXXXXXXXXXXXXXXXXXXXXXXXXXXXXX

                                      102810    102820    102830    102840    102850    102860    102870    102880

Human                        AATTAAGACTTCCAAAAGAATCTTGTTGAATAAGAGTAAAAGAAAGACCCTTACTCAGTAAAGCTGCGTAATACTTTGGC 101715
Kakapo                                                                                                        50676
GoldenEagle                                                                                                   57960
JapaneseQuail                                                                                                 43432
MediumGroundFinch                                                                                             32462
GoodesThornscrubTortoise                                                                                      82946

Majority                     XXXXXXXXXXXXXXXXXXXXXXXXXXXXXXXXXXXXXXXXXXXXXXXXXXXXXXXXXXXXXXXXXXXXXXXXXXXXXXXX

                                      102890    102900    102910    102920    102930    102940    102950    102960

Human                        AGTTTGAACCCATGGCCATGCCTCTCTGTGTGATAAGGATTTGGCAAAACCATGGAAGAAACATGCATGTTTTTGAATTG 101795
Kakapo                                                                                                        50676
GoldenEagle                                                                                                   57960
JapaneseQuail                                                                                                 43432
MediumGroundFinch                                                                                             32462
GoodesThornscrubTortoise                                                                                      82946

Majority                     XXXXXXXXXXXXXXXXXXXXXXXXXXXXXXXXXXXXXXXXXXXXXXXXXXXXXXXXXXXXXXXXXXXXXXXXXXXXXXXX

                                      102970    102980    102990    103000    103010    103020    103030    103040

Human                        TCTTTCCATGGTAACTTAAAACATTTTTTCATGAAACATGGATATATATCCTTAGAAAACTGCAAAACATAAAGCTTGTA 101875
Kakapo                                                                                                        50676
GoldenEagle                                                                                                   57960
JapaneseQuail                                                                                                 43432
MediumGroundFinch                                                                                             32462
GoodesThornscrubTortoise                                                                                      82946
```

```
Majority                    XXXXXXXXXXXXXXXXXXXXXXXXXXXXXXXXXXXXXXXXXXXXXXXXXXXXXXXXXXXXXXXXXXXXXXXXXXXXXXXX

                                      103050    103060    103070    103080    103090    103100    103110    103120

Human                       AGTTATAAGACAAGATTTATCTCTTTTTTTTTTTTTTTCTGAGAGAGTCTTGCTCTGTTGCCCAGGCCGGAGTGCAACA 101955
Kakapo                                                                                                     50676
GoldenEagle                                                                                                57960
JapaneseQuail                                                                                              43432
MediumGroundFinch                                                                                          32462
GoodesThornscrubTortoise                                                                                   82946

Majority                    XXXXXXXXXXXXXXXXXXXXXXXXXXXXXXXXXXXXXXXXXXXXXXXXXXXXXXXXXXXXXXXXXXXXXXXXXXXXXXXX

                                      103130    103140    103150    103160    103170    103180    103190    103200

Human                       GTGTGATCTCTGCTCACTGCACCCTCCGCCTCCCGGGTTCAAGCAATTCTCCTGCCTCAGCCTCCTGAGTAGCTGGGACT 102035
Kakapo                                                                                                     50676
GoldenEagle                                                                                                57960
JapaneseQuail                                                                                              43432
MediumGroundFinch                                                                                          32462
GoodesThornscrubTortoise                                                                                   82946

Majority                    XXXXXXXXXXXXXXXXXXXXXXXXXXXXXXXXXXXXXXXXXXXXXXXXXXXXXXXXXXXXXXXXXXXXXXXXXXXXXXXX

                                      103210    103220    103230    103240    103250    103260    103270    103280

Human                       ACAGGCGTGTGCCGCCAAGCCCAGCTAATTTTTTTGTATCTTTAGTAGAGACGGTTTCACCATGTTGGCCAGGCTGGTCT 102115
Kakapo                                                                                                     50676
GoldenEagle                                                                                                57960
JapaneseQuail                                                                                              43432
MediumGroundFinch                                                                                          32462
GoodesThornscrubTortoise                                                                                   82946

Majority                    XXXXXXXXXXXXXXXXXXXXXXXXXXXXXXXXXXXXXXXXXXXXXXXXXXXXXXXXXXXXXXXXXXXXXXXXXXXXXXXX

                                      103290    103300    103310    103320    103330    103340    103350    103360

Human                       CGAACTCCTGACCTCAAGTGATCTACCTGCCTCAGCCTTCTAAAGTGTTGGGATTACAGGCATGAGCCATGGCACCCGGC 102195
Kakapo                                                                                                     50676
GoldenEagle                                                                                                57960
JapaneseQuail                                                                                              43432
MediumGroundFinch                                                                                          32462
GoodesThornscrubTortoise                                                                                   82946
```

```
Majority                   XXXXXXXXXXXXXXXXXXXXXXXXXXXXXXXXXXXXXXXXXXXXXXXXXXXXXXXXXXXXXXXXXXXXXXXXXXXXXXXX

                                   103370    103380    103390    103400    103410    103420    103430    103440

Human                      CAAGATGAAATGTCAAGGCCATTTTAGCCACTATGCTTTTAAGACTACTTAAAGCTGTGCTTTCAGGTCTCCCCAGGAAT 102275
Kakapo                                                                                                      50676
GoldenEagle                                                                                                 57960
JapaneseQuail                                                                                               43432
MediumGroundFinch                                                                                           32462
GoodesThornscrubTortoise                                                                                    82946

Majority                   XXXXXXXXXXXXXXXXXXXXXXXXXXXXXXXXXXXXXXXXXXXXXXXXXXXXXXXXXXXXXXXXXXXXXXXXXXXXXXXX

                                   103450    103460    103470    103480    103490    103500    103510    103520

Human                      ACTTGTTCACTCTATACTATTGCTGCTGCTGTTCTGGCTTGAGAATTTTGAGAATCACTAAGATACAATTTTAAGTTTTG 102355
Kakapo                                                                                                      50676
GoldenEagle                                                                                                 57960
JapaneseQuail                                                                                               43432
MediumGroundFinch                                                                                           32462
GoodesThornscrubTortoise                                                                                    82946

Majority                   XXXXXXXXXXXXXXXXXXXXXXXXXXXXXXXXXXXXXXXXXXXXXXXXXXXXXXXXXXXXXXXXXXXXXXXXXXXXXXXX

                                   103530    103540    103550    103560    103570    103580    103590    103600

Human                      ACATGTAAATTATTTGGTTGAGTAGAAAGAATTAATGTAGATAAGCAAAAACCCCTTTATAAAAAATGCTTACCCTTTAA 102435
Kakapo                                                                                                      50676
GoldenEagle                                                                                                 57960
JapaneseQuail                                                                                               43432
MediumGroundFinch                                                                                           32462
GoodesThornscrubTortoise                                                                                    82946

Majority                   XXXXXXXXXXXXXXXXXXXXXXXXXXXXXXXXXXXXXXXXXXXXXXXXXXXXXXXXXXXXXXXXXXXXXXXXXXXXXXXX

                                   103610    103620    103630    103640    103650    103660    103670    103680

Human                      AAAATGTTGAATCCTCACAGTATGATTAAATACTACTTGTAAAAGTGTCTGTTAACCATAATGTATTTTTTAGATAATTT 102515
Kakapo                                                                                                      50676
GoldenEagle                                                                                                 57960
JapaneseQuail                                                                                               43432
MediumGroundFinch                                                                                           32462
GoodesThornscrubTortoise                                                                                    82946
```

```
Majority                   XXXXXXXXXXXXXXXXXXXXXXXXXXXXXXXXXXXXXXXXXXXXXXXXXXXXXXXXXXXXXXXXXXXXXXXXXXXXXXXX

                                    103690    103700    103710    103720    103730    103740    103750    103760

Human                      GAGTAGAATTATACGTGCAATATATTCTTGAGTTTGTGACCAACAAAGAAACAGATTAATGATTGAAAATTCACCATACA 102595
Kakapo                                                                                                      50676
GoldenEagle                                                                                                 57960
JapaneseQuail                                                                                               43432
MediumGroundFinch                                                                                           32462
GoodesThornscrubTortoise                                                                                    82946

Majority                   XXXXXXXXXXXXXXXXXXXXXXXXXXXXXXXXXXXXXXXXXXXXXXXXXXXXXXXXXXXXXXXXXXXXXXXXXXXXXXXX

                                    103770    103780    103790    103800    103810    103820    103830    103840

Human                      TTACTACCTATGTCTCTCCACCTGCTATCTACTTCCAATTTTTTATATATATAAAAGTCATTAAAGTCATTTGTAATTGA 102675
Kakapo                                                                                                      50676
GoldenEagle                                                                                                 57960
JapaneseQuail                                                                                               43432
MediumGroundFinch                                                                                           32462
GoodesThornscrubTortoise                                                                                    82946

Majority                   XXXXXXXXXXXXXXXXXXXXXXXXXXXXXXXXXXXXXXXXXXXXXXXXXXXXXXXXXXXXXXXXXXXXXXXXXXXXXXXX

                                    103850    103860    103870    103880    103890    103900    103910    103920

Human                      GTTTGTTTTTTTTTCTTGAGACAGGGTCTCACTCTGTCACCCAGGCTGGAGTGCAAATGGCATGATCACAGCTCACTGCA 102755
Kakapo                                                                                                      50676
GoldenEagle                                                                                                 57960
JapaneseQuail                                                                                               43432
MediumGroundFinch                                                                                           32462
GoodesThornscrubTortoise                                                                                    82946

Majority                   XXXXXXXXXXXXXXXXXXXXXXXXXXXXXXXXXXXXXXXXXXXXXXXXXXXXXXXXXXXXXXXXXXXXXXXXXXXXXXXX

                                    103930    103940    103950    103960    103970    103980    103990    104000

Human                      GCCTCAACCTCCCAGGTTCGATAGATCCTCCCACCTCAGTCCCCCACAGGCATGCATCACCATGCCTGACTAGTTTTTGT 102835
Kakapo                                                                                                      50676
GoldenEagle                                                                                                 57960
JapaneseQuail                                                                                               43432
MediumGroundFinch                                                                                           32462
GoodesThornscrubTortoise                                                                                    82946
```

```
Majority                    XXXXXXXXXXXXXXXXXXXXXXXXXXXXXXXXXXXXXXXXXXXXXXXXXXXXXXXXXXXXXXXXXXXXXXXXXXXXXXXX

                                  104010    104020    104030    104040    104050    104060    104070    104080

Human                       GTTTTTTTGTAGAGACAAGGTTTTGCCATGTTGCCCAGACTGGTCTCAAACTTGGGCCAAAGCCATCCACCTGCCTCGGC 102915
Kakapo                                                                                                       50676
GoldenEagle                                                                                                  57960
JapaneseQuail                                                                                                43432
MediumGroundFinch                                                                                            32462
GoodesThornscrubTortoise                                                                                     82946

Majority                    XXXXXXXXXXXXXXXXXXXXXXXXXXXXXXXXXXXXXXXXXXXXXXXXXXXXXXXXXXXXXXXXXXXXXXXXXXXXXXXX

                                  104090    104100    104110    104120    104130    104140    104150    104160

Human                       TTCCCAATGTGCTGGGATTACATGTGTGAGCCACCACACCCAGTCATGAGAATTATTTTAAATCAGGTAAACAAAATGTC 102995
Kakapo                                                                                                       50676
GoldenEagle                                                                                                  57960
JapaneseQuail                                                                                                43432
MediumGroundFinch                                                                                            32462
GoodesThornscrubTortoise                                                                                     82946

Majority                    XXXXXXXXXXXXXXXXXXXXXXXXXXXXXXXXXXXXXXXXXXXXXXXXXXXXXXXXXXXXXXXXXXXXXXXXXXXXXXXX

                                  104170    104180    104190    104200    104210    104220    104230    104240

Human                       TGTGGGAATATTGAGCTTTATGATAGATACCTAAGTTTTCCCAAAAATAATTGTGTTAGCATTTCAGTGAATCACTATAC 103075
Kakapo                                                                                                       50676
GoldenEagle                                                                                                  57960
JapaneseQuail                                                                                                43432
MediumGroundFinch                                                                                            32462
GoodesThornscrubTortoise                                                                                     82946

Majority                    XXXXXXXXXXXXXXXXXXXXXXXXXXXXXXXXXXXXXXXXXXXXXXXXXXXXXXXXXXXXXXXXXXXXXXXXXXXXXXXX

                                  104250    104260    104270    104280    104290    104300    104310    104320

Human                       TTAACCTAGAAATCTAGGTTACCAATAACAGTCTCTTTCAGGTTTTTTTCTTTTCCTGTGTCTTTCTTTTTACTAAAATC 103155
Kakapo                                                                                                       50676
GoldenEagle                                                                                                  57960
JapaneseQuail                                                                                                43432
MediumGroundFinch                                                                                            32462
GoodesThornscrubTortoise                                                                                     82946
```

```
Majority                   XXXXXXXXXXXXXXXXXXXXXXXXXXXXXXXXXXXXXXXXXXXXXXXXXXXXXXXXXXXXXXXXXXXXXXXXXXXXXXXXX

                                 104330    104340    104350    104360    104370    104380    104390    104400

Human                      ATTCATAACTGGGATGACTTATGGAGTTGATACCTTAGGTCAAATCCCAATTTAGAAACTCCCAAAAGCCTGTCAAATTT 103235
Kakapo                                                                                      50676
GoldenEagle                                                                                 57960
JapaneseQuail                                                                               43432
MediumGroundFinch                                                                           32462
GoodesThornscrubTortoise                                                                    82946

Majority                   XXXXXXXXXXXXXXXXXXXXXXXXXXXXXXXXXXXXXXX

                                 104410    104420    104430

Human                      TGACACATACTTTAAATGAACTGTTATTGAGTAGATATA                          103274
Kakapo                                                                                      50676
GoldenEagle                                                                                 57960
JapaneseQuail                                                                               43432
MediumGroundFinch                                                                           32462
GoodesThornscrubTortoise                                                                    82946
```
