# Supplementary material for: PTBP2 – a gene with relevance for both Anorexia nervosa and body weight regulation
Source: Transl Psychiatry. 2022 Jun 9;12:241. doi: 10.1038/s41398-022-02018-5 (PMC9184595; doi:10.1038/s41398-022-02018-5)
Supplement: Supplementary file 4 — gDNA alignment report (Rodent) [file 41398_2022_2018_MOESM4_ESM.pdf]

Alignment Report of Rodent.meg ClustalW (Slow/Accurate, IUB)

Montag, 2. Mai 2022 11:32

```
Majority                    --------------------------------------------------------------------------------
                                     10        20        30        40        50        60        70        80
Human                       TGCCTCCAAATGTTAAACTCCAACTATTGTAGCATTTTAACACATGGCTCCATTTGTGTCTATTTCCACCTAGCGTATTG 80
GuineaPig                   -------------------------------------------------------------------------------- 0
NorthernAmericanDeerMouse   -------------------------------------------------------------------------------- 0
Mouse                       -------------------------------------------------------------------------------- 0
ChineseHamsterGHOK1GS       -------------------------------------------------------------------------------- 0
LongTailedChinchilla        -------------------------------------------------------------------------------- 0

Majority                    --------------------------------------------------------------------------------
                                     90       100       110       120       130       140       150       160
Human                       TGAATATTTTCTACAGGCGCACACTAATCACCAACTGAAGTGAGAATCACATTTTCCTCCACTTGGCTTGGTACTACAGG 160
GuineaPig                   -------------------------------------------------------------------------------- 0
NorthernAmericanDeerMouse   -------------------------------------------------------------------------------- 0
Mouse                       -------------------------------------------------------------------------------- 0
ChineseHamsterGHOK1GS       -------------------------------------------------------------------------------- 0
LongTailedChinchilla        -------------------------------------------------------------------------------- 0

Majority                    --------------------------------------------------------------------------------
                                    170       180       190       200       210       220       230       240
Human                       TCTTGAAGCAAAAGCCTGTATTTCAGTCTGAGGAGTTCTACTTGCTTGAGAAAGTACTGTTGTTACCAAGTGGTCTCGTT 240
GuineaPig                   -------------------------------------------------------------------------------- 0
NorthernAmericanDeerMouse   -------------------------------------------------------------------------------- 0
Mouse                       -------------------------------------------------------------------------------- 0
ChineseHamsterGHOK1GS       -------------------------------------------------------------------------------- 0
LongTailedChinchilla        -------------------------------------------------------------------------------- 0

Majority                    --------------------------------------------------------------------------------
                                    250       260       270       280       290       300       310       320
Human                       CAGTGGGGTAAAGATTTTTGTTTTAGAGGCTGGAGCCGGTCTGCAAATAAAAGATGGCTTAGGAGAAAAGCTATCCAATG 320
GuineaPig                   -------------------------------------------------------------------------------- 0
NorthernAmericanDeerMouse   -------------------------------------------------------------------------------- 0
Mouse                       -------------------------------------------------------------------------------- 0
ChineseHamsterGHOK1GS       -------------------------------------------------------------------------------- 0
LongTailedChinchilla        -------------------------------------------------------------------------------- 0
```

Majority	--	
	330 340 350 360 370 380 390 400	
Human	TTTTTTCCTTGGCCCGCATAGGTTGACGGTAGCCAAGCTGGTACTTGGCTTGGAGTGAAAAGTGGAGGCTGCCTGGGGGC	400
GuineaPig	--	0
NorthernAmericanDeerMouse	--	0
Mouse	--	0
ChineseHamsterGHOK1GS	--	0
LongTailedChinchilla	--	0

Majority	--	
	410 420 430 440 450 460 470 480	
Human	GTCCTGCAGTCAGGGCAGAGAGAGTGTGGAGAGCTGTTGGGAGGTGTCCCACCCACGCCCCCACAAGGAGGGGAAACCAG	480
GuineaPig	--	0
NorthernAmericanDeerMouse	--	0
Mouse	--	0
ChineseHamsterGHOK1GS	--	0
LongTailedChinchilla	--	0

Majority	--	
	490 500 510 520 530 540 550 560	
Human	GTCGCCGCGGTCTGCAGCAGAACGCCAGGTTCCAGAGGACGGCAGGCGGAGGGCGGAGGGAGAAAGGCGCGCGAAGGCGC	560
GuineaPig	--	0
NorthernAmericanDeerMouse	--	0
Mouse	--	0
ChineseHamsterGHOK1GS	--	0
LongTailedChinchilla	--	0

Majority	--	
	570 580 590 600 610 620 630 640	
Human	GGAGGAGGCGTCGGCTGCGGCGCCCACTCCCCCTAGTCCCAGCGCCCTGCCGCTCGCCTCCCGCTGCCGGGCGCCGGCGC	640
GuineaPig	--	0
NorthernAmericanDeerMouse	--	0
Mouse	--	0
ChineseHamsterGHOK1GS	--	0
LongTailedChinchilla	--	0

```
Majority                     --------------------------------------------------------------------------------
                                      650       660       670       680       690       700       710       720
Human                        GCTCGGCTCTTTCCGCCGCCGCCGCTGCCGCGCGGTGGCCCGTGCGCCTCGGCACCTTCGGCAATTTCCGTCGGGCCCCA 720
GuineaPig                    -------------------------------------------------------------------------------- 0
NorthernAmericanDeerMouse    -------------------------------------------------------------------------------- 0
Mouse                        -------------------------------------------------------------------------------- 0
ChineseHamsterGHOK1GS        -------------------------------------------------------------------------------- 0
LongTailedChinchilla         -------------------------------------------------------------------------------- 0

Majority                     --------------------------------------------------------------------------------
                                      730       740       750       760       770       780       790       800
Human                        GCCGCCATTTTCTCGCCGCTTGTGTGGCTCGCTGGCTGCGTGGCTCGGTTCTTGTGAGCGAAGCTTTGTCCGGTTCGGCA 800
GuineaPig                    -------------------------------------------------------------------------------- 0
NorthernAmericanDeerMouse    -------------------------------------------------------------------------------- 0
Mouse                        -------------------------------------------------------------------------------- 0
ChineseHamsterGHOK1GS        -------------------------------------------------------------------------------- 0
LongTailedChinchilla         -------------------------------------------------------------------------------- 0

Majority                     --------------------------------------------------------------------------------
                                      810       820       830       840       850       860       870       880
Human                        ATGGACGGGTATGTAATCGGGCCGGCGAGAAGGTGTGTGTGAGAGAGGAGTTGGACCGTCCTTCGGCCCGGTCCCGGGCC 880
GuineaPig                    -------------------------------------------------------------------------------- 0
NorthernAmericanDeerMouse    -------------------------------------------------------------------------------- 0
Mouse                        -------------------------------------------------------------------------------- 0
ChineseHamsterGHOK1GS        -------------------------------------------------------------------------------- 0
LongTailedChinchilla         -------------------------------------------------------------------------------- 0

Majority                     --------------------------------------------------------------------------------
                                      890       900       910       920       930       940       950       960
Human                        GGGGAGAAACCCTCCCGCGGCCCCTCCCAGGGCTGGGCTGCCGTTACCCAACCCCCGCCCCATCGCACACACCCCTCCCT 960
GuineaPig                    -------------------------------------------------------------------------------- 0
NorthernAmericanDeerMouse    -------------------------------------------------------------------------------- 0
Mouse                        -------------------------------------------------------------------------------- 0
ChineseHamsterGHOK1GS        -------------------------------------------------------------------------------- 0
LongTailedChinchilla         -------------------------------------------------------------------------------- 0
```

```
Majority                     --------------------------------------------------------------------------------
                                      970       980       990      1000      1010      1020      1030      1040
Human                        TTGCTTCCCCCGGCGGGCTTTGGTCGAGAAAATGAGAAGAAAGCGGGCTTGGAGGCTGGGGAGGCATAGGGGCGATGGCG 1040
GuineaPig                    -------------------------------------------------------------------------------- 0
NorthernAmericanDeerMouse    -------------------------------------------------------------------------------- 0
Mouse                        -------------------------------------------------------------------------------- 0
ChineseHamsterGHOK1GS        -------------------------------------------------------------------------------- 0
LongTailedChinchilla         -------------------------------------------------------------------------------- 0

Majority                     --------------------------------------------------------------------------------
                                     1050      1060      1070      1080      1090      1100      1110      1120
Human                        GGGGTGAGGATCCCGGAGTGGGAGCGGGCACCGGCTTGGCGGCGGGGGATGGGGTGGGAACCCCATAACGTCTCCCCGGT 1120
GuineaPig                    -------------------------------------------------------------------------------- 0
NorthernAmericanDeerMouse    -------------------------------------------------------------------------------- 0
Mouse                        -------------------------------------------------------------------------------- 0
ChineseHamsterGHOK1GS        -------------------------------------------------------------------------------- 0
LongTailedChinchilla         -------------------------------------------------------------------------------- 0

Majority                     --------------------------------------------------------------------------------
                                     1130      1140      1150      1160      1170      1180      1190      1200
Human                        CTGTCCCTGCCCCCTCTAGGAGCCATTTCGATCCGTACCTTGGGACCCGACCCTTGGGTTAGCGGTGCCTGTGAGAGCGA 1200
GuineaPig                    -------------------------------------------------------------------------------- 0
NorthernAmericanDeerMouse    -------------------------------------------------------------------------------- 0
Mouse                        -------------------------------------------------------------------------------- 0
ChineseHamsterGHOK1GS        -------------------------------------------------------------------------------- 0
LongTailedChinchilla         -------------------------------------------------------------------------------- 0

Majority                     --------------------------------------------------------------------------------
                                     1210      1220      1230      1240      1250      1260      1270      1280
Human                        GTGGGATGGGCAGAGACAGGCCTTTGGATTGGGGGAGCCTCTAGGGGAAGAGGAGAGAGGCACCCCATGTGGACCCCAGC 1280
GuineaPig                    -------------------------------------------------------------------------------- 0
NorthernAmericanDeerMouse    -------------------------------------------------------------------------------- 0
Mouse                        -------------------------------------------------------------------------------- 0
ChineseHamsterGHOK1GS        -------------------------------------------------------------------------------- 0
LongTailedChinchilla         -------------------------------------------------------------------------------- 0
```

Majority	--	
	1290 1300 1310 1320 1330 1340 1350 1360	
Human	CATGAGCAACCTCTCCGGCCTCGCCCGGCCCTCCGTGGTCGGGAGAGATGCCGGTGGCGGGAGCTCCGGGGAAAGCCTAG	1360
GuineaPig	--	0
NorthernAmericanDeerMouse	--	0
Mouse	--	0
ChineseHamsterGHOK1GS	--	0
LongTailedChinchilla	--	0

Majority	--	
	1370 1380 1390 1400 1410 1420 1430 1440	
Human	TGGGAGCCGCTGGGGAAGGGGGGAGGGCGTGCGGCGGCGGGAGGAAGGGGGAGGGCAGATGTCATACTCCTTTGTTTTCA	1440
GuineaPig	--	0
NorthernAmericanDeerMouse	--	0
Mouse	--	0
ChineseHamsterGHOK1GS	--	0
LongTailedChinchilla	--	0

Majority	--	
	1450 1460 1470 1480 1490 1500 1510 1520	
Human	TTTGAGTCTGGTAGTGGGGGCGGGAGGGAGGAAAAAATGCCTTTTTGTGGGGACTGAAAACATTCAAGGCTTGGCAAAAG	1520
GuineaPig	--	0
NorthernAmericanDeerMouse	--	0
Mouse	--	0
ChineseHamsterGHOK1GS	--	0
LongTailedChinchilla	--	0

Majority	--	
	1530 1540 1550 1560 1570 1580 1590 1600	
Human	GGCCCAGAAATTAAATCATCTAAAAAACGAAGTGTTTGAAGCCGACATCAGTCACATGGGCAAAGGCCAACAGCAATGGC	1600
GuineaPig	--	0
NorthernAmericanDeerMouse	--	0
Mouse	--	0
ChineseHamsterGHOK1GS	--	0
LongTailedChinchilla	--	0

Majority	--	
	1610 1620 1630 1640 1650 1660 1670 1680	
Human	AGAAACAAGAGCAGATAGTCAACAAAGGGCTATCTCTTTAGCTACTTTGGTCAGTTTCTGGAAAAACGACTATAAAAACC	1680
GuineaPig	--	0
NorthernAmericanDeerMouse	--	0
Mouse	--	0
ChineseHamsterGHOK1GS	--	0
LongTailedChinchilla	--	0

Majority	--	
	1690 1700 1710 1720 1730 1740 1750 1760	
Human	CAGAACCATGCTGCATCTTTGCGAACCTGAGTCAAGACGGAATGCTGAGTGAGGTGAGAGGCAAGACTGTGAAATGGTAG	1760
GuineaPig	--	0
NorthernAmericanDeerMouse	--	0
Mouse	--	0
ChineseHamsterGHOK1GS	--	0
LongTailedChinchilla	--	0

Majority	--	
	1770 1780 1790 1800 1810 1820 1830 1840	
Human	GGACCGATGTCTCGCTCATATGCTTCACACCTGAAAAGACTCAGTAATTTAACTCAATGTTAGAAAGGGGGCACGCTTAA	1840
GuineaPig	--	0
NorthernAmericanDeerMouse	--	0
Mouse	--	0
ChineseHamsterGHOK1GS	--	0
LongTailedChinchilla	--	0

Majority	--	
	1850 1860 1870 1880 1890 1900 1910 1920	
Human	GGTCAAAAATACAGCAGTATGCAGCTCTTTTGGGAGTGTAAACATAGTATCATTTAACAAGTACATTTATTAAAATCAGT	1920
GuineaPig	--	0
NorthernAmericanDeerMouse	--	0
Mouse	--	0
ChineseHamsterGHOK1GS	--	0
LongTailedChinchilla	--	0

```
Majority                     --------------------------------------------------------------------------------
                                     1930      1940      1950      1960      1970      1980      1990      2000
Human                        GAGTATTTGAAGAATCAACAATTTGGTTAAGCGCCTAGAGTTTGGTGTGTTTTAAAAGGTACTAAAAACAAGATTTTATA 2000
GuineaPig                    -------------------------------------------------------------------------------- 0
NorthernAmericanDeerMouse    -------------------------------------------------------------------------------- 0
Mouse                        -------------------------------------------------------------------------------- 0
ChineseHamsterGHOK1GS        -------------------------------------------------------------------------------- 0
LongTailedChinchilla         -------------------------------------------------------------------------------- 0
```

```
Majority                     --------------------------------------------------------------------------------
                                     2010      2020      2030      2040      2050      2060      2070      2080
Human                        AAAGTTAACACAGTCTCACAGAATTCAAATCATCCTAGATGTTACCTAAAGATTATTTTCCAGATGAAACAAATCCAGAG 2080
GuineaPig                    -------------------------------------------------------------------------------- 0
NorthernAmericanDeerMouse    -------------------------------------------------------------------------------- 0
Mouse                        -------------------------------------------------------------------------------- 0
ChineseHamsterGHOK1GS        -------------------------------------------------------------------------------- 0
LongTailedChinchilla         -------------------------------------------------------------------------------- 0
```

```
Majority                     --------------------------------------------------------------------------------
                                     2090      2100      2110      2120      2130      2140      2150      2160
Human                        AAGTTCAGTATTCCCCCCAGATCAACACGGAAATTGATGTCAAAACTTGCACTAGAATTCATGTTTCCTGATTCCTGGTT 2160
GuineaPig                    -------------------------------------------------------------------------------- 0
NorthernAmericanDeerMouse    -------------------------------------------------------------------------------- 0
Mouse                        -------------------------------------------------------------------------------- 0
ChineseHamsterGHOK1GS        -------------------------------------------------------------------------------- 0
LongTailedChinchilla         -------------------------------------------------------------------------------- 0
```

```
Majority                     --------------------------------------------------------------------------------
                                     2170      2180      2190      2200      2210      2220      2230      2240
Human                        CCTGTGGAGACTACTAAAGAGTTTAAGCGTTAAATTATTAATCTCAGTATACTGGCATTGTAGAAATGGCATAGTGTTTT 2240
GuineaPig                    -------------------------------------------------------------------------------- 0
NorthernAmericanDeerMouse    -------------------------------------------------------------------------------- 0
Mouse                        -------------------------------------------------------------------------------- 0
ChineseHamsterGHOK1GS        -------------------------------------------------------------------------------- 0
LongTailedChinchilla         -------------------------------------------------------------------------------- 0
```

```
Majority                     --------------------------------------------------------------------------------
                                      2250      2260      2270      2280      2290      2300      2310      2320
Human                        GGGTTTTGTTCTTTTTGGCTATTGAATGTGTTGTTAAAAAATACCACAAGTTGTCTCACATTTGAAAGTTACTTTTAGAA 2320
GuineaPig                    -------------------------------------------------------------------------------- 0
NorthernAmericanDeerMouse    -------------------------------------------------------------------------------- 0
Mouse                        -------------------------------------------------------------------------------- 0
ChineseHamsterGHOK1GS        -------------------------------------------------------------------------------- 0
LongTailedChinchilla         -------------------------------------------------------------------------------- 0

Majority                     --------------------------------------------------------------------------------
                                      2330      2340      2350      2360      2370      2380      2390      2400
Human                        AACCAGGTTGGTAACAGTACAGGTAAGTCACGGATCATCTGTGGTATTTTTTATCCCCATCTGTGTGTGAGTGGCTGGAA 2400
GuineaPig                    -------------------------------------------------------------------------------- 0
NorthernAmericanDeerMouse    -------------------------------------------------------------------------------- 0
Mouse                        -------------------------------------------------------------------------------- 0
ChineseHamsterGHOK1GS        -------------------------------------------------------------------------------- 0
LongTailedChinchilla         -------------------------------------------------------------------------------- 0

Majority                     --------------------------------------------------------------------------------
                                      2410      2420      2430      2440      2450      2460      2470      2480
Human                        GATTTATGAATGATGGACTATCCTAAAAAGTTTTAGAGCCAAAGAGTGAGTGATTAGAAGAATAACAGGAGGTTGAAACT 2480
GuineaPig                    -------------------------------------------------------------------------------- 0
NorthernAmericanDeerMouse    -------------------------------------------------------------------------------- 0
Mouse                        -------------------------------------------------------------------------------- 0
ChineseHamsterGHOK1GS        -------------------------------------------------------------------------------- 0
LongTailedChinchilla         -------------------------------------------------------------------------------- 0

Majority                     --------------------------------------------------------------------------------
                                      2490      2500      2510      2520      2530      2540      2550      2560
Human                        ACTTATTTTTTCTTTGCAGAATCGTCACTGAGGTTGCAGTTGGCGTGAAGGTAGGAAAATACTATGTTTGAAACTGGGAT 2560
GuineaPig                    -------------------------------------------------------------------------------- 0
NorthernAmericanDeerMouse    -------------------------------------------------------------------------------- 0
Mouse                        -------------------------------------------------------------------------------- 0
ChineseHamsterGHOK1GS        -------------------------------------------------------------------------------- 0
LongTailedChinchilla         -------------------------------------------------------------------------------- 0
```

Majority	--	
	2570 2580 2590 2600 2610 2620 2630 2640	
Human	TGTTGGATCTATTATCATAATTACTGGAAAATTTTAGCTTTTGCTTTTGAAAACTATAACGTAGCTAACAAAACCCAAGT	2640
GuineaPig	--	0
NorthernAmericanDeerMouse	--	0
Mouse	--	0
ChineseHamsterGHOK1GS	--	0
LongTailedChinchilla	--	0

Majority	--	
	2650 2660 2670 2680 2690 2700 2710 2720	
Human	GTAAAATGTTTCCTTTCATTTGTAAAGTTGGACCATATAAACAGTTTGTAATCACCATAAGTTTTTCTAGAACTGTCAGT	2720
GuineaPig	--	0
NorthernAmericanDeerMouse	--	0
Mouse	--	0
ChineseHamsterGHOK1GS	--	0
LongTailedChinchilla	--	0

Majority	--	
	2730 2740 2750 2760 2770 2780 2790 2800	
Human	CTGTAAGAATAACCTTTTAAAGTAAGTATGAATGATCAAGAAATTGAAAATGCTTTCACACATGGCTTCTTGTAATATAA	2800
GuineaPig	--	0
NorthernAmericanDeerMouse	--	0
Mouse	--	0
ChineseHamsterGHOK1GS	--	0
LongTailedChinchilla	--	0

Majority	--	
	2810 2820 2830 2840 2850 2860 2870 2880	
Human	AACGATACCCCAATTTTCTGAGGGGAAATAATGATTTTAAATGGATGGTAAGTGGTTTGACCTATATCTTATAATTTTGT	2880
GuineaPig	--	0
NorthernAmericanDeerMouse	--	0
Mouse	--	0
ChineseHamsterGHOK1GS	--	0
LongTailedChinchilla	--	0

```
Majority                      --------------------------------------------------------------------------------
                                       2890      2900      2910      2920      2930      2940      2950      2960
Human                         TAATCTAAGTTTTTTGTTTTTTATTACTTTTTGTCATGAAAATGAATATATTTTGGTTAATAATAAAGCACATTAGAAAT 2960
GuineaPig                     -------------------------------------------------------------------------------- 0
NorthernAmericanDeerMouse     -------------------------------------------------------------------------------- 0
Mouse                         -------------------------------------------------------------------------------- 0
ChineseHamsterGHOK1GS         -------------------------------------------------------------------------------- 0
LongTailedChinchilla          -------------------------------------------------------------------------------- 0

Majority                      --------------------------------------------------------------------------------
                                       2970      2980      2990      3000      3010      3020      3030      3040
Human                         GAATTACACTGATCCTTAAAAAGGAAGTTGTTGAGGCAGTTAGCTTCAAATTTGAATTAGAAGTTAAGAGCTGGTTTGTC 3040
GuineaPig                     -------------------------------------------------------------------------------- 0
NorthernAmericanDeerMouse     -------------------------------------------------------------------------------- 0
Mouse                         -------------------------------------------------------------------------------- 0
ChineseHamsterGHOK1GS         -------------------------------------------------------------------------------- 0
LongTailedChinchilla          -------------------------------------------------------------------------------- 0

Majority                      --------------------------------------------------------------------------------
                                       3050      3060      3070      3080      3090      3100      3110      3120
Human                         TATTAAAGAAAGTCTTTATGGTAGAGATCCTTTGGAATCCTTCCTTTTTGGCTAAGAAAACTACTCATCGTTTAAACTTT 3120
GuineaPig                     -------------------------------------------------------------------------------- 0
NorthernAmericanDeerMouse     -------------------------------------------------------------------------------- 0
Mouse                         -------------------------------------------------------------------------------- 0
ChineseHamsterGHOK1GS         -------------------------------------------------------------------------------- 0
LongTailedChinchilla          -------------------------------------------------------------------------------- 0

Majority                      --------------------------------------------------------------------------------
                                       3130      3140      3150      3160      3170      3180      3190      3200
Human                         AGTTCTAATGCTTAACATTTTCTTGAGTACTTGCAACTTTTAATGTCATGATGTATTGTAATTTTTTTTTGTTTTTTGTT 3200
GuineaPig                     -------------------------------------------------------------------------------- 0
NorthernAmericanDeerMouse     -------------------------------------------------------------------------------- 0
Mouse                         -------------------------------------------------------------------------------- 0
ChineseHamsterGHOK1GS         -------------------------------------------------------------------------------- 0
LongTailedChinchilla          -------------------------------------------------------------------------------- 0
```

```
Majority                    --------------------------------------------------------------------------------
                                     3210      3220      3230      3240      3250      3260      3270      3280
Human                       TTTTTGAGACGGAGCCTCACTCTGTTGCCCAGGCTGGATGCAGTGGCACGATCTTGGCCCACTGCAACCTCTGCCTCCTG 3280
GuineaPig                   -------------------------------------------------------------------------------- 0
NorthernAmericanDeerMouse   -------------------------------------------------------------------------------- 0
Mouse                       -------------------------------------------------------------------------------- 0
ChineseHamsterGHOK1GS       -------------------------------------------------------------------------------- 0
LongTailedChinchilla        -------------------------------------------------------------------------------- 0

Majority                    --------------------------------------------------------------------------------
                                     3290      3300      3310      3320      3330      3340      3350      3360
Human                       GGTTCAAGCAGTTCTCCTGCCTCAGCCTCCTGAGTAGCTGGGATTACAGGTGCGCACCACCACGCCCAGCTAATTTGTAT 3360
GuineaPig                   -------------------------------------------------------------------------------- 0
NorthernAmericanDeerMouse   -------------------------------------------------------------------------------- 0
Mouse                       -------------------------------------------------------------------------------- 0
ChineseHamsterGHOK1GS       -------------------------------------------------------------------------------- 0
LongTailedChinchilla        -------------------------------------------------------------------------------- 0

Majority                    --------------------------------------------------------------------------------
                                     3370      3380      3390      3400      3410      3420      3430      3440
Human                       TTTTAGTAGAAATGGCATTTCACCCTGTTGGTCAGGCTGGTCTCAAACTCCTGACCTCATGATCTGCCTGCCTCTTATGA 3440
GuineaPig                   -------------------------------------------------------------------------------- 0
NorthernAmericanDeerMouse   -------------------------------------------------------------------------------- 0
Mouse                       -------------------------------------------------------------------------------- 0
ChineseHamsterGHOK1GS       -------------------------------------------------------------------------------- 0
LongTailedChinchilla        -------------------------------------------------------------------------------- 0

Majority                    --------------------------------------------------------------------------------
                                     3450      3460      3470      3480      3490      3500      3510      3520
Human                       TCTGCCTGCCTTGACCTCCCAAAGTGCTGGGATTACAAGTGTGAGCCACCGTGCCCGGCCTGTATTGTCATTTTTCATTT 3520
GuineaPig                   -------------------------------------------------------------------------------- 0
NorthernAmericanDeerMouse   -------------------------------------------------------------------------------- 0
Mouse                       -------------------------------------------------------------------------------- 0
ChineseHamsterGHOK1GS       -------------------------------------------------------------------------------- 0
LongTailedChinchilla        -------------------------------------------------------------------------------- 0
```

```
Majority                      --------------------------------------------------------------------------------
                                       3530      3540      3550      3560      3570      3580      3590      3600
Human                         GCAAGCCTCTCTGTCCTGAGAGGCTTTCTGCTTAGTCCAGTGATGCTGACTGTTCAAACATTTTTTCGAATTCTTAAAAT 3600
GuineaPig                     -------------------------------------------------------------------------------- 0
NorthernAmericanDeerMouse     -------------------------------------------------------------------------------- 0
Mouse                         -------------------------------------------------------------------------------- 0
ChineseHamsterGHOK1GS         -------------------------------------------------------------------------------- 0
LongTailedChinchilla          -------------------------------------------------------------------------------- 0

Majority                      --------------------------------------------------------------------------------
                                       3610      3620      3630      3640      3650      3660      3670      3680
Human                         TGTATCCATAATGGGGGGAGGGGGAAGGGATAGCATTGGGAGATATAGGGGGGAGGGGGGAGGGATAGCTTTAGGAGATA 3680
GuineaPig                     -------------------------------------------------------------------------------- 0
NorthernAmericanDeerMouse     -------------------------------------------------------------------------------- 0
Mouse                         -------------------------------------------------------------------------------- 0
ChineseHamsterGHOK1GS         -------------------------------------------------------------------------------- 0
LongTailedChinchilla          -------------------------------------------------------------------------------- 0

Majority                      --------------------------------------------------------------------------------
                                       3690      3700      3710      3720      3730      3740      3750      3760
Human                         TACCTAATGCTAAATGACAAGTTAATGGGTGCAGCACACCAACATGGCACATGTATACATATGTAACAAACCTGCACGTT 3760
GuineaPig                     -------------------------------------------------------------------------------- 0
NorthernAmericanDeerMouse     -------------------------------------------------------------------------------- 0
Mouse                         -------------------------------------------------------------------------------- 0
ChineseHamsterGHOK1GS         -------------------------------------------------------------------------------- 0
LongTailedChinchilla          -------------------------------------------------------------------------------- 0

Majority                      --------------------------------------------------------------------------------
                                       3770      3780      3790      3800      3810      3820      3830      3840
Human                         GTACTCATGTACCCTAAAACTTAAAGTATAATAATGATAATAATAATTGTATCCATAATGATGTGGTGTTCTTTCAAATA 3840
GuineaPig                     -------------------------------------------------------------------------------- 0
NorthernAmericanDeerMouse     -------------------------------------------------------------------------------- 0
Mouse                         -------------------------------------------------------------------------------- 0
ChineseHamsterGHOK1GS         -------------------------------------------------------------------------------- 0
LongTailedChinchilla          -------------------------------------------------------------------------------- 0
```

```
Majority                      --------------------------------------------------------------------------------
                                      3850      3860      3870      3880      3890      3900      3910      3920
Human                         CTTTTGGGGATGGTACTGTGTTTTCTTGCTTATTTTCTGTCCTCTTTCCTATAGTGAAATAGATATTTGCCCATTCTTTA 3920
GuineaPig                     -------------------------------------------------------------------------------- 0
NorthernAmericanDeerMouse     -------------------------------------------------------------------------------- 0
Mouse                         -------------------------------------------------------------------------------- 0
ChineseHamsterGHOK1GS         -------------------------------------------------------------------------------- 0
LongTailedChinchilla          -------------------------------------------------------------------------------- 0

Majority                      --------------------------------------------------------------------------------
                                      3930      3940      3950      3960      3970      3980      3990      4000
Human                         GGCTTGTTTCTTGAGTTCATTTTGTACAGACTAAAGGAGGAATAATACGCTCTTTTTGGTGATAGTATTTTCCAAGTTGA 4000
GuineaPig                     -------------------------------------------------------------------------------- 0
NorthernAmericanDeerMouse     -------------------------------------------------------------------------------- 0
Mouse                         -------------------------------------------------------------------------------- 0
ChineseHamsterGHOK1GS         -------------------------------------------------------------------------------- 0
LongTailedChinchilla          -------------------------------------------------------------------------------- 0

Majority                      --------------------------------------------------------------------------------
                                      4010      4020      4030      4040      4050      4060      4070      4080
Human                         GTTAGTCATGGGATTTAAAATCAGGAATAGCTAAAGATTGCAAAATGGTAGATGTTGTAGGTTCCATCCAAACAGATTTC 4080
GuineaPig                     -------------------------------------------------------------------------------- 0
NorthernAmericanDeerMouse     -------------------------------------------------------------------------------- 0
Mouse                         -------------------------------------------------------------------------------- 0
ChineseHamsterGHOK1GS         -------------------------------------------------------------------------------- 0
LongTailedChinchilla          -------------------------------------------------------------------------------- 0

Majority                      --------------------------------------------------------------------------------
                                      4090      4100      4110      4120      4130      4140      4150      4160
Human                         TGTAAATTTTGTAAAAGAATAAATGATACAAGTGTGAGTTGTCGACTGTTAGTGGAAAAGGAGAGGAGTTTGTAGACAAG 4160
GuineaPig                     -------------------------------------------------------------------------------- 0
NorthernAmericanDeerMouse     -------------------------------------------------------------------------------- 0
Mouse                         -------------------------------------------------------------------------------- 0
ChineseHamsterGHOK1GS         -------------------------------------------------------------------------------- 0
LongTailedChinchilla          -------------------------------------------------------------------------------- 0
```

```
Majority                    --------------------------------------------------------------------------------
                                     4170      4180      4190      4200      4210      4220      4230      4240
Human                       GCTCAGAATGAGTAGACAGGAAAAAACTGAGGAGACTTGGAATAGGATGTCCAGGTGTAATGTATTGGTTACACCAGTTT 4240
GuineaPig                   -------------------------------------------------------------------------------- 0
NorthernAmericanDeerMouse   -------------------------------------------------------------------------------- 0
Mouse                       -------------------------------------------------------------------------------- 0
ChineseHamsterGHOK1GS       -------------------------------------------------------------------------------- 0
LongTailedChinchilla        -------------------------------------------------------------------------------- 0

Majority                    --------------------------------------------------------------------------------
                                     4250      4260      4270      4280      4290      4300      4310      4320
Human                       TTTTTTTTTTTTTTTGAGACAGAGTCTCACTCTTCGCCCAGGTGGAGTGCAGTGGCACGATCCTGGCTCACTACAAGCTC 4320
GuineaPig                   -------------------------------------------------------------------------------- 0
NorthernAmericanDeerMouse   -------------------------------------------------------------------------------- 0
Mouse                       -------------------------------------------------------------------------------- 0
ChineseHamsterGHOK1GS       -------------------------------------------------------------------------------- 0
LongTailedChinchilla        -------------------------------------------------------------------------------- 0

Majority                    --------------------------------------------------------------------------------
                                     4330      4340      4350      4360      4370      4380      4390      4400
Human                       CGCCTTCCGGGTTCACTCCATTCTTCTGCCTCAGCCTCCCGAGTAGCTGGGACTACAGGCGCCCACCACCACGCCCGGCT 4400
GuineaPig                   -------------------------------------------------------------------------------- 0
NorthernAmericanDeerMouse   -------------------------------------------------------------------------------- 0
Mouse                       -------------------------------------------------------------------------------- 0
ChineseHamsterGHOK1GS       -------------------------------------------------------------------------------- 0
LongTailedChinchilla        -------------------------------------------------------------------------------- 0

Majority                    --------------------------------------------------------------------------------
                                     4410      4420      4430      4440      4450      4460      4470      4480
Human                       AATTTTTTGTATTTTTAGTAGAGACGGGGTTTCACCGTGTTAGCCAGGATGGTCTTGATCTCCTGACCTTGTGGTTACAC 4480
GuineaPig                   -------------------------------------------------------------------------------- 0
NorthernAmericanDeerMouse   -------------------------------------------------------------------------------- 0
Mouse                       -------------------------------------------------------------------------------- 0
ChineseHamsterGHOK1GS       -------------------------------------------------------------------------------- 0
LongTailedChinchilla        -------------------------------------------------------------------------------- 0
```

```
Majority                    --------------------------------------------------------------------------------
                                     4490      4500      4510      4520      4530      4540      4550      4560
Human                       CAGTTTTTTGTTAGTTGTTTGAGGAGAACACTTTAGAAGATGATAAAAAAAAATTGCAAATCGTTTTTAAAAATCCATTG 4560
GuineaPig                   -------------------------------------------------------------------------------- 0
NorthernAmericanDeerMouse   -------------------------------------------------------------------------------- 0
Mouse                       -------------------------------------------------------------------------------- 0
ChineseHamsterGHOK1GS       -------------------------------------------------------------------------------- 0
LongTailedChinchilla        -------------------------------------------------------------------------------- 0

Majority                    --------------------------------------------------------------------------------
                                     4570      4580      4590      4600      4610      4620      4630      4640
Human                       TTTAATATAAAAGTCTGAATAATTAAAGTTAATTTAATTTTCTGGAATAATGGTTAAGAATTTTTGCTTTGGTGTCAGGC 4640
GuineaPig                   -------------------------------------------------------------------------------- 0
NorthernAmericanDeerMouse   -------------------------------------------------------------------------------- 0
Mouse                       -------------------------------------------------------------------------------- 0
ChineseHamsterGHOK1GS       -------------------------------------------------------------------------------- 0
LongTailedChinchilla        -------------------------------------------------------------------------------- 0

Majority                    --------------------------------------------------------------------------------
                                     4650      4660      4670      4680      4690      4700      4710      4720
Human                       CTGTGTACAAGTAATTAAGTGTTCTTTAAAACTTAAAACATTTGTGAATTTGCTTTAAAGACACTGATGCATGTGCATAT 4720
GuineaPig                   -------------------------------------------------------------------------------- 0
NorthernAmericanDeerMouse   -------------------------------------------------------------------------------- 0
Mouse                       -------------------------------------------------------------------------------- 0
ChineseHamsterGHOK1GS       -------------------------------------------------------------------------------- 0
LongTailedChinchilla        -------------------------------------------------------------------------------- 0

Majority                    --------------------------------------------------------------------------------
                                     4730      4740      4750      4760      4770      4780      4790      4800
Human                       TTAAAAAAATTCAGGCCGGGCGCGGTGGGTCACACCTGTAATCCCAGCACTTTGGGAGGCCGAGGCGGGCGGATCACGAG 4800
GuineaPig                   -------------------------------------------------------------------------------- 0
NorthernAmericanDeerMouse   -------------------------------------------------------------------------------- 0
Mouse                       -------------------------------------------------------------------------------- 0
ChineseHamsterGHOK1GS       -------------------------------------------------------------------------------- 0
LongTailedChinchilla        -------------------------------------------------------------------------------- 0
```

Majority	--	
	4810 4820 4830 4840 4850 4860 4870 4880	
Human	TTCAGGAGAGACCATCCTGGCTAACACGGTGAAACCCTGTCTTTACTAAAAATACAAAAAATTAGCCGGGTGTGGTGACG	4880
GuineaPig	--	0
NorthernAmericanDeerMouse	--	0
Mouse	--	0
ChineseHamsterGHOK1GS	--	0
LongTailedChinchilla	--	0
Majority	--	
	4890 4900 4910 4920 4930 4940 4950 4960	
Human	GGTGCCTGTAGTCCCAGCTACTCGGGAGGCTGAGGCAGGAGAATGGCATGAACCTGCGAGGCAGAGGTTGCAGTAAGCGG	4960
GuineaPig	--	0
NorthernAmericanDeerMouse	--	0
Mouse	--	0
ChineseHamsterGHOK1GS	--	0
LongTailedChinchilla	--	0
Majority	--	
	4970 4980 4990 5000 5010 5020 5030 5040	
Human	AGATTGCACCACTGCACTCCAGCCTGGGCGACAGAGACAGACTCTATCTCAAAAAAAAAAAAAAAAAAAAAAAAAAAAAA	5040
GuineaPig	--	0
NorthernAmericanDeerMouse	--	0
Mouse	--	0
ChineseHamsterGHOK1GS	--	0
LongTailedChinchilla	--	0
Majority	--	
	5050 5060 5070 5080 5090 5100 5110 5120	
Human	AAAAAAATTCAAACTGCTTCACAGCTTTTTTTGCTATTTCTTAGGTGTAGGTAATTGTGTTAAAATTTTGTGCCTTTTTT	5120
GuineaPig	--	0
NorthernAmericanDeerMouse	--	0
Mouse	--	0
ChineseHamsterGHOK1GS	--	0
LongTailedChinchilla	--	0

```
Majority                      --------------------------------------------------------------------------------
                                       5130      5140      5150      5160      5170      5180      5190      5200
Human                         CTTGAAATTATTAATTTATATATTAGCACACATATAAACATCTTTATTTACGTGATTGAATTCAAACTATTGTTTTGGCC 5200
GuineaPig                     -------------------------------------------------------------------------------- 0
NorthernAmericanDeerMouse     -------------------------------------------------------------------------------- 0
Mouse                         -------------------------------------------------------------------------------- 0
ChineseHamsterGHOK1GS         -------------------------------------------------------------------------------- 0
LongTailedChinchilla          -------------------------------------------------------------------------------- 0

Majority                      --------------------------------------------------------------------------------
                                       5210      5220      5230      5240      5250      5260      5270      5280
Human                         CACATCTTGCCTTTTTTTTTTTTTTTTGAGTCGGAGTCTCCCTCTGTCATCAGGCTGGAGTGCAGTGGCATGATCTCGGC 5280
GuineaPig                     -------------------------------------------------------------------------------- 0
NorthernAmericanDeerMouse     -------------------------------------------------------------------------------- 0
Mouse                         -------------------------------------------------------------------------------- 0
ChineseHamsterGHOK1GS         -------------------------------------------------------------------------------- 0
LongTailedChinchilla          -------------------------------------------------------------------------------- 0

Majority                      --------------------------------------------------------------------------------
                                       5290      5300      5310      5320      5330      5340      5350      5360
Human                         TTACTGCAACCTCTGCCTCCTGGGTTCAGGCAATTCTCCTGCCTCAGCCTCCCTAGTAGCTGGGACTACAGGCACACGCC 5360
GuineaPig                     -------------------------------------------------------------------------------- 0
NorthernAmericanDeerMouse     -------------------------------------------------------------------------------- 0
Mouse                         -------------------------------------------------------------------------------- 0
ChineseHamsterGHOK1GS         -------------------------------------------------------------------------------- 0
LongTailedChinchilla          -------------------------------------------------------------------------------- 0

Majority                      --------------------------------------------------------------------------------
                                       5370      5380      5390      5400      5410      5420      5430      5440
Human                         GCCATGCCAGACTAATTTTTTTTTTTTTTTCGAGACGGAGTCTCGCCCGGTCTCCCAGGCTGGAGTGCAGTGTCGCGATC 5440
GuineaPig                     -------------------------------------------------------------------------------- 0
NorthernAmericanDeerMouse     -------------------------------------------------------------------------------- 0
Mouse                         -------------------------------------------------------------------------------- 0
ChineseHamsterGHOK1GS         -------------------------------------------------------------------------------- 0
LongTailedChinchilla          -------------------------------------------------------------------------------- 0
```

Majority	--	
	5450 5460 5470 5480 5490 5500 5510 5520	
Human	TCTGCTCACTGCAACCTCTGCCTCCTGGGTTCACGCCATTCTCCTGTCTCAGCCTCCCAAGTAGCTGGGACTACAGGCGC	5520
GuineaPig	--	0
NorthernAmericanDeerMouse	--	0
Mouse	--	0
ChineseHamsterGHOK1GS	--	0
LongTailedChinchilla	--	0

Majority	--	
	5530 5540 5550 5560 5570 5580 5590 5600	
Human	CTGCCACCACGCCTGGCTTCTTTTTTGTATATTCAGTAGAGACAGAGTTTCACCATGTTAGCCAGGATAGTCTCTATCTC	5600
GuineaPig	--	0
NorthernAmericanDeerMouse	--	0
Mouse	--	0
ChineseHamsterGHOK1GS	--	0
LongTailedChinchilla	--	0

Majority	--	
	5610 5620 5630 5640 5650 5660 5670 5680	
Human	CTGACCTCGTGATCTGCCTGCCTCGGCCTCCCAAAGTGCTGGGATTACAAGCGTGAGCCACCGCGCCTAGCCAAAATGCC	5680
GuineaPig	--	0
NorthernAmericanDeerMouse	--	0
Mouse	--	0
ChineseHamsterGHOK1GS	--	0
LongTailedChinchilla	--	0

Majority	--	
	5690 5700 5710 5720 5730 5740 5750 5760	
Human	AGACTAATTTTTGTATTTTTAGTAGAGACAGGGTTTCACCATTTTGGCCAGGATGGTCTTGATCTCTTGACTCCATCATC	5760
GuineaPig	--	0
NorthernAmericanDeerMouse	--	0
Mouse	--	0
ChineseHamsterGHOK1GS	--	0
LongTailedChinchilla	--	0

Majority	--	
	5770 5780 5790 5800 5810 5820 5830 5840	
Human	CGCCCGCCTTGACTTCCCAAAGTGTTGGGATTACAGGCTTGAGCCACCGCGTCCGGCCCATATCTTGCCTTTAAAAAAAA	5840
GuineaPig	--	0
NorthernAmericanDeerMouse	--	0
Mouse	--	0
ChineseHamsterGHOK1GS	--	0
LongTailedChinchilla	--	0

Majority	--	
	5850 5860 5870 5880 5890 5900 5910 5920	
Human	TAGCTTTATTGAGATATAATTTGACATAAAATAAACTACCCATATTAAATATACAATGTTTTATCTCATATATACCCTTG	5920
GuineaPig	--	0
NorthernAmericanDeerMouse	--	0
Mouse	--	0
ChineseHamsterGHOK1GS	--	0
LongTailedChinchilla	--	0

Majority	--	
	5930 5940 5950 5960 5970 5980 5990 6000	
Human	TGAAACCACTGCAATCAAGATAATGAACATCTATCACTCCCTACAGTATCATCTTGCGCCTTTATAGTCCCTCCTTTCTG	6000
GuineaPig	--	0
NorthernAmericanDeerMouse	--	0
Mouse	--	0
ChineseHamsterGHOK1GS	--	0
LongTailedChinchilla	--	0

Majority	--	
	6010 6020 6030 6040 6050 6060 6070 6080	
Human	CTTTTCTTTCCCCAAGGAACCACTGATCTGCTTTCTGTCACTATAGATTAGTTCACACTTTTTAGAATTTTAGATAAATG	6080
GuineaPig	--	0
NorthernAmericanDeerMouse	--	0
Mouse	--	0
ChineseHamsterGHOK1GS	--	0
LongTailedChinchilla	--	0

Majority	--	
	6090 6100 6110 6120 6130 6140 6150 6160	
Human	TAATCATATGGTATGTACATTTTTGGTCTGGGTTAGTCCACTCAGAATAATTATTTTGAAATTCACCCATGTTGTCTTTC	6160
GuineaPig	--	0
NorthernAmericanDeerMouse	--	0
Mouse	--	0
ChineseHamsterGHOK1GS	--	0
LongTailedChinchilla	--	0

Majority	--	
	6170 6180 6190 6200 6210 6220 6230 6240	
Human	AATAGTCCATTCTGTTTTATTGCTTAGTAATAGTACACTTTATGGCTATACAGCAATTTGTTAATCCATTCAAGTCTTGA	6240
GuineaPig	--	0
NorthernAmericanDeerMouse	--	0
Mouse	--	0
ChineseHamsterGHOK1GS	--	0
LongTailedChinchilla	--	0

Majority	--	
	6250 6260 6270 6280 6290 6300 6310 6320	
Human	GGGACATTTGTATTGTTTTCCGTTTTGAGCTAATAAAAATATAGTTGTGAACATTACTGTACAAGTCTTTGTATGGACAT	6320
GuineaPig	--	0
NorthernAmericanDeerMouse	--	0
Mouse	--	0
ChineseHamsterGHOK1GS	--	0
LongTailedChinchilla	--	0

Majority	--	
	6330 6340 6350 6360 6370 6380 6390 6400	
Human	ATGCTTTCATTTCTCTTGGTGGGGCAGGCTATGATTGGAAAGTCTGGATCATGTGATAGGCATATGTTTAACTTTTTAAG	6400
GuineaPig	--	0
NorthernAmericanDeerMouse	--	0
Mouse	--	0
ChineseHamsterGHOK1GS	--	0
LongTailedChinchilla	--	0

```
Majority                     --------------------------------------------------------------------------------
                                         6410      6420      6430      6440      6450      6460      6470      6480
Human                        GAACTGCAAACTTTTTAAAAGTAGTTGTACCATCTTACATTGCCATCAGCATGGTATGAAATTTCCAGTCCTTCCACATC  6480
GuineaPig                    --------------------------------------------------------------------------------  0
NorthernAmericanDeerMouse    --------------------------------------------------------------------------------  0
Mouse                        --------------------------------------------------------------------------------  0
ChineseHamsterGHOK1GS        --------------------------------------------------------------------------------  0
LongTailedChinchilla         --------------------------------------------------------------------------------  0

Majority                     --------------------------------------------------------------------------------
                                         6490      6500      6510      6520      6530      6540      6550      6560
Human                        CTCTTTAATGCCTGTTATCTTCTGTTTTTGATTATAGATATTGTAGTGTATATGAAGTGATATTTCACTGTGGATTTAAT  6560
GuineaPig                    --------------------------------------------------------------------------------  0
NorthernAmericanDeerMouse    --------------------------------------------------------------------------------  0
Mouse                        --------------------------------------------------------------------------------  0
ChineseHamsterGHOK1GS        --------------------------------------------------------------------------------  0
LongTailedChinchilla         --------------------------------------------------------------------------------  0

Majority                     --------------------------------------------------------------------------------
                                         6570      6580      6590      6600      6610      6620      6630      6640
Human                        TTGCATTCCTCTAATATCTATGATGTTGGACATCTTTTCGTGTGCTTTTTGTCAGCTGTTATCTTTGGTGAAGTGTCTGT  6640
GuineaPig                    --------------------------------------------------------------------------------  0
NorthernAmericanDeerMouse    --------------------------------------------------------------------------------  0
Mouse                        --------------------------------------------------------------------------------  0
ChineseHamsterGHOK1GS        --------------------------------------------------------------------------------  0
LongTailedChinchilla         --------------------------------------------------------------------------------  0

Majority                     --------------------------------------------------------------------------------
                                         6650      6660      6670      6680      6690      6700      6710      6720
Human                        TTATATCTTTAGCCCAATTTTTTATTTGGTTATTTATTTTAAATTGAGTTTTGAGAGGTCTTCATTGTGGTTACAAGTCC  6720
GuineaPig                    --------------------------------------------------------------------------------  0
NorthernAmericanDeerMouse    --------------------------------------------------------------------------------  0
Mouse                        --------------------------------------------------------------------------------  0
ChineseHamsterGHOK1GS        --------------------------------------------------------------------------------  0
LongTailedChinchilla         --------------------------------------------------------------------------------  0
```

Majority	--	
	6730 6740 6750 6760 6770 6780 6790 6800	
Human	TTTATCAGCCATAAGGCCTGCAAATATTTTCTGCCACTCATCTTCTTATTCTCGCATTGTCTTTTGAAGAGCAACAGTTA	6800
GuineaPig	--	0
NorthernAmericanDeerMouse	--	0
Mouse	--	0
ChineseHamsterGHOK1GS	--	0
LongTailedChinchilla	--	0

Majority	--	
	6810 6820 6830 6840 6850 6860 6870 6880	
Human	TTAAATCTGATGGACTCCAGTTTACCAATTTTTTCTTTTATGGATTGTAGTTTTGGGGTCTTGTCTGAGCCATTTTTGCC	6880
GuineaPig	--	0
NorthernAmericanDeerMouse	--	0
Mouse	--	0
ChineseHamsterGHOK1GS	--	0
LongTailedChinchilla	--	0

Majority	--	
	6890 6900 6910 6920 6930 6940 6950 6960	
Human	TGACTGTAAGCCATGAAGATTTTTTTCCTTTTGTTTGTTAATAATAATAATAATTTTTTTTTAGAGACAGGACCTGACTC	6960
GuineaPig	--	0
NorthernAmericanDeerMouse	--	0
Mouse	--	0
ChineseHamsterGHOK1GS	--	0
LongTailedChinchilla	--	0

Majority	--	
	6970 6980 6990 7000 7010 7020 7030 7040	
Human	TGTCACCCCAGCTGGAGTGCAGTGGTGCAATCGTAGCTCATGATAACCTTGAACTCCTGGACACAAGGGCTCCTGCTTCA	7040
GuineaPig	--	0
NorthernAmericanDeerMouse	--	0
Mouse	--	0
ChineseHamsterGHOK1GS	--	0
LongTailedChinchilla	--	0

Majority	--	
	7050 7060 7070 7080 7090 7100 7110 7120	
Human	GCCTCCCAAGTAGCTAGGACTATAGGCACACAGCACCATGCCCAGCTAACTTTTAAATTTTTTATAGACCCGGGGTCTCT	7120
GuineaPig	--	0
NorthernAmericanDeerMouse	--	0
Mouse	--	0
ChineseHamsterGHOK1GS	--	0
LongTailedChinchilla	--	0

Majority	--	
	7130 7140 7150 7160 7170 7180 7190 7200	
Human	CTGTTTTGCCCAGGCTGGTGTTGAACTCCTGGCCTCAAGCGATCTTTCTGCCTCAGCCTTCCAAAGTGTTGGGATTAAAG	7200
GuineaPig	--	0
NorthernAmericanDeerMouse	--	0
Mouse	--	0
ChineseHamsterGHOK1GS	--	0
LongTailedChinchilla	--	0

Majority	--	
	7210 7220 7230 7240 7250 7260 7270 7280	
Human	GCATGAGCCACTACACTTGGCCACCTTTGTTTTCTAATAGAAGTTTTATACTTTGGTTTAACATTTGGGTCTCTTTCATT	7280
GuineaPig	--	0
NorthernAmericanDeerMouse	--	0
Mouse	--	0
ChineseHamsterGHOK1GS	--	0
LongTailedChinchilla	--	0

Majority	--	
	7290 7300 7310 7320 7330 7340 7350 7360	
Human	TTGACTTAATATTTTGAATATGGTACAAAGTGTGGGTCAGAAGTATTTGGTTTTGCATGGGGATATCCAGTGTTTACAGC	7360
GuineaPig	--	0
NorthernAmericanDeerMouse	--	0
Mouse	--	0
ChineseHamsterGHOK1GS	--	0
LongTailedChinchilla	--	0

Sequence	7370 … 7440	Count
Majority	--	
Human	ATCATTTGTTGAAAAGACTATCCTTTCTCCATTCACTTGGCTTTGCAGCTTCATGAAAAATCACTTATGTCTGTATGTGT	7440
GuineaPig	--	0
NorthernAmericanDeerMouse	--	0
Mouse	--	0
ChineseHamsterGHOK1GS	--	0
LongTailedChinchilla	--	0

Sequence	7450 … 7520	Count
Majority	--	
Human	GATTTTATGTCTGTACTCTTTTTTGTATTGACCCATTTTCCTTTATCTAGATGCTGGAAACATACTGTCTTAATCATTGT	7520
GuineaPig	--	0
NorthernAmericanDeerMouse	--	0
Mouse	--	0
ChineseHamsterGHOK1GS	--	0
LongTailedChinchilla	--	0

Sequence	7530 … 7600	Count
Majority	--	
Human	AGCTTTATATTGAGTCTTGAAACCACTTACTTAGTCTTGGCCCTCCAACTCTGTTCTTTTTCAAAGTTGTTTTTAACTCT	7600
GuineaPig	--	0
NorthernAmericanDeerMouse	--	0
Mouse	--	0
ChineseHamsterGHOK1GS	--	0
LongTailedChinchilla	--	0

Sequence	7610 … 7680	Count
Majority	--	
Human	TCTGAGTCCTTTGTATTTTCATATGAGTCAGTTCTTAGAAAAACTGCTTGGATTTTACTGGATTTGACTTGAATCTGTAG	7680
GuineaPig	--	0
NorthernAmericanDeerMouse	--	0
Mouse	--	0
ChineseHamsterGHOK1GS	--	0
LongTailedChinchilla	--	0

```
Majority                    --------------------------------------------------------------------------------
                                     7690      7700      7710      7720      7730      7740      7750      7760
Human                       AGCAAATTTGGGGAAAATTGGCATCATGACTACAATTGATGTCTCTATTAGGTCTTTAATTTCTTTAAGCATTTTTTTTT 7760
GuineaPig                   -------------------------------------------------------------------------------- 0
NorthernAmericanDeerMouse   -------------------------------------------------------------------------------- 0
Mouse                       -------------------------------------------------------------------------------- 0
ChineseHamsterGHOK1GS       -------------------------------------------------------------------------------- 0
LongTailedChinchilla        -------------------------------------------------------------------------------- 0

Majority                    --------------------------------------------------------------------------------
                                     7770      7780      7790      7800      7810      7820      7830      7840
Human                       TTTTTTTCAGTGCACACGTTTTGTGCATTGTTTGATTAGATTTACCCATAAGTATTTTATGTATTTTGAAGTACTGTAAA 7840
GuineaPig                   -------------------------------------------------------------------------------- 0
NorthernAmericanDeerMouse   -------------------------------------------------------------------------------- 0
Mouse                       -------------------------------------------------------------------------------- 0
ChineseHamsterGHOK1GS       -------------------------------------------------------------------------------- 0
LongTailedChinchilla        -------------------------------------------------------------------------------- 0

Majority                    --------------------------------------------------------------------------------
                                     7850      7860      7870      7880      7890      7900      7910      7920
Human                       TGATATTTTAAAAACTTAATTTCCAATTGTTTGCTAGTGTATAGAAATGATTATGGCATATTGCTCATATGTTCTGAACC 7920
GuineaPig                   -------------------------------------------------------------------------------- 0
NorthernAmericanDeerMouse   -------------------------------------------------------------------------------- 0
Mouse                       -------------------------------------------------------------------------------- 0
ChineseHamsterGHOK1GS       -------------------------------------------------------------------------------- 0
LongTailedChinchilla        -------------------------------------------------------------------------------- 0

Majority                    --------------------------------------------------------------------------------
                                     7930      7940      7950      7960      7970      7980      7990      8000
Human                       TTTGCTAAACTCATTTAGTAGATCTAATAGTTTTTTTTATATATTTCATCAGATTTGCTTGAGAGGGAGTCTCAGTCACC 8000
GuineaPig                   -------------------------------------------------------------------------------- 0
NorthernAmericanDeerMouse   -------------------------------------------------------------------------------- 0
Mouse                       -------------------------------------------------------------------------------- 0
ChineseHamsterGHOK1GS       -------------------------------------------------------------------------------- 0
LongTailedChinchilla        -------------------------------------------------------------------------------- 0
```

```
Majority                    --------------------------------------------------------------------------------
                                           8010      8020      8030      8040      8050      8060      8070      8080
Human                       CAGGCTGGAGTGCAGTGACGTGATCTTGGCTCACTGCAACCTGCACCTCCTGGGTCAAGCAATTCTCCTGCCTCAGCCTC 8080
GuineaPig                   -------------------------------------------------------------------------------- 0
NorthernAmericanDeerMouse   -------------------------------------------------------------------------------- 0
Mouse                       -------------------------------------------------------------------------------- 0
ChineseHamsterGHOK1GS       -------------------------------------------------------------------------------- 0
LongTailedChinchilla        -------------------------------------------------------------------------------- 0

Majority                    --------------------------------------------------------------------------------
                                           8090      8100      8110      8120      8130      8140      8150      8160
Human                       ATAAGTAGCTGGGACTGCAGGTGCACACCACCACGCCCAGCTAATTTTTGCATTTTCAGTTGAGGCAGGGTTTCACCATG 8160
GuineaPig                   -------------------------------------------------------------------------------- 0
NorthernAmericanDeerMouse   -------------------------------------------------------------------------------- 0
Mouse                       -------------------------------------------------------------------------------- 0
ChineseHamsterGHOK1GS       -------------------------------------------------------------------------------- 0
LongTailedChinchilla        -------------------------------------------------------------------------------- 0

Majority                    --------------------------------------------------------------------------------
                                           8170      8180      8190      8200      8210      8220      8230      8240
Human                       TTGGCCAGGCTGGTCTGGAACTCCTGACCTCAAGTGATACACTTACCTAGGCCTCCCAAAGTGTTAAGATTACAGGCGTG 8240
GuineaPig                   -------------------------------------------------------------------------------- 0
NorthernAmericanDeerMouse   -------------------------------------------------------------------------------- 0
Mouse                       -------------------------------------------------------------------------------- 0
ChineseHamsterGHOK1GS       -------------------------------------------------------------------------------- 0
LongTailedChinchilla        -------------------------------------------------------------------------------- 0

Majority                    --------------------------------------------------------------------------------
                                           8250      8260      8270      8280      8290      8300      8310      8320
Human                       AGCCACCACCCCCAGCCTATGCCATTAAATTTTCTACATAGATAATTTATACCCAATTGTTTTTAAATAGTTTTCTTTCT 8320
GuineaPig                   -------------------------------------------------------------------------------- 0
NorthernAmericanDeerMouse   -------------------------------------------------------------------------------- 0
Mouse                       -------------------------------------------------------------------------------- 0
ChineseHamsterGHOK1GS       -------------------------------------------------------------------------------- 0
LongTailedChinchilla        -------------------------------------------------------------------------------- 0
```

```
Majority                      --------------------------------------------------------------------------------
                                        8330      8340      8350      8360      8370      8380      8390      8400
Human                         TTTCTATCTGGATGCCATTTTTTGTCTCCTTTTTTTGTCTGTGGTATCCAGTACAATGTTTAATAGAAGTGTTGAGAGAA 8400
GuineaPig                     -------------------------------------------------------------------------------- 0
NorthernAmericanDeerMouse     -------------------------------------------------------------------------------- 0
Mouse                         -------------------------------------------------------------------------------- 0
ChineseHamsterGHOK1GS         -------------------------------------------------------------------------------- 0
LongTailedChinchilla          -------------------------------------------------------------------------------- 0

Majority                      --------------------------------------------------------------------------------
                                        8410      8420      8430      8440      8450      8460      8470      8480
Human                         TATCTTTTCCTTGCTCCTGATATTAGGGTGAGAACTCTAAAATTTCTTCCTTAACTGACACAGTTCACTAGTGAAGCCAT 8480
GuineaPig                     -------------------------------------------------------------------------------- 0
NorthernAmericanDeerMouse     -------------------------------------------------------------------------------- 0
Mouse                         -------------------------------------------------------------------------------- 0
ChineseHamsterGHOK1GS         -------------------------------------------------------------------------------- 0
LongTailedChinchilla          -------------------------------------------------------------------------------- 0

Majority                      --------------------------------------------------------------------------------
                                        8490      8500      8510      8520      8530      8540      8550      8560
Human                         GTGGGGCCAGATGTTTGTTTTGTCGGGAGGTTTTAAACTACATATTCAACTTATTTAATAGATATAGGGCTATTCAGTTT 8560
GuineaPig                     -------------------------------------------------------------------------------- 0
NorthernAmericanDeerMouse     -------------------------------------------------------------------------------- 0
Mouse                         -------------------------------------------------------------------------------- 0
ChineseHamsterGHOK1GS         -------------------------------------------------------------------------------- 0
LongTailedChinchilla          -------------------------------------------------------------------------------- 0

Majority                      --------------------------------------------------------------------------------
                                        8570      8580      8590      8600      8610      8620      8630      8640
Human                         GTTTAGTTCTTCTTGAGTGAACTTTGGTAATTTGTATCTTCTGTAGAACTAGTCCATTTCCTTTAAGTTGCTGAATGTAT 8640
GuineaPig                     -------------------------------------------------------------------------------- 0
NorthernAmericanDeerMouse     -------------------------------------------------------------------------------- 0
Mouse                         -------------------------------------------------------------------------------- 0
ChineseHamsterGHOK1GS         -------------------------------------------------------------------------------- 0
LongTailedChinchilla          -------------------------------------------------------------------------------- 0
```

Majority	--	
	8650 8660 8670 8680 8690 8700 8710 8720	
Human	TTGTGTAGAGTTGTTCATGATACTACTATTTTTTTTTTTTTTTTTTTTTTGAGACGGAGCCTTGCTCTGTCGCCCGGGCT	8720
GuineaPig	--	0
NorthernAmericanDeerMouse	--	0
Mouse	--	0
ChineseHamsterGHOK1GS	--	0
LongTailedChinchilla	--	0

Majority	--	
	8730 8740 8750 8760 8770 8780 8790 8800	
Human	GGAGTGCAGTGGCACCATCTCGGCTCAATGCAACCTCCACGTCCCGGGTTCACGCGGTTCTCCTGCCTCAGCATCCCAAG	8800
GuineaPig	--	0
NorthernAmericanDeerMouse	--	0
Mouse	--	0
ChineseHamsterGHOK1GS	--	0
LongTailedChinchilla	--	0

Majority	--	
	8810 8820 8830 8840 8850 8860 8870 8880	
Human	TAGCTGGGATCACAGGCACACACCACCACACCTGGCTAATTTTTTGTATTTTTAGTAGAGACGGGGTTTCACTATGTTGG	8880
GuineaPig	--	0
NorthernAmericanDeerMouse	--	0
Mouse	--	0
ChineseHamsterGHOK1GS	--	0
LongTailedChinchilla	--	0

Majority	--	
	8890 8900 8910 8920 8930 8940 8950 8960	
Human	CCAGGTTGGTCTCGAACTTGTGATCTGCCCGCCCTGCCTCACAAAGTGCTGGGATTACAGGCGTGAGCCACTGCGCCCAG	8960
GuineaPig	--	0
NorthernAmericanDeerMouse	--	0
Mouse	--	0
ChineseHamsterGHOK1GS	--	0
LongTailedChinchilla	--	0

Sequence	8970 · 8980 · 8990 · 9000 · 9010 · 9020 · 9030 · 9040	
Majority	-	
Human	CCATGATACTACATTTTTACACTTTTGGTATCTGTTGAATCCTTAGTGATGTCAGCTTTCTCATTCCCAGTAGGGATAAT	9040
GuineaPig	-	0
NorthernAmericanDeerMouse	-	0
Mouse	-	0
ChineseHamsterGHOK1GS	-	0
LongTailedChinchilla	-	0

Sequence	9050 · 9060 · 9070 · 9080 · 9090 · 9100 · 9110 · 9120	
Majority	-	
Human	TTGTGTCATCTTTTTTCCTTGTCTGTCTGGATTGAAGTTTATTGATTTTATTAATCTCCAAAATACCAGCTTTTGGTTTG	9120
GuineaPig	-	0
NorthernAmericanDeerMouse	-	0
Mouse	-	0
ChineseHamsterGHOK1GS	-	0
LongTailedChinchilla	-	0

Sequence	9130 · 9140 · 9150 · 9160 · 9170 · 9180 · 9190 · 9200	
Majority	-	
Human	AGTTTTCTCTGCTGTTTTTCTGTTTTCTATTTTATTGATTTCTGCTATTTTTATTTTGTTTTGCTTTGTGGTTAGTTTGA	9200
GuineaPig	-	0
NorthernAmericanDeerMouse	-	0
Mouse	-	0
ChineseHamsterGHOK1GS	-	0
LongTailedChinchilla	-	0

Sequence	9210 · 9220 · 9230 · 9240 · 9250 · 9260 · 9270 · 9280	
Majority	-	
Human	TCTTCATTTTCTACTTAGTTAACATTGAAGTTAATGTCATTAACTTAGTACTTTTACTTATATAGGGATTTATTGCTGTA	9280
GuineaPig	-	0
NorthernAmericanDeerMouse	-	0
Mouse	-	0
ChineseHamsterGHOK1GS	-	0
LongTailedChinchilla	-	0

```
Majority                     --------------------------------------------------------------------------------
                                      9290      9300      9310      9320      9330      9340      9350      9360
Human                        TGTTTCCCTTTTAAGTACCGCTTTCACGGCATTCCTCAAATTTTGACACGTATATTTTTATTCACTTCAAAATACTTTTT 9360
GuineaPig                    -------------------------------------------------------------------------------- 0
NorthernAmericanDeerMouse    -------------------------------------------------------------------------------- 0
Mouse                        -------------------------------------------------------------------------------- 0
ChineseHamsterGHOK1GS        -------------------------------------------------------------------------------- 0
LongTailedChinchilla         -------------------------------------------------------------------------------- 0

Majority                     --------------------------------------------------------------------------------
                                      9370      9380      9390      9400      9410      9420      9430      9440
Human                        TAAATATTTCTTTGACCCATGGGTTATTGGAAAGTATGTTATTTATGTTATTTAATTTCCAAATATTTGGAGTTTTCCAT 9440
GuineaPig                    -------------------------------------------------------------------------------- 0
NorthernAmericanDeerMouse    -------------------------------------------------------------------------------- 0
Mouse                        -------------------------------------------------------------------------------- 0
ChineseHamsterGHOK1GS        -------------------------------------------------------------------------------- 0
LongTailedChinchilla         -------------------------------------------------------------------------------- 0

Majority                     --------------------------------------------------------------------------------
                                      9450      9460      9470      9480      9490      9500      9510      9520
Human                        ATATCTTTCTGTTATGTATTCATAATTTTAGTTTTATTCTGGCCAGAGAACATATTTTCTAAGACTTGGATCTTTGTAAA 9520
GuineaPig                    -------------------------------------------------------------------------------- 0
NorthernAmericanDeerMouse    -------------------------------------------------------------------------------- 0
Mouse                        -------------------------------------------------------------------------------- 0
ChineseHamsterGHOK1GS        -------------------------------------------------------------------------------- 0
LongTailedChinchilla         -------------------------------------------------------------------------------- 0

Majority                     --------------------------------------------------------------------------------
                                      9530      9540      9550      9560      9570      9580      9590      9600
Human                        CATGGAGACTTGTTTTGTGGTCCAAATATATATGTTGTTAAGTATTCACTGAATACTCTGATGATTCTCTGCAGATTTGG 9600
GuineaPig                    -------------------------------------------------------------------------------- 0
NorthernAmericanDeerMouse    -------------------------------------------------------------------------------- 0
Mouse                        -------------------------------------------------------------------------------- 0
ChineseHamsterGHOK1GS        -------------------------------------------------------------------------------- 0
LongTailedChinchilla         -------------------------------------------------------------------------------- 0
```

```
Majority                    --------------------------------------------------------------------------------
                                     9610      9620      9630      9640      9650      9660      9670      9680
Human                       AGAAATCTCTGTGCAGTTCTTTTCTCTGGTACTTTGACCTGTATGTAAACTCTAGTTACTTTGGTCTTCTCAGGCTCTTG 9680
GuineaPig                   -------------------------------------------------------------------------------- 0
NorthernAmericanDeerMouse   -------------------------------------------------------------------------------- 0
Mouse                       -------------------------------------------------------------------------------- 0
ChineseHamsterGHOK1GS       -------------------------------------------------------------------------------- 0
LongTailedChinchilla        -------------------------------------------------------------------------------- 0

Majority                    --------------------------------------------------------------------------------
                                     9690      9700      9710      9720      9730      9740      9750      9760
Human                       GCTCTTTCACAATTAAAGTAGTCTTTGAGGCTCAGCCTGCTTTCCTCATAGCTATGCTATGGCCTGGACACTCAAGGGAG 9760
GuineaPig                   -------------------------------------------------------------------------------- 0
NorthernAmericanDeerMouse   -------------------------------------------------------------------------------- 0
Mouse                       -------------------------------------------------------------------------------- 0
ChineseHamsterGHOK1GS       -------------------------------------------------------------------------------- 0
LongTailedChinchilla        -------------------------------------------------------------------------------- 0

Majority                    --------------------------------------------------------------------------------
                                     9770      9780      9790      9800      9810      9820      9830      9840
Human                       TATAAGCTGAGGCAAACATGGACTCATTTGTTTTCTAACTTTCAGGGATTATTGTCCATCATTGCCTGATGTCCAGTGTC 9840
GuineaPig                   -------------------------------------------------------------------------------- 0
NorthernAmericanDeerMouse   -------------------------------------------------------------------------------- 0
Mouse                       -------------------------------------------------------------------------------- 0
ChineseHamsterGHOK1GS       -------------------------------------------------------------------------------- 0
LongTailedChinchilla        -------------------------------------------------------------------------------- 0

Majority                    --------------------------------------------------------------------------------
                                     9850      9860      9870      9880      9890      9900      9910      9920
Human                       TTGAAAAGCAATTATTCTGTATAGTTGCTTGATTGTTTGGTGTTGTTTCGGCGAGGCAAATCTGGGTCATGTTATTCTGT 9920
GuineaPig                   -------------------------------------------------------------------------------- 0
NorthernAmericanDeerMouse   -------------------------------------------------------------------------------- 0
Mouse                       -------------------------------------------------------------------------------- 0
ChineseHamsterGHOK1GS       -------------------------------------------------------------------------------- 0
LongTailedChinchilla        -------------------------------------------------------------------------------- 0
```

Majority	--	
	9930 9940 9950 9960 9970 9980 9990 10000	
Human	CTTGACTGGAAGTAGAAGTCCACTGTTTTTTTTTAATTACATAATATGGTCTCAGCAGTTGTAGATCTACCTCATTCTTT	10000
GuineaPig	--	0
NorthernAmericanDeerMouse	--	0
Mouse	--	0
ChineseHamsterGHOK1GS	--	0
LongTailedChinchilla	--	0
Majority	--	
	10010 10020 10030 10040 10050 10060 10070 10080	
Human	GTTAATGACAATGGAGGATACTCGTGTGGCTGCACCACAGTGTAGTTACTGGACATACCACAATTTAGTTTCCTACATCG	10080
GuineaPig	--	0
NorthernAmericanDeerMouse	--	0
Mouse	--	0
ChineseHamsterGHOK1GS	--	0
LongTailedChinchilla	--	0
Majority	--	
	10090 10100 10110 10120 10130 10140 10150 10160	
Human	ATGGACATTTAGGTTTGATTCCAGTTTGTTGTTATAACATGTGGACATATTTAGAATAGAAGTAAAAATGCTGTGTCAAA	10160
GuineaPig	--	0
NorthernAmericanDeerMouse	--	0
Mouse	--	0
ChineseHamsterGHOK1GS	--	0
LongTailedChinchilla	--	0
Majority	--	
	10170 10180 10190 10200 10210 10220 10230 10240	
Human	TGAAATTTATACTTAAAGAGTTACTGAAAACCACAATGCCAGAACACTCATTTGTTTTTCACAAGTGTGTATGGGAGTGC	10240
GuineaPig	--	0
NorthernAmericanDeerMouse	--	0
Mouse	--	0
ChineseHamsterGHOK1GS	--	0
LongTailedChinchilla	--	0

Majority	--	
	10250 10260 10270 10280 10290 10300 10310 10320	
Human	CTATTTTTCCATACCCTTGGCAATTCTAGGTTTTATCAAAATTTAAGAATACTAGCTTATTTTACACTAAAAATTATTTT	10320
GuineaPig	--	0
NorthernAmericanDeerMouse	--	0
Mouse	--	0
ChineseHamsterGHOK1GS	--	0
LongTailedChinchilla	--	0
Majority	--	
	10330 10340 10350 10360 10370 10380 10390 10400	
Human	GTAATTTATATTTTGAAATGGGTGAGGTTTTTCAGATTTGGTTATTTATATTTCATTTTCTGTGAACTGCCTGTTTGTGT	10400
GuineaPig	--	0
NorthernAmericanDeerMouse	--	0
Mouse	--	0
ChineseHamsterGHOK1GS	--	0
LongTailedChinchilla	--	0
Majority	--	
	10410 10420 10430 10440 10450 10460 10470 10480	
Human	ACTGGACCTAGTTAATTCAAATATATATATTTGAATTAAGTGATTTTATTGTTTAAAGTCCATTTGATGTGTAAACATTT	10480
GuineaPig	--	0
NorthernAmericanDeerMouse	--	0
Mouse	--	0
ChineseHamsterGHOK1GS	--	0
LongTailedChinchilla	--	0
Majority	--	
	10490 10500 10510 10520 10530 10540 10550 10560	
Human	TTAGCCTCAGCTTCTTCACCTTTTAAATTAGGATTTTAATAGTTCCACAAACTAGGTCTAATGTTAATGTATTTACATTT	10560
GuineaPig	--	0
NorthernAmericanDeerMouse	--	0
Mouse	--	0
ChineseHamsterGHOK1GS	--	0
LongTailedChinchilla	--	0

```
Majority                     --------------------------------------------------------------------------------
                                      10570     10580     10590     10600     10610     10620     10630     10640
Human                        AGAATATCCAAGTTTTTATGGCTTTAGGATACAAATGAAATCTCATTTTCCATTATTTTATAAATTTGCTGTATTCAGCT 10640
GuineaPig                    -------------------------------------------------------------------------------- 0
NorthernAmericanDeerMouse    -------------------------------------------------------------------------------- 0
Mouse                        -------------------------------------------------------------------------------- 0
ChineseHamsterGHOK1GS        -------------------------------------------------------------------------------- 0
LongTailedChinchilla         -------------------------------------------------------------------------------- 0

Majority                     --------------------------------------------------------------------------------
                                      10650     10660     10670     10680     10690     10700     10710     10720
Human                        TTTCTTGATTTTAGACTTTTTTTTGTTAGCTCAGTGAGACTATTAAGAAAGAATATTTGTGAGTTTGTAATAGGAGATTT 10720
GuineaPig                    -------------------------------------------------------------------------------- 0
NorthernAmericanDeerMouse    -------------------------------------------------------------------------------- 0
Mouse                        -------------------------------------------------------------------------------- 0
ChineseHamsterGHOK1GS        -------------------------------------------------------------------------------- 0
LongTailedChinchilla         -------------------------------------------------------------------------------- 0

Majority                     --------------------------------------------------------------------------------
                                      10730     10740     10750     10760     10770     10780     10790     10800
Human                        GTTGCATTTCTTTAAAATGGCTTTTTATATTCTTCGTAATTTGTAATATCAGGGGTGTCATTTCTGTACTCTTAACCATT 10800
GuineaPig                    -------------------------------------------------------------------------------- 0
NorthernAmericanDeerMouse    -------------------------------------------------------------------------------- 0
Mouse                        -------------------------------------------------------------------------------- 0
ChineseHamsterGHOK1GS        -------------------------------------------------------------------------------- 0
LongTailedChinchilla         -------------------------------------------------------------------------------- 0

Majority                     --------------------------------------------------------------------------------
                                      10810     10820     10830     10840     10850     10860     10870     10880
Human                        TTGCCAATGTGAAATCCTTGCAAAGTAATCGGCCTATTACTTGGGAAGACAGAATTGTATGAAATGACTGTAGAATAATT 10880
GuineaPig                    -------------------------------------------------------------------------------- 0
NorthernAmericanDeerMouse    -------------------------------------------------------------------------------- 0
Mouse                        -------------------------------------------------------------------------------- 0
ChineseHamsterGHOK1GS        -------------------------------------------------------------------------------- 0
LongTailedChinchilla         -------------------------------------------------------------------------------- 0
```

```
Majority                     --------------------------------------------------------------------------------
                                      10890     10900     10910     10920     10930     10940     10950     10960
Human                        CAGGATGGTCCCCAATACGGTAAAATGTTAACTTTTTAGATGTAGATTTTAAGTATGGTGAAATTTGTAGCAGTGTTCTT 10960
GuineaPig                    -------------------------------------------------------------------------------- 0
NorthernAmericanDeerMouse    -------------------------------------------------------------------------------- 0
Mouse                        -------------------------------------------------------------------------------- 0
ChineseHamsterGHOK1GS        -------------------------------------------------------------------------------- 0
LongTailedChinchilla         -------------------------------------------------------------------------------- 0

Majority                     --------------------------------------------------------------------------------
                                      10970     10980     10990     11000     11010     11020     11030     11040
Human                        TAGTAAGTTAACTATTAATATATCAAGACCTATAACTGGACAACTAATAAATAACCTGTTTCTTAAAATGTTAGCTTTTT 11040
GuineaPig                    -------------------------------------------------------------------------------- 0
NorthernAmericanDeerMouse    -------------------------------------------------------------------------------- 0
Mouse                        -------------------------------------------------------------------------------- 0
ChineseHamsterGHOK1GS        -------------------------------------------------------------------------------- 0
LongTailedChinchilla         -------------------------------------------------------------------------------- 0

Majority                     --------------------------------------------------------------------------------
                                      11050     11060     11070     11080     11090     11100     11110     11120
Human                        CTAAATATTCTGAAAGTACTGGTTGTAAATTGCTGAACTCACAATTTAAATTATGATATGACTATTGAATTCATCCTCAT 11120
GuineaPig                    -------------------------------------------------------------------------------- 0
NorthernAmericanDeerMouse    -------------------------------------------------------------------------------- 0
Mouse                        -------------------------------------------------------------------------------- 0
ChineseHamsterGHOK1GS        -------------------------------------------------------------------------------- 0
LongTailedChinchilla         -------------------------------------------------------------------------------- 0

Majority                     --------------------------------------------------------------------------------
                                      11130     11140     11150     11160     11170     11180     11190     11200
Human                        TGGCTTGCATAGAAAATTACAATCAAATAATGCTAAATGTAACTTTTTTGGTAATAGTTGTGTGTAACTGGTTGTTCATT 11200
GuineaPig                    -------------------------------------------------------------------------------- 0
NorthernAmericanDeerMouse    -------------------------------------------------------------------------------- 0
Mouse                        -------------------------------------------------------------------------------- 0
ChineseHamsterGHOK1GS        -------------------------------------------------------------------------------- 0
LongTailedChinchilla         -------------------------------------------------------------------------------- 0
```

```
Majority                    --------------------------------------------------------------------------------
                                      11210     11220     11230     11240     11250     11260     11270     11280
Human                       ATAGATGTGTTATTCAAAACTTTGGGTTTTAGGTTTTGACTTTTCTTAAAATACTTTTCTTAAATGCTACTTAATGCTT 11280
GuineaPig                   -------------------------------------------------------------------------------- 0
NorthernAmericanDeerMouse   -------------------------------------------------------------------------------- 0
Mouse                       -------------------------------------------------------------------------------- 0
ChineseHamsterGHOK1GS       -------------------------------------------------------------------------------- 0
LongTailedChinchilla        -------------------------------------------------------------------------------- 0

Majority                    --------------------------------------------------------------------------------
                                      11290     11300     11310     11320     11330     11340     11350     11360
Human                       TCTCTAGGGTATTTTTATTAGAATTTCAATTAACAGATATTTTGATGTCTGCGAAATGACAGTACTATGTTAGAAATTGA 11360
GuineaPig                   -------------------------------------------------------------------------------- 0
NorthernAmericanDeerMouse   -------------------------------------------------------------------------------- 0
Mouse                       -------------------------------------------------------------------------------- 0
ChineseHamsterGHOK1GS       -------------------------------------------------------------------------------- 0
LongTailedChinchilla        -------------------------------------------------------------------------------- 0

Majority                    --------------------------------------------------------------------------------
                                      11370     11380     11390     11400     11410     11420     11430     11440
Human                       TGTATTGGCTCCTTGAGGGCAGTGACCCTATTTGTTATGTTTGTTCCTCAATCATTAATAGTACACATATTAAATAGTAC 11440
GuineaPig                   -------------------------------------------------------------------------------- 0
NorthernAmericanDeerMouse   -------------------------------------------------------------------------------- 0
Mouse                       -------------------------------------------------------------------------------- 0
ChineseHamsterGHOK1GS       -------------------------------------------------------------------------------- 0
LongTailedChinchilla        -------------------------------------------------------------------------------- 0

Majority                    --------------------------------------------------------------------------------
                                      11450     11460     11470     11480     11490     11500     11510     11520
Human                       ATATACACTTAATATTTAATAACTCTTAGAAGTGTCATAGGCTTTAATATGCTCATACTCTAATGAGCCATTTATCAATA 11520
GuineaPig                   -------------------------------------------------------------------------------- 0
NorthernAmericanDeerMouse   -------------------------------------------------------------------------------- 0
Mouse                       -------------------------------------------------------------------------------- 0
ChineseHamsterGHOK1GS       -------------------------------------------------------------------------------- 0
LongTailedChinchilla        -------------------------------------------------------------------------------- 0
```

```
Majority                    --------------------------------------------------------------------------------
                                      11530     11540     11550     11560     11570     11580     11590     11600
Human                       AAAAGATAAATAACAAGACTATGTCTTTTAGGACTTTAGTTAGAGAAATAAGGACTCAAGGTTTTAATTAAGTGCTATAT 11600
GuineaPig                   -------------------------------------------------------------------------------- 0
NorthernAmericanDeerMouse   -------------------------------------------------------------------------------- 0
Mouse                       -------------------------------------------------------------------------------- 0
ChineseHamsterGHOK1GS       -------------------------------------------------------------------------------- 0
LongTailedChinchilla        -------------------------------------------------------------------------------- 0

Majority                    --------------------------------------------------------------------------------
                                      11610     11620     11630     11640     11650     11660     11670     11680
Human                       GGCTACAGTAGGAAAGATTGGTTTGGGTTGGTAGGCTCAGAATAGTGGTCTGTGACCTAGCTAGATTGGGAGATAGGAGA 11680
GuineaPig                   -------------------------------------------------------------------------------- 0
NorthernAmericanDeerMouse   -------------------------------------------------------------------------------- 0
Mouse                       -------------------------------------------------------------------------------- 0
ChineseHamsterGHOK1GS       -------------------------------------------------------------------------------- 0
LongTailedChinchilla        -------------------------------------------------------------------------------- 0

Majority                    --------------------------------------------------------------------------------
                                      11690     11700     11710     11720     11730     11740     11750     11760
Human                       AAGAGTGGAGAGCAACAGGCGTTGAGAGAAGGACGTAACCAAAGTTGTGTCTATAGGTTGGCATAAATTTACAGCCATTG 11760
GuineaPig                   -------------------------------------------------------------------------------- 0
NorthernAmericanDeerMouse   -------------------------------------------------------------------------------- 0
Mouse                       -------------------------------------------------------------------------------- 0
ChineseHamsterGHOK1GS       -------------------------------------------------------------------------------- 0
LongTailedChinchilla        -------------------------------------------------------------------------------- 0

Majority                    --------------------------------------------------------------------------------
                                      11770     11780     11790     11800     11810     11820     11830     11840
Human                       ATCAGTGAGTTGAATAGTTTGGCCTTTATGGCTAACAGCTTGTATATGGATATGAGAGAAAAGATAGAATGGCAGATAGG 11840
GuineaPig                   -------------------------------------------------------------------------------- 0
NorthernAmericanDeerMouse   -------------------------------------------------------------------------------- 0
Mouse                       -------------------------------------------------------------------------------- 0
ChineseHamsterGHOK1GS       -------------------------------------------------------------------------------- 0
LongTailedChinchilla        -------------------------------------------------------------------------------- 0
```

Majority	--	
	11850 11860 11870 11880 11890 11900 11910 11920	
Human	TTATATAGAAGACCTCAATTACAGGTAGTGAAATTTGTACTTCATCTGGTAGGCATTATTCTATATACCTGTAATTTTCC	11920
GuineaPig	--	0
NorthernAmericanDeerMouse	--	0
Mouse	--	0
ChineseHamsterGHOK1GS	--	0
LongTailedChinchilla	--	0

Majority	--	
	11930 11940 11950 11960 11970 11980 11990 12000	
Human	ACATAAAAGTAGCTCATTACTCTGCTTTCTTTCTTTCTTTTTTTTTTTTTTTGCTATAGCAAATTTATTGTAACAACACA	12000
GuineaPig	--	0
NorthernAmericanDeerMouse	--	0
Mouse	--	0
ChineseHamsterGHOK1GS	--	0
LongTailedChinchilla	--	0

Majority	--	
	12010 12020 12030 12040 12050 12060 12070 12080	
Human	AAGTATTATCTTATAGTTTTGTAGGTGAGAAATCTGACACAGGTGGGCTAAAATCAAGGTGTTAGCAGGGCTGTGTTCCT	12080
GuineaPig	--	0
NorthernAmericanDeerMouse	--	0
Mouse	--	0
ChineseHamsterGHOK1GS	--	0
LongTailedChinchilla	--	0

Majority	--	
	12090 12100 12110 12120 12130 12140 12150 12160	
Human	TTTTGCAGGATCTGGGGGGAATTTGTTTCCTTGACATAACTCGGCTGTTAGCTTCATCTTCAAAGAAGGAATGGCAAAGA	12160
GuineaPig	--	0
NorthernAmericanDeerMouse	--	0
Mouse	--	0
ChineseHamsterGHOK1GS	--	0
LongTailedChinchilla	--	0

Majority	--	
	12170 12180 12190 12200 12210 12220 12230 12240	
Human	AGACTCTGTCTCACATCACATCACTCCGACACATTCTTGTGCCTCCGTTTCCCACTTACAGGGACCTTGTGATTACAATG	12240
GuineaPig	--	0
NorthernAmericanDeerMouse	--	0
Mouse	--	0
ChineseHamsterGHOK1GS	--	0
LongTailedChinchilla	--	0

Majority	--	
	12250 12260 12270 12280 12290 12300 12310 12320	
Human	GGCCCAACTACATAATAGAGGATAAATGTTTAAATTACAAATTAATGAAGTGGCAACTGAAACAAGTTTTAACCTATTCT	12320
GuineaPig	--	0
NorthernAmericanDeerMouse	--	0
Mouse	--	0
ChineseHamsterGHOK1GS	--	0
LongTailedChinchilla	--	0

Majority	--	
	12330 12340 12350 12360 12370 12380 12390 12400	
Human	CAAGAAAGGGCTTTGCTGGAATGGTGGCTCACACCTGTAATCCCAGAACTTAGGGAGGCTGAGGCAGGCAGATCACTTGA	12400
GuineaPig	--	0
NorthernAmericanDeerMouse	--	0
Mouse	--	0
ChineseHamsterGHOK1GS	--	0
LongTailedChinchilla	--	0

Majority	--	
	12410 12420 12430 12440 12450 12460 12470 12480	
Human	GCCCAGGAGTTTGAGACCAGCCTGGTCCTCATAGCAAGACCCCATCTCTACAAAAACTTTTAAAAATTAGATAGATCTGG	12480
GuineaPig	--	0
NorthernAmericanDeerMouse	--	0
Mouse	--	0
ChineseHamsterGHOK1GS	--	0
LongTailedChinchilla	--	0

```
Majority                     --------------------------------------------------------------------------------
                                       12490     12500     12510     12520     12530     12540     12550     12560
Human                        TGGCATGGGCCTGTAGTCCCAGCTACTTGGGAGGCTGAGACAAGAGGATCACCTGAGCCCAGGCGGCAGTGAGCTATGAT 12560
GuineaPig                    -------------------------------------------------------------------------------- 0
NorthernAmericanDeerMouse    -------------------------------------------------------------------------------- 0
Mouse                        -------------------------------------------------------------------------------- 0
ChineseHamsterGHOK1GS        -------------------------------------------------------------------------------- 0
LongTailedChinchilla         -------------------------------------------------------------------------------- 0

Majority                     --------------------------------------------------------------------------------
                                       12570     12580     12590     12600     12610     12620     12630     12640
Human                        TGTATCACTACACTCCAGCCTGGGCGAGAGGAGATCCAGTCTCAAAAAAAAAGGAAAAGGGCTTTCCTGAGGGTCAAATG 12640
GuineaPig                    -------------------------------------------------------------------------------- 0
NorthernAmericanDeerMouse    -------------------------------------------------------------------------------- 0
Mouse                        -------------------------------------------------------------------------------- 0
ChineseHamsterGHOK1GS        -------------------------------------------------------------------------------- 0
LongTailedChinchilla         -------------------------------------------------------------------------------- 0

Majority                     --------------------------------------------------------------------------------
                                       12650     12660     12670     12680     12690     12700     12710     12720
Human                        AAGTACAGTTGACCCTTGAACAACATGATTTTGAATTACACAGGTCCACTTATGCATAGATCCACTTATACAGAATTTTC 12720
GuineaPig                    -------------------------------------------------------------------------------- 0
NorthernAmericanDeerMouse    -------------------------------------------------------------------------------- 0
Mouse                        -------------------------------------------------------------------------------- 0
ChineseHamsterGHOK1GS        -------------------------------------------------------------------------------- 0
LongTailedChinchilla         -------------------------------------------------------------------------------- 0

Majority                     --------------------------------------------------------------------------------
                                       12730     12740     12750     12760     12770     12780     12790     12800
Human                        TTCTCCCTCTTCCACCCAAGACAGAAAGACCAACTCGTCCTCTTCCTCCTTAGCCTACTCAGCGTGAAGACAGTCAGGAT 12800
GuineaPig                    -------------------------------------------------------------------------------- 0
NorthernAmericanDeerMouse    -------------------------------------------------------------------------------- 0
Mouse                        -------------------------------------------------------------------------------- 0
ChineseHamsterGHOK1GS        -------------------------------------------------------------------------------- 0
LongTailedChinchilla         -------------------------------------------------------------------------------- 0
```

Majority	--	
	12810 12820 12830 12840 12850 12860 12870 12880	
Human	GAAGACCTTTATTATGACTGATCTCCAAAAAGATTGGCTTCAGTATTTCATGGAGTGCTATGAATTAGAATCCTGAGTCT	12880
GuineaPig	--	0
NorthernAmericanDeerMouse	--	0
Mouse	--	0
ChineseHamsterGHOK1GS	--	0
LongTailedChinchilla	--	0

Majority	--	
	12890 12900 12910 12920 12930 12940 12950 12960	
Human	CTATATTGTTAATTATTTTAGTCTCTTGGTCTACGTGTGTTCCTAAACTGTAGTAGCTTTCTTAATTTAGTTCTAGGCAT	12960
GuineaPig	--	0
NorthernAmericanDeerMouse	--	0
Mouse	--	0
ChineseHamsterGHOK1GS	--	0
LongTailedChinchilla	--	0

Majority	--	
	12970 12980 12990 13000 13010 13020 13030 13040	
Human	ACTACATTAGGGGATAAGGTGGTTAAGTGATATTTTCACTTTAAAAGTAATAAGGTGTCACAGAAAGTTATCACGAGATT	13040
GuineaPig	--	0
NorthernAmericanDeerMouse	--	0
Mouse	--	0
ChineseHamsterGHOK1GS	--	0
LongTailedChinchilla	--	0

Majority	--	
	13050 13060 13070 13080 13090 13100 13110 13120	
Human	TTTTTGAGCAGTACTTCTTTTTTAAAACTATTATAATGAATTTTAGACACAATAGTATCATGAACCATTGTGTATCTATC	13120
GuineaPig	--	0
NorthernAmericanDeerMouse	--	0
Mouse	--	0
ChineseHamsterGHOK1GS	--	0
LongTailedChinchilla	--	0

Majority	--	
	13130 13140 13150 13160 13170 13180 13190 13200	
Human	ATTCAGTCCCCAAAACCATTAGTAACCCATAGCTAAAATGATGATATCCACACTCTGCTTTTCCACTTCTGTTATTTTGA	13200
GuineaPig	--	0
NorthernAmericanDeerMouse	--	0
Mouse	--	0
ChineseHamsterGHOK1GS	--	0
LongTailedChinchilla	--	0

Majority	--	
	13210 13220 13230 13240 13250 13260 13270 13280	
Human	GATAAATTTCAGATATTCATATTGTTTTATCTGTAAAGGCTTCAGTGTATATCTTTAAAGAATAGGGACTCTCCTCTCCT	13280
GuineaPig	--	0
NorthernAmericanDeerMouse	--	0
Mouse	--	0
ChineseHamsterGHOK1GS	--	0
LongTailedChinchilla	--	0

Majority	--	
	13290 13300 13310 13320 13330 13340 13350 13360	
Human	TTTTAAAATATAATCAAAAGACTGTTAGGACACACACACAAAGTTACCTCATATTATAAAATGTCAAATTTGTATTAAAA	13360
GuineaPig	--	0
NorthernAmericanDeerMouse	--	0
Mouse	--	0
ChineseHamsterGHOK1GS	--	0
LongTailedChinchilla	--	0

Majority	--	
	13370 13380 13390 13400 13410 13420 13430 13440	
Human	TTTCTAATTGTCTCATAAAAGTCATAGTATTTTTATAGGGTTTTTTTTTTTAGTGGGGGAAGGAGTCACGATAAAATAAG	13440
GuineaPig	--	0
NorthernAmericanDeerMouse	--	0
Mouse	--	0
ChineseHamsterGHOK1GS	--	0
LongTailedChinchilla	--	0

```
Majority                     --------------------------------------------------------------------------------
                                       13450     13460     13470     13480     13490     13500     13510     13520
Human                        ATCAGCAAATTTAACAGGTCGTTATTACCTCTTAAATATCTTTTAATGTACAAGCTCTCCGTTCCTGTTTTTTGTTTTTT 13520
GuineaPig                    -------------------------------------------------------------------------------- 0
NorthernAmericanDeerMouse    -------------------------------------------------------------------------------- 0
Mouse                        -------------------------------------------------------------------------------- 0
ChineseHamsterGHOK1GS        -------------------------------------------------------------------------------- 0
LongTailedChinchilla         -------------------------------------------------------------------------------- 0

Majority                     --------------------------------------------------------------------------------
                                       13530     13540     13550     13560     13570     13580     13590     13600
Human                        TTTTAAGTTGCATTTCATTTGTTGAAGAAAACTGGTTATTGGTCTTACACAGTTGCCTATAGACTACATTTACTGATTGC 13600
GuineaPig                    -------------------------------------------------------------------------------- 0
NorthernAmericanDeerMouse    -------------------------------------------------------------------------------- 0
Mouse                        -------------------------------------------------------------------------------- 0
ChineseHamsterGHOK1GS        -------------------------------------------------------------------------------- 0
LongTailedChinchilla         -------------------------------------------------------------------------------- 0

Majority                     --------------------------------------------------------------------------------
                                       13610     13620     13630     13640     13650     13660     13670     13680
Human                        GTTATGATTTTGTAATTTAGTATGTTTTCCATATTTCCAGTAAATTGGTAGTTAGATCTAGAGACTTGCTCAGGGTCTCT 13680
GuineaPig                    -------------------------------------------------------------------------------- 0
NorthernAmericanDeerMouse    -------------------------------------------------------------------------------- 0
Mouse                        -------------------------------------------------------------------------------- 0
ChineseHamsterGHOK1GS        -------------------------------------------------------------------------------- 0
LongTailedChinchilla         -------------------------------------------------------------------------------- 0

Majority                     --------------------------------------------------------------------------------
                                       13690     13700     13710     13720     13730     13740     13750     13760
Human                        ATTTTTTTGGTAATATTTTTAAGTGGTGGGTTTTTATTTCTGTTAGACATGGTTTCTGACTTTTCGTGTATGTCATGTTT 13760
GuineaPig                    -------------------------------------------------------------------------------- 0
NorthernAmericanDeerMouse    -------------------------------------------------------------------------------- 0
Mouse                        -------------------------------------------------------------------------------- 0
ChineseHamsterGHOK1GS        -------------------------------------------------------------------------------- 0
LongTailedChinchilla         -------------------------------------------------------------------------------- 0
```

```
Majority                    --------------------------------------------------------------------------------
                                     13770     13780     13790     13800     13810     13820     13830     13840
Human                       TTCCCAGGAACATTTCTTATACTTTACCTTGTTTGTGCTAGGCATAGTCTGTGCTCAATAATTGTCTCCTTTTTTTTTCT 13840
GuineaPig                   -------------------------------------------------------------------------------- 0
NorthernAmericanDeerMouse   -------------------------------------------------------------------------------- 0
Mouse                       -------------------------------------------------------------------------------- 0
ChineseHamsterGHOK1GS       -------------------------------------------------------------------------------- 0
LongTailedChinchilla        -------------------------------------------------------------------------------- 0

Majority                    --------------------------------------------------------------------------------
                                     13850     13860     13870     13880     13890     13900     13910     13920
Human                       TTTTTTTTTTTTTTTTTTCCTGCTTTTTGAGAGACAAGAGTCTTGCCCTGTTGCCCAGGCTGGAGTGCAGTGGCCCAATC 13920
GuineaPig                   -------------------------------------------------------------------------------- 0
NorthernAmericanDeerMouse   -------------------------------------------------------------------------------- 0
Mouse                       -------------------------------------------------------------------------------- 0
ChineseHamsterGHOK1GS       -------------------------------------------------------------------------------- 0
LongTailedChinchilla        -------------------------------------------------------------------------------- 0

Majority                    --------------------------------------------------------------------------------
                                     13930     13940     13950     13960     13970     13980     13990     14000
Human                       TCAGCTCAGTGCAACCTCTGCCTCCCGGATTCAAGCGATTCTCCTGCCTCAGCTTCCCAAGTAGCTGGAACTACAGGCGC 14000
GuineaPig                   -------------------------------------------------------------------------------- 0
NorthernAmericanDeerMouse   -------------------------------------------------------------------------------- 0
Mouse                       -------------------------------------------------------------------------------- 0
ChineseHamsterGHOK1GS       -------------------------------------------------------------------------------- 0
LongTailedChinchilla        -------------------------------------------------------------------------------- 0

Majority                    --------------------------------------------------------------------------------
                                     14010     14020     14030     14040     14050     14060     14070     14080
Human                       CTGCCACTACACCTGGCTAATTTTTGTGTTTTTAGTAGGGATGGGGTTTCACCACGTTGGCCAGGCTGACTTGGAACTCC 14080
GuineaPig                   -------------------------------------------------------------------------------- 0
NorthernAmericanDeerMouse   -------------------------------------------------------------------------------- 0
Mouse                       ---------TGAAATTTAGATTTAATGATTGTTCACATAAGTGAAAAATTTTCTGTGAAAGGTCAAAACAGTTACCATTC 71
ChineseHamsterGHOK1GS       -------------------------------------------------------------------------------- 0
LongTailedChinchilla        -------------------------------------------------------------------------------- 0
```

```
Majority                      --------------------------------------------------------------------------------
                                       14090     14100     14110     14120     14130     14140     14150     14160
Human                         TGACCTCAAGTGATCTGCCCACCTTGGCTTCCCAAAGTGCTGGGATTATAGGCATGAGCCACTGTGCCTGGCCAATAAGT  14160
GuineaPig                     --------------------------------------------------------------------------------  0
NorthernAmericanDeerMouse     --------------------------------------------------------------------------------  0
Mouse                         GGATGGAAAAGAACACATTACTGTACTGAAAGAAGATCTGGGTATGAAAAGAATACTGTCCAGCCACTTCACTTTTTATT  151
ChineseHamsterGHOK1GS         --------------------------------------------------------------------------------  0
LongTailedChinchilla          --------------------------------------------------------------------------------  0

Majority                      --------------------------------------------------------------------------------
                                       14170     14180     14190     14200     14210     14220     14230     14240
Human                         GTCTCTTAATGAGTATTCTTAAAGGATATCTAAAAAGAAATGTAACTTAAAATTCACATAATAGTAATACTATACAGTAT  14240
GuineaPig                     --------------------------------------------------------------------------------  0
NorthernAmericanDeerMouse     --------------------------------------------------------------------------------  0
Mouse                         GCCCTATTCTGTTAGGACTTCATAAACATGAATTGAATCTAGTCGGCATTTACAAGGGTTGGCCATCCTCCACTGTTTAA  231
ChineseHamsterGHOK1GS         --------------------------------------------------------------------------------  0
LongTailedChinchilla          --------------------------------------------------------------------------------  0

Majority                      --------------------------------------------------------------------------------
                                       14250     14260     14270     14280     14290     14300     14310     14320
Human                         TAACATTTAATGTAAGTTGGAACTATTGATGGTGGACAAGAATGTTTTCTTGTCAAGAACATACGATTTAGAATGAAAAG  14320
GuineaPig                     --------------------------------------------------------------------------------  0
NorthernAmericanDeerMouse     --------------------------------------------------------------------------------  0
Mouse                         TAATCCAGCCCCTAGTTAACCTACACAGGCACACAGATTGACATATGGGTGGGACTTTACAAATTTGCCGGTGACATTTC  311
ChineseHamsterGHOK1GS         --------------------------------------------------------------------------------  0
LongTailedChinchilla          --------------------------------------------------------------------------------  0

Majority                      --------------------------------------------------------------------------------
                                       14330     14340     14350     14360     14370     14380     14390     14400
Human                         ATGAGGGTTTGACTTTTATAGCATTGAACAGGTTAACCTCTGAGCTTCAGTTTTATACATTTTTAAATGGTTTCAGTTGT  14400
GuineaPig                     --------------------------------------------------------------------------------  0
NorthernAmericanDeerMouse     --------------------------------------------------------------------------------  0
Mouse                         CCCTGGTTTATTCATTTGTACGAGTGCCAATCTAATTTCATTACTGACATGTTTAACCTTTGTGAAGAATCTCTGTACAG  391
ChineseHamsterGHOK1GS         --------------------------------------------------------------------------------  0
LongTailedChinchilla          --------------------------------------------------------------------------------  0
```

Majority	--	
	14410 14420 14430 14440 14450 14460 14470 14480	
Human	ACCTACTTCCAAAATTATTTTGAATGTTAGATGAGATGAGGAAAAGACTTATGCTGAAAGGATGAGAATGGTTGTTGTTG	14480
GuineaPig	--	0
NorthernAmericanDeerMouse	--	0
Mouse	ATACAGCTATACGGATACGGGCTGCCTCCGAAGTTCCAAAAGCCAAAAGTGTGGCCTGTTCCTTCTGCAGAGAATAGTAT	471
ChineseHamsterGHOK1GS	--	0
LongTailedChinchilla	--	0

Majority	--	
	14490 14500 14510 14520 14530 14540 14550 14560	
Human	TAGTTCTTTAATCTGTAAATCTACATTTGGAAGGAACACTTTTATGGAAATCAAATACAGTAAATTAATTTCTAAGAGGT	14560
GuineaPig	--	0
NorthernAmericanDeerMouse	--	0
Mouse	CAGTGACAGGAACAACAACCAATCACCAACAATCAAGGCGATAGCAGCACCGCTTCTTAAGCATTGACTGCGGCCAGGCT	551
ChineseHamsterGHOK1GS	--	0
LongTailedChinchilla	--	0

Majority	--	
	14570 14580 14590 14600 14610 14620 14630 14640	
Human	CCTGGAGTTCAGGTTAAAAATAGCCTCAGACAGCACTAAATATCTTTATTAGTGAACAAAAAAATAAAAATAACTTAGAA	14640
GuineaPig	--	0
NorthernAmericanDeerMouse	--	0
Mouse	GTCAACTGGACACCAATTTGGATGATGACGTCACTCTTAATCTCATTTTCATGAACTTCATCCTTGGGTTATGCTGACCA	631
ChineseHamsterGHOK1GS	--	0
LongTailedChinchilla	--	0

Majority	--XX---------X-X-------X	
	14650 14660 14670 14680 14690 14700 14710 14720	
Human	TTTAGCACAGAGAAGCAAAGAGATGAAAATAGGAGAAGCGTTAAAAATGTGTTGGAAAGGAAGAGAAGTTCTAACAAACA	14720
GuineaPig	--	0
NorthernAmericanDeerMouse	--	0
Mouse	TGTCCAGTGTCACGTAGCTAACAAATGGAACATCTCAAACGCGAAGCCAGGCTTTCATGGATATGTTTTTGTGGTATATA	711
ChineseHamsterGHOK1GS	---------------------------------------TACAGCAACAAACAGTCCAGGCAAGAGCAGTTTCTTGCCCA	41
LongTailedChinchilla	--	0

```
Majority                    -----------XX-X-----X---------X-----------X--X---------------------X--X---------
                                     14730     14740     14750     14760     14770     14780     14790     14800
Human                       TCTAATTAGCGTTCCAGAAATTAAAGATTGCAGAAAGGCAAAA--TTTGAAATGCTAAAGGCTGATAATTTTTCAGAATT  14798
GuineaPig                   --------------------------------------------------------------------------------  0
NorthernAmericanDeerMouse   --------------------------------------------------------------------------------  0
Mouse                       GCACACAGGGATTACAGTCTTGTCCTGCTACCAGATGCCTACAACTGCAGGTGCTAGCAGCCTCTGTATCTGTTATTTCA  791
ChineseHamsterGHOK1GS       AATGGCTAATGTTGCCATGTTGAAAGCAAACAAGTTTTTTCCA--TGCCCATCCTCCTTTCTGATGTAATTGGTGTGCCA  119
LongTailedChinchilla        --------------------------------------------------------------------------------  0

Majority                    --------------------X-X----------X--------XX--------X-----X----XX-----------X---
                                     14810     14820     14830     14840     14850     14860     14870     14880
Human                       GGTGAGCTATATGAGTTCTTAGATTTAGGAGGCACAAGAAAACCTGGACACATAACAGTAAAAGAGCAGAGCACCAAAGA  14878
GuineaPig                   --------------------------------------------------------------------------------  0
NorthernAmericanDeerMouse   --------------------------------------------------------------------------------  0
Mouse                       TTCTGTACGCCCATCTTCCCAAAACAATGTATGATAAGCTAACCTCACACTATTCGTATATTAGACATAGCCAGAGATTA  871
ChineseHamsterGHOK1GS       GGACATTTACTCTGTCAAGAAAAACCCTAAACCATTTAAATGCCATATATTCTGTGGGTCCTTGAAAGGTTTGAAGAATG  199
LongTailedChinchilla        --------------------------------------------------------------------------------  0

Majority                    ---X----------X------X-----X-X-X--XXTXAC-XAAGXXXX-XAAXTAXXXTXXXTXXXTXXXXTTXXTXXX
                                     14890     14900     14910     14920     14930     14940     14950     14960
Human                       CATAGAGATCTTTAAAGCAGCCAGAGAAAATCAGATTCCCTAAAGAATAAGAACTAGAAAGTCCTAATATGCTTCATCAG  14958
GuineaPig                   --------------------------------------------------------------------------------  0
NorthernAmericanDeerMouse   --------------------------------------------------------------------------------  0
Mouse                       ACAATACTAAAAACAGAGTAACTGCACAAAACATACTCAGGAAAGACATGGGAATATGTTTCCTGTCTTGAAATACTTGG  951
ChineseHamsterGHOK1GS       CCTAT-CCATCTCAAATACATCTTTGTATATCTAGAAAACCTAATTAACCTAATTATAATTTTTGACTATTATAGATGGC  278
LongTailedChinchilla        ----------------------------------GTTTACATAAGTGCATTAGCCACTGTACATTTGCTGTCTTGCACTT  46

Majority                    TXT-TXXXXXXXXT--A-XXXXGXAT--TAXTTTXXXTG--XXXXXXAAAXAXXXXTGCXXXACXXXXTX-T-XGXTTGC
                                     14970     14980     14990     15000     15010     15020     15030     15040
Human                       CATGGGCATCCAGAAGATAGTGGAATAATATTTTCAATGTGTTGAGTGAAAATAGCTGCC-CACCTGTTATT-TTATAGC  15036
GuineaPig                   -----------------------------------------------------------------------C-AGGTTGT  8
NorthernAmericanDeerMouse   --------------------------------------------------------------------------------  0
Mouse                       TTGCTTGTTCACCTCCAGGGAAGCATTTTACGATTTCTTGACATATGACACTTTCTTGGCATACGGATTGCCAGGAGCAC  1031
ChineseHamsterGHOK1GS       TATATAATCTGTATTTAATCATGCATATCATTTTAAAAGATCTGCATAAACACAATACCTAAACAAGAGGATATAGATAT  358
LongTailedChinchilla        TTTTTAAAGTGTGTGAACACTACAAT--TTCTTCTGCTGCCTCACAGAAGAAATGCTGCTACCCCTCGTAGT-GGTTTGC  123
```

```
Majority                    TACTCACATGATCXXX-AGGXAAGTTAACTAA-TAAXXXCTXX-XCAAXAXAAAGXCATTXAGAC--CATTATAXCAXXT
                                      15050     15060     15070     15080     15090     15100     15110     15120
Human                       CAGCCAAATTATCATATAAGGAGAAGAACAAAATAAAGACACTTTTTGAAACACGTCATTCTGCCTCCAAAAAAGCAATT 15116
GuineaPig                   TTATCACAGCAAT-----GGAAAGTTGACTAA-TGCTTCT-----CAACACAGAAACATTAAGAC--CACTGTGACAG-- 73
NorthernAmericanDeerMouse   -------------------------------------------------------------------------------- 0
Mouse                       TACTCTCGTGCTTTGGGAGTTGAGGTAACTAAGGAACTCCTTCCTGCACATACAAACCTTGAGATAACATGATAGTTAAG 1111
ChineseHamsterGHOK1GS       CACAAAATTGACCTTAAAGTTGTATCAATTAATGAAAATCTGTACCAATGCAAAGTATTCATTCCTATATCATATTCCCT 438
LongTailedChinchilla        TTTTCCCAAGATCCGGCAAGAAAGTTAATCAA-TACTGATTGAGCCAATGTGAGGGAATACAGAG--CATTA-GACAGTT 199

Majority                    TXXX--XAXTXTXXXXXCTXAXXXAXTAAAXXAATTXXTGGAGATCXTCATXXACAXXXA---XCAATATATAXXAGAAA
                                      15130     15140     15150     15160     15170     15180     15190     15200
Human                       TAGG--AAATGTATCAACAGACCCACTGAAGGAATTTCTGGAGTTTGTACTTCAGGAAGAAAGCCAATG-ACACCAGAAA 15193
GuineaPig                   -CCA--AATTGTTAAGTCACAGCTACTAAGTGACTAACTGGAGGGTACCATGTACACAC----TGATTATGCTGGACAAA 146
NorthernAmericanDeerMouse   -------------------------------------------------------------------------------- 0
Mouse                       CTGATCGAAGACACCTGTTCATTGAATAAGCTAGCTGGAAGATAGCGTGTTTAGCCTTGATACACTGAACATAGAGGTGA 1191
ChineseHamsterGHOK1GS       TT------------TTTTTTCTTTTAAAAAGAGATTGACTGTGATCATTACGATTTATAGCCAACCCCATTTAAATGAAA 506
LongTailedChinchilla        TCTG--TCCTTTGCAAACTTACACATTACACTGTTGAGTGAAAATCCACGCACAAACCCA---CAAATGTATAAGAGTAA 274

Majority                    AXAATGATXTTACTGXXGXXAGXXCXTTCAGGAA--TGATAGGXCTTTGCTTGAXAXTXX--XXTXGXATTXTGAGAAXT
                                      15210     15220     15230     15240     15250     15260     15270     15280
Human                       GGAAGTCTGATATAGAAGTGAGGCTAATCAAGAAATTGGTAGGTCTGTGAGTAAATCTAATTACTGACCATATAAGAATG 15273
GuineaPig                   AGGATGATTTTACTG----TACTTCATTCAGAAT--AGATAGGGTTTAGCTTGTACATTT-----TTTATTTCTAAAATT 215
NorthernAmericanDeerMouse   ------------------------------------------------GCATGAGA------TCTCAAGTGCTGAG-ATT 25
Mouse                       CCCATGTCCTGACTGGAGAGTGAGATGGAGGCGATTTCATTATGCTTTACCTCAGAATGG--TGCAGAATTTTAAACTAT 1269
ChineseHamsterGHOK1GS       ACAAACATTTATTTACGGTTTCTCCCTACACTACCCCCTTTGG---TTCTTTGAGA------CATAGTCCTCTGTGTAAC 577
LongTailedChinchilla        TTACTAATGGTGCTGATCACAGGTCCTTTATGTA--TGATAGGTCGATGCTTAAAGGGGA-----GGCATTGTGGGAGAT 347

Majority                    XAATCXTATTTXTT-XTAAGGC--XTGAXAXT----TTAGAGCCACTGATTAACACCAAAXTAACAGAGAGCXACATAGA
                                      15290     15300     15310     15320     15330     15340     15350     15360
Human                       TAATAGCAGTGATTGATAAGGGGTTTAAAAATAGAATTAAAAATATTAAATCATAACAATATGTGAGAGAGTGGTAGAGA 15353
GuineaPig                   TCTATTTAATATTT--CCAGGC--CATAGGTTAC--T----GCTGGTTATAAAATCCAAAAAAGCAAAACCTTCCATAAG 285
NorthernAmericanDeerMouse   AAAGC-CGTGTGCCACCACTGC--CTGAC-------TCTGTTCC-CAGTGTAGCCTTGAACTCACAGAGATCCAGATGGA 94
Mouse                       AAATCATGTCTAGATTTTACAC--TTAATATT----TCCCAGCCACGGTTTACCACTGAACAAATGGCTTGAAACAGAAA 1343
ChineseHamsterGHOK1GS       AGACC-TAACTGTC-CTGGAAC--TTGAT-------TTGTAGGC-CAGGTTGGCCTCAAACTCACTGAGATCCACCTG-- 643
LongTailedChinchilla        TAATTTTATTTTTT--TAAGGA--CTGACAATCC--CTAGGGCCAGTCAAGAATACGTAAACATCTGGGTGCTGTAAAGC 421
```

```
Majority                   CCCCXGXXTCAXAGXTXTTGXXATCTAXTGTTGXGTGCXGCT-CATCXGCATCTXXCCTAACTXTXAAXCCTT-GTXTCT
                                     15370     15380     15390     15400     15410     15420     15430     15440
Human                      CACTGGAGTTTGAGTTCTGATGATCTAGTATTGATTGGAGCTGTGTTGTCAGTTACAGTAGCTACTAGTCATTTGTTACT  15433
GuineaPig                  AGGGAAG--TAAGACCACTGTTGTATTTTGTTG-CATTAGCCTCATTTCTAATTCACATTATGCTTTGTCATT-GTAATT  361
NorthernAmericanDeerMouse  TCTCTGCCTCCCAAGTGATAGGAT-TAAGGTTGTGTAC-----CACCAGTGTCTGGCCTCTATGTCTAATCTA-GTGTCT  167
Mouse                      CCTCAGGTAGATGTCCAGAGGAGCCTATTGCTGTGTGCTGATACATCAGCATCATTCCCAACTTTAAACCCTT-ATTTCT  1422
ChineseHamsterGHOK1GS      CCCCTGCCTCCCAGGTGTTGGAAT-TAAAGGCAGGTGC-----TACACACAGCCAGGCTACATAC--ACTCT---TATTT  712
LongTailedChinchilla       TGCAGAA--CAAAGTT-TTGTTCTCTCGTTTTG-CGGTGGCTCGGTCGGCCTCCCCCCCGACGGTCA--CGCC-ATCGCC  494

Majority                   GTCXXCXXXTXTXAXATCTAGTTCATGXXXATGTGTXTXGTCXTTCA-XAXXXXACACTACCTCAAAXATTTTCTACXAA
                                     15450     15460     15470     15480     15490     15500     15510     15520
Human                      GAGCACTTGACATGTATGTAGTTTAAGTTGATCTTCTACTTAATTGAAAAATACACATTTC---TGAGCCTTAGTCTGAA  15510
GuineaPig                  CTCAGCTCACCTCATTTTTAATTTATTAATTTATGTGTCTTAATTCATTAATTTACATCTCTAGAAAGTGTTTAAGTAAG  441
NorthernAmericanDeerMouse  GTC------TTTGTCCTCTGATTCCCATATAAGTTTATTAGGGTACACAATGGTCAACTACCTCAAATATTTTCTAC-AA  240
Mouse                      TTACTACATTTTAAAATCTAGTGCTTT---ATGTGTCTTGCCTTGCG--------AACTACCTCACATATTTCCTACCGA  1491
ChineseHamsterGHOK1GS      AT-------TTTAAAATCTAGTACTTG---ATGTATCTTGACTTTTA--------GACTATCTCAAATATTTTCCAG-AA  773
LongTailedChinchilla       GCCTACCCGCCCCGCAGCTGGCCCACGGGC--ACGCACCGTC-CTGAGCGGCGGACGGC-----AAAGTCTGTGGCCAAG  566

Majority                   AGTACTGACTXAAATA-AGXCAGCATXTTGAXXXTXGTTGTATGTTAGCCCTAXXATACTTTXTTAGGAXGXGAAAAAAA
                                     15530     15540     15550     15560     15570     15580     15590     15600
Human                      AGAAGAATGTAAAATA--TCCCAAGTTTTCATATTGGTTACATGTACAGCGGATAATATTTTGGTATTTTGGGTTAAATG  15588
GuineaPig                  CATATTGACTATTGTA-TGTTTTCTGTCTTGCACTTTTTTAAAATAA-----AAAATAAAATGT------GTGCAAATAT  509
NorthernAmericanDeerMouse  AGTACTGACTTAAATA-AACCAGCATATTGATTAT-GTTGTATGTTTGTTCTCCTATACTTTATTAGGAAGAAAAATAAA  318
Mouse                      AGTGTTGACTTAAATA-AGCCAGCATATTGACTGT-GCTGTG-GTTTGCCCT--TATACTTTATTAGAAGACCAACAAAA  1566
ChineseHamsterGHOK1GS      AGTACTGACTTAAATA-AATCAGCATATTGATTATTGTTGTATGTTAGCTCTCTTATAGTTTATTAGGAAGAAAAACAAA  852
LongTailedChinchilla       -ATGCTCGCTAAGGTTCTGTCCTATGTAAAGCAGTATTTAAAAACTATACGGAAAGTACCATGTTCCGTGGTGAAATTGC  645

Majority                   GAATAXATTTTAXTGAATACCTTACACTTGAXA---XAAXTTCTGCXTXTXATGACAX-AGTTTGTCTTCCCCAAGATAA
                                     15610     15620     15630     15640     15650     15660     15670     15680
Human                      AAATACATTATGTGTGTTGCCTTTTCCTCTTTT----TTCCTTTCCTTTTTTTTAAGATAGGGTCTTGCTCTGTCCACCAG  15664
GuineaPig                  AATTTC-------TTGTTACCTCACAAAAAATT----CTGTTACCCCTTGTAGTAC---AGTTTGCTTTTGCCAAGAACA  575
NorthernAmericanDeerMouse  GAATATATTTTAATGAATACTTTACACTTGACA-AAGAAATTCTGTCCCTCATGACAC-AGTTTGTCTTCCCCAAGATAA  396
Mouse                      GGTTGTAGTTTAGTGAACATCTGACACTTGACA---GAAGTTCTGCTCTTCTTGACCC-AGATTGTCTTCCCTGAGATAA  1642
ChineseHamsterGHOK1GS      GAATGTATTTTAATGAATACTTGACACTTGAAAGAAGAAATTCTGTTTCTTATGACAT-AGTTTGTCTTCCCCAAGATAA  931
LongTailedChinchilla       CACTACCGAGGGGTGAGCGGCTTGCGGGGAGGC----GGCCCGGGGCACGCGGGGCGGGAGCNNNNNNNNNNNNNNNNNN  721
```

```
Majority                   AGCAAGAACCAATTAATCAATXTTGATTGAGCCAATGTGAGGXATATAGAGAA-----TGAATGCXGTATXCCXXXX--C
                                          15690     15700     15710     15720     15730     15740     15750     15760
Human                      GCTGGAGTGCCGTGGCATGATCATGGCAGCCTCAACCTCCTGGGCTCCAGTGACCCTCCCACCTCAGCCTCCCAAGTAGC  15744
GuineaPig                  ATCAAGAAACAGTTAATCAATCTTGATTAAGACAATGTGAGGGAATCTAGAGGATTAGTTTCTGTCCTTTGAAAACTTAC  655
NorthernAmericanDeerMouse  AGCAAGAACCAATAAATCAATATTGATTGAGCCAATGTGAGATATATAGAGAA-----TGAATACCGTATTCC-------  464
Mouse                      AACGAGAACCAATTA-TCAGTGTTGATTGTGCCAGTGCGAAGAAGATGGAGAA-----TGCATCCTGGATCCCCTCCCTC  1716
ChineseHamsterGHOK1GS      AGCAAGAACCAATTAATCAATATTGATTGAGCCAATTTGAGATATATAGAGAA-----TAAATGTTGTATTCC-------  999
LongTailedChinchilla       NNNNNNNNNNNNNNNNNNNNNNNNNNNNNNNNNNNNNNNNNNNNNNNNNNNNNNNNNNNNNNNNNNNNNNNNNNNNNNNN  801

Majority                   --TCCCTCCCXTTGGGAGCATACACATTTA----ATTGTTGAGTXGAAACXAXXTGAACAAAAXCCACAACACATGA---
                                          15770     15780     15790     15800     15810     15820     15830     15840
Human                      TGGGACTACAGTTGTGTGCCACCACACCTGGCTAATTTTTGAGTTTTTTTTTGTAGAGACAGCGTCTCGCTGTATCGCTT  15824
GuineaPig                  ATATTAAACTGTTGAGTGAAGATACTTGTA-TAAACTCACAAGTATATAAAGTAATTATTAATAGTACTTATAATAGATT  734
NorthernAmericanDeerMouse  --TCCCTCCCCTTGGGAGCATACACATTTA----ATTGTTGAGT-GAAACCACATGAACGAAA-CCACAACACATGA---  533
Mouse                      CCTCCCTCCCTCTGGGAGCATACAGATTTA----ATTGTTGAGTTGAAACTATGTGAACAAAA-CCACAACCCATGA---  1788
ChineseHamsterGHOK1GS      --TCCTTCCCTTTGGGA-CATACACATTTA----ATTGTTGAGT-GAAACCACGTGAACAAAAACCACAACACATGA---  1068
LongTailedChinchilla       NNNNNNNNNNNNNNNNNNNNNNNNNNNNNN-NNNNNNNNNNNNNNNNNNNNNNNNNNNNNNNNNNNNNNNNNNNNNNNNNN  880

Majority                   AAAACTATTATTAATGGAAATGATAATGGGXCCTTXATGTATCATAAGTXAAXAGTGTAGAXAXAAGGGAGGTXXTACTG
                                          15850     15860     15870     15880     15890     15900     15910     15920
Human                      AGGCTGGTCTCAAACTCCTGGGCTCAAGTGATCCTCCTGCTTTAGCCTTGCAAAGTGCTGGGATTACAGGACTGAGTCTG  15904
GuineaPig                  CATTATGTGTCTCAAGTCAATGCTTAAAGGGAAGTGAAGACATAGGAAATTAGATGGTATTTTTTTAAGGATTAACAATC  814
NorthernAmericanDeerMouse  AAAACCATTATTAATGGAAATTATAATGGGTCCTTCATGTATCATAAGTCAATAGTGTAGACAAAAGGGAGGCATTATAG  613
Mouse                      AAAACTATTATTAATGGAAATGATAATGGGGCCTTTACGTATCATAAGTCAAAAGTATAGATAGAAGGGAGGTGTTGCCG  1868
ChineseHamsterGHOK1GS      AAAACTATTATTAATGGAAATGATAATGGGTCCTTTATGTATCATAAGTTAGTAGTGTAGACAAAAGGGAGGC-------  1141
LongTailedChinchilla       NNNNNNNNNNNNNNNNNNNNNNNNNNNNNNNNNNNNNNNNNNNNNNNNNNNNNNNNNNNNNNNNNNNNNNNNNNNNNNNN  960

Majority                   GXAATTAGATTTTTAAAAXGAATTGACAXTCCCTGAGGCAAATCAAGATTATTTAACCXTGTGGTGGCTTGGAAAAGTTC
                                          15930     15940     15950     15960     15970     15980     15990     16000
Human                      GCCACCTTTTTCTTTCTTTCTTTTTTTTTTAGACGGAGTCTCCCTTTGTTGCCCAGGCTGGAGTACAGTGGCTCTATCTC  15984
GuineaPig                  CCTAGGCAAATCAAGAATGTTTAAACGTTTGGGTGCTTAAGAGTTCCATAACAAAGTTTTGTTCTCTTGAGGTAGGGGCT  894
NorthernAmericanDeerMouse  AAAGTTAGATTTTTAAAAAGAATTGACAGTCCCTGAGGCAAATCAAGAATATTTAACCGTGTAGTGGCTTTGAAAAGTTC  693
Mouse                      GAAATTAGATTTTTAAAAAGAATCGACAATCCCTAAGGCAAATCAAGACTATTTGACCAGGTGGTGGCTTGGAAACGTTC  1948
ChineseHamsterGHOK1GS      ----------TTTTAAAATGAATTGACAATCCCTGAGACAAATCAAGAGTGTTTGACCATGTGGTGGCTTTGAAAAGTTC  1211
LongTailedChinchilla       NNNNNNNNNNNNNNNNNNNNNNNNNNNNNNNNNNNNNNNNNNNNNNNNNNNNNNNNNNNNNNNNNNNNNNNNNNNNNNNN  1040
```

Majority	TXATAXCAACAAXXGTGTGXXXXTGTGTXCXGGTXATGX-X-X--X-TAATTGCCTTTGAAACXGGGTCTCACTAAGTTG	
	16010 16020 16030 16040 16050 16060 16070 16080	
Human	TGCTTCCTGCAAGCTCTGCCTCCTGGGTTCACGCCATTCTCCTGCCTCAGCCTCCCGAGTAGCTGGGACTACAGGCGCCT	16064
GuineaPig	CACTAATTTGCACAGGCTGGCCTCCAATTCAAGTCATCCCCTG--CCTAACTTCCCCAGCAGCTGGGACTACAGACATGC	972
NorthernAmericanDeerMouse	TTAGAGCAACAGT-GTGTGT------------------------------TTGCTTTTGAAATAGGGTCTCACTAAGTTG	742
Mouse	TTAGAGCAACAACAGTGCACATATGTGTGTGGTTGTTGT--------TGATTGCCTTTGAAACAAGGTCTCACTAAGTT-	2019
ChineseHamsterGHOK1GS	TAATAACAACACT-GTGTGTGTGTGTGGGGGGGGGGGGTATGTGTTGTATTTGCTTTTGAAAT-GGGTCTCACTAAGTTG	1289
LongTailedChinchilla	NNNNNNNNNNNNNNNNNNNNNNNNNNNNNNNNNNNNNNNNNNNN--NNNNNNNNNNNNNNNNNNNNNNNNNNNNNNNNNN	1118

Majority	TCCAGGCXGATCTCATCCTXTGGAGTAGXTXGGGCCAGGTTCTGATCTATCATTTTAAAAATXATCTXCAAXCTACCATT	
	16090 16100 16110 16120 16130 16140 16150 16160	
Human	GCCACCACACCCGGCTAATTTTTTGTATTTTTAGTAGAGATGGGTTTTACCATGGTCTCGATCTCCTGACCTCCTGATCT	16144
GuineaPig	ACCATGGCGAGTAGCTTATGACAAACTATTCTCTCTCTTTAAGGCTCTGTCCTATCTAAAGTAATGTTTTAAAAAATTAT	1052
NorthernAmericanDeerMouse	CACAGGCTGATCTCATCCTCTGGAGTAGACCGGGCCAGGTTCTGATCTACCATTTTAAAAAATATCCACAACCTACCATT	822
Mouse	T-----C----TTCATCCTCTGGAGTAGCTGGGGCCAGGTTCTGACTTATC-TTTTAAAAATAATATACAATCTCCCATT	2089
ChineseHamsterGHOK1GS	TCCAGGCTGGTCTCATCCTTTGGAGTAGATGGGGTCAGGTTCTGATCTATCATTTAGAAAATTATCCTCAACCTACCATT	1369
LongTailedChinchilla	NNNNNNNNNNNNNNNNNNNNNNNNNNNNNNNNNNNNNNNNNNNNNNNNNNNNNNNNNNNNNNNNNNNNNNNNNNNNNNNN	1198

Majority	TAGXGAAGTGAAATTCCAGXTGGTAAGGGCAAGAGGTTTTCCXGAAGGXGACCXGGCAGATCTGGAGGXGGXATTGAGAA	
	16170 16180 16190 16200 16210 16220 16230 16240	
Human	GCCCACCCTGGCCTCCCAAAGTGCTGGGATTACAGGCGTGAGCCACTGCGACCGGCCCACTTTTTCTTTTTACTTTTAAA	16224
GuineaPig	TTAGAAACTAACATTTTCTAAAGTGAAATTCCAATTAATAAAGGATAAGAGGTGTGTCAGTGAGGAGATACAGAAAAACA	1132
NorthernAmericanDeerMouse	TAGTGAAGTGAAATTCCAGCTGGTATGGGCAAGAGGTTTTCCCGAAGGAGACATGGCAGATCTGGAGGGGGTATTGAGAA	902
Mouse	TAGTGAGGTGAAATTCCAGGTGGTAAGGGCAAG-AGGCTTCTTGAAGGGGACGCGGCAGATCTGGAGGAGGTATGGAGGA	2168
ChineseHamsterGHOK1GS	TAGCGAAGTAAAATTCCAGCTGGTAAGGGCAAG-GGTTTTCCTGAAGGAGACCTGGCAGATCTGGAGGGGGCACTGAGAA	1448
LongTailedChinchilla	NNNNNNNNNNNNNNNNNNNNNNNNNNNNNNNNNNNNNNNNNNNNNNNNNNNNNNNNNNNNNNNNNNNNNNNNNNNNNNNN	1278

Majority	CAACTGGAGAAACGCAGTTGXTGAGXGTTGAAXXGTGGXX-XTGAXGCXTXAXCGAAGGAXGGTGCTGGCATTAGTGXCA	
	16250 16260 16270 16280 16290 16300 16310 16320	
Human	AATGTGGCTAATAGAAATTTATGAGATTATATTTATGGTTCATACTACGTTTCTTTTGGACAGTGCCAGAGTGAATCAGA	16304
GuineaPig	TAAGAGTTGCCACTGAGAGTAACTGGAGAAATGTGGGGAATATTAGTTATTTCCCAAGACCTCTGGT-GTGTGTTGGAAG	1211
NorthernAmericanDeerMouse	CAACTGGAGAAACGCAGTTGGTGAAGGTTGAAAAGTAGTTAGTGAAGCCTAAAAGAAAGAGGGTGTTGGCATTAGCGTCA	982
Mouse	CACCTGGACAGACACAGTTGGTAAGTGCTGGAGGAGGT------AGGGAAGAGCAAAGGGTGGTGCTGGCATTAGTGGCA	2242
ChineseHamsterGHOK1GS	CAACTGCAGGAATGCAGTTG---AGTGTTGAAAAGTAG-----GAAGAGTGAACGAAAGAGGGTTCTGGCATTAGTGTCA	1520
LongTailedChinchilla	NNNNNNNNNNNNNNNNNNNNNNNNNNNNNNNNNNNNNNNNNNNNNNNNNNNNNNNNNNNNNNNNNNNN-NNNNNNNNNNN	1357

```
Majority                      TXXAGXGAGCATTTTCCAAAACCA----AAAXTTTGXTGGXGCTATGXTGCAAATACXTTAAAGGAGAACGGAAAACATG
                                       16330     16340     16350     16360     16370     16380     16390     16400
Human                         TA-AGCTTGCATTTTAAAATCCTAAGGGTAAATGCAATAGAGATAGAACGCAAATAATTGGGGAGGGGGGTTGACTGAAA  16383
GuineaPig                     TG-AGGGTGAAGGACCTCAAAGGTAGGGTGCTGGTAAAGGAGTAACCTAGTGAGGTGTTCCAAAGTGAGAGCCTTGTGAA  1290
NorthernAmericanDeerMouse     TCCAGTGAGCATTTTCCAGAACCC----AAACTTTGTTGATGCTATGCTGCAAATACCTTAAAGGAAAACGGAAAACGTG  1058
Mouse                         TGCCGGGAACATTTTCCGAAACC-----AAATGTTGCTGGGGCTATGATACAAATACATTAAAGAAGAACGGAAAACATG  2317
ChineseHamsterGHOK1GS         TCCAGTGAGCATTTTCCAAAACCA----AAACTTAGTTGAGGCTATGCTCCAAATACATTAAAGGAAAACAGAAAACATG  1596
LongTailedChinchilla          NN-NNNNNNNNNNNNNNNNNNNNNNNNNNNNNNNNNNNNNNNNNNNNNNNNNNNNNNNNNNNNNNNNNNNNNNNNNNNNN  1436

Majority                      TXXTATGCTATTAAAXTTAAGAXCTTXAGTCXTATXGGAAAGAGAGXXAXTTTGTCTAGGCCATTTT-AACATTTTCAGC
                                       16410     16420     16430     16440     16450     16460     16470     16480
Human                         TTAAAGATGTATAATCCAAAAGAAGGCAAAAAAAAAGGAAAGACACAAAGTGAGCTTATATGTTAAT-AGCGTGGAAAGG  16462
GuineaPig                     ACTTTATCGATCATCTACTAACATGTTAAAGTCAGCGGTGTGGTTAAAATTGAGACAAGGGGCATGT-GGAAAAGGGAGT  1369
NorthernAmericanDeerMouse     TAATATGCTATTAAATTTAAGACCTTGAGTCTTACAGGAAAAAGAGCGGCTTTGTTTAGACCATTTC-AACATTT-CAGC  1136
Mouse                         TTCTATGATAT------TAAGACCTTTAGTTCTATGGAAAAGTGAT---TTTTGTCTAGGCCGTTTT-AACATTTTCATC  2387
ChineseHamsterGHOK1GS         TATTATGCTATTAAACTTAAGATCTTGAGTCCTATGGAAAAAAGAGCGACTTTGTCTAGGCCATTTTTAACATTTTCACC  1676
LongTailedChinchilla          NNNNNNNNNNNNNNNNNNNNNNNNNNNNNNNNNNNNNNNNNNNNNNNNNNNNNNNNNNNNNNNNNNN-NNNNNNNNNNNN  1515

Majority                      CTCTCTGAC-AAGGCTAACTCAATTGTCTTCGXTXXCATTCTCATCTTTCCATAXCXCCAAATGTTGGAATGTACAAACA
                                       16490     16500     16510     16520     16530     16540     16550     16560
Human                         TTCTATGAA-GGGTTGTTTAGAGCCTTTAAGGGCAAAATTTTCTTGTCTGGTTGCAGGAAAGAGTGGCATTGTTTTTGTT  16541
GuineaPig                     CAATTTGGG-TAGCCCAGTTTAATGTTTCTAGATTATAGAAATGTTTCTAGATG---------GTGTGAATTCAACTATC  1439
NorthernAmericanDeerMouse     CTCTCTGACCAAGGCTAACTCAGTTGTCTTCGCTGCCATTCTCACCTTTCCATATCCCCAAATGTTGGAATGTACAAACA  1216
Mouse                         CCCTGTAACTAATGCTAACTCAATTGGC------------CTCCTCTTTCCATATCCCCAAATGTTGGAATACAGGAACG  2455
ChineseHamsterGHOK1GS         CTCTCTGAT----GCTAACTCAATTGTCTTCATTCCCATTCTCATCTTTCCATACCTCCAAATATTGGAATGTACAAACA  1752
LongTailedChinchilla          NNNNNNNNN-NNNNNNNNNNNNNNNNNNNNNNNNNNNNNNNNNNNNNNNNNNNN---------NNNNNNNNNNNNNNNNN  1585

Majority                      CTCTXTGACACCTCGCTC----AGCGTGTGACXATTCCCATXTAGGGAACTXGGAXTATATGTTTT--------TXATTT
                                       16570     16580     16590     16600     16610     16620     16630     16640
Human                         CACTTTTAAAGCTACAAA----AACAAGTAATGATGAGGAAAATTGGGACATGTTGAGGAAGTAAG--------TAATTT  16609
GuineaPig                     TCCACTCCCATCCGTGCT----TTCTTTCACATATCCAAATGTTAGTCACTAGCTATTGATGGCAT--------TTTTTA  1507
NorthernAmericanDeerMouse     CTG---GACACCCTGCTC----AGCGTGTGACCATTCCCATTTAGGGAACTGGGATTATGTTTTTTGTTTTGTCTTATTT  1289
Mouse                         CTTTCTGCCACCTCGCTCGCTCAGCGTGTGGCCATTCCCATGTAGGGATCTTGGAATATATGTTTT------------T-  2522
ChineseHamsterGHOK1GS         CTCT--GACACCTCGTTC----AGCGTGTGACTCTTCCTATTTAGGGAACTGGGAG-ATATTTTTT--------TAATTT  1817
LongTailedChinchilla          NNNNNNNNNNNNNNNNNN----NNNNNNNNNNNNNNNNNNNNNNNNNNNNNNNNNNNNNNNNNNNN--------NNNNNN  1653
```

```
Majority                    XTTGAXTXXTAACAGATGCACAAXAATTACXACCCAAAATGAGGATCAXXTTTTCTTCCAXTTGGCCGGATTGATTCCA-
                                      16650     16660     16670     16680     16690     16700     16710     16720
Human                       TTTGGAATGTAGGTTGAGTTAGAGTAGTAGAAGACAAAATTTAAAAAGTAGTTTTTGACAGTAATATGGAGAGCTTTGAG  16689
GuineaPig                   TATAAACGCTAATACTTCCATCAATCATTCCACTCAGGCCATTGAAAATATTTCCCACAGTTATACACTTTTAACCCAAA  1587
NorthernAmericanDeerMouse   ACTGCTTTTTAACAGATGCACAGTAATTACTACCCTAAAAGAGGATCACGTTTTCTTCCACTTGGCCGGATTGATGCC--  1367
Mouse                       -------CCTAACAGATGCACAAAAATTACCAACCCACATGAGAATCACCTTTTCTTCCTCTTGGCCTGATTGATTCC--  2593
ChineseHamsterGHOK1GS       ATTGATTCTTAACAGATGCACAGTAATTACTACCCGAAATGAGGATCAGGTTTTCTTCCAGTTGGCCGGATTGATTCCAT  1897
LongTailedChinchilla        NNNNNNNNNNNNNNNNNNNNNNNNNNNNNNNNNNNNNNNNNNNNNNNNNNNNNNNNNNNNNNNNNNNNNNNNNNNNNNNN  1733

Majority                    XXAGGTTTGTAAGCAAACXCCAATCTGGTGAXTTXTCCXTGXTTXGGAXXXATTGTTXTXXXTTCTAAAG-CAGTGGATC
                                      16730     16740     16750     16760     16770     16780     16790     16800
Human                       TGCAAAGGATTTGCAGTGAGGACCTTTGAAAATTGAACCTGCTTTCTCCTATCAGTATTTATGTTTAAATAAACTTGATT  16769
GuineaPig                   CGAAGACTGTGTTCGCTTCTACTTTGCGTGATTGAGCTCAGCTTTGGAAACCAAAATCTATGTTTCGACGTGATGAGTTC  1667
NorthernAmericanDeerMouse   --GGGTTCGGAAGTAAACGCCAATCTGATGATCTCCTTGTT-TTAGAAGTAATTATTAT---TACTAAAA-CAGTGGATC  1440
Mouse                       --AGGTTTGTAAGCAAACACTAAGCTGGTGAGCAGTCCTTAATTAGGAGATGTTGTTATATTTTCCTAAG-CAATGGATC  2670
ChineseHamsterGHOK1GS       CCAGATTTGGAAATAAACGCCAATCTGATGAGTTCTCCTTG-TTGAGAAGTATTGTTTT---TACTAAG--CAGTGGGTC  1971
LongTailedChinchilla        NNNNNNNNNNNNNNNNNNNNNNNNNNNNNNNNNNNNNNNNNNNNNNNNNNNNNNNNNNNNNNNNNNNNNNNNNNNNNNNN  1813

Majority                    TTXXTTCAGTAGGGTAAXATTTTTTAGGATGAAXAXTXTGTAAATAXGGTTTAGGATGCAGCTATAAAXTXTTTTTCXCT
                                      16810     16820     16830     16840     16850     16860     16870     16880
Human                       CTTTGTGAGTATTGTTATATATGTTTCATAACTACTGTTGTAGTAACGTTTCTTTTTTTTTTACTAGCTTGCTTTCATGC  16849
GuineaPig                   TACTTGCTTTAGACAAGTGTTTATTAAGCAGTGGTCTCGTTCAATGGTAATTGTTGGGGGGCTGGAGAAGGTCTGTAATA  1747
NorthernAmericanDeerMouse   TTTATTCAGTAGGGTAA----------------CAGTCTGTAAACAGGGTATAGGATGCAGCTATTAAACATTTTTCCCT  1504
Mouse                       TT-GACCAGGGGGGTAAGCTCTTTGAGGATGAAGAATTTGTAAACGAGGTCTAGGATGCAGCTATAAAGTCTTTTTCTCT  2749
ChineseHamsterGHOK1GS       TT-GTTCAGTGGGATAAGACTTTTTAGGGTGGACAGTGTGTAAATACAGTGTAGGACGCAGCTATTAGGTATTTT-CCCT  2049
LongTailedChinchilla        NNNNNNNNNNNNNNNNNNNNNNNNNNNNNNNNNNNNNNNNNNNNNNNNNNNNNNNNNNNNNNNNNNNNNNNNNNNNNNNN  1893

Majority                    AAXGCTGAGXAGGTGAAGGGTXXCCAGGCTGXGGCXGGGACTGGCCTXTGTGGGTGGXAATAAXCCAGCTTGGGGTTGGA
                                      16890     16900     16910     16920     16930     16940     16950     16960
Human                       CACGCTGTTGATTAAATATTTATGGGCCATTTTAAGGCTTCTAATCTTCATTTGTGATGAACTTTTGAGGAAGAGTTAGG  16929
GuineaPig                   AAAGGGGGCAACTGAAGCAAGGTGCAACATTTTGGCTTCGCCCACACCTGTCGAGGTAGAGCAGCTGCAGCTGGAAAGTG  1827
NorthernAmericanDeerMouse   AGGGCTGAGTAGGTGGAGGGTCCCCAGGCTGGGGCCGGGATTGGCCTGTGTGGGTGGAAGTAACCCAGCTTGGGGTTGGA  1584
Mouse                       AGAGCTGAGAGGGCGAAGGGTGCCCGGGCCGGGCCTGGGATTGGTCCTTT---GTGGTAATAATGCAGCTTGAGGTTGGA  2826
ChineseHamsterGHOK1GS       AAGGCTAAGTGGGTGGAGGGCCACCAGGCTGAGGCTGGGACTGGCCTGTGTGGGTGGCAGTAACCCAGCTTGGGGTTGGA  2129
LongTailedChinchilla        NNNNNNNNNNNNNNNNNNNNNNNNNNNNNNNNNNNNNNNNNNNNNNNNNNNNNNNNNNNNNNNNNNNNNNNNNNNNNNNN  1973
```

```
Majority                    XGGAAXAGTGGAAGCXGCCTGXGAGCAXXAGTXCXGCXGTCCGGGCCAGCCTGXGG-XACACTXGGGX-TGCXGGCXGGA
                                     16970     16980     16990     17000     17010     17020     17030     17040
Human                       TTTTGTAAGTGTTGAACTCCAATAAACACAGTTCCACTTTCCTTAACTTCTCAGAAAGATACTTTGCTATTTGATACAGA 17009
GuineaPig                   AATAATAGGGAAAAGCAGAGGCTGCCTGGAGTCCCGAGGTCCCGGCCACCCTGG---GGCAGAAGGGGTTGGGGGTCGGG 1904
NorthernAmericanDeerMouse   GGGAAAAGTGGAGGCTGCCTGGGAGCAATC---CTGCCATCCGGGCCAGCCTAAGG-CACACCGGGGGGTGCAGGCTGGA 1660
Mouse                       AGGAAGAGTGGAAGCAGCCTGAGAGCAGCAGGGTTGCAGTCCGGGCTAGCCTGAGG-CACACACGG---TGCAGGCTGCA 2902
ChineseHamsterGHOK1GS       GGGAAAAGTGGAAACTGCCTGGGAGCAATCAATCTGCAGTCGGGGCCAGCCTGAGG-CACACTGGG---TGCAGGCTGGA 2205
LongTailedChinchilla        NNNNNNNNNNNNNNNNNNNNNNNNNNNGGGTCCTCGGGTTCTCGG--GTCCTCG---GGTCCTCGGGTCCTCGGCGCGGG 2048

Majority                    GGXTGTCCCACTCACACCGCCCACAGXXGGGAAXX-AAGACAXCXGAGCCAGC-CACTGCXGCXGGCAXGTCXCCGAGGG
                                     17050     17060     17070     17080     17090     17100     17110     17120
Human                       TAGTTGTCTAGGCAAGCTATTAGCAGGGTTTCAGGTAAGACATATGTGTTTG--CTATTTGTTTT-AAGCACTCTTCCCT 17086
GuineaPig                   TGGTGTCCCATTCACACCGCCCCGGGCGGGAACTC-AGGATGCCAGGCCCAGCAGTCCCGGCAGGTCAGGCACCCGAGAG 1983
NorthernAmericanDeerMouse   GGCTGTCCCACTCACACCGCCCACAACAGGGAACC-CAGGCAGCCCAGCCAGC-CACTGCAGCAGGCACGTCGCAGAGGG 1738
Mouse                       GGCTGTCCCACTCACACCGCCCACGGAAGAGAAAG-AACCCAGGTCAGCCAGC-CACTGCAGCGGGCTCGTCTCCGAGGG 2980
ChineseHamsterGHOK1GS       GGCTGTCCCACTCACACCGCCCACAGAAGGGAACC-CAGGACACCTAGCCAGC-CACTGCAGCAGGCACGTCGCAGAGGG 2283
LongTailedChinchilla        CCGGG-CTCGGCCGCAGCGCCCGAAGGGTCGGAGG-AGCATCTGAGTGCCGGGGCCCTTCGCCTTGAAGGACCCCGAAAG 2126

Majority                    CGGGAGG-------------------CGGXCGCGCAGGCXGGCGGAGGCAGCCAACGGCGGAGGCXCCCACTCCCCCAXX
                                     17130     17140     17150     17160     17170     17180     17190     17200
Human                       CATTTAT-------------------TCAAATTGTAGCAAACATCTACTACACCCATTTCCAGTTCTTTGTCTTAATAGT 17147
GuineaPig                   CTGCGGG-------------------CGGAGGGACTCGCGCGGCGTCGCGGGGAAGGCAACGAGGCGGCGCCCACTCCTC 2044
NorthernAmericanDeerMouse   CGGGAG--------------------CAGGCGCGCGGGCAGGCGGAGGCAGCCAACGGCGGAGGCGCCCACTCCCCCACC 1798
Mouse                       CGGGAGGCAGTGCGCGCGCGGGCGGGCGGGCGGGCAAGCGGGCGGAGGCAGCCAACGGCGGAGGCGCCCACTCCCCC--- 3057
ChineseHamsterGHOK1GS       CGGGAG--------------------CAGGCGCGCGGGCAGGCGGAGGCAGCCAACGGCGGAGGCGCCCACTCCCCC--- 2340
LongTailedChinchilla        GTGGTGG-------------------TGAATGCACAGCCGTAAAAGT-TAAACCATCCCAGAAGGCGTCTTGGAAATAGA 2186

Majority                    TCXGTCCCTGTGCCCCGCCGXTCGTCTCCXGCCGCCGCXGGGCGCCCGXGCGCTXGGCTCTXT----CCGCXGCCGCGCG
                                     17210     17220     17230     17240     17250     17260     17270     17280
Human                       TCAACTATTATAAAAAGGAG-TGGTATATATATATATAAACTCTTGCTTGGCTTTGGTTTGATTGAATTAATAAGGTGAG 17226
GuineaPig                   TTAGTC-CCGCTCGTCTCC--TGC---CGCTGGGCGCCGGCGCGCTCG-GCTCTTTCCGCCA--CCGCCGTTGCCGCGCG 2115
NorthernAmericanDeerMouse   CCGGTCCCTGTGCCCCGCCGCTCGGCTCCCGCCGCCGCCGGGCGCCCGAGCGCTCGGCTCTTT----CCGCCGCCGCGCG 1874
Mouse                       --GGTCCCTGTGCCCCGCCGCTCGTCTCCCGCCGCCGCCGGGCGCCCGCGCGCTCGGCTCTTT----CCGCCGCCGCGCG 3131
ChineseHamsterGHOK1GS       -CGGTCCCTGTGCCCCGCCGCTCGTCTCCAGCCGCCG---GGCGCCCGAGCGCTCGGCTCTTT----CCGCCGCCGCGCG 2412
LongTailedChinchilla        TTATTTTCCAGATGGAACC--TACATTCGAGGGATTTCAGTGTTCCCCCGCATCTACGGCGA--AAATTGATGGCAAGAC 2262
```

```
Majority                    CTGCCCCXTGCGCTCXGGCACCT-----TCGGCAATTTCCGTCGGGCCCTGGCC----GCCATTTTCTCGCCGCTTGTGX
                                          17290     17300     17310     17320     17330     17340     17350     17360
Human                       TTTTTTTT-GTTTGTTTGTTTTGAGATGGAGTCTCGCT--CTGTTGCCCAGGCT----GGAGTGCAGTGGTGGGATCTC-  17298
GuineaPig                   CTGCCCTTTGCGCTCTGGCACCT-----TCGGCAATTTCCGTCGGGCCCTGGCC----GCCATTTTCTCGCCGCTTGTG-  2185
NorthernAmericanDeerMouse   CTGCCCCGTGCGCTCCGGCACCT-----TCGGCAATTTCCGTCGGGCCCTGGCC----GCCATTTTCTCGCCGCTTGTGT  1945
Mouse                       CTGCCCCGTGCGCTCCGGCACCT-----TCGGCAATTTCCGTCGGGCCCTGGCC----GCCATTTTCTCGCCGCTTGTGT  3202
ChineseHamsterGHOK1GS       CTGCCCCGTGCGCTCCGGCACCT-----TCGGCAATTTCCGTCGGGCCCTGGCC----GCCATTTTCTCGCCGCTTGTGT  2483
LongTailedChinchilla        CTGGTCGT-GGATTCTAGTTCCTATAGTCCGTCTAGGTTACTAGAGTACAAGTGTTAAGTTATTAATCTGAGGGTACTA-  2340

Majority                    GGCTCGCTGGCTGCXTGXXXCGGTXCTTGGGAGCGAAGCTTTGTCXGGTTCGGCAATGGACGGGTATGXACCCGGCGC-C
                                          17370     17380     17390     17400     17410     17420     17430     17440
Human                       GGCTCACTGCAGG-CCATTCTCCTGCCTCAGCCTCCCGAGTAGCTGGGACTGCAGGTGCCTGCCACCACACCTGGCT-AA  17376
GuineaPig                   TGGCTGCGTGGCT-CGGTTCTTGAGA-GCGAAGCTTTGTCC----GGT-TCGGCAATGGACGGGTATGTAATCGGC---C  2255
NorthernAmericanDeerMouse   GGCTCGCTGGCTGCGTGGCTCGGTTCTTGGGAGCGAAGCTTTGTCCGGTTCGGCAATGGACGGGTAAGGTCCCGGCGC-C  2024
Mouse                       GGCTCGCTGGCTGCGTGGCTCGGTTCTTGGGAGCGAAGCTTTGTCCGGTTCGGCAATGGACGGGTAGGTACCGGGAGC-C  3281
ChineseHamsterGHOK1GS       GGCTCGCTGGCTGCGTGGCTCGGTTCTTGGGAGCGAAGCTTTGTCCGGTTCGGCAATGGACGGGTA-GGTACCGGAGC-C  2561
LongTailedChinchilla        GCGTTGCGGGAATGCCATTCCAGTGTTGTGGACTTTTATCCTTTTGGTATTAAAGATGTTTAAAAATACGTTCGATTTAC  2420

Majority                    CGTGAGGAGATGGGXGXGGGAGAG--GAGTTTGGCCG----GGCCGCGGXTGGGGXCGGGGXCGGGTCGGG-----AXGA
                                          17450     17460     17470     17480     17490     17500     17510     17520
Human                       TTTTTTGTATTTTTAGTAGAGATG--GGGTTTCACCGTGTTAGCCAGGATGGTCTAGATCTCCTGACCTTGT---GATCC  17451
GuineaPig                   AGAAAGGAGATGCGTGTGAGAGAG--GAGCTG---------GAGCGCCCTTGGGTCGGGTCCGGGGTCGGG-----AAGA  2319
NorthernAmericanDeerMouse   CGTGAGGGGATGGGGAAGGGAGAGAGGAGTGTGGCCG----GGCTGCGGCCGGGGCCGGGGTCGGGGTCG--------GG  2092
Mouse                       CGTGAGGAGATGGGCGAGGGAGAG--GAGGCCGGCGA----GGCGGCGGCCGGGATCCGGGTCGGGTCGGGTCCCGGGAA  3355
ChineseHamsterGHOK1GS       CGTGAGGAGATGGGGGAGGGAGAG--GAGTGTGGCCG----GGCCGCGGCTGGGGTCGGGGTCGGGTCGGA-------GA  2628
LongTailedChinchilla        CGAATTTCGTTTTCTGTTTGAAAG--TTATTTC-------TAGACGAGATTGATGATAGTGCGAAGACGTAC---CATTG  2488

Majority                    GCGXGGCTCGGGXGXCCXCTCCAAGGCTGXGCTGCCGTTACCCAACCCCCGCCCCATCACACACACCCCTCCCXXGGCTT
                                          17530     17540     17550     17560     17570     17580     17590     17600
Human                       GCCCGCCTCGGAAATACTTTGCATTGTTGGATTGATTGGGTTTGAGCCCCTGTTG--TACTTAAGTGATTATGAACAAGT  17529
GuineaPig                   AATC-CTGCGGCGGCCCCTCCCAAGGCTGGGCTGCCGTTACCCAACCCCCGCCCCATCGCACACACCCCTCCCTTGGCTT  2398
NorthernAmericanDeerMouse   CCGGGGAGCGGG---GCTCGGGNNNNNNNNNNNNNNNNNNNNNNNNNNNNNNNNNNNNNNNNNNNNNNNNNNNNNNNNNN  2169
Mouse                       GCGGGGCTCGGGCGGCCCCTCCCAGGCCGCGCCGCCGTTACCCAACCCCCGCCCCATCACACACACCCCTCCCCGGGCTT  3435
ChineseHamsterGHOK1GS       GCGGGGCTCGGGCGGCCCCTCCCAGGCCGCGCCGCCGTTACCCAACCCCCGCCCCATCACACACACCCCTCCCCGGGCTT  2708
LongTailedChinchilla        GCTC-TCAGTGTGTTTCTCTCCATGGTT--ATTGTGGGTGTCTAGAAGATTCCTGAATGTTCA-ACAATTCTTAAAAGTC  2564
```

```
Majority                    CCCTCAGCGGCCTCGGCXXCXGXCTCGXAGCXC--AGCXTXGACXXXGAXXXXXGXXAGGXCXCGGGGCTGTTGTGGAGA
                                           17610     17620     17630     17640     17650     17660     17670     17680
Human                       CTTTTA-TAATCTGAATTTCCCTTTCCTAGTTTGTAATATTGAGGATAATAATTTCTAACTTGCATGATTGTTGTGAAGA  17608
GuineaPig                   CCCCCGGCGGGCTCGGGGCGAGAGAAGGAGAAGCCGGCTTGGAGGCCGG--GGAGCCAGGGCGGGGGGCAGTGGCGGAGG  2476
NorthernAmericanDeerMouse   NNNNNNNNNNNNNNNNNNNNNNNNNNNNNNNNN--NNNNNNNNNNNNNNNNNNNNNNNNNNNNNNNNNNNNNNNNNNNNNNNN  2247
Mouse                       CCCTCGGCCGCTCCGGCTACCGGCTCGAACCGC--AGCCGCGGCTGGGAGGCCGGGGAGGCCTCGGGGCTGCTGTGGGGA  3513
ChineseHamsterGHOK1GS       CCCTCAGCGGCTTCGGCGACTAGCGCGACCCGC--AGCCGCGGCTGGGAGGCCGGGGAGGCCTCGGGGCTTCTGTGGGGA  2786
LongTailedChinchilla        GTAAGAGTGAACCAGTAACCATTACAAGAGCAA--GACATTGACACTGT--ATTTTTTCTTTACAGAATTGTCACTGAGG  2640

Majority                    X-TCXXGXXGGCXTGACGGXGGGXGXXGXXXCGXGCAGCCXXGGGCGGXCCTGXXCAATTXGGAXCCATGTCGXXGCCXC
                                           17690     17700     17710     17720     17730     17740     17750     17760
Human                       TTAAATAAGACAATGATATTGGTAGGAGCTCTTTATAATCTTTCTCTCTTATGTACAATTCAAATATATGTCGGTTTAAA  17688
GuineaPig                   AGCCCCCTTTAGGTGGGGGGAGCCGGGCAC-CTCGCGGCGGCGGG--GACCCCAAAAAGTCTCCCCC--GTCCTTGCCCG  2551
NorthernAmericanDeerMouse   N-NNNNNNNNNNNNNNNNNNNNNNNNNNNNNNNNNNNNNNNNNNNNNNNNNNNNNNNNNNNNNNNNNNNNNNNNNNNNNN  2326
Mouse                       C-GCGGGGCGGCCCGACTGCGGGGGATGGGGCGGGAAGCCCGGGGCGGCCCTGCCCCCTCGGGAGCCATGTCGACGCCGC  3592
ChineseHamsterGHOK1GS       C-TCGGTGGGGCCGGACGGCGGGGGCTGGGGCGGGGAGCCCGGTGCGGCCCTGCCCCCTCGGGAGCCATGTCGACGCGGC  2865
LongTailedChinchilla        T-TGCTGTTGGTGTGAAGGTAGGAGAAGAT-TGTACAGTATTTGAAAGAAATAAGAAAATTGGGTTT--GTTGGATCTAC  2716

Majority                    XGXCAG---ACCCGAATCAXXXGGTXGXGXCGGXGXCGACXXCGXXGGTGXXGGXGXXAGTGCGCAAGGXCACAGXGCCC
                                           17770     17780     17790     17800     17810     17820     17830     17840
Human                       TACCAAAATACCACTTTAAAAAATCTGGGCTGAAATGGACACTTCTAATGTAGG-AGTAGTGCTTTAGAAAATAATGCTT  17767
GuineaPig                   CTTCAG--GAGCCATTTCGATCGGTTTTCTGGGACCCGAACCCGAGGGTGGCGG-GGCGGGGCGCGGGGGGCGAG-GCCC  2627
NorthernAmericanDeerMouse   NNNNNN---NNNNNNNNNNNNNNNNNNNNNNNNNNNNNNNNNNNNNNNNNNNNNNNNNNNNNNNNNNNNNNNNNNNNNNNN  2403
Mouse                       GGGGAG---ACCCGAACCCTGGGGTGGCGGCGGCGGCGGCGGCGGCGGCGCGGGCGCGAGCGGGCCAAGCCGCGGAGGCC  3669
ChineseHamsterGHOK1GS       GGGGGG---ACCCGAACCTTGGGGTGGCGGCGGTGGCGGCGGCGGGCGCGCGGGCGCGAGTGGGGAACGCCACGGAGGCC  2942
LongTailedChinchilla        CGTCAT--TACCATAATTAGTAGAGAGTTTCACTTTTGAAAACTATGGTATAGCTGACAAAACTCATGCATAAAATGCTT  2794

Majority                    TGTGTXGTGCXXXT---XGXXTAX-CGAGGXCCGXCATCCCXXGCCTCGCCCGAACCACCGCGXCXGGAGAGCXGAGTGG
                                           17850     17860     17870     17880     17890     17900     17910     17920
Human                       TGTTTCGTATTCTTTTCCTAGTATTTTATGTCTAGTAACTC--ATAAGGAAAAAATGATCA-GATAAGTATATTTTTTAG  17844
GuineaPig                   GGGGC-GCGGGGCTAG-GGGGCAGCCGGGGACCGGACTGCC--GTTCGCGGCGACCCGGCCCGAACCTCCCCCTCCCCCG  2703
NorthernAmericanDeerMouse   NNNNNNNNNNNNNN---NNNNN---NNNNNNNNNNNNNNNCCGGCCTCGCCCGAACCACCGCGGCGGGAGAGCCGAGTGG  2477
Mouse                       TGTGCGGAGCGCGA---AGCCTGAGCGAGGCCCGCCATCCCCGGCCTCGCCCGAAACGCCGCGGCCGGAGCGCCGAGTGG  3746
ChineseHamsterGHOK1GS       TGTGTGGGGCAGCG---AAGCC---CGAGGCCCGCCATCCCCGGCCTCGCCCGAGCCACCGCGGCGGGAGAGCCGAGTGG  3016
LongTailedChinchilla        CGTTT-GTAAAATT---GGATTAAACAACAACCATGAAGTT--GGATAATATAAATCAAC---AGTCATAAGCTGTCCAA  2865
```

Majority	GAGCXGCGGGGAAGXGGAGGGXGCGTGGCGGCGAX-XXGAXXGXX-GXXXAXG-X-GXGAGGGAGGAGGGCACATGTCAC	
	17930 17940 17950 17960 17970 17980 17990 18000	
Human	ATGCTGATTACAGCATTATTT-AAATAGTAGAAATAACAACTGTTCAGTGATAAGACAATGAATAAATTCTGTTCATAAA	17923
GuineaPig	GTGCCGGTGGCGGGAGCCCC--GGGA-AAGCCGAGGGGGAGCCGC-GGGAAGGCGGGAGGAGGGGGAGGGCAGATGTCAC	2779
NorthernAmericanDeerMouse	GAGCCGCGGGGAAGGGGAGGGCGCGCGGCGGCGAGCGGGCGGGCGGGCGGGAGGGAGGGAGGGAGGAGGGCACATGTCAC	2557
Mouse	GAGCTGCGGGGAAGGGGAGGGCGCGTGGCGGCG------------------------GGAGGGAGGAGGGCACATGTCAC	3802
ChineseHamsterGHOK1GS	GAGCCGCGGGGAAAGGGAGGGCGCGTGGCGGCG------------------------GGAGGGAGGAGGGCACATGTCAC	3072
LongTailedChinchilla	GAACTGACAGTAAGAATAAC--ATTTTGAAGTAATTATGAATGGT-GTTCAAG---GAATTAATGCTTTGCACATGTGAC	2939

Majority	ACTCCTTTGTTTTCATCCGAGTCTGGGAGXGGGGGCGGGAG--GXCATXXAA-ATXXCTTT-ACXAXTXXXTXX----XA	
	18010 18020 18030 18040 18050 18060 18070 18080	
Human	CATTACAAATGTTTTTTAAATATTTTTCATATGTGTGAAAAGTAGGATTAAAAATAACTTTGACAAATGCATAC----AA	17999
GuineaPig	ACTCCTTTGTTTTCATCCCAGTCTGGCTGTGGGGGCGGGA---GGCATTAAA---AAAAT--GCCATTTTCTGA----GG	2847
NorthernAmericanDeerMouse	ACTCCTTTGTTTTCATCTGAGTCTGGGAGCGGGGGCGGGAG--GCCATCGAA-ATGCCTTT-AC---------------A	2618
Mouse	ACTCCTTTGTTTTCATCCGAGTCTGGGAGCGGGGGCGGGAG--GCCATCGGA-ATGCCTTT-ACCACCACCACAACGACA	3878
ChineseHamsterGHOK1GS	ACTCCTTTGTTTTCATCCGAGTCTGGGAGCGGGGGCGGGAG--GCCATCGAA-ATGCCTTC-AC---------------A	3133
LongTailedChinchilla	TTTTGTCAATATAAAATTGTATCATTTTTTGAGGGCAGGCAATGGTTTTAAATGGAAATT--GCAATTTTTTACTT--AA	3015

Majority	ACAAXAAXATTTC--AAGGCTTGGCCCAAAGGGCCCAGAAATTAAAXCXTAAACA-XAXCXAAACCCC-ATGAAGTGTT-	
	18090 18100 18110 18120 18130 18140 18150 18160	
Human	TTTATATGTGTCCCCAAAAGCTGGAGGGCAGTGTACCAAAATAGAGTAGTTTTCTCCAGGTTGATGGG-ATTAGTTTTT-	18077
GuineaPig	CCTGAGAACGTTGAAGAGGCTTG-GCAAAAAGGCCCAGAAATTAAATCATCA----AAG--AAAAAAA-AA-GTATTTG-	2917
NorthernAmericanDeerMouse	ACAA-AAAATTTC--AAGGCTTGGCCCAAAGGGCCCAGAAATTAAACCGTAAACA-CAACAGAACCCC-AGGAAGTGTTG	2693
Mouse	ACAACAAGAATTC--GGGGCTCGGCCCACAGGGCCCAGAAATTAAGCCATAAACA-AAACCAAACCCCCATGAAGTGTTG	3955
ChineseHamsterGHOK1GS	ACAA-AAAATTTC--AAGGCTTGGCCCAAAGGGCCCAGAAATGAAACCATACACA-AAACAAAACCCC-GTGAAGTGTT-	3207
LongTailedChinchilla	ATCGTGAGTTTCGGGAAGGCTTATATGCTTATTCTCAGGCTTAAAATTGCAT----CAG--TGATG------GTGTGTG-	3082

Majority	----AXGGXAGCATCAGTCACATGGGXAAAGGCCACCAAG--CCATGGCAGAGAAAAGAXTAGATAATCCACACAXGGTT	
	18170 18180 18190 18200 18210 18220 18230 18240	
Human	----TGTAATTCTTTATATATTTTCTCTATATTCCACATTTTAAGTATTTTATGATAAAGTTATTAATATTGATAAAGTT	18153
GuineaPig	----AGGTCGCCATCAGTCACATGGGTGAAGGCCAACAG---CCATGGCAGAAACAAGAGCAGATAAGTCAACGCAGGGC	2990
NorthernAmericanDeerMouse	GATGGAGGTAGCATCAATCACATGGGCAAGGGCCACCAAG--CCGTGGCAGAGAAAAGAAAAGAAATTCCACACTGGGTT	2771
Mouse	G--AGAGGCAGCATCAGTCACATGGGCAAAGGCCAC-AAG--CCACG-CAAGGCAGACAACAGATAATCCACACAGGGTT	4029
ChineseHamsterGHOK1GS	--TGAGGGTAGCATCAATCACATGGGTAGAGGCCCCCAAG--CCATGGCAAAGACAAGAATGGGTATTCCACACAGAATT	3283
LongTailedChinchilla	----AATAATTTTTTAGA-ACACTTTTGAAGGTGTGTTGTTTCCTTGTCTGATAGTGAAGTAAATAATT--GCCCATGCT	3155

```
Majority                      TTTTXTTTTXXXTXXTTXXTTXXXTXC-TGXTATXCXC-XXGCTGCTTTGGTXAGTTTXTGGGXXAAXGAAAXAXACAAA
                                               18250     18260     18270     18280     18290     18300     18310     18320
Human                         ATTAATGTTGCTTAATTGATTAAACCC-TGATATTTTC-TTGTTTCTTTAAAAAACGTTGCATAACTTGATATAAATCTT  18231
GuineaPig                     CATCATTTTAGCTACTTGCTTAGTTTCTGGGGAAAAAAACCCAGGCCAACCCAAGTACTACAAAAAG-G---TAGAAAGG  3066
NorthernAmericanDeerMouse     TTCTTTTTCTTTTCTTTCTTTCTTTCTTTTTTTTCCTC-AAGCCGCTTTGGTTAGTTTCTGGGGGAAATAAAAA--CTAA  2848
Mouse                         GGCCTTCCCC-------------------------CGC-CCCCTACTTTGATTAGTTTCTGGAGGAAAAAAAAATACAAA  4083
ChineseHamsterGHOK1GS         TTTTTTTTTT-----------------------------AAGCTACTTTCGTTAGTTTCTGGGGGAACAAAAAA--CTAA  3332
LongTailedChinchilla          TTATATTTAGGCTTATTTCTTGGGTTCATGTTATAGAAATTAGGG--GAGGAAAACATTTGGGAAAG-GGCATAGTTAAT  3232

Majority                      AATAAXXGXAAAXA------XXX-AXXXTTXACAATCCGAXCXAATATGGAAXAXX-A-XTGAGT-CGTGAXGCATCXTT
                                               18330     18340     18350     18360     18370     18380     18390     18400
Human                         TTTAATTGTACAAAAAAAC-ACATAAAATTTACTATCTTAACCATTTTTAAGTGTACACTTAAATTGTTTAATTATTCTT  18310
GuineaPig                     CACAACCGTG----------ATGCATCTTTGAGAGCCCGAGTGAAGATGGAATGGCTGAGTGAG--GTGAGACACGGCCT  3134
NorthernAmericanDeerMouse     AA-----GCAAACA-----------------GAAATCCGAGCAGACATGGAACA-------GAGT-CGTGATGCGTCTTT  2898
Mouse                         AATAAAAACAAACACCCCCCCCCCCACACCAGCCCCCCAAGCAAATATGGAACATGGATCAGAGT-CGTGTTGCATCTTT  4162
ChineseHamsterGHOK1GS         AAAACAAACAAAAA-----------------AAAATCCCAAGGAACACGGAACAA------GAGT-CGTGATGCATCTTT  3388
LongTailedChinchilla          TTTATTTGTG----------GTG-ATATTTTCCTGGTTGAAATAATACGAGATTGGAAGTTGGG--AATAGAGAGGCCGG  3299

Majority                      GXAGAGACTGGGCGTXXAGACAX---XXTGCAGAGXGCGATGXGAGXACTGTGXGAAXAXATGAGGAXXXXXXCXAATGT
                                               18410     18420     18430     18440     18450     18460     18470     18480
Human                         ATACGTACTTAATAATATTACAT-----TGCAAAACCCATCTCCAGAACTTTTTGGAGAACTGAAACTCTATACTGATTT  18385
GuineaPig                     CTGGGAAATG--ATACGGACCA-----------GTCTCTCTGGTACCAATGCTCCACATCTGAAATGACTCAGTTTAACT  3201
NorthernAmericanDeerMouse     G-AAAGCTTGGGCGTTGAGATAGA-GGATGCAGAATGAGATGGGAG--CTGTGTGAAAAGATGAGGA------CCAATGT  2968
Mouse                         GGAGAGGCTGGGCGTGAAGACCGGAGGATGCTGAGTGAGATGAGAGAGCTGTGAGAAGAAAAGAGGAGGCTCACCAATGT  4242
ChineseHamsterGHOK1GS         G-AGAGCCTGGGTGTGAAGATAA---GATGCAGAGTGTGATGAGAG--CTGTGAGAAA-GATGAGGA------CCAATGT  3455
LongTailedChinchilla          GGATATAGTT--CAGCGGCACA-----------AGCCCCTGCCTGGCAA-GCGCAAGGTCCTGAGTTTGATTCCTGATAC  3365

Majority                      CXCGCCTXTGTTAXTTCCACATCTCXAAGGACXCAGTTXAACTCATTGTTXAGAXXXXXXAGXTXAXTTXCXG-TAAAXA
                                               18490     18500     18510     18520     18530     18540     18550     18560
Human                         TAAAACTCCCTCTTTTGTCCTTCTGCTGGTAACCAGC--ATTCTATTTTCTGTGTCTATGAATTTGACTACTT-TAAATA  18462
GuineaPig                     CATTGTTAGAAGGTGGGCACA--CTGAAGCACGAAGAC-AGAGAAGTGCCCAGCTCCTTCAGAAAACTGTCAG-TTAACA  3277
NorthernAmericanDeerMouse     CGCGCCTGTGTTAACTCCACATCTCAAAGGACTCAGTTCAACTCATTGTGAAGAGAAGGGGGCACAGTTCTGGATAAAAA  3048
Mouse                         CGCGCCCTTGTTAACTCCACATCCCGAACGACCCAGTTCAACTCATTGTTCAGAG---------------------AAGG  4301
ChineseHamsterGHOK1GS         CGCGCCCGTGTTAATTCCACATCTCAAAGGACTCAGTTCAACTCATTGTTAAGAG-----GGCTCAGTTCTGGATAAAAA  3530
LongTailedChinchilla          CAAAATAAGAAAGTTGTGAAAAGTTCAAGAGTGCAAA--ATGTAGGTGATGTTGTTCTCCAAATAAATTTC---TTCCC-  3439
```

```
Majority                     CXTGACACTACTXAXAGCACAXAXAGTGGXXGXXGGATCA--------XXAAAGAAAXGTCAGGXAACXTGAACATTTAT
                                          18570     18580     18590     18600     18610     18620     18630     18640
Human                        CCTCACATAAGTGGAATTATACAGTATAGGTTAAGCATCCCTAATCTGAAAATCCAAAGTCTGAATGCTACAAAATCTGA 18542
GuineaPig                    AGTACGTGTACTGAAATCAGTGACAGTTTTTGAAGAATCA--------AAAGTAAAAAGTTCTTAGTCTTTAAGACACTG 3349
NorthernAmericanDeerMouse    CATGACACTACTCACAGCTCATTTAGGGCAAGGGGGATTA----------AAAGAAAC-TCGGGTAACATGAACATTTAT 3117
Mouse                        GG-GCCACAGTTCACAGCCCACAGAGAGGAAGGGGCACGA----------AAAGAGAC-CCAGTGAACATGAACCTTTAT 4369
ChineseHamsterGHOK1GS        TATGACACTATTCACAGCTCATATAGGGGGAGGGGGATTA----------AAAGAGACCTCAGGTAACATGAACATTTAT 3600
LongTailedChinchilla         --TGAATTTTCTGAAAAAAGAATAAATGATTTAAGGTTCTTTTGT---AAAAGAAAAAGTGATGCAAGTCTGAGTTGTGT 3514

Majority                     XAAAATXGXGTXTCXGTATTTGAXGXATCTAAXXTAGTAAAATCXXXGTTTATTXAGTXGGTTAT--ATTGAGXT-XAGT
                                          18650     18660     18670     18680     18690     18700     18710     18720
Human                        AACTGGTGTGTGTTTTTTTTTTTTTTTTTTTTTTTTTTGAGACGGAGTCTTACTCTGTCGCCCAG--GCTGGAGTGCAGT 18620
GuineaPig                    AATGATCATGTAAAAATAACAGAGCCATAAAAGTTGTATTTTCCTAGAAGTCGTCTTAGAGATAG--ATTATTTTCCAGA 3427
NorthernAmericanDeerMouse    TAAAGT-GAGTATCAGTGTTTGAGGGATCTAA---AGTAAAATCCGAGTTTATTAAGTTGATTATCAATCTAG------T 3187
Mouse                        TAAAATTGAGGGTCTGGATTTGACGAGTCTAAGAAAGTAAAATCCGAGTTTATTCAATAGGTTATCAGTCTAGAGTTAGT 4449
ChineseHamsterGHOK1GS        TAAAAG-GAGTATCTGTGTTTGAAGAATCTAA---GGTAAAATTTGAGTTTATTAAGTTGGTTAT--ATAGAG------T 3668
LongTailedChinchilla         ACTGTTGGTGGGTGAGAAGTTTATAGACAAGACCTAGAATGAAAAAGCAGAAAAAAGTGAGGAGA--CTTGGAATAAGAA 3592

Majority                     TXXCTGATGTTTTTTXAGAGATAXTXXAAAGTAXCAGXXTCXAGACATCAT-XCCAXAAATTACCTTAAXGGXXTATXAT
                                          18730     18740     18750     18760     18770     18780     18790     18800
Human                        GGCGCGATCTCTGCTCACTGCAAGCTCCACCACCCGGGTTCACGCCATCTT--CCTGAG--TAGCTGGGACTACAGGCGC 18696
GuineaPig                    TGAAATATGA--ATTCA-AAGAAGTTCA--GTATTTCCCCCATATCAATA---GTGGAAATTGATGACAAAACCTGGTCT 3499
NorthernAmericanDeerMouse    TATCTGATGTTTTTA--GAGATAATAAAAAGTAACAGATCTTAGAAATGAT-GCCACAGATTACCTTAATGGGATACCAT 3264
Mouse                        TATCTGGTGTTTTAGAAGAGATAATAAAAAG------GCTTTAAACATCATTGCCACAAATTACCTTAATGGGATATTAT 4523
ChineseHamsterGHOK1GS        TACCTAATGTTTTTAA-GAGATAATAAAAAGTAACACACTGTAGGAATCAT-GCCACAAATTACCTTAATGGTTTATCAT 3746
LongTailedChinchilla         AGGGTTGTATT-GCTTG-TACCAGTTCT--CTTTTAGTTTCAGGATAAAAT--ACTGAAAATGTTAAAAAAAATTATTAA 3666

Majority                     TTACCACAAGXAATG--TAGATTTTGACATGTAXXXAATTXTCTCCAXACAXT-GAT--XGXAXXTTGXTXAAAAATXXX
                                          18810     18820     18830     18840     18850     18860     18870     18880
Human                        CCGCCACCACGCCCGGCTAATTTTTTGTATATATATATATATTTTTTAGTAGA-GAC--GGGGTTTCACTGTAGCCAGGA 18773
GuineaPig                    -TAGATTCATGTTTC-CTGGTTCCTGTATTCTATGTAGAT-TACTAAAA----------AGTACAAGTGTTAAATCTGGT 3566
NorthernAmericanDeerMouse    TTTCCACATGAAATG--TAGATTTGGACATGTTCTGAATTCCCTCCAGACAATTGATGACAAAACCTGAGCAGAAACCCG 3342
Mouse                        TTACCACAGGAAATG--TAGCTTTAGACATGT---GAATTATCCCCAGAC-----A------------------------ 4569
ChineseHamsterGHOK1GS        TTTCCACATGAAATG--TAGATTCACACATGTTCAGAATTCCCTCCAGACAGTTGATAGCAAAACTTGAGCAGAGATCCG 3824
LongTailedChinchilla         GCATTTTAAAGGCCA-TTAAAAATTTGAGTAAAGTTAGTT-TAACATAGTTCT-TTT--AGGATATTGGTTAAGAATTTT 3741
```

```
Majority                      TGTTXTCCAATTTCCAGAT--TXTATTTAGXTTACCAXAGAACTTGAXAXATGTTGCXXATGTCAGXAXAXAAGCACTGT
                              18890 18900 18910 18920 18930 18940 18950 18960
Human                         TGGACTCTTATCTCCCGACCTCGTGATCCGCCTGC-CTCGGCCTCCCAAAGTGCTGG-GATTACAGGCCTGAGCCACCGC 18851
GuineaPig                     TGTGCTAGCATTTGGGGAA-TGACATTCTAGTGT--TTGGAACTTTTTATGTTTT-----TGTTACGAAAAA-------T 3631
NorthernAmericanDeerMouse     TGTTTTCCACTTCCCAGAT--TCTATGTAGATTACCAAAGAACCGGACTTACATTGCTAATGTCAGTAGACAAGCACTGT 3420
Mouse                         -GTTTTCCAATTTCCAGAT--TTTGTTTAGGTTACCAAAGAACTCACTCAACGTTGCTGATGTCAGTATATAAGCACGTT 4646
ChineseHamsterGHOK1GS         TGTTTTCCAAATCCCAGAT--TCTATCTAGTTTACCAAAGAACTTGAGTTATATTGCTAACGTCAGTATACAAGCACTGT 3902
LongTailedChinchilla          TGCTCTGGTATTTATGTAC-AAGTAATTAATTTTCTTTAGAAATTGAAAAAAGTT-----TTAAAGGAAATAATA--TGT 3813

Majority                      XGGXATAGAAT-XAAXGTTTCTGGATTTTCATCTTGXTGXTAAAGGTTCTTAAXAATTATAATAAACTGXCTGAAACXTX
                              18970 18980 18990 19000 19010 19020 19030 19040
Human                         GCCTGGCCAATCTGAAACTTTTTGAGCACTTACTTGATGCTCAAAGGAAGTGCTTAGTGGAGCATTTTGGATTTTAGATT 18931
GuineaPig                     GTTTAAAAAACACTAAGTTTACTGAATATC--ATT--TTAT--GTTTCATAGTTATTTCTAGAAAACTAGAT----TGAT 3701
NorthernAmericanDeerMouse     AGGAATGGCATGTAGTGTT-CTGGATTTTCATCTTGCTGTTAAAGGTTCTTAAAAATTAC-ATAAACTGACTAAAACATC 3498
Mouse                         AGGAATAGTAT--AGTGTTTCTGCATTTTCATCTTGCTGTTAAAGGTTCTTAAAAATGATCATAAACTGACTGAAACGTC 4724
ChineseHamsterGHOK1GS         AGGAATGGCAC--AATGTT-CTGGATTTTCATATTGTTGCTAAAGGTTCTTAAAAATTACCATAAACTGACTGAAACATC 3979
LongTailedChinchilla          GTTTAAAAAATTCAAAGCGAAACAGTTGTC--CTTGTTTATTAGTTTGCTTAATAAGTGTAAATAGTTAGGTGCAACGTT 3891

Majority                      XXAXGXTTCAAAX--TTGTTTXTAAAAAAAXAAAT-X--AAATAXTTXCCAAGTAXXTAATAXCTT---XTAAXTXXGTA
                              19050 19060 19070 19080 19090 19100 19110 19120
Human                         TTCAGATTAGGAATGCTCAGGTTGTGTTTGTCCTTTTGTGACTGGCTTATATTCACTTAATATAATGTCCTCAGGGTCCA 19011
GuineaPig                     AATAG-TACAAAGACGTATTGTGGGTGTCTGGAATATTCATAAATTTTC-AATTTTTTTAAAAGTT---CTAAGTGTCAA 3776
NorthernAmericanDeerMouse     TCATGTTTCAAG---TTGTTTCTAAAAA----A---------T-----CCAGGTAGATAATAGCTT----TAAA-AAGTA 3552
Mouse                         GAATGGTTCAAAA--TTATTTCTAAAAAAAAAAAA----AAACAAAAAACAAAAACAAAAAACCTC----CAGGTAAATA 4794
ChineseHamsterGHOK1GS         CCATGTTTCCAG---TTGTTTCTAAAAAAAAAAAA----AAATTAT--CCAGGTAGATAATAGCTT----TAAA-AAGTA 4045
LongTailedChinchilla          ATTAGGTGTAGATAATTGATCTAAAATAAAGTATTTTGTGCATATTTCTGAAGTTATTTATATGTTA--CCATATGTGAA 3969

Majority                      TCAXT-XGAAXAGXTTXXXACTGGTTTTCTTCCTGTATTGCXAGTGTCXGGXXAATTXATTAXAGATCTTXAAAGTTTXA
                              19130 19140 19150 19160 19170 19180 19190 19200
Human                         TCCAT-TTTGTAGCATGTGACAAGATTTCCTCCC-TTTTTAAGGCTACATAGCATTCCATTGTGGTACACAAAAGTTCAT 19089
GuineaPig                     GCAGTAAGAAGAGATTGAAACTGTATTTTTTCA--TTACAGAATTGTCACTGAGGTT-GCTGTTG-GTGTGAAGGTAGGA 3852
NorthernAmericanDeerMouse     TCACT--GAGCATCTCTCTACTTGTTTTCTGACTGTATTGC-AGTGTCTGGAGAATTTATGAAAGATCTTGAAAGTTTAA 3629
Mouse                         ATAGTTAAAAAAAATTATCACTGAGCATCTG--TGTATTGCCAGGGCCTGGGTAATTTATAAAAGATCTTAAAAGTTTTA 4872
ChineseHamsterGHOK1GS         TCACT--GAACATCTCTGTATTTGTTCTCTAACTGTATTGCTAGTGTCTGGAGAATTAATGAAAGATCTTGAAAGTTTTA 4123
LongTailedChinchilla          T--ATCTTTAAAGATTAAGACTGTTCTGCTTCACATCTTGGCCTTTTCAAAAAAATTAGCTATTG-AGATAAAATTTCCC 4046
```

```
Majority                      AAGTTAXGCACTX-TXATTTGAAGAATAAGAGGTTGAXACXTTTA---TTTTTXCCTTTTCAGAATTXTCACTGAGXTTG
                                        19210     19220     19230     19240     19250     19260     19270     19280
Human                         --TTGAAGCCCC--CTGTGTCATAACTCAGACTTCCATAACAAAATACCATAGACTGAGTGACTTAAAAAACAGAGATTC  19165
GuineaPig                     GAAGATTGTACA--TTATTTCAAAAAAAAGAAAACT-TGGCTTGT-----TGGATCT-ATTGT---CATTACCGTAATTA  3920
NorthernAmericanDeerMouse     AAGTTATGCAATGATCATTTGAAGAGTCAGAGGTTGACACTGTTA---TTTTTTCCTTTACAGAATTGTCACTGAGGTTG  3706
Mouse                         AAGTTAAGCATTGATCATTAGAAGAGTAAGAGGTTGACACTTTTA---TTTTTTCCTTTACAGAATTGTCACTGAGGTTG  4949
ChineseHamsterGHOK1GS         AAGTTAAACAGTGGTCATTAGAAGAATAAGAGGTTGACACTTCT----TTTTTTCCTTTTCAGAATTGTCACTGAGGTTG  4199
LongTailedChinchilla          --ATAATGACCC--TTATTTAAGGCATGCAATGTTT-TGGCATCTG--TACACACCT-GTGACAAACATCACCATGATCA  4118

Majority                      XTGTTGGTGTGAAGGTAGGAGAXTXTGXTTXAAAGXAAAGAGGAAACTXGXXATGTTXAATTTACTXCXATTGTAAAGTX
                                        19290     19300     19310     19320     19330     19340     19350     19360
Human                         G-TTTTCTCACAGTTCTGGAAACTGGGAAGTCCAA-GATCAAGGTACCAGC-AAGGCAACTTTCCTTCTGAGGCCACTTC  19242
GuineaPig                     GTAGAAAGTTTCACTTTGAAAACTATAATATAGCT-AATAAAACTCAAGCATAAAATAC-TTCATTTGTAAAGTTGGATT  3998
NorthernAmericanDeerMouse     CTGTTGGTGTGAAGGTAGGAGATTGTGTTTGAAAGTCAAGGGGAAACTAGTATTGTTGAACATACTACCATTATAAAG--  3784
Mouse                         CTGTTGGTGTGAAGGTAGGAGATTATGTTTGAAAGTAACGTGGAAACTGGGGATGTTGGATATGGTACCATTGTAAAGAG  5029
ChineseHamsterGHOK1GS         CTGTTGGTGTGAAGGTAGGAGATTGTGTTTCAAAGTAAAGTGGAAACTAAAAATGTTGAGTCTACTATCATTATAAAGAG  4279
LongTailedChinchilla          G-AATAAGAATAGTACGAGTGACTATTACTCTCAG-AAATACCCCCTTGCCTCTGATAAATTCCCTTCTACTCTTTCTTC  4196

Majority                      TCAAAAXTT-CAGTXAACC--TTGACAXCAAAXXCCAXXXXTAAAATATTTCXTTTAAXTTGTAAXGTCAGAXTXTATAA
                                        19370     19380     19390     19400     19410     19420     19430     19440
Human                         TCTTGAAGT-GAGGCCACCATTTTACTTTGTGTGCCTCACATGACCTTTTCTTTGTGAGCACACTTTGGGGGAGTGGGTG  19321
GuineaPig                     ACATAA-------ACAACT--TTAAAGTTAGATAATATCATCA--ACAAGAGTCATAAGCTGTTCTA---GAACTGA---  4061
NorthernAmericanDeerMouse     TTAAAATTT-CAGTTAACC--TTGACAACAAAACCCAGTTATAAGATACTTCCTTTAATTTGTAAAGTCAGAGTCTATAA  3861
Mouse                         TGGAAAATTTCAGTTAGCC--TTGACAACAAAAGCCAAGCTTAAAATATTTCCTTTAATTTGTAAAGTCAGAGTCTATAA  5107
ChineseHamsterGHOK1GS         TTGAAATTTACAATTAGCC--TTGACAACAAAACCCAGTTATAAAATATTTCCTTTAATTTGTAAAGTCAGAGTCTCTAA  4357
LongTailedChinchilla          CCACAA-------GCAACTG-CTAGTCTCCTGTAACTAGATTAGTACATTCTTTAGAAGTTCAATTAAGTGAAATCA---  4265

Majority                      ACAXCXGCACAXTXGGATATTTACTXT-AATGGCXXTAGTCT-XX-ATTATGAXATTTTTTTTTTTTTGXTTT--TTXAA
                                        19450     19460     19470     19480     19490     19500     19510     19520
Human                         GCAGAAGCTCTCTGGTATTTCTTCTCATAAAGGCATTAATCC--C-ATCATGAAGGCCTTACTCTCATGATCTCATGAAA  19398
GuineaPig                     -------CAACCTGAGAGAATAACATT---TTGAAGTA--------ATTATG-AATGCTGTTCAAGATGCTTT------G  4116
NorthernAmericanDeerMouse     ACAACTAGACCATTGGATAGTTAGTATAAATGGCTGTAGGCT-TTGATAATAAGTGTTTTTGTTTTTGTTTTT--TTTTA  3938
Mouse                         ACAACTGTACAGTTGGATATTGAT----AATGACCATGGTATGTTAATTACTTGATAATAAGCAGATTTTTTT--TTAAC  5181
ChineseHamsterGHOK1GS         ACACCCGTACAGTTGGA----------------CTATAGGGT-TGGCTAATAAGCTTTTTTTTTTTT---TTT--TTTAA  4415
LongTailedChinchilla          -------CACATTGTGAACTCTTCTTTGGTTTGGCTTATTTT----ATTCTGTAATTTTTTTTTTTGAGATTT------A  4328
```

```
Majority                      CTCXTXAAGTAG-TGTTAAXXXTGTTATTTTAAXATXGTTTGACTCT---GATTAA-AGTGAAA--TGTGXXTGTTAGGX
                                       19530     19540     19550     19560     19570     19580     19590     19600
Human                         TCCTCCAGTTAACTCCTAAAGGCCCTGTTTCAAAATTGCATCACATTTGGGGTTAGGACTTCAGCATGTGATTTTTGGGG  19478
GuineaPig                     CACATGTGACTTTTGTGAATA-TAAAATTCTA---TTATTT-----------------TTAAAG---ACAGTGGTTTCA-  4171
NorthernAmericanDeerMouse     CTTTTGAAGTAG-TGTTAACTTTGTTAGTTCAAGATAGTTTGACTCT---GATTAA-AGTGAAA--TGTGCATGTTAGGA  4011
Mouse                         CTCTTTAAGTAG-TGTAAAG--TGTTAGTTTAAAACAGTT-GACTCT---GATTAA-AGTGAAA--TGTGTGTGCTAGGC  5251
ChineseHamsterGHOK1GS         CTCATTAAGTGG-TGTTAACTTTGTTA----AAGATAGTTTGGCTCT---GATTAA-AGTGAAA--TGTGTGTATTAGGA  4484
LongTailedChinchilla          CTCAT--AACATTTATTAGTAGTCCTTTTCTG---TTGCTTAG----------TAACATTACATTGTATGGCTGTCTGGC  4393

Majority                      AT------------XAXTCACAXTXGXAAXXXXTTAGTTGXCC-TXACAXCTAAGAXXXACAXAGTTGCTATAAGXAXTT
                                       19610     19620     19630     19640     19650     19660     19670     19680
Human                         AAACATAAGCATTGAGTTGATAATACCCTGCATATAGTTGACCCATACTTCTCCACTCCAGGTAATTACTATACTAAATT  19558
GuineaPig                     --------------AATGGAAA-TAAGTAGGGTTTATTTTGT--TTTTGCTTTTACTTAAGTCATATAATGTTA-AGAT-  4232
NorthernAmericanDeerMouse     AT------------GAATCACATTGGGAG----TTAGTTTCCC-TGACACCAAAGAAGAGCAAAGTTGTTC-AAGTCCTT  4073
Mouse                         AT------------GAATCACACTGGTAA----TCAGTTGTCC-TGACATGGAAGAAGGACACAGTTGCTTTAAGTCCTT  5314
ChineseHamsterGHOK1GS         AT------------GAATCACATTGGAAATTAATTAGTTGCCC-TGACACCAAAGAA---CAAAGTTGTT--AAGTACTT  4546
LongTailedChinchilla          --------------AATTTATT-TATTCAACTCCTAATGGAC--ATTCATGTTCTTTCCAATTTATGGCTATTATAAAT-  4455

Majority                      TXAXXTTXAXTCTXTXAAGAGCTTTCTTGTCTGXTGTAGGTTTXTACATXXCTTXTTGGGTAGXAAXACTGXTCAACCTT
                                       19690     19700     19710     19720     19730     19740     19750     19760
Human                         TGATGTTTACCATTCCCATAAATTTCTCATTTACTACATGTTTATGTATTTTTATCTTAAGCAATATATGGTTTTGTTTT  19638
GuineaPig                     --ATTTTTACT------------CTTTGGTCTTATGGAAATAAAAATATCTTTATCAGTAATAAGATACACTAAAAAAAA  4298
NorthernAmericanDeerMouse     TCAGCTTGAGTCCCTAAAGAGCTGTCTTGTCTGTAGTAGGTTTTTAC-TTACTTCTTGGGTAGGAAAACTGTTCAACCTT  4152
Mouse                         CCAGCTTGAGTTGTTGAAGAGCTTTCTTGTGTGT-GTAGGTTTTTCCCTCACTTCTTGGGTAGAAAAACTGCTCAGCCCT  5393
ChineseHamsterGHOK1GS         TTACTTGGAGTCTATAAAGAGCTGTCTTGTCTG--GTAGGTTTTTAC-TCACTTCTTGGGTAGGAAAACTGCTCAACCTT  4623
LongTailedChinchilla          --AAGTTTGCTGT----GAACATTTTTGCTCTTATGTGGACATATTCATTTCTCTT-GTGGGGGCATATGACTAGAACTG  4528

Majority                      TACAXTTXACTCTATAG-----XTACCATCTGGCACTTCCTGXAAXACATXGAX-XXXXXTTTAGTG--TTGGTGTATTA
                                       19770     19780     19790     19800     19810     19820     19830     19840
Human                         GCATGTTTAAACTATAGATGTACTATCATGCTGTATGTATTCTGTAATTTGCTTTTCTTGTTCAGTG--TTATTTTTGTT  19716
GuineaPig                     AAGAGTCTG--ATCCTTGGAAAGAAAGTTTAGGCAGTTAACTTTAAATTTGAGCCAGACCTTAAAGG--ATGTTTTGTCT  4374
NorthernAmericanDeerMouse     TACAATTGCCTCCATGG-----TTACCATCTGGCGCTTCCTGGAAGACATAGAC-------CTGG----TTGGTGTATTA  4216
Mouse                         TACAATTGACTCCATGC-----CTACCATCTGGCACTTCCTTGAAGGCATAGACAGACCTTTTAGTGTCTTGGTGCATTA  5468
ChineseHamsterGHOK1GS         TACATTTGACTCTATAC-----TTACCATCTGCTGCTTCCTGGAAGACACAGAT-------CTTGTA--TTGGTGTATTA  4689
LongTailedChinchilla          GAAACTTTT--CTACAGTGGTTGTCCCATTTTACA-TTGCCGTCAACAGAGTATGAGATGTTCAGTG--CCTCCATATCC  4603
```

```
Majority                    XAGATTGXTTXTTTGTA-AGTCTTTCTGCCXTGT-----AGGXCATAT-TCCXTGTCTTTTTGCTA---XXGCCCAAGXC
                                      19850     19860     19870     19880     19890     19900     19910     19920
Human                       AAGTTTATATATATATATTTTCATTGCCATATAGTATATGATATATGAATATATCATCTTGACAAATGAGTTAATATT  19796
GuineaPig                   AATAAAGGAAATA-----GACCTTTCT--------------------------T-TCTTCTTGCTA--GGAAATGACTTA  4420
NorthernAmericanDeerMouse   CAGATTA-TTGTTTGTC-ATTCTGTCTGCCCTGTT----AGGGCATAT-TCCCTGTCTTTTAGCTT---ATGCCCAAGCC  4286
Mouse                       CAGATTGGTTGGTTGCA-AGCCTTTCCACCTTGTT----AGGACAGAT-TCTTTGTCTTTTAACTT---ATGCCCAAGCC  5539
ChineseHamsterGHOK1GS       TAGATTT-TTGTTTGCA-AGTCCATCTGCCCTGT-----AGGGCAT---TCCTTGTCTTTTTGCGC---ATGCCCAAGTC  4756
LongTailedChinchilla        TCTAATGCCTATGTCTGTGGTTTTTCTGAT---------------CATCAACAT-TCTTGTGGGTA--TGAAGCAATACT  4665

Majority                    TGXAATAAXT-GXTTXXTCCTGXXCTT----XATCXGXAATTATAAGCA-XXAATATAAAXCCCCXTAAATATGXATACC
                                      19930     19940     19950     19960     19970     19980     19990     20000
Human                       TGACGAGATTGATTTGTCAAATATTTTAAATTTTTTTCCATTACAAACTGCTACTGTAAG----CATTAATATGCATATT  19872
GuineaPig                   TCCTTTAAAC--TTTATATCTAATCCTTAGGTCTTTGGTGTCTTTATTAGTTTTTAT------TTGTAAACCTGTCTGCC  4492
NorthernAmericanDeerMouse   TGGAATAAGAAGATTGCTCATGCCCT-----CAGCCGTAATTATAAGCA-TAAATATAAAACCCCTCAAATATCAGTACC  4360
Mouse                       TGTAATAG---GATTTCTCCTGTCCCT----CAACCTTAATTATAAGTA-TAAATATAAAACCCCATGCATATAAATACC  5611
ChineseHamsterGHOK1GS       TTGAATAAGTAGATTTCTCCTGCCCTT----CAACCAAAATTATAAGCA-CAAATATAAAGCCCCTCAAATAT-------  4824
LongTailedChinchilla        TCATGTAATT--TTGATTTGTGTTTTGGTAGTATCTGCGATTTGGAACATCTTTTATGAGCTTTTCATGAGCTGTTTATT  4743

Majority                    CTXAGGAGXAATTGTTAGAATTXCTGGTTTAXTGAGGTT----TTTXATAT--TXXTTGGATXTTAAAATATTXTXTTXA
                                      20010     20020     20030     20040     20050     20060     20070     20080
Human                       CTAACGTGTTAATATTAGAGTTTCTCATAGATTGTGTCT----TTTCAAAT--CGATTGAATATTAACATATTGCTTTCA  19946
GuineaPig                   CTATTAGGTGATCCTTGGATTTTAATTTTTAAT----------TTTCATA----AGCTTGCCACAACCATGAAATCTTCA  4558
NorthernAmericanDeerMouse   CTTAGGAG-AATTGCTAGGATTCCTGGTTTAATGAGGTTGACCCTCAGTATTTTTCTTGGATCTTAAAAAATTGTGTTTT  4439
Mouse                       CTTAGGAG-AACTGTAAGTGTACCTGGTTTAGTGAAGTTGACCTTCAGTATTTTTCTTGGACTTTAAAAAATTATGTTTA  5690
ChineseHamsterGHOK1GS       ------AG-AATTGTTAGTATTCCTGGTTTAGTGAGGTT-----------------------------------------  4856
LongTailedChinchilla        TTGTGAAGTGTCCAATTAAATCTTTTGCCCATT----------TTTTAAAT--TGGGCTATTTCTTAATTGAATTTTGAG  4811

Majority                    TGGTXGXTTATTTTXXAXATT-CXXTTAGXACXXXATATAGXX--XTXTTTXTGAXAATTATCTAGAXGTTTGTACTTTA
                                      20090     20100     20110     20120     20130     20140     20150     20160
Human                       TAGGTAATTATATTAATTATA-CTGTTAGCGCTGCATGTAGATTCTTGTTTCTATACATTCTTTGAAAATCGGCATTGTT  20025
GuineaPig                   TCCCCACCCCTTTCAAACTTTCCCCTTTCAGCTGTATGTAGCC------TTGGGAAGGCTTTATTCACAATGAGACTCAC  4632
NorthernAmericanDeerMouse   TGGTGGCTTATTTGTCACATT-AGATTAGGAGATGTTATTGTGTTTTCTGTCTGATAATGAACTAGATGTTTGTACTTTA  4518
Mouse                       TGATGGTTTAATCTTCAGAAT-AGGTTAGGAGATGTTACTGTG--TTTTCTATGATAATGATCTAGAGGCCCATGCTTTA  5767
ChineseHamsterGHOK1GS       ----------------------------------------------TCTGCTTGATAATTAACTAGATGTTTGTACTTTA  4890
LongTailedChinchilla        AGGATGTTTATTATGGATATATCCCTTTTCAC-ATACATAGC-------TTACAGATGTTTTCTGCACTTTTCTATTTGC  4883
```

```
Majority                      AXACTTATGTTTTTTXXTGAXC--X-X-XTXCTTGTXTAGXACATTTAGTTTTATTXAXXGTCATXTTTXCCAAGTXGAG
                                              20170     20180     20190     20200     20210     20220     20230     20240
Human                         GGACTTTTTAATTTTGCTGATC--AAATGTACATGGGAAGGATATCTTGTGGTTTTAATTTGCATTCCCTATTAATGCAA  20103
GuineaPig                     AATTTAGTATGTGTTTAGTTTCTTAAAATTGCATCAGTGAGGCCAAGGATATAGTTCTTTGGCTCATTGCCCGCCTGGCT  4712
NorthernAmericanDeerMouse     ATACTTAGGTTATTTCTTGAGT--T---CATGTTGTATAGAACATTTAGTTTTATTTTCAGTCAT--AGCCCAAGTTGAG  4591
Mouse                         ATA-----------------------------TCGTGTAGAACATTTAGTTTCATTGACAGTCATGTTTTTCAAGTTGAG  5818
ChineseHamsterGHOK1GS         ACATTTAGGCTATTTCTTAAGT--T---CATATTGTATAGAACATTTAGTTTTATTGACAGTCAT--TTTCCAAGTTGAG  4963
LongTailedChinchilla          AAAC-AATGTCTTTTGAAGAAC----AGTTATTCTGATGAGCTCTC--ATCTGTTTTATTTTTTTTTTTCTTTTATGGAT  4956

Majority                      ATAGTTAAXAGXTTTGAAGTXXAGXCAXGGXTAGCTC---XXXAGTACAAAATGTTTTGATATTXTAXTTATTATCCAAX
                                              20250     20260     20270     20280     20290     20300     20310     20320
Human                         AAAAAAAAAAACATTGATTCCTGGGAGTTTTTATATA-CTGTGCAGACTAATTGTTTTGTTATTTTTTTTGGTTTGTTTG  20182
GuineaPig                     AGTGCAA--GGTGCTGAATTCAATTCCTGGTTACCCCTTCCTCCCCAAAAAA-ACTCAACAAAAAAATTG--CAT--CAG  4785
NorthernAmericanDeerMouse     ATAGTTAAAAGCTTTGCAGT-----CGGGGATAGCTC---AAAAGTACAAAATGTGTTGATGTTGTAGTTATTATCCAAA  4663
Mouse                         ATA-TTAAGAGCTTTGAAGT-----CAGGAATAGGTC---AAGAGTACAAAATGTCTTGATATTGTAGTTATTATCCAAA  5889
ChineseHamsterGHOK1GS         ATAGTTAAGAGTTTTGCAGTGCAGTCAGGAATAGCTT---GA--GTACAAAATGTGTTGATGT---AGTTATTATCCAAA  5035
LongTailedChinchilla          GACACTTTTGGTGTCATACCTAAGCCATTGTTGCCTAGCCTTAAGTAAAAAATATTTTACTCCTTCATTT--TCTAATAG  5034

Majority                      TAGXTXTXTTCTGTGAATTTTGTAAAAGAATAXAXXAT-XCAAGGTACTTTGTTAAXATGACTT---XXATAGTXCTGTG
                                              20330     20340     20350     20360     20370     20380     20390     20400
Human                         TTT-TGTTTTGTTTCATTTTTGTTTTTGTTTTGAGAGA--CAGAGTCTCACTGTGTCATCCAGG---CTGGAGTGCAGTG  20256
GuineaPig                     TGG-TGATGTGTAATATTTTAGAACACTTTTAGATC----CAATGTGTTGTGTTCTTGTC-------AGATAGTGAAGTA  4853
NorthernAmericanDeerMouse     TAGATTTCTTCTGTGAATTTTGTAAAAGAATAAATGAT-ATAAGGTAATTTGTAAAAATGACTT----TCTAAGACTCTG  4738
Mouse                         TAGATTTCTTCTGTGAATTATATAAAAGAATAAATGATTACAAGGTACTTTGTCAAAATGGGTTAACTGATAGCTCTGTG  5969
ChineseHamsterGHOK1GS         TAGATGTCTTCTGTGAATTTTGTTAAAGAATAAAGAAT-ATAAGGTACTTTGTAAGGATGACTT----GATAAATATGTG  5110
LongTailedChinchilla          AAG-TTTTATATTTTGGCTAAATACGGAACTTGGGC----CTGTGTTCCATTTTAAGGTTTTGA---ATATAGTACGAAG  5106

Majority                      GTXTXTTAXTAGTG-CTGAGTTTXTAAXXAXXAXGTXATTAXCAGTTXTGXXAACXXTGXX--GA-XATTAGATTAGACT
                                              20410     20420     20430     20440     20450     20460     20470     20480
Human                         GCTCGGTCACAGTCACTGCAGTCTTGACATCCCAGGCTCTAGCAATTCTCTCACCTTTGTTT-CCTGAGTAGCTGGGACT  20335
GuineaPig                     GATAATTGTCAGTG-CTTTATATTTAGCT-----TTATTTCTTGGATTC----ATGTTATG-------AAAATTAGGATA  4916
NorthernAmericanDeerMouse     GTCTTTTACTAGCAGGTAAGTTTACATACAAAACCTCATGAGCAGTCATGAAAAAAGGGAA--GAGGATTAGACTAGACT  4816
Mouse                         GTCTTTTACTAGAGGATAAGTT-ATAAAAATGAGTTAAGTAATAGCTCTGTGGTCTGTTACTTGAAAATTGTATAAAAAT  6048
ChineseHamsterGHOK1GS         GTCTCCTATTAG----TGAGTTTACATGCAAAACGTCATGAACAGTCAGGAAAAAAGGGAG--GACAGTTAGACTAGACT  5184
LongTailedChinchilla          AATGGGTGAGAATT-TTGGGTTTTAGATG-----GGAACATCCAGTTTTTCCAACGTCATT-------GTAGCTTTGTTG  5173
```

```
Majority                   XCATGXATAXGXXATTTTGXXTTTXTAATTTTTCAXT-XTTTTATXXAXATCAAGXXXXAXC-TGTTAXAAAAXTGTATX
                                          20490     20500     20510     20520     20530     20540     20550     20560
Human                      ACAGGAATACACCACTATGCCCAGCTAATTTTTCAATTTTTTTTGTAGAGATGGAGTCTCACTGTATTACCCAGGTTTGTT  20415
GuineaPig                  AGAAAAAAATTGGGAAGGGCATATTTAATTTA--------TTTGTGGTAATATTTTCCTG----GTTGAATTAATGTAAG  4984
NorthernAmericanDeerMouse  GCCTGGATAGGATATGTTGGCTTTACCAGTTTTCAGT-ATTATA----GTTCAAGGAGAAAC-TGTTAGAAAATTGTATA  4890
Mouse                      ATTC--ACTACCCATTCTAAAATGTTTAATATAAAAT-TTTGTA---AAGTTAAAGTTCATT-TTTTATCATTTTCTAC-  6120
ChineseHamsterGHOK1GS      GCCTGGATACGATATGTTGTTTTTACCAGTTTTCAGT-ATTATATGTAGTTCAAGGAGAAAC-TATTAGAAAACTGTATA  5262
LongTailedChinchilla       GAATGTCTGTGTGACTTTGTTTCTGTAGTCTCCC----CTTTTCCATTAACCCATTTCTGTC--GCTGTAGAGGCCTAAG  5247

Majority                   AGAATATTXCXGAXTTXATTAAGTAGTTTGXXXTAXXX-TXTXCAXAGTTXAAATTAAXTTGC--XTAXXTCXTTXTAXA
                                          20570     20580     20590     20600     20610     20620     20630     20640
Human                      GCGAACTCCCAGGCTCAAGCAGTTCTCCCGCATTGGCCTCCTAAAGTGCTGAGATTACA-GGC--GTGAGTCGCTGTGCC  20492
GuineaPig                  ATTAGAAGTTGGGAGTAGTTCAAGA---------------GTGCAAAATGTAGATCATGCTGT--T---GCTATCCAAAT  5044
NorthernAmericanDeerMouse  GGAATACTACCCATTTAAAAAAGTTGTTTAATATAAAAGTTTGCAAAGTTAAAATTAATTT-----TATATCCTTTTCTA  4965
Mouse                      --TATATTGGTTAAATTCTTATATTGTTTGCAGCAGTT-TGTACGGCACTTAAACTTTCTTACAAGTAAATAGTTGTAAA  6197
ChineseHamsterGHOK1GS      AAAATACTGCTCATTTTAAAAAGTAGTTTGATATAAAC-TTTGCAAAGTTAAAATTAATTT-----TATATCATTTTATA  5336
LongTailedChinchilla       AGAATATCTTAGTCACTATTAGGTATAT------------ATACAGTCTTGAAATCAAGTAGC--TTTGGTCCTCCAACT  5313

Majority                   GAACX---TTXGTTAATTAXXTTTGTT--GGXX-ATXGAAATTATGX--XGATCCATTTXXACTXXATTAAXA--GTAXX
                                          20650     20660     20670     20680     20690     20700     20710     20720
Human                      TGGCCTGTTTTGTTAGTTACATGTCCTACAGATAACTCTCTCCTCTCTCTGACCTGTTTTTTTTACACTGATGA-ATAGT  20571
GuineaPig                  AAATT---TCCCTGAATTTTCTAAAA--------ATAGAATAAGTGACTTGATTCTTTTGTAAAAGAATAA----ATGAT  5109
NorthernAmericanDeerMouse  GAACA---TTGGTTAAATATGTTTGTT--GGT--ATCAAAATTATGT--ACAAGCACTTAAACTTTCTTAAAAA-GTAAA  5035
Mouse                      GAAAG---TAATCTATGTGCGTTTTTA--AAATAATTGAAACTGTA---GTATCCATTTCTCCTTCATTCACAGTTCAGT  6269
ChineseHamsterGHOK1GS      GAACA---TTGGTTAAATACATTTGTT--GGT--GTCAGAATTATGT--ACAAGCATTTAAACTTTCTTAAAA--GTAAA  5405
LongTailedChinchilla       TGGTTCATTTTCAGAGTTGTTTTGGCTCTGGGTCACAGAAATAATGCTGGGATCTTGTTGAGTTAGACTCA----GTAGA  5389

Majority                   XGCXAATTTTAAGGGAAGTXXTATXTXTTCATGTTTAAAATXT-----ACXGAAGXX--TXCXTAXAGTATXATGCAXTT
                                          20730     20740     20750     20760     20770     20780     20790     20800
Human                      TGTAAATTTTAATGGGACATTTATGTTTTTCTTTTAAAAAAACATTTAACCTAAGGCCTTACGTATAGGCTTGTGTATTT  20651
GuineaPig                  -GCAAGTC---TGAG-----TTGTGTACTGTTGTTGAGTG------------AGAAG--TGTATAGA----TAAGACCTA  5162
NorthernAmericanDeerMouse  AAATAATTTTAAAGAAAGTCATACATATGCATGTTTAAAATG------ATTGAA----------AATGTAGCATGCA---  5096
Mouse                      TGCTTAGGTGTAGATGTGTTAAGTTTTT-CATTTTTAAAAATT-----ACTGATTTG--C----ACATTATCATGCA-TT  6336
ChineseHamsterGHOK1GS      AAATAATTTTAAGGAAAGTAATATATGTGCATGTTTAAAATATAGC--ATGAATGTC--TCCTTGATCTACCGTTCAGTT  5481
LongTailedChinchilla       -GCAAATT---GAGGAAAACTGACCTCTTACTGTTGAATATTTGT---ACCCAAGAA--TACATACAGTTTTATGTATTC  5460
```

```
Majority                      TCTTATXTATGTTTTXATAAXATXGTTTTTAAATTTTTATXAT- - AGTTTXAXXTXAAXTATTTXTTTGTXXAXTAXATA
                              20810  20820  20830  20840  20850  20860  20870  20880
Human                         TATTTTGAAAGTTTTAAAATTTTGATTTTCACATTTATGGCGT- TAATCTACCTGGAATTTATTCTTTGTGTAGCATATG 20730
GuineaPig                     GATAGAATAAGTAGGCAGAAAAAAGTGAGGAGACATGGAATAAAGAGTATTGTTTATACCAGTTTTTTGTTAGTTTCAAG 5242
NorthernAmericanDeerMouse     - CTTCTCCTTAATTAGATAA- ATTGTTTTTAAAATTCTTTCAC- - ATTTAAAAA- - AATTATTGACTTGTGTATTACATA 5170
Mouse                         TCTTATTTTTTGTAGAATCCTGACCTTTTCAAATTTTTATTTT- - GGTATGACATAAACTGCCTGTATTTAAAGTATGCA 6414
ChineseHamsterGHOK1GS         TCTTAGTCGTAGATTGATAA- ATTGTTTTTAAATTTTTTTCAT- - GTTTTGAAA- - AATTATTGACTTGCATAATATATA 5556
LongTailedChinchilla          ATTCACATAGGTCTTTCTTTCTGCAGGGTTTAGTTTTCAGTGTATAGTTTTTTTTTAACCA- TCTTTTATTAGAATCATT 5539

Majority                      TTGTATXXTTTATXATXTTTTXTATATAAATA- TAT- CXTATTATGAXCCATAXTX- XAATTXTTTAAAGTTTATXATTT
                              20890  20900  20910  20920  20930  20940  20950  20960
Human                         TTACAGGTTTAATTATTTTTTCTATATGGATAGCAAGCTTAGTATGTACTTGATT- - GAATAGCTCAGCTTTTCTCCAAT 20808
GuineaPig                     ATAAA- ACATTAG- AAAATGTCAAAAAAATTACTAACCATTTTAAAAACCATG- - - - - AAAAGTTTGAG- - - - - TGAGTA 5310
NorthernAmericanDeerMouse     TTGCAT- GCTTGCCTTATTTTTTGTACAAATA- TAT- TGTGTTATGACCCATATCTTGACCT- TTTAAAATTTTTTATTT 5246
Mouse                         ATGTCTCATTTACACACACACACACACACACA- CACACACACACTTACACACACTTACTCGTATTTAAAGTATGCAATGT 6493
ChineseHamsterGHOK1GS         TTGTAC- ACTCGTCTT- - - TCTTGTATAGATA- TGT- TGTATTATGGCCC- - - - - - - - - - - T- TTTAAAGTTTATT- - - - 5614
LongTailedChinchilla          CTTTGTATTTCAT- ATCTTTTGGAATTAACAAATGTTATTTTTGAAAATTTAGCTTCCAATTATTTGAGGCTCATATTTA 5618

Majority                      XAGXTXGTTTCXXAXXX- X- - - - - XXTCAAGTATACX- - - - GTACCTTTTXTATXCXXACTTTTXTAGCCATC- CXACAX
                              20970  20980  20990  21000  21010  21020  21030  21040
Human                         GATCTGCTTTCTCAGCTCTTCA- GGTTTCCGTATATTCACAGAACCATTTCTGTGCTATGTCTTGTAGCAGTA- CAACAG 20886
GuineaPig                     AAGTTAGTTTT- - - - - - - - - - - - - ACTCATGTATACT- - - - GTACCCTTGCTAAGCC- TATTTCAGAG- - ATC- TGATAG 5369
NorthernAmericanDeerMouse     CAGTATATGACATAATTGCCTGTATTTAAAGTATGCA- - - - ATGTTTTCTATATACATACTT- TGATGCCA- - - - - GC- A 5315
Mouse                         CTCATTTACACACACACACACA- - - CACACACACACA- - - - CACACTTACACACACTTACTCGAGTGGGCGAAGCCACTA 6566
ChineseHamsterGHOK1GS         - - - - - - - - - - - - - - - - - - - - - - - - - TTAAAGTATGCA- - - - ATGTTTTGTATATACACACTTGTAAAGCCACCACCAC- A 5664
LongTailedChinchilla          GAGCTAGTTTTTTCGTATT- - - - - GCTCCAGTATACT- - - - GTACCAGTGCTAAGCC- CATTTCATAG- - ATC- TAATAG 5685

Majority                      CTXCXXTCTAGGTXGAAXCAXXTXTTXCXCTAAGAAATGCXATCCTGCCCXXTTGTXGTXXCAXC- ACXTTCTTTXXCAC
                              21050  21060  21070  21080  21090  21100  21110  21120
Human                         TGTCTTAATTACTTATGGCA- - TCAGC- ATTAAGTTTTGGAATCTTGTACAGTGAGTGTCAAA- - - ACTTTGTTTTTCAA 20960
GuineaPig                     CTTTTTTGTAGATTTCATCAGATTTTCCAATAAGACC- - - ATTTATGCTCAGTTGTCTTGAG- - C- ATTTTCTTTCCTAT 5443
NorthernAmericanDeerMouse     CTACAATCGAGATAGAAGCTTTTGTTACTCCTAGAAATGCCATCCTGCCCCTTTGTAGTCCCACC- - CCTACTCTTTCCC 5393
Mouse                         CTACAATCGAGGTAGAAAC- - CTGTTACTCATAGGAATGCCCTCCTGCCCCTCTGTTGTCCCCTTTGCCTTGCCTCTCCC 6644
ChineseHamsterGHOK1GS         CCACAATCAAGGTAGAAACTTCTATTACTCTGAGAAATGCCATCCTGCCTCTTTGTAGTGCCCCC- - CCC- CTTTTCCTC 5741
LongTailedChinchilla          CTTTTTTGTAGGTTCTGTCAGATTTTCCACCAAGACC- - - ATTTATGCTCAGTTGTCTTGAGAGC- ATTTTCTTTCCTAT 5761
```

```
Majority                     CAGAXXCCAGTXATXTXCTT-CA-------T-TGCTTGTAXTTTTAGAX-TXTAAATGAATXGXTTCXGAGAGXGGGGTC
                                               21130     21140     21150     21160     21170     21180     21190     21200
Human                        AATTGTCTTGGTGTTTTCTT-CA-------C-TGCATTTATTTTAAGA--T-CATTTGGCTAAGTTCTGTGA-AGGCTCT  21027
GuineaPig                    CACGGTGCCATTTTTCTCTT-TC-------T-TGCTTGTGACGCCTAG--A-ATAATGTTGAAGTTGTGAG--TGGGCAG  5509
NorthernAmericanDeerMouse    CAGAAGCCAGTGATCTGCTGGCATTATAGATTTGCTTGCATTTTTAGAAGTTTAAACAAATGGAATCAGAAGGCGAGGTC  5473
Mouse                        CAGAAGCGAGTGATCTGCTTTCACTGCAGATTCGCTTGTATTTTTAGAATTTTAAATGAATGGACTCAGGGAA----GTC  6720
ChineseHamsterGHOK1GS        CAGAAGCCAGTGATCTGCTG------------------CAATTTTAGAATTTTAAATGAATGGAATCAGAGGG--AGGTC  5801
LongTailedChinchilla         CAGGGTGC--TTTTTTTCTT-TC-------T-TGCTTGTGACGTCTAG--T-ATAGTGTTGAGGTTGTGAGAGTGGGTGG  5827

Majority                     XTTTAXCTTXTTT-CACTCAXXAXAAXXTTXTAAGT-TAXTTXXAAGTGTTTTXTGTXTXXTTTXXCAAXXTTTGCTXXG
                                               21210     21220     21230     21240     21250     21260     21270     21280
Human                        TTGGGTCTTATTTATACTTGCCTTAAATTTAGTAGT-TAATTGGGAGAACATTATAGCTGCATTATCAAAATAAAATGGC  21106
GuineaPig                    CTTTACCTCCTTT---CTGATACTAAGATCAACACTGTGAATGGTATTCTTTT-TATCTGACTTAACCA--TGAATTGTG  5583
NorthernAmericanDeerMouse    TTTTAGCCTATTA-CACTCAATAGAAATTTTTAAGT-TATTTACATGTGTGTTGTGATCCTTTCC--TACCCTTGCTTGG  5549
Mouse                        TTCTGTTGTATTC-CACTCAGCAGAAGCTTTTAAGT-TATTTACACGGTGTTAGTGTTCTCTTCCCCACTGTTTGCTTAG  6798
ChineseHamsterGHOK1GS        CTCTAGCCTCTTAACACTCAATAGAAACTTTTAAGT-TATTTACACGTGTGTATTGGTTAGTATC--ATTACTTGCTTAG  5878
LongTailedChinchilla         CTTTACCTTCTTT---CTGATACTAAGGTGAACACTATAAATGGTACTGTTTT-TTTCTTTCTTAAC---------TGCG  5894

Majority                     AATXATTAXXXXXTTTAXXXXXXAAAAACTTTTAGTGGGCATTTGXXXTTGGGG--TAGTATAAGGTTGCTG---TXXTC
                                               21290     21300     21310     21320     21330     21340     21350     21360
Human                        AATTTTTTTTGAGCTGAAAGGCAGAGAACTATAACATTTCTTTGAAGAATTTTC--CACTTTCTTCTTCATGGTATTTTC  21184
GuineaPig                    AATTGTGCC--AGTT-AAAGGAATAATACTGTTATTAAGTATTTGAAAGCAAAG--TTGTATGGGCTTG-------TTTT  5651
NorthernAmericanDeerMouse    AATCATTATATTATCTAGTAATTAAGAACGTGTAGTGGGCATTTGTGTTGGGGGTATAGTATAAAGTTGCTG---TGAAC  5626
Mouse                        TGTCATCATGCTATATGGTAACTAAAAACCTGTAGTGGGCATTTGTGTTTGGGG-GTATTATAAAGTTGCTA---TGATC  6874
ChineseHamsterGHOK1GS        TATCATTACACTATCTGGTAATTAAAAACTTTTAGTGGGCATTTGTATTTGGGG-ATATTATAAGGTTTCTC---TGAAC  5954
LongTailedChinchilla         AATTGTGTC--AGCT-AAAGGAATAATACTATTATTAAGTATTTGAAAACAAAG--TTGTGTGGGTTTGATGTTTGTTTT  5969

Majority                     XTTTCTGXACATXTTTTATGTAGAXATATXXXTTTAXTTXTCTTXGGXXXXATTXTTAAXAATGGCTGAATCATATGXXT
                                               21370     21380     21390     21400     21410     21420     21430     21440
Human                        CCTAAGAGACATGCTTAATGCAGA-AAATATATTAAAAGGATTTTAAACAGCTTATATGTAAGGGATGAGGGAACAGGGA  21263
GuineaPig                    CTTTGTGGGGGGGTTTTATAGT-----ACATATTTAATTG--TTTTGATATAATAGTAATACAGGGCTAT----TGGGG-  5719
NorthernAmericanDeerMouse    ATTTCTGCACGTTTTT-GTGTAGACATATGCTTTTATTTCTCTTGGGGCCTATTGTTAAGAATGGCTGAATCATATGTTT  5705
Mouse                        ATTTCTGCACTTTTTT---GTGCACATATGCTTTTATTTCTCTTGGGGTCCATTGTTAAGAATGGCTGAATCATATGTTA  6951
ChineseHamsterGHOK1GS        ATTACTGCGCATTTTTTGTGTAGACATATGCTTTTATTTCTCTTGGGGCCCATTGTTAAGAATGGCTGAATCATATGTTT  6034
LongTailedChinchilla         CTTTGTGGAGAGGTTTTATACT-----TTATATTTAATTG--TTT-GATAAAATAATAGTATGGGGCTAT----TCAGGT  6037
```

```
Majority                    GXCATXTGCTTAACTXXTTGAGXAACTGXAGTCTCTTTTCCACAGTGXGTGTXXXXTCTTGCXAXCTGCXGGATATGAAA
                                      21450     21460     21470     21480     21490     21500     21510     21520
Human                       TACTTGGACTAATAT--TTCAATATTTCTAAAATTATTGGTTTATTGAGTACGTT-CCTAGAAAACTGTTTTAT-TGAAA  21339
GuineaPig                   ---CTG---TTTTTT--CTTGCTGAGTTTAGTGTCTTGTACAAAATTAGTCTGTT-TACTCTAAGTTGCTAGTTTTATTT  5790
NorthernAmericanDeerMouse   GGCATATGCTTAACTAATTGAGAAACTGCAGTCTCTTTTCCACAGTGGTTGTACCATCTTGCCATCAGCAGGCTATGAAA  5785
Mouse                       GGCATATGTTTAACTAATTGAGAAACTGCAGCCTCTTTTCCGCAGTGGTTGCACCATCTTGCCACCACCAGGATATGAAA  7031
ChineseHamsterGHOK1GS       GTCATATGCTTAACTAATTGAGAAACTGCAGTCTCTTTTCCACAGTGGATGTACCATCTTGCCATCCACAGGGTATGAAA  6114
LongTailedChinchilla        TACCTG---TTACTT--CTTGGTGAGTTTTGTGTCTTGTGTAGAATTAGTCTGTT-TCCTCTAAGTTGCTGAATGTATTT  6111

Majority                    XXTTCAXTTXCTCCAGAXACTTCTXAGTGCTTTTTAXTGXTTAGTGGXTGTGAXXTGATAATTTAATTXTGGTTTTAATT
                                      21530     21540     21550     21560     21570     21580     21590     21600
Human                       GCTTCAAATTTCTCAGA-ATTGTATAGCATTTTTGAATTCTCTCTTTTATTACTAATATCAAGCACCTATGTT--TTCTC  21416
GuineaPig                   GCATGCAGTTCACATTA-AAGTACTTTTGATTCTTTAGACTAAGTTGTGACATTAA-TTCTATTCCTGATACTGATCAGT  5868
NorthernAmericanDeerMouse   ATTTCAGTTCCTCCAGAGACTTCTCAGTGTTTTTTAGTGTTTAGTGGGTGTGAAGTGATAATTTAATTGTGGTTTTAATT  5865
Mouse                       ATTTCAGTTCCTCCAGAGACTTCTCAGTGCTTTTTAGTGTTTAGTGGGTGTGAAGTGATAGTTTAATTGTGGTTTTAATT  7111
ChineseHamsterGHOK1GS       ATTTCAGTTCCTCCGGAGACTTCTCCGTGCTTTTTAGTGTTTAGTGGGTGTGAAGTGATAATTTAATTGTGGTTTTAATT  6194
LongTailedChinchilla        GCCTAGAGTTGTTTACA-ATAAAATATTACTTTTTAATACTTT-TGATTTCTTTAG-ACTAGGTCATGATGC----CAAC  6184

Majority                    TTTXTTXCTGTGTGGTXTXTATTATGATTATTTTTCAGTGTXCCTTTTXCCAGCTGTTATTGTTTTXTTCCTGT--TTCC
                                      21610     21620     21630     21640     21650     21660     21670     21680
Human                       CTATTTCTTGATTTGTCTTGTCACAGAATTTATTTTACTGGTC--TTTTCAAAGAACCAACCTT-TTTACTTGT--TTTC  21491
GuineaPig                   TTTTTTCCTTTTTAGTCTTTATTTTATTGATTTCCAAATGATGAGCTTTTG---GCTTTGTGTTGTTTTCCTGT--TTCC  5943
NorthernAmericanDeerMouse   TGTACTTCTGTGTGGTATCTATTATGATCA-TTTTCAGTGTGCCTTTTGCCAGCTGTTATTGATTTCTTCCTCTTCCTCC  5944
Mouse                       CTTATTTCTGTATGGTATCTGTTATGAGTG-TTTTCAGTGTGCCTTTTGCCAGCTGTTGTTCTCTTCTTCCTTTTGTTTT  7190
ChineseHamsterGHOK1GS       TGTACTTCTGTGTGGTATCTATTATGAGCAGTTTTCAGTGTGCCTTTTACCAGCTGTTGTTGATTTCCTCCTTT--CTCC  6272
LongTailedChinchilla        TCTTTTCCTTTGCAGTCTTGATAAAAGTTTTTTTAATTTTATTAATCTCCAAAAGATCAACTTTGTTTTTCTGT--TTCG  6262

Majority                    TXTTTTGTTGXTX---TGGTTTTATTTTXXTTGAGTXAAGXT-----TTXACTXTXTATACTXGGTTTGCCTXXAAACT-
                                      21690     21700     21710     21720     21730     21740     21750     21760
Human                       TTTTTTGTTGGG-----GGCAGTGGAGGGATTCACTTTAATT-----TGATCCTGAACTCCTTTATTTTCTTTTAAA---  21558
GuineaPig                   TATTTTGTTGATT---TGCTTTTATTTTCCTTTGTTTTTGTT-----TATACTTTCCATACTTGGTTTACATTGAAACTG  6015
NorthernAmericanDeerMouse   TCCTCCTCT-------TGGTTTT--------TGAGGCAAGGT-----TTCACTGT--AGATTAGGCTGGTCTCTCAACT-  6001
Mouse                       TTTTTGTTTGTTTGTTTGTTTTTGTTTTGTTTGAGACAAGGT-----TTGACTGTATAGACCAGGTTAGTCTCC------  7259
ChineseHamsterGHOK1GS       TCCTCTCTT-------TGGTTTTA-------TGAGTCAAGGT-----TTCACTGTGTAAACCAGGTTGGCCTCCCAACT-  6332
LongTailedChinchilla        TATTTTGTTGATT---TGTTTTTATTTTCCTTTGCTTCTGTTGAGTTTGTACTTGCGATACATAGTTTACATTGAAACTG  6339
```

```
Majority                    AXGCTAGAAATXTGACTXATTXTCXXCTXXTGCXTXXGXGCTGXGTGXTXGGXTTAXAGGCTTTGA--X-CCAXCATGAC
                                     21770     21780     21790     21800     21810     21820     21830     21840
Human                       ATGCATTCAAGGTTAAAGGTAATCAGCTGATAGCTTTG-GCTTTTTTTTTTTTTTTTTACTTTTAGAGTTCTCTTTTTAT 21637
GuineaPig                   ACGTTACTAAAGTGATGCCTTT-CTACTAGGGATTTAGTGCCATGTGTTTCCTAGGTTTCCTATGTCTTTCCATGATGTC 6094
NorthernAmericanDeerMouse   GGGCTAGAGATCTGCCTGATTCTGCTTTCTTGCTTCTTTCCTGAGTGCTGGGATTAAAGGCTTGCA----CCACCATGTC 6077
Mouse                       -AGCTAGAAGTCTGACTGACTGT----CTCTGCCTC----CCCAGTGCTGGGATTAAAGGCTTAGA----TCACCATGAC 7326
ChineseHamsterGHOK1GS       AGACTAGAGATCTGCCTCACTCTGCTTCTTTGCCTC----CTGAGTGCTGGGATTAAAGGCTTGCA----CCACCTTGAC 6404
LongTailedChinchilla        ATGTCATTAATGTGAAGCCTTTTCTACTAGGAGTTTAGTGCTGTGTGTTTCCTTCTTAGGTAATG--CTCCCATGACAGT 6417

Majority                    TGTTXAATTATAGTAAGGGTTCTAXTCTAXAGGAAXAXAAXTGATAGAXXAXAXCAXTTTCTTTTTTXGGTTTXTTXATA
                                     21850     21860     21870     21880     21890     21900     21910     21920
Human                       TGTAAACTTCATTTAAAGGTTTTAAGCTACACAAAAGTAGAGGGAATGGTATATAATACACTTTTTTCTGTTGGATAATA 21717
GuineaPig                   CCTCAAATTTTAGCATGTGTTCATATATTCATTCAATTCAGA-CTTTTTAATACTGTTTTATTTTTTAGGTTTATGGATA 6173
NorthernAmericanDeerMouse   GGATGTCTTAAAGACAGGGTTCTAGTCTAGAGGAATAGAAGTGATAGAATGAATCAATTTCTCTTGGTCTTTCTTTCTTG 6157
Mouse                       TGGTGAGTTATAGTTAGGGTTCT---TTAGAGGAAGAGGATTTACAGAAAGAATCAATGCCCACCTCCCATATCTACATA 7403
ChineseHamsterGHOK1GS       TGGTGTATTATAGTCAGGGTTCTAGTCTGGAGGAATAGAACTGATAGAGTAAACCAATTTCTCTGTGTGTCTGTCTTGTG 6484
LongTailedChinchilla        CCTCAAATTTTAACATGAGTTGG---GTTCATTCAGTTGACATCTTCTTAATACTGTTTTATTTCTTGGGTTTATGGGTA 6494

Majority                    TTCXTGTXAATXTXTGATXXTTTXXG-ATGTAXATTTXAAGTATXGTAXAATTTXTXXCAGTXT----TTXAXTGTXGTC
                                     21930     21940     21950     21960     21970     21980     21990     22000
Human                       TTAATGCAAATCTCAGATCATTTC---ATATAATCTGCAGATATATCAGAATGTGTGTCTCTCTA---TTAAATGTGACA 21791
GuineaPig                   CTCTAATAAAATATTAATATTTCTAG-ATGCAGATTTTAAGTATGGTAAAATTTGCAGCAGTTT----TTAAATAAGCTA 6248
NorthernAmericanDeerMouse   TGTGTGTG---------------------------------------A-AATCTA-------------------GTTGCC 6178
Mouse                       TACATGTGTGTGTGCGTGTGTGCGCGTGTGTGACTTTAT-TATTAGAGTAGCTTAT-------------AGGTTGTTGTC 7469
ChineseHamsterGHOK1GS       TGTGTGTGTGTGTGTGTGTGTGTGTGTGTGTGTGTGTACACACTCATGGAATTTATTGGAGTAGTTCATAGGTTGTTGTC 6564
LongTailedChinchilla        CTCTAGGAAAATATTAATAATTTTAG-ATGCAGATTGTAAGTATGGTAAAATTTGTACCAGTTT----TTTAGTAAGCT- 6568

Majority                    TTGGXAXXAXTATXT--XGCXXTXAAATGAXTCCXTAXTXTTXGATAAXTTTTAXAXAGT-AAAXCATTTTXTTGXXATT
                                     22010     22020     22030     22040     22050     22060     22070     22080
Human                       TTGGTACCATTATAA---AGTTTAAAATAATTTCTTAATGTCGTCTAATCTCCAG-TATTCAGAATACTTTCTTTTCATT 21867
GuineaPig                   ATGAAATGAAGATAT--TACAGCTGGATGTTTCCCTATTTTTACATTATTTTAAAATGATGTTTACCTGTTTTTAAAATT 6326
NorthernAmericanDeerMouse   TTGGTT------C-----GCCA------GACTTTGTT-----------CTTTTTCAAAG-------------TTG----- 6212
Mouse                       ATAGAA----TTTCTATTGCTGTTAAGTTACACTGTGATGGA-GACAACTCTTATAAGGA-AAA-CATTTAATTGGGGCT 7542
ChineseHamsterGHOK1GS       TTAGTTATGGTTTCTATTGCCATGAAGAGACTCCATGGCCATTGGCAACTCTTACAAAGG-AAAGCATTTAATTGGGGCT 6643
LongTailedChinchilla        ---AAATGAAGATT-----------GATGTTTACC-ATTTTTAAATAATTTTTAAATACTGTATCCATGTTTTTAAAATT 6633
```

```
Majority                      AXXXTAAATTTTXAXX--TTTAGTTXATTATTXTTATGAX--GXXXX-XXGTXXX-TGXA-GGXAXTCATGXTXXXGX--
                                      22090     22100     22110     22120     22130     22140     22150     22160
Human                         ATTTTATTTTTCTTGCC-TTTTCTTTTTTGTTGTTGTTAGATAAAGT-GAGTCATCTGTACAATTTCCACTTTCTGGC--  21943
GuineaPig                     AAGATAATTTTTAAAG--TCGAGTTTATTATTATTATTATTTATGTT-TTGTACCGGGGATGGAATTTATGACTTTGTGC  6403
NorthernAmericanDeerMouse     ---------------------A--------------------G-----------------------TCATG---------  6219
Mouse                         GGCATAAAGATTCAAAGGTTTAGTTCATTATAGTCATGGCAGGAAGCATGGTGGCATGCA-GGTAGACATAGTGCAGGAG  7621
ChineseHamsterGHOK1GS         GGCTTACAGGTTCAGAGATTTAGCTCATTATCCTCATGGT--GGGACAGGGTGGTTTGCA-GGCAGTCATGATGCTGGTG  6720
LongTailedChinchilla          AAGCTAGATTTT------TTGAGCT--TTATTATTATGA-----------------------------------------  6664

Majority                      --GXXGCXXAGXGXXXXACAXXTG--TXXXC---CAGCAX------------------GXA-AAXCACXXXXTXAGAATG
                                      22170     22180     22190     22200     22210     22220     22230     22240
Human                         --TGGGCGTGGTGGCTCACGCCTG--TAATCC--CAGCACTTCGGGAGGCTGAGGCGGGCA-GATCACGAGGTCAGGAGA  22016
GuineaPig                     TTGCTGGGCAGGTGCTTACACAGC--TGAGCA--AATCTCCAACTCCGAGTTTTATTCTCA-AAGCACTAGCTTATAATG  6478
NorthernAmericanDeerMouse     ---------------------------------------------------------------------GA---------  6221
Mouse                         AAGAAGCCAAGAGTTCTACATCTGGATTGGCTGGCAGCAGCAGGGGGTTCGGGGGAGGGAAGGAAGAGGGAGGGAGACAG  7701
ChineseHamsterGHOK1GS         AAGGAGCTAATAGTGCGACATTTGGATTGTTGGGCAGCAG------------------GAAGAGACACAGACTGGGCCTG  6782
LongTailedChinchilla          --------------------------------------------------------------AAGCACTAGTTTATAATG  6682

Majority                      TCXXXACXAXXTXXXXXTAGAGTATTAGAAACTGGXCATTCTXAAACTGCGGAXACAXX------ATXCAXGCXTXTTAA
                                      22250     22260     22270     22280     22290     22300     22310     22320
Human                         TCAAGACCATTTTGGCTAACACAGTGAAAACCTGTCTCTACTAAAAATACGAAAAAATT------AGCCAGGCGTGGTGG  22090
GuineaPig                     TGTTTACTAGGTGACAGCATTGTATTAGTAACTGGTCATTCAAAAGCAGTGAAAATA--------ATGCATGCTTCTTAA  6550
NorthernAmericanDeerMouse     -----------------TTGAATGTGTGAAAC------------------------------------------------  6236
Mouse                         ACACACACAGAGAGACATAGAGAATCACAGACAAACCTGGTTTGAATTGAAGCCCCTCCGCATTGACACCCTTCCTCTAA  7781
ChineseHamsterGHOK1GS         GC---------------TTGAGTATTTGAAACCTCAAAGCCTGCCCCTAGAGACACACTTCCTCCAACAAGGCCTCACCA  6847
LongTailedChinchilla          TGCCTACTAAGTGACAGCATTGTATTAGTAACTGGTGATTCAGAAACAGCGGAGACAGA------ATATATGTTCTTTAA  6756

Majority                      GGGXAXXXACCTXAATAGTTTXATTTAXTAXXTCTCCATTA---AGCATGGXAATXGTGTXXACTCAGXATXC------X
                                      22330     22340     22350     22360     22370     22380     22390     22400
Human                         GGG---GCACCT--GTAGTCCCAGCTACTCGGGAGGC-TGA---GGCAGGAGAATGGTGTGAACCCGGGAGGC------G  22155
GuineaPig                     GGGCAGAGACCTTATTATTTTTATTTATTGTTTCTCCATTATGAATCATGTAAATGGTGAATACTTAGTATTT------A  6624
NorthernAmericanDeerMouse     --------------------------------------------------------------------------------  6236
Mouse                         CAAGGCCAGGAACCCTCCTTTGAAATAATAGCTTTCCCTTT--GAGCCTAGTGGCCATTTTCATTCAAATGACCACAGCT  7859
ChineseHamsterGHOK1GS         CTTAATCCTTTCAAATAATGTCACTCATTATGAGCCTATGT--GTGTGGGGGGGT-ATTTTCATTCCAACTACCACAGCT  6924
LongTailedChinchilla          GGGCAGAGACCTTAATGGCTT-ATTTACTTCTCCTCCATTA---ATCATGCAAATAGTGGATACTTAGTATT-------A  6825
```

```
Majority                    GTGATTXTAGTAGTXTCACXGG---CTTCAGCCTGX-TGAXAGTCCAAGAA--CXATTTTAGXAAAGXAAAATAAATTXC
                                     22410     22420     22430     22440     22450     22460     22470     22480
Human                       GAGCTTGCAGTGGCGCCACTGGA--CTCCAGCCTGGGTGACAGAGCAAGACT-CCGTCTTAAAAAAAAAAAAAAAATTCC  22232
GuineaPig                   ATAACCTTAGAACTATCTCAGG---CTTCAGCTTGT-TCATATTCTGTGAA--CTATTTATCAAAAGAAAGATAAATGAC  6698
NorthernAmericanDeerMouse   --------------------------------------------------------------------------------  6236
Mouse                       GTGGTTCTGTTAGTCTAAC-AGTGGTGTCTCCCAGACCAAAAGTTCAAGAATTCAGTAATAGTTAAGTCTAAGAGGCTAC  7938
ChineseHamsterGHOK1GS       GTGTTTCAACTAGTCTAACCAGTGGCAGTCTCCCAGTGGCAAGTCCAAGAAACCAATGGTGGTTCAGTTCACTAGGCTGG  7004
LongTailedChinchilla        ATGACTTTGGAAGTATCTCAGG---CTTCAATCTGT-TTATATGCTGTGAA--CTATTTTCTCAAA--AAGATAAATGCC  6897

Majority                    XCTTTCTGGAT--X--XGAXXTTAC----X--TTCTXATTGXATATGTTCT----T--CA-TGXAAAAAAGXCTGTGTXX
                                     22490     22500     22510     22520     22530     22540     22550     22560
Human                       ACTTTCTGGATTCTGCTGATTGTACCGCTGTGTTGTCATTTCATATGTTCT----T--CA-TGTAAACAAGTTTACCTGA  22305
GuineaPig                   TCTTTCTGTAC------AACATTGC-------TTTTTATTGGAGATC----------------CAAAAGAGACTGTGTTT  6749
NorthernAmericanDeerMouse   --------------------------------------------------------------------------------  6236
Mouse                       TGTTGTCAGCTGGTCATGTGCTCACATCAGAATCCTGAAGGACTAGGTTCTATAATGCCAGTGAAAGAATGCCTTT-CAA  8017
ChineseHamsterGHOK1GS       GTGTCTCAGCTGGTCTTGTGTGTATGTATGAATCCTGATGGAGTAGGCTCTG--ATACCAGTGAAGGAATGCCTCAGCAG  7082
LongTailedChinchilla        AATTTCTGTAT------GACATGAC-------TTCTTATTGGAGATCTTGG----A--GA-TCCAAAAGAAACTGTGTTT  6957

Majority                    XATAXGXGAXCTTGXCCTGGXT---------GAAGAGATAAATACCAAAAGTXTXXXXC--AXATG--XXCCATGT----
                                     22570     22580     22590     22600     22610     22620     22630     22640
Human                       AATACAGGGGTTTGATCAGACTC-------TGGTGTAATTTTTACCAAGAATATTTTGC--AGATGTACTCTATGTAA-T  22375
GuineaPig                   T-TAGGAGATACTCTCCTGCTT---------GAAGAGATAGGTACCAAAAGT---------AAATG----CTATGT----  6802
NorthernAmericanDeerMouse   GATCTG--------------------------------------------------------------------------  6242
Mouse                       CATACATGAACTTGCCCTTGAGAACGAAGGCAAACAGGCAAAAAGCAAAAGTGTCTCTCCTCCATGCCCTTCAAATTGGC  8097
ChineseHamsterGHOK1GS       GATCTGTGAAGTTGCCATTGAGAAAGAGGGCAAGCAGACAAAAAGCAAAAGCTTCCTTCCTCCATGTCCTTCATAA-GGC  7161
LongTailedChinchilla        T-TAGGAGGGCCTCATCTGGTT---------GGAGAGATAAATACCTAAACTTCTAAGG--AAATG----CCATGT----  7017

Majority                    TGCTACXGXAAGXXAGXTTACTTT-TGTTTGAXAGXGXXXATGTAXXTTTCATCTAAX----X-TAGT-AGCTAATGAXX
                                     22650     22660     22670     22680     22690     22700     22710     22720
Human                       TCCTGTTGTATGTCAGCTTGCTCCATCTATTACAGAGCGCATCATCCTTTCACCTAATAGTTTTTAGT-AGCTAATGAT-  22453
GuineaPig                   TGCTACAGTAAGAGAGATTACTTT-TGGTAGATAGGGTCAGAATACATTTCATCAAAG------TAGT-AGACTGTGACA  6874
NorthernAmericanDeerMouse   --------------------------------------------------------------------------------  6242
Mouse                       CAGCACCAGAAGTTAGGTCTTCTGACTTCAAAAGATGGAGATGTAGGATAGGTCTTCCTGCCTCCAGTGAATTAATTAAG  8177
ChineseHamsterGHOK1GS       TGGCACCAGAAAGTGTGGCCCAGATAGATTCAAGATAGGGATTTATGATGGGGCTTCCCACCTCCAATGAGCTAACCAAG  7241
LongTailedChinchilla        TGCTACAGCAAGAGAGCTTACTTT-TGTTTGATAGGGTCAGAGTAGATTTCATCAAAG------TAGT-AGGCTGTGACA  7089
```

```
Majority                   TGXAXATTXTTAXTAGGTXGGGAXAXXCXGXAGCTXGXGTTTXG-XCAGTACATGTTXXXXXXGTTAACAA------XXA
                                  22730     22740     22750     22760     22770     22780     22790     22800
Human                      TGCATACTTTTATTAGGTATTGCAAAAGTGGCGCTATTCTTTTTTTCTGTGTATATTAGAATTCTTAAGAATT----ATA  22529
GuineaPig                  TGTGCATTATGA-TAGGTTGAGAGATGCAGAACATAGTGAAGAG--CAGTACATGTTTAAGTTTTGA-AAA--------A  6942
NorthernAmericanDeerMouse  ------------------AGGAAAA---------T------------------------------TGACAG---------  6256
Mouse                      AAAAACTCCTTACGGGTCTGGTCTACCCACCAGCTTGGGTTTGGTTAACTCCAGGCTAGTCAAGTTGACAATCACGGATA  8257
ChineseHamsterGHOK1GS      AAAAATCCCTTAA----CAGGGATACCC------TTGGGTTTTCATTAATATCAGGTGTT---GTTAACAGCTAAGAATA  7308
LongTailedChinchilla       TGTGCATTAGAGCTAGGTTGAGAGATGCTGAAGCGGGCAAAGAG--CAGTACATGTTGAGAGAGTGACAAA--------A  7159
```

```
Majority                   XCXXTCAXXTGTGT--TTGTXCATTXGTAXAXXTTTATAGXCAXXX-XGTAXXGAXTTTAACTT--TGAXTTTT---X-X
                                  22810     22820     22830     22840     22850     22860     22870     22880
Human                      TCCATCAACTGTTTGGTCATACGTAAGTACCTTTTTATAAGAAAAGCAGGAGAGAATTTGACTTCTTAAGTTTTCTGATG  22609
GuineaPig                  AT--TAAGTTGTGT--TTGTGCTTTGGTGTAAATTCATAGCCATCA-AATGGTGAATCGAAC------AGTTTT------  7005
NorthernAmericanDeerMouse  --------------------------------------------------------TTTAATTC--TGAATTTT------  6272
Mouse                      GCCATCACATCTAGC-TTGTTTATTTGTCCATGTTTCTAAGATACT--GT------TTTACGTT--TGGATTTTGTCAGG  8326
ChineseHamsterGHOK1GS      GCCATCACACCTGGC-TTGTTTATTAAGTATCTGTTGAAGTCTTTT--GTACAAGTTTTACCTT--TGGATTTTGACAGG  7383
LongTailedChinchilla       AC--TCAGTTGTGT--TTATGCCCTGGTATAAATTCATAGCCACTG-AACAATGAGTTGAAC------AGTTTT------  7222
```

```
Majority                   -GTCXTTXXXXT----TCTTTATTAXTXGTCAGTAXGTGTT---TTTTGC-XCACXTACTATTGT--AXACAGXACXTAX
                                  22890     22900     22910     22920     22930     22940     22950     22960
Human                      AGTTGTTACCCTAAAACCTCCAGTATAA-TCAATATGAGTTCCTTTTTGTTGCTGTTAGTATCGTCGCAACAGCAAAGAG  22688
GuineaPig                  -GCCTTTGCTTT----TCTTTAGTATGGGTCAGTAGGTGTT---TTTTGC-ACACAAACTTACGT----CTAATATGAAG  7072
NorthernAmericanDeerMouse  -----------------CCT-ATCAAT-----G----------------------------------ACAAAATACATTT  6295
Mouse                      CTTCATTCTGGACTTGTTTTTATTAATTGAGTAGATTGCACAAATTTTCTATTACCTTCCATTCTCTATACAGCACTTGT  8406
ChineseHamsterGHOK1GS      CTTCATTC---------CTTTATTAATAGCCTGCACATTTTT--TTCCCCATCACTTTGCATTCTTTACACATTACCTTT  7452
LongTailedChinchilla       -GCCTTTGTTTT----TCTTTAATATGGGTCAGTAGGTGTT---TTTTGC-ACAAAAACT-ATGT----GTAGCACGAAG  7288
```

```
Majority                   TAA-AACAAXAXXTATXXTXAAGAGCATTXXTTTAXTT-----ACTTTTTCTTTTAXAXA--A---------T---ATCT
                                  22970     22980     22990     23000     23010     23020     23030     23040
Human                      TTT-AATAACATTTATTTTCTAGTGTATTGCAGTAATC-----ATTCTTCTTTTTTTTAA--A---------T---TTCT  22748
GuineaPig                  TAA-AACAAAACATGTAAGAAAGAGCATT---------------CTGTCACGGTGACACC--A---------T---ACCT  7122
NorthernAmericanDeerMouse  -------------------------------------------ATCTCTTCATTCACA---------------------T  6311
Mouse                      GAAGAGCAGTAGTTACGCTGCTAAACTCCATTTTAGTT----AACTTTTTCTTTTATGGATGGTTTAGTTTGTCCTATTT  8482
ChineseHamsterGHOK1GS      GAGGAATAACAGCTATACTGGTGAATTCTATTTTAGTTCACCAACTTTTTCATTTATGGATGATGTGGTTTGTCCTATCT  7532
LongTailedChinchilla       TAA-AACAAAACATGTGAGAAATAGCATTGGTTTGCTT-----TCTGTCACTGTGCCACA--A---------G---ACCT  7348
```

```
Majority                    AAGCCTTTTTXXAXAAXGXAAAAATXAXTTTGTT----GTTTTXTXAGATTTTTC-TXTTXXXTTAATATTTAGTTXXTT
                                     23050     23060     23070     23080     23090     23100     23110     23120
Human                       AAGCTGTTTTATTAAATGAAAAGAGAACAATGCTAAGCAGCTTGTATGGTGTGTG-TGTTGTGTGGGTTTTTATTTTGTA 22827
GuineaPig                   GAGAAAACATAGAGAAGGAAAGATTTATTTT-------------AGAGA-------------GTCTATGGTTGGTTGACT 7176
NorthernAmericanDeerMouse   AGGCCTTT--------------A-CATTTTTGTT----GTTCTTTGAGATTGTTC-------TTTAAGATTT-------- 6357
Mouse                       AAGCCTTTCCTACCTAAAGTAAAATGACTTTTTT----GTTTTCTAAGAGTTTTCATTCCAGTTTAATACTTAGGTCTCT 8558
ChineseHamsterGHOK1GS       AAGCCTTTTCTAACC----TAAAATGACTCCTTT----GTTTTCTAAGAGTTTTCATTTTAGTTTAATATTTAGGTCTTT 7604
LongTailedChinchilla        GAGAAAACGTGGAGGAGGGAAGATTTATTTTGGCTCATGGTTTTAGAGATTTTGT-TCTTCAGTCCATGGTTGGTTGGTT 7427

Majority                    TXXXXXXT----XTAXAAGTCTTTXGCAXXXXCXTXGCATCXTAXXA-XTTGAAXGGC-X-X-GTGGTTGXAAAAAXATG
                                     23130     23140     23150     23160     23170     23180     23190     23200
Human                       TGAATGTT-----AAAAACACGGTGGCTAAAGCCTGTAATCCCAGCATTTTGGGAGGCCA-AGGTGGTTGGATCACAAGG 22901
GuineaPig                   CACTTGCT-----TTTAGGTCTATGGC-AGGATAGAGCAACATAGCA----GAAAGGT-----GTGGTTGAAAAAAAATG 7241
NorthernAmericanDeerMouse   ----CACT----GTAAAAGTTTTATACATTA---T-------TATTAATTT----------------------------- 6390
Mouse                       TCTCCATTTGG-GAATTACAGTTTTTCAGGACCTCTGCATTTTGTTACATTGAC-ACACACACACACACACACACACACA 8636
ChineseHamsterGHOK1GS       TCTACATTTGGGGTGAAATTTTTTTCCACTGCCTTTCCATTTTGTTAGATTAAAGACCAACAAAACTCTAGAAGGACATG 7684
LongTailedChinchilla        CACTTGCT-----TTTAGGTCTGTGGC-GAGGCAGAGAAACATAGCA----GAAGGGC-----GTGGTAGAAGAAGGATG 7492

Majority                    XXAAAXAC-GTGA---CAGCCAGGA---AXTAGAGAAGXAXXXTGGAGAGAG---------GXTATAGTCCCAAAAGTXA
                                     23210     23220     23230     23240     23250     23260     23270     23280
Human                       TCAGGAGC-TTGAGACCAGCCTGGCTCTACTAAAAATAAAATGTGGTGAAACCCCGTCTCTACTAAAAATACAAAAATTA 22980
GuineaPig                   CTCAACAC-GTGA---CAGCCAAGA---AATAGGGA------CTGGAGACAG---------GGTATAGTCCCAAGGGCAT 7299
NorthernAmericanDeerMouse   -------------------------------------------GGGAG-GG----------G------------------ 6398
Mouse                       TCAAAAACCTAGAAGGAAATAAGGG-GGTAGAGAGGAGGACTACAGTATGAGAAGCAGGGAG--AAGAGAGAAAAAGTCA 8713
ChineseHamsterGHOK1GS       GGAAAGATGGTGAAGGACATGTAGA-CTACAACAAGAGAAGCAGGGAGAGGGGAGAAAAAAGTCATAGTCCCACAAATCA 7763
LongTailedChinchilla        CTCACCA--GTGA---CAGCCAGGA---AGTAGAGAAGGAGACTGGGGACAG---------GGTATAGTCCCAAGGGCAC 7555

Majority                    GCCCTC-------ATTXCACACGCCCXTAAGCTTGGXTCCACXXT--TXTGATTTAXATXACXTCATXAXXXTTTCTXAC
                                     23290     23300     23310     23320     23330     23340     23350     23360
Human                       GCCCTTT---GTTGTTGCACGCGCCTATAGTCCCAGCTACTCGGGAGGCTGAGGCAGAAAAATTGCTTGAACCTGGGGAG 23057
GuineaPig                   GCCCGC-------ATGCCCCACTCCCTTCAGTTTGGCCCCACTGT---CTAAATTACATCACCTAATAATGCTCTCAAAT 7369
NorthernAmericanDeerMouse   -----------------------------------------------TTAGATTC--A---------------TCCT--- 6411
Mouse                       AGAGTC-----CCACAAAACAGGTTGAGAACGTGGATTGAAAAAA--TTTATTTTAGATATTGACATCAGTTTTTCTAAC 8786
ChineseHamsterGHOK1GS       GAAGTTAACGATTTTTAAAAAGGGTACAAAGCATGGATTGAAAAT--TTTGATTTAAATGTGGGCATCAGTTTTTCTGGC 7841
LongTailedChinchilla        GCCCCC-------ATCCCGCACTCCCTTTAGCTTGGTCCCACTTT---CTGAGTTCCATCACCTTCTAATAAT-----AC 7620
```

```
Majority                      XAXXXXTGXGXAAAATTAX-XTGXXXTC-XXAATTAGXX-TTGCAGCTTX--X-AAAXCACGTTXCTTT--AXX-X-XGC
                                        23370     23380     23390     23400     23410     23420     23430     23440
Human                         GCTGAGGCAGAAAAATTAC-TTGAACCCGGGAGACAGAGGTTGCAGCGAGC-CGAGATTGCGCCACTGC--ACTCC-AGC 23132
GuineaPig                     TATCTATGAGTGTATTAAT-CCAGTGTT-GAAATTAGAGACCTCAAGAAC----AAATCACTTTATTTT--ATTTAAAAA 7441
NorthernAmericanDeerMouse     --------T--AAG--TA--------T--TCA-----------CAGCTT------------------------------- 6427
Mouse                         AA----TGCTAGAAAATGC-----TTTCATTAATTAGTC-TTGCAGCTTTGTCAAAAATAAGTTGCCTTAAAAAGCTTTC 8856
ChineseHamsterGHOK1GS         AACATTTGCTGAAAATAATGTTGTCTTCATTAATAAACC-TTGTAGCTTTGTCAAAAACAAGTTGCCTTTAAAAAGTTGC 7920
LongTailedChinchilla          TGCCAAGGGG----------CTGG-----GGATTTAG----CTCAGCGGC----AAAGCGCCTGCCT----------GGC 7667

Majority                      CTGXATAAXGTTTTAAGTXTGXATXCXAXTACXXX-XCAXATATXTATXTXTXTAG-XG-CAXAXXXXTXCTXT---AAX
                                        23450     23460     23470     23480     23490     23500     23510     23520
Human                         CTGGGCAACAGACTGAGATTCCATCTAAAAACAAAACCATGTATATATGTAAGTTTGAG-GAAAAGAGTTGTATGA-AAA 23210
GuineaPig                     AAAGATTAAGATAAAAGAGGGCATAACAGTACAGG-TCAGAACAGAACAATTTAAGCAA-CATAGTGTTTCACCAC-AAG 7518
NorthernAmericanDeerMouse     -------------------------------------------------------------------------------- 6427
Mouse                         CTATATATGATTTTATGTCTGTCTTCTCTGCTGTGGACT-GTGTTTATCTATCTAGATGTCAAGGGCATACTGTTGTAAG 8935
ChineseHamsterGHOK1GS         CTGAATGACGTTTTATTTCTATACTCTTTCCTGTTGACCCATTTTTATCTGTCTAGTTGCCAGAAACATACTGT---AAT 7997
LongTailedChinchilla          AAGTGCAAGGTCGTGAGTTCGGTTCCCGGTAC-----CGGAAAAAAAC-------------------------C---AAA 7714

Majority                      ---AAGTTXXXXAATAAAAGTAGAAATCAAXTTGTCTTXGTXTXCXAA-XTTTGTTAXTXTXCCAA-XTTATTTTGGXXC
                                        23530     23540     23550     23560     23570     23580     23590     23600
Human                         TTCAGGATAATTAATAAAAGTAAACGAGAACTTCTTTACATATATATATATTTTTTAATTTTCTCAGTTAATGTTTGAAT 23290
GuineaPig                     ---ACAGTCCGACATAGCAGTAGCTACCATAGTGTCTCTTTCCTT-GAAATATTTGAGTATACCTT-CATATACTATAGT 7593
NorthernAmericanDeerMouse     -----GTT----AT---------AAATCA-------TTGGTTTTCAAA-CTGTG----------------------GGC- 6458
Mouse                         CATTAGTTCCATATTAACTCTTGAAATCAAGTTGCCTTGCTCTGCCAA-CTTTGTTGTTTTTGCAAAGTTGTTTTGGCCC 9014
ChineseHamsterGHOK1GS         CTTTAGTT----ACTGTATCTTGAAATCAAGTTGCCTTAGTTCACCAA-CTTTGTT-------CAAAGTTGTTTTGGCTC 8065
LongTailedChinchilla          ---AAAAAAAAAAAAAAAAGTA-CTGCCAAATTGTCTGT-------GA-ATGTATTAATCCACTGA-TGAAGTTAGAGAC 7781

Majority                      TTCAXAXACAXAXAAAGTXTCAAXGAXXTTXXCTGXAXAXAXXXAXXAT--TTGAXGAAATTTXAXXXXXXXT---AAXX
                                        23610     23620     23630     23640     23650     23660     23670     23680
Human                         TTTTTTAACGTAGAAAATGTCAATGGGGTTATCTGGGGAGAAAGATTATAGTCCATGAGTCCCCCTGTTGTATATATACC 23370
GuineaPig                     ATCAAACACAAATCACTTTCCAAAAATCTTGCCTCAACACATGAACCTT--TTGGGGAAATTTTATTTTGGTACTGAGAA 7671
NorthernAmericanDeerMouse     -------------------------------------------------------------------------------- 6458
Mouse                         TTCTGAGTCACAAAAAGCATACTGGAATCCTTCTGTATTTGGCTTGAATTGGTGAAGCGATTTGAGAAGAACTGACAATT 9094
ChineseHamsterGHOK1GS         TTTAGAGTCATAGAAAGGATGCTGGAATCTTACTGTATTTGGCTTGAAT--GTGAAGCAATTTGAGGAAAATT---AATT 8140
LongTailedChinchilla          CTCATGAACAAATCACCTTCCAAAAATCTCACCTCAACACATGAACCTT--TTGGGGAAATTT----------------- 7842
```

```
Majority                     TX-TAXTXAAXTATXCTATXAXTGC-X-----AXXX--ATXTCTT-ATT-ACATAXXXCX--AAG-XTXGXXXXXTTGXT
                                      23690     23700     23710     23720     23730     23740     23750     23760
Human                        TG-TATTAAAATATGCAATAAATGCTATTTTAAAAA--ATGTCTT-ATT-ACATAGAACA-GAAGACCAGAATACTAGCT  23444
GuineaPig                    TG-GAAGTTAAGACCTTGCACTTGCCAGGCAGGCAC--TGTACCA-TTG-AGCTATGTCC-CTAGCCTGGAAATCTTTAT  7745
NorthernAmericanDeerMouse    ---------------C------------------------------------ATGACC-------------------ATC  6468
Mouse                        TACTATTGAATTTTCCTATCAATGACAGAATAAAGCTTATTTCTTCATTCACATAGGTCTTTAAG-TTTGGGGCATTGGT  9173
ChineseHamsterGHOK1GS        TAATACTGAATTATTCTATCATTAC-------AGTGTTATCTCTTCATTCACATAACCTTTAAAGATTTTTGGCATTGTT  8213
LongTailedChinchilla         ---------------T-------------------------------------------------------------TAT  7846

Majority                     CTTTAAGACTTAACTGTTAATCTTTGXAAATTAXX---T-----AXCXAXGXCATCXXGXXGTAXACATAXCXAXAXTTX
                                      23770     23780     23790     23800     23810     23820     23830     23840
Human                        CTCCACGTCTTATGTCTTGATCAGTGAAGATTAGC---T-----AGCAAAACAACCATAACTTAGGGATAGCTTTATTTG  23516
GuineaPig                    ATTTAAAACATAACAAATGCTCTTGGAAATATA------------ACTATGCCTTCTAGTGGAAAACATACCAATACTTA  7813
NorthernAmericanDeerMouse    CCTTAAGACT--ATTGGAAA------------A---------------------------------------CACAGATA  6495
Mouse                        TTTTATGATTTCACTGTGAAGTTTTATATGTCTTTTTGTTTTATGTTGAGCTCATCCCCCAGTTGATGTGTTTTTAAGAG  9253
ChineseHamsterGHOK1GS        CTTTAAGATTTCACTGTAAAGATTTATGCATTATTATATTTTGGAGGGGGTGGATTCATC--TCTAAGTATTCACAGGTG  8291
LongTailedChinchilla         ATTTAAAACATAA---ATACTCTTGGGAACATAGG---T-----AACTGTGTCTTCTTGTGGAAAACACACCACCACTTA  7915

Majority                     TTXAAAGTGTGATTTATTAXAGCAAAAX-TTAXAXATXTGATGTAGTCTTT--GAAXTCATXTXAATAXTTTTXXAXATT
                                      23850     23860     23870     23880     23890     23900     23910     23920
Human                        TGGGAAGTGTCATTTAGTATGTCATGTA-TTAAAAATCATTTGTAGTCTTTCAGAGAGCATCTTATCTTTCTTGTAGGTG  23595
GuineaPig                    TA--AAGTGTTATATCCCAAAAAAGAGA-GAGGAGCTCTGATCAAGTCTCT--AGATTCAACTGCCTGTAATTTAATATT  7888
NorthernAmericanDeerMouse    CTTACATTATGATTCATAACAGCAAAA--TTACAGTTATGAAGTAGTAGTA--AAAATAGTTTCAATAATTT-----AAT  6566
Mouse                        TTCA-ACTTCGATTGTTTGGGGCAAGAGTTTAGAAATATGATGTGTTCTTG--TTCTTGCTTTCTGCACTTTTACCAAAT  9330
ChineseHamsterGHOK1GS        TTTACATTATGATTCATAACAGCAAAA--TTACAGATATGAAGTACCAATG--GAAAAAATTTTAATAATTT-----CTT  8362
LongTailedChinchilla         TA--AAGTGTTAC-TACTATTAAAAAAG-GAGAAACTCTGATCAAGTCTGT--GGGTCCAACTAT-----ATATTAAATT  7984

Majority                     XCAXTTTGTGAATXXGATXAGTTTGTTTXAGATXCTAAXAXXATTTTTAXXTTTACCXTTXXTGXXCAGX-X-TGTAAAA
                                      23930     23940     23950     23960     23970     23980     23990     24000
Human                        ATAGTATTGGCTATCTATGTAGTGTCTTAAGTTCTTTAAATTATCCTTATTCTTACTTTTTGTGTACATTGTTTATAAAA  23675
GuineaPig                    ACATTATCAGAATACAGTCAGA-------AGGACCAGAAGTTGC----ATGGTGCCAGATTGTGGATAAC-TCTATAAAA  7956
NorthernAmericanDeerMouse    TCAATTTGTAAATTTGATAAGTTTGTTTTAGATTTTAACAGAATTTTTACATTTACCATTGATGCCCAGT---TGTACT-  6642
Mouse                        TCAGTTTGTTAATCTGAT--GTTTGTTTCAGATTCTAACAGAATTTTTACATTTACCATTGATGACCAGC---TGTTTGG  9405
ChineseHamsterGHOK1GS        TCAATTTGTAAGTCTGATGAGTTTGTCTTAGAGTGTAACAGAATTTTCACTTTTACCTTTGATGTTTAGT---TGTGCA-  8438
LongTailedChinchilla         ACATCTTCAGCATACAATCAGATAG---AAGAGCCAGAAGTTGC----ATGGTGCCAGACTGTGGGCAAC-TCTGTGAAA  8056
```

```
Majority                    TAXAXAGTTTTTTCTCXTXTXXCAGCTGTAXTATXXTTTGAGTAXA-----------------XGCCAGTTTX---TXTA
                                     24010     24020     24030     24040     24050     24060     24070     24080
Human                       TAATGTGATCATCTTAATCTACCATTTTGATTATGGTTTGATCATTC----------------TTTTACTATT---TCCA  23736
GuineaPig                   TCAAAAGCTAGTTCTC-TGTAACAAATAAAACATAATTTTAAAAAAA----------------GAATAGAGA-------A  8012
NorthernAmericanDeerMouse   TAG----TTTTCTCTCTT-----GGCTGT--------TTGGGT---------------------GCCATTTTTTTCTTTT  6684
Mouse                       TTGCCATTTTTTTCTTGTCTGTGAGCTGTAGTAAGTGTTAAGTTGACTGGAGGTGGTAAGAGTAGCCAGTTTTACTTGTC  9485
ChineseHamsterGHOK1GS       TAGACAGTTTTCTTTCTT-----GGCTGT--------TTGGGT---------------------GCCATTTT---CTTTT  8481
LongTailedChinchilla        TCAAAAGCTAGTTCT---GTCACAAATAAAATGTAATTCTAAAAAA-----------------GAAGAGAGA-------A  8109

Majority                    CTT--TAXTAXGACXGXTAGT--TXCAATATTAATC-TGAAATXAAXTACTATXAXXTGTCAATTTTAGTATGAATXTCX
                                     24090     24100     24110     24120     24130     24140     24150     24160
Human                       CTTGTTAGCAAAGCCTTTATACTTACTAAATTATGA-TAAAAATGATTATTATAACATGCCCGGTTTTACTTAAATGTCT  23815
GuineaPig                   CTT--TATAAAGAAACAG-----TGCAATGATAACC-TGGAGTTTGTAACAGCAAAGGGAATAGTTGAGTATGAATATTG  8084
NorthernAmericanDeerMouse   CTT--GCCTACGACTGCTAGTTAGTAAGTATTAGGT-TGAATCGAAGTGGTAAGAGTTGTCAATTTTACCATGTTCTTCC  6761
Mouse                       CTTTGATTTAGGATGAATAGTCTATCTTCCTGACTCATGAATTTAAGCTCTATGAACCATCCATTAAGGAAGGAATGCCA  9565
ChineseHamsterGHOK1GS       CTT--CCCTATGACTGGTAGT----AAGTATTAATT-TGAAAAGAAGTGGTAAGAGTTGTTAGTTTTATTGTGTTCTTCC  8554
LongTailedChinchilla        CTTG-TAGAAGAACAGTG-----TGCAATGATA-TC-TGGAATGTGTCACAGTAATGGGAAAAATTGAGTATGAATATTG  8181

Majority                    TATTXXXG-----TGAXTXTTA-------------XTTCTTTCTTATTTAXGATGTTXTXAXTXATAXATXXAGCA----
                                     24170     24180     24190     24200     24210     24220     24230     24240
Human                       TATTTAAACGTTCTGATTGTTA--------------TTCTTTCTTATTCAGGTTTCTTATTGTCATGTTTTATGTC----  23877
GuineaPig                   TATTT-------TTAGCTGTGG--------------ATTGTTAATGTTTA--TTGGCATTGATCATAGATA---------  8132
NorthernAmericanDeerMouse   CATCAGGG-----TGAATATTA-------------TTTCTCCCTTAATTATGAA-TTGTAAGTTGTACA--GAACA----  6816
Mouse                       TTTTAAAG-----TGAGGCTCTGTAGGTCTGGGGCTTGCTCTCTCTGTGGGGAAGTTTTAAGTTACATACTGAGCATCCT  9640
ChineseHamsterGHOK1GS       CATTAGGG-----TGAATTTTA-------------TTTCTTCCTTAATTATGAG-TTGTAAATTGTACA--AAACAAAGC  8613
LongTailedChinchilla        TATTT-------TTAATTATG---------------------AATGTTTA--CTGGTACTGATCATAGATACAGTA----  8227

Majority                    ---TGGTTT---GTGXXXCTXTTXTCTCTAAAATTXACTXCAAT----X-XATGACAAAAXATCTTXTTACTXTAXAXTX
                                     24250     24260     24270     24280     24290     24300     24310     24320
Human                       ---TGTTTTCAGGTGTAATTGTCCTCTCACAAATGTATCCCATTT---TGGGTGACAAAATACCTGATCACCTT--ATCC  23949
GuineaPig                   ---TGATTT---GTGAGGCTTTTGTGTCTAAAATTAACTACAAT------AATTATAAAAAGCATTGTTACTCAAAACTG  8200
NorthernAmericanDeerMouse   --------------------------------------------------------------------------------  6816
Mouse                       TTATGCTTTTTATTGTCCTTCTTTTCTTTTCTTTTCTTTTTTTTTTCCTTTTTGAGATAGGATCTTACTGTTATTTTTTT  9720
ChineseHamsterGHOK1GS       T---GGGTGGTGGTGGAACACATCCCTTTAATCCCAGCACTCAGGAGGTAGAGGCAGGCGGATCTCTGTGGTCTACCAAG  8690
LongTailedChinchilla        ---TGGTTT---GTGAGGCTTTTGTGTCTAAAATTCACTACAAT------AATTACAAAAAGTGTTGTTACTTAAAACTC  8295
```

```
Majority                      TTAXCATAATTTTTTXAGXTATTTTATTXCTATAAAAATAXAAAXATTAXTTX-CCAXTXTATCXTCXAATATAGTAGGC
                                       24330     24340     24350     24360     24370     24380     24390     24400
Human                         TTATAGTTTTCTCCCTAGAATCTTTTTTGTTATATTAAT-CAAAGTTTATTTG-TGAGAATGTTATGAAATATAAGAAAA  24027
GuineaPig                     TTGTGATAACTTTTTGAGCTATTTTATTGCTTTTAAAATAAAAATATTATTTAACCACCTTATCCTCTAATGTAGTAGGC  8280
NorthernAmericanDeerMouse     ----CACAG-----------------------------------------------------------------------  6821
Mouse                         CCAACCTAAGACTTTTATGTAGATGACACCTATAGGAGTCTCAGGTCTGCCCT-CTTGGGAAGCTGGCACTACAGTTGTG  9799
ChineseHamsterGHOK1GS         CTA-CACAGAGAAACACTGTCTTGAAAAACCAAAAAAAAAAAAAAAGAAAAAG-AAAATAAACAAACAAACAAAAAACCC  8768
LongTailedChinchilla          TTCAGATAATTTTTTGAGATATTTTATTACTTT-AAAGTACAAATATTACTTAACCACTTTATCCTCTAACATAGTATGC  8374

Majority                      C-TXXAXATAAATAACXXXTXGXXXTXAATTTCAGXXTTXAATGTGAAXTA-XXTTXGTCTTTTTTTTXTTTXCTCTGA-
                                       24410     24420     24430     24440     24450     24460     24470     24480
Human                         --TGAACATCATGAACCTGTCACTCAAATAAAAAAACTTGAATGTTACCAA-TACTATTGTATATTCCATATGCAGCAAT  24104
GuineaPig                     C-TGGAAATAAATAAA---------TATAGTTTAGTCATACATGTAGACT----TTTATCTACTTTATATGTGCTTTTA-  8345
NorthernAmericanDeerMouse     --------------------TGGAAGGAATGTCA--TTACAATGTGAAGCTCTGTAGGCCTTTGGTTTGTTTTCTCTG--  6877
Mouse                         CGCCTCTGCACTTAGCCCTTCTCCCTGCTTTCCAGGTTAGAACCTGCAGGAACTTGAGCGCTCTGCATTCTTACACTGAT  9879
ChineseHamsterGHOK1GS         CAAAGTTTTACAGAACAAATTGGAAGGAATGTCA--TTGCAAAGTGAAG-----TAGGTCTTGGTTTTGTTTTCTCTGT-  8840
LongTailedChinchilla          C-TAAAAATAAATTACGAGTACTTTTATAGTTTAGTCATACATGTAGACTA-GTTTTTTTTTTTTTTTTTTCACTTTAAC  8452

Majority                      --XXXATGTTTTTTTTTTXTTATTGTTTTGXTTTGATACA------XXXGTTCAGXTXTXCXATXTCXXCTGXCXGGTAX
                                       24490     24500     24510     24520     24530     24540     24550     24560
Human                         ----ATTGCTTTTTTTTTTTTAGAGTCTTGCTCTGATACCCACGCTGGAGTGCAGTGGTGTCATCTCGGCTCACAGCAAG  24180
GuineaPig                     ------TCTATTTTATATATT-TTGTTTGGTTTTGTGAC-------AGGGTGTGATTGTGCAGGTTAGGCAGGCCTGGAA  8411
NorthernAmericanDeerMouse     --GGAAAGTTTTAAGCTACATACTGGGTTTGTTTAATAGAGCAGG-GCTATTCAGGTTACCCATTTCTTCT--TGGGTTA  6952
Mouse                         TATTCATGTGTTGTTTTGATTATTGA-TTCATAGTATAAATGTTC-TCACTTCAGCTTCATGAACTTTTCTGTCAATTTT  9957
ChineseHamsterGHOK1GS         -GGGGAAGTTTTAAGTTACATACTGAATTTGTTTAATAGA-------CTATTCAGGTTACCTATTTCTTCT--TGGGT--  8908
LongTailedChinchilla          --TAGATCTTTGTTTTGTTTTGTTGTTTTGTTTTGAGAC-------AGGGTCCGACTGT--AGCCCAGGCTGGCCTGAAG  8521

Majority                      CTTGGA-----XGATTCXCTXTATTXTXTTGCXTCATXCXXCXGAXTXXGTGGXGTTCXXGGX----AX-ACTCTAXC--
                                       24570     24580     24590     24600     24610     24620     24630     24640
Human                         CTTCGCCTCCCTGGTTCACCCCATTCTCCTGCCTCAGCCTCCCGAGTAGCTGGGACTTCAGGC----AC-CCGCCACC--  24253
GuineaPig                     CCTGGA------GCTCCATAAGATTATGTTGCCTCACCCTCCCAAG-AAGTAGGGTTATAGGC----AT-GCACTACA--  8477
NorthernAmericanDeerMouse     CTTCCATAATTTGATTTTCTGTA--GAACTGGTGAATTTATTTGGATG-GAGTTGTTCATGGT-------ACTCTATG--  7020
Mouse                         TTTGTCTTCATTGATTCTTTTTCCTTCTTTCATTTCTTCATGTGAATGTATACTTTTCCTTTTTAAAAAAATTTTATTTT  10037
ChineseHamsterGHOK1GS         ------------GACTTTCTGTA--TAATTAGTGAATTTATTCAGATG-GTGGTGTTCGTGGT-------ACTTTATCTG  8966
LongTailedChinchilla          CCTGGA------GCTCCA-GAGATCCTCTTGCCTCAGCCTCCTGAG-AGCCTGGATTATAGGC----AT-GCCCTACC--  8586
```

```
Majority                    --AXACXTAXXTATTXTTTTXTTTCTTTAXTXGA-GXCXGGGTXTCAXTGTTX---ATTTTGGXTGGCXTXXAATTCA--
                                      24650     24660     24670     24680     24690     24700     24710     24720
Human                       --ACACCCGGCTAATTTTTTGAATTTTTAGTAGA-GACGGGGTTTCACCCTGT--TAGCCAGGATGGTTTTGATCTCATG  24328
GuineaPig                   --ATGCCTAGCC-------------CTAACTAGA-ACCTGAACTCTTTTTTTTTTTAAATTGTGTAAAAACACATTCA-G  8540
NorthernAmericanDeerMouse   --ACACTTT-ATATTGTTTTCTTTCGTT--TTGA-GGTAGGGTCTCATTGTT----ATTTTGGTTGGCCTAAAACT----  7086
Mouse                       TGATTTTTTTAAATTAATTAATTACTTTACTTGA-GACAGGTTCTCACTGTGTA--GCTCTGACTGTCTCGGAATTCA--  10112
ChineseHamsterGHOK1GS       ATACACTTA-ATATTGTTTTCTTTCTTT--TTGA-GGTGGGGTCTCACTGCT----ATTTTGGTTGGCCTAAA-------  9031
LongTailedChinchilla        --CTCCCTATCC-------------CTAAATAGATGCCTGAACTCTTTTCT-------ATTGTGTAAAAATGCATTCA-A  8643

Majority                    ATXTGTAGAXCXGGCT-GTCGAXAXCTCACAXXTXTGXTXGACTTTXGXX-T-----GXTXCTGXGTTX-XAGTTXAXGA
                                      24730     24740     24750     24760     24770     24780     24790     24800
Human                       ACCTCGTGATCCGTCT-GCCTCGGCCTCCCAAAGTGCTGGGATTATAGGTGTG---AGCTACTGCGCCC--AGCCCAATA  24402
GuineaPig                   AGTTAAAAAT----CT-GTTGATAACTGGCAATTATTTTTGACTTTAGAGAT-------CTGTTCTTCA--TTTTTAAAA  8606
NorthernAmericanDeerMouse   CTATGTAGACCAGGCCTGTCTTAAGGTCACAGATCTGCCAGCTTCAT----------GAGACTGGGATTAAAGTT-GTG-  7154
Mouse                       TTGCGTAGACCGGGCTGACCGAGAGCTCACAGTTGTGCTCCTCCCCTTGCCCCGCCAAGTGCTAGGAGTAAGGTTCATGC  10192
ChineseHamsterGHOK1GS       CTATGTAGACCAGGT-----------TCTCAGATGTGCCACCCTTAT----------GAGGCTGGGTT--AAGTT-GTG-  9086
LongTailedChinchilla        AGTTAAAGATT-ATCT-GTTGATAACTGACAATTATTTTTGACTTTAGAGTT-------TCCTTCATT---TTTCTAAAA  8711

Majority                    TTTCATTATAAAXCT--TTCATXTGXTGCXATGGGXATGTX-ATAXAAXA---GTTTXGAAXAXTXXTTCTXATXXXGAA
                                      24810     24820     24830     24840     24850     24860     24870     24880
Human                       TTGCTTTTTAATCCCCCTTCTTACCCTGCCATGGGTAGGTGGCTGCCACAA--CTTTTGCCTTATTTTTTCAATATGGAT  24480
GuineaPig                   CTTCATTATAAAAAT--TGGATACATGAAAAAGGGAATGT---TATAATA-------------AACTTTCTGTTGAAGAA  8668
NorthernAmericanDeerMouse   TGCCACTAT---------CCACT-------------------ATA--------GTTTAGAACAGTGGTTCTCAA------  7192
Mouse                       TTTCTCTACCAAGTGGTTTTACTTGCTGCTCTGTTGCTATG-ACAAACACCATGGCTGAAAAAGCAGCTTGGGGAAGAAA  10271
ChineseHamsterGHOK1GS       TGCCATTGCACTGCAGTCCCCTTTTGTACTTT-------CG-ATA--------GTTTAGAACAGTGGTTCTCAG------  9144
LongTailedChinchilla        CTTCATTATAAAAGT--TTGATATGTAGAAAAGGGGATGT---TATAATAC--CCTTTTTTTTACTTTTCTATTGGAGAA  8784

Majority                    TTTTCATGTXAATTACAA---X-ATCATXTAGGGATXTTT-CAGXAGXTACAAXCXAXAXXATTAGAAAACA------AT
                                      24890     24900     24910     24920     24930     24940     24950     24960
Human                       TTTTCATGTAAATGACAAAA-ATAT-ATTTAGTTTTTTTT-TAGTTTATGGAAACGGTATGCCTATATAATGTTTTCCAT  24557
GuineaPig                   TTTTAAAGCAAACCTTGT-----ATCATCTCCAGATATTT-CAGTAGGTACAA--GATGGGATCAGAAAT--------AC  8732
NorthernAmericanDeerMouse   ----CCTATGGGTTGCAA------TCTTTGAGGGATCTTCACAGAGGTCAC--CTAAGACCATTGGAAAACACA------  7254
Mouse                       GGTTTATTTGGCTTACAAGTTA-GTCATCAAGGGAAGCCAGGACAAGCTAAAGCCAGGAACAGAAAGAAGCAGAGACCAT  10350
ChineseHamsterGHOK1GS       ----CCTGTGAGTTATGAC-----TGCTTTGGGGGTTTTCACAGAGGTCAC--TCAAGACCATTGGAAGACA--------  9205
LongTailedChinchilla        TTTTAAAGCAAATCTCATTTCATATCATCTACAGATATTT-CAGTAGATACAA--GATGGGATCAGAAA---------AT  8852
```

```
Majority                     XX--GATXTTT-AATTXAAXAXX-XTTXTXACAXX-XXXXATATTTTGX-------TTXATAATXXTAGCAAX---TACA
                                          24970     24980     24990     25000     25010     25020     25030     25040
Human                        GTCTTGTGTTTTAATTCAATAATGATTCTAATAAATAGCCGTATTTTTTGGATAGTTTTATAATACTCATTGC---TGTG  24634
GuineaPig                    ACTGAATGTTTGAATTTAAAACT-TTTATTACAGGGCTGGGGATTTAGC----------TCAGTGACATAAG--------  8793
NorthernAmericanDeerMouse    ----GATATTT-----------------------------ACATTGTGA-------TTCATAAC--TAGCAAA---TACA  7289
Mouse                        GAGGGAGATTTCCCATAGCTTGCTCTGTCAGCTTTCTTATACATCCTGGGACCACCTTTCCAGAGGTAGCACCATCTTCA  10430
ChineseHamsterGHOK1GS        ----GATATTT-----------------------------ATATTACGA-------TTCATAACAATAGCAAAAT-TACA  9244
LongTailedChinchilla         AC--ACTGTTTAAATTTAAAAAC-ATTATTACAGTAAAAGATATATTGCCA--------TTAATCCCCTTCAG---AACA  8918

Majority                     G-----X-TGCCXTXCTTATGAAATACCAATGAAATCATTTTATGGXT--------X--X----------AXXTTTXGAG
                                          25050     25060     25070     25080     25090     25100     25110     25120
Human                        A-----TATACTCTTTTGTGAAAATACCCATTTTATCTTTTATTGGACATTTGGGTTGATGGACATTTGAATATTTAAAG  24709
GuineaPig                    --------TGCCTGCCT---GGCAAGCGTG----------------------------------------AGGTTTAG--  8820
NorthernAmericanDeerMouse    G---------------TTATGAAGTAGCAATGAAATAATTTTATGGTT----------------------------GGGG  7326
Mouse                        GGGAACTGGGCCCTTCCTGTCAATAATCAACGAAAACACCCCATAGATTT----GCTCCTAGGCCT---GTCTGATGGAG  10503
ChineseHamsterGHOK1GS        G---------------TCATGAAGTAGCAACGAGATAATTTTATGGCT----------------------------GGGG  9281
LongTailedChinchilla         C-----TTTGCCTTCCTTCAAACACACTTATCCCATCATTCTTGGCTC-------TTACTGAA-----GCAGGTTTAGAA  8981

Majority                     XTCAX-CCTCA------CCTGAGTGACTTTA--TTXXAGXXXXXGXACTTTA-X--GGCAAGGTTGA--XAAXC---AAC
                                          25130     25140     25150     25160     25170     25180     25190     25200
Human                        ATTTTGACTTAGGCCGGGCGTGGTGGCTCAAGCTTGTAATCCCAGCACTTTGGGA-GGCCAGGGCGGGCAGATCACAAGG  24788
GuineaPig                    ------------------CTCAGTGACATAA-------------GTGCCTGCCT--GGCAAGCGTG---AGGTTGTGAGT  8864
NorthernAmericanDeerMouse    -TCA--CCACAA-----CATGAGGAACTGTA--TTAAAGGGTCACAGCATTA-----GAAGGGTTGA--GAACC----AC  7385
Mouse                        ACCGTTCCTCAG-----CTTAAGTTCCTTTG--TCCTGGGCCTCTTCTCTCATGTATGTCAGATTGACAGAACCT--AAG  10574
ChineseHamsterGHOK1GS        GTCA--CCACA------CATGAGAAACTGTA--TTAAAGTATGGAAACTTTA-----GCAAGGTTGA--GAACC----AC  9340
LongTailedChinchilla         GTTCTCTTTC--------CTGAGTGGTTTTACTT----------GCACTTTGAT--GACTACTTTG---ATGTCCTGAAC  9038

Majority                     T----GXTTTAAAACCTXTXXTCXXATXAXXXT-A----CTCTXTCXTTXXTAAXXATGCTAAXATX-XCXXXTTATGGT
                                          25210     25220     25230     25240     25250     25260     25270     25280
Human                        TCAGGAGTTTGAGACCAGCCTGGCCAACATAGTGAAAC-CCCCATCTCTACTAAAAATACCATAATTAGCGGGGTATGGT  24867
GuineaPig                    TC---GATCCCCGAT--ACCCCCCCAAAATTATAA----ATCT---TTTATTAAAG----TAAAATACAC-----ATTGC  8923
NorthernAmericanDeerMouse    T----GGTTTAGAACCT-TAATCACTTGAG---------CTCTCTGCTTTCTAATAATCATAATA----ATTATTCATGT  7447
Mouse                        C----ACACACAAACCTTTTCTGATACAGGGTTTAGCTATTCTATCCCTCTTAGTGATGCTTCTATGGCATCCTCTAGAT  10650
ChineseHamsterGHOK1GS        T----GCTTTAGAACCTGTAATCATGTGAG---------CTCTCTGCTTTCTAATATTGATTATT----CATGTTTTGTT  9403
LongTailedChinchilla         T----GATTCAAAAC--ATTTACCTTTCATGGTCA----TTTTGACTTTGTGGAAGA-GCCAGAAGTTGC-----ATGGT  9102
```

```
Majority                     XTTXXCTTX-TTXTGATTGXXGAXAC--TCATXX-XXTTXXXTGAGTTTATTAATGXAXXXXXTGTXXCAGAX--XXXXA
                                          25290     25300     25310     25320     25330     25340     25350     25360
Human                        GGTGGGTGC-CTGTAGTCCCAGCTAC--TCAGGAGGCTGAGACAGGAGAATCGCTTGAACCCAGGAGGCGGAGG-TTGCA  24943
GuineaPig                    CATGAATCC-CTGTAATGGTTGACACA-TTATGTCAACTTGAAAAGTTTAGAAATTTAAGGGATTCAGCAGAA--GACTA  8999
NorthernAmericanDeerMouse    TTTACCTTG-TTTTGATTG------------------TTGATTGATTTTATTAATG------TTCTCACAG---------  7493
Mouse                        CTTGACTTGCTTTTCATTGAATATGCTGTCTTATATTTTGTTCGTATTTAATACTGCACGTTTTGTTGCAGACTTGTAAA  10730
ChineseHamsterGHOK1GS        TTTACCTTG-TTTTGATTG------------------TGAATTGATTTCATTAATG------TTCTCAC-----------  9447
LongTailedChinchilla         GCCAGATCC-A-GTGGATGAGGACACG-CCATAATGATTTTATGAGCCTGTCTTTCTGTTGGATGGTATTCAG--TAGCA  9177

Majority                     GXTXGXCAXCTTXATGACAGTTXXCTTCXGXCTGXTTXTTTGTCTTTXXXTGCTX-XTXXAAAXCAXXXTCCXTTCAACA
                                          25370     25380     25390     25400     25410     25420     25430     25440
Human                        GTGAGCTGAGACCGCGCCATTACACTCCAGCCTGTGTGACAGACTGAGACTCCAT-CTCAAAAACAAACAAAAAAAAACA  25022
GuineaPig                    GGATCTTCTCTTTGTAAAC---ATCTTCATCCTGTTTCATGTACCCTAATGGGT--------AGTAGCTTGCTTATAATA  9068
NorthernAmericanDeerMouse    --TAGTCACCTTCATGA-AGTT---TTCTGTCAAGTT-TTTGTCCTTCTCATCCATTTCTTTTTC----TCCCTTCTGCC  7562
Mouse                        TATGGCCAGATTTATAGCAGTTGGCTTTTGTTTGTTTGTTTGTTTTTGTTTATTAACTAAAAATCAAGGTCTGTTTATAA  10810
ChineseHamsterGHOK1GS        --TGGTCAACTTCATGA-AGTT---TTCTGTCAAGTT-TTTGTTGTGCCC-------------------CCCCTTCTGCT  9501
LongTailedChinchilla         GTATTCCCTATTTTTGTCA---CACCTCATCTTGGTTACTTTTCTCTTGTTGCTG-AGACAAAATACCCAGCACCCAA-A  9252

Majority                     XTAXAATXATXATAACTTXTXCTTTTCCTTAXTXXX--CXTGTTTCTTTTCGXAATTGGXTCTXGCTGTA----TAGCXC
                                          25450     25460     25470     25480     25490     25500     25510     25520
Human                        ACAACAAAAAAAGATTTTGACTTTAAGAACAATACTGCTGTGAACTTCTTTGTACTTGTTTCCTGGTGCAT---TTATGC  25099
GuineaPig                    AGGAAAGAAAAGAGGCTTG---TTCTCCCTCTTTTG--------CACTCCTTAATT----TTAGGTGTT---------G  9123
NorthernAmericanDeerMouse    TTTGTCTCATGTTAACTTCTGCTTTTCCTT--------CTCTTTTCTTTTTGAAACAGAATCTCACTG------TAGCCC  7628
Mouse                        ATAGAGTGATGATAAAGTATTCCTGTTGTTAAGAGTAGCTTGCTTTTTTACCAAATTGGATTCTGCTCTAAAAATAACAC  10890
ChineseHamsterGHOK1GS        TTTGTCTCGTGTTAACTTCTGCTTTTCCTT--------CATTTTTATTTT-GAAACAGGTTCTCACTGTG----TAGCCC  9568
LongTailedChinchilla         TTAAAAGGGGAAAGGTTTA---TTTAGCTCATTTCGTGGAGGTTTCAGTCCATAGTTGG--CTGGCTGCAA---A-GCAG  9323

Majority                     AXTXGTCTGT--CTCAGAGTTCATTATXTAXAAXAGGXAGXXTXCACTXGXXXTCAAAGTXATGCTCTTXXX-----CT-
                                          25530     25540     25550     25560     25570     25580     25590     25600
Human                        AAGCGTTTTT--GAGAGGGTATATTTAAAGGTATAGGTATATATTACTAGTGTGTAAAATAAACTTTATGAGA----CAG  25173
GuineaPig                    AGTGTTTTAT--TTCAGCA---ATAAGAGAAAAGTAAGACAGTCCCTTT----------CAAGACACTTTCC-----CTT  9183
NorthernAmericanDeerMouse    ---TGACTGT--CTCAGAATTCACTGTGTAGACCAGGCAGGCTGGACTAGAACTCAAAGTTGTGT---------------  7688
Mouse                        ATTCATCTGTAACTCACAGTTTAAGTTGTACTATAGGACTATTGAATTCTTCTTCATTGGTTTGCTTTCATGAGTAATTA  10970
ChineseHamsterGHOK1GS        ---TGACTAC--CTCAGAGCTCACTATGTATACCAGGTTGGC----CTCAAACTCACAGTTGTGCCCC------------  9627
LongTailedChinchilla         AGTGGCACGT--GACAGAGGGCATAAGAGCAAAAAGGGAGGAGCCGCAGGG----AGAGCAAGCCTCTTGCC-----CTC  9392
```

Majority	XXTTACACCXAXCCTX-CTXTXTTGXTCATX---CTAXTGTXAXCXXTXXXTXXTAAGGAAGXCXTGTGXTTTTT-ATAC	
	25610 25620 25630 25640 25650 25660 25670 25680	
Human	TACTAGATTATTTTCCAGTGTATTGTTCCTATTTATGTTCTCACCAGTAGTGCGT-AGGAGTTCTTGCTGTTTCAGATCC	25252
GuineaPig	CCTT-CAAACACACT-----TAACGATCAT-----CATTCTTGGCTCTAAGCT-TGAGTAGGT--TTAGTTTT-----TC	9244
NorthernAmericanDeerMouse	-----CCC---------CCCTTTTGTTCAT----CAAGTGC----------TATGAATAAAGGCATGTGCTTTTT-ATAC	7739
Mouse	AATAATACCAAGCATGTCTTTTTTGGTAATAGT-TGAATGTAACTTTTTTTTTTTAAAGTTAGCTTTTTTTTTTT-CCAT	11048
ChineseHamsterGHOK1GS	----ACCCCTCCCCCACCCCTTTTGCTCAT----CTAGTGC----------TGGGATTGTAGGTGTGTGGGTTTT-CTAC	9688
LongTailedChinchilla	CCTTACATGCAGGCTGGGTGTAGCACTCACACCCCTAATGTCAGCATTGGACCATGAGTCAATCATAAGCTGTAGAAGCC	9472

Majority	XXTTTXGTGX-TGXT-X--TXATTTXCTTXXCCATTGCTXXAXTAAA----XACCATGACTGATAAAXXACXXXGXX---	
	25690 25700 25710 25720 25730 25740 25750 25760	
Human	CCACCAGCACTTGGCTAG-GTATTTAATTTCTTA----ATTTTTAACAA--TTTGGTGTGTAGTATATTCTCTGGTCTTC	25325
GuineaPig	TCTTTCCTGAATGTT-------TTTAGTTGCAT------------------TATGATGATTGATTTAATGCCTTGAA---	9296
NorthernAmericanDeerMouse	TTCATGGTG----------TTATT-GCTTCTCCATTGCTGGAATAAA----CAC-ATGACTGAAAAAGCA----------	7793
Mouse	TTTTTATTAGGTATTTAGCTCATTTACATTTCCAATGCTATACCAAAAGTCCCCCATACCCACCCACCCCCACTCCTCTA	11128
ChineseHamsterGHOK1GS	CACTTGGTT----------TCATTTGCTTCTCCATTGCTTCAATAAA----CACCATGACTGAAAATGCA----------	9744
LongTailedChinchilla	CATCCCATGACTGCTCAA-GGCTTTGAGGGCACATGCAGACACAAACCA--TAACACACCTCATAAGATACTCAGAAAAA	9549

Majority	----------X-CTGXTTTTGXAGXAGGXA-------------GGGXTTATTAXGTTTACA-GTX-AGXGX-CCTTTCXT	
	25770 25780 25790 25800 25810 25820 25830 25840	
Human	CTTTGTCTTACCCTGATTATTAATGAGGTCTT---ACATCTTTTTTCCCCCCACATTTATG-GCC-ATGGA-TGTTTCTC	25399
GuineaPig	------------CTGCTTCAAAA------------------------------CATTTA-------------CCTTTCAT	9321
NorthernAmericanDeerMouse	----------------TCTTGGAGAAGGAA-------------GGGTTTATTAGGCTTACAAGTT-ACAGTCTTTGTCAT	7843
Mouse	CCCACCCACTCCCCCTTTTTGGCCCTGGCATTCCCCTGTACTGGGGCATATAAAGTTTGCGTGTCCAATGGGCCTCTCTT	11208
ChineseHamsterGHOK1GS	----------------TCTTGGGGAAGAAA-------------GGGTTTATTAGGGTTACAAGTT-AGTGT----GCTAC	9790
LongTailedChinchilla	GAACTTGCAGCACTGCTTCAGAAAGTGGCA-----AGAACAGTAGAATAAGTGTGTTTGAA-GTA-GGGGG-CAGTTCTT	9621

Majority	TGXXGTGAAGTCXXGGTAGXAGXT-----TATTCATXXAT-TGAAGCTGATAXCATGTGXXCT-XGXGTACTGCTXAXTT	
	25850 25860 25870 25880 25890 25900 25910 25920	
Human	TTTTGTGAAATTCTGTTAAATGCC-----TATTTGTGTTT-TTTGCTTGATAAAATGTGAGCTTATTATTTAACTGATTT	25473
GuineaPig	GGTCATTTAGTCTTTGTGGAAG--------AACCAGAAGT-TAAAT--GGTGCCAGATCCAGT--------GGATGA---	9379
NorthernAmericanDeerMouse	TGAAG-GAAGCCAGGGTAGGAGCTC----AAGACAAGAAC-TGAAGCAGAGACCATG----CA-AGAGTACTGCTTATTG	7912
Mouse	TGCAGTGATGGCCGACTAGGCCATC----TTTTGATACATATGCAGCTAGAGTCAAGAGCTCT-GGGGTACTGGTTAGTT	11283
ChineseHamsterGHOK1GS	TGA-G-GAAGCCAGGGCAGGA-----------------AC-TGAAGCAGAGAGCATG----CA-GGGGTACTGCTTACTG	9845
LongTailedChinchilla	TGGCAGTAAGTTTTAACAAATTTTTATTTTGCTCATCTGT-TACATCTGTTAACAGATGTACTCTCT-TAAAGGTGACAT	9699

```
Majority                     TXTAGTTTXATXXAACCXATXGXXTXXXXGG------TCAGXXATXTGA--TACXTACTXGGXCCACTXACTTXGAXTXC
                                            25930     25940     25950     25960     25970     25980     25990     26000
Human                        TGTAGTTTTAAAAAATTGCTGGGGGCGGTGGCTTGCGTCTGTAATCTCAGCTTTTGGGAGGCTGAAGGAGGAGGATTGC 25553
GuineaPig                    ------ACCATGCAACAGTCATATACCATAG----------TGATTTA-----------------GTGAGCCTGGCTTT 9425
NorthernAmericanDeerMouse    TCTGGTTT-------CCCATGGCTT-----GCTC-TGTCAGCTTTCTGA--TACATCCTGGGACCACCTTCTTAGA---- 7973
Mouse                        CATAATGTTGTTCCACCTATAGGGTTGCAGATCC-CTTTAGCTCCTTGGG-TTCTTTCTCTAGCTCCTCCATTGGGAGCC 11361
ChineseHamsterGHOK1GS        TCTGGTTT-------TCCATG----------------CCATCCTTCTGA--TACGTACTGGGACCACCTTCCTACA---- 9896
LongTailedChinchilla         TATAGCATCATATAACTACCAGAATTTAAGC----------TAATTAATTTCATGTACAACAGTAGATAACTTTGATTTC 9769

Majority                     CTGTGGXGCC---AXXAGTXXXCXGAG-CAGTXCXCTTTXGTGTCAGTCATGCXXXGTAAXAGTCTCATAGAA-AXXXCT
                                            26010     26020     26030     26040     26050     26060     26070     26080
Human                        TTGTGGGGCCC--AGGAGTTTGAGGCTGCAGTGAGCTATGATCGTTCCATTGCACTCTAGCTTGGTGACAGAGCAAGACT 25631
GuineaPig                    CTGTTGGGTG---GACGCTCAATAG---CAGTATTCACTAGTTTCAGGCATGCCTTGTAAGGATACTATGCAAAAAGAAC 9499
NorthernAmericanDeerMouse    -TGTGG---------------GCTGAG------CCCTTCCATGTCAGTCAT-CAAAGAAAAAGCCCCATAG--------- 8021
Mouse                        CTGTGATCCATCCATTAGCTGACTGTGAGCATCCACTTCTGTGTTTGCTAGGCCCCGGCATAGTCTCACAAGAGACAGCT 11441
ChineseHamsterGHOK1GS        -TGTGGCACTACCCATAGTGGGCCGAG------CCCTTTCTTGTCAATCAT-CAAAGAAAAAGCCTCATAGAT-----TT 9963
LongTailedChinchilla         CTTTCATGTC---AGGAACCAAGGAAATCAGCATGATGTGGTT--AATAATGTCTCTTAGGGTTTTNNNNNNNNNNNNNN 9844

Majority                     XCXCATAGGTCAXTCXGATAGAGXX-XGCGAGTTXCXCAXXTAGTGTTXGXXXTTXCXGTTTGTXTXTGGTTTXTGTCAA
                                            26090     26100     26110     26120     26130     26140     26150     26160
Human                        TTGTCTGAAAAAAAAAAAAAAAAAAGTGTGTGTTTTACAGTTACTGATTGTCTTTTGGTTATTTGTGTTGCTGGTGTCTT 25711
GuineaPig                    TTGCAGAACTGTTTCAGAAAGTGACAAGAGTAGTGGGCTAAGTGTGTTTGGGGGAGCAGTTTGAGGGGGATTAATGGCAG 9579
NorthernAmericanDeerMouse    GCTCATAGGCCAGTCTGATGGAG----GCCAATTCCTCAGTTAAAGTTCCCTCTTCCTGGTTGTCTCTG-----TGTCAA 8092
Mouse                        ACATCTGGGTCCTTTCGATAAAATCTTGCTAGTGTATGCAATGGTGTCAGCGTTTGGATGCTGATTATGGGGT-GGTTCC 11520
ChineseHamsterGHOK1GS        GCTCATAGGTCAGTCTGATGGAG----GC-AGTTCCACA-----AGTTCCTTCTTCCTGTGTATCTCTGGTTTGTGTTAA 10033
LongTailedChinchilla         NNNNNNNNNNNNNNNNNNNNNNNNNNNNNNNNNNNNNNNNNNNNNNNNNNNNNNNNNNNNNNNNNNNNNNNNNNNNNNNN 9924

Majority                     XTXGACXTTTXTXTCTTTACTTG-TGAAXCXTTT-X--CTAAXTCAXGACTTTGCXTCTXTATXTCX-TCCCTXXXTCCT
                                            26170     26180     26190     26200     26210     26220     26230     26240
Human                        TTCCCAGTTTGTAGCTTGCCTTG-TCACTTCTTT-ATGCTGTCTTGTCTGTCTGTCTGTCTGTCTCCCTCCCTCCCTCCC 25789
GuineaPig                    TAAGAGTTTTTTTTTTTTTTT-----GTGCTAG-----TAAGTTTTTAACTTTA----------------ACATCCATCT 9633
NorthernAmericanDeerMouse    ATAGAC------------ACATA-TGAACCTTTT----CTAATGCAGGGTTTTGCTACTATATA------CCT------- 8142
Mouse                        CTGGATATGGCAGTCTCTACATGGTCCATCCTTTCATCTCAGCTCCAAACTTTGTCTCTGTAACTCCTTCCATGGGTGTT 11600
ChineseHamsterGHOK1GS        ATGGACGGAACT-TAACGACTTG-TGAACCTTTTT---CTAATACAAGGTTTTGCTTCTATATATCTATTCCTCT-TAGT 10107
LongTailedChinchilla         NNNNNNTTTTTTTGGTTTTTCTC-CGGTACCGGG-AACCAAACTCACGACCTTGCGCTTGCCAGGCAGGCGCTTTTGCCG 10002
```

```
Majority                     CTXTTXGXXGTXC-TCTGXCCC----XATXGTGTCCTCXTTTAACTTTTCXTTCXTXCTXXXCTTCATGTGXXXATXX--
                                       26250     26260     26270     26280     26290     26300     26310     26320
Human                        CCTCCCTCCGTCCGTCTGTCCC-TCCCTCCCTCCCTCCCTCCCTCCCTCCCTCCCTCCCTCCCTTCCTTCCTTCCTTCCT 25868
GuineaPig                    CTAACAGATGTAC-TCTGTCCC----TCAAAGGTGACATTATAGCATTATATACCTACTAAAATTTAAGGT--------- 9699
NorthernAmericanDeerMouse    CTCTTAGTGGTGC-TTTG---------ATGGTATCCTC--TTAAGTTTTCATTGTTGCT---CTTCAT-TGAAGAGCA-- 8204
Mouse                        TTGTTCCCAATTC-TAAGGAGG--GGCATAGTGTCCACACTTCAGTCTTCATTCTTCTTGAGTTTCATGTGTTTAGCAA- 11676
ChineseHamsterGHOK1GS        CTCTTAGTGGTGC-TTTT---------ATGGTGTCCTC--TTAATTTTTCCTTGTTGCT---CTTCAT-TGAGTAT---- 10167
LongTailedChinchilla         CTGAGCTAAATCC-CCGGCCCCATTTTTTTTGGTCTTTTTGAGACAGGATCATCCTA-TGCAGTTCAGGTTGGCCTTG-- 10078

Majority                     AXXTTAXTTTXTX--XXAXX-TAGTCTTAAGTTT------AXXATTXTTTTATXTXTGXCTACCTAATGX--XAGXGACT
                                       26330     26340     26350     26360     26370     26380     26390     26400
Human                        TCCTTCCTTCCTACTTTACTTTAGATTCAGGGTTACATGTGCAGGTTTGTTACATGGGTATATGGCATGAAGCTGAGGTT 25948
GuineaPig                    AATTAATTATAT---CTACAATAGT----AGAT-------AAGATTTTTTT-------CCTTTCA--TG---TCAGGAAC 9753
NorthernAmericanDeerMouse    ACCCTAACC----------------CTTAAGTTT----------TTTGTTTGTTTATAGCTACCTTATAC---AGCAACT 8255
Mouse                        ATTGTATCTTATA--TCTTGGGAATCCTAGGTTTGGGGCTAATATCCACTTATCAGTGAGTACATATTGTGTGAGTTCCT 11754
ChineseHamsterGHOK1GS        ATTGT--------------------CTTAAGTTT----------TTCATTTATTTATAGCTACTTAATAC---AGCAACT 10214
LongTailedChinchilla         AGCTCACTTTGTAGCCCAAGCTGGTCTCCAGCTCGCA---ACGATCCTCCTGCCTCAGCCTACCAAGTG---CTGGGATT 10152

Majority                     TTXXGXAXATGTXAXCTTGTTXGGXXXXXXX----XCAGXTCTATXX------XC-AGXAGTTXAXT--TTACTTCXXTT
                                       26410     26420     26430     26440     26450     26460     26470     26480
Human                        TGCAATACAAGTGATCCTGTCAGCCAGAGAGT----GAGCTCAGTAC------CC-AACAGTTAGAT--TTACAATCCTT 26015
GuineaPig                    CAAGGAA---ATCATGATGATATGGTTAATA------ATGTCTCTTA------TC-AATAAACTACT--TTACCTCCC-T 9814
NorthernAmericanDeerMouse    TT----AGATGCAGACTTGTATGG-----------CCAGATTTAT-----------AGAGGTT---------GTTTTTCT 8300
Mouse                        TTGTGAATGTGTTACCTCACTCAGGATGATGCCCTCCAGGTCCATCCATTTGGCTAGGAATTTCATAAATTCATTCTTTT 11834
ChineseHamsterGHOK1GS        GT----AGATGCAGACTTGTTTGG-----------CCAGATTTAT-----------AGCAGTTTGTTTGTTTGTTGTTTT 10268
LongTailedChinchilla         ACAGGCATGTATCACCATGCCCGGCTCTTAA------ATTTCTTTTA------TC-AGTAAACCACT--TTACCTCCCCT 10217

Majority                     ACTGXGTTAATTAATAATTCATTXTGTGGXXATACCXXXXGTGXTXTATACAGTGTXCTGTTXAATGGXXTC---XTTGT
                                       26490     26500     26510     26520     26530     26540     26550     26560
Human                        TCTCCCTCCCCCCACCACTAGTTCCCAGTGCATATTGTTGCGGATTAATACAGATTCTTAATGTATAGTTTTA-ATGTGG 26094
GuineaPig                    ACTGCTTTTATTTCTTTTTCTTTGTATAGCCAGCCTG--GGTCATCTGTGCAGTTTCCTCATTACTGGATTC-----CGC 9887
NorthernAmericanDeerMouse    AGTGAGTTAATTAAAAA-CCAAGATGTGATTATAGCTGAAGTGCTG-ATAAAGTGTT--GTTAAAAG-CAGCTTGATTTT 8375
Mouse                        TAATAGCTGAGTAGTACTCCATTGTGTAGATGTACCACATTTTCTGTATCCATTCCTCTGTTGAGGGGCATCTGGGTTCT 11914
ChineseHamsterGHOK1GS        GGTGAGTTAACTAATAA-TCAGTATGTGGTTACAACTGGAGTGCT--ATAAAGTGTT--GTTAAAAG-TAAC---ATTCT 10339
LongTailedChinchilla         ACCCCATTAATTTCCTTTTCTTTCCTTTTCCT---------TGATCTGTACAGTGTCCACTTTACTGGGTTC-----TGC 10283
```

```
Majority                    TTAXXAXACCTCATT--------XCATTXTG-TATAAA---AGTAATAXATXTXGT--XATTCCTA-------CAAXTTC
                                     26570     26580     26590     26600     26610     26620     26630     26640
Human                       CCACAAATTCCCTTT--------GCATCTTA-GATAAA---AATAATTTATATGATCCCATTCTTTA------CACATTC  26156
GuineaPig                   TTATTATACCTCATT--------GCATTTTG-TATATT---CCTCATATCCTTTGT---ATTCCTA--------GATTTG  9944
NorthernAmericanDeerMouse   TTTTTTAACCAAATT--------GGATTCTGCTCTAAA---AGTAACACAT-------TAT-------------AAATTC  8424
Mouse                       TTCCAGCTTCTGGCTATTATAAATAAGGCTGCTATGAACATAGTGGAGCATGTGTCCTTCTTACCAGTTGGGGCATCTTC  11994
ChineseHamsterGHOK1GS       CCCCACCCCCAAATT--------TGATTCTGTTCTAAA---AGTAACACATAGCCAGCAGTG-----TTGGCTCATGCCT  10403
LongTailedChinchilla        TTATTATACCTCATT--------TCATTTTG-TATAAT---TGTCTTATGCTTTGT---ATTCCTAC------AAATTTG  10342

Majority                    TAAATTT-TGCXXTGGTXAGXTGXAGXXXTXTXCXGXTGX---XGG----CTXAATTTCTTXXA-------AGGCTATTT
                                     26650     26660     26670     26680     26690     26700     26710     26720
Human                       CAAATTT-TCCTTTGCTCTTCTGGACATCTTCCATGTTGA---TTG----CTGGAAGTCTTG-A-------AGGCAATCC  26220
GuineaPig                   TAAAATT-TG---TAATTAGCTGAAGACTTGTTCAGATGC---AGG----TTTAATTTTTTACA-------GGGATATTT  10006
NorthernAmericanDeerMouse   CTAATTC--ACAGTTGTAA-------------------G------------------T--TGTA----------------  8447
Mouse                       TGGATATATGCCCAGGAGAGGTATTGCTGGATCCTCCGGTAGTACTATGTCCAATTTTCTGAGGAACCGCCAGACTGATT  12074
ChineseHamsterGHOK1GS       TTAATCCCAGCACTTGGGAGGCAGAG------------GCAGGTGGATCTCTGAAAGTCTGAGGC-----TAGCCTGGTC  10466
LongTailedChinchilla        TACA-TT-TG---TGGTTAGATGAAGGCCTGTTCAGATG-----A-----TCTAATTTTTAGCA-------AGGATATTT  10400

Majority                    TXXAGA-GTAGTC-------XCTXTAAXTCTTCTATCACTGCAAGAGGAAAAGAXTXTCTAGATAXCT-C-------TTT
                                     26730     26740     26750     26760     26770     26780     26790     26800
Human                       CAAATA-ACAGT---------TTTTAGCTTTTCAACTGTTGCTG--TGATACGGTTTCTTATAGAGAT-T-------TTT  26280
GuineaPig                   TGTAGGTGTAGTC-------CCTGTAAGTCTTATATCACTGCAAGAAGAAAAGAATGTGTAGTTAACT-C-------TTT  10071
NorthernAmericanDeerMouse   --------------------------------CTATGACTG--------------T------------------------  8457
Mouse                       TCCAGA-GTGGTTGTACAAGCCTGCAATCCCACCAACAATGGAGGAGTGTTCCTCTTTCTCCACATCCACGCCATCATCT  12153
ChineseHamsterGHOK1GS       TACAGA-GCATTC-----------CAGGACAGCTAGGACTGTCACAGAAAAACCCTATCTGGAAAAAAAA------AAGT  10528
LongTailedChinchilla        TGTATGTGTAGTC-------CCTATAATTCTTGCATCACTGCAAGAGGAACAGAATGTCTAGTTGTCT-C-------TTT  10465

Majority                    GTAATXTT-XXAATTTXTCA-----GTAXTTTGAGXTGTXTTTAG-----XTXTXTGGCTTGATTTAA-GAGTATTTXCA
                                     26810     26820     26830     26840     26850     26860     26870     26880
Human                       GTAGT----AGAATTTATCA-----GTATTTTCTTTCATGTTCAGTTT--TCTTGTGTCTTAATTTAA-AAATCCTTACA  26348
GuineaPig                   GTAATGTT-AACATCAGTCA-----GTAAGTTGAGGTGTTTTTA------CTTTATGCATCCATTCTA-GAGTGTTCCCA  10138
NorthernAmericanDeerMouse   ---------TGAATTCCTCA---------------------T----------CATTGGCTTGGTTTAAGGAGTAATTA--  8495
Mouse                       GCTGTCACCTGAATTTTTGATCTTAGCCATTCTGACTGGTGTGAGGTGGAATCTCAGGGTTGTTTTGATTTGCATTTCCC  12233
ChineseHamsterGHOK1GS       GACACATTATAAATTCCTAAC--TCACAGTTTAAGTTGTACTATGACTG--TCTTTGGCTTGATTTAATGGGTAATTA--  10602
LongTailedChinchilla        GTAATGTT-AACATATATCA-----GTAGATTGAGGTGTGTTTAG-----CTTGATGCATCAGTTCTA-GAGTGTTCCCA  10533
```

Majority	T--TG--TAAGXATGTXAXXXATATTTT-----XXXTXXTXGTXTTTTXGTAXTXAAXAGTTGAATXTTXT-TATTTAXX	
	26890 26900 26910 26920 26930 26940 26950 26960	
Human	T--AC--TCAGAGT-TCATAAAGATACT-----ATATTTTCTTCT-GAAATTTTAAAAAATTTTGCTTTTTATATTTATC	26417
GuineaPig	T--TG--TAAGGTTTTCATTATCCTTTC-----ACTTAATGGTTTGTTAGTAGCTAATGACTATATTTTAT-TATTTACT	10208
NorthernAmericanDeerMouse	--------AATAATGCTGAGCATATCT----------------TTTTTGGTAAT----AGTTGAATATATA--ATTGA--	8543
Mouse	TGATGATTAAGGATGTTGAACATTTTTTCAGGTGCTTCTCTGCCATTCGGTATTCCTCAGGTGAGAATTCTTTGTTCAGT	12313
ChineseHamsterGHOK1GS	--------AATAATGGTAAGCATATCTC---------------TTTTTGGTAAT----AGTTGAATATA----ATTTA--	10649
LongTailedChinchilla	T--TG--TAAGGTTGCCATTATTCTTCT-----GTCTAATGGTCT-TTAGTAGCAAATGGTTACAGTTCAT-TGGTTATA	10602

Majority	---XAAAXXTAXTTTTTAATXTXGT-XTATGTTXGTXAGAATTC-------XT-XXTTXTGTTTGCTTTTTTAAGAXAAT	
	26970 26980 26990 27000 27010 27020 27030 27040	
Human	A--GAAAATTATTTTT--GTGTATT-GTAT-TTAGTAGGGATCTG-----GTTTCATTTTTTTGCCCTTATTAAGATAGT	26486
GuineaPig	---CAAAGATACTTT---ATCTAAC-GTATATTACTTAGAATTC-------CTCCATTAAGAAAGATCATTTCTGTGAAC	10274
NorthernAmericanDeerMouse	------------TTTTTAATAAGGT-ATGTGTTTGAAAAAAGT----------------TGTTTGCTTTTTTTAGATTTT	8594
Mouse	TCTGAGCCCCATTTTTTAATGGGGTTATTCGATTTTCTGAAGTCCTGAATGTAACTTTTTGTTTGTTTTTTAAATAAGGT	12393
ChineseHamsterGHOK1GS	------------TTTTTAATATGGT-ATGTGTTTCGCAAAGTTG---------------TGTTTGCTCTTTTAAAAAGAT	10701
LongTailedChinchilla	G--CAAAGATACTTTTTTATCCAAC-GTATATTAGTTGGAATTC-------CT--GTTAAGAAAGGTAATTTCCAAGAAC	10670

Majority	TAGTXXXATTXAAA------TCTTTTAA----------------XTTTTTAATAXCATAGXATXTACXTTGTGCXAGATG	
	27050 27060 27070 27080 27090 27100 27110 27120	
Human	CAATTAAATTGAAA------TCTCTGTAA-------T-GA----CTTTTTCATATTACAG-GTATATATATCGTAAGGCA	26547
GuineaPig	TGGTCACAGTGAAA------TACTC-------------------CCCGCCTCCATCAGAG-AAGCACA----ACAGATTG	10324
NorthernAmericanDeerMouse	GATTATTATTAAAA------TCTTTTAA-------------------TGTAATACCATAGCATATACTTTGTGCTAGATT	8649
Mouse	ATGTGTTTTTAAAAGTTGGGTGTTTTAAGATTTTGATTAAAGTCTTTTTTAGTGCCATAGCATGTACCTTGTGCTACAGG	12473
ChineseHamsterGHOK1GS	TTTGATGAATAAAA------TCTTTTAA-------------------TGTAATGCTGTAGCATATAACTTGTCCTAGATT	10756
LongTailedChinchilla	TAGTCACACTGAAG------TTTTT-------------------TTTTTTCCTATCAGAG-AAGCAGG----ACAGATTG	10720

Majority	XTTXTTCTAAAAATTTCAX---TX-XAACATAATGTXXATTXAATGGXAGCTXAGTX--ACATAGGCTTTTAAA-XXGTT	
	27130 27140 27150 27160 27170 27180 27190 27200	
Human	AAGGCTGAAAAAACTATCA---T---AACAAAATGC--AGAATATGGTGGCATAAAT--AAAAGAGTTTTTTTG---TTT	26614
GuineaPig	AGTGTTATAAAAT-------------TTTCTGATG------AGTTGGTCCCATGCCC--AC------------------CT	10366
NorthernAmericanDeerMouse	CTTTTTCTAAAAATTTCAG--CTTATAACATAATCTCTATTGAACAACAGCTCTGTTGAGCATAGGCTTTTAAAACAGTT	8727
Mouse	-TTTTTCTAAAAATGTCAAGTTTTCTAACATAATATCTATTGAGTGCCAGCTCTGTGGAACATAGACTTGTAAA-CAGTG	12551
ChineseHamsterGHOK1GS	CTTTTTCTAAAAGTTTCAG--TTTATAACATAATCTCTATTGAATGACAACTCAGT---ACATAGGCTTTTAAAGCAGTT	10831
LongTailedChinchilla	TGAGTTTTAAAAAAAAAAC---A---TTCCTGATG------AGATGGTCCCCTACCC--ACA-GAGTTTGTGTT---TTT	10782

```
Majority                     TGTTXATTGTTXTTCCATTX--XAGTGTXXATATTAXGXAGTTGTGATXGCTTGG---TTAAXGXTTAXTGACTATGXAA
                                      27210     27220     27230     27240     27250     27260     27270     27280
Human                        CTTTTTCTGTCATAGCAGAAG-TCCTGTTGGTATAAGAAAGTGGTATCAGTCTGCTCCATAAGGTTATTCACTGGCCAA 26693
GuineaPig                    CCAGTGTAATG------------AATGTGGTTGTTAGT--GTTGTGAATGCAAGGGTTTTAAGGTTTATTTTCTAATTTA 10432
NorthernAmericanDeerMouse    TGTTGATTGTTCTTCCATTTTTAAGTGTTCATATTAAGCAGTTATGATCACTCAG---TTAATACTTAATGACTCTGGAA 8804
Mouse                        TGTTGAGTTGTATTCCATTGTTGAGT-TACATATTAAGTAGTT-------CTCAG---TTAATGCATAATGACTATGAAA 12620
ChineseHamsterGHOK1GS        TGTTGATTGTTCTCCCATT---AAGTGTACATATTAAGCAGTG--GATAACTTGA---TTAATACTTAATGACTCTGGAA 10903
LongTailedChinchilla         TTGTTGTTGTTGTTGTTTTT--TATTGTGGTTGTTAGT--GTTGTGAGTGTATGGGTTTTAAGGTTTATTTTACAGTTCA 10858

Majority                     GTGTCXTG--TXTTGTTA---XXTTTATAATCCATXAACXATTTAGXATTAXGCXAAAXGG----TAXATATTAAXTTTX
                                      27290     27300     27310     27320     27330     27340     27350     27360
Human                        GTTTCTTTCATATTGTTG---CTTCACTGATCCCTAGGCT--TTTGTTCTTGGCTATGTGGTCTGTTGATGTGTGATTGT 26768
GuineaPig                    ATGCAGTG---ATTGTTC---CTTTCAGAATGTTGAAATTATTTCATCTCAGGCCAAGTGA------GATATAAAATACC 10500
NorthernAmericanDeerMouse    GTGTCTTGACCTTTAATA---TGTTTATAATCCAAGAACAATTTAGCATTAA----AAA------TAAATAATAATTTTA 8871
Mouse                        GTGTC-----TTGGGCTA---TGTTTATAATTCATGAACAATTTAGCAGTAAGTAAAAAGA----TAAATACTGATTTTA 12688
ChineseHamsterGHOK1GS        GTTTC--GAGTTTTAGTATAATGTTTATAATCCATGAACAATTTAGCATTAA----AAAGG----CAAATACTGGTTTTT 10973
LongTailedChinchilla         GTGCAGTG---ATTGTTC---CTTTCAGAATGCTTAAATTATTTCATCGTAGGCCAACTAG------GACATAAAATACC 10926

Majority                     TXTTACXXTATTCCTXTTTGXXGGXGXATTXXAXXXATCCAGTCTTTAGATGGAXCATTTCT-TTAAXAXA-AGCTCXAG
                                      27370     27380     27390     27400     27410     27420     27430     27440
Human                        GCTCTTCTCCTTGTTTAAAAAAAAAGTTTTCCCTGTATTTGTGTATTTCATTGTCTTTTTTT-TTTAAAGTCAGCTTTTG 26847
GuineaPig                    AATTAACTTGTTACTTCTTGAAGTGTCTTTTCTTGT-TTCAGATCTTACATCAACCATTTCT-TTAAAA---AGCTCTAG 10575
NorthernAmericanDeerMouse    TTTTACTACATTCCTATTTGTTGGAG-ATCCAAAAGATCCAGTCTTTAGAAGGATCATGTCTCCTAAGAAAATGTTGAAG 8950
Mouse                        TTTTACCATATTCCTATTTGTTGGGATAGACAAAAGATCCAGTCTGTGGAGGGATCCCGTCTCTTTGGAGA---CTCAAG 12765
ChineseHamsterGHOK1GS        TCTCACTATATTCCTATTCGTTGGAG-ATCTAAAAGATCCAGTCTCTAGAAGGATGAAATCT---GGGAAAATGCTTAAG 11049
LongTailedChinchilla         AGTTAATTTGTTACTTCTTGAAGTGTCCTTTCTTGT-TCCAGACTTTATATCACCCATTTCT-TGAAAA---AGTTCTGG 11001

Majority                     GTTTTA---TXXTAATTXXCATACAAX-ATGGTAGXXTCAAXXTXXAXTXAXAGTGGGXTCCATGGAAATAGTAAG-CTT
                                      27450     27460     27470     27480     27490     27500     27510     27520
Human                        ATTTTAC--TGATCCTCTTCATTGTACCATTGTTTTCTCTATTTTTATTGGTTGCGTGAGTCAGGGCTTTTGCAAGTCTT 26925
GuineaPig                    TTTCTA---TGA---TTTGAAAAGAAAGGT--TTCTTAAAAACTGTAATAAT-GTGGGTTCCAG---------------T 10631
NorthernAmericanDeerMouse    GTTTTA----AGTAAGTGCCATAC-----TGACAGGATCA-----------GAGTAGGCTCCCTGGAAATAGTAAG-CTC 9009
Mouse                        GGTTTA----AGTAAATGCCATACAGCTATGGTAGGGTTACTTTTCAGTGAGAGTGGG-TCCTTGGAAATAGTAAG-CTG 12839
ChineseHamsterGHOK1GS        GTTTTATTTAAGTAAGTGACTTAC-----TGACAGGCTCA-----------GAGTAGGCTCTCTGGAAACAGTGAG-CTG 11112
LongTailedChinchilla         TTTCTA---TGAGA-TTTGGGAAAAAAAATGGTCCTTAAAAACCACAATAAT-GTGGGTTCCAAGGTGGGTTC---TCAT 11073
```

Majority	TGCTTTGTAGTTGATXGXTXGAGAGXCATTTCXAXCAXXGAGTAGCAXATXT-XTGAXXGAGAC----------------	
	27530 27540 27550 27560 27570 27580 27590 27600	
Human	TAATAGGTAGGTGGTATTCAGTCCACTTCTTCATCATCTTGGTGGCACCTCTGTTTAAGGAGACCA--------------	26991
GuineaPig	TGCTT-GT-GTTACCGCTTAAAAAGCCCTTTCCATGGACAATTGCCTTTTTT-TTAAATAAAAA----------------	10692
NorthernAmericanDeerMouse	TTCTTTGTAGTGGATAGGTTGAGAGACAGGAAGAGCACAGAGTAGTACAAAG--TGACAGTGACAAATCCATGTGTTGAC	9087
Mouse	TTCTTCCTAGGAGATGGGTTGAGAGACATGGGAGGCAATGACAAGTCTGTGG--TGACTGGCA-----------------	12900
ChineseHamsterGHOK1GS	GGCTTTGTAGTGGATAGGTTGAGAGACAGAAAG--CACAGAGCAGAACACAC--TGATGCTGA-----------------	11171
LongTailedChinchilla	TGCTTAGT-GTTACTGCTTAAAG-GCCCTTTCCATGCACAATTAGCATATTT-TTGATAGAAACAC--------------	11136
Majority	-----GXAATGXXCTCATTTGGXTCCAAXTCTTATTTXXTGXATGAXAXTTTXXTCXXC-XTT--GATTTTT-------X	
	27610 27620 27630 27640 27650 27660 27670 27680	
Human	-----GGAATA--CTCACCTGGGTACGTACCTTTCTTCCTCTCTGATAATCTATTTTTC-CTCAGGAACTTA-------A	27056
GuineaPig	-------TATGACCTCATTTGTTTCCAATTCATATTTAAGGGACCACAGTTTGGAC-----TT--AAGTTTT-------G	10751
NorthernAmericanDeerMouse	AGGCAGAAATTCAGTTATTTAGCACTAAATTTAGAGTTTTAGTGTGGTTTTTTTTTTTTTTTTTTTTTTTTTTTTTTTTT	9167
Mouse	-----GAAATGCAGTCATTTAGCACCAAGTTTAATACTTTGAATGGTGTTTTTCTCCCCACCCACCCTCTTT--------	12967
ChineseHamsterGHOK1GS	--	11171
LongTailedChinchilla	-----ATTATGAGCTCATTTGTTTCCAATTCATATTTAAGGGTCCACAATTTGGGCCCC-TTT--GATTTTT-------A	11201
Majority	---------------------TATTTGTGTCCTTGXXAGTAXACTGAXXGTCXT--GTTTAGCAAXACTG-----ATCTX	
	27690 27700 27710 27720 27730 27740 27750 27760	
Human	---------------------TATTTGTGTCCTTGCCCGGTGTCTTTCTGTCAT--GGTTCCCTAGGCTGC---CATCTC	27110
GuineaPig	---------------------TATTTATATCTCTGTTAGTACACTGAAAATCTT--GTTTGCTAGCATTG-----ACATT	10803
NorthernAmericanDeerMouse	TTTTTTTTTTTTTTTTTTTACTGTAAAAGTGCTTACTGATCAACAGATGCTCCTCAGTCTAGCAAAACATGATGAATCAA	9247
Mouse	--------------------CTGTAAGAGTACTGGTCAACAGTTT--TTTCTCTCAGTCTAGCAAAACAAAATGAATCAA	13025
ChineseHamsterGHOK1GS	--	11171
LongTailedChinchilla	---------------------TATTTGTAACTCT---AGTACACTGAAAGTCTT--GTTTTGTAACATTG-----ACTTT	11250
Majority	AACATGAAXAAATATT-------GTTAGTATACAGACACAXACT--TAXAATGGXCCTXATXTTAXTAGAAAAAXA----	
	27770 27780 27790 27800 27810 27820 27830 27840	
Human	CACAGGAGAAAATGTTTG-----GTTAGTTCTCAGTCCCAAACC-ATAGCAGAGCCCAGAGACTCTTGTCACCCTC----	27180
GuineaPig	AGTATGTATAAACAGT-------TTTTCAAAAAAGCAGCAT-----TAGTGTTGCACTGACATTATTGAT-TAATA----	10866
NorthernAmericanDeerMouse	AACATAAAGTAATATTCTTGGTCATAAATATACACACACTTACTTGTTAAATGCACCTTATTTTAATAGAAAAAAA----	9323
Mouse	AATATAAAGGAATA---TT----GTTGGTTTTAAACATACACTTGTTAAATGAACCTTATTTTAATAGAAAAAAAACCCT	13098
ChineseHamsterGHOK1GS	------------------------TAAGTATACACACATTCACT----AAATGTACCTTATTTTAATAGAAAAAAG----	11219
LongTailedChinchilla	TACATGTATAAACATT-------GTT--------GC----------TAGTATTGCACTGACATTATTAGT-TAATA----	11300

```
Majority                      -----AXXXTTXAGXTXTXTTTCGCCTCXTXCGXATAXTTXGCATCTCT--------TCCXXGTACAAXTXAXAGXXAGX
                                       27850     27860     27870     27880     27890     27900     27910     27920
Human                         -----AGCCTTCAGTCCTTGGTTGCCTTCTTCGCAGACCTGGCAGCTCT--------TCCACCTGAAAGTGATA-CATGG  27246
GuineaPig                     -----AAGTTTAAGGTTTTTTTAGAGTCATTTTTGTCTTTAGTGTTTAT--------TCCACAAATAGTGTATAGTACAG  10933
NorthernAmericanDeerMouse     -------AATCCTGCTTAGTTACCCCCCCCCCCAAAACCATGTAAGTCT-----------GTGTAC------CAACCAGT  9379
Mouse                         ACTTAGTAACCTACCGCCATTCCACCTCACCCGCATACTTCACAACTCTAAGAAGTCTTTGTGTACCACTACCAGTCAGT  13178
ChineseHamsterGHOK1GS         -------TGCTGCTATCTCCTCCCGCCCCTCCGAAAATTCTTCATCTCT-----------GTGTACAACTACCAACCAGT  11281
LongTailedChinchilla          -----AAGTTTAAGGTTTCTTTAGACTCATTTTTGTCTTTAGCATATAT--------TCCACACACA----ATAGTATGG  11363

Majority                      TAAXTGAAATTGXAG-AGAAXATXATXA--------------XAGXAAATAATTGCAGGCTCTGTGCAGTTCTGTAAATT
                                       27930     27940     27950     27960     27970     27980     27990     28000
Human                         TATTAATAGTTGGACTAGACTATTATGATAAACA--------GAGTATCTAATAACCATGAGAATAAAAAACAAGAAATT  27318
GuineaPig                     TCCTGGAACTTGAA--AGTTTATTGAGA---------------ATTCCCAT----------------------------  10967
NorthernAmericanDeerMouse     TAACTGAAAATGCAG-AGAACAGAATA---------------CAGCAAATAAATGCATGCTCTGTGCAGTTCTGTAAATT  9443
Mouse                         TAACTGGAAATGTAG-AGAACATAATAGACCACAGTAGACTATAGCAAACAGTTGCAGGCCATGGGCAGTTCTGTAGGTT  13257
ChineseHamsterGHOK1GS         TTACTGAAAGTGCAG-AGAACAGAATA---------------TAGCAAATAAATGCAGGCTTTGTGTAGTTCTGTAAATT  11345
LongTailedChinchilla          TCATGGAACTTGAA--AGTTTATTGAGA---------------ATTCCTTTGTAGCTCTTGCTATGCAGTACAGGCTGGC  11426

Majority                      CTXTAAATGTGTATG-AGTTCTCTXTXGCAAATXAGATGTAATAATT----AXGGATGGAGXAGTTXTGGGTAAGXTGGT
                                       28010     28020     28030     28040     28050     28060     28070     28080
Human                         TGTAACATTGAAATG-AGTGCTAAATGGCCTTTAATCTGTACTTGCT------TTT--GCACATTTTTTTTAAG-TGGT  27388
GuineaPig                     -------------TGTGGTTC----TGACACCAAA---GTGATAAT--------------TAAGTTT-------------  11000
NorthernAmericanDeerMouse     CTGTAAATATGTATC-AGTTCTCTTTAGCAAATGAGATGTAATAATT---GAAGGATGGAGGACTTCTGGGTAAAAAGAT  9519
Mouse                         CTATAAATGTGTATC-AGTTCTCTGCAGCAAATGAGAGGTAATACTTA-AAAAGGGTAGAGTAGAGCCAGGCATGGTGGT  13335
ChineseHamsterGHOK1GS         CTGTAAATGTGTATG-AGTTCTCTGTAGCAAATGAGATGTAATGATTTTAAAAGGATGGAGAACTTCTGGGTAAAAAGAT  11424
LongTailedChinchilla          CTTGAGCTCACTGTGTAGCTCAGGTTGGCCTTGAACTTGTGATGATC----C-TCCTGGCTCAGCCTTCCAAATGCTGGG  11501

Majority                      GT-----TTTAAGA--XGTAATXTXTAXTTTTTXXXTXTTXGATTTTTTTGXTTTTXXGGXXTXTCTTGTCCCCTTAGTA
                                       28090     28100     28110     28120     28130     28140     28150     28160
Human                         TTCTTTCTCTGACG--TGTAGAGTGTATCTTCCTATTTCTGAATTTTCTTGTTTCTGTGGGATGT-TAGTTTCTCTTGAA  27465
GuineaPig                     -------------A--TTTAATTTTTGGTTTTAAGTTTTTAAG---GCTTGTTTTCTAT---------GTACT-------  11046
NorthernAmericanDeerMouse     GT-----TTTAAGA--GGTAATATCAACTCATTCACTGT--GACTTTTGTTACATTCTGATCTTTCTT-CCCACTTAGTA  9589
Mouse                         GCACGCCTTTAATCCCAGCACTCTGGAGGCAGAGGCAGGTGGATCTTTGAAAGTCCAAGGCCAGCCTTATCTACAAAGTG  13415
ChineseHamsterGHOK1GS         GT-----TTTAAGA--AGTAATACCAACTTGTTCACTGT--GACTTTTATTATATTTGGGCTCTTCCT-CCCCCTTAATA  11494
LongTailedChinchilla          GTGCCCAGCCCTAA--GTTAATGTTTGATTTTTTGTTTGTTTGTTTGTTTGTTTTTGGTTTTTCTCTGGTACCGGGAACT  11579
```

```
Majority                    AAACXXATGAAA--XXXXAXTACATXXXXGAAAAGTTXCAXXTXGTXGXACAGAXT---AAXTACCGA----AXTACTAA
                                     28170     28180     28190     28200     28210     28220     28230     28240
Human                       ATGGTCATTGAT------ATTACTCAGGAAGAAAAGTTTCTGTGGTCAGATTTAATAAGAGATATCTATGCTAGTCTTAA  27539
GuineaPig                   ----TTACATTC------TGTTCCT----------TACCCTGTCAC-------------AGGTATCTA-----ATACCA-  11087
NorthernAmericanDeerMouse   AACCCTATGAAA--TAGGATGACATTTGTGAAAAGTTACAAATTGTAGCACAGATTT--AACTTCCGA----AATACTAG  9661
Mouse                       AGTTCCAGAACAGCTAGGGCTACACAGAGAAACTGTCTCAAAAAGTCAAAAAGAGG---AAGAAGGGA----AAAAAAAA  13488
ChineseHamsterGHOK1GS       GAACCTATGAAA--TAGGAAAACATTCATGAAAAGTTACAAATTATAGCACAGATTT--AACTTCTGAC--CAGTACTAA  11568
LongTailedChinchilla        GAACTCACGACC------TGTGCTTGCCAGGCAGGCACTTTGCCGCTGAGCC-------AAATCCCCA-----GCCCCAA  11641

Majority                    GXA-TTAATXTCTTTTXXATAX---XCTAAAAXTGTTAT------TATTGGTTAGXTTTTGXGAXXTCXTXTTATAAAXG
                                     28250     28260     28270     28280     28290     28300     28310     28320
Human                       CAT-TTAATTTCTTTTAGTTGAAT--CTAAAATTGATAT------CATT-CTTAGATTAATTAAAAATTTTTTAAAGAGC  27609
GuineaPig                   -----TGATTTCTTTCTTA-------------------------------------------------------------  11101
NorthernAmericanDeerMouse   GTAATTACAAACTTTTAGATGT---CCTAATAGTGTTATAGCTGTTGTTGGGTGGTTTTTGAGAGTTCATGTTTTGAAAG  9738
Mouse                       GGA-TGAAGAACTTCTGGGTA------AAAAGATGTTGTGAGAGGTATTAGTCAGCTCTTACAGTGTCCTTATATAAAGT  13561
ChineseHamsterGHOK1GS       GTA-TTACAACCTTTTGTATAT---CCGAATAGTTTTAC------TGCTGGTTGATTTTTGAGAGTCCATGTTTTAAAAG  11638
LongTailedChinchilla        TGT-TTGATTTTTTTTTTAGAAATACTTTAAAATACCAA------CAAGGATTATCTGGCCCGAAGGATTATCATCCATG  11714

Majority                    XXATATTCTG--AXATATTTATAT-TGXAAACAXCXAATGTATATXGXX-TTTTXACAGTCXXTAATXTAGAAXXGACTT
                                     28330     28340     28350     28360     28370     28380     28390     28400
Human                       ATGTATTCTTTAGTATTTTGATTC-TAAGTACCACTAATTTGATTTG---TTTCAACAGTCATTAATGTGGGGCAGTCAT  27685
GuineaPig                   --CTCTTTT--------------------------CAATATGATTTT---TTTCA-----TGTAAATA----ACA-----  11136
NorthernAmericanDeerMouse   GAAAACACTG--AAACATTTACAT-AGGAAACAGCATGGGTATATGGAAGTTGTCACAGTCACTGGGGAAGAATTGATTG  9815
Mouse                       TCAGATCCTGCCAGGTAACAAACCCTGTAAACCAATAATGTTCATGAAAAGTTGCACATTG--TAATACAGACTTAACTT  13639
ChineseHamsterGHOK1GS       GAAAACACTG--AAACATTTATATTAGGAAAGAGCATAGGTATATGGAAGTTGTCACAGTCTCTGGAGGAGAGTTGAGTG  11716
LongTailedChinchilla        TTTTCTTCTTCTATATGTTTGTAT-TAAA-ATATGCAATATATATTA---TTTTAAAAGATGAAAATATGAGACAGAATT  11789

Majority                    CXGTGAGTXXCAT--XTXA--TATXAGTTCAAAATTXCTXTAGXTTXXAATAXXAXXACAGXX--------ACATTTAGG
                                     28410     28420     28430     28440     28450     28460     28470     28480
Human                       CAATCACTACCATTGATCA--T-TTATTTCATATACATTGTAAGTT-AAATGACCAGGCTGAA--------ATAATTCAG  27753
GuineaPig                   ------------------------------------------------------AAAACAG----------ACATTTAG-  11151
NorthernAmericanDeerMouse   TGGTGAGTCTCATATCTTAGCTACAGGTTCAAGATTTCTTTAGTTTCCAATATTATCACATTAAAATATAAACTTACAGG  9895
Mouse                       CTGACAGTACTA----------AGAAGTTACAAACTTTTATGTATCCGAATAGGTTTATAGCTGTT-----GTTTTTAAA  13704
ChineseHamsterGHOK1GS       TGGTGAGTCTCAT--TTTAGCTATTGGTTCAAGATTCCCATAGTTTCCGATATTATCATATT--------------CAGG  11780
LongTailedChinchilla        CTTTGATTGACTCTTCTGG--TCTCATATCCTAGTCCCTGAAGATT-AGAAAGCAAAACAACT--------ACATCTAGG  11858
```

```
Majority                      AAGAACTXCTTXTGC---GAACTCATGTGGTAACTT--TGXGATGCTTCAXTGCTXTTAAAGXAATTATTXGTAXCTTTA
                                       28490     28500     28510     28520     28530     28540     28550     28560
Human                         ATGAAATTATTCTGT---CAGTTAATGTGGTAATGT--TAACATTTATCAGTAGTGCTGAATCACTTCTTTTGGGCATAA  27828
GuineaPig                     --------CCTTCG----------ATATC----------------------------CATAGGTTTTATTTGCAT-----  11180
NorthernAmericanDeerMouse     AAGAACTGCTTGTTCGCAGAACTCCTGTGGTAACTTTTTGTGATGCTTCATTGCTTTTAAAGTGAAAA-TAGTAACTTTA  9974
Mouse                         GGAAACAACTGAAACG-TTTACCCAGGAAGCAGCAT----AGGTGCCTGGAAGTTGTTAGAGAAACTG-GAACAGATTTG  13778
ChineseHamsterGHOK1GS         AAGAACTGCTTGCTC--AGAACTCCTGTGGTAACTTTTTGTGATGCTTCATTGCTTTTAAAGTGAAAAATAGTAACTTTA  11858
LongTailedChinchilla          GATAGTTTCCTTTGT---GGGA-GATGTCATGTTTT--AGGCTCTGTTCCAGTAAACTATAAAAGTCATTTTTATGTGTG  11932

Majority                      TAGTGTXGTGCATTXXACXTATXACXAAXGXXXTXAAXAATGCTGTT---AAAXGTXTTTGTATXXXAXTAGAXTAXTAT
                                       28570     28580     28590     28600     28610     28620     28630     28640
Human                         TAGTGGTAAGCTTTTTGCTCAATGCAAAGACAGGCATAATTGCTTTTTTCAAATAAAAATGTAATTTGTAAAAATACTCC  27908
GuineaPig                     TAGTGTTTCCCTTT---------TGAAATGTTA--CGAAATGC---------CTGTGTCTGTCTTTCATT----------  11230
NorthernAmericanDeerMouse     TTCTCTAGTGCATTCCACCTGTAACCAAA----TTAAGAATGCTGCT---AAAAGTCTTGCCATACAAATAGACCATTAT  10047
Mouse                         AG-TGTGGTGAATCTCATATTTCAGCTATGTGTTCAAGAGTCCTATG---ACTAGTAATTGAATACTAATAGTTTCAGAT  13854
ChineseHamsterGHOK1GS         TTCTCTAGTGCATTCTACCTATAACCAAA----TTAAGAATGCTGTT---AAAAGT-TTGCCACACAAATAGACCCATAT  11930
LongTailedChinchilla          CAGTGTCTTTTCTTGATCTTAGCCAAAAGGCAG--AGAAGTGATCTGT---ACTGTGTCTTTAATTCATTATGTTATCCT  12007

Majority                      TTTXTCTAXTTTAAATTXACAXTTXTG-GAAATAATTTGTATTXTATCTTXAAAAX-TAXA----XAAAGTTAAGGATAX
                                       28650     28660     28670     28680     28690     28700     28710     28720
Human                         AGTAATTTTTGTAAAATCTCAAAATTGCAA-ATAATTTG-ACGTTATTCTCAGTAA-TGAA----GGCTGTTAAATCTCC  27981
GuineaPig                     ------TAATACAGATTTACACTAAT---------TTTGTATTTCA--------------------------AAAATAA-  11268
NorthernAmericanDeerMouse     TTTGTCTACTTTAAGTTGA----TCTT-GAATTATTTCGTATT-TTTCTTTAAAAG-TACA--CCAAAAGTGAAGGATCA  10118
Mouse                         AGTATCATATTCAAATACAAATTTATA-GAAAGAACTCATGTAATGACTTTATACCTCATTGCTTAAAAAGTGAGGATAC  13933
ChineseHamsterGHOK1GS         TTTGTCTACTTTAAATTGA----TTTG-GAATTCTTTTCTATT-TGTCTTAAAAAAATACA--TCCAAAGTTAAGGATGA  12002
LongTailedChinchilla          TTTTCTTAGTATTTGTGTACACTGCTGTGAGATCATTTGTGTGCTATCTTACATAC-TTTT----GA---TGAAGATGAT  12079

Majority                      TCTATTXACT-CAX-TATXXCXXTXCTTGXTAXACXTTAAAATTTA----XAGXAACTTTTAATATAATGG--XXAXTTT
                                       28730     28740     28750     28760     28770     28780     28790     28800
Human                         TCTCTTAACGGCAGGTATGAAATCATTTGAGGAAGAAAAAAGTTCATAAATACAAACTTAAAAAAAAATGGAAAGACTTT  28061
GuineaPig                     -------------------GTTGTGTTTGCTAGTTTT-------------------------TTTAATG---------GC  11295
NorthernAmericanDeerMouse     TCTATTGGTTACAT-TGACACCATTCTTTCTATACAGTAAAATTTA----CAATAACT--TAATATCATGGT-GTAATTT  10190
Mouse                         TAACTTTATT-CCC-TAGTGCAGTGATTCTCAACCTTCCTGATGT-----CAGGACCCATTAATATAATTCC-TCACGTT  14005
ChineseHamsterGHOK1GS         CCTATTGACC-----TATCACCATTCTTTTTATACAATAAAATTTA----TAGTAACTTTTAATATCATGG--ATAGTTT  12071
LongTailedChinchilla          TCTAACAACA-CATGTATTGTTTACTTGAAGGTCTTGAACTTTCATCTTGTTGCTTCTTGGGCTTAATG---------TT  12150
```

```
Majority                    TCTXTGTA------TXXTAXGXAXAAAGGAAAXCCAACATXTTXATTAATATCTXTCAAXTTTGTTTTTXTXA-X-XXTX
                                     28810     28820     28830     28840     28850     28860     28870     28880
Human                       TCCATGCAAAAAGATGCATTGCAGGAAGGAGGAAGAGTGTCCTTCATAGCACACCACAAGTCAGTATTAGAGG----CTT  28137
GuineaPig                   TATATGTA------TTG----------------------------CCAGTATTTTTCCAGTTTGTTGCTA-------GCC  11334
NorthernAmericanDeerMouse   TCTTTTTA------TATTAGGAACAAAGGAAAGCCAACATTTTGATTAATGTCTCTTAGATTACTTTTTATCAATAATTC  10264
Mouse                       GTCGTGAT------CCCTAACCATAAAATCGTTTTCAC-TGCTACTTCATAACTAT-ACTTTTGTTACTGTTA-TGAGTT  14076
ChineseHamsterGHOK1GS       TCTTTT-G------TATTAAGAACAAAGGAAAGCCAACATGTTTATTAATGCCTGTTGGATCTCTTTTTATCAATAAATC  12144
LongTailedChinchilla        TTTGTGTA------TGGATTCTTTTATAGAAATGCTTTATTTTGACTGATATTTTGCCACCTTGTCCTTG-------TTT  12217

Majority                    TCATTTXXXATTCXTTAGXCCTTGTXXTCACXATXT---TTCAXTGTTAGAGAATT-XXXGXCATCXGTTGXAXTAATAA
                                     28890     28900     28910     28920     28930     28940     28950     28960
Human                       GCTTTTCTACTTGAGTAGACCCAAGATTCCCCACCT---ATCG-TCTTCCAAAGTTACTTGTGATCCACTGCAATCACAA  28213
GuineaPig                   TTGTCAC-------TTTGCTGTTTTTTTGATGAGGT---T-------------ATTTCAGAAATACAGTTTTAATA----  11387
NorthernAmericanDeerMouse   TCATTTTCTATTCCCCACCCCTTGTAATCCCCATACCCCTTCTTTGCTAGAGAATC-TGGGTCATCTGTACAATTTCTAC  10343
Mouse                       ATAATGTAAGTATTTGATATCAGGATATCAG-ATATGTCACCCCTGTGAAAGGGTTGTTTGACCCCAAAGGGGTTAAGGA  14155
ChineseHamsterGHOK1GS       TCATTTCCTATACCCTGCCCCTTGTAAT-----------TTCATTGCTAGAGAATC-TGGGTCATCTGTACAATTTCTAC  12212
LongTailedChinchilla        TCTTCCTAGAATCTTTTGACATACTTTTCAAGATGT---GTGAATATATGTGAACTCCATGAATCTGTCTGTAATAAAAA  12294

Majority                    XCTTXAGTATTCXXXTTATTGTCTCA--XTGTTTTATCTGTTCCGXXXXTXXTCXATXTTAXTTATX-AAAXXXATAAAT
                                     28970     28980     28990     29000     29010     29020     29030     29040
Human                       GTTTTACAGTGT---GTATACTCTAGG--TGTATGGAAGGATTTGAATTCACAAATAGTCAAACATC-AGAAGAATAAGC  28287
GuineaPig                   ----------------TATGGTCACA---AGTTCT-----------CCTTGC---ATCCTAGTTAAA-AATTG-------  11426
NorthernAmericanDeerMouse   TCTTTAGTACTCTGCTGATTATATC---TTGTTTCATCTGTTCCTCGGATCTTCTGTATTAATTGTG--AACTTATAAAT  10418
Mouse                       CCCACAGGATGAGAATTACTGCTCTA-GTGCCTTCCACTGTTACGAATATGTTAAATGTTTGCCACACAGAGTCTCACAT  14234
ChineseHamsterGHOK1GS       TCTTCAGTATTCTGCTGATTATACC---TTGTTTTATCTGTTCCTTGGATTTTCTGTATTAATTGTG--AACTTATAAAT  12287
LongTailedChinchilla        GCTTGAATGTTCACAGTATTGTCTCA---TGTACTTTATATTCTGTCCTTACCCCATTATAGGTATC-TAATGCAATGAT  12370

Majority                    TTTATXTAACTXXATTXAATATXGGTTTAXTTTTTA--TXAAGA-ACCTCXTATAXTTATGATXTATGT--XTXA-AXTX
                                     29050     29060     29070     29080     29090     29100     29110     29120
Human                       TGATAATAATACATTTGAAAAGAGGTCAAGTATAT---TTTTGAAAACTAGGAGAGTTAAAGTGTTTTT--GTGA-ACTA  28361
GuineaPig                   TTTATGTGACTCCCCTCAC---------------------------CCCCCCATTTTC----------------------  11457
NorthernAmericanDeerMouse   TAGATGAAACTTGATCAAATATAGGTTTAATTTTTA--GCAAGA-ATGTCTTGTAGCTATGATCCATG----TAA-----  10486
Mouse                       TTTGTCTAATTTAAATTGATCTTGAACTATTTTTCA--TTAAAATACATCAAAAGTTAAGGATGTATATTGATTACATTG  14312
ChineseHamsterGHOK1GS       TAGATAAAACTTGATCAAATATGGGTTTGATTTTTA--GCAAGA-ATATCTTGTAGATATGATCCATGTATGTAAATGGA  12364
LongTailedChinchilla        TTTTTCTTACTCTTTTCAGTATGATTTTTTCATGTAAATAACAAAGCCTTCTATATCCATGAGTTTTGT--CTGC-ATTG  12447
```

Majority	XX-GTXTXTCTXTTGXA--TTGT--XAAXGXAXCAXTXTXTTAATXA--TTTTGTTCAGXAAXGXAXTAAXTCAXXTACT	
	29130 29140 29150 29160 29170 29180 29190 29200	
Human	AG-GAACAAATAAGGCA--TTGA--CAATATTTTACTTGATTAGGAA--TTTTGTTCAGGTGAGTAAAACTTTAAGCATC	28434
GuineaPig	---TTTGGTACAGGGATTCA--AACTCA------	11480
NorthernAmericanDeerMouse	------TTTCTGTTGTA--TTGT-CAAGAGGAACAGAACGTTAAT-----ATTGATCAGTAGGGTGAGGTGTCACATACT	10552
Mouse	ACAGTATTCTTTTTATA--CAGT--AAAAGTTAAAATAACTTAATGAGTAGTTTTCCTTTTAGGAATTAAGAAAAGCCAA	14388
ChineseHamsterGHOK1GS	GGTTTTTCTCTATTTCAACTTGTTCCACTGGACCAGTTTCTTTCCCACCCACTGGTCCTCACAGACCCTTACTACATACT	12444
LongTailedChinchilla	GT-GTTTCTCTTTTGAA--ATTT--TATGAAATGCCTGTGTCTTTCA--TTTAATACAAAAATACATTAACTCATTTTGT	12520

Majority	CATXTTXAGXXTTAATXTTGATTACXXCTATT---TXATTGGCTGATTXTTA-TACCGATTGATTCATAGXXTTTAXTTX	
	29210 29220 29230 29240 29250 29260 29270 29280	
Human	A--CTTAAGTTTTGTTTTTGTTTCTTACTTTT---AGAATATCTGATCTGGA-CTTCTGTGGATTTATGG--AAGAGCTG	28506
GuineaPig	-------AGAC-------------CTTGTACA---TGACAGGCAG-----------------------------------	11502
NorthernAmericanDeerMouse	CAT------------TACAGAATGCACCTAT----C-ATTTATTCACTTTTAGTAACGAATGA-TCATGGAATTTTATTG	10614
Mouse	CATGTTGA---TTAATGTCTCTTAGAGCTCTTTT-TTATCAGTTTATTCCTA-TACCCAGTCCTTCGTACTTTCTATTTC	14463
ChineseHamsterGHOK1GS	CATTCTGAGGCTTAATATTAATTATAATTATTTGGCCAATGGCTCAGGCATATTACTGACTAGCTCTCAGAATTAAATTA	12524
LongTailedChinchilla	AGTTTTAAAATTAAATTGTGGTTACTGGTTTT---TTATTGGCTGTTTGTGT-TGCCAGTATTTTTCCAGTTTGTTGCTA	12596

Majority	G---CTTTX-T-TTCTXT---XTGCTGTGXXTXXXXCAXATTTTXACTTCATAXATTAAXT--TTXG-X--TATAGXTXA	
	29290 29300 29310 29320 29330 29340 29350 29360	
Human	G---CTTTA-T-TTCTCT---ATCCTGTGGGAGGGGCAAACTTTGATTTTGTATGTGGAGTAACTTG-ACCTTCAGTTTT	28577
GuineaPig	---------------------GCGCTGTGCTTC-----------------TAAACCAAAT--TCC-------TGGCC--	11532
NorthernAmericanDeerMouse	G---------------------TATTGTGAAAATAACAAATTTTCTCTTCATATATTA----------------AAAT--	10655
Mouse	CACCCTTTAGTCTTCTGTTGCTTGTTTCATATGCTCCTTATATTTTCTTCATTAATTAT-------GAACATATAAATTA	14536
ChineseHamsterGHOK1GS	ACCCATTTC-TATTCTGTGTTTTAGCATGAGGTTTGTGGCTTGTTACCTCACATCTTGCTTTCTTGGTGACTACATGGCA	12603
LongTailedChinchilla	G---CCTTG-T-CACTTT---ATGCTGTGTTTTGAAGAGGTT---AACTGATAAATACAAT--TTTA-G--TATGGCAGA	12660

Majority	XXX---X------TXCTXCTTXAAX-XACTXTGTTCTXATTTXTTACXTATTTXAATTXTTC------TAGCTATXTGC-	
	29370 29380 29390 29400 29410 29420 29430 29440	
Human	TTTGGTCTGAGAACAAAGGCCAAAA--AATATGTAACAGTTAATAACGTAATTCACTTACTTAGTCAATAAATATGTGGG	28655
GuineaPig	---------------------------CTGTGATCTCACTCTTCACATATTTTAATTTTTC---TGATGTTTTTCTGT-	11580
NorthernAmericanDeerMouse	-------------TCCTCCTTTAAG-AACTTTGCTC--ATGAATTATTTGTTTACATTGT---------AGCCA------	10704
Mouse	GA-----------TGAAACTTGATCAAACTTGATACAGGTTTAATATTTAGCAAGAATAT---C----TTGCTAT--GC-	14595
ChineseHamsterGHOK1GS	TTTCCCCAGCTCCTCCTCCTTTCAGTAACTCTGTTTGGATTTTCTACCTGGTTCTATTCTGC-C----TAGCCATTGGC-	12677
LongTailedChinchilla	AGTTCTCCTTGCATGCTAGTTGAAA--ATTGTGATCTCATTTTTTACATATTTTAATTTTTC---CAGTGTTTTTCTGC-	12734

Majority	XACATCATCTTTGTTAAATXXTXGATGAAXTAGCAGAAAGCATTCC-------XTGTXXGAXAXXCAXTXTXXTTXGTAA	
	29450 29460 29470 29480 29490 29500 29510 29520	
Human	GACATTGTACTTGGTTGACACTTGGGAATATTGTAGTGAGCACTACTG-----ATGTGAGCCAGGCACTTTGCCTGGTAA	28730
GuineaPig	-ACATCTTCTTTGTTAATTGTTGGAAGACTT----TAAAGCAGTCC-------ATGTTAGAATAACAAACTGGT--TTTT	11646
NorthernAmericanDeerMouse	CACACCATTTTAAA---------AATGGA--AGCAGGATAGATTT-----------------------TATCTTTGATAG	10750
Mouse	TATGTCATTTCTGTTGTATTATTTCTGGAGGAGCAGACTGTCTTC------------A----------TTCCTTTTGTGA	14653
ChineseHamsterGHOK1GS	CAAATCAGCTTTATTCAGCAACCAGTAAA--AGCAACACATATTTGCAGTACACAGAAGGATATTCCCTATCACATGTAA	12755
LongTailedChinchilla	-ACATCTTCCTTGTTAAATGTTGGAAGACTTAGTTTAAAGCAGTCC-------GTGTTGGAAAAACAAACTGCT--ATTT	12804

Majority	TTTCTXTCAXTTTAXCAXTXAXXAXGAAXXXTTG----T-XGTXGX--------X-XCATXCCTATTAXA---TACTCAX	
	29530 29540 29550 29560 29570 29580 29590 29600	
Human	TCACTGTTCGTGTATAATTTAATCCTAAGGGCTGTGA-T--GTAGA--------TGTTATCCCTGTTTTA---CAAATA-	28795
GuineaPig	CTTCTTACACTGTAGCAGTCAACACGAAACATTTC---T--GTGA----------------CCCCAAAGA---TAAAAA-	11702
NorthernAmericanDeerMouse	TTTCATACATGT---------GTATAATGCATTG----T-AGTTGT--------TCTCATACCTATTT-----TCCTCAT	10803
Mouse	CATTCATCA-GT-------A-GGGTGAGATGTTG----TCAGTTTG----------ACATACTCATTATAGAATGCTCCT	14710
ChineseHamsterGHOK1GS	TTTCTGTCATATTATCAAGAAGAATAGAATATTAACATTTAGTAGGGTGAGGTGTTACATACCTGTTACAAAATGCTCCT	12835
LongTailedChinchilla	TT-----CACTACAGCAGTCAACGCGAAGGGTTG----T--GTGA----------------CCCTAAAGA---TACG---	12852

Majority	AAXATTTTTCCXCTTCXXCTXACXAGTAATTGTT--XCCTTAXTGGTXTTGTGAAAXTGATGTCXATTGCATTC-TXTAT	
	29610 29620 29630 29640 29650 29660 29670 29680	
Human	AAGAACTTCAGGCTTAGAATGATTAAGTACTGTT--ACCTAACTGATAAGTGGAAGCTGAGCTCCAGAGCACCC-TTTCT	28872
GuineaPig	GAGATTTTTCCTCACCAGCAGGCAAGCAAATTCT--GCAGTAGTGGATACCAGCTATTGGTGTCTAATGCAGT--TCAGT	11778
NorthernAmericanDeerMouse	TCCCCCTTCCCTCTTCTGCTA-----TAACCCT----CCTTCTTAGCTTTGTG---------------CCTTTC-----C	10854
Mouse	ATCATTTATTCACTTTCAGTAACAAGTGATTGTGTAATCCTACTGGTGTTGTGAAAATAACATGGTTTTTCTTCATATAT	14790
ChineseHamsterGHOK1GS	ATCATTTATTCACTTTTAGTAATGAGTGATTGTGTAGTTTTATTGGTATTGTGAAAATAATTT---TCCCCTTCACATAT	12912
LongTailedChinchilla	GAGATTTCTCCCCACCAGCAGGCGAGCAATTAAT--TCTGCAGT-------AGCTATTGATGTCTACTGCAGT--TCAAT	12921

Majority	TGAXAC--AXCATTCAXTA--TTTGCCCAGXXAXXXXXXCXAATTCXAXXAGXCXTXXCXCCAGTTXAXA-----AXCCA	
	29690 29700 29710 29720 29730 29740 29750 29760	
Human	TAATAC--AGTATTTTCTA--CTTGTCATGAAAACATGAAGAAAGCTAGGAAACATTTTCTGTGTTGATAAT--GAGCCA	28946
GuineaPig	TGTTAC--A---TTCATTA--TTTATTTGGAGGTAGCCTCAAATTCCAAAAGGCTTTCCTCCAGATGCCAG----AGCCC	11847
NorthernAmericanDeerMouse	TGGAA---CTCACTTGGTA-----GCCCAGG--------CTGGCC----T----------CGAACTCA----------CA	10894
Mouse	TAAAAG--TCCACTAAGAA---TTGCTCATG----------AATT--------------ACATGTTTA------------	14829
ChineseHamsterGHOK1GS	TGAAATTCCTCCTTCAATAACTTTGCCCAGGAATTATTATTAGTCTACATTGTAGCCACACCATTTTAAAAATGGAAGCA	12992
LongTailedChinchilla	TCTTAC--A---TTCATTA--TTTACCTGGAGACAGCCTCAGATTCCACAAGGCTTCACTCCAGTTACCA-----ATCCC	12989

```
Majority                    AAAATGTACCTXXGTCX--CTCTCAAACTTAAXTTTAXXTAAGAAAGAXXTXXXC---------------------XTCT
                            29770 29780 29790 29800 29810 29820 29830 29840
Human                       ATAATGTGTATCAGTGG--TTCTTAAACTTTAGCATGCATCAGAAACACTTGGGG---------------------ATTT  29003
GuineaPig                   AAATTGTACCTGTGCC---CTCGCCAATTTAAGTTTCAATTA--------------------------------------  11886
NorthernAmericanDeerMouse   GAGATCTGCCTGCCTCTGCCTCCC------GAGTTCTGGGATTAAAGGCGTGCGC------------------CACCACC  10950
Mouse                       -CACTGTAGCCACATGCAGTTTTTA-----AAACTGAAGCAGGATAGATTTTATT---------------------CTCT  14882
ChineseHamsterGHOK1GS       GGAATAGATCTTACTCTTAATTTCAAACATGAATTTATGCATTGTAGTTGTTCTCATTCCCATTTTCCTCTCTCATTCCC  13072
LongTailedChinchilla        AAATTGTACCTTTGCC---CTGACCAACTTAAATTTAGATTAGAAAAAAC---AC---------------------TTTT  13042

Majority                    GCTTGTTTCAX------GGXTXTTATAAACXTCAXTXTXATGAAXTCTXAXX----GXXXAGGTXTGGGAGXXXGXGTX-
                            29850 29860 29870 29880 29890 29900 29910 29920
Human                       TTTTTTTTAAACCT---GGGGTTTTTAGATCCCACCCTCAGAGTTTCTTATT----CAGTAGGTCTGGGATTGGACCTGA  29076
GuineaPig                   ---------AT------GGATATTACAAAAAATAGGATGAAGAGATATGAAA----GGTGAGGTATGGGAGAAAGTGTA-  11946
NorthernAmericanDeerMouse   GCCCGGCCC---------------ATAACCCTCCTTCTTATGAAGGCC-------------TTTTT-----------C--  10989
Mouse                       GATAGTTTC---------------ATA--CATGAATATAATGAATTGT------------AGTTGT--------------  14919
ChineseHamsterGHOK1GS       CCTTCTCTCTTTCTCTCTTCTGCTGTAACCCTCCTTCCCATGAAGTCCCAGTCCGTGTGTGTGTGTGTGTGTGTGTGTGT  13152
LongTailedChinchilla        ACTGGCTTAAA------GGATTTTACAAAGAAAAGGATGAAGAGATGTGTAA----GGTGAGGTATGGGAGAAAGGGTAT  13112

Majority                    X-------GXGATTXAXTGTGTXT-------X-TX-XTXTAACCXXCGGAXTTAGXXXGTCAXT-----ATGXGXCTGGX
                            29930 29940 29950 29960 29970 29980 29990 30000
Human                       G-------AACATATACTGTCAGTAAGC--TTCCAGGTCTAATCTTAGGATTAGGGACCACACT-----TTGAGAATGAT  29142
GuineaPig                   --------GAGATTCAGTGAGAGC-------------TCTGACTTGTGGACAGAG----TCAGT-----AGATGA--GGT  11994
NorthernAmericanDeerMouse   ---------TACCTTATCTTTTTT-------TGTGAGTGTAACCCACTGATTTACCTAGTT--TCTTCAATGAGCCTGGG  11051
Mouse                       ---------TATTCCTCTCTCTTT---------TAC-TGTAACCCTCCTTCCTGAGAAGTC--------CTCTCCCTGGG  14972
ChineseHamsterGHOK1GS       GTGTGTGTGTGTGTTTGTGTGTTTGTGTGTGTGTGTGTGTATCCCACTGATTTACCTTGTGTATCTTGAATGAGCCTGGG  13232
LongTailedChinchilla        G-------GAGATCCA-TGTGGGT-------------CCTGATTTGTGTACCGAGAGTCCCAGT-----AGATGA--GGT  13164

Majority                    TXGGAXGTTAATTXCTXGAATATGGXTAAAXTTXTXAXTGTCXATAXCAXTAAAGXAATXTGTTCATXXTXTCCCXXCAA
                            30010 30020 30030 30040 30050 30060 30070 30080
Human                       TAGAGTATTCAATTAGATCTTAGGGACTACACTGTGAGAATCAGTAAT-GTATACAATTCTGCTCATCATGTTCTTATAA  29221
GuineaPig                   TAGGTTGTTCATTTCAAAAAGCAA-ATGAAAAAGCAGTGGTGAAA----GGAAAGGAATTTATTCATTAAAGCCATAC--  12067
NorthernAmericanDeerMouse   TGGGAGGTTATTTACTGGAGTATGGGCAATTTTATGAATGCATATACCACTGAAGAAA-CAGATAGCCCTCTCCCAGCAA  11130
Mouse                       TGGGAGGCGACTCACTGGAGTATGGGAA-ATTTACCAGTGGCTGCAGCACTGAAAGACATGGTGCCCTCTCTCCCAGCAA  15051
ChineseHamsterGHOK1GS       TGGGAGGTTATTTACTGGAATATGGGTA-CTTTATCAATGCCTATACCACTAAAAAAATCAGATACTCCTGTCCCAGCAA  13311
LongTailedChinchilla        TAGGCTGTTTATCTCAAAAATCAA-ATGAAAAAGTAGTAGTGAAAAGTAGGAAAGGAATTTATTCAGTAAAGCCATATGG  13243
```

```
Majority                    XCATTXACT--GCTAXXXCTTCCTT-TXGGAXGGAGGXATTGGGTCTXATAAGCCXCTCCTC--ATTXCAGTTTTTCTGX
                                     30090     30100     30110     30120     30130     30140     30150     30160
Human                       AACTATGCCATGCCAGATCTTCCATATCTTGATTAGAAATTGGTT--AAGAAATTAGGCTTGGTGTAAATACAGTATCAA  29299
GuineaPig                   --------------------------TGGGAAGAGAAAAGGGGG--AGTGTCCTGGGCCT-----------------TGG  12102
NorthernAmericanDeerMouse   CCATTAACT--GCTAACACTCCCTT-TGGGAGGGAGGGATTGGGTCTCATAAGCCTCTCCCCC-ATTGCAGTTTTTCTGG  11206
Mouse                       ACACTGACT--GCTGATGCTTCCTT-GGGGAGGGAGGGCTTGGGTCTCCTAAGCCCCTCCTC--ATCACAGTTTTTCTAA  15126
ChineseHamsterGHOK1GS       CCGTTAAC---------------------GAGGTAGGGATTGGGTCTCATAAGCCCCTCCCC--ATTCCAGTTTTTTTGA  13368
LongTailedChinchilla        GCTTTTGTTTTACTATTTTCTTTTT-TTGGTACCAGGAATCGGACTCAGGGCCTCGTACTTGACAGGCAAGCATTCCTGG  13322

Majority                    TG-TCATTACTCCAGTGTAGT--TAGTGTGXGTTCCTTCTTTTTTATTXX-ATTACCATTXAXXAATGAAAAATTTTTXA
                                     30170     30180     30190     30200     30210     30220     30230     30240
Human                       TG-TGGCTTGAAACTGGTTGTATTTTTCTGTTTTTTTTTTTCTTTGTGCA-TTTGATG-CTAATAAGTAGAAAGCTCTTA  29376
GuineaPig                   CC-TCCTCACTCCTGTCT-----------------------TTTAGA--------------AATACTGAAAAAACCTTTA  12144
NorthernAmericanDeerMouse   TG-TAGTTAGTGCAGTGTAGT--TAGTGTGAGTTCCTTCTTTTTAATT---ATTACTATTATGAAATCAAGCATTTTAAT  11280
Mouse                       TGATCAAC-CTCCAGTGTAAT--TAGTGTGGCCTCCTCCTTTTTTGTTATTATTACCATTTGGAAATTAAG-ATTTTTAC  15202
ChineseHamsterGHOK1GS       TGATCAAAACTCCAGTGTAGT--TAGTTGGAGTTCCTTCTTTTTAATTGTTATTACCATTATGAAATGAGTAATTTTAAC  13446
LongTailedChinchilla        CC-CCATTAAAGCCATATGGGAAGAGAAAGGGAGATCAGGTGTCCAGAGC-CTTGCCGTCCAATACTGAAAAAAGCTTTA  13400

Majority                    GTTTATTTXATAGTCXAXTGCAXTGACTGTTTXAAAAATGTTATTAXATCTTGGGCAGGXGTAGXTXXAAATAA----XX
                                     30250     30260     30270     30280     30290     30300     30310     30320
Human                       AAATATTTAAGGGAGACAACCTATGGATATCTGTTTACTCTTCTCATATTTGTTGTAATACAGTTTATTCATTA------  29450
GuineaPig                   GG---TTTAAATGAAGAAAGGAAAAATGTGTCGAGGGGAGTTGGGAGATGAAGGTAAGAAGTATGCATAGTTAA------  12215
NorthernAmericanDeerMouse   GTTTATCTTCTAGTCCATTGCAGTGACTGTTTTAAAAATGCTATTACATCTTGGGCAGGTGGAGCTTCAAATAG----CA  11356
Mouse                       CTTTGTTTTGTAGTCTATTGTAGTGACTATTTTTAAAATGTTATTAAATCTTGGGCAAGTGGAGTTTCAAATAC----CA  15278
ChineseHamsterGHOK1GS       GTTTACCTTCTAGTCCATTACGGTGACTGTTTTAAAAATGCTATTACATCTTGGGCAGGTGTAGCTTCAAATAGCAAGCA  13526
LongTailedChinchilla        AG---TTTAAATAGAGAAGAGAAGAAAAGTGAGGAGGGAGTTGGGAAATGAGGGCAAGAAGTACACATAGTTAA------  13471

Majority                    GTAXCTT-TGATA-XXXGGTTGAAGTATXTTTGATT--------ATTAGCXATGCTTTTTAAXAGTCTCTGT--XCAAGC
                                     30330     30340     30350     30360     30370     30380     30390     30400
Human                       TGAGTTT-AAATA--CAAATTAGCGTAAAGCTTATG--------GTTAATTTGGATTTTTTGGACTTTCTGA---AATGC  29516
GuineaPig                   GCAGTCT-TGGT---CATGCTGTGGTCTGGTTGATC--------ATTATATCTTGTCTCTGAGGTCTTGCTG------GC  12277
NorthernAmericanDeerMouse   GTAACTTGTGATAATGTGGTTCAAGTATCTTTGTTTTAAAATTCATCAGCCATGCTTTT--AAAGTCTCTGT--CCAAGA  11432
Mouse                       GTAACTTTTGACA-TGTGGTTGAAGT---TT-G--------------AGCCATGCTTTTCAAAAGTCCTTGTGCCCAAGG  15339
ChineseHamsterGHOK1GS       GTAACTT-TGATAATGTGGTTGAAGTATCTTTGTTTTAAAATTAATCAGCCATGCTTTTTAAAAGTCTCTGTGCCCAAGA  13605
LongTailedChinchilla        GCAGCCT-TGGT---CAAGCTGTGGTCTGCTTGATT--------GTTATGTCTGGTCTCCAGGG---T----------GC  13526
```

```
Majority                    TGTXGGAAATXCTXTXTAGCAAXTACA-TAAT-XXGTGCCAAGGG-ATXCTTGCTCT--X-TTGTTXCTGTTTXATXAXX
                                      30410     30420     30430     30440     30450     30460     30470     30480
Human                       TTTGAT-AATTCATTGCCTCCTTTGCA-CATT-TTTTTCATTTTG-ATGGTTGCATT----TAACTTCTTTGTACTGATT 29588
GuineaPig                   TCTAGGGAAGTCTTTATCCCTGGAG-----TT-TCTTACTATGAG-ATGACTGCTCC----TGCCTGTGGTACAGTCTTC 12346
NorthernAmericanDeerMouse   TGTGGGAAATGTTCTTTAGAAACTACAGTAAT-GGGTGCCAAGGGGATACTTGCTCTAAGATTGTTACTGCTTCAGGACC 11511
Mouse                       TGTAGAAAATGCTCCCGAGCAATCACA-TAATGATGTGCCAAGGG-ATACTTGCTTTAAGGTTGTTTCTGTTGCACCACT 15417
ChineseHamsterGHOK1GS       TGTGGAAAATGTTCTTTAGAAACTACAATAATAGGGTGCCAAGGG-ATTCTTGTTCT--GATTGTTACTGCTTCAGGACC 13682
LongTailedChinchilla        TCTAGG-AGGTCTTCA--------------------------------------------------G--GTGTAGTCTTT 13553

Majority                    CTTTCAGTXAXCAGGTAGTAATTTTTXATAXTTAXXTXXXXAATXXAAATATATXATGAGXTCAXTCATTAAXGCAXCT-
                                      30490     30500     30510     30520     30530     30540     30550     30560
Human                       TTTTAAATCAGCCAGTATTAATTTAACCTATTTTCAATACTAATATCCATGTATGTAAATATCCATAACTAAGGCAAAAC 29668
GuineaPig                   GTTTCTGTCGT---GGAATAATTGCTCTTACTTAGGTAGACAGTTCAAATA-ATGAGGAGATCTACCTGCAAGGCAAC-- 12420
NorthernAmericanDeerMouse   CTTTCAGTGGACAGATAGCATTTTTTGATAGAGATTTTTTTGATAGAAATATATTATTAGCTCATTTATTGTTTTATTT- 11590
Mouse                       CTTTCAG-GAGCAGTTAGCAGTTTTTGATAGAAATATCTTATAAGTTCATTCAGTATTT-CTCATTCATACTTAAAGCTA 15495
ChineseHamsterGHOK1GS       CTTTCAGTGAACAGTTAGGATTTTTTGACAAC---------------AATATATTGTGAGCTCATTTATTAGTG-ATTT- 13745
LongTailedChinchilla        GTTTCTGTCAT---GGGGTAATTGCTCCTACTTAGGTAGACAGTTCAAATG-GTGGGGTGATCTAGCGGCAAGACACC-- 13627

Majority                    --CTGATTXTAG--XATTTAAAXXT---X-XXXTAXTGTTTTTXCTTATTTXATT-TAXTXTTCAAATTTATTXXATGTG
                                      30570     30580     30590     30600     30610     30620     30630     30640
Human                       AGTTGATTTTAGCTAGTTTAAATATTAGTCCATTCATATTTT-TGTAAGTACATAACATTTTCAAAATAGCTAAAATGTC 29747
GuineaPig                   --CTGTTCCTAG--ACTTTTGAAAA---TTTCTTAATGTTTTATCTTGTTACCTT-TAGTCCTGAAGCAG--GAGAGGAG 12490
NorthernAmericanDeerMouse   --CTGATTCA----TATTTAAAGTT-------ACACCGTTTTTGCTTATTTGATT-T--TCTTCAGATTTATTT-ATGTG 11653
Mouse                       CACTGGTTTTGA-TTATTTGAGTTTTA---AAATACTGGTTTTGTTTATTTGATT-TCTTTTTCAGATTTATTTTGTGTG 15570
ChineseHamsterGHOK1GS       --CTCATTTG----TATTTAAAGTT-------ATACTGGTTTTGCTTATTT--------TTTTCAAATTTATTT-ATGTG 13803
LongTailedChinchilla        --CTGTTCCTAG--ACTCTAGAAA----TTTCTTAATGTTTTATCTTGGTGTCTT-CAGTCTTGAA------GAGACACG 13692

Majority                    TTTXGXTG-TTTGXXCACATGTATXTTTGTX-ATCATGTGAAT--ACATCCTGGAXCTXGAATXATXGXXAGXXGTGAXT
                                      30650     30660     30670     30680     30690     30700     30710     30720
Human                       ACTGGCTGGTTTGAACAACTGTGCAGTTACCGAAGAGATGAATGTATAACCTTTTCTGTAAAGAATTTTAAGACATGACC 29827
GuineaPig                   GTTAGATA-----AATGTAGGGCTAATTACC-----TTTGTATTCAGAATAACGG---TCCTTAAGTC----ACATGTCT 12553
NorthernAmericanDeerMouse   T--------TTTACTCACATGTATGTCTGTGTATCATGTGAAT--GCATTCTGGACCTGGTATTATGGACAGTTGTGAAT 11723
Mouse                       TTTGAGTGTTTTGCTTACATGTATGTTTGTGTATCATGTA------CATCTTGGAACTGGAGTTACGGGCAGTTGAAAAT 15644
ChineseHamsterGHOK1GS       TATCAGTGCTTTGCTCACATGTATGTCTGT--ATCATGTGCAT--GCATCCTGGAACTGGAATTAAGGAGAGTTGTGAAC 13879
LongTailedChinchilla        GTTAGATA-----AACATATTGGGAGTT--------TTTAGAT--AGACCCTCAT---TAAGTAATTC----ACCTGTCT 13750
```

```
Majority                     TXATATGTGGGT-XXTXGGAATC----CAACCCXAAXTXTXTGCAAX-----GAAXXAGTTCTXTTAAACACTGXXCXAT
                                       30730     30740     30750     30760     30770     30780     30790     30800
Human                        TTATTTTTTTGCAGAAAGAAATCTCTTCATTCCAAAGATTTTGCCTT----TGCTTTCGGTGTTCTAGCCACTGAAGTAT  29903
GuineaPig                    GTGTACAGATACAATTAGGAATA----TGACCCAAAGTTTAAGGTAAATAAGGAGACAGATACTGTACAAAATGAATGGA  12629
NorthernAmericanDeerMouse    TGACGTGTGGGT-GCTAGGAATC----AAATCCCAGACCTCTGCAA------GAACGAGTGCTCTAAAACACTGGGCCAT  11792
Mouse                        TGACATGTGGGT-ACTGGGAATC----AAACGCCAAATC-CTGTAA------GAACAAGTGCTCTTAAACACTGGGCCAT  15712
ChineseHamsterGHOK1GS        TGATATGTGGGT-GTTGGGAATC----CAACCCCAGACCTGTGCAAA-----GAATAAGTTCTCTTAAACACTGGGCTAT  13949
LongTailedChinchilla         ATGTACAGAGCTGAATGGGAATC----CAACCCAAAGTTTAAGGTAC-TGAGGAGATAGATACTGCACAAAATGAATGGA  13825

Majority                     ATAXCXXGCCCCCTTXTXTTATGTTTGXATCTCTTXTCCCTXAAAAAXCTXGXTTGCXXATATXAAXCTTCAXXGACX--
                                       30810     30820     30830     30840     30850     30860     30870     30880
Human                        ATACCTGTTTAATTTTAATAATGATGAAGATATTGATATCTAACTCACCA-GAATGAAGTTCTAATTTTCTAAAGACC--  29980
GuineaPig                    AGACAGGTCAAGCCTTTATCTTGGCAG------TTACCCCTACGATGCC-----TGTAGGCATAGATTATCAGTGTTT--  12696
NorthernAmericanDeerMouse    TTCTCCAGCCCCCTGATTTTATGTTTGTATCTCTTGTCCCTGAAAAACCTTGTTTGCTAATATTAACCTT----------  11862
Mouse                        CTCTCTAGCCCCCTCATTTTATGCTTGTATCTCTTGTCCCTGAAAAATCTTGATTGCTAATATTAACCTTGGTATACCAT  15792
ChineseHamsterGHOK1GS        TTTTCCAGCCTCTTGATTTTATGTTTGCATCTCCTGTCCCTGAAAAATGTTGTTTGCTAATATTAACCTACTACTACT-C  14028
LongTailedChinchilla         AGACAGGCCACACCTTTATCTTGTCT-------TTATCCCTAGGACATC-----TGTAGGCACAAATAATCAGTGCTG--  13891

Majority                     -CTTAXTAXATGXA--XCCTCAXAXXTCACTGTXAXAAACACTAXXXTXGXAAAXTCAGAAGTXTTXTAXTXAAAGXAXX
                                       30890     30900     30910     30920     30930     30940     30950     30960
Human                        -TGAATTATCTGGAT-GAATAAAGGACCATTGGGATGGAGGATTTATAGAACGTTTCTGAGAAAGTCTAGCTGTTGTGGC  30058
GuineaPig                    -CTTACTAAAGGCA--GTCTCA-AACTTACTGTTACATAAGCTCTACTGGGGTATTCAGAAGCTTTCCAAACCTCCATCC  12772
NorthernAmericanDeerMouse    ----GGTAT-------ACCCCACATGGCACTTTAAAAAACACCAGCCTTGTAAAAAAGACACTAGTTT-TTAAAAAAAT  11930
Mouse                        GAGTAGCACTTTAAGAAACAC-TAGCTTACCACATGTAGCACTAGCTTT-TAAAAGAGACACTACTTTACTAAAAGCCAT  15870
ChineseHamsterGHOK1GS        ACTAACTACACTACTAACTACACACAGCACTTTGAAAAACAGTAGCTTTAAAAAAACACTAGTTTTTT-TTTAAAGCAGT  14107
LongTailedChinchilla         -CTTATGAAAAGCA--GCCTCTTAAGTCGCTGTTAGAGAAACTGTACAGGGCTATTCAGAAGCTTTCCAAACCGCA---C  13965

Majority                     XTTAXTATTTTACTAAAG--ATTTAAXATAATTTACT---TXTTTTGTXGACATGXTTTXGTCTCTAXXGXATATTTTAX
                                       30970     30980     30990     31000     31010     31020     31030     31040
Human                        TTTGGTTTGTTTGTAAAC--AATTGAGATAGCTTTCCAAAGCTTTTGCTTATAGAATCTAATCACTTTTTCTATTTATTG  30136
GuineaPig                    TTTTGGATTTTAATGAAG--ACTTCACACCATATGC----ACGATTGGTGAGATTAT-TGGTCTCTGCTGACCAGTTTTT  12845
NorthernAmericanDeerMouse    ATTAATATTGTACTAATTT-ATTTAATACAATTTATTA--TTTTTTGTAGCCATGTTTT-GTCTCTAAGGTATAATCTAC  12006
Mouse                        ATTAATACTATACTAACATTATTTAAAATAAAG-ATT---TTTTTAATAATCATGTTTTTGACTTTAGGGCATATTTCAT  15946
ChineseHamsterGHOK1GS        ATTAATACTGTACTAAA---ATTTAACGTAACTTACT---TTCTTTGTAGTCATGTTTT-GTCTCTAGGGTATATTCCAC  14180
LongTailedChinchilla         TTTTGGATTTTTATGGAG--ACTTCATGACGTTGGC----GCGATTGATGAAATTAT-TGGTCATTGCTGACCAACTTCG  14038
```

```
Majority                     AAAAXTG-GCXTAGTXTAATCATXGXATTTTAAAGTT-------TTXTGAAATTGXTXXTTGTTTTACXAXCAAATTAX-
                                      31050     31060     31070     31080     31090     31100     31110     31120
Human                        TCTTTTGGGCTTAGAGTAAAGAAGGGAAGGGAACACTGTTGCCTTTGTGAGAATGGGAGAGGGATAGATGGGAGAGTAGA  30216
GuineaPig                    AAACTT--------CTTCTCCAGGGTAAT--AC------------TGTAATACTGTAAGTTGCATCCCTC-TAACTTAC-  12901
NorthernAmericanDeerMouse    AAAAAAG-GCATAGTATAATCTTTGAATTTTAAAGTT-------TGTTGAAATTGCTCTTTGTTTTACCACCAAATTAA-  12077
Mouse                        AGAA----GCCAAATGTAGTTATTGAATTTTAAAGTT-------TATTGAAATTATTCTCTGTTCTACCAGCAAAATA--  16013
ChineseHamsterGHOK1GS        AAAAAAG-GCATAGTATAATCATTGCATTTTAAAGTT-------TATTGAAATTACTCTTCATTTTATCACCAAATT---  14249
LongTailedChinchilla         ACTTCTTCCCCTCTCTTTCTCAAGAGACTGGAG------------TGTGATACTGACAGTTGCGTTCCTCATACCTTGG-  14105

Majority                     ---XXXAGATTTGTTTTCTXTTTTAGXGATTAXCTTTAACAACT-TTXXCTTTACTCTCTCAXCTAXAATTCAAXTAAAT
                                      31130     31140     31150     31160     31170     31180     31190     31200
Human                        GGGATGAAGTTAATTTTTTATTATAGGGATTAACATTTTTAACTATTCCCAATAGTGTAACATTTGACATTTAAGTGAAA  30296
GuineaPig                    ---------TCTGCCTGGTCTTCCAGTGA---TCCTCCCCAACT-CTGAAGTTACTCTGTCACCTATAAGTCAACTCACT  12968
NorthernAmericanDeerMouse    --TGGTAGATTTGTTTTCATTTTTAAAAATTATTTTTAACTAAA-TTTCCTCTGACCTCTGAAACAAACTACAAGAAAAT  12154
Mouse                        ---AAGAGATTTGTTTTCAATTTT--GGATTAAATTAAAAAATT-TTTTTTTAACTATCTTTCCTCCAATTAAAAAAAAA  16087
ChineseHamsterGHOK1GS        --TGGTAGATTTGTTTCATTTTTTTAAAATTATTTTTAACTATA-TTATCTCTGGCTTCTGCAGC----TCCAA--AAAC  14320
LongTailedChinchilla         ---------TTGGTCT--TCTTCCAGTGA---ACTGCCCCCACT-CTGAAGTTAC-CCATCAGCTATAAGTCAGCTCACT  14169

Majority                     AXCTTCXAAAACAAX-XCTGXAATTTTXGAXAXTTAGXXAGX--CTXXCXC------TXXCAXXXAXXGGXTXTGXAGXC
                                      31210     31220     31230     31240     31250     31260     31270     31280
Human                        GGCTTTTAGATTAATATTTTTAAATTTAAAGATGTCTTATGCTAATTTG-TTTTTGTTTTCATAGAGAGGATCTGACGAA  30375
GuineaPig                    AGCTTACAAAAAGGT-TCTGTTGTTTTGGAGATTCT---------------------TGCCAGGAAATGGGGATGAAGAC  13026
NorthernAmericanDeerMouse    GACCTCTTCAACAAG--CTACAGTCATTGAAGCTTAGGTAGG--CTCCCACCCACCTCTACCATCCCACTTTTTGTACTC  12230
Mouse                        AAGACCCAAAACAAG--CTACATTCAAAGAAACTTAG-------CTCACCC--------------------TCTGTATTC  16138
ChineseHamsterGHOK1GS        AACCTCTTCAGCAAG--CTGCAAA----GAAGCTTAGTTAGC--CTACCAC--------------------TTTGTATTC  14372
LongTailedChinchilla         AGCTTACAAAAAGGT-TTTGTCATTTTGGAGATTTTCCAAGGATTTTAGGCTTTAAATGCTAGGAAATGGGGATGAAGAC  14248

Majority                     CTXAT-X-ATXTATTTTTCTTAATXGATTG-XTTTXTXCAAC----TCATGCATTATAGTCAXXTXTAAAGTTX--T-AT
                                      31290     31300     31310     31320     31330     31340     31350     31360
Human                        CTACTCTCAGGCAGTGTTCTCAGTAGTCCGAACTCTAATA----TGAGCAGCATGGTAGTTACAGGT-AAGTGT--T-AC  30447
GuineaPig                    CAAAT-----ACATATTTCAAAATAA--------------------TCACGTTTTACAGT--------------------  13061
NorthernAmericanDeerMouse    CTTCTAT-ATGTATTTTTCTTAATTGATTG-CTTTTTTCAGC----TCATCCATTATAGTCAAGTATAAAGTTC------  12298
Mouse                        TTTAAAT-GTGTATTTTCATTAATTGATTG-GTTTATCCAAC----TCATTCATTACATGCACATGTAAATTCT--TCAT  16210
ChineseHamsterGHOK1GS        TTT-----ATATATTTTCATTAATTGATT--CTTTATTCAAC----TCATCCATTATATTCAAGTATAAAGTTCTTTCAT  14441
LongTailedChinchilla         CAAAT-----ATATATTTCAGAATATCGCAAATTTGAATAATACTTTTAAGCTTTTTAGTTATTGTTGAAATAA--T-AT  14320
```

```
Majority                      T---XTCCXAXCAATATGTAXAAAX--CCACXTTAT---GCAXAATGTTA--XAT---ATXTX-XCATTTTAXTTAATGA
                                        31370     31380     31390     31400     31410     31420     31430     31440
Human                         T---CTCTTAGCAGTCTGTCATTTG--CCATTTTAGGGGGCAGAATGTTAAAACTCAAATTTAACCCTGTTATACCAGGA  30522
GuineaPig                     ------CATAGTCATATGTGA------CCACTTT-----GTAGAAT-TTATCAGT---ATTT---CGTTTTATGTTTTAT  13117
NorthernAmericanDeerMouse     ----TTCCCAACAATATGTAGAAAGTAGCACAGTAT---GCATAATGTTG--TAT---AGCTG-ACATTTTACTTAATGA  12365
Mouse                         TTTCCTTCCCACCATAGGTAGAAA-----ACATAAT---GCATAATGTCT--TAT---ATCTTGGCATTTTCCTAAATGA  16277
ChineseHamsterGHOK1GS         TTTCTTTCCCACAATATGTAGAAAGTAGCACAGTAT---ATATAATGGTT--TAT---ATCTGGACATTTTACTAAATGA  14513
LongTailedChinchilla          G---GACATAGATATTTGTGA------CCACTTT-----GCGGAAT-TTA-CAGT---ATTT---CCTTTTATGTTTAGT  14378

Majority                      TXCCTXXXTXXTTGXAAGACTTTGCAXGACXXTXXXAAXGAAATTTTAXXATAGTTTGTATXTAXTXATTAXAAXXXXT-
                                        31450     31460     31470     31480     31490     31500     31510     31520
Human                         AAGCCTTTCTTTTTAAATGCGTGTTGAAACATTTTAGAGTAAATTTTACTAAAGTATATATGAGCCGAGTGGAAAAAAA-  30601
GuineaPig                     TTCCTTGTGTTTTGAAAGTTTTTACCATACA------AAGAATTTTTAAAATATTTTGCATGTATTCATTGAAAAAGATT  13191
NorthernAmericanDeerMouse     TGCATATGTCCTTGGAAGACTCTGCATGACTGTAAGAAGGAAACTTTCTCATTGCTTGTATTTCATTGTTCCTACTCTTG  12445
Mouse                         ----CACCTC-CTGGAAAACAAACCATGTCTTCTGTACACTGGTTTTACTCTTGCCAG-AATGAAAAATAACTA------  16345
ChineseHamsterGHOK1GS         TGCATACCTC-TTGGAGGACACTGCATGGCTCTAATAAGGAAACATTCTAATAAGGGGTTGTTTGTTGTTA---------  14583
LongTailedChinchilla          TTCCTTGGGTCTTTAAGAGTTTA--CATACA------AAGAATTTTAAAGATATTTTACACGTATTCATTAAAAGCGTT-  14449

Majority                      --A-AAAAAXXTXXXXXTXGTA--XTTGTTXAX--AXTAXCTCTTAAATATGATTXTTTTTXGTXXXTGAATXXXATA--
                                        31530     31540     31550     31560     31570     31580     31590     31600
Human                         --AAAAAAACCTGAAGCTGGTAACTTTGGTGAACAAGTTACTCTTAAATTTGATTGCTTTTCATTTTTGAACCACAAATG  30679
GuineaPig                     G-AGAAAAATTTCCCATTTATA---TTGTT----------TTCTGTAATAGAATTATTTTTTGT----GAATTTGGTA-A  13252
NorthernAmericanDeerMouse     CTAGTGTGAAAAATCACCAGTA--TTTCTATAG--AATAGCTCCTAAATGTGATTTTTTTTGGTCAATGTGTAATATA--  12519
Mouse                         -------------------GTA--TTTATTTAG--AATAAGTCCTAAATATGAAATTGTTGGATCAATGTATAGTACA--  16400
ChineseHamsterGHOK1GS         -------------------------TTCTACAT--TGTAGCT--------------------------------------  14598
LongTailedChinchilla          --A-AAAATTTTCCTGTTTGTA---TTGGT----------CTCTGCCACAAAATTGTTTTTTGTTTGTGAATTTGGTA-G  14512

Majority                      -AATACAGTTTXAC---XXX-X-CCTTXATXTXCTCCXXXGGGXTXTXTATT-TAAXTTXAAATXTTAATGCXAGTGTCT
                                        31610     31620     31630     31640     31650     31660     31670     31680
Human                         TAATTCTGTCTCACATTATAGTCCCTTCATCTGCCCCTCTGGTATTTTTATT-TTGTTTTTATTTTTTGTGTTTGTGTGT  30758
GuineaPig                     CAATCCAATTCC-----------TTTTTGTTTACTCCAGAAAGATAGCCAA----AGTTAGTATCATCA-------GTTC  13310
NorthernAmericanDeerMouse     -AATACTGTTTTACCAGATA-TTCCTAAATGTTTTTGTGTGGGGGTTGTTTTCTATCCAGAAATATGAATGCTAGTATCT  12597
Mouse                         -AATACAGTTTTACTGGATA-TCCCTAAATGTTTTGTGTGGGGTGGTTTTTGCTACCCAGAAATACAAATGCCAGAGTCT  16478
ChineseHamsterGHOK1GS         --ACACCATTTTAA----------------------------------------------------AAATGCCAGTGTCT  14624
LongTailedChinchilla          CAATCCAGTTCC-----------CTTTTGTTTACCCCATAGAGTTAGCCAAT-TAAGTTAAAATCGTCA-------GTGC  14573
```

```
Majority                    CATA----------TXAXGTTCXTAXXGTATGTXCXTATAGCTXAAAXTXXGTXGXTGXXGXXGXXAAXAGXATCCCXAG
                                     31690     31700     31710     31720     31730     31740     31750     31760
Human                       CATA----------TAATATTTTTACTTTAAGTATTTATGCCACAAACCCTAAAACCCAAATTTTAAGTAAAAACCGAAG 30828
GuineaPig                   CATT----------TTCTCCTCATGTTACAGGTATACATATCTTAAAC--TGAACTTGAACCT----ACAAAATGCAGAG 13374
NorthernAmericanDeerMouse   CATA----------GCAAGAACACATAGTATGTGCCTATAGCTCCAAGTGGGTGG-TGGGGGAGATAAGAGGATCCCAAG 12666
Mouse                       CATATAATAGTACGAAGAGGTTTTGCAGTGTGTGCCTGTAGCCCTAAGTAGGTGG-TAGGGGAGGCAC-AGGACCTCGCT 16556
ChineseHamsterGHOK1GS       CATA----------ACAAGT-CTTACAGTGTATGCTTGTAGCTTCAAATGGGTGGGTGGGGTAGACAAGAGGATCCCAAG 14693
LongTailedChinchilla        CATT----------TTCTCTCCATATTATAGGTACATACATCTTACAA--TACAGGCTAACCT----GCAGAATGCAGAG 14637

Majority                    XACTXTXTXXCAAAGTCGTGTAGAA---TXAXTXXXGCCCTAGGTXCXAATGXGAXACCXTGTCTCAGGAAAXGAAAXXA
                                     31770     31780     31790     31800     31810     31820     31830     31840
Human                       AATTCAGCTACATAGTTGTTTAAAA---CCATTGCAACCATAGTTGCTATGGTTTTAACTAATTTTTTGCTGAAACAGTA 30905
GuineaPig                   CACACTCCCATA-AGTAGAGTAGAAGT-TGATTTCTGTCTTATAAGCAAAAGTCCTGC-TGGTGTAAAATAATGATATTA 13451
NorthernAmericanDeerMouse   GACTTACTGGCTAGCCCTTGTGGAATAATTAGTG-AGCCCCAGGTCCTAGTGAGAGACCCTGTCTCCTGAAATGAAAAAG 12745
Mouse                       G---TTGTAGAAAAATCAGGTA--------AGC-----TCTGGGTCCCAATGAGAGACCCTGTCTCAGGAAAAGAAAGAG 16620
ChineseHamsterGHOK1GS       AGCTTTCTGGCTAGTCCTTGTA--------AGT----CCCTAGGTCCCAGTTAGAGACCCTGTCTCAGGAAAAGAAAAAA 14761
LongTailedChinchilla        CACACTGGCATA-AGTAGAGTAGAAGT-TGATTTCTGTCTTATAAGCAGAAGTCCTACCTGGTGTAAGAAAGTATTGTTA 14715

Majority                    TTGXAAXTXTTXATAGTXXGGTGXGTGXGAXAXTATCCAAATACXGAATTAATTTGXXTTTXTTTTACCA-XTXAGGAXG
                                     31850     31860     31870     31880     31890     31900     31910     31920
Human                       TTTTAATTCTGAGTTGATCCTTTTATGTTGTCTACCTGCATATTTAAGATGATTTGTTTTAATCTTCAAAGTTAGAGTTA 30985
GuineaPig                   ACTTATTCCATAAT--TTCACTATGCCTAGTTTGTTCATCTTACTGCT-TTATCGGTCATTAAGCA-----TTTTGGCTA 13523
NorthernAmericanDeerMouse   TTGGAAGTGTTGCTAGTCTGGTGGGTGAGAAAGTATCCAAATACAGAATTAATTTGGGTTTGTTTTACCA--TAAGGAAG 12823
Mouse                       TTGGAAGTGTTGCTAGTCTGGTGGGTGAGAAAGTATCCAAATACAGAATTAATTTGGATTTGTTTTACTA--TGAGGAAG 16698
ChineseHamsterGHOK1GS       TTGGAAGTGTTGATAGTCTGGTGGATGAGAAAGTATCCAAATACAGAATTAATTTGGATTTGTTTTACCA--TGAGGAAG 14839
LongTailedChinchilla        GTCTACTCCATAATG-TTCACTGTGCTTTCTTTCCTC------TTGTT-TCATTGGTCCCTAAGCTCTCATTTTTGGCTG 14787

Majority                    AXTAAACXGATTTXXAGCT-XTGATXXTTXTTTTTTTTGCXAAXXXCXTXCAAGXTTTTXAGACTGTGTTTGAAXTTTXG
                                     31930     31940     31950     31960     31970     31980     31990     32000
Human                       AAAAAAATTGTATATGTCT-GAAATAATGTTGTTTGTAGCATCTGTCAGTAAAACAATATAGACCAAATTTTAGACCTTA 31064
GuineaPig                   CCTGGGCT-ATGTATAATT-GTG-TAATCTTTTTTTGAGGAGATGGGGTTTATGCTCCCTAGAGAACATTT---ATTTTG 13597
NorthernAmericanDeerMouse   AATAAACAGATTTTCAGCTCATGATTTTTATTTCCTATGCCAAAAACTTCCAAGTTTTTAAACCTGTGTTTGAAGTTTAA 12903
Mouse                       ATTAAACAGATTTTCAGCTTATGATTTATACTTCCTCTGCCAAGA-TCTTCAAGTTTTTAAGTCTGTGTTTGAAGTTTAG 16777
ChineseHamsterGHOK1GS       ATTAAACAGATTTTCAGCTTATGATTTCTCTACTT-----------CCTCCAAGTTTTTAAGTCTGT--TTGAAGTTTAG 14906
LongTailedChinchilla        CCTGGGCTGATGTATAATT-GTG-TAACTCTTTTTTTTTTGAA--GGGACGAGGCTTCCTAGAGAGTGTTT---ATTTTG 14860
```

```
Majority                   ATTTCXTTTTTGATTCAXTTAXTTXGAXTTTATT-XXXAATTTTATXTXGTTXAAC-TXXTTGAACXTGXTTGCATTTTT
                           32010  32020  32030  32040  32050  32060  32070  32080
Human                      GTTTTATGGCTGATGGGCCAAATTTTTTTTTGT------ATTTTGTTTAGTTTTGCTTGAATTGACGTGTTCTCATCTGA  31138
GuineaPig                  TTGTCTTTTTTAAAAAA-TCAACTTGGTTTTATT----GATCCTTTTTAACATGTCATTTGTGTGTGTGTGTGTGTGTGT  13672
NorthernAmericanDeerMouse  ATTTCCTTTTTGATTCATATATTTAAAATTTATTTCAAAATTTTATCTGGTTAAAC-TAATTGAACTTGATTGCATTTTT  12982
Mouse                      ATTTCTTTTTTGATTCAGTTATTTAGAATTTATTTCAAAATTGTATCTGGTTAAAC-CAGTTGAACTTGATTGCATTTTT  16856
ChineseHamsterGHOK1GS      ATTTCCTTTTTGATTCATTTATTTAAAATTT----CAAAATTTTATCTGGTTAAAC-CGGTTGAACTTGATTGCATTTTT  14981
LongTailedChinchilla       TTGTGGTTTTTAAAAAAGTCAACTTGGTCTTACT----GATCCTCTTCAGCATGTCATTTATGTATGTGT----------  14926

Majority                   CT--------TTTTCACTATGCTTXTT---ATTA-XAAATATXTTTCXGTGXTTGGCTAAACATXGAATGTAXTXXAGAA
                           32090  32100  32110  32120  32130  32140  32150  32160
Human                      ATGGGCCAAATTTTGAATTTGATTATTATTATTATCAAATGAATTTACCTCACA-ACTCCACATTTTACATTAT--TTAC  31215
GuineaPig                  GTGTGTGTATCTTT---TGTGTTTACTTTGTTA--CATACATATTTGCAATATACAATGCAAAGTGAATGCAAATATTGT  13747
NorthernAmericanDeerMouse  CT--------TCTTCACTATGCTTTTTT--ATTA-TAAGTATCTTCCTGTGTTTGGCTAAACATAGAATGTGTTGTAGAA  13051
Mouse                      CT--------TTTTCACTATGCTTTTC---ATGA-AAGGCATCTTACTGTGTTTGGATAAACATGGAATGTATTAGAGAG  16924
ChineseHamsterGHOK1GS      CT--------TTTTCACTATGCTTTTC---ATTA-AAAATATCTTTCTGTGTTTGGCTAAACATGTAATGTATTGTAGAA  15049
LongTailedChinchilla       ------------TT---TGTGTTCATT---------ATATATATTTGCCATAT-----------GGAGACAAG------  14964

Majority                   XTXAAATTXXAGTXCATTTCACTGA-XTXTA-XCATGXGACATTTAAATXAATATTAGXATGX-ATTTAXAGAAAXAAXX
                           32170  32180  32190  32200  32210  32220  32230  32240
Human                      CTGAAACCAAAGTTCCTTTTTCTGTATTTTAACTTTGAATTATTTGCTTGAAGAACAAATTAT-ATTTGGCAATAAAAGG  31294
GuineaPig                  ATTCACTTCATGGCCTTTGCACTCATCTACATCCCTCAGACCATTTT---------AGAATCT-ATTTTGAGATA---GG  13814
NorthernAmericanDeerMouse  TTCAAATTACAATGGATTTTTTT-----TTTT--CATGGG-CATTTAAATCAATATTAGGATGATGTTGAAAGAATGAAAC  13123
Mouse                      CTTATATTCCAGTAGATTTCATGGACATCTA--AATAGAATGAATAAATCAATATTAGGATGA--TTTAAGGAACAAAAC  17000
ChineseHamsterGHOK1GS      TTCATATTACAGTGAATTT--------------CATGGG-CATTTAAATGAGTATTAGGATGATGTTTAAACAATGAAAC  15114
LongTailedChinchilla       ----ACTTCATGACCTTTGCACTGAGCTACATCCCCCAGACCTTTTT---------AAAATTT-ATTTTGAGACA---GG  15027

Majority                   AXXTTXTCAGTCTACTXXXTTXXXCTXTTTTCCXTTAGCCXGCAX-GATTATTGGCXAXAXXGXTTTXTGCCCXXXCATX
                           32250  32260  32270  32280  32290  32300  32310  32320
Human                      ATCATGAAAGACTAGA-AGACATTTTGATTTCCCTTAGGTAGCTGTGCTTAATGTATACATACATCTTGACACACACAAA  31373
GuineaPig                  GTCTTGCCAATTTCCTCAGGCATCTT--CTTGCCTCAGCCTCCTG-GATTGCTGGGTTTACAACCATGTGCCAACATGTT  13891
NorthernAmericanDeerMouse  AGATTTTCAGTCTACTATTTTGAACTTTTTTCCTTTGGCCAGAAA-GGGTATGGGCCA---TGTTTTCTTCCTTTGCATA  13199
Mouse                      AGATTTTCAGGCTACTGTTTTGACCTATTTTTCTTGAGTCTTGAG-GCTCTTTGGCCAGAATGAGTATGGCCCATGCTTT  17079
ChineseHamsterGHOK1GS      AGATTTTTAGTCTACTGTTTTGAACTTTTTTCCCTTTGGCAGAAA-GAGTATGGTCCG---TGTTTT--TCCTTTGCATA  15188
LongTailedChinchilla       GTCTTGCCAAGTTGCTCAGCTATTCT--TCTGCTTCAGCTTCCCA-GATTTCTGGTTTTACAAGCTTGTGCGCTCATGTT  15104
```

```
Majority                    TTCCXGXTTXXATXTXXTXTATXTAXATTGTCTTTAAAXXATAAGX-TTXXGXXTGXATTAGT-AAGTTAXXAATACTTX
                                          32330     32340     32350     32360     32370     32380     32390     32400
Human                       CACAAACACACATCTCTGACAATTCAGTTAACTAAAAATTATAAGGACTTAACAGATAATAGT-CATGATGTGAAGATAA 31452
GuineaPig                   TTCAGGCTT-TGTCTTGTAGATTTTGATTG-CTGTGAGTTAAGTGC-TTTCCAATTTACCAGT-AGGTAAGTGGTACTCA 13967
NorthernAmericanDeerMouse   TTCCTGTTTTAAAATGGTGTGTATATATTGTCTTTATAAAACAAATCTTGGGCCTGGGTTGATGAAGTTAAGAACACTTG 13279
Mouse                       CTGCC-TTTGCATATCTCGTGCATAGATTGTCTTTTAAAAATAA---TCGA---TGACTTAGC--ACTTAAGAATGCTTG 17150
ChineseHamsterGHOK1GS       TTCCTGTTTTAAAATGTTGTTTATATATTTTCTTTAGAAAACAAATCTTGGGTGTGGATTGATGAAATTAAGAACACTTG 15268
LongTailedChinchilla        TTCCAGCTT-TGTCTTGTATATTTTAAT-------AGATTATGAGC-TTTCTTGTTCCCCAGT-AGGTCGGTAGTACTGA 15174

Majority                    CTGCXTTTCCTGAXXAXXXXAGXTTTGXTTCXGTGATTCATAXXAGXCAXAT---AXXCXXXTGXGACTTTAXCTTCAXG
                                          32410     32420     32430     32440     32450     32460     32470     32480
Human                       ATTTGTCAACAGGTCATT--ATCATTGATTTTGCTATGTAATGATTACAAAGCA-AAATCACTGGGCCTTGATTTTGATA 31529
GuineaPig                   GT--GTTGCCTGTTCAT---ACCTTTG-CTCTGTGTTTCATAGACCACAGAT---ACTCACATGGGGTTTTGCCTTCTTT 14038
NorthernAmericanDeerMouse   CTGCTCTT-CTGAGGACTCAAGTTTAGTTTCCAGGATTCATGTCAGGC--------------TGTG-CTTTAGCTCCAGG 13343
Mouse                       CTGCTCTTCCAGAGGACCCAGGTTTGGCTCCCGTGATTCATATCAGGCATCTTATAACCACCTGTAACTTCAACTTCAGG 17230
ChineseHamsterGHOK1GS       CTGCTTTTGCCGAGGACCCAAGTTTGGTTCCCAGGACTCATGTCAGGC--------------TGTAACTCCAGCTCTAGG 15334
LongTailedChinchilla        GT--GT-ATCTATTCAT---ATCTTTG-TTCTGTTTTTCATAGACCACAGAT---AGTCCTATGGGTTTTTGCCTTACTT 15244

Majority                    GXXTXXGTXCCTGGTTAACATTAT--XXAGGAAXTCXXTAGTXATTTTXAATGXXGGXACCTXGXTTXAXXAXAATX--T
                                          32490     32500     32510     32520     32530     32540     32550     32560
Human                       GTCAAAGTACCATGTACACATAAT--TGAACAAATAGGCAGAAGTGTGTAACTCTGGTTTGTCGGTTTCTCTTTTTG--T 31605
GuineaPig                   CTCTCAGAATCTGTATAACCT-------ACAAACTTGATA----TTTTTG-TCCTTGTCCCATA-TCTGTCATGAT---T 14102
NorthernAmericanDeerMouse   GGATGTGC-CCTGGCTAGTATTATCTTTAGGATGACTGTAGTTATCCTACAGGAGGGAACCTTGATTGAGAAAAATGCTT 13422
Mouse                       GGATATGT-CCTGGTTAACTTTAT---GACACAAGCTAGAGTAATATTAATTGAGGGAACCTCGGTTGAGAAAA----TG 17302
ChineseHamsterGHOK1GS       GGATGTGT-CCTTGCTAGTATTATCTTTAGGATGACTATAGTTATTCTAAAGGAGGGAACTTCAATTGAGAAAA-TGCCT 15412
LongTailedChinchilla        CTCTCAGAATCTGATTTTCCTC------GGGAACTTGGTA----TTTGTGGTCCTTGTCTCATG-TCTGTCATGAT---T 15310

Majority                    CXATAAAATCCATCTXAAGAGCATTTTXXTXAXXT----XX--XXTTXAXTTCCAAX--------------X-X------
                                          32570     32580     32590     32600     32610     32620     32630     32640
Human                       TTTTACAATGCATACTGTGATCGATAATGGGAAATTAATTTGGAATTAATTTCCAAATATTTAGGGCT---AGTGACAGA 31682
GuineaPig                   CCATAGATTTCTTCAAAGGAGAAAGTTTATGACCTGTTCTTAGCCTGGAATTCTAAAGGAGGCTAGGA---ACTCATCAT 14179
NorthernAmericanDeerMouse   CTGTAAGATCCAGTTGCAGGGCCTTTTCTTTATTA--------------------------------------------- 13457
Mouse                       CCATAAAATCAAGCTGTAGGGCATTTTCTTTTTCTTTTCTTTTTTTTTTATTACATATTTTCCTCAATTACATTTCCAAT 17382
ChineseHamsterGHOK1GS       CTATAAGATCCACCTTAAGAACATTT------------------------------------------------------ 15438
LongTailedChinchilla        CCCTAGATTTCATCA----------------------------TCTTGAGTCCCAA------------------------ 15338
```

```
Majority                     -X----------AGXXCTXXCXXXXCTXXXAGXTXXCCCXXGXAAC----------------TT-XTAX-TGXXXGTXAX
                                       32650     32660     32670     32680     32690     32700     32710     32720
Human                        GTAACTAGTAGCAGCCTTGGCCCCTTTTTCAGTCTCCCCCCAAAACCA---------AAAAGTATGTAACTGAATAAAAT  31753
GuineaPig                    CTTTAATCTTATAGTTCTGACTTGCCTTCCAGATATCCTTAGCAAC----------------TTGCTACAGGGAAATAAC  14243
NorthernAmericanDeerMouse    ---------------------------------------------------------------------------GTGAT  13462
Mouse                        GCTATCCCAAAAGTCCCCACCCATTCCCATATTTTGGCCCTGGCGTTTCCCTGTACTGGGGCATATAAAGTTTGCGTGTC  17462
ChineseHamsterGHOK1GS        -----------------------------------------------------------------------------GAT  15441
LongTailedChinchilla         -C----------AGTTCTTGCTTGCCTGCTAGATAGCCCTAGGAAC----------------TTCCTACTTGGGAGTAAC  15391

Majority                     TGATGGGAXTX-XXTTGXAXXGXTGGX-TGACCAGACCATA-----X-----------XATXXXXCXAXAXAXCAXXXXT
                                       32730     32740     32750     32760     32770     32780     32790     32800
Human                        ACGTGGAACAACTGTTGTTTGGAAGTGATGAGCAGACGGTAT---AA-----------GATCATCCTTGAGAGAAGGGGT  31819
GuineaPig                    AGATGGTAGTTATATTGAACAAGTACAGTGAAAAAAATATA-----------------AATATAAACAAAAACCATTAAT  14306
NorthernAmericanDeerMouse    TGATGGG--------------GGCGG---GACCAGCCCATT---------------------------------------  13486
Mouse                        CAATGGGCCTCTCTTTCCAGTGATGGC-CGACTAGACCATCTTTTGATACATATGCAGCTAGAGTCAAGAGCTCCGGGGT  17541
ChineseHamsterGHOK1GS        TGATGGG--------------GAAGG---ACCCAGACCATT---------------------------------------  15465
LongTailedChinchilla         TGATGGTAGTTGGATTGAACAGGTACAGTGAAAAGAATATAC---AATA---------AATATGACAAAAAACCATTAAT  15459

Majority                     AXTXXTXAXXTXAXTXXCAXGXAXCCTCTXXXTTGXA---------------------------------------ATGG
                                       32810     32820     32830     32840     32850     32860     32870     32880
Human                        ACACGTGAGGTAAGTATCATGAACACTCTGGCTTTCTG---------------------------------T---CTTGG  31863
GuineaPig                    ATTATTCAAATTAATGCCAAGTAGCCTCTAATCTGTA-------------------------------------------  14343
NorthernAmericanDeerMouse    ----------------------------------------------------------------------------ATAG  13490
Mouse                        ACTGGTTAGTTCGTAATGTTGCATCTACAGGGTTGCAGATCTCTTTAGCTCCTTGGATACTTTCTCTAACTCCTCCATTG  17621
ChineseHamsterGHOK1GS        ----------------------------------------------------------------------------ATGG  15469
LongTailedChinchilla         ATTATTGAAATTAATGCCAAATAGCCTCTAACTTGTAA---------------------------------T---TTTGC  15503

Majority                     GCXGTT-T-XTXXTCCATCCCATXGTTXXGTGTXXXXCTCAAATTG-TXTXXXGGTTXXGXCXXXC--------------
                                       32890     32900     32910     32920     32930     32940     32950     32960
Human                        ACTATTTTACTGCTCTAAACCACAGAGAAGTGGAGAGCCCAGA--G-TATACAGGTCTCACTGAGCTGA-----------  31929
GuineaPig                    -CTTTTAT-TTATGCGATCTCATTTTTTTGTTTCTTTCTTAAATTGGCATATAAATTGTGTCATCCTTT-----------  14410
NorthernAmericanDeerMouse    GTGGT--------TCCATCCCATGGTGGGATGT-----TCCATCCC-TGAGCTGGTT-----------------------  13533
Mouse                        GGGGTCCT-GTGATCCATCCAATAGTTGATTGTGAGCATCCACTTC-TGTGTTTGCTAGGCCCCGGCGGCATTTTCTTAA  17699
ChineseHamsterGHOK1GS        GTGGT--------TCCCTCCC--------------------------TGAGCTGGTT-----------------------  15492
LongTailedChinchilla         GCTTTTGT-TTATGTGATCTC-TTTTTATGTTTCTTCCTTAGATTGGCATATAAATTTCGTCATCC--------------  15567
```

```
Majority                    -----XX--------------X--------X------TXT-------X--GTCGTGGGTTCTXTAAGAAAGGAG-GCTGA
                                     32970     32980     32990     33000     33010     33020     33030     33040
Human                       -AGA-GACCA---AGAGTGGGTGTCAGGGCTGCCCAATCATTTGGAATTTATGGAGAAGTCCTGGAGAAAAGAGAGCTGT  32004
GuineaPig                   -TTG-GAC-----ATTTTCTTTTCTTTTCTTTTCTTTTTTTCTTTTCTTGGTAGCAGGGATCAAACTCATGATCTAGTGC  14483
NorthernAmericanDeerMouse   --------------------------------------------------GTCCTGGGTTCTGTAAGAAAGGAG-GCTGA  13562
Mouse                       TTGATGATTGATGGGAGAGAGTTTAAACACTGTACTATCTCTGGACTTGTGTCGGGGGTTCTGTAAGAAAGCAG-GCCGA  17778
ChineseHamsterGHOK1GS       --------------------------------------------------GTCCTGGGTTCTAT--GAAAGGA------A  15514
LongTailedChinchilla        -------------------------------------TTT----------------------------------------  15570

Majority                    GCAXGXXXTGAXGAGTAAGCCAGTAAGCAGCAXCCCXCCXTGGCCATXTCAXCXGXTXCTGCCTTTAGGXTCCTCXXTTG
                                     33050     33060     33070     33080     33090     33100     33110     33120
Human                       CTAGGGGTTTAGGAATAGAGCAGGGGGCAGAATTTA-GTATGGTCATTTCACTTCATTCACAGTTTTGGATAATGACTTG  32083
GuineaPig                   TTGCCGGGCAAATGCTTGTATGGCTGAGTTAAATCC-CTGTGGACATTTT-CTTAAGTATTTCTTTGGGATGCTAGTTTT  14561
NorthernAmericanDeerMouse   GCAAGCCATGATGAGTAAGCCAGTAAACAGCACCCCTCCATGGCCCTC-CATCAGCTCCTGCCTCCAGGTTCCTCTC--A  13639
Mouse                       GCAAGCCATGATGAGTAAGCAAGAAAGCAGCATCCCTCCGTGGCCTGCACATCAGCTCCTGCCCCTTG-TTCCTCCTGTG  17857
ChineseHamsterGHOK1GS       GCAGGT--TGAGGAGCAAGCCAGTAAGCAGCACCTCTCCATGGCCCTC-CATCAGCTCCTGCATTCAGGTTCCTCCCCTG  15591
LongTailedChinchilla        ---------------------------------------GTGGACATTTT-CCTAATT--TTCTTTAGGATGCTAGTTTT  15608

Majority                    TTTGAXAAATTAAGACXX-XXTTXACACTTTC-------------TXTAXTCXGTXXGTAXCTGTATACATTGTGGCCAT
                                     33130     33140     33150     33160     33170     33180     33190     33200
Human                       GTTACATGTGTGCAGG-----TTGAGGCTTTG-------------CCAGGTCTGTCAGAAATTGTCTGCTCTGGGGCTAA  32145
GuineaPig                   T--GAAAGGTTA---------TTGATACTACA--------------CGAG------AGGAAAAGTTT---CTGTGATCAG  14607
NorthernAmericanDeerMouse   TTTGAGGAACTAAGACAGGATTGAACATTTTTGTTGTTGTTGTTGTATAATCCGTGGGTACCTGTATACATACGGGCCAT  13719
Mouse                       TTTGAGGAAATAAGACAGTATTTAACACTTTCCTT----------TATAATCAGTGGGTACCTGTACACATGT--GCCAT  17925
ChineseHamsterGHOK1GS       TTTGAGAAATTTAGACAGAATTCAACTTTTTC-------------TATAATCAATTGGTACCTGTATACATATTTGCCAT  15658
LongTailedChinchilla        TTTGGAAAGTTG---------TTGATACC------------------CAGG------AGGAAAAGTTT---TTGTGGTCAG  15653

Majority                    AAAXACATXXAGAAXGAXACACATAAATCXGXAACAXTGAATTTXTAAG-CAAXTXXXATCTTGATGAXTXAGAGAXTXX
                                     33210     33220     33230     33240     33250     33260     33270     33280
Human                       ATGGTGATTCCATAGGTTGCACGGTACTCTGAAACATTGGAGTTCTGAG-AAGTCAGAATGCAGACATATTGCAGGGTAC  32224
GuineaPig                   AA------TTAGCAAGAGACATACTATTCTTAA-CATTTAATTTCTTTT-C---TTGAAT--AGATGAATTA---ATTTT  14671
NorthernAmericanDeerMouse   AAATACATGCAGA----CACACATAAAGTAGTAAAAATGAATTTTTAAA-CAAATGTTA-CTTGGTGAGTAAGAGAAGAG  13793
Mouse                       AAACACATGCTGA--CACATACATAAAACAGTAA--ATGAATCTTTAAAACAAGTCTTG-CTTGTTAAGTAGGAGAG---  17997
ChineseHamsterGHOK1GS       AAATACATGTAAAGGCATACACATAAAATAGTAAAAATGAATTTTTAAG-CAAGTCTTTTCTTGGTGAGTAAGAGAA---  15734
LongTailedChinchilla        --------TTAGGAAGAGACATGCCAATCTTAG-CATTTAATTTCTTAG-----TTGAAT--TGATGAATTA---ACTTT  15714
```

```
Majority                    T-XTXAXCAGXAXATG-AGXTAAXAXXXXATGTAGTXXTXAXTTGXAT---CTAXTGCTTATXAXA-TAATTTTXXGATA
                            33290     33300     33310     33320     33330     33340     33350     33360
Human                       T-TCAAACACTCCATTTAGACTACAGAAAGCCTGGACCTGAATAGAAAGGATCACACCCTATAGGA-AGTGCCATCTATA  32302
GuineaPig                   T-TTAAAGAGTATATACTCTTAGTAAAGATTGCTATTCTGAGTTTAGG----TACTGCTAATAAGA-TAATGTTTCAGCT  14745
NorthernAmericanDeerMouse   AAATGACCAGAATATG-AGGTCAATTTTTATGTAGTGTCAAGTTGTAT---CTGTTGCTTATGAAAATAATTTTGTGATA  13869
Mouse                       --ATGACCAGAACATG-AGCCAAAT-TTTATGTAGCATCAAGTTGTAT---CTGTTGCTTGTGAAA-TAATTTTGTGATG  18069
ChineseHamsterGHOK1GS       --ATGACCAGAATCCG-AGCAGA---TATATATAGTGTTAAATTGTAT---CTTTTACTTATGAAA-TAATTTTGTGATA  15804
LongTailedChinchilla        T-T-AAAGAGTACATA---TTAGTAAAGATTTTGATTCTGAATTTAAG----TACTGCTAACAAGA-TG-TGTATCAACA  15783

Majority                    GCCTTTAAAX--TXAXTCAXTTATC--------X-GAXTATXTGTATCAXGTTGTTCATXTGXACATCXXTGGX------
                            33370     33380     33390     33400     33410     33420     33430     33440
Human                       AGACTGAGATA-AAAACCAGAAACC----------TAGC-CCGATAGGAAGTGAAGGATTCAC-CAGTTAATCT------  32363
GuineaPig                   GGCACTAATTC-TAGAACAGCCATC----------AATTATTACTATTAACTCATACACACACACACACACACA------  14808
NorthernAmericanDeerMouse   ACCTTAAAATATTTATTTATTTGTTTATTTATTGGGGGTATGTGTATCATATTGTGCATGTGGAGGTCACTGGGCAACTT  13949
Mouse                       GCTTTTAAAA---CATTTATTTACTTG-----TGGGATTGTGTGCACCATGTTGTTCAT-TGTACATGTGGGGTC-----  18135
ChineseHamsterGHOK1GS       GCTTTTAAGA---TATTAATTTGG---------GAGGGTTCATGTATCATGTTGTTTATGTGGAGGTCAGTGG-------  15865
LongTailedChinchilla        GACACTAAAA--TGGAGCAGCCATC----------AATTATTACCATTAACTCGTTCATTT-------------------  15832

Majority                    ------AXCXXATAXCTXCX-CTATGTTAAATCAGTA--TCTXTTGCCTTXTAXXGTACTXCAGCAGACCTXGCCTGGAA
                            33450     33460     33470     33480     33490     33500     33510     33520
Human                       ------AACCAGTAACTGAT-GAAGGATTAATCAGTAATTTAGTTGCCTTCCAAAACAAAACAACACCCCCAAGAAAGAC  32436
GuineaPig                   ------CACACACACACACA-CTAAGTTAAATAGGTGGTAATATTTCCTGTAAAATTGTTGAGTGAGTC--GGTGTGGTA  14879
NorthernAmericanDeerMouse   CTGTAAATTTCTTATCTTCCACTTTGTTAAAGCAGTA--TCTCTTGT-TTCTATTGTACTCCAGCCTACCTGGCCTGGGA  14026
Mouse                       ------AGGAGACAACTCC-----TGGGAATATCGTA--ACTTTCACCTTCTATTGAACTTCAGCCGAGCTATCCTGGAA  18202
ChineseHamsterGHOK1GS       -----AATATTTTACCTTCCACTTTATTGAAGCAGTG--TCTCTTGT-TTTTGTTGTACATCAGTTTAACTGGCCTGGAA  15937
LongTailedChinchilla        ----------TATGTATATT-CTAAGTTAAATAAAT---CATGTTTCCTGTAAAACTGTTCCATCAGTC--AGTGTGCTA  15896

Majority                    ATTTXAGGAXAXTTXCTGTXTXAGCTTXTCXATXXTXXXGTAGXAGTACXXXGATTAT-XCTGATTXGTXXCXCCACAXA
                            33530     33540     33550     33560     33570     33580     33590     33600
Human                       ATGATATAGTTTTCCTGCAACTATCTTCCATAATATCTAGTATACCATCAAAAATTACAAGTGATGTGCAGAAGCAGGAA  32516
GuineaPig                   ATATT-GGATATAGTA---ATGAGCTTTT----TATTTACTGGAGATCCAAACATAAT-GCTTTTTTATAATAACATATG  14950
NorthernAmericanDeerMouse   ATTTCAGGACCAGTTCTGTCTCAGCTTCTCTGTCTTCCTGTACCAGTACTGGGATTAT---AGATTAGTGGCTCCACACA  14103
Mouse                       GTTGCAGGACAATTCCTGTCTCCATGT-TCCATCTTTCTGTAGCAGTACTGGGATTACTACGGATGAGTGACTCCACGTC  18281
ChineseHamsterGHOK1GS       GTTTCAGGACCATTCCTGTCTTAACCT-TCCATCTTCCTGTAGCAGTCCTGGGATTAG---AGATTAGTGGCTCCAC---  16010
LongTailedChinchilla        ATGTTTGGGTATAGTAGTTATGAGCTTTT----TATTTAGTGGAGAGACAAACTTAATTGCTTTTTTATAACACTGTACA  15972
```

```
Majority                    GTTXAC----C-ATGGATTXTGGGGAXCTACACTTXXGTCATCXXXATTGCACAATTAXCCAXT-TTAACCTXCCGXTCC
                                      33610     33620     33630     33640     33650     33660     33670     33680
Human                       AGTAAC----CCATGAAATATA-AAGAACACATTTAATAGGAACAGATTCCAAAATGACCTAGAGTTAGAATTAGCAGAC  32591
GuineaPig                   GTTGAC----AAAAGTATTCTTCAGGAATATTTCTAAATTCCCCAAATTGCATA-TAATTCAATATTAATTTTTCTGATT  15025
NorthernAmericanDeerMouse   TCCACCTTTACCATAGATTTTGGGGATCTGCACTCTTGTCATTAGGCTTTCCCTATTAGCCAGT-TT-ACCTGCTGATAC  14181
Mouse                       CACCTTTATAC-ATGGGTTCTGGGGACCTGAACTCTTGTCATCAGGATTGCCCCATT--------TT-ACCTGCTGGTCC  18351
ChineseHamsterGHOK1GS       -------------TGGGTTTTGGGGATCTAAACTATTGTCATCAGACTTGCACTATTAGCCATT-TTTACCTGCCGATCC  16076
LongTailedChinchilla        GTTGA-----------------------TACTGCTAAATTCCCCAAATTGCAAA-TGATTTGATACTAATTTTTCTGACT  16028
```

```
Majority                    CAGTTXCTGTCXTTXTXTGATATXTTAATCGAXAATATTGAXTXTAXTTTGTTATGGAXC-XTTTTTTTCXXA-AXAATT
                                      33690     33700     33710     33720     33730     33740     33750     33760
Human                       -AAGGACGATAAGTATTTTAAGCTGTAAAGGAAAAGATTGAAA-TAATGAGTGATGGAGGAGTGTGTTGAAGAGATAAAA  32669
GuineaPig                   --GGTACATTAATAATT----ATACTCTTAGCAGATATGAAA--TAAT---TTAAAGAACACCACATATGCCA-AAAAAA  15093
NorthernAmericanDeerMouse   CTGATCCTGACCTTGTGTGATACCTTAATCTACAGTATTGATTGTAGTTTGTTCTGAGTC-TTTTTTTTTCT---TACTT  14257
Mouse                       CTATTCCTGGCCTTGTGTGATATCTTAATCTTCAATTTTGATTGTAGTTTGTTCTGA-----TTTTTTTCTGA-TCATTT  18425
ChineseHamsterGHOK1GS       CAGTTCCTGGCCTTGTGTGATAATTTAATCAACAGGATTGATTGTAGTTTCTTGTGGTTC-TTTTTTTTTCTTA-TCATTT  16154
LongTailedChinchilla        CAGTTATAGTGATAATTTAATATACTCTCAGTAGATATAAAAA-TAAT---TTAAAGAGC--------------AAAATT  16090
```

```
Majority                    GATTACATATAAAX-X-GTATTATTTXACAXAAGTGACTTATTCAGGGTXATXAAAATXXCXTGTT--XATTXATTGTTT
                                      33770     33780     33790     33800     33810     33820     33830     33840
Human                       AACTATAAAGAAAGCATGTAGAAATATTCTAATTGAAA-AATACAGTTTTTGAAATAGTTGGGACT--GATTCACTGGTT  32746
GuineaPig                   AAAAAAAAAAAAAA-AGGTAAGACTTTGCCATGCGTAAAGATTCATTGAAGGAAAGAGTACGAG----AATATCTTGTAT  15168
NorthernAmericanDeerMouse   GGTATCTTATCATT---TGATTATTTCACAGAAGTGGCTTATT-GGGGTTATTAAAATGTTTTGTT---ATTCATTGTTT  14330
Mouse                       GGTTAGATG-----------TTATTTCACAGAAGTGGCTTATTCGGGGTGATTAAAATGCCTTATTTTTATTCATTGTTT  18494
ChineseHamsterGHOK1GS       GATTTCTTACCATT---TGATTATTTCACAGAAGTGACTTATT-AGGGTGATTAAAATGCCTTCTT---ATTTATTGTTT  16227
LongTailedChinchilla        GACTACACATAAAG-AAGTAAGACTTTGCCATGCATA--GATTCATGGTAGTAAAGAATATGAG----AATATCTTGTAT  16163
```

```
Majority                    TCTXCXXGXAACAXXTTXAATTCATTXGTGXXAATATTAXXAAACXX-TXTCTACTGCTXXA-AAAXTG-TTAGAXXTAX
                                      33850     33860     33870     33880     33890     33900     33910     33920
Human                       GGAACTAGTAGCAGATTGGAGACAATAGAAGAAATGTTAGTGAACT--TGAATACTGTTGAATACAGTGATTAAATCTGT  32824
GuineaPig                   ACCATAAGTCACATTCCCCAGCCATCAGCTTCCAGAATACCTAAG----GTCTGCTGGGGTGGGAAGAG-TTTGATACAT  15243
NorthernAmericanDeerMouse   TCTCCCTGGAACC--TTTAATTAATTTGTGAGAATATTATTAGCCCCTTATCTACTGCTT-A-AAAATG-T----GTTAC  14401
Mouse                       TCTCCCTGGAATCAATTAAATTTATTTGTGATAGTATTAGCAATCTCCAATCTACCACTT---AAAATG-TAAGTGCTAC  18570
ChineseHamsterGHOK1GS       TCTCCTTGGAACA--TTTAATTAATTTGTGGGAGTATTCTTAGCCACTTATCAACTACTTCA-AAAATG-TGAATGTTAC  16303
LongTailedChinchilla        AC-ATAAGTCATATTCCCCACCCATCAGCCTTTATAATTACTTAG----GATCACTGGGGTAGAAAGAG-TTCGATATAT  16237
```

```
Majority                      CAXATAXGXGXAXGCCTXTATAXXCTTTAATGXXXCX-XXTGTAAAXTXAXCACAGXTATACXCTGAAXATTTXTTGXXX
                                                33930     33940     33950     33960     33970     33980     33990     34000
Human                         CAGAGAGGAGAAAGTATATCTAATGGACAATGAAGCAGTCTGACATGTAAGTTGAATTTCAGAGTGAGAATGTGGGGGAA  32904
GuineaPig                     AAGAAAGCAGACACCTAAAGAATATCCTAGTGCTACGTATTCTAAAAGAGGCCA---TATACTTTTGAAAACTGACGTGG  15320
NorthernAmericanDeerMouse     CATTTATGTGTATGCCTATATCAGCTTTACCTCCCTT---TGTAT-CTTAACACAGATGTGCCCTGAAGATTTTTTCTTA  14477
Mouse                         -ATATATGTGTATGGCTGTATGCTCTTTTCTTTACCTACCTGGACATTTAGCACAGGTGTATGCTAAAGATTTTTT----  18645
ChineseHamsterGHOK1GS         CATATATGTGTATGCCTGTAT--GCTTTTACCTCTGC---TGTAT-CTTAACACAGGTATGCCCTGAAGATTTTTTTCTT  16377
LongTailedChinchilla          AAGAAAGCAGAAAGCCAGAGAATATCATGATGATACACATTTGAAAAGAGATCAAA-TACACTTTGGTAAACTGGTAGGG  16316

Majority                      GX--X-XCXTXXCAATXTGATTTTGTCAT----GTAAAXXAAAAAGXTTA----ATAXTTGGTCTXCXA----TXTTXAX
                                                34010     34020     34030     34040     34050     34060     34070     34080
Human                         TAAAACATCTATCTGTTTGAGATAGTAATGACTGAGAACTTCTAAAATTTGAATAAAATATATCAACTTACATTCCTAAG  32984
GuineaPig                     GAGGA-----ATCAGTTTGGTTTTG--------GCAAACTAAGGAAGT------ATAAGATGTTGAATA------TTAAG  15375
NorthernAmericanDeerMouse     C-----TCTTTCCAATATGATTTTGTCAT----GTAAATGAAAAAGATTA----ATATTTGGTCTTCCA----TATTCAT  14540
Mouse                         ------TCTTTCAAATATAATTTTGTCAT----GTAATTGAAAAAGATAAT---ACATTTGTTCTTCCA----TGCTCAT  18708
ChineseHamsterGHOK1GS         A-----CCCTTCCAATATGATTTTGTCAT----GTAAATGAAAAAGGTTA----ACATTTGGTCTTCCA----TGTTCAT  16440
LongTailedChinchilla          GAGGA-----ATCAAATTGATTTTG--------GGGAACTAAGGTGGTGTGG-TATAAGCCGTTGACTA------TCAAG  16376

Majority                      TGGCTTATGT--TCTGXCTTCAGGTAAGCXXXGAXTGAAAXXXXTXCCTTXAAXXTX-TTG----TGTGAAGACTTTT-A
                                                34090     34100     34110     34120     34130     34140     34150     34160
Human                         AAGCTCAGGT--AAACTCCTCATGTAGGCTAAATAAAAAGAACCATACTTAGACACA-TAGT---AGTCAAAATATTG-A  33057
GuineaPig                     CGGTTT-TGA--CTTTTGTTCAAATGAGTAAAGAATGAATGGTTGCTGTTTTTTTTT-TTTT---TCCTTTGTCTTTT-A  15447
NorthernAmericanDeerMouse     TAGCTTATGT--TCTGCCTCCAGGTAACCTTGGATTGAAAATACTACCTCCAAAATGTTTG----TGTGAATACTTTTTC  14614
Mouse                         TGGCTTATATGGTCTGCATTCAGGTAATCGTGTATTGAAAACCCTGCCTTTAAATTGGTTGCGTATGTGAAGACTTT---  18785
ChineseHamsterGHOK1GS         TGGCTTATGT--TCTGCCTCCAGGTAACCGTGAATTGAAAGTATTGCCTCAAA-----TTA----TGTGAAGACTTT--C  16507
LongTailedChinchilla          AGGGTTATGT--CTTCTGTTCAAGTGAGTAAAGAATGAATGGTTGCTCTT--------TTT------------CTTTT-A  16433

Majority                      ATXXTGTAAAXAAXTAXCTA---A--------TCTGTACAGTTTCTATTATATTXTGTAT---GAGTAXXCTXATXTTAT
                                                34170     34180     34190     34200     34210     34220     34230     34240
Human                         AAACTAGAAATGAAGAGCAA---A--------TCTAAACAGCTGCGAAAAAAACGTCTAA---CAGGAGACCAACACCTC  33123
GuineaPig                     ATCTTAT-----ACTATCTG---A--------TGAG--CAGGTCTGTTGA-----CTTAT---GAATGGGCTAATTTTAT  15501
NorthernAmericanDeerMouse     TTACTGTAAACAATGCATTA------------TCTCTACTGTTTATATTGTATTATGTATTTTAAGTAAT------TTAG  14676
Mouse                         -TCTTGTAAACATGTCGG-A------------TATGTACAATTTATATTATGTTTTGTA-----AGTAAC------TTAA  18840
ChineseHamsterGHOK1GS         TTACTGTAAAAAAATAAATAAATAAAAAGCATTCTCTACTGTTTCTGTTGTATTGTGTATTTTTATTTATATTGTGTTAT  16587
LongTailedChinchilla          ACCTT-------ACCATCTG---A--------TTTG--CAGTTCCATTGA-----TTTAT---GGATGGGCTGATTTTAT  16485
```

```
Majority                     XXACTT--XGTGTGTAAGAAAXTTX--ATGTGTACAXTXTXTXXCCAATTATGTXATGTXAXA-CACATGATAXGAGXAX
                             34250 34260 34270 34280 34290 34300 34310 34320
Human                        GTATTAA-AGGGGGAAAAAAAGCGAATTTCTTTAGACTGAATGGC--ATAACTTGGCTCTACAGGGGATAAGAGGAGTAC 33200
GuineaPig                    TCACT----CAGTGAAAGAAACTTC-----TCCACTCTGTATTGCTTATCAAGTGCACCTGAGTTATTTGATAGAAGAAC 15572
NorthernAmericanDeerMouse    AGACTTAATATGTGTCAAAAG------ATACGTACAGTTTGTCAGCACTTATGCCATGTCATA-CACAGGGCGTGAGCAT 14749
Mouse                        AGACTT--TATATGTTGGAAG------ATGTGTGTAGGTTATAAGCAGTTATGTCATGTCATA-CACAAGATATGAGGAT 18911
ChineseHamsterGHOK1GS        GTATTTCCAGTACTTTAGAGATTTAATATGTGTACAGTTCGTAATCAATTGTGTCATGTCA---CACAGGTCATGAGCAT 16664
LongTailedChinchilla         TCACT----GTGTGTAAGAAACTTCCACCTTCTGTCCTGTGTTGCTTATGGAGTGATTTTAAGTGATTTGATAGAAGAAC 16561

Majority                     TCACTCATTTTAATATXX-TAAGAATX-CAAAAGTTAXXTCXTCXXTGATATTTAXXTATXTGXXAGX-TTTATGTXTTT
                             34330 34340 34350 34360 34370 34380 34390 34400
Human                        TGGGAATGGTTAATATGTGTATAAATTTTAAAAGGGCTCTCTCTTTTCCTATCAGTTTCTTTGAAAGCCTTTATGTAAAC 33280
GuineaPig                    AAAAGCCAAACAATAAA-----------CGGTAGTCTTCCCTTGTTCGTGGTTTTACTTTCTGTGG---TTTATTTA--- 15635
NorthernAmericanDeerMouse    TCGCTTATTTTAATATCCCTAAGGATC-CAAAAGCTAATT-CTCCCAGATACTTAGGAACTGG--AGG-TGTGTGTGTTC 14824
Mouse                        TCACTCATTTTA-TGTCACTGAGAATCTCAAAACTTAATTTCTCCCTGATATATATATATCAGGTATA-TATATGTGTTT 18989
ChineseHamsterGHOK1GS        TCTCTTATTTTACTATTC-TAAGGATG-CAAAAG-TAATT-CTCCCAGATACTCAGGAACTGG--AGG-TGTGCATGATT 16737
LongTailedChinchilla         AAAAGCCACAAAAAAAAA--AAAAAATACAGTAGTCCTCCCTTATTTGTGGTTTTACTTTCTGTGA---TTGATTTATTT 16636

Majority                     ATXTGXTTGXXCATT----T---TXGGTTXACAAXTGTAAATXXATTTTCTTAAAATAXTCXXTXXXTACAATXTTAAXT
                             34410 34420 34430 34440 34450 34460 34470 34480
Human                        AACAACTTTGT-ATTA---T---GGGGTTTATTAT-GTAAATGGATGTTTATATGACAA-CAGTTAGTACAAAGTGAAGA 33351
GuineaPig                    --C--CTTGGTCATT---------GGTTTGAAAATACAAAATGAATATTCTTGAAATAAACAGTTGAT-CAATTTTAAAT 15701
NorthernAmericanDeerMouse    ATGTGTGTGCATA----------TAGGTACACAAATGTAA-TACATTTTCTGAAAATAGTGTACCTGTATAATGTTTTCT 14893
Mouse                        GTGTGTGTACACACAGGTATATGCAAATGTACAAATGTAA-TAGATTTTTAAAAAATAGT-TACCCATGCTTTGT----- 19062
ChineseHamsterGHOK1GS        ATGTGTATACACAGT----TACATAAGTGCACAAATGTAAATACATTTTATGAAAATAGTGTAACTGTACAATTT----- 16808
LongTailedChinchilla         ACC--CTTGGTCATTC---T---TGGTTTGAAAATAGAAAATGAATATTCTTTAAATAA-CAGTTCAT-CAGTTTTAAAT 16706

Majority                     TXXATXXXAXTXTTTAGGGGTGGGGTXA-----XAGGGTCT-CAXXCTGTGGTXTAGXCTGACTTXGAACXTAAXT-TXT
                             34490 34500 34510 34520 34530 34540 34550 34560
Human                        TAAATGGAATTCTTCAGTGGTAAGGTTCT----TATATTTTACCTGAAGTGATACAGTGTTAACTCTAACATAAA---AA 33424
GuineaPig                    TGTACCCTGTGCTG-AGCAATGTGGTAA------AGACTCT-GGTGTCTTGTTGAATCC-----ACTGACATGAAT-CAA 15767
NorthernAmericanDeerMouse    ATAATTGGCCTTTTTTTGGGGGGGATGAGGGAGCAGGGTCT-CACACTGGAGTCTAGGCTAATTTGGAACTTACTTCTGT 14972
Mouse                        ---------CTTTTTGGGGGTAGGG-------GAAGGGTCT-CACACTGTGGTTTAGACTGGCTTTGAACTCATTA-TGT 19124
ChineseHamsterGHOK1GS        -------GTCTTTTTTTGGGGGGG----------CAGGGTCT-CACACTGT--TCTAGGCTGACTTGGAACTCACTG-TGT 16867
LongTailedChinchilla         TGGATGCTATTCTG-AGCAGTGTGGTAA------AGTCTCTTGATGCCTTGCTTTATCCTGCCTGTAGACATGAAT-CAT 16778
```

```
Majority                    AGCTCXGTTXAGACTACA-----XAGXAGTCCTTCTGXATXAGCCTXTAGAXXGCTGAGATTACCTXTXTGAGXCAGTXX
                                                 34570     34580     34590     34600     34610     34620     34630     34640
Human                       GATTCTGTTAAGGTTGCATAT--CAAAATTCTAAGTGTAATCTCATTTAAAAAGATAAAAAGAAGTATAACGAAAAGCCA  33502
GuineaPig                   CCCTTCATC-AGACTATC-----AAAATGTGCATCATAACTATCTATTAGTTACTTTATAGTTGTTTTAGGTGACAGACA  15841
NorthernAmericanDeerMouse   AGTCCAGGTTGGTCTTCAACCTGTGGCAATCCTTCTGCTTCAGCCTCT-GAATGCTGAGATTACCATTGTGAGCCATTAC  15051
Mouse                       AG----------------------GCAGTCCTTCTGTCTCAGTCTCTCGAGTACTGGGATTACCCATGTAAGCCAGTAC  19181
ChineseHamsterGHOK1GS       AGCCCAGGTTGGCTTCCAACCTGTGGCAATCCTTCTGCATCAGCCCCA-AGGTGCTGAGATTACCAATGTGAGCCATTAC  16946
LongTailedChinchilla        CACTTCATCCAGACTATG-----CACATGTCTGTGA----TGCCTATTAGTCACTTAGTAGCTGTTTTATATGACAGACA  16849

Majority                    ACXTXACTAAGGXTGTGACXTCTGTTTTGATTXAXTGAXGATTTCAGTAATTAXXCAAATTXTAXGXXXAGXTGCATAAT
                                                 34650     34660     34670     34680     34690     34700     34710     34720
Human                       ATAGAGGAAAAATTGGAGCATTGACTACCAGTTTTCAAGAATTACTGCAAAGCTACGGTAATTAAAAATAGCATCATAAT  33582
GuineaPig                   TCAAATGGACTCTTGTC-CATT------TGACTCCATGGCAGTGCCTTTGTTTTAC-----TTAATAATGACTCCAAAGA  15909
NorthernAmericanDeerMouse   ACCTGACTGAGGCTGTGACTTCTGTTTTGATTCAGTGATGATTTCAGTAATTAGCCAAATTATATGTGCAGTTGCATAAT  15131
Mouse                       ACCTGACTCTGGCTGTGACTTCTGTTTTGATTCAGTGAAGATTTCAGTAATTAGCCAAATTATATGTGCAGTTGCATAAT  19261
ChineseHamsterGHOK1GS       ACCTGACTGTGGCTGTGACTTCTGTTTTGATTCACTGATGATTTCAGTAATTAGCCAAATTATATGTGCAGTTGCATAAT  17026
LongTailedChinchilla        TCACATAGAATGTTGTGGCATGG--CAGTGCTTGTGTTATGTAACCCTGATTTTAC-----TTAATAATGGCTCCAAAGA  16922

Majority                    ACTGTGGAGAXXTXTTCXGTGXAAATGTTCAAATXATCTXXTTTXGXGGXTATTTGXGATAAXGTTTTXAAACTTGAAXA
                                                 34730     34740     34750     34760     34770     34780     34790     34800
Human                       AACATAAAG--ATAAATAGGTCAGTGAGACAGAGCAGAGAGTTCAGAAGTTAACTCACACAATTATGGATAACTGATTTT  33660
GuineaPig                   ATAGAG-----AGAGAAAGTGCC-GAGTCTGAAACCTGAAGGT-ACAAATTACTTT---CTGTACTACGGAACATA--CA  15977
NorthernAmericanDeerMouse   ACTGTGGAGAGCTCTTCTGTGAAAATGTTCAAATTATCTCATTTGGTGGATATTTGGGATAAGGTTTTAAAGTTGAACA  15211
Mouse                       ACTGTGGAGAGCTCTTCTGTGAAAATGTTCAAATTATCTCATTTGGTGGATATTTGTGATAAGGATTT-AAAGTTGAACA  19340
ChineseHamsterGHOK1GS       ACTGTGGAGAGCTCTTCTGTGAAAATGCTCAAATTATCTCATTTGGTGGATATTTGGGATAAGGTTTTAAACTTGAATA  17106
LongTailedChinchilla        ATAGTGCTGC-ATAGGAAGTGCCAGGGTATAAAACAAAAAGGG-ACAGATTACTTT---TAGTATTAAATGGCAGGGTTA  16997

Majority                    GXGCTGXTATATGXATGGCCXTTTTX-TXGAGAG--TGTACXCTTAXAXTTGAXTXTGTTAAACTT-ATXAGXXXGCXGT
                                                 34810     34820     34830     34840     34850     34860     34870     34880
Human                       TGACAAAGGTGC--AAAGTCAATTCAGTGGAGAAAAAGTAGACTTCCAACAAAGGGTGCTACAGCA-GTTGGA--GCTAT  33735
GuineaPig                   GGAAAAACATAG-AATGTACAGTTTGGTACA----TTTTGAGTTTTCAA--GAATCTACTTACAGT-CTTATAACGCCTT  16049
NorthernAmericanDeerMouse   GTGCTGGTATATGTATGACCGTTTTT-T-GAGAG--TATACGCTGAGTCTTGAGTATGTTAAACTTTACCAGGCAATGGT  15287
Mouse                       GTGCTGGTGTATGCATGGCCGTTTT----GAGAG--TGTACACCAAGGCTTGAGTGTGTTAAACTTTACCTAGCAGCAGT  19414
ChineseHamsterGHOK1GS       GTGCTGGTATATGTATGGCCGTTTTT-TTGAGAG--TGTACACTAAGGTTTGAATATGTTAAACTT-ATCAGGTTGTAGT  17182
LongTailedChinchilla        TGGAGCATATAGGAAAGAACAGTTTG-TACAGGG-TTTCAAGTTTTCAG--AAATCTGTTGAAGGT-CTTGGAACACCTT  17072
```

```
Majority                     CCATXATTTXCXAGAGTXTCAT-TCTGATTTATGTCTCAGATTACTACAGAAGAGTTACATAT--AATTGTTXXTGXCAT
                             34890  34900  34910  34920  34930  34940  34950  34960
Human                        CC--G-TATGCAAAAAAATGATCTTGGATCTGTATCTCACACTGCATATGAAAATTAACTCCA--AATGGATCATGGCCT  33810
GuineaPig                    CCACGGTTAGAGGGAGTATAGTATGTCCCTAAGGTTACATAGTACTCC-------TGGCA--------TGTTGATGTCAT  16114
NorthernAmericanDeerMouse    AGATTATTTCCCAGCATTTCAT-TCTGATTTATGTCTCAGATTAATACAGAAGAGTTACATGT-CAATTCCTTCTGGCAT  15365
Mouse                        GTGTTATTTCCTAGGGTTTCAT-TCTGATTTATGTCTTGGATAAGTACAGAAGTGTTACATTTCAAATTGTTACTGACAC  19493
ChineseHamsterGHOK1GS        AGATTATTTCTCAGCGTTTCAT-TCTGAATTATATCTCAGATTAATACAGAAGGGTTACATACATGACAGATTCT----T  17257
LongTailedChinchilla         CCAGGGTAAGGAAGAGTACAGTATGTGGCTAAGGTTACACAGTACTTAATAGCAGTGGCAGAG--GCCTGTTCATGTCAT  17150

Majority                     XGTTXGAAXXCAXXAXATAGXXTAT-TXA-TXXCAXXXATXGTCTGXTGTCXTXGX-TCTCTXGTTTXTGTXTGCTTGTT
                             34970  34980  34990  35000  35010  35020  35030  35040
Human                        AATGTAAAACCAAAAATTATAAAAT-CTC-TAGAAAAAAATACAGAAAATCTTCGTGACTTTGGATTAAGCAGGAATGTA  33888
GuineaPig                    GGCTGGAAAGCAGAAGAAAGAAAA--------GGAGAAAGGGTCTGAGGTCTAA---TCCCCAGTATATGTAAACCCCTA  16183
NorthernAmericanDeerMouse    TTTTTGAATTACATATATAGTTTCT-TAA-TTTCT--GCTAGTCTGGTGT----G---------TTGTAATTT-TTTGTT  15427
Mouse                        TGTTTTATTGAGTTTCTTAATTTTTGTTAGTCTCATGGTTTGTGTTGTGTTGTGGG-TTTTTGGTTGTTGTTTGCTTGTT  19572
ChineseHamsterGHOK1GS        AGTGGCAATTCTTTATGTAGTTTTT-TAAATTTCT--GCTAATCTGGTGT----GA-TTTCTTTTTTTTCTTT-TTTCTT  17328
LongTailedChinchilla         G-CTGGGAAACAGAAGAGAGAAGA--------GGAGCAAGGGTCTGAGGTCCTAGTTTCCCCAGTAAATGTAAGCCC-TC  17220

Majority                     TTTGGTXGXTXACCXGTTATTTXCCTCTAXTTAATXGAXTGTAXATTGTTTTTAG-XX------------------XAXT
                             35050  35060  35070  35080  35090  35100  35110  35120
Human                        TTTGATA--TGACCCTAAAAGCACAATC-TATTAAAAAATGGAATTTGACATCAGAATTAAAATCTTCTGCCATTCAAAA  33965
GuineaPig                    GTGACTAGAAGACCTCCCATTGGTCTCTATGTCTTAGAATTCCACCACCTCTTAGAATTACCTTAGGTAATTCTGATAGT  16263
NorthernAmericanDeerMouse    TGTG--TGCTT----GTTATTTTCTTCTGCTTAATGGAGTGTACATTATTTTT---------------------------  15474
Mouse                        TTTGTTTTGTTCCTGGTTGTTTCCCCCTGATTAATGGAGTGTACATTGTTTTTGGCATCCATCCATCCACACACACCCAT  19652
ChineseHamsterGHOK1GS        TTTGGTTGGTT----GTTGTTTTCTCCTAATTAATGGAGTGTACATTGTTTTT---------------------------  17377
LongTailedChinchilla         GTGAGTAGAAGACCTCCCATTCGCCTCCACCTCTTAAAATTCTACCACCTCCCAG---------------------TAGT  17279

Majority                     XXAAXXXXCTGGGXAXCAAACXXTXA-CCAXX---TGGXXGAXX-----TXXXXACAAACCATA-XAXTTXTTAAXXXXX
                             35130  35140  35150  35160  35170  35180  35190  35200
Human                        GAAACTGCTAGGGAATGAAAAGACAAGCCATAAACTGGAAGAACA----TCATAACAAAGCATA-TAGCTGATAAAGGAC  34040
GuineaPig                    ACAATATGCTGGGGACCAAACCTTTA-CCACGAGGTGGGGGAGGGGGGCCTAACACAAACCATAGCATTTATTAACAAAT  16342
NorthernAmericanDeerMouse    --------------------------------------------------------------------------------  15474
Mouse                        CTACCCACCTGTCTGTCTGACTGTCTACCTACCTACCTACCTACTTACCTACCTACCTACTTACCTGGTTTTTAAAGGCA  19732
ChineseHamsterGHOK1GS        --------------------------------------------------------------------------------  17377
LongTailedChinchilla         GCCATAGGCTGGGGACCAAACCTTTA-CCACA---TGGGGGACA-----TTCACACAAACCATAGCATTTATTAATAAGT  17350
```

```
Majority                    TXXTAT-----XAACCT-------TTXGAT-----XAXAXTXGGXAXTXXTXCAXXXXAX---ACTGXXTXXXTXGGCCA
                                      35210     35220     35230     35240     35250     35260     35270     35280
Human                       TTCTATC--CAGAATATTAAAAGATTCAGTATATGTAAAATCAGCAGTCAAGCAACAAACT--ACTCAATGAATTGGCCA  34116
GuineaPig                   TCCTAT-----AGACCTGAT--GCTTTGAT-----CAATGTTGGGAATATTACAGTGAAT---ACTGCTAATGTGGGCCA  16407
NorthernAmericanDeerMouse   --------------------------------------------------------------------------------  15474
Mouse                       GGGTCTCTTTGTAGCCCTGGTTTCCTGGAACTCTGTAGACCAGGCTGGCCTTGAACTCAGAGATCTGCCTGCCTCTGCCT  19812
ChineseHamsterGHOK1GS       --------------------------------------------------------------------------------  17377
LongTailedChinchilla        TTGTAT-----GAACCT-------TTCGAT-----CATACTTGGGAATATTACAGTGAAC---ACTGTTTATATGGGCCA  17410

Majority                    XX-XAXTTTXGXA---X--X-XGCAXATATTGTX-ACAATTTTTAATAXGGAXCTTGACATXXAXGTTT--CATTTTTAA
                                      35290     35300     35310     35320     35330     35340     35350     35360
Human                       AAATATTTGAACAGGTAGTACAGCACAAAAGATACACAGATGGTAAGTAAGCACGTGAAAAAGCTGGTCAGCATCATTAG  34196
GuineaPig                   GA-CACTTTGGTAGTCACTA-TGCATATATTGTC-ACATTTAATTCTAAGGATTGTGATACAGTTGTT---CATGTTTTA  16481
NorthernAmericanDeerMouse   -----------------------------TTGGT-TTTATTTTTAATGTGGAGCTTCACATTAGCGTTT--CACTTTCAG  15522
Mouse                       CCCAAGTGTTGGGATCAAAA-GCTCCATATTTTT-AATATTTTTAATATGGAGCTTTACATTAACCTTTA-CATTTGCAA  19889
ChineseHamsterGHOK1GS       -----------------------------TT-------ATTTTTAATGTGGAACTTCACATTAACTTTT--CACTTTGAT  17419
LongTailedChinchilla        GG-CACTTTGGTA--------TGCATATGTTGTA-ACATTTAATTCTAAGGACCATGATACAGATGTTATCCATATTTTA  17480

Majority                    XAXCAAATAAATTTXATGCXCXTA---ACCGXTAAXXAXAAX-AAXAXAATTXATXACAG-AATTAGAATTAGAAAXXXA
                                      35370     35380     35390     35400     35410     35420     35430     35440
Human                       TTATTAGGGAAATGAAAATTAAAACT-ACAGTGAGATAATAT-TACAAACCTATTAATAGTGACTAAAATTAAAAAGAGT  34274
GuineaPig                   CAAATAAA---TGTAAGGCTAAGG---ATGGTTAAATATAATTAACACAGTTGATAAATAAAAACTGAATTCCAGAGCTT  16555
NorthernAmericanDeerMouse   TATCAAATATATTTCATGCACTTATCCACCACCAAGAACAAA-AAAA--ATTCATGGCAG-AATTAGAACTAGATAAACA  15598
Mouse                       CATCAGATACATTTCATGCACTTATCCAGCGCTAGGAAGAAA-AAAATGATCCAGGGCAG-AATCAGAATTAGAAAAAAA  19967
ChineseHamsterGHOK1GS       TATCAAGTAAATTTCATGCACTTA---GCCACAGAGAACAAA-AAAA--A---ATGACAA-AATTAGATATAGAAAACCA  17489
LongTailedChinchilla        CAAAAGAAGAATTTAAAACTTAGG---ATGGTTAAATATAAT-GACACAGTTGGTAAGTGAAAGTTGAATTCCAGAGCTC  17556

Majority                    AXXTTGTTTTATATTTAXCTTCTTTTGXAAATCATGTTCAXTXACTGAAATXAGTXXXXCATCATXCTX---XTXGTTAT
                                      35450     35460     35470     35480     35490     35500     35510     35520
Human                       GCCATCTT----GGAAAGCATGAGTTGGGAAGAATGTGGAAGAATGTTAAT--A-TTCACAATACTGCTG--ATGGGAAT  34345
GuineaPig                   TTTTTTTTTTTAAGTTAGAGTGTATTGAATACTTAACATGAAAACAAAAAC-AGCTAGACAGAATTTTA---GTTGATA-  16630
NorthernAmericanDeerMouse   A--TTGTTTTATATTTATCTTCTTTTGTAATTCACGTTCATTGCCTGCTTTCAGTCCTTTCTCATCC-------CCTTAC  15669
Mouse                       AAATAGCCTTATATTTATCTCCTTTTAGAACTCATGTTCATTGTCTGAATTTAGTCCATATTCATCCTCTAAATCCTTAT  20047
ChineseHamsterGHOK1GS       A--TTGCTTTATATTTATCTTCTTTTATAACTCATGTTCATTGCCTGCTCTTAGTCTTTCCTCATCC-------TTTTAT  17560
LongTailedChinchilla        ATTTTTTT--CAATTCAGCCCATATTGAATACTTAACAGGAAAACAAAAAAGAGGTAGACAAGATTTTTT--GTGGATA-  17631
```

```
Majority                    XTTXAGCCTTXATXCT--GXTCXGTAAXCXGTXACTTTACXTAT-ATXTAAATTXGTTT-----ACCCACTATGCATTTX
                                             35530     35540     35550     35560     35570     35580     35590     35600
Human                       GTAAAATCGTAGTACTTTGGAAAACAATCTGGTAGTTTCTCCATGATCCAGGTTATTT-----G-TCTCCTAAGCATTTA  34419
GuineaPig                   GAGAATGAATAATATA--GATCAGTGGTTCTTAAATTTTAGTAT-GTAAAAATTAGAATCCTGGACCCACCTTGAGTTTC  16707
NorthernAmericanDeerMouse   ATTTAGCCTTTCTTATTGT--CTGTAACATGT-ACTTTACATGC-ATGTACATTTGTTT-----ACACATAA-GCATATA  15739
Mouse                       ATTTAGCTTTTCTTGTGGTCTTTCTAACCTGTTACTTTACCTAC-ATGTACATTTGTTT-----ACCTATTATGTATTGG  20121
ChineseHamsterGHOK1GS       ATTTAGACTTTATT-------CTATTACCCAC-ACGT-ACATTT-GTTTAAACATATGC-----ACGCACTA-GC-----  17619
LongTailedChinchilla        GTGAACCAATAATACA--GATCAGTGGTTCTTAAATTTTAGTAT-ATTAAAATTAGA--------CCCACTCTGAATTTC  17700

Majority                    XTACATXGCACATATGXGAXAGATC-ATGAAGXTXXTTTXCCTXXTGAXTTTXXG--XTGATCTTXCTTAAXACTAX-XA
                                             35610     35620     35630     35640     35650     35660     35670     35680
Human                       CCCAAGAGAAAAGAAGGCATCTATTCATTAA-AAAACTCTCATGTTAATGTTCATAGCC-ATTTTATTTGTAATCAC-CA  34496
GuineaPig                   TTATTTAGTAGGTCTGGGATACATCTAAGAACATAAATTGCTGATGGATTTCCA--TCTGATGCTAGTCTAACGAAC-TA  16784
NorthernAmericanDeerMouse   -TACATTTCACATATGAGAGAGGAC-ATGCAGGGTTTTCTCCTTCTGAGTTTGTG---TGATCTAGCTTAATACTAT--A  15812
Mouse                       CTACATTTCACATGTGAGAGAGA---ATGCAGGTTTTG---TCCTGAGATTGTG---TGATCTTGCTTAATACTAT--A  20190
ChineseHamsterGHOK1GS       -TCCATTGCACATATGAGAGAGAAA-ATGAAGGCTTTTT--CTTCTGAGATTGTG---TGATCTTGCTTAATACTATATA  17692
LongTailedChinchilla        TTATTCAGTAGATCTGGGATACATCAAAGAACATAAATTGCCCATAGATTTCCAGGTCTGATGGTAGTCTAAGGGAC-CA  17779

Majority                    CATTTTXAXXCXAXTT-XX-TCXTXCAAXTTTTTXAG--TTATXTTTCTXTAGAAXTTAGTTT----------XCXATXT
                                             35690     35700     35710     35720     35730     35740     35750     35760
Human                       AAATGTGAAACAAACA----TTCATCAACAGATGAAC--TAATAA----GTAGATTCCTGTTT----------TGGCATCA  34557
GuineaPig                   CACTTTGAAAAAAA------TCTGTGATGCTTACCAG--TTATATCCCTGTAAAATGTAGTT-----------GTCATGC  16845
NorthernAmericanDeerMouse   CATTCCAAGTCCATTT-TTTTCTTGCAAATTTTTTAGGGTTTTTTTTCGTTAGACCTTAGTTT----------TCTATTT  15881
Mouse                       CATTCTAAGTCCATTTATTTTCCTGCAACATTTTTAGCTTCATTTTTCTTTAGGACTTAGTGTGTTTTCTCTCTCTCTCT  20270
ChineseHamsterGHOK1GS       CATTTCAAGTCCATTT-TT--CCTGTAAATTTT-----AGTTTTTTTCTCTAGAACTTAGTAT----------TCTATTT  17754
LongTailedChinchilla        CACTTTGAAAAAAAA-----TCTATGAAGTGTATCA---TTATATCCTTATAAAATCTAACTA---------CGTCATGC  17842

Majority                    XACAXXTTCACATACATTG-----------AXTACA-------AAAXAXACTTGGXTTTXATTATXXATTAACTXXCTGG
                                             35770     35780     35790     35800     35810     35820     35830     35840
Human                       TATGCTTTTCCATACAGTGG----------AATACA-------ACAGCCATGTAGAATGAGCTATTGATAGACACAACAG  34620
GuineaPig                   CAAATCTTCA-ATACTTTG-----------ATTACA-------AATTAGATGTGGTGTTAATAATGTATTAATG---TGG  16903
NorthernAmericanDeerMouse   TACATGTGTACATACAT---------------------------------T---TTTCATTATCCATT---TATCTGC  15920
Mouse                       CTCTCTCTCTCTCTCTCTCTCTCTCTCTCCTGTGTATGTGTGTGAACATACTTGTATATCACTATTATCCATCCTTCTGT  20350
ChineseHamsterGHOK1GS       TGCACGCACACACACACACACACACACACACACACACACACACACACACACTTAC-TTTCATTATCCATTAACTAGCTGT  17833
LongTailedChinchilla        CAAATCTTCCCATACCTTG-----------ATTACA-------AATTAGACTTGGTATAAATAATGTATTAATG----TGG  17900
```

Majority	XGAXAXACAXXTXG-TTAATTTCTATTTTTT-GTXACAGTTXACAAAGXAGXAGTAAACATGXTXTACAXAT-CXTTXAX	
	35850 35860 35870 35880 35890 35900 35910 35920	
Human	CAAGAATGAATCTC-CAAACAATTATGTTAT-GTAAAAGATGGGAGAAAAAAAGACTACATCCTGTATGATTCCATTTAT	34698
GuineaPig	TTTGAAACTCTTTT-TTCCCTTGTATTTCTC-TAAACATTTGTGGTTGATGAA-----CACTTTTTAAAA---CATTCAA	16973
NorthernAmericanDeerMouse	TGATAGGCAGCTAGGTTAATCCCTATTATTT-GTTATGATGAACAAAGCAACAGTAAACATGAGGTGCAGAT--------	15991
Mouse	CGATGGACACCTAGGTTAATTTCTGTTGTTT-GTTAAAGTAAACAAAGCAGCATTAAACATGGGGTACAGATCTTTTTAT	20429
ChineseHamsterGHOK1GS	TGATAGACAGCTAG-TTAATTCCTATTTTTTTGTTACAGTGAACAAAGCAGCAGTAAACATGGATTGCAGAT--------	17904
LongTailedChinchilla	CTTGAAATTTTTTT-TTATTTTGTATTTCTC-TAAACATTTGACACTTAGGAAGAAAACACTTTTAAAAA---CGTACAA	17975

Majority	AGXA---------X-TCXTTGGGXATXTATGCTXAGGXGTXAXATXTTC--------ATAGTTCTTT-GTTAXTTTAX--	
	35930 35940 35950 35960 35970 35980 35990 36000	
Human	ATAAA--------ACT-CTGGGAAATGCAAACTAATCTAAAGCAAATTC--------ATGGTTCTT--AGGAGTTTAAGA	34759
GuineaPig	AGGA-------------CTTATAAATTT---CTTTGGTCTACCATTTTC--------ATTTTTATT--GTTAGTTTAAAC	17027
NorthernAmericanDeerMouse	---------------TCTTTGGGTATGTATGCTGAGGAGTGAAATTCTA--------ATAGTTCTTTCGTTAACTTT---	16045
Mouse	AGAAGGATAGGGCATTCTCTGGGTATCTATGCCAAGGAGTGAAATAGTA-----------GTTCTTT-TTTAAGTT----	20493
ChineseHamsterGHOK1GS	---------------TCTCTGGGTATATATGTTGAGGAGTGAAGTAGTGGGTTATATGTAGTTCTTT-GTTAACTT----	17964
LongTailedChinchilla	GGGAG--------ATGACTTATGAATTT---CTTTTGTTTACCATTTTC--------ATATTTATTT-GTGAGTTAAAAC	18035

Majority	GTAG---AGAAATCTCXAAACXATTTXCCA-------CAXXATAGCTGTGX--TAGTTTGCATCCCTAXCAAXXGXXAAX	
	36010 36020 36030 36040 36050 36060 36070 36080	
Human	GTGGGAAAGGACAGGCAGGAGGGGTGGCAAAGGGACACAGGAAAACTCGGGAGTGATGGACATATAAATTATT-GTGATG	34838
GuineaPig	TCAGCCGATAAATTTAAACACAAATCACC----------ATAAATCTTAA-----------------------------	17067
NorthernAmericanDeerMouse	GTTG---ACAAATCTCCAAACTATTTTCCA-------CACAATAGCTGTGT--TAGTTTGCACCCCTACCAGCAGTGAAT	16113
Mouse	TTTG---AGAAATCTCCAAATGGTTTTCTA-------CA--ATAGCTGTGT--TAGTT--CACCCCAATCACTACCAACA	20557
ChineseHamsterGHOK1GS	GTAA---AGAAATCTCCAAGCCATTTCCCA-------CACAATAGCTGT----TAGTTTGCTTCCCTACCAACAGCAAAT	18030
LongTailedChinchilla	ATAG---ATGAGTTTAAGCACAAATTACCGGGGCTGGGGATTCAGCTCAGCGGCACAGTGCCTGCCTGGCAAG-TGCAAG	18111

Majority	GTGGTGAGXTGGATGTCX---X------GTCCTT--------------------XTTTXTCCACACCCTCTXCAACATTT	
	36090 36100 36110 36120 36130 36140 36150 36160	
Human	GTGGTTTTATGGATGTATGCATATGTCAAAACTTGTCAAATTTTATAATTTAA-ATACATATACACATCCTAGAATGGCC	34917
GuineaPig	---------TTTGGTTTCT---	17077
NorthernAmericanDeerMouse	GGGGTGAGGCGTCAGTCCT-TTTTCTCCGTCCTTCCTTTTTCTCCGTCCGTCCTTTTTCTCCGTACCCTCGCTAACATTT	16192
Mouse	GTGAGTAGA-------------------GTCCTC---------------------TCTTTTCACACCCTCTTCAAAATTT	20597
ChineseHamsterGHOK1GS	GGGGAGAGG-GCAGGT-------------GTCCTT--------------------TTTTCTCCTTACCCTCTCCAACATTT	18077
LongTailedChinchilla	GTCGTGAGTTTGATTCCCG-GTACCAGGGGGCGGGGGACGGGACGGGACGGGA-CGGGACGGACACGACACACAACAAC-	18188

```
Majority                   XX---TXAGAXATTTGTTAATGATAXXCATTXTTACTGAXGTGAGGTXXT---ATXTCXAAGTTGTTTTTATTTGXATTT
                                   36170     36180     36190     36200     36210     36220     36230     36240
Human                      GAACTTCAGAACATTGATAACACCAAATGCTGGCAAGGATGTGGAGCAACAGGAACTCTCACTTACTGCTCAGTGGAATG  34997
GuineaPig                  -----------------------TAGAC--TTCTTTAAATAT-----------ATTGATAGATTATC-TTTAATGAATAT  17120
NorthernAmericanDeerMouse  T----TGAGAGATTTGTTGATGTTAGCCATTGTTACTGGGGTGAGGTGGT---AT-TCAAAGTAGTTTTAATTTGCATTT  16264
Mouse                      GA---TGTGAGATTTGTTAATTATAGCCATTCTTACTGAGGTGAGGTGGT---GTCTCAAAGTAGGTTTCATTTGTGTTT  20671
ChineseHamsterGHOK1GS      T----TCAGACATTTGTTAATGGTAACCATTCTTACTGGGGTGAGGTAAT---AT-TCAAGGTGGCTTTAATTTGCATTT  18149
LongTailedChinchilla       -AACAAAACAACAACAACAAACACAAAT--TACCATAAATTTT----------AAGGTTAATTTGTT-TTTTTTGGACTT  18254

Majority                   CXT-X-TGTCTAGGGAXXTTGAAXX-CTTTTXTAACTATTTATTGGXCATTTTTATAXTTACXTTTAACAXTTTXCAXTC
                                   36250     36260     36270     36280     36290     36300     36310     36320
Human                      CAGAGTGGTACAGCCACTTTGGAAG-ACAATTTGGCAATTTCTTAGAAAACTATACGGCCGGGCACAGTGGCTTACGCCT  35076
GuineaPig                  T-------TTTCA---TTTTAATAG-TTGCCTTTTTTAT--ACTGATTAGTATAAT--TCACCTTT-----TTTATACTG  17180
NorthernAmericanDeerMouse  CTTTGGTGGCTAGGGATATTGAATA-CTTTTAAAAATATTTATTGGACATTTTTTAATTT---TTTAACATTGTTCATTC  16340
Mouse                      CCT---TTACTAGGGGCATTGGATG-CTTTTATA--TAGTTACTGGTCATTTTCATTTATTTTTTGAACACTGTTCATTC  20745
ChineseHamsterGHOK1GS      CTTTGTTGGCTAGGGATATTGAATA-CTTTTAAAACTAGTTATTGGACATTTTTAAAATTAATTTTAACACT--TCATTC  18226
LongTailedChinchilla       C-------TTTAAATGCTTTGATAAATTATCTTTACTATGTATTTTTCATTTTAATAGTTACGTCCACC--TTTATACTG  18325

Majority                   TTTXXTT----GTTTTGATXAGCTXXTXTXXXTATXTXTTTAGAXTGCAATTTCTTXAAXXCTTTGTAXATXCTATXTAT
                                   36330     36340     36350     36360     36370     36380     36390     36400
Human                      GTAATCCCAGCGCTTTGGGAAGCCAAGGCAGGCGGATCACCTGAGGTCAGAAGTTCAAAACCAGCCTGGCCAACATGGAG  35156
GuineaPig                  ATTAGTT----TTTTACATCAGCTAGTGTAAATATATTTTCAGAATAC-------TAAAACATCAGTAGTTAGTTTGAAT  17249
NorthernAmericanDeerMouse  TTTGCTTT---GTGTTGATTGGTTGTTTTTTTTATTTCTTTAGTGTGAAATTTCTACAGTTCTTTGTAAATTCTAGATAT  16417
Mouse                      TTTGCT-------------A----GTTTTTG--ATTTTCTTAGCATGTGATTTCTTCAATTCCTTGTAAATTC-AGATAT  20805
ChineseHamsterGHOK1GS      TTCGTTAT---GTGTTGATTGGTTGCTATTT--ATTTCCTTGGTGTGTAATTTCTGCAGTTCTTTGTAAATTCTACATGT  18301
LongTailedChinchilla       ATTAGTT----GTTTAAATCAGCTAGTAGAAATACATTTTTAGAATACCAGTAACTAAAACATCAATGGCTAGTTTGAGT  18401

Majority                   AAXCXCTTXTXTXAAGCATAGTXXTTAAAXA---TTTXCXX------XXXXATXTTGTTGGXTXTTXATTCTXXTXATTX
                                   36410     36420     36430     36440     36450     36460     36470     36480
Human                      AAACCCGATCTCTACTAAAA--AATACAAAA---TTAGCTAGG----CGTGGTGGTGCATGCCTGTAATCCCAGCCACTC  35227
GuineaPig                  AACTGTAGTTTGTAAA-ATG-GAATTTATAA---CTTATCT------CTAAAGGATTTTAGGACATGATTC-AGTTATTT  17317
NorthernAmericanDeerMouse  AGACACTTGTCTGAAGCATAGTTG-TAAAGA---TTTTCT----------CGTTTTGTGGGGTGTTAATCCTGCTGATTA  16483
Mouse                      AACCCCTTGTCTGAAGCATAGTTGGTAGAGATTTTCTCCCATTTTGTTGGCATTTTGTTGGCTGTTAATTCTGCTAATTG  20885
ChineseHamsterGHOK1GS      AGACACTTGTCTGACGAACAGTTGGTAAAGAATTTTCTCT----------TAATTTGTGGGCTATTGATTCTGCTGATTA  18371
LongTailedChinchilla       AACTGCAGTTTGTAAACATAAGAATTTACAA---TTTGCCC------TTAAAGGATTTTAGGCCATGATTC-AGTCATTT  18471
```

```
Majority                    TTTCXXTTGTTGXAX-AGAAACAT--TTXXTTCCATXXAGTGCCGTXTGTTGA-ACTTGXGXTTAGTTCCAATXXTGTTA
                            36490 36500 36510 36520 36530 36540 36550 36560
Human                       AGGAGGCTGAAGCAGGAGAATTAC--TTGAACCCAGGAGGTGGAGGTTGCAGTGAGCCAGGATCACGCCCATTTCACTCC 35305
GuineaPig                   TA----TTTTTGCAA-AGGAACTT--CTCATTCCATAGATTTTTGCTT--------------TTACTTTCAATTTTTTAA 17376
NorthernAmericanDeerMouse   TTTCTTTTGCTATAT-AAAAACAA-----TTTTTATGTTCTCCCATCTGTTGATACTTGAGACTAGTTCCTATGCTGTTA 16557
Mouse                       TTTCCTTTGTTGTAT-AGAAACCTAATTTTTTTCATACAGTGCCATCTGTTGATACTTGGGGTTAGTCCCCGTGTTGTTG 20964
ChineseHamsterGHOK1GS       TTTCTGTTGCTGTAC-AGAAACAC-----TTTACATGCCGTCCCCTCTGTTGA-ACTTGAAGTTAGTTCCTGTGCGGTTA 18444
LongTailedChinchilla        TA----TTTTTGCAG-AGAAATGT--TTGATTCCATAAATTGT-GCTT--------------TTATTTTTAATTTTTTTG 18529

Majority                    -XTCTXTGATTTAAAAAGTT--TTTXATTTCAAXGGTXTXTTGAATGXTXTXTCCAGTXGTTTXXTTTACAXGTATTT--
                            36570 36580 36590 36600 36610 36620 36630 36640
Human                       AACCTGGGCAACAAGAGCGAGACTCCATCTCAAATATACTTTCATTGTATGATCCAGCAGTTGTGCTCCCTGGTATTTGC 35385
GuineaPig                   -ATCAGTGATATATACCCCT-----AATTTTAAAGGTGAT---GGTGTTTAACTCATCAGTATGAAATTCAAATCTT--- 17444
NorthernAmericanDeerMouse   -GTGTTTAATTTAAAAAGTT--TTTGCTTACCCCAGCATCTTGAAGACTATCTCCTGTGGCTTCTTTTAAAGGTATTT-- 16632
Mouse                       -GTATTTTATTTCAAAAGTT--CTTTTTTACTCTGCTGTCTTGAAAGCTATCTCCCATGG-----TTTACCAGTGTTT-- 21034
ChineseHamsterGHOK1GS       -GTGTTTAATTTAAAAAGTT--TTTGTTTGCCTCAGCATCTTGAAGGCTATCTCCTGTGGCTTCTTTTAAGGGTATTT-- 18519
LongTailedChinchilla        TATCAGTGATGTATACATCTG-TTTAATTTTAATGGTGAT---GATGTTTAATTCATAAGTACGAGTTTCAAATTTT--- 18602

Majority                    -TAAAXXTGGXXAXATGTTAXATTAXXTXAATGTTAGT---XTXAAAXTTTATXXAACCXTTXXTTXCAXATTTTAAXTX
                            36650 36660 36670 36680 36690 36700 36710 36720
Human                       CTAAAGGAGTTGAAAACTTAGTCTGCGCAGAAAGTAGCAC-ATGGATGTTTATAGCACCTTTCTTTATAGTTGCCAGAAC 35464
GuineaPig                   ---AAGGTCCTGAGTT---TGAATAAATAACGCCTTTTGGGATGAAAGATAACAGAATTTTTTTTGAGA------AAATC 17512
NorthernAmericanDeerMouse   -TAAATATGGCCATGTGTTACATTATTTGCATGTTAGT----TAAAATTGTATGAACCCATTCCTTGCACATTTTAATTT 16707
Mouse                       -TAA-TATGGCCACATGTTCCATT---TGCATGTT-GT----TAAAATTATATGAACACATTTCTTGCACATTTTATTTT 21104
ChineseHamsterGHOK1GS       -TAG-TATGGCCACATGTTACATTATTTGAATGTCAGT----TAAAATTA-ATGAATCAATTCCTTGCATATTTTAATTT 18592
LongTailedChinchilla        ---AAGGACCTGATTTATCTGGATAAGTAAAGGCTCCTGGGATGGAAGATAACAGAATATTTTTTGAGA------AAATC 18673

Majority                    TTXCTCTTGTTCTTCTXG---TCTTT------GTTGATTGXTGAAAGXCCTTTCAGXAATATXTTXXTAAXAXXAXXCTA
                            36730 36740 36750 36760 36770 36780 36790 36800
Human                       TTCATAGCAACCAAGAAG---TCCTTTAGGAGGTGAATGGCCAAATAAACTGTGGTGTATATTCAGACAGTATAATGTTA 35541
GuineaPig                   TAGCTGCTGTGTTTTGAA---TTTTT-----TGTAAAAATTTGAGATAGATTTCCAAAACTTT-TGATTATGGGAC-CTA 17582
NorthernAmericanDeerMouse   TT-CTCTTGTTCTTCTGGCTATCTTT------GTTGATTGGTGAAAGCCCATTCAGCGGTATGTTTGGGAAAACAAACTC 16780
Mouse                       TTTCTCTTGTTCTTCTGGCTGTCTTTT-----GTTGATTGTTGAAAGCCTGTT--GGAATATGTTTGGAAAACAAGCTTA 21177
ChineseHamsterGHOK1GS       TT-CTCTTGTTCTTCTG----TCTTT------GTTGATTGGTGAAAGCCTGTT-AGCAATATGTTTGTAAAAACAAACTT 18660
LongTailedChinchilla        TAGCTGCTATGTTTTGAG---TTTTT-----GGTAGAAAACTGATACAGCTTTCTAAAACTTTCTGATTATGGTAC-CTA 18744
```

```
Majority                      AXXTTCTTCTTTCCCATATCCAGTGGCAXXCAAXAXAGAACATTTTACTGACXXXATXAAGTTXXAAGGCTTXTTCTTAC
                                        36810     36820     36830     36840     36850     36860     36870     36880
Human                         ATCAGCACTAAATGGAAATGAGCTATCAAGCCATGGAGAACCTTCAGTGAACATTACTAAATAAAAGAAGCCACTCTGAA  35621
GuineaPig                     ATC-------------TATATAAAGCCA-GCAAT--ATAACATTTGACATTAATTTTTAAGTGAATATTTGATATGTTAC  17646
NorthernAmericanDeerMouse     ACTTTCTTCTTTCCCATACCCAGTGGCAGTCGACACAGAAGGCTTTCCTGACCCCAGAAGGTTTGAAGGCTTCTTCCTAC  16860
Mouse                         ACTTTCTTCTTTCCCATACCCAGTGGCAGTCAACACAGAG-----TACTGACCCCATAAAGATTGAAGGCTTTTTTTTTC  21252
ChineseHamsterGHOK1GS         ACTTTCTTCTTTCCCATAACCAGTGGCAATCAACATGGAAGACTTTTCTGACCCCAGAAGGTTTGGAGGCTTCTTCCTAT  18740
LongTailedChinchilla          ATCATTTTTTCCTATATATATATTGCCA-GTAGT--ATAACATTTGACATTGATTTTTAAATGAATATATGAAATGTTAC  18821

Majority                      AAGCATXCAAGTXAT-AAXTXTTCAAATAX--A--------TGATATTCTCXAAATAACXTXXGAXXXXAXATCTGGXGA
                                        36890     36900     36910     36920     36930     36940     36950     36960
Human                         AATTCTGCATACCAT-ATGATTCCAACTAT--A--------TGACATTCTGGAAAAAGCAA--AATTATGGAGACAGTAA  35688
GuineaPig                     A----TGTAAGTCGC-AAGTTTTTAGAT-------------TAATATTTTTGAAT----------TTAAAGATGTCTTAC  17698
NorthernAmericanDeerMouse     TGGCAAACAAGTAATTAATTCTCCCATAACAGATACCAGTTGGGTGTCCTCCAAATAACTTCTGACATTGTACCTGGAGA  16940
Mouse                         CAGCAAACAA----T-AATTCTTTAATAAC--A-----T-----TATTCTCCAAATAACTTCTGACATAATCCCTGGAGA  21315
ChineseHamsterGHOK1GS         TAGCAGACAAGTAATTAATTCTTCAAATATAGA--CCAGT-GGGTGCCCTCCAAATAACATCTGACATTTTATCTGAAGA  18817
LongTailedChinchilla          A----TGTAAGTGAC-AGGTTTTCAGAT-------------TAATACTTTTGAAT----------TTAAAGATGTCTTGT  18873

Majority                      AAGXCTTXGATTGCAXAGGTTXAGGGTTCGAXXTTCAGATXAXXTTCXATCTTGCXXTGCCTXTCAGAXXXCXCTXXATX
                                        36970     36980     36990     37000     37010     37020     37030     37040
Human                         AAATCAGTGGTTGCTAGGGTTTAGGGGTGGAGGGAGGGATGAACTGGTGGAGTACAGAGGATTTTAGGGTACTGAAAATA  35768
GuineaPig                     GCTTATTTTTTTGTGTGTGTTTATAGA--GAGGATCTGACGAACTACTCTCAGGCAGTG---TTCTCAGTAGTCCAAAC-  17772
NorthernAmericanDeerMouse     AAGCCTTAGATTGCACAGGTTGAGGGTTCAGTCTTTAAATTTCTTTCCATTTTGAGATGCCTGTCAGAAGCCCCTGG-TG  17019
Mouse                         AAGCCTTAGATTCCATAGGTTGAGGGTTCCGTCTTCAGATT-CTCTCCATCTTGGGATGCCTGTCAGAAGCCCCTGGGTT  21394
ChineseHamsterGHOK1GS         AAGCCTTAGATTTCACAGGTTGAGGGTTCTATCTTCAAATTTCTTTCCATTTTGGGATACCAGTCAGAAGCCCCTGG-TT  18896
LongTailedChinchilla          GCTTGTTTTTTTGT----GTTTATAGA--GAGGATCTGACGAACTACTCTCAGGCAGTG---TTCTCAGTAGTCCAAAC-  18943

Majority                      -TTTXGCATXXCTTTXGAATGXXXGGTXCXCXXAX-XXATXTTXCTGAGXTCXTTTAATTTGXXAAAGAAXAGGACTTAX
                                        37050     37060     37070     37080     37090     37100     37110     37120
Human                         -TTCTGCATGATACTATAATGATAGATACATGTCATACATTTTTCCAAACCCATAGAA--TGTAAGACACTTGGAGTGAA  35845
GuineaPig                     -TCTAATATGAG--CGGCATGGTAGTCACAGGTAA-GCATTATTCT--------------TGTGATGTGATGGGGCACAA  17834
NorthernAmericanDeerMouse     GTTTGGCATTGCTTT-GAATACGTGGTTCCCACAG-TTATCTTACTGGGTTCGCTTAATTTACTAAAGAAAAGGACTTAG  17097
Mouse                         TTTTTGCCTTGCTTTTGGATGTGTGGTTCCCACAG-CTGTC---CTGAGATCACTTAATTTTCTAAAGAAAAAGACTTAG  21470
ChineseHamsterGHOK1GS         GTTTGGCATTGCTTT-GAATGTGTGGTTCCCACAG-TTGTCTTCTTGGGCTCCTTTAATCTACCAAAGAAAAGGACTTAG  18974
LongTailedChinchilla          -TCTAACATGAG--CGGCATGGTAGTTACAGGTAA-GCATTCTCTTAACAGCCTAGCATTTGTGATGTGATAGAACAGAA  19019
```

```
Majority                    AATTTXGTTAAXTATAGGATGX--AGAGXTGCTCAAGXTGAGAGAGX--GGXTGTAGAGCTTTTCTAXGXXTGCGAXCTC
                                     37130     37140     37150     37160     37170     37180     37190     37200
Human                       CCTGGAGGTAACTACAGACTTT--GAGT--GATTATAATGTGTCAGT--GTAGGTTCATCAGTTGTAACAATGTGCCATC  35919
GuineaPig                   AATTAAG---ACTA------------------------------------------------------------------  17845
NorthernAmericanDeerMouse   AGCTTTATTAGGGATCGGATGAAGAGAGGTACTCAGGGTGAGAGAGAAGGGCTGTAGAGCTTTTCTAGGTGTGCTGTCTC  17177
Mouse                       AA---TGTTAAGATTAAGATGA--AGAGGTGCTCAAGATGAGAGGG---GGATGTAGAACTTTTCTACGGAGCCATTCTC  21542
ChineseHamsterGHOK1GS       AGCTTTATTAAGGATAAGA-----AGAGATGCCCAAGGTGAGAGAGAAGGGTTGTAAAGCTTTTCTAGGTGTGCCACTCC  19049
LongTailedChinchilla        AATTCAT---ACTAGGGGCTGG--GGATTTAGCTCAGCGGCAAAAGC--GCCTGTCTGGCAAGCGCAAGGTCGTGA-GTT  19091

Majority                    CAGXXATCXCTACAXXTTAAAA-XXGGAAACTATCTGAGACTAGTXCTXTXXAXATTTATATGGAXX------GGCTTCA
                                     37210     37220     37230     37240     37250     37260     37270     37280
Human                       TGGTGGGAGATGTTGATAAAGG---GGAGGCTATTTGGGGCAGGAGGCAGGGAAATCTCTCTGCTTTCCTCTCCATTTTG  35996
GuineaPig                   ------------------------------------------------ACGATAATCTATACCACAA-------------  17864
NorthernAmericanDeerMouse   GGGGAATCTCTACATGTTAAGT-TTGGAAACTGTCTGAGACTAGTCCTTTT-AGAGTTTTATGGA--------GGCTTCA  17247
Mouse                       CAGAAATCTCTACACACTAAAAGTTGGAAACAATCGAAGACTAGTCCTTTTTGGATTTTTATGGACGA-----GGCTTCA  21617
ChineseHamsterGHOK1GS       CAGGGTTCACTACGTTTTAAAC-TTGGAAACTGTCTGAGACTAGTGCTTTT-AGGGTTATATGGA--------GGCTTCA  19119
LongTailedChinchilla        CAGTTCCCGGTACCGGAGAAAA---ACCAAAAAAAACCAAAAAAAAAAAAGAAAATTCATACTAAAACTAATCTACACCA  19168

Majority                    TTAXATAXGTXXXTTTGATCXAAXCATT-GGCTTTXAXTXATXAXXTXAACXTTTA--TXXCXTCXC-TXTTXATCTTTC
                                     37290     37300     37310     37320     37330     37340     37350     37360
Human                       CTGTGTACCTAAACTTACTCTGAAAAAT-GTCTTTGAAAATTTACTCATGCGCTTGCATTGCATTTC-TTTTGGATATTA  36074
GuineaPig                   -AATAGCCTGTATTTT----TAAATGCT-GTGTTGAAACATTT---TAG----------TGCATGTC-CTGTGACCTCAC  17924
NorthernAmericanDeerMouse   TTACATAGGCATGTTTGATCAAACCATTTGGCTATTGGTGATCAGCTTAACATTTA--TCCCCTCCC-TCTTCATCCTTG  17324
Mouse                       TTACATAGGTTTATT-GACCAAACCATT-GGCTGATGGTGATCAACTTAACCTTT---CCCTGTCCC-TCATCATCCTTC  21691
ChineseHamsterGHOK1GS       TTACATAGGCATGTTTGATCAAGCCATT-GGCAGTCAGTGATCAAATTAATCTTTA--CCCCCTCCCCTCTTCATCTTTT  19196
LongTailedChinchilla        CAATAGCCCTTATTTT----TAAATGCT-GACTTGAAACAGTT---TAGAGG---ACATTGTGTGTC-CTGTGACCTCAC  19236

Majority                    XTXTTCTXCCAGTGC----XTTCTATCAXXAACCTGCXTTGAXXTTTCCAXAXAXCA-GCCATXTXTTTAGTXTCATTTX
                                     37370     37380     37390     37400     37410     37420     37430     37440
Human                       CTGGTCTAAGTAGTCCA--GTTAAAAGACAAAGATTGTCAGACTTGATTAAAAAGCAAGGCCTAACTACACTGTCAATAC  36152
GuineaPig                   ACATGCTACACAAGC----ATTCCACCACAGAACTGTTAC--CTTAGCCCTAAAATA--CTATATTTTTATTTCTAGCTG  17996
NorthernAmericanDeerMouse   GTCTTCTTCCAGT------TGCTAATCTTGAACCTGCCTTGAGCCTTC--ATCA--A-GTCATCTTGTTAGTATCATTTT  17393
Mouse                       TTGTTCTTGCAGTGA---CCAACTATCTTGAACCTGCCTTGAGCTTTCCACTCATCA-GCCATCTTGGGAGTGTCAT---  21764
ChineseHamsterGHOK1GS       GTCTTCTTCCAGTGATAGCCTGCTATCATGAGCCTGCCTTGAGCCTTCCAGCCATCA-GTCATTTCATTAGTATCATTCT  19275
LongTailedChinchilla        ATATACTACACGAGC----ATTCTACCACAGAGCTATTCC--CTTAGCCCTAAAATAT-CTTTTTCTTTGTTTTTAACTG  19309
```

```
Majority                      TATGAXTA--TTTAAXAGTTTTAXGCXAAAAAATGGAAXTTAAGACXTTGTGXGTATXX--ATGTGXACAXX-GTATATX
                                       37450     37460     37470     37480     37490     37500     37510     37520
Human                         GAGGGTCA--GGTAGAAATATCAAGATGCAGGTTGAAAGTAAAAAGATGGAAAATGATA--TTCCGTGCAAA-CAGTAAG 36227
GuineaPig                     CATTATTA--GTTAATCTTTTTAAACTAAAAAATATTATTTGACACATAGCAATTATAC--ATACTTATGGG-GTATG-G 18070
NorthernAmericanDeerMouse     TATAAGGATTTTTCAGAGTTTTATGCCAAAAAATGGAAATGAAGACCATGTGTGTATGCGTATGTACATATACATATATT 17473
Mouse                         -GAG------TTTTAGAGCTGTATGCCAGACAACTGAACTGAAGACCTTGTGTGTAT----ATGTGCACATGTGTATATT 21833
ChineseHamsterGHOK1GS         AATAAGAATTTTTTC-AGTTTTATGGCAGAAGATGGAAACTAAGACCATGTGTGTATATGTATGTGCACACATACATATT 19354
LongTailedChinchilla          TGTGACTA--TTTAATTGTTTTAAATTAAAAA-TAT-ATTTGACACATA---AGTA-----ATATTTACAGG-GTATA-G 19375
```

```
Majority                      XXCAATATCATXCAXA----------------CATXTTATAATATTTAATXAXCATATTTXAG-X----------AXTXX
                                       37530     37540     37550     37560     37570     37580     37590     37600
Human                         CACAAGAAAGTTGATA----------------TGA-TTATATTACTAGACAGAAAAATTTAAG------------ACAAA 36278
GuineaPig                     CATAACATT-TCAGTA----------------AAC-ATGTAATATATGATGATCAGATCTGCGTA----------ATTGG 18122
NorthernAmericanDeerMouse     TGCAATATCATACAGA----------------CATTTAATTATCTTTAAT-----TATTGCAA----------------- 17515
Mouse                         CACAATATCACACAGAACTTATTAAACTTACTCATTTTATTAGTTTTAGTAAACTTATTAAAGAATATTGTCTTGAATTA 21913
ChineseHamsterGHOK1GS         TGCAATATCAT-CAGA----------------CCTTTATTCATTTGTAAT-----TATTGCAG----------------- 19395
LongTailedChinchilla          TGTAACATT-TCAGTA----------------AAC-ACATAATATATAATGATCAGATCTGGGTA----------ATTGG 19427
```

```
Majority                      TXXTAGXTGTTACATXAGXAXXTTTGTXACTATATAGTACTAGGATTTAATCXAXXATTTCXTCTTAAXTTTAGTTTCTT
                                       37610     37620     37630     37640     37650     37660     37670     37680
Human                         TATTACCAGATATAAAAAAGCATATTTCACAATGTTGAAGGGTCAGTTTATCAGAAAACATTTCTTAATTTGTATGCCAA 36358
GuineaPig                     CCACATCTGTCACCAGAGAA-ATGTATTATTTCATTGTGTTTAGAATGAATCGAAAA----TTCTTGATTT---TTTTTT 18194
NorthernAmericanDeerMouse     ---TAGTTTTTGCATTGGGATTTT-GTAACTACATAGTACGAGGATTTA--TCAGTGTTTCCTTTTATATTTAGTTTCCT 17589
Mouse                         TTTCAGTTATTATTTGAGGACTTT-GAGACTATGTACTGATAGGATTTTACCCAGTATTTCCTTTTAGATTTAGTTTCTT 21992
ChineseHamsterGHOK1GS         ---TAGTTTTTCCATAGGGATTTTTGTGACTATATATTACTAGGATTTA--CTAGTGTTTCCTCTTAGATTTAGTTTCTC 19470
LongTailedChinchilla          TCGTATCTGTCACCTTAGAA-ATTTATTAT-----------TAGAATACATTGAAAA----TCCTTGATTT---TATTTA 19488
```

```
Majority                      TTTTAGGGATXCTTGAAATATTGAGAXXXTTTTXAXA--XXXTTC---CTGGTGXTGTAAXA--ATGATCT---------
                                       37690     37700     37710     37720     37730     37740     37750     37760
Human                         TAATAACAAATCAAAGTATATTAAGAAAAACATCACAG-AACTAA---GTAGAGAAACAAAA--ATCCACA--------- 36423
GuineaPig                     TTTTT-----TTTTTTTTTTTTGAGATCAGT-TCTCA------------CTGTGATGTTCAG--GTTGTC---------- 18244
NorthernAmericanDeerMouse     TTGTAGGGATCCTTGAAATT----TTTTTTTTTTAAAAGAAGTTC---CTGGTGGTATAAGA--ATGA--T--------- 17649
Mouse                         TATTAGGGATCCTTGAAATAA----AAATTTTCTAAA---GTTTCCTTCTGCTGGTGTAAGAAAATGATTTCAGCAAGCC 22065
ChineseHamsterGHOK1GS         TT-TAGGGATCCTTGGAATAAAGATAATTTTTTTGAAGAAGTTTCTTTCTGGTGGTGTAAGAACATGATTTCAGCAAGCC 19549
LongTailedChinchilla          TTTTA-----TTTAAAAATGTTGAGGTGAGT-TCTCA------------CGGTGATGTCCAA--GTTGTC---------- 19538
```

```
Majority                    X----X-CTTXAXCTXXT-------TTCXTAAXTGXTTTTTCATCTTCAXTCAXTGGTTXGATGATGTGTAACTXCTAXA
                            37770 37780 37790 37800 37810 37820 37830 37840
Human                       A----T-CATAGCCTGGT-------TTTTTAAAACATAATTTATGGCAATTGATAGGATAGTACAGTCCTAGCCCCCAAA  36491
GuineaPig                   -------CTTGAAGT----------TACTCAAGTGATCTTCCATCTTCAGCCACTCAATAGCTGGAGTTGTAGGCTTATT  18307
NorthernAmericanDeerMouse   -----------------C---------CCTA--TGCTTTTTGTTCTTGGCTACTTGGTCTGATAATGTGTAACTGCTA-A  17700
Mouse                       ACATTTCTTTGATTTGCTTGTATGATCCCTGCTTGTGTTCTCCCCCCCCTCCCCCCTTGTGATGATGTGTAACTGCTGCA  22145
ChineseHamsterGHOK1GS       AAAATTCTTTCATCTTACATTTATGATCCTA--TGCTTTTTGTTCTTGGCTAATTGGTCTGATAATATGTAACTGCCA-A  19626
LongTailedChinchilla        -------CTTAAACTCCT-------TGTTCAAGTGGTCTTCCATCCTCA-TCACTAGGTAGCTGAGACTA-ATGCCTGTT  19602

Majority                    TTGTCACXTCCXXCTTXTGATTCTTXCTGXA---XTGTTTGTXTTATTCAXAGTTXTXCAAXTGTAACTTTTGXCTGTXA
                            37850 37860 37870 37880 37890 37900 37910 37920
Human                       TAAAAAGATACATAATTTGAAAAGTGCTATC---ATGTTGGCTTAACTCACATTTATAAAACTATACCCAGTAACTGT-A  36567
GuineaPig                   GTGTCACAGCCAGCTCTTGAC---TGCT--------TTT---TTTAACCACAAAAATACAGCT-GATTCTTTCATGTT-A  18371
NorthernAmericanDeerMouse   TTGTTATTTCCTTCTTGCTATTCTTCCTGCAGAGCTGTCTGTATTGTTGATAGTTCTTCAAATGTAACTTTTGGCTGTCA  17780
Mouse                       CTTTCCCTTTCTGCTTGGGATTCTTC-TGAG-----GTTTGTAGTTT---TAGTCCTTAAAATGTAACTTTTGGATGTCA  22216
ChineseHamsterGHOK1GS       TT-----TTTCTTTTTGCTATTCTTCCTGAAGGTTTGTCTGTATTGTTGATAGTTCTTCAAATGTAACTTTTGGCTGTCA  19701
LongTailedChinchilla        CTGCCACAGCCAGTTCTTGAT---TGCTGCT---TTTTT---TTTAATCACAGAAATACACCT-TTTTTTTTCATGTT-A  19671

Majority                    XAATTCXXTXATGTGTXXTTXGTXXATTT------------XTGGTXAXTGTTATGTXG-GXTTTTCTAGCATTTAATAX
                            37930 37940 37950 37960 37970 37980 37990 38000
Human                       AAATAACTTCAAGTGTACATAGAACATTCACA---------ATGATAGACCTTATATTGGATTATAAAATAACTCAATAA  36638
GuineaPig                   AAATTGCATCATGTGCCCTTTGTGTATTT-------------TTTTTATGTCTAGGTGG--TTGGTTTCGTGTGTGTGTG  18436
NorthernAmericanDeerMouse   GAATTCTGTTAGGTGTGTCGGCCATTGTTTTGTGTATTTTTATGGGCAGTGTCGTGT---GCTTTTCTAGCATTTAATAA  17857
Mouse                       GAAT-CTACTATGTTTATTGGCT-------------------G-----TGTGATTTAGTGCTTTTCTAGCCTTTAATAG  22270
ChineseHamsterGHOK1GS       GAATTCTGTTAGGTCTGTCAATT----TTTTGAGTATTTTTATGGGCAGTGTGATGTAGTGCTTTTCTAGCATTTAATAG  19777
LongTailedChinchilla        AAATCTCTTCATGTACCCTTTGTGCATTT-------------TTCTTAAGTTTAGGTTG--TTGTGTTTGTGTGCATGTA  19736

Majority                    GT-TTTAXGTACTCXTXXXXTXATXTXXTT---TCTCTXTX-XAAAATTGAXTAAGTATTCAAXTATATAXTTTTCTGAA
                            38010 38020 38030 38040 38050 38060 38070 38080
Human                       AT-TTCAAATGATTGACAGCTAATGTATCTGACTGACTATAGTGGAATTAAATTAGAAATCAAAAATAGACATCTAGGAA  36717
GuineaPig                   ---TTTA-------TTTGTATCATAAACTT-------------AAAACCTAA--AGTATTCAAGTATATAGTTTTCTTTG  18491
NorthernAmericanDeerMouse   GGGGTTA-GCACTCATACCTGTTCATCTTT---TCTCTCTGTCAAAGATGAGTAA-----------CCTCATTTTCTGAA  17922
Mouse                       GTACTTA-GTGCT-GT--------GTCCTT---TCTCTGTATCAAGGTTGAGTATGTTTTTTTTTTTTTTTTTTTTGAA  22337
ChineseHamsterGHOK1GS       GAGGTTAAGCACTCAT-----TTCGTCTTT---TCTCTGTG--AAGCATGAGTAA-----------CTTCATTTTCTGAA  19836
LongTailedChinchilla        GT-TTTAAGTATTCTTTATATCATAAACTTTAAACTATATT-TAAAATCTAA--AATATTCAAGTATATAGTTTGCTTTG  19812
```

```
Majority                    AGTXTAGT-TTTTCXCTGXAACXTXATATTXATTXTXTXXTTTXAXXTXATGA-XXTTXCTAGXCTGGCATXTTT-AGAX
                                          38090     38100     38110     38120     38130     38140     38150     38160
Human                       AGTACTAAATATTAGAAATTACACAATCGTGAACAATACA-TGGATTATAGAAGTGTTACCACAAGGACATATTTTTGAC  36796
GuineaPig                   TGTGACATGTTTTTGCTGTAACAATATTTTAATTGTTTCAGTTGATTTTCTT--TGTTATATACTTAACAAATTTAAGA-  18568
NorthernAmericanDeerMouse   AGTCTAGT-TTTTCTCAGGAACTTGGTATT-TTTCTCTTTTTCCAGGTTAGGA--TTTCCTAGACTGGCATCTTT-AGAG  17997
Mouse                       AGTCTAGC-TTTTCTCTGGAA-TTGATAGA-GTTTTCTTTTTTCAGGTCATGA--TTTCCTAGGCTTGCATCTT--AGAG  22410
ChineseHamsterGHOK1GS       AGTTTAGT-TTTTCTCTGGAACTTAGTATTGTTTCTCTTTTTCCAGGTCATGA--TTTCCTAGGCTGGCATCTTT-AGAT  19912
LongTailedChinchilla        -GCATCGT-TTTTTGCTGTAACAATATTTTAATTATTTCAGTTGATTTCTTTGATGTTACCTACTTA-CAAATTTAAGG-  19888

Majority                    TXAXXAXTGTCTTGTCTAXATG-TTXGTCTTCAAXCXAXCAGXGXXXATG-XGAXXTXTCAXXTT----TTXTAXTATXT
                                          38170     38180     38190     38200     38210     38220     38230     38240
Human                       TGAATGAAAATATATCAAAATTTGTTGGATACTACCCAAGGGTGTGTATGGTGAAAAGTATGTC-----TTCAAATACCT  36871
GuineaPig                   TGATTTATTTAGTGTTTAAA---TTTAAGTTTCTTTAAATTTTGTATATC-TGAAATATCAGTCA----TTTTATCACCT  18640
NorthernAmericanDeerMouse   TAGGAAGTGTCTTGGCTAGATG-TTAGTCTACAACCCAGCAGGGCTCATG--GACTTCTCAACTT----T-------TTT  18063
Mouse                       TAGGAAGTG-CTTG----GATG-TCAGTCTTCACTCTAGCAGGGCACATG--GACTTTTCAACTTCTGCTTACAGTTTTT  22482
ChineseHamsterGHOK1GS       TAAAAAGTG-CTTGACTAGATG-TCAGTCTTCAACCTAGCAGGGCCCATG--GACTTTTCAACTTCTGCTAAGGGT-TTT  19987
LongTailedChinchilla        TGACTTATTTAGTGTTTAAA---GTTGAGTTTCTTTAAATTCTATGTGTC-TGAAATGTCAGTTA----TTTTAACATCT  19960

Majority                    GXTTGXCAXCTTXGCAATXXTAAGAXTTCTTXTTTAXGAXTGAGT-AXTAXATGGTAXTTXXGTTCAATTATXATAAATA
                                          38250     38260     38270     38280     38290     38300     38310     38320
Human                       ATATTACAA--AAAAAGGTTTAAAACTCCCAATTTAAGACTGACCAAGGAAAAGACATAAATTACCAATACTGGAAATC  36949
GuineaPig                   GT-TAACAATTTATCAATTTTTAGACT----AGTTATGGCTGATTGGCCAAA----TCTTGAGTC---TCATTGTTAATT  18708
NorthernAmericanDeerMouse   GCTTGCCTTCTTGGCAGTCCTAGGAACTCTTCTG---AAGGGAGT-ACTAGATGGTAATTTGGTTCAGTTATGATAAGCA  18139
Mouse                       GCTTGCCTTCTTGGCAACCCTAAGAACTCTTTTTTGCCAGCGAGT-AGTAGATGATA----GGCTCAGTTATGATAGG-A  22556
ChineseHamsterGHOK1GS       GCTTGCCATCTTGGCACCCCTAGGAATTCTTCTG---CAGGGAGT-TGTAGATGGTAGTTAGGTTCAATTATGATAACCA  20063
LongTailedChinchilla        GT-TAACAATTTATAAATTTTTAGACTTT-AGTTTATGGCTGATTGACTAAA----TCTTGAGTGCGATTATTATTAATT  20034

Majority                    GAAGACTATTTAATAACXTXAGAAAAACCCATXCATAXCXXTAAXAT-AATTACAXXXGACTXX-AATTTTTTTTTAATC
                                          38330     38340     38350     38360     38370     38380     38390     38400
Human                       AAGGTCGAGATAATCACTTTCAGAGAAAGTGGATATATGCAGAGTAT-AATAGTATAACATAAA-TAATATAAAGTGGAG  37027
GuineaPig                   GA-----ATGCATTTACCTT---ATAAACCCTACATTT-CACATTGT---TTACCTGAAATTAA-AGTTCTTATTTTCCT  18775
NorthernAmericanDeerMouse   GAAGACTATTTAATAACATAAGAAAAACTCATTCAGACCTTTCAAAT-AATGACAAATGACTTG-GATTTTTTAAAAATC  18217
Mouse                       GAAGACTGATAAATAACAGAAGAAACCCTGACTCGTACCTTTGAAACTAATGGCAAATGACTTGCCATTTTTTTTTGAACC  22636
ChineseHamsterGHOK1GS       GAAGACTGTTTAATAACGAAAGAAAAACCCATTCAGACCTTTCAAATTAATTACAAGTGACTTGCAATTTTTTTTTAATC  20143
LongTailedChinchilla        GA-----ATGAATTTACCTT---ATAAGCCCTACATTT-CACAGTAT---TTACCTGAAACTAA-AGTTTTGGGGTATTT  20101
```

Majority	ATATTAXT--TTXTXACAXAAAXXTTAATA-TCTTGGGAGXAXTAXGTTAATXA--------------XTXAAATXATXA	
	38410 38420 38430 38440 38450 38460 38470 38480	
Human	ATAGCAC---TTTCAATAAAAGCAGAGATA-TATAGAATATAATAGGAAAATAA---------------GTCAAT-ATTA	37087
GuineaPig	GAAATATT--TATTTACGGAACTAACTATA-TTAGGGG-GTAATAGGATCATAA---------------AAGACA-AGAG	18835
NorthernAmericanDeerMouse	ACATTCATTCCTCCGACAAAAACTTTAATCCTCTTGGGAGCATTTTCTTAATTC--------------TTGAAATGGTCA	18283
Mouse	ATATTAAT--TTCTCTCAGAAATTTCAGT--TCTTGTGAGCCTTCTGTAGGATAC-------A-TTTTTTGAAATGATCA	22704
ChineseHamsterGHOK1GS	ATATTC----CTCTGACAAAAACTTTAATCCTCTTGGGAGCATTTTGTTAATTCAGTGGGATAATTTTTTTAAATGATTT	20219
LongTailedChinchilla	TTCCTATA--TCTTTACTGAAATACTTGCA-TTCAGTA-ATAAAAGGTTCATAA---------------AAGACT-AGAA	20161

Majority	GTTXTTTTATX---TAGATXTCAAAGTTTCXCTGXTCATACXTATXTATCXXGAAACATTCAAA-CTAATTTTGACATXT	
	38490 38500 38510 38520 38530 38540 38550 38560	
Human	GTAATGTTGTTA--AGGCTAACAAATTCAGCAACTTAGATGAAAAGTTTCTTGACAAATGCAAAACTGACTTAAGTATAT	37165
GuineaPig	GACACTTTATT------TTCTTC-AGGTGGCTGTGTTATAAATGTGCCTCTTGAAATACATACATACATTTCTGACATTT	18908
NorthernAmericanDeerMouse	TTTGTACTATA--ATAGATGTCAAAGTTTCTCTGACCATACTTATTTACCAAGAAACATTAAGA-C-AATTTTAACATGT	18359
Mouse	CTTGTAGCATA--GTAGATGTCGATGTTTCTCTGGTCATACTCATTTATCAAGAGACATTTAAAGCTAATTTTGACATAT	22782
ChineseHamsterGHOK1GS	TTTGTTCTATATAATAGATATCAAAGTTTCTCTGATCATACTTATTTACCAAGAGACATTCAAA-CTAATTTTGACATGT	20298
LongTailedChinchilla	GACATTTTATT------TTCCCCTAGGTAACTGTTGTGTATATGTGCGTCTTGAAATACACATACATATTTTTGACATTT	20235

Majority	AAATTXXX-AGTTAAATGTXAXACTGGTTXACATATTAXAX---X-TATAGTTTTAAAGATTT----TTCTTAAAGATGX	
	38570 38580 38590 38600 38610 38620 38630 38640	
Human	AGCTTAAC-AGCTATATACTACTGTATTATACAAATTGGAA---TTTATAATTAAAAAAATCTT---TCCACAAAGATTA	37238
GuineaPig	CAGTTAAC----TAAAAATTGTAAAGAAATATTTACCAAA------TATAGTTATAATGATGT---------AAAGGTGA	18969
NorthernAmericanDeerMouse	AAATTTTT-AGTTGAATGTAAGTCAGGTTGACATTTTAGATGAATGAACAGTTTTAGAGATTACATGTTCTTAAGGACAG	18438
Mouse	GAAATTTTTAGTTAAATGTAAAACCAGTTGACATCTTAAGA---TGAACAGTTTTAAAGATTACATATTCTTAGTACAGG	22859
ChineseHamsterGHOK1GS	AAATTTTTTAGTTTAATGTAAGACTGGTTGACATTTTAGAG--------AGTATTA-----------TTCTTAGGAAAGG	20359
LongTailedChinchilla	CAGTTAAC----TAAAATTTATAATGAAGTACTTACCAAA------TGTAGTTGTAATGATTT---------GAAGATGA	20296

Majority	XTTTGATTCTGAATTTXAXXAXXXATTXTATAAXTXTGATTTGTTTXTAGTAGXCACTAXAATGGAAXATGCATCAATTA	
	38650 38660 38670 38680 38690 38700 38710 38720	
Human	CTCTAGTCATAGATGGTTTCACTATTGAATTTTATGAAATTTTAAAGGAATACTAGCTGTCTTTGAAAATGGAGGAGGGA	37318
GuineaPig	ATTTATTAGTGCATCTTACTAAGGATTGCATATTACTAGTTTCTGTGTAGTGATTACAGTGC--AAAAATGGCAAAAC--	19045
NorthernAmericanDeerMouse	GTTTGAGTCTGAATTTAAG------TATTACAAGTTTGATTTGTTTTTAGCAGCCACTACAATGGAACAGCCATCAATTA	18512
Mouse	TTTTGATTCTGAATTTAAAATTTGATTCTAAAAGTTTGATTTGTTTTCAGTAGCCATCA-AATGGAACAGTCATCATTTA	22938
ChineseHamsterGHOK1GS	TTTTGATTCTGATATTAAA------TATTATAAAT--GATTTGTTTTCAGCAGCCACTACAATGGAACATCCATCAATTA	20431
LongTailedChinchilla	ATTTGTTAATATATATTACTAAGTACTAGTT--------TTCCTGTGTAGTGATTACAAAAC--AAAAATGACAAAGCCG	20366

Majority	TTATTATTATXTTCXTTTTATAAXXTCCAAAT---TXAXXXAXTAGGXTGAXXAAGXTATTCXATGTAXAXTTC----AT	
	38730 38740 38750 38760 38770 38780 38790 38800	
Human	ATACTACCCTGTTCACTTTATGAAACCACTATAACTGACAGCAAAACCTGACAAGGGTAATATATCAAGATTTCA--GAC	37396
GuineaPig	TGATTTTGATAATCAAGGTATCATGCACAAAT---TGAGCAAATAAGCT--TAAATGTCTACAAGTCTGCTCTC-----T	19115
NorthernAmericanDeerMouse	TTATCATTATGTGATTTTTATAGCATCCAAGT---TAA-TGACTAGGTTGAGTAAGATATTCCATGTAAAATTCC---AT	18585
Mouse	TTATTATTTTATGGTTTTTATAAAGTCCAAGT---TAAAGAACTAGGTTGACTAAGATAGTTCATGTAAAATTATTGTAC	23015
ChineseHamsterGHOK1GS	TTATCATTGTGTTTTTTTTATAGCATCCAAAT---TAAATGGCTAGGTTGAATAAGATATTTTATGTAAAAT------AT	20502
LongTailedChinchilla	TGATTTTGATAATCAAGGAATCATGTACAAATAATTGAGCAAATAGGCT--TAAGTGTCTTCAGCTGTGCTTTC-----T	20439

Majority	TAGTXXXT-TXATTAXXXTAAAAXCAG-TXTTTTXGAGXCAT---------------TATTGGTAAAXCXTTTTCX----	
	38810 38820 38830 38840 38850 38860 38870 38880	
Human	TAGTATTTCTCATTAACCTAAATGCATATATTCTTAAGAAAA---------------TGTTGGTAAATCACCTCC-----	37456
GuineaPig	TTTTA------ATTAGTACATA-ATAGTAGAATTTGACATGT---------------TTCTGA-AAATAATTTTC-----	19167
NorthernAmericanDeerMouse	TAGTTAATATGGTAATCTTAACATCAG-TGTTGTAGAATCAT-------------AATAGTGGTAAACCTTTTACTTAAT	18651
Mouse	TAGACAGTGTGTTAAACTGAACAGCAT-TGTTGTAGAGTCATTTCTCTTGGTCATGGTAGTGGTAAGCCTTATTCTCT--	23092
ChineseHamsterGHOK1GS	CAGT----------ATTGTAAAATCA--TATCTCATGGTCAT-------------AATATTGGTAAACTTTTTACTTT-T	20556
LongTailedChinchilla	TTTTCTTTTTTATTAGTACATA-ATAG--ATATTTGAAATGT---------------TGTTAA-AAATAATATTC-----	20495

Majority	GAAAXGXTGAGXXCAATTTTTT-----XXAGXXTAGTCTC---X-XXATTTXTCAGTX--TCTTCAXACCXTAAAATCAT	
	38890 38900 38910 38920 38930 38940 38950 38960	
Human	GGTAGAATATGAGAGACAATTC-----TGAGACTTATCGCA----GGATTGTAAGGTT--TGTTTA-ACATTTGAAAATG	37524
GuineaPig	TAAGTTATTAGAGCG-----------------TTGGCCCC--------TTTTCCAGTT--TCTTC---CCCTACAACCAA	19217
NorthernAmericanDeerMouse	GAAAAGGTGAGCACAATTTTCTTTTATAGAATGTAATCTGTATAGATAGTCCTCAGTAATTCTTCACATCTCAAAATCAT	18731
Mouse	GAAAGGGTGAGCATAATTCCTT---GTACAGAACAG-CTGTAGATACAGTCCTCAGTA---CTTGTCAGTTCCAAATAAT	23165
ChineseHamsterGHOK1GS	GAAAAGGTGAGCACAATTTTCT-----GAAATGTAGTCT----AGGTATCTCTCAGTAGTTCTTCACATCCTAAAATCAT	20627
LongTailedChinchilla	AGACTGATAAGAGTAACTGGTA-----GCAGCTTGGCCCC--------TTTTCCAGTT--TCTTC---CCCTAAAACCAG	20557

Majority	AAATA-XTGTGTAXTXTGAC----AAAATAXATAXAAAATXTAXTX--AXCAACAGXTATGAXATAATTXGAT--XXAAA	
	38970 38980 38990 39000 39010 39020 39030 39040	
Human	AACTC--AGTGTAATTTGTC----ACAATG-ATAGAATGAAGAAAG--AAGAATTTCTCTCAGAAAACTAGGA--AGAGA	37593
GuineaPig	AAAAA--TGTTTAATTGCAT----AAAGCACGTAGTACAGCTAATG--AGCAGAAACTATGAGATGAGTATCC--TTAAG	19287
NorthernAmericanDeerMouse	TAATAACTTTGTATTCTGACTGTTAAAATATATATAAAATGTACTC------ACAGGTATGAAGCAATTGGAT---AAAA	18802
Mouse	AAA---CTGTGTATTCTGAC----AGAATATGTGTAAAATGTACTCTCAACAACAGGTATGAAATAATAGGATGAAAAAA	23238
ChineseHamsterGHOK1GS	TAATACCTTTATATTCTGACTGTTAGAATAGTTGTAAAATATACTC------ACAGGTATGAAACAGTTGAAT---GAAA	20698
LongTailedChinchilla	AAAC---TGTCTAATTCAAT----AAAACACACAGTACAGCTGATG--AGCAGAAACTCTGAGATGATTATCC--TTGAG	20626

```
Majority                   AGAXXCTXXTATAXAGAA----AXTATXAGXTAXXTAAAAAGATTTTGCAXTXXA---TAXAXATTTGTTGTAGXGAXAA
                                   39050     39060     39070     39080     39090     39100     39110     39120
Human                      AGGAGCTTTTACAATGTA----AATAAGGGGTATCTACTAAGAACCTACAACAAAT--TTCATACTTAATGGAGAAATAT 37667
GuineaPig                  CAAAG----------GAA----AGCATGAGGTAAATTTTTAAATTATGCATTGGA---TACCTTATTGTTGTAG------ 19344
NorthernAmericanDeerMouse  AGTGACTGCTATATAAAA----ATTTTTAAACACTCAAAGCAACTTTGTCATACATGGTGAAGATTTGTTGTAGGGAGAA 18878
Mouse                      AAAGACTAATATACAGGGTTTTATTTTTTTAATTCCCAAAAGATTTTGCC-------AAAAAGATTTGTTGTAGAGAAAA 23311
ChineseHamsterGHOK1GS      AGTGACCATCATATAAAA----AATCTTAAAAACTTAAAACTACTTCG---------GTGAAGGTTTGTTGTAGGGAGAA 20765
LongTailedChinchilla       TGAAG----------GGA----AGCATGAGGTGAATTTCCTGATTACTCATTGGAG--TACCTTCCTGTTGTAGCTGAGG 20690

Majority                   G---GAGXG----CXTTXTGCXXGCAGAX-XAXTXA--ATTAXAGXCTTTXXTXTXAAT-XACTTAXAATTGGAATTTCG
                                   39130     39140     39150     39160     39170     39180     39190     39200
Human                      T---GAATGTTTTCTTCTTAAAAACAGAAATGAAGC--AAGGATGTCTGTTTTCTCAAT--TTTTCCCCTTAGCTTTCTG 37740
GuineaPig                  -------TG------------GAGCCCAACCAGTAC--AGGAGTCTCACTAAACTGAAGAGAATTGAGATTGGAATTTAG 19403
NorthernAmericanDeerMouse  ----GAGAG----CTTTCTGCATGCAGTG--AGCCA--GTTAGAGGCTTTCCTTTAA-----CTTAGAACAGGGATTTCC 18941
Mouse                      GGAAGAGAGTCAGCCTTGTGCATGTAGAGTTAATTACGAGTAAAGGCTTGGTTTCTCTTTAATTTAGAAGCAGTATTTCC 23391
ChineseHamsterGHOK1GS      GGAAGGGAG----CCTTCTGCGTGCAGTG--AGTCA--GTTAGAGGCTTTTCTTTAAAT-AACTTATAACAGAAATCTCT 20836
LongTailedChinchilla       G---TAGTG------------GAGCCCAAGCAAAGT--ATAGACGTCTCTGATCTGAAGAGACTTTAGGTTGGAATTCAG 20753

Majority                   TACACATTAXXXAGXAGTTG-CTTATGAGCXXGXAGGXTTGXAAAGAAAGX--AAGAXAXTTXAAXXATACAGTXXTTAT
                                   39210     39220     39230     39240     39250     39260     39270     39280
Human                      CTTACATTA--AGGCAGGTGAAATAAAAGACATAAAGATTGTAAAGGAAGA--AAGAAAACTGTCTTATTCACCATTCAA 37816
GuineaPig                  AGCACAT----AAACATCTGTGTTTTGTGG-TGAAGGATTCTAGAGAAAAG--GATC--------TTGTCCAGAAGTTTG 19468
NorthernAmericanDeerMouse  TACCAATTACTTTGTAGTTA-CTTAAGATCGTGTAGGGTGGAAAAGAAAGT---GATTAGTTTAAAGATACAGTGATAAT 19017
Mouse                      TACCCATTTCTTAGTAGTT--CCTATGAACCCCTAGGGT-GAAAAGAAATC--AAGCAACTTGAAAGATACCATGATAAT 23466
ChineseHamsterGHOK1GS      TACACATCACTTTGTAATTA-CTTATGATCTCATAGGGTTGAAAAAAAAGCCAAGAATAATTTAAAGATACACTGCTTAT 20915
LongTailedChinchilla       AACACCTC---AAGCATCTGTGTTTTGTGG-AGAAGGACCCTGGAGAAAAG--GAGA--------TTGTCCAGAAGTTTG 20819

Majority                   GATXCACTTCAXAXAGGATGATGTGTT-TACTXX--TXXXGAXGXTAGGTXATGXXTAGGTTXXTXATGTAXAGX----A
                                   39290     39300     39310     39320     39330     39340     39350     39360
Human                      CATGCTTTTTAAAGTAGAAGACCTGAAGGACTCTA--CAACAGATTTGGTAG-AACTAATAAGTGAATTTAGAAT----A 37889
GuineaPig                  GATGTTGTACAAAGAGCAGAATTTGGT-CATTAC---CAAAAGTTTAGATAATGACTCAGTTACATGTTTGCAG-----A 19539
NorthernAmericanDeerMouse  GATAGACATCTGAAAGGCTGTTGTGTT-TACT----TTTGGAAGCTAGATTATGTGTAGG--GGTAA------------C 19078
Mouse                      ---GCACTTTGGAAAGGATGCCATGTT-TACT----TTTGGAAGCTGGGCTTTGTGTGGGTTAATGGTGGATGGTTCGGC 23538
ChineseHamsterGHOK1GS      ---ACACATCTGAAGGA-TGTTGTATT-TACCTTTCTTTGGAAGCTAGGATATGTGTAGG--GGTGATGAATGTTTAGAT 20988
LongTailedChinchilla       GGTATCGTGCAAAGGGCAGAATGTGGTTCACTTCATCCAAAAGTTTGGGTAATGACTGGCTTACACATGTGCAG-----A 20894
```

```
Majority                    ATCAGACXTTGCXXCTTCTAXAXGGAXXXXTXXCXTCTTTXXGTA----AGXAAAGCATTAXAAXXTGXCXXTTTGXXXA
                            39370 39380 39390 39400 39410 39420 39430 39440
Human                       ATTATAGGATACAAGTTCTATGTACAGAAATAGTCTCCTTATACTTGGCAGCAAATTCTTAGAAAATGAAATTTTAAAAA 37969
GuineaPig                   TTAAGACTTTGCAAGGTTTATC---AGAAATCACCTTCTG-----------TAGGGATTGAGGTAAA-------TGGAGA 19598
NorthernAmericanDeerMouse   ATCAGACACTGGCCCTTCTAAAGGGCTGTGTATCTTCTTTGGGTA----AGGAAAGCACTAAGACTTGCCCCTTTT---- 19150
Mouse                       GTCAGA----AACCCTTCCAAATGGTTGTGTGGCTTGTTTGGGTA----AAGAAAGCACCAAAGCTCTCCAGCCTG---- 23606
ChineseHamsterGHOK1GS       ATCAGACATTGACCCCTCTAAACGGTTGTGTGTCTTCTTCCAGTA----AGTAAAGCAGTAAAACTTGTCTCTTTTGCCC 21064
LongTailedChinchilla        TTAAGACTTTGCAACATTTATG---AGAAATCACCTTTTC-----------CAGGG-CTGGGATAAA-------TGATGA 20952

Majority                    XTCTXT-XGTTTAXCTTTAGXATCAGATATXGXA----TGATTTAGTAAAGGGCTGATXT----TATTCAC-XXTACGAA
                            39450 39460 39470 39480 39490 39500 39510 39520
Human                       ATCTGTTTATAAAAGTATCAGACAAAATATCAGGAAAAAAATTTGACAAAAGATATGTAAGACATCTACACCAATATGAA 38049
GuineaPig                   TTCCAT-GGTTTAACAGT--GCTGAGACCGTGGAGTTCTGGGCATTTAAAATGCTGGTAC----CTTGCAG-AATACCTT 19670
NorthernAmericanDeerMouse   --CCCC-CTTTTACTTTTAGAATCAGATGTGGAA----TGATTTAGTGAAGGGCTGATTT----TATTCAC-TGTAGGAA 19218
Mouse                       ---------TTTGCCTTTAGAGTCAGATGCAGAA----TGATTGACTGATGGGCTGACTT----TATTCAC-TATACGAA 23668
ChineseHamsterGHOK1GS       CCCTCC-CCTTTACATTTAGAATCAGATATGGAA----TGATTCAGTAAAGGGCTGATTT----TATTCAC-TGTGAGAA 21134
LongTailedChinchilla        TTCTGTAGGTTGAACAGT--GTTGAGACATTAGA-----------------------CAT----TTTACAG-AGAACCTG 21002

Majority                    ACXCXCXTXXXATAGXTTGXAGAXAXCTTT--GTGXXXACATAXAXTXGATTXATAAAXX-TATXTTTXTATTTXATAAX
                            39530 39540 39550 39560 39570 39580 39590 39600
Human                       ACACTGCTAAGAGAAGTTTTAAAAAGACTTACATGAAGACGTAGACCATGTTCATGAATTAGAAGGCTCAATATGTTAAC 38129
GuineaPig                   AAGCTCTCAAGATAGACTACAGAAAACTC------AGGACGTAGA--AGAATCATAAAA-----GAATCGTTTTAATAA- 19736
NorthernAmericanDeerMouse   ACTGCCATCCTATAGGTGGAAGCCAGCTTT--GTGTGTACAACGATTTGATTT-TG-----TGTTTTTATAGTTTATAGT 19290
Mouse                       ACCCCAGTCCTGTAACTGGGAGCCAACTTTT-GTGTATACAGTAACTTGGTTTCAACTG--TCTTTTTATATTTCATAAG 23745
ChineseHamsterGHOK1GS       ACCCCTATCCTATAGTTAGAGGACAACTTTT-GTGTATTCAACAATTTTATTTCTCTAGT-TTTTTTTATATTTTATAAT 21212
LongTailedChinchilla        ATGCTCTCAAGATAGACTACAGAAAGCTT------AGGACCTAAA--AGAATCATAAAATA-AAGGATCTTTTTAAGAA- 21072

Majority                    ATXXXACTTATCTTAXAXTTTGAATGTX--X-GATXTATXAXGATXAXTXXATTTXXACAATATTTTXAAATTAXTGXXA
                            39610 39620 39630 39640 39650 39660 39670 39680
Human                       ATGCCAGTTCTCCCTAAATTGGGATGGCATTGAATCTATAAAGACTTATAAATTGAGAGTACATTCTAAAATTTATGTGG 38209
GuineaPig                   GCAACACTTGTCTAAGGAACCAAAAGTA--TAGATCCA-AAAGATGTGAAGGCTTCATCAGTAGTTTAAT-TGTCTGCCA 19812
NorthernAmericanDeerMouse   ATAA-AATTATTTTATATTTT--ATCTT----GATTT-TTGTGTTCAGTTTATT---ATAAGTTCTTTAAATCAGCC--A 19357
Mouse                       TTA----TTATCTTGATTGTTGTATTCA----GCTTTATTCTGATAAATTCATTAATCCAATATTTTTTAGTTATTTTAA 23817
ChineseHamsterGHOK1GS       ATGG-ATTCATTTTGTATTTTACATTTT----GATTT-TTGTGTTCAGCTTATTCTGATAAATTATTTAAATCAGAT--A 21284
LongTailedChinchilla        GTGCCACTTATCTCAGAAATTGAAAGTC--TAAACCTA-AAAG--GCAGAAGCTTCATCAGTAGTTTAGT-TTCCTGCCA 21146
```

```
Majority                    GXATXAAXX-G-XAXTTTAAAAATTAATATC-XX-XATTTAAATACACXCATTTTTAXAAXAGAXAAAACAXTT------
                                      39690     39700     39710     39720     39730     39740     39750     39760
Human                       AAATAAAAAGG-AGCTAGGACAATTAAAACC-----ATTTTGAAAAAGTAAATTTTGCCACAGTTCTCAAGACT------ 38277
GuineaPig                   GAATGAAACC----CTTAAAGAA--GACAAC-----AT-------GAGTTAGTTTTCCTGCAGTTATTACCAAT------ 19868
NorthernAmericanDeerMouse   GTATTATT----TATTTTAAATATTAATGTC-ATCTATGTAAATACACACATTTTTAAAGTAGACAAAACAGTTGATT-- 19430
Mouse                       ATATAAAT--GCCCTTCTATGCTAATATATACATTTTTTTAAATACACACATTGTTAAAATAGACAAAATAGTTGACTTA 23895
ChineseHamsterGHOK1GS       GTATTATTTAGTTATTTTAAATATTAATGTCCATATATTTAAATACACACATTTTTAAAATAGACAAAACAGTT------ 21358
LongTailedChinchilla        GAACCAGACTA-CTCTTAAAAAA--GACAAC-----AT-------GATTCAGTCTTCCTACAGCTAGTATCAAT------ 21205

Majority                    --XACTTTAAATGTTXXXCTAXTXAAATATATXTTTATAAAATA-----CAXGXATTTXCAAXATAXCTXAAACXTCAXT
                                      39770     39780     39790     39800     39810     39820     39830     39840
Human                       --CACTACAAACCTGTAGTTAATCCAAAACAG-TTTACTACTAG-----CAT-AGTTAGTGACACAGAATAGCGTTCAGG 38348
GuineaPig                   --CAATATCAGTAGCCTGCCATCAAAAAAGAA-ATTGGAAAATGCAATCCATAACCTAGAGAAGTAGGTTTTCTGCAGT 19945
NorthernAmericanDeerMouse   --TACTTTAAATGTTAGTCTAATGAAGTATATTTTTATGAAATA-----CAGATATTTTCAA-ATACCTAAAACATCAAT 19502
Mouse                       GTTACTTTAAATGCTTATCTAGTGAATTATATTTTTATAACATA-----CAGGTATTTTCAATATATCTAAAACATCACC 23970
ChineseHamsterGHOK1GS       --TACTTTAAATGTTAGTCTAGTTAAGTATATTTTTATAAAATA-----CAGGTGCTTTCAA-ATACCTAAAACATCAAT 21430
LongTailedChinchilla        --CAGTGTCAATAGCCTGCCATCAAAA------ATTGCAAGTCA-----TATGAAGTTGGAAAATATAATCCA-TAAACT 21271

Majority                    GAATGATTTGAACAXAAAAGTCATTTTXAXCATTCXCTXAAXXATTAXAAGX----X-AGCAGAXXTXTAXTACXCTTTX
                                      39850     39860     39870     39880     39890     39900     39910     39920
Human                       GAATAACTCATGCATTAAAGTTGCCTTGATCACTCAGTGAAGAAAGAGAAGGCCTTTCAACAGAT--GTGCTAGAACAGC 38426
GuineaPig                   AATTTAACTATTTTCAGTAGTCATTTTCTGCAGTAATTTAACTATCAGAA------TTAGCAGA----GACTATACATAT 20015
NorthernAmericanDeerMouse   GAATGATTTGAACATAAAAGACATTATAACCTTTCCCTAAAGAATTTTAAGAT-G---AGTCAATTTCTCTTTCTTTCTC 19578
Mouse                       GAGTGGTTTGAACATGTGAGACAGTATTACCTTTCCCTGTCTTATTATATAT-------ACATACATACATACCTTTTTG 24043
ChineseHamsterGHOK1GS       GAGTGATTTGAACACCCAA--CATTGTAATCGTTCCCTAAATAATTTTAAGG-------GTCAAGTTCTCTTTCTCTTT- 21500
LongTailedChinchilla        ACAGAAGCAGCACTCAATAGTCATTTTCTACAGTAACCTAGATATCAGAA------TTAGCAGACAGAGACTATACATAT 21345

Majority                    TTTTAATXTGTXXAATGAAAATXCTXAXXCCTTAAAXGTXGXTT---TTGTATTCGXTXTTGXXXTTGXTGATATXT--A
                                      39930     39940     39950     39960     39970     39980     39990     40000
Human                       TGAATATACATACAAAAAAGCAAAAGAAACTTAAACCCTACATCATTCTGTATAAAACAATGAGATTAATTATATA---A 38503
GuineaPig                   TTTTAAGCTCTTAAGGGAAAAGACTGAAATAATAAGCAAATAGGTAGAGGAATGTGTTAG-GAGATTTTAAAAATA---- 20090
NorthernAmericanDeerMouse   TTTTATTTTGTGCAATGAACTTTCTCATTCCTTAGATGTTGCTT---TTGTCTTCGATGTTCTAGTTGCTGATATTT--A 19653
Mouse                       TTTTGCTTTGCCAAATGAAAATTTTCATTCCTACCATGTTGGTT---TTATTTTCCGTGTTGTAGTTGTTGGTATTTT-A 24119
ChineseHamsterGHOK1GS       TTTTATTGTGCACAATGAACTTTCTCATTCCTTAGGTGTTGCTT---TTGTCTTCAGTGTTCTAGTTGCTGATATTT--A 21575
LongTailedChinchilla        TTTCAATCTCTTAAGGGAAAAGACTGAAATAATGAACAAACGGA-----GAATGTGTTAG-GAGATTTAAAAAATACAAA 21419
```

```
Majority                      TAXCTGATATTT-AAATATXXTAACAAXAAGCXXXXGATXT--XTGAAATTTGAATTTXATXXA-----AA-TTATCTGA
                              40010 40020 40030 40040 40050 40060 40070 40080
Human                         AAACTAATATAT-AGAGTTTTTAGGAAAAAGCATAGGATATACTTGCAACTTTGGGATAAATGA-----AG-ATTTCT-T  38575
GuineaPig                     GAAAGTATATTG-AAACATTTTAACAGAAAA--TAGGATAT--TTGAAATAAAACATTGATTGA-----AA-TTTCTCGC  20159
NorthernAmericanDeerMouse     TGCCTGTCTTTT--ATTATGGTAATGACTAGCTCCCTGGTT---TGAAGTGTGAATTTCCTAAA-ACCTGATTTATTTGA  19727
Mouse                         TACCTGATTTTT---ATATGCTAGTGACAAGCACCCCATTT---TGAAATTTGAAT------GA--------TTATCTGA  24179
ChineseHamsterGHOK1GS         TACCTGTCATTTTGATTATGGCAACAACTGGATCCCCATTT---TGAAGTTTGAATTTCCTAAAGACCTGATTTATCTGA  21652
LongTailedChinchilla          GAAAGCATATAG-AAACATTCTAACAGAAAGG-TAGGATAT--TTGAAATAAAACGTTGATCAA-----AA-TTAGCAGC  21489

Majority                      AXGAXTAAAAXTAGXAGAAXXXAAXAXATAAAXCXXACTTTAGAGAAAXGXTATCTTCTCXGTCCXACXXAXTXCXTTCT
                              40090 40100 40110 40120 40130 40140 40150 40160
Human                         A-AAATAGAAAAATCTTAAAGGAATATATAAACTTGACTCCATCAAAATGGGAAATTTCTGCTTCATCAAAAGACATTGT  38654
GuineaPig                     A-GAATGAAAACAGCAAAAGAAGTTTGATGAACCTGAATATAGATCACTAGTCTGCTAGAGGG--AACAAAATATATTTA  20236
NorthernAmericanDeerMouse     ATGAGCAAAACTAGTAGAATTAAAGATTCAAATAATGCTTTTGAGAATGGCTATCTCCTCTGTCCTATTCTTGTCTGTCT  19807
Mouse                         ATAAGTCACACTGTTAGAGCGGAAGAGA-AAATCATACTTG-GAGAAAGGCTGCCTCCTCTGACCTCCTCCTTTCTTTCC  24257
ChineseHamsterGHOK1GS         ATGAGGAAAACTAGTGAAAT-GAAGATTCAAATAATGCTTTTGAGAAAGGCTATCTTCTCTGTCTTATTCTTTTCTCTCT  21731
LongTailedChinchilla          A-GAATGAAAACAACAGAAGAAATTTGATGAACCTGAATACAGATGACTAGTCTGCTAGAGGG--AACAAAATATATTTA  21566

Majority                      TXTXATAGTAAAAAATTXAGGTATAGTCTTXXAXA-GAATAGATTATGGXXCXXTAAAXAXXTGTXTGTGXGTGTXTXXG
                              40170 40180 40190 40200 40210 40220 40230 40240
Human                         TAAGACATTGAAAAGACAAGGCACAGACTTGGAG---AATATAATATTTGTGTATCAATACATGCATATGACTGTATTAG  38731
GuineaPig                     CAGCATAATCAAGCAGTCTAA-AGAGTTCTAAAGATGAATAGAGAATGGGTTGAAGAAAAACTGCGTGTGTGTGTGTATG  20315
NorthernAmericanDeerMouse     TTT--TAGTAAAAATTTGAGGTATAACCTTTTAAA-GAATTGATTATGGAACTTTATACTTATCTTATTTGGAATTTGGG  19884
Mouse                         TCT--TGGTAAAAAATTGAGGTGTGCTCTTCCAAA-GAATCAATTATGGAACGTTA------------------------  24310
ChineseHamsterGHOK1GS         TTTTTTGGTAAAAATTTTAGGTGTAACCTTTTAAA-GAATTGATTGTGGAACTTTATGCTTTTCTTTCTTGGA-CTTGGG  21809
LongTailedChinchilla          CGAGATAGTCAAGCAGTCTGA-AGAGTTCTAGAG-TGAATAGAGAATGGGTTGAGGAAAAATTGTGTGTGTGTGTGTATG  21644

Majority                      TXXATGTXXGXAXTGA----ACACXTGXACTTGGXXT-XGAXGGGAXAGTTAXAAXAGATGAAGGA--XXXATTAXCCTX
                              40250 40260 40270 40280 40290 40300 40310 40320
Human                         TCCATTTTCATGCTGCTGATAAAGACGTACCCGAGACTGGGCGGGCAATTTATGAAAGAAACCAGT--TTAATGAATTTA  38809
GuineaPig                     TGTGTGTGTGT-GTGTGTGTA----TGAAGTTGGGAT-G-ACTGAAAACTTGTAAAATTTGAAAGA--AATACCAACATA  20386
NorthernAmericanDeerMouse     TAAAAGAAGGAAGTGA----ACACTTTCACTTGGTGG-AGATGGGAGAGTAAAAAGAGAAGAAG--------TTATCCTT  19951
Mouse                         ---------GAAATAA----ACACGTGTACTTGGTGT-AGATGG-AGAGTGAAGAGGGATGAATGAGGGTATTTATCCTT  24375
ChineseHamsterGHOK1GS         TAAAAGAAGGAAAGGA----ACACTTTCACTTGATGT-AGATGGGAGAGTAAAAAGGGGTGAAG--------TTATACTT  21876
LongTailedChinchilla          TGCATGTGTGT-TTGC------------AGTTGGGAC-GGACTGAAAACTTGTAAAATTTGGAGGA--AATATCAACATA  21708
```

```
Majority                   XXXTTXTXGGCAXCTXXGXTAAACCTCXGAGTCATGAX--XXXXACAGGAGTACCATXTCAGXTATCATGTAAATCXAGA
                                    40330     40340     40350     40360     40370     40380     40390     40400
Human                      CAATTCTATGTGACT-GGGGAAGCCTCATAATCATGGTGGAAGGCAAGGAGGAGCAAGTCACATCTTACGTAGATGGCAA 38888
GuineaPig                  CAATTCTAAGAACATCAGTTAAATCTCCTATGTAGGAT-------TATGAAAACCATGTTTTGTAATAAGAGAATACTAA 20459
NorthernAmericanDeerMouse  TGTTGTAGGGCATCAGTT-TGAATCTATGGGTCATAAC--CTTAACAG--GTCATATATCAGGTATCCTGTATATC-AGG 20025
Mouse                      TGCTTTAGGGCAGGGATTCTCAACCTGGGTGTTGTTGCAATCCCACAGTGGTCCCATATCAGATACCTTGCAAATC-ATA 24454
ChineseHamsterGHOK1GS      TGTTTTTGGGCTT--------------TGGGTCATAAC--TTTGACAGGGGTTAGATATCAGATATCCTGTATATCCAGG 21940
LongTailedChinchilla       CAGTTCTAAGAACCTCAGTTAAACCTCCCATGCAGGATAAA---CTATAAAAACCATGTTTAGGCACAT---AATATTGA 21782

Majority                   TATXTAAAGXAAAXXGXATAAAXGTAGCAAAATX---TA-TAXXTAXXAAXAAGATAATTXXATGGTCGXXGACCATTAC
                                    40410     40420     40430     40440     40450     40460     40470     40480
Human                      CAGGCAAAGAAAAGAGAGCTTGTGCGGGGAAACTCTCCCCTTTTTAAAACCATGAGACTTACTTCA-CTGTCACGAGAAC 38967
GuineaPig                  AAACTAATGATAA-AGATTAAATCTGACAAA---------TATCCGAGAAAA-GACATCTCATGGAACAAATACTTTTCT 20528
NorthernAmericanDeerMouse  TATTTACAGTATGATCCATAAAAATAGCAAAATTAGTTATGAAGTAGCAACAAAATAATTTTATGGTTGAGGATCATCAC 20105
Mouse                      TATACACATTACAATTCATAGCAGTAGCAAATT----TA-----CAGTTATAACATAATTT-AAGGTTGGCGGTCACTAC 24524
ChineseHamsterGHOK1GS      TATTTATATTATGGTCCATAATAGTAGCAAAATTAGTTATAAAGTAGCAGTTAAATAAT---ATGGT-GGGGGCCATTAC 22016
LongTailedChinchilla       AAACTAATGATAA-AGAGTAAATCTTAAAAA---------TAGCTAAGAAAAAGACATCTAACCGG-CAAATACCTTCCT 21851

Majority                   AXCAXXAGAAAX-AACTGXCTTXAX-----GGTT-ACAXXAXTAGGAAGGTGXT-XTXAGAACXXXXAXTT----TAGXG
                                    40490     40500     40510     40520     40530     40540     40550     40560
Human                      AGCATTGGAAAG-ACCTGCCCCCATGATTCAGTTAACTCCCACCAGGTCCCTCCCACAACACATGGGAATTCAAGAGGAG 39046
GuineaPig                  TGCGCTAGAAGA-AACTAACTTC---------T--ACACTGAACGGAAGGTGATTATATAGACTGGGAATT---ACAGAA 20593
NorthernAmericanDeerMouse  AACACGAGGAATTAACTGTATTAAATAAAGGGTT-ACAGCATTAGGAAGGTGGT--TGAGAACCACTATTT----TAGGG 20178
Mouse                      AATATGAGGAA----GTGCGTTAAG-----GGTC-ACAGTATTAGGGAGAT-----TGAGAAACACTTTCT----TAGGG 24585
ChineseHamsterGHOK1GS      AACATGAGAGA----CTGTATTAAA----GGGTT-TCAGCATTAGGAAGGT-----TGAGAACCACTATTT----TAGGG 22078
LongTailedChinchilla       TGCACTAAAAGA-AACTAACCTC----------TGCACACTGAATGGAATGTGATTATATAGACTGGGAACT---GCAGAA 21918

Majority                   ATXCAGTXAXGTGXACXAGT--GAAAXGTXAAXXATAAAXGATAXAXTTTTGTAXTAXTTTTX-----AAAAXC----XC
                                    40570     40580     40590     40600     40610     40620     40630     40640
Human                      ATGTGGGTGGGGACACAGCCAAACCATATCAATGACAAAGGACTTGTATCCCAAAGGACTTGTAAAAAAAAAACTCCTCC 39126
GuineaPig                  AGGCAGTAAGGATTAC----------TGGAAATGATAA-GTATATATGTAAATAAAAATTTT------AAAACCTTAGTT 20656
NorthernAmericanDeerMouse  ATTCAGTCATGTGTGGTAGTT-GAAAAGTTAGAAATAAAAGATAAGCTTTTGTATTATTTTT--------AAGT----AC 20245
Mouse                      ATTCAGTTGTGTGCAGCAGTA-GAAAAGTCAAAAACAAGAGATAAACTTTTGTATTATTTTTT-------AATC----CC 24653
ChineseHamsterGHOK1GS      ATTCAGTCATGTGCAATAGT--GAAAACTTAGAAATAAAAGATAAACTTTTGTATTATTTTTT-----TTAATT----GC 22147
LongTailedChinchilla       AGGTAGTAAGGAGTAC----------TGGAAATGGTAA-GTGTATATGTAAATAAAAATTTT------AAAACCCTGCTC 21981
```

Majority	AGTXTCTTAXXXTAATTTXTTTXTXTTTTTTATAXAGAGGAXXTGACGXGCXACTCTXAGGXXGTGTTCTXAGTAGXXCC	
	40650 40660 40670 40680 40690 40700 40710 40720	
Human	AATCAATAAATATGAGCAGCCCAATTTTTTTTTAAGTGGCAAAAGAAGTGACATTATAAGGATGGGTAACAAGCACATGA	39206
GuineaPig	TGCTGGTTTTCATTATTTTTATGTCTGTTATATGAATGA-AAACTTTGTAGCACAACATTTATGTGGTTTAT-TATGTAT	20734
NorthernAmericanDeerMouse	AGTGTCTTATGGTAATTTGTTTTTACTTTTTATAGAGAGGATCTGACGAGCTACTCTCAGGCAGTGTTCTCAGTAGTCCC	20325
Mouse	AGTGACTTACAGTAATTTGTTTTGTGTTTTTCTAGAGAGGATCTGACGAGCTACTCTCAGGCAGTGTTCTCAGCAGCCCC	24733
ChineseHamsterGHOK1GS	AGTATCTTACAGTACTGTTTTTGT-TTTTTTGTAGAGAGGATCTGACGAGCTACTCTCAGGCAGTGTTCTCAGTAGTCCC	22226
LongTailedChinchilla	AGTTTGCT--GATTTTCATTATATCTGTTACATAAACAA-AAACTGTGTAACACACCATTTATGGGTTTTAT-TATGTAT	22057
Majority	AAXTXTAATATXAGTXXCAXGGTAGTXACAXGTAAXTXTTXCTGGX-XTTAXCAGTXCGX-X-XTTTAATGTTTTXCAXG	
	40730 40740 40750 40760 40770 40780 40790 40800	
Human	AAAGATAATGTTAGTCATCAGGTGATCACAAGTCAAAACCATTAGAGATAGAATTTACACCAACTAGAATGGCTAAAATT	39286
GuineaPig	ATAAATGTGTGTATACAATAACTAACCTAAAGTAAAGATAAATGGAATTTATCAGTGAG--ATTTTTAATGTTTTACATG	20812
NorthernAmericanDeerMouse	AACTCTAATATGAGTGGCATGGTGGTTACAGGTAAGTGTTGCTGC--CTTAGCAGCCCGC---ATTTACTATTTTCCAGG	20400
Mouse	AACTCTAATATGAGTGGCATGGTAGTTACAGGTAAGTGTTGCCGT--CTTA------C-----------TGTTTACCAGG	24794
ChineseHamsterGHOK1GS	AACTCTAATATGAGTGGCATGGTAGTTACAGGTAAGTGTTGCTGT--ATTAGCAGCCCTC---ATTTACTGTTTTCCAGG	22301
LongTailedChinchilla	ATAGATGTGTATG-ACAACAGTTAGCCTGAAGTGAAGATAAATAGAATTTAACAGTAGG--ATTCTTAATGTTTTACATG	22134
Majority	XCATAAT--GXXAAXACTAAAXCXTACXTTAXGCCAXAATAGXTTXTT----GTTTTXAAXXXTXAAATTGAXXATTTTT	
	40810 40820 40830 40840 40850 40860 40870 40880	
Human	AAATAGATTGGCAATACCGAATGTTG-ATGAAGATGTGGAACTGTCTTT---A-ATTACTGCTGAGTATATAACATAGTT	39361
GuineaPig	ATGTAAT---ACAGTATTAAATCTAACATAAT----TTTTGATAACTT------GTTATATTTTAAAATGCAATGTGCTA	20879
NorthernAmericanDeerMouse	GCAAAAT--GCTAAAACTAAAGGATAATTTAGGCCAGAATAGCTTTT-----GTTTTGAAATCTGAAATTGATGATTTTT	20473
Mouse	GCAGAGT--GTTAAAGCCAAAGCATACTTTATGCCAGAATAGCTTTTTAAAAGTTTTGAAGCCTGAATTTGGTAATTTTT	24872
ChineseHamsterGHOK1GS	GCAGAAT--GTTAAAACTAAAGCATAATTTAGGCCAGAATTGCTTTT-----GTTTTGAAACCTGAAATTGATGATTTTT	22374
LongTailedChinchilla	AAGTAAT---GCAGTACTGAATCTCACATAAAAAGATTTTGATATCTTA---AGGTTACATTTTAAAATTCTATATGCTA	22208
Majority	GTGGXCAAXTAAXGTTTAA--ATXTGATTXAXTTT-XXATT-TGAXGCXAGGAXTTGAA---AA------GTCAGXAGAC	
	40890 40900 40910 40920 40930 40940 40950 40960	
Human	GGGAAAATAGTTTGCTTTT--TTTTTTTTTTTTTT-TTTTT-TGAGACAGAGTCTTGCT---CT------GTCACCAGGC	39428
GuineaPig	GCTCTGAAATAAGGAA-AA--AGGAAGT--------------AAAAGCAAGTAGTGAAA---AA------GTCAGAACAT	20933
NorthernAmericanDeerMouse	GTGGACAAGTGAAGTTTAA--ATCTGATTCAATTTGTTATTGTGATACTAGGGACTGAACTCAAGGGCTTGTAGGTAGAC	20551
Mouse	GTGGGCAAGTAGTGTTTCA--ATCTGATTGAATTT---ATT---------------------------------------	24908
ChineseHamsterGHOK1GS	GTGGGCAAGTAAGTTTAAATAATATGATTTAGCTTGTTATTGTGATGCTAGGAACTGAATTCAAACATTTGTAGGCTGAC	22454
LongTailedChinchilla	GCTGTAAAAAAAAGGATAA--AGGAAGTCGAGGT--------AAAAGCCAGTAGTGAAA---AA------ATCAGAACAT	22269

Majority	TT----ATX-XACXXGCTGGTTXTCAAATXACT--ACAAAGXCTXAXTAXTXAXTAXAAX-X--ATGCTAXTAGCTTAGX	
	40970 40980 40990 41000 41010 41020 41030 41040	
Human	TGG--AATG-CAGTGGCATGATCTCAGCTTACT--GCAACCTCCGCCTCCTGGGTTCAAGCG--ATTCTCCTGCCTCAGC	39501
GuineaPig	TT----ATT-GATTGCCTGGTTTCAGAATGACT--GTAAAACTACAGTAATCAGTTCAGC----ATGATATTAGCATAAA	21002
NorthernAmericanDeerMouse	TTTTCTGTCCTGCCAGCTGCTCCTCAAATAACCACACAGAGACTTAA------TTATAAATGTTTGGCTAATAGCTTAGG	20625
Mouse	----------TACCAACA------------------C-------------------TAA---------------------	24920
ChineseHamsterGHOK1GS	TTTTCTATTACACCAGCTGCTCACCAAATAACCACACACAGACTTAAGACTAATTATGAAAGTTTGGCTGATAGCTTAGG	22534
LongTailedChinchilla	TT----AT-----TGCCTGGTTTTAGAATTACT--ATAAAGCTGCACTAAACAATACAGC----ATGATATTGGCATAAA	22334

Majority	CT--TATXTAXXTTGATCTXTTXX-XCACX--AXA--XXACTCXXXTAAXTTAATGCATTTXTGGX-AXCACTGXGTTTX	
	41050 41060 41070 41080 41090 41100 41110 41120	
Human	CTCCTGAGTAGCTGGAACTACAGGTGCACGCCACC--ACACCCGGCTAATTTTTTGTATTTTTAGTAGAGACTGGGTTTC	39579
GuineaPig	A---AATATAAGTTGATCAGTGAA-ACACATTAAAGAGTTCAGAAGTGG-TCCATGCAATTATGG--ACCACCAAATTTT	21075
NorthernAmericanDeerMouse	CTTGTTGTTGTTTTTTTTTTTTTTTTTTTTTTTAAGCTAACTCTTATAACTTAACCCATTTCTGTTCATCTATGTGCTGC	20705
Mouse	---------------------T-----------------GCT----------A---CACAC-------------------	24930
ChineseHamsterGHOK1GS	CTTGTATTTAGCTAGCTCTTTT----------------AACT----TAAATTAACCCATTTCTATTAATCT--GTGCTGT	22592
LongTailedChinchilla	AC--AATATAAGTTGATCAGTGGG-GCACA--ACA--GTTCACAAGTAAATTCATGCAATTATGG--ACCACTAAATTT-	22404

Majority	GCCAAA----GGTXAGXAXXXXXTTGAXCTXCXGACXTTGCX----XAXXTGCTCAXGXTAXTTXCXCAGACTGCTXGXA	
	41130 41140 41150 41160 41170 41180 41190 41200	
Human	GCCATGT--TGGCCAGGATGGTCTTGCTCTCTTGACGTTGTG-----ATCTGCCCACGTCGGCCTCCCAGAGTGCTGGGA	39652
GuineaPig	GATAAA----GGCGAAAAGTCCATTCACTGGCAGACTTTGCA----AAAGTGCTATAAATAATTGGATATAATGCCAGAA	21147
NorthernAmericanDeerMouse	CCTGAAGCTCGTTTACCTCATGTATGTACTTCCCATCCTGCTTGCTCCGTGTCTCATGGCATCTCCTCAGACTCCTCTTC	20785
Mouse	-----------------------------T--------------------------------------GAACT-------	24936
ChineseHamsterGHOK1GS	CCCAAGTCTTGTTTAGCTCATGTATGAACTTCTTACCCTGAGTGCTTTTCATGTCATGGTGTTTCCTCAGACTCTTCCTC	22672
LongTailedChinchilla	GACAAA----GGTGAGAAGTCCATTCATGGGCAGACTTTGCAG--CAGAGTGCTATAAATA-TTGGATATAATGCCAGGA	22477

Majority	XTXCXX----XXXXCAXTXTXTXXGTCTXXTTTTXCT--XXTT--XCTTAXXXTGC-TAXXXATTXACCXXTX--XTXTT	
	41210 41220 41230 41240 41250 41260 41270 41280	
Human	TTACAGGCCTGAGCCACTGCGTCTGACCTAGTTTACT--ATTT--TCTTACGGAGT-TAAACATTAACCTATT---TTAT	39724
GuineaPig	AAGAGATTTTGGTCCTATATTTCTTTCTTTTTTTTTT--TTTT--TTTATCGGTAC-CAGGGATCTAACTCATGACTGTG	21222
NorthernAmericanDeerMouse	CTTCTTCTTCCCAGCATTCTCTTAGTCTGGTTCTCCCCCACTTAACCTTATCCTGCCTTGCTGTTGGCCAGTCACCTCTT	20865
Mouse	--	24936
ChineseHamsterGHOK1GS	CTTC------CCAACATTCTTTTAGTCTGGTTTTCCT--GCCTAACCTTATCCTGCCTACCCATCAACCAATCAGCTCTT	22744
LongTailedChinchilla	AAAAAAA---AAAGATTT--------------	22492

```
Majority                    TGTTXACXAXTXAG-AGTXXTAX-TXTTTACXXXGTACXAA-AT-GTTX-XXXX-TAGXTCTTXCAXATGCXXGAXAAAC
                                     41290     41300     41310     41320     41330     41340     41350     41360
Human                       GGTCCATAACTTCT-ACTTTTAGTTTTTTACCCAGTAAGAACAT-GTTT-GCTG-TAAAACTTGTATGTGTATCAAAAAC  39800
GuineaPig                   TGCTTGCAAGGCAGGAGCTTATGCCGCTGAACTAAATCCCTAGC-CCTG-GTCC-TATATTTTATA-ATGCATAAAATAC  21298
NorthernAmericanDeerMouse   TATTAACCAATGAG-AGTAATATGTATTTACAGTGTACAAAGATTGTTCCATAACAAGCTCTTGCATATGCCAGGGAAAT  20944
Mouse                       --------------------------------------CAC----------------GCTCTTCCCCACGCCAGAGAAAC  24962
ChineseHamsterGHOK1GS       TTTTAACCAATGAG-AATAATATATATTTACAGTGTACAAATATTGTTCCACAGCAAGCCCTTACACATGCCAGGGAAAT  22823
LongTailedChinchilla        GGC-------------AG-------------------------------------TGTATCTCCCA-GTGCATAAAAAAC  22521

Majority                    ---TTACXTCTXAATXGTXCXCAAXCTTAAAACXTXACCCTAATXTT--TATXATTTTTAAC-ATGGXAAAXTTXXTXTT
                                     41370     41380     41390     41400     41410     41420     41430     41440
Human                       ---TTTACTGATAATGGTCCCAAACTTGAA---ATAACCCAAATGTT--TATCAACAGGAAA-ATGGATAAATTGTGGTA  39871
GuineaPig                   ---TTAGTTCCAAATGGATCATAACTCAAAAGTATAACCTTAAATTT--TAGCACTTT------TGGAAAAAGTACAGTA  21367
NorthernAmericanDeerMouse   ATTCTACCACTGACTCATACCCGGTCTTAAAACTTTACCCTTATCTTCTTATTGTTGTTAACTATGGTACATTTTTTATT  21024
Mouse                       --ACTACTACACATTC---CTCAGTCGTAAAACTTTCCCCTTG-------ATTCTCTGTAACCATGGTACATTTCCTACT  25030
ChineseHamsterGHOK1GS       ----TACTGTTGAGTCATACGCAATCTTAAAACTTTTCCCTAATCTT---ATTGTTACTAACTATGGTAAATGTTTTATT  22896
LongTailedChinchilla        ---TTAGCTCTAAATGGATCATAACCCAAATGTACAATCTTAAATTT--TAGCACTTT------TGGAAAAAACACGGTG  22590

Majority                    TAAAX----AATXT-TTTTXAXTTTTAAXTAACAAAAAAT-----TTCTTAGTTGTXTATCXXAAAACAXTTTACTAXAX
                                     41450     41460     41470     41480     41490     41500     41510     41520
Human                       TAATCATATAGTATATTACTACTTACTAATAACAAAAGAA-----TGGTTAGTAGTACATGCGAAAACATGGGTGAATAG  39946
GuineaPig                   TAA------AATCT-TTGAGACTTTGAGTTAGGCAGAAAT-----TTCTTAGATATAACTCTAAAAGCACTTAACTCTAA  21435
NorthernAmericanDeerMouse   TAAAAAAATTATTTTTGTTTGTTTTTAAGTAACACAAATTCTATATTCTTACTTGTTTACCCTTTCACATTTTACTAAAC  21104
Mouse                       TAAAG----------CCTTGGTTTTTAA---ATG------------------TGGGTTAT------AGACTTTTCTA---  25070
ChineseHamsterGHOK1GS       TAAAA-------TTTTGTTTTTGTTTAAGCGACACACGTTCTGTATGCTTCCTTGTTTGTAGTTTGACATTTTACTAAAC  22969
LongTailedChinchilla        TAA------AACCT-TTGTGACTTTGAGTTAGGCAGAAAT-----TTCTTAGATATAACTCTAAAAGCAC----------  22648

Majority                    AXX-ACAXTXTATXXATX-AAXATAXG----X-X--XGCCTTGATXCTGTAGTTAATATTTTXXAAAXCCTT--AATTTC
                                     41530     41540     41550     41560     41570     41580     41590     41600
Human                       TAA-GCATTGTATGAAGC-AAAAGAAACC--AGAC-AGACCTGAAAGAGTACATAGTGTGATTCCATTTCTTGAAAGTTT  40021
GuineaPig                   AAGCACAATCTATAAAA--AACATATG---------GGCCTTGACATCGTAAGTAA-ATGTTTCTGCTTTTC--AAACAA  21501
NorthernAmericanDeerMouse   ACATACACTTAAGTGATCAAATCTGGGTTGTAAACTTATCTAGATCCTGTAGTCAATATTTTCAAAAGCCTT--TATTTC  21182
Mouse                       --------------GAT---------------------CCTGGATCCTGTAGTTAATATTTTCAAAAGCTTT--AATTTC  25113
ChineseHamsterGHOK1GS       ATGTACATTGTAATGATAAAATCTGGGTCGTAGATTTGTCTAGATCCTGTAGTCAATATTTTCAAAAGCCTT--AATTTC  23047
LongTailedChinchilla        ------AATCTATAAAAG-AACATATG---------GGCCTTGACATCATAAGTAA-ATATTTCTGCTCCTC--AAGCAA  22709
```

```
Majority                   ATTXXCXT--XXXTXXTCCTTTGTTXXXXXAC-AGAATXT-----XXXTATXXXGTGGAAATCTGXTACTGTTTTXCXXX
                                    41610     41620     41630     41640     41650     41660     41670     41680
Human                      AAGAGCAGATCATTAATTTTTTGTTTAGAAACCAGAATGTTGGAGATTAACAGAAAGGGAATATGGTGGCATTTTCTGGT  40101
GuineaPig                  AGTAATAAAAAGACAGGCCATTG---AGGGAC-AGAACAT-----GTTTACAAAGCATAGAGCTGATAAAGTATTTATAT  21572
NorthernAmericanDeerMouse  CTTTTCTTC-CTTTCCTCCTCCCTTCCCTTTCTGTTTTCT-----TCCTTTTCTGTGGCAGTCTT-CACTG-TGTCCAGG  21254
Mouse                      TTTTTCTT-----TCTTTATCCACCCCCCCCC--------------CCCCTTTTGTGGCAGTCTC--ACTGCTGTCCAGG  25172
ChineseHamsterGHOK1GS      CTTTGCTT-----TCCTCTTTTCTTTCCTTTCCTTTTTCT-----TCCTTTTCTGTGAAAATCTTTCACTG-TTATCCAA  23116
LongTailedChinchilla       AGTAATAA--AGACAGGCCATTG---AGCGAC-AGAACGT-----GTTCATAAAGCATATAGCTGATAAAGTATATTCA-  22777

Majority                   TTGXCGTXCACXTCCTXGGXT-AAXXXXGCAAGTX---AAXCXCATCTXTXT---XXGTXTXTCAXGTAGTAGAXXGACT
                                    41690     41700     41710     41720     41730     41740     41750     41760
Human                      GTGATGGAAACGTCCTATGTT-AATAAACTAAGTTGCAAAACAAATCCAAAA----GTTGTTTGAAGCAGTAGATTATCT  40176
GuineaPig                  TTAGAGTATATAACATCAG---AATATATAAACTC---AAACTCAGCAACAT----GATACTAACAATAATAAATTGGCC  21642
NorthernAmericanDeerMouse  TTGGCTTTCACCTCCTAGGCTCAAGTGGGCAAGT-----AGCCCCTCTGTGT---TAGTCTCTCATGTAGTTGA--GACT  21324
Mouse                      TTGACCTTCCCCTCCTGGGCTCAAGTGGGCATGT-----AGTCCTTCTGTGT---TAGTTTCCCATGTAGTGGG--GACT  25242
ChineseHamsterGHOK1GS      TTGGCCTTCACTTCCTGGGTT-AAGTAGGCAAGTA---TAGTAGTTCTGTGTTGTTAGTCTCTCATGTAGTTGG--GACT  23190
LongTailedChinchilla       -GAATGTATATAACATCAG---AATATATAAACTC---AAACTCAGCAACAT----GATGCTAACAGTAATAAATTGGCC  22846

Majority                   XTAAGXACCTTXXACTAXCTXTXGTTTAA-XXXTTTCXXTXTAATGAXAAATAXAXGCCXX-XTATAXXCATGCXAXXX-
                                    41770     41780     41790     41800     41810     41820     41830     41840
Human                      CTGAGAACTCTGAATTTCCAGTAGTTGTG----TCTTGCTGTAATGAGAAATATGAGATTGGGAATGACTAGGTGAGTC-  40251
GuineaPig                  --AAAATACTTGAACT---GATAGTT---------CAGCAGAAAAGATAATTAGATGGC---ATATAAGCATGAAAAAT-  21704
NorthernAmericanDeerMouse  ATTAGCACCTTAGACTAACTTCGTCTTAA-AACTTTCTTTTTAATTAAAAAGTAGAACCCA--TTCTCACAGGC---CTC  21398
Mouse                      CTAGGCACCTCAGACTAGCTCAGGCTTAATAACTTGCTTTTTAATCACACACACACACACA----CACACACAC------  25312
ChineseHamsterGHOK1GS      ATAGGCACCTCAGACTAACTTAGTGTTAATAACTTTCTTTTTAATTAAAAAAAAAGGCCTTGCTTTTCCCTTGCTTTCCC  23270
LongTailedChinchilla       --AAAATCCTTGAACT---GATAGTT---------CAGCAGAAAAGATAATTACAGGAC---ATATAAGCATGAAAAA--  22907

Majority                   GATGT---XTXGXXCCTAXATXTX--T-ATTX--X---TAATGTATGX--------------------------------
                                    41850     41860     41870     41880     41890     41900     41910     41920
Human                      CATGTAG-CAGGGGCCTAGATGTG--GGATT-------TGATGTGTGTG-------------------------------  40290
GuineaPig                  GATGC---TTGGCATCTAAATAAT--TTATTAGTT---AAATGTAAAC--------------------------------  21744
NorthernAmericanDeerMouse  GATTTTGCATTTTTCTTATTTTT-----TTTATTTTTTTATTTTTTG---------------------------------  21440
Mouse                      --------------------------------------------------------------------------------  25312
ChineseHamsterGHOK1GS      ATTTTCTTATATTTTTAATTTTTGTGTATATATCTTATTATTTTATACTACAAACACTATTGCCTATATTGTAAATAAAA  23350
LongTailedChinchilla       GGTGC---TTAGCACCCAAATAAT--TCATT-------AAATGCAAGT--------------------------------  22943
```

```
Majority                     -------------TGAXACTAGGXGXTXGCATGTAXATTCATXCGXXT-----XACTGAXATTTTAAC--AGAC--XXCT
                             41930     41940     41950     41960     41970     41980     41990     42000
Human                        -------------TGTGTGTGTGTGTGTGTGTGTGTGTGTGTGTGTGT-----AGCTTGGACTTTAAC--AGAT----TT 40346
GuineaPig                    -------------TGAAACTAGGAGATAGCACACACACTCATTGGAAT-----GACTGAAATTTTCAA--AGAC--TACT 21802
NorthernAmericanDeerMouse    -------GTTTTTCGAGACAGGGTTTCTCTGTGTAGCTTTGCGCCTTTCCTGGAACTCACTTGGTAGCCCAGGCTGGCCT 21513
Mouse                        ------------------------------------------------------------------AC----AC------ 25316
ChineseHamsterGHOK1GS        ACCCAGAGTTGACCTACACAATTGTCTCCCATGTATTTTCATCCTCATTTCTTTGCTGAAATGATAATTTTAATTAATTT 23430
LongTailedChinchilla         -------------TGAAATTAGGAGATAGCATGCACACCCATTAGAGT-----GACTAACATTTAAAA--AGACCATACT 23003

Majority                     AGATTTTX--GAAAGAATXTAXXTAAXATCTXXA---------XCTCTCAAGCAAXTXTGGGATT-----GT--------
                             42010     42020     42030     42040     42050     42060     42070     42080
Human                        ACATTTTA--GAAAGAATTTTTGTTCCATTTGTA---------TTTCACAGGTAGGATCAGTATT-----GT-------- 40402
GuineaPig                    AGGTATTG--GAAAGAATAT--AAAAGAA--ATG---------ACACTGAAGCATTTTTGGGAAT-----GT-------- 21854
NorthernAmericanDeerMouse    GGAACTC---ACAGAGATCCACCTG-CCTCTGC-----------CTCCCGAGTG---CTGGGATT--------------- 21560
Mouse                        -----AC-----ACACACACACACA-----CACA------------CACACACAAACCCATTCTC--------------- 25354
ChineseHamsterGHOK1GS        TTATTTTTTTGTAAGGATTTATTTAATCTCCACAGTTAAAGCTTTTCTTGGGCAAATCTGAAATATTTTAATGTCTAAAG 23510
LongTailedChinchilla         AGGTGTTG--GACAGAATGT--GAAAGAACTGTG---------ACACTGAAGCACTTTTGTGAAT-----GT-------- 23057

Majority                     AAAGXTXTGXGAXTA----------XTTCAAAAACXGTTCGGCAXTXTATTXTAAATATTTTTATT------AXCTTTAT
                             42090     42100     42110     42120     42130     42140     42150     42160
Human                        ATAGAGGGGAGAATA----------GTTTTAAAACATTACGGTAGTTCATTCCAAAGATACCAAGG------TCCTGAAT 40466
GuineaPig                    GAAATTATAGGAGTAC---------ATTCGAAAGCAGTTAGGCAATTTCTTAAAAAAAAAATTAAT------ATACTTCC 21919
NorthernAmericanDeerMouse    AAAGGCGTGCG----------------CCACCACCGC-CCGGCACTATATTTTTAATTTTTGTGTA-----TATCTTTAT 21618
Mouse                        A----CATGCC----------------TCATATTTGCATCTTTCTTAGATTGTGAGTTTTTGTGTTTGTGCCACCTTTCT 25414
ChineseHamsterGHOK1GS        AAAGGTATATTAATCTGAGAATTTAGTTTATGAATGG-TTAAGACTATATCTTGAATGTTGTTATTACTATTATTACTAT 23589
LongTailedChinchilla         GAAATTGTGGGAGTAC---------ATTGGAAAACAGTTCGGCAGTTTCTTAAAAACA---TTAAT------ACACTTAC 23119

Majority                     TATXTXAXCXTXCCATAAATCTXCXXGXXAT--TTATXTACTTXAXXXXAATGAAGTCXATXCTGTAAX-----TAAAAA
                             42170     42180     42190     42200     42210     42220     42230     42240
Human                        TTTAGGGTCTTGGAAGGAGAATGCAGGAAATGATAAAACGAGTTATCTGGAAGAAGACAAGTTTGGAATAATATTAAAGA 40546
GuineaPig                    TATGTGACCCTGCTGTTTATCTCCTAGTCAT--TTGTTTACCTAACCAAAATGAAGTCATACGTTTA---------AAAA 21988
NorthernAmericanDeerMouse    TATTTTA---TACCATAAACC--------------C--TCCT--G-----------TCTATGCTGTAAA-----TAAAAA 21661
Mouse                        TTCTTTA---TATTATAGCTC-----------------TATT--G-----------CCTGTGCTGTAAG-----TAACAA 25456
ChineseHamsterGHOK1GS        TATATTAATTTACCTTAAAACTATATTTTACACCTATTTTCTTGAAACCAGTTTCTTTTATTCTATAAAATCA-TAAAAG 23668
LongTailedChinchilla         TATGTGACCCTGCCATTTATCTCCTAGACGT--TTATGTACTTAACAAAAATGAAGACAAACGTTTATT-----TAAAAA 23192
```

```
Majority                      XXCTXACXTAAGAATC-----GTXCTTAG--TXTXXTTTXATTXAXAAXAATGXAAACXTAXAAXAX------GXTTCAT
                                       42250     42260     42270     42280     42290     42300     42310     42320
Human                         GTTTTGTCTTAGAATCACTGGGTTGGTAGAAGATGATGCCATTAACAAAGGGGGAAAAGTAGGAAAAG--GAGACTCGTT  40624
GuineaPig                     --CTCACATATGAAT------GTTCTTAG----CAACTTTATTTATAATAACTCAAACCTAAAACAC-------CCAAAG  22049
NorthernAmericanDeerMouse     TCCAAAGTTGA---CC------TACATAAT-TGTCTTCTG-----------TG------TA------------GTTTCAT  21702
Mouse                         CCTGAACCTGA---CC------TACACAG--TGTCTTCCC-----------TG------TA------------GTTTCAC  25496
ChineseHamsterGHOK1GS         GACAGAGTAAAAAATTATTTTTCAGTTAAC-TCTGTTTTGACACACTTGAATGTACACATATACACTCCTAATGTTTCAT  23747
LongTailedChinchilla          --CTCACATAGGAAT------GTTCTTAG----TAATTTTATTTGTAATAACTCAAACCTAAAACAA-------CCCAAG  23253

Majority                      TXTAXTXA-AXAXATXAATAXTTTGCTXXT---X---TGACATTTTAA--TXTXXATTTCCAGTXTAATXTTTTTXTXTA
                                       42330     42340     42350     42360     42370     42380     42390     42400
Human                         TTTGGTGGGAAAAAAGAATAATCCGCTACT---G---TTCTGTTTTTTTTTGTTTGTTTTTTGTTTTTTATTTTTTATTG  40698
GuineaPig                     GTCAGCAA-ACAGGTAAATAGCTTGCTGTT---T---TGACACTACGG--TATTTACATACAGTGTAATATTTGCAATCA  22120
NorthernAmericanDeerMouse     CCTCTT-----T-------TCTTTGCT--CTA----ATGACATTTTAA----TGAATTTCC------ATTTTGTTGTCTA  21754
Mouse                         CCTTTG-----------------TGCT----C-----TGACTTCTTAA----TGAATTTCC------ATTTTGTTGTCTA  25540
ChineseHamsterGHOK1GS         TTAACTAAAAATAATAAAGTATTTGCTAACTGCAGTATGATGATGTAAAGGATGAATTTCTAGTATAATTTATTTTTCTA  23827
LongTailedChinchilla          TCCA-TGA-ATAGATGGATAGCTTGCTGTT---T---TGACACTGTGTGCTGTTTACATACAGTGAAATATGTGCAGTCG  23325

Majority                      TXTAGAATTGAGATATTAATTACAGXXXXXGTAXTACCXXGXXXXXAXGXTTAGXXXXCAGXAACTXTTCTTAGXTAX--
                                       42410     42420     42430     42440     42450     42460     42470     42480
Human                         ATCATTCTTGGG-TGTTTCTCGCAGAGGGGGATTTGGCAGGGTCATAGGACAATAGTGGAGGGAAGGTCAGCAGAT----  40773
GuineaPig                     TATAGAATTGAGTTATTGATTACAGCAACAGAATCACATTGTGTGAAAAGTGGAGGGACAGAAAGA------------TG  22188
NorthernAmericanDeerMouse     CTTAAAATCTAAGAGGAAATT-------TTGTAATATC-------CACAGTTAG--------AACTTTTCTTAGGTAA--  21810
Mouse                         CTTATAAGTCTAAGAGCATTTA--------TTTATACC-----------TTTAG--------AGCTTTTCTTGGGTAG--  25591
ChineseHamsterGHOK1GS         TATAGCAATTATAAAACAGTCATAATGATTCTAATAATGAGCAAGCAGGTTTAGGTATCTGCAACTCTGCTTTGTCAAGT  23907
LongTailedChinchilla          TATAGAAT-GAGCTATTGATTACAGCAGCAGTATTACATTGTGTATAAGATGGGCAGACAGAAAGAGTAAGTCGTTTGTG  23404

Majority                      ATTCTATTTAAAATAAAXCT------------XGXATATTXXAA----XXATXTCTAAAGXACXATACXGXTTACTXGAA
                                       42490     42500     42510     42520     42530     42540     42550     42560
Human                         AAACAAGTGAACAAAGGTCTC-----------TGGTTTTCCTAGG-----C-----AGAGGACCCTGCGGACTTCCGCAG  40832
GuineaPig                     ATTCTATTAAATACAAAACT------------GGAAAATTCAAA------------------CAATACTGGTTACTGGGA  22238
NorthernAmericanDeerMouse     -----ATCTGAAATAA--------------------TATTT-------TAATGTCTAAAGAAATATAT-ATTAATCTGAG  21857
Mouse                         -----GTCTCAAAAA---------------------TATTT-------TAATGTCTGAAGAAG-AAAT----------AT  25627
ChineseHamsterGHOK1GS         TTTTTATTTGTAATAAATGGTAATTGGTGCTAGGAATATTTTAATAAATAATATCTATGGCTGTACACGAGTAACTAGTA  23987
LongTailedChinchilla          ATTCTATTAAATATAAAACTCC----------AGGAAATGCAAAG-----TAATCTAAAGCACATAGATGATTACTAGGA  23469
```

```
Majority                    GTTTTGAX-XCX----------XXXTXGAX--TXXGGAGXAGTXAXGXXCXTXAXGAATXTTXAGAXTXTATAXXTXTGX
                                     42570     42580     42590     42600     42610     42620     42630     42640
Human                       TGTTTGTGTCCCTGG------GTCCTTGAGATTAGGGAGTGGTGATGACTCTTAACGAGCATGCTG-CCTTCAAGCATCT  40905
GuineaPig                   GTTCTGATAGCA----------CAGTTGAA--TGTGGAGCAGTAAGTAGCACAAGGAAATTTGAGA-TCAATGGATATAT  22305
NorthernAmericanDeerMouse   ACTTT-------------------------------------------AGTTTATGAATGGTAAGACTGTAT---TTTGA  21891
Mouse                       GTTTT---------------------------------------------------AATTTTATGACTTTAT---TTTGA  25653
ChineseHamsterGHOK1GS       GCCTTTACCCCTTTTCTAGTCATCTTCCAAACCAAAGTTGATTGTTGGAATTGATGAATGGAAATGTAATATAAGCTTGA  24067
LongTailedChinchilla        GTTTTGAGGGTAAGG------ACAGTGGAG--TGTGGAGTAGCAAGGGGCACAAGGAGACTTGTCAGTGGATATATATAT  23541

Majority                    ATA--XAXA-AXXCATATTXXX-----TXTTATXXTTATXCTAATTTAXTTTXAGTCX----------------------
                                     42650     42660     42670     42680     42690     42700     42710     42720
Human                       GTT-TAACA-AAGCACATCTTG-----CACCGCCCTTAATCCATTTAACCCTGAGTGG----------------------  40956
GuineaPig                   AT----ACACACACATATG---------GATATATATATCCTAATATATTTTATGTATT---------------------  22351
NorthernAmericanDeerMouse   ATA------------TATTACTACCATTATTATTATTAGATTAATTTACCTTGAAAC--------------TT-------  21938
Mouse                       ATA-------------------------AT-ATTATTATATTAATTTATTTCAAGTC-----------------------  25684
ChineseHamsterGHOK1GS       GTGGAAGGAAATACCTGCAGTGACCCCTGTGCTCCTTCTGATTTTCTCCTTTGCAACAGTGCAGCCACAACTACACAGAC  24147
LongTailedChinchilla        ATCATAATACATGTATATTACA-----TGCTGTATATGTCCTAATATATTTTATGTATT---------------------  23595

Majority                    AXAXA-XATACATTTCAX----CACAXXGTACCCATTT-XGTCCXAAAACXAGTGGXTATAGXAXTCTTCTXX----A-X
                                     42730     42740     42750     42760     42770     42780     42790     42800
Human                       ACACAGCACATGTTTCAGAGAGCACAGGGTTGGGGGTAAGGTCACAGATCAACAGGATA-AGAATTTTTCTTA----G-T  41030
GuineaPig                   ATATATTATACATA-CA-----TACACAGTCCTCCTTT-AGTATCAA--GGATTGGTTATAGGACTCTTCCCC----A-C  22417
NorthernAmericanDeerMouse   --------GACATTTTAC------------ACCTATTT---TCCTGAAACCAGTTTCTTTT-----CTTCT---------  21981
Mouse                       --------TACATTTTAT------------ACCTATCT---TCCTGATACTAGTTTGTATTT---TCTCCT---------  25729
ChineseHamsterGHOK1GS       AAAGCAGAGAGATTTCACTGGACCTAAGAAACCCATTGGAATTCTAAACCTGGGGGCTTTGGGGGCATGGTTACTGAAGT  24227
LongTailedChinchilla        ATACACTATACATA-CAGTACACACACAGTTGCCCTTT-GGTATCAGAGGGATTGGTTATAGAACCCCTCCCC----A-C  23668

Majority                    XAAGXAXATXATAAAAAGTTGGGXATAXACAAXTTTTTTTAXGTXAAC----AXXGCT-CXAT-------ATTTTGCAXT
                                     42810     42820     42830     42840     42850     42860     42870     42880
Human                       ACAGAACAAAATGAAAAGTCTCCCATGTCTACCTCTTTCTACACAGAC----ACGGCAACCATCCG----ATTTCTCAAT  41102
GuineaPig                   CAAGGCTA---CTAAAACTTGAGAA-ACTCAAGTCCTTTTATGTAAA-----GTAGCT-CAGT-------ATNNNNNNNN  22480
NorthernAmericanDeerMouse   -ATAAAGATCATAAAAGGATGGA-ATAAACAACTATTTTTCAATTAAC--TC----TTTTGATAT-----A-CTTGGATC  22047
Mouse                       -ATAAAGGTCATAAAAGGATGGA-----ACACACTTCTTTCAGTTAAC--------------------------------  25771
ChineseHamsterGHOK1GS       GAAGGAAGTTGTACAGAGTTGGGCATATAGAATTTGTTGTAGGGTCATAATCAGAATTTCGATACTAGCTAGGTTGCATT  24307
LongTailedChinchilla        CAGGGATA---TGAAAATTTGAGAATGCTCAAGTTGTTTTATGTAAA-----ATGGCA-CAGT-------ATTTGCAGAT  23732
```

```
Majority                     TATACATXCACXA--XTCCAXTGTTXATTX-X-XXTAXCTCCCTAAXX---XATXXTAXGTATTTGCT------AXCTG-
                                     42890     42900     42910     42920     42930     42940     42950     42960
Human                        CTTTTCCCCACCTTTCCCCCCTTTCTATTCCACAAAACCGCCATTGTCA-TCATGGCCCGTTCTCAAT-----GAGCTGT  41176
GuineaPig                    NNNNNNNNNNNNNNN-NNNNNNNNNNNNNNNNNNNNNNNNNNNNNNN---NNNNNNNNNNNNNNNNNN-----NNNNNNN  22551
NorthernAmericanDeerMouse    TACACATATACAA--TTCTAATGTT---------TCATTTCCTTAAA----AATGATAAGTATTTGCT------AACTG-  22105
Mouse                        --TAAAAACGGGA-------------------------------AAG-----------TATATGCT------AATTG-  25798
ChineseHamsterGHOK1GS        TGTACAGGTGGGAACTTGCAGTGCTCATTAGGACTTACCTTTCTAAGGTCTAAGAGTTAATACATGCTCTCATAGGCTGA  24387
LongTailedChinchilla         AACCGATGCACATC-CTCCTTTATATATTAAATCATGTCTCGCTTATC---CATAATACCTATTTCAT-----TGCCTAC  23803

Majority                     XCAGTATXXTGA--CGTXGGAAT--XXX-XXX-CAGATGXATTXXTAXTXTAT-AGTA--X-XX-XTTXAXTTTTGTGTA
                                     42970     42980     42990     43000     43010     43020     43030     43040
Human                        TGGGTACACCTCCCAGACGGGGTGGTGGCCGGGCAGAGGGGCTCCTCACTTCCCAGTAGGGGCGGCCGGGCAGAGGCGCC  41256
GuineaPig                    NNNNNNNNNNNNNNNNNNNNNNNNNNNNNNNNNNNNNNNNNNNNNNNNNNNNN-NNNNNNNNNNNNNNNNNNNNNNNNNN  22630
NorthernAmericanDeerMouse    -CAATATGATGA----T-GTAA------------AGATGAATTTCTAGTATAT-AGTA--------TTTATTTTTGTGTA  22158
Mouse                        -CAGCATGATGG--CGT-GGAA------------AGCTGAATTTTTAGTACAT-ATTA--------TTTATTTTTCTGTA  25853
ChineseHamsterGHOK1GS        ACAGTGTTCTGAGACATTGCAATTCTGAGAAGTCAGATGTAGTCATCTTCTAG-AGTACCTCCAACTTAAAATTTGGATG  24466
LongTailedChinchilla         ACAACAATTCATTTCCATGGATTCAGCATAGTACTCAGGGTATGTCAACTTCA-AGTTTTGTTATTTTGAATTTACTTAA  23882

Majority                     CAXTACTTACAAATCXGTATXACA-----GTXCXXTGXTX--TCTGAX---XTCAAGAAXCXXXXXAGTTAAXXATGCCX
                                     43050     43060     43070     43080     43090     43100     43110     43120
Human                        CCTCACTCCCCAGTAGGGGCAGCC-----GGGCAGAGGCGCCCCTCAC---CTCCCGGAT-GGGGCGGCTGGCCGGGCGG  41327
GuineaPig                    NNNNNNNNNNNNNNNNNNNNNNNN-----NNNNNNNNNNNNNNNNNNN--NNNNNNNNNNNNNNNNNNNNNNNNNNNNNN  22702
NorthernAmericanDeerMouse    TAGTATTTATGAAACAGTATCATA-----GTTCCATGAT---TCTGAT--AATGAAGAAGC----AAGTTCAG-ATACCT  22223
Mouse                        CAA----TATAAAACGGTATCACA-----AGACCATGAT---TCTGAC------AATAAAC----CAATTAAA--TGTCT  25909
ChineseHamsterGHOK1GS        CAGAAAGTACAGATCTTTAGTAAATTTATGTTCTGTAGTAAGTCCCATTCATTTGAGAAACTGAAAAGTCTAACATGACA  24546
LongTailedChinchilla         ATTTTCTTTCAGATATTTAAATCA-----GTGATTGGTTGTTCTTGTA---GTCAGGGTTTGCAAGGGAAAACAATACAG  23954

Majority                     GXAXCTXAAXXTTTTGTXXTXCT-XTGCXTGX------XXX--XGCCC---XXXXXXATXXTCCGGGCAAATCTXTCXCA
                                     43130     43140     43150     43160     43170     43180     43190     43200
Human                        GGGGCTGACCCCCCCACCTCCCTCCCGGATGG----GGCAGCTGGCCGGGCGGGGGGCTGACCCCCCCACCTCCCTCCCG  41403
GuineaPig                    NNNNNNNNNNNNNNNNNNNNNNNNNNNNNNNNNNN-NNNNNNNNNNNNNNNNNNNNNNNNNNNNNNNNNNNNNNNNNNNN  22781
NorthernAmericanDeerMouse    GTAGCTTGA-GTTTTTTGGTCC---TGCCTG-------------GCCC---------ATGGTCAGGACAAATCTCTCTCA  22277
Mouse                        GCAACTCTGCTTTGTCAGATTT------TTG-----------------------------------ATGTATAACATA  25946
ChineseHamsterGHOK1GS        GTATGTGAATGCTTTGTAATTTTGTTGCCTGCTAAAAAATATAAGCCCCTAAGGAAGGCAATAGGGGTAAATCTTTCTCA  24626
LongTailedChinchilla         GGCACATAATTTATAGTATTGCTTTATAATAGTCA-CTCAAAGAGTCATATACAAAAAGAGTGCAGGAAATCTGTCCACA  24033
```

```
Majority                    GXCXTCXATACTAGXGATGCT-----CTAACCCAACCAAATX---XXAAAXAXAXAGGGACXTACXTXGCTXAAAACTTA
                                     43210     43220     43230     43240     43250     43260     43270     43280
Human                       GACGGGGCGGCTGGCCGGGCTGGGGGCTGACCCCCGCCACCTCCCTCCCGGACGGGGCGGCTGGCCGGGCTGGGGGCT--  41481
GuineaPig                   NNNNNNNNNNNNNNNNNNNNNNN---NNNNNNNNNNNNNNNNNNNNNNNNNNNNNNNNNNNNNNNNNNNNNNNNNNNNNN  22858
NorthernAmericanDeerMouse   CCCGCCAGTCCCGCAGCTGCT-----CAGACCCAACCAAGT------AAACACACAGAGACTTATATTGCTTACAAACTG  22346
Mouse                       GTAATTGATAGTAGGGATGTT-----TTAA---AACTTAAT----------ATTTAG-GCTATACCTGAGTAATAACTAT  26007
ChineseHamsterGHOK1GS       GTCATCCATAGTATGTATAGC-----CTAAAATAAACAAATCATGTGAAAAAAAGAGGAAAATACAAACCTAGAAAATGA  24701
LongTailedChinchilla        CACTAAAAGACCAAAGAATCCAG---CTAAGGGCCTGGATCCTATGGTAATATCCTAGCACGAGCTCAGCTGAAGACCTA  24110

Majority                    -GTGXCCA----GAXCAXXCTTCC--XCXGTCAAGTTXXXTGT-XXXAX--------XXXXTXACAXXXXX-X-X-CCAA
                                     43290     43300     43310     43320     43330     43340     43350     43360
Human                       -GACCCCCC---GACCTCCTTCCCGGACGGGGCGGCTGGCCGGGCAGAGGGGCTCCTCACTTCCCAGTAGGGGCGGCCGG  41557
GuineaPig                   -NNNNNNNN---NNNNNNNNNNNNNNNNNNNNNNNNNNNNNNNNNNNNN--------NNNNNNNNNNNNNNNNNN-NNNN  22925
NorthernAmericanDeerMouse   TATGGCCA----CAGTAGGCTTTT----TGTTA--TCTAGTTT------------------TTATATC------T-T-AA  22390
Mouse                       AGTAGTCT----TGACCCCTTTCC---CAGTCATCTTCCAAGC------------------CCACAAA--------CCAA  26054
ChineseHamsterGHOK1GS       TGTGTTCAGTAGGAGGAGACTTCTACACTGCCAAGTGTAGTATTAGAAAG--CAAAGGCTATTATAGCATTAAGT-TTAA  24778
LongTailedChinchilla        -GAGCCCGT---AACAATGCCCCCCACAAGTCCAGCTGGAGGTCAAGAG--------CCCATGACAATGGGCATG-CAGG  24177

Majority                    AXTXAATCGTTXAX-TACTXXCXGAAXAGAXGXXXGAXGGTXGXXGGATXXXXAACATCXT-TTACXAXCTGCATGXXX-
                                     43370     43380     43390     43400     43410     43420     43430     43440
Human                       GCAGAGGCGCCCCTCACCTCCCGGAAGGGGCGGCTGGCCGGGCGGGGGTCTGACCCCCCCACCTCCCTCCTGGATGGGGC  41637
GuineaPig                   NNNNNNNNNNNNNNNNNNNNNNNNNNNNNNNNNNNNNNNNNNNNNNNNNNNNNNNNNNNNN--NNNNNNNNNNNNNNNNN  23003
NorthernAmericanDeerMouse   ATTAACTCATTTT--TATTAGTCTATAAGTTGTCACATGGCTGGTGGCTTACCAGTATC-TTTTACATGTTGCATG----  22463
Mouse                       AGTTGATTGTTGG---ACTTGATGAACAGAAATTATAAGCTTGAATGAAAGGAAACACCCTGTCATGAGTCTCATG----  26127
ChineseHamsterGHOK1GS       ATGAAAAGGTTTAAATAATATCTGAAAAGAAGATGGAATGTCTTTAGATAAAGAACTTT-TATTTTAAACTGCATAAAAA  24857
LongTailedChinchilla        CCTGTTTCATGGATTTGCACTGGGAAGGCCCAAAGGTTAGTGGTGGAAGCTAGAAAGTCTG--TGCCAACAGGATGAAGG  24255

Majority                    -TXTG-XC-TGGCGGXAXXTGAXATCTXX-TTXCTCTXAGTCAGCAT-XXXXX-XG-XXXXXXTXAX--XX-XCTCCCAG
                                     43450     43460     43470     43480     43490     43500     43510     43520
Human                       GGCTGGCC-TGGCGGGGGCTGACCCCCACCTCCCTCCCAGACGGGGTGACTGCCGGGCGGAGACGCTCCTCACTTCCCAG  41716
GuineaPig                   NNNNNNNN-NNNNNNNNNNNNNNNNNNNNNNNNNNNNNNNNNNNNNNNNNNNNNNNNNNNNNNNNNNNNNNNNNNNNNNN  23082
NorthernAmericanDeerMouse   -T-----CATGGCGGCAGCTGGCAGCT---TCTCCCTGACTCAGCCT---------------TTCA------CCTCCCAG  22513
Mouse                       ----------A---TCATTTTAGGTTT---CTGCTTTGA---AGCAT---------------CTTA-------CTGCCAT  26166
ChineseHamsterGHOK1GS       TTATGAACAAGATGATATTTAAAATGTAAATTTTTCTAGTTGAGGTTAAAAGAGGGAAAATGTTTAATAAACTTTCATAA  24937
LongTailedChinchilla        ATCTGTAC-TAGCAGGCCGAGGGATCTGCTCTGGAGCCAGGAAGCAATGCTCAAAGTAAGAACTGGAGTTCTTCTGAAAG  24334
```

Majority	AAATCACTTC-------XTXCTCXGCCAAAXAGX---XCXACCTACCGGX--ACXXXGCAAXCAGXX-GXAGTACTACXC	
	43530 43540 43550 43560 43570 43580 43590 43600	
Human	ACGGCGTGGC--------TGC-CGGGCGGAGGGGCTCCTCACTTCTCAGATGGGGCGGCTGCTGGGCGGAGGGACTCCTC	41787
GuineaPig	NNNNNNNNNNN------NNNNNNNNNNNNNNNNNNNNNNNNNNNNNNNNN--NNNNNNNNNNNNNNNNNNNNNNNNNNNN	23154
NorthernAmericanDeerMouse	AATTCTCTTC--TCTCCTTGTCCTGCCTAT-A------CTTCCTACCTGGCTACTGGCCAATCAGT--GTTTTATTAACC	22582
Mouse	GACTGCATAG---------ACAAAGCTCAG-AG------GCTCCACTGG---ACCAAGGAAACACA--GTAGAACTAAAC	26225
ChineseHamsterGHOK1GS	AAATTAGTTAAATTTATTGACTCTGCCAAAGAAAAAGTATACCTATGGGGATACCAAGCAATAAGTAAACAGTCTTACCC	25017
LongTailedChinchilla	GAACCACATCC------ATTCTCAGCTACAATGAGGTCCCACTGTCCCGA--GCTCCTATCAGGAAATCCAGTGCAACAA	24406
Majority	XCTCCXXXCATCAGXCXXAXXXTCCXXXATATTCCACAXCGXAGAGXXGXAGXTATGCXXXXTAXAAGTXXXTATXXACA	
	43610 43620 43630 43640 43650 43660 43670 43680	
Human	GCTTCTCAGATGGGGCGGCCGGGCCGAGACGCTCCTCACCTCCCAGACGGGGTCGCGGCCGGGTAGAGGCGCTCCTCACA	41867
GuineaPig	NNNNNNNNNNNNNNNNNNNNNNNNNNNNNNNNNNNNNNNNNNNNNNNNNNNNNNNNNNNNNNNNNNNNNNNNNNNNNNNN	23234
NorthernAmericanDeerMouse	AATCAGAGCAACACACTTAACATACAAAACATCCCACAGCAGATACCTGCAACTCTGCTTTGTCAAGTTTTTTATTTATA	22662
Mouse	ATGCAGGG-GTTAGGCCATGGATCCTGAAT-----ACAGGGAAGTGGTGCAGTTTAGACAGCCAGAAGTTCATATAGTGA	26299
ChineseHamsterGHOK1GS	TCTCCCAACCCCATATTTAGATTTCTGGATAGTACATGAAGAGAAGGGAAAAATATATATAATATATATATATATATACA	25097
LongTailedChinchilla	GCTTCTGCAAGAGACTGAGCAGTGCACCATCTTCCCCTCCGGAGTCAGAAAGCCATGCTCAAAATGAGGACCAGAGCTCC	24486
Majority	CXCAAAXAXA--CX---XXA-AXXGATACTCXCXAXATXTXXG----TATAXGTTCACXGXTATGGAXGXTTGTAACTTG	
	43690 43700 43710 43720 43730 43740 43750 43760	
Human	TCCCAGACGGGGCGGCGGGGCAGCGGCGCTCCCCACATCTCAGA--CGATGGGCGGCCGGGCAGAGACGCTCCTCACTTC	41945
GuineaPig	NNNNNNNNNNNNNNNNN------NNNNNNNNNNNNNNNNNNNNN--NNNNNNNNNNNNNNNNNNNNNNNN--NNNNNNNN	23304
NorthernAmericanDeerMouse	---ATAAATA-------ATA-ATGGGTACTAGGAATAT-TT------TAAAAATTAATATCTATGGTTGTCTGTAGCTAG	22724
Mouse	C-----CATA-------GCAAAAGGATACTAGCTTCGT---------TATATTTTTACTGGTA-GGAAGCTTGCAA-T--	26354
ChineseHamsterGHOK1GS	CACACACACACACATATATATATATATACACACAATATATGCGTACATATATGTGCACATATGTGTGTATTTGAAACAGG	25177
LongTailedChinchilla	CCCAAAGTGGACCAA------AGCCATTCTCAGCAACAACAAGG--TCCTGAGCTCCCTGATGAGAACAG--CTGAGTTT	24556
Majority	ATXTAT-----XCXXGCCXGXXXXACXXGXTCXXGAGXXCTXXCTGXXACXCCCACCGGCXAX--XTATCCGXTTAXTC	
	43770 43780 43790 43800 43810 43820 43830 43840	
Human	CTAGATGGGATGGCGGCCGGGAAGAGGCGCTCCTCACTTCCTAGATGGGATGGCGGCCGGGCAGAGACCTCCTTTCTTTC	42025
GuineaPig	NNNNN------NNNNNNNNNNNNNNNNNNNNNNNNNNNNNNNNNNNNNNNNNNNNNNNNNNNNNNNNNNNNNNNNNNNNN	23378
NorthernAmericanDeerMouse	AGTTTT------CCTGCCTGGCCCACAG---TCAGGACAAATCTCTGTCACCCGCCAGTCCCA----TAGCCGCTCAGAC	22791
Mouse	ATCTAT------CAAAACTGCCTTACTGTAGGCCTGAGAGCTCTGTGGTGATGTCATAGGATG-----AACACTTT--TC	26421
ChineseHamsterGHOK1GS	AATTGTTGAACACTTAAGAGTTCTAAGAAATTCAGTAAGCCCTCCCCTTACCCCCACTCCCAACCTTTATTGGGTTAATT	25257
LongTailedChinchilla	ATCAA------GCAAGCCAGCATGACAAGCTCCTCCAGGAAAACCAACCACTCCCTGCAGCTTCTCTTCTCCAGTTCCCC	24630

Sequence	Alignment	Position
Majority	TXAAXCGTGTAAXCACAGAGAGGX------------XTXCTXATAAACCAXXX---AXXCCXTGCCAGGCXTATATGCAA	
	43850 43860 43870 43880 43890 43900 43910 43920	
Human	CAGACTGGGCAGCCAGGCAGAGGGG-----------CTCCTCACGTTCCAGACG--ATGGGCGGCCAGGCAGAGACGCTC	42092
GuineaPig	NNNNNNNNNNNNNNNNNNNNNNNN------------NNNNNNNNNNNNNNNNN---NNNNNNNNNNNNNNNNNNNNNNNN	23442
NorthernAmericanDeerMouse	CCAACCAAGTAAACACAGAGACTTATAT---------TGCTTATAAACTG----TATGACCGTGGCAGGCTTCT-TGCTA	22857
Mouse	TGAGACGTTTGAGTTCTGAGAGGT---------------CTGATAAAGCA----------CATTCCAGG-GTATCCTCAA	26475
ChineseHamsterGHOK1GS	TGAAACACAGAAACACAGACAGGCACAGAATAGCCAGTAATTGAAAACTAATGTTAAAGATATTTAAAGCATATATGCAA	25337
LongTailedChinchilla	TATTTTGTGTTACTGGCAACAAT-------------CCTCTGATGGGCCTGTC---ACACCCAGCACTGGGTTGATGCAG	24694

Sequence	Alignment	Position
Majority	XTXACXTTTA---XXX-GXXXX-XGXTGAXCATAAATTAATACCAXATXACTTTTXTAXATCTAG------GAXATTGGC	
	43930 43940 43950 43960 43970 43980 43990 44000	
Human	CTCACTTCCCA-AACGGGGTGGCGGCTGGGCAGAGGCTGCAATCTCGGCACTTTGGGAGGCCAAG------GCAGGCGGC	42165
GuineaPig	NNNNNNN------NNNNNNNNNNNNNNNNNNNNNNNNNNNNNNNNNNNNNNNNNNNNNNNNNNNN------NNNNNNNNN	23510
NorthernAmericanDeerMouse	ACTGTTCTTA-----------------TATCTTAAATTAAT-CCA--T---TTCTATAAATCTAT-------ACCTTGCT	22907
Mouse	CTTACAATTA-------------------GAATATAGAAAGCCCAGAT--CTTTAATAAAACTAG---------ATTTGG	26525
ChineseHamsterGHOK1GS	AAGACATTTAATAAGAAGTAAGGAGTTCACACTAAAAGAATACAAGCTAACCTTTTTAGATTTAATAAAAGGACATTAGC	25417
LongTailedChinchilla	TTCAGCT------GGGTGTTACAAGTTGACCACCAGTTGTCAGTTCACCACAGTTGTATATACAG------GTAAGAGAC	24762

Sequence	Alignment	Position
Majority	TGGXAGGTXX-XGXT-XXXAAGGGTCXXGAAAAXTCCTXTGXXATGGXGGXTGTGAAXAXTTCXTXXXTXAGTTAGTTXX	
	44010 44020 44030 44040 44050 44060 44070 44080	
Human	TGGGAGGTGGAGGTT-GTAACGAGCCGAGATCACGCCACTGCACTCCAGCCTGGGCACCATTGAGCACTGAGTGAACCAG	42244
GuineaPig	NNNNNNNNN--------NNNNNNNNNNNNNNNNNNNNNNNNNNNNNNNNNNNNNNNNNNNNNNNNNNNN---NNNNNNNNN	23579
NorthernAmericanDeerMouse	ACATGGCTCGTGGCT-TACCGGCGTCTTCACATGTTCCTTGTCATGGTGGCAGCTGGCAGTGTCCCCGTTCGCTTACTGT	22986
Mouse	TGGTAAGTGCCATTTGTTGAAGGAACTAGAAA-----TCTAACATGACAGACATGAAGATTTCATATGAAATTTAGTTG-	26599
ChineseHamsterGHOK1GS	TAAGAACTTAGAGCTACAGAAAGGTTATGAAAAGTACTGTGAAATAGCAAATGTAAATAATTCTTTTTTAACCCTGTTTC	25497
LongTailedChinchilla	CTGCTGGTC--------CAAAGGATCGCCTGTGCTCTTTTGTGATGTTGGTTTTGTGTATATACATAC---ATAAGTCAA	24831

Sequence	Alignment	Position
Majority	ACTX-XXX------XXXTTTGTTCXCXTCAAATATXCXTXXXTGXCCGXACXXTXGXGAXTCAGTXCTXTAGXCXX--XG	
	44090 44100 44110 44120 44130 44140 44150 44160	
Human	ACTCCGTCTG----CAATTCCGGCACCTCGGGAGGCCGAGGCTGGCGGATCACTCGCGGTTAGGAGCTGCAGACCA---G	42317
GuineaPig	NNNNNNNNN------NNNNNNNNNNNNNNNNNNNNNNNNNNNNNNNNNNNNNNNNNNNNNNNNNNNNNNN-NNNNN-----N	23646
NorthernAmericanDeerMouse	TCTT-TCTTTTCTCCTCTCTGTTAGTCCCGCCTATACTTCC-TGCCTAGCCACTAGCCAATCAGTGTTTTATTTATTGAC	23064
Mouse	----------------C----CTGGTTAAAAATATACATATATGGCCGGGCGGTGGTGGCGCACGCCTTTAATTCC--AG	26657
ChineseHamsterGHOK1GS	AGTCATCTGAATTTCTTTGTATTCTCTGCATTTCTGTATAT-TATGCATACTATAGTGAAACTGTATTATAGGCTTT-AT	25575
LongTailedChinchilla	ATTTTGTC-------AATTTGTACACATTAAATATGTGTGCATATTAGAATGGCCAAGATTCAGTACC-CTGAC-----A	24898

```
Majority                      CAXGTAGAXGACAGTGACA--X-X-XCAA-ACXXAXAGCAXTTGTXAXTTCGTXGGXAGTX-CCTTXGTXTTXTTXCAAX
                                         44170     44180     44190     44200     44210     44220     44230     44240
Human                         CCCGGCCAACACAGCGAAACCCCG-TCTCCACCAAAAAAATACGAAAACCAGTCAGGCGTG-GCGGCGCGTGCCTGCAAT  42395
GuineaPig                     NNNNNNNNNNNNNNNNNN-------NNNNNNNNNNNNNNNNNNNNNNNNNNNN-NNNNNNN-NNNNNNN-NNNNNNNNNN  23716
NorthernAmericanDeerMouse     CAATCAGAGTAATTTGACATACAGACCGT--CTCACAGCAGTTGTACATGTACTG-TAGTATCCTTGATCCTTTTTCCA-  23140
Mouse                         CACTTGGGAGGCAGAGGCA--------------GGCAGGATTTCTGAGTTCGAGGCCAG----CCTGGTCT-------A-  26711
ChineseHamsterGHOK1GS         TATGTAGATGGCATTGACA-ACAGTCCAAAAATGAAAACAGATGAGATTACTTTGATAAAATACTTAATATTTTATATAA  25654
LongTailedChinchilla          TCAGATGTTGACAGTAAA-------TAAAGATCAAGAGCTCTCATTTGTTGGT-AGGAATG-CTAAAGC-TACTTGGAAA  24968

Majority                      AXXAX-XTXAXXCXGXCXTCTAAAXCAAAAXXXTCTGATXXTXAGGATTTA-TGAAXXGAAXTXX-AGC--TXGAGCTCA
                                         44250     44260     44270     44280     44290     44300     44310     44320
Human                         AGCAG--GCACTCGGCAGGCTGAGGCAGGAGAATCAGGCAGGGAGGTTGCAGTGAGTCGAGATGGCAGCAGTACAGTCCA  42473
GuineaPig                     NNNNNNNNNNNNNNNNNNNNNNNNNNNN-NNNNNNNNNNNNNNNNNNNNNNNNNNNNNNNNNNNNNNNN--NNNNNNNNN  23793
NorthernAmericanDeerMouse     ------GTCAT-----CTTCCAAACCAAAG----TTGATTGTTACGATTGA-TGAAGAGAA-----AGT--ATGAGCTTG  23197
Mouse                         -------------------------CAAAG----TTGAGTTCCAGGAC-------A-----------GC--CAGGGCT--  26740
ChineseHamsterGHOK1GS         AGTAATGTTATGCTGACTTTAACATAAAAACATTCTGAACTTTAGGTTATA-TATACTAAAATCTTAGT--GCTAGCTCT  25731
LongTailedChinchilla          ACTAATTATACTCTGCTGTATAGTACAG-AGGTTCTGGTCCTCAGTATTTATTGAAAGGAATTGGAAAC--TTAAGCTCA  25045

Majority                      AATAAAAGXXAACA--XXXX--AGXCTGTXXTG---XXAAAAXTGGAAGACTXTGTGXAXXTXXXXXAXXCTTTGAAXTA
                                         44330     44340     44350     44360     44370     44380     44390     44400
Human                         GCTTCGGCTCGTCATCAGAGGGAGACTGTGGAAAGAGAGGGAGAGGGAGACCGTGGGGAGAGGAGGGAGGGGGAGGGGGA  42553
GuineaPig                     NNNNNNNNNNNNNNNNNNNNNNNNNNNNNNNNNNNNNNNNNNNNNNNNNNNNNNNNNNNNNNNNNNNNNNNNNNNNNNNN  23873
NorthernAmericanDeerMouse     AATGAAAGGAAAC----------CCCTGCAGTG------AGCCTTGTGCTCCTTCTGGTTTT-----CTGCTTTGAAACA  23256
Mouse                         -ATATAAAGAAAC-----------CCTGTCTCG-----AAAAACCGAAATATATGTACACAT-------------ACATA  26790
ChineseHamsterGHOK1GS         AAGAAAAGATAAAAATGTAGCTAGTATATAATGGTGAAAAAGCTGGAAGATTGTCTGATTTTGAAAATGACTATAAATTA  25811
LongTailedChinchilla          CAAAAAATTCTTCAGAATGTACATAGCAACTTTCCTAGTAATGGAGAAGACTTGGGAAAGCCCTGAGATATCTTGAAGTA  25125

Majority                      XA-TACATXGAXAX--CTGCTCXGAT-----AX--ACACAXAGGCXXXXCACTATATTAXX-XXXATXAAAXTCAAATGX
                                         44410     44420     44430     44440     44450     44460     44470     44480
Human                         GG-GGGAGGGGGAGGGCTGTTCTGTTTTTTTAACTATGCTTTAGGGATGATATATAATGTACTTCATGACCAAGGAATCA  42632
GuineaPig                     NN-NNNNNNNNNNNN-NNNNNNNNNN---------NNNNNNNNNNNNNNNNNNNNNNNNNNNNNNNNNNNNNNNNNNNNN  23942
NorthernAmericanDeerMouse     CAGTGCCTCTACA--ACTGCATAGAC-----AA-AGCAGAGAGGCT--TCCCTGAATCA------AAGAAACTCAGACTG  23320
Mouse                         CA-TACATACACA----CACACACAC-----A--CACACACACACA------CACATTA--------TATAT--ATATAT  26842
ChineseHamsterGHOK1GS         TAAAACCAAAATAGCACTGTTTGGCTTA---AA-GATAAATAGGTTGGTCAGTAGATCACAATATAGAGAATTCAAAAGT  25887
LongTailedChinchilla          GG-TAAATGGGTAAA-CAGCTCTGGT---------ACATCCAGACAATGTACTGTATTATTTCTTGTTAAAAGAAAATGG  25194
```

```
Majority                      AAGXXXTXXATATGTGXG-X------X-XXTAGGXGXGCATAAAGTCXTGXAXXAATA-AXXAATTAAATAXAXX-X---
                                       44490     44500     44510     44520     44530     44540     44550     44560
Human                         ATCTAATCTTTTTGTAATGA-----TGGCATAAAATTCCTTTAAGCCTTGTTTTAATACAAGAAGTCAGTTTATTTAACC  42707
GuineaPig                     NNNNNNNNNNNNNNNNNNN-------NNNNNNNNNNNNNNNNNNNNNNNNNNNNNNNNNNNNNNNNNNNNNNNNNNNNNN  24015
NorthernAmericanDeerMouse     GAATTCTAAACATGTGGG-A--------CTT-GGGGGGCATAA-GTCCTGAAGGAAAG--GAAATTATACAAA-------  23380
Mouse                         AATGTGTGTGTGTGTGTG------------TGTGTGTGTACAT--ACATGCACACATA------T-ATGTAAA-------  26894
ChineseHamsterGHOK1GS         AAGCCCACAATATATGGGCAGCTGATATTTTTGGGAGGTACAAAGTCTTGTAGGAAAAAAGAGATTAAATAGAATGCTAT  25967
LongTailedChinchilla          GCGGAGAAGCCATTGAAGA-------GATAAAGATGAACATTAAATGCCTACTAAGTGGAAGAAGTTAATCTGAAAAGTG  25267

Majority                      XTXXTGTTT-X---------XXAXXT--AAXXXXTXXXXX-AXXXXTTCTGATX-AXGTAAAAATXXXXATXAAXTTTAX
                                       44570     44580     44590     44600     44610     44620     44630     44640
Human                         TCCATGTACAGAGGAAATACTGAGTTTTAAGATTTTTCTATTTTTTTTCTGACAAAAATGAAAGTCTGCATATATTCTAA  42787
GuineaPig                     NNNNNNNNN-----------NNNNNNNNNNNNNNNNNNN-------NNNNNNNNNNNNNNNNNNNNNNNNNNNNNNNNNN  24077
NorthernAmericanDeerMouse     -T-----TTAGGC--------------------------AGACAGAATTTGGT----GTAGGGGTCATAATCAAATTTTT  23424
Mouse                         -----------------------------------------ACAGCCCTTAAG---GATGAAAATAGAATTTAATCTTTC  26930
ChineseHamsterGHOK1GS         ATAGTGTTTTGCCTTCCCATCCAGCTCAAAGTGATTTTTAGATTTATTCTGATTTAAGTAGAAATGAACTCCAAGTGTAC  26047
LongTailedChinchilla          TACATGTTT-----------TCAACTACAATATTTAGCA-------TTCTGGAAGAGGCAAAAATATGGAGATGGTAAAA  25329

Majority                      XATATXAGXAA--TTGCTTXX-XXXX-------------XXAXXAAATAXAAXTAGAXACTTTXXAGX---GXAAATTAA
                                       44650     44660     44670     44680     44690     44700     44710     44720
Human                         GAGACACCAAT--TTGAATACTGTTGC-------TTATAAGAGCAAATAGCATAAAAGACCTTTAAAAATTGGTGATTAT  42858
GuineaPig                     NNNNNNNNNNN--NNNNNNNNNNNNN-------------NNNNNNNNNNNNNNNNNNNNNNNNNNNN----NNNNNNNNN  24138
NorthernAmericanDeerMouse     GGTGCTAGCTAGGTTGCTTT-------------------------TATACAGGTAGGAACTTTGCAGT---GCCTATTAG  23476
Mouse                         TATATCTGTCA---TACATA--------------------------ATATCATTAG--CC--TACAAT--AAAAAATTAC  26975
ChineseHamsterGHOK1GS         TGTGTTGTAAATGTTATTCTGAAAGTTATAAAAATTTTAGAAGAAAACATAGGTAGAAATGTTGTAGGC-AGCAACTCAA  26126
LongTailedChinchilla          AAGATCAGTAG--TTGCTAGGGGTTG-------------GGATAAAGTGGAACCACAGAATTTTTAG----GGAAGTGAA  25390

Majority                      AAATXXX-G-AAXACTAAAGXATXAAACACATXX-XXX-----XTXCCTAGAXA---AX-GT-AXAXGXXCAGTXTTGTA
                                       44730     44740     44750     44760     44770     44780     44790     44800
Human                         ATTTGCCTGGAAAAATACTCCATGTAATTCAGATGACTCAAAAGGACGTTGGTAGACAATGTTACAAGGACACTTTTATG  42938
GuineaPig                     NNNNNNNNN--NNNNNNNNNNNNNNNNNNNNNNNNNNN-----NNNNNNNNNNNNNNNNNNNNNNNN---NNNNNNNNNN  24208
NorthernAmericanDeerMouse     AAATT---GCCTTACTAAGGACTGACCCACAT-----------CTTTCTAATA--------------GGCTGGTATTGTA  23529
Mouse                         AGATT---GTAAGAAGAAAGAGAAAAATAAAA--------------ACTAGAGG-------------G-TGAGGTGTACA  27024
ChineseHamsterGHOK1GS         AAATCTA-AAAAAACAAAAGGATTAAACATGTTGTGTCTGCC-CTTCCAAGACACTTAAAGTAATAAGGCAAGCCTTGTG  26204
LongTailedChinchilla          AATGCTCTG--ATACTATCACAATAAACACTCATCATC-----GTACATATCCAAGAATCGTGATAG---CAGTACTGAA  25460
```

```
Majority                     GTAXGCAGATATTTTTXXC-XXXTATXTXT-XXXX-XXAXX-T--AGATTGATXAXAAATTTTGXXTGTXA-XAXXCTX-
                                      44810     44820     44830     44840     44850     44860     44870     44880
Human                        GTTATCAGATATAGTACAGGATTTCATTTTTATGTTCCAGCTTCCAGCTTTATCTAAAATATTTCTTGTAA-TAGATTTT  43017
GuineaPig                    NNNNNNNNNNNNNNNNNNNNNNNNNNNNNNNNNNNNNNNNNNN--NNNNNNNNNNNNNNNNNNNNNNNNNN-NNNNNNNN  24285
NorthernAmericanDeerMouse    GCATGCAAG-GTTTATTGC----TAGAT----------------GAGACCATTGATACCTTTTCTCTGCCAGCAAGCT--  23586
Mouse                        ATAGGGGGAGACTTCCAAA-----ATGTCT---------------AGATAGATAGGAGAATTAGTTTGTAA--AGGCC--  27080
ChineseHamsterGHOK1GS        TTAGCCAAATATTTTTTGCAGAGTATAACTATGAAACTAGTATCGAGATTGTAAATAAATTTAGAAATTGTGCAAACTTA  26284
LongTailedChinchilla         CTCAGTGGAAACTGGGCTTTGTGTGCTTTGGATGTGTCAACGT--AGGTTCATCAGTTGCAATAAATTTGC-TACTCCGG  25537

Majority                     XGTATAGCATTAAGTTGXXAGACAXXTGTAX-XGTAGATCAAXATXTTTGTXTXTATAAXGXATGXTXX-T----TXXXX
                                      44890     44900     44910     44920     44930     44940     44950     44960
Human                        ACTGTAGAATTGTTTCCTAAGGCATCTGTCTCAACAGCCTTTCCCTTATCTGTGCAGACTTTTCTTTCTCTGTACTTGTT  43097
GuineaPig                    NNNNNNNNNNNNNNNNNNNNNN-NNNNNNNNNNNNNNNNNNNNNNNNNNNNNNNNNNNNNNNNNNNNNNNNN----NNNNN  24360
NorthernAmericanDeerMouse    -GCATAGCACTAAGTAGGGAGACAGTTCCTG--GTCAATCTAGGTTGTTTTCTCTATTTGGCAGGAT-------------  23650
Mouse                        ---ATAGAATTAAGTTTAAAGAGAA----A------GATTAAAATAAT-AACTGAACAGAGAATGGAA------------  27134
ChineseHamsterGHOK1GS        AATTTAGCAGTAAGGTGATGAACAGTTTTAGAAGTTGATCAAAATACTTGAACAGATTAGGCAAAAAAGAT--AATCACA  26362
LongTailedChinchilla         TGTAAAACGTTGATGGGGGAGA-GTCTGTATTTGTAAGGCAAGAAGTATGTATATGAAAAATCTCGTCGTT----TTGCT  25612

Majority                     XXX-AXXTX-TTTTAGTXAAAGAX-AXXAXTTCTTTTAXXTATAAAGTTGTXXTGTXTTAT------CTTXTXGCTA-XT
                                      44970     44980     44990     45000     45010     45020     45030     45040
Human                        GAATAGGTGCTTTTTGTCAAAGAA-AAAAATAATTTTACAGGTGAAAAAATTTCATGTTATTAGAAACTTAATCTCAGGT  43176
GuineaPig                    NNNNNNNNNNNNNNNNNNNNNNNN-NNNNNNNNNNNNNNNNNNNNNNNNNNNNNNNNNNNN------NNNNNNNNNNNNN  24433
NorthernAmericanDeerMouse    ----------CTTTAGTTAAAGC-----AGTACACTTAACTATAGGATTCTGCTGCTTTCT------CTCCTAGGTG-TT  23708
Mouse                        -----------TTTCTTTAAAG------ATTTCTTGTATTTTTAAAGT------ATAAAAT------ATTCTGACTG---  27182
ChineseHamsterGHOK1GS        AAACATGTAGTATTAGAAAAAGTGTAAGAGTTTTAATAACCATAAAGTTGTATTGTACAAT------TCTAAAGCTACTT  26436
LongTailedChinchilla         GTGAATTTAAAATTGCTCTGAAAA-ATGAAGTCTTTGATTACTTGTGTGGCCCTGTGTTAC------CTTTTGTAAAAGA  25685

Majority                     TAXTAACXAATTAXTXAATTTXX-XATTAAAAAXAATTTTTAX-TTATX-----GXXATTTXAAAXGXTATATATTTA--
                                      45050     45060     45070     45080     45090     45100     45110     45120
Human                        AAATAAATGTTTAATAAATTGATACAGTAAGGACATTTTCTAATGAATT----GGTTCCTTCAAGAGATATAAAAGTG--  43250
GuineaPig                    NNNNNNNNNNNNNNNNNNNNNNNNNNNNNNNNNNNNNNNNNNN-------------------NNNNNNNNNNNNNNNN--  24492
NorthernAmericanDeerMouse    TACTCAC-AATAAATGAACTT---CATTAAAAAAAAATCTTA--TTAT-----GGGAATTTTGAATAGCTTATATTT---  23774
Mouse                        --------AACAATTGAATAT-----TTAAAATGAAACATTTGCTTGTT-----GAGATTAAAACATGAAAATGTTCA--  27242
ChineseHamsterGHOK1GS        TAGGAACTAATTTGGCAGTTTGTCTTTTAAATAATATTTTTAATTTATTCTTCAGAAATTTCATATGTTTTGTGTTTTGA  26516
LongTailedChinchilla         TATTACTGGTCTAGACAATTGAGTTAAGAGGCAGAGTTTTCAG-------------------ATAGGATACAAAAGGA--  25744
```

```
Majority                    --ATATATTTCCAXAXTXCXACTXXXTXTAATXXXXATTAXAAXGT---------GAAXTTXATAXATXCX-TXTXTTGT
                                          45130     45140     45150     45160     45170     45180     45190     45200
Human                       --AAATATTTCAAAAATACAACTGTTCCTAAGTAGCATTAGAATTT---------GATTTTGTTAAATTTG-TAACTATT  43318
GuineaPig                   --NNNNNNNNNNNNNNNNNNNNNNNNN-NNNNNNNNNNNNNNNNNN---------NNNNNNNNNNNNNNNN-NNNNNNNN  24559
NorthernAmericanDeerMouse   --ATAGTAATCCAAATTTGAAACAACCTGAATGTCTGTTGGCAGGT---------GAA-TGGATAAATTCC-TGTTTTGG  23841
Mouse                       --ATAAATTTCCATA-----AATTTATTGACTACTAAACAAAAAGT----------ACACCCATGGGGCCG-GGCAGTGG  27304
ChineseHamsterGHOK1GS       TCATATGTATCCACCGTCCCTCCTGCTCTATTGCCCTTTACCCTGTCACTCTTTCTAACTTAGTGTATCCCCTTTTTTGT  26596
LongTailedChinchilla        --AGATCTTTCTACACTGCCTCTAGTT-TAGGTTGAAATACAAAGA---------CAGGTTAAAAGAGAAA-AAGAAAAT  25811

Majority                    TGTCATT-ACTATGCCCTTXGTXCAATCTAXAXTAX--XGXXTCTGTXGTTAXX-XGTGXXTTXTTGXTAXXTXCGGAGC
                                          45210     45220     45230     45240     45250     45260     45270     45280
Human                       TAGCAGTGACTATGAGGCTTGACAATTCTAAGGTAC--AGTCTCTTTAATTTTCTAGTGATTTCTTTGTAGCTACGGAAC  43396
GuineaPig                   NNNNNNN-NNNNNNNNNNNNNNNNNNNNNNNNNNNN--NNNNN-----NNNNNNNNNNNNNNNNNNNNNNNNNNNNNNNN  24631
NorthernAmericanDeerMouse   CATCAT----G-TGCTCTTTGTACAGTCTGTATTGA-TTAGCTCTGTAGTG------TGAGTTATTGATACAGCTGTAG-  23908
Mouse                       TGGCGC-----ACGCCTTTAATCCTA---ACACTTG--GGAGGCAGAGGCAG----GTGGATTTCTG--AGTTCCAGGAC  27368
ChineseHamsterGHOK1GS       TGTTATTAACAACCCCCTTAGTGCTCCCCACATTAGCTTGGTTCTGTGGCCATCCACTGGGGCATTGGCAATTTGCCAGC  26676
LongTailedChinchilla        TGTCATT-ACTGTGAAACTGTAACAATCAAAACAAA--GGAGT-----ATTACTGGATGCAAATTAAAACAATACTGAGA  25883

Majority                    XGXCATXTCTCCAAAGAAAXT------TXTATXATAACCAG--X---XXXX-ATACXXAGXGAAAGCGTGXCT-------
                                          45290     45300     45310     45320     45330     45340     45350     45360
Human                       TGGAATATCCCTTTAGGTATA------CATATTTCTGCCAGTGGTG-ATTG-TTACTCTGTAATAGCAGGATTTT-----  43463
GuineaPig                   NT------------------------------------------------------------------------------  24633
NorthernAmericanDeerMouse   GGACGAATTTCCAAGTCAAAT------TGTGTGAAAAATAG--A--------AGACAAAAATAAAGTGTGTCT-------  23965
Mouse                       AGCCTGGTCTAAAAAGTGAGT------TCCAGGAGAGCCAG--------GG-CTATTCAGAGAAACCCTGTCT-------  27426
ChineseHamsterGHOK1GS       AGACATGTCTCCAAAGAAAATGACTCTTATGTCTTAAGCAGCCATCCATCATGAGCATGAGCAACTCGTGGCTCCTCCCC  26756
LongTailedChinchilla        TTCCATCTCTCCCCAGAAAGA------TG-ATTATTATCAA--GAA-ATCA-ACCAGAAGTGCTGGTGGGGTTCT-----  25947

Majority                    -XGGAAAAXTGXAAXTATAXXTXXCXTAXT-----GTXXGX-ATGAAAXC-X---TAAAXCA-X-TXTXXAGCAGTCTXC
                                          45370     45380     45390     45400     45410     45420     45430     45440
Human                       -CTGAAAATTATTGCTAAACATGACATTTTG----CTCTAC-CTGAAAGCAGT--TAAACCATTCCATAGAGTCTGGAAC  43535
GuineaPig                   --------------------------------------------------------------------------------  24633
NorthernAmericanDeerMouse   ATAGATAAATGAGTTTATAAATATTATACT-----GTAGGAAATGAAAAC-----TAAATCA---TATTGACTGATCCAG  24032
Mouse                       -CGAAAAACCAAAAGTACATCTATGGGAAT------------ACCAAACA-----AGAAGCA--------AGCAGTCTTC  27480
ChineseHamsterGHOK1GS       ATGCTATGTTGGAATTTTGACTGGCTTAATCTTGCGTCAGTAACCACAGCCGCTGTGAGTTAATGTGTTCAACAGCCTTG  26836
LongTailedChinchilla        -GGGGAAAAGGGAACACTTCTCCAC-TGTTG----GTGAGC-ATGTAGACAGT--GAAACCACTGTTGAAATCAGTGTGC  26018
```

```
Majority                     ATTXTACCTAG-----XAX-TXTXACAA----GGTCTTCATX--X------T-X----------XATGTTTCTAAXXXCA
                                      45450     45460     45470     45480     45490     45500     45510     45520
Human                        ATTTTACTTAG-----AAC-TCTTTTAA----GATTTTCATT--T------TAACTTTTACAGACATATTTTTGAGGTGA  43597
GuineaPig                    --------------------------------------------------------------------------------  24633
NorthernAmericanDeerMouse    AATATACCCTAG----TATATATAACAA----TGTCTCCAT----------G-------------ATGATTCTAAATCCA  24081
Mouse                        CCTCCTCCTAAC------------ACTG----GGTTTGAAT-------------------------TTCTGGATAGCCCA  27519
ChineseHamsterGHOK1GS        CTTATATCTAGAAGTCTGTATTTCACAGCTTTTGTCCTCATTCATCAGCTCTTATCTTTCTGTTCATGTTTCTACACACA  26916
LongTailedChinchilla         AGATTCCTCAGTA---AAC-TAAAACTA----GAGCTTCAAT--T------TGATACAGCTGTTCCCCTTAATATCTTCC  26082

Majority                     XXGAA-------X------A--TTAXTAAGAXXATACXTAXACAX-TAXAXXTATAXGGXTAC---XXTTTAATAGTTTG
                                      45530     45540     45550     45560     45570     45580     45590     45600
Human                        TGTCATTCT---TTTTGCTATAATGTTTTGAAAGTATTCCAGTAGATTATTTCATAGTTTTACT-ATTTATAAAGGTTTT  43673
GuineaPig                    --------------------------------------------------------------------------------  24633
NorthernAmericanDeerMouse    GTGAA--------------A--TTGACAAGCTTATCCATTAACAA-TGTAAGTAAAAGGATAC----AATGGAGAATTAG  24140
Mouse                        CAGAG-----------------TGAGTGGGACTGTGCGTATGTGGGCACACACATGTGTGTCT----GTCTGTCTGTCTG  27578
ChineseHamsterGHOK1GS        CTGAGCCTCGGATAGGGGAAGGTTAATAAGATGATTCATACACAACTAAACATTAATGGTTACTTATACTTAGTACTTTG  26996
LongTailedChinchilla         TGAAACAGT---TAAAAACACCATATCACAGCAATATGCGCACATTCATGTTTTTAGTGGCACA-GTTTGTAATAGCTAA  26158

Majority                     AXCAXCTXTGCGTCTGXCA---------X------X--X---X----X--XCTXXGTGTXTCTCATXTTTT----TX---
                                      45610     45620     45630     45640     45650     45660     45670     45680
Human                        AACAACTCATCTTCTTGCATATAAAGGTAGTGAGAGCCTTATTTCATAG-TGTTACTATTTATGAGGGTTTT---AACAA  43749
GuineaPig                    --------------------------------------------------------------------------------  24633
NorthernAmericanDeerMouse    AACAG---TGGCTCTGTCA-------C------------------------CTTGGTGTAACCCATATTTA----T----  24178
Mouse                        TCTGTCTGTCTGTCTGTCT--------------------------------CTCTCTCTCTCTCTCTCTCT---------  27617
ChineseHamsterGHOK1GS        ATCAGCTGTGAGTCTGCTACACACTGCAAAAAGAAGCTTCTCTGACCAGATTTGAGAGCAACACAAATATGTGGGTATGA  27076
LongTailedChinchilla         ATCTTGGAAACGGCCCCAATGCCCAT-TAGTTAAGGAATGGATAAAGAA-TATGTGTTTTTTTGTTTTTTTA---TACAG  26233

Majority                     --AAATCTCTXXTATXTAXATGTAGAXAXACCXTT-----AAAXACATXTAGXCATGTXCAXC-XXGGCCTAGTATATTT
                                      45690     45700     45710     45720     45730     45740     45750     45760
Human                        CTTATCTTCTTGTATATAAAGGTAGTGAGAGCCTTCTCTAGGTGCCTTCTGTGTTTTTCCAGCTCATGCCTAATATGCTT  43829
GuineaPig                    --------------------------------------------------------------------------------  24633
NorthernAmericanDeerMouse    --AAAACTATACTGTGTAACTGCAGAACAACCTGT-----AAATATATATAG-CATGTTCATC-ATGACATAGTGTATTC  24249
Mouse                        --CTCTCTCT-CTCTATATATATATATATATATAG-----ACACACACACACACATGTCTATT--TGAAATAGAA-ATTA  27686
ChineseHamsterGHOK1GS        TCAAATATTTAGAAGGTACTTGGACCATGACTATTTAGCAAAACAGCAGTAGTCATCTTCCCCCAGGGCCTATGGTCTCT  27156
LongTailedChinchilla         TGGAGTGTGAGTCAGCCATAAAAAAGGAAAAGCTTGAGATATTTCCAGGTAAATGGATGGACCTTGAGAC-AGTACTTTT  26312
```

```
Majority                     TTGATXAXXAXATXAXACXACAXTTT--GTAAATAACAXGGXX--X--X----X----------TCTAXXXAG-XCTTXG
                                      45770     45780     45790     45800     45810     45820     45830     45840
Human                        TTGACAACTACACCACACAACTACTT--GGAAATACAAGAGTTAATAGTTACCTTTTACGGAAACCTGGTGAG-GCTTTT  43906
GuineaPig                    --------------------------------------------------------------------------------  24633
NorthernAmericanDeerMouse    TGGATCATAAAATAACAGTACATTTCAAGTGATTGA-AAGGGA---------------------TCCAGTGAG-AGAGGG  24306
Mouse                        TTGAACAC-----T-TGTTACAGTTT--GTGGGAAACATGAAC---------------------TTAAAGCAG--CTCAG  27735
ChineseHamsterGHOK1GS        CTGGTTATAGGCTTTTGACCAGGTTTAGGTAACAGACATGGAAT-TCCTCCTGTAGAGCAGGCTTCTAAGAAGCAGAAAG  27235
LongTailedChinchilla         AAGTGAAATAAATCAGACA--CACGT--GTAAATACCA----CATTGTTTCTCTGGTGTGAGAAACTGT---G-TCTTGG  26380

Majority                     TXAATXGTC----TT---XXAXCTGTGXTXAGAXTGCXXAAAXTXXCAGACAXXGAXTTCTA--X-GXTTXGTCTXXAXG
                                      45850     45860     45870     45880     45890     45900     45910     45920
Human                        TGCGTGGTGGCTGTTGCAGAAACAGCCAACAGAATAGCAGAAGTACCACCTATCTGCACCTACCT-GCTTTTTTTCTCTT  43985
GuineaPig                    --------------------------------------------------------------------------------  24633
NorthernAmericanDeerMouse    TTAATAGTC----CT----CAGTTGGGCTCAAGGGGCTCAAGGAGGGATACATGGATCTCCT----GGGTGGACTGGAGG  24374
Mouse                        TAAACATCC----------TCCCTATTATTAGGTTAATTTGAAAACCAAACACAGACTG-----------AGGCACAAAG  27794
ChineseHamsterGHOK1GS        TGGATGGTTATAGTTATGGCTGTTGTGCTAATATTGCATCAGTTGGCAGCCCCATCTTTCTAAATAGGTTGGTATTGTAG  27315
LongTailedChinchilla         TTATTTGTCTTCATTGCTGAGACAAAATATCCAGTGCCCAAAATTAAGGAGGGGGAGGTTTATTTAGCTCACCCTTTGTG  26460

Majority                     XAGGTAGXAXTTGXAACTTGATXGTXXTXAAXXGAGGA------XTGGATGXAGGXXXAXXXTXXTXAAAGAGAXGACTX
                                      45930     45940     45950     45960     45970     45980     45990     46000
Human                        TAACTCTTCTTTGGAATTTGTTTCTCATACAGGAAAGAGT--AGCAGCATTCATTTTTTTTTTCTTAGCTTAGAGTCTTT  44063
GuineaPig                    --------------------------------------------------------------------------------  24633
NorthernAmericanDeerMouse    CCGGTAAGGATGGGAACTTGA-GGGATTGGGTTGAGGA------TTGGATGGAGGAGGAGAGTGCTAAAAGAGATGACTG  24447
Mouse                        TACCCAGTAATTGAAAACTAATAGTAAAGATACTAGAA--------ATATCCACGCAAAAGACATTTAACAAGAAGTCCA  27866
ChineseHamsterGHOK1GS        CATGCAGGATTTATTGCTAGATGATGGTAACTTGAGGGATACGGTTGGATGGAGGAGGAGAATACTGAAAGAGATGACTG  27395
LongTailedChinchilla         GAGGTTGCAGTCCACACTTGGCTGGCTTCAAGGCAGGGTA--GTATGGGAAAGGGCAACAGTTCATGGTGGTGGAAGATA  26538

Majority                     GAA--TTX--GGXXXXXTACAAACCTGXCGCAAGGAGAAACTC--XT---XCCXCAAGGXXXACXXC-GCXAAG--XXCT
                                      46010     46020     46030     46040     46050     46060     46070     46080
Human                        TTA--TTT--TGTTTTTTGTTTTTCTAAAGCTAGAAAACACTGCTTT---GCCTTGAAGTCTAAAACTGCTTAG--AATT  44134
GuineaPig                    --------------------------------------------------------------------------------  24633
NorthernAmericanDeerMouse    GAAATTTCCAGGCCAGGTAGAAACCTGGTGCAAGGGAAAACTCCCACAAAACTACAAGGATGACCCCAGCAAAGACTCCT  24527
Mouse                        TA---C-----------TAAAAGATTGCCATATCCAGAGATCC-------ATCCCATGATCAGCTTC--CAAA---CTCT  27920
ChineseHamsterGHOK1GS        GAAATTTTGGGGTCAGGTACAAACATGGGGCAAGGAGAAACTC--ATGAATCTACAAGGATAACCCCAACTAAGACTCCT  27473
LongTailedChinchilla         GAA--CAA--GCCTCTTTGCCTCCCTTACATTGTGTGCAAACTACCT---GCCCCGGGGCAG------GTGTAG--CACT  26603
```

```
Majority                     AACACTAGTGGATATATXAXCTGAAT-----------TXTGATXAAA--GGAGXCTGATAAXXCT-----ATXTCTG-GT
                                              46090     46100     46110     46120     46130     46140     46150     46160
Human                        AAAACTTCTTTGCCTCTCAGTTTCATA----------TTTGATAAAG--AGAGTTAGGAAAACTTT---TATAAATGTGT  44199
GuineaPig                    --------------------------------------------------------------------------------  24633
NorthernAmericanDeerMouse    AGCATTAGTGGATATGTAACCTGAAC-----------TATGATGAAATTGGTGACTGATCAGTCTCAGCCATTTCTG-GT  24595
Mouse                        GACACCATTGCATACACTAGCAAGAT-----------TTTGCTGAAA--GGACCCAGATATAGCT-----GTCTCTT-GT  27981
ChineseHamsterGHOK1GS        AGCAATAGTGGATATATAGCCTGAACTGGCCATTTCCTGTGATCAGATTGGTGACTGATCAGTC------ATCTCTG-AT  27546
LongTailedChinchilla         CACACCCGTAATCGGAACATTGGACTG----------TGAATAAATC--AAAGTCTAGAAGCCCAC---CCTA------T  26662

Majority                     GA--CTATXCCAGXXTTX----X-XX---------------XXGACXGAAACXGAXGCAGAXACTT-----ACAGACAAX
                                              46170     46180     46190     46200     46210     46220     46230     46240
Human                        GT--CTCTGAAAGACTTG----GAGAA---T-----GAAGATGTACCTAAGTTTTGTCACTGATTTTCATTGGACAGAAG  44265
GuineaPig                    --------------------------------------------------------------------------------  24633
NorthernAmericanDeerMouse    GA--CTACCCCAGTTTTCATCAGAGAGCCTTCATCCAGTAACTGATGGAAACGGATGCAGAGACCTGTG-CACAGCCAAA  24672
Mouse                        GAGACTATGCCAGGGCCT------------------------AG-CAAACACAGAAGTGGATGCTC-----ACAGTCAGC  28031
ChineseHamsterGHOK1GS        GA--CTACCCCAGTTTTC-----------------------TGGAAGGAAACAGATACAGATACCT-----ACAGGCAAA  27596
LongTailedChinchilla         GA--CTGTATGAGGCTTT----GGGAG---G-----ACATGCTGACACAAACTGTAGCA--AACTGAAAGTGTAAACAAG  26726

Majority                     XATTAGGTTX-AXCXCGGGGAATATXATGAAGXTAX-AGAGXAAGGATTATAXGAGCCAA-AGAGGAXXAXXACACXACA
                                              46250     46260     46270     46280     46290     46300     46310     46320
Human                        TATAAGGTTTTGCCGTGTCGTATTTTATATTTGTAC-ACAGTTGAAAGTATAACGGTGAA-TGAGTAGGACTAAAATAAT  44343
GuineaPig                    --------------------------------------------------------------------------------  24633
NorthernAmericanDeerMouse    CATTAGGCCA-AGCTCGGGAAATGTGCTGAAGACAG-GGAGGAAGGATTGTAGGAGCCAA-AGGGGTTAAGGACACCACA  24749
Mouse                        TATTGAATGG-ACCACAGGGCTCCCAATGGAGGAACTAGAGAAAGCACCCAAGGAACTAA-AGGGAACTGCAACCCTATA  28109
ChineseHamsterGHOK1GS        CATTAGGTTG-AGCTCGGAGAATA--CTGAAGAGAG-GGAGGGAGGATTATATGAGCCAAGAGAGGTCAAGGACACCACA  27672
LongTailedChinchilla         GACCAGAATATA---TACTAGACATTATGAAATTGA-AAGAAAAGGCTCAGTTGGGAAGG-GGAACAGGGATGGGGGAGG  26801

Majority                     GGAXAAACCACAGAAXXAACTAACCXGGXTCCTAGGX-CTCAXA-AGTCTGXTCCAXXAAXXAGGGAG---GC-TGGGXC
                                              46330     46340     46350     46360     46370     46380     46390     46400
Human                        TTACAAATTATATGAGAAAGTAGGCCAGGTGCTGTGG-CTCATG-CCTGTATTTCAGCACTTTGGGAG---GC-TGAGGC  44417
GuineaPig                    --------------------------------------------------------------------------------  24633
NorthernAmericanDeerMouse    AGAAAACCCGCAGAAACAACTAACCTGGCTCCTAGGAACTCACATAGTCTGACCCAACAACCAGGGAGCCTGCATGGGAC  24829
Mouse                        GGTGGAACAACAATATGAACTAACCAGTATCCCAGA---------GCTCTTGTCT-------------------------  28155
ChineseHamsterGHOK1GS        GGAAAACCCACAGAAACAACTAACCTGGCTCATAGGAACTCACAGAGTCTGAACCAATAACCAGGTAGCCTGCATGGGAC  27752
LongTailedChinchilla         GGGGAAAGGAAGGAAGGGAAAAGAAAAGATAAAATGG-AAACGA-AGAAGCTTTTGTAAATAAGGGAC---GT-GGGGTT  26875
```

```
Majority                     -XA-CTAGXXCXXXTGCATATATGTGACAG-XGXATAGCXTGGTCXAXXTGXXGGACCXXCA--CTC-TGXXAAXAGXGG
                                       46410     46420     46430     46440     46450     46460     46470     46480
Human                        -AG-GTGGATCACTTGAGGCCAGGAGTTCG-AGACCAGCCTGGCCAACATGGTGAAACCCCAT-CTC-TACTGAAAATAG 44492
GuineaPig                    -------------------------------------------------------------------------------- 24633
NorthernAmericanDeerMouse    TGACCTAGGCCCTCTGCATGTATGTGACAGTAGTGTAGCTTGGTCTATTTGTGGGACGTCTA--ATGATGGGAGCAGGGG 24907
Mouse                        ----CTAG-----CTGCATATGTATCAAAA---GATGGCCTAGTC---------GGCCATCA--C---TGCAAAGAGAGG 28209
ChineseHamsterGHOK1GS        CAACCTAGGCCCAATACATATATGTGACAGTTGTATAGCTTGGTCTGCTTGTGGGACTCTCAAAACCATAGGAGCAGGGG 27832
LongTailedChinchilla         -GA-AGGGAGGGTAAGGGAATGGAAAAGAG-GAATTGAGATAAGTCATGTACAGGAACAAAGT-CTC-TGATAAAAGTG- 26949

Majority                     C---X------TCTTTGXCTGGCT-TTGGXAATGTATXCCTCAXATTXCAGTGCCXTGCCXXGXCTAAX---AAGGGGAA
                                       46490     46500     46510     46520     46530     46540     46550     46560
Human                        A---A------AAATTAGCTGGGT-GTGGTGGTGCACACCTGAATTCCCAGCTACATGGGAGGCTGTGG---CACGAGAA 44559
GuineaPig                    -------------------------------------------------------------------------------- 24633
NorthernAmericanDeerMouse    CTGTTTCTAATTCTTTGGCTGGCTCTTGGGAACCTGTTCCTCATGCTGCTTTGCCTTTCCCAGCCTCAATACAAGGGGAG 24987
Mouse                        C---C------CATTGGACTTGC-----AAACTTTATATGCCCCAGTACAGGGGAACGCCAGGGCCAAA---AAGGGGGA 28272
ChineseHamsterGHOK1GS        CTGTCCTTAAAGCTTTGACTGGCTCTTGGGAACATATTCCTCATATTGGATTGCCTTGCCCCATCTAACTACAAGGGGAA 27912
LongTailedChinchilla         ----A------TCATTATGTACAT-TTAA-AATGAACCAATAAACTT--AGTATTGTGGATAGGCAGGG---GAAAAAAA 27012

Majority                     GTGCTTA---XTCTTAXTAC-----GG-AAGGTCXTGXTTTGTTGA-ACCCATGG--GXC-TXCX--TTTCXGAATAGCA
                                       46570     46580     46590     46600     46610     46620     46630     46640
Human                        TCACTTA--AACCTGGGAAG-----TG-GAGGTTGTAGTCAACCAA-GATCATGC--CAC-TGCA--CTCCAGCCTGGCA 44625
GuineaPig                    -------------------------------------------------------------------------------- 24633
NorthernAmericanDeerMouse    GTGCTTATTAGTCTTACTACAACTTGATAATGTCATGCTTTGTTGACACCCATGGGAGGCCTTCCCCTTTCTGAACAGAA 25067
Mouse                        GTGGGTG------------------GGTAAGGGAGTGGGG-GTGGGTGGGTATGGGGGAC-------TTTTGGTATAGCA 28326
ChineseHamsterGHOK1GS        GTGCTTA---GTCTTACTACAACTTGA-AAGGCCATGCTTTGTTGATACCCATAG-AGGCCTGCCCCTTTCTAAACAGAA 27987
LongTailedChinchilla         --GCTTG--TTTCATAATGC-----GG-AAAGTGTCATTTTATTAA-ACCGAAAT--ACT-TAAT--TTGTATGCTAGCA 27076

Majority                     ATAGAGXAGXAXATTGXX-XXGAGGGAGAGAXAXXGAGAXAAXTGGAXAGAAAGAGAGXAXX--GGGXAAXCTGTXGTXA
                                       46650     46660     46670     46680     46690     46700     46710     46720
Human                        ATACAGCAAGACTCTGTCTTAGATAGATAGATAGATAGATAGATAGATAGATAGATAGATACT-GTGAAACCCAGTGCTA 44704
GuineaPig                    -------------------------------------------------------------------------------- 24633
NorthernAmericanDeerMouse    ACAGAGGAGAAGAATGGATTGGAGTGGGAGGCAACGAGAAGAGGGGAGGGAAAGAGAGGAGGAAGGGGAAACTGTGGTCA 25147
Mouse                        TTGGAAATGTAAAT-------GAGCTAAATACCTAATAAAAAATGGAAAAAAAAAGAGTACA--GGCTAACCTTT----- 28392
ChineseHamsterGHOK1GS        ACAGAGGAGTGGATTGGG--GGAGGGGGAGG----GAAGGGAGTGGGAGGAGAGGAAGGAGG--GGGGAAAATGTGGTCA 28059
LongTailedChinchilla         ATAACAC----CTTTCAAGTAAATGAAGAGAAAACCTGTGAACTGGAGAGAAGCAGAAATCCA-CCAAGTTGTGTATTTT 27151
```

```
Majority                    GXATX-TXAXATTTAATA--------AATAAA---X-X---X-AT-------XCAGGAAAXAXTCAACAGGXTATAGTXG
                            46730 46740 46750 46760 46770 46780 46790 46800
Human                       GAGTGTTTATATTTAATA--------AGTAAA--GATAGGTAGAGTA-----TCGGAACAAAAACAGCATGGGAAACTAA 44769
GuineaPig                   -----------------------------------------------------------------AAAAGGGTACAGTAG 24648
NorthernAmericanDeerMouse   GGATA-TAAAATATATTA--------AATAAA---------------------GAAGAAATAGTCCTCAGTCTATGGTGG 25197
Mouse                       ------TTAGATTTAATAG-------AATGAC-----------AT--------TAGCTAAAATCTCAGAGCACATAAAGG 28440
ChineseHamsterGHOK1GS       GGATG-TAAAATCAAATAAATAAATAAATAAATAAATAAATAAATAAATAAATAAGGAGATAGTCTTCAGTCAGTGGTGG 28138
LongTailedChinchilla        TCACATTCTTCAGTAATT--------CATAGG--AAAATG-AGATCC-----TCCCTCTCCAAGTAAAAGGATACAATAG 27215

Majority                    TAAXTTXGGXAXAGTACTGAGXAATXXTXGXTTXAA-CATGAATTTAXTXATTTA-----XXXXXCTCAGXAATTTGAAA
                            46810 46820 46830 46840 46850 46860 46870 46880
Human                       CAATTTTGGGGAATGAATGAGTAATCAGGATTCAAAGCAAGTATCAAATGAGTCG-----GGAAATTAGAAAAGTAGAAA 44844
GuineaPig                   AAAATT-GGAACACTACTTAGTTATCTTGGTTTAA--CATACATTTAGGAAACT--------ACACCCCGAAACTGGAAA 24717
NorthernAmericanDeerMouse   TCCCTAAAGGCTGGTCCAGAAAAGTGATAGACTCAATGATGAAGTTGCTTATTTATTTCATTTTTTTAAGTCATTTTTAA 25277
Mouse                       TTATTTTTGAATAGTACTGTGAAATGGCAGATGTAA--ATGA--TTCTTCTTTTA---ACCCTGTCTCAGTCATTTG-AA 28512
ChineseHamsterGHOK1GS       TACCTAAAGGCGGGTCCAGAAATGTGGTAGGCTCAAAGATGAAGATGCTTATTTA----ATTTTTATCATTATTTTTTAA 28214
LongTailedChinchilla        AAAATT-GGAACACTGCTTAGTCATCTTGGCTTTA--CATGCATTTAACACACT--------ACACTCAGAAACTAGAAA 27284

Majority                    XTAAXGTXCTTTGXAGTXXTXTGAXGXAAATTXAT--TXXXX-ATAGAXXATXTTTAGAATXXXTTGTTXXXTXAAAXAA
                            46890 46900 46910 46920 46930 46940 46950 46960
Human                       TTGAAGTTTTTAGGGGTGCTATTAGAGAAATTCAG--TGAG--GAAGAGAGGGTTCACAGTTCCTTGTTGACTGATGGAT 44920
GuineaPig                   GTAA---CGTTGCCAGT-TTATGTGGAATATCCAT--CATG--ATAGAGCATGTATTGGATCATAAGTTAACTCAAATGG 24789
NorthernAmericanDeerMouse   CCTAAGCGCTCTATCTTAATGTGAAGCTAACTTGTAGTGAATTATAGGAGATCTGAAGAAT--------GTTTTGAACAA 25349
Mouse                       TTTGTGTTCTCTGAATTTCTGTACATTACACTTAGTATATATAGTGGAAACTTTGTATCATAGACTTT-GTTGATTACAA 28591
ChineseHamsterGHOK1GS       ACAAGGTGCTATGTCCTAATGTGAGGATAATTTCTAGTGAATTATAGGAGATCTTAAGAATTACTTAAGATTTTGAAAAA 28294
LongTailedChinchilla        GTAG---CTTTGCAAGT-TTACATAGGATATTCAT--CATG--TTAGAGCATATTTTGGATAGTTAGCTAACTCAAATGG 27356

Majority                    TXXAXAATTTXAGATAXAXACTXTGAAGXTAGTXGATTXAXATTXAAXXXAGAAXXAXAGGX-------AAAGACTAXTX
                            46970 46980 46990 47000 47010 47020 47030 47040
Human                       GTGGTGATTTAACAAATTACCCATGATCCTTGCCGACACCCATTTATGTAAAAATTGCAGGT-------AAAAGCTAGTG 44993
GuineaPig                   TTGAAAATTTGC---ATAGACTGTGACTATAGTGGAATTAAATT------AGAAAT-TA----------AAAGACT---A 24846
NorthernAmericanDeerMouse   ACAACAATTTTAGATAGGCACTTGTGAGGAAGTTAATTACAATAGAAACCAGAGCAACAGGG---------AGAGTACTG 25420
Mouse                       TCATGAGACCATGATAGCAGCCCAAAAGTTAAGATGTTCTCATTTTACATAAAATAATAGTGT---------GACTTTTA 28662
ChineseHamsterGHOK1GS       ACAACAACTTTAGATAGACAGTTGTGAGGAAGATAATTAAAACAGAAGCCAGAGCAACATGATTCTTTCACAGGGTCCAG 28374
LongTailedChinchilla        TTGAAAGTTTGC---ATAGGCTGTGATTATAGTGGAAGTACATT------AGAAATGTA----------AAAAAATGTGA 27417
```

Majority	GAAAAXATXAGXCAXTXAGAAGTTGAAXTTAGATXTA--XAGAACACAGXTX-TGAX-TTXAXTXAAGXATTCAXGT-AT	
	47050 47060 47070 47080 47090 47100 47110 47120	
Human	GAGAACAAAGGTCAGTCAGCAGGAGAAAAGTGATGTA--CTGATCAGAGGTGAAGATGCTGGCTTTAGAATTGAGGTGAA	45071
GuineaPig	GAATATACCAGATACCTAGAAATTAAAATATACCATT--CAGAACAA----------------TTAAGAAATCAGATGAT	24908
NorthernAmericanDeerMouse	TCT----TTACTCATTAAGATGCTGATGTTAGATTGAAATAGGAAACAGATTTTGAC-TTAAGAGAAGTGTTCCAGT-AA	25494
Mouse	ACATA---AAAACACTCTGAGCTTGAGGTTAGATCC---TACAATCCTAGTACTGTC-TCTACGGAGTGATTTAAGT-GT	28734
ChineseHamsterGHOK1GS	AATAAGATTACTCATTAAGATGTTGATGTTAGATTGAAATAGGAAACAGATTGTGAA-TTAAGAGAAGTGTTCAAGT-AG	28452
LongTailedChinchilla	GAAAGCATCAGATAGCTAGAAATTAAAATACGCCGTT--CTGAACAA----------------TTAAGGAATCAGA--AT	27477

Majority	AAATAGTTTTXAAGX---TTTTAAXGGGAATATTXAAGTAGAAACTGTXTTXAAATTXTXTXXGXTTTXAATXAXA-ATX	
	47130 47140 47150 47160 47170 47180 47190 47200	
Human	AAACTGGTTTTGAGT---TTGGAAGAGAAGTATTCAAGTAGAAACCATTTTAAAATTTTATTTGCTTTGAATCCGTGATT	45148
GuineaPig	A-----------------TTAGAAGGGGCATATTTGTG-----ACTGGATGAAGTTACTATGTGTTTCAAA---------	24957
NorthernAmericanDeerMouse	AAATAGTTTTAAAATTTCATTTGTTTGTATTTTTAAAGTAGAAACTGTCCTTAAAGTGCCTCAGGACTGAGTAATACATG	25574
Mouse	AGCTAGTAGGTAAG--------AGTGAAAACACTGGAAGATAATCTGATTTTGAAA----ATAACTGTAAATTACAAAAA	28802
ChineseHamsterGHOK1GS	AAATAGTTTTAAAGTTTCATTTGTTTGGGTTTTTGAAGTAGAAGCTGTCCTTAAAGTGTCTCAGGACTGAGTAATATATG	28532
LongTailedChinchilla	A-----------------TCTTAAGGGGCATATTTCTG-----ACTGGATGAAAATACT--GTGTTTCAAA---------	27524

Majority	AXAAAXGXAXTTXTTAGXGGCXA---XGGCCTAXGAXG--TXGXTXA-XGTATGTAAAAGGXTXGGCTXXXAXXXTTXXT	
	47210 47220 47230 47240 47250 47260 47270 47280	
Human	ACATGAACATCTTCCCTAAGCAAG--AGGCTTTGAAAGAGTTGAATAATGTATGGAAAATGGTGTGCTTAAAGATTTTTT	45226
GuineaPig	--------ATTTGCTGGGTGCAA------CTCACAAAT-------TAATGTATGGGGGAGGGT---------GTGTCTTC	25007
NorthernAmericanDeerMouse	ATAAATGGTATGCTTAGTGACTT-CTGGGCCTCTGAGG--TGGCTCA--GCAGGTAAAGGCATTGGCTGCCAAGCCTAGT	25649
Mouse	TCAAAAGAGAATTATATTGGTTC--CTGACCAAATAGG--TTGGTCA--GTAGATCATAATATAGAGAATTTA--AAAGT	28874
ChineseHamsterGHOK1GS	ATAAATGGTATGATTAGAGACTTGCTGGGCCTCTGAAG--AAGCTCA--GCAAGTAAAAGCATTGGCTGCCAA-CCTAGT	28607
LongTailedChinchilla	--------ATTTGTTGGGTGCAA------CCTAGGAGG-------TA-TGTATGAGGATGGGTA--------GTGTCTTC	27574

Majority	AAAXCXXTATATXATXAATXGXGXCCAGATGXXXXXGTAAAATGXCTXACXXAA--GTTXTTATTTXAXTGAACATAXAC	
	47290 47300 47310 47320 47330 47340 47350 47360	
Human	ACATTCATATACAATGAATAAAATTTATATGCATAAATAAAA-GCATTACCTC---ATGCCTTCTTAACTGAACATAAAC	45302
GuineaPig	AGATGGCTATATTA-GAAAACAGTACAGATGAAATTTTAAAATGTCTGACTTTTATGTTTGTATTCATTTGAAAATACAT	25086
NorthernAmericanDeerMouse	AA-CCTGCTTGTGATTCCTGGGGCCCACATGGTGCAGAACACTGACTCAGGCAA--GTTGTCCTCTGACTTCACATGAAC	25726
Mouse	AAACCCACAT---ATTTATGGGCCCCTGATGCTTGTGGAAGGTG-------CAA--AGTCTTATGGGATAAAAGAGATTT	28942
ChineseHamsterGHOK1GS	AA-CCTGTATGGGATTCCTGGGGCCCACCTGGTGCAGAACGATGACTCAGGCAA--GTTGTTCTCTGACTTAATATGTAC	28684
LongTailedChinchilla	AGATTGCTGTATTA-GAAAAAAGTGCAGATGAAATTTTGAAATGTCTGACTTT-------GTATTTAGTTGAACATACAC	27646

```
Majority                     AXTX--GAATXATAAAXGAXTTXAXTTX-X-TTGXAAXXAAAXCTATGAATTTACTAG-XAAATXXAAGGXGXTGATXTT
                                  47370     47380     47390     47400     47410     47420     47430     47440
Human                        ATTT--GAATTATTACTGGTTTTACACG---CCCAAACAAAAGTTCTGGGTTTACTGGAGGAATGGAAGGGAAAGATCTC  45377
GuineaPig                    AT-----AATAATAAATGA------------GTGGAGCTAAAA---TAAATTTAC-----AAATTGAATGAGGTGATTGT  25141
NorthernAmericanDeerMouse    ACTGTGGCATCATAAAGTATTTAAATTC-TATTGTAATGT-ATTCATGAATAAATTAG-CATATATAAGGA-TTTTTCTT  25802
Mouse                        ACTAGAAAGCTATATAGCTCTTGCTTTCCTATCCCTCTCCTAGCTTGGGGTGAGCGAT-GAAAGCCAGACGGCAGAGTTT  29021
ChineseHamsterGHOK1GS        ACTG--GCATCATAAAGTACTTAATTTTATTTTAAAATGTTATCTATCAATATATTAG-CATGTATAAGGGCTTTTTCAT  28761
LongTailedChinchilla         AT-----AATGGTAAATGAA-----------GTGGGGCTAAAACAATAAATTTAC-----AAATTAAATGAGGTGATTGT  27705

Majority                     XTCAXXTXXAGTCCTAAAX-----CTTG--XATXTAGXATATTXGGACGXXXXTGXTGAGXTGXAGAACTGCCTTACAXG
                                  47450     47460     47470     47480     47490     47500     47510     47520
Human                        TTTCATTCCCCCAGTAAAC-----CTTTCCCATTCAGGATCATGGGAAGATGCTGAAGAATTGTAGAACTGTAACACAGG  45452
GuineaPig                    TGAAACTCGGGTGCTGAAT-----C------AGGTGAAATATAGGAAGGATGTTGGGGT----CAGAACAGCTT----AG  25202
NorthernAmericanDeerMouse    GTCAGTTGAAGTCTTAGAAATACACTTGAGTATATAGGAAATTACGACC-CACTGATCAGATGTAGAGATGCCTTACAAA  25881
Mouse                        G-CAGCTGGAGCCCT---------CTGG--CATCTTGAGCTTGATGGCC----TGA--GGCTTCACCAGGGCCTTGATGG  29083
ChineseHamsterGHOK1GS        GTCAGTTGCAGTCTTAGAAATACACTTG--TATCTAGGAAATTATGAC-----TGGTCAGATGTAGAAATGCCTTACAAA  28834
LongTailedChinchilla         TGAAACTCAGGTGCTGAAT-----C------AAGTGAAATACAGGAAGGACCTTGGTGT----CAGAACAGTGT----GG  27766

Majority                     CAGXXCTGAAXGATTTAXTAACTA-------AXX-TCTATATTTXXAGAXAATTTX-CTTTXTTGXTGAAGXATXATAAT
                                  47530     47540     47550     47560     47570     47580     47590     47600
Human                        CAGACCTAAT-GCTTCATAAGCTA-------ATG-TTTCCATTTTTGGACCCTTTTTCGGTGTTGCTGGAGTGTGTTTAT  45523
GuineaPig                    GAAACTCGA--GTATT-GGGAATA-------AAT-AA---ACCCCAAGGAAAT----------CAAGGAAGTAAGA----  25254
NorthernAmericanDeerMouse    CAATTCTGTATTATATATTATCTAATCTATAGGCATCTGTAATTACAGAGTGTTT--CTTTTTTGGTGAAACTTTGTAAT  25959
Mouse                        C--TTCTGCGC-ATGCACTCACTG-----------CCTTTATTT---GGCAGTTT--CTTAACT--TAAAGCAGTATACT  29142
ChineseHamsterGHOK1GS        CAGTTCTTTATGAAATACTATCTA-----CAGGCATCCATAATTACAGAGAATTTTACTTTTTTGGTAAAACTTTGTAAT  28909
LongTailedChinchilla         CAGACTTAACAGTGTTTGGGAATA-------AAT-AGGAGATTGCAAGAAGATACT---GAGTCAAAGAAGTAAGAATGT  27835

Majority                     GAAGGXAXATTTACTTXTTGAXATXXATACTGX-AGTCT----------XTGATTCAGA--GTXCATGCTTT-AXTTCXT
                                  47610     47620     47630     47640     47650     47660     47670     47680
Human                        ATTTGAAAATAGGCTTTGTTATATTATTAAAGG-AGCCC--CC-ATTCCCAGTTTCACATT-CTCATAGTATCATTTATT  45598
GuineaPig                    ------------------------------------------------------------------------------TT  25256
NorthernAmericanDeerMouse    GAAGGCAAATTTATTTATTGAAATGGATACTGCCAGTCTACTTTAAAAAATGATTCAGAA-GTGCATGCTTT-ACTTCAT  26037
Mouse                        GTAGG---ATTCTCCTGTT---ATCTCTCCTA--GGTCT-----------TTATTCACA--GTAAATGC----AGTTCAT  29197
ChineseHamsterGHOK1GS        GAAGGCAGATTTATTTATTGAAATGAATACTGCCTGTCTA----------ATGATTCAGA--GTGTGTACTTTTACTTCAT  28978
LongTailedChinchilla         AAAAGTGGGCATCCTTGGGGGCACTGAGAATAG-AGAAC--TT-CAGAATAGTTCTGGATCACTGATGGATGTGTTGATT  27911
```

Sequence	Alignment	End
Majority	TAAXATGTTGTTTXATXXTGTATTATGATTTGTTXATAGTAAXCCXTXAXTGT-XCCCAATGXAAATTAAACXTT-----	
	47690 47700 47710 47720 47730 47740 47750 47760	
Human	TAAAATGAAGACTTTTATAATATTTGGCACTAAG-ATATGAATACTTAGTAGTCACCTCATGGAAGCTATTAACTGAGG-	45676
GuineaPig	TAA---	25259
NorthernAmericanDeerMouse	TATGTTGTTGTTTCATTCTGTTTTATTATTTGTTCTTAGTAAGCTCTTACTGT-GCCCAATGTAAATTAAACCTT-----	26111
Mouse	TAAAATGTCATATTATG-TGAAGGTTGAGTAGTTTATAGTAATCCAAAATT-------AAAACAACTTGAATATC-----	29264
ChineseHamsterGHOK1GS	TATGTTGTTGTTTCATTCTGTTTTATTATTTGTTCTTAGTAAACTCTTACTGT-GCCCAATATCAATTAAACCTTACCAT	29057
LongTailedChinchilla	TAACAAAGGACTTAGGATCTTAGCAAGATCCATTTATGTGAAAACTGCAAGAAAACCCCATGGAGAACAAAGGGAAAGG-	27990

Sequence	Alignment	End
Majority	--GXTTATAACXTAGAXXAAAAATTX-CTGTTTAGTTTATT--CTAGCTGTTTXTA---XAA-AGTGGATAG----TTTG	
	47770 47780 47790 47800 47810 47820 47830 47840	
Human	--GAGTGTCAGGTAGTGTAAATGTTGATAGTTTACTACAGTAACTGGCTTGGTCTTTGGGAGAAGAGGACAG--ATCTTT	45752
GuineaPig	--	25259
NorthernAmericanDeerMouse	--GCTTATAACAAAGAAAAAAAATGC-CTGTTTATTTTATT--CTAGCTGTTTATA---------TAGATA-----T-AG	26171
Mouse	-CATCAGCAAATGAAAGGATAAATTC-CTGTTTTGGCATTA--TGTACTGTTTGTA---CAATGTTGTATTG----CTTA	29333
ChineseHamsterGHOK1GS	ATGCTTATAACATAGAAAAAAAAT---CTGTAGAGCTTATT--CCAGCTGTTTATAGCTATAGAGTGGATGGGGGCTTTG	29132
LongTailedChinchilla	--TTTTAGAATTTAGACGAAAAACTGGGTTTTGAGTTTGGAAGGTAAGTGGTTGAGTAGAAACAGTTTTTAA--ATTTTG	28066

Sequence	Alignment	End
Majority	XTTGGGXAXTTTG-----AAATGTAXCTCTT-XTXCAAACAXAAXCTT-CTAXATC----AGTTTGTXAAAAG-AXXGA-	
	47850 47860 47870 47880 47890 47900 47910 47920	
Human	TTTAGGCAACCTGTATGTAA-TGTAATGTTTGTTTTTGAGATCCTACT-ATAGTGT----AGTTGTTGAAGAGCACAGAC	45826
GuineaPig	--	25259
NorthernAmericanDeerMouse	AGTGGGGGCTTTG-----GAATGTATCTCTT--GACAAACGGGAGCTTGCTGTATCTGTTAGTCTGTGAAAAG-AATTA-	26242
Mouse	GCTCTGAAGTATG-----AGTTACTGATACAACTGCAGAGATAAACTT-CCAGGTCA---AGTTTGGTAAAAG-ATAGAC	29403
ChineseHamsterGHOK1GS	GATGGGGGCTTTG-----GAATGTATCTCTTTTAGCTAACGGAAGCTTTCTATATC----AGTTTGTAAAAAG-AATTA-	29201
LongTailedChinchilla	TTTTG--AATTTGCAAGTAAATGAAACTCTTCATATAAACAAAAAGCT-TTAAAAG----GACTGATTAATA-TATGGAA	28138

Sequence	Alignment	End
Majority	AATGGTAXAATXATTXTTXAXTTTAXTXATTXXACAGTTT-CTGTATGAAATXATAATTTAATATTTTTXXCTGXTTAXG	
	47930 47940 47950 47960 47970 47980 47990 48000	
Human	TATGGAATCATGCTCCCTGAGTTTAATCCTTTCTCTGCTA-CTTCCTGTCTATACCACTTAGTAACTATCTAATCTTGGG	45905
GuineaPig	-------TAATGATTTTTGTT---	25273
NorthernAmericanDeerMouse	AATGGTAATATACGTACATACTGTAGTTATTGGACAGTTT-CTGTATAAAATTCAATTTTAACGTTTTTG-CTGATTACT	26320
Mouse	GATTAAAAATTAATCGTATGATACATTTAT-AAATAGTAAATTGTAAGAAATGATAATTAAGTCATATTGGCTGCTAAGT	29482
ChineseHamsterGHOK1GS	AATGGTAATATATTTATGTACTTTAGTCATTGGACAGTTT-CTGTATGAAATGGGATTTTAATATTTTTTTCTGGTTACG	29280
LongTailedChinchilla	AATGGTGTAAAGATTTTTGTTGTGTATGTGGAACTAGCAT-GCATAAGTGAGCATTACTAAAGATCTTTTAA---TTGAG	28214

```
Majority                    TATTCACTX-TAAATAGACTGCAXATGG--TAATGGTAXGTCAAATT----ATAAAATAATXTCTXCAXXTTTTCXAAAG
                                    48010     48020     48030     48040     48050     48060     48070     48080
Human                       CATTTAACTAATTGTTACCTGTAAATGG--GGTTGATAGGTCATAATGATAGCAATCTGAGAGTTGTAAATTGCCCGGCA  45983
GuineaPig                   --------------------------------------------------------------------------------  25273
NorthernAmericanDeerMouse   TATTCTCTC-TGCATAGACTGCACATGGCTAAATGGTAGATTTAATT---GATTTAATAATTTCTCCATTCTTTTAAAAG  26396
Mouse                       AATTCAC----AGATAGGAAAAGGACGGTTCAGTGGCATGACAAAGA----GTGGCATGGCATAAACAGATTGGGGAAGG  29554
ChineseHamsterGHOK1GS       TATTTTCTC-TAAATAGACTGCACATGGCATAATGGTAACTTTAATTAATGATACAATAATTTCTCCATTCTTTCAAAAG  29359
LongTailedChinchilla        TGTGCAGTTCTAAAATCACTTCAGTTAT--TACTAGTTTTACATAC-----ACAAACAAAAGTGTATGGGTTTACCAAAA  28287

Majority                    TXTAATTX-AATTTXTXXAGAAXTTCAXAT-TTACTGCACCXXTGXXTTAAGXXTXTTCTTTAXTTTTCXTAAXAA-AGX
                                    48090     48100     48110     48120     48130     48140     48150     48160
Human                       TGTAGTT--AATTAGCAGTGAATATTAAATATTGATGCAAAATAAAATTACATCTTTTCTATATTTTTTCCAAGAA-GTC  46060
GuineaPig                   ------------------AGAAG------------ACACTACAAAATAG-----------------------------  25292
NorthernAmericanDeerMouse   TCTAATTTAAATTTTTCAAGAA-TTCATAT-TTACT-CACCTATGTTTTAAGGTTATTCTTAGCTTTTCATAAAAA-AGG  26472
Mouse                       ATGATTATATGTAACAGTTGAGCTTCACCT-CCATGGCAGCTTGGTTGTAGGACTGCCCCTTCTTCCTCCCAACAT-GCC  29632
ChineseHamsterGHOK1GS       CCTAATTTTAATTTTTCAAAAA-TTCATAT-TTACT-CACCTATGTTTTAAGGTTATTTTTAACTTTTCATAAAAATAAG  29436
LongTailedChinchilla        TGAAAGG--AACATGGGGAGAAGTCAGCATTTCTCTGCACTACTGAATAAT--------CATAGCTCTTTTAACTA-AGA  28356

Majority                    AAAATCTATGTATTXTTXT----CTXACXGAATTATAAXAAGTGXTX-XXX-XATATXTATXXAGXXAAXXGCXATXXTA
                                    48170     48180     48190     48200     48210     48220     48230     48240
Human                       AAGATCTTTGTCTTATCCTTTGATTCTCAGAAGTTTCCTACGTTCTTTCTGTGTTATTCAGCTATTTTACTTCTGCTTAA  46140
GuineaPig                   ----------------------------AAAGT----------------------------------TTAAGTGCC------  25306
NorthernAmericanDeerMouse   AAAATCCATGTATTTTTGT----CTTACTGAAATATAAAGAGTGTTT-CTG-AATATCTTT-CAGCCAACGGTAATGATA  26545
Mouse                       AAAGATTATGAATACT-------CCGACTTCTTTGTAAGATGTGAT-------ATTTTTATATAACCAGTGTACATCTTT  29698
ChineseHamsterGHOK1GS       GAAAATTACATATTTTTGT----CATACTAAAATATAAAGAGTGTTT-CTG-AATATCTTTTCAGCCAATGGTAATGATA  29510
LongTailedChinchilla        TAAATCTAATTCTACTTCCTTCCCTGATAGAGTAATAGTAACCTA-------GATGTGCAGTAAGTTGAGTGCTATCCT-  28428

Majority                    XTAXGAATTTTXAAGXAXAATATAAXATXXATGXAAAXCXXTXTCATXTXATTXACAT--AAAAGTXACCT--GGTAXAG
                                    48250     48260     48270     48280     48290     48300     48310     48320
Human                       TATCCACTATCCAGAAAACATCCAACACAAATCCAAA-ATAAATTATTTAATTTCCTT--AATAATAGGTTTAGGCAGGC  46217
GuineaPig                   --------------------------CATGTAAAA------------------------------------------  25315
NorthernAmericanDeerMouse   GTAAGAAGTTTAAAGGAGAAGATAAAATGGATGGGGCGCCCTCTCGTGTACTTCACATTCGAAAGTTACCT--GGTGAAG  26623
Mouse                       CTGTGCATTTTTAAGTCTTGTATATTATG-ATATCTAATTGTTACATCTCATTTGCAT--AGAGATAAGCA--GGTAGGA  29773
ChineseHamsterGHOK1GS       GTAAGAAGTTTAAAGGAGAAGATAAAATGGATGGGGCGCCCTCTCGTGTTCTTCATATTCGAAAGTTACCT--GGTGAAG  29588
LongTailedChinchilla        CGCCCTCTTCCCCAGATTGATTGTTTATATGTGAAAATGGGCTTTATTGTACGATTGC--AGGAGTCCCCATTTGCATAG  28506
```

Majority	TXXXXG-AATTGGAAGTXATTTTTTTAGGTTTACXTXTTXXTA--AXGTGATTAACATCCTTATGCTTAAAG------AT	
	48330 48340 48350 48360 48370 48380 48390 48400	
Human	TTTTGCCTTTTGGGAATTATTTTTGGAAAATCATTTG-AAAGGGGAAGCCTTTTTTCTCCTTATGGGTACAGT----GAT	46292
GuineaPig	-------------------TCTTC--	25320
NorthernAmericanDeerMouse	TGACTG-AAACGGAAGTCATTGCTTTAGGTTTACCTTTTGGTA--AGGTGACTAACATCCTTATGCTGAAAGGAAAAAAC	26700
Mouse	-------AATTCAAGGTTTTTTTTTTAAGTTTTCTTCAC---------TTATTCATATCTTTACACTTAT---------T	29828
ChineseHamsterGHOK1GS	TGACTG-AAACGGAAGTCATTGCTTTAGGTTTACCTTTTGGTA--AGGTGACTAACATCCTTATGCTCAAAGGAAAAAAC	29665
LongTailedChinchilla	TAGTAGTTATTTAAAATGATTTTCATAGCACTAAAGGTGAATACTTAGTATTTGTCCTTGTTATTTGAAAAA------AT	28580

Majority	XAGTTACAXXTXTATTAACXTT-TTXGTATXTTAAX----C-CXCAGACXAXAXAXXAXXGGACTTATCTGTCGC--AAX	
	48410 48420 48430 48440 48450 48460 48470 48480	
Human	AGGTAGTATCTTTATTGGTCTT-TTTTTTTCCTTGTCTTTCTCCCAGAAGGAAAAGGACAGACCAAATTAGGAGA--AAC	46369
GuineaPig	--	25320
NorthernAmericanDeerMouse	CAGGTACAGTTCTACAAACATTGCTAGCATGTTAAATAAAC-CCCAGACTACACAGAATGAGAATTATCTGTCACCTAAT	26779
Mouse	TATTTACTTATTTATTCAC----TTGATATCTCCAT------CACAGTCCCTCTCCTCCCGGCCTCCCCTTTCAT-----	29893
ChineseHamsterGHOK1GS	CAGGTACAGTTCTACAAACGTTGCTAGCATGTTAAA----C-CTCAGACTATACACAATGAGAGTTATCTGTCGCCTAAT	29740
LongTailedChinchilla	TAATGAATAACAAGTTAGCAGT-TTTCTGTATCAGGACTGCTCTTTTGAGAAAA------GAATATGTCTGTGGG--TGC	28651

Majority	XAGA-GATTAXTTTTCTGACXAXCTTXAXAXXA--TCTXGATCX------XXTTXTTTTTTTXXAATXGCAGAG---CCX	
	48490 48500 48510 48520 48530 48540 48550 48560	
Human	TGAA-ATTTGCTTTTTTGAGTATCCTGATACCA--TAACTATAAC-ACATGGGGCTTTAGGTTGGATTGTGAAGTATTTG	46445
GuineaPig	--	25320
NorthernAmericanDeerMouse	GAGAGGATTAATTTTCTGACAAACTTAAAAAGAAGTCTTGTTCATTGTTTTTTTTTTTTTTTCCAGTAGCAGAAA--CCA	26857
Mouse	-----GGCCACTCCCCCCAC---C-----------TCTCCATC------CCCTTCATCTCTGAGAAGGGTGGAGT--CCC	29946
ChineseHamsterGHOK1GS	GAGAGGATTAATTTTGTGACAAAATTAAAAAGAGGTCTTGTTC-------TTTTTTTTTTTTCAAATAGCAGAA---CCA	29810
LongTailedChinchilla	CAGT-GCATGTTTTAAAGGGTATATGTGTATTT--TGTAGATTA-----------TCTGATTTAACTTACACAG-----G	28712

Majority	XTCTCAGTAATGAXATCXAXTGCTGAXT--TATCTXATAGX--ATTTTTXGXAATCAGTXTTAATTTTXGGAGACTXGTC	
	48570 48580 48590 48600 48610 48620 48630 48640	
Human	TTCTCGTTATTCAAACAAACTAATGAACAAGAACAAATTATCAAGTTTCTGTACTCAGACTGCTTTGTCAAAAAAAGGTC	46525
GuineaPig	--CTTAGTAATGAAATGCAG-GGTGGGT------------------TTGTTTGTTTGTTTTTAGTTTCTGGAGAGTAGA-	25378
NorthernAmericanDeerMouse	GGGGCACTAATAGCATCTATTGCTTATT--TATCTGATAGT--GTTAGTAGAAATCAGTCTTAAGTTTTGAATACTCGTT	26933
Mouse	CTTTGGGTACTGAC--CGACTGACACCT--CAAGTCAATGC--ATAGCCAGATAT--ATCCTCTTCCACTGAGGCCATCC	30018
ChineseHamsterGHOK1GS	GGGGCACTATTAGCATCTATTGCTTATT--TATCTGGTAGC--ATTAGTAGAAATCAGTTGTAAATTTTGGATACTCCTT	29886
LongTailedChinchilla	TTCTCAGTAATGAAAGGCAG-GCTGGATAGTTTCCCATAGGTTTTTTTTTTTTTTTTAGTTTAATTTCCGGAGAGTGCAC	28791

```
Majority                    XAGXAA-AGTAXCAGXAGXAXAAGGATXCAGXXATXATAXAXACXXAATXTTGTXTXXGAATATTTTGTGXTT----TTA
                                    48650     48660     48670     48680     48690     48700     48710     48720
Human                       TTAGTCCTGTATCAGAATAATATGTGTTCCTTGTTGACTGATGCCGATTGTTGTGTGTGCCCATTATGTGTTT----TAA  46601
GuineaPig                   -------AGAACTAGTCAGCCAAAATTTTTGAAATAATTTATTCTGAA-GCTGCAGCTTAATGTTTTGCTTTA----TAA  25446
NorthernAmericanDeerMouse   CAGTAATAGTATCAGTAGCAGTAGGATCCAGGTATTTTAAGCATGACATTTTGTTTTAAAATATTTAATGGTTCTATTTA  27013
Mouse                       AAGGCAGCCTGGTTAGGGGGACAGGATCCACAGG---CAGACAACAGAGTTAGAGTAAGCCCCCACTCTGATT---ATTG  30092
ChineseHamsterGHOK1GS       CAATAAATATAACAGGAGCAGTAGGATCCAGGTATATTAAACATGACATTTTGTTTTAGAATATTTAATGATTCTCTTTA  29966
LongTailedChinchilla        TGGAG--AGAAACAGAAATCCACCAAGTTTTTCGTGATAT-TTCTGAA-GTTGCAGCTTAATGTTTTGCTGTA----CGA  28863

Majority                    XGGXAAXXTATGAAAAXTAAXTTXCXT--XCATTXAGCXXCTATTXAAATXAATCTGXCTA-------------GAGATX
                                    48730     48740     48750     48760     48770     48780     48790     48800
Human                       AGTATATGTGTATTTTTTGGATTCTGT--GCTTTAGCTTACATTTTCAATTAACGGGCCTA-------------GAGATG  46666
GuineaPig                   AAGCAGCATAAGAAAACTA--TTAGAT--GCATTGTTTGGCTATTCAAGTATATATGCCTAA------------GTGA--  25508
NorthernAmericanDeerMouse   GGAGAATTTATGAAACTTAACATTCTCTAACGGACAGCTAATGTTATAATTATTCTGGCTGTTTCACATTTTTCCCTTTA  27093
Mouse                       GGGAATCCCATGAAAACTAAGCTGCAT--ACGTGCAGAGGCTGGGGACATAGGTCCAGCC--------------CATGTG  30156
ChineseHamsterGHOK1GS       GAAGAATTTATGAAACTTAACATTCTCTAAAATATAGCTAATATTATAATTATGCTGGTC--------------------  30026
LongTailedChinchilla        AGGCAACATGAAGAAACTG--TTAGGT--GCATTGTTCGGCTATTCAAGTATATATGCCTAA------------GAGAGA  28927

Majority                    ATCTXTTTXAATGAGATATCACTGA-TGXAAATAXTXAA-ATTXAXXTGTXAGTAAATTTXTXTTTT-------XTXTTG
                                    48810     48820     48830     48840     48850     48860     48870     48880
Human                       ATTTTGTTAGATAAGATACTACAAAATGCAAA-GTTCAGTCTCTTCCTTTTGACAAATTCAGGT----------GCCCTG  46735
GuineaPig                   ATCTCTTAGAGTAAAATATGACTTA-CGTATATATAGCTTAGTAAATTATCAATAAATCTATGTATT-------ATTTTT  25580
NorthernAmericanDeerMouse   TATTTTTTAAATGAGACCTCTAGGG-TAGTAATAATACA-ATTCTGATGCTAGAAAATGTTTATTTTTCTCTAGTTTTTG  27171
Mouse                       TGCTCTTTGGTTGGAGGTTCAGTGTCTGGGAGCCCCCAA-GGGTCCATGTCAGTTGATTGTTGGTCT--------TCTTG  30227
ChineseHamsterGHOK1GS       -ACTTTTCAAATGAGACCTCTAAGG-TAATAATAATAAA-ATTCAGGTGCTAGAAAATGTTTATTTT-CTGTAGTTTTTG  30102
LongTailedChinchilla        ATCTCTTAGCATGAGATATGACTTA-TGTATA-ACA----AATAAATTGTCAGTGAGTTTATA-----------ATCTTT  28990

Majority                    TXGXXACACAGAXTCTTGTXXTGTAAT-------AAATXCAATGCAXAAGTCXTCTGT-----AAGGXTTTTACTTXXCT
                                    48890     48900     48910     48920     48930     48940     48950     48960
Human                       TGCGCACACAAATCTTTGTCAGTAGTG-------AAAGGACATGTGATAGCTTCCTGT-----AGTTTTTTCATATTTCT  46803
GuineaPig                   CAGATGTATCTTCTCTGGTCATCTAAT-------AAAATCATTGCTACCATCCTCTTC-----AAGCCTTTTA-----CT  25643
NorthernAmericanDeerMouse   TTAGCAAACAGATTAATGGGGTGTAGTAAATCATAGATGAAACACAGAAGTAGTTTGTTCTTGAGGGGGATTGCTTGACT  27251
Mouse                       TGGAATCCCTGACTCCTCTGG-GGACTTTTCCACAAATTCAAGGTAGAGTCCACATAT-----AAAGAACTTACTTATCT  30301
ChineseHamsterGHOK1GS       TTAGGAAACAGATTTATGAGGTGTAAT---TTGTAGATGGAACACAGAAGTAGTTTGTTCTCAAAGGGTATTGCTTGAAT  30179
LongTailedChinchilla        TAGATACATGTGCTCTTGTCATCTA------------ATCATTGC-ACCGTCATCTTC-----AAGCCCTTTA-----CT  29047
```

```
Majority                    AXAGCXCTXAATXTXTCTTTACAXTCAA--TATTTCTAXGAAA-TATATX---TXTTCXCAAATTATTCTTAXGTXCXXG
                                 48970     48980     48990     49000     49010     49020     49030     49040
Human                       GGAGTGGTGAA--GAGCTACCCAGCCAA--GATTTTCAGGGCAATATATTCAGAAGCCAGAGCTTACTCTTCTGTACAAA 46879
GuineaPig                   AAATCACTTAA--TCACTCTACTTTCAG--GGCGCATAAGAAATTGTAT----GGTTCACATACTGC-CTTACTC-CCAC 25713
NorthernAmericanDeerMouse   T-AGCTCTGATTGTGTATTTAAAATCAAAATATTTCTAGGATA-TATTTCAT-TATTTTCAAATTATTTATATGTGTGTG 27328
Mouse                       AGAGGGCCAACTTTTTTTTTGG--------TGGTTTCATGAAA-TATACA---TGCACTGAAATTGGGCACACA-----G 30364
ChineseHamsterGHOK1GS       T-AGCTCTAATTGTATATTTAAAATGAAAATATCTCTAAGATA-TGTTTTAT-TATGTTCAAGTTATGTGTATGTGTGTG 30256
LongTailedChinchilla        AAATCCCTTTACATCACTCTGCTTTCAG--CACTCCTACGAAATTATGT----GGTTCACTTATTATTCTTCCTCTCCCC 29121

Majority                    GATAACTGCATGTGAXTXGAGGTAGCTTCAGAAXCCAXAXXCATGXXATAXACTCTG----TAXCCXTAGXTTAAXACAX
                                 49050     49060     49070     49080     49090     49100     49110     49120
Human                       GACAACCTAAGGGGATTTTTAAATGCATTGGCTTGCCGTTCAAGTATATAAACCTAAGTGAAACTCCTAGAGTAAAATAT 46959
GuineaPig                   TA-GACTGCACTCCCACTAGACTAGTCTCAGGCATCAGACTCACCATATTCACTCTGT--ATTTCCATAGCCTAAAACA- 25789
NorthernAmericanDeerMouse   GATATATGCATGTGAGTGGAGGTACCTACAGAAGCCAAAG--GTGTCACATCCCCTG----GAATTGTAGTTTAAGGCAC 27402
Mouse                       CATATTT---TATAGGTACAGGTAATTGAATGACCCAAAG-----TCAGAACTTTGA----TACCACCAAATAGTGATGA 30432
ChineseHamsterGHOK1GS       GATAGATGCATGTGAATAGAGGTACCTACAGAAGCCAAAAACGTGTCACATTCTCTG----GAACTGTAGTTTAAGGCAT 30332
LongTailedChinchilla        TA-GACTGTAG------------AGCCTCAGACATTAGCCTCATAATATTCACTCTG----TTTCCATAGCGTGAAAC-- 29182

Majority                    TTCTXAAGCACAGXA---GGXCXXTAX-ATTXTCAA-TATXGATACX----AXX-GXAAXAAGXGXXTX-AAGXCXCXTC
                                 49130     49140     49150     49160     49170     49180     49190     49200
Human                       GACTTAAGTATAGCTTG-AGTCCATAT-ATTATCACTTAGAGATACC----TTCTCTGATCATCCAATA-AAGTCATTAC 47032
GuineaPig                   GCCCCAAACAAA--------TCTCTA--ATTCTCAG---TAAATACA----GTC---AATATGTGGATG-AAGACACTTA 25848
NorthernAmericanDeerMouse   TTGTGAGGCACCGTACATGGATATTGGG--TGTCAA-CATGGGTCC-----AAA-GCAACAAGCTCTTTTAACCGGCCAC 27473
Mouse                       TGATATAG-ACAGCA---GGACTTTAA---TACTGG-TAGGAATACT----AAATGATACAGTTGTTT---GGGGGACTT 30497
ChineseHamsterGHOK1GS       TTATGTAGCACTGTACATGGATGGTGGGGTTGTAAA-CTTGGGTCCTCTAGAAAAGCAACAAGCTTTT---AACAGCCTC 30408
LongTailedChinchilla        -TCCCAAACAAAA-------TCCCTA--ATTCTTAAGTTTATAAAAA----ATC---AGTCTGTGGATA-AAGACACTTA 29244

Majority                    ATCTXTCAAXXTTTATATAAAATACTAAATXXTTTTTATGXXXATCAT-XAATGTACATTAAAXXTTATATAXTAGXAAG
                                 49210     49220     49230     49240     49250     49260     49270     49280
Human                       TACTATCAATCTTCCAGTCCTAGACAAAGTAACTTTTAAGCACATCAC-TGTTTTACCTCACAGCATATACCGTAGAAAG 47111
GuineaPig                   AGAGATGAAGTTTTATAGAAAATGTGGAGTG-TTTTAGGGCTTATCA--TAAAGTACTTGAAATTGTATGTAAAATGAAG 25925
NorthernAmericanDeerMouse   CTCCTTCAGCCCTAA-ATAAGATACTAAATTCTTTTTATTAGAATACTGAAATGTAAATTGAGGGTTATATTCTAGACAA 27552
Mouse                       ATTTTGTATTTTCTT-ATAAAAGT---AATCATTTCCTTAAACATTGTAAAAGTTGTACTCCTTCCTGTTTATTCAAAGG 30573
ChineseHamsterGHOK1GS       CTCTTTCAGCCTTTCTGTAAGATACTAAATTCTTTTTATTAGAATACTGAAATGTAAATTGATGATTATATTCTAGGCAA 30488
LongTailedChinchilla        AAAGATCAAGTTTTATAGAGAATGTTGAATG-TTTTAGGGCTTGTCA--TAAAGTACTTGAAATTTTATATAAAATGAAG 29321
```

Majority	TATTTAATTAX--TACTAXXTTGTAXAXATACTTXAGTTTGAGGTAAAATTTXT--TTTTTAXTAAXATTTTTAAGXCAT	
	49290 49300 49310 49320 49330 49340 49350 49360	
Human	TATCTGGTTCAGGTACTAGTTAGTCCAGCTCCCT-ACTATAAGGTATCAGGAAT--CATATAATCATCTTGTTCACTCTC	47188
GuineaPig	TATACATGTGT--TTCTTAGATGTATAAAT-----------GTGG------------------CAGTGTTTTTTAGACAG	25974
NorthernAmericanDeerMouse	TGTTTAATTAGCAGACTATATTACACAGATACTTGAGTTTGAGTTAAAATTTGTAATTTTTATTTAAAAATTTAATTCAT	27632
Mouse	A----AATAAA--AATTA---TGTCTGGGGAAGGGGGAGCGACGTAAAAACCTT--CTTCTAACAAAATGTTGAAGAAAT	30642
ChineseHamsterGHOK1GS	T-TTTAATTAG---ACTG-ATTACACAAATATTTGAGATTGAGTTAAAATTTATAATTTTTATTTAAAATTTTAATTAAT	30563
LongTailedChinchilla	TATGCATGTGTATTTCTTGGATGTATAAATACTC-ATTTTAGTGGGTTTTTTTT--TTTTTT-TAATCTTTTTGAGACAG	29397
Majority	GGTATCTTXTTXX----ATTTXAAXCTGACXTTGAXXCATXTTTGGA--XCCAGCAAXXGXTXAAGCAGTTAXTXCGXCX	
	49370 49380 49390 49400 49410 49420 49430 49440	
Human	TGTACCCTCA-GA----ACGTAAAACATTGCTAGACACATCCCTGAATACCCAGTAAATGTTTAAGGAATTAATGAGTGA	47263
GuineaPig	GGTATCATTATGC----AGTTCAAGCTGGCCTTGAACTCACTTTGTA-GCCCAGACTGGTCTATAGCAGTCCTTTTGTCT	26049
NorthernAmericanDeerMouse	AGTTTTTTGTTTTGTTTTTTCTAAACTCACTTTCAGGCATTTTTGGA--ACTGGCAACAGAGGAAGCAGCTATTACTATG	27710
Mouse	GGGATGTCCTACAG-------TAGGTAGATTGATACACAGTTGTGGT--ACAACCAG--GTAAAAGTAGGTAAACTAACA	30711
ChineseHamsterGHOK1GS	AGTTTTTTGTTTT-----TGTGAAACTCACTTTTAGGCATTTTTGGA--ACTGGCAACAGAGGAAGCAGCTATTACTATG	30636
LongTailedChinchilla	GATCTCACTG-------AATTCAGGCTGGCCTTGAACTAACTTTGTA-ACCCAGGGTGGCCT-TGATCGTGCATGCGTCC	29468
Majority	XTXAXTTXCXAXTCTGCTGTGAXTXCTXATXTX--AXACCAACTGGCTTAXAXCCATTXTTGCATTTACA-AAXTACATT	
	49450 49460 49470 49480 49490 49500 49510 49520	
Human	ATTAATTTTGAAGATACGTTGGAGACCAAGAT---AATCTGACAGTCTTATAGATGTTGTTGAACATACACAAACACATT	47340
GuineaPig	--CAGCCTCCAGAGTGCTGGAATTACAGATGT---ATGCCACGTGGCTTG--GCCATTTTAGGATTTTAA-AGATACATT	26121
NorthernAmericanDeerMouse	GTTAATTACTATTCTGCTGTGACACCTCATCTTCGAAACCAACCAATTTACATCCAGTACTCCAATCACA-AAGAACTAA	27789
Mouse	AGCTGTGAAAAACCAGGGAAGGATCCTAATGCTTAGTACCTGCTGGGTT-TATCCAAAGTCTGATTTACC-ATGTTGCTC	30789
ChineseHamsterGHOK1GS	GTTAATTACTATTCTGCTGTGACACCTCATCTTCGTAACCAACCGATTTACATCCAGTATTCCAATCACA-AAGAACTAA	30715
LongTailedChinchilla	TCCTGCCTTC-----GCCTCCAGTACGGGCAT---ATGCCACCTTGCCTG--GCCATTTTAGCATTTAAA-AAATATATT	29537
Majority	XGACAGTTXXT--AXAAAATCATGATXGGTGTGGXAAXXTACATAAXTTATATAG---TTXXTAAAATATTAAGTATTTC	
	49530 49540 49550 49560 49570 49580 49590 49600	
Human	ATATAGTTTAT--AGGTTGTGTTGAATGTTTTAGGAAATACCATAAATTACCTAAAGTTTTATACAAAATGGAGTA-TAC	47417
GuineaPig	TCATTGT--GT--AGACAATTATGGTTCATCTGG---------TCTTTGAAATAGTTCTGCGTAAAACATTACTTACTGT	26188
NorthernAmericanDeerMouse	AGACAGATAATACATTAAACCAAG-TGAGTGTGTATATATAAATAAAATATGTAG---CTGATAGCATCTTAAGAATCTC	27865
Mouse	TCACAGTCTG---ATAAAACCATGATGGCAGTGGGAAACAAGGTAGTGAATGTAG---CTTGTAATA-------------	30850
ChineseHamsterGHOK1GS	AGACAGATAATACATTAAATCAAG-TGAGTGCATATATCTACATAAATAATATAG---TCGGTAGCATCTTAAGAATCTC	30791
LongTailedChinchilla	TGAGGGT--GT--AGATGATTATGATTGGTCTGGCA--TTGTGTTTTTTAAATGGTTCTTCATAAAAGATTACTTATTTT	29611

```
Majority                      ATAAATAXTXTTTA--XTXXTAXXXXAXTTGATTTTAXGTXAGTTTTGXA---AXTATCXTTAAAATXTTXTAXXXTTTA
                                 49610     49620     49630     49640     49650     49660     49670     49680
Human                         ATGTATATTTCTGAATGTGTCAAGATCCTTCATTTTAGCTAAGTCTCAGA---AATATCCTTGAGATCCTAAAAGACTAA  47494
GuineaPig                     ATAAGCATCCTTAA------TAAAGACTT--ATTTTAGGTGAGTTGTGGAATGAAGGCAAGTAAATAATTTGTTGATTCA  26260
NorthernAmericanDeerMouse     ATAAATAGTATATA--TTAATAGAAAAATTGCTTCTATATGCATTTTCTT---GTTATCTTTAAAATATTTTAGTGTGTA  27940
Mouse                         --GAGAAGTGTTTA--GAGCTGGGGTA---GAGTATGTGT-TGTACTGTA---ATGATGGATGTAATGTCAT--TGTATG  30917
ChineseHamsterGHOK1GS         ATAAATAGTATATA--TTAATAGAAAA-TAGTTTCTATAT--ATTTTCTT---GTTATCTTTAAAATGTCATAGTGTATG  30863
LongTailedChinchilla          ACAGACATTCTCAA------TAAGGTTTTT-ATTTTAGGTCAGTTGTGGAGTCAAAGCAAGTAAA---TTTGTTGATTAA  29681

Majority                      TATGTATTTATTTAGXACTCAXXGAXATAAXAATGTXGCTXXXTAATACXXXTG-----GXTXTAAATATXAXATAAXTT
                                 49690     49700     49710     49720     49730     49740     49750     49760
Human                         GAACCATTGATCTGG----CATAGTAGATATACTGCAAGTCCTTCAAACAACTG-----TATTTAAATATTACCTATTTT  47565
GuineaPig                     CATATATTTGTTTAAGGCTCAGAGCAATAATA-TGTTAAGGAGCAGTATG-GTG-----ATATAAATGACCACAGAATAT  26333
NorthernAmericanDeerMouse     TATGT----ATTAAGTATTCATTTACAGAAATATGTGGCTTCCTGATACTTATTTTC-TGGTCTAAATATTAGATTAGTT  28015
Mouse                         TTTGT----C---AGAACTTAGTGACATAAAAAGT--GCACTTCAA------AG-----GATCTAA----CAGAT--GCT  30971
ChineseHamsterGHOK1GS         TATGTACATATTAAGTATTCATTTACAAAAATATG--GCTTTCTGATACTCATTTTCATGGTCTAAATATTAGATTAGTT  30941
LongTailedChinchilla          AATATTTTTGTTTAAGGCTCAGAGAAATCATA-TGTTGGTGAGGAGAGTATGTG-----ATATTAATGGCCACAGAATAT  29755

Majority                      TTACXXTGXTAGATTAXXTAX----------TATTXTTAAT--TXTAGGTGAXGGTTTTTAATGTXGAXAGTAXXCXXAA
                                 49770     49780     49790     49800     49810     49820     49830     49840
Human                         ATA----GGCATTTTGAATAA----------TATTTTTATT--TTTTGGTGAAGTTTTGGAATGAAGACAGTTAACTAAT  47629
GuineaPig                     TTT----GATAGATTATGTAG----------TAATATTAACAAAATAGGAGAGGCTC---ATTGTAG---ATAAACTGTA  26393
NorthernAmericanDeerMouse     TTACTATATTTAAACACTTGTCAGAGATTTCTATTTCTTGT--TGCATTTTAAAGTTTTTAATATGGAAACGAGGGCTAA  28093
Mouse                         CTGCCCTGATGGAGAA---------------TGTTGATAAT--GGGGGG--GGGGTAGTTTATGTGGGAAGAAGG-----  31027
ChineseHamsterGHOK1GS         TTACTAT--TTAAACA----------------GTTCCTTGT--TACAGGTTAAAGTTTTTAATATGGAGACGAGGGCTAA  31001
LongTailedChinchilla          TTAAGTAGGTAGATTACGTAG----------TAATATTAA---ATTAGGAGAGGCTC---ATTGTAG---GTGAACGGTA  29816

Majority                      GTGTTGXTTGAACATCTTTXXXTTTCTXGXTATAXXAAGGXAXTAXTACAAAGTAXTXAAAGXXXTGAACACT--TTATA
                                 49850     49860     49870     49880     49890     49900     49910     49920
Human                         TTGTTGATTGAATTATGTTACTTTACTTTAAATCTCACGGTAACAGAATAAGGTAATTAAGTTCATGAGTGAT--TTAGA  47707
GuineaPig                     GTGTGGATTGG-CTGCATCATTTTTTTTT--ATAGAATTGAAGTATAACAGTGTTTTCCAAAT--TGAGCAGT--TTGTA  26466
NorthernAmericanDeerMouse     GGGTTAGTTGAAGATGTTTGAGTCTCTGGGTTTA---AGTCCCTAATACCAAGCAACCAAAGGAATAAACACTGGCAATC  28170
Mouse                         ---TATGT-GAAAATCTTTG---TTGTGGATCTA---AACTGAAAATTC------TTGTAAGGTCTGCATTCC----ATT  31087
ChineseHamsterGHOK1GS         GGGTTAGTTGAACATGTTTGAGGTTCTGGGTTTATATAGTCCCTAGTAC-AAGCAACAAAAGGAGCAAACACTGGCAAAT  31080
LongTailedChinchilla          GTGTGGATTGG-CAGCATCATCTTTTTT---ATAGCATTGAAGTATGACAACGTTTTGCAAAT--TGAACAGT--TTATA  29888
```

```
Majority                    ATCTXTXGTXXAAAAXTGGAXCXAAAAGTTAXXT--XXXAGAXGGCAXXXGXAAXGTTTXAAXAGXG------TTTCTAX
                                     49930     49940     49950     49960     49970     49980     49990     50000
Human                       GTATATTGATGTTAATAGTCACAAAATATTAGGTAGTTTAGACGGCAATAGTAATATATCAGGAGAG------GCTCATT  47781
GuineaPig                   ATTTCTCAGGTAAAATTATAAT------CTTAAT--TTTTGCCAGCC----TAACTTTCTAAA-----------TTCTAT  26523
NorthernAmericanDeerMouse   ATCTGTAGTCACA-GCTGGAGCTAGAAGTTAGGTG--CAGGAGGGGAGCCGGAGAGGTTGAAGAGGC------TTGATGA  28241
Mouse                       TTCTTTTGTAAAATACTGGACTATACAGTTTAAT---CAAAAGGATTATTGCCCTGGATTTAGAAAG-----------AA  31153
ChineseHamsterGHOK1GS       GTCTGTAGTCTCA-GCTGGAGCTAAAAGTTAGGTA--CAAGAGGGGAGCTGGAGAGACTGGAAAGGGAGACTGTTGATGA  31157
LongTailedChinchilla        ATTTTTCAGGGAAACTT-----------CT-AAT--TTTTGCCAACA----CAACTTTTAAAAAATA------TTTCTAT  29944

Majority                    GTXGTAGXTTXTTATTATAAATTXXA--XATTXTXATXXTTAGA-----------AACTXCAXTTXCTTTGATTGAACCT
                                     50010     50020     50030     50040     50050     50060     50070     50080
Human                       GTAGATAAGCCATAATATGGATTGACTGCATTATCATTTTTATA-----------AACTTTACATTCTTTGATCCCACCT  47850
GuineaPig                   ATAGTAGTTTTTTTTT-------------TTTTTAT--------------------GCATCATTTACCTTCCATAAACCT  26570
NorthernAmericanDeerMouse   GATGGAACTTGTTAGTATAAACTGCAC-TATTCTGCTGCTTAAATTGACGTTACGAAATGCGGTGCCTCTGATGGACTCT  28320
Mouse                       GTTGTAG-CTACTATTCTAAAGTCTA---GTTGGAACAAATAGGTT---------AATAGTAAAAG----GATGGAAAGT  31216
ChineseHamsterGHOK1GS       GATGGAG-TTGTTAGTATAAATTTTAC-TACTCTGTTGCTTAG-----------GAAATGCAGTGTCTTTGATTGATTCT  31224
LongTailedChinchilla        GTAGTAGTCCTCCCTTACGTAT------CATTTTAATTTTTGCA------------GCTTCATTTGCCTTGAGTGAGCCT  30006

Majority                    -XXGTTXTXCTTTTATXATCAAAGATTTTAGXTXTT-TTXGGXXXAAT----------X---------------------
                                     50090     50100     50110     50120     50130     50140     50150     50160
Human                       TCAAGTACATTTTTCAAATCAAACAATTTATAATTTCTCAGGTAAAATT--A------TA--------------------  47902
GuineaPig                   -CAGCTGAAAATATTTCATAGAAAATTCCAGATCTTTCTGGGGCTAAT--------------------------------  26617
NorthernAmericanDeerMouse   --TGT-GTTCTTTCATTGTCATAGTTTTTTGTTTTTATTTTTTTCCATAAAAATGTCCTAATTTCCGTAGACTGGTGGTA  28397
Mouse                       --TGT-ATTCCAT-ACAAACAGAAAACATAGTT-----------------------------------------------  31245
ChineseHamsterGHOK1GS       --TGTTATTCTTTTATTATCAAAGTTTTTG--------------------------------------------------  31253
LongTailedChinchilla        -CAGCTGAAA-TACTTCATGGGAGATTCCAGGTCTTGTTGGGGCTAATA--G------T---------------------  30055

Majority                    -----------------------------------------------XTCXCCCT---------------TXTXG-----
                                     50170     50180     50190     50200     50210     50220     50230     50240
Human                       -----------------------------------------------ATCTTCCTA-------------ATTTTGGT---  47919
GuineaPig                   -----------------------------------------------CTTTCCCT---------------TGTTGAG---  26632
NorthernAmericanDeerMouse   CATGCCTTTAATATTGGCACTTGGGAGACAGAGCCAATGAGTTTGAGGCCAGCCTGGTCTACAGAGTACATTCCAGGACA  28477
Mouse                       ------------------------------------------------TTATTT--------------------------  31251
ChineseHamsterGHOK1GS       -----------------------------------------------GTCACCC--------------------------  31260
LongTailedChinchilla        -----------------------------------------------CTCTCCCT---------------TGTCG-----  30068
```

```
Majority                      --------------CAGAXXXATTCTGTC-------------------AAXATXAXCXTGTTAXTTTACXXXAAXATAAT
                                       50250     50260     50270     50280     50290     50300     50310     50320
Human                         --------------CAAACCAGTTTTTCACTTTTTA------------AAAATGTTCATATTAGTTCATTATAAGATGAT  47974
GuineaPig                     --------------TAGATAGATTCTGTA-------------------AAGCTACTCCTGCCAGGTTACAC--ACATAAC  26677
NorthernAmericanDeerMouse     GCCAGGACTGTACACAGAGAAACCCTGTCTCAAAAAAAAACGAAAAAGAAAAAAAAAGTGTTAATTTTCTTGAGGAAAAC  28557
Mouse                         ----------------------TACTGTT----------------------GTGAGAACAGAAATTTTCAGATAAGGAAA  31287
ChineseHamsterGHOK1GS         ----------------------CCC--------------------------AAAAATGTGTTAATTTCCTTGAGAAAAAT  31292
LongTailedChinchilla          --------------CAGATGGATTCTGTGT------------------AAGCTGCCCCTGACAGTTTACAC--ACATAGT  30114

Majority                      TAAGTTTXAAGATTAXAXXATTATTGATTTCAGAXAXTXTGATAXAGAAXX-XXXTTXXAXX-AATGTTXXAATA-GATA
                                       50330     50340     50350     50360     50370     50380     50390     50400
Human                         TAAATTACAAGAT---ACAGTCATAAATCTTGGGGATAGTGATGAGGATAC--TCTCGGAGA-AATGCATTGTTA-GGTA  48047
GuineaPig                     --AGTTACAAAATTG-ACTGTGATGGATTTCAATGCTT--------------TCCT---------TCTTTTACTT-AATA  26730
NorthernAmericanDeerMouse     TAAGAATGAGGACTATATGAATGTTGGCTTACAAAAATATGATACAGCAGAATGTTTAAAATTAATGTTAAAATA-GATA  28636
Mouse                         TATCTATAGAAAATATTTCATTTTTA---TCAGAAAACCTAATATTGAACTGTGTCTGGAGC-AACCTTAAGACATGATA  31363
ChineseHamsterGHOK1GS         TAAGATTGAATACTATATGAATATTGACTTTTGAAAATATGGTATAGAAGA-TATTTAAAATTAATGTTAAAATA-GATA  31370
LongTailedChinchilla          --AGTTGTAAGATTG-ACT-TCTGGCATTGCAGTGCTTGTGTTGAAGTAAC--CCTT--------TACTTTACTT-ACTA  30179

Majority                      A------------------XCXTGTCAAA---GXAXAATGXTCTAAGXXAXTATATTTTCATXAATA---TGCTTTTA--
                                       50410     50420     50430     50440     50450     50460     50470     50480
Human                         A------------------TTTTGTCACT---GCATAAATGTC--ATAGAGTACATTTGCACAAACC---TGCTACA---  48098
GuineaPig                     G------------------TATCCTCAAA---GTGCAAGCTTTGAGGTCAGTATATTGTTACAGTTA---TTCTATTT--  26784
NorthernAmericanDeerMouse     AGAATGCTTTTAAACCCAAACATGTTAAATTGACATAATGGTCTAAGGACTTGTATTTTCTTTAATACAGTGCTTTTAAA  28716
Mouse                         A------------------ACATGACAGA--AGCACA-TGTTCT------TCATGTTTAGTTA-------TACTTTCA--  31408
ChineseHamsterGHOK1GS         A------------------ACATGTTAAATTGCTATAATGGTCTAAGAACTTATATTTTCTTTAATACAGTGCTTTTAAG  31432
LongTailedChinchilla          G------------------TCTCCTCAAA---GTGCAAGCTTTTATTTCAGTATGTTGTTATAGTTA---CTCTGT----  30231

Majority                      -------GTATATATTXTCTT-XGTXAAATATGGCTTXXTXTGCCTAXTTXXTAAATXAAAAXTTXTCXTXCA-TTGTXX
                                       50490     50500     50510     50520     50530     50540     50550     50560
Human                         --------CATGTAGGCTATA-TGGTATATGTAGCCTTTTGCTCCTAGGCAACAAATTTGACAGCATGTTACTGTTCTGA  48169
GuineaPig                     -------TTATTTTTTGTTTT-TGTTAACTT---CCTACTATGCCCAGATTATAAATTAAAATTTATGTTACATGTGT--  26851
NorthernAmericanDeerMouse     ACTTACTGTATATATTTGCTTCAGTAAGATACGGCTATATCTAGCTATTTGTTACATCAAAAACTTTCATTCC-TTGTAG  28795
Mouse                         -------GTAAACCATCTCAT-AGGAATTTATGGGACAGTTTACCTCTTTCTC---CCTAGATTATTCTTTCA-TTGTGA  31476
ChineseHamsterGHOK1GS         -CTTACTGTATATATTTGCTTCAGTAAAATATGGATGTATGTGGCTATTTGTTATATCAAAAACTTTCATTCC-TTGTAG  31510
LongTailedChinchilla          --------TATCTTTAGTTTT-TGCTAACTT---CTTACTATGCCCAGATTATAAATTAAACTTTATCATATAGGTGT--  30297
```

```
Majority                   -ATATCAXAACXXXGTGXAXAGXTACTTTAAXXTAXTXTTAATGGTTTCXGACAAA--AXCTGTAAXACTTX-GCCTXXA
                           50570 50580 50590 50600 50610 50620 50630 50640
Human                      -ATACTGGAGGCAGTTGCA-ACACAG--T-AATAAGTATTGATGTATCTAAACAGA--AAAGGTA-CAATAA-AAATATG  48240
GuineaPig                  -ATAAGAGAGCAA---------------TTTGGTACTATCCATGGTTTCAGGCATA--GTCTACAGGTCTTG-GTTTGTA  26912
NorthernAmericanDeerMouse  ATTTTCTTTACTTTGTGAAAAGTGACTTGAAATTATTTTTAACAGCTTATGAGAAATAAGCTGTAAAACTTTTGCCTAAA  28875
Mouse                      --C-TCAATACCTGGCATGGAGCAACTTAAAGGAAGGTTTACTTTTTTTTTTAATA-----TTGTAAGAA--------GAG  31540
ChineseHamsterGHOK1GS      ATTTTCCTAACTTTGTGAAAAGTTACTTGAAATTATTTTTAATAGCTTCTGAAAAATAAGCTGTAAAACTTTTGCCTAAA  31590
LongTailedChinchilla       -ATAGGAAAAGAAAATGTGTATATAGGATTGGGTACTATTCATGGTTTCAGGCCTG--CTCTGCAGCTCTTG-GGCTGTA  30373

Majority                   GXATAXTXXGCTAAGXAAAXATCTGCTATTTTTGCA-TTAGTCTGTGAXCAXXATATCATTTTXT-TTXAATXA-TGTAA
                           50650 50660 50670 50680 50690 50700 50710 50720
Human                      GTATTATAATCTTATGGGACCACCATTGTATATGTGGCCTGTCAATGACCAAAATGTAGTTATGTGGCACATGACTGTGA  48320
GuineaPig                  TGCCCTTCAGGTAAGGAAAAATCTACTATTTATCCA-TTAGTCTGTGAACAGATGATTTTTTTTTTTT-TACAA-TGTCA  26989
NorthernAmericanDeerMouse  GGATAGGGGGCTAACATACCATATGCTTTTATTGGT-TTAGTTTGTAACAAAGATCTCATTCCCT-TTGAATCAAAGAAG  28953
Mouse                      ATGTAGTTTGCTGGGACAG-GAGAGCCATGGTGACA-CAAGAGTGTCATCAGG---TCATG-----TTGAGTC--TGTAC  31608
ChineseHamsterGHOK1GS      GAATACAG--CTTACAAAC-ATAAGCATT-ATTGTT-TTAATTTGTAATGAAGATTTCATTTCCC-TTAAATTGAGGAAG  31664
LongTailedChinchilla       TCCCCTTTGGATAAGGAGAAATCTGCTATCTCTGCA-TTAGCCTGTGAACAGATGATTAATTT-------ATAA-TGTAA  30444

Majority                   TTAXAXXATXTXGTAATXGAAXXGTTTATTXTG----ATGXXATTAXATTTTXXTGTTTTTGXTTAXT---TATTX-TTT
                           50730 50740 50750 50760 50770 50780 50790 50800
Human                      TTGTATTCTTTAGTCATTGGACAGTTTCTTT------ATGAGATTACATTTTAATGTTGTTGCTTATTAATAATTACTCT  48394
GuineaPig                  TTGTATTATTTTGTGATTGAATAGTTTTTTT------ATGAAATCACATTTTAATGTTTTTGCTAATC-----CG--TTT  27056
NorthernAmericanDeerMouse  TTAGAAAATATGCTAAAAGAAATACTTATTCTGTACCTTTTGTTAA--TTTTTTTGCATTTGTTTAAT---TATCAATTG  29028
Mouse                      TCAGAGAGTA----AAGAGAGATG--AACCCCG------GTGCTTAGCTTGTTTTGTTTTTCTTTTAT---TCCT--TTC  31671
ChineseHamsterGHOK1GS      TTAGAAAGTATGCCAAAAGTACTA--TATTCTGTATCTTTTATTGAGATTTTTTTGCGTATGTTTAAT---TATCAGTTG  31739
LongTailedChinchilla       TTATAGTATTTTGTGATTGGATAGTTTTTTTTA----ATGAAATTATATTTTAATGTGTTTGCTAGTCACTTATT--TTT  30518

Majority                   GCAXA-TXCTGXTXAGAAATAT-TTATG-XACTGCAGTXTXAAGTX-TTXATAATTTTACTTXATAX-TXXTCXGXTGXX
                           50810 50820 50830 50840 50850 50860 50870 50880
Human                      TTATAATACTGTTAAGAAACAT--TA----ACAGCAATCTTAAATAGTTGATAATTTCATTTAATAA-TAATAGGTGGAT  48467
GuineaPig                  GCATA-TACTGGTAAGAAATATATT----CACTGCAATTTTAAGT----GATAAATTTATTTAATA------------GT  27115
NorthernAmericanDeerMouse  GCGAA-TCATGAATGTACATAT-TTATG-AAGTACAGTGTGATGTT-TTTATATTCGTCCTTTGTGGGTAATCAGATGAC  29104
Mouse                      GC-A---C-TC--T------AG-CTGTG------TAGTCTGG-GTC-TTTACACTTAAACTTT--------TCTGGTAGC  31721
ChineseHamsterGHOK1GS      GCAAA-TCTTGAATGGACATAC-TTGTGGAAGTACAGTGTGATGTT-TTTGTTTTTGTCCTTTGTGGGTAATCAGATGAC  31816
LongTailedChinchilla       GCATA-TACTGTTAAGAAATATATTACTTCACAGCAGTTTTAAGT----GATAATTTTACTTAATAA-T--------GGT  30584
```

```
Majority                    ATXX-----XX-CTCTAXXACTTTGAAAACTTAGT-TTXGAXATXXTAAGAATTGTTXAAATATTXTCAAXTATACXTXT
                                            50890     50900     50910     50920     50930     50940     50950     50960
Human                       ATGA--------TTTTATTCCTTTGAAAGCCTGGCATTTGAATTTTCAAGAATTATTTAAGTATTCTCAGCTATGTTTTT  48539
GuineaPig                   ATT---------GTCCATTATTTTGAAAACTTAGT-TTTAAATTTTTAGAAATTGAT----------CAACTGTGCTTTT  27175
NorthernAmericanDeerMouse   ATAAATTGACTTCTCTACCACCTGAAATACTTTAT-TTCCTTATGGTAAGAATGGGCAAAATATTTTGAAATATACAGGT  29183
Mouse                       ATC------CTACTGTACC-CTAGTCAAAGTGTGT-TTC--TATGGCGA-----TTCTAAATCCAGTCAAATCGACAAGC  31786
ChineseHamsterGHOK1GS       ACAAA---ACTTCTCTACCACCTAAAATACTTAAT-TTCTTTATGGTGAGAATGTTTAAAATATTTTGTAATATACAAGT  31892
LongTailedChinchilla        ATT---------CTCTGTTATTTTGAAAACGTAGT-TTTGAAGTTTTAGGAATTGATGTATTATT--CAACTATACTTTT  30652

Majority                    A--AGXTXTTTTXAXAGTT--TTATAGXCXXTTCAXACAAATXCXAACAAXA-XXXCATXXTXTTXXTXXTCXTATTTTX
                                            50970     50980     50990     51000     51010     51020     51030     51040
Human                       A--AGTTCTTTTTGGAAAT--TTGTAGTTTTTTTACACAAATAGGAAAATTC-ATACATTCTTTTATTGTTGATATATAA  48614
GuineaPig                   A--AGCTTTGTTT---------TACAGCCTTT--AAATAAATAACAAGG--------ATTAATGTGTTCTTAACATACAA  27234
NorthernAmericanDeerMouse   AGCTGTAGTTTTCATAGTTTGTCATAGACCACTCAGACCTACCCTGACCAAA-GATCATCCTCTAAG-CATCCT-TTTTG  29260
Mouse                       T-TAACCATTAACACTGTAAATAAGAAGCCAGGCTAGAGAATTCGAACAATAGCTTTGTCACCTTGGTGACCCTTGTTTG  31865
ChineseHamsterGHOK1GS       AGCTGTAATTTTCATAGTTTGCCATAAACAACTCAGACCTATTCTGGCCAAAAGAGCATCCTCCAAG-GATCCTATTTTG  31971
LongTailedChinchilla        A--AGCTTTGTTT---------TATAGGCATT--ACATAAATGACAAGA--------ATTAATATGTTCTTAACAT-CTA  30710

Majority                    XAACTGTTTTXXAXTGTTXTTCAGXCA---ATGGTAXTGATAGTAXGAXATTTXXAGGAXXAGATAAXATXGXTGGTGXC
                                            51050     51060     51070     51080     51090     51100     51110     51120
Human                       GGACTGTTTTTTAATGTCTTTCAGCCA---ATGGTAATGATAGTAAAAAATTTAAAGGAGAAGATAAAATGGATGGTGCT  48691
GuineaPig                   AAACTATTTTTAAATGCTTTTCAGCAA---ATGGTAATGATAGCAAGAAATTTAAAGGAGAAGATAAAATGGATGGTGCC  27311
NorthernAmericanDeerMouse   CCA-TGGGAGCCAGTCTTCTTGATACTTCTATGGTTTTGATTATATGAGATTCTCTCTGTGAGATCATGTACTGCATGTC  29339
Mouse                       TAAAACTATACTGGGAAACTATAGACCAGCCTGCAATTA--TGTGTAGGATGTTCATCATGATACAGTCTATTCTGGGTT  31943
ChineseHamsterGHOK1GS       CCACTGGAAGCCAGTCTTCTTGCTACTTTTATGAGCTTGATGATGTGAGATTCTCTCTATGAGATCATGTACTGCATGTC  32051
LongTailedChinchilla        AAACTATTTTTAAATGTCTTTCAGCCA---ATGGTAATGATAGCAAGAAATTTAAAGGAGAAGATAAAATGGATGGTGCC  30787

Majority                    CXTTCTCGTGTACTTCA-XXCATTXXAAAATTACCX--------GGXGXAGTGACAGAAAXTXAAXTATTXCTTTAGXC
                                            51130     51140     51150     51160     51170     51180     51190     51200
Human                       CCTTCTCGTGTACTTCA---TATTCGAAAATTACCT--------GGGGAAGTAACAGAAACTGAAGTTATTGCTTTAGGC  48760
GuineaPig                   CCTTCTCGTGTTCTTCA---CATTCGAAAATTACCC--------GGTGAAGTGACAGAAACTGAAGTTATTGCTTTAGGC  27380
NorthernAmericanDeerMouse   TTTCTCTATGCATTTTATTATACTTAGTAAA--G-------------AGATTTACCCCAGTTTTATTTATTATTTTAATT  29404
Mouse                       ATA----AAATAACTCAGTAAATTTAAAAAT-----------------GATTGGAAGAAATCTAATAAGGGACGGTAAT-  32001
ChineseHamsterGHOK1GS       TTTTTATGTGCATTTTATTACACTTAGTAAACTGTCTCCTTAAAAAGAGACTTATCCCAGTTTTATTTATTACTTTAGTT  32131
LongTailedChinchilla        CCTTCTCGTGTTCTTCA---CATTCGAAAATTACCT--------GGTGAAGTGACAGAAACTGAAGTTATTGCTTTAGGC  30856
```

```
Majority                    TT--AXCTTTTGGTXAXGTGACCAXCATCXTTAT--GCTGAAAGGAAAAAAXCA-GGTACAXXTCXTTCAAGGTXTATAA
                                     51210      51220      51230      51240      51250      51260      51270      51280
Human                       TT--ACCTTTTGGTAAGGTGACCAACATCCTTAT--GCTGAAAGGAAAAAATCA-GGTACACTTCTTTCAGGGTTTATGA  48835
GuineaPig                   TT--GCCTTTTGGTAAGGTGACCAACATCCTTAT--GCTGAAAGGAAAAAACCA-GGTACAACTCTTTCAAGACTTATGA  27455
NorthernAmericanDeerMouse   TTTGAGATTCTGAACACATTATCATTCATTTTATGTAGTGCTCTGGATGAA-----TTA-----AATCAAAGGTCTTAAA  29474
Mouse                       ----AGTTCTTAGTCACTGGTGGGTGGTCTCTAAAGGCTGACCCAGAAAAG---CAATA-----GATTCAATATGATTAT  32069
ChineseHamsterGHOK1GS       TTTGAGATTCTGATCACGCTATTATTTATTCTATTAAATGAATTAAATCCAAGGCCTTATTGCTGATCTATGTTGTCCAT  32211
LongTailedChinchilla        TT--GCCTTTTGGTAAGGTGACCAACATCCTTAT--GCTGAAAGGAAAAAACCA-GGTACAGTTCTTTCAAAGCCTATAA  30931

Majority                    XATG--X--TTAAACXCXAAAX----------TXTXXAGCXXATGXGTTTTAXTTXXAXXTXAAGXAAAXTAGTA-----
                                     51290      51300      51310      51320      51330      51340      51350      51360
Human                       AATG-----TTAAACCCCAAAC----------TATCCAGCAGGTGTGAATTAATTGTAAAAGGGAGAAAATAGTAG----  48896
GuineaPig                   AACA-----ATAAATCCCAAAT----------TATACACC---TGTGTAT----TGAGACTTAATAAAATTATTA-----  27508
NorthernAmericanDeerMouse   CATG-GTAGTTGAGCACTGAA-----------TCACTGGGTTATGCCTTCCATCTTCATTTCTAGAAGATTAAAA--AGA  29540
Mouse                       TTTA--TTTTTTATCAGTTAT----------TGTTTTTAACCGAAGTGCTCTGTTTTAATGTGACGGTCACTTGTA-----  32133
ChineseHamsterGHOK1GS       TTTGAGTTTTAGAAGATTGAAAAGGCTGGGCGTTGGTGGCAAATGCCTTTTAGTCCCATCACTCGGGAGGCAGAGGCAGG  32291
LongTailedChinchilla        C--------ATAAACCCCAGAT----------TGTACATC---TGCATGTGAGTTATCATGTAATAAGAGAATTA-----  30985

Majority                    XXGATTTTTXXAAX----------------------AGAACAGAAGTCTX-------XTTCTXAA--X---AAGTAGA-
                                     51370      51380      51390      51400      51410      51420      51430      51440
Human                       GTTATTTTTCTTAA----------------------AGAACAGAAGTCTT-------ATTCTCACTTTTTCGAGTAGA-  48945
GuineaPig                   ---CTTTTCTGAAA----------------------AAAACTGAAGTCTA-------ATCTT----------AGTAGA-  27544
NorthernAmericanDeerMouse   AAGAACTATATAAG------------------AAAGGGAACAAAAATA----GGGAGTCTCTAGA--T---ACATA-A-  29590
Mouse                       CTGAATTATAGGAG----------------------AGCTTAGGAATG---------TTTCTGAAG-----AAGCAGC-  32175
ChineseHamsterGHOK1GS       CAGATCTCTGTGAGTTCAAGGCCAGCCTGGTCTACAGAGAGCGAGAGTGCCAGGATAGACTCCAAAGCTACTACACAGAG  32371
LongTailedChinchilla        ---ATTTTATGAAA----------------------AAACTTGAAGTCTT-------TTCTT----------AGTAAA-  31021

Majority                    -----TXXTTXT-------------AGGAAACTAXTACTXXX-------TAXTXAAAG-XAX-TGAXTXXGGAXAGTTTX
                                     51450      51460      51470      51480      51490      51500      51510      51520
Human                       -----TGTCTGT-------------GGGAAACTAATGCTAGG-------TAGCCAAAGTGAG-TGAATCACTATAGTTTT  48999
GuineaPig                   -----TAAGTGT-------------AAGAAACTAATACTAAT-------TACCCAAAAAAAAATGAGTTGTGATAGTTTT  27599
NorthernAmericanDeerMouse   --------TTTT-----------------AAGTAGTGATGGC-------TACTTAAAG-------A--G-GGGGACTTTA  29628
Mouse                       --A----GTTTT-------------AGAGAGACAGTTGTGAG--AAAGGTAATTACAG-------------TAGAAAGCA  32221
ChineseHamsterGHOK1GS       AAACCCTGTCTTGAAAAACCAAAAAAAAAAAAAAGAAAAGACAGAAATATATTTAAAGGTAC-AAATAGAGGGGACTTTA  32450
LongTailedChinchilla        -----TGAATGT-------------AGGAAACTAATACTAGT-------TATTCAAAACAAA-TGAGTTGACATAGCTTT  31075
```

```
Majority                    GAAAAXTXATCTTXTAAXX--XXTTTAGAGAATXAATT-ACTTATTXTTAXCAATTTG---XATATACATAXXAXGXATG
                                     51530     51540     51550     51560     51570     51580     51590     51600
Human                       TAAACTTTGTCTTTTGAATTGACTTTAGGGCTCTAATTGAATAATTGTTATGAAATTGTGGAAAATACAAATGAAGTATT  49079
GuineaPig                   CAAATTTTATTTTTAAAAA-TACTTTT--AAATTAATT---TTATTGGTA-CAATTTG---GATATGTATATTAATCAT-  27668
NorthernAmericanDeerMouse   GAATAATGAACTTCCAAGT---GGTTAGAGAATAAATC-ACTTAGTCTAGTCTATTTG---ATTACACACAGAAGGAATG  29701
Mouse                       GAGAAATGGTTTGCTCAGA---GTCCAGCCTG-AGATT-ACTT-TCCTTACTCATGAA---GATGTTGCTGTTGGAGATG  32292
ChineseHamsterGHOK1GS       GAATAATGAACTTCAAAGT---AGTTAGAGAATAAATC-ACCTATTCTAAACTATTTG---ATTATACACAGAAGGAATG  32523
LongTailedChinchilla        CAAATTTTATCTTTTAAAA-AATTTTCTAAAATTAATT---TTATTGATA-GAATTTA---GACATACATAGCGATCATA  31147

Majority                    ---TTCATAGTXAAXCTXXTXTTTXATXTXAAAA--ATCTTTAGTGTTXATATTT-TXTXTTTXXXTXAXXTX------T
                                     51610     51620     51630     51640     51650     51660     51670     51680
Human                       TCTTTTACTGTGGCTCTGTTCTTTGGTATCAGAA--ATCAGCAGGGGTACTAGTCGCATCTTTCCTTAAGCTT------T  49151
GuineaPig                   -CCACCATAGAGAACTTGTGCTTATATATGATAC--ATCTTTAGTTTTCAAATTT-TATCTTT--TTAAATTCA-----T  27737
NorthernAmericanDeerMouse   ---TTCAAAGTAAGTCAAGTTTTAATTTCCAAA-GCCCTTTAGTGTTTG-------GTTTCACTGTTGTC----------  29761
Mouse                       ---GACATAGAAAAC--AGGTTTGACTTAAAGAGAAGTGTTGGTGTAGAAATA---GCTTTCAGGTTT-C----------  32354
ChineseHamsterGHOK1GS       ---TTCAAATTAAGTCAAGTTTTTAATTTTTAAAAGTCTTTTAGTGTTTTTTTTTTTGTTTTGGGGTTGTTTGTTTGTTT  32600
LongTailedChinchilla        TTCATCATAGGGAACCTGTACCTGTACATGATA----TCTTTAGTTTTCATATTT-TATCTTT--TTAA-TTCA-----C  31214

Majority                    XTTAGAXT--XATGTGTTTTTAXATAGTAGTAGTGXAXAAXACGAAX-TTGAAGTTXTTTTXXA-------XXTGXTGXC
                                     51690     51700     51710     51720     51730     51740     51750     51760
Human                       ATGAGAAT---ATGAAATTTTATTTAAAAATGTTAAATGATACCATT-TAGAAGAATTTATGAA-------ATTTAACAC  49220
GuineaPig                   TTTAGACT--GATGT--TTTTGAAAACTAGTAGTGGAAAATACAAAT-GGAATGTTTCTTTT----------ATGATGGC  27802
NorthernAmericanDeerMouse   -----------ATCTGTTGTTACATAGTATTTTGGCACAGAAAGAACATTGCAGTTCTTGGA------------------  29812
Mouse                       -----------ATTTGTTTGGATAT--T--TAAAGTAGAAACTGTAC-TTAAAGTGCCTTAGGA-------C--------  32403
ChineseHamsterGHOK1GS       GTTTGTTTTTGGTCTGTTTTTACATTGTAGTTGGGTACAGAAAGAACATTGCAGTTCTTTGAGATTTTAACTTAGCTGAG  32680
LongTailedChinchilla        TTTAGACTCTGATGAG-TTTTGAAAAGTAGTAGTGGAAAATACAAAT-GAGAAGTTTTTTTTTT-------TATGGTGGC  31285

Majority                    TCTGAGTAXTXTATCATA-AXTATTAGGTGTTAXTTTXXCXTGTTXTTTTTCXXTTTXTTGCTCTTTATTTTTAT-XATA
                                     51770     51780     51790     51800     51810     51820     51830     51840
Human                       TCCC-------TTTTATA---TACTTGAGGTTAATCTGACATTTAATTTGTAATTTTGTTC---TTTGTCTCTAT--ATA  49285
GuineaPig                   TCTG------GTATCAGA--TCAGTAGGTATGCTTTTAGCATCATTTTTTAAAATTTTTTTATTTTTAATTTTTT--ATT  27872
NorthernAmericanDeerMouse   ----AGTAGTCTGTCCTTCAGTTTTACCTTTTAGTTTATCTTGTTCTTATTCTCTTTATTGCTCTTTATTTTTATTCATA  29888
Mouse                       --TGAGTAATATATGATA-AA--TGGTGTGC-AACAAGACTTGTGGGCCTCC--TGGGTGGCTCAGCAGGTAAAGGCATT  32475
ChineseHamsterGHOK1GS       TTTAAGTGGTCTCTCCTTTAGTTTTATCTTTTAGTTTGTCTTGTTCGTGCTC-CTTCCTTGCTCTTCATTTTTATTCATA  32759
LongTailedChinchilla        TCTGTTTATGGTATCAGAGATTAGTAGGTGCACTATTAGCATCTTTTTTTTTTTTTTTTTTTTTTTTGTCTTTGA--GAC  31363
```

```
Majority                     ATXXXXXAAGCATAAT--GATXTTAAXTTGAXTTCXGGXAXTTTCXCAXATAXTAGXGTAAAXTXGTXXTGGAXXTCTXA
                                      51850     51860     51870     51880     51890     51900     51910     51920
Human                        AT--------TATACT--GCTTTTAAAATGACTT--GGAAATCTTACATTCTCTAGGGTAGA--AGTGGTAAAATTCTGA 49351
GuineaPig                    AT-----TAACATCTT--AATCTTAAGTTTATTTCAGGAAGATACTGATCTAATAGTGGAAATTAGTCATTGAGCTTGAA 27945
NorthernAmericanDeerMouse    ATTGAGAATGCACAATTAGAATTTTGTTAGACTCACATGATTTTCCCCCAGATTAAAGTAATGAGGATTTGTATGTCATG 29968
Mouse                        GGCTGCCAAGCCTAAT--GACCTGCACTTGATTGCTGGGA----CACACATGGTAGGAAAACATGACTCTGGCAAGCTGT 32549
ChineseHamsterGHOK1GS        GTTGAGAATGCACAATTAGAATTTTGTTAGACTCCTGTGATTTTCCCCCAGATTAAAGTAATGAGGAATTGGATGCCTCT 32839
LongTailedChinchilla         ATCCTCCTAGCATCTT--ATTCTTAAGTTTATTTCAGGAAGATACTGATCTAGTAGTAGAAATTAGTCATTGAGTCTAAA 31441

Majority                     TXXX-X-CATXCAXTATTTACACXTXTGCCAGAGAAXCATATXTXTTGXAAGAATAAATTTGTGTTTTAGAATTTTTAAT
                                      51930     51940     51950     51960     51970     51980     51990     52000
Human                        TGC----TAGAAAATGTTTACATTTTCTCTAG-------CATTTGTTGCAAGAATAGATTTGTGGGT-----------GT 49409
GuineaPig                    TATCTTTCATTCAGTATGTACAACTATATGAGTAAAGCAGAGGTGTATTGAGTATGAAATTTTGTTTTAAAATTTCTAAT 28025
NorthernAmericanDeerMouse    G-------------TAGTTCCGCTGCTGCCAAAGAGACTTAT--TTTGATCTAACAAATATGCCTTTTAGGACTTTGGAT 30033
Mouse                        C---------------CTCTGACT-TTAACATAGAATCATAA----------AATATTTTACTTTTATAGAGTATTTAAT 32603
ChineseHamsterGHOK1GS        GCCCCTGCATCCCATCTTTCACCCCCGGCCAAAGAGACATATATTTTGAACTAACAAATATGCCTTTTAGGACTTTTAAT 32919
LongTailedChinchilla         TGTCTTTCATTCAGTAGCTACAACTATGTCAGTAAAGCACAGGTATAATAAGAATGAAATTTTGTTTTAAAATTTCTAAT 31521

Majority                     GAT----TTTAAXATXXXATXTXGAAGTTATTA-XTATTGA-GTAATGAGTCXTAXXTXTGAGXTXAGCT-CTXATTGXA
                                      52010     52020     52030     52040     52050     52060     52070     52080
Human                        GA--------AAAATCATAGGTGGAAGGCAGAA-GCAGCGT-GTACTCGGTTTT-CTTCTGAGGTTAGCT-TTAACTGTA 49477
GuineaPig                    GATACTACTTACTAGTAAATATGAAATTTAACATATAATAA---AGTTAATCCT-CTTCTGAGGTTAGCT-GTAATT-CA 28099
NorthernAmericanDeerMouse    TCT----TTTAAGGGATGATTTTGTTGTTTTTA-GTGTTGAGGTGATGAGTCAGAAGCAAGAATTGAGTTGCTTTTTCAA 30108
Mouse                        C------TTTAAAATGTAACCATGAATATATTA-ACATGTA--TAAGGATTTTTTA-TATTACTTGAAGT-CTTAACAAG 32672
ChineseHamsterGHOK1GS        TCT----TTTAAGGTATGGTTTTGTTGTTTTTA-GTGTTGAAGTGATGAGTCAGAAGCAAAAATTGAGTTGCTTGTTGGT 32994
LongTailedChinchilla         GATACTATTTAATATTATATGTGGAACTTAACATATAATATTGTAGTTAATCCTACATCTGAGGTTAGCT-CTAATAGCA 31600

Majority                     TAC-CTAAACT--CXAGTAAGAXXTX---------------------XXCTAATTCTAXXTXXTAGXATATXTA--AATG
                                      52090     52100     52110     52120     52130     52140     52150     52160
Human                        TAC-CTAAACT--TCAACAAGAGATT----------------------TCTAATTCTGCTTATCAGAACATTTA--AAAG 49530
GuineaPig                    TAC-----ACT--CTAGTAAGAGATT----------------------TCTAATTCTACATATTAGAATATTTA--AATG 28148
NorthernAmericanDeerMouse    TAT-CTAAACTTCCCATTAATCTGTGTTCGTGCTTCCTGTCCTGTGAACCTGCCAGTTGCTGCACTGATACCTGTTTGTG 30187
Mouse                        TACACTTGAGTATCTAGTACATTATG---------------------ACCAACTGATAGATGTAGAGATGGCT------- 32724
ChineseHamsterGHOK1GS        TAT-CTAGACTTCCTGCTAATCTGTG--------------------AACCTGGCAGTCGCTGCTCTGATACCTG-----G 33048
LongTailedChinchilla         TAC-TTAAACT--CCAGTAAGAGGTT----------------------TCTAATTCTACTTACTAGAATATTTA--AATG 31653
```

```
Majority                     TXATXTGAAGG-GTTATXTTXTXGATAATTGATXXX----X--------AAACTAGCTGAGTAG--ATTGXATCXATAGT
                                       52170     52180     52190     52200     52210     52220     52230     52240
Human                        TAACCTGAAGG-ATTATATTCTAGATAATTGATTA--------------AAATCATCTGAATAG--ATTGCTTAGATA--  49591
GuineaPig                    TAATTTGAAGG--TTATATTTTGGGTAATTGATTTTG---TT-------AAACTAGCTGAGTAG--GTTACACAGATAGT  28214
NorthernAmericanDeerMouse    TTTTTTGTTGTTGTTGTTGTTAGTATAATTCCCAATTATATATAATTCCCAACTTGCAGTGTGCTCACTGTGTCCTTAGT  30267
Mouse                        TT--C-----A-GTTATGTATGAAATAATCTATAG----------------------------------GCATCCATAAT  32762
ChineseHamsterGHOK1GS        TTTTT----GTTGTTGTTGCCAGTATAACTCCTAG-----------------CTTGCTGTGTGTTCACTGTGTCCTTAGT  33107
LongTailedChinchilla         TAATCTGAAGG--TTATGTTCTAGGTAATTGATTTTT---TTT------AAACTAGCTGAACAA--ATTATATGGATAGT  31720

Majority                     X----TACA--XXXTCATTXATTTTXTTTXXXTTTXGAXTTTTAXAAAXTG-GCTACTTAAAXTTTXAGXXATTXTTXGA
                                       52250     52260     52270     52280     52290     52300     52310     52320
Human                        -----------------TTAATTTTATTTGAATTTATAGTATTAAAAATTGTGCTACTTAAATTTTCAGGCATTTTTGGA  49654
GuineaPig                    TACATTACAGTTCCTGATTTGTTTGATTCTAATTCAAAGTGTTAAAAATTG--CTACTTAAAATTTCAGGCATTTTTGGA  28292
NorthernAmericanDeerMouse    C----TGT----TTTCATTCACTTTCTCTGTTCTTGGAATTTAATAAAATT-CATTCTTAGCCTATTGTTTAGTATTTGA  30338
Mouse                        -----TACA------CAGTGTTTTTCTTT-TTGATGGAACTTTGTAATGAA-GGCAT--A-TTTATTTATTGAA--TGA  32824
ChineseHamsterGHOK1GS        C----TGTCTGTTTCCATTCATTTTCTCCATTCTTGGGATTTGATAAAAAA-GATTCTCAGTCTGTTGCTTAGTATTTGT  33182
LongTailedChinchilla         TACATTACAATTCCTGATTTGTTTGATTTTAATTCATAGTGTTAAAATCTG--CTACTTAAAATTTCAGGCATTTTTGGA  31798

Majority                     ACTAXCAXCXXAAGAXGXAGCCATTACTATGXTTAATXAXTATXCTGCTGTGAXXCCTXATCTXCXTAACXAXCCAXTCT
                                       52330     52340     52350     52360     52370     52380     52390     52400
Human                        ACTAGCAACCGAGGAAGCAGCTATTACTATGGTTAATTACTATTCTGCTGTGACACCTCATCTTCGTAACCAACCAATAT  49734
GuineaPig                    ACTAGCAACAGAAGAAGCAGCCATTACTATGGTTAATTACTATTCTGCTGTGACACCTCATCTTCGTAACCAACCAATTT  28372
NorthernAmericanDeerMouse    TACATTTTACTTATTTATAGTCATTTTGATATTTGATAATAGAAGAGAGGCAAGTTAATATGTGTTTAACTATAAATTCT  30418
Mouse                        ACACTACTGGTCTAATGTAATGATCCAAAAGTAT-ATACTTCTACTTCATTGTGTTGTTTT--GTTTTATTATTTATTCT  32901
ChineseHamsterGHOK1GS        TAAATTTTATTCACGTATAGGCATTTTGATATTTCATAATAGAAGAGAG-CAAGTAAATAT-TGCTTAACTATAAATTCT  33260
LongTailedChinchilla         ACTAGCAACAGAGGAAGCAGCCATTACTATGGTTAATTACTATTCTGCTGTGACACCTCATCTTCGTAACCAACCAATTT  31878

Majority                     ATATCXAGTACTXXX--AATCATAAAGAXCTAAXX-XACAXATAXTACATTXAACCAAXTAXXTXTGTATAGXTACATAA
                                       52410     52420     52430     52440     52450     52460     52470     52480
Human                        ATATCCAGTACTCG---AATCACAAAGAACTAAAG--ACAGATAATACATTAAACCAAGTAAGTATGTGTAGGTACATAA  49809
GuineaPig                    ATATCCAGTACTCT---AATCATAAAGAACTAAAG--ACAGATAATACATTAAACCAAGTGAGTATGTATAGCTACATAA  28447
NorthernAmericanDeerMouse    ATTGCAAACATTTATTAAATCACTGAGTCCTATCAGTTCACTTTTTATACTTACCTATATACTTCTCTATAGATGTAACC  30498
Mouse                        -TAGTAAGGTCCTGTGTCCTGTTTAAGCCTTACCA-TATGCATGTAACATATAGAAAAAT-CTTCTATA-----------  32967
ChineseHamsterGHOK1GS        ATTATAATCACTTATTAAATCG--GAGTCCTATCAATTTGCTTTTTATAATTACCTCTATACTTCTCT------------  33326
LongTailedChinchilla         ATATCCAGTACTCC---AATCATAAAGAACTAAAG--ACAGATAATACATTAAACCAAGTGAGTATGCATAGGTACATAA  31953
```

```
Majority                      XTAAAATGXXXTAGA--------------------------------------------------------AXATXXXA-
                                       52490     52500     52510     52520     52530     52540     52550     52560
Human                         ATAAAATGGCCTAGA--------------------------------------------------------ACATATTA-  49832
GuineaPig                     GTAAAATGACTTAGACCA-AA-----------------------------------------------TTAAGATTCAAA  28479
NorthernAmericanDeerMouse     AACCGTCTTATTAAATAAGAAACACAGAAACAATGCAAAAGAGAAAGCCGAGAGGTCAGAGCTCAGAGCTGAAATCTCAC  30578
Mouse                         --------------------------------------------------------------------------------  32967
ChineseHamsterGHOK1GS         --------------------------------------------------------------------------------  33326
LongTailedChinchilla          GTAAAATGATGTAGA--------------------------------------------------------ACATCCAA-  31976

Majority                      ----------XXAAXXXXXTAXCXXTTCAXAAGA-X--XXX-X-------------------------------------
                                       52570     52580     52590     52600     52610     52620     52630     52640
Human                         ----------TGAATCTCATAAACATTAATAGGA----------------------------------------------  49856
GuineaPig                     TATTA-GAGAAGAAATGTTTAACTGTTCAGAAGATGTTTCTGT-------------------------------------  28521
NorthernAmericanDeerMouse     CCTTCCGCCTGCGGTGTCCCAGCTTCCCGAATCAGGGCTCTATTTCCTGTCTGTTAGTCTATTTAAAGAGACAGAACAAG  30658
Mouse                         ------------------------------GAG-----------------------------------------------  32970
ChineseHamsterGHOK1GS         --------------------------------------------------------------------------------  33326
LongTailedChinchilla          ---------AGAAAATGTTTACCCATTCAAAAGATGTTTCTGT-------------------------------------  32010

Majority                      --------------------------------------------------------------------------------
                                       52650     52660     52670     52680     52690     52700     52710     52720
Human                         --------------------------------------------------------------------------------  49856
GuineaPig                     --------------------------------------------------------------------------------  28521
NorthernAmericanDeerMouse     CCACAGCTATCTCACCTCACCAGTTCCTCAGCTGGTCCTGTTTCCTCAGACTGGAAGCTTCTGTGTCCTCATCCCAATAG  30738
Mouse                         --------------------------------------------------------------------------------  32970
ChineseHamsterGHOK1GS         --------------------------------------------------------------------------------  33326
LongTailedChinchilla          --------------------------------------------------------------------------------  32010

Majority                      --------------------------------------------------------------------------------
                                       52730     52740     52750     52760     52770     52780     52790     52800
Human                         --------------------------------------------------------------------------------  49856
GuineaPig                     --------------------------------------------------------------------------------  28521
NorthernAmericanDeerMouse     CTCTCAGCTGAACTGTGTTGCTCCAAAGCCTGAAAGCTTAACCAGCCAAATGCTTCTAGTTTCTGGTCCTCACGCCTTAT  30818
Mouse                         --------------------------------------------------------------------------------  32970
ChineseHamsterGHOK1GS         --------------------------------------------------------------------------------  33326
LongTailedChinchilla          --------------------------------------------------------------------------------  32010
```

```
Majority                    ----------------------------------------------------------AGG-AXTXTXTXACTXTTXAGX
                                      52810     52820     52830     52840     52850     52860     52870     52880
Human                       ----------------------------------------------------------AGGAAATGTTTACCTGTTCAGA  49878
GuineaPig                   ----------------------------------------------------------AGGTATTTTCTTATTTTTAAGG  28543
NorthernAmericanDeerMouse   ATATCTTTTTGCTTTCTACCACCACTCCCTGGGATTAAAGGCTGGCTTTCTGGGATTAAAGGCGTGTGCCACTACCGCCA  30898
Mouse                       --------------------------------------------------------------------------------  32970
ChineseHamsterGHOK1GS       --------------------------------------------------------------------------------  33326
LongTailedChinchilla        ----------------------------------------------------------AGGCATTTTCTTACTTTTAAGG  32032

Majority                    TGTTXTTX----X-------------------------------------------------------------------
                                      52890     52900     52910     52920     52930     52940     52950     52960
Human                       TGTTCCT-------------------------------------------------------------------------  49885
GuineaPig                   TGTTTTTG---GG-------------------------------------------------------------------  28553
NorthernAmericanDeerMouse   CACTCTTGCTATGGCTCTAATAGCTCTGACCCCCGGGCAACTTTATTTATTAACATACAATCAAAATCACATTTCAGTAT  30978
Mouse                       --------------------------------------------------------------------------------  32970
ChineseHamsterGHOK1GS       --------------------------------------------------------------------------------  33326
LongTailedChinchilla        TGTTTTTG---TG-------------------------------------------------------------------  32042

Majority                    -------------------T--X-XXTAXTCCTAGCTATTTCX-TTGXTTTAXTGAAAXXXTT-TTCATGTTGATTTCTT
                                      52970     52980     52990     53000     53010     53020     53030     53040
Human                       --------------------------TA----TAGGCATTTTC-TTGTTATTTTTAAGGTATT-TTCATGCAGAATGTTT  49933
GuineaPig                   -------------------TATTGTGTATTATTAAATATTGCC-TTTAATTACTAAAACAATG-TGCA-GTTTTATTATT  28611
NorthernAmericanDeerMouse   AATTAGATTACCACCACACTTCTCTCTAATCCTTGCTACTTCTATTGCTTAGATGACA--GTTATTTGTGTTGATTTCCA  31056
Mouse                       -------------------T-----TTGATTCTAGCTGTTTAT-CCATGTGAGTGGGG--GCT-TTGGGATATATCTCTT  33022
ChineseHamsterGHOK1GS       -------------------------CTGTTCCTTGCTACTTCT-TTGCTTAGATGACATGGTTATTCATGTTGATTCCCA  33380
LongTailedChinchilla        -------------------TATTATGTACTGTTAAACATTGCC-TTCATTTACTGAAACAATA-TGCA-GTTGATTGTTT  32100

Majority                    XTAA-GTTXTXAATXXGTGXTATATCATTTX-ATTXTXXATTACXTXAAAGCCCXGGTCA-AATATTGTTTT--TCTTGT
                                      53050     53060     53070     53080     53090     53100     53110     53120
Human                       CTAA-GTATTAAAT---------ACTATGTC-ATTTTGAATTCCTTTAATCCCCTGGTCA-AATGTTCTTTT--TCTTGT  49999
GuineaPig                   TTCATGTTCTGAATTAGTGTTCAATTAATTA-ATTTTGAAATTCTTTAAAGTCTTGGTCAGAAGATTTCTTT--TACTGA  28688
NorthernAmericanDeerMouse   CTAA-GTTGTTATTCTACATCATGTCCTTTCTGTGCTTGGTTAGATAAAGGCCCAGAACA-ACTATTGTTTG--TCTCCT  31132
Mouse                       TTGG-CTGAGGGGAGCCTGCTGTATCACTTA---ACTTG--TACAAAGAATTCAATGGTA-ATTTTAATTTA---CTTGT  33092
ChineseHamsterGHOK1GS       CTAA-ATTATTCCTCTGTGACATGTCCTTTCTATGCTTGGTTAGATACAGGCCCAGAACA-GCAACTGTTTGTGTCTCCT  33458
LongTailedChinchilla        TTGATGTTCTAAATTAGTGCTAGATGAATTA-ATTTTGAAATTCTTTAAAGCCCTGGTCAGAAGGGGGTTTT--TTCTGA  32177
```

```
Majority                     TXCAXGTTXAXATTTXTTTGTXGAXACTTCT--------------------------XAX--GAATXAXATTTATTXTXX
                                      53130     53140     53150     53160     53170     53180     53190     53200
Human                        TGCAAGTTAAAATTTTTGTGTAGAAACTTAT-------------C-----------------AGGTAGGATTTATTATTA  50049
GuineaPig                    TACAAGTTAAAATTTGTATATAGAAACTATT-------------TA-----------TAG--GATTAAAATTTACTA---  28739
NorthernAmericanDeerMouse    TCCTTATGGCCATCTATTTGCCTATTTTTCTGTCTCAGAAGTAATGAGTTAATCCTGTAACTGAATGAGATTTCTCTTGC  31212
Mouse                        GTTCTTTAGTCA----TTGGACAGTTTTGGT---------------------------------ATGAAGTTGAATTTTA  33135
ChineseHamsterGHOK1GS        TCCTTATGGGCATCTATTTGCCTATACTTCT---------------------------AACTGGATGAGATTTCTCTTCT  33511
LongTailedChinchilla         TGCAAGTTAAAATTTGTGTGTAGAAACTA----------------------------TAG--AAATAGAATTTACTA---  32224

Majority                     GXATAAXCTGTT---ACXACXATTCTGCTTXGACXTTAAGXTGGGATGT-TXGTXXXGTTAGTT----GAXTATXTXTTX
                                      53210     53220     53230     53240     53250     53260     53270     53280
Human                        GCATAAACTGTTTATACAACTCTCCTGCTTAGAATTTAAGGTAAGAAGTTCAGTGCCTTTGGTTATTTGATTTTTTTTAT  50129
GuineaPig                    GCATAAACTGT----ACAACAGTACTTGGTAGACTTTAAGTTGTGAAATGTAGTGCTGTTTGTT----GACTGTTTAT--  28809
NorthernAmericanDeerMouse    TTGAAGTCTTTT---GCTGCCATTT-GCTCTCACCTATGGATGGAATGT-TGATAAGGCCAATCTG--GAAAATGTAGTG  31285
Mouse                        GTATTTTTGCTA---ATTATTATTCTACA-TAGACTGCACATGGCTTGA-TGGTAACCTTAGTT-------GATGGTTTA  33203
ChineseHamsterGHOK1GS        TTGAAGTCTTTT---GCTGCCATTTTACTCTCACCTATTTGTGGAATGT-TGATAAGGCCAATCTG--GAATATGTATTA  33585
LongTailedChinchilla         GCAGAAACTGT----ACAACATTGCTGGATAGACTTTAAATTACGATATGTAGTGCTGTTGGTT----GACTCTTTTTT-  32295

Majority                     AXGXCTTAXTTTCXTACGAAXAXA-TTAATTTXXAAXATGXGTTAXTGGAXTTCCTA-GGXAAAGAAXTXTTAAXXATXA
                                      53290     53300     53310     53320     53330     53340     53350     53360
Human                        AGGTCTTAATTTCTTACGGAAAAT-TTTTTTTTGAAGATGTGTTAATGGTTTTCCT--GGAAAAGAAATCTTAAGGATGA  50206
GuineaPig                    --GTCTTAATTTCCTAGGAAAAAA-TTCCTTTTGAAGATGTGTTAATAGCTTTCCTAAGAGGAAGAAATCTTAAGAATGA  28886
NorthernAmericanDeerMouse    AAGACCTATCTCTAAACAAATAGACAAAACAACTAATAACAGACGTTGGAGTTAATA-TGCAAGGAAGCTTGAGTGCTCA  31364
Mouse                        ATGATTTTTCTCCTTTCCATT-G----AAAGCCTAATTTTAGCTTTTCAAGC----------ATTCAGATT---TACTCA  33265
ChineseHamsterGHOK1GS        AAGACTTGTATATAAACAAGTAGACAAAACACCTAATAGCAGATGTTGGAGTTAATA-TGAAAGGAAGCTTAAATGTTCA  33664
LongTailedChinchilla         AGGTCTTAATTTCCTGTGGAAAA--TTCCTTTTGAGGATGTGTTAATGATTTTCCTAAGGGGAAGAAATCTTAAGAATGA  32373

Majority                     AXXATATATGAATGTA---TXAATXTTTTAAATAXTTTXTAXAAAX-GATATTGXTCAAATTGAXTATATXCAAXATTTX
                                      53370     53380     53390     53400     53410     53420     53430     53440
Human                        AGAATATGTGAATGTA---TTGATGTTTTAAAAATGTAATATAGAA-GATATTGTTTAAAATTAATGTATTCAAAAATAG  50282
GuineaPig                    ACAGTATATAAATGTC---TTGATGTTTTACATA-------------------------------------------TTA  28920
NorthernAmericanDeerMouse    TATATAGATGACTGGAATGTAAATAGGTAGAGTCATTTTTGAAAACTGAAATAGCTCAAGTTGAGCATATGCATGTTCTG  31444
Mouse                        CCTATGTTT---T--A----AAGTATTTTTAACTTTTCATAGGAATCCATTTTTTTCATACTGATTATAAAGAATATTT-  33335
ChineseHamsterGHOK1GS        TATATAGGTGACCAGAATGTAAATAGGTAGAATCATTTTTGAAAATTGAAATAGCTCAAGTTGAGCATATGCATGTTTT-  33743
LongTailedChinchilla         AGAGTATATAAATATA---TTAATGTTTTAAATACGTTGCATAAAA-GATATT---TAAATTCAATACATTAAAAAATAA  32446
```

```
Majority                    TATTXXAATATTTTCTGACXTXXAT-TATXXGXTTXCXXTAATAXTCT-AAAXCXXAAGXTXTXAAAATAAAATTTAXGA
                                      53450     53460     53470     53480     53490     53500     53510     53520
Human                       CATTAGAATGTTTTCCGACCTACAT-TATAAGACTGCTGTAATGATCT-AAAACTTTAGCTGTTTTAATATAGTTTTAAA  50360
GuineaPig                   TATAACTATATTTCTAGACCTATGTATGTGGGATTACTGTTGTAGTCTTAAAAGTTAATCTCTTAAAATACTATTTAAAT  29000
NorthernAmericanDeerMouse   TATT-TAATATTTTCTGCTTCTAGT-TATGTGGTTGAGAAAATATACACGAATCAAAAGATAGGCAAAGAAAATTTATGA  31522
Mouse                       ----------CTGTATGTCTTTCAG-CCAACGGTAACGATAGTAAGA--AATTTAAAGGA---GAAGATAAAATGGATGG  33399
ChineseHamsterGHOK1GS       -ATT-TAATATTTTCTGTTTCTAAT-TATGTGGCTGTGAAAACATGT--GAATCAAAAGATATGCAAAGAAAATTCATCA  33818
LongTailedChinchilla        TATAAGCATGTTTTTAGACCTAGATATGTAGGATTATTGTAATGGTCT-AAAAGTTTATCTCTTAAAATACTGTTTAAGT  32525

Majority                    AXXATCCATAXCXTX-X-GTATCTCAAAGCTXXAAATGACCTXGAAATXAAXTTGTAA-XAXAAGXTXTTXXXCATX-XT
                                      53530     53540     53550     53560     53570     53580     53590     53600
Human                       ACTAATGATATCTTTCT-CTGTCAGTAAAATACAAACTTTTTCTTAATAAAAATGTAATGGAAAACTGTTCCTCATAGAT  50439
GuineaPig                   ACTACAAGTAG--------TATTTAAGTGCCATAAATGACATTCAAATAAGTTTA------AATGCTATAAATCACA--T  29064
NorthernAmericanDeerMouse   AGCAACCATTACATTGTAGTAGCCCAAAGCTGGAAATGACCCAGATGTGCAGCAGTAA-AAGAAGTTGTTATGTATCCAT  31601
Mouse                       GGC-TCCCTCTCGT----GTACTTCACATTCGAAAGTTACCTGGTGAAGTGACTGAAA-CAGAAGTTATTGC-------T  33466
ChineseHamsterGHOK1GS       AGCATCCATTACATTGTAGTAGCCCAAAGTTGGAAATGACCCAGATGTGCAGTAGTAA-AAGAAGTTGCCGTGCATCCAT  33897
LongTailedChinchilla        ACTATAAATAG--------TATCTGAACACTATCAATACTCTTTAAATAAATTTA------AATACTAT----------T  32581

Majority                    TTA--GGTATXTTTATGGXXA-----XAXTXAATAATATXTGTATTTCAXXAAAACAXAAGXATATAT-ATAAAATXGTG
                                      53610     53620     53630     53640     53650     53660     53670     53680
Human                       TTT--TGTCACTTTACAAAGT-----GACAAAATCATTTTGTTAGTTTATGGAAAATAA---GCTTGT-AAAACTTTTTA  50508
GuineaPig                   TGA--AATAGCTAAATATTTA-----AGTTAAATATTATTTATATTTAAATACTATTGGTGTAAATAC-ATAGAATCAAG  29136
NorthernAmericanDeerMouse   TTAGTGGAGTATTTATGGCCA-AAAAGAATGAACACGCTCTGTGTATCTTCAAAGCATAAGCATACATCATAAAATGGTG  31680
Mouse                       TTA--GGTTTACCTTTTGGTA-----AGGTGACTAACATCCTTATGCTGAAAGGAAAAAACCAGGTACAGTGCACAAGCG  33539
ChineseHamsterGHOK1GS       TTAGTGGCATGTTTATGGCCATAAAAGAATGAACAAATACTGTGTATCTGCAAAGCATAAGTGTACATCATAAAATGGTG  33977
LongTailedChinchilla        -----------TAAATGCT-------AATTAAATACTATTGGT--TTCAGTAAAAC--------ATAT-ATAGCATCAGG  32632

Majority                    TTT-CTTGXXAXAAATGAAAXTTTAGTAGAATGAXTTTTATXXTTTTT------TXTTAATTTTTTTTGAAAGXTGAAAX
                                      53690     53700     53710     53720     53730     53740     53750     53760
Human                       CCTAAAAGATAGGACTGAAATTTCAGCTTTTTTAATTTGATGATGAGT------TTTTAATTTCTTTTGAAAAAGAATGT  50582
GuineaPig                   TT----TTTTTTCAATGAAAATTTAATGGAAATAGTTC---AACTTTT------TATTACTTTGTTCTTAAAG-------  29196
NorthernAmericanDeerMouse   TTT-CTTGAAAGAAGCTAGACCAAAGTAGAATGGTTTTTATTTTTATTATTTATTTATTATTTTTGTTGGGAGGTGAAAT  31759
Mouse                       TTA-CTACTAGCATGTTAAATTACA-CAGAAGGAGGCTTATCTGTCACCTAAAGGATTCATTGTCTGACAAAATTAAAAG  33617
ChineseHamsterGHOK1GS       TTTTCTTGAAAAAAACCAGGCCTTAGTAGAATGCTTTTTGTTCTGTTT------TTCTTTTTTTTGT-GGAAGGTGATAC  34050
LongTailedChinchilla        TTCTTGTTTTTTCAAAGAAAATTTAATGGAAGCAATTCCTCAGCTTTT------CATTACTTTATTTAAAAAGAAGAAAG  32706
```

```
Majority                    TAGCTXAGAXTXATTXXTTXTGXGGXATAXTACXGAXGTAAAAXAX-XTAXTTGCCTAATTTTTTACG-TAAATATXTTA
                                      53770     53780     53790     53800     53810     53820     53830     53840
Human                       ATGCTTCTAATAATTTATCAAGAGGAAGAATACCAAAAGAAAATAT-CTGCTCTTCTTTCTTTTTACT-TAGATTTTTTT  50660
GuineaPig                   TGACATGAAATCATTGTTTATAA---AAAATATAGTTGTAAAA----CTTTTTCCCTAAAAGGTAAGG-TTATCCTTTCA  29268
NorthernAmericanDeerMouse   TAGTTCAGACAAGTTCAATTTGTGGCATTTTACTAAGGTCTTTTATAGCATTTGCTTCCTTTTCCATG-TATATATAATA  31838
Mouse                       AAGTTCAGTTTCCTGTTTGTTTTGTTTTGTAATAGCAGAAACCAGGGGTACTAGTAGAATCTATTACA-TGAGTTTATTC  33696
ChineseHamsterGHOK1GS       TAGATCAGATAAGTCCAATTTTTGGTATTTTACTGAGGTCTCTCACAGCATTTGCTTCCTTTTCTTTGGTATATATAATA  34130
LongTailedChinchilla        TGACCTGAAGTCATTGTTTATGA---AAAATACGGTTGTAAAA----CTTCTTGCCTAAAAGATGGCG-TTAACATTTCA  32778

Majority                    GXTTXTTXAXXTTACTTGAAATC-XGATXATCAATTTXACTG---CTCATGTAXXA--XXAAATGTAXTXTCAXXTATTX
                                      53850     53860     53870     53880     53890     53900     53910     53920
Human                       GCATTTTTAACTTATTTTTAACA--AATAATTGTTTATACTT---ATGGTGTACAA--TGTGATGTTTTAATACATATAT  50733
GuineaPig                   GCTGTTTTTG-TTGATTGAATTT--GATAATCAACTTCATTC---CTTTTGAACAA--GACAATGAACTTTTAAGAATTC  29340
NorthernAmericanDeerMouse   GTTTGAAAATGTTACATAAAATCTAGATTGCCAAACTAACTGTGCTTCATGTATTACAACAAATGTAAAGTCATTTGTTA  31918
Mouse                       ATCTGGTAGCATTA---GAAATC-AGGCTTAAATTTTGGATG---CTCATTCATTA--ACAGTAGCAGTAGGACCTATTC  33767
ChineseHamsterGHOK1GS       TTTTGAAAATGTCACATAAAATCTAGATTGGCACTCTAGCTGTGCTTCATGTATTA----AATGTAAAGTCATTTGTTA  34205
LongTailedChinchilla        GCTGTTTTTG-TTGGTTGAATTT--GATAATAAACTTCATTC---CTTTTGAACAA--GAAAATGTGCTCTCAAGAATTT  32850

Majority                    ATAATXX------ACXAXXAGACATXX-TGGTTXACAAGXTXAXAGAXTTAGTXCTTGXXXAATTXAXATAGXATTAATG
                                      53930     53940     53950     53960     53970     53980     53990     54000
Human                       ATTATGGA-----ATGATCAAATCTGGCTGGTTAACATATTCATCTCTTCAGTACTTACAATTTCTTTATGGTAAGAACA  50808
GuineaPig                   ATCATG-------ACAGCCAAGCATAG-TGGA--ACATGTCTGTAATCCTAGAACTTGGGAGGCTGAGTCAGGAGGATTG  29410
NorthernAmericanDeerMouse   CTACAAT-----------GAGAGATCA-TAATTGTCAAGCTAAAAGAAATGGTTAATGATGAATAAGCATT--ATTAATG  31984
Mouse                       ATATTAA---------AAATGACATTA-TGTTTTAAAATTTCCAAAAATTTTATTTAGAAGAATTTATTTAATATTAG-A  33836
ChineseHamsterGHOK1GS       CTACTATTTGTTATCTATGAGAGATCA-CAGTTGGTAAGCTAAA-GAGATAGTTAACAGTGAATCAGCATATAATTAATC  34283
LongTailedChinchilla        ATCATGG------ACTGCTGGGCATGG-TGGT--GCACACCTATAGTCTTAGCACTTGCGAGGCTGAGGCAGGAGAATCG  32921

Majority                    XXATTAATTCXATXAXXAAGXATGXXX------ATAAXATGXGTTXTTXTAAXGXXXTTTTXXXTCTGX------AGCXA
                                      54010     54020     54030     54040     54050     54060     54070     54080
Human                       TTTAAAATTCCTTTAGCTATTTTGGAAGT----ATACAGTGGAATGTGGAAAAACTGTTTTTCCTCTGCTCACACCACAA  50884
GuineaPig                   CTATGAGACAGA--GGCTAGTTTGAGTT-----ACAAAATGAGTTG----AAGGTCATCCT-------------------  29460
NorthernAmericanDeerMouse   GAATTAATTCTATGAAAAAGCAT------GGAATTAATATGTGTTTTTATATTGA-TTTTTATTTCTGAAAAGATAGCCA  32057
Mouse                       AAATTAATATTCTCTAAACGTAG----------CTAATATA-ATAATTATTCCGTCCTTTTAATTTTTC------AGTTG  33899
ChineseHamsterGHOK1GS       GAATTTATTCCATGAAAAGGCATATTACTGTAATTATTACGTTTGCTTTTATAGAGCTTTTGTCTCTGA-----TAGCCA  34358
LongTailedChinchilla        CTGCAAGTTCAG--GGCCCGCCTGGGCA-----ATGTAATGAGTTC----AAGACTGGTCT-------------------  32971
```

```
Majority                     CAATCTXXATA-GTGAGACCTTGXGXACXXXAAAGGAAAXTAAX-TXAXXAAGXTGCAAGAAAAAATTCAXCTTTTGTA-
                                      54090     54100     54110     54120     54130     54140     54150     54160
Human                        CAATCAACACA-GAAGGCTTCTGTGGCCTCAAATGTGGGGGAATTTTTTTCCACGCCAAGCAAGCAGTCAGGTCTGCAG-  50962
GuineaPig                    -GAGCTTCATA-GTGAGGCCCTGT---CTCAAAAAGACCATAAATAAATAAATAAGAAAAAAAGAAAATGTATCATGAA-  29534
NorthernAmericanDeerMouse    CAATCTGTATT-GTGAGAGATT-AGTACAGGAAAGGAAACTCCC-TGACGAAGTTGCAGGGAAAACTTCACCTTTTGTA-  32133
Mouse                        TATTTTTTATA--TGAGACCTTTAGGATAGTAACATAAAATTTT--------GGTGCTAGAAAATGCTTATTTTCTCTAG  33969
ChineseHamsterGHOK1GS        CAGTCCATATTCGTGAGGGATT-AGTACAGGAAAGGAAACTTAGGTGACAAAGTTGCAGGGAAAGCTTCACCTTTTGTA-  34436
LongTailedChinchilla         -GAACTGCGTA-ATGAGACCTTGT---CTC--------CTTAAA-------AAAAAAAAAAAAAAA--------------  33017

Majority                     -TTTXCTXAAATTTA---XGCTGTGATCTAATAATTCCTGGXXXXAAAXXACATXXGAATXXXXXX----------TAGT
                                      54170     54180     54190     54200     54210     54220     54230     54240
Human                        -TGTTCTCCAACTTA----GCTCAGTTTCAACACTGTCTGCCGGGAGATAGCATCAGATCCCACAGGTTGAGGGCTCAGT  51037
GuineaPig                    -GTACACAGTATGGT----GGTACATTCCTATAATTCCAGCACATAGGCTGTGGCAGGA----------GATTTGCTAGG  29599
NorthernAmericanDeerMouse    -TTTACTGAATTTTA---TGCTGTGATCTAATGGTTCCTAGTTTCAAAAGACATTTGAATTTGTAACAGATGTTTATTGT  32209
Mouse                        TTTTTTTAAAAAACAGATTTATGGGATGTAGTAAGTTGTAGATGGAACCAACA---AAATCGAAGG------------GT  34034
ChineseHamsterGHOK1GS        -TTTACTGAAATTTAA--TGCTGTGATCTTATAATTCCTGGTCTTAAAAGACATTTAAATTTGTTA---------GTAGT  34504
LongTailedChinchilla         --------------------------------------------------------------------------------  33017

Majority                     AXTACTTGAT-------X-----X--X-----TXAAXGXXXXTAATATTTXTAAG-XXTXX-TATTXAX--TXGTATTXA
                                      54250     54260     54270     54280     54290     54300     54310     54320
Human                        CCCACAAGATC---TCCTGAGACACCAGC-AAGTCAGGCCTCTGGAACTTCTGATTGACCAGCTTCAAGT-TGGGGTTCC  51112
GuineaPig                    ACTTCAAGGCC---AGCTTGGGCAGCA------TAATGAATTTAAGACTGACCAGAACTACATAATGGCC-TTGACTCCC  29669
NorthernAmericanDeerMouse    GGTATTTAATGCTGTCATTGTAGATAAATGTGTGTAGGGAAATGATGTTCTTAAGGTCTCTTTATTTTCTATAGTATTAA  32289
Mouse                        ATTACTTGAA----------------------TCAACCCCAATATTATTTTAAAA--------ATTAAG--TGATTTCTA  34082
ChineseHamsterGHOK1GS        TGTATTTAAT--------------------TGTGGATGGTCTTAATGTCTCTTTG-TTTTTCTATAGAA-GTAGTATTAA  34562
LongTailedChinchilla         --------------------------------------------------------------------------------  33017

Majority                     AGXAXCAXGTA-TTATGGTXTTXAAAXXXT--XTXTCATTTGAGCACACTAAACTXATAGAAACATTTACTCTGXAX-TT
                                      54330     54340     54350     54360     54370     54380     54390     54400
Human                        CACAACCCCCT-TTTTGGATTTCATTAATT---TGCTAGAGCAGCACACAGAACTCAAGGAAACATTTACTGAGAT--TT  51186
GuineaPig                    CCCACCCCAAA-TTTTTTCATTTAAAAATT---TATCATGA-AATATACCAAA-----AGAAATATTTATTCTGT----T  29735
NorthernAmericanDeerMouse    AGTATCAAGTAGTTACGGTCTTAAAATGCT--GTTTCATTTGAGCTCCTTGTATTGATACATGCATTTACAGTTCAAATT  32367
Mouse                        AGA--CATGTT-TTATTATTTTCAAATGATATGTCTTGTTTGAGG---ATACGCGCATGTGAGCTAAGGCCCCAGCAGAA  34156
ChineseHamsterGHOK1GS        AGTGTCAAGTAGTTATGGTCTTAAAATGCTGAGTTTCATTTGAGCTCCTTATGCTGATATATACATTTACCATTCAAATT  34642
LongTailedChinchilla         ----------------------AA-AAAT---TATCATGA-AGTATACCAAA-----AGAAATACTTACTCTGTG--TT  33062
```

```
Majority                    ATTAXTTXGCXTT-ATAXXATXTTTAT-----TGAGXTGTCTGTTXTGA-TGTTTTGAXXXTT---GXGXTXXGTGAACX
                                      54410     54420     54430     54440     54450     54460     54470     54480
Human                       ACTAGTTTATTAT-ATAGGATATTAAAAAGGATAATAAGCCTCTAATCA-TGCCTTGATCTTTCAGGTGACCAGTCCCCA  51264
GuineaPig                   TTTACTTTG-----ATAATGCATACAT----TTGAGGAGTACATTGTGA-TGTTCTGAT----------ATACATATACA  29795
NorthernAmericanDeerMouse   ATTAATTAGCATTTAAACAATGTTTAT-----TTAGATGTGTGTTTTTAACTTTTTGAGTTTT---GAGCTGTGTGAACC  32439
Mouse                       GCCTGTGGGCGTCCATTCCTTGGG-AT-----TGGAGTTCCAGGCAAGGCTTCTGTAAGCCTT---GTGCATGGTGATGC  34227
ChineseHamsterGHOK1GS       ATTAATTAGCATT-AAATGATGTTTAT-----TTAGATATCTGTGTTGA--CTTTTGAGGATT---AAGCTTTGTGATCC  34711
LongTailedChinchilla        TTTACTTGG-----ATAATATATATATATATTTGAGGGGTACATTGTGC-TGTTTTGAT----------ACACGTACACA  33126

Majority                    TTXGAXXTTGGTXTTGXCTGXCATXAGXXXAXTAATTACTTTA--AAXTXTACTATTTTCAATAXTTXGTAXTTATXTT-
                                      54490     54500     54510     54520     54530     54540     54550     54560
Human                       TCTTAAAGTGGGGCTGCCAGCCATCAGTCAACTCATTAGCATACAAAATATATCATTTTGGAGATTTATTAGGAATTTAG  51344
GuineaPig                   TT-----GTGGTATTGTCCG--ATCAG---ACTGCTTACTATA-----TCTACTACTTCCAATACTTGGCA------TTT  29854
NorthernAmericanDeerMouse   CTGGATTTTCTTC---ACTCTCATAGGCATATTAATTCTTTTG--TATTTTTCTTTTTTCACTTAGTTGTATTTATATTA  32514
Mouse                       TGTGAACTTAGGT----CCTCTGGAGAAGCATCAGGTGCTTGT--AGCAACCATCTCTTCA-------GCACTTCAGTC-  34293
ChineseHamsterGHOK1GS       CTCGATATTCTTCTTTATTCTCATAAGCATATTAATTCTTTTG--AATTTTTCTTTTATCACTTAGTTATAGTTATATT-  34788
LongTailedChinchilla        T--------GGTATCGCCTG--ATCAG---ACTAATTACTGTA-----TCTACCACCTCCAACACTTAGTA------T--  33180

Majority                    --TTXTXTGCXAAXAAAX-TTXGTGAGAACAXAXXGXTXATAXTGAA--GTXAXAATXTXGT-TTXTATTTCAXTATTGT
                                      54570     54580     54590     54600     54610     54620     54630     54640
Human                       AGTTGTATGCCAGGAAATGGGGTTGAGGACCAAATGTCTATTTTACAATGTCACAATACAGTATTATGCATTACAGTAGT  51424
GuineaPig                   CTTTGT---------------GGTGAGAAC--AGTGTTCACGATGG---ATCATAAT------TTGTATTT-AAGACTGT  29907
NorthernAmericanDeerMouse   -TTTATCTGAAAATAAAGATTTGTCAGATCATTAAGATAATATGAAA--GTTAAATAATGGT-GTATTTTTCAGTATGGT  32590
Mouse                       ----AGATGCTAAGTTCTCTTTGTTAGAGCACTGAGACGGAAATGAG--GTTACAGTCTAGG-CAGTATTTCAAT-----  34361
ChineseHamsterGHOK1GS       ---TATCTGCAAATAAACTTTTGTGAAATCATGAAAATAATATGGAA--GTTAAATACTGGT-GTATTTTGCAGTATTGA  34862
LongTailedChinchilla        --TTGT---------------GGTGAGAAC--AGTGTTCATGATGG---GCCATAAT------TTGTATGT-ACGGCTGC  33231

Majority                    TGGCAXAXTX-CTGCAATAGAACAXCACTXXATATTCCTTXCXXATTAAATXTGGAAXTTTXXTXXXCAXTTAGGCTTXA
                                      54650     54660     54670     54680     54690     54700     54710     54720
Human                       TGCCATGCT--GTGCGGTAGATCACCAGAACCTATTCTTCCTGTCTGAAACTTTGTACTTTGACCATCATCACCCCTTCC  51502
GuineaPig                   TGGCA-ACT--CTTTATTTGAACAT---ACTATATTCCTACTGGATTGAGTACCAG----TGGTACTTGATGAGGCTTGA  29977
NorthernAmericanDeerMouse   TGGCGAATTATCTACCATAAA----CACTGAAAATATCCTTCACAGTATATCTGGAAATTTTCCCGACAGTTATAATAGA  32666
Mouse                       TAGCAGATTAGCTTAAACAGA----CACTTGAGTTTGAGTTAAAAAT-----TGTAATTTTTATTTAAGTTT--AATTCA  34430
ChineseHamsterGHOK1GS       TGGTATATTAACTGCCATAAAATAACACTGAAAATTTCCTTCACAGTATATCTGGAAATTTTCTTGACCCCTCAGGTTCC  34942
LongTailedChinchilla        TGGCA-GCT--CTG-ATTTGAACAT---GTTATATTCCTCCCGGCTTGAACATCGG----TGGTGTTCAGTGAGGCTTGA  33300
```

```
Majority                    TGXTATAXTCATCCXTXTXCXXXTCTTTGGTTXXTXCTTTTXTGXACXTXATAAA-TCTGXXAXTTTAGCTXTTTTX---
                                     54730     54740     54750     54760     54770     54780     54790     54800
Human                       CTGTTCACTCCTCCCTCCCCCAGCCTCTGGTGACTATTTTTCTACTCTCTACTAA---TGTGAGTTCAGCTTTTTTA---  51576
GuineaPig                   TG------TCACACGTGTGCCG-ACAGTGTTTCAGAGTATTGAGAACGTGTTAAAATCTGTTATTTTAGCTGTTTTG---  30047
NorthernAmericanDeerMouse   AACTATATTAAT--TTATTTTTATTTTAAATTTTTTAATTCATCTATTTTATA---TCAGAGCCACAGCCTCCCCCC---  32738
Mouse                       TGGTATCTTTTTT-AAAAACTCATTTTCAGGC-----ATTTTTGGAACTGGCAA---CAGAGGAAGCAGCTATTACT---  34498
ChineseHamsterGHOK1GS       TACCCTCCTCCTCCTCCTCCTTCTCTTCCTCCTCCTCCCTCCTCCCTCCTCTAAGCTGGGATCCTCTACTTACTCTCCTG  35022
LongTailedChinchilla        CGAGGCAGTCATGCATGTACCA-CCAGTGGTTCAAGCTAGTGGGGACATGATAAAATCTATTATTTTAGGTGTTTTG---  33376

Majority                    ---GATTATACAXTTXAATTCTTTTAXCTGXCATTX----TCCTCCTGTGX---TXACXAACXXACATAXCACXTXCAGT
                                     54810     54820     54830     54840     54850     54860     54870     54880
Human                       ---GATTACATATGTAAGTGAGATAATGTGTTATTTG---TCTTTCTTTGTT--TGACTTATTCACTTAGCACACACAGT  51648
GuineaPig                   ---GAATATACA-TTAAATTATTTTAAATGCAATTC----CCCTGCTGTGC-----ACTAGAACACAGAAAAC------T  30108
NorthernAmericanDeerMouse   ----ATTCTTCCCTCCCAGTCCCTT-CCTGCCATT----TTCCCCCTCC-----TTCCCCAC---CCTCTCCTCTTCAGT  32801
Mouse                       ----ATGGTTAATTACTATTCTGCT--GTGACACC-----TCATCTTCG-----TAACCAAC---CAATTTACATCCAGT  34559
ChineseHamsterGHOK1GS       GGAGATTCCCCTGTGCCAGTCTTTCACCTGACCTTGAAATGCACCCCGCAAGAGTACCCCACAGCCCTTCCCCCTCTGGG  35102
LongTailedChinchilla        ---AAATATACA-GTAAACTGTTTTAACTGTAATTC----CCCTGCTGTGC-----AGTAGATTACAGAAAAC------T  33437

Majority                    GXXCCXTXXAAATAGAATTXAATACCXXXTTTAGAAXCCXCTCATACCTAXTT-XC---TCXCXXAXCCXCAGTCTTXTT
                                     54890     54900     54910     54920     54930     54940     54950     54960
Human                       GTCCTCTTAAAATGACATTTAATATCCCATTTCTAGTTCGTTATTATTTAAGTAAT-----TTGCAAGTACATTCTCATT  51723
GuineaPig                   GTCCCTTCTAACTAGAAGTTTATACC---TTTAGGACCTCCTCATTCCCCACTTACTGCTCCCATAGCCACAGTCTTCTT  30185
NorthernAmericanDeerMouse   -CTCCATTCA-GGAGAATGAATGACTTTCAGGAGAATGAG---AGACCTAGTC------TCTCAGGGTCTGTGGGTTGTA  32870
Mouse                       ACTCCAATCACAAAGAACTAAAGACAGATAATACATTAAACCAAGTGAGTGTT------TTTAGATACATAAATAATATG  34633
ChineseHamsterGHOK1GS       -CTTCCCCAA-ACCTGATCACTCATTTTTTTTCATACCCACCCATCCTCAGTCGAC---TCCCAAAATCTATTTCCCGTT  35177
LongTailedChinchilla        GTCCCTTCTAAATGAAAGCTTGTACC---TTTAGGACCCCCTCGTACCTTACT--C-----CTGTAGCCACAGTCCTCTC  33507

Majority                    XXXAAXAGTTTCXTGAGTTXA-CATTTAGAGTATACTTTTXAGGXXTXGACTTXXTG--T------TXXXATATTTXTTX
                                     54970     54980     54990     55000     55010     55020     55030     55040
Human                       TTAAAAGATTAAACGAAAGGAACATGTAAAGAATAAAGCTGGGGGCTGGGCTTGGTGGCTTATACTGGTAATCCCAGCAC  51803
GuineaPig                   -----TAGTTTTATGAGTT-----TTTAGAGTATACGTTTAAGG--CAGACTTTATGGAATTTC------ACTTTGCTTC  30247
NorthernAmericanDeerMouse   GCATAGTTAGCCTTTATTTTA-CAGCTAGTATCCACTTATGAGTGAT-TAC--------------ATACCATGTTTGTCT  32934
Mouse                       GCCAATAGCATCTTAAGACTAATAAGTAGTATATGTTAATAGAAAAT--AC--------T------TCTTATATGCATTT  34697
ChineseHamsterGHOK1GS       CTCAGGGAGATCTTGGTGTCC-CCATGAACCCTCCTTGTTGTCTAGTCTCTCTGGGT-CTGGATTGTAGCATAGTTAGCT  35255
LongTailedChinchilla        -----AAGTTTTATGAGTTGTGTTTTTAGAGTGTACATTTAAGG--CAGGTTATATG-------------A--TT---TC  33562
```

```
Majority                    TTTGTGXT-CTGAGTCATXTCACT-TACGXATGTXXXGTTXTXTTAXATTCXCXTATXX--ATATGXTAAXX--TTGAXT
                                      55050     55060     55070     55080     55090     55100     55110     55120
Human                       TTTGGGAGGCTGAGGCGGGTGGAT-TACCTGAGGTCAGGAGTTCAAGACCCGCCTGACCA-ACATGGTGAAACCCTGAGT  51881
GuineaPig                   TCTGTGC--CTAACTCATTTCACT-TACC---ATACTGTTCTCTTAAA-TGACAT--TT--ATATGCCAA----TTGTAT  30312
NorthernAmericanDeerMouse   TTATTGGT-CTGGGTTACCTCACT-CA-GGATGTTTTTTTTTTCTAGTTCCCTCCAT---------TTGCCT--TCAAAT  33000
Mouse                       TTTGTTAT-CTTTAAAATATTTTTGTATGTATATATACATGTTAAGTATTCTTTTAT--------AAAAATAGGT--GGC  34766
ChineseHamsterGHOK1GS       TTTATTTT-ACAGCTAATACCACT-TATGAATGACTACATACCTTGTTTGCCCTTATCAGCCTGGGTTACCTCACCCAGA  35333
LongTailedChinchilla        TCTGTGC--TTGAGGCATTTCACT-TACT---GTGCTGTTCTCTTAAAATGGCAT--TTT-ATATCCCAG----TTGTAT  33629

Majority                    TTACTGXTACT----XTTTAXAAAACTAAXTAAT----XATTGXGTTATXXT---TXXXTCXATTTTXCATTAXCXATTT
                                      55130     55140     55150     55160     55170     55180     55190     55200
Human                       CTACTAAAAAT-----ACAAAAAAAAAAAAAAAT----TAGCCAGGCATGGTG-CCAGGTGCCTGTAATCCCAGCTACTT  51951
GuineaPig                   TTATTAATACT-----TCTACAAAACTAATTC-------AACACGTTCTCATT-TTACTTTTATTCTACTTTAGAGGTAT  30379
NorthernAmericanDeerMouse   TTCCTGGTGTC---ATTTTTAACAGCTGAGTAATACTCCATTGTGTAA-A-----TGTACCACATTTTCATTATCCATTT  33071
Mouse                       TTTCTGATACTC--ATTTTCCTGATCTAAATTAT----TATTAGATTA---------------GTTTCACTGTTT-TTT  34823
ChineseHamsterGHOK1GS       TTCCTGGTGTCCTTGTTTTTAACAGCTGAGCAATATGCCATTGTACCACATTTTCTTTATCCATTTCTCAATATCCATTT  35413
LongTailedChinchilla        TTACTGGTACT-----TTTACTAAACTAATTCTT----GAATGCGTTCTCAT-------TCTATTCTACGTTAGTGATAT  33693

Majority                    XGXXGATGAAXXAXAXCXXXXTTXTTTXCACGTTCTGXGCTAGTXCXXXTXAAX-X-XXATGXXCATCAXTGAGCTAXAG
                                      55210     55220     55230     55240     55250     55260     55270     55280
Human                       GGAGGCTGAGGCAAGAGAGAATTGCTTGAACCCGGGGGGTGGAGCTTGCGGTGATCCCAGATGCGTCACTGCACTCCAG  52031
GuineaPig                   TGGGGGTGAAACCCAAC------TTTCATGCATACTGAGCAAGTGCT----------------GTATCAGTGAGCTGTAC  30437
NorthernAmericanDeerMouse   CTCAATTGAAGGATATCTAGGTTGTTTTCAGGTTCTG-GTTATTACAGTTAAAGCTGCTATGAACATAGTTGAGCAACTG  33150
Mouse                       TAAAGACGGCAGAGATTGCTACTTTTTGCAGGTTTAAAGTTAAAATATTTTAA-----CATGGAAATTGG-GACTGAGGG  34897
ChineseHamsterGHOK1GS       CTCAAATGAAGGATATCTAGGTTGTTTTCATGTTTTG-GCTAGTACACATAAAGGTGCTATGAACATAATTGAGCAAGTG  35492
LongTailedChinchilla        GGGGGATCAAACCCAAGG-----TCTCACACATACTAGGCAAGTGCT----------------GTATCACTGAGCTGTAC  33752

Majority                    XCCTTGTGXCATAXTXGXATTTXATCTGAAXAAXXGAXCAAXAXTXXXAAAXXTGXAXXCATGXGGTCTGTTAATXGTXX
                                      55290     55300     55310     55320     55330     55340     55350     55360
Human                       CCTGGGTGACAGAGCGAGACTCTATCTCAAAAAAAAAAAAAAA-AAAAAAAAGAGTAAAGCTGAGGCCTACACATGGATG  52110
GuineaPig                   CACCAGCCTCACATTCTTATTTTACATTCAAAAGAAAGGAATG-TGTGAAGTATAAAAATAGGGAGTCTATAATGAGTTG  30516
NorthernAmericanDeerMouse   TCCTTGTGGTATGACTGAGCATCCTCTGAGTATGTGACCAAGAGTGGTATAGCTG-GGTCTTGTGGTAGGTTGATCCCCA  33229
Mouse                       TTATTTAGACACTGTAGCA-AGCAACAGAGTGAATGAACACTGGCAAGAGATGTGTAGTCACAACTAGAGCTGGTGGTCA  34976
ChineseHamsterGHOK1GS       TCCTTGTGCTATGATTGAGTGTCCTCTGGATATATGTCCAGGAGTGGTAAAGCTG-AGTCTTGTGGTAGGTTAATTCCCA  35571
LongTailedChinchilla        CACCAGCCTCATATTCTTATTTTAGATTCAAAAGAGAGGAACA-TACAAAAAATAAAAACAGGGAGTCTGTGAGGAGTTG  33831
```

Majority	GXTGTXGGAGAGAX-AXXXXAGTXAXTT-XA-AGTGGXGATGAXAACTXGAAGCCCXAXTGAXAXGGGAXXTTTGXATAA	
	55370 55380 55390 55400 55410 55420 55430 55440	
Human	GATGTGGAGGAGGG-------GCACTT---------AGGATGACTAGTGGAAGCCCCATTGAGAGGGGATATTTGAATAA	52174
GuineaPig	TATGTAGGAGAGA-----CTTGTAATTTTTA-AGTAGAGATGACCACTGGAAGCCCTATTGAGAAGGAACATTAGGATAA	30590
NorthernAmericanDeerMouse	GTTTTCTGAGAAACCACCACACTGACTTCCAAAGTGTCTATACAAATTTGCACTCCCACCATCAGTGGAGGAGTGTTCCC	33309
Mouse	GGTGCAGGAGGGG--AGGGGAATGGCTT--A-GGTGG--AGGAAAGCTTGGA--ACTGCTGTCAGGGGAAGGA-A-----	35041
ChineseHamsterGHOK1GS	GTTTTCTGAGAAACTGCCACACTGACTTCCAAAGTGGCTGTACAAGTTTACACCACCACCAGCAATGGAGGAGTGTTCCC	35651
LongTailedChinchilla	CGTGTGGGAGAGAG-AGACTTGTAATT--TA-AATAGGGTTGACCACTGGAAGCCCTATTGAGAAGGAACATTAGAATAA	33907

Majority	T-AACTTCAXAGTCXTTAGGGXATXAGCXX-CACTXXXGXXTTTGXXXTATXCCATXCTG-AXAGATAXTXAATGCAAAX	
	55450 55460 55470 55480 55490 55500 55510 55520	
Human	TGAACTTCAAAGTGGTTAGGGAATAAGCCA-C---------------TTATTCCAGACAGAAGGAATAATCAATGCAAAG	52238
GuineaPig	T-AATTTCAGAGTTGTTAGGAAGTAAGCCA-CACCACCCAATTTGGATTATTCCATA----ACAGACAATCAATGCAGAT	30664
NorthernAmericanDeerMouse	CTTGCTCCACATCCTCTCTTGCACGAGCTGTCACTTGTGTTTTTGATCTTAGCCATTCTG-ACAGGTGTAAGATGGAATC	33388
Mouse	-------------CTTGGTAACATGAATTG-CACT---G--------CTCTGCC--TCTG-GGACATAGGAAATCCAAGG	35093
ChineseHamsterGHOK1GS	TTTGCTCCACATCCTCTTCAGCATGAGCTGTTGCTTGTGTTTTTTATCTTAGCCATTCTG-ATAGATGTAAGATGGAATC	35730
LongTailedChinchilla	T-AATTTCAAAGTGGTTAGGGAGTAAGCCA-CACCACCCAGCATGGGTTATTCCATACAGAGAGAACAGTCAACGCAAAT	33985

Majority	TT---XCTXXTTTTGXAXAXXATTTTTTGAXXTC--AGAXTXTTAA---TXTTCTTATGTTTT---------TCAXTGT	
	55530 55540 55550 55560 55570 55580 55590 55600	
Human	TT---CCTTCTGTTGAAAAAAATGCTTTTATAATT--TTATTTTTCA---TCTTCTAAAGCCCT---------TCAGTCT	52301
GuineaPig	TA---CCTGCTATTGCACAAAGTTTTTTTGAAATC--AGATTTTTAA---TTTTCATATGCTAT---------TCATTGT	30727
NorthernAmericanDeerMouse	TC---AGTCATTTTGATTTGTATTTCCCCGATGGCTAAGGATGTTGAAGGTCTCCTTACGTGTT---------TCTCGGC	33456
Mouse	CCATGATTGATTCTCG---GTATTCTTTCATTAC---AGAAGGTT-----TTTTTTTTTTTTTC---------CTAATGG	35153
ChineseHamsterGHOK1GS	TTAAGAGTCATTTTGGTTTGCATTTCCCTGATGG-TAAGGATGTTAAGCATCTTCTTAAGTGTT---------TCACGGC	35800
LongTailedChinchilla	TT---CCTTCTGTTGCAAAACAGTTTTTTGAAGTC--AGATTTTTAA---CTTTTAAATGGTATAAATGGTAGTCAGTAT	34057

Majority	CXTTTGATCXTCXTT--TTXAGAXTXXAGTT-XGXTT-XTXCCTXATGTTTTTATXGGAXTTTGAGGXTTCTXGXTACAG	
	55610 55620 55630 55640 55650 55660 55670 55680	
Human	CCTTTGGTCATCTTT--TTT---TTGTAGTT--GGTT-ATGCCTTAACAACA----GTACTTTGAGGATACTAGTTACAG	52369
GuineaPig	TCTTTAATCATCCTT--TTTACATTAGAGTT--GGTC-ATACCTTAGGATTTTAT-GTTCTTTGAAAATTCTGATTATAG	30801
NorthernAmericanDeerMouse	CATTTGAGATTCTTTGATTGAGAATTCACTTTAGATTAGTACCTCATTTTTTAATTCGATTATTTGGTTTGTTGATACCT	33536
Mouse	CATATTAACTTTCTC--TTGAGAA--AAACTAAGACT-GAAGAACATGTGAATGTTGGCTTTTGAAGTATACAGATAC--	35226
ChineseHamsterGHOK1GS	CATTTGAGATTCTTCAGTTGAGAATTAGATTTAGATTAGTGCTCCATGTTTTAATTGGATTGTTTGGTTTGTTGACAACT	35880
LongTailedChinchilla	TCTTTAATCATCCTT--TTTACGTTACAGTT--GGTC-ATGTCTTAGGCTTTTAT-GCTCTTTGAGGATTCTGATTACAG	34131

```
Majority                     XATTTTXAAAXTXCXTCA-TXTACTXTXX-AXATXXGTTTTCTXTCAGAX--X-XTTTTGTGXXTATCTTGTTCTCTXTT
                                       55690     55700     55710     55720     55730     55740     55750     55760
Human                        TATTTAAAAAATACGTCACTATATGCTGAGTTATTTTTTCAGTGTTAGC-----ATT-TATGTTTATCTTGTTCTCA-TT  52442
GuineaPig                    CATTTTACAACTAGCTCAGTGTACTGTATAAATTATGTTTTTTTTCACTGTTGACTTCTGTGTTTGTCTTGT-CTCCCTT  30880
NorthernAmericanDeerMouse    TATTTCTTGAGTTCTTTA-TATACTTTGG-AAATGAGTCCTCTGTCAGATAGGGGGTTGGTAAAGATCTTATTCTTTTTT  33614
Mouse                        --TATTTAAAATTAATGT-TAAAAT--A--ATATAAGTATACTTTTAGAC-----------CAAAATATGTTAATTTGCT  35288
ChineseHamsterGHOK1GS        AGTTTCTTGAGTTCTTTA-TGTATTTTGG-AAATCAGTCTTCTGTCAGATGTGAGGTTGGTGAAGATCTTTTCCCATTCG  35958
LongTailedChinchilla         CATTTTAAAACTACCTCAGTGTACTCTAAATTGTCTGTTTCTTTT-ACT-----CTTTTGTGTTTATCTTGTTCTCACTT  34205

Majority                     XTATXCTG-----ATGCCCTXTTGGXTGTCTTTTXTTGXXTCAGAATG-ACXTXAATTTXATXAXXTXCTXTTTATAGAT
                                       55770     55780     55790     55800     55810     55820     55830     55840
Human                        CTCTTC-------ATCCCCCCTTTGTTGTCTTTTCGTGTTTCAGAATGGACAATTA---GATTTTGTTAAAGTTGTATGC  52512
GuineaPig                    CCCTCCTT-----ACACATATTTGGTTGTCTGTTCATAGATCAGAATGCACAGTGATGTACTTACATATTTATTCTAGA-  30954
NorthernAmericanDeerMouse    GTAGGCTGTCATTTTGCCCTATTGACAGTATTCCTTCGTCTTACAGAA-ACTTCAATTTTATGAGATCCCATTTATTAAT  33693
Mouse                        GTAA----------TGGTCTTTT--CTGTGTATATT-GATTCAGTATG-ATTTTCATTCTATGTAGTGCTGTGGGTGGAT  35354
ChineseHamsterGHOK1GS        GTAGGTTGCCATTTTGTCCTATTGACTGTATTCTTT-GCCTTATAGAA-ACTTCAATTTCATGAAGTCCCGTTTATTAAT  36036
LongTailedChinchilla         CTTTTCCG-----ACCCATACTTGGTTGTCTATTCATAGACCAGAATGGACAACAA---ACTTACATATTTCTTATAGAG  34277

Majority                     TGT-AXTCTAAXXGCCTGXGX-AT-XXTGTTXTG-----AAAXTTGTXTACTATXCCAATGXGTTC-AXX---TXXTTCC
                                       55850     55860     55870     55880     55890     55900     55910     55920
Human                        TTT-CCTCTAG--G--------AT-AAAGTAATG-----AAGAGCGTTTACTAAGCTGTTACCCCC-A--------TTCC  52566
GuineaPig                    --------------------------------------------------------------------------------  30954
NorthernAmericanDeerMouse    TGTTGATCTTAGTGCCTGTGTTATTGGTGTTCTGTTCAGAAAATTGTCTCCTGTACCAATGTGTTCAAGAT--TATTTCC  33771
Mouse                        CA--AATCCAAGGCCTTGAGC-----ATGTT----------AGTTGATCACTCCATCACTGAGCT----G---TGCCTTC  35410
ChineseHamsterGHOK1GS        TGTCAGTCTTAATGCCTGTGCTATTGTTGTTCTATTCATAAAGTTGTCTTCTATGTCAATGCCTTCGAGAG--TATTTCC  36114
LongTailedChinchilla         TGT-TGTAGAGAGGGCTGGGG-AT-ATAGCTGAG-----TGGCATAAGCACTTGCCTGGCAAGTCTGAGGTCATGAGTTC  34349

Majority                     CACTTTCXXTXC--XAAXGTTCAXXGTAAXXAGATTTXTATAGAGGTXXXTGATCAACTTGXACTTXAAGTXXGTGCXTG
                                       55930     55940     55950     55960     55970     55980     55990     56000
Human                        --CCCCAAATAC--CAAAGAGGCTCTTATTCTGGGT-CAACAAAACTCCCTTTAGAATTTCTGGTACCAGGCTGTGCGTG  52641
GuineaPig                    -----------------------------------TGTTATGGAATTGGATGCTCATCTGGTCCTCTCAGCCAGTGT-TA  30998
NorthernAmericanDeerMouse    CACTTTCTCTTCTTTTGGGTTCAGTGTAACTGGATTTATGGTGAGGTCTTTGATCCGCTTGGACTTGA-CTTTGTACAGG  33850
Mouse                        CATCTTC-------AAAGATTCAAAGGAAAGAAATATATATAAAGGAG---AACAAACCTAGATTTTAAATTAGTCTCAT  35480
ChineseHamsterGHOK1GS        CACTTTCTCTTCTATTGCGTTCAGTGTAACTCAATTTGTTTTGAGGCCTTTGATCCACTTGGACTTGAGTTTTGTGCAGG  36194
LongTailedChinchilla         AGTTCCCGGTAC--AAAAAAAAAAACCAAACAGAGTGTTGTAGAGTTGGATGCTGATCTGGTCCTTCTAGCCAGCAC-TA  34426
```

```
Majority                   GTGATAXATX--GATTAAGTTGCAATXXTXXGCCTXCTXGAAXCTXGTTT-X-GGGAGXAXTTXTAXGTXAGXTTTTTTT
                                     56010     56020     56030     56040     56050     56060     56070     56080
Human                      GTGGCTCACA--CTTGTAATCCCAGCA------CTTTGGGAGGCTGAGGT---GGGCGGATCACGAGGTCAGGAGATCGA  52710
GuineaPig                  AAGATATATT--TATTCTGACTCAATCTT-CCCCACCCAGAACTTGTTTTAA-GGGAGGAATTTTATGTTGGTTTGTTTT  31074
NorthernAmericanDeerMouse  GTGATAAA----GATCAAGTTGCAAGCTTCTGCATGCTGACATCTAGTTAGACAGAACCATTTGTTGAAGATGCTTTTTT  33926
Mouse                      ---ATAGA----GGGTACTTTGCTATAATGAACTTCAGAGTGGCTAG--------------------ACAATAAATTACT  35533
ChineseHamsterGHOK1GS      GTGATAGATATGGATCAATTTGCATTCCTCTGCATGCTGACATCCAGT-------------------GAGAGCTTTTCTT  36255
LongTailedChinchilla       GCGATACATT--TGTTCTGAGCCAACAAAACTCCCTTTAGAACTTGATTTAA-GGGAGAAATTGTATGTTGGTTTGTTGT  34503

Majority                   TTTXCCATXGTGGAAXTCXGXCXXTXTXXTAXXXACTXAAXTX--TTCXTAGGXXTAXTAACTGACAXCT-XGAGTTXAA
                                     56090     56100     56110     56120     56130     56140     56150     56160
Human                      --GACCATGGTGAAACCCCGTCTGTACTAAAAATACGAAAAT---TAGCTGGGCGTGGTGGCGGGCGCCT-GTAGTCCCA  52784
GuineaPig                  TTTATGATGAGGGGAGTCAGTCAGTGCCTTACTTTCCTCTCTTTTTTCTGAGTCAAAATACCTGACACCC-AAAGTTAAA  31153
NorthernAmericanDeerMouse  TTTTCCATTGTACAATTTTGGGTTTCTTGTCAAAAATCAGGAG--TTTGTAGGTGCGTGAATTTACATCTTGGTCTTCGA  34004
Mouse                      TATTCTATTATGGAA----GGAATGTTCAAATTAAGTAAAGTT--TTTA---GTTTTCAAAGGCCCCTCAGAGTGTTTAG  35604
ChineseHamsterGHOK1GS      TTTTCCATTGTATAATTTTGGCTTCCTTGTAGAAAATCGGATG--TTCATAGGTTTAGGAATTTACATCT-GGCTTTTGA  36332
LongTailedChinchilla       TTTATGATGGGGGCTGTC-GTCAGTGTCTTAGTTACTTTACT---TGCTGAGACAAAATACCTGACACCC-AAAGTTAAA  34578

Majority                   XGXX----GXXAGGTTTAXTTAGXTXATTGXT-X--XCXCXG-GXGGTTTAXATTACAATXX--TGGCTCXGTXGXAGGG
                                     56170     56180     56190     56200     56210     56220     56230     56240
Human                      GCTACTC-GGGAGGCTGAGGCAGGAGAATGGTGCAGACCCAG-GAGGCGGAGCTTGCAGTGAGCCGAGATCGCGCCA---  52859
GuineaPig                  GGGG----GAAAGGTTTATTTAGCTCATAGTT-----CGTGGAGGGTTTCAAATTATAATTGACTGGCTCCACGGCAAGG  31224
NorthernAmericanDeerMouse  TTCAATCCATTGATTTGCCTCTGTTTTTTTATGCCAATACCATGTGGTTTTTATTACTAT-----AGCTCTGTAGTAGAG  34079
Mouse                      TGTG------------------------------------------TTTT----TA-----------CCTTTTAGTTGGG  35627
ChineseHamsterGHOK1GS      TTCA----------TTTCCATCGTCTTTTTACACCAATACCATGTGGTTTTTATTACTATGA--TAGTTCTGTAATAGAG  36400
LongTailedChinchilla       GGAG----GAAAGGTTTATTTAGCTCATAGTT-----CCTAG-GGACTTCAGTCCATAATTGGTTGGCTCCAAGGCAAGG  34648

Majority                   CTGXAAAXCAGGCAGGX--------CTTXCAGAGXAXAAAAXXTTCTTAGTXAACATAAATTXTCAAAXTX----X-XCG
                                     56250     56260     56270     56280     56290     56300     56310     56320
Human                      CTGCACTTCAGCCTGG---------GCGACAGAGCGAGACTCTGTCTCAAAAAAAAAAAAAAAAAAGAATT------CCT  52924
GuineaPig                  TGGTGTGGCAGACAGGC--------ATTGCAGAGGAGAAAAAGTTCATAGTAGACAGAAGATAGCAAAGCA------CCA  31290
NorthernAmericanDeerMouse  CTTGAAATCAGGGATGGTGATACTTCTTTTATTTTACATAATTATTTGAGCTATCCTGGGTTTTATTTTTT--CCATATG  34157
Mouse                      C---AAAGAAAGAAC----------CTTGCAGTTCTTTGAAGGTTCTAAGTGCAGTTTTATTTTCAAGATAGCTTAGCAG  35694
ChineseHamsterGHOK1GS      CTTGAAATCAGGCATAGTGAT----CTTTTATAGTACAGGATTATTTTAGCTACACTGGATTTTTGTTTTTTCCCATATG  36476
LongTailedChinchilla       TGGCATGGAAGACAGGC--------ATTACAGAGGAGAAAAAGTTCATGGTGGGCAGAAAGTAGCAAAGCA------ACA  34714
```

```
Majority                     AGGTTXAXXACTGXTCTTT-----XCCCTTAXGTCTTTXXTCXGXXTACTTAXGGXGXTTGXCXX----XAXAXXCTTGX
                                      56330     56340     56350     56360     56370     56380     56390     56400
Human                        GGTACCAGCAGGGCACTT----TG--GCTCATGCCTGTAATCCCAGCACTTTGAGAAGCTGAGGT-----AAATTCAGGG  52993
GuineaPig                    AGGGACAACA--GCTCTA-------TCCTTGTGTC-TTGTCCAGGCTACTTAAGATGTGTAGCACT---CAAACCCTTAA  31357
NorthernAmericanDeerMouse    AAGTTGAGTACTGTTCTTTCAAAGTCTGTAAAGAATTGTGTTGGGATTTTGATGGGGATTGCCCTGATTCGGTAGATTGC  34237
Mouse                        AGTTTGAATGCTATCTCCT-------C-TTGAGTTTTCCCT-----------------TTG----------------TGT  35733
ChineseHamsterGHOK1GS        AGGTTGAGTATCGTATTGT-----TCTTTCAAGGTTTGTG-------------AAGAATTG-----------TAGATTGC  36527
LongTailedChinchilla         AGGTCCAACACAGCTCTTAC--TATCCCTTATGTC-GTATCCAGACTACTTAGGGTGGGTAACACT---CACACCCTTAA  34788

Majority                     TTXTAGTGAXCTTGATCTTATXACTXTAXTAXTCC-TXXATTCXXXAGCATGAXAGATTXT-ACATCTAXXXAXXTCTGA
                                      56410     56420     56430     56440     56450     56460     56470     56480
Human                        CAGCAGTGAACT-GGTCACACCACTGCACTCCAGCCTCAGCAACAAAGCAAGACACAGTCT-CTTAAAAAAAAAAAAAGG  53071
GuineaPig                    TCCTAGTGTCCTTGATGCTAGTATTGTACTATAAA-TCAGTTTTA--GCATCACATGATAC-AGATCTAGCCTCCTCTGA  31433
NorthernAmericanDeerMouse    TTTTGATAAGATGGCCATTTTGACTATTTTAATCCTATGATCCATTAGCATGTGAGATCTTTGCATCTGCTGATATCTTT  34317
Mouse                        TTGTCGTGCTCTCACTCTCCTG-CCATGCTCGTC---TGTTTCATTCTTATTTGAGAA--T-ACACAC------------  35794
ChineseHamsterGHOK1GS        TTTCAGTAAGATTGACATTTTTACTATGTTAATCCCATGATCCGTGAGCATGGGAGATCTTTCCATCT----------TC  36597
LongTailedChinchilla         TCCTAGTGCCCTTGATCTTAGCATTGCATCATGAA-TCAATTCTA--GCATCACACATTCT-AGATGTAGCCATCTCTGA  34864

Majority                     AAXAXTXTCTXXTTCCAXXGAATGAGAXTTTTTGTCATACACTXTTXTCAXTGGCTTGXCTAXAAGTXCAXCAGAXTAGX
                                      56490     56500     56510     56520     56530     56540     56550     56560
Human                        AAGAAATTCTGGTTCCTTTAAGGGAGATTTGTTGTTGTTTTCTTCTTTTAATGGTGAGGGTACAAGTTGGTCACAGGAGG  53151
GuineaPig                    AAAATGCTCACCTGTGACCTTATGAGGGTTTGAGGAAACACCTAGGCACAA-GCCATAACAGATAGTTCTTTAGAGAAGG  31512
NorthernAmericanDeerMouse    TTCAATATCTTTCTTCAAAGAATGGAATTTCTTGTCATACAT-TTTTTCACTTGCTTGGTTAGAGGTACACCAAGATATT  34396
Mouse                        ----------------TTGGAATT---CTGTTGGACTCACATAATTCCCCCTAGATTAAAGTAATGAGGACTTGGATGCC  35855
ChineseHamsterGHOK1GS        TTCAGT----TTCTCCAAAGAATTGCAGTTCTTGTCATACAAGTTTTTCACTTGTTTGGCTAGAGCTACACCAATATATT  36673
LongTailedChinchilla         AAATGCCTCTCCTACGACCACATGAGGCTTTGGGGGGACATCTAGGCACAA-GCCGTGACAGTCAGTCCTTCAGAGAAGA  34943

Majority                     AXATAXTAXAGGAGXTTAGCTXTTXX--XGTTGATTCACTGXATTTTXTCTTAGXCCXT---TT-ATCACTXGCXCXTCX
                                      56570     56580     56590     56600     56610     56620     56630     56640
Human                        -GAGA--AGAGAAATTTAGCTCTTTT---GTTAATGAACTAGACTTTCTGTTCGTCTGTTTTTCACCCACTCCCTCCTCC  53225
GuineaPig                    ACAAG-GAGAGGAGTTCAGCTGTTT----GTTCATTACCTGTATTTTCTATTAATCCCTTTATT-TTCACACTCTCTTCT  31586
NorthernAmericanDeerMouse    TTATAATATTTGAGGCTATTTTAAAGGGTGTTGATTCTCTGATTTCTTTCTAAGCCCAT---TT-ATCGTTTGCATATTG  34472
Mouse                        ATGT-TTGTCCCCGGTCAACCCCCAA-ATGCCGAAGGACC---TTTGTTCTGAGC-------T--AACAAATGTGACTTT  35921
ChineseHamsterGHOK1GS        TCATATTGTTTGAGGCTATTGTAAAGGATGTTGATTCTCTGGTTTCTTTCTAAGCCTGT---TT-ATCATTTGTGTATGG  36749
LongTailedChinchilla         AGAGGAGAGAGTAGTTTAGCTGTTT----GTTCGTCCACTGAACTTTCTGTTAATCC-----TT-TTCACTCTGCCTTCC  35013
```

```
Majority                     XXXGGACTTGXXATTTTCTTAAXTGATTCXTGTGTCXA-----------------------CTATTAGTXXAACXTCTGT
                                      56650     56660     56670     56680     56690     56700     56710     56720
Human                        TGTGAACCTGCCAATTTCTGATTTGGTTCCTGTGGCTG-----------------------CTACCAGTATAACACCTGT  53282
GuineaPig                    -GTGAACTGGCCAGTTGCTTAGCTGGTTCCTGTGACTT-----------------------CTATCAGTACAACCCCTGT  31642
NorthernAmericanDeerMouse    GAGGG-CTACTGATTTTGTTGAGTTAATCTTGTATCCA---------------------GTCACTTGACTAAAGGTGTTT  34530
Mouse                        TAGGG-CTTTTGATTCTTTTAAGGATTTATTGGTTTTAA---------------------------TGTTGAGATGATGA  35973
ChineseHamsterGHOK1GS        GGGGGGCTACTGATTTTTTTTATGCAATTTTGTATTCAAATTACATAAAAAAGTATTCAGCCACTTGACTAAAGGTGTTT  36829
LongTailedChinchilla         -ATGCACTTGCCAGTTTCTTAGCTGATTCCTGTGACCT-----------------------CTACTAGTATAACCTCTAT  35069

Majority                     TTCXTGTGTAXGAACTGTCTXGT-CAGTTCXTTTGGTTCCTTATXTXTXCTGTCTTXXAXGAATTTGATAATXTTAXTXT
                                      56730     56740     56750     56760     56770     56780     56790     56800
Human                        TTGCTGTGTGTTCACTGTGTCTC-CAGTCCATTTGGTTCCATTCATTTTCTGTGTTCCAGGAATTTGATAAAATTATTCT  53361
GuineaPig                    TCCCTGTGT---TACTGTCTT---CATTGCATTTGGTTCCATATATTTTCTCTATTTCAGGAATTTGATAAGATTATTCT  31716
NorthernAmericanDeerMouse    ATCAGCTAAAGGAATTACCTGGTAGAGTTTTTGGGGTTGTTTATGTATACTATCATATCATCTGGGAATAATGATACTTT  34610
Mouse                        GTCAAAGGCAAGAACT--------GAGTTGTTT--GTTCATTATCCAGACT-TCGCGTTAATCTGGGTCTGTGCTTCCT-  36041
ChineseHamsterGHOK1GS        ATCAACTGGAGGAATT-CCTGGTAAAGATTTTGGGGTTGCTTATGTATACTGTCTTGTTATATGTAGATAATGATACTTT  36908
LongTailedChinchilla         TTCCTGCATATTTACTGTCTTGT-CTTTCCATTTGGTTCTGTATTTTTTCTGTATTTCAGGAATTTGATAAGATTATTCT  35148

Majority                     CAGXTAAXXATXXCTACTTTGXCACCCTTTGGTGTXTXXTCTXTTT-XXTTXTTXATTXAGTT--AGAXATTTTAATAGA
                                      56810     56820     56830     56840     56850     56860     56870     56880
Human                        CAGCTAATGATA---ACCTTCCCTCCCTTCAGTGTTTGTT-TATTT--TATATTCATTCACTTCTAGGCATTTTAATAGA  53435
GuineaPig                    CAGGTAAAAATGACTACCTGACCAGTGTTT-GAGTTTATTCTGTTT--TGTGTTCACTCAAT---ATGCATTTTTACAGA  31790
NorthernAmericanDeerMouse    GACTT----CTTTCCAATTTGTAACCCCTTGGT--CTCCTTTAGTTACCTTGTTGCTCCAGTT--AGAATTTCAAGTACA  34682
Mouse                        AG--C-----TCCCTGTTTTGTGAACCTTTCTTTGCTGCTCTGATT-CCTGGTTTTTTTTGTT--TGTATTTTTGTTTTG  36111
ChineseHamsterGHOK1GS        GATTTCTTCCTTTCTAATTTGTAACCCCTTGAT--CTCCTTTAGTTGCCTTATT---CTAGCT--AGAACTTCGAGTAAT  36981
LongTailedChinchilla         CAGCTAAAGATGAATACTTGACCAGTGTTTTGAGTTTATTCTGTTT--TATATTTACTTAAT---ACACATTTTCATAGG  35223

Majority                     ATXTTXTAATXGTAG------A-XXAGTGXXTAAGTTTGTXXTXGCTGTXXATTTTATTXX-----------CTGTTTCT
                                      56890     56900     56910     56920     56930     56940     56950     56960
Human                        GTTTCATAATAGAAG------A-CATGCAAATGAGTATGCATTAACCATGAATTCTTTCACA-T--------CCGTTCAT  53499
GuineaPig                    ATTTCATAGCAGGAG------A-CAAATGAGTGAGTGTGTTTTATCTGTA----GTTTTTT-----------CTGTTTCC  31848
NorthernAmericanDeerMouse    ATATTGAGTAGATAC------AGAGAGTGGACAACCTTGT-CTTGTTCCTGATTTTAGTGGAATTGCTTTAAGTTTCTCT  34755
Mouse                        TTATTGTCATGTTGT--------TGCCAGGATAAGTTTGAACTTGCTGTGTATTTTCAT--------------------  36162
ChineseHamsterGHOK1GS        ATATTGAATAGATAGATATGAAGAGAGTGGGCAACCTTGC-CTTGTTCCTGATTTTAGT----------------TTTCT  37044
LongTailedChinchilla         ATTTTATAATAGGAG------A-TAAATGAGTAAATGTGTTTTATCTGTAAATAGTATTTC-----------CTGTTTC-  35284
```

```
Majority                    --ATTTX--X-TTGTXXTTAGTTATCGCXTTTGAXXTAAAT-GCCTT-XXX---TTXAG-X---X-CCTGXTAXT-TXXA
                                     56970     56980     56990     57000     57010     57020     57030     57040
Human                       GAAATCACCAGGTCCTATTAGTTCACACTTTTTAATTATCT-GCCTG-GTTTTCTCCATCT---TTCCTGCTACT-TGTA  53573
GuineaPig                   --------------TAATTAGTTACCTCCTT------------------------------------GTGTTACT-TGCA  31877
NorthernAmericanDeerMouse   TCATTTA--ATTTGATGTTGGCTATAGG-TTTGGTGTAAATTGCCTTTATT--GTTTAGGTATGTTCCTGGTATCCTTGA  34830
Mouse                       --TTTT-----TTGTTGTTATTCCTGGAATTTGACAAAAAT--CATT-------TTCAG-------CCTAATGT--TTAG  36217
ChineseHamsterGHOK1GS       CCATTTA--ATTTGAAGTTGGCTATAGA-TTTGATGCAAATGGCCTTCGTTATGTTTAGGTATGTCCCTGGTATCCTTGA  37121
LongTailedChinchilla        --------------TAATTAGTTACCTCTTTCTAATTAGTT-ACCTC-A------------------GTGTTACT-TGTG  35329

Majority                    TTGXTCTAAXAXXTTXAXTXATTAXCXGXTATTA----CTGTXAAAGXTXTTTTCATAATCTAXXXXATTATTATGTTAX
                                     57050     57060     57070     57080     57090     57100     57110     57120
Human                       TTGCTCAAACACACTCACCTGTTGGCTAT-ATTA----CTACGACAGTTCTCTTTATAAACTACTTTCTTTTTCTTTTCC  53648
GuineaPig                   TTATTCTGAAGTATTCACATTTTAGCTCTTATTA----CTGTGATAGCTCTTATAC-AGTCTGCTTTATTATT---TTTG  31949
NorthernAmericanDeerMouse   TTGCTGTAAGACTTTTTATCATGAAAGGGTGTTGGGTTTTGTCAAAAGCTTTTTCACGATTAAGAAGATGATTATGTGAG  34910
Mouse                       TGTTTGTTACATTATTATTCACTTACAAGTATT-----TTGGCA-----TT--TCATAATAGAGAGG-----CAAGTAAA  36280
ChineseHamsterGHOK1GS       TCTCTCTAAGACTTTT-GTCATAAAGGGGTGTTGGGTTATGTCAAAGGCTTTTTCAGCATTAAGAAGAT---CATGTGA-  37196
LongTailedChinchilla        TTGTTTAGAAGTATTCATTTTTTGGCAGT-ATTA----CTGTGAGAACTCTTATACTAGTCTACTGTATTATT---TTCC  35401

Majority                    CTXXTX----AXTTTTTTTXTTXCTTGTATAGCCXXGTXXGTTTCTTTATA-AXTGCACAAGXCCXACTAAXTTACTXCT
                                     57130     57140     57150     57160     57170     57180     57190     57200
Human                       CTTCTGG---AATTTTTTTCTCGCTAGTACAGCC-AGTACGTATCTTTCTCAAATGCACATATC----TAAGTTACTCCC  53720
GuineaPig                   CTCTTC----AGTTCTTTCACC-CTGCTACAACCAAGTAGGTATCTAT----ATTGCACAAGTCCATGTAAGTTACTCCT  32020
NorthernAmericanDeerMouse   GTTTTTTGTTTGTTTGTTTGTTTGTTTTGTTTTTCTTTCAGTTTGTTTATA-GGTGGATTAAATTGACAGATTTTTTTTT  34989
Mouse                       ---------------------TACATGTTTGGCTATAGCCATTT-CTTATA---TGATCAAGACCTATGAGTTCACTCTT  36335
ChineseHamsterGHOK1GS       --------------------TTTTTTTTTTTCCCCTCTCAGTTTGTTTATATGGTAGATTACAATGACAGGTTTTCAT--  37254
LongTailedChinchilla        CTCCTG----AATTTTTTTAATGCTAGTATAGCCGAGTATATGTCTTTCTAAATTGCACAAGTCCAGCTAAGTTACTTCC  35477

Majority                    XTATAGTXXGXCXTXXTXXXXTXTCT---AX--X--XTCCTTGCTCAXXGTXAAXGCXCXTTTTGAXGTGX-CTTGXATT
                                     57210     57220     57230     57240     57250     57260     57270     57280
Human                       ATGCAGTAGGTCCTGTT----------------------CATGCTCAGGCTAAAGGCCCTTTCTGAGCTTA-CTACTTTT  53777
GuineaPig                   GTACAACAGGTTTTTTTAAATTTTTATTTATTTA--TTTTTTGCTCAGGGTAAAAGCCCATTTGGAGATGA--TTGCATC  32096
NorthernAmericanDeerMouse   TTATGTTGAACCATCCCTGCATTTCTGAGATGAAGCCTCCTTGGTCATAGTGA-TAATTTTTTTGATGTGTTCTTGGATT  35068
Mouse                       T-ATTGTGACCTGTACACTTTTCTCT---A------TTCCTTGCTGCTACTG-----TCGCTTAGATG---------ACC  36391
ChineseHamsterGHOK1GS       --ATGTTGAACCATCCCTGTGTCTCTAGGAT-AAGCCTGCTTGGTTATAGTGGCTAATCCTTTTGATGTGTTCTTGGGTT  37331
LongTailedChinchilla        ATATAGGAGGTCCTTTT---------------------CCTTACTCAGGATAAAAGCCCATTTGGAGATGGGCTTACATT  35536
```

```
Majority                    TGXTTT-XCTTGTGTTTTATTGAGTACTATTA-XCCTATGCTX-CTGTCTCA--AAAXTGGTGXXTAATXCTCTXXCXTX
                                      57290     57300     57310     57320     57330     57340     57350     57360
Human                       TGTCTTGTCTTATGACTACTCCCACCCTATTT-GCTTATGCTT-CTGTCCCA--AAAGTAATGGTGCATCCTGTAACCTG  53853
GuineaPig                   TGTT----TTTTTGCTTTGTTGACATCTATT--GCCTGTGCTTTCTGTCTCA--GAAGTGGTGGTAAAGCCTTTGACCTG  32168
NorthernAmericanDeerMouse   TGGTTT-GCCAGTATTGTATTGAGTATTTTTACATCTATGTTC-ATGAGTA---AAATTGGTCTGTAATTATCATTCTTT  35143
Mouse                       CAGTT--A-----TTTGCATTGAGTCCCACTA-AGTTATTCTC-CTGCATC---ATGTCCTTTTTTTTTTTTTTTTTTTT  36459
ChineseHamsterGHOK1GS       TGGTTT-GCCAGCCTTTCATTGAGTAGTTTTACATCTATGTTC-ATGAATAATGAAATTGGTCTGTATTTCTCTCTCTTT  37409
LongTailedChinchilla        ACTTACATTTTCTGTTTTATGGACAACTGTTT-GCCTGTGCTT-CTGTCTCA--TAAGTAGTGGGAGATCTTCTAACCTG  35612

Majority                    XT-X-XTTTCTCTTXATTTCTGTGGTTTGXCTXCCAXTXTGAXXCTGGCCTCATACATGGAATATTGXGTTXTGTCCTCC
                                      57370     57380     57390     57400     57410     57420     57430     57440
Human                       GCATGTTTTCTCTTGATGTCAGTCGTTTG-CTGCCATTATGTCACTGCTCTCATGCATGGAATATTGTGGGTTGGCCTGC  53932
GuineaPig                   A-----TTTCCCTTGATTGCAGTATTTTG-CTGCCACTTTGTT-CT-----TACACATGGAATATTCTGTTCTG-CATGC  32235
NorthernAmericanDeerMouse   GT--------TGGGTCTTTGTGTGGTTTGGATATCAGGGTGACTGTGGCCTCATAG------TTTGGCAATGTTTCTTCT  35209
Mouse                       TTTTTTTTT-TTTTTTTTTTTTTGGCTTGGTTA-------GATAAAGGCCTAGAACA------CCTACTGTGTCTCCTCC  36525
ChineseHamsterGHOK1GS       TTTTTTTTTTTAGTTCTTTGTGTGGTTTGGGTATCATGGTGACTGTGGCCTCATAAAACTAATTTGGCATTTTTTTTTCT  37489
LongTailedChinchilla        A-----TTTCTCTTGATTTCGATCTTTTG-CTGCCACTTTACT-CTCCT-TTACTCATGGAATATTTTGTCCTG-CCTAC  35683

Majority                    XTTTTGTAGAACXATTXXXXXAGT-XATTCTTXTCATG-CTAXTTTXTAAATATCTTAAGATCCCGCTXT-X-AGCXATT
                                      57450     57460     57470     57480     57490     57500     57510     57520
Human                       CTTTTGCACCACCCTTCCTCCACCTCACACTTCACATGGCCAGT-GTTAAATATCTTGAGGTCTCCCTTT---AGCTATT  54008
GuineaPig                   -TTTTGTAGTTCTGTTCC---ATTCCATACTCCTCATG-TTAGTTATTAAATATCTTCAGATCTCTCT-----AGCTATT  32305
NorthernAmericanDeerMouse   ATTTTGTGGAATAATTTGAAGAGT--ATTGGTAGTAGCTCTTCTTTGAAAGTATGGTAGGATTCTGCACTATAAACCATC  35287
Mouse                       CTTACAGCCATCTGTTTGCCTAG---ACTTTTACC-G-----CAGTGTAA-TTTCAAAAAGTAACGATGTGTTAGTCCTC  36595
ChineseHamsterGHOK1GS       ATTTTGTGGAATAATTTGAAGAGT--ATTGGTATTAA---TTCTTTGGAAGTCTGGTAGAATTCTGCACTGTAAACCATT  37564
LongTailedChinchilla        -TTTTACAGCACCATTCC---ACTCCATTCTTCTCATGGCTAGTTGTTAAATATCTTCAGATCCCTTTTT---AGCTATT  35756

Majority                    TCTTCTTACXXGAAATTTXTCXXGTXAX--GCXXGGAGTXATTTAG-XTXTGXXTXXATXTCCTTAAGTGTXA-------
                                      57530     57540     57550     57560     57570     57580     57590     57600
Human                       TCTTCT--CCTAAAAGCTATCCCCTGATAAGCAAAGAGTAGATTAG-GTTTGGATTGAG-------AGTGTTA-------  54071
GuineaPig                   TCTTGT-ACTGGAAATATGTCCCCTAAG--CCACTGAGTGAGAGAG-TTTTGGGTTGAAATCTTTTAATGCAG-------  32374
NorthernAmericanDeerMouse   T--TCTGGCCCTAGACTCTTTTTG------GTTGGGAGACATTTAATAACTGCTTCTATTTCCTTAGGGGTTATCGGTCT  35359
Mouse                       T--AGCTGGATGAGGTTTCTCTTG-------CTTGAAGTTTTT-----GCTGCCACTGTGCTCTCACTTTT---------  36652
ChineseHamsterGHOK1GS       TGGCCCTAGGCAATATTTTTTTTGTGGGGGGGTGGGAGACTTTTAATAACTGCTTCTATTTCCTTATGTGT---------  37635
LongTailedChinchilla        TCTTTTTACTGGAAAGATGTCCCATAAT--GCACTGAGCAAGTGAG-GTTTGGGTTGAGGTCTTTCAATGCAA-------  35826
```

```
Majority                     ATTXAAATTG-TXATXXGATXCTGXTXTGXATAXAACAAGTGXXGXCTAXAATTAAAAXGATAX---XXATCXXTXATGG
                                      57610     57620     57630     57640     57650     57660     57670     57680
Human                        ACTCCGA------ATGAGATACTGCTATGCACATAACCAGAGT-GGCTAAAATTAAAAGGATA---------------GG  54129
GuineaPig                    ATTAAAATTA-TGGTAAGATACTGCCATGCATATACCAGGTG--G-CTAAAATTAA---GATACATAATATCAAATGTTG  32447
NorthernAmericanDeerMouse    ATTTAAATTGTTTATCTGATCTTGGATTGAGAAAATTGTCCATTTCTTATATTTTTGGTTGTGAGCCTAGCCTTTAATGG  35439
Mouse                        --AGATGTGGGTAAGGCCAGTCTGGGCTATGTAACAAAAGACCTATCTATAAAGAAATACACAA----GATGCCTAATAG  36726
ChineseHamsterGHOK1GS        ATTTAATTTGTTTCTTTGATCCTGGTTTAACTTAGGTTAGTGGTATCTATTAAGAAAATTGT-----CAATTTTTTATTA  37710
LongTailedChinchilla         ATTCAAACCA-TAATAGGA--CTGCTGTCTACATAACAGGTG--GGCTAAAATTAAAAAGATGCATATTATCCATGTTGG  35901
```

```
Majority                     TAXTAATATATTGX-AX--XX---T-TTACTCTXAGATXXTTGAATTT--TXAXXTAXAGGTGXXTGGAATXTAAATTG-
                                      57690     57700     57710     57720     57730     57740     57750     57760
Human                        TAATAAAATACTG-----------------------------G--------------------TG---------------  54145
GuineaPig                    TATTAATATAGAGA-AG--CT---T-TAACTC--ATGCCAGTG-------------------GAATGGAATATAAATTGA  32499
NorthernAmericanDeerMouse    CTGAGCCATCTCTCCAGCCCTGTCCATTTCTTTTAGATTTTCCAATTTTATGGAAGAGAGGTATTTGAAGTCTGACCTG-  35518
Mouse                        CAG----ATGTTGGAA-------TTGATATCCAAGGAACCTTGAATTC--TCATATAGAGGTGACTGGCATGTAAATTG-  36792
ChineseHamsterGHOK1GS        TTA--TTATTTTT--------------TTTTTTTTAGATTTTCCAATTT-GTGAAGTACAGGTTTTTGAAGTATGACCTA-  37773
LongTailedChinchilla         TGTTAATATACAGA-AG--CTGTAT-TAACTCTCATATAAATGAACTT--TCATTTACAAGTGAATGGGATGTAAATTGA  35975
```

```
Majority                     -ATAACAGTTTTT-AAAACTXGAAATATXT-XXCTAAAGTTGAGCATATGCATXXTXX-TTTATTTA---ATXATTCXTT
                                      57770     57780     57790     57800     57810     57820     57830     57840
Human                        -ACAACCATTTTTGAAAACTCGAAATATC--TACTAAAGTTGAGCATATAGCTATTCT-TTGATGTAG--TAAACTCGCT  54219
GuineaPig                    AAGAACAGTTTTTCAAAACTTGAATCATC--TACTAAAGTGGAGCATATGCACATTC-----------------------  32554
NorthernAmericanDeerMouse    -ATGATTCTCTGGATATCCTCAGTGTCTGT---TGTTATGTCCCCTTTTCATTTCTGATTTTATTTGAATATTCTTTCTC  35594
Mouse                        -GTAGAATCAT------TTTTGAAATATAT---CTCAAGTTGAGCATATGCATGCTG--TATACTTA---ATAATTCCCC  36857
ChineseHamsterGHOK1GS        -ATGATTGTTT------CCTCAGAGTCTGT---TGTTATATCCCCTTTTAGTTCTGATTTTTTTTTT--TATTTATAATT  37841
LongTailedChinchilla         AAGAACAGTTTTTTAAAACTTGAAAAATACGTACTAAAGTTGAGCATATGCATGTTCT-TTAATTTAG--TCCATTCATT  36052
```

```
Majority                     TXCTXXTTXGTXTXCTTGGAAAAACATATAXATATGAXXATGAAAAGATATGXAXAAXAAAXT-----TXXCAGAAGACT
                                      57850     57860     57870     57880     57890     57900     57910     57920
Human                        C-CTAGTTAACATACCTG--AGAACATGTACATAGGTATGTTAAAAGATATGTACAAGAAAGT-----TCATGGAACACT  54291
GuineaPig                    --CTACTTT-TGTACTTGGAAAAATACTTACATATGAGCATGAAAAGATACATACAAGAAAGT-----TCACAGAAAACT  32626
NorthernAmericanDeerMouse    TGCCTTTTAGTTAGTTTGGATAAGGGTTTGTCTATCC--TTGATATT-CTTGAAGAACCAACTCTTTGTTTC-ATTGATT  35670
Mouse                        TCC-TAATTGCATGCTTGAGAAAGCATATATATGTGA--ATAAAAAGATATGCAAAAAAAAC---CAAATTCCAGGGAGC  36931
ChineseHamsterGHOK1GS        TGC-TTTTAGTTAGTTTGGATAAAGGTATATTTATTTTGTTGATTTTTCTTGAAGAACCAACTCTCTGTTTTTGTTGATT  37920
LongTailedChinchilla         CCCTAGTTT-TGTACTTGGGAAAACATACACATATAAACATGAAAAGGT-CA---AAAAAAGT-----TCACACAAAACT  36122
```

```
Majority                    GTATGTAATATTCXAAAXGTXTATAAATXATCXXAATGTCCXXCAGCAGTTXXATX---TGXTGXATTXTACTCATTTAX
                                     57930     57940     57950     57960     57970     57980     57990     58000
Human                       GTATGTAATAGCCCAAAAAACTATAAATCATCCAAATGTTCAACAGCAGCAGAATA---TGTTGTGTTGTATTCATTTAG  54368
GuineaPig                   GTA----ATATCCCAAAG--CTAGAAATGATCCAAGAGTTCAGCAGCAGTTGAAT-----GTTGTAT---GCTCATTCAG  32692
NorthernAmericanDeerMouse   CTTTGTATTGTTTTCTTTGTTTCTATTTTGTT--GATTTCAGCCTTCAGTTTGATTATTTCCTGCAATCTACATCTCTTT  35748
Mouse                       AACTGTTACATTCTAGGAGTTTAAAGCTAGA---AAT----GACCCAAGTGTG--------CAGCAATAAGAGAAGTTGT  36996
ChineseHamsterGHOK1GS       CTTTCTTTTGTTCTCTTTGTTTCTACTTTATT--GATTTCAGCCCTCAGTTTCATTATTTCCTGCAGACTACTCCGCTGT  37998
LongTailedChinchilla        GTATGTAATATTCCAAAG--CTGGAAATGACCCAAGTGTCCAGCAGCAGTTGTATA---AGTTGTGT---ATTGATTCAG  36194

Majority                    GGXXTXTXTGTATCGTAATAAXAATGAACTAGCTA-XTGTGCTTTTAAAACATXAGTXTTXTATCTCAXAAXXX-----X
                                     58010     58020     58030     58040     58050     58060     58070     58080
Human                       TGGAATATTGTATAGTAATAAAAATGAACAAGCTACTGCTGCCTGCAAAACATGGGTAGTGTACATCATAA---------  54439
GuineaPig                   C-------TGTATGGCAGTAAGAATGAATTAG--ATACTTGTATTTGCAACATAACTGTTTTATGTCATAAAATA----A  32759
NorthernAmericanDeerMouse   GGTGTGTCTGTTTCTTTTTGTTCTGGTGCTTTCAG-GTGTGCTGTTAGAGCACTAGTATGGGATCTCTCCAAATTCTTCA  35827
Mouse                       GGAATATCTGTAGCCAAAAAGGACTGAACAAGCTCTGTGTATTTGTAAAGCATAAGTGTA----CATCC-----------  37061
ChineseHamsterGHOK1GS       GGTGTGTTTGCTTCTTTTTATTTCAGAGCTTTCTG-ATGTGCTGTTAAGTTTCTAGTATGAGATCATTC-----------  38066
LongTailedChinchilla        T-------TGTATGGCAAGAAAAATAGCTTGG--A--ACTG--T--GAAACAT---TGCTATCTGTCATAAAATA----A  36252

Majority                    XX-------TTAXTTXXXXGAAXTTXCAXCAAAGCAGAACXTXCA--------TTGXATTXXATAAXTGTGXGGATAXAX
                                     58090     58100     58110     58120     58130     58140     58150     58160
Human                       ----------TTATTGGAAGAAGCCAAACCAAAGTAGAAT----A-----------CATCT------------GATATGA  54482
GuineaPig                   TG-------TTCATTGAAAGAAATCAGACCAAAAGAGAACATGCAAGATAAAATTCCATTTAATAATTGGGAGGATGCAA  32832
NorthernAmericanDeerMouse   TGAAGGCGCTTAGGTCTGTGAACTTTCCTCTTAACACGACTTTCA--------TTGTGTTCCATAAATTTGGGTATATTG  35899
Mouse                       ----------TAAAACGGT-A--TTTCTTGAAAGCCAGAC---CA--------AAGTAGACCATAGATGT-----TACAG  37112
ChineseHamsterGHOK1GS       ---------TTAGTTCTGTGAACTTTCCTCTTAGCACCACTTTCA--------TTGTGTTCTATAAGTTTGGGTATGTTG  38129
LongTailedChinchilla        TG-------TTCGCTGAAAGAAACTAGACCAAAGGAGAACGTGTAAGATAAAATTTCATTTAATAATTGGGAGGATACAA  36325

Majority                    TTCCXTTTATTTAACTGGGAGGAAGCATGAGTXTAGCTTCTAGXXTATTGTTATTGTXCCXXTTTXTAX--------X-X
                                     58170     58180     58190     58200     58210     58220     58230     58240
Human                       TTCCATTTATGTAACTTGGAGGAAGCGTGAGTATGGCTTCTAGAGTGTTGATAATATACTGTTTTTTATCTGGGT--AAT  54560
GuineaPig                   TTCCCTTTATATAACTGGGAAGAAACATGAGTGTAGCTTCTAAAGTACTGATGTTAC-TGTTTTTATGCGGAGGAAGAGT  32911
NorthernAmericanDeerMouse   TGCATTCATTTTCACTGAATTCTAG-AAGTCTTTAACTTCTT-TATTTCTTCCTTGACCCCG-TGGTAA-----------  35965
Mouse                       TTCCATTTCTTTATCTGGGAGAGGGCGTGGTTCTGGTTTCTGGTACATTGGTAGTGTTACAGGTCTTTG-----------  37181
ChineseHamsterGHOK1GS       TGCATTCATTTTCATTGAATTTTAGGAAGTCTTTAAGTTCTT-TATTTCTTTCTTGACCCAG-CGATAA-----------  38196
LongTailedChinchilla        TTCCCTTTATGTAATTGGGAGGAAACATAAGTGTAGCTTCTAGAGTACTGTTAATGT-TGGTTTTGGGT--------AGT  36396
```

Majority	CTTXTCATAGAGXTGTCCAATTTGTGTTAXTTTXTTGXGTTXTTXTXXTXGAAATTGTXTCTTTA-------XTXXXTGG	
	58250 58260 58270 58280 58290 58300 58310 58320	
Human	TATTTCACAGAGATGTCCAATTTGTGGTATTTTATGGAGCTTTATACTTAGGTTTTGTGTCCTTTTGTGTGTGTGTGTAA	54640
GuineaPig	CATTTCTTTGAGATATCCAATTTGTGATATCTTACTGAGTTATTTTCACAAAACTAGTGTAATT----------------	32975
NorthernAmericanDeerMouse	CTCAGTAGAGAGTTGTTCAGTTTTCATGAGTTTGTAGGCTTTCTATTGTTGAAAATGCAGCTTTA-------ATCCATGG	36038
Mouse	TTTTGGATAGTGTTACAGGTTTTTGTTTTGTTTGTTGGGTTAATATTTTTTTCA-GATAAGTTTA-------ATTTGTGG	37253
ChineseHamsterGHOK1GS	CTCAATAGAAAGTTGTTGAATTTCCATGAATTTGTAGGCTTTCTGTTGTTGAAA-TCCAGCTTCA-------GTCCCTGG	38268
LongTailedChinchilla	CGTATCATAGAGATGTCTAATTTGTGATAATTTATTGAGTTATGCTCACAAAATTATTGTAATT----------------	36460

Majority	TATTTTGATAAGAX----G----------TTTXGTTAAXATXTAGGTTGTXAXXXCAXTACTTCXXTXXXTAAATTATGX	
	58330 58340 58350 58360 58370 58380 58390 58400	
Human	TATTTTGATAAGAA----G----------TTTTATTAAAATCTAGGTTTGCAAGCCAACACTGC--TCCATGAATTATAA	54704
GuineaPig	-ATTTTGATACGAA----G----------TTCAGTTAAAATCCAGGTTGTCAGACCAGTAATATGGTGAACAAAGTATG-	33039
NorthernAmericanDeerMouse	TGGTGTGATAAGATAGAAAGG--------TTAGGGAGTTATTTCAGTTGTTTTTTGTTTGTTTGTTTAGTTTTTTTTTTT	36110
Mouse	TATTTTACTGAGGT--------------CTCTTCCAAGTATTTGAGTCCTTTTCTGTGTACAT-----GTAATATTTTGA	37314
ChineseHamsterGHOK1GS	TGATCTGATAAGATACAGGGAATCCTTTCTTTCTCTGTTCCTTCCTTCCTTCCTTCCTTCCTTCCTTCCTTCCTTCCTTC	38348
LongTailedChinchilla	-ATTTTGATATGAA----G----------TTCAGTTAAAATCTAGGTTGTCAGGCCAATAATACGGTGAACAAAGTATG-	36524

Majority	GAACAXATATXTGXAX-TAT-XXTXXXTAT-----GTTGAXACTAACTCTGTGAXGAXGTXTATGXTTAATTTTGGAGAA	
	58410 58420 58430 58440 58450 58460 58470 58480	
Human	GAAAAAATATATGAAGTCATTAAAAATGAA-----ATCATAAAGAAATGTATGAAGTAATTTGTTACTGATGTGAGAGAA	54779
GuineaPig	GAATAAATATGTGCATATATGCCTGCTTAC-----GTAGATGGTAACTCTGGAAAGATCCCTAAAATTAATATTGGTAAT	33114
NorthernAmericanDeerMouse	AATC-------------------------T-----GTTGAGACATGCTTTGTGGCTGCGTATATGGTCAATTTTGGAGAA	36160
Mouse	AAAC--------------------------------CTAAAAC---CTCTAT-----------TGGCAAACTGTG-----	37343
ChineseHamsterGHOK1GS	CTTCCTTCCTTCCTTCCTTCCTTTTTGTATCTGTTGTTGAGACTTGCTTTGTGGCCAAGTATATGGTCAATTTTGGAGAA	38428
LongTailedChinchilla	GAATAGATATGTGCACGTATACTTGCATAT-----GTAGATGGTAACTCTGGAAGGATGCCTAGCATTAATAGTGGTGAT	36599

Majority	GG-TTXCXTGTAGAGCTGAAAAAAGXGTXXAXXXATTTGT----GXXTAAAAXCTTCXGTAXXTGTATTXGTTAGXTXGG	
	58490 58500 58510 58520 58530 58540 58550 58560	
Human	CA--TGATTGTTAAGCCATAAAAGGAAGATAGAGAACAAT----GAATAGTA--TTAAATAAAAGTATGAATAGTATTGA	54851
GuineaPig	CGGTCAGTAATAGAATGCAAACTTGAGTGACAAGATTTGC----AAAAAAAAGCTTTCTTCTTTGTATTAATTGGATTGG	33190
NorthernAmericanDeerMouse	GG-TTCCATGCAGTGCTGATAAGATA----ATTCTTTTGTGTTTGGGTGAAATAGTCTGTAGATATATCTGTTAGGTCCA	36235
Mouse	---CTTCATGTA---CTAAAAGAAAG------TCATTTGT-------TACTACTATTAGAAATTGTAATTGTCAAG-C--	37401
ChineseHamsterGHOK1GS	GG-TTCCATGCAGAGCTGAGAAAAAGGTATATTCTTTTGTGTTTGGTTGACATGTTCTGTAGATAT--CTGTTAGGCCCG	38505
LongTailedChinchilla	GGGTCAATCAGGGAAAGGAAACTTGGGTGACAAGATTTAT----GCGAAGAA-CTTCCTTCTTTGTATTAGTTTGATTGG	36674

```
Majority                    XTAX-XTAAAAAAATATGXTTAGTGXTXTT-------AAAXAAATTTCTGACXGGATAATTXTXXTATTXXTAAAAATGTG
                            58570 58580 58590 58600 58610 58620 58630 58640
Human                       ATA------AAAATATGAATAGTATTG---------AAAAAAAT-----ACAGACTAAGTATAATATTTATATATATATG 54911
GuineaPig                   GTAT-TCGGATCGTGTGATTCTTGTTTTTTTTT---AAAGACATTTCTAATAGTACATTGTTAATGTTTTTAAAGATGTG 33266
NorthernAmericanDeerMouse   TTTGAGTAATAATTTCTGTTATTTCT-TT---------GTTTAGTTTCTGTCTGGATGACCTGTCCATTGGTGAGAATGGG 36306
Mouse                       ------TAAAAAAGATGGTTAGCGCT--------------AA--ATAAGCCTGGA--ATTAATTCCATGA-AAAAGTGTG 37456
ChineseHamsterGHOK1GS       TTTGAATCATAGCATTTGTTAGTTCTCTTGTTTCACAGTTTAGTTTATGTCTGGATGGCCTGTCTATTGGTGAGAATGAG 38585
LongTailedChinchilla        ATAC-GCTGATCATATGATTTCTGTTTTTC------AAAGACATTTCTAACAGTGGAGTTATAGTGTTTTTTAAGATGTG 36747
```

```
Majority                    ATTTTAAAT---------XTX----TAATAXXATXTCXGGXTCTATXTXAGATATAAXXXTXAGTGXGGXXTTTXTXACAT
                            58650 58660 58670 58680 58690 58700 58710 58720
Human                       CTTGTAGAT---------GTA-----GCTAGTATCTCTGGAAGGATATCGAAGATAATACTGTGAGGGG-----------T 54967
GuineaPig                   ATTGTTACTGTAGATGAATATGCATAAGAAAATTTCAGAATCCAAATTAGATAAAAAGCAACATGGGAAATTCATCTCAT 33346
NorthernAmericanDeerMouse   ATGTTAAATT-------CTCCCACTATTATGATGTGGGGTTCTATGTATGATTTAAACTTTAGTAATGTTTCTTTTACAA 36379
Mouse                       GGATTAA------------------TAATATGCT------TTTTCTGGAAGGAC---------AGTGAGGGGATTGGTACAG 37505
ChineseHamsterGHOK1GS       ATGTTAAATT-------CTCCC--TATTACTGTGTGGGGTTCTATGGGTGATTTAAGCTTTAGTAATGTTTCTTTGACAA 38656
LongTailedChinchilla        ATT----TT---------GTG----TAAGAAAATC-CAGAATCCAAATTAGGAAAAGTGGAACATGGGAAACTCAACTCAT 36810
```

```
Majority                    TXATGGXGGXXCATACACTXGG-GTGACAGAXXTXCAGAAG-GAAAXATXAXTTTCTTGGATTXATCCTTXXTX-GATXA
                            58730 58740 58750 58760 58770 58780 58790 58800
Human                       TGGTAGGGGAAAATAGACTGG--GTGACAGAGTTGCAGGGG-A---ATTTTTAATCTTTAATACCACTTTTATACCATGA 55041
GuineaPig                   GGTCTCTGGATCATGCTCAATATATGGCAGAACCCAAGAAG-GTACAATAGCTGCCCTGGGCCCAACCTAACTGTGCTGA 33425
NorthernAmericanDeerMouse   TTATGGGTGCCCTTACATTTGG-GGCATAGATGTTCAGAATTGAGACATTATTTTGTTGGATTTTTCCTTCGAT-GAGTA 36457
Mouse                       GAAAGG--------AAACTGAG-GTGACAAAGTTGCAGGG--AAAACTTCACCTT-TTGTATTTATC--G------AATT 37565
ChineseHamsterGHOK1GS       ATATG----CCCTTATATTTGG-GGCATAGATGTTCAGAATTGAGACATCATTTTGTTGGATTTTTCCTT---T-GAATA 38727
LongTailedChinchilla        TATCTCTGGGTCATGCTCCAGCTATGGCAGAACCCAAGAAG-GCACAATGGCTGCCCTGGGCCCAGCCTAACTG--CTGA 36887
```

```
Majority                    TTAAATGGACTCCXTXXXCTXT-------CTAXTTTTAGTTTGCAXTXTAX-XTTTGXXGTAGATGTTAGXGTAGGX-TA
                            58810 58820 58830 58840 58850 58860 58870 58880
Human                       TC--ATGGATTC-CTA-------------------TTTTAGAAAACATTTTATTATTTGAAGCAGATGTTAGTG-------- 55093
GuineaPig                   CTTACTGTACTCTCTAGACTATAAGCACTCTACCCGTTCCTTGCTCCTAAACATTGGAGGTGGAAACCTGAGCAGGTGGA 33505
NorthernAmericanDeerMouse   TGAAATGGCCTTCTTCATCTCT----TGATTAATTTTGGTTGGAAGTCTGT--TTTGT--TAAATATTAGGATAGCC-TA 36528
Mouse                       TTATGTGGTTAACTTGCAGTTT-------CTAGTTTTAA--AAGATTTGAA--TTTGTAACAGATGTTAG-TTATGG-TA 37632
ChineseHamsterGHOK1GS       TGAAATGCCCTTCTCCATCTCTC--TTGACTAATTTTAGTTTGAAGTCTAT--TTTGT--TAGATGTTAG-ATAGC--TA 38798
LongTailedChinchilla        CT-GTTGCAGTCTCTG-------------------TCTTTGTTTGCCCCCAAACATCTGAGGTGGAGGTCTGAGCAGGTGGA 36949
```

```
Majority                   CACAAXCC---TXTTTCTXAGATCTXTTTGGXTXXGXAAATGTTXTTCCXXACCCCTTTACTXTG----------CAXCT
                                   58890     58900     58910     58920     58930     58940     58950     58960
Human                      -----------TTTTTCTAAGTTGTGGTAGTCTATGCT--TGTTATTATAGATAAATACAGTAAG----------AACAT  55150
GuineaPig                  CACAATCCTGCTTTGGCCACAATCTGTTTGCTTGCCTCTAAATGCCACCCCACCCCTAAACAAGG----------CAGCT  33575
NorthernAmericanDeerMouse  TGCCAGCC---TGCTTCTTAGGTCCAT-TTGATTAGAAAATCTTTTTCC--AACCCTTTACTCT-----------TTACT  36591
Mouse                      TTTAATGC---TGTCATTTAGATAAATGTCTGTAGGGAAATGTTGCTCTTAAGGTCTTTATTTT-----------CCACA  37698
ChineseHamsterGHOK1GS      CACCAGCT---TGCTTTTTAGATCTAT-TTGATTGGAAAATCTATTTCC--ACCCCTTTATTCTGAGGTAATGTCTATCT  38872
LongTailedChinchilla       CACAACCCCACCTTGACCAAGGCCTGTTTGGCTGCCTT--AGTGCTACCCGACCCCTGCACCTGG----------CAGCT  37017

Majority                   XTGCTGTTAAGXTGXGGXTXAXXTATCGCAGAXXCATGTXGXATCATGTTTTGXTACXXXXTTTXTXCXCXTGXGTCGTT
                                   58970     58980     58990     59000     59010     59020     59030     59040
Human                      GTTCACTAATGAAGAAGATAATATTGCAATGAGCTACTTAAAAT---ATTTAAATACAT---TTAAACATTTGAATAG--  55222
GuineaPig                  CTGCAGTTAAGA-GAGGCTG-GAAGTCCCAAAGCCATGTAGCATCAGGTTTTGCCAGGCTGCTTTGCCAGGTGAGTCACG  33653
NorthernAmericanDeerMouse  TTGATGTTGAGGTGTGTTTCTTGTAT-GCAGCAAAATGTTGGATGCTGTTTTCGTACCCAATCTGTTAGCTCATGTCTTT  36670
Mouse                      GTAGTATTAAAGTATCAAATAGTTATTGTTGTAAAATGCTGTTTCAT-TTGAGGTTCTTGTGATATATGCATGTGTAGTT  37777
ChineseHamsterGHOK1GS      TTGATATTGAGATGTGGTTCTTTTAT-GCAGCAGCATATTGGATCCTGTTTTCATACCTAATCTGTTAGCCTGTGTCTTT  38951
LongTailedChinchilla       CGTCTGTGATGG-GAGGCTGAGGGGTCACAGAGGCACGGAGCATCAGATTTTGCCAGGCGGATTTGCCAAGTGAGTTG--  37094

Majority                   XXTAXTGCTCAATTTAGAXCAXTTATAATGXTXTTTATTXAXGXTTXXTCAATGXTAGTX-CCAATTXCTXTATXA----
                                   59050     59060     59070     59080     59090     59100     59110     59120
Human                      -ATACTGCTCTA-TAAAACTAATTTTACCAATTTACAATTA--TTTTTTCAATAATAGTA-TCAAGATGTCTACTA----  55293
GuineaPig                  CATGCAGCACACCTTGCAGAAATTTGAAAGGATTTTTGCCAAACTTGGACCATGACAGAAACCAAGTTCACTATAA----  33729
NorthernAmericanDeerMouse  T-TATTGGTGAATTGAGTTCATTGATAATGAGGTTAATTAATGACCAATGATTGTTAATT-CCTGTTACTTTGTTGGTGG  36748
Mouse                      CATATTGTT--ATTTAG--CATTTA-AATGGTGTTTATT---------TAGATGTGTGTG-CCG---ACTTT--------  37831
ChineseHamsterGHOK1GS      T-TATTGATGAATTGAGTCCATTAATTTTGAGGGCTATTAATGACCAATTATTGTTAATT-CCTATTACTTT--------  39021
LongTailedChinchilla       --TGCAGCTCAGCTTGGAGGAATTGGAAGAGTTTTCTTCCAAGCTTGGGCCATGACAGT--CCAATTTCACCATAAA---  37167

Majority                   --------------------------------------------------------------------------------
                                   59130     59140     59150     59160     59170     59180     59190     59200
Human                      --------------------------------------------------------------------------------  55293
GuineaPig                  --------------------------------------------------------------------------------  33729
NorthernAmericanDeerMouse  TGGTGGTTGTGTGTGTGTGTTGTGTGTCTGTGTGTGTGTTTCTGTCTGTCTTTGGATTGTGCTGGTGTTTTTGCGAGTGT  36828
Mouse                      --------------------------------------------------------------------------------  37831
ChineseHamsterGHOK1GS      --------------------------------------------------------------------------------  39021
LongTailedChinchilla       --------------------------------------------------------------------------------  37167
```

```
Majority                    ---XAAXAXXCTAXXXXXXX---XAXTTXA-------------------------------X-AAATGTTXXXXTXTAA-
                            59210 59220 59230 59240 59250 59260 59270 59280
Human                       ---AAATAATCTATCAC-------ATTTCA---------------------------------AAATGTTGAGTTTTAA-  55329
GuineaPig                   ---GAAAAGTCTACAATGAGT--TACTTAAAGTACTTTAAATATATCCAAACATTTA---AGTAGATAAACTTTTATAAA  33801
NorthernAmericanDeerMouse   AGTTAACTTCCTGTTGGGAGTTTTCTTTCTAGTACTTTTTGTGGGGCTGTGTTTGTGGATAGACATTGTTTAAATTTGAT  36908
Mouse                       --------------------------------------------------------------------------------  37831
ChineseHamsterGHOK1GS       --------------------------------------------------------------------------------  39021
LongTailedChinchilla        ---GAAAAGCCTACAGTGAGC--TACTTGA-------------------------------GTAAATGTTCCTCTATAAA  37211

Majority                    XXTXTX-TXAXXTATTTTXXXTXC---------------AXXTAXATTTXCXX------XAXXXTTAAGTTGXCTTXTXT
                            59290 59300 59310 59320 59330 59340 59350 59360
Human                       -TTAG--TAAGTCATTTTGACTAAT--------------ACATAAATTTATAG-----ATAAAATTAAGTAGCATTTAAA  55387
GuineaPig                   TCTTTCTTTACCTATTTTCTTTGCC--------------AACTGTATTTTCAACA---TTAGTATTAAGTTGTCTTATAT  33864
NorthernAmericanDeerMouse   TTTGTCATAAAATACCTTGTTCTCTCCATCTATGGTAATTGACAGTTTTGCTGGATATAGTAGTCTGGGCTGACATTTGT  36988
Mouse                       --------------------------------------------------------------------------------  37831
ChineseHamsterGHOK1GS       --------------------------------------------------------------------------------  39021
LongTailedChinchilla        TCTATCTTTAGCTATTTTCCCTAC---------------AACTATATTTTCGA------GAGAGTTAAGTTGTCTTCTGT  37270

Majority                    TGTTTXT-------X-----XXATXTAXTTXXATTXTXAXXX--------XGTXTXA---X---------X--TXTGTXG
                            59370 59380 59390 59400 59410 59420 59430 59440
Human                       TGATTGTTTAG---------ATATATATCTGGACTCTCAAGC--------CGTGTGA----------------TCTGTGG  55434
GuineaPig                   TTTTTTC-------TT----GCATTTAATTAAATTATGAGATT-------TATTTAAA--T---------GAAAATGTAG  33915
NorthernAmericanDeerMouse   GGTCTCTTAGAGTTTGTAAGACATCAATTTGGGTCTCCATCGAGAAGTTGAGTGTGATTCTAATAGGTCTGCCTTTATAT  37068
Mouse                       --------------------------------------------------------------------------------  37831
ChineseHamsterGHOK1GS       --------------------------------------------------------------------------------  39021
LongTailedChinchilla        TGTTTCTCAAAATGTT----GGGTTTAATTAAATTATGAGATA-------TGTTTAAA--T---------GACTATGTGG  37328

Majority                    -XTXTTTGA-TXTTXAGCXAXGTGATXXXTX-TTX---------------------------------------------
                            59450 59460 59470 59480 59490 59500 59510 59520
Human                       -TTATATAATATTTTAGTAAAATGACTTTTC-TTG---------------------------------------------  55467
GuineaPig                   ACTTTTTGAGTCTTAAGCTAAGTGATCCCTGGCTA---------------------------------------------  33950
NorthernAmericanDeerMouse   GTTACTTGCCTTTTTTCCTTTGCTATTATTCTTTCTTTGTACTGTATGTTTAGTGTTTTGATTGTTAAGAGGCAAGGGGA  37148
Mouse                       --------------------------------------------------------------------------------  37831
ChineseHamsterGHOK1GS       --------------------------------------------------------------------------------  39021
LongTailedChinchilla        ACTTTTTGAATCTCAAGCCACGTGATCCCTAGTTAC-------A---GTTTAGTA----------------------AA-  37375
```

```
Majority                    -------------TTTATTCTC-TXAGTATXCTAATTXXTTTXTXTTCXT------------------------------
                                     59530     59540     59550     59560     59570     59580     59590     59600
Human                       -------------TTTATTCTCCTGAGTGTCCTAATTCTTTTGTATTCTC------------------------------ 55504
GuineaPig                   -------------TTTATTCTCATAAGTATTCTAATTCCTTTCTCTTCTTTCA--------------------------- 33990
NorthernAmericanDeerMouse   TTTTCTTTTCTGATCCAATCTCTTTGGTATTCTATAAGCTTCTTGTACCTTTAAAGGCATGTCCTTACTTTGGTTGAGAA 37228
Mouse                       -------------------------------------------------------------------------------- 37831
ChineseHamsterGHOK1GS       -------------------------------------------------------------------------------- 39021
LongTailedChinchilla        -TGTCCATTCTTTTTTATTTTCGTAAGTATCCTAATTTTTCTCTGTTCAT------------------------------ 37424

Majority                    -------TXTCATTXT-CTGTXTATATATXXTTXXTXAXCTGXAAAXXXX------------TXTXXAXXTAT--X--TX
                                     59610     59620     59630     59640     59650     59660     59670     59680
Human                       -------TTTCATTAACCTGTTGATATAGATTTTTCAAACTGTAAACCTA------------TGTATAGGTAT-----TT 55560
GuineaPig                   ------TTGTCTCTGTACTGTGTATATATTCTTACTTACCTGCAAATACAAATA--------TTTCAAATTACAGTG-TC 34055
NorthernAmericanDeerMouse   ACTTTTCTTCTATGATTTTGTTTAAATGTATTCTCTGGATTTTGAGCTGGGTTTCTTCTTCTTCTATTCCTATTATTCTT 37308
Mouse                       -------------------------------------------------------------------------------- 37831
ChineseHamsterGHOK1GS       -------------------------------------------------------------------------------- 39021
LongTailedChinchilla        ------TTGTCATTGTGCTATGTATGTATTCTTATTTATCTGCAAATACT------------CTTCAAACTATCATG-TC 37485

Majority                    ATGGTATXTGTTAX-----------------------XXXX-X-X-XX-AXAXTTXTXTX---TXTXTAXTTXXXTTG--
                                     59690     59700     59710     59720     59730     59740     59750     59760
Human                       GTGGTATTTGTCAT-----------------------TGTTATAT-TTTAGAACTATTTGC-CTTTGTATGTTTTTTGA- 55614
GuineaPig                   ATGGTATATGTTA------------------------------------ACAGTTATATG---TGTATAGTTGCCTTGA- 34095
NorthernAmericanDeerMouse   ATGGTTGGTCTTTTCTTAGTGTCCCAGATTTCCTGGATGTTTTGTGTTAGGAATTTTTTAGATTTAACATTTTCTTTGGC 37388
Mouse                       -------------------------------------------------------------------------------- 37831
ChineseHamsterGHOK1GS       -------------------------------------------------------------------------------- 39021
LongTailedChinchilla        ATGGTATATGTTAT-----------------------TGTTTTAT-TTAACAGTCGTGTA---TGTGTAGTTGTCCTG-- 37536

Majority                    ---------------------------------------------------------------CCXCAGXXGAGATAGAA
                                     59770     59780     59790     59800     59810     59820     59830     59840
Human                       ---------------------------------------------------------------CCACAGATAAGGTAGAA 55631
GuineaPig                   ---------------------------------------------------------------CCCCAGAAGAGACAGAA 34112
NorthernAmericanDeerMouse   TGATGAATCTATTTCTTCTGTTGTATCTTCAGTGCTTGAGATATCTCTTCCACCTCTTGTGTTCTGTTGGTGATATTTGG 37468
Mouse                       -------------------------------------------------------------------------------- 37831
ChineseHamsterGHOK1GS       -------------------------------------------------------------------------------- 39021
LongTailedChinchilla        ---------------------------------------------------------------CCCCAGGAGGGATAGAA 37553
```

Majority	AGAAGTTA----------CTTATCAAAGA-TXT-XTCATAATAA-------------------------GAAAGXXATAX	
	59850 59860 59870 59880 59890 59900 59910 59920	
Human	AGAAATTATTTC------CTTATCATAGACTGTCATCATAATAAC-----------------------AGGAAAGGATAA	55683
GuineaPig	AGAAGTTA----------CTTATCAAAGAATAT-GTCATAATAG-------------------------GAAAGGCATAG	34156
NorthernAmericanDeerMouse	ATCTGTAGTTCCTGTTCGCTTACCCAGATTTTCCATTTCCAGGATTCCCTCTGTTTGTGTTTCCTTTATTGATTCTATTT	37548
Mouse	--	37831
ChineseHamsterGHOK1GS	--	39021
LongTailedChinchilla	AGAAGTTA----------CTTATCAAAGAGTAT-GTCATAATAA-------------------------GAAAGACACAG	37597

Majority	XXATXXTXAXX------------------------------------AXTCXTTTGGAGCATTTAACAT-----------	
	59930 59940 59950 59960 59970 59980 59990 60000	
Human	TAGTAATAAAGGC----------------------------------AGTCTTTTGGAGCATTTAACATAATA-------	55722
GuineaPig	CGATAGTGAA-------------------------------------AATCATTTGGAGCATTTAACAT-----------	34188
NorthernAmericanDeerMouse	CCATTTTCAGGTCTTGAACAGTTTTCTCTATCTGTTTTTTTTTCTTTTCTTCTTTTAGGGATTTACTGCTTTCTTTCAGT	37628
Mouse	--	37831
ChineseHamsterGHOK1GS	--	39021
LongTailedChinchilla	TAACGATGAGGGG----------------------------------AATCTTTTGGAGCATTTAACAT-----------	37632

Majority	---TTGAX-X---------CAGAXTTTTTAXAAATACTGTXXXT-------X------------TXXXTTTTATAXGTTX	
	60010 60020 60030 60040 60050 60060 60070 60080	
Human	--TTTGATTGA--------CAGATTTCCTACAAATACTGTGTTTA------T------------TCAATTTTATGGGTAT	55774
GuineaPig	---TTGA------------CAGAATTTTTAAAAATACTGTAACTTT----TT------------TTTGTTTTATATGTTG	34237
NorthernAmericanDeerMouse	TTTTTGTTTGTTTTTTCCTCAGTTTCTTTAAGAGATTTGTTCATATCTTCTTTAAGGACCTCTATCATCTTCATAAAGTC	37708
Mouse	--	37831
ChineseHamsterGHOK1GS	--	39021
LongTailedChinchilla	---TTGATGGA--------CAGAATTTTTTCAAATACTGTAAAT--------------------TTTGTTTTATATGTTG	37681

Majority	AXTTTTTCX----------XTTTXAAXXTXXCAAAAATXX---------------------XCTTXTAATX---------	
	60090 60100 60110 60120 60130 60140 60150 60160	
Human	ATTTTTTCGC---------ATTTTAAAATCTCAGAAATTTGATGT----------G-----TCCTGCAATAGTCTAT---	55827
GuineaPig	ACTTTTCCA----------ATTTAAAGATACCAAAAATCA----------------------CTTATAATT---------	34276
NorthernAmericanDeerMouse	ATTTTCTTGTGCTTCAGCTGTATTGGATTGTTCAAGTCTTGCTGTTGTAGGATAGGAGGGCTCTGGTGATGCCATATTGC	37788
Mouse	--	37831
ChineseHamsterGHOK1GS	--	39021
LongTailedChinchilla	ACTTTTTCA----------GTTTAAAGGTACCAAAAATCAAA-------------A-----TCTTATAATA---------	37724

```
Majority                      --TTTATAXTTTAXTTXGTXACATCTTAXCATTTATACXTAAAATAATATTXXGTCTTGXAGTTG-TTGGTGTATTTGXT
                                            60170     60180     60190     60200     60210     60220     60230     60240
Human                         CTTCCATAGTTCAATTGGCAACATCTTATTAGTCATACATAAAACAATAGTGCATTTTATAATTA-ATGGTGTATTTGAT  55906
GuineaPig                     --ATAAAAGTTTAATTGACAACATATTATCACTGATACATAAATTAATGCTGTATCTTATAGTTG-TTGATGTATTAAAT  34353
NorthernAmericanDeerMouse     CTTTTCTGTTATTGATTGTGGCACTTAGGCATCTGGATTTGGGATGATTTTAGGTCTAGGTGTTGATTCCTGTGTTTGTT  37868
Mouse                         ---TTGAATCTTAGGTTGTGTGATCCTTG-GTTTATTCTTACAAACATATTAATTCT---------TTTGTGTTTTTCTT  37898
ChineseHamsterGHOK1GS         ---TTTTGTTATTGATTGTGGCATTTAGGCATCTAGATTTGGGATAATTTTAGGTCTAGGTGTTGATTCCTATATTTGTC  39098
LongTailedChinchilla          --TTTATAGTTTAATTGGTAACATCTTATTACTCATACATAAAATAATACTGCGCTTTGCAGTTG-TTGGTGTATTAAAT  37801

Majority                      XTXATXAAATGTGTAXTXTGTXTXXTTXCXTXTTATXTCTXGAXXATTTTCTG-X--AAAAXTTTAAGXTT-TTTGXXAX
                                            60250     60260     60270     60280     60290     60300     60310     60320
Human                         ACCATAAAATGTATGGGAAATATTCTT-CATATTATATCTTGAAGTTTACCTG----AGAATTTTGTGGTT-TTGGACTA  55980
GuineaPig                     ATTATAAAATGTGCACAAAGTTTTCTT-CATGTTATAACTTGAAAATTTCCTG----AAAAATATAAGCTT-TATGGTAC  34427
NorthernAmericanDeerMouse     TTTGTTGGATGGGTGTTTTGTTCCTTGGCTTCTCTTTTCTCTATGGTCTTCTGGCCTAAATGGCCAAGGGTTCTGGTGAA  37948
Mouse                         TTCTTTCAGAACTTAATT-GTATTTTTGTTATTTATTTACCAATAACGATTTGTC--AAACCATTAAGATAATTTGAAAT  37975
ChineseHamsterGHOK1GS         TTTGCTAGATAGGTATTTTGCTCCTTTGCTTCTATTTTCTCTCTGGTCTTCTGGCC-AAGGGATCAAGTAA-CTAGTGTG  39176
LongTailedChinchilla          ACCACAAAATGTCCACAATATATCCTT-CATATTACAGCTTGAAAATTTTCTG----AAAAATTTAAACTT-GATGGTAG  37875

Majority                      TTTTXTGTGTT-XXATXCTXTAXTGTTGATATTTTX--X-XTTXXGXXXXGTGTXAGAAGCTAXATATAGTTT---XXXT
                                            60330     60340     60350     60360     60370     60380     60390     60400
Human                         GACTACACGTT-GGTTTCTA--TTCTAAAAATTT----TG----------GTGTCAGAAGTTAAGTGTAGTAT-------  56036
GuineaPig                     TTTTTTGGGAT-AATTAATATCTTGTTGAGATTTTCCATGTCTCAGTTTTGTGG-ATAAGATAATGATAGTTTT--GATA  34503
NorthernAmericanDeerMouse     TAGCCTGTCTCTGGGTACAGTAGGGTGTCTGCTCTGGGT-TTTTGGGGGGCTATAGGACCCTAGATCTGGCCTGGCCTTT  38027
Mouse                         TTAAATATGAT-GCATTTTGCAGGGTTGATTGTATA-----TTATCTACCATAAAATATACTGAAAATAATCC--TTCAT  38047
ChineseHamsterGHOK1GS         TTTCCAGATTC-AAATTCAGTAGTCC-CCTGCTTTG----TTTTTGGGGGCTGTGGGTCCCTAGATCCGGCTTGGTCTCT  39250
LongTailedChinchilla          TTTTTTTTTTT-ATAAACTACGTTGTTGAGATTT----------------GTGG-AAAAGATAGTGATAGTTT-------  37930

Majority                      XGTAAAGCTTXAXTCTTTXAAXATXGXTXT-TTAAAGXATATXXTXGTTAXA----------------------------
                                            60410     60420     60430     60440     60450     60460     60470     60480
Human                         --GAAAAATGTATTTTCTGAATGTTCCTAT-TTTAAGATTGTATTAGAAATA----------------------------  56085
GuineaPig                     ACAAAAGTTTTATTCTTTAAATATTTCTGT-TTAAAACATTTTCTGGTTACAT---------------------------  34555
NorthernAmericanDeerMouse     GGTAAGGCCTAAGTCTTCTTCCAAAGTTGTGGACCAGGATATGGAAGCCAGGGGAGGGGGTACACACTGGGGTCCCTAGA  38107
Mouse                         AGTATATCTGAAATTGTTCCTGCTAGTTATACTAAAGACCACATTAGTTTT-----------------------------  38098
ChineseHamsterGHOK1GS         GGTAAGGCCAAAGTCTTCTTCCAAAGTTATAGACCAGGGCATGGAGGCCAG-----------------------------  39301
LongTailedChinchilla          ---AAAGTTTTAATCCTTAAAAGTTTCTGT-TTAAAACATTTCCTGGTTACAT---------------------------  37979
```

Majority	--------------AXAXXXGTCAXAXXA--X--X-----GXTCTTTTXAXAATAXTXCXGTAAAXXXCTXXTTGXTTTT	
	60490 60500 60510 60520 60530 60540 60550 60560	
Human	--------------ATAAGCTTTATGGGA-----------GTTCTTTT--GGATAATTCAGTAACCCCCTTGTTGAGATT	56138
GuineaPig	--------------ACATCTGTCAAACCACATCTT-----GTTCTTTCAAGAATAAATCAGTTTTCCTCCCTTTGCTCTT	34616
NorthernAmericanDeerMouse	TCTGGTCTGGCCTCTGGGAGACTACATTAATTTCTGGTCAGAAAATTTTATAGTATTAATATAAAAAAATACTTGTTTTT	38187
Mouse	---TG---------------GTCAGA-------------------TTTCATATTATTAGTGTAAAAAA----CTGTTTTA	38137
ChineseHamsterGHOK1GS	--------------------ACCACG-----------------------ATAGTATTAATATGAAAAG-TACTTGGTTTT	39337
LongTailedChinchilla	--------------ATATCTGTCAAACTATGTCTT-----GCTCTTTCAAGAATAAATCACTTGGCCTCTCTTTGCTCTT	38040

Majority	A--AXCXXCAGTGTCXAATXGCTAXXTTAATATTA-GAAATAATXAGXTTAATGATAAXTTTTTTGAAAGATXATC--TG	
	60570 60580 60590 60600 60610 60620 60630 60640	
Human	TACAACGTACCTGTTTTATGGAGTGATTAATGTTA-AAGTTGTATTCTCTAAATTTC---TGTTTAAAACATTTTC--TG	56212
GuineaPig	A-CAA--TTAGTGTGAAA-GCCTAGATCACTTTTT-AAAATAA-----ATACCATTAGAATACTTGAATTACAAAC--TG	34684
NorthernAmericanDeerMouse	A--ATCCCCAGTATCCAATTGCTAAGTTCATATTA-GATATAATAAGGTTAATGATAATTTTTGTGAAAGATCAT---TA	38261
Mouse	A--ATCTCCTGTGTCCATTTTTTAAGATTGTATTA-GGAATAATAAACTTAGTGATAATTTTTCTGAAAGATTAT---TG	38211
ChineseHamsterGHOK1GS	A--ATCCCCAGTGTCTAATTGCTAAGATTATATTA-GAAATAATTAGGTTAATGATAACTTTTGTGAAAGATCACCACCA	39414
LongTailedChinchilla	T-TAACGTGAGTGCCAAA-GGCAAGATCACTTTTTTGAAATGACCAGTAGAGTACTCAAGTACTTGAGTTACAAAC--TG	38116

Majority	ATAGTCCXTTTXXTGCAXAXXACCTXTTTCATGGAGX-XTTTXTGAATAATXTGCATTTAAAGTTTTXXCATXCTXAX--	
	60650 60660 60670 60680 60690 60700 60710 60720	
Human	ATGGTACATGTATTTATAA--AACTACATCTTG--TTCTTTTGAGAATAAATTACATATCATACCTTTGCATGTTCAAC-	56287
GuineaPig	ACAGTAT-TACATAGCAAA--ATGTAAGTTATGATGTATTTTTCAACTACTTTTCCTTTTAATTTTCAGCGTGCTCAA--	34759
NorthernAmericanDeerMouse	ATAGTCCCTTTCCTGCAGCGTACCTGTTTCATAGAG-------TGAATAATATGGAGTGAAAGTTTTGTAATTCTGAG--	38332
Mouse	GTAGTCCATTTCCTGTAGTGTGCCTGTTTCATAGAGGGGATAATGTATAATATGAAGCTAAAGTTTTTTTATTGTGTAAA	38291
ChineseHamsterGHOK1GS	GTAGTCCCTTTCCTGCAGTGTACCTGTTTCATGGAG-------TGAATATTATGAAATTAAGGTTTTCTAATTCTGTGAT	39487
LongTailedChinchilla	ATAGTGT-TACATAGCAAA--ATGTAAGTGATGAATGTGTTTTCAACTACTTTTCCTTTTAATTTTCAGCGTGCCCAG--	38191

Majority	XTTXXTCTTCAAXCXGTXX-X----TTCTGXTTGTAAATAXXTATC-CAGGXXCAT---CACATTTATTCAXAATX-AXT	
	60730 60740 60750 60760 60770 60780 60790 60800	
Human	ATTGATCCCAAAGCCTGGATAATTTTTTTGAATG-AAATACTAGT----AGAATAT---TTGATTTATTAAACAGGTAGT	56359
GuineaPig	GCAGTTCTTCAAGCTGTGACAGCCGTCCAGACAGCAAACACTCCTCTTAGTGGCAC---CACAGTTAGTGAGAGTGCAGT	34836
NorthernAmericanDeerMouse	-TTTGTCTTTAAATGGT--------CTCTGGTTGTAAATATCTATC-CAGGTGTATTCCCCCCCCCCCCCCAAATA-AAT	38401
Mouse	-TTTCTCATCTAACAGT--------TTCTAGTTATAAACATCTATC-CAAGTAAAT---CATATTCTTTCAGAATA-AAT	38357
ChineseHamsterGHOK1GS	TTTTCTCTTTGAATGGT--------TTCTGGTTGTAAATATCTATC-CAGGCACAT---CATATTTTTTCAAAATA-AAT	39554
LongTailedChinchilla	GCAGTTCTTCAAGCTGTGACAGCTGTCCAGACCGCAAATACTCCTCTCAGTGGCAC---CACAGTTAGTGAGAGTGCAGT	38268

```
Majority                    GGCAXTXGCTXAXXGTXCCAGXAXTGXTXAXXAAGTGTTXAGGXX---XACTATTTTGXAACACATGXAXXXAAXTTCTT
                                          60810     60820     60830     60840     60850     60860     60870     60880
Human                       A--CCTGGCTCATGG--CAGGTGCTTAAAAATATCTGTTGAGGGGCCATTTAATTTTGTTTAATCTGTG--TAAGTTCTA  56433
GuineaPig                   GACTCCAGCCCAGAGT-CCAGTGCTGAGAA-TAATCATTGACAATATGTACTACCCTGTAACACTTG-----ACGTTCTT  34909
NorthernAmericanDeerMouse   GGCATTGCCTATCTGTGTCAGCATTGGTGCCAAAGTGCTTAGGTG----ACTATGTTGAAACACATACAGTATAATACTT  38477
Mouse                       GGCATGAGCTA----TGTCAGCATCGGTGTTAAAGTGTTTAGGTA----ACTATTTGGAAACAAATGTAGTGGAATTCTT  38429
ChineseHamsterGHOK1GS       GGCATTGCCTATTTGTGATAGCATTGGTGTCAAAGTGTTTAGCCA----TCTGTTTTGAAACAAATACAGTAGAATAATT  39630
LongTailedChinchilla        GACTCCAGCCCAGAGT-CCAGTACTTAGAA-TAATTATTGACAATATGTACTACCCTGTAACACTTG-----ATGTTCTT  38341

Majority                    GAAAXAAAXTGAGATAGTAGTATAATATGTA--AXAATGTAAGTXGAAATAATGGXTATGTTACTAAACTACATXXXXTT
                                          60890     60900     60910     60920     60930     60940     60950     60960
Human                       GGAACAAAGTGAACTAGTAGTGTAACAGGTT--GCAAAGTATGTGACAATAATGGGTATGTTTCTAAACTACATAATCTT  56511
GuineaPig                   C--ACCAAGTAAGTTG--AATTTAATTTGCA--TACTTGTACATATACATTAAATAAATAATAA---AGTAAATAATGTT  34980
NorthernAmericanDeerMouse   AAAGTATAAAGAGATAGTAGTATAATATGTAACAAAATGCAAGTGAAAATAATGGCTAAGCTGCTAACCTCCTTGTCTGT  38557
Mouse                       GAGCTACAAGGAGACAGTAGTAAAACATGTA--ACAATGTAAGTAGAAATAATGGGTGTGTTACT----TGTCCGTTCCT  38503
ChineseHamsterGHOK1GS       GAACTACAAAGAGACAGTAGTATAATATGTA-CAAAATGTAAGTGGAAATAATGGCTGAGTTACTAAACTCCTTGTCTGT  39709
LongTailedChinchilla        C--ACCAAGTAAGTT------TTAATTTGCA--CCCATTTATATAGACATTGGACAAATAATAATACAGTAAATAACATT  38411

Majority                    TXXTTXCAGCGTGCTCAAGCAGTTCTTCAAGCTGTGACAGCTGTCCAGACAGCAAATACACCTCTTAGTGGCACCACAGT
                                          60970     60980     60990     61000     61010     61020     61030     61040
Human                       AATTTCCAGCGTGCTCAGGCAGTTCTTCAAGCTGTGACAGCTGTCCAGACAGCAAATACTCCTCTTAGTGGCACCACAGT  56591
GuineaPig                   TTGATCTACTGTTTCAACAGATATTTTCTAAGTTTGGT-GCTGTATTGAAGATAATCACATTTACAAAAAATAAC-CAGT  35058
NorthernAmericanDeerMouse   CAATTTCAGCGTGCTCAAGCAGTTCTTCAAGCTGTGACAGCTGTCCAGACAGCAAATACACCTCTTAGTGGCACCACAGT  38637
Mouse                       TTTTTTCAGCGTGCGCAAGTAGTTCTTCAAGCTGTGACAGCGGTCCAGACAGCAAATACACCTCTTAGTGGCACCACAGT  38583
ChineseHamsterGHOK1GS       CAATTTCAGCGTGCTCAAGCAGTTCTTCAAGCTGTGACAGCTGTCCAGACAGCAAATACACCTCTTAGTGGCACCACAGT  39789
LongTailedChinchilla        TTGATCTGATATTTTAACAGATATTTTCTAAGTTTGGT-GCTGTATTGAAGATAATCACATTTACAAAAAATAAC-CAGT  38489

Majority                    X---AGXGAGAGTGCAGTGACTCCAGCCCAGAGTCCAGTACTTAGAATAATTATTGACAATATGTACTACCCTGTAACAC
                                          61050     61060     61070     61080     61090     61100     61110     61120
Human                       T---AGCGAGAGTGCAGTGACTCCAGCCCAGAGTCCAGTACTTAGAATAATTATTGACAACATGTACTACCCTGTAACAC  56668
GuineaPig                   TTCAAGCTTTGCTCCAGTACGGTGATCCAGTAAATGCTCAACAAGCAAAACTAGTAA--GTCTAGAGTGTCTTTTGAGAT  35136
NorthernAmericanDeerMouse   C---AGTGAGAGTGCGGTGACTCCAGCCCAGAGTCCAGTACTTAGAATAATTATTGACAATATGTACTACCCTGTAACAC  38714
Mouse                       C---AGTGAGAGTGCGGTGACTCCAGCCCAGAGTCCAGTACTTAGAATAATTATTGACAATATGTACTACCCTGTAACAC  38660
ChineseHamsterGHOK1GS       C---AGTGAGAGTGCAGTGACTCCAGCCCAGAGTCCAGTACTTAGAATAATTATTGACAATATGTACTACCCTGTAACAC  39866
LongTailedChinchilla        TTCAAGCTTTGCTCCAGTATGGTGATCCAGTAAATGCTCAACAAGCAAAACTAGTAA--GTCTAGAGCGCCTTTTGAGAT  38567
```

Majority	T-TGATGTXCTTCACCAAGTAAG--X--TGTAATTTGCATACTTXCTTATAACTTXX-AGAAAX---TAAXATAGTAGGT	
	61130 61140 61150 61160 61170 61180 61190 61200	
Human	T-TGATGTTCTTCACCAAGTAAG-----TTTAATCTGCATAATTACCTATAAATTAG-AGAAATA-ATAATATAGTAAAT	56740
GuineaPig	AGTAATATTTTT---CATGGAGA-----TGTAACTTA-ATCCTAA--TGTAAGTCAGCAGTGCCATAAAGCATTATAGGT	35205
NorthernAmericanDeerMouse	T-TGATGTCCTTCACCAAGTAAG--TAGTGTAATTTGCATACTTGCTTAGAGCTTGA-AGAAA----TATTGTAGTTGGT	38786
Mouse	T-TGATGTCCTTCACCAAGTAAGTGTCATTTGCTTGGCATAGTTGCTTATAGCTTGA-AGAAA----TAATTTAGTAGGT	38734
ChineseHamsterGHOK1GS	T-TGATGTCCTTCACCAAGTAAGTGTAATGTAGTATGCATGCTTGCTTATAACTTGA-AAAAAA---TAACATA-TTGGT	39940
LongTailedChinchilla	AGTAATGTTTTTT--CATGCAGA-----TGTAACTTA-ATCCTAA--TGTAAGTCAGCAGTGCTGTGTAGCGTTGTAGAT	38637

Majority	XA-XTAATGXXTTGATTTTAATGTTTT-CACAGATATTTTCTAAGTTTGGTGCTGTATTGAAGATAATCACATTTACAAA	
	61210 61220 61230 61240 61250 61260 61270 61280	
Human	AA-GTAATGTTTTGATTTTAATGTTTT-AACAGATATTTTCTAAGTTTGGTGCTGTATTGAAGATAATCACATTTACAAA	56818
GuineaPig	GGTGGAAATTTATGCCATTAAGGTAAT-TTTAGGAAATCATTCTAAATTGTCATAAAAATTAAATGTTTATTTTTTTAAA	35284
NorthernAmericanDeerMouse	AA--TAATGCTTTGATTTAAATGTTTT-CACAGATATTTTCTAAGTTTGGTGCTGTATTGAAGATAATCACATTTACAAA	38863
Mouse	GA--TAATGCCTTGATTTTAATGTTTT-CACAGATATTTTCTAAGTTTGGTGCTGTATTGAAGATAATCACATTTACAAA	38811
ChineseHamsterGHOK1GS	AA--TAATGCCTTGATTTAAATGTTTTTCACAGATATTTTCCAAGTTTGGTGCTGTATTGAAGATAATCACATTTACAAA	40018
LongTailedChinchilla	GGTGGAAATTCATGCCACTAAGGTAGT-TTTAGGAAAACATTGTAATTGTTTTAAAAAATAAAATGTTTATTTTTTTAAA	38716

Majority	AAATAACCA--GTTTCAGGCTTTGCTCCAGTATGGTGATCCAGTAAAXGCTCAACAAGCAAAXCTAGTAAGTTXXGGX-X	
	61290 61300 61310 61320 61330 61340 61350 61360	
Human	AAATAACCA--GTTTCAAGCTTTGCTCCAGTATGGTGATCCAGTAAATGCTCAACAAGCAAAACTAGTAAGTCTTTCT--	56894
GuineaPig	GACCAATCATGGCTATAGTATCCATACTGTAAGATTGTATTAATTTACGCTTTTGATTTAGGTGCAGAGTTTTGTGAT--	35362
NorthernAmericanDeerMouse	AAATAACCA--GTTTCAGGCTTTGCTCCAGTACGGTGATCCAGTAAATGCTCAACAAGCAAAACTAGTAAGTTTGAGCCT	38941
Mouse	AAACAACCA--GTTTCAGGCTTTGCTCCAGTATGGTGATCCGGTAAACGCTCAACAAGCCAAGCTAGTAAGTCGGGGC-T	38888
ChineseHamsterGHOK1GS	AAATAACCA--GTTTCAGGCTTTGCTCCAGTATGGTGATCCAGTAAATGCCCAACAAGCAAAGCTAGTAAGTTTGAGCCT	40096
LongTailedChinchilla	GAC-AATCATAGCTGTAGTAGTCATAACACAAGCTTGTATTAGTTTACACTTCTAGTTCAGGTGCGGAGTTTTGTGTT--	38793

Majority	XTTGAGATXGTGATGXGTTTTTX------XX--XATGXATTT--X-X--XXX-XXXTGGTAT-------X-X--T--XXX	
	61370 61380 61390 61400 61410 61420 61430 61440	
Human	TTTGAGATGGTGATTTTTTTTTA------TTGAAATGTATATGTAGAAAAATATATATATAT----G-TATGTAT-ACTT	56962
GuineaPig	TTTCAGAAGACAGTAGAATTTTAAGTAGTTTTCAGTGAGTTT-CAAAGTAATTTACTGGTTCTACCAAAAAGTGTCACTT	35441
NorthernAmericanDeerMouse	CTTGGGATAGTGATGTGTTTTTC-----------ATTGACTT-------------TTAGTAT-----------T-----	38980
Mouse	CGTGAGACAGTGATGGGTTTGTC-----------ATGCATTC-------------CTGGGGT-----------G-----	38927
ChineseHamsterGHOK1GS	CTTGGAATAGTGATGTGTTTCTC-----------ATTGACAT-------------TCAGTAT-----------T-----	40135
LongTailedChinchilla	TTTGAGAAGGTAGTTGAATTTGAAGTAGTTTGCAGTGAGTTT-CAAAGTAATTTACTGGTTT----GAAATGCATCGCTT	38868

```
Majority                     XX--X-X------X-XX----X--T--XXX-XATTTAXXTXAGXAATTTTAXTAAA---CAATAAXTGTAXTXA--XXX-
                             61450 61460 61470 61480 61490 61500 61510 61520
Human                        AATGATATCTTGTAGCATTACAGATAGTAGAAATTGATGGCATTAATATTAAGAAACCTTAATATTTGTCATAA--AAGA 57040
GuineaPig                    AACAAGA----ACATCAACATAA-TGATAGGAATTCATGTCAGTGATTTTAGTAAA---CAATAATTGTTATGACAAAGG 35513
NorthernAmericanDeerMouse    ------------------------A-------ATTTAACTTAGAATTGACTTTTAA---GAATAACTATAGCTG------ 39020
Mouse                        ------------------------T-------GTTTAACTTAGAAC--------AA---CTGTAGCTCTAG--------- 38956
ChineseHamsterGHOK1GS        ------------------------A-------ATTTAACTTAGAGTTTATTTTTAA---GAATAACTGTAG--------- 40172
LongTailedChinchilla         AACAAGA----GCATCACTGTAAATGATAGAAGTTCATGTCAGTAATTTTAGTAG----CAGTAATTGTCATGA-AAAGG 38939

Majority                     XXX-XXX-XXXXX---A---X-X-XXXX---TAX-GTXATCTXTAACATAGGCTTXT-XXX-GTTACATXTTTGTTC---
                             61530 61540 61550 61560 61570 61580 61590 61600
Human                        AATATTTATTTTT---A-GGAAAATCATAGTTGTAGTTAACCATGATGTGGGTTTCTATTATTTTACTTTTTTTTTTTT 57116
GuineaPig                    AATGTTTATTTTTTT-AAAAACAGTCATGAATA--GCTGTCTATAGCATAGGCTTATATTAGTTTACATGTTTGTT---- 35586
NorthernAmericanDeerMouse    ----------------T--------------TATAGTCATCTGTAACATAGGCTTGT-----GTTATATATTTTTGC--- 39062
Mouse                        -------------------------------------TCAACTGTAACATAGGCTTGT-----CTTAGATGTTTGTTC--- 38992
ChineseHamsterGHOK1GS        -------------------------------------TCATCTGT------------------GTTATGTATTTGTTC--- 40195
LongTailedChinchilla         AATGTTTTTTTTTTTTAAGAACAATCATGACTATTGCTATCTATAGCATAGGCTTATCTTAGGTTACATTTTTGTT---- 39015

Majority                     ----AATTTXGG----AAAXTTTGTGCTCTTGAGAAGATAGXGGGATXGTATTGTG-------------------T--X
                             61610 61620 61630 61640 61650 61660 61670 61680
Human                        TTTTAATTTAAGGACAAAAGTTTGTACTCTTAAGAAAAAAGCAGGACTATATTGTG-----------A-----------A 57174
GuineaPig                    ----AATTTTAG----AAGTTTTGTGCTTTTGAGAAGATAGCTGCATAGGATTGTC---------------------CA 35636
NorthernAmericanDeerMouse    ----AATTTGGT-ACAAAACTCTCCGATGTTGGTAAGACAGTGGGGTTGTGTTATG-------------------T--- 39114
Mouse                        ----AGTTTGGT-TGTAAAC--TGTGACCTTGCAAAGACAGTGGG-TAGCCTTGTG-------------------C--- 39041
ChineseHamsterGHOK1GS        ----AATTT--------------CTGGTCTTGTTA-GCTGCTGGGATTGTGTTGTT-------------------T--- 40233
LongTailedChinchilla         ----AATTTTGG----AAGTTTTGTACTTGCGAGAAGATAGCTGGATAGGATTGTATACCTGTGTATACCTGTGTATACA 39087

Majority                     -GTGCACXTX--XXAGXT-XTTAXGTAXCTTTTGXTXGGAGTTTCATAGTAAXT--XTTTCAAGXTAA-TTTGGTTGAGA
                             61690 61700 61710 61720 61730 61740 61750 61760
Human                        TGTGCACCAGCATAAGAT-TTGAAGTGGCTTTTGCTGGGAATTTCAAAGTAATTAGTTTTCAAAAGAG-TTTGGCTGAAA 57252
GuineaPig                    AGTATACCTGCATAAGATTTTTAAATAGCTTGCAATGGGAGTTTCAAAGTAATTTATTTGTGAGATAA-TTTGGTTGAA- 35714
NorthernAmericanDeerMouse    -GTGCACAT-----AGTG--TTATGTACCTTTTGGTAGCGATCTGGTAGTAACT---TTTCAAGGTAA-TTTAGTTCAGA 39182
Mouse                        -ATGCACAC-----AGTT--CAGTGCGCCTTTTGCTAGGAGTGTCATAGTAACT---TTTCAAGGTAT-TTTGGTTTACA 39109
ChineseHamsterGHOK1GS        -GTGCACAT-----AGT---TTATGTACTTTCTGATAGGAGTTTGTTAGTAACT---TTTCAAGGTAG-TTTGGTTGAGA 40300
LongTailedChinchilla         TGTATACCTGTGTAAGATGTTTAAATAGCTTGCAGTGGGAGTTTCAGAGTAATTTATTTTTGAGATAAGTTTGGCTGAG- 39166
```

```
Majority                      TAXXTCAXT-XX-----XATGTXGTXCAXATGTTCTGATXXGCTCTAG--TTATATXXTACXTTACATTGTTTTTAXXGX
                                       61770     61780     61790     61800     61810     61820     61830     61840
Human                         TATACAGAT-----------GTGGCTCAAATGCTATGATAGGCTCTCAGTTTATTTAAAACTATATATTCTCTTTAATGT  57321
GuineaPig                     TATATTAGTGTATTAATATAGATGTACAAATGTTGTGACAGACTCTAG-----TGTAAGACTGTATCTTATGTATA----  35785
NorthernAmericanDeerMouse     TACTTGGATATA-----GATGTAGTGCAGACATTCTGATCATCTCTAG--TTATATTGTACCTTACACAGTTTTAAAAGA  39255
Mouse                         TACTTCAGT---------AT-------------TCTGGTCAGCTCTAG--TTACATTGTGTCTTACA-GGTTTTGTGGGG  39164
ChineseHamsterGHOK1GS         TACTTCAATATA-----GATGTAGTTCACATATTCGGATCAGCTCTAG--TTATATTGTATCTTACACAGTTATTAGAGA  40373
LongTailedChinchilla          TATATCAGT--------ACAGATGTACAGTTGCTGTGACAGGCTCTAG-----TGTAAGACTGTACCTTATATATA----  39229

Majority                      XTTTTTAAGACXTXTTTT-X-XXXAGGTTXTTAGTCTXTTXTTTATTCATATCATTCACXTTTTTTGTCATTXTTAXATA
                                       61850     61860     61870     61880     61890     61900     61910     61920
Human                         CTTTTTTTTAAAGGTTTTATGAATAGGTTTCTAGTCA-CTTTTTATTCATATCATTTATCTTTTTTGTCATTGTTACATA  57400
GuineaPig                     TTTTTAAAGGCATGTTTTCTCAATAGGTTTGTTACCTGTTTTTTATTCATATCATTCACCTTTTTTGTCATTGTTACGTA  35865
NorthernAmericanDeerMouse     ATTCTTAAGACTTTTTTT------AGATTCTTAGTCTGTGCTTTATTCATATCATTCACTTTTTTTTTCATTTTTAAATA  39329
Mouse                         --TTTTTGTTTTGTTTTT------AGTTTCTTA----ATGCTTT-------TTATT---TTTTTAAATCATTTTTAGATA  39222
ChineseHamsterGHOK1GS         ATTGTTAAGACTTTTTTT------AGATTCTTAGTCTATATTTTAGTCAGATCATTCATTTTTTAAATCATTTTTAGATA  40447
LongTailedChinchilla          TTTTTAAAGGCATGTTTTCTAAATAGGTTTGTGATCT-TTCTTCATTCGTATCATTCACCTTTTTTGTCATGGTTATGCA  39308

Majority                      TGGAXGGTAAGAGACTATTGAGAXXTAAA---GTTXTCXCXT-GTCATGA-TGCCATTGAAAATT-XATACXACATCTTT
                                       61930     61940     61950     61960     61970     61980     61990     62000
Human                         TGGAGGATGTAAGGTCATTGAGAGATGTAA--AATTTCCCA---TCAAGGCTGCCACTGAAAACTGCGCACCACATCTTT  57475
GuineaPig                     TGGAGATTAAGAGATTATTAGAAGAAAAAAATGTTTTCATAT-GTCATGACTACTATTGAATATTATGTACCACAACTTT  35944
NorthernAmericanDeerMouse     TGGAAGGTAAGAGACTATAGAGAAGTAAA---ATGACCACTT-GTTGTGA-TGCCATTGGAAATG-CATAC-ACATCTTT  39402
Mouse                         TGTAAGGTACGAAACCGTAGAGAAGCAAG---GTTACCCCTTTGTCATGA-TGCTGTTGAAAATT-TATAA-ATCCCTTT  39296
ChineseHamsterGHOK1GS         TGGAAGGTAAGAGACTATTGAGAAGTAAA---GTTATCACTT-GTCTTGA-TGCCATTGAAAACT-CATAA-A-ATCTTT  40519
LongTailedChinchilla          TGGAGGTTAAGAGACTATTAGAAGAAAA----GTTTTCCCAT-GTCATGATTACCATTGAAATTTATACCCCACAGCTTT  39383

Majority                      GCTTCXAAATGCXTAAATCTTTCTGTXXAATTTATTAAATTX---XXTGTTTTTTCTGTT---XAACTTCTTXTGCCATA
                                       62010     62020     62030     62040     62050     62060     62070     62080
Human                         GCTTCCAAGTACCTTAATCTTCTGTTTGTATATCTTAAATCTACTTTTGCTCTGTCTCTT---TAACTTTTTGTGCCATA  57552
GuineaPig                     GTTTCGGAATGCTTACATCTTGCAGT-GCCTT-GTTAACATAACTTTAATTCTGTTTGCA--CTAACATCTTGTACTATA  36020
NorthernAmericanDeerMouse     TGTTCCAAATGCTTAAATCTTTCTGTGTAATTTATTCAATG-------GTTTTTACTGTTT---AACTTCTTTTGACTTA  39472
Mouse                         GCTTCTTAATGCCTAAAGCCTTCTCTTTAATTTATTCAATTC-----TGTTTTTACTGTTA---AACTTCTTTTGCCAGG  39368
ChineseHamsterGHOK1GS         T--TCTGAATGCCTAAATCTTTCTGGGTAATTTATTTCATTC-----TGTGTTTCCTGTT----AGCTTCTTTTGTCTTA  40588
LongTailedChinchilla          GCTTCAAAATGCTTACGTCTTCCAGT-GCCTCCGTTAATATA-TCTTAGTTGTTTCCACTTTCTAACTTCTTGTACTGTA  39461
```

Majority	TTATCT------CATT--ATTTTTAAATTATTA-------XAAAATTCATXATTAAXTCAT-TGTTACXAAAXCATTAAA	
	62090 62100 62110 62120 62130 62140 62150 62160	
Human	GTATCTCATTATTATT--ACTATTCCATTATTAAGCCATTAAAAATTCATTATTAAGCCAT-TATTATGAAACCATTTAA	57629
GuineaPig	TTACCT------CATT--AGTTATTTATTAAAAC-----AAATTATTCATTAGTAAATCAT-TGTTACAAAGTCATTAAA	36086
NorthernAmericanDeerMouse	TTATCT------CATTTAATTTTTAAACGATTA--------AAAATTCAT-ATTGACTTTCATGTTACAAAACTTTTAAG	39537
Mouse	TTATTT------CATT--A---TTAAATCATTA--------AAAATGAAT-ATTAAGTAA--TTTTATGAAACTATTGAG	39426
ChineseHamsterGHOK1GS	TTATCT------CATTTAATTATTAAACCATTA--------ACTATTCAT-ACTGACTTA-ATG----AAAATATTCAGT	40648
LongTailedChinchilla	TCATCTT-----CATT--ATTTTTCCATTATTA------AAGCCATTCATTATTAAACCAT-TATTACGAAGTCGTTAAA	39527
Majority	TCTGTTTTT-TTTATTTAGCXTXGTCTTATTTCTT--TTTTTCAXTTTTTXTTXTTTAAATXTTCATA--GATTGTXTCT	
	62170 62180 62190 62200 62210 62220 62230 62240	
Human	TAAGCTTACATTTATCTTAACTGGTTTTCTTCCTT--TCATTCATTCTTTTACTTTCAGATTAAT-----TATTGTCTCT	57702
GuineaPig	TCTGAT----TTTATTTAGCTTTGTCTTACCTCTT-----TTCCTTTTTTTTTTCTTAAATGTTCAGA--CATTGTTTCT	36155
NorthernAmericanDeerMouse	TCTGTCTAG-TTTATCTAGTCTCAACCTAATTTTT--TTTTTTACTTTTTATTCTTTTATT-TTCATATTGATCATGTTC	39613
Mouse	TCAGTTTTT-TATGT-TAGTCT-TACCTAAT---T--TTTT--ACTTTCGATTCTTTTATT-------------------	39477
ChineseHamsterGHOK1GS	TCTGTGTTT-ACTGTTTAACTTGTTCATATTATCT--CTTTTAATTATTAAACCATTAAATATTCATACTGA-------C	40718
LongTailedChinchilla	TCTGCTGTT-TTTACTGACCTTTGTCTTATCTCTTGTCTTTTCCCTTTTTTGTTTTTAAACAAAAATA--GACTGTTTCT	39604
Majority	AGTACTTTAXTGCTTCCTXTAAATCAAGATTACTTATXTGAAXXTCAXACCATTTTTT-X---XTGTTTAXTTGTATTTT	
	62250 62260 62270 62280 62290 62300 62310 62320	
Human	AATACTTAAATGCTTCCTCCAGATCAAGATTACTTCTGTGGAGCCCAGACCATTTTTT-ACAATTGTTTCATTGCATTTC	57781
GuineaPig	AGTACCTTAGTACTTCCTATAAATCAGTGTTACTTATATGAA-------CCATTTTTTCATTATTATTTAATTGTATTTG	36228
NorthernAmericanDeerMouse	TCTACTACACTGATTCCTTCAGATCAAGATTACCCATGTAAAAGTTGGACCAGTTTTT------TGTTTTCATTTCCTTT	39687
Mouse	--TT	39479
ChineseHamsterGHOK1GS	TTTAATGTAACAAAAATGTTAAATCAA--TTCTTTATTTTAGTCTCA-ACCTATTATT------TTTTTACTTTTATTTT	40789
LongTailedChinchilla	AGTACCTTGGTGCTTCCTCTAAATCAAGATTACTTATATGGAATTCAGACCATTTTTT-ATTGTTGTTTAGATGCATTTG	39683
Majority	CATXCTXATTATXTXATTTGGGXATTAXTTGAATTCTT----AACAGAAAGGCTXTXXX----XGTAATAXTXGXAXAAT	
	62330 62340 62350 62360 62370 62380 62390 62400	
Human	CATCCACACAACCTCATTTTGGGAGCAAAATTAGTTGTAGGTAGCAGAAAGGCTATACA----AGTAATAATAGCATAAT	57857
GuineaPig	TTACCTCATTATCTTATTTGGGGATTACTGAAATCTTT----AACAGAAAGGCTGT--------GTAGT-----TACAAC	36291
NorthernAmericanDeerMouse	CATATCAAATTTGGCCAATAAAAAATACTTGAGATAGC----AACAAAAAGGCTATCTT----AGTAATAATGGCATAGT	39759
Mouse	CATACTAATCATGTTTTCTAGTATTCTATTGATTTCTC----TAGGTCAAGATTGCCCG----TCTGAAAGTTGGACTGG	39551
ChineseHamsterGHOK1GS	CATACTAATCATGTTCTCTGCTACTTCATTGACTTCCT----CAGATCAAGGTTATTTATCCGTGTGAAAGTTGGACCAG	40865
LongTailedChinchilla	TCTCCTCACTGTCTCATTTGGGGATTACTGAAATCCTTCAGTAGCAGAAAGGCTGTA-------GTAGT-----TATAAT	39751

```
Majority                    TGTXATAGTXT-XTCATTTXCTTTAAXTTA---X-XTATCATTTTTXTTGAXXAXTAXTXTTTCTTGTXAXCAAAACAGG
                                      62410     62420     62430     62440     62450     62460     62470     62480
Human                       TGTAATAGTTT-ACCAAGTGCCGTAGTC----------TCATTTTTTCTGACCACTACTCTATATTGTCAACAAAACAGG  57926
GuineaPig                   -GTGATCGTTT-ATAGTTTACTATATTTAC-----TGCCCTTTTTTCTTGACTGTTACTCTGTCTTATAAAGAGAATAGG  36364
NorthernAmericanDeerMouse   TCTAATATAGCATCTTCTCACTTTAATATAAAACAGTATTAAGATT-TTAAAGAGGTATATTACTTTTCAGCTTTTCAGG  39838
Mouse                       TTTTTTATTGT-TTCATTTCCTTTGACCTA---CCCTATCAGATTTGGCCAGTAACCATGCTTCCAGT-AGCAAA---CA  39623
ChineseHamsterGHOK1GS       TTTTTTACGGTTTTCATTTCCTTTTACTTA---CAGTATCAGATTGGCCAGTAAAAAATGCTACAGAT-AGCAAA--AAG  40939
LongTailedChinchilla        TGTCATAGTTT-ATCAGGTGTTTTATCT---------CCCTTTTTTCTTGATCATTACTTTAGCTTGTAAAGAGAACAGG  39821

Majority                    GTTTTXTTCXTXA-GTXATAXXAGGATGGTAATATXTAAXGAGXTCAXTTTCTT------AXAGAAAACAXT-XXTATGA
                                      62490     62500     62510     62520     62530     62540     62550     62560
Human                       GTTTTCCCCTGC--GTTACAAGAGGATGCTAAGATTTAAAGAGGTGAATTACTT------ACAGTGAACACCACCTATAA  57998
GuineaPig                   GTTTTTTTCAGT--GTTATAAAAGGATGCCAACATTTAAAGAGGTAAATTGCTT------ACAATTTACAGT--CTTTGT  36434
NorthernAmericanDeerMouse   TTTTTAGTCTTAACCTACTTGTTCTCAGGTTATATAATCTTATTTCACTATGCTTTTCAGATACCAGTTAACCATTAAAA  39918
Mouse                       GCTCTGTTAGTGA--TAATGGCATGATTGCAATACAGCATCTCTGCACTTTCTTG-----ATAGAAAACAGT-GTTAGGA  39695
ChineseHamsterGHOK1GS       GCTTTATTAGTAA--TAATGGCATAATTGTAATCTAGCATCTTTTCACTTTCTTT-----ATATAAAATAAT-GTTAAGA  41011
LongTailedChinchilla        TTTTTCCTCCCTTTGTTACAAAAGGATG-----ATTTAAAGAGGTAAATTACTT------ACAGAGTACACT--CTGTGA  39888

Majority                    XXTXGACATTTTXXAXAAGTATATTXCTTAXXXXTAGXTTTTXTXXC-----XAGT-ATTTTGGTTTTC-GXTTACTXGT
                                      62570     62580     62590     62600     62610     62620     62630     62640
Human                       GGTAGACATTTTTTC--AGTCT------------TAGCCTTTCTCTC-----CAGT-ATGCTGCTTCTC-----ACTGGT  58053
GuineaPig                   GATAGACATTTTTTTTAAATATCTTCCTTTCACCTAGTTTTTTTTCCC----TAGTTATAGTGGTTCTCAGGTGATACAA  36510
NorthernAmericanDeerMouse   TTTTCTTGTCCAAAATAATTATTCTTCACATGCCTTGCTTCCCAGCTTTGGAAGGT-TTTTTTTTTTTT-GTTTGTTTGT  39996
Mouse                       TGT---------AAAGAAGTCCATTACTTATCTTTTTTTTTTTCCAG-------GT-ATTTTAGTCTTA-GCCTACTAGT  39757
ChineseHamsterGHOK1GS       TTT---------AAAGAGGTATATTTGTCA------GCTTTTCAG---------GC-ATTTCAGACTTA-ACTTACTTGT  41065
LongTailedChinchilla        GATCGACATTTTTTC--AGTGTATGACTTTCAGATAGTCTTTTTTTC-----TAGT-ATATTGGTTTTCTAGTCACTGGT  39960

Majority                    TCTCAG-GGTTATATAATCTTATTXTGCTGTGTTXCCTTTX-XAGATATCAGTTATCTA----XXXTXATTATTCTTTXX
                                      62650     62660     62670     62680     62690     62700     62710     62720
Human                       TCTCGGGTATAATCTAATCTTACTGTGC--TTTCCGCTTC---AGGCATAGGTTAATAA----TTAGAATTCTTTTTTTT  58124
GuineaPig                   TCTCATT-GTGTGTTACTTTTCTGGTGTAGGAAAACAATTG-AAATTTTTTCTCTTCTT----CAGAAATTAATATTCAC  36584
NorthernAmericanDeerMouse   TTGTTT-GTTTTTGAGACAGGATTTCTCTGTGTAGCCTTTCCTGGAACTCACTCCTGTAGCCCAGGCTGGCCTGCCTTTG  40075
Mouse                       TCTCA--GGTTATATAATCTTATTTTACTATGCTTCCTTTT-CAGACACCAGTTAACTA-------TTAAAATTCTCTCT  39827
ChineseHamsterGHOK1GS       TGTCAG-GGTTATATAATTTTATTTCACTATGCTTTCCTTTGCAGATACCAGTTATCTA-------TTAAAATTCTTTCA  41137
LongTailedChinchilla        TCTCAG--GTGATACAATCTCATTGTGT-GTTTTGGAATT---ATTTTTTGTTTTTCAA----AAATAATTATTCTTCAC  40030
```

```
Majority                    TGTCCCAAXTATTXXXATTCTXXACATXCA-TXCXTCXTXTAGTTGCXAAAATTXAATATTGA-XTGAGGTTTTATX---
                                     62730     62740     62750     62760     62770     62780     62790     62800
Human                       TGTCCAAAAAATGATTATCCTCCTTTCACA-TGC----CCTGGCTACCAAAA-TCAATGTGGT-ATGAGGTTTTAT----  58193
GuineaPig                   ATTCCCTCGTCTCCCAGCTTTGGAAATACA-AATTTTATTTAGTTGCCAAAATTCAGCATAGAAGTCATGGTTTGTATG-  36662
NorthernAmericanDeerMouse   CCTCCCAAGTGCTGGGATTAAAGGCGTGTGCTGCCGCCACTGGCTGGAAAATTTTAATATTTA-TTAAGATACCAAAAAT  40154
Mouse                       TGCCCAAAATACTT--ATTCTTCGCATGCATTACCTCCTGTATTTATAAAGCTTTTATATT-------------------  39886
ChineseHamsterGHOK1GS       TGCCCCAAATAATT--ATTCTTCACATGCCTTGCAACTGAACTTGGGAAAGTTTTTATATTTA-TCGAGTTACCAAAAAC  41214
LongTailedChinchilla        ATTCCCTAGTCTCCCAACTTTGGAAATACA-AACATTATTTAGTTGCCAAAAATCAGTGTGGT-GTGAGGTTTTAT----  40104

Majority                    XAATGTGGTAXAAXXTTAXACTATTGGAX-TGAXTAGAXACTX-XGTTTTGAXGXCATTTT-X-GXCAATGTXATATGTX
                                     62810     62820     62830     62840     62850     62860     62870     62880
Human                       GAATAGGAGACAA--------TCT-------------A------------GAAGTCAT------GGT----TTCTGTGAC  58230
GuineaPig                   ACATTTTTCACAAG-TTAACTTTTTTTTTTTTTTTGTGGAACCGGGGATCAAACTCACTTGCCAGGCAGGCGCTTATGCC  36741
NorthernAmericanDeerMouse   CGATGTGGTATGAAATTATACTATTGGAG-TGACTAGAAACTGTGGTTTTGATGGCATTTTTC-CACAATGTCAGATGTA  40232
Mouse                       -AATGTGGTATAAGATTATACTATTGGAAATGACTAGAGACTT--ATTTTGATAGCATTTT----ACAATGTTAAATGTA  39959
ChineseHamsterGHOK1GS       AAATGTGGTATGAAATTACACTATTGGAAATGATTAGAAACTGTGATTTTGATGGCATTTT-C-TACCATGTCAAATGTA  41292
LongTailedChinchilla        -CATTGTGTACAA--------TCT---------------------G----AAAGTCAT------GGT----TTGTATGAC  40140

Majority                    XTTGTA----AXTGGTXTXCCXTTTXTTAAXGTGTATTAXXAATXTAXXTTTXTTTGTGTXTGTAXXXXTXTCTTTAXTX
                                     62890     62900     62910     62920     62930     62940     62950     62960
Human                       ATT------------TTTCACAACTTCAAACGTTTAAATATAGTCTACATTTTTCTTGGTGTGGAATATTTTCCTTGCTC  58298
GuineaPig                   ACTGAACTAAATCCCTGGCCCACAACTTAACATATTTTAAAATGGTACACATTTCTTGATGTGAAATAGTTTCTTTACTC  36821
NorthernAmericanDeerMouse   TTTGTA----AATGGTATACATTTTCTTGGTGTGTAATAT--ATATTTGTTTGTTTGTGTCTGTGTGTTTCTCTTAATCT  40306
Mouse                       TTTGTA----AGTGGTGTACTTTTTTAAAATGTGTATTTTCTATATCTGTTGGGTTGTGTCTGTATGTATCTCTT-ATCT  40034
ChineseHamsterGHOK1GS       TTTATA----AATGGTATACATTTTCTTGCTGTGTATTAT--AT--TTGTTTGTTTGTGTCTGTATGTATCTCTTTATTT  41364
LongTailedChinchilla        ATT------------TTTCACACCTTACAACATATTTTAAAATGGTACACATTTGTGAATGTGCAATAGTTTCTTTCCTC  40208

Majority                    XTCTGATCCAGAGGAAAAXAX-TTGXCAAAGGAXCAXTGAAXTGGAXTTTGATTAGAAT-------TTATACTACATTTG
                                     62970     62980     62990     63000     63010     63020     63030     63040
Human                       TTCTGACCCCAAAGAAAAGTATTTGTTAGAGGACCAATTAAATGAAATTTGAGTGGGG-------ATTATACTGTGTTTG  58371
GuineaPig                   TTCTGATCGAGAGGAAAAGGACTTGTCAAAAGACCAGTGAAATGGAATTTGATTAGCA-------GTTGTACTATATTTT  36894
NorthernAmericanDeerMouse   GCCTAATCCAGAGGAAAATA--TTGACAAAGGATCGATGAACTGGAGTTTGATTAGAAT-------TTATGCTACATTTG  40377
Mouse                       GTTTGATCCAGAGGAAAGTA--TTGACAAAGGATCAGTGAACTGGAGTTTGTTTAGAAT-------TTATGTTCCATTTG  40105
ChineseHamsterGHOK1GS       GCCTAATGCAGAGGAAAATA--CTGACAAAGGATCAGTGAACTGAAGTTTGATTAAAAT-------TTGTTCTACGTTTG  41435
LongTailedChinchilla        TTCTGATCCAGAGGAAAAGGACTTGTTAAAGGACCAATGAAATGGAATTTGATTGGCATTGTGGAGTTATATTGCATTGG  40288
```

```
Majority                    ----------TTATACXXAATXXGXGAAAAGTTGX------TGTGTGTATCTTGT----CTGAAXTAATCTATAGTTA-C
                                     63050     63060     63070     63080     63090     63100     63110     63120
Human                       AG--------CTATAATTCTTAAGGGGAAAATTGTTTA---TATGTGTATCTTGT----CTGAAGTAATACATAGTTA-C  58435
GuineaPig                   GG-------CTAATACTTATCTGGAGAAAAGTTTA------TGTGTACAACTTGT----GTGAAATAATTTATAGGTA-C  36956
NorthernAmericanDeerMouse   ----------TTACAGAGAA-----AAATAGGTGT------TCTGTATATCTTGT----CTGAAGTAATCTATAGTTAAC  40432
Mouse                       ----------TTAAAGAAAA------AACAGTTGA------TGTATGTATCTTGT----C-AAAATAATCTATAGTTATC  40158
ChineseHamsterGHOK1GS       ----------TAGAACAAAATCAGAAAATAGCTGT------TCTGTGTATCTTGT----CCGAAGTAATCTATAGTTGAC  41495
LongTailedChinchilla        GAGAAATTTTTCATACTGCATTGGGGCTAATTCTACTGCATTGGGGCTAATTCTTACCTGTAGAATAGCTTATGTGTA-C  40367

Majority                    ATTTAATXTTATGXTA-XAAGTACCACAATTGTTT-X--X----------------------------------------
                                     63130     63140     63150     63160     63170     63180     63190     63200
Human                       ATTAAATCTCA---TA-AAAGTACCTCAATTGTGTCAACT----------------------------------------  58471
GuineaPig                   ATTTAATCTTATGATA-AAAATAATGGTACCTCAT---------------------------------------------  36990
NorthernAmericanDeerMouse   ATTTAATATCATGGTA--AAGAACCACAATTGTTTTATGTGTGTGTGTGTGTATGTGTGTGTGTGTGTGTGTGTGTGTGT  40510
Mouse                       ATGTAATATTATGATA--AAGTATCACAATTGTTT---------------------------------------------  40191
ChineseHamsterGHOK1GS       ATTTAATATTATGTTG--AAGAACCACAGTTGTT----------------------------------------------  41527
LongTailedChinchilla        ATTTAATCTTAGTTTATAAAAGGATTATACTTCGTTACATT-------------------------------------AT  40410

Majority                    ---X-XX-----------X-ATXTXTA-TTTACTTGCCTXTXXXCXTATA-ATAAGCATCCAAXTATAXXXXTTTXTGAX
                                     63210     63220     63230     63240     63250     63260     63270     63280
Human                       --GTTTG-----------ACATTTTTG-TTTAATCTTCTGTGAACAGATA-CTAAGCATCCAACTATGA----T-ATTGA  58531
GuineaPig                   --------------------ATTTTCACTTTACTCGCCTGCATGCTTATA-ATGAGCATCAAATTGT------TTGCGAG  37043
NorthernAmericanDeerMouse   GTGTGTGTGTGTGTATATATATATATA-TATACATACATATACATATATACATAAGCATCCAGCCATATACATTTGTGAT  40589
Mouse                       ---------------------TACATA-TTTGTGTG--------GGTGTACATAAGCACCCGATTATATGTGTTTGTGAC  40241
ChineseHamsterGHOK1GS       --------------------ACATATA-TGTACTTT--------TGTGCAGATAAGCATTTGATAATACACACTTATGAT  41578
LongTailedChinchilla        ACATTTG-----------AGGTTTTCATTTTACTAGCCTATGTGCTGATA-ATAAGCATCAAGCTGTAAGTGTTCATGAG  40478

Majority                    TXTTTA-TATAXGCTTA-CTAAXGGTTXXGGTXXTXT-XACAXAAATCTAAAGTXATAATTXTAAGAG-AATAGAXXXTT
                                     63290     63300     63310     63320     63330     63340     63350     63360
Human                       TGTTTG-TGCACAGGTA-TG--C-ACTCAGGTCTTCTTGACAAAGATCTAAAGTTATAATTCTAAGAG-AGTAGATAATT  58605
GuineaPig                   TGTTCACTATACTACTA-CTACTTGTTCAGGTTCTCT--ACAAAAATGAAAAAAT-TAATTCTAAGAG-AACAGATAATT  37118
NorthernAmericanDeerMouse   TATTTA-CATATGCTCAACTAAAGTTTTCACTAAAGAAGATAGAAGTCTAAAGTGAAAATTTTAAAAGTAATACACTTTT  40668
Mouse                       TATTTA-CGTATGCTTG-CTGACGGTTTCTAT-------ACAGAAGTCTAAAGTGATAATTTTAAGAGGAATATACTTTC  40312
ChineseHamsterGHOK1GS       TAT-----ATATGCTCA-CTAAAGTTTTCTGTATAGAAGACAGAAATCTAAAGTGATAAATTTAAGAGTAATATACTTTT  41652
LongTailedChinchilla        TGTTTAATATACAGTTA-TCACTTGTTCAGGTCCTTT--ACAAAGATGTAAAA-T-TAATTCTAAGAT-AATAGATAATT  40552
```

```
Majority                     XTTTTATT-----------------AXGTTTXT----------------ATCCTGGAA----------------------
                                      63370     63380     63390     63400     63410     63420     63430     63440
Human                        GTTTTACT-----------------AAGTACTT----------------ATTCTGGTA----------------------  58630
GuineaPig                    GTTTTATT-----------------ATATTTAT----------------ATCCTAACA----------------------  37143
NorthernAmericanDeerMouse    TTTTTTTTTTTTTTTTTTTGAGACAAGGTTTCTCTGTGTAGCCCTGGCTGTCCTGGAACTCACTCTGTAGACCAGGCTAA  40748
Mouse                        T----------------------------------------------------T--AA----------------------  40316
ChineseHamsterGHOK1GS        TCTTTTT------------GAGACAGGGTTTCTCTGTGTAGCTCTGGCTATCCTGGAACTCACTTTGCAGACCAGGCTGG  41720
LongTailedChinchilla         GTTTTATT-----------------ATATTTAT----------------ATTCTAAGA----------------------  40577

Majority                     -----------XCAGA-AXXCAC---------------------------------------------------------
                                      63450     63460     63470     63480     63490     63500     63510     63520
Human                        -----------TCAGA-ACTCAC---------------------------------------------------------  58641
GuineaPig                    -----------GTTGA-CCTCA----------------------------------------------------------  37153
NorthernAmericanDeerMouse    CCTCAAA-CTCACAGAGATCCACCTTCCTCTGCCTCTAT-------GTGCTAGGATTAAAGGCGTGGGCCACAACTGCCC  40820
Mouse                        --------------G---TCCAC---------------------------------------------------------  40322
ChineseHamsterGHOK1GS        CCTCAAAACTCACAGAGATCCACCTGCCTCTGCCTCCACCTCCCAAGTGCTGGGAATAAAGGCATGCACAACCACT-CCC  41799
LongTailedChinchilla         -----------GTTGA-CCTCA----------------------------------------------------------  40587

Majority                     -----------------------------X-X-XXXXXGCTXAACCTATTAGAGAAGGAAAXGG-TATTTACTTTCCACA
                                      63530     63540     63550     63560     63570     63580     63590     63600
Human                        --------------------------------------ACTTCACTTATTACAGAAGGAAAAAGAAATTTGTTTCCTAAA  58683
GuineaPig                    --------------------------------------GCCTTA--TTTCACTTA-------------CTACTTTCCAGA  37180
NorthernAmericanDeerMouse    AGCTGAGTAATAAACTTTCTTAAATATGCATTCTGGTAGCTGAACCTATTAGAGAAGGAAAAGGGTATTCACTTTCCACA  40900
Mouse                        -----------------------------ATTCTGGTAGCTGAGCCTGTTAGCAAAGGAAATGA-TATTGACTTTCCACA  40372
ChineseHamsterGHOK1GS        AGCTGAGTAATACACTTT---AAGTACATACTATGGTAGCCGAACCTATTAGAGAAGGAAAGGGGTATACACTTTCCACA  41876
LongTailedChinchilla         --------------------------------------ACTTTA--TGTCAGTTA-------------TTACTTTGCAGA  40614

Majority                     C--XTGCXTTGCCCXATTTTCTTGTXATATAGACXACTTGGTAAAACTT------TTGATGATTCTAGCAATCTGCATTC
                                      63610     63620     63630     63640     63650     63660     63670     63680
Human                        C--TTGCTTTGCCCCATTACCTTGTAATCTAGACCCTTTGGTAAAATTTGCATTTTTGATAACTACAGTAATGTTCATTT  58761
GuineaPig                    C--TTGTTCT--CCCATTGACTTGTAATATAGACCACTTGCTAGAACTT------TTGATGATTC-AGTAATCTGCATTC  37249
NorthernAmericanDeerMouse    ---CTAACTTGCCCTATTTTCTTGCCATATAAACAACATGGAAAACCTT------TTGGTGATTCTAGCAATCTACATTC  40971
Mouse                        ATACTTCCTTGTCCTATATTCCTGGCATGTAGACTGCTTTGGCAATGTT------TTGATGAGCATTGCAGTCTGCATTC  40446
ChineseHamsterGHOK1GS        ---CTTACTTACCCTGTTTTCTTGCCATATTAATGACTTGGGAAACCTT------CTGGTGATTCTAGCAGTCAACATCC  41947
LongTailedChinchilla         C--TTGCTCT--CCCATTAACTTGTAATCTAGACTGCTTGGTAGAACTT------CTGATGATTCCAGGAATCTGCATTC  40684
```

```
Majority                      TTCATGCCTTCAXCXATGCTGTACCTAGAGAATATXACATAXTATXATATCATCTGACAATTGGTAACAGTTTAAATTXA
                                      63690     63700     63710     63720     63730     63740     63750     63760
Human                         TACTTGCCTGCATCTGGGCTCTACTTAGAGAATATCACATAATATTACATAATCTGACAATTGGTACCAGTTTAAATCCA  58841
GuineaPig                     TTTATTCCT---AA-ATGCTGTGCCTAGAGAATATCACATAA---------GTCTGACAGTTGGTAACAGCTTAAATTCA  37316
NorthernAmericanDeerMouse     TTCATGCCTTCAA---TGGTGTACGTAGAGAATATTACATACTATTATATCATCTGACAGTTGAAAATGCTTTACATTTT  41048
Mouse                         TTTGTGCCTTTATCAAGGGTGTAACTAGAGAACATTGCTTAGTATCCTATCCTCTGACA------------------TTT  40508
ChineseHamsterGHOK1GS         TTCATGCCTTCAAGGATGATGTACCTAGAGAATATTA-ATACTATCATATCATCTGACAATTGAAAATGCTTTACATTTA  42026
LongTailedChinchilla          TTCATGCCT---TTTATGCTGTGCCTAAGGAATGTCATGTA----------GTCTGACAATCGGTAACAGTTTAAATTCA  40751

Majority                      GTXAXTXTGAXTAXAAAGTAGXCATXC-TTCXAXXXGAAAXA--TCTCTTCAC-ATTXTTTCAGTCAGCAG-TAXTCTGT
                                      63770     63780     63790     63800     63810     63820     63830     63840
Human                         TCAACGTCAACTACAAGTAAGCCCTCTACTCTACTTTAAAAA--TCTCTTCCC-ATTTCTTCAGTCAACAGTTTTTCTGT  58918
GuineaPig                     GGAGCATTGCCTACAAAGTAGCCAACC-TTTGACTTGAAAAA--TTTCTTCA--GTTTTTGCAGTCAGCA--TGTTCTGT  37389
NorthernAmericanDeerMouse     GTTGGTGTGATGAGAAAGTAGTCATTC-AT---GGGGAAAT----CTTTTCAC-ATTCTTTTAGTCAGTAG-TACTTCAT  41118
Mouse                         TTGAGTGTCATCAGAAAGCAGTCATTC-TTCCTGGGGAAATAAATCCCTTCAT-GTTCCTTTTTTAGTCAG-CACTGCTT  40585
ChineseHamsterGHOK1GS         GTTAGTGTGATCAGAAAGTAGTCATTC-TT---GGGGAAAT----CTTTTCAC-ATTCTTT-AGTCAGTAG-TACTCTGT  42095
LongTailedChinchilla          GGCACATTGCCTACAA-GTAACC--CC-CTCCACTTGAAAAA--GTTCTCCACAAGTTTTCAGTCAGCA--TGTTCTGC  40823

Majority                      TTTTC---XXXXACXGTATCAGAXAXAATTXTTTACTXXTACCXXTTCTCXCCCTCATTT-TCXCTTAATTTTAAXXXAA
                                      63850     63860     63870     63880     63890     63900     63910     63920
Human                         TTTTC-ACAATGCCTATGTCAGATAGAAATTTTCCCTTCTACCATATCTCTCC-TCACTC-TCACTTGCTTGAAGAGGAA  58995
GuineaPig                     TTTTTTATAATGACTGTATCAGA---AATTT----CTCCTACCATGTCTTTTC-TCATTT-TTGCTTAATTACAAGAAAG  37460
NorthernAmericanDeerMouse     TTTAC-------ACAGTATCAGAGTTTACACTTTACTG-TGCCTCTCCTCACCCTCGTGTGTCACAGGTTTTTAA---AA  41187
Mouse                         TGTTT-------ACAGAGTTGGCAATGATGCTTTACTC-GGTCTTTTCTCACTCTCATTTGTGGCTTTTTTTTTTTTTAA  40657
ChineseHamsterGHOK1GS         TTTAC-------ACAGTATCAGAGAATATACTTTACT-----CTCTCCTCACTCTAATTTATCAAAAAAAATTAA---AA  42160
LongTailedChinchilla          TTTTC-GTAATGACTGTATCAGA---AATTTTCCCCTTCCACCACATCTTTTCCTCGTGC-TTGCTTAATTCCAGGAAAA  40898

Majority                      TXTCAGATGGAAAXTCXAXTAGTTTX-XTAGCAAAATCTXATATT---TAXACCTGTCTTTCTCATT-------TAXT--
                                      63930     63940     63950     63960     63970     63980     63990     64000
Human                         AATCACATGGAAGCTTCATCAGCTTTTCTTGCAAAATCTGCTAATACTCACACTTGTCTTTCTCTTT-------TAAT--  59066
GuineaPig                     TATCAGATAGAATCTCCATTCATTTT-CTTGCAAAGCCCATTATC-------CAGGTCCTTCTCATT-------TACT--  37523
NorthernAmericanDeerMouse     TGTCAGATGATTATTCTAGTAGTTTC--TAGCAAAATCTGATACT---TACACCTGTCTTTCTAATAAAAGGCATATTTC  41262
Mouse                         AGTCAGATGGGAATTTTAGAAGTCTC--TAGCAAAATCTAATCTG---TATCCCTATGTTTAAGTATACATGCCTTTTTT  40732
ChineseHamsterGHOK1GS         -GTCAAATGGGAATTCTAGTAGTTTC--TAGCAAAATCTGATACT---TATACCTATCTTTCTAA---------------  42219
LongTailedChinchilla          TATCAGATGAAATCTCCATTAGTTTTTCTCATGAAACCTATTATC-------CAGGTCTTTCTCAT--------------  40957
```

```
Majority                    A--AAAGTCATAGATX-TTXATCATTXAAGAACAGATCXATAAAXXTCAXXCCXTXCTXX----CTCXX-CTGXCTTAXX
                                              64010     64020     64030     64040     64050     64060     64070     64080
Human                       A--AAAGGCATAGATGTTTAGTTATCTAA-----GACCAATAAACACCAAAGCCTATCGT----CTT---CTGCCTGTCA  59132
GuineaPig                   A--AAAGTCACAGATCATTTTTCTTTTAA-----GTTCAGTAAGCAACAGATCCCAGATT----CTC---CTGCCTCCTA  37589
NorthernAmericanDeerMouse   AGCACTTTTCTAAA------ATCAGTAAAGAACAGATCCATAGATCTCATTCCTTGCTAC----CTCAGACTGTATTAGT  41332
Mouse                       AAAGAAGGCATATATG-TTCATCAATAAAGAACAGATCCATAAAT-TCATCCTTTGCTTCTTTCCTCAGGCTATCTTAGT  40810
ChineseHamsterGHOK1GS       -------------A------ATCAGAAAAGAACAGATGCATAGATTTCAATCTTTGCTAC----CTTAGACTGTATTAAT  42276
LongTailedChinchilla        ---AAAGTCACAGATTATTTTTCTTTTAATCTAAGCCTAGTAAACCACAGGTCCCATTCT----CTC---CTGCCTCCCA  41027

Majority                    AGAXATGGCAXAATXGTCT-TATCTXCTTTGACTTTTATTTTTXXATTTTXACTXCTT-ATCACCAXXATTTCAAXTATT
                                              64090     64100     64110     64120     64130     64140     64150     64160
Human                       AGAGACTGCACTATCTGTCATCCCCTTTCTCATTTATTTCTCTCTATATTGGCCACTT-TTCACCA--GTTTCAAGCATT  59209
GuineaPig                   AGAGCTGGCACTGTCATCTCTGGTTTCTTTTCTCTTAGTT--TTTCTGTTT--CACTA-ATACT----TTGTGTTTAAGT  37660
NorthernAmericanDeerMouse   AATAATGCCATAATTGTCT-TATCTACTTTGACTTTTATTTTTTCATTTCAACTGCTTAATCACCAGGATTTCAAGTGTG  41411
Mouse                       AGAAATGGCATAATTGTCT-TATCTACTTTGACT--TATTTTTCCATTCTGACTGCTT-ATCACCAGGATTTCTAATATG  40886
ChineseHamsterGHOK1GS       AATAATGGCATAATTGTTT-TATCTACTTTGACTTTTATTTTTCCATTTCAGCTGGT--GTCACCAGGATTTCAAATATT  42353
LongTailedChinchilla        CGAGTTGGCACCATCATCTCTGGTTTCTCTTACTTTCGTTATTTTTCACTTAATACTT-AAAGT----GTATCCATAAAC  41102

Majority                    CTCAGG-TTCTXGTXXATTXACTTCAGATXXTGC------TXAXTCTAXTXCTCTACATGTCTCXCCATCTXX--CCATC
                                              64170     64180     64190     64200     64210     64220     64230     64240
Human                       CTCAGC-TTCTTTCATTTTAAATGAATTTACCTTCAACTTTCAATCCATCCATCTATAACTCTACGTACTTTCTATCACC  59288
GuineaPig                   CTCAGG-TTT----ATCTCATCTCCAGATACTAC------TCCATCTATAACTCTGCAA-TCTTCCCATCTCTTTTTACC  37728
NorthernAmericanDeerMouse   CTTGG--TTCTAGTGAATTTACTTCAGATCTTGC------TGATTCTACTGTTCTACATGTCTCTCTATCCA---CCATC  41480
Mouse                       CGGAAGTTTCTCGTGAATTTACTTCAAATCTTGC------TGATTCT-CTTCTGTACATGTCTCTCCATCTTCCTCCGTC  40959
ChineseHamsterGHOK1GS       CTCGG--TTCTAGTGAATTTACTTCAGATCTTGC------TGATTCTACTGCTGTACATGTCTCTCCATCC----CCATT  42421
LongTailedChinchilla        CTCAGG-TTT----ATCTTAACTCCAGATACCAC------TCCATCTATAACTCTACAG-TCTTCCCATCTCT---TATC  41167

Majority                    CTTTCTXCGXTTCCCTTTTAA---XTATAAXCAGAXTTCTTAAXTAAGCTGTTTXXXXXTTATATCTTT-XXGTCXTXC
                                              64250     64260     64270     64280     64290     64300     64310     64320
Human                       CTGCCTCCCCTCTCCCTTTTCA--CTCATAACCAGACTTCTTAAATAGTCTATCT----TTGTTGT-TT---A--TTATC  59356
GuineaPig                   TTACCTCTGCTTTCCTTTTCAAAATTTTAACTGCATTTCTTAAATAGAATATTT----TTT----------GAAGCTTGC  37795
NorthernAmericanDeerMouse   CTTTCCTCCTGTTTTCCTTTG-------TAATCAGACTTCTTAAGAAAGCTGTTTGCTGCTTATATCTTTTCTGTCCTCC  41553
Mouse                       CTTTCTTCTTCTTGACTTTTAAG---TATAATCAGGTTTCTTAAGCAAGCTTTTTGCTGCTCATATCTTT---GTTCTTC  41033
ChineseHamsterGHOK1GS       CTTTCTTCGTGTTTTCATTTA-------TAATCAGACTTCTTAAGAAAGCTGTTTGCTGCTTATATCTTTTCTGTCCTCC  42494
LongTailedChinchilla        TTAGCTCTGCTTTCCCTTTTAAAATTTATAACCAGATACCTTAAATAAGGTGTCT----TTTTTTTCTTA-AGAGCTTGC  41242
```

```
Majority                   TCATTTCTCTGTCTACTGTAATTTGGTTTCATCCCTGTTTACCCAAATTACXCTTCXAXTGTTXXXAXTGGXCAXTAXAG
                           64330 64340 64350 64360 64370 64380 64390 64400
Human                      TCATTTCTCTACTTACTATTATTTGGTTTTAGCACTGTTGACCCAAATTGCTCTTC------------------------ 59412
GuineaPig                  TTATTTTACTG---TCTGTAATTTCATTTCATCCCTGTATACACAAATTATTCTTC------------------------ 37848
NorthernAmericanDeerMouse  TGATTTCTCTGTCTACTGTAATTTGGTTTCATCCCTGTTTACCCAAACTACCCTTCAACTGTTAGCACTGGTCATTAGAG 41633
Mouse                      TCGTTTCTCTGTGCACCGTAGTTTGGTTTCATCCATAATTCCCTAAACTACCCTTTTAACTGTGATAGTGGGCACTAAAG 41113
ChineseHamsterGHOK1GS      TCATTTCTTTGTCTACTATTACTTGGTTTCATCCCTGCATACCTAAAGTACCCTTCAACTGTTAGCAGTGGTCTTTAGAG 42574
LongTailedChinchilla       TTATTTCTCTAACTTCTGTAATTTCATTTCAGCCCTGTTAACACAGATTATTCTTTTTTTGTCTTTTTGAGACAGGGTCT 41322

Majority                   TAGT--------AGTAGTXXXTTTCAGTXTTXAXCTTGCT-TAAXCT--XXGTAXTACTXGAXXCTGTGA--CCCCAC-T
                           64410 64420 64430 64440 64450 64460 64470 64480
Human                      -CTC---------------------------------------------------------------------------- 59415
GuineaPig                  -------------------------------------------------------------------------------- 37848
NorthernAmericanDeerMouse  TAGTTCAGTTTCAGTAGTAGTTTTCAGTCTTTGTCTTGCT-TAACCTTTAGGTAATACTGTATACTGTGAA-CCCCACCT 41711
Mouse                      TTGG--------AGTAGTCAGTTTCAGTTTTAAACTTGCTGTAATCT----GTACTATTTGACACTCTGA--CCCCACTT 41179
ChineseHamsterGHOK1GS      TAGTTCAATTTCAGTAGTAGTTTTCAGTGGTTAACTTGCT-TAACCTGTAGGTAATACTTGACTCGGTGAA-CCCCAC-T 42651
LongTailedChinchilla       CGCT---A----TGCAGTTCAGGCTGGCCTTGAGCTCACTGTGTAGCCCAGGTTGGTCTCGAACTTGCAATGATCCTCCT 41395

Majority                   XXCTXAGXTTTTX-------CCXXCXGXTXTCTGACACTTXXX-XXXAGXTTTC----TCCTXXXXTTXCAAXTGTCAXX
                           64490 64500 64510 64520 64530 64540 64550 64560
Human                      -----------------------------------------------------------------------AGCGTCACC 59424
GuineaPig                  ---------------------------------------------------------------------AGAATGTCACC 37859
NorthernAmericanDeerMouse  TTCTTAGTTTTTTGTTTTTTCCCTCTGGTATCTGACACTTCTTACCTAGGTTTC----TCCTAACTTTCCAACTCTCTTT 41787
Mouse                      TGTTAAGGTTTTC-------CCTCCTACTTTCTGATACTTTCC-CTTAGATTTCACCTTCCTTTCTTTTCAATTACCCTT 41251
ChineseHamsterGHOK1GS      CTCTCTGTTTTTT-----TTCCTCCGGGTCTCTGACTCTTTTTATGCAGATTTC----TCCTCCTCCTCCAACTGTCTTT 42722
LongTailedChinchilla       GCCTCAGCCTCCAGAG----TACTGAGATTACAGGCATGCGCC-ACCATGCCCGGCCAGATGATTCTTTAGAACATCACC 41470

Majority                   AXCGATT--XXXXXXCAAXTXCTXXXTGXCAGXCGT-XATTXXTACXTCTTAGTXXTACTTAATXCTTAXGTTAACTTXX
                           64570 64580 64590 64600 64610 64620 64630 64640
Human                      AGCGATT----AGTAGAGTAGCTAAATGCTAGTGAT--ATGATTTAGTCCTTGTCTTACTTAATACCTAGATAATATTTG 59498
GuineaPig                  AGTGAT---------AAATAGTTAAATGCTAGTAGT--ATTATTA-GTCTTAACTTTACTTGATATATA----ACATTTG 37923
NorthernAmericanDeerMouse  TCCCCTTCCCATACCCCACTTCTGTTTGGCAGACGTCTACTCACACAACTTAGTCATAT--AATGCTTACATTAACTTCT 41865
Mouse                      CCCCTTT--CACATTCTGCTTGTGTTTGGCAGACTT-TATTCATACAACTTAGTTATCCTTAGTGCTTTTGTTAACTTCT 41328
ChineseHamsterGHOK1GS      TCCTATTTCCATGCTCCACTTCTGTTTGGCAGACTTGTACTCACACAGCTTACTTACATT-AGTGCTTATGTTAATTTCT 42801
LongTailedChinchilla       AGTGAT---------GAATTGTTAAATGCCAGTAGT--ATCATTTCGTCTTAACCTTACTTAACATGTAGGTCACCTTTG 41539
```

```
Majority                     GXAXXXXAGAACXTTAT------XTCAAXAXTXTXATTXXAGCXAGTCXTTXGACACTTXXXAXCT-AGATTXXTCXTAC
                                         64650     64660     64670     64680     64690     64700     64710     64720
Human                        GCACTGTGCACCATTGTC------TTAAAATTCTCTTTCTTCCTGGTCT-CTGATACTTCTCACCT-GGATTTATCCTAC  59570
GuineaPig                    ACACTGTGTAGTACTCT-------TCAAAATTTTCATT-----TTATTTCATGACATTTCTCA-------TTTATCCTGC  37984
NorthernAmericanDeerMouse    GTAGCTCAGAACTTCA-----AGACCCAGACTAGGACTCTAGCCAGTCATTGTATACTTTCAAACTTAGAATGTTC--AG  41938
Mouse                        CTAGCTCAGAACTTTACTAAAAGACCCAGACTAGGAATCTAGCCAATCATTGTACACTTTCAAACTTAGACTGTTCTCCT  41408
ChineseHamsterGHOK1GS        CTAACTCAGGACTTTA-----CTAAA-AGACCCAGACT--AGCCATTAATTGCACACTCTCAA--------TGTTC--CA  42863
LongTailedChinchilla         GCATTGTATAGTACTCT-------TAAAAATTTTCATT-----TGGTCT-GTGACATTTCTCACCT-AGATTTATCTTAC  41605

Majority                     TXCAAXXTTTXCTTCCTTGTXTTXC-TGTGTCTCXTAGTACXXXXGAAGCCXXXCCTXCTCTC-XTAA-CAXGXCAG---
                                         64730     64740     64750     64760     64770     64780     64790     64800
Human                        CTCTCTA-TTTCTTCCTTGTCTTG--TAACTCCCCTTCTACCTGGGAGGCCTCACCTGCTGT----CA-CAGCTCCA---  59639
GuineaPig                    TTTGAGGGTTTCTTACTTGCTACA--TTCTTCCTCTATTTTCTGGGAAATCTCGCCTACTCCC--CAG-CAGTTGAG---  38056
NorthernAmericanDeerMouse    TGCAGCTTCTGATTCCTTGTATTCCCTGTGTCTCATCGCAC--AAGATGCCAGCATTGTTCTCATTAA-CAAGCC-----  42010
Mouse                        TGCAACTTGTGGTTCCTAGTGTTGC-TGTGTCTCATAGTGT--AATAAGCCAGTGCCACTGTTCTTAA-CAAGCCAGAAA  41484
ChineseHamsterGHOK1GS        TGCAATCTCTGGTTCCTAGTATTCCCTGTGTCTCAAAGGAC--AAGATGCCAGCATTGTTTTCATTAAACAGGCCAGAAA  42941
LongTailedChinchilla         TTTGAGGGTTTCTTCCTTGCTACACATGCTTCTCCTACTACCTGGGAAGCCTCACCTACTCT----AA-CAATTGAG---  41677

Majority                     --TTAXACTTATTXXT------X-----XXTGTTXTCAG-CACTTXTTCTGXCTCX-GAXCXC----------TTCAXXX
                                         64810     64820     64830     64840     64850     64860     64870     64880
Human                        --TTAAACCTCTTATGAT----TCAGACAGAGTTCACAT-CACATTTTCTAGCTCA-GACCTCTCCTAAGAGACCCAC--  59709
GuineaPig                    --TTATACCT------------------AGTGTTCTTAC-CACTTTTTTGGACTA-------------------------  38090
NorthernAmericanDeerMouse    --TCATAAAAATTTCT-------------CAATTTT----TGTTCAGTGTGTC---------C----------TTCAACT  42052
Mouse                        ACTTGGAGTCATTACTCCTTCCTTCCATCCTGATTTCAGACATTGATTCGGATTCCTGAGTTCCTGTTTGTCCTTCAGCT  41564
ChineseHamsterGHOK1GS        ACTTGGAGTTATTTTTTC---CTGCCCTCCTGTTTCCAGATACTCATTCTGTTTCCTGAACTCCTG-------TTGAACT  43011
LongTailedChinchilla         --TTACACTT------------------GGTGTTCTTAG-CACTTTTTGTAGCTCA-GACC--------------CAG--  41719

Majority                     GXTXCGAXCCTAGTTTAAAXAXGGATAXCTXXAAATXXTTT---------------X---GTTXTTTTTAXCTTXTGTTX
                                         64890     64900     64910     64920     64930     64940     64950     64960
Human                        ACTAAGAACCTGGCGGACTAATGAATATCTTCAAACAGTGT-------------------GTTCAATCTAGCTCCTGTT-  59769
GuineaPig                    ---GGGAGTCCAGTAGCACATTGGTCACCTTCAGAAT-TAG-------------------GTTCTGTTCAGCTTCTGTT-  38146
NorthernAmericanDeerMouse    GTTTCCATCCTAGTTTAAACAGGTGTAGCTCAAAATCCTTT-------------TTTCTGTTTTTTTTTAACTTATACTA  42119
Mouse                        GTTACAACCCTATTTTGAACAGGTATTACTCAAAAAGTTTT---------------TCTGATTTTTTTTAACTTATGTCA  41629
ChineseHamsterGHOK1GS        GATTCTAACCTAGTTTAAACAGGGATTGTTCAAAATGCTTTGTTGTCTGTTTTGTTTTGTTTTTTTTTTAATTTATGCCA  43091
LongTailedChinchilla         ACCGGGAGTTCAGTAAAACATTGGACATCTTCAAATT-TAG-------------------GTTCAGTGCAGCTTCTGTT-  41778
```

```
Majority                     -TXTG-XAXATTAC---TXTGTAGCTXTAGTTCAA-------XGATXCXAATX---TGTTXGTCTCATTAACAAACAAGT
                                  64970     64980     64990     65000     65010     65020     65030     65040
Human                        -TTTG-GTGTCCGC---TGTGTTAGGAAAGTGCAA--------GATACCAA-----CTTCAGTCTCATTAACAAGCCAGT  59831
GuineaPig                    -TCTG-GCGATTCT---TGTGC-ATCTCCATACAA--------GGTACCAA-----TGTCCGTCTCATTGTCA-------  38200
NorthernAmericanDeerMouse    ATGTGCAAAATCA----TTTGTAGCTATAGTTTAC------TTAGTGCTGATG---T----------TTAA-AAACAAGT  42175
Mouse                        GTATA-AAAATAACATATTTGTAGCTATAGTTTACTTAGTGCTGATGCTAATAGAATGTTTTGTGTGTTTACAAACAAGC  41708
ChineseHamsterGHOK1GS        ATGTGCAAAATTACATATTTGTAGCTTTAGTTAGTG-----TTGATGCAGATAGAATGTTTTGTGTGTTTA-AAACAAGT  43165
LongTailedChinchilla         -TCTA-GAGTTTCT---TGTG---TCTCCATGCAA--------GTTACAAAGT---CAGTCGTCTCATCAGTAGGCCAGA  41839

Majority                     A-ACXTGXTATXTTTTCTCTTTCTTXTXTTXXTTAXACATATAGTCTTCATTAXAXCXXXTXAATXXCTGTXTAXAATXT
                                  65050     65060     65070     65080     65090     65100     65110     65120
Human                        AAACATGGTATTTTTCCCTTTCCTTCCAACCTGTCATCAGATATTATTGATTATACCTCCTAAATTTTTGT-TG-AATCT  59909
GuineaPig                    --ACATGAAATTATTTTTCTTTCTTCTA-----CTGAGTATTTGTTCTGA------CTCCTAAATTGCT-----------  38256
NorthernAmericanDeerMouse    A-AAGGCATATATTTTCTCT--CTGGTTTTGATTATACGTACAGCCTTCATTACAGCATTTTCATACCTGTATATAATGT  42252
Mouse                        A-ATGCTGTATATTTTCTCTATTTAGTTTTAGTTACAGATATAGGCTTCATTATATTATTT--ATACCTGTATATAATAT  41785
ChineseHamsterGHOK1GS        A-ATGCTGTATAATTTCTCT--CTGGTTTTAATTTTACATATTGCCTTCATTACAGCATTTTCATATCTGTATATAATGT  43242
LongTailedChinchilla         AAACATGAAACTGTTACTCTTTCTTCTA-----TAATCAAATATTCTTAG------CTCCTGAATTGCTGT-TG-AATAT  41906

Majority                     ATTTTXXTXXXACCCACCCTTTCTTACTACCCXATCXTGXTCXXCTATTATTCXXGXTTAXXCXTXTCTTX---------
                                  65130     65140     65150     65160     65170     65180     65190     65200
Human                        GGCCT-TTTTTCTCCAACTCTTGCTACCATTGCAGTCTAAGCCAGTGTTATTCAAGA-TGCTGGTCTCTC----------  59977
GuineaPig                    ------TTTTGACCTTCGTTCTCTTGCTTC--CATCCTGAACAGGTATTATTAAAAATT-CTGGTCTCTT----------  38317
NorthernAmericanDeerMouse    ATTTTAATCATACTCACCCATTCTTATTACCCTTTCTTGTTCCTCTATCATTCCTGTTTATCCCTTTCTACTTC-ATGTC  42331
Mouse                        ATTTTAATCACAGCCATGCTTATTAATTATCCTTTCTTGTTCTCCTACAATTCCTGTTGATCCCTTTCCAG---------  41856
ChineseHamsterGHOK1GS        ATTTTAATCACATGCACACATTCTTA---CCCTATCTTGTTCTCCTACCATTCCTGTTGATCCCTTTTCTCTTCCATAGT  43319
LongTailedChinchilla         GTCCT-TTTTGACTCTACCTCTCTTGCTGC--CATCCTGAACAGCTATTATTAAAAAATACTAGCCTCTTT---------  41974

Majority                     --------------------------------------------------------------------------GXGACA
                                  65210     65220     65230     65240     65250     65260     65270     65280
Human                        --------------------------------------------------------------------------AAGGTA  59983
GuineaPig                    --------------------------------------------------------------------------GAGACT  38323
NorthernAmericanDeerMouse    AATCTTCTTG----------T-------GTGTGTGCATTCAAGAGCATGTATGTTCCATGTGTGAGTAT-ATTATGGTCA  42393
Mouse                        ---------------------------------------------------------------------------GAATA  41861
ChineseHamsterGHOK1GS        AGTCCCCTTTCTACTTCCAGTCAGTCTTCTGTGTGCATGTGTGTGCACATATTTATCATGTATGTGTATCATTAGGATCA  43399
LongTailedChinchilla         --------------------------------------------------------------------------GAGACT  41980
```

Majority	TTTAXGXGAXTGTGT-XAAAGXGTAA-T---XXCAGCATAXTTCTTAGXTXCTTTXTG-TTATTGTGAATA--GAXACAA	
	65290 65300 65310 65320 65330 65340 65350 65360	
Human	GTAGTGTG----TGT-CAAACTGAAA-T-----CAGCATACTTCT-AGCAACAGTATGCTTAATGTCAATATTGGCATAA	60051
GuineaPig	TTTATGTGGTTGTGC-CAAATTATAT---------GCATATTTTTTAGCTGTGATATG-TCAGTGTTGATTCTGATGCAA	38392
NorthernAmericanDeerMouse	CTTACAGGAGTGTGTGTGAAGGGTTATTTACAGTAACAAGGGCCTTAATTTCTTTTCTAGT-TTGTGAACA--GACACAA	42470
Mouse	TTTGAGGGATTAT---TACAGG---------AGCAACAACAGTCTTAGTTAGTTTTTCATTGTTATGAACA--GATACCA	41927
ChineseHamsterGHOK1GS	CTTACAGGAGTGTGTGTGAAGGGTTATTTACAGGAGCAGGGATCTTATTTTCTTTTTC----TTGTGAATA--GACACCA	43473
LongTailedChinchilla	TTAAAGTGGCTGTTT-CACATTACAAAT-----CAGCATATTTCT-AGCTGTAATATG-TCACTGTTGATGGCGATACAA	42052

Majority	ATXCTAAXXCAACTTXCXXAXXXCXTTAXXGAXTXCCTTGAGXTCCTGGXXXXTCXAXXAGX-X-CXTGAXCATCA----	
	65370 65380 65390 65400 65410 65420 65430 65440	
Human	ATTTTGACACAGACTTCTTATATTCATGCAGAGTAGCCTGGGCTTCTGGACCGTCAATCATA--ATTTAAGTAGCA----	60125
GuineaPig	ATTCTGACACAAGATC-TTAT--CCAT-TAGAGTGAGGTAGGCTGCTGG-TGGTC----------CACGAACAACC----	38453
NorthernAmericanDeerMouse	TGACCAGGGCAACTTGTAGAAGACTTTACTGAAAACTTTTAGTTCCAGAGGATTAGCGTAGAGTCCGTGACCATCATGGC	42550
Mouse	CAACGAAGGCAACTTATAGAAAACTTTATTGGAGGCCTT--ATTCTTACCCTTTCCAACAG--T---TGGGAATCA----	41996
ChineseHamsterGHOK1GS	TGATCAGGGCAACTTACAGAAGTCTTTATTGAAGACTTAGAGTTCTAGAGGATTCGAGTAGAGTCCATGGCCATCATGGT	43553
LongTailedChinchilla	ATTCTCACACAAAGTCCTTAT--CCGTGCAGGGTGACCTCAGCTGCTGG-TGGTC----------CTTGAACAGCC----	42115

Majority	----AXCATGCAGCAATGAXXXXA----X--XATXXGGGCTTCA--CTGATCT--XTGACTAAAXTX-CCATTTGXACAT	
	65450 65460 65470 65480 65490 65500 65510 65520	
Human	----TTGATGTAGTAATAATTCTC--TTTCATATCTCCACTTCC--TTGGCCTT-CTGACTGAACTCACCATTTTGACAT	60196
GuineaPig	----ATTTTG-AACAA----------------GTCTGTACTTCA--TGGATCTT-CTGACTAAACTTTCCACTCTGACGT	38509
NorthernAmericanDeerMouse	AGGGAGCATGCAGCAAGGAAGGCAGGTGTGATACTGGGGCACAAAGCAGAGAGAGTTAACTGGGAATGGCATGTGCTTTT	42630
Mouse	----AGCATTCAAACATGAGCCTA----------TGGGGT------CCGA-----TTTACCAC-ACT---ATGTGCCTAC	42047
ChineseHamsterGHOK1GS	CCGGAGCATGCAGTAGTGAGGGCA----TGGTGCTGGGGC-TGTAGCTGAAAC--TTACATATGATC--CATAAGCACAA	43624
LongTailedChinchilla	----ATTTTG-AGCAA----------------ATCTGTGCTTCA--CTGGTCT--CTGACTAAACTCCCCACTCAGACAG	42170

Majority	---A------------------------------------X----TCCCCCAXXGCCATTATXTXTCCACAXAGTACTAA	
	65530 65540 65550 65560 65570 65580 65590 65600	
Human	---CT--TCCCCTACCTCCATTATTTTTCCACAGAGTACTAG	60233
GuineaPig	---TG--TCCCTTACCACTGTTCTTTATCCACAAAGTACTAA	38546
NorthernAmericanDeerMouse	GAAACAAAACATGAACCTGTGAGGGC-C---CTTCTCAAACCA---CCACCGGGGGCAACTTCAGTACCTACACTACTGA	42703
Mouse	---A------------------------------------CT-------CCTGAGGAAATGCCTCTCCCTA-GTTATCAT	42080
ChineseHamsterGHOK1GS	AGCAAAGAGTGTTAACTGGGAATGGCATGTGCTTTTGAAACAAAGCCCACCCGTGACA-CACCTCTTCTGACAAGAGAAC	43703
LongTailedChinchilla	---C---TTCCTCACTGCTGTTATTTATCCACAAAGTACTAA	42206

```
Majority                     XAATGA-TCTCAXXATAAATXAACAXTACXTCXAXTCTCAAXXTTCT--TTAXTGAXXTXXTTGXAGGXXCA-XXX-ATA
                                       65610     65620     65630     65640     65650     65660     65670     65680
Human                        GAATCA-TCTCAACGTCAAGTCATATTGCTTCTAATCACAATACCCT--TTAGTGACTTTTTTGTCCTTGCA-----ATA  60305
GuineaPig                    GAATGA-TCTCAGTTTAAAGCAACAGTACTTCCATTCTCAAC-------TCAGTGATTTACTT----------------  38601
NorthernAmericanDeerMouse    AGATGTCTTTTCTTAGTAATCATAAATGCCTGTAGATTCTACTTTCTCTTTGATAGAATGTTGGCAGGTTCAATCTCATA  42783
Mouse                        AAGTG------CCCATAGATTCTCACTACCC-----TCTTTTTTTTCTGGTTAATGGAATGTCGGCAGGCCTAGTCTTGTA  42149
ChineseHamsterGHOK1GS        ACCTAATTCTTTTCAACAGTTTCCACCAACTAGGAACCAAGTATTCA----AATGTGAGCCTATGAGGGCCATTCTCATT  43779
LongTailedChinchilla         GAATGA-TCTCAGTTTAAAACAACATTACTTCCATTCTCAAC-------TCAGTGACTGACTTTTATTCTTA-----GAA  42273

Majority                     XAGATC---CACAGXT--TATTACAGXTXXTAAGXAXCTA--------A-X-----XTCATTCCC------CAGTTTGCA
                                       65690     65700     65710     65720     65730     65740     65750     65760
Human                        -AAGTC---CACAGTCCTTATCATGGCTTATAAGACTCTA--------A-------ATCAGTCCT------CAGCTTGCC  60360
GuineaPig                    --------------------------------------------------------------------------------  38601
NorthernAmericanDeerMouse    CAGATCTTGTGCAAAT--AATTGCAGATACTGAGTGCTCA-ATAATGGAAGGCCATGCCATGCCCAGAAGACAGTTTTCA  42860
Mouse                        TAGATCTTGCACAGGT--AATTACAGATGCTAAGCACTCA--------A-GA----GTCATGCCCAGAAGACAGTTTGAA  42214
ChineseHamsterGHOK1GS        GAAACC----ACCATC--TGGAGCAACTTACCAGTATCTATGCTACTGAAGAAAATACCACTTCC------TAGTAATCA  43847
LongTailedChinchilla         TAGGTC---CACAGGTCGT-TGATAGGGATTAAGAAACTA--------T-------CTCATTCTT------TAACTTGCT  42328

Majority                     TXX--XTCTACCCCTTTCXCXXGC-CTTXTTTGCTGTXATGX-CT-T----ATTTXAGTAXCTXGXATAXXGCTGTXXTT
                                       65770     65780     65790     65800     65810     65820     65830     65840
Human                        T----CTCCATTCTCCTCTCATGC-CTTTTTAGCTATATTGGACT-T----CTTTAAATAGCTTGCATAT-ACTGTATTT  60429
GuineaPig                    ----------------------------------------GAGCT-T----ATTTAAGTAGCTTGCGTAT-GCTGAGGCT  38635
NorthernAmericanDeerMouse    TAACACTCTACCCCTTCAACTGGCTCTTATATCTTGTGATGGTCCCTGAGCATTGGAGAGAATGATACAGTTTTGTTATT  42940
Mouse                        TAACATTCTACCCCTTTCCTTGGC-TCTCTCTGCTGTGATC---CCTCAGCCTTTGAGAGAGTGATACAGTTCTTTTCTT  42290
ChineseHamsterGHOK1GS        TAAGTGCCTGTAGATTCTCACTGC-CTTCTTTCTTTCTATG-----------TCAGTGTCAGTGGTATTCTGCTAAAATC  43915
LongTailedChinchilla         T----TTCTACCTCTATCTC----------TAGCTGTATTGACCT-T----ACTGAAGTAGCTTGCATA--GCTGTGGTT  42387

Majority                     TXTT---TGAGCTCTTGTGCTTXCAG----XATTGCT--TTTCCAXTTGXTGATACTXXTTT----------TXXTCTTX
                                       65850     65860     65870     65880     65890     65900     65910     65920
Human                        TCTC---TGATGTCTCGATCTTACAG-----ATTGCT--GTTCCATTTGCTAATACTATTTT----------TTCTCTTC  60489
GuineaPig                    TCTT---TTG------AGGCTTCCAA-----TTTGCT--ATTGCATTTGTTAATACTGTGTT----------TCCTCTTC  38689
NorthernAmericanDeerMouse    TAAT-GCTGAGCACTTGTGTGTGCAG---TAA-TGCTCCTTACTGCCAAAAGAAGCTTCTTTAAGCCATAGGTGACAGCA  43015
Mouse                        AGAA--CTGAGCACTTGTGTCTCTGTAGTTAACTACTGCTTGCTGCCAGAAGAAGCTTCTC-----------TAAC--TA  42355
ChineseHamsterGHOK1GS        CACCAGTCAAGATCTTATGCTGTTCC---CAA-TACT--TTGCCACTGACTGA--CTACAT---------GATGATTCCA  43978
LongTailedChinchilla         TTTT---TGATCTTCCGGGCTTTCGG-----CTTGCT--ATTCCATTTC---GTACTGTGTT----------TTCTCTTC  42444
```

```
Majority                      ATCTXXTTAGGTCCXAC---------------------T-TCTGATTATCTTXXTCTTTTAA----X--XGAATCXGTGX
                              65930 65940 65950 65960 65970 65980 65990 66000
Human                         ATCTCTTTATGCCCAAC---------------------T-TCTGCTTCTCTTTGTCTTTCAA--------GTATCAGTTT  60539
GuineaPig                     ATCTCTT---GTCCAAC---------------------T-TCTGGTTATCTTCCTCTTTTAA--------AGATCAG---  38733
NorthernAmericanDeerMouse     AAATACTTAGGTGACATTAAAAAATACTTAGAAGACAGTTAGAGAATAGCATGTCCATTTGGTACTGACAGAATTTTTGA  43095
Mouse                         AGCCAG--AGGTGGCAA--------------------GTTCAAGAACAGCACGGTCGTTTAAGACTGGTAGACTATGTGA  42413
ChineseHamsterGHOK1GS         ATTTAGTCAGATTACTT----ATGTTCCTA-ATGTCCTTCAGTGACTACCTTTTGTCTTTAG----GATAAAATCTGTAC  44049
LongTailedChinchilla          ATCTCCTTAGGTCCAAC---------------------T-TCTGGTTATCTTCCTCTTTTAA--------GTATCAGTGT  42494

Majority                      -XACAAXT-CXCTTTATG-AGAXTGATCTGX----CTTXTCCTTTC-----AXCTAGATTAGAATCXTTCTGTTCTTAGT
                              66010 66020 66030 66040 66050 66060 66070 66080
Human                         --AGAAGC-CTCTTCCTG-AGATAAGTCTTC----TGTAACATTTC-----AACTAGATTAGATCCCTTCCTTTCTTAGT  60606
GuineaPig                     -------------------------ATCTCT----CTTACCCTTTC-----AACTAGATTAAAATC--TTTGTGATTATT  38777
NorthernAmericanDeerMouse     -CACAAATTCATTTTATGCAGAATGAACTGAGCTACTGGTCCTTGACCAAAAGTCAGAGTAGCATTGGTCTGGTCTAAGC  43174
Mouse                         -CACAAAT-CATTTCATGCAGAATGAGCTG-----CTTGTCCTTTCCCAGCAGC--GCATAGAAGTTTTCTGATCTGAGC  42484
ChineseHamsterGHOK1GS         TCATTAGTGTGCTTTAGG-AGATTAAATTGA-CTGCTTGTTTCTCA-----AGTCTGATTCCCTCCTTTTTCATCTATGT  44122
LongTailedChinchilla          --ACACTT-CTCTTTTC------AGATCTCC----CTTACCCTTTC-----AACTACATTAAAATC--TCTGTGATTATT  42554

Majority                      TATCAXXACXTCAXX--------X----AAXATCTXTATTTT-TTAGACTTGTAGGTTTTCTGCAG-GXCTXXXXXXTTT
                              66090 66100 66110 66120 66130 66140 66150 66160
Human                         TCTCAGTATCACACA--------T----AAAATTTATAATTTGTTAAATGTGTATATTTTCTGTCG-GACTGTAAGTTCT  60673
GuineaPig                     TGTTA-----------------------AGTGTCTATATTTT-TTAGAC-TATAAGTTACACGAAA-GG-----------  38820
NorthernAmericanDeerMouse     CATCAAGACATCATC-------CTCTTGGAAATCTCTACTT---CAGTGTCGGTGGTTTTCTGCTGAAACCCACCAGTTC  43244
Mouse                         CATCAGGACATCATCTGAAGGTACTTGGCTGAAATCCATTCCTTCAGGATCTTAGGTTGTTCCCAGTGGGTTTTTTACTT  42564
ChineseHamsterGHOK1GS         TAGACTTACTTAAAT-------ACTGTACAGATATGCATTTTT-TGATCTTGGAGGTTTTCAACC---ACTTAATAATTT  44191
LongTailedChinchilla          TGTTA-----------------------AGTGTCTGTATTTT-TTAGAC-TATAGGTTATATAAAA-GG-----------  42597

Majority                      GAGTACCATTTXTGCTTTXTTTTTTGXTGCACTT-----TTTCAXXXTTGACATTTXGXTTGXTAATCAGAXGT--XTTA
                              66170 66180 66190 66200 66210 66220 66230 66240
Human                         GAGTACCATGTCTGCTTTGCTTTCTGCTGTATTT-----ATTAAACATTGACATTCAGGGTGAAAATCAGAAGT--CATA  60746
GuineaPig                     GAATACCATCTTTACTTTGTTCTTTGATGCAGTT-----TTTGAGTGTTGACATTTAGA-TGTTAATCAGAATT--ATTA  38892
NorthernAmericanDeerMouse     AAGATCGTATGCCGTTCTATTTTTTCACAGAC--------TACATGATGAATCAATTACTTACACTCCTAATGTCCTTCA  43316
Mouse                         CGGTTTTGTTGTTTGCTTATTTTTCAGACTACATGATGATTTCAACTTAGACACACTACTTGCTTTCTCGGTGTCCTTCA  42644
ChineseHamsterGHOK1GS         -----------TCTCTTCATCTTTTATACCAC--------TTCA-ACTGGGTTTTTTGTTTGTTTGTTTGTTTTAACCAA  44251
LongTailedChinchilla          GAATACCATTTCTGCTTTGTTCTTTGCTGCAGTT-----TTTGAGTGTTGACATTTAGGGTGATAATCAGAAC---ATTA  42669
```

```
Majority                      GTAACAATCT-TCATTATCATTAAGAXXAA--CTTXAGTXATCATCATG-TTTXTG--AXAXXAAAXACCXTX--XTATX
                                       66250     66260     66270     66280     66290     66300     66310     66320
Human                         GTAACAATCT-TAATTATCAATAAAAAGTA-TTTTATGCGATCCTCATA-TTTATT--AAAGAAAACACCTTA--ATACC  60819
GuineaPig                     GTAACAATTT-TGATTATCAGAAAG---------TAAATGATCGTCATG-TTTGTG--AAAGAAAACACCCTG--ATACC  38957
NorthernAmericanDeerMouse     GTGACTGACTACCTTTGTCCTTAGGATAAAGCCTGTAGTCATTAGCATGGTTTAGG--AGATTAAATGGCCTC--TTATT  43392
Mouse                         GTGACTATCT---TTTGTCCTTAGGATAAAGTCTGTAGTCATCAGCATGATTTAGG--AGATTAAATGTTTGC--TTGTT  42717
ChineseHamsterGHOK1GS         ATTCCAGCCTAGTATTTTCTTCGTGGAGAA--CTATTTTCAT-AATGTACTTTGAATTAGATTATATCTTTATGATTATT  44328
LongTailedChinchilla          GTAACAATTC-TCATTATCAAAAAG---------TAAATGATTCTCATG-TTTGTG--AAAGAAAACACCCTG--ATATC  42734

Majority                      TXTTAGAAXAXCTTTC-----XTXATTTXTATTGGCG---XXCA-XTXTATCXXCTATATAGATG-TGXTTTCTXTGXGC
                                       66330     66340     66350     66360     66370     66380     66390     66400
Human                         TTTCAGAAAATCTTGT------TTATTT--ATTGGAA---AGTA-GTGTATTAGGCAAA--GATG-CGTCTTCTCTTGGC  60884
GuineaPig                     TTTTAGAAAATCTTAC------TGATAT----TGGCT---TGGA-GTGTATCAGTTATATAAAGA-TATTTTTCTTCAGT  39022
NorthernAmericanDeerMouse     TCTCAGGTCTCTTTTC----CTACTTTTTTATCTATGTTAGACT-CTTTAAATACTGTGTAGGTG-TGCATTCTCTGATC  43466
Mouse                         TCTTAAGTCTCATTTCATGACTCCCTTTTCATTTGCGTCAAACTTCTTTAAATACTTAATAGGTG-TGCTTTCTGTGATC  42796
ChineseHamsterGHOK1GS         TCTTAGAGCACTGTTT----TTCTGTTAGTATGAGTG---GATA-CTATGTCTACTTTTTCTTTGCTGCATTTAATGGGG  44400
LongTailedChinchilla          TTTTAGAAAATCTTAC------TGATATCTATTGGCT---TGCA-GTATATCAGGCATATAAAGA-TGTTTTCCTTTGGC  42803

Majority                      TTTXTAATTXACCTTTTXTTXTTXXATTXXTTAX-A--XXXAATAATTGTT-----A-CXT-AAATATAXXXATTTGTTT
                                       66410     66420     66430     66440     66450     66460     66470     66480
Human                         TTTCTAATTCAGCTTTTAT----------------------AATG-TTG----------AG-GAATATAATTACTTGAT-  60929
GuineaPig                     TTTCTAATCCACCTTTTTT----------------------AATG-TTGGT-----A-CAT-AGAAATACCCATCTGTTC  39072
NorthernAmericanDeerMouse     TTGGAGGCTTACAGT-TGCTATTCCGTTTGCTAATACTTCTTTTAATCTTTTGCACAACTTCAACTTTTTTTGTTTGTTT  43545
Mouse                         TTTGAGGCTTACACTGTGCTGTTTCATTGGTTAGGA-CGGCTTTCCTCTTC-----A-CTT--ACTTTTACTTTTTCCTT  42867
ChineseHamsterGHOK1GS         ATTGCTATTTAAGGT-GATAAACTGAAGACTTAACAACAAACCTAATTATT---ATAAAGTAAATGGTCTTCATAACTTT  44476
LongTailedChinchilla          TTTCTAATGCACCTTTTTTTTTTATATTTTTGAGAA--AAAAATAATTGGT-----A-CGT-AGAAATAGCCACCTATTT  42874

Majority                      TTAXXAAXTTXCAGXTTXXAXAXTXXXXCAXGXAXXAXXCT----ACXTTG-ACTTTX-XTT-------XX-X-X----X
                                       66490     66500     66510     66520     66530     66540     66550     66560
Human                         --------------------------------------------------------------------------------  60929
GuineaPig                     TTC-----------------------------------------------------------------------------  39075
NorthernAmericanDeerMouse     TAACCAAGTTGCAGCTTAGAAATTTCTTCGTGGAGAACTCTTCCCATATTGTACTTTTAATTAGATTAGTTCTTCATAAT  43625
Mouse                         TAATCAAATTCCAGTTTAGAAAATTCTTCACGGAGAACTCA----ACTTTGTACTTTGAATT------------------  42925
ChineseHamsterGHOK1GS         GTAAGAAAATTCATGTAGTATACTGGGAAAGCAAAGATACTCTTTACATTG-ACTTTT--TT---TTTCTTTTTCTTGTT  44550
LongTailedChinchilla          TTTTTTTTTTTTGGTTTTTCTCCGGTACCAGGAACTGAACTCACGACCTTGCGCTTGCCAGGCAGGCGCTTTTGC-CGCT  42953
```

```
Majority                    --------------------AGACAXG-TTTTTTXACATAAAXATA-------TTCC-----------------------
                                    66570     66580     66590     66600     66610     66620     66630     66640
Human                       -----------------------------------GCATAAAAATA-------T-C------------------------ 60942
GuineaPig                   -----------------------------------ATAGCAAGACA-------TTCC----------------------- 39090
NorthernAmericanDeerMouse   TATTTTTGTTTTGTTTATTGAGACAGGGTTTCTCTGTGTAGTTTTGGCTCCTGTCCTGGAGCTCACTTGGTAGCCCAGGC 43705
Mouse                       --------------------AGAATGGATTTTTTTAAATAATTAT--------TTCT----------------------- 42954
ChineseHamsterGHOK1GS       TTTTATTAGTTCAAAT-TAGGAACAAGCTTGTTTCACATGTCAATCCCTTCTCCCTCTCCTTCCCCTCAACCCTCCTACC 44629
LongTailedChinchilla        GAGCTAAATCCCCAGCCCCAAGCCACCTATTTTTAACAGCAAGATA-------TTCC----------------------- 43003

Majority                    ---------------------------------T--------------------------------------------X-
                                    66650     66660     66670     66680     66690     66700     66710     66720
Human                       -------------------------------------------------------------------------------- 60942
GuineaPig                   ---------------------------------T---------------------------------------------- 39091
NorthernAmericanDeerMouse   TGGCCTCGAACT--CACAGAGATCCACCTGCCTCTGCCTCCTGAGTGCTGGGAGTGCCCACCACCACCCAGCTTCGTAAT 43783
Mouse                       ---------------------------------T--------------------------------------------A- 42956
ChineseHamsterGHOK1GS       CCACCCCGACATACCCCCCCACCCCATCCACCCTCCACTCCCCAGGCAGAGTAGGGCCCTCAACAGG--GGCTCCGCAA- 44706
LongTailedChinchilla        ---------------------------------T---------------------------------------------- 43004

Majority                    ---------XTTCACTGXXTTTTTCTTXGTATGXXX-XAXACTGTTTCTTTTTTGTTCTTGTCTGTATX------GCATT
                                    66730     66740     66750     66760     66770     66780     66790     66800
Human                       ---------A-----------------------ATC----------TTTTTAATA---------GTAT------------ 60959
GuineaPig                   ---------ATTCACTGGGCTTTTTGTTTTAGGGTT---GTTTGTTTGTTTTATGTTTTTGTTCTTGT------------ 39147
NorthernAmericanDeerMouse   TATTTCTTAGAGGACTGTTTTTCCATTAGTATGAGTAGATACTGTGTCCACTTTGTTCTTTGCTGTATTTATTGAGCATT 43863
Mouse                       ---------GATAACTGT-TTTCTGTTAGTATGAGTGGATACTATGTCTACTTTGTAGTAGTCTATTGA------GCATT 43020
ChineseHamsterGHOK1GS       --------AGTCCACCACATCATCCTGGGC-TGGGCCTAGACCCTTCCCCATGTGTCCAGGGCAAGAGT------GCATC 44771
LongTailedChinchilla        ---------ATTCACTGGGCTTTTCGTTTTAGGGTCTTGATCTGTTTTGTTTTTGTTCTTGTGTGCGTGGTACCGGGGGT 43075

Majority                    XACATTTAXGG---TXXACTGAA--CATAXXAACXXTCXTAAXTXTCXTAAGGXAAAXTCTTXAXXTAXTT---TTGXXA
                                    66810     66820     66830     66840     66850     66860     66870     66880
Human                       -A---ATAAGA--------------CATATAA------TGTAATCTC------------CTT------------TTCACC 60991
GuineaPig                   TAAGTATGTGG--------------CACACAACCCTTCTAAAACTTT--GAGTCATGGTCTTGAGTTGCTCGTGTTGGTT 39211
NorthernAmericanDeerMouse   GACATTTATGGTGATAAACTGAA---ATAACAACAATCCTAGTTATCATAAAGTAAATGGTCTTTATAATT---TTGTAA 43937
Mouse                       GACATTTGAGGTGATAAACTGAAGCCATAACAACAATCCTAATTCTCATAAAGTAAA-GGTCTTCATATTT---TTGTAA 43096
ChineseHamsterGHOK1GS       --CCTTCACG----TGGGATGGG---CTCTCAAAGTCCCTTCTTACACCAGGGAAAAATACTAATCCA------CTACCA 44836
LongTailedChinchilla        TGAATTTAGGGGTTGGCACTGAATACACACAGCCCCTCTGAAATTTTTTGAGACACGGTCTTGAGTTGCCTGGGTTGGTT 43155
```

```
Majority                    XGGAACTXAXXGAGTXTATTGGCXXAXXCXXAXAXAGTCCT-----TGGCTTAXAAAXAACAGTTTTXAXACTXGAXTGA
                                     66890     66900     66910     66920     66930     66940     66950     66960
Human                       TGGGATTTA-AAAATTGTTT---------TTACATAGTCCT-----TACATTATAA-----------CACACATAAGTAG  61045
GuineaPig                   TGGAACTGA-AGATTTTATTGCCTCACCCTCTCAGAGTGCT-----GGGTTTACAGGCAAATGCCACCATGCCTGGCTGA  39285
NorthernAmericanDeerMouse   GGAAACTCATGGAGTATACTGGGAAAAGCAAAGATACTCTTTCCATTGGCTTCCTAAGAACAGTTTTAAAACTGGAGTGA  44017
Mouse                       GAAAACTAATGCAGTATACTGGCAAA-GCAAAGAAACTCTTTCC-TTGTCTTCTCAATAACA-TTTTAACATTGGAATGT  43173
ChineseHamsterGHOK1GS       GGGGCCCCCTGGAGTGCAGAGGCCTCTTCATTGACATCC---------ACTTTCTAATAATAGTTTTAAAACTGGAATGA  44907
LongTailedChinchilla        TGGAACTGG-AGAGCCTCTTGCCTCACCCTCCCAGAGTACT-----GGGGTTATAGGCAAGTGCCACCATGCCTAGCTGA  43229

Majority                    AXAAXAXTAATTG-ATGCATGTXAATAXCX----------ATXA----------TTAGAAXCXXCTTTTTA-XX-----X
                                     66970     66980     66990     67000     67010     67020     67030     67040
Human                       GAA--AT-----G-GTTCTTTTTGGTTTTT----------GTTA----------TTAGAAACAGCTTTT-----------  61086
GuineaPig                   AAGTTGTTTTGTGCAGCTACATTTTTATGT----------ATTAG---------TTGGAAATGACACTTAATTTCTATCT  39346
NorthernAmericanDeerMouse   ATAACACTAATTG-ATGCATGTAAATACCC---TTTTAAATAGCAAGATATATGATAGAATCTTCTTTTTACTTGGGCCT  44093
Mouse                       TTAAAGCTAATTG-AAGCCTGTAAATACCC-------------CA-------G-------------TTTTA---------  43210
ChineseHamsterGHOK1GS       ATAAAACTAATTG-ATGCTTGTAAATACCCCAGTTTTAAATAGCAAGATATGTGATAGAATCT-CTTTTTACTTGGGCTT  44985
LongTailedChinchilla        AAA---T-----G--ACAATTTTTGTTTTT----------ATTA----------TTAGAGGTAACATTT-----------  43268

Majority                    X-XAAGGTTX--T--AXXXTTGCATCXTATTXGTAAXATCXATX-XTGAXTAXCAXATXTXXTTTTGTTTTTGTTXGAAA
                                     67050     67060     67070     67080     67090     67100     67110     67120
Human                       --CAGA---------AACATGTCTT--TATTAGGAATATCTTTA-GTTGACACTGGACAGTATATTGTGTCTTCTGGAAG  61152
GuineaPig                   T-TATTATTAGAAGTAACATTGCATCCTGTTAGAAATATCTAAA-ATAGATAAAAGATACTG-GTAGTAT------GAAA  39417
NorthernAmericanDeerMouse   TTAAAGGTGG--T---TTTTTGCATCTAAGTTATAACACCAGTGCATGAGTAGCAACTGTCCTTTTGTTTTTCTTGGAAA  44168
Mouse                       ---AAGGTTA--T---TTTTTGCAGCAGAGTTGTAACAACAACGCGTGAGTAGCCAATGTCCTTTTGCTTTTGTTATTTG  43282
ChineseHamsterGHOK1GS       TTAAAGGTTGATTTTTTTTTTGCATCTAATTTATAACATTAATGTGTGAGTAACAACTGTCTTTTTATTTTTATTAGAAA  45065
LongTailedChinchilla        --CATG---------AACATTACCTCCTGCTAGAAATATCTAAA-ATACACTCAGGATACTG-GTAGTATATGTTTGGAA  43335

Majority                    XAGT---X-X-GTATATXTXTTCATTTXXATATACTATATAATXGXGXTGTXTXTTCTAGAATXTTTT------------
                                     67130     67140     67150     67160     67170     67180     67190     67200
Human                       TCCT-------GTTTATATGATCATTTTATTTTAACATATAAGTGGGTTGTTTCTTATTCTTTATTTC------------  61213
GuineaPig                   ATG----------ATATATGATCATTTTAATTTAGTGAACAT-TGGATTATTTTTA------TATTAT------------  39468
NorthernAmericanDeerMouse   TAGCTTCTCATGAACATGTCTTC-CTTGTAGACACTAGATCATGGTGCTGTGTCTTCTAGAATCTTCT------------  44235
Mouse                       AAGTCCATGAACTGTATGTCTTTGTTTGTAGATGCTAGATAATGGTGCTGTGTTGTCTAGAATCTTTTTCATATTGTCAT  43362
ChineseHamsterGHOK1GS       TAGCTTCTCATGAACATGTCTTT-CTTGTAAATACTATATAATGATGCTGTGTCTTCGAGAATCTTTTA-----------  45133
LongTailedChinchilla        ATGT-------GTATATATGGTCATTTTAATGTAGTGTACATGTGGATTCTTTTTT------TATTCC------------  43390
```

```
Majority                   -----------XTXCXXGTGGGTTACATTTCA--GATTTAATXAAAXTAAXAAXAGAAGTAAAXTAAGATGTTTGXAGCA
                                       67210     67220     67230     67240     67250     67260     67270     67280
Human                      ------------TTCCAGGAGATTCCATTTCATC-ACTAACTGATAATAAAAATAAATATGAGATAAAACATTTGCAGCA  61280
GuineaPig                  ------------TT-TAGA---TTTTGTATCA---AGTTACTAGCAGTTAAAAATGAAGTAAAATA----CTTTGCAACA  39525
NorthernAmericanDeerMouse  ----------CATACTTGTGGGTTACATTTCA--GCTTT-ATTAAGACAGCAACAGTA--AAACTGAGATGGTTGTAGCA  44300
Mouse                      TTTGTTTTAGCATACCTGTGGGTTACATTTTA--GGTTCTAAGAAAGCAACAGCAACAGTAAACTCAGATGCTTGTAGCA  43440
ChineseHamsterGHOK1GS      ----------TATACTTGTGGGTTACTTTTCA--GCTTTGAATAAGATAGCAGTAGTA--AAACTGAGATGGTTGTAGCA  45199
LongTailedChinchilla       ------------TTACAGG---TTTTATGTCATTAAGTTAGTAGTTGTTAAAAATGAGGTAAAATAAATACTTTGCAGCA  43455

Majority                   CTAAGATCATGTCXTTCAXT-CXTTCTTACAGAGXACTXACTGXTTTATGCAGATGTTTTGAGAGAXCTAATGTGATCAT
                                       67290     67300     67310     67320     67330     67340     67350     67360
Human                      GTAAGATTATGTCATTCATTTCCTTCTCACAGAGTACACATTGCTTTATGCAGATTCTTTGAGAGACCTAATGGAATTAT  61360
GuineaPig                  ATAAAATCTTGTGATGTATT-ATTTCTTACAGCCTGCTTACTACTTTATGGAGAAATTCGGAGAGAT-------------  39591
NorthernAmericanDeerMouse  CTAAGATTGTGTCCCTCAGT-CCTTCTTACAAAGAACTTACTGTTTTATATAGATGTTTTGATAGACCTAATGTGATCAT  44379
Mouse                      CTAAAATCATGCCCGTCAGT-CTTTCTTACACAGAAGTCCCTGTTCTATGCAGATGGTTTGAAAACTGCGATGTGGTGGT  43519
ChineseHamsterGHOK1GS      CTAAGATCATGTCCCTCAGT-CCTTCTTACAAAGAACTCACTGTTTTATGCAGATGTTGTGAGAGACCTAATGTGATCAT  45278
LongTailedChinchilla       GTAAGATGTTGTCATATATT-CTTTCTTTCAGCCTGCTTACCACTTTATGGAGAAATTTGGAGAGCTCTAATATACTCAG  43534

Majority                   ---X---------ACTGTGXGGACAGTCTXXTTGAAXTT--GCTTAATT--ACTT---TTTTA--ATTTATAA------A
                                       67370     67380     67390     67400     67410     67420     67430     67440
Human                      CCTTTGCTAATTTCTTGGGCAAAGATTCTCCTTGACATC-TGCTTAATT----------TACA--ATCTGTAT------A  61421
GuineaPig                  ----------------GTACGAACACTCTTTTTGAGATAACACTTAGTTCTACTT-CCTTTTAGCATTTAGAATTTTGTA  39654
NorthernAmericanDeerMouse  -------------ACTTTGTGGGCAGTTTGCTTAAACTT--GCTTAA-T--ACTT---CTTTTCAGTTTATAAC-----A  44433
Mouse                      TGGT---------ACTCTGTGGACAGTG-GCTAGAACTT--GTTTCACT--ACCT---TTT----GACTTTTAC-----A  43573
ChineseHamsterGHOK1GS      -------------ACTTTGTGGGCAGTTTATTTAAACTT--GCTTAA-T--ACC--------------------------  45314
LongTailedChinchilla       CCTTTACT---TTCCTGTACAGACATTCTCTTTGAGATA-TGCCTAATTCTGCTTACCTTTTAGCATTCATAA------G  43604

Majority                   TTTTACAT---TTTATATTAATXGAAAATTTATAATGAA-TCAGTGGTTTTAXAAXX-XXXX---XAATTXTXCATAATC
                                       67450     67460     67470     67480     67490     67500     67510     67520
Human                      TTTTACA----TTCATATTAATGGAGAATTTATGATGAAATCAGTGGTTTTAGGATGCTTTAGGAAAATCCTGCATAATC  61497
GuineaPig                  TTCCATGTTTTTTTATATGAATAGTAAAGTTTTAATAAAACTAATGCTTTCAGAATGATTTACAAAAATTCTGCATAATC  39734
NorthernAmericanDeerMouse  TTATGCAT---TCCACCTTTCTTAAAAATTCATAATGGA-TCAG---TTTTAAAG-----------AATTATACATAATC  44495
Mouse                      TTTGACAT---TTTGCATTTAT-AGGAGTTCACAGTGGA-TCAGGAGTTTTAAAA-----------GATTATATATCGTC  43637
ChineseHamsterGHOK1GS      --------------------------AATTTATAATGCA-TCAA---TTTTAAAG-----------AATTGTACATAATC  45353
LongTailedChinchilla       TTTTATATTTTTTTATATGAACGGAAGATTTTTGATAAAGCCA-TGGTTTTAGAATGTTTTATGCAAAGCTTGCATAATC  43683
```

```
Majority                   TACCAGTGTGGAGTTTT---XTGTTGXXAAATAXTTGGTTTGAAXTAXTTAATTAXTGTTAAAAXXCXAATTTTATXTTC
                                            67530     67540     67550     67560     67570     67580     67590     67600
Human                      TACCAGGGTGGGGTTATTTGATATTGGTAAATAATTAGTCTGAAGTATTACATTAGTATTATAAGCTTAATTTTGTGTTG 61577
GuineaPig                  TGCCATTGTGGAGTTTT---ATGTTGGTAAATAATTGGCA-AAAGTATTTTATTAGTGTTACAAACCTAATTT-ATGTTC 39809
NorthernAmericanDeerMouse  TACTAGTGCAGAGTTTT-----GTTG-CAGATAGTTGATTTGAACTACTTAATTATTGTTAAAAGTCCTATTTTATATTC 44569
Mouse                      TACCAGTGTGGAATTTTA---TGTTG-CAAATAGTTGATTTGAACTACTTAATTACTGTTAAAAGTCCAAG--AGAACTT 43711
ChineseHamsterGHOK1GS      TA---ATGAAGAGTTTT-----GTTA-CAGATAGTTGTTTTGAACTACATA----CTGTTAAAAATCCAATTTTATATTC 45420
LongTailedChinchilla       A---AGTGTGGAGTTTT---ATGTTGGTAAATAATTGGTA-ATAGTATTTTATTAATGTTATAAACCTAATAT-ATGTTC 43755

Majority                   TTGATTTTGATCTTTTTTCXT--G-ACTTGGTAAXAAXTAG--AXAGXAXX---TATTTAAAGAATTTGGA-XGAXTTXT
                                            67610     67620     67630     67640     67650     67660     67670     67680
Human                      TTGATTTTGATAATTTTTCAT--G-ACTTGCCAAATATTAA--AAAGTAGA---TATTTAAAGAATTTGGA--AG-TTA- 61645
GuineaPig                  T-GGTTTTGATGTGTTTTCCT--G-ACTTGGTAAAAATTAG--A---CAGA---TAATTAAAGAATTTGGA--GC-TTTC 39874
NorthernAmericanDeerMouse  TTCATGAGTAACTTTTTTTTC-CTCACTTGGTAACAACTGG--ACAGTAAG---TAATCAGAGAACTTGGAGTAAACT-T 44642
Mouse                      GGAACTTAGAGCTTCCCTCAAGAATGCCCAACAACCTCTCACTAAAGCAAGTTATATATAAATAGTGCTCATTGAAGTAT 43791
ChineseHamsterGHOK1GS      TTGATGGGTAACTTTTTTTTTTCTCACTTGTTAGCAACTGG--ACAGTAAGC--TATTCAGAGAACTCGGAGTGAACT-T 45495
LongTailedChinchilla       TTGGTTTTGATGTGTTTTCCT--G-ACTTGGTAAATATTAG--A---CAGA---TATATAAAGAATTTGGA--GA-TTTC 43821

Majority                   --X-AGGTTTCTXTAAGCACCTG-TTAAATCAGATTCC----CAACAXTCTC-TTCAXAAAAXTAAGTTXTATAXAXXAA
                                            67690     67700     67710     67720     67730     67740     67750     67760
Human                      ----AGGTTTCTCTCAGTGTGTG-TTAAATCTGATCCC----CAACAATCTC-TTCAAAAAAGTAAGTTATCAATAGTA- 61714
GuineaPig                  ----AGGTTTCTCTACGCGCCTG-TTAAATCAGATTTC----CAACAGTCTC-TTCAGAAAAGTAAGTCATAGAAAGAAG 39944
NorthernAmericanDeerMouse  TTAGAGCCTTCCTAAAGCACCTG-CTAACTCTTATGCC----CAACAGTTTC--TCATTAAAAC---TTATGT---ACAA 44709
Mouse                      CCACATGTTGCATTGTGTTCTTG-TAATTGTATATTCTGTCTTAACATTTTCCTTCATTTTGATGC-TTGTGTTTTATAA 43869
ChineseHamsterGHOK1GS      --AGAGTCTCCCTAAAGCATCTG-CTACTTC-----------CAACAATCTC--TCACAAAAACAAGTTGCAT---ACAA 45556
LongTailedChinchilla       ----AGGTTTCTCTACGCACCTGGTTAAATCAGACTCC----CAACACTCTC-TTCAAAAAAGTAAGTTGTAGAAAGAAA 43892

Majority                   -ATTXAAAATTAACATGGAAATAXXTXXATX----XX-XTTTCATTXTATTTAXTXTATATXC--XTTTTCATTGTXCTX
                                            67770     67780     67790     67800     67810     67820     67830     67840
Human                      -AACGATGAGAAAAATGGAAATAGTTTTATT---TTGGCTTTTCATGTTTTCAGTCCATATGCAGTGTTTCATTGTGCTG 61790
GuineaPig                  AAATAAACATTAACATAAAAATAGAAATATTTTTGTGGCTCTTAATCTATTTAACATATGTGT--TTCTCCATTGTGCGT 40022
NorthernAmericanDeerMouse  -AT----AGTGCACATGGAAGTGTCTGCATA--------TTTCATTGTATTG--------------TTGTTATTGTACA- 44761
Mouse                      -ATTTTAATTTATTGTGGCAAATTTTTCCTA--------ATTATTTCTACTTAGTGTCTAAAC---TTTTTTTTTTATTG 43937
ChineseHamsterGHOK1GS      -AT----ATTGTACATGGAAGTATCTGCATA--------TTTCATTGTACTG--------------TTGTCACTATATAA 45609
LongTailedChinchilla       TAGTAAACATTGACATTGAAATAGAAATATT---GTGACTCTCAGTCTTTTTAATGTATATACGATGCTTCATTGTGCTT 43969
```

```
Majority                      -TTXTTTTATXXXTTTXTGTTCXXAATATT------X-TXAXTGCATTTTGATGXTCATGTTTTAGCXATAAATTTTAAA
                              67850 67860 67870 67880 67890 67900 67910 67920
Human                         CTTTAATTATGC-TTTCTGTGCCTAATATC------T-TTCCTGTGTTTTGATTTCCATGTTTTAGCCATAAACTTTAAA 61862
GuineaPig                     ---------------TGTGT-CTTAGCATA------C-TTCTTGCAATGTGATGATCATATTTTAGCCATAAATTTTAAA 40079
NorthernAmericanDeerMouse     ----TTCTA--------TGTTTTAACAATT--------TAATTTCATTTCGATGCTTGTGTTTGATCTATAAATTTTAAT 44821
Mouse                         GTTATTTTATTTATTTACATTTCAAATGTTATCCTCCCCAGTTTCCCCTCCACAACCCCCCTATATCTCCCTCTACCCCC 44017
ChineseHamsterGHOK1GS         -CTGTTCTAACTATAAAAGTTCTAACTTTT--------TAACTGCATTTTGATGCTTTTGTT----CTATAAATTTTAAT 45676
LongTailedChinchilla          GTATAATTGTGCATTTGTGT-CCTAACATC------T-TTACTGCATTTTGATGTTCATGTTTTAGTCACAAATTTTAAA 44041

Majority                      TTTCC--XXGCAAATTTTTTXCTGATTGTTTTTGC-----------TTAXTATCTAAACTTTXATTGTXAG-TAGGXACA
                              67930 67940 67950 67960 67970 67980 67990 68000
Human                         TTTACATGAAAACTTTTTTTTCTGATTGTTTTTGC-----------TTACTGTCTAAACCTTCACTGTGAG-TAGGCACA 61930
GuineaPig                     CATT----------TTTTTTTCTGATTGTTTTTGC-----------TTTCTATCTAAACTTTCATTGTGAGGTAACCATG 40138
NorthernAmericanDeerMouse     TTTCTG-TGGCAAATTTTT-CCTGATTATTTTTGC-----------TTAGTATCTAAACTTTTACTAGAAG-TAGGAACA 44887
Mouse                         ACCCCC-CAGCACACTCCTGCCTCACTGCCCTAGCATTCCCCCACCCTGGGTCATAAACTGTTTTTGAAAG-TAGGAATA 44095
ChineseHamsterGHOK1GS         TTCCT--TGGCAAATTTTT-CCTGATTATTTTTGC-----------CTAGTGACTAAACTTTT-TTGAAAG-TAGGAACA 45740
LongTailedChinchilla          TGTACTTCTAAAAATTTTTTTGTGATTGTTTTTGC-----------TTGCTATCTAAACTTTCTTTGTGAG-TAAGCACA 44109

Majority                      TTTGAAAAGTCTXXXXXTGCTACAAATTATCTTGAGCTTTTACTGTCAGXAATCTTTGCTCCAGCAAGATCAXTGCATTC
                              68010 68020 68030 68040 68050 68060 68070 68080
Human                         TTTGGAAGGTCT-----TTCTGCAAATTATCTTGAGCTTTTAATGTCAGAAATCTTTGCTCCAACAAGATCACCACATTC 62005
GuineaPig                     TTCAAAA-GTCT-----TGGAGCAAATTATTTGGCG-TTTTATTGTCAGAAATCTTT-CTCCAGCATGTTCACTGTATTC 40210
NorthernAmericanDeerMouse     TTTGAG-AGTCTTTGCTTGCTACACATTATCTTGAACTTTAACTGCCAGTAATCTTTGCTCAAGCCAGATCATTGCATTC 44966
Mouse                         TTTGAGAAGCCTTTGCTTGTTACAAATAATGTTTAGCTTTCACTGACAGTAGCCTTTGTTCTAGCGAGTTCATTGTGGTC 44175
ChineseHamsterGHOK1GS         TGAGA--AGTCTTTGCTTGCTACACATTATCTTGAGCTTTAACTGCCAATAATCTTTGCTCAAGCCAGATTATTGCATTC 45818
LongTailedChinchilla          TTCAAAAAGTCC-----TGTTACAAATTATTTGGAGCTTTTATTATCAGAAATTTTTGCTTCAGTATGATCACCACATTC 44184

Majority                      C-TGATXGCTGAACC----XXAGGACXGGTTTGTGTXXXACT-TTXTTTCTTGGGATT--GTAAATACA---GCTTGAAA
                              68090 68100 68110 68120 68130 68140 68150 68160
Human                         C-TGATTGCTGAACCA----CAGGACTGGTTTGTGTGCTACTGCTATTTCTTGGGATTTTGCAAATACATGAGCTTGAAG 62080
GuineaPig                     CCTGATTGCCAAACT----------ACAATTTATGTGCTATTTTTATCTCCTGGGGTTACATTAATT------------- 40267
NorthernAmericanDeerMouse     C-TGAAGGCTGCACC----ATAGGATGGGTTTGTGTATCACT--TCTTTCTTGGA-TT--GTAAATTCA---GCTTGAAA 45033
Mouse                         --TGCTGGCTGCACCCACCATACGACTGGTTTGTGTATCACTATTCTTTCTTGGGATT--GTAAATACA---GCTTGAAA 44248
ChineseHamsterGHOK1GS         C-TGATGGCTGTACC----ACAGAACAGATTTGTGTATCACT--TCTTTCTTAGAATT--GTACATATA---ACTCGAAA 45886
LongTailedChinchilla          C-TGATTGCCAAAATC---------ACAGTTTGTGTGCTATTCTTACCTCCTGGGATTGTGTAAATACATGAG------- 44247
```

```
Majority                      TGAXGCTTGCATA-ATTTTTTXAAXATGAATTTTCTCTCTXCCTTACTTXTAGTCAC-TTACTTXAACCAACTXTTGAAA
                                       68170     68180     68190     68200     68210     68220     68230     68240
Human                         TGA-GCTTGCATATATTTTTTAAATTTGATTTTACTCCCTTTCTTATTTGTGGTCAC-CTCCTTTAACCAACTATTTAAA  62158
GuineaPig                     -------------------TTCATCCCGAATTGTA--------------GTCATC---TTACTTGAACCAACTATTGAAA  40311
NorthernAmericanDeerMouse     TGAAGCTTGCATA-ATTTTTTTAATATGAATTTTCTTTCTCCCTTATTTATAGTCACTTTACTTTGGGCACCTTTTGA--  45110
Mouse                         TGAAGCTTGCATA-ATTTTTTCAACATGAATT--CTTTCTGCCTTACTTACAGTCACCTTACTTCATACAAATTTTAAA-  44324
ChineseHamsterGHOK1GS         TGAAGCTTGCATA-ATTTTTT-AACATGAATTTTCTCTTTTCCTTACTTATAGTCACTTTACTTAGAGCACCTTTTGAAA  45964
LongTailedChinchilla          -------------------TTTAATTTGATTTTTGTCCCT-----ACTTGTTGTC---TTACCTGAGCTAACTATTGAAA  44300

Majority                      -ATACCTXGATTTCTGTTTTXXX------XXXGCTCTTCTTTGTTXXAX----X------X-ACAATTATAATTTAXATT
                                       68250     68260     68270     68280     68290     68300     68310     68320
Human                         -ATACCTGGATTTTTGCTCTCATACACCCTCAGCTCCTATTTCTTTCCTCTTTTGCCAACAGATGATT--AATTTACATC  62235
GuineaPig                     -ATACCTTAATTTCTGTTTTCATGTACATTCAGCTCTTATTTTTCATATTCTCTGTTGTTAAACTATTTTAATTTACATT  40390
NorthernAmericanDeerMouse     ----------TTGCTTTTTT------------GCACTTCTTTGTTGTCC-------------ACAATTATAATTTAAATT  45155
Mouse                         -TTAACTAGATTGCTATTTT------------CCTCTCCTT-GTATGAC-------------AAAATTATA---TAAAAT  44374
ChineseHamsterGHOK1GS         AATGTCTGGATTCCTGTTTT------------GCTCTCCTTTGTTCCAC-------------ACAATTATAATTTAAATT  46019
LongTailedChinchilla          -ATCCCTTGATTTCTGTTTCCATGTGTATTCACCTCCTCTTTCTCGAATCCCCTCTGGTTAAACTGTTTCAATTGCCATC  44379

Majority                      TTGTXTGXTTXTTTCTCCAGCTCTTACCXGTX-AXGTGTAATTGTGTATCTTCTACCTXCATAXAG-----ATGTTTTT-
                                       68330     68340     68350     68360     68370     68380     68390     68400
Human                         TTGTTTGCTTATTTCTCTAGCTCTTGTACCTT-AGGTATACTTCTGCATCTTCCACCTACATAAAGTTTTTGTTTTTGTT  62314
GuineaPig                     TTGTTAGCTCACTTCTCTAGCTTTTACCCTTT-AGGTATAATCGTGTATCTTCTACCTACATGAAA-----CTGTTTTTC  40464
NorthernAmericanDeerMouse     TTGCCTGGGTGTTTCTCCACCTCTTACCAGTCTATGTGTAACTGTGTATCTTT-ACCTGCTTACAG-----ATATTTT--  45227
Mouse                         TTGACTAGTTGTTTCTCCAGCTCTTCCCAGTC--TGTGTGGCTGTGTGTCTTGTACTTGTGTACAG-----ATATTTTT-  44446
ChineseHamsterGHOK1GS         TTGCCTGGCTGTTTCTCCAGGTCTTACTAGTCTGTGTGTGATTGTGTATCTTTCACCTGCATACAG-----ATGTTTT--  46092
LongTailedChinchilla          TTGTTGGCTCATTTCTCCAGCTCTTACACGTT-AGATGTAATTGTGTAACTTCTACCTACGCGAAG-----GTGTTTTTC  44453

Majority                      CTAAAAGTTAAAATXGAAAATATCAAAGGTTTCTGCAGGGTTTTGTAACAATTGGXACATTGATTG-TXGATAATTTGGC
                                       68410     68420     68430     68440     68450     68460     68470     68480
Human                         TTTAATGTTCAGAGGGAAAATTTCAAAGGGTCCTGGAGGGCTTTCTAACAATTGGCATATTCATTGTTAGATAATTTGGC  62394
GuineaPig                     TGAATATTTAAAAAGGAAAATGTCAGATGTTT-TGCAGGATTTT---TCTGTCAGT-----------TAGATAATTTGGC  40529
NorthernAmericanDeerMouse     CTAAAAGTTAAAATTGAGAATATCAAAGGTTTCTACAGGGTATTGAAACAATTGCTACATTGATTG-TGGATAATTTGGA  45306
Mouse                         CTAAAAGTTAAAATTGAAATTATCAAAGGTTTCTAAAGGGTGTTGTAGTAATTGGCACATTGATTG-TAGATAATTTGGC  44525
ChineseHamsterGHOK1GS         CTAAACGTTAAAGTTGAAAATATTAAAGGTTTCTGTAGGGTGTTGTAACAATTGCTACATTGATTG-TGGATAACTTTTA  46171
LongTailedChinchilla          CTAAAAGTTAAAAAGGAAAATGTCAACTGTCTCTGCAAGGATTTCTATCAGTCAGCAAATTCATTA-TGGATAATTTGGC  44532
```

```
Majority                    AGTCTX-CCTXAXTATTGCTTXXAAAT--AATACAGCTTXGAAAAGACT---T-TATTG-TAGATTTAGTAATTTXCTAT
                                      68490     68500     68510     68520     68530     68540     68550     68560
Human                       AGTCGGACCTCAGTATTGCTGGGAAAG--AATTCAACTCCTAAAAAAC----T-TAGTG-TGTGTTTAGTAATTTTGTAT  62466
GuineaPig                   AGTCTA-CTTCCGTATTGTTTGGAAAT--AACCCAGTTTAGAAAAGAC--CATTTATTG-AGGAGTTAGTAGTTTCCT-C  40602
NorthernAmericanDeerMouse   ACTCTA-TCTTAATATTGCTTTAATTTCAAATAATGCTTCAGAAGTCCTT--A-GATTTGTAGGTTTGGTAATTT-CTAT  45381
Mouse                       ATTCTG-TCTTAATATCACATTAA-----AATATCACTTTGAAAAGTCT------ATTG-TAGATTTAGTAATTTTCTGT  44592
ChineseHamsterGHOK1GS       ACTCTA-CCGTAATATTGCTTTAATTT--AATAATGCTTTCAAAAGTCT------ATTT-TAGATTTGATAATTT-CTAT  46240
LongTailedChinchilla        AGTCCG-CCTCTGCATTGTTTGGAAAT--AACCCAGTTTAGAAGAGACTTCATTTATTG-TTGAGTTAGCAGTTTGCTGT  44608

Majority                    CAGTACTTTTAGCATTAACCTAGXXATXGTGAXTCA-AGCCAGCTCTTXT----X-----TTT-CTCTTTTXTAATAAGX
                                      68570     68580     68590     68600     68610     68620     68630     68640
Human                       CAGCACTTTTAGCATTAACCTAGATACTGTGACTCAAACCCAG---------------------CTCTTTTCTCATAAGG  62525
GuineaPig                   GAGTGCTTTTAGCATTAACCTTGATATTGTTTCTCAGAGCCATGCCTTTGT---TTTTTGTTT-TTTTTTTTTTACAAGA  40678
NorthernAmericanDeerMouse   CAGTACTGTTAGCA----CCTAGGGATGGTGATTCA-AGCCAGCTCTTCTC--GTGACATTTT-CTCTTTAATAATAATC  45453
Mouse                       CAGTTCTGTTAGCATTAACCTAGGGATGGTGATTCA-AGCCAGCTCT--------------------------AATGAGG  44645
ChineseHamsterGHOK1GS       CAGTATCATTAGTATGAACCTAGGGATGGTGATTCA-AGCCAGCTCTTTTAAGATGATATTTTTCTCTTTAATAATAATC  46319
LongTailedChinchilla        CAGTACTTTGAGCATTAACCTTGATACTGTTTCTCAAAGCCATGTCTTCT----------TTT-TTTTTTTTTAATAAGA  44677

Majority                    TAAX-XTT----TTCTCTTTGAT--------------AXATATXXAAATA--------------------GTTACCTA--
                                      68650     68660     68670     68680     68690     68700     68710     68720
Human                       TATC-ATT----TTCTCTTTGAT--------------AAC-ATTGAAGTA--------------------GTCACCTA--  62563
GuineaPig                   TAACCATT----TTCTCTTTGATGTTATCCAAAATATAAAGATCAGAGTAAAAGTTTGCTAACTGTGAAGGAGACCAAGA  40754
NorthernAmericanDeerMouse   AAATAGTTACAGTGCTCTCTGGT--------------A-ATATTTAAATG--------------------TTTACCTA--  45496
Mouse                       TAAT-G----TGTACTCCTTGAT--------------A-GTATTTAATTA--------------------GTTACCTA--  44683
ChineseHamsterGHOK1GS       AAATAGTTACCATGCTCTCTGAT--------------A-ATATCAAAATG--------------------TTTGCCTA--  46362
LongTailedChinchilla        TAGC-ATT----TTGTCTTTGATGTTACCGGAAATACAAAGAGCGAGATAAAAGGTTGATAACTGGAAAGGGCACCAAGA  44752

Majority                    ---XGAGTAAAGTT----------------------------TG-GCTACTXAT------X--CTXCTGAT-----AXAX
                                      68730     68740     68750     68760     68770     68780     68790     68800
Human                       ---AGAGTGAATTT----------------------------TG--CTACTTAT------C---TTTTGAT-----ATTT  62596
GuineaPig                   AATGGAGTGAAGTTGGGGAGAGTTTAAAAAAAAAAATTAAGATGTGTTTTGTACACATAGCAATACCCCATGGAGAATGT  40834
NorthernAmericanDeerMouse   ---AGAGTAAAATT----------------------------TG-GCTACTCAT---------CTACTAGT-----AGAC  45530
Mouse                       ---AGGGTAAAGTT----------------------------TT-GCTACTCAT---------CTTCTGAT-----AGAC  44717
ChineseHamsterGHOK1GS       ---GGAGTAAAGTA----------------------------TT-GCTACTCAT---------CCACTGGT-----AGAC  46396
LongTailedChinchilla        AATGGAGTAGGGTTGGGGGGA----AAGAAAAAGAATTGAGATGTGTTACGTACACATACCACCTCCCCATGGAGAATGT  44828
```

```
Majority                     ATTTXXXACXXX------------GATATT-AACA-----------------GTGCCATTXAGXXTTCA-----TAXXAX
                                      68810     68820     68830     68840     68850     68860     68870     68880
Human                        ATCTATTGTGTA------------G--A---AACA------------------------TCAGT--TC------TATTAC  62627
GuineaPig                    AATCATTATGTATTACAAACATATAGTACTGAAAAAATGTTAAAATAAATTAGTAAATTTTGGTGTTCATCTTTTAATAC  40914
NorthernAmericanDeerMouse    ATTTGCAACACT------------GATATT-AACA-----------------ATGCCATTCAGACTGCA-----TAGAAA  45575
Mouse                        ATTTGCAGGACT------------GATATT-CACA-----------------GTGCCATTTAGATTGCA-----TAGAAA  44762
ChineseHamsterGHOK1GS        ATTTGCAACACT------------GATATT-AGCA-----------------GTGCCATTCAGA-------------AAA  46433
LongTailedChinchilla         AGTCATTACGTACTACAGCTGTATAGTATTAAGCA---GTTAAAGCGA-TGAGTACATTTTGCTGTTCATCTTTTGATAC  44904

Majority                     TCX----AAATTCTGTCACX----XT-XXTGGCTCACATTXTCCCTACAGXAATGCXXCAACXG----TAAATGAATACC
                                      68890     68900     68910     68920     68930     68940     68950     68960
Human                        TT----------------------AT------CTCACATTTTCCCTATAGGAATGCTACATTAG----TAAATGAATACC  62675
GuineaPig                    TCATTTAATGTGTTGAAACAGATTGTTTTTGACTCAAATTTTCCTTACAGATATGTTACATGAG----TTAGTGAATGTC  40990
NorthernAmericanDeerMouse    TCAC---AAATTCTGTCAC---------CTGGCTCACATTCTCCCTACAGTAATGCCTTTACTG----TAAACGAATACC  45639
Mouse                        TC-----AAATTCTGTCAC----------TGGTTTACATTCTCCCAACAGGAATGACTCAACTTACTGTAAATTAATAGC  44827
ChineseHamsterGHOK1GS        TCAC---AAATTCTGTCACAAA-CATGTCTGGCTCACATTCTCCCTACAGTAATGCCTTTACTTAAACTAAACAAATACC  46509
LongTailedChinchilla         TT-----GTGTGTTAAAACA---CGTTTTTGACTCACGTTTTCCCTATAGAAATGTTACGGGAG----TAAATGCATGCC  44972

Majority                     TATTTTCAGTGTTTTXTTTGCCTXXACAGXAGGXTATT--------G---XX-X-XGGT-----------------TATX
                                      68970     68980     68990     69000     69010     69020     69030     69040
Human                        TATCTTCAGTGCTTTCTTCACCTTTACACCAGGGTATT----------CAATTAATAGT-----------------TATG  62728
GuineaPig                    TATTCACAGTGTTTTGTTTGCCTTTATAGCAGAGTATTGGATTAGTTTGTATCAGTGGTGATGAACCACTGC----TGTG  41066
NorthernAmericanDeerMouse    TATTTTTAATGTGTTATTTGCCTCCACAGGAGGATATT--------G---------GAT-----------------TATT  45685
Mouse                        TATTA-----GTGGTTTTTGCCTCCACAGGAAGAAACA--------G---------AGT-----------------TATT  44868
ChineseHamsterGHOK1GS        TGTTTTTAGTGTTTTATTTGCCTCCACAGGAGGATGTT--------G---------GAT-----------------T-TT  46554
LongTailedChinchilla         TGTTTTCACTGTTCTGTTGGCCTTTATAACAGGGCATT---------CATATCAGTGGTGGCGAACCTGTGGCCCATGTG  45043

Majority                     TTACATXAAAXTGT-----------------------------T--XGATAAAXTTGATXT--ATTTGGTGXXTA--XTT
                                      69050     69060     69070     69080     69090     69100     69110     69120
Human                        TTATT-----CTGG-------------------------------CTGATAAA--------------GTTGATTG-----  62753
GuineaPig                    CCACAGCAGACTGTTGGTATCATTGAAAGCTCTAAAAGATTCACCTTCACTGGTTTATATTATACTGGGTGATAA--TTG  41144
NorthernAmericanDeerMouse    TTACATTAAATTGT-----------------------------T---GATAAAGTTGATAT--ATTTGGTGTCTA--CTT  45729
Mouse                        TTACATAAAATTCT-----------------------------T---GATAAAATTGATATT-ATTTGGTGTCT-----T  44910
ChineseHamsterGHOK1GS        TTACATTAAATTGT-----------------------------T---GATAAAGTTTATGT--ATTTGATGTCTA--CTT  46598
LongTailedChinchilla         CCACAGCAGACTGT------CACTGAAAGCTCTAAAAGGTTCCCCATCACTGGTTTGTATTACACTGGGTGATAAAATCC  45117
```

Majority	XTAXXTGGXXTAXATTTTTATGTGX-GATAGGTXXXTTGCTTXAXCCAAATGTAATTCAAATCATXATGTGTATGAAATC	
	69130 69140 69150 69160 69170 69180 69190 69200	
Human	ATACATGGCTTCCACTTTTATGTGATGATAGGTACCTTGCCTAACCCAAATGTAATTCAAATCCTGATGTGTATGAACTC	62833
GuineaPig	ATACCTGGCTTCTATTTTTATGTGACGATAGGTACCATGCTTAACTCAAATGTAATTCAAAACTCGATGT--GTGAAGTC	41222
NorthernAmericanDeerMouse	TTATGTGGTATAGACTATTTTATGT-GATTGAT---TTGCTTGATCCAAATGTAATTTG--TCATAATGTGTATGAAATG	45803
Mouse	TTATTTGGTATA---TTTTTTGTCT-AAT-GGT---TTGCTTGATCTAAATGTAATTCAAATAATAATGCTTATGAAATG	44982
ChineseHamsterGHOK1GS	TTATGTGGTATAGATTATTG-ATGT-GAT-GAT---TTGCTTGATCCAAATGTAATTTG--TCATAATGTGTATGAAGTA	46670
LongTailedChinchilla	ATACCCGACTTGTATTTTTATGGAA--ATAGGTACCTTGCTTAACCCAAATGTCATGCAAAACTTGATGTCTGTGAAATC	45195

Majority	CCTTTAAGTTAXTXXTAXXCCXGCCXTGTAATXXAXXXXXTTCTGXTG-XTTAXTTTTTAGGTTX-TTTTCA----GGTX	
	69210 69220 69230 69240 69250 69260 69270 69280	
Human	TCTTTCAGTTTTTACCAGTGCTGTATTGAAGTGGATGAATCTGTGTTGAATTATTTTCTAGGTTAGTTTTTAGGTAGGTA	62913
GuineaPig	-CTGTGAGTTTTAACAAATACTGTGTTGTAATGGGTGAAATTCTGTTGAGTTATTTT-TAGGTTAGTGTTCAGCTTGGTA	41300
NorthernAmericanDeerMouse	CCTTTATGTTAGTTTTC-ACCAGCCCTGTGATCT-------TCTGATG-ATGAATTTTTTGCTGC-TTTTCA----GATC	45869
Mouse	CCTTTAAGTTAGTTTTTTACCAGGCCTGTAATCTA----GTTGTGATG-GTGAATTTTT--------TTTCA----GCTC	45045
ChineseHamsterGHOK1GS	CCTTTAAGTTAGTTTTC-ACCAGCCCTGTGAACT-------TCTGATG-ATCAATTTTT-ACTGC-TTTTCA----GCTC	46735
LongTailedChinchilla	-CTTT--GTTATAACAAATACTGCATTGTAATGGATGAAATTCTGTT--GTTATTTT-TAGGTT------------GGTA	45257

Majority	AGTAXAXXCXTGTAAAATTT-----TTGTATATTXAGGGXAXAGGXTT-ATTTTTCCAGAGXATGAGAATATTT-XXXXX	
	69290 69300 69310 69320 69330 69340 69350 69360	
Human	GATTAGTCCATAAAAAAATT------CGTATATTAAGAGAAAAGGTTTTATATTCCCAGAG-GTGAGAATATTT------	62980
GuineaPig	AGTATACTCCTGTAAAACTTGATGGTTTTATATTAAGGGAAGAGATTT-TTTTTTAGAGA--ATGAAGATATTT------	41371
NorthernAmericanDeerMouse	AGTA----GTCGTAAAATTT-----CTGTAGATTGAGGAGACAGAATT-ATTTTTCTAGAGAATGATAATATTTTAATGT	45939
Mouse	AGTA----------AAATTT-----TTGTATATTGAGGGGA----ATT-ATTGTTCTAGAGAGTAAGAATATTTCAATGT	45105
ChineseHamsterGHOK1GS	AGTAAAGAGTCGTAAAATTT-----TTGTAGATTGAGGGGAAAGGATT-ATTGTACCTGAGAATAAGACTATTTTAATGT	46809
LongTailedChinchilla	AGTATAGTCATATAAAAATT-----TTTTATATTAAGGGAAGAGGTTT-ATTTTTCCAGAG-AGGAGGATATTT------	45324

Majority	XX-XXXXGAATTXCAAGCTTTATATACTTXA-XC----AAXXA--TAGAGXTTTACTTTTTCCA-TAACAAGACATXTTT	
	69370 69380 69390 69400 69410 69420 69430 69440	
Human	-------GAATTAAAAGCTTTATATACTTAA-GC----AACTG--TAGAGGTTTACTTTTTCCA-TAACACGACATGTTT	63045
GuineaPig	-------GAAGTATAAACTACATACCC-------------------AGAGATTTACTTTCTCCA-TAAAAGGACATGTTT	41424
NorthernAmericanDeerMouse	TTTGAATGAATTCCAAACTTTATATACTTTATACACCTCACCAACTATAGGTTTACTTTTTCCATTAATAAGACATTTTC	46019
Mouse	TT-GAATGAATTCCAATCTTAATATACTTAA-GC----AAATA--TAGATGTTTACTTTTCC-ATTAATAAGACCTTTTT	45176
ChineseHamsterGHOK1GS	TTTGAATGAATTCCAAGCTTTAAATATTTTATAC--CTAAGCAATTAGAAATTTACTTTTTC-ACTAACGACACGTT---	46883
LongTailedChinchilla	-------GAATTATAAGCTTTGTAT---------------------AGAGATTTAGCTTTTCCA-TAACAGGACATGTTT	45375

Sequence	Alignment	Position
Majority	ATTCTTCTAATGAAAA-TTAACXCTXTGTTCCCXTXXTTTATAGTTTGTTXCTAXXTTATATCXXGTTGGACTAXAAATG	
	69450 69460 69470 69480 69490 69500 69510 69520	
Human	ATTCTTCTAATGAAAAGTTAACAGTATGTTCACCTGTTCTCTAGTTTGTTTCCAGCTTATATCCAGCTGAACTAGAAATG	63125
GuineaPig	ATTTTTCTAGTGAAAAATTAACCTTGTATTTCCTTGTTTTATAGTTTGTTCCTAGCTTATATCCAGATGGACTAATGATG	41504
NorthernAmericanDeerMouse	ATTCTTTTAATGAAAA-TTAACTCACTGTTCC---CATTTATAGTTTGTTTCTATATTACATCTTATTAGACT-TGTTAG	46094
Mouse	ATTCTTCTGATGAAAA-GTAATATTATGTTCC---CATTTATA-TTTTTTCCTATATG---CTTTGTTGGACTAGAAATA	45248
ChineseHamsterGHOK1GS	---CTTCTAATGAAAA-TTAACTCATTGTTCCACCCATTTATAGTTTGTTTCTATATT-----TTGTTAGTCTATAAATG	46954
LongTailedChinchilla	ATTTTTGTAATGAAAAATTAACCCTGTGTTTCCTTGTTTTATAGTTTGTTCCTAGCTTATATCCAGATGGACTAACGATG	45455

Sequence	Alignment	Position
Majority	ATXGATAT---ACAGCAGCAAAA----X--XXAAXCATAAXXXTAXXATAGTXXTXAATAATTCATGATXXACTTTCACA	
	69530 69540 69550 69560 69570 69580 69590 69600	
Human	GTGATTATATAGGAGCAGAAAAAGTACAAGATAATCATAGCGGTAACATAGTCCTTTATAATTCATGATGTATTTTCACA	63205
GuineaPig	ATGAT------GCAGCAGCAGAA---AAC-ATAACCATAACTGCAGCATAGTTTTCCATAATTCATGATGTACTTTCACA	41574
NorthernAmericanDeerMouse	ATTGATAT---ACAACAGCAATA---------AATCATAATAATAATAG------TAATAATTCATGATAAACTTTCA--	46154
Mouse	ATTGATAT---ACAG-AGCAAAA---------ACCTATAATCATAGT--------CAATAATTCATGATAAACTTCCACA	45307
ChineseHamsterGHOK1GS	ATTGATGG---ACAGCAACAACA---------AATCATAATTATAATAACATAGTTAATAATTCATGATAAACTTTCACA	47022
LongTailedChinchilla	ATGCC------ACAGCAGAAAAA---CATTATAACCATAACAGCAGCATAGTTTCCTATAACTCATGATGTACTTTCACA	45526

Sequence	Alignment	Position
Majority	TGTGTTXTCCTTTATTTAATCCXCATAATAACCCT-TAAGGAAGCXTGGTAGCCXTTATCATT---XCTGTTTTCTAXAT	
	69610 69620 69630 69640 69650 69660 69670 69680	
Human	TATGTTGTCTTTAATTTACTCCTCTTAATAACCCT-TGAAGAAGCATGACAGCCATTGTCATCATTCCTGTTTTCCCGAT	63284
GuineaPig	TATGCTGTTCTTTATTTAATCCTCA-----ACACT------GTGCATGTTAGCCATTATCATT-----TGTTTTCTAGAT	41638
NorthernAmericanDeerMouse	TGTGTCATCCTTTATTTAATCCCAGCAATAACTCTGTAAGTAAGCTTGGTGGCCCTTATCATT---TCTATTTTCTGTAT	46231
Mouse	TGTATGGTCTTTTATTTAGTTCCCATAGCAACCTT-TAAGGAAATTTGTTGACCCTTA---TT---CCTACTTTCTATAT	45380
ChineseHamsterGHOK1GS	TGTGTCATCCATTGTTTAATCCCAATAATGACCCTGTAAGGAATCTTGGTTGCCCTTATCTTT---TCTGTTTTCTATTT	47099
LongTailedChinchilla	--TGTTATTCTTTATTTAATCCTCATAATAACACT------GTGCATGGTAGCCAGTATTGTT-----TGTCTTC-AGAT	45592

Sequence	Alignment	Position
Majority	TACXAAATTGAXACTXAGAXXX-TTAAATAXXXXTC-TAAGXATGAXCTAATTA------AGTCTAGGCTTTTAACT---	
	69690 69700 69710 69720 69730 69740 69750 69760	
Human	TAAGAAATTGAGATTCAGAGAGGTCAAATAACTTGC-TTAAGATGACCGAAAAG------AGTCTAGGCTTTTAATTAAT	63357
GuineaPig	TATGAAATCGAGTATTAGAGAG-TTACATAACTT------GAATGACCTAACTA------ATCCTAGACTTTCAGC----	41701
NorthernAmericanDeerMouse	TACTATGCTGATACTCAGGA---TTAAATAGTGGT---AGGGTTGATATAATTA------AGTCTAGGCTTTTAACA---	46296
Mouse	TTCTACAATAATACTCAAAA---TT-AGTAGTGGTC-TAACAGTGATGTCATTA------AGTCTAGACTTTTAACT---	45446
ChineseHamsterGHOK1GS	TACTATACTGCTACTTAGGA---TTGAATAGTGGTCATAGGGGT-ATGTAATTACATACAAGTCTAGGCTTTTAGTT---	47172
LongTailedChinchilla	TAAGAAATTGAGTATTAGAGAG-TTATATAACTTCC-TTAGAATGACCTAATTA------ATCCTG--------------	45650

```
Majority                    - CTAGXCCTGAXACTCTTXT- XATTCCAGAA- TTTCCTGGAATGACA- TTGGCTTTXCTAATTTCATCAAGATATTCTGA
                                      69770     69780     69790     69800     69810     69820     69830     69840
Human                       CCTAGACCTGGCACTCGTCA- - GTTTCAGAACTTTTCGGGAATGACA- TTGGATTTGCTAATTTCATCAAGATAATCTGA  63434
GuineaPig                   CCTAGGCCTGATACTCTTCT- - ATTCCAGAAATTTCCTGGAAT- - - - - - TGGGTTTG- TAATCACATCAAAGGATTCTGA  41772
NorthernAmericanDeerMouse   - CTAGACTTGACGCTCTTTT- TATTCCAGAA- TTTCCTGTAATGACA- TTGGCTTTTCTAATTCCATCAAGATATTCTAA  46372
Mouse                       - GTAGA- - TAGCACTCTTTTCTATTCCAGAA- TTTCCTGGAATGATAATTGGCTTTTCTAATTTCATCAAGATATTCTGA  45522
ChineseHamsterGHOK1GS       - CTAGGCTTGATGATCTTTT- TATTCCAGAA- TTTCCTGGAATGACA- TTTGCCTTTCTAATTTCATCATTATATTCTGA  47248
LongTailedChinchilla        - - TAGGCCTGATACTCTTCT- - ATTCCAACAATTTCCTGGAGT- - - - - - TGGGTTTGCTAATTTCATCAAGGGATTCTGA  45720

Majority                    TTCAGGCXGAACAXTTCATCTTAAGGAA- XTACTXXTTTTTA- - - - - - - - - CXTCATTTCATGGGAATTGX- - TTAXACA
                                      69850     69860     69870     69880     69890     69900     69910     69920
Human                       TTGGGGGTGAACATTTCAGGTTAAGGAAAATACTAGTTTTTTTCTTTTTTGCCTCATTTTATGGGAATTGCTATTATGTG  63514
GuineaPig                   TTCAGGCTGAATATTTCATGTTAAG- - - - ATAGTAATTTTTA- - - - - - - - - CCACATTTCATAGAAATCGCTATTATACA  41839
NorthernAmericanDeerMouse   TTCAGGTCAAACAGTTCATCTTATGGAA- GTACTTATTTTTA- - - - - - - - - CTTCATTTCATGGGAATTG- - - TTTCAAA  46439
Mouse                       TTCAGGCCCAACAGTTCATCTTAAGGAA- GTACTTGTTCTTA- - - - - - - - - CTTCATTTCATGGCAATTG- - - TTACACA  45589
ChineseHamsterGHOK1GS       TTCAGGTCAAAAAGTTCATTTTAAGGAA- GAACTTATTTTTA- - - - - - - - - CTTCATTTTATGGGAATTG- - - TTGCAAG  47315
LongTailedChinchilla        TTCAGGCTGAACATTTTATCTTAAG- - - - ATACTAGTTTTTAA- - - - - - - - CCATATTTCATAGGAATTTCAGTTATGCA  45788

Majority                    TTXTGAXGXTACATTTATTTGGTATGAGGCAACTTCATACAGTGTGGCAXCCAGTAGTAGTTXX- XTATCAAXXXXTA- -
                                      69930     69940     69950     69960     69970     69980     69990     70000
Human                       TTAGGATAAT- - - - - - - - TTGGTTTGAAGCAACTTTGTACAATGTGGCATCAAATAGCAGTTACCTTCTAAAACTGTACT  63586
GuineaPig                   TTATAATGATACATTTATTTGATCTGAGGCAACTTCATACAATATGACTCCCACCAATAGTTACCTTATAAAGCTGT- - -  41916
NorthernAmericanDeerMouse   TCTTGGGGCTGCATTTATTTGGTATGAGGCAACTTCATACAGTGTGGCATTTGGTTGTAATTG- - - TGTCAGTTCATA- -  46514
Mouse                       TTTTGGGGCTGCATTTCTTTG- TATAAGGCAACTTCCTACAGTGTGGTATCCAGGAGGAGTTG- - - AATCAGCTCATT- -  45663
ChineseHamsterGHOK1GS       TCTTGAGGCTACATTTATTTGGCATGAGGCAACTTCATACAGTGTAGCACCTGGTACTATTTG- - - TATCAACTCATA- -  47390
LongTailedChinchilla        TTACAATGATACATTTATTTGGTCTGAGGCAACTTCATACAGTGTGACACCCTACAGCAGTTACTTTATGAAACTGT- - -  45865

Majority                    - - - - - - - - TXAGTGTXATCATT- AGAAXXAAATXTGTGTATAATAXXXTTA- XTTXATTGXGXAAATTAXTX- AGTXCTG
                                      70010     70020     70030     70040     70050     70060     70070     70080
Human                       GAATCATATAAGTACTGCCATCCAGCAATAAATATATGTATAATAAAATTAACCTTATTGAGCAGAGTAGTGTGGTACT-  63665
GuineaPig                   - - - - - - - - TAA- - - - - - TTTGTGTAAA- - - - - TACTGTCATAATAAAGTTA- CTTTATTGTGCAAATTAATA- AATACTA  41975
NorthernAmericanDeerMouse   - - - - - - - - TTAGTGTCATCCT- - - GAATAAAATGTGTGTATAATA- TATTG- TTCAAATGAGTAAAC- - - - - - AGGGCTG  46575
Mouse                       - - - - - - - - TTAGTGTTACCATT- AGGGCTGACTGTGTGTGTGAC- - TGTTA- TCAGTTTGTTTGCTT- - - - - - - - - GTTG  45722
ChineseHamsterGHOK1GS       - - - - - - - - TTAGTGTCATCCT- - - GAATAAAATGTACATATAACA- TATTG- TTCAAATGAGTAAATATTATTAGGGTTG  47457
LongTailedChinchilla        - - - - - - - - TAA- - - - - - TCATTCAGCG- - - - - TATGA- - ATAATAAAGTTAACTTGATTGTGCAAATTAGTA- AATACTG  45923
```

```
Majority                    ACTTTGGTAGX----CXCTATTTXTGXXXCTTAXTXXTTTTT--AAAAXTXACXTATTAGXXTTTTXTTTXATTTXAAAG
                                           70090     70100     70110     70120     70130     70140     70150     70160
Human                       ACTTTGGTAGG-ACTTCCTGTTTGTGATCCTTATTAGTTTTTTTAAAACTAACCTATTTGCAATTTTTAAAAATTAAAAG  63744
GuineaPig                   ACTTTGGTAGATGGACTGTATTTGTGACCCTTACAAGTTTTC--AAAGATTACATTTTCATAGTTCTTTTAG-TTAAAAG  42052
NorthernAmericanDeerMouse   AA-------------AACTATTATCAGTT-----T--TTTTT----AA-------AGCAGTTTTTATTTTATTAGAAAG  46624
Mouse                       TTT--------------TTATTTTAGGCTG-------TTTT---------------ACAGCTTTTTATTTTATTTAAAAG  45766
ChineseHamsterGHOK1GS       ACTGTATGTTT----GACTGTTATCAGTTGGGCTT--TTTTT--TTAAGCCGTTTAATAGCTTTTTATTTTATTAGAAAG  47529
LongTailedChinchilla        ACTTTGGTAGACAGGCTGTACTTGTGACCCTTACAAGTTTTT--AAAGATTACACTTTTGTAGTTTTTTTAA-TTGAAAA  46000

Majority                    TXTTAGCATTGATGATXAAATGXTTGTATTTTAGAGTTAAGTAGCATTTXCTAXTTAAXGAAGGXAGTGTTGGTGGAAA
                                           70170     70180     70190     70200     70210     70220     70230     70240
Human                       TATTAGCATGGATGCTTAGCTGGTTGTATTTTAGGTTGAAGTAGCATTTTCTATTTAAGGAAGATAATATTTGGGGGAAA  63824
GuineaPig                   TATTAGCATTAATTATTAAAAATTTTAA----AAGGTTAAGTAGCATTTTCTAATCAAAGAAGATAGTGTTTGGTGGAAA  42128
NorthernAmericanDeerMouse   TGTTAGCATTGATGATGAAATG-TTGTATTTTAGAGTTAAGTCGTATTTCCTACTTAATGAAGGCAGTGAATGATAGAAA  46703
Mouse                       TGTTA-TATTGATGATGAAATGGTTATATTTTAGAGTTAAATAGCATTGCCTACTTAAGGAAGGCAGTGAGTGGTAGAAA  45845
ChineseHamsterGHOK1GS       TGTCAGCATTGATGAGGAAATG-TTGTATTTTAGAATTAAGTAGCATTTCCTAGTTAATGAAAGCAGTT-----TCAAAC  47603
LongTailedChinchilla        TATTAGCATGCATTATTAAAATTTTAAA----AGACTTAAGTAGCGTTTTGTAATTAAAGAAGGTAGTATTTGGTGGAAA  46076

Majority                    CTAACACATTTAXAXC--XAXXAGCATXGXTTGATXTXTGAAATTTGAAT-CCTTTTCC---------------------
                                           70250     70260     70270     70280     70290     70300     70310     70320
Human                       GTAATACATGGACAGCTGGAACAGCAAAATTTGATATATGAAATTTGAATCCTTTTCCTGGTAGCTTTTAAGTTCGAGTT  63904
GuineaPig                   CTAACACATTTACAGCAGGAACAGTGCAGTGTGATATGTGAAATTTGAAAACCTTTTCC---------------------  42187
NorthernAmericanDeerMouse   CTAACACACTTAGAA-----GTAGCATGGCTTGATGTATGAAATTTTAAT-CCTTTTCC---------------------  46756
Mouse                       CTAACACATTTAGAAC---AGTACCATGGCTTGATATGTGAAATTTTAAT-CCTTTTCC---------------------  45900
ChineseHamsterGHOK1GS       TTAGAACA----GAA-----GTAGCATGGCTTGATGTATGTAATTT-----------CC---------------------  47642
LongTailedChinchilla        CTAATCCATTTACAGAACGAACAGTACAGTGTGATGTGTGAAATTTGAAA-CATTCTCC---------------------  46134

Majority                    -----------------------------TATTTTTGCTAATTTTCAXATTTT----XAGATXGCTGATATAXTTTATTC
                                           70330     70340     70350     70360     70370     70380     70390     70400
Human                       TTTGTTGTTGAGTTTTATTACTAAAATTATACTTTCACTAATTTTTATTTTTTGTTTAAGATTGCTGATATGCTTTATTC  63984
GuineaPig                   -----------------------------CATTTTTACATGTTTTTATGCCTT----AAGATTGCTAATGTACTTCATTC  42234
NorthernAmericanDeerMouse   -----------------------------TATTTTTGCTAATTATCAAATTGC-----AAATGGCTGATACATTTTATTC  46802
Mouse                       -----------------------------TGTTTTTGCTAATTTTCAAATTTA-----TGATGGCTGATATATTTTATTC  45946
ChineseHamsterGHOK1GS       -----------------------------TGTTTTTGCTTATTTTCAAATTTA-----AAATGGCTGATACATTTTATTC  47688
LongTailedChinchilla        -----------------------------CATTTTTGCAAATTTTCATGCCTT----AAGATTGCTAATATACTTCATTC  46181
```

```
Majority                    ACTTTTACAGGCCCTAGATGGTCAXAATATTTATAATGCTTGCTGTACCCTAAGGATTGATTTTTCCAAACTTGTGAATT
                                     70410     70420     70430     70440     70450     70460     70470     70480
Human                       ACTTTTACAGGCCCTAGATGGTCAGAATATTTATAATGCCTGCTGTACCCTAAGGATTGATTTTTCCAAACTTGTGAATT 64064
GuineaPig                   ACTTTTACAGGCCCTAGATGGTCAGAATATTTATAATGCTTGCTGTACACTAAGGATTGATTTTTCAAAACTTGTGAATT 42314
NorthernAmericanDeerMouse   ACTTTCACAGGCCCTAGATGGTCAAAATATTTATAATGCTTGCTGTACCCTAAGGATTGATTTTTCCAAACTTGTGAATT 46882
Mouse                       ACTTTTACAGGCCCTAGATGGTCAAAATATTTATAATGCTTGCTGTACCCTAAGGATTGATTTTTCCAAACTTGTGAATT 46026
ChineseHamsterGHOK1GS       ACTTTCACAGGCCCTAGATGGTCAAAATATTTATAATGCTTGCTGTACCCTAAGGATTGATTTTTCCAAACTTGTGAATT 47768
LongTailedChinchilla        ACTTTTACAGGCCCTAGATGGTCAGAATATTTATAATGCTTGCTGTACACTAAGGATTGATTTTTCAAAACTTGTGAATT 46261

Majority                    TGAATGTAAAATACAACAATGATAAAAGTAGGGATTATACTCGACCTGATCTTCCATCTGGAGATGGXCAGCCTGCATTX
                                     70490     70500     70510     70520     70530     70540     70550     70560
Human                       TGAATGTAAAATACAACAATGATAAAAGTAGGGATTATACTCGACCTGATCTTCCATCTGGGGATGGACAACCTGCATTG 64144
GuineaPig                   TGAATGTAAAATACAACAATGATAAAAGTAGGGATTATACTCGACCTGATCTCCCATCTGGAGATGGACAACCTGCATTG 42394
NorthernAmericanDeerMouse   TGAATGTAAAATACAACAATGATAAAAGTAGGGATTATACTCGACCTGACCTTCCATCTGGAGATGGCCAGCCTGCATTA 46962
Mouse                       TGAATGTAAAATACAACAATGATAAAAGTAGGGATTATACTCGACCTGATCTGCCATCTGGAGACGGCCAGCCTGCGTTA 46106
ChineseHamsterGHOK1GS       TGAATGTAAAATACAACAATGATAAAAGTAGGGATTATACACGACCTGATCTTCCATCTGGAGATGGCCAGCCTGCATTA 47848
LongTailedChinchilla        TGAATGTAAAATACAACAATGATAAAAGTAGGGATTATACTCGACCTGATCTCCCATCTGGAGATGGACAGCCTGCCTTG 46341

Majority                    GACCCAGCTATTGCTGCAGCATTTGCCAAGGAGACATCCCTTTTAGGTATGATTTTTAXTGTCT-ATACCACXTTTCTXC
                                     70570     70580     70590     70600     70610     70620     70630     70640
Human                       GACCCAGCTATTGCTGCAGCATTTGCCAAGGAGACATCCCTCTTAGGTATGATTTTTATTGTCTTA-ACCACTTTTCTCC 64223
GuineaPig                   GACCCAGCTATTGCTGCAGCATTTGCCAAGGAGACATCCCTTTTAGGTATGATTTTTATTGTCTAATACCACTTTTCTCC 42474
NorthernAmericanDeerMouse   GACCCAGCTATTGCTGCAGCATTTGCCAAGGAGACATCCCTTTTAGGTATGATTTTTACTGTCT-ATACCACCTTTCTTC 47041
Mouse                       GACCCAGCCATTGCTGCAGCATTTGCCAAGGAGACATCCCTACTAGGTAGGACTTTTACTGTCT-GTAGCACCTTTCCTC 46185
ChineseHamsterGHOK1GS       GATCCAGCTATTGCTGCAGCATTTGCCAAGGAGACATCCCTTTTAGGTATGATTTTTACTGTCT-ATACCACCTTTCTTC 47927
LongTailedChinchilla        GACCCAGCTATTGCTGCAGCATTTGCCAAGGAGACATCCCTTTTAGGTATGATTTTTATTGTCTAATACCACTCTTTTCC 46421

Majority                    CAT-XTGCTXAATGGGAAAGTXCCTGTTXAACTXCTGTAAGTATXATTAATCXTXT-ATTTTXGACCTTAACAAATTXTG
                                     70650     70660     70670     70680     70690     70700     70710     70720
Human                       CATTTTGCCAAATGGAAAAG-----------TACCAGTAAGTATAATGAATCCCCCCATTTTGGACCTTACCAAATTGTG 64292
GuineaPig                   CATTTTGCTAAATGGGAAGG--CTTATTTGACTTCTGTAAGTATGATAAATTCTTT-ATTTTGGACTTTAATAAATTGT- 42550
NorthernAmericanDeerMouse   CAT--TGTTCGGTGGGAAAGTACCTGTTAAACTGCTGTAAGTATAATTAATTTTAT-ATATTAAACCTTAACAAATTATG 47118
Mouse                       CAT--TGCTCTCTGGGAAAGTGCCTGGTGACCTACTGTAAGTATGATTGACCTTGG-ACTTCATACCTTAACAAATGACA 46262
ChineseHamsterGHOK1GS       CAT--TGTTCGTTGGGAAAGTACCTGATAA-CTGCTGTAAGTATAATTATTCTTAT-ATATTAAACCTTAACAAATTATG 48003
LongTailedChinchilla        CATGTTGCTAAATGGGAAAGTGCCTATTTAACTTCAGTAAGTATGATGAATCCTTT-ATTTTGGACTTGAACAAATTGT- 46499
```

```
Majority                    XTTAXATTTTATGAGTTTT-X-TTTGTCXGGTGAGAAGGTXATATGAATTCTGTTACAAGGAAXCCT-XXTTXXCTTGXT
                                      70730     70740     70750     70760     70770     70780     70790     70800
Human                       -TTAGGTTTCGTGAGTTTTCTTTTTCTCAAGTGAGAAGGC-ATATGAATACTGTTAAGAAAAACCCAAAGTATTCTTTAT  64370
GuineaPig                   -TTCAATTTTATGAGTTCT-----TTTAATGTGAAAAAA--ATGTGA-------------------AGTATTCTTGAT  42601
NorthernAmericanDeerMouse   TTCATATTTTATGACTTTTGTTTTTGTCTGTTTAGAAGGTTATATGAGTTCTGTTACAAGGAAGCCT---TTAACTTGCT  47195
Mouse                       TGGAGAGTTTATGAGTTTT---CTTGCATATTCAGAGGGTTGTATGAATTCCATTACAAGGAAGCCT---TTAACTTCCT  46336
ChineseHamsterGHOK1GS       TTCATATGTTATGAGTTGT---TTTGTCTGATCAGAATGTTATATGAATTCCATTACAAGGAAATCT---TTAACTTGCT  48077
LongTailedChinchilla        -TTAAATTTCATGTGTTTTGT---TCTCAGGTGAAAAAGC-ATGTGAGTACTGTTAAAAA-----CCAAGTGTTCTTCAT  46569

Majority                    AAGTATAATTAA----AAGTXTATTXGTTTTGTTCC---TTTAXTACCT--AXCTTXTTXCTCATACXACTCTCCXAAAA
                                      70810     70820     70830     70840     70850     70860     70870     70880
Human                       AAGTCTAATTAAA--ATTATATGTTAGTTTTGTTCCT--TTTAATACCTTCAGCTTCTCTTCCATGCCACTCTCCCAGAA  64446
GuineaPig                   AAATGTGATTAA---------ATTTAGTTTTGTTCTG--TTTACTACAT-TACCTTCTTTCTTACACCGCTGTCCCAAAA  42669
NorthernAmericanDeerMouse   AAGTATAATTAA----AAGTGTATTCTT----------TTTTATCACCT--CATTTTGTCCCCATTCAACTCTCCTAAAA  47259
Mouse                       GAGGATAACTAACTAAAAGTATCTTCATCTTACCCCTCTTTTATCACCT--CTCTTTTCCCACATATAACTCCCCTAAAA  46414
ChineseHamsterGHOK1GS       AAGTATAAATAA----AAGTGTATTC-------------------------ATTTTATCCTCATGCAACTCTGCTAAAA  48127
LongTailedChinchilla        AAGTGTAATTAG---------TATTAGTTGTGTTCC---TTTACTACCT-CACCTTCTTTCTTATACCACTGTCCCAAAA  46636

Majority                    CAAAAGAAGAAAAGCTGGAATGAAAA-XAXAXTXTTGAGTX-TTATTAAAATATTTATGCTTCAXATTTGXTGTXAT--X
                                      70890     70900     70910     70920     70930     70940     70950     70960
Human                       CAAAAGAAGAAAAGCTCTAATGAAGA-AATACTGTTGAATTCTTAATGCCATATTTACACTTTAAACTTGATGTCACTAT  64525
GuineaPig                   GAAAA--------GCTGAAATGAAAA-AGTACTTTTGAGATTTTAGGTAAATACTTCTGCTTCAAATTTGTTGTCAA--T  42738
NorthernAmericanDeerMouse   TGAAAGAAGAAAGACTGGAATGAAAA--AGAATGTTAAGT--TTATTAAAATATATATGTTTCAGATTTG-----CT---  47327
Mouse                       TAAATGAAGAAAAACTGGAATGAAAA--AGAATG--------TTATTAAAATATGTATGCTTCAGATTTGGCGTTCT---  46481
ChineseHamsterGHOK1GS       CAAAAGAAGAAAACCTGGAGTGAAAA--AGAATTTTAAGT--TTATTAAAAGATGTATGCTTCAGATTTG-----AT---  48195
LongTailedChinchilla        CAAAATAAGAACAGCTGTAATGAAAAGAATACTTCCGAGTTTTTAATAAAATATTTTCGCTTTAAACTTGTTGTTA---T  46713

Majority                    -XXX-XGTCTAAGCCTAAGGXCXTTXTGCXTAXTTGTTAGGGTTAATXTGATTGX-----TTTAGCAGTGTGXAGAAACA
                                      70970     70980     70990     71000     71010     71020     71030     71040
Human                       GCCTGAGTCTAGGCCTAAAGTTCTTGTGTGTGTTTTTCAGTGCTGATCTGATTGTTTTAATTTAGCATTGCATGGAAACA  64605
GuineaPig                   ACCTGAGTCTCAGCCAAAGGCCCTTGTGCCTATTTGTTAGGGTCAAAATGATTG----AATTTTGCACTTCATAGGAACA  42814
NorthernAmericanDeerMouse   ------GTCTAAGCCTAAGGCCTTTATGCCTACTTTTTAGGGTTGATCTGATTGT-----TTTAGCAGTGTGCAGAAACA  47396
Mouse                       ------GTCTAAACCAAAGGTCTTTATGTGTTCTTGTTAGGGTGATTTTGTTTGC-----TTTAGCAGCATGCAGAAATA  46550
ChineseHamsterGHOK1GS       ------GTCTGAGCCTAAGGCCTTTATGCTTACTTCTTAAGGTTAATTTGATTGC-----TTTAGCAGTGTGCAAAAACA  48264
LongTailedChinchilla        ACCTAAACCTAAGCCTAAGGTCCTTGTGCTTATTTGTTAGGGTTAAAATGATT-------TTTAAC------TAGAAATA  46780
```

Majority	XXAAAXTATTAACAGATAXAAXA---TAXGXGTGTGGXGXTAXATGGXATTTTTTT-AAAAGCATATTGTAXXACAXXTT	
	71050 71060 71070 71080 71090 71100 71110 71120	
Human	GTGAAATGTTAAGAGACAAAACATACATAATGGGTGGAGGCATATGGGATTTTTTT-TGAAGCATATTGTAGTGGCCTTT	64684
GuineaPig	GTGAAATGTTAATAGATAAAACA-----GGTGATGGGAATTGTAAATGGATTTTTTTCAAAGCCCAT-ATTATTCTCTCT	42888
NorthernAmericanDeerMouse	ACAAAGTATTAACATGGGTAAAG---TAAGAGTGTGAG--CACATGGAGTTTTT---AAAAGCATATTGTATCATAAATC	47468
Mouse	ACAAAGTATTTACGAAGATGAAAA--TAGGAGTCCATGGGTGCATGGAATTTTTTAAAAAAGTATAGTCCACCACAAATG	46628
ChineseHamsterGHOK1GS	ACAAAGTATTAACAAATATAAAAA--TAAGAGTGTGGGG-TACATGGAATTTTT---AAAAGCATAATGTACCATAAATC	48338
LongTailedChinchilla	GTAAAATACTAGCAGATAAAACA-----GGTGATGGGAATTATATGTGAGTTTTTT-CAAAGCCTAT-AT-ATACCCTCT	46852

Majority	TTXAAA---AATTTXAAXTAGAAXXAXTTTTCTXTAAAATTTATXTTGCTXTTXXTTXGCATTXT-X--XCATTA-TTAT	
	71130 71140 71150 71160 71170 71180 71190 71200	
Human	TAAAAAAACAATTTAAACTAGAAGAAATTTTCTGTAAAATTAATTTTGCTATTTCTTTGCATTTTGTCTTAATCATTTAT	64764
GuineaPig	TTTAAG--TAATTTAAACTAGAAGAAATTTTCTGTATAACTTACTTTGCTATTCATTCACATTAT-----------TTAT	42955
NorthernAmericanDeerMouse	TTAAAA---AATTATAATTGAGG--ATTTTTCCATAAAATTTGTATTGTTCTTTCTT---------TAGGCTTTG-TT--	47531
Mouse	TTGAAA---AATTGTACATGAAG--ATTTTTCTAGAACATGCTTATTGCTCTTCTTTGGATGTGG-TTATCATTA-TTTT	46701
ChineseHamsterGHOK1GS	TTGAAA---AATGCTCTTCAT-----TTGTACTGTGTTATTTATATATTACCTT---------------GCCCTG-CA--	48392
LongTailedChinchilla	TTTAAG--TAATTTAAAGTAGAAGAAATTTTCTATAAAATTTATTTTGCTATTCATTTGCATTATC-----ATTATTTAT	46925

Majority	ATTTXACCTTGTXXXXXGXTGGATTTGXGCCACXTAAXTAAAAT-ACAGTGTAAAAATAXGCAXCTGX------------	
	71210 71220 71230 71240 71250 71260 71270 71280	
Human	ATTTTACTTTGTACCACAATGGACTTGAGCCACCTAATAAAAAT-ACAGTGCAAACATATGCAACTTAT------AGTTG	64837
GuineaPig	ATTTCACCTTGTCTTGTGGTAGAATTGAGCCACCTTAGAAAAAT-ACAATATAAAAATATACAACTGA------------	43022
NorthernAmericanDeerMouse	------------------ATGGATTTATGTTACTTATGTAAAACTATAGTGTAAAGATAAGCAGCTAG------------	47581
Mouse	AATTTACCCCTAAAATGGATGGATCTGTGCTACTTACATAAAGT-GCAGTATAAAAACAAGCAGCTGG------------	46768
ChineseHamsterGHOK1GS	------------------GTGGATTTGTGCCACTTACATAAAAC----ATGTAAAAATAAGCAGCTGG------------	48438
LongTailedChinchilla	ATTTCACCTTGTCCTATGGTAGAATTGAACCACCTAATTAAAAT-ACAGTGTAAAAATATACAACTGATGGAGTCAGTTG	47004

Majority	--------TAGTTGATGGAXTXAAGATXAAGGAAAAAX-X-X-XXX-X--XX-XTTGTAAGTXAAAATXAGCAAAXTGAT	
	71290 71300 71310 71320 71330 71340 71350 71360	
Human	-------ATAGC--GTGGAATCAAGATGAAGAAAAAAATAAGGAAAATCTTACTTTGTAAGT-AAAATGTGCAAAGTGAT	64907
GuineaPig	--------CAGTTGACAGAATCAAGATGAAGAAAATATAAGGAAAAATTGTATTTTATAAGT-AAAATGAACAAAGGGAT	43093
NorthernAmericanDeerMouse	--------TAGTTGAGGGACTGAAGATTGGAGAAAAAT----------------TTGTAAGTTAGAATAAGCAAACTGAT	47637
Mouse	--------AAGTTGATTGACCGAAGAGTAGGGGAAA------------------T-GTAAGTTAGAATAAGCAAACTGAT	46821
ChineseHamsterGHOK1GS	--------TAGTTGATGGGCTGAAGATTGTAGAAAAAA----------------TTGTAAGTTATACTAAGCAAACTGAT	48494
LongTailedChinchilla	ATGGAATACAGTTGATGGAATCAAGATGAAGGAAATACAAGGGAAA-TGGTATTTTGTAAGT-AAAATGAACAAAGGGAT	47082

```
Majority                      TTATAAXXX-XGTXAGAATTTXATAGTTTXCTTTTATTTGXTGTGTTTTGGAAA------CTTTTXTTXTGGTATTXG--
                                         71370     71380     71390     71400     71410     71420     71430     71440
Human                         TTATAAGTTAAGATGGAATTTTAGAGTTTCCTTTTATTTGATGTATTT------------CTTGATTTCTAGTATTGG--  64973
GuineaPig                     TTACGAGTTTAGATAGCATTTTACAGGCACCTTTTACTTGCTGTTTTTGAGAAATTACTTCTTTTTTGCTGGTATTGACA  43173
NorthernAmericanDeerMouse     TTAT-G----TGTCAGAATTTAATAGTTTTATTGGATTTGGTGTGTTTTGAGGA------TTTTTATTTTGGAATTTG--  47704
Mouse                         --AA------TGTCAAATTTTAATCGTTTTCTTTAATTTGTCCTGTTTTGAAA--------TTTCACTTTGATATTTT--  46883
ChineseHamsterGHOK1GS         TTGTAG----TGTCAGTATTTAATAGTTTTCTTGAATTTGGTGTGTTTTGGAGA------TTTTTATTTTGGTATTTG--  48562
LongTailedChinchilla          TTACAAGTTCAAGTGGCATTTTACAGGCTCCTTCTATTTGATGGGTTTGAGAAAGTTTTACTTACTTACTGGTGTTGACA  47162

Majority                      ---TAAXATTATCTTTAX-TXXTTATTTXTGTXTXTATGXXXTTCXAGAXTGTXTGTTXXXATACXAGXTXTGT--XXXA
                                         71450     71460     71470     71480     71490     71500     71510     71520
Human                         ---TAAGATTATCTTTAAGTCATGATTTTTATATATATGGAACTCAAAATTGATAGTTGACAAACTAGCTTTTACAATTT  65050
GuineaPig                     GTATAAGATTTTGTTTAAATCAAAGTGT--GTCCATGGGGAATCCAAGATTGGTGGTCGACACACTAGTTCTA-----TA  43246
NorthernAmericanDeerMouse     ---TAGTATTACCTTT-----TTTATTTATGTGTGAATATTTTTTCTGTATGTATGTTTGTATACCATATATGT--GCAA  47774
Mouse                         ---TAATACTATCTTCA--TTTTTATTTCTGTGTGGATGTTTTCTCTGCATGTATGTTTGTATACCACCTGTGT--AGAG  46956
ChineseHamsterGHOK1GS         ---TAGTGCTGTCTTTA--TTTTTATTTATGTTTGGATGTTTTTCCCTTATGTATGTTTGTATACCATATGTGT--GCAA  48635
LongTailedChinchilla          GTATAAGATTTTCTTCAAACAAGCATTTCTGTCCATATGGAATTCAAGATTGGTGCTTGACACACTAGTTCTT-----TA  47237

Majority                      XGTXTGTGXXXGTCATTXXXXAAXAXCAGATACTXTXX--AAXTGGXGTTTXXAXATACTTGTTAGCTGXCATXTAACXT
                                         71530     71540     71550     71560     71570     71580     71590     71600
Human                         AGTTTATGTCTGTCATTTCAAAAGAAAAAATAGTATAATTAAGTAGTTATTGGAAATATTTACTTCCCAATATATAACAG  65130
GuineaPig                     AATTCATGTCCATCATTCTTTAAAAAAA-ATGTTATAACAAAGTGGTGCTTGGAAATACTTGAGT-TGGATGTATAACAA  43324
NorthernAmericanDeerMouse     TGTCTGTAAAGGGCA----------TCAGATACTCTGG--AACTGGAGTTTACAGACAATTGTTAGCTCCCATGTGAATT  47842
Mouse                         TGCCTGTGAAGGGCAGAAGAGGGCATCTGATTCTCTGG--AACTGGAGTTTACAG----TTGTTAGCTGCCACATGAGTT  47030
ChineseHamsterGHOK1GS         TGCCTGTAAAAGCCG----------TCAGATCCCCTGG--AACTGGAGTTTACAGACAGTTGTTAGCTGCCATGTAAATT  48703
LongTailedChinchilla          AGTT-GTGTCCATCATTTTTTAAAAA---GTATTA-AATGCAGTGATTCTTGGAAATACTT-----TGGACGTGTAACAG  47307

Majority                      XXATGXXCTGAACXXXTGTCT------TCTGTAAGTG--XCXATAAGTGCTCXXAXCTACTG--AGCCA-----TXTCTX
                                         71610     71620     71630     71640     71650     71660     71670     71680
Human                         TCATGCCATCAGTTTGTGAATATGAAATCTGTAAGTG-GTATATATGTAGAGAGAGATACTCTAAGGCAGTTGGTATTTG  65209
GuineaPig                     TCATGCTACTTACTTGT-----------AAGTATTTA-ATCTTTAAGT-----GAGGTATAAACCTTTAAGGTCTATGTA  43387
NorthernAmericanDeerMouse     CTA--AGCTGAACCCAGGTCC------TCTGCAAGAG---CAACAAGTGCTCCTAACCACTG--AGCCA-----TCTCTA  47904
Mouse                         CTGGGACCCGAGCCCATGTCT------TCAACAGGT----CACTAAGTGCTCATGACTACTG--AGCCA-----TCTCTA  47093
ChineseHamsterGHOK1GS         CTAGGAACTGAATCCAGGTCT------TCTGTAAGAG---CAACAAGTGCTCTTAACCACTG--AGCTC-----TCTCTG  48767
LongTailedChinchilla          TCATTCTGTTTACTTGT-----------CTTTAGGTGTGTGTGTGTGT-----GAGATATGA-TATTCA-----TATATG  47365
```

Majority	TCCCCAAXTAX-TACAATCTTXAAATAACGGTTAXTATCTXGAG--AXAAXC----CAGAXCCTTTGTTXGAGAAAGTAG	
	71690 71700 71710 71720 71730 71740 71750 71760	
Human	ATTCTGGTAACTAATAAGAATTAACTACCTGTTAATTCTGGCAGCTGCAAACAAATCTGACCCTTTATTCGAATTTTCTG	65289
GuineaPig	TACATACATACACACAAATATGCAAGAGCTGTTAGTATTTGAAATTACAG------TGGTACCTTGTTTCATGAAGTTT-	43460
NorthernAmericanDeerMouse	TCCCCCACTAA-TACTATCTTTAATTCAGGATTACTATCTAGAG--AGAATC----CAA-------GATTGAGAAAGTAG	47970
Mouse	TCCCCAAGTA--TAGTATCTTGAAATGAAGGTTACTGGCTAGAG--AGAATC----CAGA-TTGATGGTTGAGAAAGAAG	47164
ChineseHamsterGHOK1GS	GCCCCAACTAA-TACAATCTTTAATTCAGGAGTATTATCTAGA---AGAACC----CAAAGTTGATGGTTGAGAAAGGAG	48839
LongTailedChinchilla	TATGGGATGAGACACAAGTGTGCAAGAGCAGCCAGTCCCTGAAGTCACGG------CGGGGCCTTGTTCCAGGGAGCTCG	47439

Majority	AT---TXTCTGCTAAAGTTTTAGT--TTATXXCCAXXXTTTTAGAAAX----AATCATXXGTGTTX-------AAAATAC	
	71770 71780 71790 71800 71810 71820 71830 71840	
Human	GTGTATCCTAGCAGAAGTCATAGTGTTTATTATGACTGTAGTAGATGCTTTAGGTCACTTACACTCTTTCCTGACAGAAT	65369
GuineaPig	AT---TATCTGCACAATTG-------TGGTTGCAACTGAGTTGGAC------AATCATCTGAGTT----------AATGG	43514
NorthernAmericanDeerMouse	A------TC--TTAAAATTTTAGT--TTATGTCCATCATTTTAAAAA---------ACAGATGTT--------AAAATAC	48023
Mouse	AAAG-TAGCTTCTAAAGTTTTAGT--TTATGCCCATCATTTTACAAAA----CAG-ATGAGTGTTG-------AAAATAC	47229
ChineseHamsterGHOK1GS	AAG----TCTCTTAAAAATTTACT--TTATGTTCATCATTTAAAAAAA----AAACATGAGTATTG-------AAAATAC	48902
LongTailedChinchilla	AT---TCTTTGCCCAGGCC-------TGGTTGCAGCTGAGTTGGACGGG--AAGCGAGCCCAGTTC---------AGTGG	47498

Majority	TTATXATXGATATGTXACTXXCXXXXTXAXTTGGAAATTTGT-X----AAAATXGAXTGGTTC---GTATGXGA------	
	71850 71860 71870 71880 71890 71900 71910 71920	
Human	CTCTACTACCTCTGTGTGTGTCTAGCTTAATTCGTAATTCAAGTTAATAAGATTGAATATTTCACAGTGTGCAGTTTTTA	65449
GuineaPig	TTATAATGGTTTAGTGT-TGAC---CTTAAGGGGAAATTCGATTTG--GTTCTCATCTAGTTC---GTTTGCGACT---G	43582
NorthernAmericanDeerMouse	TTACTATAGATATGTAACTACCACATCCACTTGTAAATATGT------AAAAT--ATTGGTTT---GTATGTGA------	48086
Mouse	TTATTACAAACATGTAACAACTCCATTCACTTGTAAATGTAT------AAAAATGATTGGTAT---GAATGCAA------	47294
ChineseHamsterGHOK1GS	TTACTATGGATATGTAACTATCATGTCCACTTACAAATATGT------AAAAC--GATTGTAC---ATATGTGA------	48965
LongTailedChinchilla	TTCTAGTGGTTCAGTGC-TGGC---CTTAAGAGGAAGTTTGCTT----GCCCTCGTCCGTTGC---GTGTGTGACC---A	47564

Majority	-XXAXAAGXCTAATXTTGAXT--XXGTXXTTXXAXGTCAXTXCTTATXACTACAAATAAAXCXXXTGACCCXTTXTXXTT	
	71930 71940 71950 71960 71970 71980 71990 72000	
Human	AAGGCAAAGTCAGGAATGAAA--GAGTGAAACATGGTTTTTGCTTATAATTATTACTATAGATTATTAAACTCTTTACTG	65527
GuineaPig	GAGTCATGGAGAAAATTGAGT--CTGTGAACCAAGGTAGCA--CTGTAATTGAC-TTCAAACAAATCAGTTATTCTAATT	43657
NorthernAmericanDeerMouse	--AATGAGTCTAATATTTATT----GTTGTTTGAAGTCAGTGCTAATGACTACAAAAAA-----TTGACCCCT------T	48149
Mouse	--AATGAGTCTAATGCTCAAGAGCAGTTGTTTGCAGTTAATTTTCAGGAGGACATACAAAACTGGTGACCCTTTATTAGC	47372
ChineseHamsterGHOK1GS	--AATTAATCTAATGTTAAT-----GTTATTTGAAGTCATTTCTAATGACCACAAAAAAA----CTGACCCCTA----GC	49030
LongTailedChinchilla	GAGGCATGG-GAGAGCTGGGT--TCATGGGCCAGGGCCCC---CTGCAACTGAT-CTTCAGCAAGTCACTCACTCTGCTT	47637

```
Majority                    ATTTGGTGTCTXT-AGCACCAXXCXCTGCTGTTTGT-GTTGATACXATAGTAGATA----CTTTAGGTT---------GC
                                     72010     72020     72030     72040     72050     72060     72070     72080
Human                       ATAGACATTGTTTTAGAAAGAATCAAAATTTATTGA-GTAGCTTCTAGGTCATATT----CATTCAGCTAGGCACTTGGC  65602
GuineaPig                   TTCTAGTATATTT-GGCACCAGCCGTAGCTGTTTG---TTGTTACTGTAATAGATG----CTTTAGGTT---------GC  43720
NorthernAmericanDeerMouse   ATTTGGTGTCTCT-AGCAGAAC-CATTGCTGCTTATTGT-GACTGAATAGTAGATAG---CTTTAGGTT---------GC  48214
Mouse                       ATTTGCTGTCTCC-AGCACCATGCGCTGCTGTTTGTGACTAA--AAATAGTAGGTAGATGGTTTAGGTTCTTCCT---AT  47446
ChineseHamsterGHOK1GS       ATTTGGTGTCTCC-AGCACAAC-CACTGATGTTTATTGTAGATAAAATAGTAGATA----TTTTAGGTT---------GC  49095
LongTailedChinchilla        TTCTGGCGTGTTT-GGCACCAGCCGCGGCTCTCTGG--TTGTTACTGTGATAGGTG----CTTTAGGTT---------GC  47701

Majority                    TXXCAXTXTTCTTTGACAGAATCTTATTTGCTXTGTAGGTA-TGXTTXAAGXXTTACTTXXAAATTXXCAAXGTTTAATG
                                     72090     72100     72110     72120     72130     72140     72150     72160
Human                       AGATACATTACTTTGTTAAAATAGATGGTCTCATCAAGATTTTACATATAGTTT--CTAAGAAGTTGAAAAATTCACATG  65680
GuineaPig                   TAACACTTTCTTTTGACAGAATGTCTATTGGTCTGTAGTTAATTCCCTCAATTCAACTTGCAAATTGATAACATTGACTA  43800
NorthernAmericanDeerMouse   GGGCAGTGTTCTTT-ACAGAATCTTATTTGCTGTGTGGGTC-TGGTTAAAGCCTTATT----AATT--CATTGTTTAGTG  48286
Mouse                       TAGCAGTGTTCCTG-ACAGAATTTTATTTGCACTGTAGGTT-TGGTTGAAGCCTTGGTTTATAGTTAGCAGTTTTTAATG  47524
ChineseHamsterGHOK1GS       TAGCAGTGTTCCTTGACAGCATCTTATTTGCTATGTGGGTA-TGGTTGAAGCCTTACT----AATT--CATAGCTTAATG  49168
LongTailedChinchilla        TGACGCTTTCTTCGGACAGAACCTCCGTTGGTGTGTAGTTAATTCTCCCAGTTCAGCTTGCAG-TTAGCAACGTTGACTG  47780

Majority                    A---X--------X-----X-CAAAGTCAAGATX----GAGACTATAATAAATGAT----XXX--AGCTTTTGCTTTTAA
                                     72170     72180     72190     72200     72210     72220     72230     72240
Human                       CAACTCATCAAAGAAACAATTTAAAGATAAAATACCAAGAAACTCCAGCTAAAGATAAAATACCAAGAAACTCCAGCTAG  65760
GuineaPig                   ATTTTTGGTGTGCAACTTTTTCAAGGAATAGTTA-----AGATTGAAAAAAATAATT-GATACGTGGTTTTTGCGTATAA  43874
NorthernAmericanDeerMouse   A--------------------CAAAGTCAAGATT----GAGACCATAATAAGTGAT---------AGCTTTTGCTTTTAA  48333
Mouse                       G--------------------CAGTGTCAAGATT----GAGATGATAATAAATGAC---------A--TGCAGCTTTTAA  47569
ChineseHamsterGHOK1GS       A--------------------TAAAATCAAAATT----GAGACCATAATAAATGAT---------AGCTTTTGCTTTTAA  49215
LongTailedChinchilla        TGTCTTGGTGTACAGATTTT-CAAGGGAAAGTTA-----AGACTGAAAGAA-TAAT--GTTACATGGCTTTTGCTTACAG  47851

Majority                    CTCTTXXAXTXGACX----XGAACTXTT------A---XXXXXAXAAAAGTAAXXTTXXAXAAAGXCCTXXAATTGAGTA
                                     72250     72260     72270     72280     72290     72300     72310     72320
Human                       TATTAACAATGGACTACAATAATCACTTCTTTGGACCCTACTGAGGTGTGTGTATTTGCCCATCTGGATAT-ACTTAGAT  65839
GuineaPig                   ATCTTACTTTAGATT---TTAAACTCTT----------TACTGAGAAAAGTTGCATTGTAGAAAAGAATAA-----GGTA  43936
NorthernAmericanDeerMouse   CTCTT-TACTTGACA-----GAACTTGT------A------CAAAAGAAGAAA--------AAGGACCTGCAATTGAGTA  48387
Mouse                       CTC---TGTTGGACA-----TAACTTTT------A------CAAAAAGAGTAG--------AAAGACCTGTAATTG--TA  47619
ChineseHamsterGHOK1GS       CTCTT-TACTTGACA-----GAACTTGT------A------CAAAAAAAAAAAAAAAAAACAAAGACCTGCAATCAAATA  49277
LongTailedChinchilla        TTCTTACTATAGGTTA--TTGAACTCTT----------TACTGAGAAAAGTTACGTTACAGAAAAGAGTAACATTGAGTA  47919
```

Majority	GXTTCTXGGTCTTAG---GCATTCTTCTX-XAACACTTGGCAGXTAXAT-TAGTGAATCXATAA--ATAGCCAAATTAAG	
	72330 72340 72350 72360 72370 72380 72390 72400	
Human	GTGCTTGGATCCTGGGTGGGAGGCTTGGTTAGAAGTCACGTGGATTCTTCTAGGGAATTTATCACCATTTTTCTTGTGAT	65919
GuineaPig	ATTTCTATGTCTTTG---ACATTCTCCTATAGACACTTGG-GGATACAT-TATAGAATCTCTAAAAGTACCCAAATTTAG	44011
NorthernAmericanDeerMouse	GCTTGTGGGTCTTAG---GCATTCTTCTA--AGTCTTTGACAGGTATAT-TAGTGAAACCATAA--ATAGCGAAATAAAG	48459
Mouse	GCTTCTGGTTTTTAG---GCATTCTTCT----ACACTTCCCCGGTATGT-TAATGAAACCTTAA--GTAGCCAAACTAAA	47689
ChineseHamsterGHOK1GS	GCTTGTAGGTCTTAG---GCATTCATCTT--AATACTTGACAGGTATAT-TAGTGAAGCCATAA--ATAGCCAAATAAAG	49349
LongTailedChinchilla	GTTTCTAGGTTTTTGTTGACATTCTCCT--AAGCACTTGGCAGATACAT-TATTGAATCT----------------TAAG	47980

Majority	TAGGTXGCAGXXTAAAGATTTTATAGTCTCTAAGAAATTTAAAAAXTCA---TTTAX------XACTXATTAAAAAA---	
	72410 72420 72430 72440 72450 72460 72470 72480	
Human	CTTATGACCTTGCTGAAACTTTTAAAACAATAATAAAGTCATTTTTTTTCTCTTTGAGATGGAATCTCACTCTGTCGCCC	65999
GuineaPig	TAGGGGGCAGTGTCAAGATTTTATAGTTTATAAGAAGTTTTAAAATTTT---TTTA-------AACTCATTAAAAAAACA	44081
NorthernAmericanDeerMouse	TAGGTAG-GGCATAAAGATTTTATAGTCTCTAAGAAAGTTAAAAAATCA---TTTAT------CATTTCTTAAAGAA---	48526
Mouse	TGGGTAGTAGCAAAGA--TTTTATAGTCT---AGAAAATTAAACAATCA---CATAT------CGCTTTGTAAAAAA---	47752
ChineseHamsterGHOK1GS	TAGGTAGTGGCATAAA--TTTTATAGTCTCTAAGAAATCTAATAAATCA---TATAC------CATTTCTTTAA------	49412
LongTailedChinchilla	TAAATGGCAGTGTCAAGATTTTATAGTTACTGAGAAGTTTAAAACTTCA---TTTGC------AACTCATTAAAAAA---	48048

Majority	----------------------------------AXAAATTGAGGGTAA--AATACCAAAGAA-XXXXXGTXC---CATT	
	72490 72500 72510 72520 72530 72540 72550 72560	
Human	GTGTCAGAGTGTGGTGGCGCAGTCTCGGCTCACTGCAACTTCCGCCTCCCGGGTTCAAGCGATTCTCCCGCCTCAGCCTC	66079
GuineaPig	GTTTAAGAATAAATTAGCAGAAAACTGTATCANNNNNNNNNNNNNNNNN-NNNNNNNNNNNNNNNNNNNNNNN-NNNNNN	44159
NorthernAmericanDeerMouse	----------------------------------AAAAATTGAGGGTAA--AATACCAAAA------GATTTC---CATT	48561
Mouse	----------------------------------AAAAGTTATGGGTAA--AATACGAAAGAA----ATGTGC---CATT	47789
ChineseHamsterGHOK1GS	----------------------------------AGGAATTGAGGGTAA--AATACCAAAAAAAAAAGACTTC---CACT	49453
LongTailedChinchilla	-----------------------------------CCAATTGAAGAATA-AAATACCAGAGAAGCTCCCGTCA-AGCATC	48091

Majority	--GAAACATTGTTXATATAT-AAXATGAXAGGATTCTAAGAAGCTXTGTGTGTGTTTGCATXXGGXTGXXCTTXXXXT--	
	72570 72580 72590 72600 72610 72620 72630 72640	
Human	CTGAGCATCTGGGACTACAGGCGTGTACCACCACCCCTGGCTAACTTTTGTCTTTTTTCGTAGAGATGAGATTTCACCA-	66158
GuineaPig	--NNNNNNNNNNNNNNNNNNNNNNNNNNNNNNNNNNNNNNNNNNNNNNNNNNNNNNCCCATCTGGTTATACTTGGGTTG-	44236
NorthernAmericanDeerMouse	--AAAACATTGTTTATATAT-AATATGATAGGATTCTAAGAAGCTGTGTGTGTGTGTGTGTATGCGCGCGCGCCTTATCT	48638
Mouse	--AAAACACTGTTGATA----A-CATGAAAGGATTCTAAGAAACTATGTGTATGTTTGGAT-------------------	47843
ChineseHamsterGHOK1GS	--GAAACATTATTAATATATTAACATGATAGGATTCTAAGAAGCTATGTGTGTGTTTGAAT-------------------	49512
LongTailedChinchilla	--GAACAGTGGACTGTCGTAATTACTTCCTTGACCCTCGGCGGCTGTGTGTGTTTGCCCATCGGGTTGTACTTGGATT--	48167

```
Majority                     XXTGAX-----------TXGAXXCAXTTTTXXXAGGTC--------XTTTGGAXTTTTCTAAG----GAAXTTX-X----
                             72650  72660  72670  72680  72690  72700  72710  72720
Human                        TGTTGGCCAGGCTGGTCTGGAACTCCTGATCTCAGGTGATCTGCCCGCCTCAGCCTCCCAAAGTGCTGAGATTACAGGTG  66238
GuineaPig                    TGTT-------------TGGA--TCCTATGCAAAGGCA-------TGTTTTAT-TTTATGTAG----GAAATT-------  44282
NorthernAmericanDeerMouse    CCTGAG-----------TAGAGGCATATTTTG-AAGTC--------ATCTGGAATTTTCTGTG----GAATGTACAA---  48691
Mouse                        CCTAAA-----------TAGAGGCATATTTTTTAGGTC--------ATTTGGAATTTTCTAGG----GAA----------  47890
ChineseHamsterGHOK1GS        CCTGAA-----------TAGAAACATGTTTTG-AAGTC--------ATTTGGAGTTTTCTATG----GAATGTATAA---  49565
LongTailedChinchilla         TGTG-------------TGGA--CGCTACGCACAGACG-------TGTTTAGGGTTCCTCTAG----GGAGTT-------  48214
```

```
Majority                     ---------XXGCATAACTXTTTXTGTTGATXXCTTAAGGCTTTGGTGAGAXAA-TATTAXAGXTGTAXAAATAAATX--
                             72730  72740  72750  72760  72770  72780  72790  72800
Human                        TGAGCCACCATGCCTGGCCGCTTTTGTTCATTTTTTGAGCGTTGTACATTTTTA-CATTAAAGGCGTGTAAATTAAGTTG  66317
GuineaPig                    ------------CATTACTATTTTTATTGTGATCTTTAGGTTTTGGTGAGAT---TTTTACAGATGTAGTAATAAAAT-A  44346
NorthernAmericanDeerMouse    --------TTCGTATAACTTTTTGTGAT-----CTTAAGGCTCTGGTGAAAAAAGTGTT------TTACACACAAATC--  48750
Mouse                        ---------CCGAATAACTCTTTGTTGTGAT--CTTAAGGCTTTGGTTAGAAAA-TATAA-----AGAAAAAAAAATC--  47951
ChineseHamsterGHOK1GS        --------CTTGTGTAACTTTTTGTGATGATC-CCTTAGGCTTTGGTGAAAAAAGTTTTGTTTTTTCATCCAAAAATC--  49634
LongTailedChinchilla         ------------CATCGCTATTTTTATTACGATTTTAAGGGTTTGGTGAGAT---CATCATAGATGTAGTAATAAAAT-G  48278
```

```
Majority                     -XX--TTTCATAAA-AXGXATCTAGGAXCTAGCTCAGXTTTTAA-XTGC-TTTXTCAGT--XTXGCATTXTT--------
                             72810  72820  72830  72840  72850  72860  72870  72880
Human                        CTCGCTTTGCCAAAAAATTGTCTAGTGTCTTTTTCCACTTCTGTGTTGAATTTTTTAGTATAACGCATTTTTGTTTCTTC  66397
GuineaPig                    TTC---TTCATAAA-GATTATGAAGC-CCTAGCTATGCTTTT---TT---TTTTTCAGTATATTACATTTTT--------  44407
NorthernAmericanDeerMouse    -----TTTCATACA-ATGAACCGAGGATCTGACTCAGTTGGTAAAGTGC-TTTCTCTGT---TGGCAT------------  48808
Mouse                        -----TTTAACAGC-ATGGAACT-GGAGATGGCTCAATTTTTAAAGTGC-TTGCTCTGT---TGGCATGATC--------  48012
ChineseHamsterGHOK1GS        -----TTTCATAAA-ATGAATCTAGGAGATAGCTCATTTGGTCA-GTGC-CTTCTGAG--------------------  49684
LongTailedChinchilla         TTC---TTCATAAAAGACGATGAAGC-CCTAGCTATGCTTTTA--TT---TTTTCCAGTGCATTACATTGTT--------  48341
```

```
Majority                     --------XTXXTAAGXXTGAATCXXAAGTAXTTXX---XTAAGAAGC--CTTCAXATTTXTXTCXXTTTT--TXACXGX
                             72890  72900  72910  72920  72930  72940  72950  72960
Human                        ATCTCTGCCTCATAAGAAAAAGGAAAAAGTGTTTAGCCTTTCCTAAGTGACTTCTTTTTTTTATCTGTTTTAATGTTTTT  66477
GuineaPig                    ---------CCACTA----------AAAGCTTTTG----------------CTGAGCTTTCATTCTGTTTT---GCATGT  44449
NorthernAmericanDeerMouse    --------GTACTAAGCATGAATCCCAAGTACCTTG---GTAAGAAGC--CTTAACAATGATGGCCATTTG--TAACCGC  48873
Mouse                        -------TATACTAAGTTTGAATCTCAAGTACTT-----ATAGGAAGC--CTGCATAGTAGTCACCATTTT--TAACCCC  48076
ChineseHamsterGHOK1GS        --------GTACGAAGTTTGAATCCCAAGTACCTTT---GCAAGAAGC--CTGCACA-TGATGGCCATTTT--TAACCCC  49748
LongTailedChinchilla         ---------CCATGA----------AAAGCTTTTGTTCTATTAAAAG----CTCAGGTTTTGTTCTGTTTT---GCATGT  48395
```

```
Majority                     XXGTTXGXTAGTAGXXAXGXACXXGAXC--ATGXXAGGXAGXAXCXCTGAAGCX-XTGAGCXAA----XXXXTAAGXTCC
                                       72970     72980     72990     73000     73010     73020     73030     73040
Human                        GCATCTTGTTATAACTGGGCTTTTAACT-ATACTCAAAAATAGGTTAAAAGTAGGTGAGTTAAAG--GTTTTAAAAATC  66554
GuineaPig                    GCCTTAGATACAAGCCAAACACTTTAGC--ATGGTAGGCAGTCATTCTTAC------GAGCTAA----ACCTTAGGCCCT  44517
NorthernAmericanDeerMouse    AGGGTT-AGAGTAGAAAGGGGCGGGA----AGAGATGGGAGGATCCCTGGAGCTTATTAGCCAG-------GTGAGGTCC  48941
Mouse                        AGGGTGGGGAGTAGAGAAGAAGGGGACAGTAGGAAATGGAGGACCCCTGGAGCTCATTAGCCAACCTCATTGGTGAGTCC  48156
ChineseHamsterGHOK1GS        AAGAT--ATAGTGTAGAGAAGTGGGC----AAGAAGAGATGGAGCCCATTAGCCAGCCTGTTTG-------GGGAGTTCC  49815
LongTailedChinchilla         GTGTTAGGTAGAGGCCAAGGACTTGAGC--ATGCTAGGCAGTCACTCTGACAC---CGAGGCAA----GCCTTAA-CCCT  48465

Majority                     XGGTTATGXTTXAXGAXTTXAGCTTGXXTT--------ACCAAATXCTTTCATCTTT-AXXTGTXXXAAX-TGXX-C---
                                       73050     73060     73070     73080     73090     73100     73110     73120
Human                        AGATAATGGACCCTGGTTTTGATAATTCACAAGAGATAACCATTTATTTTCATATTTTTTATGTAAATACATGTTTCTGT  66634
GuineaPig                    TGCT--TGGTTCTTAATT--AACTTGTCT-------------AATGTTTCCTTCCCA-----------------------  44557
NorthernAmericanDeerMouse    TGGTTAGATGTGAGGAATTCAGCTTGCTTT--------AC-ATGTTCTGTCATCTTG-ACTTGTTTTAAT-TGTT-C---  49006
Mouse                        AGGTTAAATTTGAGGAACTAGGCTTGCTTT--------ACCAAACTCT--------T-----------------------  48197
ChineseHamsterGHOK1GS        AGGTTAAGTGTGAGGAATTAAGTTTGCTTT--------CTTAAATCCTCTCATCTTTTACTTGTTTTAAT-TGTT-C---  49882
LongTailedChinchilla         TGCT--TGGTTCTTAATA--AGCTTTTCTTTTTTGAAAACCAAATGATAAAATACCACAAGTATAACAACAAG---CTGC  48538

Majority                     -TAXAXTTGTAAGTG------GTXTTTTXACTTGAATTXTTTTCXAATGGXATTXAAAXT--AXXTX-AGTTTATXTTTX
                                       73130     73140     73150     73160     73170     73180     73190     73200
Human                        ACAAAATTAAAATTGCTCTGTATTTGCAGATTTGCATTATCTTTTATTGTAATTGACATTTTACTTAGAATTTGTTTTAA  66714
GuineaPig                    -TTGTGTGGAAACT-------TTTTAATAGAGTATATT--TTATTTCTA-----------------------TATTT---  44601
NorthernAmericanDeerMouse    -TGCATTTGTAAGTG------GGCTTTTGACTTGAACTGTTCTCAAATGGTTTTAAAAGT--AGGTG-AGTGTAACTTTC  49076
Mouse                        -CA------TAAGTG------GACTTTTTACTTTAACTCTTCTCAAATTGGTTTTAAA----------AGTTTAACTTTC  48254
ChineseHamsterGHOK1GS        -TACGTTTGTAAGTG------GGCTTTTTACTTTAATGATATTCAAATGCTATTAAAAAT--AGGTG-AGTTTACCTTTT  49952
LongTailedChinchilla         ATCAAGCAAGAGCT-------ATTTAAAAATATGAATTGTTTACTTCTGAAATT----TTTCATTTA----CTATTTTTA  48603

Majority                     AAAA---CTCCAGTA-----ATAGXXT-CTAA-------GCXAT-TCAT-AAXAXATAACATTCA---TATX-XXTGCTT
                                       73210     73220     73230     73240     73250     73260     73270     73280
Human                        AAGATAGCTTTAATACCTGCGTAGCATGTTCATGGGTATGCCATACCTTTGAAAGTGGACATGTAGGCTATTCCATACTT  66794
GuineaPig                    --------------------------------------------------------------------------CTGCCT  44607
NorthernAmericanDeerMouse    AAAA---CTTTAGTA-----ATAGCCT-GTAA-------GCTAT-TCAT-AAAAGATAACATTCA---TGTATTTTGTTC  49135
Mouse                        AAAA---CTCCAATA-----ATAGATT-CTG--------------T----AAGTTACGGTATTCA---TAAG--------  48296
ChineseHamsterGHOK1GS        AAAA---CTCCAGTA-----ATAGACT-CTAAAAGGTGTGGTAT-TCAT-AAGGAATAACATTCA---TATATTTTGTTC  50018
LongTailedChinchilla         GA-----CTCCAGTACC---ACAAGTAACTAAAACAAAAGCAAAACGATGAATAAGAAGCAGTTTCTGTA---CCTACTT  48672
```

```
Majority                     TATATTXAAX-----XTGTACATTTTTGX-TXXXAAATTAAAAXX--AXTTT-AXXTTTTGXXTTTXXXXXTAATXGXC-
                                      73290     73300     73310     73320     73330     73340     73350     73360
Human                        TTTATTAAAACACCCTTGTACTTTTTTGCCTCTCAAATTATTTTCTTAGACTAGATTCCTAGAATTAGAATTATTGGGTC 66874
GuineaPig                    GACAAGAAAA-----TGGGATACTGTG-------AAA------------------------------------------- 44632
NorthernAmericanDeerMouse    TGTCTTCTGTT----ATATACATTTTTGG-TACAAAATTAAAACC--ATTTT-ACCTTTTGTGTTTTAGTGTAATTGCC- 49206
Mouse                        ---------------AGATACATTCCTAC-TAACCAGTTAAA-----G--------TTTTGACTTTAGGAAAAAAAAAC- 48346
ChineseHamsterGHOK1GS        TATTTTCTATT----AAGAACATTTTTGA-TGCAAAATTAAAACC--ATTTTTAGCTTTTGTGTTTTACTATAATTGCC- 50090
LongTailedChinchilla         CATATGGAAG-----TTGAATATGGTT-------AAATAAGGAA-TGAGTTAAAATTTTAAAAT-------------GCT 48727

Majority                     -AXTTAXATTTTGAA--CTTTTAAATAGAXATGATAATTTTAATAATTTATXAX--AATGAATATATATATAXXXT-ATT
                                      73370     73380     73390     73400     73410     73420     73430     73440
Human                        AAAGAAAATGTTAAAGCCTGTTTACTTTATTTGCAAATTTCTTCATCTAAAGGTGATATTAACTTAAACTAACATTAATT 66954
GuineaPig                    -----ATATATTGAG----------------------------------------------------------------- 44642
NorthernAmericanDeerMouse    -ATTTA-ACTTTGAA--CTTTTAAATAGAGATAATAATTTTAATAATTTATTAC--AATGGCTATATATATATATATATC 49280
Mouse                        -AGTTATATTTAGAA--CTTATAAGAAGAAATGATAATTTTAATAACTTAT-----AATGAATATACTTATA-----ATC 48413
ChineseHamsterGHOK1GS        -ATTTA-ACTTAGAA--CTTTCAAATAGAAATGATAATTTTAATAATTTATTAT--AATGGATATATATATATCATCATT 50164
LongTailedChinchilla         TAGAAGAATGTTGAGCATGTTGTAAGACATGTGAAATGAATAAACATTAAAAACCCTGAGAGTGTATTGTTAGGCTGGTT 48807

Majority                     X-X-TCTTTGGXTTTTTTTTTXAC-XTGXTGTAGTXC-ATTGAATCTTTGAAAGTXXACATXAAAXTATTTTXTXTTXTA
                                      73450     73460     73470     73480     73490     73500     73510     73520
Human                        A-TATATGAGGCTGGGTGTCCTAC-TTTTTCCCCTGCCAGCATACTATTTTTTAATTTCACAAGATTAATTTGATAGGCA 67032
GuineaPig                    -------------------TTCAC-CTGT-----------------------CTTTCC---AAAATGATTTCATCTTTTG 44676
NorthernAmericanDeerMouse    A---TCTTTGGTTTTTTTTTTCAAACTGCTGCAGTAC-ATTGAATCTTTGAAAGTGTACATTAAAGTATATT-------A 49349
Mouse                        T---TCATTGGTTATTTTTGTTTTTTTGCTGTAGTAC-ATTGAATCTTTGAAAGTGGACATTAAAGTATTTTTTATTA-A 48488
ChineseHamsterGHOK1GS        GGTGTCTTTGGGTTTTTTCTTTA--CGACTGTAGAGC-ATTGAATCTTTGAAAGTGGACATTAAAATGTATTTTTTTTTA 50241
LongTailedChinchilla         T-TTTT-----CTTACTTTTTCAC-TTATTATAATCTGAATATATCATTACTCTTTCCCACAAGACTGTTTTGTATTATG 48880

Majority                     AAAXTXTTACCTTAAATTATTTTXTAAXATXATAGTTXTAXAACTATTGA--TAAAAACATTAAAGACTA-TTGATA---
                                      73530     73540     73550     73560     73570     73580     73590     73600
Human                        AATGCCTATTT-TAATTTCTGTTAAGATTTA---GTTTACAATCTTTAAATGTTTATTTGATAGTCACAATTTGCTAAAT 67108
GuineaPig                    CA-------------------------------TGTTTTAAA------------------------------TGGT---- 44691
NorthernAmericanDeerMouse    AAAATGTTACCTTAAATTATTTCCTAAGGCTATATTCCTAGAACTATTGC--TAAAAACATTTAAGACTA-TTGATA--- 49423
Mouse                        AAATCTTTACCTTAAATTATTTTCTAAGATGATACTTCTAGAACTATTGAA-TAAAAATATTAAAGATTA-TTAATA--- 48563
ChineseHamsterGHOK1GS        AAAGTGTTACCTTAAATTATTTCTTAAAACTATATTCCAAGAACTATTGA--TAAAAGCATTAAAGATTA-TTGATA--- 50315
LongTailedChinchilla         TACATCGAACAGTGACTTCTATTTGAATATAAATGTTTTAAAACTATTGCCATTTAAACAATGGGATCTTATTGGTGCAC 48960
```

```
Majority                  -GX---TXTXXATTTTTCTTCCTGGAAGATACGXATTAATTTGA-AATAATATA----AATGGTGXAGAAGXXXGTATTT
                                  73610     73620     73630     73640     73650     73660     73670     73680
Human                     TGCTTGCCTGCATTCTTTCTGTTAGGATGTACG-GTAATTCTTATAA-AATGCATAGTAAGGATATTAATATGCATATTT 67186
GuineaPig                 ------TCTGCATCT----------------------------------------------------------------- 44700
NorthernAmericanDeerMouse -GT---TTTAAATTT--CTTTCTGGAAGATACGTATTAATTTGA-AATAACATA----AATGGTGAAGAAGGTAGTACTT 49492
Mouse                     -GC---TTTACAAGTTTCTTCCTGGAAGATATGTATTAATTTGA-GATAATATA----AATGGTGTAGAAGGTTGTATTT 48634
ChineseHamsterGHOK1GS     -GT---TTTAAATTTTTCTTCCTGGAAGATACATATTAGTTTGA-AATAACATA----AATGGTGAAGAAGACAGTATTT 50386
LongTailedChinchilla      TG----TCTGAATTTCTACCAATGTACAGTCCG-ATCAACTAAATGATAGTGTACTGTTTCCATACAAGC-TGTGCACAC 49034

Majority                  TTGCC--------CTGTCAGTA-----CACCTTTCTTGG--------TTGAGACAATGTTATTGTGT-X------TXTTA
                                  73690     73700     73710     73720     73730     73740     73750     73760
Human                     TGGATGAGCCAAGCCTTCAGCTTTATTCACTTATGTTAGGTCAGTTTTTGGAAAAGTATTTTTCAATAATCTCTCTTTTT 67266
GuineaPig                 ---------------------------------------------------------------------------TGTTA 44705
NorthernAmericanDeerMouse TTGCC--------CTGTCAGTA-----CAGCTTTCTTGG--------TTGAGACAATGTTATGGTGT------------- 49538
Mouse                     TTGCC--------CTGTCAGCA-----CACCTTTCTTGG--------TTGAGACAATGTCACTGTGTCAAGAGAACTCTG 48693
ChineseHamsterGHOK1GS     TTGCC--------CTGTCAGTA-----TAGCTTTCTTGG--------TTGAGACAATGTTACTGTGTAA---------CC 50436
LongTailedChinchilla      TAGATA---CAGAATTTAAGTATTGAGCACTTTTTGT------GTTAATGAAAAAACATTTTAA-----------TGTTA 49094

Majority                  XAA--AXXXGCXTXXXXCCCA--XT-TXXXXXTGTAXT-C-TXGAGXXXX---XXXCTGXXTXA-AC-TTTXCTGGX-XT
                                  73770     73780     73790     73800     73810     73820     73830     73840
Human                     AAATAATTGATCTCAATCCCAAAGTATTCATTTGTAGGACATGAAGACACAATTTTCTTCTTCATACGTTTGGTGAATAT 67346
GuineaPig                 CAA---------------------------------------------------------------------CTGGA-TT 44715
NorthernAmericanDeerMouse -------------------------------------------------------------------------------- 49538
Mouse                     GAA--ATTTGCTTTCCTCCCAG-GT-TAGCCCTGTACT-C-TTGACTTTTTTTTTTCTGCCTCA-AC-TTTACTAAT-GC 48764
ChineseHamsterGHOK1GS     CAG--AAGTGCCCTACACCCTC-GT-TGACCCTGTACT-C-TGGAGTTTT------CTGTTTTA-GC-TTCACTAGT-GT 50501
LongTailedChinchilla      AAAATAAGGGCTTGGAGGTTA---TGTGGAATAGACGTACATAGAGAGAGAACTTTAAGGGAAGAATAAGTGCTGGG-AT 49170

Majority                  TTXGXTX-XTATTTTXCTT--XTATAXXTG-CTTX--ATTTCTTTTTXTTXTG-TATXTTXXATTXC-TXXGXXX----X
                                  73850     73860     73870     73880     73890     73900     73910     73920
Human                     TTTGATC-TCAGTTTCCACTTCTCTTTTTGCTTTTTAAACATTGTTTATGGTGATATCCAATATTGCATGAGTGAGGTCA 67425
GuineaPig                 TT-------TATTTATTTT------------------ATTTATTTATATTTTG-TACT---------------------- 44747
NorthernAmericanDeerMouse -----------------------------------------------------------------------G-------- 49539
Mouse                     TGAAACCACTGTTTTGTTC--TTATACTTGACTTC--ATTACTTTTTTTCAAAGCATATTAAATTAC-TGGGGAA----A 48835
ChineseHamsterGHOK1GS     TGAGCTTATTGGTTTGCTTCATTACACCTGGCTTT--ATTTCTTTACTTTAGGGCATATTCATTTAC-TACGGGA----A 50574
LongTailedChinchilla      TTGGGTT-GTATTTATCCTTGAAATAAGTGTCTAGCAATTTCTTTTTGTTTTG-TACTTTTCATTGTACCAGTT------ 49242
```

73930 73940 73950 73960 73970 73980 73990 74000

Majority	TGTXTCXXXXAXTAXXAAXTXAXTTXTXXXTTXXAAXTAXTTXXTXATXXXXXTTX--TAAACTTATXXCTTTGTG-XXX	
Human	TGCCTTTTAGAATAATAACTGAATCTTTTCTTCTAATTATTTAATCAGATGACTTCAGCAAACTAATATTGGTGGGATTA	67505
GuineaPig	-------------GGGAAT---------------------------------------TGAACTCAGGACCTTGT-----	44770
NorthernAmericanDeerMouse	--	49539
Mouse	TGTATCATTAATTAGAAACTATTTTGTAGTTTTAAAATACTTGTTTATAATTTTTCAGTTGTCTGGAGTCTTTCAG-CTG	48914
ChineseHamsterGHOK1GS	TGTCTCCATCATTGTAAAATAATTTGTGGTTTTTAAATACTTGGTACTTATAACTGCTCAGTCATCCAG-TCTCTG-AGG	50652
LongTailedChinchilla	TGTATCAAAGTGCAAGAGTATAACTATGCAAGCCACTCAGCGTATTATCTGTATTT--TAAATCTATCACATTGTTTCCT	49320

74010 74020 74030 74040 74050 74060 74070 74080

Majority	XXXTXXTXXXTAGTXCTT---XAA-AXTACTTXXTXXAGAXX-XAX-XTTGXTXXGGXTXTXAGXX---XAATTTTXX--	
Human	ACTTCCTGCTCAGTCTTCCAGTAACAGTACTTGTTCAAAATGAAATAATTGATAGAGTATTTATTTCAAAAATTTTTAAG	67585
GuineaPig	---ACTTGT---	44776
NorthernAmericanDeerMouse	--	49539
Mouse	GTGTGTTAGGTAGTTCTTA-ACAA-AGTACTTACTAGAGATT-TAGGTTTGATTAGGATATGAGCT---GAATCTTGAAC	48988
ChineseHamsterGHOK1GS	TGGTGTTAGGTAGTTCTT---CAA-AATACTGACTAGAGACT-TAACATTCTTTAGGATTTGAGGT---GAGTCTTCAGC	50724
LongTailedChinchilla	TTCTCATCATTAGTCCCTGCATAAAGCTATGTCATCAGGAGGACAGTCCTGGCCTGCTTCTTAGT----AAACTTAC---	49393

74090 74100 74110 74120 74130 74140 74150 74160

Majority	----CTXGTXXGXGCTGTTACTTTTXAGXTAAATXTTTTXXGXXAXX-TTX-X----CT--TTTXTXAXXXXXAXXTXAX	
Human	AACACAAATATAAGGTGATACTCTTTTGCTAAAATTTGTGGGGGGCT-TCACTTTACCT--TGTATGATTTTTATATTAG	67662
GuineaPig	----CAGGCAGCTGTTTGTACTGCTGAGCTATATCCC---	44809
NorthernAmericanDeerMouse	---G--------------------------------------	49540
Mouse	----TTGCTTAGGTCTGTTACTTTTCAGAAGAGTATTTTTAGTAACTATT-------CTCATTTTTTAAAAAAAAATAAA	49057
ChineseHamsterGHOK1GS	T-CATTTTTTAGAACTGTTACCTTTCAGTAATACCACTGAGGAAATAATTATTCTCACTAGTTTTTAACATGCATTTGAT	50803
LongTailedChinchilla	----CTAGTGTTTCCTTCCACCTCTGTGTTAAATTTTTTAATATATC-TTATTTCTGCA--TTTCCGCTTGACAAGAAAA	49466

74170 74180 74190 74200 74210 74220 74230 74240

Majority	XT-TTXTXA-XX----GAA-XTAA-GAXXTTX--ATC-TTXCXTTAAXXXXTA-TTGXXXTXTTXXA--TAXTXAGXTTX	
Human	ATCTAGCTACTTGACTGAATTTAATTACTTTAGAATCAGTACTATAAATTTTAATTGATTTATTTTACACAGTAATTTTT	67742
GuineaPig	--	44809
NorthernAmericanDeerMouse	--	49540
Mouse	GT-TTATGTCTTTTTAAAA-CTAA-GGTCTTG--ATC-TTGCCTTAATACCTA-TTGCAGTCTTGAAA-TAGTGAGACTG	49129
ChineseHamsterGHOK1GS	GTCTTATTAGTTTCTGAG-ACAA-GACCTTG--ATC-TTGCTTTAACTCGTA-TTGCAGCCTTGAAGGTGATGAGATTA	50877
LongTailedChinchilla	ACACTGTGA-------GAATATGATGAATTCACCTTTGTTTCCTAAATAATTTCCTCTTCTACATCGTCTAAATGGTTCT	49539

```
Majority                    TAAGXXTX---XXTATGTCTAGCTTTT-----X-XTACTTTAAXXXT-TTCXT-ACAGTTTXTXAGAGCATGT-GAGCTX
                                      74250     74260     74270     74280     74290     74300     74310     74320
Human                       TAAGACTAACATTTATGTCTGGCTTTTC--TGT-TTCCTTTAATGCTAGGTATCACAGTGTCTCAGAAAATGT-AATTTG 67818
GuineaPig                   ---------------CAACTAGATTTT--------TATTTTAAGCAT---------AGTTTTTGAGAATAGG--GAGCTA 44855
NorthernAmericanDeerMouse   -------------------------------------------------------------------------------- 49540
Mouse                       TAAGTCTGTGCTGTGTGTCCAGCTCAGTGCTATGTTTCTGAAAAGTTGTTCTTGACACTTTCCAGGGGCATCTAGTGCTC 49209
ChineseHamsterGHOK1GS       CAGGTGTGTGCTGTATGTCCAGCTCAATGCCATGTTGCT-------CTTTCCATGCACTTCACAGGAGCATCCTGTATTG 50950
LongTailedChinchilla        TCATCTTA----TTACAACTGGCTTTT--------TATTTTAAGCATCTTCAT-ATGGTTTTTGAAAGGAGGT-GAGCTA 49605
```

```
Majority                    T---AAXXAAGAAX-XTGAXTAX---GXXXTGXGTXTTXTATCTCAXTTXTAXAACACATGATXXCTAAT-XGGCTGAXX
                                      74330     74340     74350     74360     74370     74380     74390     74400
Human                       TTCAAAGAAAGGAGACTGAGTGCTTTGTAATGAGTTTAAAATCTGACATCTAAAATACATAATCACAAATGTGGCCGACT 67898
GuineaPig                   T---AACTTAAAAAATTGAATAG---GGGTTGGGGGCTGTAACTCAGTAAAAGAACACTTGACTACTAT---GCCTGA-- 44924
NorthernAmericanDeerMouse   -------------------------------------------------------------------------------- 49540
Mouse                       CATTAGTGATGTCGCCACTATAAAATGATAACTGTCTTTTCTCTATTTTTTGCCTCATAGGATATTTTAT-CGGGTGACT 49288
ChineseHamsterGHOK1GS       CATTAGTGATGCC-------------------TGTCTTTTCTCTCATCTTTGCCTCTTAGGATATTTAAT-CAGGTTACT 51010
LongTailedChinchilla        T---AACTTAAAAA-TTGAGTAA---GGACTGGGG-CTGTAGCTCAGTCAAAGAACACTTGCCTGCCATCATGCCTGA-- 49675
```

```
Majority                    ---GGTTXTXXXCAXAGTCXTT-X-----AACACCXTXATTAAAAXTXCTTTAXAATXXAGAXXXACAXATAXAAATGAT
                                      74410     74420     74430     74440     74450     74460     74470     74480
Human                       ATTAGTCATTCACATATTCTGTTTCAAAGAACACAGTGATCAAAAATCCTTCAGATTATAAATTGAGACATCATTATAAT 67978
GuineaPig                   ---GGTTCTGGGTTCAGTCCTT-------AGCACCATAACTAAAA-TAAATTAAAATTAAATGGAACAAATAGAAATAAA 44993
NorthernAmericanDeerMouse   -------------------------------------------------------------------------------- 49540
Mouse                       TCAGAGTACAACCAAAGTAATAGTGGCGAGATTCCGTTCTTGGTCAGTCTTTCTGATGCAGAACTCCTGAAATAATTGCT 49368
ChineseHamsterGHOK1GS       TCAGAAT-TAACCAAAGTAATAGTGACAAGATTCCTTTCTTAGCCATTCTTTCTCAGGCAGAACTTCAGAAATAATTGCT 51089
LongTailedChinchilla        ---GGTTCTGGGCTCAGTCCCT-------AGCACTATAACTAAAA-TCAATTAAAATAAAGT---A--AGTTAACATGAA 49739
```

```
Majority                    ACAGTACTCTXTXXTTTTAA----X-TATXTAAXX---XX--------ATAAATTTTAGXGGAATXXXX----TCTXTTT
                                      74490     74500     74510     74520     74530     74540     74550     74560
Human                       GGATCATTTTATCATGTTAAGAGGTGTAGTTAATTTATTAAGGGGTAGATCACTTTTAGAAAAATTGCTGGAAGTAATTT 68058
GuineaPig                   ACAGTACTCTCTAGTTTTGA------TGTCCAACC-------------ATGTATTTTAGTGTAAT--------TCTTTTT 45046
NorthernAmericanDeerMouse   -------------------------------------------------------------------------------- 49540
Mouse                       ACCGTGCCCCTTTACTGAAAAGACTATACTTA------TATT---CTTTCAAATATATGAGGGTTCACTTT--GCT-TTT 49436
ChineseHamsterGHOK1GS       ACAATATTCCCTTCCTTAAAAGATTATATATAGTTAAATATT---CTGCCAAATATTTGGGGGTTTACTTT--TCAATTT 51164
LongTailedChinchilla        ATAGTACTCTTTAGTTTTGA------TATCCAACC-------------ATAAATTTTAATGCAATGC------TCTTTTT 49794
```

```
Majority                      TTXATXTAXATGTTTTXTACAATGTTAAAATX-----X-X---------CAXAGTGTATTTXT------XATTTXXTTAX
                                       74570     74580     74590     74600     74610     74620     74630     74640
Human                         TTCATGATCATGTTATCTACATTCTAAAAATTAGGAGAGAGACTGTGTACAAAGAGTGTTTATTTTAGAGCTTTCCTTGT 68138
GuineaPig                     TTAATAAATATTTTTTGTACCAAGTTAAAAT------------------AATAGTGTATCTAT------AGTTTTGTTAC 45102
NorthernAmericanDeerMouse     -------------------------------------------------------------------------------- 49540
Mouse                         TATATTTAGCTGCTTCAATGAATGTCTTTACTTTTCAAAA----------AGATTTTATTTTAAACA-CTATTT--TTAA 49503
ChineseHamsterGHOK1GS         AATGTTTAGCTGCTTCAGTGAGTGTTGTTACTTTTTAAAATCAGTAATTCAGATTTCATTTTAAATG-ATAATTATTTCT 51243
LongTailedChinchilla          TTAATGTACATTTTTTGTACCAAGTTAAAAT------------------CATAGTGTATTTGT------AATTTTGTTAC 49850

Majority                      ATT-TXACTX--XXX-X-XGCATTTXTATCXXAXAGXTTTTTTT---AAXAAAAATCACAXTATCTGXA--XX--TATTG
                                       74650     74660     74670     74680     74690     74700     74710     74720
Human                         ATTTCAAATTGAATAACAGGCATTCTCATCATAAAGTTTTTAAAAGAAAGGCAAAGCAGACTTTCTGTAGGAAATCATTG 68218
GuineaPig                     ATTGTTACT----------GCAGTTGTATCTTAGAGGTTTTTT----GAAAACAATTTTAATATCTGCA------TATTG 45162
NorthernAmericanDeerMouse     -------------------------------------------------------------------------------- 49540
Mouse                         ACTATCACTT--ATACCTGGTTTCCCTCC--------CTCTTTT---ACTGAGAATCACAGTATTTTGGTAAA--TGTAG 49568
ChineseHamsterGHOK1GS         AAACTCACTT--ATACCTGGTTTCTCTTTTACTAGTTCTCTTTT---ACTAGAAATCACACTGTTTTGGAAAA--TGTAG 51316
LongTailedChinchilla          ATT-CAACT----------GCAGTTGTATCTGAGAGGTTTTTTTGAAGAAAATAATTTCAATATCTGCA------CATTA 49913

Majority                      ----XX----XXTTAXGX-XATGAACAX-XXX----X-TATCCTTGGAAGTGAAXATXTAG-TCTCATX--XXXTTXTGT
                                       74730     74740     74750     74760     74770     74780     74790     74800
Human                         ACGTTAAAATAGTTATAATTGTGAACAGATACAACATTTATTCATGAAGGTAAACATGTAGGTCTTATAGAATATTGTTT 68298
GuineaPig                     ------------TTACGCCAATGAGTA-----------TGTCTTTGGAGGTGAATATTTAG-TCTCATG----TTTTTGT 45214
NorthernAmericanDeerMouse     -------------------------------------------------------------------------------- 49540
Mouse                         TTAATATT-TAGAGAAGG-AAAACACAG-TACTTTGTAGATACTTAGAATGTAACATCTAAATAACATA--ATCATAAGC 49643
ChineseHamsterGHOK1GS         TT--TATT-CAGAGAAGA-GAGAGACAG-TACTTTGTAGTTGGTTAGAAATGAAACATCTGAATACATG--ATCATAAGC 51389
LongTailedChinchilla          ------------TTATGC---TGAGTA-----------TATCCTTGAAAGTGGATATGTAG-TCTCAT-----TTTTTTT 49961

Majority                      TTXAXAATATX-TTGTXTACT--------XX-XTXCTXCCCCAGA----XXX---------XX--X-XXX-X-------T
                                       74810     74820     74830     74840     74850     74860     74870     74880
Human                         CTCAAAATTTTGCTGCACGTTGAAGTCACCAGATTCTACCCCAGGCTGAGAATAGTTAAAGAGTGGGTCACAGGCATTCC 68378
GuineaPig                     GTTATAATATC-CTGTGTACT------------TCATGCCCCAT-----------------------------------T 45246
NorthernAmericanDeerMouse     -------------------------------------------------------------------------------- 49540
Mouse                         TTCACAGTATATTTATTCACTCA------CATAC-CTCTTTCAGA----GAACA-------AGTTGGTCAAAA------T 49699
ChineseHamsterGHOK1GS         TTGGCAGTCCTTTTATGTACTGG------CATACTCTATTCCAGA----GAATG-------AGGTGATCAAAAA-----T 51447
LongTailedChinchilla          ATTAAAATATC-TTGTATACT------------TCATGCCCCAAA----------------------------------T 49994
```

```
Majority                     TATTXXTTTAGAXT----AGAXX-TTXXAAT----XTTGTGAGTXGAXAAATATTXTT--X--XX-GAXTXTGTAACTTT
                                     74890     74900     74910     74920     74930     74940     74950     74960
Human                        TATGTTTTTAGGATCTCAAGATGGTTTTACTATGTAGCAAGAGTTGAGAATTACTGCTACAGAAAAGAGCTTGTAACTTA  68458
GuineaPig                    TATTGCCTTAGACT----AGATTTTTGGAACTGAAATTGTGAGTCAAAGAAAATTTTT--------GATACTGCCATTTT  45314
NorthernAmericanDeerMouse    --------------------------------------------------------------------------------  49540
Mouse                        TATTTACTATAAGT----ACACA-TTAAAAT-----TTTTGTCAAGGTAAATGTTAATATATTAAGAGGTGGGTAGCTTT  49769
ChineseHamsterGHOK1GS        TATCGATTGTAAATT--AAGACA-TTATAATGATCATTGTGTCAAGGTAAATGTTAGTGTACTAAGAGATGGATAGCTTT  51524
LongTailedChinchilla         TATTCTTTTAGACT----AGA---T------------TGTGAGTCAAAAAAGACTGTT--------GAAGTTGTCATTTT  50047

Majority                     ATXXAATTXAXAT--X----GGTA---TTTTTTTAXXATXATXTTAXTXXTTTATXAAATTAGAX--X-XAATTTTC-AT
                                     74970     74980     74990     75000     75010     75020     75030     75040
Human                        ATGTCATTTAAATCTATTTTGGTACAGTTCTCCTGGCACAATTTTAATTAGCTGTTAAACTGAATTTA-TAATTTTCTAT  68537
GuineaPig                    ATCCTAGAAAGAT-------GGTA---TTTATTTAAATTAACT--AGTGGTTTATGAAATTAGCC-----AATTTTC-AT  45376
NorthernAmericanDeerMouse    --------------------------------------------------------------------------------  49540
Mouse                        AGAAAATTGTTAG--A----AATG--ATTTTT-CATGATCATGTTGCCACTTAAAAATATTAGAGCAACTAATCTGTCAA  49840
ChineseHamsterGHOK1GS        AGAAAATTGCTGG--A----AATA--ATTTTTTCATGATCATATTGCCACATAAAAGTATTAAAGTGACTAATTTATCAG  51596
LongTailedChinchilla         GTCCAAGAAAGAT-------GGTA---TTTGTTTAAATTGACATGAATGGTTTATGAAATTGGTT-----GATTTTC-AT  50111

Majority                     AXGAXTXTXX----X-AAGTTXAXTGGAAAACXTTC---TXATTTCXXT-----XX---TAGGGGTTATTCTXCTGXT--
                                     75050     75060     75070     75080     75090     75100     75110     75120
Human                        ATGTTTGTGATACATAAAATTAACTGGAAAACTTTGATTTTCTTTCTTTGTTACATTTGTATGGGTTATTCATTTGGTAA  68617
GuineaPig                    AGGATTGT--------AAGTTGATTGGAAAGTGTTC-----ATTTCTGT----------TAGGTTATACTTTACTGTTTT  45433
NorthernAmericanDeerMouse    --------------------------------------------------------------------------------  49540
Mouse                        AAGAATAGAGT---TTGAATAAAGTAGCACCCATTC---TTAGAATAACA--GTATTAGTAGAGGTTGTGCAGTTAGT--  49910
ChineseHamsterGHOK1GS        AAGAATAGAGC---TTTTG---------ATACAGTT---TAAATTGAATA--AAATATCAATTCTTCGTTCTTCAAAT--  51657
LongTailedChinchilla         AGGACTCTTT------AAGTTTATAGGAAAATGTTACATTAATTTCCGT----------TAGGAGTTACTTTGCTGTTTT  50175

Majority                     TAAAGGXTTAGTTGATATTXAAXATTTX-GX-ATTXGGTTXTXT--XGXATTCCTTA-----XX--X-----X--AATXX
                                     75130     75140     75150     75160     75170     75180     75190     75200
Human                        TATTTATTAATTTATTATTAGAGATTTCAGCTATTTGCTAATATAATGAATCCCATACACCTGTCATCTTGGTTCAATAG  68697
GuineaPig                    TAAAGGCTTATTTGCTATTTACAGTTTCTG--ATTTAGTTGTGT---GGGTTCTTTA------------------AATGT  45490
NorthernAmericanDeerMouse    --------------------------------------------------------------------------------  49540
Mouse                        TGATGGTCATGAGGAGATAGAACATTTT-TCATGAAGGTTAATT--TGTACTTCTTG----TGTAATGGTGCTTTTCCAA  49983
ChineseHamsterGHOK1GS        GACAGAATTAGCAGAGACTGAACAGTTG-ACGA----------T--TGTACTCCTTG----CGTAGTGTACTTACT----  51716
LongTailedChinchilla         TAAATGCTTAGTTAATATTTACAGTTT--G--ATTCGGTTGTGT---GGATTCTTTA------------------AATGG  50230
```

```
Majority                     XXXTGTXGATTATTTTTAXCXXTAXAATTTTXTXXTTXTGGTCCGAACTXAXTCXTXAAXTTTATX-CATXTCCCXXAAA
                                      75210     75220     75230     75240     75250     75260     75270     75280
Human                        CGCTGACACTTAGTTTTATCTATTCAGTTTTTCTTTTGTGTTCTAAAGCAAATCCTTGACATTGTGACATGTCTTCTCAA  68777
GuineaPig                    GAATGTACATTATTTTTAAATGAATTATGTTCACATT-TGGTCTGAGCTGAGCCTTTAGTTTTATT-CACGTACTTTAAA  45568
NorthernAmericanDeerMouse    --------------------------------------------------------------------------------  49540
Mouse                        ATGTGTTGGCTGTGGACATCACTGAAAATCAGTGGGAGTGGGCCCAACACATTCATAACGTTTTTA-AATATCCCAAAAT  50062
ChineseHamsterGHOK1GS        ----GGCTATTGAAATCACCACTAAAAATCAGTGGGAGTAGGTCTAAGGCATTCATAGAGTTTATA-A-TATCCCAAAAT  51790
LongTailedChinchilla         GAATGTAGAATATTTTTAAAGGCATTATATTCGTATT-TGATACGAGCTGAGCCTTCAACTTCATT-CACTTACCC---A  50305

Majority                     ATTTTAGTXAAXXAXCAXTTXAA-A-ATTACATXTTATTX--XAAXCXTTTTAXXXXTATX-CATTTXTAAGXXTTGA-A
                                      75290     75300     75310     75320     75330     75340     75350     75360
Human                        ATATTACCATAGTATCTCTAAAATA-ATTACATTTTCTTACCAAACCATAATATGATTATTACACTTACCAGAATTGACA  68856
GuineaPig                    ATGTCTTTTAAAAAACATTTTAAAGTAATTCATCTCTTTA--AATACATGTTAATTGTATC-CATTTATAAGACCTGA-A  45644
NorthernAmericanDeerMouse    --------------------------------------------------------------------------------  49540
Mouse                        ATTTTAGTGAAGCAGGAGTTGAA-A-ATTAAATGCTATTG--GAATTTTTATAC-----CA-AATTTTAGGGTTTTAGTA  50132
ChineseHamsterGHOK1GS        ATTTTAATGATACAAGAGTTGAA-A-ATTACCTGCTATTG--GAATTTTTTTCTGTATACT-AAATTTTAGGGATTAGTA  51865
LongTailedChinchilla         TTTTAAGTAATTTATCTCTTTA----AATTTATCTTATT-------CCCTTTAAAAGTATC-CATTTGTAAGGCCTGA-A  50372

Majority                     GTTATTTXTTA-XXTCCTCATAAAXXGAXXTTATXTTAXXXXXTTXC--XXACTATTTXXAXXTTXGCTTTTAAAXXTTG
                                      75370     75380     75390     75400     75410     75420     75430     75440
Human                        ATGATTTCTTAATGTCCTCAAATACCCAGCTTACATTAAAATTTCCC--CAATTATTTTGAAAAT-GCTTTTAAAGTTTG  68933
GuineaPig                    GTTTTTTTTT----CCCCTAGAAATTGGATGTATATTACTATCTTTTAGTGACTACTTACTACTTCGCTTTTAAAAGTTG  45720
NorthernAmericanDeerMouse    --------------------------------------------------------------------------------  49540
Mouse                        ATTACTTATTA-TGTCCTCATAAAAAGACATC----------ATTGC--ACAGTCT----ATTTTAATATTCATGTCATA  50195
ChineseHamsterGHOK1GS        GTGACTTCTGA-TGTCCTCATAAACAGACATCCTTCACAACCATTGC--CCACTATTTTAATATTTTTATTCATCTCATA  51942
LongTailedChinchilla         GTTTTTTTTTTT--CCCCTACACATTTGGTGTATGTTATGTCCCTTGAGTAACTACTTCATGCTTTGCTTTTAAAAGTTG  50450

Majority                     ATTT---CA----AAXATTCTXTTXAXGG--XCATXCAXTATTGXXTTXXTAAAT-TCATX-GTXT-TTXXAXXTTXTAA
                                      75450     75460     75470     75480     75490     75500     75510     75520
Human                        ATTTGTTCAGAACAAGGTCCTATGGAGGGCCGCACATAGTATTTGGTTGTTACATCTCATTAGTCTGTTTCAGTCTGTAA  69013
GuineaPig                    GCTT---CA----GATACTCTTTATAAGG--ACATCCAGTATTGCATTAGTGAAG-TCAT--GTCT-TT-GAAATAAAAA  45786
NorthernAmericanDeerMouse    --------------------------------------------------------------------------------  49540
Mouse                        ATTTAC-TTGG-TATAATTCTGATAAAATG--------TTATTGTTAATCTAGGT-TTACT-ATGT-TTCCCCCTTTTAT  50262
ChineseHamsterGHOK1GS        ATTTACCTTAA-CATAATTCTAATGATAAAATATTACTTTATTGTTAAGCTCTGT-TTACT-GTGT-TTCTTCCTTTTAT  52018
LongTailedChinchilla         GCTT---CA----AATACCCTTTCTAGGG--ACATCCAATACTGCATTAGTAAAG-TCAT--GTTT-TTTGAGATAAAAA  50517
```

```
Majority                    XTGTA-TCTTXXX------ATTXCCAAXC----A-TTATTTXXXAAXXX-XAXXXTTTTXCTATTGTTXCTTTTCAXTGG
                                      75530     75540     75550     75560     75570     75580     75590     75600
Human                       TAGTCCTCTCCCTCTCTCTACTCCCAATCTCTCATTCATTTACTTATGAAGTGATGTTTCCTTTTAGAAAGTTTCACAGA  69093
GuineaPig                   CTGAA-TCTTTTGCTTGTAATTGATAATC----AGATAAATTCAAAGAACAACCATTCTAATATTGACAGCATTCATTGT  45861
NorthernAmericanDeerMouse   -------------------------------------------------------TTTTGTTATTGTTGCTTTTGAATGG  49565
Mouse                       GTGTAATCCTAAA------ATTACCAAGT------TTATTTAAAACCTTGGAAGCTTTTTCTATTGTTGCTTTTAAGTGG  50330
ChineseHamsterGHOK1GS       GTGTTATCCTAAA------ATTACCAAGTAA--GCTTATTTCAGAATTCTAAAGCTTTTTCTATTATTGCTTTTGAATGG  52090
LongTailedChinchilla        CTGAA-TCTTCTG------ATTGATAACC----A-----------------ACCTGTCTAGTTGTGATAGCATTCATT-T  50568

Majority                    XTCXTTCAXATGGTXATATTTTXXAAATTGX-------AXTTXTXXXTCAXTCTTXXATXXTCTTAAAATXAAXCCCACA
                                      75610     75620     75630     75640     75650     75660     75670     75680
Human                       TTAGACTAGTCTGGCTGTTTCTTTAGAGTGTCATGTTAACTTGTTCCTCTATCTGCTGTGTTTCTTATATAAACTTAAAG  69173
GuineaPig                   TTTGCTCAG-----------TCTTAAACAGT-------ACTTGTTCA---GAATGAAATAAT----A-TTACACCCC---  45912
NorthernAmericanDeerMouse   GTCATTCACATGGTAATATTTTACAATTTGC-------ATTTATATTTCAATTGTTG----TCTTACAATGAATCCTGCA  49634
Mouse                       GTCATTCACATGGTAATATTTTATAAATTAC-------ATTTATATGTCAGTCTTCCAT-GTCTTAGAATGAATCCCACA  50402
ChineseHamsterGHOK1GS       GTCATTCACATGGTTATATTTTACAATTTCC-------ATTTATATTTCAATCATTGTTTGTCTTAGAATGAATCCTACA  52163
LongTailedChinchilla        TTTGCTCAGGCT-------TTCTCAAACAGT-------ACCTGTTCA---GAACAAAATGATTGATA-ATACACCCC---  50627

Majority                    TTTCTXTXATAXGTTTTXTAXXAATCACAXTGXXTT-----TXTXAAXTATTTXT----TTTTCTATTGTGTTTTAXAXX
                                      75690     75700     75710     75720     75730     75740     75750     75760
Human                       TTAGTTCCAAAGGCTTGATAAGATTTAGAGCCTTTTTTTTTTTTGAAGTGTTTCTTGTGCTTCCTATTGTGTCTCAGTAG  69253
GuineaPig                   CTTATTTCATAGGTTTTATAAGAACGCCAA-----------TATAAAATATTAC-----TTTTCTGCTCAATATTGGGAG  45976
NorthernAmericanDeerMouse   TTTCTGTAATCATATCAGTAGTGATCACTGTGGATT------------TACTTTT----TTTTCTATTGTGTTTTAAATC  49698
Mouse                       TTTCTGTAATAAGATTAGTAGTGATCACTGTGAATT--------TACTTGTTTTT----ATTTCTATTGTGTTTTAAATC  50470
ChineseHamsterGHOK1GS       TTTCTGTAATCACTTCTGTAGTAATCACCATGGTTTCCTT-AGTTTTTTTTTTTT----TTTTCTATTATGTTTTAAATC  52238
LongTailedChinchilla        TTTATTTCAAAGATTTTAGAAGAACACAAA-----------TATAAAGTATTAC-----CTTTCTGCTCACTATTGGGAG  50691

Majority                    AAXGCATTXATT--X---TTACXCATATTTTAACTATXXA--------XXTA-----------------------CAATA
                                      75770     75780     75790     75800     75810     75820     75830     75840
Human                       GAGACATCTAATATCTGGTTGCCCCACTTTTAGTGATTTTAATGATGCTTTAAGACTGATTGTGGATTTGGGTGTTGACA  69333
GuineaPig                   AGGAGGTTCAGT------TTACCTTTTTTTTTTTTTTTTT--------TTT-----------------------------  46013
NorthernAmericanDeerMouse   AAAGCATTGATA-CC--A-CAGTCACATCTCAGCTAAC-A---------CCA-----------------------CAATG  49741
Mouse                       AAAG------------------TCATATCTTAACTATC-A---------CAA-----------------------CAATA  50499
ChineseHamsterGHOK1GS       AAAGCATTGTTA-TC--AGTTGTCACATTTCAACTGTCCA---------CCA-----------------------CACTA  52283
LongTailedChinchilla        AGGGAGTTCACT------TTACCTGTTTTTTAACTAGTCA--------TTTGGGTGAATGTAATTACTTTAGAATTAGTA  50757
```

```
Majority                   TCCCAAAXT---------XTTTTTTTCAAAAXATXATTTXXXTCTACCACATXAAXAATTXC--TXXXXAATTT-AXTAG
                                     75850     75860     75870     75880     75890     75900     75910     75920
Human                      TTCTGATCCTTTCGTTACTTTTTTTTCTACTAATGATTTTAGTATCCAATAATGGTTGCTGCCTTAATTAATTTCACTAG  69413
GuineaPig                  ------------------TTTTTTTTTGACTAGTCATTTGGGTG----AGTTT---------------------------  46044
NorthernAmericanDeerMouse  TCCCAAAATG--------TTGTTAACAAAACAATATAT----CTACCACATCAAGAATTATC-TGGTTAATTT-AATAG  49806
Mouse                      TCCCCAGAT---------TTTTTACCAAAACAAAATTTA--TCTACCACATCAAGAATTTC--TGATTAATTG-AGTAG  50564
ChineseHamsterGHOK1GS      TCCCAGATT---------TTCTTGCCAAAACATTATATGGCCCTACCACATCAAAAGTTAC-------------------  52335
LongTailedChinchilla       TTACAGA-----------TTTTATTTTAAACAGTAATTTTAAAG----AGATTAAAAAATGGTACAG--AATTTCAAAAC  50820

Majority                   -AAAC-A-AGGGTCTCATTA-TGTGGTCCAXTTT--ACAXTCTXGCCTTGXAAAT--XTATXTAGCXCAGACTGCCCTTA
                                     75930     75940     75950     75960     75970     75980     75990     76000
Human                      TGGGTTGCAAATATTGCTTTTTCTCCCCTAATTCACATATTCCT-TCTCTACA----TTAATTAGCTGAAAATCTTTATA  69488
GuineaPig                  -------------------------------------CATTACT-------------TTAGTCAGTTTAC----------  46064
NorthernAmericanDeerMouse  -AAAC-A-AGGGTCTCATTAATGTGGTCCAGTCT--ACAGTCTGGCCTTAGGAAT--CTATGTAGCACAGGCTGCCCTCA  49879
Mouse                      -AAAC-A-AGAGTCTCACTA-TGTGGTCCAGGTT--CCAGTATGGCCTTGCAAATTGCTATGTAGAACAGACTGCCCTTA  50638
ChineseHamsterGHOK1GS      ---------GGGTCTCATTA-TGTGGTCCATTCT--ACAGTCTGGCCTTGGAAAT--CTATGTAGCACAGACTGCCCTCA  52401
LongTailedChinchilla       AAAACGATAAGAAATCAACAGTGGTGGTTATGTTGCATATTGTTATCTTCAAAGTAGTTGCTCAGATTACCAGATATATG  50900

Majority                   XACTXAAAATXTTTTXTTXT---TCAGCCTCXTXTATGC---TXTTXXAAXC----CTGTXTCACXAXXACAXXCAXTGG
                                     76010     76020     76030     76040     76050     76060     76070     76080
Human                      -AAGTAGAATTTTCCTTCATCAACTAGAATTATCAACCTTG-TAATTCAATTTGTGCAGGACAATTAGTTAAATTCTTGC  69566
GuineaPig                  ------AAATT-------------------------------TGTTTTAAAC----AGTAATTATTAGGACCATCAT---  46100
NorthernAmericanDeerMouse  AACTCAAAATGTTTTGTTCT---TCAGCCTCCTTTATGC-----------------CTCTGTCACCCAGACAGGCAGTGG  49939
Mouse                      GACT-AAAATGTTTTATTCT---TCAGCCTCCTGCATGCTGGGATTCCATGCCCTCCTGTGTCACCAAACCAGGCAGAGG  50714
ChineseHamsterGHOK1GS      AACTCAAAATGTTATTTTTT---TCAGCCTCTTGTATGC-----------------CTATGTCACCCAACCAGGCAGTGG  52461
LongTailedChinchilla       CATGTAAAATTGCTAAAATAATACAAGCACTAGCTAGTCCT-TGTAGTAAACTAATAGGAATCAGTAGTACAATTATCTT  50979

Majority                   ATTTTTXTTCTTXTT---TXTTTAXTTTTATTTTTTXTTTTTTTGAAAXXAXTTXTTTCXCTTAXATAAXACATXGXGAT
                                     76090     76100     76110     76120     76130     76140     76150     76160
Human                      ATTTGTTTGCTAATTTTAAGTTGGTGCCCAGCTTTTGTTTTCAGGTAAGTACTTAAACCAGCT-TATGA-ACAGGTACAG  69644
GuineaPig                  --------------------------------------------------------------------------------  46100
NorthernAmericanDeerMouse  ATTTTCGTTCTT-------------TTTTAATTTTTTTTATTGAGAATAGATTTGTTTTTCTCACATAATACATCGTGAT  50006
Mouse                      GTTTTCTTTCTCCCTC-GTTTTTAAATTTATTTATTCTTTCATTGAGAGCAGTT-TTTCGCTTAGATAGTATATCCTGAT  50792
ChineseHamsterGHOK1GS      ATTTTTGTTCTTCTT---TTTTTACTTTTATTTTTTCTTTTGTTGAAAAGATTCGTTTTTCTCACATAATACAT-GAGGT  52537
LongTailedChinchilla       AGTTATCTTGTGTTTGTATATATATTTTTTTCCTCTTCTTTTCTCCTTTCCCCCATCCCAGTTGTTTGC-TTTTGAGCAG  51058
```

Majority	TATAXTTTCTTCXCCCTCTXCTTATXCAAGTTCX----CTGTCXCTXCCTXTCAGAXTCXAGTCACTTACTGTXTXT---	
	76170 76180 76190 76200 76210 76220 76230 76240	
Human	TATAGATTCTTCCCACTGTCTGCTTTAAACTTTTGAAACTGTCAGTTGTTTGCAGGTTCTGGTAGCCCAGAGTGGTTTGA	69724
GuineaPig	-------------------------TCAATTTCT----CTGTTGCTTTCTTTTAG-TCCTAGAGAATCACAATGTTT---	46147
NorthernAmericanDeerMouse	TATTTTTTCTTCTCCCTCTACTTATCCCAGTTCC-------TCTACCCCAATCTGAATCCACTCCCTTTCTGTCTCT---	50076
Mouse	TATGCTTTCCCCTCCCTCTCCTCCTCCTAGTTCCATC-CTACCCTCCCCCAGTCAAATCCAGTCCCTTTTTGTCTCT---	50868
ChineseHamsterGHOK1GS	TATAGTTTCTCTACCCTCTACTTATCCCAGTTCC-------TCT--CCCAATCTGAATCCACTCACTTCCTGTCTCT---	52605
LongTailedChinchilla	TTTAT-TTCATACTATTATTCATAATTAATTTCT----CTGCTGCTTTCTTTCAG-TTCTAGGGA-TAACGGTGTTT---	51128

Majority	----------------CAGXAGXAXGXAAXTAGXXT-------------------TCXAAAGXAXAAT------------	
	76250 76260 76270 76280 76290 76300 76310 76320	
Human	AATGGTGACCTTAGCCCAGAAGTATGTAATTATTTAATTTGGCCTTGACCAGTGGCCCAAAAAAGGAAGGGGGTGGGGTG	69804
GuineaPig	----------------CAGAGT-ATGTAATTTATT----------------------CAAAGAAG---------------	46173
NorthernAmericanDeerMouse	----------------CAGTAGAAAGCAAATAGGCT-------------------TTTAGGGGATAAT------------	50109
Mouse	----------------CAGTAGTAAACAAAAAGGCT-------------------TCTAAAGGATAGT------------	50901
ChineseHamsterGHOK1GS	----------------TAGTAGAAAGCAAACAGGCT-------------------TCTAAGAGATAAT------------	52638
LongTailedChinchilla	----------------CAGAAA-ATGTAATTTATT----------------------CAAAGAAGAAA------------	51157

Majority	XATAAAGXXXTATATAA-ATXAXXXAAAAAXXAGXAATTT----------A--XAXAXATG--XACAAAACAAA------	
	76330 76340 76350 76360 76370 76380 76390 76400	
Human	GATAAAGGACTAAACAAAATTAATTAAAATTTAGAAATTTTTCTCTCAGAAGGAATTCATGCCCATAAAACAAAGACCAG	69884
GuineaPig	GATACAGAGTTTTGTAA--TTAGTTAGAAATCTGACATCT------------AAATACATAATCACAAC-----------	46229
NorthernAmericanDeerMouse	AATAAAGTAAAATATAAGATAAGACAAAACCCAACAAATC----------A---TCATAGG---ATAAAACAAACA---G	50170
Mouse	ACCAAG------------ATAAAACAAAAACTATCAAATC----------A---GCATACG---ACAAAACTAG------	50947
ChineseHamsterGHOK1GS	AATAAA-----ATATAAGGTAAAACAAAAACTAACA--TT----------A---GCATAGG---ACAAAACAGA------	52689
LongTailedChinchilla	GATAGAGTGCTCTGTAA--TTAGTTAGAAGTCTGACATTT------------AAATACATAGTCACAAACC---------	51214

Majority	--------X--------XAAAGXXAXACCAAAAGAXXATAAXTC----------AXTCAXAXACAGXAXTTCAAAGAXXA	
	76410 76420 76430 76440 76450 76460 76470 76480	
Human	GATACATTCGTATTTTATCGGTAAATACCAATAGATTATAAATCAC----TTTTCTCGTATACATAATTGTGAAAAAAAA	69959
GuineaPig	----------------------------TGGCAGACTACTAGTCC---------ATTCACATACTGTGTTTCAAAGAACA	46272
NorthernAmericanDeerMouse	AAAAAGCTCAAAGAA-AAAAAGGCATGCCAAAAAAAAAAAACCCCAAAAAACAGAGATAGACACAGGAATTCACTCATTC	50249
Mouse	-----------------TAAAGAAAAATCAAAAGGTGTAAG-----------------AAACAC----ATGTA--GATAT	50987
ChineseHamsterGHOK1GS	-----GCCCAAG-----AAAAGGCACACACAAAAAAGATAA-----------------AGACACAGAAACTCACTGATTC	52742
LongTailedChinchilla	----------------------------TGGCAGACCATTAGTC----------ATTCACATATTCTGTTTCAAAGAACA	51256

```
Majority                     AGA--TACTCAGGAATC-CATXAAAXTCAXAAAXXXAGAAACXATA------XAAAGGXCATXTXGTCTAATAAAAAAAA
                                      76490     76500     76510     76520     76530     76540     76550     76560
Human                        AAAACCATCCAGGTATG-CATGTTATTTATTCATTGAGAGGATATACATCTCTCAGGTGTTTTTTGTCGTATGTGTACTT  70038
GuineaPig                    AGG--TGGTCAAAAATT-CTTGAGTTT-ATAAATTGAAACATTGTA--------GTGATCATTTTGTCATATTAAAAT--  46338
NorthernAmericanDeerMouse    ACA--CACTCAGGAATCACATAAAAA-CACAAAACTAAAAACCATAA-----CAAAAGACATGTAGTATAATAAAATAAA  50321
Mouse                        AGA--AACTTATTAATCCCATAAAAAACACAAAACTGGAAACCATA------TAAAGGCCATGTAGTATAATAAAAGAAA  51059
ChineseHamsterGHOK1GS        ATA--TACTCAGGAATCCCATAAAAAACGCAAAACTGGAAACCATAA-----CAAAAGACATGTAGTGTAATAAAAGAAA  52815
LongTailedChinchilla         AGG--TGGTCAAAAATT-CTTGAGATT-ATAAATTGAGACACTATA--------ATGATCATTTTGTCACGTTAAAAAG-  51323

Majority                     ---XXTAXTXAATTTAX---------------X--AAGGAAXGTAAAAXTTAAAXAAX--X---GAGAAAAAXAAXATGC
                                      76570     76580     76590     76600     76610     76620     76630     76640
Human                        TTGTGTAGTCTGCCTGTTTATTCTTTTTGAATAGGATGATATGTACAATTAAAAAAAATCTTTTGTGAAATGTGGTTTTC  70118
GuineaPig                    ---TGTATTTAATTTAG------------------AAGGGGTATACTATGTAGAGA------------------------  46373
NorthernAmericanDeerMouse    T----AAATAGAATAAAGTA------------AAAAATGAAAGTGAAAATTAAAAAAAAATAA-GAGAAAAATAAAAGGC  50384
Mouse                        -----AAATAAATTTAA---------------AAAAGTAAAAATAAAAATTAAAAGG--------AGGAAAAGACCATGA  51111
ChineseHamsterGHOK1GS        ---------------------------------------AAAGTAAAACTTACAGAA-------GTGGAAAAGAAAAGGC  52849
LongTailedChinchilla         ---TGTAGTTAATTTATT-----------------AAGGGGTGTATTACTTAGAGAAAAATTGCTAGAAATA-ATTTTTC  51382

Majority                     CTTGAXATGACAT-------------TATAAATTAAGGAAXX--XT-TAGAGATAGTGX--TTGTXTTTT-----TTXXX
                                      76650     76660     76670     76680     76690     76700     76710     76720
Human                        TTAAATACATTATTGCATTTCATACACAGTGATGAAGGGAAAAACTACAAAGCTAGACAGATTGCAAATTAGTATTGCAG  70198
GuineaPig                    --------------------------TAAAAACT----------------------------------------------  46381
NorthernAmericanDeerMouse    CTTGAAGTGACAT-------------TATAAAACAAGGAAC------TGGAGATACTG---TTGTTTGTT-----TTGGC  50437
Mouse                        CTTGACATGACATG-----------ACATGAGACAGGAACTT--CT-TAAAGACAGTGAGTTCATTTTCTA----TTGGC  51173
ChineseHamsterGHOK1GS        CTTGAAGTGACAT-------------TATAAATTAAGGAACC--TC-TAGAAATACTG---TTGAGTTCC-----CCTTG  52905
LongTailedChinchilla         ATGATCATGTTGTC------TACATTTAAAAATTAGATAGTATATTAAAGAAATAGTTACTATAGATCTTCCAGTGTTTT  51456

Majority                     TATTTA---XACTGAGCX-TX---AX---TTXCCTTTAAX---GXGTAGTTXX---TTTTTTCAGTGAGAXTCXTTTGGA
                                      76730     76740     76750     76760     76770     76780     76790     76800
Human                        TAGTGAGGTAATAGAGCAATACCCAAATGTTTTCTTAAAATATATGTAGGTGATGCCTGTGTTCTTTACTTTTGTGTGTG  70278
GuineaPig                    --------------------------------------------------------------------------------  46381
NorthernAmericanDeerMouse    TGTTTAC--TGCTGAGCA-CA---AGG--CTACCCTTAT----GAGTAGT-------TTCCCCAGTAAGACTCCCTTGGA  50498
Mouse                        CATCTAT--GGCTGAGCC-TG---GGGCTTTGCCCTTAAA---GAGTGCTTTG---TGTTTTCAATGAGACTCCCTTGAA  51241
ChineseHamsterGHOK1GS        GGCTCA---TACTCAGC------------CTACCTTTAT----GGGTGGTTTG---TTTCTCCAGTGAGAATCTTTTAGA  52963
LongTailedChinchilla         AAATTAAATAA--GAGCCATTCTCAT-TGTTGTCTTCAAACAAAGGCAAAACAGTCTCTTTTTCCTGATAATTGTTAAGA  51533
```

```
Majority                   GA-----------AAACAAATTTTCATXTA--CAAGAAGTTATCX-XTAXGAXATT---GCTTCT--GGGTT--AGGXXC
                                       76810     76820     76830     76840     76850     76860     76870     76880
Human                      TGTATGTGTTTTTAAACAAGATTCATTGTAGTCTTTTAGATATAATTTAATAAATTTAGGCTGTTCAGGGTTTCAGTATC  70358
GuineaPig                  ------------------------------------------------------------------------------TC  46383
NorthernAmericanDeerMouse  GA-----------AAACTAATTTTCATATG--CAAGAAGTTAACCTATTGGAGATT---GCTTCT--GGGTT--AGGGAT  50558
Mouse                      GA-----------AAACTAATTTTCATTCA--CCAGAGGTTATCA-GTAGGAGATT---GCTTTT--AGGTT--AGGAAT  51300
ChineseHamsterGHOK1GS      GA-----------AAACAAATTTTCATTTG--CAAGAAGTTATCT--------ATT---GCTTCT--GGGTT--AGGGAC  53015
LongTailedChinchilla       CATAC-----CGCAAACAGTTTTTCATATAGGTAAATGTGTAGTTCTTAAGAACATAGCGTTTCTCAAACTTT-----TC  51603

Majority                   CXAXACATGTAXAXGX--X-XCTAXGCXCTXXGGXTTTTGGTXCTAGGACXXXGX--TGGTXXAGACATGGGCTGAXXXT
                                       76890     76900     76910     76920     76930     76940     76950     76960
Human                      CTACAAAGAAGAACGCTGCTTCTAAGTTTTTGGGTTTCTTGAACCAGAGCTTAGAAAGAACAAAAATATGGCATGGTATT  70438
GuineaPig                  CTACACATTGAAATCA-----CCAAGCACTGAGATCGGTGGGAGTAGGACTTGGG-----------CATTCA-TAGTTT-  46445
NorthernAmericanDeerMouse  AGGAACATGTATAGGG--CATATGTTCCCATTTGCTTTTAGTTCTAGAACCCCATC-TGGTTTAGACACGTGCCGACACT  50635
Mouse                      -GAGACGTGTCTA----------CTTC-----TGCTTTCGGTTCTAGGACCC-----TGGTGCAGACCTGGGCAGACCCT  51359
ChineseHamsterGHOK1GS      AGAGGCATGTATAGGG--CATGTGTCC------ACTTTTAGTTCTAGGACTCCATC-TGGTGCAGACACATGCTGATGCT  53086
LongTailedChinchilla       CTGAACGTTGAAATAA-----CCAAGCACTGAGGTCAGTGGGAGTAGGTCCTGGA-----------CATTGT-TACCTTC  51666

Majority                   TTXAAGGXTX-CTAAGGCTXXTTXTA-CTGATXXTTGTGATTTAGAAGXTTX-----GCTTCCTTGGXAACATXXTXCCC
                                       76970     76980     76990     77000     77010     77020     77030     77040
Human                      TTCATGGTTTAGTAAGGCTACTTTTAGTTAATTATTGTGATGTAGGCCATTAAGTAGACTTTTCTAAAACGAAACCCCAG  70518
GuineaPig                  TTTAAGATTC--CAGAG-TGCTTTTA-CTATATAATGAGAGTTGGAA-ATTA------CTGCCATTGAAAAA-GTT----  46509
NorthernAmericanDeerMouse  GTGCATGCTGCCTTGTTCTATGTGTG-CTGATCCTTCTGGTTTAGAAGGCCTT----GCTTCCTTGGTATCCTTCTTCCC  50710
Mouse                      C------C---CTCAGTCTGTGTGCA-TTGATCCTGTTGATTTACAGGGTTTT----GTGTCCTCGGTAACCTTCTTCCC  51425
ChineseHamsterGHOK1GS      GTGCATGC---CTTATACTATATGTG-CTGGTCCTTTTGATTTAGAGGGACT-----GTATCCTTGGTATCTTCTTCCCC  53157
LongTailedChinchilla       TTTAAGATTC--CAAGG-TGCTTTTA-CTGTATAGTGAGATTTGGAA-ATTA------CTGCTGTAGAAAAAAGTT----  51731

Majority                   ---CXCXGGCTTTT-XAAXTXXXTACATXTTTXCTTCTXAGTXXXXTGAGCCXX-AGXAGTXAXGGXXTTTGAAAXAGAC
                                       77050     77060     77070     77080     77090     77100     77110     77120
Human                      AGGAGAGGGGTTTTGAAGATACATATATACTGGTTTCTAAGAAC--TAATCTTTTTGGATCATTGGTATTGGTAGAAGGG  70596
GuineaPig                  ---TGTAAATTTTT--AGATGC-TAAATTTTAGCTTTT-AGAG----GAGTA------ACTAATAGTATCTTCAAAAGAC  46572
NorthernAmericanDeerMouse  ---CTCTGGCTCTTAACACTTTCTACCAGCCTTCTG---AGTTTCCTGAGCCTTGAGCGGTGAGGGATTT-GATTGAGGC  50783
Mouse                      ---CTCCGGCTTTTAA-ACTCTGCCCACCTCTTCTACTGAGTGCCCTGAGCCC--AGAGGTGAGGGATTTTGACTGAGAC  51499
ChineseHamsterGHOK1GS      ---CTCTTACATT-----CTTTCCACCTGTTTTCTGCTGAGTTTCCCGAGCCCTCAAGAGGGAGGGATTT-GATAGAGAC  53228
LongTailedChinchilla       ---GGTAAGGTTTT--AAATGC-TAAATTTTAGCTTTTTAGTA----AAGTT------ACTAGTGGTATCTTCAAAAAAT  51795
```

```
Majority                    ATT-CCTTTTAXXGCTGXAXXTTXCXAGXTCTCTXTAC----TCTXTXCATATTXTCTXAXTGTAXATXT--ATGXTTTT
                                      77130     77140     77150     77160     77170     77180     77190     77200
Human                       ATT-CGCTATGTTTTCTGCCACCATTGGATCTCTGTATAAAGTTTGGCTCCACTATCAAAATGCTTAGATAGATGCCTTT  70675
GuineaPig                   ATT-CATGGTAGTCATTGA----ACTGTATCTCAATAC----TTTAT----GTCATTTAAATCTATATA---ATTCTATT  46636
NorthernAmericanDeerMouse   ATC-CCTTTTATGGCTGAATGTTCTAAGGTCTCT-GAC----TC--TGCGTATTGTCTGGCTGTAGGTCT--CTGTATTT  50853
Mouse                       ATAACCTTTTGGGGCTGAACATTTCAAGGTCTCTTCCC----TCTCTCCATATTGTCTCACTGTTGGCCT--ATGTTTTT  51573
ChineseHamsterGHOK1GS       ATA-CCTTTTATGGCTGAATGTTCCAAAGTCCTT-CAC----TCTCTGCATATTGTCTGGCTGTAGATCT--CTGTATTT  53300
LongTailedChinchilla        ATT-CATTATAGTTATGGT----GCTATATCTTAATAT----TCTATTCACATCATTTCAATCTCTATA---ATTCTGTT  51863

Majority                    GTTCCAGTCT----------GCTAGGCAXXAAXTT-X--XXTTAATGTTXCTTACTX---TXCAACCTXTGTTGCXTXTX
                                      77210     77220     77230     77240     77250     77260     77270     77280
Human                       GCAATAGTTGCCACCCTAGGAATAGCTCAAAAATTATTAACTTAATACTGGTTACTTAAATTCCACCTCTGTTGCTTGGA  70755
GuineaPig                   AATGCAGT------------GTTAGTCATTAAGTTC--AGTTTACTTTTACTTTCCT---TCATACATTTGATGCATATG  46699
NorthernAmericanDeerMouse   GTTCACATCT----------GCCAGGCAACA---------GTTAATGTGACCAAATA---TCTAACCTACCTTAATT---  50908
Mouse                       GTTCCTGGCT----------GCTAGGCAGAAAT---T--TCTTAATGTGACGAAATA---TTTAACCTGCATCAAAATT-  51634
ChineseHamsterGHOK1GS       GTTCACTTCT----------TCCAGGCAGCAATTTTT--TGTTGTTGTTGTTGGCCA---TTCAGCTTTGTTTTTTTGTT  53365
LongTailedChinchilla        GATACAGT------------AATAGCCGTTAAGTT----GTTTACTTTCGCTTTCTT---TCCTACCTGTGATGCATATG  51924

Majority                    XTTXXXXXXXXXXTXTTTXTTTTT--------XAGXGXGTTTXTTTXTGTTTGXTATTTTTAXATGXX----GTAXXXTA
                                      77290     77300     77310     77320     77330     77340     77350     77360
Human                       GTTGTGCTTTTTTTTTTTTTTTTTTTTTTTGAGATAGAGTCTGGCTCTGTCACCCAGGCTGGAGTGCA----GTGGCATG  70831
GuineaPig                   ATTAATACAAACCTAAGTAATTT----------AGAGAGCTT---TTTCTTAGTTACTTTCAAATG------ATAGTTTA  46760
NorthernAmericanDeerMouse   ---------------T---TTTTT--------AAGGGTATTTATTCATGTTTGGTATTTTTAGGCCG-------A-----  50950
Mouse                       ---TC----------TTAGTTTA---------AAAGGTATTTATTTATGTTTGGTCTTTTTAGATACA------A-----  51682
ChineseHamsterGHOK1GS       TGTTTGTTTGTTTGTTTGTTTTTT--------TGGGGTTTTTTTTTGGTTTTTTTTTTTTTAGACCAATTAGGTACCTTA  53437
LongTailedChinchilla        ATTAACAAAAACCTAAGTGATTT----------AGAGAGCTT---TTTGTTTGCTACTTTTGAATG------GTAATCTA  51985

Majority                    XAAT--------XXTX-XTXAAXAXTTCTXTTXCXXXGTAC-CGXXAGXATXA-XXXTCA------CXTAX-XXTXAXXT
                                      77370     77380     77390     77400     77410     77420     77430     77440
Human                       CAATCTCG----GCTCACTGCAAGCTCCGCCTCCTGGGTTCACGCCATTCTCCTGCCTCAG-----CCTCCCAAGTAGCT  70902
GuineaPig                   AAAT--------TTTTTTTGAAGATTTCCATTACCATGTAC-CGATAGAATAA-ATTTCA------CATATGTATCATTT  46824
NorthernAmericanDeerMouse   -----------------------G--TCTT------ATTA----------------------------------------  50959
Mouse                       --------------------------TCTC------AATA----------------------------------------  51690
ChineseHamsterGHOK1GS       GGCAGACAAGGTAAAACATCAGCACATCTTTACAGAGTTAAACAAATGCAGCATAAATAAAAGGAACACATAATTAAGCA  53517
LongTailedChinchilla        AAAT--------GTTTGTTAAAGATTTCCGTTACCGTGTAC-TGGTAGAATGA-GTCTCA------CGTACTCGTCATTT  52049
```

```
Majority                     XGGXXXA-XAGTGTACCTCACXXXXXXXAXTTXXTTTXTTAATXTATTTTCCAGTXXA-XXXGXX-XTTTXXXXTGXXAX
                                      77450     77460     77470     77480     77490     77500     77510     77520
Human                        GGGACTACAGGCGCACGCCACCACGCCCAGCTAATTTCTTCATATTTTTTTTAGTAGA-GACGGGGTTTTACTGTGTTAG  70981
GuineaPig                    TGGTTCAACAGTGTTCAC-ACTTGTTGTATTTTTTTTTCTATTGTATTTTAAAGCAGATCTTGACATTGTAACATGGCAT  46903
NorthernAmericanDeerMouse    -----------TGTAGCTCAGGC---------------TGAATTGCTCTGCCAGTCC---------CTTC----------  50994
Mouse                        -----------TGTAACTCAGGG--------------------TCCTGTGCCAGTCC---------CTAT----------  51720
ChineseHamsterGHOK1GS        AGTTAAATAAATGTAGCATAAACACATCTCTAAATAGTTGAACAAATACTCCAATCCACAACATGTCATCTTTTGGCATA  53597
LongTailedChinchilla         TGGCTCAGCAGTGTGCAT-ACTTGTTTTAGTTTTTTTCCTACTGTATTTTAAAGCAGATGTTGGCCTTATGACATGTCAT  52128

Majority                     XXXAXXTXTTXXTG----XXXGATXTXXAAAAXA-ATTTXCTAAG-----CAAAGTT-X-----TXCATGXXTTXAXXAT
                                      77530     77540     77550     77560     77570     77580     77590     77600
Human                        CCAGGATGGTCTTGATCTCCTGACCTCCTGATCCTCCTGCCTCGGCCTCCCAAAGTGCTGGGATTACGGGCGTGAGCCAC  71061
GuineaPig                    TTCAAATATTACTG----TAAGATCTCTAAAACACATTTTCTTA------CAAAGT--------TATATG-----ATAAG  46960
NorthernAmericanDeerMouse    ----------------------ATTAAAAAAAAA--------AAA-----AAAAGTT-------TTT---TTTTAAGGGT  51029
Mouse                        ----------------------ATTTAAAAAAAA--------AAG-----TGGCTTTTTGTTTGTTCATCTTTTTAAGGT  51765
ChineseHamsterGHOK1GS        TGGATTATTTTGAGATA-AGTAATTGAGAAACAACATATGCCAAG-----AAGAGTTCTATGCTTTC---CCTTCCATTT  53668
LongTailedChinchilla         CTCAAGTATTCCTA----TAAGATCTCTAAAAC--ATTTTCTTA------CAAAGT--------TACATG-----GCTAT  52183

Majority                     AGCXTTGAGCC-GAXCAGGATT--------GXTTXXTTXGTGXCA------CTTAAAAAAAXACTAGCXXA-AXXXXAXT
                                      77610     77620     77630     77640     77650     77660     77670     77680
Human                        CGCGCCTAGCCTGCTTAGAGTTTTTTATGTAATTATTTTGTGACACAAAC-ATTTAAAATACTGTAGCTTAGAATGAAAT  71140
GuineaPig                    TTGATGGAGCC-----AAGATT--------GGTTACTGAGTGTCA--------TCAACAAACCCCAGTCCATATGAAAGT  47019
NorthernAmericanDeerMouse    AGCTTTGTGCA-GAACAGGATC-----------------------------CTGAAAAAGATACTAG-------------  51066
Mouse                        AGCCTTGTACA-GAGCAAGATT-----------------------------CTGAGAGAGATAATTG-------------  51802
ChineseHamsterGHOK1GS        AGCCTGAAGAG-GAACAGAAATATTTAGAGGGCAGTTTGGCAACATAACCACTTAGCAAAACAATAGCAGTACCCGCAGC  53747
LongTailedChinchilla         TGCACTGAGCC-----AGGATT--------GATTGCTTAATGTCA--------TCAAAAAATCC-AGCCCACATTAAAAT  52241

Majority                     XTACC--XTXAXXXGAX--XXATGTTTXTTGAATCTGTCTXCXXT--TXXTAAXXXXTTXT-XXTXCTGTGXCAGGCCTT
                                      77690     77700     77710     77720     77730     77740     77750     77760
Human                        TGTCTCAATCCTAGGAGCCAAAAGTTTGATGAATATGAATACTAA--TATTAACCATTTTTGTGTTCTGTGTCAGGCACT  71218
GuineaPig                    CTCCC--ATAATGTGAA--AAATGTTTTTTAAGGC-----TTTTT--TGAGAACAGGATCC--------TACC-------  47073
NorthernAmericanDeerMouse    -------------------TGATGGTTGTTGTTTCTGTCTCCA-------TAATTCCTTTT-CCTACTGTGGCAGTCCTT  51119
Mouse                        --A----------------TGTTAGTTGTTA--TTTATCTGCA-------TAATTTCTTTT-TGTACTGTGATAAGCCCT  51854
ChineseHamsterGHOK1GS        CTACCTTCTCAGGACCTG-TGATGTCTTTTGACTCTGTTTACAGTGCTGGAAATGAATTCC-CCCTGTAAGGCAGACCTT  53825
LongTailedChinchilla         TTACCCAGTTACCTGAA--AAATGTTTTTTTAAGGGTTGATTTGT--TCAGAACAGGATCT--------TACCGAGGGTG  52309
```

```
Majority                     -X---TAGCXTTTX-X---XTAAXXCAA---XXCTTCATCTXT-------------CXAXTATXGAXTXCXXTTXXTXXX
                                       77770     77780     77790     77800     77810     77820     77830     77840
Human                        GTTCTTAGCCCTTTAC-AAGTAAGTTAA---TACATCATCTCTA--AATGTAGTTACCATTATCGATCCCATTTTATAGA  71292
GuineaPig                    -----AAGTCTTTC-------AATTGAA---CCTTTTCTCCCT-------------CTACTACAGATTTCGGTTC-CATT  47124
NorthernAmericanDeerMouse    -----TATCTCCT------TTGCACCAC-----CTTCACTTTT--------------------------------GTTGA  51151
Mouse                        --------CTTTT------CTGTCCCAA-----CTTCAGTTTT--------------------------------ATTGG  51883
ChineseHamsterGHOK1GS        ATG--TATCTAATAACTGGTTACACCATAGAGACTTCACCTTTCTCCACATCTTTGCCAGCATTTGATATCATTTGTTTC  53903
LongTailedChinchilla         ATGTTAAGTCTTTTTC-AA--AATTTAA---CCTCTTCTCCCT-------------CTACTCTAGACTTCAGTTC-TATT  52369

Majority                     CTTGATAAATAGAXXXXAAAX-TCAGTT-----AGAXTTX--------XAGAX------------AGTTXCTAXTTTTAG
                                       77850     77860     77870     77880     77890     77900     77910     77920
Human                        TTTGGAAAGTAGGGTACAAAGAACAGTTTGCCCAGATTCCTTTAACTTTTGAGTGGCCGAGCCCTAGCACTGAAAGCCAG  71372
GuineaPig                    CTT----AATAGAT--------TCAGTT-----AGTTGTA-------CTTGAG------------A------AAAGTTAG  47162
NorthernAmericanDeerMouse    CTTGATAAAGAAGCCTTAAAT-TAAGGT----------TT---------AAAA------------AGTTTCTAGTTTTAC  51199
Mouse                        TTTGATAAAAAGACAAAAAACATTAAATTAAG----CTTT-----T---AGAA------------AGTTGCTAGTTATAC  51939
ChineseHamsterGHOK1GS        CTTGATGATAGCCATTCTAAC-TGGGATGAGAGAGAATCT-----C---AAAGT-----------AGTTTTTTGTTTTGT  53963
LongTailedChinchilla         CTT----AATGGAG--------ACAGTT-----ACACTT--------CTAGAA------------AGTTCCCAAGTTCAG  52412

Majority                     AT----TGGTTT---XGTTX-----CXTGXXTXXCXTGTAGTG--------TCATATTXXCTTXACXT---CTTTGTTCX
                                       77930     77940     77950     77960     77970     77980     77990     78000
Human                        GTAGTCTGGTTCCAAAGTCCGTACTCCTGACTAATAAATAGTGGAATAAAATTAGATTAGCTTGACAT---TTTAGCCTG  71449
GuineaPig                    AC----TGGTTT----GTTT-----CTTGG--------TAGTG--------TCATATTAACATGATTT---CTCTGTTCT  47210
NorthernAmericanDeerMouse    GT----TGCTCT---GATT----------ATTTCCTTGTAGTA--------TCATAATTTGTTC-------CTCTCTTCT  51247
Mouse                        AC----TGCTCT---GGTT----------GTTTCCCTGCGGGG--------TCATACATTCTTC-------CTGTGTGCA  51987
ChineseHamsterGHOK1GS        TT----TGTTTT---TATTTAAATTAGAAACAAGCTTGTTTTACATGCCAATCCCAGTTCCCTCTCCCTCCCCTCCTCCC  54036
LongTailedChinchilla         AT----TGGTTTCATTGTTC-----CCTGG--------TAGTG--------TCATGTTAAT-TGACTT---CTTTGTTCA  52463

Majority                     CTGXATTTTX----------------------------------------TAAXXXXTTACGT---CCATCXTTTAAGCT
                                       78010     78020     78030     78040     78050     78060     78070     78080
Human                        TCGTCTTCTCACCTGTAGAATAGTTATCAAGAATAGTAATATACATATAGTAAAAATTTAAGT--CCCATCATTTTAGCT  71527
GuineaPig                    CTGCATTTTT----------------------------------------AAAAAATACAGG----CTAACATGTAAACT  47246
NorthernAmericanDeerMouse    CTGTATTT---------------------------------------------GGGCTTACAT-----ATCTCTAAAGAC  51277
Mouse                        CTGCGTTT---------------------------------------------GGGCTTACTG----CATCTCTGAAGAA  52018
ChineseHamsterGHOK1GS        CTGTCTCCCACCAACCCCTATCCCATCCCCTTTCTGCTCTCCAGGGGAGGTGAGGCCTTCCATGGGGCATCTTCAAAGTC  54116
LongTailedChinchilla         TTGCATTTTT----------------------------------------TAAAA-TGTAAG----CTAACATATAAACT  52498
```

```
Majority                    XACATTATCAT----X-CAAAGGXAT--------X-TAXGTTCCTXXXXTATXT------------TTATTGACTXAGAX
                                      78090     78100     78110     78120     78130     78140     78150     78160
Human                       TGCTTTTTTTTTACATACTAAGATATTTTTTGTTACCAAGTCCATTAAGTATTTAGCCTGATTATCTTATTGATTAAAAT  71607
GuineaPig                   AACATGCTC--------CAAAGGTAT--------AATAAGTGTCTTAGTTACTT--------------GTTG-CTAAGGC  47295
NorthernAmericanDeerMouse   A--CATACCAT----------G----------------GGTTCTTCC---ATGT------------TTACTGACTTA---  51311
Mouse                       GACACTAGCATC---T----------------------GGTTCCTCC---ATGG------------CTCCTCACTTG---  52055
ChineseHamsterGHOK1GS       TGTCGTATCATT---TGGAGCAGGGC------C---TAGGCTCTTCCCCCATGT------------GTCTAGGCTGAGAG  54172
LongTailedChinchilla        GACATGCTC--------CAAAGGGAT--------AATAAGTATCTTAGGTACTTTGC---------TCATTG-CTGAGAT  52552

Majority                    A----XAATXXXTGXXAXT----------X--TAAAA-----TATACTAGAGXTTGAT-TGTXACTCACTGXTXGTAXAC
                                      78170     78180     78190     78200     78210     78220     78230     78240
Human                       ACCAAAAATGTGTATTGTTCATGCATT-GATTCAAAATTGGGTACAAAACGGGTAGATATGTAACATGCAAATAATAAAT  71686
GuineaPig                   A----GAATACTTGATACTGA--------AAGTAAAA------AGAGGAGAGGTTTAT-TGTAACACACAGTTGGTAAA-  47355
NorthernAmericanDeerMouse   -------------G------------------T---------TATACTGGAGATTGAT-TGTGACTCAC-----GTGGTC  51345
Mouse                       -------------G------------------T---------TGTGCTGGAGAGTGGT-TGTGACTTACT----ATTACC  52090
ChineseHamsterGHOK1GS       AGTAACCCTCTATGTGAAGAGGGCTCCCAAAGTCCATTCC--TATACTAGGGATAAAT-ACTGATTCACTGCCAGAGGCC  54249
LongTailedChinchilla        A----GAATACTTGACACT-------------CAAAA------AGAGGAGAGGTTTAT-TGTAGCTCACTGTTTGTAGA-  52607

Majority                    -XCATTTXXXTXT-TAX----TTAACXGACXCCCAGXXAXGAT----GXCA---------TXXCA---------X-----
                                      78250     78260     78270     78280     78290     78300     78310     78320
Human                       TGAACTTATATTTTTATCTTTTTGACAAACAACACGTTAAAATTTATGTCAATCTTATTTTTTCAAAATCCTTTTCTATA  71766
GuineaPig                   --GATTTCAGTTTATAG----TTAGCTGACTCCCAGGCAGGAT----GGCA---------TCACA---------------  47401
NorthernAmericanDeerMouse   -ACATG------------------AA------------------------------------------------------  51352
Mouse                       -ACATG-----------------AA-------------------------------------------------------  52097
ChineseHamsterGHOK1GS       -TCATAGATTGCCCAGTCCTCCTAACTGACACCCATGAAGGGGGGGGGGGGCGTCTGGGTGGGTCCTATGCTGCTTTCCT  54328
LongTailedChinchilla        --GGTTTCAGTCTGTAA----TTGGCCGGCTCCCAGTCACGAT----GTCA---------TGGCAGAGGGGCGCTGCAGA  52668

Majority                    -----------CXGXTXATGGCAGXXAXXAGXCAXXXAAGXAX-C-AXXXAAXCX-------GCAGXXXXGTCTTXAXGC
                                      78330     78340     78350     78360     78370     78380     78390     78400
Human                       TTTTATTAAGACTAATTATAGCAAATAATAGCCATAAAATTAAACCAAAAAACTCATCTACTGCAGACATATTTGAAAGC  71846
GuineaPig                   -----------CAGTTAATGGCAGGCAGAAGGCAACTAAGCAG-CTAGGAAGCA--------GCAGGTGACT----AGGC  47457
NorthernAmericanDeerMouse   ----------------------------------------------------------------------GCCTTGATTC  51362
Mouse                       ----------------------------------------------------------------------GCCTTCATTC  52107
ChineseHamsterGHOK1GS       AGCTATCAGTCTGGGGTCTGTGAGCTCCCCCTTGTTCAGGTCAGCTGTTTCTGTGCATTTCTCCAGCCTGGTCTTGACCC  54408
LongTailedChinchilla        ----GGGGAAGCAGTTCATGGCGGGCAGAAGGCAGCAGAGCAG-C-AAGAAACA--------GCAGATGACT----AAGC  52730
```

```
Majority                      TXTT------GAXXXXC--AXGTXTCTGCAAATGGXTXXXXXTCCXTXXATXXXCTAGXGAC-XX-----TACCTGXCTT
                                       78410     78420     78430     78440     78450     78460     78470     78480
Human                         TGTCCCCCAAGAAAGACCAAAGTAATCACACATACCTAAACATGCTAGTATTTCCCAGAAATATTAAAAATACATAGATT 71926
GuineaPig                     TGT-------GAACAGC--AGGC-------------TGTGTCTCCCTTTGTCACTTCCGGAC--------TACCTGCCTT 47507
NorthernAmericanDeerMouse     TATT------------------TTTCTGCTAATG---------------ATCTAGTA-------------------A-TG 51389
Mouse                         TAGT------------------TTTCTGCTAACCGGTTA---GT-----ATTCACTAGTGAC-TT--CT--GCCTTAGTA 52156
ChineseHamsterGHOK1GS         CATTGCCCATCACTTCT--CCCTCTCTGCAACTGGATTGCAGGAGTTCAGCTCAATATTTAT-TTATCTCTGGGTGTCTG 54485
LongTailedChinchilla          AGT-------GAGCAGC--AGGCCACAACAGAAGGCTGTATCTCCCTTAATC-CCCAAAGAC--------TACCTGTCTT 52792

Majority                      TTXX------TTCATCAGXXCCAGAATGAXGCXAAAXGAAGXAXTXXAX-TAAXTTAAXAATXACA---XTATC-TXAXX
                                       78490     78500     78510     78520     78530     78540     78550     78560
Human                         TTGAAGCACATTGCTGAATGCCATAGTAACACCAAACTAAAAAATACATGTAAATTAAAAATTAATATTTTATTTTCAAC 72006
GuineaPig                     TTGGC------------CACCCAGAACTATGCAAAATCAATCAGTGGAC-TAGTTTAATAATCACAC----ATCCTTAAT 47570
NorthernAmericanDeerMouse     TCT-------TTCATTAG------AGTGA--------GAGG----------------------------TTAT------- 51413
Mouse                         TCT-------TTCATCAG------AATGA--------GGAG-----------------------------TTT------- 52179
ChineseHamsterGHOK1GS         CTTCT--GCTTCCATCAGCTACTGGATGAAGTTTGAGGATGGCATTTAAGGTAGTCATCAATCTCA---TTATCAGGGAA 54560
LongTailedChinchilla          TTGGGTGGGATTATCCACACCCAGAACTGTGCCAAACCAGTCAGTGGAC-TAATTTAATAATTACA-----GTCGTTA-T 52865

Majority                      XTX-XX----AXXACXXT----------------XAATXXCXGXXTXA--XXTX--XTAXXTXXAGCXAXXX----TGAX
                                       78570     78580     78590     78600     78610     78620     78630     78640
Human                         ATAAATCAGAAATACTTGGTTAGAGGTCACATGATAATACCTGTCCTAACTGTCCCATAACTTCGGCTTTCTCATTTCAT 72086
GuineaPig                     CTACACGC--AACACACT----------------TAATCTCAGCTTCA--CATTCTCTAGATTGAGCCTC------TGAA 47624
NorthernAmericanDeerMouse     -----------------------------------AA------------------------------------------- 51415
Mouse                         -----------------------------------GA-------------------------------A----------- 52182
ChineseHamsterGHOK1GS         GGACATTTAAGGTAGCCTCTCCACTGTTGCTT--AGATTGTTAGTTGG--GGTC--ATCCTTGTAGATATCT----CGAC 54630
LongTailedChinchilla          CT---C----AGCACTTT----------------CAATCTCGGCCTCA--TATA-TCTAGATCCAGCCACCTG---TGAA 52916

Majority                      AXGXCXTA---XCTXTXXATTGAXXACXCTTTGAAGXX-XCATGTXXACAXXXGCXATXXTAA-XTATTTTCXXATTCTT
                                       78650     78660     78670     78680     78690     78700     78710     78720
Human                         GTGTTTTAATATCTAAGAAGTAAAAAATAGTTGGAGCATGCCTCTTTATATGTGCAATTTTAAATTATGTTGACTTCTTT 72166
GuineaPig                     AAGCCTCA---CCTGTGACTCCAGGAGGCTTTGAAGGGGACATGTAGACACAAGCCATAATAA-TTAGTATCAGATACTT 47700
NorthernAmericanDeerMouse     ----------------AGATTGCCCACCCTTTGAA------A--------------A-------CTCTTTTCTTTTTTTT 51452
Mouse                         ----------------AGATTGTCCTCCTTTCACA--------------------TA-------CTTCTTTTTCATTAGT 52219
ChineseHamsterGHOK1GS         ATTTCCTA---GTGCTAGATTGATTTCTCTTTAAACCTGTAATGGCTCCCTCTATTATGGTAT-CTGTTTTCTTGTTCTC 54706
LongTailedChinchilla          AAGCCCTA---CCTGTGACCCCATGAGGCTCTGGAGGG-ACATGTAGACACAAGCCGTAACAA-TTATT-TCAGATGCTT 52990
```

```
Majority                    GTGTT-TTTAACXXCTXXXXXXTTTXXCXTXXXXXXXXCTXTXXXA-GTXXXTTT----GTXX----AXXT-XCTXTC--
                                    78730     78740     78750     78760     78770     78780     78790     78800
Human                       GGATTATTGAACTGTCCTCTGCTTTGCTATAGCTGAATTTTTAAAATGTTAATTTTGAAGTCTTAAAATGTGTCTTTTGG  72246
GuineaPig                   GAGTT-TTTAACCACTTGACAGTTTACCACAAGCAGCACTCTGACA-GTGTGTTT----GTTT----ACATGACTAACC-  47769
NorthernAmericanDeerMouse   TTTT---TTTTTT-------------------------------------------------------------------  51462
Mouse                       TAGCA-AAAACTT-------------------------------------------------------------------  52231
ChineseHamsterGHOK1GS       CTCTA-TTCTTCCCCTGACTCAATTTTCCTGCTCCCTCCTGTCCTCCTCTGCCCTGCTCTTCTCCCCCTCTCTTTCTCCT  54785
LongTailedChinchilla        GTGTT-TT-AACCACTTAACAGTTTATCCTGATTGGCACTCAGGCA-GTGTGTTT----GTT-----ACGT-ACTATC--  53055

Majority                    ----AXCATTG------CTAXXXXXGXTX-------TCATTTCAACXXXTTXXTTXTTAXXTT-----GTXXTTTTTA--
                                    78810     78820     78830     78840     78850     78860     78870     78880
Human                       AAAAAACATTGGAAGAGCTAAAGTAGATAGAGTTGTTCATTTAAATTGTTTATTTTTTATTTTATTTTATTTTTTTTAGT  72326
GuineaPig                   --ATAGCATTG------CTAGGACTGGTG-------TCATTTCAACCAATTAATTCTTACATTGATT-GTTGTTTTTA--  47831
NorthernAmericanDeerMouse   --------------------------------------------------------------------------------  51462
Mouse                       --------------------------------------------------------------------------------  52231
ChineseHamsterGHOK1GS       AGCTCCCTCTCCCCTCCCCCCATGCTCCCAATTTGCTCAGGACATCTTGTCCCTTTCTCTTTCTCCGGGGGACCATCTAT  54865
LongTailedChinchilla        ----AGCATTG------CTAAGGCAGGTG-------TCATTTCAACCAGTTGATTCTTACGTT-----GTCATTTTTA--  53111

Majority                    -----TATT--XXCTXTTXTX----X----AX-XAXTXXCXXXAXGXXTGXXCAXTTXXTG-TCXTX----CTXTXTTTA
                                    78890     78900     78910     78920     78930     78940     78950     78960
Human                       TGGAGTACTGGCTCTGTTGCCTGGGCTGGAGTGCAGTGGCGGGATCTCTGCTCACTGCAAGCTCCGCCTC-CTGGGTTCA  72405
GuineaPig                   -----TATT--TCCTGTTATATTATCTTACAAAAATTAACAAAAAGAATGTGCAATTTTTGTTCATTA---TTGTATTTA  47901
NorthernAmericanDeerMouse   --------------------------------------------------------------------------------  51462
Mouse                       --------------------------------------------------------------------------------  52231
ChineseHamsterGHOK1GS       GTCTCTCTTAGGGTCCTCTTTGTTTCCTAGAGTCTCTGGTGGTGTGGGTGGTAGGCTAGTGATCCTTTGCTCTATGTCTA  54945
LongTailedChinchilla        -----TATT--TCCTATTATA---------AA-AATTAACAAAAAGAATGTGCACTTTTTGGTCATC----CTATATTTA  53170

Majority                    AAXXATT----------XGXCTX-TXAXTGXCTGXX---------------------ACXTXXXTXGTTTTTTTTGTXA
                                    78970     78980     78990     79000     79010     79020     79030     79040
Human                       CGCCATTCTGCCGCCTCAGCCTCCTGAGTAGCTGGGGCTACAGGTGCCCTCCACCAC-ACCTGGCTAATTTTTTTTGTAG  72484
GuineaPig                   AATAATT----------GGACTGATAACTGACTGAAC---------------------ACTTTATAAATGTTCTTTGT-A  47949
NorthernAmericanDeerMouse   -----------------------------------------------------------------TGGTTTTTTCA----  51473
Mouse                       -------------------------------------------------------------A---AGGTTTTTTTTTTCC  52247
ChineseHamsterGHOK1GS       AAATCCATTTATGAGTGAGTATATACCATGTTTGTCTTTTTGTGACTGGGTTACCTCACTCAGGATGGTTTCTTCTGTTT  55025
LongTailedChinchilla        AATAATT----------GGACTGGTAAGTGGCTAAA---------------------ACATTATTAGTGTTTTTTGTTA  53218
```

```
Majority                     CTTT----------------XTCA----GTXAXXAAGXAT---XT---AXXTAXTAXTXTTGTX---XTGXCTAXXTXTG
                                      79050     79060     79070     79080     79090     79100     79110     79120
Human                        TTTTAG--TAGAGACAGGCTTTCACCGTGTTAGCCAGGATGGTCTC-AATCTCCTGACCTCGTGA-TCCGCCCACCTTGG  72560
GuineaPig                    TTTT----------------CTCA----GTAAATAAGAATTATTT---ACATATCACTTCTGTCT-GATGGCTAGTTGTG  48005
NorthernAmericanDeerMouse    --------------------------------------------------------------------------------  51473
Mouse                        CCCTC-----------------------------------------------AATA------------------------  52256
ChineseHamsterGHOK1GS        CCATCTATTCAGGAAGCAATTTCTTAATGTGACCAATTACCTAACCTGTACTAATATTTTTGTTTGTTTGTTTAAAGATA  55105
LongTailedChinchilla         CTTT----------------CTCA----GTAAATAAGAAT---AT---ATAAACTGTTCTTATT---ATGGCTAGTTGTG  53269

Majority                     ----------AX------AXATAXAGTTTCGXXTTXX----X----------------TCAGTTA-----TTTXA-----
                                      79130     79140     79150     79160     79170     79180     79190     79200
Human                        TCT--CCCAAAGTGCTGGGATTACAGTCATGAGCCACCG--T-GCCTGGCC-------TCATTTAAAATTTTTTAACA--  72626
GuineaPig                    ----------AA------AAATACAAC--CGAATTAC---------------------TCAGTTA-----TTTTA-----  48036
NorthernAmericanDeerMouse    ------------------AGACAGGGTTTCTCTGTGT-----------------------AG------------------  51494
Mouse                        ------------------AGCTAGACCTTTGCCTTGTCA--T------------------AG------------------  52280
ChineseHamsterGHOK1GS        TATAAGTTTGGTATTTTTAGGCAGAGTCTCACTATGTTGCTTAGGCTGAATTGTTCTGCCAGTCCCTGTATTAAAAAAGG  55185
LongTailedChinchilla         ----------AGGGGAAGAAATACAGGTACGAGTTAC---------------------TCAGTTA-----TTTCACG---  53310

Majority                     ------------------XXTXTTCTTG---XACAAATTTXTGTG-------------------TXTATTCTXG-----G
                                      79210     79220     79230     79240     79250     79260     79270     79280
Human                        ------CTGGGTAAAATTTTTATTTTTGAGTCACAAATTAATTTGA--AGCTG-----------TGTCTTTTAGA--GAG  72685
GuineaPig                    ------------------GATGTTCTTA---TACAAATATATATG-------------------TATATTCTAT-----G  48071
NorthernAmericanDeerMouse    ------------------------------------CTTTGCGC------------------------------------  51502
Mouse                        -----------------------------------AGTGTGTGTGCGAAAT---------------AATTCTGGT-----  52305
ChineseHamsterGHOK1GS        GGTAGCTTTGTGCAGATCAGGATTCTGGAAAAGATACTTGGTGTTGGTTGTTGTGTCTGTTTCCATAATTCCTTTTCATA  55265
LongTailedChinchilla         -----------------TGTTGTTCTTG---TACAAATCTATTTG-------------------TGTATTCTGG-----G  53346

Majority                     CXTXTTXAGXCCTTTCTTXCATTTTX-AXGAXTTGTTAAGXACTX------------GAAGCAGTTTTTXT----X--GX
                                      79290     79300     79310     79320     79330     79340     79350     79360
Human                        CTTATTCAACTCTTAATTCCATTATAAATAATATTTGAATGAATGTGTTTTACATCTGGAATGATTTTTATCATCTGAGT  72765
GuineaPig                    CCTTTTTGGTCTTTTTGAACTTTCTGCAAGATGGGTTAAATAA---------------AAATACTTTTGGT---------  48127
NorthernAmericanDeerMouse    -----------CTTTCCTGGAGCTC-----ACTTGGTAG--CCCA------------G--GCTG--------------GC  51536
Mouse                        -----------CTTTCTTTCA-CTT-----ACTTTTGGGG--CTG------------GAAGCAGT-ACTGCAGACTAAGC  52353
ChineseHamsterGHOK1GS        GTGTGACAGCCCTTTCTTTCAGTTCTGCTGACTTGATAAGCCTTAAATT--AAGCTTTAAGAAGTTGCTTAGTTTTATGT  55343
LongTailedChinchilla         CCTGTTTAGTCTTTTTGAGCCTTCTGTAAGATGGGTTAACGAC---------------AAGCACCTTTTAT---------  53402
```

```
Majority                      - - CXCGAAATA- X- TXTXATXTXTGCTXT- AXXTXT- - - - - - - - XATTACAGACXACTXATGGGAAAAAGX- - - - TXTGA
                                        79370       79380       79390       79400       79410       79420       79430       79440
Human                         - AAAGGAAATA- TGTTTGATGGTAGTTGGGAACTTTGAA- - - GGTATTACGGGCTACAGATGAGAAAAAAA- - - - TGTTA  72836
GuineaPig                     - - - - - AAAATA- - - TCCTATATATGCTGTCATATTT- - - - - - - - TATTACAGTGGAGTAATGGGAAAAATG- - - - C- TAA  48186
NorthernAmericanDeerMouse     - - CTCGAA- - - - - - - - - - - - - - - - - - - - - - - - - - CT- - - - - - - - - - - - - C- - A- - - - - - - CAGAGATCCG- - - - - CCTGG  51561
Mouse                         - - CCCGAA- - - - - - C- - - - - - - - - - - - - - - - - - - - CT- - - - - - - - - - - - - CAGACC- CTTTTGGGCATATGC- - - - TATGA  52388
ChineseHamsterGHOK1GS         TGCTCTGATTATTTCATAATTTGTTCTCTATTCTCTGCATTTGGGTTTACGTACCTCTGAAGAAGACCTGGAACATCTGG  55423
LongTailedChinchilla          - - - - - GAAATACTGTCTTATATATGCTATTACATTT- - - - - - - - CATTGCAGTGGAGTAATGGGAAAAAGC- - - - TGTAA  53465

Majority                      CTCTGXC- - - - - - - - - - - - - - - - XCAXACT- - - - - - - - - - - - - - - - TGAAATTAGC- TTXTXGCTAXXGGATTXTAXAGC
                                        79450       79460       79470       79480       79490       79500       79510       79520
Human                         CTACGTCAT- - - - - TTCATGTTTACATACTGACCAGC- ATTTTCAATGAAATAAGC- TTATTACTATAGGACTATAAAAT  72909
GuineaPig                     GATTGT- - - - - - - - - - - - - - - - - GCAAATT- - - - - - - - - - - - - - - - TGAAATTAGCATTGTAGCNNNNNNNNNNNNNNNN  48233
NorthernAmericanDeerMouse     CTCTGCC- - - - - - - - - - - - - - - - - - - - - - - - - - - - - - - - - - - - - - - - - - - - - - TCCCGAGTGCTGGGATTAAAGGC  51591
Mouse                         CTCAGCC- - - - - - - - - - - - - - - - - - - - - - - - - - - - - - - - - - - - - - - - - - - - - - - TATCTTTAAGGCAGTGTAGAGC  52417
ChineseHamsterGHOK1GS         TTCTTCCATGTTTACTGACATAGTTATACTGGAGATTGATTGTGATTTACTCTTATTTTTCTGCTAATGGGTTTTGTAGT  55503
LongTailedChinchilla          GATCGT- - - - - - - - - - - - - - - - - ACAAACT- - - - - - - - - - - - - - - - TGAAATTAGCATTGTGGCAGTGAAATCATAGAGC  53512

Majority                      GXXXACCXCC- - ATTXTCTGTX- - - - - AXGX- XXXTAATXAAGACT- - - XX- C- ATCCAT- - - - TTXTTTTXCATTTGGT
                                        79530       79540       79550       79560       79570       79580       79590       79600
Human                         GTTTTTCAAAGGTTTAACTGTC- - - C- AGGGCAGATACTTAAGACTATCTGATCATCCATTA- AAAACTTTTCACATAGT  72984
GuineaPig                     NNNNNNN- - - - - - NNNNNNNNN- - - N- NNNNNNNNNNNNNNNNNN- - - - - - - - - NNNNNN- - - - NNNNNNNNNNNNNNNN  48290
NorthernAmericanDeerMouse     GTGCGCCACC- - AACGCCCG- - - - - - - - - GC- - - - - - - TGAAAACT- - - - - - C- - - - - - - - - - - - - - - TTTTCATTAGGT  51632
Mouse                         AAAAAATTCCTCAGTTTCTT- - - T- - - - - GC- - - - TAACAAATACAGT- TG- CTAGACAAAACATTTGCATGTATTTAGT  52483
ChineseHamsterGHOK1GS         GAGTACTGCCTTATTGTCTGTCATCAGAGTGAGGGTTATAAAGACTGC- TGTCCCTTCACATGCTTTTTTCCCATTAGGT  55582
LongTailedChinchilla          AGTACCC- - - - - - CTATGTATT- - - T- AAAATAAATGTAGGGGAC- - - - - - - - - ACCTAT- - - - GTATTTTGTGTGTGTA  53569

Majority                      TXTAAXAAXTA- GATTCATTXT- - XTXCCT- XTXAATAXGTTAGAAXTTTAXCTTTTAGAATAGAGTXTXCXCXAGAXCA
                                        79610       79620       79630       79640       79650       79660       79670       79680
Human                         CTTGCTTAAATGGATCCATTATGTTTACCCATTATAT- TGTTCTCAGCTGTAGTTTTAAGAAATTTATTTCATTTGTTAA  73063
GuineaPig                     NNNNNNNNNNNNNNNNNNNNNNNN- - - NNNNNNNNNNNNNNNNNNNNNNNNNNNNNNNNNNNNNNNNNNNNNNNNNNNNNN  48367
NorthernAmericanDeerMouse     GATAACAACTA- GATTTATTTT- - TTCCCT- - - - AATAAGCTAGACTTTTACCTTGTAGAATAGAGTGTGTGC- AGAACA  51704
Mouse                         TGTTTTAATTATGACTGGGTAC- - AAGCAT- - - TCATTGTTGTGAAGTTTATGAATTGGTACAGGGTATAGGCTAGATTA  52558
ChineseHamsterGHOK1GS         AGTCAAAACTA- GATTATTTTT- - CCCCCTCCTTAATAAGCTAGACCTTTAACTTGTATAATAGAGTGTGCAC- AGAGCA  55658
LongTailedChinchilla          TTCAGAAAACAAAATTCATTGT- - - TGTCCTTTAGATATGTAATAACTTGTTCGTTCAGAATTTCAATATCCTAGGAAGA  53646
```

```
Majority                     TTXCTGGTTTXCCTTTCACTTACTTTTTGGGCXAAAGXCXGTAXXTXAXATTAGACCXX---GAXACTCA-XCCTTGGTA
                                      79690     79700     79710     79720     79730     79740     79750     79760
Human                        TACTTACTTTCCATTACCTTCCCCTTTTCTGTGACAATCCGTTGATACTTGAAGACCTCTCTTGTATTCATCTTAGGGTT  73143
GuineaPig                    NNNNNNNNNNNNNNNNNNNNNNN-NNNNNNNNNNNNNNNNNNNNNNNNNNNNNNNNNN----NNNNNNNNNNNNNNNNNN  48442
NorthernAmericanDeerMouse    GTTCTGGTTTTCCTTTCACTTACTCTTGGGGCTGAAGGCAGTACTAGAGATTAAACTCA---GAACCTCACTCCTTATTA  51781
Mouse                        TTCCCAGTATCTGCTTTA--TACTTTAGGAGCCATTGCTTCCAGGTTCTAGTAGCCTGC---GAGAGTA--GCCTAGGGA  52631
ChineseHamsterGHOK1GS        GTTCTGGTTTTCCTTTCACATACTTTTTGGGCTGAAGGCTGAACTAGAGATTAAACCCA---GGACCTC--CCCTTTTTA  55733
LongTailedChinchilla         TAAGTGCCATGTCTTAGGGTTTT-TTTTCCCCCAGAACCACTCAGTAACCTGAGACCT----TAGAAAGAAGAAATGGTA  53721

Majority                     GXCATXTXCXAX-TCAXXCAGCTXTATTCCTATGXXATTCX----GAGCXATATGTXCTTTACTTXXTTTCCTAAAGAAA
                                      79770     79780     79790     79800     79810     79820     79830     79840
Human                        AATATTTTTAAGGCCAAACAGCCTAAATTCTATGGTAATCAACTCCAGCCTTGTGTAATGAAATCTTCTGCATAAAGATA  73223
GuineaPig                    NNNNNNNNNNNN----NNNNNNNNNNNNNNNNNN--NNNNN---------NNNNNNNNNNNNNNNNNNNNNNNNNNNNNN  48507
NorthernAmericanDeerMouse    GACATATGCTATGTCACTCAGCTGTATCCCTAGGCCTCTCC----GAGCAATAAATTCCTTACTTGCTTTGCTAAAGAAA  51857
Mouse                        GTCAT--------TGATGCAGTT-TATTCATGTGTGATTGTT---GACTCACTTGCCCTTTCTCTGACTCCAGAATGGAA  52699
ChineseHamsterGHOK1GS        GGCATATGCTGTATCACTTAGCGGTATCCTTAGGCCTTTATA---GAGCAATACATTTCTCACTTTCTTTGCTAAAGAAA  55810
LongTailedChinchilla         CTTATTTTCAAG----AGTAGTATGGCTACTTTG--AGTTA---------TTAGGCAGTGGAGTACTTTTCCAAAAAGGA  53786

Majority                     XXAXTTG-X------XGAXAXXGXATTTGATXXAAXTAGTTATGXX--TAAGATXXXTCAAA-----GAXTXGXTXTATG
                                      79850     79860     79870     79880     79890     79900     79910     79920
Human                        GGTTTAAATCAAATCAGATTGCAGATTTTATTGAAGAAATTGTGTTTTTAAGAGTTGACAAA-----TATATGTTGTATG  73298
GuineaPig                    ------------------------NNNNNNNNNNNNNNNNNNNN-----NNNNN---NNNNN-----NNNNNNNN-----  48545
NorthernAmericanDeerMouse    ACAGTTGAT------AGACACAGCATTTGCTGTAAGTAGTTATGTTC-TTACATCAATTTAA-----GAATGGGTATAAG  51925
Mouse                        GGAACTG---------GGCAGGGGACTTAACAAAGCTACTAAT-----CAAAGTTTATCCCA-----GGTTAGATATATT  52760
ChineseHamsterGHOK1GS        ACAGTTGCT------AGATACAGCATTTGCTGTAAATAGTTGTGTTC-TTATAACAGTTGAACACAGGACTAAGTTTATG  55883
LongTailedChinchilla         ------------------------ACCCCATTGAAAAAGCTATG-----AAGAC---ACAAA-----TACATGTT-----  53824

Majority                     XCTTAXXCXXTGGXAXCACXTCACATCXTTXXTCAXATAXAATAXGAAAXCAXAXATATTTAXXTXT-ACTXTGTAXTAX
                                      79930     79940     79950     79960     79970     79980     79990     80000
Human                        GCTAAAACAAAGAAAATACTTCTGTTGCTTCTGCATTTAGTAGAAGAAAAACTATATATGTTTGTGA-CCAAAGTA-TAA  73376
GuineaPig                    -NNNNNNNNNNNNNNNNNNNNNNNNNNNNNNNNNNNNNNNNNNNNNNNNNNNNNNNNNNNNNNNNNN-NNNTTCTA----  48619
NorthernAmericanDeerMouse    TGCCAGGCGGTGGTGGT---TCACGCCTTTAATCCCA-GCACTCGGGAGGCAGAGCCAGGCAGATCT--CTGTGAGTTCG  51999
Mouse                        TCTTTCTCCAAAGAAACGCATCCC--CATGAATCAAAAAAGATCAGAAAGCAATAGTATTT---TCTTAATAAATAGCAG  52835
ChineseHamsterGHOK1GS        AATTGGTTCTTAATATCACATCACATCTCTCTTACCATGAATTATGGGAACAAAAAGACTCAGGTGTGACTGTGAGCTAA  55963
LongTailedChinchilla         -CCTAAGAAGTGGGATCTTTTTGGATCATTGGTTAATGAGAGAAGGAATTAGTTGATATTTGTATAA-ACATTTGACTCC  53902
```

```
Majority                    AXTATAAXACTTTCTAXC----GTAAXXGTTGTXXAAATXXTTTCCXXCCXTAGXXXXAGAAAGCXAAXXXXAAXTAATX
                                      80010     80020     80030     80040     80050     80060     80070     80080
Human                       AATATGATTCTTTCCAGGGAG-GTAAAGGTTATGCAGAAGATTTTCACTAGCAGCTCTAAAAGGCTACCCTCAATTAATT 73455
GuineaPig                   -----------------------------------------TTTCTGT-------------------------------- 48626
NorthernAmericanDeerMouse   AGGCCAGCCTGGTCTACC----GAGCAAGATCCAAGANNNNNNNNNNNNNNNNNNNNNNNNNNNNNNNAAAAAAAAAAAA 52075
Mouse                       AAGATAATACATTATACCTCTTGTCATAATTGTGAGAACAGAAACAGACCCTGGTGTGACTGAGGCAGTGAGAAGTTTTC 52915
ChineseHamsterGHOK1GS       AGTATAAAAGTTAGTAAT----GTAATTGAATTATAATTGATTTGGCCCTTTATTGCTTGCTACTCCATCCCCATCCCTC 56039
LongTailedChinchilla        ACTATCAAAATTCCCAGATAG-ATTCCTTTTG---AAATAGTTTCCATCCATAACT--AGAAAGCTAA-------TAGTT 53969

Majority                    XATATXAAXATAAXAAA---------GXATXXTTGTACXTTCXTATXTTTXCXTAGAXTXXTTTTTGTTTT---TTATTA
                                      80090     80100     80110     80120     80130     80140     80150     80160
Human                       GCCATGAACATTTCATAGCCCTAGAAGGATGTTGGCTCATTTCAGTGTCATCCTGGTTTATTCTTTATTGTA--TTATTC 73533
GuineaPig                   ------------------------------------------------TTTCTTAAAGTTCTTTGTGTCATC--TTAGTT 48656
NorthernAmericanDeerMouse   AAAAAAAAAAAAAAAAAA--------GAATGGGTATAAGCACTAATTCTTGCCAGGTTTATGAATTGGTTT---TTAATA 52144
Mouse                       AGTTTAGTCATAATAAATT-------GGACCTTTTTACTTTCTTTGCTTGGCCCTTCTAGCCTTTCCCTTTCACTTCTCA 52988
ChineseHamsterGHOK1GS       TTTTTCTTTCCAAATAG---------GAATTCTTCTACTTTCATATCTCTGCTTAGAGTCTAATCCATATT---ATGACA 56107
LongTailedChinchilla        TATAAGAA-ATTACTTA---------------TGGTCCACTTCTA---TTTCTTAAAGTTCTTTGTGTCAT---TTATTT 54027

Majority                    AATAGXX---TXTATACTAXTXTGAA---TTTAGCCATTCAXAAAAXTAACXGTGTTTXXGXXXCXGTXXAGCTTGAXAX
                                      80170     80180     80190     80200     80210     80220     80230     80240
Human                       AGCAGTCATTTTAACACTATGCTAGACACTTTAGAGATTCAGAAGAGTAACAGGGTTTCTGTTCTCATGAAGCTTATCAG 73613
GuineaPig                   TTGGG-------GAAAAAACAGTAAA---TTCAGCCATTCATCAAA-TAACATTGTTTTAGTTACT-----GGTTGATGT 48720
NorthernAmericanDeerMouse   AATAGCA---TCTCTCTTATTGTGAA---TTTTGAGAAAAAAAGAGCCAGGTGTGACTGTGAGCTAGTAAAGTGATAAAA 52218
Mouse                       AATAGTC---TTTGTTCTACT-TCCA---CGTCGCTTCTTAGACTGTAGAGTGTCCACATAGCACAGTACAACTGGGGAG 53061
ChineseHamsterGHOK1GS       GA--ACA---TCTGAGCT-----GGA---TCTTTTCATCTCCTCCACAGAATCCTTCATAGAGCAGGTTCATCATTTTAA 56174
LongTailedChinchilla        TGTGG-------AATAACGGTCTGAA---TTCAGCCAGTCACCAAA-TAACAGTGTTTCAGCCACT-----GGTTGACAT 54091

Majority                    GTAXGTAAACCATCTXXCTTAXATXTXATTGGTGCX---------TXAAXTATATTXTTGXTXAXGX-TATCT-------
                                      80250     80260     80270     80280     80290     80300     80310     80320
Human                       AGACAGAAAACATATGAGTTAGATCTAATTGGAGGCAAACTGAAATATATAGTGGAGTTAGTGTGGT-TATCAGCACATA 73692
GuineaPig                   GTATGTAGACCATGTATATAAG----AATTGGTCCT---------TAAATTATATTAGTGATAAAA--TTCCT------- 48778
NorthernAmericanDeerMouse   GTTGATAATATTTCGACCTTTTATTTCCCTGGTG------------GTGCTAGATT----GTAAAGG-TGTCC------- 52274
Mouse                       TCTCTTCCATCCCTCAGCAGCAGCTTCCATAGCACA----------GACCCATCATTTTGATGAGGTCTAATT------- 53124
ChineseHamsterGHOK1GS       TGAGGCCAACTTCCTGGCTTTTATCTCAGTGATGTAT--------GGAGCTAGATTTGGGGTGGGGGGTATCC------- 56239
LongTailedChinchilla        GTATGTAGACCATCTGTATGA-----AGTTGGTTCC---------TAGATAATAATGTTGATAAAAA-TTCCTG------ 54150
```

```
Majority                    ------TCATCTAGXAACTTTAGTAGAXT----GTXXXXTTTTATCXTCCAXXTXXAGXCATXXTTGTAXGAGXAATGXT
                                    80330     80340     80350     80360     80370     80380     80390     80400
Human                       AATGAGTGATCCATCAACAAAAGGAGAAATT-GGGAGGGTTTTATGGGCCAAAAACAG-CATGATTAAATGTGATAGAGT 73770
GuineaPig                   --------ATCTAGTAACTTTAGCACAGC---------ATTTTACC-------------CATGATTGTGG------TGAT 48822
NorthernAmericanDeerMouse   ------TCTCCTAGCCAGTTCAGTTG------AGTTTCATTTCATTATCCATTTTGAGTTACTTTTGTATGAGGAATGAG 52342
Mouse                       ------TACTGT-CTTGTCTTAGTGGTTT----GTGAGGTTCAATCGTGAAGGTGTGGTCTCTTTTCTAGCACTTTTGGT 53193
ChineseHamsterGHOK1GS       ------TCTTCTACCCATTTCAGTTATTTTTTAGTTTAATTTCATTATCCATTTTGAGTCATTTTTGTATGAGAAATGAA 56313
LongTailedChinchilla        ------TCATCTAGTAACTGTAGCATAAT---------GCATTACC-------------CATGATTGTGGCAATACTGGT 54202

Majority                    ATXXATAAXXTTGTAGTTXTXXT-XATAGTTGTTT---TTXAACTAGCAGATT--CTCCXAXTCTAAXCAAGGTTXAAXX
                                    80410     80420     80430     80440     80450     80460     80470     80480
Human                       ATATGTCATGTTTTAGGTGTGATGAACATTCAGTTATGTGTGACGAATAGGATAATTGAAAAAATATGAAAGGCTATGAT 73850
GuineaPig                   ATAAA-----CTGTAGTTG-----AATATACCTTC---TTCTCCATGTG-------------CTGCACAGGCT------ 48869
NorthernAmericanDeerMouse   GCTTAGGA---CA--GTTT------GTTGTTGTTT---TTCAACTAGCAGATT--CTCCAAATCCAACCAAGATTGAATG 52406
Mouse                       TTTAGTTTTTTCGAAAATTTTTT--GTCGTTGTTT---C-AAACTAGCCGATT--CTCCCAGTCTAACCAAGGCTGAACC 53265
ChineseHamsterGHOK1GS       ACTTAAAAGTTTGTTGTTTTGCT--ATTGTTGTTT---TTAAACTAGCAGATT--CTCCCAGTCCAATGGAGAACAAATG 56386
LongTailedChinchilla        ATAAATAAACCAGTGGTCGGACCAAATAACTGTTC---TATTACAGACATATTTTTTCT----CTATAAAAAGT------ 54269

Majority                    TCXAXXXAATXACATX---GXTCATTTTCAAATAXXXATTACXXAXAATGTTTGAATTTX-TXTX---------------
                                    80490     80500     80510     80520     80530     80540     80550     80560
Human                       GCCAGAAAGTATTATGGGACAAGATCTTAAAACCAGTGTTACCCAGGGAGTATGAATTTAATATGGGAAT---------- 73920
GuineaPig                   --TAGAAAATG-----------ATGTTGAG-------TCCTTTACATTACTAAACTACCTGTG--------------- 48912
NorthernAmericanDeerMouse   TCTATTGAATCACATTT--GTTCTTTTTCAGATAATAATTAGAAATACTGTTTGGATTT-------------------- 52463
Mouse                       TCTATTGAGTTACATCC--GTTCTTTTTCAAATA---------------------------------------------- 53297
ChineseHamsterGHOK1GS       TCCATTGAATTACATT---GCTCCTTTTCAAGTAATAATTAGAAATATTGTTTGAATTTATTCTCTCTCTCTCTCTCTCT 56463
LongTailedChinchilla        --CAGAATATATCCA-----AACATGTTAGAA---ATATTTCCTAGAAATTTTAAACTATGTATA--------------- 54324

Majority                    -------------T--CTTTXT--------------------------------GAXXATXTGA--ATXTTAXXTXX-
                                    80570     80580     80590     80600     80610     80620     80630     80640
Human                       -----TCTTAAACTC-CTTTATGACTGGAA-----GATGAGCATCAGAGTGTCTGCGACCATTTGATGATATTATGTACC 73989
GuineaPig                   ----------------CTTTGCT--------------------------------TGAATATTTGAA-ATGTTAATTTTT 48943
NorthernAmericanDeerMouse   ------------TTTTTCTTTTT--------------------------------------------------------- 52474
Mouse                       -------------T---TTTCT---------------------------------------------------------- 53303
ChineseHamsterGHOK1GS       CTCTCTCTCTCTCTCTCTCTCTCTCTCTCTCTCTCTCTGTGTGTGTGTGTGTGTGTGTGTGTGTGTGTGTGTGATGTATG 56543
LongTailedChinchilla        ----------------TTTTA----------------------------------GAACATATCAC-ATAGTAGTTTCA 54352
```

Majority	AAXT------XXTGTGTGTXTXTTTGXTCAXTTCATGCTTXCATGTTXTTXGTXAAXACCX-ACTXCTTTGTTXTTTTTA	
	80650 80660 80670 80680 80690 80700 80710 80720	
Human	AAGTT---T-TAGATGTTTGGCTTTTTTCAGGTTATGAAAGCGGGGATGAGTTAAGAACC-ACTGCTGTGAAGGATTCA	74064
GuineaPig	AAGT-----------TGTAAAATATTTCTTA---GAAAAGCAGTTTTAAGAGTGTACCAGAT-AGAACTGTCTAGTTTTAA	49009
NorthernAmericanDeerMouse	-----------GTGTATGTGTGTGTGGTTCATTCAAGCTTTCATATTTTT-GTCGATACCAAACTGCTTTGGTCTTTCTA	52542
Mouse	------------GGTGTGTGTTTATGTTCCATTCATGCTTGCGTGTTTCT-ATCGGTACCTTACTACCTTGTTTACTCTA	53370
ChineseHamsterGHOK1GS	TATGTATGTATGTATGTGTATGTGTGGTCACTTCATGCTTCCATGTTTTTTGTCAGCACCAGATTGCTTTGGTTGTTTTA	56623
LongTailedChinchilla	AAATA---CATATGTACATAACATTCATCA---TACGTACATATATAACAAGTTAAAAACC-ATTACTTTGTTATGTTCA	54425

Majority	GC-TCTATATT-AAGTATTTATATAAAAATCTT---TCXATCATTTTGTCATTTTCTTT---TCAAAATTATTTTGGATX	
	80730 80740 80750 80760 80770 80780 80790 80800	
Human	TCAACTATTTTTAGGCAGTTGGGTAAAAATGACCAATTTAGTTTTAAGAAACTGACTGTGGCTCCAGAGTATGTTGGAGA	74144
GuineaPig	A---------------ATTTATTGGTAGAT---------------------ATATTT------TTAATTTATTCATAAGT	49047
NorthernAmericanDeerMouse	GT-TCTGTATT-AGGTAT-AATATAAAAAGCTT---TCCATCATTTTGTCATTTTCTTTT--TTAAGATAACCTTGGCTG	52614
Mouse	GC-TCTATATT-CAGTAT---TATAAACATCTT---TCCATCATTTTGTCATTTTCTTT----CAAAATTATTTTGAATA	53438
ChineseHamsterGHOK1GS	GC-TCTATATT-AAGTATTAATATAAAAAGGTT---TCTATCATTTTGTCATTTTCTTTT--TCAAAATTATTTTGGATG	56696
LongTailedChinchilla	ACATAAGTA---AAGTATTTGATGAGAAGTCAC------------ATGAAAATATCTGT---CCTAATTCATTGTTTAAC	54487

Majority	ATTTT------------------------A-XXGTXXXTTTCTGTCAGTCTTATGXTTTTAAAAAAX-----XCAXTTCC	
	80810 80820 80830 80840 80850 80860 80870 80880	
Human	AGTGAAAATGGAGACTAGGAATAACAGGTGGGAGACTATTAGTCTAATTAAGATGTAATTATAAATCTAAGCTAGGAACG	74224
GuineaPig	AATTT--------------------------GAACTTACATCCTTAA-----AAGAGCTTACTCAAT-----TGAGTCC-	49090
NorthernAmericanDeerMouse	-TTTT------------------------A---GTACCTTTCTGTCAGTCTTCTGCTTTTAAAAAG-------CATTTCC	52659
Mouse	ATTTT------------------------A---GTGCCTTTCTGTCAGTCTTCTGCTTTTAAAAAAA------CATTTCC	53485
ChineseHamsterGHOK1GS	-TTTT------------------------A---GTGCCTTTCTGTCAGTCTTGCTTTTTAAAAAAG-------CATTTCC	56741
LongTailedChinchilla	AATTTAAGAAGAAACTGTAGTT----------GAATATATCTCCTCCATGTGCACAGGCTTAGAAAATGATGTTGAGTTCT	54558

Majority	TGAAATTACATT-TGXXATATGXTTXACTTTTTTGTGATTTTXTA---ACAAATGXCAGA---XAAAATTTAA-------	
	80890 80900 80910 80920 80930 80940 80950 80960	
Human	TAAAATGAGAATGCAAAGTAAGAAACAAATATGGGGAAAATTATATGTAAAAGTAATAGGACTTGGCATCTTACTGATGT	74304
GuineaPig	---------------AAATAATATGTGAAT-GAATTGG--TTTTA-----AAATAGAA-----TGACTTTT---------	49133
NorthernAmericanDeerMouse	TGAAATTACATT-TGCCAAATGTTTTGCTTTTTTATGATTCTGTA---ACACCTG-CAGA----AAAATTTAA-------	52723
Mouse	TGAAATTACATT-TGCCAT-TGGTTCCCTTTTTTGTGATTCTATA---ACTTATG-CAGA----AAAATGTAA-------	53548
ChineseHamsterGHOK1GS	TGAAATTACATT-TGCCAAATGGTTCACTTTTTTGTGATTTTGTA---ACACATGGCAGA----AAAATTTAA-------	56806
LongTailedChinchilla	TTGGATTAC----TAAACTGTCCTGTACTT-TGGCTAAATTTTTA-----AAGTGTTAATTTTTAAGTTTTAA-------	54621

```
Majority                     -ACXACTTTTXTGAGACATXAAXX-ATATGT---GXTXTATTXGAT-------ATTTTAXAACAGCACCXGGAXXAXAA-
                             80970 80980 80990 81000 81010 81020 81030 81040
Human                        GATTGATTATGAGAAAAATGAAGC-ATGTGGAGGAGTCCACTGGACAGTAGGAAATTCAGCCTAAGACTTGGGTAAGAGT 74383
GuineaPig                    ---TTCTCTTCTGGTGTAAGGAA--ATAT------GTTTTGTGGT--------AGTT--GAAAA---------------- 49176
NorthernAmericanDeerMouse    -ACAACTGCTCTGAGACATTATGTTGTATGT---GTTGTATTACAT-------ATTTTATAACAGCACCGGGAAGGAAA- 52791
Mouse                        -ACAGCTTTTACAAGACATTCCATTATACAT---GTTATATTAAGT-------ATTTTATCATAGCACCAGGAGAAAAA- 53616
ChineseHamsterGHOK1GS        -ACAACCGTTATGAGACATTAAAC-ATATGT---GTTATATTGCGT-------ATTTTATAACAGCACCAGGAGGAGAA- 56873
LongTailedChinchilla         -AATACTTCTTTCAGAAAAGAAG--ATGTAA--GAGCTCAGCAGATAG-----AATT--GTCCA---------------- 54673

Majority                     TCTGXAAAXTC----X-TXXXAXGTGAGXGTTGTAXCAXTXAX--T-----AATAAAGXAXTXTTGTCAT-XGTXTXCAA
                             81050 81060 81070 81080 81090 81100 81110 81120
Human                        TCTGTGGAGTTGTGAATTCAGAGGCCAGAGATGTGATATTTAAAATTTTGGTTCAAGATTTCCCAGGTATAAGAAAGCAA 74463
GuineaPig                    CTTGAAATGTA------TTATGGGCTATGGCTGTGACAT----------------AAGCAA-------------CAGAA 49220
NorthernAmericanDeerMouse    ACTGCAAATGCAGACAGTAGCAAATGAGCATTGTAGCAGTGAG--T-----AATCGGGC--TCTTCTCAT-CATTTCCAA 52861
Mouse                        ACT---------------GTAAGTGAGTATTGTAGCAGT-----------AATAGAGC--TGTTTTCAT-TGTTTCCAT 53666
ChineseHamsterGHOK1GS        TCTACAAATTCAAACAGTAGCAAATGAACGTTGGAGCAGTGAG--T-----AATAATGTAGTTTCGTCGT-CGTCTTGAA 56945
LongTailedChinchilla         TTTTTAAAATG------TCAGTGGATAAATTTTTAACTTTTTACTCA------CAAAGTAATTTGAACCTATGTCCTTAA 54741

Majority                     AATATXTG---TATTA-TAXCTXAGCTTTAATTTGTGXATXTTTAAA--AXACATAAATTAAAAXXXXTXXXTXXX-XTX
                             81130 81140 81150 81160 81170 81180 81190 81200
Human                        GAGGATTAAAGCATTG-TAATTAAACTTTAAGCAGTGCATATTTATGTTATAGATAAGATAAACAAGAAATCTAGGGATC 74542
GuineaPig                    AAAAATT----CATT--TAACAA----CCAAGT--TACATACCTGCA--GCTATATCATTAAATTAATTGGTTGAAATTA 49286
NorthernAmericanDeerMouse    AATATATG---TGCTAGTACCTGAGCTTTCATTTGTGTGTGT-------------------------------------- 52900
Mouse                        AATATATGC--TAGTAAGACCTGGTTTTTCATCTGTGTATGTTCAAA--AAACATGAATTACTG-------------TA 53728
ChineseHamsterGHOK1GS        AAT-TATG---TATTAGTACCTATGCTTTTATTTATGTGTATTAAGA--AAACAAAAGTTGTCACTTTTTAGATATTATT 57019
LongTailedChinchilla         AAGAGTT----TATT--CAACTGAG-TCCAAATAATACTTGAATGAATTGCTTTTCAAATGGAATGACTTTTTGCTCTTC 54814

Majority                     XAATTTTAGTAAATTTAGXXTTTTTXATTCAGTACA--XXX---X-XXTGCTACTTXTAA-AGTGTXTXXCXTGAACCAT
                             81210 81220 81230 81240 81250 81260 81270 81280
Human                        AAATAGGATTAAAATTAGTAGTGATCATTCAGTACAGTAGTTACGTACTGTTATTCACAAGAGTATATAAATCAAATTAC 74622
GuineaPig                    TATAT--AGTACATATA----TCATAATACAGTA------------------CTTATAACAAAGAAATGGTTGAAGGAA 49341
NorthernAmericanDeerMouse    --GTGTTAGTTTGTCCGGTGTTTTGGAGTCC-T----------------GCTACTTGTAA-AGTGTCTTCC-TGAACCTT 52959
Mouse                        -GTTTTTAATATTTTTAACATTTTTTAATTTGTTCA-TAGT---G-GTTTCTACTTCTAA-AGTTTCTAT--TGAACCAT 53799
ChineseHamsterGHOK1GS        CAATGTTTTTTAGCTTGGGGTTTTGGAGTCC-TCCA-AAGT---G-ACAGCTACTTCTAA-AATGTCTTTC-TGAACCAT 57091
LongTailedChinchilla         TGATTTAAGTAAATCT-----TTTTTTTTCAGTAAA---------TCTTGT--TTTGTGGTAGTTGAAAATTTGAAATAT 54878
```

```
Majority                    XAG---XTAXXACC---AATGT-TTAGAAAGACAGAAAXG---CTGXTTGTT--AGTTTTGXGCATAAXTXAGGTTCATT
                            81290 81300 81310 81320 81330 81340 81350 81360
Human                       AAGGAATTAAGGATATAAACGTGATAAGAAAGTATGCACTGTACTCTTTGAGGAAGTTTGCCATAGAAAGGAAGAAGAAA 74702
GuineaPig                   AC----TTAAGCTT---GATG-----------------------ACTTTGC---TTTTATTTGCATAACTGTAATGTAC- 49387
NorthernAmericanDeerMouse   TAG----TAGAACC---AGTGC-TTAGAAAGACAGAAATG---CTGCTTGTT--AGTTTTG----TAAGTAAGGCTCCTT 53022
Mouse                       TAG----TAAAAC-----ATGC-TTAGAAAGATGGAAACG---CTGCTTGTT--AATTTTGTGAATCAA-AAGGTTCCCT 53863
ChineseHamsterGHOK1GS       TAG----TAGAACC---AGTCT-TTAGGGAGACATAAATG---CTGCTTGTT--AGTTTGGGGGGTAAGTAAGACTCTTT 57158
LongTailedChinchilla        AT----TTTGGGCCACAGATGTGTCATAAAAACAGGAAAGA---AGTTTACAGACATTGTTCACTTAACGGAGGTACATG 54951

Majority                    T-AATTAGTTAAXTCAXTXCTXGAAT------------AAGTACXTTTTT-AAAXAGA-TAGTAXXAGTX-------AAA
                            81370 81380 81390 81400 81410 81420 81430 81440
Human                       T-AGGATGGTAGATCAGAAGTAAAGCAGGACCCAGTGGGGGGAGTGTTTGCAGTGAGG-CAGTATGTATAATCATTTAAA 74780
GuineaPig                   ------AGCAACATAA--AATTAT---------------AGTACTATATAG--------TACAATCAGCA------AAAA 49430
NorthernAmericanDeerMouse   TCAATTAATTAAGACAGTGCTGGAATT-----------AAGTACCTTCCT-AAAAAGA--AGTGCCAGTG-------AAA 53081
Mouse                       TCAATTA-TTAAGGCGTTGCTAGAATT-----------AAGTACCTTTTTTAAAGAGACTAGTGCTAGTG-------AA- 53923
ChineseHamsterGHOK1GS       T-AATTAATTAAGACAATGCTGGAAT------------AAGTGCCTTTCT-AAAAAGA--AGCAC-AGTG-------AAA 57214
LongTailedChinchilla        T-CTGTAGCTAGATCATTAATTGTTT-------------GGAATTGTATA---------TAGTATGTATA------TAAA 55002

Majority                    X----GAGTTGTAGXCCXXTGX------ATXTTX--XAT-XAXAXAAXGATXAGXA---CTXTCTGTXCTGTCXCTCATX
                            81450 81460 81470 81480 81490 81500 81510 81520
Human                       ACATGGGTTTGGAGTCCTCTCAGGTTCCATGTTTGTAATGGACATAATGATAATAATCCCTTTCATTTAAGGCTGTTGTG 74860
GuineaPig                   --------TTGCAGTACT----------CTAT---------AAATA-----AGGTG----------TGCTTTCCAAAAAG 49468
NorthernAmericanDeerMouse   A----GAGTTGTAGACACATGG------ATCTTAGGTATAGGTACAAAGATTAGCA---CTCTCTGTCCTGTCTCTCATT 53148
Mouse                       -----GAGTTGTGAACGCGTGA------AT-------------ACAAGGATTAGCAG--CACTCTGTCCTGTCTCCCATT 53977
ChineseHamsterGHOK1GS       A----GAGCTGTAGACACCTGG------ATCTTT--CTTAGGTACAAGGATTAGTG---CTTTCTGTTCTGTCCCTCATT 57279
LongTailedChinchilla        --------TTATAGTCCT----------GTATTCAGAAC--AAATAGTCAAAGGAAACTCCA-CGTTGATGTCCAAGAAG 55061

Majority                    ATXXAATTXCXXTTTXAXXXTTTXACTCCACTGXAXAXTACCTA------AACAXATXCTTTCAXG--XATAGTTTXCXX
                            81530 81540 81550 81560 81570 81580 81590 81600
Human                       AGGATTAAATGTGTTAATGTGCAAATAACTTTACACAGTGCCTGGTATATAATAAATGCTTGCTACCTATTAACTAGTAT 74940
GuineaPig                   AAGACATTGAGGCTTTATAAGTGAGCTGTACAGCACTGTACACACACACACACACACACACACAGGGTAGTAGGTAATA- 49547
NorthernAmericanDeerMouse   ATTTAATTACTGTTTTAAGTTTTGACCCCACTGTTAAATACCTA------AATA--TCCTTTCGGT--GACAGTTT-CTA 53217
Mouse                       ATTTAGTTCCTATTTAAAAATTTGACTCCACTGTCAAATACCTA------GACAGATTATTTTAAG--GATAGTTT-CTC 54048
ChineseHamsterGHOK1GS       ATTTAACTCCTATTTATAGTTTTGACCCAAACCTTAAATACTTA------GACAGATCCTTTTGGG--GATAGTTTTCTA 57351
LongTailedChinchilla        AAGATACTGAGTCTTTATAAACAAACTGTACAGCACTGTACAAA-----AAAAAAGTAAGTCCAAG-CAGTGGGTCGGA- 55134
```

```
Majority                    C-CTTXAGAATAACTAGAAAAXTXCCAG------TTTAAAXAA----ATAAXGAXTTXAGXXTACAXCT---XATTTAAA
                            81610 81620 81630 81640 81650 81660 81670 81680
Human                       TTGTTTCTAAGGCTAATTTAAGTCCTAGAATTGATTGCAAGGATTAGATCAGGAGTATAGTGGACATGTTGGGATTTAAA 75020
GuineaPig                   --ATTCTCAAC---------AGTCC----------------------ATAAAAAGTTTATTGAACA-----AGAAACAGC 49589
NorthernAmericanDeerMouse   C-CTGCAGAATAACTAGAAAAATACCAG------TTTAAAAAA----ATAATGGCTTAAGATTCCACCTC--TGTATAAA 53284
Mouse                       C-CTACAGAATAACTAGAAAAATACCAG------TTTAAAGAA----A-AACTACTTCAGATTTTATCTC--TACTTAAA 54114
ChineseHamsterGHOK1GS       C-CTGTAGAATAACTAGAAAAATACCAG------TTTAAAAAA----ATAGTAACTTAAGATTTCACCT---TATTTAAA 57417
LongTailedChinchilla        --ATTTTCAAC---------AGTCC----------------------ATAAAGAGGTCATTGAACA-----AGGTGTACA 55176

Majority                    XTTCT-XTXATGXCAXTTGTTTGTXGXAGCAXCATTTGAACTAGAA----CTXCTTACTXAGTGTTTXAATCAXCG----
                            81690 81700 81710 81720 81730 81740 81750 81760
Human                       TATTTAAATATAGAGATGCTTTTTAGGACCATTGTTAGAACCAGAAGAGATTTTTTACCAAGTTCACACAGAAATGTAGG 75100
GuineaPig                   TGT---ATCATTGTAAGAGTTTATAAGTAC-TCATTCCCTCTAAA-----CTTCTTACTG------TAGAACAACG---- 49650
NorthernAmericanDeerMouse   GTTCT--TCATGTCATTTTTTTGTGGCAGAAACATATGAAATAGAA----CTACTTAGTGAGTGTTTCAGTCAGCA---- 53354
Mouse                       GTTCT--TTATGTCATTTGTT-GTGGCAGGAACATTTAAAATACAA----CCATTTACTAAGTGTTTCAGTCAGAG---- 54183
ChineseHamsterGHOK1GS       GTTCT--TTAAGCTGTTTGTT-GTGGCAGAAACATATGAACTAGAA----CCACTTA-TGAGTGTTTCATTCAGCA---- 57485
LongTailedChinchilla        TGTT--ATCATAACAAGAGTTTATAAGTAC-TCATTCTCTCTAAAGTAAGCTTGTTACTA------TAGAACAATG---- 55243

Majority                    ---ATGGACTXATCTTXTXAGGAXXTTCXTATA--X--AGATTXTXATXTXGCTAAXGAATTXTAX-TGTTTAGTAACAT
                            81770 81780 81790 81800 81810 81820 81830 81840
Human                       TGCATTGGCTGGGCATGGTGGCTCACACCTGCAATCCCAGCACTTGGGAAGGCTGAGGCAGAAGAACTGCTTGAGGCCAA 75180
GuineaPig                   ---ATAG--TGAACCTTTTAGAACATTTATTGATACAAACAGTGTGCTGTGGCTCATGTGCTATAG--GTTTTCCATCAC 49723
NorthernAmericanDeerMouse   ---GTGGACTCATCTCATGAGGATGTTCCTATA-----AGATTATAATGTAGCTAAGAAATTCTAC-TGTCTAGTAATAT 53425
Mouse                       ---ATGGATTCATCTAGAGAAGATGTTCTCATT-----AGATTGTAATATAGGTAAAAAATGCTAC-TGTATAGTAACAT 54254
ChineseHamsterGHOK1GS       ---ATGAACTCATCTTATGAGGATGTTCTTATA-----AGATTATAATGTAGCTAA--AGTTCTAA-AGTCTAGTAATAT 57554
LongTailedChinchilla        ---ATTG--TGAACCTTTTAGAGCATTCAAAGAAACAAACAGCCTGCGATGGCGTGTGTACCATAG--GTTTGCCACCAC 55316

Majority                    TGTTTTX----CAXXCTXXATXATGXAXCAGTXGTCC-TACCCAXXGAATAAAGXXXTATTXXAXATAGTTAATXAXCXG
                            81850 81860 81870 81880 81890 81900 81910 81920
Human                       CATTTTGAGACCAGCCTGGGCAACATATTAAGACCCCGTCTCCACCAAAAAAAAAAAAAAAAGAAGTAGGTGCAGAGCTG 75260
GuineaPig                   TGCTATG----TAACCTAAATGAAGT-TCATAAGTCCTTAGCTATCTAGGATAGGAGTTTTATAA--AGTTCTTAA--TG 49794
NorthernAmericanDeerMouse   TGTATGTTA--CACAGTGCATTATCAAACAGTTGTAA-TGCCCACTGTATAAAG-TACAGTGGGTATAGTTAATGTGAAG 53501
Mouse                       TGTCT------CATGAGAGAC-ATGAAACAGTTGTGC-TGCCTACTGTATAAACATATAGTGAATATAGTTAATGTACAG 54326
ChineseHamsterGHOK1GS       TGTAT------CACAGTGCATTCTAAAACAGTAGTGC-TACCCATTGGATGAAC-TGCATTG--TATAGATAATACACAG 57624
LongTailedChinchilla        TGCTGTA----CAATCTAAATGTTGT-TCATATGTCCTTAGCCATCTAGGG-AGGAGTTTCATAA--AGTTGTTAA--TG 55386
```

```
Majority                    XATXXA--AXCTGGTATTGXTXXTXXAAGAXTTAXTXXXTTTTXTXGTTA----TXATXGXTTTTAAAXATTATTTTAGA
                            81930 81940 81950 81960 81970 81980 81990 82000
Human                       GAAGCAG-AACCGAAATCATCAGTGTTACAGTCATTATTCTTTCCTGTCACCATTATATGTCTTTATGAAGCAAGGAGA 75339
GuineaPig                   GGTG----CTCTGGTCTTACTTAAATGAGATACATTATTTTTACTTGTTA-----AATTGTTCTTA-------------- 49851
NorthernAmericanDeerMouse   TACACAATACTTGATAATGATGGT-AATGGCTTCCTGGCTTATATAGTTG----TCATAGCTTTTAAACATTATTTTAGA 53576
Mouse                       TATATAATACTTGGTAATGTTGTTGAATGACTTGCTGGCTT-TATA--------TCATACCTTTTAAACATTATTTTAGG 54397
ChineseHamsterGHOK1GS       TACAGA-------------------------TTCCTGGCTTATATAGTTG----TCATACCTTTTAAATATTATTTTTGA 57675
LongTailedChinchilla        GATG----ATCTGGTCTTGCTTAGACCAGATTTATTATTTTTACTTGTTA-----AATTGTTCTTA-------------- 55443

Majority                    AAAGACTCCAGTTXC------------TTAX--XAAATTTXCTGTAAGATXTXATTTXATTTTXTTCXAGXAX-------
                            82010 82020 82030 82040 82050 82060 82070 82080
Human                       AAGAAGAACAGATGAAAGAAGTGAGGATTTT--GAAGTTGGTTGAAAGATTTGATTGAATTCTGATCTAAAAATTATAAG 75417
GuineaPig                   ----ACTATAGTT--------------TTAA--GAAATTTGCTGTTAATTTTGTATTTCCTTTGCTTTT----------- 49900
NorthernAmericanDeerMouse   AAAGACTCCAGTTAC------------TTAC--AAAACTTACTATAAGATAATATGT-GTGTTTTTCCAGCAT------- 53634
Mouse                       AAAGACTCCAGTAAC------------TTGG--AAAACTTACTGTACCATAGTGTCCCATGTTATTCCAGGAG------- 54456
ChineseHamsterGHOK1GS       AAAGATTCCAGTTTC------------TTTTTTATAAGTTACTAGAAGATAGTATGCCATTTTATTCCAGCAG------- 57736
LongTailedChinchilla        ----ACTGTATTT--------------TTAG--GAAATTGGCTGGTAAGTTTGTATTTCCTTT-TTCTT----------- 55491

Majority                    ---CAGTXTTAATGAXTT----XTXTGTXXXX----ATG---------TXTXXTTXAAGATGXTCTCAGAAAXTGAC--T
                            82090 82100 82110 82120 82130 82140 82150 82160
Human                       GCACTTGTTTAACAAGTTGAAAGTAGGAAAGTAGACATAAGACTCTACTAGATTTGGGGAAACTCTCAAAAATGGAC--T 75495
GuineaPig                   ---CCCTTTTTAACAGTT--------------------------------ACTTGAAGATCCTGTCTCATATTTAC--C 49942
NorthernAmericanDeerMouse   ---CAGTCTTAATGTACCTCCTGTCTGTGAGT----ATG---------TTTGCGAAAAGATGATCTTAGAAAATGATG-T 53697
Mouse                       ---CAGTCTTAATGCA-------TATATA-------ATG---------TATGTTCAAAGATGATCTGAGAAAATG----T 54506
ChineseHamsterGHOK1GS       ---CAGTCTTAATGCATTTTCTGTTTGTGAGTGTG-ATG---------TTTGCAGAAAGATGATCTTAGAAAATGATGGT 57803
LongTailedChinchilla        ---CCTCTTTTCTGAGTT--------------------------------ATTTGAAGACCCTTTCTTATATTTAC--C 55533

Majority                    GGAATCTTXGTT---------TAXTAAXXTXTTTTTXATXTTA----CTXTAGT-----TGAATTTTAACXCTAGCTTXX
                            82170 82180 82190 82200 82210 82220 82230 82240
Human                       GGAAATTCAGCTAAAAGTGGATAACAAAATATTTCTAGAATTAGCATTTGTGGGGTGTGTGTGTTTTCACTCTAGTATTT 75575
GuineaPig                   CTAACCTTA-------------------GTATTTTTAATGCCAACCAGTATGAATTCTGTGATAATTAGCTCTAGCTTTT 50003
NorthernAmericanDeerMouse   GGAGTCTTGGTT---------TATTAACTTGTCTTCTATTTTA----CTGTAGT-----TGAATTTTAAAATT--CTTAA 53757
Mouse                       GGATTCTTGGTC---------TATTAATCTGTGTCTTATATTA----CTGTAGT-----TGAATTTTTGTATACTCTTAA 54568
ChineseHamsterGHOK1GS       GGAGTCTTGGTT---------TACTAATCTGTCTACTATTTTA----CTATAGT-----TCAAATTTAAAACTA-TTTAA 57864
LongTailedChinchilla        CTAAACTTA-------------------ATATTTT-AATGC-------TA----------------T-AACTCTAGCTTTT 55570
```

```
Majority                    TGTTTAAXTCXXAATTXXTTTATAAAXGAAATTXXGGAA-CXAXTXTXGTGACTTTAGTAX---XTAAXXXTAXXAXX-T
                                    82250     82260     82270     82280     82290     82300     82310     82320
Human                       --GTCAAGCCCAGATGAAAGCATAGACAGAATGTAAGACTGGATTTATCTAAGTCTGGAATTGTGTAACATTAAAGGAAT 75653
GuineaPig                   TGATAGAAGACAAATCAAATTGTACATTTTATTTAGAAG-AGATTTTTGTGGTTTTAACAG---ACAACAGTTATATG-T 50078
NorthernAmericanDeerMouse   TGTTTAAGTCTTAACTCTTT-AAAAACGAAATTGGGGGA-CAACTGTGGTGACTTGAGTCG---ATTATCCCAGGA---- 53828
Mouse                       TGTTTACATCTTAATTCTTTTAGAAAAGAAATTGGGGAA-CAACTGTCATGACACCAGTCT---GTAATCTCAGAACA-G 54643
ChineseHamsterGHOK1GS       TGTTTAAGTCTTAACTCTTTTAGAAAAGAAATTGGGGAA-CACCTGTGCTGACTTGAGTTT---GTAATCCTAGGA---- 57936
LongTailedChinchilla        TGATAGCAGACAA-TCAAGTTGTAGATTTTATTTAGAAG-GGATTTT-GTATTTTTAAGAG---ATAACAGTTACGTG-T 55643

Majority                    -GTCAAGAATAAXXTGAXTATTTTCXXAAGXAAATXCCTGXXXXTAATXTCXXTTTTATAXTAXACTXTGGXXAAXGAAG
                                    82330     82340     82350     82360     82370     82380     82390     82400
Human                       AGTAGCAAATGAGCAGAGTGTTGGCTCAAGCCTAAGCTTGAGCCTAAGCTTGACTCTATGGTAAAGTCAAGTCAAGGGAG 75733
GuineaPig                   AGTAT-GAATAAAACGAAAA-TTTCTGAAGTACATTCCTG----TTATTTC--TTTAATAATAGACTTTGG--AAGTAGG 50148
NorthernAmericanDeerMouse   -ATCAAGAATAGCCTTAATTCCATGATAGTGCACGTTATGGCTAGAAAAGATCTATTAAAATGAAACCAGGCTACAGAAA 53907
Mouse                       GACCAAGAAGAT--TGAGGATTTTAAGACTGAATAGCCTGAATTCTATAACAGTTCTATAATAGCCTTTGGCTCTAGAAG 54721
ChineseHamsterGHOK1GS       -CTCAAGGAGAG--TTAGTATATTTAAA----ACTGGGTAGCCTGAAT---TCCATGACAGTGTACTCTGTGGCTAGAAA 58006
LongTailedChinchilla        -GTGG-ATATAACAGAAAAGCTTTCTAAAGTACATTCCTG----TAACTTC--TTTAATAGTATACTTTGG--AAGTGTG 55713

Majority                    AAXTXTT---XXGATACCAXAC---TCAXAAATXAXTTTAXTTTTXTCGTAGTCATXGGC------XXXXTAGAATCGAA
                                    82410     82420     82430     82440     82450     82460     82470     82480
Human                       AATAGAAAGGGGGTCACCATAAAGGTCAAAAGTGGGTTTAGTGGTTGTGTGGGAATAGGCAGATCAAGAAAAGAAT-GAA 75812
GuineaPig                   AACTTTTT-----ATACCACAC---TTACAAATGTAGTAGTTTTTCTCTTAACTGAAG-------AGAACAGAATAGAA 50212
NorthernAmericanDeerMouse   A---------ATGAAACCAGGCT--ACAGACATAATTTTAAATTCATCGTGGTCATGAAC-------ACCTA-AATCCCA 53968
Mouse                       ACTTGTTACAATGAAACCATGCT--GCAGACTTAACTATAAGCTTACTATAGTCATGTGC-------ACCTGTAATCCTA 54792
ChineseHamsterGHOK1GS       A---------GATTTGTTA--------AAATGAAACTT----------GTACTCATGAAC-------ACCTGTAATCTGA 58052
LongTailedChinchilla        AACTTTT------ATACCACAC---TTACAAATGTACATAGTTTTCTCCTAACTGAAGG-------AGAAAAGAGTAGAA 55777

Majority                    GTTXGGAGAXTGAGGCAXGAGTGTX--ATGA--XTTTCAAXAACXGTCTGXAX-------------AXCTAXCAXXAGA
                                    82490     82500     82510     82520     82530     82540     82550     82560
Human                       GTTAGGAAA-GGAGATATAAGTGTTGAATGACCATTACAAAAAGAGACAGAGGAAAGAAAAATGAAGATGTATCAAAAGA 75891
GuineaPig                   GGTAGGAAA-TAAAGAATGAAT-TT---------TTTCAGAAAA---------------------------CTGTAGC 50252
NorthernAmericanDeerMouse   CTTGGGATTCTGAGGCAGGAGTGTC--ATGA--GTTTCAAGAACTGTCTGGAC-------------AGCAAATG--AGA 54028
Mouse                       TTTGGGAGGCTGAGGCAGGAATGTC--ATGA--ATT-CAAGAACTGTCTGAAGC------------ACCTAGTGTGAGA 54854
ChineseHamsterGHOK1GS       CTTGGGAGGCTGAGGCAGGAGTTTC--ATGA--GTT-CAAGAACCGTCTGGACT------------ACCTAGAA--AGA 58112
LongTailedChinchilla        GGTAGGTGA-TAAAGAATGAGTATT---------TTTCAGAAAA---------------------------CAGTAGC 55818
```

```
Majority                     AXTTGTCXCXCCXGAATAXCAAAACAX---XTTTXXA-XXXT-XAAAGTTGACAGCXXXXG---------ATGATAATGC
                                      82570     82580     82590     82600     82610     82620     82630     82640
Human                        AGTTGCTAATAT-GGATGGCAAAGTAGATGTTTTTAAGAAATCATGAGACCAGAGTCTTGGAAAAGTCATAGGATGATGC 75970
GuineaPig                    AGTTGCCATT--------------------CTTTGTACTTGT-------TTA---CCTT----------CAA-TTAGTAC 50291
NorthernAmericanDeerMouse    CCTTGTCTCACCAGAAAAGCAAAACAGAAATTTTGTATTTTT-AAAAGTTGACAGCTACAG---------ATGATAATGC 54098
Mouse                        CCTTGTCTCACCAGAATAATAAAACAG-----------------AAAGATGACAGCTACTG---------ATGAC-ATGT 54907
ChineseHamsterGHOK1GS        TCTTGTCTCACCAGAATAACAAAACA------------------AAAGTTGACAGTTACAG---------ATAATTATGT 58165
LongTailedChinchilla         AGTTGTCATT--------------------CTTTTCAAAAGTAAAAATTTTA---CCTT----------CAGGTTAGTGC 55865

Majority                     ATXAAATXTAGAAAAGXTTXCTAAAGATXXACA-TGAXCTCTXTXTGTGXAGTXAXXG------XCXAXAAXCCAXTGGC
                                      82650     82660     82670     82680     82690     82700     82710     82720
Human                        AGGGAATGGAGAAGAGGGAAATAAAGCCAGGTGCTGAAGTCTTTATGTAATGGGAGGAGATGTTCCAGTAATCCAATGGC 76050
GuineaPig                    ATGAAATCTAA------------------------GAACTCCCTTTCTCA--------------CCTGGGATCCCTGTGC 50333
NorthernAmericanDeerMouse    ATAATATATAGAAAAAGTTTCTAAAGATGCACA-TAACCTCT--ATATGCAGTTGAAG------ATGATAGGTCACTGCC 54169
Mouse                        ATGAAACTCAGAAAAGCTTCCTAAAGATTTACA-TCACATCTGTGTGTGCAGTTAGTGC-----AACAGAAGAAGGTAGC 54981
ChineseHamsterGHOK1GS        GTAAAATATAGAAAAGATTACTAAAGATGCACA-TTACCTCTCTGTGTGCAGTCAAAG------ATCACACGCCATTGGC 58238
LongTailedChinchilla         ATAAAATCTAA------------------------GAACTCCTTTTCTCA--------------CCTACGATCCCCATGC 55907

Majority                     TXTXXXAATXAGAAXGAXXXT------X---XXTGGAATTXXCACCGGAAAXXTTCATCA---X-X--------XXX---
                                      82730     82740     82750     82760     82770     82780     82790     82800
Human                        TATTTTGATGGGAAAGAGTGTGGTATGATTGGGTGGCATTGACATCGGAAGCCATCCTCATTGATGGTGGTGGAACAGCA 76130
GuineaPig                    TTTAAGAATTAGAAAGAACCT------------TGGAATCC-CACCACCATTACTCTTAA-------------------- 50380
NorthernAmericanDeerMouse    TATGGTAGAAAGTCTTCCAGCAGCTTGA---GGTGAAGATTATGCCGGAGAGTTTTACCAACTA-GAAAAT---ACAA-G 54241
Mouse                        TTG------------------------A---GGTGAAG-TTATACAGGAGAGTTTCATCAACGA-GAAAAC---ACAA-G 55028
ChineseHamsterGHOK1GS        CAT--------------------------------------------------TTTAT---------------------- 58246
LongTailedChinchilla         TTTAAGAATTAGAAGGAACCT------------TGGAATCC-CACCACAAACCCTCTTCT-------------------- 55954

Majority                     ATACAXAAXTAACXTTXCA--------------AXXAGATXXTTGATTTTAAXXATTTCTTTTTTTGXATXXCTGTGXXA
                                      82810     82820     82830     82840     82850     82860     82870     82880
Human                        GTTTGAAAGTAACATTGTGCGGTGAGGTAGAGTGGCACATGATGCATCCTTATTCTTACCTTTGGAGAAAAGTTGAGGGA 76210
GuineaPig                    ATATAGAAGTAATG---------------------CATAAGATAATTATTAACTGTTGCATGTTTTGAACAG-----GGA 50434
NorthernAmericanDeerMouse    ATGCA-ACAGAACATTCCA--------------ATTAGATTCTTGATTTTGAACATTTTTTTCTTTGTATTACTGTGAAC 54306
Mouse                        ATACATACAGAAGGTTTCA--------------ATTAGGTTCTTGATTTTAAGCA--TATTTTTTCATATTACTTTGAAC 55092
ChineseHamsterGHOK1GS        --------AAAACGTTCCA--------------ACTAGATTCTTGATTTTAAGCATTTATTTCTTCGTATTACTGTGAA- 58303
LongTailedChinchilla         GTACAGAAGTAATA---------------------CATAAGAT---TGTTACCTATTACGTGTTGTGAACAG-----GGA 56005
```

```
Majority                    AGTAAAAAAAGXTTTCT----GATATTATAGAXTTTAXXTTCAAGXXXXAGGX-GTTTCXXAGXATAXXTTTAATTXGAG
                                      82890     82900     82910     82920     82930     82940     82950     82960
Human                       GACCAAAAATGACTTTTTGAGGGAATTGTAGAAGTTTCATTAGAAGAAAAGTAAGTTTTTAATTAAAAAGTTAATCTGAG  76290
GuineaPig                   AGTGAAAAAAGAT-----------ATCATCTAAATCACTTTGAATGAAA-----GTTTCAGAATAGAAAATAAA-----A  50493
NorthernAmericanDeerMouse   AGAAAAATAAGGTTTCT----GATCTTACAGATTTTATTGTCAAGTGGGAGGT--TTAACAAGAATAGTTATAATTAGAG  54380
Mouse                       AGAATACCAAGGTGTCT----GATGATA----TTTTATATTCTAGTGGGAGGTGATTCCCAAGAATAGTTTTGATTTAAG  55164
ChineseHamsterGHOK1GS       -GTATAACATGGTTTCT----GATCTTATAGATTT-GTATTCAAGTGGGAGGAGGTTAATGAGAATAGTTGTAATTGGAG  58377
LongTailedChinchilla        GGTGAAAAAAGACCA---AAAGAAATTATCTAATTGACTTTGGATGAAA-----GTTTCAGAATAGGAAATAAA-----G  56072

Majority                    GAAAA----XAAACATAXTXTAGTTCTTXXXGXTXXA------X--XTTTXA--GXXXXAAAAXX----------TXTGG
                                      82970     82980     82990     83000     83010     83020     83030     83040
Human                       GAACAGGTAGAATAAAAGTGTAGTTGTTAGTGGTAGAAGAGAATGGATTCCATAGGGCAAAATAAGAACTCAAGGGAAGG  76370
GuineaPig                   GAATT-----AACCAGATTCTACCTCTTTCAGAATCA-------------------------------------------  50525
NorthernAmericanDeerMouse   GAAAA----CAAACATA-CATAGTCCATTTTACTGTAGT----TGAATTTTA--GAATAAAACAAACATACATAGTGTGG  54449
Mouse                       ATAA------TAAATGTAGTGTTGTTT--------------------TCTGA--GAGTGAAATC-----------TGTGA  55205
ChineseHamsterGHOK1GS       GTAAA----CAAACATA-AATAGT----------------------------------------------------ATGG  58400
LongTailedChinchilla        GAACT-----AAACAGATTCTACATCTTGCAGATTCAT---AGTAAACTTCGACAGTGAAGAGTTGCTTTAGGCATTATG  56144

Majority                    TT--XTGAATAGAATGAXAXXXGTGXTTXTTXAAAA--GX---X--------AAXCTXGAGXXXATAAXGAGCTTTAGGT
                                      83050     83060     83070     83080     83090     83100     83110     83120
Human                       TTGGTGGAAGAGGAAGAGGATTGAATTGTTTCAAGAAAGAATAGCAGTTGTCATCCTTATGAAAAGTAAAATTTTTATTT  76450
GuineaPig                   ----CTGAATAGAGTAAGA---------TGCTAAA-------------------CCT----AAAAAAACGATTGTTAGGT  50569
NorthernAmericanDeerMouse   TT--TTGAATACACTGAAATGAGTGATTCTTCCAAA-GGAA--G--------AATTGTGAGGGTTTTGTTTGCCAAGGAC  54516
Mouse                       TT--CATGATCAGAAATAGCTGATGTATCTTCTAAA--G-------------AATCAGGAGGGTCTAAGGAGCTCTGTGT  55268
ChineseHamsterGHOK1GS       TT--TTGAGTATAGTGAAATGAGTGATTCCTTCAAAAGGAG--G--------AATCAGGAGGGTGTGATGAGCCAAAGAT  58468
LongTailedChinchilla        TTGACTAAATAGAAGGAGA---------TCCTAAAA------------------CCT----AAAAAAAGAAACTTCAGGT  56193

Majority                    ACACT--TAAAGAAATXXAXXATXAXGCTTGXAXACXTXTAGTTXATAXGGGTCAAATATTX-TTGAAXGGCAXATAXAA
                                      83130     83140     83150     83160     83170     83180     83190     83200
Human                       TCAAA--TCAGGAAATGTAAAATGTGCCTTCCAGACCCCTTGGTGGTATACATGGGAGATTGGTTCTAGGACACACACAG  76528
GuineaPig                   ATACT--CTAAGGAA------ATGTGATTGGTGAACTT-TAGTTACTATGTCT---ATATTGATT---GATTAATTACAA  50634
NorthernAmericanDeerMouse   ATAGTATTAAAAAAATACACTATAAAGCCTGT----TTAGAACTTGTAAGGGGCAAATATT--TTGAATGGCATATAGAA  54590
Mouse                       GCAGT-GTAAGCAAAGGTGACACAAAGTCTGCG---CTGTGTTTCACAAGGGACAAATATT--TTGAATGGCACATAGAA  55342
ChineseHamsterGHOK1GS       ACACT-TTAAACAAATACACTATAAAGCTTATAAAACTGTGTTTTATGAGGGTCAAATATT--TTGAATGACATATAGAA  58545
LongTailedChinchilla        ACACT--CCAAGGAA------ATGAGAATGGCAGACTT-TAGTTAATATGTAT---ATGCTGGCT---GGGTAATTGCAA  56258
```

```
Majority                    TAA--------X-------A-TXXGXATGACATGGTGXTAXAAAAGXTTACXGAXXAAXATXTTAXGXATX-AAAXTAXT
                                     83210     83220     83230     83240     83250     83260     83270     83280
Human                       TCCCATCCCCCACCCTCTGACCCCATACACCCCCTGGATACTCAAATCCACTGATGCTCAAGTTCCTTGCATAAAATGGT  76608
GuineaPig                   C------------------------TATATCATGTTCATATATGATGTTAATAATGA---------------AAAGGAAC  50675
NorthernAmericanDeerMouse   TAAT---TGGGA------AAATTTGAATGAAATGGTGCCACAAAAGTTTACAGAGCAAGATCTTAGGAAT------TACT  54655
Mouse                       TAA----------------A-TAGGAATGAGATGGTGCTACAAAAGGTTACAGAGCAAGATCTTAAGGATAATGTTATCT  55405
ChineseHamsterGHOK1GS       TAAT---TGAAA------AAATATGAATGAAATGATGCCATAAAAGTTTATAGAGCAATATGTTAGGAATA-----TTAT  58611
LongTailedChinchilla        TAA---------------------ATATGCCATGGTAATGTACAATGTTAATAATGA---------------AAAGGAGC  56302

Majority                    ATGGGXT----GTGXAXTTTATACAGGAXTTATTA-----ACTTCTTAXCATCAAXAG-TTXCTTAXA------AGXTAA
                                     83290     83300     83310     83320     83330     83340     83350     83360
Human                       ATAGTGTTTGCATGTGACCTATACACAACCTCTTATGTGTACTTTAAATCATCTCTAGATTACTTATATTACCCAGTACA  76688
GuineaPig                   ATTGGGT----GCCTAATTTAGGTAGGA------------ACT--GTAATGTCTTCAGTTTTCTTAGA-----------A  50726
NorthernAmericanDeerMouse   GTGAAAT----GTGAATTTTATACAGGAATTATTA-----GCCTCTTAGCATCAAAAG----------------AGGTAA  54710
Mouse                       GTGAAAT----GTGAATTTTATACTGGAGTTATTA-----GCGTCTTAGCATCAAAAGCTTACCAAAA------AAATAA  55470
ChineseHamsterGHOK1GS       CTGTGAT----GTGAATTTTCTAATGGAATTATTT-----ACCTCTTA-CATCAAGAG----------------AGATAA  58665
LongTailedChinchilla        ATAGGGT----GCCTGATTTATGTAGGA------------ACTCCATAATGTCCTCAAGTTTCTTAGA-----------A  56355

Majority                    XTTTXAAAGTATTCTAXXTAGXTATTTXATAAAGATAXTTAGXTATAATTAACXAGAATAATTGTTTAA--XCATTTGXX
                                     83370     83380     83390     83400     83410     83420     83430     83440
Human                       ATATAAATGTTATGTAAATGGTTGTTATAGTGTATTGTTTAGGGAATAATGACAAGAACAACTTTCTAT--ACATTTGCA  76766
GuineaPig                   ATCCAAAACCATTGCCAAAAGTTAATTAAAAACCATAAGTAGTAAGATGAATTAAGAAAAATAGTTT-----TACTCTTA  50801
NorthernAmericanDeerMouse   GTTTCTAAGTATTCTA--TAACTATCTCATAAAGATAATAAGATATAATTAACCAAAATAATTGGTTAAGAACCTTTGTT  54788
Mouse                       GCTTCCAGGCATTCT------CTATTTTATGAAGATAG--AT-TATAATTATCCAAAATG-TTAATTAA---TGTTTGCT  55537
ChineseHamsterGHOK1GS       GTTTCCAAGTATTCTG--TAACTATCTCATGAAGATAGTAAGATATAATTAACCAGAATAATTGGTTAAGAACCTTTGCT  58743
LongTailedChinchilla        ATCTAAAACTGTTCCAAAAAGTTAATTAAAAACCATA-GTGGTAAGAT-----AAGAAAAATTGTTT-----CATTTTTA  56424

Majority                    GTGAAGGATCTATXAXXTGTCGXAXACATTTGATTAXAG-X--GXTXAXTXTAGTCXTXAAGAAACTGXGTXCAGXGTAT
                                     83450     83460     83470     83480     83490     83500     83510     83520
Human                       GTACACCATTGTTTTACCCCCAAATATTTTTGATCCAAGGTTGGTTGAATCGGAACCCAGAGATACAGAGGGCTGACTAT  76846
GuineaPig                   AATTAGAATGTGCAAACTTTGGAATGCCTGTGATTTCAG------TGAAACATAGCCTGAAGGGAAGAAGGATTGA---T  50872
NorthernAmericanDeerMouse   GTGAAGGATCAATCAGTTGTCGGA-ACAGTTGAATA-------GATCAGTTTAGTTTT----AAACTGGTTCCAGGGGAT  54856
Mouse                       G--AAGGATCTATTGGTTGTCTGA-ATAATTGAGTAAGGAT--GGTCAGTTTAGTTTTTAAGAAACTGGCTTCATGGTGT  55612
ChineseHamsterGHOK1GS       GTGAAGGATCTATCAGTTGTTGGA-ACAGTGAATAAGG-AT--GATCAGTTTAGTGTT----AAACTGGTTCCAGGGTAT  58815
LongTailedChinchilla        AGCTGAAATGTGCAAACTTTGGAATGCCTGTGGTTTCAA------TGAAACATGACCCAAACAGAAGAAGTGTAGA---T  56495
```

```
Majority                     AATTGXGGXXTAAAAATXXGCTCACTATXA--XXX-GTXTCT------TCAGTTTTTAXGGAGG----XAAGAGTTTAAA
                                     83530     83540     83550     83560     83570     83580     83590     83600
Human                        ACTTTAAGAATTAGAATTAGCTGGGTGTGGTGGTGGGTGCCTGTAGTCCCAGCTACTCGGGAGGC----TGACGCAGGAG  76922
GuineaPig                    AAATAATTTATATAATTTTCCTCTACATGA--GTGAGTATCTT----GTCAGTTTCAGTGGAGGG----AAGGGTTAATA  50942
NorthernAmericanDeerMouse    GATTGGGAGCTAAAAA-ACGAGTACTAAAG------GCTTCT------TAAACTCTTA----------AAAGAGTCTAAA  54913
Mouse                        ATCTGGGGACTAAAGACAGGTTCACTAAAT------GCTTTTAT--TTTTATTTTTTATTTTTGTCTCAAAGAGTCTAAG  55684
ChineseHamsterGHOK1GS        ATTTGGGAGCTAAAAACAAGACTACTATAA------GGCTTT------TAG--TCTTA----------AAAGAGTTTAAA  58871
LongTailedChinchilla         AAATAATGTATATAATTTCTCTCTACATGA--GTGGGTATCT-----ATCAGTTTTAGGGGAGG-----AAGGGTTAATA  56563

Majority                     AT--AAGCATGAXAX--------GXAATXTGGAXAAAXTXAATATAAAAGCACTGTATTTTAATXATAATGXCTGXXXAA
                                     83610     83620     83630     83640     83650     83660     83670     83680
Human                        AA--AGGCGTGAACCCGGGAGGTGGAGCTTGCAGTGAGCCGAGATCGTGCCACTGCACTCCAGCCTGGGCGACAGAGCGA  77000
GuineaPig                    AT--AATATTGTATC--------AGAAATAATAA--AAAC---ATAAAATTGCTGT----TACTCAAAATGTCTGGATAG  51003
NorthernAmericanDeerMouse    AT---AGCATGAGATGAA-----GAAATGTGGGGAAAATAAAC--TAAAGCATCCTATTTTAATGATAATTACTTACAAA  54983
Mouse                        AT---AGCATGAAAT--------GAAATGTGGCAAAAGTAACT--AAATGCAGTATATTTTAATGATAG-----------  55740
ChineseHamsterGHOK1GS        ATGGGAGCATGAGATTAA-----GAAATGTGGGGAAAGTAAATGTCAGAGCATTGTATCTTAATGATAA----------A  58936
LongTailedChinchilla         A---AATATTGTGTC--------AGAACTAAAAATGAAACCTTATAAAATAGCTGTAT--TACTCAAAATGTCTGGGTAG  56630

Majority                     XXCTCAGXTGXTAAAA---XTATXX-------AATAGXXAATTTXXTXTXGAATATXTT-TGCCT-TGATACXXGXCTTC
                                     83690     83700     83710     83720     83730     83740     83750     83760
Human                        GACTCTGTCTCTAAAAAAAAAAAAAAAAAATTAGAAGGGGCTGTCAGGCGTGGTGTCTCATGCCTATAATCCCAGTCATG  77080
GuineaPig                    --CCCAGCTGATGAAA--------------------GATATTTTGATATGGAAAAGATT---CCT-TGATATTTTCTTTC  51057
NorthernAmericanDeerMouse    GAATGAAATGTGAAGATCTCTATCAGG----CAATAGGAAATTTAGTATAGAATTTTGTTTGTTT-TGAGACAAGGTCTC  55058
Mouse                        -------------------TTATT--------TATAAAGAATGTGGCGCA----------CGCCT-T-----------T-  55770
ChineseHamsterGHOK1GS        GAATGAAATGTGAAGATCTCTATCAGC----CAATAGGAAATTTAGTGTAGAATCTTGTGTGTTT-TGAGACAGGGCTTC  59011
LongTailedChinchilla         --CCCAGCTGCTGAA---------------------GATGTTTTCATATGGAAAAGCTT---CCTGTGATCTTTCCCTTC  56684

Majority                     XCTGTAGTACC-----CATGCAAGCTTAGGAAGAXXACXTTXTAXAXXAXXCTGXCTTCAAAXGCAXXCTGXTX-X--XG
                                     83770     83780     83790     83800     83810     83820     83830     83840
Human                        CCTGTAATCCCAGCACTTTGGGAGGCTAAGGTGGGCATATCACTTGAGATCATGAGTTCAAAACCAGCCTGGCCAACATG  77160
GuineaPig                    TCTTAAGTA-------AATGTCAATTTATGAAGAAGCTGTTTTGAAATACTCTGTTCTTATGCTGATTCTGG-------A  51123
NorthernAmericanDeerMouse    ACTGTGTAACC-----CATGCAGGCTTAGAA--TTTACTTTATAAAGAAGGCTGGCTTCCAATGCACAAAGATCCATCTG  55131
Mouse                        -------AATC-----CCAGCA--CTTGGGA--------------GGCA-----------AAGGCAGGTGGAT-------  55804
ChineseHamsterGHOK1GS        ATTGTGCCACC-----CAGACAGGCTTAGAAAACTTACTCTGTAGACCAGGCTAGCTTCCAAAGCACAAAGATCCATCTG  59086
LongTailedChinchilla         TCTGAAGTA-------TATGGCAGTTTATGAAGAAGCCGTTTTATATTATTGTGTCCTTATACTGGTCCTGG-------A  56750
```

```
Majority                      XTXXXXXXTTXTAXGTAXTXGGXTTACXX--XTXCACCAXXTXTGXTXXTXXGXXGXTXTXXTTTTXTXX-CT-------
                                       83850     83860     83870     83880     83890     83900     83910     83920
Human                         GTGAAACACTGTCTCTACTAAAAATACAAATATTAGCCAGGTGTGGTGGTGGGCGCCTGTAATCTTAGCTACTCAGGAGG 77240
GuineaPig                     ATA------GGTATTTAT-----TTAC-----CTCATCATCTCTATTAATAAGTAGTTTTTTTTTTTTTC-CT------- 51179
NorthernAmericanDeerMouse     CCTCTGTCTTCCAAGTGCTGGGATTAAAAGCATGCACCAGCCGGGCGGTGGTGGCGCACGCCTTTAATCCCAGCACTCGG 55211
Mouse                         --------TTCTGAGTTTGGGG--------------CCA----------------------------------------- 55821
ChineseHamsterGHOK1GS         CCTGTGTCTTCCAAGTGCTAGGATTTAAAGCATGCACCA----------------------------------------- 59125
LongTailedChinchilla          GTACAACA-GGTATTTAT-----GTAC-----CCCATCATGTCTGTTTGTAAGTAGTTTTTTTTTTTTTTTCT------- 56812

Majority                      -------AGXAXAXXTGCT--XXCTXAG----TGXXGGTTXXXXTGXGXT----TXXXAXCXXXAXGXTXXG---TGTGT
                                       83930     83940     83950     83960     83970     83980     83990     84000
Human                         CTGAGTCAGGAGAATTGCTTGAACCCAGGAGGTGGAGGTTGCAGTGAGCTGAGATCATGCCATTGCACTCCAGCCTGTGT 77320
GuineaPig                     -------AGAATACCTGCT--ATCTTAG----TGATAGTTTACGTATGAT----TCGCATCTGAAGGGTTAG---TGTGT 51239
NorthernAmericanDeerMouse     GAGGCAGAGCCAGGCGGATCGCTGTGAGT---TTGAGGCCAGCCTGGGCTACCAAGTGAGCTCCAGGAAAGGCGCAAAAC 55288
Mouse                         -------------------------------------------------------------------------------- 55821
ChineseHamsterGHOK1GS         -------------------------------------------------------------------------------- 59125
LongTailedChinchilla          -------AGCATACTTGCT--GCCTTAG----TGATGGTTTGGATGTGGT----TTGTACCCCAACGGTTAG---TGTGT 56872

Majority                      TXXAXAGTXAXXCCC-AXXXXGXXAAT-CXAAXAXAXXTTGXXAXXTXT-AAGAX---------------------XXXX
                                       84010     84020     84030     84040     84050     84060     84070     84080
Human                         GACAGAGTGAAACTCCATCTCAAAAATACAAAAAGAATTAGAAAGGGCTTTAGAGTCCCACCACTAATGCTCTTCTGTTC 77400
GuineaPig                     TGAAAAGTTAGTCCCCAGTGTGGCGATGCTAAGAGATGTTGGGACCTCT-AAGAT---------------------AGAG 51297
NorthernAmericanDeerMouse     TACGCAGAGAAACCCTGTCTCGAAAAACCAAAAAAAAAATAAAAAATAAAAAAATAAAAAAATAAAAAAAAAAAAAAAGC 55368
Mouse                         -------------------------------------------------------------------------------- 55821
ChineseHamsterGHOK1GS         -------------------------------------------------------------------------------- 59125
LongTailedChinchilla          TGAAAAGTTAGTCCC-AGTATGGTAGTTCTAAGAAGTGTTGGGACCTTTTAAGAG---------------------GTGG 56930

Majority                      AG----AXXCATGXCTXXTCTAT--GAGTTXTTXTGAGTGTTXATCT-X--X----CXXAAAGAXTTXAXAXAGTAXTTX
                                       84090     84100     84110     84120     84130     84140     84150     84160
Human                         AGTAGTACACAAGACTAATAACTACGAGATGTTATGAACAGGGAAAAGAAGTAAGACCAAAAGAAATCATAAATTACTTT 77480
GuineaPig                     AG----ATTCGTGAGAGATTGTT--GAGTTTTTAAGAGTGCTGAGCT---------CTGAAAGAATGTATTTCTTATTTC 51362
NorthernAmericanDeerMouse     ATGCACCACCATGCCTAGTCTAA-TCTGTTATTTTGAGTTTTAGTCTAAATTTT--GGCAA--GTTTTAGAGAGTTGA-G 55442
Mouse                         ------------GCCTGGTCTA-------------CAATGTGAGT-------------------TCCAAGACAGCCAGGG 55857
ChineseHamsterGHOK1GS         --------CTATGCCTAGTCTA----------TTTGAGTTTTAATCTAACTTTT--GGAAAGAGTTTAAGGGAGTTGTTA 59185
LongTailedChinchilla          AG----ACTGGTGAGTGATTACT--GAGTTTTTGAGACTGCCGAGCT---------CTGAAAGAATTGATGT---ATTTC 56992
```

```
Majority                    TTATXGAAGTTXXAGATTGGTXCTTAGAXXGA-X---XX-ACAXTGTTXAAAATAAXAGAGXAXAXTXTXAAAT-T--G-
                                      84170     84180     84190     84200     84210     84220     84230     84240
Human                       GGATGGAACTTTTAGAATGGCAGATGGAGGGATAAATTTGACATTGTTCAAAATCATAGAGTAAACTCTCAAAAGTAAGG  77560
GuineaPig                   TCATGGGAGTTTT-GAATGGTT----GGGAGA--------AAATTGTTTAAAAGAATGGAACTGATTGT-----------  51418
NorthernAmericanDeerMouse   TTTTAGAAGCCAGAGATTAAAACTTAAAACTTCA--CTT-ACAATTTTCAAAATATAAGAGAACAAGATTAAAT-T--GT  55516
Mouse                       TTATACAG---AGAAACCCTGTCTCAAAA-------------AATCTTTAAAAAAAAAAA----------AAA-----G-  55905
ChineseHamsterGHOK1GS       CTTTAGAAGCCAGGGGTTAATACTTAGAACTTCA--CTTTACAATTTTCATAGTATAAGAGAACTAGATTAAAT-C--GT  59260
LongTailedChinchilla        TCATGGGAGTTTT-GATTGGTTCT--GAGAGA--------AGGTTGTTTAAAAGAATGGAACTGATTCTCCCCTCT----  57057

Majority                    -XAACCTAAA----------GTTTGGAXGTAC--------XCTXXXACTXTGAAXCA--------AAATGCAATTXAXGC
                                      84250     84260     84270     84280     84290     84300     84310     84320
Human                       GGAACCTAAGGAGACAAGATGATTAAATGTAATAGGAGATACTGGAACAACAAAAAAGGGACATTAGATGCAATTTGAGG  77640
GuineaPig                   ---TCCT-------------GGCTTG----------------------------------------CGTACACTTCCACC  51442
NorthernAmericanDeerMouse   TGAA-TTAAA----------TTTTGGAAGTACA------CATTCATACTGTGTATCA--------AAATGAGATTGAGGC  55571
Mouse                       --AA--TGAA----------ATGTGAAGATAC---------------CTATGAGACA--------AAAGGGAATTTAG--  55946
ChineseHamsterGHOK1GS       TGAAATTAAA----------TTTTGGAAGTACA------TACTCATATTTTATATCA--------AAATGAGATTGAGGC  59316
LongTailedChinchilla        ---TCCT-------------GGCTTGGTGT-----------CTTGCCCTGTGATCGCTCCGTCTCGCATGCACTTCCACA  57110

Majority                    ---TXTGAXXAT-X----TTGTTGG--X-XGCTTXXTTA------AAXTACAAGGXC---TTXATTTGAXTCCCAAACAG
                                      84330     84340     84350     84360     84370     84380     84390     84400
Human                       AAATCTGATAATGGACTTTTGTTGATTTATCGATATTGGTTGATTAATTGCAAGAACTAATAAATTGTAATACCATACTA  77720
GuineaPig                   ----CTAATG--------TTGTTG-----CTCTTACT---------GTAACAAGGAGGTTCTCATTAGAG-ATAAAAAAG  51495
NorthernAmericanDeerMouse   ---TGGGGAAATAGTTCATTGGTAGAGT-TGCTTGCTTACT----AAGTACAAGGCC---TTGGTTTCAATCCCCAGTAT  55640
Mouse                       ---TGTAAAGAT---------CT-------GTTTGTTTA------AAG-ACAGGGTC---TCACTGTGAAACTTAGGCAG  55997
ChineseHamsterGHOK1GS       ---TGGGAAAATAGTTCGGTAGTAGAGT--GCTTGCTTACC----AAGTTCAAGGCC---CTGATTTCAGTCCTCAAAAT  59384
LongTailedChinchilla        ----CTGATG--------CCATTGG----CCCCTATT---------ATGACAA--------TCACCAGAG-GCCAAACAG  57156

Majority                    AXGAX-XXXTGCCAATAATATGAGAXXTGGA-TXTCCAXAXCAGAAAGGAACA----AAXGTXXX--ATTTGTTTGTAAX
                                      84410     84420     84430     84440     84450     84460     84470     84480
Human                       ATGTA-TGATGCTAATAATGGGGGAACTGCGGTTTCCAGGTTATATAAGAACTCTGTAATGTCTTAAATTTGTTTGTAAA  77799
GuineaPig                   ATGAG-CCCTCCCAGT--TTTGAATGTCTTAGTTTCCAAAATGGAACCAAATA----AAC--------TTTGTGTGTAAG  51560
NorthernAmericanDeerMouse   TCAGA---------ATGAAACTTGA--TGG---CTGTAA--CAGTTAGGATTA----AAAGTCAA--AGTGGCCCCTCAA  55698
Mouse                       GCTTG----------CAATATCAGG--CTGC-TCTCCAGCTCACAAAGATCCA----TGTGTGTC----TGACTTCCAAG  56056
ChineseHamsterGHOK1GS       GAAAAGTGGTGGCTATAAAATTAGAAGTGGA-TCTTCAATGCAGTATGTGTCAT---AAAATTATTCAAAAAAAATAAAA  59460
LongTailedChinchilla        AGGAG-GCCAGCCAGT--TTGGGGCTTTGCACTTTACAGAACTGAGCTGAACA----AACGT----ACTTTGTTGGTAAG  57225
```

Majority	TGXATAGCXXCAXGAGTA-TTXAXXAXAATAAX-X-XX-----AAATAAAXTAAT----------------X--XXX---	
	84490 84500 84510 84520 84530 84540 84550 84560	
Human	TCTAAACCTTTTAAAATAGCTAAAAATTATAGATACAATTTCTAAATGATTTAATTAACAAAACCAGTTTAATTCTTCAG	77879
GuineaPig	TTTACAGCTTCAGGTTTT-TT------AACCAA----------AAACAGACTAAT----------------ACACTTACC	51607
NorthernAmericanDeerMouse	TGCATAGGAGCCATAGGA-TTATTCACAAAAACAAGAA-----CAATCAATTAATA-------------T----------	55749
Mouse	TGC-TAGAAGCAAAAGCA-TGCACTCCCATACC-------------TAATCTAAT-------------------------	56096
ChineseHamsterGHOK1GS	AGCATAGTAGGTGGAGTA-GATGATAAAAAAACAATAA-----CAATAAGTTAATA-------------C----------	59511
LongTailedChinchilla	TTTGCAGCTTCAGGTGTT-TTCACAGTAATAGA----------AAATGGACTAAT----------------ATACTTATG	57278

Majority	TAAXAATGTGAATXXTTAAXG--A-------X-X-XXGTATG-----X-AAAGTAAAACATTAGAXATAXA-XGTATAT-	
	84570 84580 84590 84600 84610 84620 84630 84640	
Human	TTGAAATGAGCAAGGTTAGGGATACCTGTGTGTTTCAGTGTAATATCTGAAAGGAAAAGAATAGATACATAATTTATATC	77959
GuineaPig	TAAAGATGTAAATCGTTAA--------------TCCTCTAGG-----TAAAAATAAT-TAATGGAGATATA-TGTATAA-	51665
NorthernAmericanDeerMouse	TAAGAATA-GG--A----GAA--A-------T----GGCATG---A---TGAGTAAAACATTCCCCGTGGG-G-AATGT-	55800
Mouse	CTAGAATT-----T-------------------------------------A---AAATTTT------A---G-----T-	56116
ChineseHamsterGHOK1GS	CCAGAAGATGTTTGTCATAGG--A-------A----GGAGGT---A---TAAGTAAAACATTACCCAAGGG-GGAATGT-	59570
LongTailedChinchilla	TAAAGATGTAAATCATTAAAGTTAC---TATTTTTCTGTATG-----TAAAAACAATGCATTAGAGATACA-TGTATATA	57349

Majority	------------------------TTGCATTXTGGXA---------------AXATTCATAXGGAGATA-------AATT	
	84650 84660 84670 84680 84690 84700 84710 84720	
Human	ACTTCTCTCTGGACTTAGGGTCTCTTGTCATATACATGACTGGGCATCCATCAGCTTCAGTGGAGGAAAGGTTAGGAATA	78039
GuineaPig	-------------------------TATGTTTTACA----------------AATTTCACAAGGAGACA--------ATG	51696
NorthernAmericanDeerMouse	------------------------TTGCAGTGTGGCA---------------GAATTCATATGGATTTG-------AAGT	55834
Mouse	------------------------CTGAATTTTGGCA---------------AGAGCTATAGGGAGCTGT------GAAT	56151
ChineseHamsterGHOK1GS	------------------------TTGCAGTGTGGCA---------------GAATTTATATGGAGTTC-------AAGT	59604
LongTailedChinchilla	----------------------TATTACTTTATGTA----------------AGTTTCAGAAAGGGACA--------ATG	57383

Majority	XTAGAATTA---CXAAATTGAAAATTX----AA--TATAGXTTTACT--XTAATXTAXXX-X--------TGTXXA---X	
	84730 84740 84750 84760 84770 84780 84790 84800	
Human	ATAGTGTCAGAACTAACATGAGAATTTTAGGAGATTACTGAGTCACT-CAAAATGTCTGGGTGACCCAGCTGATGAGGGC	78118
GuineaPig	GTAAAATTATCTTATAGTTGAGAATT-----AA--TATGGAAAAAC-----AATGCAAAG----------TATATA----	51750
NorthernAmericanDeerMouse	CTAGCATTT---CCACATTGAAAATGT----GA--TGTAGCTTTACTGTCTGGCTTGGTTTTTGTTTTTTTGTTTTT-TT	55904
Mouse	TTAGAAACC---AGAGATTAATACT----------TAAAACTTTGCT--TTAATTTA----------------------C	56194
ChineseHamsterGHOK1GS	CTAGCATTG---CCACACTGAAAATGT----GA--CTTAGCTTTACTATCTGGCTTA---------------------TA	59654
LongTailedChinchilla	GTAAAATTA---CATAGTTGAAAATT-----AAGGTATGAAAATAC-----AATGCAGAAGTA------TTGTAGA----	57440

```
Majority                    XATTTXCAATAAAAATXAT----GCA--X----XX-X--X-XX----------GAXXATXAAATTXXXTXATAXATTTTX
                                     84810     84820     84830     84840     84850     84860     84870     84880
Human                       CATTTCACATGAAAAAGATTCCTGCAACACCTTCTTTTCTTTAGCACATGATCGTTTATGAAGCTCTTTTACATTGTTTT  78198
GuineaPig                   -ATTTGTTATAAAAATG-------CA-----------------------------TTATAAAA-TATTTCAGAAGTTTTT  51792
NorthernAmericanDeerMouse   TTTTTTCAGTGAACATAACA---GTAATAATCCCTATCATCTAAGGTTGTTTGGAGAATTAAATTCAATGATGCATATGA  55981
Mouse                       AATTTTCA--AAATATAA-----------------------------------GAGAACAAGATTAAATTGTTGAAGTAA  56237
ChineseHamsterGHOK1GS       CATTTGCAGTAGATATAATG---GTGTTAATTCCTGTCATCTAAAGTTGTTGTGAGAATTAAGTACAATGATGCATGTGA  59731
LongTailedChinchilla        -ATTTATTATAAAAATGGT----GCA-----------------------------TTATGAAA-TATTTCATAGGTTTTT  57485

Majority                    AXXXTTTTAXX-A-X-XTTAGAATATAXCAAACATTTXTXTTXTATTTTTATGAGTTTGATT-TXAGGXTXAXAGXAXAG
                                     84890     84900     84910     84920     84930     84940     84950     84960
Human                       ATCTTTATAGC-AATCCTCAGAATAGATCAAGCTTTTTTTTTTTTTTTTTTTGAGATGGAGT-CTTGCTCTGTCGCCCAG  78276
GuineaPig                   GTATTTTTA--------TTGCAACAAAC--A---TTTAGATTCTATTTTTAAAAATTTGATA-CACA-TTTTCAGAAAAG  51857
NorthernAmericanDeerMouse   AGGATTTTACCCAGTGCTTAGCATGTAACAAATGTCTGTTACATATTAGCAGGTGTTTGTTTTTCAGGCTAAAATTACAG  56061
Mouse                       A--ATTTTGG--A------AGTACATAATACACATTATTATTGTAGTAAAATGAGATTG------AGGCTGGGGACATAG  56301
ChineseHamsterGHOK1GS       AGGATTTTGCCCAGTGCTTATCATGTAGCAGATATCTGTTATATGCTGGCATGTGTTTGTTTTTCAGGCTAAAATTATAG  59811
LongTailedChinchilla        ACATTTTTA--------TTGCAGTAGAC--AGCATTTAGATTCTGATTTTTTAA-TTCGAT--TTTG-TACATAGAAAAG  57551

Majority                    AATXGXTTGTA---GAXTGGTTTAGG---X-X---AATTAAAXXTCXGAGGCTTXX---XX-------X-----XXT-TT
                                     84970     84980     84990     85000     85010     85020     85030     85040
Human                       GCTGGAGTGCAATGGTGCGATCTCGGCTCACTGCAAACTCGGCCTCCCGGGTTCGTGCCATTCTTCTGCCTCAGCCTCCC  78356
GuineaPig                   AATATATTGAA---GGACAGTTTTGG---------AATTTAATCTCAGAGGCTT-------------------------T  51900
NorthernAmericanDeerMouse   AATTGGTTGTA---GATTGGACCAAGA--AAT---AATGAAAATATTGAGGTTTAGAAAAT------AC----ACCTTTT  56123
Mouse                       C-TCAGTGGTA---GAGTGCTT-GCT-----------TAACAGTTGCAAAGCCTGG--------------------C-TT  56344
ChineseHamsterGHOK1GS       GATTGGTTGTA---GATTGGATCAAGA--AAT---AATGAAAATATTAAGGCATAAAAAAT------GC----ACCTTTT  59873
LongTailedChinchilla        ACTGCATTGAA---GGATAATTT-GG---------AGTTTAATCTCAGAGGCTT-------------------------T  57593

Majority                    XAGTAGXC-CTATTAXAAGA-CCTAAAACGAX---CAGTTA---XTCATGTCTGT---XXAGAGAXX--X----------
                                     85050     85060     85070     85080     85090     85100     85110     85120
Human                       GAGTAGCTGCTACTACAGGTGCCTGCCACCACGCCCAGCTAATTTTTTTGTATGTTTAGTAGAGATGGGGTTTCATCGTG  78436
GuineaPig                   GAGAAGTC-TGATTAAAAAC--TTAAGACTGC-TTCAGTGA---TTCAGAACTGT---GTAAAGGCATG-----------  51959
NorthernAmericanDeerMouse   TAATAGGA-CTGTTATGAGA-CCTAGAACGAA---TTT--A----TCATGTTCAT---ACAGAAA----G----------  56175
Mouse                       TAGT--------CTCCAGGA-CCTAAAATGAA---AAG-------TGACGGCTGTG--ACAGCCA---------------  56388
ChineseHamsterGHOK1GS       TAATAGGC-TCATTATGAGA-CCTAGAAGGAA---TTTTTA----TCATGTTCAT---ACAGAAAACTAG----------  59931
LongTailedChinchilla        GAGAAATC-CAATTAAAAAT--TTAAGACTAC-TCCAGTTA---TTTATAACTGT---GTAAAGGCA-------------  57650
```

```
Majority                     --GGCCAXGAXTGXCTXXAGCTX--------GTGATAGXTXGAXTG-TGTXA--------XXXXAAXXXTGTXTXX----
                                      85130     85140     85150     85160     85170     85180     85190     85200
Human                        TTAGCCAGGATGGTCTCGATCTCCTGACCTTGTGATAGATCAAGTATTTTTATCCCTC--TCATCAACATGTGTAAACAG  78514
GuineaPig                    -TAGCCTGGGTTTTCTTAA------------GTTATGGATCCAGGG----------------------------------  51992
NorthernAmericanDeerMouse    --GGCCAAAACTGACAGTAGCTA--------GTGATAGGTTGTATGTTGTAGGTTGTATGTTGTAAGTTTGTGTGTTGGA  56245
Mouse                        --GATTAAAACTCACTGTGGCTC--------TTGAC----TGCAC--TGTCA--------CCACAAGCTTGTCCAC----  56440
ChineseHamsterGHOK1GS        --GGCCAACACTGACAACAGGTA--------GTGATAGTTTGGATGATGTCATTT-----CTGCAAAGGTTTTTGTTAGA  59996
LongTailedChinchilla         ---GCCTGGGTTTTTTTAA------------GTTATAGGTCCAGAG----------------------------------  57681

Majority                     ---XX-----XX---X-X---XX--XX---AXAGATX--GAAATCATTXAAGTAAAGXCTA--------GTAGGAGG---
                                      85210     85220     85230     85240     85250     85260     85270     85280
Human                        TTTTTTCCTCACATATCTGCTGCCTTAGCTAAAGATG--CAAATCATTAAAGTTAAAATTATAATTTATGTAGCTAAAAG  78592
GuineaPig                    ------------------------------AAGAAT----AAGTAAAAGAAAAAATCACTA--------GTAGT------  52024
NorthernAmericanDeerMouse    GACTT----AACCCCTTTGTGGCAGTATTAAGAGGTGATGAAACCTTTTAAGTGGAGTCTA--------GTAGGAGGT--  56311
Mouse                        ------------------------------AGAAATA--G-------------AAAGCGTA--------GCAGGAGG---  56464
ChineseHamsterGHOK1GS        GACTTGATCAACCCTTCTATGGCAGTATTAAGAGATAATGAGACCTTTTAAGTAAGGTCTA--------GTAGGAGGT--  60066
LongTailedChinchilla         ------------------------------AAGGA------AGTAATGGAAGGAAAGGACA--------GTGGC------  57711

Majority                     X-XTCXGTGAXAGCCXGTGAAXACAXTATGTTTA-XGXTXTTTGXGAAAXTGTCTCXXAAAAA----XX-X---------
                                      85290     85300     85310     85320     85330     85340     85350     85360
Human                        TAATCTGTTAGAGCCAGTGTATACAATATGTGTAATATGCTTTCTATAACTTTCTCAAAGGGCCCTTGGTAAGTTTATTG  78672
GuineaPig                    --GTCAAGGACAGATGATGGCTGCCCCATGTTTG------TCTGTTTGTCTGTCTC------------------------  52072
NorthernAmericanDeerMouse    TCTTCGGTGATGTCCTGTGAAAAAAATTGTTTTAGTGGTTCTTGAGAAAAGGTTTCTGAAAAA--ACGGGA---------  56380
Mouse                        --------------------AAATA-----------GATTATACAGAAAAAACAAACAAAAAA-----------------  56496
ChineseHamsterGHOK1GS        TCTTAGGTGATGTCCTGTGAAAAAA-TTGTTTTAGTGGTCCTTGAGAAAAGGTTTCTGAAAAAAGATGGGA---------  60136
LongTailedChinchilla         --GTCAGGGACAGATGGTGACTACCCCATGTCTG------TCTGTCCGTCTGTCTCC-----------------------  57760

Majority                     CTXXCCCCTTGTACTGGG--TTXAXAATATGACXTTXT------XXACATXXA-X-TXX--------CA--AAXAAXTAX
                                      85370     85380     85390     85400     85410     85420     85430     85440
Human                        TTTTCTCATTTAGCTGGGAGTTAAGAACAGAACTATTTTCTAGAATGTATTAATTATATAAATAGTGCATCATGAAATAT  78752
GuineaPig                    ---------TGTACTGGGATTAAAACTAAGACCTTC----------ACACTAA------------------AGTAAAACT  52116
NorthernAmericanDeerMouse    CTGGCCCCTCTTACTTAC--CTCCCTGTGTGGTTTCTTT-----ACACATGTGCT-TCTTCCT----TAA-CACCAGTAC  56447
Mouse                        CCAAACCCACACAATAA-----------ATAACTGTCCA-----AAAGATGT----TGC--------CA-----TAGGAT  56543
ChineseHamsterGHOK1GS        CTGGCCCCTTGCACTCGT--CTCCCCATGTGATCTCTT-------CACATGTCCT-TCCTTAA----CA--CACCAGTAC  60200
LongTailedChinchilla         TTTTGTCTTTGTGCTGGGGATTGAAATCAGGGCCTTC---------ACACTAA------------------ACTAAAGCC  57813
```

```
Majority                    CTAGXXGXGCTTTTAXTTTXTATTAXT--ACCCAAAAXCTAXATTAX----------XGTACCTAGTTX-XCAAGTACTT
                                     85450     85460     85470     85480     85490     85500     85510     85520
Human                       TTTGTAGGTTTTTATATTTTTATTGCAGCAGACAGCATTTAGATTAAACCTAAA---AATACCTGATTGACAAAGTAGTT  78829
GuineaPig                   CTGGTAGTCCATTTAATTTTTATTTTG--ACGCACAGTCTTGATTAG------------TTACTAGTTGCCCAGGTACTC  52182
NorthernAmericanDeerMouse   CTAAAGGAGTTCTTACCAGATGGGACT--GCCTAAAAGCTAAATCACTTTCAAAGCCAGTACGTAGTT--TCAAGTACTT  56523
Mouse                       GGAGAAATGGCATG---ATAAATAATT--ACCCAAGAGGGAA-T--------------GTTTGCAGTG--TGGCAGAATT  56601
ChineseHamsterGHOK1GS       CTAAAGGAGCTCTT--CTGATGTGACT--GCCTAAAAGCTAAATAAACTTTC---TCAGTACCTAGTT--TCAAGTCGTT  60271
LongTailedChinchilla        CTAGTGGCCC-TTTATTTTTTATTTTG--AGACAAGATCTTGATTAG------------T-GCTAGTTGCCCAGGTACTC  57877

Majority                    TTXXACXXXXAAGXATXXTGTCTTX-------GAXCXTXCXXAAXTTTTGGAAXTCCAGXXAXXTGCTA--AXTATGXCX
                                     85530     85540     85550     85560     85570     85580     85590     85600
Human                       TTTCAGACAAAAGTATATTGAGGGAAATTGGAGAATCTTCAGAGGCTTTAAAAGTCCAGGAAAATCTTAG-ACTACTCCA  78908
GuineaPig                   TTGAACTTGGGAGCATCCTGCCT---------GAGCTTCCTAGAGTTCTGGAATTACAGACATGTGCTA--ATTATGCCC  52251
NorthernAmericanDeerMouse   TGTTACAGTAATGGAAGATGTATTAGT-----GCTCGTAGCTAGAGTTTGTAAATCAGTAAAGTTACAGTTTATCTTTCT  56598
Mouse                       C------------------ATAATG-----------------ATAATGTTGAAGTC----------------------CA  56624
ChineseHamsterGHOK1GS       T-----------------TATCTTC---------------CTAAATTTTAAAACTTA-------CGTAACATATGTGAC-  60311
LongTailedChinchilla        TTGAACTTGGAATCATCCTGCCTT--------GAACTTCCAAGAGTTCTGGAATTACAGGGATGTGCTA--ACTATGTCT  57947

Majority                    XXXTTXTXG---------TTTAAGTTTTXCXAAGGAGCXTTTATCTTGTAXAATACGCGAGTTGAAAXTTATGXAAGAGA
                                     85610     85620     85630     85640     85650     85660     85670     85680
Human                       GTGATTTAGAACG---GTGTAAAGATAGCCTAAGTAGTTTTTCTAAGTTATCAGCCGCGGGGAGGAAATAATGAAAGAGA  78985
GuineaPig                   AGCCCCTTG----------------TCTCTTGCAGTGCTTTTGCCTTGTGTTATACACTATTTAAAAATGC----AGAAA  52311
NorthernAmericanDeerMouse   AAATTTTAAAACTTACGTTTTATTTTTTACAAAGGAGCAATTATCTTGTAAAATAATTGAGTTGATATTTATGTAGACGT  56678
Mouse                       GCATTCCTA---------TGTAAGTTTTACAAAGGAGCAGCTATCTTGTAAAAT---TGACTTAACATTTATACA-----  56687
ChineseHamsterGHOK1GS       ATGTTTTA----------TTTAAGTTTTACAAAGGAACAGTTATCGTGTAAAATAACCGAGTTGACATTTATGTAGGAGT  60381
LongTailedChinchilla        GGCCTCTTG----------------TCTCTTAAAATGCTTTAGCATCGTGTTACACGCTGTT-GAAAACAC----AGAAA  58006

Majority                    XACACTTTT----TTCATAXAGXTXATXXXXTGTAATATTTTXT----XXTTTTCTTAGTAXCCXXAAXTACTTATGTGA
                                     85690     85700     85710     85720     85730     85740     85750     85760
Human                       AATACATACGAGTGGCATTAAGGACAGGTGGTGGCTTCTTTTAC------CTATTAAAGCATTCTTACACCATACTGTGA  79059
GuineaPig                   AATCCTTTT----ATCATATAGGTCACTTGATTTAGTATCTCGG------T------AGTCTGTTCAAAATCTGTTCTCA  52375
NorthernAmericanDeerMouse   TGCAGTTTTA---TTAATAAAGATAATTCATTGTAAAACTTTTTTTTAGGTTTTCTTAACAGCCACAAGTACTTAGGTGA  56755
Mouse                       -------------TTTATGTAGAAATTCCACT--AACATTTTGT---AGGTTTTCTTG-TTGCCACAAGTACTTATGTAA  56748
ChineseHamsterGHOK1GS       TTCAGTTTTA---TTGATAAAGATAATGCATTGTGAAACTTTTT----GGTTTTCTTA-TAGCCGTAAGTACTTAG-TAA  60452
LongTailedChinchilla        GACCCTTT-----GTCATATAGGTCACCTGATGCAGTAGCTC---------------AGTCTGTGTAGAGTCCATTCTGG  58066
```

```
Majority                     AXTCTXXAAAAXAXXXX----TGTTGXTCAAAXTTAATTT-TCXGAGAAGXXXATTTGGXATXAXAAACTGAAATTTAXX
                                  85770     85780     85790     85800     85810     85820     85830     85840
Human                        ACTGTGTAAAAGGAAGGA--TCATTGGACATAATAGATTA-TCTGACAGGGTGGTCCGGAATGGTGGAAGTAAATCGACA  79136
GuineaPig                    TCT-AGTAGTCCAGCA--------TGGCAGAAATACAT------GGATAAGTGAT--GGAATTTTTAATTAGCACTTA--  52436
NorthernAmericanDeerMouse    AATCTAAAAAACTTCTA----TGTTGATCAAAGTTAAATTCTCAGAGAAGAGTATTTTGGGGGAAAAGCTGAAGTCCATT  56831
Mouse                        AATCTAAAAAGTTTCTGT--GTGATGA-CAAAGTTAATTTTTCAGAGAAGAATATTTGG--AAAAAAGCTGATATTTGTC  56823
ChineseHamsterGHOK1GS        AATCTAAAAAAAAAAAAAAAGTGTTGATCAAAGTTAATTTTTCTGAGAATACTATTTGGTAAAAAAATCTGAAGTTCACC  60532
LongTailedChinchilla         ACTCAGTAGGCGAGAG--------TGGTGGTAATGCAT------AGAAAAGCGAT--GGGATTTTGAATTT-CATTTA--  58127

Majority                     CAXXGAGGCXXTXA--X--CXTACAG-CAGTXXGAGCTATXCATCTXGT-TTAGATXTTXXGGTAXXTTT-XXTATTACC
                                  85850     85860     85870     85880     85890     85900     85910     85920
Human                        GGTGAGGACACTGAATTAGCATTCAGACAGTGGGGACCTCACACCTTGTATTCCCTTTTTTGGTAAGATT-TTTATTACC  79215
GuineaPig                    ------GACAGT-------TATACAGACAATGGAAGCTGTACAGTTTATATTACATTTTTTGGTAAGGTT-TTTATTAAC  52502
NorthernAmericanDeerMouse    CACAGAGGCTTTAA--------A----GAGTTTAAGCTATGTCTCTGGT-TTAGAAGTGAAGGTAGTTTT-CCAGTAACC  56897
Mouse                        CACAGAGGTTTTCAAGT--CTCATGG-AAGCTTGCGCTGTGCCTCTGGT-TTAGAAGTGAAGGTAGTTTTTCCTGTTAGC  56899
ChineseHamsterGHOK1GS        CATGGAGGGTATAACAT--CTGATAA-GAGGTTCAGTTATGTTTCTGGT-TTAGAGGTAAAGGTAGTTTT-CCTATTACC  60607
LongTailedChinchilla         ------GGCAGT-------TGTACAG-CAGTGGGGGCTATACAGTTTATGTTCTATTTTTTAGTAAGATT-TTTATTATC  58192

Majority                     XCCXTAG----XGGXGXXAAATTACAXCTGATTAGAGTCTAACAXG--T--ATTTAAGGGXAATATTXGTXGCCXTAGC-
                                  85930     85940     85950     85960     85970     85980     85990     86000
Human                        TCCTTAG---AAAGAGGTAAATT-CATCTATTTCGAGCCCAAA-----TTTTTCTGTCTAGAAGATTGGTAGTCTTATTT  79286
GuineaPig                    TCCTTAATATAAGTAGGTAAATTGCATCTGTTTTGAGTCTCAC----------CTTATGGGAAAATTAACAGACTT----  52568
NorthernAmericanDeerMouse    AACCTGG-----GGCAAGAAATTACAGAGGAGAAAAGCATAGCATGT-T--ATTAAGGGGAAGTAGTGGTTGCCACAGC-  56968
Mouse                        AGCCTAG-----GG-GAGAAATAACTG-----CACAGTGGAACATGT-T--AGTAAGGAGAAATGCTGTTTGCCATAGC-  56964
ChineseHamsterGHOK1GS        AACCTAG-----GGGAAGAAATTACAGAAGAGAAAAGTATAGCAGG-----ATTTAAGGGAAGTAGTAGTTGCCACAGC-  60676
LongTailedChinchilla         TCCTTAATATGAGGGGGTAAATTGCATCTTATTTGAGTCCCAAGGGACTCAAATTCATGGGAAAGTTAGCAGACTT----  58268

Majority                     -TXXTAAAGXACAXTXXTXXXAXATAXAAGXTAGCTTXTXXXXTAXXTXTTTXXGTCCTTTXACATAATTTXACTXACCT
                                  86010     86020     86030     86040     86050     86060     86070     86080
Human                        TTACTAAAGTACAAGATTATTTAGTAAAAGGTAGCATTCTC--TGGCTTTTTTTTTTTTTTTTTTTAATGTTTCAGATCT  79364
GuineaPig                    -TACCAAAGGACAGTATAATTCATTAAAATACAGCTTTTTC--TCGCTTTTTTACCACCTTTG-----------------  52628
NorthernAmericanDeerMouse    -TCTTAAA--GCACTGGTTCCATATAGCAGATTACTCATGACATAATCATCTAAGTCCTTTGACATAATTTGACTCACCT  57045
Mouse                        -TCTTAAAAACCAGTGGTTCCATATGGCAGGTGGCTTAGGACACAATTATGCAGG-CCTTTGACATCATTTCACTAACCT  57042
ChineseHamsterGHOK1GS        -CCTTAA---GCACTGGTTCCATATAGTAGGTTGCTTAAGGCATAATTATCCAGGTCCTTTGACATAGTTTGACTAACCT  60752
LongTailedChinchilla         -TACTAAAGGACAATATCATTTATTAAAATATAGCTTTTT---TAGCATTTTT-CCCCCTTTG-----------------  58326
```

```
Majority                    GCTTTGTAGCAXGGTXGXTXAGGAGTXGTGATAXTXAATAGATTTTGXATC--X-XX-TTXAACAGXTTTAAXTAXGAG-
                                    86090     86100     86110     86120     86130     86140     86150     86160
Human                       GTCACACTAAAAAGGGCTTTAGACCAGGTGTTGTGGCTCACACCTGTAATCCCAGCACTTTGACAGGTCAAGGTGGGAG-  79443
GuineaPig                   --------GGAAGAAGGTTAAGGTTATTTTGGTTGGAATGGGTTTTGGGTC-------TTGCACAGTTTTTAAAAAGGC-  52692
NorthernAmericanDeerMouse   GCTTTGTAGCTTGGTAGCTGAGGAGTAGTGATAATAAATAGACTGTGAATT--ATCATTTAAGTAGGTAGAAATACTATT  57123
Mouse                       GCTTTGTAGCCTCTTAGCC-AGGAGTAGTGATAATACATAGATTGTGGATT--AGCATTTAAACAAATGTAATAAGCAGC  57119
ChineseHamsterGHOK1GS       ACTTTGTATCCTGGTAGCCAAGGAGT---GATAATAAATAAA-----AAT------------------------------  60794
LongTailedChinchilla        --------GGAAGAAGGTTGAGGTT---TTGTGTTGAATGGGTTTGGGGTC-------TTTTACACTTTTTTTTAAAAG-  58387

Majority                    ---GGXTCXTXXAAGCTTACATTTTXC-----XTTTTGGTXAXATTXTXA-XXCTTTCTTCCTXCT--AAXAXGAAAXAA
                                    86170     86180     86190     86200     86210     86220     86230     86240
Human                       ---GATTGCTGGAGGCCAGGAGTTTGAGACCAGCCTGAGCAACATAGTGAGATCTCAGTCTCTACTA-AAAATAAAAAAA  79519
GuineaPig                   ---TGTTTTT------------TTG--------------------------AGATAGGATCTCACTG-----TGTAACTC  52726
NorthernAmericanDeerMouse   G--AGGTCCTCAAAGCTTACATTTTACC----GTTTTGGTGATATTTTCATTACCTCCTTCCT-----AAGAGGTTGGAA  57192
Mouse                       ACTGGGGCTTGCAAGCTTACATTTTCC-----ATTTTGGTAAGGTTCCTTATCTCTTCTTACT-----AAAAGAGAATTC  57189
ChineseHamsterGHOK1GS       ----GGTCCTCAAAGCTTACATTTTAC-----ATTTTGGTGATATTTTCACAACTCTCTTCCTTACTTAAGAGGAGATAA  60865
LongTailedChinchilla        ---GGTTTTT------------TTT--------------------------TTTTTTTTTTTTTCT------TGAGACAA  58420

Majority                    ATTGXTXCTGXTTTGTAACXCAXXCTTTTCTTCCXTGAXAXTTG-GCAGTCT---------------XTTTA--XTAXA-
                                    86250     86260     86270     86280     86290     86300     86310     86320
Human                       ATTAGCCATGCCTGGTGGCATGTGCCTGTAGTCCCAGCTACTTA-GTAGGCTGAGGTGGGAGGATTGCCTGAGCCCAGAA  79598
GuineaPig                   A---ACCTGACCT--TAACTCATGATGGTCTTCCTAGCT-------CAGTCT----------------------CTAGA-  52771
NorthernAmericanDeerMouse   ATTGCTTCTGTTTTG-AACCCCAACTTTTCTTTTATGAAAGTTG-GCTATCT---------------CTTTA--TTAAA-  57252
Mouse                       ATTGCTTCTGTTTTG------AAACTTTTCTTGGGTGAAATTTGAGCAGTCT---------------TTTTA--TTAAA-  57245
ChineseHamsterGHOK1GS       ATTGTATCTGTTTTTCAACCTAAACTTTTCTTTCATAAAATTTG-GCTGTCT---------------TTTTA--TTAAA-  60926
LongTailedChinchilla        G---GTCTCAC-T--TGACTCATGGTGATCTTCCTGGAT-------CAGCCT----------------------CCAGA-  58464

Majority                    ---------------GGGXAATXXTXATTAAAATGCAXXTTAGACT---------TXTAAACTGTCTCTXXXATXTAXAA
                                    86330     86340     86350     86360     86370     86380     86390     86400
Human                       GATAGAAACAGCAGTGAGCCATGATCATGCCACTGCACTCTAGCCTGGGTGACAGAGCAAACCTTGTCTCAAAAAATAAA  79678
GuineaPig                   ---------------GTGCTGAGATCATAGGCATGAAC----------------------------TCCCAGCTA-----  52803
NorthernAmericanDeerMouse   ---------------GGATAATATTTATTAAAATGTAGATTAGACTA--------TTTAAACTGCCTCTTGAATCTATAA  57309
Mouse                       ---------------AGGTAATATTTATTAAAATTCAGCTTAGACT---------TTTAAACTGTCTCTTGAATCTATAA  57301
ChineseHamsterGHOK1GS       ---------------GGATAATATGTATTAAAATGCAGCTTAGACTG--------TGTAAACTGTCTCTTGGATCTACAG  60983
LongTailedChinchilla        ---------------GTGCTGGGATCATAGGCGTGCACAACCAC----------------------TCCCAGCTA-----  58502
```

```
Majority                      CXA--------XTTTAXCXTXGCCTTTTAATTCTCAATTAXXXTXG---XTTCATTTXAA--------TAATTCTTTTAA
                                    86410     86420     86430     86440     86450     86460     86470     86480
Human                         GGTGGGGAAGCTTTAGCCTAGGCTTTTAATTCTCAATTACCATTATAAATTTATTTTAATTATTAACTAATTACTTTAA  79758
GuineaPig                     -----------CTTTAACCTACACTTTCAGTTCTCAGTTACCATT----------------------TTAATTCTTGCAA  52850
NorthernAmericanDeerMouse     CAA---------TTAATGTTTGCCTTTTAATTCTCAATTATTTTAG---ATTCATTTAAA--------TAAGTCTTTTAG  57369
Mouse                         CAA---------TTTACATTTGCCTTTTAATTCACAATTATTTTAG---GTTCATTTAAA--------TAATTCTCTT--  57359
ChineseHamsterGHOK1GS         CTA---------TTAACATTTACCTTTTAATTCTCAATTATTTTAG---GTTCATTTTAA--------TAATTCTTTTAG  61043
LongTailedChinchilla          -----------CTTTAACCTACACTTCTAGTTCTCAGTTACCATT----------------------TTAATTCTTGTAA  58549

Majority                      TTT--AATGAXXTTTTTTCXTC--TAAXGCTTTA---TCTAAAT---ATTTTACCACCAAGTTXXCTCXCXTXTAAAAAC
                                    86490     86500     86510     86520     86530     86540     86550     86560
Human                         TTCTCAATTACTATTTTTCCATGAAAAGGCTTGATGTTCTAAACAGTATTATACCACCAAGTTATCTCT-ACTGGAAAAC  79837
GuineaPig                     TTTTGAGTGATTGATTTTCCTAAATAAGACTTAATGTTCTCATTTA-GTATCGCTATCAAGTTATCTCTTACTGAAAAAC  52929
NorthernAmericanDeerMouse     TTT--AATGATATTTCATAATC--TAAAGCTTTA---TCTAAAT---ATTTTACCACTAAGTTTGTTCCCTGATAAAAAC  57439
Mouse                         -----AATGACATTTTATGACC--TAAAGCTTTA---TCTAAAT---ATTTAAC-ATCAAATTTGCTCCCTTATAAAA-C  57424
ChineseHamsterGHOK1GS         TTT--AATGACATTTCGTGATC--TAAAGCTTTA---TCTAAGT---ATTTTACTACCAAGTTTGCTCCCTTATAAAA-C  61112
LongTailedChinchilla          TT----GTGGTTGTTTTTCCTA-----GGCTTGA---TCTCAGT-G-ATACTGCCACTAAGTTATCTCT-ATTAAAACAC  58614

Majority                      CCAGATTTXAAXACXXXAAG--TATXTTTTTAAAACAATTATGCXT-AAATATTT---ATAXTTTTGAAXAAXTTT-TAA
                                    86570     86580     86590     86600     86610     86620     86630     86640
Human                         CCAGATTTCAAGAAAAAAAAAATGTATTTTGAAAACAATTATGTCTTAAATATTTAAAATTCTTTTAAACATGTTTTTAA  79917
GuineaPig                     TCAG---TGAAGGGAAAA------TATTTTAAAAACAATTGTGCCT------------GTATTTTTGAACAAGTTTTCTA  52988
NorthernAmericanDeerMouse     CCAGATTTAAAAACCTGGAG--TATTT-TTTTAAATTATTATGCTT-AAATATTT---AGAATTTTGAATAAATTT-TAA  57511
Mouse                         ACAGATGTAAAAACCTGGAG--TATTTCTTTTAAACAATTATGTTT-TAATATTT---AGTATTTTGAATAAATTC-TCT  57497
ChineseHamsterGHOK1GS         CCAGGTTTCAAAACCTGAAG--TATTT-TTTAAAAT----ATGCTT-AAATATTT---ACAGTTTTAAATAAATTT-TAA  61180
LongTailedChinchilla          CCAG---TGAAGGAAAAAA-----TATTTTAAAATCAATTGTGCCTTAAATATTTAAAGTATTTTTGAACAAGTTTGCTA  58686

Majority                      CTATCTTTAAATTTTTX-XX-XXXTAATTTT-GTTAGATTATTGAATTGTXTATX---ACGGTAGAAA--TGTATXTTAA
                                    86650     86660     86670     86680     86690     86700     86710     86720
Human                         CCATCTTTAAATTTTTTTGGTAAGTAATTTTCATTAGATTGTTAAAATGTATAGTGGCACGGTAGGGT--TGTATCTCAA  79995
GuineaPig                     CTATCTTTGAATTTTCT-GGTAAGTAGTTTTCATTAGAT-----------ATATT-------TAATTA--TATATTG---  53044
NorthernAmericanDeerMouse     TTAGTTTTAAATTTTTG------ATAATTTT-GTTAGATTATTGAATCATTTGTC---ACAGTAGAAA--TGTATCTTAA  57579
Mouse                         ACTTATTAATTTTTTTTG------ATAATTTT-GTTAGAGTATTGAATTGTT-ATC---ATGGGAAAAAAATGTATCTTAA  57566
ChineseHamsterGHOK1GS         TTAGTTTTAAATTTTTG------ATAATTTT-GTTAGATTATTGAATCATT-GTC---ATGGTAGAAA--TGTATTTTGA  61247
LongTailedChinchilla          CTACCTTTGAATTTTCT-GGCAAGTAGTTTTTGTTAGATT----AATTGTATATTAATACAATAGAAA--CGTATTT---  58756
```

```
Majority                    AATAAGAAXCAGT-TXTTXXCAAAAATAAA-----TTAAXXXGAGGXAXGTATATTTAGATAGAXXXXAAATTTAAATAG
                            86730 86740 86750 86760 86770 86780 86790 86800
Human                       GATATGAAGCAGT-TTTTCATAAAAATAAA--GTATTAACCTTAGGCTGGTATGTTTAGATCAATACTGTATTTAAATAG 80072
GuineaPig                   -ATAAGG-----------TAGAAATGTATA-TACATCAACCTGAGGCCAGTATATATACATTGATACTAAATTTAAATAA 53111
NorthernAmericanDeerMouse   AAGAAGCCTCC---CCCCCCCAAAAAAAAG-----CAAA---GGGGTAAGTATATTTAGATAGA----AAATTTAAATAG 57644
Mouse                       AAGAAGAAAAAGAGTTTTCCCAAAAGTGAA-----GTAA---GTACTAAGTATACTTAGATAGA----AATCT--TGTTA 57632
ChineseHamsterGHOK1GS       AATAGGCTTTTTT-TCCCTCCCAAAAAAAG-----TAAA---GTAGTAGCTGTATTTAGATAGA----AAATT-AAGTAG 61313
LongTailedChinchilla        -ATAAAAA-CAGT-CATTTACAAAAATAAAGTATGTTAACCTGAGGCTGGTATATTTAGATTGATACTAAGTTTAAATAG 58833
```

```
Majority                    AT----AATTTT---TATXTTTTATGAATXATTXX--TTTT--------------GAGAA-----AGXAGGAAA------
                            86810 86820 86830 86840 86850 86860 86870 86880
Human                       ATTGATAATTTTTTATATATTTTATGAATTTATACTTTTTCCTGAGATTTCAGGAGGGAAAAATGAGTAGGAGATGACTG 80152
GuineaPig                   AT----AATTTT--ATGTGTTTAATGAATTAGTAA--TTTT--------------GAGA--------------------- 53148
NorthernAmericanDeerMouse   AT----TTTGTT---TATATTTTAAGGATAATTTG--TTTT--------------GAGATA--TCAGCAGGAAA------ 57693
Mouse                       AT----AGT-TT---AATGATTTATTATTATTTT---TTTA--------------AATAA-----AACAGGAAA------ 57676
ChineseHamsterGHOK1GS       AT----ATTGTT---TATATTTTAAGAATAATTT---TTTT--------------GAGA------ACAGGAAAA------ 61357
LongTailedChinchilla        AT----AATTT----TGTGTTTAATGAATTAGTAAATTTTT--------------GAGAAGCAGTAGTAATAAATTACT- 58890
```

```
Majority                    --------------------------------------------------ATGAGTAAXA--------------------
                            86890 86900 86910 86920 86930 86940 86950 86960
Human                       AGAGCTTAAAGTTTGGGAGTGTCAATTAACTCAGCATTCTTTTAAAAAACATGTGTCATATATTACAGCATTTTCTTTTA 80232
GuineaPig                   -------------------------------------------------------------------------------- 53148
NorthernAmericanDeerMouse   --------------------------------------------------ATGAGTAAGA-------------------- 57703
Mouse                       --------------------------------------------------ACAAGTAA---------------------- 57684
ChineseHamsterGHOK1GS       --------------------------------------------------ATGTGTAAAA-------------------- 61367
LongTailedChinchilla        -------AGTTTTTGGG--------------CAGTCTACATGTATAAAGCATCACTAATGGAGTCTA------------- 58936
```

```
Majority                    ----AXGTGA-------------TTTAGA-T-----XTX-----------------------------TXTGAATA----
                            86970 86980 86990 87000 87010 87020 87030 87040
Human                       TTTGAAGTGAGTAAATGTATCTTTTTAAATTCCTTAGTAATTTTTGAGCACTCCATATGTATAAAGCATGTGAATATTTG 80312
GuineaPig                   --------------------------------------------------------------------CGTGAACA---G 53157
NorthernAmericanDeerMouse   ----A-GTGA-------------TTTAGA----------------------------------------CTGAGTA---- 57721
Mouse                       ------GTGA-------------TTAGGACT-----GTC-----------------------------ACTAGTCT---- 57707
ChineseHamsterGHOK1GS       ----AAGGGA-------------ATTAGAAC------TG-----------------------------TCTGAGTA---- 61391
LongTailedChinchilla        --CAAAGTCAG-------ATATCTTCAGTGTTTACAGTATCTTGC-----------------------TGTGAATG---A 58981
```

```
Majority                    GTA--ATTTTAAX-ACATX-----------------CCCTGATA-TCCTTTTXAGATGTC--------------------
                                     87050     87060     87070     87080     87090     87100     87110     87120
Human                       GTAGCATTTTACAAATGTCCAGAGATTTGTGAGAGTTCCTGAGA-TCTTCATAGGGGGCCCACAAAGTTTAGTATTACTT  80391
GuineaPig                   GTA--GTATTAAA-------------------------------------------------------------------  53168
NorthernAmericanDeerMouse   GTA--ATTTTAAC-ACATA-----------------CCCTGATA-TCCTTTTCAGGTGTC--------------------  57760
Mouse                       GTA--ATTTTAACTACATA-----------------CCTTGATACTCCTTTTTAAATGTC--------------------  57748
ChineseHamsterGHOK1GS       GTA--ATTTTATC-CCAC------------------CCTGGTTT-TACTTTTCAGATGTC--------------------  61429
LongTailedChinchilla        GTAT-ATTTTACAGCTCTTTG----------------CCCAGGG-CATTTTTAGGACAC---------------------  59022

Majority                    -----------ACTACAGXA---------TTTTCTTXCTTT---------------------------GAAAXXAAXT--
                                     87130     87140     87150     87160     87170     87180     87190     87200
Human                       TTCACGGTAATACTAAAGTGTATTTTGCCTCTTTTTACTTTTTCTCTTAATAGCATACAGTGGTAACTGAAGGCTAATAG  80471
GuineaPig                   ------------------------------TTTCTTACT-----------------------------------------  53177
NorthernAmericanDeerMouse   -----------ACTACAGCA---------TGTCTTCTCTTT---------------------------GCAAGGAGTTG-  57792
Mouse                       -----------ATTACAGCA-----------TTCTTTCTTT---------------------------GAAATGAATT--  57777
ChineseHamsterGHOK1GS       -----------ACCACAGAAC--------TTTCCTTTCTTT---------------------------GAAAAAAAAA--  61461
LongTailedChinchilla        --------------------------ATCCTTTCTTATTTTCCC------------------------------------  59040

Majority                    -ATGTCTXTTAA-------------ATT-----TCTTAGT-----------------------------AACXTTTTXTX
                                     87210     87220     87230     87240     87250     87260     87270     87280
Human                       TATGTGTGTTTATGTGCTTTAAAAAGTTCGTGGTTTTGGCCAGGCGCAGTGGCTCAGACCTGTAATCCCAGCACTTTGGG  80551
GuineaPig                   -ATTTTTTTT-----------------------------------------------------------AACAATCCATA  53197
NorthernAmericanDeerMouse   TTTGTCTTTTAA-------------ATT-----TCTTAGT-----------------------------A---TTTTCT-  57821
Mouse                       ---GTCTGTTAA-------------ATA-----CCTTAGT-----------------------------A---CTTTTT-  57803
ChineseHamsterGHOK1GS       --TGTCTTTTAA-------------ATT-----CCTTAGT-----------------------------AAAGTGTACTG  61492
LongTailedChinchilla        -ATTTTTGTTTA-------------ATTTTTATTTTTATTTTGGAA-----------------------AACGTTTAAAA  59083

Majority                    TATATAAAGTXTX--------------------------------------ACTGTCTA------TAAAAT------XTT
                                     87290     87300     87310     87320     87330     87340     87350     87360
Human                       AGGCTGAGGCAGGTGGATCACCTGAGGTCAGGAGTTCAAGACCAAAACCAGCCTGGCCAACATGGTGAAACCCCATCTCT  80631
GuineaPig                   TATATAAAGAAAG-------------------------------------------------------------------  53210
NorthernAmericanDeerMouse   TATGTAAAGTGT---------------------------------------ACTGTTTA------CAAAAT------GTT  57850
Mouse                       AATATTAAGGGT---------------------------------------ACTATTTA------TAAAAT------ATT  57832
ChineseHamsterGHOK1GS       TTTATAAAATGTT--------------------------------------AGGGTCTA------CAAAATCAAA--ATT  61526
LongTailedChinchilla        TAAACAAAGTAGAAGA--------------------------------------GACTAGAGTATTGAACTTCC--CTGT  59123
```

```
Majority                    ACTA------------TTAXXCAGX--TXGTAGGGAATXCCTTAATGXC----TGATCGXGTXC-TC-------------
                                    87370     87380     87390     87400     87410     87420     87430     87440
Human                       ACTAAAAATACAAAAATTAGCCAGGCATGGTAGTGGGTGCCTGTAGTCTCAGCTACTCGAGATGCTGAGGCAGGAGAATC  80711
GuineaPig                   --CA------------TCAGTCAAG-------------------------------------------------------  53221
NorthernAmericanDeerMouse   CCTG--------------A---AGA--TTGTAGGCCATCTCCTAATGCC----TGTTGGTGTCC----------------  57891
Mouse                       ACCA--------------A---AGA--TTGTAGGGAATCTCTTAATGTC----TGATAGGGAGC-TC-------------  57875
ChineseHamsterGHOK1GS       TCTGTCA---------TTAACAATA--AAATAGGTTTTGCCTTTTTGACT--TTGATTTTCTTTACCT----------TT  61583
LongTailedChinchilla        ACTA------------TTAATCAGGTGTAACAAGGAACACCCAATGGACAATCTCATCCACTCACTCA-----------T  59180

Majority                    --XX---X--------------X---XX--A--CAAAGTCTXGX--X--------------------------XAAATTX
                                    87450     87460     87470     87480     87490     87500     87510     87520
Human                       ACTTGAAGCTGGGAGGTGGAGGTTGCAGTGAGCCAAAATCTCGCCATTACACTCCAGCCTGGGGGACAAGAGCAAGACTC  80791
GuineaPig                   ----------------------------------AAGTCTTGT------------------------------GAAGTCA  53237
NorthernAmericanDeerMouse   ------------------------------A--CAAAATCC---------------------------------AAATTT  57906
Mouse                       ---------------------------------CACAGATC---------------------------------AAAATA  57889
ChineseHamsterGHOK1GS       TTTTGGGGGGGGT-------GGTTGGAG--A--CAGGGTTTCTCTGTG-------------------------TAGCTTT  61627
LongTailedChinchilla        TTTTAAAGT----------AAATCACAG-GAACAAGGGTCTTGTCATTTTAAAACAG----------------GAAATCC  59233

Majority                    XATXTC--------------------T--TGXCATTAATTCTX-AAA---XX-------------X--------------
                                    87530     87540     87550     87560     87570     87580     87590     87600
Human                       CATCTCAAAAAAAAAAAAAAAAAAGTTATTGGTTTTAATTTTGGAAATGACAACCATTAATAGAACCCACATTAGGCCGG  80871
GuineaPig                   AATATT--------------------T-----------------------------------------------------  53244
NorthernAmericanDeerMouse   ----C------------------------TGACATTAATACT--AAA---------------------------------  57923
Mouse                       ----T-------------------------GACATTAATACT--AAA---------------------------------  57905
ChineseHamsterGHOK1GS       GGAGCCTA------------------TCCTGGCACTTGCTCTGGAGAC--CAGGCTGGCCTCAAACTCAC----------  61677
LongTailedChinchilla        CATGTC--------------------TGCTGTTTATTACTACG-------CAGTGGTCTTCAGTGCCTTT----------  59276

Majority                    ATAGGTT--T-TGTCTTTXXAXT---AXTACT----------------------TX---------------XAAATGCAC
                                    87610     87620     87630     87640     87650     87660     87670     87680
Human                       GCGCGGTGGCTCATGCCTGTAATCCTAGCACTTTGGGAGGCCGAGACGGGTGGATCACGAGGTCAGGAGATCAAGACCAT  80951
GuineaPig                   -------------TCTTCACACT---AATACT---------------------------------------GAAATGCAC  53269
NorthernAmericanDeerMouse   ATAGGCT--T-TGCCTTT------------------------------------TT----------------------GC  57942
Mouse                       ATAGGTT--T-TGAGTTT------------------------------------TG----------------------AT  57924
ChineseHamsterGHOK1GS       AGAGATCCACCTGCCTCTGCCTCCCGAGTGCTG------------------GGATTA------------AAGGCATGCGC  61727
LongTailedChinchilla        GAGTGTTATTGTGTCTTTATAAT---AATACT---------------------------------------CAAATGTAC  59314
```

```
Majority                     XTTGGATX-----------XCXXTTTTCTAXTTXGTATXXA-------------------TAGXGGXT--TTGTTTTCTX
                                      87690     87700     87710     87720     87730     87740     87750     87760
Human                        CTTGGCTAACACGGTGAAACCCCGTTTCTACTAAAAATACAAAAAATTAGCCGGGCGTGTTGGCGGGCGCCTGTAGTCCC  81031
GuineaPig                    TTTGCCTT-----------TCCTACTGTTATGTGGCATATA---------------------GCGGA-----GTTTTCTT  53312
NorthernAmericanDeerMouse    CTTTGAT----------------TTTTCTTACTAGTATGTT-------------------TAGGGGTT--TTGTTTTCTA  57985
Mouse                        TCTTGAT----------------TTTTCTCATTGGTATGCA-------------------TAGGAGCT--TTATTCTGTG  57967
ChineseHamsterGHOK1GS        CACCAATGCCTGG------CTGATTTTCTTCTTGATATGTT-------------------TAGTGGTT--TTGTTTTCTA  61780
LongTailedChinchilla         TTCGCTTT-----------TCTTACTGTTATTTAGCATACA---------------------GTGGA-----GTTTTCTT  59357

Majority                     CGTXACTCTXAAGGCT-----AXTAGAXTG-CATGATTGTXTG---------TTCTTXTGXTTT----TXAXTTTATTXX
                                      87770     87780     87790     87800     87810     87820     87830     87840
Human                        AGCTACTTGGGAGGCTGAGGCAGGAGAATGGCATGAACCTGGGAGGCGGAGCTTGCAGTGAGCCGAGATCACGCCACTGC  81111
GuineaPig                    TGTTATTCAGAAAACT-----AGTAGAATG-TGTGATTGTGTG---------TTCTTGTG-----------CTCTATAAA  53366
NorthernAmericanDeerMouse    CATCACCCTTAAGTCT-----AATAGAG-G-CATAATTGTATG---------TTCTCTTATTTT----TAATTTTATT--  58043
Mouse                        CATCAGGCTTAAGGCT-----AATAGAGTA-CATGATTATATG---------TTCTCTTGGTTT----TAATTTGGTT--  58026
ChineseHamsterGHOK1GS        CGTCACCCTTAAGGCT-----AATAGAGTG-CATAACTGTATG---------TTTTTTTGTTTT----TGTTTTTGTTTT  61841
LongTailedChinchilla         TGTTACTCTGAAAGCT-----AGTAGAATG-TGTGATTGTGTG---------TTCTTGTG-----------CTTTACAGA  59411

Majority                     AXTTXXXXTT-------------XGXAXXTGTCAGXAXAAATATAAXTCAGXXAAA-X---X--X---X--XAAXAAXAG
                                      87850     87860     87870     87880     87890     87900     87910     87920
Human                        ACTCCAACCTGAGAGACACAGCGAGACTCCGTCTCGAAAAAAAAAAAAAAAAAAAAAAGAACCCACATTAACAAAAACTC  81191
GuineaPig                    AGTTTGTTTT-----------------TCTATAATGACAAATATCAGTACCTAGAA---------------CCAGAAGCC  53414
NorthernAmericanDeerMouse    ---------------------ATAGCAAATGTCAGT--ACATATAATTCAGGTAAATA---CTTATA-T--AAACAAAAG  58094
Mouse                        ------------------------GTAAAAGTCAGT--AGGTATAACTCAGATAAA----------------------AG  58058
ChineseHamsterGHOK1GS        TTTTTTACTTTTAGTTATATAATAGCAAATGTCAGTAGATATATAATTCAGGTAAATA---CTTAAGGT--AAACTAAAG  61916
LongTailedChinchilla         AGTTCATTTT-----------------TCTGTCATGTAAAATATCAGTGGATAGAA---------------TGAGAAGCT  59459

Majority                     CTTGTXXTXCTCAGTAATTTTAXAAGTGTAAAXGGG-GXCATGAXACCAAAAGGTTTCAXAAXTTCTTTAGXGTXGGXTA
                                      87930     87940     87950     87960     87970     87980     87990     88000
Human                        CTTGTGATTCTCCATAGTTTTAAGACTATAAAGGGG-AGC-TGAGACCAAAAAGTTTGAGAACTTTTTCACTGTGGGATA  81269
GuineaPig                    CTTTTGGTTTTTAGTG-----AACAATATAAAAGTG-GTCCTGGTACCAAAACTTTTCAGAGCTTCTTTATTGTGGGATA  53488
NorthernAmericanDeerMouse    CTTGTTTTCCTCTATAATTTTT-AAGTGTAAGGTGG-GACATGAAACCAAAAGGTTTCAAAATTCCTTTAGCTTAGG-TC  58171
Mouse                        CTTGTTCTCCTCAGTCATTTT--AAGTGTAAAAGGGAGACATGAGAGCAAAAGGTTTCAATTTTGTTTTAGCCTAGT-TA  58135
ChineseHamsterGHOK1GS        CTTGTTTTCCTCAGTAATTTTT-AAGTGTAAAGGGG-GGCATGAAGCCAAAAGGTTTCAAAATTTCTTTAGCTTAGG-TA  61993
LongTailedChinchilla         CTTCTGGTTCTTAGTA-----AAGAGTGTAAAAGTG-ATCCTGATACCAAAACGTTTCAGAACTTCTTTATTGTGGGATA  59533
```

```
Majority                     CA-AAGTTCAAGAXAAXTCTAXT-XCATAAACAXGAG--------XATXTTAATAAA--------G-X--X-AGAGTXAA
                                             88010      88020      88030      88040      88050      88060      88070      88080
Human                        CA-AAGTTGAAGACAATTATAGTTAAATAAGCAGGAGATACAGAGTATTTTAATGAACAGTAGATGGTTATAAGAGTCAA  81348
GuineaPig                    CACAGGTTCAAGACAATTCTCAT-ACATGAAAATGAG--------TAATTTAACAGA----------TGATACGAGTCAA  53549
NorthernAmericanDeerMouse    CA-AAGCTCAAGAGAACTGCAGT--ATTTAACAGAAG---------ATGCTGATTAA--------G------AGACTGGA  58225
Mouse                        CA-AAGCTCAAG-GAGCTCGGAT--CCTAAACAAGAG---------ATGTTAAGAGT--------T------AGAG-GTA  58187
ChineseHamsterGHOK1GS        TG-TAGTTCAAGAGAACTCTAGT---TTGGAAACAGG---------AGGATGCTACA--------G------AGA--AAA  62044
LongTailedChinchilla         TA-AATTTCAAGACAATTCTCAT-ACATAAACATGAAA------ATAATTTAACAAACAGT------TGCTCAGAGTTAA  59599

Majority                     GAGATAXACAGAGAAAATGCTXGTGATTCGTG-----AGATTGTTTTAXTTTT-GTXTTXAGTTAT----XXTTXTTCAG
                                             88090      88100      88110      88120      88130      88140      88150      88160
Human                        GAGATGTACAGAGAAAATGCTAGTGATTAGAGTTTTTAGATTATTTTAATTTTGGTAACTAATTAT----AATTCCTCAG  81424
GuineaPig                    GAGATACACAGAGAAAACGCTAGTGATTTGAA-----AGATTGTTTAATTTT--GTACTTAGCTAT----AATTCTTCAG  53618
NorthernAmericanDeerMouse    TATTTAGAGAAAATAGTGTTTGGGGATTCTTG-----AGGTTGTTTTACTTTT-GTGTTAAGTTAT----GGTTTTTCAG  58295
Mouse                        CAGAGAAAAGACAAGAGATCTGAAAATTCCTG-----AGATTGTTTTATTTTT-GTGTTAAGTTATGGCTGGGTTTTCAG  58261
ChineseHamsterGHOK1GS        TACTAGGGG---------CTTGGGGACTCTTG-----AGGTTATTTTACTTTT-GTGTTAAGTTATAGTTGGCTTTTCAG  62109
LongTailedChinchilla         GAGGTACACAGAGAAAATGCTAGTGATTTGCA-----AGATTGTTTTAATTT--GTACTTAGCTAT----AATTCTTCAG  59668

Majority                     TXAAAGTTTTTTGGGTTAATG-XCTTTGAGAXXCXXTTCTTACCTGTTAGAATTGAAAGT--AXXCCA----TAAATGAA
                                             88170      88180      88190      88200      88210      88220      88230      88240
Human                        TTAAGATTTTTTGGCTTAGTGGGCCCTTAGAATTACTTCTTC-CTGTTAGAATTAAAAGT-GAAACCA----GAAATTAA  81498
GuineaPig                    TTA-----CCAT----------ACTATCAGAATCATTTCTCAACTGTTACAATGGAAAAATAAAACCATTAAAAAGTGAA  53683
NorthernAmericanDeerMouse    GAAAAGTTTTCTGGGTTAATG-GCTTTGAGTGCCTTTTCTTGCCTGTTAGAATTGAAAGT--AGCCCA----TAAATGAA  58368
Mouse                        TAAAAGTTTTTTGAGTTACTG-GCCTTGAGAGCATCTCCTTACCTGTTAAAATTGAAAAT--AGCCCA----TAAATGAA  58334
ChineseHamsterGHOK1GS        GAAAAGTTTTCTGGGTTAATG-ACGTTGAGAGCGTTTCCTTGCCTCTTAGAATTAAAAGT--AGCCC-----TAAATGAA  62181
LongTailedChinchilla         TTA-----TTTTG--TTATTGGACTGTCAGAATCACTTCTTAACTGTGAGAATTGAAATA-AAAACCA----AAAATGAA  59736

Majority                     ATCGATTTXAT-TTXTTATTGTGGXAAAGAGCTTATC-AAATATCTGTTT--GA-TTTTTGAAX-------TCTTTATAT
                                             88250      88260      88270      88280      88290      88300      88310      88320
Human                        AGTGATTTAATTTTTTTATTGTGGCAAAGATCTAGTCCAAATATCTTTGATGGACTTTTTGAAAACAGACTTTATTGT-T  81577
GuineaPig                    ATCAATTCAGTCTTTTTACTATGGGAAAATGCCAAAT-ACCTATTTT-----GCTTTTTTAAGTTTTTTTTTTTTTATAT  53757
NorthernAmericanDeerMouse    GTCGACTTCAT-TTGTTATTGTGGGAAAGAACTTATC-AAATAATTGTTT--GA-ATTTTGAAG-------TCTTTATAT  58436
Mouse                        GTTGATTTCAT-TTGTTGTAGGAA--AAGAGCTTACC-AAATACCTGTTT---------TGAAG-------TCGTTACGT  58394
ChineseHamsterGHOK1GS        ------TCCAT-TTGTTATTGTGG---AGAACTTATC-GAATAACTGTTTCTGA-TCTTTGAAA-------TCTTTACAT  62242
LongTailedChinchilla         ATCAGTTTAATCTTTTTGCTGTGGAAAAGTGCTGATC-AAATAT--------------------------------G---  59780
```

Majority	AAAAXXXGXATXTGAGGCTTAACAXXGAACTXTTTTTTCXTATXXC----X--GTATTGXAGTTGGTGGTTTXXTTTTTG	
	88330 88340 88350 88360 88370 88380 88390 88400	
Human	AAAAAGGGAATTTGAGGCTTGGTACTGAACTTCTTCTGCATGCCA---------TATTGAAGTTGGTGGTTT----TTTG	81644
GuineaPig	AAAATGGATTTTTGAGGCTTTGAACTGAAGTCTTTTT-CACATCAT-------GTATTGTAGTTGGTGTTTTGTTTTTTG	53829
NorthernAmericanDeerMouse	AAAA-----ATGTGAGGCTTAACATGGAACTTTTTTTTTCTATTGCACCCAGTTTATTGCAGTTGGTGGCTTTGTTTTTG	58511
Mouse	AAAAG-AGAACGTGAGGCTTAACATGGAACTTTTTTTTGCTGTTGCTTTTAGAGTATTAAGTAAGGTTGGTGGCTTTGTT	58473
ChineseHamsterGHOK1GS	AAAA-----ATGTGAGACTTAACATGGAACACTTGTTTTCTATTGCACTCACTGTATTGCATTTGGTGGCTTTGTTTTTG	62317
LongTailedChinchilla	AAAATGAGCTTTTAAGGCTTCAAACTGAAGTCTCTTTGCATATCAT-------GTGTTGTAGTTCGTGTTTT-TGTTTGA	59852

Majority	TTTGTTTX-XTTTGCCA--XTXAATTCTGXXTCTCATAAAGACCATGGAATTTAACAGXATTTT-AAGTTACTTTTTX-T	
	88410 88420 88430 88440 88450 88460 88470 88480	
Human	TTT--G----TTTGCCA---TCAGT--GGGATCTCATACAGACTATGGAATTTAATAGAATTTTTAAGATACTGTTTT-T	81712
GuineaPig	TTTGGTT---TTTGCTA---TCAGCTCTGGATCTTCTAACAAGTATGAAGTTTAACAGAATTA--AAGTTACTTTTTTGT	53901
NorthernAmericanDeerMouse	TTTGTTTAATTTTGCCAGGATTAATTCCGAGTCTCATAAAGACCATGGAATTTAACAGCATTTT-AAATTAGTTTTCC-T	58589
Mouse	TTTGTTTAGTTTTCCCAATATTAATTCTGAGTCTCATAAAGACCATAGAATTTAGCAACAT-----A-TTACTTTTTC-T	58546
ChineseHamsterGHOK1GS	TTTGTTTAATTTTGCCAGGATTAATTCTGAGTCTCATAAAAACCATGGAATTTAACAGCATTTT-AAATTAGTTTTTC-T	62395
LongTailedChinchilla	TTT--TT---TTTACCA---TCAACTGTGGATCTTCTCAAGACCATGGAGTTTAACGGAATTTT-AAGTTACTTTTTTCT	59923

Majority	TCXAAGAGGTAGCCAATTAAAGXCACXXAAACAAA-AAATGATTTGACAATATAATTACTACATCTGGXATTTCCACTAX	
	88490 88500 88510 88520 88530 88540 88550 88560	
Human	CCTAAAAGGTAGCTAACTAAAGGCAGGAAAATAGA---ATGATGTAACAATATAATGACTACATCTGGAGTTTCTGCTAC	81789
GuineaPig	GCTAAGAGATAACCAG--AAAGGCAGGGCAAGAAA---------------TTATACTGACTACATCTGAAAATTCCA---C	53962
NorthernAmericanDeerMouse	TCCAAGAGGTAGCCAATTAAAGCCACTAAAACAAAGAAATGGTTTGACAATATAATTACTACATCTGGTATTTCCACTAT	58669
Mouse	TCCAAGAGGTAGCCAATTAAAGCTACTAAAACAAAGAAATGATTTGACAATATAATTACTACATCTGGTATTTCCACTAT	58626
ChineseHamsterGHOK1GS	TCCAAGAGGTAGCCAATTTAAGCCACTGAAACAAA-AAATGATTTGACAATATAATTACTACATCTGGTATTTCCACTAT	62474
LongTailedChinchilla	GCTGAGAGATAGCCAGTTAAAGGGAAGGCAGGAAACAAGTTATTTGGTATTTTACCAGTTAAATCTGAAACTTCCAGTAC	60003

Majority	TXTTAAATAATTGTATTGTCAACTTAAGTTGAATGTTXTGTXCATTCAGCCAAA-CTGCCTGAAAATGCTGXG-X-XXXX	
	88570 88580 88590 88600 88610 88620 88630 88640	
Human	TAATAAATAATTGTATTGTCAACTTTAGTCAAATATTCTGTGCATTCAGTCAGAGCTATCTGAAAATACTGAC-------	81862
GuineaPig	AGTTAAATAATTGTCTTGTAAACTTAAGTTGAATCTTATAT--ATTCATCTAAAGCTGTCTGAAAATGCTGAA-------	54033
NorthernAmericanDeerMouse	TATTAAATAATTGTATTGTCAACCTAAGTTGAATGCTTTGTACATTCAGCCAAA-CTGCCTGAAGATGCTGGGTGGTTTC	58748
Mouse	TATTAAATAATTGTATTGTCA---------------------------GCCAAA-CCGCCTAAAAATGCTGGGTGTTTTC	58678
ChineseHamsterGHOK1GS	TGTTAAATAATTGTGTTGTCAACCTAAGTTGAGTGCTTAGTACATTCAGCCAAA-CTGCCTGAAAATGCTGGGCGTTTTC	62553
LongTailedChinchilla	TGTTAAATAATTGTGTTGTCAACTTAAGTTGAATGTTACATGCATTCATCTAAAACTG--TAAAATTGCTGAA-------	60074

```
Majority                    XXXGTXTAG------XXXXXTTTXXAAXCATXGAGAGGTCACTTXCCAAGA--GAATGTAAGAAAAAAATTAC---TGAG
                            88650 88660 88670 88680 88690 88700 88710 88720
Human                       ---GAATAG-----------TATTACAATATGGGGAGGTCACTTCTCAAGACTGCAAGAAAAAAAATTGTTGTGTGTAGG 81928
GuineaPig                   ---GTATGG-----------TGTTAA----GTGTAAGGTTACTTTGCAAGA--GAATGCAGAAG---------------- 54077
NorthernAmericanDeerMouse   CTTGTGTTGTTGTTACTTTGTTTCTAATCATTCAGAGGTCACTTACCAAGA--GGATGTAAGAAAAAAATTAC---CGAG 58823
Mouse                       CTTGTGTAG------CTTTGTTTGTAAGCATGGAGAGGTCACTTACCAAGA--GAATATAAGAGAAAAAATAC---TGAG 58747
ChineseHamsterGHOK1GS       CTTGTGTTGTTGTTACTTTGTTTCTAATCATGGAGAGGTCA-------AGA--CAATGTAAGGAAAAAATTAC---TGAG 62621
LongTailedChinchilla        ---GCATAG-----------TGTAAAAAAAGTGTCAGGTTATTTTCCAAGA--GAATGCAAGAAAAAAACT--------- 60129

Majority                    TGTGAXTGTCTXXAXXTTAXAAXTGTATATXTX---TCCATTGACAAAAAGTAAAATTATXCXTTGAATGTTAAATGGAA
                            88730 88740 88750 88760 88770 88780 88790 88800
Human                       AATGTTTGCCTAGGAATTAGGAATGTATACTTG----ACAACAAGTGAACG------TATCCATTGAATGTTAAATGAGA 81998
GuineaPig                   ------------GGAATTAGAAATGTATCTTTACTTTGCATGCAAAAAAAATGAAAATATCCATCAAATGGTAAATGGAA 54145
NorthernAmericanDeerMouse   TATGAATGTCTGTAGTTTATAAGTGTATATACG---TCCCTTGACAAAAAATAAAATTACACTTTGAACATTAAATGGAA 58900
Mouse                       TGTGAGTGTC--TAGTTTATAAGTGTACATATG---TCCTCTGACAAAAAGTAAAGTTATTCTTAAAATGTTAAATGGAA 58822
ChineseHamsterGHOK1GS       TGTAAGCATCTGTAGTTTATAAGTGTATATACA---TCCCTTGACAAAAAGTAAAATTACACTTTGAATGTTAAAAGGAA 62698
LongTailedChinchilla        AGTGCTTTCCTAGAAATTAGAAATGTATCTTTA-TTTGCATGCAGAAAAA-------CAT--ATTGAATGTTCAATAGAA 60199

Majority                    AXAACCTXTGA--XGCXTXATGAGAAGTATAGTATTTAATATGGAAA----AGTXAGXXXTCAAGXAGAATACTTGGTXG
                            88810 88820 88830 88840 88850 88860 88870 88880
Human                       ACAACTTATGGGGAGGATAGTGAGAGGTATAGTATTTAACATGGAAA----AGTTAGTAATATAGTAGAATATTTGGTTG 82074
GuineaPig                   GCAATCTAGGA--AGCATAATGAGAACTATAGTATTTAATATGGAAA----AGTTAGTAATGCAATTTAATAGTTGGTT- 54218
NorthernAmericanDeerMouse   ATAACCTGTGA--GATGTGATGAGAAGTATTATATTTAATA----------AGTCAG-GGCCAAGGAGACAGCTTAGTGG 58967
Mouse                       ATAACGTGTGA--GGTGTGGTGAGAAGTACTGTATTTAATATGAAGAAGTAAGTCGG-GGTCAAGGAGATGTCCTGTTGG 58899
ChineseHamsterGHOK1GS       ATAACCTGTGA--GACGTGATGAGAA-----ATATTTAATA----------AGTCAG-GGCCAAGGAGACCGCTTAGTGG 62760
LongTailedChinchilla        GCAATCTATGA--AGCATAATTAAAACTATAGCATTTAACATGGAAA----AGTTAGTAATGCGGTTGAATAGTTGGTT- 60272

Majority                    GXAAXXTCC---CCATXX-GTCAAXCXXAAGXAAC-----ACTGAXXACCAGXATGCATXTXAXXAAGCXTGXAACXXGA
                            88890 88900 88910 88920 88930 88940 88950 88960
Human                       AAAAGATCC---ACATTG-GTGTATTCAAAGACAC-----ACTGAAGACTATTGTG--CATTAGTAAGTATTAAACATGA 82143
GuineaPig                   GAAAGACCC---TCATTG-GTCAATT--ACAAGAC-----ACTGAAGACTACTATGCATATTAATAAACAATTGACATGA 54287
NorthernAmericanDeerMouse   GTAAAGTGC---CTGTCA-GTCAAGCCCAAGGAAT--------GATCCCCAGAATGCAGGTGAAAAAGCCTGCAGTGGCA 59035
Mouse                       GTAAAGACAGTACTGTCATGCCAGGCATAAGGACCTAAGCGCCGATCCCCAGAACCAGGGTGAGAAAGCCTGGAAGGGCT 58979
ChineseHamsterGHOK1GS       GTAAAGTGC---CCATCATGTCAAGCACAAGGAACTAGGTTCAGATCTCCAGAACCCATGTGAGAAAGTCTGGAGTGGTA 62837
LongTailedChinchilla        AAAAGATCC---GCACTG-GTCGATGTAAAAAGGT-----ACTGAAGACCACTGTG--TAGTAATAAACAGCAGACATGG 60341
```

```
Majority                   GXATTCC--TAATTXTAXAXXTA-XXGATCCTXGTTXXTGXGATXTXCTXTXC-AGTCAGTCTGG-XX---XAATGAGAT
                                          88970     88980     88990     89000     89010     89020     89030     89040
Human                      GAATTCT--TAATTTTAAAATTG---GACCCTAATTTCTGAGATTTACTTTAAGAATAAAAGGGATG---AAACGAGAT  82215
GuineaPig                  GAGTTTC--TA-TTTTAAAATGAGAAGATCCTTATTCTTGTGATTTACTTTAA-AATGAGTGCGT------GAATGAGAT  54357
NorthernAmericanDeerMouse  GCACTCCTGTAAACCTAGAGCTA-GGGGTCAGGGGTTGTGGAAGCTTATCTGCCACCCAGCCTAGCTGAGTCAATTAGCT  59114
Mouse                      GCACCATCCTAACTCTCGTGCC--------------CG------CCTCGCTG--AGTCAGCCTGG------------GGT  59025
ChineseHamsterGHOK1GS      CCAATCCTATAACCCTAGTGCTA-GGGGTT-GAGGTTATGGGAGCTTATCTGCAAGCCAGTCTAGATGTGTCAGTGAACT  62915
LongTailedChinchilla       GATTTCC--TA-TTTTAAAATGGCAAGATCCTTGTTCCTGTGATTTACTTCAC-AGTGAGT----------GGTTGAGAT  60407

Majority                   TXAGXXTXAGAXXXX---TXXTAAACTTXAATCAT--AGCXAXAGAGXAGTXCAXTAXTTTGGXXXCXTTXACXXTTGXA
                                          89050     89060     89070     89080     89090     89100     89110     89120
Human                      TGGCCATGAGTTGATCATTGTTAAAGTTGGGTGAT--GGCTACTTAGAGTTGCATTATTTTAGTCACTTTTACTTTTGTA  82293
GuineaPig                  TGTCC-TTAAGTGTT---TGTTATACCTGATTAAT--AG-TATATTAAAGTTAATTATTCAAGTCTCTTTTACTTTTTT-  54429
NorthernAmericanDeerMouse  TCAGGTTCAGAGAGA---TACTCA-CTAAAAACATCAAGCCAAAGAGCAATGCAAAACATTGG--GCCTACACACATGCG  59188
Mouse                      TCAAG---AGAGACAG--TACCCAACTCAGAGCACAAAGGCAAAGGGCAGTATGTGAT-T------CCTCCACACGTGCA  59093
ChineseHamsterGHOK1GS      TCAGGTTCAGAGAGA---CACTTAACTTAAAACTT--AACCAGAGAGCAGCACAAAACATTGG--GTCTACACACGTGCA  62988
LongTailedChinchilla       TGTGCATTAGGTGTTCCTTGTTACACCTGATTAAT--GG-TACAGGGAAGTTCAATACTTAGGTCTCTTTTACTTTTAT-  60483

Majority                   CATTT-X-X-ATXTTXTAAAXT---AAA------CATAAXAXAAACAAXGAAAAATAAGAXAGAATXGX-----XAACAA
                                          89130     89140     89150     89160     89170     89180     89190     89200
Human                      TATGTTGGAAATCTTCCAAAACAAAATGTATAGCCATAAAAAAAAGGGAGCAAAGTAAGAAAAAAAGGAGAGCCTAAAAA  82373
GuineaPig                  --TTTTGAAGGTTTTCTAAAATTTAAAA-------AAAAAAAAAATCTAGAAAAATAAGAAAGAATGTG-----TGCCAA  54495
NorthernAmericanDeerMouse  CACTT-----ACATTATCAGGT---AAA------CATACCACATACAAGAATGAGTAAAATGGATTAGT------AGCTG  59248
Mouse                      CAATT-----ACCTTATCAAGC---ATA------CATACCACATACAAGAAGAAATAAAATGGGCTAAT------AACTA  59153
ChineseHamsterGHOK1GS      CACTT------TATTATTGAGT---A--------CATTACATAGTCCAATACACATTCCATATACAAG-------AATGA  63044
LongTailedChinchilla       --TCT-GAAGGTTTTCCAAAAT---AAA-------ATTAAATAAAAATGGGAAAATAAGAAAGGATGTG-----TCCTAA  60545

Majority                   AAAXXAXATXGGCAAAGXGTTAT-XAAXTAXAXTXXXTGATAXXAAGAGTXTTATXATTXXAXTCXTCATACXTTTGATT
                                          89210     89220     89230     89240     89250     89260     89270     89280
Human                      AAAAGGTACAGGCAAAAAGGTGTGAAAAGAAACTCATCAAGAGCAAGAGTATTAA-ATGAGAAACA-TATGCCACTGACA  82451
GuineaPig                  ACATGCCATGGGCAAAGATCTAT-AAAGTATACACATTCAGAGCAAGAATCTCA------GTATTAACATTCCACTGATG  54568
NorthernAmericanDeerMouse  ATAAAACATTGTGTCAGGATTAC-TAAACATGTGTGATGGTACTTGGTGTTATGTTATTCTAGTCTTCTTATTTTTCTTT  59327
Mouse                      AAATAATATTGGTTCATGATTGT-TCAGAAGGTATGATGATACTACTGGTTTTATTATTTTAGCCTTTGTAATTTTATTT  59232
ChineseHamsterGHOK1GS      ATAAAAT---------GGGTTAC-TAAATACATTTGATGGTACTTGGTGGATTGTTATTCTAGTCTTCTTATTTTTCATT  63114
LongTailedChinchilla       AAATGCCATAGACAAAGAGCTAT-AAAGTGCACTCATTCAAAGCAAGAATGTTAA-GTAAGAAACAAAACACCGGTGCTG  60623
```

```
Majority                     XAGTTTGAACX-XXAGAXACAAAGGCTXAATTXCAXGGAXAGTGGXCAXAAAGTTXTAXAAXXCAXAXAXTTAG--XAAG
                                       89290     89300     89310     89320     89330     89340     89350     89360
Human                        GAGTTCTCA---GAAGAGATAAAGGCTGAATTAAAAAGAGATGGAGGGGAAAGTTTTATAAGTCAAAAACTTAGGTTAAG 82528
GuineaPig                    GATTTTGAGA--GAAAGGATTAAGGCTGAATTAAGTGGAGACTGGAGAGAAAATTTTAT--GTCATAAGTTT----TAAG 54640
NorthernAmericanDeerMouse    AAGCTGGAACATTCTGAAACAAAAACTAACAACCGAGGAAAGTTGGCAAAAAGCTGTAAAATACACCCACCCAG---AAC 59404
Mouse                        AAGTTAGAACATTCAGAAACAAATG-TAATCCCCAAGGAAAGTGGACAAAAAACTGCAAAATACACTCACCCAG---AAC 59308
ChineseHamsterGHOK1GS        AAGCCTGAACACTCTGAAACAAAAAATAACAACTATGTAAAGTAGGCAAAAGG-TGCAAAATACAGCCATGGAG---AAT 63190
LongTailedChinchilla         GATTCTGAG---GAAAGGGCTAAGGCTGAATTACATGGAGATTGGATGGAAATTTTTAT--GTCAGAAATTT----TAAG 60694

Majority                     AATTTTXXAXATTAXAA---TCTTCAGXCTXCX-----XTGAXAGGXXAXXCAXAGAAXXXXXXGTAAAGCT-AATGA--
                                       89370     89380     89390     89400     89410     89420     89430     89440
Human                        GTTTGTGAATATTAAAGAGCGCTTCAGATTGCCA----ATGAAAGGTCAT----AGTAGTTTCTATAAAGTCAAAAGATC 82600
GuineaPig                    AATTTTGAGTCTTAAC----TCTTTAGACTGCCA----GTGAAAGGTCATACGTAATACTTTCTGTAAA----------- 54701
NorthernAmericanDeerMouse    AGTCTTATAAATGAGAG---TCTTCATTCTAG-------TGTTAGAGTACTCACAGAAAAGGAAGGAAAGCTGAATGAG- 59473
Mouse                        AATTTTATAATTGAGAA---TCTTTTGTGTACCTACCTTTAGTAGGGTGCACACAGAAGAGGAAGAAAGCCTGAATGAT- 59384
ChineseHamsterGHOK1GS        AATTTTATAAATAAGAA---TCTTCATTGTAG-------TGATAGAGTACTCAAAGAAAAGGAAGAAAGGCT-AATGA-- 63257
LongTailedChinchilla         AATTGTGAGTCTTAACA--CTCTTCAGACTGC------GTGAAAGGTCAT----AACATTTTCTATAAA----------- 60751

Majority                     ----GXTXXACTTXXAAGAGTA---TCTXTCAAXXCAXAXXCXAXXGAAXTTTGTXTTTAAATX---TAGAAACTTGCAG
                                       89450     89460     89470     89480     89490     89500     89510     89520
Human                        AAAAACTTGACTTAAAAGAGTAAGATCTCTAAAACCAAAGGTAACAGCAGATTCTTATGAAATGCACCAGAAAGCTGGAA 82680
GuineaPig                    ----GTTGGACT-AAATGAATA---TCTCTGAAACTAAAGGTAACAAATGTTTGAGTTTAAATGAATTC----------- 54762
NorthernAmericanDeerMouse    ----G---AAATTGGAAGGGGA---TGATTCATGTCAGAAGCTTAGGAATTTTGTCTTTAACAC---TAGAAACTTGCAG 59540
Mouse                        ----GAGGAAATTGGAGGAGAT---GTTTTCACGT-AGAAACTTAGGAATTTTCACTCTAAATTG--TTGAAACTTCCAG 59454
ChineseHamsterGHOK1GS        --------AATTTAGGAGGG-----TGGTTCATGTCAGAAACTCAG-AACTTTGTTTTTAAGTC---TAGAAACTGGCAG 63320
LongTailedChinchilla         ----ACTTGACT-GAAAAAGTA---TCTCTGAAACTAAAGACAACAAA-------------------------------- 60791

Majority                     TGAAAGGT-----------------------------------AGXGXXXXCTCTGTAAAXCXAT-X---GAAAXCTXAX
                                       89530     89540     89550     89560     89570     89580     89590     89600
Human                        AGAAAGAAGAAATAAAATGTGAACTTATGGAACAAGAGTGCTAAGGGCCTGTTCTACCCAGGTATGTTATGAAATCAAAT 82760
GuineaPig                    -------------------------------------------------------------------------------- 54762
NorthernAmericanDeerMouse    TGAAAGGTC----------------------------------AGAGCAGTCTCTGTAAAGCTAA-----GAAACCTGCT 59581
Mouse                        TGAAAGGT-----------------------------------GGTTTTACCTCCGTAAACCCATGT---AAAAGCTGAC 59496
ChineseHamsterGHOK1GS        TGAAAGGTC----------------------------------AGAGTAGCCTCTGTAAAACCGTCT---GAAACCTAAG 63363
LongTailedChinchilla         -------------------------------------------------------------------------------- 60791
```

```
Majority                    AXXX--------AGAXTXXGGTCXCTXXXX---TGAAG-TAGC------X--X-X----XXXXCATAAX------AACXC
                            89610 89620 89630 89640 89650 89660 89670 89680
Human                       TCATTTAGTTTGGGTGTTTGTTCCTTTATGTTGGGAAGATAAAAAAGAACATATAATTCGTTTCTTGAGTGAACCAGTAT 82840
GuineaPig                   -------------------------------------------------------------------------------- 54762
NorthernAmericanDeerMouse   AAGA-------AAGAATAAGGTCTCTGCCCC--TGAAGGTAGC----------------AGCACATAAG------AACAC 59630
Mouse                       AGGC--------AGAGTACGGTCCCGACTAG--TGAAG-TAGCAGTAGACCCAGAGT--GATGCATAAGAACAAGAACGC 59563
ChineseHamsterGHOK1GS       AAAACC----TAAGAAAGAGGTCTCTGACC---TGAAGGTAGCT-CAGCCCCACAGC--AACACATAA-------AACTC 63426
LongTailedChinchilla        -------------------------------------------------------------------------------- 60791

Majority                    TAXXAAGAGATAA-------------------------TXXX--T-TTACAAAGXCAXAGX-X-----XXXGTXGXTAAX
                            89690 89700 89710 89720 89730 89740 89750 89760
Human                       TTATGAGAAATAAGCTGGGTGTGTAAGAATTTAAGGGATGAAAATATTCCAAGTTGATTGGTAATTGAAAAGTAGATGAA 82920
GuineaPig                   --------------------------------------------TTTTAATACTTGTAAACTATGTCAATAATAGGCAAA 54798
NorthernAmericanDeerMouse   TACAAAGAGATAA-------------------------T-------TTACACAGCCACAG-------------------- 59658
Mouse                       TATCAAAAGAAGAG--------------AAACACAGTGTGAA--T-TTACAAAGGCACAGTGAA--GGACTGTTTCTAAG 59624
ChineseHamsterGHOK1GS       TACAAAGAGATAA-------------------------TGAA--T-TTACACAGCCAAGGG--------CGGTTGCTAAG 63470
LongTailedChinchilla        -------------------------------------------------------------------------------- 60791

Majority                    CXAXTXTXXCAAXAGA------TCTGTCT---------------------X-XX-AAGATAGXAGGCTGGCACTX----X
                            89770 89780 89790 89800 89810 89820 89830 89840
Human                       CGGTGCTAACACAAAAGGGAATTCTGTCTAGAATAAATGAAAATATGGTGACTTAAAGATAGTAGGACAGCAATAAGACT 83000
GuineaPig                   CTATTTTAAGAAAAAATAAAAGTCCGT------------------------------GATA--AGAGTGACACT------ 54840
NorthernAmericanDeerMouse   --------CCAAGAGC------TGTTTCT--------------------------GAGCAAGTATGCTG----------- 59687
Mouse                       CAAGTATGTCATTAGA------TCCGGATC--------------------ATTT-AAG-TATAGGGCTGGGGCCA---TT 59673
ChineseHamsterGHOK1GS       CGAGTATGTCAGGAGA------TCTTTCTT--------------------ATTT-AAGGTAGAGTACTGGTGCCA---TT 63520
LongTailedChinchilla        -------------A--------TCTGT------------------------------GATA--AGGACGACAAT------ 60812

Majority                    -----XX-------XX---X---XX-X-----------XX-X-X--------------CTGTACTGXTXXGXATTXTAC-
                            89850 89860 89870 89880 89890 89900 89910 89920
Human                       GGAAATTAGAACAATAAAATAAGAAGGAAATGATAACATAGATTGGGAAAGGATAGGAAAATACTGAAACAAAATCTGTG 83080
GuineaPig                   -------------------------------------------------------------------------------- 54840
NorthernAmericanDeerMouse   ----------------------------------------------------------CTGTACTGTTAAGCATTGTAC- 59708
Mouse                       -----TT-------TA---TGTCAAGG---------TGTAAACT--------------CTGTACTGATTTGGATTATAC- 59714
ChineseHamsterGHOK1GS       -----TT-------TA---TGGGAAAG---------CCTAAATT--------------CTGTACTGTTTAGCATTGTAC- 63561
LongTailedChinchilla        -------------------------------------------------------------------------------- 60812
```

```
Majority                      TTXX--A---X--GTAATXXGTXCTAGGXAAATGXGAATTXAAGGGTTGAAGGXAXTTGXGXTTXATTXTAXT--TACTT
                                        89930     89940     89950     89960     89970     89980     89990     90000
Human                         TTGTATAGTGATGGTTTCTTGTTCTTTCAACCGGGAGAGTGTTGTGTTGAAGGAACTTGTA-TTCATTTTAAT--TACTT  83157
GuineaPig                     TTGT-----------TCTTTCTACTGGAGATTGT----------AGTTTCAGGAACTTGTG-TTTATTGTAAT--TACTT  54896
NorthernAmericanDeerMouse     TT----AATGA--GTAATAAGTAATAGG-AAATCAGAATTGAAGGGTTGAAAGTATTTGGGGTTGTTGTTT---CATTT  59778
Mouse                         TTGT-CAACAA--ATAATAAATACTAGGTAAATGGGAATTTAAGGGTTGAAAATGTTTGGGGTTCATTTTGTTTCTACTT  59791
ChineseHamsterGHOK1GS         TT----A------GTAATAAAATATAGGTAAATAAGAATTTAAGGGTTGAATGTATTTGGGGTTCATTGTTTTAACACTT  63631
LongTailedChinchilla          TT---------------TTTGTTCTG-----------------AGTTTGAGGAACTTGTG-TGTATTTTAAC--TATTT  60856

Majority                      GAXTGTGAAATTXAGAAGGCAAAXXXGTXTTGTTAA-ATTCTTTAGXXCA-XTTGXXTGAATTG------TTTAGGGTGA
                                        90010     90020     90030     90040     90050     90060     90070     90080
Human                         GAGTGTATAATTAAGAAGGCAAGCACATTTTGCTAAGATTTTCTTTATTTGCATGAATTAAATAATGATGTTCAGAGTGA  83237
GuineaPig                     AAATGTAGAATGAAGAAGGCAAACACGTTTTGTTAA-ATTCTTTAGATTA-CTAGAGTGACTT-------------GTGA  54961
NorthernAmericanDeerMouse     GAGTCTGAAATTGAGAAGGCAAAT-TGTCCTATCAT-TTTCTTTTGGACA-TTTGTGTGAATTG------TTTAGGAGGA  59849
Mouse                         CAGTATGAAATTGAGAATGCAACT-TGTCACAT-----TT--TTAGGACA-TTTATATGAATTG------TTTAGGTGGA  59856
ChineseHamsterGHOK1GS         AAATCTGAAATCGAGAAGGCCAGT-TGTCCTCTGGT-TTTCTTCAGGACA-TTTGTGTGAATTG------TTTAGGA---  63699
LongTailedChinchilla          GAATGTGGAATTAAGAAGGCAAACACACTTTGTTAA-ATTTTTTAGATCT-CTTGAATGGATTAAAGAC--TCAGGGTGA  60932

Majority                      TTTGTCTXTAGGAACTGAAXXXXXXXACTAAAXXAAATTTGTTTA-XXXXXCTXAXCTXTAXTTGTT---TAXTGCA----
                                        90090     90100     90110     90120     90130     90140     90150     90160
Human                         TTTGTCTCTAGGAGCTGAA----AGACT--ACTACCTTTGTTTC------ATAAGCTGTAATTATT--TGATTTCA----  83299
GuineaPig                     TTTGTCTCTAGAAACTAAG----GGACTTAACTCTATTTGTTTT------GTAAGCAGTAATT------TATTCCA----  55021
NorthernAmericanDeerMouse     TTTGTTTGTAGGAGCTGAATTTTATAGAAAAACAATCTTGTTTATAGGAACTGAACTATATTTGTTAAGTGCTGCATGGA  59929
Mouse                         TTTGTCTGTACTAACTGAATTTTGTATGAAAACAAATTTGTGTATAGGAAGTGAACTATGTTTGTT-----CTGTA----  59927
ChineseHamsterGHOK1GS         TTTGTCTGTACGAACTGAATTTTATAGGAAAACAATTTTGTTTA-AGGAACTGAACTATATTTGTTAAGTGCTGAATGGT  63778
LongTailedChinchilla          TTTGTCTCTGGAAACTAAA----GGACTTAACTATATTTGTTTC------CTAAGTAGTAACT------GATTTCA----  60992

Majority                      ---AGTGTGAGGTTATGAAGXAG---ATAGCACTTGTAGAAAAGGGX--XXAXTTACCATTATGTTGAAGGX-X-X-TTT
                                        90170     90180     90190     90200     90210     90220     90230     90240
Human                         ---AGTGTGAGTTTATGAAGAAG---ATAGCACTTGCGGAAAAAAG---TGAATTACCATTATGTCGAAAGTGATACTTT  83370
GuineaPig                     ---AGTGTGAGCTTACGAAGAAGATGATAGCACTTGTAGAAAAAGGA--CAATTTACCATTATGCTGAAGGCAACA-TTT  55095
NorthernAmericanDeerMouse     TTCAAAATTAGGTTATGAAGGAG---ACAATACTTGTAGAAATGGGATGACGAGTACCATTGTCTTGAAGGT-----TTT  60001
Mouse                         -----------GCAGTGA--GAA---GTATCACT-GTGGTAAAGGG-------------------------------TTT  59959
ChineseHamsterGHOK1GS         TTCAAAATTAGGTTGTGAAGGAG---ACAACACTTGTAGAAATGGA------------------TGAAGGG-----TTT  63831
LongTailedChinchilla          ---AGTGTGAGCTCTTGAAGATGCTGATAGTACTTTTAGAAAATAGA--TGATTTACCATTATGTTGAAGGCAACA-TAT  61066
```

Majority XTTATATTTXCCT-------------X-TCACTGTAAATCATGGAAXTGAAXXX--XXX--XX-X-GXXXXXXXATTAXA

90250 90260 90270 90280 90290 90300 90310 90320

Human TTTACATTTGGCC-------------TTTTACTGTAAATCGTGGAAGTGAATTTTCCAAGCCTGT-GACTTGGAATTATA 83436
GuineaPig TTCATACTTGAATTTTCATATTTGACTTTCACTGTAAACCACGGAA-TCAATTTGTCAAATCTTTTGACTTGGAAATACA 55174
NorthernAmericanDeerMouse CTTATATTTACCT-------------C-TCACTGTAAATCCTGGAATTGAA---------------A-------ATTTTA 60045
Mouse CTTATACCTACCT-------------C-TCACTGTAAATGGTGGAAATGA------------------------------ 59995
ChineseHamsterGHOK1GS CTTATATTTACCT-------------C-TTACTGTAAATCATGGAAGTGAC---------------A-------ATTT-A 63874
LongTailedChinchilla TTCAT----GACT-------------TCTCACCGTCAACCATGGAAATGAATTTGTCAAGTCTTTTGACTTGGAAATACA 61129

Majority XTCTTCATGCTTTGTTTGCTTTTCCATAGG--AGATAXAGTA-T--XATXTATXTAATATXTTAACATAATACAGGTATG

90330 90340 90350 90360 90370 90380 90390 90400

Human TTTTTCATGCTTTGTTTGCTTTTCCAGAGG--AAATAGAGTA-T--TATATATTTGATATTTTAACATAATACAGGTATG 83511
GuineaPig TTCTTCATGCTTTATTTGCTTTTCCATAGG--AGGTGGAGTA-T--TTTGTATTTAATTTTTTAACATAATACAGGTATG 55249
NorthernAmericanDeerMouse CTCTTCATGCTTTGTTAGCTTTTCCATAGGGAAGATACAGGACT--CATATATGTAATATGCTAACAAAATACAGGTATG 60123
Mouse -------------GT------------------GATAC--------CATGTATGTAATATGTTAACATTATACAGGTATG 60036
ChineseHamsterGHOK1GS CTCTTCATGCTTTGTTAGCTTTTCCATAGGGAAGATACAGGACTATCATGTATGCAATATGCTAACATTATACAGGTATG 63954
LongTailedChinchilla ATCTTTGTGCTTCGTTTGCTTTTCCATAGG--AGGTGGAATA-T--TGTATATTTAATTTTTAAAC--AATACAGGTATG 61202

Majority TACCTTAGTTCAAATAATTAATGACT--GTTGAAATCAGTTATTTTTCTATAA----CTXCTCCACAXCAGCAAAXCATT

90410 90420 90430 90440 90450 90460 90470 90480

Human TACTTTAGTTCAAATAATTAATGGTTTTGTTGAAATCAATTATTTTTCT--------TTACTACACATCAGCAAACAATT 83583
GuineaPig TATATTAGTTCGAATAGT----AACTCCATTAAAACCAGATACTTTTCTGTAA----CTACTGCACATCAGCAAACCATT 55321
NorthernAmericanDeerMouse TACCTTAGTTCAAATAATTAATGACT--GTTGAAATCAGTTATTTTTTTATAA----CTGCTCCACACCAACAAAACATT 60197
Mouse TACCTTAGTTCAAATAATAAACGACT--GTTGAAATCGATTATTTTTCTATAA----CTGCTCCACACCAACAAAACACT 60110
ChineseHamsterGHOK1GS TACCTTAGTTCAAATGATAAATGACT--GTTGAAATCAGTTATTTTTCTATAACTAACTGTTCTATACCAGCAAAACAGT 64032
LongTailedChinchilla TATATTACTTCAAATAATTACCAACTTTGTTGAAACCAGCTACTTCTCTGTAA----CTACTGCGCATCAGCAAACCGTT 61278

Majority TTTCTAAACATTATAAAAATTAACTTXCAGGAACCATTTGGAAXGGTCAGCCXTAAGCCATGCAGCCATCGTGCTTAAAT

90490 90500 90510 90520 90530 90540 90550 90560

Human TTTCTAAACACTATAAAAATTAACTTTCAGGAACCATTTGGAATGGTCAGCCGTAAGCCATGCAGCCATCGTGCTTAAAT 83663
GuineaPig TTTCTAAACATTGTAAAAATTAATTTTCAGGAATCGTTTGGAGTGGTCGGC-GTAAGCCACGCAGGCTTCGTGCTTAAAT 55400
NorthernAmericanDeerMouse TTTCTAAACATA---AAAATTAACTTACAGGAACCATTTGGAACGGTCAGCCATAAGCCATGCAGCCATCGTGCTTAAAT 60274
Mouse TTTCTAAACATCAAAAAAATTGACTTACAGGAACCATTTGGAACGGTCACCCATAAGCCATGCAGCCATTCTGCTTAAAT 60190
ChineseHamsterGHOK1GS TTTCTAAATATCAT-AAAATTAACTTACAGGAACCATTTGGAACGGTCAGCCATAAGCCATGCAGCCATCATGCTTAAAT 64111
LongTailedChinchilla TTTCTAAACATTACAAACATTAACTTTCAGGAACCGTTGGGAGTGGTCGGCTGCAAGCCCTGCAGCCATCGTGCTTAAAT 61358

```
Majority                     TGGT-TTTGTAAACGACTTGTGTGTGTTTCGTAAAATATGCTTTATTTTAAAATTAGGGCTTCCTGTTGCAGCTGTTCCA
                             90570 90580 90590 90600 90610 90620 90630 90640
Human                        TGGTTTTTGTAAACGACTTGTGTGTGTTTCGTAAAATATGCTTTATTTTAAAATTAGGGCTTCCTGTTGCAGCTGTTCCA 83743
GuineaPig                    TGGTCTTTATAAACGACTTGTGTGTGTTTCGTAAAATATGCTTTATTTTAAAATTAGGGCTTCCTGTTGCAGCTGTTCCA 55480
NorthernAmericanDeerMouse    TGGT-TTTGTAAACGACTTGTGTGTGTTTCGTAAAATATGCTTTATTTTAAAATTAGGGCTTCCTGTTGCAGCTGTTCCA 60353
Mouse                        TGGT-TTTGTAAACGACTTGTGTGTGTTTCGTAAAATATGCTTTATTTTAAAATTAGGGCTTCCTGTTGCAGCTGTTCCA 60269
ChineseHamsterGHOK1GS        TGGT-TTTGTAAACGACTTGTGTGTGTTTCGTAAAATATGCTTTATTTTAAAATTAGGGCTTCCTGTTGCAGCTGTTCCA 64190
LongTailedChinchilla         TGGTCTTTGTAAACGACTTGTGTGTGTTTCGTAAAATATGCTTTATTTTAAAATTAGGGCTTCCTGTTGCAGCTGTTCCA 61438

Majority                     GGAGCTCTXAGTCCTTTGGCTATTCCAAATGCTGCTGCAGCAGCTGCTGCAGCTGCXGCTGGCCGAGTGGGTATGCCTGG
                             90650 90660 90670 90680 90690 90700 90710 90720
Human                        GGAGCTCTGAGTCCTTTGGCCATTCCAAATGCTGCTGCAGCAGCTGCTGCAGCTGCTGCTGGCCGAGTGGGTATGCCTGG 83823
GuineaPig                    GGAGCTCTGAGTCCTTTGGCTATTCCAAATGCTGCTGCAGCCGCTGCAGCAGCTGCCGCTGGCCGAGTGGGTATGCCTGG 55560
NorthernAmericanDeerMouse    GGAGCTCTCAGTCCTTTGGCTATTCCAAATGCTGCTGCAGCAGCTGCTGCAGCTGCTGCTGGCCGAGTGGGTATGCCTGG 60433
Mouse                        GGGGCTCTCAGTCCTTTGGCTATTCCAAATGCTGCTGCAGCAGCTGCTGCCGCTGCCGCTGGCCGAGTGGGCATGCCTGG 60349
ChineseHamsterGHOK1GS        GGAGCTCTCAGTCCTTTGGCTATTCCAAATGCTGCTGCAGCAGCTGCTGCAGCTGCTGCTGGCCGAGTGGGTATGCCTGG 64270
LongTailedChinchilla         GGAGCGCTGAGTCCTTTGGCTATTCCAAATGCCGCTGCAGCCGCTGCAGCAGCTGCCGCTGGCCGAGTGGGTATGCCTGG 61518

Majority                     AGTCTCAGCTGGTGGCAATACAGTCCTGTTGGTTAGCAATTTAAATGAAGAGGTTAGTGAAATAATTT-XTAATGTXXXT
                             90730 90740 90750 90760 90770 90780 90790 90800
Human                        AGTCTCAGCTGGTGGCAATACAGTCCTGTTGGTTAGCAATTTAAATGAAGAGGTTAGTAAAATAATCT-CTAATGTTTAT 83902
GuineaPig                    AGTTTCAGCTGGTGGCAATACTGTCCTGTTGGTTAGCAATTTAAATGAAGAGGTTAGTGCAATAATTTTCTAATGTTTAT 55640
NorthernAmericanDeerMouse    AGTCTCAGCTGGTGGCAATACAGTCCTGTTGGTTAGCAATTTAAATGAAGAGGTTAGTGAAATAATTT-TTAATG---CT 60509
Mouse                        AGTCTCAGCTGGTGGCAATACAGTCCTGTTGGTTAGCAATTTAAATGAAGAGGTTAGTGAAATCATTT-TTAATGG--CT 60426
ChineseHamsterGHOK1GS        AGTCTCAGCTGGTGGCAATACAGTCCTGTTGGTTAGCAATTTAAATGAAGAGGTTAGTGAAATACTTT-TTAATG---CC 64346
LongTailedChinchilla         AGTCTCAGCTGGTGGCAATACAGTCCTGTTGGTTAGCAATTTAAATGAAGAGGTTAGTAAAATAATTTCCTAATGTTTAT 61598

Majority                     TXTTXAXCTCCATTTCATTTGTGXAAGTTTTCXTGTTTATTTCATTTTGCACTTGCCTTTCTTTTTX-----ATGTAXA
                             90810 90820 90830 90840 90850 90860 90870 90880
Human                        TCTTTAACTCCATTTCATTTGTGAAAGTTTTCATGTTTATTTCATTTTGCACTTGCCTTTCTTTTT------ATGTACA 83976
GuineaPig                    TCTTTAACTCCATTTCATTTGTGGAAGTTTTCATGTTTATTTCATTTTGCACTTGCCTTTTTTTTTTTTTTAATGTACA 55720
NorthernAmericanDeerMouse    TTTTCATCTCCATTTCATTTGTGGAAGTTTCA-TATTTATTTCATTTTGCACTTGCCTTTCCTTTT------ATGTATA 60582
Mouse                        TTTTCATCTCCATTTCATTTGTGAAAGTTTT----CATATTTCATTTTGCACTTGCCTTTCTTTTT------ATGTATA 60496
ChineseHamsterGHOK1GS        TTTTCATCTCCATTTCATTTGTGAAAGTTTCA-TATTTATTTCATTTTGCACTTGCCTTTCCTTTTT-----ATGTATA 64420
LongTailedChinchilla         TCTTTAACTTCATTTCATTTGTGGAAGTTTTCATGTTTATTTCATTTTGCACTTGCCCTTTTTTTTT----ATGTACA 61674
```

```
Majority                     TTCATTAGCCAAAXTATXTTXTGT--XTTTCTXCTTCTGAATAAAATCTACGTACTTGTTTAGGTAXTACTXXGTTTCXT
                                       90890     90900     90910     90920     90930     90940     90950     90960
Human                        TTCATTAGTCAAAGTATTTTCTGTATGTTTCTACTTCTGAATAAAATCTACATA-TTGTTTAGGTAATACTTTATTTCCT  84055
GuineaPig                    TTCATTAGCCAGAATATTTTCTGT--GTTTCTACTTCTGAATAAAATCTGTGTACTTGTTTAGGTAATATTTTGTTTCCT  55798
NorthernAmericanDeerMouse    TTCATTAGCCAAAATATCTTTTGT--ATTTCTGCTTCTGAATAAAATTTACGCACTTGTTTAGGTAGTACT--GTTTTTT  60658
Mouse                        TTCATTAGCCAAAATATCTTTTGT--ATTTCTGCTTCTGAATAAAATCTCCGTACTTGTTTAGTTAGTGCT--GTTTCTC  60572
ChineseHamsterGHOK1GS        TTCATTAGCCAAAGTATCTTTTGT--ATTTCTGCTTCTGAATAAAATTTACGTACTTGTTTAGGTAGTACT--GTTTTTT  64496
LongTailedChinchilla         TTCATTAGCCAGAGTATTTTCTGT--GTTTCTACTTCTGAATAAAATCTGTGTACTTGTTTAGGTAATGTTTTGTTTCCT  61752

Majority                     TAGACAGTCATATXTCTTTATAT--------ATGTATATATA--TTTTAAXXTAXCTACCTATTTTTCCTAAGAAAAATA
                                       90970     90980     90990     91000     91010     91020     91030     91040
Human                        TAAACAGTCATGTCTCTTTATATCTATATCTATATATGTATATTTTTTAACCTAA---CCTATTTTTCCTAAGAAAAATA  84132
GuineaPig                    TAGACAGTCATATCTCTTTATAT--------ATGTATGTAT---TTTTAACCTAAGTACCTATTTTTCCTAAGAAAAATA  55867
NorthernAmericanDeerMouse    TAGACAGTCATATATCTTTATAT--------ATGTATATATA--TTTTAATATAGCTACCTATTTTTCCTAAGAAAAATA  60728
Mouse                        TAGACAGTCATATATCTTTATAT--------ATGTATATAGA--TTCTAATATAGCTACCTATTTTTCCTAAGAAAAATA  60642
ChineseHamsterGHOK1GS        -AGACAGTCATATATCCTTATAT--------ATGTATATATA--TTTTAATATAGCTACCTATTTTTCCTAAGAAAAATA  64565
LongTailedChinchilla         TAGACAGTCATATCTCTTTATAT--------ATGTATATAT---TTTTAACCTAAGTACCTATTTTTCCTAAGAAAAATA  61821

Majority                     TGGTGAAGTACTGTAGTTTGGCTTTTTATXTTTT-TGTTGTTCTCAAAGGCAAATGTGATCTA-ATTTGCAAATXTAACT
                                       91050     91060     91070     91080     91090     91100     91110     91120
Human                        TGGTGAAGTACTGTAGTTTGGCTT---ATCTTTT-TGTTGTTCTCAAAGGCAAATGTGATCTA-ATTTGCAAATTTAACT  84207
GuineaPig                    TGGTGAAGTACTATAGTTTGGCTTTGTATCATTT-TGTTGTTCTCAAAGGCAAATGTGATGTA-ATTTGCAAATTTAACT  55945
NorthernAmericanDeerMouse    TGGTGAAGTACCGTAGTCTGGCTTTTTATTTTTC-TGTTGTTCTCAAAGGCAAATGTGGTCTAATTTTGCAAATGTATCT  60807
Mouse                        TGGTGAAGTACCGTAGTCTGGCTTTTTATTTTTC-TGTTATTCTCAAAGGCAAACGTGATCTA-CTTTGCAAGTGTAACT  60720
ChineseHamsterGHOK1GS        TGGTGAAGTACTGTAGTTTGGCTTTTTATTTTTTGTGTTGTTCTCAAAGGCAAATGTGATCTAATTTTGCAAATGTAACT  64645
LongTailedChinchilla         TGGTGAAGTACTATAGTTTGGCTTTGTATCATTT-TGTTGTTCTCAAAGGCAAATGTGATCTA-ATTTGCAAATTTAACT  61899

Majority                     GTAAAACAATTTTGCTCTTTGCCTTTTCTAATXATTTGCTTGTTCATTTCATGCTTATGTGTCATTGCATTTTTTTT-XA
                                       91130     91140     91150     91160     91170     91180     91190     91200
Human                        GTAAAACAATTTTGCTCTTTGCCTTTTCTAATTATTTGCTTGTTCATTTCATGCTTATATGTCATTGCATTTTTTTT-AA  84286
GuineaPig                    ATAAAACAATTTTGCTCTTTGCCTTTTCTAATTATTTGCTTGTTCATTTCATGCTTATGTGTCATTGCATTTTTTTTTAA  56025
NorthernAmericanDeerMouse    AGAAAACAGTTTTGCTCTTTGCCTTTTCTAATGATTTGCTTGTTCATTTCATGCTTATGTGTCATTGCATTTTTTT---A  60884
Mouse                        GTGAAATGATTTTGCTCTTTGCCTTTTCTAATGATTTGCTTGTTCATTTCATGCTTATGTGTCATTGCGTTTTTTTT--A  60798
ChineseHamsterGHOK1GS        GGAAAACAATTTTGCTCTTTGCCTTCTCTAATGATTTGCTTGTTCATTTCATGCTTATGTGTCATTGCATTTTTTTT--A  64723
LongTailedChinchilla         GTAAGAAAATTTTGCTCTTTGCCTTTTCTAATTATTTGCTTGTTCATTTCATGCTTATGTGTCATTGCATTTTTTTT-AA  61978
```

```
Majority                    ATCTTATTTTATGTGAACTACTCTAATACATTTTTCTTTGAAGGTTCTTCCAAAGATCTTGACGAGGCACTCTTCCAGTC
                                    91210     91220     91230     91240     91250     91260     91270     91280
Human                       ATCTTATTTTATGTGAACTACTCTAATACATTTTTCTTTGAAGGTTCTTCCAAAGATCTTGACGAGGCACTCTTCCCGTC  84366
GuineaPig                   ATCTTATTTTATGTGAACTACTCTAATACATTTTTCTTTGAAGGTTCTCCCAAAGATCTTGACGAGGCACTCTTCCAGTC  56105
NorthernAmericanDeerMouse   ATCTTATTTTGTGTGAACTACTCTAATACATTTTTCTTTGAAGGTTCTCCCAAAGATCTTGACGAGGCACTCTTCCAGTC  60964
Mouse                       ATCTTATTTTATGTGAACTACTCTAATACATTTTTCTTTGAAGGTTCTTCCAAAGATCTTGACGAGGCACTCTTCCAGTC  60878
ChineseHamsterGHOK1GS       ATCTTATTTTATGTGAACTACTCTAATACATTTTTCTTTGAAGGTTCTTCCAAAGATCTTGACGAGGCACTCTTCCAGTC  64803
LongTailedChinchilla        ATCTTATTTTATGTGAACTACTCTAATACATTTTTCTTTGAAGGTTCTTCCAAAGATCTTGACGAGGCACTCTTCCAGTC  62058

Majority                    TTTCTTAGTAATTTTTTCTTTGCAGTTXTTAGTCATTGTCTTCTAXAAATATXT--TTCAACTTTAXCCXXCT--XCCXT
                                    91290     91300     91310     91320     91330     91340     91350     91360
Human                       TTTCTTAGTAATTTTTTCTTTGCAGTTATTAGTCATTGTCTTCTAAAAATATTT--TTTAACTTTATTCCCCACCCCTT  84444
GuineaPig                   TTTCTTAGTAATTTTTTCTTTGCAGTTATTAGTCATTGTCTTCTAAAAATATTT--TTCAACTTTATCTCCCT--CCCTT  56181
NorthernAmericanDeerMouse   TTTCTTAGTAATTTTTTCTTTGCAGTTTTTAGTCATTGTCTTCTACAAATATAT--TTCAACTTTACCCTGCC---CCCT  61039
Mouse                       TTTCTTAGTAATTTTTTCTTTGCAGTTTTTAGTCATTGTCTTCTACAAATATATATTTCAACTTTACCCTGCA---CCCT  60955
ChineseHamsterGHOK1GS       TTTCTTAGTAATTTTTTCTTTGCAGTTTTTAGTCATTGTCTTCTACAAATATAT--TTCAACTTTACCCTGCT---CCCT  64878
LongTailedChinchilla        TTTCTTAGTAATTTTTTCTTTGCAGTTATTAGTCATTGTCTTCTAAAAATATTT--TTCAACTTTATCTCCCT--CCCTT  62134

Majority                    TCTT---------------------GTTAAATAAAXTT----GCT--GTTATTTTCTTTAGTTGTACATTTTATGTCTGX
                                    91370     91380     91390     91400     91410     91420     91430     91440
Human                       CCTTCCTTACCACCCCCTCCACCCAGTTAAATAAAATTTATTGCTAAGTTATTTTCTTTAGTTGTACATTTTATGTCTGG  84524
GuineaPig                   TCTT---------------------GTTAAATAAAATT----GCT--GTTATTTTCTTTAGTTGTACATTTTATGTCTGA  56234
NorthernAmericanDeerMouse   TCTT---------------------GTTAAATAAAGTT----GCT--GTTATTTTCTTCAGTTGTACATTTTATGTCTGG  61092
Mouse                       T-TT---------------------GTTAAATATAGTT----GCT--GTTATTTTCTTCAGTTGTACATTTTATGTCTGA  61007
ChineseHamsterGHOK1GS       TCTT---------------------GTTAAATAAAGTT----GCT--ATTATTTTCTTTAGCTGTACGTTTTATGTCTGG  64931
LongTailedChinchilla        TCTT---------------------GTTAAATAAAATT----GCT--GTTATTTTCTTTAGTTGTACATTTTATGTCTGA  62187

Majority                    TATATTTTATTTTGATTGCTATGAAAGCTGGTATGAAATGTGGGAXXCTCAAAXGTATCAGTTTATGXTGTCTAATTTGA
                                    91450     91460     91470     91480     91490     91500     91510     91520
Human                       TATATTTTATTTTGAATGCTATGAAAGCTGGTATGAAATGTGGGAAGCTCAGT-GTATCAGTTTATGATGTCTAATTTGA  84603
GuineaPig                   TATATTTTATTTTGATTGCTATGAAAGCTGGTATGAAATGTGGGAAGCTTAAA-GTATTAGTTAATGATGTCTAATTTAA  56313
NorthernAmericanDeerMouse   TATATTTTATTTTGATTGCTATGAAAGCTGGTATGAAATGTGGGGCCCTCAGATGTATCAGTTTATGGTGTCTAATTTGA  61172
Mouse                       TATATTTTATTTTGATTGCTATGAAAGCTGGTATGAAATGTGGGACCCTCAAATGTATCAGTTTATTATGTCTAATTTGA  61087
ChineseHamsterGHOK1GS       TATATTTTATTTTGATTGCTATGAAAGCTGGTATGAAATGTGGGACCCTCAAATGTATCAGTTTATGGTGTCTAATTTGA  65011
LongTailedChinchilla        TATATTTTATTTTGATTGCTATGAAAGCTGGTATGAAATGTGGGAAGCTCAAA-GTATTAGTTAATGGTGT-----TTAA  62261
```

Majority	ATATTTGTXTXATTTTTXATTGGCCTCTGTTAATATXAXXXXTAAXCTAATTTTATC-ATTTAATACTGTTATCATTCAC	
	91530 91540 91550 91560 91570 91580 91590 91600	
Human	ATATTTGTTTCATTTTTAATTGGCCTCTGTTAATATGAACATTA-GCTTATTTTATCCATTTAATACTATTATCATTCAC	84682
GuineaPig	ACAT--GTTTTA-TTTTAATTGGCCTCTGTTAATATGAGCATTAAGCTTATTTTATC-ATTTAATATTGTTA-------C	56382
NorthernAmericanDeerMouse	ATATTTGTCTCATTTTTGATTGGCCTCTGTTAATAATGGATATGACCTAATTTTATC-ATTTAATACTGTTATCATTCAC	61251
Mouse	GTATTTGTCTTATTTTTGGTTGGCCTCTGTTAATAATAAACATAACCTAATTTTGTC-GTTTAGTACTGTTATCATTCAC	61166
ChineseHamsterGHOK1GS	ATATTTGTCTCATTTTTGATTGGCCTCTGTAAATAGTGGATATAACCTAATTTTATC-ATTTAATATTGTTATCATTCAC	65090
LongTailedChinchilla	ACAT--GTTAT--TTTTAATTGGCCTCTGTTAATATGAACCTTAAGCCCATTTTATC-ATTTAATACTATTATTATTCAC	62336

Majority	AGTTCTGCATGCTAAAATGTTTGGATCAGAGTG------XXTTTXTTTTAAGACTT--GCCTGCATTTCATAACCAGCCA	
	91610 91620 91630 91640 91650 91660 91670 91680	
Human	AGTTCTGCATGCTAAAATGTTTGAATCAGAGTGCCTTTGTTTTTATTTTAAGACTTTTGCCTGCATTTCATAACCAGCCA	84762
GuineaPig	AGTTCTGCATACTAAAATGTTTGGATCAGAGT-------TT-AATTTTTAAGACTT--GCCTGCATTTCATAACCAGCCA	56452
NorthernAmericanDeerMouse	AGTTCTGCATGCTAAAGTGTTTGGATCAGAGTG------CCTTTGTTTTAAGACTT--GCCTGCATTTCATAACCAGCCA	61323
Mouse	AGTTCTGCATGCTAAAATGTTTGAATCAGAGTG------CCT-----TTACGACTT--GCCTGCATTTCATAACCAGCCA	61233
ChineseHamsterGHOK1GS	AGTTCTGCATGCTAAAGTGCTTGGATCAGAGTG------CCTTTGTTTTAAGACTTTTGCCTGCATTTCATAACCAGCCA	65164
LongTailedChinchilla	AGTACTTCATGCTAAAATGTTTGGATTACAGT-------TTTAATTTTTAAGACTT--GCCTGCATTTCATAACCAGCCA	62407

Majority	TGCTTATGCAGTTAAAGTTCAAAGTTTAAAATTCCTATGCATGCTTTCCTTCCCTATGTTGAGATGAAATGCTGTAATTT	
	91690 91700 91710 91720 91730 91740 91750 91760	
Human	TGCTTATGCAGTTAAAGTTCAAAGTTTAAAATTCCTATGCATGCTTTCCTTCCCTATGTTGAGATGAAATGCTGTAATTT	84842
GuineaPig	TGCTTATGCAGTTAAAGTTCAAAGTTTAAAATTCCTATGCATGCTTTCCTTCCCTATGTTGAGATGAAATGCTGTAATTT	56532
NorthernAmericanDeerMouse	TGCTTATGCAGTTAAAGTTCGAAGTTTAAAATTCCTATGCATGCATTCCTTCCCTATGTGGAGATGAAATGCTGTAATTT	61403
Mouse	TGCTTATGCAGTTAAAGTTCAAAGTTTAAAGC-CCTATGCATGCGTCCCTTCCC-ATGTTGAGATGAAATGCTGTA-TTT	61310
ChineseHamsterGHOK1GS	TGCTTATGCAGTTAAAGTTCAAAGTTTAAAATTCCTATGCATGCGTTCCTTCCCTATGTTGAGATGAAATGCTGTAATTT	65244
LongTailedChinchilla	TGCTTATGCAGTTAAAGTTCAGAATTTAAAATTCCTATGCATGCTTTCCTTCCCTATGTTGAGATGAAATGCTGTAATTT	62487

Majority	ACTCTTTGATATAGATGTACTTTACCCATATTTGTCTTGGATGACTACATTTTXCTCAGTTTTTCTTGTACAGTACATAA	
	91770 91780 91790 91800 91810 91820 91830 91840	
Human	ACTCTTTGATATAGATGTACTTTACCCATATTTGTCTTGGATGACTACATTTTACTCAGTTTTTCTTGTACAGTACATAA	84922
GuineaPig	ACTCTTTGATATAGATGTACTTTATCCATATTTGTCTTGGATGACTACATTTTACTCAGTTTTTCTTGTACAGTACATAA	56612
NorthernAmericanDeerMouse	ACTCTTTGATATAGATGTACTTTACCCATATTTGTCTTGGATGACTACATTTTGCTCAGTTTTTCTTGTACAGTACATAA	61483
Mouse	ACTCTTTGATAGATATGTACTTTACCCATATTTGTCTTGGATGACTACATTTTGCTCAGTTTTTCTTGTACAGTACATAA	61390
ChineseHamsterGHOK1GS	ACTCTTTGATATAGATGTACTTTACCCATATTTGTCTTGGATGACTACATTTTGCTCAGTTTTTCTTGTACAGTACATAA	65324
LongTailedChinchilla	ACTCTTTGATATAGATGTACTTTACCCATATTTGTCTTGGATGACTACATTTTACTCAGTTTTTCTTGTACAGTACATAA	62567

Majority	ACCAACCATTTT-CTGACCAAATTCCTGCATTTCCTATGTACTGACCTATATTTTATTTTTTTTTTGTTCCCCXATTCCT	
	91850 91860 91870 91880 91890 91900 91910 91920	
Human	ACCAACCATTTT-CTGACCAAATTCCTGCATTTCCTATGTACTGACCTATATTTTATTTTGTTTTTGTTCCCCAATTCCT	85001
GuineaPig	ACCAACCATTTTTTCTGACCAAATTCCTGCATTTCCTATGTACTGACCTATTTTAT-TTTTTTTTTTGTTCCCCGATTCCT	56691
NorthernAmericanDeerMouse	ACCAACCATTTT-CTGACCAAATTCCTGCATTTCCTATGTACTGACCTATATTTTATTTTTTTTTTGTTCCCCAATTCCT	61562
Mouse	ACCAACCATTTT-CTGACCAAATTCCTGCATTTCCTATGTACTGACCTATATTTTATTTTTTTTTTGTTCCCCAATTCCT	61469
ChineseHamsterGHOK1GS	ACCAACCATTTT-CTGACCAAATTCCTGCATTTCCTATGTACTGACCTATATTTTATTTTTTTTT-GTTCCCCGATTCCT	65402
LongTailedChinchilla	ACCAACCATTTTTTCTGACCAAATTCCTGCATTTCCTATGTACTGACCTATATTTTATTTTTTTTTTGTTCCCCGATTCCT	62647

Majority	TATTTTTTT-CTTCTGCATTGCTGTTTCCCTTCCCCATTTCATCCTTTTCCCTGTGTGTTCACCTXCCCTTTCCTTGTCT	
	91930 91940 91950 91960 91970 91980 91990 92000	
Human	TATTTTTTT-CTTCTGCATTGCTGTTTCCCTTCCCCATTTCATCCTTTTCCCTGTGTGTTCACCTTCCCTTTCCTTGTCT	85080
GuineaPig	TATTTTTTT-CTTCTGCATTGCTGTTTCCCTTCCCCATTTCATCCTTTTCCCTGTGTGTTCACCTTCCCTTTCCTTGTCT	56770
NorthernAmericanDeerMouse	TATTTTTTTTCTTCTGCATTGCTGTTTCCCTTCCCCATTTCATCCTTTTCCCTGTGTGTTCACCTCCCCTTTCCTTGTCT	61642
Mouse	TATTTTTTT-CTTCTGCATTGCTGTTTCCCTTCCCCATTTCATCCTTTTCCCTGTGTGTTCACCTCCCCTCTCCTTGTCT	61548
ChineseHamsterGHOK1GS	TATTTTTTT-CTTCTGCATTGCTGTTTCCCTTCCCCATTTCATCCTTTTCCCTGTGTGTTCACCTCCCCTTTCCTTGTCT	65481
LongTailedChinchilla	TATTTTTTT-CTTCTGCATTGCTGTTTCCCTTCCCCATTTCATCCTTTTCCCTGTGTGTTCACCTTCCCTTTCCTTGTCT	62726

Majority	TTTCCCAAATGCCCATTCCCTTCCCTGTCTTATCCTTTATTTTCCTTGTCCTTGTCTTCATTCCCTGTCTCCATTCCCTA	
	92010 92020 92030 92040 92050 92060 92070 92080	
Human	TTTCCCAAATGCCCATTCCCTTCCCTGTCTTATCCTTTATTTTCCTTGTCCTTGTCTTCATTCCCTGTCTCCATTCCCTA	85160
GuineaPig	TTTCCCAAATGCCCATTCCCTTCCCTGTCTTATCCTTTATTTTCCTTGTCCTTGTCTTCATTCCCTGTCTCCATTCCCTA	56850
NorthernAmericanDeerMouse	TTCCCCAAATGCCCATTCCCTTCCCTGTCTTATCCTTTATTTTCCTTGTCCTTGTCTTCATTCCCTGTCTCCATTCCCTA	61722
Mouse	TCTCCCAAATGCCCGTTCCCTTCCCTGTCTTATCCTT-ATTTTCCTTGTCCTTGTCTTCATTCCCTGTCTCCATTCCCTA	61627
ChineseHamsterGHOK1GS	TTCCCCAAATACCCATTCCCTTCCCTGTCTTATCCTTTATTTTCCTTGTCCTTGTCTTCATTCCCTGTCTCCATTCCCTA	65561
LongTailedChinchilla	TTTCCCAAATGCCCATTCCCTTCCCTGTCTTATCCTTTATTTTCCTTGTCCTTGTCTTCATCCCCTGTCTCCATTCCCTA	62806

Majority	TGTTCATGCTTCTGTGCTTGAACAAATGTTCCTCGGACCAACTTGCCCCAATTAACCGCCTTGAACCATGATCCATGACC	
	92090 92100 92110 92120 92130 92140 92150 92160	
Human	TGTTCATGCTTCTGTGCTTGAACAAATGTTCCTCGGACCAACTTGCCCCAATTAACCGCCTTGAACCATGATCCATGACC	85240
GuineaPig	TGTTCATGCTTCTGTGCTTGAACAAATGTTCCTCGGACCAACTTGCCCCAATTAACCGCCTTGAACCATGATCCATGACC	56930
NorthernAmericanDeerMouse	TGTTCATGCTTCTGTGCTTGAACAAATGTTCCTCGGACCAACTTGCCCCAATTAACCGCCTTGAACCATGATCCATGACC	61802
Mouse	TGTTCATGCTTCTGTGCTTGAACAAATGTTCCTCGGACCAACTTGCCCCAATTAACCGCCTTGAACCATGATCCATGACC	61707
ChineseHamsterGHOK1GS	TGTTCATGCTTCTGTGCTTGAACAAATGTTCCTCGGACCAACTTGCCCCAATTAACCGCCTTGAACCATGATCCATGACC	65641
LongTailedChinchilla	TGTTCATGCTTCTGTGCTTGAACAAATGTTCCTCGGACCAACTTGCCCCAATTAACCGCCTTGAACCATGATCCATGACC	62886

```
Majority                     ACCTCACCATTCTGCGGGAACCACCCTTCGTTATGGATGATCTGTTCATCTCCGCTCTTCCTCGACTCTTCTCTCTTCTT
                                       92170     92180     92190     92200     92210     92220     92230     92240
Human                        ACCTCACCATTCTGCGGGAACCACCCTTCGTTATGGATGATCTGTTCATCTCCGCTCTTCCTCGACTCTTCTCTCTTCTT  85320
GuineaPig                    ACCTCACCATTCTGCGGGAACCACCCTTCGTTATGGATGATCTGTTCATCTCCGCTCTTCCTCGACTCTTCTCTCTTCTT  57010
NorthernAmericanDeerMouse    ACCTCACCATTCTGCGGGAACCACCCTTCGTTATGGATGATCTGTTCATCTCCGCTCTTCCTCGACTCTTCTCTCTTCTT  61882
Mouse                        ACCTCACCATTCTGCGGGAACCACCCTTCGTTATGGATGATCTGTTCATCTCCGCTCTTCCTCGACTCTTCTCTCTTCTT  61787
ChineseHamsterGHOK1GS        ACCTCACCATTCTGCGGGAACCACCCTTCGTTATGGATGATCTGTTCATCTCCGCTCTTCCTCGACTCTTCTCTCTTCTT  65721
LongTailedChinchilla         ACCTCACCATTCTGCGGGAACCACCCTTCGTTATGGATGATCTGTTCATCTCCGCTCTTCCTCGACTCTTCTCTCTTCTT  62966

Majority                     GTCTTACGCTGCTTGCTCTTCTCTCCTTCTAAAGATGGTTACGCCCCAAAGTCTGTTTACCCTCTTCGGTATGTTATTGT
                                       92250     92260     92270     92280     92290     92300     92310     92320
Human                        GTCTTACGCTGCTTGCTCTTCTCTCCTTCTAAAGATGGTTACGCCCCAAAGTCTGTTTACCCTCTTCGGTATGTTATTGT  85400
GuineaPig                    GTCTTACGCTGCTTGCTCTTCTCTCCTTCTAAAGATGGTTACGCCCCAAAGTCTGTTTACCCTCTTCGGTATGTTATTGT  57090
NorthernAmericanDeerMouse    GTCTTACGCTGCTTGCTCTTCTCTCCTTCTAAAGATGGTTACGCCCCAAAGTCTGTTTACCCTCTTCGGTATGTTATTGT  61962
Mouse                        GTCTTACGCTGCTTGCTCTTCTCTCCTTCTAAAGATGGTTACGCCCCAAAGTCTGTTTACCCTCTTCGGTATGTTATTGT  61867
ChineseHamsterGHOK1GS        GTCTTACGCTGCTTGCTCTTCTCTCCTTCTAAAGATGGTTACGCCCCAAAGTCTGTTTACCCTCTTCGGTATGTTATTGT  65801
LongTailedChinchilla         GTCTTACGCTGCTTGCTCTTCTCTCCTTCTAAAGATGGTTACGCCCCAAAGTCTGTTTACCCTCTTCGGTATGTTATTGT  63046

Majority                     TAGCACTATACTTTTATTATTGATTTGATTTTTXX-GTTTCACCTTAATTCTTATTTGTAGCTAGCACTTTGGCTTAAAG
                                       92330     92340     92350     92360     92370     92380     92390     92400
Human                        TAGCACTATACTTTTATTATTGATTTGATTTTT---GTTTCACCTTAATTCTTATTTGTAGCTAGCACTTTGGCTTAAAG  85477
GuineaPig                    TAGCACTATACTTTTATTATTGATTTGATTTTT---GTTTCACCTTAATTCTTATTTGTAGCTAGCACTTTGGCTTAAAG  57167
NorthernAmericanDeerMouse    TAGCACTATACTTTTATTATTGATTTGATTTTTTT-GTTTCACCTTAATTCTTATTTGTAGCTAGCACTTTGGCTTAAAG  62041
Mouse                        TAGCACTATACTTTTATTATTGATTTGATTTTTTTTGTTTCACCTTAATTCTTATTTGTAGCTAGCACTTTGGCTTAAAG  61947
ChineseHamsterGHOK1GS        TAGCACTATACTTTTATTATTGATTTGATTTTTTTTGTTTCACCTTAATTCTTATTTGTAGCTAGCACTTTGGCTTAAAG  65881
LongTailedChinchilla         TAGCACTATACTTTTATTATTGATTTGATTTTT---GTTTCACCTTAATTCTTATTTGTAGCTAGCACTTTGGCTTAAAG  63123

Majority                     TTGAATAGTAAATCTTTTGCTATTTTTCTTTGCTATTTAAAACTCTCCATAGACACAAAXTTTGTTTTAATGCATGCTGA
                                       92410     92420     92430     92440     92450     92460     92470     92480
Human                        TTGAATAGTAAATCTTTTGCTATTTTTCTTTGCTATTTAAAACTCTCCATAGACACAAAATTTGTTTTAATGCATGCTAA  85557
GuineaPig                    TTGAATAGTAAATCTTTTGCTATTTTTCTTTGCTATTTAAAACTCTCCATAGACACAAAATTTGTTTTAATGCATGCTGA  57247
NorthernAmericanDeerMouse    TTGAATAGTAAATCTTTTGCTATTTTTCTTTGCTATTTAAAACTCTCCATAGACACAAAGTTTGTTTTAATGCATGCTGA  62121
Mouse                        TTGAATAGTAAATCTTTTGCTATTTTTCTTTGCTATTTAAAACTCTCCATAGACACAAAGTTTGTTTTAATGCATGCTGA  62027
ChineseHamsterGHOK1GS        TTGAATAGTAAATCTTTTGCTATTTTTCTTTGCTATTTAAAACTCTCCATAGACACAAAGTTTGTTTTAATGCATGCTGA  65961
LongTailedChinchilla         TTGAATAGTAAATCTTTTGCTATTTTTCTTTGCTATTTAAAACTCTCCATAGACACAAAATTTGTTTTAATGCATGCTGA  63203
```

```
Majority                     TTTATTTTGCATGGTXTTTAATTTAATATCATTCACATAGCTTTGAGGGTTTATCAXAAATTATTCTTTTCAAAATTCAC
                                      92490     92500     92510     92520     92530     92540     92550     92560
Human                        TTTATTTTGCATGGTCTTTAATTTAATATCATTCACATAGCTTTGAGGGTTTATCAAAAATTATTCTTTTCAAAATTCAC  85637
GuineaPig                    TTTATTTTGCATGTTCTTTAATTTAATATCATTCACATAGCTTTGAGGGTTTATCAGAAATTATTCTTTTCAAAATTCAC  57327
NorthernAmericanDeerMouse    TTTATTTTGCATGGTTTTTAATTTAATATCATTCACATAGCTTTGAGGGTTTATCAAAAATTATTCTTTTCAAA-TTCAC  62200
Mouse                        TTTATTTTGCATGGTTTTTAATTTAATATCATTCACATAGCTTTGAGGGTTTATCAAAAATTATTCTTTTCAAAATTCAC  62107
ChineseHamsterGHOK1GS        TTTGTTTTGCATGGTTTTTAATTTAATATCATTCACATAGCTTTGAGGGTTTATCAGAAATTATTCTTTTCAAAATTCAC  66041
LongTailedChinchilla         TTTATTTTGCATGGTCTTTAATTTAATATCATTCACATAGCTTTGAGGGTTTATCAGAAATTATTCTTTTCGAAATTCAC  63283

Majority                     TGTTCAAAATCTTGXTCTTCTTTATTCATTTGTGAXAATGXTGAGATTGAGTGAGTGATCATGTTGATGTCTGAATGTTT
                                      92570     92580     92590     92600     92610     92620     92630     92640
Human                        TGTTCAAAATCTTGATCTTCTTTATTCATTTGTGAGAATGATGAGAT----TGAGTGATCATGTTGATGTCTGAATGTTT  85713
GuineaPig                    TGTTCAAAATCTTGATCTTCTTTATTCATTTGTGAGAATGATGAGATTGAATGAGTGATCATGTTGATGTCTGAATGTTT  57407
NorthernAmericanDeerMouse    TGTTCAAAATCTTGTTCTTCTTTATTCATTTGTGCAAATGGTGAGATTGAGTGAGTGATCATGTTGATGTCTGAGTGTTT  62280
Mouse                        TGTTCAAAATCTTGTTCTTCTTTATTCATTTGTGTAAATGGTGAGATTGAGTGAGTGATCATGTTGATGTCTGAATGTTT  62187
ChineseHamsterGHOK1GS        TGTTCAAAATCTTGTTCTTCTTTATTCATTTGTGCAAATGGTGAGATTGAGTGAGTGATCATGTTGATGTCTGAATGTTT  66121
LongTailedChinchilla         TGTTCAAAATCTTGATCTTCTTTATTCATTTGTGAGAATGATGAGATTGAATGAGTGATCATGTTGATGTCTGAATGTTT  63363

Majority                     CATTGATXTGTCAGAAAGATAATCT----TTAGXTGACTTTCATATAAAATTAATTCCATTTXTGGATTACCTCTCTGTG
                                      92650     92660     92670     92680     92690     92700     92710     92720
Human                        CATTGATATGTCAGAAAGATAATCT----TTAGGTGACTTCCACATAAAATTAATTCCATTTTTGGATTACCTCTCTGTG  85789
GuineaPig                    CATTGATATGTCAGAAAGATAGTCT----TCAG----CTTTAATGTAAAATTAATTCCATTTTTGGATTACCTCTCTGTG  57479
NorthernAmericanDeerMouse    CATTGATGTGTCAGAAATATAATCT----TTAGATGACTTGCATATAAAATTAATTCCATTTCTGGATTACCTCTCTGTG  62356
Mouse                        CATTGATGTGTCAGAAAGATAATCAATCGTTAGCTGACTTTCACATAAAATTAATTCCATTTCTGGATTACCTCTCTGTG  62267
ChineseHamsterGHOK1GS        CATTGATGTGTCAGAAAGATAATCT----TTAGCTGACTTGCATATAAAATTAATTCCATTTCTGGATTACCTCTCTGTG  66197
LongTailedChinchilla         CATTAATATGTCAGAAAGATAGTCT----TCCG----CTCTCATGTAAAATTAATTCCATTTT-GGATTACCTCTCTGTG  63434

Majority                     XCTCCAXAAAAAGGTGTTTATGGAGATGTGCAGCGCGTGAAGATTTTGTACAATAAGAAAGACAGTGCTCTXATACAGAT
                                      92730     92740     92750     92760     92770     92780     92790     92800
Human                        GCTCCA-AAAAAGGTGTTTATGGAGATGTGCAGCGTGTGAAGATTTTATACAATAAGAAAGACAGCGCTCTAATACAGAT  85868
GuineaPig                    ACTCCA-AAAAAGGTGTTTATGGAGATGTGCAGCGCGTGAAGATTTTATACAATAAGAAAGACAGTGCTCTAATACAGAT  57558
NorthernAmericanDeerMouse    ACTCCACAAAAAGGTGTTTATGGAGATGTGCAGCGCGTGAAGATTTTGTACAATAAAAAAGACAGTGCTCTGATACAGAT  62436
Mouse                        GCTCCACCAAAAGGTGTTTATGGAGATGTGCAGCGCGTGAAGATTCTGTACAATAAGAAAGACAGTGCTCTGATACAGAT  62347
ChineseHamsterGHOK1GS        GCTTCACAAAAAGGTGTTTATGGAGATGTGCAGCGCGTGAAGATTTTGTACAATAAGAAAGACAGTGCTCTGATACAGAT  66277
LongTailedChinchilla         ACTCCA-AAAAAGGTGTTTATGGAGATGTGCAGCGCGTGAAGATTTTGTACAATAAGAAAGACAGTGCTCTAATACAGAT  63513
```

Majority	GGCTGATGGXAACCAATCACAGCTTGGTAAGAXXAAAC---X--TAXCTTXXGTATACATTTTCAXTTCXXATTTCAAXT	
	92810 92820 92830 92840 92850 92860 92870 92880	
Human	GGCTGATGGAAACCAATCACAACTTGGTAAGATTAAAC---T-ATGTTTTATCTATACATCTTCACTTCTGCTTTCAAAT	85944
GuineaPig	GGCTGATGGAAATCAATCACAGCTTGGTAAGATTAAACTACTTGTTACCTACATGCACATTTTCAGTTCTCAATTCAAA-	57637
NorthernAmericanDeerMouse	GGCTGATGGGAACCAATCACAGCTTGGTAAGAGGAATA------TATATTCCGAATTTGTT-----TTAAGATTTGTATT	62505
Mouse	GGCTGATGGGAACCAGTCCCAGCTCGGTAAGAGGGGGC------TACCTTCCCTATACACTGAAATTCTACATTTAAATG	62421
ChineseHamsterGHOK1GS	GGCTGATGGGAACCAGTCACAGCTCGGTAAGAGGAATA------CACATTCTGAATTCATT-----TTAAGATTTCTATT	66346
LongTailedChinchilla	GGCTGATGGAAACCAATCACAGCTTGGTAAGATTAAAC---TTGTTACCTATGTGTATATTTTCAGTTCTCAATTCAAA-	63589

Majority	CCAXAGTXTAXATCTXTXAATAAAAATAAACTXXTTTATATAAGTCAX--AAATCAXTTGX----TTAATGXCCTGGXXT	
	92890 92900 92910 92920 92930 92940 92950 92960	
Human	GCATAATGTGAATGTGCGAATAAAAATAAACTCCTTTACATCAGTAAG--AAATTATTT-------TAATGGCCAGGGCT	86015
GuineaPig	-CACCATATAAACCT-TGAATAACAATAAACCACTTTATGTGACTTAA-GAAATCGCTTATACTTTTAATTGCCTGTGTT	57714
NorthernAmericanDeerMouse	C--CTGATTATTTCCTGAATTAGAAAAGAA-TTGTTCATAAAGGTCAT--AAAAACCCTGA----TTAATGTCCTTGTAT	62576
Mouse	CAGTAGTCCATATCTGTGACTAAAAACAGA----CCCTTGTAATTCAG--AGATCATTTG------TGACTTTTTAATGA	62489
ChineseHamsterGHOK1GS	C--CTGGTTATTTCCTGAATTAGAAAAAAAATTGTTTAAATAGGTCAT--AAAA-CCCTGA----TTGATGTCCTTGTAT	66417
LongTailedChinchilla	-CATAGTGTAAGCCT-TAAATGAAAATAAGCCATATTATATATATGACTAAAATCATTTATATTTCTAGTGGCCAGTGGT	63667

Majority	XXAAX----XXTCTAAXATTAATTTXTAXXTXTTG-XTTCATTTTXGXAXTGAXATXGXATCTTCCTGXTGTATTTTAAG	
	92970 92980 92990 93000 93010 93020 93030 93040	
Human	CAAAATGTCATTTTAATGTGAATAGTTGTATGTGACGAACATATTTGTATTTTTCTGCTATCCTCCTACTGTTTTTCAAA	86095
GuineaPig	CAAAA----ATCCTCATTTTAATTT--ACCTCCC-----CTCCCCCGCACTGAGATAGGTTCTTGCTG-TGTATTCTAGG	57782
NorthernAmericanDeerMouse	TCT--------TCTAAAAATCATTTATAGGTATTG-ATTCAATTTAGCAATGGAAAGGAAAATTCCTTACAGATATTAAG	62647
Mouse	TCAAG------TCTGAAAATGTTAT-TAACTTTTA-TATTTTTATAGTTTATACATAGTGTGTCCTTGTTTTGCTTTATT	62561
ChineseHamsterGHOK1GS	TCT--------TCTAAAA-TCATGTGTAGGTATTG-ATTGAACTTGGCAGTGGAAAGGAAAAT-CCTTAGATATGTGAAG	66486
LongTailedChinchilla	CAAAG----ATC-TCATTTTAATTTTCACATTTTGGGTTCCTTTTTTTTCTGAGATAGGGTCTTGCTG-GGTAGTCCAGG	63741

Majority	TTTXCXAAAATGAGAA--X-AGCTTXAXCXATXXXXXXGTTAGCTCAXAGACATA-GAXTXTAG-XXTCAGTXACCTTXC	
	93050 93060 93070 93080 93090 93100 93110 93120	
Human	TGCACTCATAATAGGAA-GAGATTTTAGCTTCAAAATTGTAATAATAGTTTCACATGAGAATTA-TATCATTTATATTGA	86173
GuineaPig	CTGGCTTC----AGAT--GTTGCTAACATCCTACCTCACCCACCTCAGTG--CTGGGGTTATAG-TGTCCACTACCATGC	57853
NorthernAmericanDeerMouse	TTTTACAAAACAGAAA----ATCTTCACCAATG-----GTTAGTTCAAAGGCATA-AAATGTAG--TTTAGTCACGTTAC	62715
Mouse	TCTAACAAATTGAGCAC---AGCTTTATGTAAAAGGATGTTAGCCCCAAAACTGA-A---GTGA--CTT--TTGCTTAAG	62630
ChineseHamsterGHOK1GS	TCTTGTAAGATGAAGA----ATCTTCACCAATG-----TTTAGTTCAAAGACAT------GTAG--TTCAGTCAGCTTAC	66549
LongTailedChinchilla	CTGGCCTCCCTCAGATTTGTGGCCAACATCCTGCCTCAGCCTTCACAGACACCTGCGATTACAGGTGTCAGCCACCATGC	63821

```
Majority                    --AXXTGXATXATTTTTTTTXATAGATGXXXXAAGTXTCTAGTGTATXTCTTTATATTCAGCTTGTATA------AXTCT
                            93130 93140 93150 93160 93170 93180 93190 93200
Human                       AGATTCATAGTGATTTTTATTCTAGGTACTCCTTGTTAATAGAGGCTAACTGTTCATACAGTCCATAGA-ATCCTTCTTA 86252
GuineaPig                   -----TGAATGCATTTGTTTTTCTGTTTTCCTAAGTGTTTAGTGTATGTTTTCATTTTCAGTTTGTGTATACAGAATTCT 57928
NorthernAmericanDeerMouse   --ATCTGTATCATT--TTTTCATACATGCTTCCA---TCTAG----------TATACTCAGCATGTAAT------AATCT 62772
Mouse                       --AGATTAGAAATAATTGTTCATATAGGTCATAAAATCCTATTCAATGTCCTTGTGTTTTTCTAAAATC------ACTTT 62702
ChineseHamsterGHOK1GS       --AAATATATCATTGTTTTTCATACATGCTTCAA---TCAAGAATACATCTATATACACAGCATGTAAT------AATCT 66618
LongTailedChinchilla        CCAGCTGAATACGTTTGTTTTTCTGCTTTCATAAGTGTCTAGTGTTTCATTTCCTTTTCAACTTGGGTATGCAGAGTTCT 63901

Majority                    ATXCTCT-XXGAGXCTATTTATXACTXXAGTCTT--------AGXTT---AGXATTTGAXXTACTXAXAXTXAAAXCAXT
                            93210 93220 93230 93240 93250 93260 93270 93280
Human                       ATGCCAT-GTTATTTTAATTCTT-CTAAAGTCTTGCGATACCAGGTTCTTAGAAAATGAAAAGCTTGTGCTAAAAACACT 86330
GuineaPig                   GTGCTCTAGTGAGCATAATCATTACTGTGACC------------------AGCATTTGACATACTCA-AC--AAAGCAGT 57987
NorthernAmericanDeerMouse   ATAATCT---GCATC--TCTGTCACCAGGGTCTT--------AGCTT---ACAATTTAATTCATTAACATTCA-AAAATG 62835
Mouse                       AGATTTT---GACTCAGTTTGGCAGTGGAAGGGG--------AAAT----ATCCTTTGAGTTGCTGAGTTTTACAAAACA 62767
ChineseHamsterGHOK1GS       ATACTCT---GTGCC--TGTATCACTAAACTCTT--------AGCTT---ATAGTTTAATTCATTAAGATTCA-AGTATG 66681
LongTailedChinchilla        GTGCTCTGGTGAGCATAGTCATTGCTGTTGTTTTTGTGTGTACTGTAGGAAGCATTTGACATAGTCA-GC--AAAGCAGT 63978

Majority                    CTAAXTCTTCAXTTTGXTTTTATTCAX-X-XATTTT--TTAAGATXTXGATTXTAXCATTTCTT----------------
                            93290 93300 93310 93320 93330 93340 93350 93360
Human                       GTTGTTAATCCCTTTGGGAATCTTAAAAGGCACTTTAGTCAGGTTACACTTTATATCATTACTTAAATAAGTAATTGATT 86410
GuineaPig                   CTAAAGT----ATTTGATTTTGTTTTTTGTCATTAT--TTGAGATATAGATGGTTTTCCCTCATG--------------- 58046
NorthernAmericanDeerMouse   AGGCTACTTTATTCTGATTGCATTCAC----ATTTT--TTAATCTTTGAAATTTACCATTTTTG---------------- 62893
Mouse                       AAAAATCTTCACCAATGCTTAATTCA-----AAGGC--ATAAAATGTAGTTCAGTCAAGTTCCA---------------- 62824
ChineseHamsterGHOK1GS       CTACTACCTTATTCTGATTGCATTCA-----GTCTT--TTAAAATTTGAAATTTACCATTTTTG---------------- 66738
LongTailedChinchilla        CTAAATTTGCTATTTGGTTTT-TTCCTTGTCACCGC--TTGAGGTGTGGGTGGTCTTACCTCATGAT--GCTGAGTCATC 64053

Majority                    G---X--TGAAATTACTGTCXX---XTTTCA-AXATGTTXGAXTTTXTTXTAXAAAXAXATTXTXTTAAGT------TAG
                            93370 93380 93390 93400 93410 93420 93430 93440
Human                       ATTTTAATTAAACTTATGTCGTTTGACTTCATACATCCTCGAATACATAAATCTAGAACACACCTGCACATTCACACCAG 86490
GuineaPig                   ---GTGGAGAAGTACCTGAGGTAGAATCTCAGAAGCCTTAGGTTTTGTTTTAGGTAAAGATTCTTTTAAGT----GATAG 58119
NorthernAmericanDeerMouse   A------TGGGATTAATGTT------TTTTG---ATGTT--AAACCCATCTGAAACTCTGTGAACTTAGCT------CAG 62950
Mouse                       G------GTATATCACTGTC------TTTCACACATGCTTCAGTGTAGAATACAACTGCATGCTCAGAATG------TGA 62886
ChineseHamsterGHOK1GS       G------TG---TTATAGCC------ATCTG---AAGT-----------CTGTAAATGTAGTAGCTCAGGT------GAA 66783
LongTailedChinchilla        GTGGTGGAGAATTACCTGAGGTAGAATTTCAAAAGTTTTTGGTTTTGTTTTATATAAAGACTATTTTAAGTAAGTGATAT 64133
```

```
Majority                     T--XTAXTTGTAAXXATXACCXXAXTCTTXGATTT---TAAAXTGATACTXTGCXXTXXXGATTXXXCAGXAXAAAX-XX
                                      93450     93460     93470     93480     93490     93500     93510     93520
Human                        TGATTGACTGTAAATGCCAGGATGTTCTTACATTTCTGTTCATTGAGATTCAGGGATAATGGTGCCTCAGACTGATTGTA  86570
GuineaPig                    T--TTATTTTTTATCATAACAGTATG-----------ATACCAAAGTACTTTGCTGTGGGGCTTGAACATCAGAAATTTA  58186
NorthernAmericanDeerMouse    C---TGATAGAAA--ATTATCTGACTTTCTGATTT---TAAAGTGACAGTATCT-------AT-----------------  62998
Mouse                        T---TATTTGTAACTATCACCAGGCTCTTAGCTTC---TAATTTGATTCATTGCA------ATT--CAAGAACGAC----  62948
ChineseHamsterGHOK1GS        ------ATAGAAA--ACTACCTGACTTTCTGATTT---TAAAGTGACAATACCCATGTTAAATGTGCTAGTCTATA----  66848
LongTailedChinchilla         T--TTATTTTTTATCATAACAGTATTCTTGGACTACCATAACAAAATACTGTATGGTGGAGTTTGAACAGTAGAAATTTA  64211

Majority                     XXCAAAXGTXTTTGGXXTGTAGTTTTCXX-XTTAGTTTGTXAGCATGTCTXTXTTTTXGAGGXGACTCAATGCTXTTXTT
                                      93530     93540     93550     93560     93570     93580     93590     93600
Human                        TTCACA--TTGTTAATTTGTAAATTTGCCATTTTTGATGTAAGGACGAGTGTATTTTTGCTCAGAGATGAACAACTTGCT  86648
GuineaPig                    TTTAAAAATATTTGGATGGTAGAGTTCCAGATCAGGGTATAAGCAGATTTATATTTTGGAAAGGGCTCCATTCTCTTTTT  58266
NorthernAmericanDeerMouse    -----AGGTGTTTGGTCTATCATTTTCT----TGGTTTGTGTGCATGTCTCTTTTTTTGAGGTGACTCACTGCCATTATT  63069
Mouse                        -GCTACCTTTTTCTGACTGCATTCACATT-TTTAACTTTGGAATTTACCATTTTT---GAAGGGATTAATTGTTACAGCA  63023
ChineseHamsterGHOK1GS        -ACACAGGTGTTTGGTCTATTGTTTTCTC-CTTGGTTTGTGTGAGTGTCTCTTTT---GAGGTGACTCATTGCCATTATT  66923
LongTailedChinchilla         TGAAAAAGT-TTTGAATGGTAGGGTCCCAGACCAGTGTACAAGCAGGTTTGTATTTTAGAGAAGGCTTTATGCTCTTTTT  64290

Majority                     ATTX-TTATCCCTXTGTACTTGGTAGGXTTTXTACTGTXXAAXXXXAATTXTXXGXAXCAAAT-----------------
                                      93610     93620     93630     93640     93650     93660     93670     93680
Human                        ATAT--TAGACCAATTTTGGCCTTTGACATCACATAGAAAATTTTGAATTT---CAAACACATAATAGGTTTGAATTTTA  86723
GuineaPig                    CTTC-TTACCCCCATGTCCTTG-TCAGCTTTCCACTGTGAAAGCAGAGTGGGGGAAAAAATATCCCTTCCTTTTGTTTTT  58344
NorthernAmericanDeerMouse    -TTG-TTAT-------CACTGAGTAGTTTTTGTGGTGTTTTTTGGTAATTCTTCGGATCAAAT-----------------  63123
Mouse                        ATCA-GGAGCTCTGTGAACTTGGTAGGTCAGATACAAAACTA-TCTGATTTTTCTGGTTTAA------------------  63083
ChineseHamsterGHOK1GS        ATTG-TTATTATTTATCACTGAGTAGCTTTTGTGTTGTTT---GGTAGTTCTGTGGATCAAAT-----------------  66982
LongTailedChinchilla         CTTCCTTTTCCTTGAGTCCTTGCTTAGCTTTCTACTGTGCAAGCAGAATG-----AAAAAAA------------------  64347

Majority                     ------------------------------X---------X-----------------------CC--------------
                                      93690     93700     93710     93720     93730     93740     93750     93760
Human                        GT---GTTTCTTGGCAATAGTCTTAGCAACTCAGATGAGGTATAAAACTATCCATCTGGCTGT-CCATTTTTAAAATGGT  86799
GuineaPig                    TTTTTGTTTGTTTGTTTTTGTTTTTGTTTTTGTTTTTGTTTTTGTCCTTTCGTTTTTATCAGTACCGGGAATCAAACTCA  58424
NorthernAmericanDeerMouse    ----------------------------------------------------------------CC--------------  63125
Mouse                        --------------------------------------------------------------------------------  63083
ChineseHamsterGHOK1GS        ----------------------------------------------------------------CC--------------  66984
LongTailedChinchilla         ------------------------------T---------T---------------------------------------  64349
```

```
Majority                    --------------AGAGA----------------------------------------XCCTTCATATTXTAAACAXXX
                            93770 93780 93790 93800 93810 93820 93830 93840
Human                       GGTATGTGTTTTAAAGAGATACGAAAGTGCTGATTATGTCAAGATACTTAGTACATATTTTCCTCTTTCTCTGTGTGCAC 86879
GuineaPig                   CGACTGCCTGCTTACAAGGCAGGCACTTATGCCACTGAGCTAAATCCCCAGCCACTTATCCCTTCCTTTTGTAAAGACAG 58504
NorthernAmericanDeerMouse   --------------AGAGA----------------------------------------TCTTGCATATACTAGACAGGT 63151
Mouse                       --------------AGTGG----------------------------------------CAATACATATTTTAAATGTGC 63109
ChineseHamsterGHOK1GS       --------------AGAGA----------------------------------------TCTTGTATATGTTAGGCAGGT 67010
LongTailedChinchilla        -----------------------------------------------------------CCCTTTCTCTTGTAAACATAG 64370

Majority                    TCCXGXT---XTTATGXXXTXGATATTA-GXACTGXXTTTXTCTTCAXXTXTXTTTTAAGAXGXXXGTGTCTCTGCATAG
                            93850 93860 93870 93880 93890 93900 93910 93920
Human                       TTCTGT----ACTCTGGTTGGAATAGT---CATTGCTGTTGTTTTCATATATGTTGTAAGTAGCACTTAACAGTACTTCA 86952
GuineaPig                   TCCTATT-AGATTATGACCTTGCCATTATGAACTTACTTTACCTTCAT-TGCCTCTTTAAAG---TCTGTCTTTGCATAG 58579
NorthernAmericanDeerMouse   CCTCTG--------------AGCTA-TA-GACCTGCCTCACTGCCTAGGGGTGTTTCAAGACAAGAGTTTTTCTGCATAG 63215
Mouse                       TAGTAGC----TTAAACA-TTGATATTT-GGTATGTCATTTTCTCCTGGGTT-TATGAACATGCT-TTTCTTCTGAGGTG 63181
ChineseHamsterGHOK1GS       CCTCGGT----TTTTTCAACAGTAACTA-GTTTTTGTTTACTTTTTTGTTCT-TTTCGAGACAGG-GTGTCTCTGTGAAG 67083
LongTailedChinchilla        TCCCGTTTACATTATGATTTCAACATTATGCACTCATTTTATCTTCAT-TACCTCTTCAAAGG--TGTGTCTATGCATAA 64447

Majority                    AATTGTCAGTXTTTTGATATXTGTGAAT--X-X--XXXTAATTTTXTAAGXXAX-------X-XXTTTXAXTTGAATTCT
                            93930 93940 93950 93960 93970 93980 93990 94000
Human                       TAATAGGAGTATTCTCAAATATGTCATT--TTTTTCCCTAATTGTTTGAGGTATGGGTGGCATTGTGTCATTTAAATAA- 87029
GuineaPig                   AATCATCACAGTTTTTATACATGTAAATCATGTAACCCCTGTTTCATAAGGTA---TCACTAAAATTTTACTTGTTTTTT 58656
NorthernAmericanDeerMouse   TCCTGGCAGT-CCTGGCTGTCCTGGAAC----------TCACTTTGTAGACCAG----------ACTGGCCTTAAACTCA 63274
Mouse                       AGTTGTTGCTATTTTGTTATCACTGAAT----------TAATTAATTAATTCATT--------CATTCATTTTTAATGCT 63243
ChineseHamsterGHOK1GS       ------CAGT-CCTGGTTATCCTGGAAC----------TTGCTCTGTAGACCAG----------GCTTGCCTTGAACTCA 67136
LongTailedChinchilla        AATTGTTATAGTTTTTAAATATGTCAATCGTGTATCCCCAATTTCATAAGATACTGTCACTAAAGTTTTATTTGACTTTT 64527

Majority                    GGG----ATATXTAGXXTTTTGTXTTTGCTTATCAX-AGTGCTAGGAT----X----X-X-TTAXATXTTTGTX------
                            94010 94020 94030 94040 94050 94060 94070 94080
Human                       GGG----ATGCATAGTCTTT-ATGGTTGGTAATTACCTGTGGTAGAATTACCTCAGATCTTCCAGATAGTTTTT----GT 87100
GuineaPig                   GGGC--TATATGTAGCATT--GTTTTCATTTATCACTGCTGCTAGGATAGATTTTGTTTTCTTAGAAATATGTATAATAC 58732
NorthernAmericanDeerMouse   GAG----ATCCTTC----TCTGCCTCTGCCTCCCA--AGTGCTGGGA--------------TTAAAGGCTTGTG------ 63324
Mouse                       GGGTATCAAATCCAGAGATTTGTACATGCTAGGCA--AGTTCTCTGAG-------------CTAAAGCCCCAGC------ 63302
ChineseHamsterGHOK1GS       CAG----AGATCCT----CTTGCCTCTGCCTCCCA--AGTGTTGGGA--------------TTAAATGTGTGTA------ 67186
LongTailedChinchilla        GGAC--TATATTTAGAATTAAGTTTTTCTTTATCACTGCTGCTAGGATAGATTT--TTTTCTTGGATTTTTTTT------ 64597
```

```
Majority                      -X--------------CCXCTG----X--XTGATCXTXTACAXTACXTGXXTTTTGTATGXAGCATT-TXATAXTXTTXC
                                             94090      94100      94110      94120      94130      94140      94150      94160
Human                         TTTATGTAAAGACTGTTTTATGTAATATTTTAATTTTTTGCCATGCTCGTAGTCTGCTTGGGCTGCTGTAACCAAAATAC  87180
GuineaPig                     ATACATCTTAGAAATACCTGTGATACATCTTGATCGTTCACAAAACTAGGATTTGTTATGGAGAAGAATAAAGAGACTTA  58812
NorthernAmericanDeerMouse     ----------------CCACCA-------CTGCTCAGCTACAGTAACT-ATTTTTGTATACAGCATT-TGATAGTGTTCC  63379
Mouse                         ----------------TCACTG-------TTGTTTATGTATAGTGCCT-TTCTTTGTAAGTAACATT-TGACAGTGTTAC  63357
ChineseHamsterGHOK1GS         ----------------CCACCA-------CCAACAGGCTACAGTAACTTATTTTTGTATGTAGCATT-TGATAGTGTTCC  67242
LongTailedChinchilla          -T--------------CTTGTG---TACCCTGATCCTACACAAAACTAGGATTTGCTGTGAAGGAGAATAATGAGACTTA  64659

Majority                      XAG---TXAACAXXAATXXTTT-----TCXXGTACTTXCTTTTXTTAATTXTXTTXGAATTGAAXATGGCTTTXATCATC
                                             94170      94180      94190      94200      94210      94220      94230      94240
Human                         CA----TAGAC-TGAGTAGATGAAACAACAAAAATGTATTTCTTCAAAGTTTTGAGAAATAAAACTTTGGGGTCTAGAAG  87255
GuineaPig                     CAAGATTAAATATGCATTGTTTCTGTGCTCTGTTCTCACCCTTTTTGAGTTTTATGGAACTATATATGGAAATACTCATT  58892
NorthernAmericanDeerMouse     TAG---TGAACAAAAAC-ATTC-----TCAGGTACTTCCTTTTCTTACCAGTATTTGAATTGAACATGGCTTTTATCTTC  63450
Mouse                         TCA---TGAACAAAAAACATTT-----TCAGGTACTTCCTTCTCTTATTTGTATTTGAATTGAGTATGGCTTT-ATCTTC  63428
ChineseHamsterGHOK1GS         TAG---TGAGCAAAAG--ATTC-----TCCAGTACTTCCTTTTCTTAATAGGATTTGTATTGAACATGGCTTT-GTCATC  67311
LongTailedChinchilla          CAGAATTAAATATGCTTCGTTTATGTGCTCTGTTCTTACCCTTTTTGGTTTTTATGGAACTGTTTATGGAAATA-TTATT  64738

Majority                      XTXTAXGT-XX--X-TXTAXCAAXTXXTXCATAXTGAGAAXT-TX-TGTXGTATAGTGACCAAT-XXTTTATTCTGAATA
                                             94250      94260      94270      94280      94290      94300      94310      94320
Human                         TCCCAGAT-GAAACTGCTAGCAGATTGTTTCTGGTGAGGTCTCTCTTGGCTTGCAGCCAACCAT-CTTCCCCC-TGTGTC  87332
GuineaPig                     TTATAGGCAGATGCCTTGATTAAGAAGTACGTAGTGAAAAAATTA-AGATATATAGTAGCAAAT-TTTTAAATATGAGTG  58970
NorthernAmericanDeerMouse     CTGTAAGT-------TGTACCAAATTAATCATATTGAGAATTGTC-TGTGGTATAGTGATCAACAAATTTATTCTGAACA  63522
Mouse                         CTTTAATT-------AATAACAAGT----CATCATGAGA---------T---ATGGGGACTAACACATTTATTCGGAACA  63485
ChineseHamsterGHOK1GS         CTGTAAGT-------AGTACCAAAT----CATATTGAGAATTATA-TGTGGTATAGGGATCAA---ATTTTTTCTGAATA  67376
LongTailedChinchilla          TTATAGGCAGATGCCTTCATTAAGAAATGCACAATGAGAAA-----AGATGTATAGTTCCTAAT-TTTTTAAT--GAATG  64810

Majority                      CTTXTTG----TTTTXATTTTAXAXGX-----AAGACTXXXTT-----XX-TAAXCAX----GTGGTACT-XXTTXAXCT
                                             94330      94340      94350      94360      94370      94380      94390      94400
Human                         CTCATGTGGCCTTTCCACTGTGCAAGCACGTTAAGAGTCTCTT-CCCCTTGTAAGGAT---CCTAGTCC--TGTTGAATC  87406
GuineaPig                     CTGATTA---TTTTGTGTCTTAGAAGCTTTGCTAAATCAATTT-TAGTTTGCAAGCATTAAGATGTTTCTTTGATGAACT  59046
NorthernAmericanDeerMouse     ATTTTTG----T-TTCATTTTATATG------AAGACT----T--------TAAACAA----GTGGTATT---TTAATCT  63572
Mouse                         CTTTTTG----TTTTCATTTTATATA------AAGACTCACTT--------TAAACAA----GTGGTACC---TTAATCC  63540
ChineseHamsterGHOK1GS         ATTTTTG----TCTTTATTTTACATA------CTTACT----T--------TAAACAA----GTGGTATT---TTAATCT  67427
LongTailedChinchilla          GTAATTA---TTTTATGTTTTAGAAGCTTTACTAAATTATTTTACAGTTTTCAAGCATTAACATGTTTCTTTGATGAACT  64887
```

```
Majority                    TTGT--X--TXTGCTTXXTTACCACAXCAAXATXCTTAATTX-AXXXTTTTAAXGATAXXATTTXTAAXTXXXXXTGTTT
                                      94410     94420     94430     94440     94450     94460     94470     94480
Human                       AGGG--CTTTACCCTTAATGACCACATT--TAACCTTAATT--ACCTCCTTAAAGGTCCTGTTTTTAAGTACAGTTACAT 87480
GuineaPig                   TTGT-ACCGTAACCCTATTTAACAGATTGATATTTTTAATTG-GACATTTTAAGGATATAATTTGTAAATTTTTTTCTTT 59124
NorthernAmericanDeerMouse   TTAT-----TGTGTTTGGGTGCCACAACAAAATGCTCA-------------------------------------AGTTA 63610
Mouse                       TTAT-----TGTGCTTGATTGTCACAACAAAATACTATGATGTAAAGTTGAAACAACAGAAAGTAGTTTTCTCGAAGTTG 63615
ChineseHamsterGHOK1GS       TCGT-----TGTGCTTGGATACCACAACAAAATGCTC---------------------------------------GTTA 67463
LongTailedChinchilla        TTGTTACTGTAAACCTATTTAACTGATCAGTATTTTTAATTG-AGTATTTTGAGGATATTATTTGTAAATTCTCGTCTTT 64966

Majority                    TGGATGATAGAGXTCCAXATXAAXXTGCTGAXXTGAATGXAAGXCXXTC-X-X-XXTXGTTGAGXTTGGAGAXAXXC-AX
                                      94490     94500     94510     94520     94530     94540     94550     94560
Human                       TGGGGGTTAGGACTTCAACATATAAATTTGGGGAGGACACAATTCAGT--TAATAGTACTTAAATTTGTAAATCATTTAT 87558
GuineaPig                   -AAAAAGTAGTACACTGATTTTCAAAGATGAAGTGAATTCTAGTAAAACGTGAGAGTAGTTTCTTTATGATAAAATAGAT 59203
NorthernAmericanDeerMouse   TGGATGATAGAGTTCCAGATCAAGGTGCTAACATAAATGGAAGGCTATC-------TTGTTGAGCTTGGAGACAGCC-AC 63682
Mouse                       TGGCTGACATATTTGCAGATCAAGGTGCTGATACCCATGGAAGGCTGTC-------TTGTTGAGCCTCGTGACAGC--AC 63686
ChineseHamsterGHOK1GS       TGGATGATAGAGTTCCAGATCAAGGGGCTAACATGCATGGAAGGCTGTT-------TTGTTGAGCTTGGAGAGAGCC-AC 67535
LongTailedChinchilla        TAAAAAATAGTGCACTGATTTCCAATGATGAAGTGAATTCTAGTGAAATGTGAGAGTAATTTCTTTATGGTAAAATGCAT 65046

Majority                    CCXTACXTXTXCTATTCAT-GTTTTTA---CXXAXT-TTAXXAXTXTXXCTXTTXXXXTTTXXXXTATGAGXATAXTGAA
                                      94570     94580     94590     94600     94610     94620     94630     94640
Human                       CCCAAATTTTATAAGATATTGTCACTAAAGTTTTGACTTGCTAGTAGGGCTTATAATTTTTGGACTATCTGTAGTGGTAA 87638
GuineaPig                   ACCTAGTTTTTCATTTGT-GTTTTTAGGAATTACAGTTAAAAATATCCCCATTTTAATTTAATATACAATTGTCATCTA 59282
NorthernAmericanDeerMouse   CCTTACCTGTGCTAGTCAT-GCTTTTG---CCCAATATGAGCATATTGAATATGAGCATATTGAATATGAGCATATTGAA 63758
Mouse                       TTTTACCTG--CTATTCCT-GTTTGTA---CCCAGC------------------------------CTGAGCATACTGAA 63730
ChineseHamsterGHOK1GS       CCTTACCTGTGCTACTCAT-GTTTTTA---CCCCAT-----------------------------TATGAGCATATTGAA 67582
LongTailedChinchilla        ACCTAGTTTTTCCATTCAT-GTTTTCAAGAATTACTTTTAAAAATGTCCCTGTTTTTGTTTAATGTACAATTGCCATCTA 65125

Majority                    XAAATTXAC---X--TTCTTGCTX-X---CX-X-TTTGCTTCAATAAAXTATCTGACCAXAGA-----AAXTTAXTXGAG
                                      94650     94660     94670     94680     94690     94700     94710     94720
Human                       ATAATTCATCATTTCTCATTGCTGCTAGGTAGATTTTGCTTTCTTGAACTGTGTGACCATAAA------AGTATGTACTG 87712
GuineaPig                   AAAATTTAC--GTAGTTCTTGATCATGA-CAGACCTTT----ACTAAATTATATCAAAATATA-----AAGTGAGTAGAT 59350
NorthernAmericanDeerMouse   TATATTCGC------TTCTTTCTT-----T----TTTGCTTCAACGCAATATCTGACCAGAGA-----AACTTAAGGGGG 63818
Mouse                       TGGATTCAC------TTCTTTCTT-----C----TTTGCTTCAACAGAGTATCTGACT-GAGA-----AACTTAAAGAAA 63789
ChineseHamsterGHOK1GS       TATATTTGC------TGCTTC-------------TTTGCTTCAATAGAATATCTGACCAGAGA-----AACTTAAGGGAG 67638
LongTailedChinchilla        AAAATTTAC--TTAGTACTTGATCATAA-CAAACCTTTTTCTACTAAATTATATCAAAATATAGTATAAAGTGAGTAGAA 65202
```

```
Majority                     AXAGGATTACTTTTGT-CTTACA------ATTTGAAXXTTT--AGXCXATCAXAATGXGCTAT-------X-TTCCATCX
                                       94730     94740     94750     94760     94770     94780     94790     94800
Human                        ACACCATCATTTCCTTTAAAACA-CACACACACAAACACACACACA-CACACGCATACACTCCCACACAAAACTCTATGG 87790
GuineaPig                    GTTATAGTACTTTTGTGTTTACA-----TATTTGAGCATTTTTAAAGCATGTGTTTGGGCTTT-------ATTTCCATAG 59418
NorthernAmericanDeerMouse    AAAGGGTTTATTTTGG-CTTACA------ATTTAAAGGTGC--AGTCTATCACAATGAGGTAC---------TCACATCA 63880
Mouse                        ATAGGTTTACTTTTAG-CTTACA------GTTTGAAGGTTT--GGTCCATCACAATGAGGCAG---------TCCCATCA 63851
ChineseHamsterGHOK1GS        AAAGGGTTTATTTTG--CTTACA------GTTTGAAGGT----AGTCTATCACAATGCCATAT---------TTGTATCA 67697
LongTailedChinchilla         ACTACATTACTCTTGTGTTTATAGTATGCATTTGAGCAATTTTAAAGTATGTGTATGGGCTTTC------ATTTTCACTG 65276

Majority                     XTAAXAACT---TGXGGTAGCT--XAGXCAC-ATTGAA---------------------------TTTTTAGCXAG--
                                       94810     94820     94830     94840     94850     94860     94870     94880
Human                        TGTACTATTTAGTATGGTGTACTAAAAAGATTATCAAACTTGGTATTTAAAAAGTTAGCTATATACTTTTTACAAGAGAC 87870
GuineaPig                    TTTAAAAGT--ATTTGATAGCAGTAAGTCAC-ATT------------------------------TTTGAGATAT-- 59460
NorthernAmericanDeerMouse    GTAAGAACT---TGAAGTG------A------ATTGAA---------------------------TTGTTGGCCAG-- 63914
Mouse                        GCAAACACT---TGAGGTAGCT---GGCCAC-AGTGAG---------------------------GTCATAGCCAG-- 63893
ChineseHamsterGHOK1GS        GTAAGAACT---TGAGGCAGCT---GGCCAC-ATTGTA---------------------------TTCTTAGCAAG-- 67739
LongTailedChinchilla         TT-ACAAGT--ATTTGACAGATGAAAGTCAT-ATTACATTTGCTATTTGGTTTTTTTCCTTGTCACCGTTTGAGGTGTGG 65352

Majority                     ------------G-X-AAGCXGAGA-A---TGGTGAAT--GTTAGCTGGGX-----X-TTXAGXXTGXTTTATATAATCT
                                       94890     94900     94910     94920     94930     94940     94950     94960
Human                        ATG-----------TAAAGCAGAAGAAT----ATGAAA-GATTGAAAGGAAAAGGATGTAAAGAGATACTAATATACTGG 87934
GuineaPig                    --------------TACTGCTGGTC----------------------------TCTTAAGAGTAATAGGATTGATTT 59495
NorthernAmericanDeerMouse    ------------G---AAGCAGAGA-A---CAGTGAAT--GCTAGCTGGGG-------TTCAACTTGCTTTATATAGCCT 63966
Mouse                        ------------G---AATCAGAG------TGGTGAATCAGTTAGCTGGGA-------TACAGCTTGCTTTTTATAATCT 63945
ChineseHamsterGHOK1GS        ------------G---AAGCTGAGA-A---TGATGAAT--GTTAGCTCAG--------TTCAGCTTGGTTTATATGACCT 67790
LongTailedChinchilla         GTGGTCTTACCTCATGATGCTGAATCATCATGGTGGAG-AATTACCTGAGGTAGAATTTCAAAAGTTTTTGGTTTTGTTT 65431

Majority                     -TATXTAGT-------CTAGX--------A---X-TGXXAGCCCATGXCAT-ATTGCTGT----CCATAGT----TATTG
                                       94970     94980     94990     95000     95010     95020     95030     95040
Human                        ACAGATAGTAAGCAAGACAAAACTTAAATACC-TTTATTAACTTAAGACAGAATTGATAAAG-GATTTAGTTCACTA--G 88010
GuineaPig                    -T-TATAA------------------------TGTTTTTCCCCAAAACAA---------------------------A 59520
NorthernAmericanDeerMouse    -TGACTAGT-------CTGGG--------A-----TGCAAGCCCATGGCAT-GTTGCTGT----CCATAGT----TATTG 64016
Mouse                        -TATGTTGT-------CTAGA--------A-----TGCAAGTCCATGGGAT-ATTGCTGT----CCTTAGT----TATTG 63995
ChineseHamsterGHOK1GS        -TGTCTAGT-------CTAG---------A-----TGCAAGCCCATGGCAT-GTTGCTGT----CCATAGT----TATTG 67839
LongTailedChinchilla         -TATGTAAAGACTATTTTAAGTAAGTGATACTTTATTTTTTATCATGACAGTATTCTTGGACTGCCATAACAGAATACTA 65510
```

```
Majority                    TAAGTCTTX--------------------CCAXCTGAGXTAATCTXXATXXATGCT---------GCAAATCA--A----
                                     95050     95060     95070     95080     95090     95100     95110     95120

Human                       AAAGATATAACAGTTTATGTATATGTGTGCCTAGTGAAATAGCCTCAAAATA--T----------ATAAATAATAA---G  88075
GuineaPig                   TAAG-------------------------------GAAAGGAGCTAAAAATA-------------GCAAATGA-------  59549
NorthernAmericanDeerMouse   TCGGTCTTC--------------------CCAACTGAGTTCATCTTGATCAGTGCC---------TCTACCC--------  64059
Mouse                       TGTGCCTTT--------------------CCATCTGAGTTCATCTAGATCCCTGCT---------GTGACTCT--A----  64040
ChineseHamsterGHOK1GS       TGGGTCTTC--------------------CCATCAGAGTTAATCTTGATCAATGCT---------GCCATCCTTTA----  67886
LongTailedChinchilla        TAAGTCATACTTCGAAGTACTGTTGCTGATCTCTTAAGAGAGACTGAAGAGACCTTTTCCCCCAAACAAATAAGGAAATG  65590

Majority                    ---ATAAAGTTXCXXATGXTGTGGXGAXAAXCAAXXATAAAATTTXXXTXXAAXXXXXTX-XX-XGXATXAXTTATCATX
                                     95130     95140     95150     95160     95170     95180     95190     95200

Human                       TAGATAGAACTACAGGTAGACTTGAGCAAATCTATTATAGTGGTGGATTTCAATTCACTTTTC-TGAATTACATGTCAAA  88154
GuineaPig                   ---------TAGGAGATGA--GGGGGAAAACATAGTATAAAATTTAGATAAAAAAATTTTGAGATGTATCAAAAATAATA  59618
NorthernAmericanDeerMouse   ---GGGAAGTTCCTCGTGCTGTGGTGACCCCCAACCATAACATTA---------------------------TTTTCGTT  64109
Mouse                       ---ATACAGTTTATTATATCGTGACC-CTTTCAATCATAAAATTTTGTTGTTACTTCACAACT--AATTTTGTTATTGCT  64114
ChineseHamsterGHOK1GS       ---ATGTAGTTCCTCATGTTGTGCTGACCCCCAACCATAATTGT----------------------------TTTTCATT  67935
LongTailedChinchilla        AGGGAAAAATAGCAAATGATAGGGGGAAAATAGAGTATAAAACTTATATAAAATAAATTCCAGATGCATCAAGAATAATA  65670

Majority                    GXAAATAXAAAXGTATAAATTTGACAXXXAXAAAATTX-TXXGATXXAXXTTTXTGAAACXXTCXTTTGAGCCCXAXAXA
                                     95210     95220     95230     95240     95250     95260     95270     95280

Human                       CTGACCAAAAAAAGACAAAATCAACAAAGATACAAATGATACAATACAATTACCTTA-ACAGAATTATGTGTCTAAGTTA  88233
GuineaPig                   GCAAATAAAAGAGAGTAAGTTTCTCAATTAAAAAATTAGTCAGATTTGGCTTTCTAACACCTTCCTGCTGGCTCGAGCCA  59698
NorthernAmericanDeerMouse   GCTATTTCGTATCTGTAGTTTTGCTGC----------------------TGTTATGAAT------TGTAA----TAAAAA  64157
Mouse                       ATAAATCGTAATCTAAATACTTGACATGCAGGATATCT-GATGTATGACTTCTGTGAAACGATCATTTGACCCCCAAAGG  64193
ChineseHamsterGHOK1GS       ATGAATCTTAATGTAAATATCTGATACGCA-----------------ACCTCTGTGAAAA--TCATTTGACCCCTAAAAG  67996
LongTailedChinchilla        GCAAATATAAAAG--TAAATTTATCAGTTAGAAAATTA-TCAGATCTGGCTTTTTGACACATTCCTTCAGGCTTAAGCTA  65747

Majority                    CXTXTCAXXXACATAGGTTXAGAAC-CATTGATC-----TXXATAAXCCXTCAGXXXCTTXTCXXXTAGGX-XGTTXXA-
                                     95290     95300     95310     95320     95330     95340     95350     95360

Human                       TGCATCCAACACAGTTTTTAAACGCATATGGAACATTTTTGAAAAATCAGTCACATACTAAACCATAAAGCAAGCTTCAG  88313
GuineaPig                   CTGAT--GGTACATGCGTGAGTGTCCCATTGGGTGAGCAGGTTGAACTAGATG-GTACCGTCCCACCTGCCCAGTGACA-  59774
NorthernAmericanDeerMouse   CATCTGATATGCACAGGTTGAGAAC-CACTGATC-----TACATAACCCTTCAGAAGCTTCTCTCCTAGGT--GTTCTA-  64228
Mouse                       TGTGACA-GCCGACAGGTTGAGAAG-TACTGATC-----TAGTTAATCCTTCAGGGGCTTGTCTCTTAGGT--GTTCTA-  64263
ChineseHamsterGHOK1GS       GATCACA-ACCCATAGGTTGAGAAC-CATTATTC-----TAGATAATCCTTCAGAAGCTTGTCTCCTAGGT--GTTGTA-  68066
LongTailedChinchilla        CTGGT--GGTACATGCATGAATGTCCCGTTGGGC-AGCTTGAACCACCCGATG-GGACTGTCCCATGTGCCCAGTGACA-  65822
```

```
Majority                   -XXATCCTGTAGXGTXTAACTGTTXGTXTXAACCATTGTGXAATTAACXATTCAXXACAAA-X-GXXAAGAXCCTTA--T
                                   95370     95380     95390     95400     95410     95420     95430     95440
Human                      CTAATTTCAAAGAGTATCA-TACAGACCTCATTCTCTATTGCACAAGTAAGTTAGAATTAA-TAACAAAAAGATGCA--T  88389
GuineaPig                  -TAGTCATGCAT-GTATTGCTGATAGTTAGAGCCAGTGGGAATGTGACAATTCAGAGCTACCTGGCAGTGAGACATACTC  59852
NorthernAmericanDeerMouse  --GATTCTGTTGTGT-TAAAGTTTGGTGTTAACCATTGCGTTGTTGAACTTTCAATACAAA---GTGAAGAACCTA---T  64299
Mouse                      --GATCCTGTTGTGT-TAACGCTCAGTATAAACCATTGTGTAA--AACCCTTTAATACAAA---GTGAAGGATCTTT--T  64333
ChineseHamsterGHOK1GS      --GATCCTGTCAGGT-TAACTGTCAGTATTAACCATCGTGCTGTTAGACCTTCAATACAAA---GTGAAGAACCTAC--T  68138
LongTailedChinchilla       -TAGTCGTACAG-GTACCACTGTTGGTTCGAGCCAGTGGAAAAATAACAAATCAGAACCACCTGGCAGGGAGCCGTAACC  65900

Majority                   AAXAATAGCCXXXXTTCXAXAGCTXXX-XATAX-X----XGAAXXXCAAGAAXCAXAAA-AXXX--XGXXXGGTTAATAT
                                   95450     95460     95470     95480     95490     95500     95510     95520
Human                      -ATAAAATCTGTATCCTGAGATTTAAA-AATAAGTC-TCAGTATTGCATGAGTCAGAAGTTTGTT-TGTTTGTTTGTTTT  88465
GuineaPig                  CATTGTGAACATATTT-AAAAGCTGAGTAATACTTAGTAGGAAACACAAGAAGCAAAAAAAGAAA-GGAAAGGTTAAAAG  59930
NorthernAmericanDeerMouse  AAAAACAGCCCCCCTTC---------------------------------------------------------TAATAT  64322
Mouse                      AAAAATAGCCTCCCTCCCCCCGCCTCCCTTAATGTGCCTAGACTTGTGACTAAAGAAAACACTTAACTCTTTGCTAATAT  64413
ChineseHamsterGHOK1GS      AAAAACAGCCCCCCTTC---------------------------------------------------------TAATAT  68161
LongTailedChinchilla       CATTGTGAATGTATTTTAAAAGCTATCTGATAC-----GGGAAACACAAGAAGCACAGAGATAC--GGAAAGGTTAAAAG  65973

Majority                   TAXGCAAGXTCTCTXCTTTGAGCAACATCXT-XXCTAXAGAAXXXTAAXTTTAXXXCXAXGTATXTTAAAAXAXTTXX-C
                                   95530     95540     95550     95560     95570     95580     95590     95600
Human                      TGAGCCAGTGTCTTGCTCTGTCCCCCAGGGTGGAGTGCAGTGGTGCTATTGTGGCTCACTGCAGCCTCGACCTCCCAG--  88543
GuineaPig                  TAATGTATA----TATATTGGGCAA-ATAGTAATCTAAAGAAACTTAAATTTAGTGTGATGAATATTAAAAGATTTAGTT  60005
NorthernAmericanDeerMouse  TAGGCAAGATCTCTTTTTTGAATGACATCCT-GAC-----------------------AGGTACCTCAAGAAG------C  64372
Mouse                      TAGGCAAGTTCTCAGACTGGAGCAATGGCTC-AGCCATTAAAGGCTAGGTTAACAACCAAATATATTAGCCAAGTT---C  64489
ChineseHamsterGHOK1GS      TAGGCAAGTTCTCATCTTTGAATGACAACCT-GGC-----------------------AGGTACCTTAAG---------C  68208
LongTailedChinchilla       TAATGTAGAGAGATACATTGGGCAA-ATA---ATCTAAAGAAATTTAAATTTAACACAACAAATATTAAAAGATTTAGTT  66049

Majority                   CCTTXGGAAGATAXC-XXCTTT-AXXTATCTAAGTAXXXAGTGTAATATGCXCAXACXACXTXCTG---TXXTTTXTCTT
                                   95610     95620     95630     95640     95650     95660     95670     95680
Human                      GCTTAAGCAGTCCTTGCACTTAAGCCTCC-AAAGTAGCTAGGACTAAAAGCATGCACCACCTGCCCGGCTAATTTATTAT  88622
GuineaPig                  CATTAGGAAGATAA--CAGTTGTATATAT-TATGTTCCTAGTGAAATACACTCAAACTACATACTG---TAAATCATAAA  60079
NorthernAmericanDeerMouse  CCTATT---GATGTC---CTAT----TTTCTTAGCA--CTATGTGCTGTGTAGAGACAGT---------TGTTTTGTCTT  64431
Mouse                      TCTTTGGAAGACAAC---CTGACAGGTACCTAAGGAAGCCATATGATATCTTCTTACCATGTGCTG---TGTTTTGTCTT  64563
ChineseHamsterGHOK1GS      CCTGT----GATATC---TTAT----TTTCACAGTA--CTGTGTACTGTGCAGAGAC-----------TGTTTTGTCTT  68263
LongTailedChinchilla       GATTAGGAAGATAAAACAGTTTGAGGTATGTGTGTGCTTAGTGAAATACACTCAAACTACATACTG---TAAATCATACA  66126
```

Majority	TATXXGTATTATAAXTXGAAGGGAXAAAATAATXAAXTGAT----TTGAATXXAT----TATXGTGAATXTAAAX-GXAT	
	95690 95700 95710 95720 95730 95740 95750 95760	
Human	TATTATTATTTTTTGTAGAGATGAGTTCTCACTGTGTTGCCCAGACAGAAGTTAT--TTTAATGGGAATATAAAATACTT	88700
GuineaPig	TGGTAGAACTGTAGGTAGACTAGCAAATCTGTCATAGTGG---ATTTCAAACTTT--CATATTGAGCACAAAACA-GCAG	60153
NorthernAmericanDeerMouse	TATGTGTATTACAATTTGAAGGGAGAAAATAAT-AAATGAT----TTGAATGAAT----TTTGGTGAATTTAAAG-ATAT	64501
Mouse	CATGTGTCCTAGAATTTGAAGGGAAAAAATAATCAAATGAT----TTGAATAAAT----TATGGTGACTTTAAAG-GTAC	64634
ChineseHamsterGHOK1GS	TATGTGTATTACAATTTGAAGGGAGAAAATAAT-A--TGAT----TTGAATAAAT----TTTGGTGAATTTAAAG-GTAT	68331
LongTailedChinchilla	TGGTAGAAGTGTAGGTAGACTAGCACATCTGGCACAGTGGTAGATTTCAAGCTTTTCCATGTTTTGAGCATATCA-GCAT	66205

Majority	AAG--X-------ATAXXCATAXAGAXTATT--TAGTGATATATGAAAXXTTXCAACATATGATXAAAAGG----XXTTT	
	95770 95780 95790 95800 95810 95820 95830 95840	
Human	AAAATTAAGCGATAAAGTACTATATACAAAT--TAGATATATGTGGTACAGTGAAAGAGAAGTCTTGACGGGAAGTT-TT	88777
GuineaPig	AA---TTGATAGAACAATT-TGTAGAGTATTTTTAAGCATGTAAGAGACATTTTAAAATTTGCTCACTTA-----TTATC	60224
NorthernAmericanDeerMouse	AGG----------GTATCCATAAAGAATATT--TAGTGATAACTGAACTTTTACAGCATATGATAAAAAGG----CCTTT	64565
Mouse	AGG----------GTACCCATAGATGATATT--TAGTGAGATATGAACTTCTGCAACAGATGGTTAAGATG----CCTCT	64698
ChineseHamsterGHOK1GS	AAGT---------ATATCCATAAAG-----------TGAGATCTGAATTTTTACAGCCTAAGATAAAAAGG----CCTTT	68387
LongTailedChinchilla	AACAATTGATAAAACAGTTGTGCAGAGTATTTTTAAGTACATATGGAACATTTTAAAATTTGATCAGCTAATAAATTATC	66285

Majority	AAGTAGAA---X-X-X---X-GXXACTTCAXAAAAATGATAT-------GACAAAAXTXAGAX--AAX-GTATXAAAATA	
	95850 95860 95870 95880 95890 95900 95910 95920	
Human	AACTTTAA--GAATAACAGTGTTACCCCCAGG-GAAGGAGATA-TTAAAGACAAGACTGGAAATCAATGGTATAAAAG-A	88852
GuineaPig	AAGTAAGCTTCAGTTAATATCACAGTGCCATA--AATCTTGT---CTGTTTCACAAGTTAGA------------------	60281
NorthernAmericanDeerMouse	TTGTAGAA-------------GAGACTTCA-AAAAATGCTAT-------GACAAAACTAAGAG--AAA-GTATCATTATG	64621
Mouse	--GTAGAA-------------GTGACTTCAGAAAAATTATTT-------GACAAAATTGAGAG--AAGTGTTAGAGAATA	64754
ChineseHamsterGHOK1GS	TTGTAGAA-------------GAGACTTCA-AAAAATGATAT-------GACAAAATTAAGA---------ATCGAAATG	68437
LongTailedChinchilla	AAGTAAACTTCAGTTAACATCAGAGTGTCATACAAATCTCATTCTCTGTTTCACAAGTTAGAATTAATGGCACAAAAATA	66365

Majority	C----------------------------------TTTCT----AGCCAGAGAG--XX----------------------	
	95930 95940 95950 95960 95970 95980 95990 96000	
Human	CAGTTGAGAGAAAAAAACATGCC-ACAAGTTGGATTCTTTGAAAAGACTAAAAAAATAGACAAACA----TCTAAAGATT	88927
GuineaPig	--	60281
NorthernAmericanDeerMouse	C----------------------------------TTTCT----AAGCAGAGAGTGTAT---------------------	64642
Mouse	C----------------------------------TTTTT----AGCCAGAGAGC-------------------------	64771
ChineseHamsterGHOK1GS	C----------------------------------TTTCT----AACCAGAGAG--------------------------	68453
LongTailedChinchilla	CATTTAAAATCTCCATGTATCTAGACATTTCCCAGTATCTCACTGGCCAGAGAAGTTATTTTAATAGTAGTTTAAAAATA	66445

```
Majority                    -----XAATAT---------------AAAGAAT-----------------------------------------------
                                     96010     96020     96030     96040     96050     96060     96070     96080
Human                       GATTAAAAAATGAGAGTGGTAACACAAAAAAATTTTTTTG-AATTATAAAGGGGAGAAGCATTGATAAAGCTCCAGTTTC 89006
GuineaPig                   -------------------------------------------------------------------------------- 60281
NorthernAmericanDeerMouse   -----AAATAT---------------AAGGAAT----------------------------------------------- 64655
Mouse                       ------ATTAT---------------AAAAAAT----------------------------------------------- 64783
ChineseHamsterGHOK1GS       ------AATAT---------------AAGGAAT----------------------------------------------- 68465
LongTailedChinchilla        CTTAAAAGTATTTTAAAAAGCATTTAAAAGTATTTTAAAGTAAATGCTAATGAAAGTAGCATACACAAATTAGATATACT 66525

Majority                    ------------ACAGTACT------------------------------------------------------------
                                     96090     96100     96110     96120     96130     96140     96150     96160
Human                       CACATCATATAAACACCACTATGTCTGTAAATTTCTTGAGGT--CTTTTAAAATTTTTATT--------ATTCGTTAATA 89076
GuineaPig                   -------------------------------------------------------------------------------- 60281
NorthernAmericanDeerMouse   ------------ACAGTTCT------------------------------------------------------------ 64663
Mouse                       ------------ACACTACT------------------------------------------------------------ 64791
ChineseHamsterGHOK1GS       ------------ACAGTACT------------------------------------------------------------ 68473
LongTailedChinchilla        TGGTACAGTAAAATAGTGCTCAATGAAAAGATTTCACTGTCTAGCAATTGAAACGCAAAAAAAGGAGAAATTTTTTAAAA 66605

Majority                    --------------------------------------------------AAGA--TGXCATTXCA--------------
                                     96170     96180     96190     96200     96210     96220     96230     96240
Human                       ATCTCTTTCATAGATGGATTAAGGGAAATGTAAAAAATTACATTAAATCAAAAA--TGTCATTTCAATTGTGAAACATTG 89154
GuineaPig                   -------------------------------------------------------------------------------- 60281
NorthernAmericanDeerMouse   --------------------------------------------------AAGA--TGGGATTCCA-------------- 64677
Mouse                       --------------------------------------------------AAGA--TGCCATTACA-------------- 64805
ChineseHamsterGHOK1GS       --------------------------------------------------AAGA--TGCCATTCCA-------------- 68487
LongTailedChinchilla        AGACTAGAAATCAGTAATGTATATGAAAGCTGAGAGCACAAAACAAATCCAAAAGTTGTTCTTTGAAAAGACTAATAAAA 66685

Majority                    --------------------------------------------------------------------------------
                                     96250     96260     96270     96280     96290     96300     96310     96320
Human                       ACTTTTTCCTTTATTATTGCCTTGT-CCATTTATGCCATATTTCTTGGAATAGGAGAGTTCAAGTGTAAGATTCACATTG 89233
GuineaPig                   -------------------------------------------------------------------------------- 60281
NorthernAmericanDeerMouse   -------------------------------------------------------------------------------- 64677
Mouse                       -------------------------------------------------------------------------------- 64805
ChineseHamsterGHOK1GS       -------------------------------------------------------------------------------- 68487
LongTailedChinchilla        AGTAGATCTGTCTGTAACGATTGATGTTGTTTAAAGAACAAGAGCAGCACTACAGTATTTTGAGTTACAGTGTGATTAAC 66765
```

```
Majority                    ------------------------TAATAGCTTGGATA---------------------------GTGTCTT--------
                            96330 96340 96350 96360 96370 96380 96390 96400
Human                       TTTAAAATTTTATTCATTTAAAATTAGTAATTCAGGTAATTTCCTTCTGC-TCTGTTTACTCCTTGTGTACTTTCAGTTT 89312
GuineaPig                   -------------------------------------------------------------------------------- 60281
NorthernAmericanDeerMouse   ------------------------TAATAGCTTGGATA---------------------------GTATCTT-------- 64698
Mouse                       ------------------------TTATGGCTTGGATA---------------------------GTGTCTT-------- 64826
ChineseHamsterGHOK1GS       ------------------------TAATGGCTTGGATA---------------------------TTGTCT--------- 68507
LongTailedChinchilla        TATAAACTCCTTTTCCATATTATCTAAAATAATAGGTAAACATATTATATATTTTTATTTTTTTGGTATTTTTCCGGTCC 66845

Majority                    -----AC--AACT-------------------------------------------------------------------
                            96410 96420 96430 96440 96450 96460 96470 96480
Human                       TTG--ACAAAGCTTTTGTTTTCTTTTTTACTTAAAAATTTTGGTATT-CTAATTATCATCCCCCTTCCCCTTTAGAT-CA 89388
GuineaPig                   -------------------------------------------------------------------------------- 60281
NorthernAmericanDeerMouse   -----AC--TACT------------------------------------------------------------------- 64704
Mouse                       -----AC--AGCA------------------------------------------------------------------- 64832
ChineseHamsterGHOK1GS       ----------ACT------------------------------------------------------------------- 68510
LongTailedChinchilla        CGGGAACCGAACTCACGACCTTGCACTTGCCAGGCAGGCGCTGTGCCGCTGAGCTAAATCCCCAGCCCCCGGTAAACATA 66925

Majority                    ----------------------------------------------------------TACTTCCC--------------
                            96490 96500 96510 96520 96530 96540 96550 96560
Human                       CTTCAGATCCTA--TTGTCAGCAA--T-CAGCTGGCTAGAACTCTCTTGTTACCTGGCTACTTCCTGAGGTAGCTGTTTG 89463
GuineaPig                   -------------------------------------------------------------------------------- 60281
NorthernAmericanDeerMouse   ----------------------------------------------------------TACTTCCC-------------- 64712
Mouse                       ----------------------------------------------------------TCATTCCC-------------- 64840
ChineseHamsterGHOK1GS       ----------------------------------------------------------TGCTTCCC-------------- 68518
LongTailedChinchilla        TTATAAACATTAAATTCTCTATAAGTTTCTTGTGTTCTGAATTTTCATTCTTCATTAATATTTTCTTGAGAGGTGGATTA 67005

Majority                    ----------------AGCATTXA---X--------------------------------------XX-X----------
                            96570 96580 96590 96600 96610 96620 96630 96640
Human                       G---------TTATTTAGCTTTATTTTAATCCCATTCCTCTATCACTCATCTGTTCTGTATGCAGTTACTATGCCTGGGA 89534
GuineaPig                   -------------------------------------------------------------------------------- 60281
NorthernAmericanDeerMouse   ----------------AGCACTCATATAG-------------------------------------TAGT---------- 64729
Mouse                       ----------------CACAAGCT-------------------------------------------------------- 64848
ChineseHamsterGHOK1GS       ----------------AGCATTAA-------------------------------------------------------- 68526
LongTailedChinchilla        AAGGAAAGGCAAAAATAGCATTTAGCCAAACATGCCATTTCTGCAGCACTTTCCTTTATCATTGCTTAGTCCACTTAGGT 67085
```

```
Majority                     ----------------------------------------GXTXTT------------------A---------------
                                      96650     96660     96670     96680     96690     96700     96710     96720
Human                        CTTATTTTCTTATTCTTTCCCTCCATTATCTCAAAGCTCTGCTGTTTGTGCTGTCTTTTTATTTAGACAGCTTTTTTTCT  89614
GuineaPig                    --------------------------------------------------------------------------------  60281
NorthernAmericanDeerMouse    ---------------------------------------TGGTGTT------------------A---------------  64737
Mouse                        ----------------------------------------GTTAAT------------------G---------------  64855
ChineseHamsterGHOK1GS        --------------------------------------------------------------------------------  68526
LongTailedChinchilla         CAT-TTTTTTGAATAGTAGACTTCAAAGCACAAGGTTCTCGTTATTAAAAACCTGACTCATTTGAAATTATTTTTATTAC  67164

Majority                     -----------------------------------------------CACACCT--------------------------
                                      96730     96740     96750     96760     96770     96780     96790     96800
Human                        GACAACGTTAAGTCTACTTATTTATATCAC--TTTATTTCTCAAACTCACACTTAATAACACATTTTATCTGAGGTAGTT  89692
GuineaPig                    --------------------------------------------------------------------------------  60281
NorthernAmericanDeerMouse    -----------------------------------------------CATACCA--------------------------  64744
Mouse                        -----------------------------------------------CATCACT--------------------------  64862
ChineseHamsterGHOK1GS        -----------------------------------------------CACACCT--------------------------  68533
LongTailedChinchilla         TTAATCATTGTGTACTTTAATTTTTGCTAATGTTTTCTTTTAAACTTCGCTATTCTTATTGTCCCTCCAGTCTCCCTCCT  67244

Majority                     ---------------------------------------------------------TCAXT------------------
                                      96810     96820     96830     96840     96850     96860     96870     96880
Human                        TATCTAAACAAAAGTTTGTTCACTTAAAATAGTTATAGAACTTTAAAATTTTATAATTCAGTGGGTTTTATTGTAAAAGG  89772
GuineaPig                    --------------------------------------------------------------------------------  60281
NorthernAmericanDeerMouse    ---------------------------------------------------------TCAGT------------------  64749
Mouse                        ---------------------------------------------------------TCAAT------------------  64867
ChineseHamsterGHOK1GS        ---------------------------------------------------------TTAAT------------------  68538
LongTailedChinchilla         TAGATCACTTCAGATTTTTTGTCAGCAGTCAGCTGACTGAAGTAGTGGGAATTTATCTCACTACGTTGCTATGGCGTAGC  67324

Majority                     --------------------------------------------------------------------------------
                                      96890     96900     96910     96920     96930     96940     96950     96960
Human                        ATTTTATGTACTGTCACTGTAGATAATTGGTTTTACAGGTGTAAGCGTAAATTTGCAAGTATATG-AAATTAAGTATGTG  89851
GuineaPig                    --------------------------------------------------------------------------------  60281
NorthernAmericanDeerMouse    --------------------------------------------------------------------------------  64749
Mouse                        --------------------------------------------------------------------------------  64867
ChineseHamsterGHOK1GS        --------------------------------------------------------------------------------  68538
LongTailedChinchilla         TCTTTGGTTACT-TTGCTTTACCTTACTTCTGTCGCCCGTCTCTTCTTTATATGGTTACTGTGCCTGGACTTTTTGTTTT  67403
```

```
Majority                   --------------------------------------------------------------------------------
                                    96970     96980     96990     97000     97010     97020     97030     97040
Human                      AAATGCAGTGTGAGAAACAAATGAGAAAATTCAGACTTGTGAGAAGATTGTTTTGTTT--TTTTTCTGTGCTTAGCCTTG 89929
GuineaPig                  -------------------------------------------------------------------------------- 60281
NorthernAmericanDeerMouse  -------------------------------------------------------------------------------- 64749
Mouse                      -------------------------------------------------------------------------------- 64867
ChineseHamsterGHOK1GS      -------------------------------------------------------------------------------- 68538
LongTailedChinchilla       CTTATTCTTGCCTTCCATATCTCAAAATGCAGTTGTTTGTACCGTCTTTCATTTAGCTAGCTTTTTTATGATGACTTTAG 67483

Majority                   ---AAATATC-----------------TGCAA----------AGAAGCXXGAAG--------------------------
                                    97050     97060     97070     97080     97090     97100     97110     97120
Human                      GCGAAAAATCTGAGAAAACTTAAAATATATAACACTGGAGTGACAATCAGGAAGAGTGGACTCTTTTTTTTTTTAATTTT 90009
GuineaPig                  -------------------------------------------------------------------------------- 60281
NorthernAmericanDeerMouse  ---AAATATC-----------------TGCAA----------AGAAGCCAGAAG-------------------------- 64773
Mouse                      ---TAATATTC--------------TGTGCAG----------AGAGGCTGGGAG-------------------------- 64894
ChineseHamsterGHOK1GS      ---AAATATC-----------------TGCAA----------AGAAGCCAGAAG-------------------------- 68562
LongTailedChinchilla       TCATAACATACAGTGATTTTTATCTGATGTAAAACAGCATTTAGAACTTTAAAAAT--ACTTGACAAATCCGTGGGTTTC 67561

Majority                   --------------------------------------------------------------GTATATAAGGGTCAX---
                                    97130     97140     97150     97160     97170     97180     97190     97200
Human                      TTTAAACAAATTAGGGAAGTAAGTTCAATGGCTAGTGGTTATCATAATAAGAAAAATTTAAGGAAGTTATAGGTAAAATA 90089
GuineaPig                  -------------------------------------------------------------------------------- 60281
NorthernAmericanDeerMouse  --------------------------------------------------------------GTATATAAGGGGCAG--- 64788
Mouse                      --------------------------------------------------------------GAATGCAAGGGTCAG--- 64909
ChineseHamsterGHOK1GS      --------------------------------------------------------------GTATATAAGGGTCA---- 68576
LongTailedChinchilla       ATTATAAATGTATTTCAAATTATATGTAGAGATTTTAATTTTTATATGTACAAGTATAAATTGTATATGAAAGTATAGTG 67641

Majority                   ---------------------------AXTGGGA----------------------------------------------
                                    97210     97220     97230     97240     97250     97260     97270     97280
Human                      AGA-ATTTTACTTTTGTTTCTTTTCTGAATGGTACCTCATATTTTGACAGCATTATCCTTTTCAGATCTGTTTTATATTT 90168
GuineaPig                  -------------------------------------------------------------------------------- 60281
NorthernAmericanDeerMouse  ---------------------------ACTGGGA---------------------------------------------- 64795
Mouse                      ---------------------------GCTGGGA---------------------------------------------- 64916
ChineseHamsterGHOK1GS      ---------------------------ATTGGGA---------------------------------------------- 68583
LongTailedChinchilla       TGAGAGACTAGGGAGAAGAAATCTAGGCTTGGGATATGAAATTCTTATTTTTCTATTGCCTCTGTTGAAATTCTAAGAGA 67721
```

```
Majority                     -------------------------------------------AATGTAG-------------------------CTATG
                                         97290     97300     97310     97320     97330     97340     97350     97360
Human                        AATTGGTTTATTATACATGTAAGTATGATTA----GATTCATGGATTTA------AC--CAGTTCTTTGTATCTTCTGTG 90236
GuineaPig                    -------------------------------------------------------------------------------- 60281
NorthernAmericanDeerMouse    -------------------------------------------AATGTAG-------------------------CTATG 64807
Mouse                        -------------------------------------------AATGTAG-------------------------CTACA 64928
ChineseHamsterGHOK1GS        -------------------------------------------AATGTAA-------------------------CTATG 68595
LongTailedChinchilla         AAATGGTTTCAAATATGTAACACTAGTGTTTCTGCAAGATAGGGAAGTAGTAAGAACATCACTTTCGTGTTTTTTCTTCA 67801

Majority                     TTAAAAXGTA------A---X-XXGTTTAGATTX----------------------------------------------
                                         97370     97380     97390     97400     97410     97420     97430     97440
Human                        TCAAAATGTATACTGTAGTATACTGTTTATATT-AGAAACTCTTTAAAAGTGGATGTTCTATTCTTAATTTCCTGATCTT 90315
GuineaPig                    --------------------T-CTTTTTGGAT------------------------------------------------ 60292
NorthernAmericanDeerMouse    TTAAAACAGA------A-------GCTTAGATTT---------------------------------------------- 64828
Mouse                        TTAAAGAGTA------A-------GTTTAGGTTT---------------------------------------------- 64949
ChineseHamsterGHOK1GS        TTAAAAAGTA------A-------ACTTAGCTT----------------------------------------------- 68615
LongTailedChinchilla         TGGTAACTCATACAGCATTAT-CTTTTTGGATTTATTTTGTGTATTGTATACAAAACCAGATTCATAAGTGAACAGATTC 67880

Majority                     ---------------------------------------------TGT--------------------------------
                                         97450     97460     97470     97480     97490     97500     97510     97520
Human                        CCACATTGTCTTTATCTGT-AACTCTACTGTTTTTTGTTGGTTTTTGTTTTTGTTTTGAGACAGAGTCTCGCTCTGTCAC 90394
GuineaPig                    -------------------------------------------------------------------------------- 60292
NorthernAmericanDeerMouse    ---------------------------------------------TGG-------------------------------- 64831
Mouse                        ---------------------------------------------TGT-------------------------------- 64952
ChineseHamsterGHOK1GS        -------------------------------------------------------------------------------- 68615
LongTailedChinchilla         TAATGTGTACTCTGTACACGAATTGTTAAAAAACTTTTAAGAGGTTGTGTTTTCAGTTTCTCGCTCTTTTATATTGTCTT 67960

Majority                     -------------------------------------T------------------------------------------
                                         97530     97540     97550     97560     97570     97580     97590     97600
Human                        CCAGGCTGGAGTGCACTGTGGCACCATCTCAGCTCACT---GTAACCTCCGCCTCCTGGGTACAAGCAATTCTGCCTCAG 90471
GuineaPig                    -------------------------------------------------------------------------------- 60292
NorthernAmericanDeerMouse    -------------------------------------T------------------------------------------ 64832
Mouse                        -------------------------------------T------------------------------------------ 64953
ChineseHamsterGHOK1GS        -------------------------------------------------------------------------------- 68615
LongTailedChinchilla         CTCTGCTGTT-CATACTTCTTTCCAGTTTGAGTATAGTCAGATAATCAGTCTAGTTTAAATATGACTTACTCAAAATGAT 68039
```

Majority	--	
	97610 97620 97630 97640 97650 97660 97670 97680	
Human	CCTCCTGAGTAACTGGGAC-CACAGGCACGAGCCACCACGCCCAGCTAATTTTTGTATTTTTAGTAGAGATGGGGTGTTA	90550
GuineaPig	--	60292
NorthernAmericanDeerMouse	--	64832
Mouse	--	64953
ChineseHamsterGHOK1GS	--	68615
LongTailedChinchilla	CTTTCTTAGTTCTTATGACGTGTGAGTAAAAAGTAGAAGACATAACTT-CTGTAGTAGTAGACCAGTTTATTTCATTTAA	68118
Majority	---CCC----------------------------	
	97690 97700 97710 97720 97730 97740 97750 97760	
Human	CCATATTG-GCCAGGCTGGTCTCGAACT---CCTAGACCTTGTGATCCGCCCACCTCAGCCTCCCAAAGTGTTGGGATTA	90626
GuineaPig	--	60292
NorthernAmericanDeerMouse	---CCT----------------------------	64835
Mouse	---CCC----------------------------	64956
ChineseHamsterGHOK1GS	--	68615
LongTailedChinchilla	AAATAACATGCCAAGTACCTCCAGAAATAGCCATGTAGTGTTTCGTGTGCCCACACCACTGTATGCTGGCATAGACTTAA	68198
Majority	--	
	97770 97780 97790 97800 97810 97820 97830 97840	
Human	CAGGCGTGAGCCAACGCGCCCGGCATCTACTGT---TAATACTTTATTATCTAGTTGATT----ATAGTCATTTAGACCT	90699
GuineaPig	--	60292
NorthernAmericanDeerMouse	--	64835
Mouse	--	64956
ChineseHamsterGHOK1GS	--	68615
LongTailedChinchilla	TGAATTCTGTCTAAGTACCTTAGAATCTGAAGTAAGAAATAATGGATAATTTCATTAAGTTGTGATAGTCTTTTAAAAAG	68278
Majority	--X-	
	97850 97860 97870 97880 97890 97900 97910 97920	
Human	TCAGTTTAGC-CAAAGTGCATGACATATTAGAAATGGTCTTTTTTAGTTATTTTGACTGGTGAATAAAACATATAACTTT	90778
GuineaPig	--	60292
NorthernAmericanDeerMouse	--A-	64836
Mouse	--A-	64957
ChineseHamsterGHOK1GS	--	68615
LongTailedChinchilla	TCGTAGTAGAGTACAACATGAAAAATATCTAGTGAGATTTCAAACCTTTGCAAGGATGATAAAAAGACCTTTTTTGGCA	68358

```
Majority                     --GTGATCTTXAXXAATXAXTXXXCTXAXAAGAXA--TAXAAGXTAT---XGGAAATCXTTAAXX--XXXAXCTXTATAT
                                       97930     97940     97950     97960     97970     97980     97990     98000
Human                        CTAGCATCAGACCAGTTCATTTAGTTTAAAAATGATCCAGCAGCTATGTCAAGAAATAGTCATGT-AATGTTCTGTTTTC 90857
GuineaPig                    ACGTGATCTTGAAGAATGAGTAGACTGAGAGAAAA--TAAAGGGTAT---ACGAAATTATTAGAA--AAAACATGTACAT 60365
NorthernAmericanDeerMouse    --GTTACTTGTACTAAGTAATGACCGAATCAGGTT--CATAACATGT----GAAAGTCTTTTACT---G---CTCTGTGG 64902
Mouse                        --GGGATGTTTATTAAGTGATGCCATGGTCAGAGT--TATAACCTGT----GAAAGTCTTTTAAC---TGATCTCTCTCT 65026
ChineseHamsterGHOK1GS        -------------------------------------------------------------------------------- 68615
LongTailedChinchilla         AAATGACCTTGAAGAATGAGTAGGCTAAGAGGAAAAGTAAAGGATAT---AGGAAATGGTTAAGAGAAAAACATAGACAT 68435

Majority                     AXGXATAGAAX----X---XCTGGGGTXXAGAAAAAGGTTT----X-XGXAAGCAAGGCATAXAAGGAATTG-AGTTACC
                                       98010     98020     98030     98040     98050     98060     98070     98080
Human                        ACACACTGCACT---ATGTTCTGGGGTAGAGACAAAGGTTTGGATTTTGGAATATTTGCATTATACCAATTG-AGCTTCC 90933
GuineaPig                    AAGAATAAAAGTAAGAGAAACTAGGGTCAGGATAAATGTTT----TCTGCAGTCAGAGCATAAAAT-GCAAG-GAGTACA 60439
NorthernAmericanDeerMouse    ATTCAGTGGGT--T-----GGGGGGAAATAGAAGAAGATTA-------AACAGAAAGGCACATGAAGAATTA-CGTTACC 64967
Mouse                        GTGTATAGAAT--G-----GATGGGGGTTAGAAACAGATCC-------AACAGCAAGGCAGGTAAGGAATTGGAGTTACC 65092
ChineseHamsterGHOK1GS        -----------------------------AGAAGAAGGTTC-------GGTAGGAAGGCAGATAAGGAATTG-AGTTACC 68658
LongTailedChinchilla         AAGAATAAAAG----AGAAACTAGTGTCAGGATATATGTTT----TCTGTAGCCAAAGCATAAAA--GAGTA-TGGTGCC 68504

Majority                     CAGAT----CTTXAATAXTXXXXXXAA---X--X-X----XXTXATXCTGACCAXTGAX-X-GAAATXGAGGGAACATAA
                                       98090     98100     98110     98120     98130     98140     98150     98160
Human                        CAAAT----CTGAAATGTTCCAATGAGCATTTCCCTTGAGCATCATGTTGGCCCTCAGTTTTGGATTTTGGAACATTTCA 91009
GuineaPig                    GAGATACTTCTTCCCTAGTGCCTACAAAGGTATCTTACTACTTTATTCCCAACACTGATGTAGTGGATGATGCTGCATAT 60519
NorthernAmericanDeerMouse    CAGAT----TTTTAAAAAA-----------------------TGACAGTGAGCAATGA----GAAGTAGAGGGAACATAA 65016
Mouse                        CAGAT----CT-----A------------------------------GTGACTAGTGA----GAAACAGAGGGAACATGA 65129
ChineseHamsterGHOK1GS        CAGAT----CTTTAAAAAAAAAAAAAA--------------ATGACACTGACCAAGGA----GAAATAAAGGGA-CATAA 68715
LongTailedChinchilla         AAGATACTTCTTCCATAGTGACTGGGATAGTACCTTACTACTTTATGCCCAACACTGATGTAGCGATTGAG-----ATAT 68579

Majority                     GTTTGATAXG--X-GAATTGAGXAGAXTCAXXCCTXACCCXGCTGAXAAXGXGATAAGACACXXTAXXXAXT-GAGXXTC
                                       98170     98180     98190     98200     98210     98220     98230     98240
Human                        GATTTCAGATTTCTGCATTAAGGATACTCAACC-TGTACAGACTGGGAAAGGTATAAGCCATGTTAAAGAAT-GAGTTTC 91087
GuineaPig                    CATTGATAAATACCAGTTCAGAGAGATGGAGATATGTAAGGCTTAAGAAAGGCATAATTTATGATAAAGAAT-GAATTTA 60598
NorthernAmericanDeerMouse    ATTTGCTAGG----GAACT-AGACAAATCATTGCAAACCCTGCTGACTAGGAGATAGAACACAGTAGGAAGT-GAGCAGC 65090
Mouse                        ATTTGATAGG----GAATTGAGAGGA--CAGTCCTAACCCTGCTGCCCAGGAGTTATAACACACTAGGAAGCCGAGCAGC 65203
ChineseHamsterGHOK1GS        GTTTGATAGG----TAACTGAGAGGAATAATCACAAACCCTGTTGACCAGGAGATAGGAGACATTAGGAAGT-AAGCAAG 68790
LongTailedChinchilla         GTCTGTTCAG-ACAGTCTGGGAGATATATAAA---GCTCAGTCTGGGAAAGGCATAAGCTACGATAAAGGAT-GAGTTTA 68654
```

```
Majority                    AXCXTTXT-----XGGATTXTAAGCAXATAXTGAC-GTGACXX---TTXXAATXXAX-GXXXGTCATCAGGXTTXTX--X
                                     98250     98260     98270     98280     98290     98300     98310     98320
Human                       AACTTTGTCCTTAAGGGTTTTAAGCAGGTATTGAC-GTGATCAGATTTGTAATTTAT-GAAGGTCATCAAGCTGCTG--T  91163
GuineaPig                   GACTTTA------AGGATTTTAAGCAAATAGTGAC-AA--------TTGTAATTTATTGGAAGTCATTTTAGTAGTTTGT  60663
NorthernAmericanDeerMouse   AGCAGGAT-----GGGAACATTAGCA----------GTGAC-------AAAACCCA--GCTTATCATCAGGGTTGTC---  65143
Mouse                       AGGAAGGA-----GGGATGACAACTATATTTTTCTTGTGGCCA---TTAAAATCCA--GTTCATCATCAGGATTCTT---  65270
ChineseHamsterGHOK1GS       AGCAG-AT-----GGGAACACTAGCA----------GTGAC-------AAAACCCA--GTTTGTCATCAGGATTGTA---  68842
LongTailedChinchilla        GACTTTG------AGGATTTTAAGCAGATAGTGAC-ATAATCAGATTTGTAATTTAT-GAAAGTCACTTAACTGCTGTCT  68726

Majority                    ATAGAAXAT-GGATTGXAGAXAT---------TATTCXX-XXXXXGXXXAAT--XAGAGXXAGTTATTTG----------
                                     98330     98340     98350     98360     98370     98380     98390     98400
Human                       ATAGAAAAT-GGATTGGAAAAAT---------TATCCAACAGGAAGACAAATATGAGACTGAGTTATCCAGATAAAAAAT  91233
GuineaPig                   GTATTGAAT-GAATTGGAGA------------TATCCAATAGGAAGACAAAT--GAGAGTGAGTTATGTG----------  60718
NorthernAmericanDeerMouse   ACAGAAGACAGGTTTGTAGAGGT---------TTCTCTT------GTTCTGT---GGAAGAACTACCTTGT---------  65196
Mouse                       ATAGTAGAT--GCTTGTAGACAT---------TGTTCTT------GTTCAAT---GAAGGAAGTTTTTG-----------  65320
ChineseHamsterGHOK1GS       ACAGAAGACAGCTTTCTAGAGGT---------TATTCTT------GTTCTA-----GAAGAAGCTTTTTG----------  68892
LongTailedChinchilla        GTATAGAAT-GGATTGGAGAAAAATATGGAGATACTGAATAGGAAGACAAAT--GAGAGTGAGCTATCTGAAT-------  68796

Majority                    -------------------------X-XTXTXAGTTTGAGAGA-AATTAGXAGGXAXXXTATXACAATATTTXGTGX---
                                     98410     98420     98430     98440     98450     98460     98470     98480
Human                       AATGTCAGCGAGAAGTAGAGAAACAGATCATGAATTTGAGGGACAATTAGGAGGAAGGCTATCACAATATTTAGCAATTA  91313
GuineaPig                   -------------------------GTTTTTTTGTTTGAGAGATAATTAGGAGGGAAAATATCACAGGATTTAATGGTTA  60773
NorthernAmericanDeerMouse   -----------------------C----TAGTAGAAATGGAAAATGAAAGTTAAGTCTTAACTACCAACTCTTGTGC---  65246
Mouse                       -------------------------------------------------------------GTGCAATACTTTGT-----  65334
ChineseHamsterGHOK1GS       -------------------------------------------------------------CTACAATACCTTGT-----  68906
LongTailedChinchilla        -------------------------GTTTTTGAGTTTGAGAGAGAATTAGAAGGAAGAATATCACAGGATTTAGTGG---  68848

Majority                    ------------XXGAAGGXAGAXAXX-----X-X-XTXXXTATTXAXTTTCTTTCTTGXXTAGCTXA--X-XTXATXXX
                                     98490     98500     98510     98520     98530     98540     98550     98560
Human                       GGA--TGTGTAACAGAAGGGAGAAAAAGAGCTAGGGATGCTTATTAAGTTTCTTTCTTGTGTAGCTCACTATACGATTTG  91391
GuineaPig                   AATATGGAGCAGCAGAAGGTAGACAGA-----AAGAATGAATATTAAATTTCTTTCTTGGATAGCTAA--AAATGATGAT  60846
NorthernAmericanDeerMouse   ----------T---GAATTCATTACTT---A------TTTTTTTTTTTTTTTTTTTTTTTTTTTTTTT-----TTTTTGA  65299
Mouse                       --------------------------------------------------------------------------------  65334
ChineseHamsterGHOK1GS       --------------------------------------------------------------------------------  68906
LongTailedChinchilla        ------------CAGAAGGTAGAGAAG-----AGGAACAACTACTCAGTTTCTTTCTTGGGTAGCTAA--AAATAATGAT  68909
```

```
Majority                     XTACXAGXXTTGXTXTGGAAGATAGA--AXTATAATCTXGATTTT--XTAAATGAAAGTAGCTGGCTTTTTXGCCA-TAA
                                      98570     98580     98590     98600     98610     98620     98630     98640
Human                        TTAGCAGGATTGTTGAGGAAGATAGATATTCATAATCTGGATTTTCATTCTATGAAAGTAGCTGGCTTTTTTGCCA-GAA  91470
GuineaPig                    TTATCAGGATTGTTGGGGAAGACAGAGGAGTATAATCCAAATTTT-GTTATATGAAAGTAGCTGGCTTTTTGGCCAGTAA  60925
NorthernAmericanDeerMouse    GTCCTACTGTGTATATGGGAGGCTAG--NNNNNNNNNNNNNNNNN---NNNNTTGGTAGCCCAGGCTGGCCTCGAACTCA  65374
Mouse                        ---CTA--GTAGAAATAAAAAATGGT--AATAAATTCATGATTTT---TAAGT---------------------------  65377
ChineseHamsterGHOK1GS        ---CTA--GTAGAAATGGAAAGTGAA--ATTTAAATCTTAACTAC---CAATT---------------------------  68949
LongTailedChinchilla         GTATCAGTATTGTTGGGGAAGATAG----GCATAATCTAGATTTT-GTTAAATGAAAGTAGCTGGCTTTTTGGCCC-TAA  68983

Majority                     XXXXXTGTCTXXTXCAAATGAAAATXXA-XXTTAAGTCTXAA-TCATCAGTGGCCAXGXT-----AAATTCATGATTTAT
                                      98650     98660     98670     98680     98690     98700     98710     98720
Human                        TGCCTTGTCTAGTTCAAATGAAAATGTA--CTTAAGTCTTA--TCACCAGTGGCTATGGT-----AAATTGGTGATTTAT  91541
GuineaPig                    TACATTGTCTTATAAAAATGAAAATAGAATTTTAATTCTTAA-TCATCAGTGGGCACGATAAGATAAATCCATGGTTTGT  61004
NorthernAmericanDeerMouse    CAGAGATCCGCCTGCCTCTGCCTCCCGAGTGCTGGGATTAAAGGCGTGCGCCACCACGCC----CGGCTACATTACTTAT  65450
Mouse                        ------------------TGTAA-----------G----AAA-----------------------------ATTCAGAAT  65395
ChineseHamsterGHOK1GS        --------------CTTATGC-----------------TAAA--------------------------TTCATGATTTAT  68972
LongTailedChinchilla         CTTCCTGTCTTGTTAAAATGATAATACAGTGTTAAGTCTTAA-TCATCAATGGCCATGAT-----AAATTCATGGTTTGT  69057

Majority                     TTTAAAATCATAAGAAAATTCAAATTTT-XX-XXXTTAXATTAXAAGAATTATTTTCATXT-XXX---XXX---X-TTT-
                                      98730     98740     98750     98760     98770     98780     98790     98800
Human                        TTTAAAATCATAAGAAAATTCAAATTTTTAAACTGTTACGTTAAAAGAATTATGTTTGTTTTGAGCTTTGATATTGTTTG  91621
GuineaPig                    TTTAAAATCCTAAGAAAATACAGATTTT-AAGCTGTTTTAAAATTAGAATTATTTTCATTT-GAGAGCTGATAGTTTTTG  61082
NorthernAmericanDeerMouse    TTTTAAATTGTAAGAAAATTCAAACTTT-------TCAAATTAGAAATACTACCTTCATAT---------------CTT-  65507
Mouse                        TTCCAAATTAGAAGTA---------TTT-------TTATCCTAAA------CTTTTGATAT---------------TTT-  65437
ChineseHamsterGHOK1GS        TTTCAAATCATAAGAAAATTCAAAATTT-------TCAATTTAGAAATATTGCTTTCATAT---------------TTT-  69029
LongTailedChinchilla         TTTAAAGTCCTAAGAAAATACATATTTT-AAGCTGTTTCAAAATTAGAATTATTTTCATTTTGAGAGCTGA-GGTTTTT-  69134

Majority                     --XXAATAGXCATTCTTTXTTTTTCTAGCXATGAATCATCTTAATGGXCAGAAAATGTATGGAAAAATTATTCGTGTTAC
                                      98810     98820     98830     98840     98850     98860     98870     98880
Human                        --TTAATAGACATGCTTTCTTTTTATAGCCATGAATCATCTTAATGGACAGAAAATGTATGGAAAAATTATTCGTGTTAC  91699
GuineaPig                    TCTTAATATGCGTTTTTTCTCCTT-TAGCTATGAATCATCTTAATGGACAGAAAATGTATGGAAAAATTATTCGTGTTAC  61161
NorthernAmericanDeerMouse    ----AATAGACATTCTTTATTTTTCTAGCTATGAACCATCTTAATGGGCAGAAAATGTATGGAAAAATTATTCGTGTTAC  65583
Mouse                        ----AATAGGTATTCTTTATTTTCCTAGCCATGAATCATCTTAATGGGCAGAAGATGTATGGAAAAATTATTCGTGTTAC  65513
ChineseHamsterGHOK1GS        ----AATAGACATTCTTCATTTTTCTAGCCATGAATCATCTTAATGGGCAGAAAATGTATGGAAAAATTATACGTGTTAC  69105
LongTailedChinchilla         --TTAATATGCACTCTTTCTCCTTATAGCTATGAATCATCTTAATGGACAGAAAATGTATGGAAAAATTATTCGTGTTAC  69212
```

```
Majority                    TCTCTCTAAACATCAGACTGTXCAACTACCTCGAGAGGGACTTGATGATCAAGGGCTAACAAAAGATTTTGGTAATTCCC
                                      98890     98900     98910     98920     98930     98940     98950     98960
Human                       TCTGTCTAAACATCAGACTGTACAGCTACCTCGAGAGGGACTTGATGATCAAGGGCTAACAAAAGATTTTGGTAATTCCC  91779
GuineaPig                   TCTCTCTAAACATCAGACCGTACAACTACCTCGAGAGGGTCTTGATGATCAAGGGCTAACAAAAGATTTTGGCAATTCCC  61241
NorthernAmericanDeerMouse   TCTCTCTAAACATCAGACTGTGCAACTACCTCGAGAGGGGCTTGACGATCAAGGGCTAACAAAAGATTTTGGTAATTCCC  65663
Mouse                       TCTCTCTAAACATCAGACTGTGCAACTACCTCGAGAGGGACTTGATGATCAAGGGCTAACAAAAGATTTTGGGAATTCAC  65593
ChineseHamsterGHOK1GS       TCTCTCTAAACATCAGACTGTGCAACTACCTCGAGAGGGACTTGATGATCAAGGGCTAACAAAAGATTTTGGTAATTCCC  69185
LongTailedChinchilla        TCTCTCTAAACATCAGACCGTACAACTACCTCGAGAGGGTCTTGATGATCAAGGGCTAACAAAAGATTTTGGTAATTCCC  69292

Majority                    CATTGCAXCGTTTTAAXAAACCTGGATCCAAAAATTTTCAXAACATTTTTCCTCCTTCTGCCACCCTTCACCTGTCTAAT
                                      98970     98980     98990     99000     99010     99020     99030     99040
Human                       CATTGCATCGTTTTAAGAAACCTGGATCCAAAAATTTTCAAAACATTTTTCCTCCTTCTGCCACCCTTCACCTATCTAAT  91859
GuineaPig                   CATTGCATCGTTTTAAGAAGCCTGGATCCAAAAATTTTCAGAACATTTTTCCTCCTTCTGCAACTCTTCACCTGTCTAAT  61321
NorthernAmericanDeerMouse   CATTGCACCGTTTTAAAAAACCTGGATCCAAAAATTTTCAAAACATTTTTCCTCCTTCTGCCACCCTTCACCTGTCCAAT  65743
Mouse                       CACTGCACCGTTTTAAAAAACCGGGATCCAAAAACTTTCAGAACATTTTCCCTCCTTCTGCTACCCTTCACCTGTCTAAC  65673
ChineseHamsterGHOK1GS       CATTGCACCGTTTTAAAAAACCTGGATCCAAAAATTTTCAAAACATTTTTCCTCCTTCTGCCACCCTGCACCTGTCTAAT  69265
LongTailedChinchilla        CTTTGCATCGTTTTAAGAAGCCCGGATCCAAAAATTTTCAGAACATCTTTCCTCCTTCTGCAACCCTTCACCTATCTAAT  69372

Majority                    ATCCCGTXAGTAT----TTXAGCTGA-TGXCTAXXGAGATXCATATTATTTXAXCAAAXXATAAXATTTATTATTAATCT
                                      99050     99060     99070     99080     99090     99100     99110     99120
Human                       ATCCCGTAAGTAT----ATAAGCTAG-AGTGTATTGAGATACATTCTATTTGATAAAATATGAAATTTATTCTTAATCT  91934
GuineaPig                   ATTCCGTAAGTTT----TAAAACTA--TGTTTATTCAGA-ATATAT-ATTTTAAGAAAATACAAAATGGATTATTAATCT  61393
NorthernAmericanDeerMouse   ATCCCGTGAGTAT----TTCAGCTGATTCACCAAGGATATTCATCTTGTTT-AGCAAAGCATAAGATTCATTATTGGTCT  65818
Mouse                       ATCCCGTGAGTATCTATTTCAGCTCA-TGACTGAGCACAGTCATATTATC--AGCCAAGC---AGGTCTATGGTT-ATCT  65746
ChineseHamsterGHOK1GS       ATCCCGTGAGTAT----TTCAGCTGACTTACTGAGGAT-TTCATCTTGTTT-AGCAAAGCGTGAGAATTATGATTGATTT  69339
LongTailedChinchilla        ATCCCGTAAGTTT----TTATACTGG-TGTTTATTAAGA-ATATAC-ATACTAAGAAAATATAAAATTTATCATTAACCT  69445

Majority                    XTAXTTXTTTXXACTCTTCCTGTCXAATTTTXCTAGTCCTTCXGTAGCAGAAGAGGATCTGCGAACXCTGTTTGCTAACA
                                      99130     99140     99150     99160     99170     99180     99190     99200
Human                       TCACTTTTTCT-----T-CCCATTCAATTTTCCTAGTCCATCAGTAGCAGAAGAGGATCTACGAACACTGTTCGCTAACA  92008
GuineaPig                   TTACTTTTTTTCCCCCTTCCCGTCCAATTTTCCTAGTCCTTCAGTAGCAGAAGAGGATCTCCGAACACTGTTTGCTAACA  61473
NorthernAmericanDeerMouse   GTATTAATTTGTACTCTTTCTGCCTAATTTTTTTAGTCCTTCTGTAGCAGAAGAGGATCTGCGAACTCTATTTGCTAACA  65898
Mouse                       GTATTCATCTGGACTCTCCCTGTCTTCTTTTTCTAGCCCTTCTGTAGCAGAAGAGGATCTGCGAACTCTGTTTGCCAACA  65826
ChineseHamsterGHOK1GS       GTATTAATTTGTACTCTTT-TGCCTAATTTTTT-AGTCCTTCTGTAGCAGAAGAGGATCTGCGAACTCTATTTGCTAACA  69417
LongTailedChinchilla        TTACCTTTTTT-------CCTGTCCAATTT-CCCAGTCCTTCAGTAGCAGAAGAGGATCTCCGAACACTGTTCGCTAACA  69517
```

```
Majority                    CXGGGGGCACTGTGAAAGCATTTAAGTTTTTTCAGTAAGCAAGCTTCXTTXTCTT-TAAATTXGTGACTTGATAGAAXTX
                                      99210     99220     99230     99240     99250     99260     99270     99280
Human                       CTGGGGGCACTGTGAAAGCATTTAAGTTTTTTCAGTAAGCAAGCTTCCTTATCTT-TAAATTAGTGACCTGATAAAA---  92084
GuineaPig                   CTGGGGGCACTGTGAAAGCATTTAAATTTTTTCAGTAAGCAAGTTTCCTTATCTTTTAAATTAGTGACTTGATAGAAGTA  61553
NorthernAmericanDeerMouse   CCGGGGGCACTGTGAAAGCATTTAAGTTTTTTCAGTAAGCAAGCTTCTTTGTCTT-TACATTTGTGACTTGAT----A--  65971
Mouse                       CCGGGGGCACTGTGAAAGCATTTAAGTTTTTTCAGTAAGCAAGCTTCTTTGTCTT-AAAATGTGTGACTTGATAGAACTT  65905
ChineseHamsterGHOK1GS       CCGGAGGCACTGTGAAAGCATTTAAGTTTTTTCAGTAAGCAAGCTTCTTTGTCTT-TAAATTTGTGACTTGATAGAAATT  69496
LongTailedChinchilla        CTGGAGGCACTGTGAAAGCATTTAAATTTTTTCAGTAAGCAAGCTTCCTTATCTTCTAAGTTAGTGACTTGATAGAAGTA  69597

Majority                    AAAATTTTXTTCAAGTAGTT-TXTAXTCTTTTTGTTT-XXXXXX-XX-X-AAXCTCAAATGGGXAAAAXTCAAGTATTTA
                                      99290     99300     99310     99320     99330     99340     99350     99360
Human                       -------TTTTTAAGTAGTT-TTTGTTCTTTTCGTTTATAGAAATTTTTGATTCCGGAATGGCCAAAATTCAAGTATTTA  92156
GuineaPig                   AAAATTTTCCTCAAGTAGTTAATCATTCTCTTGGATTTTAGAAAATTGTTAATCTAAAGTTAGCACAATTCGAGTATTTA  61633
NorthernAmericanDeerMouse   ---------------------TA----CTTTTTGTTT-------------AACCTCAAATAGGTAAAAATCAAATATTTA  66013
Mouse                       AAATTTTTTTTTCAAA-----TATA-TCTATTTATTT-------------A-CCTCAAATGGGTGAAAATCAG-TGTTTA  65964
ChineseHamsterGHOK1GS       AAAATTTTGTTCAAATATTTTTATAGTCTTTATGTTT-------------AACCTCAAATGGGTAAAAATCAAATGTTTA  69563
LongTailedChinchilla        AAAATTTTCTTCAAGTAGTTACTCAGTCTCTTTGATTTTAGAAAATTCTTAATGTGGAATGAGCAAAATTCAAGTATTTA  69677

Majority                    AA--XX-XXX-CTXTTXT-TATGAAGTATCTAATTTCATAATTTTGTTTCAGAAGAGATCACAAAATGGCTCTTCTTCAG
                                      99370     99380     99390     99400     99410     99420     99430     99440
Human                       ATAAGCCCTTTCTAATTTGTATGAAGTGTCTAATTTTATAATTTTGTTTCAGAAGAGATCACAAAATGGCTCTTCTTCAG  92236
GuineaPig                   A---GCACTTCCTACCTT-TATGAAGTGTCTAATTTCATAATTTTGTTTCAGAAGAGATCACAAAATGGCTCTTCTTCAG  61709
NorthernAmericanDeerMouse   AA---------CTCTTCT-TTTGAAGTATCTAATTTCATAATTTTGTTTCAGAAGAGATCACAAAATGGCTCTTCTTCAG  66083
Mouse                       AA---------CTCTTCT-TATGAAGTATCTAATTTCATAATTTTGTTTCAGAAGAGATCACAAAATGGCTCTTCTTCAG  66034
ChineseHamsterGHOK1GS       AA---------CTCTTCT-TATGAAGTATCTAATTTCATAATTTTGTTTCAGAAGAGATCACAAAATGGCTCTTCTTCAG  69633
LongTailedChinchilla        A---GCACTTCCTACCTT-TATGAAGTATCTAATTTCATAATTTTGTTTCAGAAGAGATCACAAAATGGCTCTTCTTCAG  69753

Majority                    ATGGCAACAGTGGAXGAAGCTATTCAGGCXTTGATTGATCTXCATAATTATAACCTTGGTGAAAACCATCATCTGAGAGT
                                      99450     99460     99470     99480     99490     99500     99510     99520
Human                       ATGGCAACAGTGGAAGAAGCTATTCAGGCCTTGATTGATCTTCATAATTATAACCTTGGAGAAAACCATCATCTGAGAGT  92316
GuineaPig                   ATGGCCACAGTGGAAGAAGCTATTCAGGCCTTGATTGATCTGCATAATTATAACCTTGGTGAAAACCATCATCTGAGAGT  61789
NorthernAmericanDeerMouse   ATGGCGACAGTGGAGGAAGCTATTCAGGCTTTGATCGATCTTCATAATTATAACCTTGGTGAAAACCATCATCTGAGAGT  66163
Mouse                       ATGGCAACAGTGGAGGAAGCTATTCAGGCTTTGATTGATCTTCATAATTATAACCTTGGAGAAAACCATCATCTGAGAGT  66114
ChineseHamsterGHOK1GS       ATGGCAACAGTGGAGGAAGCTATTCAGGCTTTGATTGATCTGCATAATTATAACCTTGGTGAAAACCATCATCTGAGAGT  69713
LongTailedChinchilla        ATGGCAACAGTCGAAGAAGCCATCCAGGCCTTGATTGATCTGCATAATTATAACCTTGGTGAGAACCATCATCTGAGAGT  69833
```

```
Majority                     GTCTTTCTCCAAGTCAACAATTTAAGAAAGGGAAGATG-AAGATTGXGGGXXAATCACATTGTTTGGTGTCATCACCTAT
                                      99530     99540     99550     99560     99570     99580     99590     99600
Human                        GTCTTTCTCCAAGTCAACAATTTAAAAATGGGAAGATG-AAGATTGGGGGTGAATCACATTGTTCAATGTCATCACCTAT  92395
GuineaPig                    GTCTTTCTCCAAATCAACAATTTAAGAAAAGAAAGA----AGATTGAGGGTGAATC-CATTGTT-GGTGTCATCACCTAT  61863
NorthernAmericanDeerMouse    GTCTTTCTCCAAGTCAACAATTTAAGGAAGGGAAGATG-AAGATTGTGGGCAAATCACATTGTTTGGTGTCATCACCTAT  66242
Mouse                        GTCTTTCTCCAAGTCAACAATCTAAGCACGGGA-GATG-AAGATGGCGGGCAGATCCCATTGTT-GGTGTCATCACCTAT  66191
ChineseHamsterGHOK1GS        GTCTTTCTCCAAGTCAACAATTTAAGGAAGGGAAGATGGAAGATTATGGGCAAATCACATTGTTTGGTGTCATCACCTAT  69793
LongTailedChinchilla         GTCTTTCTCCAAGTCAACAATTTAAGAAAAGAAAGA----AGATTGGGGGTGAATCACATTGTTTGGTGTCATCACCTAT  69909

Majority                     T-GACTGTTCAGAAAA-GTGGGGACCAGAGTTTGATT--------XXX-XXX-XXX-----TX--XXXX-XTTTTXCATG
                                      99610     99620     99630     99640     99650     99660     99670     99680
Human                        TTGACTGTTCAGAAAA-GTGGGGACCAGAGTTTGATTTTTTTTGTTTTTGTTTTTTTGGGGTTTCTTTTTTTTTTCCATG  92474
GuineaPig                    T-GACTGTTCAGAAAA-GTGGGGACCAGAGTTTGATTTTC-----TTTCGTTGTTTC----TTGTTTTTGTTTTTACATG  61932
NorthernAmericanDeerMouse    T-GACTGTTCAGAAAA-GTGGGGACCAGAGTTTGATT------------------------T---------TTTT-CATG  66286
Mouse                        T-GACTGTTCAGAAAA-GTGGGGACCAGAGTTTGATT------------------------T---------TTTT-CATG  66235
ChineseHamsterGHOK1GS        T-GACTGTTCAGAAAAAGTGGGGACCAGAGTTTGATT------------------------T---------TTTTTCATG  69839
LongTailedChinchilla         T-GACTGTTCAGAAAA-GTGGGGACCAGAGTTTGATT--------TTTTGTTGTTTC----TTGTTTTTGTTTTTACATG  69975

Majority                     CTGTTATCATTCCTTGGTTATAAAATGAAATGGCATATGTAAAGGCAGAGTTACTAACTGCTXTXTTTCATCTGTTCXAT
                                      99690     99700     99710     99720     99730     99740     99750     99760
Human                        CTGTTATCATTCCTTGGTTATAAAATGAAATGGCATATGTAAAGGCAGAGTTGTTAACTGCTATATTTCATCTGTTCTAT  92554
GuineaPig                    CTGTTATCATTCCTTGGTTGTAAAATGAAATGGCATATGTAAAGGCAGAGTTATGAACTGCTATACATCATCTGTTCAAT  62012
NorthernAmericanDeerMouse    CTGTTATCATTCCTTGGTTATAAAATGAAATGGCATATGTAAAGGCAGAGTTACTAACTGCTGTGTTTCATCTGTTCTAT  66366
Mouse                        CTGTTATCATTCCTTGGTTATAAAATGAAATGGCATATGTAAAGGCAGAGTTGCTAACTGCTGTGTTTCATCTGTTCAAT  66315
ChineseHamsterGHOK1GS        CTGTTATCATTCCTTGGTTATAAAATGAAATGGCATATGTAAAGGCAGAGTTACTAACTGCTATGTTTCATCTGTTCTAT  69919
LongTailedChinchilla         CTGTTATCATTCCTTGGTTGTAAAGTGAAATGGCATATGTAAAGGCAGAGTTAGGAACTGCTGTACGTCACCTGTTCAGT  70055

Majority                     AGGGAAGCCATTTT-ATTGTCTGTTTAAAXTTTXAGTTTAATTTTGCTTTXTTTTTTT-----XXX---TTTTCAACTTA
                                      99770     99780     99790     99800     99810     99820     99830     99840
Human                        AGGGAAGCCATTTT----GTCTGTTTAAAATTTCAGTTTAATTTTGCTTTTTTTTTTTTTTTTTTTTTCCTTTCAACTTA  92630
GuineaPig                    AGGGAAGCCATTTTGATTGTCTGTTTAAAATTTCAGTTTAATTTTGCTTTCTTTATT----------------CAACTTA  62076
NorthernAmericanDeerMouse    AGGGAAGCCATTTT-ATTGTCTGTTTAAATTTTTAGTTTAATTTTGCTTTCTTTTTTTCTTTATTT---TTTTCAACTTA  66442
Mouse                        AGGGAAGCCATTTT---TGTCTGTTTAAATTTTTAGTTTAATTTTGCTTTTTTTTTTT-----------TTTCCAACTTA  66381
ChineseHamsterGHOK1GS        AGGGGAGCCATTTTTATTGTCTGTTTATATTTTTAGTTTGATTTTGCTTTTTTTTTTT-----TTT---TTTTTAACTTA  69991
LongTailedChinchilla         AGGAAAGCCATTTTTATTGTCTGTTTGATATTTCAGTTTAATTTTGCTTTCTTTTTT----------------CAACTTA  70119
```

```
Majority                    GTTGACATACGTGCCTTAXAAAGGAAAACTAGTGTTGCTATTGTGCATTTACTAGXAAAAAGGAATTGGTTGTTTAGGGC
                                          99850     99860     99870     99880     99890     99900     99910     99920
Human                       GTTGACATACGTGCCTTAAAAAGGAAAACTAGTGTTGCTATTGTGCATTTACTAGAAAAAAGGAATTGGTTGTTTAGGGC  92710
GuineaPig                   GTTGACATACGTGCCTTAAAAAGGAAAACTAGTGTTGCTATTATGCATTTACTAGAAAAAAGGAATTGGTTGTCTAGGGC  62156
NorthernAmericanDeerMouse   GTTGACATACGTGCCTTACAAAGGAAAACTAGTGTTGCTGTTCTGCATTTACTAGGAAAAAAGAATTGGTTGTTTAGGGC  66522
Mouse                       GTTGACATACGTGCCTTACAAAGGAAAACTAGTGTTGCTATTCTGCATTTACTAGGAAAAAGGAATTGGTTGTTTAGGGC  66461
ChineseHamsterGHOK1GS       GTTGACATACGTGCCTTACAAAGGAAAACTAGTGTTGCTGTTGTGCATTTACTAGGACAAAAGAATTGGTTGTTTAGGGC  70071
LongTailedChinchilla        GTTGACATATGTGCCTTAAAAAGGAAAACTAGTGTTGCTATTGTGCATTTACTAGAAAAAAGGAATTGGTTGTCTAGGGC  70199

Majority                    ACAXXGTTATXTGGGAATTAAAATATGTTTAGGCAGGGGTGTGTXAAAAGGTTAAGTTTTTGTTTCTCCTGCTTGGAACT
                                          99930     99940     99950     99960     99970     99980     99990    100000
Human                       ACACTGTTATATGGGAATTAAAATATGTTTAGGCAGGGGTGTGTAAAAAGGTTAAGTTTTTGTTTCTCCTGCTTGGAACT  92790
GuineaPig                   ACACTGTTATAAGGGAATTAAAATATGTTTAGGCAGGGGTGTGTAAAAAGGTTAAGTTTTTGTTTCTCCTGCTTGGAACT  62236
NorthernAmericanDeerMouse   ACATCGTTATGTGGGAATTAAAATATGTTTAGGCAGGGGTGTGTCAAAAGGTTAAGTTTTTGTTTCTCCTGCTTGGAACT  66602
Mouse                       ACATCGTTATGTGGGAATTAAAATATGTTTGGGCAGGGGTGTGTCAAAAGGTTAAGTTTTTATTTCTCCTGCTTGGAACT  66541
ChineseHamsterGHOK1GS       ACATCGTTATGTGGGAATTAAAATATGTTTTGGCAGGGGTGTGTCAAAAGGTTAAGTTTTTGTTTCTCCTGCTTGGAACT  70151
LongTailedChinchilla        ACACTGTTATAAGGGAATTAAAATATGTTTAGGCAGGGGTGTGTAAAAAGGTTAAGTTTTTGTTTCTCCTGCTTGGAACT  70279

Majority                    TATTTTGAATTATTGGCTTATCACATTTCTTTCTATTTAATCXAATAAGATACTTGATACTGAAAGAATAAAGCAGCATT
                                         100010    100020    100030    100040    100050    100060    100070    100080
Human                       TATTTTGAATTACTGGCTTGTCACCTTTTTTTCTATTTAATCAAATAAGATACATGATATTGAAAGAATAAAGCAGCATT  92870
GuineaPig                   TACTTTGAATTATTGGCTTATTACATTTCTTT--ATTTAATCA-----GATACTGGATATTGAAAGAATAAAGCAGCATT  62309
NorthernAmericanDeerMouse   TATTTTGAATTATTGGCTTATCACATTTCTTTCTATTTAATCTAATAAGATACTTGATACTGAAAGAATAAAGCAGCATT  66682
Mouse                       TTCTTTGAATTATTGGCTTATCACATTTCTTTCTATTTAATCTAATAAGATACTTGATACTGAGAGTATAAAACAGCATT  66621
ChineseHamsterGHOK1GS       TATTTTGAATTATTGGCTTATCACATTTCTTTCTATTTAATCTAATAAGATACTTGATACTGAAAGAATAAAGCAGCATT  70231
LongTailedChinchilla        TATTTTGAATTATTGGCTTATCGTATTTCTTTCTATTTAATCAAATAAGATACTGGATACTGAAAGAATAAAGCAGCATT  70359

Majority                    TTTAGTTTTTACTAXCTTAGGCTTTATTGCTTTTGAAA-XACATTGGCCTTTTGTATCTCACAAXTCTGGTCTAGATTCA
                                         100090    100100    100110    100120    100130    100140    100150    100160
Human                       TTTAGTTTTTACTACCTTAGGCTTTATTGCTTTGAAAACAACATTGGCCTTTTGTATCTCACAATTCTGGTCTAGATTCA  92950
GuineaPig                   TTTAATTTTTAATACCTTAGGCTTTGTTGCTTTTTAAAAAACATTGGCCTTTTGTATCTCAGAATTCTGGTCTAGATTCA  62389
NorthernAmericanDeerMouse   TTTAGTTTCTACTATCTTAGGCTTTATTGCTTTTGAAA--ACATTGGCCTTTTGTATCTCACAAATCTGGTCTAGATTCA  66760
Mouse                       TTTAGTTTTTACTATCTTAGGCTTTATTGCTTTTGAAA--ACATTGGCCTTTTGTATCTCACAAATCTGGTCTAGATTCA  66699
ChineseHamsterGHOK1GS       TTTGGTTTCTACTATCTTAGGCTTTATTGCTTTTGAAA--ACATTGGCCTTTTGTATCTCACAAATCTGGTCTAGATTCA  70309
LongTailedChinchilla        TTTAGTTTTTACTACCTTAGGCTTTATTGCTTTTTAAAAAACATTGGCCTTTTGTATCTCACAATTCTGGTCTAGATTCA  70439
```

```
Majority                    GTTATGAATGTAGGCATTAGTTAAAATTAACAAGATGCAGAGTATTAATTTCTTAAGACAA-CAAAGTGATTTCTGTAAG
                                      100170    100180    100190    100200    100210    100220    100230    100240
Human                       GTTATGAATGTAGGCATTAGTTAAAATTAACAAGATGCAGAGTATTAATTTCTTAAGACAA-CAAAGTGATTTCTGTAAG 93029
GuineaPig                   GTTATGAATGTAGGCATTAGTTAAAGTTAATAAGATGCAGCGTATTAATTTCTTAAGACAA-CAAAGTGATTTCTGTAAG 62468
NorthernAmericanDeerMouse   GTTATGAATGTAGGCATTAGTTAAAATT                                                     66788
Mouse                       GTTATGAATGTAGGCATTAGTTAAAATTAACAAGATGCAGAGTATTAATTTCTTAAGACA-----AGTGATTTCTGTAAG 66774
ChineseHamsterGHOK1GS       GTTATGAATGTAGGCATTAGTTAAAATTAACAAGATGCAGAGTATTAATTTCTTAAGACAA--CAAGTGATTTCTGTAAG 70387
LongTailedChinchilla        GTTATGAATGTAGGCATTAGTTAAAGTTAACAAGATGCAGAGTATTAATTTCTTAAGAAAAACAAAGTGATTTCTGTAAG 70519

Majority                    TTTGAGCCCTATGTGGAAAGCATTGTGGAATCTTAACCTTTTTGTACACACTCTTGTGGGACGTATCATATAAATGTCAG
                                      100250    100260    100270    100280    100290    100300    100310    100320
Human                       TTTGAGCCCTATGTGGAAAGCATTGTGGAATCTTAACCTTTTCGTACACACTCTTGTGGGACGTATCATATAAATGTCAG 93109
GuineaPig                   TTTGAGCCCTATGTGGAAAGCATTGTGGAATCTTAACCTTTTTGTACACACTCTTGTGGGACGTATCATATAAATGTCAG 62548
NorthernAmericanDeerMouse                                                                                    66788
Mouse                       TTTGAGCCCTATGTGGAAAGCATTGTGGAACCTTAACCTTTTTGTACACACTCTTGTGGGACGTGTCATATAAATGTCAG 66854
ChineseHamsterGHOK1GS       TTTGAGCCCTATGTGGAAAGCGTTGTGGAACCTTAACCTTTTTGTACACACTCTTGTGGGACGTATCATATAAATGTCAG 70467
LongTailedChinchilla        TTTGAGCCCTACGTGGAAAGCATTGTGGATTCTTAACCTTTTTGTACACACTCTTGTGGGACGTATCATATAAATGTCAG 70599

Majority                    CACTAAGTAATGTCTTGTTTGTGGCTGAATATTTTTCGTAGATGTTTTTGAAGTTGACATGACTTACGTGCATTTAAATA
                                      100330    100340    100350    100360    100370    100380    100390    100400
Human                       CACTAAGTAATGTCTTGTTTGTGGCTGAATATTTTTCGTAGATGTTTTTGAAGTTGACATGACTTACGTGCATTTAAATA 93189
GuineaPig                   CACTAAGTAATGTCTTGTTTGTGGCTGAATATTTTTCGTAGATGTTTTTGAAGTTGACATGACTTACGTGCATTTAAATA 62628
NorthernAmericanDeerMouse                                                                                    66788
Mouse                       CACTAAGTAATGTCTTGTTTGTGGCTGAATATTTTTCGTAGATGTTTTTGAAGTTGACATGACTTACGTGCATTTAAATA 66934
ChineseHamsterGHOK1GS       CACTAAGTAATGTCTTGTTTGTGGCTGAATATTTTTCGTAGATGTTTTTGAAGTTGACATGACTTATGTGCATTTAAATA 70547
LongTailedChinchilla        CACTCAGTAATGTCTTGTTTGTGGCTGAATACTTTTCGTAGATGTTTTTGAAGTTGACATGACTTACGTGCATTTAAATA 70679

Majority                    TATATTGCCATCCTTAGTTTGTAATTAAGATTTGGAATATGGTTGTGGATTTCTGAGCATGTGCAGACTGGTCTAGCTAG
                                      100410    100420    100430    100440    100450    100460    100470    100480
Human                       TATATTGCCATCCTTAGTTTGTAATTAAGATTTGGAATATGGTTGTGGATTTCTGAGCATGTGCAGACTGGTCTAGCTAG 93269
GuineaPig                   TATATTGCCATCCTTAGTTTGTAATTAAGATTTGGAATATGGTTGTGGATTTCTGAGCATGTGCAGACTGGTCTAGCTAG 62708
NorthernAmericanDeerMouse                                                                                    66788
Mouse                       TATATTGCCATCCTTAGTTTGTAATTAAGATTTGGAAAATGGTTGTGGATTTCTGAGCATGTGCAGACTGGTCTAGCTAG 67014
ChineseHamsterGHOK1GS       TATATTGCCATCCTTAGTTTGTAATTAAGATTTGGAAAATGGTTGTGGATTTCTGAGCATGTGCAGACTGGTCTAGCTAG 70627
LongTailedChinchilla        TATATTGCCATCCTTAGTTTGTAATTAAGATTTGGAATATGGTTGTGGATTTCTGAGCATGTGCAGACTGGTCTAGCTAG 70759
```

```
Majority                     TTCAGGAACTGGTGCATGTATTTTTCAAAGACAAAGAAAGTGTACTGCGAAAATTTGCAGGAAGATTAATTTTGTGGCAG
                                       100490    100500    100510    100520    100530    100540    100550    100560
Human                        TTCAGGAACTGGTGCATGTATTTTTCAAAGATAAAGAAAGTGTACTGCGAAAATATGCAGGAAGATTAATTTTGTGGCAG  93349
GuineaPig                    TTCAGGAACTGGTGCATGTATTTTTCAAAGACAGAGAAAGTGAACTGCGAAAATTTGCAGGAAGATTAATTTTGTGGCAG  62788
NorthernAmericanDeerMouse                                                                                      66788
Mouse                        TTCAGGAACTGGTGCATGTATTTTTCAAAGACAAAGAAAGTGTACTGCGAAAACTTGCAGGAAGATTAATTTTGTGGCAG  67094
ChineseHamsterGHOK1GS        TTCAGGAACTGGTGCATGTATTTTTCAAAGACAAAGAAAGTGTACTGCGAAAACTTGCAGGAAGATTAATTTTGTGGCAG  70707
LongTailedChinchilla         TTCAGGAACTGGTGCATGTATTTTTCAAAGACAAAGAAAGTGTACTGCGAAAATTTGCAGGAAGATTAATTTTGTGGCAG  70839

Majority                     TTTTCTAAAACTGACAACCAGGTGGGACCAAAGTTTATGTGCCTTTAGTCTTAATTTACCTTGCATTGTAATATTCAGTT
                                       100570    100580    100590    100600    100610    100620    100630    100640
Human                        TTTTCTAAAACTGACAACCAGGTGGGACCAAAGTTTATGTGCCTTTAGTCTTAATTTACCTTGCATTGTAATATTCAGTT  93429
GuineaPig                    TTTTGGAAAACTGACAACCAGGTGGGACCAAAGTTTATGTGCCTTTAGTCTTAATTTACCTTGCATTGTAATATTCAGTT  62868
NorthernAmericanDeerMouse                                                                                      66788
Mouse                        TTTTCTAAAACTGACAACCAGGTGGGACCAAAGTTTATGTGCCTTTAGTCTTAATTTACCTTGCATTGTAATATTCAGTT  67174
ChineseHamsterGHOK1GS        TTTTCTAAAACTGACAACCAGGTGGGACCAAAGTTTATGTGCCTTTAGTCTTAATTTACCTTGCATTGTAATATTCAGTT  70787
LongTailedChinchilla         TTTTTGAAAACTGACAACCAGGTGGGACCAAAGTTTATGTGCCTTTAGTCTTAATTTACCTTGCATTGTAATATTCAGTT  70919

Majority                     TTAATAAATCTTCAAAATATTTTGTATTTAGGAATAGATCTGACTTTAATAAAAACATGGCTCAGAATCTACAGGTCAAA
                                       100650    100660    100670    100680    100690    100700    100710    100720
Human                        TTAATAAATCTTCAAAATATTTTGTATTTAGGAATAGATCTGACTTTAATAAAAACATGGCTCAGAATCTACAGGTCAAA  93509
GuineaPig                    TTAATAAATCTTCAAAATATTTTGTATTTAGGAATAGATCTGACTTTAATAAAAACATGGCTCAGAATCTACAGGTCAAA  62948
NorthernAmericanDeerMouse                                                                                      66788
Mouse                        TTAATAAATCTTCAAAATATTTTGTATTTAGGAATAGATCTGACTTTAATAAAAACATGGCTCAGAATCTACAGGTCAAA  67254
ChineseHamsterGHOK1GS        TTAATAAATCTTCAAAATATTTTGTATTTAGGAATAGATCTGACTTTAATAAAAACATGGCTCAGAATCTACAGGTCAAA  70867
LongTailedChinchilla         TTAATAAATCTTCAAAATATTTTGTATTTAGGAATAGATCTGACTTTAATAAAAACATGGCTCAGAATCTACAGGTCAAA  70999

Majority                     TTTATTTGAACAGTTCTTGTCAATCTGAATTGTTGATTCTGTT-AAATGACCAATACTTTTTGAAATTGATGTACTTAGT
                                       100730    100740    100750    100760    100770    100780    100790    100800
Human                        TTAATTTGAACAGTTCTTGTCAATCCGAATTGTTGATTCTGTTTAAATGACCAATACTTTTTGAAATTGATGTACTTAGT  93589
GuineaPig                    TTTATTTGAACAGTTCTTGTCAATCTGAATTGTTGATTCTGTT-AAATGACCAATACTTTTTGAAATTGATGTACTTAGT  63027
NorthernAmericanDeerMouse                                                                                      66788
Mouse                        TTTATTTGAACAGTTCTTGTCAATCTGAATTGTTGATTCTGTT-AAATGACCAATACTTTTTGAAATTGATGTACTTAGT  67333
ChineseHamsterGHOK1GS        TTTATTTGAACAGTTCTTGTCAATCTGAATTGTTGATTCTGTT-AAATGACCAATACTTTTTGAAATTGATGTACTTAGT  70946
LongTailedChinchilla         TTTATTTGAACAGTTCTTGTCAATCTGAATTGTTGATTCTGTT-AAATGACCAATACTTTTTGAAATTGATGTACTTAGT  71078
```

Majority	TTCAAGATTCATAGATTCTGTTATCTATGTAGACAGAATGGTCATGTATATTTTCTATTAGTTGAGTTTTTACATCTTTA	
	100810 100820 100830 100840 100850 100860 100870 100880	
Human	TTCAAGATTCATAGATTCTGTTATCTATGTAGACAGAATGGTCATGTATATTTTCTATTAGTTGAGTTTTTACATCTTTA	93669
GuineaPig	TTCAAAATTCATAGATTCTGTTATCTATGTAGACAGAATGGTCATGTATATTTTCTATTAGTTGAGTTTTTACATCTTTA	63107
NorthernAmericanDeerMouse		66788
Mouse	TTCAAGATTCATAGATTCTGTTATCTATGTAGACAGAATGGTCATGTATATTTTCTATTAGTTGAGTTTTTACATCTTTA	67413
ChineseHamsterGHOK1GS	TTCAAGATTCATAGATTCTGTTATCTATGTAGACAGAATGGTCATGTATATTTTCTATTAGTTGAGTTTTTACATCTTTA	71026
LongTailedChinchilla	TTCAAGATTCATAGATTCTGTTATCTATGTAGACAGAATGGTCATGTATATTTTCTATTAGTTGAGTTTTTACATCTTTA	71158

Majority	GAAATGTAAAATTCAGTATAGTTTGAAAGCGGCACAATTAAAAATTAATTTTCTAACAAAGTTGGGAGGTTTGATGGTTG	
	100890 100900 100910 100920 100930 100940 100950 100960	
Human	GAAATGTAAAATTCAGTATAGTTTGAAAGCGGCACAATTAAAAATTAATTTTCTAACAAAGTTGGGAGGTTTGATGGTTG	93749
GuineaPig	GAAATGTAAAATTCAGTATAGTTTGAAAGCGGCACAATTAAAAATTAATTTTCTAACAAAGTTGGGAGGTTTGATGGTTG	63187
NorthernAmericanDeerMouse		66788
Mouse	GAAATGTAAAATTCAGTATAGTTTGAAAGCGGCACAATTAAAAATTAATTTTCTAACAAAGTTGGGAGGTTTGATGGTTG	67493
ChineseHamsterGHOK1GS	GAAATGTAAAATTCAGTATAGTTTGAAAGCGGCACAATTAAAAATTAATTTTCTAACAAAGTTGGGAGGTTTGATGGTTG	71106
LongTailedChinchilla	GAAATGTAAAATTCAGTATAGTTTGAAAGCGGCACAATTAAAAATTAATTTTCTAACAAAGTTGGGAGGTTTGATGGTTG	71238

Majority	TTTAATTTCATTTTGTGTGTACTCTGCTTACCTCTGTAGCATGCTCAATAAACACTTCTGTAGCTCTGTATTCACCTTTT	
	100970 100980 100990 101000 101010 101020 101030 101040	
Human	TTTAATTTCATTTTGTGTGTACTCTGCTTACCCCTGTAGCATGCTCAATAAACACTTCTGTAGCTCTATATTCACCTTTT	93829
GuineaPig	TTTAATTTCATTTTGTGTGTACTCTGCTTACCTCTGTAGCATGCTAAATAAACACTTCTGTAGCTCTGTATTCACCTTTT	63267
NorthernAmericanDeerMouse		66788
Mouse	TTTAATTTCACTTTGTGTGTACTCTGCTTACCTCTGTAGCATGCTCAATAAACACTTCTGTAGCTCTGTATTCACCTTTT	67573
ChineseHamsterGHOK1GS	TTTAATTTCATTTTGTGTGTACTCTGCTTACCTCTGTAGCATGCTCAATAAACACTTCTGTAGCTCTGTATTCACCTTT-	71185
LongTailedChinchilla	TTTAATTTCATTTGGTGTGTACTCTGCTTACCTCTGTAGCATGCGAAATAAACACTTCTGTAGCTCTGTGTTCACCTTTT	71318

Majority	CTGTCTTTCTCTGCTGCCTTTTCTCTCTCCTCTTCTTTGTTTTCACTCCACTGTGCTTCTGAATTCATGTTTATTCTCTG	
	101050 101060 101070 101080 101090 101100 101110 101120	
Human	CTGTCTTTCTCTGCTGCCTTTTCTCTCTCCTCTTCTTTGTTTTCACTCCACTGTGCTTCTGAATTCATGTTTATTCTCTG	93909
GuineaPig	CTGTCTTTCTCTGCTGCCTTTTCTCTCTCCTCTTCTTTGTTTTCACTCCACTGTGCTTCTAAATTCATGTTTATTCTCTG	63347
NorthernAmericanDeerMouse		66788
Mouse	CTGTCTTTCTCTGCTGCCTTTTCTCTCTC---TCCTTTGTTTTCACTCCACTGTGCTTCTGAGTTCATGTTTACTCT--G	67648
ChineseHamsterGHOK1GS	CTGTCTTTCTCTGCTGCCTTTTCTCTCTC---TCCTTTGTTTTCACTCCACTGTGCTTCTGAATTCATGTTTACTCTCTG	71262
LongTailedChinchilla	CTGTCTCTCTCTGCTGCCTTCTCTCTCTCCTCCTCTTTGTTTTCACTCCACTGTGCTTCTGAATTCATGTTTATTCTCTG	71398

```
Majority                     CCAGGGTGGGAAAGGAGTAATAATATTACAATTCTATGGCTTTATACCATAAATAAATCTAGATGCTGTGAAAATACACC
                             101130 101140 101150 101160 101170 101180 101190 101200
Human                        CCAGGGTGGGAAAGGAGTAATAATATTACAATTCTATGGCTTTATACCATAAATAAATCTAGATGCTGTGAAAATATACC 93989
GuineaPig                    CCAGGGTGGGAAAGGAGTAATAATATTACAGTTCTATGGCTTTATACCATAAATAAATCTAGATGCTGTGAAAATATACC 63427
NorthernAmericanDeerMouse                                                                                     66788
Mouse                        CCAGGGTGGGAAAGGAGTAATACTATCACAATCCTATGGCTTTATACCATAAATAAATCCAGATGCTGTGAAAATGCACC 67728
ChineseHamsterGHOK1GS        CCAGGGTGGGAAAGGAGTAATAGTATTACAATCCTATGGCTTTATACCATAAATAAATCTAGATGCTGTGAAAATGCACC 71342
LongTailedChinchilla         CCAGGGTGGGAAAGGAGTACTAATACTAGAGTTCTATGGCTTTCTACCATAAATAAATGTAGATGCTGTGAAAATACACC 71478

Majority                     AGCTGTTTTT------TTTTTTAATGTAAAAGAXGGTAACTGCTTTT-CAGGAGGACACATATTAAACATTTCCCACCCT
                             101210 101220 101230 101240 101250 101260 101270 101280
Human                        AGCTGGTTT--------TTTTTAATTTAAAAGATGGTAACTGCTTTT-CAGGAGGACACATATTAAACATTTCCCACCCT 94060
GuineaPig                    AGCTGTTTTTTTTTTTTTTTTTAATGTAAAAGACGATGACTGCTCTT-CAGGAGGACACATATTAAACATTTCCCACCCT 63506
NorthernAmericanDeerMouse                                                                                     66788
Mouse                        AGCTGTTCCT------TTTTTTAATGCAAAAGTCGGTAACTGCTTTTTCAGGAGGACACATATTAAACATTTCCCACCCT 67802
ChineseHamsterGHOK1GS        AGCTGTTCCT------TTTTTTAATGCAAAAGAGGGTAACTGCTTTTTCAGGAGGACACATATTAAACATTTCCCACCCT 71416
LongTailedChinchilla         AGCGGGGTT-------TTATTTAATGTAAAAGATGATAACTGCTCTT-CAGGAGGACACATATTAAACATTTCCCACCCT 71550

Majority                     GTTTATAATCTXCTGCTTTAAAGACATAACTTT-TATTGTAGCTTGTTAATTCTXCTATCTCTTTTGTTGTTGTTGTTG
                             101290 101300 101310 101320 101330 101340 101350 101360
Human                        GTT-ATAATCTACTGCTTTAAAGACATAACTTT-TATTGTAGCTTGTTAATTCTATCTCT-TTTGTTCTTGTTGTTTTTT 94137
GuineaPig                    GTTTATAATCTACTGCTTTAAACACATAACTTTCTATTGTAGCTTGTTAATTCTTTCTATCTTTTTTGTTATTGTTGTTG 63586
NorthernAmericanDeerMouse                                                                                     66788
Mouse                        GTTTATAATCT---GCTTTAAAGACATAACTTC-TATTGTAGCTTGTTAATTCTCTCTATCTCTTTTGTTGTTGTTGTTG 67878
ChineseHamsterGHOK1GS        GTTTAAAATCT---GCTTTAAAGACATAACTTC-TATTGTAGCTCGTTAATTCTCTCTATCTCTTTTGTTGTTGTTGTTG 71492
LongTailedChinchilla         GTTTACAACCTCCTGCTTTACAAACGTAACTTTCTATTGTAGCTTGTTAACCCTTTCTATCCCTTTTGTTCTTGCTGTTG 71630

Majority                     TTXTTTTTCCAGTAGATTTATGCACTAATAGATCTTTTGGATTTGCCATGCTXTCTTGCTGCAGTTTCATCTTTCATCTT
                             101370 101380 101390 101400 101410 101420 101430 101440
Human                        TTTTTTTTCCAGTAGATTTATGCACTAATAGATCTTTTGGATTTGCCATGCTCTCTTGCTGCAGTTTCATCTTTCATCTT 94217
GuineaPig                    TTGTTTTTCTAGTAGATTTATGCACTAGTAGATCTTTTGGACTTGCCATGCTGTTTTGCTGCAGTTTCATCTTTCATCTT 63666
NorthernAmericanDeerMouse                                                                                     66788
Mouse                        TT----TTCCCGTAGATTTATGCACTAATAGGTCTTTTGGATTTGCCATGCTCTCTTGCTGCAGTTTCATCTTTCATCTT 67954
ChineseHamsterGHOK1GS        TT----TTCCCGTAGATTTATGCACTAATAGATCTTTTGGATTTGCCATGCTTTCTTGCTGCAGTTTCATCTTTCATCTT 71568
LongTailedChinchilla         TTGTTTTCCTAGTAGATTTATGCACTAGTAGATCTTTTGGACTTGCCATGCTGTTTTGCTGCAGTTTCATCTTCCATCTT 71710
```

```
Majority                    TTGTGTCTGCTXAAGATTTCCTACTAATCTTAG-ACTACCTTGTGAGTTTCAACAAAGAGXACTTX---TXTTACTXGGA
                            101450  101460  101470  101480  101490  101500  101510  101520
Human                       TTGTGTCTGCTAAAGATTTCTTACTAATCTTAG-ACTACCTTGTGAGTT--AACAAAAAGAACTTGA--TATTACTGGGA  94292
GuineaPig                   TTGTGTCTGCTGAAAATTTCCTACTAATCTTAA-ACTACCTTGTGAGTTTCAACAAAGAGCACTTA---TTGTCATTGGA  63742
NorthernAmericanDeerMouse                                                                                     66788
Mouse                       T-GTGTCTGCTCTAGATTTCCTACTAATTTTAG-ACTAC-TACTGAGTTTTAACAAAGGGTTCTGC---TGTTACTGGAA  68028
ChineseHamsterGHOK1GS       T-GTGTCTGCTCAAGATTTCCTACTAATTTTAG-ACTACCTTCTGAGCTACAACAAAGGGCTCTAG---TGTGACTAGAA  71643
LongTailedChinchilla        TTGTGTCCGCTAAAAATTTCCTACTAATCTTAAGACTACCTTGTGAGTTTCAACAAAGAGTACTTAACGTTTTTATTGGA  71790

Majority                    AGAAAGAGXAGXATATC-------------ATCTGCTTTTGTTTTGGGAXGGAAX----ATAXACATTCAGGTATTTAAA
                            101530  101540  101550  101560  101570  101580  101590  101600
Human                       AGAAAGAGCAGCATATCTGCATATCAGCATATCTGCTTTTGCCTTGGGTGGGAAGAATGATATACATTCAAGTATTTAAA  94372
GuineaPig                   AATAAGAGTAGAATACC-------------ATTTGTTTTTGTTTTGGGAAGAAA-----ATACACATTCCGATA------  63798
NorthernAmericanDeerMouse                                                                                     66788
Mouse                       AGAAAGG--ACTATGGC-------------ATTGGCTTCTATTTGAGTGCCATA---------G------GGTATATACA  68078
ChineseHamsterGHOK1GS       AGAAAGG--AGTGTGTC-------------ACCTGCTTTTAGTTTGGTAGGGGAT----ATATACATGTTGGTATTCCGA  71704
LongTailedChinchilla        AATGAGAGCAGCATATC-------------ATCTGTTTTTGTTTTGGGAAGGAAG----ATACACATTCAGGTATTGAAA  71853

Majority                    AAC------------ACACTTXXCAACTGCGGATGTTXCTACTTTCACTXTTTAXXCXGTTACAATTAXGGTGCTXTTT-
                            101610  101620  101630  101640  101650  101660  101670  101680
Human                       AACTTAGAGTAATTTGCATTTAACAACTGAGGATGTTACTACTGTAACTTGTTAAGCAGTTACAATTAGGGTGCTATTTA  94452
GuineaPig                   -----------------------------------------CTTT-----------TGGTTACAATTAAGGTGCTATTT-  63825
NorthernAmericanDeerMouse                                                                                     66788
Mouse                       TGC------------AGGCTTTTAAACCGCAGGTGCTGTCCTCCTCACTCTTTATACTGATGCTGTCATCAAG---CTA-  68142
ChineseHamsterGHOK1GS       CAA------------ACAGCATTTAACTGCTGATGTTACAGTGTTCACTCTTTGTACTGATGTGGTCATCGGGGTGCTA-  71771
LongTailedChinchilla        AAC-------AGTTTACACTTAACACCTGGGTATGTTGCTACTTTCACTTTTCA--TGGTTACAATTAGGTTGCTGTTT-  71923

Majority                    --XTCAGAATATTTTTGGTXACTTTTCTAAACAXAXATATTGXTATCAAATAACTTTGGAGTTA---CATXTGTTCCTTA
                            101690  101700  101710  101720  101730  101740  101750  101760
Human                       TATTCAAAATATTTCGGTCAGTTTTTCTAAACAGATATATTGGTATCAAATAACTTTGGAGTTA---CACATGTTTCTTA  94529
GuineaPig                   ----CAGAGTATTTTTGGACACTTTTCTAAACAAGGGTATTGATATCAGACAAGTTTGTAATTA---TAAATGTTTCTT-  63897
NorthernAmericanDeerMouse                                                                                     66788
Mouse                       --TTTTCTTTTTTTTTTTGGTAACTTTATATATACAAATACTGGTATCAATTAACTTTGGTGTTAATACATGTATACCTCA  68220
ChineseHamsterGHOK1GS       --GTTGTTACTTCTTTGGTTACTTTTTTATATACAAATATTGCTACCGATTAATTTTGGAGTTG-CACATCTATACCTCA  71848
LongTailedChinchilla        ----CAGAATATTTTTGGTCACTTT-CTAAACAAGTGTGTTGATGTCAGATAAATTTGTAGTTA---CATGTGTTCCTT-  71994
```

```
Majority                       XXXTTATTCT--TATAAAAXTTAACAXAAXTTTATAAAACTXAXXAATAAGATXATAAAAAXTXAATGAAGGCACAXAAA
                                         101770    101780    101790    101800    101810    101820    101830    101840
Human                          GGTTTGTTCTGTTATAAAATATCCCACACTTATGCAAAACTTACACATAGGATAATAAAAAGTAAATGAAGGAACATAAA  94609
GuineaPig                      ----TGTTCT--TATAAAACATCTCAAAACTTACGAAAACTATGCAGTAGGATGATTAAAA---AATGAAGGCACAATAG  63968
NorthernAmericanDeerMouse                                                                                        66788
Mouse                          AAGTCACACA-CTG--GACATTAGGGTCATTTTATAAAAATGAAGAATCATGTTTAAAAGTATTGTTCTTTTCACA-AGA  68296
ChineseHamsterGHOK1GS          AAGTCACACA-ATGTAGACATTAAAATGACTTTTTAAAA-TGAAGGAGCATGTTTCAAAGTATTGTTCTAAT-GCA-AGA  71924
LongTailedChinchilla           ----TATTCT--TATAAAACTTAACAAAAATAAAAAAAATTATCCAGTAAGATGATTTAAA---AATGAAGGCACGTAAG  72065

Majority                       ATATATTXAACTXTACTGAAXXTAATACXXXATXAXXXAXXTXXAXXAAAXXXXXXXATAXTATXTAAAXXXXXXXXXXX
                                         101850    101860    101870    101880    101890    101900    101910    101920
Human                          ATATGTTTTACTATACTTAATGTAGTAAT---TCAGTTACCTCCAAGAAAGAAGAATATAATAAGTAAAATAATTTTGTT  94686
GuineaPig                      ATACATTAAA-TGTACTGAACATAATACTTTATAAAGAAAATATATAAAA-------ATTTTGTTTACATTAG-------  64033
NorthernAmericanDeerMouse                                                                                        66788
Mouse                          GTCTACTCAACTCCAAGAAGTACAGTAAG                                                    68325
ChineseHamsterGHOK1GS          ATTCACTCAACTCCCAAGAAATAAATACAGTAAGTAACAATGATTCTGTTGTAGAGTTAAGAATTTGAGTGTTGCTTGGA  72004
LongTailedChinchilla           ATATGTTAAA-TGTACTGTACGTAATACA--ATCATTCACTTCAAAGAAA-------ATAATATATAAA-----------  72124

Majority                       XXXXXXAGTXXXXXXXXXXXXATTTXAXGAXGXXXXXTTXGAXXCTAXXXXXXXXGATGXXTTGGXCXCXXXXTGTTXXX
                                         101930    101940    101950    101960    101970    101980    101990    102000
Human                          TACACTAGTGTCACTGTTGTCATCTGAAGAAGAACCATTAGAGTCAACAGTCTGGGATGATTTGGAAGCTAGGTGATCTG  94766
GuineaPig                      ------AGTCA-------ACAATTTAAGGATGCT---TTGGAAACTA--------GATGA-TTAGTCTCC-CATGTTA--  64085
NorthernAmericanDeerMouse                                                                                        66788
Mouse                                                                                                            68325
ChineseHamsterGHOK1GS          AGCCATGGCTGATCATGCATGTTTTCTCTTAAAGTGGTGTTGCAGTACAGTGACAGCATTTAAGAACATCAACTGTGCCT  72084
LongTailedChinchilla           ------AAT-----------AAGTTAAAGATGGT---TTAGAAGCTA--------GATG--TTGGGCTCT-GATGTT---  72170

Majority                       XXTTTTXTTCTTTAAATAXCATXXTXGXXCXGAXXTAXTXTAXCAGCATCXXXXTAXXTXXXXXXXXXXXXXXXXXXXTX
                                         102010    102020    102030    102040    102050    102060    102070    102080
Human                          TGTTTTTTTCTTAAAATAACATCGTAGCACAGAGGAACTCTACCAGCATC-AGATAAGTCTAAAGTCTAAAAAACCAATA  94845
GuineaPig                      --TTTTGTTCTTTAAATAGCATTGTAGTACAGACATAATGTAGCAACATCCAAGTATGTCTGAAAAAAAACAGTACCATG  64163
NorthernAmericanDeerMouse                                                                                        66788
Mouse                                                                                                            68325
ChineseHamsterGHOK1GS          TAATTCTGTAGTTTATAACCATCATTGGGACAGAATACTTTAATTGGGTTTTCCTCAAT-----------------ACTG  72147
LongTailedChinchilla           --TTTTATTCTATAAATAGCATTATG---CGGACCTCGTCTAGCAGCATCCCCATATAT--------------------  72224
```

Majority	CAXXGGTTAXTGAATAXGTAATTGAACAXAXXTTTXXXAAGTGGAGTTTTTCTCAGTAXTTXXTXGTXGXTXXAAXAXTT	
	102090 102100 102110 102120 102130 102140 102150 102160	
Human	CCAGGGTTAGTGAATA-GTAATTGAACAAAGCTTCTCAAGGTGAAGTTTTTCTCAGTAATTAGTGGTGGCTATAATATTT	94924
GuineaPig	CATTGGTTAGTGAGTA-GTAATTTAACACATTTTT---AAGTGGAGTTTTTCTCAGTGTTGCTTGGTAGTTACAA-ACTT	64238
NorthernAmericanDeerMouse		66788
Mouse		68325
ChineseHamsterGHOK1GS	CACGGTAGAACCAATACTTGAAAGTAGATTTTTATCTCCATCTGGGTAGTTATTTGTAGATGACAGTTTGCTTGAGAGAA	72227
LongTailedChinchilla	-ATTGGTTAATGAATAAGTAATTGAACATACCTTT---AAGTGGAGTTTTTCTCAGTATTTCTTACTGGTTGCAATACTT	72300

Majority	GAAAXTAGGTTXTXTCXCXXXTXXXXTTXXTXXXXGGGXXXXGGATTGXXTXXXGAXGGAGAAACXAAXGTCCAGXAGCT	
	102170 102180 102190 102200 102210 102220 102230 102240	
Human	GAAAGTAGGTTCTGTCACCTTTTCAGGTGCTCATTGTGGGTGGGATTGCCTTGGGAAGGAGAAACTAA-GTCCTG-AGCT	95002
GuineaPig	GAAAATAGGTTTTATCCCTGAT---ATTTTTTGAAGGGTCTGGGATTGCCT--AGAGGGAGAAACAAAAGTCCAGGAGCT	64313
NorthernAmericanDeerMouse		66788
Mouse		68325
ChineseHamsterGHOK1GS	AAGGTTCTGAGCTGGGATCATTCCACTTGATGATTTGGAAGTAATTCCTGATCTCTGTTTAATGAGGATAACCAGAGACA	72307
LongTailedChinchilla	GAAAGTAGGTTTTATCCCTGA-----TTTTTCGGGGGGATATGGAGTGTTT--AGAAGGAGAAACAAAAGTCCAGAAGTT	72373

Majority	AXGTGTGTXAXAXAXXXGTTGXXXXAGXTGCTXXXCTGXXXXAXXXAGTXTCTXXTTXXXXXXG-XXXXGXXGTXACCAA	
	102250 102260 102270 102280 102290 102300 102310 102320	
Human	GTGTGTGTGG-ACTTAGTTTGTGCCAGTTGCTAAGCTGGTGGG---AGTTCCCAGTCTCCCTAG--TGGGAGATAACCAG	95076
GuineaPig	ATGTGTGTAA---AGGAGTTGCTT-AGCTGCTGAACTGGCAAAGTTAGTATCTCCTTATCGAGG-TCCAGATGTGATCAA	64388
NorthernAmericanDeerMouse		66788
Mouse		68325
ChineseHamsterGHOK1GS	AGTTCTCCCACACAATTGGTGGCT-GGTTTTAATGGTTTTCCACCAACAGTCTTATTTTAAAAA--TATGGAGCCACTGA	72384
LongTailedChinchilla	ACGTGTGTAATAAAGGAGTTGCTCCAGCTGCTGCACTGAACAA---AGTATCTCCTTGCTGGGGGTCTGGGTGTGACCAA	72450

Majority	AATCCAAAXTTCTXXCTGCXTXAGGACXTAXAGCAXATXXXXTAGTTTCTTACXXTXTXXTCXXXTXXXXCCTTXCTTXG	
	102330 102340 102350 102360 102370 102380 102390 102400	
Human	AAACCA--GCTCTTCCTGCCTAAGGAC-TACAGCAGATTCTATACTGTCTTACAGTATGCCCTAATACGGCCTTCCTTCC	95153
GuineaPig	AATCCAAAATTCTACCTGCATTAGGACAGA--GCAGATT--GTGGTTTCTTACTCTGTATTCCTCTGTAACCTCACTTTG	64464
NorthernAmericanDeerMouse		66788
Mouse		68325
ChineseHamsterGHOK1GS	AGTGGGAATTTTGATACTCTGGAATTCCTCTATTCAATGCATTAGATTACAATAGCCCACTTTGGCAAGAAAATATTAGG	72464
LongTailedChinchilla	AATCCAAAGTTCTTTCTGTATAAGGATATATAGCACATC--ATAGTTTCTTAC--TGTGTTCCTCTGTTGCCTTGCTTTG	72526

```
Majority                     AAAGCACCAAGTCAXTXXAGTXGGAXXXXXXXXXX--XXXXXATTTTXXXTTCAXXACATTAAXTTAACCCAGGCXXTGG
                                     102410    102420    102430    102440    102450    102460    102470    102480
Human                        AAAGCACCAAGTCACTAAAGGGGGATTTTGACATTCTAGAATATTTTTAATTCAGTACATCAAATTAACCCAGGCCTTGG  95233
GuineaPig                    AAAGCACCAAGTCAGTACAGTAGGA-----------------ATTTT--GTTCAACACATTAAGTTAACCCAAGCCATGG  64525
NorthernAmericanDeerMouse                                                                                      66788
Mouse                                                                                                          68325
ChineseHamsterGHOK1GS        TAAAAGTACTTTCAGTGGTATATTATCACTGCCTC--GACCTTTCCTCTCCCAGTGCTGTTTTCAAAATATTGTTAGTGA  72542
LongTailedChinchilla         AAAGCACCAAGTCAATGAAGTGGGA-----------------ATTTT--ATTCAGCACATTAAGTTAACCCAGGCAATGG  72587

Majority                     XATATAXTXAATTGXXXAXTAXXXAXGXTAGXTAXXAXXXAGXACAXTXXGCTTTGTCATXTAXXXXXXXXXXXXXXXXX
                                     102490    102500    102510    102520    102530    102540    102550    102560
Human                        CATATAGTGAATTGCTTATTAA--AAGTTAGGTAAAAATAAAAACA----GCTTTGTCGTATATTAGTACTCTATGACTC  95307
GuineaPig                    AATACAGTAAATTGTGCAGTAGTAATGTTAGGTACAGGTGAGTACAGTCAGCTTCGTCAT-TACAATTGTTCT--G--TC  64600
NorthernAmericanDeerMouse                                                                                      66788
Mouse                                                                                                          68325
ChineseHamsterGHOK1GS        TAGCTGCTTTGTTAGAAGATTTGCCTTGGAATTTAGAAGCAGATAACTTGACTTTTTCATATAT                  72606
LongTailedChinchilla         AATATAATAAATTGTGTAGTAGTCAAGGTCGATAGCAG-AAGTACA-TCAGCTTTGTCAT-TACAACTGTTCTATG--TC  72662

Majority                     XXXXXXXXXXXXXXXXXXXXXXXXXXXXXXXXXXXXXXXXXXXXXXXXXXXXXXXXXXXXXXXXXXXXXXXXXXXXXXXX
                                     102570    102580    102590    102600    102610    102620    102630    102640
Human                        TTAGAGTGAACAAGCTAACAAGTATTCTTAGGACTTAGCTGCTTTGTTAATAGAGGTCTTAACTTGAATATTTAGAAATA  95387
GuineaPig                    TCAGATT--------------------------CTTATCTTTTTT---AACAATGATCTAGCATT-AACACT--------  64642
NorthernAmericanDeerMouse                                                                                      66788
Mouse                                                                                                          68325
ChineseHamsterGHOK1GS                                                                                          72606
LongTailedChinchilla         TCAGAGT--------------------------TTTACCTTTTT----AACAATGATCTTGTGCT-AACATT--------  72703

Majority                     XXXXXXXXXXXXXXXXXXXXXXXXXXXXXXXXXXXXXXXXXXXXXXXXXXXXXXXXXXXXXXXXXXXXXXXXXXXXXXXX
                                     102650    102660    102670    102680    102690    102700    102710    102720
Human                        ATCAGAAAAATTATTTTTTGGTATTGAAGATGTATTTTGGAAATGGGTTTTAAAGTACCTACTTAAGGAAGAGATATCTA  95467
GuineaPig                    ----GAAGTATTATGATTCAGC----------TGCTCTATTAATAAAATTTTGAATATTTA--------G-AAGTAACT-  64698
NorthernAmericanDeerMouse                                                                                      66788
Mouse                                                                                                          68325
ChineseHamsterGHOK1GS                                                                                          72606
LongTailedChinchilla         ----GAAGTATTATGATTCAGC----------TGCTCTATTAATA---CTTTGAATATTTA--------GGAAATAACTG  72758
```

```
Majority                    XXXXXXXXXXXXXXXXXXXXXXXXXXXXXXXXXXXXXXXXXXXXXXXXXXXXXXXXXXXXXXXXXXXXXXXXXXXXXXXX
                                     102730    102740    102750    102760    102770    102780    102790    102800
Human                       CAGTTTATATTTAACGTTAGCATTTGAAAGCAATGAAGAAAATTTTTCCTTAACCAGGAAGTGTAATGAATCATTATATT 95547
GuineaPig                   ---TTTTTCTGTGAC-TTGAGA--GAAAAAC------------------------------------------------- 64723
NorthernAmericanDeerMouse                                                                                    66788
Mouse                                                                                                        68325
ChineseHamsterGHOK1GS                                                                                        72606
LongTailedChinchilla        GA-TTTCTTTGTGAC-TAAGGATGGAAAAACTTTATGATTTATTTTTATTTTT--------TATGATAAAACTTTTTA-- 72826

Majority                    XXXXXXXXXXXXXXXXXXXXXXXXXXXXXXXXXXXXXXXXXXXXXXXXXXXXXXXXXXXXXXXXXXXXXXXXXXXXXXXX
                                     102810    102820    102830    102840    102850    102860    102870    102880
Human                       AGAGATTAAATTAACACTTTGGAAGCATTTTAGCTCCTAGAGTTAAGGTTAGTATAGTGAAATTATGGTGATTAGCTTAA 95627
GuineaPig                   ---------------ATTTTGGAAATGTTTCAAAGCTTGGTCTAAAAG-------GGTAGATTCATG----------CAG 64771
NorthernAmericanDeerMouse                                                                                    66788
Mouse                                                                                                        68325
ChineseHamsterGHOK1GS                                                                                        72606
LongTailedChinchilla        -------AAATATTTATTTTGGAAATGTTTCAAAGCTCCTTCTTAAAG-------AGTAGGTTCATG----------CAA 72882

Majority                    XXXXXXXXXXXXXXXXXXXXXXXXXXXXXXXXXXXXXXXXXXXXXXXXXXXXXXXXXXXXXXXXXXXXXXXXXXXXXXXX
                                     102890    102900    102910    102920    102930    102940    102950    102960
Human                       TTCATTCATGTACATCTATTTGTATCACCATATAATTAC-TAGGGAAAGGACAAATCACTTTTCAAAGCCTCAATGTTAT 95706
GuineaPig                   TTTGTTCTTGTAGTTCTGTTAGGATCACCATAAAGTTTTACAGGGAAAAGAAAAATCACTTC-CCAAGGCTCAGTGTCTT 64850
NorthernAmericanDeerMouse                                                                                    66788
Mouse                                                                                                        68325
ChineseHamsterGHOK1GS                                                                                        72606
LongTailedChinchilla        TTTGTTCTTGTGGTTCTGCTTGGATCACCGTAAAGTTTTACAGGGAAAAGAGAAATCACTTCTCCAAGGCTCAGTGTTTT 72962

Majority                    XXXXXXXXXXXXXXXXXXXXXXXXXXXXXXXXXXXXXXXXXXXXXXXXXXXXXXXXXXXXXXXXXXXXXXXXXXXXXXXX
                                     102970    102980    102990    103000    103010    103020    103030    103040
Human                       T-TGTAAAATGGAGGCAATAAGTTCTGCTCTGCTCCCCTCACAGGATTATTACAGAGGCTCAAATAGGATAAGTGAAGGA 95785
GuineaPig                   TATCTAAAATGGAGGCAATAAATTTGGTTGTGTTCACTCCAAAGGGTTATTGC-AAGGCTCAAATAGGGTAAATGTATGA 64929
NorthernAmericanDeerMouse                                                                                    66788
Mouse                                                                                                        68325
ChineseHamsterGHOK1GS                                                                                        72606
LongTailedChinchilla        TATCCAAAATGGAGGCAATAAATTTGGCTATGCTTACTCCAAAGGGTTATTGT-GAGGCTCAGGTATGGTAAAGGTAGGA 73041
```

Majority	XXXXXXXXXXXXXXXXXXXXXXXXXXXXXXXXXXXXXXXXXXXXXXXXXXXXXXXXXXXXXXXXXXXXXXXXXXXXXXXX	
	103050 103060 103070 103080 103090 103100 103110 103120	
Human	ATGCTATGCAATTAGTTAACTA------------------ATGCCAGTTCC--CAGACTGCCAGATTTGCCCACAGTAAA	95845
GuineaPig	ATGATGTGCAATTGATAAACCACTCTGAATAAAAATTACAGTGCCAGAGT--GCAGCCTGCTAAATTTCCCTAAAATATG	65007
NorthernAmericanDeerMouse		66788
Mouse		68325
ChineseHamsterGHOK1GS		72606
LongTailedChinchilla	A---TGTGCAGCTGATCAACTACACTAAATAAAAATTACAATACCAGAGCTCGCAGCCTGCTAAATTTCCCTAAAATAAA	73118

Majority	XXXXXXXXXXXXXXXXXXXXXXXXXXXXXXXXXXXXXXXXXXXXXXXXXXXXXXXXXXXXXXXXXXXXXXXXXXXXXXXX	
	103130 103140 103150 103160 103170 103180 103190 103200	
Human	CTTCATTATCCACAATACATGTTATCCTAATATGTCTTTTTGCTACAAAGAAATACTAATGTTTTAAAATTATTTA----	95921
GuineaPig	CTTAGTTACTCACAATAAAT---GTCCTA-------TTTGCTTTTTCTTATAAAGATT-TGTTATTAAATTATTTAAATA	65076
NorthernAmericanDeerMouse		66788
Mouse		68325
ChineseHamsterGHOK1GS		72606
LongTailedChinchilla	CTTAGTTATCCACAATAAAC---ATCTTA-------TTTTTCTTATAAAGAATGATTT-TGTTGTTAAATTATTTAAACA	73187

Majority	XXXXXXXXXXXXXXXXXXXXXXXXXXXXXXXXXXXXXXXXXXXXXXXXXXXXXXXXXXXXXXXXXXXXXXXXXXXXXXXX	
	103210 103220 103230 103240 103250 103260 103270 103280	
Human	CTAGTTTCATAAAGCAGAAAGATGTAAAATGCAGTTATAGAGAGCATCCCAAGCCAGATGAAAAGAAACCATGGATTTAG	96001
GuineaPig	ATAATCTGGTAAAGCAGAAAGATATATAATGCAGTGAT-----GTACCCTGTGGCCAAAGGAAAGAGCCTTTGGATTTGA	65151
NorthernAmericanDeerMouse		66788
Mouse		68325
ChineseHamsterGHOK1GS		72606
LongTailedChinchilla	TTAATCTGGTAAAGTAGCAAGATGTATAGTGCCGTAAT-----GCACCACACAGCGAAAGGAAAGAGCCTTTGGATTTGG	73262

Majority	XXXXXXXXXXXXXXXXXXXXXXXXXXXXXXXXXXXXXXXXXXXXXXXXXXXXXXXXXXXXXXXXXXXXXXXXXXXXXXXX	
	103290 103300 103310 103320 103330 103340 103350 103360	
Human	CTTTATATTTTCTGTTTGAACTTGATAGCCCAATCATAAAATGCACATATTTAATTATAGACTGCACTTAAGATTTCAT-	96080
GuineaPig	CCTCGTATAT-CTGAATTGTTAAGACAACTCAATGATAAAATGTACATACTTGATTTCAGATTTCATGTAAAAATTAAAT	65230
NorthernAmericanDeerMouse		66788
Mouse		68325
ChineseHamsterGHOK1GS		72606
LongTailedChinchilla	CCTTGTATGT-CTGAAATATTT-GACAACTCAATAATAAAATGTACATACTTAGTAACAGACTTCATGTAAAAATTAA--	73338

```
Majority                    XXXXXXXXXXXXXXXXXXXXXXXXXXXXXXXXXXXXXXXXXXXXXXXXXXXXXXXXXXXXXXXXXXXXXXXXXXXXXXXX
                                     103370    103380    103390    103400    103410    103420    103430    103440
Human                       -ATGTATAAAAAAGTTGCTGTGAAACAGGTAATTTTAACACTCAGGAATTTCAAGGCTAAGGAAGAAAGACATACTCTCA 96159
GuineaPig                   GGAATATTTGTAAATCTTTAGAAGACAG----TT                                               65260
NorthernAmericanDeerMouse                                                                                    66788
Mouse                                                                                                        68325
ChineseHamsterGHOK1GS                                                                                        72606
LongTailedChinchilla        --AGTATTTATAAATCTTTAGAAGACAG----TTGTAAAACCTGAAGACTTTAAACGTAAAGTAGGAAAAAAGGTCTTCA 73412

Majority                    XXXXXXXXXXXXXXXXXXXXXXXXXXXXXXXXXXXXXXXXXXXXXXXXXXXXXXXXXXXXXXXXXXXXXXXXXXXXXXXX
                                     103450    103460    103470    103480    103490    103500    103510    103520
Human                       TGAGCAGCTCTGAACTTCAAACAGGATTTTCTGGCTGCCTTAATATATTAAAATGGAGCCTAAATCTGAAAACGTTTTTT 96239
GuineaPig                                                                                                    65260
NorthernAmericanDeerMouse                                                                                    66788
Mouse                                                                                                        68325
ChineseHamsterGHOK1GS                                                                                        72606
LongTailedChinchilla        GGTAAGATTCCCTGTCTTACATTAAAAAGAAACATAAATTTGAAATTGTTTCGTTAACTGT---TCTGTCTACTTAAAAG 73489

Majority                    XXXXXXXXXXXXXXXXXXXXXXXXXXXXXXXXXXXXXXXXXXXXXXXXXXXXXXXXXXXXXXXXXXXXXXXXXXXXXXXX
                                     103530    103540    103550    103560    103570    103580    103590    103600
Human                       TCATTCAGAAGAAAGTACAGAATT--CAGTGAAATTGAGTATACTAGGAAATCTGATGGCAAGAATTCAGC-----AGAA 96312
GuineaPig                                                                                                    65260
NorthernAmericanDeerMouse                                                                                    66788
Mouse                                                                                                        68325
ChineseHamsterGHOK1GS                                                                                        72606
LongTailedChinchilla        TTATTTAGGGGGTGTCCTGTTATTTTCAGTAGTCTTAAATTTCCCTGTCTGGTTAGCAAAAATACTGCAACTTCCAGCAG 73569

Majority                    XXXXXXXXXXXXXXXXXXXXXXXXXXXXXXXXXXXXXXXXXXXXXXXXXXXXXXXXXXXXXXXXXXXXXXXXXXXXXXXX
                                     103610    103620    103630    103640    103650    103660    103670    103680
Human                       AGTCTTAAT-TTTAAACCGTAATATTCA----TTGATTATGCAAAACC-TTAGTTTTATAGTTATTCTTTGTTATTTTGA 96386
GuineaPig                                                                                                    65260
NorthernAmericanDeerMouse                                                                                    66788
Mouse                                                                                                        68325
ChineseHamsterGHOK1GS                                                                                        72606
LongTailedChinchilla        TGTTTTGTTGTCCAGACTCGAGCATACACTTCTTTGCTTCACATAACCACCATTCTTACAGCTTCCGCCCTTTCTTCCAT 73649
```

```
Majority                    XXXXXXXXXXXXXXXXXXXXXXXXXXXXXXXXXXXXXXXXXXXXXXXXXXXXXXXXXXXXXXXXXXXXXXXXXXXXXXXX
                                      103690    103700    103710    103720    103730    103740    103750    103760
Human                       ATAGCTTCCCTTTAAATTTTCTAGTTTCAGATTTTGCAAATAAAAGTGTACATGTTAAAGTTATAGAGTATTAGATTATT  96466
GuineaPig                                                                                                    65260
NorthernAmericanDeerMouse                                                                                    66788
Mouse                                                                                                        68325
ChineseHamsterGHOK1GS                                                                                        72606
LongTailedChinchilla        TCTACCTTTCTCTG---TTCCTCACTACCAGTTCAGTGGGTACAAA-GCGCATTTGGCATCTCTGTTAACCTGGGGCAA-  73724

Majority                    XXXXXXXXXXXXXXXXXXXXXXXXXXXXXXXXXXXXXXXXXXXXXXXXXXXXXXXXXXXXXXXXXXXXXXXXXXXXXXXX
                                      103770    103780    103790    103800    103810    103820    103830    103840
Human                       CACATTAAGAACTGTTCAAATACGCGGAAAAATAAAACCGTAATGAACACCTATGCACTTCACTCAACTATA--TCACTA  96544
GuineaPig                                                                                                    65260
NorthernAmericanDeerMouse                                                                                    66788
Mouse                                                                                                        68325
ChineseHamsterGHOK1GS                                                                                        72606
LongTailedChinchilla        -GTGTTGTGCAGGATTTTGAGTCCAGAGATGGTAACTTTTTAGTAGAATATAACATAGCTCTATCAGCTTTAGTTACCGA  73803

Majority                    XXXXXXXXXXXXXXXXXXXXXXXXXXXXXXXXXXXXXXXXXXXXXXXXXXXXXXXXXXXXXXXXXXXXXXXXXXXXXXXX
                                      103850    103860    103870    103880    103890    103900    103910    103920
Human                       CCTAACATATTCCATATT--TTAAACGTAATTATATATTAGTTGAAAGAAAACGTTACAGAATGAACTGAAATCCCATAT  96622
GuineaPig                                                                                                    65260
NorthernAmericanDeerMouse                                                                                    66788
Mouse                                                                                                        68325
ChineseHamsterGHOK1GS                                                                                        72606
LongTailedChinchilla        CTTAACACCTTTCACGTTCCCTGCACCTGTTTCTCATTCCACATATTAATACTTATCTCGGACATACAGTATTTCATCAT  73883

Majority                    XXXXXXXXXXXXXXXXXXXXXXXXXXXXXXXXXXXXXXXXXXXXXXXXXXXXXXXXXXXXXXXXXXXXXXXXXXXXXXXX
                                      103930    103940    103950    103960    103970    103980    103990    104000
Human                       GAACCTCTTTCTGATCTTAGTTCAAACCTTATTCTTTCCCAAGAGGTAATTGTTACCTTCAGTTTAGTATTTTATTATTC  96702
GuineaPig                                                                                                    65260
NorthernAmericanDeerMouse                                                                                    66788
Mouse                                                                                                        68325
ChineseHamsterGHOK1GS                                                                                        72606
LongTailedChinchilla        AAAACT---TCCCAGCACATTTCTCCTACAGTTTTATTCCTGGTTGTAGTCGCTATTCTCTGGTGATACCTGAATTGGCC  73960
```

Majority	XXXXXXXXXXXXXXXXXXXXXXXXXXXXXXXXXXXXXXXXXXXXXXXXXXXXXXXXXXXXXXXXXXXXXXXXXXXXXXXX	
	104010 104020 104030 104040 104050 104060 104070 104080	
Human	AGTGGTTGCTTTTCAAGAGGAATTTGTCCTATATTCTGTAGGCTAAATTTCTTGTTTTGCCTAGTTATAAAAATGGCAAA	96782
GuineaPig		65260
NorthernAmericanDeerMouse		66788
Mouse		68325
ChineseHamsterGHOK1GS		72606
LongTailedChinchilla	-----TCGCTCTCCATAT	73973

Majority	XXXXXXXXXXXXXXXXXXXXXXXXXXXXXXXXXXXXXXXXXXXXXXXXXXXXXXXXXXXXXXXXXXXXXXXXXXXXXXXX	
	104090 104100 104110 104120 104130 104140 104150 104160	
Human	TAATATGATATTGGGTGTTTAAATAGTACAATATTGTTCAAAACACACTTTATAATTTTTAACAGACTGTTTGGACATTG	96862
GuineaPig		65260
NorthernAmericanDeerMouse		66788
Mouse		68325
ChineseHamsterGHOK1GS		72606
LongTailedChinchilla		73973

Majority	XXXXXXXXXXXXXXXXXXXXXXXXXXXXXXXXXXXXXXXXXXXXXXXXXXXXXXXXXXXXXXXXXXXXXXXXXXXXXXXX	
	104170 104180 104190 104200 104210 104220 104230 104240	
Human	ATAATTCCCCTATCAAATCTCATGACATTTAACCAATTTTTAACAAGGATTTTAAATTGTGTTTAAGAAATAAGTCATTT	96942
GuineaPig		65260
NorthernAmericanDeerMouse		66788
Mouse		68325
ChineseHamsterGHOK1GS		72606
LongTailedChinchilla		73973

Majority	XXXXXXXXXXXXXXXXXXXXXXXXXXXXXXXXXXXXXXXXXXXXXXXXXXXXXXXXXXXXXXXXXXXXXXXXXXXXXXXX	
	104250 104260 104270 104280 104290 104300 104310 104320	
Human	GGGCCTCAGGTTTCTCTTAAGGATGTAAACAAGGAGCTATATCATTGGTATTTTAGACGCTGAATTATGTGTAAGGTCCT	97022
GuineaPig		65260
NorthernAmericanDeerMouse		66788
Mouse		68325
ChineseHamsterGHOK1GS		72606
LongTailedChinchilla		73973

```
Majority                      XXXXXXXXXXXXXXXXXXXXXXXXXXXXXXXXXXXXXXXXXXXXXXXXXXXXXXXXXXXXXXXXXXXXXXXXXXXXXXXX
                              104330 104340 104350 104360 104370 104380 104390 104400
Human                         TAAATACAAACATACCCTTTGTAGCTTCACAGAGCCACCCGTTTTGCACTTCTCTCGCTTTTCTTCCTCCCTCTTTGGCT 97102
GuineaPig                     65260
NorthernAmericanDeerMouse     66788
Mouse                         68325
ChineseHamsterGHOK1GS         72606
LongTailedChinchilla          73973

Majority                      XXXXXXXXXXXXXXXXXXXXXXXXXXXXXXXXXXXXXXXXXXXXXXXXXXXXXXXXXXXXXXXXXXXXXXXXXXXXXXXX
                              104410 104420 104430 104440 104450 104460 104470 104480
Human                         CCCATTTCAATGGACAGAAAGACTATGTGGCATCTCTATTTTCATTCACTTGGTTTATTGTCTTGGTATGTTGTATAGGT 97182
GuineaPig                     65260
NorthernAmericanDeerMouse     66788
Mouse                         68325
ChineseHamsterGHOK1GS         72606
LongTailedChinchilla          73973

Majority                      XXXXXXXXXXXXXXXXXXXXXXXXXXXXXXXXXXXXXXXXXXXXXXXXXXXXXXXXXXXXXXXXXXXXXXXXXXXXXXXX
                              104490 104500 104510 104520 104530 104540 104550 104560
Human                         TTTTGAATCAGAAAATTTTAACATTTTTAGATATTTCAAACATACAAAAAATAGAAGAATATTTAACAAACCCTTGTCAG 97262
GuineaPig                     65260
NorthernAmericanDeerMouse     66788
Mouse                         68325
ChineseHamsterGHOK1GS         72606
LongTailedChinchilla          73973

Majority                      XXXXXXXXXXXXXXXXXXXXXXXXXXXXXXXXXXXXXXXXXXXXXXXXXXXXXXXXXXXXXXXXXXXXXXXXXXXXXXXX
                              104570 104580 104590 104600 104610 104620 104630 104640
Human                         CTTCAGTTAGCAACTTAGATCTTCCTCGTTTCCTCCACCCAATTCTCATCTCCTTTATTAATGCAGATCCCAGATATATT 97342
GuineaPig                     65260
NorthernAmericanDeerMouse     66788
Mouse                         68325
ChineseHamsterGHOK1GS         72606
LongTailedChinchilla          73973
```

```
Majority                    XXXXXXXXXXXXXXXXXXXXXXXXXXXXXXXXXXXXXXXXXXXXXXXXXXXXXXXXXXXXXXXXXXXXXXXXXXXXXXXX
                                     104650    104660    104670    104680    104690    104700    104710    104720
Human                       TTATCAAGAAATTTTGTAGCATATATTTGTGTTGTTTTATTCGTGGTCATAGTTGCTTTCTCTAATATCTAGACTAGCCT  97422
GuineaPig                                                                                                     65260
NorthernAmericanDeerMouse                                                                                     66788
Mouse                                                                                                         68325
ChineseHamsterGHOK1GS                                                                                         72606
LongTailedChinchilla                                                                                          73973

Majority                    XXXXXXXXXXXXXXXXXXXXXXXXXXXXXXXXXXXXXXXXXXXXXXXXXXXXXXXXXXXXXXXXXXXXXXXXXXXXXXXX
                                     104730    104740    104750    104760    104770    104780    104790    104800
Human                       AGCTGGTCAATACTACTTTTCTTCAAATCCTAGAACCTCCCTATTAAAAATATAGTCACCAGACCAGTGGCAAATTAGTA  97502
GuineaPig                                                                                                     65260
NorthernAmericanDeerMouse                                                                                     66788
Mouse                                                                                                         68325
ChineseHamsterGHOK1GS                                                                                         72606
LongTailedChinchilla                                                                                          73973

Majority                    XXXXXXXXXXXXXXXXXXXXXXXXXXXXXXXXXXXXXXXXXXXXXXXXXXXXXXXXXXXXXXXXXXXXXXXXXXXXXXXX
                                     104810    104820    104830    104840    104850    104860    104870    104880
Human                       TCACCTAGGAGTTTATTAGAAACTCAGTCTATGAGCCTACTGAATCTACATTTTGACAAGATTGGCAGGTGATGCATTTG  97582
GuineaPig                                                                                                     65260
NorthernAmericanDeerMouse                                                                                     66788
Mouse                                                                                                         68325
ChineseHamsterGHOK1GS                                                                                         72606
LongTailedChinchilla                                                                                          73973

Majority                    XXXXXXXXXXXXXXXXXXXXXXXXXXXXXXXXXXXXXXXXXXXXXXXXXXXXXXXXXXXXXXXXXXXXXXXXXXXXXXXX
                                     104890    104900    104910    104920    104930    104940    104950    104960
Human                       GACATAAGTTGGAGAAGCACTCAATGCATTTTTTCAGTTCTATAAAAATCAGCTTCAAAGTTTTTTCCTGAGTGTGGAAA  97662
GuineaPig                                                                                                     65260
NorthernAmericanDeerMouse                                                                                     66788
Mouse                                                                                                         68325
ChineseHamsterGHOK1GS                                                                                         72606
LongTailedChinchilla                                                                                          73973
```

```
Majority                   XXXXXXXXXXXXXXXXXXXXXXXXXXXXXXXXXXXXXXXXXXXXXXXXXXXXXXXXXXXXXXXXXXXXXXXXXXXXXXXX
                                    104970    104980    104990    105000    105010    105020    105030    105040
Human                      TTATTTCATTAGTAATTTTAAGTTATGCAGATATAGTTAATACCAAGAAATAATTGAACTGAAAAGGAACCCGGATATCT  97742
GuineaPig                                                                                                   65260
NorthernAmericanDeerMouse                                                                                   66788
Mouse                                                                                                       68325
ChineseHamsterGHOK1GS                                                                                       72606
LongTailedChinchilla                                                                                        73973

Majority                   XXXXXXXXXXXXXXXXXXXXXXXXXXXXXXXXXXXXXXXXXXXXXXXXXXXXXXXXXXXXXXXXXXXXXXXXXXXXXXXX
                                    105050    105060    105070    105080    105090    105100    105110    105120
Human                      AGTTTTAACCAGCTGTTTTAAAGATAAACAGTTCTAGAGATCAAATGAATAGTTTAGCAACATATTTATTGTAACTTACC  97822
GuineaPig                                                                                                   65260
NorthernAmericanDeerMouse                                                                                   66788
Mouse                                                                                                       68325
ChineseHamsterGHOK1GS                                                                                       72606
LongTailedChinchilla                                                                                        73973

Majority                   XXXXXXXXXXXXXXXXXXXXXXXXXXXXXXXXXXXXXXXXXXXXXXXXXXXXXXXXXXXXXXXXXXXXXXXXXXXXXXXX
                                    105130    105140    105150    105160    105170    105180    105190    105200
Human                      TGTAGTTAGAATCCAAGTCCCTGGCTTTCCAATCCAGTCTTCAATGTATAATACTGATCAAAAGAAATACAGGCCTGCTA  97902
GuineaPig                                                                                                   65260
NorthernAmericanDeerMouse                                                                                   66788
Mouse                                                                                                       68325
ChineseHamsterGHOK1GS                                                                                       72606
LongTailedChinchilla                                                                                        73973

Majority                   XXXXXXXXXXXXXXXXXXXXXXXXXXXXXXXXXXXXXXXXXXXXXXXXXXXXXXXXXXXXXXXXXXXXXXXXXXXXXXXX
                                    105210    105220    105230    105240    105250    105260    105270    105280
Human                      TTATTCATAAATTTAAACTATTATGTACAAGTTGATAGCTTATAATAGAAATCAGTATGATTAGTGTTAAAAGTCAAATT  97982
GuineaPig                                                                                                   65260
NorthernAmericanDeerMouse                                                                                   66788
Mouse                                                                                                       68325
ChineseHamsterGHOK1GS                                                                                       72606
LongTailedChinchilla                                                                                        73973
```

Majority	XXXXXXXXXXXXXXXXXXXXXXXXXXXXXXXXXXXXXXXXXXXXXXXXXXXXXXXXXXXXXXXXXXXXXXXXXXXXXXXX	
	105290 105300 105310 105320 105330 105340 105350 105360	
Human	TAGTCTGACAGGGAAGGAATGTCCATGTGAACTGATTTTTTTTAATAGAATGCAAATTAATACTCAATTCCTGATTTGTT	98062
GuineaPig		65260
NorthernAmericanDeerMouse		66788
Mouse		68325
ChineseHamsterGHOK1GS		72606
LongTailedChinchilla		73973
Majority	XXXXXXXXXXXXXXXXXXXXXXXXXXXXXXXXXXXXXXXXXXXXXXXXXXXXXXXXXXXXXXXXXXXXXXXXXXXXXXXX	
	105370 105380 105390 105400 105410 105420 105430 105440	
Human	TATTACATTTTGTGGTTTAATGAAATGATACTCAGGTCATTTTCATAAATAGCTTTTCTATTAATGCTTCCTCAGTCACA	98142
GuineaPig		65260
NorthernAmericanDeerMouse		66788
Mouse		68325
ChineseHamsterGHOK1GS		72606
LongTailedChinchilla		73973
Majority	XXXXXXXXXXXXXXXXXXXXXXXXXXXXXXXXXXXXXXXXXXXXXXXXXXXXXXXXXXXXXXXXXXXXXXXXXXXXXXXX	
	105450 105460 105470 105480 105490 105500 105510 105520	
Human	TTCTCTATTCCTATTGCATAGCACAGTCTAACAGTTGAAATGCCTAAGCCTTACTGAATGATGACTGATATTCTCTGTGA	98222
GuineaPig		65260
NorthernAmericanDeerMouse		66788
Mouse		68325
ChineseHamsterGHOK1GS		72606
LongTailedChinchilla		73973
Majority	XXXXXXXXXXXXXXXXXXXXXXXXXXXXXXXXXXXXXXXXXXXXXXXXXXXXXXXXXXXXXXXXXXXXXXXXXXXXXXXX	
	105530 105540 105550 105560 105570 105580 105590 105600	
Human	GAGTTAGAGCTGTACTATAAATATACATTACCTGTGCAAAGTATGCCTTCAGGGGCTATTCCCCATAACCTTAGCAAAAT	98302
GuineaPig		65260
NorthernAmericanDeerMouse		66788
Mouse		68325
ChineseHamsterGHOK1GS		72606
LongTailedChinchilla		73973

Majority	XXXXXXXXXXXXXXXXXXXXXXXXXXXXXXXXXXXXXXXXXXXXXXXXXXXXXXXXXXXXXXXXXXXXXXXXXXXXXXXX	
	105610 105620 105630 105640 105650 105660 105670 105680	
Human	TTTAAAATATTCTTATGGCTAGCAATCTTATCTATTGTCATGTCTTACTATTTCCTGCTTACCACCCCCATTTCTCTCTC	98382
GuineaPig		65260
NorthernAmericanDeerMouse		66788
Mouse		68325
ChineseHamsterGHOK1GS		72606
LongTailedChinchilla		73973

Majority	XXXXXXXXXXXXXXXXXXXXXXXXXXXXXXXXXXXXXXXXXXXXXXXXXXXXXXXXXXXXXXXXXXXXXXXXXXXXXXXX	
	105690 105700 105710 105720 105730 105740 105750 105760	
Human	AACCTTGTTGCTGCATTAGCTGTCCATCACTTAACACTGTTTATTTCTCTGTTTTTCTTCCATCATTCTCTTCTTTGCAA	98462
GuineaPig		65260
NorthernAmericanDeerMouse		66788
Mouse		68325
ChineseHamsterGHOK1GS		72606
LongTailedChinchilla		73973

Majority	XXXXXXXXXXXXXXXXXXXXXXXXXXXXXXXXXXXXXXXXXXXXXXXXXXXXXXXXXXXXXXXXXXXXXXXXXXXXXXXX	
	105770 105780 105790 105800 105810 105820 105830 105840	
Human	CTAAATGATTTAGACTTTTTATAACCATAATCAACCAGTTAGATTCTTTTTATTTGTGGTTATTGAGTTGATAAATGATA	98542
GuineaPig		65260
NorthernAmericanDeerMouse		66788
Mouse		68325
ChineseHamsterGHOK1GS		72606
LongTailedChinchilla		73973

Majority	XXXXXXXXXXXXXXXXXXXXXXXXXXXXXXXXXXXXXXXXXXXXXXXXXXXXXXXXXXXXXXXXXXXXXXXXXXXXXXXX	
	105850 105860 105870 105880 105890 105900 105910 105920	
Human	GTGCATTTTATGTAATATTCAGGTGTTATGCTATTTTTATCTGGTTCACTTTAAAACCTCCCTCCCCCCACTCCCTTTTT	98622
GuineaPig		65260
NorthernAmericanDeerMouse		66788
Mouse		68325
ChineseHamsterGHOK1GS		72606
LongTailedChinchilla		73973

Majority	XXXXXXXXXXXXXXXXXXXXXXXXXXXXXXXXXXXXXXXXXXXXXXXXXXXXXXXXXXXXXXXXXXXXXXXXXXXXXXXX	
	105930 105940 105950 105960 105970 105980 105990 106000	
Human	TTGTGGGTTTTTTTTTTATTATTATTATTTTCCAGTTTTGTGTTGTTTTGCTTTTTAACTTCTGAGATTGCAGTGGTTCC	98702
GuineaPig		65260
NorthernAmericanDeerMouse		66788
Mouse		68325
ChineseHamsterGHOK1GS		72606
LongTailedChinchilla		73973

Majority	XXXXXXXXXXXXXXXXXXXXXXXXXXXXXXXXXXXXXXXXXXXXXXXXXXXXXXXXXXXXXXXXXXXXXXXXXXXXXXXX	
	106010 106020 106030 106040 106050 106060 106070 106080	
Human	AGATGGTTGAAATCACTAGTAGTGATTTACATGAAGCATTCATGTATTTATTACTGGTATATTCAACTATGGTGGAGGGA	98782
GuineaPig		65260
NorthernAmericanDeerMouse		66788
Mouse		68325
ChineseHamsterGHOK1GS		72606
LongTailedChinchilla		73973

Majority	XXXXXXXXXXXXXXXXXXXXXXXXXXXXXXXXXXXXXXXXXXXXXXXXXXXXXXXXXXXXXXXXXXXXXXXXXXXXXXXX	
	106090 106100 106110 106120 106130 106140 106150 106160	
Human	AATTGGTCGAAGAATTACGGTTAAAGAGCTCCTTTAAAAAGGAGATTTGTAGAATTCTAAAATGTGATTCTGATTCATAT	98862
GuineaPig		65260
NorthernAmericanDeerMouse		66788
Mouse		68325
ChineseHamsterGHOK1GS		72606
LongTailedChinchilla		73973

Majority	XXXXXXXXXXXXXXXXXXXXXXXXXXXXXXXXXXXXXXXXXXXXXXXXXXXXXXXXXXXXXXXXXXXXXXXXXXXXXXXX	
	106170 106180 106190 106200 106210 106220 106230 106240	
Human	AGTCATGATTCTTAGAAATTAGCTACAGGAAGAGATAGAAACATTGCTATAGTTGTCTTTGCCCTAAGTTTGGAGTTTTA	98942
GuineaPig		65260
NorthernAmericanDeerMouse		66788
Mouse		68325
ChineseHamsterGHOK1GS		72606
LongTailedChinchilla		73973

Majority	XXXXXXXXXXXXXXXXXXXXXXXXXXXXXXXXXXXXXXXXXXXXXXXXXXXXXXXXXXXXXXXXXXXXXXXXXXXXXXXX	
	106250 106260 106270 106280 106290 106300 106310 106320	
Human	TAGTGTAATATATATATAATAGCTCTAATTATAAATACACTTTTACTCTGGAAAAAGCATAGACATTGACATAAGAGACA	99022
GuineaPig		65260
NorthernAmericanDeerMouse		66788
Mouse		68325
ChineseHamsterGHOK1GS		72606
LongTailedChinchilla		73973

Majority	XXXXXXXXXXXXXXXXXXXXXXXXXXXXXXXXXXXXXXXXXXXXXXXXXXXXXXXXXXXXXXXXXXXXXXXXXXXXXXXX	
	106330 106340 106350 106360 106370 106380 106390 106400	
Human	TTTAAAAACCATTATTTTCTCATCTGTAAAATGGAGAGAATTACTTCATATGTTCACTTATAAGGATTATGTGAAATACT	99102
GuineaPig		65260
NorthernAmericanDeerMouse		66788
Mouse		68325
ChineseHamsterGHOK1GS		72606
LongTailedChinchilla		73973

Majority	XXXXXXXXXXXXXXXXXXXXXXXXXXXXXXXXXXXXXXXXXXXXXXXXXXXXXXXXXXXXXXXXXXXXXXXXXXXXXXXX	
	106410 106420 106430 106440 106450 106460 106470 106480	
Human	GTTCCATTCCATTAGTAGTTTTTCTTTTCCTCTAATTGGGGGTGGGGGCAGTTTTTGGCATTCTATTTATGGAAGGTGTT	99182
GuineaPig		65260
NorthernAmericanDeerMouse		66788
Mouse		68325
ChineseHamsterGHOK1GS		72606
LongTailedChinchilla		73973

Majority	XXXXXXXXXXXXXXXXXXXXXXXXXXXXXXXXXXXXXXXXXXXXXXXXXXXXXXXXXXXXXXXXXXXXXXXXXXXXXXXX	
	106490 106500 106510 106520 106530 106540 106550 106560	
Human	TTTTTCCCTAAGATCATTTTGATGTCTCAGAAGACATTTGCTATGTACGTTGGACATTTGCCATGTACATTGTAGAGAAT	99262
GuineaPig		65260
NorthernAmericanDeerMouse		66788
Mouse		68325
ChineseHamsterGHOK1GS		72606
LongTailedChinchilla		73973

```
Majority                    XXXXXXXXXXXXXXXXXXXXXXXXXXXXXXXXXXXXXXXXXXXXXXXXXXXXXXXXXXXXXXXXXXXXXXXXXXXXXXXX
                                    106570    106580    106590    106600    106610    106620    106630    106640
Human                       ACAAAGATAAACAGAACCACAGTACAGCTGGTTCTATTAGACATTGAAAATGCATATTCTAGCATATTTAATATATTAGG 99342
GuineaPig                                                                                                    65260
NorthernAmericanDeerMouse                                                                                    66788
Mouse                                                                                                        68325
ChineseHamsterGHOK1GS                                                                                        72606
LongTailedChinchilla                                                                                         73973

Majority                    XXXXXXXXXXXXXXXXXXXXXXXXXXXXXXXXXXXXXXXXXXXXXXXXXXXXXXXXXXXXXXXXXXXXXXXXXXXXXXXX
                                    106650    106660    106670    106680    106690    106700    106710    106720
Human                       GAACAATTTAAGCATAATGTGAATTTCATGTTTACTGAAGATTTCATCCTGAGAAACAAATGAAAGCAGAAAATTACAAC 99422
GuineaPig                                                                                                    65260
NorthernAmericanDeerMouse                                                                                    66788
Mouse                                                                                                        68325
ChineseHamsterGHOK1GS                                                                                        72606
LongTailedChinchilla                                                                                         73973

Majority                    XXXXXXXXXXXXXXXXXXXXXXXXXXXXXXXXXXXXXXXXXXXXXXXXXXXXXXXXXXXXXXXXXXXXXXXXXXXXXXXX
                                    106730    106740    106750    106760    106770    106780    106790    106800
Human                       CAACTGAACCAGCTGTGTAAGAAATATATATATGGTGTGCACACATGTACATACCTCAGACATCTATTAGCATTCTCAGT 99502
GuineaPig                                                                                                    65260
NorthernAmericanDeerMouse                                                                                    66788
Mouse                                                                                                        68325
ChineseHamsterGHOK1GS                                                                                        72606
LongTailedChinchilla                                                                                         73973

Majority                    XXXXXXXXXXXXXXXXXXXXXXXXXXXXXXXXXXXXXXXXXXXXXXXXXXXXXXXXXXXXXXXXXXXXXXXXXXXXXXXX
                                    106810    106820    106830    106840    106850    106860    106870    106880
Human                       TTTCCACAAGTTAAAAGCCACGCTCATCCATATCTGGTGCTAATAACTTCCTATCCAATTTCAGATAGCTGTTTTCAACA 99582
GuineaPig                                                                                                    65260
NorthernAmericanDeerMouse                                                                                    66788
Mouse                                                                                                        68325
ChineseHamsterGHOK1GS                                                                                        72606
LongTailedChinchilla                                                                                         73973
```

Majority	XXXXXXXXXXXXXXXXXXXXXXXXXXXXXXXXXXXXXXXXXXXXXXXXXXXXXXXXXXXXXXXXXXXXXXXXXXXXXXXX	
	106890 106900 106910 106920 106930 106940 106950 106960	
Human	CATCTGACACCTTCTTTTTTTCAAGCAAACTTCAGGTCTTTTTCAAGATAAAGTCATTTATAGTAGTGTCTATATATTTC	99662
GuineaPig		65260
NorthernAmericanDeerMouse		66788
Mouse		68325
ChineseHamsterGHOK1GS		72606
LongTailedChinchilla		73973

Majority	XXXXXXXXXXXXXXXXXXXXXXXXXXXXXXXXXXXXXXXXXXXXXXXXXXXXXXXXXXXXXXXXXXXXXXXXXXXXXXXX	
	106970 106980 106990 107000 107010 107020 107030 107040	
Human	TTAACCATTTAACATGTAAACTGTGCTGCCTTTTAAGAAATTAATTCTATCCTTTTTAGTGTCAGAGTTGAAGATTTTGA	99742
GuineaPig		65260
NorthernAmericanDeerMouse		66788
Mouse		68325
ChineseHamsterGHOK1GS		72606
LongTailedChinchilla		73973

Majority	XXXXXXXXXXXXXXXXXXXXXXXXXXXXXXXXXXXXXXXXXXXXXXXXXXXXXXXXXXXXXXXXXXXXXXXXXXXXXXXX	
	107050 107060 107070 107080 107090 107100 107110 107120	
Human	GTGTTGTGCCCTAACCTTATCATTTTCCTCATAAGTCCTGTGGTTTTTATTGTATGATTTTGCATAGTGTGGCAATTCTT	99822
GuineaPig		65260
NorthernAmericanDeerMouse		66788
Mouse		68325
ChineseHamsterGHOK1GS		72606
LongTailedChinchilla		73973

Majority	XXXXXXXXXXXXXXXXXXXXXXXXXXXXXXXXXXXXXXXXXXXXXXXXXXXXXXXXXXXXXXXXXXXXXXXXXXXXXXXX	
	107130 107140 107150 107160 107170 107180 107190 107200	
Human	GGAAATGCTTATGTTGTGTTATAGTATAACTGACTTACTACTTTCAAATAGCTTCTAGTCTGATTCATGTTATGACAGTA	99902
GuineaPig		65260
NorthernAmericanDeerMouse		66788
Mouse		68325
ChineseHamsterGHOK1GS		72606
LongTailedChinchilla		73973

Majority	XXXXXXXXXXXXXXXXXXXXXXXXXXXXXXXXXXXXXXXXXXXXXXXXXXXXXXXXXXXXXXXXXXXXXXXXXXXXXXXX	
	107210 107220 107230 107240 107250 107260 107270 107280	
Human	TACAGTATAGCAAAATAAGGACATCAGGGAACAGGAGGCTGAGCATATCACCATTTACCAATGGTAGCTTGAGCAAGGCA	99982
GuineaPig		65260
NorthernAmericanDeerMouse		66788
Mouse		68325
ChineseHamsterGHOK1GS		72606
LongTailedChinchilla		73973

Majority	XXXXXXXXXXXXXXXXXXXXXXXXXXXXXXXXXXXXXXXXXXXXXXXXXXXXXXXXXXXXXXXXXXXXXXXXXXXXXXXX	
	107290 107300 107310 107320 107330 107340 107350 107360	
Human	CTTCATTTATCGAAGTCCATTTTCTCATCTGTACAATGGAGGTGATGATAGTACATGTCTTTTCTGTCTTGTGTTATTTG	100062
GuineaPig		65260
NorthernAmericanDeerMouse		66788
Mouse		68325
ChineseHamsterGHOK1GS		72606
LongTailedChinchilla		73973

Majority	XXXXXXXXXXXXXXXXXXXXXXXXXXXXXXXXXXXXXXXXXXXXXXXXXXXXXXXXXXXXXXXXXXXXXXXXXXXXXXXX	
	107370 107380 107390 107400 107410 107420 107430 107440	
Human	TAGTTTCAAGTAAGATATGAAATATGCTGTAGAAATACAGGGTTGGGAATATCTAGTGGTACAAAAATCAACTTTTGGCC	100142
GuineaPig		65260
NorthernAmericanDeerMouse		66788
Mouse		68325
ChineseHamsterGHOK1GS		72606
LongTailedChinchilla		73973

Majority	XXXXXXXXXXXXXXXXXXXXXXXXXXXXXXXXXXXXXXXXXXXXXXXXXXXXXXXXXXXXXXXXXXXXXXXXXXXXXXXX	
	107450 107460 107470 107480 107490 107500 107510 107520	
Human	CAATTTTAAGGTGAGATAATTATGAGACATTTTTCGACCTCAAAGTCTTTGTTCTTTTCTCTCTACACCATTGTGAATAA	100222
GuineaPig		65260
NorthernAmericanDeerMouse		66788
Mouse		68325
ChineseHamsterGHOK1GS		72606
LongTailedChinchilla		73973

Majority	XXXXXXXXXXXXXXXXXXXXXXXXXXXXXXXXXXXXXXXXXXXXXXXXXXXXXXXXXXXXXXXXXXXXXXXXXXXXXXXX	
	107530 107540 107550 107560 107570 107580 107590 107600	
Human	CACAGTGTATATATGAGATTAATTTATTACCTCGACTTTCCATTTTTGTTTTGTAAAGTAGTTTAATTTTTTATATCTAT	100302
GuineaPig		65260
NorthernAmericanDeerMouse		66788
Mouse		68325
ChineseHamsterGHOK1GS		72606
LongTailedChinchilla		73973

Majority	XXXXXXXXXXXXXXXXXXXXXXXXXXXXXXXXXXXXXXXXXXXXXXXXXXXXXXXXXXXXXXXXXXXXXXXXXXXXXXXX	
	107610 107620 107630 107640 107650 107660 107670 107680	
Human	TTATAAAGTTAACTGCAGGAGTGTACCTGCAGTTAACTTTATACAGTTAAGAAGAGGTTGGTTTCAGTTTTAAAGATTGT	100382
GuineaPig		65260
NorthernAmericanDeerMouse		66788
Mouse		68325
ChineseHamsterGHOK1GS		72606
LongTailedChinchilla		73973

Majority	XXXXXXXXXXXXXXXXXXXXXXXXXXXXXXXXXXXXXXXXXXXXXXXXXXXXXXXXXXXXXXXXXXXXXXXXXXXXXXXX	
	107690 107700 107710 107720 107730 107740 107750 107760	
Human	ATGTCAGTGTAAAACATCCATTATTATGCTTTCAAGTAAAATAGCTCCTAAAAATATGGATTACTATCAACTTTCAAGGA	100462
GuineaPig		65260
NorthernAmericanDeerMouse		66788
Mouse		68325
ChineseHamsterGHOK1GS		72606
LongTailedChinchilla		73973

Majority	XXXXXXXXXXXXXXXXXXXXXXXXXXXXXXXXXXXXXXXXXXXXXXXXXXXXXXXXXXXXXXXXXXXXXXXXXXXXXXXX	
	107770 107780 107790 107800 107810 107820 107830 107840	
Human	AAATAGCTCCTAAAAATATGGATTACTACCAACTTTCAAGGAAAATAGCTCCTAGAAATATGGATTACTAGCCACATAAA	100542
GuineaPig		65260
NorthernAmericanDeerMouse		66788
Mouse		68325
ChineseHamsterGHOK1GS		72606
LongTailedChinchilla		73973

Majority	XXXXXXXXXXXXXXXXXXXXXXXXXXXXXXXXXXXXXXXXXXXXXXXXXXXXXXXXXXXXXXXXXXXXXXXXXXXXXXXX	
	107850 107860 107870 107880 107890 107900 107910 107920	
Human	TTGCTAACATTTGCTTTTATTATTATTATTATTATTATTTTAGACACAGTCTTGCTCTGTCACCCAGGCTGGAGTGCGGT	100622
GuineaPig		65260
NorthernAmericanDeerMouse		66788
Mouse		68325
ChineseHamsterGHOK1GS		72606
LongTailedChinchilla		73973

Majority	XXXXXXXXXXXXXXXXXXXXXXXXXXXXXXXXXXXXXXXXXXXXXXXXXXXXXXXXXXXXXXXXXXXXXXXXXXXXXXXX	
	107930 107940 107950 107960 107970 107980 107990 108000	
Human	GGTATTATCTCGGCTCACTGCAACCTCCACCTCTCGGGTTCAAGCAATTCTCCTGCCTCAGCCTTCCGAGTAGCTGGAAT	100702
GuineaPig		65260
NorthernAmericanDeerMouse		66788
Mouse		68325
ChineseHamsterGHOK1GS		72606
LongTailedChinchilla		73973

Majority	XXXXXXXXXXXXXXXXXXXXXXXXXXXXXXXXXXXXXXXXXXXXXXXXXXXXXXXXXXXXXXXXXXXXXXXXXXXXXXXX	
	108010 108020 108030 108040 108050 108060 108070 108080	
Human	TACAGGCACCCACCACCACACCCAGCTAATTTTTGTATTTTTAGTAGAGACAGGGTTTTACCATGTGACCAGGCTGGTTT	100782
GuineaPig		65260
NorthernAmericanDeerMouse		66788
Mouse		68325
ChineseHamsterGHOK1GS		72606
LongTailedChinchilla		73973

Majority	XXXXXXXXXXXXXXXXXXXXXXXXXXXXXXXXXXXXXXXXXXXXXXXXXXXXXXXXXXXXXXXXXXXXXXXXXXXXXXXX	
	108090 108100 108110 108120 108130 108140 108150 108160	
Human	CGAACTCCTGACCTCAAATGATCCACCTGCCTCAGCCTCCCAAACTGCTGGGATTACAGGTGTGAGCCACCGCTCCCAGC	100862
GuineaPig		65260
NorthernAmericanDeerMouse		66788
Mouse		68325
ChineseHamsterGHOK1GS		72606
LongTailedChinchilla		73973

Majority	XXXXXXXXXXXXXXXXXXXXXXXXXXXXXXXXXXXXXXXXXXXXXXXXXXXXXXXXXXXXXXXXXXXXXXXXXXXXXXXX	
	108170 108180 108190 108200 108210 108220 108230 108240	
Human	CTATTTGCTAATATTTAACCTCTTGAGAGTCTTTAATTCTTTTTTTCAACAAGTGTTCATTACCTGCTATGTGCCAGCTT	100942
GuineaPig		65260
NorthernAmericanDeerMouse		66788
Mouse		68325
ChineseHamsterGHOK1GS		72606
LongTailedChinchilla		73973

Majority	XXXXXXXXXXXXXXXXXXXXXXXXXXXXXXXXXXXXXXXXXXXXXXXXXXXXXXXXXXXXXXXXXXXXXXXXXXXXXXXX	
	108250 108260 108270 108280 108290 108300 108310 108320	
Human	AGATGCTTTGGATGAAATACAGGTTGGTGCGAAAGTAATTGCGATTTTTGCCGTTAAAATTAGAAAAACCACAATTACTT	101022
GuineaPig		65260
NorthernAmericanDeerMouse		66788
Mouse		68325
ChineseHamsterGHOK1GS		72606
LongTailedChinchilla		73973

Majority	XXXXXXXXXXXXXXXXXXXXXXXXXXXXXXXXXXXXXXXXXXXXXXXXXXXXXXXXXXXXXXXXXXXXXXXXXXXXXXXX	
	108330 108340 108350 108360 108370 108380 108390 108400	
Human	TTGCACCAACCTAAATAACTAAAACACAATTTCTTCCTATCATGGGGCTTTCAGTTCAGTATGAGATAAGTAAAGAATTA	101102
GuineaPig		65260
NorthernAmericanDeerMouse		66788
Mouse		68325
ChineseHamsterGHOK1GS		72606
LongTailedChinchilla		73973

Majority	XXXXXXXXXXXXXXXXXXXXXXXXXXXXXXXXXXXXXXXXXXXXXXXXXXXXXXXXXXXXXXXXXXXXXXXXXXXXXXXX	
	108410 108420 108430 108440 108450 108460 108470 108480	
Human	TATGGGTTGATAAATTTTCTGGAGGAACTATTGGGTGTTATTACAGTGTGCTCAATCTTTAGCAGTTAGGAAAGGATTCT	101182
GuineaPig		65260
NorthernAmericanDeerMouse		66788
Mouse		68325
ChineseHamsterGHOK1GS		72606
LongTailedChinchilla		73973

```
Majority                    XXXXXXXXXXXXXXXXXXXXXXXXXXXXXXXXXXXXXXXXXXXXXXXXXXXXXXXXXXXXXXXXXXXXXXXXXXXXXXXX
                                     108490    108500    108510    108520    108530    108540    108550    108560
Human                       TGAAGGTAGTTGTATTACCTGGGTTGGAGGTAAGTGTTGGGGACACAAGTGTTAGAAACAGAACAGTTTGTTTAAAAGTT 101262
GuineaPig                                                                                                    65260
NorthernAmericanDeerMouse                                                                                    66788
Mouse                                                                                                        68325
ChineseHamsterGHOK1GS                                                                                        72606
LongTailedChinchilla                                                                                         73973

Majority                    XXXXXXXXXXXXXXXXXXXXXXXXXXXXXXXXXXXXXXXXXXXXXXXXXXXXXXXXXXXXXXXXXXXXXXXXXXXXXXXX
                                     108570    108580    108590    108600    108610    108620    108630    108640
Human                       GGGGACAAAAAAGGACCAGAATCAAGATTACGGCACCCTGGAGAAAGTTAAGCATGGCTCGGTCATAAATTAGGAGACAA 101342
GuineaPig                                                                                                    65260
NorthernAmericanDeerMouse                                                                                    66788
Mouse                                                                                                        68325
ChineseHamsterGHOK1GS                                                                                        72606
LongTailedChinchilla                                                                                         73973

Majority                    XXXXXXXXXXXXXXXXXXXXXXXXXXXXXXXXXXXXXXXXXXXXXXXXXXXXXXXXXXXXXXXXXXXXXXXXXXXXXXXX
                                     108650    108660    108670    108680    108690    108700    108710    108720
Human                       GGAACAAATAGAGAAATACGTTTGGATAGAATAGTCAGATTATAGGCTGTGCTAAGTTGTTTGGGCTCCACTAGGGTTTC 101422
GuineaPig                                                                                                    65260
NorthernAmericanDeerMouse                                                                                    66788
Mouse                                                                                                        68325
ChineseHamsterGHOK1GS                                                                                        72606
LongTailedChinchilla                                                                                         73973

Majority                    XXXXXXXXXXXXXXXXXXXXXXXXXXXXXXXXXXXXXXXXXXXXXXXXXXXXXXXXXXXXXXXXXXXXXXXXXXXXXXXX
                                     108730    108740    108750    108760    108770    108780    108790    108800
Human                       TAAATGCAGTTATTGGTAAACTGGACTTAGGAGAAGGGACAGGGTTGACATGACAAATCTAGTGAATTTATGTTCATTAA 101502
GuineaPig                                                                                                    65260
NorthernAmericanDeerMouse                                                                                    66788
Mouse                                                                                                        68325
ChineseHamsterGHOK1GS                                                                                        72606
LongTailedChinchilla                                                                                         73973
```

```
Majority                      XXXXXXXXXXXXXXXXXXXXXXXXXXXXXXXXXXXXXXXXXXXXXXXXXXXXXXXXXXXXXXXXXXXXXXXXXXXXXXXX
                                       108810    108820    108830    108840    108850    108860    108870    108880
Human                         AATATCCCTTTTGAATTGATTTAGTTTTTGTATGTTTCTTTTTTATAATTCCAAAAAATCATACAAATAAGTTAAACTTA 101582
GuineaPig                                                                                                      65260
NorthernAmericanDeerMouse                                                                                      66788
Mouse                                                                                                          68325
ChineseHamsterGHOK1GS                                                                                          72606
LongTailedChinchilla                                                                                           73973

Majority                      XXXXXXXXXXXXXXXXXXXXXXXXXXXXXXXXXXXXXXXXXXXXXXXXXXXXXXXXXXXXXXXXXXXXXXXXXXXXXXXX
                                       108890    108900    108910    108920    108930    108940    108950    108960
Human                         TCCATCTAAAAGCTGCACACAAAACTTGATGTTAGATTCTTTTAAAATTACAAAATTAAGACTTCCAAAAGAATCTTGTT 101662
GuineaPig                                                                                                      65260
NorthernAmericanDeerMouse                                                                                      66788
Mouse                                                                                                          68325
ChineseHamsterGHOK1GS                                                                                          72606
LongTailedChinchilla                                                                                           73973

Majority                      XXXXXXXXXXXXXXXXXXXXXXXXXXXXXXXXXXXXXXXXXXXXXXXXXXXXXXXXXXXXXXXXXXXXXXXXXXXXXXXX
                                       108970    108980    108990    109000    109010    109020    109030    109040
Human                         GAATAAGAGTAAAAGAAAGACCCTTACTCAGTAAAGCTGCGTAATACTTTGGCAGTTTGAACCCATGGCCATGCCTCTCT 101742
GuineaPig                                                                                                      65260
NorthernAmericanDeerMouse                                                                                      66788
Mouse                                                                                                          68325
ChineseHamsterGHOK1GS                                                                                          72606
LongTailedChinchilla                                                                                           73973

Majority                      XXXXXXXXXXXXXXXXXXXXXXXXXXXXXXXXXXXXXXXXXXXXXXXXXXXXXXXXXXXXXXXXXXXXXXXXXXXXXXXX
                                       109050    109060    109070    109080    109090    109100    109110    109120
Human                         GTGTGATAAGGATTTGGCAAAACCATGGAAGAAACATGCATGTTTTTGAATTGTCTTTCCATGGTAACTTAAAACATTTT 101822
GuineaPig                                                                                                      65260
NorthernAmericanDeerMouse                                                                                      66788
Mouse                                                                                                          68325
ChineseHamsterGHOK1GS                                                                                          72606
LongTailedChinchilla                                                                                           73973
```

Majority	XXXXXXXXXXXXXXXXXXXXXXXXXXXXXXXXXXXXXXXXXXXXXXXXXXXXXXXXXXXXXXXXXXXXXXXXXXXXXXXX	
	109130 109140 109150 109160 109170 109180 109190 109200	
Human	TTCATGAAACATGGATATATATCCTTAGAAAACTGCAAAACATAAAGCTTGTAAGTTATAAGACAAGATTTATCTCTTTT	101902
GuineaPig		65260
NorthernAmericanDeerMouse		66788
Mouse		68325
ChineseHamsterGHOK1GS		72606
LongTailedChinchilla		73973

Majority	XXXXXXXXXXXXXXXXXXXXXXXXXXXXXXXXXXXXXXXXXXXXXXXXXXXXXXXXXXXXXXXXXXXXXXXXXXXXXXXX	
	109210 109220 109230 109240 109250 109260 109270 109280	
Human	TTTTTTTTTTCTGAGAGAGAGTCTTGCTCTGTTGCCCAGGCCGGAGTGCAACAGTGTGATCTCTGCTCACTGCACCCTCC	101982
GuineaPig		65260
NorthernAmericanDeerMouse		66788
Mouse		68325
ChineseHamsterGHOK1GS		72606
LongTailedChinchilla		73973

Majority	XXXXXXXXXXXXXXXXXXXXXXXXXXXXXXXXXXXXXXXXXXXXXXXXXXXXXXXXXXXXXXXXXXXXXXXXXXXXXXXX	
	109290 109300 109310 109320 109330 109340 109350 109360	
Human	GCCTCCCGGGTTCAAGCAATTCTCCTGCCTCAGCCTCCTGAGTAGCTGGGACTACAGGCGTGTGCCGCCAAGCCCAGCTA	102062
GuineaPig		65260
NorthernAmericanDeerMouse		66788
Mouse		68325
ChineseHamsterGHOK1GS		72606
LongTailedChinchilla		73973

Majority	XXXXXXXXXXXXXXXXXXXXXXXXXXXXXXXXXXXXXXXXXXXXXXXXXXXXXXXXXXXXXXXXXXXXXXXXXXXXXXXX	
	109370 109380 109390 109400 109410 109420 109430 109440	
Human	ATTTTTTTGTATCTTTAGTAGAGACGGTTTCACCATGTTGGCCAGGCTGGTCTCGAACTCCTGACCTCAAGTGATCTACC	102142
GuineaPig		65260
NorthernAmericanDeerMouse		66788
Mouse		68325
ChineseHamsterGHOK1GS		72606
LongTailedChinchilla		73973

```
Majority                     XXXXXXXXXXXXXXXXXXXXXXXXXXXXXXXXXXXXXXXXXXXXXXXXXXXXXXXXXXXXXXXXXXXXXXXXXXXXXXXX
                                      109450    109460    109470    109480    109490    109500    109510    109520
Human                        TGCCTCAGCCTTCTAAAGTGTTGGGATTACAGGCATGAGCCATGGCACCCGGCCAAGATGAAATGTCAAGGCCATTTTAG 102222
GuineaPig                                                                                                     65260
NorthernAmericanDeerMouse                                                                                     66788
Mouse                                                                                                         68325
ChineseHamsterGHOK1GS                                                                                         72606
LongTailedChinchilla                                                                                          73973

Majority                     XXXXXXXXXXXXXXXXXXXXXXXXXXXXXXXXXXXXXXXXXXXXXXXXXXXXXXXXXXXXXXXXXXXXXXXXXXXXXXXX
                                      109530    109540    109550    109560    109570    109580    109590    109600
Human                        CCACTATGCTTTTAAGACTACTTAAAGCTGTGCTTTCAGGTCTCCCCAGGAATACTTGTTCACTCTATACTATTGCTGCT 102302
GuineaPig                                                                                                     65260
NorthernAmericanDeerMouse                                                                                     66788
Mouse                                                                                                         68325
ChineseHamsterGHOK1GS                                                                                         72606
LongTailedChinchilla                                                                                          73973

Majority                     XXXXXXXXXXXXXXXXXXXXXXXXXXXXXXXXXXXXXXXXXXXXXXXXXXXXXXXXXXXXXXXXXXXXXXXXXXXXXXXX
                                      109610    109620    109630    109640    109650    109660    109670    109680
Human                        GCTGTTCTGGCTTGAGAATTTTGAGAATCACTAAGATACAATTTTAAGTTTTGACATGTAAATTATTTGGTTGAGTAGAA 102382
GuineaPig                                                                                                     65260
NorthernAmericanDeerMouse                                                                                     66788
Mouse                                                                                                         68325
ChineseHamsterGHOK1GS                                                                                         72606
LongTailedChinchilla                                                                                          73973

Majority                     XXXXXXXXXXXXXXXXXXXXXXXXXXXXXXXXXXXXXXXXXXXXXXXXXXXXXXXXXXXXXXXXXXXXXXXXXXXXXXXX
                                      109690    109700    109710    109720    109730    109740    109750    109760
Human                        AGAATTAATGTAGATAAGCAAAAACCCCTTTATAAAAAATGCTTACCCTTTAAAAAATGTTGAATCCTCACAGTATGATT 102462
GuineaPig                                                                                                     65260
NorthernAmericanDeerMouse                                                                                     66788
Mouse                                                                                                         68325
ChineseHamsterGHOK1GS                                                                                         72606
LongTailedChinchilla                                                                                          73973
```

Majority	XXXXXXXXXXXXXXXXXXXXXXXXXXXXXXXXXXXXXXXXXXXXXXXXXXXXXXXXXXXXXXXXXXXXXXXXXXXXXXXX	
	109770 109780 109790 109800 109810 109820 109830 109840	
Human	AAATACTACTTGTAAAAGTGTCTGTTAACCATAATGTATTTTTTAGATAATTTGAGTAGAATTATACGTGCAATATATTC	102542
GuineaPig		65260
NorthernAmericanDeerMouse		66788
Mouse		68325
ChineseHamsterGHOK1GS		72606
LongTailedChinchilla		73973

Majority	XXXXXXXXXXXXXXXXXXXXXXXXXXXXXXXXXXXXXXXXXXXXXXXXXXXXXXXXXXXXXXXXXXXXXXXXXXXXXXXX	
	109850 109860 109870 109880 109890 109900 109910 109920	
Human	TTGAGTTTGTGACCAACAAAGAAACAGATTAATGATTGAAAATTCACCATACATTACTACCTATGTCTCTCCACCTGCTA	102622
GuineaPig		65260
NorthernAmericanDeerMouse		66788
Mouse		68325
ChineseHamsterGHOK1GS		72606
LongTailedChinchilla		73973

Majority	XXXXXXXXXXXXXXXXXXXXXXXXXXXXXXXXXXXXXXXXXXXXXXXXXXXXXXXXXXXXXXXXXXXXXXXXXXXXXXXX	
	109930 109940 109950 109960 109970 109980 109990 110000	
Human	TCTACTTCCAATTTTTTATATATATAAAAGTCATTAAAGTCATTTGTAATTGAGTTTGTTTTTTTTTCTTGAGACAGGGT	102702
GuineaPig		65260
NorthernAmericanDeerMouse		66788
Mouse		68325
ChineseHamsterGHOK1GS		72606
LongTailedChinchilla		73973

Majority	XXXXXXXXXXXXXXXXXXXXXXXXXXXXXXXXXXXXXXXXXXXXXXXXXXXXXXXXXXXXXXXXXXXXXXXXXXXXXXXX	
	110010 110020 110030 110040 110050 110060 110070 110080	
Human	CTCACTCTGTCACCCAGGCTGGAGTGCAAATGGCATGATCACAGCTCACTGCAGCCTCAACCTCCCAGGTTCGATAGATC	102782
GuineaPig		65260
NorthernAmericanDeerMouse		66788
Mouse		68325
ChineseHamsterGHOK1GS		72606
LongTailedChinchilla		73973

Majority	XXXXXXXXXXXXXXXXXXXXXXXXXXXXXXXXXXXXXXXXXXXXXXXXXXXXXXXXXXXXXXXXXXXXXXXXXXXXXXXX	
	110090 110100 110110 110120 110130 110140 110150 110160	
Human	CTCCCACCTCAGTCCCCCACAGGCATGCATCACCATGCCTGACTAGTTTTTGTGTTTTTTTGTAGAGACAAGGTTTTGCC	102862
GuineaPig		65260
NorthernAmericanDeerMouse		66788
Mouse		68325
ChineseHamsterGHOK1GS		72606
LongTailedChinchilla		73973

Majority	XXXXXXXXXXXXXXXXXXXXXXXXXXXXXXXXXXXXXXXXXXXXXXXXXXXXXXXXXXXXXXXXXXXXXXXXXXXXXXXX	
	110170 110180 110190 110200 110210 110220 110230 110240	
Human	ATGTTGCCCAGACTGGTCTCAAACTTGGGCCAAAGCCATCCACCTGCCTCGGCTTCCCAATGTGCTGGGATTACATGTGT	102942
GuineaPig		65260
NorthernAmericanDeerMouse		66788
Mouse		68325
ChineseHamsterGHOK1GS		72606
LongTailedChinchilla		73973

Majority	XXXXXXXXXXXXXXXXXXXXXXXXXXXXXXXXXXXXXXXXXXXXXXXXXXXXXXXXXXXXXXXXXXXXXXXXXXXXXXXX	
	110250 110260 110270 110280 110290 110300 110310 110320	
Human	GAGCCACCACACCCAGTCATGAGAATTATTTTAAATCAGGTAAACAAAATGTCTGTGGGAATATTGAGCTTTATGATAGA	103022
GuineaPig		65260
NorthernAmericanDeerMouse		66788
Mouse		68325
ChineseHamsterGHOK1GS		72606
LongTailedChinchilla		73973

Majority	XXXXXXXXXXXXXXXXXXXXXXXXXXXXXXXXXXXXXXXXXXXXXXXXXXXXXXXXXXXXXXXXXXXXXXXXXXXXXXXX	
	110330 110340 110350 110360 110370 110380 110390 110400	
Human	TACCTAAGTTTTCCCAAAAATAATTGTGTTAGCATTTCAGTGAATCACTATACTTAACCTAGAAATCTAGGTTACCAATA	103102
GuineaPig		65260
NorthernAmericanDeerMouse		66788
Mouse		68325
ChineseHamsterGHOK1GS		72606
LongTailedChinchilla		73973

Majority	XXXXXXXXXXXXXXXXXXXXXXXXXXXXXXXXXXXXXXXXXXXXXXXXXXXXXXXXXXXXXXXXXXXXXXXXXXXXXXXX	
	110410 110420 110430 110440 110450 110460 110470 110480	
Human	ACAGTCTCTTTCAGGTTTTTTTCTTTTCCTGTGTCTTTCTTTTTACTAAAATCATTCATAACTGGGATGACTTATGGAGT	103182
GuineaPig		65260
NorthernAmericanDeerMouse		66788
Mouse		68325
ChineseHamsterGHOK1GS		72606
LongTailedChinchilla		73973

Majority	XXXXXXXXXXXXXXXXXXXXXXXXXXXXXXXXXXXXXXXXXXXXXXXXXXXXXXXXXXXXXXXXXXXXXXXXXXXXXXXX	
	110490 110500 110510 110520 110530 110540 110550 110560	
Human	TGATACCTTAGGTCAAATCCCAATTTAGAAACTCCCAAAAGCCTGTCAAATTTTGACACATACTTTAAATGAACTGTTAT	103262
GuineaPig		65260
NorthernAmericanDeerMouse		66788
Mouse		68325
ChineseHamsterGHOK1GS		72606
LongTailedChinchilla		73973

Majority	XXXXXXXXXXXX	
	110570	
Human	TGAGTAGATATA	103274
GuineaPig		65260
NorthernAmericanDeerMouse		66788
Mouse		68325
ChineseHamsterGHOK1GS		72606
LongTailedChinchilla		73973
