# Supplementary material for: PTBP2 – a gene with relevance for both Anorexia nervosa and body weight regulation
Source: Transl Psychiatry. 2022 Jun 9;12:241. doi: 10.1038/s41398-022-02018-5 (PMC9184595; doi:10.1038/s41398-022-02018-5)
Supplement: Supplementary file 5 — gDNA alignment report (Fish) [file 41398_2022_2018_MOESM5_ESM.pdf]

```
Majority                   --------------------------------------------------------------------------------
                                   10        20        30        40        50        60        70        80
Human                      TGCCTCCAAATGTTAAACTCCAACTATTGTAGCATTTTAACACATGGCTCCATTTGTGTCTATTTCCACCTAGCGTATTG 80
AsianBonytongue            -------------------------------------------------------------------------------- 0
Reedfish                   -------------------------------------------------------------------------------- 0
NorthernPike               -------------------------------------------------------------------------------- 0
SpottedGar                 -------------------------------------------------------------------------------- 0
ParamormyropsKingsLeyae    -------------------------------------------------------------------------------- 0

Majority                   --------------------------------------------------------------------------------
                                   90       100       110       120       130       140       150       160
Human                      TGAATATTTTCTACAGGCGCACACTAATCACCAACTGAAGTGAGAATCACATTTTCCTCCACTTGGCTTGGTACTACAGG 160
AsianBonytongue            -------------------------------------------------------------------------------- 0
Reedfish                   -------------------------------------------------------------------------------- 0
NorthernPike               -------------------------------------------------------------------------------- 0
SpottedGar                 -------------------------------------------------------------------------------- 0
ParamormyropsKingsLeyae    -------------------------------------------------------------------------------- 0

Majority                   --------------------------------------------------------------------------------
                                  170       180       190       200       210       220       230       240
Human                      TCTTGAAGCAAAAGCCTGTATTTCAGTCTGAGGAGTTCTACTTGCTTGAGAAAGTACTGTTGTTACCAAGTGGTCTCGTT 240
AsianBonytongue            -------------------------------------------------------------------------------- 0
Reedfish                   -------------------------------------------------------------------------------- 0
NorthernPike               -------------------------------------------------------------------------------- 0
SpottedGar                 -------------------------------------------------------------------------------- 0
ParamormyropsKingsLeyae    -------------------------------------------------------------------------------- 0

Majority                   --------------------------------------------------------------------------------
                                  250       260       270       280       290       300       310       320
Human                      CAGTGGGGTAAAGATTTTTGTTTTAGAGGCTGGAGCCGGTCTGCAAATAAAAGATGGCTTAGGAGAAAAGCTATCCAATG 320
AsianBonytongue            -------------------------------------------------------------------------------- 0
Reedfish                   -------------------------------------------------------------------------------- 0
NorthernPike               -------------------------------------------------------------------------------- 0
SpottedGar                 -------------------------------------------------------------------------------- 0
ParamormyropsKingsLeyae    -------------------------------------------------------------------------------- 0

Majority                   --------------------------------------------------------------------------------
                                  330       340       350       360       370       380       390       400
Human                      TTTTTTCCTTGGCCCGCATAGGTTGACGGTAGCCAAGCTGGTACTTGGCTTGGAGTGAAAAGTGGAGGCTGCCTGGGGGC 400
AsianBonytongue            -------------------------------------------------------------------------------- 0
Reedfish                   -------------------------------------------------------------------------------- 0
NorthernPike               -------------------------------------------------------------------------------- 0
SpottedGar                 -------------------------------------------------------------------------------- 0
ParamormyropsKingsLeyae    -------------------------------------------------------------------------------- 0

Majority                   --------------------------------------------------------------------------------
                                  410       420       430       440       450       460       470       480
Human                      GTCCTGCAGTCAGGGCAGAGAGAGTGTGGAGAGCTGTTGGGAGGTGTCCCACCCACGCCCCCACAAGGAGGGGAAACCAG 480
AsianBonytongue            -------------------------------------------------------------------------------- 0
Reedfish                   -------------------------------------------------------------------------------- 0
NorthernPike               -------------------------------------------------------------------------------- 0
SpottedGar                 -------------------------------------------------------------------------------- 0
ParamormyropsKingsLeyae    -------------------------------------------------------------------------------- 0
```

```
Majority                   --------------------------------------------------------------------------------
                                     490       500       510       520       530       540       550       560
Human                      GTCGCCGCGGTCTGCAGCAGAACGCCAGGTTCCAGAGGACGGCAGGCGGAGGGCGGAGGGAGAAAGGCGCGCGAAGGCGC 560
AsianBonytongue            -------------------------------------------------------------------------------- 0
Reedfish                   -------------------------------------------------------------------------------- 0
NorthernPike               -------------------------------------------------------------------------------- 0
SpottedGar                 -------------------------------------------------------------------------------- 0
ParamormyropsKingsLeyae    -------------------------------------------------------------------------------- 0

Majority                   --------------------------------------------------------------------------------
                                     570       580       590       600       610       620       630       640
Human                      GGAGGAGGCGTCGGCTGCGGCGCCCACTCCCCCTAGTCCCAGCGCCCTGCCGCTCGCCTCCCGCTGCCGGGCGCCGGCGC 640
AsianBonytongue            -------------------------------------------------------------------------------- 0
Reedfish                   -------------------------------------------------------------------------------- 0
NorthernPike               -------------------------------------------------------------------------------- 0
SpottedGar                 -------------------------------------------------------------------------------- 0
ParamormyropsKingsLeyae    -------------------------------------------------------------------------------- 0

Majority                   --------------------------------------------------------------------------------
                                     650       660       670       680       690       700       710       720
Human                      GCTCGGCTCTTTCCGCCGCCGCCGCTGCCGCGCGGTGGCCCGTGCGCCTCGGCACCTTCGGCAATTTCCGTCGGGCCCCA 720
AsianBonytongue            -------------------------------------------------------------------------------- 0
Reedfish                   -------------------------------------------------------------------------------- 0
NorthernPike               -------------------------------------------------------------------------------- 0
SpottedGar                 -------------------------------------------------------------------------------- 0
ParamormyropsKingsLeyae    -------------------------------------------------------------------------------- 0

Majority                   --------------------------------------------------------------------------------
                                     730       740       750       760       770       780       790       800
Human                      GCCGCCATTTTCTCGCCGCTTGTGTGGCTCGCTGGCTGCGTGGCTCGGTTCTTGTGAGCGAAGCTTTGTCCGGTTCGGCA 800
AsianBonytongue            -------------------------------------------------------------------------------- 0
Reedfish                   -------------------------------------------------------------------------------- 0
NorthernPike               -------------------------------------------------------------------------------- 0
SpottedGar                 -------------------------------------------------------------------------------- 0
ParamormyropsKingsLeyae    -------------------------------------------------------------------------------- 0

Majority                   --------------------------------------------------------------------------------
                                     810       820       830       840       850       860       870       880
Human                      ATGGACGGGTATGTAATCGGGCCGGCGAGAAGGTGTGTGTGAGAGAGGAGTTGGACCGTCCTTCGGCCCGGTCCCGGGCC 880
AsianBonytongue            -------------------------------------------------------------------------------- 0
Reedfish                   -------------------------------------------------------------------------------- 0
NorthernPike               -------------------------------------------------------------------------------- 0
SpottedGar                 -------------------------------------------------------------------------------- 0
ParamormyropsKingsLeyae    -------------------------------------------------------------------------------- 0

Majority                   --------------------------------------------------------------------------------
                                     890       900       910       920       930       940       950       960
Human                      GGGGAGAAACCCTCCCGCGGCCCCTCCCAGGGCTGGGCTGCCGTTACCCAACCCCCGCCCCATCGCACACACCCCTCCCT 960
AsianBonytongue            -------------------------------------------------------------------------------- 0
Reedfish                   -------------------------------------------------------------------------------- 0
NorthernPike               -------------------------------------------------------------------------------- 0
SpottedGar                 -------------------------------------------------------------------------------- 0
ParamormyropsKingsLeyae    -------------------------------------------------------------------------------- 0
```

```
Majority                 --------------------------------------------------------------------------------
                                  970       980       990       1000      1010      1020      1030      1040
Human                    TTGCTTCCCCCGGCGGGCTTTGGTCGAGAAAATGAGAAGAAAGCGGGCTTGGAGGCTGGGGAGGCATAGGGGCGATGGCG 1040
AsianBonytongue          -------------------------------------------------------------------------------- 0
Reedfish                 -------------------------------------------------------------------------------- 0
NorthernPike             -------------------------------------------------------------------------------- 0
SpottedGar               -------------------------------------------------------------------------------- 0
ParamormyropsKingsLeyae  -------------------------------------------------------------------------------- 0

Majority                 --------------------------------------------------------------------------------
                                  1050      1060      1070      1080      1090      1100      1110      1120
Human                    GGGGTGAGGATCCCGGAGTGGGAGCGGGCACCGGCTTGGCGGCGGGGGATGGGGTGGGAACCCCATAACGTCTCCCCGGT 1120
AsianBonytongue          -------------------------------------------------------------------------------- 0
Reedfish                 -------------------------------------------------------------------------------- 0
NorthernPike             -------------------------------------------------------------------------------- 0
SpottedGar               -------------------------------------------------------------------------------- 0
ParamormyropsKingsLeyae  -------------------------------------------------------------------------------- 0

Majority                 --------------------------------------------------------------------------------
                                  1130      1140      1150      1160      1170      1180      1190      1200
Human                    CTGTCCCTGCCCCCTCTAGGAGCCATTTCGATCCGTACCTTGGGACCCGACCCTTGGGTTAGCGGTGCCTGTGAGAGCGA 1200
AsianBonytongue          -------------------------------------------------------------------------------- 0
Reedfish                 -------------------------------------------------------------------------------- 0
NorthernPike             -------------------------------------------------------------------------------- 0
SpottedGar               -------------------------------------------------------------------------------- 0
ParamormyropsKingsLeyae  -------------------------------------------------------------------------------- 0

Majority                 --------------------------------------------------------------------------------
                                  1210      1220      1230      1240      1250      1260      1270      1280
Human                    GTGGGATGGGCAGAGACAGGCCTTTGGATTGGGGGAGCCTCTAGGGGAAGAGGAGAGAGGCACCCCATGTGGACCCCAGC 1280
AsianBonytongue          -------------------------------------------------------------------------------- 0
Reedfish                 -------------------------------------------------------------------------------- 0
NorthernPike             -------------------------------------------------------------------------------- 0
SpottedGar               -------------------------------------------------------------------------------- 0
ParamormyropsKingsLeyae  -------------------------------------------------------------------------------- 0

Majority                 --------------------------------------------------------------------------------
                                  1290      1300      1310      1320      1330      1340      1350      1360
Human                    CATGAGCAACCTCTCCGGCCTCGCCCGGCCCTCCGTGGTCGGGAGAGATGCCGGTGGCGGGAGCTCCGGGGAAAGCCTAG 1360
AsianBonytongue          -------------------------------------------------------------------------------- 0
Reedfish                 -------------------------------------------------------------------------------- 0
NorthernPike             -------------------------------------------------------------------------------- 0
SpottedGar               -------------------------------------------------------------------------------- 0
ParamormyropsKingsLeyae  -------------------------------------------------------------------------------- 0

Majority                 --------------------------------------------------------------------------------
                                  1370      1380      1390      1400      1410      1420      1430      1440
Human                    TGGGAGCCGCTGGGGAAGGGGGGAGGGCGTGCGGCGGCGGGAGGAAGGGGGAGGGCAGATGTCATACTCCTTTGTTTTCA 1440
AsianBonytongue          -------------------------------------------------------------------------------- 0
Reedfish                 -------------------------------------------------------------------------------- 0
NorthernPike             -------------------------------------------------------------------------------- 0
SpottedGar               -------------------------------------------------------------------------------- 0
ParamormyropsKingsLeyae  -------------------------------------------------------------------------------- 0
```

```
Majority                  --------------------------------------------------------------------------------
                                   1450      1460      1470      1480      1490      1500      1510      1520
Human                     TTTGAGTCTGGTAGTGGGGGCGGGAGGGAGGAAAAAATGCCTTTTTGTGGGGACTGAAAACATTCAAGGCTTGGCAAAAG 1520
AsianBonytongue           -------------------------------------------------------------------------------- 0
Reedfish                  -------------------------------------------------------------------------------- 0
NorthernPike              -------------------------------------------------------------------------------- 0
SpottedGar                -------------------------------------------------------------------------------- 0
ParamormyropsKingsLeyae   -------------------------------------------------------------------------------- 0

Majority                  --------------------------------------------------------------------------------
                                   1530      1540      1550      1560      1570      1580      1590      1600
Human                     GGCCCAGAAATTAAATCATCTAAAAAACGAAGTGTTTGAAGCCGACATCAGTCACATGGGCAAAGGCCAACAGCAATGGC 1600
AsianBonytongue           -------------------------------------------------------------------------------- 0
Reedfish                  -------------------------------------------------------------------------------- 0
NorthernPike              -------------------------------------------------------------------------------- 0
SpottedGar                -------------------------------------------------------------------------------- 0
ParamormyropsKingsLeyae   -------------------------------------------------------------------------------- 0

Majority                  --------------------------------------------------------------------------------
                                   1610      1620      1630      1640      1650      1660      1670      1680
Human                     AGAAACAAGAGCAGATAGTCAACAAAGGGCTATCTCTTTAGCTACTTTGGTCAGTTTCTGGAAAAACGACTATAAAAACC 1680
AsianBonytongue           -------------------------------------------------------------------------------- 0
Reedfish                  -------------------------------------------------------------------------------- 0
NorthernPike              -------------------------------------------------------------------------------- 0
SpottedGar                -------------------------------------------------------------------------------- 0
ParamormyropsKingsLeyae   -------------------------------------------------------------------------------- 0

Majority                  --------------------------------------------------------------------------------
                                   1690      1700      1710      1720      1730      1740      1750      1760
Human                     CAGAACCATGCTGCATCTTTGCGAACCTGAGTCAAGACGGAATGCTGAGTGAGGTGAGAGGCAAGACTGTGAAATGGTAG 1760
AsianBonytongue           -------------------------------------------------------------------------------- 0
Reedfish                  -------------------------------------------------------------------------------- 0
NorthernPike              -------------------------------------------------------------------------------- 0
SpottedGar                -------------------------------------------------------------------------------- 0
ParamormyropsKingsLeyae   -------------------------------------------------------------------------------- 0

Majority                  --------------------------------------------------------------------------------
                                   1770      1780      1790      1800      1810      1820      1830      1840
Human                     GGACCGATGTCTCGCTCATATGCTTCACACCTGAAAAGACTCAGTAATTTAACTCAATGTTAGAAAGGGGGCACGCTTAA 1840
AsianBonytongue           -------------------------------------------------------------------------------- 0
Reedfish                  -------------------------------------------------------------------------------- 0
NorthernPike              -------------------------------------------------------------------------------- 0
SpottedGar                -------------------------------------------------------------------------------- 0
ParamormyropsKingsLeyae   -------------------------------------------------------------------------------- 0

Majority                  --------------------------------------------------------------------------------
                                   1850      1860      1870      1880      1890      1900      1910      1920
Human                     GGTCAAAAATACAGCAGTATGCAGCTCTTTTGGGAGTGTAAACATAGTATCATTTAACAAGTACATTTATTAAAATCAGT 1920
AsianBonytongue           -------------------------------------------------------------------------------- 0
Reedfish                  -------------------------------------------------------------------------------- 0
NorthernPike              -------------------------------------------------------------------------------- 0
SpottedGar                -------------------------------------------------------------------------------- 0
ParamormyropsKingsLeyae   -------------------------------------------------------------------------------- 0
```

```
Majority                 --------------------------------------------------------------------------------
                                  1930      1940      1950      1960      1970      1980      1990      2000
Human                    GAGTATTTGAAGAATCAACAATTTGGTTAAGCGCCTAGAGTTTGGTGTGTTTTAAAAGGTACTAAAAACAAGATTTTATA 2000
AsianBonytongue          -------------------------------------------------------------------------------- 0
Reedfish                 -------------------------------------------------------------------------------- 0
NorthernPike             -------------------------------------------------------------------------------- 0
SpottedGar               -------------------------------------------------------------------------------- 0
ParamormyropsKingsLeyae  -------------------------------------------------------------------------------- 0

Majority                 --------------------------------------------------------------------------------
                                  2010      2020      2030      2040      2050      2060      2070      2080
Human                    AAAGTTAACACAGTCTCACAGAATTCAAATCATCCTAGATGTTACCTAAAGATTATTTTCCAGATGAAACAAATCCAGAG 2080
AsianBonytongue          -------------------------------------------------------------------------------- 0
Reedfish                 -------------------------------------------------------------------------------- 0
NorthernPike             -------------------------------------------------------------------------------- 0
SpottedGar               -------------------------------------------------------------------------------- 0
ParamormyropsKingsLeyae  -------------------------------------------------------------------------------- 0

Majority                 --------------------------------------------------------------------------------
                                  2090      2100      2110      2120      2130      2140      2150      2160
Human                    AAGTTCAGTATTCCCCCCAGATCAACACGGAAATTGATGTCAAAACTTGCACTAGAATTCATGTTTCCTGATTCCTGGTT 2160
AsianBonytongue          -------------------------------------------------------------------------------- 0
Reedfish                 -------------------------------------------------------------------------------- 0
NorthernPike             -------------------------------------------------------------------------------- 0
SpottedGar               -------------------------------------------------------------------------------- 0
ParamormyropsKingsLeyae  -------------------------------------------------------------------------------- 0

Majority                 --------------------------------------------------------------------------------
                                  2170      2180      2190      2200      2210      2220      2230      2240
Human                    CCTGTGGAGACTACTAAAGAGTTTAAGCGTTAAATTATTAATCTCAGTATACTGGCATTGTAGAAATGGCATAGTGTTTT 2240
AsianBonytongue          -------------------------------------------------------------------------------- 0
Reedfish                 -------------------------------------------------------------------------------- 0
NorthernPike             -------------------------------------------------------------------------------- 0
SpottedGar               -------------------------------------------------------------------------------- 0
ParamormyropsKingsLeyae  -------------------------------------------------------------------------------- 0

Majority                 --------------------------------------------------------------------------------
                                  2250      2260      2270      2280      2290      2300      2310      2320
Human                    GGGTTTTGTTCTTTTTGGCTATTGAATGTGTTGTTAAAAAATACCACAAGTTGTCTCACATTTGAAAGTTACTTTTAGAA 2320
AsianBonytongue          -------------------------------------------------------------------------------- 0
Reedfish                 -------------------------------------------------------------------------------- 0
NorthernPike             -------------------------------------------------------------------------------- 0
SpottedGar               -------------------------------------------------------------------------------- 0
ParamormyropsKingsLeyae  -------------------------------------------------------------------------------- 0

Majority                 --------------------------------------------------------------------------------
                                  2330      2340      2350      2360      2370      2380      2390      2400
Human                    AACCAGGTTGGTAACAGTACAGGTAAGTCACGGATCATCTGTGGTATTTTTTATCCCCATCTGTGTGTGAGTGGCTGGAA 2400
AsianBonytongue          -------------------------------------------------------------------------------- 0
Reedfish                 -------------------------------------------------------------------------------- 0
NorthernPike             -------------------------------------------------------------------------------- 0
SpottedGar               -------------------------------------------------------------------------------- 0
ParamormyropsKingsLeyae  -------------------------------------------------------------------------------- 0
```

```
Majority                     --------------------------------------------------------------------------------

                                      2410      2420      2430      2440      2450      2460      2470      2480

Human                        GATTTATGAATGATGGACTATCCTAAAAAGTTTTAGAGCCAAAGAGTGAGTGATTAGAAGAATAACAGGAGGTTGAAACT 2480
AsianBonytongue              -------------------------------------------------------------------------------- 0
Reedfish                     -------------------------------------------------------------------------------- 0
NorthernPike                 -------------------------------------------------------------------------------- 0
SpottedGar                   -------------------------------------------------------------------------------- 0
ParamormyropsKingsLeyae-------------------------------------------------------------------------------- 0

Majority                     --------------------------------------------------------------------------------

                                      2490      2500      2510      2520      2530      2540      2550      2560

Human                        ACTTATTTTTTCTTTGCAGAATCGTCACTGAGGTTGCAGTTGGCGTGAAGGTAGGAAAATACTATGTTTGAAACTGGGAT 2560
AsianBonytongue              -------------------------------------------------------------------------------- 0
Reedfish                     -------------------------------------------------------------------------------- 0
NorthernPike                 -------------------------------------------------------------------------------- 0
SpottedGar                   -------------------------------------------------------------------------------- 0
ParamormyropsKingsLeyae-------------------------------------------------------------------------------- 0

Majority                     --------------------------------------------------------------------------------

                                      2570      2580      2590      2600      2610      2620      2630      2640

Human                        TGTTGGATCTATTATCATAATTACTGGAAAATTTTAGCTTTTGCTTTTGAAAACTATAACGTAGCTAACAAAACCCAAGT 2640
AsianBonytongue              -------------------------------------------------------------------------------- 0
Reedfish                     -------------------------------------------------------------------------------- 0
NorthernPike                 -------------------------------------------------------------------------------- 0
SpottedGar                   -------------------------------------------------------------------------------- 0
ParamormyropsKingsLeyae-------------------------------------------------------------------------------- 0

Majority                     --------------------------------------------------------------------------------

                                      2650      2660      2670      2680      2690      2700      2710      2720

Human                        GTAAAATGTTTCCTTTCATTTGTAAAGTTGGACCATATAAACAGTTTGTAATCACCATAAGTTTTTCTAGAACTGTCAGT 2720
AsianBonytongue              -------------------------------------------------------------------------------- 0
Reedfish                     -------------------------------------------------------------------------------- 0
NorthernPike                 -------------------------------------------------------------------------------- 0
SpottedGar                   -------------------------------------------------------------------------------- 0
ParamormyropsKingsLeyae-------------------------------------------------------------------------------- 0

Majority                     --------------------------------------------------------------------------------

                                      2730      2740      2750      2760      2770      2780      2790      2800

Human                        CTGTAAGAATAACCTTTTAAAGTAAGTATGAATGATCAAGAAATTGAAAATGCTTTCACACATGGCTTCTTGTAATATAA 2800
AsianBonytongue              -------------------------------------------------------------------------------- 0
Reedfish                     -------------------------------------------------------------------------------- 0
NorthernPike                 -------------------------------------------------------------------------------- 0
SpottedGar                   -------------------------------------------------------------------------------- 0
ParamormyropsKingsLeyae-------------------------------------------------------------------------------- 0

Majority                     --------------------------------------------------------------------------------

                                      2810      2820      2830      2840      2850      2860      2870      2880

Human                        AACGATACCCCAATTTTCTGAGGGGAAATAATGATTTTAAATGGATGGTAAGTGGTTTGACCTATATCTTATAATTTTGT 2880
AsianBonytongue              -------------------------------------------------------------------------------- 0
Reedfish                     -------------------------------------------------------------------------------- 0
NorthernPike                 -------------------------------------------------------------------------------- 0
SpottedGar                   -------------------------------------------------------------------------------- 0
ParamormyropsKingsLeyae-------------------------------------------------------------------------------- 0
```

```
Majority                   --------------------------------------------------------------------------------
                                   2890      2900      2910      2920      2930      2940      2950      2960
Human                      TAATCTAAGTTTTTTGTTTTTTATTACTTTTTGTCATGAAAATGAATATATTTTGGTTAATAATAAAGCACATTAGAAAT 2960
AsianBonytongue            -------------------------------------------------------------------------------- 0
Reedfish                   -------------------------------------------------------------------------------- 0
NorthernPike               -------------------------------------------------------------------------------- 0
SpottedGar                 -------------------------------------------------------------------------------- 0
ParamormyropsKingsLeyae    -------------------------------------------------------------------------------- 0

Majority                   --------------------------------------------------------------------------------
                                   2970      2980      2990      3000      3010      3020      3030      3040
Human                      GAATTACACTGATCCTTAAAAAGGAAGTTGTTGAGGCAGTTAGCTTCAAATTTGAATTAGAAGTTAAGAGCTGGTTTGTC 3040
AsianBonytongue            -------------------------------------------------------------------------------- 0
Reedfish                   -------------------------------------------------------------------------------- 0
NorthernPike               -------------------------------------------------------------------------------- 0
SpottedGar                 -------------------------------------------------------------------------------- 0
ParamormyropsKingsLeyae    -------------------------------------------------------------------------------- 0

Majority                   --------------------------------------------------------------------------------
                                   3050      3060      3070      3080      3090      3100      3110      3120
Human                      TATTAAAGAAAGTCTTTATGGTAGAGATCCTTTGGAATCCTTCCTTTTTGGCTAAGAAAACTACTCATCGTTTAAACTTT 3120
AsianBonytongue            -------------------------------------------------------------------------------- 0
Reedfish                   -------------------------------------------------------------------------------- 0
NorthernPike               -------------------------------------------------------------------------------- 0
SpottedGar                 -------------------------------------------------------------------------------- 0
ParamormyropsKingsLeyae    -------------------------------------------------------------------------------- 0

Majority                   --------------------------------------------------------------------------------
                                   3130      3140      3150      3160      3170      3180      3190      3200
Human                      AGTTCTAATGCTTAACATTTTCTTGAGTACTTGCAACTTTTAATGTCATGATGTATTGTAATTTTTTTTTGTTTTTTGTT 3200
AsianBonytongue            -------------------------------------------------------------------------------- 0
Reedfish                   -------------------------------------------------------------------------------- 0
NorthernPike               -------------------------------------------------------------------------------- 0
SpottedGar                 -------------------------------------------------------------------------------- 0
ParamormyropsKingsLeyae    -------------------------------------------------------------------------------- 0

Majority                   --------------------------------------------------------------------------------
                                   3210      3220      3230      3240      3250      3260      3270      3280
Human                      TTTTTGAGACGGAGCCTCACTCTGTTGCCCAGGCTGGATGCAGTGGCACGATCTTGGCCCACTGCAACCTCTGCCTCCTG 3280
AsianBonytongue            -------------------------------------------------------------------------------- 0
Reedfish                   -------------------------------------------------------------------------------- 0
NorthernPike               -------------------------------------------------------------------------------- 0
SpottedGar                 -------------------------------------------------------------------------------- 0
ParamormyropsKingsLeyae    -------------------------------------------------------------------------------- 0

Majority                   --------------------------------------------------------------------------------
                                   3290      3300      3310      3320      3330      3340      3350      3360
Human                      GGTTCAAGCAGTTCTCCTGCCTCAGCCTCCTGAGTAGCTGGGATTACAGGTGCGCACCACCACGCCCAGCTAATTTGTAT 3360
AsianBonytongue            -------------------------------------------------------------------------------- 0
Reedfish                   -------------------------------------------------------------------------------- 0
NorthernPike               -------------------------------------------------------------------------------- 0
SpottedGar                 -------------------------------------------------------------------------------- 0
ParamormyropsKingsLeyae    -------------------------------------------------------------------------------- 0
```

```
Majority                    --------------------------------------------------------------------------------
                                     3370      3380      3390      3400      3410      3420      3430      3440
Human                       TTTTAGTAGAAATGGCATTTCACCCTGTTGGTCAGGCTGGTCTCAAACTCCTGACCTCATGATCTGCCTGCCTCTTATGA 3440
AsianBonytongue             -------------------------------------------------------------------------------- 0
Reedfish                    -------------------------------------------------------------------------------- 0
NorthernPike                -------------------------------------------------------------------------------- 0
SpottedGar                  -------------------------------------------------------------------------------- 0
ParamormyropsKingsLeyae     -------------------------------------------------------------------------------- 0

Majority                    --------------------------------------------------------------------------------
                                     3450      3460      3470      3480      3490      3500      3510      3520
Human                       TCTGCCTGCCTTGACCTCCCAAAGTGCTGGGATTACAAGTGTGAGCCACCGTGCCCGGCCTGTATTGTCATTTTTCATTT 3520
AsianBonytongue             -------------------------------------------------------------------------------- 0
Reedfish                    -------------------------------------------------------------------------------- 0
NorthernPike                -------------------------------------------------------------------------------- 0
SpottedGar                  -------------------------------------------------------------------------------- 0
ParamormyropsKingsLeyae     -------------------------------------------------------------------------------- 0

Majority                    --------------------------------------------------------------------------------
                                     3530      3540      3550      3560      3570      3580      3590      3600
Human                       GCAAGCCTCTCTGTCCTGAGAGGCTTTCTGCTTAGTCCAGTGATGCTGACTGTTCAAACATTTTTTCGAATTCTTAAAAT 3600
AsianBonytongue             -------------------------------------------------------------------------------- 0
Reedfish                    -------------------------------------------------------------------------------- 0
NorthernPike                -------------------------------------------------------------------------------- 0
SpottedGar                  -------------------------------------------------------------------------------- 0
ParamormyropsKingsLeyae     -------------------------------------------------------------------------------- 0

Majority                    --------------------------------------------------------------------------------
                                     3610      3620      3630      3640      3650      3660      3670      3680
Human                       TGTATCCATAATGGGGGGAGGGGGAAGGGATAGCATTGGGAGATATAGGGGGGAGGGGGGAGGGATAGCTTTAGGAGATA 3680
AsianBonytongue             -------------------------------------------------------------------------------- 0
Reedfish                    -------------------------------------------------------------------------------- 0
NorthernPike                -------------------------------------------------------------------------------- 0
SpottedGar                  -------------------------------------------------------------------------------- 0
ParamormyropsKingsLeyae     -------------------------------------------------------------------------------- 0

Majority                    --------------------------------------------------------------------------------
                                     3690      3700      3710      3720      3730      3740      3750      3760
Human                       TACCTAATGCTAAATGACAAGTTAATGGGTGCAGCACACCAACATGGCACATGTATACATATGTAACAAACCTGCACGTT 3760
AsianBonytongue             -------------------------------------------------------------------------------- 0
Reedfish                    -------------------------------------------------------------------------------- 0
NorthernPike                -------------------------------------------------------------------------------- 0
SpottedGar                  -------------------------------------------------------------------------------- 0
ParamormyropsKingsLeyae     -------------------------------------------------------------------------------- 0

Majority                    --------------------------------------------------------------------------------
                                     3770      3780      3790      3800      3810      3820      3830      3840
Human                       GTACTCATGTACCCTAAAACTTAAAGTATAATAATGATAATAATAATTGTATCCATAATGATGTGGTGTTCTTTCAAATA 3840
AsianBonytongue             -------------------------------------------------------------------------------- 0
Reedfish                    -------------------------------------------------------------------------------- 0
NorthernPike                -------------------------------------------------------------------------------- 0
SpottedGar                  -------------------------------------------------------------------------------- 0
ParamormyropsKingsLeyae     -------------------------------------------------------------------------------- 0
```

```
Majority                   --------------------------------------------------------------------------------
                                    3850      3860      3870      3880      3890      3900      3910      3920
Human                      CTTTTGGGGATGGTACTGTGTTTTCTTGCTTATTTTCTGTCCTCTTTCCTATAGTGAAATAGATATTTGCCCATTCTTTA 3920
AsianBonytongue            -------------------------------------------------------------------------------- 0
Reedfish                   -------------------------------------------------------------------------------- 0
NorthernPike               -------------------------------------------------------------------------------- 0
SpottedGar                 -------------------------------------------------------------------------------- 0
ParamormyropsKingsLeyae    -------------------------------------------------------------------------------- 0

Majority                   --------------------------------------------------------------------------------
                                    3930      3940      3950      3960      3970      3980      3990      4000
Human                      GGCTTGTTTCTTGAGTTCATTTTGTACAGACTAAAGGAGGAATAATACGCTCTTTTTGGTGATAGTATTTTCCAAGTTGA 4000
AsianBonytongue            -------------------------------------------------------------------------------- 0
Reedfish                   -------------------------------------------------------------------------------- 0
NorthernPike               -------------------------------------------------------------------------------- 0
SpottedGar                 -------------------------------------------------------------------------------- 0
ParamormyropsKingsLeyae    -------------------------------------------------------------------------------- 0

Majority                   --------------------------------------------------------------------------------
                                    4010      4020      4030      4040      4050      4060      4070      4080
Human                      GTTAGTCATGGGATTTAAAATCAGGAATAGCTAAAGATTGCAAAATGGTAGATGTTGTAGGTTCCATCCAAACAGATTTC 4080
AsianBonytongue            -------------------------------------------------------------------------------- 0
Reedfish                   -------------------------------------------------------------------------------- 0
NorthernPike               -------------------------------------------------------------------------------- 0
SpottedGar                 -------------------------------------------------------------------------------- 0
ParamormyropsKingsLeyae    -------------------------------------------------------------------------------- 0

Majority                   --------------------------------------------------------------------------------
                                    4090      4100      4110      4120      4130      4140      4150      4160
Human                      TGTAAATTTTGTAAAAGAATAAATGATACAAGTGTGAGTTGTCGACTGTTAGTGGAAAAGGAGAGGAGTTTGTAGACAAG 4160
AsianBonytongue            -------------------------------------------------------------------------------- 0
Reedfish                   -------------------------------------------------------------------------------- 0
NorthernPike               -------------------------------------------------------------------------------- 0
SpottedGar                 -------------------------------------------------------------------------------- 0
ParamormyropsKingsLeyae    -------------------------------------------------------------------------------- 0

Majority                   --------------------------------------------------------------------------------
                                    4170      4180      4190      4200      4210      4220      4230      4240
Human                      GCTCAGAATGAGTAGACAGGAAAAAACTGAGGAGACTTGGAATAGGATGTCCAGGTGTAATGTATTGGTTACACCAGTTT 4240
AsianBonytongue            -------------------------------------------------------------------------------- 0
Reedfish                   -------------------------------------------------------------------------------- 0
NorthernPike               -------------------------------------------------------------------------------- 0
SpottedGar                 -------------------------------------------------------------------------------- 0
ParamormyropsKingsLeyae    -------------------------------------------------------------------------------- 0

Majority                   --------------------------------------------------------------------------------
                                    4250      4260      4270      4280      4290      4300      4310      4320
Human                      TTTTTTTTTTTTTTTGAGACAGAGTCTCACTCTTCGCCCAGGTGGAGTGCAGTGGCACGATCCTGGCTCACTACAAGCTC 4320
AsianBonytongue            -------------------------------------------------------------------------------- 0
Reedfish                   -------------------------------------------------------------------------------- 0
NorthernPike               -------------------------------------------------------------------------------- 0
SpottedGar                 -------------------------------------------------------------------------------- 0
ParamormyropsKingsLeyae    -------------------------------------------------------------------------------- 0
```

```
Majority                    --------------------------------------------------------------------------------

                                      4330      4340      4350      4360      4370      4380      4390      4400

Human                       CGCCTTCCGGGTTCACTCCATTCTTCTGCCTCAGCCTCCCGAGTAGCTGGGACTACAGGCGCCCACCACCACGCCCGGCT 4400
AsianBonytongue             -------------------------------------------------------------------------------- 0
Reedfish                    -------------------------------------------------------------------------------- 0
NorthernPike                -------------------------------------------------------------------------------- 0
SpottedGar                  -------------------------------------------------------------------------------- 0
ParamormyropsKingsLeyae-------------------------------------------------------------------------------- 0

Majority                    --------------------------------------------------------------------------------

                                      4410      4420      4430      4440      4450      4460      4470      4480

Human                       AATTTTTTGTATTTTTAGTAGAGACGGGGTTTCACCGTGTTAGCCAGGATGGTCTTGATCTCCTGACCTTGTGGTTACAC 4480
AsianBonytongue             -------------------------------------------------------------------------------- 0
Reedfish                    -------------------------------------------------------------------------------- 0
NorthernPike                -------------------------------------------------------------------------------- 0
SpottedGar                  -------------------------------------------------------------------------------- 0
ParamormyropsKingsLeyae-------------------------------------------------------------------------------- 0

Majority                    --------------------------------------------------------------------------------

                                      4490      4500      4510      4520      4530      4540      4550      4560

Human                       CAGTTTTTTGTTAGTTGTTTGAGGAGAACACTTTAGAAGATGATAAAAAAAAATTGCAAATCGTTTTTAAAAATCCATTG 4560
AsianBonytongue             -------------------------------------------------------------------------------- 0
Reedfish                    -------------------------------------------------------------------------------- 0
NorthernPike                -------------------------------------------------------------------------------- 0
SpottedGar                  -------------------------------------------------------------------------------- 0
ParamormyropsKingsLeyae-------------------------------------------------------------------------------- 0

Majority                    --------------------------------------------------------------------------------

                                      4570      4580      4590      4600      4610      4620      4630      4640

Human                       TTTAATATAAAAGTCTGAATAATTAAAGTTAATTTAATTTTCTGGAATAATGGTTAAGAATTTTTGCTTTGGTGTCAGGC 4640
AsianBonytongue             -------------------------------------------------------------------------------- 0
Reedfish                    -------------------------------------------------------------------------------- 0
NorthernPike                -------------------------------------------------------------------------------- 0
SpottedGar                  -------------------------------------------------------------------------------- 0
ParamormyropsKingsLeyae-------------------------------------------------------------------------------- 0

Majority                    --------------------------------------------------------------------------------

                                      4650      4660      4670      4680      4690      4700      4710      4720

Human                       CTGTGTACAAGTAATTAAGTGTTCTTTAAAACTTAAAACATTTGTGAATTTGCTTTAAAGACACTGATGCATGTGCATAT 4720
AsianBonytongue             -------------------------------------------------------------------------------- 0
Reedfish                    -------------------------------------------------------------------------------- 0
NorthernPike                -------------------------------------------------------------------------------- 0
SpottedGar                  -------------------------------------------------------------------------------- 0
ParamormyropsKingsLeyae-------------------------------------------------------------------------------- 0

Majority                    --------------------------------------------------------------------------------

                                      4730      4740      4750      4760      4770      4780      4790      4800

Human                       TTAAAAAAATTCAGGCCGGGCGCGGTGGGTCACACCTGTAATCCCAGCACTTTGGGAGGCCGAGGCGGGCGGATCACGAG 4800
AsianBonytongue             -------------------------------------------------------------------------------- 0
Reedfish                    -------------------------------------------------------------------------------- 0
NorthernPike                -------------------------------------------------------------------------------- 0
SpottedGar                  -------------------------------------------------------------------------------- 0
ParamormyropsKingsLeyae-------------------------------------------------------------------------------- 0
```

```
Majority                   --------------------------------------------------------------------------------
                                    4810      4820      4830      4840      4850      4860      4870      4880
Human                      TTCAGGAGAGACCATCCTGGCTAACACGGTGAAACCCTGTCTTTACTAAAAATACAAAAAATTAGCCGGGTGTGGTGACG 4880
AsianBonytongue            -------------------------------------------------------------------------------- 0
Reedfish                   -------------------------------------------------------------------------------- 0
NorthernPike               -------------------------------------------------------------------------------- 0
SpottedGar                 -------------------------------------------------------------------------------- 0
ParamormyropsKingsLeyae    -------------------------------------------------------------------------------- 0

Majority                   --------------------------------------------------------------------------------
                                    4890      4900      4910      4920      4930      4940      4950      4960
Human                      GGTGCCTGTAGTCCCAGCTACTCGGGAGGCTGAGGCAGGAGAATGGCATGAACCTGCGAGGCAGAGGTTGCAGTAAGCGG 4960
AsianBonytongue            -------------------------------------------------------------------------------- 0
Reedfish                   -------------------------------------------------------------------------------- 0
NorthernPike               -------------------------------------------------------------------------------- 0
SpottedGar                 -------------------------------------------------------------------------------- 0
ParamormyropsKingsLeyae    -------------------------------------------------------------------------------- 0

Majority                   --------------------------------------------------------------------------------
                                    4970      4980      4990      5000      5010      5020      5030      5040
Human                      AGATTGCACCACTGCACTCCAGCCTGGGCGACAGAGACAGACTCTATCTCAAAAAAAAAAAAAAAAAAAAAAAAAAAAAA 5040
AsianBonytongue            -------------------------------------------------------------------------------- 0
Reedfish                   -------------------------------------------------------------------------------- 0
NorthernPike               -------------------------------------------------------------------------------- 0
SpottedGar                 -------------------------------------------------------------------------------- 0
ParamormyropsKingsLeyae    -------------------------------------------------------------------------------- 0

Majority                   --------------------------------------------------------------------------------
                                    5050      5060      5070      5080      5090      5100      5110      5120
Human                      AAAAAAATTCAAACTGCTTCACAGCTTTTTTTGCTATTTCTTAGGTGTAGGTAATTGTGTTAAAATTTTGTGCCTTTTTT 5120
AsianBonytongue            -------------------------------------------------------------------------------- 0
Reedfish                   -------------------------------------------------------------------------------- 0
NorthernPike               -------------------------------------------------------------------------------- 0
SpottedGar                 -------------------------------------------------------------------------------- 0
ParamormyropsKingsLeyae    -------------------------------------------------------------------------------- 0

Majority                   --------------------------------------------------------------------------------
                                    5130      5140      5150      5160      5170      5180      5190      5200
Human                      CTTGAAATTATTAATTTATATATTAGCACACATATAAACATCTTTATTTACGTGATTGAATTCAAACTATTGTTTTGGCC 5200
AsianBonytongue            -------------------------------------------------------------------------------- 0
Reedfish                   -------------------------------------------------------------------------------- 0
NorthernPike               -------------------------------------------------------------------------------- 0
SpottedGar                 -------------------------------------------------------------------------------- 0
ParamormyropsKingsLeyae    -------------------------------------------------------------------------------- 0

Majority                   --------------------------------------------------------------------------------
                                    5210      5220      5230      5240      5250      5260      5270      5280
Human                      CACATCTTGCCTTTTTTTTTTTTTTTTGAGTCGGAGTCTCCCTCTGTCATCAGGCTGGAGTGCAGTGGCATGATCTCGGC 5280
AsianBonytongue            -------------------------------------------------------------------------------- 0
Reedfish                   -------------------------------------------------------------------------------- 0
NorthernPike               -------------------------------------------------------------------------------- 0
SpottedGar                 -------------------------------------------------------------------------------- 0
ParamormyropsKingsLeyae    -------------------------------------------------------------------------------- 0
```

```
Majority                    --------------------------------------------------------------------------------

                                     5290      5300      5310      5320      5330      5340      5350      5360
Human                       TTACTGCAACCTCTGCCTCCTGGGTTCAGGCAATTCTCCTGCCTCAGCCTCCCTAGTAGCTGGGACTACAGGCACACGCC 5360
AsianBonytongue             -------------------------------------------------------------------------------- 0
Reedfish                    -------------------------------------------------------------------------------- 0
NorthernPike                -------------------------------------------------------------------------------- 0
SpottedGar                  -------------------------------------------------------------------------------- 0
ParamormyropsKingsLeyae     -------------------------------------------------------------------------------- 0

Majority                    --------------------------------------------------------------------------------

                                     5370      5380      5390      5400      5410      5420      5430      5440
Human                       GCCATGCCAGACTAATTTTTTTTTTTTTTTCGAGACGGAGTCTCGCCCGGTCTCCCAGGCTGGAGTGCAGTGTCGCGATC 5440
AsianBonytongue             -------------------------------------------------------------------------------- 0
Reedfish                    -------------------------------------------------------------------------------- 0
NorthernPike                -------------------------------------------------------------------------------- 0
SpottedGar                  -------------------------------------------------------------------------------- 0
ParamormyropsKingsLeyae     -------------------------------------------------------------------------------- 0

Majority                    --------------------------------------------------------------------------------

                                     5450      5460      5470      5480      5490      5500      5510      5520
Human                       TCTGCTCACTGCAACCTCTGCCTCCTGGGTTCACGCCATTCTCCTGTCTCAGCCTCCCAAGTAGCTGGGACTACAGGCGC 5520
AsianBonytongue             -------------------------------------------------------------------------------- 0
Reedfish                    -------------------------------------------------------------------------------- 0
NorthernPike                -------------------------------------------------------------------------------- 0
SpottedGar                  -------------------------------------------------------------------------------- 0
ParamormyropsKingsLeyae     -------------------------------------------------------------------------------- 0

Majority                    --------------------------------------------------------------------------------

                                     5530      5540      5550      5560      5570      5580      5590      5600
Human                       CTGCCACCACGCCTGGCTTCTTTTTTGTATATTCAGTAGAGACAGAGTTTCACCATGTTAGCCAGGATAGTCTCTATCTC 5600
AsianBonytongue             -------------------------------------------------------------------------------- 0
Reedfish                    -------------------------------------------------------------------------------- 0
NorthernPike                -------------------------------------------------------------------------------- 0
SpottedGar                  -------------------------------------------------------------------------------- 0
ParamormyropsKingsLeyae     -------------------------------------------------------------------------------- 0

Majority                    --------------------------------------------------------------------------------

                                     5610      5620      5630      5640      5650      5660      5670      5680
Human                       CTGACCTCGTGATCTGCCTGCCTCGGCCTCCCAAAGTGCTGGGATTACAAGCGTGAGCCACCGCGCCTAGCCAAAATGCC 5680
AsianBonytongue             -------------------------------------------------------------------------------- 0
Reedfish                    -------------------------------------------------------------------------------- 0
NorthernPike                -------------------------------------------------------------------------------- 0
SpottedGar                  -------------------------------------------------------------------------------- 0
ParamormyropsKingsLeyae     -------------------------------------------------------------------------------- 0

Majority                    --------------------------------------------------------------------------------

                                     5690      5700      5710      5720      5730      5740      5750      5760
Human                       AGACTAATTTTTGTATTTTTAGTAGAGACAGGGTTTCACCATTTTGGCCAGGATGGTCTTGATCTCTTGACTCCATCATC 5760
AsianBonytongue             -------------------------------------------------------------------------------- 0
Reedfish                    -------------------------------------------------------------------------------- 0
NorthernPike                -------------------------------------------------------------------------------- 0
SpottedGar                  -------------------------------------------------------------------------------- 0
ParamormyropsKingsLeyae     -------------------------------------------------------------------------------- 0
```

```
Majority               --------------------------------------------------------------------------------
                             5770      5780      5790      5800      5810      5820      5830      5840
Human                  CGCCCGCCTTGACTTCCCAAAGTGTTGGGATTACAGGCTTGAGCCACCGCGTCCGGCCCATATCTTGCCTTTAAAAAAAA 5840
AsianBonytongue        -------------------------------------------------------------------------------- 0
Reedfish               -------------------------------------------------------------------------------- 0
NorthernPike           -------------------------------------------------------------------------------- 0
SpottedGar             -------------------------------------------------------------------------------- 0
ParamormyropsKingsLeyae-------------------------------------------------------------------------------- 0

Majority               --------------------------------------------------------------------------------
                             5850      5860      5870      5880      5890      5900      5910      5920
Human                  TAGCTTTATTGAGATATAATTTGACATAAAATAAACTACCCATATTAAATATACAATGTTTTATCTCATATATACCCTTG 5920
AsianBonytongue        -------------------------------------------------------------------------------- 0
Reedfish               -------------------------------------------------------------------------------- 0
NorthernPike           -------------------------------------------------------------------------------- 0
SpottedGar             -------------------------------------------------------------------------------- 0
ParamormyropsKingsLeyae-------------------------------------------------------------------------------- 0

Majority               --------------------------------------------------------------------------------
                             5930      5940      5950      5960      5970      5980      5990      6000
Human                  TGAAACCACTGCAATCAAGATAATGAACATCTATCACTCCCTACAGTATCATCTTGCGCCTTTATAGTCCCTCCTTTCTG 6000
AsianBonytongue        -------------------------------------------------------------------------------- 0
Reedfish               -------------------------------------------------------------------------------- 0
NorthernPike           -------------------------------------------------------------------------------- 0
SpottedGar             -------------------------------------------------------------------------------- 0
ParamormyropsKingsLeyae-------------------------------------------------------------------------------- 0

Majority               --------------------------------------------------------------------------------
                             6010      6020      6030      6040      6050      6060      6070      6080
Human                  CTTTTCTTTCCCCAAGGAACCACTGATCTGCTTTCTGTCACTATAGATTAGTTCACACTTTTTAGAATTTTAGATAAATG 6080
AsianBonytongue        -------------------------------------------------------------------------------- 0
Reedfish               -------------------------------------------------------------------------------- 0
NorthernPike           -------------------------------------------------------------------------------- 0
SpottedGar             -------------------------------------------------------------------------------- 0
ParamormyropsKingsLeyae-------------------------------------------------------------------------------- 0

Majority               --------------------------------------------------------------------------------
                             6090      6100      6110      6120      6130      6140      6150      6160
Human                  TAATCATATGGTATGTACATTTTTGGTCTGGGTTAGTCCACTCAGAATAATTATTTTGAAATTCACCCATGTTGTCTTTC 6160
AsianBonytongue        -------------------------------------------------------------------------------- 0
Reedfish               -------------------------------------------------------------------------------- 0
NorthernPike           -------------------------------------------------------------------------------- 0
SpottedGar             -------------------------------------------------------------------------------- 0
ParamormyropsKingsLeyae-------------------------------------------------------------------------------- 0

Majority               --------------------------------------------------------------------------------
                             6170      6180      6190      6200      6210      6220      6230      6240
Human                  AATAGTCCATTCTGTTTTATTGCTTAGTAATAGTACACTTTATGGCTATACAGCAATTTGTTAATCCATTCAAGTCTTGA 6240
AsianBonytongue        -------------------------------------------------------------------------------- 0
Reedfish               -------------------------------------------------------------------------------- 0
NorthernPike           -------------------------------------------------------------------------------- 0
SpottedGar             -------------------------------------------------------------------------------- 0
ParamormyropsKingsLeyae-------------------------------------------------------------------------------- 0
```

```
Majority                    --------------------------------------------------------------------------------
                                     6250      6260      6270      6280      6290      6300      6310      6320
Human                       GGGACATTTGTATTGTTTTCCGTTTTGAGCTAATAAAAATATAGTTGTGAACATTACTGTACAAGTCTTTGTATGGACAT 6320
AsianBonytongue             -------------------------------------------------------------------------------- 0
Reedfish                    -------------------------------------------------------------------------------- 0
NorthernPike                -------------------------------------------------------------------------------- 0
SpottedGar                  -------------------------------------------------------------------------------- 0
ParamormyropsKingsLeyae     -------------------------------------------------------------------------------- 0

Majority                    --------------------------------------------------------------------------------
                                     6330      6340      6350      6360      6370      6380      6390      6400
Human                       ATGCTTTCATTTCTCTTGGTGGGGCAGGCTATGATTGGAAAGTCTGGATCATGTGATAGGCATATGTTTAACTTTTTAAG 6400
AsianBonytongue             -------------------------------------------------------------------------------- 0
Reedfish                    -------------------------------------------------------------------------------- 0
NorthernPike                -------------------------------------------------------------------------------- 0
SpottedGar                  -------------------------------------------------------------------------------- 0
ParamormyropsKingsLeyae     -------------------------------------------------------------------------------- 0

Majority                    --------------------------------------------------------------------------------
                                     6410      6420      6430      6440      6450      6460      6470      6480
Human                       GAACTGCAAACTTTTTAAAAGTAGTTGTACCATCTTACATTGCCATCAGCATGGTATGAAATTTCCAGTCCTTCCACATC 6480
AsianBonytongue             -------------------------------------------------------------------------------- 0
Reedfish                    -------------------------------------------------------------------------------- 0
NorthernPike                -------------------------------------------------------------------------------- 0
SpottedGar                  -------------------------------------------------------------------------------- 0
ParamormyropsKingsLeyae     -------------------------------------------------------------------------------- 0

Majority                    --------------------------------------------------------------------------------
                                     6490      6500      6510      6520      6530      6540      6550      6560
Human                       CTCTTTAATGCCTGTTATCTTCTGTTTTTGATTATAGATATTGTAGTGTATATGAAGTGATATTTCACTGTGGATTTAAT 6560
AsianBonytongue             -------------------------------------------------------------------------------- 0
Reedfish                    -------------------------------------------------------------------------------- 0
NorthernPike                -------------------------------------------------------------------------------- 0
SpottedGar                  -------------------------------------------------------------------------------- 0
ParamormyropsKingsLeyae     -------------------------------------------------------------------------------- 0

Majority                    --------------------------------------------------------------------------------
                                     6570      6580      6590      6600      6610      6620      6630      6640
Human                       TTGCATTCCTCTAATATCTATGATGTTGGACATCTTTTCGTGTGCTTTTTGTCAGCTGTTATCTTTGGTGAAGTGTCTGT 6640
AsianBonytongue             -------------------------------------------------------------------------------- 0
Reedfish                    -------------------------------------------------------------------------------- 0
NorthernPike                -------------------------------------------------------------------------------- 0
SpottedGar                  -------------------------------------------------------------------------------- 0
ParamormyropsKingsLeyae     -------------------------------------------------------------------------------- 0

Majority                    --------------------------------------------------------------------------------
                                     6650      6660      6670      6680      6690      6700      6710      6720
Human                       TTATATCTTTAGCCCAATTTTTTATTTGGTTATTTATTTTAAATTGAGTTTTGAGAGGTCTTCATTGTGGTTACAAGTCC 6720
AsianBonytongue             -------------------------------------------------------------------------------- 0
Reedfish                    -------------------------------------------------------------------------------- 0
NorthernPike                -------------------------------------------------------------------------------- 0
SpottedGar                  -------------------------------------------------------------------------------- 0
ParamormyropsKingsLeyae     -------------------------------------------------------------------------------- 0
```

```
Majority                --------------------------------------------------------------------------------
                                 6730      6740      6750      6760      6770      6780      6790      6800
Human                   TTTATCAGCCATAAGGCCTGCAAATATTTTCTGCCACTCATCTTCTTATTCTCGCATTGTCTTTTGAAGAGCAACAGTTA 6800
AsianBonytongue         -------------------------------------------------------------------------------- 0
Reedfish                -------------------------------------------------------------------------------- 0
NorthernPike            -------------------------------------------------------------------------------- 0
SpottedGar              -------------------------------------------------------------------------------- 0
ParamormyropsKingsLeyae -------------------------------------------------------------------------------- 0

Majority                --------------------------------------------------------------------------------
                                 6810      6820      6830      6840      6850      6860      6870      6880
Human                   TTAAATCTGATGGACTCCAGTTTACCAATTTTTTCTTTTATGGATTGTAGTTTTGGGGTCTTGTCTGAGCCATTTTTGCC 6880
AsianBonytongue         -------------------------------------------------------------------------------- 0
Reedfish                -------------------------------------------------------------------------------- 0
NorthernPike            -------------------------------------------------------------------------------- 0
SpottedGar              -------------------------------------------------------------------------------- 0
ParamormyropsKingsLeyae -------------------------------------------------------------------------------- 0

Majority                --------------------------------------------------------------------------------
                                 6890      6900      6910      6920      6930      6940      6950      6960
Human                   TGACTGTAAGCCATGAAGATTTTTTTCCTTTTGTTTGTTAATAATAATAATAATTTTTTTTTAGAGACAGGACCTGACTC 6960
AsianBonytongue         -------------------------------------------------------------------------------- 0
Reedfish                -------------------------------------------------------------------------------- 0
NorthernPike            -------------------------------------------------------------------------------- 0
SpottedGar              -------------------------------------------------------------------------------- 0
ParamormyropsKingsLeyae -------------------------------------------------------------------------------- 0

Majority                --------------------------------------------------------------------------------
                                 6970      6980      6990      7000      7010      7020      7030      7040
Human                   TGTCACCCCAGCTGGAGTGCAGTGGTGCAATCGTAGCTCATGATAACCTTGAACTCCTGGACACAAGGGCTCCTGCTTCA 7040
AsianBonytongue         -------------------------------------------------------------------------------- 0
Reedfish                -------------------------------------------------------------------------------- 0
NorthernPike            -------------------------------------------------------------------------------- 0
SpottedGar              -------------------------------------------------------------------------------- 0
ParamormyropsKingsLeyae -------------------------------------------------------------------------------- 0

Majority                --------------------------------------------------------------------------------
                                 7050      7060      7070      7080      7090      7100      7110      7120
Human                   GCCTCCCAAGTAGCTAGGACTATAGGCACACAGCACCATGCCCAGCTAACTTTTAAATTTTTTATAGACCCGGGGTCTCT 7120
AsianBonytongue         -------------------------------------------------------------------------------- 0
Reedfish                -------------------------------------------------------------------------------- 0
NorthernPike            -------------------------------------------------------------------------------- 0
SpottedGar              -------------------------------------------------------------------------------- 0
ParamormyropsKingsLeyae -------------------------------------------------------------------------------- 0

Majority                --------------------------------------------------------------------------------
                                 7130      7140      7150      7160      7170      7180      7190      7200
Human                   CTGTTTTGCCCAGGCTGGTGTTGAACTCCTGGCCTCAAGCGATCTTTCTGCCTCAGCCTTCCAAAGTGTTGGGATTAAAG 7200
AsianBonytongue         -------------------------------------------------------------------------------- 0
Reedfish                -------------------------------------------------------------------------------- 0
NorthernPike            -------------------------------------------------------------------------------- 0
SpottedGar              -------------------------------------------------------------------------------- 0
ParamormyropsKingsLeyae -------------------------------------------------------------------------------- 0
```

```
Majority                   --------------------------------------------------------------------------------

                                   7210      7220      7230      7240      7250      7260      7270      7280

Human                      GCATGAGCCACTACACTTGGCCACCTTTGTTTTCTAATAGAAGTTTTATACTTTGGTTTAACATTTGGGTCTCTTTCATT 7280
AsianBonytongue            -------------------------------------------------------------------------------- 0
Reedfish                   -------------------------------------------------------------------------------- 0
NorthernPike               -------------------------------------------------------------------------------- 0
SpottedGar                 -------------------------------------------------------------------------------- 0
ParamormyropsKingsLeyae    -------------------------------------------------------------------------------- 0

Majority                   --------------------------------------------------------------------------------

                                   7290      7300      7310      7320      7330      7340      7350      7360

Human                      TTGACTTAATATTTTGAATATGGTACAAAGTGTGGGTCAGAAGTATTTGGTTTTGCATGGGGATATCCAGTGTTTACAGC 7360
AsianBonytongue            -------------------------------------------------------------------------------- 0
Reedfish                   -------------------------------------------------------------------------------- 0
NorthernPike               -------------------------------------------------------------------------------- 0
SpottedGar                 -------------------------------------------------------------------------------- 0
ParamormyropsKingsLeyae    -------------------------------------------------------------------------------- 0

Majority                   --------------------------------------------------------------------------------

                                   7370      7380      7390      7400      7410      7420      7430      7440

Human                      ATCATTTGTTGAAAAGACTATCCTTTCTCCATTCACTTGGCTTTGCAGCTTCATGAAAAATCACTTATGTCTGTATGTGT 7440
AsianBonytongue            -------------------------------------------------------------------------------- 0
Reedfish                   -------------------------------------------------------------------------------- 0
NorthernPike               -------------------------------------------------------------------------------- 0
SpottedGar                 -------------------------------------------------------------------------------- 0
ParamormyropsKingsLeyae    -------------------------------------------------------------------------------- 0

Majority                   --------------------------------------------------------------------------------

                                   7450      7460      7470      7480      7490      7500      7510      7520

Human                      GATTTTATGTCTGTACTCTTTTTTGTATTGACCCATTTTCCTTTATCTAGATGCTGGAAACATACTGTCTTAATCATTGT 7520
AsianBonytongue            -------------------------------------------------------------------------------- 0
Reedfish                   -------------------------------------------------------------------------------- 0
NorthernPike               -------------------------------------------------------------------------------- 0
SpottedGar                 -------------------------------------------------------------------------------- 0
ParamormyropsKingsLeyae    -------------------------------------------------------------------------------- 0

Majority                   --------------------------------------------------------------------------------

                                   7530      7540      7550      7560      7570      7580      7590      7600

Human                      AGCTTTATATTGAGTCTTGAAACCACTTACTTAGTCTTGGCCCTCCAACTCTGTTCTTTTTCAAAGTTGTTTTTAACTCT 7600
AsianBonytongue            -------------------------------------------------------------------------------- 0
Reedfish                   -------------------------------------------------------------------------------- 0
NorthernPike               -------------------------------------------------------------------------------- 0
SpottedGar                 -------------------------------------------------------------------------------- 0
ParamormyropsKingsLeyae    -------------------------------------------------------------------------------- 0

Majority                   --------------------------------------------------------------------------------

                                   7610      7620      7630      7640      7650      7660      7670      7680

Human                      TCTGAGTCCTTTGTATTTTCATATGAGTCAGTTCTTAGAAAAACTGCTTGGATTTTACTGGATTTGACTTGAATCTGTAG 7680
AsianBonytongue            -------------------------------------------------------------------------------- 0
Reedfish                   -------------------------------------------------------------------------------- 0
NorthernPike               -------------------------------------------------------------------------------- 0
SpottedGar                 -------------------------------------------------------------------------------- 0
ParamormyropsKingsLeyae    -------------------------------------------------------------------------------- 0
```

```
Majority               --------------------------------------------------------------------------------

                                7690      7700      7710      7720      7730      7740      7750      7760

Human                  AGCAAATTTGGGGAAAATTGGCATCATGACTACAATTGATGTCTCTATTAGGTCTTTAATTTCTTTAAGCATTTTTTTTT 7760
AsianBonytongue        -------------------------------------------------------------------------------- 0
Reedfish               -------------------------------------------------------------------------------- 0
NorthernPike           -------------------------------------------------------------------------------- 0
SpottedGar             -------------------------------------------------------------------------------- 0
ParamormyropsKingsLeyae-------------------------------------------------------------------------------- 0

Majority               --------------------------------------------------------------------------------

                                7770      7780      7790      7800      7810      7820      7830      7840

Human                  TTTTTTTCAGTGCACACGTTTTGTGCATTGTTTGATTAGATTTACCCATAAGTATTTTATGTATTTTGAAGTACTGTAAA 7840
AsianBonytongue        -------------------------------------------------------------------------------- 0
Reedfish               -------------------------------------------------------------------------------- 0
NorthernPike           -------------------------------------------------------------------------------- 0
SpottedGar             -------------------------------------------------------------------------------- 0
ParamormyropsKingsLeyae-------------------------------------------------------------------------------- 0

Majority               --------------------------------------------------------------------------------

                                7850      7860      7870      7880      7890      7900      7910      7920

Human                  TGATATTTTAAAAACTTAATTTCCAATTGTTTGCTAGTGTATAGAAATGATTATGGCATATTGCTCATATGTTCTGAACC 7920
AsianBonytongue        -------------------------------------------------------------------------------- 0
Reedfish               -------------------------------------------------------------------------------- 0
NorthernPike           -------------------------------------------------------------------------------- 0
SpottedGar             -------------------------------------------------------------------------------- 0
ParamormyropsKingsLeyae-------------------------------------------------------------------------------- 0

Majority               --------------------------------------------------------------------------------

                                7930      7940      7950      7960      7970      7980      7990      8000

Human                  TTTGCTAAACTCATTTAGTAGATCTAATAGTTTTTTTTATATATTTCATCAGATTTGCTTGAGAGGGAGTCTCAGTCACC 8000
AsianBonytongue        -------------------------------------------------------------------------------- 0
Reedfish               -------------------------------------------------------------------------------- 0
NorthernPike           -------------------------------------------------------------------------------- 0
SpottedGar             -------------------------------------------------------------------------------- 0
ParamormyropsKingsLeyae-------------------------------------------------------------------------------- 0

Majority               --------------------------------------------------------------------------------

                                8010      8020      8030      8040      8050      8060      8070      8080

Human                  CAGGCTGGAGTGCAGTGACGTGATCTTGGCTCACTGCAACCTGCACCTCCTGGGTCAAGCAATTCTCCTGCCTCAGCCTC 8080
AsianBonytongue        -------------------------------------------------------------------------------- 0
Reedfish               -------------------------------------------------------------------------------- 0
NorthernPike           -------------------------------------------------------------------------------- 0
SpottedGar             -------------------------------------------------------------------------------- 0
ParamormyropsKingsLeyae-------------------------------------------------------------------------------- 0

Majority               --------------------------------------------------------------------------------

                                8090      8100      8110      8120      8130      8140      8150      8160

Human                  ATAAGTAGCTGGGACTGCAGGTGCACACCACCACGCCCAGCTAATTTTTGCATTTTCAGTTGAGGCAGGGTTTCACCATG 8160
AsianBonytongue        -------------------------------------------------------------------------------- 0
Reedfish               -------------------------------------------------------------------------------- 0
NorthernPike           -------------------------------------------------------------------------------- 0
SpottedGar             -------------------------------------------------------------------------------- 0
ParamormyropsKingsLeyae-------------------------------------------------------------------------------- 0
```

```
Majority                  --------------------------------------------------------------------------------

                                   8170      8180      8190      8200      8210      8220      8230      8240

Human                     TTGGCCAGGCTGGTCTGGAACTCCTGACCTCAAGTGATACACTTACCTAGGCCTCCCAAAGTGTTAAGATTACAGGCGTG 8240
AsianBonytongue           -------------------------------------------------------------------------------- 0
Reedfish                  -------------------------------------------------------------------------------- 0
NorthernPike              -------------------------------------------------------------------------------- 0
SpottedGar                -------------------------------------------------------------------------------- 0
ParamormyropsKingsLeyae   -------------------------------------------------------------------------------- 0

Majority                  --------------------------------------------------------------------------------

                                   8250      8260      8270      8280      8290      8300      8310      8320

Human                     AGCCACCACCCCCAGCCTATGCCATTAAATTTTCTACATAGATAATTTATACCCAATTGTTTTTAAATAGTTTTCTTTCT 8320
AsianBonytongue           -------------------------------------------------------------------------------- 0
Reedfish                  -------------------------------------------------------------------------------- 0
NorthernPike              -------------------------------------------------------------------------------- 0
SpottedGar                -------------------------------------------------------------------------------- 0
ParamormyropsKingsLeyae   -------------------------------------------------------------------------------- 0

Majority                  --------------------------------------------------------------------------------

                                   8330      8340      8350      8360      8370      8380      8390      8400

Human                     TTTCTATCTGGATGCCATTTTTTGTCTCCTTTTTTTGTCTGTGGTATCCAGTACAATGTTTAATAGAAGTGTTGAGAGAA 8400
AsianBonytongue           -------------------------------------------------------------------------------- 0
Reedfish                  -------------------------------------------------------------------------------- 0
NorthernPike              -------------------------------------------------------------------------------- 0
SpottedGar                -------------------------------------------------------------------------------- 0
ParamormyropsKingsLeyae   -------------------------------------------------------------------------------- 0

Majority                  --------------------------------------------------------------------------------

                                   8410      8420      8430      8440      8450      8460      8470      8480

Human                     TATCTTTTCCTTGCTCCTGATATTAGGGTGAGAACTCTAAAATTTCTTCCTTAACTGACACAGTTCACTAGTGAAGCCAT 8480
AsianBonytongue           -------------------------------------------------------------------------------- 0
Reedfish                  -------------------------------------------------------------------------------- 0
NorthernPike              -------------------------------------------------------------------------------- 0
SpottedGar                -------------------------------------------------------------------------------- 0
ParamormyropsKingsLeyae   -------------------------------------------------------------------------------- 0

Majority                  --------------------------------------------------------------------------------

                                   8490      8500      8510      8520      8530      8540      8550      8560

Human                     GTGGGGCCAGATGTTTGTTTTGTCGGGAGGTTTTAAACTACATATTCAACTTATTTAATAGATATAGGGCTATTCAGTTT 8560
AsianBonytongue           -------------------------------------------------------------------------------- 0
Reedfish                  -------------------------------------------------------------------------------- 0
NorthernPike              -------------------------------------------------------------------------------- 0
SpottedGar                -------------------------------------------------------------------------------- 0
ParamormyropsKingsLeyae   -------------------------------------------------------------------------------- 0

Majority                  --------------------------------------------------------------------------------

                                   8570      8580      8590      8600      8610      8620      8630      8640

Human                     GTTTAGTTCTTCTTGAGTGAACTTTGGTAATTTGTATCTTCTGTAGAACTAGTCCATTTCCTTTAAGTTGCTGAATGTAT 8640
AsianBonytongue           -------------------------------------------------------------------------------- 0
Reedfish                  -------------------------------------------------------------------------------- 0
NorthernPike              -------------------------------------------------------------------------------- 0
SpottedGar                -------------------------------------------------------------------------------- 0
ParamormyropsKingsLeyae   -------------------------------------------------------------------------------- 0
```

```
Majority                    --------------------------------------------------------------------------------

                                     8650      8660      8670      8680      8690      8700      8710      8720

Human                       TTGTGTAGAGTTGTTCATGATACTACTATTTTTTTTTTTTTTTTTTTTTTGAGACGGAGCCTTGCTCTGTCGCCCGGGCT 8720
AsianBonytongue             -------------------------------------------------------------------------------- 0
Reedfish                    -------------------------------------------------------------------------------- 0
NorthernPike                -------------------------------------------------------------------------------- 0
SpottedGar                  -------------------------------------------------------------------------------- 0
ParamormyropsKingsLeyae     -------------------------------------------------------------------------------- 0

Majority                    --------------------------------------------------------------------------------

                                     8730      8740      8750      8760      8770      8780      8790      8800

Human                       GGAGTGCAGTGGCACCATCTCGGCTCAATGCAACCTCCACGTCCCGGGTTCACGCGGTTCTCCTGCCTCAGCATCCCAAG 8800
AsianBonytongue             -------------------------------------------------------------------------------- 0
Reedfish                    -------------------------------------------------------------------------------- 0
NorthernPike                -------------------------------------------------------------------------------- 0
SpottedGar                  -------------------------------------------------------------------------------- 0
ParamormyropsKingsLeyae     -------------------------------------------------------------------------------- 0

Majority                    --------------------------------------------------------------------------------

                                     8810      8820      8830      8840      8850      8860      8870      8880

Human                       TAGCTGGGATCACAGGCACACACCACCACACCTGGCTAATTTTTTGTATTTTTAGTAGAGACGGGGTTTCACTATGTTGG 8880
AsianBonytongue             -------------------------------------------------------------------------------- 0
Reedfish                    -------------------------------------------------------------------------------- 0
NorthernPike                -------------------------------------------------------------------------------- 0
SpottedGar                  -------------------------------------------------------------------------------- 0
ParamormyropsKingsLeyae     -------------------------------------------------------------------------------- 0

Majority                    --------------------------------------------------------------------------------

                                     8890      8900      8910      8920      8930      8940      8950      8960

Human                       CCAGGTTGGTCTCGAACTTGTGATCTGCCCGCCCTGCCTCACAAAGTGCTGGGATTACAGGCGTGAGCCACTGCGCCCAG 8960
AsianBonytongue             -------------------------------------------------------------------------------- 0
Reedfish                    -------------------------------------------------------------------------------- 0
NorthernPike                -------------------------------------------------------------------------------- 0
SpottedGar                  -------------------------------------------------------------------------------- 0
ParamormyropsKingsLeyae     -------------------------------------------------------------------------------- 0

Majority                    --------------------------------------------------------------------------------

                                     8970      8980      8990      9000      9010      9020      9030      9040

Human                       CCATGATACTACATTTTTACACTTTTGGTATCTGTTGAATCCTTAGTGATGTCAGCTTTCTCATTCCCAGTAGGGATAAT 9040
AsianBonytongue             -------------------------------------------------------------------------------- 0
Reedfish                    -------------------------------------------------------------------------------- 0
NorthernPike                -------------------------------------------------------------------------------- 0
SpottedGar                  -------------------------------------------------------------------------------- 0
ParamormyropsKingsLeyae     -------------------------------------------------------------------------------- 0

Majority                    --------------------------------------------------------------------------------

                                     9050      9060      9070      9080      9090      9100      9110      9120

Human                       TTGTGTCATCTTTTTTCCTTGTCTGTCTGGATTGAAGTTTATTGATTTTATTAATCTCCAAAATACCAGCTTTTGGTTTG 9120
AsianBonytongue             -------------------------------------------------------------------------------- 0
Reedfish                    -------------------------------------------------------------------------------- 0
NorthernPike                -------------------------------------------------------------------------------- 0
SpottedGar                  -------------------------------------------------------------------------------- 0
ParamormyropsKingsLeyae     -------------------------------------------------------------------------------- 0
```

```
Majority                     --------------------------------------------------------------------------------
                                      9130      9140      9150      9160      9170      9180      9190      9200
Human                        AGTTTTCTCTGCTGTTTTTCTGTTTTCTATTTTATTGATTTCTGCTATTTTTATTTTGTTTTGCTTTGTGGTTAGTTTGA 9200
AsianBonytongue              -------------------------------------------------------------------------------- 0
Reedfish                     -------------------------------------------------------------------------------- 0
NorthernPike                 -------------------------------------------------------------------------------- 0
SpottedGar                   -------------------------------------------------------------------------------- 0
ParamormyropsKingsLeyae      -------------------------------------------------------------------------------- 0

Majority                     --------------------------------------------------------------------------------
                                      9210      9220      9230      9240      9250      9260      9270      9280
Human                        TCTTCATTTTCTACTTAGTTAACATTGAAGTTAATGTCATTAACTTAGTACTTTTACTTATATAGGGATTTATTGCTGTA 9280
AsianBonytongue              -------------------------------------------------------------------------------- 0
Reedfish                     -------------------------------------------------------------------------------- 0
NorthernPike                 -------------------------------------------------------------------------------- 0
SpottedGar                   -------------------------------------------------------------------------------- 0
ParamormyropsKingsLeyae      -------------------------------------------------------------------------------- 0

Majority                     --------------------------------------------------------------------------------
                                      9290      9300      9310      9320      9330      9340      9350      9360
Human                        TGTTTCCCTTTTAAGTACCGCTTTCACGGCATTCCTCAAATTTTGACACGTATATTTTTATTCACTTCAAAATACTTTTT 9360
AsianBonytongue              -------------------------------------------------------------------------------- 0
Reedfish                     -------------------------------------------------------------------------------- 0
NorthernPike                 -------------------------------------------------------------------------------- 0
SpottedGar                   -------------------------------------------------------------------------------- 0
ParamormyropsKingsLeyae      -------------------------------------------------------------------------------- 0

Majority                     --------------------------------------------------------------------------------
                                      9370      9380      9390      9400      9410      9420      9430      9440
Human                        TAAATATTTCTTTGACCCATGGGTTATTGGAAAGTATGTTATTTATGTTATTTAATTTCCAAATATTTGGAGTTTTCCAT 9440
AsianBonytongue              -------------------------------------------------------------------------------- 0
Reedfish                     -------------------------------------------------------------------------------- 0
NorthernPike                 -------------------------------------------------------------------------------- 0
SpottedGar                   -------------------------------------------------------------------------------- 0
ParamormyropsKingsLeyae      -------------------------------------------------------------------------------- 0

Majority                     --------------------------------------------------------------------------------
                                      9450      9460      9470      9480      9490      9500      9510      9520
Human                        ATATCTTTCTGTTATGTATTCATAATTTTAGTTTTATTCTGGCCAGAGAACATATTTTCTAAGACTTGGATCTTTGTAAA 9520
AsianBonytongue              -------------------------------------------------------------------------------- 0
Reedfish                     -------------------------------------------------------------------------------- 0
NorthernPike                 -------------------------------------------------------------------------------- 0
SpottedGar                   -------------------------------------------------------------------------------- 0
ParamormyropsKingsLeyae      -------------------------------------------------------------------------------- 0

Majority                     --------------------------------------------------------------------------------
                                      9530      9540      9550      9560      9570      9580      9590      9600
Human                        CATGGAGACTTGTTTTGTGGTCCAAATATATATGTTGTTAAGTATTCACTGAATACTCTGATGATTCTCTGCAGATTTGG 9600
AsianBonytongue              -------------------------------------------------------------------------------- 0
Reedfish                     -------------------------------------------------------------------------------- 0
NorthernPike                 -------------------------------------------------------------------------------- 0
SpottedGar                   -------------------------------------------------------------------------------- 0
ParamormyropsKingsLeyae      -------------------------------------------------------------------------------- 0
```

```
Majority                    --------------------------------------------------------------------------------

                                     9610      9620      9630      9640      9650      9660      9670      9680

Human                       AGAAATCTCTGTGCAGTTCTTTTCTCTGGTACTTTGACCTGTATGTAAACTCTAGTTACTTTGGTCTTCTCAGGCTCTTG 9680
AsianBonytongue             -------------------------------------------------------------------------------- 0
Reedfish                    -------------------------------------------------------------------------------- 0
NorthernPike                -------------------------------------------------------------------------------- 0
SpottedGar                  -------------------------------------------------------------------------------- 0
ParamormyropsKingsLeyae-------------------------------------------------------------------------------- 0

Majority                    --------------------------------------------------------------------------------

                                     9690      9700      9710      9720      9730      9740      9750      9760

Human                       GCTCTTTCACAATTAAAGTAGTCTTTGAGGCTCAGCCTGCTTTCCTCATAGCTATGCTATGGCCTGGACACTCAAGGGAG 9760
AsianBonytongue             -------------------------------------------------------------------------------- 0
Reedfish                    -------------------------------------------------------------------------------- 0
NorthernPike                -------------------------------------------------------------------------------- 0
SpottedGar                  -------------------------------------------------------------------------------- 0
ParamormyropsKingsLeyae-------------------------------------------------------------------------------- 0

Majority                    --------------------------------------------------------------------------------

                                     9770      9780      9790      9800      9810      9820      9830      9840

Human                       TATAAGCTGAGGCAAACATGGACTCATTTGTTTTCTAACTTTCAGGGATTATTGTCCATCATTGCCTGATGTCCAGTGTC 9840
AsianBonytongue             -------------------------------------------------------------------------------- 0
Reedfish                    -------------------------------------------------------------------------------- 0
NorthernPike                -------------------------------------------------------------------------------- 0
SpottedGar                  -------------------------------------------------------------------------------- 0
ParamormyropsKingsLeyae-------------------------------------------------------------------------------- 0

Majority                    --------------------------------------------------------------------------------

                                     9850      9860      9870      9880      9890      9900      9910      9920

Human                       TTGAAAAGCAATTATTCTGTATAGTTGCTTGATTGTTTGGTGTTGTTTCGGCGAGGCAAATCTGGGTCATGTTATTCTGT 9920
AsianBonytongue             -------------------------------------------------------------------------------- 0
Reedfish                    -------------------------------------------------------------------------------- 0
NorthernPike                -------------------------------------------------------------------------------- 0
SpottedGar                  -------------------------------------------------------------------------------- 0
ParamormyropsKingsLeyae-------------------------------------------------------------------------------- 0

Majority                    --------------------------------------------------------------------------------

                                     9930      9940      9950      9960      9970      9980      9990     10000

Human                       CTTGACTGGAAGTAGAAGTCCACTGTTTTTTTTTAATTACATAATATGGTCTCAGCAGTTGTAGATCTACCTCATTCTTT 10000
AsianBonytongue             -------------------------------------------------------------------------------- 0
Reedfish                    -------------------------------------------------------------------------------- 0
NorthernPike                -------------------------------------------------------------------------------- 0
SpottedGar                  -------------------------------------------------------------------------------- 0
ParamormyropsKingsLeyae-------------------------------------------------------------------------------- 0

Majority                    --------------------------------------------------------------------------------

                                    10010     10020     10030     10040     10050     10060     10070     10080

Human                       GTTAATGACAATGGAGGATACTCGTGTGGCTGCACCACAGTGTAGTTACTGGACATACCACAATTTAGTTTCCTACATCG 10080
AsianBonytongue             -------------------------------------------------------------------------------- 0
Reedfish                    -------------------------------------------------------------------------------- 0
NorthernPike                -------------------------------------------------------------------------------- 0
SpottedGar                  -------------------------------------------------------------------------------- 0
ParamormyropsKingsLeyae-------------------------------------------------------------------------------- 0
```

```
Majority                     --------------------------------------------------------------------------------

                                      10090     10100     10110     10120     10130     10140     10150     10160

Human                        ATGGACATTTAGGTTTGATTCCAGTTTGTTGTTATAACATGTGGACATATTTAGAATAGAAGTAAAAATGCTGTGTCAAA 10160
AsianBonytongue              -------------------------------------------------------------------------------- 0
Reedfish                     -------------------------------------------------------------------------------- 0
NorthernPike                 -------------------------------------------------------------------------------- 0
SpottedGar                   -------------------------------------------------------------------------------- 0
ParamormyropsKingsLeyae------------------------------------------------------------------------------------- 0

Majority                     --------------------------------------------------------------------------------

                                      10170     10180     10190     10200     10210     10220     10230     10240

Human                        TGAAATTTATACTTAAAGAGTTACTGAAAACCACAATGCCAGAACACTCATTTGTTTTTCACAAGTGTGTATGGGAGTGC 10240
AsianBonytongue              -------------------------------------------------------------------------------- 0
Reedfish                     -------------------------------------------------------------------------------- 0
NorthernPike                 -------------------------------------------------------------------------------- 0
SpottedGar                   -------------------------------------------------------------------------------- 0
ParamormyropsKingsLeyae------------------------------------------------------------------------------------- 0

Majority                     --------------------------------------------------------------------------------

                                      10250     10260     10270     10280     10290     10300     10310     10320

Human                        CTATTTTTCCATACCCTTGGCAATTCTAGGTTTTATCAAAATTTAAGAATACTAGCTTATTTTACACTAAAAATTATTTT 10320
AsianBonytongue              -------------------------------------------------------------------------------- 0
Reedfish                     -------------------------------------------------------------------------------- 0
NorthernPike                 -------------------------------------------------------------------------------- 0
SpottedGar                   -------------------------------------------------------------------------------- 0
ParamormyropsKingsLeyae------------------------------------------------------------------------------------- 0

Majority                     --------------------------------------------------------------------------------

                                      10330     10340     10350     10360     10370     10380     10390     10400

Human                        GTAATTTATATTTTGAAATGGGTGAGGTTTTTCAGATTTGGTTATTTATATTTCATTTTCTGTGAACTGCCTGTTTGTGT 10400
AsianBonytongue              -------------------------------------------------------------------------------- 0
Reedfish                     -------------------------------------------------------------------------------- 0
NorthernPike                 -------------------------------------------------------------------------------- 0
SpottedGar                   -------------------------------------------------------------------------------- 0
ParamormyropsKingsLeyae------------------------------------------------------------------------------------- 0

Majority                     --------------------------------------------------------------------------------

                                      10410     10420     10430     10440     10450     10460     10470     10480

Human                        ACTGGACCTAGTTAATTCAAATATATATATATTTGAATTAAGTGATTTTATTGTTTAAAGTCCATTTGATGTGTAAACATTT 10480
AsianBonytongue              -------------------------------------------------------------------------------- 0
Reedfish                     -------------------------------------------------------------------------------- 0
NorthernPike                 -------------------------------------------------------------------------------- 0
SpottedGar                   -------------------------------------------------------------------------------- 0
ParamormyropsKingsLeyae------------------------------------------------------------------------------------- 0

Majority                     --------------------------------------------------------------------------------

                                      10490     10500     10510     10520     10530     10540     10550     10560

Human                        TTAGCCTCAGCTTCTTCACCTTTTAAATTAGGATTTTAATAGTTCCACAAACTAGGTCTAATGTTAATGTATTTACATTT 10560
AsianBonytongue              -------------------------------------------------------------------------------- 0
Reedfish                     -------------------------------------------------------------------------------- 0
NorthernPike                 -------------------------------------------------------------------------------- 0
SpottedGar                   -------------------------------------------------------------------------------- 0
ParamormyropsKingsLeyae------------------------------------------------------------------------------------- 0
```

```
Majority                      --------------------------------------------------------------------------------
                                       10570     10580     10590     10600     10610     10620     10630     10640
Human                         AGAATATCCAAGTTTTTATGGCTTTAGGATACAAATGAAATCTCATTTTCCATTATTTTATAAATTTGCTGTATTCAGCT 10640
AsianBonytongue               -------------------------------------------------------------------------------- 0
Reedfish                      -------------------------------------------------------------------------------- 0
NorthernPike                  -------------------------------------------------------------------------------- 0
SpottedGar                    -------------------------------------------------------------------------------- 0
ParamormyropsKingsLeyae       -------------------------------------------------------------------------------- 0

Majority                      --------------------------------------------------------------------------------
                                       10650     10660     10670     10680     10690     10700     10710     10720
Human                         TTTCTTGATTTTAGACTTTTTTTTGTTAGCTCAGTGAGACTATTAAGAAAGAATATTTGTGAGTTTGTAATAGGAGATTT 10720
AsianBonytongue               -------------------------------------------------------------------------------- 0
Reedfish                      -------------------------------------------------------------------------------- 0
NorthernPike                  -------------------------------------------------------------------------------- 0
SpottedGar                    -------------------------------------------------------------------------------- 0
ParamormyropsKingsLeyae       -------------------------------------------------------------------------------- 0

Majority                      --------------------------------------------------------------------------------
                                       10730     10740     10750     10760     10770     10780     10790     10800
Human                         GTTGCATTTCTTTAAAATGGCTTTTTATATTCTTCGTAATTTGTAATATCAGGGGTGTCATTTCTGTACTCTTAACCATT 10800
AsianBonytongue               -------------------------------------------------------------------------------- 0
Reedfish                      -------------------------------------------------------------------------------- 0
NorthernPike                  -------------------------------------------------------------------------------- 0
SpottedGar                    -------------------------------------------------------------------------------- 0
ParamormyropsKingsLeyae       -------------------------------------------------------------------------------- 0

Majority                      --------------------------------------------------------------------------------
                                       10810     10820     10830     10840     10850     10860     10870     10880
Human                         TTGCCAATGTGAAATCCTTGCAAAGTAATCGGCCTATTACTTGGGAAGACAGAATTGTATGAAATGACTGTAGAATAATT 10880
AsianBonytongue               -------------------------------------------------------------------------------- 0
Reedfish                      -------------------------------------------------------------------------------- 0
NorthernPike                  -------------------------------------------------------------------------------- 0
SpottedGar                    -------------------------------------------------------------------------------- 0
ParamormyropsKingsLeyae       -------------------------------------------------------------------------------- 0

Majority                      --------------------------------------------------------------------------------
                                       10890     10900     10910     10920     10930     10940     10950     10960
Human                         CAGGATGGTCCCCAATACGGTAAAATGTTAACTTTTTAGATGTAGATTTTAAGTATGGTGAAATTTGTAGCAGTGTTCTT 10960
AsianBonytongue               -------------------------------------------------------------------------------- 0
Reedfish                      -------------------------------------------------------------------------------- 0
NorthernPike                  -------------------------------------------------------------------------------- 0
SpottedGar                    -------------------------------------------------------------------------------- 0
ParamormyropsKingsLeyae       -------------------------------------------------------------------------------- 0

Majority                      --------------------------------------------------------------------------------
                                       10970     10980     10990     11000     11010     11020     11030     11040
Human                         TAGTAAGTTAACTATTAATATATCAAGACCTATAACTGGACAACTAATAAATAACCTGTTTCTTAAAATGTTAGCTTTTT 11040
AsianBonytongue               -------------------------------------------------------------------------------- 0
Reedfish                      -------------------------------------------------------------------------------- 0
NorthernPike                  -------------------------------------------------------------------------------- 0
SpottedGar                    -------------------------------------------------------------------------------- 0
ParamormyropsKingsLeyae       -------------------------------------------------------------------------------- 0
```

```
Majority                --------------------------------------------------------------------------------

                                 11050     11060     11070     11080     11090     11100     11110     11120

Human                   CTAAATATTCTGAAAGTACTGGTTGTAAATTGCTGAACTCACAATTTAAATTATGATATGACTATTGAATTCATCCTCAT 11120
AsianBonytongue         -------------------------------------------------------------------------------- 0
Reedfish                -------------------------------------------------------------------------------- 0
NorthernPike            -------------------------------------------------------------------------------- 0
SpottedGar              -------------------------------------------------------------------------------- 0
ParamormyropsKingsLeyae-------------------------------------------------------------------------------- 0

Majority                --------------------------------------------------------------------------------

                                 11130     11140     11150     11160     11170     11180     11190     11200

Human                   TGGCTTGCATAGAAAATTACAATCAAATAATGCTAAATGTAACTTTTTTGGTAATAGTTGTGTGTAACTGGTTGTTCATT 11200
AsianBonytongue         -------------------------------------------------------------------------------- 0
Reedfish                -------------------------------------------------------------------------------- 0
NorthernPike            -------------------------------------------------------------------------------- 0
SpottedGar              -------------------------------------------------------------------------------- 0
ParamormyropsKingsLeyae-------------------------------------------------------------------------------- 0

Majority                --------------------------------------------------------------------------------

                                 11210     11220     11230     11240     11250     11260     11270     11280

Human                   ATAGATGTGTTATTCAAAACTTTGGGTTTTTAGGTTTTGACTTTTCTTAAAATACTTTTCTTAAATGCTACTTAATGCTT 11280
AsianBonytongue         -------------------------------------------------------------------------------- 0
Reedfish                -------------------------------------------------------------------------------- 0
NorthernPike            -------------------------------------------------------------------------------- 0
SpottedGar              -------------------------------------------------------------------------------- 0
ParamormyropsKingsLeyae-------------------------------------------------------------------------------- 0

Majority                --------------------------------------------------------------------------------

                                 11290     11300     11310     11320     11330     11340     11350     11360

Human                   TCTCTAGGGTATTTTTATTAGAATTTCAATTAACAGATATTTTGATGTCTGCGAAATGACAGTACTATGTTAGAAATTGA 11360
AsianBonytongue         -------------------------------------------------------------------------------- 0
Reedfish                -------------------------------------------------------------------------------- 0
NorthernPike            -------------------------------------------------------------------------------- 0
SpottedGar              -------------------------------------------------------------------------------- 0
ParamormyropsKingsLeyae-------------------------------------------------------------------------------- 0

Majority                --------------------------------------------------------------------------------

                                 11370     11380     11390     11400     11410     11420     11430     11440

Human                   TGTATTGGCTCCTTGAGGGCAGTGACCCTATTTGTTATGTTTGTTCCTCAATCATTAATAGTACACATATTAAATAGTAC 11440
AsianBonytongue         -------------------------------------------------------------------------------- 0
Reedfish                -------------------------------------------------------------------------------- 0
NorthernPike            -------------------------------------------------------------------------------- 0
SpottedGar              -------------------------------------------------------------------------------- 0
ParamormyropsKingsLeyae-------------------------------------------------------------------------------- 0

Majority                --------------------------------------------------------------------------------

                                 11450     11460     11470     11480     11490     11500     11510     11520

Human                   ATATACACTTAATATTTAATAACTCTTAGAAGTGTCATAGGCTTTAATATGCTCATACTCTAATGAGCCATTTATCAATA 11520
AsianBonytongue         -------------------------------------------------------------------------------- 0
Reedfish                -------------------------------------------------------------------------------- 0
NorthernPike            -------------------------------------------------------------------------------- 0
SpottedGar              -------------------------------------------------------------------------------- 0
ParamormyropsKingsLeyae-------------------------------------------------------------------------------- 0
```

```
Majority               --------------------------------------------------------------------------------
                                11530     11540     11550     11560     11570     11580     11590     11600
Human                  AAAAGATAAATAACAAGACTATGTCTTTTAGGACTTTAGTTAGAGAAATAAGGACTCAAGGTTTTAATTAAGTGCTATAT 11600
AsianBonytongue        -------------------------------------------------------------------------------- 0
Reedfish               -------------------------------------------------------------------------------- 0
NorthernPike           -------------------------------------------------------------------------------- 0
SpottedGar             -------------------------------------------------------------------------------- 0
ParamormyropsKingsLeyae-------------------------------------------------------------------------------- 0

Majority               --------------------------------------------------------------------------------
                                11610     11620     11630     11640     11650     11660     11670     11680
Human                  GGCTACAGTAGGAAAGATTGGTTTGGGTTGGTAGGCTCAGAATAGTGGTCTGTGACCTAGCTAGATTGGGAGATAGGAGA 11680
AsianBonytongue        -------------------------------------------------------------------------------- 0
Reedfish               -------------------------------------------------------------------------------- 0
NorthernPike           -------------------------------------------------------------------------------- 0
SpottedGar             -------------------------------------------------------------------------------- 0
ParamormyropsKingsLeyae-------------------------------------------------------------------------------- 0

Majority               --------------------------------------------------------------------------------
                                11690     11700     11710     11720     11730     11740     11750     11760
Human                  AAGAGTGGAGAGCAACAGGCGTTGAGAGAAGGACGTAACCAAAGTTGTGTCTATAGGTTGGCATAAATTTACAGCCATTG 11760
AsianBonytongue        -------------------------------------------------------------------------------- 0
Reedfish               -------------------------------------------------------------------------------- 0
NorthernPike           -------------------------------------------------------------------------------- 0
SpottedGar             -------------------------------------------------------------------------------- 0
ParamormyropsKingsLeyae-------------------------------------------------------------------------------- 0

Majority               --------------------------------------------------------------------------------
                                11770     11780     11790     11800     11810     11820     11830     11840
Human                  ATCAGTGAGTTGAATAGTTTGGCCTTTATGGCTAACAGCTTGTATATGGATATGAGAGAAAAGATAGAATGGCAGATAGG 11840
AsianBonytongue        -------------------------------------------------------------------------------- 0
Reedfish               -------------------------------------------------------------------------------- 0
NorthernPike           -------------------------------------------------------------------------------- 0
SpottedGar             -------------------------------------------------------------------------------- 0
ParamormyropsKingsLeyae-------------------------------------------------------------------------------- 0

Majority               --------------------------------------------------------------------------------
                                11850     11860     11870     11880     11890     11900     11910     11920
Human                  TTATATAGAAGACCTCAATTACAGGTAGTGAAATTTGTACTTCATCTGGTAGGCATTATTCTATATACCTGTAATTTTCC 11920
AsianBonytongue        -------------------------------------------------------------------------------- 0
Reedfish               -------------------------------------------------------------------------------- 0
NorthernPike           -------------------------------------------------------------------------------- 0
SpottedGar             -------------------------------------------------------------------------------- 0
ParamormyropsKingsLeyae-------------------------------------------------------------------------------- 0

Majority               --------------------------------------------------------------------------------
                                11930     11940     11950     11960     11970     11980     11990     12000
Human                  ACATAAAAGTAGCTCATTACTCTGCTTTCTTTCTTTCTTTTTTTTTTTTTTGCTATAGCAAATTTATTGTAACAACACA 12000
AsianBonytongue        -------------------------------------------------------------------------------- 0
Reedfish               -------------------------------------------------------------------------------- 0
NorthernPike           -------------------------------------------------------------------------------- 0
SpottedGar             -------------------------------------------------------------------------------- 0
ParamormyropsKingsLeyae-------------------------------------------------------------------------------- 0
```

```
Majority                   --------------------------------------------------------------------------------
                                   12010     12020     12030     12040     12050     12060     12070     12080
Human                      AAGTATTATCTTATAGTTTTGTAGGTGAGAAATCTGACACAGGTGGGCTAAAATCAAGGTGTTAGCAGGGCTGTGTTCCT 12080
AsianBonytongue            -------------------------------------------------------------------------------- 0
Reedfish                   -------------------------------------------------------------------------------- 0
NorthernPike               -------------------------------------------------------------------------------- 0
SpottedGar                 -------------------------------------------------------------------------------- 0
ParamormyropsKingsLeyae    -------------------------------------------------------------------------------- 0

Majority                   --------------------------------------------------------------------------------
                                   12090     12100     12110     12120     12130     12140     12150     12160
Human                      TTTTGCAGGATCTGGGGGGAATTTGTTTCCTTGACATAACTCGGCTGTTAGCTTCATCTTCAAAGAAGGAATGGCAAAGA 12160
AsianBonytongue            -------------------------------------------------------------------------------- 0
Reedfish                   -------------------------------------------------------------------------------- 0
NorthernPike               -------------------------------------------------------------------------------- 0
SpottedGar                 -------------------------------------------------------------------------------- 0
ParamormyropsKingsLeyae    -------------------------------------------------------------------------------- 0

Majority                   --------------------------------------------------------------------------------
                                   12170     12180     12190     12200     12210     12220     12230     12240
Human                      AGACTCTGTCTCACATCACATCACTCCGACACATTCTTGTGCCTCCGTTTCCCACTTACAGGGACCTTGTGATTACAATG 12240
AsianBonytongue            -------------------------------------------------------------------------------- 0
Reedfish                   -------------------------------------------------------------------------------- 0
NorthernPike               -------------------------------------------------------------------------------- 0
SpottedGar                 -------------------------------------------------------------------------------- 0
ParamormyropsKingsLeyae    -------------------------------------------------------------------------------- 0

Majority                   --------------------------------------------------------------------------------
                                   12250     12260     12270     12280     12290     12300     12310     12320
Human                      GGCCCAACTACATAATAGAGGATAAATGTTTAAATTACAAATTAATGAAGTGGCAACTGAAACAAGTTTTAACCTATTCT 12320
AsianBonytongue            -------------------------------------------------------------------------------- 0
Reedfish                   -------------------------------------------------------------------------------- 0
NorthernPike               -------------------------------------------------------------------------------- 0
SpottedGar                 -------------------------------------------------------------------------------- 0
ParamormyropsKingsLeyae    -------------------------------------------------------------------------------- 0

Majority                   --------------------------------------------------------------------------------
                                   12330     12340     12350     12360     12370     12380     12390     12400
Human                      CAAGAAAGGGCTTTGCTGGAATGGTGGCTCACACCTGTAATCCCAGAACTTAGGGAGGCTGAGGCAGGCAGATCACTTGA 12400
AsianBonytongue            -------------------------------------------------------------------------------- 0
Reedfish                   -------------------------------------------------------------------------------- 0
NorthernPike               -------------------------------------------------------------------------------- 0
SpottedGar                 -------------------------------------------------------------------------------- 0
ParamormyropsKingsLeyae    -------------------------------------------------------------------------------- 0

Majority                   --------------------------------------------------------------------------------
                                   12410     12420     12430     12440     12450     12460     12470     12480
Human                      GCCCAGGAGTTTGAGACCAGCCTGGTCCTCATAGCAAGACCCCATCTCTACAAAAACTTTTAAAAATTAGATAGATCTGG 12480
AsianBonytongue            -------------------------------------------------------------------------------- 0
Reedfish                   -------------------------------------------------------------------------------- 0
NorthernPike               -------------------------------------------------------------------------------- 0
SpottedGar                 -------------------------------------------------------------------------------- 0
ParamormyropsKingsLeyae    -------------------------------------------------------------------------------- 0
```

```
Majority                    --------------------------------------------------------------------------------
                                      12490     12500     12510     12520     12530     12540     12550     12560
Human                       TGGCATGGGCCTGTAGTCCCAGCTACTTGGGAGGCTGAGACAAGAGGATCACCTGAGCCCAGGCGGCAGTGAGCTATGAT 12560
AsianBonytongue             -------------------------------------------------------------------------------- 0
Reedfish                    -------------------------------------------------------------------------------- 0
NorthernPike                -------------------------------------------------------------------------------- 0
SpottedGar                  -------------------------------------------------------------------------------- 0
ParamormyropsKingsLeyae-------------------------------------------------------------------------------- 0

Majority                    --------------------------------------------------------------------------------
                                      12570     12580     12590     12600     12610     12620     12630     12640
Human                       TGTATCACTACACTCCAGCCTGGGCGAGAGGAGATCCAGTCTCAAAAAAAAAGGAAAAGGGCTTTCCTGAGGGTCAAATG 12640
AsianBonytongue             -------------------------------------------------------------------------------- 0
Reedfish                    -------------------------------------------------------------------------------- 0
NorthernPike                -------------------------------------------------------------------------------- 0
SpottedGar                  -------------------------------------------------------------------------------- 0
ParamormyropsKingsLeyae-------------------------------------------------------------------------------- 0

Majority                    --------------------------------------------------------------------------------
                                      12650     12660     12670     12680     12690     12700     12710     12720
Human                       AAGTACAGTTGACCCTTGAACAACATGATTTTGAATTACACAGGTCCACTTATGCATAGATCCACTTATACAGAATTTTC 12720
AsianBonytongue             -------------------------------------------------------------------------------- 0
Reedfish                    -------------------------------------------------------------------------------- 0
NorthernPike                -------------------------------------------------------------------------------- 0
SpottedGar                  -------------------------------------------------------------------------------- 0
ParamormyropsKingsLeyae-------------------------------------------------------------------------------- 0

Majority                    --------------------------------------------------------------------------------
                                      12730     12740     12750     12760     12770     12780     12790     12800
Human                       TTCTCCCTCTTCCACCCAAGACAGAAAGACCAACTCGTCCTCTTCCTCCTTAGCCTACTCAGCGTGAAGACAGTCAGGAT 12800
AsianBonytongue             -------------------------------------------------------------------------------- 0
Reedfish                    -------------------------------------------------------------------------------- 0
NorthernPike                -------------------------------------------------------------------------------- 0
SpottedGar                  -------------------------------------------------------------------------------- 0
ParamormyropsKingsLeyae-------------------------------------------------------------------------------- 0

Majority                    --------------------------------------------------------------------------------
                                      12810     12820     12830     12840     12850     12860     12870     12880
Human                       GAAGACCTTTATTATGACTGATCTCCAAAAAGATTGGCTTCAGTATTTCATGGAGTGCTATGAATTAGAATCCTGAGTCT 12880
AsianBonytongue             -------------------------------------------------------------------------------- 0
Reedfish                    -------------------------------------------------------------------------------- 0
NorthernPike                -------------------------------------------------------------------------------- 0
SpottedGar                  -------------------------------------------------------------------------------- 0
ParamormyropsKingsLeyae-------------------------------------------------------------------------------- 0

Majority                    --------------------------------------------------------------------------------
                                      12890     12900     12910     12920     12930     12940     12950     12960
Human                       CTATATTGTTAATTATTTTAGTCTCTTGGTCTACGTGTGTTCCTAAACTGTAGTAGCTTTCTTAATTTAGTTCTAGGCAT 12960
AsianBonytongue             -------------------------------------------------------------------------------- 0
Reedfish                    -------------------------------------------------------------------------------- 0
NorthernPike                -------------------------------------------------------------------------------- 0
SpottedGar                  -------------------------------------------------------------------------------- 0
ParamormyropsKingsLeyae-------------------------------------------------------------------------------- 0
```

```
Majority                 --------------------------------------------------------------------------------
                                  12970     12980     12990     13000     13010     13020     13030     13040
Human                    ACTACATTAGGGGATAAGGTGGTTAAGTGATATTTTCACTTTAAAAGTAATAAGGTGTCACAGAAAGTTATCACGAGATT 13040
AsianBonytongue          -------------------------------------------------------------------------------- 0
Reedfish                 -------------------------------------------------------------------------------- 0
NorthernPike             -------------------------------------------------------------------------------- 0
SpottedGar               -------------------------------------------------------------------------------- 0
ParamormyropsKingsLeyae-------------------------------------------------------------------------------- 0

Majority                 --------------------------------------------------------------------------------
                                  13050     13060     13070     13080     13090     13100     13110     13120
Human                    TTTTTGAGCAGTACTTCTTTTTTAAAACTATTATAATGAATTTTAGACACAATAGTATCATGAACCATTGTGTATCTATC 13120
AsianBonytongue          -------------------------------------------------------------------------------- 0
Reedfish                 -------------------------------------------------------------------------------- 0
NorthernPike             -------------------------------------------------------------------------------- 0
SpottedGar               -------------------------------------------------------------------------------- 0
ParamormyropsKingsLeyae-------------------------------------------------------------------------------- 0

Majority                 --------------------------------------------------------------------------------
                                  13130     13140     13150     13160     13170     13180     13190     13200
Human                    ATTCAGTCCCCAAAACCATTAGTAACCCATAGCTAAAATGATGATATCCACACTCTGCTTTTCCACTTCTGTTATTTTGA 13200
AsianBonytongue          -------------------------------------------------------------------------------- 0
Reedfish                 -------------------------------------------------------------------------------- 0
NorthernPike             -------------------------------------------------------------------------------- 0
SpottedGar               -------------------------------------------------------------------------------- 0
ParamormyropsKingsLeyae-------------------------------------------------------------------------------- 0

Majority                 --------------------------------------------------------------------------------
                                  13210     13220     13230     13240     13250     13260     13270     13280
Human                    GATAAATTTCAGATATTCATATTGTTTTATCTGTAAAGGCTTCAGTGTATATCTTTAAAGAATAGGGACTCTCCTCTCCT 13280
AsianBonytongue          -------------------------------------------------------------------------------- 0
Reedfish                 -------------------------------------------------------------------------------- 0
NorthernPike             -------------------------------------------------------------------------------- 0
SpottedGar               -------------------------------------------------------------------------------- 0
ParamormyropsKingsLeyae-------------------------------------------------------------------------------- 0

Majority                 --------------------------------------------------------------------------------
                                  13290     13300     13310     13320     13330     13340     13350     13360
Human                    TTTTAAAATATAATCAAAAGACTGTTAGGACACACACACAAAGTTACCTCATATTATAAAATGTCAAATTTGTATTAAAA 13360
AsianBonytongue          -------------------------------------------------------------------------------- 0
Reedfish                 -------------------------------------------------------------------------------- 0
NorthernPike             -------------------------------------------------------------------------------- 0
SpottedGar               -------------------------------------------------------------------------------- 0
ParamormyropsKingsLeyae-------------------------------------------------------------------------------- 0

Majority                 --------------------------------------------------------------------------------
                                  13370     13380     13390     13400     13410     13420     13430     13440
Human                    TTTCTAATTGTCTCATAAAAGTCATAGTATTTTTATAGGGTTTTTTTTTTTTAGTGGGGAAGGAGTCACGATAAAATAAG 13440
AsianBonytongue          -------------------------------------------------------------------------------- 0
Reedfish                 -------------------------------------------------------------------------------- 0
NorthernPike             -------------------------------------------------------------------------------- 0
SpottedGar               -------------------------------------------------------------------------------- 0
ParamormyropsKingsLeyae-------------------------------------------------------------------------------- 0
```

```
Majority                    --------------------------------------------------------------------------------

                                    13450     13460     13470     13480     13490     13500     13510     13520

Human                       ATCAGCAAATTTAACAGGTCGTTATTACCTCTTAAATATCTTTTAATGTACAAGCTCTCCGTTCCTGTTTTTTGTTTTTT 13520
AsianBonytongue             -------------------------------------------------------------------------------- 0
Reedfish                    -------------------------------------------------------------------------------- 0
NorthernPike                -------------------------------------------------------------------------------- 0
SpottedGar                  -------------------------------------------------------------------------------- 0
ParamormyropsKingsLeyae     -------------------------------------------------------------------------------- 0

Majority                    --------------------------------------------------------------------------------

                                    13530     13540     13550     13560     13570     13580     13590     13600

Human                       TTTTAAGTTGCATTTCATTTGTTGAAGAAAACTGGTTATTGGTCTTACACAGTTGCCTATAGACTACATTTACTGATTGC 13600
AsianBonytongue             -------------------------------------------------------------------------------- 0
Reedfish                    -------------------------------------------------------------------------------- 0
NorthernPike                -------------------------------------------------------------------------------- 0
SpottedGar                  -------------------------------------------------------------------------------- 0
ParamormyropsKingsLeyae     -------------------------------------------------------------------------------- 0

Majority                    --------------------------------------------------------------------------------

                                    13610     13620     13630     13640     13650     13660     13670     13680

Human                       GTTATGATTTTGTAATTTAGTATGTTTTCCATATTTCCAGTAAATTGGTAGTTAGATCTAGAGACTTGCTCAGGGTCTCT 13680
AsianBonytongue             -------------------------------------------------------------------------------- 0
Reedfish                    -------------------------------------------------------------------------------- 0
NorthernPike                -------------------------------------------------------------------------------- 0
SpottedGar                  -------------------------------------------------------------------------------- 0
ParamormyropsKingsLeyae     -------------------------------------------------------------------------------- 0

Majority                    --------------------------------------------------------------------------------

                                    13690     13700     13710     13720     13730     13740     13750     13760

Human                       ATTTTTTTGGTAATATTTTTAAGTGGTGGGTTTTTATTTCTGTTAGACATGGTTTCTGACTTTTCGTGTATGTCATGTTT 13760
AsianBonytongue             -------------------------------------------------------------------------------- 0
Reedfish                    -------------------------------------------------------------------------------- 0
NorthernPike                -------------------------------------------------------------------------------- 0
SpottedGar                  -------------------------------------------------------------------------------- 0
ParamormyropsKingsLeyae     -------------------------------------------------------------------------------- 0

Majority                    --------------------------------------------------------------------------------

                                    13770     13780     13790     13800     13810     13820     13830     13840

Human                       TTCCCAGGAACATTTCTTATACTTTACCTTGTTTGTGCTAGGCATAGTCTGTGCTCAATAATTGTCTCCTTTTTTTTTCT 13840
AsianBonytongue             -------------------------------------------------------------------------------- 0
Reedfish                    -------------------------------------------------------------------------------- 0
NorthernPike                -------------------------------------------------------------------------------- 0
SpottedGar                  -------------------------------------------------------------------------------- 0
ParamormyropsKingsLeyae     -------------------------------------------------------------------------------- 0

Majority                    --------------------------------------------------------------------------------

                                    13850     13860     13870     13880     13890     13900     13910     13920

Human                       TTTTTTTTTTTTTTTTTTCCTGCTTTTTGAGAGACAAGAGTCTTGCCCTGTTGCCCAGGCTGGAGTGCAGTGGCCCAATC 13920
AsianBonytongue             -------------------------------------------------------------------------------- 0
Reedfish                    -------------------------------------------------------------------------------- 0
NorthernPike                -------------------------------------------------------------------------------- 0
SpottedGar                  -------------------------------------------------------------------------------- 0
ParamormyropsKingsLeyae     -------------------------------------------------------------------------------- 0
```

```
Majority                   --------------------------------------------------------------------------------
                                    13930     13940     13950     13960     13970     13980     13990     14000
Human                      TCAGCTCAGTGCAACCTCTGCCTCCCGGATTCAAGCGATTCTCCTGCCTCAGCTTCCCAAGTAGCTGGAACTACAGGCGC 14000
AsianBonytongue            -------------------------------------------------------------------------------- 0
Reedfish                   -------------------------------------------------------------------------------- 0
NorthernPike               -------------------------------------------------------------------------------- 0
SpottedGar                 -------------------------------------------------------------------------------- 0
ParamormyropsKingsLeyae    -------------------------------------------------------------------------------- 0

Majority                   --------------------------------------------------------------------------------
                                    14010     14020     14030     14040     14050     14060     14070     14080
Human                      CTGCCACTACACCTGGCTAATTTTTGTGTTTTTAGTAGGGATGGGGTTTCACCACGTTGGCCAGGCTGACTTGGAACTCC 14080
AsianBonytongue            -------------------------------------------------------------------------------- 0
Reedfish                   -------------------------------------------------------------------------------- 0
NorthernPike               -------------------------------------------------------------------------------- 0
SpottedGar                 -------------------------------------------------------------------------------- 0
ParamormyropsKingsLeyae    -------------------------------------------------------------------------------- 0

Majority                   --------------------------------------------------------------------------------
                                    14090     14100     14110     14120     14130     14140     14150     14160
Human                      TGACCTCAAGTGATCTGCCCACCTTGGCTTCCCAAAGTGCTGGGATTATAGGCATGAGCCACTGTGCCTGGCCAATAAGT 14160
AsianBonytongue            -------------------------------------------------------------------------------- 0
Reedfish                   -------------------------------------------------------------------------------- 0
NorthernPike               -------------------------------------------------------------------------------- 0
SpottedGar                 -------------------------------------------------------------------------------- 0
ParamormyropsKingsLeyae    -------------------------------------------------------------------------------- 0

Majority                   --------------------------------------------------------------------------------
                                    14170     14180     14190     14200     14210     14220     14230     14240
Human                      GTCTCTTAATGAGTATTCTTAAAGGATATCTAAAAAGAAATGTAACTTAAAATTCACATAATAGTAATACTATACAGTAT 14240
AsianBonytongue            -------------------------------------------------------------------------------- 0
Reedfish                   -------------------------------------------------------------------------------- 0
NorthernPike               -------------------------------------------------------------------------------- 0
SpottedGar                 -------------------------------------------------------------------------------- 0
ParamormyropsKingsLeyae    -------------------------------------------------------------------------------- 0

Majority                   --------------------------------------------------------------------------------
                                    14250     14260     14270     14280     14290     14300     14310     14320
Human                      TAACATTTAATGTAAGTTGGAACTATTGATGGTGGACAAGAATGTTTTCTTGTCAAGAACATACGATTTAGAATGAAAAG 14320
AsianBonytongue            -------------------------------------------------------------------------------- 0
Reedfish                   -------------------------------------------------------------------------------- 0
NorthernPike               -------------------------------------------------------------------------------- 0
SpottedGar                 -------------------------------------------------------------------------------- 0
ParamormyropsKingsLeyae    -------------------------------------------------------------------------------- 0

Majority                   --------------------------------------------------------------------------------
                                    14330     14340     14350     14360     14370     14380     14390     14400
Human                      ATGAGGGTTTGACTTTTATAGCATTGAACAGGTTAACCTCTGAGCTTCAGTTTTATACATTTTTAAATGGTTTCAGTTGT 14400
AsianBonytongue            -------------------------------------------------------------------------------- 0
Reedfish                   -------------------------------------------------------------------------------- 0
NorthernPike               -------------------------------------------------------------------------------- 0
SpottedGar                 -------------------------------------------------------------------------------- 0
ParamormyropsKingsLeyae    -------------------------------------------------------------------------------- 0
```

```
Majority                  --------------------------------------------------------------------------------

                                   14410     14420     14430     14440     14450     14460     14470     14480

Human                     ACCTACTTCCAAAATTATTTTGAATGTTAGATGAGATGAGGAAAAGACTTATGCTGAAAGGATGAGAATGGTTGTTGTTG 14480
AsianBonytongue           -------------------------------------------------------------------------------- 0
Reedfish                  -------------------------------------------------------------------------------- 0
NorthernPike              -------------------------------------------------------------------------------- 0
SpottedGar                -------------------------------------------------------------------------------- 0
ParamormyropsKingsLeyae   -------------------------------------------------------------------------------- 0

Majority                  --------------------------------------------------------------------------------

                                   14490     14500     14510     14520     14530     14540     14550     14560

Human                     TAGTTCTTTAATCTGTAAATCTACATTTGGAAGGAACACTTTTATGGAAATCAAATACAGTAAATTAATTTCTAAGAGGT 14560
AsianBonytongue           -------------------------------------------------------------------------------- 0
Reedfish                  -------------------------------------------------------------------------------- 0
NorthernPike              -------------------------------------------------------------------------------- 0
SpottedGar                -------------------------------------------------------------------------------- 0
ParamormyropsKingsLeyae   -------------------------------------------------------------------------------- 0

Majority                  --------------------------------------------------------------------------------

                                   14570     14580     14590     14600     14610     14620     14630     14640

Human                     CCTGGAGTTCAGGTTAAAAATAGCCTCAGACAGCACTAAATATCTTTATTAGTGAACAAAAAAATAAAAATAACTTAGAA 14640
AsianBonytongue           -------------------------------------------------------------------------------- 0
Reedfish                  -------------------------------------------------------------------------------- 0
NorthernPike              -------------------------------------------------------------------------------- 0
SpottedGar                -------------------------------------------------------------------------------- 0
ParamormyropsKingsLeyae   -------------------------------------------------------------------------------- 0

Majority                  --------------------------------------------------------------------------------

                                   14650     14660     14670     14680     14690     14700     14710     14720

Human                     TTTAGCACAGAGAAGCAAAGAGATGAAAATAGGAGAAGCGTTAAAAATGTGTTGGAAAGGAAGAGAAGTTCTAACAAACA 14720
AsianBonytongue           -------------------------------------------------------------------------------- 0
Reedfish                  -------------------------------------------------------------------------------- 0
NorthernPike              -------------------------------------------------------------------------------- 0
SpottedGar                -------------------------------------------------------------------------------- 0
ParamormyropsKingsLeyae   -------------------------------------------------------------------------------- 0

Majority                  --------------------------------------------------------------------------------

                                   14730     14740     14750     14760     14770     14780     14790     14800

Human                     TCTAATTAGCGTTCCAGAAATTAAAGATTGCAGAAAGGCAAAATTTGAAATGCTAAAGGCTGATAATTTTTCAGAATTGG 14800
AsianBonytongue           -------------------------------------------------------------------------------- 0
Reedfish                  -------------------------------------------------------------------------------- 0
NorthernPike              -------------------------------------------------------------------------------- 0
SpottedGar                -------------------------------------------------------------------------------- 0
ParamormyropsKingsLeyae   -------------------------------------------------------------------------------- 0

Majority                  --------------------------------------------------------------------------------

                                   14810     14820     14830     14840     14850     14860     14870     14880

Human                     TGAGCTATATGAGTTCTTAGATTTAGGAGGCACAAGAAAACCTGGACACATAACAGTAAAAGAGCAGAGCACCAAAGACA 14880
AsianBonytongue           -------------------------------------------------------------------------------- 0
Reedfish                  -------------------------------------------------------------------------------- 0
NorthernPike              -------------------------------------------------------------------------------- 0
SpottedGar                -------------------------------------------------------------------------------- 0
ParamormyropsKingsLeyae   -------------------------------------------------------------------------------- 0
```

```
Majority                  --------------------------------------------------------------------------------

                                14890     14900     14910     14920     14930     14940     14950     14960

Human                     TAGAGATCTTTAAAGCAGCCAGAGAAAATCAGATTCCCTAAAGAATAAGAACTAGAAAGTCCTAATATGCTTCATCAGCA 14960
AsianBonytongue           -------------------------------------------------------------------------------- 0
Reedfish                  -------------------------------------------------------------------------------- 0
NorthernPike              -------------------------------------------------------------------------------- 0
SpottedGar                -------------------------------------------------------------------------------- 0
ParamormyropsKingsLeyae   -------------------------------------------------------------------------------- 0

Majority                  --------------------------------------------------------------------------------

                                14970     14980     14990     15000     15010     15020     15030     15040

Human                     TGGGCATCCAGAAGATAGTGGAATAATATTTTCAATGTGTTGAGTGAAAATAGCTGCCCACCTGTTATTTTATAGCCAGC 15040
AsianBonytongue           -------------------------------------------------------------------------------- 0
Reedfish                  -------------------------------------------------------------------------------- 0
NorthernPike              -------------------------------------------------------------------------------- 0
SpottedGar                -------------------------------------------------------------------------------- 0
ParamormyropsKingsLeyae   -------------------------------------------------------------------------------- 0

Majority                  --------------------------------------------------------------------------------

                                15050     15060     15070     15080     15090     15100     15110     15120

Human                     CAAATTATCATATAAGGAGAAGAACAAAATAAAGACACTTTTTGAAACACGTCATTCTGCCTCCAAAAAAGCAATTTAGG 15120
AsianBonytongue           -------------------------------------------------------------------------------- 0
Reedfish                  -------------------------------------------------------------------------------- 0
NorthernPike              -------------------------------------------------------------------------------- 0
SpottedGar                -------------------------------------------------------------------------------- 0
ParamormyropsKingsLeyae   -------------------------------------------------------------------------------- 0

Majority                  --------------------------------------------------------------------------------

                                15130     15140     15150     15160     15170     15180     15190     15200

Human                     AAATGTATCAACAGACCCACTGAAGGAATTTCTGGAGTTTGTACTTCAGGAAGAAAGCCAATGACACCAGAAAGGAAGTC 15200
AsianBonytongue           -------------------------------------------------------------------------------- 0
Reedfish                  -------------------------------------------------------------------------------- 0
NorthernPike              -------------------------------------------------------------------------------- 0
SpottedGar                -------------------------------------------------------------------------------- 0
ParamormyropsKingsLeyae   -------------------------------------------------------------------------------- 0

Majority                  --------------------------------------------------------------------------------

                                15210     15220     15230     15240     15250     15260     15270     15280

Human                     TGATATAGAAGTGAGGCTAATCAAGAAATTGGTAGGTCTGTGAGTAAATCTAATTACTGACCATATAAGAATGTAATAGC 15280
AsianBonytongue           -------------------------------------------------------------------------------- 0
Reedfish                  -------------------------------------------------------------------------------- 0
NorthernPike              -------------------------------------------------------------------------------- 0
SpottedGar                -------------------------------------------------------------------------------- 0
ParamormyropsKingsLeyae   -------------------------------------------------------------------------------- 0

Majority                  --------------------------------------------------------------------------------

                                15290     15300     15310     15320     15330     15340     15350     15360

Human                     AGTGATTGATAAGGGGTTTAAAAATAGAATTAAAAATATTAAATCATAACAATATGTGAGAGAGTGGTAGAGACACTGGA 15360
AsianBonytongue           -------------------------------------------------------------------------------- 0
Reedfish                  -------------------------------------------------------------------------------- 0
NorthernPike              -------------------------------------------------------------------------------- 0
SpottedGar                -------------------------------------------------------------------------------- 0
ParamormyropsKingsLeyae   -------------------------------------------------------------------------------- 0
```

```
Majority                    --------------------------------------------------------------------------------
                                     15370     15380     15390     15400     15410     15420     15430     15440
Human                       GTTTGAGTTCTGATGATCTAGTATTGATTGGAGCTGTGTTGTCAGTTACAGTAGCTACTAGTCATTTGTTACTGAGCACT 15440
AsianBonytongue             -------------------------------------------------------------------------------- 0
Reedfish                    -------------------------------------------------------------------------------- 0
NorthernPike                -------------------------------------------------------------------------------- 0
SpottedGar                  -------------------------------------------------------------------------------- 0
ParamormyropsKingsLeyae     -------------------------------------------------------------------------------- 0
```

```
Majority                    --------------------------------------------------------------------------------
                                     15450     15460     15470     15480     15490     15500     15510     15520
Human                       TGACATGTATGTAGTTTAAGTTGATCTTCTACTTAATTGAAAAATACACATTTCTGAGCCTTAGTCTGAAAGAAGAATGT 15520
AsianBonytongue             -------------------------------------------------------------------------------- 0
Reedfish                    -------------------------------------------------------------------------------- 0
NorthernPike                -------------------------------------------------------------------------------- 0
SpottedGar                  -------------------------------------------------------------------------------- 0
ParamormyropsKingsLeyae     -------------------------------------------------------------------------------- 0
```

```
Majority                    --------------------------------------------------------------------------------
                                     15530     15540     15550     15560     15570     15580     15590     15600
Human                       AAAATATCCCAAGTTTTCATATTGGTTACATGTACAGCGGATAATATTTTGGTATTTTGGGTTAAATGAAATACATTATG 15600
AsianBonytongue             -------------------------------------------------------------------------------- 0
Reedfish                    -------------------------------------------------------------------------------- 0
NorthernPike                -------------------------------------------------------------------------------- 0
SpottedGar                  -------------------------------------------------------------------------------- 0
ParamormyropsKingsLeyae     -------------------------------------------------------------------------------- 0
```

```
Majority                    --------------------------------------------------------------------------------
                                     15610     15620     15630     15640     15650     15660     15670     15680
Human                       TGTGTTGCCTTTTCCTCTTTTTTCCTTTCCTTTTTTTAAGATAGGGTCTTGCTCTGTCCACCAGGCTGGAGTGCCGTGGC 15680
AsianBonytongue             -------------------------------------------------------------------------------- 0
Reedfish                    -------------------------------------------------------------------------------- 0
NorthernPike                -------------------------------------------------------------------------------- 0
SpottedGar                  -------------------------------------------------------------------------------- 0
ParamormyropsKingsLeyae     -------------------------------------------------------------------------------- 0
```

```
Majority                    --------------------------------------------------------------------------------
                                     15690     15700     15710     15720     15730     15740     15750     15760
Human                       ATGATCATGGCAGCCTCAACCTCCTGGGCTCCAGTGACCCTCCCACCTCAGCCTCCCAAGTAGCTGGGACTACAGTTGTG 15760
AsianBonytongue             -------------------------------------------------------------------------------- 0
Reedfish                    -------------------------------------------------------------------------------- 0
NorthernPike                -------------------------------------------------------------------------------- 0
SpottedGar                  -------------------------------------------------------------------------------- 0
ParamormyropsKingsLeyae     -------------------------------------------------------------------------------- 0
```

```
Majority                    --------------------------------------------------------------------------------
                                     15770     15780     15790     15800     15810     15820     15830     15840
Human                       TGCCACCACACCTGGCTAATTTTTGAGTTTTTTTTTGTAGAGACAGCGTCTCGCTGTATCGCTTAGGCTGGTCTCAAACT 15840
AsianBonytongue             -------------------------------------------------------------------------------- 0
Reedfish                    -------------------------------------------------------------------------------- 0
NorthernPike                -------------------------------------------------------------------------------- 0
SpottedGar                  -------------------------------------------------------------------------------- 0
ParamormyropsKingsLeyae     -------------------------------------------------------------------------------- 0
```

```
Majority                 --------------------------------------------------------------------------------

                                  15850     15860     15870     15880     15890     15900     15910     15920

Human                    CCTGGGCTCAAGTGATCCTCCTGCTTTAGCCTTGCAAAGTGCTGGGATTACAGGACTGAGTCTGGCCACCTTTTTCTTTC 15920
AsianBonytongue          -------------------------------------------------------------------------------- 0
Reedfish                 -------------------------------------------------------------------------------- 0
NorthernPike             -------------------------------------------------------------------------------- 0
SpottedGar               -------------------------------------------------------------------------------- 0
ParamormyropsKingsLeyae  -------------------------------------------------------------------------------- 0

Majority                 --------------------------------------------------------------------------------

                                  15930     15940     15950     15960     15970     15980     15990     16000

Human                    TTTCTTTTTTTTTTAGACGGAGTCTCCCTTTGTTGCCCAGGCTGGAGTACAGTGGCTCTATCTCTGCTTCCTGCAAGCTC 16000
AsianBonytongue          -------------------------------------------------------------------------------- 0
Reedfish                 -------------------------------------------------------------------------------- 0
NorthernPike             -------------------------------------------------------------------------------- 0
SpottedGar               -------------------------------------------------------------------------------- 0
ParamormyropsKingsLeyae  -------------------------------------------------------------------------------- 0

Majority                 --------------------------------------------------------------------------------

                                  16010     16020     16030     16040     16050     16060     16070     16080

Human                    TGCCTCCTGGGTTCACGCCATTCTCCTGCCTCAGCCTCCCGAGTAGCTGGGACTACAGGCGCCTGCCACCACACCCGGCT 16080
AsianBonytongue          -------------------------------------------------------------------------------- 0
Reedfish                 -------------------------------------------------------------------------------- 0
NorthernPike             -------------------------------------------------------------------------------- 0
SpottedGar               -------------------------------------------------------------------------------- 0
ParamormyropsKingsLeyae  -------------------------------------------------------------------------------- 0

Majority                 --------------------------------------------------------------------------------

                                  16090     16100     16110     16120     16130     16140     16150     16160

Human                    AATTTTTTGTATTTTTAGTAGAGATGGGTTTTACCATGGTCTCGATCTCCTGACCTCCTGATCTGCCCACCCTGGCCTCC 16160
AsianBonytongue          -------------------------------------------------------------------------------- 0
Reedfish                 -------------------------------------------------------------------------------- 0
NorthernPike             -------------------------------------------------------------------------------- 0
SpottedGar               -------------------------------------------------------------------------------- 0
ParamormyropsKingsLeyae  -------------------------------------------------------------------------------- 0

Majority                 --------------------------------------------------------------------------------

                                  16170     16180     16190     16200     16210     16220     16230     16240

Human                    CAAAGTGCTGGGATTACAGGCGTGAGCCACTGCGACCGGCCCACTTTTTCTTTTTACTTTTAAAAATGTGGCTAATAGAA 16240
AsianBonytongue          -------------------------------------------------------------------------------- 0
Reedfish                 -------------------------------------------------------------------------------- 0
NorthernPike             -------------------------------------------------------------------------------- 0
SpottedGar               -------------------------------------------------------------------------------- 0
ParamormyropsKingsLeyae  -------------------------------------------------------------------------------- 0

Majority                 --------------------------------------------------------------------------------

                                  16250     16260     16270     16280     16290     16300     16310     16320

Human                    ATTTATGAGATTATATTTATGGTTCATACTACGTTTCTTTTGGACAGTGCCAGAGTGAATCAGATAAGCTTGCATTTTAA 16320
AsianBonytongue          -------------------------------------------------------------------------------- 0
Reedfish                 -------------------------------------------------------------------------------- 0
NorthernPike             -------------------------------------------------------------------------------- 0
SpottedGar               -------------------------------------------------------------------------------- 0
ParamormyropsKingsLeyae  -------------------------------------------------------------------------------- 0
```

```
Majority                    --------------------------------------------------------------------------------

                                     16330     16340     16350     16360     16370     16380     16390     16400

Human                       AATCCTAAGGGTAAATGCAATAGAGATAGAACGCAAATAATTGGGGAGGGGGGTTGACTGAAATTAAAGATGTATAATCC 16400
AsianBonytongue             -------------------------------------------------------------------------------- 0
Reedfish                    -------------------------------------------------------------------------------- 0
NorthernPike                -------------------------------------------------------------------------------- 0
SpottedGar                  -------------------------------------------------------------------------------- 0
ParamormyropsKingsLeyae     -------------------------------------------------------------------------------- 0

Majority                    --------------------------------------------------------------------------------

                                     16410     16420     16430     16440     16450     16460     16470     16480

Human                       AAAAGAAGGCAAAAAAAAAGGAAAGACACAAAGTGAGCTTATATGTTAATAGCGTGGAAAGGTTCTATGAAGGGTTGTTT 16480
AsianBonytongue             -------------------------------------------------------------------------------- 0
Reedfish                    -------------------------------------------------------------------------------- 0
NorthernPike                -------------------------------------------------------------------------------- 0
SpottedGar                  -------------------------------------------------------------------------------- 0
ParamormyropsKingsLeyae     -------------------------------------------------------------------------------- 0

Majority                    --------------------------------------------------------------------------------

                                     16490     16500     16510     16520     16530     16540     16550     16560

Human                       AGAGCCTTTAAGGGCAAAATTTTCTTGTCTGGTTGCAGGAAAGAGTGGCATTGTTTTTGTTCACTTTTAAAGCTACAAAA 16560
AsianBonytongue             -------------------------------------------------------------------------------- 0
Reedfish                    -------------------------------------------------------------------------------- 0
NorthernPike                -------------------------------------------------------------------------------- 0
SpottedGar                  -------------------------------------------------------------------------------- 0
ParamormyropsKingsLeyae     -------------------------------------------------------------------------------- 0

Majority                    --------------------------------------------------------------------------------

                                     16570     16580     16590     16600     16610     16620     16630     16640

Human                       ACAAGTAATGATGAGGAAAATTGGGACATGTTGAGGAAGTAAGTAATTTTTTGGAATGTAGGTTGAGTTAGAGTAGTAGA 16640
AsianBonytongue             -------------------------------------------------------------------------------- 0
Reedfish                    -------------------------------------------------------------------------------- 0
NorthernPike                -------------------------------------------------------------------------------- 0
SpottedGar                  -------------------------------------------------------------------------------- 0
ParamormyropsKingsLeyae     -------------------------------------------------------------------------------- 0

Majority                    --------------------------------------------------------------------------------

                                     16650     16660     16670     16680     16690     16700     16710     16720

Human                       AGACAAAATTTAAAAAGTAGTTTTTGACAGTAATATGGAGAGCTTTGAGTGCAAAGGATTTGCAGTGAGGACCTTTGAAA 16720
AsianBonytongue             -------------------------------------------------------------------------------- 0
Reedfish                    -------------------------------------------------------------------------------- 0
NorthernPike                -------------------------------------------------------------------------------- 0
SpottedGar                  -------------------------------------------------------------------------------- 0
ParamormyropsKingsLeyae     -------------------------------------------------------------------------------- 0

Majority                    --------------------------------------------------------------------------------

                                     16730     16740     16750     16760     16770     16780     16790     16800

Human                       ATTGAACCTGCTTTCTCCTATCAGTATTTATGTTTAAATAAACTTGATTCTTTGTGAGTATTGTTATATATGTTTTCATA 16800
AsianBonytongue             -------------------------------------------------------------------------------- 0
Reedfish                    -------------------------------------------------------------------------------- 0
NorthernPike                -------------------------------------------------------------------------------- 0
SpottedGar                  -------------------------------------------------------------------------------- 0
ParamormyropsKingsLeyae     -------------------------------------------------------------------------------- 0
```

```
Majority                    --------------------------------------------------------------------------------
                                     16810     16820     16830     16840     16850     16860     16870     16880
Human                       ACTACTGTTGTAGTAACGTTTCTTTTTTTTTACTAGCTTGCTTTCATGCCACGCTGTTGATTAAATATTTATGGGCCATT 16880
AsianBonytongue             -------------------------------------------------------------------------------- 0
Reedfish                    -------------------------------------------------------------------------------- 0
NorthernPike                -------------------------------------------------------------------------------- 0
SpottedGar                  -------------------------------------------------------------------------------- 0
ParamormyropsKingsLeyae     -------------------------------------------------------------------------------- 0

Majority                    --------------------------------------------------------------------------------
                                     16890     16900     16910     16920     16930     16940     16950     16960
Human                       TTAAGGCTTCTAATCTTCATTTGTGATGAACTTTTGAGGAAGAGTTAGGTTTTGTAAGTGTTGAACTCCAATAAACACAG 16960
AsianBonytongue             -------------------------------------------------------------------------------- 0
Reedfish                    -------------------------------------------------------------------------------- 0
NorthernPike                -------------------------------------------------------------------------------- 0
SpottedGar                  -------------------------------------------------------------------------------- 0
ParamormyropsKingsLeyae     -------------------------------------------------------------------------------- 0

Majority                    --------------------------------------------------------------------------------
                                     16970     16980     16990     17000     17010     17020     17030     17040
Human                       TTCCACTTTCCTTAACTTCTCAGAAAGATACTTTGCTATTTGATACAGATAGTTGTCTAGGCAAGCTATTAGCAGGGTTT 17040
AsianBonytongue             -------------------------------------------------------------------------------- 0
Reedfish                    -------------------------------------------------------------------------------- 0
NorthernPike                -------------------------------------------------------------------------------- 0
SpottedGar                  -------------------------------------------------------------------------------- 0
ParamormyropsKingsLeyae     -------------------------------------------------------------------------------- 0

Majority                    --------------------------------------------------------------------------------
                                     17050     17060     17070     17080     17090     17100     17110     17120
Human                       CAGGTAAGACATATGTGTTTGCTATTTGTTTTAAGCACTCTTCCCTCATTTATTCAAATTGTAGCAAACATCTACTACAC 17120
AsianBonytongue             -------------------------------------------------------------------------------- 0
Reedfish                    -------------------------------------------------------------------------------- 0
NorthernPike                -------------------------------------------------------------------------------- 0
SpottedGar                  -------------------------------------------------------------------------------- 0
ParamormyropsKingsLeyae     -------------------------------------------------------------------------------- 0

Majority                    --------------------------------------------------------------------------------
                                     17130     17140     17150     17160     17170     17180     17190     17200
Human                       CCATTTCCAGTTCTTTGTCTTAATAGTTCAACTATTATAAAAAGGAGTGGTATATATATATATAAACTCTTGCTTGGCTT 17200
AsianBonytongue             -------------------------------------------------------------------------------- 0
Reedfish                    -------------------------------------------------------------------------------- 0
NorthernPike                -------------------------------------------------------------------------------- 0
SpottedGar                  -------------------------------------------------------------------------------- 0
ParamormyropsKingsLeyae     -------------------------------------------------------------------------------- 0

Majority                    --------------------------------------------------------------------------------
                                     17210     17220     17230     17240     17250     17260     17270     17280
Human                       TGGTTTGATTGAATTAATAAGGTGAGTTTTTTTTGTTTGTTTGTTTTGAGATGGAGTCTCGCTCTGTTGCCCAGGCTGGA 17280
AsianBonytongue             -------------------------------------------------------------------------------- 0
Reedfish                    -------------------------------------------------------------------------------- 0
NorthernPike                -------------------------------------------------------------------------------- 0
SpottedGar                  -------------------------------------------------------------------------------- 0
ParamormyropsKingsLeyae     -------------------------------------------------------------------------------- 0
```

```
Majority                   --------------------------------------------------------------------------------

                                    17290     17300     17310     17320     17330     17340     17350     17360

Human                      GTGCAGTGGTGGGATCTCGGCTCACTGCAGGCCATTCTCCTGCCTCAGCCTCCCGAGTAGCTGGGACTGCAGGTGCCTGC 17360
AsianBonytongue            -------------------------------------------------------------------------------- 0
Reedfish                   -------------------------------------------------------------------------------- 0
NorthernPike               -------------------------------------------------------------------------------- 0
SpottedGar                 -------------------------------------------------------------------------------- 0
ParamormyropsKingsLeyae    -------------------------------------------------------------------------------- 0

Majority                   --------------------------------------------------------------------------------

                                    17370     17380     17390     17400     17410     17420     17430     17440

Human                      CACCACACCTGGCTAATTTTTTGTATTTTTAGTAGAGATGGGGTTTCACCGTGTTAGCCAGGATGGTCTAGATCTCCTGA 17440
AsianBonytongue            -------------------------------------------------------------------------------- 0
Reedfish                   -------------------------------------------------------------------------------- 0
NorthernPike               -------------------------------------------------------------------------------- 0
SpottedGar                 -------------------------------------------------------------------------------- 0
ParamormyropsKingsLeyae    -------------------------------------------------------------------------------- 0

Majority                   --------------------------------------------------------------------------------

                                    17450     17460     17470     17480     17490     17500     17510     17520

Human                      CCTTGTGATCCGCCCGCCTCGGAAATACTTTGCATTGTTGGATTGATTGGGTTTGAGCCCCTGTTGTACTTAAGTGATTA 17520
AsianBonytongue            -------------------------------------------------------------------------------- 0
Reedfish                   -------------------------------------------------------------------------------- 0
NorthernPike               -------------------------------------------------------------------------------- 0
SpottedGar                 -------------------------------------------------------------------------------- 0
ParamormyropsKingsLeyae    -------------------------------------------------------------------------------- 0

Majority                   --------------------------------------------------------------------------------

                                    17530     17540     17550     17560     17570     17580     17590     17600

Human                      TGAACAAGTCTTTTATAATCTGAATTTCCCTTTCCTAGTTTGTAATATTGAGGATAATAATTTCTAACTTGCATGATTGT 17600
AsianBonytongue            -------------------------------------------------------------------------------- 0
Reedfish                   -------------------------------------------------------------------------------- 0
NorthernPike               -------------------------------------------------------------------------------- 0
SpottedGar                 -------------------------------------------------------------------------------- 0
ParamormyropsKingsLeyae    -------------------------------------------------------------------------------- 0

Majority                   --------------------------------------------------------------------------------

                                    17610     17620     17630     17640     17650     17660     17670     17680

Human                      TGTGAAGATTAAATAAGACAATGATATTGGTAGGAGCTCTTTATAATCTTTCTCTCTTATGTACAATTCAAATATATGTC 17680
AsianBonytongue            -------------------------------------------------------------------------------- 0
Reedfish                   -------------------------------------------------------------------------------- 0
NorthernPike               -------------------------------------------------------------------------------- 0
SpottedGar                 -------------------------------------------------------------------------------- 0
ParamormyropsKingsLeyae    -------------------------------------------------------------------------------- 0

Majority                   --------------------------------------------------------------------------------

                                    17690     17700     17710     17720     17730     17740     17750     17760

Human                      GGTTTAAATACCAAAATACCACTTTAAAAAATCTGGGCTGAAATGGACACTTCTAATGTAGGAGTAGTGCTTTAGAAAAT 17760
AsianBonytongue            -------------------------------------------------------------------------------- 0
Reedfish                   -------------------------------------------------------------------------------- 0
NorthernPike               -------------------------------------------------------------------------------- 0
SpottedGar                 -------------------------------------------------------------------------------- 0
ParamormyropsKingsLeyae    -------------------------------------------------------------------------------- 0
```

```
Majority               --------------------------------------------------------------------------------
                               17770     17780     17790     17800     17810     17820     17830     17840
Human                  AATGCTTTGTTTCGTATTCTTTTCCTAGTATTTTATGTCTAGTAACTCATAAGGAAAAAATGATCAGATAAGTATATTTT 17840
AsianBonytongue        -------------------------------------------------------------------------------- 0
Reedfish               -------------------------------------------------------------------------------- 0
NorthernPike           -------------------------------------------------------------------------------- 0
SpottedGar             -------------------------------------------------------------------------------- 0
ParamormyropsKingsLeyae-------------------------------------------------------------------------------- 0

Majority               --------------------------------------------------------------------------------
                               17850     17860     17870     17880     17890     17900     17910     17920
Human                  TTAGATGCTGATTACAGCATTATTTAAATAGTAGAAATAACAACTGTTCAGTGATAAGACAATGAATAAATTCTGTTCAT 17920
AsianBonytongue        -------------------------------------------------------------------------------- 0
Reedfish               -------------------------------------------------------------------------------- 0
NorthernPike           -------------------------------------------------------------------------------- 0
SpottedGar             -------------------------------------------------------------------------------- 0
ParamormyropsKingsLeyae-------------------------------------------------------------------------------- 0

Majority               --------------------------------------------------------------------------------
                               17930     17940     17950     17960     17970     17980     17990     18000
Human                  AAACATTACAAATGTTTTTTAAATATTTTTCATATGTGTGAAAAGTAGGATTAAAAATAACTTTGACAAATGCATACAAT 18000
AsianBonytongue        -------------------------------------------------------------------------------- 0
Reedfish               -------------------------------------------------------------------------------- 0
NorthernPike           -------------------------------------------------------------------------------- 0
SpottedGar             -------------------------------------------------------------------------------- 0
ParamormyropsKingsLeyae-------------------------------------------------------------------------------- 0

Majority               --------------------------------------------------------------------------------
                               18010     18020     18030     18040     18050     18060     18070     18080
Human                  TTATATGTGTCCCCAAAAGCTGGAGGGCAGTGTACCAAAATAGAGTAGTTTTCTCCAGGTTGATGGGATTAGTTTTTTGT 18080
AsianBonytongue        -------------------------------------------------------------------------------- 0
Reedfish               -------------------------------------------------------------------------------- 0
NorthernPike           -------------------------------------------------------------------------------- 0
SpottedGar             -------------------------------------------------------------------------------- 0
ParamormyropsKingsLeyae-------------------------------------------------------------------------------- 0

Majority               --------------------------------------------------------------------------------
                               18090     18100     18110     18120     18130     18140     18150     18160
Human                  AATTCTTTATATATTTTCTCTATATTCCACATTTTAAGTATTTTATGATAAAGTTATTAATATTGATAAAGTTATTAATG 18160
AsianBonytongue        -------------------------------------------------------------------------------- 0
Reedfish               -------------------------------------------------------------------------------- 0
NorthernPike           -------------------------------------------------------------------------------- 0
SpottedGar             -------------------------------------------------------------------------------- 0
ParamormyropsKingsLeyae-------------------------------------------------------------------------------- 0

Majority               --------------------------------------------------------------------------------
                               18170     18180     18190     18200     18210     18220     18230     18240
Human                  TTGCTTAATTGATTAAACCCTGATATTTTCTTGTTTCTTTAAAAAACGTTGCATAACTTGATATAAATCTTTTTAATTGT 18240
AsianBonytongue        -------------------------------------------------------------------------------- 0
Reedfish               -------------------------------------------------------------------------------- 0
NorthernPike           -------------------------------------------------------------------------------- 0
SpottedGar             -------------------------------------------------------------------------------- 0
ParamormyropsKingsLeyae-------------------------------------------------------------------------------- 0
```

```
Majority                   --------------------------------------------------------------------------------

                                    18250     18260     18270     18280     18290     18300     18310     18320

Human                      ACAAAAAAACACATAAAATTTACTATCTTAACCATTTTTAAGTGTACACTTAAATTGTTTAATTATTCTTATACGTACTT 18320
AsianBonytongue            -------------------------------------------------------------------------------- 0
Reedfish                   -------------------------------------------------------------------------------- 0
NorthernPike               -------------------------------------------------------------------------------- 0
SpottedGar                 -------------------------------------------------------------------------------- 0
ParamormyropsKingsLeyae-------------------------------------------------------------------------------- 0

Majority                   --------------------------------------------------------------------------------

                                    18330     18340     18350     18360     18370     18380     18390     18400

Human                      AATAATATTACATTGCAAAACCCATCTCCAGAACTTTTTGGAGAACTGAAACTCTATACTGATTTTAAAACTCCCTCTTT 18400
AsianBonytongue            -------------------------------------------------------------------------------- 0
Reedfish                   -------------------------------------------------------------------------------- 0
NorthernPike               -------------------------------------------------------------------------------- 0
SpottedGar                 -------------------------------------------------------------------------------- 0
ParamormyropsKingsLeyae-------------------------------------------------------------------------------- 0

Majority                   --------------------------------------------------------------------------------

                                    18410     18420     18430     18440     18450     18460     18470     18480

Human                      TGTCCTTCTGCTGGTAACCAGCATTCTATTTTCTGTGTCTATGAATTTGACTACTTTAAATACCTCACATAAGTGGAATT 18480
AsianBonytongue            -------------------------------------------------------------------------------- 0
Reedfish                   -------------------------------------------------------------------------------- 0
NorthernPike               -------------------------------------------------------------------------------- 0
SpottedGar                 -------------------------------------------------------------------------------- 0
ParamormyropsKingsLeyae-------------------------------------------------------------------------------- 0

Majority                   --------------------------------------------------------------------------------

                                    18490     18500     18510     18520     18530     18540     18550     18560

Human                      ATACAGTATAGGTTAAGCATCCCTAATCTGAAAATCCAAAGTCTGAATGCTACAAAATCTGAAACTGGTGTGTGTTTTTT 18560
AsianBonytongue            -------------------------------------------------------------------------------- 0
Reedfish                   -------------------------------------------------------------------------------- 0
NorthernPike               -------------------------------------------------------------------------------- 0
SpottedGar                 -------------------------------------------------------------------------------- 0
ParamormyropsKingsLeyae-------------------------------------------------------------------------------- 0

Majority                   --------------------------------------------------------------------------------

                                    18570     18580     18590     18600     18610     18620     18630     18640

Human                      TTTTTTTTTTTTTTTTTTTTGAGACGGAGTCTTACTCTGTCGCCCAGGCTGGAGTGCAGTGGCGCGATCTCTGCTCACTG 18640
AsianBonytongue            -------------------------------------------------------------------------------- 0
Reedfish                   -------------------------------------------------------------------------------- 0
NorthernPike               -------------------------------------------------------------------------------- 0
SpottedGar                 -------------------------------------------------------------------------------- 0
ParamormyropsKingsLeyae-------------------------------------------------------------------------------- 0

Majority                   --------------------------------------------------------------------------------

                                    18650     18660     18670     18680     18690     18700     18710     18720

Human                      CAAGCTCCACCACCCGGGTTCACGCCATCTTCCTGAGTAGCTGGGACTACAGGCGCCCGCCACCACGCCCGGCTAATTTT 18720
AsianBonytongue            -------------------------------------------------------------------------------- 0
Reedfish                   -------------------------------------------------------------------------------- 0
NorthernPike               -------------------------------------------------------------------------------- 0
SpottedGar                 -------------------------------------------------------------------------------- 0
ParamormyropsKingsLeyae-------------------------------------------------------------------------------- 0
```

```
Majority                    --------------------------------------------------------------------------------

                                     18730     18740     18750     18760     18770     18780     18790     18800
Human                       TTGTATATATATATATATTTTTTAGTAGAGACGGGGTTTCACTGTAGCCAGGATGGACTCTTATCTCCCGACCTCGTGAT 18800
AsianBonytongue             -------------------------------------------------------------------------------- 0
Reedfish                    -------------------------------------------------------------------------------- 0
NorthernPike                -------------------------------------------------------------------------------- 0
SpottedGar                  -------------------------------------------------------------------------------- 0
ParamormyropsKingsLeyae     -------------------------------------------------------------------------------- 0

Majority                    --------------------------------------------------------------------------------

                                     18810     18820     18830     18840     18850     18860     18870     18880
Human                       CCGCCTGCCTCGGCCTCCCAAAGTGCTGGGATTACAGGCCTGAGCCACCGCGCCTGGCCAATCTGAAACTTTTTGAGCAC 18880
AsianBonytongue             -------------------------------------------------------------------------------- 0
Reedfish                    -------------------------------------------------------------------------------- 0
NorthernPike                -------------------------------------------------------------------------------- 0
SpottedGar                  -------------------------------------------------------------------------------- 0
ParamormyropsKingsLeyae     -------------------------------------------------------------------------------- 0

Majority                    --------------------------------------------------------------------------------

                                     18890     18900     18910     18920     18930     18940     18950     18960
Human                       TTACTTGATGCTCAAAGGAAGTGCTTAGTGGAGCATTTTGGATTTTAGATTTTCAGATTAGGAATGCTCAGGTTGTGTTT 18960
AsianBonytongue             -------------------------------------------------------------------------------- 0
Reedfish                    -------------------------------------------------------------------------------- 0
NorthernPike                -------------------------------------------------------------------------------- 0
SpottedGar                  -------------------------------------------------------------------------------- 0
ParamormyropsKingsLeyae     -------------------------------------------------------------------------------- 0

Majority                    --------------------------------------------------------------------------------

                                     18970     18980     18990     19000     19010     19020     19030     19040
Human                       GTCCTTTTGTGACTGGCTTATATTCACTTAATATAATGTCCTCAGGGTCCATCCATTTTGTAGCATGTGACAAGATTTCC 19040
AsianBonytongue             -------------------------------------------------------------------------------- 0
Reedfish                    -------------------------------------------------------------------------------- 0
NorthernPike                -------------------------------------------------------------------------------- 0
SpottedGar                  -------------------------------------------------------------------------------- 0
ParamormyropsKingsLeyae     -------------------------------------------------------------------------------- 0

Majority                    --------------------------------------------------------------------------------

                                     19050     19060     19070     19080     19090     19100     19110     19120
Human                       TCCCTTTTTAAGGCTACATAGCATTCCATTGTGGTACACAAAAGTTCATTTGAAGCCCCCTGTGTCATAACTCAGACTTC 19120
AsianBonytongue             -------------------------------------------------------------------------------- 0
Reedfish                    -------------------------------------------------------------------------------- 0
NorthernPike                -------------------------------------------------------------------------------- 0
SpottedGar                  -------------------------------------------------------------------------------- 0
ParamormyropsKingsLeyae     -------------------------------------------------------------------------------- 0

Majority                    --------------------------------------------------------------------------------

                                     19130     19140     19150     19160     19170     19180     19190     19200
Human                       CATAACAAAATACCATAGACTGAGTGACTTAAAAAACAGAGATTCGTTTTCTCACAGTTCTGGAAACTGGGAAGTCCAAG 19200
AsianBonytongue             -------------------------------------------------------------------------------- 0
Reedfish                    -------------------------------------------------------------------------------- 0
NorthernPike                -------------------------------------------------------------------------------- 0
SpottedGar                  -------------------------------------------------------------------------------- 0
ParamormyropsKingsLeyae     -------------------------------------------------------------------------------- 0
```

```
Majority                    --------------------------------------------------------------------------------
                                      19210     19220     19230     19240     19250     19260     19270     19280
Human                       ATCAAGGTACCAGCAAGGCAACTTTCCTTCTGAGGCCACTTCTCTTGAAGTGAGGCCACCATTTTACTTTGTGTGCCTCA 19280
AsianBonytongue             -------------------------------------------------------------------------------- 0
Reedfish                    -------------------------------------------------------------------------------- 0
NorthernPike                -------------------------------------------------------------------------------- 0
SpottedGar                  -------------------------------------------------------------------------------- 0
ParamormyropsKingsLeyae     -------------------------------------------------------------------------------- 0

Majority                    --------------------------------------------------------------------------------
                                      19290     19300     19310     19320     19330     19340     19350     19360
Human                       CATGACCTTTTCTTTGTGAGCACACTTTGGGGGAGTGGGTGGCAGAAGCTCTCTGGTATTTCTTCTCATAAAGGCATTAA 19360
AsianBonytongue             -------------------------------------------------------------------------------- 0
Reedfish                    -------------------------------------------------------------------------------- 0
NorthernPike                -------------------------------------------------------------------------------- 0
SpottedGar                  -------------------------------------------------------------------------------- 0
ParamormyropsKingsLeyae     -------------------------------------------------------------------------------- 0

Majority                    --------------------------------------------------------------------------------
                                      19370     19380     19390     19400     19410     19420     19430     19440
Human                       TCCCATCATGAAGGCCTTACTCTCATGATCTCATGAAATCCTCCAGTTAACTCCTAAAGGCCCTGTTTCAAAATTGCATC 19440
AsianBonytongue             -------------------------------------------------------------------------------- 0
Reedfish                    -------------------------------------------------------------------------------- 0
NorthernPike                -------------------------------------------------------------------------------- 0
SpottedGar                  -------------------------------------------------------------------------------- 0
ParamormyropsKingsLeyae     -------------------------------------------------------------------------------- 0

Majority                    --------------------------------------------------------------------------------
                                      19450     19460     19470     19480     19490     19500     19510     19520
Human                       ACATTTGGGGTTAGGACTTCAGCATGTGATTTTTGGGGAAACATAAGCATTGAGTTGATAATACCCTGCATATAGTTGAC 19520
AsianBonytongue             -------------------------------------------------------------------------------- 0
Reedfish                    -------------------------------------------------------------------------------- 0
NorthernPike                -------------------------------------------------------------------------------- 0
SpottedGar                  -------------------------------------------------------------------------------- 0
ParamormyropsKingsLeyae     -------------------------------------------------------------------------------- 0

Majority                    --------------------------------------------------------------------------------
                                      19530     19540     19550     19560     19570     19580     19590     19600
Human                       CCATACTTCTCCACTCCAGGTAATTACTATACTAAATTTGATGTTTACCATTCCCATAAATTTCTCATTTACTACATGTT 19600
AsianBonytongue             -------------------------------------------------------------------------------- 0
Reedfish                    -------------------------------------------------------------------------------- 0
NorthernPike                -------------------------------------------------------------------------------- 0
SpottedGar                  -------------------------------------------------------------------------------- 0
ParamormyropsKingsLeyae     -------------------------------------------------------------------------------- 0

Majority                    --------------------------------------------------------------------------------
                                      19610     19620     19630     19640     19650     19660     19670     19680
Human                       TATGTATTTTTATCTTAAGCAATATATGGTTTTGTTTTGCATGTTTAAACTATAGATGTACTATCATGCTGTATGTATTC 19680
AsianBonytongue             -------------------------------------------------------------------------------- 0
Reedfish                    -------------------------------------------------------------------------------- 0
NorthernPike                -------------------------------------------------------------------------------- 0
SpottedGar                  -------------------------------------------------------------------------------- 0
ParamormyropsKingsLeyae     -------------------------------------------------------------------------------- 0
```

```
Majority                   --------------------------------------------------------------------------------

                                    19690     19700     19710     19720     19730     19740     19750     19760

Human                      TGTAATTTGCTTTTCTTGTTCAGTGTTATTTTTGTTAAGTTTATATATATATATATTTTCATTGCCATATAGTATATGAT 19760
AsianBonytongue            -------------------------------------------------------------------------------- 0
Reedfish                   -------------------------------------------------------------------------------- 0
NorthernPike               -------------------------------------------------------------------------------- 0
SpottedGar                 -------------------------------------------------------------------------------- 0
ParamormyropsKingsLeyae    -------------------------------------------------------------------------------- 0

Majority                   --------------------------------------------------------------------------------

                                    19770     19780     19790     19800     19810     19820     19830     19840

Human                      ATATGAATATATCATCTTGACAAATGAGTTAATATTTGACGAGATTGATTTGTCAAATATTTTAAATTTTTTTCCATTAC 19840
AsianBonytongue            -------------------------------------------------------------------------------- 0
Reedfish                   -------------------------------------------------------------------------------- 0
NorthernPike               -------------------------------------------------------------------------------- 0
SpottedGar                 -------------------------------------------------------------------------------- 0
ParamormyropsKingsLeyae    -------------------------------------------------------------------------------- 0

Majority                   --------------------------------------------------------------------------------

                                    19850     19860     19870     19880     19890     19900     19910     19920

Human                      AAACTGCTACTGTAAGCATTAATATGCATATTCTAACGTGTTAATATTAGAGTTTCTCATAGATTGTGTCTTTTCAAATC 19920
AsianBonytongue            -------------------------------------------------------------------------------- 0
Reedfish                   -------------------------------------------------------------------------------- 0
NorthernPike               -------------------------------------------------------------------------------- 0
SpottedGar                 -------------------------------------------------------------------------------- 0
ParamormyropsKingsLeyae    -------------------------------------------------------------------------------- 0

Majority                   --------------------------------------------------------------------------------

                                    19930     19940     19950     19960     19970     19980     19990     20000

Human                      GATTGAATATTAACATATTGCTTTCATAGGTAATTATATTAATTATACTGTTAGCGCTGCATGTAGATTCTTGTTTCTAT 20000
AsianBonytongue            -------------------------------------------------------------------------------- 0
Reedfish                   -------------------------------------------------------------------------------- 0
NorthernPike               -------------------------------------------------------------------------------- 0
SpottedGar                 -------------------------------------------------------------------------------- 0
ParamormyropsKingsLeyae    -------------------------------------------------------------------------------- 0

Majority                   --------------------------------------------------------------------------------

                                    20010     20020     20030     20040     20050     20060     20070     20080

Human                      ACATTCTTTGAAAATCGGCATTGTTGGACTTTTTAATTTTGCTGATCAAATGTACATGGGAAGGATATCTTGTGGTTTTA 20080
AsianBonytongue            -------------------------------------------------------------------------------- 0
Reedfish                   -------------------------------------------------------------------------------- 0
NorthernPike               -------------------------------------------------------------------------------- 0
SpottedGar                 -------------------------------------------------------------------------------- 0
ParamormyropsKingsLeyae    -------------------------------------------------------------------------------- 0

Majority                   --------------------------------------------------------------------------------

                                    20090     20100     20110     20120     20130     20140     20150     20160

Human                      ATTTGCATTCCCTATTAATGCAAAAAAAAAAAAACATTGATTCCTGGGAGTTTTTATATACTGTGCAGACTAATTGTTTT 20160
AsianBonytongue            -------------------------------------------------------------------------------- 0
Reedfish                   -------------------------------------------------------------------------------- 0
NorthernPike               -------------------------------------------------------------------------------- 0
SpottedGar                 -------------------------------------------------------------------------------- 0
ParamormyropsKingsLeyae    -------------------------------------------------------------------------------- 0
```

```
Majority               --------------------------------------------------------------------------------
                                20170     20180     20190     20200     20210     20220     20230     20240
Human                  GTTATTTTTTTTGGTTTGTTTGTTTTGTTTTGTTTCATTTTTGTTTTTGTTTTGAGAGACAGAGTCTCACTGTGTCATCC 20240
AsianBonytongue        -------------------------------------------------------------------------------- 0
Reedfish               -------------------------------------------------------------------------------- 0
NorthernPike           -------------------------------------------------------------------------------- 0
SpottedGar             -------------------------------------------------------------------------------- 0
ParamormyropsKingsLeyae-------------------------------------------------------------------------------- 0

Majority               --------------------------------------------------------------------------------
                                20250     20260     20270     20280     20290     20300     20310     20320
Human                  AGGCTGGAGTGCAGTGGCTCGGTCACAGTCACTGCAGTCTTGACATCCCAGGCTCTAGCAATTCTCTCACCTTTGTTTCC 20320
AsianBonytongue        -------------------------------------------------------------------------------- 0
Reedfish               -------------------------------------------------------------------------------- 0
NorthernPike           -------------------------------------------------------------------------------- 0
SpottedGar             -------------------------------------------------------------------------------- 0
ParamormyropsKingsLeyae-------------------------------------------------------------------------------- 0

Majority               --------------------------------------------------------------------------------
                                20330     20340     20350     20360     20370     20380     20390     20400
Human                  TGAGTAGCTGGGACTACAGGAATACACCACTATGCCCAGCTAATTTTTCAATTTTTTTGTAGAGATGGAGTCTCACTGTA 20400
AsianBonytongue        -------------------------------------------------------------------------------- 0
Reedfish               -------------------------------------------------------------------------------- 0
NorthernPike           -------------------------------------------------------------------------------- 0
SpottedGar             -------------------------------------------------------------------------------- 0
ParamormyropsKingsLeyae-------------------------------------------------------------------------------- 0

Majority               --------------------------------------------------------------------------------
                                20410     20420     20430     20440     20450     20460     20470     20480
Human                  TTACCCAGGTTTGTTGCGAACTCCCAGGCTCAAGCAGTTCTCCCGCATTGGCCTCCTAAAGTGCTGAGATTACAGGCGTG 20480
AsianBonytongue        -------------------------------------------------------------------------------- 0
Reedfish               -------------------------------------------------------------------------------- 0
NorthernPike           -------------------------------------------------------------------------------- 0
SpottedGar             -------------------------------------------------------------------------------- 0
ParamormyropsKingsLeyae-------------------------------------------------------------------------------- 0

Majority               --------------------------------------------------------------------------------
                                20490     20500     20510     20520     20530     20540     20550     20560
Human                  AGTCGCTGTGCCTGGCCTGTTTTGTTAGTTACATGTCCTACAGATAACTCTCTCCTCTCTCTGACCTGTTTTTTTTACAC 20560
AsianBonytongue        -------------------------------------------------------------------------------- 0
Reedfish               -------------------------------------------------------------------------------- 0
NorthernPike           -------------------------------------------------------------------------------- 0
SpottedGar             -------------------------------------------------------------------------------- 0
ParamormyropsKingsLeyae-------------------------------------------------------------------------------- 0

Majority               --------------------------------------------------------------------------------
                                20570     20580     20590     20600     20610     20620     20630     20640
Human                  TGATGAATAGTTGTAAATTTTAATGGGACATTTATGTTTTTCTTTTAAAAAAACATTTAACCTAAGGCCTTACGTATAGG 20640
AsianBonytongue        -------------------------------------------------------------------------------- 0
Reedfish               -------------------------------------------------------------------------------- 0
NorthernPike           -------------------------------------------------------------------------------- 0
SpottedGar             -------------------------------------------------------------------------------- 0
ParamormyropsKingsLeyae-------------------------------------------------------------------------------- 0
```

```
Majority               --------------------------------------------------------------------------------

                                20650     20660     20670     20680     20690     20700     20710     20720

Human                  CTTGTGTATTTTATTTTGAAAGTTTTAAAATTTTGATTTTCACATTTATGGCGTTAATCTACCTGGAATTTATTCTTTGT 20720
AsianBonytongue        -------------------------------------------------------------------------------- 0
Reedfish               -------------------------------------------------------------------------------- 0
NorthernPike           -------------------------------------------------------------------------------- 0
SpottedGar             -------------------------------------------------------------------------------- 0
ParamormyropsKingsLeyae-------------------------------------------------------------------------------- 0

Majority               --------------------------------------------------------------------------------

                                20730     20740     20750     20760     20770     20780     20790     20800

Human                  GTAGCATATGTTACAGGTTTAATTATTTTTTCTATATGGATAGCAAGCTTAGTATGTACTTGATTGAATAGCTCAGCTTT 20800
AsianBonytongue        -------------------------------------------------------------------------------- 0
Reedfish               -------------------------------------------------------------------------------- 0
NorthernPike           -------------------------------------------------------------------------------- 0
SpottedGar             -------------------------------------------------------------------------------- 0
ParamormyropsKingsLeyae-------------------------------------------------------------------------------- 0

Majority               --------------------------------------------------------------------------------

                                20810     20820     20830     20840     20850     20860     20870     20880

Human                  TCTCCAATGATCTGCTTTCTCAGCTCTTCAGGTTTCCGTATATTCACAGAACCATTTCTGTGCTATGTCTTGTAGCAGTA 20880
AsianBonytongue        -------------------------------------------------------------------------------- 0
Reedfish               -------------------------------------------------------------------------------- 0
NorthernPike           -------------------------------------------------------------------------------- 0
SpottedGar             -------------------------------------------------------------------------------- 0
ParamormyropsKingsLeyae-------------------------------------------------------------------------------- 0

Majority               --------------------------------------------------------------------------------

                                20890     20900     20910     20920     20930     20940     20950     20960

Human                  CAACAGTGTCTTAATTACTTATGGCATCAGCATTAAGTTTTGGAATCTTGTACAGTGAGTGTCAAAACTTTGTTTTTCAA 20960
AsianBonytongue        -------------------------------------------------------------------------------- 0
Reedfish               -------------------------------------------------------------------------------- 0
NorthernPike           -------------------------------------------------------------------------------- 0
SpottedGar             -------------------------------------------------------------------------------- 0
ParamormyropsKingsLeyae-------------------------------------------------------------------------------- 0

Majority               --------------------------------------------------------------------------------

                                20970     20980     20990     21000     21010     21020     21030     21040

Human                  AATTGTCTTGGTGTTTTCTTCACTGCATTTATTTTAAGATCATTTGGCTAAGTTCTGTGAAGGCTCTTTGGGTCTTATTT 21040
AsianBonytongue        -------------------------------------------------------------------------------- 0
Reedfish               -------------------------------------------------------------------------------- 0
NorthernPike           -------------------------------------------------------------------------------- 0
SpottedGar             -------------------------------------------------------------------------------- 0
ParamormyropsKingsLeyae-------------------------------------------------------------------------------- 0

Majority               --------------------------------------------------------------------------------

                                21050     21060     21070     21080     21090     21100     21110     21120

Human                  ATACTTGCCTTAAATTTAGTAGTTAATTGGGAGAACATTATAGCTGCATTATCAAAATAAAATGGCAATTTTTTTTGAGC 21120
AsianBonytongue        -------------------------------------------------------------------------------- 0
Reedfish               -------------------------------------------------------------------------------- 0
NorthernPike           -------------------------------------------------------------------------------- 0
SpottedGar             -------------------------------------------------------------------------------- 0
ParamormyropsKingsLeyae-------------------------------------------------------------------------------- 0
```

```
Majority                   --------------------------------------------------------------------------------
                                   21130     21140     21150     21160     21170     21180     21190     21200
Human                      TGAAAGGCAGAGAACTATAACATTTCTTTGAAGAATTTTCCACTTTCTTCTTCATGGTATTTTCCCTAAGAGACATGCTT 21200
AsianBonytongue            -------------------------------------------------------------------------------- 0
Reedfish                   -------------------------------------------------------------------------------- 0
NorthernPike               -------------------------------------------------------------------------------- 0
SpottedGar                 -------------------------------------------------------------------------------- 0
ParamormyropsKingsLeyae    -------------------------------------------------------------------------------- 0

Majority                   --------------------------------------------------------------------------------
                                   21210     21220     21230     21240     21250     21260     21270     21280
Human                      AATGCAGAAAATATATTAAAAGGATTTTAAACAGCTTATATGTAAGGGATGAGGGAACAGGGATACTTGGACTAATATTT 21280
AsianBonytongue            -------------------------------------------------------------------------------- 0
Reedfish                   -------------------------------------------------------------------------------- 0
NorthernPike               -------------------------------------------------------------------------------- 0
SpottedGar                 -------------------------------------------------------------------------------- 0
ParamormyropsKingsLeyae    -------------------------------------------------------------------------------- 0

Majority                   --------------------------------------------------------------------------------
                                   21290     21300     21310     21320     21330     21340     21350     21360
Human                      CAATATTTCTAAAATTATTGGTTTATTGAGTACGTTCCTAGAAAACTGTTTTATTGAAAGCTTCAAATTTCTCAGAATTG 21360
AsianBonytongue            -------------------------------------------------------------------------------- 0
Reedfish                   -------------------------------------------------------------------------------- 0
NorthernPike               -------------------------------------------------------------------------------- 0
SpottedGar                 -------------------------------------------------------------------------------- 0
ParamormyropsKingsLeyae    -------------------------------------------------------------------------------- 0

Majority                   --------------------------------------------------------------------------------
                                   21370     21380     21390     21400     21410     21420     21430     21440
Human                      TATAGCATTTTTGAATTCTCTCTTTTATTACTAATATCAAGCACCTATGTTTTCTCCTATTTCTTGATTTGTCTTGTCAC 21440
AsianBonytongue            -------------------------------------------------------------------------------- 0
Reedfish                   -------------------------------------------------------------------------------- 0
NorthernPike               -------------------------------------------------------------------------------- 0
SpottedGar                 -------------------------------------------------------------------------------- 0
ParamormyropsKingsLeyae    -------------------------------------------------------------------------------- 0

Majority                   --------------------------------------------------------------------------------
                                   21450     21460     21470     21480     21490     21500     21510     21520
Human                      AGAATTTATTTTACTGGTCTTTTCAAAGAACCAACCTTTTTACTTGTTTTCTTTTTTGTTGGGGGCAGTGGAGGGATTCA 21520
AsianBonytongue            -------------------------------------------------------------------------------- 0
Reedfish                   -------------------------------------------------------------------------------- 0
NorthernPike               -------------------------------------------------------------------------------- 0
SpottedGar                 -------------------------------------------------------------------------------- 0
ParamormyropsKingsLeyae    -------------------------------------------------------------------------------- 0

Majority                   --------------------------------------------------------------------------------
                                   21530     21540     21550     21560     21570     21580     21590     21600
Human                      CTTTAATTTGATCCTGAACTCCTTTATTTTCTTTTAAAATGCATTCAAGGTTAAAGGTAATCAGCTGATAGCTTTGGCTT 21600
AsianBonytongue            -------------------------------------------------------------------------------- 0
Reedfish                   -------------------------------------------------------------------------------- 0
NorthernPike               -------------------------------------------------------------------------------- 0
SpottedGar                 -------------------------------------------------------------------------------- 0
ParamormyropsKingsLeyae    -------------------------------------------------------------------------------- 0
```

```
Majority                   --------------------------------------------------------------------------------

                                     21610     21620     21630     21640     21650     21660     21670     21680

Human                      TTTTTTTTTTTTTTTACTTTTAGAGTTCTCTTTTTATTGTAAACTTCATTTAAAGGTTTTAAGCTACACAAAAGTAGAGG 21680
AsianBonytongue            -------------------------------------------------------------------------------- 0
Reedfish                   -------------------------------------------------------------------------------- 0
NorthernPike               -------------------------------------------------------------------------------- 0
SpottedGar                 -------------------------------------------------------------------------------- 0
ParamormyropsKingsLeyae    -------------------------------------------------------------------------------- 0

Majority                   --------------------------------------------------------------------------------

                                     21690     21700     21710     21720     21730     21740     21750     21760

Human                      GAATGGTATATAATACACTTTTTTCTGTTGGATAATATTAATGCAAATCTCAGATCATTTCATATAATCTGCAGATATAT 21760
AsianBonytongue            -------------------------------------------------------------------------------- 0
Reedfish                   -------------------------------------------------------------------------------- 0
NorthernPike               -------------------------------------------------------------------------------- 0
SpottedGar                 -------------------------------------------------------------------------------- 0
ParamormyropsKingsLeyae    -------------------------------------------------------------------------------- 0

Majority                   --------------------------------------------------------------------------------

                                     21770     21780     21790     21800     21810     21820     21830     21840

Human                      CAGAATGTGTGTCTCTCTATTAAATGTGACATTGGTACCATTATAAAGTTTAAAATAATTTCTTAATGTCGTCTAATCTC 21840
AsianBonytongue            -------------------------------------------------------------------------------- 0
Reedfish                   -------------------------------------------------------------------------------- 0
NorthernPike               -------------------------------------------------------------------------------- 0
SpottedGar                 -------------------------------------------------------------------------------- 0
ParamormyropsKingsLeyae    -------------------------------------------------------------------------------- 0

Majority                   --------------------------------------------------------------------------------

                                     21850     21860     21870     21880     21890     21900     21910     21920

Human                      CAGTATTCAGAATACTTTCTTTTCATTATTTATTTTTCTTGCCTTTTCTTTTTTTGTTGTTGTTAGATAAAGTGAGTCAT 21920
AsianBonytongue            -------------------------------------------------------------------------------- 0
Reedfish                   -------------------------------------------------------------------------------- 0
NorthernPike               -------------------------------------------------------------------------------- 0
SpottedGar                 -------------------------------------------------------------------------------- 0
ParamormyropsKingsLeyae    -------------------------------------------------------------------------------- 0

Majority                   --------------------------------------------------------------------------------

                                     21930     21940     21950     21960     21970     21980     21990     22000

Human                      CTGTACAATTTCCACTTTCTGGCTGGGCGTGGTGGCTCACGCCTGTAATCCCAGCACTTCGGGAGGCTGAGGCGGGCAGA 22000
AsianBonytongue            -------------------------------------------------------------------------------- 0
Reedfish                   -------------------------------------------------------------------------------- 0
NorthernPike               -------------------------------------------------------------------------------- 0
SpottedGar                 -------------------------------------------------------------------------------- 0
ParamormyropsKingsLeyae    -------------------------------------------------------------------------------- 0

Majority                   --------------------------------------------------------------------------------

                                     22010     22020     22030     22040     22050     22060     22070     22080

Human                      TCACGAGGTCAGGAGATCAAGACCATTTTGGCTAACACAGTGAAAACCTGTCTCTACTAAAAATACGAAAAAATTAGCCA 22080
AsianBonytongue            -------------------------------------------------------------------------------- 0
Reedfish                   -------------------------------------------------------------------------------- 0
NorthernPike               -------------------------------------------------------------------------------- 0
SpottedGar                 -------------------------------------------------------------------------------- 0
ParamormyropsKingsLeyae    -------------------------------------------------------------------------------- 0
```

```
Majority                   --------------------------------------------------------------------------------

                                   22090     22100     22110     22120     22130     22140     22150     22160

Human                      GGCGTGGTGGGGGGCACCTGTAGTCCCAGCTACTCGGGAGGCTGAGGCAGGAGAATGGTGTGAACCCGGGAGGCGGAGCT 22160
AsianBonytongue            -------------------------------------------------------------------------------- 0
Reedfish                   -------------------------------------------------------------------------------- 0
NorthernPike               -------------------------------------------------------------------------------- 0
SpottedGar                 -------------------------------------------------------------------------------- 0
ParamormyropsKingsLeyae    -------------------------------------------------------------------------------- 0

Majority                   --------------------------------------------------------------------------------

                                   22170     22180     22190     22200     22210     22220     22230     22240

Human                      TGCAGTGGCGCCACTGGACTCCAGCCTGGGTGACAGAGCAAGACTCCGTCTTAAAAAAAAAAAAAAAAATTCCACTTTCTG 22240
AsianBonytongue            -------------------------------------------------------------------------------- 0
Reedfish                   -------------------------------------------------------------------------------- 0
NorthernPike               -------------------------------------------------------------------------------- 0
SpottedGar                 -------------------------------------------------------------------------------- 0
ParamormyropsKingsLeyae    -------------------------------------------------------------------------------- 0

Majority                   --------------------------------------------------------------------------------

                                   22250     22260     22270     22280     22290     22300     22310     22320

Human                      GATTCTGCTGATTGTACCGCTGTGTTGTCATTTCATATGTTCTTCATGTAAACAAGTTTACCTGAAATACAGGGGTTTGA 22320
AsianBonytongue            -------------------------------------------------------------------------------- 0
Reedfish                   -------------------------------------------------------------------------------- 0
NorthernPike               -------------------------------------------------------------------------------- 0
SpottedGar                 -------------------------------------------------------------------------------- 0
ParamormyropsKingsLeyae    -------------------------------------------------------------------------------- 0

Majority                   --------------------------------------------------------------------------------

                                   22330     22340     22350     22360     22370     22380     22390     22400

Human                      TCAGACTCTGGTGTAATTTTTACCAAGAATATTTTGCAGATGTACTCTATGTAATTCCTGTTGTATGTCAGCTTGCTCCA 22400
AsianBonytongue            -------------------------------------------------------------------------------- 0
Reedfish                   -------------------------------------------------------------------------------- 0
NorthernPike               -------------------------------------------------------------------------------- 0
SpottedGar                 -------------------------------------------------------------------------------- 0
ParamormyropsKingsLeyae    -------------------------------------------------------------------------------- 0

Majority                   --------------------------------------------------------------------------------

                                   22410     22420     22430     22440     22450     22460     22470     22480

Human                      TCTATTACAGAGCGCATCATCCTTTCACCTAATAGTTTTTAGTAGCTAATGATTGCATACTTTTATTAGGTATTGCAAAA 22480
AsianBonytongue            -------------------------------------------------------------------------------- 0
Reedfish                   -------------------------------------------------------------------------------- 0
NorthernPike               -------------------------------------------------------------------------------- 0
SpottedGar                 -------------------------------------------------------------------------------- 0
ParamormyropsKingsLeyae    -------------------------------------------------------------------------------- 0

Majority                   --------------------------------------------------------------------------------

                                   22490     22500     22510     22520     22530     22540     22550     22560

Human                      GTGGCGCTATTCTTTTTTTCTGTGTATATTAGAATTCTTAAGAATTATATCCATCAACTGTTTGGTCATACGTAAGTACC 22560
AsianBonytongue            -------------------------------------------------------------------------------- 0
Reedfish                   -------------------------------------------------------------------------------- 0
NorthernPike               -------------------------------------------------------------------------------- 0
SpottedGar                 -------------------------------------------------------------------------------- 0
ParamormyropsKingsLeyae    -------------------------------------------------------------------------------- 0
```

```
Majority                    --------------------------------------------------------------------------------
                                     22570     22580     22590     22600     22610     22620     22630     22640
Human                       TTTTTATAAGAAAAGCAGGAGAGAATTTGACTTCTTAAGTTTTCTGATGAGTTGTTACCCTAAAACCTCCAGTATAATCA 22640
AsianBonytongue             -------------------------------------------------------------------------------- 0
Reedfish                    -------------------------------------------------------------------------------- 0
NorthernPike                -------------------------------------------------------------------------------- 0
SpottedGar                  -------------------------------------------------------------------------------- 0
ParamormyropsKingsLeyae     -------------------------------------------------------------------------------- 0

Majority                    --------------------------------------------------------------------------------
                                     22650     22660     22670     22680     22690     22700     22710     22720
Human                       ATATGAGTTCCTTTTTGTTGCTGTTAGTATCGTCGCAACAGCAAAGAGTTTAATAACATTTATTTTCTAGTGTATTGCAG 22720
AsianBonytongue             -------------------------------------------------------------------------------- 0
Reedfish                    -------------------------------------------------------------------------------- 0
NorthernPike                -------------------------------------------------------------------------------- 0
SpottedGar                  -------------------------------------------------------------------------------- 0
ParamormyropsKingsLeyae     -------------------------------------------------------------------------------- 0

Majority                    --------------------------------------------------------------------------------
                                     22730     22740     22750     22760     22770     22780     22790     22800
Human                       TAATCATTCTTCTTTTTTTTAAATTTCTAAGCTGTTTTATTAAATGAAAAGAGAACAATGCTAAGCAGCTTGTATGGTGT 22800
AsianBonytongue             -------------------------------------------------------------------------------- 0
Reedfish                    -------------------------------------------------------------------------------- 0
NorthernPike                -------------------------------------------------------------------------------- 0
SpottedGar                  -------------------------------------------------------------------------------- 0
ParamormyropsKingsLeyae     -------------------------------------------------------------------------------- 0

Majority                    --------------------------------------------------------------------------------
                                     22810     22820     22830     22840     22850     22860     22870     22880
Human                       GTGTGTTGTGTGGGTTTTTATTTTGTATGAATGTTAAAAACACGGTGGCTAAAGCCTGTAATCCCAGCATTTTGGGAGGC 22880
AsianBonytongue             -------------------------------------------------------------------------------- 0
Reedfish                    -------------------------------------------------------------------------------- 0
NorthernPike                -------------------------------------------------------------------------------- 0
SpottedGar                  -------------------------------------------------------------------------------- 0
ParamormyropsKingsLeyae     -------------------------------------------------------------------------------- 0

Majority                    --------------------------------------------------------------------------------
                                     22890     22900     22910     22920     22930     22940     22950     22960
Human                       CAAGGTGGTTGGATCACAAGGTCAGGAGCTTGAGACCAGCCTGGCTCTACTAAAAATAAAATGTGGTGAAACCCCGTCTC 22960
AsianBonytongue             -------------------------------------------------------------------------------- 0
Reedfish                    -------------------------------------------------------------------------------- 0
NorthernPike                -------------------------------------------------------------------------------- 0
SpottedGar                  -------------------------------------------------------------------------------- 0
ParamormyropsKingsLeyae     -------------------------------------------------------------------------------- 0

Majority                    --------------------------------------------------------------------------------
                                     22970     22980     22990     23000     23010     23020     23030     23040
Human                       TACTAAAAATACAAAAATTAGCCCTTTGTTGTTGCACGCGCCTATAGTCCCAGCTACTCGGGAGGCTGAGGCAGAAAAAT 23040
AsianBonytongue             -------------------------------------------------------------------------------- 0
Reedfish                    -------------------------------------------------------------------------------- 0
NorthernPike                -------------------------------------------------------------------------------- 0
SpottedGar                  -------------------------------------------------------------------------------- 0
ParamormyropsKingsLeyae     -------------------------------------------------------------------------------- 0
```

```
Majority                    --------------------------------------------------------------------------------
                                     23050     23060     23070     23080     23090     23100     23110     23120
Human                       TGCTTGAACCTGGGGAGGCTGAGGCAGAAAAATTACTTGAACCCGGGAGACAGAGGTTGCAGCGAGCCGAGATTGCGCCA 23120
AsianBonytongue             -------------------------------------------------------------------------------- 0
Reedfish                    -------------------------------------------------------------------------------- 0
NorthernPike                -------------------------------------------------------------------------------- 0
SpottedGar                  -------------------------------------------------------------------------------- 0
ParamormyropsKingsLeyae     -------------------------------------------------------------------------------- 0

Majority                    --------------------------------------------------------------------------------
                                     23130     23140     23150     23160     23170     23180     23190     23200
Human                       CTGCACTCCAGCCTGGGCAACAGACTGAGATTCCATCTAAAAACAAAACCATGTATATATGTAAGTTTGAGGAAAAGAGT 23200
AsianBonytongue             -------------------------------------------------------------------------------- 0
Reedfish                    -------------------------------------------------------------------------------- 0
NorthernPike                -------------------------------------------------------------------------------- 0
SpottedGar                  -------------------------------------------------------------------------------- 0
ParamormyropsKingsLeyae     -------------------------------------------------------------------------------- 0

Majority                    --------------------------------------------------------------------------------
                                     23210     23220     23230     23240     23250     23260     23270     23280
Human                       TGTATGAAAATTCAGGATAATTAATAAAAGTAAACGAGAACTTCTTTACATATATATATATTTTTTAATTTTCTCAGTTA 23280
AsianBonytongue             -------------------------------------------------------------------------------- 0
Reedfish                    -------------------------------------------------------------------------------- 0
NorthernPike                -------------------------------------------------------------------------------- 0
SpottedGar                  -------------------------------------------------------------------------------- 0
ParamormyropsKingsLeyae     -------------------------------------------------------------------------------- 0

Majority                    --------------------------------------------------------------------------------
                                     23290     23300     23310     23320     23330     23340     23350     23360
Human                       ATGTTTGAATTTTTTTAACGTAGAAAATGTCAATGGGGTTATCTGGGGAGAAAGATTATAGTCCATGAGTCCCCCTGTTG 23360
AsianBonytongue             -------------------------------------------------------------------------------- 0
Reedfish                    -------------------------------------------------------------------------------- 0
NorthernPike                -------------------------------------------------------------------------------- 0
SpottedGar                  -------------------------------------------------------------------------------- 0
ParamormyropsKingsLeyae     -------------------------------------------------------------------------------- 0

Majority                    --------------------------------------------------------------------------------
                                     23370     23380     23390     23400     23410     23420     23430     23440
Human                       TATATATACCTGTATTAAAATATGCAATAAATGCTATTTTAAAAAATGTCTTATTACATAGAACAGAAGACCAGAATACT 23440
AsianBonytongue             -------------------------------------------------------------------------------- 0
Reedfish                    -------------------------------------------------------------------------------- 0
NorthernPike                -------------------------------------------------------------------------------- 0
SpottedGar                  -------------------------------------------------------------------------------- 0
ParamormyropsKingsLeyae     -------------------------------------------------------------------------------- 0

Majority                    --------------------------------------------------------------------------------
                                     23450     23460     23470     23480     23490     23500     23510     23520
Human                       AGCTCTCCACGTCTTATGTCTTGATCAGTGAAGATTAGCTAGCAAAACAACCATAACTTAGGGATAGCTTTATTTGTGGG 23520
AsianBonytongue             -------------------------------------------------------------------------------- 0
Reedfish                    -------------------------------------------------------------------------------- 0
NorthernPike                -------------------------------------------------------------------------------- 0
SpottedGar                  -------------------------------------------------------------------------------- 0
ParamormyropsKingsLeyae     -------------------------------------------------------------------------------- 0
```

```
Majority                   --------------------------------------------------------------------------------
                                   23530     23540     23550     23560     23570     23580     23590     23600
Human                      AAGTGTCATTTAGTATGTCATGTATTAAAAATCATTTGTAGTCTTTCAGAGAGCATCTTATCTTTCTTGTAGGTGATAGT 23600
AsianBonytongue            -------------------------------------------------------------------------------- 0
Reedfish                   -------------------------------------------------------------------------------- 0
NorthernPike               -------------------------------------------------------------------------------- 0
SpottedGar                 -------------------------------------------------------------------------------- 0
ParamormyropsKingsLeyae    -------------------------------------------------------------------------------- 0

Majority                   --------------------------------------------------------------------------------
                                   23610     23620     23630     23640     23650     23660     23670     23680
Human                      ATTGGCTATCTATGTAGTGTCTTAAGTTCTTTAAATTATCCTTATTCTTACTTTTTGTGTACATTGTTTATAAAATAATG 23680
AsianBonytongue            -------------------------------------------------------------------------------- 0
Reedfish                   -------------------------------------------------------------------------------- 0
NorthernPike               -------------------------------------------------------------------------------- 0
SpottedGar                 -------------------------------------------------------------------------------- 0
ParamormyropsKingsLeyae    -------------------------------------------------------------------------------- 0

Majority                   --------------------------------------------------------------------------------
                                   23690     23700     23710     23720     23730     23740     23750     23760
Human                      TGATCATCTTAATCTACCATTTTGATTATGGTTTGATCATTCTTTTACTATTTCCACTTGTTAGCAAAGCCTTTATACTT 23760
AsianBonytongue            -------------------------------------------------------------------------------- 0
Reedfish                   -------------------------------------------------------------------------------- 0
NorthernPike               -------------------------------------------------------------------------------- 0
SpottedGar                 -------------------------------------------------------------------------------- 0
ParamormyropsKingsLeyae    -------------------------------------------------------------------------------- 0

Majority                   --------------------------------------------------------------------------------
                                   23770     23780     23790     23800     23810     23820     23830     23840
Human                      ACTAAATTATGATAAAAATGATTATTATAACATGCCCGGTTTTACTTAAATGTCTTATTTAAACGTTCTGATTGTTATTC 23840
AsianBonytongue            -------------------------------------------------------------------------------- 0
Reedfish                   -------------------------------------------------------------------------------- 0
NorthernPike               -------------------------------------------------------------------------------- 0
SpottedGar                 -------------------------------------------------------------------------------- 0
ParamormyropsKingsLeyae    -------------------------------------------------------------------------------- 0

Majority                   --------------------------------------------------------------------------------
                                   23850     23860     23870     23880     23890     23900     23910     23920
Human                      TTTCTTATTCAGGTTTCTTATTGTCATGTTTTATGTCTGTTTTCAGGTGTAATTGTCCTCTCACAAATGTATCCCATTTT 23920
AsianBonytongue            -------------------------------------------------------------------------------- 0
Reedfish                   -------------------------------------------------------------------------------- 0
NorthernPike               -------------------------------------------------------------------------------- 0
SpottedGar                 -------------------------------------------------------------------------------- 0
ParamormyropsKingsLeyae    -------------------------------------------------------------------------------- 0

Majority                   --------------------------------------------------------------------------------
                                   23930     23940     23950     23960     23970     23980     23990     24000
Human                      GGGTGACAAAATACCTGATCACCTTATCCTTATAGTTTTCTCCCTAGAATCTTTTTTGTTATATTAATCAAAGTTTATTT 24000
AsianBonytongue            -------------------------------------------------------------------------------- 0
Reedfish                   -------------------------------------------------------------------------------- 0
NorthernPike               -------------------------------------------------------------------------------- 0
SpottedGar                 -------------------------------------------------------------------------------- 0
ParamormyropsKingsLeyae    -------------------------------------------------------------------------------- 0
```

```
Majority                    --------------------------------------------------------------------------------

                                     24010     24020     24030     24040     24050     24060     24070     24080

Human                       GTGAGAATGTTATGAAATATAAGAAAATGAACATCATGAACCTGTCACTCAAATAAAAAAACTTGAATGTTACCAATACT 24080
AsianBonytongue             -------------------------------------------------------------------------------- 0
Reedfish                    -------------------------------------------------------------------------------- 0
NorthernPike                -------------------------------------------------------------------------------- 0
SpottedGar                  -------------------------------------------------------------------------------- 0
ParamormyropsKingsLeyae-------------------------------------------------------------------------------- 0

Majority                    --------------------------------------------------------------------------------

                                     24090     24100     24110     24120     24130     24140     24150     24160

Human                       ATTGTATATTCCATATGCAGCAATATTGCTTTTTTTTTTTTAGAGTCTTGCTCTGATACCCACGCTGGAGTGCAGTGGTG 24160
AsianBonytongue             -------------------------------------------------------------------------------- 0
Reedfish                    -------------------------------------------------------------------------------- 0
NorthernPike                -------------------------------------------------------------------------------- 0
SpottedGar                  -------------------------------------------------------------------------------- 0
ParamormyropsKingsLeyae-------------------------------------------------------------------------------- 0

Majority                    --------------------------------------------------------------------------------

                                     24170     24180     24190     24200     24210     24220     24230     24240

Human                       TCATCTCGGCTCACAGCAAGCTTCGCCTCCCTGGTTCACCCCATTCTCCTGCCTCAGCCTCCCGAGTAGCTGGGACTTCA 24240
AsianBonytongue             -------------------------------------------------------------------------------- 0
Reedfish                    -------------------------------------------------------------------------------- 0
NorthernPike                -------------------------------------------------------------------------------- 0
SpottedGar                  -------------------------------------------------------------------------------- 0
ParamormyropsKingsLeyae-------------------------------------------------------------------------------- 0

Majority                    --------------------------------------------------------------------------------

                                     24250     24260     24270     24280     24290     24300     24310     24320

Human                       GGCACCCGCCACCACACCCGGCTAATTTTTTGAATTTTTAGTAGAGACGGGGTTTCACCCTGTTAGCCAGGATGGTTTTG 24320
AsianBonytongue             -------------------------------------------------------------------------------- 0
Reedfish                    -------------------------------------------------------------------------------- 0
NorthernPike                -------------------------------------------------------------------------------- 0
SpottedGar                  -------------------------------------------------------------------------------- 0
ParamormyropsKingsLeyae-------------------------------------------------------------------------------- 0

Majority                    --------------------------------------------------------------------------------

                                     24330     24340     24350     24360     24370     24380     24390     24400

Human                       ATCTCATGACCTCGTGATCCGTCTGCCTCGGCCTCCCAAAGTGCTGGGATTATAGGTGTGAGCTACTGCGCCCAGCCCAA 24400
AsianBonytongue             -------------------------------------------------------------------------------- 0
Reedfish                    -------------------------------------------------------------------------------- 0
NorthernPike                -------------------------------------------------------------------------------- 0
SpottedGar                  -------------------------------------------------------------------------------- 0
ParamormyropsKingsLeyae-------------------------------------------------------------------------------- 0

Majority                    --------------------------------------------------------------------------------

                                     24410     24420     24430     24440     24450     24460     24470     24480

Human                       TATTGCTTTTTAATCCCCCTTCTTACCCTGCCATGGGTAGGTGGCTGCCACAACTTTTGCCTTATTTTTTCAATATGGAT 24480
AsianBonytongue             -------------------------------------------------------------------------------- 0
Reedfish                    -------------------------------------------------------------------------------- 0
NorthernPike                -------------------------------------------------------------------------------- 0
SpottedGar                  -------------------------------------------------------------------------------- 0
ParamormyropsKingsLeyae-------------------------------------------------------------------------------- 0
```

```
Majority                    --------------------------------------------------------------------------------

                                     24490     24500     24510     24520     24530     24540     24550     24560

Human                       TTTTCATGTAAATGACAAAAATATATTTAGTTTTTTTTTAGTTTATGGAAACGGTATGCCTATATAATGTTTTCCATGTC 24560
AsianBonytongue             -------------------------------------------------------------------------------- 0
Reedfish                    -------------------------------------------------------------------------------- 0
NorthernPike                -------------------------------------------------------------------------------- 0
SpottedGar                  -------------------------------------------------------------------------------- 0
ParamormyropsKingsLeyae     -------------------------------------------------------------------------------- 0

Majority                    --------------------------------------------------------------------------------

                                     24570     24580     24590     24600     24610     24620     24630     24640

Human                       TTGTGTTTTAATTCAATAATGATTCTAATAAATAGCCGTATTTTTTGGATAGTTTTATAATACTCATTGCTGTGATATAC 24640
AsianBonytongue             -------------------------------------------------------------------------------- 0
Reedfish                    -------------------------------------------------------------------------------- 0
NorthernPike                -------------------------------------------------------------------------------- 0
SpottedGar                  -------------------------------------------------------------------------------- 0
ParamormyropsKingsLeyae     -------------------------------------------------------------------------------- 0

Majority                    --------------------------------------------------------------------------------

                                     24650     24660     24670     24680     24690     24700     24710     24720

Human                       TCTTTTGTGAAAATACCCATTTTATCTTTTATTGGACATTTGGGTTGATGGACATTTGAATATTTAAAGATTTTGACTTA 24720
AsianBonytongue             -------------------------------------------------------------------------------- 0
Reedfish                    -------------------------------------------------------------------------------- 0
NorthernPike                -------------------------------------------------------------------------------- 0
SpottedGar                  -------------------------------------------------------------------------------- 0
ParamormyropsKingsLeyae     -------------------------------------------------------------------------------- 0

Majority                    --------------------------------------------------------------------------------

                                     24730     24740     24750     24760     24770     24780     24790     24800

Human                       GGCCGGGCGTGGTGGCTCAAGCTTGTAATCCCAGCACTTTGGGAGGCCAGGGCGGGCAGATCACAAGGTCAGGAGTTTGA 24800
AsianBonytongue             -------------------------------------------------------------------------------- 0
Reedfish                    -------------------------------------------------------------------------------- 0
NorthernPike                -------------------------------------------------------------------------------- 0
SpottedGar                  -------------------------------------------------------------------------------- 0
ParamormyropsKingsLeyae     -------------------------------------------------------------------------------- 0

Majority                    --------------------------------------------------------------------------------

                                     24810     24820     24830     24840     24850     24860     24870     24880

Human                       GACCAGCCTGGCCAACATAGTGAAACCCCCATCTCTACTAAAAATACCATAATTAGCGGGGTATGGTGGTGGGTGCCTGT 24880
AsianBonytongue             -------------------------------------------------------------------------------- 0
Reedfish                    -------------------------------------------------------------------------------- 0
NorthernPike                -------------------------------------------------------------------------------- 0
SpottedGar                  -------------------------------------------------------------------------------- 0
ParamormyropsKingsLeyae     -------------------------------------------------------------------------------- 0

Majority                    --------------------------------------------------------------------------------

                                     24890     24900     24910     24920     24930     24940     24950     24960

Human                       AGTCCCAGCTACTCAGGAGGCTGAGACAGGAGAATCGCTTGAACCCAGGAGGCGGAGGTTGCAGTGAGCTGAGACCGCGC 24960
AsianBonytongue             -------------------------------------------------------------------------------- 0
Reedfish                    -------------------------------------------------------------------------------- 0
NorthernPike                -------------------------------------------------------------------------------- 0
SpottedGar                  -------------------------------------------------------------------------------- 0
ParamormyropsKingsLeyae     -------------------------------------------------------------------------------- 0
```

```
Majority                    --------------------------------------------------------------------------------

                                    24970     24980     24990     25000     25010     25020     25030     25040
Human                       CATTACACTCCAGCCTGTGTGACAGACTGAGACTCCATCTCAAAAACAAACAAAAAAAAACAACAACAAAAAAAGATTTT 25040
AsianBonytongue             -------------------------------------------------------------------------------- 0
Reedfish                    -------------------------------------------------------------------------------- 0
NorthernPike                -------------------------------------------------------------------------------- 0
SpottedGar                  -------------------------------------------------------------------------------- 0
ParamormyropsKingsLeyae     -------------------------------------------------------------------------------- 0

Majority                    --------------------------------------------------------------------------------

                                    25050     25060     25070     25080     25090     25100     25110     25120
Human                       GACTTTAAGAACAATACTGCTGTGAACTTCTTTGTACTTGTTTCCTGGTGCATTTATGCAAGCGTTTTTGAGAGGGTATA 25120
AsianBonytongue             -------------------------------------------------------------------------------- 0
Reedfish                    -------------------------------------------------------------------------------- 0
NorthernPike                -------------------------------------------------------------------------------- 0
SpottedGar                  -------------------------------------------------------------------------------- 0
ParamormyropsKingsLeyae     -------------------------------------------------------------------------------- 0

Majority                    --------------------------------------------------------------------------------

                                    25130     25140     25150     25160     25170     25180     25190     25200
Human                       TTTAAAGGTATAGGTATATATTACTAGTGTGTAAAATAAACTTTATGAGACAGTACTAGATTATTTTCCAGTGTATTGTT 25200
AsianBonytongue             -------------------------------------------------------------------------------- 0
Reedfish                    -------------------------------------------------------------------------------- 0
NorthernPike                -------------------------------------------------------------------------------- 0
SpottedGar                  -------------------------------------------------------------------------------- 0
ParamormyropsKingsLeyae     -------------------------------------------------------------------------------- 0

Majority                    --------------------------------------------------------------------------------

                                    25210     25220     25230     25240     25250     25260     25270     25280
Human                       CCTATTTATGTTCTCACCAGTAGTGCGTAGGAGTTCTTGCTGTTTCAGATCCCCACCAGCACTTGGCTAGGTATTTAATT 25280
AsianBonytongue             -------------------------------------------------------------------------------- 0
Reedfish                    -------------------------------------------------------------------------------- 0
NorthernPike                -------------------------------------------------------------------------------- 0
SpottedGar                  -------------------------------------------------------------------------------- 0
ParamormyropsKingsLeyae     -------------------------------------------------------------------------------- 0

Majority                    --------------------------------------------------------------------------------

                                    25290     25300     25310     25320     25330     25340     25350     25360
Human                       TCTTAATTTTTAACAATTTGGTGTGTAGTATATTCTCTGGTCTTCCTTTGTCTTACCCTGATTATTAATGAGGTCTTACA 25360
AsianBonytongue             -------------------------------------------------------------------------------- 0
Reedfish                    -------------------------------------------------------------------------------- 0
NorthernPike                -------------------------------------------------------------------------------- 0
SpottedGar                  -------------------------------------------------------------------------------- 0
ParamormyropsKingsLeyae     -------------------------------------------------------------------------------- 0

Majority                    --------------------------------------------------------------------------------

                                    25370     25380     25390     25400     25410     25420     25430     25440
Human                       TCTTTTTTCCCCCCACATTTATGGCCATGGATGTTTCTCTTTTGTGAAATTCTGTTAAATGCCTATTTGTGTTTTTTGCT 25440
AsianBonytongue             -------------------------------------------------------------------------------- 0
Reedfish                    -------------------------------------------------------------------------------- 0
NorthernPike                -------------------------------------------------------------------------------- 0
SpottedGar                  -------------------------------------------------------------------------------- 0
ParamormyropsKingsLeyae     -------------------------------------------------------------------------------- 0
```

```
Majority                  --------------------------------------------------------------------------------

                                   25450     25460     25470     25480     25490     25500     25510     25520

Human                     TGATAAAATGTGAGCTTATTATTTAACTGATTTTGTAGTTTTAAAAAATTGCTGGGGGCGGTGGCTTGCGTCTGTAATCT 25520
AsianBonytongue           -------------------------------------------------------------------------------- 0
Reedfish                  -------------------------------------------------------------------------------- 0
NorthernPike              -------------------------------------------------------------------------------- 0
SpottedGar                -------------------------------------------------------------------------------- 0
ParamormyropsKingsLeyae   -------------------------------------------------------------------------------- 0

Majority                  --------------------------------------------------------------------------------

                                   25530     25540     25550     25560     25570     25580     25590     25600

Human                     CAGCTTTTTGGGAGGCTGAAGGAGGAGGATTGCTTGTGGGGCCCAGGAGTTTGAGGCTGCAGTGAGCTATGATCGTTCCA 25600
AsianBonytongue           -------------------------------------------------------------------------------- 0
Reedfish                  -------------------------------------------------------------------------------- 0
NorthernPike              -------------------------------------------------------------------------------- 0
SpottedGar                -------------------------------------------------------------------------------- 0
ParamormyropsKingsLeyae   -------------------------------------------------------------------------------- 0

Majority                  --------------------------------------------------------------------------------

                                   25610     25620     25630     25640     25650     25660     25670     25680

Human                     TTGCACTCTAGCTTGGTGACAGAGCAAGACTTTGTCTGAAAAAAAAAAAAAAAAAAAAGTGTGTGTTTTACAGTTACTGATT 25680
AsianBonytongue           -------------------------------------------------------------------------------- 0
Reedfish                  -------------------------------------------------------------------------------- 0
NorthernPike              -------------------------------------------------------------------------------- 0
SpottedGar                -------------------------------------------------------------------------------- 0
ParamormyropsKingsLeyae   -------------------------------------------------------------------------------- 0

Majority                  --------------------------------------------------------------------------------

                                   25690     25700     25710     25720     25730     25740     25750     25760

Human                     GTCTTTTGGTTATTTGTGTTGCTGGTGTCTTTTCCCAGTTTGTAGCTTGCCTTGTCACTTCTTTATGCTGTCTTGTCTGT 25760
AsianBonytongue           -------------------------------------------------------------------------------- 0
Reedfish                  -------------------------------------------------------------------------------- 0
NorthernPike              -------------------------------------------------------------------------------- 0
SpottedGar                -------------------------------------------------------------------------------- 0
ParamormyropsKingsLeyae   -------------------------------------------------------------------------------- 0

Majority                  --------------------------------------------------------------------------------

                                   25770     25780     25790     25800     25810     25820     25830     25840

Human                     CTGTCTGTCTGTCTCCCTCCCTCCCTCCCCCTCCCTCCGTCCGTCTGTCCCTCCCTCCCTCCCTCCCTCCCTCCCT 25840
AsianBonytongue           -------------------------------------------------------------------------------- 0
Reedfish                  -------------------------------------------------------------------------------- 0
NorthernPike              -------------------------------------------------------------------------------- 0
SpottedGar                -------------------------------------------------------------------------------- 0
ParamormyropsKingsLeyae   -------------------------------------------------------------------------------- 0

Majority                  --------------------------------------------------------------------------------

                                   25850     25860     25870     25880     25890     25900     25910     25920

Human                     CCCTCCCTCCCTTCCTTCCTTCCTTCCTTCCTTCCTTCCTACTTTACTTTAGATTCAGGGTTACATGTGCAGGTTTGTTA 25920
AsianBonytongue           -------------------------------------------------------------------------------- 0
Reedfish                  -------------------------------------------------------------------------------- 0
NorthernPike              -------------------------------------------------------------------------------- 0
SpottedGar                -------------------------------------------------------------------------------- 0
ParamormyropsKingsLeyae   -------------------------------------------------------------------------------- 0
```

```
Majority                     --------------------------------------------------------------------------------

                                     25930     25940     25950     25960     25970     25980     25990     26000

Human                        CATGGGTATATGGCATGAAGCTGAGGTTTGCAATACAAGTGATCCTGTCAGCCAGAGAGTGAGCTCAGTACCCAACAGTT 26000
AsianBonytongue              -------------------------------------------------------------------------------- 0
Reedfish                     -------------------------------------------------------------------------------- 0
NorthernPike                 -------------------------------------------------------------------------------- 0
SpottedGar                   -------------------------------------------------------------------------------- 0
ParamormyropsKingsLeyae      -------------------------------------------------------------------------------- 0

Majority                     --------------------------------------------------------------------------------

                                     26010     26020     26030     26040     26050     26060     26070     26080

Human                        AGATTTACAATCCTTTCTCCCTCCCCCCACCACTAGTTCCCAGTGCATATTGTTGCGGATTAATACAGATTCTTAATGTA 26080
AsianBonytongue              -------------------------------------------------------------------------------- 0
Reedfish                     -------------------------------------------------------------------------------- 0
NorthernPike                 -------------------------------------------------------------------------------- 0
SpottedGar                   -------------------------------------------------------------------------------- 0
ParamormyropsKingsLeyae      -------------------------------------------------------------------------------- 0

Majority                     --------------------------------------------------------------------------------

                                     26090     26100     26110     26120     26130     26140     26150     26160

Human                        TAGTTTTAATGTGGCCACAAATTCCCTTTGCATCTTAGATAAAAATAATTTATATGATCCCATTCTTTACACATTCCAAA 26160
AsianBonytongue              -------------------------------------------------------------------------------- 0
Reedfish                     -------------------------------------------------------------------------------- 0
NorthernPike                 -------------------------------------------------------------------------------- 0
SpottedGar                   -------------------------------------------------------------------------------- 0
ParamormyropsKingsLeyae      -------------------------------------------------------------------------------- 0

Majority                     --------------------------------------------------------------------------------

                                     26170     26180     26190     26200     26210     26220     26230     26240

Human                        TTTTCCTTTGCTCTTCTGGACATCTTCCATGTTGATTGCTGGAAGTCTTGAAGGCAATCCCAAATAACAGTTTTTAGCTT 26240
AsianBonytongue              -------------------------------------------------------------------------------- 0
Reedfish                     -------------------------------------------------------------------------------- 0
NorthernPike                 -------------------------------------------------------------------------------- 0
SpottedGar                   -------------------------------------------------------------------------------- 0
ParamormyropsKingsLeyae      -------------------------------------------------------------------------------- 0

Majority                     --------------------------------------------------------------------------------

                                     26250     26260     26270     26280     26290     26300     26310     26320

Human                        TTCAACTGTTGCTGTGATACGGTTTCTTATAGAGATTTTTGTAGTAGAATTTATCAGTATTTTCTTTCATGTTCAGTTTT 26320
AsianBonytongue              -------------------------------------------------------------------------------- 0
Reedfish                     -------------------------------------------------------------------------------- 0
NorthernPike                 -------------------------------------------------------------------------------- 0
SpottedGar                   -------------------------------------------------------------------------------- 0
ParamormyropsKingsLeyae      -------------------------------------------------------------------------------- 0

Majority                     --------------------------------------------------------------------------------

                                     26330     26340     26350     26360     26370     26380     26390     26400

Human                        CTTGTGTCTTAATTTAAAAATCCTTACATACTCAGAGTTCATAAAGATACTATATTTTCTTCTGAAATTTTAAAAAATTT 26400
AsianBonytongue              -------------------------------------------------------------------------------- 0
Reedfish                     -------------------------------------------------------------------------------- 0
NorthernPike                 -------------------------------------------------------------------------------- 0
SpottedGar                   -------------------------------------------------------------------------------- 0
ParamormyropsKingsLeyae      -------------------------------------------------------------------------------- 0
```

```
Majority                   --------------------------------------------------------------------------------

                                   26410     26420     26430     26440     26450     26460     26470     26480

Human                      TGCTTTTTATATTTATCAGAAAATTATTTTTGTGTATTGTATTTAGTAGGGATCTGGTTTCATTTTTTTGCCCTTATTAA 26480
AsianBonytongue            -------------------------------------------------------------------------------- 0
Reedfish                   -------------------------------------------------------------------------------- 0
NorthernPike               -------------------------------------------------------------------------------- 0
SpottedGar                 -------------------------------------------------------------------------------- 0
ParamormyropsKingsLeyae-------------------------------------------------------------------------------- 0

Majority                   --------------------------------------------------------------------------------

                                   26490     26500     26510     26520     26530     26540     26550     26560

Human                      GATAGTCAATTAAATTGAAATCTCTGTAATGACTTTTTCATATTACAGGTATATATATCGTAAGGCAAAGGCTGAAAAAA 26560
AsianBonytongue            -------------------------------------------------------------------------------- 0
Reedfish                   -------------------------------------------------------------------------------- 0
NorthernPike               -------------------------------------------------------------------------------- 0
SpottedGar                 -------------------------------------------------------------------------------- 0
ParamormyropsKingsLeyae-------------------------------------------------------------------------------- 0

Majority                   --------------------------------------------------------------------------------

                                   26570     26580     26590     26600     26610     26620     26630     26640

Human                      CTATCATAACAAAATGCAGAATATGGTGGCATAAATAAAAGAGTTTTTTTGTTTCTTTTTCTGTCATAGCAGAAGTCCTG 26640
AsianBonytongue            -------------------------------------------------------------------------------- 0
Reedfish                   -------------------------------------------------------------------------------- 0
NorthernPike               -------------------------------------------------------------------------------- 0
SpottedGar                 -------------------------------------------------------------------------------- 0
ParamormyropsKingsLeyae-------------------------------------------------------------------------------- 0

Majority                   --------------------------------------------------------------------------------

                                   26650     26660     26670     26680     26690     26700     26710     26720

Human                      TTGGTATAAGAAAGTGGTATCAGTCTGCTCCATAAGGTTATTCACTGGGCCAAGTTTCTTTCATATTGTTGCTTCACTGA 26720
AsianBonytongue            -------------------------------------------------------------------------------- 0
Reedfish                   -------------------------------------------------------------------------------- 0
NorthernPike               -------------------------------------------------------------------------------- 0
SpottedGar                 -------------------------------------------------------------------------------- 0
ParamormyropsKingsLeyae-------------------------------------------------------------------------------- 0

Majority                   --------------------------------------------------------------------------------

                                   26730     26740     26750     26760     26770     26780     26790     26800

Human                      TCCCTAGGCTTTTGTTCTTGGCTATGTGGTCTGTTGATGTGTGATTGTGCTCTTCTCCTTGTTTAAAAAAAAAGTTTTCC 26800
AsianBonytongue            -------------------------------------------------------------------------------- 0
Reedfish                   -------------------------------------------------------------------------------- 0
NorthernPike               -------------------------------------------------------------------------------- 0
SpottedGar                 -------------------------------------------------------------------------------- 0
ParamormyropsKingsLeyae-------------------------------------------------------------------------------- 0

Majority                   --------------------------------------------------------------------------------

                                   26810     26820     26830     26840     26850     26860     26870     26880

Human                      CTGTATTTGTGTATTTCATTGTCTTTTTTTTTTAAAGTCAGCTTTTGATTTTACTGATCCTCTTCATTGTACCATTGTTT 26880
AsianBonytongue            -------------------------------------------------------------------------------- 0
Reedfish                   -------------------------------------------------------------------------------- 0
NorthernPike               -------------------------------------------------------------------------------- 0
SpottedGar                 -------------------------------------------------------------------------------- 0
ParamormyropsKingsLeyae-------------------------------------------------------------------------------- 0
```

```
Majority                    --------------------------------------------------------------------------------

                                     26890     26900     26910     26920     26930     26940     26950     26960

Human                       TCTCTATTTTTATTGGTTGCGTGAGTCAGGGCTTTTGCAAGTCTTTAATAGGTAGGTGGTATTCAGTCCACTTCTTCATC 26960
AsianBonytongue             -------------------------------------------------------------------------------- 0
Reedfish                    -------------------------------------------------------------------------------- 0
NorthernPike                -------------------------------------------------------------------------------- 0
SpottedGar                  -------------------------------------------------------------------------------- 0
ParamormyropsKingsLeyae     -------------------------------------------------------------------------------- 0

Majority                    --------------------------------------------------------------------------------

                                     26970     26980     26990     27000     27010     27020     27030     27040

Human                       ATCTTGGTGGCACCTCTGTTTAAGGAGACCAGGAATACTCACCTGGGTACGTACCTTTCTTCCTCTCTGATAATCTATTT 27040
AsianBonytongue             -------------------------------------------------------------------------------- 0
Reedfish                    -------------------------------------------------------------------------------- 0
NorthernPike                -------------------------------------------------------------------------------- 0
SpottedGar                  -------------------------------------------------------------------------------- 0
ParamormyropsKingsLeyae     -------------------------------------------------------------------------------- 0

Majority                    --------------------------------------------------------------------------------

                                     27050     27060     27070     27080     27090     27100     27110     27120

Human                       TTCCTCAGGAACTTAATATTTGTGTCCTTGCCCGGTGTCTTTCTGTCATGGTTCCCTAGGCTGCCATCTCCACAGGAGAA 27120
AsianBonytongue             -------------------------------------------------------------------------------- 0
Reedfish                    -------------------------------------------------------------------------------- 0
NorthernPike                -------------------------------------------------------------------------------- 0
SpottedGar                  -------------------------------------------------------------------------------- 0
ParamormyropsKingsLeyae     -------------------------------------------------------------------------------- 0

Majority                    --------------------------------------------------------------------------------

                                     27130     27140     27150     27160     27170     27180     27190     27200

Human                       AATGTTTGGTTAGTTCTCAGTCCCAAACCATAGCAGAGCCCAGAGACTCTTGTCACCCTCAGCCTTCAGTCCTTGGTTGC 27200
AsianBonytongue             -------------------------------------------------------------------------------- 0
Reedfish                    -------------------------------------------------------------------------------- 0
NorthernPike                -------------------------------------------------------------------------------- 0
SpottedGar                  -------------------------------------------------------------------------------- 0
ParamormyropsKingsLeyae     -------------------------------------------------------------------------------- 0

Majority                    --------------------------------------------------------------------------------

                                     27210     27220     27230     27240     27250     27260     27270     27280

Human                       CTTCTTCGCAGACCTGGCAGCTCTTCCACCTGAAAGTGATACATGGTATTAATAGTTGGACTAGACTATTATGATAAACA 27280
AsianBonytongue             -------------------------------------------------------------------------------- 0
Reedfish                    -------------------------------------------------------------------------------- 0
NorthernPike                -------------------------------------------------------------------------------- 0
SpottedGar                  -------------------------------------------------------------------------------- 0
ParamormyropsKingsLeyae     -------------------------------------------------------------------------------- 0

Majority                    --------------------------------------------------------------------------------

                                     27290     27300     27310     27320     27330     27340     27350     27360

Human                       GAGTATCTAATAACCATGAGAATAAAAAACAAGAAATTTGTAACATTGAAATGAGTGCTAAATGGCCTTTAATCTGTACT 27360
AsianBonytongue             -------------------------------------------------------------------------------- 0
Reedfish                    -------------------------------------------------------------------------------- 0
NorthernPike                -------------------------------------------------------------------------------- 0
SpottedGar                  -------------------------------------------------------------------------------- 0
ParamormyropsKingsLeyae     -------------------------------------------------------------------------------- 0
```

```
Majority                    --------------------------------------------------------------------------------
                                     27370     27380     27390     27400     27410     27420     27430     27440
Human                       TGCTTTTGCACATTTTTTTTTAAGTGGTTTCTTTCTCTGACGTGTAGAGTGTATCTTCCTATTTCTGAATTTTCTTGTTT 27440
AsianBonytongue             -------------------------------------------------------------------------------- 0
Reedfish                    -------------------------------------------------------------------------------- 0
NorthernPike                -------------------------------------------------------------------------------- 0
SpottedGar                  -------------------------------------------------------------------------------- 0
ParamormyropsKingsLeyae     -------------------------------------------------------------------------------- 0

Majority                    --------------------------------------------------------------------------------
                                     27450     27460     27470     27480     27490     27500     27510     27520
Human                       CTGTGGGATGTTAGTTTCTCTTGAAATGGTCATTGATATTACTCAGGAAGAAAAGTTTCTGTGGTCAGATTTAATAAGAG 27520
AsianBonytongue             -------------------------------------------------------------------------------- 0
Reedfish                    -------------------------------------------------------------------------------- 0
NorthernPike                -------------------------------------------------------------------------------- 0
SpottedGar                  -------------------------------------------------------------------------------- 0
ParamormyropsKingsLeyae     -------------------------------------------------------------------------------- 0

Majority                    --------------------------------------------------------------------------------
                                     27530     27540     27550     27560     27570     27580     27590     27600
Human                       ATATCTATGCTAGTCTTAACATTTAATTTCTTTTAGTTGAATCTAAAATTGATATCATTCTTAGATTAATTAAAAATTTT 27600
AsianBonytongue             -------------------------------------------------------------------------------- 0
Reedfish                    -------------------------------------------------------------------------------- 0
NorthernPike                -------------------------------------------------------------------------------- 0
SpottedGar                  -------------------------------------------------------------------------------- 0
ParamormyropsKingsLeyae     -------------------------------------------------------------------------------- 0

Majority                    --------------------------------------------------------------------------------
                                     27610     27620     27630     27640     27650     27660     27670     27680
Human                       TTAAAGAGCATGTATTCTTTAGTATTTTGATTCTAAGTACCACTAATTTGATTTGTTTCAACAGTCATTAATGTGGGGCA 27680
AsianBonytongue             -------------------------------------------------------------------------------- 0
Reedfish                    -------------------------------------------------------------------------------- 0
NorthernPike                -------------------------------------------------------------------------------- 0
SpottedGar                  -------------------------------------------------------------------------------- 0
ParamormyropsKingsLeyae     -------------------------------------------------------------------------------- 0

Majority                    --------------------------------------------------------------------------------
                                     27690     27700     27710     27720     27730     27740     27750     27760
Human                       GTCATCAATCACTACCATTGATCATTTATTTCATATACATTGTAAGTTAAATGACCAGGCTGAAATAATTCAGATGAAAT 27760
AsianBonytongue             -------------------------------------------------------------------------------- 0
Reedfish                    -------------------------------------------------------------------------------- 0
NorthernPike                -------------------------------------------------------------------------------- 0
SpottedGar                  -------------------------------------------------------------------------------- 0
ParamormyropsKingsLeyae     -------------------------------------------------------------------------------- 0

Majority                    --------------------------------------------------------------------------------
                                     27770     27780     27790     27800     27810     27820     27830     27840
Human                       TATTCTGTCAGTTAATGTGGTAATGTTAACATTTATCAGTAGTGCTGAATCACTTCTTTTGGGCATAATAGTGGTAAGCT 27840
AsianBonytongue             -------------------------------------------------------------------------------- 0
Reedfish                    -------------------------------------------------------------------------------- 0
NorthernPike                -------------------------------------------------------------------------------- 0
SpottedGar                  -------------------------------------------------------------------------------- 0
ParamormyropsKingsLeyae     -------------------------------------------------------------------------------- 0
```

```
Majority                    --------------------------------------------------------------------------------
                                     27850     27860     27870     27880     27890     27900     27910     27920
Human                       TTTTGCTCAATGCAAAGACAGGCATAATTGCTTTTTTCAAATAAAAATGTAATTTGTAAAAATACTCCAGTAATTTTTGT 27920
AsianBonytongue             -------------------------------------------------------------------------------- 0
Reedfish                    -------------------------------------------------------------------------------- 0
NorthernPike                -------------------------------------------------------------------------------- 0
SpottedGar                  -------------------------------------------------------------------------------- 0
ParamormyropsKingsLeyae     -------------------------------------------------------------------------------- 0

Majority                    --------------------------------------------------------------------------------
                                     27930     27940     27950     27960     27970     27980     27990     28000
Human                       AAAATCTCAAAATTGCAAATAATTTGACGTTATTCTCAGTAATGAAGGCTGTTAAATCTCCTCTCTTAACGGCAGGTATG 28000
AsianBonytongue             -------------------------------------------------------------------------------- 0
Reedfish                    -------------------------------------------------------------------------------- 0
NorthernPike                -------------------------------------------------------------------------------- 0
SpottedGar                  -------------------------------------------------------------------------------- 0
ParamormyropsKingsLeyae     -------------------------------------------------------------------------------- 0

Majority                    --------------------------------------------------------------------------------
                                     28010     28020     28030     28040     28050     28060     28070     28080
Human                       AAATCATTTGAGGAAGAAAAAAGTTCATAAATACAAACTTAAAAAAAAATGGAAAGACTTTTCCATGCAAAAAGATGCAT 28080
AsianBonytongue             -------------------------------------------------------------------------------- 0
Reedfish                    -------------------------------------------------------------------------------- 0
NorthernPike                -------------------------------------------------------------------------------- 0
SpottedGar                  -------------------------------------------------------------------------------- 0
ParamormyropsKingsLeyae     -------------------------------------------------------------------------------- 0

Majority                    --------------------------------------------------------------------------------
                                     28090     28100     28110     28120     28130     28140     28150     28160
Human                       TGCAGGAAGGAGGAAGAGTGTCCTTCATAGCACACCACAAGTCAGTATTAGAGGCTTGCTTTTCTACTTGAGTAGACCCA 28160
AsianBonytongue             -------------------------------------------------------------------------------- 0
Reedfish                    -------------------------------------------------------------------------------- 0
NorthernPike                -------------------------------------------------------------------------------- 0
SpottedGar                  -------------------------------------------------------------------------------- 0
ParamormyropsKingsLeyae     -------------------------------------------------------------------------------- 0

Majority                    --------------------------------------------------------------------------------
                                     28170     28180     28190     28200     28210     28220     28230     28240
Human                       AGATTCCCCACCTATCGTCTTCCAAAGTTACTTGTGATCCACTGCAATCACAAGTTTTACAGTGTGTATACTCTAGGTGT 28240
AsianBonytongue             -------------------------------------------------------------------------------- 0
Reedfish                    -------------------------------------------------------------------------------- 0
NorthernPike                -------------------------------------------------------------------------------- 0
SpottedGar                  -------------------------------------------------------------------------------- 0
ParamormyropsKingsLeyae     -------------------------------------------------------------------------------- 0

Majority                    --------------------------------------------------------------------------------
                                     28250     28260     28270     28280     28290     28300     28310     28320
Human                       ATGGAAGGATTTGAATTCACAAATAGTCAAACATCAGAAGAATAAGCTGATAATAATACATTTGAAAAGAGGTCAAGTAT 28320
AsianBonytongue             -------------------------------------------------------------------------------- 0
Reedfish                    -------------------------------------------------------------------------------- 0
NorthernPike                -------------------------------------------------------------------------------- 0
SpottedGar                  -------------------------------------------------------------------------------- 0
ParamormyropsKingsLeyae     -------------------------------------------------------------------------------- 0
```

```
Majority                    --------------------------------------------------------------------------------
                                     28330     28340     28350     28360     28370     28380     28390     28400
Human                       ATTTTTGAAAACTAGGAGAGTTAAAGTGTTTTTGTGAACTAAGGAACAAATAAGGCATTGACAATATTTTACTTGATTAG 28400
AsianBonytongue             -------------------------------------------------------------------------------- 0
Reedfish                    -------------------------------------------------------------------------------- 0
NorthernPike                -------------------------------------------------------------------------------- 0
SpottedGar                  -------------------------------------------------------------------------------- 0
ParamormyropsKingsLeyae     -------------------------------------------------------------------------------- 0

Majority                    --------------------------------------------------------------------------------
                                     28410     28420     28430     28440     28450     28460     28470     28480
Human                       GAATTTTGTTCAGGTGAGTAAAACTTTAAGCATCACTTAAGTTTTGTTTTTGTTTCTTACTTTTAGAATATCTGATCTGG 28480
AsianBonytongue             -------------------------------------------------------------------------------- 0
Reedfish                    -------------------------------------------------------------------------------- 0
NorthernPike                -------------------------------------------------------------------------------- 0
SpottedGar                  -------------------------------------------------------------------------------- 0
ParamormyropsKingsLeyae     -------------------------------------------------------------------------------- 0

Majority                    --------------------------------------------------------------------------------
                                     28490     28500     28510     28520     28530     28540     28550     28560
Human                       ACTTCTGTGGATTTATGGAAGAGCTGGCTTTATTTCTCTATCCTGTGGGAGGGGCAAACTTTGATTTTGTATGTGGAGTA 28560
AsianBonytongue             -------------------------------------------------------------------------------- 0
Reedfish                    -------------------------------------------------------------------------------- 0
NorthernPike                -------------------------------------------------------------------------------- 0
SpottedGar                  -------------------------------------------------------------------------------- 0
ParamormyropsKingsLeyae     -------------------------------------------------------------------------------- 0

Majority                    --------------------------------------------------------------------------------
                                     28570     28580     28590     28600     28610     28620     28630     28640
Human                       ACTTGACCTTCAGTTTTTTTGGTCTGAGAACAAAGGCCAAAAAATATGTAACAGTTAATAACGTAATTCACTTACTTAGT 28640
AsianBonytongue             -------------------------------------------------------------------------------- 0
Reedfish                    -------------------------------------------------------------------------------- 0
NorthernPike                -------------------------------------------------------------------------------- 0
SpottedGar                  -------------------------------------------------------------------------------- 0
ParamormyropsKingsLeyae     -------------------------------------------------------------------------------- 0

Majority                    --------------------------------------------------------------------------------
                                     28650     28660     28670     28680     28690     28700     28710     28720
Human                       CAATAAATATGTGGGGACATTGTACTTGGTTGACACTTGGGAATATTGTAGTGAGCACTACTGATGTGAGCCAGGCACTT 28720
AsianBonytongue             -------------------------------------------------------------------------------- 0
Reedfish                    -------------------------------------------------------------------------------- 0
NorthernPike                -------------------------------------------------------------------------------- 0
SpottedGar                  -------------------------------------------------------------------------------- 0
ParamormyropsKingsLeyae     -------------------------------------------------------------------------------- 0

Majority                    --------------------------------------------------------------------------------
                                     28730     28740     28750     28760     28770     28780     28790     28800
Human                       TGCCTGGTAATCACTGTTCGTGTATAATTTAATCCTAAGGGCTGTGATGTAGATGTTATCCCTGTTTTACAAATAAAGAA 28800
AsianBonytongue             -------------------------------------------------------------------------------- 0
Reedfish                    -------------------------------------------------------------------------------- 0
NorthernPike                -------------------------------------------------------------------------------- 0
SpottedGar                  -------------------------------------------------------------------------------- 0
ParamormyropsKingsLeyae     -------------------------------------------------------------------------------- 0
```

```
Majority                    --------------------------------------------------------------------------------

                                     28810     28820     28830     28840     28850     28860     28870     28880

Human                       CTTCAGGCTTAGAATGATTAAGTACTGTTACCTAACTGATAAGTGGAAGCTGAGCTCCAGAGCACCCTTTCTTAATACAG 28880
AsianBonytongue             -------------------------------------------------------------------------------- 0
Reedfish                    -------------------------------------------------------------------------------- 0
NorthernPike                -------------------------------------------------------------------------------- 0
SpottedGar                  -------------------------------------------------------------------------------- 0
ParamormyropsKingsLeyae-------------------------------------------------------------------------------- 0

Majority                    --------------------------------------------------------------------------------

                                     28890     28900     28910     28920     28930     28940     28950     28960

Human                       TATTTTCTACTTGTCATGAAAACATGAAGAAAGCTAGGAAACATTTTCTGTGTTGATAATGAGCCAATAATGTGTATCAG 28960
AsianBonytongue             -------------------------------------------------------------------------------- 0
Reedfish                    -------------------------------------------------------------------------------- 0
NorthernPike                -------------------------------------------------------------------------------- 0
SpottedGar                  -------------------------------------------------------------------------------- 0
ParamormyropsKingsLeyae-------------------------------------------------------------------------------- 0

Majority                    --------------------------------------------------------------------------------

                                     28970     28980     28990     29000     29010     29020     29030     29040

Human                       TGGTTCTTAAACTTTAGCATGCATCAGAAACACTTGGGGATTTTTTTTTTTTAAACCTGGGGTTTTAGATCCCACCCTCA 29040
AsianBonytongue             -------------------------------------------------------------------------------- 0
Reedfish                    -------------------------------------------------------------------------------- 0
NorthernPike                -------------------------------------------------------------------------------- 0
SpottedGar                  -------------------------------------------------------------------------------- 0
ParamormyropsKingsLeyae-------------------------------------------------------------------------------- 0

Majority                    --------------------------------------------------------------------------------

                                     29050     29060     29070     29080     29090     29100     29110     29120

Human                       GAGTTTCTTATTCAGTAGGTCTGGGATTGGACCTGAGAACATATACTGTCAGTAAGCTTCCAGGTCTAATCTTAGGATTA 29120
AsianBonytongue             -------------------------------------------------------------------------------- 0
Reedfish                    -------------------------------------------------------------------------------- 0
NorthernPike                -------------------------------------------------------------------------------- 0
SpottedGar                  -------------------------------------------------------------------------------- 0
ParamormyropsKingsLeyae-------------------------------------------------------------------------------- 0

Majority                    --------------------------------------------------------------------------------

                                     29130     29140     29150     29160     29170     29180     29190     29200

Human                       GGGACCACACTTTGAGAATGATTAGAGTATTCAATTAGATCTTAGGGACTACACTGTGAGAATCAGTAATGTATACAATT 29200
AsianBonytongue             -------------------------------------------------------------------------------- 0
Reedfish                    -------------------------------------------------------------------------------- 0
NorthernPike                -------------------------------------------------------------------------------- 0
SpottedGar                  -------------------------------------------------------------------------------- 0
ParamormyropsKingsLeyae-------------------------------------------------------------------------------- 0

Majority                    --------------------------------------------------------------------------------

                                     29210     29220     29230     29240     29250     29260     29270     29280

Human                       CTGCTCATCATGTTCTTATAAAACTATGCCATGCCAGATCTTCCATATCTTGATTAGAAATTGGTTAAGAAATTAGGCTT 29280
AsianBonytongue             -------------------------------------------------------------------------------- 0
Reedfish                    -------------------------------------------------------------------------------- 0
NorthernPike                -------------------------------------------------------------------------------- 0
SpottedGar                  -------------------------------------------------------------------------------- 0
ParamormyropsKingsLeyae-------------------------------------------------------------------------------- 0
```

```
Majority                --------------------------------------------------------------------------------

                                 29290     29300     29310     29320     29330     29340     29350     29360
Human                   GGTGTAAATACAGTATCAATGTGGCTTGAAACTGGTTGTATTTTTCTGTTTTTTTTTTTCTTTGTGCATTTGATGCTAAT 29360
AsianBonytongue         -------------------------------------------------------------------------------- 0
Reedfish                -------------------------------------------------------------------------------- 0
NorthernPike            -------------------------------------------------------------------------------- 0
SpottedGar              -------------------------------------------------------------------------------- 0
ParamormyropsKingsLeyae -------------------------------------------------------------------------------- 0

Majority                --------------------------------------------------------------------------------

                                 29370     29380     29390     29400     29410     29420     29430     29440
Human                   AAGTAGAAAGCTCTTAAAATATTTAAGGGAGACAACCTATGGATATCTGTTTACTCTTCTCATATTTGTTGTAATACAGT 29440
AsianBonytongue         -------------------------------------------------------------------------------- 0
Reedfish                -------------------------------------------------------------------------------- 0
NorthernPike            -------------------------------------------------------------------------------- 0
SpottedGar              -------------------------------------------------------------------------------- 0
ParamormyropsKingsLeyae -------------------------------------------------------------------------------- 0

Majority                --------------------------------------------------------------------------------

                                 29450     29460     29470     29480     29490     29500     29510     29520
Human                   TTATTCATTATGAGTTTAAATACAAATTAGCGTAAAGCTTATGGTTAATTTGGATTTTTTGGACTTTCTGAAATGCTTTG 29520
AsianBonytongue         -------------------------------------------------------------------------------- 0
Reedfish                -------------------------------------------------------------------------------- 0
NorthernPike            -------------------------------------------------------------------------------- 0
SpottedGar              -------------------------------------------------------------------------------- 0
ParamormyropsKingsLeyae -------------------------------------------------------------------------------- 0

Majority                --------------------------------------------------------------------------------

                                 29530     29540     29550     29560     29570     29580     29590     29600
Human                   ATAATTCATTGCCTCCTTTGCACATTTTTTTCATTTTGATGGTTGCATTTAACTTCTTTGTACTGATTTTTTAAATCAGC 29600
AsianBonytongue         -------------------------------------------------------------------------------- 0
Reedfish                -------------------------------------------------------------------------------- 0
NorthernPike            -------------------------------------------------------------------------------- 0
SpottedGar              -------------------------------------------------------------------------------- 0
ParamormyropsKingsLeyae -------------------------------------------------------------------------------- 0

Majority                --------------------------------------------------------------------------------

                                 29610     29620     29630     29640     29650     29660     29670     29680
Human                   CAGTATTAATTTAACCTATTTTCAATACTAATATCCATGTATGTAAATATCCATAACTAAGGCAAAACAGTTGATTTTAG 29680
AsianBonytongue         -------------------------------------------------------------------------------- 0
Reedfish                -------------------------------------------------------------------------------- 0
NorthernPike            -------------------------------------------------------------------------------- 0
SpottedGar              -------------------------------------------------------------------------------- 0
ParamormyropsKingsLeyae -------------------------------------------------------------------------------- 0

Majority                --------------------------------------------------------------------------------

                                 29690     29700     29710     29720     29730     29740     29750     29760
Human                   CTAGTTTAAATATTAGTCCATTCATATTTTTGTAAGTACATAACATTTTCAAAATAGCTAAAATGTCACTGGCTGGTTTG 29760
AsianBonytongue         -------------------------------------------------------------------------------- 0
Reedfish                -------------------------------------------------------------------------------- 0
NorthernPike            -------------------------------------------------------------------------------- 0
SpottedGar              -------------------------------------------------------------------------------- 0
ParamormyropsKingsLeyae -------------------------------------------------------------------------------- 0
```

```
Majority                  --------------------------------------------------------------------------------

                                   29770     29780     29790     29800     29810     29820     29830     29840

Human                     AACAACTGTGCAGTTACCGAAGAGATGAATGTATAACCTTTTCTGTAAAGAATTTTAAGACATGACCTTATTTTTTTGCA 29840
AsianBonytongue           -------------------------------------------------------------------------------- 0
Reedfish                  -------------------------------------------------------------------------------- 0
NorthernPike              -------------------------------------------------------------------------------- 0
SpottedGar                -------------------------------------------------------------------------------- 0
ParamormyropsKingsLeyae-------------------------------------------------------------------------------- 0

Majority                  --------------------------------------------------------------------------------

                                   29850     29860     29870     29880     29890     29900     29910     29920

Human                     GAAAGAAATCTCTTCATTCCAAAGATTTTGCCTTTGCTTTCGGTGTTCTAGCCACTGAAGTATATACCTGTTTAATTTTA 29920
AsianBonytongue           -------------------------------------------------------------------------------- 0
Reedfish                  -------------------------------------------------------------------------------- 0
NorthernPike              -------------------------------------------------------------------------------- 0
SpottedGar                -------------------------------------------------------------------------------- 0
ParamormyropsKingsLeyae-------------------------------------------------------------------------------- 0

Majority                  --------------------------------------------------------------------------------

                                   29930     29940     29950     29960     29970     29980     29990     30000

Human                     ATAATGATGAAGATATTGATATCTAACTCACCAGAATGAAGTTCTAATTTTCTAAAGACCTGAATTATCTGGATGAATAA 30000
AsianBonytongue           -------------------------------------------------------------------------------- 0
Reedfish                  -------------------------------------------------------------------------------- 0
NorthernPike              -------------------------------------------------------------------------------- 0
SpottedGar                -------------------------------------------------------------------------------- 0
ParamormyropsKingsLeyae-------------------------------------------------------------------------------- 0

Majority                  --------------------------------------------------------------------------------

                                   30010     30020     30030     30040     30050     30060     30070     30080

Human                     AGGACCATTGGGATGGAGGATTTATAGAACGTTTCTGAGAAAGTCTAGCTGTTGTGGCTTTGGTTTGTTTGTAAACAATT 30080
AsianBonytongue           -------------------------------------------------------------------------------- 0
Reedfish                  -------------------------------------------------------------------------------- 0
NorthernPike              -------------------------------------------------------------------------------- 0
SpottedGar                -------------------------------------------------------------------------------- 0
ParamormyropsKingsLeyae-------------------------------------------------------------------------------- 0

Majority                  --------------------------------------------------------------------------------

                                   30090     30100     30110     30120     30130     30140     30150     30160

Human                     GAGATAGCTTTCCAAAGCTTTTGCTTATAGAATCTAATCACTTTTTCTATTTATTGTCTTTTGGGCTTAGAGTAAAGAAG 30160
AsianBonytongue           -------------------------------------------------------------------------------- 0
Reedfish                  -------------------------------------------------------------------------------- 0
NorthernPike              -------------------------------------------------------------------------------- 0
SpottedGar                -------------------------------------------------------------------------------- 0
ParamormyropsKingsLeyae-------------------------------------------------------------------------------- 0

Majority                  --------------------------------------------------------------------------------

                                   30170     30180     30190     30200     30210     30220     30230     30240

Human                     GGAAGGGAACACTGTTGCCTTTGTGAGAATGGGAGAGGGATAGATGGGAGAGTAGAGGGATGAAGTTAATTTTTTATTAT 30240
AsianBonytongue           -------------------------------------------------------------------------------- 0
Reedfish                  -------------------------------------------------------------------------------- 0
NorthernPike              -------------------------------------------------------------------------------- 0
SpottedGar                -------------------------------------------------------------------------------- 0
ParamormyropsKingsLeyae-------------------------------------------------------------------------------- 0
```

```
Majority                    --------------------------------------------------------------------------------

                                     30250     30260     30270     30280     30290     30300     30310     30320

Human                       AGGATTAACATTTTTAACTATTCCCAATAGTGTAACATTTGACATTTAAGTGAAAGGCTTTTAGATTAATATTTTTTAAA 30320
AsianBonytongue             -------------------------------------------------------------------------------- 0
Reedfish                    -------------------------------------------------------------------------------- 0
NorthernPike                -------------------------------------------------------------------------------- 0
SpottedGar                  -------------------------------------------------------------------------------- 0
ParamormyropsKingsLeyae     -------------------------------------------------------------------------------- 0

Majority                    --------------------------------------------------------------------------------

                                     30330     30340     30350     30360     30370     30380     30390     30400

Human                       TTTAAAGATGTCTTATGCTAATTTGTTTTTGTTTTCATAGAGAGGATCTGACGAACTACTCTCAGGCAGTGTTCTCAGTA 30400
AsianBonytongue             -------------------------------------------------------------------------------- 0
Reedfish                    -------------------------------------------------------------------------------- 0
NorthernPike                -------------------------------------------------------------------------------- 0
SpottedGar                  -------------------------------------------------------------------------------- 0
ParamormyropsKingsLeyae     -------------------------------------------------------------------------------- 0

Majority                    --------------------------------------------------------------------------------

                                     30410     30420     30430     30440     30450     30460     30470     30480

Human                       GTCCGAACTCTAATATGAGCAGCATGGTAGTTACAGGTAAGTGTTACTCTCTTAGCAGTCTGTCATTTGCCATTTTAGGG 30480
AsianBonytongue             -------------------------------------------------------------------------------- 0
Reedfish                    -------------------------------------------------------------------------------- 0
NorthernPike                -------------------------------------------------------------------------------- 0
SpottedGar                  -------------------------------------------------------------------------------- 0
ParamormyropsKingsLeyae     -------------------------------------------------------------------------------- 0

Majority                    --------------------------------------------------------------------------------

                                     30490     30500     30510     30520     30530     30540     30550     30560

Human                       GGCAGAATGTTAAAACTCAAATTTAACCCTGTTATACCAGGAAAGCCTTTCTTTTTAAATGCGTGTTGAAACATTTTAGA 30560
AsianBonytongue             -------------------------------------------------------------------------------- 0
Reedfish                    -------------------------------------------------------------------------------- 0
NorthernPike                -------------------------------------------------------------------------------- 0
SpottedGar                  -------------------------------------------------------------------------------- 0
ParamormyropsKingsLeyae     -------------------------------------------------------------------------------- 0

Majority                    --------------------------------------------------------------------------------

                                     30570     30580     30590     30600     30610     30620     30630     30640

Human                       GTAAATTTTACTAAAGTATATATGAGCCGAGTGGAAAAAAAAAAAAAACCTGAAGCTGGTAACTTTGGTGAACAAGTTAC 30640
AsianBonytongue             -------------------------------------------------------------------------------- 0
Reedfish                    -------------------------------------------------------------------------------- 0
NorthernPike                -------------------------------------------------------------------------------- 0
SpottedGar                  -------------------------------------------------------------------------------- 0
ParamormyropsKingsLeyae     -------------------------------------------------------------------------------- 0

Majority                    --------------------------------------------------------------------------------

                                     30650     30660     30670     30680     30690     30700     30710     30720

Human                       TCTTAAATTTGATTGCTTTTCATTTTTGAACCACAAATGTAATTCTGTCTCACATTATAGTCCCTTCATCTGCCCCTCTG 30720
AsianBonytongue             -------------------------------------------------------------------------------- 0
Reedfish                    -------------------------------------------------------------------------------- 0
NorthernPike                -------------------------------------------------------------------------------- 0
SpottedGar                  -------------------------------------------------------------------------------- 0
ParamormyropsKingsLeyae     -------------------------------------------------------------------------------- 0
```

```
Majority                   --------------------------------------------------------------------------------
                                    30730     30740     30750     30760     30770     30780     30790     30800
Human                      GTATTTTTATTTTGTTTTTATTTTTTGTGTTTGTGTGTCATATAATATTTTTACTTTAAGTATTTATGCCACAAACCCTA 30800
AsianBonytongue            -------------------------------------------------------------------------------- 0
Reedfish                   -------------------------------------------------------------------------------- 0
NorthernPike               -------------------------------------------------------------------------------- 0
SpottedGar                 -------------------------------------------------------------------------------- 0
ParamormyropsKingsLeyae    -------------------------------------------------------------------------------- 0

Majority                   --------------------------------------------------------------------------------
                                    30810     30820     30830     30840     30850     30860     30870     30880
Human                      AAACCCAAATTTTAAGTAAAAACCGAAGAATTCAGCTACATAGTTGTTTAAAACCATTGCAACCATAGTTGCTATGGTTT 30880
AsianBonytongue            -------------------------------------------------------------------------------- 0
Reedfish                   -------------------------------------------------------------------------------- 0
NorthernPike               -------------------------------------------------------------------------------- 0
SpottedGar                 -------------------------------------------------------------------------------- 0
ParamormyropsKingsLeyae    -------------------------------------------------------------------------------- 0

Majority                   --------------------------------------------------------------------------------
                                    30890     30900     30910     30920     30930     30940     30950     30960
Human                      TAACTAATTTTTTGCTGAAACAGTATTTTAATTCTGAGTTGATCCTTTTATGTTGTCTACCTGCATATTTAAGATGATTT 30960
AsianBonytongue            -------------------------------------------------------------------------------- 0
Reedfish                   -------------------------------------------------------------------------------- 0
NorthernPike               -------------------------------------------------------------------------------- 0
SpottedGar                 -------------------------------------------------------------------------------- 0
ParamormyropsKingsLeyae    -------------------------------------------------------------------------------- 0

Majority                   --------------------------------------------------------------------------------
                                    30970     30980     30990     31000     31010     31020     31030     31040
Human                      GTTTTAATCTTCAAAGTTAGAGTTAAAAAAAATTGTATATGTCTGAAATAATGTTGTTTGTAGCATCTGTCAGTAAAACA 31040
AsianBonytongue            -------------------------------------------------------------------------------- 0
Reedfish                   -------------------------------------------------------------------------------- 0
NorthernPike               -------------------------------------------------------------------------------- 0
SpottedGar                 -------------------------------------------------------------------------------- 0
ParamormyropsKingsLeyae    -------------------------------------------------------------------------------- 0

Majority                   --------------------------------------------------------------------------------
                                    31050     31060     31070     31080     31090     31100     31110     31120
Human                      ATATAGACCAAATTTTAGACCTTAGTTTTATGGCTGATGGGCCAAATTTTTTTTTGTATTTTGTTTAGTTTTGCTTGAAT 31120
AsianBonytongue            -------------------------------------------------------------------------------- 0
Reedfish                   -------------------------------------------------------------------------------- 0
NorthernPike               -------------------------------------------------------------------------------- 0
SpottedGar                 -------------------------------------------------------------------------------- 0
ParamormyropsKingsLeyae    -------------------------------------------------------------------------------- 0

Majority                   --------------------------------------------------------------------------------
                                    31130     31140     31150     31160     31170     31180     31190     31200
Human                      TGACGTGTTCTCATCTGAATGGGCCAAATTTTGAATTTGATTATTATTATTATCAAATGAATTTACCTCACAACTCCACA 31200
AsianBonytongue            -------------------------------------------------------------------------------- 0
Reedfish                   -------------------------------------------------------------------------------- 0
NorthernPike               -------------------------------------------------------------------------------- 0
SpottedGar                 -------------------------------------------------------------------------------- 0
ParamormyropsKingsLeyae    -------------------------------------------------------------------------------- 0
```

```
Majority                    --------------------------------------------------------------------------------
                                     31210     31220     31230     31240     31250     31260     31270     31280
Human                       TTTTACATTATTTACCTGAAACCAAAGTTCCTTTTTCTGTATTTTAACTTTGAATTATTTGCTTGAAGAACAAATTATAT 31280
AsianBonytongue             -------------------------------------------------------------------------------- 0
Reedfish                    -------------------------------------------------------------------------------- 0
NorthernPike                -------------------------------------------------------------------------------- 0
SpottedGar                  -------------------------------------------------------------------------------- 0
ParamormyropsKingsLeyae     -------------------------------------------------------------------------------- 0

Majority                    --------------------------------------------------------------------------------
                                     31290     31300     31310     31320     31330     31340     31350     31360
Human                       TTGGCAATAAAAGGATCATGAAAGACTAGAAGACATTTTGATTTCCCTTAGGTAGCTGTGCTTAATGTATACATACATCT 31360
AsianBonytongue             -------------------------------------------------------------------------------- 0
Reedfish                    -------------------------------------------------------------------------------- 0
NorthernPike                -------------------------------------------------------------------------------- 0
SpottedGar                  -------------------------------------------------------------------------------- 0
ParamormyropsKingsLeyae     -------------------------------------------------------------------------------- 0

Majority                    --------------------------------------------------------------------------------
                                     31370     31380     31390     31400     31410     31420     31430     31440
Human                       TGACACACACAAACACAAACACACATCTCTGACAATTCAGTTAACTAAAAATTATAAGGACTTAACAGATAATAGTCATG 31440
AsianBonytongue             -------------------------------------------------------------------------------- 0
Reedfish                    -------------------------------------------------------------------------------- 0
NorthernPike                -------------------------------------------------------------------------------- 0
SpottedGar                  -------------------------------------------------------------------------------- 0
ParamormyropsKingsLeyae     -------------------------------------------------------------------------------- 0

Majority                    --------------------------------------------------------------------------------
                                     31450     31460     31470     31480     31490     31500     31510     31520
Human                       ATGTGAAGATAAATTTGTCAACAGGTCATTATCATTGATTTTGCTATGTAATGATTACAAAGCAAAATCACTGGGCCTTG 31520
AsianBonytongue             -------------------------------------------------------------------------------- 0
Reedfish                    -------------------------------------------------------------------------------- 0
NorthernPike                -------------------------------------------------------------------------------- 0
SpottedGar                  -------------------------------------------------------------------------------- 0
ParamormyropsKingsLeyae     -------------------------------------------------------------------------------- 0

Majority                    --------------------------------------------------------------------------------
                                     31530     31540     31550     31560     31570     31580     31590     31600
Human                       ATTTTGATAGTCAAAGTACCATGTACACATAATTGAACAAATAGGCAGAAGTGTGTAACTCTGGTTTGTCGGTTTCTCTT 31600
AsianBonytongue             -------------------------------------------------------------------------------- 0
Reedfish                    -------------------------------------------------------------------------------- 0
NorthernPike                -------------------------------------------------------------------------------- 0
SpottedGar                  -------------------------------------------------------------------------------- 0
ParamormyropsKingsLeyae     -------------------------------------------------------------------------------- 0

Majority                    --------------------------------------------------------------------------------
                                     31610     31620     31630     31640     31650     31660     31670     31680
Human                       TTTGTTTTTACAATGCATACTGTGATCGATAATGGGAAATTAATTTGGAATTAATTTCCAAATATTTAGGGCTAGTGACA 31680
AsianBonytongue             -------------------------------------------------------------------------------- 0
Reedfish                    -------------------------------------------------------------------------------- 0
NorthernPike                -------------------------------------------------------------------------------- 0
SpottedGar                  -------------------------------------------------------------------------------- 0
ParamormyropsKingsLeyae     -------------------------------------------------------------------------------- 0
```

```
Majority               --------------------------------------------------------------------------------
                                31690     31700     31710     31720     31730     31740     31750     31760
Human                  GAGTAACTAGTAGCAGCCTTGGCCCCTTTTTCAGTCTCCCCCCAAAACCAAAAAGTATGTAACTGAATAAAATACGTGGA 31760
AsianBonytongue        -------------------------------------------------------------------------------- 0
Reedfish               -------------------------------------------------------------------------------- 0
NorthernPike           -------------------------------------------------------------------------------- 0
SpottedGar             -------------------------------------------------------------------------------- 0
ParamormyropsKingsLeyae-------------------------------------------------------------------------------- 0

Majority               --------------------------------------------------------------------------------
                                31770     31780     31790     31800     31810     31820     31830     31840
Human                  ACAACTGTTGTTTGGAAGTGATGAGCAGACGGTATAAGATCATCCTTGAGAGAAGGGGTACACGTGAGGTAAGTATCATG 31840
AsianBonytongue        -------------------------------------------------------------------------------- 0
Reedfish               -------------------------------------------------------------------------------- 0
NorthernPike           -------------------------------------------------------------------------------- 0
SpottedGar             -------------------------------------------------------------------------------- 0
ParamormyropsKingsLeyae-------------------------------------------------------------------------------- 0

Majority               --------------------------------------------------------------------------------
                                31850     31860     31870     31880     31890     31900     31910     31920
Human                  AACACTCTGGCTTTCTGTCTTGGACTATTTTACTGCTCTAAACCACAGAGAAGTGGAGAGCCCAGAGTATACAGGTCTCA 31920
AsianBonytongue        -------------------------------------------------------------------------------- 0
Reedfish               -------------------------------------------------------------------------------- 0
NorthernPike           -------------------------------------------------------------------------------- 0
SpottedGar             -------------------------------------------------------------------------------- 0
ParamormyropsKingsLeyae-------------------------------------------------------------------------------- 0

Majority               --------------------------------------------------------------------------------
                                31930     31940     31950     31960     31970     31980     31990     32000
Human                  CTGAGCTGAAGAGACCAAGAGTGGGTGTCAGGGCTGCCCAATCATTTGGAATTTATGGAGAAGTCCTGGAGAAAAGAGAG 32000
AsianBonytongue        -------------------------------------------------------------------------------- 0
Reedfish               -------------------------------------------------------------------------------- 0
NorthernPike           -------------------------------------------------------------------------------- 0
SpottedGar             -------------------------------------------------------------------------------- 0
ParamormyropsKingsLeyae-------------------------------------------------------------------------------- 0

Majority               --------------------------------------------------------------------------------
                                32010     32020     32030     32040     32050     32060     32070     32080
Human                  CTGTCTAGGGGTTTAGGAATAGAGCAGGGGGCAGAATTTAGTATGGTCATTTCACTTCATTCACAGTTTTGGATAATGAC 32080
AsianBonytongue        -------------------------------------------------------------------------------- 0
Reedfish               -------------------------------------------------------------------------------- 0
NorthernPike           -------------------------------------------------------------------------------- 0
SpottedGar             -------------------------------------------------------------------------------- 0
ParamormyropsKingsLeyae-------------------------------------------------------------------------------- 0

Majority               --------------------------------------------------------------------------------
                                32090     32100     32110     32120     32130     32140     32150     32160
Human                  TTGGTTACATGTGTGCAGGTTGAGGCTTTGCCAGGTCTGTCAGAAATTGTCTGCTCTGGGGCTAAATGGTGATTCCATAG 32160
AsianBonytongue        -------------------------------------------------------------------------------- 0
Reedfish               -------------------------------------------------------------------------------- 0
NorthernPike           -------------------------------------------------------------------------------- 0
SpottedGar             -------------------------------------------------------------------------------- 0
ParamormyropsKingsLeyae-------------------------------------------------------------------------------- 0
```

```
Majority                    --------------------------------------------------------------------------------

                                     32170     32180     32190     32200     32210     32220     32230     32240

Human                       GTTGCACGGTACTCTGAAACATTGGAGTTCTGAGAAGTCAGAATGCAGACATATTGCAGGGTACTTCAAACACTCCATTT 32240
AsianBonytongue             -------------------------------------------------------------------------------- 0
Reedfish                    -------------------------------------------------------------------------------- 0
NorthernPike                -------------------------------------------------------------------------------- 0
SpottedGar                  -------------------------------------------------------------------------------- 0
ParamormyropsKingsLeyae     -------------------------------------------------------------------------------- 0

Majority                    --------------------------------------------------------------------------------

                                     32250     32260     32270     32280     32290     32300     32310     32320

Human                       AGACTACAGAAAGCCTGGACCTGAATAGAAAGGATCACACCCTATAGGAAGTGCCATCTATAAGACTGAGATAAAAACCA 32320
AsianBonytongue             -------------------------------------------------------------------------------- 0
Reedfish                    -------------------------------------------------------------------------------- 0
NorthernPike                -------------------------------------------------------------------------------- 0
SpottedGar                  -------------------------------------------------------------------------------- 0
ParamormyropsKingsLeyae     -------------------------------------------------------------------------------- 0

Majority                    --------------------------------------------------------------------------------

                                     32330     32340     32350     32360     32370     32380     32390     32400

Human                       GAAACCTAGCCCGATAGGAAGTGAAGGATTCACCAGTTAATCTAACCAGTAACTGATGAAGGATTAATCAGTAATTTAGT 32400
AsianBonytongue             -------------------------------------------------------------------------------- 0
Reedfish                    -------------------------------------------------------------------------------- 0
NorthernPike                -------------------------------------------------------------------------------- 0
SpottedGar                  -------------------------------------------------------------------------------- 0
ParamormyropsKingsLeyae     -------------------------------------------------------------------------------- 0

Majority                    --------------------------------------------------------------------------------

                                     32410     32420     32430     32440     32450     32460     32470     32480

Human                       TGCCTTCCAAAACAAAACAACACCCCCAAGAAAGACATGATATAGTTTTCCTGCAACTATCTTCCATAATATCTAGTATA 32480
AsianBonytongue             -------------------------------------------------------------------------------- 0
Reedfish                    -------------------------------------------------------------------------------- 0
NorthernPike                -------------------------------------------------------------------------------- 0
SpottedGar                  -------------------------------------------------------------------------------- 0
ParamormyropsKingsLeyae     -------------------------------------------------------------------------------- 0

Majority                    --------------------------------------------------------------------------------

                                     32490     32500     32510     32520     32530     32540     32550     32560

Human                       CCATCAAAAATTACAAGTGATGTGCAGAAGCAGGAAAGTAACCCATGAAATATAAAGAACACATTTAATAGGAACAGATT 32560
AsianBonytongue             -------------------------------------------------------------------------------- 0
Reedfish                    -------------------------------------------------------------------------------- 0
NorthernPike                -------------------------------------------------------------------------------- 0
SpottedGar                  -------------------------------------------------------------------------------- 0
ParamormyropsKingsLeyae     -------------------------------------------------------------------------------- 0

Majority                    --------------------------------------------------------------------------------

                                     32570     32580     32590     32600     32610     32620     32630     32640

Human                       CCAAAATGACCTAGAGTTAGAATTAGCAGACAAGGACGATAAGTATTTTAAGCTGTAAAGGAAAAGATTGAAATAATGAG 32640
AsianBonytongue             -------------------------------------------------------------------------------- 0
Reedfish                    -------------------------------------------------------------------------------- 0
NorthernPike                -------------------------------------------------------------------------------- 0
SpottedGar                  -------------------------------------------------------------------------------- 0
ParamormyropsKingsLeyae     -------------------------------------------------------------------------------- 0
```

```
Majority                  --------------------------------------------------------------------------------

                                  32650     32660     32670     32680     32690     32700     32710     32720

Human                     TGATGGAGGAGTGTGTTGAAGAGATAAAAAACTATAAAGAAAGCATGTAGAAATATTCTAATTGAAAAATACAGTTTTTG 32720
AsianBonytongue           -------------------------------------------------------------------------------- 0
Reedfish                  -------------------------------------------------------------------------------- 0
NorthernPike              -------------------------------------------------------------------------------- 0
SpottedGar                -------------------------------------------------------------------------------- 0
ParamormyropsKingsLeyae   -------------------------------------------------------------------------------- 0

Majority                  --------------------------------------------------------------------------------

                                  32730     32740     32750     32760     32770     32780     32790     32800

Human                     AAATAGTTGGGACTGATTCACTGGTTGGAACTAGTAGCAGATTGGAGACAATAGAAGAAATGTTAGTGAACTTGAATACT 32800
AsianBonytongue           -------------------------------------------------------------------------------- 0
Reedfish                  -------------------------------------------------------------------------------- 0
NorthernPike              -------------------------------------------------------------------------------- 0
SpottedGar                -------------------------------------------------------------------------------- 0
ParamormyropsKingsLeyae   -------------------------------------------------------------------------------- 0

Majority                  --------------------------------------------------------------------------------

                                  32810     32820     32830     32840     32850     32860     32870     32880

Human                     GTTGAATACAGTGATTAAATCTGTCAGAGAGGAGAAAGTATATCTAATGGACAATGAAGCAGTCTGACATGTAAGTTGAA 32880
AsianBonytongue           -------------------------------------------------------------------------------- 0
Reedfish                  -------------------------------------------------------------------------------- 0
NorthernPike              -------------------------------------------------------------------------------- 0
SpottedGar                -------------------------------------------------------------------------------- 0
ParamormyropsKingsLeyae   -------------------------------------------------------------------------------- 0

Majority                  --------------------------------------------------------------------------------

                                  32890     32900     32910     32920     32930     32940     32950     32960

Human                     TTTCAGAGTGAGAATGTGGGGGAATAAAACATCTATCTGTTTGAGATAGTAATGACTGAGAACTTCTAAAATTTGAATAA 32960
AsianBonytongue           -------------------------------------------------------------------------------- 0
Reedfish                  -------------------------------------------------------------------------------- 0
NorthernPike              -------------------------------------------------------------------------------- 0
SpottedGar                -------------------------------------------------------------------------------- 0
ParamormyropsKingsLeyae   -------------------------------------------------------------------------------- 0

Majority                  --------------------------------------------------------------------------------

                                  32970     32980     32990     33000     33010     33020     33030     33040

Human                     AATATATCAACTTACATTCCTAAGAAGCTCAGGTAAACTCCTCATGTAGGCTAAATAAAAAGAACCATACTTAGACACAT 33040
AsianBonytongue           -------------------------------------------------------------------------------- 0
Reedfish                  -------------------------------------------------------------------------------- 0
NorthernPike              -------------------------------------------------------------------------------- 0
SpottedGar                -------------------------------------------------------------------------------- 0
ParamormyropsKingsLeyae   -------------------------------------------------------------------------------- 0

Majority                  --------------------------------------------------------------------------------

                                  33050     33060     33070     33080     33090     33100     33110     33120

Human                     AGTAGTCAAAATATTGAAAACTAGAAATGAAGAGCAAATCTAAACAGCTGCGAAAAAAACGTCTAACAGGAGACCAACAC 33120
AsianBonytongue           -------------------------------------------------------------------------------- 0
Reedfish                  -------------------------------------------------------------------------------- 0
NorthernPike              -------------------------------------------------------------------------------- 0
SpottedGar                -------------------------------------------------------------------------------- 0
ParamormyropsKingsLeyae   -------------------------------------------------------------------------------- 0
```

```
Majority                   --------------------------------------------------------------------------------

                                    33130     33140     33150     33160     33170     33180     33190     33200

Human                      CTCGTATTAAAGGGGGAAAAAAAGCGAATTTCTTTAGACTGAATGGCATAACTTGGCTCTACAGGGGATAAGAGGAGTAC 33200
AsianBonytongue            -------------------------------------------------------------------------------- 0
Reedfish                   -------------------------------------------------------------------------------- 0
NorthernPike               -------------------------------------------------------------------------------- 0
SpottedGar                 -------------------------------------------------------------------------------- 0
ParamormyropsKingsLeyae    -------------------------------------------------------------------------------- 0

Majority                   --------------------------------------------------------------------------------

                                    33210     33220     33230     33240     33250     33260     33270     33280

Human                      TGGGAATGGTTAATATGTGTATAAATTTTAAAAGGGCTCTCTCTTTTCCTATCAGTTTCTTTGAAAGCCTTTATGTAAAC 33280
AsianBonytongue            -------------------------------------------------------------------------------- 0
Reedfish                   -------------------------------------------------------------------------------- 0
NorthernPike               -------------------------------------------------------------------------------- 0
SpottedGar                 -------------------------------------------------------------------------------- 0
ParamormyropsKingsLeyae    -------------------------------------------------------------------------------- 0

Majority                   --------------------------------------------------------------------------------

                                    33290     33300     33310     33320     33330     33340     33350     33360

Human                      AACAACTTTGTATTATGGGGTTTATTATGTAAATGGATGTTTATATGACAACAGTTAGTACAAAGTGAAGATAAATGGAA 33360
AsianBonytongue            -------------------------------------------------------------------------------- 0
Reedfish                   -------------------------------------------------------------------------------- 0
NorthernPike               -------------------------------------------------------------------------------- 0
SpottedGar                 -------------------------------------------------------------------------------- 0
ParamormyropsKingsLeyae    -------------------------------------------------------------------------------- 0

Majority                   --------------------------------------------------------------------------------

                                    33370     33380     33390     33400     33410     33420     33430     33440

Human                      TTCTTCAGTGGTAAGGTTCTTATATTTTACCTGAAGTGATACAGTGTTAACTCTAACATAAAAAGATTCTGTTAAGGTTG 33440
AsianBonytongue            -------------------------------------------------------------------------------- 0
Reedfish                   -------------------------------------------------------------------------------- 0
NorthernPike               -------------------------------------------------------------------------------- 0
SpottedGar                 -------------------------------------------------------------------------------- 0
ParamormyropsKingsLeyae    -------------------------------------------------------------------------------- 0

Majority                   --------------------------------------------------------------------------------

                                    33450     33460     33470     33480     33490     33500     33510     33520

Human                      CATATCAAAATTCTAAGTGTAATCTCATTTAAAAAGATAAAAAGAAGTATAACGAAAAGCCAATAGAGGAAAAATTGGAG 33520
AsianBonytongue            -------------------------------------------------------------------------------- 0
Reedfish                   -------------------------------------------------------------------------------- 0
NorthernPike               -------------------------------------------------------------------------------- 0
SpottedGar                 -------------------------------------------------------------------------------- 0
ParamormyropsKingsLeyae    -------------------------------------------------------------------------------- 0

Majority                   --------------------------------------------------------------------------------

                                    33530     33540     33550     33560     33570     33580     33590     33600

Human                      CATTGACTACCAGTTTTCAAGAATTACTGCAAAGCTACGGTAATTAAAAATAGCATCATAATAACATAAAGATAAATAGG 33600
AsianBonytongue            -------------------------------------------------------------------------------- 0
Reedfish                   -------------------------------------------------------------------------------- 0
NorthernPike               -------------------------------------------------------------------------------- 0
SpottedGar                 -------------------------------------------------------------------------------- 0
ParamormyropsKingsLeyae    -------------------------------------------------------------------------------- 0
```

```
Majority                    --------------------------------------------------------------------------------
                                    33610     33620     33630     33640     33650     33660     33670     33680
Human                       TCAGTGAGACAGAGCAGAGAGTTCAGAAGTTAACTCACACAATTATGGATAACTGATTTTTGACAAAGGTGCAAAGTCAA 33680
AsianBonytongue             -------------------------------------------------------------------------------- 0
Reedfish                    -------------------------------------------------------------------------------- 0
NorthernPike                -------------------------------------------------------------------------------- 0
SpottedGar                  -------------------------------------------------------------------------------- 0
ParamormyropsKingsLeyae     -------------------------------------------------------------------------------- 0

Majority                    --------------------------------------------------------------------------------
                                    33690     33700     33710     33720     33730     33740     33750     33760
Human                       TTCAGTGGAGAAAAAGTAGACTTCCAACAAAGGGTGCTACAGCAGTTGGAGCTATCCGTATGCAAAAAAATGATCTTGGA 33760
AsianBonytongue             -------------------------------------------------------------------------------- 0
Reedfish                    -------------------------------------------------------------------------------- 0
NorthernPike                -------------------------------------------------------------------------------- 0
SpottedGar                  -------------------------------------------------------------------------------- 0
ParamormyropsKingsLeyae     -------------------------------------------------------------------------------- 0

Majority                    --------------------------------------------------------------------------------
                                    33770     33780     33790     33800     33810     33820     33830     33840
Human                       TCTGTATCTCACACTGCATATGAAAATTAACTCCAAATGGATCATGGCCTAATGTAAAACCAAAAATTATAAAATCTCTA 33840
AsianBonytongue             -------------------------------------------------------------------------------- 0
Reedfish                    -------------------------------------------------------------------------------- 0
NorthernPike                -------------------------------------------------------------------------------- 0
SpottedGar                  -------------------------------------------------------------------------------- 0
ParamormyropsKingsLeyae     -------------------------------------------------------------------------------- 0

Majority                    --------------------------------------------------------------------------------
                                    33850     33860     33870     33880     33890     33900     33910     33920
Human                       GAAAAAAATACAGAAAATCTTCGTGACTTTGGATTAAGCAGGAATGTATTTGATATGACCCTAAAAGCACAATCTATTAA 33920
AsianBonytongue             -------------------------------------------------------------------------------- 0
Reedfish                    -------------------------------------------------------------------------------- 0
NorthernPike                -------------------------------------------------------------------------------- 0
SpottedGar                  -------------------------------------------------------------------------------- 0
ParamormyropsKingsLeyae     -------------------------------------------------------------------------------- 0

Majority                    --------------------------------------------------------------------------------
                                    33930     33940     33950     33960     33970     33980     33990     34000
Human                       AAAATGGAATTTGACATCAGAATTAAAATCTTCTGCCATTCAAAAGAAACTGCTAGGGAATGAAAAGACAAGCCATAAAC 34000
AsianBonytongue             -------------------------------------------------------------------------------- 0
Reedfish                    -------------------------------------------------------------------------------- 0
NorthernPike                -------------------------------------------------------------------------------- 0
SpottedGar                  -------------------------------------------------------------------------------- 0
ParamormyropsKingsLeyae     -------------------------------------------------------------------------------- 0

Majority                    --------------------------------------------------------------------------------
                                    34010     34020     34030     34040     34050     34060     34070     34080
Human                       TGGAAGAACATCATAACAAAGCATATAGCTGATAAAGGACTTCTATCCAGAATATTAAAAGATTCAGTATATGTAAAATC 34080
AsianBonytongue             -------------------------------------------------------------------------------- 0
Reedfish                    -------------------------------------------------------------------------------- 0
NorthernPike                -------------------------------------------------------------------------------- 0
SpottedGar                  -------------------------------------------------------------------------------- 0
ParamormyropsKingsLeyae     -------------------------------------------------------------------------------- 0
```

```
Majority                   --------------------------------------------------------------------------------

                                    34090     34100     34110     34120     34130     34140     34150     34160

Human                      AGCAGTCAAGCAACAAACTACTCAATGAATTGGCCAAAATATTTGAACAGGTAGTACAGCACAAAAGATACACAGATGGT 34160
AsianBonytongue            -------------------------------------------------------------------------------- 0
Reedfish                   -------------------------------------------------------------------------------- 0
NorthernPike               -------------------------------------------------------------------------------- 0
SpottedGar                 -------------------------------------------------------------------------------- 0
ParamormyropsKingsLeyae    -------------------------------------------------------------------------------- 0

Majority                   --------------------------------------------------------------------------------

                                    34170     34180     34190     34200     34210     34220     34230     34240

Human                      AAGTAAGCACGTGAAAAAGCTGGTCAGCATCATTAGTTATTAGGGAAATGAAAATTAAAACTACAGTGAGATAATATTAC 34240
AsianBonytongue            -------------------------------------------------------------------------------- 0
Reedfish                   -------------------------------------------------------------------------------- 0
NorthernPike               -------------------------------------------------------------------------------- 0
SpottedGar                 -------------------------------------------------------------------------------- 0
ParamormyropsKingsLeyae    -------------------------------------------------------------------------------- 0

Majority                   --------------------------------------------------------------------------------

                                    34250     34260     34270     34280     34290     34300     34310     34320

Human                      AAACCTATTAATAGTGACTAAAATTAAAAAGAGTGCCATCTTGGAAAGCATGAGTTGGGAAGAATGTGGAAGAATGTTAA 34320
AsianBonytongue            -------------------------------------------------------------------------------- 0
Reedfish                   -------------------------------------------------------------------------------- 0
NorthernPike               -------------------------------------------------------------------------------- 0
SpottedGar                 -------------------------------------------------------------------------------- 0
ParamormyropsKingsLeyae    -------------------------------------------------------------------------------- 0

Majority                   --------------------------------------------------------------------------------

                                    34330     34340     34350     34360     34370     34380     34390     34400

Human                      TATTCACAATACTGCTGATGGGAATGTAAAATCGTAGTACTTTGGAAAACAATCTGGTAGTTTCTCCATGATCCAGGTTA 34400
AsianBonytongue            -------------------------------------------------------------------------------- 0
Reedfish                   -------------------------------------------------------------------------------- 0
NorthernPike               -------------------------------------------------------------------------------- 0
SpottedGar                 -------------------------------------------------------------------------------- 0
ParamormyropsKingsLeyae    -------------------------------------------------------------------------------- 0

Majority                   --------------------------------------------------------------------------------

                                    34410     34420     34430     34440     34450     34460     34470     34480

Human                      TTTGTCTCCTAAGCATTTACCCAAGAGAAAAGAAGGCATCTATTCATTAAAAAACTCTCATGTTAATGTTCATAGCCATT 34480
AsianBonytongue            -------------------------------------------------------------------------------- 0
Reedfish                   -------------------------------------------------------------------------------- 0
NorthernPike               -------------------------------------------------------------------------------- 0
SpottedGar                 -------------------------------------------------------------------------------- 0
ParamormyropsKingsLeyae    -------------------------------------------------------------------------------- 0

Majority                   --------------------------------------------------------------------------------

                                    34490     34500     34510     34520     34530     34540     34550     34560

Human                      TTATTTGTAATCACCAAAATGTGAAACAAACATTCATCAACAGATGAACTAATAAGTAGATTCCTGTTTTGGCATCATAT 34560
AsianBonytongue            -------------------------------------------------------------------------------- 0
Reedfish                   -------------------------------------------------------------------------------- 0
NorthernPike               -------------------------------------------------------------------------------- 0
SpottedGar                 -------------------------------------------------------------------------------- 0
ParamormyropsKingsLeyae    -------------------------------------------------------------------------------- 0
```

```
Majority               --------------------------------------------------------------------------------
                                 34570     34580     34590     34600     34610     34620     34630     34640
Human                  GCTTTTCCATACAGTGGAATACAACAGCCATGTAGAATGAGCTATTGATAGACACAACAGCAAGAATGAATCTCCAAACA 34640
AsianBonytongue        -------------------------------------------------------------------------------- 0
Reedfish               -------------------------------------------------------------------------------- 0
NorthernPike           -------------------------------------------------------------------------------- 0
SpottedGar             -------------------------------------------------------------------------------- 0
ParamormyropsKingsLeyae-------------------------------------------------------------------------------- 0

Majority               --------------------------------------------------------------------------------
                                 34650     34660     34670     34680     34690     34700     34710     34720
Human                  ATTATGTTATGTAAAAGATGGGAGAAAAAAAGACTACATCCTGTATGATTCCATTTATATAAAACTCTGGGAAATGCAAA 34720
AsianBonytongue        -------------------------------------------------------------------------------- 0
Reedfish               -------------------------------------------------------------------------------- 0
NorthernPike           -------------------------------------------------------------------------------- 0
SpottedGar             -------------------------------------------------------------------------------- 0
ParamormyropsKingsLeyae-------------------------------------------------------------------------------- 0

Majority               --------------------------------------------------------------------------------
                                 34730     34740     34750     34760     34770     34780     34790     34800
Human                  CTAATCTAAAGCAAATTCATGGTTCTTAGGAGTTTAAGAGTGGGAAAGGACAGGCAGGAGGGGTGGCAAAGGGACACAGG 34800
AsianBonytongue        -------------------------------------------------------------------------------- 0
Reedfish               -------------------------------------------------------------------------------- 0
NorthernPike           -------------------------------------------------------------------------------- 0
SpottedGar             -------------------------------------------------------------------------------- 0
ParamormyropsKingsLeyae-------------------------------------------------------------------------------- 0

Majority               --------------------------------------------------------------------------------
                                 34810     34820     34830     34840     34850     34860     34870     34880
Human                  AAAACTCGGGAGTGATGGACATATAAATTATTGTGATGGTGGTTTTATGGATGTATGCATATGTCAAAACTTGTCAAATT 34880
AsianBonytongue        -------------------------------------------------------------------------------- 0
Reedfish               -------------------------------------------------------------------------------- 0
NorthernPike           -------------------------------------------------------------------------------- 0
SpottedGar             -------------------------------------------------------------------------------- 0
ParamormyropsKingsLeyae-------------------------------------------------------------------------------- 0

Majority               --------------------------------------------------------------------------------
                                 34890     34900     34910     34920     34930     34940     34950     34960
Human                  TTATAATTTAAATACATATACACATCCTAGAATGGCCGAACTTCAGAACATTGATAACACCAAATGCTGGCAAGGATGTG 34960
AsianBonytongue        -------------------------------------------------------------------------------- 0
Reedfish               -------------------------------------------------------------------------------- 0
NorthernPike           -------------------------------------------------------------------------------- 0
SpottedGar             -------------------------------------------------------------------------------- 0
ParamormyropsKingsLeyae-------------------------------------------------------------------------------- 0

Majority               --------------------------------------------------------------------------------
                                 34970     34980     34990     35000     35010     35020     35030     35040
Human                  GAGCAACAGGAACTCTCACTTACTGCTCAGTGGAATGCAGAGTGGTACAGCCACTTTGGAAGACAATTTGGCAATTTCTT 35040
AsianBonytongue        -------------------------------------------------------------------------------- 0
Reedfish               -------------------------------------------------------------------------------- 0
NorthernPike           -------------------------------------------------------------------------------- 0
SpottedGar             -------------------------------------------------------------------------------- 0
ParamormyropsKingsLeyae-------------------------------------------------------------------------------- 0
```

```
Majority                    --------------------------------------------------------------------------------

                                     35050     35060     35070     35080     35090     35100     35110     35120

Human                       AGAAAACTATACGGCCGGGCACAGTGGCTTACGCCTGTAATCCCAGCGCTTTGGGAAGCCAAGGCAGGCGGATCACCTGA 35120
AsianBonytongue             -------------------------------------------------------------------------------- 0
Reedfish                    -------------------------------------------------------------------------------- 0
NorthernPike                -------------------------------------------------------------------------------- 0
SpottedGar                  -------------------------------------------------------------------------------- 0
ParamormyropsKingsLeyae     -------------------------------------------------------------------------------- 0

Majority                    --------------------------------------------------------------------------------

                                     35130     35140     35150     35160     35170     35180     35190     35200

Human                       GGTCAGAAGTTCAAAACCAGCCTGGCCAACATGGAGAAACCCGATCTCTACTAAAAAATACAAAATTAGCTAGGCGTGGT 35200
AsianBonytongue             -------------------------------------------------------------------------------- 0
Reedfish                    -------------------------------------------------------------------------------- 0
NorthernPike                -------------------------------------------------------------------------------- 0
SpottedGar                  -------------------------------------------------------------------------------- 0
ParamormyropsKingsLeyae     -------------------------------------------------------------------------------- 0

Majority                    --------------------------------------------------------------------------------

                                     35210     35220     35230     35240     35250     35260     35270     35280

Human                       GGTGCATGCCTGTAATCCCAGCCACTCAGGAGGCTGAAGCAGGAGAATTACTTGAACCCAGGAGGTGGAGGTTGCAGTGA 35280
AsianBonytongue             -------------------------------------------------------------------------------- 0
Reedfish                    -------------------------------------------------------------------------------- 0
NorthernPike                -------------------------------------------------------------------------------- 0
SpottedGar                  -------------------------------------------------------------------------------- 0
ParamormyropsKingsLeyae     -------------------------------------------------------------------------------- 0

Majority                    --------------------------------------------------------------------------------

                                     35290     35300     35310     35320     35330     35340     35350     35360

Human                       GCCAGGATCACGCCCATTTCACTCCAACCTGGGCAACAAGAGCGAGACTCCATCTCAAATATACTTTCATTGTATGATCC 35360
AsianBonytongue             -------------------------------------------------------------------------------- 0
Reedfish                    -------------------------------------------------------------------------------- 0
NorthernPike                -------------------------------------------------------------------------------- 0
SpottedGar                  -------------------------------------------------------------------------------- 0
ParamormyropsKingsLeyae     -------------------------------------------------------------------------------- 0

Majority                    --------------------------------------------------------------------------------

                                     35370     35380     35390     35400     35410     35420     35430     35440

Human                       AGCAGTTGTGCTCCCTGGTATTTGCCTAAAGGAGTTGAAAACTTAGTCTGCGCAGAAAGTAGCACATGGATGTTTATAGC 35440
AsianBonytongue             -------------------------------------------------------------------------------- 0
Reedfish                    -------------------------------------------------------------------------------- 0
NorthernPike                -------------------------------------------------------------------------------- 0
SpottedGar                  -------------------------------------------------------------------------------- 0
ParamormyropsKingsLeyae     -------------------------------------------------------------------------------- 0

Majority                    --------------------------------------------------------------------------------

                                     35450     35460     35470     35480     35490     35500     35510     35520

Human                       ACCTTTCTTTATAGTTGCCAGAACTTCATAGCAACCAAGAAGTCCTTTAGGAGGTGAATGGCCAAATAAACTGTGGTGTA 35520
AsianBonytongue             -------------------------------------------------------------------------------- 0
Reedfish                    -------------------------------------------------------------------------------- 0
NorthernPike                -------------------------------------------------------------------------------- 0
SpottedGar                  -------------------------------------------------------------------------------- 0
ParamormyropsKingsLeyae     -------------------------------------------------------------------------------- 0
```

```
Majority                    --------------------------------------------------------------------------------

                                    35530     35540     35550     35560     35570     35580     35590     35600

Human                       TATTCAGACAGTATAATGTTAATCAGCACTAAATGGAAATGAGCTATCAAGCCATGGAGAACCTTCAGTGAACATTACTA 35600
AsianBonytongue             -------------------------------------------------------------------------------- 0
Reedfish                    -------------------------------------------------------------------------------- 0
NorthernPike                -------------------------------------------------------------------------------- 0
SpottedGar                  -------------------------------------------------------------------------------- 0
ParamormyropsKingsLeyae     -------------------------------------------------------------------------------- 0

Majority                    --------------------------------------------------------------------------------

                                    35610     35620     35630     35640     35650     35660     35670     35680

Human                       AATAAAAGAAGCCACTCTGAAAATTCTGCATACCATATGATTCCAACTATATGACATTCTGGAAAAAGCAAAATTATGGA 35680
AsianBonytongue             -------------------------------------------------------------------------------- 0
Reedfish                    -------------------------------------------------------------------------------- 0
NorthernPike                -------------------------------------------------------------------------------- 0
SpottedGar                  -------------------------------------------------------------------------------- 0
ParamormyropsKingsLeyae     -------------------------------------------------------------------------------- 0

Majority                    --------------------------------------------------------------------------------

                                    35690     35700     35710     35720     35730     35740     35750     35760

Human                       GACAGTAAAAATCAGTGGTTGCTAGGGTTTAGGGGTGGAGGGAGGGATGAACTGGTGGAGTACAGAGGATTTTAGGGTAC 35760
AsianBonytongue             -------------------------------------------------------------------------------- 0
Reedfish                    -------------------------------------------------------------------------------- 0
NorthernPike                -------------------------------------------------------------------------------- 0
SpottedGar                  -------------------------------------------------------------------------------- 0
ParamormyropsKingsLeyae     -------------------------------------------------------------------------------- 0

Majority                    --------------------------------------------------------------------------------

                                    35770     35780     35790     35800     35810     35820     35830     35840

Human                       TGAAAATATTCTGCATGATACTATAATGATAGATACATGTCATACATTTTTCCAAACCCATAGAATGTAAGACACTTGGA 35840
AsianBonytongue             -------------------------------------------------------------------------------- 0
Reedfish                    -------------------------------------------------------------------------------- 0
NorthernPike                -------------------------------------------------------------------------------- 0
SpottedGar                  -------------------------------------------------------------------------------- 0
ParamormyropsKingsLeyae     -------------------------------------------------------------------------------- 0

Majority                    --------------------------------------------------------------------------------

                                    35850     35860     35870     35880     35890     35900     35910     35920

Human                       GTGAACCTGGAGGTAACTACAGACTTTGAGTGATTATAATGTGTCAGTGTAGGTTCATCAGTTGTAACAATGTGCCATCT 35920
AsianBonytongue             -------------------------------------------------------------------------------- 0
Reedfish                    -------------------------------------------------------------------------------- 0
NorthernPike                -------------------------------------------------------------------------------- 0
SpottedGar                  -------------------------------------------------------------------------------- 0
ParamormyropsKingsLeyae     -------------------------------------------------------------------------------- 0

Majority                    --------------------------------------------------------------------------------

                                    35930     35940     35950     35960     35970     35980     35990     36000

Human                       GGTGGGAGATGTTGATAAAGGGGAGGCTATTTGGGGCAGGAGGCAGGGAAATCTCTCTGCTTTCCTCTCCATTTTGCTGT 36000
AsianBonytongue             -------------------------------------------------------------------------------- 0
Reedfish                    -------------------------------------------------------------------------------- 0
NorthernPike                -------------------------------------------------------------------------------- 0
SpottedGar                  -------------------------------------------------------------------------------- 0
ParamormyropsKingsLeyae     -------------------------------------------------------------------------------- 0
```

```
Majority                --------------------------------------------------------------------------------
                                 36010     36020     36030     36040     36050     36060     36070     36080
Human                   GTACCTAAACTTACTCTGAAAAATGTCTTTGAAAATTTACTCATGCGCTTGCATTGCATTTCTTTTGGATATTACTGGTC 36080
AsianBonytongue         -------------------------------------------------------------------------------- 0
Reedfish                -------------------------------------------------------------------------------- 0
NorthernPike            -------------------------------------------------------------------------------- 0
SpottedGar              -------------------------------------------------------------------------------- 0
ParamormyropsKingsLeyae -------------------------------------------------------------------------------- 0

Majority                --------------------------------------------------------------------------------
                                 36090     36100     36110     36120     36130     36140     36150     36160
Human                   TAAGTAGTCCAGTTAAAAGACAAAGATTGTCAGACTTGATTAAAAAGCAAGGCCTAACTACACTGTCAATACGAGGGTCA 36160
AsianBonytongue         -------------------------------------------------------------------------------- 0
Reedfish                -------------------------------------------------------------------------------- 0
NorthernPike            -------------------------------------------------------------------------------- 0
SpottedGar              -------------------------------------------------------------------------------- 0
ParamormyropsKingsLeyae -------------------------------------------------------------------------------- 0

Majority                --------------------------------------------------------------------------------
                                 36170     36180     36190     36200     36210     36220     36230     36240
Human                   GGTAGAAATATCAAGATGCAGGTTGAAAGTAAAAAGATGGAAAATGATATTCCGTGCAAACAGTAAGCACAAGAAAGTTG 36240
AsianBonytongue         -------------------------------------------------------------------------------- 0
Reedfish                -------------------------------------------------------------------------------- 0
NorthernPike            -------------------------------------------------------------------------------- 0
SpottedGar              -------------------------------------------------------------------------------- 0
ParamormyropsKingsLeyae -------------------------------------------------------------------------------- 0

Majority                --------------------------------------------------------------------------------
                                 36250     36260     36270     36280     36290     36300     36310     36320
Human                   ATATGATTATATTACTAGACAGAAAAATTTAAGACAAATATTACCAGATATAAAAAAGCATATTTCACAATGTTGAAGGG 36320
AsianBonytongue         -------------------------------------------------------------------------------- 0
Reedfish                -------------------------------------------------------------------------------- 0
NorthernPike            -------------------------------------------------------------------------------- 0
SpottedGar              -------------------------------------------------------------------------------- 0
ParamormyropsKingsLeyae -------------------------------------------------------------------------------- 0

Majority                --------------------------------------------------------------------------------
                                 36330     36340     36350     36360     36370     36380     36390     36400
Human                   TCAGTTTATCAGAAAACATTTCTTAATTTGTATGCCAATAATAACAAATCAAAGTATATTAAGAAAAACATCACAGAACT 36400
AsianBonytongue         -------------------------------------------------------------------------------- 0
Reedfish                -------------------------------------------------------------------------------- 0
NorthernPike            -------------------------------------------------------------------------------- 0
SpottedGar              -------------------------------------------------------------------------------- 0
ParamormyropsKingsLeyae -------------------------------------------------------------------------------- 0

Majority                --------------------------------------------------------------------------------
                                 36410     36420     36430     36440     36450     36460     36470     36480
Human                   AAGTAGAGAAACAAAAATCCACAATCATAGCCTGGTTTTTTAAAACATAATTTATGGCAATTGATAGGATAGTACAGTCC 36480
AsianBonytongue         -------------------------------------------------------------------------------- 0
Reedfish                -------------------------------------------------------------------------------- 0
NorthernPike            -------------------------------------------------------------------------------- 0
SpottedGar              -------------------------------------------------------------------------------- 0
ParamormyropsKingsLeyae -------------------------------------------------------------------------------- 0
```

```
Majority                    --------------------------------------------------------------------------------

                                     36490     36500     36510     36520     36530     36540     36550     36560

Human                       TAGCCCCCAAATAAAAAGATACATAATTTGAAAAGTGCTATCATGTTGGCTTAACTCACATTTATAAAACTATACCCAGT 36560
AsianBonytongue             -------------------------------------------------------------------------------- 0
Reedfish                    -------------------------------------------------------------------------------- 0
NorthernPike                -------------------------------------------------------------------------------- 0
SpottedGar                  -------------------------------------------------------------------------------- 0
ParamormyropsKingsLeyae-------------------------------------------------------------------------------- 0

Majority                    --------------------------------------------------------------------------------

                                     36570     36580     36590     36600     36610     36620     36630     36640

Human                       AACTGTAAAATAACTTCAAGTGTACATAGAACATTCACAATGATAGACCTTATATTGGATTATAAAATAACTCAATAAAT 36640
AsianBonytongue             -------------------------------------------------------------------------------- 0
Reedfish                    -------------------------------------------------------------------------------- 0
NorthernPike                -------------------------------------------------------------------------------- 0
SpottedGar                  -------------------------------------------------------------------------------- 0
ParamormyropsKingsLeyae-------------------------------------------------------------------------------- 0

Majority                    --------------------------------------------------------------------------------

                                     36650     36660     36670     36680     36690     36700     36710     36720

Human                       TTCAAATGATTGACAGCTAATGTATCTGACTGACTATAGTGGAATTAAATTAGAAATCAAAAATAGACATCTAGGAAAGT 36720
AsianBonytongue             -------------------------------------------------------------------------------- 0
Reedfish                    -------------------------------------------------------------------------------- 0
NorthernPike                -------------------------------------------------------------------------------- 0
SpottedGar                  -------------------------------------------------------------------------------- 0
ParamormyropsKingsLeyae-------------------------------------------------------------------------------- 0

Majority                    --------------------------------------------------------------------------------

                                     36730     36740     36750     36760     36770     36780     36790     36800

Human                       ACTAAATATTAGAAATTACACAATCGTGAACAATACATGGATTATAGAAGTGTTACCACAAGGACATATTTTTGACTGAA 36800
AsianBonytongue             -------------------------------------------------------------------------------- 0
Reedfish                    -------------------------------------------------------------------------------- 0
NorthernPike                -------------------------------------------------------------------------------- 0
SpottedGar                  -------------------------------------------------------------------------------- 0
ParamormyropsKingsLeyae-------------------------------------------------------------------------------- 0

Majority                    --------------------------------------------------------------------------------

                                     36810     36820     36830     36840     36850     36860     36870     36880

Human                       TGAAAATATATCAAAATTTGTTGGATACTACCCAAGGGTGTGTATGGTGAAAAGTATGTCTTCAAATACCTATATTACAA 36880
AsianBonytongue             -------------------------------------------------------------------------------- 0
Reedfish                    -------------------------------------------------------------------------------- 0
NorthernPike                -------------------------------------------------------------------------------- 0
SpottedGar                  -------------------------------------------------------------------------------- 0
ParamormyropsKingsLeyae-------------------------------------------------------------------------------- 0

Majority                    --------------------------------------------------------------------------------

                                     36890     36900     36910     36920     36930     36940     36950     36960

Human                       AAAAAGGTTTAAAACTCCCAATTTAAGACTGACCAAGGAAAAAGACATAAATTACCAATACTGGAAATCAAGGTCGAGAT 36960
AsianBonytongue             -------------------------------------------------------------------------------- 0
Reedfish                    -------------------------------------------------------------------------------- 0
NorthernPike                -------------------------------------------------------------------------------- 0
SpottedGar                  -------------------------------------------------------------------------------- 0
ParamormyropsKingsLeyae-------------------------------------------------------------------------------- 0
```

```
Majority                    --------------------------------------------------------------------------------

                                     36970     36980     36990     37000     37010     37020     37030     37040

Human                       AATCACTTTCAGAGAAAGTGGATATATGCAGAGTATAATAGTATAACATAAATAATATAAAGTGGAGATAGCACTTTCAA 37040
AsianBonytongue             -------------------------------------------------------------------------------- 0
Reedfish                    -------------------------------------------------------------------------------- 0
NorthernPike                -------------------------------------------------------------------------------- 0
SpottedGar                  -------------------------------------------------------------------------------- 0
ParamormyropsKingsLeyae     -------------------------------------------------------------------------------- 0

Majority                    --------------------------------------------------------------------------------

                                     37050     37060     37070     37080     37090     37100     37110     37120

Human                       TAAAAGCAGAGATATATAGAATATAATAGGAAAATAAGTCAATATTAGTAATGTTGTTAAGGCTAACAAATTCAGCAACT 37120
AsianBonytongue             -------------------------------------------------------------------------------- 0
Reedfish                    -------------------------------------------------------------------------------- 0
NorthernPike                -------------------------------------------------------------------------------- 0
SpottedGar                  -------------------------------------------------------------------------------- 0
ParamormyropsKingsLeyae     -------------------------------------------------------------------------------- 0

Majority                    --------------------------------------------------------------------------------

                                     37130     37140     37150     37160     37170     37180     37190     37200

Human                       TAGATGAAAAGTTTCTTGACAAATGCAAAACTGACTTAAGTATATAGCTTAACAGCTATATACTACTGTATTATACAAAT 37200
AsianBonytongue             -------------------------------------------------------------------------------- 0
Reedfish                    -------------------------------------------------------------------------------- 0
NorthernPike                -------------------------------------------------------------------------------- 0
SpottedGar                  -------------------------------------------------------------------------------- 0
ParamormyropsKingsLeyae     -------------------------------------------------------------------------------- 0

Majority                    --------------------------------------------------------------------------------

                                     37210     37220     37230     37240     37250     37260     37270     37280

Human                       TGGAATTTATAATTAAAAAAATCTTTCCACAAAGATTACTCTAGTCATAGATGGTTTCACTATTGAATTTTATGAAATTT 37280
AsianBonytongue             -------------------------------------------------------------------------------- 0
Reedfish                    -------------------------------------------------------------------------------- 0
NorthernPike                -------------------------------------------------------------------------------- 0
SpottedGar                  -------------------------------------------------------------------------------- 0
ParamormyropsKingsLeyae     -------------------------------------------------------------------------------- 0

Majority                    --------------------------------------------------------------------------------

                                     37290     37300     37310     37320     37330     37340     37350     37360

Human                       TAAAGGAATACTAGCTGTCTTTGAAAATGGAGGAGGGAATACTACCCTGTTCACTTTATGAAACCACTATAACTGACAGC 37360
AsianBonytongue             -------------------------------------------------------------------------------- 0
Reedfish                    -------------------------------------------------------------------------------- 0
NorthernPike                -------------------------------------------------------------------------------- 0
SpottedGar                  -------------------------------------------------------------------------------- 0
ParamormyropsKingsLeyae     -------------------------------------------------------------------------------- 0

Majority                    --------------------------------------------------------------------------------

                                     37370     37380     37390     37400     37410     37420     37430     37440

Human                       AAAACCTGACAAGGGTAATATATCAAGATTTCAGACTAGTATTTCTCATTAACCTAAATGCATATATTCTTAAGAAAATG 37440
AsianBonytongue             -------------------------------------------------------------------------------- 0
Reedfish                    -------------------------------------------------------------------------------- 0
NorthernPike                -------------------------------------------------------------------------------- 0
SpottedGar                  -------------------------------------------------------------------------------- 0
ParamormyropsKingsLeyae     -------------------------------------------------------------------------------- 0
```

```
Majority                    --------------------------------------------------------------------------------
                                     37450     37460     37470     37480     37490     37500     37510     37520
Human                       TTGGTAAATCACCTCCGGTAGAATATGAGAGACAATTCTGAGACTTATCGCAGGATTGTAAGGTTTGTTTAACATTTGAA 37520
AsianBonytongue             -------------------------------------------------------------------------------- 0
Reedfish                    -------------------------------------------------------------------------------- 0
NorthernPike                -------------------------------------------------------------------------------- 0
SpottedGar                  -------------------------------------------------------------------------------- 0
ParamormyropsKingsLeyae     -------------------------------------------------------------------------------- 0

Majority                    --------------------------------------------------------------------------------
                                     37530     37540     37550     37560     37570     37580     37590     37600
Human                       AATGAACTCAGTGTAATTTGTCACAATGATAGAATGAAGAAAGAAGAATTTCTCTCAGAAAACTAGGAAGAGAAGGAGCT 37600
AsianBonytongue             -------------------------------------------------------------------------------- 0
Reedfish                    -------------------------------------------------------------------------------- 0
NorthernPike                -------------------------------------------------------------------------------- 0
SpottedGar                  -------------------------------------------------------------------------------- 0
ParamormyropsKingsLeyae     -------------------------------------------------------------------------------- 0

Majority                    --------------------------------------------------------------------------------
                                     37610     37620     37630     37640     37650     37660     37670     37680
Human                       TTTACAATGTAAATAAGGGGTATCTACTAAGAACCTACAACAAATTTCATACTTAATGGAGAAATATTGAATGTTTTCTT 37680
AsianBonytongue             -------------------------------------------------------------------------------- 0
Reedfish                    -------------------------------------------------------------------------------- 0
NorthernPike                -------------------------------------------------------------------------------- 0
SpottedGar                  -------------------------------------------------------------------------------- 0
ParamormyropsKingsLeyae     -------------------------------------------------------------------------------- 0

Majority                    --------------------------------------------------------------------------------
                                     37690     37700     37710     37720     37730     37740     37750     37760
Human                       CTTAAAAACAGAAATGAAGCAAGGATGTCTGTTTTCTCAATTTTTCCCCTTAGCTTTCTGCTTACATTAAGGCAGGTGAA 37760
AsianBonytongue             -------------------------------------------------------------------------------- 0
Reedfish                    -------------------------------------------------------------------------------- 0
NorthernPike                -------------------------------------------------------------------------------- 0
SpottedGar                  -------------------------------------------------------------------------------- 0
ParamormyropsKingsLeyae     -------------------------------------------------------------------------------- 0

Majority                    --------------------------------------------------------------------------------
                                     37770     37780     37790     37800     37810     37820     37830     37840
Human                       ATAAAAGACATAAAGATTGTAAAGGAAGAAAGAAAACTGTCTTATTCACCATTCAACATGCTTTTTAAAGTAGAAGACCT 37840
AsianBonytongue             -------------------------------------------------------------------------------- 0
Reedfish                    -------------------------------------------------------------------------------- 0
NorthernPike                -------------------------------------------------------------------------------- 0
SpottedGar                  -------------------------------------------------------------------------------- 0
ParamormyropsKingsLeyae     -------------------------------------------------------------------------------- 0

Majority                    --------------------------------------------------------------------------------
                                     37850     37860     37870     37880     37890     37900     37910     37920
Human                       GAAGGACTCTACAACAGATTTGGTAGAACTAATAAGTGAATTTAGAATAATTATAGGATACAAGTTCTATGTACAGAAAT 37920
AsianBonytongue             -------------------------------------------------------------------------------- 0
Reedfish                    -------------------------------------------------------------------------------- 0
NorthernPike                -------------------------------------------------------------------------------- 0
SpottedGar                  -------------------------------------------------------------------------------- 0
ParamormyropsKingsLeyae     -------------------------------------------------------------------------------- 0
```

```
Majority                   --------------------------------------------------------------------------------
                                     37930     37940     37950     37960     37970     37980     37990     38000
Human                      AGTCTCCTTATACTTGGCAGCAAATTCTTAGAAAATGAAATTTTAAAAAATCTGTTTATAAAAGTATCAGACAAAATATC 38000
AsianBonytongue            -------------------------------------------------------------------------------- 0
Reedfish                   -------------------------------------------------------------------------------- 0
NorthernPike               -------------------------------------------------------------------------------- 0
SpottedGar                 -------------------------------------------------------------------------------- 0
ParamormyropsKingsLeyae    -------------------------------------------------------------------------------- 0

Majority                   --------------------------------------------------------------------------------
                                     38010     38020     38030     38040     38050     38060     38070     38080
Human                      AGGAAAAAAATTTGACAAAAGATATGTAAGACATCTACACCAATATGAAACACTGCTAAGAGAAGTTTTAAAAAGACTTA 38080
AsianBonytongue            -------------------------------------------------------------------------------- 0
Reedfish                   -------------------------------------------------------------------------------- 0
NorthernPike               -------------------------------------------------------------------------------- 0
SpottedGar                 -------------------------------------------------------------------------------- 0
ParamormyropsKingsLeyae    -------------------------------------------------------------------------------- 0

Majority                   --------------------------------------------------------------------------------
                                     38090     38100     38110     38120     38130     38140     38150     38160
Human                      CATGAAGACGTAGACCATGTTCATGAATTAGAAGGCTCAATATGTTAACATGCCAGTTCTCCCTAAATTGGGATGGCATT 38160
AsianBonytongue            -------------------------------------------------------------------------------- 0
Reedfish                   -------------------------------------------------------------------------------- 0
NorthernPike               -------------------------------------------------------------------------------- 0
SpottedGar                 -------------------------------------------------------------------------------- 0
ParamormyropsKingsLeyae    -------------------------------------------------------------------------------- 0

Majority                   --------------------------------------------------------------------------------
                                     38170     38180     38190     38200     38210     38220     38230     38240
Human                      GAATCTATAAAGACTTATAAATTGAGAGTACATTCTAAAATTTATGTGGAAATAAAAAGGAGCTAGGACAATTAAAACCA 38240
AsianBonytongue            -------------------------------------------------------------------------------- 0
Reedfish                   -------------------------------------------------------------------------------- 0
NorthernPike               -------------------------------------------------------------------------------- 0
SpottedGar                 -------------------------------------------------------------------------------- 0
ParamormyropsKingsLeyae    -------------------------------------------------------------------------------- 0

Majority                   --------------------------------------------------------------------------------
                                     38250     38260     38270     38280     38290     38300     38310     38320
Human                      TTTTGAAAAAGTAAATTTTGCCACAGTTCTCAAGACTCACTACAAACCTGTAGTTAATCCAAAACAGTTTACTACTAGCA 38320
AsianBonytongue            -------------------------------------------------------------------------------- 0
Reedfish                   -------------------------------------------------------------------------------- 0
NorthernPike               -------------------------------------------------------------------------------- 0
SpottedGar                 -------------------------------------------------------------------------------- 0
ParamormyropsKingsLeyae    -------------------------------------------------------------------------------- 0

Majority                   --------------------------------------------------------------------------------
                                     38330     38340     38350     38360     38370     38380     38390     38400
Human                      TAGTTAGTGACACAGAATAGCGTTCAGGGAATAACTCATGCATTAAAGTTGCCTTGATCACTCAGTGAAGAAAGAGAAGG 38400
AsianBonytongue            -------------------------------------------------------------------------------- 0
Reedfish                   -------------------------------------------------------------------------------- 0
NorthernPike               -------------------------------------------------------------------------------- 0
SpottedGar                 -------------------------------------------------------------------------------- 0
ParamormyropsKingsLeyae    -------------------------------------------------------------------------------- 0
```

```
Majority                    --------------------------------------------------------------------------------

                                     38410     38420     38430     38440     38450     38460     38470     38480

Human                       CCTTTCAACAGATGTGCTAGAACAGCTGAATATACATACAAAAAAGCAAAAGAAACTTAAACCCTACATCATTCTGTATA 38480
AsianBonytongue             -------------------------------------------------------------------------------- 0
Reedfish                    -------------------------------------------------------------------------------- 0
NorthernPike                -------------------------------------------------------------------------------- 0
SpottedGar                  -------------------------------------------------------------------------------- 0
ParamormyropsKingsLeyae     -------------------------------------------------------------------------------- 0

Majority                    --------------------------------------------------------------------------------

                                     38490     38500     38510     38520     38530     38540     38550     38560

Human                       AAACAATGAGATTAATTATATAAAAACTAATATATAGAGTTTTTAGGAAAAAGCATAGGATATACTTGCAACTTTGGGAT 38560
AsianBonytongue             -------------------------------------------------------------------------------- 0
Reedfish                    -------------------------------------------------------------------------------- 0
NorthernPike                -------------------------------------------------------------------------------- 0
SpottedGar                  -------------------------------------------------------------------------------- 0
ParamormyropsKingsLeyae     -------------------------------------------------------------------------------- 0

Majority                    --------------------------------------------------------------------------------

                                     38570     38580     38590     38600     38610     38620     38630     38640

Human                       AAATGAAGATTTCTTAAAATAGAAAAATCTTAAAGGAATATATAAACTTGACTCCATCAAAATGGGAAATTTCTGCTTCA 38640
AsianBonytongue             -------------------------------------------------------------------------------- 0
Reedfish                    -------------------------------------------------------------------------------- 0
NorthernPike                -------------------------------------------------------------------------------- 0
SpottedGar                  -------------------------------------------------------------------------------- 0
ParamormyropsKingsLeyae     -------------------------------------------------------------------------------- 0

Majority                    --------------------------------------------------------------------------------

                                     38650     38660     38670     38680     38690     38700     38710     38720

Human                       TCAAAAGACATTGTTAAGACATTGAAAAGACAAGGCACAGACTTGGAGAATATAATATTTGTGTATCAATACATGCATAT 38720
AsianBonytongue             -------------------------------------------------------------------------------- 0
Reedfish                    -------------------------------------------------------------------------------- 0
NorthernPike                -------------------------------------------------------------------------------- 0
SpottedGar                  -------------------------------------------------------------------------------- 0
ParamormyropsKingsLeyae     -------------------------------------------------------------------------------- 0

Majority                    --------------------------------------------------------------------------------

                                     38730     38740     38750     38760     38770     38780     38790     38800

Human                       GACTGTATTAGTCCATTTTCATGCTGCTGATAAAGACGTACCCGAGACTGGGCGGGCAATTTATGAAAGAAACCAGTTTA 38800
AsianBonytongue             -------------------------------------------------------------------------------- 0
Reedfish                    -------------------------------------------------------------------------------- 0
NorthernPike                -------------------------------------------------------------------------------- 0
SpottedGar                  -------------------------------------------------------------------------------- 0
ParamormyropsKingsLeyae     -------------------------------------------------------------------------------- 0

Majority                    --------------------------------------------------------------------------------

                                     38810     38820     38830     38840     38850     38860     38870     38880

Human                       ATGAATTTACAATTCTATGTGACTGGGGAAGCCTCATAATCATGGTGGAAGGCAAGGAGGAGCAAGTCACATCTTACGTA 38880
AsianBonytongue             -------------------------------------------------------------------------------- 0
Reedfish                    -------------------------------------------------------------------------------- 0
NorthernPike                -------------------------------------------------------------------------------- 0
SpottedGar                  -------------------------------------------------------------------------------- 0
ParamormyropsKingsLeyae     -------------------------------------------------------------------------------- 0
```

```
Majority                    --------------------------------------------------------------------------------

                                     38890     38900     38910     38920     38930     38940     38950     38960

Human                       GATGGCAACAGGCAAAGAAAAGAGAGCTTGTGCGGGGAAACTCTCCCCTTTTTAAAACCATGAGACTTACTTCACTGTCA 38960
AsianBonytongue             -------------------------------------------------------------------------------- 0
Reedfish                    -------------------------------------------------------------------------------- 0
NorthernPike                -------------------------------------------------------------------------------- 0
SpottedGar                  -------------------------------------------------------------------------------- 0
ParamormyropsKingsLeyae     -------------------------------------------------------------------------------- 0

Majority                    --------------------------------------------------------------------------------

                                     38970     38980     38990     39000     39010     39020     39030     39040

Human                       CGAGAACAGCATTGGAAAGACCTGCCCCCATGATTCAGTTAACTCCCACCAGGTCCCTCCCACAACACATGGGAATTCAA 39040
AsianBonytongue             -------------------------------------------------------------------------------- 0
Reedfish                    -------------------------------------------------------------------------------- 0
NorthernPike                -------------------------------------------------------------------------------- 0
SpottedGar                  -------------------------------------------------------------------------------- 0
ParamormyropsKingsLeyae     -------------------------------------------------------------------------------- 0

Majority                    --------------------------------------------------------------------------------

                                     39050     39060     39070     39080     39090     39100     39110     39120

Human                       GAGGAGATGTGGGTGGGGACACAGCCAAACCATATCAATGACAAAGGACTTGTATCCCAAAGGACTTGTAAAAAAAAAAC 39120
AsianBonytongue             -------------------------------------------------------------------------------- 0
Reedfish                    -------------------------------------------------------------------------------- 0
NorthernPike                -------------------------------------------------------------------------------- 0
SpottedGar                  -------------------------------------------------------------------------------- 0
ParamormyropsKingsLeyae     -------------------------------------------------------------------------------- 0

Majority                    --------------------------------------------------------------------------------

                                     39130     39140     39150     39160     39170     39180     39190     39200

Human                       TCCTCCAATCAATAAATATGAGCAGCCCAATTTTTTTTTAAGTGGCAAAAGAAGTGACATTATAAGGATGGGTAACAAGC 39200
AsianBonytongue             -------------------------------------------------------------------------------- 0
Reedfish                    -------------------------------------------------------------------------------- 0
NorthernPike                -------------------------------------------------------------------------------- 0
SpottedGar                  -------------------------------------------------------------------------------- 0
ParamormyropsKingsLeyae     -------------------------------------------------------------------------------- 0

Majority                    --------------------------------------------------------------------------------

                                     39210     39220     39230     39240     39250     39260     39270     39280

Human                       ACATGAAAAGATAATGTTAGTCATCAGGTGATCACAAGTCAAAACCATTAGAGATAGAATTTACACCAACTAGAATGGCT 39280
AsianBonytongue             -------------------------------------------------------------------------------- 0
Reedfish                    -------------------------------------------------------------------------------- 0
NorthernPike                -------------------------------------------------------------------------------- 0
SpottedGar                  -------------------------------------------------------------------------------- 0
ParamormyropsKingsLeyae     -------------------------------------------------------------------------------- 0

Majority                    --------------------------------------------------------------------------------

                                     39290     39300     39310     39320     39330     39340     39350     39360

Human                       AAAATTAAATAGATTGGCAATACCGAATGTTGATGAAGATGTGGAACTGTCTTTAATTACTGCTGAGTATATAACATAGT 39360
AsianBonytongue             -------------------------------------------------------------------------------- 0
Reedfish                    -------------------------------------------------------------------------------- 0
NorthernPike                -------------------------------------------------------------------------------- 0
SpottedGar                  -------------------------------------------------------------------------------- 0
ParamormyropsKingsLeyae     -------------------------------------------------------------------------------- 0
```

```
Majority                   --------------------------------------------------------------------------------
                                    39370     39380     39390     39400     39410     39420     39430     39440
Human                      TGGGAAAATAGTTTGCTTTTTTTTTTTTTTTTTTTTTTTTGAGACAGAGTCTTGCTCTGTCACCAGGCTGGAATGCAGTG 39440
AsianBonytongue            -------------------------------------------------------------------------------- 0
Reedfish                   -------------------------------------------------------------------------------- 0
NorthernPike               -------------------------------------------------------------------------------- 0
SpottedGar                 -------------------------------------------------------------------------------- 0
ParamormyropsKingsLeyae    -------------------------------------------------------------------------------- 0

Majority                   --------------------------------------------------------------------------------
                                    39450     39460     39470     39480     39490     39500     39510     39520
Human                      GCATGATCTCAGCTTACTGCAACCTCCGCCTCCTGGGTTCAAGCGATTCTCCTGCCTCAGCCTCCTGAGTAGCTGGAACT 39520
AsianBonytongue            -------------------------------------------------------------------------------- 0
Reedfish                   -------------------------------------------------------------------------------- 0
NorthernPike               -------------------------------------------------------------------------------- 0
SpottedGar                 -------------------------------------------------------------------------------- 0
ParamormyropsKingsLeyae    -------------------------------------------------------------------------------- 0

Majority                   --------------------------------------------------------------------------------
                                    39530     39540     39550     39560     39570     39580     39590     39600
Human                      ACAGGTGCACGCCACCACACCCGGCTAATTTTTTGTATTTTTAGTAGAGACTGGGTTTCGCCATGTTGGCCAGGATGGTC 39600
AsianBonytongue            -------------------------------------------------------------------------------- 0
Reedfish                   -------------------------------------------------------------------------------- 0
NorthernPike               -------------------------------------------------------------------------------- 0
SpottedGar                 -------------------------------------------------------------------------------- 0
ParamormyropsKingsLeyae    -------------------------------------------------------------------------------- 0

Majority                   --------------------------------------------------------------------------------
                                    39610     39620     39630     39640     39650     39660     39670     39680
Human                      TTGCTCTCTTGACGTTGTGATCTGCCCACGTCGGCCTCCCAGAGTGCTGGGATTACAGGCCTGAGCCACTGCGTCTGACC 39680
AsianBonytongue            -------------------------------------------------------------------------------- 0
Reedfish                   -------------------------------------------------------------------------------- 0
NorthernPike               -------------------------------------------------------------------------------- 0
SpottedGar                 -------------------------------------------------------------------------------- 0
ParamormyropsKingsLeyae    -------------------------------------------------------------------------------- 0

Majority                   --------------------------------------------------------------------------------
                                    39690     39700     39710     39720     39730     39740     39750     39760
Human                      TAGTTTACTATTTTCTTACGGAGTTAAACATTAACCTATTTTATGGTCCATAACTTCTACTTTTAGTTTTTTACCCAGTA 39760
AsianBonytongue            -------------------------------------------------------------------------------- 0
Reedfish                   -------------------------------------------------------------------------------- 0
NorthernPike               -------------------------------------------------------------------------------- 0
SpottedGar                 -------------------------------------------------------------------------------- 0
ParamormyropsKingsLeyae    -------------------------------------------------------------------------------- 0

Majority                   --------------------------------------------------------------------------------
                                    39770     39780     39790     39800     39810     39820     39830     39840
Human                      AGAACATGTTTGCTGTAAAACTTGTATGTGTATCAAAAACTTTACTGATAATGGTCCCAAACTTGAAATAACCCAAATGT 39840
AsianBonytongue            -------------------------------------------------------------------------------- 0
Reedfish                   -------------------------------------------------------------------------------- 0
NorthernPike               -------------------------------------------------------------------------------- 0
SpottedGar                 -------------------------------------------------------------------------------- 0
ParamormyropsKingsLeyae    -------------------------------------------------------------------------------- 0
```

```
Majority                   --------------------------------------------------------------------------------
                                    39850     39860     39870     39880     39890     39900     39910     39920
Human                      TTATCAACAGGAAAATGGATAAATTGTGGTATAATCATATAGTATATTACTACTTACTAATAACAAAAGAATGGTTAGTA 39920
AsianBonytongue            -------------------------------------------------------------------------------- 0
Reedfish                   -------------------------------------------------------------------------------- 0
NorthernPike               -------------------------------------------------------------------------------- 0
SpottedGar                 -------------------------------------------------------------------------------- 0
ParamormyropsKingsLeyae    -------------------------------------------------------------------------------- 0

Majority                   --------------------------------------------------------------------------------
                                    39930     39940     39950     39960     39970     39980     39990     40000
Human                      GTACATGCGAAAACATGGGTGAATAGTAAGCATTGTATGAAGCAAAAGAAACCAGACAGACCTGAAAGAGTACATAGTGT 40000
AsianBonytongue            -------------------------------------------------------------------------------- 0
Reedfish                   -------------------------------------------------------------------------------- 0
NorthernPike               -------------------------------------------------------------------------------- 0
SpottedGar                 -------------------------------------------------------------------------------- 0
ParamormyropsKingsLeyae    -------------------------------------------------------------------------------- 0

Majority                   --------------------------------------------------------------------------------
                                    40010     40020     40030     40040     40050     40060     40070     40080
Human                      GATTCCATTTCTTGAAAGTTTAAGAGCAGATCATTAATTTTTTGTTTAGAAACCAGAATGTTGGAGATTAACAGAAAGGG 40080
AsianBonytongue            -------------------------------------------------------------------------------- 0
Reedfish                   -------------------------------------------------------------------------------- 0
NorthernPike               -------------------------------------------------------------------------------- 0
SpottedGar                 -------------------------------------------------------------------------------- 0
ParamormyropsKingsLeyae    -------------------------------------------------------------------------------- 0

Majority                   --------------------------------------------------------------------------------
                                    40090     40100     40110     40120     40130     40140     40150     40160
Human                      AATATGGTGGCATTTTCTGGTGTGATGGAAACGTCCTATGTTAATAAACTAAGTTGCAAAACAAATCCAAAAGTTGTTTG 40160
AsianBonytongue            -------------------------------------------------------------------------------- 0
Reedfish                   -------------------------------------------------------------------------------- 0
NorthernPike               -------------------------------------------------------------------------------- 0
SpottedGar                 -------------------------------------------------------------------------------- 0
ParamormyropsKingsLeyae    -------------------------------------------------------------------------------- 0

Majority                   --------------------------------------------------------------------------------
                                    40170     40180     40190     40200     40210     40220     40230     40240
Human                      AAGCAGTAGATTATCTCTGAGAACTCTGAATTTCCAGTAGTTGTGTCTTGCTGTAATGAGAAATATGAGATTGGGAATGA 40240
AsianBonytongue            -------------------------------------------------------------------------------- 0
Reedfish                   -------------------------------------------------------------------------------- 0
NorthernPike               -------------------------------------------------------------------------------- 0
SpottedGar                 -------------------------------------------------------------------------------- 0
ParamormyropsKingsLeyae    -------------------------------------------------------------------------------- 0

Majority                   --------------------------------------------------------------------------------
                                    40250     40260     40270     40280     40290     40300     40310     40320
Human                      CTAGGTGAGTCCATGTAGCAGGGGCCTAGATGTGGGATTTGATGTGTGTGTGTGTGTGTGTGTGTGTGTGTGTGTGTGTG 40320
AsianBonytongue            -------------------------------------------------------------------------------- 0
Reedfish                   -------------------------------------------------------------------------------- 0
NorthernPike               -------------------------------------------------------------------------------- 0
SpottedGar                 -------------------------------------------------------------------------------- 0
ParamormyropsKingsLeyae    -------------------------------------------------------------------------------- 0
```

```
Majority               --------------------------------------------------------------------------------
                                40330     40340     40350     40360     40370     40380     40390     40400
Human                  TGTGTAGCTTGGACTTTAACAGATTTACATTTTAGAAAGAATTTTTGTTCCATTTGTATTTCACAGGTAGGATCAGTATT 40400
AsianBonytongue        -------------------------------------------------------------------------------- 0
Reedfish               -------------------------------------------------------------------------------- 0
NorthernPike           -------------------------------------------------------------------------------- 0
SpottedGar             -------------------------------------------------------------------------------- 0
ParamormyropsKingsLeyae-------------------------------------------------------------------------------- 0

Majority               --------------------------------------------------------------------------------
                                40410     40420     40430     40440     40450     40460     40470     40480
Human                  GTATAGAGGGGAGAATAGTTTTAAAACATTACGGTAGTTCATTCCAAAGATACCAAGGTCCTGAATTTTAGGGTCTTGGA 40480
AsianBonytongue        -------------------------------------------------------------------------------- 0
Reedfish               -------------------------------------------------------------------------------- 0
NorthernPike           -------------------------------------------------------------------------------- 0
SpottedGar             -------------------------------------------------------------------------------- 0
ParamormyropsKingsLeyae-------------------------------------------------------------------------------- 0

Majority               --------------------------------------------------------------------------------
                                40490     40500     40510     40520     40530     40540     40550     40560
Human                  AGGAGAATGCAGGAAATGATAAAACGAGTTATCTGGAAGAAGACAAGTTTGGAATAATATTAAAGAGTTTTGTCTTAGAA 40560
AsianBonytongue        -------------------------------------------------------------------------------- 0
Reedfish               -------------------------------------------------------------------------------- 0
NorthernPike           -------------------------------------------------------------------------------- 0
SpottedGar             -------------------------------------------------------------------------------- 0
ParamormyropsKingsLeyae-------------------------------------------------------------------------------- 0

Majority               --------------------------------------------------------------------------------
                                40570     40580     40590     40600     40610     40620     40630     40640
Human                  TCACTGGGTTGGTAGAAGATGATGCCATTAACAAAGGGGGAAAAGTAGGAAAAGGAGACTCGTTTTTGGTGGGAAAAAAG 40640
AsianBonytongue        -------------------------------------------------------------------------------- 0
Reedfish               -------------------------------------------------------------------------------- 0
NorthernPike           -------------------------------------------------------------------------------- 0
SpottedGar             -------------------------------------------------------------------------------- 0
ParamormyropsKingsLeyae-------------------------------------------------------------------------------- 0

Majority               --------------------------------------------------------------------------------
                                40650     40660     40670     40680     40690     40700     40710     40720
Human                  AATAATCCGCTACTGTTCTGTTTTTTTTTGTTTGTTTTTTGTTTTTTATTTTTTATTGATCATTCTTGGGTGTTTCTCGC 40720
AsianBonytongue        -------------------------------------------------------------------------------- 0
Reedfish               -------------------------------------------------------------------------------- 0
NorthernPike           -------------------------------------------------------------------------------- 0
SpottedGar             -------------------------------------------------------------------------------- 0
ParamormyropsKingsLeyae-------------------------------------------------------------------------------- 0

Majority               --------------------------------------------------------------------------------
                                40730     40740     40750     40760     40770     40780     40790     40800
Human                  AGAGGGGGATTTGGCAGGGTCATAGGACAATAGTGGAGGGAAGGTCAGCAGATAAACAAGTGAACAAAGGTCTCTGGTTT 40800
AsianBonytongue        -------------------------------------------------------------------------------- 0
Reedfish               -------------------------------------------------------------------------------- 0
NorthernPike           -------------------------------------------------------------------------------- 0
SpottedGar             -------------------------------------------------------------------------------- 0
ParamormyropsKingsLeyae-------------------------------------------------------------------------------- 0
```

```
Majority                    --------------------------------------------------------------------------------

                                    40810     40820     40830     40840     40850     40860     40870     40880

Human                       TCCTAGGCAGAGGACCCTGCGGACTTCCGCAGTGTTTGTGTCCCTGGGTCCTTGAGATTAGGGAGTGGTGATGACTCTTA 40880
AsianBonytongue             -------------------------------------------------------------------------------- 0
Reedfish                    -------------------------------------------------------------------------------- 0
NorthernPike                -------------------------------------------------------------------------------- 0
SpottedGar                  -------------------------------------------------------------------------------- 0
ParamormyropsKingsLeyae     -------------------------------------------------------------------------------- 0

Majority                    --------------------------------------------------------------------------------

                                    40890     40900     40910     40920     40930     40940     40950     40960

Human                       ACGAGCATGCTGCCTTCAAGCATCTGTTTAACAAAGCACATCTTGCACCGCCCTTAATCCATTTAACCCTGAGTGGACAC 40960
AsianBonytongue             -------------------------------------------------------------------------------- 0
Reedfish                    -------------------------------------------------------------------------------- 0
NorthernPike                -------------------------------------------------------------------------------- 0
SpottedGar                  -------------------------------------------------------------------------------- 0
ParamormyropsKingsLeyae     -------------------------------------------------------------------------------- 0

Majority                    --------------------------------------------------------------------------------

                                    40970     40980     40990     41000     41010     41020     41030     41040

Human                       AGCACATGTTTCAGAGAGCACAGGGTTGGGGGTAAGGTCACAGATCAACAGGATAAGAATTTTTCTTAGTACAGAACAAA 41040
AsianBonytongue             -------------------------------------------------------------------------------- 0
Reedfish                    -------------------------------------------------------------------------------- 0
NorthernPike                -------------------------------------------------------------------------------- 0
SpottedGar                  -------------------------------------------------------------------------------- 0
ParamormyropsKingsLeyae     -------------------------------------------------------------------------------- 0

Majority                    --------------------------------------------------------------------------------

                                    41050     41060     41070     41080     41090     41100     41110     41120

Human                       ATGAAAAGTCTCCCATGTCTACCTCTTTCTACACAGACACGGCAACCATCCGATTTCTCAATCTTTTCCCCACCTTTCCC 41120
AsianBonytongue             -------------------------------------------------------------------------------- 0
Reedfish                    -------------------------------------------------------------------------------- 0
NorthernPike                -------------------------------------------------------------------------------- 0
SpottedGar                  -------------------------------------------------------------------------------- 0
ParamormyropsKingsLeyae     -------------------------------------------------------------------------------- 0

Majority                    --------------------------------------------------------------------------------

                                    41130     41140     41150     41160     41170     41180     41190     41200

Human                       CCCTTTCTATTCCACAAAACCGCCATTGTCATCATGGCCCGTTCTCAATGAGCTGTTGGGTACACCTCCCAGACGGGGTG 41200
AsianBonytongue             -------------------------------------------------------------------------------- 0
Reedfish                    -------------------------------------------------------------------------------- 0
NorthernPike                -------------------------------------------------------------------------------- 0
SpottedGar                  -------------------------------------------------------------------------------- 0
ParamormyropsKingsLeyae     -------------------------------------------------------------------------------- 0

Majority                    --------------------------------------------------------------------------------

                                    41210     41220     41230     41240     41250     41260     41270     41280

Human                       GTGGCCGGGCAGAGGGGCTCCTCACTTCCCAGTAGGGGCGGCCGGGCAGAGGCGCCCCTCACTCCCCAGTAGGGGCAGCC 41280
AsianBonytongue             -------------------------------------------------------------------------------- 0
Reedfish                    -------------------------------------------------------------------------------- 0
NorthernPike                -------------------------------------------------------------------------------- 0
SpottedGar                  -------------------------------------------------------------------------------- 0
ParamormyropsKingsLeyae     -------------------------------------------------------------------------------- 0
```

```
Majority                   --------------------------------------------------------------------------------
                                41290     41300     41310     41320     41330     41340     41350     41360
Human                      GGGCAGAGGCGCCCCTCACCTCCCGGATGGGGCGGCTGGCCGGGCGGGGGGCTGACCCCCCCACCTCCCTCCCGGATGGG 41360
AsianBonytongue            -------------------------------------------------------------------------------- 0
Reedfish                   -------------------------------------------------------------------------------- 0
NorthernPike               -------------------------------------------------------------------------------- 0
SpottedGar                 -------------------------------------------------------------------------------- 0
ParamormyropsKingsLeyae    -------------------------------------------------------------------------------- 0

Majority                   --------------------------------------------------------------------------------
                                41370     41380     41390     41400     41410     41420     41430     41440
Human                      GCAGCTGGCCGGGCGGGGGGCTGACCCCCCCACCTCCCTCCCGGACGGGGCGGCTGGCCGGGCTGGGGGCTGACCCCCGC 41440
AsianBonytongue            -------------------------------------------------------------------------------- 0
Reedfish                   -------------------------------------------------------------------------------- 0
NorthernPike               -------------------------------------------------------------------------------- 0
SpottedGar                 -------------------------------------------------------------------------------- 0
ParamormyropsKingsLeyae    -------------------------------------------------------------------------------- 0

Majority                   --------------------------------------------------------------------------------
                                41450     41460     41470     41480     41490     41500     41510     41520
Human                      CACCTCCCTCCCGGACGGGGCGGCTGGCCGGGCTGGGGGCTGACCCCCCGACCTCCTTCCCGGACGGGGCGGCTGGCCGG 41520
AsianBonytongue            -------------------------------------------------------------------------------- 0
Reedfish                   -------------------------------------------------------------------------------- 0
NorthernPike               -------------------------------------------------------------------------------- 0
SpottedGar                 -------------------------------------------------------------------------------- 0
ParamormyropsKingsLeyae    -------------------------------------------------------------------------------- 0

Majority                   --------------------------------------------------------------------------------
                                41530     41540     41550     41560     41570     41580     41590     41600
Human                      GCAGAGGGGCTCCTCACTTCCCAGTAGGGGCGGCCGGGCAGAGGCGCCCCTCACCTCCCGGAAGGGGCGGCTGGCCGGGC 41600
AsianBonytongue            -------------------------------------------------------------------------------- 0
Reedfish                   -------------------------------------------------------------------------------- 0
NorthernPike               -------------------------------------------------------------------------------- 0
SpottedGar                 -------------------------------------------------------------------------------- 0
ParamormyropsKingsLeyae    -------------------------------------------------------------------------------- 0

Majority                   --------------------------------------------------------------------------------
                                41610     41620     41630     41640     41650     41660     41670     41680
Human                      GGGGGTCTGACCCCCCCACCTCCCTCCTGGATGGGGCGGCTGGCCTGGCGGGGGCTGACCCCCACCTCCCTCCCAGACGG 41680
AsianBonytongue            -------------------------------------------------------------------------------- 0
Reedfish                   -------------------------------------------------------------------------------- 0
NorthernPike               -------------------------------------------------------------------------------- 0
SpottedGar                 -------------------------------------------------------------------------------- 0
ParamormyropsKingsLeyae    -------------------------------------------------------------------------------- 0

Majority                   --------------------------------------------------------------------------------
                                41690     41700     41710     41720     41730     41740     41750     41760
Human                      GGTGACTGCCGGGCGGAGACGCTCCTCACTTCCCAGACGGCGTGGCTGCCGGGCGGAGGGGCTCCTCACTTCTCAGATGG 41760
AsianBonytongue            -------------------------------------------------------------------------------- 0
Reedfish                   -------------------------------------------------------------------------------- 0
NorthernPike               -------------------------------------------------------------------------------- 0
SpottedGar                 -------------------------------------------------------------------------------- 0
ParamormyropsKingsLeyae    -------------------------------------------------------------------------------- 0
```

```
Majority                   --------------------------------------------------------------------------------
                                   41770     41780     41790     41800     41810     41820     41830     41840
Human                      GGCGGCTGCTGGGCGGAGGGACTCCTCGCTTCTCAGATGGGGCGGCCGGGCCGAGACGCTCCTCACCTCCCAGACGGGGT 41840
AsianBonytongue            -------------------------------------------------------------------------------- 0
Reedfish                   -------------------------------------------------------------------------------- 0
NorthernPike               -------------------------------------------------------------------------------- 0
SpottedGar                 -------------------------------------------------------------------------------- 0
ParamormyropsKingsLeyae    -------------------------------------------------------------------------------- 0

Majority                   --------------------------------------------------------------------------------
                                   41850     41860     41870     41880     41890     41900     41910     41920
Human                      CGCGGCCGGGTAGAGGCGCTCCTCACATCCCAGACGGGGCGGCGGGGCAGCGGCGCTCCCCACATCTCAGACGATGGGCG 41920
AsianBonytongue            -------------------------------------------------------------------------------- 0
Reedfish                   -------------------------------------------------------------------------------- 0
NorthernPike               -------------------------------------------------------------------------------- 0
SpottedGar                 -------------------------------------------------------------------------------- 0
ParamormyropsKingsLeyae    -------------------------------------------------------------------------------- 0

Majority                   --------------------------------------------------------------------------------
                                   41930     41940     41950     41960     41970     41980     41990     42000
Human                      GCCGGGCAGAGACGCTCCTCACTTCCTAGATGGGATGGCGGCCGGGAAGAGGCGCTCCTCACTTCCTAGATGGGATGGCG 42000
AsianBonytongue            -------------------------------------------------------------------------------- 0
Reedfish                   -------------------------------------------------------------------------------- 0
NorthernPike               -------------------------------------------------------------------------------- 0
SpottedGar                 -------------------------------------------------------------------------------- 0
ParamormyropsKingsLeyae    -------------------------------------------------------------------------------- 0

Majority                   --------------------------------------------------------------------------------
                                   42010     42020     42030     42040     42050     42060     42070     42080
Human                      GCCGGGCAGAGACCTCCTTTCTTTCCAGACTGGGCAGCCAGGCAGAGGGGCTCCTCACGTTCCAGACGATGGGCGGCCAG 42080
AsianBonytongue            -------------------------------------------------------------------------------- 0
Reedfish                   -------------------------------------------------------------------------------- 0
NorthernPike               -------------------------------------------------------------------------------- 0
SpottedGar                 -------------------------------------------------------------------------------- 0
ParamormyropsKingsLeyae    -------------------------------------------------------------------------------- 0

Majority                   --------------------------------------------------------------------------------
                                   42090     42100     42110     42120     42130     42140     42150     42160
Human                      GCAGAGACGCTCCTCACTTCCCAAACGGGGTGGCGGCTGGGCAGAGGCTGCAATCTCGGCACTTTGGGAGGCCAAGGCAG 42160
AsianBonytongue            -------------------------------------------------------------------------------- 0
Reedfish                   -------------------------------------------------------------------------------- 0
NorthernPike               -------------------------------------------------------------------------------- 0
SpottedGar                 -------------------------------------------------------------------------------- 0
ParamormyropsKingsLeyae    -------------------------------------------------------------------------------- 0

Majority                   --------------------------------------------------------------------------------
                                   42170     42180     42190     42200     42210     42220     42230     42240
Human                      GCGGCTGGGAGGTGGAGGTTGTAACGAGCCGAGATCACGCCACTGCACTCCAGCCTGGGCACCATTGAGCACTGAGTGAA 42240
AsianBonytongue            -------------------------------------------------------------------------------- 0
Reedfish                   CCTCCCGATCGCTCAGTTTAGATGGCCGGCCAGCTCTAGGAAGAGTCCTGGTGGTTTTGAACTTCTTCCACTTACAGGTG 80
NorthernPike               -------------------------------------------------------------------------------- 0
SpottedGar                 -------------------------------------------------------------------------------- 0
ParamormyropsKingsLeyae    -------------------------------------------------------------------------------- 0
```

```
Majority                   --------------------------------------------------------------------------------

                                   42250     42260     42270     42280     42290     42300     42310     42320

Human                      CCAGACTCCGTCTGCAATTCCGGCACCTCGGGAGGCCGAGGCTGGCGGATCACTCGCGGTTAGGAGCTGCAGACCAGCCC 42320
AsianBonytongue            -------------------------------------------------------------------------------- 0
Reedfish                   ATGGAGGCCACTGTGCTCATTGGGACCTTCAAAGAAGCAAAAATTTTTCTGTAACCTTCCCCAGATTTGTGCCTCGAGAC 160
NorthernPike               -------------------------------------------------------------------------------- 0
SpottedGar                 -------------------------------------------------------------------------------- 0
ParamormyropsKingsLeyae-------------------------------------------------------------------------------- 0

Majority                   --------------------------------------------------------------------------------

                                   42330     42340     42350     42360     42370     42380     42390     42400

Human                      GGCCAACACAGCGAAACCCCGTCTCCACCAAAAAAATACGAAAACCAGTCAGGCGTGGCGGCGCGTGCCTGCAATAGCAG 42400
AsianBonytongue            -------------------------------------------------------------------------------- 0
Reedfish                   AATCCTGTCTCTGAGGTCTACAGACAATTCCTTTGACTTCATGCTTGGTTTGTGCTCTGACATGAACTGTCAACTGTGGG 240
NorthernPike               -------------------------------------------------------------------------------- 0
SpottedGar                 -------------------------------------------------------------------------------- 0
ParamormyropsKingsLeyae-------------------------------------------------------------------------------- 0

Majority                   --------------------------------------------------------------------------------

                                   42410     42420     42430     42440     42450     42460     42470     42480

Human                      GCACTCGGCAGGCTGAGGCAGGAGAATCAGGCAGGGAGGTTGCAGTGAGTCGAGATGGCAGCAGTACAGTCCAGCTTCGG 42480
AsianBonytongue            -------------------------------------------------------------------------------- 0
Reedfish                   ACCTTATATAGACAGGCGTGTGCCTTTCCAAATCATGTGCAATCAACTGAATTTACCACAGGTGGACTCCAATTAAGCTG 320
NorthernPike               -------------------------------------------------------------------------------- 0
SpottedGar                 -------------------------------------------------------------------------------- 0
ParamormyropsKingsLeyae-------------------------------------------------------------------------------- 0

Majority                   --------------------------------------------------------------------------------

                                   42490     42500     42510     42520     42530     42540     42550     42560

Human                      CTCGTCATCAGAGGGAGACTGTGGAAAGAGAGGGAGAGGGAGACCGTGGGGAGAGGAGGGAGGGGGAGGGGGAGGGGGAG 42560
AsianBonytongue            -------------------------------------------------------------------------------- 0
Reedfish                   CAGAAACATCTCAAGGATGATCAGGGGAAACAGGATGCACCTGAGCTCAATTTTGAGCTTCATGGCAAAGGCTGTAAATA 400
NorthernPike               -------------------------------------------------------------------------------- 0
SpottedGar                 -------------------------------------------------------------------------------- 0
ParamormyropsKingsLeyae-------------------------------------------------------------------------------- 0

Majority                   --------------------------------------------------------------------------------

                                   42570     42580     42590     42600     42610     42620     42630     42640

Human                      GGGGAGGGCTGTTCTGTTTTTTTAACTATGCTTTAGGGATGATATATAATGTACTTCATGACCAAGGAATCAATCTAATC 42640
AsianBonytongue            -------------------------------------------------------------------------------- 0
Reedfish                   CATGTGTAAATGCATGTGCTGTACATGTGCTTTCTCAGTTTTTTTATTTTTAATGAATTTGCAAAAATCTCAAGTAAACT 480
NorthernPike               -------------------------------------------------------------------------------- 0
SpottedGar                 -------------------------------------------------------------------------------- 0
ParamormyropsKingsLeyae-------------------------------------------------------------------------------- 0

Majority                   --------------------------------------------------------------------------------

                                   42650     42660     42670     42680     42690     42700     42710     42720

Human                      TTTTTGTAATGATGGCATAAAATTCCTTTAAGCCTTGTTTTAATACAAGAAGTCAGTTTATTTAACCTCCATGTACAGAG 42720
AsianBonytongue            -------------------------------------------------------------------------------- 0
Reedfish                   TTTTTCACGTTGTCATTATGGGATGTTGTGTGTAGAATTCTGAGGAAAAAAATAAATTTAATCCATTTTGCAATAAGGTT 560
NorthernPike               -------------------------------------------------------------------------------- 0
SpottedGar                 -------------------------------------------------------------------------------- 0
ParamormyropsKingsLeyae-------------------------------------------------------------------------------- 0
```

```
Majority                   --------------------------------------------------------------------------------

                                    42730     42740     42750     42760     42770     42780     42790     42800

Human                      GAAATACTGAGTTTTAAGATTTTTCTATTTTTTTTCTGACAAAAATGAAAGTCTGCATATATTCTAAGAGACACCAATTT 42800
AsianBonytongue            -------------------------------------------------------------------------------- 0
Reedfish                   GCAACATAACAAAATGTGGAAAAAGTGATGTGCTGTGAATACTTTCCGGATGCACTGTATGTATGTATGATAACCTCCAA 640
NorthernPike               -------------------------------------------------------------------------------- 0
SpottedGar                 -------------------------------------------------------------------------------- 0
ParamormyropsKingsLeyae    -------------------------------------------------------------------------------- 0

Majority                   --------------------------------------------------------------------------------

                                    42810     42820     42830     42840     42850     42860     42870     42880

Human                      GAATACTGTTGCTTATAAGAGCAAATAGCATAAAAGACCTTTAAAAATTGGTGATTATATTTGCCTGGAAAAATACTCCA 42880
AsianBonytongue            -------------------------------------------------------------------------------- 0
Reedfish                   ACATTTCTTAAACCCATGTATAAGCTCCTTGCATCTTTCTGCTGGTAGTTTCTCCAGCTCTTTAGTTCTGGCTCGAGTGC 720
NorthernPike               -------------------------------------------------------------------------------- 0
SpottedGar                 -------------------------------------------------------------------------------- 0
ParamormyropsKingsLeyae    -------------------------------------------------------------------------------- 0

Majority                   --------------------------------------------------------------------------------

                                    42890     42900     42910     42920     42930     42940     42950     42960

Human                      TGTAATTCAGATGACTCAAAAGGACGTTGGTAGACAATGTTACAAGGACACTTTTATGGTTATCAGATATAGTACAGGAT 42960
AsianBonytongue            -------------------------------------------------------------------------------- 0
Reedfish                   TTGCAGGTATTTTGTTTGCAAAAAGCAGATTTTAGCTCCATTCAAGGATGTTTAATTTGGTTAAGATCATGACTGTTTAT 800
NorthernPike               -------------------------------------------------------------------------------- 0
SpottedGar                 -------------------------------------------------------------------------------- 0
ParamormyropsKingsLeyae    -------------------------------------------------------------------------------- 0

Majority                   --------------------------------------------------------------------------------

                                    42970     42980     42990     43000     43010     43020     43030     43040

Human                      TTCATTTTTATGTTCCAGCTTCCAGCTTTATCTAAAATATTTCTTGTAATAGATTTTACTGTAGAATTGTTTCCTAAGGC 43040
AsianBonytongue            -------------------------------------------------------------------------------- 0
Reedfish                   CTATACCCAGTGATTTGTTTGATGTTGGCTTTATTTATCCTACCAAAAATTCTGTCACTACAATATGTAAAACATTGATT 880
NorthernPike               -------------------------------------------------------------------------------- 0
SpottedGar                 -------------------------------------------------------------------------------- 0
ParamormyropsKingsLeyae    -------------------------------------------------------------------------------- 0

Majority                   --------------------------------------------------------------------------------

                                    43050     43060     43070     43080     43090     43100     43110     43120

Human                      ATCTGTCTCAACAGCCTTTCCCTTATCTGTGCAGACTTTTCTTTCTCTGTACTTGTTGAATAGGTGCTTTTTGTCAAAGA 43120
AsianBonytongue            -------------------------------------------------------------------------------- 0
Reedfish                   ACGTCCTTAAGAGTCTTTATAGATGATGGGAAAGTATTTACTCACATTATGAAAAACATCAATTTCAAATACTTTGCTAG 960
NorthernPike               -------------------------------------------------------------------------------- 0
SpottedGar                 -------------------------------------------------------------------------------- 0
ParamormyropsKingsLeyae    -------------------------------------------------------------------------------- 0

Majority                   --------------------------------------------------------------------------------

                                    43130     43140     43150     43160     43170     43180     43190     43200

Human                      AAAAAATAATTTTACAGGTGAAAAAATTTCATGTTATTAGAAACTTAATCTCAGGTAAATAAATGTTTAATAAATTGATA 43200
AsianBonytongue            -------------------------------------------------------------------------------- 0
Reedfish                   TTTAGTATCTTTATGTTTAAATGTGTCCTTGCTGTGCCTAAAGCACTTGACCTCCTCTTTCTGTATTTAATACTATTTCT 1040
NorthernPike               -------------------------------------------------------------------------------- 0
SpottedGar                 -------------------------------------------------------------------------------- 0
ParamormyropsKingsLeyae    -------------------------------------------------------------------------------- 0
```

```
Majority               --------------------------------------------------------------------------------

                                43210     43220     43230     43240     43250     43260     43270     43280

Human                  CAGTAAGGACATTTTCTAATGAATTGGTTCCTTCAAGAGATATAAAAGTGAAATATTTCAAAAATACAACTGTTCCTAAG 43280
AsianBonytongue        -------------------------------------------------------------------------------- 0
Reedfish               CTCTAACTACCTTTTAGCATTCTTAATTCAGACACTTCTGTAATAAATTACTATTACATAATGTTCTGAGTCTTTCTGGG 1120
NorthernPike           -------------------------------------------------------------------------------- 0
SpottedGar             -------------------------------------------------------------------------------- 0
ParamormyropsKingsLeyae-------------------------------------------------------------------------------- 0

Majority               --------------------------------------------------------------------------------

                                43290     43300     43310     43320     43330     43340     43350     43360

Human                  TAGCATTAGAATTTGATTTTGTTAAATTTGTAACTATTTAGCAGTGACTATGAGGCTTGACAATTCTAAGGTACAGTCTC 43360
AsianBonytongue        -------------------------------------------------------------------------------- 0
Reedfish               TAACCCTAGTATAATTTTATGTTTGTCTGTTCCTTGTGTGTTTTTAAATAAGAAGGTAATTTTTAAAAAGTCTTGCTGGG 1200
NorthernPike           -------------------------------------------------------------------------------- 0
SpottedGar             -------------------------------------------------------------------------------- 0
ParamormyropsKingsLeyae-------------------------------------------------------------------------------- 0

Majority               --------------------------------------------------------------------------------

                                43370     43380     43390     43400     43410     43420     43430     43440

Human                  TT-TAATTTTCTAGTGATTTCTTTGTAGCTACGGAACTGGAATATCCCTTTAGGTATACATATTTCTGCCAGTGGTGATT 43439
AsianBonytongue        -------------------------------------------------------------------------------- 0
Reedfish               TAGTAGAATTGCATCTTCAGCAACTCTGTTATCTAATTCCAGTATGACTTTGTCAACAAACATCTTTATATATAATCTTC 1280
NorthernPike           -------------------------------------------------------------------------------- 0
SpottedGar             -------------------------------------------------------------------------------- 0
ParamormyropsKingsLeyae-------------------------------------------------------------------------------- 0

Majority               --------------------------------------------------------------------------------

                                43450     43460     43470     43480     43490     43500     43510     43520

Human                  GTTACTCTGTAATAGCAGGATTTTCTGAAAATTATTGCTAAACATGACATTTTGCTCTACCTGAAAGCAGTTAAACCATT 43519
AsianBonytongue        -------------------------------------------------------------------------------- 0
Reedfish               ATTTGGATCTTGATCTTTGTTTGTCCGCGACTTCATGTTCCCCTGACACTGCCTTGGTTGTTACTTTTGTTTACAAACAC 1360
NorthernPike           -------------------------------------------------------------------------------- 0
SpottedGar             -------------------------------------------------------------------------------- 0
ParamormyropsKingsLeyae-------------------------------------------------------------------------------- 0

Majority               --------------------------------------------------------------------------------

                                43530     43540     43550     43560     43570     43580     43590     43600

Human                  CCATAGAGTCTGGAACATTTTACTTAGAACTCTTTTAAGATTTTCATTTTAACTTTTACAGACATATTTTTGAGGTGATG 43599
AsianBonytongue        -------------------------------------------------------------------------------- 0
Reedfish               TGTCGATACCACTCTTTGTGTTTAGATCTGGCGTCGACACCTGCTTCATTGTCGAGACTGTGTTTTTTTCTGTTTCTATC 1440
NorthernPike           -------------------------------------------------------------------------------- 0
SpottedGar             -------------------------------------------------------------------------------- 0
ParamormyropsKingsLeyae-------------------------------------------------------------------------------- 0

Majority               --------------------------------------------------------------------------------

                                43610     43620     43630     43640     43650     43660     43670     43680

Human                  TCATTCTTTTTGCTATAATGTTTTGAAAGTATTCCAGTAGATTATTTCATAGTTTTACTATTTATAAAGGTTTTAACAAC 43679
AsianBonytongue        -------------------------------------------------------------------------------- 0
Reedfish               GACTGTCGTTGGCAGCACTTCGTCCAAATCAAATGTTGACCCGCTTCTTGGAGTCGGCAGTCAGAGTATATAAGTCGGAC 1520
NorthernPike           -------------------------------------------------------------------------------- 0
SpottedGar             -------------------------------------------------------------------------------- 0
ParamormyropsKingsLeyae-------------------------------------------------------------------------------- 0
```

```
Majority                --------------------------------------------------------------------------------

                                43690     43700     43710     43720     43730     43740     43750     43760

Human                   TCATCTTCTTGCATATAAAGGTAGTGAGAGCCTTATTTCATAGTGTTACTATTTATGAGGGTTTTAACAACTTATCTTCT 43759
AsianBonytongue         -------------------------------------------------------------------------------- 0
Reedfish                ACACTTCTGTGATTTTCCATCAGTCGGCGTTTGGAGTTGAAGAAAGAAGCATTGGTTCTTCCTTACTCTTTGGACTGCCA 1600
NorthernPike            -------------------------------------------------------------------------------- 0
SpottedGar              -------------------------------------------------------------------------------- 0
ParamormyropsKingsLeyae -------------------------------------------------------------------------------- 0

Majority                --------------------------------------------------------------------------------

                                43770     43780     43790     43800     43810     43820     43830     43840

Human                   TGTATATAAAGGTAGTGAGAGCCTTCTCTAGGTGCCTTCTGTGTTTTTCCAGCTCATGCCTAATATGCTTTTGACAACTA 43839
AsianBonytongue         -------------------------------------------------------------------------------- 0
Reedfish                CAAAGCAACCTGCGAGATTGGAGAAAGGTTGAGAAGAGATCGTGAGAGGAAACGACAGCGTCGTGAAAACGAGACGGACT 1680
NorthernPike            -------------------------------------------------------------------------------- 0
SpottedGar              -------------------------------------------------------------------------------- 0
ParamormyropsKingsLeyae -------------------------------------------------------------------------------- 0

Majority                --------------------------------------------------------------------------------

                                43850     43860     43870     43880     43890     43900     43910     43920

Human                   CACCACACAACTACTTGGAAATACAAGAGTTAATAGTTACCTTTTACGGAAACCTGGTGAGGCTTTTTGCGTGGTGGCTG 43919
AsianBonytongue         -------------------------------------------------------------------------------- 0
Reedfish                GTGAACAGAGACAAGCAGAAATGCTCCTCCACCACCACATAATTACTATTCGGACAGTGATTCCGAATAGGCCGTTCCTA 1760
NorthernPike            -------------------------------------------------------------------------------- 0
SpottedGar              -------------------------------------------------------------------------------- 0
ParamormyropsKingsLeyae -------------------------------------------------------------------------------- 0

Majority                --------------------------------------------------------------------------------

                                43930     43940     43950     43960     43970     43980     43990     44000

Human                   TTGCAGAAACAGCCAACAGAATAGCAGAAGTACCACCTATCTGCACCTACCTGCTTTTTTTCTCTTTAACTCTTCTTTGG 43999
AsianBonytongue         -------------------------------------------------------------------------------- 0
Reedfish                TCGAATCAATGTCCAAGGGTTTTGTTTGTAATTTTGTTTTCCCTTATAAAAAATCATAATGCTGTGCGACGAAGGGCCCA 1840
NorthernPike            -------------------------------------------------------------------------------- 0
SpottedGar              -------------------------------------------------------------------------------- 0
ParamormyropsKingsLeyae -------------------------------------------------------------------------------- 0

Majority                --------------------------------------------------------------------------------

                                44010     44020     44030     44040     44050     44060     44070     44080

Human                   AATTTGTTTCTCATACAGGAAAGAGTAGCAGCATTCATTTTTTTTTTCTTAGCTTAGAGTCTTTTTATTTTGTTTTTTGT 44079
AsianBonytongue         -------------------------------------------------------------------------------- 0
Reedfish                GTTCACGACTGGTAGCCGCGTTTAAACAGGGAGCCCTTCACAGACAACTTTAACACGCGCAACGTAGTCGGGCGCACATG 1920
NorthernPike            -------------------------------------------------------------------------------- 0
SpottedGar              -------------------------------------------------------------------------------- 0
ParamormyropsKingsLeyae -------------------------------------------------------------------------------- 0

Majority                --------------------------------------------------------------------------------

                                44090     44100     44110     44120     44130     44140     44150     44160

Human                   TTTTCTAAAGCTAGAAAACACTGC-TTTGCCTTGAAGTCTAAAACTGCTTAGAATTAAAACTTCTTTGCCTCTCAGTTTC 44158
AsianBonytongue         -------------------------------------------------------------------------------- 0
Reedfish                GCTAGTTTTATAATATTTTGCTATATTTTCCTATTTGCAAAAAAATAAAAAAAGCTGTACATTCTGACCTACACTTCAAA 2000
NorthernPike            -------------------------------------------------------------------------------- 0
SpottedGar              -------------------------------------------------------------------------------- 0
ParamormyropsKingsLeyae -------------------------------------------------------------------------------- 0
```

```
Majority                     --------------------------------------------------------------------------------
                                     44170     44180     44190     44200     44210     44220     44230     44240
Human                        ATATTTGATAAAGAGAGTTAGGAAAACTTTTATAAATGTGTGTCTCTGAAAGACTTGGAGAATGAAGATGTACCTAAGTT 44238
AsianBonytongue              -------------------------------------------------------------------------------- 0
Reedfish                     ATGTCTTTTGTGCTTTGGTTTCAGAGAGGATCTGATGAACTCCTATCTAGCAGTTTATACAACAGTCCCAACTCTAATAT 2080
NorthernPike                 -------------------------------------------------------------------------------- 0
SpottedGar                   -------------------------------------------------------------------------------- 0
ParamormyropsKingsLeyae      -------------------------------------------------------------------------------- 0

Majority                     --------------------------------------------------------------------------------
                                     44250     44260     44270     44280     44290     44300     44310     44320
Human                        TTGTCACTGATTTTCATTGGACAGAAGTATAAGGTTTTGCCGTGTCGTATTTTATATTTGTACACAGTTGAAAGTATAAC 44318
AsianBonytongue              -------------------------------------------------------------------------------- 0
Reedfish                     GAGTGGAATGGTGGTAACTGGTAAGTGTATTTGGCAGTTTTTGTATGTTCTGTAGATCTGACAGTTGGCAAGTCAAATTT 2160
NorthernPike                 -------------------------------------------------------------------------------- 0
SpottedGar                   -------------------------------------------------------------------------------- 0
ParamormyropsKingsLeyae      -------------------------------------------------------------------------------- 0

Majority                     --------------------------------------------------------------------------------
                                     44330     44340     44350     44360     44370     44380     44390     44400
Human                        GGTGAATGAGTAGGACTAAAATAATTTACAAATTATATGAGAAAGTAGGCCAGGTGCTGTGGCTCATGCCTGTATTTCAG 44398
AsianBonytongue              -------------------------------------------------------------------------------- 0
Reedfish                     TAACGGGGTACTAGTGCACAAAAATAGTTCTACAGGATTACAAGCAATCTTTTGGCTAAAATATAAATACATTTAAATAA 2240
NorthernPike                 -------------------------------------------------------------------------------- 0
SpottedGar                   -------------------------------------------------------------------------------- 0
ParamormyropsKingsLeyae      -------------------------------------------------------------------------------- 0

Majority                     --------------------------------------------------------------------------------
                                     44410     44420     44430     44440     44450     44460     44470     44480
Human                        CACTTTGGGAGGCTGAGGCAGGTGGATCACTTGAGGCCAGGAGTTCGAGACCAGCCTGGCCAACATGGTGAAACCCCATC 44478
AsianBonytongue              -------------------------------------------------------------------------------- 0
Reedfish                     TTATATAGCCAATTACATTTAGAATGCCTTTTGAGCTGTCTAGATTATGAC-ATTTTCCTCTAAATAATTGTTTTAATTA 2319
NorthernPike                 -------------------------------------------------------------------------------- 0
SpottedGar                   -------------------------------------------------------------------------------- 0
ParamormyropsKingsLeyae      -------------------------------------------------------------------------------- 0

Majority                     --------------------------------------------------------------------------------
                                     44490     44500     44510     44520     44530     44540     44550     44560
Human                        TCTACTGAAAATAGAAAAATTAGCTGGGTGTGGTGGTGCACACCTGAATTCCCAGCTACATGGGAGGCTGTGGCACGAGA 44558
AsianBonytongue              -------------------------------------------------------------------------------- 0
Reedfish                     TCTGCATATACAGAAAAAAAACTTAGATGTGGTGTTAAATTTTCTCATTCTCCAGCTAATGGAAATGATAGTAAGAAGTT 2399
NorthernPike                 -------------------------------------------------------------------------------- 0
SpottedGar                   -------------------------------------------------------------------------------- 0
ParamormyropsKingsLeyae      -------------------------------------------------------------------------------- 0

Majority                     --------------------------------------------------------------------------------
                                     44570     44580     44590     44600     44610     44620     44630     44640
Human                        ATCACTTAAACCTGGGAAGTGGAGGTTGTAGTCAACCAAGATCATGCCACTGCACTCCAGCCTGGCAATACAGCAAGACT 44638
AsianBonytongue              -------------------------------------------------------------------------------- 0
Reedfish                     ACGAGTAGAAGATAGGATGGACTGTCCACCATCACGTGTTCTTCACATCAGAAAGCTGCCTAATGAAGTTTCAGAAACAG 2479
NorthernPike                 -------------------------------------------------------------------------------- 0
SpottedGar                   -------------------------------------------------------------------------------- 0
ParamormyropsKingsLeyae      -------------------------------------------------------------------------------- 0
```

```
Majority                   --------------------------------------------------------------------------------
                                   44650     44660     44670     44680     44690     44700     44710     44720
Human                      CTGTCTTAGATAGATAGATAGATAGATAGATAGATAGATAGATAGATACTGTGAAACCCAGTGCTAGAGTGTTTATATTT 44718
AsianBonytongue            -------------------------------------------------------------------------------- 0
Reedfish                   AAGTAATTGCCTTAGGTTTACCATTTGGCAAAGTTACAAATATTCTGATGCTGAAAGGAAAAAATCAGGTATGTTAACTA 2559
NorthernPike               -------------------------------------------------------------------------------- 0
SpottedGar                 -------------------------------------------------------------------------------- 0
ParamormyropsKingsLeyae    -------------------------------------------------------------------------------- 0

Majority                   --------------------------------------------------------------------------------
                                   44730     44740     44750     44760     44770     44780     44790     44800
Human                      AATAAGTAAAGATAGGTAGAGTATCGGAACAAAAACAGCATGGGAAACTAACAATTTTGGGGAATGAATGAGTAATCAGG 44798
AsianBonytongue            -------------------------------------------------------------------------------- 0
Reedfish                   TACTGTAATTTTTTTTTCTTATTAATAAAGTGCTACTGCTTATTGGTAAAATTACATTTTAATTGGGACAGTGACTGGGT 2639
NorthernPike               -------------------------------------------------------------------------------- 0
SpottedGar                 -------------------------------------------------------------------------------- 0
ParamormyropsKingsLeyae    -------------------------------------------------------------------------------- 0

Majority                   --------------------------------------------------------------------------------
                                   44810     44820     44830     44840     44850     44860     44870     44880
Human                      ATTCAAAGCAAGTATCAAATGAGTCGGGAAATTAGAAAAGTAGAAATTGAAGTTTTTAGGGGTGCTATTAGAGAAATTCA 44878
AsianBonytongue            -------------------------------------------------------------------------------- 0
Reedfish                   GGATATATGCTAATGTAGGTGATATTCAAAGAAAACAAAACTTGGAATTTTAATTATGTTGACATTGGAGCTCATTGTTG 2719
NorthernPike               -------------------------------------------------------------------------------- 0
SpottedGar                 -------------------------------------------------------------------------------- 0
ParamormyropsKingsLeyae    -------------------------------------------------------------------------------- 0

Majority                   --------------------------------------------------------------------------------
                                   44890     44900     44910     44920     44930     44940     44950     44960
Human                      GTGAGGAAGAGAGGGTTCACAGTTCCTTGTTGACTGATGGATGTGGTGATTTAACAAATTACCCATGATCCTTGCCGACA 44958
AsianBonytongue            -------------------------------------------------------------------------------- 0
Reedfish                   ACATTAAAATTCACTTTTCTGTCATGTTATTAAATATAAGAATTTATTATGTACTAGAATGAATATTACATATATGGAAA 2799
NorthernPike               -------------------------------------------------------------------------------- 0
SpottedGar                 -------------------------------------------------------------------------------- 0
ParamormyropsKingsLeyae    -------------------------------------------------------------------------------- 0

Majority                   --------------------------------------------------------------------------------
                                   44970     44980     44990     45000     45010     45020     45030     45040
Human                      CCCATTTATGTAAAAATTGCAGGTAAAAGCTAGTGGAGAACAAAGGTCAGTCAGCAGGAGAAAAGTGATGTACTGATCAG 45038
AsianBonytongue            -------------------------------------------------------------------------------- 0
Reedfish                   ATTATTAAACACAAAATGTAATCTTATTAGATGCTTAGCAACTTTTTGTTAGCATTCTACATAAAGCATTTTATGCCCTT 2879
NorthernPike               -------------------------------------------------------------------------------- 0
SpottedGar                 -------------------------------------------------------------------------------- 0
ParamormyropsKingsLeyae    -------------------------------------------------------------------------------- 0

Majority                   --------------------------------------------------------------------------------
                                   45050     45060     45070     45080     45090     45100     45110     45120
Human                      AGGTGAAGATGCTGGCTTTAGAATTGAGGTGAAAAACTGGTTTTGAGTTTGGAAGAGAAGTATTCAAGTAGAAACCATTT 45118
AsianBonytongue            -------------------------------------------------------------------------------- 0
Reedfish                   GCAAGCAAGTGTTGGTCTCTTTCTCTCTGCACTTTAATGGAGTTGTACTTTGAGTTCAAATTTCATTGATTTAATTAATC 2959
NorthernPike               -------------------------------------------------------------------------------- 0
SpottedGar                 -------------------------------------------------------------------------------- 0
ParamormyropsKingsLeyae    -------------------------------------------------------------------------------- 0
```

```
Majority                     --------------------------------------------------------------------------------
                                      45130     45140     45150     45160     45170     45180     45190     45200
Human                        TAAAATTTTATTTGCTTTGAATCCGTGATTACATGAACATCTTCCCTAAGCAAGAGGCTTTGAAAGAGTTGAATAATGTA 45198
AsianBonytongue              -------------------------------------------------------------------------------- 0
Reedfish                     AAACAATTTAATTACATTTATGTAGCACTTTTCTAA---CCTACTCAAAGCACTTTACATAGGCAGTGGGGAAGCACCTT 3036
NorthernPike                 -------------------------------------------------------------------------------- 0
SpottedGar                   -------------------------------------------------------------------------------- 0
ParamormyropsKingsLeyae      -------------------------------------------------------------------------------- 0

Majority                     --------------------------------------------------------------------------------
                                      45210     45220     45230     45240     45250     45260     45270     45280
Human                        TGGAAAATGGTGTGCTTAAAGATTTTTTACATTCATATACAATGAATAAAATTTATATGCATAAATAAAAGCATTACCTC 45278
AsianBonytongue              -------------------------------------------------------------------------------- 0
Reedfish                     AACCACCAACCAGTATGTGGCATCCACCAGCATGATGCGACCACATACATTCTTGGACTTGTATACTTACCACATATTAC 3116
NorthernPike                 -------------------------------------------------------------------------------- 0
SpottedGar                   -------------------------------------------------------------------------------- 0
ParamormyropsKingsLeyae      -------------------------------------------------------------------------------- 0

Majority                     --------------------------------------------------------------------------------
                                      45290     45300     45310     45320     45330     45340     45350     45360
Human                        ATGCCTTCTTAACTGAACATAAACATTTGAATTATTACTGGTTTTACACGCCCAAACAAAAGTTCTGGGTTTACTGGAGG 45358
AsianBonytongue              -------------------------------------------------------------------------------- 0
Reedfish                     CTATTAAATGATGAAGGAGTAAAATAGCCCATTAGAGATAGGGGGTGATTAGAATGAACAAGGCCGTGATGGGCAATTTA 3196
NorthernPike                 -------------------------------------------------------------------------------- 0
SpottedGar                   -------------------------------------------------------------------------------- 0
ParamormyropsKingsLeyae      -------------------------------------------------------------------------------- 0

Majority                     --------------------------------------------------------------------------------
                                      45370     45380     45390     45400     45410     45420     45430     45440
Human                        AATGGAAGGGAAAGATCTCTTTCATTCCCCCAGTAAACCTTTCCCATTCAGGATCATGGGAAGATGCTGAAGAATTGTAG 45438
AsianBonytongue              -------------------------------------------------------------------------------- 0
Reedfish                     GCCAGGACATTGGGAAACACCCTACTCTCTTCGATCCCTAGGGTTGTTTTATGACCATAGAGAGTCAAGACCTCTGTTTT 3276
NorthernPike                 -------------------------------------------------------------------------------- 0
SpottedGar                   -------------------------------------------------------------------------------- 0
ParamormyropsKingsLeyae      -------------------------------------------------------------------------------- 0

Majority                     --------------------------------------------------------------------------------
                                      45450     45460     45470     45480     45490     45500     45510     45520
Human                        AACTGTAACACAGGCAGACCTAATGCTTCATAAGCTAATGTTTCCATTTTTGGACCCTTTTTCGGTGTTGCTGGAGTGTG 45518
AsianBonytongue              -------------------------------------------------------------------------------- 0
Reedfish                     ACATCTCATTCGCATGACAATGCCAATTTTTACAGTACAGCATTCCCATAATTTCACTGGGGCATTAGGGTTAACACACA 3356
NorthernPike                 -------------------------------------------------------------------------------- 0
SpottedGar                   -------------------------------------------------------------------------------- 0
ParamormyropsKingsLeyae      -------------------------------------------------------------------------------- 0

Majority                     --------------------------------------------------------------------------------
                                      45530     45540     45550     45560     45570     45580     45590     45600
Human                        TTTATATTTGAAAATAGGCTTTGTTATATTATTAAAGGAGCCCCCATTCCCAGTTTCACATTCTCATAGTATCATTTATT 45598
AsianBonytongue              -------------------------------------------------------------------------------- 0
Reedfish                     GACTACAGTGAGAGCCCCCCCCAACCCCCCACTGGCCTAACTAAGATATTTGCTCAATATATGCAATTGCACTTTGAGCA 3436
NorthernPike                 -------------------------------------------------------------------------------- 0
SpottedGar                   -------------------------------------------------------------------------------- 0
ParamormyropsKingsLeyae      -------------------------------------------------------------------------------- 0
```

```
Majority              --------------------------------------------------------------------------------

                               45610     45620     45630     45640     45650     45660     45670     45680

Human                 TAAAATGAAGACTTTTATAATATTTGGCACTAAGATATGAATACTTAGTAGTCACCTCATGGAAGCTATTAACTGAGGGA 45678
AsianBonytongue       -------------------------------------------------------------------------------- 0
Reedfish              TATAAGGCAGAAGTGTCAAACTTAAGGCCTGCATGTTTTTTAAAATGACTTTCATCCTTTTATTAATGTGTTATATACAG 3516
NorthernPike          -------------------------------------------------------------------------------- 0
SpottedGar            -------------------------------------------------------------------------------- 0
ParamormyropsKingsLeyae-------------------------------------------------------------------------------- 0

Majority              --------------------------------------------------------------------------------

                               45690     45700     45710     45720     45730     45740     45750     45760

Human                 GTGTCAGGTAGTGTAAATGTTGATAGTTTACTACAGTAACTGGCTTGGTCTTTGGGAGAAGAGGACAGATCTTTTTTAGG 45758
AsianBonytongue       -------------------------------------------------------------------------------- 0
Reedfish              CCCATTGTCACTTTTATGTTTTACAAAAACTCTTTGATGTGTGTTTCATATAGATTGTATCGGTATATGATTTAACTAAA 3596
NorthernPike          -------------------------------------------------------------------------------- 0
SpottedGar            -------------------------------------------------------------------------------- 0
ParamormyropsKingsLeyae-------------------------------------------------------------------------------- 0

Majority              --------------------------------------------------------------------------------

                               45770     45780     45790     45800     45810     45820     45830     45840

Human                 CAACCTGTATGTAATGTAATGTTTGTTTTTGAGATCCTACTATAGTGTAGTTGTTGAAGAGCACAGACTATGGAATCATG 45838
AsianBonytongue       -------------------------------------------------------------------------------- 0
Reedfish              TTACTTTTACACCTGGCATTACAGCACATCTCTAGTGTCCAGTAGCTCAAATCTTCACTCATGGACACCCTGTGTTTATG 3676
NorthernPike          -------------------------------------------------------------------------------- 0
SpottedGar            -------------------------------------------------------------------------------- 0
ParamormyropsKingsLeyae-------------------------------------------------------------------------------- 0

Majority              --------------------------------------------------------------------------------

                               45850     45860     45870     45880     45890     45900     45910     45920

Human                 CTCCCTGAGTTTAATCCTTTCTCTGCTACTTCCTGTCTATACCACTTAGTAACTATCTAATCTTGGGCATTTAACTAATT 45918
AsianBonytongue       -------------------------------------------------------------------------------- 0
Reedfish              TAGAACATATATCATATCATTCAGAATATTCTAATTTGATCTAATTTCTGTGTCTAGAGGACACCATAAAAAGATGGGTT 3756
NorthernPike          -------------------------------------------------------------------------------- 0
SpottedGar            -------------------------------------------------------------------------------- 0
ParamormyropsKingsLeyae-------------------------------------------------------------------------------- 0

Majority              --------------------------------------------------------------------------------

                               45930     45940     45950     45960     45970     45980     45990     46000

Human                 GTTACCTGTAAATGGGGTTGATAGGTCATAATGATAGCAATCTGAGAGTTGTAAATTGCCCGGCATGTAGTTAATTAGCA 45998
AsianBonytongue       -------------------------------------------------------------------------------- 0
Reedfish              TCAAATCACAGTGACCCTTTTTTCATTTTATATATTGCTTTGTATTACAATAGTAGTTTTAGGTTGCTGACAGCTAGTAC 3836
NorthernPike          -------------------------------------------------------------------------------- 0
SpottedGar            -------------------------------------------------------------------------------- 0
ParamormyropsKingsLeyae-------------------------------------------------------------------------------- 0

Majority              --------------------------------------------------------------------------------

                               46010     46020     46030     46040     46050     46060     46070     46080

Human                 GTGAATATTAAATATTGATGCAAAATAAAATTACATCTTTTCTATATTTTTTCCAAGAAGTCAAGATCTTTGTCTTATCC 46078
AsianBonytongue       -------------------------------------------------------------------------------- 0
Reedfish              ATTCCCAGTGTAAGTTAATATATAGGACATTTTAGCCTTGAGATAATACTAAGACTGAAGCTGATGTTTTTCAGAAATTG 3916
NorthernPike          -------------------------------------------------------------------------------- 0
SpottedGar            -------------------------------------------------------------------------------- 0
ParamormyropsKingsLeyae-------------------------------------------------------------------------------- 0
```

```
Majority                     --------------------------------------------------------------------------------

                                      46090     46100     46110     46120     46130     46140     46150     46160

Human                        TTTGATTCTCAGAAGTTTCCTACGTTCTTTCTGTGTTATTCAGCTATTTTACTTCTGCTTAATATCCACTATCCAGAAAA 46158
AsianBonytongue              -------------------------------------------------------------------------------- 0
Reedfish                     TTTTGGCTGGAATGTATTTTGGCAATGTAACAACACAACCCTTTAAAATGGAAATGAATCAGTTGTTAAAAAGCAGCTTA 3996
NorthernPike                 -------------------------------------------------------------------------------- 0
SpottedGar                   -------------------------------------------------------------------------------- 0
ParamormyropsKingsLeyae-------------------------------------------------------------------------------- 0

Majority                     --------------------------------------------------------------------------------

                                      46170     46180     46190     46200     46210     46220     46230     46240

Human                        CATCCAACACAAATCCAAAATAAATTATTTAATTTCCTTAATAATAGGTTTAGGCAGGCTTTTGCCTTTTGGGAAT-TAT 46237
AsianBonytongue              -------------------------------------------------------------------------------- 0
Reedfish                     ATGAATAACGAGGTTACTTTGTAATAATTTAATTGATTATATGGTTCACAGATGCTGTAAATAAAATTATTGTAATGTAA 4076
NorthernPike                 -------------------------------------------------------------------------------- 0
SpottedGar                   -------------------------------------------------------------------------------- 0
ParamormyropsKingsLeyae-------------------------------------------------------------------------------- 0

Majority                     --------------------------------------------------------------------------------

                                      46250     46260     46270     46280     46290     46300     46310     46320

Human                        TTTTGGAAAATCATTTGAAAGGGGAAGCCTTTTTTCTCCTTATGGGTACAGTGATAGGTAGTATCTTTATTGGTCTTTTT 46317
AsianBonytongue              -------------------------------------------------------------------------------- 0
Reedfish                     AGTTGGTCAACTGTTTGGCAAATATTTAAATGTTTACTTCTGTACTTTTTTTACTTCAATTAGGCCTTTTTAGAGCTTGG 4156
NorthernPike                 -------------------------------------------------------------------------------- 0
SpottedGar                   -------------------------------------------------------------------------------- 0
ParamormyropsKingsLeyae-------------------------------------------------------------------------------- 0

Majority                     --------------------------------------------------------------------------------

                                      46330     46340     46350     46360     46370     46380     46390     46400

Human                        TTTTCCTTGTCTTTCTCCCAGAAGGAAAAGGACAGACCAAATTAGGAGAAACTGAAATTTGCTTTTTTGAGTATCCTGAT 46397
AsianBonytongue              -------------------------------------------------------------------------------- 0
Reedfish                     AACAGAAGAAGCTGCAATCACCATGGTTAATTATTACACAGCTGTAACACCTCATGTTCGTAATGTGCCTGTTTTCATAC 4236
NorthernPike                 -------------------------------------------------------------------------------- 0
SpottedGar                   -------------------------------------------------------------------------------- 0
ParamormyropsKingsLeyae-------------------------------------------------------------------------------- 0

Majority                     --------------------------------------------------------------------------------

                                      46410     46420     46430     46440     46450     46460     46470     46480

Human                        ACCATAACTATAACACATGGGGCTTTAGGTTGGATTGTGAAGTATTTGTTCTCGTTATTCAAACAAACTAATGAACAAGA 46477
AsianBonytongue              -------------------------------------------------------------------------------- 0
Reedfish                     AGTATTCAAATCATAAAGAACTGAAGACAGACAGTGCTCTCAACCAGGTATGCTTTTATATCAGTAATTGCAGTATTGCT 4316
NorthernPike                 -------------------------------------------------------------------------------- 0
SpottedGar                   -------------------------------------------------------------------------------- 0
ParamormyropsKingsLeyae-------------------------------------------------------------------------------- 0

Majority                     --------------------------------------------------------------------------------

                                      46490     46500     46510     46520     46530     46540     46550     46560

Human                        ACAAATTATCAAGTTTCTGTACTCAGACTGCTTTGTCAAAAAAAGGTCTTAGTCCTGTATCAGAATAATATGTGTTCCTT 46557
AsianBonytongue              -------------------------------------------------------------------------------- 0
Reedfish                     ACATTGTACAAAGTAGATAAATTGAAATATTAGACTTGAAATTTTGCACTGAAAATATTAAATATTTTTGTTTTGTGCAA 4396
NorthernPike                 -------------------------------------------------------------------------------- 0
SpottedGar                   -------------------------------------------------------------------------------- 0
ParamormyropsKingsLeyae-------------------------------------------------------------------------------- 0
```

```
Majority                      --------------------------------------------------------------------------------
                                   46570     46580     46590     46600     46610     46620     46630     46640
Human                         GTTGACTGATGCCGATTGTTGTGTGTGCCCATTATGTGTTTTAAAGTATATGTGTATTTTTTGGATTCTGTGCTTTAGCT 46637
AsianBonytongue               -------------------------------------------------------------------------------- 0
Reedfish                      TTACTATCATGTGGTTTGATATTCCTGATGGTTTGTATTTTTACAGATTAGAGGCATAAGCAAGATTTCACTATTTTTTG 4476
NorthernPike                  -------------------------------------------------------------------------------- 0
SpottedGar                    -------------------------------------------------------------------------------- 0
ParamormyropsKingsLeyae       -------------------------------------------------------------------------------- 0

Majority                      --------------------------------------------------------------------------------
                                   46650     46660     46670     46680     46690     46700     46710     46720
Human                         TACATTTTCAATTAACGGGCCTAGAGATGATTTTGTTAGATAAGATACTACAAAATGCAAAGTTCAGTCTCTTCCTTTTG 46717
AsianBonytongue               -------------------------------------------------------------------------------- 0
Reedfish                      GATAAAAGTACTAAACTATCATCGTTTTTGATGCAAAATTAAAGGCATTCAGTGTTTTGTCATTGATCTCAATTAGAATA 4556
NorthernPike                  -------------------------------------------------------------------------------- 0
SpottedGar                    -------------------------------------------------------------------------------- 0
ParamormyropsKingsLeyae       -------------------------------------------------------------------------------- 0

Majority                      --------------------------------------------------------------------------------
                                   46730     46740     46750     46760     46770     46780     46790     46800
Human                         ACAAATTCAGGTGCCCTGTGCGCACACAAATCTTTGTCAGTAGTGAAAGGACATGTGATAGCTTCCTGTAGTTTTTTCAT 46797
AsianBonytongue               -------------------------------------------------------------------------------- 0
Reedfish                      GGACAATGTCTAAAGGAAATTTTCCCTTGATGTGAAATTCAGTTTGTCACATGCCTTAAAGTTGAACAACAAAAACATCA 4636
NorthernPike                  -------------------------------------------------------------------------------- 0
SpottedGar                    -------------------------------------------------------------------------------- 0
ParamormyropsKingsLeyae       -------------------------------------------------------------------------------- 0

Majority                      --------------------------------------------------------------------------------
                                   46810     46820     46830     46840     46850     46860     46870     46880
Human                         ATTTCTGGAGTGGTGAAGAGCTACCCAGCCAAGATTTTCAGGGCAATATATTCAGAAGCCAGAGCTTACTCTTCTGTACA 46877
AsianBonytongue               -------------------------------------------------------------------------------- 0
Reedfish                      GCCTTTTTTGAAGAGGGCTAAATGCTTTCTGTTGGTAGAGCACAGTTTGCATTTTATTTCTTTTTGTCCCTTCTAAATTT 4716
NorthernPike                  -------------------------------------------------------------------------------- 0
SpottedGar                    -------------------------------------------------------------------------------- 0
ParamormyropsKingsLeyae       -------------------------------------------------------------------------------- 0

Majority                      --------------------------------------------------------------------------------
                                   46890     46900     46910     46920     46930     46940     46950     46960
Human                         AAGACAACCTAAGGGGATTTTTAAATGCATTGGCTTGCCGTTCAAGTATATAAACCTAAGTGAAACTCCTAGAGTAAAAT 46957
AsianBonytongue               -------------------------------------------------------------------------------- 0
Reedfish                      TTATTTTTGTCTTTATTGAGAACTTTGTGTGTATGTGTCTGATTTTGATTTCTGCCCTGGTCGAATAGGTGGATTTAAGA 4796
NorthernPike                  -------------------------------------------------------------------------------- 0
SpottedGar                    -------------------------------------------------------------------------------- 0
ParamormyropsKingsLeyae       -------------------------------------------------------------------------------- 0

Majority                      --------------------------------------------------------------------------------
                                   46970     46980     46990     47000     47010     47020     47030     47040
Human                         ATGACTTAAGTATAGCTTGAGTCCATATATTATCACTTAGAGATACCTTCTCTGATCATCCAATAAAGTCATTACTACTA 47037
AsianBonytongue               -------------------------------------------------------------------------------- 0
Reedfish                      ATAAATAAATGAATGGAAATTGTTTTTTAGCTTATCTTAACCCTTTAACCGCCAACTCCCTAAATATTCCCCACGCCAGG 4876
NorthernPike                  -------------------------------------------------------------------------------- 0
SpottedGar                    -------------------------------------------------------------------------------- 0
ParamormyropsKingsLeyae       -------------------------------------------------------------------------------- 0
```

```
Majority                    --------------------------------------------------------------------------------

                                    47050     47060     47070     47080     47090     47100     47110     47120

Human                       TCAATCTTCCAGTCCTAGACAAAGTAACTTTTAAGCACATCACTGTTTTACCTCACAGCATATACCGTAGAAAGTATCTG 47117
AsianBonytongue             -------------------------------------------------------------------------------- 0
Reedfish                    CGAAATCTGAACAATTTTCGTTTTTTTACTTTTTTACTGTAAGCGGCCCTTCCACAACAATAAACTTTCATCAACTGCAA 4956
NorthernPike                -------------------------------------------------------------------------------- 0
SpottedGar                  -------------------------------------------------------------------------------- 0
ParamormyropsKingsLeyae-------------------------------------------------------------------------------- 0

Majority                    --------------------------------------------------------------------------------

                                    47130     47140     47150     47160     47170     47180     47190     47200

Human                       GTTCAGGTACTAGTTAGTCCAGCTCCCTACTATAAGGTATCAGGAATCATATAATCATCTTGTTCACTCTCTGTACCCTC 47197
AsianBonytongue             -------------------------------------------------------------------------------- 0
Reedfish                    CTGACGGTCCTGGCATGTAGGGCAACTGAAATGCTTCAAATAAATGATCAATCAAAGGACGTAGCTTGAACAAGCGGTCG 5036
NorthernPike                -------------------------------------------------------------------------------- 0
SpottedGar                  -------------------------------------------------------------------------------- 0
ParamormyropsKingsLeyae-------------------------------------------------------------------------------- 0

Majority                    --------------------------------------------------------------------------------

                                    47210     47220     47230     47240     47250     47260     47270     47280

Human                       AGAACGTAAAACATTGCTAGACACATCCCTGAATACCCAGTAAATGTTTAAGGAATTAATGAGTGAATTAATTTTGAAGA 47277
AsianBonytongue             -------------------------------------------------------------------------------- 0
Reedfish                    CGGTTTGGATCTTTCTTATCTGGCTCATTTCTGTTGTCATTCAAATGAAAGAATTTCAGCAGCAAAGAGAATCGGTTACG 5116
NorthernPike                -------------------------------------------------------------------------------- 0
SpottedGar                  -------------------------------------------------------------------------------- 0
ParamormyropsKingsLeyae-------------------------------------------------------------------------------- 0

Majority                    --------------------------------------------------------------------------------

                                    47290     47300     47310     47320     47330     47340     47350     47360

Human                       TACGTTGGAGACCAAGATAATCTGACAGTCTTATAGATGTTGTTGAACATACACAAACACATTATATAGTTTATAGGTTG 47357
AsianBonytongue             -------------------------------------------------------------------------------- 0
Reedfish                    TGTCATGACAGCTGCAAAAATAGGTGTTGCATACATAGGATCTGTAGACCAGTACATCTCAATATCTGGTTTTCTGATTA 5196
NorthernPike                -------------------------------------------------------------------------------- 0
SpottedGar                  -------------------------------------------------------------------------------- 0
ParamormyropsKingsLeyae-------------------------------------------------------------------------------- 0

Majority                    --------------------------------------------------------------------------------

                                    47370     47380     47390     47400     47410     47420     47430     47440

Human                       TGTTGAATGTTTTAGGAAATACCATAAATTACCTAAAGTTTTATACAAAATGGAGTATACATGTATATTTCTGAATGTGT 47437
AsianBonytongue             -------------------------------------------------------------------------------- 0
Reedfish                    TTCCCATCAACATCAAAATCCCAATGAATTTTTTCATTTCGTTTTCATCAGTGTCAAACCAAGCACGAACACGGGAATGT 5276
NorthernPike                -------------------------------------------------------------------------------- 0
SpottedGar                  -------------------------------------------------------------------------------- 0
ParamormyropsKingsLeyae-------------------------------------------------------------------------------- 0

Majority                    --------------------------------------------------------------------------------

                                    47450     47460     47470     47480     47490     47500     47510     47520

Human                       CAAGATCCTTCATTTTAGCTAAGTCTCAGAAATATCCTTGAGATCCTAAAAGACTAAGAACCATTGATCTGGCATAGTAG 47517
AsianBonytongue             -------------------------------------------------------------------------------- 0
Reedfish                    GGAGGTAAATTGGGATTTTTCTCAATAAACTATGCTGCATACAGATTTGTCTGATGAACAAAATGTCTGATCAAATCAGG 5356
NorthernPike                -------------------------------------------------------------------------------- 0
SpottedGar                  -------------------------------------------------------------------------------- 0
ParamormyropsKingsLeyae-------------------------------------------------------------------------------- 0
```

```
Majority                   --------------------------------------------------------------------------------
                                   47530     47540     47550     47560     47570     47580     47590     47600
Human                      ATATACTGCAAGTCCTTCAAACAACTGTATTTAAATATTACCTATTTTATAGGCATTTTGAATAATATTTTTATTTTTTG 47597
AsianBonytongue            -------------------------------------------------------------------------------- 0
Reedfish                   TGACACAAACAGCTCATAAAACTGCTCAGCAGTGTAATTGTTTACATCAACAATAAAGCCAGACGTTGCCTCAAACGGAT 5436
NorthernPike               -------------------------------------------------------------------------------- 0
SpottedGar                 -------------------------------------------------------------------------------- 0
ParamormyropsKingsLeyae    -------------------------------------------------------------------------------- 0

Majority                   --------------------------------------------------------------------------------
                                   47610     47620     47630     47640     47650     47660     47670     47680
Human                      GTGAAGTTTTGGAATGAAGACAGTTAACTAATTTGTTGATTGAATTATGTTACTTTACTTTAAATCTCACGGTAACAGAA 47677
AsianBonytongue            -------------------------------------------------------------------------------- 0
Reedfish                   GCAGAAAAGGTAGTTCACCTCGGGCAGCAGTCCAGCTGAGGTGCTGGGGATACACCCACTCAGCGCCGTCATCCGATGCG 5516
NorthernPike               -------------------------------------------------------------------------------- 0
SpottedGar                 -------------------------------------------------------------------------------- 0
ParamormyropsKingsLeyae    -------------------------------------------------------------------------------- 0

Majority                   --------------------------------------------------------------------------------
                                   47690     47700     47710     47720     47730     47740     47750     47760
Human                      TAAGGTAATTAAGTTCATGAGTGATTTAGAGTATATTGATGTTAATAGTCACAAAATATTAGGTAGTTTAGACGGCAATA 47757
AsianBonytongue            -------------------------------------------------------------------------------- 0
Reedfish                   TCTTCATTCACAGTATCATGCAGCGCACGTTGCTGCTCATCATTGTCGCTAAAATCTTCTTCAGAACTGCTACAATCATG 5596
NorthernPike               -------------------------------------------------------------------------------- 0
SpottedGar                 -------------------------------------------------------------------------------- 0
ParamormyropsKingsLeyae    -------------------------------------------------------------------------------- 0

Majority                   --------------------------------------------------------------------------------
                                   47770     47780     47790     47800     47810     47820     47830     47840
Human                      GTAATATATCAGGAGAGGCTCATTGTAGATAAGCCATAATATGGATTGACTGCATTATCATTTTTATAAACTTTACATTC 47837
AsianBonytongue            -------------------------------------------------------------------------------- 0
Reedfish                   ATCAGAATTGTCCAAAATCGCCTGCAAAGCCTCACTTGAAGTCAGTTTAC-GTTTCGCCATATTCACAGCTGTCACATGC 5675
NorthernPike               -------------------------------------------------------------------------------- 0
SpottedGar                 -------------------------------------------------------------------------------- 0
ParamormyropsKingsLeyae    -------------------------------------------------------------------------------- 0

Majority                   --------------------------------------------------------------------------------
                                   47850     47860     47870     47880     47890     47900     47910     47920
Human                      TTTGATCCCACCTTCAAGTACATTTTTCAAATCAAACAATTTATAATTTCTCAGGTAAAATTATAATCTTCCTAATTTTG 47917
AsianBonytongue            -------------------------------------------------------------------------------- 0
Reedfish                   GAATCACGTCACATGACCGGCCAAAACAACCACAGACTTGTCGAAATGCAATGTAGTAATAATACCCACGCCAAACCGTC 5755
NorthernPike               -------------------------------------------------------------------------------- 0
SpottedGar                 -------------------------------------------------------------------------------- 0
ParamormyropsKingsLeyae    -------------------------------------------------------------------------------- 0

Majority                   --------------------------------------------------------------------------------
                                   47930     47940     47950     47960     47970     47980     47990     48000
Human                      GTCAAACCAGTTTTTTCACTTTTTAAAAATGTTCATATTAGTTCATTATAAGATGATTAAATTACAAGATACAGTCATAA 47997
AsianBonytongue            -------------------------------------------------------------------------------- 0
Reedfish                   AGTTATACTACTTGCCAGGCATTCATATAACCACAGGCAAATGTGCCGGATAATTCCGGCAGTATGGCGTTAGCAATAAA 5835
NorthernPike               -------------------------------------------------------------------------------- 0
SpottedGar                 -------------------------------------------------------------------------------- 0
ParamormyropsKingsLeyae    -------------------------------------------------------------------------------- 0
```

```
Majority                     --------------------------------------------------------------------------------

                                     48010     48020     48030     48040     48050     48060     48070     48080

Human                        ATCTTGGGGATAGTGATGAGGATACTCTCGGAGAAATGCATTGTTAGGTAATTTTGTCACTGCATAAATGTCATAGAGTA 48077
AsianBonytongue              -------------------------------------------------------------------------------- 0
Reedfish                     ACAGCGGTGCCGAGGGGGTTAATAGTCATGCTTGAATCCAAGCAAAGTTATAAATGAGACCCCTGGTGTGGAATTTTCCA 5915
NorthernPike                 -------------------------------------------------------------------------------- 0
SpottedGar                   -------------------------------------------------------------------------------- 0
ParamormyropsKingsLeyae      -------------------------------------------------------------------------------- 0

Majority                     --------------------------------------------------------------------------------

                                     48090     48100     48110     48120     48130     48140     48150     48160

Human                        CATTTGCACAAACCTGCTACACATGTAGGCTATATGGTATATGTAGCCTTTTGCTCCTAGGCAACAAATTTGACAGCATG 48157
AsianBonytongue              -------------------------------------------------------------------------------- 0
Reedfish                     CTTGTAGTGTGTTTTTTTTTTTTTTTTTTTTTTGTGTTAGTGTGTTTTTTTTTTTGTGTGTGTGTGTGTGCCCATGCAG 5995
NorthernPike                 -------------------------------------------------------------------------------- 0
SpottedGar                   -------------------------------------------------------------------------------- 0
ParamormyropsKingsLeyae      -------------------------------------------------------------------------------- 0

Majority                     --------------------------------------------------------------------------------

                                     48170     48180     48190     48200     48210     48220     48230     48240

Human                        TTACTGTTCTGAATACTGGAGGCAGTTGCAACACAGTAATAAGTATTGATG-TATCTAAACAGAAAAGGTACAATAAAAA 48236
AsianBonytongue              -------------------------------------------------------------------------------- 0
Reedfish                     CACTCTTGAAAATACATGGGTGTTGTCATTTGCATGTACAGCATATTGAAACTGTCACATTTTATGGGGGATCCCTTTAA 6075
NorthernPike                 -------------------------------------------------------------------------------- 0
SpottedGar                   -------------------------------------------------------------------------------- 0
ParamormyropsKingsLeyae      -------------------------------------------------------------------------------- 0

Majority                     --------------------------------------------------------------------------------

                                     48250     48260     48270     48280     48290     48300     48310     48320

Human                        TATGGTATTATAATCTTATGGGACCACCATTGTATATGTGGCCTGTCAATGACCAAAATGTAGTTATGTGGCACATGACT 48316
AsianBonytongue              -------------------------------------------------------------------------------- 0
Reedfish                     GCCTCTTTATTCCATATAGTTGATTGAGTGATGGTGCTACGTGTTACTCACTACTTCACGAGAGTCCCCAACCCCAGTTG 6155
NorthernPike                 -------------------------------------------------------------------------------- 0
SpottedGar                   -------------------------------------------------------------------------------- 0
ParamormyropsKingsLeyae      -------------------------------------------------------------------------------- 0

Majority                     --------------------------------------------------------------------------------

                                     48330     48340     48350     48360     48370     48380     48390     48400

Human                        GTGATTGTATTCTTTAGTCATTGGACAGTTTCTTTATGAGATTACATTTTAATGTTGTTGCTTATTAATAATTACTCTTT 48396
AsianBonytongue              -------------------------------------------------------------------------------- 0
Reedfish                     TTGATAGCATGTCTTAAATATATATTGTCTCACAGTCCACCCTGCATGGGGAGTGCTTTGAGTAGTGAGAAAAGCACTAT 6235
NorthernPike                 -------------------------------------------------------------------------------- 0
SpottedGar                   -------------------------------------------------------------------------------- 0
ParamormyropsKingsLeyae      -------------------------------------------------------------------------------- 0

Majority                     --------------------------------------------------------------------------------

                                     48410     48420     48430     48440     48450     48460     48470     48480

Human                        ATAATACTGTTAAGAAACATTAACAGCAATCTTAAATAGTTGATAATTT---CATTTAATAATAATAGGTGGATATGATT 48473
AsianBonytongue              -------------------------------------------------------------------------------- 0
Reedfish                     ATACAGTGGTGCAAAAAAGTATTTAGTCAGCCACCAATTGTGCAAGTTCTCCCACTTAAAAAGATGAGAGAGGCCTGTAA 6315
NorthernPike                 -------------------------------------------------------------------------------- 0
SpottedGar                   -------------------------------------------------------------------------------- 0
ParamormyropsKingsLeyae      -------------------------------------------------------------------------------- 0
```

```
Majority                    --------------------------------------------------------------------------------
                                    48490     48500     48510     48520     48530     48540     48550     48560
Human                       TTATTCCTTTGAAAGCCTGGCATTTGAATTTTCAAGAATTATTTAAGTATTCTCAGCTATGTTTTTAAGTTCTTTTTGGA 48553
AsianBonytongue             -------------------------------------------------------------------------------- 0
Reedfish                    TTTTCATCATAGGTATACCTCAACTATGAGAGACAAAATGAGAAAAAAAAATCCAGAAAATCACATTGTCTGATGTTAAAG 6395
NorthernPike                -------------------------------------------------------------------------------- 0
SpottedGar                  -------------------------------------------------------------------------------- 0
ParamormyropsKingsLeyae     -------------------------------------------------------------------------------- 0

Majority                    --------------------------------------------------------------------------------
                                    48570     48580     48590     48600     48610     48620     48630     48640
Human                       AATTTGTAGTTTTTTTACACAAATAGGAAAATTCATACATTCTTTTATTGTTGATATATAAGGACTGTTTTTTAATGTCT 48633
AsianBonytongue             -------------------------------------------------------------------------------- 0
Reedfish                    AATTTATTTGCAAATTATGGTGGAAAATACAGTGGGGCAAAAAAGTATTTAGTCAGCCACCAATTGTGCAAGTTCTCCCA 6475
NorthernPike                -------------------------------------------------------------------------------- 0
SpottedGar                  -------------------------------------------------------------------------------- 0
ParamormyropsKingsLeyae     -------------------------------------------------------------------------------- 0

Majority                    --------------------------------------------------------------------------------
                                    48650     48660     48670     48680     48690     48700     48710     48720
Human                       TTCAGCCAATGGTAATGATAGTAAAAAATTTAAAGGAGAAGATAAAATGGATGGTGCTCCTTCTCGTGTACTTCATATTC 48713
AsianBonytongue             -------------------------------------------------------------------------------- 0
Reedfish                    CTTAAAAAGATGAGAGAGGCCTGTAATTTTCATCATAGGTATACCTCAACTATGAGAGACAAAATGAGAAAAAAAAATCCA 6555
NorthernPike                -------------------------------------------------------------------------------- 0
SpottedGar                  -------------------------------------------------------------------------------- 0
ParamormyropsKingsLeyae     -------------------------------------------------------------------------------- 0

Majority                    --------------------------------------------------------------------------------
                                    48730     48740     48750     48760     48770     48780     48790     48800
Human                       GAAAATTACCTGGGGAAGTAACAGAAACTGAAGTTATTGCTTTAGGCTTACCTTTTGGTAAGGTGACCAACATCCTTATG 48793
AsianBonytongue             -------------------------------------------------------------------------------- 0
Reedfish                    GAAAATCACATTGTCTGATGTTAAAGAATTTATTTGCAAATTATGGTGGAAAATACAGTGGGGCAAAAAAGTATTTAGTC 6635
NorthernPike                -------------------------------------------------------------------------------- 0
SpottedGar                  -------------------------------------------------------------------------------- 0
ParamormyropsKingsLeyae     -------------------------------------------------------------------------------- 0

Majority                    --------------------------------------------------------------------------------
                                    48810     48820     48830     48840     48850     48860     48870     48880
Human                       CTGAAAGGAAAAAATCAGGTACACTTCTTTCAGGGTTT--A------TGAAATGTTAAACCCCAAACTATCCAGCAGGTG 48865
AsianBonytongue             -------------------------------------------------------------------------------- 0
Reedfish                    AGCCACCTATTGTGCAAGTTCTCCCACTTAAAAAGATGAGAGATGCCTGTAATTTTCATCATAGGTATACCTCAACTATG 6715
NorthernPike                -------------------------------------------------------------------------------- 0
SpottedGar                  -------------------------------------------------------------------------------- 0
ParamormyropsKingsLeyae     -------------------------------------------------------------------------------- 0

Majority                    --------------------------------------------------------------------------------
                                    48890     48900     48910     48920     48930     48940     48950     48960
Human                       TGAATTAATTGTAAAAGGGAGAAAATAGTAGGTTATTTTTCTTAAAGAACAGAAGTCTTATTCTCACTTTTTCGAGTAGA 48945
AsianBonytongue             -------------------------------------------------------------------------------- 0
Reedfish                    AGAGACAAAATGAGAAAAAAAAAATTCAGAAAATCACATTGTCTGAGTTTTTAAAGAATTTATTTGCAAATTATGGTGGAA 6795
NorthernPike                -------------------------------------------------------------------------------- 0
SpottedGar                  -------------------------------------------------------------------------------- 0
ParamormyropsKingsLeyae     -------------------------------------------------------------------------------- 0
```

```
Majority                 --------------------------------------------------------------------------------

                              48970     48980     48990     49000     49010     49020     49030     49040

Human                    TGTCTGTGGGAAACTAATGCTAGGTAGCCAAAGTGAGTGAATCACTATAGTTTTTAAACTTTGTCTTTTGAATTGACTTT 49025
AsianBonytongue          -------------------------------------------------------------------------------- 0
Reedfish                 AATAAGTATTTGGTCACCTACAAACAAGCAAGATTTCTGGCTCTCACAAACCTGTAACTTCTTCTGTAAGAGGCTCCTCT 6875
NorthernPike             -------------------------------------------------------------------------------- 0
SpottedGar               -------------------------------------------------------------------------------- 0
ParamormyropsKingsLeyae  -------------------------------------------------------------------------------- 0

Majority                 --------------------------------------------------------------------------------

                              49050     49060     49070     49080     49090     49100     49110     49120

Human                    AGGGCTCTAATTGAATAATTGTTATGAAATTGTGGAAAATACAAATGAAGTATTTCTTTTACTGTGGCTCTGTTCTTTGG 49105
AsianBonytongue          -------------------------------------------------------------------------------- 0
Reedfish                 GTCCTCCACTCGTTACCTGTATTAATGGCACCTATTTGAGGTTGTGGACAGGTATCTTTTATATTGATAACGAGTTCAAA 6955
NorthernPike             -------------------------------------------------------------------------------- 0
SpottedGar               -------------------------------------------------------------------------------- 0
ParamormyropsKingsLeyae  -------------------------------------------------------------------------------- 0

Majority                 --------------------------------------------------------------------------------

                              49130     49140     49150     49160     49170     49180     49190     49200

Human                    TA-TCAGAAATCAGCAGGGGTACTAGTCGCATCTTTCCTTAAGCTTTATGAGAATATGAAATTTTATTTAAAAATGTTAA 49184
AsianBonytongue          -------------------------------------------------------------------------------- 0
Reedfish                 CAGTCACACTCCAAACTCTACTATGGCCAAGACCAAAGAGCTGTCAAAGGACACCAGAAACAAAATTGTAGACCTGCACC 7035
NorthernPike             -------------------------------------------------------------------------------- 0
SpottedGar               -------------------------------------------------------------------------------- 0
ParamormyropsKingsLeyae  -------------------------------------------------------------------------------- 0

Majority                 --------------------------------------------------------------------------------

                              49210     49220     49230     49240     49250     49260     49270     49280

Human                    ATGATACCATTTAGAAGAATTTATGAAATTTAACACTCCCTTTTATATACTTGAGGTTAATCTGACATTTAATTTGTAAT 49264
AsianBonytongue          -------------------------------------------------------------------------------- 0
Reedfish                 AGGCTGGGAAGACTGAATCTGCAATAGGTAAGCAGCTTGGTGTGAAGAAATCAACTGTGGGAGCAATTATTAGAAAATGG 7115
NorthernPike             -------------------------------------------------------------------------------- 0
SpottedGar               -------------------------------------------------------------------------------- 0
ParamormyropsKingsLeyae  -------------------------------------------------------------------------------- 0

Majority                 --------------------------------------------------------------------------------

                              49290     49300     49310     49320     49330     49340     49350     49360

Human                    TTTGTTCTTTGTCTCTATATAATTATACTGCTTTTAAAATGACTTGGAAATCTTACATTCTCTAGGGTAGAAGTGGTAAA 49344
AsianBonytongue          -------------------------------------------------------------------------------- 0
Reedfish                 AAGACATATAAGACCACTGATAATCTCCCTCAATCTGGGGCTCCACGCAAGATCTCATCCCGTGGGGTCAAAATGATCAC 7195
NorthernPike             -------------------------------------------------------------------------------- 0
SpottedGar               -------------------------------------------------------------------------------- 0
ParamormyropsKingsLeyae  -------------------------------------------------------------------------------- 0

Majority                 --------------------------------------------------------------------------------

                              49370     49380     49390     49400     49410     49420     49430     49440

Human                    ATTCTGATGCTAGAAAATGTTTACATTTTCTCTAGCATTTGTTGCAAGAATAGATTTGTGGGTGTGAAAAATCATAGGTG 49424
AsianBonytongue          -------------------------------------------------------------------------------- 0
Reedfish                 AAGAACGGTGAGCAAAACTCCCAGAACCACAGGGGGGGACCTAGTGAATGACCTGCAGAGAGCTGGGACCAAAGTAACAA 7275
NorthernPike             -------------------------------------------------------------------------------- 0
SpottedGar               -------------------------------------------------------------------------------- 0
ParamormyropsKingsLeyae  -------------------------------------------------------------------------------- 0
```

```
Majority                  --------------------------------------------------------------------------------

                                   49450     49460     49470     49480     49490     49500     49510     49520

Human                     GAAGGCAGAAGCAGCGTGTACTCGGTTTTCT----TCTGAGGTTAGCTTTAACTGTATACCTAAACTTCAACAAGAGATT 49500
AsianBonytongue           -------------------------------------------------------------------------------- 0
Reedfish                  AGGCTACCATCAGTAACACACTACGCCGCCAGGAATTCAAATCCTGCAGTGCCAGACGTGTCCCCCTGCTTAAGCCAGTA 7355
NorthernPike              -------------------------------------------------------------------------------- 0
SpottedGar                -------------------------------------------------------------------------------- 0
ParamormyropsKingsLeyae   -------------------------------------------------------------------------------- 0

Majority                  --------------------------------------------------------------------------------

                                   49530     49540     49550     49560     49570     49580     49590     49600

Human                     TCTAATTCTGCTTATCAGAACATTTAAAAGTAACCTGAAGGATTATATTCTAGATAATTGATTAAAATCATCTGAATAGA 49580
AsianBonytongue           -------------------------------------------------------------------------------- 0
Reedfish                  CATGTGCAGGCCCATCTGAAGTTTGCTAGAGAGCATTTGGATGATCCAGAAAAGGATTGGGAGAGTGTCATATGGTCAGA 7435
NorthernPike              -------------------------------------------------------------------------------- 0
SpottedGar                -------------------------------------------------------------------------------- 0
ParamormyropsKingsLeyae   -------------------------------------------------------------------------------- 0

Majority                  --------------------------------------------------------------------------------

                                   49610     49620     49630     49640     49650     49660     49670     49680

Human                     TTGCTTAGATATTAATTTTATTTGAATTTATAGTATTAAAAATTGTGCTACTTAAATTTTCAGGCATTTTTGGAACTAGC 49660
AsianBonytongue           -------------------------------------------------------------------------------- 0
Reedfish                  TGAAACCAAAATAGAACTTTTTGGTAAAAACTCTACTCGTCGTGTTTGGAAGAGAACGAATACTGAGTTGCATCCAAAGA 7515
NorthernPike              -------------------------------------------------------------------------------- 0
SpottedGar                -------------------------------------------------------------------------------- 0
ParamormyropsKingsLeyae   -------------------------------------------------------------------------------- 0

Majority                  --------------------------------------------------------------------------------

                                   49690     49700     49710     49720     49730     49740     49750     49760

Human                     AACCGAGGAAGCAGCTATTACTATGGTTAATTACTATTCTGCTGTGACACCTCATCTTCGTAACCAACCAATATATATCC 49740
AsianBonytongue           -------------------------------------------------------------------------------- 0
Reedfish                  ACACCATACCTACTGTAAAGCATGGGGGTGGAAACATCATGCTTTGGGGCTGTTTTTCTGCAAAGGGACCATGACGACTG 7595
NorthernPike              -------------------------------------------------------------------------------- 0
SpottedGar                -------------------------------------------------------------------------------- 0
ParamormyropsKingsLeyae   -------------------------------------------------------------------------------- 0

Majority                  --------------------------------------------------------------------------------

                                   49770     49780     49790     49800     49810     49820     49830     49840

Human                     AGTACTCGAATCACAAAGAACTAAAGACAGATAATACATTAAACCAAGTAAGTATGTGTAGGTACATAAATAAAATGGCC 49820
AsianBonytongue           -------------------------------------------------------------------------------- 0
Reedfish                  ATCCGTGTAAAGGAAAGAATGTATGGGTCCATGTATCGTCAGATTTTGAGTGAAAACCTCCATCTTCAATGCCCTTGCTG 7675
NorthernPike              -------------------------------------------------------------------------------- 0
SpottedGar                -------------------------------------------------------------------------------- 0
ParamormyropsKingsLeyae   -------------------------------------------------------------------------------- 0

Majority                  --------------------------------------------------------------------------------

                                   49850     49860     49870     49880     49890     49900     49910     49920

Human                     TAGAACATATTATGAATCTCATAAACATTAATAGGAAGGAAATGTTTACCTGTTCAGATGTTCCTTATAGGCATTTTCTT 49900
AsianBonytongue           -------------------------------------------------------------------------------- 0
Reedfish                  ATGGAAATGTGGCTGGGTCTTTCAGCATGACAATGATCCCAAACACACCACCCGGGTAACGAAGGAGTGGCTTCATAAGA 7755
NorthernPike              -------------------------------------------------------------------------------- 0
SpottedGar                -------------------------------------------------------------------------------- 0
ParamormyropsKingsLeyae   -------------------------------------------------------------------------------- 0
```

```
Majority                    --------------------------------------------------------------------------------
                                     49930     49940     49950     49960     49970     49980     49990     50000
Human                       GTTATTTTTAAGGTATTTTCATGCAGAATGTTTCTAAGTATTAAATACTATGTCATTTTGAATTCCTTTAATCCCCTGGT 49980
AsianBonytongue             -------------------------------------------------------------------------------- 0
Reedfish                    AGCATTTCAAGGTCCTGGAGCGGCCTAACCAGTCTCCAGATCTCAACTCCATGGAAAATCTTTGGAGGGAGTTGAAAGTC 7835
NorthernPike                -------------------------------------------------------------------------------- 0
SpottedGar                  -------------------------------------------------------------------------------- 0
ParamormyropsKingsLeyae     -------------------------------------------------------------------------------- 0

Majority                    --------------------------------------------------------------------------------
                                     50010     50020     50030     50040     50050     50060     50070     50080
Human                       CAAATGTTCTTTTTCTTGTTGCAAGTTAAAATTTTTGTGTAGAAACTTATCAGGTAGGATTTATTATTAGCATAAACTGT 50060
AsianBonytongue             -------------------------------------------------------------------------------- 0
Reedfish                    CATGTTGCCCAGCGACAGCCCCAAAACATCACTGCTCTAGAAGAGATCTACATGGAGGAATGGGCCAAAATATCAGCAAC 7915
NorthernPike                -------------------------------------------------------------------------------- 0
SpottedGar                  -------------------------------------------------------------------------------- 0
ParamormyropsKingsLeyae     -------------------------------------------------------------------------------- 0

Majority                    --------------------------------------------------------------------------------
                                     50090     50100     50110     50120     50130     50140     50150     50160
Human                       TTATACAACTCTCCTGCTTAGAATTTAAGGTAAGAAGTTCAGTGCCTT--TGGTTATTTGATTTTTTTTATAGGTCTTAA 50138
AsianBonytongue             -------------------------------------------------------------------------------- 0
Reedfish                    AGGGTGTGAAAACCTTGTGAAGACTTACAGAAAACGTTTGACCTCTGTCATTGCCAACAAAGGTATATAACAAAATATTG 7995
NorthernPike                -------------------------------------------------------------------------------- 0
SpottedGar                  -------------------------------------------------------------------------------- 0
ParamormyropsKingsLeyae     -------------------------------------------------------------------------------- 0

Majority                    --------------------------------------------------------------------------------
                                     50170     50180     50190     50200     50210     50220     50230     50240
Human                       TTTCTTACGGAAAATTTTTTTTTGAAGATGTGTTAATGGTTTTCCTGGAAAAGAAATCTTAAGGATGAAGAATATGTGAA 50218
AsianBonytongue             -------------------------------------------------------------------------------- 0
Reedfish                    AGATGAACTTTTGTTATTGACCAAATACTTTTTTTCCACCATAATTTGCAAATAAATTCTTTAAAATCAGACAATGTGAT 8075
NorthernPike                -------------------------------------------------------------------------------- 0
SpottedGar                  -------------------------------------------------------------------------------- 0
ParamormyropsKingsLeyae     -------------------------------------------------------------------------------- 0

Majority                    --------------------------------------------------------------------------------
                                     50250     50260     50270     50280     50290     50300     50310     50320
Human                       TGTATTGATGTTTTAAAAATGTAATATAGAAGATATTGTTTAAAATTAATGTATTCAAAAATAGCATTAGAATGTTTTCC 50298
AsianBonytongue             -------------------------------------------------------------------------------- 0
Reedfish                    TTTTCTGGATTTTTTTTTCCTCATTTTGTCTCTCATAGTTGAGGTATACCTATGATGAAAATTACAGGCCTCTCTCATCT 8155
NorthernPike                -------------------------------------------------------------------------------- 0
SpottedGar                  -------------------------------------------------------------------------------- 0
ParamormyropsKingsLeyae     -------------------------------------------------------------------------------- 0

Majority                    --------------------------------------------------------------------------------
                                     50330     50340     50350     50360     50370     50380     50390     50400
Human                       GACCTACATTATAAGACTGCTGTAATGATCTAAAACTTTAGCTGTTTTAATATAGTTTTAAAACTAATGATATCTTTCTC 50378
AsianBonytongue             -------------------------------------------------------------------------------- 0
Reedfish                    TTTTAAGTGGGAGAACTTGCACAATTGGTGGCTGACTAAATACTTTTTTGCCCCACTGTATGTGAATGTATGGATATTGC 8235
NorthernPike                -------------------------------------------------------------------------------- 0
SpottedGar                  -------------------------------------------------------------------------------- 0
ParamormyropsKingsLeyae     -------------------------------------------------------------------------------- 0
```

```
Majority                  --------------------------------------------------------------------------------
                                   50410     50420     50430     50440     50450     50460     50470     50480
Human                     TGTCAGTAAAATACAAACTTTTTCTTAATAAAAATGTAATGGAAAACTGTTCCTCATAGATTTTTGTCACTTTACAAAGT 50458
AsianBonytongue           -------------------------------------------------------------------------------- 0
Reedfish                  GGGAGTTTTTCCCTTTACTTTTGTATGAGAGCCGTATATTTGCCTATATTGAAGGTCAGTGCCACAGATATTTTGGTTTT 8315
NorthernPike              -------------------------------------------------------------------------------- 0
SpottedGar                -------------------------------------------------------------------------------- 0
ParamormyropsKingsLeyae--------------------------------------------------------------------------------- 0

Majority                  --------------------------------------------------------------------------------
                                   50490     50500     50510     50520     50530     50540     50550     50560
Human                     GACAAAATCATTTTGTTAGTTT---ATGGAAAATAAGCTTGTAAAACTTTTTACCTAAAAGATAGGACTGAAATTTCAGC 50535
AsianBonytongue           -------------------------------------------------------------------------------- 0
Reedfish                  CTTCAGACCACTTTATTTGGTCTGCAGGCTTTTCATAATTGTTTTCGTTAGTATAGCAGCTACTTGCTGAAAATAGTAAA 8395
NorthernPike              -------------------------------------------------------------------------------- 0
SpottedGar                -------------------------------------------------------------------------------- 0
ParamormyropsKingsLeyae--------------------------------------------------------------------------------- 0

Majority                  --------------------------------------------------------------------------------
                                   50570     50580     50590     50600     50610     50620     50630     50640
Human                     TTTTTTAATTTGATGATGAGTTTTTAATTTCTTTTGAAAAAGAATGTATGCTTCTAATAATTTATCAAGAGGAAGAATAC 50615
AsianBonytongue           -------------------------------------------------------------------------------- 0
Reedfish                  CGCATCAATTAAAACTTCAATATTTCCTTAAGCATGAAATACTATTAGCACCTTGTAAAGAAGAGCAGCCTGGATGTTAC 8475
NorthernPike              -------------------------------------------------------------------------------- 0
SpottedGar                -------------------------------------------------------------------------------- 0
ParamormyropsKingsLeyae--------------------------------------------------------------------------------- 0

Majority                  --------------------------------------------------------------------------------
                                   50650     50660     50670     50680     50690     50700     50710     50720
Human                     CAAAAGAAAATATCTGCTCTTCTTTCTTTTTACTTAGATTTTTTTGCATTTTTAACTTATTTTTAACAAATAATTGTTTA 50695
AsianBonytongue           -------------------------------------------------------------------------------- 0
Reedfish                  AGTACTTAACTATAGATCCATTTCAAATCTTCCTTTTATAGGTAAGATTATTGAAAAAGCTGTCATCTTACAACTCAACA 8555
NorthernPike              -------------------------------------------------------------------------------- 0
SpottedGar                -------------------------------------------------------------------------------- 0
ParamormyropsKingsLeyae--------------------------------------------------------------------------------- 0

Majority                  --------------------------------------------------------------------------------
                                   50730     50740     50750     50760     50770     50780     50790     50800
Human                     TACTTATGGTGTACAATGTGATGTTTTAATACATATATATTATGGAATGATCAAATCTGGCTGGTTAACATATTCATCTC 50775
AsianBonytongue           -------------------------------------------------------------------------------- 0
Reedfish                  CATTTTTAAACTCCAGTGGCTGCCTTGATGATTTACACTCTTGCCTTCTACCAAAATTATAGCACTGAGACAG-CATTTA 8634
NorthernPike              -------------------------------------------------------------------------------- 0
SpottedGar                -------------------------------------------------------------------------------- 0
ParamormyropsKingsLeyae--------------------------------------------------------------------------------- 0

Majority                  --------------------------------------------------------------------------------
                                   50810     50820     50830     50840     50850     50860     50870     50880
Human                     TTCAGTACTTACAATTTCTTTATGGTAAGAACATTTAAAATTCCTTTAGCTATTTTGGAAGTATACAGTGGAATGTGGAA 50855
AsianBonytongue           -------------------------------------------------------------------------------- 0
Reedfish                  TTAAAGTTTTAAATGATATTGTTTATACACAGACTGTTTAAAATAACAGCTGTGGTATTGCTACACCTCAGTGCTGCCTT 8714
NorthernPike              -------------------------------------------------------------------------------- 0
SpottedGar                -------------------------------------------------------------------------------- 0
ParamormyropsKingsLeyae--------------------------------------------------------------------------------- 0
```

```
Majority                    --------------------------------------------------------------------------------
                                     50890     50900     50910     50920     50930     50940     50950     50960
Human                       AAACTGTTTTTCCTCTGCTCACACCACAACAATCAACACAGAAGGCTTCTGTGGCCTCAAATGTGGGGGAATTTTTTTCC 50935
AsianBonytongue             -------------------------------------------------------------------------------- 0
Reedfish                    TGTTACTGTTGACCATGGAATACTTTTAGAAAGGTTTGATAACTGGGTTGGATTATCAGAAGTAGTTCCCAGTTTGTTTT 8794
NorthernPike                -------------------------------------------------------------------------------- 0
SpottedGar                  -------------------------------------------------------------------------------- 0
ParamormyropsKingsLeyae     -------------------------------------------------------------------------------- 0

Majority                    --------------------------------------------------------------------------------
                                     50970     50980     50990     51000     51010     51020     51030     51040
Human                       ACGCCAAGCAAGCAGTCAGGTCTGCAGTGTTCTCCAACTTAGCTCAGTTTCAACACTGTCTGCCGGGAGATAGCATCAGA 51015
AsianBonytongue             -------------------------------------------------------------------------------- 0
Reedfish                    AGGACATATTTTAAAGGCAGGGGGTTTCTTTGTTGAAACTGGTATTTATTGGTCAAAGGCGACAGAAATAACACGTGGAT 8874
NorthernPike                -------------------------------------------------------------------------------- 0
SpottedGar                  -------------------------------------------------------------------------------- 0
ParamormyropsKingsLeyae     -------------------------------------------------------------------------------- 0

Majority                    --------------------------------------------------------------------------------
                                     51050     51060     51070     51080     51090     51100     51110     51120
Human                       TCCCACAGGTTGAGGGCTCAGTCCCACAAGATCTCCTGAGACACCAGCAAGTCAGGCCTCTGGAACTTCTGATTGACCAG 51095
AsianBonytongue             -------------------------------------------------------------------------------- 0
Reedfish                    TACCACAAGGATCTATTTTGGGTCCTCTTTTATTTAATCTTTACATGCTTCCATTGGGTAGATTGTTACAAGTAATCAGT 8954
NorthernPike                -------------------------------------------------------------------------------- 0
SpottedGar                  -------------------------------------------------------------------------------- 0
ParamormyropsKingsLeyae     -------------------------------------------------------------------------------- 0

Majority                    --------------------------------------------------------------------------------
                                     51130     51140     51150     51160     51170     51180     51190     51200
Human                       CTTCAAGTTGGGGTTCCCACAACCCCCTTTTTGGATTTCATTAATTTGCTAGAGCAGCACACAGAACTCAAGGAAACATT 51175
AsianBonytongue             -------------------------------------------------------------------------------- 0
Reedfish                    CTTAATTACCACAGCTACTCAGACGATACCCAGATTTATTTGCCTCTTCCTCCAAATGATTTCAGTTCTGTTGATTCACT 9034
NorthernPike                -------------------------------------------------------------------------------- 0
SpottedGar                  -------------------------------------------------------------------------------- 0
ParamormyropsKingsLeyae     -------------------------------------------------------------------------------- 0

Majority                    --------------------------------------------------------------------------------
                                     51210     51220     51230     51240     51250     51260     51270     51280
Human                       TACTGAGATTTACTAGTTTATTATATAGGATATTAAAAAGGATAATAAGCCTCTAATCATGCCTTGATCTTTCAGGTGAC 51255
AsianBonytongue             -------------------------------------------------------------------------------- 0
Reedfish                    GTGTGATTGCCTACAGCTAACTAACATCTGGATGCAGCAAAATTTCCTTCAATTAAACAAAGATATAACTAAAATGATAC 9114
NorthernPike                -------------------------------------------------------------------------------- 0
SpottedGar                  -------------------------------------------------------------------------------- 0
ParamormyropsKingsLeyae     -------------------------------------------------------------------------------- 0

Majority                    --------------------------------------------------------------------------------
                                     51290     51300     51310     51320     51330     51340     51350     51360
Human                       CAGTCCCCATCTTAAAGTGGGGCTGCCAGCCATCAGTCAACTCATTAGCATACAAAATATATCATTTTGGAGATTTATTA 51335
AsianBonytongue             -------------------------------------------------------------------------------- 0
Reedfish                    TATATATCAATAAACAGGAAAGACTTAAAATTGTTTCTTATTTAGAGTCCAAGGCACTAAAGGCTGAAGTCCAAGTCAAA 9194
NorthernPike                -------------------------------------------------------------------------------- 0
SpottedGar                  -------------------------------------------------------------------------------- 0
ParamormyropsKingsLeyae     -------------------------------------------------------------------------------- 0
```

```
Majority                  --------------------------------------------------------------------------------

                                   51370     51380     51390     51400     51410     51420     51430     51440

Human                     GGAATTTAGAGTTGTATGCCAGGAAATGGGGTTGAGGACCAAATGTCTATTTTACAATGTCACAATACAGTATTATGCAT 51415
AsianBonytongue           -------------------------------------------------------------------------------- 0
Reedfish                  AATCTTCAGGTACTTCTGGATGCAGATCTTTGTTTCAGTAAGAATATTAAAGCAATAACTATATCTGCATTCTGTAATGT 9274
NorthernPike              -------------------------------------------------------------------------------- 0
SpottedGar                -------------------------------------------------------------------------------- 0
ParamormyropsKingsLeyae-------------------------------------------------------------------------------- 0

Majority                  --------------------------------------------------------------------------------

                                   51450     51460     51470     51480     51490     51500     51510     51520

Human                     TACAGTAGTTGCCATGCTGTGCGGTAGATCACCAGAACCTATTCTTCCTGTCTGAAACTTTGTACTTTGACCATCATCAC 51495
AsianBonytongue           -------------------------------------------------------------------------------- 0
Reedfish                  GAAAAACATTTCTAAAAGCAGACGCTTGATGTCAAAAACAGATATGAAAAAAAATTATCCATGCCTTTGTTTTCAGCTGA 9354
NorthernPike              -------------------------------------------------------------------------------- 0
SpottedGar                -------------------------------------------------------------------------------- 0
ParamormyropsKingsLeyae-------------------------------------------------------------------------------- 0

Majority                  --------------------------------------------------------------------------------

                                   51530     51540     51550     51560     51570     51580     51590     51600

Human                     CCCTTCCCTGTTCACTCCTCCCTCCCCCAGCCTCTGGTGACTATTTTTCTACTCTCTACTAATGTGAGTTCAGCTTTTTT 51575
AsianBonytongue           -------------------------------------------------------------------------------- 0
Reedfish                  GTAGATTACTGCAGTGTCCTTTTCTCAGGTCTACCTGAAAGAGTCTTAAAGTTGCAATTTGTTCAGAATGCTGCTGCCAG 9434
NorthernPike              -------------------------------------------------------------------------------- 0
SpottedGar                -------------------------------------------------------------------------------- 0
ParamormyropsKingsLeyae-------------------------------------------------------------------------------- 0

Majority                  --------------------------------------------------------------------------------

                                   51610     51620     51630     51640     51650     51660     51670     51680

Human                     A--GATTACATATGTAAGTGAGATAATGTGTTATTTGTCTTTCTTTGTTTGACTTATTCACTTAGCACACACAGTGTCCT 51653
AsianBonytongue           -------------------------------------------------------------------------------- 0
Reedfish                  AATGCTTGCCAAAATTACTCCAGCATTAAGATCACTACATTGGTTTCCAGTTAGCTGTAGGATTGATTTTAAGGTAGCAG 9514
NorthernPike              -------------------------------------------------------------------------------- 0
SpottedGar                -------------------------------------------------------------------------------- 0
ParamormyropsKingsLeyae-------------------------------------------------------------------------------- 0

Majority                  --------------------------------------------------------------------------------

                                   51690     51700     51710     51720     51730     51740     51750     51760

Human                     CTTAAAATGACATTTAATATCCCATTTCTAGTTCGTTATTATTTAAGTAATTTGCAAGTACATTCTCATTTTAAAAGATT 51733
AsianBonytongue           -------------------------------------------------------------------------------- 0
Reedfish                  TTTTAGTATACAAGTCACTAAACAACTTAGGACCAGTAAACCTCTCCGAGATACTAACAGTTTACAAACCTTTTAGATCC 9594
NorthernPike              -------------------------------------------------------------------------------- 0
SpottedGar                -------------------------------------------------------------------------------- 0
ParamormyropsKingsLeyae-------------------------------------------------------------------------------- 0

Majority                  --------------------------------------------------------------------------------

                                   51770     51780     51790     51800     51810     51820     51830     51840

Human                     AAACGAAAGGAACATGTAAAGAATAAAGCTGGGGGCTGGGCTTGGTGGCTTATACTGGTAATCCCAGCACTTTGGGAGGC 51813
AsianBonytongue           -------------------------------------------------------------------------------- 0
Reedfish                  TTAGGCGCTGGGCAGCTGGTTATACTGTGTTATTATTTTATTTATTTTATGTGTTATTTTTACTTTCTGCCATTTGACTG 9674
NorthernPike              -------------------------------------------------------------------------------- 0
SpottedGar                -------------------------------------------------------------------------------- 0
ParamormyropsKingsLeyae-------------------------------------------------------------------------------- 0
```

Majority --

51850 51860 51870 51880 51890 51900 51910 51920

Human TGAGGCGGGTGGATTACCTGAGGTCAGGAGTTCAAGACCCGCCTGACCAACATGGTGAAACCCTGAGTCTACTAAAAATA 51893
AsianBonytongue -- 0
Reedfish TGTTTCTAATTATCTTTTGATTGCAACTTTTTTATTGTATTTTATTCTTATTATGTAAAGCACTTTGAATTGCCTAATGG 9754
NorthernPike -- 0
SpottedGar -- 0
ParamormyropsKingsLeyae-- 0

Majority --

51930 51940 51950 51960 51970 51980 51990 52000

Human CAAAAAAAAAAAAAAAATTAGCCAGGCATGGTGCCAGGTGCCTGTAATCCCAGCTACTTGGAGGCTGAGGCAAGAGAGAAT 51973
AsianBonytongue -- 0
Reedfish TATAAAATGTGCTATATAAATAACTTGCTTTGCTTTGTACTGCCTTGCTATGACTATTGTTTTGAGAACCAACCACGTTT 9834
NorthernPike -- 0
SpottedGar -- 0
ParamormyropsKingsLeyae-- 0

Majority --

52010 52020 52030 52040 52050 52060 52070 52080

Human TGCTTGAACCCGGGGGGGTGGAGCTTGCGGTGATCCCAGATGCGTCACTGCACTCCAGCCTGGGTGACAGAGCGAGACTC 52053
AsianBonytongue -- 0
Reedfish AGCCATTTTTCAAGCTAGCACCAAGTTTATTTTTAAATATTAATTATCTTTGAGAGCATATACAAGTCTCAAAGATGCCT 9914
NorthernPike -- 0
SpottedGar -- 0
ParamormyropsKingsLeyae-- 0

Majority --

52090 52100 52110 52120 52130 52140 52150 52160

Human TATCTCAAAAAAAAAAAAAAAAAAAAAAAGAGTAAAGCTGAGGCCTACACATGGATGGATGTGGAGGAGGGGCACTTAGG 52133
AsianBonytongue -- 0
Reedfish TAACATGTTTCCAAGTTTTTAAACTGAGTGATGCACCACTGATACTCTGTGAAAAGCAAAACAATGTAGCATGAAACAAA 9994
NorthernPike -- 0
SpottedGar -- 0
ParamormyropsKingsLeyae-- 0

Majority --

52170 52180 52190 52200 52210 52220 52230 52240

Human ATGACTAGTGGAAGCCCCATTGAGAGGGGATATTTGAATAATGAACTTCAAAGTGGTTAGGGAATAAGCCACTTATTCCA 52213
AsianBonytongue -- 0
Reedfish AGTATCAACAACTGATTTTGAAATGGTTGCAAGAATATAAAATCACCTGAATCACAACATCCCAAGTCTATTGTCAAGTT 10074
NorthernPike -- 0
SpottedGar -- 0
ParamormyropsKingsLeyae-- 0

Majority --

52250 52260 52270 52280 52290 52300 52310 52320

Human GACAGAAGGAATAATCAATGCAAAGTTCCTTCTGTTGAAAAAAATGCTTTTATAATTTATTTTTCATCTTCTAAAGCCC 52293
AsianBonytongue -- 0
Reedfish GTATGCAAATAATTTTGCTTGTTTGTATTTATTATTATTTAGTTCTATTTTATGCAATGAATAGCATTACTAAGCACACC 10154
NorthernPike -- 0
SpottedGar -- 0
ParamormyropsKingsLeyae-- 0

```
Majority                --------------------------------------------------------------------------------
                                 52330     52340     52350     52360     52370     52380     52390     52400
Human                   TTCAGTCTCCTTTGGTCATCTTTTTTTTGTAGTTGGTTATGCCTTAACAACAGTACTTTGAGGATACTAGTTACAGTATT 52373
AsianBonytongue         -------------------------------------------------------------------------------- 0
Reedfish                ATTGAAATGCCTTTGTGATTACACGTGGGTATTTTAAATGTTTAAAGTTGTAGGAGCTGTAGAATGCCTACTAAAAAAAG 10234
NorthernPike            -------------------------------------------------------------------------------- 0
SpottedGar              -------------------------------------------------------------------------------- 0
ParamormyropsKingsLeyae-------------------------------------------------------------------------------- 0

Majority                --------------------------------------------------------------------------------
                                 52410     52420     52430     52440     52450     52460     52470     52480
Human                   TAAAAAATACGTCACTATATGCTGAGTTATTTTTTCAGTGTTAGCA-TT--------TATGTTTATCTTGTTCTCATTCT 52444
AsianBonytongue         -------------------------------------------------------------------------------- 0
Reedfish                ACCCTTTCTTATCGAGGCAACTGTAGTTGCATTACAGGTTTAGGCAACACAGCATGCTTAACCTTTTAAATTCATGCTTT 10314
NorthernPike            -------------------------------------------------------------------------------- 0
SpottedGar              -------------------------------------------------------------------------------- 0
ParamormyropsKingsLeyae-------------------------------------------------------------------------------- 0

Majority                --------------------------------------------------------------------------------
                                 52490     52500     52510     52520     52530     52540     52550     52560
Human                   CTTCATCCCCCCTTTGTTGTCTTTTCGTGTTTCAGAATGGACAATTAGATTTTGTTAAAGTTGTATGCTTTCCTCTAGGA 52524
AsianBonytongue         -------------------------------------------------------------------------------- 0
Reedfish                GGATAAATAACCCTAAATGCAGCATTAAAAATCGCAGGAATTGATCTCCTTGCAAGTAATACATACAAAAAGACCTGTCC 10394
NorthernPike            -------------------------------------------------------------------------------- 0
SpottedGar              -------------------------------------------------------------------------------- 0
ParamormyropsKingsLeyae-------------------------------------------------------------------------------- 0

Majority                --------------------------------------------------------------------------------
                                 52570     52580     52590     52600     52610     52620     52630     52640
Human                   TAAAGTAATGAAGAGCGTTTACTAAGCTGTTACCCCCATTCCCCCCAAATACCAAAGAGGCTCTTATTCTGGGTCAACAA 52604
AsianBonytongue         -------------------------------------------------------------------------------- 0
Reedfish                TTTCTGTAATCATAGACTAGCTGAAATACCCAGTGTTGCCCTGGAGGAAAATGGTGTGTTTTTTTTTTTTTGTTTGAGAA 10474
NorthernPike            -------------------------------------------------------------------------------- 0
SpottedGar              -------------------------------------------------------------------------------- 0
ParamormyropsKingsLeyae-------------------------------------------------------------------------------- 0

Majority                --------------------------------------------------------------------------------
                                 52650     52660     52670     52680     52690     52700     52710     52720
Human                   AACTCCCTTTAGAATTTCTGGTACCAGGCTGTGCGTGGTGGCTCACACTTGTAATCCCAGCACTTTGGGAGGCTGAGGTG 52684
AsianBonytongue         -------------------------------------------------------------------------------- 0
Reedfish                AAATATACTGTATGTACTCACATATAAGTCGGGTCTTGAAATCCAAAAAATCGATCATAAAATCAGTCCCCGACTTATAT 10554
NorthernPike            -------------------------------------------------------------------------------- 0
SpottedGar              -------------------------------------------------------------------------------- 0
ParamormyropsKingsLeyae-------------------------------------------------------------------------------- 0

Majority                --------------------------------------------------------------------------------
                                 52730     52740     52750     52760     52770     52780     52790     52800
Human                   GGCGGATCACGAGGTCAGGAGATCGAGACCATGGTGAAACCCCGTCTGTACTAAAAATACGAAAATTAGCTGGGCGTGGT 52764
AsianBonytongue         -------------------------------------------------------------------------------- 0
Reedfish                GCCTGTTCAAAAATGCTACACTTAAATTTCCTACTCCAATCTCGCATCAGTTTCTCAGATGCATTGAATTTTGTTGCAGC 10634
NorthernPike            -------------------------------------------------------------------------------- 0
SpottedGar              -------------------------------------------------------------------------------- 0
ParamormyropsKingsLeyae-------------------------------------------------------------------------------- 0
```

```
Majority                  --------------------------------------------------------------------------------
                                   52810     52820     52830     52840     52850     52860     52870     52880
Human                     GGCGGGCGCCTGTAGTCCCAGCTACTCGGGAGGCTGAGGCAGGAGAATGGTGCAGACCCAGGAGGCGGAGCTTGCAGTGA 52844
AsianBonytongue           -------------------------------------------------------------------------------- 0
Reedfish                  AGTGCATTTACCAGTTTCTTTCGCTGCTTCAGTGACGTTTAATTTAAAACCAGACTTCATATTTTCTTCTGATCGAACGC 10714
NorthernPike              -------------------------------------------------------------------------------- 0
SpottedGar                -------------------------------------------------------------------------------- 0
ParamormyropsKingsLeyae   -------------------------------------------------------------------------------- 0

Majority                  --------------------------------------------------------------------------------
                                   52890     52900     52910     52920     52930     52940     52950     52960
Human                     GCCGAGATCGCGCCACTGCACTTCAGCCTGGGCGACAGAGCGAGACTCTGTCTCAAAAAAAAAAAAAAAAAAAGAATTCCT 52924
AsianBonytongue           -------------------------------------------------------------------------------- 0
Reedfish                  TCCATCGTAGATAAGGGATGCTCTTACAGTAAAGGTGTATGAAGGTGTGAGATACAAAAAACACAAATCAGTGCAAACGT 10794
NorthernPike              -------------------------------------------------------------------------------- 0
SpottedGar                -------------------------------------------------------------------------------- 0
ParamormyropsKingsLeyae   -------------------------------------------------------------------------------- 0

Majority                  --------------------------------------------------------------------------------
                                   52970     52980     52990     53000     53010     53020     53030     53040
Human                     GGTACCAGCAGGGCACTTTGGCTCATGCCTGTAATCCCAGCACTTTGAGAAGCTGAGGTAAATTCAGGGCAGCAGTGAAC 53004
AsianBonytongue           -------------------------------------------------------------------------------- 0
Reedfish                  TGCTTTGGAATAATTTGGGTATTACTGCGTGGTCCTGTAGGCACAATAGGGAGAGAGAGAGAGGTTAGGAGAATATACTG 10874
NorthernPike              -------------------------------------------------------------------------------- 0
SpottedGar                -------------------------------------------------------------------------------- 0
ParamormyropsKingsLeyae   -------------------------------------------------------------------------------- 0

Majority                  --------------------------------------------------------------------------------
                                   53050     53060     53070     53080     53090     53100     53110     53120
Human                     TGGTCACACCACTGCACTCCAGCCTCAGCAACAAAGCAAG---ACACAGTCTCTTAAAAAAAAAAAAAGGAAGAAATTCT 53081
AsianBonytongue           -------------------------------------------------------------------------------- 0
Reedfish                  ATACAGCGCAATGCCGCACCCACATAGAAAATAAAGGCAGTGTGCTCCGTGGTTACTCTCTCTGGTGAGTGTTAGCATAT 10954
NorthernPike              -------------------------------------------------------------------------------- 0
SpottedGar                -------------------------------------------------------------------------------- 0
ParamormyropsKingsLeyae   -------------------------------------------------------------------------------- 0

Majority                  --------------------------------------------------------------------------------
                                   53130     53140     53150     53160     53170     53180     53190     53200
Human                     GGTTCCTTTAAGGGAGATTTGTTGTTGTTTTCTTCTTTTAATGGTGAGGGTACAAGTTGGTCACAGGAGGGAGAAGAGAA 53161
AsianBonytongue           -------------------------------------------------------------------------------- 0
Reedfish                  CATAATTTCTTGGACCAATAGCGTGAGTTTTCCGCATTCGACTTATACGACCGACATTATAAAATACCAGAAATTATACA 11034
NorthernPike              -------------------------------------------------------------------------------- 0
SpottedGar                -------------------------------------------------------------------------------- 0
ParamormyropsKingsLeyae   -------------------------------------------------------------------------------- 0

Majority                  --------------------------------------------------------------------------------
                                   53210     53220     53230     53240     53250     53260     53270     53280
Human                     ATTTAGCTCTTTTGTTAATGAACTAGACTTTCTGTTCGTCTGTTTTTCACCCACTCCCTCCTCCTGTGAACCTGCCAATT 53241
AsianBonytongue           -------------------------------------------------------------------------------- 0
Reedfish                  GTAAAATCAACTCCCGACTTAACCGTGGGAGAACTTATCCGCAAGTATATATGGTAATAAAAAAAACACAACTTTAAAAA 11114
NorthernPike              -------------------------------------------------------------------------------- 0
SpottedGar                -------------------------------------------------------------------------------- 0
ParamormyropsKingsLeyae   -------------------------------------------------------------------------------- 0
```

```
Majority                   --------------------------------------------------------------------------------
                                    53290     53300     53310     53320     53330     53340     53350     53360
Human                      TCTGATTTGGTTCCTGTGGCTGCTACCAGTATAACACCTGTTTGCTGTGTGTTCACTGTGTCTCCAGTCCATTTGGTTCC 53321
AsianBonytongue            -------------------------------------------------------------------------------- 0
Reedfish                   AGCACAAAGGAGTGGCTAAGGGACAATGATGAGAAAGTTCTTGAGAATGCACAGTCAGAGCCTGAACCTGTCTCTGTAGA 11194
NorthernPike               -------------------------------------------------------------------------------- 0
SpottedGar                 -------------------------------------------------------------------------------- 0
ParamormyropsKingsLeyae    -------------------------------------------------------------------------------- 0

Majority                   --------------------------------------------------------------------------------
                                    53370     53380     53390     53400     53410     53420     53430     53440
Human                      ATTCATTTTCTGTGTTCCAGGAATTTGATAAAATTATTCTCAGCTAATGATAACCTTCCCTCCCTTCAGTGTTTGTTTAT 53401
AsianBonytongue            -------------------------------------------------------------------------------- 0
Reedfish                   GGCCTGAAAAAAACCTGACAGAGCTTGAGAGGATCTGCAGAGAAGAATGGCAGAAAATCTCCAAATCCAGGTTTATGGAG 11274
NorthernPike               -------------------------------------------------------------------------------- 0
SpottedGar                 -------------------------------------------------------------------------------- 0
ParamormyropsKingsLeyae    -------------------------------------------------------------------------------- 0

Majority                   --------------------------------------------------------------------------------
                                    53450     53460     53470     53480     53490     53500     53510     53520
Human                      TTTATATTCATTCACTTCTAGGCATTTTAATAGAGTTTCATAATAGAAGACATGCAAATGAGTATGCATTAACCATGAAT 53481
AsianBonytongue            -------------------------------------------------------------------------------- 0
Reedfish                   CTTGCTGCGTCATATCCTAGAAGACCCTAGACTGGGGTCGCTACCAAAGGGGCATGAACTA--AATCACTAACTAAAGAT 11352
NorthernPike               -------------------------------------------------------------------------------- 0
SpottedGar                 -------------------------------------------------------------------------------- 0
ParamormyropsKingsLeyae    -------------------------------------------------------------------------------- 0

Majority                   --------------------------------------------------------------------------------
                                    53530     53540     53550     53560     53570     53580     53590     53600
Human                      TCTTTCACATCCGTTCATGAAATCACCAGGTCCTATTAGTTCACACTTTTTAATTATCTGCCTGGTTTTCTCCATCTTTC 53561
AsianBonytongue            -------------------------------------------------------------------------------- 0
Reedfish                   TTGTATAGTTGTGACAATGTTACATTTGGGTTTTTTAAAATTTTATTTTAATACATATTCCAGAAATTCTAAAATCCTTT 11432
NorthernPike               -------------------------------------------------------------------------------- 0
SpottedGar                 -------------------------------------------------------------------------------- 0
ParamormyropsKingsLeyae    -------------------------------------------------------------------------------- 0

Majority                   --------------------------------------------------------------------------------
                                    53610     53620     53630     53640     53650     53660     53670     53680
Human                      CTGCTACTTGTATTGCTCAAACACACTCACCTGTTGGCTATATTACTACGACAGTTCTCTTTATAA-ACTACTTTCTTTT 53640
AsianBonytongue            -------------------------------------------------------------------------------- 0
Reedfish                   TTTCTGTTAGTCATTTTGACGTATTGAGTATTGTTTGATGAATGGGGGGAAAAAAAATAAGATTAGTACAAGGCTGCAAC 11512
NorthernPike               -------------------------------------------------------------------------------- 0
SpottedGar                 -------------------------------------------------------------------------------- 0
ParamormyropsKingsLeyae    -------------------------------------------------------------------------------- 0

Majority                   --------------------------------------------------------------------------------
                                    53690     53700     53710     53720     53730     53740     53750     53760
Human                      TCTTTTCCCTTCTGGAATTTTTTTCTCGCTAGTACAGCCAGTACGTATCTTTCTCAAATGCACATATCTAAGTTACTCCC 53720
AsianBonytongue            -------------------------------------------------------------------------------- 0
Reedfish                   ATAGCAAAATGTGAAAACAGTGAAGGGGCCTGAATGTACAGTATGTTACTTTATACAAATTGCAGGTAAAATGACTGTTT 11592
NorthernPike               -------------------------------------------------------------------------------- 0
SpottedGar                 -------------------------------------------------------------------------------- 0
ParamormyropsKingsLeyae    -------------------------------------------------------------------------------- 0
```

```
Majority                 --------------------------------------------------------------------------------
                                 53770     53780     53790     53800     53810     53820     53830     53840
Human                    ATGCAGTAGGTCCTGTTCATGCTCAGGCTAAAGGCCCTTTCTGAGCTTACTACTTTTTGTCTTGTCTTATGACTACTCCC 53800
AsianBonytongue          -------------------------------------------------------------------------------- 0
Reedfish                 AAGTAAATAAGCAAGTATAAGTGAAAGAGTGAGACAGTAGATAGGAAGACTAGAAGAAAAAATCCCACTTTCTCAATCAG 11672
NorthernPike             -------------------------------------------------------------------------------- 0
SpottedGar               -------------------------------------------------------------------------------- 0
ParamormyropsKingsLeyae  -------------------------------------------------------------------------------- 0

Majority                 --------------------------------------------------------------------------------
                                 53850     53860     53870     53880     53890     53900     53910     53920
Human                    ACCCTATTTGCTTATGCTTCTGTCCCAAAAGTAATGGTGCATCCTGTAACCTGGCATGTTTTCTCTTGATGTCAGTCGTT 53880
AsianBonytongue          -------------------------------------------------------------------------------- 0
Reedfish                 TAGTTAAAGTGAAAATAAAAAGGGATGGAGATGGAAGAGTAAATTGAGATTGAAGAAATGGAAACTTCCCACTCCTGATG 11752
NorthernPike             -------------------------------------------------------------------------------- 0
SpottedGar               -------------------------------------------------------------------------------- 0
ParamormyropsKingsLeyae  -------------------------------------------------------------------------------- 0

Majority                 --------------------------------------------------------------------------------
                                 53930     53940     53950     53960     53970     53980     53990     54000
Human                    TGCTGCCATTATGTCACTGCTCTCATGCATGGAATATTGTGGGTTGGCCTGCCTTTTGCACCACCCTTCCTCCACCTCAC 53960
AsianBonytongue          -------------------------------------------------------------------------------- 0
Reedfish                 CAGCCCACGATAGCAGTGTCGTTAGCGAACTTTTGCACGTGACAGGACTCTGAGTTGTATTGGAAGTCCAATGTATATAG 11832
NorthernPike             -------------------------------------------------------------------------------- 0
SpottedGar               -------------------------------------------------------------------------------- 0
ParamormyropsKingsLeyae  -------------------------------------------------------------------------------- 0

Majority                 --------------------------------------------------------------------------------
                                 54010     54020     54030     54040     54050     54060     54070     54080
Human                    ACTTCACATGGCCAGTGTTAAATATCTTGAGGTCTCCCTTTAGCTATTTCTTCTCCTAAAAGCTATCCCCTGATAAGCAA 54040
AsianBonytongue          -------------------------------------------------------------------------------- 0
Reedfish                 GCTGAACAGGACCGGAGAAAGTACAGTCCCCTGCGGCACTCCTGTGTTGCTGACCACAGTGTCACACCTGCAGTTCCCGA 11912
NorthernPike             -------------------------------------------------------------------------------- 0
SpottedGar               -------------------------------------------------------------------------------- 0
ParamormyropsKingsLeyae  -------------------------------------------------------------------------------- 0

Majority                 --------------------------------------------------------------------------------
                                 54090     54100     54110     54120     54130     54140     54150     54160
Human                    AGAGTAGATTAGGTTTGGATTGAGAGTGTTAACTCCGAATGAGATACTGCTATGCACATAACCAGAGTGGCTAAAATTAA 54120
AsianBonytongue          -------------------------------------------------------------------------------- 0
Reedfish                 GATGCACATACTGAGGACTGTCTTTAAGATAGTCTACGATCAATGCTACCAGGTATGAATCTACTCCCATCTCTGTCAGC 11992
NorthernPike             -------------------------------------------------------------------------------- 0
SpottedGar               -------------------------------------------------------------------------------- 0
ParamormyropsKingsLeyae  -------------------------------------------------------------------------------- 0

Majority                 --------------------------------------------------------------------------------
                                 54170     54180     54190     54200     54210     54220     54230     54240
Human                    AAGGATAGGTAATAAAATACTGGTGACAACCATTTTTGAAAACTCGAAATATCTACTAAAGTTGAGCATATAGCTATTCT 54200
AsianBonytongue          -------------------------------------------------------------------------------- 0
Reedfish                 TTGACCCTAAGGAACAGAGGTTGGATGTTGTTGAAGGTGCTAGAGAAGTCCAGAAACATAATTCTTACAGCACCACTGCC 12072
NorthernPike             -------------------------------------------------------------------------------- 0
SpottedGar               -------------------------------------------------------------------------------- 0
ParamormyropsKingsLeyae  -------------------------------------------------------------------------------- 0
```

```
Majority                    --------------------------------------------------------------------------------

                                     54250     54260     54270     54280     54290     54300     54310     54320

Human                       TTGATGTAGTAAACTCGCTCCTAGTTAACATACCTGAGAACATGTACATAGGTATGTTAAAAGATATGTACAAGAAAGTT 54280
AsianBonytongue             -------------------------------------------------------------------------------- 0
Reedfish                    TCTGTCCAAGTGGGAGAGGGATCGGTGTAGCATGTAGATGATGGCATCCTCGGCTCCCACCTTCTCCTGGTATGCGAACT 12152
NorthernPike                -------------------------------------------------------------------------------- 0
SpottedGar                  -------------------------------------------------------------------------------- 0
ParamormyropsKingsLeyae     -------------------------------------------------------------------------------- 0

Majority                    --------------------------------------------------------------------------------

                                     54330     54340     54350     54360     54370     54380     54390     54400

Human                       CATGGAACACTGTATGTAATAGCCCAAAAAACTATAAATCATCCAAATGTTCAACAGCAGCAGAATATGTTGTGTTGTAT 54360
AsianBonytongue             -------------------------------------------------------------------------------- 0
Reedfish                    GCAGAGAGTCGAGAGCGTGGTGGACCTGTGGCCTCAGGTGGTGAAGCAGCAGCCGCTCCATGGTCTTCATCACATGTGAC 12232
NorthernPike                -------------------------------------------------------------------------------- 0
SpottedGar                  -------------------------------------------------------------------------------- 0
ParamormyropsKingsLeyae     -------------------------------------------------------------------------------- 0

Majority                    --------------------------------------------------------------------------------

                                     54410     54420     54430     54440     54450     54460     54470     54480

Human                       TCATTTAGTGGAATATTGTATAGTAATAAAAATGAACAAGCTACTGCTGCCTGCAAAACATGGGTAGTGTACATCATAAT 54440
AsianBonytongue             -------------------------------------------------------------------------------- 0
Reedfish                    GTCAGAGCGACAGGCCAGAAGTCATTCAGCGCACCTGGACGTGATACCTTTGGGACTGGGGTGATGAAAGATGTTTTCCA 12312
NorthernPike                -------------------------------------------------------------------------------- 0
SpottedGar                  -------------------------------------------------------------------------------- 0
ParamormyropsKingsLeyae     -------------------------------------------------------------------------------- 0

Majority                    --------------------------------------------------------------------------------

                                     54490     54500     54510     54520     54530     54540     54550     54560

Human                       TATTGGAAGAAGCCAAACCAAAGTAGAATACATCTGATATGATTCCATTTATGTAACTTGGAGGAAGCGTGAGTATGGCT 54520
AsianBonytongue             -------------------------------------------------------------------------------- 0
Reedfish                    AAGCCTTGGGACTCTCCCCTGTTCCAGGCTCAGGTTGAAGATGTGCTGTAGAGGACTCCCCAGCTCCAACGCACAGGCCT 12392
NorthernPike                -------------------------------------------------------------------------------- 0
SpottedGar                  -------------------------------------------------------------------------------- 0
ParamormyropsKingsLeyae     -------------------------------------------------------------------------------- 0

Majority                    --------------------------------------------------------------------------------

                                     54570     54580     54590     54600     54610     54620     54630     54640

Human                       TCTAGAGTGTTGATAATATACTGTTTTTTATCTGGGTAATTATTTCACAGAGATGTCCAATTTGTGGTATTTTATGGAGC 54600
AsianBonytongue             -------------------------------------------------------------------------------- 0
Reedfish                    TCAGCAGTCGTGGCGATACTCCATCTGGACCCGCTGCTTTGCTGGTACAAAGTCTCCTCAGCTCTCTGCTTACCTGGGCC 12472
NorthernPike                -------------------------------------------------------------------------------- 0
SpottedGar                  -------------------------------------------------------------------------------- 0
ParamormyropsKingsLeyae     -------------------------------------------------------------------------------- 0

Majority                    --------------------------------------------------------------------------------

                                     54650     54660     54670     54680     54690     54700     54710     54720

Human                       TTTATACTTAGGTTTTGTGTCCTTTTGTGTGTGTGTGTAATATTTTGATAAGAAGTTTTATTAAAATCTAGGTTTGCAAG 54680
AsianBonytongue             -------------------------------------------------------------------------------- 0
Reedfish                    GCTGTAATTCTGGGTTGGGGTAAACTCTCACCTATGCTGGTATCAGCAGAAGGATGGGTGA-AGAGTGCAGTACTCCGAA 12551
NorthernPike                -------------------------------------------------------------------------------- 0
SpottedGar                  -------------------------------------------------------------------------------- 0
ParamormyropsKingsLeyae     -------------------------------------------------------------------------------- 0
```

```
Majority                  --------------------------------------------------------------------------------

                                   54730     54740     54750     54760     54770     54780     54790     54800

Human                     CCAACACTGCTCCATGAATTATAAGAAAAAATATATGAAGTCATTAAAAATGAAATCATAAAGAAATGTATGAAGTAATT 54760
AsianBonytongue           -------------------------------------------------------------------------------- 0
Reedfish                  GTGAGAGTGGGTAAGAGTGGTCAAACCGGTTTAAAAAAAAAAAAAAAAAAAGAGGTTCATCTGGTTTGCTCTCTCCACAT 12631
NorthernPike              -------------------------------------------------------------------------------- 0
SpottedGar                -------------------------------------------------------------------------------- 0
ParamormyropsKingsLeyae-------------------------------------------------------------------------------- 0

Majority                  --------------------------------------------------------------------------------

                                   54810     54820     54830     54840     54850     54860     54870     54880

Human                     TGTTACTGATGTGAGAGAACATGATTGTTAAGCCATAAAAGGAAGATAGAGAACAATGAATAGTATTAAATAAAAGTATG 54840
AsianBonytongue           -------------------------------------------------------------------------------- 0
Reedfish                  CTCTCTTGATGGTGGCGCCCTGCTTCGAACTGCAGCCAGTGATGATCTTCATCCCATCCCACACTTCCTTCATGCTGTTA 12711
NorthernPike              -------------------------------------------------------------------------------- 0
SpottedGar                -------------------------------------------------------------------------------- 0
ParamormyropsKingsLeyae-------------------------------------------------------------------------------- 0

Majority                  --------------------------------------------------------------------------------

                                   54890     54900     54910     54920     54930     54940     54950     54960

Human                     AATAGTATTGAATAAAAATATGAATAGTATTGAAAAAAATACAGACTAAGTATAATATTTATATATATATGCTTGTAGAT 54920
AsianBonytongue           -------------------------------------------------------------------------------- 0
Reedfish                  TTCTGCAACTTCTGCTCCTTCGCCACCCTGAGCTGGACTCGGAGTTCCTTCTGCACGCGCTTGAGCTCATGCTGATCACC 12791
NorthernPike              -------------------------------------------------------------------------------- 0
SpottedGar                -------------------------------------------------------------------------------- 0
ParamormyropsKingsLeyae-------------------------------------------------------------------------------- 0

Majority                  --------------------------------------------------------------------------------

                                   54970     54980     54990     55000     55010     55020     55030     55040

Human                     GTAGCTAGTATCTCTGGAAGGATATCGAAGATAATACTGTGAGGGGTTGGTAGGGGAAAATAGACTGGGTGACAGAGTTG 55000
AsianBonytongue           -------------------------------------------------------------------------------- 0
Reedfish                  GCCTTTAAAAGCCCTTTTTTTCTGGTTCAATAGGCCCTTGATATGTCTTGTAATCCATGGCTTGTTGT-TAGCATAGCAG 12870
NorthernPike              -------------------------------------------------------------------------------- 0
SpottedGar                -------------------------------------------------------------------------------- 0
ParamormyropsKingsLeyae-------------------------------------------------------------------------------- 0

Majority                  --------------------------------------------------------------------------------

                                   55050     55060     55070     55080     55090     55100     55110     55120

Human                     CAGGGGAATTTTTAATCTTTAATACCACTTTTATACCATGATCATGGATTCCTATTTTAGAAAACATTTTATTATTTGAA 55080
AsianBonytongue           -------------------------------------------------------------------------------- 0
Reedfish                  TGTATTGTTCTTACTGGAACTACAATGTCCATACAGAAGTTGATCTAGTCAGTAGTGCAGTCAACAACCTCCTCAATGTT 12950
NorthernPike              -------------------------------------------------------------------------------- 0
SpottedGar                -------------------------------------------------------------------------------- 0
ParamormyropsKingsLeyae-------------------------------------------------------------------------------- 0

Majority                  --------------------------------------------------------------------------------

                                   55130     55140     55150     55160     55170     55180     55190     55200

Human                     GCAGATGTTAGTGTTTTTCTAAGTTGTGGTAGTCTATGCTTGTTATTATAGATAAATACAGTAAGAACATGTTCACTAAT 55160
AsianBonytongue           -------------------------------------------------------------------------------- 0
Reedfish                  CTCACTATGTGATCCCTGCAGGATATCCCAGGCCGTAGCTCCAAAGCAGTCTCCTGCTCTGCCTCAGGGGACCACTTCCT 13030
NorthernPike              -------------------------------------------------------------------------------- 0
SpottedGar                -------------------------------------------------------------------------------- 0
ParamormyropsKingsLeyae-------------------------------------------------------------------------------- 0
```

```
Majority                   --------------------------------------------------------------------------------
                                    55210     55220     55230     55240     55250     55260     55270     55280
Human                      GAAGAAGATAATATTGCAATGAGCTACTTAAAATATTTAAATACATTTAAACATTTGAATAGATACTGCTCTATAAAACT 55240
AsianBonytongue            -------------------------------------------------------------------------------- 0
Reedfish                   GAATGAGCATGTTTTTGTAGGTAGCTCCCTCACTCTTGGTTTGTAGTGCGGCTGAAGCACAACCAGGTTATGATCTGCTT 13110
NorthernPike               -------------------------------------------------------------------------------- 0
SpottedGar                 -------------------------------------------------------------------------------- 0
ParamormyropsKingsLeyae    -------------------------------------------------------------------------------- 0

Majority                   --------------------------------------------------------------------------------
                                    55290     55300     55310     55320     55330     55340     55350     55360
Human                      AATTTTACCAATTTACAATTATTTTTTCAATAATAGTATCAAGATGTCTACTAAAATAATCTATCACATTTCAAAATGTT 55320
AsianBonytongue            -------------------------------------------------------------------------------- 0
Reedfish                   TCCCAAGCGCAGGCAGCGGGGTGGAGCTGTATGCGTCTTTAACGTTTGCATACAGTAGGTCGATAGTCCTATTTCCCCGG 13190
NorthernPike               -------------------------------------------------------------------------------- 0
SpottedGar                 -------------------------------------------------------------------------------- 0
ParamormyropsKingsLeyae    -------------------------------------------------------------------------------- 0

Majority                   --------------------------------------------------------------------------------
                                    55370     55380     55390     55400     55410     55420     55430     55440
Human                      GAGTTTTAATTAGTAAGTCATTTTGACTAATACATAAATTTATAGATAAAATTAAGTAGCATTTAAATGATTGTTTAGAT 55400
AsianBonytongue            -------------------------------------------------------------------------------- 0
Reedfish                   GTGTTACAATCCACATACTGGGAGAAGGCAGGCAATGTTTTGTCCAGCGTCACATGGTAAAAGTCTCCAGCGATTAGCAC 13270
NorthernPike               -------------------------------------------------------------------------------- 0
SpottedGar                 -------------------------------------------------------------------------------- 0
ParamormyropsKingsLeyae    -------------------------------------------------------------------------------- 0

Majority                   --------------------------------------------------------------------------------
                                    55450     55460     55470     55480     55490     55500     55510     55520
Human                      ATATATCTGGACTCTCAAGCCGTGTGATCTGTGGTTATATAATATTTTAGTAAAATGACTTTTCTTGTTTATTCTCCTGA 55480
AsianBonytongue            -------------------------------------------------------------------------------- 0
Reedfish                   AAGCGCCTCGGGGTGCTGTGTTTGCAATTTAGTAGCTGCGGAATGGATGACATCACCCGCTATCTCCACGTTCGCCTGAG 13350
NorthernPike               -------------------------------------------------------------------------------- 0
SpottedGar                 -------------------------------------------------------------------------------- 0
ParamormyropsKingsLeyae    -------------------------------------------------------------------------------- 0

Majority                   --------------------------------------------------------------------------------
                                    55530     55540     55550     55560     55570     55580     55590     55600
Human                      GTGTCCTAATTCTTTTGTATTCTCTTTCATTAACCTGTTGATATAGATTTTTCAAACTGTAAACCTATGTATAGGTATTT 55560
AsianBonytongue            -------------------------------------------------------------------------------- 0
Reedfish                   GAGGGATGTAAACAGTAGCAACAATGACGTGTCCAAACTCTCTGGGCAAGTAATAGGGATGCAGACTTACGGCCAACAGT 13430
NorthernPike               -------------------------------------------------------------------------------- 0
SpottedGar                 -------------------------------------------------------------------------------- 0
ParamormyropsKingsLeyae    -------------------------------------------------------------------------------- 0

Majority                   --------------------------------------------------------------------------------
                                    55610     55620     55630     55640     55650     55660     55670     55680
Human                      GTGGTATTTGTCATTGTTATATTTTAGAACTATTTGCCTTTGTATGTTTTTTGACCACAGATAAGGTAGAAAGAAATTAT 55640
AsianBonytongue            -------------------------------------------------------------------------------- 0
Reedfish                   GCGATGTCCCTGCAGCAAGTGGAGATTTTAACGTTAACATGTCCAGAGTTGCAACACCTTGTATTGACATAGAGTGCAAG 13510
NorthernPike               -------------------------------------------------------------------------------- 0
SpottedGar                 -------------------------------------------------------------------------------- 0
ParamormyropsKingsLeyae    -------------------------------------------------------------------------------- 0
```

```
Majority                  --------------------------------------------------------------------------------
                                   55690     55700     55710     55720     55730     55740     55750     55760
Human                     TTCCTTATCATAGACTGTCATCATAATAACAGGAAAGGATAATAGTAATAAAGGCAGTCTTTTGGAGCATTTAACATAA 55720
AsianBonytongue           -------------------------------------------------------------------------------- 0
Reedfish                  TCCTCCTCCTTTGTGCTTCCCGCAGGTACTTGTGTCTCTGTCCGCTCTAACTCTGCTAAACCCGGGTAGCTCCACATTAG 13590
NorthernPike              -------------------------------------------------------------------------------- 0
SpottedGar                -------------------------------------------------------------------------------- 0
ParamormyropsKingsLeyae   -------------------------------------------------------------------------------- 0

Majority                  --------------------------------------------------------------------------------
                                   55770     55780     55790     55800     55810     55820     55830     55840
Human                     TATTTGATTGACAGATTTCCTACAAATACTGTGTTTATTCAATTTTATGGGTATATTTTTTCGCATTTTAAAATCTCAGA 55800
AsianBonytongue           -------------------------------------------------------------------------------- 0
Reedfish                  CATCTAGAATGATAGTTGATTGCCATGTTTCACTAAAACACAACAAACTGCATTCTCTGTAGGTCCTGACATTTTTCACA 13670
NorthernPike              -------------------------------------------------------------------------------- 0
SpottedGar                -------------------------------------------------------------------------------- 0
ParamormyropsKingsLeyae   -------------------------------------------------------------------------------- 0

Majority                  --------------------------------------------------------------------------------
                                   55850     55860     55870     55880     55890     55900     55910     55920
Human                     AATTTGATGTGTCCTGCAATAGTCTATCTTCCATAGTTCAA--TTGGCAACATCTTATTAGTCATACATAAAACAATAGT 55878
AsianBonytongue           -------------------------------------------------------------------------------- 0
Reedfish                  AGTGCAGCCAGTTCGTCAATTTTATTTGGTAGTGAGTTCACATTTCCCAGGATCACAGAAGGCACCGAAGGTTTGTAGCG 13750
NorthernPike              -------------------------------------------------------------------------------- 0
SpottedGar                -------------------------------------------------------------------------------- 0
ParamormyropsKingsLeyae   -------------------------------------------------------------------------------- 0

Majority                  --------------------------------------------------------------------------------
                                   55930     55940     55950     55960     55970     55980     55990     56000
Human                     GCATTTTATAATTAATGGTGTATTTGATACCATAAAATGTATGGGAAATATTCTTCATATTATATCTTGAAGTTTACCTG 55958
AsianBonytongue           -------------------------------------------------------------------------------- 0
Reedfish                  CCACTTTCTCGCTAAACACTTGGCTTTTATCTTAGCACCGGCTCTGCTGTCGCGATACCACCTTCTTACCTCGTCAAGTA 13830
NorthernPike              -------------------------------------------------------------------------------- 0
SpottedGar                -------------------------------------------------------------------------------- 0
ParamormyropsKingsLeyae   -------------------------------------------------------------------------------- 0

Majority                  --------------------------------------------------------------------------------
                                   56010     56020     56030     56040     56050     56060     56070     56080
Human                     AGAATTTTGTGGTTTTGGACTAGACTACACGTTGGTTTCTATTCTAAAAATTTTGGTGTCAGAAGTTAAGTGTAGTATGA 56038
AsianBonytongue           -------------------------------------------------------------------------------- 0
Reedfish                  AATAGGGAACCACTCCGGCGCGGGCATTTGTTCTCAGTGCTTGAAGTTGACTACTTGAATAGACGAGTCTCAGTGTGTAA 13910
NorthernPike              -------------------------------------------------------------------------------- 0
SpottedGar                -------------------------------------------------------------------------------- 0
ParamormyropsKingsLeyae   -------------------------------------------------------------------------------- 0

Majority                  --------------------------------------------------------------------------------
                                   56090     56100     56110     56120     56130     56140     56150     56160
Human                     AAAATGTATTTTCTGAATGTTCCTATTTTAAGATTGTATTAGAAATAATAAGCTTTATGGGAGTTCTTTTGGATAATTCA 56118
AsianBonytongue           -------------------------------------------------------------------------------- 0
Reedfish                  AAATCCATGTCCATAGTAAAAAGTGTCCAGGGAACGAGGTAAAAAAATGTAGAAAAATAGTCATACCCGGAGCTCCTGGA 13990
NorthernPike              -------------------------------------------------------------------------------- 0
SpottedGar                -------------------------------------------------------------------------------- 0
ParamormyropsKingsLeyae   -------------------------------------------------------------------------------- 0
```

```
Majority                    --------------------------------------------------------------------------------
                                    56170     56180     56190     56200     56210     56220     56230     56240
Human                       GTAACCCCCTTGTTGAGATTTACAACGTACCTGTTTTATGGAGTGATTAATGTTAAAGTTGTATTCTCTAAATTTCTGTT 56198
AsianBonytongue             -------------------------------------------------------------------------------- 0
Reedfish                    ATAGCTGCCACACGCGGCGCGCCATTAATTCATTTATGATCAAATCATAAAAATTCATAAAATTATAAAAAAGGATCATT 14070
NorthernPike                -------------------------------------------------------------------------------- 0
SpottedGar                  -------------------------------------------------------------------------------- 0
ParamormyropsKingsLeyae     -------------------------------------------------------------------------------- 0

Majority                    --------------------------------------------------------------------------------
                                    56250     56260     56270     56280     56290     56300     56310     56320
Human                       TAAAACATTTTCTGATGGTACATGTATTTATAAAACTACATCTTG----TTCTTTTGAGAATAAATTACATATCATACCT 56274
AsianBonytongue             -------------------------------------------------------------------------------- 0
Reedfish                    GCACAGAATTTTACCAGCAACCAGCTGCTTTATATTCAGGGTACAACACCTGCTGCTTTAGGAAGTCAAACTTAGTTGAT 14150
NorthernPike                -------------------------------------------------------------------------------- 0
SpottedGar                  -------------------------------------------------------------------------------- 0
ParamormyropsKingsLeyae     -------------------------------------------------------------------------------- 0

Majority                    --------------------------------------------------------------------------------
                                    56330     56340     56350     56360     56370     56380     56390     56400
Human                       TTGCATGTTCAACATTGATCCCAAAGCCTGGATAATTTTTTTGAATGAAATACTAGTAGAATATTTGATTTATTAAACAG 56354
AsianBonytongue             -------------------------------------------------------------------------------- 0
Reedfish                    TTTCTCACTTCCTCCTTCTACCAAACAGTATAGGACCATTGGCACTCAACAGTATATTGAGAGATAGTTTCTACTCTTAA 14230
NorthernPike                -------------------------------------------------------------------------------- 0
SpottedGar                  -------------------------------------------------------------------------------- 0
ParamormyropsKingsLeyae     -------------------------------------------------------------------------------- 0

Majority                    --------------------------------------------------------------------------------
                                    56410     56420     56430     56440     56450     56460     56470     56480
Human                       GTAGTACCTGGCTCATGGCAGGTGCTTAAAAATATCTGTTGAGGGGCCATTTAATTTTGTTTAATCTGTGTAAGTTCTAG 56434
AsianBonytongue             -------------------------------------------------------------------------------- 0
Reedfish                    GTTGTAAGGTTGCTTAACAGTCATTCATTAAAAATTCTGATATTGGAAGTAATATATGTTGCATGCTGTGTATACTTTAT 14310
NorthernPike                -------------------------------------------------------------------------------- 0
SpottedGar                  -------------------------------------------------------------------------------- 0
ParamormyropsKingsLeyae     -------------------------------------------------------------------------------- 0

Majority                    --------------------------------------------------------------------------------
                                    56490     56500     56510     56520     56530     56540     56550     56560
Human                       GAACAAAGTGAACTAGTAGTGTAACAGGTTGCAAAGTATGTGACAATAATGGGTATGTTTCTAAACTACATAATCTTAAT 56514
AsianBonytongue             -------------------------------------------------------------------------------- 0
Reedfish                    TTTTTGTGCTGTATTTTAGTTTAGTGTTCTCAAGAGGCATGCAAGTAAGAATTTCATTATATTAGTTACCAATGACAATA 14390
NorthernPike                -------------------------------------------------------------------------------- 0
SpottedGar                  -------------------------------------------------------------------------------- 0
ParamormyropsKingsLeyae     -------------------------------------------------------------------------------- 0

Majority                    --------------------------------------------------------------------------------
                                    56570     56580     56590     56600     56610     56620     56630     56640
Human                       TTCCAGCGTGCTCAGGCAGTTCTTCAAGCTGTGACAGCTGTCCAGACAGCAAATACTCCTCTTAGTGGCACCACAGTTAG 56594
AsianBonytongue             -------------------------------------------------------------------------------- 0
Reedfish                    GCCAATACTCTTGACTTGAGAGAGGCTGGAATAGTGTTTGTTTGTATGCAACATCTTCCAACAGGTGCAAGAGTTTTTCT 14470
NorthernPike                -------------------------------------------------------------------------------- 0
SpottedGar                  -------------------------------------------------------------------------------- 0
ParamormyropsKingsLeyae     -------------------------------------------------------------------------------- 0
```

```
Majority                   --------------------------------------------------------------------------------
                                   56650     56660     56670     56680     56690     56700     56710     56720
Human                      CGAGAGTGCAGTGACTCCAGCCCAGAGTCCAGTACTTAGAATAATTATTGACAACATGTACTACCCTGTAACACTTGATG 56674
AsianBonytongue            -------------------------------------------------------------------------------- 0
Reedfish                   ACATAGTACCATTTTATGACATGGGAGTTGACAGAATACCTTAAAGAAAAAAAAAGGTGGAGAAGGAAAAGTAGATTCTG 14550
NorthernPike               -------------------------------------------------------------------------------- 0
SpottedGar                 -------------------------------------------------------------------------------- 0
ParamormyropsKingsLeyae--------------------------------------------------------------------------------- 0

Majority                   --------------------------------------------------------------------------------
                                   56730     56740     56750     56760     56770     56780     56790     56800
Human                      TTCTTCACCAAGTAAGTTTAATCTGCATAATTACCTATAAATTAGAGAAATAATAATATAGTAAATAAGTAATGTTTTGA 56754
AsianBonytongue            -------------------------------------------------------------------------------- 0
Reedfish                   TAAAGAGCAATTGATGTGTTTGTGGGAAGGAGGACATAGCCAAATAAGATGGAGAATGGAAAACCAATAGTATAATGTCT 14630
NorthernPike               -------------------------------------------------------------------------------- 0
SpottedGar                 -------------------------------------------------------------------------------- 0
ParamormyropsKingsLeyae--------------------------------------------------------------------------------- 0

Majority                   --------------------------------------------------------------------------------
                                   56810     56820     56830     56840     56850     56860     56870     56880
Human                      TTTTAATGTTTTAACAGATATTTTCTAAGTTTGGTGCTGTATTGAAGATAATCACATTTACAAAAAATAACCAGTTTCAA 56834
AsianBonytongue            -------------------------------------------------------------------------------- 0
Reedfish                   AGGTCAGGAAGAGACACCCCAGCACTCAAGTCTGTCACAAACTAGGGTTGAATGATTAAGATTAGGTCTCTTACATTCCA 14710
NorthernPike               -------------------------------------------------------------------------------- 0
SpottedGar                 -------------------------------------------------------------------------------- 0
ParamormyropsKingsLeyae--------------------------------------------------------------------------------- 0

Majority                   --------------------------------------------------------------------------------
                                   56890     56900     56910     56920     56930     56940     56950     56960
Human                      GCTTTGCTCCAGTATGGTGATCCAGTAAATGCTCAACAAGCAAAACTAGTAAGTCTTTCTTTTGAGATGGTGATTTTTTT 56914
AsianBonytongue            -------------------------------------------------------------------------------- 0
Reedfish                   AGGAAACTTAAAACTGATGACTTCAGCCCAAGAAAGGAGAAAACAGGACCATAGATTGCATGGTGTATACTGCTCATTTT 14790
NorthernPike               -------------------------------------------------------------------------------- 0
SpottedGar                 -------------------------------------------------------------------------------- 0
ParamormyropsKingsLeyae--------------------------------------------------------------------------------- 0

Majority                   --------------------------------------------------------------------------------
                                   56970     56980     56990     57000     57010     57020     57030     57040
Human                      TTATTGAAATGTATATGTAGAAAAATATATATATATGTATGTATACTTAATGATATCTTGTAGCATTACAGATAGTAGAA 56994
AsianBonytongue            -------------------------------------------------------------------------------- 0
Reedfish                   AACAGTGGCAAGATGAGATTGAATAAAGAGAGAAAGCTTAGACCAGGGGTCTTCAACATGTCAGATTGAATAAAGAGAGA 14870
NorthernPike               -------------------------------------------------------------------------------- 0
SpottedGar                 -------------------------------------------------------------------------------- 0
ParamormyropsKingsLeyae--------------------------------------------------------------------------------- 0

Majority                   --------------------------------------------------------------------------------
                                   57050     57060     57070     57080     57090     57100     57110     57120
Human                      ATTGATGGCATTAATATTAAGAAACCTTAATATTTGTCATAAAAGAAATATTTATTTTTAGGAAAATCATAGTTGTAGTT 57074
AsianBonytongue            -------------------------------------------------------------------------------- 0
Reedfish                   AAGCTTAGACCAGGGGTCTTCAGCATGTCAAGTAGCTCGCCAAAGGGTTAATGAATCCTATATAAATTTGAAAACTTGAT 14950
NorthernPike               -------------------------------------------------------------------------------- 0
SpottedGar                 -------------------------------------------------------------------------------- 0
ParamormyropsKingsLeyae--------------------------------------------------------------------------------- 0
```

```
Majority                   --------------------------------------------------------------------------------

                                   57130     57140     57150     57160     57170     57180     57190     57200

Human                      AACCATGATGTGGGTTTCTATTATTTACTTTTTTTTTTTTTTTTTAATTTAAGGACAAAAGTTTGTACTCTTAAGAAAA 57154
AsianBonytongue            -------------------------------------------------------------------------------- 0
Reedfish                   TAGTCAAATTAGGGGTGTGCGATCTTTCCAAAAAATCATATCACAATCCTTTTAACACAAAGTCACAATCCACAATCTGA 15030
NorthernPike               -------------------------------------------------------------------------------- 0
SpottedGar                 -------------------------------------------------------------------------------- 0
ParamormyropsKingsLeyae    -------------------------------------------------------------------------------- 0

Majority                   --------------------------------------------------------------------------------

                                   57210     57220     57230     57240     57250     57260     57270     57280

Human                      AAGCAGGACTATATTGTGAATGTGCACCAGCATAAGATTTGAAGTGGCTTTTGCTGGGAATTTCAAAGTAATTAGTTTTC 57234
AsianBonytongue            -------------------------------------------------------------------------------- 0
Reedfish                   ATTGCAATCTATCTTTTCAATGTGTCATACAAGAGAGAATATCCGGACTCAAACTCATCAAGACCTAAGTAACACTTTAT 15110
NorthernPike               -------------------------------------------------------------------------------- 0
SpottedGar                 -------------------------------------------------------------------------------- 0
ParamormyropsKingsLeyae    -------------------------------------------------------------------------------- 0

Majority                   --------------------------------------------------------------------------------

                                   57290     57300     57310     57320     57330     57340     57350     57360

Human                      AAAAGAGTTTGGCTGAAATATACAGATGTGGCTCAAATGCTATGATAGGCTCTCAGTTTATTTAAAACTATATATTCTCT 57314
AsianBonytongue            -------------------------------------------------------------------------------- 0
Reedfish                   TTTAAATATCAAACAAAGTTGAATTCAGTTGTTCTCTCGTTCGCTAGCTAAGTGGAGTTAAGGAACACGACCCAAAGCTG 15190
NorthernPike               -------------------------------------------------------------------------------- 0
SpottedGar                 -------------------------------------------------------------------------------- 0
ParamormyropsKingsLeyae    -------------------------------------------------------------------------------- 0

Majority                   --------------------------------------------------------------------------------

                                   57370     57380     57390     57400     57410     57420     57430     57440

Human                      TTAATGTCTTTTTTTTAAAGGTTTTATGAATAGGTTTCTAGTCACTTTTTATTCATATCATTTATCTTTTTTGTCATTGT 57394
AsianBonytongue            -------------------------------------------------------------------------------- 0
Reedfish                   GCGAGCGAGTGAGGAACGCCCCTCCCCTCGGCCCACTGCATATTTCCCAGATTCGCACAAAGAAATCAGTACCACAAGTG 15270
NorthernPike               -------------------------------------------------------------------------------- 0
SpottedGar                 -------------------------------------------------------------------------------- 0
ParamormyropsKingsLeyae    -------------------------------------------------------------------------------- 0

Majority                   --------------------------------------------------------------------------------

                                   57450     57460     57470     57480     57490     57500     57510     57520

Human                      TACATATGGAGGATGTAAGGTCATTGAGAGATGTAAAATTTCCCATCAAGGCTGCCACTGAAAACTGCGCACCACATCTT 57474
AsianBonytongue            -------------------------------------------------------------------------------- 0
Reedfish                   AACTATGATAAATAGCGAAATAAGAGAAGTCGCAGAATTAACCGGAATGTTCAAGCAGATTATAGAAAAAAACCCGCTCT 15350
NorthernPike               -------------------------------------------------------------------------------- 0
SpottedGar                 -------------------------------------------------------------------------------- 0
ParamormyropsKingsLeyae    -------------------------------------------------------------------------------- 0

Majority                   --------------------------------------------------------------------------------

                                   57530     57540     57550     57560     57570     57580     57590     57600

Human                      TGCTTCCAAGTACCTTAATCTTCTGTTTGTATATCTTAAATCTACTTTTGCTCTGTCTCTTTAACTTTTTGTGCCATAGT 57554
AsianBonytongue            -------------------------------------------------------------------------------- 0
Reedfish                   AAAACCGTTAAGTAGTTCTCTCATGAAAAACAGACAGAGACATTGGATATTATATATTAGTAAACCTTCAAAATAATATG 15430
NorthernPike               -------------------------------------------------------------------------------- 0
SpottedGar                 -------------------------------------------------------------------------------- 0
ParamormyropsKingsLeyae    -------------------------------------------------------------------------------- 0
```

```
Majority                   --------------------------------------------------------------------------------

                                   57610     57620     57630     57640     57650     57660     57670     57680

Human                      ATCTCATTATTATTACTATTCCATTATTAAGCCATTAAAAATTCATTATTAAGCCATTATTATGAAACCATTTAATAAGC 57634
AsianBonytongue            -------------------------------------------------------------------------------- 0
Reedfish                   CAGTTAAAGTCTCAATAGCACGCTCAGCATTTCTGGAGCTTAGTAGAGCCAGATAACTACAGTATAAGAATCTTCACCAA 15510
NorthernPike               -------------------------------------------------------------------------------- 0
SpottedGar                 -------------------------------------------------------------------------------- 0
ParamormyropsKingsLeyae    -------------------------------------------------------------------------------- 0

Majority                   --------------------------------------------------------------------------------

                                   57690     57700     57710     57720     57730     57740     57750     57760

Human                      TTACATTTATCTTAACTGGTTTTCTTCCTTTCATTCATTCTTTTACTTTCAGATTAATTATTGTCTCTAATACTTAAATG 57714
AsianBonytongue            -------------------------------------------------------------------------------- 0
Reedfish                   GCAGCTCTGAAAATGTCTGCTTTGTTTGGGTCTATGTACCTCTGTGAGTCTGCCTTTTAAGTGGCTACACTCCACCTATA 15590
NorthernPike               -------------------------------------------------------------------------------- 0
SpottedGar                 -------------------------------------------------------------------------------- 0
ParamormyropsKingsLeyae    -------------------------------------------------------------------------------- 0

Majority                   --------------------------------------------------------------------------------

                                   57770     57780     57790     57800     57810     57820     57830     57840

Human                      CTTCCTCCAGATCAAGATTACTTCTGTGGAGCCCAGACCATTTTTTACAATTGTTTCATTGCATTTCCATCCACACAACC 57794
AsianBonytongue            -------------------------------------------------------------------------------- 0
Reedfish                   CACCTCTCTTGCAGTGCCAGTCATCTCACTAACTAAAAAACACATCACACATGCAACTGGACATGTGAATACTCTTG--- 15667
NorthernPike               -------------------------------------------------------------------------------- 0
SpottedGar                 -------------------------------------------------------------------------------- 0
ParamormyropsKingsLeyae    -------------------------------------------------------------------------------- 0

Majority                   --------------------------------------------------------------------------------

                                   57850     57860     57870     57880     57890     57900     57910     57920

Human                      TCATTTTGGGAGCAAAATTAGTTGTAGGTAGCAGAAAGGCTATACAAGTAATAATAGCATAATTGTAATAGTTTACCAAG 57874
AsianBonytongue            -------------------------------------------------------------------------------- 0
Reedfish                   -TGAAGTGAAACCGTGACATGTAACAAAATGAATGACAAATGATTAAGTAATATCTGTGTATTTGTAGTTGCATTGTTTT 15746
NorthernPike               -------------------------------------------------------------------------------- 0
SpottedGar                 -------------------------------------------------------------------------------- 0
ParamormyropsKingsLeyae    -------------------------------------------------------------------------------- 0

Majority                   --------------------------------------------------------------------------------

                                   57930     57940     57950     57960     57970     57980     57990     58000

Human                      TGCCGTAGTCTCATTTTTTCTGACCACTACTCTATATTGTCAACAAAACAGGGTTTTCCCCTGCGTTACAAGAGGATGCT 57954
AsianBonytongue            -------------------------------------------------------------------------------- 0
Reedfish                   GTTTTAACAGCATTCATGTTTTAATTCAATAGTGTGAGATACGCTAGAAAGTGCATACAGACATGCAAAAATGTATTTGC 15826
NorthernPike               -------------------------------------------------------------------------------- 0
SpottedGar                 -------------------------------------------------------------------------------- 0
ParamormyropsKingsLeyae    -------------------------------------------------------------------------------- 0

Majority                   --------------------------------------------------------------------------------

                                   58010     58020     58030     58040     58050     58060     58070     58080

Human                      AAGATTTAAAGAGGTGAATTACTTACAGTGAACACCACCTATAAGGTAGACATTTTTTCAGTCTTAGCCTTTCTCTCCAG 58034
AsianBonytongue            -------------------------------------------------------------------------------- 0
Reedfish                   AGGTGAACTAAATATTTTTGTGATTTTTCTAATGCAAAATGTGAGTTGTGGACACCAACATTTTGTAAATGTGCAGGCAA 15906
NorthernPike               -------------------------------------------------------------------------------- 0
SpottedGar                 -------------------------------------------------------------------------------- 0
ParamormyropsKingsLeyae    -------------------------------------------------------------------------------- 0
```

```
Majority                    --------------------------------------------------------------------------------
                                     58090     58100     58110     58120     58130     58140     58150     58160
Human                       TATGCTGCTTCTCACTGGTTCTCGGGTATAATCTAATCTTACTGTGCTTTCCGCTTCAGGCATAGGTTAATAATTAGAAT 58114
AsianBonytongue             -------------------------------------------------------------------------------- 0
Reedfish                    AACAAACTCATTCAGTTTGTTTGGGTTGAAATAAGCTATGAGAATAAACGTTAGAAAACATGAGTAACTCTCGTCCATTT 15986
NorthernPike                -------------------------------------------------------------------------------- 0
SpottedGar                  -------------------------------------------------------------------------------- 0
ParamormyropsKingsLeyae     -------------------------------------------------------------------------------- 0

Majority                    --------------------------------------------------------------------------------
                                     58170     58180     58190     58200     58210     58220     58230     58240
Human                       TCTTTTTTTTTGTCCAAAAAATGATTATCCTCCTTTCACATGCCCTGGCTACCAAAATCAATGTGGTATGAGGTTTTATG 58194
AsianBonytongue             -------------------------------------------------------------------------------- 0
Reedfish                    TCATTTTGTTAAAGTAGCTCGGGGGGATGTTGGAGACCTCTGGTTTAGACCCATTATTAAGAGGTTCTTGCATTAGAAAA 16066
NorthernPike                -------------------------------------------------------------------------------- 0
SpottedGar                  -------------------------------------------------------------------------------- 0
ParamormyropsKingsLeyae     -------------------------------------------------------------------------------- 0

Majority                    --------------------------------------------------------------------------------
                                     58250     58260     58270     58280     58290     58300     58310     58320
Human                       AATAGGAGACAATCTAGAAGTCATGGTTTCTGTGACATTTTTCACAACTTCAAACGTTTAAATATAGTCTACATTTTTCT 58274
AsianBonytongue             -------------------------------------------------------------------------------- 0
Reedfish                    GACCCAAATGCAGTTATTAAAGAATATATCTGAAGATGGAATAGAGAGCTCAACTCTGAAAACCAAAATCTGAAACAGGC 16146
NorthernPike                -------------------------------------------------------------------------------- 0
SpottedGar                  -------------------------------------------------------------------------------- 0
ParamormyropsKingsLeyae     -------------------------------------------------------------------------------- 0

Majority                    --------------------------------------------------------------------------------
                                     58330     58340     58350     58360     58370     58380     58390     58400
Human                       TGGTGTGGAATATTTTCCTTGCTCTTCTGACCCCAAAGAAAAGTATTTGTTAGAGGACCAATTAAATGAAATTTGAGTGG 58354
AsianBonytongue             -------------------------------------------------------------------------------- 0
Reedfish                    ACAGGAGTAAAAGAGAGGATGGAGCCTTGGAGACAAAACTCTCAGAGGAAAAAGGGAGGGTTTAGTCTAATTAATTTTGT 16226
NorthernPike                -------------------------------------------------------------------------------- 0
SpottedGar                  -------------------------------------------------------------------------------- 0
ParamormyropsKingsLeyae     -------------------------------------------------------------------------------- 0

Majority                    --------------------------------------------------------------------------------
                                     58410     58420     58430     58440     58450     58460     58470     58480
Human                       GGATTATACTGTGTTTGAGCTATAATTCTTAAGGGGAAAATTGTTTATATGTGTATCTTGTCTGAAGTAATACATAGTTA 58434
AsianBonytongue             -------------------------------------------------------------------------------- 0
Reedfish                    ACCACATACAAAGTCCTAAATAGATTTAAAATGTGATGGGCATCCTCTGCATATGAGCCTATTATGCTAATTCAGAATTT 16306
NorthernPike                -------------------------------------------------------------------------------- 0
SpottedGar                  -------------------------------------------------------------------------------- 0
ParamormyropsKingsLeyae     -------------------------------------------------------------------------------- 0

Majority                    --------------------------------------------------------------------------------
                                     58490     58500     58510     58520     58530     58540     58550     58560
Human                       CATTAAATCTCATAAAAGTACCTCAATTGTGTCAACTGTTTGACATTTTTGTTTAATCTTCTGTGAACAGATACTAAGCA 58514
AsianBonytongue             -------------------------------------------------------------------------------- 0
Reedfish                    GAACAAGTAATATAAACCGCAAATTTTACTTGTCTACTGTTAGTGGTTTAAGACATAAATTAAAAAGCAGTGGTAAAGAA 16386
NorthernPike                -------------------------------------------------------------------------------- 0
SpottedGar                  -------------------------------------------------------------------------------- 0
ParamormyropsKingsLeyae     -------------------------------------------------------------------------------- 0
```

```
Majority                   --------------------------------------------------------------------------------
                                   58570     58580     58590     58600     58610     58620     58630     58640
Human                      TCCAACTATGATATTGATGTTTGTGCACAGGTATGCACTCAGGTCTTCTTGACAAAGATCTAAAGTTATAATTCTAAGAG 58594
AsianBonytongue            -------------------------------------------------------------------------------- 0
Reedfish                   TGCAGCCTTGCT-TGGCTATTCCTCTGTTAATAGAGAAAGGGCGTGAGATGTTGGAATTATTAAGGCGGTTCCATCCCTG 16465
NorthernPike               -------------------------------------------------------------------------------- 0
SpottedGar                 -------------------------------------------------------------------------------- 0
ParamormyropsKingsLeyae    -------------------------------------------------------------------------------- 0

Majority                   --------------------------------------------------------------------------------
                                   58650     58660     58670     58680     58690     58700     58710     58720
Human                      AGTAGATAATTGTTTTACTAAGTACTTATTCTGGTATCAGAACTCACACTTCACTTATTACAGAAGGAAAAAGAAATTTG 58674
AsianBonytongue            -------------------------------------------------------------------------------- 0
Reedfish                   GGGCCTTGCTTATGTTCATGAGGCCCAGCACCTCTGAGTTTAGCTATAGAAAAGTGGAATCTAAAATAGAAGCCTCTTTC 16545
NorthernPike               -------------------------------------------------------------------------------- 0
SpottedGar                 -------------------------------------------------------------------------------- 0
ParamormyropsKingsLeyae    -------------------------------------------------------------------------------- 0

Majority                   --------------------------------------------------------------------------------
                                   58730     58740     58750     58760     58770     58780     58790     58800
Human                      TTTCCTAAACTTGCTTTGCCCCATTACCTTGTAATCTAGACCCTTTGGTAAAATTTGCATTTTTGATAACTACAGTAATG 58754
AsianBonytongue            -------------------------------------------------------------------------------- 0
Reedfish                   TCTGTGTGATGTGGAAGGGGACGTTTATCAAGAAATAATCTAATATTAAAGGGGCTGGTGCTGAGATAATCACTAAATAA 16625
NorthernPike               -------------------------------------------------------------------------------- 0
SpottedGar                 -------------------------------------------------------------------------------- 0
ParamormyropsKingsLeyae    -------------------------------------------------------------------------------- 0

Majority                   --------------------------------------------------------------------------------
                                   58810     58820     58830     58840     58850     58860     58870     58880
Human                      TTCATTTTACTTGCCTGCATCTGGGCTCTACTTAGAGAATATCACATAATATTACATAATCTGACAATTGGTACCAGTTT 58834
AsianBonytongue            -------------------------------------------------------------------------------- 0
Reedfish                   TTTGTTGTAGTTATTAAAAAAAAAAAAAAAAAATTTGTTGGAGCTGTTTGACATCTAGTTTACATCCTTAATCTGCAGAT 16705
NorthernPike               -------------------------------------------------------------------------------- 0
SpottedGar                 -------------------------------------------------------------------------------- 0
ParamormyropsKingsLeyae    -------------------------------------------------------------------------------- 0

Majority                   --------------------------------------------------------------------------------
                                   58890     58900     58910     58920     58930     58940     58950     58960
Human                      AAATCCATCAACGTCAACTACAAGTAAGCCCTCTACTCTACTTTAAAAATCTCTTCCCATTTCTTCAGTCAACAGTTTTT 58914
AsianBonytongue            -------------------------------------------------------------------------------- 0
Reedfish                   TATGTCTAAGCAAAATAAGAATCCCGCACATCTTGGATTCAGCAGAGGAATAAGCTGCATCCAATATTTTTTTTTTTTAA 16785
NorthernPike               -------------------------------------------------------------------------------- 0
SpottedGar                 -------------------------------------------------------------------------------- 0
ParamormyropsKingsLeyae    -------------------------------------------------------------------------------- 0

Majority                   --------------------------------------------------------------------------------
                                   58970     58980     58990     59000     59010     59020     59030     59040
Human                      CTGTTTTTCACAATGCCTATGTCAGATAGAAATTTTCCCTTCTACCATATCTCTCCTCACTCTCACTTGCTTGAAGAGGA 58994
AsianBonytongue            -------------------------------------------------------------------------------- 0
Reedfish                   CAGATGTGACTCCTGATTTAAGGCCATATCAATATTGTGACAGCAGAGAAGGCTGAACATGCTTAAGTGCTGCTGACCAG 16865
NorthernPike               -------------------------------------------------------------------------------- 0
SpottedGar                 -------------------------------------------------------------------------------- 0
ParamormyropsKingsLeyae    -------------------------------------------------------------------------------- 0
```

```
Majority                   --------------------------------------------------------------------------------
                                    59050     59060     59070     59080     59090     59100     59110     59120
Human                      AAATCACATGGAAGCTTCATCAGCTTTTCTTGCAAAATCTGCTAATACTCACACTTGTCTTTCTCTTTTAATAAAAGGCA 59074
AsianBonytongue            -------------------------------------------------------------------------------- 0
Reedfish                   AGTTAAAATCCCTGACAATCCAGTAAATAATATTAAACAAAGCCATAGTCAGTATTAGAAGAGAGGTAGCTACAACAGAA 16945
NorthernPike               -------------------------------------------------------------------------------- 0
SpottedGar                 -------------------------------------------------------------------------------- 0
ParamormyropsKingsLeyae    -------------------------------------------------------------------------------- 0

Majority                   --------------------------------------------------------------------------------
                                    59130     59140     59150     59160     59170     59180     59190     59200
Human                      TAGATGTTTAGTTATCTAAGACCAATAAACACCAAAGCCTATCGTCTTCTGCCTGTCAAGAGACTGCACTATCTGTCATC 59154
AsianBonytongue            -------------------------------------------------------------------------------- 0
Reedfish                   TATTGGAAAACTTGGGGGAATGAAACTGGTGTATTATGGCACCATATCCAGTGAATTCAAAAATCTACATCCCCGAAGTT 17025
NorthernPike               -------------------------------------------------------------------------------- 0
SpottedGar                 -------------------------------------------------------------------------------- 0
ParamormyropsKingsLeyae    -------------------------------------------------------------------------------- 0

Majority                   --------------------------------------------------------------------------------
                                    59210     59220     59230     59240     59250     59260     59270     59280
Human                      CCCTTTCTCATTTATTTCTCTCTATATTGGCCACTTTTCACCAGTTTCAAGCATTCTCAGCTTCTTTCATTTTAAATGAA 59234
AsianBonytongue            -------------------------------------------------------------------------------- 0
Reedfish                   CACTCAACTTAGTTACCCATCCCTGATCTATGCCCCCAGCTGATACTTCCAAATCTGACTAACCACCAGCAGGAGAAAGG 17105
NorthernPike               -------------------------------------------------------------------------------- 0
SpottedGar                 -------------------------------------------------------------------------------- 0
ParamormyropsKingsLeyae    -------------------------------------------------------------------------------- 0

Majority                   --------------------------------------------------------------------------------
                                    59290     59300     59310     59320     59330     59340     59350     59360
Human                      TTTACCTTCAACTTTCAATCCATCCATCTATAACTCTACGTACTTTCTATCA-----CCCTGCCTCCCCTCTCCCTTTTC 59309
AsianBonytongue            -------------------------------------------------------------------------------- 0
Reedfish                   CACTCTTTATGTTGTAAAATACTAAGTAACAGTTTATTAGGAAAAACAAGCAATGGCCACAAAAGTACCTCAAATCATTA 17185
NorthernPike               -------------------------------------------------------------------------------- 0
SpottedGar                 -------------------------------------------------------------------------------- 0
ParamormyropsKingsLeyae    -------------------------------------------------------------------------------- 0

Majority                   --------------------------------------------------------------------------------
                                    59370     59380     59390     59400     59410     59420     59430     59440
Human                      ACTCATAACCAGACTTCTTAAATAGTCTATCTTTGTTGTTTATTATCTCATTTCTCTACTTACTATTATTTGGTTTTAGC 59389
AsianBonytongue            -------------------------------------------------------------------------------- 0
Reedfish                   ATCCACTCCCCATATAATTAAGCACGAGCAATAACCAAATGCCTATTAGAGTACAATTGTATGAGTAATTGGGCAAATAA 17265
NorthernPike               -------------------------------------------------------------------------------- 0
SpottedGar                 -------------------------------------------------------------------------------- 0
ParamormyropsKingsLeyae    -------------------------------------------------------------------------------- 0

Majority                   --------------------------------------------------------------------------------
                                    59450     59460     59470     59480     59490     59500     59510     59520
Human                      ACTGTTGACCCAAATTGCTCTTCCTCAGCGTCACCAGCGATTAGTAGAGTAGCTAAATGCTAGTGATATGATTTAGTCCT 59469
AsianBonytongue            -------------------------------------------------------------------------------- 0
Reedfish                   ACTGTAACAAATCACAGGATGATATTAAATGCAGCATTCAACTATTAAAGGTAATGCTGTGATTGTCAAATGGCACCTGC 17345
NorthernPike               -------------------------------------------------------------------------------- 0
SpottedGar                 -------------------------------------------------------------------------------- 0
ParamormyropsKingsLeyae    -------------------------------------------------------------------------------- 0
```

```
Majority               --------------------------------------------------------------------------------
                              59530     59540     59550     59560     59570     59580     59590     59600
Human                  TGTCTTACTTAATACCTAGATAATATTTGGCACTGTGCACCATTGTCTTAAAATTCTCTTTCTTCCTGGTCTCTGATA-C 59548
AsianBonytongue        -------------------------------------------------------------------------------- 0
Reedfish               TGTAGCCCCTACACCATCCCCAGCCCACAAGCCAATATAACAAGAGGAAAAAAAAATAAGGCAGTGGTGCTTAAATTAAC 17425
NorthernPike           -------------------------------------------------------------------------------- 0
SpottedGar             -------------------------------------------------------------------------------- 0
ParamormyropsKingsLeyae-------------------------------------------------------------------------------- 0

Majority               --------------------------------------------------------------------------------
                              59610     59620     59630     59640     59650     59660     59670     59680
Human                  TTCTCACCTGGATTTATCCTACCTCTCTATTTCTTCCTTGTCTTGTAACTCCCCTTCTACCTGGGAGGCCTCACCTGCTG 59628
AsianBonytongue        -------------------------------------------------------------------------------- 0
Reedfish               ATAGGTGCACAAAATGAAGCATAATACCAAGAATCCATAACAAACTAAAGGTTATAAAAGTTAGGACTTTGAAGCAGATC 17505
NorthernPike           -------------------------------------------------------------------------------- 0
SpottedGar             -------------------------------------------------------------------------------- 0
ParamormyropsKingsLeyae-------------------------------------------------------------------------------- 0

Majority               --------------------------------------------------------------------------------
                              59690     59700     59710     59720     59730     59740     59750     59760
Human                  TCACAGCTCCATTAAACCTCTTATGATTCAGACAGAGTTCACATCACATTTTCTAGCTCAGACCTCTCCTAAGAGACCCA 59708
AsianBonytongue        -------------------------------------------------------------------------------- 0
Reedfish               TTAAAATACTAAAGCTGAGGAGTCAGTCAATCTGAAGATAAACGATTGTAAGCTTGGCCACAACTTGAACTAGTTTAACA 17585
NorthernPike           -------------------------------------------------------------------------------- 0
SpottedGar             -------------------------------------------------------------------------------- 0
ParamormyropsKingsLeyae-------------------------------------------------------------------------------- 0

Majority               --------------------------------------------------------------------------------
                              59770     59780     59790     59800     59810     59820     59830     59840
Human                  CACTAAGAACCTGGCGGACTAATGAATATCTTCAAACAGTGTGTTCAATCTAGCTCCTGTTTTTGGTGTCCGCTGTGTTA 59788
AsianBonytongue        -------------------------------------------------------------------------------- 0
Reedfish               ACTGAATGAAATAGCTGCTGATAGTGAAGGATAGGTGCTAATTGTAGTGAAATTGTCTGTTTGGGCAAGCGTCTACAGTG 17665
NorthernPike           -------------------------------------------------------------------------------- 0
SpottedGar             -------------------------------------------------------------------------------- 0
ParamormyropsKingsLeyae-------------------------------------------------------------------------------- 0

Majority               --------------------------------------------------------------------------------
                              59850     59860     59870     59880     59890     59900     59910     59920
Human                  GGAAAGTGCAAGATACCAACTTCAGTCTCATTAACAAGCCAGTAAACATGGTATTTTTCCCTTTCCTTCCAACCTGTCAT 59868
AsianBonytongue        -------------------------------------------------------------------------------- 0
Reedfish               GCGAATTGGGGGGGCGGGAGTGCACTAATCTGAAAGTTGTTTTTCTCTTTATTAGGACTAAGTGAAATATGAAGCTTTAC 17745
NorthernPike           -------------------------------------------------------------------------------- 0
SpottedGar             -------------------------------------------------------------------------------- 0
ParamormyropsKingsLeyae-------------------------------------------------------------------------------- 0

Majority               --------------------------------------------------------------------------------
                              59930     59940     59950     59960     59970     59980     59990     60000
Human                  CAGATATTATTGATTATACCTCCTAAATTTTTGTTGAATCTGGCCTTTTTTCTCCAACTCTTGCTACCATTGCAGTCTAA 59948
AsianBonytongue        -------------------------------------------------------------------------------- 0
Reedfish               AGACTTTTTTTGACCAGGATGGTAGCATTTCTATATTGGGTCATAATCAGACAAGTCCTGCAGCTCCCAATACCCTTCAC 17825
NorthernPike           -------------------------------------------------------------------------------- 0
SpottedGar             -------------------------------------------------------------------------------- 0
ParamormyropsKingsLeyae-------------------------------------------------------------------------------- 0
```

```
Majority                   --------------------------------------------------------------------------------
                                   60010     60020     60030     60040     60050     60060     60070     60080
Human                      GCCAGTGTTATTCAAGATGCTGGTCTCTCAAGGTAGTAGTGTGTGTCAAACTGAAATCAGCATACTTCTAGCAACAGTAT 60028
AsianBonytongue            -------------------------------------------------------------------------------- 0
Reedfish                   ATATAATATTCTGAACCTGACACACCTTCAGAATTAAGCCTGCAGTCTGAGAACCTCAGAAACTTAATAAAAGGGCAGGA 17905
NorthernPike               -------------------------------------------------------------------------------- 0
SpottedGar                 -------------------------------------------------------------------------------- 0
ParamormyropsKingsLeyae-------------------------------------------------------------------------------- 0

Majority                   --------------------------------------------------------------------------------
                                   60090     60100     60110     60120     60130     60140     60150     60160
Human                      GCTTAATGTCAATATTGGCATAAATTTTGACACAGACTTCTTATATTCATGCAGAGTAGCCTGGGCTTCTGGACCGTCAA 60108
AsianBonytongue            -------------------------------------------------------------------------------- 0
Reedfish                   ACCTGATGAATCTGCACAACCTGTATACACTAATGCACGTGTAATATTGGGGACAAAGTCTGGGAGACTTGGGAAAAACA 17985
NorthernPike               -------------------------------------------------------------------------------- 0
SpottedGar                 -------------------------------------------------------------------------------- 0
ParamormyropsKingsLeyae-------------------------------------------------------------------------------- 0

Majority                   --------------------------------------------------------------------------------
                                   60170     60180     60190     60200     60210     60220     60230     60240
Human                      TCATAATTTAAGTAGCATTGATGTAGTAATAATTCTCTTTCATATCTCCACTTCCTTGGCCTTCTGACTGAACTCACCAT 60188
AsianBonytongue            -------------------------------------------------------------------------------- 0
Reedfish                   ATTTCCTGCTTTTTGTATGAGAAAAGAGATCAGATTGTCTTTCCTGGCATCTCTTAAAGCCCTAGAATTGTTAAGTTGTC 18065
NorthernPike               -------------------------------------------------------------------------------- 0
SpottedGar                 -------------------------------------------------------------------------------- 0
ParamormyropsKingsLeyae-------------------------------------------------------------------------------- 0

Majority                   --------------------------------------------------------------------------------
                                   60250     60260     60270     60280     60290     60300     60310     60320
Human                      TTTGACATCTTCCCCTACCTCCATTATTTTTCCACAGAGTACTAGGAATCATCTCAACGTCAAGTCATATTGCTTCTAAT 60268
AsianBonytongue            -------------------------------------------------------------------------------- 0
Reedfish                   TTTGGTGAATGCATTCTTCTGATTGATTAAATTTGTTTGTATAGTGGCAGCCTCATTATGCAGCACGTTTATTTATAAAT 18145
NorthernPike               -------------------------------------------------------------------------------- 0
SpottedGar                 -------------------------------------------------------------------------------- 0
ParamormyropsKingsLeyae-------------------------------------------------------------------------------- 0

Majority                   --------------------------------------------------------------------------------
                                   60330     60340     60350     60360     60370     60380     60390     60400
Human                      CACAATACCCTTTAGTGACTTTTTTGTCCTTGCAATAAAGTCCACAGTCCTTATCATGGCTTATAAGACTCTAAATCAGT 60348
AsianBonytongue            -------------------------------------------------------------------------------- 0
Reedfish                   CATGGCAGCATACACTGGATTGCAGAGCCTGATTAATTTTGTCTGCTCCTCCTTAATGCCAGACAGTTCAACACATGTCC 18225
NorthernPike               -------------------------------------------------------------------------------- 0
SpottedGar                 -------------------------------------------------------------------------------- 0
ParamormyropsKingsLeyae-------------------------------------------------------------------------------- 0

Majority                   --------------------------------------------------------------------------------
                                   60410     60420     60430     60440     60450     60460     60470     60480
Human                      CCTCAGCTTGCCTCTCCATTCTCCTCTCATGCCTTTTTAGCTATATTGGACTTCTTTAAATAGCTTGCATATACTGTATT 60428
AsianBonytongue            -------------------------------------------------------------------------------- 0
Reedfish                   CTGTAAAGTTGATCAGCATAGTTGTATTACTCGTGGTGTTATGTTTAAGAAATTTCTCTTTGAATTTCCCAATATTTATT 18305
NorthernPike               -------------------------------------------------------------------------------- 0
SpottedGar                 -------------------------------------------------------------------------------- 0
ParamormyropsKingsLeyae-------------------------------------------------------------------------------- 0
```

```
Majority                 --------------------------------------------------------------------------------

                                 60490     60500     60510     60520     60530     60540     60550     60560

Human                    TTCTCTGATGTCTCGATCTTACAGATTGCTGTTCCATTTGCTAATACTATTTTTTCTCTTCATCTCTTTATGCCCAACTT 60508
AsianBonytongue          -------------------------------------------------------------------------------- 0
Reedfish                 AATGTTTTTGCAGGATTCTGATGATGGGTGCTTTAGCTAAAAAGAAAAAAGACTAATTCTTTATGCCTTATGCCAG---T 18382
NorthernPike             -------------------------------------------------------------------------------- 0
SpottedGar               -------------------------------------------------------------------------------- 0
ParamormyropsKingsLeyae  -------------------------------------------------------------------------------- 0

Majority                 --------------------------------------------------------------------------------

                                 60570     60580     60590     60600     60610     60620     60630     60640

Human                    CTGCTTCTCTTTGTCTTTCAAGTATCAGTTTAGAAGCCTCTTCCTGAGATAAGTCTTCTGTAACATTTCAACTAGATTAG 60588
AsianBonytongue          -------------------------------------------------------------------------------- 0
Reedfish                 GAGGTTTACCTTCAAACGCATCCTGCTTATTTATCATCACAAACAAGGGCAGCCCCACAACCACATCTGATTCTCTTAGA 18462
NorthernPike             -------------------------------------------------------------------------------- 0
SpottedGar               -------------------------------------------------------------------------------- 0
ParamormyropsKingsLeyae  -------------------------------------------------------------------------------- 0

Majority                 --------------------------------------------------------------------------------

                                 60650     60660     60670     60680     60690     60700     60710     60720

Human                    ATCCCTTCCTTTCTTAGTTCTCAGTATCACACATAAAATTTATAATTTGTTAAATGTGTATATTTTCTGTCGGACTGTAA 60668
AsianBonytongue          -------------------------------------------------------------------------------- 0
Reedfish                 TATTGTCATTTGGTCTGCCAAAGTTGTTAATAATTGACCTGTCTAACTGAATCTACTAAAGCTCATGTCAATATTATGCA 18542
NorthernPike             -------------------------------------------------------------------------------- 0
SpottedGar               -------------------------------------------------------------------------------- 0
ParamormyropsKingsLeyae  -------------------------------------------------------------------------------- 0

Majority                 --------------------------------------------------------------------------------

                                 60730     60740     60750     60760     60770     60780     60790     60800

Human                    GTTCTGAGTACCATGTCTGCTTTGCTTTCTGCTGTATTTATTAAACATTGACATTCAGGGTGAAAATCAGAAGTCATAGT 60748
AsianBonytongue          -------------------------------------------------------------------------------- 0
Reedfish                 ATTTTTGTCATGGGGTATGCCCGCTGCACCATGTCAAATTAAACAGCTGAAAATCATAGCCTGTGTTACATTTCCCGATT 18622
NorthernPike             -------------------------------------------------------------------------------- 0
SpottedGar               -------------------------------------------------------------------------------- 0
ParamormyropsKingsLeyae  -------------------------------------------------------------------------------- 0

Majority                 --------------------------------------------------------------------------------

                                 60810     60820     60830     60840     60850     60860     60870     60880

Human                    AACAATCTTAATTATCAATAAAAAGTATTTTATGCGATCCTCATATTTATTAAAGAAAACACCTTAATACCTTTCAGAAA 60828
AsianBonytongue          -------------------------------------------------------------------------------- 0
Reedfish                 TGAGCACCAATCTTCCAGTGCTCACCCTTTTTTCTGATAGCTAATGTTCTGTTGCCTGACCAATGCCACTTTACAAATTA 18702
NorthernPike             -------------------------------------------------------------------------------- 0
SpottedGar               -------------------------------------------------------------------------------- 0
ParamormyropsKingsLeyae  -------------------------------------------------------------------------------- 0

Majority                 --------------------------------------------------------------------------------

                                 60890     60900     60910     60920     60930     60940     60950     60960

Human                    ATCTTGTTTATTTATTGGAAAGTAGTGTATTAGGCAAAGATGCGTCTTCTCTTGGCTTTCTAATTCAGCTTTTATAATGT 60908
AsianBonytongue          -------------------------------------------------------------------------------- 0
Reedfish                 ACAGCTGCAGTTAGTTCAGCAGCAATCTGTGCCCTTTATTTTTCTCCTTCTGGTAAGATAAAATATTAGGCATCAGACAC 18782
NorthernPike             -------------------------------------------------------------------------------- 0
SpottedGar               -------------------------------------------------------------------------------- 0
ParamormyropsKingsLeyae  -------------------------------------------------------------------------------- 0
```

```
Majority                 --------------------------------------------------------------------------------

                                 60970     60980     60990     61000     61010     61020     61030     61040

Human                    TGAGGAATATAATTACTTGATGCATAAAAATATCAATCTTTTTAATAGTATAATAAGACATATAATGTAATCTCCTTTTC 60988
AsianBonytongue          -------------------------------------------------------------------------------- 0
Reedfish                 ACAAGATTTAGTTCAGCTTGTGAAGTTGAAAACAAATGTGTTCCTTATTCCTCGAAACTGGATTCTATTCATTGTATTTC 18862
NorthernPike             -------------------------------------------------------------------------------- 0
SpottedGar               -------------------------------------------------------------------------------- 0
ParamormyropsKingsLeyae  -------------------------------------------------------------------------------- 0

Majority                 --------------------------------------------------------------------------------

                                 61050     61060     61070     61080     61090     61100     61110     61120

Human                    ACCTGGGATTTAAAAATTGTTTTTACATAGTCCTTACATTATAACACACATAAGTAGGAAATGGTTCTTTTTGGTTTTTG 61068
AsianBonytongue          -------------------------------------------------------------------------------- 0
Reedfish                 TAGAAAGGAATTCATTCGTTCATTTGGTTGTCGGCACTCTTGCATATACAGCCTGCTCCTAGGCTAACTGGAAAATCAAA 18942
NorthernPike             -------------------------------------------------------------------------------- 0
SpottedGar               -------------------------------------------------------------------------------- 0
ParamormyropsKingsLeyae  -------------------------------------------------------------------------------- 0

Majority                 --------------------------------------------------------------------------------

                                 61130     61140     61150     61160     61170     61180     61190     61200

Human                    TTATTAGAAACAGCTTTTCAGAAACATGTCTTTATTAGGAATATCTTTAGTTGACACTGGACAGTATATTGTGTCTTCTG 61148
AsianBonytongue          -------------------------------------------------------------------------------- 0
Reedfish                 TATATCTAGGCAGGCGCAGACTAGTTGGCCTCAATTCTTAGACATGCCTCATTAGACAATCAGGTTACTGGGAGTGTGAC 19022
NorthernPike             -------------------------------------------------------------------------------- 0
SpottedGar               -------------------------------------------------------------------------------- 0
ParamormyropsKingsLeyae  -------------------------------------------------------------------------------- 0

Majority                 --------------------------------------------------------------------------------

                                 61210     61220     61230     61240     61250     61260     61270     61280

Human                    GAAGTCCTGTTTATATGATCATTTTATTTTAACATATAAGTGGGTTGTTTCTTATTCTTTATTTCTTCCAGGAGATTCCA 61228
AsianBonytongue          -------------------------------------------------------------------------------- 0
Reedfish                 TCAGACTGGCCAAGATAATGTAAATGTGACATAGGCTTTACTTGCT-TCATAAAATATGTGTTCCTAGTTGAGCCTTTCA 19101
NorthernPike             -------------------------------------------------------------------------------- 0
SpottedGar               -------------------------------------------------------------------------------- 0
ParamormyropsKingsLeyae  -------------------------------------------------------------------------------- 0

Majority                 --------------------------------------------------------------------------------

                                 61290     61300     61310     61320     61330     61340     61350     61360

Human                    TTTCATCACTAACTGATAATAAAAATAAATATGAGATAAAACATTTGCAGCAGTAAGATTATGTCATTCATTTCCTTCTC 61308
AsianBonytongue          -------------------------------------------------------------------------------- 0
Reedfish                 TCCTTTGTGGTAGAGGGCTTCATTCCAGCAAAATGTACAGAGCCTGACAAAAAATATTTATGGTCTGAAGATGACACTCC 19181
NorthernPike             -------------------------------------------------------------------------------- 0
SpottedGar               -------------------------------------------------------------------------------- 0
ParamormyropsKingsLeyae  -------------------------------------------------------------------------------- 0

Majority                 --------------------------------------------------------------------------------

                                 61370     61380     61390     61400     61410     61420     61430     61440

Human                    ACAGAGTACACATTGCTTTATGCAGATTCTTTGAGAGACCTAATGGAATTATCCTTTGCTAATTTCTTGGGCAAAGATTC 61388
AsianBonytongue          -------------------------------------------------------------------------------- 0
Reedfish                 CAATTTTTTTTTTTTTTTAACTTTAGAAACTGCAAACGTGCATAATTCTCCAGATTAAATCATTCAGTAAAACTGGTTAT 19261
NorthernPike             -------------------------------------------------------------------------------- 0
SpottedGar               -------------------------------------------------------------------------------- 0
ParamormyropsKingsLeyae  -------------------------------------------------------------------------------- 0
```

```
Majority                    --------------------------------------------------------------------------------
                                     61450     61460     61470     61480     61490     61500     61510     61520
Human                       TCCTTGACATCTGCTTAATTTACAATCTGTATATTTACATTCATATTAATGGAGAATTTATGATGAAATCAGTGGTTTT 61468
AsianBonytongue             -------------------------------------------------------------------------------- 0
Reedfish                    TCTTATACATTCTTCAGGTTGGCTACAGGCATGGCATGTACATAATCACATTCATTTATGGTAGTGATGTTCTGTTCAGT 19341
NorthernPike                -------------------------------------------------------------------------------- 0
SpottedGar                  -------------------------------------------------------------------------------- 0
ParamormyropsKingsLeyae     -------------------------------------------------------------------------------- 0

Majority                    --------------------------------------------------------------------------------
                                     61530     61540     61550     61560     61570     61580     61590     61600
Human                       AGGATGCTTTAGGAAAATCCTGCATAATCTACCAGGGTGGGGTTATTTGATATTGGTAAATAATTAGTCTGAAGTATTAC 61548
AsianBonytongue             -------------------------------------------------------------------------------- 0
Reedfish                    CGGTCACTATTCTAAGTAGGCCAGTTTTCACACCAAATGACTAAATGTTCTGTAAATCTTAATAACTTTTTTCAGTATCT 19421
NorthernPike                -------------------------------------------------------------------------------- 0
SpottedGar                  -------------------------------------------------------------------------------- 0
ParamormyropsKingsLeyae     -------------------------------------------------------------------------------- 0

Majority                    --------------------------------------------------------------------------------
                                     61610     61620     61630     61640     61650     61660     61670     61680
Human                       ATTAGTATTATAAGCTTAATTTTGTGTTGTTGATTTTGATAATTTTTCATGACTTGCCAAATATTAAAAAGTAGATATTT 61628
AsianBonytongue             -------------------------------------------------------------------------------- 0
Reedfish                    TTTTTTACAATACGTTGCCTGAGGAAGTGCATGTGTACTTTTGCTGTCGGAACTTACTGCAACTCGCTGTATTCTGGGAT 19501
NorthernPike                -------------------------------------------------------------------------------- 0
SpottedGar                  -------------------------------------------------------------------------------- 0
ParamormyropsKingsLeyae     -------------------------------------------------------------------------------- 0

Majority                    --------------------------------------------------------------------------------
                                     61690     61700     61710     61720     61730     61740     61750     61760
Human                       AAAGAATTTGGAAGTTAAGGTTTCTCTCAGTGTGTGTTAAATCTGATCCCCAACAATCTCTTCAAAAAAGTAAGTTATCA 61708
AsianBonytongue             -------------------------------------------------------------------------------- 0
Reedfish                    TAACAAATCCTTGATACACAGGTTACAGTTGGTACAAAATGCTGCTGCTCGCTTTCTGGTTGGGGCAAGAAAGTATGACT 19581
NorthernPike                -------------------------------------------------------------------------------- 0
SpottedGar                  -------------------------------------------------------------------------------- 0
ParamormyropsKingsLeyae     -------------------------------------------------------------------------------- 0

Majority                    --------------------------------------------------------------------------------
                                     61770     61780     61790     61800     61810     61820     61830     61840
Human                       ATAGTAAACGATGAGAAAAAATGGAAATAGTTTTATTTTGGCTTTTCATGTTTTCAGTCCATATGCAGTGTTTCATTGTGC 61788
AsianBonytongue             -------------------------------------------------------------------------------- 0
Reedfish                    CTCTCTCCTATTTTAGCTTCTTTACACTGGCTGCCTGTCAGTTTTCGAATTGATTTTAAAATCCTGCTGCTAGTTTTTAA 19661
NorthernPike                -------------------------------------------------------------------------------- 0
SpottedGar                  -------------------------------------------------------------------------------- 0
ParamormyropsKingsLeyae     -------------------------------------------------------------------------------- 0

Majority                    --------------------------------------------------------------------------------
                                     61850     61860     61870     61880     61890     61900     61910     61920
Human                       TGCTTTAATTATGCTTTCTGTGCCTAATATCTTTCCTGTGTTTTGATTTCCATGTTTTAGCCATAAACTTTAAATTTACA 61868
AsianBonytongue             -------------------------------------------------------------------------------- 0
Reedfish                    ATTTTTACATGGGCTTGCTCCTGCCTATTTATCTGAACTGTGTGTTTTACACCAGCCATCCAGAGTGCTTAGATCTTCTG 19741
NorthernPike                -------------------------------------------------------------------------------- 0
SpottedGar                  -------------------------------------------------------------------------------- 0
ParamormyropsKingsLeyae     -------------------------------------------------------------------------------- 0
```

```
Majority               --------------------------------------------------------------------------------
                            61930     61940     61950     61960     61970     61980     61990     62000
Human                  TGAAAACTTTTTTTTCTGATTGTTTTTGCTTACTGTCTAAACCTTCACTGTGAGTAGGCACATTTGGAAGGTCTTTCTGC 61948
AsianBonytongue        -------------------------------------------------------------------------------- 0
Reedfish               GTCAGCTGTCTCTGGTTGTCCCTCGTACCAAGTGTAAAACCAAGGGGGACAGGTCTTTTGCAGCTGTTGCACCTCGCCTG 19821
NorthernPike           -------------------------------------------------------------------------------- 0
SpottedGar             -------------------------------------------------------------------------------- 0
ParamormyropsKingsLeyae-------------------------------------------------------------------------------- 0

Majority               --------------------------------------------------------------------------------
                            62010     62020     62030     62040     62050     62060     62070     62080
Human                  AAATTATCTTGAGCTTTTAATGTCAGAAATCTTTGCTCCAACAAGATCACCACATTCCTGATTGCTGAACCACAGGACTG 62028
AsianBonytongue        -------------------------------------------------------------------------------- 0
Reedfish               TGGAACTCTTTACCTCATCATATAAAGGAGTCGTCTACAATTGAACTGTTTAAAACAAGATTAAAGACTCATTTCTACTC 19901
NorthernPike           -------------------------------------------------------------------------------- 0
SpottedGar             -------------------------------------------------------------------------------- 0
ParamormyropsKingsLeyae-------------------------------------------------------------------------------- 0

Majority               --------------------------------------------------------------------------------
                            62090     62100     62110     62120     62130     62140     62150     62160
Human                  GTTTGTGTGCTACTGCTATTTCTTGGGATTTTG-CAAATACATGAGCTTGAAGTGAGCTTGCATATATTTTTTAAATTTG 62107
AsianBonytongue        -------------------------------------------------------------------------------- 0
Reedfish               ACTTGCATTCAGTTACCTTCAGTAATACTGATGGCTTCCTCTTTGTGATTATATTACATTACTTCTATTTTTTATGTATA 19981
NorthernPike           -------------------------------------------------------------------------------- 0
SpottedGar             -------------------------------------------------------------------------------- 0
ParamormyropsKingsLeyae-------------------------------------------------------------------------------- 0

Majority               --------------------------------------------------------------------------------
                            62170     62180     62190     62200     62210     62220     62230     62240
Human                  ATTTTACTCCCTTTCTTATTTGTGGTCACCTCCTTTAACCAACTATTTAAAATACCTGGATTTTTGCTCTCATACACCCT 62187
AsianBonytongue        -------------------------------------------------------------------------------- 0
Reedfish               TAACATTACTTCTATTTATTATGTATTTTATTTTATGTTCAAATATTTTATTTCTATTCATGTTTTATGTAAATTTGTTC 20061
NorthernPike           -------------------------------------------------------------------------------- 0
SpottedGar             -------------------------------------------------------------------------------- 0
ParamormyropsKingsLeyae-------------------------------------------------------------------------------- 0

Majority               --------------------------------------------------------------------------------
                            62250     62260     62270     62280     62290     62300     62310     62320
Human                  CAGCTCCTATTTCTTTCCTCTTTTGCCAACAGATGATTAATTTACATCTTGTTTGCTTATTTCTCTAGCTCTTGTACCTT 62267
AsianBonytongue        -------------------------------------------------------------------------------- 0
Reedfish               TATTTTTGTAAAGCACTTTGGCCACAGCATTACTATGTTTGTTTAAATGTGCTATATAAATAAATTGACATTGACATAAC 20141
NorthernPike           -------------------------------------------------------------------------------- 0
SpottedGar             -------------------------------------------------------------------------------- 0
ParamormyropsKingsLeyae-------------------------------------------------------------------------------- 0

Majority               --------------------------------------------------------------------------------
                            62330     62340     62350     62360     62370     62380     62390     62400
Human                  AGGTATACTTCTGCATCTTCCACCTACATAAAGTTTTTGTTTTTGTTTTTAATGTTCAGAGGGAAAATTTCAAAGGGTCC 62347
AsianBonytongue        -------------------------------------------------------------------------------- 0
Reedfish               CTGAAAACAGAGCATACAGGAGAGCGAGAAAAGACTGGAATATTGGCCTTTACATTAACTCTTTTAGGGCTAATTTTTTT 20221
NorthernPike           -------------------------------------------------------------------------------- 0
SpottedGar             -------------------------------------------------------------------------------- 0
ParamormyropsKingsLeyae-------------------------------------------------------------------------------- 0
```

```
Majority                    --------------------------------------------------------------------------------
                                     62410     62420     62430     62440     62450     62460     62470     62480
Human                       TGGAGGGCTTTCTAACAATTGGCATATTCATTGTTAGATAATTTGGCAGTCGGACCTCAGTATTGCTGGGAAAGAATTCA 62427
AsianBonytongue             -------------------------------------------------------------------------------- 0
Reedfish                    TTTCCATATCTCCCAGGGCTGAATATTTTTCCAAAAACTAACATTTTTAAAAAAAGAACACAAAGCAATTGTTTAACATA 20301
NorthernPike                -------------------------------------------------------------------------------- 0
SpottedGar                  -------------------------------------------------------------------------------- 0
ParamormyropsKingsLeyae     -------------------------------------------------------------------------------- 0

Majority                    --------------------------------------------------------------------------------
                                     62490     62500     62510     62520     62530     62540     62550     62560
Human                       ACTCCTAAAAAACTTAGTGTGTGTTTAGTAATTTTGTATCAGCACTTTTAGCATTAACCTAGATACTGTGACTCAAACCC 62507
AsianBonytongue             -------------------------------------------------------------------------------- 0
Reedfish                    TCAAATCAAATCAACAAAAAAATATTTACTTTTGACAAATGTTACTGTCTTGCATGTTGTATGAGCCTGCATACTATATAT 20381
NorthernPike                -------------------------------------------------------------------------------- 0
SpottedGar                  -------------------------------------------------------------------------------- 0
ParamormyropsKingsLeyae     -------------------------------------------------------------------------------- 0

Majority                    --------------------------------------------------------------------------------
                                     62570     62580     62590     62600     62610     62620     62630     62640
Human                       AGCTCTTTTCTCATAAGGTATCATTTTCTCTTTGATAACATTGAAGTAGTCACCTAAGAGTGAATTTTGCTACTTATCTT 62587
AsianBonytongue             -------------------------------------------------------------------------------- 0
Reedfish                    TTCACATACATATCACATACATTTTACACAGCAAAGTCCTGCAAAGTCTGATCTCGCTCAAAGCAGCCAATTTCAGTCAT 20461
NorthernPike                -------------------------------------------------------------------------------- 0
SpottedGar                  -------------------------------------------------------------------------------- 0
ParamormyropsKingsLeyae     -------------------------------------------------------------------------------- 0

Majority                    --------------------------------------------------------------------------------
                                     62650     62660     62670     62680     62690     62700     62710     62720
Human                       TTGATATTTATCTATTGTGTAGAAACATCAGTTCTATTACTTATCTCACATTTTCCCTATAGGAATGCTACATTAGTAAA 62667
AsianBonytongue             -------------------------------------------------------------------------------- 0
Reedfish                    TGCCCCATTGCACTCCTTACAATATGTGTTGCTTTGGTGCCTACTTTTCAATTGTCTGTTGCTGCACTTTCTCACATATA 20541
NorthernPike                -------------------------------------------------------------------------------- 0
SpottedGar                  -------------------------------------------------------------------------------- 0
ParamormyropsKingsLeyae     -------------------------------------------------------------------------------- 0

Majority                    --------------------------------------------------------------------------------
                                     62730     62740     62750     62760     62770     62780     62790     62800
Human                       TGAATACCTATCTTCAGTGCTTTCTTCACCTTTACACCAGGGTATTCAATTAATAGTTATGTTATTCTGGCTGATAAAGT 62747
AsianBonytongue             -------------------------------------------------------------------------------- 0
Reedfish                    TTGTTAGTGTGTACTGTAGAGAGACAAGTCACCCATTTGCCATCGTGCCATGCCACTGCCACCAAGTTTTCTGCCCGCAT 20621
NorthernPike                -------------------------------------------------------------------------------- 0
SpottedGar                  -------------------------------------------------------------------------------- 0
ParamormyropsKingsLeyae     -------------------------------------------------------------------------------- 0

Majority                    --------------------------------------------------------------------------------
                                     62810     62820     62830     62840     62850     62860     62870     62880
Human                       TGATTGATACATGGCTTCCACTTTTATGTGATGATAGGTACCTTGCCTAACCCAAATGTAATTCAAATCCTGATGTGTAT 62827
AsianBonytongue             -------------------------------------------------------------------------------- 0
Reedfish                    GAAAACCGGATTGTCACCTCTTTTCATCTTCTGAAACTTTATGAACTTTATGCATGGCATTATAGCCTGGCTCACCCCAT 20701
NorthernPike                -------------------------------------------------------------------------------- 0
SpottedGar                  -------------------------------------------------------------------------------- 0
ParamormyropsKingsLeyae     -------------------------------------------------------------------------------- 0
```

```
Majority                   --------------------------------------------------------------------------------
                                     62890     62900     62910     62920     62930     62940     62950     62960
Human                      GAACTCTCTTTCAGTTTTTACCAGTGCTGTATTGAAGTGGATGAATCTGTGTTGAATTATTTTCTAGGTTAGTTTTTAGG 62907
AsianBonytongue            -------------------------------------------------------------------------------- 0
Reedfish                   GGGATTTGCTTCTGTTTATTACAGAAGTGAATAAAACTTTGCAGCAGCACGTACCTATCACCACGCTGCATAACCTGTCC 20781
NorthernPike               -------------------------------------------------------------------------------- 0
SpottedGar                 -------------------------------------------------------------------------------- 0
ParamormyropsKingsLeyae    -------------------------------------------------------------------------------- 0

Majority                   --------------------------------------------------------------------------------
                                     62970     62980     62990     63000     63010     63020     63030     63040
Human                      TAGGTAGATTAGTCCATAAAAAAATTCGTATATTAAGAGAAAAGGTTTTATATTCCCAGAGGTGAGAATATTTGAATTAA 62987
AsianBonytongue            -------------------------------------------------------------------------------- 0
Reedfish                   AAAGCCACTAGGGGACAAAGCACGTTTGGACCAATGCTCCCTGAAGTTATATCACCAGTTCTGTCCCATCTCTATTTGTA 20861
NorthernPike               -------------------------------------------------------------------------------- 0
SpottedGar                 -------------------------------------------------------------------------------- 0
ParamormyropsKingsLeyae    -------------------------------------------------------------------------------- 0

Majority                   --------------------------------------------------------------------------------
                                     63050     63060     63070     63080     63090     63100     63110     63120
Human                      AAGCTTTATATACTTAAGCAACTGTAGAGGTTTACTTTTTCCATAACACGACATGTTTATTCTTCTAATGAAAAGTTAAC 63067
AsianBonytongue            -------------------------------------------------------------------------------- 0
Reedfish                   ATGCCACAGCACGCTTCATCTCGTCTTTTGTTGTGGGTTTCCACTTTGAAAAATGAGAATGCGAAAAAACCGCAGCCCGC 20941
NorthernPike               -------------------------------------------------------------------------------- 0
SpottedGar                 -------------------------------------------------------------------------------- 0
ParamormyropsKingsLeyae    -------------------------------------------------------------------------------- 0

Majority                   --------------------------------------------------------------------------------
                                     63130     63140     63150     63160     63170     63180     63190     63200
Human                      AGTATGTTCACCTGTTCTCTAGTTTGTTTCCAGCTTATATCCAGCTGAACTAGAAATGGTGATTATATAGGAGCAGAAAA 63147
AsianBonytongue            -------------------------------------------------------------------------------- 0
Reedfish                   GATTCAAAAAATTTCTCTGCC-TACCTGTTTGTCTCATCTGACAGTAGCTGAAAAGCAGCATCAGGAGAGAGCAGCCTGA 21020
NorthernPike               -------------------------------------------------------------------------------- 0
SpottedGar                 -------------------------------------------------------------------------------- 0
ParamormyropsKingsLeyae    -------------------------------------------------------------------------------- 0

Majority                   --------------------------------------------------------------------------------
                                     63210     63220     63230     63240     63250     63260     63270     63280
Human                      AGTACAAGATAATCATAGCGGTAACATAGTCCTTTATAATTCATGATGTATTTTCACATATGTTGTCTTTAATTTACTCC 63227
AsianBonytongue            -------------------------------------------------------------------------------- 0
Reedfish                   AGTAGTTCAACAGCTGGTGATCTGTCGTGTCCAGTAGCAAGCCATGCCTTCTTGTGAAGTCAGGTAGCCAGATCGGCTCC 21100
NorthernPike               -------------------------------------------------------------------------------- 0
SpottedGar                 -------------------------------------------------------------------------------- 0
ParamormyropsKingsLeyae    -------------------------------------------------------------------------------- 0

Majority                   --------------------------------------------------------------------------------
                                     63290     63300     63310     63320     63330     63340     63350     63360
Human                      TCTTAATAACCCTTGAAGAAGCATGACAGCCATTGTCATCATTCCTGTTTTCCCGATTAAGAAATTGAGATTCAGAGAGG 63307
AsianBonytongue            -------------------------------------------------------------------------------- 0
Reedfish                   CATGGATCAATTTCTGGGTATTCCTCCCATGCGAACCTTGTCGTAGATGTACCAGCAGCGCAAATGCGCTCAGCTGCCAG 21180
NorthernPike               -------------------------------------------------------------------------------- 0
SpottedGar                 -------------------------------------------------------------------------------- 0
ParamormyropsKingsLeyae    -------------------------------------------------------------------------------- 0
```

```
Majority                  --------------------------------------------------------------------------------
                                   63370     63380     63390     63400     63410     63420     63430     63440
Human                     TCAAATAACTTGCTTAAGATGACCGAAAAGAGTCTAGGCTTTTAATTAATCCTAGACCTGGCACTCGTCAGTTTCAGAAC 63387
AsianBonytongue           -------------------------------------------------------------------------------- 0
Reedfish                  CCGACCAGCTGGGGCGGCATCGGCTGGTCTCCGATCGGCTGATGCCGGCTCCTCAATCTCTTCCTCAATCTCCTGATCAC 21260
NorthernPike              -------------------------------------------------------------------------------- 0
SpottedGar                -------------------------------------------------------------------------------- 0
ParamormyropsKingsLeyae   -------------------------------------------------------------------------------- 0

Majority                  --------------------------------------------------------------------------------
                                   63450     63460     63470     63480     63490     63500     63510     63520
Human                     TTTTCGGGAATGACATTGGATTTGCTAATTTCATCAAGATAATCTGATTGGGGGTGAACATTTCAGGTTAAGGAAAATAC 63467
AsianBonytongue           -------------------------------------------------------------------------------- 0
Reedfish                  TGTCTATGAAATCC---GACTCTGAAAAATCAGAGTCAGACTCCGCGATAATGCGCAAAATGTCATCTGCCGAGTATTTT 21337
NorthernPike              -------------------------------------------------------------------------------- 0
SpottedGar                -------------------------------------------------------------------------------- 0
ParamormyropsKingsLeyae   -------------------------------------------------------------------------------- 0

Majority                  --------------------------------------------------------------------------------
                                   63530     63540     63550     63560     63570     63580     63590     63600
Human                     TAGTTTTTTTCTTTTTTGCCTCATTTTATGGGAATTGCTATTATGTGTTAGGATAATTTGGTTTGAAGCAACTTTGTACA 63547
AsianBonytongue           -------------------------------------------------------------------------------- 0
Reedfish                  CTTTTCTCGACTCGCTTAGCTCCCTCGTGAGATGTTGATGACATGTTGTCTTTGTTTACATTTCGCAACTCACGCACACG 21417
NorthernPike              -------------------------------------------------------------------------------- 0
SpottedGar                -------------------------------------------------------------------------------- 0
ParamormyropsKingsLeyae   -------------------------------------------------------------------------------- 0

Majority                  --------------------------------------------------------------------------------
                                   63610     63620     63630     63640     63650     63660     63670     63680
Human                     ATGTGGCATCAAATAGCAGTTACCTTCTAAAACTGTACTGAATCATATAAGTACTGCCATCCAGCAATAAATATATGTAT 63627
AsianBonytongue           -------------------------------------------------------------------------------- 0
Reedfish                  CAAGGATCATTTGTTGAGTCAACGAGTCTAGCATTCCTCCAAGCACAGAGGGAATGCCAGTGACGTGACAATGAGTTTTG 21497
NorthernPike              -------------------------------------------------------------------------------- 0
SpottedGar                -------------------------------------------------------------------------------- 0
ParamormyropsKingsLeyae   -------------------------------------------------------------------------------- 0

Majority                  --------------------------------------------------------------------------------
                                   63690     63700     63710     63720     63730     63740     63750     63760
Human                     AATAAAATTAACCTTATTGAGCAGAGTAGTGTGGTACTACTTTGGTAGGACTTCCTGTTTGTGATCCTTATTAGTTTTTT 63707
AsianBonytongue           -------------------------------------------------------------------------------- 0
Reedfish                  TCGCCATTAA---CAGCTGATTGTTGCCCTCTATCCCTGGATGTCAACTTTTGTCGACATCCACCCTCAACCCCTCCTGT 21574
NorthernPike              -------------------------------------------------------------------------------- 0
SpottedGar                -------------------------------------------------------------------------------- 0
ParamormyropsKingsLeyae   -------------------------------------------------------------------------------- 0

Majority                  --------------------------------------------------------------------------------
                                   63770     63780     63790     63800     63810     63820     63830     63840
Human                     TAAAACTAACCTATTTGCAATTTTTAAAAATTAAAAGTATTAGCATGGATGCTTAGCTGGTTGTATTTTAGGTTGAAGTA 63787
AsianBonytongue           -------------------------------------------------------------------------------- 0
Reedfish                  CGACAAAAGTCGACATCCGCCCTAAAAGAGTTAAAGAAAGTGAAGTAAAAATCTCATGAAAAGAAATGATCCTCTGCATA 21654
NorthernPike              -------------------------------------------------------------------------------- 0
SpottedGar                -------------------------------------------------------------------------------- 0
ParamormyropsKingsLeyae   -------------------------------------------------------------------------------- 0
```

```
Majority                    --------------------------------------------------------------------------------
                                     63850     63860     63870     63880     63890     63900     63910     63920
Human                       GCATTTTCTATTTAAGGAAGATAATATTTGGGGGAAAGTAATACATGGACAGCTGGAACAGCAAAATTTGATATATGAAA 63867
AsianBonytongue             -------------------------------------------------------------------------------- 0
Reedfish                    GAGAGGAACAGCAGATGTATATGTTGTCTGGCTGAAAGATTGAAGAGGTGGAAGAAAAGAGGGTAATCTTTGACAAACAT 21734
NorthernPike                -------------------------------------------------------------------------------- 0
SpottedGar                  -------------------------------------------------------------------------------- 0
ParamormyropsKingsLeyae-------------------------------------------------------------------------------- 0

Majority                    --------------------------------------------------------------------------------
                                     63930     63940     63950     63960     63970     63980     63990     64000
Human                       TTTGAATCCTTTTCCTGGTAGCTTTTAAGTTCGAGTTTTTGTTGTTGAGTTTTATTACTAAAATTATACTTTCACTAATT 63947
AsianBonytongue             -------------------------------------------------------------------------------- 0
Reedfish                    TATGAGAAGCTGGTCTAATGAGTTCAGACCTTTTAATTTTGTTCCTTAATATAAATGCATGCTCTGTGTGTGTCAAGCAT 21814
NorthernPike                -------------------------------------------------------------------------------- 0
SpottedGar                  -------------------------------------------------------------------------------- 0
ParamormyropsKingsLeyae-------------------------------------------------------------------------------- 0

Majority                    --------------------------------------------------------------------------------
                                     64010     64020     64030     64040     64050     64060     64070     64080
Human                       TTTATTTTTTGTTTAAGATTGCTGATATGCTTTATTCACTTTTACAGGCCCTAGATGGTCAGAATATTTATAATGCCTGC 64027
AsianBonytongue             -------------------------------------------------------------------------------- 0
Reedfish                    GTATGATCTTTATAAATTCCTTATAAACATTTTATTACAATTGTGACTTGTAATTAGGGTTGCCACAATATAAGAACTGT 21894
NorthernPike                -------------------------------------------------------------------------------- 0
SpottedGar                  -------------------------------------------------------------------------------- 0
ParamormyropsKingsLeyae-------------------------------------------------------------------------------- 0

Majority                    --------------------------------------------------------------------------------
                                     64090     64100     64110     64120     64130     64140     64150     64160
Human                       TGTACCCTAAGGATTGATTTTTCCAAACTTGTGAATTTGAATGTAAAATACAACAATGATAAAAGTAGGGATTATACTCG 64107
AsianBonytongue             -------------------------------------------------------------------------------- 0
Reedfish                    AGTAGTCTATGGCAGTACCAGTGAACTTTCACAATACTAGATATCGTTCAACACCAGTGTAACAGCAAGTGGTGGAAATT 21974
NorthernPike                -------------------------------------------------------------------------------- 0
SpottedGar                  -------------------------------------------------------------------------------- 0
ParamormyropsKingsLeyae-------------------------------------------------------------------------------- 0

Majority                    --------------------------------------------------------------------------------
                                     64170     64180     64190     64200     64210     64220     64230     64240
Human                       ACCTGATCTTCCATCTGGGGATGGACAACCTGCATTGGACCCAGCTATTGCTGCAGCATTTGCCAAGGAGACATCCCTCT 64187
AsianBonytongue             -------------------------------------------------------------------------------- 0
Reedfish                    TTATAATGTTGAAGAGATTTTGGCATAGGGCCTATAAAGGATGATATTAGCATGGACTGCTTAGTTCACTAGACAGTTAC 22054
NorthernPike                -------------------------------------------------------------------------------- 0
SpottedGar                  -------------------------------------------------------------------------------- 0
ParamormyropsKingsLeyae-------------------------------------------------------------------------------- 0

Majority                    --------------------------------------------------------------------------------
                                     64250     64260     64270     64280     64290     64300     64310     64320
Human                       TAGGTATGATTTTTATTGTCTTAACCACTTTTCTCCCATTTTGCCAAATGGAAAAGTACCAGTAAGTATAATGAATCCCC 64267
AsianBonytongue             -------------------------------------------------------------------------------- 0
Reedfish                    CTTACATGTACACCTTCCATGTTTGTTCTCTGACATGTACTTCTCCACTAACTAAAAAATATTGCTTACTAAACATTAAA 22134
NorthernPike                -------------------------------------------------------------------------------- 0
SpottedGar                  -------------------------------------------------------------------------------- 0
ParamormyropsKingsLeyae-------------------------------------------------------------------------------- 0
```

```
Majority                    --------------------------------------------------------------------------------

                                     64330     64340     64350     64360     64370     64380     64390     64400

Human                       CCATTTTGGACCTTACCAAATTGTGTTAGGTTTCGTGAGTTTTCTTTTTCTCAAGTGAGAAGGCATATGAATACTGTTAA 64347
AsianBonytongue             -------------------------------------------------------------------------------- 0
Reedfish                    GTTTATTAAAGTACAGTTATTCTATCTGTACACCTGGATTTTCTTGGAATTCATTTTTCAAGATATTTTAAT----TGAA 22210
NorthernPike                -------------------------------------------------------------------------------- 0
SpottedGar                  -------------------------------------------------------------------------------- 0
ParamormyropsKingsLeyae     -------------------------------------------------------------------------------- 0

Majority                    --------------------------------------------------------------------------------

                                     64410     64420     64430     64440     64450     64460     64470     64480

Human                       GAAAAACCCAAAGTATTCTTTATAAGTCTAATTAAAATTATATGTTAGTTTTGTTCCTTTTAATACCTTCAGCTTCTCTT 64427
AsianBonytongue             -------------------------------------------------------------------------------- 0
Reedfish                    ACATTCTTTAAATCTGTTTTTGTAACAACATGCTTTTTAAAGCCTGGTTTCTCATGGAGTCACTCTATTAGGGTTCTAAT 22290
NorthernPike                -------------------------------------------------------------------------------- 0
SpottedGar                  -------------------------------------------------------------------------------- 0
ParamormyropsKingsLeyae     -------------------------------------------------------------------------------- 0

Majority                    --------------------------------------------------------------------------------

                                     64490     64500     64510     64520     64530     64540     64550     64560

Human                       CCATGCCACTCTCCCAGAACAAAAGAAGAAAAGCTCTAATGAAGAAATACTGTTGAATTCTTAATGCCATATTTACACTT 64507
AsianBonytongue             -------------------------------------------------------------------------------- 0
Reedfish                    ATTTGCTAAAGAGTAACAGATAAGTCAAACTGTCCCTCCTTTTTTATTTTTTTACATTTGTCCAAACCTTTGATTTCATG 22370
NorthernPike                -------------------------------------------------------------------------------- 0
SpottedGar                  -------------------------------------------------------------------------------- 0
ParamormyropsKingsLeyae     -------------------------------------------------------------------------------- 0

Majority                    --------------------------------------------------------------------------------

                                     64570     64580     64590     64600     64610     64620     64630     64640

Human                       TAAACTTGATGTCACTATGCCTGAGTCTAGGCCTAAAGTTCTTGTGTGTGTTTTTCAGTGCTGATCTGATTGTTTTAATT 64587
AsianBonytongue             -------------------------------------------------------------------------------- 0
Reedfish                    TAAGAAATTATCTTGTCAGCATTACTTTAATCC---ATTATTTCAGACCACCTGTAAAACACTCTTTGTTCAGCCATTTT 22447
NorthernPike                -------------------------------------------------------------------------------- 0
SpottedGar                  -------------------------------------------------------------------------------- 0
ParamormyropsKingsLeyae     -------------------------------------------------------------------------------- 0

Majority                    --------------------------------------------------------------------------------

                                     64650     64660     64670     64680     64690     64700     64710     64720

Human                       TAGCATTGCATGGAAACAGTGAAATGTTAAGAGACAAAACATACATAATGGGTGGAGGCATATGGGATTTTTTTTGAAGC 64667
AsianBonytongue             -------------------------------------------------------------------------------- 0
Reedfish                    CAAAGTTTCGTATGTCTTTTTTTGCTTAGCCTGTGTACAAGTCCAGAATTAGAACATTAACTGGGCCTTTAGTTCAAATGG 22527
NorthernPike                -------------------------------------------------------------------------------- 0
SpottedGar                  -------------------------------------------------------------------------------- 0
ParamormyropsKingsLeyae     -------------------------------------------------------------------------------- 0

Majority                    --------------------------------------------------------------------------------

                                     64730     64740     64750     64760     64770     64780     64790     64800

Human                       ATATTGTAGTGGCCTTTTAAAAAAACAATTTAAACTAGAAGAAATTTTCTGTAAAATTAATTTTGCTATTTCTTTGCATT 64747
AsianBonytongue             -------------------------------------------------------------------------------- 0
Reedfish                    AAAACTACATTATTCTCACAAATGTACCTTTAAAATATGACATATGTGCTTTCACTATGTATGTTACATATTTCTGTCTT 22607
NorthernPike                -------------------------------------------------------------------------------- 0
SpottedGar                  -------------------------------------------------------------------------------- 0
ParamormyropsKingsLeyae     -------------------------------------------------------------------------------- 0
```

```
Majority                   --------------------------------------------------------------------------------
                                    64810     64820     64830     64840     64850     64860     64870     64880
Human                      TTGTCTTAATCATTTATATTTTACTTTGTACCACAATGGACTTGAGCCACCTAATAAAAATACAGTGCAAACATATGCAA 64827
AsianBonytongue            -------------------------------------------------------------------------------- 0
Reedfish                   TAGCAATGAAGTGTTTCATTTCGCTCATCCTGCTAACTGGCATAATTGCAGTGCTTTGAAGCACTACATTTTTATTTTGG 22687
NorthernPike               -------------------------------------------------------------------------------- 0
SpottedGar                 -------------------------------------------------------------------------------- 0
ParamormyropsKingsLeyae    -------------------------------------------------------------------------------- 0

Majority                   --------------------------------------------------------------------------------
                                    64890     64900     64910     64920     64930     64940     64950     64960
Human                      CTTATAGTTGATAGCGTGGAATCAAGATGAAGAAAAAAATAAGGAAAATCTTACTTTGTAAGTAAAATGTGCAAAGTGAT 64907
AsianBonytongue            -------------------------------------------------------------------------------- 0
Reedfish                   TATTTAACAACTTGAGCAGCTCGATTCATCTCCAGTGTAGGCAAGCCAAATAACCACTTTGTTTAGAAACTTACCACAAT 22767
NorthernPike               -------------------------------------------------------------------------------- 0
SpottedGar                 -------------------------------------------------------------------------------- 0
ParamormyropsKingsLeyae    -------------------------------------------------------------------------------- 0

Majority                   --------------------------------------------------------------------------------
                                    64970     64980     64990     65000     65010     65020     65030     65040
Human                      TTATAAGTTAAGATGGAATTTTAGAGTTTCCTTTTATTTGATGTATTTCTTGATTTCTAGTATTGGTAAGATTATCTTTA 64987
AsianBonytongue            -------------------------------------------------------------------------------- 0
Reedfish                   TTTTACTTGCTACAGCTGTTTGTGCATTACAGTGCATGTGACATTTTCCACGCAACCAAGAACTGTACAAAACCAGTTAC 22847
NorthernPike               -------------------------------------------------------------------------------- 0
SpottedGar                 -------------------------------------------------------------------------------- 0
ParamormyropsKingsLeyae    -------------------------------------------------------------------------------- 0

Majority                   --------------------------------------------------------------------------------
                                    65050     65060     65070     65080     65090     65100     65110     65120
Human                      AGTCATGATTTTTATATATATGGAACTCAAAATTGATAGTTGACAAACTAGCTTTTACAATTTAGTTTATGTCTGTCATT 65067
AsianBonytongue            -------------------------------------------------------------------------------- 0
Reedfish                   AAAATAGACAGATGCTTAAAAGTACATTCGTGGACATGTTTGACGTAATCGGACATGGTTTTAAAAAGCGATTCATTACA 22927
NorthernPike               -------------------------------------------------------------------------------- 0
SpottedGar                 -------------------------------------------------------------------------------- 0
ParamormyropsKingsLeyae    -------------------------------------------------------------------------------- 0

Majority                   --------------------------------------------------------------------------------
                                    65130     65140     65150     65160     65170     65180     65190     65200
Human                      TCAAAAGAAAAAATAGTATAATTAAGTAGTTATTGGAAATATTTACTTCCCAATATATAACAGTCATGCCATCAGTTTGT 65147
AsianBonytongue            -------------------------------------------------------------------------------- 0
Reedfish                   CCTTCATTTTGTCTCCTTTTACAACATCAAGACTGTACAAGTTGAGTGATTAAGACTCAACATCTGATCAGTCACATCAT 23007
NorthernPike               -------------------------------------------------------------------------------- 0
SpottedGar                 -------------------------------------------------------------------------------- 0
ParamormyropsKingsLeyae    -------------------------------------------------------------------------------- 0

Majority                   --------------------------------------------------------------------------------
                                    65210     65220     65230     65240     65250     65260     65270     65280
Human                      GAATATGAAATCTGTAAGTGGTATATATGTAGAGAGAGATACTCTAAGGCAGTTGGTATTTGATTCTGGTAACTAATAAG 65227
AsianBonytongue            -------------------------------------------------------------------------------- 0
Reedfish                   GGCACTCGCAAAGCATGCATGAAGCTATTTATGAATTAATTCTCTAATGGGATAAAAGAGGAAATACCAAAAGCCTGTGT 23087
NorthernPike               -------------------------------------------------------------------------------- 0
SpottedGar                 -------------------------------------------------------------------------------- 0
ParamormyropsKingsLeyae    -------------------------------------------------------------------------------- 0
```

```
Majority                  --------------------------------------------------------------------------------
                                   65290     65300     65310     65320     65330     65340     65350     65360
Human                     AATTAACTACCTGTTAATTCTGGCAGCTGCAAACAAATCTGACCCTTTATTCGAATTTTCTGGTGTATCCTAGCAGAAGT 65307
AsianBonytongue           -------------------------------------------------------------------------------- 0
Reedfish                  TTGTGTTTTGACTTTTTTAAAACAATAATAATCATGATTCTTGCAGTCGTGTACTGAAATTCTCCTGTCGTAAGGGCAGT 23167
NorthernPike              -------------------------------------------------------------------------------- 0
SpottedGar                -------------------------------------------------------------------------------- 0
ParamormyropsKingsLeyae-------------------------------------------------------------------------------- 0

Majority                  --------------------------------------------------------------------------------
                                   65370     65380     65390     65400     65410     65420     65430     65440
Human                     CATAGTGTTTATTATGACTGTAGTAGATGCTTTAGGTCACTTACACTCTTTCCTGACAGAATCTCTACTACCTCTGTGTG 65387
AsianBonytongue           -------------------------------------------------------------------------------- 0
Reedfish                  TTTCTAGATAAGTTTTTTCTTTTGAATATATAGTGCTGATCATGGAGTGCTGTATCATCATCACCATGAGCCATTCGTGT 23247
NorthernPike              -------------------------------------------------------------------------------- 0
SpottedGar                -------------------------------------------------------------------------------- 0
ParamormyropsKingsLeyae-------------------------------------------------------------------------------- 0

Majority                  --------------------------------------------------------------------------------
                                   65450     65460     65470     65480     65490     65500     65510     65520
Human                     TGTCTAGCTTAATTCGTAATTCAAGTTAATAAGATTGAATATTTCACAGTGTGCAGTTTTTAAAGGCAAAGTCAGGAATG 65467
AsianBonytongue           -------------------------------------------------------------------------------- 0
Reedfish                  AGGTAGCTCTTATTCACATTTATCTCACATTACTTTGCCTACTGGAATAATTTAAAGTTTATTTATTTCAGCATTTTATG 23327
NorthernPike              -------------------------------------------------------------------------------- 0
SpottedGar                -------------------------------------------------------------------------------- 0
ParamormyropsKingsLeyae-------------------------------------------------------------------------------- 0

Majority                  --------------------------------------------------------------------------------
                                   65530     65540     65550     65560     65570     65580     65590     65600
Human                     AAAGAGTGAAACATGGTTTTTGCTTATAATTATTACTATAGATTATTAAACTCTTTACTGATAGACATTGTTTTAGAAAG 65547
AsianBonytongue           -------------------------------------------------------------------------------- 0
Reedfish                  TGGTCACTGAACAATAAGCCTACAGAATTTCATTTTGCTTATTGTAAAAGACAAGGGGTCCCAACAAAGCCTTAACACAC 23407
NorthernPike              -------------------------------------------------------------------------------- 0
SpottedGar                -------------------------------------------------------------------------------- 0
ParamormyropsKingsLeyae-------------------------------------------------------------------------------- 0

Majority                  --------------------------------------------------------------------------------
                                   65610     65620     65630     65640     65650     65660     65670     65680
Human                     AATCAAAATTTATTGAGTAGCTTCTAGGTCATATTCATTCAGCTAGGCACTTGGCAGATACATTACTTTGTTAAAATAGA 65627
AsianBonytongue           -------------------------------------------------------------------------------- 0
Reedfish                  CACACTAAAACAGTCCCTGGTGTAAAGTAAACATTTTTATTTTACAAAAATTCCTTTATACAGACCAATCCACAAACTTC 23487
NorthernPike              -------------------------------------------------------------------------------- 0
SpottedGar                -------------------------------------------------------------------------------- 0
ParamormyropsKingsLeyae-------------------------------------------------------------------------------- 0

Majority                  --------------------------------------------------------------------------------
                                   65690     65700     65710     65720     65730     65740     65750     65760
Human                     TGGTCTCATCAAGATTTTACATATAGTTTCTAAGAAGTTGAAAAATTCACATGCAACTCATCAAAGAAACAATTTAAAGA 65707
AsianBonytongue           -------------------------------------------------------------------------------- 0
Reedfish                  CAGTATGCAGTCCGCTCCATTTGAGCTTTGCCTTCTGCTTCCTGACTCTGACTCAACTGGCTGGATGGATTGCTTTTATG 23567
NorthernPike              -------------------------------------------------------------------------------- 0
SpottedGar                -------------------------------------------------------------------------------- 0
ParamormyropsKingsLeyae-------------------------------------------------------------------------------- 0
```

```
Majority                      --------------------------------------------------------------------------------
                                        65770     65780     65790     65800     65810     65820     65830     65840
Human                         TAAAATACCAAGAAACTCCAGCTAAAGATAAAATACCAAGAAACTCCAGCTAGTATTAACAATGGACTACAATAATCACT 65787
AsianBonytongue               -------------------------------------------------------------------------------- 0
Reedfish                      TTAGATCCTAGAATACTTCCGGTATCGACATAGCCCAACCAAAGCACTTCTGGGTCAAATGGAACCCTCCTGAAACATGG 23647
NorthernPike                  -------------------------------------------------------------------------------- 0
SpottedGar                    -------------------------------------------------------------------------------- 0
ParamormyropsKingsLeyae-------------------------------------------------------------------------------- 0

Majority                      --------------------------------------------------------------------------------
                                        65850     65860     65870     65880     65890     65900     65910     65920
Human                         TCTTTGGACCCTACTGAGGTGTGTGTATTTGCCCATCTGGATATACTTAGATGTGCTTGGATCCTGGGTGGGAGGCTTGG 65867
AsianBonytongue               -------------------------------------------------------------------------------- 0
Reedfish                      AGTCCTTCTCTGGCAGTATCCAAAGGCCCCGACAAGGTTGTACCTCGGGACTACGATTCCTATGAATCCCAGCGGGTGTC 23727
NorthernPike                  -------------------------------------------------------------------------------- 0
SpottedGar                    -------------------------------------------------------------------------------- 0
ParamormyropsKingsLeyae-------------------------------------------------------------------------------- 0

Majority                      --------------------------------------------------------------------------------
                                        65930     65940     65950     65960     65970     65980     65990     66000
Human                         TTAGAAGTCACGTGGATTCTTCTAGGGAATTTATCACCATTTTTCTTGTGATCTTATGACCTTGCTGAAACTTTTAAAAC 65947
AsianBonytongue               -------------------------------------------------------------------------------- 0
Reedfish                      TATACTGGGTCCAATCCAGCAGAGCACTGCCATCTACTGTACTGGGGGAAGAACTGCTCTTGATGGGCCAACTCCATCAC 23807
NorthernPike                  -------------------------------------------------------------------------------- 0
SpottedGar                    -------------------------------------------------------------------------------- 0
ParamormyropsKingsLeyae-------------------------------------------------------------------------------- 0

Majority                      --------------------------------------------------------------------------------
                                        66010     66020     66030     66040     66050     66060     66070     66080
Human                         AATAATAAAGTCATTTTTTTTCTCTTTGAGATGGAATCTCACTCTGTCGCCCGTGTCAGAGTGTGGTGGCGCAGTCTCGG 66027
AsianBonytongue               -------------------------------------------------------------------------------- 0
Reedfish                      ACTATCTGCTAATTTTTCCCTTTGCATGAGGTATTGATCCAGTCCTATCCCGGCTGAGTTATGCCTCCTTCCATACTATC 23887
NorthernPike                  -------------------------------------------------------------------------------- 0
SpottedGar                    -------------------------------------------------------------------------------- 0
ParamormyropsKingsLeyae-------------------------------------------------------------------------------- 0

Majority                      --------------------------------------------------------------------------------
                                        66090     66100     66110     66120     66130     66140     66150     66160
Human                         CTCACTGCAACTTCCGCCTCCCGGGTTCAAGCGATTCTCCCGCCTCAGCCTCCTGAGCATCTGGGACTACAGGCGTGTAC 66107
AsianBonytongue               -------------------------------------------------------------------------------- 0
Reedfish                      CTTCCGTTATGGCTTCCCGACTGGGAAAGGGTCCAACACCGCTCTTAAATTATAAAACAAGTTTGTAAAACCTCTATAAT 23967
NorthernPike                  -------------------------------------------------------------------------------- 0
SpottedGar                    -------------------------------------------------------------------------------- 0
ParamormyropsKingsLeyae-------------------------------------------------------------------------------- 0

Majority                      --------------------------------------------------------------------------------
                                        66170     66180     66190     66200     66210     66220     66230     66240
Human                         CACCACCCCTGGCTAACTTTTGTCTTTTTTCGTAGAGATGAGATTTCACCATGTTGGCCAGGCTGGTCTGGAACTCCTGA 66187
AsianBonytongue               -------------------------------------------------------------------------------- 0
Reedfish                      AGTAAAAATACCAGAGTTAATACCTTAATTGTAGAAGTTAAGGTTAAACATTATTATTACTATAGGAGCTTAGTGTAAAA 24047
NorthernPike                  -------------------------------------------------------------------------------- 0
SpottedGar                    -------------------------------------------------------------------------------- 0
ParamormyropsKingsLeyae-------------------------------------------------------------------------------- 0
```

```
Majority                    --------------------------------------------------------------------------------
                                    66250     66260     66270     66280     66290     66300     66310     66320
Human                       TCTCAGGTGATCTGCCCGCCTCAGCCTCCCAAAGTGCTGAGATTACAGGTGTGAGCCACCATGCCTGGCCGCTTTTGTTC 66267
AsianBonytongue             -------------------------------------------------------------------------------- 0
Reedfish                    AGTCTTAACACTAAAATCCCTGGAGCCTATGAAAAAAATCATAATGCCAGGCCACCTCAAATTCCTTCGCACCTCTTCAT 24127
NorthernPike                -------------------------------------------------------------------------------- 0
SpottedGar                  -------------------------------------------------------------------------------- 0
ParamormyropsKingsLeyae     -------------------------------------------------------------------------------- 0

Majority                    --------------------------------------------------------------------------------
                                    66330     66340     66350     66360     66370     66380     66390     66400
Human                       ATTTTTTGAGCGTTGTACATTTTTACATTAAAGGCGTGTAAATTAAGTTGCTCGCTTTGCCAAAAAATTGTCTAGTGTCT 66347
AsianBonytongue             -------------------------------------------------------------------------------- 0
Reedfish                    CAGCGTCTTTGTTTCGCAAATGTGTCAATCATCACAAGCAGCAAGCAGTTTGCTGTCCCATACCTCAGTGACACGGATGA 24207
NorthernPike                -------------------------------------------------------------------------------- 0
SpottedGar                  -------------------------------------------------------------------------------- 0
ParamormyropsKingsLeyae     -------------------------------------------------------------------------------- 0

Majority                    --------------------------------------------------------------------------------
                                    66410     66420     66430     66440     66450     66460     66470     66480
Human                       TTTTCCACTTCTGTGTTGAATTTTTTAGTATAACGCATTTTTGTTTCTTCATCTCTGCCTCATAAGAAAAAGGAAAAAGT 66427
AsianBonytongue             -------------------------------------------------------------------------------- 0
Reedfish                    AGTGAGATGCAAAGTTCTCCCAGCTCAAGTCTGTTTATCTGGGTGTGAGGTGCCTGGAATTGTATAGGGTAAATAATGTA 24287
NorthernPike                -------------------------------------------------------------------------------- 0
SpottedGar                  -------------------------------------------------------------------------------- 0
ParamormyropsKingsLeyae     -------------------------------------------------------------------------------- 0

Majority                    --------------------------------------------------------------------------------
                                    66490     66500     66510     66520     66530     66540     66550     66560
Human                       GTTTAGCCTTTCCTAAGTGACTTCTTTTTTTTTATCTGTTTTAATGTTTTTGCATCTTGTTATAACTGGGCTTTTTAACTA 66507
AsianBonytongue             -------------------------------------------------------------------------------- 0
Reedfish                    TCTTTATTTGGAGTACATGCATTTCATGTCTGTTCCGTGTCTACAATGATCTAGGTAAATGTAGGAGGACAGGAAATGTG 24367
NorthernPike                -------------------------------------------------------------------------------- 0
SpottedGar                  -------------------------------------------------------------------------------- 0
ParamormyropsKingsLeyae     -------------------------------------------------------------------------------- 0

Majority                    --------------------------------------------------------------------------------
                                    66570     66580     66590     66600     66610     66620     66630     66640
Human                       TACTCAAAAATAGGTTAAAAGTAGGTGAGTTAAAGGTTTTAAAAATCAGATAATGGACCCTGGTTTTGATAATTCACAAG 66587
AsianBonytongue             -------------------------------------------------------------------------------- 0
Reedfish                    AGGCAAGAAATGTTGAACACATAACTAAAACAAACTTTATGTTAGAGTAATAATTACAAAATGCTGACGTGAAGTGTATA 24447
NorthernPike                -------------------------------------------------------------------------------- 0
SpottedGar                  -------------------------------------------------------------------------------- 0
ParamormyropsKingsLeyae     -------------------------------------------------------------------------------- 0

Majority                    --------------------------------------------------------------------------------
                                    66650     66660     66670     66680     66690     66700     66710     66720
Human                       AGATAACCATTTATTTTCATATTTTTTATGTAAATACATGTTTCTGTACAAAATTAAAATTGCTCTGTATTTGCAGATTT 66667
AsianBonytongue             -------------------------------------------------------------------------------- 0
Reedfish                    ATGTGTGAAGACTGATGTCCAAATATCAAGTAAACACTTTCACAAAAGGTATAATGAAACAAGTGTGCTTTTATTCAAGA 24527
NorthernPike                -------------------------------------------------------------------------------- 0
SpottedGar                  -------------------------------------------------------------------------------- 0
ParamormyropsKingsLeyae     -------------------------------------------------------------------------------- 0
```

```
Majority                --------------------------------------------------------------------------------
                                66730     66740     66750     66760     66770     66780     66790     66800
Human                   GCATTATCTTTTATT-GTAATTGACATTTTACTTAGAATTTGTTTTAAAAGATAGCTTTAATACCTGCGTAGCATGTTCA 66746
AsianBonytongue         -------------------------------------------------------------------------------- 0
Reedfish                AAATAACAAAAGAAAAGGAAATCGTTCAATATGCATATTGATGTCAATGAGTAAAAACCCAAGCCCAAATATCAATCATC 24607
NorthernPike            -------------------------------------------------------------------------------- 0
SpottedGar              -------------------------------------------------------------------------------- 0
ParamormyropsKingsLeyae -------------------------------------------------------------------------------- 0

Majority                --------------------------------------------------------------------------------
                                66810     66820     66830     66840     66850     66860     66870     66880
Human                   TGGGTATGCCATACCTTTGAAAGTGGACATGTAGGCTATTCCATACTTTTTATTAAAACACCCTTGTACTTTTTTGCCTC 66826
AsianBonytongue         -------------------------------------------------------------------------------- 0
Reedfish                AGAAAGCCTGCTTGGTTGTTGCAGAGGTGAGAACTGCTGCCTCATAAGTAAAAGGTTGCAGTTTCGAGCCTGTTTGTTTT 24687
NorthernPike            -------------------------------------------------------------------------------- 0
SpottedGar              -------------------------------------------------------------------------------- 0
ParamormyropsKingsLeyae -------------------------------------------------------------------------------- 0

Majority                --------------------------------------------------------------------------------
                                66890     66900     66910     66920     66930     66940     66950     66960
Human                   TCAAATTATTTTCTTAGACTAGATTCCTAGAATTAGAATTATTGGGTCAAAGAAAATGTTAAAGCCTGTTTACTTTATTT 66906
AsianBonytongue         -------------------------------------------------------------------------------- 0
Reedfish                CCCTGTTTTCAGTAGTGAGCTGCTATTAGATAGATAGATAGATAGATAGATAGATAGATAGATAGATAGATAGATAGATA 24767
NorthernPike            -------------------------------------------------------------------------------- 0
SpottedGar              -------------------------------------------------------------------------------- 0
ParamormyropsKingsLeyae -------------------------------------------------------------------------------- 0

Majority                --------------------------------------------------------------------------------
                                66970     66980     66990     67000     67010     67020     67030     67040
Human                   GCAAATTTCTTCATCTAAAGGTGATATTAACTTAAACTAACATTAATTATATATGAGGCTGGGTGTCCTACTTTTTCCCC 66986
AsianBonytongue         -------------------------------------------------------------------------------- 0
Reedfish                CTTTATTAATCCCAATGGGAAATTCACATTCTTCAGCAGCAGCATACTGATACAATAAATAATATTAAATTAAAGAATGA 24847
NorthernPike            -------------------------------------------------------------------------------- 0
SpottedGar              -------------------------------------------------------------------------------- 0
ParamormyropsKingsLeyae -------------------------------------------------------------------------------- 0

Majority                --------------------------------------------------------------------------------
                                67050     67060     67070     67080     67090     67100     67110     67120
Human                   TGCCAGCATACTATTTTTTAATTTCACAAGATTAATTTGATAGGCAAATGCCTATTTTAATTTCTGTTAAGATTTAGTTT 67066
AsianBonytongue         -------------------------------------------------------------------------------- 0
Reedfish                TAATAATGCAGGTGAAAAACAGACAATAACTATTATATAATAAAAACATACATTTGATTTGAGTCTGTAACACCTGGTGT 24927
NorthernPike            -------------------------------------------------------------------------------- 0
SpottedGar              -------------------------------------------------------------------------------- 0
ParamormyropsKingsLeyae -------------------------------------------------------------------------------- 0

Majority                --------------------------------------------------------------------------------
                                67130     67140     67150     67160     67170     67180     67190     67200
Human                   ACAATCTTTAAATGTTTATTTGATAGTCACAATTTGCTAAATTGCTTGCCTGCATTCTTTCTGTTAGGATGTACGGTAAT 67146
AsianBonytongue         -------------------------------------------------------------------------------- 0
Reedfish                AAATTTTTGCTACCTGTAAAAGTTAGCGATTTTTTTTTTTAATTGTTTTATTCTCTGTCACATTAACGTAGTACAATGAAC 25007
NorthernPike            -------------------------------------------------------------------------------- 0
SpottedGar              -------------------------------------------------------------------------------- 0
ParamormyropsKingsLeyae -------------------------------------------------------------------------------- 0
```

```
Majority                   --------------------------------------------------------------------------------
                                   67210     67220     67230     67240     67250     67260     67270     67280
Human                      TCTTATAAAATGCATAGTAAGGATATTAATATGCATATTTTGGATGAGCCAAGCCTTCAGCTTTATTCACTTATGTTAGG 67226
AsianBonytongue            -------------------------------------------------------------------------------- 0
Reedfish                   TTACCTCTCCCTTAATGAATTGACGATGTTTATCTCCATTCTTTTCACAAGAGCAGTGATGACCACAGTTGCTGCTGTGG 25087
NorthernPike               -------------------------------------------------------------------------------- 0
SpottedGar                 -------------------------------------------------------------------------------- 0
ParamormyropsKingsLeyae    -------------------------------------------------------------------------------- 0

Majority                   --------------------------------------------------------------------------------
                                   67290     67300     67310     67320     67330     67340     67350     67360
Human                      TCAGTTTTTGGAAAAGTATTTTTCAATAATCTCTCTTTTTAAATAATTGATCTCAATCCCAAAGTATTCATTTGTAGGAC 67306
AsianBonytongue            -------------------------------------------------------------------------------- 0
Reedfish                   TTGATGCCTGGTCAGAATTATTTGTTATACCGGCAATCAAATATATGATGCCCCGTGACCGCTATCAAGAGTATCATGCA 25167
NorthernPike               -------------------------------------------------------------------------------- 0
SpottedGar                 -------------------------------------------------------------------------------- 0
ParamormyropsKingsLeyae    -------------------------------------------------------------------------------- 0

Majority                   --------------------------------------------------------------------------------
                                   67370     67380     67390     67400     67410     67420     67430     67440
Human                      ATGAAGACACAATTTTCTTCTTCATACGTTTGGTGAATATTTTGATCTCAGTTTCCACTTCTCTTTTTGCTTTTTAAACA 67386
AsianBonytongue            -------------------------------------------------------------------------------- 0
Reedfish                   TCATTTGCGCTTTGACAACAGACTCCCTTGCAGAATGAGTGAAAAACAACAGATTTGCTGTGATCTCGGACATCAGGCAA 25247
NorthernPike               -------------------------------------------------------------------------------- 0
SpottedGar                 -------------------------------------------------------------------------------- 0
ParamormyropsKingsLeyae    -------------------------------------------------------------------------------- 0

Majority                   --------------------------------------------------------------------------------
                                   67450     67460     67470     67480     67490     67500     67510     67520
Human                      TTGTTTATGGTGATATCCAATATTGCATGAGTGAGGTCATGCCTTTTAGAATAATAACTGAATCTTTTCTTCTAATTATT 67466
AsianBonytongue            -------------------------------------------------------------------------------- 0
Reedfish                   CATTTTTGCTGAGAACTGTGTTTTGAGTTACAACCCAGGTCAACATATTACTGTTGATGGGCTCCTGTTTCCATCCAAGG 25327
NorthernPike               -------------------------------------------------------------------------------- 0
SpottedGar                 -------------------------------------------------------------------------------- 0
ParamormyropsKingsLeyae    -------------------------------------------------------------------------------- 0

Majority                   --------------------------------------------------------------------------------
                                   67530     67540     67550     67560     67570     67580     67590     67600
Human                      TAATCAGATGACTTCAGCAAACTAATATTGGTGGGATTAACTTCCTGCTCAGTCTTCCAGTAACAGTACTTGTTCAAAAT 67546
AsianBonytongue            -------------------------------------------------------------------------------- 0
Reedfish                   TCAGTTGTCCTTTCTTGCAATCTCAACCAAGCCTGACAAATGTGGCATAAAGTTTTGAGTTGCGGCAGATTTGGAGACAA 25407
NorthernPike               -------------------------------------------------------------------------------- 0
SpottedGar                 -------------------------------------------------------------------------------- 0
ParamormyropsKingsLeyae    -------------------------------------------------------------------------------- 0

Majority                   --------------------------------------------------------------------------------
                                   67610     67620     67630     67640     67650     67660     67670     67680
Human                      GAAATAATTGATAGAGTATTTATTTCAAAAATTTTTAAGAACACAAATATAAGGTGATACTCTTTTGCTAAAATTTGTGG 67626
AsianBonytongue            -------------------------------------------------------------------------------- 0
Reedfish                   AGTACATGTGCAATGCCACACCTCACTTAGGAACAGATCCCAGTTGTCCCAATTCCAGTTACATGAAATCAGAGATCAGT 25487
NorthernPike               -------------------------------------------------------------------------------- 0
SpottedGar                 -------------------------------------------------------------------------------- 0
ParamormyropsKingsLeyae    -------------------------------------------------------------------------------- 0
```

```
Majority                   --------------------------------------------------------------------------------
                                   67690     67700     67710     67720     67730     67740     67750     67760
Human                      GGGGCTTCACTTTACCTTGTATGATTTTTATATTAGATCTAGCTACTTGACTGAATTTAATTACTTTAGAATCAGTACTA 67706
AsianBonytongue            -------------------------------------------------------------------------------- 0
Reedfish                   AGTGATGAG---TGAACTTCACGCTGTTCACCTTGCCTCAAGTTCAGAGAAATCATGAAAGTCTTTATGAATATCACCGA 25564
NorthernPike               -------------------------------------------------------------------------------- 0
SpottedGar                 -------------------------------------------------------------------------------- 0
ParamormyropsKingsLeyae    -------------------------------------------------------------------------------- 0

Majority                   --------------------------------------------------------------------------------
                                   67770     67780     67790     67800     67810     67820     67830     67840
Human                      TAAATTTTAATTGATTTATTTTACACAGTAATTTTTTAAGACTAACATTTATGTCTGGCTTTTCTGTTTCCTTTAATGCT 67786
AsianBonytongue            -------------------------------------------------------------------------------- 0
Reedfish                   ACTTAGCAAAATGGTTTTAAGTCAATGGGGAAGGACTAACTGAACTAGATTTGACTGGATTATAATTGTGCAATCACCTT 25644
NorthernPike               -------------------------------------------------------------------------------- 0
SpottedGar                 -------------------------------------------------------------------------------- 0
ParamormyropsKingsLeyae    -------------------------------------------------------------------------------- 0

Majority                   --------------------------------------------------------------------------------
                                   67850     67860     67870     67880     67890     67900     67910     67920
Human                      AGGTATCACAGTGTCTCAGAAAATGTAATTTGTTCAAAGAAAGGAGACTGAGTGCTTTGTAATGAGTTTAAAATCTGACA 67866
AsianBonytongue            -------------------------------------------------------------------------------- 0
Reedfish                   CTAAATGTCCCTGAGGGCTAGGGAAATGTCTGCCAACTACACTGGACTAATTATTTTGGGCAAAAAGTTGACCTGAGCCA 25724
NorthernPike               -------------------------------------------------------------------------------- 0
SpottedGar                 -------------------------------------------------------------------------------- 0
ParamormyropsKingsLeyae    -------------------------------------------------------------------------------- 0

Majority                   --------------------------------------------------------------------------------
                                   67930     67940     67950     67960     67970     67980     67990     68000
Human                      TCTAAAATACATAATCACAAATGTGGCCGACTATTAGTCATTCACATATTCTGTTTCAAAGAACACAGTGATCAAAAATC 67946
AsianBonytongue            -------------------------------------------------------------------------------- 0
Reedfish                   TGCAGCAACAGTGTCGACCACTGCACCGCTGTGTCTCAGTGAGATCAAAGAAAAAAGAATGCTGTAAGATGAGCACAATC 25804
NorthernPike               -------------------------------------------------------------------------------- 0
SpottedGar                 -------------------------------------------------------------------------------- 0
ParamormyropsKingsLeyae    -------------------------------------------------------------------------------- 0

Majority                   --------------------------------------------------------------------------------
                                   68010     68020     68030     68040     68050     68060     68070     68080
Human                      CTTCAGATTATAAATTGAGACATCATTATAATGGATCATTTTATCATGTTAAGAGGTGTAGTTAATTTATTAAGGGGTAG 68026
AsianBonytongue            -------------------------------------------------------------------------------- 0
Reedfish                   ATATATCTCGTCAAAACTGAGCAGAAATGCCAAAATCATAGTCCGTACCTAGGTCTTTTAAAGGCAGAAAATTCAAAGTT 25884
NorthernPike               -------------------------------------------------------------------------------- 0
SpottedGar                 -------------------------------------------------------------------------------- 0
ParamormyropsKingsLeyae    -------------------------------------------------------------------------------- 0

Majority                   ------------------------------------T----A--T---C---A---T-----------------------
                                   68090     68100     68110     68120     68130     68140     68150     68160
Human                      ATCACTTTTAGAAAAATTGCTGGAAGTAATTTTTCATGATCATGTTATCTACATTCTAAAAATTAGGAGAGAGACTGTGT 68106
AsianBonytongue            -------------------------------------------------------------------------------- 0
Reedfish                   GCGTGTCAAGATTGAAACTGCTGACTCTTTTCTTTTTATGAAGTTGCTCTGAAGGCTTTCCGGACTGTGCATTATCAAAA 25964
NorthernPike               ------------------------------------TGTCCATCTTAGCGGGACTGTGTATTGTCCCATAGTGGCATCAA 44
SpottedGar                 -------------------------------------------------------------------------------- 0
ParamormyropsKingsLeyae    -------------------------------------------------------------------------------- 0
```

```
Majority                  A-A-AGA----T--T-------------------T----AT----T-A-------T------------T-----------

                                  68170     68180     68190     68200     68210     68220     68230     68240

Human                     ACAAAGAGTGTTTATTTTAGAGCTTTCCTTGTATTTCAAATTGAATAACAGGCATTCTCATCATAAAGTTTTTAAAAGAA 68186
AsianBonytongue           -------------------------------------------------------------------------------- 0
Reedfish                  AGATAGAAATGTCTTGTAAATAATGATTAATCTTTCAGTATTATTTCATGGGGTCTCCTGTTTTTGTATTTCAGGCTCCT 26044
NorthernPike              ATACAGAACCCTTCTTGGTCAAAACATCTCTTCATTAAGATCTCCTCAGACAATCT---ACTTGCGGATGGACATGGTCA 121
SpottedGar                -------------------------------------------------------------------------------- 0
ParamormyropsKingsLeyae-------------------------------------------------------------------------------- 0

Majority                  -------------T-T---------T---T-----T-A----------------A---------C-T--A----T--A

                                  68250     68260     68270     68280     68290     68300     68310     68320

Human                     AGGCAAAGCAGACTTTCTGTAGGAAATCATTGACGTTAAAATAGTTATAATTGTGAACAGATACAACATTTATTCATGAA 68266
AsianBonytongue           -------------------------------------------------------------------------------- 0
Reedfish                  TCAAGGTTTTTTTTTTTTTTTTAATCTCCATGTGGCTAATTATGCATATGGCATGCATCTTCTTCGCTTATAAAGATCGA 26124
NorthernPike              ATATTCATCCGTGTGTAAAGTCAGTTTTCCTCCTTCTGATCGACAAACTCAAAACAAACCTAATGCCATTAAATGTTTTA 201
SpottedGar                -------------------------------------------------------------------------------- 0
ParamormyropsKingsLeyae-------------------------------------------------------------------------------- 0

Majority                  --T---CA--T-------A-----T-----T--T-----------T------T---------A-----------G--T-

                                  68330     68340     68350     68360     68370     68380     68390     68400

Human                     GGTAAACATGTAGGTCTTATAGAATATTGTTTCTCAAAATTTTGCTGCACGTTGAAGTCACCAGATTCTACCCCAGGCTG 68346
AsianBonytongue           -------------------------------------------------------------------------------- 0
Reedfish                  CCTGCACATTTGATGACAAGCAGATATAATTTGTTACTTATGCTGTATATGTTTCATAAAAAAATTTGAGAACCTGCATT 26204
NorthernPike              CTTGTGCAATTGCTTTTCATTACTTCGTCATAATATCACTATATTTAGCACATTTCATATCTAGAAGCAAATTAAGAGTA 281
SpottedGar                -------------------------------------------------------------------------------- 0
ParamormyropsKingsLeyae-------------------------------------------------------------------------------- 0

Majority                  ---------A---------T-----------T--------------C------------------------------

                                  68410     68420     68430     68440     68450     68460     68470     68480

Human                     AGAATAGTTAAAGAGTGGGTCACAGGCATTCCTATGTTTTTAGGATCTCAAGATGGTTTTACTATGTAGCAAGAGTTGAG 68426
AsianBonytongue           -------------------------------------------------------------------------------- 0
Reedfish                  ATTTACCTAAGTCCCACAGTCTGTAATGCAAATGATCAGCAATATATACGCAGGGACCGGTCCTATCCAGTGTTGGCAGT 26284
NorthernPike              CATTTGACCAGAGACAGTATTATCAATACTCCTGAAAGCAAGATCCCCCTTTGGCCCCCTCTAACCTTGATAATAATTTT 361
SpottedGar                -------------------------------------------------------------------------------- 0
ParamormyropsKingsLeyae-------------------------------------------------------------------------------- 0

Majority                  ------T----------------T--------A----AT-------------------G--------G------T-----

                                  68490     68500     68510     68520     68530     68540     68550     68560

Human                     AATTACTGCTACAGAAAAGAGCTTGTAACTTAATGTCATTTAAATCTATTTTGGTACAGTTCTCCTGGCACAATTTTAAT 68506
AsianBonytongue           -------------------------------------------------------------------------------- 0
Reedfish                  TTCAGCTCCGGTTGATGATGTACTGGTCTCGCAAAGTATTATGTATAGGGTGCTCGGGGGGAAAAAAGTTCCCCTAATCG 26364
NorthernPike              TAATGATGGAAAAACCTGGGGATTAATTCCATAAGGGATAGATAGGAAGAAGGAGCTTGCCTTGTTCGATTTGCTAAGGG 441
SpottedGar                -------------------------------------------------------------------------------- 0
ParamormyropsKingsLeyae-------------------------------------------------------------------------------- 0

Majority                  ---C--------------T-----------------------A--T---A---------A-----TG-------T-C--T

                                  68570     68580     68590     68600     68610     68620     68630     68640

Human                     TAGCTGTTAAACTGAATTTATAATTTTCTATATGTTTGTGATACATAAAATTAACTGGAAAACTTTGATTTTCTTTCTTT 68586
AsianBonytongue           -------------------------------------------------------------------------------- 0
Reedfish                  AGCCAAATTATCAGTAAATAATTCGACAAACAAGAGTGAACCAAATTCAAATGCAGCAAATTCGCTGCAAATGATCCTTT 26444
NorthernPike              AAGCCTTCCCAGAAATATTTTGTCAGATACTGACATATTACAAACTGTTAAAAAACAATAAAGCATGTGACAAATACAAT 521
SpottedGar                -------------------------------------------------------------------------------- 0
ParamormyropsKingsLeyae-------------------------------------------------------------------------------- 0
```

```
Majority                  --T--A--T---TG----A---------T-----TT-T--ATT-AAGACA--AGATGGGATCTCATTACTTTATGAATGA

                                  68650     68660     68670     68680     68690     68700     68710     68720

Human                     GTTACATTTGTATGGGTTATTCATTTGGTAATATTTATTAATTTATTATTAGAGATTTCAGCTATTTGCTAATATAATGA 68666
AsianBonytongue           -------------------------------------------------------------------------------- 0
Reedfish                  GGTGAAATTCGCTGATCAATACTAGTGATCAGTATTGTCCCTAAGAGAAAGTGATTGGGATATCCTTAGTCTATGACTGT 26524
NorthernPike              TCTAGATGTTCATGTCTCAGCATGAATATGTGTATTCTAATGTCCACTCACCATATAAGGTGCAAAACCTTTAGAAAGTA 601
SpottedGar                ----------------------------------------ACAGAGGACGTAAGAGGGCAACTCAAAACCTTGTGGAGAA 40
ParamormyropsKingsLeyae   -------------------------------------------------------------------------------- 0

Majority                  ATTTTATA-ACATGA-ATCTT-TTTCGACCGAGCTGACATGT-ATCAAATA-ATT-AGCATTTCTATTTTGTTG-AGTAC

                                  68730     68740     68750     68760     68770     68780     68790     68800

Human                     ATCCCATACACCTGTCATCTTGGTTCAATAGCGCTGACACTTAGTTTTATCTATTCAGTTTTTCTTTTGTGTTCTAAAGC 68746
AsianBonytongue           -------------------------------------------------------------------------------- 0
Reedfish                  ATTTTAGATAAAGAA-ATGTTTTGGAAACAGAGCTATAATGTGAACCAATGCAGTAATCCCTCCTCCATCGCGGGGGTTG 26603
NorthernPike              AATTACCAAATGTGATAACTTCTGCTGTGCATACAGACTTAACAGCAATGAGGTCTCAAATATGTAATCTGCTGCAGTAC 681
SpottedGar                CCACTGTAGACATAG-ATTTCACTTCGCCCAAGAAAAACCGGTATGAAACAATATGAGCATATTCATGTTATGAAAGAAA 119
ParamormyropsKingsLeyae   -------------------------------------------------------------------------------- 0

Majority                  -GATCCTTGGTGTT-TGAGAAGTCAGAATATCTATTCACTAAAACTCACTTATATAAATA-TTTTTATTACACAATTATA

                                  68810     68820     68830     68840     68850     68860     68870     68880

Human                     AAATCCTTGACATTGTGACATGTCTTCTCAAATATTACCATAGTATCTCTAAAATAATTACATTTTCTTACCAAACCATA 68826
AsianBonytongue           -------------------------------------------------------------------------------- 0
Reedfish                  CGTTCCAGAGCCACCCGCGAAATAAGAAAATCCGCGAAGTAGAAACCATATGTTTATATGGTTATTTTTATATATTTTAA 26683
NorthernPike              GAAACCTTGTTGTTTAAACACATCAGACTACCTTGTCTCTATCCCTTACTAATAAAAGTATTATTTATACCACTGTAGTC 761
SpottedGar                TGGAACATGGTGGTATCAGAAGTTGCAATGTAAATTCATAAAAACGCACATAAAGGGACAACCAACAATGCACCATTATT 199
ParamormyropsKingsLeyae   -------------------------------------------------------------------------------- 0

Majority                  GTCTTTTTAT-ACATT--CCATTATT-ACTACGTTTTACTTAT-TGATTAAAGAATAAGCTTT-ATTAA--TTTTACAAA

                                  68890     68900     68910     68920     68930     68940     68950     68960

Human                     ATATGATTATTACACTTACCAGAATTGACAATGATTTCTTAATGTCCTCAAATACCCAGCTTACATTAAAATTTCCCCAA 68906
AsianBonytongue           -------------------------------------------------------------------------------- 0
Reedfish                  GCCCTTATACACTCTCCCACACTATTATAAACATTTCACGTACAATTATACAGCATAAACCCTTTGTATTCTCTTAGATA 26763
NorthernPike              TGCAGGATGTCACTTTAGCTGTTATTTCCTGAGCGCTGCTTGACTGATAAACGGTGGTGATTTGGTGAGCGTTTGACAAT 841
SpottedGar                GTTTTTTGTTGTGATG--GCTTTGTTCAATACCTTGTAAGACTTAGAATGAGAAATACATTTTAAAAAAGTACATTAAAG 277
ParamormyropsKingsLeyae   -------------------------------------------------------------------------------- 0

Majority                  TTATTTTTTCTTCATTT-ATCTGTGTGTTTTAATCAG-TCATGTTACCATGGCAGGCCGCAGATACT-TATTGGTGTTAG

                                  68970     68980     68990     69000     69010     69020     69030     69040

Human                     TTATTTTGAAAATGCTTTTAAAGTTTGATTTGTTCAGAACAAGGTCCTATGGAGGGCCGCACATAGTATTTGGTTGTTAC 68986
AsianBonytongue           -------------------------------------------------------------------------------- 0
Reedfish                  TTAGGTAAGATTCATTGAAATTATGTATGTAAACACAGTTTACATACAGTAAAACCTAAATATTATTTTAAAGATATCGA 26843
NorthernPike              TTGCTTATTCCCCTTCTGCTCAGAGAGCTCTCATCAGCTAATTTCAACATGGCAGGCCGCGGAAGCACAACTAGTGTTTG 921
SpottedGar                TGTTCCTTTCTTCAATACATCTATCTCTCGTAGTTTGTTCCTGTTGTCTATGCTTCCTACAGATACAGAATTAGATTGAG 357
ParamormyropsKingsLeyae   -------------------------------------------------------------------------------- 0

Majority                  GGGTCATT-ATATTTTATTTACTGTA-AAGTCCTTTCAGTCA-TCAATTAGCAATAACTCATTCCTATG-ACGTGAAGTT

                                  69050     69060     69070     69080     69090     69100     69110     69120

Human                     ATCTCATTAGTCTGTTTCAGTCTGTAATAGTCCTCTCCCTCTCTCTACTCCCAATCTCTCATTCATTTACTTATGAAGTG 69066
AsianBonytongue           -------------------------------------------------------------------------------- 0
Reedfish                  GCGTCTCCGATATCACATATGTTACAGCCATTACGACAGACAGGCCACCAGCAATAAATACATACAATGCAAGAAAAATT 26923
NorthernPike              GGGAAATGTCATTTTTAATTAAATCCCAGGTCCTTTCAGATAAGCAGTTAGGCATTAATTTCACCTAGG-GCGTGAAGTT 1000
SpottedGar                GGGGCATTCAAATTTCATTTACTGTATAAATCCTTTATATCCTTTAATATACAAAAACACATTTCTAGATACTAACCTGG 437
ParamormyropsKingsLeyae   -------------------------------------------------------------------------------- 0
```

```
Majority                  GTGTATCGTTA-AGATG-TTACCGTGATTCTATTAA-A-TACTTTTTTT-AAAACTTTAATGCTATATTGATGCTTATTC

                                   69130     69140     69150     69160     69170     69180     69190     69200

Human                     ATGTTTCCTTTTAGAAAGTTTCACAGATTAGACTAGTCTGGCTGTTTCTTTAGAGTGTCATGTTAACTTGTTCCTCTATC 69146
AsianBonytongue           -------------------------------------------------------------------------------- 0
Reedfish                  GTATACAGTAAAATGTGTGTACAGTGACACTAAACTATGTACATGTAATAAGTACTGTACGTAAATAATTATGGTTACTC 27003
NorthernPike              GTATAGAGCCAGAGTAGCATGACGTATGTCTATCAAGACAAATTATATGCATAAACTCTGTACTATGCCGATCCTTCTAG 1080
SpottedGar                TAGTATCTTTACTAATGATTAACATCATGATATTAACAATAGCTTATTTGCAATCATAAATGCTTTATTAATGCACATTT 517
ParamormyropsKingsLeyae-------------------------------------------------------------------------------- 0

Majority                  -ACTATAATGATCTTATGAAATTTATGTTTGTTACAAGTTTATGTTAA---AATTACA-ATTTTAGCTTTATAGATCTCA

                                   69210     69220     69230     69240     69250     69260     69270     69280

Human                     TGCTGTGTTTCTTATATAAACTTAAAGTTAGTTCCAAAGGCTTGATAA--GATTTAGAGCCTTTTTTTTTTTTGAAGTGT 69224
AsianBonytongue           -------------------------------------------------------------------------------- 0
Reedfish                  ACCAACAATGACACGACGACTTGTCCGATAACGATGAGTTTAATTTTACTGCATAACAAAGGATAGCGTTACAGCTCTTC 27083
NorthernPike              CATGAAAAAGATCTTACAGAAGCTTTATGTCTGTCAAGTTTCTATAAT---ACTCCCACATTTTGACAGTGTATTACACA 1157
SpottedGar                GAGTATTAACAGCTTGTGTTAATAATGATTGTTACAGATTTATGAAACACATATTTTATGTTATAGCTTTATCAATAACA 597
ParamormyropsKingsLeyae-------------------------------------------------------------------------------- 0

Majority                  TAAAGGTGCTTCTTCTTGCGATTCATTAGGAGAGCT-TAATCTTT-TT-GGTCCATATTTAGTAATATTCATAAAGTTAT

                                   69290     69300     69310     69320     69330     69340     69350     69360

Human                     TTCTTGTGCTTCCTATTGTGTCTCAGTAGGAGACATCTAATATCTGGTTGCCCCACTTTTAGTGATTTTAATGATGCTTT 69304
AsianBonytongue           -------------------------------------------------------------------------------- 0
Reedfish                  TAAAGGAGCCTCTTCAGGCGACTGTTTAGCACCGCCGTTGTTCTTCTTCCATCACTCTTCAATCCAAATCCCTAAAGCAG 27163
NorthernPike              TATGGTTAGTTGTGAATAGACTACATGCCGATGGATAAATTCCATTTTAGGTAAATAGTTAGCAATAGACAAACATTTAA 1237
SpottedGar                GAAAGATAATCATGCTTGCTATGCATTTATGGAGCTTTAAACTTCAGTGTGTACTTAAATAAAAGCATACATAGAGTCAT 677
ParamormyropsKingsLeyae-------------------------------------------------------------------------------- 0

Majority                  ATTACAGATTGTATATT-TCTTTTTGCCTCTT-GAATGTTTCGTTTCTTT-CCTTGTTGTAATTGATTGCAGATCTA-AT

                                   69370     69380     69390     69400     69410     69420     69430     69440

Human                     AAGACTGATTGTGGATTTGGGTGTTGACATTCTGATCCTTTCGTTACTTTTTTTTCTACTAATGATTTTAGTATCCA-AT 69383
AsianBonytongue           -------------------------------------------------------------------------------- 0
Reedfish                  ATTCCATCCATACTACTGCCTTATCACGTCCACTTGCAACTCGTTTTGCGCCCTGGTTAAAGGAAACTGCAGCCGTAGAT 27243
NorthernPike              GTTACAGGGCAGATTTTCTCTTTTTGAATCTTGGGATGAACCCAGTCTTCAGAAGAGTGTCACTGATTGGACAGCCCTTT 1317
SpottedGar                ATTAAAAATTGTAGCTGATTATTTTACCTATTACAATGTTTTAAAAATGTGCCTTGTGGAATTTGATTCCAGATCTA-AT 756
ParamormyropsKingsLeyae-------------------------------------------------------------------------------- 0

Majority                  TTTA-ATGCTTCATTAATTAGTTACATAAAAAGCTTGAAGGTCTTGTTTTT-TTCTCAT-GTGACCTTACTT-TTATATA

                                   69450     69460     69470     69480     69490     69500     69510     69520

Human                     AATGGTTGCTGCCTTAATTAATTTCACTAGTGGGTTGCAAATATTGCTTTTTCTCCCCTAATTCACATATTCCTTCTCTA 69463
AsianBonytongue           -------------------------------------------------------------------------------- 0
Reedfish                  CTTACATGCTTTTCCTCCTTTTTAAATAAAAAGAATCGTGGACTCATTTATGCTGTAATGGTGTCCTGCAGTGGTGTAGC 27323
NorthernPike              TTTTTCTGGATCACTGATTGGCCAACTCATCCTCCTGAGGAGTATGACATCATACTGTATGACACCATACTTTTTAAATA 1397
SpottedGar                TAAAAATAAATGATTAAAAAGTTGCATAGAAACCTTAAAAGTCAAATAACCCTGCACATCTAGAAATAACTGAATATGGT 836
ParamormyropsKingsLeyae-------------------------------------------------------------------------------- 0

Majority                  GTTTTATTAGCTTAAACA-TTCAAAACGTTGTACTTTTTTTTGCTCT-CTTTAAATATCCA-TT-GTGCT-----------

                                   69530     69540     69550     69560     69570     69580     69590     69600

Human                     CATTAATTAGCTGAAAATCTTTATAAAGTAGAATTTTCCTTCATCAACTAGAATTATCAACCTTGTAATTCAATTTGTGC 69543
AsianBonytongue           -------------------------------------------------------------------------------- 0
Reedfish                  CGTTTCCTTCCTTCAACATATCCAAAACTTTTACCTTTTCTGCAATCATTTGAACCTTCTGTTGGCGCTTGGGCACGTTA 27403
NorthernPike              GTCTATTTGATTAAAAAAAAAAAAAATCCATGAAATATCTGAGATCCTTCAGAAATGTCCCATACATGC------------ 1465
SpottedGar                GTGTTACTAGCGTATACAGTACTGTACGGTATTCTACTATTCCTCTGCCTTTAATAAAAATTTAAAACTGCAATCCTAGA 916
ParamormyropsKingsLeyae-------------------------------------------------------------------------------- 0
```

```
Majority                     -----GAATAGGTGAGTTTTTACAT----GTGAAATTTGTA-TTCGTGACTTAGCATTTGTTTACCACCCAGTA-TTTAA

                                     69610     69620     69630     69640     69650     69660     69670     69680

Human                        AGGACAATTAGTTAAATTCTTGCATTTGTTTGCTAATTTTAAGTTGGTGCCCAGCTTTTGTTTTCAGGTAAGTACTTAAA 69623
AsianBonytongue              -------------------------------------------------------------------------------- 0
Reedfish                     ATGCTGAATGAGTGAGATTACACTTCCTGGTTAATGCAGCACTCCGTCAATGAGCCAATCAGCAGCACACAGGAACTTAA 27483
NorthernPike                 -----AAAAAGCTTAGTTCTCACAT----GTGAAATGCATAGATGGGGACCTAATATGTATTTAAATTCACTAATATATG 1536
SpottedGar                   GCCTGGTGAATGTGCTGGTGTACCT----GTGGAATTTGCTTTGCCTGCTTTGGGATTTGAAAGCCACCCTGTGGTTTCT 992
ParamormyropsKingsLeyae      -------------------------------------------------------------------------------- 0

Majority                     C-GGGT--TTTGCACGGGTAGAGTATAGGCCTTTCGCATTTACTGTTTT-TTTTTTAGTTACAGTGAGTC-CTTGTGTGG

                                     69690     69700     69710     69720     69730     69740     69750     69760

Human                        CCAGCT--TATGAACAGGTACAGTATAGATTCTTCCCACTGTCTGCTTTAAACTTTTGAAACTGTCAGTTGTTTGCAGGT 69701
AsianBonytongue              -------------------------------------------------------------------------------- 0
Reedfish                     CTGCGTGCTCTTATTGGGTAGCTTCTCAGCCATCCGCCAATAGCATCCCTTGTTTGAATTCAAATGCGTCCCTTGTTTGA 27563
NorthernPike                 AGCAGTAACTAACAAAAATCGTATTATTGCATGTTGGATTTATATTTTTGTTCAGTGCATATAGTGACTCTCAAAAGTAG 1616
SpottedGar                   TAGGCTGACTTGCACGGCCACAGGAGAGTACTGAAGAATGAGCTGTCAGCATTGCCAGTGACAGTTTGTAAGAATTGTGG 1072
ParamormyropsKingsLeyae      -------------------------------------------------------------------------------- 0

Majority                     TTTCA-ATTGGCGCAT-AATTGTGGAG-TGAACTTATT-GAGACCTCTGAAATTATTTATTTTG-GA-TAAACAGCGCTA

                                     69770     69780     69790     69800     69810     69820     69830     69840

Human                        TCTGGTAGCCCAGAGTGGTTTGAAATGGTGACCTTAGCCCAGAAGTATGTAATTATTTAATTTGGCCTTGACCAGTGGCC 69781
AsianBonytongue              -------------------------------------------------------------------------------- 0
Reedfish                     ATTCAAATGGGCAAATCAACTGAGGAAACGCACGTACTGTAGACCGCAGACATCCGCGAACCAGTGAAAAATCCGCGATA 27643
NorthernPike                 TCACCCCTTTGTGCATTAAATTTAAAACTGAATTAATTAGGATTATTTAATTTTACTAATTTTGCCACTAAACGACTCAA 1696
SpottedGar                   ATTCAGAGTGACACACACATTCTGGCGTTATTAATATCTGACACCTCTGGACATATTTATATTGAGTGTATATATAGCTA 1152
ParamormyropsKingsLeyae      -------------------------------------------------------------------------------- 0

Majority                     CAAAATCAAAAATGT-TACGTTTAAAATACGACATAAAGAAAATACTCAAAA-TTACAAATTTTTATCTCAGGAGATAAT

                                     69850     69860     69870     69880     69890     69900     69910     69920

Human                        CAAAAAAGGAAGGGGGTGGGGTGGATAAAGGACTAAACAAAATTAATTAAAATTTAGAAATTTTTCTCTCAGAAGGAATT 69861
AsianBonytongue              -------------------------------------------------------------------------------- 0
Reedfish                     TATATTCACATATGCTTACATTTAAAATCCGCGATGGAGTGAAGCCGCGAAAGATGAAGCGCGATATAGCGAGGGATCAC 27723
NorthernPike                 CAAAGTCCATAATGTCAAAGTGAAAAATTCAATCTACAAATTGTTCTAAACAATTACACATTTTAAACTTAAAATAATGT 1776
SpottedGar                   GAACACCAAAAAGATATTCACCTACAGAAACTCAGAAAGAAGAAAAGCAAAGCATACAATTTTGAAGATAAGGCCATAAG 1232
ParamormyropsKingsLeyae      -------------------------------------------------------------------------------- 0

Majority                     AATGCTTATAAATGAAA--ACAG-ATATATTAGTTTATATTGATTATATACATGTTTTTAATTGGT-TTTTTTGT-GTAT

                                     69930     69940     69950     69960     69970     69980     69990     70000

Human                        CATGCCCATAAAACAAAGACCAGGATACATTCGTATTTTATCGGTAAATACCAATAGATTATAAATCACTTTTCTCGTAT 69941
AsianBonytongue              -------------------------------------------------------------------------------- 0
Reedfish                     TGTATTTGATAGTGGAATTAAAATATATATTTGTTTATCATGAGTTTATTCATGATTTTAATTTGTTTTTGAGTTTGTTT 27803
NorthernPike                 ATTGACATTAGAATAAAC-ACAAAACAAAGTAAGAAAAATGTATTGTCCAGAGGTTGTTAGATGGAAAGATTAGAAGAAC 1855
SpottedGar                   AAAACTTATAAATGATAA-GAAGCCTGTTCGAACTGAGATGGATTAGGTGCTTTTATTCCTGTGATGTTGGATGTGATAT 1311
ParamormyropsKingsLeyae      -------------------------------------------------------------------------------- 0

Majority                     AGATTGTGGTG-ATGCAAAAA-CT-TT-AG-TTTG-TTTTTATTT-TTCAACTGTACTATTTTAAT-GC-CAGGTGTTTT

                                     70010     70020     70030     70040     70050     70060     70070     70080

Human                        ACATAATTGTGAAAAAAAAAAAACCATCCAGGTATGCATGTTATTTATTCATTGAGAGGATATACATCTCTCAGGTGTTTT 70021
AsianBonytongue              -------------------------------------------------------------------------------- 0
Reedfish                     GGGTTGGAGTTTCTGCTGAGTTTTTTTTTTTTTTTTTTTTTTTTTTTGAACACTTTCCTCATTTAATAGCATTTGAATTTT 27883
NorthernPike                 ATCCAGTGGAAGATGCAAAAAGCTGTCAGCATCTGATGCACAGTCCTGAAAGGGCACTACCTTGAGGGACAGAATGAAAT 1935
SpottedGar                   TGATTTTGTTGCATGGGAAAACAGCATGAGCTTGAGTTTTACAAAGTTCACCTGTATGTTTCCATCTGTGCAGATGTGGC 1391
ParamormyropsKingsLeyae      -------------------------------------------------------------------------------- 0
```

```
Majority                  CA-GCTGTTG-ATTGTTCAG-GT-GCC-AACAGTTAT-CTTTATTAA-TAGAATA--ACTGTCAGGTAAGATA--TATAT
                                   70090     70100     70110     70120     70130     70140     70150     70160
Human                     TTGTCGTATGTGTACTTTTGTGTAGTCTGCCTGTTTATTCTTTTTGAATAGGATGATATGTACAATTAAAAAAAATCTTT 70101
AsianBonytongue           -------------------------------------------------------------------------------- 0
Reedfish                  CACCCTGTGAAAATGATCAGGACGACAGAACACCAAAGCAATAAAATGTGGCATATGACTGTCAGATCAGTTCTGTACAG 27963
NorthernPike              CATGTTAAGTGCTTGCTCAGCAGCAAGCAAAAGCTGTAGTTGATTTATTCAACAACAGCTGTATAGCATGGTAGCTTTAT 2015
SpottedGar                CAAGAAGTTGCAAGTTACACAGTTGCCAAACAATAATCCTTAAATAGCAATAATCGCAATCTAAGGAAATAGTCTTACCT 1471
ParamormyropsKingsLeyae-------------------------------------------------------------------------------- 0

Majority                  T-TAAATTGTTTTTATTTTGCATGCA-TTTTGTTATCTTTT-AAAGTGATTTCACTTAAAAATACAATCTCCATTGG-GT
                                   70170     70180     70190     70200     70210     70220     70230     70240
Human                     TGTGAAATGTGGTTTTCTTAAATACATTATTGCATTTCATACACAGTGATGAAGGGAAAAACTACAAAGCTAGACAGATT 70181
AsianBonytongue           -------------------------------------------------------------------------------- 0
Reedfish                  ACTTATTTGTTTTACTTCTGCATGCAGTTAGGTTCTCTTGTTTGCAAGTGGTCCCTTAAAAGACTGCTCTCCATTGGTGC 28043
NorthernPike              TTCAAATAGCTGTGATTTCAAAGTGGCTACTACTAACTTGGAAAACTAAATACAATTAAGAATACAAAAGCTATATTTGT 2095
SpottedGar                TACAGGTTGTTTTTAAAATGCTTGCAAGTTAATTAACAATTGCATGCTGTTTAACTCTTTCATACAATCTCCTTTGG-AT 1550
ParamormyropsKingsLeyae-------------------------------------------------------------------------------- 0

Majority                  A-GTGTTA-TCTTCCACTGGCTAG-TACACTATTTAAC-CTTAATGTTTTCTTTCATTTTAAATGCGTAATCATTC-ATA
                                   70250     70260     70270     70280     70290     70300     70310     70320
Human                     GCAAATTAGTATTGCAGTAGTGAGGTAATAGAGCAATACCCAAATGTTTTCTTAAAATATATGTAGGTGATGCCTGTGTT 70261
AsianBonytongue           -------------------------------------------------------------------------------- 0
Reedfish                  AGGTATTTCTCGTCCCATGGCTCCATAAACTCTAAACCACTTGTTTTGTGATATCTTTTTAAATTTGTAATCTTTCAAAA 28123
NorthernPike              CTCTGGTCATTTTTTACAGGCTGGTTGCATTTGTTCATGCTTTA-GTTTGCCTCCAAGATGAACGCGCATCCATTCCCTA 2174
SpottedGar                AAGTGTGATTCAACAACTCAACAGCCACACTATTTAACTTGCAATACATTTGGTGATTTTTCATGCACAGATATTTGAGC 1630
ParamormyropsKingsLeyae-------------------------------------------------------------------------------- 0

Majority                  CTTTACTTTTATGTTTGTTTCA-AGTTTTTTCTTACAAGGTT-T-TG-C-GAAATTAGCTATAT-TTCAA-AATTTTGT-
                                   70330     70340     70350     70360     70370     70380     70390     70400
Human                     CTTTACTTTTGTGTGTGTGTAT--GTGTTTTTAAACAAGATTCATTGTAGTCTTTTAGATATAATTTAATAAATTTAGGC 70339
AsianBonytongue           -------------------------------------------------------------------------------- 0
Reedfish                  CACAGCTGATATGTTTCTTTCATATTTTTTCCTTCCCTGGTATTATAGCACAAAGTGGCTTTATCTTTTAGAACTCCCAG 28203
NorthernPike              CGTCAATTTGATGTCGACTTGAAAGCTCTCTATTATTAGGAAATGTGAACGAAATTTACCATGTAGACGATGGGTGTGTT 2254
SpottedGar                CTTTCCCAATAAATTGGGGCCATACTTCATTCCATCAAAACTGGCAGCCTGTCAGCAATAACAAGTACAACATTTTTTTA 1710
ParamormyropsKingsLeyae-------------------------------------------------------------------------------- 0

Majority                  TATGAATGGTATTCTTATTCTAC-TACTGGCTATTTTA-TATAGTTTTTTGCATGTATTT-TT-TGAGCTTATTATGAT-
                                   70410     70420     70430     70440     70450     70460     70470     70480
Human                     TGTTCAGGGTTTCAGTATCCTACAAAGAAGAACGCTGCTTCTAAGTTTTTGGGTTTCTTGAACCAGAGCTTAGAAAGAAC 70419
AsianBonytongue           -------------------------------------------------------------------------------- 0
Reedfish                  TATGTGTGTCATTTTTATTATGCTCAATGAGTATCCAAGCAAAAATATTTACATGTATCACTTGTGTGCCTGTTACTTTG 28283
NorthernPike              CCAGAAAAGTATGCTCATACCATCTACTGGCTGATATGGTTATGTCGATTGCAAGTAATTGTTTTAAACGCATTATGAA- 2333
SpottedGar                TACAACTGATATTCCCACTGTACGTACCTACAATTTTA-TATGGTTTAAAACACATTAATTAAATAACTTCAATATAATC 1789
ParamormyropsKingsLeyae-------------------------------------------------------------------------------- 0

Majority                  ---AT-A------G--ATTTTCTGTTTTTAGTAAGTCTACTTAGAT-AAATACATTTAATG-CAATGATTTAATAGAG-A
                                   70490     70500     70510     70520     70530     70540     70550     70560
Human                     AAAAATATGGCATGGTATTTTCATGGTTTAGTAAGGCTACTTTTAGTTAATTATTGTGATGTAGGCCATTAAGTAGACTT 70499
AsianBonytongue           -------------------------------------------------------------------------------- 0
Reedfish                  CACTTCATTGTTACACAAATTTCAACTTTCTCAAGTCTGCTTAGTTAAAATACGTTTAAATCCACTTTTAGCATCTAGCC 28363
NorthernPike              ---AT--------GTGATTGTGTGTTATTAACCATCTGACCTGAATGATTTGGACCACTCAGAAATGAAATAATGGTGAA 2402
SpottedGar                TTCATGAAACTAGACAATTAGCTGTTTTCAGTTCCTCCTGCAAGCTCACATACACTGAATGACTATGGTTTAACAGCAGA 1869
ParamormyropsKingsLeyae-------------------------------------------------------------------------------- 0
```

```
Majority                   A-AGAGTAGTA-AACCCTGAGGAGAGGGGCTT-ATATATACATAT-G-TTAAATACAGAGAT-TAATGTT-TT-G-TTTC
                                   70570     70580     70590     70600     70610     70620     70630     70640
Human                      TTCTAAAACGAAACCCCAGAGGAGAGGGGTTTTGAAGATACATATATACTGGTTTCTAAGAACTAATCTTTTTGGATCAT 70579
AsianBonytongue            -------------------------------------------------------------------------------- 0
Reedfish                   ACAAAGTTGTCTATCATTACGACCTGGGGCCTCATGTATAAACGGTGCATACGTACAAAAATGTTGCGTAAGCCCGTTTC 28443
NorthernPike               AAAGGTTATTTCAACCCTGATAATAGATTCTTGATATATGCTTTTGGGTTAAATAACGCATTTAAATGCTGTTACCTTAC 2482
SpottedGar                 GGTGAGTGGAAGAAGAAAAAGGAGAGGAAAGAAATAACATTGTAACGTTTTAACAGAGGGACAACTGATTCTCTGTACTC 1949
ParamormyropsKingsLeyae    -------------------------------------------------------------------------------- 0

Majority                   TGGTTTTAGTAGGCGTTCTATA-AA-GTTTATTT--CC-AAAGCGATTCTGA-TAGA-T-TTGCC-CA-G-TC-GACTTT
                                   70650     70660     70670     70680     70690     70700     70710     70720
Human                      TGGTATTGGTAGAAGGGATTCGCTATGTTTTCTGCCACCATTGGATCTCTGTATAAAGTTTGGCTCCACTATCAAAATGC 70659
AsianBonytongue            -------------------------------------------------------------------------------- 0
Reedfish                   CACGCTCAAATCGCGATGTATAAAACCTAAACTTGGCGTAAAGCCACGCACATTTCCACGGTAGCTCCAACCCTGGCGTT 28523
NorthernPike               TGATTTTTATCAGGTTTTCTAGATCGAAATAATGAATACAAAACTGGTTTTTACTCGACTATTTCCGCTTGGTTCGACTAT 2562
SpottedGar                 CCGGTGGTGTACACCTCCCATAGACGGTTTATTTTACTGCCATCGATTCGGCGCAGGTTCCCGTCAGAGGTTCGGCTATG 2029
ParamormyropsKingsLeyae    -------------------------------------------------------------------------------- 0

Majority                   TTCGGCCATTTTCTTCA-ACT-TCTGCCACCATTGTAAAGGCT-GAATACTGTT--CTTAAGA-TTGTTTTTTAGATATC
                                   70730     70740     70750     70760     70770     70780     70790     70800
Human                      TTAGATAGATGCCTTTGCAATAGTTGCCACCCTAGGAATAGCTCAAAAATTATTAACTTAATACTGGTTACTTAAATTCC 70739
AsianBonytongue            -------------------------------------------------------------------------------- 0
Reedfish                   CTCCGCTCTGTTTTGCAAACTGGCTGCACCCAGTGTCAAAGCAGTGTTACTGTTCCAGTGTGGTTTCCCTTTTTTAGATC 28603
NorthernPike               TTCCGCCAAGGTCTCCAGTCCCTCCATTTTAGGAGAACAGTGCAGAAATCGGTGTTGTTAAGAAGGATTTTCTGGCAATG 2642
SpottedGar                 TTCGGCCATTTCCGTCCTGGTTTCCGCCACCATTTTGTCGACTTGCGTGCAGCTGGCTGAGGTGCTGTACTGTAGATCAC 2109
ParamormyropsKingsLeyae    -------------------------------------------------------------------------------- 0

Majority                   A-CGT-GATGTTTCGGCTCT-TCAATT-TCTG-TATTTTTTTTTTTTAGTTTGTTAATTGTACGCATCT-AATTGTCGTT
                                   70810     70820     70830     70840     70850     70860     70870     70880
Human                      ACCTCTGTTGCTTGGAGTTGTGCTTTTTTTTTTTTTTTTTTTTTTTGAGATAGAGTCTGGCTCTGTCACCCAGGCTGGAG 70819
AsianBonytongue            -------------------------------------------------------------------------------- 0
Reedfish                   CGCATCCCAGATGCGGCTCTATAAATACACTGAAATTAACTGCATATTGTTTATTAGTTTAATGCATCT-GATTGTAATT 28682
NorthernPike               GACGGGTATGTATTGGCCATCTTAAATATGAACACCGTTTTTGCTTTAAAACATGAATTGTGCGTGGCG-AGATGTCGTT 2721
SpottedGar                 ATCGTAGAGATAGCTAATCCGGCAATGGACGGGTAAGGTGCTTTTTGGTTATGTTAATTATAAACATTTTAATTGTCATA 2189
ParamormyropsKingsLeyae    -------------------------------------------------------------------------------- 0

Majority                   TATTAGCGAAAGCATAAT-G-TTCACTACAGGTTCAGCTAA-CTGG-TATGA-CACTCT--TG-GCGTGTTACTTTAGCT
                                   70890     70900     70910     70920     70930     70940     70950     70960
Human                      TGCAGTGGCATGCAATCTCGGCTCACTGCAAGCTCCGCCTCCTGGGTTCACGCCATTCTCCTGCCTCAGCCTCCCAAGTA 70899
AsianBonytongue            -------------------------------------------------------------------------------- 0
Reedfish                   AACCTGTAACAATATAATGGTCCAAAGAATAGTCAAACTATTCTAAATACCATGGCTGCTTTAGCGTTGTTACTCTCACT 28762
NorthernPike               TATTAACTGAAGAGTATCTACTTCACCTCAGATGCTAGACAGATATCTATGAACACGTTAGCTAGCGAATTACTTTAGCT 2801
SpottedGar                 TCTTAGCGATCACTGCAAAGATACAGTAGAGACTAAGATAAACTGGGAATGAGAAGTCGGACGTGCGTGTCCAGTTAACA 2269
ParamormyropsKingsLeyae    -------------------------------------------------------------------------------- 0

Majority                   ACCGGTCTTTCTGGCTCTTTCCCCCACTTCCGGTAGATGT-GTCATTTTTGGTTTTGTAATGTCGGGTTTTTTTTTT-TT
                                   70970     70980     70990     71000     71010     71020     71030     71040
Human                      GCTGGGACTACAGGCGCACGCCACCACGCCCAGCTAATTTCTTCATATTTTTTTAGTAGAGACGGGGTTTTACTGTGTT 70979
AsianBonytongue            -------------------------------------------------------------------------------- 0
Reedfish                   ACACCTTCTTCTTCCTCTTTCAGCTTCTTCCGTTAGGGGTTGCCATAGCAGATCATCTTTTTCCATATTACTCTCACTGC 28842
NorthernPike               AGCTAACTAGCCAGCTAGCTAGCCACTTTAGCGTGGCTAAAGTGAGTTAACGCCTGGCAACATCGGGTGTACTGATAAAT 2881
SpottedGar                 AACGGTCTGTGTGTTTTTTCACCCCAGCTGAGAAAGAAGTGATTTTTCTTGGTTATCTGATGTTAGGGCTTTTTTTTCTC 2349
ParamormyropsKingsLeyae    -------------------------------------------------------------------------------- 0
```

```
Majority                   ACCAGGGATGTT-TTTATATCGTTAT-TC-TGAACG-G-TA-CTCGGCAAACCAA-TAA--ATTGTTACTAGCATT--AT

                                  71050     71060     71070     71080     71090     71100     71110     71120

Human                      AGCCAGGATGGTCTTGATCTCCTGACCTCCTGATCCTCCTGCCTCGGCCTCCCAAAGTGCTGGGATTACGGGCGTG--AG 71057
AsianBonytongue            -------------------------------------------------------------------------------- 0
Reedfish                   ACCAGTCGGAATATTTATATCGCTGTATC-TGAGTGGGGAATCACAGCTCTACAACAGATGATTGGAAAGAGAATT--AT 28919
NorthernPike               ACGAAGCAAGTTTGCTACACGGCTATTTAGTTAACTAGTTCAATAAGTAAACGAAGTACGCTTTCTAGCTAACATGTAAT 2961
SpottedGar                 CTTTGGGTTATAGCGTGAATTGTAAGGGT-TGAAAGCAACAGAGTGTGAAAAATGTTAAAAATGGTTCTTTGTTTT--GT 2426
ParamormyropsKingsLeyae    -------------------------------------------------------------------------------- 0
```

```
Majority                   T-GACAAGTGTATCATGCTTAG-GCTTTTTT-GTAAT-ATTTT-ATTGAAATAGTCTTGTACGACTGTAGCTT-GGAT--

                                  71130     71140     71150     71160     71170     71180     71190     71200

Human                      CCACCGCGCCTAGCCTGCTTAGAGTTTTTTATGTAATTATTTGTGACACAAACATTTAAAATACTGTAGCTTAGAATGA 71137
AsianBonytongue            -------------------------------------------------------------------------------- 0
Reedfish                   CGGAATACAGCATCAAGCACAC-GCTGCCTCAGCCATGCTGTCTATTGAACTGCTCTCATACGGCAAACACTTCAGAGCC 28998
NorthernPike               TTAGCATGTGTCTAATAGTTCGTGACTTGTTGGCTATCAAGCTAATGAATGTAGTGCTGTGCAATCGTAGGCCGGGGGTT 3041
SpottedGar                 TAGAGAAATAAGGCGTGTTTATTGCTGCTTTCGTAGAAAGTAGCGCTGAAAAAGCCCTGGCGGTCTCTTGTCGTGGATAG 2506
ParamormyropsKingsLeyae    -------------------------------------------------------------------------------- 0
```

```
Majority                   TATTGTG-CAGTT-TAGGAGC-TATTGTTTCATGACTATGGATACTGATTT-AACCGTTTTTTTATTGC-TCTGGTGCAC

                                  71210     71220     71230     71240     71250     71260     71270     71280

Human                      AATTGTCTCAATCCTAGGAGCCAAAAGTTTGATGAATATGAATACTAATATTAACCATTTTTGTGTTCTGTGTCAGGCAC 71217
AsianBonytongue            -------------------------------------------------------------------------------- 0
Reedfish                   TTTTCTCGCAGTT-CAGAAACAGTTTCATCCCAAGAACTATAAACGCACTCAATCAGTCCATCAAGTGCTCCTTGTAGAA 29077
NorthernPike               TGTCAAGCATGAGACTAGGGTGTGTCGTCACTTGTCGAGGGATCTTGAGTTGAATCCTTTGTTGATTGAAAATGGAGCTA 3121
SpottedGar                 CACTGGGATCATT-TGTGTGCTTATTATTTTAAAACTAAGGTTACGGATTTCTGCCGCACTTTTACAACCTCTGATGCAC 2585
ParamormyropsKingsLeyae    -------------------------------------------------------------------------------- 0
```

```
Majority                   -GTTCTTAGCTATAGA-AGGT-TCGTGTTA-A-CAG-TCCAATCGTCGTTATCTTTCTC-ATCTGATTGACTA-AT-T-T

                                  71290     71300     71310     71320     71330     71340     71350     71360

Human                      TGTTCTTAGCCCTTTACAAGTAAGTTAATACATCATCTCTAAATGTAGTTACCATTATCGATCCCATTTTATAGATTTGG 71297
AsianBonytongue            -------------------------------------------------------------------------------- 0
Reedfish                   CGCTTTGTACTTAAGTACAATTACCTCACTGTAAACTTGCGATAGTTATAATATTGCACAACCTGAGCCACTTTATAAAG 29157
NorthernPike               GTTGGCTAGCTAACGCTAGCTGTCGGGTCAAAGCCGAAACAATCAGCGCTGTCTAGCTGCAAATCTATGGCTAATTGACT 3201
SpottedGar                 AATCCAAACGAATAAAGAGGTCTTGGGTTCTACTTGGTCCTACCTACAGTTTCTTTCTTTTTCCGGTGGAGAGCATCTTT 2665
ParamormyropsKingsLeyae    -------------------------------------------------------------------------------- 0
```

```
Majority                   TGCGTA--GTCATATGGACG--TAGTCTAGTATTTTTTAA-TTAAATGTGGTGTAAT-TTTG-ACTGTTA-ATA-GTTGT

                                  71370     71380     71390     71400     71410     71420     71430     71440

Human                      AAAGTAGGGTACAAAGAACAGTTTGCCCAGATTCCTTTAACTTTTGAGTGGCCGAGCCCTAGCACTGAAAGCCAGGTAGT 71377
AsianBonytongue            -------------------------------------------------------------------------------- 0
Reedfish                   TGCATATTTACATATGATAA--TAATATATCATTTTTAAGATGAAATGCTGGAAAATGTTTATTATGTTATACACATAGA 29235
NorthernPike               TGCACTCCCAAATACCGCTG--TGGTCGAATAATTTTAAAGCCAGTGGCCATGTTTTTGTTG-ACTGTTTAGTTAGCTAG 3278
SpottedGar                 CCTGTCAAGTCACGTAGACG--TATCTTTGTACTTTGTAATCTAAATATGGTGTGATATAAGCGGTGCTACATGTGCTTT 2743
ParamormyropsKingsLeyae    -------------------------------------------------------------------------------- 0
```

```
Majority                   TTATTTTAAAACTTTTA-GTTGTTATTGATAATAATTGAA-ATGAGATTACACTGTGCT-C-ATTTTAGCCTGTCGT-TG

                                  71450     71460     71470     71480     71490     71500     71510     71520

Human                      CTGGTTCCAAAGTCCGTACTCCTGACTAATAAATAGTGGA-ATAAAATTAGATTAGCTTGACATTTTAGCCTGTCGTCTT 71456
AsianBonytongue            -------------------------------------------------------------------------------- 0
Reedfish                   TTATTTAAATGCTGTTAGCATTTTATTGTTAATAATTAAACATGTGAGGACACTGTGCCACAGTGCTAGCAAGGCGCTGG 29315
NorthernPike               CTAATGTAACACATTTGTGTTGGTGTTGAAAATAAACGCTTGCTAGATCCTTCTGTGGTTTTATTTACTATCTAGTAAG 3358
SpottedGar                 TTTCATACAAATTAAACGTTGGTTCTAATACAAATCAAACCTGACATTGCAAAGACCCCCGTTATTATACGCTGATGTG 2823
ParamormyropsKingsLeyae    -------------------------------------------------------------------------------- 0
```

```
Majority                  CTCACCTGTTACGTAGTGTTCAAGACTAGCGCTGTAT----------GTATAGAGGACTTTTCAGTGACATAG-T-TAG-

                                   71530     71540     71550     71560     71570     71580     71590     71600

Human                     CTCACCTGTAGAATAGTTATCAAGAATAGTAATATAC----------ATATAGTAAAAATTTAAGTCCCATCATTTTAGC 71526
AsianBonytongue           -------------------------------------------------------------------------------- 0
Reedfish                  CACTCCATTCACGGATTGTTCCTGCCTCACGCTGTATTCTTGCTGGGGCTGGCACGACACTGGAAGGATAGATGGATAGA 29395
NorthernPike              CTAGCTTGTTAGCTACGGTACAGTAATAGCAATGTAG-CTACTAGCGATGTTGCGCTGTTTACGTTTAGTTAGCTGTGTT 3437
SpottedGar                GAAATGAGTTACGCGGTATTGGAGGCGAAGGCCATAT----------GGAAACAGGCCTGTTCAGTGGCGTGGAGCCAGG 2893
ParamormyropsKingsLeyae   -------------------------------------------------------------------------------- 0

Majority                  TT-CTTTTTTTT-GACTACAAAGCTATTTCTTCGTGCTAAGTA-AAACAGTTTTTGACT-GATGTGCTTATTGAAGTAAA

                                   71610     71620     71630     71640     71650     71660     71670     71680

Human                     TTGCTTTTTTTTTACATACTAAGATATTTTTTGTTACCAAGTCCATTAAGTATTTAGCCTGATTATCTTATTGATTAAAA 71606
AsianBonytongue           -------------------------------------------------------------------------------- 0
Reedfish                  ATAATTAAACATGTACTAAAAAGATATTTCTGAGCGTGAAGTATAAACCGGCCCTAAGTGTAAATGGACAATAAAGTTAA 29475
NorthernPike              AGCCGGTTGACTCGTCTACACTGCTGTATCTACGTGCTACATTACAACAGTTGGGGATTCGATGCACATACTGTAGTGAC 3517
SpottedGar                TTTATGCCTTTGAGAGTAGGGCCCTATTGATTCACGCTGTAGAGGAGTTGTTTTTGTCGAAGTGTGCTTCTCGGAGAAAA 2973
ParamormyropsKingsLeyae   -------------------------------------------------------------------------------- 0

Majority                  TT-CATAACTGTA-ATTTTGCATGCTATGTATACA-TTGGGATCCTATCTGAGTTTATATACAACTCGTTTATG-TAGTC

                                   71690     71700     71710     71720     71730     71740     71750     71760

Human                     TACCAAAAATGTGTATTGTTCATGCATTGATTCAAAATTGGGTACAAAACGGGTAGATATGTAACATGCAAATAATAAAT 71686
AsianBonytongue           -------------------------------------------------------------------------------- 0
Reedfish                  TTACTTAACCACAGTTATTGTGACAGATGTAAATACCAGAAATTATGTCTTTGTTTAATTACAAATGTTTTGAGTTGGGA 29555
NorthernPike              TTTAAATAACGTTACTTTTGCTAGCTGGGTATACAGTCAGGAGCTAAGTGGAGTTGAGGAACCATTCGTGTATGCATCTC 3597
SpottedGar                GTGGATCTCTGTACATAATACATTTTAAGAATGCTTTTGAGTGCCTTTCTGAGGTTGTATCCAGCGCCATTTAAGCAGTC 3053
ParamormyropsKingsLeyae   -------------------------------------------------------------------------------- 0

Majority                  GTAACTCATTTAAATCTTTTTTTCTCTATCAACTTTTCTAAAATTAAGT-AT-ATTAGGTTATAATTAT-CTTTAATATT

                                   71770     71780     71790     71800     71810     71820     71830     71840

Human                     TGAACTTATATTTTTATCTTTTTGACAAACAACACGTTAAAATTTATGTCAATCTTATTTTTTCAAAATCCTTTTCTATA 71766
AsianBonytongue           -------------------------------------------------------------------------------- 0
Reedfish                  GTAAACAATTAAAATCTTTTTAACTATATCAATATTTCTTTTATTAAGTTATGTAGAGATAATGAATAAAATTTAATTCT 29635
NorthernPike              CGACGTCACTTGCGAATTTTATAACCAATAGAATTTGCCAAAATGTGTAGATAATTAAGTTATATTGGTGCTGAAATGTT 3677
SpottedGar                GTGACGCTCATAAACCTCTCCCTCTGTAATTGCTAGGCTGCGGTGAAGAACACAAAAGGATGTAATTTTTGTAGCAAATA 3133
ParamormyropsKingsLeyae   -------------------------------------------------------------------------------- 0

Majority                  TTTGATTA--AAACTAACTCAAGCTG-TTCT-GTCACTGCATTGTA-GAGCCTTTTGAGTTAATATTGACTGATTTTAAA

                                   71850     71860     71870     71880     71890     71900     71910     71920

Human                     TTTTATTA--AGACTAATTATAGCAAATAATAGCCATAAAATTAAACCAAAAAACTCATCTACTGCAGACATATTTGAAA 71844
AsianBonytongue           ----------------------------------------ATTGTA-GGGCCTGTCAAGTCAAGATTGAATGGTCATGAC 39
Reedfish                  TTTGAGTTATAAACTTCATTAACCTGCCACTTATTGCAGATTGTTATAAGACTTTGAAATATATAATGGGTGATTTGAAA 29715
NorthernPike              TTTATTTAA-CAAGAATCACATGTTTGTTGACGTCAGTGCTTCGCGCGCACTCATCGCGGTGTACCCAGATACTCATGCT 3756
SpottedGar                ACTGCCCGTTAACAAAACACCAGAGGTCTCAGACAACTACATTGTA-GTGCCGTTTGTAATAACATTGACCAAATTCAAA 3212
ParamormyropsKingsLeyae   -------------------------------------------------------------------------------- 0

Majority                  TATTTTAGGTGTTTAAGCTTCAAGTGGGCATATACCTC--TGTATGCTGGA---TTGCAGTATTAT--ATAATGCATTGA

                                   71930     71940     71950     71960     71970     71980     71990     72000

Human                     GCTGTCCCCCAAGAAAGACCAAAGTAATCACACATACCTAAACATGCTAGTATTTCCCAGAAATATTAAAAATACATAGA 71924
AsianBonytongue           AAATATAGCCCCCCCACCCCCAAGTGGGCTTATACCTT--AGTATTTAGGA---TGACAAAGAAAT---CAATGTGTTCA 111
Reedfish                  TGTTTTAAGTCTTTAACCTTAAAGAGAGATTCTACCTCC-TGTGTACTAAA---ATGTACGATTTTTAAAAATGTAAAAA 29791
NorthernPike              TTATTATCGTGTACAGAATTGGTTTTGCTAATGACATTAATAATTGTTTCCTTTTTTCAGTATTGGAGATGTTGCAGTTG 3836
SpottedGar                CAGGGCGGTTGCTTAAGTTACAC-TGGCCAGATGTGTC--TCTAAACTGAG---TTGTAATGGA-----TGATGCTTTGT 3281
ParamormyropsKingsLeyae   -------------------------------------------------------------------------------- 0
```

```
Majority                    TTTTTCAGGAAAGTTCTGGTTTTTAGAGTCA--GAGGACCCTTG--TATTGAGTACTGAATTGTAAAGTTATGTTTCTTT
                                    72010     72020     72030     72040     72050     72060     72070     72080
Human                       TTTTGAAGCACATTGCTGAATGCCATAGTAA--CACCAAACTAA--AAAATACATGTAAATTAAAAATTAATATTTTATT 72000
AsianBonytongue             AGTTTCCACAGAGCTCAAGATTTTAAATTCA--GAATCCCCTT---TAATAA--ACTGACTAGAAACCTTAAAAGTCTGT 184
Reedfish                    TATATGTTGCATGTTTCATGTTTCAGAACCA--AATGAACATGGA-TATAGATAACAGAACGGCAAAGTAACGTAGATCA 29868
NorthernPike                GCGTAAAGGTAAGGATTGGTCCATAGCCAAATCCAGGACAAGTAAATGTTGTGTACTTGTTTTTAAAGTGTTGATGATTT 3916
SpottedGar                  TTTCTCAGGATAAAGCAGGTTGGTCGCGTGG--GAGGGTCTTTGTTTGCTGGGTACCATACAGTAACACTGACTTTCTCC 3359
ParamormyropsKingsLeyae-------------------------------------------------------------------------------- 0

Majority                    TTTTATATAGATGTGTAGTAATCAGTGTGACTGCATATGGTAATAAA-GTTCTGATTATGATTGGTTGTACACGTG---T
                                    72090     72100     72110     72120     72130     72140     72150     72160
Human                       TTCAACATAAATCAGAAATACTTGGTTAGAGGTCACATGATAATACCTGTCCTAACTGTCCCATAACTTCGGCTTTCTCA 72080
AsianBonytongue             TTTGGTAGAGAAGTTTTGAAATCTGAGTGAATGCTTTTGGCACTGAA-GGTTTGATTCTCACAAGGTGAACAC------T 257
Reedfish                    TTGAAAGTATAAATCTAGGAAACAGTACAGCTTTAAAGGTTAATTTA-GTCCAGTGAACGTTGATCAGAACCTGTCCCCT 29947
NorthernPike                TTTTCTAAAGTTCAGTAGTAATGCTTGTGACTGCTTATTGTTGGAAAAAGTACTGACATGATTGATTGGATATCTG---A 3993
SpottedGar                  TTTTTCTGTGTTGTAGCATTGTCACTGATGTTGCAGTTGGCGTAAAG-GTAAGGATTATTGTTGGTTATAAACGTGTGAT 3438
ParamormyropsKingsLeyae-------------------------------------------------------------------------------- 0

Majority                    TTTAATGTTTTTCAATTACTGTGAGGTAGC-TTATTTTTTTGTGGATGCCTATGTGTATATGCAAGTTGCTGCTATGCCG
                                    72170     72180     72190     72200     72210     72220     72230     72240
Human                       TTTCATGTGTTTTAATATCTAAGAAGTAA--AAAATAGTTGGAGCATGCCTCTTTATATGTGCAATTTTAAATTATGTTG 72158
AsianBonytongue             TTCAGCGTTTTACAATGGTTGGAATGCAGCCTTACAATCTGAAGGTTGCCAGTGTGAATATCACTCTTGCTGCAGTACCC 337
Reedfish                    TTTAATGCCA--TAGTCAGAAGAGGAGAGCAGAATTCTTGTGTGGAAGGACATCTCTCAAAGAGGATTCAATGTATGGTT 30025
NorthernPike                AATGGGATTGTTCAAATACTGTGTGCTGA--TTGTTTGAAAGGCACAACATATGTGGAGTGCCTAGACAGTGCTTAGCCG 4071
SpottedGar                  TTTAATGTTT-ACACTTCCTGTGCCAGGCCTTTATTTTCATGTGAGTGCTCATCTTCACAAAGAAGTGGCTCAAATAACC 3517
ParamormyropsKingsLeyae-------------------------------------------------------------------------------- 0

Majority                    TGGTCTTTAGGTAATTTACCAAAACCTGACGTAGCA--TAGAAAATGGTTTAATGTGTAATTTGTTAAAT-CTATATTAT
                                    72250     72260     72270     72280     72290     72300     72310     72320
Human                       ACTTCTTTGGATTATTGAACTGTCCTCTGCTTTGCTA-TAGCTGAATTTTTAAAATGTTAATTTTGAAGTCTTAAAATGT 72237
AsianBonytongue             TTGATT--AAGGCATTTACCATGAACTAACGCAGTA-----AAAATACTCTACTGTATAAATGGGTAAAT-CAGTATTAG 409
Reedfish                    GGCCATTGACATAGATGACAGAAGCCTGAAGTGGCAGGCAGTATAGAAGTTAGTTGTCGGTCAGTTGAAGTATATTTTTC 30105
NorthernPike                TAGTATATTGGAAATATATCACAACCCGAGATCGTGCTTATAAACCGGTTACAAACGTAATCAGAGCAGTACAACGTCAT 4151
SpottedGar                  TGCACCCCATGAAGACAAAAAAAAACTCTCACAACAT-TAGAAAGTGGTTTGAGGCATCACTTGTCTAATGCTTTCCTCT 3596
ParamormyropsKingsLeyae-------------------------------------------------------------------------------- 0

Majority                    GTGCAT-TGTACCCAAAATTGAAAGT-G-GACATAGTTAGAGCTTTTAATGTTAATTTTTTATGATTTATAT-GAATTGT
                                    72330     72340     72350     72360     72370     72380     72390     72400
Human                       GTCTTT-TGGAAAAAACATTGGAAGAGCTAAAGTAGATAGAGTTGTTCATTTAAATTGTTTATTTTTTATTTTATTTTAT 72316
AsianBonytongue             ATGCAT---TACCTTAATATGCTAGT---GAAACAATTATAACTATTAACATT---TTTTTTTAACTTA----GGAAATG 476
Reedfish                    ATGGATATTTAAACATAAGAAAAAGTCGGGACACAGTGGGTGCCTAGAATGTTGACTTTTCAAGGAGAATCTTGAGCTAA 30185
NorthernPike                GTGATGTCATACCCATGGTATACAGT-ATTACATACCACGACTGTCAGCCAATCATCATTAAGGATTCATACCACCTGGT 4230
SpottedGar                  GTTCATCTGTAGGCTAAAATGTAATTTG-GATGTGGAAAGGACTGCTTATGTTAGTTATTTTAGAGTTGTAT-GAACAGG 3674
ParamormyropsKingsLeyae-------------------------------------------------------------------------------- 0

Majority                    TTATTTGGGTAGGTACTG--TTGATGATGTGTTGATTTG-----TTGTGGTTTTGTCTTTGAATACAAA--GGTTTAAGC
                                    72410     72420     72430     72440     72450     72460     72470     72480
Human                       TTTTTTTAGTTGGAGTACTGGCTCTGTTGCCTGGGCTGG-AGTGCAGTGGCGGGATCTCTGCTCACTGCA-AGCTCCGCC 72394
AsianBonytongue             TCAGCCATCTAGGGGCTG--TTGATAATGTATTGATTAG--------AGCTATTGCCTTTGGACACAAA--GGTTCAAGG 544
Reedfish                    TTAATTCGGACTGGAAGAAATTCAAGAAGTGTTTAATAA-----TTGAGATTGAGACTTGAAATAATAACTGATGTAAAC 30260
NorthernPike                TTATGAGACCATATACCA---TGGGTATGATCTCACATACTTTTTTGTGTTATAGTTGTTGGTAACATGTTTATTATATC 4307
SpottedGar                  ACAGGTGGATAGGTAATGAATTCAAGGTGAGTTGGTTTGTAAGGTTTGGGTTATGTCGTAGAGTACAAA--GACATAACC 3752
ParamormyropsKingsLeyae-----------------------------------------------------GCCCTGCAATTAAAC--TGCTTAGGC 24
```

```
Majority                  --TTAAATCTTCACCATT-TGCAGCTTCAGTATGCTT-ATATGTTGTAGTTATTTTTGTTAGCTGCTCAACTGTGCTAAT

                                 72490     72500     72510     72520     72530     72540     72550     72560

Human                     TCCTGGGTTCACGCCATTCTGCCGCCTCAGCCTCCTG-AGTAGCTGGGGCTACAGGTGCCCTCCACCACACCTGGCTAAT 72473
AsianBonytongue           --TTCAAACTCCCCCAA----CAGCTATAGTATGTTT-AAATGAGGTACTTATCCCTAAAAAGTGCTCAGCTGTGCAAAT 617
Reedfish                  AATGGAAGAATGGTCATTCTCTAACACCTTCACACTTCATAAGCTATACTAATTATTTTGAGAAAAAGTAATGCCACATT 30340
NorthernPike              A-GTAAGGCTTCATGGTT---CCGTGCATGTATTATTTGAATGTTATGAATATAATAGGTACTGACTCA---ATGCCAAT 4380
SpottedGar                --TTAAATCATCACAGATGTGCACTTAAAATAAGCTT-ATAAAAAGCAGACTTTTGTTCAAGATG--TAACTCTCCTACT 3827
ParamormyropsKingsLeyae   --TTAACTTTACAAGAA---AAAGCATGAATGCGGTT-GTGTGTTTATAGTATGTTTGTTACCCG-------ATATTAAT 91

Majority                  AATTTTGGTTA--TTAATACT-GCCAGTTGTCTT---GGAAGTTAGGTTAATTTCAATAACTCATTCTAC----CAGTTA

                                 72570     72580     72590     72600     72610     72620     72630     72640

Human                     TTTTTTTGTAGTTTTAGTAGAGACAGGCTTTCACCGTGTTAGCCAGGATGGTCTCAATCTCCTGACCTCGTGATCCGCCC 72553
AsianBonytongue           AATTGTAGGTAGATTAACACT-GCAAGTTGCTTT---GGAGAAGAGGGTCATCTAAACAAATAAATGTAA------GTTA 687
Reedfish                  CATCCTGCCCT--CCAAATCTGGCCCTTTGTAAC---TACCACTTGGACTACTTCTCTGAGCCATTCTATG--CCAGTCA 30413
NorthernPike              T-TCAAGGAGATAGCATTAATATTCTTCTGCCTT-GTGGAAGTTAGCTGAATTCCAACAATTCAGCATTCTGATCAACTC 4458
SpottedGar                A-TTTTGGTTT--TCACTACT-CACAGTTGTCT-----GAGGTGATTTTAATTGAAGTGCCTCGGCTTC--------TTG 3890
ParamormyropsKingsLeyae   AAAACTACATA--TTAAAAC--GCCAACTCTTTA---TTCACTCAAACTAAACAGAGCAAATCATTCTAC----CAGCGA 160

Majority                  CACT----TAGGCTATTTAGGTGTGTGAATAAGAATTTCAAGT--TGCCCTTGAATAGC-GTATACAAGGTTTTTCGGTA

                                 72650     72660     72670     72680     72690     72700     72710     72720

Human                     ACCT----TGGTCTCCCAAAGTGCTGGGATTACAGTCATGAGC--CAC-CGTGCCTGGCCTCATTTAAAATTTTTTAACA 72626
AsianBonytongue           CAGT----AAAGAATTTTAGATGAGTAAGTGGTGACTCCAACT--TGCCCTTGAGGTGT-GTGTACAAGGGGGTGCGGTG 760
Reedfish                  AAGT----GTAAATAGTAAAGTGGTTGCACAAGCAAGACAAAA--TATTTTACTTTAGTGGAATGTAATTTTTTTATTTA 30487
NorthernPike              CCCTCTTATAGGCACACTTGCTATGCTGACAAATGCTTTAAAAATTCTATATTAAAATCTTTGAAAAAGGGATTTAGATT 4538
SpottedGar                TGCT----TAGTATATCTAGG-ATGTCACTTTCAATTTAAAG----GCCCATGAAGAACAGTAGACAACATTTTTCGGTG 3961
ParamormyropsKingsLeyae   CACC----GCCGCCATTTAGC---GCAAATAAGAAACGCGTTT--TGCACCTGTATCAC---CAGCAAGCCGGTTCAGCA 228

Majority                  ATACAATGAATTTTTGAA--T-----TTGGGTCCT---TTTTAATAGGTTTGGATTACCACTTCGT--A----TGATTTA

                                 72730     72740     72750     72760     72770     72780     72790     72800

Human                     CTGGGTAAAATTTTTATT--T-----TTGAGTCAC------AAATTAATTTGAAGCTGTGTCTTTTAGAG----AGCTTA 72689
AsianBonytongue           ACACAGTGGGT---TGGA--------CCGGGTCCTGCTCTCCGGTGGGTCTGGGGTTCGAGTCCA----------ACTTG 819
Reedfish                  AAAAATCAAGTTTATCAGCAGTCAA-TGGGGTGAT-CGCCCCAAAATATTTCGATCACCACTTTGTATATCACTATTTAA 30565
NorthernPike              GCACAATGAATACCCTATGTTT----TTCAGTGCT---TGTTGTTAAGCTTGGACAGAAATTGAGTGAAC---TGAATGA 4608
SpottedGar                -TACAATGAATATTTGAAATCAAAGTTTACGTTTTTTATTTTAAAAGTCCTCTAAAACAACCTCAT---------ATTTA 4031
ParamormyropsKingsLeyae   ATAAAAAAAAA-TGTGGA--------CTCGGTCCT----TTAAGCGGCTTTGATTTATCGCCCCGC-------TGCTTCG 288

Majority                  GGTTGTCTTGAGAGCGATTGTCATTCTGTCTTGACTGTGTCTGTTTTTCTTCGT-----GAAAGCTTTTTGACATCTTAG

                                 72810     72820     72830     72840     72850     72860     72870     72880

Human                     TTCAACTCTTAATTCCATTATAAATAATATTTGAATGAATGTGTTTTACATCTG-----GAATGATTTTTATCATCTGAG 72764
AsianBonytongue           GGGTGCCTTGCGACGGACTGGCGTCCAGTCCTGGGTGTGTCCCCTCCCCCTCG---------AGCCTTGTGCCCTCT--G 888
Reedfish                  AATTGTAATAAGGGCAATTAATAATATGTCTATATTGACTTTAATTTTATTTAT-----TTTCTTTTTTAAACATCTTAG 30640
NorthernPike              TGGCATATAGGAAATGACTGGCACTTTG-CTTGACCATGTCTGCTATTCTTTGTTTAAAGAGAGATGAGTGACACCAGTG 4687
SpottedGar                GCTGGTCCCAACAAAAATAGTAATTCTGTCTTGACTGTTTCTTACTCTTATCGC-----CAAAGCATTACAAAAACG--A 4104
ParamormyropsKingsLeyae   GATTCAGTCCACAGAGCCGGTCTTCCTGCACAAACAGCACGTCTGTCATCCGGA-----GCCAGCCATCTGTCAACGTGA 363

Majority                  CGGGCGGA---TGTTGGACTGCGGTTCGT-CCTTCGGTGCAATTTGGGACTACCGGTGGAGAAGAAGTG---TTAGTGAG

                                 72890     72900     72910     72920     72930     72940     72950     72960

Human                     TAAAGGAAATATGTTTGATGGTAGTTGGGAACTTTGAAGGTATTACGGGCTACAGATGAGAAAAAAATG---TTACTACG 72841
AsianBonytongue           CTGCCGG-----GTTAGGCTCCGGCTC---CCCGCGACCCCGTATGGGACAAGCGGTTCA---GAAATG---TATGTAAT 954
Reedfish                  CGAGCTCA---AGCAGTGCTGCAAGCCGTGTCAGCTGTGCAAGCAGCAAGCACCCCTATATCAGGAGCA---GCCGTGAG 30714
NorthernPike              GTGAAGCATGGTGCTGCTTTTCTGTTTGT-CCATCAGTGTAATTTATCAAATTTGGTGGAGAAGGGTTGGCTTAACTGAA 4766
SpottedGar                TGGTTTG-----GTCGTACACTGTTTC---TGTTAAATCAATTTTAAGTTTGCTGCTGGAA--GAGGTG---TTAGTGGG 4171
ParamormyropsKingsLeyae   CGGGCGGAG--AGCAAGACTGAGGGTATGGCGTGCGGGGCTGTCAGAGACGGGCGGCGGGGGTACAGTG---TCCGTGGT 438
```

```
Majority                  TC--TCTGTGATGACA-CCTACCCAGTGCTTCGT---GTATATGCTTATTGCTTTAGT---ATTCTGGTGT-CTACACTA
                                   72970     72980     72990     73000     73010     73020     73030     73040
Human                     TCATTTCATGTTTACATACTGACCAGCATTTTCAATGAAATAAGCTTATTACTATAGG---ACTATAAAATGTTTTTCAA 72918
AsianBonytongue           TA---CTACAATGATC-CCCACAGGGAAATTCA----GTAAATG--TGATGCTGCATT---GCCCAGGCAT-CAAACCTG 1020
Reedfish                  TGAATGTGCAATAACA-CCTGCCCAGAGCCCCGTTCTGAGAATAATTATTGACAACAT---GTTCTACCCTGTTACATTA 30790
NorthernPike              GCCATTCTTGAGTAAAATGCATACAGTGCCTTCTGTATTCTGACCCCATTGCTTTATCCTGATTTTGTTGTTCTAGACTT 4846
SpottedGar                GC---CAGCAGGGGGCGCTCACCCTGTGGTCCAC---GTGGGTCC-TAATGCCCCAGT---ATAGTGATGG-GGACACTA 4240
ParamormyropsKingsLeyae   G----CGGTGGTGACACACTACTCCACACCCCGA---AAAGAAAAATAATCCCTTGGT---ATCCTCGTGC-CTAAACTG 507

Majority                  GGCTT---CTTTCAAG-GCAGTT-TTTATAAGGATTTTATCATTTATGCCTTAGTGCTCTG-TGGTCTT---TAAAAATT
                                   73050     73060     73070     73080     73090     73100     73110     73120
Human                     AGGTTTAACTGTCCAGGGCAGATACTTAAGACTATCTGATCATCCATTAAAAACTTTTCACATAGTCTTGCTTAAATGGA 72998
AsianBonytongue           GGCCT---CTTACAGG-GCAGGC------AAGAATTTTACCACTGAAGCAGCAGTGCACAGGTATACAT---AAAAAAAT 1087
Reedfish                  GATGT---CCTTCAAC---AGGTAAATTTATAGTTTTTATTATTTATGCCTTTGTGGATG--TGATTTC---CAGTATTT 30859
NorthernPike              AATCT--GTACTTGAATACATTT-TGTATAAGGTTTTCTTCCTTTCAGTTTTTTTGCTCTGTTCAGCCT-----TCCCTC 4918
SpottedGar                TACTG---TAAACAGGTGCCGTCCTTCGGATGAGCCGTAAAACCGAGGTCCTGACTCTCTG-TGGTCAT---TAAAAA-T 4312
ParamormyropsKingsLeyae   -GCAT---CGCACAAA--TAGA-------AGGGAACTTCAAATTATTGCGACAGTGCCCT--T--TC------AGGGAAG 564

Majority                  TTTATTGTGTTTATCTA--AAATTGTTGTCGTGTAGCTTTATCATTTAATTTATTTTTTTCTTCACACTTACTTATTAT
                                   73130     73140     73150     73160     73170     73180     73190     73200
Human                     TCCATTATGTTTACCCATTATATTGTTCTCAGCTGTAGTT-TTAAGAAATTTATTTCATTTGTTAATACTTACTTTCCAT 73077
AsianBonytongue           GTTATTGCATGCAAGTA--AATTTACTGACATGTACATTCATCAATTTAGCTGATGCTTTTCTTCAAAATAACTTACAAT 1165
Reedfish                  TTGTTTCTAGGTATTTG--TTGGTGGTGT-GGGTTGTTTTTTAATTTAAGACCTTTTTTTTCATCTCAAAGAT--ATTTT 30934
NorthernPike              CCTGATTGGTCTCCCCAG-TCCCTGTTGCCGAGAAGCATCCCCATAGAATGATGCACTCACCATCATGCTTCACCATAGG 4997
SpottedGar                CCCAGGGCGTTTCTCGA--AAAGAGTAAGGGTGTAACCCCAGCATCCTGGCCAAATTTCCCATTGGCCCTTACCAATCAT 4390
ParamormyropsKingsLeyae   TTTAATAAGTTTGATGG--AAACAAACAG-GTGTGACGTTAACATTAAATTGTACTTAAATGTTCACGTGTAC--AGTAT 639

Majority                  TACCTTCTGGTAATCTCTGTTAACCCATTGGTTCTGTAAGTC-ACTCTCTTTTTCATTTTTAACTGAAGTTTCTTGAGCC
                                   73210     73220     73230     73240     73250     73260     73270     73280
Human                     TACCTTCCCCTTTTCTGTGACAATCCGTTGATACTTGAAGACCTCTCTTGTATTCATCTTAGGGTTAATATTTTTAAGGC 73157
AsianBonytongue           ATTAGCCTTTTTACACTTATTCACTCATTTATACAGCTGGGC-ATTTTTTTTTTCTTTTTTAACTGGAGCAACTTAAGGG 1244
Reedfish                  CAAAGTTTGGAACTGTCCTAAAGATTATTACTTTCACAAAGA-ACAATCAATTTCAAGCTTTACTCCAGTTTAGTGACCC 31013
NorthernPike              TATTAGCTGGTGATGAGTGGTACCCCCCCACCCCACTAGTTGTATTGCCTGGTATTCAGGCCTTTTACAAACCCGATTCC 5077
SpottedGar                GGCCTCCTAATAATCCCCATCTATGAATTGGCTTCATTACTCTGCTCTCCTCCCCACTAATAGCTGATGTGTGGTGAGCA 4470
ParamormyropsKingsLeyae   TGCCCTGTGAAAGCCTCCGGTCACCCTGCGGCTATGACAAAA------CAAGTCCAATGGAAACGGACATGTATAGCGCC 713

Majority                  TAAGTAGT----GCTCTATGGTTGTT-ACTGTAGCG---TCCAAGTGAATTTTATACCTGTATGTTT---TTGGCTCA--
                                   73290     73300     73310     73320     73330     73340     73350     73360
Human                     CAAACAGCCTAAATTCTATGGTAATCAACTCCAGCCTTGTGTAATGAAATCTTCTGCATAAAGATAGG-TTTAAATCAAA 73236
AsianBonytongue           TAAGTAGC----ATGCT-CAGATGT--ACTGTACC----TACAAGTGAGATTTAAACCTGTAACCTT---TGGGCTCA-- 1308
Reedfish                  AATG-AAT----GCTCAACAAGCCAA-ACTGGTAAG---TACTTGAGAATTTTATTTTTATTTTTTT---TTGCCTGT-- 31079
NorthernPike              AAAAAAGTTGGGACGCTGTACAAATTGAGTAAAAAAGGAATGGAATAATTTACAAATCTCAAACTTATATTTAATTCACA 5157
SpottedGar                TTC-TGGT----GCACTATGGCTGCC-GTCGCATCA---TCCAGGTGGATGCTGCACATTGGTGGTG---GTGGAGGG-- 4536
ParamormyropsKingsLeyae   TCT---AT----GCAAA-TGGTCGCTCGCCTTAGCG---TCTGTTTGATGATGGTGCCTGGGGGTT-----AAGGTTA-- 775

Majority                  -TAGATTTCATAATTTA--TTAAACGTTTTGTTTTGAGCATCTGATAGTGTTAGCTTTTATGAATTG--TCATTATGTTA
                                   73370     73380     73390     73400     73410     73420     73430     73440
Human                     TCAGATTGCAGATTTTATTGAAGAAATTGTGTTTTTAAGAGTTGACAAATATATGTTGTATGGCTAAAACAAAGAAAATA 73316
AsianBonytongue           -CAGGCATCACTACT------AACCACTATGCTGCCAGCTGCCCCTTGTCTGAACTGCCGTGTCTT---CCACTGTGTCA 1378
Reedfish                  -TCCTGTTTATAATTAAGTTTAAATGTCTGCTTTTGTCAAACACATAAAATTAGCTTTGATTATTTT--ACATTTTTTTC 31156
NorthernPike              ATAGAATATAGATAACATATCGAATGTTGAAAGTGAGACATTTTGTAATGTCA--TGCCAAATATTGGCTCATTTTGGAT 5235
SpottedGar                -GAGACCCCATTACCTG--TAAAGCGCTTTGAGTGCAGTGTCCAGAAGTGTAAGCAATTATTAATTA--TTATTATAATT 4611
ParamormyropsKingsLeyae   -GGCTTTTCATAGTTTA--TTGGGCAATTAACTCTGACCGCAGGAAAGTCTGG--CAGAATGAACTG--TCAGTATCTGA 848
```

```
Majority                  CTTA--------GTATA-----TAGTTTCACAAAAAGTTCA-TGTCAGATCGCAATACAG-ATATGA-------AAACTA
                                   73450     73460     73470     73480     73490     73500     73510     73520
Human                     CTTCTGTTGCTTCTGCATTTAGTAGAAGAAAAACTATATATGTTTGTGACCAAAGTATAAAATATGATTCTTTCCAGGGA 73396
AsianBonytongue           CTTA--------GCATG-----TAGAAGCCCAAAGATTTC--TCTAAAATGG-AGTACAGCAGATGA-------CAACTA 1435
Reedfish                  ATGAATTAGAAGTTATAGGTGATACTTTCAGAGGTGGTTTAATGTCAACCCCAGATTCAG--TGTGG-------AAACTG 31227
NorthernPike              TTCA---------TGAGAGCTATACATTCCAAAAAAGTTGG--GACAGGTAGCAATAAGAGGCCGGAAAAGTTAAGGATA 5304
SpottedGar                TTTAAAATTACTGCATT-----TTTTTTCACAAAAAGCTCAATCTCAATTCGCAATTCAG-AGATGA-------AGACAG 4678
ParamormyropsKingsLeyaeCT-----------ATA-----CAGTTTTTCAGAAAATCC---GTGAGACTTTTTTGTAA-CCATTA-------TCAGTA 900

Majority                  TGTTACAGCTGTAGCAA------TCA-T--AATTTAAAATTTGCAATTC--TTAAGTAATTAGAATGTATAAAATTATAG
                                   73530     73540     73550     73560     73570     73580     73590     73600
Human                     GGTAAAGGTTATGCAGAAGATTTTCACTAGCAGCTCTAAAAGGCTACCC--TCAATTAATTGCCATGAAC-ATTTCATAG 73473
AsianBonytongue           AGTTCAAGCTGCTCCA-------CTA-T--GATTTAACATTGGCACTAC--ATATGCACTAAGAATGTATTAAACTCCAG 1503
Reedfish                  TCTGAAAACTGTCACAGAATTGGCCT-CAAAAAAAAAAAAAAACATTTT--TCCAGTAGACAAGGACCGGAAGCTGGTAC 31304
NorthernPike              AGGAACAGCTGGAGGA--------CC----AATTTGCAACTTATTATGTCAATTGGCAACATGATTGGGTATAAATAGAG 5372
SpottedGar                CATTCCCATTTTAGCAACAAC--TTA-TCAAATTAAACGTGTGAGGTTC--TTATACAAATAAAATATCTAATATCATAG 4753
ParamormyropsKingsLeyaeTTTGACAGCCATAGCAA------------AATTCATATTGTGCAATA---ATAAATAGCC-GAAGGAACAAAATGGCAA 963

Majority                  T--TTCAGAAGGGTGTTTGTGTCTT-CAGTATTAGGGTGTATTAGAGT-AAGTCATCCATT---TGTTCAATGTTGC--A
                                   73610     73620     73630     73640     73650     73660     73670     73680
Human                     C--CCTAGAAGGATGTTGGCTCATTTCAGTGTCATCCTGGTTTATTCTTTATTGTATTATTCAGCAGTCATTTTAACACT 73551
AsianBonytongue           C--TTCAGTGAAACTATGATTTATT-CACTAATAGCATATATTACAGCGAAGTCCGCCATT---TGTTCAAGACTAATTA 1577
Reedfish                  TCAGTTGGTAATGTGTCATGGCCTG-CGATTTTCCGTTATGTTTGACTTGGTACAGCGATCAA-TGACTGTAGTCCGCAA 31382
NorthernPike              CCCTTC---AGAGTGGCAGTGTCTCTCAGAAGTCAAGATGGGCAGAGG---ATCACCCATT---CCCCCAATGCTGC--- 5440
SpottedGar                TAGTTCAGATGGGTAGCTGCGTCAG-CATGCGTAGGCTGCAAAGGAAC-AAGTTATAGGTT---TATTCCATGCTGA--A 4826
ParamormyropsKingsLeyaeT------GAACGATGATTAAAACTT-CACAAATAAAGTGCATTTTAAT---GGCATTCGTT---TTTTCACCCTTCC--- 1027

Majority                  AT--TAGGCAGATCAGTGGCACAATATGTTG-ACAGTGTTTTCTTTTCCATGTGTTTTATAGCTTATGAAGATCATATGA
                                   73690     73700     73710     73720     73730     73740     73750     73760
Human                     ATGCTAGACACTTTAGAGATTCAGAAGAGTA-ACAGGGTTTCTGTTCTCATGAAGCTTATCAGAGACAGAAAACATATGA 73630
AsianBonytongue           AG--TAGGCAGATCAACAGGTCAAGGCCTAG-AAGGAGCAGTCTTTTCCAC-TGGATTATGTCTTCTGGAG------TGA 1647
Reedfish                  AT--CTGACATACCCCCACAATAAAAAGTTGTGCAATGTGATATCTGCTTTA-GTGTGAAAGCGAGTGAATAAAATGCTG 31459
NorthernPike              ----GGTGAAAAATAGTGGAGCAATATCAG--AAAGGAGTTTCTCAGAGAAAAATTGCAAAGAGTTTGTAGTTATCATCA 5514
SpottedGar                AG--AAAGAAGAAAGAGAACACAACGTTTCG-GCCGTGGAGCCTTCTTCAGGTGTGTCACACCTGAAGAAGGCTCCACGG 4903
ParamormyropsKingsLeyae-------------CACTGTCACAATATGTC--ATTATGTTCCCTTTTCTGC--ATTTTGGCAACAAAGAAGATCAAACTA 1090

Majority                  TTTAAATTATATTAT---CTATTTCAAATGTTTTGTGAATATAGTGTAGTTTTTTT-ATTTAATTGTTAGGTCTCTAAAA
                                   73770     73780     73790     73800     73810     73820     73830     73840
Human                     GTTAGATCTAATTGGAGGCAAACTGAAATATATAGTGGAGTTAGTGTGGTTATCAGCACATAAATGAGTGATCCATCAAC 73710
AsianBonytongue           GGAAAAGGATGTTAC---GTGTTTTATGTGTTCCATGAATAAAGGGCTGTCAGGTG-ATGTAACAGTTAAGGTCATAA-- 1721
Reedfish                  TCCTAGTTATCTTACA--CCATATCTGCTCTTTTCTTAATA-AGCCTGCTTCCTTT-GTCTCAGAGCAATTTCTCTGGAT 31535
NorthernPike              TCTACAGTGCATAAT---ATAATTCAAAGATTCAGAGAATCTGGAACAATCTCTGT-GCGTAAGGGTCAAGGCCGGAAAA 5590
SpottedGar                CTGAAACGTTGTGTT---CTCTTTCTTCTTTTTTTTCAGCATGGAATAAACCTATT-ACTTGTTCCTTTGCTATCTAACA 4979
ParamormyropsKingsLeyaeAAAGCACAAAATGA----GAAAGTCAAAGGCTGCATTACTATAATCTAGTTTTTAT-ATTTGATTGTCA--TATTTAA-- 1161

Majority                  ACACGTTTAATTTTAG--GGCTTTATT--------ATTTCATGTAGAACTGTT----ATGATATGTTATGATTC---TGT
                                   73850     73860     73870     73880     73890     73900     73910     73920
Human                     AAAAGGAGAAATTGGGAGGGTTTTATGGGCCAAAAACAGCATGATTAAATGTGATAGAGTATATGTCATGTTTTAGGTGT 73790
AsianBonytongue           --ACGTGTAATGTAAA--GG-TTCATC-------AAT--CATGTATAACCT--------AAAGTCATATGAACC---TGT 1776
Reedfish                  AAGCATTTAGTCCCATTTAGCTTTATTT-------TTTTGATGTAGAAGTGATTTGGTTGATTTAGTGTAAGTG---ATT 31605
NorthernPike              CCATACTGGATGCTCGTGATCTTTGGG--------CCCTCAGACGGCACTGC-----ATCACATACAGGAATGATACTGT 5657
SpottedGar                TCATGTCTTGTTTAAG--GGCTTTATTCT-----GATTCCTTGAAGGACCCCTTACCAGAAGGTGTTAGGATCC---TGC 5049
ParamormyropsKingsLeyae-GGCAACTATTATTGA--GGAATGATT--------ATCTTATCGAAAATTGTT------GAAATGTTCAGCTTC---ACT 1221
```

```
Majority                  AATCTAAATCTTATTAT-GTTTAAGTAATACTACTATTGAA-AC---TTTGAGTAACTTATTTCCTTGAAATTATTGTA-
                                   73930     73940     73950     73960     73970     73980     73990     74000
Human                     GATGAACATTCAGTTAT-GTGTGACGAATAGGATAATTGAAAAAA--TATGAAAGGCTATGATGCCAGAAAGTATTATG- 73866
AsianBonytongue           AACCTGAAGGTTGTTA--GTTCAAGTCCTGCTGCTGCTGTA-GCACCTTTGATCAAGGTACTTTCTCAAAATTACTCTA- 1852
Reedfish                  AAGCA-AACCGCAGTAT-TCCTATAATCAAACACTATAATAGAA---TTCTGTTAATATACTACAGTTACATTACTATA- 31679
NorthernPike              AATGGAAATCACAACATGGGCTCAGGAATACTTCCAGAAAACAT---TGTTGGTGAACACAATCCACCGTGCCA-TTCGC 5733
SpottedGar                TGTCTTGACCTGAGTAT-GTTCTTCCTGTTTCCCAAATGAATGCCCTTCTGAGCAACTGATCTCCATATCAGTATTGTAA 5128
ParamormyropsKingsLeyaeGGATT--AATTTATCAG-GATAAAGTAACACTACTATTGCA------TTGGAGTAAAAT-TCTCCTTGTAACTA--GAAA 1289

Majority                  -TGTGGCAGTTATAACTATTTATGTAACCTATTAAATATTGTTTAAATATGA-GAAGTTTTTTATGCTTTT--TCTTTTA
                                   74010     74020     74030     74040     74050     74060     74070     74080
Human                     --GGACAAGATCTTAAAACCAGTGTTACCCAGGGAGTATGAATTTAATATGG-GAATTCTTAAACTCCTTT--ATGACTG 73941
AsianBonytongue           --GTGG-GAATGCACCGATTTCTG-AATATGCTCAATATGCTTTGCATGCAA-GGAGTCCGCTAAGCAAAC--ACACTCA 1925
Reedfish                  -TCTATCTATCAAGTATGCTGATG-ACACTATGGCACTATGGCTAACTATGG-TTAGCTCGTCATCTTTTGGCTCGTTTC 31756
NorthernPike              CGTTGCCGGCTAAAACTCTATAGGTCAAAAAAGAAGCCGTATCTAAACAGGATCCAGAAGCGCAGGCGTTTTCTCTGGGC 5813
SpottedGar                ATGTGA-ATTTGTCTCAGTTGACATAACTGGCTATAAACTGATTAAATAAAA-AATACATCTTAAACTTATT-GCATGCA 5205
ParamormyropsKingsLeyaeTTGTAGCAGCTACAATTATTACAGTGGCCCTTTAAAAAATTGTTAAATAAAA-CAAGTTATATATCTATGC---ATTTTA 1365

Majority                  AAAGAACAATACTGTTTTGTATGT--CAAGGTTTGAAAAAATTTTAGAGGGACGTATAGATAATTA--CTT-CAGTAGAT
                                   74090     74100     74110     74120     74130     74140     74150     74160
Human                     GAAGATGAGCATCAGAGTGTCTGC--GACCATTTGATGATATTATGTACCAAGTTTAGATGTTTG-GCTTTTTTCAGGT 74018
AsianBonytongue           TAAGAGCAACACCGTTTT---CGC--CACGACGCGGCGGAAATTTCCGGCCACGTTCGGCCATTTC------CGGCAGAT 1994
Reedfish                  ACATCTCATTTCTGTTTGTCATTTAATAAAGAAACGAATCAATTCAGAGGTCTGAATCCTTAAAAACAGGGCTATTAAAA 31836
NorthernPike              CAAGGATAATTTAAAATGGACTGTGGCTAAGTTGAAAAAAGTGTTCTGTGGTCAGACAAATCAAAA--TTTGAAGTTAAT 5891
SpottedGar                AAAGAACTAGACTGTAAAAGATGT--TAAGGTGCCAAAAAAGTGTAGAAGGACATACAAATAATTAAGCAAACAACAGAA 5283
ParamormyropsKingsLeyaeAACAAATTGTGCTGATTTGTATGTAACAGGCTCTTAGAGGGGCTTAGAGTGGAGGCTGTATTGTTG------CAGTACAA 1439

Majority                  TTAGAACACATGATGCTTGCTCGAATCCGCACTTGAGGAGG--TTATTGACTGAAGTT--GATCTGTGTTGAGTACCGAC
                                   74170     74180     74190     74200     74210     74220     74230     74240
Human                     TATGAAAGCGGGGGATGAGTTAAGAACCACTGCTGTGAAGGATTCATCAACTATTTTTAGGCAGTTGGGTAAAAATGACC 74098
AsianBonytongue           TCCG--CACTCGCTTCCGCCTCCATTTTGTCCTCCTGGATG--TTGTTGGATGAA-----GAACCGAAACGAAGA-CGAC 2064
Reedfish                  TGAAGGGAAATGAAGTTAACTAGCAGTGGTCACTGATTAGG--AAAAGGGTTAGAATGAAAACCTGCAGCCACTGCGGCC 31914
NorthernPike              TTTGGAAAACTGGGACGCCATGTAATCCGGACTAAAGAGGACAAGGACAACCCAAGTTGTTATCAGCGCTCAGTTCAGAA 5971
SpottedGar                TGAGAATACATGATGCATGGTCAGTACAGCAGTGCAAGCAG--GTATAGTTCGAACC---GGTGTGTGTTGAGGAGCTGG 5358
ParamormyropsKingsLeyaeTTAAACCACACCATGGTTG--TGAGTCATCACTTGAAGAGG--TCAATGACTTATGCT--ACTCTGTATTTGCACCCCAC 1513

Majority                  GACTTGGCCTCAGATGATATACTCCTGCATCAGTG--------GGTAGTGTGACTAGACCTCTGGAATGTCAGGTTGGAA
                                   74250     74260     74270     74280     74290     74300     74310     74320
Human                     AATTTAGTTTTAAGAAACTGACTGTGGCTCCAGAGTATGTTGGAGAAGTGAAAATGGAGACTAGGAATAACAGGTGGGAG 74178
AsianBonytongue           GACGTCGC-ACGGA-AATATCCTCCGGCAATGGAC--------GGGTATGTG--TTGCTCTCCGGATT-----------A 2121
Reedfish                  CTCCAGGCCCGAAGTTTGACACCCCTGTCTTAGTG--------GGTAGAGAAAAAAGACAAGGAGTGACTAATGTAAACA 31986
NorthernPike              GCCTGCATCTCTGATGGTATGGGGTTGCATGAGTGTGTGT-GGTATGGGCAGCCTACACATCTGGAAAGGCACCATTAAT 6050
SpottedGar                AAGGTGGCTATGGATGATGTAGTCCTGCAGCTTCA--------GGGTGTCTGACTTCACCACTGAAATGCAAGGGGGGGA 5430
ParamormyropsKingsLeyaeCCCCATTCCCCAACACACACCTTTCATTCACAATA--------GACAGTCTTTTCATGGCCATGGAAGGTCTAGTTGTAA 1585

Majority                  ACTGATCGTTTAATTATTGTGGAGTACTTA---TTAGTGAGTAAGTGAAATGGTGTGTGTAAAGAAAGAAAGTGATATTG
                                   74330     74340     74350     74360     74370     74380     74390     74400
Human                     ACTATTAGTCTAATTAAGATGTAATTATAAATCTAAGCTAGGAACGTAAAATGAGAATGCAAAGTAAGAAACAAATATGG 74258
AsianBonytongue           ATTAAACGTGTGCATATTGTGGGATGCTTC---CTTGAGTATGAGTAGAACGTTTTGTGG---AAGCGAAAGTGATACTG 2195
Reedfish                  ACCAGTAAGTTATTTTTTATGAAGGAGTTAGGGTCAAAAAGGGAGAGAAATATGATTTGTACAGAAAGGAAGTAACGTCA 32066
NorthernPike              GCTGACAGTTATATCCAAGTTCTATGCTTGGATATGCTCCCATCCAGACGTCGTCTCTTTCAGGGAAGACCTTGCATTTT 6130
SpottedGar                AATGAGCAGGCTAGGAGTGTAGAGAAGTAA---CTACTGAGTAAGTAGGTTAGTGGGTGAGAAAAGGGAAATTTACATAG 5507
ParamormyropsKingsLeyaeGACGACCATTTAGCATTTATGAAGCACTT---------GAATAACTGCAGTGGTGTATTGTATGACTGAAAGAGATTCAT 1656
```

```
Majority                    CTAAA----TATTACGAAGTTACAGGCTGCAGCA-TTCTGG--ATGTGGCAGACTATAAG-AAGACTGGGGTAGTGTATG

                                   74410     74420     74430     74440     74450     74460     74470     74480

Human                       GGAAAATTATATGTAAAAGTAATAGGACTTGGCA-TCTTACTGATGTGATTGATTATGAGAAAAATGAAGCATGTGGAGG 74337
AsianBonytongue             AGAGA----AAACAGGGAGCTCCTGTCTGTGACA-CAGTGC--GTGTCACACAGCGTA---GAGCCGCCGTTCGTGTGTG 2265
Reedfish                    TTAAC----TATGTCGAGTGTGATCCCCTCTATT-TTCTGGCCATGAGGTATCCCTTAAC-TGAGCTGCGATAGTATTTG 32140
NorthernPike                CCAACATGACGATGCCAAACCACATACTGCATCAATTACAATGTCATGGCTGCATAGAAGAAGGATTTGGGTACTGAAAT 6210
SpottedGar                  ATAAG----TAACATGAAGTCAGAGGTAGCAGAG-CACTGA--AGACAGCAGTATATAAG-GAGAGAGGGATCATAGGTT 5579
ParamormyropsKingsLeyaeCCATA----TTTTAAACAGTTACCCAGTACAGGG-TCATGG--AAGGTAGGGACTGGAGC-ATATCTGAGGCAGTGTAGG 1728

Majority                    AGCC------GGGTCAGGA--TTTTGCTCAATACATGTGTATTGTTTATGTATTGTTGTCAGATTAGAGGCAAGAGTTGT

                                   74490     74500     74510     74520     74530     74540     74550     74560

Human                       AGTCCACTGGACAGTAGGAAATTCAGCCTAAGACTTGGGTAAGAGTTCTGTGGAGTTGTGAATTCAGAGGCCAGAGATGT 74417
AsianBonytongue             ATGC------GCATCACGCCTCTCTGCTCTGTCTGTGTGTTTTGTTTATTTATTTTTGTCTGTGTCGGGGAGGGGGGTGC 2339
Reedfish                    AACT------GGCCAACAG--ACATTGTCAGTCCTGGCATATCTTTTCCCCAAAGATGCCACATTCTCCTCTACTTTTGT 32212
NorthernPike                GGCCAGCCTGCAGTCCAGATCTTTCCCCCAAAGAAAACATTTGGCAGAGGAAGGTGCAACAAAGAAGACCCAAGACAGTT 6290
SpottedGar                  GGAT------GTGTTTTGG----TAGCTAAAGACAAGTGCAAAGATGAGGGGTTGCTAGCAGAAGAGGGGTATGGATTTT 5649
ParamormyropsKingsLeyaeACAC------AGGGCAGGAGGACAGCCTGGACACATGTACAATGGACAATTTAGAAATGCAGAAATTATGCAGAAACTAT 1802

Majority                    G--TTTCAGTATTTGAGTTCTGG------CAGGTTTAAGACA-CAT------TA-AGAGTTATAAATGCAATTTAATCAC

                                   74570     74580     74590     74600     74610     74620     74630     74640

Human                       GATATTTAAAATTTTGGTTCAAGATTTCCCAGGTATAAGAAAGCAAGAGGATTAAAGCATTGTAATTAAACTTTAAGCAG 74497
AsianBonytongue             ---CGCGAGTACCTGAGTTTGGG------CAGGTTTAAAAAA-CT-----------GTGTTACACACACAAAATAAACAG 2398
Reedfish                    C--TCCCTCCCTTTTTGCTCCTA------GCGAAGCAACACC-TG-----------CAATCAGTCACAGCATCTGG-CAC 32271
NorthernPike                G-----AGCTACTAGAAGCCTGTATTAGATAAGATTGGGACAACATT---CCTATTCATAAACTTGAGCAATTTGTCTCC 6362
SpottedGar                  A--TTTCCATCCAGAAATCCTGA------CTAGTTCAATACA-CAATATACCTAAAGTGTTAAAAATGCAAATTCTTTTT 5720
ParamormyropsKingsLeyae--------GTATTTGGGCTCAGG------AGGA---AACACA-CAT---------AGAGCACTGAGAGGAACTCAATCCC 1855

Majority                    TCAAACTTGG----ATATATAAGGTTTGATAGATATCT---GGTCTTTCTTTATTGAAGCAAGCAGTTATAATTTAAGAG

                                   74650     74660     74670     74680     74690     74700     74710     74720

Human                       TGCATATTTATGTTATAGATAAGATAAACAAGAAATCTAGGGATCAAATAGGATTAAAATTAGTAGTGATCATTCAGTAC 74577
AsianBonytongue             ATAAA--TAA----ATAAATAATGGCTGTTTTTTTTTT---TTTTTTTTTTAACTGAAACTTGTAGGAAGAGAGCAAGAG 2469
Reedfish                    CCAAACTTGA----GTTTTTTAGTTCAGTTGCTTACCA---CAACTTACTTAATT--AGCCAGCTGCTTTTCCTTTTGAT 32342
NorthernPike                TCAGTCCCCGAGACGTTTGCAGTCTTTTATAAAAAGAA-GAGGGGATGCCACACAGTGGTAAACATGGACT-TGTCCCAA 6440
SpottedGar                  TTAAAGTTGG----TGAAATCGAATTTAAAAAACATCA---GGACTTTCCTTGTCGAAACA-GCAGATAAAATGTAAGAG 5792
ParamormyropsKingsLeyaeACAACCATGG----AGGTGTGAGGCAACAGCGCTATCT---GCCTTGCGTATATCTGAGCA-GCAGTTATA-TTTAAGA- 1925

Majority                    ATTTTTTTGGATATATTGTTACCTTGTGATTATAAGTCTTATTATATTTATTTTTAGGTCTAAG------------GGTA

                                   74730     74740     74750     74760     74770     74780     74790     74800

Human                       AGTAGTTACGTACTGTTATTCACAAGAGTATATAAATCAAATTACAAGGAATTAAGGATATAAACGTGA-TAAGAAAGTA 74656
AsianBonytongue             GG------GAACAGAATGG-AGCAGGTGGCCA-GAGCCCCACTTGAGTTTTTCTTAAGC-------------------TT 2522
Reedfish                    TAGGTTATGTTTTGCATGCAAATTTGCAATAGCAAAACTTATTATAAACAGGTATTTGGCTTTG------------GGCG 32410
NorthernPike                CTTTTTTGAGATGTGTTGATGCCATGACATTTAAAATCAACTTATTTTTCCCTTAAAGTGAAAGATTTTCTCAGTTTAAA 6520
SpottedGar                  ATCTAAATAAACAAATTTTTATCTGATGTTTATAAGTATTAAAACATTTTGTTATAGGTCGAGG------------GATT 5860
ParamormyropsKingsLeyae-------------AATGGGGCCTTGGGAACG-ACGTTTTGCTGGTGGTATTTTAAGGAC------------------AA 1972

Majority                    TGTTTTTTTTTATTTGTAGAGTTTTAAAATAAATGAGGAGTATT--AATTT-ATGATAAGTTTT---TAAATTGGGAATT

                                   74810     74820     74830     74840     74850     74860     74870     74880

Human                       TGCACTGTACTCTTTGAGGAAGTTTGCCATAGAAAGGAAGAAGA--AATAGGATGGTAGATCAGAAGTAAAGCAGGACCC 74734
AsianBonytongue             TCTTTTTTATTATTTAAAAAAAAAAAAAAATTAAAGAGAGAC-----AGTGA-ATGATACCTTGT---TGAGCAGGGAATA 2593
Reedfish                    TTTAAGTTTTTTTTTGGGGTGTGTTAAAAGAAAAGAGGAGGATTCTCATTTT-CAGTAACTTTT---T-TACTAGTAATA 32485
NorthernPike                CATTTAATGTCATCTGTTGTGTTCTGAATAAAATATAGACATTTTAAACTTACACATTGTTTTTATTCACAATTGTACAG 6600
SpottedGar                  TGTTATCTTTTAGTTTCAAAGTTTTAAAACAAATGAGTGATGCCTTAGTAACGCAATTAGTTAC---CAAATTGGGTGTT 5937
ParamormyropsKingsLeyaeTCCAGCCGCATAATTCTAAAGAAATATAGTTCATGTTCATT-----ATTTT-----TAAGTTTT---CATTTTGGAAATT 2039
```

```
Majority                   TGTGTAAT---TTTTTTTAGAATCGCATT-GTGTATAATTATTTTTTTAAG-CATTTTTTATTGTTCTCAGGTTGAGTG
                                   74890     74900     74910     74920     74930     74940     74950     74960
Human                      AGTGGGGGGAGTGTTTGCAGTGAGGCAGT-ATGTATAATCATTTAAAACATG--GGTTTGGAGTCCTCTCAGGTTCCATG 74811
AsianBonytongue            TATGCTCT---TTCACCCATATTATTAAA-GTGAAGGACCATTTTCTTTCCCACATTCTTTGTTTTGCTCTAGTTAGGCA 2669
Reedfish                   AATATCAT---CTTTTCTTGAATTTAATTAGTGGGTTATACATTTTATTTATTCAGTATTTATTACAGTAAATTGGAGGA 32562
NorthernPike               TGTCCAAA---CTTTTTTGGAATCGGGTTTGTAGTTAGATTTTGGTCTCATGAGACTTGTTAATGTTCTCATGCTCAGAG 6677
SpottedGar                 GGTGTAATAAATTTGTTTAAAATCCAAATTATAATTAAGTAGTTTTTTTAAC-TATTTCTTCTCAAAAACACATTA--CA 6014
ParamormyropsKingsLeyaeGTTTTAGTG--CTTATTGTAGACAGCTGT--TATATGATTTCTATTTTTAAA--GTCTTATGTCGAGCTACGGTTG--TG 2111

Majority                   TTTTCAATGTTTGTCAATGCAATATGCTTTTTAAT--ATAGTTGTTTTTCTCTTTCCA-T-GA-AATG-AGGGTTTTCTT
                                   74970     74980     74990     75000     75010     75020     75030     75040
Human                      TTTGTAATGGACATAATGATAATAATCCCTTTCATTTAAGGCTGTTGTGAGGATTAAATGTGTTAATGTGCAAATAACTT 74891
AsianBonytongue            GTGTCTATTTTTGCCAGTCCAATATGTGGTTAGAC--ACAGGTGTCTTTTTTTTT------------------TTTTTTT 2729
Reedfish                   ATGTCAGGGTTAG------CAAAATATTTATAAAC--ATAATCCTCATCCTCTCTGCAGTGAACGATT-ACCAGATTATG 32633
NorthernPike               TCCTTCAGGCGGC---ACGTCATATGCCTTTTACTCAGGAGTGGCTTCTGTCTAGCCGCTCAACCATAAAGGCTGGATTG 6754
SpottedGar                 GTATCAATCCTTTTGCATGCATTATGCTCATTAAA--ATTGTCATTTTTATCTCTCATACAGA-ACAA-ATAGTTTTCTT 6090
ParamormyropsKingsLeyaeTCTAAGAATTAAGACAATACACTAAGGTTTTTCG---GCTATAGTCGTTCAATGT------GG-ACCG-GGGGCTTTCCG 2180

Majority                   TCCACGCTAGTCTGGTAT-TGTTATATTTC-GGCTTGCTGCTAACTATTATT----TTGTATTAAAGAAGATTTGGTTC
                                   75050     75060     75070     75080     75090     75100     75110     75120
Human                      TACACAGT-GCCTGGTATATAATAAATGCTT--GCTACCTATTAACTAGTATT----TGTTTCTAAGGCTAATTTAAGTC 74964
AsianBonytongue            TACACATTAGTCTAGTAT-TGTCATACTT----AGATGTGAGTAAGAGTTTGT----TTG-----AAGAAGATGAGGTTT 2795
Reedfish                   TGAACAAGACTCTAATTTATGATGTACAGGTCCACAGTCTGTTATCAATAACC----CCAAATCAAAAAAGCTTTGAAAA 32709
NorthernPike               ATGGTGCTGCAGAGATAGTGGTCCTCCTGGCAGGTTCTCTGCTGAGAAAGGTTGAAGCTCTGTTAGTGGCCATTGGGTTC 6834
SpottedGar                 TCTATGATAAAAAGGGAT-TGATACATTTTCTGAATTGTTTCCAGATATATTT----TTGGACTTAAGAACTCTTGGTTC 6165
ParamormyropsKingsLeyaeTCATCGCTCGGCTCTGTT-CGTC-TATTTCCGTGCAGGCCCCCGGCTGCCATT----TTGT-TGTCTGACTTGTTGGCTG 2253

Majority                   TTAGG------GTTTGTTATTTTTAGATTATATT--TAAAGTGGATACTTT----TT-----CTATCAATAGAGAGATGC
                                   75130     75140     75150     75160     75170     75180     75190     75200
Human                      CTAGAATT---GATTGCAAGGATTAGATCAGGAG--TATAGTGGACATGTTGGGATTTAAATATTTAAATATAGAGATGC 75039
AsianBonytongue            TT----------TTTTTTTTTTAAATTTAAATT--TAAAGCAGTCAGTGG----TT-----CTGAGAAAAGGACTCTTC 2853
Reedfish                   ACAAAATTTCCCTTCCCTACCTTTTGTCTAGGCT--TTACATCAACACCTT----TTACACATTAGCGATCAAGAAATGC 32783
NorthernPike               TTGGTCACCTAACTCATTACTTACACATTCTATTAATAAAAAGAATCCACCTTTCTCCAGCGCTAAACGCTGTGACTGCC 6914
SpottedGar                 TTAAG------GTTTCTGGTTTCTTTATCATAAC--TAATGCCACTTGTTT----TT-----CTATTAAAATAATGAAAT 6228
ParamormyropsKingsLeyaeAGGCG------CGGTGCTGTAGTTAGCAGGAACC--CAGCGTAGATACGTT----CT-----CCGGCAATGGACGGGTAC 2316

Majority                   TTTTTATGGCTTATGTTG----CCGAGGTGATTTTATAGCAGCTTTTCACGA----TTAGGTCTTTTTGTTGAGCATGTT
                                   75210     75220     75230     75240     75250     75260     75270     75280
Human                      TTTTTAGGACCATTGTTAGAACCAGAAGAGATTTTTTACCAAGTTCACACAGAAATGTAGGTGCATTGGCTGGGCATGGT 75119
AsianBonytongue            TTGCCATGACTCATGGAG----CAAAGGTGGTCTGACC-CAGCAGTTCAGGA----TGGGGGCTTTTGGATGAACACACA 2924
Reedfish                   TTCTCATAATTGTTTTTG----CCATCA-GTTTTCAAATGAATAGGCTGCAG----TCTGCTCTTCCTGTTGAGTGTGTT 32854
NorthernPike               CTGTTCTGGCACATTATG----CTGGGGTGAACCAATCACAAAGCTGTCACAGAACTGATGCCCGTTAGTTTAACATGTT 6990
SpottedGar                 AATTTAAAGAATAAGAGC----AGAAGATAAAATTATAGCAGCTTATAAAGA----TTAAAATGTTTTAATTGTGTTAAA 6300
ParamormyropsKingsLeyaeGTATCGAGGTCTGTGTCG----CCGGAG-------ACAGCGGCGTCTCA-------TTCGGTTTATTCCTTGACGTCTCC 2378

Majority                   GTCATA-CACTCTCAATATCGGAACTCGG-AATGTCGCG-TACATGGGTCACTTGG---TGCCAGAAGCTAAACCAGAGC
                                   75290     75300     75310     75320     75330     75340     75350     75360
Human                      GGCTCA-CACCTGCAATCCCAGCACTTGGGAAGGCTGAGGCAGAAGAACTGCTTGA---GGCCAACATTTTGAGACCAGC 75195
AsianBonytongue            CACACA-CACACACACCATCTGAAACCGC--TTGTCCCA-TACG-GGGTCGCGGGG---AGCCGGAGCCTAGCACAGGGC 2996
Reedfish                   ACAATA--TCTATCCATTTTCCAACCCGCTGAATCCGAA-CACA-GGGTCACGGGGGTCTGCTGGAGCCAATCCCAGCCA 32930
NorthernPike               GACATT----TCTGGACAGGGAAACTCGA-AATTCAACTTTACATGGATCAGTAAA---GGCCAGTAGAAAAGCTAGAGC 7062
SpottedGar                 ATTGTATCAGAAGCAATATACAGGTTGATAATCATTGTG-TCCATGGATGATTTTT---TTTCAGTCACTAAATCATATT 6376
ParamormyropsKingsLeyaeGTTAAG-CGCTCACAATATAGCCAGTCGG----GTCGCG-AACTTGGGTTACT-------TTTAAAAGTGAAACGGCAAT 2445
```

```
Majority                  CTGCAGGAC----GGAGGGGACAAATCCAGGTTAGGGGTTCAGTTTGTCTGAAGGCGC---CC--TAGATGGCAAACTCA

                                   75370     75380     75390     75400     75410     75420     75430     75440

Human                     CTGGGCAACATATTAAGACCCCGTCTCCACCAAAAAAAAAAAAAAAAGAAGTAGGTGCAGAGC--TGGAAGCAGAACCGA 75273
AsianBonytongue           GTG-AGGGG----GGAGGGGACAAACCCAGGACGGGACGCCAGTCCGTCGCAAGGCAC---CC--CAAGTGG--GACTCG 3064
Reedfish                  ACACAGGGCACAAGGCAGGAAACAATCCTGGGCAGGGTGCCAACCCACCGCAGGACACACACC--CACACACCAAGCACA 33008
NorthernPike              AAACAACAATGCTGCTAGTGTCTGATTACAGGTTGTTGTTTGATTTGACTGAGTTTGCTATCTAGTAGGTGAAAAGATCA 7142
SpottedGar                TTGCAGTAC----AGATGGACTAAAATGAACTTGTAGGTTCAGTTAATCTTGAA-CGT---CC--CTTATGT--GATTCA 6444
ParamormyropsKingsLeyaeCTG----------GA--GGGACACAGCTTGCTTAGCCCTTTAGCATGTTAGCATGCGA---GC--TAGCTGGCCAAACTG 2508

Majority                  AACGCCAGCTGTTTTGGAAATCATGATTTGCTTAAACCC-A---CTTTTATCTGTGGGTAGACACCCTCGATGACTGGTG

                                   75450     75460     75470     75480     75490     75500     75510     75520

Human                     AATCATCAGTGTTACAGTCATTATTCTTTCCTGTCACCATTATATGTCTTTATGAAGCAAGGGAGAAAGAAGAACAGATG 75353
AsianBonytongue           AACGCCAGACCCCCTGGAGAGCAGGACTCGATCAAACCC-A---CCGCGCCCCCTGGATGAACAC-----AGGTTTTGTA 3135
Reedfish                  CACTAGGGCCAATTTAGAATCGCCAATCCACCTAACCTGCATGTCTTTGGACTGTGGGAGGAAACCCACGCAGACACGGG 33088
NorthernPike              GTAGCCAGCCTGCATGGCAACCACG-TTTAATAGAATTA----ACAATTATTTTCGCATTGCAACATTCAAAAAATAATG 7217
SpottedGar                GA------ATGTTTTGTTGTTCGTTACCTGCTTATTTCCTA---CTGTCATTCATAATTAAACTGCCTTGATAGTTCATA 6515
ParamormyropsKingsLeyaeAACGGAACTTCTCCTGGAAATCATG--CTGGTGGGAGGC-----TT-TTATCGCTGGGGAAACGCTGG-GATGGCAGGTG 2579

Majority                  AAGTGAATGTGG----TGGCACAAGTTAAGAGCTATG-AATTGAACTTGTATCTATTAA--TTATTGGGTAGCT-CGATA

                                   75530     75540     75550     75560     75570     75580     75590     75600

Human                     AAAGAAGTGAGGATTTTGAAGTTGGTTGAAAGATTTG--ATTGAATTCTGATCTAAAAA--TTATAAGGCACTTGTTTAA 75429
AsianBonytongue           AAAT-AAATTAT----TAGAACAAACTAAGAACTATA-AACTG---TTGTACAGATCAA--TAAATGGATAATT-AAATA 3203
Reedfish                  GAGAACATGCAAA---CTCCACGCAGGGAGGACCCGGGAACCGAACCCAGGTCTCCAAA--CTGCGAGGCAGCAGCGCTA 33163
NorthernPike              ACCCGGCTGTGTGTATTGGCACATGACAAAAGGAATG-AATGAAAATTGTATGAATTGACTTTAATGAAAATCTTCCAGT 7296
SpottedGar                GTGTTAAAGTGG----TGGGAGAAGTCATGTGCATTGCAATTAACCTTTTGGTGTTTAA--TTATTGAATACCT-AGAAA 6588
ParamormyropsKingsLeyaeCTGAGCACATGG----TCGTGTAACTTTAATGATAGAAAACAAA----GTACAAGTGCG--GTTCTGAG--GCT-CCCGT 2646

Majority                  C----CTGTTGCACATAGGATAGTGTTTAT--GT----CTGCACTGCTTTTTT--GAATTCAGATAGCCCAATTGTTAAT

                                   75610     75620     75630     75640     75650     75660     75670     75680

Human                     C----AAGTTGAAAGTAGGAAAGTAGACATAAGA----CTCTACTAGATTTGGGGAAACTCTCAAAAATGGACTGGAAAT 75501
AsianBonytongue           C----CTGTAGCAGGTAGTGTACTGTGTA-----------GCACTACCACTGT--GTAGTCAAAGGGCTCACGTTTGAAT 3266
Reedfish                  C----CCACTGCGC-CACCATGCCGCCCGT--GT-----TACAATATGCTTTT--AAATAATTATAATCAAATTCATAAT 33229
NorthernPike              CATTGTTATTTTACATATGTTAAGGTTTTATAAAGGCCCGGCAATGCTGTATCATGAATACAGTCAGTCAAATTGTTTGC 7376
SpottedGar                T----TAGCAGTGTAAAATACTTTGATTAT--TG----ATGTACTGTTTTCAT--TTCATCAGATCACATAAGGATTTTT 6656
ParamormyropsKingsLeyaeC----CTTTAGCTAATGGGTTAGCGGTTGT--GT----CTGCGATGCGAGCA---GCCGGCGGAGAGCCCGACGGGAGGC 2713

Majority                  GTTGCTTATTGT-----ATCTTTTA-TTGTTTATCTAAGGTA-ATTAGCT-TGTGTTTCTTCATTATGCTATTAGTCAA-

                                   75690     75700     75710     75720     75730     75740     75750     75760

Human                     TCAGCTAAAAGTGGATAACAAAATATTTCTAGAATTAGCATTTGTGGGGTGTGTGTGTTTTCACTCTAGTATTTGTCAAG 75581
AsianBonytongue           GCCACCTCCTGC------TCTAGTA-TCGTCAATCTAAAGTA-CTTACCC-TGAATTGCTGCAGT-TGAAATTACTCAG- 3335
Reedfish                  ATTGGTAATTGTAAGAAATCATTTAATTGTAAAGCTAAGTTGTTTCATAT-AGACAGTCATCATTTTGACAAGAGTAAT- 33307
NorthernPike              CTGGTCTGGTT---GTTATTGTTTACTTTTTTGTAGGAGGTAGACAAACTGTGGGATGTTTTTAGATCCTGTTTTTAGAC 7453
SpottedGar                CTTAAGCATTCT------TCTTCCAGTTGGCTGTCTAACATA-ATCTCCC-ACTTATCACACATTATGTTGCTACTCTG- 6727
ParamormyropsKingsLeyaeGCGGCGCTCCG-------TCTCGCA--TGCTTTGTTATGG---ATTAGCC-TGGGTTTCTTTGTT-TACTATTGGGAAA- 2778

Majority                  --CTATATGAAAGTTTAGTAAGAATTTAGGACTTGGTTTCTTTTGTGTCTGGTATAATGTATTATGTAAATGAGTAATAG

                                   75770     75780     75790     75800     75810     75820     75830     75840

Human                     C-CCAGATGAAAGCATAGACAGAATGTAAGACTGGAT-TTATCTAAGTCTGGAATTGTGTAACAT-TAAAGGAATAGTAG 75658
AsianBonytongue           --CTGAATGAAGGATAAATAAGTGTAAATAGCTTAACACCAGTTTTATTTAGAGAAAAGAGTAATCTAAATAAGTAAAAG 3413
Reedfish                  --ATTTAAAAAAAAAAAAAAAAAAATCAGGAGTGTGCTCCCCTCGATTTTCTTGTACTATATACTGAGATAGAGGAAAAG 33385
NorthernPike              CCTTTTACAGTGGGGCTGTTAAACTTAGGTTCTGGGTGGCTTCTGTAGCAGCCAGAACTATTTATAAGAAAGTGTTGTAC 7533
SpottedGar                --CCATGGCTTTCTTTGGCTACGGCACAGACTCTGGTATCTTTCAAGGCTAATATTGTGGATTATGTCAGGATATGATAG 6805
ParamormyropsKingsLeyae--TAAATCCCCATTTTAACATTTATTTTCGATTTTTTTTCTCCTCTCCCATGTAACACCCCATATCTATTTAAGCAATAA 2856
```

```
Majority                   GCAACGAGTAGAGTGTTGG--GTTACGCTATGTTCG----ATCTCACATTTCTCTTTCTGGTTAAAATCA-----GGTCA
                                   75850     75860     75870     75880     75890     75900     75910     75920
Human                      CAAATGAGCAGAGTGTTGG-CTCAAGCCTAAGCTTG----AGCCTAAGCTTGACTCTATGG-TAAAGTCA-----AGTCA 75727
AsianBonytongue            ATAGCAGGTTGGGTGTTAG--GTCACGGCGTGAAC-----ATATAACATGAAGGTAGAAGGTTAAAATCT-----GA--- 3478
Reedfish                   GTCACCAGAACCGCTTCCA--GTTGCACAATATTTCTCAAATATCATATTTCTGTTTCACGTCATAACTAATTGAGAATA 33463
NorthernPike               TCTGCAAGTTGGCCTCTGGGCATTCTGGTATGTTCTCGTAATCCTAAACGTATCCTACAACTGTTGAGAAGACTTGGT-- 7611
SpottedGar                 GCACTGCACACAGTGTCAG-TATTTCTTAGTATTA-----ATGTCAC-TGCTGTCCAATGGCTCAAATAA-----GACAG 6873
ParamormyropsKingsLeyaeGCCACGTTTCGAGGGT-----GTTACCCTATCCCCG----ATTTCGCTTTTCCCCGTCTGGTTGCTGCCG-----AGCCT 2922

Majority                   TGGAAGTCAGCAATA--ATGCTCCTTTGTATTTTA--CTTTCTTTGTCTGTTATG---AAATTGTTAGTTCACTAATATT
                                   75930     75940     75950     75960     75970     75980     75990     76000
Human                      AGGGAGAATAGAAAGGGGGTCACCATAAAGGTCAAAAGTGGGTTTAGTGGTTGTGTGGGAATAGGCAGATCAAGAAAA-- 75805
AsianBonytongue            TGGAAGTCTGCTGTG--GTGCTCATCTGAAAGGTA--CTTAATTCGATTATTGTG---AAATTTTTCTGTCGAAAAAATT 3551
Reedfish                   TAGATATACATAATATTATATTTTTTTTTTTTTTGGTCAAAATTTGTCTGTAAAACTCAAAGTGCTTGTTCATTGTTATT 33543
NorthernPike               ATCAGGTCATCAAGATAAGGCTACATTTTAAGTTG--TCATTTCTAGCTCTTATG---ATATTAGAGCAACAGTAATTAA 7686
SpottedGar                 TAGAAATAAGCAACA--AAACTT-TCAGTATAATA--CATGCTATGAAAGCAAGGC--AAAATATAATTTTACTTAGCCT 6946
ParamormyropsKingsLeyaeCGCCTGTCAGCC------CGCCC-GCCGAGCATCA--CTTTCCCCGTCGGTGAGG-----------GGCTGCGGCCGTT 2981

Majority                   TTATGGATTTATGAATGGCCATTTCAGTATGTTATAAG-C-TGTTCTTGAATGTTAAAGAGTTTTGATTGTGTATACATG
                                   76010     76020     76030     76040     76050     76060     76070     76080
Human                      GAATGAAGTTAGGAAAGGAGATATAAGTGTTGAATGAC---CATTACAAAAAGAGACAGAGGAAAGAA-AAATGAAGATG 75881
AsianBonytongue            GCGTGTATCTATTTACAGTGAATTTGATATGTTTCG-----TATTCTGGAACTTGAAAAAGTGTATGTTA-GCAGAAATA 3625
Reedfish                   TAAAAGACACGTGGGGAGCTAATCCAGCCCTTGATAAAAC-AGTGTTTCACAGATAAAAGGTTTACTGTGAGTATAAAGT 33622
NorthernPike               ATAGGAAAAAACAAGTGTCCATTCCAGTTAGGACTAAGCC-TGTTCCCTTTTATAAATGTGTTCTGATCTTGTACCCTCC 7765
SpottedGar                 TTTCACATTGGTGAATAATCATTTTTGTATGCTTTAGTTTTTATTGTTAAAGTTTTAGGAACATTAATTGTATAGTCTTG 7026
ParamormyropsKingsLeyaeTTGTGGGCGGACG---GCCCACCTCGACATGACACAGGGC-TGTGCTCGGGTGCTCTGTGCTGTTCGT-GTGCATGCATG 3056

Majority                   TATTTTATGTTTT---TTAAATAGGTGTGTCAGTCTCTATTTTAAAGTA--TTCAC----TGAAACCTTTAT-GTTGCAT
                                   76090     76100     76110     76120     76130     76140     76150     76160
Human                      TATCAAAAGAAGTTGCTAATATGGATGGCAAAGTAGATGTTTTTAAGAA--ATCAT-----GAGACCAGAGTCTTGGAAA 75954
AsianBonytongue            TATTTTTTATGTT---TTCAACAGCTTTTTCAGTGT--ATACTAAAGTT--TCAAC-------AGTCTTCACCGCTGCAT 3691
Reedfish                   AATTCAGTAGCCTACATAAGAAAGGCATATAAAATCACTTGTGAATGCA--GTCATT-AATGAAACTTGTTT-TTTTCGC 33698
NorthernPike               TAATCC--AGTTTGTGTCCAACAACTGTGTTTCTCTCTGCTGCAAAACAAATTAACTTCCTCTGAGGGAAATACTTTTAA 7843
SpottedGar                 TATTTTATGATTG---TTAATTAAGTGCATGAGTCATAATGTCAAAATT--CAAAGC-CATAAAACCATCAT-GTAATAT 7099
ParamormyropsKingsLeyaeTATGGC-TGTCAT---TTGAATAGTTACGTCGTTCAC-ACATGCATATA--TGCAC--------ACGCATAT-GTCGCAG 3120

Majority                   AATCA-AGGTTGCTGT-GGCAGTTGTTTTT-GAT-AATGAGTGTGGGTGTTTAATTCTTTATTTTATATGAAAT----AG
                                   76170     76180     76190     76200     76210     76220     76230     76240
Human                      AGTCATAGGATGATGCAGGGAATGGAGAAGAGGGAAATAAAGCCAGGTGCTGAAGTCTTTATGTAATGGGAGGAGATGTT 76034
AsianBonytongue            TCTGG-ACATT-ATGT-AGCACTCATTTT------GGTGACTTTTTGGATTTCATTCCCCATTTTGTATGAATT-----G 3757
Reedfish                   AGCTA-TATTTTCTGTTTGTACAAGGTTTT-GATTCACCAGTGTGGATGCATTTATTTTTAATTTTTGTTAAAC----AG 33772
NorthernPike               AAACAAACAGTCCTTT-GATAGTGTTATTGGGCTTTGCCTTTGTGTTTATTTAAT-CAATATTCCATATCCAAAAGAAGG 7921
SpottedGar                 TATCA-AGGGTGATGTTAGCAGCTGTTTTT-TAA-AATAAATATGGGTGATTATTTTCTCTGTATATACAGAATCTGGAA 7176
ParamormyropsKingsLeyaeAACTG-CTGTT-CTGC---CATTTAATTT-------ACGGGTGTGAACAGTTAAT----------ATTTAAAAC------ 3172

Majority                   TTAGTTGTTTATAATCTGCTTTGCATTGT-TATATTTTTATACCAGGGTGTTACATTGAC---CTAGTCATCCTTCATGA
                                   76250     76260     76270     76280     76290     76300     76310     76320
Human                      CCAGTAATCCAATGGCTATTTTG-ATGGGAAAGAGTGTGGTATGATTGGGTGGCATTGACA---TCGGAAGCCATCCTCA 76110
AsianBonytongue            CTAGCAGTTTATAATGTGTATTGCAGTAT-TATATTTTAAT-CCAGGATGTTACACTGT----CCAGGTGACCTCAGCAA 3831
Reedfish                   GTAAATTTTTAGAGTCTACTTTAAATTTTGTATTATTTTGGAGCAAATTGTTTTATTGATTAGTTGCTGATCACTGACTA 33852
NorthernPike               TTAGTTTAGATAAACATTCTTTACAGT---TTTATTCCCAG-CCCGGGTGTGAGTTAGACCTAGTTGTCACGTTTTATGT 7997
SpottedGar                 TTGCCTGTTGATCATCTGAGTT-CATTGC-TGTGGCTCTTTACCAGGACAACATAGATTGGTACCAAACATCTCTCAGAG 7254
ParamormyropsKingsLeyaeCTGAACGTGTATAACGTGCTTC--------CCCGCCCCCAA-CCAGGCCGTTACAGGCG----------GGCCTTCGGGC 3233
```

```
Majority                  TTGATTGTTTTTTCTTAACAGTATT-GTCGTTTATGGTCCTGTGGGGGA--AGTGGTAAGCTCTATTTTGTTTATTTTTAT

                                  76330     76340     76350     76360     76370     76380     76390     76400

Human                     TTGATGGTGGTGGAACAGCAGTTTGAAAGTAACATTGTGCGGTGAGGTAG-AGTGGCACATGATGCATCCTTATTCTTAC 76189
AsianBonytongue           TGACTTTTTTTTCCTTAACAGCATT-GTCACTGATGTTCCTGTGGGTGT--AAAGGTAAGCTCACTTCTGTTTATGTTAA 3908
Reedfish                  TCATGTATGTTTTGTTTATAGAATATGGCGTTTGAACTAGAGAAGTAAAACTGTGACATGTTTTAACTTTTTTATTTTTT 33932
NorthernPike              CAAAGACAACTTTATTTCCCGCAAT--TTATTTGTGGACTCATGGCCCCACGGGGGTGCTCTGTGTTTTATCCACTCTAT 8075
SpottedGar                CTGTCCAATAGTTCTAAGCCCTGTA-GTTCACATGTTCCCTGTAGGAG---AATGATGAGATAAATTTGGGGAGTGTTGT 7330
ParamormyropsKingsLeyae   TTGATGGA---TCCTTAACGCATCG-GGCGTGTACGGTCCAGTGATGCA--CGTAGGCAGCTCGACATCGGCTTTCTTAA 3307

Majority                  AAATGTAGTAAAG-AAAGGTGTAAGATAAATAGCTTTTCAAAGAG-TTCTGTATGTTTGAT---CGGATTTATTTGGTTA

                                  76410     76420     76430     76440     76450     76460     76470     76480

Human                     CTTTGGAGAAAAGTTGAGGGAGACCAAAAATGACTTTTTGAGGGAATTGTAGAAGTTTCATTAGAAGAAAAGTAAGTTTT 76269
AsianBonytongue           AAAGAAAAAAAA--AAAGATGGAAAATAAATAG-TACATAAACAG-TAATGAAGGATTGAC---AGATATCATTTAGTTA 3981
Reedfish                  TTATCTCTCAAAA-GGCTTTGGATGGTCAGAATATTTACAATGCT-TGCTGTACATTA------CGCATTGACTTTTCAA 34004
NorthernPike              AAATGTTGTAGTGATAACACATTCCAACAAAAGCATTTCAAATAAAATTGCAATGTTTGGT----AGATTTTTCTTATCT 8151
SpottedGar                AGCTCTAGCTGAC-AAACGTGTAAGCCATACAGGTGCCCAACAAG-TCCTACAGGCCTGAA---GAGACTGATGAGGTCA 7405
ParamormyropsKingsLeyae   AATTTACGTTTTG-ACAAGTGTTATGCTTGCGT---TTCAAAC---TCCTGTGTGTTT--C---CGCCGTTGCTCGGCTC 3375

Majority                  TACTTGTTTAGTTAAATTGGA-AATATGTATATTTGAATATTCGTGA----GTAGGACTAGAGTTGACTG-ATTACTTA-

                                  76490     76500     76510     76520     76530     76540     76550     76560

Human                     TAATTAAAAAGTTAATCTGAGGAACAGGTAGAATAAAAGTGTAGTTG----TTAGTGGTAGAAGAGAATGGATTCCATA- 76344
AsianBonytongue           CTCTTAGTGAGTGAAATTACA-GAATTGTGGACGGGGTTATTTGTGA----ATGGCAGAGCAGTTGCTTG-ATTAA--A- 4052
Reedfish                  AACTTGTGAATTTAAATGTGA-AATACAACAATGAGAAAAGCCGTGACTATACACGGCCAGAGCTTCCTGCAGGAGATG- 34082
NorthernPike              TACCGGGTTTTTTAACTAACAATGTATCCTTATTTGCAGCAGTGACTTGGGGAGTGATTTGGATAAACTACATTACTCAT 8231
SpottedGar                TACTTGCACAGTCTGGTAGT--GCTCAGCAAGTTGGGATCTACTCGA----GAAGTCCTAAAGCTGACTA-ATGATCCA- 7477
ParamormyropsKingsLeyae   TGTTCGTCTAACGCGATCGGA-ATCTCTTATATTTGATCACGCGCAG----GTTTGACATGACATAGGCG-GTTTCT-A- 3447

Majority                  GTCCATCATAAGTGGCTAAACCTGTAGTTTT----TGTAATGAGGTTTTATTTATTCATAGTAAGT-TAAAGGTATATTT

                                  76570     76580     76590     76600     76610     76620     76630     76640

Human                     GGGCAAAATAAGAACTCAAGGGAAGGTTGGTGGAAGAGGAAGAGGATTGAATTGTTTCAAGAAAGAATAGCAGTTGTCAT 76424
AsianBonytongue           AGGCAGGACAAGTGGTGATAGTTGAAGTCA------GTGATGA-----AGCGAATGCACAGTAGGT-CTGAGGTACAGGA 4120
Reedfish                  GGCAGCCAGCTATAGATCCATCTGTAGCTGCAGCATTTAATAAGGATTCGTCTCTTCTTGGTAAGAATCAAGGTATAATA 34162
NorthernPike              ATACATTATGGTTGCCTACACCTTGGGATTTG--TTCTAGCAGCCTTTTAGTTACCTGTCATGTGGTCTTACTGCTGCTT 8309
SpottedGar                ATCCAGCAAGAATGACTCAACCTGTACCCTT----TATAATGTCTTTTAATTTCACAAAATAAAC-TAAAGGTATATTT 7552
ParamormyropsKingsLeyae   GTCAGTCCCACGTGACGGAGCTGATTTTTTT----CGTCATAT-----TTGGCATACAGACTTAGT-CAAAGTAACGTGT 3517

Majority                  TTTTCTTACCAGT----TTTTAAATGTTATATTTAGTAATGTATATTATGTCTTTTTGTTTATTGCATGATATCTGTGTT

                                  76650     76660     76670     76680     76690     76700     76710     76720

Human                     CCTTATGAAAAGTAAAATTTTTATTTTCAAATCAGGAAATGTAAAATGTGCCTTCCAGACCCCTTGGTGGTATACATGGG 76504
AsianBonytongue           ATTCCTTGAGAGC----TTTTAGCGGATATTTTTTGTTGTGTATAGAACATAATTTTGTTTTTTGTATGATATTTGTGTT 4196
Reedfish                  ATTTTGTTCCAAA----TATTAAATGTTAGCATCAGAAATGTTTAGTAAGTGATTTCTATTTTTACTTAATAACAGTATC 34238
NorthernPike              CCAGCTAACCTGTGTAATTCTAAATAATAAAAAAAATAGTTGAGATGAAGTCTGATTATCGACCAAGCTATGTTTGTAAA 8389
SpottedGar                TCATCTTCGTTTT----TTTCAAAAGTTATAGTTCATATTGTATGTATTGTA-TAGTATTTATGGCAAGGTATCCGTGCA 7627
ParamormyropsKingsLeyae   TTTTCCG----------TTACAAAAACTA--------AATGTATTTGTTTTTCCCCTGTT-ACAGCAT--TGTCAGTGAT 3576

Majority                  ATTTCTGTTCTAGAGTGTGGAAGGTGTTATATATATCT-CGTACTATGACCTAATACACAGCAAGGATAGTAATTACCTC

                                  76730     76740     76750     76760     76770     76780     76790     76800

Human                     AGATTGGTTCTAGGACACACACAGTCCCATCCCCCACC-----CTCTGACCCCATACACCCCCTGGATACTCAAATCCAC 76579
AsianBonytongue           ATTTCTCTTCTAAAGTATGTAAGAAGTTATTCATAAAC-CGTACAAGAACCTAGCACAAAGCAGGAA-AGCACATGCGTT 4274
Reedfish                  AGTCCATTTAAATACTGTGCTGTCCTCTTTACATTTCTTTGAACTATATTCTAATTTACTTTAATGTAAAGGGGTA---- 34314
NorthernPike              GTTTCATGACCTGGTCATGGAGGCTGTTCAATGTATCTTATTTGAAGGGCTCACATTAGAGCAAAGTTATTTGTATCATG 8469
SpottedGar                ATACATGTGC-ATAATGTAGCAGGTATCACATACACATGCAAACAGTCACATAAAGCTATGAGTGTATAGTAAGCAGCTC 7706
ParamormyropsKingsLeyae   GTTGCTGTTG--GAGTG---AAGGTAAGATTTGTTCCT-----CTGTTGCATCACACATAACGATTGAAGTAATTA---- 3642
```

```
Majority                   TGTTATAGATGGTCTTTGTGT-TGATTTGTTTAGTTTTTTTTTTTCAAGTTTACATATTCCCAAATGTTTAATTGAATGT
                                   76810     76820     76830     76840     76850     76860     76870     76880
Human                      TGATGCTCAAGTTCCTTGCAT-AAAATGGTATAGTGTTTGCATGTGACCTATACACAACCTCTTATGTGTACTTTAAATC 76658
AsianBonytongue            GGTGTTAGAAGAGCTTCGT-----ACATATTTGACAGTTGTTTAGCAAGCTTGCATAAGGCCTAGCATGCAGAGAAATGC 4349
Reedfish                   ---TATATCCAGTTTCAGTGT-TGGTTTATTCCATTTTTTTTTTTAAAGAGTATACTTTTCCATATTTTCAAATTTATAA 34390
NorthernPike               TGAGAATGGTAAGCCAAGAGAATGATTGGCACAGGCAATTCTGATTAGGTTCGGCTATACCACAGTCTTACATTGAAACA 8549
SpottedGar                 ATTTGTACAGACACTTCATCA-TGATCAGTCCAACATTTTTATTACAATATAATATTTCAGAAAATATTTAAGAGAATGT 7785
ParamormyropsKingsLeyae    -TTATGGATGGATTGTGTC----GTTAATGTAGATGCATTATTACTGT-TCACTTACTACCAAATGCCAGACTGAATGT 3715

Majority                   TTAAGT-TTT-TTTTA-ATTACAT--TATACAG--TATGCATATTTATGT-A-TTATTGATAGTTCGTTTTAAGTACTG-
                                   76890     76900     76910     76920     76930     76940     76950     76960
Human                      ATCTCTAGATTACTTATATTACCC--AGTACAA--TATAAAT-GTTATGTAAATGGTTGTTATAGTGTATTGTTTAGGGA 76733
AsianBonytongue            TCA---------TTCA-ATGAAAC--TTTACC---TCTGCGTAGTTATGC-----ACTGACACTAATAATGATGCACTG- 4408
Reedfish                   TTAAAATTTGAATTTATATTAAATAGTAAACATT-TATGCAAATTAATTTGA-ATATTGATTAACAGTTTTGAACACTT- 34467
NorthernPike               GTAAGGCTTTGTTTGTCATTGCACCTTATAATGCGGAAGACTATTTGTCTCTCTCTCTGACTGTTCGATTTTACTGTTG- 8628
SpottedGar                 TGAGGTGTGACTTTCA-ATACGGT--TTCTCTA--TATGCATTTTTTTGTTCCATATTGAAAGTTCAGTTCAAGGATTG- 7859
ParamormyropsKingsLeyae    CTA----------TTG-ATGCTTT--TAAGCAGT-CCTGCTTTTTTGCGT-----GTTGCAAACACC-TTAACATCCCG- 3774

Majority                   -TAATTTCTAGTATAGCCTGT-GTATATTTTCAGTACAT-ATTATTTTAACCCATAATATTATTGATCTTCATTTAGTTT
                                   76970     76980     76990     77000     77010     77020     77030     77040
Human                      ATAATGACAAGAACAACTTTCTATACATTTGCAGTACACCATTGTTTTACCCCCAAATATTTTTGATCCAAGGTTGGTTG 76813
AsianBonytongue            -TAATTTATACTATAGCCTGT-GTAAAT-TACAGTACAT-ATTAATTGGACCCATTGTATTGTTTTTGGTCATCTTGCCT 4484
Reedfish                   -TAACTACTAATTTGTACTGT-ACATGTTTTTTTTATAA-ATATTCAAATTATATCTGGATAGTGACCTTCACAAATACA 34544
NorthernPike               -T-GGTTTTAAAAC-GTTTCTTGCAGATTGGCAGGACTATTAAAGGGGAACATACAAACTGATCATCAGTAATGAAGTAT 8705
SpottedGar                 -TAACATTTCTTGT-GCATAT-GTCTGTCTTTCTTCCAT-CTTTGTTCATACAAGGATAGAACTGAGCTTTACAGAGATG 7935
ParamormyropsKingsLeyae    -GGTGGTCTAGTACAACCTG--ATGGAT--ACAACAGAT-GTTATTTTAAAGCACAGTCTCACC--TGTTGAGTTTACTT 3846

Majority                   CTTAAAA---C-GAAAGAAACGGCGTACAGATAATCC-TTCAGCATATAAATTTA-AGATATGTAT-AGTGTTAAATGTG
                                   77050     77060     77070     77080     77090     77100     77110     77120
Human                      AATCGGAA--CCCAGAGATACAGAGGGCTGACTATAC-TTTAAGAATTAGAATTA--GCTGGGTGT-GGTGGTGGGTG-C 76886
AsianBonytongue            CCTGAAA-----GAAGCAAACGGGGTAAAGATGTTCC-CTCAGTTTTTAGATTTA-AAAAAAAAAA-AAAAAAAAATA-G 4555
Reedfish                   CTTAAAG---CAAAATGGTATCCAATAAAGATATTGTACTGAGCACAAAAGTTGAAGAATGTGTATTAATGATAAATGTG 34621
NorthernPike               GCTAACT-------ATGAAACACTGTTCAAATAAACCTATCCAATCAGAAAATGTTTGTCACAGAA-AATGCGGTTTGTA 8777
SpottedGar                 CATTAAAATTCTGGGAGAATCGGCCTTCATGCAATAC-TTCAGCAAATATTTTTA-ATACATGTAT-AGCATTAAAAATG 8012
ParamormyropsKingsLeyae    CTTAAGA------AAAGGCATCTCTAATGCTTAATCC-TGCAGAATGTGATATGACAGAAATGTTT-GGAGTCATGTGAG 3918

Majority                   CTATTGTGTTCACATCCAGAACATCTG--TTCAACATATATTTAT-A--ACTGGATG--TGTAGTTGCACTTTAGGAAAG
                                   77130     77140     77150     77160     77170     77180     77190     77200
Human                      CTGTAGTCCCAGCTACTCGGGAGGCTGA-CGCAGGAGAAAGGCGTGAACCCGGGAGG--TGGAGCTTGCAGTGAGCCGAG 76963
AsianBonytongue            TTTTTGTGTCCATGTCCGTAACA--TA--TTCAGCATTTGTCC-------CTGGATA--TATAATTGCTCTTCTAAAATG 4622
Reedfish                   AAATATAGTAATAATTAATAACATTTGG-TTCAAATTCTAAATATTACAATTTAAGG--AATGGGTGCATTTTAGGGAAC 34698
NorthernPike               GCATGGATTAGAGATTGAGATTATCTGATTGCGACCAATTTTCTTTATTACTTGATAATTCTCACAGTTGCTGGGGAAAA 8857
SpottedGar                 CTATTCTATTTTCTTCCAAAGCTACTT--TTTAACACATAGGTAT----ATTGATTG--TGTGGTTATACATTAGAAGGG 8084
ParamormyropsKingsLeyae    ATATGGAAATCAAAACCGGCATA-CCT--CATAACCTTTAATTCT----GCCGGTTT--AGGAAGTACA---AGAGGATC 3986

Majority                   TTTGTGTTTTTATAGTT--GTTTGATC-CTACCTAGACAATTTGTGTTTGAATAAATTTATTTGTGGTTG-----TAATT
                                   77210     77220     77230     77240     77250     77260     77270     77280
Human                      ATCGTGCCACTGCACTCCAGCCTGGGCGACAGAGCGAGACTCTGTCTCTAAAAAAAAAAAAAAAAAATTAGAAG-GGGCT 77042
AsianBonytongue            TCT---TTATTAAAATT--GTTTAATT-TTACCTGGA---------TTGGGGTAAGTGTACTTGT-------------TT 4674
Reedfish                   CCTGAAATTTTCTGCCT--TTTTGATCACTGCCAGGACATTTAGTGTCTTGATTTTTTTTTTTTTGGTTACCTGCTACCT 34776
NorthernPike               CTTGAAATTTCACAGGTATCTAAGACTAGTCCATAAACAATTTCTGTACCAACAGAATACAATACATCAGTGTTTCTATT 8937
SpottedGar                 TTTGGGTCATTGTAGTA--AACTACTA-CTGCCAAGAAGATATTGCCTTGAACAAACCTACCAGTGGAAG-----TAATT 8156
ParamormyropsKingsLeyae    TGTCTGTATTTAAAG----ACTTAGTC-CTACTTAGT-----------------AGT---TCTGTG------------TT 4029
```

```
Majority                  ATTTAGTGTTTTTT-TGCTGC--ATTTTTT------TGGCTTTGCTTTCAGAAGTTT---ATCCTTAGAA--G-CT-TTA
                                 77290     77300     77310     77320     77330     77340     77350     77360
Human                     GTCAGGCGTGGTGTCTCATGCCTATAATCCCAGTCATGCCTGTAATCCCAGCACTTTGGGAGGCTAAGGTGGGCATATCA 77122
AsianBonytongue           GGTTACTGTTAT---TGCTGC--ATTCTTT------TGTCCTTGTTTTCAAAAG--------TCATA--------T-TCA 4726
Reedfish                  ATTTAGTCCCTTTT-TGCTGC--AGTATGTGGAAGAAAATGTGGTTATCAGCCACATTGAATCCTTGGAAAAGCCT-GTG 34852
NorthernPike              ACTATGTTTTTTTTAATTTGTCAAATTGTT------TTGCATTCCACTCACATGAGTGTGATCTATAATCAGGTAACACA 9011
SpottedGar                AATGACTGATTTG--TCTTGC--GATTCTT------TGACTTGGCTAATTAAAGGA----GTTCATAAAA----CT-CTA 8217
ParamormyropsKingsLeyaeATGCAACGTTGTT--TACCG-----T----------TGGCCTAACGTTTGTATATT----AGCCTTG----------GTG 4078

Majority                  CTTTTG--TCCTGGTTTAAGAAGTGT--TCTGT---ACTT--GTTTACAC--ATTTTATTAATAACACTAATGTCA----
                                 77370     77380     77390     77400     77410     77420     77430     77440
Human                     CTTGAG-ATCATGAGTTCAAAACCAG--CCTGGCCAACATGGTGAAACACTGTCTCTACTAAAAATACAAATATTAGCCA 77199
AsianBonytongue           CTTTC-----CTGATTCAATAAATGT--TCTA----ATTT--GCCTATTT--GTATAATC--TGGCTTTAATGCCA---- 4785
Reedfish                  GTGTCT-ATTTGGGCGTGGGAAGTCTCTTTTAAGATACTTTAGTTTATGCCCATATTACCAGCCACACAAAGCTCAATT- 34930
NorthernPike              CTTTATTGTCATACAAAAATAAGGGCGGAGCATGAAAATGGGGTCCCCGCCCAAGGTAATATTAAGAGTAGGATTG---- 9087
SpottedGar                ATTTTG---GCAGGTGCAAGAAGGGT--TCTGC---ACTT--GTTAAGAG--ATTTGGTT--TCAGCT-GATGCCA---- 8278
ParamormyropsKingsLeyaeCTGTTG---CCTGGTTTATGAA------TTTGT---AGTT--GCTCACAA--ATGAAGTTCATAACCCCAAT-CCA---- 4137

Majority                  --TATAGGCTTTGGCGTTTC----ATTTTTTACTTATTTCGTTGACCACAGAACAGTTGT--TTCAACATATGT----AA
                                 77450     77460     77470     77480     77490     77500     77510     77520
Human                     GGTGTGGTGGTGGGCGCCTGTAATCTTAGCTACTCAGGAGGCTGAGT-CAGGAGAATTGC--TTGAACCCAGGAGGTGGA 77276
AsianBonytongue           --TTTAGGTTTTCAAAATT------TTCACAACATATTTCTGTAATCAGATGACAGCTGT--TTCAAAATAAAT----AT 4851
Reedfish                  --TATTAACACTAGAATCCCTGAAGCTTATGATTTTTTTTGTTGTCCCTTACCTATTTTT--CCTGACATATGTCAAGAA 35006
NorthernPike              ---ATAGGGTTAAACTGATTATGTACTATTATGCCATTGTATTAAAAAAACAATTATAGCAGTACAACATCAGTAACCTG 9164
SpottedGar                --GGAAGGCCTGTGTGTTTA----ATTTGTTACCTCTGTC-ATATCCACACAGCA-CTGT--GCCAGAATGT-------- 8340
ParamormyropsKingsLeyae--CATCAGCACTGACCTTTC----ATTCTTCCGGTATTTC--TGAAAACCGAACTGTCCC--GTTGACATCC-------- 4199

Majority                  GTTCATCACTGTGCTGAGAGGCTGTT--TATATTGCTAT-TGTGT--TTTTTTGTAAGTCAGTTTTGTAGAGGAAAAACA
                                 77530     77540     77550     77560     77570     77580     77590     77600
Human                     GGTTGC-AGTGAGCTGAGATCATGCCATTGCACTCCAGCCTGTGTGACAGAGTGAAACTCCATCTCAAAAATACAAAAAG 77355
AsianBonytongue           GTTACTTATTTAGTTAATGGGCAGT----ATCTTGTTAT-TGTGT--CTCTTTGTAACTCATTTTTTTAGAGGAAAAAC- 4923
Reedfish                  GCTCATAGCTGCGCTAATAATCTTTTTTTGTTTTGCAAA-TGTGTGGTTTATCCGAACACAGCTTGCTACACCCTCAGCA 35085
NorthernPike              ATGAACCACTGTTCTTGGTAGTAGTG---ATATTCCTGTTTGGCAAATAATGGACTTGTAGACGTAGAACAGGAGAAAAT 9241
SpottedGar                -TTGATGATTGTGTTGAAAGACTGCT--CACAAAGAGAT-TGAGT--TGATAGTAAAGTCAGTGTAGGGTAGGAATATCA 8414
ParamormyropsKingsLeyae-TCGTCACCGGGCACAGTGGCAGTGG-CTCATTTTCTC-TGTGT--TTTATAATAAGTCTGTTTTAGGGGGTTGTAAT- 4272

Majority                  ATTTTGT-----CTTTACATTCCCCCC--CATT-----------GACGAATACATAAGTCT--ATAA-----AGATTATA
                                 77610     77620     77630     77640     77650     77660     77670     77680
Human                     AATTAGAAAGGGCTTTAGAGTCCCACCACTAATGCTCTTCTGTTCAGTAGTACACAAGACTA-ATAACTACGAGATGTTA 77434
AsianBonytongue           -TTTTTT-----TTTT-----CCCCAC--CATT-----------AGCGGATAAATAATTCT--TTAA-----AGTATATA 4972
Reedfish                  ATTCCCC-----CCCCCCCCTCCCCCTTTCATTCA---------GATGAAGGCATTGCCCTG-ATGC-----AGTCCTCA 35145
NorthernPike              AGTCCCATCTGTCATGACATCCACCCTGCTTGTTCTGAGTAATTGACTCATTCATTGAAAAAGGCATGTTCAAAATAACA 9321
SpottedGar                ATTGTGT-----TTTTAAATATCTACC--CATT-----------GTTCATCATATAAAGCT--ATAA-----AGATTAAG 8469
ParamormyropsKingsLeyae-GGGTGT-----CTGG---------------------------AACGCACTTGGAAGTCA--TTAA-----AGCTGAAG 4311

Majority                  TGATT----A-TTCAGGTATA-GCACTAAAGCTTATAA----ATTACTTCGGATGG----T-TATATTGGCAATGTGATA
                                 77690     77700     77710     77720     77730     77740     77750     77760
Human                     TGAACAGGGAAAAGAAGTAAGACCAAAAGAAATCATAA----ATTACTTTGGATGGAACTTTTAGAATGGCAGATGGAGG 77510
AsianBonytongue           TTTTT------TTCAGTT-TA-GCACTGAAGCATTTA---------TTTCAAATGA------AATATTAGC--TGTGATA 5027
Reedfish                  CAGTTCAACAGTTCAAGTCTGTGCAGAAGTGTTTATGTGGCGATACGTTTGTAAACA---TTTATATAGGTAATGAAATG 35222
NorthernPike              ACAGTGTGGAGTTTAAGTAGA-GGATACAGTCATTTGGTGAGAAAACTGGTGTCATTAATTGTCCATTATCAAGGAAGAA 9400
SpottedGar                TCAAT------TTTAGGTGTATGGAATAAAGCATATAAGCCTACTGCTCCTCATCG------TGTTTTGACACTGTTATA 8537
ParamormyropsKingsLeyaeCGGGC------TGCAGATATC-CCCCTTCATTTTGTA----------TTCGAGGGC------TGTAGCCTTAAGGCGATC 4368
```

```
Majority                   GT---ATTTTAATTA------TTTCACATA-----CGT---AAATGAAATGTTCTTTTATAAAATAATATGATTGTATTC
                                   77770     77780     77790     77800     77810     77820     77830     77840
Human                      GATAAATTTGACATTGTTCAAAATCATAGAGTAAACTCTCAAAAGTAAGGGGAACCTAAGGAGACAAGATGATTAAATGT 77590
AsianBonytongue            -----ATGTAGAGTA------CTGCAGCAA-----AGT---AAGGAAAATGTCTGTTAATAAACCAGCATCTTCCTATTA 5088
Reedfish                   CTGTGATTTGAAATGCATACATTTCATGTGTGGTCCGT---ATCTGCAATGATCTGTGTAAATATAGAATGACAGAGGAC 35299
NorthernPike               GA--GATGCAAATG-------TTTCACAAA--A--TGTC--AAAAGATATATTCCCTTCTGAATTG--TCCATTCCAAAC 9463
SpottedGar                 GG---ATTTTCACTA------TTTCACCTG-----CTT---TATTCCATTGTCATTTGGGCAAATAATATAATTTTGTTT 8600
ParamormyropsKingsLeyaeGT---GTTTTGATT-------TATCACAGA-----CGT---CAAAATCGGGGGGTGATAAAGAACAATTTAGTGGGATTC 4430

Majority                   ATTCTTTGATAGAGTTACTTAATACATTTAAACTTTGGA--GATTGGGGGTATTTTGTTCATGGACTCACGATTGTGAAT
                                   77850     77860     77870     77880     77890     77900     77910     77920
Human                      AATAGGAGATACTGGAACAACAAAAAAGGGACATTAGATGCAATTTGAGGAAATCTGATAATGGACTTTTGTTGATTTAT 77670
AsianBonytongue            AAGATGGAAGGGTTTGA-GTGACACATCTTTGCTTTCCA--GATTGGGACAACATAGTTC----ACTAATGATTGAGAAT 5161
Reedfish                   ATGCAAGGCAAGAAATGCTGAAAACATCTAAAACAGAAAATGTTTTCATATGTTATATTTATAATGACACAATTTTGACA 35379
NorthernPike               ATTGTTTGGAGGAGCAACTTAAGTGGATTAAACGCTTGA---TTGGAGGGGAAAATGTATAAAGAAGTGCAGCAAAGTAT 9540
SpottedGar                 TTTTTTTACTCACCTCACACAATTGCTTTATCCTTTAGG--GAATGTGTATATGTTTTTCCAGGACTCAGAAAAGTGAAT 8678
ParamormyropsKingsLeyaeATTCTCAGACAAAACTGAGGCATCCAATTAGAAAGTGGA--GATTGCAGCGGCCCTGTGC----GCACCCGTCCGTCCGT 4504

Majority                   CTAATTGTTAGCTGTGT---TCTTAGCTTAAATTTGTT-GCAATTTCGTAAA-ATGCTGCTTTATGCGGATAGGAGGG--
                                   77930     77940     77950     77960     77970     77980     77990     78000
Human                      CGA--TATTGGTTGATTAATTGCAAGAACTAATAAATT-GTAATACCATAC---TAATGTATGATGCTAATAATGGGGGA 77744
AsianBonytongue            CAACCTGCTGGCAGGGT---TCTTAGCTTAAAATTGTG-ACAATTTTG-----------CTCAATGGAAACAGGAC---- 5222
Reedfish                   CAAAGTGTAAGATGTGTGAAACTGAAGCCCAGATATCCAAGAAACACTTTAACAAAATGTGTAACACAACAAGGATGTTT 35459
NorthernPike               AGGATGCTTAGCTAAAA-----TTATATCAAATGCTTT-AAAGCAGCGACAAAAAGCTGAAACACGCGGATGAAAACG-A 9613
SpottedGar                 CTGATTTTGAACCTTGCA--TCTTGGTTTAATTTTGTTTGCACGTTTGAATAGATGCCACTTTATGAGGTTAGGAGGA-- 8754
ParamormyropsKingsLeyaeCTATCAGTCACTTTTAG---CATCGCCCTGAGCGGGTCGGCAGCCGCA-----------CGTTGTCTGGGGAGGAAG--- 4567

Majority                   --AGCTAATGCTAAGTTAGATAAGA-AATTGTCTTCTCTGTTAATATGTTG-TAATCCTCAGCTGT----AATAGATAAA
                                   78010     78020     78030     78040     78050     78060     78070     78080
Human                      ACTGCGGTTTCCAGGTTATATAAGA-ACTCTGTAATGTCTTAAATTTGTTTGTAAATCTAAACCTTTTAAAATAGCTAAA 77823
AsianBonytongue            ----CTAATGCTAAATTACACAAGA-AATGGCATCATCTATAAATA----A-CGATTGTTGGGTGG----AAAAAAT--- 5285
Reedfish                   TTGTTAAAGATTAAAATCGAAGAAA-AAGACACTGCTCAATTCACATATTGTTGAAGCCCAACTATC---ATTCTGTAAA 35535
NorthernPike               GCAACTACTGCTAAAATTAATAGAAGAATTGTCAGAATGGCCAAGATGCAGCCAGTCATCACCTCC---AGAAAGATCAG 9690
SpottedGar                 --AGCTGAAGCTAAGCCAGGCTTGA-GGTATTTTTCACTGTGGATGTACTG-TAGTCTTTAGATGT----AAGAAGTGAA 8826
ParamormyropsKingsLeyae---GGAGGGGGGGGCTTCCGATAA-AATTGGCCTGGCTGGTAATTT-----GACGCATCGGGCAT----GCGAGAT--- 4630

Majority                   AAATA-AACAATATTTTCTGGATTGTGTTATTACAGGTTTCT-TTT--AACTTATGTTTTTATGGAGAGGTTTTTTTATC
                                   78090     78100     78110     78120     78130     78140     78150     78160
Human                      AATTATAGATACAATTTCTAAATGATTTAATTAACAAAACCAGTTTAATTCTTCAGTTGAAATGAGCAAGGTTAGGGATA 77903
AsianBonytongue            ------AACAGGGCTTTAAGGATCATTCTAAAATAGGGT---------GAATTATTTTGTTATAGAGAGATTTGAGTCTC 5350
Reedfish                   AAAGTAAATGATATTCGCATATGTGTGTTTTTGCGTGTTTCATTTTCAACCAGTTGTCACAGTGGTAACACTGCTCCATC 35615
NorthernPike               GGATG-ATCTCAAATTACCTGTGAGTGCTGTTACAGTCA----------AAAGACGTTTGAACGAAGCGGAGTTATCAGC 9759
SpottedGar                 CAGTAAAACATTTTTTTCGGAAACGTGCTATTACAAATCCCTATTTTCAGTCTACGACATTTTGAAAAAGTTTTTTTTTC 8906
ParamormyropsKingsLeyae--------TAAAACAACATGGCCTGTGTT-TTATGCGCCTGTCTC------CTCTGCTTTTATGGACAGAGTTGTATTTC 4695

Majority                   ACAGATCTGCTTTTTTGGTATAATGTCTCATG-TC----CAGAATTCATATTGTAGTATG-TATTACTCCTTCATTGCCT
                                   78170     78180     78190     78200     78210     78220     78230     78240
Human                      CC---TGTGTGTTTC-AGTGTAATATCTGAAAGGAAA---AGAATAGATAC-ATAATTTA-TATCACTTCTCTCTGGACT 77974
AsianBonytongue            ACAAGTCTGCTTATTTGGGATGCTAACTCTGG-TC----CAG--TCCAAATTGTAGTAAA-CAGAAAAAATGCTTTGCAT 5422
Reedfish                   ACAGATCTGCGCAAATGTCACAGTGAATAAGCTTCAGTTTAGTAATCTTGAGATAATAGG-TTTTAATCCCGCATTGCCT 35694
NorthernPike               AAAAAGCCCCATTGCTGAAAAAATGGCATTTG-------CAGAAT--AGGTTGAACTTTGCCAAGGCACATTGATTGGCC 9830
SpottedGar                 CCAGAAGTGCTCTTTTCATGTAACATCTCAAAATG----CGGCATTCCAACAGTAGCAAAATTTTTGTTGTTCACTACAT 8982
ParamormyropsKingsLeyaeAC---TCTGTCCAAT-AGGATGCTGCGTCATG--------AG-------AG---AGTATG-CCTTATTCTTCTGTGTCAT 4752
```

```
Majority                      TCCCG--T-TAGTCGT-TACCTTACTGAGCTTCTATTTG-TATTGTTGAGGTCTTTTTAAGAGTC-TGGT---TTTAATA
                                        78250     78260     78270     78280     78290     78300     78310     78320
Human                         TAGGGTCTCTTGTCATATACATGACTGGGCATCCATCAGCTTCAGTGGAGGAAAGGTTAGGAATAATAGTGTCAGAACTA 78054
AsianBonytongue               TCC---------TCGT-TACTGTACAAAGCTCCTGTGTG-TAATGCAGAG-TCTTGTTAAAAG---------------TA 5475
Reedfish                      CTGCA--TTTAGTGCT-TTGGGTAGTGACCTGCTATTAT-TATTGTTACTGTATAATAACATGTT-TGATGAGTCTATTA 35769
NorthernPike                  CAAAGAG--AAATGGCGTAACATACTGCGCACTGATGAG-AGTAAGATTGTTCTTTTCGAGTACAGGGGT--CGTTGACA 9905
SpottedGar                    TCCAAAGTTAAGCTTT-TGCCTCTGTGAACTGCTATCTA-TAATGTGGAGCTCATACCAATGGGC-TGTT---TTCTATA 9056
ParamormyropsKingsLeyaeTCCC--------TCCC-ACCCCCATCAAACTTTTCTTTT-TGTGGTTCCTCATTTTCAGAGAGGC-------------TC 4809

Majority                      ATATGG-AGTT----GATATCAATGAGTT-CTTATTCTAGCTTGGGAACAGTTTTGATGAGGGATTGTACAGTAGAAATT
                                        78330     78340     78350     78360     78370     78380     78390     78400
Human                         ACATGAGAATTTTAGGAGATTACTGAGTCACTCAAAATGTCTGGGTGACCCAGCTGATGAGGGCCATTTCACATGAAAAA 78134
AsianBonytongue               AGCTGC-AGTA----AATACCAAAATGTTGCTTGTTCCAGCCTCAGAAATGGTTTTACAGTG--TTGTATAGTAGTTA-T 5547
Reedfish                      GCGCAGTGATA----AGGAGCTACGAGCTTCTTGGCATATGTTAGAAAAACTTGAGGTCAGGGACAGTG-GATAAAAATC 35844
NorthernPike                  GTACGTCAGAC------GACCCCCGGGTACTGAATTCAAGCCACAGAACACTGGAGATGAAGGATGGTAGCGCAACGATC 9979
SpottedGar                    ATGTTG-AGCT----CATGTCAATGGGCT-CTTTTCTTTACTTTGAATTAGTTTTTCAATAATTCATGCAGTGGAAGCT 9130
ParamormyropsKingsLeyaeAGACG--AGCT----GCTGTCAGGCAGCC-ACTACACCAGTCCGAGCACTGGTATGAGTGGGA-TTGTA-ACTGGTAAGG 4880

Majority                      ATTGACTGCTGG--TTT--TTCTTAATTTACTTATTTGATAATTAAGTTTTT--AAA-TAGTTTTATTTTTGT-------
                                        78410     78420     78430     78440     78450     78460     78470     78480
Human                         GATTCCTGCAACACCTTCTTTTCTTTAGCACATGATCGTTTATGAAGCTCTTTTACA-TTGTTTTATCTTTAT-----AG 78208
AsianBonytongue               ATAGTTCAATGG--TTT--TTACTAATTTACATATTTTTTAAGG-AGTAGGT--AAA--GGTCTTATTTTGGA------- 5611
Reedfish                      ATAAACTTAAGGCATTCTAGTGTTAATTTACTGTTATCATCAATACATTTACCAAAAATAGCTTTGTTTTTATTCATAAT 35924
NorthernPike                  GTGGTATGGGGATGTTT--TTCATACTATGGTGATGGGCCTAGTTATCCTATACCGGGGAACATCAGTTTGAATACGTCA 10057
SpottedGar                    ATTAATGACTGA--TTTGTCTAGTGGTTCATTGACTTGATAATTCAGTTTTT--AAA--ATTCAAATTCTTG-------- 9196
ParamormyropsKingsLeyaeAAGGACC-CCGT--TTT--TCCTCGATTGGGATATTTGATATTGATGTCTGT--ACATTAGTGTAATTTGTGT------- 4946

Majority                      ATATGTTCAGAATAAATTAT--TCATTTGTTTTTATATGTGGTGATTGAGTAATTCAATGACTTCTAACATGGCGAGCAA
                                        78490     78500     78510     78520     78530     78540     78550     78560
Human                         CAATCCTCAGAATAGATCAAGCTTTTTTTTTTTTTTTTTTTGAGATGGAGTCTTGCTCTGTCGCCCAGGCTGGAGTGCAA 78288
AsianBonytongue               GTTTATTCTGCATAAACT-------CTTGATGATGAATGTG-------AGTAAT-TAAGGACCTCTTA-ATGTCAAACAA 5675
Reedfish                      ACTTCTATGGCTTTAAGTCCAGTCAGGTCTTATTATATGTGGGGATTATGTTCCTGAAAAACCCAGTGGAAACCGAAAAC 36004
NorthernPike                  AAATACTT-GAAGAGATTGTTGCCATATGCCTGAAGATGAAATGCCCCTGAAAT--GGGTGTTTTCAACAAGATGA-CAA 10133
SpottedGar                    GCATGTAGAGGAACAACTTT--GCACTTGTTAACAGATTTGGTTTCAGAGGAATGCAAAGACTGCTCACATAGCAATTGA 9274
ParamormyropsKingsLeyaeATATGTGCAAATCCATGCAT--CCCTTTGATGTTACATCCATAAAATAAAAAGCTATGTTATTTCTAACATGCAAAGTAA 5024

Majority                      TTTGTGAATCTTGA-TCTCTGTGCATCTT--------CAGTCCAA-AACATTATTTTGGCATAA---CTGACGTAGCTTC
                                        78570     78580     78590     78600     78610     78620     78630     78640
Human                         TGGTGCGATCTCGGCTCACTGCAAAC-TCGGCCTCCCGGGTTCGT-GCCATTCTTCTGCCTCAGCCTCCCGAGTAGCTGC 78366
AsianBonytongue               CTTGCAAAATGTA--TTTCTGTTGATATC--------CAGT-----AACAAAATTTTGGCACCA----TAAGACAATATT 5736
Reedfish                      ATGGTGGACCCTAT-TCTCTGTACATTTTGCTACCCTTTACCATA-AACACACATTTTGAATAA---CAGAAATCACCTC 36079
NorthernPike                  CCCCCAACACACAAGCAAGTGAGCAACGTCTTGGTTCCAGTCAAAAAGGATTGGAGTGGCCTGCTCAATCCCCTGGCCTC 10213
SpottedGar                    GTTAATACTCCTG--GCAGTGTGCCACTC--------TAGGCCAA-AATATCATTTTAAAATAAATACTGACTGGTCATC 9343
ParamormyropsKingsLeyaeTATATGAATTTTGAGGATTTGTCCATATT--------CAGCCCAA-GAGGTTACATGAAAAGAT------ATGCAGATTG 5089

Majority                      ACCTCTAGGTAGAAAGTGTGGAGTTCAGTTCATTTTGTAGTATATTT-TTATTGTTGGGG-TTTCTTAATATTTTT----
                                        78650     78660     78670     78680     78690     78700     78710     78720
Human                         TACTACAGGTGCCTGCCACCACGCCCAGCTAATTTTTTTGTATGTTTAGTAGAGATGGGG-TTTCATCGTGTTAGCCAGG 78445
AsianBonytongue               ACCCTTTCCAAGAAATT------TTCAGCTCCCAGTGTAGTACAACT-GTCCTTTT--------CTTCACATTTCC---- 5797
Reedfish                      AGCTCAACATAGCACATTTGCACTTAGGTACACTTGGCAACATACTT-TCATTATCACTA-TGCTTTGCTATTTTTGAAA 36157
NorthernPike                  AACCCCATTGAAAACCTGTGGGGTGACATTAAATATACAGTGTCTGAATCAAAACCCAAAACTTCTCAAGGACTGTGCAA 10293
SpottedGar                    ATTGCTGGGGAGAAAGTGTTGACTTGAGTTAAAGGCATAAAATCAAT-ATTCTGTTGAGG-TATTTTAACAATGTT---- 9417
ParamormyropsKingsLeyaeGCTTCTTGATACTAAGC------TT--GTCCCTGTTCTGAGGCATTT-TGCTTGTCAG-----TATTAGTCTAGAT---- 5151
```

```
Majority                     ---ATCACATTCATC----------ATTGCTTTACTA-CTTTATTCTAGATGCCA-ATATTTGTAGAA------------
                                      78730     78740     78750     78760     78770     78780     78790     78800
Human                        ATGGTCTCGATCTCCTGACCTTGTGATAGATCAAGTATTTTTATCCCTCTCATCA-ACATGTGTAAACAGTTTTTTCCTC 78524
AsianBonytongue              ---ACCACAGTGAG-----------ATTGTTTTG-----TTAATGTTTGATGCAC-ACTTTTATAGTA------------ 5845
Reedfish                     TTTAATACATTTATCTGA----AAAAGTGATACACAGAGAAAACAGTACATGTA--ATATTTTTAGAACCTGGAGACTTG 36231
NorthernPike                 TGTAGTCCAGTCATCC---------TGTGCTGAAATA-CCGGTTTCTAGGTGCCAGAGGTTGGTGGAC------T----- 10352
SpottedGar                   ---ACAGGATTTTC-----------ACTACTTTACCTGCTTTATTCTAGGTTCCT-GAATTGATGAAA------------ 9470
ParamormyropsKingsLeyae      ---GTCACATCCAT-----------ATTGGTGGAA----TTTCCCATACATGGAG-ATATGACCAGGA------------ 5200

Majority                     -CAATTTAAGTGCTTTTCTTAAACGTTTA----ACCTTTTTTAAATTTAAAATAGTTTTATTTAAGAGTTTTTTGTTAAA
                                      78810     78820     78830     78840     78850     78860     78870     78880
Human                        ACATATCTGCTGCCTTAGCTAAAGATGCAAATCATTAAAGTTAAAATTATAATTTATGTAGCTAAAAGTAATCTGTTAGA 78604
AsianBonytongue              --AGTTT---TTCTTTTTTTAAACTTTT-----CACTTTTTGGAAAGCAAAATAGTTTTCTTTTT---TTTTTTTTT--- 5909
Reedfish                     TCAACATAAGTCTCAAACTGAAGTCATTAAACTTCCATTTGCAAAGCAGAAAGAGAGGTACCTTGGCCCTTGTCACCAAA 36311
NorthernPike                 -CGATGCAA-CGCAGATGCGCAGCAGTTATCAAAAACAAAGGTTATGCAACTAAGTATTAGTTAAGTACTGTTCAGTGAA 10430
SpottedGar                   --AATTTAATTTCTTGGCTTTCAAGTTT-----GCCTTTTTTAAATTTAATGTAGAATTTTTCATGAATTCGCTGTTAGG 9543
ParamormyropsKingsLeyae      --AATGGGTGAGTTCTGCTGGAACGAT-------GTTCCAGCAAATATAAAGCAGTGTTATATGAGGGTGTGTACTTAAG 5271

Majority                     G-AGG-----A------------TCACATGTTTTCCATTCCTTTTT-------------TTGCAGCGTTTATTGGTTTA-
                                      78890     78900     78910     78920     78930     78940     78950     78960
Human                        GCCAGTGTATAC---AATATGTGTAATATGCTTTCTATAACTTTCTCAAAGGGCCC---TTGGTAAGTTTATTGTTTTCT 78678
AsianBonytongue              -----------------------TCCCTTTCC------CCCTCCTC-------------TCACAGGATCTACTGGTGTA- 5946
Reedfish                     AAAGGAAGGAAGGGAAGCAAAGAGCACATAGTGACCATTCCGGTTTAAGTAGGAACAGTTGGCAGGGCTTATGAGGTTGA 36391
NorthernPike                 GTTGG-----AA-----------CTAGAAGATTTTGTTTCCGTTTT---A--------TATAGTACATTTTTGGGTTTG- 10482
SpottedGar                   GGAC-------------------TCACTTGTTAAACATTTTTTTTT-------------CTGCAGCTTACATTGGCTTA- 9590
ParamormyropsKingsLeyae      G----------------------CCAGGTGTT---------------------------TGGAACCGTGAATTCGTGAAG 5302

Majority                     AATTGAGGAAGGTTC-TAGAATGGAGTTATTTTTTAACACTTAAT--TTATATG---TGTGT----TGTGATCTTCTGAA
                                      78970     78980     78990     79000     79010     79020     79030     79040
Human                        CATTTAGCTGGGAGTTAAGAACAGAACTATTTTCTAGAATGTATTAATTATATAAATAGTGCATCATGAAATATTTTGTA 78758
AsianBonytongue              AGTCGTCAAAGGTTC-TTGTCTGGAGTAACTTTATTACGCTCAAT--TTATTTG---TGGGT----TGTGGCCTTAAG-- 6014
Reedfish                     AAGCAGGGGAGATGGGTTGGACGAGATGCCAGCTCGAGTCCCATCC-TTGTACC---TGTCCCACCTCTAACATCCCCCA 36467
NorthernPike                 AATTGAACAATGTC--CACATTGCAATTTTTTTGAACAGCTTAAT--TC-CCT----TCTCT----TTTGTTCCTGTGAA 10549
SpottedGar                   AATCCAGTAAGATT--TAGATTGTAGCTATATTTTGACATTTAAT--TTATATG---TGGGC----TCTGACCTTCTG-- 9657
ParamormyropsKingsLeyae      AAATATGGGTCACTC-TCGAATGGGGTGCCAGGCAAACAATTGAA--TAACGC----TGTGT----AATAATCTCACGAC 5371

Majority                     GGTCTC-ACATTTATTTTATTGCATCCATAAATAG-------TGTAGCCTATAAATAACA-TTTG-TGTAATTCTCTCTT
                                      79050     79060     79070     79080     79090     79100     79110     79120
Human                        GGTTTTTATATTTTTATTGCAGCAGACAGCATTTAGA-----TTAAACCTAAAAATACCTGATTGACAAAGTAGTTTTTC 78833
AsianBonytongue              GGTCTC-ACATTTAATTTATTGTAACCATAAAAA--------TGTAAGCGATAAAGAACAATTTAGGGGAATTCGCTCAC 6085
Reedfish                     GGGTCC-ACTTTCTCACCCTTGAAGTTCAGTAGTGATACTTCCGCAGCTTCATACAGAAACCTTGTTATGACTTTCTCTA 36546
NorthernPike                 AGAATA-ACGCAGACGTAATAAAATCCATAATTAAGGTCTGATATAGCTTTGAAATATGATGTT--TGTAAGTGTTCAAT 10626
SpottedGar                   GGGCTT-GCATTTAATTTATTGCTTCCATAAAAAG-------TGAAGCCTATAAAGAACA-TTTGCAGGAATGCACGCTT 9728
ParamormyropsKingsLeyae      GACCGC-CCACATGTGTCGGTGCACACTCGTGCAG-------CACAGCCCAGCACAACCA-------GTGATCCTC-CAG 5435

Majority                     AGATATAAGCA--AGGTGTCATATTGGATGGTCTG-GCGG-TTTCAAGTTTCTGTAATAGTGTTGTGTTTTACCATCTAT
                                      79130     79140     79150     79160     79170     79180     79190     79200
Human                        AGACAAAAGTATATTGAGGGAAATTGGAGAATCTTCAGAGGCTTTAAAAGTCCAGGAAAATCTTAGACTACTCCAGTGAT 78913
AsianBonytongue              AGACACAACCA--AGATGTCCTATTAAAAAGT--------------TAGTCCTGTAATGGCCCGGCGTTTGCCCATCTGC 6149
Reedfish                     GATAGTCTTCT--TGGCACACTACTGGAGGTCATGAGCGACAACGGAGGGCCGATCTGAGTACCTCACTGTACCATCCAA 36624
NorthernPike                 TGATACCAGC---AGGTGTTAGATTCAGTGATTTC----G-TTTCCGGTTCCTTGATTAGTGTGGGGTCAAACTTTGTAT 10698
SpottedGar                   AGATGAAACTG--AGGTGTCACATTAGATAGTGTA-GTGGATAACACAATTCAGTAGTCAATTTGTGTTTGACCACCCAT 9805
ParamormyropsKingsLeyae      AAATCTAA-----ACGTGTCGTGTCGACAGGTCAG-GCGG-------GTGTTAGTGAACATGCCATGGTTTGCAAGGTTT 5502
```

```
Majority                  TTGTCAGT--CATTGTTTGCTTTGCTTTGGGGGCACTTCTGACTGTATCGGAAACAGTTTGGACGCTTTGAGGAGGACAT

                                   79210     79220     79230     79240     79250     79260     79270     79280

Human                     TTAGAA----CGGTGTAAAGATAGCCTAAGTAGTTTTTCTAAGT-TATCAGCCGCGGGGAGGAAATAATGAAAGAGAAAT 78988
AsianBonytongue           GCATCAGT--CACTTTTAGCGGTGCTCTGAGCGCACTGCTGACTGCGTTGCAGACAGCTTGGGGCCTTTGGGGAGAACAG 6227
Reedfish                  TTGGTAGAAGCAACGTGTAGTGTGAATAATGGGCAGGGACCTAAGCAGTGTAAGCTTGTGGCCTGCACTTACTGGGAGAG 36704
NorthernPike              TTGAAAGGAAAGCAGTTTTTTTTTTTGTCCTTTAAAATCAAATT-GATCAGAAATAGTGTTGACATTTTGTTAATGTTGT 10777
SpottedGar                CTATCAGT--CATTTTAAGCACAGCTCTTAG-GAGCTTCTGACTGAAGCAATGACTGTTCTGAATCTTTAGGGAGAACAT 9882
ParamormyropsKingsLeyaeCTGCCGAATGCATCGTGTGCCTGGTATCGGGGGGTCTGGAGCCTATCCCGGAATCAATGGGCACGAGGCAAGGAACAACC 5582

Majority                  AAGTTCTGGTTGCA---ATTACA--T-------TCATTTGACT---------TTTTTAGA-CATAGACTTACACGTGTAA

                                   79290     79300     79310     79320     79330     79340     79350     79360

Human                     ACATACGAGTGGCATTAAGGACAGGTGGTGGCTTCTTTTACCTATTAAAGCATTCTTACACCATACTGTGAACTGTGTAA 79068
AsianBonytongue           AGGCTCTGGATGAA---ATTACG----------CCATCTGAC----------ATTTCG------AGACTTGCGAGATTAA 6278
Reedfish                  GAGTTCTGGTTGCC---ATCACAAATATAA--TTCAGGTGGCTTACCTACGCCTCTTGGTGCCAGGTTTGACACACGTTA 36779
NorthernPike              AAATTATTATTGTA--GCTGGCAACTGCTA---CTTTTTAATG------GAATATCTATA---TAGGCTTACAGAGGCCC 10843
SpottedGar                GAGGTCCAGATGAA---ATTACA----------CCATCTGGT---------ATTTTAGATCTTAGACTTACAAGATTAA 9940
ParamormyropsKingsLeyaeCAGGACAGGAGGCC---AGCCCA----------TCGCAGGACA---------CACTCACA-CATTCACTCACACATGCAC 5639

Majority                  AACAATGG--------ACAATTTTGCATTTCAG-GCCGTGTTGTTTTATGTCC-----TTATTTCTACGA---ACTGTCT

                                   79370     79380     79390     79400     79410     79420     79430     79440

Human                     AAGGAAGGATCATTGGACATAATAGATTATCTG-ACAGGGTGGTCCGGAATGGTGGAAGTAAATCGACAGGTGAGGACAC 79147
AsianBonytongue           AACAACAC--------AAAAGGCTGCGATGAAG-CCGGTGTT---TTATGTCC-----TCGTTTCCTCAA---ACTGTCT 6338
Reedfish                  ATCCATTCAGTGT--AGCATTTTTGCATTGCAG-CCCCTGATATCTAGCACACAACAGACATTTTTTTGC---TCAGTCT 36853
NorthernPike              ATTATCAG--CAACCATCAGTCCTGTGTTCCAATGGCGTGTTGTTTGCTAATCCAAGTTTATTATTACAA--AATCCTTT 10919
SpottedGar                AACAGTG---------AAAAGAGAGCAATACAG-GTTGTGTTGTTTTATGTCT-----CTCTATCTGTGT---ACTGTCT 10002
ParamormyropsKingsLeyaeACCTATGG--------GCAATTTAGTAACTCCA-ATCAGCCTGA-GCATGTC------TTGTGTCCACG----ACGACAC 5699

Majority                  TG-AGAGAGCTGTTGCACAGT------CTCATATTTGTTTTT---CTTTTGGGTATGACCGAAGCTGCCTTCTAG-AGAC

                                   79450     79460     79470     79480     79490     79500     79510     79520

Human                     TG-AATTAGCATTCAGACAGTGGGGACCTCACACCTTGTATTCCCTTTTTTGGTAAGATTTTTATTACCTCCTTAGAAAG 79226
AsianBonytongue           TG-AGAGAGCTGT-------------------ATTTCATTTT---CTTGCCAGGACAATGGAAGGTGTCTTTTAG----- 6390
Reedfish                  TACATGGTGCTACTGCACGATAAGCTTCTCATAAATGTTTTTG--CTTTTGTGCATGTCCGTAAATAGGCACTAGTAGAC 36931
NorthernPike              TGCAATTATGT-TAGCACAG-------CTGAAAACTGTTGTGCTGATTAAAGAAGCGACAAAACCGGCCTTCTTTAGACT 10991
SpottedGar                TA-AGAGATCTGTTCAATATTTACGATTTCCTATTTACTTTT---TCAGAGAGGATCAGACGAGCTACTGTCTAGCAGTC 10078
ParamormyropsKingsLeyaeAG-GGAGAAC----------------------ATGCAAACTC---CACACACATGTAACCCAGGCGGAGACTTGA----- 5748

Majority                  AGTA-AAT----C-ATCTCGAGCACAAGTTTTTTTGTCT--A------GTGATTTTAAGTTAATT-AATTTCGTAACCAG

                                   79530     79540     79550     79560     79570     79580     79590     79600

Human                     AGGTAAATTCATCTATTTCGAGCCCAAATTTTTCTGTCTAGAAGATTGGTAGTCTTATTTTTACTAAAGTACAAGATTAT 79306
AsianBonytongue           ----------------------ATGAGTGC-----------------GTGTCTTTAAATTATTT--ATTTCTTTTCCAG 6428
Reedfish                  AGCA-GGTTTTGCAAAATAGGTTACACATTTTTAATACCAGAAA---AGGGAATCCTGGGGAATT-GAATCAGCAAATAG 37006
NorthernPike              AGTGATAT------ATCTGGAGCACCAGCAGTTTTGAGT---------TCGATTACAGGCTTAAAATGTCCAGAAAGAAA 11056
SpottedGar                TGTACAACAGCCCCAACTCTAATATGAGTGGCATGGTTGTAACG---GGTGAGTGTAAATCAATTTAAATTTGTGACCCA 10155
ParamormyropsKingsLeyae--------------------ACCCAGGTCCC----------------AGAAATGTGAGGCAACA----GTGCTAACCAC 5787

Majority                  TGAGTATCTACTGTAATTCTGTTTTGTATTTTTTTTT-AATATAATAGTGTA-GACTGT----CATTCTCTAAGTATCT

                                   79610     79620     79630     79640     79650     79660     79670     79680

Human                     TTAGTAAAAGGTAGCATTCTCTGGCTTTTTTTTTTTTTTTTTAATGTTTCAGATCTGT----CACACTAAAAAGGGCT 79382
AsianBonytongue           AGAGGATCAGACGAGCTGCTGTCTGGAAGTCTTT--------ATAATAGTCCA-AACT-------CTGGTATAAGCAGTA 6492
Reedfish                  TGAGGATATACTGTACTGTTTTT--AAAATCTTACTGTAAATATAGAAGTAAATGACTATTTTTCAAAGTCAAAGTGAAC 37084
NorthernPike              GGACTGTCTCCTGAAACTCATCAGTCTATTCTTGTTCTGAGAAATGAAGGCTATTCCATGCAAGGAATTTCTAAGAAACT 11136
SpottedGar                AATAAATTGAACTTAATTTTGTTTTGTACTTTTTGCTTCAACATTATAGTGAG-GACTGGGTGATCTTCTTTAATTATCG 10234
ParamormyropsKingsLeyaeTGCACCACCACGTCGGCCCTGT---GTATATATG-------CACCACCACGTC-GGCC----------CTGTATATATGC 5846
```

```
Majority                    TCCGTCCTGGTGTTCCTGTTC---TATATGATTCCACCACATTGGCACGGT-A-ATTTAACCATGCATTACTGGGCTGTG

                                     79690     79700     79710     79720     79730     79740     79750     79760

Human                       TTAGACCAGGTGTTGTGGCTCACACCTGTAATCCCAGCACTTTGACA-GGTC--AAGGTGGGAGG-ATTGCTGGAGGCCA 79458
AsianBonytongue             ACGTCTCTGGTATGTCCCTCC---CCCATG---CCCCCACAAAACTA-------ATTTGATCGTG-ATTACTG------- 6551
Reedfish                    TTTGTCATCTCAACCATATACAAGTATACAGATAGACAAAATTGCGAAGCTCAGGGTCCACAGTGTAACAACATGATGTG 37164
NorthernPike                GAAGATCTCGTGTACGCTTTGTACTACTTGTTTTACAGAACCGCGCATGCTTACTCTAACCCACGAGTCACCGACCCGTC 11216
SpottedGar                  GCCGTCATGATAATTCTGTGC---TAAATGATTTTTTCAAAGAAAGACAGA-AGATTCAAACAAGCACTACTAAACTTTG 10310
ParamormyropsKingsLeyae     ACCACCAGGTCGGCCCTGTA----TATATG-CACCACCACGTCGGCCC-------TGTATATATGCACCACCAGG----- 5909

Majority                    -GT-TTTGACAT--------GTAACATGTTGAAATTTCAGTAT-------AAATGTAGTAAATAATCAATATATGTTATT

                                     79770     79780     79790     79800     79810     79820     79830     79840

Human                       GGAGTTTGAGACCAGCCTGAGCAACATAGTGAGATCTCAGTCTCTACTAAAAATAAAAAAAATTAGCCATGCCTGGTGGC 79538
AsianBonytongue             ----TTCTATCT--------GT---ATGTTAAAAGTGCAGAAT-------AACTCTGTTG--TGATCAGCATATGT---- 6603
Reedfish                    CAAATCATAAATTAA---AAATAGAATTTTAAAATTTTAATTT-------AAAATTAAAACACAAACAAGACAAGACATT 37234
NorthernPike                GATCTTGGAAGC--------ATTTTAAGTCGATCGCGAATAGTTT-TCAAAATCAGAGGGAAAAATAAATATATATTTGT 11287
SpottedGar                  -GTTTTTCACAT--------GTCACATGCAGTAACAACAGTAT-------AATTGGGCCAACTGAATAGGATGCTTCATA 10374
ParamormyropsKingsLeyae     ----TCGGCCCT--------GT---ATATAAAATAAAAAGCAT-------AAATGTAGTT--TAAGTGATAGATGG---- 5961

Majority                    ATTCATCTGCAATG--AAGTATTAGGAACAATTTGTAGGCATGATATAT-TAGTCCA--ATTTGTAAATAACAG--AGTT

                                     79850     79860     79870     79880     79890     79900     79910     79920

Human                       ATGTGCCTGTAGTCCCAGCTACTTAGTAGGCTGAGGTGGGAGGATTGCCTGAGCCCAGAAGATAGAAACAGCAGTGAGCC 79618
AsianBonytongue             ATTCATCTGCAAGG--AAGAT---------ATTTGCAGTTAC------------------TGTGCAAATAACC---CCTT 6651
Reedfish                    GTGCAAAGACAAGACAAAGAAGTAGCAGCAATATCGATGTGTAAGATATGTAATATA--ATTAATAAATAATAG-ATATA 37311
NorthernPike                ATTCATTATTTATT-AATTTAATATGCACCATGTGTGAGCCTATTGTGTGCAGTAAATGGTGTCTAGGGGAGGGAGAGGC 11366
SpottedGar                  ATTTAGTGGCATTA--AAGTCTTAGTAACAATTCATAGACATGAGAGAT-TGTTCCC--ATATCCAAATAAAA---ACTT 10446
ParamormyropsKingsLeyae     --CCATCAAACATG--AAATGT---------TTTGTGTGCAT------------------TTTG--A-TAAA------TT 6001

Majority                    GATATTATGGAAATGCGCTGTCGTATTTTT---AAAGTTTGCGTGTTCTTGTACAATATCTGTTGGAGAGGTATTACAAC

                                     79930     79940     79950     79960     79970     79980     79990     80000

Human                       ATGATCATGCCACTGCACTCTAGCCTGGGTGACAGAGCAAACCTTGTCTCAAAAAATAAAGGGTGGGGAAGCTTTAGCCT 79698
AsianBonytongue             GATCTTTTGTAGGTGTGCACCCACACATG----AAAGTATGCGCCTGAAAGTGTAAT-TCTGTGTGCCAGGCATTTCAAT 6726
Reedfish                    GATAATACAGAAATTATCAGT-GTATGGTA---ATAGTTAAGACATGTGTAAACAATAACAGGTCAGAATGTTTCACAGC 37387
NorthernPike                TGT-CCAATCAAATAGCCTATAGTTTACATATGAAAAGCTCTCTCTATGTTTTCGGT-TCTGATGGAGTGGTGAC---GC 11441
SpottedGar                  G-TGTCCTGGAGATCCATTGTCATTCTTAT---AAAATTTGAGTGAACTTGTGTAGTATTTTTGGGAGGAATACTCTGAC 10522
ParamormyropsKingsLeyae     G---GTCTGAAAATAAGT----GCACTTT----AAATTTTCAGTGTTCAT-CACAAC-CCTCAATGACACAAAT----AC 6064

Majority                    AATCGTGTAAAT----ATTACCA--AATAATTTTTTTGATAATT---TAATGTATTTA--ATACTAATGTAATATATTT

                                     80010     80020     80030     80040     80050     80060     80070     80080

Human                       AGGCTTTTAATTCTCAATTACCATTATAAATTTATTTAATTATTAACTAATTACTTTA--ATTCTCAATTACTATTTTT 79776
AsianBonytongue             AA-TGTGTA--------TCAGAG--GATAAGCCTTTCTTGTTTTT---CAATGATTTT-------CTCTAGAGTGGACTT 6785
Reedfish                    AGAAATGGAACA---AAATAACT--GCTCCTTTCCTTGGGTCATTGCATAATATATTGAA-ACACCACTGTCAGAGATTT 37461
NorthernPike                GTTCATGTAAAC----GGCACCT--AAAAGCTGTCTCAAAGAACCAGCAAAAGTAATGATTATGAAAATGTAAAATATTT 11515
SpottedGar                  AACTGTATGCAT---TTTCACTA--AAAAAGGATATTGGATATTT---CAAGACTGTTGC-AAAGTTTGAAAAAAAACTC 10593
ParamormyropsKingsLeyae     AATCGTTTG---------TACCA--ATTA---TTAGTCCATAACT---TGAGGGACTG-------TAAGCCAGCCTCTTT 6120

Majority                    GCATGAAATGG--T-ATAGTCTGATT-----TGTATTTTCTTGTTTTTTTA-TAGAAAGTTAATACTT-ATGAG--AAA

                                     80090     80100     80110     80120     80130     80140     80150     80160

Human                       CCATGAAAAGGCTTGATGTTCTAAACAGTATTATACCACCAAGTTATCTCTACTGGAAAACCCAGATTTCAAGAAAAAAA 79856
AsianBonytongue             GCATGGAA-------ACAGTGTGAA-------AGCTTTCCTGGTTTCTTTTG-TTGTTCTTTCTCACTG--TGAG--ACA 6846
Reedfish                    GTGTTATAACGGAT-ATAATTTGTCTG----TGTAATTTGTTAATTTATTAA-TAAAATGTCAGTAATTTCAGTG--AAT 37533
NorthernPike                CAATGAGACGTTTTAATTGTTTATTAATAAACAAATGTTTTTTTGTGTTGGCTGCAAAGATGAAGTTG-ATGATACTGA 11594
SpottedGar                  TCATCAATTTC--C-ATACTCTGATT-----TGAATATTCTAATACTTTTTT-AAGAATAGAAATACTACTTGTA--CAA 10662
ParamormyropsKingsLeyae     GCTCCCAGT----C-ACAGCCCAGTC-----AGTGTTATGTGGTTCCGTTTA-TAGTGAGAAAACACTC--------GGC 6181
```

```
Majority                  AAATTTATTTTAAAAGTATTCATCAC-CATATATTACAAATTTTTATTGTCATAA-GTTGTCAGTCTTTACTTTAAGTCA

                                   80170     80180     80190     80200     80210     80220     80230     80240

Human                     AAATGTATTTTGAAAACAATTATGTCTTAAATATTTAAAATTCTTTTAAACATGTTTTTAACCATCTTTAAATTTTTTTG 79936
AsianBonytongue           AAGATCATTTTAATTGTGTTGATCA---GCAAACTGC-GACGTTAAATGCTGAAA-GTCGGTAGGAACTACTTGAAGTTC 6921
Reedfish                  GTGCCAGTTCTTATTATAAGCAGTGC-CATATTTAGCAGCTTTCAGTTGAGCCACTATCTTAAGTCACTGCTTTAGGAGA 37612
NorthernPike              CCCGCGATCTTCGCAGAACGCGCCG---GAGAGTTATATACTATTTGTATAATAAATATTTTGCTAGTGACTAACAGGCA 11671
SpottedGar                AAAATTATTTCAGAACTATTCTTTACTCATAAATTACTAAAGGTGATCATAGCAA-GTTGTCACATGTTAAAATAAATCA 10741
ParamormyropsKingsLeyaeTTATTCCTTCCACAATTGCAGTACATGCACATA-TACAATAATCCAGTGCCAGGG-GAAGCCCTTCTCTGGCCTAG--CA 6257

Majority                  TTAAGTAAAGTTCAATTGAACGTTAAACTG-ATTTTGTCAGTATTTAAGGC-A-CTTATTATCTTCAGTTACTTTTTATA

                                   80250     80260     80270     80280     80290     80300     80310     80320

Human                     GTAAGTAATTTTCATTAGATTGTTAAAATGTATAGTGGCACGGTAGGGTTGTATCTCAAGATATGAAGCAGTTTTTCATA 80016
AsianBonytongue           TTAAGCAGAGTGCCATTGGAC-TTAAACT---TCTGAGCTGAATCTGAG-----CCTAGAAAGTCCAGT-ACTTTTAAAA 6991
Reedfish                  TTTGTTAAACCTAGATGAAACGATTTGTTG-AAATTGTCATTGTAAAAGAA-A--GATTTTCTTTTAGGTACTCCATCTG 37688
NorthernPike              ACAAAGAATGTCAAACTACATTGTGGCCTC-GTTTTTTCATAATTGTTTGCAAGTGGTAGATCTTGGTTTACATTTGGAC 11750
SpottedGar                TAAAGAGAAACAAAAGAGATCCTTTGACTA-ATTTTGCCTGCATTTACGGC-ATCTTATTTGCTTAAGTGATTTTTAATG 10819
ParamormyropsKingsLeyaeTAGAATCTACCCCGTTTACCC---AAACT---CCACACCAGTATGCAAAG------AATAAGCCCCAATTCCTTTGTCTA 6325

Majority                  TTGTTGAATTGTCATACTTTAGCAGAGATGG--ATGTGACT-C--TTATGATCTAGCTTGTATAATTTGAATATGTTTAG

                                   80330     80340     80350     80360     80370     80380     80390     80400

Human                     AAAATAAAGTATTAACCTTAGGCTGGTATGTTTAGATCAATACTGTATTTAAATAGATTGATAATTTTTTATATATTTTA 80096
AsianBonytongue           C-----AGAAGCTACACATTAAAAAAAAAAA--AAAAAA-------AAAGAACTCCCTTGTATAATTTGACCATATGAAG 7057
Reedfish                  GAATTGTACCTTCTTACTCTAGCGGTGGTGG--ATTTCCAT----CTTCCCTTGGCTTTGCTCAATCTGCAGGTGCCTAA 37762
NorthernPike              TTGGGATGTGATCTTAGGCTTGAAAAGGTTG----GTGACC-----TCTGCTCTAACCAGAATAAAAAGAGTGGGAGGCC 11821
SpottedGar                TTGTCGAATAGCCAGAACTTAGAAAAGATGG--ATGAGATTTCTATGAGAAAAGGACTTTTAATATGTAAATATATTATT 10897
ParamormyropsKingsLeyaeCT-----TATGTCACAAATTAGCTGAGTTGG--ATGTTACCTCCACCAAGGTCAAGCTTGCATCAGTTTTGCTTGCTGAG 6398

Majority                  TGGTTTTATAATTTATGGAAAAAATAAAAATTATTTCATTGAGTTGGAGACAGATATTGTTTTAGATTCTTCACGTGTTG

                                   80410     80420     80430     80440     80450     80460     80470     80480

Human                     TGAATTTATACTTTTTCCTGAGATTTCAGGAGGGAAAAATGAGTAGGAGATGACTGAGAGCTTAAAGTTTGGGAGTGTCA 80176
AsianBonytongue           GGGTGCTGAAATAAAAAAAAAAAAAAAAAAGAATTTGACCAAGCTAAAGAAGAATATTATTTTAAACCCTTAACACTTTG 7137
Reedfish                  TGTGTTTTACATTGTATGAAAAAACAGTTTTCATTACAA-ATGTTATCATATGCAACTTTTGTATGTTAATCACCAGTAG 37841
NorthernPike              CCGGTGCACAACTGAGCAGGAGAATAAATATTAGTGTCTAGTTTGAGAAACAGATG-TCTCACAGATCCTCAACTGGCAG 11900
SpottedGar                TTTTTTTACAGCTAATGGAAATGATAGCAAAAGTTAA--GAGTTGAAGACAGA-ATGGATTGTCCCCCTTCACGTGTTC 10974
ParamormyropsKingsLeyaeGAGACTGGTGCATTCTGGGATACACTATAGTTCTCTCTTTGCTCTGGCAACAGG---TGAACTTG--TTCTTACACTTT- 6472

Majority                  ATTACTTTAG-AT--TTTTTTAATAAATATTCGT--A-----ACATTATTAGTTTTTGTTAGGCGTTTGTGAATGTAACT

                                   80490     80500     80510     80520     80530     80540     80550     80560

Human                     ATTAACTCAGCAT--TCTTTTAAAAAACATGTGTCATATATTACAGCATTTTCTTTTATTTGAAGTGAGTAAATGTATCT 80254
AsianBonytongue           ATCACTTTAA-----TTGCTATATAATTTTT--------------TTATTAATTTTGGTAATGAAGGTGGATATGTAGCT 7198
Reedfish                  GTTAGAGTAGAGTTATTTTTAAATGTGCATACAATTGTGATCATATTATAAGCTGCTGTCAGGGCATTCAAAATA-AACT 37920
NorthernPike              CTTCATTAAATAA---TACCCGCAAAATACCAGTCT-------CAACATCAGTTGTTAAGAGGCAACTCTGGGAT--GCT 11968
SpottedGar                TCCACATCAGGAA-GCTGCCCAATGAAGTTACAG--AGACAGAAGTTATTGCCTTGGGTTTACCGTTTGGGAAAGTCACT 11051
ParamormyropsKingsLeyaeATCATGCAAC------T-TTTTATCATTATTC----A--------TTGTTGCTCCATGACATGTTTTCCTGGACCTAAAT 6533

Majority                  ATCT-------TTCGTGATAGTTAAGAAGTAAA-ATCGGTAAATCATGTCAATTTTGATTTTTATTGTTCT--GAATACA

                                   80570     80580     80590     80600     80610     80620     80630     80640

Human                     TTTTAAATTCCTTAGTAATTTTTGAGCACTCCATATGTATAAAGCATGTGAATATTTGGTAGCATTTTACAA-ATGTCCA 80333
AsianBonytongue           AAC--------CAC-TGACAGCAAAAAGGTAAG-ACAATTAAAGCAAGTTAATGTAGATTTTAACTGGT----GGATACA 7264
Reedfish                  AGCT-------TTATCTGCAAACAAGAATTACC-AGTGGAAAATTATGCCAGTCAGCAGTTATGTTGAGCT--ATATCCA 37990
NorthernPike              GGCC-----TTCTAGGCACAGTTGCAAAGAAAA-AGCCGCAACTCGGACTGAGTCAGACTGAGATGGGCAAAAGAAAACA 12042
SpottedGar                AACA-------TTC-TCATGCTTAAAGGAAAAA-ATCAGGTGTGCTTGTCACTTGTGTTTTTTATTATTTTG-GAAAACA 11121
ParamormyropsKingsLeyaeTTTA-------TGCATGATCGTGAAGATGCAACCACAGTTGGTTCGGTTCAGTTTGGAAG--GGTTGACAT--GAAAACA 6602
```

```
Majority                  GATATTTGTGTTTGTTTCACTGACTGTCTTAGGT--------CAGTCTTGTAA-TAT-ATTTTATGTTACT-----CGTG

                                80650     80660     80670     80680     80690     80700     80710     80720

Human                     GAGATTTGTGAGAGTTCCTGAGATCTTCATAGGGGGCCCACAAAGTTTAGTAT-TAC-TTTTCACGGTAATACTAAAGTG 80411
AsianBonytongue           GT-------------ACACGTACCAGCTCA-----------CAGCCTTGCAA-TAT-ATCATACGTTAC------CATA 7311
Reedfish                  TCTAAATCAGTGTTTCTCAACCTCGGTCCTGGGGA-------CCCCCTGAGGC-TGCAGGTTTTGTTCC-------AAC 38055
NorthernPike              GACACTTTATTTTGTTTTACTGCCAGAAACATATAGCCTTTTATTTTTTGTAAACGGAAAACCAAATCGCGTTTATGGTG 12122
SpottedGar                GATCTTCGTGATTCTGACATGTATTGTTTCAAGT--------TAGTGTTATAA-TATTATTTAAAGTTACTTGGTCCTGA 11192
ParamormyropsKingsLeyae   GT---------------CAATGACTGTTCTG-------------GCCTGACAC-CAA-GCTTTCTAATGGG--------- 6643

Majority                  TATTTTGCCTATTTTTTGCT-TACACTTAAAAC-ATA-AGTGG-AACTGTCAGCTGGTATCAATGTTTTTTTATCTTGCT

                                80730     80740     80750     80760     80770     80780     80790     80800

Human                     TATTTTGCCTCTTTTTACTTTTTCTCTTAATAGCATACAGTGGTAACTGAAGGCTAATAGTA-TGTGTGTTTAT-GTGCT 80489
AsianBonytongue           TATTATGTCCATATATTGC---ACAGTT-----------GCAA-AGCTCTCAGCTGACATCAAAGTATGCTTGCATTGCT 7376
Reedfish                  CAGATTCCTAATCAGTGACAACAC-CTGATAGCACTGATCTCA-TTTAATTAGATGGTATTATTTTTCTTTTATCCTGCA 38133
NorthernPike              TACTCCTCCCTCCTTAAGTCCTTGACACCAAACTTGATAGTGGAAACAAAAGTCTGGAGCCAATATTCACATAAATCACC 12202
SpottedGar                TATTTTGTTTTTTTTCTCCTGAATACTTTAGCTGAAAGAGTGA-AACTGTCAGCCATTAGCAAGAATTTTTTATTTAACT 11271
ParamormyropsKingsLeyae   -GTTCTGCGGATTTGTCAG--------------------GTAG-AGCAGTGAGCTGGTGTCACTGTCCCACGGCCGTATC 6701

Majority                  TT----A-A-CGTAGCTGTAGGCATTCATAATTGGTAAGGCCTTTTGT-A-AGTACT--TTTTTCAATAGAAGTAAGTGC

                                80810     80820     80830     80840     80850     80860     80870     80880

Human                     TTAAAAAGTTCGTGGTTTTGGCCAGGCGCAGTGGCTCAGACCTGTAATCCCAGCACT--TTGGGAGGCTGAGGCAGGTGG 80567
AsianBonytongue           T---------CGTAGCTGTAACCCTTCATAGTGTGTAGTGTATTCTCT---------------------GGAAGAAGCAC 7426
Reedfish                  TTCAGAAAAGCACAGCAGCATGATTTTTACATTTATAAGACATTTAGAAATATTTCTGCTTTTTCTATAGATTTAAATGC 38213
NorthernPike              AGTGTGACCCTGGTGCCGGGGTAAAGCACCAGTGGAAATGCTGCATGAGTGACCCCAAACTTTTGAATGGTAGTGTATGG 12282
SpottedGar                GA------AATAGACCTTTAAACATTCAAAATAAGTTTGGCCATCTTTTA-AGTAGTGAATGGACAACAGAAGGAAATGC 11344
ParamormyropsKingsLeyae   T---------GTTATAAAGAGGTGTCTTTGCTTGGGCTAAGCCTGTGC---------------------GAATCAGGTAC 6751

Majority                  TTTATTTTATTTTTGGCGTT-TTAGAGTTAAACATTTCTTGTCTATGTAGT-TCTTCCCTTTTCTGCTAATAGTATTAAT

                                80890     80900     80910     80920     80930     80940     80950     80960

Human                     ATCACCTGAGGTCAGGAGTT-CAAGACCAAAACCAGCCTGGCCAACATGGTGAAACCCCATCTCTACTAAAAATACAAAA 80646
AsianBonytongue           ACTAATTCATTTTT-----T-GCACAATTAAGCATTT--------TGCATT-TCTTCACGTTACAGCTAATGGAAATGAC 7491
Reedfish                  TTAATACTCTTTTGTTGATT-TCATCATATTTTGCCCTTTCTCTGTGCAGT-TTTTCCCCTTTGT-TTTATCTTATTAAT 38290
NorthernPike              ATGAAAGCAAATTCATCTCTATTGAACCAAAACATTGAGAGAATTTCTAAAGCCCTAATTTGTTGTGGGAGAGTGTCATT 12362
SpottedGar                TTTTTTTTTTTTTTGGAGCT-GTAGAGTTAAATTTTAGATATTCATGTATG-TCTTCATTTTCCC-CCAACAGGCCTTCC 11421
ParamormyropsKingsLeyae   TCTGTTTGACATGCCGCA-T-TAACGGCTTTGCACCTC-------TGCTGT-AATCACCATCACAGCTAATGGAAATGAT 6821

Majority                  ATTAATT-GAAATTGA-GAGTGGAAGACT-ATGGTGGACGCCACTCACTCTG-----CTGTGGCATC---CACATTAGTA

                                80970     80980     80990     81000     81010     81020     81030     81040

Human                     ATTAGCC-AGGCATGG-TAGTGGGTGCCT-GTAGTCTCAGCTACTCGAGATG-----CTGAGGCAGGAGAATCACTTGAA 80718
AsianBonytongue           AGTAA---AAAATTAA-GAGTGGAAGAA--AGGATGGACTGCCCTCCCTC-------CCGTGTCATC---CACATCCGTA 7555
Reedfish                  GAGAATTAAAAATGAG-CAGCGCAGACACACTGGCAAACAAAACTGAATAAG-----CAAAGGCTGCATCTACTTTAGCA 38364
NorthernPike              GTTAATCTGAAATTGCTGAATTCAAAACCAACGGCTGGTGCCACCAACTGTGAAAGTCTGTGGCACC---AGCTTCACTA 12439
SpottedGar                TTGAACTTGGAACTGA-GGAAGCAGCTATTACAATGGTTAACTATTACACAG-----CTGTGACTCCT--CATGTTCGTA 11493
ParamormyropsKingsLeyae   AGCAA---GAAACTGA-GAGTGGAAGAC--AGAATGGACGCCACCCCTTC-------CCGTGTCCTC---CACATAAGGA 6885

Majority                  ACGTGCCCATTT-TTATGAAGT-ATTCGATACAGAGCAATTT-A--ACAGTCATTGC-ATA--A-ACTGGGCTTGCCTCT

                                81050     81060     81070     81080     81090     81100     81110     81120

Human                     GCTGGGAGGTGGAGGTTGCAGTGAGCCAAAATCTCGCCATTACACTCCAGCCTGGGGGACA--AGAGCAAGACTCCATCT 80796
AsianBonytongue           AATTGCCCA------ATGAAGT-CTCCGAGACGGAA-------------GTCATTGC--------ACTGGGCTTGCCTTT 7607
Reedfish                  TCGGATCCACTAATTAGTAAAT-AATGGATTAATTAAACAATTAGAACACCTAGAAAAGTA--G-AATGAAAAT-CAAGA 38439
NorthernPike              AAGTGAAATGTTTGTCTGGAGC-CCTGGATAAAGAGCAATTTGTTCAAATTCACCCACAAAGCAAAAAAAGCCTAAGACT 12518
SpottedGar                ATGTGCCCGTTT-TCATCCAGT-ATTCAAATCACAAAGAATTAAAGACTGACAATGCTCTT--A-ACCAGGTATGTTCCG 11568
ParamormyropsKingsLeyae   AGCTGCCCA------ATGAAGT-TTCCGAGACGGAG-------------GTCATCGC--------CTTGGGCTTACCTTT 6937
```

```
Majority                  TGGGAATGTTACAAATATC-ATAT----CTAATGCTTCCAGGAAATAACCAGGTATCTAATTGGTCTGTTGTCGAGATTT

                                  81130     81140     81150     81160     81170     81180     81190     81200

Human                     CAAAAAAAAAAAAAAAAAAGTTATTGGTTTTAATTTTGGAAATGACAACCATTAATAGAACCCACATTAGGCCGGGCGCG 80876
AsianBonytongue           TGGGAAAGTTACAAATATC---------CTGACGCTAAAAGGAAAAAATCAGGTGCTTGGCTTGTCTGTGAAGCAGCTTT 7678
Reedfish                  TGAAAATATTGTTAAAAAGAAAAAAAT-ACATTATTCCCATATAACGTCTTGGTACAT-TTTAATATACAGTATAGATAT 38517
NorthernPike              CAGGCCTGTTCGATAGTTTCCTCT----GTAATGACTCCAGGCCTTGCCTGACTGTCTCATCAGTCAGTTTTAGCGGTGC 12594
SpottedGar                CTAGATTGCTAAAAATGACTAGGACAG-CTAACTCCACCTGCATCTAAAAAGACCTCAAACTGGTGTTGTAACCTAATTA 11647
ParamormyropsKingsLeyaeTGGGAAGGTCACCAATATC---------CTGATGCTTAAGGGAAAGAATCAGGTAC---GTCGGTCTGCTG-CAAGATGT 7004

Majority                  GTGTATCTTGTCTTTCTT--AA------TTGTTTGTCAAGATTCGGT-ATATGGCCAAATCAGA---CTTGGGTCATGAC

                                  81210     81220     81230     81240     81250     81260     81270     81280

Human                     GTGGCTCATGCCTGTAATCCTAGCACTTTGGGAGGCCGAGACG-GGT-GGATCACGAGGTCAGGAGATCAAGACCATCTT 80954
AsianBonytongue           CTGTACAATGCATTTCTG--AA------TTGTTAGTTAAGATAAGGTAAAGTAATCAAATCAGT---CTTGAGTCCTCCC 7747
Reedfish                  ATATATTTTTTTCTTTTT--AACCAAGCTTAATTTTCTAATTTCTATATTATTCCCAAAACACAGAACTTGGGAAATAAC 38595
NorthernPike              TTGGAGCATGTCAGCCACCAACTGCAGCCTAACGGACAGAGGACAGC-TCAGAGCCTACCGGGAAGCACTGGGGAACTCC 12673
SpottedGar                AGGAATGTCGTATTTAT---AA------TGGTTTCTTAAGATT-GGTGAATTGTGTTAATCCCG---CTTGCGTCATGAC 11714
ParamormyropsKingsLeyaeGACTTTATCCTCTATGCT--GG------ATGTCATTCTGGGCTCCGT-ATCTGGCCCGGTAGTA---ATAGAG--ATGAT 7070

Majority                  TGATAAATT--TTTGCCCTGGATTCT---GAAAA---GCAACATTAGCTTGATGTAAATACTGAATTTTATAGTCAGC-G

                                  81290     81300     81310     81320     81330     81340     81350     81360

Human                     GGCTAACACGGTGAAACCCCGTTTCTACTAAAAATACAAAAAATTAGCCGGGCGTGTTGGCGGGCGCCTGTAGTCCCA-G 81033
AsianBonytongue           T-ATAGACT--TTTGAATTGG--------GAACT---GTCACCTTAACATGAAGTGAAAAATTAATTTTGTCTTCAG--- 7810
Reedfish                  AGTTCACTTAATTAGCCCAGGAGTCCAATTAAAA---ACAGAAGCTGGTTGGAACAAAAACCTGCAGCCACAGTGGGTCC 38672
NorthernPike              AGATGAAATGACATGTCTGACATCTT---GAGACTCTGCAAGATTAAACTGACGAAACTAGAAAAAGAACCAACCTACCT 12750
SpottedGar                TGATACATT--TTCACGGTAGCGTCTTCAAAACT---GTTATATCTGTTAAATGTACATAATGAGCTTAAAAGTGAAA-- 11787
ParamormyropsKingsLeyaeC--TGTATG--TCCACCCGGG---------GAGA---GCCACAGC----TAATGCATATGCTGCATTTTATGCCAAGCAG 7130

Majority                  GCATTTTTGAGGCTGGG-----------------GT-----TGACAGAGC-TTGAGGCAGCCGTTACCATGGTGAATTAT

                                  81370     81380     81390     81400     81410     81420     81430     81440

Human                     CTACTTGGGAGGCTGAGGCAGGAGAATGGCATGAACCTGGGAGGCGGAGCTTGCAGTGAGCCGAGATCACGCCACTGCAC 81113
AsianBonytongue           GCCTTCTTGGAGCTTGG-------------------------GACAGAG----GAAGCAGCCATGACAATGGTCAACTAC 7861
Reedfish                  CCAGGACCGAGGTTGAGAAACACTGATCTAAATGGT---GTTGGTATAGC-TTGTCATAGTAGTGCCCAAAGTGAATTAT 38748
NorthernPike              GGTGTTTTATGTCTCCCCTCCA------T--TTCCT----CAAACTGTCCATAGAGGCCTGTATTTCATTGTCTTTTTAA 12818
SpottedGar                GAACTATTTACATTTGAT----------------GC---AATGACAAAATACTGAGGAAAGTGTTACAATTAAAAATTAA 11848
ParamormyropsKingsLeyaeGCATTCCTGGAGCTGGG-------------------------GACAGAG----GAAGCCGCCATTACCATGGTGAATTAT 7181

Majority                  TACACGGTTGTTGCCACT--GACACCTCATGTCTGT-AAATGAAGATA-AATATACCAGTCTTTATTTATT---ACTC--

                                  81450     81460     81470     81480     81490     81500     81510     81520

Human                     TCCAACCTGAGAGACACAGCGAGACTCCGTCTCGAAAAAAAAAAAAAAAAAAAAAAAGAACCCACATTAACAAAAACTCCT 81193
AsianBonytongue           TACACAGCTGT---------GACACCTCATGTTCGT-AA------------CGTTCCTGTTTTTATTCAGT---ATTC-- 7914
Reedfish                  TATTCCATTATAACATATGACATAAACAAAAAATATTAACTGAAAATATTATACACAAGACTTTATTTGTTTT-GCTC-- 38825
NorthernPike              TGGGCTCTGCTTGCTGCA--AACGGAACATGTCTGT-TCTTGGAGAGCCATACCATAAGCCTTTATCTATTT--GTTTCT 12893
SpottedGar                CGAATGTTTGTTGCCAATTCGACACCTTTCACATGT-AAACCTGGATAGAATATAATAGAACTTATTTGCA---AATA-- 11922
ParamormyropsKingsLeyaeTACACGGCCGT---------GACACCTCATGTCCGC-AA------------CGTCCCCGTCTTCATCCAGT---ACTC-- 7234

Majority                  ---GATTCTCAATTATCTGAAGACTGACAATG----------CCATCAACTAGGTAATCTTTTACACTGTAAGACCTGTC

                                  81530     81540     81550     81560     81570     81580     81590     81600

Human                     TGTGATTCTCCATAGTTTTAAGACTATAAAGGGGAGCTGAGACCAAAAAGTTTGAGAACTTTTTCACTGTGGGATACAAA 81273
AsianBonytongue           ---AAATCACAAAGAGCTGAAGACTGACAATG----------CAGGAAACCAGGTAAGGTTTTACACTAAAAAGCACATC 7981
Reedfish                  ---AAATTTTAATTTCCAAACACATCACAGT-----------CAAACAACTAATTAATGTGA-ATGTACGAAGACATAAC 38890
NorthernPike              TGTTTTCTCCTTTCTCCAGAGAGGATCTGATG---------AGCTGTTATCAAGCAGTCTCT-ACAACAGCAGCTCTGAC 12963
SpottedGar                ---GTTTCCTCAATATGTGTGTGCTAACCCTG----------CAATGGACT--GCCGTCCCTTCCAAAGTGTAGCCTGTC 11987
ParamormyropsKingsLeyae---CAATCACAAAGAGCTGAAGACCGATAATG----------CCCTCAACCAGGTGAG-----------AGGACCAGTC 7289
```

```
Majority                  CT-----ATATCTGTGGTTAGTTTATCTGGAA-TGTGTGGTGCTCCAATTATCAGTATTTGA-AAGATGTTTAAATAGAA
                                   81610     81620     81630     81640     81650     81660     81670     81680
Human                     GTTGAAGACAATTATAGTTAAATAAGCAGGAGATACAGAGTATTTTAATGAACAGTAGATGGTTATAAGAGTCAAGAGAT 81353
AsianBonytongue           CA----CACATTTGTCATTAGTT-------AA-------GAGCGGCAGTTATC-----------AAATATCCCAATACCA 8032
Reedfish                  AA-----TTTTCTGAGCTTTGCTGATATAGAATTGCTTGGTTATACAATTACTGGGATTCAA-AAGATGTTTATACAGTG 38964
NorthernPike              ATGAGCAGTATCAGTGGTAAGCTTTTTTTTGGGGGTGGGATCCCCCTTTTCTCACTCTGTCA-GCCCAGTCTAAATGGAG 13042
SpottedGar                TTGTGTTTTGTCTGCCCTTTGTTTCGCTGCAACCCTGTTGTGCTCCAATCGTCTGTATTGGACAAAGTGGTTAAGAATAA 12067
ParamormyropsKingsLeyaeCG-----A-GCGTGAAGGCAGT-------------------G------TCATC-----------AGAGG--CTGGCATAA 7325

Majority                  GTG-AGTGAATATGT-TGTGTTTTGAGGATTTGTAATTATAATCATTTATATAGTTTTTAGTTGTTCCTACTTTGATGTT
                                   81690     81700     81710     81720     81730     81740     81750     81760
Human                     GTACAGAGAAAATGCTAGTGATTAGAGTTTTT-AGATTATTTTAATTTTGGTAACTAATTATAATTCCTCAGTTAAGATT 81432
AsianBonytongue           GTG-TGTACCCA-------ATCCAGAGGTGCTCTAGTGAAAATGGTC---AGAGGTTTAAGCTGATTCCACCTTGCTGTT 8101
Reedfish                  GAGGAATAATTATTTGATCCCCTGCTGAATTTGTAAGTTTGCTCACTTACAAAGAAATGAGCAGTCTCTATTTTTATGGT 39044
NorthernPike              ACTTCATGAATGTAT-TTTGATCGGCAAGACAATAAGCAGAAGGATTAATGTATTTGTACGTATTCCTTGCGGGGGGATA 13121
SpottedGar                GTG-AATGGACACGGGTGTATTTTGGAGACTTGTATTGAAAACCTTTTCAAAAGTTTTTAGATGTTGGCATTTTGAAGTC 12146
ParamormyropsKingsLeyaeGC--CGTAGCTA----TGTTCTCTGAGGAGC--TGGTCATGCTCGTTTGTTCAGTTCTGTCGTTTTCTTCCTCCCTCGTC 7397

Majority                  TTTTTG-ATTTTAATGGAAGCGTGCACAAG-TCAGTCC-------ATCCAGGCTGTAACAGCTGTCCAGGCAGGTGGTAC
                                   81770     81780     81790     81800     81810     81820     81830     81840
Human                     TTTTGG---CTTAGTGGGCCCTTAGAATTACTTCTTCCTGTTAGAATTAAAAGTGAAACCAGAAATTAAAGTGATTTAAT 81509
AsianBonytongue           TTTTG----TTCTGTGGAAGCGTGCACAGG--CTGTCC--------TCCAGGCGGTCTCAGCGGTTCAGGCAGGTGGCAC 8167
Reedfish                  AGTTTC-ATTTTAATGGAGAGAGACTCAATATCAATCAAA----AATCCAGAAAAAAAACACATTACATAAAAATTGTAA 39119
NorthernPike              TTTCCGTATCTAAATAACTTCAGGCTCTGCATTAGTTTGGCACCCACACACAAGCTAGCATGTTACTGGAATAGTGATGC 13201
SpottedGar                CATTCA-ATTTTCATTGTAGCGTGCGCAAG--CAGTTC--------TTCAAGCTGTAACTGCTGTCCAGGCAGCCAGTAC 12215
ParamormyropsKingsLeyaeATTCT----GTCTTTCGAAGCGTGCACAAG--CGGTCC--------TCCAGGCCGTCACAGCTGTCCAGGCAGGAGGCAC 7463

Majority                  TCTGTTTTCGGGGACGACTGCTAGTGAGAGTGCG----TT-----GACTCC---AGCGCCAAGTCTAGTGCTCA-AGAAT
                                   81850     81860     81870     81880     81890     81900     81910     81920
Human                     TTTTTATTGTGGCAAAGATCTAGTCCAAATATC----TTTGATGGACTTTTTGAAAACAGACTTTATTGTTAAAAAGGG 81585
AsianBonytongue           GCCCTCTACGGGGACGACTGCCAGTGAGAGCGCC----TT-----GACCCC---CGCACCCAGCCCAGTGCTCA--GAAT 8233
Reedfish                  ATTGATTTGCATGTCATTGATTGAAATAAGTAT-----TT-----GATCCC---AGCCAGAATACTGGCTCCCACAGATT 39186
NorthernPike              TGTGTTCTCTAGGAGGATTTGTCTTTTTAGCACGGACCGTGTCTAATCTTTTAAAGAGGAAAGTATGGTACGTT-AGATC 13280
SpottedGar                CCCAACATCAGGGACTACAGCAAGTGAGAGTGCA----TT-----AACACC---AGCTCCAAGTCCAGTGCTCA--GAAT 12281
ParamormyropsKingsLeyaeCCCGACGTCGGGGACGACGGCCAGCGAGAGTGCG----CT-----GACGCC---GGCGCCCAGCCCCGTGCTGC--GGAT 7529

Majority                  AATT----AT-TGACA----ATATGTTCTA-----TCCTGTCACTCTGGATGTTCTTCAA-CAGGTATGGCTTTAGTTGT
                                   81930     81940     81950     81960     81970     81980     81990     82000
Human                     AATTTGAGGCTTGGTACTGAACTTCTTCTGCATGCCATATTGAAGTTGGTGGTTTTTTGT--TTGTTTGCCATCAGTGGG 81663
AsianBonytongue           AATC----AT-TGACA----ACATGTTCTA-----TCCCGTCACTCTGGATGTCCTTCAA-CAGGTCAGGCTGCAATT-- 8296
Reedfish                  GACT----GTGTGCCC----ATATGGCACACAGATTACAATCAGTCAATCAATCAATCAATCACAGATACTCCTGATCTC 39258
NorthernPike              CTGTGGAGATGTTCATCAGTGTTCATTTTA-----TTTCCAAACTTAAAATATTTTGCATTAAGATCCTAATTAGGTTGT 13355
SpottedGar                AATC----AT-TGACA----ATATGTTTTA-----CCCTGTTACACTGGATGTTCTTCAG-CAGGTAAGTTGGTATTTTA 12346
ParamormyropsKingsLeyaeAATC----AT-CGACA----ATATGTTCTA-----TCCTGTCACCCTGGACGTCCTTCAA-CAGGTATGGCACTCGTGGT 7594

Majority                  CACTCATTATTTCTATCAACTTTAA--G--TGGTTA------TGTTTATCTTGA--CGTT--AACTTTTTCACAGGAAGC
                                   82010     82020     82030     82040     82050     82060     82070     82080
Human                     ATCTCATACAGACTATGGAATTTAATAGAATTTTTAAGATACTGTTTTTCCTAAAAGGTAGCTAACTAAAGGCAGGAAAA 81743
AsianBonytongue           --------ATTTCTGTTTCCCTTC------C--CTA------CCTTTATCCTG---CAC---AACTTTTTCACAAAAA-- 8346
Reedfish                  AACTCATTATGTGTATAAAGCAGCCTTG--TCCACA------GAATCAATTTCTTCCATTCCAACTTCTCCACCACCATG 39330
NorthernPike              CCTAGCCTAATGTTTTCAGCTTTAA--A--CACGTATTGGTGGGACTGACTCAATGTGTT--GCCTTCTCCTGAACTAAC 13429
SpottedGar                TACTCTTAACTTTTGTCTGGTTTA------TGGTTA------TTTTTAACTTGA--CATTA-AATTTTATTTCAGGCAGT 12411
ParamormyropsKingsLeyaeCATGCGGCCTCGCTATAAACCACAGAGG--CGGCCG------CGTTCGTCTTCT--CGT----ATGTGCGGATAGGAAGC 7660
```

```
Majority                    -TTGAAAGATCTAAGAGTTAGATGACATTTTTT-TGACTTGTGTTTTTA---GTACAAGC-TGTATTGGA----G-TGTC

                                     82090     82100     82110     82120     82130     82140     82150     82160

Human                       -TAGAATGATGTAACAATATAATGACTACATCTGGAGTTTCTGCTACTA---ATAAATAATTGTATTGTCAACTTTAGTC 81819
AsianBonytongue             ------AAATCTAA----CAGATGTCTTTTGCT-TGATTTTTATTTTAA---TAGCAAGC-TGTGCTAG--------GTC 8403
Reedfish                    --GGCAAGACCAAAGAGCTATCAAAGGATTTCCAGGACAAGATTGTAGACCTGCACAAGGCTGGAATGGGCTACAAGACC 39408
NorthernPike                TTTTAATGAGTTGTGAGTTTAGTTACATGCTTT--AACTTTGGTACTCA--CCACTACAT-TGTGAGAGA----GCATTC 13500
SpottedGar                  GTTCTTAGATCCCAAGCACAGGTACCATTTTTTATGTCTTGTTCTTTAA---GCACAAGAGTGAATCAGA----GGTGTC 12484
ParamormyropsKingsLeyae-CAGACGGGGCTGAGGACTGCAAGGCAGTGCTA--GGCCTGGGTACTTA---GTTTATGC-TTTACCGAA------TTTC 7727

Majority                    ATGAAGTCT---CAGTTTGTTAATG-----TTATGTGCATAGTTTGACAGATATTTTCAAAATG----GAAGACAGTTTT

                                     82170     82180     82190     82200     82210     82220     82230     82240

Human                       AAATATTCTGTGCATTCAGTCAGAG-----CTATCTGAAAATACTGACGAATAGTATTACAATATGGGGAGGTCACTTCT 81894
AsianBonytongue             AACAAGACT---TACTTTCAAAAT-----------------ATTCAAAGAATTTTTTCGAAAT------AACATTATTTC 8457
Reedfish                    ATCAGCAAG---AAGCTTGGTGAGA-----AGTTGAGAACTGTTGGTGTGATTATCCAGAAATG----GAAGAAATATAA 39476
NorthernPike                AAGTGGTGCTGGGAGTTTCTCAATCGGCCCTTGCTCACTTGGTTTGCTACACGTAGTAACTATAGAAACATGTCACTTTG 13580
SpottedGar                  ATTAAATGT---AGCTTTTCTAACC-----AGACCTGTTTCAATTGACAGATATTTTCAAAATT----TGGAACTGTTAT 12552
ParamormyropsKingsLeyaeATGGTGTCG---CAACCAAATGGAG-----TTAGGGACAAAGGATAGCAGATAGTGCCAGTGTG----GGAAGCGGTACA 7795

Majority                    TATGACCGTTA--CATCCTCAAACTGTA-GTCA-TGTGTTTGGCTTTGCATTTGGTGAATGTAATGGTGGATACGGTGAA

                                     82250     82260     82270     82280     82290     82300     82310     82320

Human                       CAAGACTGCAAGAAAAAAAAATTGTTGTGTGTAGGAATGTTTGCCTAGGAATTAGGAATGTATACTTGACAACAAGTGAAC 81974
AsianBonytongue             TACGTGCGTTA--CA------GACAGCA-GT----GTTT---GTTATGCACCTGCTGCTTTCATTGGCAGATCTTCT--- 8518
Reedfish                    AATGACCATCAGTCGCCCTCGGTCTGGA-GC-ACCATGCAGGATCTTGCATAGGGTGAGGATGATCATGAGAAAGGTGAG 39554
NorthernPike                AATGTGAGTGCTAAATCCGCAAAAAGGTAACAATTATGTTT-TCTTTTTAGATGGTACATCTAACGGGAGTGACAGTAAA 13659
SpottedGar                  GAAGATCATTA--CATTCACAAAGAACA-ATCA--GTTTCAAGCTTTACTTCAGTTTAATGACCCAGTGAATGCCCAGCA 12627
ParamormyropsKingsLeyaeTAGGGCAGCTA--CAGGTTGAAACTATC-ATCGATGTGTTGGGCAATGTGATGGC--AATGTAACAGTGGAGGGCGCT-- 7868

Majority                    GGCCTAATTGTGAAGTACACGATAGAAACTTGTCACTTTTATATTC-GAAGATGTGGTGAT-GTGACGAAAGAGACTATT

                                     82330     82340     82350     82360     82370     82380     82390     82400

Human                       GTATCCATTGAATGTTAAATGAGAACAACTTATGGGGAGGATAGTG-AGAGGTATAGTATTTAACATGGAAAAGTTAGTA 82053
AsianBonytongue             --CCAAATTTGGAACTGT-CAT--GAAAATCATCACATTTACAAA--GAACAACCAGTTC------CAAGCTTTACTGC- 8584
Reedfish                    GGATCAGCCCAAAACTACACGGTAGGAGCTTGTTAATGATCTTAAG-GCAGTTGGGACCACAGTCACCAAGAACACCATT 39633
NorthernPike                AAGCTGAGAGTGGAAGACAGGATGGAGGCTGCTCCCTCTCGTGTTCTGCACATCAGAAAAC--TTCCCAATGAAACTACA 13737
SpottedGar                  AGCCAAATTGGTGAGTGA-AGACAGAAAATGGCTATTTTTATGTTC-AAACAGTTGGTGTGCATGTCGAAAGAGACTAGC 12705
ParamormyropsKingsLeyae-ATCTGGTCTTGACGGAC-CCACCCAAAATCTCCAAGGTT-TAGTC-AATGACGAGGTGATGGGGACAAAACAAGCCATT 7944

Majority                    AAGTTATAGATTATTTCAT---GGTAAATGCAAATTGGGCTA-T-AAAGCCAGCCTGGTG---ATT-TGCTTTA-TGAGC

                                     82410     82420     82430     82440     82450     82460     82470     82480

Human                       ATATAGTAGAATATTTGGT-TGAAAAGATCCACATTGGTGTATTCAAAGACACACTGAAGACTATTGTGCATTAGTAAGT 82132
AsianBonytongue             -AGTTCAA--TGATCCC------GTGAATGCCCAGC----------AAGCCAAACTGGTG---AGT-CGCCTTG-CGTAC 8640
Reedfish                    -GGCAACACACTACTGCATACAGGTCTGTCTTAAGTTTGCTAGTGAACGTCTAAGTGATTCAGAGA-AGGCTTG-GGAGA 39710
NorthernPike                GAAACGGAGGTCATTGCCT---TGGGGTTACCTTTTGGGAAAGTCACAAATATCCTGACGCTAAAGGGAAAAAACCAGGT 13814
SpottedGar                  AAGTTATG--CTATCTT------TAAAATGCAAAATTGACTT----GATCCAGCATGGTC---AAT-GGCTTTA-AGAAC 12768
ParamormyropsKingsLeyaeCAGGTACAGTTTATCTAA---AGGTAACTGCAAACTGAACTG----CTTCATGCGCAGTT---CTT-TGTTTT--TGTGC 8011

Majority                    ATTTAATGTTAGAATGCTGTAATTGTATT--ATTTGCTGTTAATTGTATATATTTGTTTTAAGT-TATTAGGGGTTATGA

                                     82490     82500     82510     82520     82530     82540     82550     82560

Human                       ATTAAACATGAGAATTCT-TAATTTTAAA--ATTGGACCCTAATTTCTGAGATTTACTTTAAGAATAAAAGGGGATGAAA 82209
AsianBonytongue             ------------AATGCT-TAATTCTACT--ACTTGCTGATAAT-GTATATATTTCTTTTGAAT-CCCCAGACTGTATGA 8703
Reedfish                    AAGTGCTGTGAAAGTGCTCAGATGAAACCAAAATCGATCTCTTTGGAATCAACTTGACCCGCCG-TGTTTGGAGGAAGAG 39789
NorthernPike                GAGTGAGAATA-AACGAAGCAGCCATTTTAGAATTTCTGTTACGGACATTCCCCTATCACAAATATAAACGCAACATGCA 13893
SpottedGar                  CTTTACTTTTGTATTGTTGCAATTGTATTTGATTTAAGATCAATAGTATGTACTTGCTTTAAGT-CATTAGTCTTTTTGG 12847
ParamormyropsKingsLeyaeATTTAATGA-GGAAAGTGATGACAGCATC--AGTCCCT--CGTTTGTATAGATCTTTTCCAAGT-TCGGAACGGTCATGA 8085
```

```
Majority                   AGATCATGA-CAT-TGTTCATTATTGTTAATGTTCTT---TAGGCTCTGAGGGTGG----AT-----TCAGT-----ATT
                                   82570     82580     82590     82600     82610     82620     82630     82640
Human                      CGAGATTGGCCATGAGTTGATCATTGTTAAAGTTGGGTGATGGCTACTTAGAGTTGCATTATTTAGTCACTTTT--ACT 82287
AsianBonytongue            CGTTCACG------TGTTCATTAATGTTTTCTTTCC-----AGGCTCTA--GATGG-----------TCAGA-----ACA 8754
Reedfish                   AAATGCTGAGTAT-GATACAAAGAGCACCATCCCCACAGTCAAGCAATGGAGGTGGAAACATTATGCTTTGG-----GAC 39863
NorthernPike               AAATATTGATCCTTGTTTCATGCTGTGAAAAGATCCTCAATGTATTCCCAGTCATGTGAAATCCAGTTAAGTTCTTAATT 13973
SpottedGar                 GGAATATGAAAAA-TACTGATTATTCCTTCTGTTCTC---AAGGCTCTG--GATGG-----------TCAGA-----ATA 12905
ParamormyropsKingsLeyaeAGATCATCA-----CGTTCACCAAGAACAATCAGTTT---CAAGCCCTGCTGCAGT-----------TCAGC-----GAC 8141

Majority                   TCTAT-AATGC----------CTACTGCACATTGCGTATTGACTTTTCTAAGCTGGTAAATTTTAATGTCAAGTACAGC-
                                   82650     82660     82670     82680     82690     82700     82710     82720
Human                      TTTGTATATGTTGGAAATCTTCCAAAACAAAATGTATAGCCATAAAAAAAAGGGAGCAAAGTAAGAAAAAAAGGAGAGCC 82367
AsianBonytongue            TCTAT-AATGC----------CTGCTGCACATTGCGCATCGACTTCTCCAAGCTGGTAAACCTCAATGTCAAGTACAAC- 8822
Reedfish                   TGTTTTTCTGC----------TAAGGGTACAGAACGACTTCACCTCATTGAGGGGCCAATGGATGGAGCCATGTACCAT- 39932
NorthernPike               TCAATCAGCAGATGTATTTTTTTACAACAATTAAAAAAAAGATTTTTTTATTTTTGTTCAATATACTAT----TTTTGTT 14049
SpottedGar                 TTTAT-AATGC----------CTGCTGCACTCTACGCATTGACTTTTCTAAACTGGTCAATTTAAATGTTAAATACAAC- 12973
ParamormyropsKingsLeyaeCCCGTGAACGC----------CCAGCAAGCAAAGC-TGGTGAGTTACCTTAGCGACCCGCTCTGAATTC----TGCAGC- 8205

Majority                   --AATTACAAGAGTCAGGACTACACTTGTCCCTAGCACCCTACTGGAGAT-GGGCGGCCTGGTATGGACCTTTCAGTATT
                                   82730     82740     82750     82760     82770     82780     82790     82800
Human                      TAAAAAAAAAGGTACAGGCAAAAAGGTGTGAAAAGAAACTCATCAAGAGCAAGAGTATTAAATGAGAAACATATGCCACT 82447
AsianBonytongue            --AATGACAAGAGTCGGGACTACACAAGACCAGAGCTCCCTGCTGGAGAT-GGGCAGCCTGCCATGGACCCCTCAGTGGC 8899
Reedfish                   --AAAATCTTGGATGAAAACC-TCCTTCCCTCTGCTACAACACTGAAGAT-GGGTCG--TGGAATGGTCTTC-CAGCATG 40005
NorthernPike               TTCCTTTTAAACCAGTGATTTTCTTTTAAGTATTGCATAATACTGGATGCGTGCCGGATTTTTGTTTCTGGTTCATTAAT 14129
SpottedGar                 --AACGACAAGAGCCGTGACTACACCCGTCCCGAGCTTCCAGCTGGAGAT-GGTCAGCCCAGCATGGACCCTTCTGTAGC 13050
ParamormyropsKingsLeyae--ATGTGTAAGAAGCAGGTTT-TAAAGATCCCATGAACCCAAACAGAGA--GCCAGGCCCGGTTTAACTTTCACCCTGTT 8280

Majority                   TGCAGGATTTAATAAGGATTCATCATTTCTACTAAGTATGAACTGAGGTGTAA--------AGTCAATGCTCTAA-GCTG
                                   82810     82820     82830     82840     82850     82860     82870     82880
Human                      GACAGAGTTCTCAGAAGAGATAAAGGCTGAATTAAAAAGAGATGGAGGGGAAAGTTTTATAAGTCAAAAACTTAG-GTTA 82526
AsianBonytongue            AGTAGCTTTCAACAAGGATTCATCATCTTTACTTGGTAAGAACTGAGGTGGAG--------AGGTGTTGCT-----GCTC 8966
Reedfish                   ACAATGACCCAAAACATAACCACCAATTCAACAAAGGAGTGGCTAAAGAAGAAGCACATTAAGGTAATGGAGTGG-CCTA 40084
NorthernPike               TACCGTATTTCGTTGATTTGTATTCTTGTAATTCAGT-TGAACTGCCCTGTAA--------ACTCAATCATACGACTCTG 14200
SpottedGar                 TGCTGCATTTAATAAGGATTCCTCG---CTGCTTGGTAAGAGTCAAGGTATAAT-------AAACTTTGTTCCAA-ACTT 13119
ParamormyropsKingsLeyaeTTCCAGTCCTTGGATGGCCAGAACAT--CTACAATGCCTGTTGCACGCTGCGC--------ATTGACTTCTCTAA-GCTG 8349

Majority                   GAGAATGTCAATGT-CATATAGCTCAAGAAGATGCGCGAAAGTGCTTTTCCCTTTTTGTGGCT-TTCTGACGGTCGCGAC
                                   82890     82900     82910     82920     82930     82940     82950     82960
Human                      AGGTTTGTGAATAT-TAAAGAGCGCTTCAGATTGCCAATGAAAGGTCATAGTAGTTTCTATAAAGTCAAAAGATCAAAAA 82605
AsianBonytongue            CATAGGCCCTCTGG-CCTAT----CAGGCAGATGCAC------GCTCTCCTCTGTTTGCGG---TTCTGAATGGGGTG-- 9030
Reedfish                   GCCAGTCTCCATAT-CTCAATCCTATAGAAAATTTGTGGAGGGAGCTAAATCTTCGAGTTGCC-ACGTGGCAGCCAAGAA 40162
NorthernPike               CAGAACGTCATTGGGTATGTAGCACAACGGGGAACTTGAAATTGCTCTTCCTGTCCTCAGGCTTTCTTGGAGCTCGGGAC 14280
SpottedGar                 TAAAAGGGGTGGGG-GATATGGGTCAGGAAGTTGCACAACAATGCCTTTCCCTTTTCGTGGCCCTTCTGACAGATGCAAC 13198
ParamormyropsKingsLeyaeCTCAACCTTAATGT-CAAGTACAACAACGACAAGAGCC---GGGACTACACCAGGCCGGAGCT-TCCTGCCGGAGACG-- 8422

Majority                   -----CTTGGAGGACTTAG----TTATTCACAG-TGCGACTGCC-ATTTCACTGAGATG--------ACCTGGTCACC--
                                   82970     82980     82990     83000     83010     83020     83030     83040
Human                      CTTGACTTAAAAGAGTAAGATCTCTAAAACCAAAGGTAACAGCAGATTCTTATGAAATGCACCAGAAAGCTGGAAAGAAA 82685
AsianBonytongue            ------CAGGAGCTCTTAT----TTCTCCACTG-TGAGAAGGCC--TTTCACTGCTATG--------ACTT--TCAG--- 9084
Reedfish                   ----ACCTGCAGGATTTAGAG-TTTATGTAAAGATGAGTGGGCCAAAATCCCTGAGATGTGT-GCAAACCTGGTGACC-- 40234
NorthernPike               -----AGAGGAAGCCGCAA-----TAACCATGG--TCAACTACT-ACTCCACTGTCACA---------CCCCATGTCC-- 14336
SpottedGar                 ----TTTTGATATACTTAG----TTGTTTTCAG-TT-GTGTGCC--TTTGACCACAAAG--------ATCTGATCAC--- 13255
ParamormyropsKingsLeyae-------GACAGCCCTCCG----TCGATCCCAC-TGCGGCAGCCGGCTTCAGCAAGGAT--------ACCTCCTCCCT-- 8480
```

```
Majority                  -ACTACGTCAAATGTG---TTATGAAATGTTCGTGCCATAAAC-ATTTCTACACA-GTTTGCT-TGTGATCACTTACATG
                                83050     83060     83070     83080     83090     83100     83110     83120
Human                     GAAGAAATAAAATGTGAACTTATGGAACAAGAGTGCTAAGGGCCTGTTCTACCCAGGTATGTTATGAAATCAAATTCATT 82765
AsianBonytongue           -AAAAAGTCTAC-------ACATGGAATGTTC------TAAAA-ATGTCTAAGTA-GTTTGCT-TAAGATGACTGACAAA 9147
Reedfish                  -AACACAAGAAACGTC---TTACCAC-TGTGCTTGCCAAAAAGGGTTTCTCCACC-AAGTACTAAGTCATGTTTTGCTTG 40308
NorthernPike              -GCAACGTCCCAGTGT---TCATTCAATACTCCAACCACAAAG-AACTCAAAAC--AGATGCT---GGA-AACCAACGGG 14405
SpottedGar                -ACTTTGCCCGATGTA---AAATGACATGTTCGTGTTGTAAAC-ATTGTTAAAT--GTTTGCC-AGTGGGCACAAAGAAG 13327
ParamormyropsKingsLeyae -GCTCGGTGAGGGAGG---GTTTGAGACGAGAGTTGCCTAAACCAATGTTTCCCA-ACCTGCTCTTTGGGCACCCCCAGA 8555

Majority                  GAGTTAGAATGTTT----TGTTGTGTCAGTG----GGTAAGGATCTTGTAACT-----CTTATGTGAGCCAGTTTATATG
                                83130     83140     83150     83160     83170     83180     83190     83200
Human                     TAGTTTGGGTGTTTGTTCCTTTATGTTGGGAAGATAAAAAAGAACATATAATTCGTTTCTTGAGTGAACCAGTATTTATG 82845
AsianBonytongue           AAC--ATAATTGTT----TGAAGAATCAATA----GGTGAGAAGCATGAAAC------CGGATTTAGGCTCATTTCTCCT 9211
Reedfish                  GGTATCAAATACTT----ATTTGTCTCAGTGTCATGCAAGGGAATTTATAACT-----TTTATGTAATGCGTTTTTTCTG 40379
NorthernPike              CCCAGGCAGTCCTG--CAGGCAGTGTCAGCG----GTCCAGGGGGGCGGTACC-----CCCACATCAGGC--TCCACAGT 14472
SpottedGar                GAATGAGAATATAA----TGGAATTTGGGTT----GATTTAAATCTTGCTTCT-----CACACGCCAACAAAGTGATAAG 13394
ParamormyropsKingsLeyae CAGT--CCATGTTT----TCTTGTGGGAGGG----AGCACAAGTGTAGACGGT-----CTGGGGGGTCCCTGAGGACTGG 8620

Majority                  GGTTATTGGTTTAGTATCTAAAGCTTCTTTGGGGGAA------TCTAGTGTAGTAATGATTGAAATAT--ATTATACTTG
                                83210     83220     83230     83240     83250     83260     83270     83280
Human                     AGAAATAAGCTGGGTGTGTAAGAATTTAAGGGATGAAAATATTCCAAGTTGATTGGTAATTGAAAAGTAGATGAACGGTG 82925
AsianBonytongue           ATTTCTTG--CAAACAGCAGAAGCTGCTTAGAGGGAA------TCTATTGTAGTGCTTGTTGAATTAC--ATTAAACTTG 9281
Reedfish                  GGTTTTTGATTTGATATTCTGTCTCTCTCCATTAAAA-----------TGAAACTACCATGAAATTAG--ATACTGTTCA 40446
NorthernPike              AGATCTGGCTCTAACAGCTGCATCCAGCCCAGTGCTC------------AGAATAATAATTGACA-ACATGTTCTACCCA 14539
SpottedGar                GGTAATTGGGTAAGCATTTAAGGAGTCTTTGGGGGAA------CATAGTGTAGCACTGATTGAAA-----ATTATCCTTG 13463
ParamormyropsKingsLeyae GTTCAGAAACCCTGTGTCTAAAGGTCTTTTGTGTGAAG-----TATGAAGAAGAAATGAAGATACAGT--ACAGCATATG 8693

Majority                  TTTGTTTTGTTGGTTCTTCATCATATATTGTGGTAGATCTGTATTGAATTCTCTGT-CTGTGGACAGAATTTAATGTACA
                                83290     83300     83310     83320     83330     83340     83350     83360
Human                     CTAACACAAAAGGGAATTCTGTCTAGAATAA-ATGAAAATATGGTGACTTAAAGAT-AGTAGGACAGCAATAAGACTGGA 83003
AsianBonytongue           -TGATTTTATTTTTTCCTCTTTCTGTAATGTCGTAGGCGTGTATAAGATCCTCTG-----TGAAAAGAATTTAATGTACA 9355
Reedfish                  TTTGT-TCGTAAGTGAGCAAACATAAATTCAGCAGGAGATCAAATTATTTCCCTCA-CTGTACATGTAATGTAAAACAGG 40524
NorthernPike              GTCACTCTGGATGTACTGCAGCAGGTAGTGT--ACGGTCTCTACCGACTCAGATATGCAGTGCACATTCTTTGTTGCACT 14617
SpottedGar                GCTGTTGAACTGCTTTCTCACAGTATATTGTTGTAGATTTA-ATGGCATTGTCTG-----TTAAAAGAATTTAATGTACA 13537
ParamormyropsKingsLeyae TCTGT-GTGCTGCTTCTCAGTCATTGGGTGAGTTACACCTGTGTAGGACTTACTGA-TTGTCGACAGAGCTGACCACCCC 8771

Majority                  CATTTTCCCTATGTACTCCATCTGGAATGGTATCTCC--CTGTTCTAGTGTTGGAGGGTTTCCATCTTCTCTCTGTTTT-
                                83370     83380     83390     83400     83410     83420     83430     83440
Human                     AATTAGAACAATAAAATAAGAAGGAAATGATAACATAGATTGGGAAAGGATAGGAAAATACTGAAACAAAATCTGTGTT- 83082
AsianBonytongue           CATTTTCCTCAGGTACTCCATCTGGAATGGTAGCTCC--CTATTCTAGTGGTGGAGGGTTTCCATCTTCTCTTGGCTTT- 9432
Reedfish                  TTTTATTAATATTTAATAAAAG-AGTATAATGTGTAG--TAGAAATGTTTTAATAAAGCTTCAGCTTTGTATCAGTCTT- 40600
NorthernPike              CCGTTGTGC-ATAAATGACAGCTACTTCTGCCCTTGAAAGTGGTTTAATATTGAAATCTTCACAGCTCAGTTGTATTTTC 14696
SpottedGar                AATTTTCCTTAGGTACTCCTTCTGGAATGGTATCTCC--CTATTCTGGCGGTGGAGGGTTTCCATCCTCTCTTGGTTTT- 13614
ParamormyropsKingsLeyae CCTCCCCTCTAGGAACTCCATCTGGAATGGTGACTCC--CTACACCAGTGGCGCGGCATTCCCATCCACCCTCAGCTTT- 8848

Majority                  -GCACAGGGTGG-----AGGTACTTGTACTTTCATTT---CTGATTTTTTGTTTATGTTGTTTTTA----------TTTG
                                83450     83460     83470     83480     83490     83500     83510     83520
Human                     -GTATAGTGATG------GTTTCTTGTTCTTTCAACCGGGAGAGTGTTGTGTTGAAGGAACTTGTATTCATTTTAATTAC 83155
AsianBonytongue           -GCACAGGGTGG-----AGGTACCTATACTTCCATTT---ATGATTTTTTGTTTCTGTTGCTAACACCATTCCT--TTCA 9501
Reedfish                  -AAACATAGTGCCTGTCAGGTACGTCTTGTAGTGTTA---CTGTTAGTTTGCTTTGGTCTTTTTTAGAACAGTC--CTTT 40674
NorthernPike              AATGTGGGGCGAGAGAAAAAATCCTGTACTGTTATTT---CCCCTTTTGTCTGCAAGATCTTCTCA-----------AAG 14762
SpottedGar                -GCCCAGGGTGG-----AGGTACATGTACTTTCATT-------ATGGTCTGTTCAGGCTGTTCGTG----------TTCA 13671
ParamormyropsKingsLeyae -GCACAGAGCGG-----AGGTATTTAT-CTTCCATCT---TTAATTTCACCTCTTTGTTG-TAATA----------TGTG 8907
```

```
Majority                   TTTAG-GTA-GGTTATGAAGAGTAGCA-A-TTTTGCAAGGAA---TTCTGTATTTG-CAGT-TT-TTGCACAATTAACTA

                                   83530     83540     83550     83560     83570     83580     83590     83600

Human                      TTGAGTGTATAATTAAGAAGGCAAGCACA-TTTTGCTAAGAT--TTTCTTTATTTGCATGAATTAAATAATGATGTTCAG 83232
AsianBonytongue            TTTGG-GAA-GAATATAAAGAGTAGCC---TTCTATATGCAAAGTACATATAATTGAAAGGTATCTGGGAAAAGAAAAAA 9576
Reedfish                   TTTAAAATACACCCACTAGAAGTAATGAAATTGTGGAAGTAATTATTTTGTTTGTGTCAATCTTTGTGCAGAAATAATTT 40754
NorthernPike               TTTGG--TACGGTCATGAAGATAATCACA-TTCACCAAGAATA--ATCAGTTCCAGGCCCTGCTGCAGTTCAATGACCCA 14837
SpottedGar                 CTGAG-GTA-GCTTAAGCAGATTGGC----TTGTTTATGCAG------T--AATTG---------TTGCAAAAGAACACC 13728
ParamormyropsKingsLeyaeCTCAGTGTT-GGAAGTCTGAGGTGACA---TCTTGCAGAGGA---------ACCTG-------------GCAATTTACTA 8961

Majority                   ACTGCTG-GTATCTAGTATCAGTT----TTAGATGTGACTCTATTA--AAGTAATGGTTGTGTTTCAGGT----GTTTAT

                                   83610     83620     83630     83640     83650     83660     83670     83680

Human                      AGTGATTTGTCTCTAGGAGCTGAAAGACTACTACCTTTGTTTCATAAGCTGTAATTATTTGATTTCAAGTGTGAGTTTAT 83312
AsianBonytongue            AAAAAAA-AAAACTTGTATCAGCT----TTGCATCTTGCTCAGGTGTGAAAAAATGGTTGTGTTTCAGTT----GTATAT 9647
Reedfish                   AATACTT-GTATAATTTACTAGCCA---TCCCCTACGGCTCCACCA--GCGTAGTAGTTAAACAGGACAAA---ATTTAA 40825
NorthernPike               TCTACCGCACAGCAAGCCAAAATTGTGAGTAGTCGCGACTATATCA--ATCTGATTTAAGAGACCCCGGCC---TTCCTC 14912
SpottedGar                 ACTGCTG-GTTTTCAGCATACATC----TTAGGTTTGAGACAATTA--AAAAAATGTTTTTCTTTAGAAC----GTTTAT 13797
ParamormyropsKingsLeyaeACTGAGG-AAGCCTGGT---TGGG----TTAGATGTAAAACTGGCC---ATTAAGGGTTGGGATTCAGG-----GCTTCT 9025

Majority                   GAATCTGTAACCGCTTG--CAAGCTCAG--GCAACGTTTGCATTGAAC---TTA-GTTGTACTATTTCATATTTGGCATG

                                   83690     83700     83710     83720     83730     83740     83750     83760

Human                      GAAGAAGATAGCACTTGCGGAAAAAAAGTGAATTACCATTATGTCGAAA-------GTGATACTTTTTTACATTTGGCCTT 83385
AsianBonytongue            GAAAATGTAGCAGTTTGTTTAGACTCAG--GCAACTTTGGCATTAAACCTTTTAAGTTGCACTAATCCAAAATATTCATG 9725
Reedfish                   AAATCACAAACCGGTATCGCTAGCTAAGCTGCAGGGTACACTCCAAAACGCAGAGGTTGACCGATTCAAACGGAGGCTGG 40905
NorthernPike               GGACCCCTGATGGCTGC--TCATCTCACCGCCAGAGTTTGGAATGAACAGTTT--TCTTTATAATGTCATTGCTCGCTGG 14988
SpottedGar                 AAAT---TAACTGCATG--CAAGATTAG--ACAA-----GCTGTAAAG----TAACTTACCCTATGGCACATGTGCCATG 13861
ParamormyropsKingsLeyaeGGCCCT-CAATCGTTGG--CGAGATCAT--TGTC-----GTAATGAGT---TT--TTTGAACGGTTTGATTTTCTGAATA 9090

Majority                   T---TATATAATTATGCAG-----GAGTTTTCTAATCTTGCTTTATGAAGTCCT-ATCTTTATGCTTGATGTTTGCTTTG

                                   83770     83780     83790     83800     83810     83820     83830     83840

Human                      TTACTGTA-AATCGTGGAAGT---GAATTTTCCAAGCCTGTGACTTGGAATTATATTTTTCATGCT--TTGTTTGCTTTT 83459
AsianBonytongue            T---TAAATGATTATTCAGTTTAGAAATATCCAGATGTTACTGGATGAAATCCC-ATCAGTAGACTTGAGTTTTGCCAAA 9801
Reedfish                   TGCATGAGTGAGGATGGCTCCACCTGGCTCCCTAATCCTGCCGTCATGCCTCCTCCTCCCTTGGCCCACAGCCT-CTGTC 40984
NorthernPike               TTTATTTGTATACTTGCTG-------CTTTTAATGTTTTACTTTATGAAGAGCA--CTGCTGAGTCTGCTCTCCACGTGC 15059
SpottedGar                 T---TATATTCCTGTGCAG----AGACCATGGTAATCTTGATTA--GAAGTCCTGAGCTTTATGTGTGAAGCTTGCGTGG 13932
ParamormyropsKingsLeyaeT---TAAACAGTGATGAAA-----GTGCATTGCCGTAGCAGCTG----TGTGTT--TCTGTAAAGG-GAGTTCAGCCGTG 9155

Majority                   TTAGAGAAGGGAAAGTGCCATTTCTTTTGTATTT--GCTCAGGTTCTG-AC--G----CAGGAGTCTTACTAATG-TCTT

                                   83850     83860     83870     83880     83890     83900     83910     83920

Human                      CCAGAGGAAATAGAGTATTATATATTTGATATTTTAACATAATACAGGTATGTACTTTAGTTCAAATAATTAATGGTTTT 83539
AsianBonytongue            TTTGCCTCGGGAAGTTGCCATTTATTTTGCATGAAAGGTCAGATTAACCACATG----CAAGAGCCTTATTAACT-TCCC 9876
Reedfish                   TCGGAATAGCGTAAATAT-ATCGCTCCTGCAAGTGTACTATGATTCTTAGCGCGATGAGAGAAGTCGCAAAATCA-TCTG 41062
NorthernPike               AAATAAAATCGCAACTCACGGTTCCCCTTTCTTT---CACAGGCTCTGGAC--G--GCCAGAACATTTACAACTCCTGTT 15132
SpottedGar                 CTAGATAAAGGAAAGCTCCAAGGCTTCTG-------GTTCAAGT-----AC-------CCAGCATCCGACGAATG-TTTC 13992
ParamormyropsKingsLeyaeTAGTAGAGCTGTAGACGCAGTGTGGTTTT---------CCCAGC-----AC------------CCCCTACTACCC-TCTG 9208

Majority                   GTTTAACCCGG-TTTTTTTCTCCAGGGGCTCTCA------T---ACA-GTAGTTCTCT-GAGTGCTGCAGCAGCTG----

                                   83930     83940     83950     83960     83970     83980     83990     84000

Human                      GTTGAAATCAATTATTTTTCTTTACTACACATCA----------GCAAACAATTTTTCTAAACACTATAAAAATTAACTT 83609
AsianBonytongue            ATCTAACCCTGCTTCTTTTCTGCAGCTGTTGTCACTGCTGTGTAACA-GTTGTGTTCT-GAGAACTAGAGAGTCTGGTTT 9954
Reedfish                   GAATATTCAAG-TAAATTACAGAAAAAAACCTGATTTAAATCCGTTAAGTAGTTCTCTCGTTTGCTAGCTAAGTGG---- 41137
NorthernPike               GTACACTCCGCATCGACTACTCCAAACTGGTCAACCTGAATGTCAAGTACAACAACGACAAGAGCCGCGACTACACTCGG 15212
SpottedGar                 TTCTAACCAGGGCTTGCCTTTCCAGGGGCTCTCA-----------------GTCCTCT-GAGTGCAGCAGCAGCTG---- 14050
ParamormyropsKingsLeyaeATTGGCCC----CTTCTGTCCCCAGGGGCTCTGA-----------------GTCCGCT-GAGCGCCGCGGCGGCAG---- 9262
```

```
Majority                  -CGGCGACCGCTGCCGGGGGGGTGCCGCTGTCTT-GGGGTCTCAG--TGGGCAGTGGTTTC---GGTGAGTGCTCCA--T

                                  84010     84020     84030     84040     84050     84060     84070     84080

Human                     TCAGGAACCATTTGGAATGGTCAGCCGTAAGCCATGCAGCCATCGTGCTTAAATTGGTTTT---TGTAAACGACTTG--T 83684
AsianBonytongue           TTGGCCACAGGTGCCGTGGGAGTGCTCTTTTCTT-GGTGTGGCAGGGTGGGCCGTAGGGTC---GCTGCTCGCTCCA--T 10028
Reedfish                  -AAGTAAAGTACGCCCCAAGGCTGAAGCTGTTTT-GCGTTTTTAG--TAGTCTGTAATA-C---ATAAAATGATTTA--T 41207
NorthernPike              CCGGAGCTCCCGGCCGGGGACGGCCAGCCGGCCGTG-GATCCCAACATGGCCGCTGCTTTCCAAGGCAAGGACTCCAGTT 15291
SpottedGar                -CAGCCGCAGCCGCAGGGCGGGTAGCGCTGTCT--GGGGTCTC-----GGGCACCAG---C-----AGCGTGCTCC---- 14110
ParamormyropsKingsLeyae-CGGCGGCTGCAGCCGGGAGGGTGGCGCTGTCT----GGTCACAC---GGGCAGCGG--------CGGTGTGCTGC---- 9322

Majority                  GT-TGGTCGGTAACCTCTTAT--GTACGGAGGTTAGTACTCCTCTTTACTGTTTTCCT-----CTGTTGCAGGGGCTCTC

                                  84090     84100     84110     84120     84130     84140     84150     84160

Human                     GTGTGTTTCGTAAAATATGCTTTATTTTAAAATTAGGGCTTCCTGTTGCAGCTGTTCCAGGAGCTCTGAGTCCTTTGGCC 83764
AsianBonytongue           ATTTGCCCGGTACCCTCCTAC--GTAGGTAGCTTCCCACTCCTCTTGACCGCCCTCTTT----CTGTTGCAGGGGCTCTC 10102
Reedfish                  G--TAGAAGATACAGTTTTAT--GTACAGATTTTTGTTTTCTAATGGATTTTTTTCATCGTAGCTGTTCCAGGAGCCCTT 41283
NorthernPike              CTCTGCTCGGTAAGATCCCAG--GTACATCCCTCTCTTTACCTCTTTCCTCTTGTCATG------GCATTGGGGGCTCTT 15363
SpottedGar                ---TGGTCAGCAATCTCA-----ATGAGGAGGTCAGTAATCC-----ACTGCTTTCCC-----CAGCCGACTGCGTTCCC 14172
ParamormyropsKingsLeyae---TAGTCAGCAACCTCA-----ATGAGGAGGTCAGTACGCC-------CGTCACCCT---------GGAA---ACCCCC 9375

Majority                  AGTCCTTTGAGTGCGGCTGCAGCTGCAGCTGCTGCGGCTGGCAGAGTGGCACTGTTTGGCTTTTCAGCCTCCTGTAGCAG

                                  84170     84180     84190     84200     84210     84220     84230     84240

Human                     ATTCCAAATGCTGCTGCAGCAGCTGCTGCAGCTGCTGCTGGCCGAGTGGGTATGCCTGGAGTCTCAGCTGGTGGCAATAC 83844
AsianBonytongue           AGCCCATTGAGTGCAGCGGCAGCAGCAGCAGCTGCAGCTGGGAGGGTGGCACTGTCAGGGCATTCAGGCACCAGT-GGAG 10181
Reedfish                  AGTCCTTTGAGTGCAGCTGCAGCTGCAGCTGCTGCAGCAGGTCGAGTGGCACTTTCTGGAGTGTCTGCATCAAACAGCAG 41363
NorthernPike              GGGGTTAGGGGTCCGGGTGGTGTTGGCGG-CTTGCCGCTCGCTTAAGGGGGAGGCTGTGCTTTGTA-CATATTGTAAAAG 15441
SpottedGar                TTTCTGTTGAGTGAGGTGACCATTGCAT--ATTGTGCTTTTTATTGTGTTGCTCTTTTGCCAT-----CGTCTGT-GCGG 14244
ParamormyropsKingsLeyaeCCCCCCCCCAACA-GGCATACACAGCCAGTCACATGATCACCACACT-CCATTCTTAATTTAAGTTGGGTCCTGT-GCCG 9452

Majority                  TGTTCTTTTGGTCAGCAATCTTAATGAAGAGGTTAGTAA----------TT-AA-TCATTCTGA-------CAGCTTGTT

                                  84250     84260     84270     84280     84290     84300     84310     84320

Human                     AGTCCTGTTGGTTAGCAATTTAAATGAAGAGGTTAGTAA-------------AA--TAATCTCT--------AATGTTTA 83901
AsianBonytongue           TACT--CCTGGTCAGCAACCTCAACGAAGAGGTCAGTAC----------TCACTGCCAGCCCCA-------CAGCGTGAT 10242
Reedfish                  TGTTCTTTTGGTTAGCAACCTTAATGAAGAGGTCAGTGAGAACCATTCATTCAGCTCATTCTGGTTTCTTTCTGCTGAGT 41443
NorthernPike              TCTGCTTCTGTCCAGTC-TCTACTCATGTCGTTTAACAACA--CATGTTTAGAATTTGTGTGGAATCGGTCTGACTGGAC 15518
SpottedGar                TCTTGATATAGTGTGTAGTATTTCAAATGAGGTGAACA-------------------AATCTGA-------CATCTATTT 14298
ParamormyropsKingsLeyaeTTTCT-TGCTCCCTGCCGCATGTTAATACAGATTAGCGT----------AT----TCGTATTGG-------TGGCCTGGA 9510

Majority                  GTGTTTTACTTTTTTTTATTTTTTTAAGTTTTTT-TATTTTTTGCGTTTTGTGTCTGTCTGTCCATCTAGGTGCTTTTTT

                                  84330     84340     84350     84360     84370     84380     84390     84400

Human                     TTCTTTAACTCCATTTCATTTGTGAAAGTTTTTCATGTTTATTTCATTTTGCACTTGCCTTTCTTTTTATGTACATTCAT 83981
AsianBonytongue           GCGTACCGCTGCATACTGTGCTCCTG--TTGTTT-CTCCCTTCACGCCAT-TGTCTGTCTGTATATATAGGCAAAATGTT 10318
Reedfish                  GAGTTAAACATTGCATAATTATTTTAAGTTGCTC-TTTTTGTTGTTTTTGATGTCGTACTTTGCATGTATGTGTTATTTT 41522
NorthernPike              ATGTTTTTTTTGTGTGGACCTGAGTAAAATATACCGACGACTGGCGCTTGGTGAC-ATCTGTCCGCCCGAGCGCCGTCGT 15597
SpottedGar                GGGTTTTAGTCTTATTTGTAGCATTA--ACTCTT-CATCCTGAAAATCAAATATACGTTAGCTAATATAGGTGTTTTTTT 14375
ParamormyropsKingsLeyaeATATTCTAATTATTATTTTTTTGATAGAATCATT-GATTTGCAGCGTTAA-TGTCTCACCCCCCAGCCTGCCACGGTGGC 9588

Majority                  TAT---TCATTGTAAGCTCTTTGT-AGTTCCTGT-----TCCATAATCACAT-----ATATTT--------GTAGGTATT

                                  84410     84420     84430     84440     84450     84460     84470     84480

Human                     TAG---TCAAAGTATTTTCTGTAT--GTTTCTAC-----TTCTGAATAAAATC-TACATATTGT-------TTAGGTAAT 84043
AsianBonytongue           TAT---ACATTGTCAGCTACAAATTAGTGCCTGT-----TTCATCACAACTT-----AGTTTT--------GCATGTTTT 10377
Reedfish                  TTTTTTTCCTTACAATTTATTTGAAAGTTATTTTGAATTTCTACAGTCGCTTTGTTTACATTTCTGAAAGGCTAGATATA 41602
NorthernPike              CCCTAACCGGCCTGACCCCTCTCT--CTCCAGGTG----CCCTGAGCCCTCT---GAATGCT---------GCGGCTGCG 15659
SpottedGar                TA----ACACTTGAAGAGATAAG--AGTTCTTGT-----C-CATAATTATAT-----ATATTT--------ACAGATTCT 14430
ParamormyropsKingsLeyaeAAG---GCCCTCTATGCTCTTTGT--GTGCCCGC-----TCTCTAATCACAC-----ACACT---------ATACGCATT 9644
```

```
Majority                   GTTATTTATGCTGCAGTAATCGTATCTCTGTTACATCACTCAGAATTTATTTAGACATTATTGGCTT-------------

                                   84490     84500     84510     84520     84530     84540     84550     84560

Human                      ACTTTATTTCCTTAAACAGTCATGTCTCT-TTATATCTATATCTATATATGTATATTTTTTAACCTA------------- 84109
AsianBonytongue            AGTATTCACGCTTCAGTTAAAGTAACTTTGCTAAGTTATTTTAAACATAAACCAACAGATTTTCCTG------------- 10444
Reedfish                   GTTATTTTTAATGT-GTAAACCCATGCTAGTTACATCATTCAGAATTTCTTTAATCTTTATTACCTTAGAGGTTAAACAA 41681
NorthernPike               GCAGCTGCTGCTGCAGGGAGGGTGGCCCTGTCAGGCCACTCCGGCTCCAGCGGGGTGCTCCTGGCCTCCAACCTCAACGA 15739
SpottedGar                 GTGATGTA-ACTGCATTAATATTATGTGTAGCACATTTCCCAAAAGTGCTTCTGACATAAGGGGCTT------------- 14496
ParamormyropsKingsLeyaeAACATTCA--------TGAGCACTGCTCTGCAGAATGCCTCAGCTACCATTTAGTCAGCACTGGCTC------------- 9703

Majority                   --TGCTAAATG-CCTTTGCAATTATCTCTGCAGT---ATTCTAGTTTGTC-AACAATCTATTCTGTTAACTTAACATATA

                                   84570     84580     84590     84600     84610     84620     84630     84640

Human                      --ACCTATTTTTCCTAAGAAAAATATGGTGAAGT---ACTGTAGTTTGGC--TTATCTT--TTTGTTGTTCTCAAAGGCA 84180
AsianBonytongue            --TTCTGAAGG-GCTTGGCTTGTATCTCTGGAGT---TTTCTATTTTCTCTAACAATCCATTCTGATATCTTAATGTGAA 10518
Reedfish                   TGTGTTAAATGTATTATGTAATTATCCATATCAGGTAATTTAAGTTTATAGTAAAATGCTCTCAGTTAATTTAATCAAAA 41761
NorthernPike               AGAGGTCAGTGCCCCTCTCAACTGCCCCCACCTC----CACACATAGGCCTAACACCCTCCCCCGTCTAGTTCTCATTTG 15815
SpottedGar                 --TGCTT------CTTTGGAATCACCTAAGCAGG---TCTCGGCTTATTC-AACAATATATTACACCCACTTAACGTATA 14564
ParamormyropsKingsLeyae--AGCTAA-----CTCTTCCTGACTCACTGATGC---ATTCTAATTTCCC--ATAACAT-TGTTGTTACCCAGCCATACA 9770

Majority                   AT---AATTATATAGTTAATTT-TAGCTA-------TTGTTTTTCATTTTTTTGTTTTTTCAA--TTTTTGTCTTTCTTT

                                   84650     84660     84670     84680     84690     84700     84710     84720

Human                      AATGTGATCTAATTTGCAAATT-TAACTGTA-----AAACAATTTTGCTCTTTGCCTTTTCTA--ATTATTTGCTTGTTC 84252
AsianBonytongue            AT---AACT-TATAGATAA------GTTA-------ATAATGTTCAGTGGCTTGTTTTTT------TTTTGGCTTGCTTT 10575
Reedfish                   TCCTAAATAAAATAGTAAAACT-GTATTAGTTCAATTTTGCTTTCACTTTTTTGTTAGTTAAATCTGTGTATTTTGGTTT 41840
NorthernPike               ATTCCCCTTCTGAACTCTCTCGCTCGCTCTCTCTTTGTGTTTCTCTCTTTTTTTTTCTCTCGCGCTCTCTCTCCCTCGCT 15895
SpottedGar                 AC---AAAAATATAGTTGATTG-TAGCTC-------TTGTTGTTCATTGTTTTATTTTCTAAACTATTTTGTTTGCCTTT 14633
ParamormyropsKingsLeyaeGG---GATG--ATAGGAAA-------CAA-------TGCGGATTTATTTTCCTACCCCTCCA-----TCCCCCCACACTC 9826

Majority                   GTTTT--TATGCTTGCATAT-ATCGTTT-TTGTTAATTTCTTGCTTTTTGG-CTGTTCTACTGAAATCCGTTTTTCTTTT

                                   84730     84740     84750     84760     84770     84780     84790     84800

Human                      ATTT---CATGCTTATATGTCATTGCATTTTTTTTAAATCTTATTTTATGT---GAACTACTCTAATACATTTTTCTTTG 84326
AsianBonytongue            GTTTA--TATGCTGGCACATTATCGCTT-CTGTCCTTTTCTTGCTTTAAAATGTATTCTACTGAAATCCGTTTTTCTT-C 10651
Reedfish                   TTGTTGCTATGCTTGCTTAT-ATCATTTGTTAACACTTTCCTGCTTTTTTGTCATTTCTATTGATGCACATTTTTCTTTT 41919
NorthernPike               CTCTCTTTCTGTTTCTCTCCTGCTCCATGATGTCCAGGTCTGCCAGTTCAGACTGAGCCCCTCTCCCCAGATATATCAAA 15975
SpottedGar                 GTATT--TGTGCTTACATAT-GTCATAT-TTGTTAAATCTTGGCTTTGTGG-AAATTCTATTGAAATACCTCTTTCTTTC 14708
ParamormyropsKingsLeyaeATTT-----TGCTTTCAAAT-AACGTTC-TCATTAAGCTCCAGACTTGTGC-TCGTATATCTTACGTCCGTTTCCTTGCT 9898

Majority                   AAGGTTCTC-TTCC---AAAAAAATTGATGAGGAGCTCTTTCCAGTCTT-TTAGC--TGGTTTTTTTC---TTTTTGCCC

                                   84810     84820     84830     84840     84850     84860     84870     84880

Human                      AAGGTTCT---TCC---AAAGATCTTGACGAGGCACTCTTCCCGTCTTTCTTAGT---AATTTTTTC------TTTGC-- 84389
AsianBonytongue            AAGGTTTTC-GTC----AAAAGAATGGATGGGCAGCTCATTCTAGTCTT-TTAGCCATGGTTTTTTTCTTTTTTTTTTCC 10725
Reedfish                   AAGGGTCCC-CCC----AAAGAGCTTGATGAGGTGCTCTATCCAGTCTT-TTAGT---AGTG--T-TC-----TCTATAC 41982
NorthernPike               AGGGACACAAGTCTATAAACTAATTTGACTTTGACTCCTTTCTAACCTC-TCTGCCATGTTGAACTGACTGAAGCTGGTT 16054
SpottedGar                 AAGGTTCTC-TCCC---AAGAGAATTGATGAGGGGCACTTTCCAGTCTC-TTAGC---AGTA--TTT----TCTTTGC-- 14772
ParamormyropsKingsLeyaeAAAACTGTG-TTCA---TGCATGATTCTCAAAGAGGGGACCCCCCTCCCCTTTGCTCTGTTTTCTTTACAGATTTGACCA 9974

Majority                   ------------T--AGTTTTTGGTCACTGTCTTCTAAACTCATTCTTTTACATTCTT---TTCTTTCTCTATCCTACTT

                                   84890     84900     84910     84920     84930     84940     84950     84960

Human                      ---------------AGTTATTAGTCATTGTCTTCTAAAATATTTTTTAACTTTATT--CCCCCACCCCTTCCTTCCTT 84452
AsianBonytongue            TTTTTTCCTTTCTTAGCATATTGGTCACTTTTTCCTAAACACACTCTCACACACTCTT---TCCTTCCTCTCTCCCACTT 10802
Reedfish                   ---------------AGATCTTGGTCACTGTCTTTTGTACTCATTCTTTTACACCTTG---TTATTAACT------AAAT 42038
NorthernPike               AACTTTTTTTCTTCTCTTCTCTAAGCTTTCCTATCTGGCTCAATGTGTCATGATGGTTTTGTCTTGTCTGAATCACTCTC 16134
SpottedGar                 ---------------AGATTTTGGCCACTGTCTTCTAAACTCATCCTTCTGTACTCCT---TTTTTTGTC------AACT 14828
ParamormyropsKingsLeyaeCTTCG-----TCTCATCTCTTTCTTTGCTGTCATGTAAACTGACACTTGTTAATTCTTGCTCTCTCTCTCTATGCTATGT 10049
```

```
Majority                   TTTACT-CGCTCCTCCTTTT-A-ATGTTTTTTCTTTCTATGC------TCTGGGTCGTTTGCCTTT--ACTCTC-CTGTC

                                   84970     84980     84990     85000     85010     85020     85030     85040

Human                      ACCACC-CCCTCCACCCAGTTAAATAAAATTTATTGCTAAGTTA----TTT----TCTTTAGTTGT--ACATTTTATGTC 84521
AsianBonytongue            TTCCCC-TGCTCTGCCTTGT-GTGTACTGCTGCTTGTTATGC------TCTGAATCGTCTGCCTTTGAGCTCTCGCTGGC 10874
Reedfish                   CTCTAT-AACTCTTGGTCT-------TTTTCTCTCTCTATGC------TCT-AGTTATTTGCTTTT--ACTGTC-CTGAT 42100
NorthernPike               ACTATTATTCTCCCGCTTTCATTTTGCAATACATAACTAAAACTAAATTGTGGTTCTTTTCTCTCTTCACTCTC-CTGGC 16213
SpottedGar                 GATACT-CGTTAATTCTTG------GTCTTTTCTCTCTAAGC------TCT----CGTTTGCCTTT--TCTCTC-CTGAC 14888
ParamormyropsKingsLeyaeTTTTTC-CCCCCCTCCTCTC-A-ATGTCGTGAACATTTATGAAG----TGTGGGACATATGCCTCTC-TCTCTTTCTGTC 10121

Majority                   TTT-----TCTTTCTTCTTTTCT-GTT-GCTATGAACGCTGG------TATGAAATGTGGGGGGCTCATTATTCCCCTTA

                                   85050     85060     85070     85080     85090     85100     85110     85120

Human                      TGG-------TATATTTTATTTT-GAATGCTATGAAAGCTGG------TATGAAATGTGGGAAGCTCAGTGTATCAGTTT 84587
AsianBonytongue            TTTTAACGTCCTGACTCTTCTCT-GT--GATATCAACGCTGG------TATGAAATGTGGAGGCCACATCACTTTAGAAA 10945
Reedfish                   CTC-------TTTCTACTGTTCT-ATT-GCTTTGAAAGCTGG------TATGAAATGTAGGGTGCTTTCTATTGCCCATT 42165
NorthernPike               TTGAGCACTCTGTCTCCCTTCCTAGTATCAAATCTCCGACGGAGAGACCCTAAATTGTGTGTGTATCCATATACACATGC 16293
SpottedGar                 TTT------CTCTACTGTTTTCT-GTT-GCTATGAACGCTGG------TATGAAATGTGGGAGC---GTTACCCCACTTA 14951
ParamormyropsKingsLeyaeTCT--GTCTCTCTCTCTCTCTCT-GTC-TCTCTCTCCCTCCC------CCCTCCCTTT-----------TACCCCCCGGA 10180

Majority                   GTGGATTATATGATTTAAATTTGTTATCAT--TTTCCCAAACGCCTTGT-GGTTTGTGGATTTAA--CATGATTTGCTTT

                                   85130     85140     85150     85160     85170     85180     85190     85200

Human                      ATGATGTCTAATTTGAATATTTGTT-TCAT--TTTTAATTGGCCTCTGTTAATATGAACATTAGC----TTATTTTATCC 84660
AsianBonytongue            GCAGCAAACCGGATTGAAATTTCTTATTAT--TCCCTCAAAAGCCTTG--GGTATCAAAATTCGA--TGAGATTTTGTTT 11019
Reedfish                   AAAGATTACATGATGTCAGTTTATCATGAT--TGTCCCTGAAACCT-----GTTTGTGGAATTAAA-CATATTCTGCTTT 42237
NorthernPike               AAAGACATTAT-AAATATATTTGCCCTCAT-ATGACCCACATGTACAGT--CTGTCTGTGTTTAA-------CATGACTT 16362
SpottedGar                 GGGGTATATCA-ATTTAAATTTGCCATGAT--TTTCCCAAACTCTTTGTGAGTTTGTGGATATAA--CATGATGTGCTTT 15026
ParamormyropsKingsLeyaeGTGGATATTACGAGTTGCATTTTCTATCATGATTTCCCAAACGCCCCC--GGTTTGTGGATTTAAAACATGATGTGCTGT 10258

Majority                   GTT-TAATACCATAACTTTGCAAGGTGGTTTT-CATGTCTACACTTGCATGTCTG----AAACGGACTGACCTTGT--CT

                                   85210     85220     85230     85240     85250     85260     85270     85280

Human                      ATT-TAATACTATTATCATTCA---CAGTTCT------GCATGCTAAAATGTTTG----AATCAGAGTGCCTTTGT--TT 84724
AsianBonytongue            ATA-CAATGT--TTACTAGGCA----GATTTT-CAAGTCTGCGTCTGCAAG-CCA----AAACCGGCCAAACTTGAGACA 11086
Reedfish                   GTTCTGTTACCATAACCTTGCAAGGTGGTTTTTCATCAGCACACCTGCATGCTTG----CCGTGTTTGATCCTTGG---- 42309
NorthernPike               GTG--AATGCCATAACTCTACCAGGTGGTTTC--ATGTCTGTTCTCTCATGTCCGTGCTATAAAAACAGATTTGACTGCT 16438
SpottedGar                 GTG-TAGTACCATAACTTTGCAAGGTGGTTTT-CATTTCTACACTTGCATG-CTA----AAACCGATTGAACCAGTG-CA 15098
ParamormyropsKingsLeyaeTTA-TACCAT---AACTTCGCAAGGTGGTTTT-CATGTCCAAACCCGCATGTCTG----ACACGGACTGTGCTTGC---- 10325

Majority                   TGGTCTTTAAA-----ATTTTGCCTGCATTTCATACTCTG-----CCATGCTTAAGTTGCTAAAT-TTAAAACTTAATAT

                                   85290     85300     85310     85320     85330     85340     85350     85360

Human                      TTATTTTAAGA-----CTTTTGCCTGCATTTCATAACCAG-----CCATGCTTATGCAGTTAAAGTTCAAAGTTTAAAAT 84794
AsianBonytongue            TGGCCTTTCA------AGTTTGCCTGCATTTCATACCTTT-----CTTCGCTTCACTTGCTAGATCCTAAAACTTAAGAT 11155
Reedfish                   CAGCTTCAGAATAGTTTATTTGTTTGCATTTCATACTCAG-----CCATGCTTAA---GCAG----TTAAAATT--AAA- 42374
NorthernPike               TGGTCTTTCGAC----TAATTGTCTTCATTTCATACTCTGTATTGCCACACTTCAGATGACATTTCCTGAACGTTAATCT 16514
SpottedGar                 CCTTCTTTAAC-----AGTTTGCCTGCATTTCATACTTTG-----CCATGCTTAA---GCTA----TTAAAACGTCAGAT 15161
ParamormyropsKingsLeyaeCAGCCTCGACA-----ATGTGGCTTGCATCCCATACTCCG-----CTGTGCCTGACTTACTAAAT-CTTAAACTGTGTAT 10394

Majority                   T----CTTTTGCATGCCC-TTCTTCCCTTCATT--GTTAG-----TGTTCTGTA-TTTATTCTTTGATATA-AATGTA-C

                                   85370     85380     85390     85400     85410     85420     85430     85440

Human                      T----CCTATGCATGCTT-TCCTTCCCTATGTTGAGATGA-----AATGCTGTAATTTACTCTTTGATATA-GATGTA-C 84862
AsianBonytongue            T----CTGCTGCATACCCTCCCCCCCCCTCATT--ATTACTACATTATATTGTA-TGTATTCTTTAATATA-AATGTA-C 11226
Reedfish                   -----TT-TTGCATGTTA-TTTTTCCTTTCATTCAGTCAG-----TGCACTGAA-GGTGTTCTTTAATATA-AATGTA-C 42439
NorthernPike               CTTGCCTACTGCATGCGT-TTGTTTTCTTCATT--GCTAGT---TAGTTCTATG-ATTACGGTGTGATTTATGATTTGAC 16587
SpottedGar                 T----CTTTTGCATGCCC-CTTTTTTCCTCATT--GTTA------TATGCTGTA-ATTATTCTTTGATATG-AATGTA-C 15225
ParamormyropsKingsLeyaeC----CTATTGCATGGCC-TCCTCCCTCTCATT--GTTA------TGCTGTGAT-TTTATTCTTTGATGTG-AATGTAAC 10459
```

```
Majority                  TTTGCCCATGTTTGTCTTGGG--TGACTACATTTTGCACAGTTA-TTTTCTTGTATA-------A--A-ATACATAAACC

                                   85450     85460     85470     85480     85490     85500     85510     85520

Human                     TTTACCCATATTTGTCTTGGA--TGACTACATTTTACTCAGTT---TTTCTTGTACA------------GTACATAAACC 84925
AsianBonytongue           TTTGCCCATGTTTGTCTTGGG--TGACTACATTTGGAACACATA-TTTTCTTGTATAT-----AAAAACAAACATAAACC 11298
Reedfish                  TTTGCCCATGTTTGTCTTGGG--TGACTACATTTTGCACTTTT--TTTTCTTGTTAA------------ATACATAAACC 42503
NorthernPike              ATTGA--ATGTTTGTCCAGGGCTTGATTACATTTGTCACAGTTTATTTGCTTGTTTAGTTCT-GACTAAGTACATGAACC 16664
SpottedGar                TTTGCCCACGTTTGTCATGGG--TGACTACATTTGGCACAATTA-TTTTCTTGTAAA------------AAACATAAACC 15290
ParamormyropsKingsLeyaeTCTGCCCAAGTTTCACTTGGG--TGGCTACATTTT-TGCAGTTA-ATTTCTCGTGTGTTTTTAAACGAAAAGAGCAAAAC 10535

Majority                  ---AGCCATTTTCTGACCAAACTCCTGCATTTCCTATGTACTGACCTGTATTTTATTTTT-----GTTCCCCAACTCCTT

                                   85530     85540     85550     85560     85570     85580     85590     85600

Human                     ---AACCATTTTCTGACCAAATTCCTGCATTTCCTATGTACTGACCTATATTTTATTTTGTTTTTGTTCCCCAATTCCTT 85002
AsianBonytongue           ---AGCCATTTTCTGACCAAATTCCTGCATTTCCAGTGTACTGACCTGTATTTTATTTT------GTTCCCCAACT---- 11365
Reedfish                  ---AGCCATTTTTCTGACCAACTCCTGCATTTCCTATGTACTGACCTATATTTTATTTTT-----GTTCCCCAACTTCTT 42575
NorthernPike              ---AGCCAT--TCTGACC----TCCTGCATTTCCAGTGTACTGACCTGTTTTTTTGTCCC-----CCTCCCTTATT-TTT 16729
SpottedGar                ---AGCCATTTTCTGACCAAACTCCTGCATTTCCTATGTACTGACCTATATTTTATTTTT-----GTTCCCCTACTCCTT 15362
ParamormyropsKingsLeyaeGAAAGCCATTTTCTGACCAAACTCCTGCATTTCCTATGTACTGACCTGTTTTATTTTTT------GTTCCCCCCCACTTT 10609

Majority                  GT---TTTATTCTGCATTGCTGTTCCCCTTC----------CTCCTTTT-----CATTCCTTTCCCCT-----TTTTTCT

                                   85610     85620     85630     85640     85650     85660     85670     85680

Human                     ATTTTTTTCTTCTGCATTGCTGTTTCCCTTCCCCATTTCATCCTTTTCCCTGTGTGTTCACCTTCCCTTTCCTTGTCTTT 85082
AsianBonytongue           -----TTTATTTCGCATTGCTGCCCTCCCCTAT------A-CTCCTTTT-----CATTCCTCTCTCCT-----TTTTTCT 11423
Reedfish                  GT---TTTATTCTGCATTGCTACTCCCCTT-----------CCCTTTTC-----CATACCTTTACCCT-----TTTTTTT 42631
NorthernPike              AT---TTTTTTATACATTTGCATTTCCCTCC----------CTCCGTCT------GCCCCCGTCCCCT------CTGTCC 16784
SpottedGar                GT---TTTATTCTGCATTGCTACCCCCCTT-----------CCCCTTTC-----CATTCCTTTCCCCT-----CCTTTCT 15418
ParamormyropsKingsLeyaeGT---TTTCTTCTGCATTGCTGTTCCCCCCCCT------CCCTCCTTTTTTTGTCATTCCTGTCCCCTCCCCGTTTGCCT 10680

Majority                  GTCTCTTTGCCCT-T---TTTCCTGTCTTCCCCTTCCTTTTC-CCCTCTTCATTATCTT--CCCCCTCCC---CCCT--G

                                   85690     85700     85710     85720     85730     85740     85750     85760

Human                     TCCCAAATGCCCATTCCCTTCCCTGTCTTATCCTTTATTTTCCTTGTCCTTGTCTTCATTCCCTGTCTCCATTCCCTATG 85162
AsianBonytongue           GCCTCTT-GCCCCAT---TATCCCCTCTATCCCTTCTTTTCC-TCTTCTTTACTATCTG--CCCCCACCC---CACT--G 11491
Reedfish                  GTC------------------------ATCCCTTCCCTTTC--CCTCTTCAATACCTT----TCCTCCT---------G 42671
NorthernPike              GTCCGTTTGTCTG--------TCTGTCTGCCCCCCCCCCCCCCCCCTTTCCCTGTAAC-----CCTCCT---------G 16842
SpottedGar                TTCTCTT-GCCCTCTGTTTGCCCTTTGTTCCCCTGCCCCCTC-CCCTCTTCATTACCTGTCCCCCCTCCC---TCCT--G 15491
ParamormyropsKingsLeyaeGTCTTTTTGTTTTGTTTTTTTTCCCGCCCCCCCTTTTTCTTTACCCACCCCCTCGCCCCGACCCCCTCCCTTTCCCC--G 10758

Majority                  TTCATGCTT-TGTGCTTGAACAAAA--TGTT-CCTTGGACCAACTTG----CCCCAATTAACTGCCTTGAACCATGATCC

                                   85770     85780     85790     85800     85810     85820     85830     85840

Human                     TTCATGCTTCTGTGCTTGAACAAA---TGTT-CCTCGGACCAACTTG----CCCCAATTAACCGCCTTGAACCATGATCC 85234
AsianBonytongue           TTCATGCTT-TATGCTTGAACAAAA--TATA-TCTTGGACCAACTTG----CCCCAATTAACTACCTTGAACCATGATCC 11563
Reedfish                  TTCATGCTT-TGTGCTTGAACAAAAA-TGTT-CCTCGGACCAACTTG----CCCCAATTAACTGCCTTGAACCATGATCC 42744
NorthernPike              TTCATGCTT-GGTGCTTGAACAAAACCTGTTTCCTTGGACCTACCTAAAACCCCAAATTAACTGCCTTGAACCATGCTCC 16921
SpottedGar                TTCATGCTT-TGTGCTTGAACAAAA--TGTT-CCTCGGACCAACTTG----CCCCAATTAACTGCCTTGAACCATGATCC 15563
ParamormyropsKingsLeyaeTTCATGCTT-TGTGCTTGAACAAAA--TGTT-CCTTGGACCGACTTG----CCCCAATTAACTGCCTTGAACCATGATCC 10830

Majority                  ATGACCACCT-CACCATTCTACGGGAAACCACCCTTCGTTATGGATGATCTGTTCATCTCCGCTCTT--------CCTCG

                                   85850     85860     85870     85880     85890     85900     85910     85920

Human                     ATGACCACCT-CACCATTCTGCGGGAA-CCACCCTTCGTTATGGATGATCTGTTCATCTCCGCTCTT--------CCTCG 85304
AsianBonytongue           ATGACCACCT-CACCATTCTGCGGGAAACCACCCTTCGTTATGGATGATCTGTTCATCTCCGCTCTT--------CCTCG 11634
Reedfish                  ATGACCACCT-CACCATTCTACGGGAAACCACCCTCCGTTATGGATGATCTGTTCATCTCCGCTCTT--------CCTCG 42815
NorthernPike              ATGACCACCTTCACCATTCTACGGGAAACCACCCTCCGTTATGGATGATCTGTCAATCTCCGCTCTCTACCTCCGCTCCG 17001
SpottedGar                ATGACCACCT-CACCATTCTACGGGAAACCACCCTTCGTTATGGATGATCTGTTCATCTCCGCTCTT--------CCTCG 15634
ParamormyropsKingsLeyaeATGACCACCT-CACCATTCTACGGGAAACCACTCTTCGTTATGGATGATCTGTTCATCTTCGCTCTT--------CCTCG 10901
```

```
Majority                     ACTCTTCT----CTCTTCCTGTCTTACGCTGCTTGCTCTTCTCTCCTTCTAAAGATGGTTACGCCCCAAAGTCTGTTTAC
                                   85930     85940     85950     85960     85970     85980     85990     86000
Human                        ACTCTTCT----CTCTTCTTGTCTTACGCTGCTTGCTCTTCTCTCCTTCTAAAGATGGTTACGCCCCAAAGTCTGTTTAC 85380
AsianBonytongue              ACTCTTCT----TTCTTCCTGTCTTACGCTGCTTGCTCTTCTCTCCTTCTAAAGATGGTTACGCCCCAAAGTCTGTTTAC 11710
Reedfish                     ACTCTTCT----CTCTTCCTGTCTTATGCTGCTTGCTCTTCTCTCCTTCTAAAGATGGTTACGCCCCAAAGTCTGTT-AC 42890
NorthernPike                 ACTCTTCTACCTCTCTACCTGTCTTCCGCTGCTTGCTCTTCTCTCCTTCTAAAGATGGTTACGCCCCAAAGTCTGTTTAC 17081
SpottedGar                   ACTCTTCT----CTCTTCCTGTCTTACGCTGCTTGCTCTTCTCTCCTTCTAAAGATGGTTACGCCCCAAAGTCTGTTTAC 15710
ParamormyropsKingsLeyaeACTCTTCT----CTCTTCCTGTCTTACGCTGCTTGCTCTTCTCTCCTTCTAAAGATGGTTACGCCCCAAAGTCTGTTTAC 10977

Majority                     CCTCTTCGGTATGTTATTGTTAGCACTCTACTTTTT--T------TTTATTTATTTTA--TTTATTTGTT-ATTCATGTT
                                   86010     86020     86030     86040     86050     86060     86070     86080
Human                        CCTCTTCGGTATGTTATTGTTAGCACTATACTTTTA----------TTATTGATTTGA----TTTTTGT---TTCACCTT 85443
AsianBonytongue              CCTCTTCGGTATGTTATCGTTAGCACTCTACTTTTTATTTA----TTTATCTATCTTATGTTTATTTATT-ATTAATGTT 11785
Reedfish                     CCTCTTCGGTATGTTATCGTTAGCACTATACTTTTTATTATTGATTTTTTTTATTTTGATTTTAATTTTTATTTTATATT 42970
NorthernPike                 CCTGTTCGGTATGTTAATGTTAGCTCTCTTTTGATG---------TTTATTTCTGTTA---CAAAGAGATCATTCAAGGT 17149
SpottedGar                   CCTCTTCGGTATGTTATTGTTAGCACTCTACTTTTT---------TTTAT-TATTTT---TTTAAATGTT-TTTCATCTT 15776
ParamormyropsKingsLeyaeCCTCTTCGGTATGTTATCATTAGCACTCTACCTTTTTATCG----TTTAT--ATCTA-----TATGCGC--ACACACACG 11044

Majority                     TTCACTTTTTATT-----TTATTGCTAGTATTTTGGCTTG-TAGTTGATTAGTA------------------------T
                                   86090     86100     86110     86120     86130     86140     86150     86160
Human                        ---AATTCTTATT-----T-GTAGCTAGCACTTTGGCTTA-AAGTTGAATAGTA------------------------- 85487
AsianBonytongue              TTCACTTTTTGCT-----TTATTGGTAGTGTTTTGGCTTG-TAGTTCAATAGTATTTTGCTATTTTCATTGTTTTCTCCT 11859
Reedfish                     TCCACCTTTAATTCTTTCTTATAGCTAGCTTTTTGGCTTA-TAGTTGATTGGTAACTCTTTGGCTACTCTCTCTCTCTCT 43049
NorthernPike                 T--GTTTTTTTTT-----TTCTTTCTGTTATGTTGGTTTGGTCGTTTTTCAGTC------------------------T 17197
SpottedGar                   TTGAATTCT-GAT-----TTATACCTAGCATTTTGGCTTA-CAGTT--GTAGTA------------------------T 15822
ParamormyropsKingsLeyaeCACACGTTTTACG-----TTAATGTTAATATTAGTGGTTG-GCCTT-GGTACGG------------------------T 11092

Majority                     CTTTTTTTTTTTACTTTTTTTTTTTTTCTTT----------------GAA------ATTTCC----TTCTCCATAGATAGT
                                   86170     86180     86190     86200     86210     86220     86230     86240
Human                        -AATCTTTTGCTATTTTTCTTTGCTATTTA----------------AAA------CTCTC------CATAGACACAAAAT 85538
AsianBonytongue              TTTTTTTGTTTTACCTCTATCTCTATCTTTCACTCTCTCTTGCTCACAAACACACGTTTTCAAAATTTCTCCTAGGTAAT 11939
Reedfish                     CTCTCTTTCTTTTCTCTCTTTTCTCTCTCTC--TCCTCTTTGCTGTGAA------ATCTT-----TTCTCCATAGACAGT 43116
NorthernPike                 CTCTTTAAATTGATCCATAGCGTTCTGTTA--------------ATGCATGGTAAATTGGCA---TGAACAGTAGTTGGA 17260
SpottedGar                   CTTTTTTTTTTTACTTCTCTCTTTGGCTTT----------------AAA------GTTTC------TCTCCATAGAAAGT 15874
ParamormyropsKingsLeyaeCTTCTCTATGTTTGTTTTTTTTTCTCTTTT----------------GAAATCTCCATCTCC----TTCTCCATTGATGAT 11152

Majority                     TTATGTTAATGCATGGTTAAATTATTTTGCATGTA-T--TTATTTTAACTTTA-TCACA-AAGCTTGTTTT-GGAGGGTT
                                   86250     86260     86270     86280     86290     86300     86310     86320
Human                        TTGTTTTAATGCATGCTA-ATTTATTTTGCATGGTCT--TTAATTTAATATCATTCACA-TAGC----TTT--GAGGGTT 85608
AsianBonytongue              TTATTTCAGTGCATGGTTAAATTATTTTGCATGTG-----TGTTTTTACCAT--TCACA-AAGCTTGTTTT-AAAGGGTG 12010
Reedfish                     T-ATGTTAATGCATGGTATACTTATTTTGCATGTAATTTTTAATTTTATCATT-TCATG-TAGC--ATTTCTGAAGGCTT 43191
NorthernPike                 TTTGCCAAACATCTCACAAAGCTTTTTTAACTTCTTTCAGTCTCCCACATACACTCGCACAAGCACACCTTGGCAACTCT 17340
SpottedGar                   TTATGTTAATGCATGGTTAAATTATTTTGCATGTA-----TTTTTTATCATT--TCACA-AAGCTTGTTTT-GGAGGGTG 15945
ParamormyropsKingsLeyaeTTATGTTAATGCATGGTT--ATTATTTTGCATGTAGTGCTTAATTTACCTTTC-TCATG-AAGGCTGTTTCCGGAGGGTT 11228

Majority                     TATCAGAATTTTTTCTTT-------TCTCTATTC-AAATCTTGAT-TTGTTTATTCATTTGGATGAATGTTCTATCTTTT
                                   86330     86340     86350     86360     86370     86380     86390     86400
Human                        TATCAAAAATTATTCTTTTCAAAATTCACTGTTCAAAATCTTGATCTTCTTTATTCATTTGTGAGAATGATGAGATTGAG 85688
AsianBonytongue              TACCAGAACTGTGTTCTC-------------TTC-AAATCT-------GTTTATTCCTTTTGATGAATGTTCTCTCTATT 12069
Reedfish                     TCTTCCAATCTTTATTTC-----------------AAATCTTGAT-TTGTTTATTCATTTGGATGAATGTCCTATCTTTT 43253
NorthernPike                 TGTTTATTTTTTGCCAATTTTAA--TTCTGATGAATGATCTCTAT-ATAGCCAGTC-TCTACACTCTCTCTCAATCTTTC 17416
SpottedGar                   TATCAGAATGTTCTCTTCAAA----TCTCTATTC-AAATCTTGAT-TTATTTATTCATTTGGACGAATGTGCTATCTTTT 16019
ParamormyropsKingsLeyaeTATCGCAATAGATTCCGT--------CTCTAAGC-TACTGTTCG---GCTCTATTTGATTGGATGAATGTTCTTTCTTTT 11296
```

```
Majority                 C----ACTTTTATGTTTAT-TGTTAGTTTGTGA--T---AACTGT-TTCTATTGAT--CTTCCAC------TTAA-TTCC
                                 86410     86420     86430     86440     86450     86460     86470     86480
Human                    TGATCATGTTGATGTCTGAATGTTTCATTGATATGTCAGAAAGATAATCTTTAGGTGACTTCCACATAAAATTAATTCCA 85768
AsianBonytongue          -----ATTTTTAT-TTTGT-TGTTAATTGGT----------CTG--CTAAATTGA---ATTCCA-------TCAT-TTAA 12119
Reedfish                 C----ACATTTCT--TAAT------GTTTGT----------CTG---TAAATTGAGAAATTTCAC------TTTAATTCC 43302
NorthernPike             CTACGACTCTTGT-CCTAACTGTT--TTCCTCA--------CTGTCTGCTCTTTCTCCCCTCCTCCT----CCTCCCTCC 17481
SpottedGar               C----ACTTTGATGTCTATATATCAGTTTCTGAAATGTTAACTG--TTCTCTTAA---CTTCTAA------TTAA-TTTC 16083
ParamormyropsKingsLeyaeT---CACTTTCACGTTGAG-TATCAAATTCTGAAGTACTAACTGTTCTCAATCAAT--CCTCTCT------TTGTGTTCC 11364

Majority                 TTTTTCCCTTGGCTGCTTTTCGCTCCAAAACAGGTGTTTATGGTGATGCTCAGCGTGTGAAGATTCTATACAATAAGAAA
                                 86490     86500     86510     86520     86530     86540     86550     86560
Human                    TTTTTGGATTACCTCTCTGTGGCTCCAAAAAAGGTGTTTATGGAGATGTGCAGCGTGTGAAGATTTTATACAATAAGAAA 85848
AsianBonytongue          TTTTTGCTCTGGAAACTTTTACCTTCAAAACAGGCGTTTATGGAGATGCTCAACGTGTGAAGATACTATATAATAAGAAA 12199
Reedfish                 CTTTTCATTTAACTGCTTTTCAATCCAAAA-AGGTGTTTATGGTGATGCTCAGCGTGTGAAGATTCTCTATAATAAAAAA 43381
NorthernPike             GCTTCCTCTTGTCTGCTCTTGGCTTTAACACAGGGGTTTACGGGGATTGCCAGAGGGTGAAGATTCTCTACAATAAAAAG 17561
SpottedGar               TTTTCCCCCTGACTGCTTTTCACTCTGAAA-AGGTGTTTATGGTGATGCTCAGCGCGTGAAGATACTTTACAATAAGAAA 16162
ParamormyropsKingsLeyaeCCATCTCCCTGGCCATCTCGCTGGGGGAAAAAGGAGTTTACGGCGATGCGCAGCGTGTGAAGATACTATACAATAAAAAA 11444

Majority                 GACAGTGCTCTGATACAGATGGCTGATGGAAATCAAGCTCAGCTTGGTAAGATTT---TATTTTTAAGATATTTAAGATT
                                 86570     86580     86590     86600     86610     86620     86630     86640
Human                    GACAGCGCTCTAATACAGATGGCTGATGGAAACCAATCACAACTTGGTAAGATTAAACTATGTTTTATCTATACATCTTC 85928
AsianBonytongue          GACAGTGCACTCATACAAATGGCTGATGGAAATCAAGCCCAACTGGGTGAGTTGCAGATATTTTTAAAATATCTTAAAAT 12279
Reedfish                 GACAGTGCTCTGATTCAGATGGCAGATGGAAATCAGGCTCAGCTTGGTAAGA---------ATCTAAGGGACCCCTGCAT 43452
NorthernPike             GACAGTGCTCTGATACAGATGGCTGATGCCAACCAAGCCCAGCTAGGTAAGATAC---T-CATCCATGATGCTT---C-T 17633
SpottedGar               GACAGTGCTCTGATACAAATGGCAGATGCAAATCAAGCTCAGCTTGGTAAGATTT-G--ATTTTTAGGAT-TTTAAGATT 16238
ParamormyropsKingsLeyaeGACAGCGCGCTCATACAAATGGCCGACGCCAATCAAGCACAGCTTGGTGAG--AT------TGTCGTAGC-----AAAGC 11511

Majority                 ACTTTTTCTTTATTATATTAAAAATGATTCTAAGTATCTAACTGTACTG--TTTTCATTACTATCAAGTTTTTTTCTTGC
                                 86650     86660     86670     86680     86690     86700     86710     86720
Human                    ACTTCTGCTTTCAAATGCATAATGTGAATGTGCGAATAAAAATAAACTCC-TTTACATCAGTAAGAAATTATTTTAATGG 86007
AsianBonytongue          ACTTTGACCTCAATAAAAAAAAAAAAATTAAAAGCATTCCACTGGAAGGAACTAGCATT-CCATCAGGAGTGTTCCTTGC 12358
Reedfish                 ACAATATGTATAGGATATCATATAAAATGTTTATTGTGAAAGTGTTTATG-TATTCATTTATCTTAAGTACTCGTCTTCT 43531
NorthernPike             GTTTCCCCAACTGTCTGTTCAGAGTGGATCTACATGTCTAATGAATCTC--CACAGGCTAATTTAGTAACTGTTGATGAA 17711
SpottedGar               AAGATTTTTTTGTAGGATTACCAATGATTCTAA--ACCTAAATTGAAGG--TTGTCATC-CACCCGAGAAAAAAATATGGC 16313
ParamormyropsKingsLeyaeACTTAATCCCCATTAGATTAGCCTCATTGCTAA--TGCTGGCTGTCGTG--CTGCCATCGCGATCTTGTTTCCTTCTGCA 11587

Majority                 CTTGTGT-CCTGATGTTTTTTTTATTTG-----TTGTATGGCAA----------GTATTTTTTTACTATGTTTAATTTGT
                                 86730     86740     86750     86760     86770     86780     86790     86800
Human                    CCAGGGCTCAAAATGTCATTTTAATGTGAATAGTTGTATGTGACGAACATATTTGTATTTTTCTGCTATCCTCCTACTGT 86087
AsianBonytongue          CTTGTGT-CCTG-TGCTTTTGTAATAGG-----TTCCAGACCA-----------CCATTACCCTGCATTGGTCAAGCAGT 12420
Reedfish                 ATAGCATATTTGATCTTTTTTTTTTCCA-----CTGCTTAGCAA----------GGCTAATATAACCTTAAATAATTTAA 43596
NorthernPike             CAGGCTACCGTGTTAGCATGGATATTTGGGTAGTTTTGGGGAAA---------TGTATCAGGTTACAACCTATGTTTACT 17782
SpottedGar               CATGTCT-GCAAACGTTTCTTAGATCTG-----AAGTGTATTAA----------CTAGTTTTCCACCATGCGTTAGTTCT 16377
ParamormyropsKingsLeyaeCTTATGA-TACAGTATCTTTAATACTTG-----TGGCAGGGAAA----------AAATTTTTTTTTCTTTTCAGCCATG 11651

Majority                 TATT-------ACAAATAAGGGTAGAAGA-----GGTTTGTAATTTTATTTC-AGTTTGATCTG------------TGTT
                                 86810     86820     86830     86840     86850     86860     86870     86880
Human                    TTTTCAAATGCACTCATAATAGGAAGAGATTTTAGCTTCAAAATTGTAATAATAGTTTCACATGAGAATTATATCATTTA 86167
AsianBonytongue          TGTT-------GCTAATGGTGATAGAATA-----ACTTTGTAATTTTTTCAA-AACTTTACCTGAGG---------TGTT 12478
Reedfish                 TACT-------GCACTTAAGGGGGGGGGG-----GGTATGTAATTCTGTTTC-TGTGGGTCATAGAGAA------GTAAT 43657
NorthernPike             TACT--------AAAAAGACAGTAAAAAA------GTTTACATTTCCATTGTTATTTCGGTGAT--------------TT 17834
SpottedGar               TACC-------ACAATTAAGAATAATAAA-----GGGATGGCATATACTTTC-ATTTCCATTCA------------TGAT 16432
ParamormyropsKingsLeyaeAGTC-------ATCTGAATGGCCAGAAGA-----TGTACGGAAAGATAATCC-GGGTGACTCTG------------T--- 11703
```

```
Majority                   TACCGAACGTGAATCTTGGCTTTTAGTTGAGGGATGTCTTTTTG--------TGACTGTTCGTATCTTCCATTGAATC-T
                                   86890     86900     86910     86920     86930     86940     86950     86960
Human                      TATTGAAGATTCATAGTGATTTTTATTCTAGGTACTCCTTGTTAATAGAGGCTAACTGTTCATACAGTCCATAGAATCCT 86247
AsianBonytongue            TACAGAACGTAACCCCTG--TGTAAGTAGAGGGACATCTCTTTG--------TGGCTTTTGTTGTCTGTCACTGGGG--- 12545
Reedfish                   CTGCTTAACTGAATCTCAGTGATCACCTGCTAGAAATGTATTTG--------GGATTGTAAGTATTGCCCACTAATTGAG 43729
NorthernPike               CAACCACCTGGAAGCTCGACCAATGGAAAAAACATGTTTGTTTAAT------TGATTGTTCCCCAGCTGTCTTATAC--T 17906
SpottedGar                 TG-AAAATGCCATCCATAGCTTTCAGATCAGAAATGCCTTCTTG--------CCAAATTTTACAAAAGACAACGAAACAT 16503
ParamormyropsKingsLeyae -CGAAACATCAGACGGTGCAGCTGCCCAGGGAAGGTCT-------------GGACGACCAGGGGCTCACAAAGGACT-T 11767

Majority                   GTATAATTCACTTTTAT---ATTTACTCTAGATTCTTGCTTTAATATATACTTGCAATTTCATCTAT-TTGTCGTTTGAT
                                   86970     86980     86990     87000     87010     87020     87030     87040
Human                      TCTTAATGCCATGTTATTTTAATTCTTCTAAAGTCTTGCGATACCAGGTTCTTAGAAAATGAAAAGC-TTGTGCTAAAAA 86326
AsianBonytongue            GTAAAACTGACTTTTAG---ACTTACAAATAAATTGTGTTATGA-ACAGACTTCCAGTCCCATCTGT-TTATAGTTTGAG 12620
Reedfish                   GAAGGGCACATAAATGT---AGTCAGAGTAACTGAATGATTTATTCAAAAACAGTCTTCACATATAG-GTGTCATTTAAT 43805
NorthernPike               GCAGTATCAACTATTAT---GATGCCCAGCGATGTTCAATTGCTAATATGTCTGATATTTTATTAATACCACAGATTCAT 17983
SpottedGar                 GTCCAATAAACCTTTT-----TTTTCTCTTGATTCCTGCGAAAATATGGCACTGAAAATGGATGGAT-TTGTTTTTTTGG 16577
ParamormyropsKingsLeyae CACTAATTCCCCTCTGC---ATCGATTCAAGAAGCCCGGCTCGA--AAAACTTCCAGA-ACATCTTT-CCGCCGTCTGCC 11840

Majority                   TATTTTTAGTATTCCC---AAGTATC---ACAGTTATATTATTCAGTGTTTATTGTTTATTATTATTTAAATGTGTG---
                                   87050     87060     87070     87080     87090     87100     87110     87120
Human                      CACTGTTGTTAATCCCTTTGGGAATCTTAAAAGGCACTTTAGTCAGGTTACACTTTATATCATTACTTAAATAAGTAATT 86406
AsianBonytongue            TATGAGA-ACAGCCCC---AAGTACC---ACATTAAAAGAATTTACTGGTTGCAGTTTGGTA-GATCTAAGGGAAAGA-- 12690
Reedfish                   ATTTGTTAGTGTTTCC---CTATTTTTAAACAATTTGATTATTCGTATATTTTCATTTTTTTTTTTTTTAATACAAGT-- 43880
NorthernPike               TGTTATCAATATGCTT----AGTGCC---GCACTTATAAAATAGAATGTACACTGTGTACCAACTGTTAAAAGTGGA--- 18053
SpottedGar                 GACGTGTTGAAAAATG---AAGTATT---ACGGTTGTAAGCCCCCAAGTTTATAGGAAACTATAATTTCATAGTAGG--- 16648
ParamormyropsKingsLeyae ACTCTCCACCTTTCCA---ACATCCC---GTGAGTAATGGGCAGAGTGGGCCTCAGCCGTTT---GCTAATTGTGTG--- 11908

Majority                   -CTGTTTTTAGTTTAAGTTA--TTGT--GGTTTTGCACATC-------ACTTACCACTAGTTCTTTTTT-TTTTTTAATA
                                   87130     87140     87150     87160     87170     87180     87190     87200
Human                      GATTATTTTAATTAAACTTATGTCGTTTGACTTCATACATCCTCGAATACATAAATCTAGAACACACCTGCACATTCACA 86486
AsianBonytongue            ACAGACCCCATTTAAAATCATGTTGT--AATCTTGCAATTA-------ACTTGCTAGTGTTTTTTTTTT-TTTTTTAATA 12760
Reedfish                   AATGTTTTTGTGTGCTGTAA--GTAA--AGTGTGAATGACT-------AGCCCTACTAACAATACTTTT-TTTTTGAATA 43948
NorthernPike               -CTGTGTGGAGTAGGACTT----TGTGAGGTATATGCCATGG------ACCCAGCAGGCCTTCCTCTGT-GGACCCAGGT 18121
SpottedGar                 -CATTTTTTAGTCTAGATTA--ATGT--GTTTTTGAAATCC-------ATTTCCCCCCAATACTTTCCT-TATTCCA-CA 16714
ParamormyropsKingsLeyae -CTCTCTTCTGCTTTAGTCG-ATCAT--AGGCTTGCTTCTC-------ACATAAGACGAGGTCTAACAT-GGCCTTTGTG 11976

Majority                   ACAGTTTATGGCTGTAGAATCTTTGCTGT--TT-TATTTTT--------A-TGAATTTCATGGTTAAAAAATTACTAAAA
                                   87210     87220     87230     87240     87250     87260     87270     87280
Human                      CCAGTGATTGACTGTAAATGCCAGGATGTTCTTACATTTCT----GTTCATTGAGATTCAGGGATAATGGTGCCTCAGAC 86562
AsianBonytongue            ATTATTTTTGGCTTTAAAAACTTTGCTGT-----TAATTTT--------AATGAACTTCATGTTTAAAAAAAAAAAAAAA 12827
Reedfish                   ACAATTTACAGTAGT-GTGTGTTTCAGGCTGTTTTGTTTTCTTAATCGTA-TGTATAACATTAGCCAGTTATTACAAAAA 44026
NorthernPike               CCGGTCCATGGACCTGAAAGCAAGATTAT------AATTTT----------CTTATTTCATAGTCCCTGAAATGCTGAGC 18185
SpottedGar                 ATTCCTTATTTCAGTTGAAAGTTTCCTGTCCTTGTATTTCTTAGCGATGAGTCATCTGAATGGCCAGAAGATGTATGGGA 16794
ParamormyropsKingsLeyae AGAGGCTGTTGCCAT-GGATTTTCACAGC--------TTTC--------A-CGAATACTATTGGTGAAAAGTCACCCAAT 12038

Majority                   TGATC-AT------G-TGTTCATTTAAAGATTAGGCTGTTTATTTGTA-GGTGGAGTGGTTTGATGATCATGGACTTATA
                                   87290     87300     87310     87320     87330     87340     87350     87360
Human                      TGATT-GTATTCACATTGTTAATTTGTAAATTTGCCATTTTTGATGTAAGGACGAGTGTATTTTTGCTCAGAGATGAACA 86641
AsianBonytongue            AAATC-AT------G-TCTTCATTAACATTTTAACATGTCCA-CTGT--AGTTAAAGGGTATGATGTGTGAAGTCTTGTC 12896
Reedfish                   TGTTCTACATTATTGCTGTTCATTACTGGAGAAAGAAATTTGTTTTCAGGATGATTAGATTAGAAAATCATTACTCTGTA 44106
NorthernPike               TTGTT---------TCAGATGGATGGAAGAGAAGGCTATCTCTTCGC--AGGGAGGTGGGGTTATGGATAGGCAGCGATT 18254
SpottedGar                 AGATC-ATTCGAGTG-ACCTTATCTAAACATCAGACTGTACAGCTGCC-AAGAGAGGGGCTTGATGATCAAGGGCTCACA 16871
ParamormyropsKingsLeyae AGGTG-GC------G-TGCTAGTAGAAACGCT----TG------TGTT-GGTGGACAGTCAACATGAG-ATCAACTTATG 12098
```

```
Majority                   AATGGATTAGTCAATTCGCTATTAGACCTTTGCAT-A------CTGTTTTAAATTTTATTTGAATTATATTTATTTATTT

                                     87370     87380     87390     87400     87410     87420     87430     87440

Human                      ACTTGCTATATTAGACCAATTTTGGCCTTTGACATCACATAGAAAATTTTGAATTTCAAACACATAATAGGTTTGAATTT 86721
AsianBonytongue            AATGGATT-GCCAATTT-CTGCTTGACCTTTG------------TGCTTTAAAAATTATTTTATTTAT-TTTACATTTTC 12961
Reedfish                   TTTTGAATATCCAGTTG--TACAAAAACTTTGAAGCA------TTGTTGTAACTTTTAGTGCAGTTGGAATTATATTATT 44178
NorthernPike               A--GCACTAGTCCATGAGCAACTG-ACGTTGGCAT-G-------TGTTTTTTATTTTTGGTGAATTTC-AGTCTGTACGA 18322
SpottedGar                 AAGGATTTCACCAACTCGCCATTACATCGTTTCAAGA---AGCCTGGGTCAAAGAACTTCCAGAATATATTTCCTCCTTC 16948
ParamormyropsKingsLeyaeAAAGAGTC-CTTGATTAGTCATTA-ACTTTTATAG--------CCCTTGCAGATCTCCACTGAGCCATGCGTAGTTACAT 12168

Majority                   TGGAAT-GCTTGACCTGACAAATAGCCG-TAAGGTTAATTAAATAACTATC---CAGGTTATTTTGTTGAAACATG----

                                     87450     87460     87470     87480     87490     87500     87510     87520

Human                      TAGTGTTTCTTGGCAATAGTCTTAGCAACTCAGATGAGGTATAAAACTATCCATCTGGCTGTCCATTTTTAAAATGGTGG 86801
AsianBonytongue            AGCAAT-GAGTCACCTT---AATGGCCA-AAAGATGTATGGAAAGATCATC---CGGGTCACGTTGTCGAAACATC---- 13029
Reedfish                   GGAAATAGTGTTACAAGCAAGATGCTTG-TAACAGAAATTTAACTGGTAGTGAATAAGCCACTTCTCTTCAGAATG---- 44253
NorthernPike               TGGGGA-GCTTGTGCAGACAAGCTGTAGTCAAGGTTGATCAGTTCTCCAGC----AGGAGGTGCTGTCCAGCCATGCTTT 18397
SpottedGar                 AGCAAC-CCTTCATCTGTCAAACATCCC-GTAAGTAAATAAAATAACTGTC---CCATTTTTTGGGTTGATA-ATT---- 17018
ParamormyropsKingsLeyaeTTTAAA-GCTTGGAGTA---AATAGC---AAAGGTTAACTATATAA-C---------ATTATATACTTGGAG-ATG---- 12226

Majority                   -ATTCAGTTTAAATATAAACAGAAGTCCTTTTTACTCATACCAGTGAATGCATATTTGACTATTTT--T-CGTTTGT-A

                                     87530     87540     87550     87560     87570     87580     87590     87600

Human                      TATGTGTTTTAAAGAGATACGAAAGTGCTGATTATGTCAAGATACTTAGTACATATTTCCTCTTTCTCTGTGTGCACTT 86881
AsianBonytongue            -AAACAGTTCAGCTGCCAAGAGAAGGCCTTGATGACCAAGGCCTTACAAAGGAC--TTTACTAACTC--T-CCCTTGC-A 13102
Reedfish                   -CCTTATACATTACACAAGAACAGTAAGATTGAGCATGGCACTGGGAAATGCTTGTTCCACTAGTTTGGT-CGTTAATCA 44331
NorthernPike               TTTCCTGACTTGATAT-AATAGAAGTACTTAATTATTCATA---G-GGTTGCCCAATTGACATTTTT--------TGTTA 18464
SpottedGar                 -TTACAGCTGAAAGGT-AACCTGAGCTTTTTTTCCCCATTTACAGACAAGACATAACCGAAGAGGAC--C-TCAGAGT-A 17092
ParamormyropsKingsLeyae-AATAAATTTGAGATTAAACAGAATTCCTTTGTTAATTAAACCAGTGTTTACCT--TTGTGCACTTT--T-CACTGGT-A 12299

Majority                   CTGTTTTAAGATT-CC--TGGCACTATGAATTTTTTCA-ACATTTTTCAAGTAGCTTTTTTTATTAACTTA------GTC

                                     87610     87620     87630     87640     87650     87660     87670     87680

Human                      CTGTACTCTGGTTGGAATAGTCATTGCTGTTGTTTTCATATATGTTGTAAGTAGCACTTAACAGTACTTCATAATAGGAG 86961
AsianBonytongue            TCGTTTCAAGAAA-CC--TGGCTCCAAGAATTTCCAGA-ACATATTTCCACCGTCTGCAACTCTTCACCT-------GTC 13171
Reedfish                   CTGCTGTAGAATTGTCATTTGCACCATCACCCTCC-CT-CCATCCTTGCATCGTAATTGACTATATACTTA------TAC 44403
NorthernPike               CCGTTATGACAAT-----CCTCCGGTTGTTTTTTTGCCAATTTGTTTCTGGTTCCTGCATTGATTAAACCAGT---TGGC 18536
SpottedGar                 CTGTTCTCAAACT-CCGGTGGCACTGTGAAAGCCTTCA-AGTTTTTCCAGTAAGATTCTTATCTTAATTTT------ATC 17164
ParamormyropsKingsLeyaeTCGGTTTA--A-------TGACATTAAGAATCTTT----ACATTTCCCAAGGAGTTTTTTTTAATTACTT---------- 12356

Majority                   TATCATCAGGTGTGTAAATTTTTTTACTTGT-GTTGGATGTATGCTTTTAATTATGTTATTTACTTCTGGGATGTTTAGT

                                     87690     87700     87710     87720     87730     87740     87750     87760

Human                      TATTCTCAAATATGTCATTTTTTTCCCTAATTGTTTGAGGTATGGGTGGCATTGTGTCATTTAAATAAGGGATGCATAGT 87041
AsianBonytongue            AAACATCCCGTGAGCAAAGG-GATTACTTGT-GTTGTATTCACCCTTTTATGTATGCTACCAAA--ATAGTAACATTGGT 13247
Reedfish                   TAACG--ATATATGCAGTAGTTATCACTGGA-AAGCAGTGTATTATTTGAATGGTTGTTGAAACAACTGGATACATAAAT 44480
NorthernPike               ACCCATTGGTTGCTTAAATGATTATTCCATTAATTTGAATTATATTCTGTCCTAGAACATCT--TTCTGGGTTGTCTCCA 18614
SpottedGar                 GATTTTCAGGTCCCTTAACTTGCTTATATGT-GGGGGAAGTTTCCTTTTAAA-AGCTTATTGACTTGTATGAGTTTTAGC 17242
ParamormyropsKingsLeyae---------TTGGAACAGTTGCATAATGAT-GCTGGA-GTATGCATTTATGTATGTTTT---------T-----TTTTT 12410

Majority                   CTAAATTGTTGTTTTGTAGTCAAGATGTAACTGAAGATGATCTT--CGATTTTTTTTCTTTAGTATTTCGGTTGCACTGT

                                     87770     87780     87790     87800     87810     87820     87830     87840

Human                      CTTTATGGTTGGTAATTACCTGTGGTAGAATTACCTCAGATCTTCCAGATAGTTTTTGTTTTATGTAAAGACTGTTTTAT 87121
AsianBonytongue            CTAATTTATTATTTTGCAGACAAGACATAACTGAAGATGACCTT--CGGGCCCTCTTCTCCA--ACTCCGGTGGCACTGT 13323
Reedfish                   CTGTATTGT--GTCAACTGGCATTGCACAGGTCAGCAAGATTTTTTCCATTTTTTTTAATTGTAAATTTGGCTATAGCTT 44558
NorthernPike               CTTATTCAGTGTTCCACATTCAAGAAGCAAAT---TCTAATATTTAGACCTAACCTCAATTTGAAAGACGTTTTCCTGGA 18691
SpottedGar                 TCAACAT-CTATTTTGTTTTTACAATGTAACC--AGATGCCTTA--TAAATTATTTTTAAAAGTACTTTGTTTTCACTTA 17317
ParamormyropsKingsLeyaeTCAAATT-------TGTAGACAAAATGTAACTGAAGATGACCTC--CGACTGCTGTTCTCGA--ATTCCGGTGGCACTGT 12479
```

```
Majority                 GTAAGCATTTAAGTTTTTGCA---GTAATTGCCTCTTTAAGAA-C--CAATTTAGATTTTGTA--TTAGGTTGATTAGAG

                                 87850     87860     87870     87880     87890     87900     87910     87920

Human                    GTAATATTTTAATTTTTTGCCATGCTCGTAGTCTGCTTGGGCTGCTGTAACCAAAATACCATAGACTGAGTAGATGAAAC 87201
AsianBonytongue          GAAAGCCTTCAAGTTTTTTCA---GTAAGAGCTTCCTTAGGAAAC--CAGTTTGGTGGTTGCA--TTAGGGTAAAGAGAG 13396
Reedfish                 AATATC--TTAAGTGTGTACTT--TGCATTGCCACTTAAT-------CATTCTAGTGCAT-----TTAGTGTGAGCTGAG 44622
NorthernPike             GTGGGGA--ACGGTCTTGGCA---ATAAACTACAGTGGTAAAACACCTAATACAAATACTGTGCTTC--------TAGCC 18758
SpottedGar               GTTAAAAAGCTATTTTAACCAT--GCAAATCCTTATTTAAGTGTT--AAATTTATATTTTGCACATTAGATGGGTTAATT 17393
ParamormyropsKingsLeyaeGAAAGCATTTAAGTTTTTCCA---GTAAGTGTCTCTTAAA-------CAGCTTTGATTTT-------------------- 12529

Majority                 AAAATTCATCTGATTAGACAGAATATT-ACGAGTAAAAT-GTCAGGTTTAGCCTGCCC---TTG-ATTTCCAACT-ATTG

                                 87930     87940     87950     87960     87970     87980     87990     88000

Human                    AACAAAAATGTATTTCTTCAAAGTTTTGAGAAATAAAACTTTGGGGTCTAGAAGTCCCAGATGAAACTGCTAGCAGATTG 87281
AsianBonytongue          AGAATCCAGAAGA-GAGAGAGAAAATT-ACATGTACAAT-GTCAGATGAAGTCAGACAGGGTTA-ATTGGCAGTC-CTTG 13471
Reedfish                 AAAA--GGGCTAGCTAGATATTATTCT-CTGATTTAAAT-GTCAATTCAGAACAGCCAC--TTGCATTTCCAACA-ACTG 44695
NorthernPike             ATGAGTCATCTGAACGGTCAGAAAATGTACGGG-AAGGTGATCAGGGTGACCCTGTCC-------AAGCACACCTCGGTG 18830
SpottedGar               AAAGTTTATTTTATGATAAAATATACT-ACCAAAACATTTGTCCAGTTTAGCATTATTTTGCTGCATTTACCATT-AACG 17471
ParamormyropsKingsLeyae----------------------------C------AAT--------------------------TCTCTAACT-CCTG 12546

Majority                 GAACATGTCAAGTATGACC--------A--TGAATTTCTTTGACCTCT-A-CATCTTAGGCTTTTTCACCGTGCATTTTC

                                 88010     88020     88030     88040     88050     88060     88070     88080

Human                    TTTCTGGTGAGGTCTCTCTTGGCTTGCAGCCAACCATCTTCCCCCTGTGTCCTCATGTGGCCTTTCCACTGTGCAAGCAC 87361
AsianBonytongue          GCACAGGTCAAATATGAAC--------ATATGACCACATTTGACTTCTCAGTATTATACGCTTATTTACCTAGCATAAAC 13543
Reedfish                 A----AGACAAGTGTGCAC-----------CATATTTCTTCCATTATCTA-TATCTAGCACTTTTTCTGTGTGGACTTTC 44759
NorthernPike             CAGCTTCCCAGGGACGGCC-----------TGGATGACCAGGGCCTCA---C--CAAAGACTTCACCAAC---T------ 18885
SpottedGar               AAA-ACGTTAATTACCATT--------A-ATGAATTTCTTTAAAAACT-GGCAT-GTAGGCTTTAAAGTCATGCATTTTT 17539
ParamormyropsKingsLeyaeC---ATGTGAATTGTAAC-----------TG----GCTTTGACCAAC-------------------------------- 12575

Majority                 --T--GAATCTCGTTCCTTTGTA------CAGGGATCACAGAAT---GGCACTGCTGCAGATGTCCACTGTTGA-----A

                                 88090     88100     88110     88120     88130     88140     88150     88160

Human                    GTTAAGAGTCTCTTCCCCTTGTAAGGATCCTAGTCCTGTTGAATCAGGGCTTTACCCTTAATGACCACATTTAACCTTAA 87441
AsianBonytongue          AAT--GCATCTTGTTTCTTTGCA------CAGGGATCGCAAAAT---GGCACTGCTGCAGATGTCCACTGTTGA-----A 13607
Reedfish                 CCT--GGCTCTCACTCATATGTGG-----TCCCCTTCACATATT---GGTACCCCTGATAAAGATGAGTAATCA-----A 44824
NorthernPike             --------CCCCGCTCCACCGCTT-----CAAGAAGCCCGGCTCCA-AGAACTTCCA-GAACATCTTCCCTCCATCC--A 18948
SpottedGar               --T--GAAAACTATTCCTTTTTA------CAGGGATCACAAAAT---GGCACTGCTGCAGATGGCAACTGTTGA-----A 17601
ParamormyropsKingsLeyae------------TGTCGTTGCC------CAGAGACCACAGAAT---GGCACTGCTGCAGATGTCAACGGTGGA-----A 12628

Majority                 GAAGCG-ATTCAGGCCTTGATTGACCTGTACAATTACA--------ATATGGGTGACA----ACCATCACCTGAGG--GT

                                 88170     88180     88190     88200     88210     88220     88230     88240

Human                    TTACCTCCTTAAAGGTCCTGTTTTTAAGTACAGTTACATT--GGGGGTTAGGACTTCAAC--ATATAAATTTGGGGAGGA 87517
AsianBonytongue          GAGGCG-ATTCAGGCCTTGATTGACCTGCACAATTACA--------ATATGGGTGACA----ACCATCACCTGCGG--GT 13672
Reedfish                 GATTAATATTCAGTTTTTGGTGGATTACTTTAATCTCACTCTGAAAATATGGGAGAAATCCCACCTTTAACTGAAGTAAC 44904
NorthernPike             CCACCC--TCCACCTCTCCAACATCCCGTAAGTCTACGAC---ACAGCCCAGAGATCAGATATCTGTTCCCCGAAAATAA 19023
SpottedGar               GAAGCA-ATTCAGGCCTTGATTGACCTTCACAATTACA--------ACATGGGAGATA----ATCATCACCTGAGA--GT 17666
ParamormyropsKingsLeyaeGAAGCG-ATCCAGGCCTTGATCGACCTCCACAATTACA--------ACATGGGTGACA----ACCATCACTTGAGG--GT 12693

Majority                 CTCC--TTCTCTAAGTCGA----CAATCTGAAAATACTGTTTGGAAAGG-ATTTTTTCCTTTATGG---TCTCTAGAGGC

                                 88250     88260     88270     88280     88290     88300     88310     88320

Human                    CACAA-TTCAGTTAATAGTACTTAAATTTGTAA--ATCATTTATCCCAA-ATTTTATAAGATATTG---TCACTAAAGTT 87590
AsianBonytongue          CTCC--TTCTCCAAGTCAA----CCATCTGAACACACTGACGGGGAAGG-ATTCGTTTCTTTGTGG---TCTCAGGAGGC 13742
Reedfish                 TTTA--TTCTTAGAAAGAAATCCTAATCAAAAAATAATTTTAACAAAACCACACATGCCACAATCA---TTTGCAATTAT 44979
NorthernPike             ATCCTCTGGTTTGGGTTGGCTGTGCACCCGGAAAGCTAAGTTGACATGTTGTTTTGTCCAGTGCGGATGTAACCGAGGAA 19103
SpottedGar               CTCC--TTCTCTAAATCAA----CAATTTGAACATGCTCGCAGGATAAA-ATGTG----TTTACGC---TCTTTAGAAGC 17732
ParamormyropsKingsLeyaeCTCC--TTCTCGAAGTCGA----CTATCTGAA---GCAGTTGAGAGTGG-CTCCT---CTTAATGC---TTTCGAGTGGC 12757
```

```
Majority                  GTTACCTGTTGGCTGTTTTTGTATACTGGGGAC---------T---CACCATTTTGACA--T-T----------------

                                   88330     88340     88350     88360     88370     88380     88390     88400

Human                     TTGACTTGCTAGTAGGGCTTATAATTTTTGGACTATCTGTAGTGGTAAATAATTCATCATTTCTCATTGCTGCTAGGTAG 87670
AsianBonytongue           CATGCCTGTTGGCTCTTTCCAAATAT--GGGAC-------------CACAGTTTTGAGA--------------------- 13786
Reedfish                  GATTTACATTTAATAATTTTGTACAACCAGCACAGAGTTTGTTTTATAACATCTTAAAAGGTTGGAGAACATAGAGCAGG 45059
NorthernPike              GACCTCCGACTGCTGTTCTCCAATGCCGGAGGC-------ACTGTGAACGCGTTCAAGT----T---------------- 19156
SpottedGar                GTTACCTATTGACTGTTTCCGGACACTGGGGAC-------------CACAGTTT-GACA--------------------- 17777
ParamormyropsKingsLeyaeGTTACCTGTTGACAGTTTCTCCATACTGGGGAC-------------CACCATTTTGGCTCTTGTGT-----TCTTTGTTT 12819

Majority                  -TTTTTATT----TTTATCTGTTTTTCCTTCAGTGTATATGGTTG-GTTATT-CT--ACTAAAAATAATCATGAAAAAAC

                                   88410     88420     88430     88440     88450     88460     88470     88480

Human                     ATTTTGCTTTC--TTGAACTGTGTGACCATAAAAGTATGTACTGACACCATCATTTCCTTTAAAACACACACACACAAAC 87748
AsianBonytongue           -TTTTTGGT----TTTATCAGTCATTCCTTAAGAGAACATGGATA-GTGA-------AGTAAAACTGATCTTGTGTGAAA 13853
Reedfish                  GTTTTTAATGGGATTTGGGTCCAGGAACTACAGTATGTATGGGAG-GCATTTTCTGTAAAAAAAAAAAAAAATGGAAATGA 45138
NorthernPike              -TTTCCAGTAA--GTCTTCTCTTCTTCCATCACTGATAGTGACTGAGCTTGAGC---TCTGGATTTTGTTGTGAACGAAC 19230
SpottedGar                -TTTTAAAT----TTTATCAATCATTCCTTTA-AGAATATGATGG-ATTA-------TCAGAAAAGAAACATTACACAGC 17843
ParamormyropsKingsLeyaeTTTTTTTT----TTTTCCTTTTTTTTTTTCCTTTTGTTTGTTCA-GTCATTCCT-TAATATGACGGATTCTGAAAAAAA 12893

Majority                  AGATTATCATTCAAGCTATTACTCTTTTACGATATTTCTTTCTTCATTTTTT----TGTTC--GCAAGCCATTAACTTTT

                                   88490     88500     88510     88520     88530     88540     88550     88560

Human                     ACACACACACACACGCATACACTCCCACACAAAACTCTATGGTGTACTATTT--AGTATG---GTGTACTAAAAAGATTA 87823
AsianBonytongue           AGATTAT-------GCTATTACCCCTTTACCATAATTCTTTCCCTTCTCTTT----TCTT-----------TTAACTTTT 13911
Reedfish                  AAATTAAAATTTATAAAATGAAAATGTAGTGTCATTTGCAATTTCATTTTTCAAAGTCTGCACGCAAGAAGGCTGCAGAT 45218
NorthernPike              TTGAATTCATTCAAATGATTTCCGTTGTGCTTTAGGGCTTGTTTCAGGGGTAC---TGTAC---CATG--GCTGACTCCG 19302
SpottedGar                AGAGTCT-------ACTAGTCTGCTTTGAAGACTTTCCCCACCCCATTTATA----TGTTAG-GCAAGCCATTAACGTTT 17911
ParamormyropsKingsLeyaeAAATAATTTGGAGAGATACTGCTCTGCTTTGGAGATACTGTCCTCATTTGTC----TGTTAG-GCAAGCCATTAAC-TCT 12967

Majority                  TTCTACTTCTTCATAAGAAGGTTGGATATGAACTTTTTTTCTTAG------TAAAATTGAAGTAGATGAAAAGTTAAGTG

                                   88570     88580     88590     88600     88610     88620     88630     88640

Human                     TCAAACTTGGTATTTAAAAAGTTAGCTATATACTTTTTACAAGAGACAT-GTAAAGCAGAAGAATATGAAAGATTGAAAG 87902
AsianBonytongue           TTCCACTGCTTCCT------------------CTTCCTTTCTTAT------TATAATT---------GACAAGTTATGTG 13958
Reedfish                  ATTAAAATTAAAATGAAACAACCCCATTTGAATTTTCAGCCTGAA------CCAAAATGAAGCTGCAAAAATGAAAAGTA 45292
NorthernPike              TGCTCCTCTCTCATCGCAATGTTTGTCACCATCTTGTCTTCTCAGGGATCACAAAATGGCGCTGCTCCAGATGTCAACGG 19382
SpottedGar                TTCCATTTCATCATAAGAAGGAAGTAAGTAAATTCCTCTTTTTAT------TATAATTTGATTAGATGACAAGATATGTG 17985
ParamormyropsKingsLeyaeTTCTATTTCTTCAAAAGAAGGATG-----GAAATCTTTTTATTAG------TG-AGTT---------GAAAAGACACGTG 13026

Majority                  CCTTACTTGACA-AAATGGTATCAACAC-TTTTACATCTGTT-CATTTACATAAA---TCATTTCAGTTGTCTATTTAGG

                                   88650     88660     88670     88680     88690     88700     88710     88720

Human                     GAAAAGGATGTA-AAGAGATACTAATATACTGGACAGATAGT-AAGCAAGACAAAACTTAAATACCTTTATTAACTTAAG 87980
AsianBonytongue           CCTTACTTGACA-AAATGTTATCAACAG-TTTTGCATCTGTT-TATTTACATAAA---TGACTGAAGTTGGCAGATAAGG 14032
Reedfish                  AAATGCAAAT---AAACACTGGTGCCAC-CTGCTGCTCGTTG-AATTTTCATTTT---CCATTTTGCATTTCAACATGAA 45364
NorthernPike              TCGAAGAGGCCATACAGGGTCTGATTGACCTCCACAACTACAACATGGGAGACAACCACCACCTGAG-AGTCTCTTTTTC 19461
SpottedGar                CCTTACTTGACA-AAATAGTATCAACAC-TTTTACATCAGTA-CATTTACATGAT---CCATTACAGTTGACTATTTAGG 18059
ParamormyropsKingsLeyaeCCTTACTT-----ATACAGCACCAACAC-TTCAGTTTTTG---CATTTAAGTAGG---TTA-------TATCTGTTTAGG 13087

Majority                  CACATTGTTTCTTATGGTGT--GTTGAGTGGAGATGTATAAATATTTATTGCCTT-TTTGTCT-GTGTATATTCTCTGAA

                                   88730     88740     88750     88760     88770     88780     88790     88800

Human                     -ACAGAATTGATAAAGGATTTAGTTCACTAGAAAGATATAACAGTTTATGTATATGTGTGCCTAGTGAAATAGCCTCAAA 88059
AsianBonytongue           CATGTTTCTTTT--TGGTG---GTTAAGTGAAATTGTAATTTTTTTCCTTCCCTCTTACTCCC-TTGTGTA-TCTCTGAA 14105
Reedfish                  AATAGCAAGGGTCTTTGGGC--GGGGCTTGGCGATGTTCCATAATTTTTTGTCC--TTCATAG-CTGTAGAGTCACTTTG 45439
NorthernPike              CAAGTCCACCATT-TAAAC----CTGCATAACGAGTCCTCAACATCCATCGCGTTGTTTCCCAAGCAGCTCTTGGCTGGT 19536
SpottedGar                CACATTGGTTCTGATGGTGT--AAGAAGCAGAGATGTAAAAATAGGTATTTCTG--TATGTAT-TTGTACAGTTTGTGGA 18134
ParamormyropsKingsLeyaeCACATTGTTTCTTATGGTCT--GTGGGGTGGATTTGTTTAAATGTCTCCTGTCTC---CCTCT-GTGTCTGTTATTTGAC 13161
```

```
Majority                  ATTCTTT-CTCCTCAATGTGTAGTCCT-T-ATTAGCTTTTAG--AATTTAATTTTT--GTGTATTTTTATT-----TTTT

                                 88810     88820     88830     88840     88850     88860     88870     88880

Human                     ATATATAAATAATAAGTAGATAGAACTACAGGTAGACTTGAGCAAATCTATTATAGTGGTGGATTTCAATTCACTTTTCT 88139
AsianBonytongue           ATTCTG----CCTCAA-GTTTATTCCT-T-AAAAA-----AC--AATTTAATATGA--GTGAGGCTATATT-----TTTT 14164
Reedfish                  ATCGATAGATACTTCTGACTTGGACTG-TGTTTAGTTCCCCG--AGTTGGATCTTT--TCATTTCCGTACT-----GCTA 45509
NorthernPike              GTTCGTT--TACTCTGTCCGGACTCCGATGGACCACATTTTGACTTTTTGACCTCA--TTGT-TTTTCATTCTACTTTCT 19611
SpottedGar                GTTCTCTACCCCTCAAAGTGTAGTCCT-T-ACTAGCTGTGAC--TTATTAATTTCT--GCAGATTTCCTTC-----CTTT 18203
ParamormyropsKingsLeyaeATGCTTC-CCCCTTAA-GTGTAGACC----ATTAACTCCTAG--AATTCAATTTTTTTGTGAATTTTTTCT-----TTTT 13228

Majority                  GATTTACATCTAAAATTTATCTTAATAGTTAAAGACTAAGAAT-A----A-TTGTGGAAGAC-------CTTAGATGAAT

                                 88890     88900     88910     88920     88930     88940     88950     88960

Human                     GAATTACATGTCAAACTGACCAAAAAAAGACAAAATCAACAAAGATACAAATGATACAATACAAT-TACCTTAACAGAAT 88218
AsianBonytongue           GATCTA---------TTTATCTTGGTTGTTATGGAATGAGTAT--------TGGCAGAAGAT-------CTGAGATGAGT 14220
Reedfish                  GCTTCACAGCAAAACTGTCTCTCAAATTCCGATGACCACGCATAATTGTAGTTCTGAAGAAG-------TTAATGTGCAA 45582
NorthernPike              AAATGTTATCAGTCATTCCTTCAGAGAATCACAAACTAGAAGAAA--AAAAAAACGGGAAATTTTATCCCTTTCATTTGA 19689
SpottedGar                AGTTGAAAATTACATTTTATTTTATTAGTTA-AGATTCAGAGT--------TTTTGTAAGAC-------CTAAGGTGAAC 18267
ParamormyropsKingsLeyaeGTTTTAG---CAAAGTTCATATGAGGAGTAA-GGACCCACA----------TAGCACCAGGC-------CTGA-ATGGTT 13286

Majority                  TTTGAATCTA-GTTTTGCAAACATTGAAGTTTTGCGTTTTATATTGAAC-TT-----ACATTTAGTCATTCTGTTGTCCG

                                 88970     88980     88990     89000     89010     89020     89030     89040

Human                     TATGTGTCTAAGTTATGCATCCAACACAGTTTTTAAACGCATATGGAACATTTTTGAAAAATCAGTCACATACTAAACCA 88298
AsianBonytongue           TTTGA------GTTCTCCAAACATGGAAACTTTAATTTTTATGATGA--------------TTAGTCATCCTGACAACCA 14280
Reedfish                  TTTAATTACA-ATATTTAACATGGTGGAAATTAGCCTTTTACATCATAGTTT-----ACATTTATTTACACTGAATTGGG 45656
NorthernPike              TCCAGACCGATGTGTAAGTCCCATTCCCCATTTGTATAGAAGGCAAAGCCTTTTCCACCGTCTCCTCATTATGGTGGGTG 19769
SpottedGar                AAAGGACATC-GTTCTGCTAAGATTGAGGCTG-GGGTGATGTATTGA--------------TTAATCAGTAAATTGTCAG 18331
ParamormyropsKingsLeyaeTGTGA-----------GGAAACAGTGAGTTTA-ACGTCATTTAT                                     13318

Majority                  TACGGTAAGCTTTTGATGATTTTTTATATTATCGTCAGTGCCTCATTCTTTTTTTTCCCTGTCA----------------

                                 89050     89060     89070     89080     89090     89100     89110     89120

Human                     TAAAGCAAGCTTCAGCTAATTTCAAAGAGTATCATACAGACCTCATTCTCTATTGCACAAGTAAGTTAGAATTAATAACA 88378
AsianBonytongue           -ACGATAAAGCTTTAATGATTGTTTA-ATTTATG--AACGTTTTAATAATTTTTTTCCTTCTT----------------- 14339
Reedfish                  AACGGGATACTTTTATTTTTGTAGTACTGTACCGTCGGCGCCAAGCACCACAATGGCTCTTTGG---------------- 45720
NorthernPike              GATGAATGGATGTTGGCGCCTCTTTTTATTGTAATAAGTTAAACAAAATGTGCCTTACCTGACAATATCAACTC-T---- 19844
SpottedGar                TACATTTCCTTTTTGATCATTATCGATATCCAGGCCAGTGCATCATTTATTTTTTCCCCTCACA---------------- 18395
ParamormyropsKingsLeyae                                                                                 13318

Majority                  -TAAGGCAGTTACAAATCCT-TACCTCCTGATTTATATATTTGTGTGCTTGTTGCATGTGACGTAAATTGATTTATTTGT

                                 89130     89140     89150     89160     89170     89180     89190     89200

Human                     AAAAGATGCATATAAAATCTGTATCCTGAGATTTAAAAATAAGTCTCAGTATTGCATGAGTCAGAAGTTTGTTTGTTTGT 88458
AsianBonytongue           ---------TTACACATC----ACCTGCTG-TTAGTGTATTTGTACACGTGTTGCTTGTGACTGAATATACTTTAAGTAT 14405
Reedfish                  -TGGAGGTGCTGGAGAGCCTCGAGGCCCAGATCTATATCCTGGAGTGCTTTTTGCAACAGCAGTACAGGGATGAAGACGG 45799
NorthernPike              -TTTTACATCTAAATATTACATAAATCCCTGCAGTCTGATTTTGAGGCACATTAATTCTGATGTAAAGTGTTGTATTTTC 19923
SpottedGar                -AAAGGCAGTCACAAATCT--GACATCTTAATTCAAATACATTTGGGTTAGTTAGATGTGGAGCAGATTAAATCAAACAG 18472
ParamormyropsKingsLeyae                                                                                 13318

Majority                  T----------CCAGAATCTGACTCCATTTTTGCGTGTGCATTGCCAT----TTACTTTGTCTTTTGTTATTTAAATTGG

                                 89210     89220     89230     89240     89250     89260     89270     89280

Human                     TTGTTTTTGAGCCAGTGTCTTGCTCTGTCCCCCAGGGTGGAGTGCAGTGGTGCTATTGTGGCTCACTGCAGCCTCGACCT 88538
AsianBonytongue           T----------CTTGAACCT---TCCTCTTGTGCATTTATACTGCCAT----TTACTTTGTATTTGGTTATTTAAAATGG 14468
Reedfish                  C----------CGAGAAACTGACACCATTTTTGCAGGTGCATGGACAA----TGGCAGAGCTTCAGGCTCATGAAATTGG 45865
NorthernPike              T----------CCCATGTGTGATTGTATTTTTGTTTTACCACCCAAGATGTTTTGCCTTTTCAATTTTTGCTTAAATTCG 19993
SpottedGar                A----------GCATAAAGA---AGCCCAAATAAGAGTACATTACCA-------ACATAGACATTTGTTATCTCTGGCAG 18532
ParamormyropsKingsLeyae                                                                                 13318
```

```
Majority                  ATGTGGTTTTTGCAGAGCATGTACTTATTGGTCTAATGTTAGTCGTATTAAAAACGGGTATTGTTTGCACTGCTGAATGA
                                   89290     89300     89310     89320     89330     89340     89350     89360
Human                     CCCAGGCTTAAGCAGTCCTTGCACTTAAGCCTCCAAAGTAGCTAGGACTAAAAGCATGCACCACCTGCCCGGCTAATTTA 88618
AsianBonytongue           ATGTGGTTTTTCC-GAGCATGTACTGACTGGTCTAGT--TAGCCCTGGGTCAAACTGGTGTTGCATGCACTGTAAAAAGC 14545
Reedfish                  AGAAAAGTGTGGCAGATCAAATTGCCACTGACCCCAAGTGAGTCGTGAAAAGCAGGGCTGGTGATTAAAATGCAGAAGGA 45945
NorthernPike              CTGTGGTTGTTGTGGAGTGGGGATCAATAGGAGGTTTGTTCATCAGATGAACAA-ACACATGGTTTGTTCATCTGAATTT 20072
SpottedGar                ATAGGGTTTTAGT-TTGCCTAAAAGTATTTGCCTAATG-CAGGGATATTATTAAAGCAAATTATATGGAAGTTGGATGGA 18610
ParamormyropsKingsLeyae                                                                                    13318

Majority                  TTATTTAT-TTAG--TCTGTAGAGTTGTAGTCTCATTCTGTTGTACTGAGTGCAGTAATTTTG--GGGTATATAACATAT
                                   89370     89380     89390     89400     89410     89420     89430     89440
Human                     TTATTATTATTATTTTTTGTAGAGATGAGTTCTCACTGTGTTGCCCAGACAGAAGTTATTTTAATGGGAATATAAAATAC 88698
AsianBonytongue           TGACCGAAGTGAG--TCTACAGGAGCGCCATCACATGCCGGATTTCTGAAGACAGCAACCCGGT-GGGACCAAAGTTTAT 14622
Reedfish                  CAAGGCAG-------ATCATAGAGGCATTGTGAAGCACTGGTGAAGGGAGAGCAACAAAGTTG--AGATCCAGACCATGA 46016
NorthernPike              TTTTTTTTTTTTAACCCTTTTTTTTTTTAGATTACTTGGACCATGATGTGTAAAGTTATTCGC--AGACAGACAACCAGT 20150
SpottedGar                TGGCTTAC-CAAG--CCAGAAGAATAGTAATATGTTGCAGTTGTAATAAGTTTAGTAGTTTTGG-TTGTATATAACAGAA 18686
ParamormyropsKingsLeyae                                                                                    13318

Majority                  TTGCCATTTAGTGCTAATTTTCAATATATAAATTAAAAATTAGTTGTACATT----TTGAACTTTTTACCATAAGTTTTA
                                   89450     89460     89470     89480     89490     89500     89510     89520
Human                     TTAAAATTAAGCGATAAAGTACTATATACAAATTAGATATATGTGGTACAGTGAAAGAGAAGTCTTGACGGGAAGTTTTA 88778
AsianBonytongue           GTGCC-TTTAGTCTTAATTTACATTGCATT-GCAAAAAATCAGTTGTA--------TTGAACTTTTTGAAATATTTTGTA 14692
Reedfish                  ACGTGGTTTGCAGCACCTGTTCAATAAATGAAACCGCAAAGAAACGTACATC----TGCACCTCCACACCAGGAGGTTCG 46092
NorthernPike              GTTCC-TTTAATCCTTTTTTCATATGTCCAGTTTAAAAATTGTTTCCCCATTTTAGTTGACACATCAACCATTTACTAAA 20229
SpottedGar                TTGCCATTCCAAGGCAGTCCTGATTAAA---AAAAAAAAATAGTTGTGGCTC----TTGAATGTGTTAAAGAAAAGTATA 18759
ParamormyropsKingsLeyae                                                                                    13318

Majority                  TT-TTTTAAGTATAACATTTTTA-A-TGAGGAAAGGTGATTTGCTAAACAAAAGTA--ATTACATTGTATTXCAGTTGTT
                                   89530     89540     89550     89560     89570     89580     89590     89600
Human                     AC--TTTAAGAATAACAGTGTTACCCCCAGGGAAGGAGATATTAAAGACAAGACTGGAAATCAATGGTATAAAAGACAGT 88856
AsianBonytongue           TT-TCATAAAGACAACATTTATA------GCAAAAATGACTTGATCAATACAGA----ATTACATAAGGCT---GTTGTA 14758
Reedfish                  CCTGCTTGCCTACAGCTGATCAGTATTGAACACACCTACTGCGCCAAACAACAGCAC-AGTACTTAACATCCCGGTTGTG 46171
NorthernPike              ATAGTCTAAATGTTTCAATTCTT-------TATAGGTGTTTTCCTTTCCTTATC------TGCTTTCTATTGCAGTTTAT 20296
SpottedGar                TTCTTTTTGGGGTGATGTCTTTAAAATGGGGAAGAAACACTTGTTGCTTAAAAGTATTGTCTTATTGAATTTAAGTTGAG 18839
ParamormyropsKingsLeyae                                                                                    13318

Majority                  TGATGTACCTGCCTGTGCAGCAAGT-GGTTTCCTTAATGTGTACATTCAAATAGCAGGACA--TAAAGAAACATAAAAAA
                                   89610     89620     89630     89640     89650     89660     89670     89680
Human                     TGAGAGAAAAAAACATGCCACAAGTTGGATTCTTTGAAAAGACTAAAAAAATAGACAAACATCTAAAGATTGATTAAAAA 88936
AsianBonytongue           CCATGACCTCACCTGTACATCCCG--GGTTTCAAGAATGGTTACATTCTTGGAACAG------TGGTCAAACTTAGACCA 14830
Reedfish                  CCA----CCTGTCAGTGCAGCATGC-AGTATCCCTGCTGTGCCATCTGACAACACAGCACTCCTAAACCCTAATGATGAG 46246
NorthernPike              TGATGTACATGGGTAGGAAGTGAGT-GGTTTTCTAAGCATGTACTGCCTGGTTCATGGA-----ATAAAAAAAAAAGACA 20370
SpottedGar                TATTGTTCCCTCTTGTATAAT-----GGTGTCATTAATCAGCACATTAAAAGAGCAAGATA--TTACGTGACCTAGAAAA 18912
ParamormyropsKingsLeyae                                                                                    13318

Majority                  CTGTCACTCGTCACTCTAT-ATTCTTTTATATGTTATCACAGGTATAATGTTTCA-A-AAATTTGGTTTCTGTGTCATA-
                                   89690     89700     89710     89720     89730     89740     89750     89760
Human                     ATGAGAGTGGTAACACAAAAAAATTTTTTTGAATTATAAAGGGGAGAAGCATTGATAAAGCTCCAGTTTCCACATCATAT 89016
AsianBonytongue           CCCTCACTCCTCACTCT----TGCTGATGGATGT-ACCTAAAATATGGGGG-------GAAAAAAGATCCTGTGTCAAA- 14897
Reedfish                  CCATCAACACTGGCTGCATGACTGAAGCAGGAGGGAAGACCGCTCAGCTGTTTCA-ACTAGTTTGTGCACTGTTGCAAGG 46325
NorthernPike              TTGTGATACTTAACTCAT---TTAAACTATATGTCATCACTGTCATAATTA--------AACATTATGACCATGTTATG- 20438
SpottedGar                CTGTCACTAGCCCCTGTATTGTGCTTTCCCATGTTATTTCATGTATATTGTTCCC-ATAAAATAGAATTTTGCCCCACAC 18991
ParamormyropsKingsLeyae                                                                                    13318
```

```
Majority                  AGTCATGAGTAGTTCTCTAGGTTTGGTGGGTTTTTATATTTTTTTAAATG--CATAATCTAATACTTTCGAATTTGTATG
                                   89770     89780     89790     89800     89810     89820     89830     89840
Human                     AAACACCACTATGTCTGTAAATTTCTTGAGGTCTTTTAAAATTTTTATTATTCGTTAATAATCTCTTTCATAGATGGATT 89096
AsianBonytongue           AGTCATGTAT-ATTTTCTGTATTAGTTGGGTTTTTACATCTTTACAAATG--TAAAATTTAGTAGTTTGAAATTGGCACG 14974
Reedfish                  AGTTGGAAGT-GGCCGCTGGGCACAGTGACTTTGAAGGGAGGGCTAAAAGATCAAAATCCCATACAGTCGAAGTGGGAAG 46404
NorthernPike              ----ATGTACAGTTCAAAAGGTTTTGCATAATCTTCTGTTGCATGCATTGG-AAGAGGCAAACAGGAGAGAACCTGTAGT 20513
SpottedGar                AGTCAGAAGTCATGAAATAAGCATGGAAGGCTTTAAAATTTAATTAAATA--CATT-TCTGCTTCCATATCTTCCATATG 19068
ParamormyropsKingsLeyae                                                                                    13318

Majority                  AATGGTAGTTTAAATG-GGATCTCAAAAGATGAAGAATTTGAGATTGGTTTGAATTCTATGTCTATTTCC-TTTATTTTA
                                   89850     89860     89870     89880     89890     89900     89910     89920
Human                     AAGGGAAATGTAAAA---AATTACATTAAATCAAAAATGTCATTTCAATTGTGAAACATTGACTTTTTCC-TTTATTATT 89172
AsianBonytongue           ATTAAAAGTTGATTT-----TCTAACAAGTTGAGAACTTTGACAGTCCTTTGATTTCTCTGTCTGTGTAC-TCTGTTTTA 15048
Reedfish                  TATTCTATCGACAAAGTGGCTCTACAGCTATGAAGAACA-AAGAATTGTGGGAAATCGAGGTACAAAGCCCCTCCCATTA 46483
NorthernPike              GATGTCAAATGTGTTGCAGTTTACTGAAGAGGGATAACCCTGGTTGGGACCAAAGTTTATGTGCCTTTAGTCTTAATTTA 20593
SpottedGar                ACCAGTGGTTTGCACCAGGATCCCAAAAGATAAAGACTT-GACAATGATTTAAGTATGACACTGATGTTCATATAATTGG 19147
ParamormyropsKingsLeyae                                                                                    13318

Majority                  CTTCTGATCATTTACGCAATAAGTACTTGTTTAG-CTGTGTTGATCTGTTTTATTTGTGTTTTTTA-ATCTTCTACTGTA
                                   89930     89940     89950     89960     89970     89980     89990     90000
Human                     GCCTTGTCCATTTATGCCATATTTCTTGGAATAG-GAGAGTTCAAGTGTAAGATTCACATTGTTTA-AAATTTTATTCAT 89250
AsianBonytongue           CTTCTG-TAGCATGCTCAATAAACACTTCTGTAG-CTCTGTTCACCTCTTCTGTCTGCGCTGCCTC--TCTTCTGCTGTG 15124
Reedfish                  ACCCACACTATTTACATGTTGAGAACTTGAAAATGCAACGTGGAAATGTTTTACTTTTCATTTTTGCAGTTTAGGTTGAA 46563
NorthernPike              CATTACATCATCAAGGAAAGGAATATAATTTCA--ATAATCTGGTCTTTTGTATATCTGGAAATGAGATCCAGTACCTTA 20671
SpottedGar                ATACTGGTATTTCACAGAATATGTACACCCTTT--TTGTGTTGATTTCTTTTGATGGAATTTTCTAAACATTCTACAGTA 19225
ParamormyropsKingsLeyae                                                                                    13318

Majority                  TTCTAATTTGCAATTTGGG--ATTTCATTTTTTTTTATTTAGTAGTAATGCACCTTTATTTTATGAAAAAGCTTCCAAAA
                                   90010     90020     90030     90040     90050     90060     90070     90080
Human                     TTAAAATTAGTAATTCAGGTAATTTCCTTCTGCTCTGTTTACTCCTTGTGTACTTTCAGTTTTTGACAAAGCTTTTGTTT 89330
AsianBonytongue           CTCCACCTCACTTTCTG----ATGTCATATTCTTTCACAGGGTGGGAAAGCTACACCACTTTATGGAAATGGTTCCGGAA 15200
Reedfish                  GATGAAATTGCAAATGGGGGTATTTCATTTTCATTTTTGCAGCATGAATGCATGTAGACTTTGAAAACGAAATTCCAAAA 46643
NorthernPike              TTCTGAAACACAAATTCCAC-AGTTATTTTATGCTGATATACATGTCATACCCACTTGTTTTATGAAGAATCTCACAAGC 20750
SpottedGar                GCATACTTTGAATTT------ATGCAGTATTTTGTCCTTAAGAAATAACATGAC-TTATTTCATCAATAGGCCTACAAAA 19298
ParamormyropsKingsLeyae                                                                                    13318

Majority                  TAAAGTTTGCATTTACATTTTTATATTTTAATTTTCATCACTTTACCCATTTTGTATCA-TTTAAGATCAAATTGTTCTT
                                   90090     90100     90110     90120     90130     90140     90150     90160
Human                     TCTTTTTTACTTAAAAATTTTGGTATTCTAATTATCATCCCCCT-TCCCCTTTAGATCA-CTTCAGATCCTATTGTCAGC 89408
AsianBonytongue           AA----TTGAACCAAAATGAACAGACTTGACTGCAAAGCACTTT-CCCACCCTGTATTA-CTCTGGCTTCTTCTGGTTTT 15274
Reedfish                  GAGAGATTGCATTTTCATTTTGTTGTTTTCATTTTCACTTTTGCAGAAAATGCCTCCCA-TACAGGAACAAAGGTAGCCT 46722
NorthernPike              TCAAGTTTCTGGATAGATGTATCTAAAAAAAAAAAAAAAAAACATCAGATTCTCTGTCAGTTTAAGAGAAAAGTCATGTA 20830
SpottedGar                TAAC-ATTGAATTTACTGTTTTACTCTCTTCTTTTTATCAAGTTACTTATTTTGTTTTA-ACTAAAATAAATTAATACAG 19376
ParamormyropsKingsLeyae                                                                                    13318

Majority                  TATCAG-TGGCTTGGATTTTTTTTGCATCTAGCTATTTAGTATGCTTTGAGCCTCTGTTTTT---TTTATTATAATCCCA
                                   90170     90180     90190     90200     90210     90220     90230     90240
Human                     AATCAGCTGGCTAGAACTCTCTTGTTACCTGGCTACTTCCTGAGGTAGCTGTTTGGTTATTTAGCTTTATTTTAATCCCA 89488
AsianBonytongue           TAACCC-TGTTTTGTAC-CTAGCTGTAGCTAC-TAATCAGTCTGCTCTGAGCCTCACTCTTT---TCTTCTGCAAATCCA 15348
Reedfish                  TGGCAG-GAGCTTGATTTTGTGTTCCTTAAACCATTTCTGTTTGATTTGGAGATATGTTTTT---TGGATCATTGTCCCA 46798
NorthernPike              TATTTTCTATATTAGTTAATTTTTACATCTAGAAATGTACGATTCAGATATAGTTTGTAAGTGGCACAATTAAAATAATT 20910
SpottedGar                TGCCAG-TGGAGCAGATATTAATTGCATATGTGTGGGAAGAAAGCATACAACCTCCAGTTTT---TTTTAACTGGAACCA 19452
ParamormyropsKingsLeyae                                                                                    13318
```

```
Majority                  TTCTTTTATCTTCTATTTGTTAGGTTTTGATTTGCTGTTACTGA-A-T-ATTTTCTTTTTCAT-CCTCTTCTCATGTAAX
                                   90250     90260     90270     90280     90290     90300     90310     90320
Human                     TTCCTCTATCACTCATCTGTTCTGTATGCAGTTACTATGCCTGGGACTTATTTCTTATTCTTTCCCTCCATTATCTCAA 89568
AsianBonytongue           -TCTTTCACCTTCTCTCTGCT---TACAGACCCCTTTTTACTAA-----ATCCTCTGTCTCAG-CCAATGCTCATACAAC 15418
Reedfish                  CTGGAAGATACAACAATGACCAGGTTTTAGTTTCCTGTCAGAGGCAGCCAGATTTAATTTTAA-AATCTCCTGGTATTTC 46877
NorthernPike              TTCTAACAAAGTCGGGAGGTTTGGTCTGACTTGGTTGTG--TGATTTTGATTTTCTTTTTCCTCCCTCCTCTCTCGTGT  20987
SpottedGar                CTCTGTTACTTAATTTTTATTAC-TTTTGATTGGCTATTGCTTA-----ATTTTTTGCGTGA---CAGGTGTAATGTAAA 19523
ParamormyropsKingsLeyae                                                                                    13318

Majority                  AXXXXXXCXXXGXXXXCTXXCXXTXXAATXAXXXXXXTXTXTTXXTXTXAAXXXTAAAXXXXXXXTXAXXXXAXTXAXTT
                                   90330     90340     90350     90360     90370     90380     90390     90400
Human                     AGCTCTGCTGTTTGTGCTGTCTTTTTATTTAGACAGCTTTTTTTCTGACAACGTTAAGT--CTACTTATTTATATCACTT 89646
AsianBonytongue           ACACGACCAGAGAAAACTGACTCTTGAATGAAGCTTTTGTCTTCTTCTGA--CCTAAAC----TGTTAAACCATTTGTTT 15492
Reedfish                  ATGAAGCCCATGATGCCATGTATCCTAACAAAGTTCCTAGGCCCTTTTAGAGCTAAAACAGCCCCCAGCATCACAGAACA 46957
NorthernPike                                                                                               20987
SpottedGar                AGGTGAGATTAGTGAATTATCCATACAATGTGTGATTAATCTTTACCTGAAATCTCAATATTTTATAAACAGATTAAATT 19603
ParamormyropsKingsLeyae                                                                                    13318

Majority                  XXXAXCXXTAXXXXXXAXTTXXXXAXAXAXXTXTTCXXXXXXAXXTTCXTXAXXXXAXXXXXXXXTTXXXTXXXXAAXXGX
                                   90410     90420     90430     90440     90450     90460     90470     90480
Human                     TATTTCTCAAACTCACACTTAATAACACATTTTATCTGAGGTAGTTTATCTAAACAA-AAGTTTGTTCACTTAAAATAGT 89725
AsianBonytongue           CCAATT--TATTCTGAATTTAT-TATATA-GTCTTCAAGACCTATTTCTTGG----------ACATTTTCTTTCAACTGA 15558
Reedfish                  CACACCATTCTTGACGGTGGGGATCAGGTTCTTTTCAGTATAACCATCCTTATACAACTTGAGTTTTTGTTGCCAAAAGG 47037
NorthernPike                                                                                               20987
SpottedGar                TGGACCAGTAGCCAGCAGTTGTACAGATAAGTCTGCCAGGTGAGCTCCCTAACC--ATGCACACATTCAAACAGAACTGA 19681
ParamormyropsKingsLeyae                                                                                    13318

Majority                  TXX----------AXTTXTXXATTTCAXXXGAXXXTXXXXXXXXXX-XXXXTTTGXXXTCCCATTXTXXATXXXXAXXAX
                                   90490     90500     90510     90520     90530     90540     90550     90560
Human                     TATAGAACTTTAAAATTTTATAATTCAGTGGGTTTTATTGTAAAAGGATTTTATGTACTGTCACTGTAGATAATTGGTTT 89805
AsianBonytongue           T------------ATTTGT--ATTTCGGCAAAGTTTTAACC---------CTTTGTGATCCCATGGTATACATAAAGTAG 15615
Reedfish                  TTC----------AATTTTT-GTTTCATCTGACCATAGAACACTGT-TCCATTTAAAGTCCCATTA-GCGTTTAGAAAAT 47104
NorthernPike                                                                                               20987
SpottedGar                TGC----------ATTAGTTCATTTTACTCGAGGATCAGTTTGGAG-AGACTTTGACAACCCTTTCTTTATCCTCAACAC 19750
ParamormyropsKingsLeyae                                                                                    13318

Majority                  XXCAGGXGTXXGCXTX----TAXXXGTXTTTXXXXTTTXXTXTXXXAAXTXXA---XXXXXXXCXAATGXXXXAAXTXXX
                                   90570     90580     90590     90600     90610     90620     90630     90640
Human                     TACAGGTGTAAGCGTAAATTTGCAAGTATATGAAATTAAGTATGTGAAATGCAGTGTGAGAAACAAATGAGAAAATTCAG 89885
AsianBonytongue           GACAGAATTATGCTT-----TAAGGGCATTTGGTGTCTACATAGTAAATTACA---CAA----CTAATGAGGAAAATTGT 15683
Reedfish                  TGCAGGTGCTTACAT-----TAAGGCTTTTTTTTTTTTTTTTTGCTGGTGTA---CCTTCCAGAAATGCTTGGAGTGGA 47176
NorthernPike                                                                                               20987
SpottedGar                ATCCTGAGTGCGCCCA----TAGCTGTGATTAACATTTTTTCTACTAATTCTT---TGGAATCCTAACTTTAGAAACCCA 19823
ParamormyropsKingsLeyae                                                                                    13318

Majority                  XCXXGTGXXXXXXXXXXXXXXXXXXXXXXXXXXXXXXXXXXXXXXXXXXXXXXXXXXXXXXXXXXXXXXXXXXXXXXXXX
                                   90650     90660     90670     90680     90690     90700     90710     90720
Human                     ACTTGTGAGAAGATTGTTTTGTTTTTTTTCTGTGCTTAGCCTTGGCGAAAAATCTGAGAAAACTTAAAATATATAACACT 89965
AsianBonytongue           GCAAGGG                                                                          15690
Reedfish                  GGTGGTGTCTGATGGTAGTTATGTATTTCTTCATCAGTGATCTTTAGGGGTTTATTTTACTTCTTACTCCTGTTCACTGT 47256
NorthernPike                                                                                               20987
SpottedGar                ACAGATGTTGTCGGAATACTGACAGTTTCACCTACAGTAGCTTGTGGGACATGGTACCAATCAGTTTCTAATCACAAGGG 19903
ParamormyropsKingsLeyae                                                                                    13318
```

```
Majority                  XXXXXXXXXXXXXXXXXXXXXXXXXXXXXXXXXXXXXXXXXXXXXXXXXXXXXXXXXXXXXXXXXXXXXXXXXXXXXXXX
                                   90730     90740     90750     90760     90770     90780     90790     90800
Human                     GGAGTGACAATCAGGAAGAGTGGACTCTTTTTTTTTTTAATTTTTTTAAACAAATTAGGGAAGTAAGTTCAATGGCTAGT 90045
AsianBonytongue                                                                                            15690
Reedfish                  GCTTGGGGGCAAAATAAACGTC-------TTCTTCCAGGCAAACTGGTAACACTTCCTGTTGATTTGAACTTTT-TAATT 47328
NorthernPike                                                                                               20987
SpottedGar                TATGTGGTTTTTCAGAAGCATTGGAA---CTGTTAATTAAGTTTCGTTCTCTAGTCCAGTTTGTATGTTTGGTT-TCACT 19979
ParamormyropsKingsLeyae                                                                                    13318

Majority                  XXXXXXXXXXXXXXXXXXXXXXXXXXXXXXXXXXXXXXXXXXXXXXXXXXXXXXXXXXXXXXXXXXXXXXXXXXXXXXXX
                                   90810     90820     90830     90840     90850     90860     90870     90880
Human                     GGTTATCATAATAAGAAAAATTTAAGGAAGTTATAGGTAAAATAAGAATTTTACTTTTGTTTCTTTTCTGAATGGTACCT 90125
AsianBonytongue                                                                                            15690
Reedfish                  ATTGCTCTATCAAAGCTTTATGCAAGCTTTGGACAACCTATTAAAATATTATCATTGTATATATGTAGCTAGAGAGAGAT 47408
NorthernPike                                                                                               20987
SpottedGar                ATAATCCCCCACTGGCTTTACTGACAATTTGCTAAAGTGAAAGAACAAATTTATCAATATCTCAATTTTATGGGAAACAA 20059
ParamormyropsKingsLeyae                                                                                    13318

Majority                  XXXXXXXXXXXXXXXXXXXXXXXXXXXXXXXXXXXXXXXXXXXXXXXXXXXXXXXXXXXXXXXXXXXXXXXXXXXXXXXX
                                   90890     90900     90910     90920     90930     90940     90950     90960
Human                     CATATTTTGACAGCATTATCCTTTTCAGATCTGTTTTATATTTAATTGGTTTATTATACATGTAAGTATGATTAGATTCA 90205
AsianBonytongue                                                                                            15690
Reedfish                  GAAAAACATA-GCGTTTTATTTTTTTTTTTTTTTGATGCTTGTTCTTTTTGGGATGTGTCCTCAGGTTTCATTAATCACA 47487
NorthernPike                                                                                               20987
SpottedGar                TATATACAGATAAAAAGTCCTCGCCCCAGCCTTGGCTAAAAATGATCATTTTGCATTTGTTTCACTATTAACTTGCTTTA 20139
ParamormyropsKingsLeyae                                                                                    13318

Majority                  XXXXXXXXXXXXXXXXXXXXXXXXXXXXXXXXXXXXXXXXXXXXXXXXXXXXXXXXXXXXXXXXXXXXXXXXXXXXXXXX
                                   90970     90980     90990     91000     91010     91020     91030     91040
Human                     T-GGATTTAACCAGTTCTTTGTATCTTCTGTGTCAAAATGTATACTGTAGTATACTGTTTATATTAGAAACTCTTTAAAA 90284
AsianBonytongue                                                                                            15690
Reedfish                  TTAAATTTTATTAAACTTGTGCTACATTTGCTCTATTACTAAGAGGGAATTAATTTTGAAATCTTTAGAACTGTAGAGAG 47567
NorthernPike                                                                                               20987
SpottedGar                TCCAGACAGCCAAAGATAAAGCTTTAATCTTATCTGAAACTCGGTAAAACCATCTAAAATGTCTGTTATACACTGTTGAG 20219
ParamormyropsKingsLeyae                                                                                    13318

Majority                  XXXXXXXXXXXXXXXXXXXXXXXXXXXXXXXXXXXXXXXXXXXXXXXXXXXXXXXXXXXXXXXXXXXXXXXXXXXXXXXX
                                   91050     91060     91070     91080     91090     91100     91110     91120
Human                     GTGGATGTTCTATTCTTAATTTCCTGATCTTCCACATTGTCTTTATCTGTAACTCTACTGTTTTTTGTTGGTTTTTGTTT 90364
AsianBonytongue                                                                                            15690
Reedfish                  TGACATTTGATTGTTTATGTCACAGTCTGTTTGCAAAAGTCTTTAATCTGAACACATTAATTGGGTTATATTGGTTGGTG 47647
NorthernPike                                                                                               20987
SpottedGar                GAATTTAGTGTAAGTGTTATGACTAAATATACTTTGTAGTTGTT--TTTTGACCTTACTTATGTGCATTTATATTTTACC 20297
ParamormyropsKingsLeyae                                                                                    13318

Majority                  XXXXXXXXXXXXXXXXXXXXXXXXXXXXXXXXXXXXXXXXXXXXXXXXXXXXXXXXXXXXXXXXXXXXXXXXXXXXXXXX
                                   91130     91140     91150     91160     91170     91180     91190     91200
Human                     TTGTTTTGAGACAGAGTCTCGCTCTGTCACCCAGGCTGGAGTGCACTGTGGCACCATCTCAGCTCACTGTAACCTCCGCC 90444
AsianBonytongue                                                                                            15690
Reedfish                  GCAATTATTATGTCCATCTCCACACTTTCTTTCCATTGACATGCTGAATTCAGTGCTGTAATCCATCTTGAATTTCAAGG 47727
NorthernPike                                                                                               20987
SpottedGar                ATGTTTAGTTTATATTTGAGGATTTAAAATGACTGTGGATTTCTGAGCATGTACTGACTGGTCTAGTTAGTTCTGGGACA 20377
ParamormyropsKingsLeyae                                                                                    13318
```

```
Majority                  XXXXXXXXXXXXXXXXXXXXXXXXXXXXXXXXXXXXXXXXXXXXXXXXXXXXXXXXXXXXXXXXXXXXXXXXXXXXXXXX

                                   91210     91220     91230     91240     91250     91260     91270     91280

Human                     TCCTGGGTACAAGCAATTCTGCCTCAGCCTCCTGA-GTAACTGGGACCACAGGCACGAGCCACCACGCCCAGCTAATTTT 90523
AsianBonytongue                                                                                             15690
Reedfish                  CATATGTAATGAAGATTTCATAAACACTGATGTGAAGAAATTGGTCAGACAAGTCAATATTAGTGAATATTACATATGAA 47807
NorthernPike                                                                                                20987
SpottedGar                AACTGCTGTTGCATGCATTATAAAAAGCTAACAGAAGTGAGCTGTGCAGTCAGCCTACAGTAACAGTGACAGTTTTTGTT 20457
ParamormyropsKingsLeyae                                                                                     13318

Majority                  XXXXXXXXXXXXXXXXXXXXXXXXXXXXXXXXXXXXXXXXXXXXXXXXXXXXXXXXXXXXXXXXXXXXXXXXXXXXXXXX

                                   91290     91300     91310     91320     91330     91340     91350     91360

Human                     TGTATTTTTAGTAGAGATGGGGTGTTACCATATTGGCCAGGCTGGTCTCGAACTCCTAGACCTTGTGATCCGCCCACCTC 90603
AsianBonytongue                                                                                             15690
Reedfish                  AAATGGTCTAAATCTGTTC----TCAAAAGTCCTAATTCCATGTGTTTTTTGCTGCTGAATGTACTGATGGCATGAAACC 47883
NorthernPike                                                                                                20987
SpottedGar                TGAAGAGGACAGCCTGGTGGG--ACCAAAGTTTATGTGCCTTTAGTCTTAATTTTCCTTGCATTGTAATATTCAGTTTTA 20535
ParamormyropsKingsLeyae                                                                                     13318

Majority                  XXXXXXXXXXXXXXXXXXXXXXXXXXXXXXXXXXXXXXXXXXXXXXXXXXXXXXXXXXXXXXXXXXXXXXXXXXXXXXXX

                                   91370     91380     91390     91400     91410     91420     91430     91440

Human                     AGCCTCCCAAAGTGTTGGGATTACAGGCGTGAGCCAACGCGCCCGGCATCTACTGTTAATACTTTATTATCTAGTTGATT 90683
AsianBonytongue                                                                                             15690
Reedfish                  CCTAAGCACGTTTTTTATTTGTTTTATTACATTTTACAACATCAATAGTGCTGTGCCAGTGTGTGGCATTCAGAGTCTGA 47963
NorthernPike                                                                                                20987
SpottedGar                ATGAACCTATGAAATTTTTTGTATTTTAAAATCCAGACTTTATAACAAACATGCCTCAGAATCTACATATGAATCCTTCA 20615
ParamormyropsKingsLeyae                                                                                     13318

Majority                  XXXXXXXXXXXXXXXXXXXXXXXXXXXXXXXXXXXXXXXXXXXXXXXXXXXXXXXXXXXXXXXXXXXXXXXXXXXXXXXX

                                   91450     91460     91470     91480     91490     91500     91510     91520

Human                     ATAGTCATTTAGACCTTCAGTTTAGCCAAAGTGCATGACATATTAGAAATGGTCTTTTTTAGTTATTTTGACTGGTGAAT 90763
AsianBonytongue                                                                                             15690
Reedfish                  ATGAATTCTTTTAATCTCCTTT--AAAAAAAAAAAAAAAAAGTTGGTCAAAGCGTCTCAATGAAAAATGGAATCTAAGTG 48041
NorthernPike                                                                                                20987
SpottedGar                AGGATATTTACTACATGAGCTC--ACCTGTGATCACCATCAGATGTTGAAAATCCTTTCCCCTCAATCCCCCCCCCCACC 20693
ParamormyropsKingsLeyae                                                                                     13318

Majority                  XXXXXXXXXXXXXXXXXXXXXXXXXXXXXXXXXXXXXXXXXXXXXXXXXXXXXXXXXXXXXXXXXXXXXXXXXXXXXXXX

                                   91530     91540     91550     91560     91570     91580     91590     91600

Human                     AAAACATATAACTTTCTAGCATCAGACCAGTTCATTTAGTTTAAAAATGATCCAGCAGCTATGTCAAGAAATAGTCATGT 90843
AsianBonytongue                                                                                             15690
Reedfish                  GCATAAAATCCTAACTTTTTCTCATGTCTTATGAATGTTTTAAGATTAAAATCAGAAGTTAGTCAAACAGTAACTTCAGA 48121
NorthernPike                                                                                                20987
SpottedGar                CCCCAATAGATGTTCTTAGTTAAAAAATAGATTCTGTGGTCTATGTTTAAAAAATCATGTATATTTTCTATATTAGTTGA 20773
ParamormyropsKingsLeyae                                                                                     13318

Majority                  XXXXXXXXXXXXXXXXXXXXXXXXXXXXXXXXXXXXXXXXXXXXXXXXXXXXXXXXXXXXXXXXXXXXXXXXXXXXXXXX

                                   91610     91620     91630     91640     91650     91660     91670     91680

Human                     AATGTTCTGTTTTCACACACTGCACTATGTTCTGGGGTAGAGACAAAGGTTTGGATTTTGGAATATTTGCATTATACCAA 90923
AsianBonytongue                                                                                             15690
Reedfish                  TAAGCTACCAGTGGATAAGACCTGCTTAACTTCAGGTTTGTTTTTTCAAA---AATCTAAATTGATCAGTCTTCAAAAAT 48198
NorthernPike                                                                                                20987
SpottedGar                GTTTTTACATCTTTAGAAATGTAAAATTCAGTATAGTTTGGGAGCGGCAC---AATTAAAAATTAATTTTCTAACAAGTC 20850
ParamormyropsKingsLeyae                                                                                     13318
```

```
Majority                    XXXXXXXXXXXXXXXXXXXXXXXXXXXXXXXXXXXXXXXXXXXXXXXXXXXXXXXXXXXXXXXXXXXXXXXXXXXXXXXX
                                    91690     91700     91710     91720     91730     91740     91750     91760
Human                       TTGAGCTTCCCAAATCTGAAATGTTCCAATGAGCATTTCCCTTGAGCATCATGTTGGCCCTCAGTTTTGGATTTTGGAAC 91003
AsianBonytongue                                                                                              15690
Reedfish                    CAAAGTAACATAAAATGCAGTGGTATGAACTAACAGATGTCTAATGGAAATTTATTTCAAAAATGTTTCAGTATATTTGA 48278
NorthernPike                                                                                                 20987
SpottedGar                  GGGAGGTTTGATGGTTGTGTTAATTTCACCCTGCGTGTACTCTGCTTACCTCTGTAGCATGCTCAATAAACACTTCTGTA 20930
ParamormyropsKingsLeyae                                                                                      13318

Majority                    XXXXXXXXXXXXXXXXXXXXXXXXXXXXXXXXXXXXXXXXXXXXXXXXXXXXXXXXXXXXXXXXXXXXXXXXXXXXXXXX
                                    91770     91780     91790     91800     91810     91820     91830     91840
Human                       ATTTCAGATTTCAGATTTCTGCATTAAGGATACTCAACCTGTACAGACTGGGAAAGGTATAAGCCATGTTAAAGAATGAG 91083
AsianBonytongue                                                                                              15690
Reedfish                    ATGATGTTTTTCTGATTTTTTTTTTTTTTTTTTTTTT-TTTTTTAAGCCATGAGCCATCTTAATGGTCAAAAAATGTATG 48357
NorthernPike                                                                                                 20987
SpottedGar                  GCTCTATATTCACCTCTTCTGTCTCTATTCTGCCTC-TCTCTCTTCTCTTGTATTTCACCTCACTATGCTTCTGATGTCA 21009
ParamormyropsKingsLeyae                                                                                      13318

Majority                    XXXXXXXXXXXXXXXXXXXXXXXXXXXXXXXXXXXXXXXXXXXXXXXXXXXXXXXXXXXXXXXXXXXXXXXXXXXXXXXX
                                    91850     91860     91870     91880     91890     91900     91910     91920
Human                       TTTCAACTTTGTCCTTAAGGGTTTTAAGCAGGTATTGACGTGATCAGATTTGTAATTTATGAAGGTCATCAAGCTGCTGT 91163
AsianBonytongue                                                                                              15690
Reedfish                    GAAAAATAATTCGTGTTACCTTATCAAAGCATCAGACAGTACAGTTGCCAAGAGAAGGGCTTGATGATCAAGGCTTAACA 48437
NorthernPike                                                                                                 20987
SpottedGar                  TGATCTTTGTCAGGGTGGGAAAATGCAGTTATTTGAAAGCTTTTACATTTCGGAATCCTAATCGGGAAAAGAAAGCCAAA 21089
ParamormyropsKingsLeyae                                                                                      13318

Majority                    XXXXXXXXXXXXXXXXXXXXXXXXXXXXXXXXXXXXXXXXXXXXXXXXXXXXXXXXXXXXXXXXXXXXXXXXXXXXXXXX
                                    91930     91940     91950     91960     91970     91980     91990     92000
Human                       ATAGAAAATGGATTGGAAAAATTATCCAACAGGAAGACAAATATGAGACTGAGTTATCCAGATAAAAAATAATGTCAGCG 91243
AsianBonytongue                                                                                              15690
Reedfish                    AAGGATTTTACAAACTCGCCATTACATCGATTTAAAAAGCCCGGCTCAAAGAATTTTCAAAACATATTTCCTCCTTCTGC 48517
NorthernPike                                                                                                 20987
SpottedGar                  TAAGAAATGAACCAACAAAAACCCCACTGTCGAATCTTAAAAACCTGTTTCCCACCCTGTATTGTACTCTGCTGCGAAAC 21169
ParamormyropsKingsLeyae                                                                                      13318

Majority                    XXXXXXXXXXXXXXXXXXXXXXXXXXXXXXXXXXXXXXXXXXXXXXXXXXXXXXXXXXXXXXXXXXXXXXXXXXXXXXXX
                                    92010     92020     92030     92040     92050     92060     92070     92080
Human                       AGAAGTAGAGAAACAGATCATGAATTTGAGGGACAATTAGGAGGAAGGCTATCACAATATTTAGCAATTAGGATGTGTAA 91323
AsianBonytongue                                                                                              15690
Reedfish                    AACTCTTCACCTCTCCAATATACCGTAAGTAAACAGGAAAGGCTATAAAATTGTTTTTAAGGCTTTTTTTTTTTTTTTTA 48597
NorthernPike                                                                                                 20987
SpottedGar                  AAATCTGCCCTTTCTGATTTTTAACTCTTTGTTCTGTCTCCGTAGATGTTTATACTAATCAATCTTTCAGGGTTTCATGC 21249
ParamormyropsKingsLeyae                                                                                      13318

Majority                    XXXXXXXXXXXXXXXXXXXXXXXXXXXXXXXXXXXXXXXXXXXXXXXXXXXXXXXXXXXXXXXXXXXXXXXXXXXXXXXX
                                    92090     92100     92110     92120     92130     92140     92150     92160
Human                       CAGAAGGGAGAAAAAGAGCTAGGGATGCTTATTAAGTTTCTTTCTTGTGTAGCTCACTATACGATTTGTTAGCAGGATTG 91403
AsianBonytongue                                                                                              15690
Reedfish                    AATGAATATAATATGTTTGCTTTTACCTTCTTTTACAGACAAAATATTACAGAAGAAGATCTACGTTCACTGTTTTCAAA 48677
NorthernPike                                                                                                 20987
SpottedGar                  TTCTTTACTGCAAATTCATCTTTCACCTTCTATCTGCTATAGAGCCCTTTTCTACTAAATCTTTTCTCTTCACAAAATGC 21329
ParamormyropsKingsLeyae                                                                                      13318
```

```
Majority                 XXXXXXXXXXXXXXXXXXXXXXXXXXXXXXXXXXXXXXXXXXXXXXXXXXXXXXXXXXXXXXXXXXXXXXXXXXXXXXXX

                                 92170     92180     92190     92200     92210     92220     92230     92240

Human                    TTGAGGAAGATAGATATTCATAATCTGGATTTTCATTCTATGAAAGTAGCTGGCTTTTTTGCCAGAATGCCTTGTCTAGT 91483
AsianBonytongue                                                                                           15690
Reedfish                 CTCCGGTGGTACTGTGAAAGCCTTCAAGTTTTTCCAGTAAGCACTTTATACTAAACACAGTTCTGTTCTTTATAGCTCCT 48757
NorthernPike                                                                                              20987
SpottedGar               TCATACAGTGCATTTGTCTATAATGTATTTTGATTGGCAGTCACTCTTGATGCAGATTTATCCTCGCTTATTTGTTGAGA 21409
ParamormyropsKingsLeyae                                                                                   13318

Majority                 XXXXXXXXXXXXXXXXXXXXXXXXXXXXXXXXXXXXXXXXXXXXXXXXXXXXXXXXXXXXXXXXXXXXXXXXXXXXXXXX

                                 92250     92260     92270     92280     92290     92300     92310     92320

Human                    TCAAATGAAAATG-TACTTAAGTCTTATCACCAGTGGCTATGGTAAATTGGTGATTTATTTTAAAATCATAAGAAAATTC 91562
AsianBonytongue                                                                                           15690
Reedfish                 GTTTCTGTAATTGCATATTATAGTTATTATATAGCACAGGTTATTGTGCTGGTGATATCCTCAGATAATAATATTTTTCC 48837
NorthernPike                                                                                              20987
SpottedGar               TGCTTCTGCAACAGTGCAGCAAAACTGTCTGTAAGCACTGCTATCCAGATGTTCCCAGCATTTATTTTTATAAAATGTTG 21489
ParamormyropsKingsLeyae                                                                                   13318

Majority                 XXXXXXXXXXXXXXXXXXXXXXXXXXXXXXXXXXXXXXXXXXXXXXXXXXXXXXXXXXXXXXXXXXXXXXXXXXXXXXXX

                                 92330     92340     92350     92360     92370     92380     92390     92400

Human                    AAATTTTTAAACTGTTACGTTAAAAGAATTATGTTTGTTTTGAGCTTTGATATTGTTTGTTAATAGACATGCTTTCTTTT 91642
AsianBonytongue                                                                                           15690
Reedfish                 TTTTTGGTTTTAAGGAGTGTTTCTTTTGTTTCTGATGCAGAAATTATGTTTGTTTCAAACTGTTGGACTTTCATCAGTTC 48917
NorthernPike                                                                                              20987
SpottedGar               CAGATCCGGCAAAGAAAATTATAATTGGTTACTTCGCAATCTACTTTTCATATTATAGAAGTTTATCCCTCTAATTATTG 21569
ParamormyropsKingsLeyae                                                                                   13318

Majority                 XXXXXXXXXXXXXXXXXXXXXXXXXXXXXXXXXXXXXXXXXXXXXXXXXXXXXXXXXXXXXXXXXXXXXXXXXXXXXXXX

                                 92410     92420     92430     92440     92450     92460     92470     92480

Human                    TATAGCCATGAATCATCTTAATGGACAGAAAATGTATGGAAAAATTATTCGTGTTACTCTGTCTAAACATCAGACTGTAC 91722
AsianBonytongue                                                                                           15690
Reedfish                 AAAGATTTGATCAACGTGCTACATACTGTGTGAAGTGTATTGTTATGGTATTTTTATTCCTTTTATACAGTGATTTCCTA 48997
NorthernPike                                                                                              20987
SpottedGar               GACGTTTTTTAAATGGATTTACATTTTGTATACAATTTAACAAGTATGAAGGTTTGCATCAGATTCGAGAAATCTTATTG 21649
ParamormyropsKingsLeyae                                                                                   13318

Majority                 XXXXXXXXXXXXXXXXXXXXXXXXXXXXXXXXXXXXXXXXXXXXXXXXXXXXXXXXXXXXXXXXXXXXXXXXXXXXXXXX

                                 92490     92500     92510     92520     92530     92540     92550     92560

Human                    AGCTACCTCGAGAGGGACTTGATGATCAAGGGCTAACAAAAGATTTTGGTAATTCCCCATTGCATCGTTTTAAGAAACCT 91802
AsianBonytongue                                                                                           15690
Reedfish                 GAAGTTCCAAAGATTTTTCATGTGAACACATCTTCAGTTAATTTATTTAACAAAAATGCTTGTACCAAATAGAAGGGTGT 49077
NorthernPike                                                                                              20987
SpottedGar               CATAAAATGAAAAAAATAAATTGGCATTCAATGAAA---AATATGATGTACCAGTGTTAATATCTTCCTTTGTTTTTCTT 21726
ParamormyropsKingsLeyae                                                                                   13318

Majority                 XXXXXXXXXXXXXXXXXXXXXXXXXXXXXXXXXXXXXXXXXXXXXXXXXXXXXXXXXXXXXXXXXXXXXXXXXXXXXXXX

                                 92570     92580     92590     92600     92610     92620     92630     92640

Human                    GGATCCAAAAATTTTCAAAACATTTTTCCTCCTTCTGCCACCCTTCACCTATCTAATATCCCGTAAGTATATAAGCTAGA 91882
AsianBonytongue                                                                                           15690
Reedfish                 TTATTTCAGTATGATTTATATATTTTTTTAATTAATCAAGTGATATAATAATGGTGAAGATGTTAGTAACTGGGAGGTAC 49157
NorthernPike                                                                                              20987
SpottedGar               TTTAAAGTGATTTCATTAGGTGCCTTTTCTCTTCTACACATGCTCTTTGCTTAATTGAACAGTTAGAAGTATCATTTGGA 21806
ParamormyropsKingsLeyae                                                                                   13318
```

```
Majority                   XXXXXXXXXXXXXXXXXXXXXXXXXXXXXXXXXXXXXXXXXXXXXXXXXXXXXXXXXXXXXXXXXXXXXXXXXXXXXXXX

                                     92650     92660     92670     92680     92690     92700     92710     92720

Human                      GTGTA-TTGAGATACATTCTATTTTGATAAAATATGAAATTTATTCTTAATCTTCACTTTTTCTTCCCATTCAATTTTCC 91961
AsianBonytongue                                                                                             15690
Reedfish                   AAGGATTTGACTTGCATAATAATACTGTCCCTTTTAGCAGTGGTTTATACAAATTATATTCTATCTACTACAGTGGAAAA 49237
NorthernPike                                                                                                20987
SpottedGar                 ATGTAATTACAGGATTTGCAAACAGGAAAAAATAGTAAAATGTATCTTTTATAAAAAGTGTCATACACTATAATATTAAG 21886
ParamormyropsKingsLeyae                                                                                     13318

Majority                   XXXXXXXXXXXXXXXXXXXXXXXXXXXXXXXXXXXXXXXXXXXXXXXXXXXXXXXXXXXXXXXXXXXXXXXXXXXXXXXX

                                     92730     92740     92750     92760     92770     92780     92790     92800

Human                      TAGTCCATCAGTAGCAGAAGAGGATCTACGAACACTGTTCGCTAACACTGGGGGCACTGTGAAAGCATTTAAGTTTTTTC 92041
AsianBonytongue                                                                                             15690
Reedfish                   TATAATGCTGTTGTTTATAAAATAAGTTTATTCCATTACAGCTTAGATATATGAACCTAGAGTCTATTTGTTATTTGCCT 49317
NorthernPike                                                                                                20987
SpottedGar                 TACTAGGTACTTAGTAGTATAGTCTTTGTGACACTTTGCTGGT--AATGGGTTGTGTCAGAGGCTCATTCATATATAGAA 21964
ParamormyropsKingsLeyae                                                                                     13318

Majority                   XXXXXXXXXXXXXXXXXXXXXXXXXXXXXXXXXXXXXXXXXXXXXXXXXXXXXXXXXXXXXXXXXXXXXXXXXXXXXXXX

                                     92810     92820     92830     92840     92850     92860     92870     92880

Human                      AGTAAGCAAGCTTCCTTATCTTTAAATTAGTGACCTGATAAAATTTTTAAGTAGTTTTTGTTCTTTTCGTTTATAGAAAT 92121
AsianBonytongue                                                                                             15690
Reedfish                   GTGAATTTTTCAAAATAGTAAATTTATTGTAAAATGAACTGTTTGTATTGAAATTTTTATATAGCCTGTTATAGGTCATT 49397
NorthernPike                                                                                                20987
SpottedGar                 GGTGTATGTGTGGTAGTCCAAGCGTATATAAAGGTAATCTACACTATTTTAGAAGTTTTATGAATGTGTGTTGCACATAG 22044
ParamormyropsKingsLeyae                                                                                     13318

Majority                   XXXXXXXXXXXXXXXXXXXXXXXXXXXXXXXXXXXXXXXXXXXXXXXXXXXXXXXXXXXXXXXXXXXXXXXXXXXXXXXX

                                     92890     92900     92910     92920     92930     92940     92950     92960

Human                      TTTTGATTCCGGAATGGCCAAAATTCAAGTATTTAATAAGCCCTTTCTAATTTGTATGAAGTGTCTAATTTTATAATTTT 92201
AsianBonytongue                                                                                             15690
Reedfish                   GCAATTCCACTGTTCATGATGCATTACTTTAGCACTATTTTTTTTTTTTTTTTTTTGCATGGGTTGCGTAACTTTTAAA 49477
NorthernPike                                                                                                20987
SpottedGar                 ATTGAGGTAGATGGCAAATCAGCCTGCATTGGAAGTTGGTCACCTATAACTGCCCATGAAGATGTGGAAGTTCATAACTG 22124
ParamormyropsKingsLeyae                                                                                     13318

Majority                   XXXXXXXXXXXXXXXXXXXXXXXXXXXXXXXXXXXXXXXXXXXXXXXXXXXXXXXXXXXXXXXXXXXXXXXXXXXXXXXX

                                     92970     92980     92990     93000     93010     93020     93030     93040

Human                      GTTTCAGAAGAGATCACAAAATGGCTCTTCTTCAGATGGCAACAGTGGAAGAAGCTATTCAGGCCTTGATTGATCTTCAT 92281
AsianBonytongue                                                                                             15690
Reedfish                   AATATATTCCAGATTGTGAAATATTCACCTTTTATTTTGT--CAATTAAAAAGCCTTGTCCTCTATTTGCAGAGATCACA 49555
NorthernPike                                                                                                20987
SpottedGar                 GGGGAATGCTGACCGTGCACGGCAAACTGTGGAACACAAT--GAAGAATAATAGCTGATGCTCAGTGCTTCAAGGTTATC 22202
ParamormyropsKingsLeyae                                                                                     13318

Majority                   XXXXXXXXXXXXXXXXXXXXXXXXXXXXXXXXXXXXXXXXXXXXXXXXXXXXXXXXXXXXXXXXXXXXXXXXXXXXXXXX

                                     93050     93060     93070     93080     93090     93100     93110     93120

Human                      AATTATAACCTTGGAGAAAACCATCATCTGAGAGTGTCTTTCTCCAAGTCAACAATTTAAAAATGGGAAGATGAAGATTG 92361
AsianBonytongue                                                                                             15690
Reedfish                   AAATGGCACTTCTCCAGATGTCAACTGTGGAGGAAGCAATTCAAGCCTTGATTGACCTTCATAATTACAATCTCGGAGAT 49635
NorthernPike                                                                                                20987
SpottedGar                 TACTGTATGCTATGGGAATTTTTTTTGATCAGTCTGCTCAAAAAGGAATACACAAGGATGGCTTTTGGATTCTTGAATTC 22282
ParamormyropsKingsLeyae                                                                                     13318
```

```
Majority                  XXXXXXXXXXXXXXXXXXXXXXXXXXXXXXXXXXXXXXXXXXXXXXXXXXXXXXXXXXXXXXXXXXXXXXXXXXXXXXXX

                                   93130     93140     93150     93160     93170     93180     93190     93200

Human                     GGGGTGAATCACATTGTTCAATGTCATCACCTATTTGACTGTTCAGAAAAGTGGGGACCAGAGTTTGATTTTTTTTGTTT 92441
AsianBonytongue                                                                                            15690
Reedfish                  AATCACCATCTGAGAGTTTCATTCTCAAAATCTACCATTTAAGCT-TTTCTCCCCTTTTTTATTTTTCCTTTTTAAACGC 49714
NorthernPike                                                                                               20987
SpottedGar                GCCGATTATT--GAGGTATGGAGAAGGGAATCATCTGTCGGAGTT-ATGAGTACTCAAAGAATTCTTTGGTTTGCAGTGC 22359
ParamormyropsKingsLeyae                                                                                    13318

Majority                  XXXXXXXXXXXXXXXXXXXXXXXXXXXXXXXXXXXXXXXXXXXXXXXXXXXXXXXXXXXXXXXXXXXXXXXXXXXXXXXX

                                   93210     93220     93230     93240     93250     93260     93270     93280

Human                     TTGTTTTTTTGGGGTTTCTTTTTTTTTTCCATGCTGTTATCATTCCTTGGTTATAAAATGAAATGGCATATGTAAAGGCA 92521
AsianBonytongue                                                                                            15690
Reedfish                  TGTTTAGCGTGGCATTACTTGTTGACTGTTTCAGGACACTGGGACCACAGTTTGGCATATAATTTTGAATTTAGTCATTC 49794
NorthernPike                                                                                               20987
SpottedGar                AATCTTTGATTAAGCGTGGTGATGACAATATGTTTATATGCATACATTTCTCATGACTTGGACTAGAAGCATT        22432
ParamormyropsKingsLeyae                                                                                    13318

Majority                  XXXXXXXXXXXXXXXXXXXXXXXXXXXXXXXXXXXXXXXXXXXXXXXXXXXXXXXXXXXXXXXXXXXXXXXXXXXXXXXX

                                   93290     93300     93310     93320     93330     93340     93350     93360

Human                     GAGTTGTTAACTGCTATATTTCATCTGTTCTATAGGGAAGCCATTTTGTCTGTTTAAAATTTCAGTTTAATTTTGCTTTT 92601
AsianBonytongue                                                                                            15690
Reedfish                  CTAATACAATACATTATGGTAAAGCTGTAAATGAAAATATGTTAAAGCCATGATTTGGGGCTTTTATTTCAATGTTTTCC 49874
NorthernPike                                                                                               20987
SpottedGar                                                                                                 22432
ParamormyropsKingsLeyae                                                                                    13318

Majority                  XXXXXXXXXXXXXXXXXXXXXXXXXXXXXXXXXXXXXXXXXXXXXXXXXXXXXXXXXXXXXXXXXXXXXXXXXXXXXXXX

                                   93370     93380     93390     93400     93410     93420     93430     93440

Human                     TTTTTTTTTTTTTTTTTCCTTTCAACTTAGTTGACATACGTGCCTTAAAAAGGAAAACTAGTGTTGCTATTGTGCATTTA 92681
AsianBonytongue                                                                                            15690
Reedfish                  CCTTTACCTATACCATTGGAAAGTCATTAACAATTTTATCACTTTGTAAAAAAAAAAAAAACAGAAGATAATTCATTTCT 49954
NorthernPike                                                                                               20987
SpottedGar                                                                                                 22432
ParamormyropsKingsLeyae                                                                                    13318

Majority                  XXXXXXXXXXXXXXXXXXXXXXXXXXXXXXXXXXXXXXXXXXXXXXXXXXXXXXXXXXXXXXXXXXXXXXXXXXXXXXXX

                                   93450     93460     93470     93480     93490     93500     93510     93520

Human                     CTAGAAAAAAGGAATTGGTTGTTTAGGGCACACTGTTATATGGGAATTAAAATATGTTTAGGCAGGGGTGTGTAAAAAGG 92761
AsianBonytongue                                                                                            15690
Reedfish                  TTATGATAAACGTACAAGTCGACAAGACGTGCCTTACCTGACAAAACAGTATCAACACTTTTCCATTAATGTGTTTGTAT 50034
NorthernPike                                                                                               20987
SpottedGar                                                                                                 22432
ParamormyropsKingsLeyae                                                                                    13318

Majority                  XXXXXXXXXXXXXXXXXXXXXXXXXXXXXXXXXXXXXXXXXXXXXXXXXXXXXXXXXXXXXXXXXXXXXXXXXXXXXXXX

                                   93530     93540     93550     93560     93570     93580     93590     93600

Human                     TTAAGTTTTTGTTTCTCCTGCTTGGAACTTATTTTGAATTACTGGCTTGTCACCTTTTTTTCTATTTAATCAAATAAGAT 92841
AsianBonytongue                                                                                            15690
Reedfish                  CAGTTTATTTACAGTTTTACAGTTTGGAATATAGGCACATTGGTTCTGGTATTTAAATGAGTGGCTGTGTGAAAAGGCTT 50114
NorthernPike                                                                                               20987
SpottedGar                                                                                                 22432
ParamormyropsKingsLeyae                                                                                    13318
```

```
Majority                XXXXXXXXXXXXXXXXXXXXXXXXXXXXXXXXXXXXXXXXXXXXXXXXXXXXXXXXXXXXXXXXXXXXXXXXXXXXXXXX
                                 93610     93620     93630     93640     93650     93660     93670     93680
Human                   ACATGATATTGAAAGAATAAAGCAGCATTTTTAGTTTTTACTACCTTAGGCTTTATTGCTTTGAAAACAACATTGGCCTT 92921
AsianBonytongue                                                                                          15690
Reedfish                TTGTTTCCTTTAAGTAATCGTACAGTTTCTAAAGTTGGTAATGCCCTCATAAAGGATATTCATAGTTTTACCTAGTATAA 50194
NorthernPike                                                                                             20987
SpottedGar                                                                                               22432
ParamormyropsKingsLeyae                                                                                  13318

Majority                XXXXXXXXXXXXXXXXXXXXXXXXXXXXXXXXXXXXXXXXXXXXXXXXXXXXXXXXXXXXXXXXXXXXXXXXXXXXXXXX
                                 93690     93700     93710     93720     93730     93740     93750     93760
Human                   TTGTATCTCACAATTCTGGTCTAGATTCAGTTATGAATGTAGGCATTAGTTAAAATTAACAAGATGCAGAGTATTAATTT 93001
AsianBonytongue                                                                                          15690
Reedfish                TGAAAAATATTAAAGTATTTTTGTTTACCATGTCTTATTTTGCTTTTTGGGCAATGTTACAAAACATTTTACCTTCATAA 50274
NorthernPike                                                                                             20987
SpottedGar                                                                                               22432
ParamormyropsKingsLeyae                                                                                  13318

Majority                XXXXXXXXXXXXXXXXXXXXXXXXXXXXXXXXXXXXXXXXXXXXXXXXXXXXXXXXXXXXXXXXXXXXXXXXXXXXXXXX
                                 93770     93780     93790     93800     93810     93820     93830     93840
Human                   CTTAAGACAACAAAGTGATTTCTGTAAGTTTGAGCCCTATGTGGAAAGCATTGTGGAATCTTAACCTTTTCGTACACACT 93081
AsianBonytongue                                                                                          15690
Reedfish                AACTGCCATCTTTTTCATTTTTAGATATAACCTGCTTTATCCTATAACCAAAGATAAAGCTTTAACCCTAGTTATCATTT 50354
NorthernPike                                                                                             20987
SpottedGar                                                                                               22432
ParamormyropsKingsLeyae                                                                                  13318

Majority                XXXXXXXXXXXXXXXXXXXXXXXXXXXXXXXXXXXXXXXXXXXXXXXXXXXXXXXXXXXXXXXXXXXXXXXXXXXXXXXX
                                 93850     93860     93870     93880     93890     93900     93910     93920
Human                   CTTGTGGGACGTATCATATAAATGTCAGCACTAAGTAATGTCTTGTTTGTGGCTGAATATTTTTCGTAGATGTTTTTGAA 93161
AsianBonytongue                                                                                          15690
Reedfish                GTGATCATTGTTACCTTTGTTTGCACAACCACTGCTGAAGAACTGGTATTATTTGAGTAAATATTCTTTGTAGCTGTTCT 50434
NorthernPike                                                                                             20987
SpottedGar                                                                                               22432
ParamormyropsKingsLeyae                                                                                  13318

Majority                XXXXXXXXXXXXXXXXXXXXXXXXXXXXXXXXXXXXXXXXXXXXXXXXXXXXXXXXXXXXXXXXXXXXXXXXXXXXXXXX
                                 93930     93940     93950     93960     93970     93980     93990     94000
Human                   GTTGACATGACTTACGTGCATTTAAATATATATTGCCATCCTTAGTTTGTAATTAAGATTTGGAATATGGTTGTGGATTT 93241
AsianBonytongue                                                                                          15690
Reedfish                TTGTCATTG---CATATGTGCATTTATTTATATTGCCATCTTTCATTTGTACCTTGAGGATTTAAATGGCTGTGGACTTT 50511
NorthernPike                                                                                             20987
SpottedGar                                                                                               22432
ParamormyropsKingsLeyae                                                                                  13318

Majority                XXXXXXXXXXXXXXXXXXXXXXXXXXXXXXXXXXXXXXXXXXXXXXXXXXXXXXXXXXXXXXXXXXXXXXXXXXXXXXXX
                                 94010     94020     94030     94040     94050     94060     94070     94080
Human                   CTGAGCATGTGCAGACTGGTCTAGCTAGTTCAGGAA------CTGGTGCATGTATTTTTCAAAGATAAAGAAAGTGTACT 93315
AsianBonytongue                                                                                          15690
Reedfish                CTAAGCATGTACTGACTGGTATACTTAGTTTTGGAAAGACTGCTGTTGCATGCATTTTAAAAAGCTAGCAGAAGTGAGCT 50591
NorthernPike                                                                                             20987
SpottedGar                                                                                               22432
ParamormyropsKingsLeyae                                                                                  13318
```

```
Majority                XXXXXXXXXXXXXXXXXXXXXXXXXXXXXXXXXXXXXXXXXXXXXXXXXXXXXXXXXXXXXXXXXXXXXXXXXXXXXXXX

                                 94090     94100     94110     94120     94130     94140     94150     94160

Human                   GCGAAA-ATATGCAGGAAGATTAATTTTGTGGCAGTTTTCTAAAACTGACAACCAGGTGGGACCAAAGTTTATGTGCCTT 93394
AsianBonytongue                                                                                          15690
Reedfish                GCACAGTGAACAGCAGCAAAGTAATAGTATGGCAGTTTTCTAAAGCTAACAACCTGGTGGGACCAAAGTTTATGTGCCTT 50671
NorthernPike                                                                                             20987
SpottedGar                                                                                               22432
ParamormyropsKingsLeyae                                                                                  13318

Majority                XXXXXXXXXXXXXXXXXXXXXXXXXXXXXXXXXXXXXXXXXXXXXXXXXXXXXXXXXXXXXXXXXXXXXXXXXXXXXXXX

                                 94170     94180     94190     94200     94210     94220     94230     94240

Human                   TAGTCTTAATTTACCTTGCATTGTAATATTCAGTTTTAATAAATCTTCAAAATATTTTGTATTTAGGAATAGATCTGACT 93474
AsianBonytongue                                                                                          15690
Reedfish                TAGTCTTAATTTACCTTGCATTGTAATAATTAAGTTTTGTTAAACCTGGAATATTTTATATTTCAAAAAAAAAAAAAAAC 50751
NorthernPike                                                                                             20987
SpottedGar                                                                                               22432
ParamormyropsKingsLeyae                                                                                  13318

Majority                XXXXXXXXXXXXXXXXXXXXXXXXXXXXXXXXXXXXXXXXXXXXXXXXXXXXXXXXXXXXXXXXXXXXXXXXXXXXXXXX

                                 94250     94260     94270     94280     94290     94300     94310     94320

Human                   ---TTAATAAAAACATGGCTCAGAATCTACAGGTCAAATTAATTTGAACAGTTCTTGTCAATCCGAATTGTTGATTCTGT 93551
AsianBonytongue                                                                                          15690
Reedfish                AGACTTTTTAACTAATGGTTCAGAATCTACCAGTTAGTTTAAAGGCCATTCATGTGTAAGCTCAGTTGTTACAGTGTACA 50831
NorthernPike                                                                                             20987
SpottedGar                                                                                               22432
ParamormyropsKingsLeyae                                                                                  13318

Majority                XXXXXXXXXXXXXXXXXXXXXXXXXXXXXXXXXXXXXXXXXXXXXXXXXXXXXXXXXXXXXXXXXXXXXXXXXXXXXXXX

                                 94330     94340     94350     94360     94370     94380     94390     94400

Human                   TTAAATGACCAATACTTTTTGAAATTGATGTACTTAGTTTCAAGATTCA--T--------AGATTCTGTTATCTATGTAG 93621
AsianBonytongue                                                                                          15690
Reedfish                TTGTTTGAAAGGACCCACCCCCCATTACCCCCTCCCATTGATGTATTTAGGTTAAAACTGATAGATTCTGTGGTCTGTTA 50911
NorthernPike                                                                                             20987
SpottedGar                                                                                               22432
ParamormyropsKingsLeyae                                                                                  13318

Majority                XXXXXXXXXXXXXXXXXXXXXXXXXXXXXXXXXXXXXXXXXXXXXXXXXXXXXXXXXXXXXXXXXXXXXXXXXXXXXXXX

                                 94410     94420     94430     94440     94450     94460     94470     94480

Human                   ACAGAATGGTCATGTATATTTTCTATTAGTTGAGTTTTTACATCTTTAGAAATGTAAAATTCAGTATAGTTTGAAAGCGG 93701
AsianBonytongue                                                                                          15690
Reedfish                CAGAAAATCATGTATATTTTCTATATTAGTTGAGTTTTTACATCTTCAGAAATGTAAAATTAAGTCTAGTTTGGAAGCGG 50991
NorthernPike                                                                                             20987
SpottedGar                                                                                               22432
ParamormyropsKingsLeyae                                                                                  13318

Majority                XXXXXXXXXXXXXXXXXXXXXXXXXXXXXXXXXXXXXXXXXXXXXXXXXXXXXXXXXXXXXXXXXXXXXXXXXXXXXXXX

                                 94490     94500     94510     94520     94530     94540     94550     94560

Human                   CACAATTAAAAATTAATTTTCTAACAAAGTTGGGAGGTTTGATGGTTGTTTAATTTCATTTTGTGTGTACTCTGCTTACC 93781
AsianBonytongue                                                                                          15690
Reedfish                CACAATTAAAAATTAATTTTCTAACAAGTTGGAAGGTTTGATGGTTGTGTTAATTTCACGTTGTGTGTACTCTGCTTACC 51071
NorthernPike                                                                                             20987
SpottedGar                                                                                               22432
ParamormyropsKingsLeyae                                                                                  13318
```

```
Majority                XXXXXXXXXXXXXXXXXXXXXXXXXXXXXXXXXXXXXXXXXXXXXXXXXXXXXXXXXXXXXXXXXXXXXXXXXXXXXXXX

                                 94570     94580     94590     94600     94610     94620     94630     94640

Human                   CCTGTAGCATGCTCAATAAACACTTCTGTAGCTCTATATTCACCTTTTCTGTCTTTCTCTGCTGCCTTTTCTCTCTCCTC 93861
AsianBonytongue                                                                                          15690
Reedfish                TCTGTAGCATGCTCAATAAACACTTCTGTAGCTCTATATTCACCTCTTCTGTCTCTATGCTGCCTCTCTCTCTTTTC--- 51148
NorthernPike                                                                                             20987
SpottedGar                                                                                               22432
ParamormyropsKingsLeyae                                                                                  13318

Majority                XXXXXXXXXXXXXXXXXXXXXXXXXXXXXXXXXXXXXXXXXXXXXXXXXXXXXXXXXXXXXXXXXXXXXXXXXXXXXXXX

                                 94650     94660     94670     94680     94690     94700     94710     94720

Human                   TTCTTTGTTTTCACTCCACTGTGCTTCTGAATTCATGTTTATTCTCTGCCAGGGTGGGAAAGGAGTAATAATATTACAAT 93941
AsianBonytongue                                                                                          15690
Reedfish                -TCTTATGTTTCACCTCACTTTGCTTCTGAAATCATGTTCTTTGTCAGGGTGGAAAATCTTTCAGATTTTTATTTCCAT 51227
NorthernPike                                                                                             20987
SpottedGar                                                                                               22432
ParamormyropsKingsLeyae                                                                                  13318

Majority                XXXXXXXXXXXXXXXXXXXXXXXXXXXXXXXXXXXXXXXXXXXXXXXXXXXXXXXXXXXXXXXXXXXXXXXXXXXXXXXX

                                 94730     94740     94750     94760     94770     94780     94790     94800

Human                   TCTATGGCTTTATACCATAAATAAATCTAGATGCTGTGAAAATATACCAGCTGGTTTTTTTTAATTTAAAAGATGGTAAC 94021
AsianBonytongue                                                                                          15690
Reedfish                TGATTTAAATATTAAAATGACAGTACTTCAAGCTTTTGTTAAAAATCTAACTT-TATTTCCCACCCTGTGTTATTCTCCT 51306
NorthernPike                                                                                             20987
SpottedGar                                                                                               22432
ParamormyropsKingsLeyae                                                                                  13318

Majority                XXXXXXXXXXXXXXXXXXXXXXXXXXXXXXXXXXXXXXXXXXXXXXXXXXXXXXXXXXXXXXXXXXXXXXXXXXXXXXXX

                                 94810     94820     94830     94840     94850     94860     94870     94880

Human                   TGCTTTTCAGGAGGACACATATTAAACATTTCCCACCCTGTTATAATCTACTGCTTTAAAGACATAACTTTTATTGTAGC 94101
AsianBonytongue                                                                                          15690
Reedfish                TCCAATTATAACTGAGTTCTGTGAACTTTCTTTTCTGTCTGCATAGATGTATCTACCAAATCCGCCTTTCAGGGTTTCAT 51386
NorthernPike                                                                                             20987
SpottedGar                                                                                               22432
ParamormyropsKingsLeyae                                                                                  13318

Majority                XXXXXXXXXXXXXXXXXXXXXXXXXXXXXXXXXXXXXXXXXXXXXXXXXXXXXXXXXXXXXXXXXXXXXXXXXXXXXXXX

                                 94890     94900     94910     94920     94930     94940     94950     94960

Human                   TTGTTAATTCTATCTCTTTTGTTCTTGTTGTTTTTTTTTTTTTTCCAGTAGATTTATGCACTAATAGATCTTTTGGATTT 94181
AsianBonytongue                                                                                          15690
Reedfish                GCTTTTCTGCTGCAAATCCATTTCTCTCCTTCTGTCTGCTGTAGATACTCTAATTCTGTAATGTCTGAAGTGCAATTCCC 51466
NorthernPike                                                                                             20987
SpottedGar                                                                                               22432
ParamormyropsKingsLeyae                                                                                  13318

Majority                XXXXXXXXXXXXXXXXXXXXXXXXXXXXXXXXXXXXXXXXXXXXXXXXXXXXXXXXXXXXXXXXXXXXXXXXXXXXXXXX

                                 94970     94980     94990     95000     95010     95020     95030     95040

Human                   GCCATGCTCTCTTGCTGCAGTTTCATCTTTCATCTTTTGTGTCTGCTAAAGATTTCTTACTAATCTTAGACTACCTTGTG 94261
AsianBonytongue                                                                                          15690
Reedfish                AGGTGGATATTTTCTTTATATTTCTTAAAGATCCACTATCAAATTTAAGCAAATGTTTTTAAGTGTAAATAACAACTAGT 51546
NorthernPike                                                                                             20987
SpottedGar                                                                                               22432
ParamormyropsKingsLeyae                                                                                  13318
```

```
Majority                XXXXXXXXXXXXXXXXXXXXXXXXXXXXXXXXXXXXXXXXXXXXXXXXXXXXXXXXXXXXXXXXXXXXXXXXXXXXXXXX
                                 95050     95060     95070     95080     95090     95100     95110     95120
Human                   AGTTAACAAAAAGAACTTGATATTACTGGGAAGAAAGAGCAGCATATCTGCATATCAGCATATCTGCTTTTGCCTTGGGT 94341
AsianBonytongue                                                                                          15690
Reedfish                AATTATGTTCAGTACATTACCAAAACATTCGACTTTTGCATATTTTTTGCCACACTGCAATTTTTTTTCAAGCCTAAAGC 51626
NorthernPike                                                                                             20987
SpottedGar                                                                                               22432
ParamormyropsKingsLeyae                                                                                  13318

Majority                XXXXXXXXXXXXXXXXXXXXXXXXXXXXXXXXXXXXXXXXXXXXXXXXXXXXXXXXXXXXXXXXXXXXXXXXXXXXXXXX
                                 95130     95140     95150     95160     95170     95180     95190     95200
Human                   GGGAAGAATGATATACATTCAAGTATTTAAAAACTTAGAGTAATTTGCATTTAACAACTGAGGATGTTACTACTGTAACT 94421
AsianBonytongue                                                                                          15690
Reedfish                TTAAAACTAAAATATGGATGAAAAAGACAAGGATATTCAACAACTGCAGAGGGCAAAATCAGAAGGTATGTGGACAAAGA 51706
NorthernPike                                                                                             20987
SpottedGar                                                                                               22432
ParamormyropsKingsLeyae                                                                                  13318

Majority                XXXXXXXXXXXXXXXXXXXXXXXXXXXXXXXXXXXXXXXXXXXXXXXXXXXXXXXXXXXXXXXXXXXXXXXXXXXXXXXX
                                 95210     95220     95230     95240     95250     95260     95270     95280
Human                   TGTTAAGCAGTTACAATTAGGGTGCTATTTATATTCAAAATATTTCGGTCAGTTTTTCTAAACAGATATATTGGTATCAA 94501
AsianBonytongue                                                                                          15690
Reedfish                CAAACCA                                                                          51713
NorthernPike                                                                                             20987
SpottedGar                                                                                               22432
ParamormyropsKingsLeyae                                                                                  13318

Majority                XXXXXXXXXXXXXXXXXXXXXXXXXXXXXXXXXXXXXXXXXXXXXXXXXXXXXXXXXXXXXXXXXXXXXXXXXXXXXXXX
                                 95290     95300     95310     95320     95330     95340     95350     95360
Human                   ATAACTTTGGAGTTACACATGTTTCTTAGGTTTGTTCTGTTATAAAATATCCCACACTTATGCAAAACTTACACATAGGA 94581
AsianBonytongue                                                                                          15690
Reedfish                                                                                                 51713
NorthernPike                                                                                             20987
SpottedGar                                                                                               22432
ParamormyropsKingsLeyae                                                                                  13318

Majority                XXXXXXXXXXXXXXXXXXXXXXXXXXXXXXXXXXXXXXXXXXXXXXXXXXXXXXXXXXXXXXXXXXXXXXXXXXXXXXXX
                                 95370     95380     95390     95400     95410     95420     95430     95440
Human                   TAATAAAAAGTAAATGAAGGAACATAAAATATGTTTTACTATACTTAATGTAGTAATTCAGTTACCTCCAAGAAAGAAGA 94661
AsianBonytongue                                                                                          15690
Reedfish                                                                                                 51713
NorthernPike                                                                                             20987
SpottedGar                                                                                               22432
ParamormyropsKingsLeyae                                                                                  13318

Majority                XXXXXXXXXXXXXXXXXXXXXXXXXXXXXXXXXXXXXXXXXXXXXXXXXXXXXXXXXXXXXXXXXXXXXXXXXXXXXXXX
                                 95450     95460     95470     95480     95490     95500     95510     95520
Human                   ATATAATAAGTAAAATAATTTTGTTTACACTAGTGTCACTGTTGTCATCTGAAGAAGAACCATTAGAGTCAACAGTCTGG 94741
AsianBonytongue                                                                                          15690
Reedfish                                                                                                 51713
NorthernPike                                                                                             20987
SpottedGar                                                                                               22432
ParamormyropsKingsLeyae                                                                                  13318
```

```
Majority                 XXXXXXXXXXXXXXXXXXXXXXXXXXXXXXXXXXXXXXXXXXXXXXXXXXXXXXXXXXXXXXXXXXXXXXXXXXXXXXXX

                                  95530     95540     95550     95560     95570     95580     95590     95600

Human                    GATGATTTGGAAGCTAGGTGATCTGTGTTTTTTTCTTAAAATAACATCGTAGCACAGAGGAACTCTACCAGCATCAGATA 94821
AsianBonytongue                                                                                           15690
Reedfish                                                                                                  51713
NorthernPike                                                                                              20987
SpottedGar                                                                                                22432
ParamormyropsKingsLeyae                                                                                   13318

Majority                 XXXXXXXXXXXXXXXXXXXXXXXXXXXXXXXXXXXXXXXXXXXXXXXXXXXXXXXXXXXXXXXXXXXXXXXXXXXXXXXX

                                  95610     95620     95630     95640     95650     95660     95670     95680

Human                    AGTCTAAAGTCTAAAAAACCAATACCAGGGTTAGTGAATAGTAATTGAACAAAGCTTCTCAAGGTGAAGTTTTTCTCAGT 94901
AsianBonytongue                                                                                           15690
Reedfish                                                                                                  51713
NorthernPike                                                                                              20987
SpottedGar                                                                                                22432
ParamormyropsKingsLeyae                                                                                   13318

Majority                 XXXXXXXXXXXXXXXXXXXXXXXXXXXXXXXXXXXXXXXXXXXXXXXXXXXXXXXXXXXXXXXXXXXXXXXXXXXXXXXX

                                  95690     95700     95710     95720     95730     95740     95750     95760

Human                    AATTAGTGGTGGCTATAATATTTGAAAGTAGGTTCTGTCACCTTTTCAGGTGCTCATTGTGGGTGGGATTGCCTTGGGAA 94981
AsianBonytongue                                                                                           15690
Reedfish                                                                                                  51713
NorthernPike                                                                                              20987
SpottedGar                                                                                                22432
ParamormyropsKingsLeyae                                                                                   13318

Majority                 XXXXXXXXXXXXXXXXXXXXXXXXXXXXXXXXXXXXXXXXXXXXXXXXXXXXXXXXXXXXXXXXXXXXXXXXXXXXXXXX

                                  95770     95780     95790     95800     95810     95820     95830     95840

Human                    GGAGAAACTAAGTCCTGAGCTGTGTGTGTGGACTTAGTTTGTGCCAGTTGCTAAGCTGGTGGGAGTTCCCAGTCTCCCTA 95061
AsianBonytongue                                                                                           15690
Reedfish                                                                                                  51713
NorthernPike                                                                                              20987
SpottedGar                                                                                                22432
ParamormyropsKingsLeyae                                                                                   13318

Majority                 XXXXXXXXXXXXXXXXXXXXXXXXXXXXXXXXXXXXXXXXXXXXXXXXXXXXXXXXXXXXXXXXXXXXXXXXXXXXXXXX

                                  95850     95860     95870     95880     95890     95900     95910     95920

Human                    GTGGGAGATAACCAGAAACCAGCTCTTCCTGCCTAAGGACTACAGCAGATTCTATACTGTCTTACAGTATGCCCTAATAC 95141
AsianBonytongue                                                                                           15690
Reedfish                                                                                                  51713
NorthernPike                                                                                              20987
SpottedGar                                                                                                22432
ParamormyropsKingsLeyae                                                                                   13318

Majority                 XXXXXXXXXXXXXXXXXXXXXXXXXXXXXXXXXXXXXXXXXXXXXXXXXXXXXXXXXXXXXXXXXXXXXXXXXXXXXXXX

                                  95930     95940     95950     95960     95970     95980     95990     96000

Human                    GGCCTTCCTTCCAAAGCACCAAGTCACTAAAGGGGGATTTTGACATTCTAGAATATTTTTAATTCAGTACATCAAATTAA 95221
AsianBonytongue                                                                                           15690
Reedfish                                                                                                  51713
NorthernPike                                                                                              20987
SpottedGar                                                                                                22432
ParamormyropsKingsLeyae                                                                                   13318
```

```
Majority                  XXXXXXXXXXXXXXXXXXXXXXXXXXXXXXXXXXXXXXXXXXXXXXXXXXXXXXXXXXXXXXXXXXXXXXXXXXXXXXXX

                                   96010     96020     96030     96040     96050     96060     96070     96080

Human                     CCCAGGCCTTGGCATATAGTGAATTGCTTATTAAAAGTTAGGTAAAAATAAAAACAGCTTTGTCGTATATTAGTACTCTA 95301
AsianBonytongue                                                                                            15690
Reedfish                                                                                                   51713
NorthernPike                                                                                               20987
SpottedGar                                                                                                 22432
ParamormyropsKingsLeyae                                                                                    13318

Majority                  XXXXXXXXXXXXXXXXXXXXXXXXXXXXXXXXXXXXXXXXXXXXXXXXXXXXXXXXXXXXXXXXXXXXXXXXXXXXXXXX

                                   96090     96100     96110     96120     96130     96140     96150     96160

Human                     TGACTCTTAGAGTGAACAAGCTAACAAGTATTCTTAGGACTTAGCTGCTTTGTTAATAGAGGTCTTAACTTGAATATTTA 95381
AsianBonytongue                                                                                            15690
Reedfish                                                                                                   51713
NorthernPike                                                                                               20987
SpottedGar                                                                                                 22432
ParamormyropsKingsLeyae                                                                                    13318

Majority                  XXXXXXXXXXXXXXXXXXXXXXXXXXXXXXXXXXXXXXXXXXXXXXXXXXXXXXXXXXXXXXXXXXXXXXXXXXXXXXXX

                                   96170     96180     96190     96200     96210     96220     96230     96240

Human                     GAAATAATCAGAAAAATTATTTTTTGGTATTGAAGATGTATTTTGGAAATGGGTTTTAAAGTACCTACTTAAGGAAGAGA 95461
AsianBonytongue                                                                                            15690
Reedfish                                                                                                   51713
NorthernPike                                                                                               20987
SpottedGar                                                                                                 22432
ParamormyropsKingsLeyae                                                                                    13318

Majority                  XXXXXXXXXXXXXXXXXXXXXXXXXXXXXXXXXXXXXXXXXXXXXXXXXXXXXXXXXXXXXXXXXXXXXXXXXXXXXXXX

                                   96250     96260     96270     96280     96290     96300     96310     96320

Human                     TATCTACAGTTTATATTTAACGTTAGCATTTGAAAGCAATGAAGAAAATTTTTCCTTAACCAGGAAGTGTAATGAATCAT 95541
AsianBonytongue                                                                                            15690
Reedfish                                                                                                   51713
NorthernPike                                                                                               20987
SpottedGar                                                                                                 22432
ParamormyropsKingsLeyae                                                                                    13318

Majority                  XXXXXXXXXXXXXXXXXXXXXXXXXXXXXXXXXXXXXXXXXXXXXXXXXXXXXXXXXXXXXXXXXXXXXXXXXXXXXXXX

                                   96330     96340     96350     96360     96370     96380     96390     96400

Human                     TATATTAGAGATTAAATTAACACTTTGGAAGCATTTTAGCTCCTAGAGTTAAGGTTAGTATAGTGAAATTATGGTGATTA 95621
AsianBonytongue                                                                                            15690
Reedfish                                                                                                   51713
NorthernPike                                                                                               20987
SpottedGar                                                                                                 22432
ParamormyropsKingsLeyae                                                                                    13318

Majority                  XXXXXXXXXXXXXXXXXXXXXXXXXXXXXXXXXXXXXXXXXXXXXXXXXXXXXXXXXXXXXXXXXXXXXXXXXXXXXXXX

                                   96410     96420     96430     96440     96450     96460     96470     96480

Human                     GCTTAATTCATTCATGTACATCTATTTGTATCACCATATAATTACTAGGGAAAGGACAAATCACTTTTCAAAGCCTCAAT 95701
AsianBonytongue                                                                                            15690
Reedfish                                                                                                   51713
NorthernPike                                                                                               20987
SpottedGar                                                                                                 22432
ParamormyropsKingsLeyae                                                                                    13318
```

```
Majority                  XXXXXXXXXXXXXXXXXXXXXXXXXXXXXXXXXXXXXXXXXXXXXXXXXXXXXXXXXXXXXXXXXXXXXXXXXXXXXXXX

                                   96490     96500     96510     96520     96530     96540     96550     96560

Human                     GTTATTTGTAAAATGGAGGCAATAAGTTCTGCTCTGCTCCCCTCACAGGATTATTACAGAGGCTCAAATAGGATAAGTGA 95781
AsianBonytongue                                                                                            15690
Reedfish                                                                                                   51713
NorthernPike                                                                                               20987
SpottedGar                                                                                                 22432
ParamormyropsKingsLeyae                                                                                    13318

Majority                  XXXXXXXXXXXXXXXXXXXXXXXXXXXXXXXXXXXXXXXXXXXXXXXXXXXXXXXXXXXXXXXXXXXXXXXXXXXXXXXX

                                   96570     96580     96590     96600     96610     96620     96630     96640

Human                     AGGAATGCTATGCAATTAGTTAACTAATGCCAGTTCCCAGACTGCCAGATTTGCCCACAGTAAACTTCATTATCCACAAT 95861
AsianBonytongue                                                                                            15690
Reedfish                                                                                                   51713
NorthernPike                                                                                               20987
SpottedGar                                                                                                 22432
ParamormyropsKingsLeyae                                                                                    13318

Majority                  XXXXXXXXXXXXXXXXXXXXXXXXXXXXXXXXXXXXXXXXXXXXXXXXXXXXXXXXXXXXXXXXXXXXXXXXXXXXXXXX

                                   96650     96660     96670     96680     96690     96700     96710     96720

Human                     ACATGTTATCCTAATATGTCTTTTTGCTACAAAGAAATACTAATGTTTTAAAATTATTTACTAGTTTCATAAAGCAGAAA 95941
AsianBonytongue                                                                                            15690
Reedfish                                                                                                   51713
NorthernPike                                                                                               20987
SpottedGar                                                                                                 22432
ParamormyropsKingsLeyae                                                                                    13318

Majority                  XXXXXXXXXXXXXXXXXXXXXXXXXXXXXXXXXXXXXXXXXXXXXXXXXXXXXXXXXXXXXXXXXXXXXXXXXXXXXXXX

                                   96730     96740     96750     96760     96770     96780     96790     96800

Human                     GATGTAAAATGCAGTTATAGAGAGCATCCCAAGCCAGATGAAAAGAAACCATGGATTTAGCTTTATATTTTCTGTTTGAA 96021
AsianBonytongue                                                                                            15690
Reedfish                                                                                                   51713
NorthernPike                                                                                               20987
SpottedGar                                                                                                 22432
ParamormyropsKingsLeyae                                                                                    13318

Majority                  XXXXXXXXXXXXXXXXXXXXXXXXXXXXXXXXXXXXXXXXXXXXXXXXXXXXXXXXXXXXXXXXXXXXXXXXXXXXXXXX

                                   96810     96820     96830     96840     96850     96860     96870     96880

Human                     CTTGATAGCCCAATCATAAAATGCACATATTTAATTATAGACTGCACTTAAGATTTCATATGTATAAAAAAGTTGCTGTG 96101
AsianBonytongue                                                                                            15690
Reedfish                                                                                                   51713
NorthernPike                                                                                               20987
SpottedGar                                                                                                 22432
ParamormyropsKingsLeyae                                                                                    13318

Majority                  XXXXXXXXXXXXXXXXXXXXXXXXXXXXXXXXXXXXXXXXXXXXXXXXXXXXXXXXXXXXXXXXXXXXXXXXXXXXXXXX

                                   96890     96900     96910     96920     96930     96940     96950     96960

Human                     AAACAGGTAATTTTAACACTCAGGAATTTCAAGGCTAAGGAAGAAAGACATACTCTCATGAGCAGCTCTGAACTTCAAAC 96181
AsianBonytongue                                                                                            15690
Reedfish                                                                                                   51713
NorthernPike                                                                                               20987
SpottedGar                                                                                                 22432
ParamormyropsKingsLeyae                                                                                    13318
```

```
Majority                   XXXXXXXXXXXXXXXXXXXXXXXXXXXXXXXXXXXXXXXXXXXXXXXXXXXXXXXXXXXXXXXXXXXXXXXXXXXXXXXX
                                    96970     96980     96990     97000     97010     97020     97030     97040
Human                      AGGATTTTCTGGCTGCCTTAATATATTAAAATGGAGCCTAAATCTGAAAACGTTTTTTTCATTCAGAAGAAAGTACAGAA 96261
AsianBonytongue                                                                                             15690
Reedfish                                                                                                    51713
NorthernPike                                                                                                20987
SpottedGar                                                                                                  22432
ParamormyropsKingsLeyae                                                                                     13318

Majority                   XXXXXXXXXXXXXXXXXXXXXXXXXXXXXXXXXXXXXXXXXXXXXXXXXXXXXXXXXXXXXXXXXXXXXXXXXXXXXXXX
                                    97050     97060     97070     97080     97090     97100     97110     97120
Human                      TTCAGTGAAATTGAGTATACTAGGAAATCTGATGGCAAGAATTCAGCAGAAAGTCTTAATTTTAAACCGTAATATTCATT 96341
AsianBonytongue                                                                                             15690
Reedfish                                                                                                    51713
NorthernPike                                                                                                20987
SpottedGar                                                                                                  22432
ParamormyropsKingsLeyae                                                                                     13318

Majority                   XXXXXXXXXXXXXXXXXXXXXXXXXXXXXXXXXXXXXXXXXXXXXXXXXXXXXXXXXXXXXXXXXXXXXXXXXXXXXXXX
                                    97130     97140     97150     97160     97170     97180     97190     97200
Human                      GATTATGCAAAACCTTAGTTTTATAGTTATTCTTTGTTATTTTGAATAGCTTCCCTTTAAATTTTCTAGTTTCAGATTTT 96421
AsianBonytongue                                                                                             15690
Reedfish                                                                                                    51713
NorthernPike                                                                                                20987
SpottedGar                                                                                                  22432
ParamormyropsKingsLeyae                                                                                     13318

Majority                   XXXXXXXXXXXXXXXXXXXXXXXXXXXXXXXXXXXXXXXXXXXXXXXXXXXXXXXXXXXXXXXXXXXXXXXXXXXXXXXX
                                    97210     97220     97230     97240     97250     97260     97270     97280
Human                      GCAAATAAAAGTGTACATGTTAAAGTTATAGAGTATTAGATTATTCACATTAAGAACTGTTCAAATACGCGGAAAAATAA 96501
AsianBonytongue                                                                                             15690
Reedfish                                                                                                    51713
NorthernPike                                                                                                20987
SpottedGar                                                                                                  22432
ParamormyropsKingsLeyae                                                                                     13318

Majority                   XXXXXXXXXXXXXXXXXXXXXXXXXXXXXXXXXXXXXXXXXXXXXXXXXXXXXXXXXXXXXXXXXXXXXXXXXXXXXXXX
                                    97290     97300     97310     97320     97330     97340     97350     97360
Human                      AACCGTAATGAACACCTATGCACTTCACTCAACTATATCACTACCTAACATATTCCATATTTTAAACGTAATTATATATT 96581
AsianBonytongue                                                                                             15690
Reedfish                                                                                                    51713
NorthernPike                                                                                                20987
SpottedGar                                                                                                  22432
ParamormyropsKingsLeyae                                                                                     13318

Majority                   XXXXXXXXXXXXXXXXXXXXXXXXXXXXXXXXXXXXXXXXXXXXXXXXXXXXXXXXXXXXXXXXXXXXXXXXXXXXXXXX
                                    97370     97380     97390     97400     97410     97420     97430     97440
Human                      AGTTGAAAGAAAACGTTACAGAATGAACTGAAATCCCATATGAACCTCTTTCTGATCTTAGTTCAAACCTTATTCTTTCC 96661
AsianBonytongue                                                                                             15690
Reedfish                                                                                                    51713
NorthernPike                                                                                                20987
SpottedGar                                                                                                  22432
ParamormyropsKingsLeyae                                                                                     13318
```

```
Majority                      XXXXXXXXXXXXXXXXXXXXXXXXXXXXXXXXXXXXXXXXXXXXXXXXXXXXXXXXXXXXXXXXXXXXXXXXXXXXXXXX

                                       97450     97460     97470     97480     97490     97500     97510     97520

Human                         CAAGAGGTAATTGTTACCTTCAGTTTAGTATTTTATTATTCAGTGGTTGCTTTTCAAGAGGAATTTGTCCTATATTCTGT 96741
AsianBonytongue                                                                                                15690
Reedfish                                                                                                       51713
NorthernPike                                                                                                   20987
SpottedGar                                                                                                     22432
ParamormyropsKingsLeyae                                                                                        13318

Majority                      XXXXXXXXXXXXXXXXXXXXXXXXXXXXXXXXXXXXXXXXXXXXXXXXXXXXXXXXXXXXXXXXXXXXXXXXXXXXXXXX

                                       97530     97540     97550     97560     97570     97580     97590     97600

Human                         AGGCTAAATTTCTTGTTTTGCCTAGTTATAAAAATGGCAAATAATATGATATTGGGTGTTTAAATAGTACAATATTGTTC 96821
AsianBonytongue                                                                                                15690
Reedfish                                                                                                       51713
NorthernPike                                                                                                   20987
SpottedGar                                                                                                     22432
ParamormyropsKingsLeyae                                                                                        13318

Majority                      XXXXXXXXXXXXXXXXXXXXXXXXXXXXXXXXXXXXXXXXXXXXXXXXXXXXXXXXXXXXXXXXXXXXXXXXXXXXXXXX

                                       97610     97620     97630     97640     97650     97660     97670     97680

Human                         AAAACACACTTTATAATTTTTAACAGACTGTTTGGACATTGATAATTCCCCTATCAAATCTCATGACATTTAACCAATTT 96901
AsianBonytongue                                                                                                15690
Reedfish                                                                                                       51713
NorthernPike                                                                                                   20987
SpottedGar                                                                                                     22432
ParamormyropsKingsLeyae                                                                                        13318

Majority                      XXXXXXXXXXXXXXXXXXXXXXXXXXXXXXXXXXXXXXXXXXXXXXXXXXXXXXXXXXXXXXXXXXXXXXXXXXXXXXXX

                                       97690     97700     97710     97720     97730     97740     97750     97760

Human                         TTAACAAGGATTTTAAATTGTGTTTAAGAAATAAGTCATTTGGGCCTCAGGTTTCTCTTAAGGATGTAAACAAGGAGCTA 96981
AsianBonytongue                                                                                                15690
Reedfish                                                                                                       51713
NorthernPike                                                                                                   20987
SpottedGar                                                                                                     22432
ParamormyropsKingsLeyae                                                                                        13318

Majority                      XXXXXXXXXXXXXXXXXXXXXXXXXXXXXXXXXXXXXXXXXXXXXXXXXXXXXXXXXXXXXXXXXXXXXXXXXXXXXXXX

                                       97770     97780     97790     97800     97810     97820     97830     97840

Human                         TATCATTGGTATTTTAGACGCTGAATTATGTGTAAGGTCCTTAAATACAAACATACCCTTTGTAGCTTCACAGAGCCACC 97061
AsianBonytongue                                                                                                15690
Reedfish                                                                                                       51713
NorthernPike                                                                                                   20987
SpottedGar                                                                                                     22432
ParamormyropsKingsLeyae                                                                                        13318

Majority                      XXXXXXXXXXXXXXXXXXXXXXXXXXXXXXXXXXXXXXXXXXXXXXXXXXXXXXXXXXXXXXXXXXXXXXXXXXXXXXXX

                                       97850     97860     97870     97880     97890     97900     97910     97920

Human                         CGTTTTGCACTTCTCTCGCTTTTCTTCCTCCCTCTTTGGCTCCCATTTCAATGGACAGAAAGACTATGTGGCATCTCTAT 97141
AsianBonytongue                                                                                                15690
Reedfish                                                                                                       51713
NorthernPike                                                                                                   20987
SpottedGar                                                                                                     22432
ParamormyropsKingsLeyae                                                                                        13318
```

```
Majority                   XXXXXXXXXXXXXXXXXXXXXXXXXXXXXXXXXXXXXXXXXXXXXXXXXXXXXXXXXXXXXXXXXXXXXXXXXXXXXXXX

                                    97930     97940     97950     97960     97970     97980     97990     98000

Human                      TTTCATTCACTTGGTTTATTGTCTTGGTATGTTGTATAGGTTTTTGAATCAGAAAATTTTAACATTTTTAGATATTTCAA 97221
AsianBonytongue                                                                                             15690
Reedfish                                                                                                    51713
NorthernPike                                                                                                20987
SpottedGar                                                                                                  22432
ParamormyropsKingsLeyae                                                                                     13318

Majority                   XXXXXXXXXXXXXXXXXXXXXXXXXXXXXXXXXXXXXXXXXXXXXXXXXXXXXXXXXXXXXXXXXXXXXXXXXXXXXXXX

                                    98010     98020     98030     98040     98050     98060     98070     98080

Human                      ACATACAAAAAATAGAAGAATATTTAACAAACCCTTGTCAGCTTCAGTTAGCAACTTAGATCTTCCTCGTTTCCTCCACC 97301
AsianBonytongue                                                                                             15690
Reedfish                                                                                                    51713
NorthernPike                                                                                                20987
SpottedGar                                                                                                  22432
ParamormyropsKingsLeyae                                                                                     13318

Majority                   XXXXXXXXXXXXXXXXXXXXXXXXXXXXXXXXXXXXXXXXXXXXXXXXXXXXXXXXXXXXXXXXXXXXXXXXXXXXXXXX

                                    98090     98100     98110     98120     98130     98140     98150     98160

Human                      CAATTCTCATCTCCTTTATTAATGCAGATCCCAGATATATTTTATCAAGAAATTTTGTAGCATATATTTGTGTTGTTTTA 97381
AsianBonytongue                                                                                             15690
Reedfish                                                                                                    51713
NorthernPike                                                                                                20987
SpottedGar                                                                                                  22432
ParamormyropsKingsLeyae                                                                                     13318

Majority                   XXXXXXXXXXXXXXXXXXXXXXXXXXXXXXXXXXXXXXXXXXXXXXXXXXXXXXXXXXXXXXXXXXXXXXXXXXXXXXXX

                                    98170     98180     98190     98200     98210     98220     98230     98240

Human                      TTCGTGGTCATAGTTGCTTTCTCTAATATCTAGACTAGCCTAGCTGGTCAATACTACTTTTCTTCAAATCCTAGAACCTC 97461
AsianBonytongue                                                                                             15690
Reedfish                                                                                                    51713
NorthernPike                                                                                                20987
SpottedGar                                                                                                  22432
ParamormyropsKingsLeyae                                                                                     13318

Majority                   XXXXXXXXXXXXXXXXXXXXXXXXXXXXXXXXXXXXXXXXXXXXXXXXXXXXXXXXXXXXXXXXXXXXXXXXXXXXXXXX

                                    98250     98260     98270     98280     98290     98300     98310     98320

Human                      CCTATTAAAAATATAGTCACCAGACCAGTGGCAAATTAGTATCACCTAGGAGTTTATTAGAAACTCAGTCTATGAGCCTA 97541
AsianBonytongue                                                                                             15690
Reedfish                                                                                                    51713
NorthernPike                                                                                                20987
SpottedGar                                                                                                  22432
ParamormyropsKingsLeyae                                                                                     13318

Majority                   XXXXXXXXXXXXXXXXXXXXXXXXXXXXXXXXXXXXXXXXXXXXXXXXXXXXXXXXXXXXXXXXXXXXXXXXXXXXXXXX

                                    98330     98340     98350     98360     98370     98380     98390     98400

Human                      CTGAATCTACATTTTGACAAGATTGGCAGGTGATGCATTTGGACATAAGTTGGAGAAGCACTCAATGCATTTTTTCAGTT 97621
AsianBonytongue                                                                                             15690
Reedfish                                                                                                    51713
NorthernPike                                                                                                20987
SpottedGar                                                                                                  22432
ParamormyropsKingsLeyae                                                                                     13318
```

```
Majority                   XXXXXXXXXXXXXXXXXXXXXXXXXXXXXXXXXXXXXXXXXXXXXXXXXXXXXXXXXXXXXXXXXXXXXXXXXXXXXXXX
                                     98410     98420     98430     98440     98450     98460     98470     98480
Human                      CTATAAAAATCAGCTTCAAAGTTTTTTCCTGAGTGTGGAAATTATTTCATTAGTAATTTTAAGTTATGCAGATATAGTTA 97701
AsianBonytongue                                                                                             15690
Reedfish                                                                                                    51713
NorthernPike                                                                                                20987
SpottedGar                                                                                                  22432
ParamormyropsKingsLeyae                                                                                     13318

Majority                   XXXXXXXXXXXXXXXXXXXXXXXXXXXXXXXXXXXXXXXXXXXXXXXXXXXXXXXXXXXXXXXXXXXXXXXXXXXXXXXX
                                     98490     98500     98510     98520     98530     98540     98550     98560
Human                      ATACCAAGAAATAATTGAACTGAAAAGGAACCCGGATATCTAGTTTTAACCAGCTGTTTTAAAGATAAACAGTTCTAGAG 97781
AsianBonytongue                                                                                             15690
Reedfish                                                                                                    51713
NorthernPike                                                                                                20987
SpottedGar                                                                                                  22432
ParamormyropsKingsLeyae                                                                                     13318

Majority                   XXXXXXXXXXXXXXXXXXXXXXXXXXXXXXXXXXXXXXXXXXXXXXXXXXXXXXXXXXXXXXXXXXXXXXXXXXXXXXXX
                                     98570     98580     98590     98600     98610     98620     98630     98640
Human                      ATCAAATGAATAGTTTAGCAACATATTTATTGTAACTTACCTGTAGTTAGAATCCAAGTCCCTGGCTTTCCAATCCAGTC 97861
AsianBonytongue                                                                                             15690
Reedfish                                                                                                    51713
NorthernPike                                                                                                20987
SpottedGar                                                                                                  22432
ParamormyropsKingsLeyae                                                                                     13318

Majority                   XXXXXXXXXXXXXXXXXXXXXXXXXXXXXXXXXXXXXXXXXXXXXXXXXXXXXXXXXXXXXXXXXXXXXXXXXXXXXXXX
                                     98650     98660     98670     98680     98690     98700     98710     98720
Human                      TTCAATGTATAATACTGATCAAAAGAAATACAGGCCTGCTATTATTCATAAATTTAAACTATTATGTACAAGTTGATAGC 97941
AsianBonytongue                                                                                             15690
Reedfish                                                                                                    51713
NorthernPike                                                                                                20987
SpottedGar                                                                                                  22432
ParamormyropsKingsLeyae                                                                                     13318

Majority                   XXXXXXXXXXXXXXXXXXXXXXXXXXXXXXXXXXXXXXXXXXXXXXXXXXXXXXXXXXXXXXXXXXXXXXXXXXXXXXXX
                                     98730     98740     98750     98760     98770     98780     98790     98800
Human                      TTATAATAGAAATCAGTATGATTAGTGTTAAAAGTCAAATTTAGTCTGACAGGGAAGGAATGTCCATGTGAACTGATTTT 98021
AsianBonytongue                                                                                             15690
Reedfish                                                                                                    51713
NorthernPike                                                                                                20987
SpottedGar                                                                                                  22432
ParamormyropsKingsLeyae                                                                                     13318

Majority                   XXXXXXXXXXXXXXXXXXXXXXXXXXXXXXXXXXXXXXXXXXXXXXXXXXXXXXXXXXXXXXXXXXXXXXXXXXXXXXXX
                                     98810     98820     98830     98840     98850     98860     98870     98880
Human                      TTTTAATAGAATGCAAATTAATACTCAATTCCTGATTTGTTTATTACATTTTGTGGTTTAATGAAATGATACTCAGGTCA 98101
AsianBonytongue                                                                                             15690
Reedfish                                                                                                    51713
NorthernPike                                                                                                20987
SpottedGar                                                                                                  22432
ParamormyropsKingsLeyae                                                                                     13318
```

```
Majority                XXXXXXXXXXXXXXXXXXXXXXXXXXXXXXXXXXXXXXXXXXXXXXXXXXXXXXXXXXXXXXXXXXXXXXXXXXXXXXXX
                                 98890     98900     98910     98920     98930     98940     98950     98960
Human                   TTTTCATAAATAGCTTTTCTATTAATGCTTCCTCAGTCACATTCTCTATTCCTATTGCATAGCACAGTCTAACAGTTGAA 98181
AsianBonytongue                                                                                          15690
Reedfish                                                                                                 51713
NorthernPike                                                                                             20987
SpottedGar                                                                                               22432
ParamormyropsKingsLeyae                                                                                  13318

Majority                XXXXXXXXXXXXXXXXXXXXXXXXXXXXXXXXXXXXXXXXXXXXXXXXXXXXXXXXXXXXXXXXXXXXXXXXXXXXXXXX
                                 98970     98980     98990     99000     99010     99020     99030     99040
Human                   ATGCCTAAGCCTTACTGAATGATGACTGATATTCTCTGTGAGAGTTAGAGCTGTACTATAAATATACATTACCTGTGCAA 98261
AsianBonytongue                                                                                          15690
Reedfish                                                                                                 51713
NorthernPike                                                                                             20987
SpottedGar                                                                                               22432
ParamormyropsKingsLeyae                                                                                  13318

Majority                XXXXXXXXXXXXXXXXXXXXXXXXXXXXXXXXXXXXXXXXXXXXXXXXXXXXXXXXXXXXXXXXXXXXXXXXXXXXXXXX
                                 99050     99060     99070     99080     99090     99100     99110     99120
Human                   AGTATGCCTTCAGGGGCTATTCCCCATAACCTTAGCAAAATTTTAAAATATTCTTATGGCTAGCAATCTTATCTATTGTC 98341
AsianBonytongue                                                                                          15690
Reedfish                                                                                                 51713
NorthernPike                                                                                             20987
SpottedGar                                                                                               22432
ParamormyropsKingsLeyae                                                                                  13318

Majority                XXXXXXXXXXXXXXXXXXXXXXXXXXXXXXXXXXXXXXXXXXXXXXXXXXXXXXXXXXXXXXXXXXXXXXXXXXXXXXXX
                                 99130     99140     99150     99160     99170     99180     99190     99200
Human                   ATGTCTTACTATTTCCTGCTTACCACCCCCATTTCTCTCTCAACCTTGTTGCTGCATTAGCTGTCCATCACTTAACACTG 98421
AsianBonytongue                                                                                          15690
Reedfish                                                                                                 51713
NorthernPike                                                                                             20987
SpottedGar                                                                                               22432
ParamormyropsKingsLeyae                                                                                  13318

Majority                XXXXXXXXXXXXXXXXXXXXXXXXXXXXXXXXXXXXXXXXXXXXXXXXXXXXXXXXXXXXXXXXXXXXXXXXXXXXXXXX
                                 99210     99220     99230     99240     99250     99260     99270     99280
Human                   TTTATTTCTCTGTTTTTCTTCCATCATTCTCTTCTTTGCAACTAAATGATTTAGACTTTTTATAACCATAATCAACCAGT 98501
AsianBonytongue                                                                                          15690
Reedfish                                                                                                 51713
NorthernPike                                                                                             20987
SpottedGar                                                                                               22432
ParamormyropsKingsLeyae                                                                                  13318

Majority                XXXXXXXXXXXXXXXXXXXXXXXXXXXXXXXXXXXXXXXXXXXXXXXXXXXXXXXXXXXXXXXXXXXXXXXXXXXXXXXX
                                 99290     99300     99310     99320     99330     99340     99350     99360
Human                   TAGATTCTTTTTATTTGTGGTTATTGAGTTGATAAATGATAGTGCATTTTATGTAATATTCAGGTGTTATGCTATTTTTA 98581
AsianBonytongue                                                                                          15690
Reedfish                                                                                                 51713
NorthernPike                                                                                             20987
SpottedGar                                                                                               22432
ParamormyropsKingsLeyae                                                                                  13318
```

```
Majority                      XXXXXXXXXXXXXXXXXXXXXXXXXXXXXXXXXXXXXXXXXXXXXXXXXXXXXXXXXXXXXXXXXXXXXXXXXXXXXXXX

                                       99370     99380     99390     99400     99410     99420     99430     99440

Human                         TCTGGTTCACTTTAAAACCTCCCTCCCCCCACTCCCTTTTTTTGTGGGTTTTTTTTTTATTATTATTATTTTCCAGTTTT 98661
AsianBonytongue                                                                                               15690
Reedfish                                                                                                      51713
NorthernPike                                                                                                  20987
SpottedGar                                                                                                    22432
ParamormyropsKingsLeyae                                                                                       13318

Majority                      XXXXXXXXXXXXXXXXXXXXXXXXXXXXXXXXXXXXXXXXXXXXXXXXXXXXXXXXXXXXXXXXXXXXXXXXXXXXXXXX

                                       99450     99460     99470     99480     99490     99500     99510     99520

Human                         GTGTTGTTTTGCTTTTTAACTTCTGAGATTGCAGTGGTTCCAGATGGTTGAAATCACTAGTAGTGATTTACATGAAGCAT 98741
AsianBonytongue                                                                                               15690
Reedfish                                                                                                      51713
NorthernPike                                                                                                  20987
SpottedGar                                                                                                    22432
ParamormyropsKingsLeyae                                                                                       13318

Majority                      XXXXXXXXXXXXXXXXXXXXXXXXXXXXXXXXXXXXXXXXXXXXXXXXXXXXXXXXXXXXXXXXXXXXXXXXXXXXXXXX

                                       99530     99540     99550     99560     99570     99580     99590     99600

Human                         TCATGTATTTATTACTGGTATATTCAACTATGGTGGAGGGAAATTGGTCGAAGAATTACGGTTAAAGAGCTCCTTTAAAA 98821
AsianBonytongue                                                                                               15690
Reedfish                                                                                                      51713
NorthernPike                                                                                                  20987
SpottedGar                                                                                                    22432
ParamormyropsKingsLeyae                                                                                       13318

Majority                      XXXXXXXXXXXXXXXXXXXXXXXXXXXXXXXXXXXXXXXXXXXXXXXXXXXXXXXXXXXXXXXXXXXXXXXXXXXXXXXX

                                       99610     99620     99630     99640     99650     99660     99670     99680

Human                         AGGAGATTTGTAGAATTCTAAAATGTGATTCTGATTCATATAGTCATGATTCTTAGAAATTAGCTACAGGAAGAGATAGA 98901
AsianBonytongue                                                                                               15690
Reedfish                                                                                                      51713
NorthernPike                                                                                                  20987
SpottedGar                                                                                                    22432
ParamormyropsKingsLeyae                                                                                       13318

Majority                      XXXXXXXXXXXXXXXXXXXXXXXXXXXXXXXXXXXXXXXXXXXXXXXXXXXXXXXXXXXXXXXXXXXXXXXXXXXXXXXX

                                       99690     99700     99710     99720     99730     99740     99750     99760

Human                         AACATTGCTATAGTTGTCTTTGCCCTAAGTTTGGAGTTTTATAGTGTAATATATATATAATAGCTCTAATTATAAATACA 98981
AsianBonytongue                                                                                               15690
Reedfish                                                                                                      51713
NorthernPike                                                                                                  20987
SpottedGar                                                                                                    22432
ParamormyropsKingsLeyae                                                                                       13318

Majority                      XXXXXXXXXXXXXXXXXXXXXXXXXXXXXXXXXXXXXXXXXXXXXXXXXXXXXXXXXXXXXXXXXXXXXXXXXXXXXXXX

                                       99770     99780     99790     99800     99810     99820     99830     99840

Human                         CTTTTACTCTGGAAAAAGCATAGACATTGACATAAGAGACATTTAAAAACCATTATTTTCTCATCTGTAAAATGGAGAGA 99061
AsianBonytongue                                                                                               15690
Reedfish                                                                                                      51713
NorthernPike                                                                                                  20987
SpottedGar                                                                                                    22432
ParamormyropsKingsLeyae                                                                                       13318
```

```
Majority                     XXXXXXXXXXXXXXXXXXXXXXXXXXXXXXXXXXXXXXXXXXXXXXXXXXXXXXXXXXXXXXXXXXXXXXXXXXXXXXXX

                                      99850     99860     99870     99880     99890     99900     99910     99920

Human                        ATTACTTCATATGTTCACTTATAAGGATTATGTGAAATACTGTTCCATTCCATTAGTAGTTTTTCTTTTCCTCTAATTGG 99141
AsianBonytongue                                                                                               15690
Reedfish                                                                                                      51713
NorthernPike                                                                                                  20987
SpottedGar                                                                                                    22432
ParamormyropsKingsLeyae                                                                                       13318

Majority                     XXXXXXXXXXXXXXXXXXXXXXXXXXXXXXXXXXXXXXXXXXXXXXXXXXXXXXXXXXXXXXXXXXXXXXXXXXXXXXXX

                                      99930     99940     99950     99960     99970     99980     99990    100000

Human                        GGGTGGGGGCAGTTTTTGGCATTCTATTTATGGAAGGTGTTTTTTTCCCTAAGATCATTTTGATGTCTCAGAAGACATTT 99221
AsianBonytongue                                                                                               15690
Reedfish                                                                                                      51713
NorthernPike                                                                                                  20987
SpottedGar                                                                                                    22432
ParamormyropsKingsLeyae                                                                                       13318

Majority                     XXXXXXXXXXXXXXXXXXXXXXXXXXXXXXXXXXXXXXXXXXXXXXXXXXXXXXXXXXXXXXXXXXXXXXXXXXXXXXXX

                                     100010    100020    100030    100040    100050    100060    100070    100080

Human                        GCTATGTACGTTGGACATTTGCCATGTACATTGTAGAGAATACAAAGATAAACAGAACCACAGTACAGCTGGTTCTATTA 99301
AsianBonytongue                                                                                               15690
Reedfish                                                                                                      51713
NorthernPike                                                                                                  20987
SpottedGar                                                                                                    22432
ParamormyropsKingsLeyae                                                                                       13318

Majority                     XXXXXXXXXXXXXXXXXXXXXXXXXXXXXXXXXXXXXXXXXXXXXXXXXXXXXXXXXXXXXXXXXXXXXXXXXXXXXXXX

                                     100090    100100    100110    100120    100130    100140    100150    100160

Human                        GACATTGAAAATGCATATTCTAGCATATTTAATATATTAGGGAACAATTTAAGCATAATGTGAATTTCATGTTTACTGAA 99381
AsianBonytongue                                                                                               15690
Reedfish                                                                                                      51713
NorthernPike                                                                                                  20987
SpottedGar                                                                                                    22432
ParamormyropsKingsLeyae                                                                                       13318

Majority                     XXXXXXXXXXXXXXXXXXXXXXXXXXXXXXXXXXXXXXXXXXXXXXXXXXXXXXXXXXXXXXXXXXXXXXXXXXXXXXXX

                                     100170    100180    100190    100200    100210    100220    100230    100240

Human                        GATTTCATCCTGAGAAACAAATGAAAGCAGAAAATTACAACCAACTGAACCAGCTGTGTAAGAAATATATATATGGTGTG 99461
AsianBonytongue                                                                                               15690
Reedfish                                                                                                      51713
NorthernPike                                                                                                  20987
SpottedGar                                                                                                    22432
ParamormyropsKingsLeyae                                                                                       13318

Majority                     XXXXXXXXXXXXXXXXXXXXXXXXXXXXXXXXXXXXXXXXXXXXXXXXXXXXXXXXXXXXXXXXXXXXXXXXXXXXXXXX

                                     100250    100260    100270    100280    100290    100300    100310    100320

Human                        CACACATGTACATACCTCAGACATCTATTAGCATTCTCAGTTTTCCACAAGTTAAAAGCCACGCTCATCCATATCTGGTG 99541
AsianBonytongue                                                                                               15690
Reedfish                                                                                                      51713
NorthernPike                                                                                                  20987
SpottedGar                                                                                                    22432
ParamormyropsKingsLeyae                                                                                       13318
```

```
Majority                   XXXXXXXXXXXXXXXXXXXXXXXXXXXXXXXXXXXXXXXXXXXXXXXXXXXXXXXXXXXXXXXXXXXXXXXXXXXXXXXX
                                  100330    100340    100350    100360    100370    100380    100390    100400
Human                      CTAATAACTTCCTATCCAATTTCAGATAGCTGTTTTCAACACATCTGACACCTTCTTTTTTTCAAGCAAACTTCAGGTCT 99621
AsianBonytongue                                                                                             15690
Reedfish                                                                                                    51713
NorthernPike                                                                                                20987
SpottedGar                                                                                                  22432
ParamormyropsKingsLeyae                                                                                     13318

Majority                   XXXXXXXXXXXXXXXXXXXXXXXXXXXXXXXXXXXXXXXXXXXXXXXXXXXXXXXXXXXXXXXXXXXXXXXXXXXXXXXX
                                  100410    100420    100430    100440    100450    100460    100470    100480
Human                      TTTTCAAGATAAAGTCATTTATAGTAGTGTCTATATATTTCTTAACCATTTAACATGTAAACTGTGCTGCCTTTTAAGAA 99701
AsianBonytongue                                                                                             15690
Reedfish                                                                                                    51713
NorthernPike                                                                                                20987
SpottedGar                                                                                                  22432
ParamormyropsKingsLeyae                                                                                     13318

Majority                   XXXXXXXXXXXXXXXXXXXXXXXXXXXXXXXXXXXXXXXXXXXXXXXXXXXXXXXXXXXXXXXXXXXXXXXXXXXXXXXX
                                  100490    100500    100510    100520    100530    100540    100550    100560
Human                      ATTAATTCTATCCTTTTTAGTGTCAGAGTTGAAGATTTTGAGTGTTGTGCCCTAACCTTATCATTTTCCTCATAAGTCCT 99781
AsianBonytongue                                                                                             15690
Reedfish                                                                                                    51713
NorthernPike                                                                                                20987
SpottedGar                                                                                                  22432
ParamormyropsKingsLeyae                                                                                     13318

Majority                   XXXXXXXXXXXXXXXXXXXXXXXXXXXXXXXXXXXXXXXXXXXXXXXXXXXXXXXXXXXXXXXXXXXXXXXXXXXXXXXX
                                  100570    100580    100590    100600    100610    100620    100630    100640
Human                      GTGGTTTTTATTGTATGATTTTGCATAGTGTGGCAATTCTTGGAAATGCTTATGTTGTGTTATAGTATAACTGACTTACT 99861
AsianBonytongue                                                                                             15690
Reedfish                                                                                                    51713
NorthernPike                                                                                                20987
SpottedGar                                                                                                  22432
ParamormyropsKingsLeyae                                                                                     13318

Majority                   XXXXXXXXXXXXXXXXXXXXXXXXXXXXXXXXXXXXXXXXXXXXXXXXXXXXXXXXXXXXXXXXXXXXXXXXXXXXXXXX
                                  100650    100660    100670    100680    100690    100700    100710    100720
Human                      ACTTTCAAATAGCTTCTAGTCTGATTCATGTTATGACAGTATACAGTATAGCAAAATAAGGACATCAGGGAACAGGAGGC 99941
AsianBonytongue                                                                                             15690
Reedfish                                                                                                    51713
NorthernPike                                                                                                20987
SpottedGar                                                                                                  22432
ParamormyropsKingsLeyae                                                                                     13318

Majority                   XXXXXXXXXXXXXXXXXXXXXXXXXXXXXXXXXXXXXXXXXXXXXXXXXXXXXXXXXXXXXXXXXXXXXXXXXXXXXXXX
                                  100730    100740    100750    100760    100770    100780    100790    100800
Human                      TGAGCATATCACCATTTACCAATGGTAGCTTGAGCAAGGCACTTCATTTATCGAAGTCCATTTTCTCATCTGTACAATGG 100021
AsianBonytongue                                                                                             15690
Reedfish                                                                                                    51713
NorthernPike                                                                                                20987
SpottedGar                                                                                                  22432
ParamormyropsKingsLeyae                                                                                     13318
```

```
Majority                  XXXXXXXXXXXXXXXXXXXXXXXXXXXXXXXXXXXXXXXXXXXXXXXXXXXXXXXXXXXXXXXXXXXXXXXXXXXXXXXX

                                100810    100820    100830    100840    100850    100860    100870    100880

Human                     AGGTGATGATAGTACATGTCTTTTCTGTCTTGTGTTATTTGTAGTTTCAAGTAAGATATGAAATATGCTGTAGAAATACA 100101
AsianBonytongue                                                                                            15690
Reedfish                                                                                                   51713
NorthernPike                                                                                               20987
SpottedGar                                                                                                 22432
ParamormyropsKingsLeyae                                                                                    13318

Majority                  XXXXXXXXXXXXXXXXXXXXXXXXXXXXXXXXXXXXXXXXXXXXXXXXXXXXXXXXXXXXXXXXXXXXXXXXXXXXXXXX

                                100890    100900    100910    100920    100930    100940    100950    100960

Human                     GGGTTGGGAATATCTAGTGGTACAAAAATCAACTTTTGGCCCAATTTTAAGGTGAGATAATTATGAGACATTTTTCGACC 100181
AsianBonytongue                                                                                            15690
Reedfish                                                                                                   51713
NorthernPike                                                                                               20987
SpottedGar                                                                                                 22432
ParamormyropsKingsLeyae                                                                                    13318

Majority                  XXXXXXXXXXXXXXXXXXXXXXXXXXXXXXXXXXXXXXXXXXXXXXXXXXXXXXXXXXXXXXXXXXXXXXXXXXXXXXXX

                                100970    100980    100990    101000    101010    101020    101030    101040

Human                     TCAAAGTCTTTGTTCTTTTCTCTCTACACCATTGTGAATAACACAGTGTATATATGAGATTAATTTATTACCTCGACTTT 100261
AsianBonytongue                                                                                            15690
Reedfish                                                                                                   51713
NorthernPike                                                                                               20987
SpottedGar                                                                                                 22432
ParamormyropsKingsLeyae                                                                                    13318

Majority                  XXXXXXXXXXXXXXXXXXXXXXXXXXXXXXXXXXXXXXXXXXXXXXXXXXXXXXXXXXXXXXXXXXXXXXXXXXXXXXXX

                                101050    101060    101070    101080    101090    101100    101110    101120

Human                     CCATTTTTGTTTTGTAAAGTAGTTTAATTTTTTATATCTATTTATAAAGTTAACTGCAGGAGTGTACCTGCAGTTAACTT 100341
AsianBonytongue                                                                                            15690
Reedfish                                                                                                   51713
NorthernPike                                                                                               20987
SpottedGar                                                                                                 22432
ParamormyropsKingsLeyae                                                                                    13318

Majority                  XXXXXXXXXXXXXXXXXXXXXXXXXXXXXXXXXXXXXXXXXXXXXXXXXXXXXXXXXXXXXXXXXXXXXXXXXXXXXXXX

                                101130    101140    101150    101160    101170    101180    101190    101200

Human                     TATACAGTTAAGAAGAGGTTGGTTTCAGTTTTAAAGATTGTATGTCAGTGTAAAACATCCATTATTATGCTTTCAAGTAA 100421
AsianBonytongue                                                                                            15690
Reedfish                                                                                                   51713
NorthernPike                                                                                               20987
SpottedGar                                                                                                 22432
ParamormyropsKingsLeyae                                                                                    13318

Majority                  XXXXXXXXXXXXXXXXXXXXXXXXXXXXXXXXXXXXXXXXXXXXXXXXXXXXXXXXXXXXXXXXXXXXXXXXXXXXXXXX

                                101210    101220    101230    101240    101250    101260    101270    101280

Human                     AATAGCTCCTAAAAATATGGATTACTATCAACTTTCAAGGAAAATAGCTCCTAAAAATATGGATTACTACCAACTTTCAA 100501
AsianBonytongue                                                                                            15690
Reedfish                                                                                                   51713
NorthernPike                                                                                               20987
SpottedGar                                                                                                 22432
ParamormyropsKingsLeyae                                                                                    13318
```

```
Majority                  XXXXXXXXXXXXXXXXXXXXXXXXXXXXXXXXXXXXXXXXXXXXXXXXXXXXXXXXXXXXXXXXXXXXXXXXXXXXXXXX
                                101290    101300    101310    101320    101330    101340    101350    101360
Human                     GGAAAATAGCTCCTAGAAATATGGATTACTAGCCACATAAATTGCTAACATTTGCTTTTATTATTATTATTATTATTATT 100581
AsianBonytongue                                                                                            15690
Reedfish                                                                                                   51713
NorthernPike                                                                                               20987
SpottedGar                                                                                                 22432
ParamormyropsKingsLeyae                                                                                    13318

Majority                  XXXXXXXXXXXXXXXXXXXXXXXXXXXXXXXXXXXXXXXXXXXXXXXXXXXXXXXXXXXXXXXXXXXXXXXXXXXXXXXX
                                101370    101380    101390    101400    101410    101420    101430    101440
Human                     TTAGACACAGTCTTGCTCTGTCACCCAGGCTGGAGTGCGGTGGTATTATCTCGGCTCACTGCAACCTCCACCTCTCGGGT 100661
AsianBonytongue                                                                                            15690
Reedfish                                                                                                   51713
NorthernPike                                                                                               20987
SpottedGar                                                                                                 22432
ParamormyropsKingsLeyae                                                                                    13318

Majority                  XXXXXXXXXXXXXXXXXXXXXXXXXXXXXXXXXXXXXXXXXXXXXXXXXXXXXXXXXXXXXXXXXXXXXXXXXXXXXXXX
                                101450    101460    101470    101480    101490    101500    101510    101520
Human                     TCAAGCAATTCTCCTGCCTCAGCCTTCCGAGTAGCTGGAATTACAGGCACCCACCACCACACCCAGCTAATTTTTGTATT 100741
AsianBonytongue                                                                                            15690
Reedfish                                                                                                   51713
NorthernPike                                                                                               20987
SpottedGar                                                                                                 22432
ParamormyropsKingsLeyae                                                                                    13318

Majority                  XXXXXXXXXXXXXXXXXXXXXXXXXXXXXXXXXXXXXXXXXXXXXXXXXXXXXXXXXXXXXXXXXXXXXXXXXXXXXXXX
                                101530    101540    101550    101560    101570    101580    101590    101600
Human                     TTTAGTAGAGACAGGGTTTTACCATGTGACCAGGCTGGTTTCGAACTCCTGACCTCAAATGATCCACCTGCCTCAGCCTC 100821
AsianBonytongue                                                                                            15690
Reedfish                                                                                                   51713
NorthernPike                                                                                               20987
SpottedGar                                                                                                 22432
ParamormyropsKingsLeyae                                                                                    13318

Majority                  XXXXXXXXXXXXXXXXXXXXXXXXXXXXXXXXXXXXXXXXXXXXXXXXXXXXXXXXXXXXXXXXXXXXXXXXXXXXXXXX
                                101610    101620    101630    101640    101650    101660    101670    101680
Human                     CCAAACTGCTGGGATTACAGGTGTGAGCCACCGCTCCCAGCCTATTTGCTAATATTTAACCTCTTGAGAGTCTTTAATTC 100901
AsianBonytongue                                                                                            15690
Reedfish                                                                                                   51713
NorthernPike                                                                                               20987
SpottedGar                                                                                                 22432
ParamormyropsKingsLeyae                                                                                    13318

Majority                  XXXXXXXXXXXXXXXXXXXXXXXXXXXXXXXXXXXXXXXXXXXXXXXXXXXXXXXXXXXXXXXXXXXXXXXXXXXXXXXX
                                101690    101700    101710    101720    101730    101740    101750    101760
Human                     TTTTTTTCAACAAGTGTTCATTACCTGCTATGTGCCAGCTTAGATGCTTTGGATGAAATACAGGTTGGTGCGAAAGTAAT 100981
AsianBonytongue                                                                                            15690
Reedfish                                                                                                   51713
NorthernPike                                                                                               20987
SpottedGar                                                                                                 22432
ParamormyropsKingsLeyae                                                                                    13318
```

```
Majority                  XXXXXXXXXXXXXXXXXXXXXXXXXXXXXXXXXXXXXXXXXXXXXXXXXXXXXXXXXXXXXXXXXXXXXXXXXXXXXXXX

                                   101770    101780    101790    101800    101810    101820    101830    101840

Human                     TGCGATTTTTGCCGTTAAAATTAGAAAAACCACAATTACTTTTGCACCAACCTAAATAACTAAAACACAATTTCTTCCTA 101061
AsianBonytongue                                                                                            15690
Reedfish                                                                                                   51713
NorthernPike                                                                                               20987
SpottedGar                                                                                                 22432
ParamormyropsKingsLeyae                                                                                    13318

Majority                  XXXXXXXXXXXXXXXXXXXXXXXXXXXXXXXXXXXXXXXXXXXXXXXXXXXXXXXXXXXXXXXXXXXXXXXXXXXXXXXX

                                   101850    101860    101870    101880    101890    101900    101910    101920

Human                     TCATGGGGCTTTCAGTTCAGTATGAGATAAGTAAAGAATTATATGGGTTGATAAATTTTCTGGAGGAACTATTGGGTGTT 101141
AsianBonytongue                                                                                            15690
Reedfish                                                                                                   51713
NorthernPike                                                                                               20987
SpottedGar                                                                                                 22432
ParamormyropsKingsLeyae                                                                                    13318

Majority                  XXXXXXXXXXXXXXXXXXXXXXXXXXXXXXXXXXXXXXXXXXXXXXXXXXXXXXXXXXXXXXXXXXXXXXXXXXXXXXXX

                                   101930    101940    101950    101960    101970    101980    101990    102000

Human                     ATTACAGTGTGCTCAATCTTTAGCAGTTAGGAAAGGATTCTTGAAGGTAGTTGTATTACCTGGGTTGGAGGTAAGTGTTG 101221
AsianBonytongue                                                                                            15690
Reedfish                                                                                                   51713
NorthernPike                                                                                               20987
SpottedGar                                                                                                 22432
ParamormyropsKingsLeyae                                                                                    13318

Majority                  XXXXXXXXXXXXXXXXXXXXXXXXXXXXXXXXXXXXXXXXXXXXXXXXXXXXXXXXXXXXXXXXXXXXXXXXXXXXXXXX

                                   102010    102020    102030    102040    102050    102060    102070    102080

Human                     GGGACACAAGTGTTAGAAACAGAACAGTTTGTTTAAAAGTTGGGGACAAAAAAGGACCAGAATCAAGATTACGGCACCCT 101301
AsianBonytongue                                                                                            15690
Reedfish                                                                                                   51713
NorthernPike                                                                                               20987
SpottedGar                                                                                                 22432
ParamormyropsKingsLeyae                                                                                    13318

Majority                  XXXXXXXXXXXXXXXXXXXXXXXXXXXXXXXXXXXXXXXXXXXXXXXXXXXXXXXXXXXXXXXXXXXXXXXXXXXXXXXX

                                   102090    102100    102110    102120    102130    102140    102150    102160

Human                     GGAGAAAGTTAAGCATGGCTCGGTCATAAATTAGGAGACAAGGAACAAATAGAGAAATACGTTTGGATAGAATAGTCAGA 101381
AsianBonytongue                                                                                            15690
Reedfish                                                                                                   51713
NorthernPike                                                                                               20987
SpottedGar                                                                                                 22432
ParamormyropsKingsLeyae                                                                                    13318

Majority                  XXXXXXXXXXXXXXXXXXXXXXXXXXXXXXXXXXXXXXXXXXXXXXXXXXXXXXXXXXXXXXXXXXXXXXXXXXXXXXXX

                                   102170    102180    102190    102200    102210    102220    102230    102240

Human                     TTATAGGCTGTGCTAAGTTGTTTGGGCTCCACTAGGGTTTCTAAATGCAGTTATTGGTAAACTGGACTTAGGAGAAGGGA 101461
AsianBonytongue                                                                                            15690
Reedfish                                                                                                   51713
NorthernPike                                                                                               20987
SpottedGar                                                                                                 22432
ParamormyropsKingsLeyae                                                                                    13318
```

```
Majority                    XXXXXXXXXXXXXXXXXXXXXXXXXXXXXXXXXXXXXXXXXXXXXXXXXXXXXXXXXXXXXXXXXXXXXXXXXXXXXXXX

                                     102250    102260    102270    102280    102290    102300    102310    102320

Human                       CAGGGTTGACATGACAAATCTAGTGAATTTATGTTCATTAAAATATCCCTTTTGAATTGATTTAGTTTTTGTATGTTTCT 101541
AsianBonytongue                                                                                              15690
Reedfish                                                                                                     51713
NorthernPike                                                                                                 20987
SpottedGar                                                                                                   22432
ParamormyropsKingsLeyae                                                                                      13318

Majority                    XXXXXXXXXXXXXXXXXXXXXXXXXXXXXXXXXXXXXXXXXXXXXXXXXXXXXXXXXXXXXXXXXXXXXXXXXXXXXXXX

                                     102330    102340    102350    102360    102370    102380    102390    102400

Human                       TTTTTATAATTCCAAAAAATCATACAAATAAGTTAAACTTATCCATCTAAAAGCTGCACACAAAACTTGATGTTAGATTC 101621
AsianBonytongue                                                                                              15690
Reedfish                                                                                                     51713
NorthernPike                                                                                                 20987
SpottedGar                                                                                                   22432
ParamormyropsKingsLeyae                                                                                      13318

Majority                    XXXXXXXXXXXXXXXXXXXXXXXXXXXXXXXXXXXXXXXXXXXXXXXXXXXXXXXXXXXXXXXXXXXXXXXXXXXXXXXX

                                     102410    102420    102430    102440    102450    102460    102470    102480

Human                       TTTTAAAATTACAAAATTAAGACTTCCAAAAGAATCTTGTTGAATAAGAGTAAAAGAAAGACCCTTACTCAGTAAAGCTG 101701
AsianBonytongue                                                                                              15690
Reedfish                                                                                                     51713
NorthernPike                                                                                                 20987
SpottedGar                                                                                                   22432
ParamormyropsKingsLeyae                                                                                      13318

Majority                    XXXXXXXXXXXXXXXXXXXXXXXXXXXXXXXXXXXXXXXXXXXXXXXXXXXXXXXXXXXXXXXXXXXXXXXXXXXXXXXX

                                     102490    102500    102510    102520    102530    102540    102550    102560

Human                       CGTAATACTTTGGCAGTTTGAACCCATGGCCATGCCTCTCTGTGTGATAAGGATTTGGCAAAACCATGGAAGAAACATGC 101781
AsianBonytongue                                                                                              15690
Reedfish                                                                                                     51713
NorthernPike                                                                                                 20987
SpottedGar                                                                                                   22432
ParamormyropsKingsLeyae                                                                                      13318

Majority                    XXXXXXXXXXXXXXXXXXXXXXXXXXXXXXXXXXXXXXXXXXXXXXXXXXXXXXXXXXXXXXXXXXXXXXXXXXXXXXXX

                                     102570    102580    102590    102600    102610    102620    102630    102640

Human                       ATGTTTTTGAATTGTCTTTCCATGGTAACTTAAAACATTTTTTCATGAAACATGGATATATATCCTTAGAAAACTGCAAA 101861
AsianBonytongue                                                                                              15690
Reedfish                                                                                                     51713
NorthernPike                                                                                                 20987
SpottedGar                                                                                                   22432
ParamormyropsKingsLeyae                                                                                      13318

Majority                    XXXXXXXXXXXXXXXXXXXXXXXXXXXXXXXXXXXXXXXXXXXXXXXXXXXXXXXXXXXXXXXXXXXXXXXXXXXXXXXX

                                     102650    102660    102670    102680    102690    102700    102710    102720

Human                       ACATAAAGCTTGTAAGTTATAAGACAAGATTTATCTCTTTTTTTTTTTTTTTTCTGAGAGAGTCTTGCTCTGTTGCCCAG 101941
AsianBonytongue                                                                                              15690
Reedfish                                                                                                     51713
NorthernPike                                                                                                 20987
SpottedGar                                                                                                   22432
ParamormyropsKingsLeyae                                                                                      13318
```

```
Majority                   XXXXXXXXXXXXXXXXXXXXXXXXXXXXXXXXXXXXXXXXXXXXXXXXXXXXXXXXXXXXXXXXXXXXXXXXXXXXXXXX
                                  102730    102740    102750    102760    102770    102780    102790    102800
Human                      GCCGGAGTGCAACAGTGTGATCTCTGCTCACTGCACCCTCCGCCTCCCGGGTTCAAGCAATTCTCCTGCCTCAGCCTCCT 102021
AsianBonytongue                                                                                             15690
Reedfish                                                                                                    51713
NorthernPike                                                                                                20987
SpottedGar                                                                                                  22432
ParamormyropsKingsLeyae                                                                                     13318

Majority                   XXXXXXXXXXXXXXXXXXXXXXXXXXXXXXXXXXXXXXXXXXXXXXXXXXXXXXXXXXXXXXXXXXXXXXXXXXXXXXXX
                                  102810    102820    102830    102840    102850    102860    102870    102880
Human                      GAGTAGCTGGGACTACAGGCGTGTGCCGCCAAGCCCAGCTAATTTTTTTGTATCTTTAGTAGAGACGGTTTCACCATGTT 102101
AsianBonytongue                                                                                             15690
Reedfish                                                                                                    51713
NorthernPike                                                                                                20987
SpottedGar                                                                                                  22432
ParamormyropsKingsLeyae                                                                                     13318

Majority                   XXXXXXXXXXXXXXXXXXXXXXXXXXXXXXXXXXXXXXXXXXXXXXXXXXXXXXXXXXXXXXXXXXXXXXXXXXXXXXXX
                                  102890    102900    102910    102920    102930    102940    102950    102960
Human                      GGCCAGGCTGGTCTCGAACTCCTGACCTCAAGTGATCTACCTGCCTCAGCCTTCTAAAGTGTTGGGATTACAGGCATGAG 102181
AsianBonytongue                                                                                             15690
Reedfish                                                                                                    51713
NorthernPike                                                                                                20987
SpottedGar                                                                                                  22432
ParamormyropsKingsLeyae                                                                                     13318

Majority                   XXXXXXXXXXXXXXXXXXXXXXXXXXXXXXXXXXXXXXXXXXXXXXXXXXXXXXXXXXXXXXXXXXXXXXXXXXXXXXXX
                                  102970    102980    102990    103000    103010    103020    103030    103040
Human                      CCATGGCACCCGGCCAAGATGAAATGTCAAGGCCATTTTAGCCACTATGCTTTTAAGACTACTTAAAGCTGTGCTTTCAG 102261
AsianBonytongue                                                                                             15690
Reedfish                                                                                                    51713
NorthernPike                                                                                                20987
SpottedGar                                                                                                  22432
ParamormyropsKingsLeyae                                                                                     13318

Majority                   XXXXXXXXXXXXXXXXXXXXXXXXXXXXXXXXXXXXXXXXXXXXXXXXXXXXXXXXXXXXXXXXXXXXXXXXXXXXXXXX
                                  103050    103060    103070    103080    103090    103100    103110    103120
Human                      GTCTCCCCAGGAATACTTGTTCACTCTATACTATTGCTGCTGCTGTTCTGGCTTGAGAATTTTGAGAATCACTAAGATAC 102341
AsianBonytongue                                                                                             15690
Reedfish                                                                                                    51713
NorthernPike                                                                                                20987
SpottedGar                                                                                                  22432
ParamormyropsKingsLeyae                                                                                     13318

Majority                   XXXXXXXXXXXXXXXXXXXXXXXXXXXXXXXXXXXXXXXXXXXXXXXXXXXXXXXXXXXXXXXXXXXXXXXXXXXXXXXX
                                  103130    103140    103150    103160    103170    103180    103190    103200
Human                      AATTTTAAGTTTTGACATGTAAATTATTTGGTTGAGTAGAAAGAATTAATGTAGATAAGCAAAAACCCCTTTATAAAAAA 102421
AsianBonytongue                                                                                             15690
Reedfish                                                                                                    51713
NorthernPike                                                                                                20987
SpottedGar                                                                                                  22432
ParamormyropsKingsLeyae                                                                                     13318
```

```
Majority                 XXXXXXXXXXXXXXXXXXXXXXXXXXXXXXXXXXXXXXXXXXXXXXXXXXXXXXXXXXXXXXXXXXXXXXXXXXXXXXXX

                                  103210    103220    103230    103240    103250    103260    103270    103280

Human                    TGCTTACCCTTTAAAAAATGTTGAATCCTCACAGTATGATTAAATACTACTTGTAAAAGTGTCTGTTAACCATAATGTAT 102501
AsianBonytongue                                                                                           15690
Reedfish                                                                                                  51713
NorthernPike                                                                                              20987
SpottedGar                                                                                                22432
ParamormyropsKingsLeyae                                                                                   13318
```

```
Majority                 XXXXXXXXXXXXXXXXXXXXXXXXXXXXXXXXXXXXXXXXXXXXXXXXXXXXXXXXXXXXXXXXXXXXXXXXXXXXXXXX

                                  103290    103300    103310    103320    103330    103340    103350    103360

Human                    TTTTTAGATAATTTGAGTAGAATTATACGTGCAATATATTCTTGAGTTTGTGACCAACAAAGAAACAGATTAATGATTGA 102581
AsianBonytongue                                                                                           15690
Reedfish                                                                                                  51713
NorthernPike                                                                                              20987
SpottedGar                                                                                                22432
ParamormyropsKingsLeyae                                                                                   13318
```

```
Majority                 XXXXXXXXXXXXXXXXXXXXXXXXXXXXXXXXXXXXXXXXXXXXXXXXXXXXXXXXXXXXXXXXXXXXXXXXXXXXXXXX

                                  103370    103380    103390    103400    103410    103420    103430    103440

Human                    AAATTCACCATACATTACTACCTATGTCTCTCCACCTGCTATCTACTTCCAATTTTTTATATATATAAAAGTCATTAAAG 102661
AsianBonytongue                                                                                           15690
Reedfish                                                                                                  51713
NorthernPike                                                                                              20987
SpottedGar                                                                                                22432
ParamormyropsKingsLeyae                                                                                   13318
```

```
Majority                 XXXXXXXXXXXXXXXXXXXXXXXXXXXXXXXXXXXXXXXXXXXXXXXXXXXXXXXXXXXXXXXXXXXXXXXXXXXXXXXX

                                  103450    103460    103470    103480    103490    103500    103510    103520

Human                    TCATTTGTAATTGAGTTTGTTTTTTTTTCTTGAGACAGGGTCTCACTCTGTCACCCAGGCTGGAGTGCAAATGGCATGAT 102741
AsianBonytongue                                                                                           15690
Reedfish                                                                                                  51713
NorthernPike                                                                                              20987
SpottedGar                                                                                                22432
ParamormyropsKingsLeyae                                                                                   13318
```

```
Majority                 XXXXXXXXXXXXXXXXXXXXXXXXXXXXXXXXXXXXXXXXXXXXXXXXXXXXXXXXXXXXXXXXXXXXXXXXXXXXXXXX

                                  103530    103540    103550    103560    103570    103580    103590    103600

Human                    CACAGCTCACTGCAGCCTCAACCTCCCAGGTTCGATAGATCCTCCCACCTCAGTCCCCCACAGGCATGCATCACCATGCC 102821
AsianBonytongue                                                                                           15690
Reedfish                                                                                                  51713
NorthernPike                                                                                              20987
SpottedGar                                                                                                22432
ParamormyropsKingsLeyae                                                                                   13318
```

```
Majority                 XXXXXXXXXXXXXXXXXXXXXXXXXXXXXXXXXXXXXXXXXXXXXXXXXXXXXXXXXXXXXXXXXXXXXXXXXXXXXXXX

                                  103610    103620    103630    103640    103650    103660    103670    103680

Human                    TGACTAGTTTTTGTGTTTTTTTGTAGAGACAAGGTTTTGCCATGTTGCCCAGACTGGTCTCAAACTTGGGCCAAAGCCAT 102901
AsianBonytongue                                                                                           15690
Reedfish                                                                                                  51713
NorthernPike                                                                                              20987
SpottedGar                                                                                                22432
ParamormyropsKingsLeyae                                                                                   13318
```

```
Majority                  XXXXXXXXXXXXXXXXXXXXXXXXXXXXXXXXXXXXXXXXXXXXXXXXXXXXXXXXXXXXXXXXXXXXXXXXXXXXXXXX

                                103690    103700    103710    103720    103730    103740    103750    103760

Human                     CCACCTGCCTCGGCTTCCCAATGTGCTGGGATTACATGTGTGAGCCACCACACCCAGTCATGAGAATTATTTTAAATCAG 102981
AsianBonytongue                                                                                            15690
Reedfish                                                                                                   51713
NorthernPike                                                                                               20987
SpottedGar                                                                                                 22432
ParamormyropsKingsLeyae                                                                                    13318

Majority                  XXXXXXXXXXXXXXXXXXXXXXXXXXXXXXXXXXXXXXXXXXXXXXXXXXXXXXXXXXXXXXXXXXXXXXXXXXXXXXXX

                                103770    103780    103790    103800    103810    103820    103830    103840

Human                     GTAAACAAAATGTCTGTGGGAATATTGAGCTTTATGATAGATACCTAAGTTTTCCCAAAAATAATTGTGTTAGCATTTCA 103061
AsianBonytongue                                                                                            15690
Reedfish                                                                                                   51713
NorthernPike                                                                                               20987
SpottedGar                                                                                                 22432
ParamormyropsKingsLeyae                                                                                    13318

Majority                  XXXXXXXXXXXXXXXXXXXXXXXXXXXXXXXXXXXXXXXXXXXXXXXXXXXXXXXXXXXXXXXXXXXXXXXXXXXXXXXX

                                103850    103860    103870    103880    103890    103900    103910    103920

Human                     GTGAATCACTATACTTAACCTAGAAATCTAGGTTACCAATAACAGTCTCTTTCAGGTTTTTTTCTTTTCCTGTGTCTTTC 103141
AsianBonytongue                                                                                            15690
Reedfish                                                                                                   51713
NorthernPike                                                                                               20987
SpottedGar                                                                                                 22432
ParamormyropsKingsLeyae                                                                                    13318

Majority                  XXXXXXXXXXXXXXXXXXXXXXXXXXXXXXXXXXXXXXXXXXXXXXXXXXXXXXXXXXXXXXXXXXXXXXXXXXXXXXXX

                                103930    103940    103950    103960    103970    103980    103990    104000

Human                     TTTTTACTAAAATCATTCATAACTGGGATGACTTATGGAGTTGATACCTTAGGTCAAATCCCAATTTAGAAACTCCCAAA 103221
AsianBonytongue                                                                                            15690
Reedfish                                                                                                   51713
NorthernPike                                                                                               20987
SpottedGar                                                                                                 22432
ParamormyropsKingsLeyae                                                                                    13318

Majority                  XXXXXXXXXXXXXXXXXXXXXXXXXXXXXXXXXXXXXXXXXXXXXXXXXXXXX

                                104010    104020    104030    104040    104050

Human                     AGCCTGTCAAATTTTGACACATACTTTAAATGAACTGTTATTGAGTAGATATA                            103274
AsianBonytongue                                                                                            15690
Reedfish                                                                                                   51713
NorthernPike                                                                                               20987
SpottedGar                                                                                                 22432
ParamormyropsKingsLeyae                                                                                    13318
```
