# Supplementary material for: PTBP2 – a gene with relevance for both Anorexia nervosa and body weight regulation
Source: Transl Psychiatry. 2022 Jun 9;12:241. doi: 10.1038/s41398-022-02018-5 (PMC9184595; doi:10.1038/s41398-022-02018-5)
Supplement: Supplementary file 1 — gDNA alignment report (Primate) [file 41398_2022_2018_MOESM1_ESM.pdf]

```
Majority         --------------------------------------------------------------------------------
                          10        20        30        40        50        60        70        80
Human            -------------------------------------------------------------------------------- 0
Coquerel'sSifaka -------------------------------------------------------------------------------- 0
Ma'sNightMonkey  -------------------------------------------------------------------------------- 0
VervetAGM        -------------------------------------------------------------------------------- 0
Gorrilla         -------------------------------------------------------------------------------- 0
GrabEatingMacaque-------------------------------------------------------------------------------- 0
Macaque          -------------------------------------------------------------------------------- 0
PigTailedMacaque GCTTAAATAAGCAAGGAGATTAACTACTGTATGTTTGTGGTTTTGTACTGTATTAGGAAAAAAATAAAGAAAAATGTGTT 80
Bonobo           -------------------------------------------------------------------------------- 0
OliveBaboon      -------------------------------------------------------------------------------- 0
Orangutan        -------------------------------------------------------------------------------- 0

Majority         --------------------------------------------------------------------------------
                          90       100       110       120       130       140       150       160
Human            -------------------------------------------------------------------------------- 0
Coquerel'sSifaka -------------------------------------------------------------------------------- 0
Ma'sNightMonkey  -------------------------------------------------------------------------------- 0
VervetAGM        -------------------------------------------------------------TTGTCTTTGCTAAGGTAAA 19
Gorrilla         ------------------------------------------------------------------TTTGCCAAGATAAA 14
GrabEatingMacaque-------------------------------------------------------------------------------- 0
Macaque          -------------------------------------------------------------------------------- 0
PigTailedMacaque TTTGTGAACACTGTAATTTCTTCTGCTGCCTAAGGACAAGCTTCCCTCCTCATAGAATGGTTTGTCTTTGCTAAGGTAAA 160
Bonobo           -------------------------------------------------------------------------------- 0
OliveBaboon      -------------------------------------------------------------------------------- 0
Orangutan        -------------------------------------------------------------------------------- 0

Majority         --------------------------------------------------------------AGTTCC-GCCCTTTGGAA
                         170       180       190       200       210       220       230       240
Human            -------------------------------------------------------------------------------- 0
Coquerel'sSifaka --------------------------------------------------------------------------AGGCCC 6
Ma'sNightMonkey  -------------------------------------------------------------------------------- 0
VervetAGM        GTGAGAACCAATCAATCAATATTGATCAAGCGAATGTGAGTAACACCGAGAATTAATACCATAGTTCCTGCCCTTTGGAA 99
Gorrilla         GTGAGAACCAATCAATCAATATCGATCGAGCGAATGTGGGGAATACCGAGAATTAATACCATAGTTCCGGCCCTTTGGTA 94
GrabEatingMacaque------------------------------------------------------AATACCATAGTTCCTGCCCTTTGGAA 26
Macaque          --------------------------------------------------------------AGTTCCTGCCCTTTGGAA 18
PigTailedMacaque GTGAGAACCAATCAATCAATATTGATCAAGCGAATGTGAGTAATACCGAGAATTAATACCATAGTTCCTGCCCTTTGGAA 240
Bonobo           -----------------------------------------AATACCGAGAATTAATACCATAGTTCCCGCCCTTTGGTA 39
OliveBaboon      -------------------------------------------------------------------------------- 0
Orangutan        -------------------------------------------------------------------------------- 0
```

```
Majority          GCTTGCATATTTAAGTGTTGAGTGAAAACACATGTACGAAACCACAA-TATG--AAAAGTGATTATTA-ATGGCACCAAT
                           250       260       270       280       290       300       310       320
Human             -------------------------------------------------------------------------------- 0
Coquerel'sSifaka  TATTTTTAATCCTATTTTCAGTAAAGGAAACTTTTTCCAGGAAACATTAATGAACATACCTAATGTCACACAGTTATTAA 86
Ma'sNightMonkey   ---------CTGACCTCGTGATCCACCCGCCTTGGCCTCCTAAAGTGCTGGGCTTACAGGCGTGAGCT-ACAGCGCCCAG 70
VervetAGM         GCTTGCATATTTAAGTGTTGAGTGAAAACACATGTACGAAACCACAA-CATGTGAAAAGTGATTATTA-ATGGCACCAAT 177
Gorrilla          GCTTGCACATTTAATTGTTGAGTGAAAACACATGTATGAAACCACAAATATGTGAAAAGTGATTATTA-GTGGCACTAAT 173
GrabEatingMacaqueGCTTGCATATTTAAGTGTTGAGTGAAAACACATGTACGAAACCACAA-TATG--AAAAGTGATTATTA-ATGGCACCAAT 102
Macaque           GCTTGCATATTTAAGTGTTGAGTGAAAACACATGTACGAAACCACAA-TATG--AAAAGTGATTATTA-ATGGCACCAAT 94
PigTailedMacaque  GCTTGCATATTTAAGTGTTGAGTGAAAACACATGTACGAAACCACAA-TATG--AAAAGTGATTATTA-ATGGCACCAA- 315
Bonobo            GCTTGCACATTCAATTGTTGAGTGAAAACACATGTATGAAACCACAAATATGTGAAAAGTGATTATTA-GTGGCACTAAT 118
OliveBaboon       -------------GACACAGCGAGACTCCGTCTCAAAAAAAAAAAAA-AAAG--AAAAGTGATTATTA-ATGGCACCAAT 63
Orangutan         --------------------------------------------------TGTGAAAAGTGATTATTA-GTGGCACTAAT 29

Majority          AACAGATCCTTTATTTTAAGGTTTAAGGTGAAG-----------------------------------------------
                           330       340       350       360       370       380       390       400
Human             -------------------------------------------------------------------------------- 0
Coquerel'sSifaka  ATTAAAGAGCTCAAATCTGAACCCAAACTTAAATCAAATATTCCCTGTATCTCTCCAGTTGTGTATACTTTGTTGCATTA 166
Ma'sNightMonkey   CCCGAAGACTCTATTTTCAG---CAAAAGAAAGT-----------------------------------------TTCCA 106
VervetAGM         AACAGATCCTTTATTTTAAGGTTTAAGGTGAAG----------------------------------------------- 210
Gorrilla          AACAGATCCTTTATTTTAAGGTTTAAGGTGAAG----------------------------------------------- 206
GrabEatingMacaqueAACAGATCCTTTATTTTAAGGTTTAAGGTGAAG----------------------------------------------- 135
Macaque           AACAGATCCTTTATTTTAAGGTTTAAGGTGAAG----------------------------------------------- 127
PigTailedMacaque  AACAGATCCTTTATTTTAAGGTTTAAGGTGAAG----------------------------------------------- 348
Bonobo            AACAGATCCTTTATTTTAAGGTTTAAGGTGAAGTGGTTATTGTAGGAAATTTGATATGTGGTTTTTTTTTTTNTTTTTTT 198
OliveBaboon       AACAGATCCTTTATTTTAAGGTTTAAGGTGAAG----------------------------------------------- 96
Orangutan         AACAGAACCTTTATTTTAAGGTTTAAGGTGAAG----------------------------------------------- 62

Majority          ------G-GGGTTATTGTAGGAAATTTGACGTGTGTGTGTGTTTGTGTGTGTG------T----------------TTTC
                           410       420       430       440       450       460       470       480
Human             -------------------------------------------------------------------------------- 0
Coquerel'sSifaka  GCCTCAATAGACTCATGTTATGC-TTTGTCCTCTTGTTTTATTCTTATTTCACTTCATTTTTTTAATCTAGTACTTTATA 245
Ma'sNightMonkey   AGA---G-AGGTTAATG-AACATGTCTAATGTCACACAGGTATTAAATGAAAG-----------A-----------GCTC 159
VervetAGM         ------G-GGGTTATTGTAGGAAATTTGATGTGTGTGTGTG--T---------------T----------------TT-C 249
Gorrilla          --------GGGTTATTGTAGGAAATTTGATATGTGGTTTTTTTTTTGTTTTT------------------------TTTT 254
GrabEatingMacaque------G-GGGTTATTGTAGGAAATTTGACGTGTGTGTGTGTGTGTGTGTGTGTGTGTGTGTGTGT------TTTC 202
Macaque           ------G-GGGTTATTGTAGGAAATTTGACGTGTGTGTGTGTGTGTGTGTGTGTGTGTGTGTGTGTGTGTGTGTGTTTTC 200
PigTailedMacaque  ------G-GGGTTATTGTATGAAATTTGACGTGTGTGTGTGTGTGTGTGTGTGTGTGTGTGTGTGTGT------TTTC 415
Bonobo            GTTTTTA-GGNNNNNNNNNNNNNNNNNNNNNNNNNNNNNNNNNNNNNNNNNN------------------------NNNN 253
OliveBaboon       ------G-GGATTATTGTAGGAAATTTGACGTGTGTGTGTGTGTGTGTGTGTGTGT--------------TTTC 155
Orangutan         ------G-GGGTTATTGTAGGCAATTTGATATGTGTTTTTTTTTGTTTGTTT------------------------GTTT 111
```

```
Majority          TTTCTTGAGGCG-GAGTTTCACTCTTGTTGCCCAGGCTGGAGTGCAATGGCACTATCTCGGCTCACCGCAACCTCCGCCT

                          490       500       510       520       530       540       550       560

Human             -------------------------------------------------------------------------------- 0
Coquerel'sSifaka  TGCCTTAACTTGTAAACTCCCTTACATCTTTTCTAGAAAGTGTTGGCTTACATAAACAAGCATATTAACTATTGCATGTT 325
Ma'sNightMonkey   AAATCTGAACCC-AGGCTCTAATCAATATGCTTCTGT--GTACACTGTTGCGTTAGCCTCATTCCTGACTCATGCTATGC 236
VervetAGM         TTTCTTGAGGCG-GAGTTTCGCTCTTGTTGCCCAGGCTGGAGTGCAATGGCACTATCTCGGCTCACCGCAACCTCCGCCT 328
Gorrilla          TTTTTTGAGGCA-GAGTTTCGCTCTTGTTGCCCAGGCTGGAGTGCAATGGCGTGATCTTGGCTCACCGCAACCTCGGCCT 333
GrabEatingMacaqueTTTCTTGAGGCG-GAGTTTCACTC--GTTGCCCAGGCTGGAGTGCAATGGCACTATCTCGGCTCACCGCAACCTCCGCCT 279
Macaque           TTTCTTGAGGCG-GAGTTTCACTC--GTTGCCCAGGCTGGAGTGCAATGGCACTATCTCGGCTCACCGCAACCTCCGCCT 277
PigTailedMacaque  TTTCTTGAGGCG-GAGTTTCACTCTTGTTGCCCAGGCTGGAGTGCAATGGCACTATCTCGGCTCACCGCAACCTCCGCCT 494
Bonobo            NNNNNNNNNNNN-NNNNNNNNNNNNNNNNNNNNNNNNNNNNNNNNNNNNNNNNNNNNNNNNNNNNNNNNNNNNNNNNNNN 332
OliveBaboon       TTTCTTGAGGCG-GAGTTTCACTCTTGTTGCCCAGGCTGGAGTGCAATGGCACTATCTCGGCTCACCGCAACCTCCGCCT 234
Orangutan         TTTTTTGAGACA-GAGTTTCGCTCTTGTTGCCCAGGCTGGAGTGCAATGGCGCGATCTTGGCTCACCGCAACCTCGGCCT 190

Majority          CCTGGGTTCAAGCGATTCTCCTGCCTCAG-CCTCCCGAGTAGCTGGGATTACAGGCATGCACCACCACCCTGGCTAATTT

                          570       580       590       600       610       620       630       640

Human             -------------------------------------------------------------------------------- 0
Coquerel'sSifaka  TGTAGTCTCGTACTTTATTAGGAGGAAAAAAAAAGTGTGTTTTTGTGAACACTATAATATCTT-CTGCCACAGAAGGACA 404
Ma'sNightMonkey   TTTGTCCTCTTGTTTTATTCTTGTTTCA--CTTCTTGTTTTAATCTAGTACTTTATGTGCTTTGACTTGTTAACTA-CCT 313
VervetAGM         CATGGGTTCAAGCGATTCTCCTGCCTCAG-CCTCCCGAGTAGCTGGGATTACAGGCATGCACCACCACCCTGGCTAATTT 407
Gorrilla          CCCAGGTTCAAGCGATTCTCCTGCCTCAG-CCTCCCGAGTAGCTGGGATTACAGACATGCACCACCATCCCGGCTAATTT 412
GrabEatingMacaqueCCTGGGTTCAAGCGATTCTCCTGCCTCAG-CCTGCCGAGTAGCTGGGATTACAGGCATGCACCACCACCCTGGCTAATTT 358
Macaque           CCTGGGTTCAAGCGATTCTCCTGCCTCAG-CCTCCCGAGTAGCTGGGATTACAGGCATGCACCACCACCCTGGCTAATTT 356
PigTailedMacaque  CCTGGGTTCAAGCGATTCTCCTGCCTCAG-CCTCCCGAGTAGCTGGGATTACAGGCATGCACCACCACCCTGGCTAATTT 573
Bonobo            NNNNNNNNNNNNNNNNNNNNNNNNNNNNN-NNNNNNNNNNNNNNNNNNNNNNNNNNNNNNNNNNNNNNNNNNNNNNNNNN 411
OliveBaboon       CCTGGGTTCAAGCGATTCTCCTGCCTCAG-CCTCCCGAGTAGCTGGGATTACAGGCATGCACCACCACCCTGGCTAATTT 313
Orangutan         CCCGGGTTCAAGCGATTCTCCTGGCTCAG-CCTCCCGAGTAGCAGGGATTACAGACATGCACCACCACCCCAGCTAATTT 269

Majority          TGTATTTTTAGTAGAAACGTGGTTTCTCCATTTT-GGTCAGGCTGGTCTTGAACTCCCGACCTCAGGTGATCCACTTGCC

                          650       660       670       680       690       700       710       720

Human             -------------------------------------------------------------------------------- 0
Coquerel'sSifaka  GGCTTCCCCCCTCATGGAACAGTTTCTCTTTGCCAAGATAGAATGACAACCAATCAAAATCCACCAAGTGAATGTGAGGA 484
Ma'sNightMonkey   TGCATTTTTTCAAGAAA-GTGTTATCTTAA-AT--AAGCAAGCAGATTAACTATTGTATGTTTGTGGTCTTGTACT-GTG 388
VervetAGM         TGTATTTTTAGTAGAAACGTGGTTTCTCCATTTT-GGTCAGGCTGGTCTTGAACTCCCGACCTCAGGTGATCCACTTGCC 486
Gorrilla          TGTATTTTTAGTAGAGACGGGGTTTCTCCATGTTTGGTCAGGCTGGTCTCGAACTCCCGAGCTCAGGTGATCTGCTTGCC 492
GrabEatingMacaqueTGTATTTTTAGTAGAAACGTGGTTTCTCCATTTT-GGTCAGGCTGGTCTTGAACTCCCGACCTCAGGTGATCCACTTGCC 437
Macaque           TGTATTTTTAGTAGAAACGTGGTTTCTCCATTTT-GGTCAGGCTGGTCTTGAACTCCCGACCTCAGGTGATCCACTTGCC 435
PigTailedMacaque  TGTATTTTTAGTAGAGACGTGGTTTCTCCATTTT-GGTCAGGCTGGTCTTGAACTCCCGACCTCAGGTGATCCACTTGCC 652
Bonobo            NNNNNNNNNNNNNNNNNNNNNNNNNNNNNNNNNNNNNNNNNNNNNNNNNNNNNNNNNNNNNNNNNNNNNNNNNNNNNNNN 491
OliveBaboon       TGTATTTTTAGTAGAAACGTGGTTTCTCCATTTT-GGTCAGGCTGGTCTTGAACTCCCGACCTCAGGTGATCCACTTGCC 392
Orangutan         TGTATGTTTAGTAGAGACGGGGTTTCCCCATGTT-GGTCAGGCTGGTCTTGAACTCCCGACCTCAGGTGATCTGCTTGCC 348
```

```
Majority          TCAGCCTCCCAAAGTGCTGGCATTACAGG----AGTGAGCCACCGCTCCTGGTCTTT-----------------------
                           730       740       750       760       770       780       790       800
Human             -------------------------------------------------------------------------------- 0
Coquerel'sSifaka  ATACAGAGAATTAATGCTATAGTTCCTGTGCTTTGGTAGCTTACATATTTAATTGTTAAGTGAAAATACACGTACAAGCT 564
Ma'sNightMonkey   TTAGAAAAAAAAAAAAAAAGAAAAATGTG----TGAACACTGATATTTCTTCTGCTAAGGACAAGCTTCCCTCTCA---- 460
VervetAGM         TCAGCCTCCCAAAGTGCTGGCATTACAGG----AGTCAGCCAGCGCTACCTGTCTTT----------------------- 539
Gorrilla          TCGGCCTCCCAAAATGCTGGGATTACAGG----CGGGAGCCACCGTGCCCGGCCTTTTTTTTTTTTTTTTTTTTTTTTTT 568
GrabEatingMacaque TCAGCCTCCCAAAGTGCTGGCATTACAGG----AGTGAGTCACCGCTCCTGGTCTTT----------------------- 490
Macaque           TCAGCCTCCCAAAGTGCTGGCATTACAGG----AGTGAGTCACCGCTCCTGGTCTTT----------------------- 488
PigTailedMacaque  TCAGCCTCCCAAAGTGCTGGCATTACAGG----AGTGAGCCACCGCTCCCGGTCTTT----------------------- 705
Bonobo            NNNNNNNNNNNNNNNNNNNNNNNNNNNNN----NNNNNNNNNNNNNNNNNNNNNNNNNNNNNNNNNNNNNNNNNNNNNNNNN 567
OliveBaboon       TCAGCCTCCCAAAGTGCTGGCATTACAGG----AGTGAGCCACCGCTCCTGGTCTTT----------------------- 445
Orangutan         TCGGCCTCCCAAAGTGCTGGGATTACAGG----CGTGAGCCACTGTGCCCGGCATTT------T---------------- 402

Majority          ---T-------TTTTTTTTT--------GACAATCCCATAGGCAAA-TCAATAATTTGTAAAAATGTGGGTGCTTTGAAG
                           810       820       830       840       850       860       870       880
Human             -------------------------------------------------------------------------------- 0
Coquerel'sSifaka  GGGCGTGGCGGCTAACACCTGTAATCCTAGCACTTTGGAAGGCCAAGGTAGGAGGAGGGCTTGAGCCCAGGAGTTTGAGG 644
Ma'sNightMonkey   ---TAGAACGGTTTGTCTTTGCCA-A--GACAAAGTGAGAAGCAA--TCAATCAATACTGATGGAGCGAATG--TGGGGA 530
VervetAGM         ---T-------TTTTTTTTTTTTTTTTTGACAATCCCATAGGCAAA-TCAATAATTTGTAAAAATGTGGGTGCTTTGAAG 608
Gorrilla          T--TTTTTTTTTTTTTTTTTTAAGGATTGACAATCCCATAGGCAAA-TCAAGAATTTGTAAAAATTTGGGTGGTTTGAAG 645
GrabEatingMacaque ---T-------TTTTTTT----------GACAATCCCATAGGCAAA-TCAATAATTTGTAAAAATGTGGGTGCTTTGAAG 549
Macaque           ---T-------TTTTTTTT---------GACAATCCCATAGGCAAA-TCAATAATTTGTAAAAATGTGGGTGCTTTGAAG 548
PigTailedMacaque  ---T-------TTTTTTTTTTT------GACAATCCCATAGGCAAA-TCAATAATTTGTAAAAATGTGGGTGCTTTGAAG 768
Bonobo            N--NNNNNNNNNNNNNNNNNNNNNNNNNGACAATCCCATAGGCAAA-TCAAGAATTTGTAAAAATTTGGGTGCTTTGAAG 644
OliveBaboon       ---T-------TTTTTTTT---------GACAATCCCATAGGCAAA-TCAATAATTTGTAAAAATGTGGGTGCTTTGAAG 505
Orangutan         -----TTTGTTTGTTTGTTTGAAGGATTGACAATCCCATAGGCAAA-TCAAGAATTTGTAAAAATTTGGGTGCTTTGAAG 476

Majority          --AGTTCTATAGCAAAGTGTTTGGACAA-ATGCTCTTTAAGGGTCTA----TCCAATTTAAAAGTAATATTATTTACTGC
                           890       900       910       920       930       940       950       960
Human             -------------------------------------------------------------------------------- 0
Coquerel'sSifaka  TTAAAGCTATGATAATACCACTGCACTCCAGCCCGTGTGACAGAGAGAGACCCTGTCTCAAAAACAAAACAAAACAAAA- 723
Ma'sNightMonkey   --ATACCGAGAATTAATAGCATAGTTCC--TGCCCTTTGGTAGCTTA----CATATTTAATTATTGAGAGAAAACA-G-- 599
VervetAGM         --AGTTCTATAGCAAAGTGTTTGGACAA-ATGTTCTTTAAGTGTCTA----TCCAATTTAAAAGTAATATTATTTACTGC 681
Gorrilla          --AGTTCTGTAGCAAAGTGTTTGGACAAGATGCTCTTTAAGGGTCTA----TCCAATTTAAAAGTAATATTATTTATGGC 719
GrabEatingMacaque --AGTTCTATAGCAAAGTGTTTGGACAA-ATGCTCTTTAAGGGTCTA----TCCAATTTAAAAGTAGTATTATTTACTGC 622
Macaque           --AGTTCTATAGCAAAGTGTTTGGACAA-ATGCTCTTTAAGGGTCTA----TCCAATTTAAAAGTAATATTATTTACTGC 621
PigTailedMacaque  --AGTTCTATAGCAAAGTGTTTGGACAA-ATGTTCTTTAAGGGTCTA----TCCAATTTAAAAGTAATATTATTTACTGC 841
Bonobo            --AGTTCTATAGCAAAGTGTTTGGACAAGATGCTCTTTAAGGGTCTA----TCCAATTTAAAAGTAATATTATTTATGGC 718
OliveBaboon       --AGTTCCATAGCAAAGTGTTTGGACAA-ATGCTCTTTAAGGGTCTA----TCCAATTTAAAAGTAATATTATTTACTGC 578
Orangutan         --AGTTCTATAGCAAAGTGTTTGGACAAGATGTTCTTTAAGGGTCTA----TCCAATTTAAAAATAATATTATTTATGGC 550
```

```
Majority          CGGGCGCGGTGGCTCACAGCTGTAATCCCAGCACTTTGGGAGGCCGAGGAGGGCGGATCACCTGAGGTCCGGAGTTCGAG

                           970       980       990      1000      1010      1020      1030      1040

Human             -------------------------------------------------------------------------------- 0
Coquerel'sSifaka  -AAGTACAAAACCACAAATATGTGAA--AAGTGATTATTAATGGCACTGAAAACAGGTCCTTTATGTATCGTAAGTCAAT 800
Ma'sNightMonkey   -GAGCACAAAACCTCAAATATGTGAA--AGGTGATTATTAATGGCACTAATAACAGAT--CCTTTGTTTTAAGATTTAAG 674
VervetAGM         CGGGCGCGGTGGCTCACAGCTGTAATCCCAGCACTTTGGGAGGCCGAGGAGGGCGGATCACCCGAGGTCCGGAATTCGAG 761
Gorilla           CGGGCACGGTGGCTTACGCCTGTAATCCCAGCACTTTGGGAGGCCGAGGAGGGCGGATCACCTGAGGTCAGGAGTTCGAG 799
GrabEatingMacaque CGGGCGCGGTGGCTCACAGCTGTAATCCCAGCACTTTGGGAGGCCGAGGAGGGCGGATCACCTGAGGTCCGGAGTTCGAG 702
Macaque           CGGGCGCGGTGGCTCATAGCTGTAATCCCAGCACTTTGGGAGGCCGAGGAGGGCGGATCACCTGAGGTCCGGAGTTCGAG 701
PigTailedMacaque  CGGGCGCGGTGGCTCACAGCTGTAATCCCAGCACTTTGGGAGGCCGAGGAGGGCGGATCACCTGAGGTCCGGAGTTCGAG 921
Bonobo            CGGGCACGGTGGCTCACGCCTGTAATCCCAGCACTTTGGGAGGCCGAGGAGGGCGGATCACCTGAGGTCAGGAGTTCGAG 798
OliveBaboon       CGGGCGCGGTGGCTCACAGCTGTAATCCCAGCACTTTGGGAGGCCGAGGAGGGCGGATCACCTGAGGTCCGGAGTTCGAG 658
Orangutan         CGGGCGCGGTGGCTCACGCCTGTAATCCCAGCACTT-GGGAGGCCGAGGAGGGCGGATCACCTGAGGTCAGGAGTTCGAG 629

Majority          ACCGGCCTCA----------------------------------------------------------------------

                          1050      1060      1070      1080      1090      1100      1110      1120

Human             -------------------------------------------------------------------------------- 0
Coquerel'sSifaka  GGTGAAGGTGA--------------------------------------------AGGAGAGGTATTGTAGGAAGTTTGT 836
Ma'sNightMonkey   GTGAAGGGGG----------------------------------------TAATTGTAGGAGATTTGACAGGTGGGTTTT 714
VervetAGM         ACCGGCCTCA---------------------------------------------------------------------- 771
Gorilla           ACCAACCTGACCAACATGGAGAAACCCTGTCTCTACTAAAAATACAAAGTTAGCTGGGCGTGGTGTCACGTGCCTGTAAT 879
GrabEatingMacaque ACCGGCCTCA---------------------------------------------------------------------- 712
Macaque           ACCGGCCTCA---------------------------------------------------------------------- 711
PigTailedMacaque  ACCGGCCTCA---------------------------------------------------------------------- 931
Bonobo            ACCAACCTGACCAACATGGAGAAACCCTGTCTCTACTAAAAATACAAAGTTAGCTGGGCGTGGTGGCGCGTGCCTGTAAT 878
OliveBaboon       ACCGGCCTCA---------------------------------------------------------------------- 668
Orangutan         ACCAACCTGACCAACATGGAGAAACCCTGTCTCTACTAAAAATACAAAATTAGCCGGGCGTGGTGGCGCGTGCCTGTAAT 709

Majority          --------------------------------------------GGAGGCGGAAGTTGCGGTTAGCCGA-GATCGCGACA

                          1130      1140      1150      1160      1170      1180      1190      1200

Human             -------------------------------------------------------------------------------- 0
Coquerel'sSifaka  TTGATTTCCTTTTAAGGACTGACAATCTGTGGGGCAAATCAA-GAATTGTAAACATTTGGGTGCTTTGA-GAGATCCATA 914
Ma'sNightMonkey   TTTTTTTTTTTTTAAGGATTGACAATCCCAGAGGCAAATCAAGAATTTGTAAAAATTTGGGTGCTTTGAAGAGTTCTCTA 794
VervetAGM         --------------------------------------------GGAGGCGGAAGTTGCGGTTAGCCGA-GATCGCGACA 806
Gorilla           CCCAGCTACTCGGGAGGCTGAGGCAGGAGAATCGCTTGAACCAGGGAGGCCGAGGTTGCAGTGAGCCGA-GATTGCGCCA 958
GrabEatingMacaque --------------------------------------------GGAGGAGGAAGTTGCGGTTAGCCGA-GATCGCGACA 747
Macaque           --------------------------------------------GGAGGCGGAAGTTGCGGTTAGCCGA-GATCGCGACA 746
PigTailedMacaque  --------------------------------------------GGAGGCGGAAGTTACGGTTAGCCGA-GATCGCGACA 966
Bonobo            CCCAGCTACTCGGGAGGCTGAGGCAGGAGAATCGCTTGAACCAGGGAGGCCGAGGTTGCAGTGAGCCGA-GATTGCGCCA 957
OliveBaboon       --------------------------------------------GTAGGCGGAAGTTGCGGTTAGCCGA-GATCGCGACA 703
Orangutan         CCCAGCTACTCGGGAGGCTGAGGCAGGAGAATCGCTTGAACCCGGGATGCCGAGGTTGCAGTGAGCCGA-GATTGCGCCA 788
```

```
Majority          TTGCACTCCAGCCCGGGCAACAAGAGC---GAGACTC--------AAAAATATATATGTATTTTAAGAATTATATAGAA-

                           1210      1220      1230      1240      1250      1260      1270      1280

Human             -------------------------------------------------------------------------------- 0
Coquerel'sSifaka  CCAAAGT---ATTTGGACAAGATGCTCTTCAAGGTTTTGTTCAATTTAAAGTAAT----ATTTTAAAAATTATACAGACA 987
Ma'sNightMonkey   ACAAAGT---GTCTGGACAAGATGCTCTTTAAGGGTCTATCCAATTTAAAAGTAATATTATTTTAAGAATTATACAGAA- 870
VervetAGM         TTGCACTCCAGCCCAGGCAACAAGAGC---GAAACTC--------AAAAATATATATGTATTTTAAGAATTATATAGAA- 874
Gorrilla          TTGCACTTCAGCCCGGGCAACAAGAGC---GAGACTCGGTCTCAAAAAAAAAAATA----TTCTAAAAATTATACACAA- 1030
GrabEatingMacaqueTTGCACTCCAGCCCAGGCAACAAGAGC---GAAACTC--------AAAAATATATATGTATTTTAAGAATTATATAGAC- 815
Macaque           TTGCACTCCAGCCCAGGCAACAAGAGC---GAAACTC--------AAAAATATATATGTATTTTAAGAATTATATAGAC- 814
PigTailedMacaque  TTGCACTCCAGCCCAGGCAACAAGAGT---GAAACTC--------AAAAATATATATGTATTTTAAGAATTATATAGAC- 1034
Bonobo            TTGCACTTCAGCCTGGGCAACAAGAGC---AAGACTCGGTCTACAAAAAAAAAAT------TCTAAAAATTATACACAA- 1027
OliveBaboon       TTGCACTCCAGCCCAGGCAACAAGAGC---GAAACTC--------AAAAATATATATGTATTTTAAGAATTATATAGAA- 771
Orangutan         TTGCACTTCAGCCTGGGCAACAAGAGA---GAGACTACGTCTCAAAAAAAAAAAAAAAAATTCTAAGAATTATACAGAA- 864

Majority          -TATAATTTAGTATAGTGAAATTCCAATTAGTAAAGGATAAGCGCTTTGCCTGAAGGAGATATGGAAAAACCCAGAGT--

                           1290      1300      1310      1320      1330      1340      1350      1360

Human             -------------------------------------------------------------------------------- 0
Coquerel'sSifaka  CTACAATTTAGTATGGTGAAATTCCAATTAGTAAAGAGTAAGAGGTTTGTCTGAAGGAGATCTGGGAAAACACAGAGTGG 1067
Ma'sNightMonkey   -TACCATTTGATATAGTGAAATTCCAATTAGTAAAGGGTGAGAGCTTTGCCTGAAGGAGATGTGAGAAACCCCGGAGTGA 949
VervetAGM         -TATAATTTAGCATAGTGAAATTCCAATTAGTAAAGTATAAGCGCTTTGCCTGAAGAAGATATGGAAAAACCCAGAGT-- 951
Gorrilla          -TATCATTTAGTATA-TGAAATTCCAATTAGCAAAGGATAAGAGCTCTGCCTGAAGGAGATACGGAAAAACCCAGAGTGA 1108
GrabEatingMacaque-TATAATTTAGTATAGTGAAATTCCAATTAGTAAAGGATAAGCGCTTTGCCTGAAGAAGATATGGAAAAACCCAGAGT-- 892
Macaque           -TATAATTTAGTATAGTGAAATTCCAATTAGTAAAGGATAAGCGCTTTGCCTGAAGAAGATATGGAAAAACCCAGAGT-- 891
PigTailedMacaque  -TATAATTTAGTATAGTGAAATTCCAATTAGTAAAGGATAAGCGCTTTGCCTGAAGAAGATATGGAAAAACCCAGAGT-- 1111
Bonobo            -TATCATTTAGTATA-TGAAATTCCAATTAGCAAAGGATAAGAGCTTTGCCTGAAGGAGATACAGAAAAACCCAGAGTGA 1105
OliveBaboon       -TATAATTTAGTATACTGAAATTCCAATTAGTAAAGGATAAGCGCTTTGCCTGAAGAAGATATGGAAAAACCCAGAGT-- 848
Orangutan         -TATCATTTAGTATAGTGAAATTCCAATTAGCAAAGGATAAGAGCTTTGCCTGAAGGAGACACGGAAAAACCCAGAGTGA 943

Majority          --ATTGAGAGTAACTGGAGGGACACAGGGAGTATTTGTCATTTTTCAGAATTATAAGTGCCTTTGTGCTTGTGTGTGTTG

                           1370      1380      1390      1400      1410      1420      1430      1440

Human             -------------------------------------------------------------------------------- 0
Coquerel'sSifaka  GTAAGGAGAACAACTGGAGAGAAATACTGGGAATATTTGTAATTTCTCAGGACTGGAAACACGTTTGTGTGC-------CGG 1140
Ma'sNightMonkey   GTATTGAGAGTAACTGGAGGGACGCAGGG-GTATTTGTCATTTTTCTGGATTAGAAGTGCATTTGTGCTTGTG--TGTTG 1026
VervetAGM         --ATTGAGAGTAACTGGAGGGACACAGGGAGTATTTGTCATTTTTCAGAATTATAAGTGCCTTTGTGCTT------GTTG 1023
Gorrilla          GTATTGAGAGTAACTGGAGGGACACAGGGAGTATTTGTCATTTTTCAGAATTAGAAGTGCATTTGTGCTTGTGTGCGTTG 1188
GrabEatingMacaque--ATTGAGAGTAACTGGAGGGACACAGGGAGTATTTGTCATTTTTCAGAATTATAAGTGCCTTTGTGCTTGTGTGTGTTG 970
Macaque           --ATTGAGAGTAACTGGAGGGACACAGGGAGTATTTGTCATTTTTCAGAATTATAAGTGCCTTTGTGCTTGTGTGTGTTG 969
PigTailedMacaque  --ATTGAGAGTAACTGGAGGGACACAGGGAGTATTTGTCATTTTTCAGAATTATAAGTGCCTTTGTGCTTGTGTGTGTTG 1189
Bonobo            GTATTGAGAGTAACTGGAGGGACACAGGGAGTATTTGTCATTTTTCAGAATTAGAAGTGCATTTGTGCTTGTGAGTGTTG 1185
OliveBaboon       --ATTGAGAGTAACTGGAGGGACACAGGGAGTATATGTCATTTTTCAGAATTATAAGTGCCTTTGTGCTTGTGTGTGTTG 926
Orangutan         GTATTGAGAGTAACTGGAGGGACACAGGAAGTATTTGTCATTTTTCAGAATTAGAAGTGCATTTGTGCTTGTGTATGTTG 1023
```

```
Majority         GGGGTGGAGGTGAAGGGTGCAGGAGAAGGATGGGTACTGGCAAAGTCGTTACTCACTGAGCAGATTTTCAAGAAGTGGGT

                          1450      1460      1470      1480      1490      1500      1510      1520

Human            -------------------------------------------------------------------------------- 0
Coquerel'sSifaka GAGGTGG--GTGAAAGGTGCATAA--AGGAAACGTGCTGGCAAAGAAGTCACCCACTGAGCAAATTTTCCAGAAATGGTT 1216
Ma'sNightMonkey  GGGGTGGAAGTGAAGGGTGCAGGAGAAGGATGGATACTGGCAAAGTCATTACTCACTGAGCACATTTTCAAGAAGTGGGT 1106
VervetAGM        GGGGTGGAGGTGAAGGGTGCAGGAGAAGGAAGGGTACTGGCAAAGTCGTTACTCACTGAGCAGATTTTCAAGAAGTAGGT 1103
Gorilla          GGGGTGGAGGTGAAGGGTGCAGGAGAAGGATGGGTACTGGCAAAGTCGCTACTCACTGAGCAAATTTTCAAG---CGGGT 1265
GrabEatingMacaqueGGGGTGGAGGTGAAGGGTGCAGGAGAAGGATGGGTACTGGCAAAGTCGTTACTCACTGAGCAGATTTTCAAGAAGTAGGT 1050
Macaque          GGGGTGGAGGTGAAGGGTGCAGGAGAAGGATGGGTACTGGCAAAGTCGTTACTCACTGAGCAGATTTTCAAGAAGTAGGT 1049
PigTailedMacaque GGGGTGGAGGTGAAGGGTGCAGGA---GGATGGGTACTGGCAAAGTCGTTACTCACTGAGCAGATTTTCAAGAAGTAGGT 1266
Bonobo           GGG-TGGAGGTGAAGGGTGCAGGAGAAGGATGGGTACTGGCAAAGTCGCTACTCACTGAGCAAATTTTCAAG---CGGGT 1261
OliveBaboon      GGGGTGGAGGTGAAGGGTGCAGGAGAAGGATGGGTACTGGCAAAGTCGTTACTCACTGAGCAGATTTTCAAGAAGTAGGT 1006
Orangutan        GGGGTGGAGGTGAAGGGTGCAGGAGAAGGATGGGTACTGGCAAAGTCGTTACTCACTGAACAAATTTTCAAG---CGGGT 1100
```

```
Majority         GCCTCTCCCAATTTTGTTGAGCCTCTGGTAAAAACACGTTAAAGTAAAATGTCCACTATAGTATCAAGTAAAAATTGAGA

                          1530      1540      1550      1560      1570      1580      1590      1600

Human            -------------------------------------------------------------------------------- 0
Coquerel'sSifaka GCCTTTTCAAACTTTGTTGAGCCTCTGACAAAAA---------------TGT-----------TAAAATTAAAATTGAGA 1270
Ma'sNightMonkey  TCCCCTCTCGATTTTATTGAGCCTCTGATAAAAACACCTCAAGGTAAAATGTCCA--------TCAAGTTAAAATTGAGA 1178
VervetAGM        GCCTCTCCCAATTTTGTTGAGCCTCTGGTAAAAACACGTTAAAGTAAAATGTCCACTATAGTATCAAGTAAAAATTGAGA 1183
Gorilla          GCCTTTCCCAATTTTGTTGAGCCTCTGATAAAAACACGTTGAAGTAAAATATCCACTATAGTGTCAAGTAAAAATTGAGA 1345
GrabEatingMacaqueGCCTCTCCCAATTTTGTTGAGCCTCTGGTAAAAACACTTTAAAGTAAAATGTCCACTATAGTATCAAGTAAAAATTGAGA 1130
Macaque          GCCTCTCCCAATTTTGTTGAGCCTCTGGTAAAAACACTTTAAAGTAAAATGTCCACTATAGTATCAAGTAAAAATTGAGA 1129
PigTailedMacaque GCCTCTCCCAATTTTGTTGAGCCTCTGGTAAAAACACTTTAAAGTAAAATGTCCACTATAGTGTCAAGTAAAAATTGAGA 1346
Bonobo           GCCTTTCCCAATTTTGTTGAGCGTCTGATAAAAACACGTTGAAGTAAAATGTCCACTATAGTATCAAGTAAAAATTGAGA 1341
OliveBaboon      GCCTCTCCCAATGTTGTTGAGCCTCTGGTAAAAACACGTTAAAGTAAAATGTCCACTATAGTATCAAGTAAAAATTGAGA 1086
Orangutan        GCCTCTCCCAATTTTGTTGAGCCTCTGATAAAAACACGTTAAAGTAAAATGTCCACTATAGTATCAAGTAAAAATTGAGA 1180
```

```
Majority         CCGTAAGATAT---AGCAAAGGGAGTCAATTTGCATAGCCCATTTTAATGCTTCTACCTTCTCTAAACAATCTG--AAGA

                          1610      1620      1630      1640      1650      1660      1670      1680

Human            -------------------------------------------------------------------------------- 0
Coquerel'sSifaka CCGTGAGACATTACAGAAAAGGCAGCCAATTTGTGTGGCCCATTTTAATGTTTCTACCCTCTCTCGTCAATGAGGAAAGA 1350
Ma'sNightMonkey  CTGTAAAATCT---AGCAAAGGGAGTCAATTTGCATAGCCCATTTTAATGCTTCTACTCTCTCTAATCAATCTG--AAGA 1253
VervetAGM        CCGTAAGATAT---AGCAAAGGGAGTCAATTTGCATAGCCCATTTTAATGCTTCTACCTTTTCTAAACAATCTG--AAGA 1258
Gorilla          CCGTAAGATAT---AGCAAAGGGAGTCAATTTGCATAGCCCATTTTAATGCTTCTACTTTCTCTAACCAATCTG--AAGA 1420
GrabEatingMacaqueCCGTAAGATAT---AGCAAAGGGAGTCAATTTGCATAGCCCATTTTAATGCTTCTACCTTCTCCAAACAATCTG--AAGA 1205
Macaque          CCGTAAGATAT---AGCAAAGGGAGTCAATTTGCATAGCCCATTTTAATGCTTCTACCTTCTCTAAACAATCTG--AAGA 1204
PigTailedMacaque CCGTAAGATAT---AGCAAAGGGAGTCAATTTGCATAGCCCATTTTAATGCTTCTACCTTCTCTAAACAATCTG--AAGA 1421
Bonobo           CCGTAAGATAT---AGCAAAGGGAGTCAATTTGCATAGCCCATTTTAATGCTTCTACTTTCTCTAACCAATCTG--AAGA 1416
OliveBaboon      CCGTAAGATAT---AGCAAAGGGAGTCAATTTGCATAGCCCATTTTAATGCTTCTACCTTCTCTAAACAATCTG--AAGA 1161
Orangutan        CCGTAAGATAT---AGCAAAGGGAGTCAATTCGCATAGCCCATTTTAATGCTTCTACTTTCTCTAACCAATCTG--AAGA 1255
```

```
Majority          CTCAGTTGCTCCCACTCCCATCTCCACTTTCCCTTTCATGCCTCCAAATGTTAAA-CTCCAACTATTG-TAGCATTTTAA

                          1690      1700      1710      1720      1730      1740      1750      1760

Human             -------------------------------------TGCCTCCAAATGTTAAA-CTCCAACTATTG-TAGCATTTTAA 40
Coquerel'sSifaka  CTCAGTTGCCTCCACACCCACCCCCACTTTCCCTTTCAAGCCCCCAAATGTTAGC-CTCTAACAATTGGTAGTATTTTAA 1429
Ma'sNightMonkey   CTCAGTTGCTGTCACTCCCATCTCCACTTTCCCTTTCACGCCCCTAAATGTTAAA-TTCCAACTATTGGTAGCATTGCAA 1332
VervetAGM         CTCAGTTGCTCCCACTCCCATCTCCACTTTCCCTTTCATGCCTCCAAATGTTAAA-CTCCAACTATCG-TAGCATTTTAA 1336
Gorrilla          CTTAGTTGTTCCCACTCCCATCTCCACTTTCCCTTTCATGCCTCCAAATGTTAAA-CTCCAACTATTG-TAGCATTTTAA 1498
GrabEatingMacaqueCTCAGTTGCTCCCACTCCCATCTCCACTTTCCCTTTCATGCCTCCAAATGTTAAA-CTCCAACTATCG-TAGCATTTTAA 1283
Macaque           CTCAGTTGCTCCCACTCCCATCTCCACTTTCCCTTTCATGCCTCCAAATGTTAAA-CTCCAACTATCG-TAGCATTTTAA 1282
PigTailedMacaque  CTCAGTTGCTCCCACTCCCATCTCCACTTTCCCTTTCGTGCCTCCAAATGTTAAA-CTCCAACTATCG-TAGCATTTTAA 1499
Bonobo            CTCAGTTGTTCCCACTCCCATCTCCACTTTCCCTTTCATGCCTCCAAATGTTAAAACTCCAACTATTG-TAGCATTTTAA 1495
OliveBaboon       CTCAGTTGCTCCCACTCCCATCTCCACTTTCCCTTTCATGCCTCCAAATGTTAAA-CTCCAACTATCG-TAGCATTTTAA 1239
Orangutan         CTCAGTTGTTCCCACTCCCATCTCCACTTTCCCTTTCATGCCTCCAAATGTTAAA-CTCCAACTATTG-TAGCATTTTAA 1333
```

```
Majority          CACATCGCTCCATTTGTGTCTATTT--CCACCTAGGGTATTGTGAATATTTTCTACAGGTGCACACTAATCACCAACTGA

                          1770      1780      1790      1800      1810      1820      1830      1840

Human             CACATGGCTCCATTTGTGTCTATTT--CCACCTAGCGTATTGTGAATATTTTCTACAGGCGCACACTAATCACCAACTGA 118
Coquerel'sSifaka  CACACCGTTCAACCTCTGTCCGTTT--CCACCGAGGGTAGTGTGAATACTTTCTACTGGTGCATAGTAATCA---ACTCA 1504
Ma'sNightMonkey   CACATCGCTCCATTTGTATCCGTTTTTCCACCCAGGGTATTGCGAATATTTTCTACAGGTGCACACTAATCACCAACTGA 1412
VervetAGM         CACATCGCTCCATTTGTGTCTATTT--CCACCTAGGGTATCGTCAATATTTTCTACAGTTGTACACTAATCACCAACTGA 1414
Gorrilla          CACATGGCTCCATTTGTGTCTATTT--CCACCTAGCGTATTGTGAATATTTTCTACAGGCGCACACTAATCACCAACTGA 1576
GrabEatingMacaqueCACATCGCTCCATTTGTGTCTATTT--CCACCTAGGGTATCGTGAATATTTTCTACAGTTGCACACTAATCACCAACTGA 1361
Macaque           CACATCGCTCCATTTGTGTCTATTT--CCACGTAGGGTATCGTGAATATTTTCTACAGTTGCACACTAATCACCAACTGA 1360
PigTailedMacaque  CACATCGCTCCATTTCTGTCTATTT--CCACCTAGGGTATCGTGAATATTTTCTACAGTTGCACACTAATCACCAACTGA 1577
Bonobo            CACATGGCTCCATTTGTGTCTATTT--CCACCTAGCGTATTGTGAATATTTTCTACAGGCGCACACTAATCACCAACTGA 1573
OliveBaboon       CACATCGCTCCATTTGTGTCTATTT--CCACCTAGGGTATCGTGAATATTTTCTACAGTTGCACACTAATCACCAACTGA 1317
Orangutan         CACATGGCTCCATTTGTGTCTATTT--CCACCTAGCGTATTGTGAATATTTTCTACAGGCGCACACTAATCACCAACTGA 1411
```

```
Majority          AGTGAGAATCACATTTTCCTCCACTTGGCTTGGTACTACAGGTCTTGAAGCAAAAGCCT-ATATTTCAGTCTGAGGAGTT

                          1850      1860      1870      1880      1890      1900      1910      1920

Human             AGTGAGAATCACATTTTCCTCCACTTGGCTTGGTACTACAGGTCTTGAAGCAAAAGCCT-GTATTTCAGTCTGAGGAGTT 197
Coquerel'sSifaka  GGTGAGAATCACATTTTT-TCTCTTTGGCGTGGTACTACAGGTCTGGAAGCAAAAGCCT-GTATTTCAGTCTGATGAGTT 1582
Ma'sNightMonkey   AGTGAGAATCACATTTTCCTCCACTTGGCTTGGTACTACAGGTCTTGAAGCAAAAGGCT-GCATTTCAGTCTGAGGAGTC 1491
VervetAGM         AGTGAGGATCACATTTTCCTCCACGTGGCTTGGTACTACAGGTCTTGAAGCAAAAGCCTTATATTTCAGTCTGAGGAGTT 1494
Gorrilla          AGTGAGAATCACATTTTCCTCCACTTGGCTTGGTACTACAGGTCTTGAAGCAAAAGCCT-ATATTTCAGTCTGAGGAGTT 1655
GrabEatingMacaqueAGTGAGGATCACATTTTCCTCCACGTGGCTTGGTACTACAGGTCTTGAAGCAAAAGCCTTATATTTCAGTCTGAGGAGTT 1441
Macaque           AGTGAGGATCACATTTTCCTCCACGTGGCTTGGTACTACAGGTCTTGAAGCAAAAGCCTTATATTTCAGTCTGAGGAGTT 1440
PigTailedMacaque  AGTGAGGATCACATTTTCCTCCACGTGGCTTGATACTACAGGTCTTGAAGTAAAAGCCTTATATTTCAGTCTGAGGAGTT 1657
Bonobo            AGTGAGAATCACATTTTCCTCCACTTGGCTTGGTACTACAGGTCTTGAAGCAAAAGCCT-GTATTTCAGTCTGAGGAGTT 1652
OliveBaboon       AGTGAGGATCACATTTTCCTCCACGTGGCTTGGTACTACAAGTCTTGAAGCAAAAGCCTTATATTTCAGTCTGAGGAGTT 1397
Orangutan         AGTGAGAATCACATTTTCCTCCACTTGGCTTGGTACTACAGGTCTTGAAGCAAAAGCCT-ATATTTCAGTCTGAGGAGTT 1490
```

```
Majority          CTACTTGCTTGAGAAAGTACTGTTGTTACCAAG---TGGTCTTGCTCAGTGGGGTAAAGATTTTTGTTTTAGAGGCTGGA
                           1930      1940      1950      1960      1970      1980      1990      2000
Human             CTACTTGCTTGAGAAAGTACTGTTGTTACCAAG---TGGTCTCGTTCAGTGGGGTAAAGATTTTTGTTTTAGAGGCTGGA 274
Coquerel'sSifaka  CTACTGGCATTAGAAAGTACTGTTTTTACTAAGCAGTGGTCTTGTTCAATGAGATAAAGATTTTTGTTTTTGTGCATGGA 1662
Ma'sNightMonkey   CTACTTGCTTGAGAAAGTACTGTTTCTACCAAG---TGGTCTTGCTCAATGGGGTAAAGGTTTTTGTTTTAGAGTTTGGA 1568
VervetAGM         CTACTTGCTTGAGAAAGTACTGTTGTTACCAAG---TGGTCTTGCTCAGTGGGGTAAAGATTTTTGTTTTAGAGGCTGGA 1571
Gorrilla          CTACTTGCTTGAGAAAGTACTGTTGTTACCAAG---TGGTCTCGTTCAGTGGGGTAAAGATTTTTGTTTTAGAGGCTGGA 1732
GrabEatingMacaqueCTACTTGCTTGAGAAAGTACTGTTGTTACCAAG---TGGTCTTGCTCAGTGGGGTAAAGATTTTTGTTTTAGAGGCCGGA 1518
Macaque           CTACTTGCTTGAGAAAGTAGTGTTGTTACCAAG---TGGTCTTGCTCAGTGGGGTAAAGATTTTTGTTTTAGAGGCCGGA 1517
PigTailedMacaque  CTACTTGCTTGAGAAAGTACTGTTGTTACCAAG---TGGTCTTGCTCAGTGGGGTAAAGATTTTTGTTTTAGAGGCCGGA 1734
Bonobo            CTACTTGCTTGAGAAAGTACTGTTGTTACCAAG---TGGTCTCGTTCAGTGGGGTAAAGATTTTTGTTTTAGAGGCTGGA 1729
OliveBaboon       CTACTTGCTTGAGAAAGTACTGTTGTTACCAAG---TGGTCTTGCTCAGTGGGGTAAAGATTTTTGTTTTAGAGGCCGGA 1474
Orangutan         CTACTTGCTTGAGAAAGTACTGTTGTTACCAAG---TGGTCTTGTTCAGTGGGGTAAAGATTTTTGTTTTAGAGGCTGGA 1567

Majority          GCCGGTCTGCAAATAAAAGATGGCTTAGGAGAAAAGCTATCCAATGTTTTTT-CCTTGGCCCGCATAGGTTGACGGTAGC
                           2010      2020      2030      2040      2050      2060      2070      2080
Human             GCCGGTCTGCAAATAAAAGATGGCTTAGGAGAAAAGCTATCCAATGTTTTTT-CCTTGGCCCGCATAGGTTGACGGTAGC 353
Coquerel'sSifaka  GTGGGTTTGC--ATAAAAGATGGCATAGGAGGAAAGCTATAAATTTTTTTTTTCCTTGGCCCACTTAGGTCGAGGGTAGC 1740
Ma'sNightMonkey   GTCGGTCTGCAAATAAAAGATGGCTTAGGAGAA--GCTATCCAATTTTTTTCCCTTGGCCCGCATAGGTTGACGGCAGA 1646
VervetAGM         GCCAGTCTGCAAATAAAAGATGGCTTAGGAGAAAAGCTATCCAATGTTTTTTTCCTTGGCCCGCATAGGTTGACGGTAGC 1651
Gorrilla          GCCGGTCTGCAAATAAAAGATGGCTTAGGAGAAAAGCTATCCAGTGTTTTTT-CCTTGGCCCGCATAGGTTGACGGTAGC 1811
GrabEatingMacaqueGCCAGTCTGCAAATAAAAGATGGCTTAGGAGAAAAGCTATCCAACGTTTTTT-CCTTGGCCCGCATAGGTTGACGGTAGC 1597
Macaque           GCCAGTCTGCAAATAAAAGATGGCTTAGGAGAAAAGCTATCCAACGTTTTTT-CCTTGGCCCGCATAGGTTGACGGTAGC 1596
PigTailedMacaque  GCCAGTCTGCAAATAAAAGATGGCTTAGGAGAAAAGCTATCCAACGTTTTTT-CCTTGGCCCGCATAGGTTGACGGTAGC 1813
Bonobo            GCCGGTCTGCAAATAAAAGATGGCTTAGGAGAAAAGCTATCCAATGTTTTTT-CGTTGGCCCGCATAGGTTGACGGTAGC 1808
OliveBaboon       GCCAGTCTGCAAATAAAAGATGGCTTAGGAGAAAAGCTATCCAACGTTTTTT-CCTTGGCCCGCATAGGTTGACGGTAGC 1553
Orangutan         GCCGGTCTGCAAATAAAAGATGGCTTAGGAGAAAAGCTATCCAATGTTTTTT-CCTTGGCCCGCATAGGTTGACGGTAGT 1646

Majority          CAAGCTGGTACTTGGCTTGGAGTGAAAAGTGGAGGCTGCTTGGGGGCCGTCCTGCAGTCAGGGC----------------
                           2090      2100      2110      2120      2130      2140      2150      2160
Human             CAAGCTGGTACTTGGCTTGGAGTGAAAAGTGGAGGCTGCCTGGGGGC-GTCCTGCAGTCAGGGC---------------- 416
Coquerel'sSifaka  CAAGCTGGTACTTGGATTGGAGGGAAAAGTGGAGGCTGCCTGGGGGCAGTGCTTCAGCCCCAGGGCCACCGTCCTGAGGT 1820
Ma'sNightMonkey   CAAGTTGGTACTTGGCTTGGAGGGAAAAGTGGAGGCTGCTTGGGGCCCGTCCCGTAGTCAGGGCCATCCCCCTCAGGTCC 1726
VervetAGM         CAAGCTGGTACTTGGCGTGGAGTGAAAAGTGGAGGCTGCTTGGGGGCCGTCCTGCAGTCAGGGA---------------- 1715
Gorrilla          CAAGCTGGTACTTGGCTTGGAGTGAAAAGTGGAGGCTGCCTGGGGGC-GTCCTGCAGTCAGGGC---------------- 1874
GrabEatingMacaqueCAAGCTGGTACTTGGCGTGGAGTGAAAAGTGGAGGCTGCTTAGGGGCCGTCCTGCAGTCAGGGA---------------- 1661
Macaque           CAAGCTGGTACTTGGCGTGGAGTGAAAAGTGGAGGCTGCTTAGGGGCCGTCCTGCAGTCAGGGA---------------- 1660
PigTailedMacaque  CAAGCTGGTACTTGGCGTGGAGTGAAAAGTGGAGGCTGCTTAGGGGCCGTCCTGCAGTCAGGAA---------------- 1877
Bonobo            CAAGCTGGTATTTGGCTTGGAGTGAAAAGTGGAGGCTGCCTGGGGGC-GTCCTGCAGTCAGGGC---------------- 1871
OliveBaboon       CAAGCTGGTACTTGCCGTGGAGTGAAAAGTGGAGGCTGCTTGGGGGCCGTCCTGCAGTCTGGGA---------------- 1617
Orangutan         CAAGCTGGTACTTGGCTTGGAGTGAAAAGTGGAGGCTGCTTGGGGGC-GTCCTGCAGTCAGGGC---------------- 1709
```

```
Majority         -----AGAGACAGTGTGGAGGGCTGTTGGGAGGTGTCCCACCCACGCCCCCC---AAGGAGGGGAAACCAGGACGCCGCT

                          2170      2180      2190      2200      2210      2220      2230      2240

Human            -----AGAGAGAGTGTGGAGAGCTGTTGGGAGGTGTCCCACCCACGCCCCCAC--AAGGAGGGGAAACCAGGTCGCCGCG 489
Coquerel'sSifaka CCAAGGCAGACGGTGTAGAGG---GTTGGGAGGTGTCCCACTCACACCGCTC---AGGGAGGTGAACCCAGGACGCCACG 1894
Ma'sNightMonkey  AGGGCAGATAGAGCGTAGAGTGCTGTTGGGAGGT-TCCCACCCACACCCCTC---AAGGAGGGGAAACCAGGACGCCGGA 1802
VervetAGM        -----AGAGACAGTGTGGAGGGCTGTTGGGAGGTGTCCCACCCACGCCCCCC---AAGGAGTGGAAACCAGGACGCCGCT 1787
Gorrilla         -----AGAGAGAGTGTGGAGAGCTGTTGGGAGGTGTCCCACCCACGCCCCCCCCCAAGGAGGGGAAACCAGGACGCCGCG 1949
GrabEatingMacaque-----AGAGACAGTGTGGAGGGCTGTTGGGAGGTGTCCCACCCACGCCCCTC---AAGGAGTGGAAACCAGAACGCCGCT 1733
Macaque          -----AGAGACAGTGTGGAGGGCTGTTGGGAGGTGTCCCACCCACGCCCCTC---AAGGAGTGGAAACCAGAACGCCGCT 1732
PigTailedMacaque -----AGAGACAGTGTGGAGGGCTGTTGGGAGGTGTCCCACCCACGCCCCCC---AAGGAGTGGAAACCAGAACGCCGCT 1949
Bonobo           -----AGAGAGAGTGTGGAGAGCTGTTGGGAGGTGTCCCACCCACGCCCCCCC--AAGGAGGGGAAACCGGGACGCCGCG 1944
OliveBaboon      -----AGAGACAGTGTGGAGGGCTGTTGGGAGGTGTCCCACCCACGCCCCCC---AAGGAGTGGAAACCAGGACGCCGCT 1689
Orangutan        -----ACAGAGAGTGTGGAGAGCTGTTGGGAGGTGTCCCACCCACGCTCCCCCC-AAGGAGGGGAAACCGGGACGCCGCG 1783

Majority         GTCTGCAGCAG---CAGGCCAGGTTCCAGAGGGCGG-------CAGGCGGAGGGCGGAGGGAGGAAGGCGC--GCGGAGG

                          2250      2260      2270      2280      2290      2300      2310      2320

Human            GTCTGCAGCAG---AACGCCAGGTTCCAGAGGACGG-------CAGGCGGAGGGCGGAGGGAGAAAGGCGC--GCGAAGG 557
Coquerel'sSifaka CTATGCAGCAGTAGCAGGCCAGGTCCCAGAGAGCGGAGGAAGCTAGGAGCACGGCGGCGCGGAGGAGGCGACGGCGACGG 1974
Ma'sNightMonkey  GTCTGCAGCAG---TAGGCCAGGTTTCAGAGGGCGG-------CAGGCGGAGGGCGGAGGGAGGAAGGCGC--GCGGAGG 1870
VervetAGM        GTCTGCAGCAG---CAGGCCAGGTTCCAGAGGGCGG-------CAGGCGGAGGGCGGAGGGAGGAAGGCGC--TCGGAGG 1855
Gorrilla         GTCTGCAGCAG---AAGGCCAGGTTCCAGAGGACGG-------CAGGCGGAGGGCGGAGGGAGAAAGGCGC--GCGGAAG 2017
GrabEatingMacaqueGTCTGCAGCAG---CAGGCCAGGTTCCAGAGGGCGG-------CAGGCGGAGGGCGGAGGGAGGAAGGCGC--GCGGAGG 1801
Macaque          GTCTGCAGCAG---CAGGCCAGGTTCCAGAGGGCGG-------CAGGCGGAGGGCGGAGGGAGGAAGGCGC--GCGGAGG 1800
PigTailedMacaque GTCTGCAGCAG---CAGGCCAGGTTCCAGAGGGCGG-------CAGGCGGAGGGCGGAGGGAGGAAGGCGC--GCGGAGG 2017
Bonobo           GTCTGCAGCAG---AACGCCAGGTTCCAGAGGANNN-------NNNNNNNNNNNNNNNNN-------------------- 1994
OliveBaboon      GTCTGCAGCAG---CAGGCCAGGTTCCAGAGGGCGG-------CAGGCGGAGGGCGGAGGGAGGAAGGCGC--GCGGAGG 1757
Orangutan        GTCTGCAGCAG---CAGGCCAGGTTCCAGAGGGCGG-------CAGGCGGAGGGCGGAAAGAGGAAGGCGC--GCGGAGG 1851

Majority         CG-CGGAGGAGGCGTCGGCGGCGGCGCCCACTCCCCCTAGTCCCCGCGCCCCGCCGCTCGCCTCCCGCTGCCGGGCGCCG

                          2330      2340      2350      2360      2370      2380      2390      2400

Human            CG-CGGAGGAGGCGTCGGCTGCGGCGCCCACTCCCCCTAGTCCCAGCGCCCTGCCGCTCGCCTCCCGCTGCCGGGCGCCG 636
Coquerel'sSifaka CGGCGGCGGCGGCGGCCGCGGCGGCGCCCACTCCCCCTCGTCCACGCGCCCAGCAGCTCGCCTCCCGCTGCCGGGCGCCG 2054
Ma'sNightMonkey  CG-CGGAGGAGGCGTCGGCGGCGGCGCCCACTCCCCCTAGTCCCCGCGCCCCGCCACTCACCTCCCGCTGCCGGGCGCCG 1949
VervetAGM        CG-CGGAGGTGGCGTCGGCGGCGGCGCCCACTCCCCCTAGTCCCCGCGCCCCGCCGCTCGCCTCCTGCTGCCGGGCGCCG 1934
Gorrilla         CG-CGGAGGAGGCGTCGGCTGCGGCGCCCACTCCCCCTAGTCCCAGCGCCCCGCCGCTCGCCTCCCGCTGCCGGGCGCCG 2096
GrabEatingMacaqueCG-CGGAGGAGGCGTCGGCGGCGGCGCCCACTCCCC-TAGTCCCCGCGCCCCGCCGCTCGCCTCCCGCTGTCGGGCGCCG 1879
Macaque          CG-CGGAGGAGGCGTCGGCGGCGGCGCCCACTCCCCCTAGTCCCCGCGCCCCGCCGCTCGCCTCCCGCTGTCGGGCGCCG 1879
PigTailedMacaque CG-CGGAGGAGGCGTCGGCGGCGGCGCCCACTCCCCCTAGTCCCCGCGCCCCGCCGCTCGCCTCCAGCTGTCGGGCGCCG 2096
Bonobo           ------------------------------------------------------C------------------------- 1995
OliveBaboon      CG-CGGAGGAGGCGTCGGCGGCGGCGCCCACTCCCCCTAGTCCCCGCGCCCCGCCGCTCGCCTCCCGCTGCCGGGCGCCG 1836
Orangutan        CG-CCGAGGAGGCGTCGGCGGCGGCGCCCACTCCCCCTCGTCCCAGCGCCCCGCCGCTCGCCTCCCGCTGCCGGGCGCCG 1930
```

```
Majority          GCGCGCTCGGCTCTTTCCGCCGCCGCCGCTGCCGCGCGCTGCCCCGTGCGCCTCGGCACCTTCGGCAATTTCCGTCGGGC

                          2410      2420      2430      2440      2450      2460      2470      2480

Human             GCGCGCTCGGCTCTTTCCGCCGCCGCCGCTGCCGCGCGGTGGCCCGTGCGCCTCGGCACCTTCGGCAATTTCCGTCGGGC 716
Coquerel'sSifaka  GCGCGCTCGGCTTTTTCCGTCGCCGCCGCTGCCGCGCGCTGCCCCGTGCGCCTCGGCTCGTTCGGCAATTTCCGTCGGGC 2134
Ma'sNightMonkey   GCGCGCTCGGCTCTTTCCGCCGCCGTCGCTGCCGCGCGCTGCCCCGTGCGCCTCGGCACCTTCGGCAATTTCCGTCGGGC 2029
VervetAGM         GCGCGCTCGGCTCTTTCCGCCGCCGCCGCTGCCGCGCGCTGCCCCGTGCGCCTCGGCACCTTCGGCAATTTCCGTCGGGC 2014
Gorrilla          GCGCGCTCGGCTCTTTCCGCCGCCGCTGCTGCCGCGCGGTGGCCCGTGCGCCTCGGCACCTTCGGCAATTTCCGTCGGGC 2176
GrabEatingMacaqueGCGCGCTCGGCTCTTTCCGCCGCCGCCGCTGCCGCGCGCTGCCCCGTGCGCCTCGGCACCTTCGGCAATTTCCGTCGGGC 1959
Macaque           GCGCGCTCGGCTCTTTCCGCCGCCGCCGCTGCCGCGCGCTGCCCCGTGCGCCTCGGCACCTTCGGCAATTTCCGTCGGGC 1959
PigTailedMacaque  GCGCGCTCGGCTCTTTCCGCCGCCGCCGCTGCCGCGCGCTGCCCCGTGCGCTTCGGCACCTTCGGCAATTTCCGTCGGGC 2176
Bonobo            ----------------------------------------------------TCGGCACCTTCGGCAATTTCCGTCGGGC 2023
OliveBaboon       GCGCGCTCGGCTCTTTCCGCCGCCGCCGCTGCCGCGCGCTGCCCCGTGCGCCTCGGCACCTTCGGCAATTTCCGTCGGGC 1916
Orangutan         GCGCGCTCGGCTCTTTCCGCCGCCGCCGCTGCCGCGCGCTGGCCCGTGCGCCTCGGCACCTTCGGCAATTTCCGTCGGGC 2010

Majority          CCCAGCCGCCATTTTCTCGCCGCTTGTGTGGCTCGCTGGCTGCGTGGCTCGGTTCTTGTGAGCGAAGCTTTGTCCGGTTC

                          2490      2500      2510      2520      2530      2540      2550      2560

Human             CCCAGCCGCCATTTTCTCGCCGCTTGTGTGGCTCGCTGGCTGCGTGGCTCGGTTCTTGTGAGCGAAGCTTTGTCCGGTTC 796
Coquerel'sSifaka  CCTAGCCGCCATTTTCTCGCCGCTTGTGTGGGTCGCTGGCTGCGTGGCTCGGTTCTTGTGAGCGAAGCTTTGTCCGGTTC 2214
Ma'sNightMonkey   CCTAGCCGCCATTTTCTCGCCGCTTGTGTGGCTCGCTGGCTGCGTGGCTCGGTTCTTGTGAGCGAAGCTTTGTCCGGTTC 2109
VervetAGM         CCCAGCCGCCATTTTCTCGCCGCTTGTGTGGCTCGCTGGCTGCGTGGCTCGGTTCTTGTGAGCGAAGCTTTGTCCGGTTC 2094
Gorrilla          CCCAGCCGCCATTTTCTCGCCGCTTGTGTGGCTCGCTGGCTGCGTGGCTCGGTTCTTGTGAGCGAAGCTTTGTCCGGTTC 2256
GrabEatingMacaqueCCCAGCCGCCATTTTCTCGCCGCTTGTGTGGCTCGCTGGCTGCGTGGCTCGGTTCTTGTGAGCGAAGCTTTGTCCGGTTC 2039
Macaque           CCCAGCCGCCATTTTCTCGCCGCTTGTGTGGCTCGCTGGCTGCGTGGCTCGGTTCTTGTGAGCGAAGCTTTGTCCGGTTC 2039
PigTailedMacaque  CCCAGCCGCCATTTTCTCGCCGCTTGTGTGGCTCGCTGGCTGCGTGGCTCGGTTCTTGTGAGCGAAGCTTTGTCCGGTTC 2256
Bonobo            CCCAGCCGCCATTTTCTCGCCGCTTGTGTGGCTCGCTGGCTGCGTGGCTCGGTTCTTGTGAGCGAAGCTTTGTCCGGTTC 2103
OliveBaboon       CCCAGCCGCCATTTTCTCGCCGCTTGTGTGGCTCGCTGGCTGCGTGGCTCGGTTCTTGTGAGCGAAGCTTTGTCCGGTTC 1996
Orangutan         CCCAGCCGCCATTTTCTCGCCGCTTGTGTGGCTCGCTGGCTGCGTGGCTCGGTTCTTGTGAGCGAAGCTTTGTCCGGTTC 2090

Majority          GGCAATGGACGGGTATGTAATCGGGCCGGCGAGAAGGTGTGTGTGAGAGAGGAGTTGGACCGTCCTTCGGCCCTGTCCCG

                          2570      2580      2590      2600      2610      2620      2630      2640

Human             GGCAATGGACGGGTATGTAATCGGGCCGGCGAGAAGGTGTGTGTGAGAGAGGAGTTGGACCGTCCTTCGGCCCGGTCCCG 876
Coquerel'sSifaka  GGCAATGGACGGGTATGTAATCGGGCCGGCGAAGAGGTGTGTGTGGGAGAGGAGTTGGACTGTCCTTCCGTCCCGTCCGG 2294
Ma'sNightMonkey   GGCAATGGACGGGTATGTAATCGGGCCGGCGAGAAGGTGTGTGTGAGAAAGGAGTTGGACAGTCCTTCGGCCCGATCCCG 2189
VervetAGM         GGCAATGGACGGGTATGTAATCGGGCCGGCGAGAAGGTGTGTGTGAGAGAGGAGTTGGACCGTCCTTCGGGCCTGTCCCG 2174
Gorrilla          GGCAATGGACGGGTATGTAATCGGGCCGGCGAGAAGGTGTGTGTGAGAGAGGAGTTGGACCGTCCTTCGGCCCGGTCCCG 2336
GrabEatingMacaqueGGCAATGGACGGGTATGTAATCGGGTCGGCGAGAAGGTGTGTGTGAGAGAGGAGTTGGACCGTCCTTCGGCCCTATCCCG 2119
Macaque           GGCAATGGACGGGTATGTAATCGGGCCGGCGAGAAGGTGTGTGTGAGAGAGGAGTTGGACCGTCCTTCGGCCCTATCCCG 2119
PigTailedMacaque  GGCAATGGACGGGTATGTAATCGGGCCGGCGAGAAGGTGTGTGTGAGAGAGGAGTTGGACCGTCCTTCGGCCCTATCCCG 2336
Bonobo            GGCAATGGACGGGTATGTAATCGGGCCGGCGAGAAGGTGTGTGTGAGAGAGGAGTTGGACCGTCCTTCGGCCCGGTCCCG 2183
OliveBaboon       GGCAATGGACGGGTATGTAATCGGGCCGGCGAGAAGGTGTGTGTGAGAGAGGAGTTGGACCGTCCTTCGGCCCTATCCCG 2076
Orangutan         GGCAATGGACGGGTATGTAATCGGGCCGGCGAGAAGGTGTGTGTGAGAGAGGAGTTGGACTGTCCTTCGGCCCGGTCCCG 2170
```

```
Majority          GGCCGGGGAGAAACCCTCCCGCGGCCCCTCCCAGGGCTGGGCTGCCGTTACCCAACCCCCGCCCCATCGCACACACCCCT

                          2650      2660      2670      2680      2690      2700      2710      2720

Human             GGCCGGGGAGAAACCCTCCCGCGGCCCCTCCCAGGGCTGGGCTGCCGTTACCCAACCCCCGCCCCATCGCACACACCCCT 956
Coquerel'sSifaka  GATCGGGCAGAAACCCTTCAGCCGCCCCTCCCAGGGTTGGGCTGCCGTTACCCAACCCCCGCCCCATCGCACACACCCCT 2374
Ma'sNightMonkey   GGCCGGCGAGAAACCCTCCCGCGGCCCCTCCCAGGGCTGGGCTGCCGTTACCCAACCCCCGCCCCATCGCACACACCCCT 2269
VervetAGM         GGCCGGGGAGAAACCCTCCCGCGGCCCCTCCCAGGGCTGGGCTGCCGTTACCCAACCCCCGCCCCATCGCACACACCCCT 2254
Gorrilla          GGCCGGGGAGAAACCCTCCCGCGGCCCCTCCCAGGGCTGGGCTGCCGTTACCCAACCCCCGCCCCATCGCACACACCCCT 2416
GrabEatingMacaqueGGCCGGGGAGAAACCCTCCCGCGGCCCCTCCCAGGGCTGGGCTGCCGTTACCCAACCCCCGCCCCATCGCACACACCCCT 2199
Macaque           GGCCGGGGAGAAACCCTCCCGCGGCCCCTCCCAGGGCTGGGCTGCCGTTACCCAACCCCCGCCCCATCGCACACACCCCT 2199
PigTailedMacaque  GGCCGGGGAGAAACCCTCCCGCGGCCCCTCCCAGGGCTGGGCTGCCGTTACCCAACCCCCGCCCCATCGCACACACCCCT 2416
Bonobo            GGCCGGGGAGAAACCCTCCCGCGGCCCCTCCCAGGGCTGGGCTGCCGTTACCCAACCCCCGCCCCATCGCACACACCCCT 2263
OliveBaboon       GGCCGGGGAGAAACCCTCCCGCGGCCCCTCCCAGGGCTGGGCTGCCGTTACCCAACCCCCGCCCCATCGCACACACCCCT 2156
Orangutan         GGCCGGGGAGAAACCCTCCCGCGGTCCCTCCCAGGGCTGGGCTGCCGTTACCCAACCCCCGCCCCATCGCACACACCCCT 2250

Majority          CCCTTTGCTTCCCCCGGCGGGCTTTGGTCGAGAAAATGAGAAGAAAGCGGGCTTGGAGGCTGGGGAGGCATTGGGGCGAT

                          2730      2740      2750      2760      2770      2780      2790      2800

Human             CCCTTTGCTTCCCCCGGCGGGCTTTGGTCGAGAAAATGAGAAGAAAGCGGGCTTGGAGGCTGGGGAGGCATAGGGGCGAT 1036
Coquerel'sSifaka  CCCTTTGCTTCCCCCGGCGAGCTGTGGCCGAGAAAATGAGGAGAAAGCGGGCTTGGAGGCAGGGGAGGCATTGGGGCGAT 2454
Ma'sNightMonkey   CCCTTTGCTTCCCCCGGCGGGCTTTGGTCGAGAAAATGAGAAGAAAGCGGGCTTGCAGGCTGGGGAGGCATTGGGGAGAT 2349
VervetAGM         CCCTTTGCTTCCCCCGGCGGGCTTTGGTCGAGAAAATGAGAAGAAAGCGGGCTTGGAGGCTGGGGAGGCACTGGGGCGAT 2334
Gorrilla          CCCTTTGCTTCCCCCGGCGGGCTTTGGTCGAGAAAATGAGAAGAAAGCGGGCTTGGAGGCTGGGGAGGCATTGGGGCGAT 2496
GrabEatingMacaqueCCCTTTGCTTCCCCCGGCGGGCTTTGGTCGAGAAAATGAGAAGAAAGCGGGCTTGGAGGCTGGGGAGGCACTGGGGCGAT 2279
Macaque           CCCTTTGCTTCCCCCGGCGGGCTTTGGTCGAGAAAATGAGAAGAAAGCGGGCTTGGAGGCTGGGGAGGCACTGGGGCGAT 2279
PigTailedMacaque  CCCTTTGCTTCCCCCGGCGGGCTTTGGTCGAGAAAATGAGAAGAAAGCGGGCTTGGAGGCTGGGGAGGCACTGGGGCGAT 2496
Bonobo            CCCTTTGCTTCCCCCGGCGAGCTTTGGTCGAGAAAATGAGAAGAAAGCGGGCTTGGAGGCTGGGGAGGCATTGGGGCGAT 2343
OliveBaboon       CCCTTTGCTTCCCCCGGCGGGCTTTGGTCGAGAAAATGAGAAGAAAGCGGGCTTGGAGGCTGGGGAGGCACTGGGGCGAT 2236
Orangutan         CCCTTTGCTTCCCCCGGCCGGCTTTGGTCGAGAAAATGAGAAGAAAGCGGGCTTGGAGGCTTGGGAGGCATTGGGGCGAT 2330

Majority          GGCGGGG-GTGAGGATCCCGGAGTGGGAGCGGGCACCGGCTTGGCGGCGGGGGATGGGGTGGGAACCCCATAACGTCTCG

                          2810      2820      2830      2840      2850      2860      2870      2880

Human             GGCGGGG-GTGAGGATCCCGGAGTGGGAGCGGGCACCGGCTTGGCGGCGGGGGATGGGGTGGGAACCCCATAACGTCTCC 1115
Coquerel'sSifaka  GGCGGGGTGGGACGACCCCGGAGAGGGAGCGGGCATCGGCTTGGCGGCGGGG-ATGGGGTGGGAACCCCAGAACGTCTCT 2533
Ma'sNightMonkey   GGCGGGG-GTGAGGATCCCAGAGTGGCAGCGGGCTCCGGCTTGGCGGCGGGGGATGGGGTGGGAACCTCATAACGTCTTG 2428
VervetAGM         GGCGGGG-GTGAGGATCCCGGAGTGGGAGCGGGCACCGGCTTGGCGGCGGGGGATGGGGTGGGAACCCCCTAACGTCTCG 2413
Gorrilla          GGCGGGG-GTGAGGATCCCGGAGTGGGAGCGGGTACCGGCTTGCCGGCGGGGGATGGGGTGGGAACCCCATAACGTCTCG 2575
GrabEatingMacaqueGGCGGGG-GTGAGGATCCCGGAGTGGGAGCGGGCACCGGCTTGGCGGCGGGGGATGGGGTGGGAACCCCATAACGTCTCG 2358
Macaque           GGCGGGG-GTGAGGATCCCGGAGTGGGAGCGGGCACCGGCTTGGCGGCGGGGGATGGGGTGGGAACCCCATAACGTCTCG 2358
PigTailedMacaque  GGCGGGG-GTGAGGATCCCGGAGTGGGAGCGGGCACCGGCTTGGCGGCGGGGGATGGGGTGGGAACCCCATAACGTCTCG 2575
Bonobo            GGCGGGG-GTGAGGATCCCGGAGTGGGAGCGGGCACCGGCTTGGCGGCGGGGGATGGGGTGGAAACCCCGTAACGTCTCG 2422
OliveBaboon       GGCGGGG-GTGAGGATCCCGGAGTGGGAGCGGGCACCGGCTTGGCGGCGGGGGATGGGGTGGGAACCCCATAACGTCTCG 2315
Orangutan         GGCGGGG-GTGAGGATCCCGGAGTGGGAGCGGGCACCGGCTTGGCGGCGGGGGATGGGGTGGGAACCCCATAACGTCTCG 2409
```

```
Majority          CCGGTCCGTCCCTGCCCCCTCTAGGAGCCATTTCGATCCGTACCCTGGGACCCGACCCTTGGGTTAGCGGTGCCTGTGAG

                          2890      2900      2910      2920      2930      2940      2950      2960

Human             CCGGTCTGTCCCTGCCCCCTCTAGGAGCCATTTCGATCCGTACCTTGGGACCCGACCCTTGGGTTAGCGGTGCCTGTGAG 1195
Coquerel'sSifaka  CATGTCCCTGCC---CCCCTCTAGGAGCCATTTCGATCTGCACCCTGGGACCCGGACCCTGGGGTAGCGGTGGCTGTGGG 2610
Ma'sNightMonkey   CCGGTCCGTCCCTGCCCCCTCTAGGAGCCATTTCGATCCGTACCCTGGGACCCGACCCTTGGGGTAGCGGTGCCTATGAG 2508
VervetAGM         CCGGTCCGTCCCTGCCCCCTCTAGGAGCCATTTCGATCCGTACCCTGGGACCCGACCCTTGGGTTAGCGGTGCCTGTGAG 2493
Gorrilla          CCGGTCTGTCCCTGCCCCCTCTAGGAGCCATTTCGATCCGTACCCTGGGACCCGACCCTTGGGTTAGCGGTGCCTGTGAG 2655
GrabEatingMacaqueCCGGTCCGTCCCTGCCCCCTCTAGGAGCCATTTCGATCCGTACCCTGGGACCCGACCCTTGGGTTAGCGGTGCCTGTGAG 2438
Macaque           CCGGTCCGTCCCTGCCCCCTCTAGGAGCCATTTCGATCCGTACCCTGGGACCCGACCCTTGGGTTAGCGGTGCCTGTGAG 2438
PigTailedMacaque  CCGGTCCGTCCCTGCCCCCTCTAGGAGCCATTTCGATCCGTACCCTGGGACCCGACCCTTGGGTTAGCGGTGCCTGTGAG 2655
Bonobo            CCGGTCTGTCCCTGCCCCCTCTAGGAGCCATTTCGATCCGTACCCTGGGACCCGACCCTTGGGTTAGCGGTGCCTGTGAG 2502
OliveBaboon       CCGGTCCGTCCCTGCCCCCTCTAGGAGCCATTTCGATCCGTACCCTGGGACCCGACCCTTGGGTTAGCGGTGCCTGTGAG 2395
Orangutan         CTGGTCTGTCCCTGCCCCCTCTAGGAGCCATTTCGATCCGTACCCTGGGACCCGACCCTTGGGTTAGCGGTGCCTGTGAG 2489

Majority          AGCGAGTGGGATGGGCAGAGACAGGCCTTTGGAGTGGGGG-AGCCTCTAGGGGAAGAGGAGAGAGGCACCCCTTGTGGAC

                          2970      2980      2990      3000      3010      3020      3030      3040

Human             AGCGAGTGGGATGGGCAGAGACAGGCCTTTGGATTGGGGG-AGCCTCTAGGGGAAGAGGAGAGAGGCACCCCATGTGGAC 1274
Coquerel'sSifaka  ATCGAGTGAGGTGGGCAGAGACAGGCCC--GGGGCGGGGGGAGGCCCT-GGGGAAGAGGAGGGAGGCACCGCTTGTGGAC 2687
Ma'sNightMonkey   AGCCAGTGGGATGGGCAGACACAGGCCTGTGGGGTGGGGG-AGGCCCTAGGGGAAGAGGAGAGAGGCACCCCATGTGGAC 2587
VervetAGM         AGCGAGTGGGATGGGCAAAGACAGGCCTTTGGAGTGGGGG-AACCTCTAGGGGAAGAGAAGAGAGCCACCCCTTGTGGAC 2572
Gorrilla          AGCGAGTGGGATGGGCAGAGACAGGCCTTTGGAGTGGGGG-AGCCTCTAGGGGAAGAGGAGAGAGGCACCCCTTGTGGAC 2734
GrabEatingMacaqueAGCGAGTGGGATGGGCAAAGACAGGCCTTTGGAGTGGGGG-AACCTCTAGGGGAAGAGAAGAGAGCCACCCCTTGTGGAC 2517
Macaque           AGCGAGTGGGATGGGCAAAGACAGGCCTTTGGAGTGGGGG-AACCTCTAGGGGAAGAGAAGAGAGCCACCCCTTGTGGAC 2517
PigTailedMacaque  AGCGAGTGGGATGGGCAAAGACAGGCCTTTGGAGTGGGGG-AACCTCTAGGGGAAGAGAAGAGAGCCACCCCTTGTGGAC 2734
Bonobo            AGCGAGTGGGATGGGCAGAGACAGGCCTTTGGATTGGGGG-AGCCTCTAGGGGAAGAGGAGAGAGGCACCCCATGTGGAC 2581
OliveBaboon       AGCGAGTGGGATGGGCAAAGACAGGCCTTTGGAGTGGAGG-AACCTCTAGGGGAAGAGAAGAGAGCCACCCCTTGTGGAC 2474
Orangutan         AGCGAGTGGGATGGGCAGAGACAGGCCTTTGGAGTGGGGGGAGCGTCTAGGGGAAGAGGAGAGAGGCACCCCTTGTGGAC 2569

Majority          CCCAGCCATCCGC-AGCCTCTCCGGCCTCGCCCGGCCCGCCGTGGTCGGGAGAGATGCCGGTGGCGGGAGCTCCGGGGAA

                          3050      3060      3070      3080      3090      3100      3110      3120

Human             CCCAGCCATGAGC-AACCTCTCCGGCCTCGCCCGGCCCTCCGTGGTCGGGAGAGATGCCGGTGGCGGGAGCTCCGGGGAA 1353
Coquerel'sSifaka  CCCAGCCATCCCCTAGCCTGCCGGGCCTCGCCCGGCCCGCC-TGGTTGGGAGAGATGCAGGTGGCGGGAGCTCGGGGGAA 2766
Ma'sNightMonkey   CCCAGCCATCTGC-AACCTCCCCGGCCTCGCCCGGCCCGCCGTGGTCTGGAGAGATGCGGGTGGCGGGAGCTCCGGGGAA 2666
VervetAGM         CCCAGCCGTCCGC-AGCCTCTCCGGCCTCGCCCGGCCCGCCGTGGTCGGGAGAGATGCCGGTGGCGGGAGCTCCGGGGAA 2651
Gorrilla          CCCAGCCATGAGC-AACCTCTCCGGCCTCGCCCGGCCCGCCGTGGTCGGGAGAGATGCCGGTGGCGGGAGCTCCGGGGAA 2813
GrabEatingMacaqueCCCAGCCGTCCGC-AGCCTCTCCGGCCTCGCCCGGCCCGCCGTGGTCGGGAGAGATGCGGGTGGCGGGAGCTCCGGGGAA 2596
Macaque           CCCAGCCGTCCGC-AGCCTCTCCGGCCTCGCCCGGCCCGCCGTGGTCGGGAGAGATGCCGGTGGCGGGAGCTCCGGGGAA 2596
PigTailedMacaque  CCCAGCCGTCCGC-AGCCTCTCCGGCCTCGCCCGGCCCGCCGTGGTCGGGAGAGATGCCGGTGGCGGGAGCTCCGGGGAA 2813
Bonobo            CCCAGCCATGAGC-AACCTCTCCGGCCTCGCCCGGCCTTCCGTGGTCGGGAGAGATGCCGGTGGCGGGAGCTCCGGGGAA 2660
OliveBaboon       CCCAGCCGTCCGC-AGCCTCTCCGGCCTCGCCCGGCCCGCCGTGGTCGGGAGAGATGCCGGTGGCGGGAGCTCCGGGGAA 2553
Orangutan         CCCAGCCATGCGC-AACCTCTCTGGCCTCGCCCGGCCCGCCGTGGTCGGGAGAGATGCCGGTGGCGGGAGCTCCGGGGAA 2648
```

```
Majority          AGCCTAGTGGGAGCCGCTGGGGAAGGGGGGAGGGCGTGCGGCGGCGGGAGGAAGGGGGAGGGCAGATGTCATACTCCTTT
                          3130      3140      3150      3160      3170      3180      3190      3200
Human             AGCCTAGTGGGAGCCGCTGGGGAAGGGGGGAGGGCGTGCGGCGGCGGGAGGAAGGGGGAGGGCAGATGTCATACTCCTTT 1433
Coquerel'sSifaka  AGCCGAGTGGGAGCCGCTGGGGAAGTGGGG--------------------GA-------GGGCAGATGTCATACTCCTTT 2819
Ma'sNightMonkey   AGCCTAGTGGGAGCCGCTGGGGAAGGGGGGAGGGCGTGCGGCGGCGGGAGGAAGGGGGAGGGCAGATGTCATACTCCTTT 2746
VervetAGM         AGCCTAGTGGGAGCCGCTGGGGAAGGGGGGAGGGCGTGCGGCGGCGGGAGGAAGGGGGAGGGCAGATGTCATACTCCTTT 2731
Gorrilla          AGCCTAGTGGGAGCCGCTGGGGAAGGGGGGAGGGCGTGCGGCGGCGGGAGGAAGGGGGAGGGCAGATGTCATACTCCTTT 2893
GrabEatingMacaque AGCCTAGTGGGAGCCGCTGGGGAAGGGGGGAGGGCGTGCGGCGGCGGGAGGAAGGGGGAGGGCAGATGTCATACTCCTTT 2676
Macaque           AGCCTAGTGGGAGCCGCTGGGGAAGGGGGGAGGGCGTGCGGCGGCGGGAGGAAGGGGGAGGGCAGATGTCATACTCCTTT 2676
PigTailedMacaque  AGCCTAGTGGGAGCCGCTGGGGAAGGGGGGAGGGCGTGCGGCGGCGGGAGGAAGGGGGAGGGCAGATGTCATACTCCTTT 2893
Bonobo            AGCCTAGTGGGAGCCGCTGGGGAAGGGGGGAGGGCGTGCGGCGGCGGGAGGAAGGGGGAGGGCAGATGTCATACTCCTTT 2740
OliveBaboon       AGCCTAGTGGGAGCCGCTGGGGAAGGGGGGAGGGCGTGCGGCGGCGGGAGGAAGGGGGAGGGCAGATGTCATACTCCTTT 2633
Orangutan         AGCCTAGTGGGAGCCGCTGGGGAAGGGGGGAGGGCGTGCGGCGGCGAGAGGAAGGGGGAGGGCAGATGTCATACTCCTTT 2728

Majority          GTTTTCATTTGAGTCTGGTAGTGGGGGCGGGAGGGAGGAAAAAATGCCTTTTTGTGGGGACTGAAAACATTCAAGGCTTG
                          3210      3220      3230      3240      3250      3260      3270      3280
Human             GTTTTCATTTGAGTCTGGTAGTGGGGGCGGGAGGGAGGAAAAAATGCCTTTTTGTGGGGACTGAAAACATTCAAGGCTTG 1513
Coquerel'sSifaka  GTTTTCATTCGAGTCTGGAAGTGGGGGCGGGAGGCAGAAAAAA-TGCCTTTTTGTGGGGCATGAGAACATTCAAGGCTTG 2898
Ma'sNightMonkey   GTTTTCATTTGAGTCTGGTAGTGGGGGCGGGAGAGAGGAAAAAATGCCTTTTTGTGGGGACTTAAAACATTCAAGGCTTG 2826
VervetAGM         GTTTTCATTTGAGTCTGGTAGTGGGGGCGGGAGGGAGGAAAAA-TGCCTTTTTGTGGGGACTGAAGACATTCAAGGCTTG 2810
Gorrilla          GTTTTCATTTGAGTCTGGTAGTGGGGGCGGGAGGGAGGAAAAAATGCCTTTTTGTGGGGACTGAAAACATTCAAGGCTTG 2973
GrabEatingMacaque GTTTTCATTTGAGTCTGGTAGTGGGGGCGGGAGGGAGGAAAAA-TGCCTTTTTGTGGGGACTGAAGACATTCAAGGCTTG 2755
Macaque           GTTTTCATTTGAGTCTGGTAGTGGGGGCGGGAGGGAGGAAAAA-TGCCTTTTTGTGGGGACTGAAGACATTCAAGGCTTG 2755
PigTailedMacaque  GTTTTCATTTGAGTCTGGTAGTGGGGGCGGGAGGGAGGAAAAAATGCCTTTTTGTGGGGACTGAAGACATTCAAGGCTTG 2973
Bonobo            GTTTTCATTTGAGTCTGGTAGTGGGGGCGGGAGGGAGGAAAAAATGCCTTTTTGTGGGGACTGAAAACATTCAAGGCTTG 2820
OliveBaboon       GTTTTCATTTGAGTCTGGTAGTGGGGGCGGGAGGGAGGAAAAA-TGCCTTTTTGTGGGGACTGAAGACATTCAAGGCTTG 2712
Orangutan         GTTTTCATTTGAGTCTGGTAGTGGGGGTGGGAGGGAGGAAAAAATGCCTTTTTGTGGGGACTGAAAACATTCAAGGCTTG 2808

Majority          GCAAAAGGGCCCAGAAATTAAATCATCTAAAAAACGAAGTGTTTGAGGCCGACATCAGTCACATGGGCAAAGGCCAACAG
                          3290      3300      3310      3320      3330      3340      3350      3360
Human             GCAAAAGGGCCCAGAAATTAAATCATCTAAAAAACGAAGTGTTTGAAGCCGACATCAGTCACATGGGCAAAGGCCAACAG 1593
Coquerel'sSifaka  GCAAAAGGGCCCAGAAATTAAATCATCTAAAAAACGAAGTGTTTGAGGTCCACATCAGTCACATGGGCAAAGGCCAACGG 2978
Ma'sNightMonkey   GCAAAAGGGCCCAGAAATTAAATCATCTAAAAAACGAAGTGTTTGAGGCCGACATCAGTCACATGGGCAAAGGCCAACAG 2906
VervetAGM         GCAAAAGGGCCCAGGAATTAAATCATCTAAAAAGCGAAGTGTTTGAGGCCGACATCAGTCACATGGGCAAAGGCCAACAG 2890
Gorrilla          GCAAAAGGGCCCAGAAATTAAATCATCTAAAAAACGAAGTGTTTGAAGCCGACATCAGTCACATGGGCAAAGGCCAACAG 3053
GrabEatingMacaque GCAAAAGGGCCCAGAAATTAAATCATCTAAAAAGCGAAGTGTTTGAGGCCGACATCAGTCACATGGGCAAAGGCCAACAG 2835
Macaque           GCAAAAGGGCCCAGAAATTAAATCATCTAAAAAGCGAAGTGTTTGAGGCCGACATCAGTCACATGGGCAAAGGCCAACAG 2835
PigTailedMacaque  GCAAAAGGGCCCAGAAATTAAATCATCTAAAAAGCGAAGTGTTTGAGGCCGACATCAGTCACATGGGCAAAGGCCAACAG 3053
Bonobo            GCAAAAGGGCCCAGAAATTAAATCATCTAAAAAACGAAGTGTTTGAAGCCGACATCAGTCACATGGGCAAAGGCCAACAG 2900
OliveBaboon       GCAAAAGGGCCCAGAAATTAAATCATCTAAAAAGCGAAGTGTTTGAGGCCGACATCAGTCACATGGGCAAAGGCCAACAG 2792
Orangutan         GCAAAAGGGCCCAGAAATTAAATCATCTAAAAAACGAAGTGTTTGAAGCCGACATCAGTCACATGGGCAAAGGCCAACAG 2888
```

```
Majority          CAATGGCAGAAACAAGAGCAGATAGTCAACACAGGGCTATCTCTTT-AGCTACTTTGGTCAGTTTCTGGAAAAACGACTA

                           3370      3380      3390      3400      3410      3420      3430      3440

Human             CAATGGCAGAAACAAGAGCAGATAGTCAACAAAGGGCTATCTCTTT-AGCTACTTTGGTCAGTTTCTGGAAAAACGACTA 1672
Coquerel'sSifaka  CCATGGCCAAAACAAGAGCAGATAGTCAACACAGGGCCATCTTTTTTAGCTACTTCGGCCAGTTTCTGGAAAAACAAAAA 3058
Ma'sNightMonkey   CAATGGCAGAAACAAGAGCAGATAGTCAACACAGGGCCATCTCTTT-AGCTACTTTGGTCAGTTTCTGGAATAACGACTA 2985
VervetAGM         CAATGGCAGAAACAAGAGCAGATAGTCAACACAGGGCTATCTCTTT-AGCTACTTTGGTCAGTTTCTGGAAAAATGACTA 2969
Gorrilla          CAATGGCAGAAACAAGAGCAGATAGTCAACAAAGGGCTATGTCTTT-AGCTACTTTGGTCAGTTTCTGGAAAAACGACTA 3132
GrabEatingMacaqueCAATGGCAGAAACAAGAGCAGATAGTCAACACAGGGCTATCTCTTT-AGCTACTTTGGTCAGTTTCTGGAAAAATGACTA 2914
Macaque           CAATGGCAGAAACAAGAGCAGATAGTCAACACAGGGCTATCTCTTT-AGCTACTTTGGTCAGTTTCTGGAAAAATGACTA 2914
PigTailedMacaque  CAATGGCAGAAACAAGAGCAGATAGTCAACACAGGGCTATCTCTTT-AGCTACTTTGGTCAGTTTCTGGAAAAATGACTA 3132
Bonobo            CAATGGCAGAAACAAGAGCAGATAGTCAACAAAGGGCTATCTCTTT-AGCTACTTTGGTCAGTTTCTGGAAAAACGACTA 2979
OliveBaboon       CAATGGCAGAAACAAGAGCAGATAGTCAACACAGGGCTATCTCTTT-AGCTACTTTGGTCAGTTTCTGGAAAAATGACTA 2871
Orangutan         CAATGGCAGAAACAACAGCAGATAGTCAACAAAGGGCTATCTCTTT-AGCTACTTTGGTCAGTTTCTGGAAAAACGACTA 2967

Majority          CAA--AAACCCAGAACCATGCTGCATCTTTGCGAACCT--GAGTGAAGACAGAATGCTGAG-TGAGGTGAGAGGCAAGA-

                           3450      3460      3470      3480      3490      3500      3510      3520

Human             TAA--AAACCCAGAACCATGCTGCATCTTTGCGAACCT--GAGTCAAGACGGAATGCTGAG-TGAGGTGAGAGGCAAGA- 1746
Coquerel'sSifaka  CTACAAAAGCGAGAACCATGCTGCATCTTTGAGAGCATAAGAGTGAAGATAGAATGCTGAGGTGAGGCTAGGGCTCAGG- 3137
Ma'sNightMonkey   CAA--AAAGCCAGAACCATGCTGCATCTTTGTGAACCT--GAGTGAAGACAGAATGCTGAG-TGAGGTGAGAGGCAAGAG 3060
VervetAGM         CAA--AAACCCAGAACCATGCTACATCTTTGCGAACCT--GAGTGAAAACAGAATGCTGAG-TGAGGTGAGAGGCAAGA- 3043
Gorrilla          TAA--AAACCCAGAACCATGCTGCATCTTTGCGAACCT--GAGTGAAGACGGAATGCTGAG-TGAGGTGAGAGGCAAGA- 3206
GrabEatingMacaqueCAA--AAACCCAGAACCATGCTACATCTTTGCGAACCT--GAGTGAAAAC-------TGAG-TGAGGTGAGAGGCAAGA- 2981
Macaque           CAA--AAACCCAGAACCATGCTACATCTTTGCGAACCT--GAGTGAAAACAGAATGCTGAG-TGAGGTGAGAGGCAAGA- 2988
PigTailedMacaque  CAA--AAACCCAGAACCATGCTACATCTTTGCGAACCT--GAGTGAAAACAGAATGCTGAG-TGAGGTGAGAGGCAAGA- 3206
Bonobo            TAA--AAACCCAGAACCATGCTGCATCTTTGCGAACCT--GAGTGAAGACGGAATGCTGAG-TGAGGTGAGAGGCAAGA- 3053
OliveBaboon       CAA--AAACCCAGAACCATGCTACATCTTTGCGAACCT--GAGTGAAAACAGAATGCTGAA-TGAGGTGAGAGGCAAGA- 2945
Orangutan         CAA--AAACCCAGAACCATGCTGCATCTTTGCGAACCT--GAGTGAAGACAGGATGCTGAG-TGAGGTGAGAGGCAAGA- 3041

Majority          --CTGTGAAATGGTAGGGACCAATGTCTCTCTGAT----ACGCTTCACACCTGAAAAGACTCAGTAATTTAACTCATCGT

                           3530      3540      3550      3560      3570      3580      3590      3600

Human             --CTGTGAAATGGTAGGGACCGATGTCTCGCTCAT----ATGCTTCACACCTGAAAAGACTCAGTAATTTAACTCAATGT 1820
Coquerel'sSifaka  ------GAAATGATACGGACCAATGTCTCGCTGGTGCTAACGCTCCACATCTGAACACACTCAGTAGTTTAACTCACCCT 3211
Ma'sNightMonkey   CTCCGGGAAATGATAGGGACCGATGTCTCGCTGAT----AAGCTTCACACCTGAAAAGAGTAATTTATTTAACTCCTCCT 3136
VervetAGM         --CTGTGAAATGGTAGGGACCAATGTCTCTCTGAC----ACGCTTCACACCTGAAAAGACTCAGTAATTTAAGTCATCGT 3117
Gorrilla          --CTGTGAAATGGTAGGGACCGATGTCTCTCTCAT----ACGCTTCACACCTGAAAAGACTCAGTAATTTAACTCATTGT 3280
GrabEatingMacaque--CTGTGAAATGGTAGGGACCAATGTCTCTCTGAC----ACGCTTCACACCTGAAAAGACTCAGTAATTTAAGTCATCGT 3055
Macaque           --CTGTGAAATGGTAGGGACCAATGTCTCTCTGAC----ACGCTTCACACCTGAAAAGACTCAGTAATTTAAGTCATCGT 3062
PigTailedMacaque  --CTGTGAAATGGTAGGGACCAATGTCTCTCTGAC----ACGCTTCACACCTGAAAAGACTCAGTAATTTAAGTCATCGT 3280
Bonobo            --CTGTGAAATGGTAGGGACCGATGTCTCTCTCAT----ACGCTTCACACCTGAAAAGACTCAGTAATTTAACTCATTGT 3127
OliveBaboon       --CTGTGAAATGGTAGGGACCAGTGTCTCTCTGAC----ACGCTTCACACCTGAAAAGACTCAATAATTTAAGTCATCGT 3019
Orangutan         --CTGTGAAATGGTAGGGACCGATGTCTCTCTGAT----AGGCTTCACACCTGAAAAGATTCAGTAATTTAACTCATTGT 3115
```

```
Majority          TAGAAA--GGGGGCACGCTTAAGGTCGAAAATA-TAGCAGTATGCAGCTCTTTTGGGAGTGTAAACATAGTATCATTTAA

                          3610      3620      3630      3640      3650      3660      3670      3680

Human             TAGAAA--GGGGGCACGCTTAAGGTCAAAAATA-CAGCAGTATGCAGCTCTTTTGGGAGTGTAAACATAGTATCATTTAA 1897
Coquerel'sSifaka  TAGAAA--GGGAGCACGCTTTAAGGCGAAAATA-CAGCAGTA---AGCCCGTTTAGGGGTATAAAAACAGTACCATTTAA 3285
Ma'sNightMonkey   TATAAA--CGGGGCACGCTTAGGGGCGAAAATA-TAGCAGTATGCAGCTCTTTTGGGAGTGTAAACGTAGTATCATTTAA 3213
VervetAGM         TAGAAA--GGGGGCAGACTTAAGGTCGAAAATA-TAGCAGTATGCAGCTCTTTTGGGAGTGTAAACATAATATCATTTAG 3194
Gorrilla          TAGAAA--GGGGGCACGCTTAAGGTCAAAAATA-CAGCAGTATGCAGCTCTTTTGGGAGTGTAAAC--AGTATCATTTAA 3355
GrabEatingMacaqueTAGAAA--GGGGGCAGGCTTAAGGTCGAAAATA-TAGCAGTATGCAGCTCTTTTGGGAGTGTAAACATAATATCATTTAG 3132
Macaque           TAGAAA--GGGGGCAGGCTTAAGGTCGAAAATA-TAGCAGTATGCAGCTCTTTTGGGAGTGTAAACATAATATCATTTAG 3139
PigTailedMacaque  TAGAAA--GGGGGCAGGCTTAAGGTCGAAAATA-TAGCAGTATGCAGCTCTTTTGGGAGTGTAAACATAATATCATTTAG 3357
Bonobo            TAGAAA--GGGGGCACGCTTAAGGTCAAAAATA-CAGCAGTATGCAGCTCTTTTGGGAGTGTAAACATAGTATCATTTAA 3204
OliveBaboon       TAGAAA--GGGGGCAGGCTTAAGGTCGAAAATA-TAGCAGTATTCAGCTCTTTTGGGAGTGTAAACATAATATCATTTAG 3096
Orangutan         TAGAAACCGGGGGCACGCTTAAGGTCAAAAATAACAGCAGTATGCAGCTCTTTTGGGAGTGTAAACACAGTATCATTTAA 3195

Majority          CAAGTACATTTATTAAAATCAGTGA--GTATTTGAAGAATCAAAAATTTGGTTAAGC------GCCTA------GAGTTT

                          3690      3700      3710      3720      3730      3740      3750      3760

Human             CAAGTACATTTATTAAAATCAGTGA--GTATTTGAAGAATCAACAATTTGGTTAAGC------GCCTA------GAGTTT 1963
Coquerel'sSifaka  CAAGTACATTTATTGAAATCAATGACAATATTTGAAGAGTCAAAAGTAAAATGAATTTATTTTGCTTAAGGGTTGAGTTT 3365
Ma'sNightMonkey   CAAGCACATTTATTAAAATCAGCGACAGTATTTGAAGAATCAAAAATTTGGTTAAGC------GCCTA------GAGTTT 3281
VervetAGM         CAAATACATTTATTAAAATCAGTGA--GTATTTGAAGAATCAAAAATTTGGTTAAGC------GCCTA------GAGTTT 3260
Gorrilla          CAAGTACATTTATTAAAATCAGTGA--GTATTTGAAGAATCAAAAATTTGGTTAAGC------GCCTA------GAGTTT 3421
GrabEatingMacaqueCAAATACATTTATTAAAATCAGTGA--GTATTTGAAGAATCAAAAATTTGGTTAAGC------GCCTA------GAGTTT 3198
Macaque           CAAATACATTTATTAAAATCAGTGA--GTATTTGAAGAATCAAAAATTTGGTTAAGC------GCCTA------GAGTTT 3205
PigTailedMacaque  CAAATACATTTATTAAAATCAGTGA--GTATTTGAAGAATCAAAAATTTGGTTAAGC------GCCTA------GAGTTT 3423
Bonobo            CAAGTACATTTATCAAAATCAGTGA--GTATTTGAAGAATCAAAAATTTGGTTAAGC------GCCTA------GAGTTT 3270
OliveBaboon       CAAATACATTTATTAAAATCAGTGA--GTATTTGAAGAATCAAAAATTTGGTTAAGC------GCCTA------GAGTTT 3162
Orangutan         CAAGCACATTTATTAAAATCAGTGA--GTATTTGAAGAATCAAAAATTTGGTTAAGC------GCCTA------GAGTCT 3261

Majority          GGTG-TTTTTTAAAAGATATTAAAAAGAAGATTTTATAAAAGTTAACACAGTCTTACAGAATTCAAATCGTCCTAGATGT

                          3770      3780      3790      3800      3810      3820      3830      3840

Human             GGTG-TGTTTTAAAAGGTACTAAAAACAAGATTTTATAAAAGTTAACACAGTCTCACAGAATTCAAATCATCCTAGATGT 2042
Coquerel'sSifaka  GGTGATTTTTTTTAAGATATTG-AATGAAGATCTTATAAAAGTAAAC--AGACTTATACAATTTAAATCTTCTTTGAGGT 3442
Ma'sNightMonkey   GGTG-TTTTTTAAAATATATTAAAAAGATGGTTTTATAAAAGTTAAC--GGTCTTACAGAATTTAAATCATCCTAGATGT 3358
VervetAGM         GGTG-TGTTTTAAAAGATGTTAAAAAGAAGATTT-ATAAAAGTTAACACAGTTTTACAGAATTCAAATCGTCCTAGATGT 3338
Gorrilla          GGTG-TGTTTTAAAAGGTACTAAAAACAAGATTTTATAAAAGTTAACACAGTCTTACAGAATTCAAATCATCCTAGATGT 3500
GrabEatingMacaqueGGTG-TTTTTTAAAAGATGTTATAAAGAAGATTTTATAAAAGTTAACACAGTTTTACAGAATTCAAATCGTCCTAGATGT 3277
Macaque           GGTG-TTTTTTAAAAGATGTTATAAAGAAGATTTTATAAAAGTTAACACAGTTTTACAGAATTCAAATCGTCCTAGATGT 3284
PigTailedMacaque  GGTG-TTTTTTAAAAGATGTTATAAAGAAGATTTTATAAAAGTTAACACAGTTTTACAGAATTCAAATCGTCCTAGATGT 3502
Bonobo            GGTG-TGTTTTAAAAGGTACTAAAAACAAGATTTTATAAAAGTTAACACAGTCTTACAGAATTCAAATCATCCTAGATGT 3349
OliveBaboon       GGTG-TTTTTAAAAAGATGTTATAAAGAAGATTTTATAAAAGTTAACACAGTTTTACAGAATTCAAATCGTCCTAGATGT 3241
Orangutan         GGTG-TGTTTTAAAAGGTACTAAAAACAAGATTTTATAAAAGTTAACACAGTCTTACAGAATTCAAATCATCCTAGATGT 3340
```

```
Majority          TACCTAAAGATTATTTTCCAGATGAAACA--GATCCAGAGAAGTTCAGTATTCCCCCCAGATCAACACGGAAATTGATGT
                          3850      3860      3870      3880      3890      3900      3910      3920
Human             TACCTAAAGATTATTTTCCAGATGAAACA--AATCCAGAGAAGTTCAGTATTCCCCCCAGATCAACACGGAAATTGATGT 2120
Coquerel'sSifaka  TATCTAGAGATTATTTTTTTGGTGACACA--ACTCCAGAGAAGTTTTGTATTCCTCCC-GATCAACA-GAAGATTGAT-- 3516
Ma'sNightMonkey   TATCTAAAGATTATTTGCCAGATGAAACATAGATCCAGAGAAGTTCAGTATTCCCCTCAGATCAACACAGAAATTGATGG 3438
VervetAGM         TACCTTA-GATTATTTTCCAGATGAAACA--GATCCAGAGAATTTCAGTTTTCCCCCCAGATCAACACGGCAATTGATGT 3415
Gorrilla          TACCTAAAGATTATTTTCCAGGTGAAACA--GATCCAGAGAAGTTCAGTATTCCCCCCAGGTCAACACGGAAATTGATGT 3578
GrabEatingMacaqueTACCTTAAGATTATTTTCCAGATGAAACA--GATCCAGAGAATTTCAGTTTTCCCCCCAGGTCAACACGGCAATTGATGT 3355
Macaque           TACCTTAAGATTATTTTCCAGATGAAACA--GATCCAGAGAATTTCAGTTTTCCCCCCAGGTCAACACGGCAATTGATGT 3362
PigTailedMacaque  TACCTTAAGATTATTTTCCAGATGAAACA--GATCCAGAGAATTTCAGTTTTCCCCCCAGGTCAACACGGCAATTGATGT 3580
Bonobo            TACCTAAAGATTATTTTCCAGATGAAACA--GATCCAGAGAAGTTCAGTATTCCCCCCAGATCAACACGGAAATTGATGT 3427
OliveBaboon       TACCTTAAGATTATTTTCCAGATGAAACA--GATCCAGAGAATTTCAGTTTTCCACTCAGGTCAACACGGCAATTGATGT 3319
Orangutan         TACCTAAAGATTATTTTCCAGATGAAACA--GATCGAGAGAAGTTCAGTATTCCCCCCAGATCAACACGGAAATTGATGT 3418

Majority          CAAAACTTGCACTAGAATTCATGTTTCCTGATTCCTGGTTCCTATGGAGACTACTAAAGAGTTTAAGCGTTAAATTATTA
                          3930      3940      3950      3960      3970      3980      3990      4000
Human             CAAAACTTGCACTAGAATTCATGTTTCCTGATTCCTGGTTCCTGTGGAGACTACTAAAGAGTTTAAGCGTTAAATTATTA 2200
Coquerel'sSifaka  -------TGCACTGGAATTCATGTTTCCTGATTCATAGATTCTATCCAGACTACTAAAGATTTTATGTGTTGCATTATAA 3589
Ma'sNightMonkey   CAAAACTTGCACTAGAATTCATGTTTCCTGATTCCTGGCTCCTATGGAGACAAGTAAAGAATTGAAGAGTTAAATTATTA 3518
VervetAGM         CAAAACTTGCACCAGAATTCATGTTTCCTGATTCCTGGTTCCTATGGAAACTACTAAAGAGTTTAAGCGTTAAATTATTA 3495
Gorrilla          CAAAACTTGCACTAGAATTCATGTTTCCTGATTCCTGGTTCCTGTGGAGACTACTAAAGAGTTTAAGCGTTAAATTATTA 3658
GrabEatingMacaqueCAAAACTTGCACCAGAATTCATGTTTCCTGATTCCTGGTTCCTATGGAAACTACTAAAGAGTTTAAGCGTTAAATTATTA 3435
Macaque           CAAAACTTGCACCAGAATTCATGTTTCCTGATTCCTGGTTCCTATGGAAACTACTAAAGAGTTTAAGCGTTAAATTATTA 3442
PigTailedMacaque  CAAAACTTGCACCAGAATTCATGTTTCCTGATTCCTGGTTCCTATGGAAACTACTAAAGAGTTTAAGCGTTAAATTATTA 3660
Bonobo            CAAAACTTGCACTAGAATTCATGTTTCCTGATTCCTGGTTCCTGTGGAGACTACTAAAGAGTTTAAGCGTTAAATTATTA 3507
OliveBaboon       CAAAACTTGCACCAGAATTCATGTTTCCTGATTCCTGGTTCCTATGGAAACTACTAAAGAGTTTAAGCGTTAAATTATTA 3399
Orangutan         CAAAACTTGCACTAGAATTCATGTTTCCTGATTCCTGGTTCCTATGGAGACTACTAAAGAGTTTAAGTGTTAAATTATTA 3498

Majority          ATCTCAGTATACTGGCATTGTAGAAATGGCA---TAGTGTTTTGGGTTTTGTTC-TTTTGGCTATTGAATGTGTTGTTAA
                          4010      4020      4030      4040      4050      4060      4070      4080
Human             ATCTCAGTATACTGGCATTGTAGAAATGGCA---TAGTGTTTTGGGTTTTGTTCTTTTTGGCTATTGAATGTGTTGTTAA 2277
Coquerel'sSifaka  ATTTAAGTGTACTAACATTGTAGAAATGGCACTATAGTGTGTTGGGTTTTGTTC-TTTTTGCTTTTGAAAGTCTTGCTAA 3668
Ma'sNightMonkey   ATCTCAGTATACTGGCATTGTAGAAATGGCA---TGGTGTTTTGGGTTTTGTTCTTTTTGTCTATTGAATGTGTTGTTAA 3595
VervetAGM         ATTTCGGTATACTGGCATTGTGGAAATGGCA---TAGTGTTTTGGGTTTTGTTC-TTTTGGCTATTGAATATGTTGTCAA 3571
Gorrilla          ATCTCAGTATACTGGCATTGTAGAAATGGCA---TAGTGTTTTGGGTTTTGTTCTTTTTGGCTATTGAATGTGTTGTTAA 3735
GrabEatingMacaqueATCTCAGTGTATTGGTATTGTGGAAATGGCA---TAGTGTTTTGGGTTTTGTTC-TTTTGGCTATTGAATATGTTGTCAA 3511
Macaque           ATCTCAGTGTATTGGTATTGTGGAAATGGCA---TAGTGTTTTGGGTTTTGTTC-TTTTGGCTATTGAATATGTTGTCAA 3518
PigTailedMacaque  ATCTCAGTGTATTGGCATTGTGGAAATGGCA---TAGTGTTTTGGGTTTTGTTC-TTTTGGCTATTGAATATGTTGTCAA 3736
Bonobo            ATCTCAGTATACTGGCATTGTAGAAATGGCA---TAGTGTTTTGGGTTTTGTTCTTTTTGGCTATTGAATGTGTTGTTAA 3584
OliveBaboon       ATCTCAGTGTATTGGCATTGTGGAAATGGCA---TAGTGTTTTGGGTTTTGTTC-TTTTGGCTATTGAATATGTTGTCAA 3475
Orangutan         ATCTCAGTATACTGGCATTGTAGAAATGGCA---TAGTGTTTTGGGTTTTGTTCCTTTTGGCTATTGAATGTGTTGTTAA 3575
```

```
Majority          AA--GATACCACAAGGTTACTGAATTT-TGTCTCACGTTTGAAAGTTACTTTTAGAAAACCAGGTTGATAACAGTAC--A

                           4090      4100      4110      4120      4130      4140      4150      4160

Human             AA--AATACCACAAG---------T---TGTCTCACATTTGAAAGTTACTTTTAGAAAACCAGGTTGGTAACAGTAC--A 2341
Coquerel'sSifaka  AAAAGATAGCGTAAGTTTACTGAGTTT-CATTTCATATT-GAAAGTTATTTCTAGAAAGTCAAGTAGATAATAGTGCTAA 3746
Ma'sNightMonkey   AA--TATACCACAAGTTTATTGAATTT-TGTCTCAGGTTTGAAAGTTATTTTTAGTAA-CCAGGTTGATAACAGTA---- 3667
VervetAGM         AA--GATACCACGAGGTTACTGAATTTTTGCCTCACGTTTGAAAGTTACTTTTAGAAAACCAGGTTGATAACAGTAC--A 3647
Gorrilla          AA--AATACCACAAG---------T---TGTCTCACATTTGAAAGTTACTTTTAGAAAACCAGGTTGGTAACAGTAC--A 3799
GrabEatingMacaqueAA--GATACCACGAGGTTACTGAATTTTTGCCTCACGTTTGAAAGTTACTTTTAGAAAACCAGGTTGATAACAGTAC--A 3587
Macaque           AA--GATACCACGAGGTTACTGAATTTTTGCCTCACGTTTGAAAGTTACTTTTAGAAAACCAGGTTGATAACAGTAC--A 3594
PigTailedMacaque  AA--GATACCACGAGGTTACTGAATTTTTGCCTCACGTTTGAAAGTTACTTTTAGAAAACCAGGTTGATAACAGTAC--A 3812
Bonobo            AA--AATACCACAAG---------T---TGTCTCACATTTGAAAGTTACTTTTAGAAAACCAGGTTGGTAACAGTAC--A 3648
OliveBaboon       AA--GATACCATGAGGTTACTGAATTTTTGCCTCACGTTTGAAAGTTACTTTTAGAAAACCAGATTGATAACAGTAC--A 3551
Orangutan         AA--AATACCACAAG---------T---TGTCTCACATTTGAAAGTTAGTTTTAGAAAACCAGGTTGGTAACAGTAC--A 3639

Majority          GGTAAGTCACTGACC-ATCTGTGGTATTTTTTTT---CCCCATCTGTGTGTGAGTGGCTGGAAGATTTATGAATGATGAA

                           4170      4180      4190      4200      4210      4220      4230      4240

Human             GGTAAGTCACGGATC-ATCTGTGGTATTTTTTAT---CCCCATCTGTGTGTGAGTGGCTGGAAGATTTATGAATGATGGA 2417
Coquerel'sSifaka  GAAGTGTCACTGACC-ATCTGTGTGGTTTTTTC-------CATCTGCAC---TGTGTCTG-AAGATTTATGAACGGTGAG 3814
Ma'sNightMonkey   GGTAAGTCACTGACCCATCTGTGGTATTTTTTTTTTTCCCCATCTGTATGTGAGTGGCTGGAAGACTTATGAATGATGAA 3747
VervetAGM         GGTAAGTCACTGACC-ATCTGTGGTATTTTTTTT---CCCCATCCGTGTGTGAGTGGCTGGAAGATTTATGAATGATGAA 3723
Gorrilla          GGCAAGTCACTGATC-ATCTGTGGTATTTTTTTTT--CCCCATCTGTGTGTGAGTGGCTGGAAGATTTATGAATGGTGGA 3876
GrabEatingMacaqueGGTAAGTCACTGACC-ATCTGTGGTATTTTTTT----CCCCATCTGTGTGTGAGTGGCTGGAAGATTTATGAATGATGAA 3662
Macaque           GGTAAGTCACTGACC-ATCTGTGGTATTTTTTT----CCCCATCTGTGTGTGAGTGGCTGGAAGATTTATGAATGATGAA 3669
PigTailedMacaque  GGTAAGTCACTGACC-ATCTGTGGTATTTTTTTT---CCCCATCTGTGTGTGAGTGGCTGGAAGATTTATGA-TGATGAA 3887
Bonobo            GGTAAGTCACTGATC-ATCTGTGGTATTTTTTTT---CCCCATCTGTGTGTGAGTGGCTGGAAGATTTATGAATGATGGA 3724
OliveBaboon       GGTAAGTCACTGACC-ATCTGTGGTATTTTTTTT---CCCCATCTGTGTGTGAGTGGCTGGAAGATTTATGAATGATGAA 3627
Orangutan         GGTAAGTCACTGATC-ATCTGTGGTATTTTTTTTT--CCCCATCTGTGTGTGAGTGGCTGGAAGATTTATGAATGATGAA 3716

Majority          CTATCCTAAAAAGTTTTAGAGCCAAAGAG----TGATTAGAAGAATAACAAGAAGTTGAAACTATTTATTTTTTCTTTGC

                           4250      4260      4270      4280      4290      4300      4310      4320

Human             CTATCCTAAAAAGTTTTAGAGCCAAAGAGTGAGTGATTAGAAGAATAACAGGAGGTTGAAACTACTTATTTTTTCTTTGC 2497
Coquerel'sSifaka  AGCTACCCTAAAGTTTTAGAGTCAAACAG----TGATCAGTAGAAAAATAATGAGTTGAAACTATTTATTTTTTCTTTTC 3890
Ma'sNightMonkey   CTGTCCTAAAAAGTTTTAGAGCCA--G------TGATTAGAAGAATAACAAGAAGTTGAAACTATTTATTTTTTCTTTAC 3819
VervetAGM         CTATCCTAAAAAGTTGTAGAGCCAAAGAG----TGATTAGAAGAATAACAAGAAGTTGAAACTATTTATTTTTTCTTTGC 3799
Gorrilla          CTATCCTAAAAAGTTTTAGAGCCAAAGAGTGAGTGATTAGAAGAATAACAGGAAGTTGAAACTACTTGTTTTTTCTTTGC 3956
GrabEatingMacaqueCTATCTTAAAAAGTTGTAGAGCCAAAGAG----TGATTAGAAGAATAACAAGAAGTTGAAACTATTTATTTTTTCTTTGC 3738
Macaque           CTATCTTAAAAAGTTGTAGAGCCAAAGAG----TGATTAGAAGAATAACAAGAAGTTGAAACTATTTATTTTTTCTTTGC 3745
PigTailedMacaque  CTATCTTAAAAAGTTGTAGAGCCAAAGAG----TGATTAGAAGAATAATAAGAAGTTGAAACTATTTATTTTTTCTTTGC 3963
Bonobo            CTATCCTAAAAAGTTTTAGAGCCAAAGAGCGAGTGATTAGAAGAATAACAGGAAGTTGAAACTACTTATTGTTTCTTTGC 3804
OliveBaboon       CTATGCTAAAAAGTTGTAGAGCCAAAGAG----TGATTAGAAGAATAACAAGAAGTTGAAACTATTTATTTTTTCTTTGC 3703
Orangutan         CTATCCTAAAAAGTTTTAGAGCCAAAGAGTGAGTGATTAGAAGAATAACAGGAAGTTGAAACTATTTATTTTTTCTTTGC 3796
```

```
Majority          AGAATCGTCACTGAGGTTGCAGTTGGTGTGAAGGTAGGAAAAGATTATGTTTGAAACTG-----------GGATTGTTGG

                           4330      4340      4350      4360      4370      4380      4390      4400

Human             AGAATCGTCACTGAGGTTGCAGTTGGCGTGAAGGTAGGAAAATACTATGTTTGAAACTG-----------GGATTGTTGG 2566
Coquerel'sSifaka  AGAATTGTCACTGAGGTTGCAGTTGGTGTGAAGGTAGGAAAAGATTATGTTTGAAACTAATACGAAAACTGGATTGTTGA 3970
Ma'sNightMonkey   AGAATCGTCACTGAAGTTGCAGTTGGTGTGAAGGTAGGAAAAGATTATGTTTGAAACTG-----------GGATTGTTGG 3888
VervetAGM         AGAATCGTCACTGAGGTTGCAGTTGGTGTGAAGGTAGGAAAAGATTATGTTTGAAACTG-----------GGATCGTTGG 3868
Gorrilla          AGAATCGTCACTGAGGTTGCAGTTGGCGTGAAGGTAGGAAAATACTATGTTTGAAACTG-----------GGATTGTTGG 4025
GrabEatingMacaqueAGAATCGTCACTGAGGTTGCAGTTGGTGTGAAGGTAGGAAAAGATTATGTTTGAAACTG-----------GGATCGTTGG 3807
Macaque           AGAATCGTCACTGAGGTTGCAGTTGGTGTGAAGGTAGGAAAAGATTATGTTTGAAACTG-----------GGATCGTTGG 3814
PigTailedMacaque  AGAATCGTCACTGAGGTTGCAGTTGGTGTGAAGGTAGGAAAAGATTATGTTTGAAACTG-----------GGATCGTTGG 4032
Bonobo            AGAATCGTCACTGAGGTTGCAGTTGGCGTGAAGGTAGGAAAATACTATGTTTGAAACTG-----------GGATTGTTGG 3873
OliveBaboon       AGAATCGTCACTGAGGTTGCAGTTGGTGTGAAGGTAGGAAAAGATTCTGTTTGAAACTG-----------GGATCGTTGG 3772
Orangutan         AGAATCGTCACTGAGGTTGCAGTTGGCGTGAAGGTAGGAAAATACTATGTTTGAAACTG-----------GGATTGTTGG 3865

Majority          ATCTACTGTTATCATAATTACTGGAAAATTTTAGCTTTTGCTTTTGAAAACTATAATGTAGCTAACAAAACCCAAGTGTA

                           4410      4420      4430      4440      4450      4460      4470      4480

Human             ATCTA---TTATCATAATTACTGGAAAATTTTAGCTTTTGCTTTTGAAAACTATAACGTAGCTAACAAAACCCAAGTGTA 2643
Coquerel'sSifaka  ATCTACTCTTA---TAATTAATGGAAAATTTCAG--TTAGCTTTTGAAAACTATAAGGTACCTAATAAAACCCAAGTATA 4045
Ma'sNightMonkey   ATCTATTATTATGATAATTACTGGAAAATTTTAG--TTAGCTTTTGAAAACTATAATGTAGCTAACAAAACCCAAGTGTA 3966
VervetAGM         ATCTACTGTTA---TAATGACTGGAAAATTTTAGCGTTTGCTTTTGAAAACTATACTGTAGCTAACAAAACCCAAGTGTA 3945
Gorrilla          ATCTA---TTATCATAATTACTGGAAAATTTTAGCTTTTGCTTTTGAAAACTATAACGTAGCTAACAAAACCCAAGTGTA 4102
GrabEatingMacaqueATCTACTGTCATCATAATTACTGGAAAATTTTAGCTTTTGCTTTTGAAAACTATACTGTAGCTAACAAAACCCAAGTGTA 3887
Macaque           ATCTACTGTCATCATAATTACTGGAAAATTTTAGCTTTTGCTTTTGAAAACTATACTGTAGCTAACAAAACCCAAGTGTA 3894
PigTailedMacaque  ATCTACTGTCATCATAATTACTGGAAAATTTTAGCTTTTGCTTTTGAAAACTATACTGTAGCTAACAAAACCCAAGTGTA 4112
Bonobo            ATCTA---TTATCATAATTACTGGAAAATTTCAGCTTTTGCTTTTGAAAACTATAACGTAGCTAACAAAACCCAAGTGTA 3950
OliveBaboon       ATCTACTGTTATCATAATTACTGGAAAATTTTAGCTTTTGCTTTTGAAAACTATACTGTAGCTAACAAAACCCAAGTGTA 3852
Orangutan         ATCTACTATTATCATAATTACTGGAAAATTTTAGCTTTTGCTTTTGAAAACTATAATGTAGTTAACAAAACCCAAGTGTA 3945

Majority          AAATGTTTCCTTTCATTTGTAAAGTTGGACCATATAA---ACAGTTTGTAATCACCA-TAAGTTTTTCTAGAACTGGCAA

                           4490      4500      4510      4520      4530      4540      4550      4560

Human             AAATGTTTCCTTTCATTTGTAAAGTTGGACCATATAA---ACAGTTTGTAATCACCA-TAAGTTTTTCTAGAACTGTCAG 2719
Coquerel'sSifaka  AAATGTTTCCTTTAATTTGTAAAGTTGC-CTATATAGTTAATAATCTAAATAAACAACTAAGTTTTTCTAGAATTGGTAA 4124
Ma'sNightMonkey   AAATGTTTCCTTTAATTTGTAAAGTTGGACCATATAA---AGAGTTTGCAGTCACCA-TAAGTTTTTCTAGAACTGTCAC 4042
VervetAGM         AAATGTTTCCTTTCATTTCTAAAGTTGGACCATATTA---ACAGTTTGTAATCACCA-TAAGTTTTTCTAGAACTGGCCA 4021
Gorrilla          AAATGTTTCCTTTCATTTGTGAAGTTGGACCATATAA---ACAGTTTGTAATCACCA-TAAGTTTTTCTAGAACTGGCAG 4178
GrabEatingMacaqueAAATGTTTC-----ATTTCTAAAGTTGGACCATATTA---ACAGTTTGTAATCACCA-TAAGTTTTTCTAGAACTGGCCA 3958
Macaque           AAATGTTTC-----ATTTCTAAAGTTGGACCATATTA---ACAGTTTGTAATCACCA-TAAGTTTTTCTAGAACTGGCCA 3965
PigTailedMacaque  AAATGTTTT-----ATTTCTAAAGTTGGACCATGTTA---ACAGTTGGTAATCACCA-TAAGTTTTTCTAGAACTGGCCA 4183
Bonobo            AAATGTTTCCTTTCATTTGTAAAGTTGGACCATATAA---ACAGTTTGTAATCACCA-TAAGTTTTTCTAGAACTGTCAG 4026
OliveBaboon       AAATGTTTC-----ATTTCTAAAGTTGGACCATATTA---ACAGTTTGTAATCACCA-TAAGTTTTTCTAGAACTGGCCA 3923
Orangutan         AAGTATTTCCTTTCATTTGTAAAGTTGGACCATATA-----CAGTTTGTAATCACCA-TAAGTTTTTCTAGAACTGGCAG 4019
```

```
Majority          TCTGTAAGAATAACCTTT-TAAAGTAAGTATGAATGAT---CAGGAAATTAAAAATGCTTT-CACACATGGCTTCTTGT-

                           4570      4580      4590      4600      4610      4620      4630      4640

Human             TCTGTAAGAATAACCTTT-TAAAGTAAGTATGAATGAT---CAAGAAATTGAAAATGCTTT-CACACATGGCTTCTTGT- 2793
Coquerel'sSifaka  TCTGTAAGAGCATTTTAAACAAAGTATGTATGAATGGTGTTCAGTGAATTAAAAATGCTTTTCATACATGGCTTCTTGTG 4204
Ma'sNightMonkey   TGTGTAAGAATAACCTTT-TAAAGTAAGTATGAATGAT---CAAGGAATTAAAAATGCTTT-CACACATGGCTTCTTGTG 4117
VervetAGM         TCTGTAAGAATAACCTTT-TAAAGTAAGTATGAATGAT---CAGGAAATTAAAAATGCTTT-CACACATGGCTTCTTGT- 4095
Gorrilla          TCTGTAAGAATAACCTTT-TAAAGTAAGTATGAATGAT---CAAGAAATTGAAAATGCTTT-TACACATGGCTTCTTGT- 4252
GrabEatingMacaqueTCTGCAAGAATAACCTTT-TAAAGTAAGTATGAATGAT---CAGGAAATTAAAAATGCTTT-CATACATGGCTTCTTGT- 4032
Macaque           TCTGCAAGAATAACCTTT-TAAAGTAAGTATGAATGAT---CAGGAAATTAAAAATGCTTT-CATACATGGCTTCTTGT- 4039
PigTailedMacaque  TCTGCAAGAATAACCTTT-TAAAGTAAGTATGAATGAT---CAGGAAATTAAAAATGCTTT-CATACATGGCTTCTTGT- 4257
Bonobo            TCTGTAAGAATAACCTTT-TAAAGTAAGTATGAATGAT---CAAGAAATTGAAAATGCTTT-CACACATGGCTTCTTGT- 4100
OliveBaboon       TCTGCAAGAATAACCTTT-TAAAATAAGTATGAATGAT---CAGGAAATTAAAAATGCTTT-CATACATGGCTTCTTGT- 3997
Orangutan         TCTGTAAGAATAACCTTT-TAAAGTAAGTATGAATGAT---CAAGAAATTGAAAATGCTTT-CACACATGGCTTCTTGT- 4093

Majority          AATATAAAATGATACCCCAATTTTCTGAGGGGAAATAATG-ATTTTAAATGGATGGTAAGTGGTTTGACCTATATCTTAT

                           4650      4660      4670      4680      4690      4700      4710      4720

Human             AATATAAAACGATACCCCAATTTTCTGAGGGGAAATAATG-ATTTTAAATGGATGGTAAGTGGTTTGACCTATATCTTAT 2872
Coquerel'sSifaka  AACACAAAATAATATCCTGGTTTTTTGAGTGGAAACAATGGAGTTTGAATGGACGATAAGTGGTTTGACTTAAATCATAT 4284
Ma'sNightMonkey   AATATAAAATGATATCGCAGTTTTCTGAGGGGAAATAATG-ATTTTAAGTGGATGGTAAGTGGTTTGACTTGTATCTTAT 4196
VervetAGM         AATATAAAATTATACCCCAATTTTCTGAGGGGAAATAATG-ATTTTAAATGGATGGTAAGTGGTTTGACCTATATCTTAT 4174
Gorrilla          AATATAAAACGATACCCCAGTTTTCTGAGGGGAAATAATG-ATTTTAAATGGATGGTAAGTGGTTTGACCTATATCTTAT 4331
GrabEatingMacaqueAATATAAAATGATACCCCAATTTTCTGAGGGGAAATAATG-ATTTTAAATGGATGGTAAGTGGTTTGACCTATATCTTAT 4111
Macaque           AATATAAAATGATACCCCAATTTTCTGAGGGGAAATAATG-ATTTTAAATGGATGGTAAGTGGTTTGACCTATATCTTAT 4118
PigTailedMacaque  AATATAAAATGATACCCCAATTTTCTGAGGGGAAATAATG-ATTTTAAATGGATGGTAAGTGGTTTGACCTATATCTTAT 4336
Bonobo            AATATAAAACGATACCCCAATTTTCTGAGGGGAAATAATG-ATTTTAAATGGATGGTAAGTGGTTTGACCTATATCTTAT 4179
OliveBaboon       AATATAAAATGATACCCCAATTTTCTGAGGGGAAATAATG-ATTTTAAATGGATGGTAAGTGGTTTGACCTATATCTTAT 4076
Orangutan         AATATAAAACGATACCCCAATTTTCTGAGGGGAAATAATG-ATTT-AAAGGGACGGTAAGGGGTTTGACCTATATCTTAT 4171

Majority          AATTTTGTTAATCTAAGTTTTTTGTTT-------------TTTGTTTTTTTTTTTACTTTTTGTCATATGAAAATGAAT

                           4730      4740      4750      4760      4770      4780      4790      4800

Human             AATTTTGTTAATCTAAGTTTTTTGTTT-------------TTTATT---------ACTTTTTGTCAT--GAAAATGAAT 2927
Coquerel'sSifaka  AATTTTGTTAATCTAAGATTTTT---------------------TTTT--------ACTCCTGTGTTATGAAAATGAAA 4334
Ma'sNightMonkey   AATTTTGTTAATCTAAGATTTTTTCTT----------------TTTTTTTTTCTACTTTTTGTCTTATGAAAATGAAT 4259
VervetAGM         AATTTTGTTAATCTGT-TTTTTTGTT-----------------GTTTTTTTTTAACTTTTTGTCATATGAAAATGAAT 4235
Gorrilla          AATTT-GTTAATCTAAGTTTTTTGTTT-------------TTTGTTTTTTTT--ACTTTTTGTCAT--GAAAATGAAT 4392
GrabEatingMacaqueAATTTTGTTAATCTAAGTTTTTT---------------------TTTTTTTTTTTACTTTTTGTCATATGAAAATGAAT 4169
Macaque           AATTTTGTTAATCTAAGTTTTTTGTT------------------TTTTTTTTTTTACTTTTTGTCATATGAAAATGAAT 4179
PigTailedMacaque  AATTTTGTTAATCTAAGGTTTTTTTTT------------GTTTGTTTTTTGTTTTACTTTTTGTCATATGAAAATGAAT 4403
Bonobo            AATTTTGTTAATCTAAGTTTTTTGTTT-------------TTTGTT---------ACTTTTTGTCAT--GAAAATGAAT 4234
OliveBaboon       AATTTTGTTAATCTAAGTTTTTTTTTTTTGTTTTTTTTTTGTTTTTTTTTTTTTTACTTTTTGTCATATGAAAATGAAT 4156
Orangutan         AATTTTGTTAATCTAAGTTTTTTGTTT-------------TTTGTTTTTTTTTTTACTTTTTGTCATATGAAAATGAAT 4237
```

```
Majority          A-TATTTTGGTCAATAATAAGGCACATTAGAAATGAATTATCCTGATCCTTAAAAA-----GAAAGTTGTTGAGGCAATT

                         4810      4820      4830      4840      4850      4860      4870      4880

Human             A-TATTTTGGTTAATAATAAAGCACATTAGAAATGAATTACACTGATCCTTAAAAA-----GGAAGTTGTTGAGGCAGTT 3001
Coquerel'sSifaka  AATATTTTGGTCAATAATAAGGCACATTAGAAACGAGTTGTCCTGACCCTTAAAAAGACAAGAAAGTTGTTTAGGCAATT 4414
Ma'sNightMonkey   A-TATTTTAGTCAATAATAAGGCACATTAGAAATGAATTATCCTGATCCTTAAAAA-----GAAAGTTGTTGAGGCAATT 4333
VervetAGM         A-TATTTTGGTCAATAAGAAGGCACATTAGAAATGAATTATCCTGATCCTTAAAAA-----GAAAGTTGTTGAGGCAGTT 4309
Gorrilla          A-TATTTTGGTTAATAATAAAGCACATTAGAAATGAATTATACTGAGCCTTAAAA------GGAAGTTGTTGAGGCAGTT 4465
GrabEatingMacaqueA-TATTTTGGTCAATTAGAAGGCACATTAGAAATGAATTATCCTGATCCTTAAAAA-----GAAAGTTGTTGAGGCAATT 4243
Macaque           A-TATTTTGGTCAATTAGAAGGCACATTAGAAATGAATTATCCTGATCCTTAAAAA-----GAAAGTTGTTGAGGCAATT 4253
PigTailedMacaque  A-TATTTTGGTCAATAAGAAGGCACATTAGAAATGAATTATCCTGATCCTTAAAAA-----GAAAGTTGTTGAGGCAATT 4477
Bonobo            A-TATTTTGGTTAATAATAAAGCACATTAGAAATGAATTATACTGATCCTTAAAAA-----GGAAGTTGTTGAGGCAGTT 4308
OliveBaboon       A-TATTTTGGTCAATAAGAAGCCACATTAGAAATGAATTATCCTGATCCTTAAAAA-----GAAAGTTGTTGAGGCAATT 4230
Orangutan         A-TATTTTGGTCAATAATAAGGCACATTAGAAATGAATTATCCTGATCCTTAAAAA-----GAAAGTTGTTGAGGCAGTT 4311

Majority          AGCTTCAAATTTGAATCAGAAGTTAAGAGCTGGTTTGTCT--------ATTAAAGAAAGGCTTTATGGTAGAGATCCTTT

                         4890      4900      4910      4920      4930      4940      4950      4960

Human             AGCTTCAAATTTGAATTAGAAGTTAAGAGCTGGTTTGTCT--------ATTAAAGAAAGTCTTTATGGTAGAGATCCTTT 3073
Coquerel'sSifaka  ACCTTCAAATTTGAGTCAGAAGGTAAGAGTTGATTTGTCTTTCTATTAATGAAAGATAGACCTTATGGTAGAGATCCTTT 4494
Ma'sNightMonkey   AGCTTCAAATTTGAAACAGAAGTTGAGAGCTGGTTTG------------TTAAAGAAAGGCTTTATGGTAGAGATCGTTT 4401
VervetAGM         AGCTTCAAATTTGAATCAGAAGTTAAGAGCTGGTTTGTCT--------ATTAAAGAAAGGCTTTATGGTAGAGATCCTTT 4381
Gorrilla          AGCTTCAAATTTGAATTAGAAGTTAAGAGCTGGTTTGTCA--------ATTAAAGAAAGTCTTTATGGTAGAGATCCTTT 4537
GrabEatingMacaqueAGCTTCAAATTTGAATCAGAAGTTAAGAGCTGGTTTTTCT--------ATTAAAGAAAGGCTTTATGGTAGAGATCCTTT 4315
Macaque           AGCTTCAAATTTGAATCAGAAGTTAAGAGCTGGTTTTTCT--------ATTAAAGAAAGGCTTTATGGTAGAGATCCTTT 4325
PigTailedMacaque  AGCTTCAAATTTGAATCAGAAGTTAAGAGCTGGTTTTTCT--------ATTAAAGAAAGGCTTTATGGTAGAGATCCTTT 4549
Bonobo            AGCTTCAAATTTGAATTAGAAGTTAAGAGCTGGTTTGTCT--------ATTAAAGAAAGTCTTTATGGTAGAGATCCTTT 4380
OliveBaboon       AGCTTCAAATTTGAATCAGAAGTTAAGAGCTGGTTTTTCT--------ATTAAAGAAAGGCTTTATGGTAGAGATCCTTT 4302
Orangutan         AGCTTCAAATTTGAATTAGAAGTTAAGAGCTGGTTTGTCT--------ATTAAAGAAAGTCTTTATGGTAGAGATCCTTT 4383

Majority          GGAATCCTTCCT---------TTTGGCCAAGAAAACTACTCATCGTTTAAACTTTAGTTCTAATGCTTAACATTTTCTTG

                         4970      4980      4990      5000      5010      5020      5030      5040

Human             GGAATCCTTCCT--------TTTTGGCTAAGAAAACTACTCATCGTTTAAACTTTAGTTCTAATGCTTAACATTTTCTTG 3145
Coquerel'sSifaka  GAAATCCTTCCTCACTCACTTTTTGGCCAGGAAAACTATTTATCCTTTAAACTTTAGTTGTAATGCTTAGGTTTTTC--- 4571
Ma'sNightMonkey   GGAATCTTTCCT--------TTTTGGCTAAGAAAACTACTCATCCTTTAAACTTTAGTTCTAATGCTTACCATTTCCTTT 4473
VervetAGM         GGAATCCTTCCT---------TTTGGCCAAGAAAACTACTCATCGTTTAAACTTTAGTTCTAATGCTTAACATTTTCTTG 4452
Gorrilla          GGAATCCTT------------TTTGGCTAAGAAAACTACTCATCGTTTAAACTTTAGTTCTAATGCTTAACATTTTCTCG 4605
GrabEatingMacaqueGGAATCCTTCCT---------TTTGGCCAAGAAAACTACTCATCGTTTAAACTTTAGTTCTAATGCTTAACATTTTCTTG 4386
Macaque           GGAATCCTTCCT---------TTTGGCCAAGAAAACTACTCATCGTTTAAACTTTAGTTCTAATGCTTAACATTTTCTTG 4396
PigTailedMacaque  GGAATCCTTCCT---------TTTGGCCAAGAAAACTACTCATCGTTTAAACTTTAGTTCTAATGCTTAACATTTTCTTG 4620
Bonobo            GGAATCCTTCCT--------TTTTGGCTAAGAAAACTACTCATCGTTTAAACTTTAGTTCTAATGCTTAACATTTTCTTG 4452
OliveBaboon       GGAATCCTTCCT---------TTTGGCCAAGAAAACTACTCATCGTTTAAACTTTAGTTCTAATGCTTAACATTTTCTTG 4373
Orangutan         GGAATCCTTCCT--------TTTTGGCTAAGAAAACTACTCATCATTTAAACTTTAGTTCTAATGCTTAACATTTTCTTG 4455
```

```
Majority          AGTACTTGCAAGTTTTAATGTCA-TGATGTATTGTAATTTTTTTTTTTTTTTTG--------------------------

                          5050      5060      5070      5080      5090      5100      5110      5120

Human             AGTACTTGCAACTTTTAATGTCA-TGATGTATTGTAATTTTTTTTTGTTTTTTGTT------------------------ 3200
Coquerel'sSifaka  -----------ATTTTAACATCA-TGATATATTATAA---TTTTT---TGTTTG-------------------------- 4607
Ma'sNightMonkey   AGTACTTAAAACTTTTAATGTCA-TAATGTATTGTAATTTTTCTTCCTCTTTTTTT------------------------ 4528
VervetAGM         AGTACTTGCAAGTTTTAATGTCAACGATGTATTGTAATTTTTTTTTTTTTTTTG-------------------------- 4506
Gorrilla          AGTACTTGCAACTTTTAATGTCA-TGATGTATTGTAATTTTTTTTTTTTTTTTTTTGAGATGGAGCCTCACTGTGTTGCC 4684
GrabEatingMacaque AGTACTTGCAAGTTTTAATGTCA-CGATGTATTGTAA-TTTTTTTTTTTTTTTG-------------------------- 4438
Macaque           AGTACTTGCAAGTTTTAATGTCA-CGATGTATTGTAA-TTTTTTTTTTTTTTTG-------------------------- 4448
PigTailedMacaque  AGTACTTGCAAGTTTTAATGTCA-CGATATATTGTAA-TTTTTTTTTTTTTTTG-------------------------- 4672
Bonobo            AGTACTTGCAACTTTTAATGTCA-TGATGTATTGTAATTTTTTTTTTTTTTTTTTT------------------------ 4507
OliveBaboon       AGTACTTGCAAGTTTTAATGTCA-CGATGTATTGTAAATTTTTTTTTTTTTTTG-------------------------- 4426
Orangutan         AGTACTTGCAACTTTTAATGTCA-TGATGTATTGTAATTTTTTTTAGTTTTTTTTT------------------------ 4510

Majority          --------------------------------------------------------------------------------

                          5130      5140      5150      5160      5170      5180      5190      5200

Human             -------------------------------------------------------------------------------- 3200
Coquerel'sSifaka  -------------------------------------------------------------------------------- 4607
Ma'sNightMonkey   -------------------------------------------------------------------------------- 4528
VervetAGM         -------------------------------------------------------------------------------- 4506
Gorrilla          CAGGCTGGATGCAGTGGCACGATCTTGGCCCACTGCAACCTCTGCCTCCTGGGTTCAAGCAGTTCTCCTGCCTCAGCCTC 4764
GrabEatingMacaque -------------------------------------------------------------------------------- 4438
Macaque           -------------------------------------------------------------------------------- 4448
PigTailedMacaque  -------------------------------------------------------------------------------- 4672
Bonobo            -------------------------------------------------------------------------------- 4507
OliveBaboon       -------------------------------------------------------------------------------- 4426
Orangutan         -------------------------------------------------------------------------------- 4510

Majority          --------------------------------------------------------------------------------

                          5210      5220      5230      5240      5250      5260      5270      5280

Human             -------------------------------------------------------------------------------- 3200
Coquerel'sSifaka  -------------------------------------------------------------------------------- 4607
Ma'sNightMonkey   -------------------------------------------------------------------------------- 4528
VervetAGM         -------------------------------------------------------------------------------- 4506
Gorrilla          CTGAGTAGCTGGGATTACAGGTGCGCACCACCACGCCCAGCTAATTTGTATTTTTAGTAGAAATGGCATTTCACCCTGTT 4844
GrabEatingMacaque -------------------------------------------------------------------------------- 4438
Macaque           -------------------------------------------------------------------------------- 4448
PigTailedMacaque  -------------------------------------------------------------------------------- 4672
Bonobo            -------------------------------------------------------------------------------- 4507
OliveBaboon       -------------------------------------------------------------------------------- 4426
Orangutan         -------------------------------------------------------------------------------- 4510
```

```
Majority        --------------------------------------------------------------------------------

                         5290      5300      5310      5320      5330      5340      5350      5360

Human           -------------------------------------------------------------------------------- 3200
Coquerel'sSifaka -------------------------------------------------------------------------------- 4607
Ma'sNightMonkey -------------------------------------------------------------------------------- 4528
VervetAGM       -------------------------------------------------------------------------------- 4506
Gorilla         GGTCAGGCTGGTCTCAAACTCCTGACCTCATGATCTGCCTGCCTCTTATGATCTGCCTGCCTTGACTTCCCAAAGTGCTG 4924
GrabEatingMacaque-------------------------------------------------------------------------------- 4438
Macaque         -------------------------------------------------------------------------------- 4448
PigTailedMacaque -------------------------------------------------------------------------------- 4672
Bonobo          -------------------------------------------------------------------------------- 4507
OliveBaboon     -------------------------------------------------------------------------------- 4426
Orangutan       -------------------------------------------------------------------------------- 4510

Majority        --------------------------------------------------------------------------------

                         5370      5380      5390      5400      5410      5420      5430      5440

Human           -------------------------------------------------------------------------------- 3200
Coquerel'sSifaka -------------------------------------------------------------------------------- 4607
Ma'sNightMonkey -------------------------------------------------------------------------------- 4528
VervetAGM       -------------------------------------------------------------------------------- 4506
Gorilla         GGATTACAAGTGTGAGCCACCGTGCCCGGCCTGTATTGTCATTTTTCATTTGCAAGTCTCTCTGTCCTCAGAGGCTATCT 5004
GrabEatingMacaque-------------------------------------------------------------------------------- 4438
Macaque         -------------------------------------------------------------------------------- 4448
PigTailedMacaque -------------------------------------------------------------------------------- 4672
Bonobo          -------------------------------------------------------------------------------- 4507
OliveBaboon     -------------------------------------------------------------------------------- 4426
Orangutan       -------------------------------------------------------------------------------- 4510

Majority        --------------------------------------------------------------------------------

                         5450      5460      5470      5480      5490      5500      5510      5520

Human           -------------------------------------------------------------------------------- 3200
Coquerel'sSifaka -------------------------------------------------------------------------------- 4607
Ma'sNightMonkey -------------------------------------------------------------------------------- 4528
VervetAGM       -------------------------------------------------------------------------------- 4506
Gorilla         GCTTAGTCCAGTGATGCTGACTGTTCAAACATTTTTTTGAATTCTTAAAATTGTATCCATAATGGGGGAGATGGGTAAGA 5084
GrabEatingMacaque-------------------------------------------------------------------------------- 4438
Macaque         -------------------------------------------------------------------------------- 4448
PigTailedMacaque -------------------------------------------------------------------------------- 4672
Bonobo          -------------------------------------------------------------------------------- 4507
OliveBaboon     -------------------------------------------------------------------------------- 4426
Orangutan       -------------------------------------------------------------------------------- 4510
```

```
Majority           --------------------------------------------------------------------------------

                            5530      5540      5550      5560      5570      5580      5590      5600

Human              -------------------------------------------------------------------------------- 3200
Coquerel'sSifaka   -------------------------------------------------------------------------------- 4607
Ma'sNightMonkey    -------------------------------------------------------------------------------- 4528
VervetAGM          -------------------------------------------------------------------------------- 4506
Gorrilla           CATTGCCTTGAAAGATATAGGGAAAAACATTTTCAGTATCCAAGAATCCTTGTTTTAGTGGATGGGGAGAAGGGAGTTGT 5164
GrabEatingMacaque  -------------------------------------------------------------------------------- 4438
Macaque            -------------------------------------------------------------------------------- 4448
PigTailedMacaque   -------------------------------------------------------------------------------- 4672
Bonobo             -------------------------------------------------------------------------------- 4507
OliveBaboon        -------------------------------------------------------------------------------- 4426
Orangutan          -------------------------------------------------------------------------------- 4510

Majority           --------------------------------------------------------------------------------

                            5610      5620      5630      5640      5650      5660      5670      5680

Human              -------------------------------------------------------------------------------- 3200
Coquerel'sSifaka   -------------------------------------------------------------------------------- 4607
Ma'sNightMonkey    -------------------------------------------------------------------------------- 4528
VervetAGM          -------------------------------------------------------------------------------- 4506
Gorrilla           CTGTGTTGAGAATATTACTGCAGTTTAGATCTGCAAGAAATCTTTAAAATCAGTCATTGCAAACCTCTTTTTGTTTTTCA 5244
GrabEatingMacaque  -------------------------------------------------------------------------------- 4438
Macaque            -------------------------------------------------------------------------------- 4448
PigTailedMacaque   -------------------------------------------------------------------------------- 4672
Bonobo             -------------------------------------------------------------------------------- 4507
OliveBaboon        -------------------------------------------------------------------------------- 4426
Orangutan          -------------------------------------------------------------------------------- 4510

Majority           --------------------------------------------------------------------------------

                            5690      5700      5710      5720      5730      5740      5750      5760

Human              -------------------------------------------------------------------------------- 3200
Coquerel'sSifaka   -------------------------------------------------------------------------------- 4607
Ma'sNightMonkey    -------------------------------------------------------------------------------- 4528
VervetAGM          -------------------------------------------------------------------------------- 4506
Gorrilla           ATGAAGGAGGATGAGGTATAGCATTTCCTTTCTTTCTATCCCTCACACCTCTGAAAAATAGGACCGACACCAACATTCTT 5324
GrabEatingMacaque  -------------------------------------------------------------------------------- 4438
Macaque            -------------------------------------------------------------------------------- 4448
PigTailedMacaque   -------------------------------------------------------------------------------- 4672
Bonobo             -------------------------------------------------------------------------------- 4507
OliveBaboon        -------------------------------------------------------------------------------- 4426
Orangutan          -------------------------------------------------------------------------------- 4510
```

```
Majority        --------------------------------------------------------------------------------

                        5770      5780      5790      5800      5810      5820      5830      5840

Human           -------------------------------------------------------------------------------- 3200
Coquerel'sSifaka -------------------------------------------------------------------------------- 4607
Ma'sNightMonkey -------------------------------------------------------------------------------- 4528
VervetAGM       -------------------------------------------------------------------------------- 4506
Gorrilla        TAATGAGAGTCATGTAATTTAAAAAATATTTTGTTAAGTTTTATAAGAAAAAAGTCATTTTGGAATTTGAAGATAAAACA 5404
GrabEatingMacaque-------------------------------------------------------------------------------- 4438
Macaque         -------------------------------------------------------------------------------- 4448
PigTailedMacaque -------------------------------------------------------------------------------- 4672
Bonobo          -------------------------------------------------------------------------------- 4507
OliveBaboon     -------------------------------------------------------------------------------- 4426
Orangutan       -------------------------------------------------------------------------------- 4510

Majority        --------------------------------------------------------------------------------

                        5850      5860      5870      5880      5890      5900      5910      5920

Human           -------------------------------------------------------------------------------- 3200
Coquerel'sSifaka -------------------------------------------------------------------------------- 4607
Ma'sNightMonkey -------------------------------------------------------------------------------- 4528
VervetAGM       -------------------------------------------------------------------------------- 4506
Gorrilla        TTTAAACAAAGTCGACCTAAGTAAAATGGAACATCAAACCCTAGATGATAAGAAACACCAAAGTAAATAGCATGAGATTG 5484
GrabEatingMacaque-------------------------------------------------------------------------------- 4438
Macaque         -------------------------------------------------------------------------------- 4448
PigTailedMacaque -------------------------------------------------------------------------------- 4672
Bonobo          -------------------------------------------------------------------------------- 4507
OliveBaboon     -------------------------------------------------------------------------------- 4426
Orangutan       -------------------------------------------------------------------------------- 4510

Majority        --------------------------------------------------------------------------------

                        5930      5940      5950      5960      5970      5980      5990      6000

Human           -------------------------------------------------------------------------------- 3200
Coquerel'sSifaka -------------------------------------------------------------------------------- 4607
Ma'sNightMonkey -------------------------------------------------------------------------------- 4528
VervetAGM       -------------------------------------------------------------------------------- 4506
Gorrilla        GAGTAGGAAGTGAATGCTTTCATAATACTTTTCCTGCCTTTTCTTCCTGGAGCTGCTTCTTGAGAATGAAATAGCTTTTC 5564
GrabEatingMacaque-------------------------------------------------------------------------------- 4438
Macaque         -------------------------------------------------------------------------------- 4448
PigTailedMacaque -------------------------------------------------------------------------------- 4672
Bonobo          -------------------------------------------------------------------------------- 4507
OliveBaboon     -------------------------------------------------------------------------------- 4426
Orangutan       -------------------------------------------------------------------------------- 4510
```

```
Majority         --------------------------------------------------------------------------------
                          6010      6020      6030      6040      6050      6060      6070      6080
Human            -------------------------------------------------------------------------------- 3200
Coquerel'sSifaka -------------------------------------------------------------------------------- 4607
Ma'sNightMonkey  -------------------------------------------------------------------------------- 4528
VervetAGM        -------------------------------------------------------------------------------- 4506
Gorilla          AACTGNNNNNNNNNNCTGAGGGGAAATAATGATTTTAAATGGATGGTAAGTGGTTTGACCTATATCTTATAATTTGTTAA 5644
GrabEatingMacaque-------------------------------------------------------------------------------- 4438
Macaque          -------------------------------------------------------------------------------- 4448
PigTailedMacaque -------------------------------------------------------------------------------- 4672
Bonobo           -------------------------------------------------------------------------------- 4507
OliveBaboon      -------------------------------------------------------------------------------- 4426
Orangutan        -------------------------------------------------------------------------------- 4510

Majority         --------------------------------------------------------------------------------
                          6090      6100      6110      6120      6130      6140      6150      6160
Human            -------------------------------------------------------------------------------- 3200
Coquerel'sSifaka -------------------------------------------------------------------------------- 4607
Ma'sNightMonkey  -------------------------------------------------------------------------------- 4528
VervetAGM        -------------------------------------------------------------------------------- 4506
Gorilla          TCTAAGTTTTTTGTTTTTTGTTTTTTTTTACTTTTTGTCATGAAAATGAATATATTTTGGTTAATAATAAAGCACATTAG 5724
GrabEatingMacaque-------------------------------------------------------------------------------- 4438
Macaque          -------------------------------------------------------------------------------- 4448
PigTailedMacaque -------------------------------------------------------------------------------- 4672
Bonobo           -------------------------------------------------------------------------------- 4507
OliveBaboon      -------------------------------------------------------------------------------- 4426
Orangutan        -------------------------------------------------------------------------------- 4510

Majority         --------------------------------------------------------------------------------
                          6170      6180      6190      6200      6210      6220      6230      6240
Human            -------------------------------------------------------------------------------- 3200
Coquerel'sSifaka -------------------------------------------------------------------------------- 4607
Ma'sNightMonkey  -------------------------------------------------------------------------------- 4528
VervetAGM        -------------------------------------------------------------------------------- 4506
Gorilla          AAATGAATTATACTGAGCCTTAAAAGGAAGTTGTTGAGGCAGTTAGCTTCAAATTTGAATTAGAAGTTAAGAGCTGGTTT 5804
GrabEatingMacaque-------------------------------------------------------------------------------- 4438
Macaque          -------------------------------------------------------------------------------- 4448
PigTailedMacaque -------------------------------------------------------------------------------- 4672
Bonobo           -------------------------------------------------------------------------------- 4507
OliveBaboon      -------------------------------------------------------------------------------- 4426
Orangutan        -------------------------------------------------------------------------------- 4510
```

```
Majority          --------------------------------------------------------------------------------

                          6250      6260      6270      6280      6290      6300      6310      6320

Human             -------------------------------------------------------------------------------- 3200
Coquerel'sSifaka  -------------------------------------------------------------------------------- 4607
Ma'sNightMonkey   -------------------------------------------------------------------------------- 4528
VervetAGM         -------------------------------------------------------------------------------- 4506
Gorilla           GTCAATTAAAGAAAGTCTTTATGGTAGAGATCCTTTGGAATCCTTTTTGGCTAAGAAAACTACTCATCGTTTAAACTTTA 5884
GrabEatingMacaque -------------------------------------------------------------------------------- 4438
Macaque           -------------------------------------------------------------------------------- 4448
PigTailedMacaque  -------------------------------------------------------------------------------- 4672
Bonobo            -------------------------------------------------------------------------------- 4507
OliveBaboon       -------------------------------------------------------------------------------- 4426
Orangutan         -------------------------------------------------------------------------------- 4510

Majority          -------------------------------------------------------------------------------G

                          6330      6340      6350      6360      6370      6380      6390      6400

Human             -------------------------------------------------------------------------TTTTT-G 3206
Coquerel'sSifaka  -------------------------------------------------------------------------------- 4607
Ma'sNightMonkey   -----------------------------------------------------------------------------TTG 4531
VervetAGM         -------------------------------------------------------------------------------A 4507
Gorilla           GTTCTAATGCTTAACATTTTCTCGAGTACTTGCAACTTTTAATGTCATGATGTATTGTAATTTTTTTTTTTTTTTTTTTG 5964
GrabEatingMacaque -------------------------------------------------------------------------------A 4439
Macaque           -------------------------------------------------------------------------------A 4449
PigTailedMacaque  -------------------------------------------------------------------------------A 4673
Bonobo            -------------------------------------------------------------------------TTTTTTG 4514
OliveBaboon       -------------------------------------------------------------------------------A 4427
Orangutan         ---------------------------------------------------------------------------TT--G 4513

Majority          -GATGGTGCTTCACTCTGTTGCCCAGGCTGGA-TGCAGTGGCGCGATCTTGGCCCGCTGCAACCTCTGCCTCCTGGGTTC

                          6410      6420      6430      6440      6450      6460      6470      6480

Human             AGACGGAGCCTCACTCTGTTGCCCAGGCTGGA-TGCAGTGGCACGATCTTGGCCCACTGCAACCTCTGCCTCCTGGGTTC 3285
Coquerel'sSifaka  -------------------------------------------------------------------------------- 4607
Ma'sNightMonkey   AGACTGAGTCTCCCTCTGTTGCCCAGGCTGGAGTGCAGTGATGCAGTTTCGCCTCACTGCGGCCTCCGCCTCCCAGGTTC 4611
VervetAGM         -GATGGTGCTTCACTCTATCGCCCAGGCTGGA-TGCAGTGGCGCGATCTTGGCCCGCTGCAACCTCTGCCTCCTGGGTTC 4585
Gorilla           AGATGGAGCCTCACTGTGTTGCCCAGGCTGGA-TGCAGTGGCACGATCTTGGCCCACTGCAACCTCTGCCTCCTGGGTTC 6043
GrabEatingMacaque -GATGGTGCTTCACTCTGTCGCCCAGGCTGGA-TGCAGTGGCGCGATCTTGGCCCGCTGCAACCTCTGCCTCCTGGGTTC 4517
Macaque           -GATGGTGCTTCACTCTGTTGCCCAGGCTGGA-TGCAGTGGCGCGATCTTGGCCCGCTGCAACCTCTGCCTCCTGGGTTC 4527
PigTailedMacaque  -GATGGTGCTTCACTCTGTCGCCCAGGCTGGA-TGCAGTGGCACGATCTTGGCCCGCTGCAACCTCTGCCTCCTGGGTTC 4751
Bonobo            AGACGGAGCCTCACTCTGTTGCCCAGGCTGGA-TGCAGTGGCACGATCTTGGCCCACTGCGACCTCTGCCTCCTGGGTTC 4593
OliveBaboon       -GATGGTGCTTCACTCTGTCGCCCAGGCTGGA-TGCAGTGGCGCGATCTTGGCCCGCTGCAACCTCTGCCTCCTGGGTTC 4505
Orangutan         AGACGGAGCCTCGCTCGGTCACCCAGGCTGGA-TGCAGTGGCGCGATCTTGGCCCACTGCAACCTCTGCATCCTGGGTTC 4592
```

```
Majority          AAGCAATTCTCCTGCCTCAGCCTCCTGAGTAGCTGGGATTACAGGTGCTCACCACCACGCCC-AGCTAATTT--GTATTT

                          6490      6500      6510      6520      6530      6540      6550      6560

Human             AAGCAGTTCTCCTGCCTCAGCCTCCTGAGTAGCTGGGATTACAGGTGCGCACCACCACGCCC-AGCTAATTT--GTATTT 3362
Coquerel'sSifaka  ------------------------------------------------------CAAG---------------------- 4611
Ma'sNightMonkey   AGGCAATTCTCCTGCCTTAGCCTCCTGAGTAGCTGGGATTACAGGCACATGCCATTACACCCTGGCTAATTTTTGTATTT 4691
VervetAGM         AAGCAATTCTCCTGCCTCAGCCTCCTGAGTAGCTGGGATTACAGGTGCTCACCACCACGCCC-AACTAATTT--GTATTT 4662
Gorrilla          AAGCAGTTCTCCTGCCTCAGCCTCCTGAGTAGCTGGGATTACAGGTGCGCACCACCACGCCC-AGCTAATTT--GTATTT 6120
GrabEatingMacaqueAAGCAATTCTCCTGCCTCAGCCTCCTGAGTAGCTGGGATTACAGGTGCTCACCACCACGCCC-AACTAATTT--GTATTT 4594
Macaque           AAGCAATTCTCCTGCCTCAGCCTCCTGAGTAGCTGGGATTACAGGTGCTCACCACCACGCCC-AACTAATTT--GTATTT 4604
PigTailedMacaque  AAGCAATTCTCCTGCCTCAGCCTCCTGAGTAGCTGGGATTACAGGTGCTCACCACCACGCCC-AACTAATTT--GTATTT 4828
Bonobo            AAGCAATTCTCCTGCCTCAGCCTCCTGAGTAGCTGGGATTACAGGTGCGCACCACCACGCCC-AGCTAATTT--GTATTT 4670
OliveBaboon       AAGCAATTCTCCTGCCTCAGCCTCCTGAGTAACTGGGATTACAGGTGCTCACCACCACGCCC-AACTAATTT--GTATTT 4582
Orangutan         AAGCAGTTCTTCTGCCTCAGCCTCCTGAGTAGCTGGGATTACAGGTGCGCACCACCACGCCC-AGCTAATTT--GTATTT 4669

Majority          TTAGTAGAGATTGGGTTTCACTCTGTTGGTCAGGCTGGTCTCGAACTCCTTACCTCATGATCTGCCTGCCTC--------

                          6570      6580      6590      6600      6610      6620      6630      6640

Human             TTAGTAGAAATGGCATTTCACCCTGTTGGTCAGGCTGGTCTCAAACTCCTGACCTCATGATCTGCCTGCCTCTTATGATC 3442
Coquerel'sSifaka  -------------------------CCT----------------------------------GTCTGCTCT-------- 4623
Ma'sNightMonkey   TTAGTAGAGGCGAGGGTTCACCATCTTGGTCAGGCTGGTCTCGAACTCCTGACCTTGTGATCCGCCCACCTT-------- 4763
VervetAGM         TTAGTAGAGATTGGGTTTCACTCTGTTGGTCAGGCTGGTCTCGAACTCCTTACCTCATGATCTGCCTGCCTC-------- 4734
Gorrilla          TTAGTAGAAATGGCATTTCACCCTGTTGGTCAGGCTGGTCTCAAACTCCTGACCTCATGATCTGCCTGCCTCTTATGATC 6200
GrabEatingMacaqueTTAGTAGAGATTGGGTTTCACTCTGTTGGTCAGGCTGGTCTCGAACTCCTTACCTCATGATCTGCCTGCCTC-------- 4666
Macaque           TTAGTAGAGATTGGGTTTCACTCTGTTGGTCAGGCTGGTCTCGAACTCCTTACCTCATGATCTGCCTGCCTC-------- 4676
PigTailedMacaque  TTAGTAGAGATTGGGTTTCACTCTGTTGGTCAGGCTGGTCTCGAACTCCTTACCTCATGATCTGCCTGCTTC-------- 4900
Bonobo            TTAGTAGAAATGGCATTTCACCCTGTTGGTCAGGCTGGTCTCAAACTCCTGACCTCATGATCTGCCTGCCTCTTATGATC 4750
OliveBaboon       TTAGTAGAGATTGGGTTTCACTCTGTTGGTCAGGCTGGTCTCGAACTCCTTACCTCATGATCTGCCTGCCTC-------- 4654
Orangutan         TTAGTAGAGACGGCGTTTCACCCTGTTGGTCAGGCTGGTCTCAAACTCCTGACCTCATGATCTGCCTGCCTCTCATGATC 4749

Majority          ----------GACCTCCCAAAGTTTTGGGATTACAGGTGTGAGCCACCGTGCCCAGCCTGTATTGTCATTTTTCATTTGC

                          6650      6660      6670      6680      6690      6700      6710      6720

Human             TGCCTGCCTTGACCTCCCAAAGTGCTGGGATTACAAGTGTGAGCCACCGTGCCCGGCCTGTATTGTCATTTTTCATTTGC 3522
Coquerel'sSifaka  --------------------------------------GAGAGGCTGTGTGCTTAGTCCA----GTGATGTT-------- 4653
Ma'sNightMonkey   ----------GGCCTCCCAAAGTGCTGGGATTACAGGTGTGAGCTGCGGCGTCTGGCCTATATTGTAATTTTTCATTTGC 4833
VervetAGM         ----------GACCTCCCAAAGTTTTGGGATTACAGGCCTGAGCCACTGTGCCCAGCCTGTATTGTCATTTTTCATTTGC 4804
Gorrilla          TGCCTGCCTTGACTTCCCAAAGTGCTGGGATTACAAGTGTGAGCCACCGTGCCCGGCCTGTATTGTCATTTTTCATTTGC 6280
GrabEatingMacaque----------GACCTCCCAGTGTTTTGGGATTACAGGCGTGAGCCACCGTGCCCAGCCTGTATTGTCGTTTTTCATTTGC 4736
Macaque           ----------GACCTCCCAGTGTTTTGGGATTACAGGCGTGAGCCACCGTGCCCAGCCTGTATTGTCATTTTTCATTTGC 4746
PigTailedMacaque  ----------GACCTCCCAATGTTTTGGGATTACAGGCGTGAGCCACCGTGCCCAGCCTGTATTGTCATTTTTCATTTGC 4970
Bonobo            TGCCTGCCTTGACCTCCCAAAGTGCTGGGATTACAAGTGTGAGCCACCGTGCCCGGCCTGTATTGTCATTTTTCATTTGC 4830
OliveBaboon       ----------GACCGCCCAAAGTTTTGGGATTACAGGCGTGAGCCACTGTGCCCAGCCTGTATTGTCATTTTTCATTTGC 4724
Orangutan         TGCCTGTCTCGACCTCCCAAAGTGCTGGGATTACAGATGTGAGCCACCGTGCCCGGCCTGTATTGTCATTTTTCATTTGC 4829
```

```
Majority           AAGCCTGTCTGTCCTGAGAGGCTATCTGCTTAGTCCAGTGATGCTGACTGTTCAA-ACATTTTTTGGAATTCTTAAAATT

                            6730      6740      6750      6760      6770      6780      6790      6800

Human              AAGCCTCTCTGTCCTGAGAGGCTTTCTGCTTAGTCCAGTGATGCTGACTGTTCAA-ACATTTTTTCGAATTCTTAAAATT 3601
Coquerel'sSifaka   --------------------------------GACCGGTGATGCTGACCGTTCAGTATATTTATTGTAATTCTTAAAATT 4701
Ma'sNightMonkey    CAGCCTTTCTGCCTTGAGAGGCTGTCTGCTTAGTCCAGTGATGCTAACTGTTCAA-ACACTTTTTGGAACTCTTGAAATT 4912
VervetAGM          AAACCTGTCTGTCCTGAGAGGCTATCTGCTTAGTCCATTGATGCTGACTGTTCAA-ACATTTTTTGGAATTCTTTTTTTT 4883
Gorrilla           AAGTCTCTCTGTCCTCAGAGGCTATCTGCTTAGTCCAGTGATGCTGACTGTTCAA-ACATTTTTTTGAATTCTTAAAATT 6359
GrabEatingMacaque  AAACCTGTCTGTCCTGAGAGGCTATCTGCTTAGTCCATTGATGCTGACTGTTCAA-ACATTTTTTGGAATTCTTAAAATT 4815
Macaque            AAACCTGTCTGTCCTGAGAGGCTATCTGCTTAGTCCATTGATGCTGACTGTTCAA-ACATTTTTTGGAATTCTTAAAATT 4825
PigTailedMacaque   AAACCTGTCTGTCCTGAGAGGCTATCTGCTTAGTCCATGGATGCTGACTGTTCAA-ACATTTTTTGGAATTCTTAAAATT 5049
Bonobo             AAGCCTCTCTGTCCTGAGAGGCTATCTGCTTAGTCCAGTGATGCTGACTGTTCAA-ACAGTTTTTCGAATTCTTAAAATT 4909
OliveBaboon        AAACCTGTCTGTCCTGAGAGGCTATCTGCTTAGTCCATTGATGCTGACTGTTCAA-ACATTTTTTGGAATTCTTAAAATT 4803
Orangutan          AAGCCTCTGTGTCCGGAGAGGCTATCTGCTTAGTCCAGTGATGCTGACTGTTCAA-ACATTTTTTGGAATTCTTAAAATT 4908

Majority           GTA-TCCAT-----AATGGTGTGATG-TTGTTTCGAATA-CTTTTGGGGATAT--TAGTGTGTTTTCTTGCTTATTTTCT

                            6810      6820      6830      6840      6850      6860      6870      6880

Human              GTA-TCCAT-----AATGGGGGG-AG-GGGGAAGGGATAGCATTGGGAGATATAGGGGGGAGGGGGGAGGGATAGCTTTA 3673
Coquerel'sSifaka   GCAATTCAT-----AGTGGTGTGATAATTCTTCAGAATA-CTTTTGGAGATAT--TATTGTGTTTTCTTGCTTATTTTCT 4773
Ma'sNightMonkey    GCA-TCCAT-----AATGATGTGATG-TTTTTTAGAATA-CTTTTGGGGATGG--TACTGTGTTTTCTTGCTTATTTTCT 4982
VervetAGM          TTT-TTTTTTTGAGACGGAGTCTCG-CTCTGTCGCCCA-GGCTGGAGTGTAG--TGGCCGGATCTCA-GCTNNNNNNNN 4957
Gorrilla           GTA-TCCAT-----AATGGGGGGGAG-GGGGAAGGGATAGCATTGGGAGATATAGGGGGGAGGGGGGAGGGATAGCTTTA 6432
GrabEatingMacaque  GTA-TCCAT-----AATGATGTTATG-TTCTTTCGAATA-CTTTTGGGGATGT--TACTGTGTTTTCTTGCTTATTTTCT 4885
Macaque            GTA-TCCAT-----AATGATGTTATG-TTCTTTCGAATA-CTTTTGGGGATGT--TACTGTGTTTTCTTGCTTATTTTCT 4895
PigTailedMacaque   GTA-TCCAT-----AATAATGTTATG-TTGTTTCGAATA-CTTTTGGGGATGT--TACTGTGTTTTCTTGCTTATTTTCT 5119
Bonobo             GTA-TCCAT-----AATGGGGGGGAG-GGGGAAGGGATAGCATTGGGAGATATAGGGGGGAGGGGGGAGGGATAGCTTTA 4982
OliveBaboon        GTA-TCCAT-----AATGATGTTATG-TTCTTTCGAATA-CTTTTGGGGATGT--TACTGTGTTTTCTTGCTTATTTTCT 4873
Orangutan          GTA-TCCAT-----AATGGGGGG------GGGAGGGATAGCATTAGGAGATAC--GGGGGAGAGGGGAGGGATAGCTTTA 4974

Majority           GTCGTCTTACCTA-TAGTGAACTAGATATTTGTCCATTCTTTAG-ACCTGTA--TG-GTTCTTGAATTCATATTTTACAG

                            6890      6900      6910      6920      6930      6940      6950      6960

Human              GGAGATATACCTAATGCTAAATGACAAGTTAATGGGTGCAGCAC-ACCAACA--TG-GCACATGTATACATATGTAACAA 3749
Coquerel'sSifaka   GTCCTCTGCCCTA-CAGTGAACTAGATACTTGTCCATCCTTTAATATTTAGCCTTGTTTTTTTGAATTTATATTTTGCAT 4852
Ma'sNightMonkey    GTGAACTGACCTA-TAGTGAACTGGATACTTGTCCATTCTTTAATATCTAGGCTTG-TTTCTTGAGTTCAGATTATACAG 5060
VervetAGM          NNTCCCAAAGTGC-TGGGATTACAGGCTTGAGCCACCGCGCCCG-GCCTGGA-----ATTCTTAAA---ATTGTATCCAT 5027
Gorrilla           GGAGATATACCTAATGCTAAATGACAAGTTAATGGATGCAGCAC-ACCAACA--TG-GCACATGTATACATATGTAACAA 6508
GrabEatingMacaque  GTCGTCTTACCTA-TAGTGAACTAGATATTTGTCCATTCTTTAG-GCTTGT-------TTCTTTAGTTCATTTTGTACAG 4956
Macaque            GTCGTCTTACCTA-TAGTGAACTAGATATTTGTCCATTCTTTAG-GCTTGT-------TTCTTTAGTTCATTTTGTACAG 4966
PigTailedMacaque   GTCGTCTTACCTA-TAGTGAACTAGATATTTGTCCATTCTTTAG-GCTTGT-------TTCTTTAGTTCATTTTGTACAG 5190
Bonobo             GGAGATATACCTAATGCTAAATGACAAGTTAATGGGTGCAGCAC-ACCAGCA--TG-GCACATGTATACATATGTAACAA 5058
OliveBaboon        GTCGTCTTACCTA-TAGTGAACTAGATATTTCTCCATTCTTTAG-GCTTGT-------TTCTTTAGTTCATTTTGTACAG 4944
Orangutan          GGAGATACACCTAATGCTAAATGACGAGTTAATGGGTGCAGCAC-ACCAACA--TG-GCACATGTATACATATGTAGCAA 5050
```

```
Majority          ACTGACGGAGAAATAATAAGCATCTTTTTGGTGATA-GTATTATAATTTTTTT--T-----------TATCCATAATGTT

                            6970      6980      6990      7000      7010      7020      7030      7040

Human             ACCTGCACGTTGTACTCATGTACCCTAAAACTTAAA-GTATAATAATGATAATAATAAT------TGTATCCATAATGAT 3822
Coquerel'sSifaka  ACTGGGAGAGAAATAACAAACTGTTTTGGAGAG----------------------------------------------- 4885
Ma'sNightMonkey   ACTGAGGGAAAAATAATAAGC-TCTTTGTGGTG----------------------------------------------- 5092
VervetAGM         AATGATGTTATGTTCTTTCGAATACTTTTGGGGATG-TTACTGTGTTTTCTT----------------------GCTTTT 5084
Gorilla           ACCTGCACGTTGTACACATGTACCCTAAAACTTAAA-GTATAATAATAATAATAATAATAATTGTATCCATAATGAT 6587
GrabEatingMacaqueACTGAAGGAGAAATAATAAGC-TCTTTTTGGTGTTA-GTATTTTTCTTTTTTT--------------GAGCCGGAGTCTT 5020
Macaque           ACTGAAGGAGAAATAATAAGC-TCTTTTTGGTGTTA-GTATTTTTCTTTTTTTTTTT----------GAGCCGGAGTCTC 5034
PigTailedMacaque  ACTGAAGGAGAAATAATAAGC-TCTTTTTGGTGATA-GTATTTTTCTTTTTTTTTTTTTTTT--TTTTTGCCGGAGTCTC 5266
Bonobo            ACCTGCACGTTGTACTCATGTACCCTAAAACTTAAA-GTATAATAATAATAATAATAAT---TGTATCCATAATGAT 5134
OliveBaboon       ACTGAAGGAGAAATAATAAGC-TCTTTTTGGTG----------------------------------------------- 4976
Orangutan         ACCTGCACATTGTGCACATGTACCCTAAAACTTAAAAGTATAATAATAATAATAATAAAC----TTGTATCCATAATGAT 5126

Majority          GTTCTGTTGTTTAGA--ATAGTTTTGGGGCTGGTTCTGTGTT---T------------------TCTTGCTTATTTTCTG

                            7050      7060      7070      7080      7090      7100      7110      7120

Human             GTGGTGTTCTTTCAA--ATACTTTTGGGGATGGTACTGTGTT---T------------------TCTTGCTTATTTTCTG 3879
Coquerel'sSifaka  -------------------------------------------------------------------------------- 4885
Ma'sNightMonkey   -------------------------------------------------------------------------------- 5092
VervetAGM         TTTCTGTCGTCTTACCTATAGTG-AA---CTAGATATTTGTA-----------------------------CATTCT--- 5128
Gorilla           GTGATGTTCTTTCGA--ATACTTTTGGGGATGGTACTGTGTT---T------------------TCTTGCTTATTTTCTG 6644
GrabEatingMacaqueGCTCTGTCGCCCAGGCTGGAGTGCAGTGGCCGGATCTCAGCTCACTGCAAGCTCTGCCTCCAGGTTCACGCCATTCTCCT 5100
Macaque           GCTCTGTCGCCCAGGCTGGAGTGCAGTGGCCGGATCTCAGCTCACTGCAAGCTCTGCCTCCGGGTTCACGCCATTCTCCT 5114
PigTailedMacaque  GCTCTGTCGCCCAGGCTGGAGTGCAGTGGCTGGATCTCAGCT--C--------CGCCTCCTGGGTTCACGCCATTCTCCT 5336
Bonobo            GTGATGTTCTTTCAA--ATACTTTTGGGGATGGTACTGTGTT---T------------------TCTTGCTTATTTTCTG 5191
OliveBaboon       ------------------------------------------------------------------------A------- 4977
Orangutan         GTGATGTTCTTTAGA--ATACTTTTAGGAATGGTACTGTGTT---T------------------TCTTGCTTATTTTCTG 5183

Majority          TCCTC----TT-CCTATAGTTAA-TA-ATATTTGCCC---ATTTTTTAGGCTTGTTTCTTTAGTTCATTTTGTACAGA--

                            7130      7140      7150      7160      7170      7180      7190      7200

Human             TCCTC----TTTCCTATAGTGAAATAGATATTTGCCC---ATTCTTTAGGCTTGTTTCTTGAGTTCATTTTGTACAGA-- 3950
Coquerel'sSifaka  -------------------------------------------------------------AGCACATTTAGT------- 4897
Ma'sNightMonkey   -------------------------------------------------------------------------------- 5092
VervetAGM         ---TTAG-----------GCT--------------------TGT-----------TTCTTTAGTTCATTTTGTACAGA-- 5161
Gorilla           TCCTC----TTACCTATAGTGAAATAGATATTTGCCC---ATTCTTTAGGCTTGTTTCTTGAGTTCATTTTGTACAGA-- 6715
GrabEatingMacaqueGCCTCAGCCTCCCGAGTAGCTGGGACTACAGGCGCC----ACCTCGCCCGGCTAGTTTTTTGTATTTTTTAGTAGAGATG 5176
Macaque           GCCTCAGCCTCCCGAGTAGCTGGGACTACAGGCGCCCGCCACCTCGCCCGGCTAGTTTTTTGTATTTTTTAGTAGAGACG 5194
PigTailedMacaque  GCCTCAGCCTCCCGAGTAGCTGGGACTACAGGCGCCCGCCACCTCTCCCGGCTAGTTTTTTGTATTTTTTAGTAGAGACG 5416
Bonobo            TCCTC----TTACCTATAGTGAAATAGATATTTGCCC---ATTCTTTAGGCTTGTTTCTTGAGTTCATTTTGTACAGA-- 5262
OliveBaboon       -------------------------------------------------------------------------------- 4977
Orangutan         TCCTC----TTACCTATAGTGAACTACATATTTGTCC---ATTCTTTAGGCTTGTTTCTTGAGTTCATTTTGTACAGA-- 5254
```

```
Majority          ------------------CTGAAGGA-----------------------------------------------G---GAA
                           7210      7220      7230      7240      7250      7260      7270      7280
Human             ------------------CTAAAGGA-----------------------------------------------G---GAA 3962
Coquerel'sSifaka  -------------------------------------------------------------------------------- 4897
Ma'sNightMonkey   -------------------------------------------------------------------------------- 5092
VervetAGM         ------------------CTGAAGGA-----------------------------------G---------------AAA 5173
Gorrilla          ------------------CTAAAGGA-----------------------------------------------G---GAA 6727
GrabEatingMacaqueGGGTTTCACCGTGTTAGCCAGGACGGTCTCGATCTCCTGACCTCGTGATCCGCCCATCTCAGCCTCCCAAAGTGCTGGGA 5256
Macaque           GGGTTTCACCGTGTTAGCCAGGACGGTCTCGATCTCCTGACCTCGTGATCCGCCCATCTCAGCCTCCCAAAGTGCTGGGA 5274
PigTailedMacaque  GGGTTTCACCGTGTTAGCCAGGATGGTCTCGATCTCCTGACCTCGTGATCCGCCCATCTCAGCCTCCCAAAGTGCTGGGA 5496
Bonobo            ------------------CTAAAGGA-----------------------------------------------G---GAA 5274
OliveBaboon       -------------------------------------------------------------------------------- 4977
Orangutan         ------------------CTGAAGGA-----------------------------------------------G---AAA 5266

Majority          TAATAGGCT-------------CTTTTTGGTGATAGTATTTTCCAAGTTGAGTTAGTCATGGGATTTAAAATCAGGAATA
                           7290      7300      7310      7320      7330      7340      7350      7360
Human             TAATACGCT-------------CTTTTTGGTGATAGTATTTTCCAAGTTGAGTTAGTCATGGGATTTAAAATCAGGAATA 4029
Coquerel'sSifaka  --------T-------------TTAGTTGGCAATTATATTTTCCAGGTTGGGATAGCTATAGGATTAGAAATCAGGAATA 4956
Ma'sNightMonkey   ------------------------------A--TACTATTTTCCCAGTTGAGATAGTCATGGGATTTAAAATCAGGAATA 5140
VervetAGM         TAATAAGCT-------------CTTTTTGGTGATAGTATTTTCCAAGTTGAGTTAGTCATGGGGTTTAAAATCAGGAATA 5240
Gorrilla          TAATACGCT-------------CTTTTTGGTGATAGTATTTTCCAAGTTGAGTTAGTCATGGGATTTAAAATCAGGAATA 6794
GrabEatingMacaqueTTACAGGCTTGAGCCACCGCGCCCGGCCGGTGATAGTATTTTTCAAGTTGAGTTAGTCATGGGATTTAAAATCAGGAATA 5336
Macaque           TTACAGGCTTGAGCCACCGCGCCCGGCCGGTGATAGTATTTTTCAAGTTGAGTTAGTCATGGGATTTAAAATCAGGAATA 5354
PigTailedMacaque  TTACAGGCTTGAGCCGCCGCGCCTGGCCGGTGATAGTATTTTTCAAGTTGAGTTAGTCATGGGATTTAAAATCAGGAATA 5576
Bonobo            TAATACGCT-------------CTTTTTGGTGATAGTATTTTCCAAGTTGAGTTAGTCATGGGATTTAAAATCAGGAATA 5341
OliveBaboon       ------------------------------T---AGTATTTTTCAAGTTGAGTTAGTCATGGGATTTAAAATCAGGAATA 5024
Orangutan         TAATATGCT-------------CTTTTTGGTGATAATATTTTCCAAGTTGAGTTAGTCATGGGATTTAAAATCAGGAATA 5333

Majority          GCTAAAGAGTGCAAAATGGTAGATGTTGTAGTTTCCATCCAAACAGATTTCTTCTGTGAATTTTGTAAAAGAATAAATGA
                           7370      7380      7390      7400      7410      7420      7430      7440
Human             GCTAAAGATTGCAAAATGGTAGATGTTGTAGGTTCCATCCAAACAGATTTC---TGTAAATTTTGTAAAAGAATAAATGA 4106
Coquerel'sSifaka  GCTGAAGAGTGCAAAATG-TAGATGTTGTAGTTACCATCCAAACAGATTTCCTCTGTGAATTTTATGAAAGAATAAATGA 5035
Ma'sNightMonkey   GCTAAAGAGTGCAAAATGGTAGATGTTGTAGTTACCATCCAAACAGATTTCTCGTCTAAATTTTGTAAAAGAATAAATAA 5220
VervetAGM         GCTAAAGAGTACAAAATGGTAGATGTTGTAGTTTCCTTCCAAACAGATTTCTTCTGTGAATTCTGGAAAATAATAAATGA 5320
Gorrilla          GCTAAAGAGTGCAAAATGGTAGATGTTGTAGGTTCCATCCAAACAGATTTC---TGTAAATTTTGTAAAAGAATAAATGA 6871
GrabEatingMacaqueGCTAAAGAGTGCAGAATGGTAGATGTTGTAGTTTCCATCCAAACAGATTTCTTCTGTGAATTCTGGAAAATAGTAAATGA 5416
Macaque           GCTAAAGAGTGCAGAATGGTAGATGTTGTAGTTTCCATCCAAACAGATTTCTTCTGTGAATTCTGGAAAATAGTAAATGA 5434
PigTailedMacaque  GCTAAAGAGTGCAGAATGGTAGATGTTGTAGTTTCCATCCAAACAGATTTCTTCTGTGAATTCTGGAAAATAGTAAATGA 5656
Bonobo            GCTAAAGAGTGCAAAATGGTAGATGTTGTAGGTTCCATCCAAACAGATTTC---TGTAAATTTTGTAAAAGAATAAATGA 5418
OliveBaboon       GCTAAAGAGTGCAAAATGGTAGATGTTGTAGTTTCCGTCCAAACAGATTTCTTCTGTGAATTCTGGAAAATAGTAAATGA 5104
Orangutan         GCTAAAGAGTGCAAAATGGTAGATGTTGTAGGTTCCATCCAAACAGATTTC---TGTAAATTTTGTAAAAGAATAAATGA 5410
```

```
Majority          TACAAGTGTGAGTTGTCCACTGTTAGTGGAAAAGGAGAGTAGTTTGTAGACAAGGCTCAGAATGAGTAGACAGAAAAAAA
                           7450      7460      7470      7480      7490      7500      7510      7520
Human             TACAAGTGTGAGTTGTCGACTGTTAGTGGAAAAGGAGAGGAGTTTGTAGACAAGGCTCAGAATGAGTAGACAGGAAAAAA 4186
Coquerel'sSifaka  TACAAGTCTAAGTTGTCCACTGTTAGTGGATAAGGAGAG-AGTTAATAGACAAGACCTAGAGTGAGTAGGCAGAAAAAGG 5114
Ma'sNightMonkey   TACAAGTGTGACTTGTCCACTATTAGTGGAAAAGGAAAGGAGGTTGTAGGCAAGGCTCAGAATGAGTAGACAGAAGAAAA 5300
VervetAGM         TACAAGTGTTAGT-GTCCACTGTTAGAGGAAAAGGAGAGTAGTTTGTAGATAACGCTCAGAATAAGTAGACAGAAAAAAA 5399
Gorrilla          TACAAGTGTGAGTTGTCGACTGTTAGTGGAAAAGGAGAGGAGTTTGTAGACAAGGCTCAGAATGAGTAGACAGGAAAAAA 6951
GrabEatingMacaqueTACAAGTGTGAGT-GTCCACTGTTAGAGGAAAAGGAGAGTAGTTTGTAGATAACGCTCAGAATGAGTAGACAGAAAAAAA 5495
Macaque           TACAAGTGTGAGT-GTCCACTGTTAGAGGAAAAGGAGAGTAGTTTGTAGATAACGCTCAGAATGAGTAGACAGAAAAAAA 5513
PigTailedMacaque  TACAAGTGTGAGT-GTCCACTGTTAGAGGAAAAGGAGAGTAGTTTGTAGATAACGCTCAGAATGAGTAGACAGAAAAAAA 5735
Bonobo            TACAAGTGTGAGTTGTCGACTGTTAGTGGAAAAGGAGAGGAGTTTGTAGACAAGGCTCAGAATGAGTAGACAGGAAAAAA 5498
OliveBaboon       TACAAGTGTGAGT-GTCCACTGTTAGAGGAAAAGGAGAGTAGTTTGTAGATAACGCTCAGAATGAGTAGACAGAAAAAAT 5183
Orangutan         TACAAGTGTGAGTTGTCGACTATTAGTGGAAAAGGAGAAGAGTTTGTAGACAAGACTCAGAATGAGTAGACAGAAAAAAA 5490

Majority          -CTGAGGAGACTAGGAATAGGATGTCCAGATG---TAATGTATTGGTTACACCAGTTTT---------------------
                           7530      7540      7550      7560      7570      7580      7590      7600
Human             -CTGAGGAGACTTGGAATAGGATGTCCAGGTG---TAATGTATTGGTTACACCAGTTTTTTTTTTTTTTTTTT--GAGAC 4260
Coquerel'sSifaka  --TGAGGAGACTCCGAAGAGAATGTCCAGATACAATGATGTATTGGTTACATCAATTTT--------------------- 5171
Ma'sNightMonkey   -CTGAGGAGACTCAGAATAGGATGTCCAGATG---TAATACATTGGTTACACCAGTTTT--------------------- 5355
VervetAGM         -CCGAGGAGACTAGGAGTAGGATGTCCAGATG---TAATGTATTGGTTACACCAGTTT---------------------- 5453
Gorrilla          -CTGAGGAGACTTGGAATAGGATGTCCAGGTG---TAATGTATTGGTTACACCAGTTTTTTTTTTTTTTTTTTTTGAGAC 7027
GrabEatingMacaque-CTGAGGAGACTAGGAGTAGGATGTCCAGATG---TAATGTATTGGTTACACCAGTTT---------------------- 5549
Macaque           -CTGAGGAGACTAGGAGTAGGATGTCCAGATG---TAATGTATTGGTTACACCAGTTT---------------------- 5567
PigTailedMacaque  ACTGAGGAGACTAGGAGTAGGATGTCCAGATG---TAATGTATTGGTTACACCAGTTT---------------------- 5790
Bonobo            -CTGAGGAGACTTGGAATAGGATGTCCAGGTG---TAATGTATTGGTTACACCAGTTTTTTTTTTTTTTTT----GAGAC 5570
OliveBaboon       -CTGAGGAGACTAGGAGTAGGATGTCCAGATG---TAATGTATTGGTTACACCAGTTT---------------------- 5237
Orangutan         -CTGAGGAGACTCGGAATAGTATGTCCAGATG---TTATGTATTGGTTACGCCAGTTGTTTTTTTTTTTTTT-------- 5558

Majority          --------------------------------------------------------------------------------
                           7610      7620      7630      7640      7650      7660      7670      7680
Human             AGAGTCTCACTCT-TCGCCCAGG-TGGAGTGCAGTGGCACGATCCTGGCTCACTACAAGCTCCGCCTTCCGGGTTCACTC 4338
Coquerel'sSifaka  -------------------------------------------------------------------------------- 5171
Ma'sNightMonkey   -------------------------------------------------------------------------------- 5355
VervetAGM         -------------------------------------------------------------------------------- 5453
Gorrilla          GGAGTCTCACTCTGTCGCCCAGG-TGGAGTGCAGTGGCACAATCCCGGCTCACTACAAGCTCCGCCTTCCGGGTTCACTC 7106
GrabEatingMacaque-------------------------------------------------------------------------------- 5549
Macaque           -------------------------------------------------------------------------------- 5567
PigTailedMacaque  -------------------------------------------------------------------------------- 5790
Bonobo            GGAGTCTCACTCT-TCGCCCAGG-TGGAGTGCAGTGGCACGATCCTGGCTCACTACAAGCTCCGCCTTCCGGGTTCACTC 5648
OliveBaboon       -------------------------------------------------------------------------------- 5237
Orangutan         -------T--TTTGTCGCCCAGGCTGGAGTGCAGTGGCACGATCCCAGCTCACTACAAGCTCCGCCTTCTGGGTTCACTC 5629
```

```
Majority        -----------------------------------------------------------------------

                        7690      7700      7710      7720      7730      7740      7750      7760

Human           CATTCTTCTGCCTCAGCCTCCCGAGTAGCTGGGACTACAGGCGCCCACCACCACGCCCGGCTAATTTTTTGTATTTTTAG 4418
Coquerel'sSifaka -------------------------------------------------------------------------------- 5171
Ma'sNightMonkey -------------------------------------------------------------------------------- 5355
VervetAGM       -------------------------------------------------------------------------------- 5453
Gorrilla        CATTCTTCTGCCTCAGCCTCCCGAGTAGCTGGGACTACAGGTGCCCACCACCACGCCCGGCTAATTTTTTGTATTTTTAG 7186
GrabEatingMacaque-------------------------------------------------------------------------------- 5549
Macaque         -------------------------------------------------------------------------------- 5567
PigTailedMacaque -------------------------------------------------------------------------------- 5790
Bonobo          CATTCTTCTGCCTCAGCCTCCCGAGTAGCTGGGACTACAGGCGCCCACCACCACGCCCGGCTAATTTTTGTATTTTTTAG 5728
OliveBaboon     -------------------------------------------------------------------------------- 5237
Orangutan       CATTCTCCTGCCTCAGCCTCCCGAGTTGCTGGGACTACAAGCGCCCGTCACCACGCCTGGCTAATTTTTTGTATTTTTAG 5709

Majority        -------------------------------------------------T---------------------TTATGTTAG

                        7770      7780      7790      7800      7810      7820      7830      7840

Human           T-AGAGAC-GGGGTTTCACCGTGTTAGCCAGGATGGTCTTGATCTCCT--GACCTTGTGGTTACACCAGTTTTTGTTAG 4494
Coquerel'sSifaka -----------------------------------------------------------------------GTATATTAG 5180
Ma'sNightMonkey -----------------------------------------------------------------------TTATGTTAG 5364
VervetAGM       -------------------------------------------------T---------------------TTATGTTAA 5463
Gorrilla        T-AGAGAC-CGGGTTTCACCGTGTTAGCCAGGATGGTCTTGATCTCCT--GACCTTGTGGTTACACCAGTTTTCTGTTAG 7262
GrabEatingMacaque-------------------------------------------------T---------------------TTATGTTAG 5559
Macaque         -------------------------------------------------T---------------------TTATGTTAG 5577
PigTailedMacaque -------------------------------------------------T---------------------TTATGTTAG 5800
Bonobo          TTAGAGAC-CGGGTTTCACCGTGTTAGCCAGGATGGTCTTGATCTCCT--GACCTTGTGGTTACACCAGTTTTTTGTTAG 5805
OliveBaboon     -------------------------------------------------T---------------------TTTTGTTAG 5247
Orangutan       T-AGAGACAGGGGTTTCACCGTGTTAGCCAGGATGGTCTCGATCTCCTCTGACCTTGTGGTTACACGAGTTTTTTGTTAG 5788

Majority        TTGTTTGAGGAGAACACTTTAGAAGATGATAAAAAAAAG-TTGCAAACCATTTTTAAAAATCCATTGTTTAATATAAAAG

                        7850      7860      7870      7880      7890      7900      7910      7920

Human           TTGTTTGAGGAGAACACTTTAGAAGATGATAAAAAAAAA-TTGCAAATCGTTTTTAAAAATCCATTGTTTAATATAAAAG 4573
Coquerel'sSifaka TTGTTTGAGGGAAAAACATTAGAAAATGATAAAAAAAAA-TTGCTTACCATTT-AAAAAATCCATTGTTTA-----AGAG 5253
Ma'sNightMonkey TTGTTTGAGGAGAATACTTTAGAAGATGATATAAAAATAATTGCACATCATTTTTTAAAATCCATTGTTTAATATAAAAG 5444
VervetAGM       TTGTTTGAGGAGAACACTTTAGAAGATGATAAAAAAAAG-TTGCAAACCATTTTTAAAAATTCATTGTTTAATATAAAAG 5542
Gorrilla        TTGTTTGAGGAGAACACTTTAGAAGATAATAAAAAAAAA-TTGCAAACCATTTTTAAAAATCCATTGTTTAATATAAAAG 7341
GrabEatingMacaqueTAATTTGAGGAGAACACTTTAGAAGATGATTAAAAAAAG-TTGCAAACCATTTTTAAAAATCCATTGTTTAATATAAAAG 5638
Macaque         TAATTTGAGGAGAACACTTTAGAAGATGATAAAAAAAAG-TTGCAAACCATTTTTAAAAATCCATTGTTTAATATAAAAG 5656
PigTailedMacaque TAATTTGAGGAGAACACTTTAGAAGATGATAAAAAAAAG-TTGCAAACCATTTTAAAAAATCCATTGTTTAATATAAAAG 5879
Bonobo          TTGTTTGAGGAGAACACTTTAGAAGATGATTAAAAAAA--TTGCAAATCGTTTTTAAAAATCCATTGTTTAATATAAAAG 5883
OliveBaboon     TAATTTGAGGAGAACACTTTAGAAGATGATAAAAAAAAG-TTGCAAACCATTTTTAAAAATCCGTTGTTTAATATAAAAG 5326
Orangutan       TTGCTTGA---GAACACTTTAGAAGATGATAAAAAAAAAAATTGCAAACCATTTTAAAAAATCCATTGTTTAATATAAAAG 5865
```

```
Majority         TCTGAATAATTAAAGTT-AATTTAATTTTCTGGAATAATGGTTAAGAATTTTTGCTTTGGTGTCAGGCCTGTGTACAAGT
                          7930      7940      7950      7960      7970      7980      7990      8000
Human            TCTGAATAATTAAAGTT-AATTTAATTTTCTGGAATAATGGTTAAGAATTTTTGCTTTGGTGTCAGGCCTGTGTACAAGT 4652
Coquerel'sSifaka TTTGAATAATTAAAGTTTTATCTAATTTTCTAGAGTAATGGTTAAGAATTTTTGCTGTGGTGTCAGGCCTATGTACAAGT 5333
Ma'sNightMonkey  TCTGAATAATTAAAGTT-AATTTAATTTTCTAGAATAATGGTTAAGAATTTTTGCTTTAGTGTCAGGCCTGTGTACAAGC 5523
VervetAGM        TCTGAATAATTAAAGTTTAATTTAATTTTCTGGAATAATGGTTAA---TTTTTGCTTTGGTGTCGGGCCTGTGTACAAGT 5619
Gorrilla         TCTGAATAATTAAAGTT-AATTTAATTTTCTGGAATAATGGTTAAGAATTTTTGCTTTGGTGTCAGGCCTGTGTACAAGT 7420
GrabEatingMacaqueTCTGAATAATTAAAGTT-AATTTAATTTTCTGGAATAATGGTTAAGAATTTTTGCTTTGGCGTCAGGCCTGTGTACAAGT 5717
Macaque          TCTGAATAATTAAAGTT-AATTTAATTTTCTGGAATAATGGTTAAGAATTTTTGCTTTGGCGTCAGGCCTGTGTACAAGT 5735
PigTailedMacaque TCTGAATAATTAAAGTT-AGTTTAATTTTCTGGAATAATGGTTAAGAATTTTTGCTTTGGCGTCAGGCCTGTGTACAAGT 5958
Bonobo           TCTGAATAATTAAAGTT-AATTTAATTTTCTGGAATAATGGTTAAGAATTTTTGCTTTGGTGTCAGGCCTGTGTACAAGT 5962
OliveBaboon      TCTGAATAATTAAAGTT-AATTTAATTTTCTGGAATAATGGTTAAGAATTTTTGCTTTGGCGTCAGGCCTGTGTACAAGT 5405
Orangutan        TCTGAATAATTGAAGTT-AATTTAATTTTCTGGAATAATGGTTAAGAATTTTTGCTTTGGTGTCAGGCCTGTGTACAAGT 5944

Majority         AATTAAGTGTTCTTTAAAA------ACATTTGTG-----GC------G--CA----TG-A----CA----T-----A---
                          8010      8020      8030      8040      8050      8060      8070      8080
Human            AATTAAGTGTTCTTTAAAACTTAAAACATTTGTGAATTTGCTTTAAAG-ACACTGATGCATGTGCATATTTAAAAAAATT 4731
Coquerel'sSifaka AATTGAGCTTGCTTTAAAA------AAGTTTTT----------------------------------------------- 5360
Ma'sNightMonkey  AACTAAGTGTTCTTTAAAA-------CATTCGTA---------------------------------------------- 5550
VervetAGM        AGTTAAGTGTTCTTTAAAA------ACATTTGT----------------------------------------------- 5646
Gorrilla         AATTAAGTGTTCTTTAAAACTTAAAACATTTGTGAATTTGCTTTAAAG-TCACTGATGCACGTGCATATTTAAAAAAATT 7499
GrabEatingMacaqueAGTTAAGTGTTCTTTAAAA------ACATTTGTGGCCGGGCACGGTGGCTCAAGCCTGTAATCCCAGCACTTTGGGAGGC 5791
Macaque          AGTTAAGTGTTCTTTAAAA------ACATTTGTGGCCGGGCACGGTGGCTCAAGCCTGTAATCCCAGCACTTTGGGAGGC 5809
PigTailedMacaque AGTTAAGTGTTCTTTAAAA------ACATTTGT----------------------------------------------- 5985
Bonobo           AATTAAGTGTTCTTTAAAACTTAAAACATTTGTGAATTTGCTTTAAAG-ACACTGATGCATGTGCATATTTAAAAAAATT 6041
OliveBaboon      AGTTAAGTGTTCTTTAAAA------ACATTTGT----------------------------------------------- 5432
Orangutan        AATGAAGTGTTCTTTAAAACTTAAAACATTTGTGAATTTGCTTTAAAG-ACACTGATGCATGTGCATATTTAAAAAAATT 6023

Majority         C--G-CGGGCG------G----------A-----A-CA---TGG----C---G-----C----------T-A--A-A--
                          8090      8100      8110      8120      8130      8140      8150      8160
Human            CAGGCCGGGCGCGGTGGGTCACACCTGTAATCCCAGCACTTTGGGAGGCCGAGGCGGGCGGATCACGAGTTCAGGAGA-- 4809
Coquerel'sSifaka -------------------------------------------------------------------------------- 5360
Ma'sNightMonkey  -------------------------------------------------------------------------------- 5550
VervetAGM        -------------------------------------------------------------------------------- 5646
Gorrilla         CAGGCCGGGCCCGGTGGGTCACGCCTGTAATCCCAGCACTTTGGGANNNNNNNNNNNNNNNNNNNNNNNNNNNNNNNN-- 7577
GrabEatingMacaqueCGAGACGGGCGGATCACGAGGTCAGGAGATCGAGACCAGCCTGGCTAACACGGTGAAACCCCGTCTCTACTAAAAATATA 5871
Macaque          CGAGACGGGCGGATCACGAGGTCAGGAGATCGAGACCAGCCTGGCTAACACGGTGAAACCCCGTCTCTACTAAAAATATA 5889
PigTailedMacaque -------------------------------------------------------------------------------- 5985
Bonobo           CAGGTCGGGCGCGGTGGGTCACGCCTGTAATCCCAGCACTTTGGGAGGCCGAGGCGGGCGGATCACGAGTTTAGGAGATT 6121
OliveBaboon      -------------------------------------------------------------------------------A 5433
Orangutan        CAGGCTGGGCGCAGTGGGTTACGCCTGTAATCCCAGCACTCTGGGAGGCTGAGGCGGGCAGATCACGAGTTCAGGAGATC 6103
```

```
Majority          -------------------------------GT-----C----A-T----A---T-AG-C-GG------G-------C--
                           8170      8180      8190      8200      8210      8220      8230      8240
Human             --GACCATCCTGGCTAACACGGTGAAACCCTGTCTTTACTAAAAATACAAAAAATTAGCCGGGTGTGGTGACGGGTGCCT 4887
Coquerel'sSifaka  -------------------------------------------------------------------------------- 5360
Ma'sNightMonkey   -------------------------------------------------------------------------------- 5550
VervetAGM         -------------------------------------------------------------------------------- 5646
Gorrilla          -------------------------------NNN---NNNNNNNNNNNNNNNNNNNNNNNNNNNNNNNNNNNNNNNNNNN 7623
GrabEatingMacaqueAAAA-CTAGCCGGGCGAGGTGGCGAA-GCCTGTAGTCCCAGCTATTTGGGAGGCTGAGGCAGGAGAATGGC--AAACCTG 5947
Macaque           AAAAACTAGCCGGGCGAGGTGGCAGGCGCCTGTAGTCCCAGCTATTTGGGAGGCTGAGGCAGGAGAATGGCGTAAACCTG 5969
PigTailedMacaque  -------------------------------------------------------------------------------- 5985
Bonobo            GAGACCATCCTGGCTAACACGGTGAAACCCTGTCTTTACTAAAAATACAAAAAATTAGCCAGGTGTGGTGACGGGTGCCT 6201
OliveBaboon       -------------------------------------------------------------------------------- 5433
Orangutan         GAGACCATCCTGGCTAACACAGTGAAACCCCGTCTCTACTAAAAATACAAAAAATTAGCCAGGCATGGTGGCGGGCGCCT 6183

Majority          G-AG-C--AGCT------------------------------------TG-G-GAC-GAG--------------------
                           8250      8260      8270      8280      8290      8300      8310      8320
Human             GTAGTCCCAGCTACTCGGGAGGCTGAGGCAGGAGAATG-GCATGAACCTGCGAGGCAGAGGTTGCAGTAAGCGGAGATTG 4966
Coquerel'sSifaka  -------------------------------------------------------------------------------- 5360
Ma'sNightMonkey   -------------------------------------------------------------------------------- 5550
VervetAGM         ------------------------------------------------------A------------------------- 5647
Gorrilla          NNNNTTTTTTTTTTT------------------------T--------TTTGAGACGGAG-------------------- 7651
GrabEatingMacaqueGGAGGCAGAGCT-----------GAGATCCG-CCACTGCACTCCAGCCTGGGTGACAGAGCGAGACTCTGTCTCAAAAAA 6015
Macaque           GGAGGCAGAGCTTGTAGTGACCTGAGATCCGGCCACTGCACTCCAGCCTGGGTGACAGAGCGAGACTCTGTCTCAAAAAA 6049
PigTailedMacaque  -------------------------------------------------------------------------------- 5985
Bonobo            GTAGTCCCAGCTACTCGGGAGGCTGAGGCAGGAGAATG-GCATGAACCTGCGAGGCAGAGGTTGCAGTAAGCGGAGATTG 6280
OliveBaboon       -------------------------------------------------------------------------------- 5433
Orangutan         GTAGTCCCAGCTACTCGGGAGGCTGAGGCGGGAGAATG-GCGTGAACCCGGGAGGCGGAGGTTGCAGTAAGCCGAGATTG 6262

Majority          ---------A--------------AATTTGCTTTAAAGACACTGA-TGCATGTTTAAAAAAATTCAAACTGATTCA----
                           8330      8340      8350      8360      8370      8380      8390      8400
Human             CACCACTGCA----------CTCCAGCCTGGGCGACAGAGACAGACTCTATCTCAAAAAAAAAAAAAAAAAAAAAAAAAA 5036
Coquerel'sSifaka  ------------------------AATTTTCTTTAAAGA---------------------------AAGTAA-------- 5381
Ma'sNightMonkey   ------------------------AATTTGCTTTACAGA---------------------------AACTGA-------- 5571
VervetAGM         ------------------------AATTTGCTTTAAAGGCACTGA-TGCATGTTTAAAAATATTCAAACTGATTCA---- 5698
Gorrilla          ---------------------TCTCGCCCGG-------------------TCTC-------------------------- 7665
GrabEatingMacaqueAAAAAAAAAT---------TTGTAAATTTGCTTTAAAGACACTGA-TGCATGTTTAAAAAAATTCAAACTGATTCA---- 6081
Macaque           AAAAAAAAAAAAAAAAAAATTGTAAATTTGCTTTAAAGACACTGA-TGCATGTTTAAAAAAATTCAAACTGATTCA---- 6124
PigTailedMacaque  ---------A--------------AATTTGCTTTAAAGACACTGA-TGCATGTTTAAAAAAATTCAAACTGATTCA---- 6037
Bonobo            CACCACTGCA----------CTCCAGCCTGGGCGACAGAGACAGACTCTATCTCAAAAAAAAAAATAATAANN------- 6343
OliveBaboon       ------------------------AATTTGCTTTAAAGACACTGA-TGCATGTTTAAAAAAATTCAAACTGATTCA---- 5484
Orangutan         TGCCACTGCA----------CTCCAGCCTGGGCAACAGAGCGAGACTCCATCTCAAAAAAAAGAAAAAAAATTTCAAACT 6332
```

```
Majority         -----------------------CAGGTTTTTT----TTATTTCTTAGGTGTAGGTAATTGTGTTAAAATTTTGTGCCTT

                          8410      8420      8430      8440      8450      8460      8470      8480

Human            AAAAAAAAAAATTCAAACTGCTTCACAGCTTTTTTTGCTATTTCTTAGGTGTAGGTAATTGTGTTAAAATTTTGTGCCTT 5116
Coquerel'sSifaka -------------------------------------------------------------------------------- 5381
Ma'sNightMonkey  -------------------------------------------------------------------------------- 5571
VervetAGM        -----------------------CAGGTTTTTT----TTATTTCTTAGGTGTAGGTAATTGTGTTAAAATTTTGCGCCTT 5751
Gorrilla         -------------------------------------------------------------------------------- 7665
GrabEatingMacaque-----------------------CAGGTTTTTT----TTATTTCTTAGGTGTAGGTAATTGTGTTAAAATTTTGTGCCTT 6134
Macaque          -----------------------CAGGTTTTTT----TTATTTCTTAGGTGTAGGTAATTGTGTTAAAATTTTGTGCCTT 6177
PigTailedMacaque -----------------------CAGGTTTTTT----TTATTTCTTAGGTGTAGGTAATTGTGTTAAAATTTTGTGCCTT 6090
Bonobo           -------------------------------------------------------------------------------- 6343
OliveBaboon      -----------------------CAGGTTTTTT----TAATTTCTTAGGTGTAGGTAATTGTGTTAAAATTTTGTGCCTT 5537
Orangutan        GATTCA-----------------CAGTTTTTTTAAATTTATTTGTTAGGTGTAGGTAATTGTGTTAAAATTTTGTGCCTT 6395

Majority         TTTACCTGAAATTATTGAT----ATATTACCACACATATAAACATCTTTATTTACATAATTGGATTTAAACTATTGTTCT

                          8490      8500      8510      8520      8530      8540      8550      8560

Human            TTTCTTGAAATTATTAATTTATATATTAGCACACATATAAACATCTTTATTTACGTGATTGAATTCAAACTATTGTTTT 5196
Coquerel'sSifaka -------------------------------------------------------------------------------- 5381
Ma'sNightMonkey  -------------------------------------------------------------------------------- 5571
VervetAGM        TTTACCTGAAATTATTGAT----ATATTACCACACATATAAACATCTTTATTTACATAATTGGATTCAAACTATTGTTCT 5827
Gorrilla         -------------------------------------------------------------------------------- 7665
GrabEatingMacaqueTTTACCTGAAATTATTGAT----ATATTACCACACATATAAACATCTTTATTTACATAATTGGATTTAAACTATTGTTCT 6210
Macaque          TTTACCTGAAATTATTGAT----ATATTACCACACATATAAACATCTTTATTTACATAATTGGATTTAAACTATTGTTCT 6253
PigTailedMacaque TT-ACCTGAAATTATTGAT----ATATTACCACACATATAAACATCTTTATTTACATAATTGGATTTAAACTATTGTTCT 6165
Bonobo           -------------------------------------------------------------------------------- 6343
OliveBaboon      TTTACCTGAAATTATTGAT----ATATTACCACACATATAAACATCTTTATTTACATAATTGGATTTAAACTATTGTTCT 5613
Orangutan        TTTTCTTGAAATCATTAATTTATATATTAGCACACATATAAACATCTTTATTTACGTGATTGAATTCAAACTATAGTTTT 6475

Majority         GGCCCACATCTTGCCTTTTTTTTTT--TTTTTTGAATGGGAGTC-CCCTCTGTCACCAGGCTGGAGTGCAGTGGCACGAT

                          8570      8580      8590      8600      8610      8620      8630      8640

Human            GGCCCACATCTTGCCTTTTTTTTTT--TTTTTTGAGTCGGAGTCTCCCTCTGTCATCAGGCTGGAGTGCAGTGGCATGAT 5274
Coquerel'sSifaka ----------------------------------------------------TACATGTGTATGTTTAAAAAA----AAT 5405
Ma'sNightMonkey  ----------------------------------------------------TGGACGTGCATGTTTAAAAAAGTTCAAA 5599
VervetAGM        GGCCCACATCTTGCCTTTTTTTTTT--TTTTTTGAATGGGAGTC-CCCTCTGTCACCAGGCTGGAGTGCAGTGGCACGAC 5904
Gorrilla         -------------------------------------------------------CCAGGCTGGAGTGCAGTGGCGCGAT 7690
GrabEatingMacaqueGGCCCACATCTTGCCTTTTTTTTTT-GTTTTTTGAATGGGAGTC-CCCTCTGTCACCAGGCTGGAGTGCAGTGGCACGAT 6288
Macaque          GGCCCACATCTTGCCTTTTTTTTTT-GTTTTTTGAATGGGAGTC-CCCTCTGTCACCAGGCTGGAGTGCAGTGGCACGAT 6331
PigTailedMacaque GGCCCACATCTTGCCTTTTTTTTTT-GTTTTTTGAATGGGAGTC-CCCTCTGTCACCAGGCTGGAGTGCAGTGGCACGAT 6243
Bonobo           -------------------------------------------------NNNNNNNNNNNNNNNNNNNGCAGTGGCGCGAT 6374
OliveBaboon      GGCCCACATCTTGCCTTTTTTTTTTTGTTTTTTGAATGGGAGTC-CCCTCTGTCACCAGGCTGGAGTGCAGTGGCACGAT 5692
Orangutan        GGCCCACATCTTGCCTTTTTTTTTT--TTTTT-GAGTCGGAGTCTCCTTCTGTCATCAGGCTGGAGTGCAGTGGCACGAT 6552
```

```
Majority          CTCAGC--------------------------------------------------------------------------

                            8650      8660      8670      8680      8690      8700      8710      8720

Human             CTCGGCTTACTGCAACCTCTGCCTCCTGGGTTCAGGCAATTCTCCTGCCTCAGCCTCCCTAGTAGCTGGGACTACAGGCA 5354
Coquerel'sSifaka  TTGA---------------------------------------------------------------------------- 5409
Ma'sNightMonkey   CTGA---------------------------------------------------------------------------- 5603
VervetAGM         CTCCGC-------------------------------------------------------------------------- 5910
Gorrilla          CTCTGC-------------------------------------------------------------------------- 7696
GrabEatingMacaque CTCAGC-------------------------------------------------------------------------- 6294
Macaque           CTCAGC-------------------------------------------------------------------------- 6337
PigTailedMacaque  CTCAGC-------------------------------------------------------------------------- 6249
Bonobo            CTCG---------------------------------------------------------------------------- 6378
OliveBaboon       CTCCGC-------------------------------------------------------------------------- 5698
Orangutan         CTTGGCTCACTGCAACCTCTGCCTCCTGGGTTCAGGCGATTCTCCTGCCTCAGCCTCCCTAGTAGCTGGGACTACAGGCG 6632

Majority          --------------------------------------------------------------------------------

                            8730      8740      8750      8760      8770      8780      8790      8800

Human             CACGCCGCCATGCCAGACTAATTTTTTTTTTTTTTTC-GAGACGGAGTCTCGCCCGGTCTCCCAGGCTGGAGTGCAGTGT 5433
Coquerel'sSifaka  -------------------------------------------------------------------------------- 5409
Ma'sNightMonkey   -------------------------------------------------------------------------------- 5603
VervetAGM         -------------------------------------------------------------------------------- 5910
Gorrilla          -------------------------------------------------------------------------------- 7696
GrabEatingMacaque -------------------------------------------------------------------------------- 6294
Macaque           -------------------------------------------------------------------------------- 6337
PigTailedMacaque  -------------------------------------------------------------------------------- 6249
Bonobo            -------------------------------------------------------------------------------- 6378
OliveBaboon       -------------------------------------------------------------------------------- 5698
Orangutan         CACGCCACCATGCCAGACTAATTTTTTTTTTTTTTTTTGAGATGGAGTCTCGCCCTGTCTCCCAGGCTGGAGTGCAGTGG 6712

Majority          ------------TCACTGCAACCTCCGCCTCCC-----------------------------------------------

                            8810      8820      8830      8840      8850      8860      8870      8880

Human             CGCGATCTCTGCTCACTGCAACCTCTGCCTCCTGGGTTCACGCCATTCTCCTGTCTCAGCCTCCCAAGTAGCTGGGACTA 5513
Coquerel'sSifaka  --------------AGTGAAA----------------------------------------------------------- 5416
Ma'sNightMonkey   -------------------------------------------------------------------------------- 5603
VervetAGM         ------------TCACTGCAACCTCCGCCTCCCAGGGTTCAAGTGATTCTC----------------------------- 5949
Gorrilla          ------------TCACTGCAACCTCTGCCTCCTGGGTTCACGCCATTCTCCTATCTCAGCCTCCCGAGTAGCTGGGACTA 7764
GrabEatingMacaque ------------TCACTGCAACCTCCGCCTCCC----------------------------------------------- 6315
Macaque           ------------TCACTGCAACCTCCGCCTCCC----------------------------------------------- 6358
PigTailedMacaque  ------------TCACTGCAACCTCCGCCTCCC----------------------------------------------- 6270
Bonobo            ------------TCACTGCAACCTCTGCCTCCTGGGTTCACGCCATTCTCCTGTCTCAGCCTCCCAAGTAGCTGGGACTA 6446
OliveBaboon       ------------TCACTGCAACCTCTGCTTCCC----------------------------------------------- 5719
Orangutan         TGCGATCT--GCTCACTGCAACCTCCACCTCCTGGGTTCATGCCATTCTCC--TCTCAGCCTCCCGAGTAGCTGGGACTA 6788
```

```
Majority           ----------------------------------------------------GGGGTTCAA--------------------

                            8890      8900      8910      8920      8930      8940      8950      8960

Human              CAGGCGCCTGCCACCACGCCTGGCTTCTTTTTTGTATATTCAGTAGAGACAGAGTTTCACCATGTTAGCCAGGATAGTCT 5593
Coquerel'sSifaka   -------------------------------------------------------------------------------- 5416
Ma'sNightMonkey    -------------------------------------------------------------------------------- 5603
VervetAGM          -------------------CTGCCTCAGCCTCCCGAATAACTGGACTACAGGGGTTCAA--------------------- 5990
Gorrilla           CAGGCGCCCGCCACCACGCCTGGCTTATTTTTTGTATTTCAGTAGAGACAGAGTTTCACCATGTTAGCCAGGATAGTCT 7844
GrabEatingMacaque---------------------------------------------------GGGGTTCAA-------------------- 6324
Macaque            ---------------------------------------------------GGGGTTCAA-------------------- 6367
PigTailedMacaque   ---------------------------------------------------GGGGTTCAA-------------------- 6279
Bonobo             CAGGCGCCTGCCACCACGCCTGGCTTCTTTTTTGTATATTCAGTAGAGACAGAGTTTCACCATGTTAGCCAGGATAGTCT 6526
OliveBaboon        ---------------------------------------------------GGGGTTCAA-------------------- 5728
Orangutan          CAGGCACCTGCCACCATGCCCAGCTAAGTTTTTGTATTTCAGTAAAGACAGAGTTTCACCATGTTAGCCAGGATAGTTT 6868

Majority           ---------------GTGATTCTCCTGCCTCAGCCTCCCAAATAACTGGACTACAGGCGCCCGCCACC-----------

                            8970      8980      8990      9000      9010      9020      9030      9040

Human              CTATCTCCTGACCTCGTGATCTGCCTGCCTCGGCCTCCCAAAGTGCTGGGATTACAAGCGTGAGCCACCGCGCCTAGCCA 5673
Coquerel'sSifaka   -----------------GATATTTCTCCCT-G--------A-------------------------TT----------- 5433
Ma'sNightMonkey    ------------------------------------------------------------------TT----------- 5605
VervetAGM          ---------------GTGATTCTCCTGCCTCAGCCTCCCGAATAACTGGACTACAGGCGCCCGCCACC----------- 6044
Gorrilla           CGATCTCCTGACCTCGTGATCTGCCTGCCTCGGCCTCCCAAAGTGCTGGGATTACAAGCGTGAGCCACCGCGCCTAGCCA 7924
GrabEatingMacaque---------------GTGATTCTCCTGCCTCAGCCTCCCGAATAACTGGACTACAGGCGCCCGCCACC----------- 6378
Macaque            ---------------GTGATTCTCCTGCCTCAGCCTCCCGAATAACTGGACTACAGGCGCCCGCCACC----------- 6421
PigTailedMacaque   ---------------GTGATTCTCCTGCCTCAGCCTCCCGAATAACTGGACTACAGGCGCCCGCCACC----------- 6333
Bonobo             CGATCTCCTGACCTCGTGATCTGCCTGCCTCGGCCTCCGAAAGTGCTGGGATTACAAGCGTGAGCCACCGCGCCTAGCCA 6606
OliveBaboon        ---------------GTGATTCTCCTGCCTCAGCCTCCCAAATAACTGGACTACAGGCGCCCGCCACC----------- 5782
Orangutan          CGATCTCCTGACCTCATGATCCGTCTGCCTCAGCCTCCCAAAGTGCTGGGATTACAAGCGTGAGCCACCACGCCTGGCCA 6948

Majority           --ACGCCAGACTAATTTTTGTATTTTTAGTACTGACGGGGTTTC--ACCATTTGGCCAGGATGGTCTTGATCTCTTGAC

                            9050      9060      9070      9080      9090      9100      9110      9120

Human              AAATGCCAGACTAATTTTTGTATTTTTAGTAGAGACAGGGTTTC--ACCATTTGGCCAGGATGGTCTTGATCTCTTGAC 5751
Coquerel'sSifaka   ------CA---TTGTATTTATTTCTTAGGTATAGATAATTTTGTTAAAATTTTTTGTGCATTTTTTTCTGAAATTATTGA 5504
Ma'sNightMonkey    ------CACAGTTTTTTCTTTTTATTAGGTGTGGGTAATTTTGT--GCCTTTTT-----------TCTTGAAATTATTAA 5666
VervetAGM          --ACGCCAGACTAACTTTTGTATTTTTAGTACTGACGGGGTTTC--ACCATGTTGGCCAGGATGGTCTTGTTCTCTTGAC 6120
Gorrilla           AAACGCCAGACTAATTTTTGTATTTTTAGTAGAGACAGGGTTTC--ACCATTTGGCCAGGATGGTCTTGATCTCTTGGC 8002
GrabEatingMacaque--ACGCCAGACTAACTTTTGTATTTTTAGTACTGACGGGGTTTC--ACCATGTTGGCCAGGATGGTCTTGTTCTCTTGAC 6454
Macaque            --ACGCCAGACTAACTTTTGTATTTTTAGTACTGACGGGGTTTC--ACCATGTTGGCCAGGATGGTCTTGTTCTCTTGAC 6497
PigTailedMacaque   --ATGCCAGACTAACTTTTGTATTTTTAGTACTGACGGGGTTTC--ACCATGTTGGCCAGGATGGTCTTGTTCTCTTGAC 6409
Bonobo             AAACGCCAGACTAATTTTTGTATTTTTAGTAGAGACAGGGTTTC--ACCATTTGGCCAGGATGGTCTTGATCTCTTGAC 6684
OliveBaboon        --ACGCCAGACTAACTTTTGTATTTTTAGTACTGACGGGGTTTC--ACCATGTTGGCCAGGATGGTCTTGTTCTCTTGAC 5858
Orangutan          AAACGCCAGACTAATTTTTGTATTTTTAGTAGAGACGGGGTTTC--ACCATTTGGCCAGGGTAGTCTTGATCTCTTGAC 7026
```

```
Majority          TCCATG-AT-CCGCC-CGCCTTGACCTCCCAAAGTGTTGGGATTACAGGCTTGAGCCACTGTGCCCGGCCCATATCTTGC

                          9130      9140      9150      9160      9170      9180      9190      9200

Human             TCCATC-AT-CCGCC-CGCCTTGACTTCCCAAAGTGTTGGGATTACAGGCTTGAGCCACCGCGTCCGGCCCATATCTTGC 5828
Coquerel'sSifaka  TTTATGTATTACGTCACATATGAACACCTT----CGTTTATACAAGGGATTCAAACTATTTGTTCTGGCTCACATTTTGC 5580
Ma'sNightMonkey   TTTATG-TTACCATC-CATATAAACATCTTTACATAATTAGATT-------CAAACTATTGT-TCTGGCCCACATCTTGC 5736
VervetAGM         TCCATG-AT-CCGCC-CGCCTTGACCTCCCAAAGTGCTGGGATTACAGGCGTGAGCTGCTGCGCCCAGCCCGTCTCTTG- 6196
Gorrilla          TCCATC-AT-CCGCC-CGCCTTGACTTCCCAAAGTGTTGGGATTACAGGCTTGAGCCACCGCGCCCGGCCCACATCTTGC 8079
GrabEatingMacaqueTCCTTG-AT-TCGCC-CGCCTTGACCTCCCAAAGTGCTGGGATTACAGGCGTGAGCCGCTGTGCCCAGCCCGTCTCTTG- 6530
Macaque           TCCTTG-AT-TCGCC-CGCCTTGACCTCCCAAAGTGCTGGGATTACAGGCGTGAGCCGCTGTGCCCAGCCCGTCTCTTG- 6573
PigTailedMacaque  TCCATG-AT-CCGCC-CGCCTTGACCTCCCAAAGTGCTGGGATTACAGGCGTGAGCCGCTGTGCCCAGCCTGTCTCTTG- 6485
Bonobo            TCCATC-AT-CCGCC-CGCCTTGACTTCCCAAAGTGTTGGGATTACAGGCTTGAGCCACCGCGTCCGGCCCATATCTTGC 6761
OliveBaboon       TCCATG-AT-CCGCC-CGCCTTGACCTCCCAAAGTGCTGGGATTACAGGCGTGAGCCGCTGTGCCCAGCCCGTCTCTTG- 5934
Orangutan         CCCATG-AT-CCGCC-CGCCTTGATCTCCCAAAGTGTTGGGATTACAGGCTTGAGCCACCGCGCCTGGCCCACATCTTGC 7103

Majority          CTTT----------------TTGAGATATAATTTGACATAAAATAAACTACCCATATTAAATATACAATGTTTTGTCTCA

                          9210      9220      9230      9240      9250      9260      9270      9280

Human             CTTTAAAAAAAATAGCTTTATTGAGATATAATTTGACATAAAATAAACTACCCATATTAAATATACAATGTTTTATCTCA 5908
Coquerel'sSifaka  CTCT----------------TTTAAAAAAAA--TAGCTTTACATACACTACTCATTTACCGTATATATTGTTTTGACATA 5642
Ma'sNightMonkey   CTTTTTAAAAAATAGCTTTATTGAGATATAATTTGACATAAAATAAAGTACCCATATTAAATATACAATGTTTTATCTCA 5816
VervetAGM         -----------------------AGAGATAATTTGACATAAAATAAACTACTCATATTAAATATACAATGTTTTGTCTCA 6253
Gorrilla          CTTTAAAAAAAATAGCTTTATTGAGATATAATTTAACATAAAGTAAACTACCCATATTAAATATACAATGTTTTATCTCA 8159
GrabEatingMacaque-----------------------AGATATAATTTGACATAAAATAAACTACCCATATTAAATATACAATGTTTTGTCTCA 6587
Macaque           -----------------------AGATATAATTTGACATAAAATAAACTACCCATATTAAATATACAATGTTTTGTCTCA 6630
PigTailedMacaque  -----------------------AGATATAATTTGACATAAAATAAACTACCCATATTAAATATACAATGTTTTGTCTCA 6542
Bonobo            CTTTAAAAAAA-TAGCTTTATTGAGATATAATTTGACATAAAATAAACTACCCATATTAAATATACAATGTTTTATCTCA 6840
OliveBaboon       -----------------------AGATATAATTTGACATAAAATAAACTACCTATATTAAATATACAATGTTTTGTCTCA 5991
Orangutan         CTTTAAAAAAA-TAGGTTTATTGAGATACAATGTGACATAAAATAAACTACCCATATTAAATATACAGTGTTTTGTCTCA 7182

Majority          TATATACTCTTGTGAAACCATCACCACAATCAAGATAATGAACATCTATCACTCCCCACAATATCATCTTGCGCCTTTGT

                          9290      9300      9310      9320      9330      9340      9350      9360

Human             TATATACCCTTGTGAAACCA---CTGCAATCAAGATAATGAACATCTATCACTCCCTACAGTATCATCTTGCGCCTTTAT 5985
Coquerel'sSifaka  TGTACACATCTGTGAAATCA---CCACAATCAAGATAATGAACAT----CACTCCCAAAAATATCATCTTGCCCCTTTGT 5715
Ma'sNightMonkey   TGTATACCCTTGTGAAACCATCACCACAATGAAGATAATGAACATCTATCACTGCCCACAATATCATCTTGCGCCTTTGT 5896
VervetAGM         TACATACTCTTGTGAAACCATCACCACAATCAAGATAATGAACATCAATCACTCCCCACAATATCATCTTGCACCTTTGT 6333
Gorrilla          TATATACCCTTGTGAAACCA---CCACAATGAAGATAATGAACATCTATCACTCCCCACAGTATCATCTTGCGCCTTTAT 8236
GrabEatingMacaqueTACATACTCTTGTGCAACCATCACCACAATCAAGATAATGAACATCAGTCACTCCGCACAATATCATCTTGCACCTTTGT 6667
Macaque           TACATACTCTTGTGCAACCATCACCACAATCAAGATAATGAACATCAGTCACTCCCCACAATATCATCTTGCACCTTTGT 6710
PigTailedMacaque  TACATACTCTTGTGCAACCATCACCACAATCAAGATAATGAACATCAGTCACTCCCCACAATATCATCTTGCACCTTTGT 6622
Bonobo            TATATACCCTTGTGAAACCA---CCACAATCAAGATAATGAACATCTATCACTCCCTACAGTATCATCTTGCGCCTTTAT 6917
OliveBaboon       TACATACTCTTGTGAAACCATCACCACAATCAAGATAATGAACATCAGTCACTCCCCACAATATCATCTTGCACCTTTGT 6071
Orangutan         TATATACCCTTGTGAAACCATCACCACAATCAAGATAATGAACATCTATCACTCCCCGCAGTATCGTCTTGCGCCTTTAT 7262
```

```
Majority          AGTCCCTCCCTTC-TGCTTTTCTTTCCCCAAGCAACCACTGATCTGCTTTCTGTCACTGTAGATTAGTTCACACTTTTTA

                          9370      9380      9390      9400      9410      9420      9430      9440

Human             AGTCCCTCCTTTC-TGCTTTTCTTTCCCCAAGGAACCACTGATCTGCTTTCTGTCACTATAGATTAGTTCACACTTTTTA 6064
Coquerel'sSifaka  AATTCCTCTCTTT-TGACTTTCTTCCCCCAAACAACCACTGATCTGCTTTCTGTCACTATAGATTAGTTTGTATTTTTTA 5794
Ma'sNightMonkey   AGTCCCTCCCTTCCTGCTTCTCTTTCCCCAAGCAGCCACTGATCTGCTTTCTGTCACTATAGATTAGTTCACAGTGTTTA 5976
VervetAGM         ACTCCATCCCTTC-TGCTTTTCTTTCCCCAAGCAACCACTGATCTGCTTTCTGTCACTGTAGATTAGTTCACACTTTTTA 6412
Gorrilla          AGTCCCTCCTTTC-TGCTTTTCTTTCCCCAAGGAACCACTGATCTGCTTTCTGTCACTATAGATTAGTTCACACTTTTTA 8315
GrabEatingMacaqueACTCCATCCCTTC-TGCTTTTCTTTCCCCAAGCAACCACTGATCTGCTTTCTGTCACTGTAGATTAGTTCACACTTTTTA 6746
Macaque           ACTCCATCCCTTC-TGCTTTTCTTTCCCCAAGCAACCACTGATCTGCTTTCTGTCACTGTAGATTAGTTCACACTTTTTA 6789
PigTailedMacaque  ACTCCATCCCTTC-TGCTTTTCTTTCCCCAAGCAACCACTGATCTGCTTTCTGTCACTGTAGATTAGTTCACACGTTTTA 6701
Bonobo            AGTCCCTCCTTTC-TGCTTTTCTTTCCCCAAGGAACCACTGATCTGCTTTCTGTCACTATAGATTAGTTCACACTTTTTA 6996
OliveBaboon       ACTCCATCCCTTC-TGCTTTTCTTTCCCCAAGCAACCACTGATCTGCTTTCTGTCACTGTAGATTAGTTCACACTTTTTA 6150
Orangutan         AGTCCCTCCTTTC-TGCTTTTCTTTCCCCAAGGAACCACTGATCTGCTTTCTGTCACTGTAGATTAGTTCACACTTTTTA 7341

Majority          GAATTTTAGGTAAATGTAATCATATGGTATGTACATTTTT-GGTCTGGGTTAGTCCACTCAGAATAATTATTTTGAGATT

                          9450      9460      9470      9480      9490      9500      9510      9520

Human             GAATTTTAGATAAATGTAATCATATGGTATGTACATTTTT-GGTCTGGGTTAGTCCACTCAGAATAATTATTTTGAAATT 6143
Coquerel'sSifaka  GAATTTTAAATAACTGTAAT--TATGATATGTACCCTTTTTTGTCTAGGTTATTTCATGCAGCGTAATTATTTTGAAATT 5872
Ma'sNightMonkey   GAATTTTAGGTAAATGTAATCATATAGTATGTACTTTTTTTGGTCTAGGTAAGTCCACTCAGAATAATTATTTTGAGATT 6056
VervetAGM         GAATTTTAGGTAAATGTGATCATGTGGTATGTACATTTTT-GGCCTGGGTT------------------ATTTTGAGATT 6473
Gorrilla          GAATTTTAGGTAAATGTAATCATATGGTATGTACATTTTT-GGTCTGGGTTAGTCCACTCAGAATAATTATTTTGAAATT 8394
GrabEatingMacaqueGAATTTTAGGTAAATGTAATCATGTGGTATGTACATTTTT-GGCCTGTGTTAGTCCACTCAGAAT---TATTTTGAGATT 6822
Macaque           GAATTTTAGGTAAATGTAATCATGTGGTATGTACATTTTT-GGCCTGTGTTAGTCCACTCAGAAT---TATTTTGAGATT 6865
PigTailedMacaque  GAATTTTAGGTAAATGTAATCATGTGGTATGTACATTTTT-GGCCTGTGTTAGTCCACTCAGAAT---TATTTTGAGATT 6777
Bonobo            GAATTTTAGATAAATGTAATCATATGGTATGTACATTTTT-GGTCTGGGTTAGTCCACTCAGAATAATTATTTTGAAATT 7075
OliveBaboon       GAATTTTAGGTAAATGTAATCATGTGGTATGTACATTTTT-GGCCTGTGTTAGTCCACTCAGAAT---TATTTTGAGATT 6226
Orangutan         GAATTTCAGGTAAATGTAATCATATGGTATGTACATTTTT-GGTTTGGGTTAGTCCACTCAGAATAATTATTTTGAGATT 7420

Majority          CACCCATGTTGTGTTTCAATAGTCCATTCTCTTTTATTGCTTAGTAATATTACACTTTATGGCTATACAGCAATTTGTTA

                          9530      9540      9550      9560      9570      9580      9590      9600

Human             CACCCATGTTGTCTTTCAATAGTCCATTCTGTTTTATTGCTTAGTAATAGTACACTTTATGGCTATACAGCAATTTGTTA 6223
Coquerel'sSifaka  -ACCTACGCTGTGTATCTCTAGTCCATTCCTTTTTATTGCTTAATAATATTACACTGTATGGCTATATGGG-------CA 5944
Ma'sNightMonkey   CACCCATGTCGTGTTTCAATAGTCCATTCTCTTTTGTTGCTTAGTAATAATACACTTTATGGCTATACGGCAGTTTGTTA 6136
VervetAGM         CACCCATGTTGTGTTTCAATAGTCCATTCTCTTTTATTGCTTAGTAATATTACACTTAATGGCTATACAGCAATTTGTTA 6553
Gorrilla          CAGCCATGTTGTGTTTCAATAGTCCATTCTCTTTTATTGCTTAGTAATATTATACTTTATGGCTATACAGCAATTTGTTA 8474
GrabEatingMacaqueCACCCATGTTGTGTTTCAATAGTCCATTCTCTTTTATTGCTTAGCAATATTACACTTAATGGCTATACAGCAATTTGTTA 6902
Macaque           CACCCATGTTGTGTTTCAATAGTCCATTCTCTTTTATTGCTTAGCAATATTACACTTAATGGCTATACAGCAATTTGTTA 6945
PigTailedMacaque  CACCCATGTTGTGTTTCAATAGTCCATTCTCTTTTATTGCTTAGCAATATTACACTTAATGGCTATACAGCAATTTGTTA 6857
Bonobo            CACCCATGTTGTGTTTCAATAGTCCATTCTCTTTTATTGCTTAGTAATAGTACACTTTATGGCTATACAGCAATTTGTTA 7155
OliveBaboon       CACCCATGTTGTGTTTCAATAGTCCATTCTCTTTTATTGCTTAGCAATATTACACTTAATGGCTATACAGCAATTTGTTA 6306
Orangutan         CACCCATGTTGTGTTTCAATAGTCCATTCTCTTTTATTGCTTAGTAATATTACACTTTATGGCTATACAGCAATTTGTTA 7500
```

```
Majority          ATCCATTCAAGTCTTGAGGGACATTTGTATTGTTTTCTGTTTTGAGCTA-TAAAA-TATAGTTGT---------------
                           9610      9620      9630      9640      9650      9660      9670      9680
Human             ATCCATTCAAGTCTTGAGGGACATTTGTATTGTTTTCCGTTTTGAGCTAATAAAAATATAGTTGT--------------- 6288
Coquerel'sSifaka  ATTTGTGCAACTCTTGATGGACGTGTATATTGTTTCCAGTTTTGGCTATTAAAA-TATAGTTAC--------------- 6008
Ma'sNightMonkey   ATCCATTCAAGTCTTGAGGGATATTTGTATTGTTTTCCATTTTGGGCTATTAAAAATATAGTTGTCAACGCACTGGTGGC 6216
VervetAGM         ATCCATTCAAGTCTTGAGGGACATTTGT-----TTTCTGTTTTGAGCTA------------------------------- 6597
Gorilla           ATCCATTCAAGTCTTGAGGGACATTTGTATTGTTTTCCGTTTTGAGCTAATAAAAATATAGTTGT--------------- 8539
GrabEatingMacaque ATCCATTCAAGTCTTGAGGGACATTTGT-----TTTCTGTTTTGAGCTA------------------------------- 6946
Macaque           ATCCATTCAAGTCTTGAGGGACATTTGT-----TTTCTGTTTTGAGCTA------------------------------- 6989
PigTailedMacaque  ATCCATACAAGTCTTGAGGGACATTTGT-----TTTCTGTTTTGAGCTA------------------------------- 6901
Bonobo            ATCCATTCAAGTCTTGAGGGACATTTGTATTGTTTTCCGTTTTGAGCTAATAAAAATATAGTTGT--------------- 7220
OliveBaboon       ATCCATTCAAGTCTTGAGGGACAGTTGT-----TTTCTGTTTTGAGCTA------------------------------- 6350
Orangutan         ATCCATTCAAGTCTTGAGGGACATTTGTATTGTTTTCCGTTTTGAGCTAATAAAAATATAGTTGT--------------- 7565

Majority          -------------------------------------------------------TTCCTGTACAAGTCTTTGTATGGAC
                           9690      9700      9710      9720      9730      9740      9750      9760
Human             --------------------------------------------------GAACATTACTGTACAAGTCTTTGTATGGAC 6318
Coquerel'sSifaka  -------------------------------------------------------TGTTCATATAAGTCCTTTTGTGTAT 6033
Ma'sNightMonkey   GCAGTGGGTAACGAGTCTGACCGTAGATCAAAAAAAAATATAGTTGTTGTGAGCATTCCTATACCAGTCTTTGTGTGGAC 6296
VervetAGM         -------------------------------------------------------TACCTGTACAAGTCTTTGTATGGAC 6622
Gorilla           --------------------------------------------------GAACATTACTGTACAAGTCTCTGTATGGAC 8569
GrabEatingMacaque -------------------------------------------------------TACCTGTACAAGTCTTTGTATGGAC 6971
Macaque           -------------------------------------------------------TACCTGTACAAGTCTTTGTATGGAC 7014
PigTailedMacaque  -------------------------------------------------------TACCTGTACAAGTCTTTGTATGGAC 6926
Bonobo            --------------------------------------------------GAACATTACTGTACAAGTCTTTGTATGGAC 7250
OliveBaboon       -------------------------------------------------------TACCTGTACAAGTCTTTGTATGGAC 6375
Orangutan         --------------------------------------------------GAACATTCCTGTACAAGTCTTTGTATGGAC 7595

Majority          ATATGCTTTCATTTCTCTTGGTGGGGGGGG-CTATGATTGGAAAGTCTGGATCATGTGGTAGGCATATGTTTAACTTTTT
                           9770      9780      9790      9800      9810      9820      9830      9840
Human             ATATGCTTTCATTTCTCTTGGTGGGGCAGG-CTATGATTGGAAAGTCTGGATCATGTGATAGGCATATGTTTAACTTTTT 6397
Coquerel'sSifaka  ATATGCTTTCATTTCTTTT----GGGGAGGCCTGTGATTAGAATGGTTGAATCATATGGTAGGCATATGTTTAACTTTTT 6109
Ma'sNightMonkey   ATATGCTT-CATTTCTCTTGGTGGGGCAG--CTGTGATTGGAAAGTCTGGATCTTATGGTAGGCATATGTTTAACTTTTT 6373
VervetAGM         ATAGGCTTTCATTTCTCTTCGTCGGGGGAG-CTATGATTGGAAAGTCTGGATCATGTGGTAGGCATATGTTTAACTTTTT 6701
Gorilla           ATATGCTTTCATTTCTCTTGGTGGGGCAGG-CTATGATTGGAAAGTCTGGATCATGTGATAGGCATATGTTTAACTTTTT 8648
GrabEatingMacaque ATAGGCTTTCATTTCTCTTCGTCGGCGGGG-CTATGATTGGAAAGTCTGGATCATGTGGTAGGCATATGTTTAACTTTTT 7050
Macaque           ATAGGCTTTCATTTCTCTTCGTCGGCGGGG-CTATGATTGGAAAGTCTGGATCATGTGGTAGGCATATGTTTAACTTTTT 7093
PigTailedMacaque  ATAGGCTTTCATTTCTCTTCGTCGGCGGGG-CTATGATTGGAAAGTCTGGATCATGTGGTAGGCATATGTTTAACTTTTT 7005
Bonobo            ATATGCTTTCATTTCTCTTGGTGGGGCAGG-CTATGATTGGAAAGTCTGGATCATGTGATAGGCATATGTTTAACTTTTT 7329
OliveBaboon       ATAGGCTTTCATTTCTCTTCGTCGGCGGGG-CTATGATTGGAAAGTCTGGATCATGTGGTAGGCATATGTTTAACTTTTT 6454
Orangutan         ATATGCTTTCATTTCTCTTGGTGGGGGGGGGCTATGATTGGAAAGTCTGGATCATGTGGTAGGCATATGTTTAACTTTTT 7675
```

```
Majority          AAGGAACTGCAAACT--TTTTAAAAGTAGTTGTACCATCTTACATTGCCATCAGCAGGGTATGAAATTTCCAGTCCCTCC
                           9850      9860      9870      9880      9890      9900      9910      9920

Human             AAGGAACTGCAAACT--TTTTAAAAGTAGTTGTACCATCTTACATTGCCATCAGCATGGTATGAAATTTCCAGTCCTTCC 6475
Coquerel'sSifaka  AAGGAACTGAAAATTATTTTTTAAAATAGTTGTACCATTTTATATTGTCATCAGTAGTGTATGAGATTTCCAGTCCCTCC 6189
Ma'sNightMonkey   A---AACTGCGAACT--TTTTAGAAGTAGTTGTTCCATCTTACATTGCCATCAGCAGGGTATGAAATTTCCAGTCCCTCC 6448
VervetAGM         AAGGAACTGCAAACT--TTTTAAAAGTAGTTCTACCATCTTACATTGCCATCAGCGGGGTATGAAATTTCCAGTCCCTCC 6779
Gorrilla          AAGGAACTGCAAACT--TTTTAAAAGTAGTTGTACCGTCTTACATTGCCATCAGCACGGTATGAAATTTCCAGTCCTTCC 8726
GrabEatingMacaqueAAAGAACTGCAAACT--TTTTAAAAGTAGTTCTACCATCTTACATTGCCATCAGCGGGGTATGAAATTTCCAGTCCCTCC 7128
Macaque           AAGGAACTGCAAACT--TTTTAAAAGTAGTTCTACCATCTTACATTGCCATCAGCGGGGTATGAAATTTCCAGTCCCTCC 7171
PigTailedMacaque  AAGGAACTGCAAACT--TTTTAAAAGTAGTTCTACCATCTTACATTGCCATCAGCGGGGTATGAAATTTCCAGTCCCTCC 7083
Bonobo            AAGGAACTGCAAACT--TTTTAAAAGTAGTTGTACCATCTTACATTGCCATCAGCACGGTATGAAATTTCCAGTCCTTCC 7407
OliveBaboon       AAGGAACTGCAAACT--TTTTAAAAGTAGTTCTACCATCTTACATTGCCATCAGCGGGGTATGAAATTTCCAGTCCCTCC 6532
Orangutan         AAGGAACTGCAAACT--TTTTAAAAATAGTTGTACTATCTTGCACTGCCATCAGCACGGTATGAAATTTCCAGTCCTTCC 7753

Majority          ACATCCTCTCTAATGCCTGTTAT---CTTGTCTTTTTGATTATAGACATTCTAGTGTATATGAAGTGATATTTCATTGTG
                           9930      9940      9950      9960      9970      9980      9990     10000

Human             ACATCCTCTTTAATGCCTGTTAT---CTTCTGTTTTTGATTATAGATATTGTAGTGTATATGAAGTGATATTTCACTGTG 6552
Coquerel'sSifaka  ACATCCTCTCTAGTGCCTGTTATTGTCTTGTCTATTTGGTTATAGACATTCTAGTGGGTGTGAAGTGGTATCTTACTGTG 6269
Ma'sNightMonkey   ACATCCTCTCTAGTGCCTGTTGT---CTTGTCTTTTTGATTATAGGCATTCTAGTATATATATAGTGATAGTTCATTGCG 6525
VervetAGM         ACATCCTTTCTAATGCCTGTTAT---CTTGTCTTTTTGATTGCAGACGTTCTCGTGTATATGAAGTGATATTTCATTGTG 6856
Gorrilla          ACATCCTCTTTAATGCCTGTTAC---CTTGTCTTTTTGATTATAGATATTGTGGTGTATATGAAGTGATATTTCACTGTG 8803
GrabEatingMacaqueACATCCTTTCTAATGCCTGTTAT---CTTGTCTTTTTGATTGCAGACATTCTAGTGTATATGAAGTGATATTTCATTGTG 7205
Macaque           ACATCCTTTCTAATGCCTGTTAT---CTTGTCTTTTTGATTGCAGACATTCTAGTGTATATGAAGTGATATTTCATTGTG 7248
PigTailedMacaque  ACATCCTTTCTAATGCCTGTTAT---CTTGTCTTTTTGATTGCAGACATTCTAGTGTATATGAAGTGATATTTCATTGTG 7160
Bonobo            ACATCCTCTTTAATGCCTGTTAT---CTTCTCTTTTTGATTATAGATATTGTAGTGTATATGAAGTGATATTTCACTGTG 7484
OliveBaboon       ACATCCTTTCTAATGCCTGTTAT---CTTGTCTTTTTGATTGCAGACATTCTAGTGTATATGAAGTGATACTTCATTGTG 6609
Orangutan         ACATCCTCTCTAATGCCTGTTAT---CTTGTCTTTTTGATTATAGACATTGTAGTGTATATGAAGTGATATTTCATTGTG 7830

Majority          GTTTTAATTTGCATTCCTCTAATATCTATGATGTTGAACATCTTTTCATGTGCTTTTTGTCAACTGTTACCTTTGGTGAA
                          10010     10020     10030     10040     10050     10060     10070     10080

Human             GATTTAATTTGCATTCCTCTAATATCTATGATGTTGGACATCTTTTCGTGTGCTTTTTGTCAGCTGTTATCTTTGGTGAA 6632
Coquerel'sSifaka  GTTTTCATTTGCATTTCTCTAATATCTGTGATATTGAACAGCTTTTTATGCACTTTTGGTCAGCTATTACCTTTGATGAA 6349
Ma'sNightMonkey   -TTTTAATTTGCTTTCCTCTCATGGCTGTGATGTTGAACATCTTTTCATGTGCTTTTTGTCAACTGTTAGCTTTGGTGAA 6604
VervetAGM         GTTTTAATTTGCATTCCTCTAATATCTATGATGTTGAACATCTTTTCATGTGCTTTTTATCAACTGTTACCTTTGGTGAA 6936
Gorrilla          GATTTAATTTGCATTCCTCTAGTATCTATGATGTTGGACATCTTTTCGTGTGCTTTTTGTCAGCTGTTATCTTTGGTGAA 8883
GrabEatingMacaqueGTTTTAATTTGCATTCCTCTAATATCTATGATGTTGAACATCTTTTCATGTGCTTTTTGTCAACTGTTACCTTTGGTGAA 7285
Macaque           GTTTTAATTTGCATTCCTCTAATATCTATGATGTTGAACATCTTTTCATGTGCTTTTTGTCAACTGTTACCTTTGGTGAA 7328
PigTailedMacaque  GTTTTAATTTGCATTCCTCTAATATCTATGATGTTGAACATCTTTTCATGTGCTTTTTGTCAACTGTTACCTTTGGTGAA 7240
Bonobo            GATTTAATTTGCATTCCTCTAATATCTATGATGTTGGACATCTTTTCGTGTGCTTTTTGTCAGCTGTTATCTTTGGTGAA 7564
OliveBaboon       GTTTTAATTTGCATTCCTCTAATATCTATGATGTTGAACATCTTTTCATATGCTTTTTGTCAACTGTTACCTTTGGTGAA 6689
Orangutan         GATTTAATTTGCATTCCTCTAATATCTATGATGTTGAACATCTTTTCGGGTGCTTTTTGTCAACTGTTATCTTTGGTGAA 7910
```

```
Majority          GTGTCTGTTTATATCTTTTGCCCAATTTTTTATTTGGTTATTTATTTTTAATTGAGTTTTGAGAGGTCTTCATTGTGGTT

                           10090     10100     10110     10120     10130     10140     10150     10160

Human             GTGTCTGTTTATATCTTTAGCCCAATTTTTTATTTGGTTATTTATTTTAAATTGAGTTTTGAGAGGTCTTCATTGTGGTT 6712
Coquerel'sSifaka  GTGTCTGTTTAAAT-T----CCCCACTTCTTAT-GGGTTATTTCTTTTTTCTTGAGTTTTGACAGGTCTTCATTGTGGTT 6423
Ma'sNightMonkey   GTGTCTGTTTATATCTTTTGCCCAATTTTTTATTGGGTTATTTATTTTTAAATGTGTTTTGAGAGGTCTTCATTCTGGTT 6684
VervetAGM         GTGTGTGTTTATATCTTTTGCCCAATTTTTTATTTGGTTGTTTATTTTTAATTGAGTTTTGAGAGGTCTTCATTGTGGTT 7016
Gorrilla          GTGTCTGTTTATATCTTTAGCCCAATTTTTTATTTGGTTATTTATTTTAAATTGAGTTTTGAGAGGTCTTCATTGTGGTT 8963
GrabEatingMacaqueGTGTGTGTTTATATCTTTTGCCCAATTTTTTATTTGGTGGTTTATTTTTAATTGAGTTTTGAGAGGTCTTCATTGTGGTT 7365
Macaque           GTGTGTGTTTATATCTTTTGCCCAATTTTTTATTTGGTGGTTTATTTTTAATTGAGTTTTGAGAGGTCTTCATTGTGGTT 7408
PigTailedMacaque  GTGTGTGTTTATATCTTTTGCCCGATTTTTTATTTGGTGGTTTATTTTTAATTGAGTTTTGAGAGGTCTTCATTGTGGTT 7320
Bonobo            GTGTCTGTTTATATCTTTAGCCCAATTTTTTATTTGGTTATTTATTTTAAATTGAGTTTTGAGAGGTGTTCATTGTGGTT 7644
OliveBaboon       GTGTCTGTTTATATCTTTTGCCCAATTTTTTATTTGGTTGTTTATTTTTAATTGAGTTTTGAGAGGTCTTCATTGTGGTT 6769
Orangutan         GTGTCTGTTTATATCTTTTGCCCAATTTATTATTTGGTTATTTATTTTAAATTGAGTTTTGAGAGGTCTTCATTGTGGTT 7990

Majority          ATAAGTCCTTTATCAGATATATGGCCTGCAAATATTTTCTGCCACTCGTCTTCTCGTTCTCAGCATTGTCTTTTGAAGAG

                           10170     10180     10190     10200     10210     10220     10230     10240

Human             ACAAGTCCTTTATCAGCCATAAGGCCTGCAAATATTTTCTGCCACTCATCTTCTTATTCTC-GCATTGTCTTTTGAAGAG 6791
Coquerel'sSifaka  ACAAGTCCTGTGCCAGATAAATGGTTTGCATATATTTTCTGCCACTTGTCTTTTCTTTCTCAGCAGTGTCTTTTGAAGAG 6503
Ma'sNightMonkey   GTAAGTCCTTTATCAGATATATGGCCTGCAAATATTTTCTGCCACTTATCTTTTCATTCTCAGCATTGTCTTTTGAAGAA 6764
VervetAGM         ATAAGTCCTTTATCAGATATATGGCCTGCAAATCTTTTCTGCCACTCGTCTTCTCGTTCTCAGCATTGTCTTTTGAAGAG 7096
Gorrilla          ATAAGTCCTTTATCAGCTATATGGCCTGCAAATATTTTCTGCCACTCATCTTCTTCTTCTC-GCATTGTCTTTTGAAGAG 9042
GrabEatingMacaqueATAAGTCCTTTATCAGATATATGGCCTGCAAATCTTTTCTGCCACTCGTCTTCTCGTTCTCAGCATTGTCTTTTGAAGAG 7445
Macaque           ATAAGTCCTTTATCAGATATATGGCCTGCAAATCTTTTCTGCCACTCGTCTTCTCGTTCTCAGCATTGTCTTTTGAAGAG 7488
PigTailedMacaque  CTAAGTCCTTTATCAGATATATGGCCTGCAAATCTTTTCTGCCACTCGTCTTCTCGTTCTCAGCATTGTCTTTTGAAGAG 7400
Bonobo            ACAAGTCCTTTATCAGCTATATGGCCTGCAAATATTTTCTGCCACTCATCTTCTTATTCTC-GCATTGTCTTTTGAAGAG 7723
OliveBaboon       ATAAGTCCTTTATCAGATATATGGCCTGCAAATCTTTTCTGCCACTCGTCTTCTCGTTCTCAGCATTGTCTTTTGAAGAG 6849
Orangutan         ATAAGTCCTTTATCAGATATATGGCCTGGAAATATTTTCTGCCACTCATCTTCTCATTCTCAGCATTGTCTTTTGAAGAG 8070

Majority          CAACAGTTATTAATTCTTATGGACTCCAGTTTACCAATTTTTTCTTTTATGGATTGTGGTTTTGGGGTCTTGTCTGAGCC

                           10250     10260     10270     10280     10290     10300     10310     10320

Human             CAACAGTTATTAAATCTGATGGACTCCAGTTTACCAATTTTTTCTTTTATGGATTGTAGTTTTGGGGTCTTGTCTGAGCC 6871
Coquerel'sSifaka  CAGCAGTTA----TTCTCATGGACTCCGGTTTACCAGTTTTTTCTTTTGTGGATTGTGCTTTTGGTGTCACATCTAAAAC 6579
Ma'sNightMonkey   CAACAGTTATTAATTATGATGGAGTCCAGTTTACCAATTTTTTCTTTTATGGATTATACTTTCGTGGTTTTGTCTGAGCC 6844
VervetAGM         CAACTGTTACTAATTCTTGTGGACTCCAGTTTACCAGGTTTTTCTTTTATGGATTGTGGTTTTGGGGTCTTGCCTGAGCC 7176
Gorrilla          CAACAGTTATTAATTCTGATGGACTCCAGTTTACCAATGTTTTCTTTTATGGATTGTAGTTTTGGGGTCTTGTCTGAGCC 9122
GrabEatingMacaqueCAACTGTTACTAATTCTTGTGGACTCCAGTTTACCAAGTTTTTCTTTTATGGATTGTGGTTTTGGGGTCTTGCCTGAGCC 7525
Macaque           CAACTGTTACTAATTCTTGTGGACTCCAGTTTACCAAGTTTTTCTTTTATGGATTGTGGTTTTGGGGTCTTGCCTGAGCC 7568
PigTailedMacaque  CAACTGTTACTAATTCTTGTGGACTCCAGTTTACCAAGTTTTTCTTTTATGGATTGTGGTTTCGGGGTCTTGCCTGAGCC 7480
Bonobo            CAACAGTTATTAATTCTGATGGACTCCAGTTTACCAATTTTTTCTTTTATGGATTGTAGTTTTGGGGTCTTGTCTGAGCC 7803
OliveBaboon       CAACTGTTACTAATTCTTGTGGACTCCAGTTTACCAAGTTTTTCTTTTATGGATTGTGGTTTTGGGGTCTTGCCTGAGCC 6929
Orangutan         CAACAGTTATTAATTCTGATGGACTCCAGTTTACCAATTTTTTCTTTTATGGATTGTAGTTTTGGGGTCTTGTCTGAGCC 8150
```

```
Majority          AT-TTTTGCCTAACTGTAAGCCATGAAGATTTTTT-CCTTTTGTTTGTTAATAATAATA---A--A---TAATT--TTTT

                          10330     10340     10350     10360     10370     10380     10390     10400

Human             AT-TTTTGCCTGACTGTAAGCCATGAAGATTTTTTTCCTTTTGTTTGTTAATAATAATA---A------TAATT--TTTT 6939
Coquerel'sSifaka  TTCTTTTGCCTAACCTAAATTCACAAAGATTTTCT--CTTTTGTTTTCTAATA--------------------------- 6630
Ma'sNightMonkey   AT-CTTTGCCTAACTGTAAGTCATGAAGATTTTTT-CCTTTTGTT-AATAATAATAATT------T---TTTTTTTTTTT 6912
VervetAGM         AT-TTTTGCCTAACTGTAAGCCATGAAGATTTTTT-CCTTTTGTTTGTTAATAATAATAAAAATAATAATAATAATTTTT 7254
Gorrilla          AT-TTTTGCCTGACTGTAAGCCATGAAGATTTTTTTCCTTTTGTTTGTTAATAATAATA---A------TAATT--TTTT 9190
GrabEatingMacaqueAT-TTTTGCCTAACTGTAAGCCATGAAGATTTTTT-CCTTCTGTTTGTTAATAATAATAAAAATAATAATAATAATATTT 7603
Macaque           AT-TTTTGCCTAACTGTAAGCCATGAAGATTTTTT-CCTTCTGTTTGTTAATAATAATAAAAATAATAATAATAATATTT 7646
PigTailedMacaque  AT-TTTTGCCTAACTGTAAGCCATGAAGATTTTTT-CCTTCTGTTTGTTAATAATAATAAAAATAATAATAATT---TTT 7555
Bonobo            AT-TTTTGCCTGACTGTAAGCCATGAAGATTTTTTTCCTTCTGTTTGTTAATAATAATA---A------TAATT--TTTT 7871
OliveBaboon       AT-TTTTGCCTAACTGTAAGCCATGAGGATTTTTT-CCTTCTGTTTGTTAATAATAATAAAAATAATAATAATAATTTTT 7007
Orangutan         AT-TTTTGCTTGACTGTAAGCCATGAAGATTTTTTTCCTTTTGTTTGTTAATAATAAT-------------TT--TTTT 8213

Majority          TTTAGAGACAGGGCCTGACT--GTCACCCAGGCTGGAGTGCAGTGGTGCAATCGTAGCTCAGGATAATCTTGAACTCCTG

                          10410     10420     10430     10440     10450     10460     10470     10480

Human             TTTAGAGACAGGACCTGACTCTGTCACCCCAGCTGGAGTGCAGTGGTGCAATCGTAGCTCATGATAACCTTGAACTCCTG 7019
Coquerel'sSifaka  --------------------------------------GCA---GT-------------------------------- 6635
Ma'sNightMonkey   TTAAGAGACAGGGTCTGACTCTGTCATTCAGGCTGGAGTACAGTGGTGCAATCATAGCTCACAATTACCTTGAACTCCTG 6992
VervetAGM         TTTAGAGACAGGGCCTG------TCACCCAGGCTGAAGTGCAATGGTGCAATCGTAGCTCAGAATAATCTTGAACTCCTG 7328
Gorrilla          TTTAGAGACAGGACCTGACT--GTCACCCCAGCTGGAGTGCAGTGGTGCAATCGTAGCTCATGATAACCTTGAACTCCTG 9268
GrabEatingMacaqueTTTAGAGACAGGGCCTG------TCACCCAGGCTGAAGTGCAATGGTGCAATCGTAGCTCAGGATAATCTTGAACTCCTG 7677
Macaque           TTTAGAGACAGGGCCTG------TCACCCAGGCTGAAGTGCAGTGGTGCAATCGTAGCTCAGGATAATCTTGAACTCTTG 7720
PigTailedMacaque  TTTAGAGACAGGGCCTGACT--GTCACCCAGGCTGAAGTGCAGTGGTGCAATCGTAGCTCAGGATAATCTTGAACTCCTG 7633
Bonobo            TTTAGAGACAGGACCTGACTCTGTCACCCCAGCTGGAGTGCAGTGGTGCAATCGTAGCTCATGATAACCTTGAACTCCTG 7951
OliveBaboon       TTTAGAGACAGGGCCTGACT--GTCACCCAGGCTGAAGTGCAATGGTGCAGTCGTAGCTCAGGATAATCTTGAACTCCTG 7085
Orangutan         TTTAGAGACAGGGCCTGACTCTGTCACCCAGGCTGGAGTGCAGTGGTGCAGCTGTAGCTCACGATAACCTTGAACTCCTG 8293

Majority          GACACAAGGGCTCCTGCTTCAGCCTCCCAAGTAGCTAGTACTACAGGCACACAGCACCATGCTCAGCTAATTTTTAAATT

                          10490     10500     10510     10520     10530     10540     10550     10560

Human             GACACAAGGGCTCCTGCTTCAGCCTCCCAAGTAGCTAGGACTATAGGCACACAGCACCATGCCCAGCTAACTTTTAAATT 7099
Coquerel'sSifaka  -------------------------------------------------------------------------------- 6635
Ma'sNightMonkey   G------G--------CTTTGGCCTCCCAAGTAGCTAGGACTACAGGCACACAGCACCAAGCCCAGCTCATTTTTACTTT 7058
VervetAGM         GACACAAGGGCTTCTGCTTCAGCCTCCCAAGTAGCTAGTACTACAGGCACACAGCACCATGCTCAGCTGATTTTTAAATT 7408
Gorrilla          GACACAAGGGCTCCTGCTTCAGCCTCCCAAGTAGCTAGGACTATAGGCACACAGCACCATGCCCAGCTAATTTTTAAATT 9348
GrabEatingMacaqueGACACAAGGGCTCCTGCTTCAGCCTCCCAAGTAGCTAGTACTACAGGCACACAGCACCATGCTCAGCTAATTTTTAAATT 7757
Macaque           GACACAAGGGCTCCTGCTTCAGCCTCCCAAGTAGCTAGTACTACAGGCACACAGCACCATGCTCAGCTAATTTTTAAATT 7800
PigTailedMacaque  GACACAAGGGCTCCTGCTTCAGCCTCCCAAGTAGCTAGTACTACAGGCACACAGCACCATGCTCAGCTAATTTTTAAATT 7713
Bonobo            GACACAAGGGCTCCTGCTTCAGCCTCCCAAGTAGCTAGGACTATAGGCACACAGCACCATGCCCAGCTAATTTTTAAATT 8031
OliveBaboon       GACACAAGG-CTCCTGCTTCAGCCTCCCAAGTAGCTAGTACTACAGGCACACAGCACCATGCTCAGCTAATTTTTAAATT 7164
Orangutan         GACACAAGGGCTCCTACTTCAGCCTCCCAAGTAGGTAGGACTACAGGCACACACCACCATGCCCAGCTAATTTTTAAATT 8373
```

```
Majority          TTTTATAGACCTGGTGTCTGTCTGTTTTGCCCAGGCTGGTCTTGAACTCCTGGCCTCAAGCAGTCTTTCTGCCTCAGCCT
                          10570     10580     10590     10600     10610     10620     10630     10640
Human             TTTTATAGACCCGGGGTCTCTCTGTTTTGCCCAGGCTGGTGTTGAACTCCTGGCCTCAAGCGATCTTTCTGCCTCAGCCT 7179
Coquerel'sSifaka  -------------------------------------------------------------------------------- 6635
Ma'sNightMonkey   TTTTATAGACCTGGTGTCTCTATATTTTGCCCAGGCTAGTCTTGAACCCCTGGCCTCAAGCCATCTTTCTGCCTCAGCCT 7138
VervetAGM         TTTTATAGACCTAGTGTCTGTCTGTTTTGCCCAGGCTGGTCTTGAACTCCTGGCCTCAAGCAGTCTTTCTGCCTCAGCCT 7488
Gorrilla          TTTTATAGACCTAGGGTCTCTCTGTTTTGCCCAGGCTGGTGTTGAACTCCTGGCCTCAAGCGATCTTTCTGCCTCAGCCT 9428
GrabEatingMacaqueTTTTATAGACCTGGTGTCTGTCTGTTTTGCCCAGGCTGGTCTTGAACTCCTGGCCTCAAGCAGTCTTTCTGCCTCAGCCT 7837
Macaque           TTTTATAGACCTGGTGTCTGTCTGTTTTGCCCAGGCTGGTCTTGAACTCCTGGCCTCAAGCAGTCTTTCTGCCTCAGCCT 7880
PigTailedMacaque  TTTTATAGACCTGGTGTCTGTCTGTTTTGCCCAGGCTGGTCTTGAACTCCTGGCCTCAAGCAGTCTTTCTGCCTCAGCCT 7793
Bonobo            TTTTATAGACCCGGGGTCTCTCTGTTTTGCCCAGGCTGGTGTTGAACTCCTGGCCTCAAGTGATCCTTCTGCCTCAGCCT 8111
OliveBaboon       TTTTATAGACCTGGTGTCTGTCTGTTTTGCCCAGGCTGGTCTTGAACTCCTGGCCTCAAGCAGTC--TCTGCCTCAGCCT 7242
Orangutan         TTTTATAGACCCGGGGTCTCT--GTTTTGCCCAGGCTGGTGTTGAACTCCTGGCCTCAAGCGATCTTTCTGCCTCAGCCT 8451
```

```
Majority          TCCAAAGTGTTGGGATTAAAGGCATGAGCCACTGCCCTTGGCCACCTTT-TTTTCTAATAGAAGTTTTATACTTTGGTTT
                          10650     10660     10670     10680     10690     10700     10710     10720
Human             TCCAAAGTGTTGGGATTAAAGGCATGAGCCACTACACTTGGCCACCTTTGTTTTCTAATAGAAGTTTTATACTTTGGTTT 7259
Coquerel'sSifaka  ------------------------------------TT-----------------------TTTGTATTCTGATGT 6652
Ma'sNightMonkey   TCCAAAGTGTTGGGATTATAGGCATGAGCCACTGCACTTGGCCACCTTTGTTTTCTAATGGAAGTTTTATACTTCGGTTT 7218
VervetAGM         TCCAAAGTGTTGGGATTAAAGGAATGAGCCACTGCCCTTGGCCACCTTT-TTTTCTAATAGAAGTTTTTTACTTTGGTTT 7567
Gorrilla          TCCAAAGTGTTGGGATTAAAGGCATGAGCCACTACACTTGGCCACCTTTGTTTTCTAATAGAAGTTTTATGCTTTGGTTT 9508
GrabEatingMacaqueTCCAAAGTGTTGGGATTAAAGGAATGAGCCGCTGCCCTTGGCCACCTTT-TTTTCTAATAGAAGTTTTATACTTTGGTTT 7916
Macaque           TCCAAAGTGTTGGGATTAAAGGAATGAGCCGCTGCCCTTGGCCACCTTT-TTTTCTAATAGAAGTTTTATACTTTGGTTT 7959
PigTailedMacaque  TCCAAAGTGTTGGGATTAAAGGAATGAGCCGCTGCCCTTGGCCACCTTT-TTTTCTAATAGAAGTTTTATACTTTGGTTT 7872
Bonobo            TCCAAAGTGTTGGGATTAAAGGCATGAGCCACTACACTTGGCCACCTTTGTTTTCTAATAGAAGTTTTATACTTTGGTTT 8191
OliveBaboon       TCCAAAGTGTTGGGATTAAAGGAATGAGCCACTGCCCTTGGCCACCTTT-TTTTCTAATAGAAGTTTTATACTTTGGTGT 7321
Orangutan         TCCAAAGTGTTGGGATTAAAGGCATGAGCCACTATACTTGGCCACCTTTGTTTTCTAATAGAAGTTTTATACTTTGGTTT 8531
```

```
Majority          AACATTTAGG-TCTCTTTCATTTTGACTTAATATTTTGAATATGGTACAAAGTGTGGGTCAGAAGTATTTGGTTTTGCAT
                          10730     10740     10750     10760     10770     10780     10790     10800
Human             AACATTTGGG-TCTCTTTCATTTTGACTTAATATTTTGAATATGGTACAAAGTGTGGGTCAGAAGTATTTGGTTTTGCAT 7338
Coquerel'sSifaka  AACATTAGGTCTCTGTTCCATTTTGAGTTAATATTTTGGATATGGTACAAAG---GGGTC--------TTGGTTTGAGAT 6721
Ma'sNightMonkey   AGCATTTAGG-TCTCTTTCATTTTGACTTAATATTTTGAATATGGTACAAAATTTGGGTCAGAAGTATTTAGTTTTGCAT 7297
VervetAGM         AACATTTAGG-TCTCTTTCATTTTGACTTAATATTTTGAATATGGCACAAAGTGTGGGTCAGAAGTATTTGGTTTTGCAT 7646
Gorrilla          AACATTTGGG-TCTCTTTCATTTTGACTTAATATTTTGAATATGGTACAAAGTGTGGGTCAGAAGTATTTGGTTTTGCCT 9587
GrabEatingMacaqueAACATTTAGG-TCTCTTTCATTTTGACTTAATATTTTGAATATGGTACAAAGTGTGGGTCAGAAGTATTTGGTTTTGCAT 7995
Macaque           AACATTTAGG-TCTCTTTCATTTTGACTTAATATTTTGAATATGGTACAAAGTGTGGGTCAGAAGTATTTGGTTTTGCAT 8038
PigTailedMacaque  AACATTTAGG-TCTCTTTCATTTTGACTTAATATTTTGAATATGGTACAAAGTGTGGGTCAGAAGTATTTGGTTTTGCAT 7951
Bonobo            AACATTTGGG-TCTCTTTCATTTTGACTTAATATTTTGAATATGGTACAAAGTGTGGGTCAGAAGTATTTGGTTTTGCAT 8270
OliveBaboon       AACATTTAGG-TCTCTTTCATTTTGACTTAATATTTTGAATATGGTACAACGTGTGGGTCAGAAGTATTTGGTTTTGCAT 7400
Orangutan         AACATTTAGG-TCTCTTTCATTTTGACTTAATATTTTGAATATGGTACAAAGTGTGGGTCAGAAGTATTTGGTTTTGCAT 8610
```

```
Majority          GGGGACATCCAGTGTTTACAGCATCATTTGTTGAAAAGACTATCCTTTCTCCATTCACTTGGCTTTGCAGCTTCATGAAA

                          10810     10820     10830     10840     10850     10860     10870     10880

Human             GGGGATATCCAGTGTTTACAGCATCATTTGTTGAAAAGACTATCCTTTCTCCATTCACTTGGCTTTGCAGCTTCATGAAA 7418
Coquerel'sSifaka  GTGGACATCTAGTGTTTCCAGCATCATTTGGTAAAAAGACTATCCTTTCTCCATTGAATTGGCTTTGCAGCTTTGTCAAA 6801
Ma'sNightMonkey   GTTGACATCCAGTGTTTACAACGTCATTTATTGAAAAGACTGTCCTTTCTCCATTCACTTGTCTTTGCAACTTCATGAAA 7377
VervetAGM         GGGGACATCCAGTGTTTACAGCATTATTTGTTGAAAAGACTATCCTTTCTCCATTCACTTGGCTTTGCAGCTTCATGAAA 7726
Gorrilla          GGGGATATCCAGTGTTTACAGCATCATTTGTGGAAAAGACTATCCTTTCTCCATTCACTTGGCTTTGCAGCTTCATGAAA 9667
GrabEatingMacaqueGGGGACATCCAGTGTTTACAGCATTATTTGTTGAAAAGACTATCCTTTCTCCATTCACTTGGCTTTGCAGCTTCATGAAA 8075
Macaque           GGGGACATCCAGTGTTTACAGCATTATTTGTTGAAAAGACTATCCTTTCTCCATTCACTTGGCTTTGCAGCTTCATGAAA 8118
PigTailedMacaque  GGGGACATCCAGTGTTTACAGCATTATTTGTTGAAAAGACTATCCTTTCTCCATTCACTTGGCTTTGCAGCTTCATGAAA 8031
Bonobo            GGGGATATCCAGTGTTTACAGCATCATTTGTTGAAAAGACTATCCTTTCTCCATTCACTTGGCTTTGCAGCTTCATGAAA 8350
OliveBaboon       GGGGACATCCAGTGTTTACAGCATTATTTGTTGAAAAGACTATCCTTTCTCCCTTCACTTGGCTTTGCAGCTTCATGAAA 7480
Orangutan         GGGGACATCCAGTATTTACAGCATCATTTGTTGAAAAGACTATCCTTTCTCCATTCACTTAGCTTTGCAGCTTCATGAAA 8690

Majority          AATCAGTTATCTCTGTATGTATGATTATATGTCTGTACTCTATTCTTTTGTATTGACCCATTTTCCTTTATCTAGATGTT

                          10890     10900     10910     10920     10930     10940     10950     10960

Human             AATCACTTATGTCTGTATGTGTGATTTTATGTCTGTACTCT---TTTTTGTATTGACCCATTTTCCTTTATCTAGATGCT 7495
Coquerel'sSifaka  AATCAAT--TCTCTATACGT---ATTGTATTTCTGTACTCT----TTTTCCATTGACTCATTTTTCTTTACCTAGATGTC 6872
Ma'sNightMonkey   AATCGGTTATCTCTATATGTATGATTATGTGTCTGTACTCTACTCTTTTGTGTTGACCCATTTTCCTTTATCTAGATGCT 7457
VervetAGM         AATCAGTTATCTCTGTATGTATGATTATATGTCTGTACTCTATTCTTTTGTATTGACCCATTTTCCTTTA----GATGTT 7802
Gorrilla          AATCAGTAATCTCTGTATGTGTGATTTTATGTCTGTACTCTACTTTTTTGTATTGACCCATTTTCCTTTATCTAGATGCT 9747
GrabEatingMacaqueAATCAGTTATCTCTGTATGTATGATTATATGTCTGTACTCTATTCTTTTGTATTGACCCATTTTCCTTCA----GATGTT 8151
Macaque           AATCAGTTATCTCTGTATGTATGATTATATGTCTGTACTCTATTCTTTTGTATTGACCCATTTTCCTTCA----GATGTT 8194
PigTailedMacaque  AATCAGTTATCTCTGTATCTATGATTATATGTCTGTACTCTATTCTTTTGTATTGACCCATTTTCCTTTA----GATGTT 8107
Bonobo            AATCAGTTATCTCTGTATGTGTGATTTTATGTCTGTACTCTACTTTTTTGTATTGACCCATTTTCCTTTATCTAGATGCT 8430
OliveBaboon       AATCAGTTATCTCTGTATGTATGATTATATGTCTGTACTCTATTCTTTTGTATTGACCCATTTTCCTTTA----GATGTT 7556
Orangutan         AATCAGTTATCTGTGTGTGTATGATTTTATGTCTGTACTCTACTTTTTTGTATTGACCCATTTTCCTTTATCTAGATGCT 8770

Majority          AGAAACATACTGTCTTAATCATTGTAGCTTTATATTGAGTCTTGAAACCACTTA--CTTAGTCTTGGCCCTCCAACTCTG

                          10970     10980     10990     11000     11010     11020     11030     11040

Human             GGAAACATACTGTCTTAATCATTGTAGCTTTATATTGAGTCTTGAAACCACTTA--CTTAGTCTTGGCCCTCCAACTCTG 7573
Coquerel'sSifaka  AAAAACATACTGTCTTAATCACTGTAGCTTTATATTGAGTCTTGAAACCAGTTGATTTGATTTTGGTCCTTCAACTTAG 6952
Ma'sNightMonkey   AGAAACATACTGTCTTAATCATTGTAGCTTTGTATTGAGTCTTGAAA----CCA--CTTAGTCTGGGACCTCCAGCTCTG 7531
VervetAGM         AGAAACATACTGTCTTAATCATTGTAGCTTTATGTCGAGTCTTGAA-----CCA--CTTAGTCTTGGCCCTCCAACTCTG 7875
Gorrilla          GGAAACATACTGTCTTAATCATTGTAGCTTTATATTGAGTCTTGAAACCACTTA--CTTAGTCTTGGCCCTCCAACTCTG 9825
GrabEatingMacaqueAGAAACATACTGTCTTAATCATTGTAGCTTTATATCAAGTCTTGAAACCACTTA--CTTAGTCTTGGCCCTCCAACTCTG 8229
Macaque           AGAAACATACTGTCTTAATCATTGTAGCTTTATATCAAGTCTTGAAACCACTTA--CTTAGTCTTGGCCCTCCAACTCTG 8272
PigTailedMacaque  AGAAACATACTGTCTTAATCATTGTAGCTTTATATCAAGTCTTGAAACCACTTA--CTTAGTCTTGGCCCTCCAACTCTG 8185
Bonobo            GGAAACATACTGTCTTAATCATTGTAGCTTTATATTGAGTCTTGAAACCACTTA--CTTAGTCTTGGCCCTCCAACTCTG 8508
OliveBaboon       AGAAACATACTGTCTTAATCATTGTAGCTTTATATCAAGTCTTGAAACCACTTA--CTTAGTCTTGGCCCTCCAACTCTG 7634
Orangutan         GGAAACATACTGTCTTAATCATTGTAGCTTTATATTGAGTCTTGAAACCACTTA--CTTAGTCTTGGCCCTCCAACTCTG 8848
```

```
Majority         TTCTTTTTCAAAGTTGTTTTTAACTCTTCTGAGTCCTTTGTATTTTCATATGAGTCAGTTTTTACAAAAACTGCTTGGAT

                         11050     11060     11070     11080     11090     11100     11110     11120

Human            TTCTTTTTCAAAGTTGTTTTTAACTCTTCTGAGTCCTTTGTATTTTCATATGAGTCAGTTCTTAGAAAAACTGCTTGGAT 7653
Coquerel'sSifaka TTCTTTTTCAAAGTTATTTT-GACTCTTCTGAGTCCTTCGTATTTTCTTATGAGTAAGTTTTTAGAAAAACTGTTGGAAT 7031
Ma'sNightMonkey  TTGTTTTTCAAAATTGTTTTTAACTCTTCTGAGTCCTTGGTATTTTCAT--GAGTCAGTTTTTACAAAAACTACTTGGAT 7609
VervetAGM        TTCTTTTTCAAAGTTGTTTTTAACTCTTCTGAGTCCTTTGTGTTTTCATATGAGTCAGTTTTTACAAAAACTGCTTTGAT 7955
Gorrilla         TTCTTTTTCAAAGTTGTTTTTAACTCTTCTGAGTCCTTTGTATTTTCATATGAGTCAGTTTTTACAAAAACTGCTTGGAT 9905
GrabEatingMacaqueTTCTTTTTCAAAGTTGTTTTTAACTCTTCTGAGTCCTTTGTATTTTCATATGAGTCAGTTTTTACAAAAACTGCTTGGAT 8309
Macaque          TTCTTTTTCAAAGTTGTTTTTAACTCTTCTGAGTCCTTTGTACTTTCATATGAGTCAGTTTTTACAAAAACTGCTTGGAT 8352
PigTailedMacaque TTCTTTTTCAAAGTTGTTTTTAACTCTTCTGAGTCCTTTGTATTTTCATATGAGTCAGTTTTTACAAAAACTGCTTGGAT 8265
Bonobo           TTCTTTTTCAAAGTTGTTTTTAACTCTTCTGAGTCCTTTGTATTTTCATATGAGTCAGTTCTTACAAAAACTGCTTGGAT 8588
OliveBaboon      TTCTTTTTCAAAGTTGTTTTTAACTCTTCTGAGTCCTTTGTGTTTTCATATGAGTCAGTTTTTACAAAAACTGCTTGGAT 7714
Orangutan        TTCTTTTTCAAAGTTGTTTTTAACTCTTCTGAATCCTTTATATTTTCATATGAGTCAGTTTTTACAAAAACTGCTTGGAT 8928

Majority         TTTACTGGATTTGACTTGAATCTGTAGAGCAA-TTTGGGGAAAATTGTCATCTTAACAAGTGAGTCTTTCTAACCATGAC

                         11130     11140     11150     11160     11170     11180     11190     11200

Human            TTTACTGGATTTGACTTGAATCTGTAGAGCAAATTTGGGGAAAATTGGCATC-----------------------ATGAC 7710
Coquerel'sSifaka TTTATTGGATTTGACTTGAATCTTCAGAGCAA-TTTGGGGAAAATAGACATCTTAACAA-TGTTGAGTCTTATTCATGA- 7108
Ma'sNightMonkey  TTTAGTGGATTTGACTTGAATCTGTAGAGCAA-TTTGAGGAAAATTGTCATCTTAACAACTGAGTCTTTCTAACTATGAC 7688
VervetAGM        TTTACCGGATTTGACTTGAATCTGTAGAGCAA-TTTGGGGAAAATCGTCATCTTAACAAGTGAGTCTTTCTAACCATGAC 8034
Gorrilla         TTTACTGGATTTGACTTGAATCTGTAGAGCAA-TTTGGGGAAAATTGGCATC-----------------------ATGAC 9961
GrabEatingMacaqueTTTACCGGATTTGACTTGAATCTGTAGAGCAA-TTTGGGGAAAATTGTCATCTTAACAAGTGAGTCTTTCTAACCATGAC 8388
Macaque          TTTACCGGATTTGACTTGAATCTGTAGAGCAA-TTTGGGGAAAATTGTCATCTTAACAAGTGAGTCTTTCTAACCATGAC 8431
PigTailedMacaque TTTACCGGATTTGACTTGAATCTGTAGAGCAA-TTTGGGGAAAATTGTCATCTTAACAAGTGAGTCTTTCTAACCATGAC 8344
Bonobo           TTTACTGGATTTGACTTGAATCTGTAGAGCAAATTTGGGGAAGATTGGCATC-----------------------ATGAC 8645
OliveBaboon      TTTACCGGATTTGACTTGAATCTGTAGAGCAA-TTTGGGGAAAATTGTCATCTTAACAAGTGAGTCTTTCTAACCATGAC 7793
Orangutan        TTTACTGGATTTGACTTGAATCTGTAGAGCAA-TTTGGGGAAAATTGGCATC-----------------------ATGAC 8984

Majority         TACAGTTGATGTCTCTATTA--------GGTCTTTAATTTCTTT-AAGCAGTGTTTTTTTTTTT---CAGTGCACAGGTT

                         11210     11220     11230     11240     11250     11260     11270     11280

Human            TACAATTGATGTCTCTATTA--------GGTCTTTAATTTCTTT-AAGCATTTTTTTTTTTTTTTT-CAGTGCACACGTT 7780
Coquerel'sSifaka -ACAGTATATCTCTCCATTTACTTAAGTCTTCTTTAATTTCTTT-AAGCAATGTTTCTAGTTTT---CAGCATACAGATT 7183
Ma'sNightMonkey  TACAATTTATGTCTCTATTA--------GATCTTTAATTTCTTT-AAGCAGTGTTTTTATTTTT---CAGTGCACAGGTT 7756
VervetAGM        TACAGTTGATGTCTCTATT---------GGTCTTTAATTTCTTTTAAGCAGTGTTTTTTTT------CAGTGCACAGGTT 8099
Gorrilla         TACAATTGATGTCTCTATTA--------GGTCTTTAATTTCTTT-AAGCATTGTTTTTTTTTTTTTTCAGTGCACACGTT 10032
GrabEatingMacaqueTACAGTTGATGTCTCTATT---------GGTCTTTAATTTCTTT-AAGCAGTGTTTTTTTTT-----CAGTGCACAGGTT 8453
Macaque          TACAGTTGATGTCTCTATT---------GGTCTTTAATTTCTTT-AAGCAGTGTTTTTTTTT-----CAGTGCACAGGTT 8496
PigTailedMacaque TACAGTTGATGTCTCTATT---------GGTCTTTAATTTCTTT-AAGCAGTGTTTTTTTTT-----CAGTGCACAGGTT 8409
Bonobo           TACAATTGATGTCTCTATTA--------GGTCTTTAATTTCTTT-AAGCATTGTTTTTTTTTTT---CAGTGCACACGTT 8713
OliveBaboon      TACAGTTGATGTCTCTATT---------GGTCTTTAATTTCTTT-AAGCAGTGTTTTTTTTT-----CAGTGCACAGGTT 7858
Orangutan        TAAAATTGATGTCTCTGTTA--------GGTCTTTAATTTCTTT-AAGCATTGTTTTTTTTTTC----ATTGCACATGTT 9051
```

```
Majority          TTGTGCATTGTTTGATTAGATTTATCCATAAGTATTTTATATATTTTGATGTTATTGTAAATGATATTTTAAAAACTT-A

                          11290     11300     11310     11320     11330     11340     11350     11360

Human             TTGTGCATTGTTTGATTAGATTTACCCATAAGTATTTTATGTATTTTGAAGT-ACTGTAAATGATATTTTAAAAACTT-A 7858
Coquerel'sSifaka  TTGCACATTTTTTGGTCAGATTTAACCTTGAGTATGTCATACCTTTTGATGT---TGTAAATAGTATTTAAAAAAATTCA 7260
Ma'sNightMonkey   TTGTTCATTGAT-----AGATTTATCCATAAGTATTTTATATATTTTGATATTATTGTAAAT---ATTTTAAAAACTT-A 7827
VervetAGM         TTGTGCATTGTTTGATTAGATTTATCCATAAGTATTTTATATATTTTGATGTTATTGTAAATGATATTTTAAAAACTT-A 8178
Gorrilla          TTGTGCACTGTTTGATTAGATTTACCCATAAGTATTTTATATATTTTGAAGT-ACTGTAAATGATATTTTAAAAACTT-A 10110
GrabEatingMacaqueTTGTACATTGTTTGATTAGATTTATCCATAAGTATTTTATATATTTTGATGTTATTGTAAATGATATTTTAAAAACGT-A 8532
Macaque           TTGTACATTGTTTGATTAGATTTATCCATAAGTATTTTATATATTTTGATGTTATTGTAAATGATATTTTAAAAACGT-A 8575
PigTailedMacaque  TTGTGCATTGTTTGATTAGATTTATCCATAAGTATTTTATATATTTTGATGTTATTGTAAATGATATTTTAAAAACGT-A 8488
Bonobo            TTGTGCATTGTTTGATTAGATTTACCCATAAGTATTTTATGTATTTTGAAGT-ACTGTAAATGATATTTTAAAAACTT-A 8791
OliveBaboon       TTGTGCATTGTTTGATTAGATTTATCCATACGTATTTTATATATTTTGATGTTATTGTAAATGATATTTTAAAAACGT-A 7937
Orangutan         TTGTGCATTGTTTGATTAGATTTATCCATAAGTATTTTATGTATTTTGAAGT-ACTGTAAATGATATTTAAAAAACTT-A 9129

Majority          ATTTCCAATTGTTTG---CTAGTATATAGAAA-------TGATTATGGCATATTGCTCTTGTGTTCTGATCCTTTGCTAA

                          11370     11380     11390     11400     11410     11420     11430     11440

Human             ATTTCCAATTGTTTG---CTAGTGTATAGAAA-------TGATTATGGCATATTGCTCATATGTTCTGAACCTTTGCTAA 7928
Coquerel'sSifaka  AGTTCCAATTGTTTGGTACTGATGTATAGAAGAAATAAGTGATTTTTGCATATTGTTCTTGTATTCTGCATCCTGGCTAA 7340
Ma'sNightMonkey   ATTTCCAATTGTTTG---CTAGTATATAGAAA-------TGATTATGGCATATTGCTCTTCTGTTTT------------- 7884
VervetAGM         ATTTCCAGTGGTTTA---CTAGTATATAGAAA-------TGATTATGGCATATCGCTCTTGTGTTCTGATCCTTTGCTAA 8248
Gorrilla          ATTTCCAATTGTTTG---CTAGTGTATAGAAA-------TGGTTATGGCATATTGCTCTTATGTTCTGAACCTTTGCTAA 10180
GrabEatingMacaqueATTTCCAGTGGTTTA---CTAGTATATAGAAA-------TGATTATGGCATATCGCTCTTGTGTTCTGATCCTGTGGGAA 8602
Macaque           ATTTCCAGTGGTTTA---CTAGTATATAGAAA-------TGATTATGGCATATCGCTCTTGTGTTCTGATCCTTTGCGAA 8645
PigTailedMacaque  ATTTCCAGTGGTTTA---CTAGTATATAGAAA-------TGATTATGGCATATCGCTCTTGTGTTCTGATCCTTTGCGAA 8558
Bonobo            ATTTCCAATTGTTTG---CTAGTGTATAGAAA-------TGATTATGGCATATTGCTCATATGTTCTGAACCTTTGCTAA 8861
OliveBaboon       ATTTCCAGTGGTTTA---CTAGTATATAGAAA-------TGATTATGGCATATCGCTCTTGTGTTCTGGTCCTTTGCGAA 8007
Orangutan         ATTTCCAATTGTTTG---CTAGTGTATAGAAA-------TGATTATGGCATATTGCTCTTATGCTCTGAACCTTTGCTAA 9199

Majority          ACTCATTTAGTAGGTCTAATAGTTTTTTTT-ATATGTTCCATCAGATTTTCTTGAGAGGGA-GTCTCA----GTCACCCA

                          11450     11460     11470     11480     11490     11500     11510     11520

Human             ACTCATTTAGTAGATCTAATAGTTTTTTTT-ATATATTTCATCAGATTTGCTTGAGAGGGA-GTCTCA----GTCACCCA 8002
Coquerel'sSifaka  ACTCATTTAGTAGAGCTAATAGATTTTTTAAATATGTTCTATTGGATTTTCTACATAGACA-ATTTAT----GTCCAATT 7415
Ma'sNightMonkey   --------A----------------------TATATTCCATCAGATTTTCTTGAGAGGGGCGTCTCACTGAGTCACCCA 7933
VervetAGM         ACTCATTTAGTAGGTCTAATAGTTTTTTTT-ATATGTCCCATCAGATTTTCTTGAGAGGGA-GTCTCA----GTCACCCA 8322
Gorrilla          ACTCATTTAGTAGATCTAATAGTTTTTTT--ATGTATTTCATCAGATTTGCTTGAGAGGGA-GTCTCA----GTCACCCA 10253
GrabEatingMacaqueACTCATTTAGTAGGTCTAATAGTTTTTTTT-ATATGTCCCATCAGATTTTCTTGAGAGGGA-GTCTCA----GTCACCCA 8676
Macaque           ACTCATTTAGTAGGTCTAATAGTTTTTTTT-ATATGTCCCATCAGATTTTCTTGAGAGGGA-GTCTCA----GTCACCAA 8719
PigTailedMacaque  ACTCATTTAGTAGGTCTAATAGTTTTTTTT-ATATGTCCCATCAGATTTTCTTGAGAGGGA-GTCTCA----GTCACCCA 8632
Bonobo            ACTCATTTAGTAGATCTAATAGTTTTTTT--ATATATTTCATCAGGTTTGCTTGAGAGGGA-GTCTCA----GTCACCCA 8934
OliveBaboon       ACTCATTTAGTAGGTCTAATAGTTTTTTTT-ATATGTCCCATCAGATTTTCTTGAGAGGGA-GTCTCA----GTCACCCA 8081
Orangutan         ACTCATTTAGTAGATCTAATAGTTTTTTTT-ATATATTCCATCAGATTTTCTTGAGAGGGA-GTCTCA----GTCACCCA 9273
```

```
Majority          GGCTGGAGTGCAGTGACACGATCTTGGCTCACTGCAACCTCTGCCTCCTGGGT-CAAGCAATTCTCCTGCCTCAGCCTCC
                          11530     11540     11550     11560     11570     11580     11590     11600
Human             GGCTGGAGTGCAGTGACGTGATCTTGGCTCACTGCAACCTGCACCTCCTGGGT-CAAGCAATTCTCCTGCCTCAGCCTCA 8081
Coquerel'sSifaka  GTCTTTAG-ACAGTTCCTT--TCCTGTCTGGATGCCATTTTTTTCTTTT-----T------CTTGCCTGTGGCA-TCTAG 7480
Ma'sNightMonkey   GGCTGGAGTGCAGTGGCACGGTCTTGGCTCACTGCAGCCTCTGCCTACTGGGTTCAAGCAATTCTCCTGCCTCAGCCTCC 8013
VervetAGM         GGCTGGAGTGCAGTGACACGATCTTGGCTCACTGCAACCTCTGCCTCCTGGGT-CAAGAAATTCTCCTGCCTCAGGCTCC 8401
Gorrilla          GGCTGGAGTGCAGTGACGTGATCTTGGCTCACTGTAACCTGCACCTCCTGGGT-CAAGCAATTCTCCTGCCTCAGCCTCA 10332
GrabEatingMacaqueGGCTGGAGTGCAGTGACACGATCTTGGCTCACTGCAACTTCTGCCTCCTGGGT-CAAGAAATTCTCCTGCCTCAGCCTCC 8755
Macaque           GGCTGGAGTGCAGTGACACGATCTTGGCTCACTGCAACTTCTGCCTCCTGGGT-CAAGAAATTCTCCTGCCTCAGCCTCC 8798
PigTailedMacaque  GGCTGGAGTGCAGTGACACGATCTTGGCTCACTGCAACCTCTGCCTCCTGGGT-CAAGAAATTCTCCTGCCTCAGCCTCC 8711
Bonobo            GGCTGGAGTGCAGTGACGTGATCTTGGCTCACTGCAACCTGCACCTCCTGGGT-CAAGCAATTCTCCTGCCTCAGCCTCA 9013
OliveBaboon       GGCTGGAGTGCAGTGACACGATCTTGGCTCACTGCAACCTCTGCCTCCTGGGT-CAAGAAATTCTCCTGCCTCAGCCTCC 8160
Orangutan         GGCTGCAGTGCAGTGACGTGATCTTGGCTCACTGCAACCTCCGCCTCGTGGGT-CAAGCAGTTCTCCTGCCTCAGCCTCC 9352

Majority          CAAGTAGCTGGGACTACTGGTGCA--TACCACCACGCC-TGGCTAATTTTTGTATTTTCAGTTGAGGCAGGGTTTCACCA
                          11610     11620     11630     11640     11650     11660     11670     11680
Human             TAAGTAGCTGGGACTGCAGGTGCA--CACCACCACGCC-CAGCTAATTTTTGCATTTTCAGTTGAGGCAGGGTTTCACCA 8158
Coquerel'sSifaka  TACGATGTTGAAGCTATTAGAGTAGGTATCTTTACATTGTTCCTTATTTTAGGGTGAACACTCAAAATGGTATTATTTCT 7560
Ma'sNightMonkey   CAAGTAGCTGGGACTACAGGTGTG--CACCACCACACC-CTGCTAATTTTTGTGTTTTTAGTTGAGACAGAGTTTCCCCA 8090
VervetAGM         CAAATAGCTGGGACTACTGGCGCA--TACCACCATGCC-TGGCTAATTTTTGTATTTTCAGTTGAGGCAGGGTTTCACCA 8478
Gorrilla          TAAGTAGCTGGGACTGCAGGTGCA--CACCACCACGCC-CAGCTAATTTTTGCATTTTCAGTTGAGGCAGGGTTTCACCA 10409
GrabEatingMacaqueCAAATAGCTGGGACTACTGGTGCA--TACCACCATGCC-TGGCTAATTTTTGTATTTTCAGTTGAGGCAGGGTTTCACCA 8832
Macaque           CAAATAGCTGGGACTACTGGTGCA--TACCACCATGCC-TGGCTAATTTTTGTATTTTCAGTTGAGGCAGGGTTTCACCA 8875
PigTailedMacaque  CAAATAGCTGGGACTACTGGTGCA--TACCACCATGCC-TGGCTAATTTTTGTATTTTCAGTTGAGGCAGGGTTTCACCA 8788
Bonobo            TAAGTAGCTGGGACTGCAGGTGCA--CACCACCACGCC-CAGCTAATTTTTGCATTTTCAGTTGAGGCAGGGTTTCACCA 9090
OliveBaboon       CAAATAGCTGGGACTACTGGTGCA--TACCACCATGCC-TGGCTAATTTTTGTATTTTCAGTTGAGGCAGGGTTTCACCA 8237
Orangutan         TAAGTAGCTGGGACTACAGGTTCA--CACCACCACAGC-CAGCTAATTTTTCTATTTTCAGTTGAGGCAGGGTTTCACCA 9429

Majority          TG-TTGGC------G-TCTCGAACTCTTGACC--TCAAGTGATCTG--CCTGCCTAGGCCTCCCAAAGTGCTAAGATTAT
                          11690     11700     11710     11720     11730     11740     11750     11760
Human             TG-TTGGCCAGGCTGGTCTGGAACTCCTGACC--TCAAGTGATACA--CTTACCTAGGCCTCCCAAAGTGTTAAGATTAC 8233
Coquerel'sSifaka  TCCTTAGTT-----G-TTTGGTACAATTCACCAGTGAAGCCATGTGAGCCTGATGTTTGTTTTCCTTGTGGGGAGGTTTT 7634
Ma'sNightMonkey   T--TTGGCCAGGCTGGTCTTGAACTCCTGACC--TCAAGAGATCTG--CCTGCCTAGGTCTCCCAAAATGCTGGGATTTC 8164
VervetAGM         TG-TTGGC---------CTCAAACTCTTGACC--TCAAGTGATCTG--CCTGCCTAGGCCTCCCAAAGTGCTAAGATTAT 8544
Gorrilla          TG-TTGGCCAGGCTGGTCTGGAACTCCTGACC--TCAAGTGATACA--CTTACCTAGGCCTCCCGAAGTGTTAAGATTAC 10484
GrabEatingMacaqueTG-TTGGC---------CTCAAACTCTTGACC--TCAAGTGATCTG--CCTGCCTAGGCCTCCCAAAGTGCTAAGATGAT 8898
Macaque           TG-TTGGC---------CTCAAACTCTTGACC--TCAAGTGATCTG--CCTGCCTAGGCCTCCCAAAGTGCTAAGATGAT 8941
PigTailedMacaque  TG-TTGGC---------CTCAAACTCTTGACC--TCAAGTGATCTG--CCTGCCTAGGCCTCCCAAAGTGCTAAGATTAT 8854
Bonobo            TG-TTGGCCAGGCTGGTCTGGAACTCCTGACC--TCAAGTGATACA--CTTACCTAGGCCTCCCAAAGTGTTAAGATTAC 9165
OliveBaboon       TG-TTGGC---------CTCAAACTCTTGACC--TCAAGTGATCTG--CCTGCCTAGGCCTCCCAAAGTGCTAAGATTAT 8303
Orangutan         TG-TTGGCTAGGCTGGTCTCGAACTCCTGACC--TCAAGTGATCCA--CTTGCCTAGGCCTCCCAAAGTGCTAAGATTAC 9504
```

```
Majority          AGGCGTGAGCCT-TACCATTATTACCTATACCAGCCTATGCCATTAAATT--TTCTACATAGACAATTTATACCCAATTG
                          11770     11780     11790     11800     11810     11820     11830     11840
Human             AGGCGTGAGCC---------ACCACCC---CCAGCCTATGCCATTAAATT--TTCTACATAGATAATTTATACCCAATTG 8299
Coquerel'sSifaka  AAACTACATATTTAATTTATTTAATAGATACAGGGCTATTCAGGTTATCTATCTCTTCTTGGGTGAGCTTTGGTAATTTG 7714
Ma'sNightMonkey   AGGCGTGAGCC---------ACCATCC---CCAGCCTATTCCATTAAATT--TTCTACATAGATGATATATACCCAATTG 8230
VervetAGM         AGGCGTCAGCCTATTCCATTATTACCTATACCAGCCTATGCCATTAAATT--TTCTACATAGACAATTTATACCCAATTG 8622
Gorrilla          AGGCGTGAGCC---------ACCACCC---CCAGCCTATGCCATTAAATT--TTCTACATAGACAATTTATACCCAATTG 10550
GrabEatingMacaqueAGGCGTGAGTCTATACCATTATTACCTATACCAGCCTATGCCATTAAATT--TTCTACATAGACAATTTATACCCAATTG 8976
Macaque           AGGCGTGAGTCTATACCATTATTACCTATACCAGCCTATGCCATTAAATT--TTCTACATAGACAATTTATACCCAATTG 9019
PigTailedMacaque  AGGCGTGAGTCTGTACCATTATTACCTATACCAGCCTATGCCATTAAATT--TTCTACATAGACAATTTATACCCAATTG 8932
Bonobo            AGGCGTGAGCC---------ACCACCC---CCAGCCTATGCCATTAAATT--TTCTACATAGACAATTTATACCCAATTG 9231
OliveBaboon       AGGCGTGAGCCTATACCATTATTACCTATACCAGCCTATGCCATTAAATT--TTCTACATAGACAATTTATACCCAATTG 8381
Orangutan         AGGCGTGAGCC---------ACCACCC---CCAGCCTATGCCATTAAATT--TTCTACATAGACAATTTATACCCAATTG 9570

Majority          TTT--TTAAATAGTTTTCTTCCTTTTCTATCTGGGTGCCATTTTTTGTCTCCATTTTCTTGTCTGTGGTATC-CAGTACA
                          11850     11860     11870     11880     11890     11900     11910     11920
Human             TTT--TTAAATAGTTTTCTTTCTTTTCTATCTGGATGCCATTTTTTGTCTCCTTTTT-TTGTCTGTGGTATC-CAGTACA 8375
Coquerel'sSifaka  TGTCCTCTTGTAGAATTAGTCCATTTCTTCTAAGTTGCCAAATTT-ATTTGCACAGGGTTGTTCATGATATTACCTTTTT 7793
Ma'sNightMonkey   TTT--TCAGACAGTTTTCTTCCTTTTCTATCTGGGTGCCATTTTTGGTCATCTTTTTCTTGTCTGTGGCATC-CAGTACA 8307
VervetAGM         TTT--TTAAATAGTTTTCTTCCTTTTCTATCTGGGTGCCATTTTTTGTCTCCATTTTCTTGTCTGTGGTATC-CAGTACA 8699
Gorrilla          TTT--TTAAATAGTTTTCTTCCTTTTCTATCTGGATGCCATTTTTTGTCTCCTTTTTCTTGTCTATGGTATC-CAGTACC 10627
GrabEatingMacaqueTTT--TTAAATAGTTTTCTTCCTTTTCTATCTGGGTGCCATTTTTTGTCTCCATTTTCTTGTCTGTGGTATC-CAGTACA 9053
Macaque           TTT--TTAAATAGTTTTCTTCCTTTTCTATCTGGGTGCCATTTTTTGTCTCCATTTTCTTGTCTGTGGTATC-CAGTACA 9096
PigTailedMacaque  TTT--TTAAATAGTTTTCTTCCTTTTCTATCTGGGTGCCATTTTTTGTCTCCATTTTCTTGTCTGTGGTATC-CAGTACA 9009
Bonobo            TTT--TTAAATAGTTTTCTTCCTTTTCTATCTGGATGCCATTTTTTGTCTCCTTTTT-TTGTCTATGGTATC-CAGTACA 9307
OliveBaboon       TTT--TTAAATAGTTTTCTTCCTTTTCTATCTGGGTGCCATTTTTTGTCTCCATTTTCTTGTCTGTGGTATC-CAGTACA 8458
Orangutan         TTT--TTAAATAGTTTTCTTCCTTTTCTATCTGGATACCATTTTTTGTCTCCTTTTTCTTGTCTGTGGTATC-CAGTACA 9647

Majority          ATGTTTAATAGAAGTGCTGAGAGAATATCTTTACCTTGCTCCTGATATT-GGGTGAAAACTCTAAAATTTCTTCCTTAAC
                          11930     11940     11950     11960     11970     11980     11990     12000
Human             ATGTTTAATAGAAGTGTTGAGAGAATATCTTTTCCTTGCTCCTGATATTAGGGTGAGAACTCTAAAATTTCTTCCTTAAC 8455
Coquerel'sSifaka  ATACTTTCGATATCTGTAGTGATAACAGCT----CTCATTCCTGATATT--GATAATTTGTTTCATCTTTTTTTCTTG-- 7865
Ma'sNightMonkey   ATGTTGAATAGAAGTGCTCAGAAGGTATCTTTACCTTGCTCCTGATATTGGGGTGAACACTCTAAAATTTCTTCCTTAAC 8387
VervetAGM         ATGTTTAATAGAAGTGCTGAGAGAATATCTTTACCTTGCTCCTGATACT-GCGTGAAAACTCTAAAATTTCTTCCTTAAC 8778
Gorrilla          ATGTTTAATAGAAGTGCTGAGAGAATATCTTCACCTTGCTCCTGATATTAGGGTGAGAACTCTAAAATTTCTTCCTTAAC 10707
GrabEatingMacaqueATGTTTAATAGAAGTGCTGAGAGAATATCTTTACCTTGCTCCTGATACT-GGGTGAAAACTCTAAAATTTCTTCCTTAAC 9132
Macaque           ATGTTTAATAGAAGTGCTGAGAGAATATCTTTACCTTGCTCCTGATACT-GGGTGAAAACTCTAAAATTTCTTCCTTAAC 9175
PigTailedMacaque  ATGTTTAATAGAAGTGCTGAGAGAATATCTTTACCTTGCTCCTGATACT-GGGTGAAAACTCTAAAATTTCTTCCTTAAC 9088
Bonobo            ATGTTTAATAGAAGTGTTGAGAGAATATCTTTTCCTTGCTCCTGATATTAGGGTGAGAACTCTAAAATTTCTTCCTTAAC 9387
OliveBaboon       ATGTTTAATAGAAGTGCTGAGAGAATATCTTTACCTTGCTCCTGATACT-GGGTGAAAACTCTAAAATTTCTTCCTTAAC 8537
Orangutan         ATGTTTAATAGAAGTGCTAAGAGAATATCTTTAACTTGCTCCTGATATTAGGGTGAGAACTCTAAAATTTCTTCCTTAAC 9727
```

```
Majority          TGATACAATTCACTAGTGAAGCCATGTGGGGCCAGATGTTTGTTTTCTTTGTTGGGAGGTTTTAAACTACATATTTAACT
                           12010     12020     12030     12040     12050     12060     12070     12080
Human             TGACACAGTTCACTAGTGAAGCCATGTGGGGCCAGATGTTTGTTTT----GTCGGGAGGTTTTAAACTACATATTCAACT 8531
Coquerel'sSifaka  ---------TCATT-GTGGGTAGATGTTAATTGATTTCATTAATGTCTAAAAGGTGAGCTTTTGGTTT---CATTGAGTT 7932
Ma'sNightMonkey   TGATACAATTCACTAGTGAAGCCATGTGGGGCCAGATGTTTGTTTTCTTTGTTGTGAGGTTTTAAACTACATATTCAACT 8467
VervetAGM         TGATACAATTCACTAGTGAAGCCATGTGGGGCCAGATGTTTGTTTTCTATGTTGGGAGGTTTTAAACTACATATTTAACT 8858
Gorrilla          TGATACAGTTCACTAGTGAAGCCATGTGGGGCCAGATGTTTGTTTTCTTTGTCGGGAGGTTTTAAACTACATATTCAACT 10787
GrabEatingMacaqueTGATACAATTCACTAGTGAAGCCATGTGGGGCCAGATGTTTGTTTTCTTTGTTGGGAGGTTTTAAACTACATATTTAACT 9212
Macaque           TGATACAATTCACTAGTGAAGCCATGTGGGGCCAGATGTTTGTTTTCTTTGTTGGGAGGTTTTAAACTACATATTTAACT 9255
PigTailedMacaque  TGATACAATTCACTAGTGAAGCCATGTGGGGCCAGATGTTTGTTTTCTTTGTTGGGAGGTTTTAAACTACATATTTAACT 9168
Bonobo            TGATACAGTTCACTAGTGAAGCCATGTGGGGCCAGATGTTTGTTTT----GTCGGGAGGTTTTAAACTACATATTCAACT 9463
OliveBaboon       TGATACAATTCACTAGTGAAGCCATGTGGGGCCAGATGTTTGTTTTCTTTGTTGGGAGGTTTTAAACTACATATTTAACT 8617
Orangutan         TGATACAGTTCACTAATGAAGCCATGTGGGGCCAGATGTTTGTTTTCTTTGTCGGGAGGTTTTAAACTACATATTCAATT 9807

Majority          TATTTAATAGATATAGGGCTATTCAGTTTGTTTAGTTCTT---GAGTGAACTTTGGTAATTTGTCTCTTCTGTAGAACTA
                           12090     12100     12110     12120     12130     12140     12150     12160
Human             TATTTAATAGATATAGGGCTATTCAGTTTGTTTAGTTCTTCTTGAGTGAACTTTGGTAATTTGTATCTTCTGTAGAACTA 8611
Coquerel'sSifaka  TTCTCTGTTGTTTTTTGTTTCCT--ATTTTATTGATTTCT-----GTTATTTTTATTTTTTTCTGCTTTGTGTTTAATTT 8005
Ma'sNightMonkey   TATTTAGTAGGTATAGGGCTA---------G--------A----------------GG--T-------TCTGTAGAACTA 8505
VervetAGM         TATTTAATAGATACAGGGCTATTCAGTTTGTTTAGTTCTT---GAGTGAACTTTGGTAATTTGTCTCTTCTGTAGGACTA 8935
Gorrilla          TATTTAATAGATATAGGGCTATTCAGTTTGTTTAGTTCTTCTTGAGTGAACTTTGGTAATTTGTATCTTCTGTAGAACTA 10867
GrabEatingMacaqueTATTTAATAGATACAGGGCTATTCAGTTTGTTTAGTTCTT---GAGTGAACTTCGGTAATTTGTCTCTTCTGTAGGACTA 9289
Macaque           TATTTAATAGATACAGGGCTATTCAGTTTGTTTAGTTCCT---GAGTGAACTTCGGTAATTTGTCTCTTCTGTAGGACTA 9332
PigTailedMacaque  TATTTAATAGATACAGGGCTATTCAGTTTGTTTAGTTCTT---GAGTGAACTTTGGTAATTTGTCTCTTCTGTAGGACTA 9245
Bonobo            TATTTAATAGATATAGGGCTATTCAGTTTGTTTAGTTCTTCTTGAGTGAACTTTGGTAATTTGTATCTTCTGTAGAACTA 9543
OliveBaboon       TATTTAATAGATACAGGGCTATTCAGTTTGTTTAGTTCTT---GAGTGAACTTTGGTAATTTGTCTCTTCTGTAGGACTA 8694
Orangutan         TATTTAATAGATATAGGGCTATTCAGTTTGTTTAGTTCTTCTTGAGTGAACTTCGGTAATTTGTAGCTTCTGTAGAACTA 9887

Majority          GTCCATTTCCTTT-AAGTTGCTGAATGTATTTGGGTAGA-GTTGTTCATGATACTACTGTTTTTTTTTGTTTTT------
                           12170     12180     12190     12200     12210     12220     12230     12240
Human             GTCCATTTCCTTT-AAGTTGCTGAATGTATTTGTGTAGA-GTTGTTCATGATACTACTATTTTTTTTTTTTTTTTTTTTT 8689
Coquerel'sSifaka  GCTC-TTCCATTT----CCACTTAGTATCATTGACATGTTACCTTTTCTTATAGAGGGGTTTGGTGCTGTGTATTTCC-- 8078
Ma'sNightMonkey   GTCTACTTCCTCT-GAGTTGCTGAATGTATTTGCATAGA-GTTGTTCATGCTACTGCTTTTTTTTTTTTTTTTTTTTTTT 8583
VervetAGM         GTCCATTTCCTTTTAAGTTGCTGAATGTATTTGGGTAGA-GTTGTTCATGATACTACAGTTTTTTTTGG----------- 9003
Gorrilla          GTCCATTTCCTTT-AAGTTGCTGAATGTATTTGCGTAGAAGTTGTTCATGATACTACTATTTTTTTTTTTTTTTTTTTTT 10946
GrabEatingMacaqueGTCCATTTCCTTTTAAGTTGCTGAATGTATTTGGGTAGA-GTTGTTCATGATACTACAGTTTTTTTTTG----------- 9357
Macaque           GTCCATTTCCTTTTAAGTTGCTGAATGTATTTGGGTAGA-GTTGTTCATGATACTACAGTTTTTTTTTG----------- 9400
PigTailedMacaque  GTCCATTTCCTTTTAAGTTGCTGAATGTATTTGGGTAGA-GTTGTTCATGATACTACAGTTTTTTTTTG----------- 9313
Bonobo            GTCCATTTCCTTT-AAGTTGCTGAATGTATTTGTGTAGA-GTTGTTCATGATACTACTATTTTTTTTTTTTTTTG--T-- 9617
OliveBaboon       GTCCATTTCCTTTTAAGTTGCTGAATGTATTTGGGTAGA-GTTGTTCATGATACTACAGTTTTTTTTTG----------- 8762
Orangutan         GTCCATTTCCTTT-AAGTTGCTGAATGTATTTGCGTAGA-GTTGTTGATGATACTACTATTTTTTTTTTTTTTTG----- 9960
```

```
Majority        ---AGACGGAGTCTTGCTCTGTCGCCCGGG-CTGGAGTGCAGTGGCGCAATCTCGGCTCACTGCAACCTCCGCGTCCCGG
                         12250     12260     12270     12280     12290     12300     12310     12320
Human           T-GAGACGGAGCCTTGCTCTGTCGCCCGGG-CTGGAGTGCAGTGGCACCATCTCGGCTCAATGCAACCTCCACGTCCCGG 8767
Coquerel'sSifaka ---CTATTAAGTACTGCTTTCATGGCATCCCTCAAATTTTGATGTGTGCATTTTCATTCAGTTCAAAAT--ACTTTCTAA 8153
Ma'sNightMonkey --GAGACAAAGTCTCATACTGTTGCCTGGG-CTGAAGTGCAATGGGGCAATCTCGTCTCACTGTAACCTCTGCGTCCCAG 8660
VervetAGM       ---AGACGGAGTCTTGATCTGTCGCCCAGG-CTGGAGTGCAGTAGCGCAATCTCGGCTCACTGCAACCTTCGCATCCTGG 9079
Gorilla         TTGAGACGGAGCCTCACTCTGTCGCCTGGGGCTGGAGTGCAGTGGCACCATCTCGGCTCAATGCAACCTCCATGTCCCGG 11026
GrabEatingMacaque---AGACGGAGTCTTGATCTGTCGCCCAGG-CTGGAGTGCAGTAGCGCAGTCTCCGCTCACTGCAACCTCCGCGTCCCGG 9433
Macaque         ---AGACGGAGTCTTGATCTGTCGCCCAGG-CTGGAGTGCAGTAGCGCAATCTCTGCTCACTGCAACCTCCGCGTCCCGG 9476
PigTailedMacaque ---AGACGGAGTCTTGATCTGTCGCCCAGG-CTGGAGTGCAGTAGCGCAATCTCTGCTCACTGCAACCTCCGCGTCCCGG 9389
Bonobo          ---AGACGGAGCGTCGCTCTGTCACCTGGG-CTGGAGTGCAGTGGCACCATCTCGGCTCAATGCAACCTCTACGTCCCGG 9693
OliveBaboon     ---AGACGAAGTCTTGATCTGTCGCCCAGG-CTGGAGTGCAGTAGCGCAATCTCCGCTCACTGCAACCTCCGCGTCCCGG 8838
Orangutan       ---AGACGGAGCCTCGCTCTGTCGCCCGGG-CTGGAGTACAGTGGCACCATCTCGGCTCAGTGCAACCTCCACGTCCCGG 10036

Majority        GTTCACGCGATTCTCCTGCCTCAGCATCCCAAGTAGCTGGGATCACAGGCACACACCACCACACCTGGCTAATTTTTTGT
                         12330     12340     12350     12360     12370     12380     12390     12400
Human           GTTCACGCGGTTCTCCTGCCTCAGCATCCCAAGTAGCTGGGATCACAGGCACACACCACCACACCTGGCTAATTTTTTGT 8847
Coquerel'sSifaka TCTT---TGCTTTTATTTCTTTGACCTATCAGTTA-TTGGGATGTATGTAATTTAATA--ATGTTTGG--AATTTTTCAC 8225
Ma'sNightMonkey GTTCATGCGATTCTCCTGCCTCAGCCTCCTGAGTAGCTGGGATTACAGGCACCCAACACCACACCT---------TTAGT 8731
VervetAGM       GTTCACGTGATTCTCCTGCCTCAGCATCCCAAGTAGCTGGGATCGCAGGCATACACCACCACACCTGGCTAATATTTTGT 9159
Gorilla         GTTCACGCGGTTCTCTTGCCTCAGCATCCCAAGTAGCTGGGATCACAGGCACACACCACCACACCTGGCTAATTTTTTGT 11106
GrabEatingMacaqueGTTCACGCGATTCTCCTGCCTCAGCATCCCAGGTAGCTGGGATCACAGGCACACACCACCACACCTGGCTAATATTTTGT 9513
Macaque         GTTCACGCGATTCTCCTGCCTCAGCATCCCAGGTAGCTGGGATCACAGGCACACACCACCACACCTGGCTAATATTTTGT 9556
PigTailedMacaque GTTCACGCGATTCTCCTGCCTCAGCATCCCAGGTAGCTGGGATCACAGGCACACACCACCACACCTGGCTAATATTTTGT 9469
Bonobo          GTTCACGCGGTTCTCCTGCCTCAGCATCCCAAGTAGCTGGGATCACAGGCACACACCACCACACCTGGCTAATTTTTTGT 9773
OliveBaboon     GTTCACGCGATTCTCCTGCCTCAGCATCCCAGGTAGCTGGGATCACAGGCACACACCACCACACCTGGCTAATATTTTGT 8918
Orangutan       GTTCACGTGATTCTCCTGCCTCAGCATCCCAAGTAGCTGGGATCACAGGCACATACCACCACACCTGGCTAATTTTTTGT 10116

Majority        ATTTTTAGTAGAGATGG-GGTTTCACTATG-TTGGCCAGGCTGGTCTCGAACTCCTGACCTTGTG-ATCTGCCTGCCCTG
                         12410     12420     12430     12440     12450     12460     12470     12480
Human           ATTTTTAGTAGAGACGG-GGTTTCACTATG-TTGGCCAGGTTGGTCTCGAACT--------TGTG-ATCTGCCCGCCCTG 8916
Coquerel'sSifaka AAATCTGTTATGCATTTTAATTCCATTCTGACCAGAGAACATATTTTATAAAACTTGAATCTTTGTAGATACAAGACTTG 8305
Ma'sNightMonkey ATTTTTAGTA-AGACGG-GGTTTCACTGTG-TTGGCCAGGATGGTTTTGAACTCTTGACCTCGTG-ATCTGCCCACCTCG 8807
VervetAGM       ATTTTTAGGAGAGATGG-GGTTTCACTATG-TTGGCCAGGCTGGTCTCGAACTCCTGACCTTGCG-ATCTGCCTGCCCTG 9236
Gorilla         ATTTTTAGTAGAGACGG-GGTTTTACTATG-TTGGCCAGGTTGGTCTCGAACT-----------------------CCTG 11161
GrabEatingMacaqueATTTTTAGTAGAGATGG-GGTTTCACTATG-TTGGCCAGGCTGGTCTCGAACTCCTGACCTTGCG-ATCTGCCTGCCCTG 9590
Macaque         ATTTTTAGTAGAGATGG-GGTTTCACTATG-TTGGCCAGGCTGGTCTCGAACTCCTGACCTTGCG-ATCTGCCTGCCCTG 9633
PigTailedMacaque ATTTTTAGTAGAGATGG-GGTTTCACTATG-TTGGCCAGGCTGGTCTCGAACTCCTGACCTTGCG-ATCTGCCTGCCCTG 9546
Bonobo          ATTTTTAGTAGAGACGG-GGTTTCACTATG-TTGGCCAGGTTGGTCTCGAACTCCTGAC-TTGTG-ATCCGCCCGCCCTG 9849
OliveBaboon     ATTTTTAGTAGAGATGG-GGTTTCACTATG-TTGGCCAGGCTGGTCTCGAACTCCTGACCTTGCG-ATCTGCCTGCCCTG 8995
Orangutan       ATTTTTACTAGAGACGG-GGTTTCACTATA-TTGGCCAGGTTGGTCTCGAACTCCTGAC-TTGTG-ATCCGCCCACCTTG 10192
```

```
Majority         GCCTCCCAAAGTGCTGGGATTACAGGCGTGAGCCACTGTGCCCAGCCATGATACTACATTTTTACACTTTTGATA-TCTG

                         12490     12500     12510     12520     12530     12540     12550     12560

Human            -CCTCACAAAGTGCTGGGATTACAGGCGTGAGCCACTGCGCCCAGCCATGATACTACATTTTTACACTTTTGGTA-TCTG 8994
Coquerel'sSifaka GTTTATGGTCCAAATATAGTATATCTTGTTAAATGTCTTAGATATCTTTGATAAGGGTGT-----GTATTCGGTTGTTGG 8380
Ma'sNightMonkey  GCCTCCCGAAGTGCTGGGATCACAGGTGTGAGCCGCCACACCCAGCCATGATACTACATTTTTACACTTTTGATAATCTG 8887
VervetAGM        GCTTCCCAAAGTGCTGGGATTACAGGCGTGAGCCACTGTGCCCAGCCATGATACTACATTTTTACACTTTTGATA-CCTG 9315
Gorrilla         -ACTTGTGAT---CCGC------------------CCGCACCCAGCCATGATACTACATTTTTACACTTTCGATA-TCTG 11218
GrabEatingMacaqueGCCTCCCAAAGTGCTGGGATTACAGGCGTGAGCCACTGTGCCCAGCCATGATACTACA--------CTTTTGATA-CC-G 9660
Macaque          GCCTCCCAAAGTGCTGGGATTACAGGCGTGAGCCACTGTGCCCAGCCATGATACTACA--------CTTTTGATA-CC-G 9703
PigTailedMacaque GCCTCCCAAAGTGCTGGGATTACAGGCGTGAGCCACTGTGCCCAGCCATGATACTACATTTTTACACTTTTGATA-CC-A 9624
Bonobo           -CCTCACGAAGTGCTGGGATTACAGGCGTGAGCCACTGCGCGCAGCCATGATACTGTATTTTTACACTTTTGATA-TCTG 9927
OliveBaboon      GCCTCCCAAAGTGCTGGGATTACAGGCGTGAGCCACTGTGCCCAGCCATGATACTACATTTTTACACTTTTGATA-CC-G 9073
Orangutan        GCCTCACAAAGTGCTGGGATTACAGGCGTGAGCCACTGTGCCCAGCCATGATACTGCATTTTTACACTTTTGATA-TCTG 10271

Majority         TTGAATCCTTAGTGATGTCAGCTCTCTCATTCCCAGTAGGGATAATTTGTGTCATCTTTTTTCCTTGTCTGTCTGGATTG

                         12570     12580     12590     12600     12610     12620     12630     12640

Human            TTGAATCCTTAGTGATGTCAGCTTTCTCATTCCCAGTAGGGATAATTTGTGTCATCTTTTTTCCTTGTCTGTCTGGATTG 9074
Coquerel'sSifaka GTGGTAGTCTATAAATATCATTTAGGTCATTTTTATTGATAATG--TTGTTTCTTTCTCTTTACTGACTT--TTGGTCTA 8456
Ma'sNightMonkey  TCGAATGCTT-GTGATGTCAGCTCTCTCATTCCCAACAGTGGTAATTTGTGTTATCGTTTTTCCTTGTCTGTCTGGATTG 8966
VervetAGM        TTGAATCCTTAGTGATGTCAGCTCTCTCATTCCCAGTAGGGATAATTTATGTCATCTTTTTTCCTTGTCTGTCTGGATTG 9395
Gorrilla         TTGAATCCTTAGTGATGTCAGCTCTCTCATTCCCAATAGGGATAATTTGTGTCATCTTTTTTCCTTGTCTGTCTGGATTG 11298
GrabEatingMacaqueTTGAATCCTTAGTGATGTCAGCTCTCTCATTCTCAGTAGGGATAATTTATGTCATTTTTTTTCCTTGTCTGTCTGGATTG 9740
Macaque          TTGAATCCTTAGTGATGTCAGCTCTCTCATTCTCAGTAGGGATAATTTATGTCATTTTTTTTCCTTGTCTGTCTGGATTG 9783
PigTailedMacaque TTGAATCCTTAGTGATGTCAGCTGTCTCATTCCCAGTAGGGATAATTTATGCCATTTTTTTTCCTTGTCTGTCTGGATTG 9704
Bonobo           TTGAATCCTTAGTGATGTCAGCTCTCTCATTCCCAATAGGGATAATTTGTGTCATCTTTTTTCCTTGTCTGTCTGGATTG 10007
OliveBaboon      TTGAATCCTTAGTGATGTCAGCTCTCTCATTCCCAGTAGGGATAATTTATGTCATTTTTTTCCCTTGTCTGTCTGGATTG 9153
Orangutan        TTGAATCCTTAGTGATGTCAGCTCTCTCATTCCCAATAGGGAGAATTTGTGTCATCTTTTTTCCTTGTCT----GGATTG 10347

Majority         AAGTT-TATTGATTTTATTAAT--CTCCAAAATATCAGC------TTTTGGTTTCATTGAGTTTTCTCTGTGGTTTTTCT

                         12650     12660     12670     12680     12690     12700     12710     12720

Human            AAGTT-TATTGATTTTATTAAT--CTCCAAAATACCAGC------TTTTGGTTT----GAGTTTTCTCTGCTGTTTTTCT 9141
Coquerel'sSifaka TTGTTATATCAGTTGAAGAGAGGATATTAAAATTTCTGCCTATACTTGTGGATTTGTCTATTTCTCTTTGCAGTTCTACT 8536
Ma'sNightMonkey  AAGTT-TATTGATTTTATTAGT--CTCCAAAATAACAGC------TTTTGGTTTCATTAAGTTTTCTCT-----TTTTCT 9032
VervetAGM        AAGTT-TATTGATTTTATTAAT--CTCCAAAATGTCAGC------TTTTGGTTTCATTGAGTTTTCTCTGTGGTTTTTCT 9466
Gorrilla         AAGTT-TATTGATTTTATTAAT--CTCCAGAATACCAGC------TTTTGGTTT----GAGTTTTCTCTGCTGTTTTTCT 11365
GrabEatingMacaqueAAGTT-TATTGATTTTATTAAT--CTCCAAAATATCAGC------TTTTGGTTTCATTGAGTTTTCTCTGTGGTTTTTCT 9811
Macaque          AAGTT-TATTGATTTTATTAAT--CTCCAAAATATCAGC------TTTTGGTTTCATTGAGTTTTCTCTGTGGTTTTTCT 9854
PigTailedMacaque AAGTT-TATTGATTTTATTAAT--CTCCAAAATATCAGC------TTTTGGTTTCATTGAGTTTTCTCTGTGGTTTTTCT 9775
Bonobo           AAGTT-TATTGATTTTATTAAT--CTCCAAAATACCAGC------TTTTGGTTT----GAGTTTTCTCTGCTGTTTTTCT 10074
OliveBaboon      AAGTT-TATTGATTTTATTAAT--CTCCAAAATATCAGC------TTTTGGTTTCATTGAGTTTTCTCTGTGGTTTTTCT 9224
Orangutan        AAGTT-TATTGATTTTATTAAT--CTCCAAAATACCAGC------TTTTGATTTCATTGAGTTTTCTCTGTTGTTTTTTT 10418
```

```
Majority          GTTTTCTATTTTATTGATTTCTGCTGTTTTTATTTTGTTTTGCTTTGTGTTTAGT-TTGATCTTCATTTTCTACTTAGTT
                          12730     12740     12750     12760     12770     12780     12790     12800
Human             GTTTTCTATTTTATTGATTTCTGCTATTTTTATTTTGTTTTGCTTTGTGGTTAGT-TTGATCTTCATTTTCTACTTAGTT 9220
Coquerel'sSifaka  AGTTTTTGATTTATGTATTTTGA--AGTTTTATTATTAGTACCTAAATGTTTAGGATTACTATATCCCCTTGATGCATTT 8614
Ma'sNightMonkey   GATTTC-----TATTGATTTCTCCTGTTTTTATTTTGTTTTGCTTTCTGTTTAGT-TTGATCTTTGTTTTCTACTTAGTT 9106
VervetAGM         GTCTTCTATTTTATTGATTTCTGCTGTTTTTATTTTGTTTTGCATTGTGTTTAGT-TTGATCTTCATTTTCTACTTAGTT 9545
Gorrilla          GTTTTCTATTTTATTGATTTCTGCTATTTTTATTTTGTTTTGCTTTGTGGTTAGT-TTGATCTTCATTTTCTACTTAGTT 11444
GrabEatingMacaqueGTCTTCTATTTTATTGATTTCTGCTGTTTTTATTTTGTTTTGCATTGTGTTTAGC-TTGATCTTCATTTTCTACTTAGTT 9890
Macaque           GTCTTCTATTTTATTGATTTCTGCTGTTTTTATTTTGTTTTGCATTGTGTTTAGC-TTGATCTTCATTTTCTACTTAGTT 9933
PigTailedMacaque  GTCTT-----TTATTGATTTCTGCTGTTTTTATTTTGTTTTGCATTGTGTTTAGC-TTGATCTTCATTTTCTACTTAGTT 9849
Bonobo            GTTTTCTATTTTATTGATTTCTGCTATTTTTATTTTGTTTTG-----TGGTTAGT-TTGATCTTCATTTTCTACTTAGTT 10148
OliveBaboon       GTCTTCTGTTTTATTGATTTCTGCTGTTTTTATTTTGTTTTGCATTGTGTTTAGT-TTGATCTTCATTTTCTACTTAGTT 9303
Orangutan         GTTTTCTATTTAATTGATTTCTGCTGTTTTTATTTTGTTTTGCTTTGTGTTTAGC-TTGATCTTCATTTTCTACTTAGTT 10497

Majority          AACATTGAAGTTGATGT-CATTAACTTGATACTTTTACTTATGTAGGAGTTTAGTGCTGTATGTTTCCCTTTTAAGTACC
                          12810     12820     12830     12840     12850     12860     12870     12880
Human             AACATTGAAGTTAATGT-CATTAACTTAGTACTTTTACTTATATAGGGATTTATTGCTGTATGTTTCCCTTTTAAGTACC 9299
Coquerel'sSifaka  ATCATTTTTAGTATTTTGCAGTGACCATAT--TTATCCCTGATAATAGTCTTTGCTTTAAAATCTACTTTTTCTAATATT 8692
Ma'sNightMonkey   AACATTGAAGTTAATAT-CACTAACTTGA------TACTTATGTAGGGGTTTAGTGCTATATGTTTCCCTTTTAAGTACT 9179
VervetAGM         AACATTGAAGTTGATGT-CATTAACTTGATACTTTTACTTATGTAGGAGTTTAGTGCTGTATGTTTCCCTTTGAAGTACC 9624
Gorrilla          AACATTGAAGTTAATGT-CATTAACTTGGGACTTTTACTTATATAGGGATTTATTGCTGTATGTTTCCCTTTTAAGTACC 11523
GrabEatingMacaqueAATGTTGAAGTTGATGT-CATTAACTTGATACTTTTACTTATGTAGGAGTTTAGTGCTGTAAGTTTCCCTTTGAAGTACC 9969
Macaque           AATGTTGAAGTTGATGT-CATTAACTTGATACTTTTACTTATGTAGGAGTTTAGTGCTGTAAGTTTCCCTTTGAAGTACC 10012
PigTailedMacaque  AACGTTGAAGTTGATGT-CATTAACTTGATACTTTTACTTATGTAGGAGTTTAGTGCTGTAAGTTTCCCTTTGAAGTACT 9928
Bonobo            AACATTGAAGTTAATGT-CATTAACTTGGTACTTTTACTTATATAGGGATTTATTGCTGTATGTTTCCCTTTTAAGTACC 10227
OliveBaboon       AACGTTGAAGTTGATGT-CATTAACTTGATACTTTTACTTATGTAGGAGTTTAGTGCTGTAAGTTTCCCTTTGAAGTACC 9382
Orangutan         AACGTTGAAGTTGATGT-CATTAACTTGGTACTTTTACTTATATAGGAGTTTAGTGCTGTATGTTTCCCTTTTAAGTACC 10576

Majority          GCTTTCATGGCATTCCTCAAATTTTGACATGTATATTTTTATTCATTTCAAAATACTTATTAAAAAATTTTTTTT-GACC
                          12890     12900     12910     12920     12930     12940     12950     12960
Human             GCTTTCACGGCATTCCTCAAATTTTGACACGTATATTTTTATTCACTTCAAAATACTTTTTTAAATA-TTTCTTT-GACC 9377
Coquerel'sSifaka  AATACAACTGTCTCTGCCTTGTTTTGAATAGG----TTATGTCTATTTTCATCCTTTTATTTTAAACTTATTTTT--ATC 8766
Ma'sNightMonkey   GCTTTCATGGCATTCCTCAAATTTTGATGAAT---TTTTCATTCAGTTCAAAATACTTTTTTAAACAATTTCTTT-GACC 9255
VervetAGM         GCTTTCATGGCATTCCTCAAATTTTGACATGTATATTTTTATTCATTTCAAAATACTTAAAAAAAAATTTTTTTTTGACC 9704
Gorrilla          GTTTTCACGGCATTCCTCAAATTTTGACACGTATATTTTTATTCACTTCAAAATACTTTTTTAAAAAATTTCTTT-GACC 11602
GrabEatingMacaqueGCTTTCATGGTATTCCTCAAATTTTGACATGTATATTTTTATTCATTTCAGAATACTTAAAAAAAATTTTTTTT-GACC 10048
Macaque           GCTTTCATGGTATTCCTCAAATTTTGACATGTATATTTTTATTCATTTCAGAATACTTAAAAAAAATTTTTTTTTGACC 10092
PigTailedMacaque  GCTTTCATGGCATTCCTCAAATTTTGACATGTATATTTTTATTCATTTCAGAATACTTAAAAAAAATTTTTTTTTGACT 10008
Bonobo            GCTTTCACGGCATTCCTCAAATTTTGACACGTATATTTTTATTCACTTCAAAATACTTTTTTAAATA-TTTCTTT-GACC 10305
OliveBaboon       GCTTTCATGGCATTCCTCAAATTTTGACATGTGTATTTTTATTCATTTCAGAATACTTAAAAAAAATTTTTTTTTGACC 9462
Orangutan         GCTTTCATGGCATTCCTCAAATTTTGACACATACATTTTTATTCACTTCAAAATACTTTTTAAAAAAATTTCTTT-GACC 10655
```

```
Majority        CATGGGTTATTGGGAAGTATGTTATTTATGTTATTTAATTTCCAAATATTTGGAGTTTTCTATATATCTTT-CTGTTATG

                        12970     12980     12990     13000     13010     13020     13030     13040

Human           CATGGGTTATTGGAAAGTATGTTATTTATGTTATTTAATTTCCAAATATTTGGAGTTTTCCATATATCTTT-CTGTTATG 9456
Coquerel'sSifaka CTTAGATTTAAAGTATGTTTCTTGTAGGCAGCATAAAGTTGGAAATTGCTTTT--TTTCCTACATCTTTTTTTTTTTTT 8844
Ma'sNightMonkey CATGGGTTATTGGGAAGCATGTTATTTA---------ATTTCTAAATATTTGGAGTTTTCTACATATCTTT-CTGTTGTA 9325
VervetAGM       CAGGAGTTATTGACAAGTATGTTATTTATGTTATTTAATTTCCAAATATTTGGAGTTTTCTATATATCT-----GTTATG 9779
Gorrilla        CATGGGTTATTGGAAAGTATGTTATTTATGTTAATTAATTTCCAAATATTTGGAGTTTTCTGTATATCTTT-CTGTTATG 11681
GrabEatingMacaqueCAGGAGTTATTGGGAAGTATGTTATTTATGTTATTTAATTTCCAAATATTTGGAGTTTTCTATATATCT-----GTTATG 10123
Macaque         CAGGAGTTATTGGGAAGTATGTTATTTATGTTATTTAATTTCCAAATCTTTGGAGTTTTCTATATATCT-----GTTATG 10167
PigTailedMacaque CAGGAGTTATTGGGAAGTATGTTATTTATGTTATTTAATTTCCAAATATTTGGAGTTTTCTATATATCT-----GTTATG 10083
Bonobo          CATGGGTTATTGGAAAGTATGTTATTTATGTTA----ATTTCCAAATATTTGGAGTTTTCCATATATCTTT-CTGTTACG 10380
OliveBaboon     CAGGAGTTATTGGGGAGTATGTTATTTATGTTATTTAATTTCCAAATATTTGGAGTTTTCTATATATCT-----GTTATG 9537
Orangutan       CATGGGTTATTGGGAAGTATGTTATTTATGTTATTTAATTTCCAAATATTTGGAGTTTTCTGTATATCTTTTCTGTTATG 10735

Majority        TATTCTTAATTTTAGTTT--TATTCTGGCCAGAGAATGTATTTTCTAAGACTTGGATCTTTGTAAACATGGAGACTTGTT

                        13050     13060     13070     13080     13090     13100     13110     13120

Human           TATTCATAATTTTAGTTT--TATTCTGGCCAGAGAACATATTTTCTAAGACTTGGATCTTTGTAAACATGGAGACTTGTT 9534
Coquerel'sSifaka GAGACAGAGTCTCACTCTGTTGCCCTGGCTAGAGTGAGTG----CCGCGGCGTCAGCCTAGCTCACAGCAACCTCAAACT 8920
Ma'sNightMonkey TATTTGTAATTTTAATTT--TATTCTGGCCAGAGAACATATTTTCTAAGACTTGGATCTTTGTAAACATGGAGACTTACT 9403
VervetAGM       TATTCTTAATTTTTGTTT--TATTCTGGCCAGAGAATGTATTTTCTAAGACTTGGATCTTTGTAAACATGGAGACTTGTT 9857
Gorrilla        TATTCATAATTTTAGTTT--TATTCTGGCCAGAGAACATATTTTCTAAGACTTGGATCTTTGTAAACATGGAGACTTGTT 11759
GrabEatingMacaqueTATTCTTAATTTTTGTTT--TATTCTGGCCAGAGAATGTATTTTCTAAGACTTGAATCTTTGTAAACATGGAGACTTGTT 10201
Macaque         TATTCTTAATTTTTGTTT--TATTCTGGCCAGAGAATGTATTTTCTAAGACTTGAATCTTTGTAAACATGGAGACTTGTT 10245
PigTailedMacaque TATTCTTAATTTTTGTTT--TATTCTGGCCAGAGAATGTATTTTCTAAGACTTGAATCTTTGTAAACATGGAGACTTGTT 10161
Bonobo          TATTCATAATTTTAGTTT--TATTCTGGCCAGAGAACATATTTTCTAAGACTTGGATCTTTGTAAACATGGAGACTTGTT 10458
OliveBaboon     TATTCTTAATTTTTGTTT--TATTCTGGCCAGAGAATGTATTTTCTAAGACTTGGATCTTTGTAAACATGGAAACTTGTT 9615
Orangutan       TATTCGTAATTTTAGTTT--TATTCTGGCCAGAGAACGTATTTTCTAAGACTTGGATCTTTGTAAACATGGAGACTTGTT 10813

Majority        TTATGGTCCAAATATTATATATCTTGTTAAGTATTCACTGAATACTCTGAT--TTCTCTGCAGATTTGGAGAGATCTCTG

                        13130     13140     13150     13160     13170     13180     13190     13200

Human           TTGTGGTCCAAAT---ATATATGTTGTTAAGTATTCACTGAATACTCTGATGATTCTCTGCAGATTTGGAGAAATCTCTG 9611
Coquerel'sSifaka CCTGGGCTCAAGTG--ATCCTTTTGCCTCAGCCTC--CTGAGTAGCTGGGA----CTACAGGCATGTGCCACCATGCCCG 8992
Ma'sNightMonkey TTATGGTCCAAATATTAT-TATCTTGTGAAGTATTTACTGAATACTCTGACG----ATTGCAGATTTGGAAAGCTCTTTG 9478
VervetAGM       TTATGGTCCAAATATTATATATCTTGTTAAGTATTCACTGAATACTCTGAT--TTCTCTGCAGATTTGGAGAGATCTCTG 9935
Gorrilla        TTGTGGTCCAAAT---ATATATGTTGTTAAGTATTCACTGAATACTCTGATGATTCTCTGCAGATCTGGGGAAATCTCTG 11836
GrabEatingMacaqueTTATGGTCCAAATATTATATATCTTGTTAAGTATTCACTGAATACTCTGAT--TTCTCTGCAGATTTGGAGAGATCTCTG 10279
Macaque         TTATGGTCCAAATATTATATATCTTGTTAAGTATTCACTGAATACTCTGAT--TTCTCTGCAGATTTGGAGAGATCTCTG 10323
PigTailedMacaque TTATGGTCCAAATATTATATATCTTGTTAAGTATTCACTGAATACTCTGAT--TTCTCTGCAGATTTGGAGAGATCTCTG 10239
Bonobo          TTGTGGTCCAAAT---ATATATGTTGTTAAGTATTCACTGAATACTCTGATGATTCTCTGCAGATTTGGAGAAATCTCTG 10535
OliveBaboon     TTATGGTCCAAATATTATATATCTTGTTAAGTATTCACTGAATACTCTGAT--TTCTCTGCAGATTTGGAGAGATCTCTG 9693
Orangutan       TTATGGTCCAAAT---ATATATGTTGTTAAGTATTCACTGAATACTCTGATGATTCTCTGCAGATTTGGAGAAATCTCTG 10890
```

```
Majority        -GCAGCTCTTTTCTCTGGTACTTTGACCTGTA----AACTCTAGTTACTTTGGTCTTCTCAGGCTCTTGGC---TCTTTC
                         13210     13220     13230     13240     13250     13260     13270     13280
Human           TGCAGTTCTTTTCTCTGGTACTTTGACCTGTATGTAAACTCTAGTTACTTTGGTCTTCTCAGGCTCTTGGC---TCTTTC 9688
Coquerel'sSifaka -GCTAATTTTTTCTATA-TATTTTTAGTTGTC----CAGATAATTTCTTTCTATTTTTTTGTAGTAGAGACGG-GGTCTC 9065
Ma'sNightMonkey TGCAACTCTTTTCTCTGGTACTTTGACATGTA----AACTCTAGTTACTTTGGTCTTCTC------T------------C 9536
VervetAGM       --CAGCTCTTTTCTCTGGTACTTTGACCTGTA----AACTCTAGTTACTTTGGTCTTCTCAGGCTCTTGGC---TGTTTC 10006
Gorrilla        TGCAGCTCTTTTCTCTGGTACTTTGACCTGTATGTAAACTCTAGTTACTTTGGTTTTCTCAGGCTCTTGGCGGCTCTTTC 11916
GrabEatingMacaque--CAGCTCTTTTCTCTGGTACTTTGACCTGTA----AACTCTAGTTACTTTGGTCTTCTCAGGCTCTTGGC---TCTTTC 10350
Macaque         --CAGCTCTTTTCTCTGGTACTTTGACCTGTA----AACTCTAGTTACTTTGGTCTTCTCAGGCTCTTGGC---TCTTTC 10394
PigTailedMacaque --CAGCTCTTTTCTCTGGTACTTTGACCTGTA----AACTCTAGTTACTTTGGTCTTCTCAGGCTCTTGGC---TCTTTC 10310
Bonobo          TGCAGCTCTTTTCTCTGGTACTTTGACCTGTATGTAAACTCTAGTTACTTTGGTCTTCTCAGGCTCTTGGC---TCTTTC 10612
OliveBaboon     --CAGCTCTTTTCTCTGGTACTTTGACCTGTA----AACTCTAGTTACTTTGGTCTTCTCAGGCTCTTGGT---TCTTTC 9764
Orangutan       TGCAGCTCTTTTCTTTGGTATTTTGACCTGTA----AACTCTAGTTACTTTGGTCTTCTCAGGCTCTTGGC---TCTTTC 10963

Majority        ACAATTAAAGTAGTCTTTGAGGCTCAGCCTGGTTTCCTCATAGCTGTGCTGTGGCCTGGACACTCAAGGGAGTA--AGCT
                         13290     13300     13310     13320     13330     13340     13350     13360
Human           ACAATTAAAGTAGTCTTTGAGGCTCAGCCTGCTTTCCTCATAGCTATGCTATGGCCTGGACACTCAAGGGAGTATAAGCT 9768
Coquerel'sSifaka GCTCTTGGT-CAGGCT---GGTCTCGAACTCCTGAGCTCA-AACAATCCGCCCGCCTCGGCCTCCCAGAGTGCT---GGG 9137
Ma'sNightMonkey ACAGTTAAAGTAGTCTTTGAAACTCAGCCTGGTTTCCTCATAGCTGTGCTATGGCCTGGACACTCAAGGGAGTA--AGCT 9614
VervetAGM       ACAATTAAAGTAGACTTTGAGGCTCGGCGTGGTTTCCTCATAGTTGTGCTGTGGCCTGGACACTTAAGGGAGTA--AGCT 10084
Gorrilla        ACAATTAAAGTAGTCTTAGAGGCTCAGCCTGCTTTCCTCATAGCTGTGCTATGGCCTGGACACTCAAGGGAGTATAAGCT 11996
GrabEatingMacaqueACAATTAAAGTAGACTTTGAGGCTCAGTGTGGTTTCCTTATAGTTGTGCTGTGGCCTGGACACTTAAGGGAGTA--AGCT 10428
Macaque         ACAATTAAAGTAGACTTTGAGGCTCAGTGTGGTTTCCTTATAGTTGTGCTGTGGCCTGGACACTTAAGGGAGTA--AGCT 10472
PigTailedMacaque ACAATTAAAGTAGACTTTGAGGCTCAGTGTGGTTTCCTTATAGTTGTGCTGTGGCTTGGACACTTAAGGGAGTA--AGCT 10388
Bonobo          ACAATTAAAGTAGTCTTTGAGGCTCAGCCTGCTTTCCTCATAGCTGTGCTATGGCCTGGACACTCAAGGGAGTATAAGCT 10692
OliveBaboon     ACAATTAAAGTAGACTTTGAGGCTCAGCATGGTTTCCTTATAGTTGTGCTGTGGCCTGGACACTTAAGGGAGTA--AGCT 9842
Orangutan       ACAATTAAAGTAGTCTTTGAGGCTCAGCCTGCTTTCCTCATAGCTGTGCTATGGCCTGGACACTCAAGGGAGTG--AGCT 11041

Majority        GAGGCAAACATGGACT--CATTTGTTTTCTAACTTTCAGGGATTATTGTCCATCATTGCCTGATCTCCAGTGTCTTGAAA
                         13370     13380     13390     13400     13410     13420     13430     13440
Human           GAGGCAAACATGGACT--CATTTGTTTTCTAACTTTCAGGGATTATTGTCCATCATTGCCTGATGTCCAGTGTCTTGAAA 9846
Coquerel'sSifaka ATTATAGGTGTGAGCC--ACCGCGCCGGCCATTTTTCCTACATCTTAACTCTTTTTTTTTTTTGGTTAAGACCATTTAC 9215
Ma'sNightMonkey GAGGCAAACATGGACTTACATTTGTTTTCTGACTTTCAGGGATTGTTGTCCATCATTGCCTGATGTCCAGTGTCTTGAAA 9694
VervetAGM       GAGGCAAACATGGACT--CATTTGTTTTCTAACTTTCAGGGACTATTGTCCATCATTGCCTGATCTCCAGTGTCTTGAAA 10162
Gorrilla        GAGGCAAACATGGACT--CATTTGTTTTCTAACTTTCAGGGATTACTGTCCATCATTGCCTGATGTCCAGTGTCTTGAGA 12074
GrabEatingMacaqueGAGGCAAACATGGACT--CATTTGTTTTCTAACTTTCAGGGACTATTGTCCATCATTGCCTGATCTCCAGTGTCTTGAAA 10506
Macaque         GAGGCAAACATGGACT--CATTTGTTTTCTAACTTTCAGGGACTATTGTCCATCATTGCCTGATCTCCAGTGTCTTGAAA 10550
PigTailedMacaque GAGGCAAACATGGACT--CATTTGTTTTCTAACTTTCAGGGACTATTGTCCATCATTGCCTGATCTCCAGTGTCTTGAAA 10466
Bonobo          GAGGCAAACATGGACT--CATTTGTTTTCTAACTTTCAGGGATTATTGTCCATCATTGCCTGATGTCCAGTGTCTTGAAA 10770
OliveBaboon     GAGGCAAACATGGACT--CATTTGTTTTCTAACTTTCAGGGGCTGTTGTCCATCATTGCCTGATCTCCAGTGTCTTGAAA 9920
Orangutan       GAGGCAAACATGGACT--CATTTGTTTTCTAACCTT-AGGGATTATTGTCCATCATTGCCTGATCTCCAGTGTCTTGAAA 11118
```

```
Majority         ACCCATTGTTCTGTATATT-TGCTTGATTGTTT----GTTGTTGTTTCGGGAAGGCAAATCTGGGTCATGTTATTCTATC
                         13450     13460     13470     13480     13490     13500     13510     13520
Human            AGCAATTATTCTGTATAGT-TGCTTGATTGTTT----GGTGTTGTTTCGGCGAGGCAAATCTGGGTCATGTTATTCTGTC 9921
Coquerel'sSifaka ATTTAGTGTTGTTACTACTATGGTTAATTTTGAA---ACTACCACTTTGCTATTTTTAATTTGT-TCTTGGTGTTCTTTG 9291
Ma'sNightMonkey  ACACATTGTTCTGTATATT-TGTTTGATTTTTTTGTTGCTGTTGTTTCAGCAAGGCAAATCTGGGTCATCTTATTCCATC 9773
VervetAGM        ACCCATTGTTCTGTGTATT-TGCTTGATTGTTT----GTTGTTGTTTCGGGAAGGCAAATCTGGGTCATGTTATTCTATC 10237
Gorrilla         AGCAATTGTTCTGTATATT-TGCTTGATTGTTT----GGTGTTGTTTCGGCAAGGCAAATCTGGGTCATGTTATTCTGTC 12149
GrabEatingMacaqueACCCATTGTTCTGTATATT-TGCTTGATTGTTT----GTTGTTGTTTCGGGAAGGCAAATCTGGGTCATGTTATTCTATC 10581
Macaque          ACCCATTGTTCTGTATATT-TGCTTGATTGTTT----GTTGTTGTTTCGGGAAGGCAAATCTGGGTCATGTTATTCTATC 10625
PigTailedMacaque ACCCATTGTTCTGTATATT-TGCTTGATTGTTT----GTTGTTGTTTCGGGAAGGCAAATCTGGGTCATGTTATTCTATC 10541
Bonobo           AGCAATTGTTCTGTATATT-TGCTTGATTGTTT----GGTGGTGTTTCGGCAAGGCAAATCTGGGTCATGTTATTCTGTC 10845
OliveBaboon      ACCCATTGTTCTGTATATT-TGCTTGATTGTTT----GTTGTTGTTTCGGGAAGGCAAATCTGGGTCATGTTATTCTATC 9995
Orangutan        AGCGATTGTTCTGTATATT-TGCTTGATTGTTT----GGTGTTGTTTCGGCAAGGCAGATCTGGGTGATGTTATTCTATG 11193

Majority         TTGACTGGAAGTAGAAGTCCACTGTTTTTTTT-----------------T--TT------AT------------------
                         13530     13540     13550     13560     13570     13580     13590     13600
Human            TTGACTGGAAGTAGAAGTCCACTGTTTTTTTTT----------------------------------------------- 9954
Coquerel'sSifaka TTCCCT--TTTTCCTCCTTTTCTGCTTTCTTG-----------------T--GT------AT------------------ 9326
Ma'sNightMonkey  TTGACTGGAAGTAGAAGTCCACTGTTTTTTTC------------------------------------------------ 9805
VervetAGM        TTGACTGGAAGTAGAAGTCCACTGTTTTTTTTTTTTATTATTTTAATTTTTTTTTTATTATACTTTAAGTTCTAGGGTA 10317
Gorrilla         TTGACTGGCAGTAGAAGTCCACTGTTTTTTTT------------------------------------------------ 12181
GrabEatingMacaqueTTGACTGGAAGTAGAAGTCCACTGTTTTTTTTAAA--ATTATTTTAATTTTTTTT--ATTATACATTAAGTTCTAGGGTA 10657
Macaque          TTGACTGGAAGTAGAAGTCCACTGTTTTTTTTAAA--ATTATTTTAATTTTTTTT--ATTATACATTAAGTTCTAGGGTA 10701
PigTailedMacaque TTGACTGGAAGTAGAAGTCCACTGTTTTTTTTAAA--ATTATTTTAATTTTTTTT--ATTATACATTAAGTTCTAGGGTA 10617
Bonobo           TTGACTGGAAGTAGAAGTCCACTGTTTTTTTT------------------------------------------------ 10877
OliveBaboon      TTGACTGGAAGTAGAAGTCCACTGTTTTTTTTTAA--ATTGTTTTAATTTTTTTT--ATTATACTTTAAGTTCTAGGGTA 10071
Orangutan        TTGACTGGAGGTAGAAGTCAACTGTTTTTTTT------------------------------------------------ 11225

Majority         CATGTG-ACAACGTGCAGGTTTGTTGCTTATGTATAC-TGTGCCATGTTGGTGTGCTGCACCCATTAACTCGTCATTTAC
                         13610     13620     13630     13640     13650     13660     13670     13680
Human            -------------------------------------------------------------------------------- 9954
Coquerel'sSifaka CGAGCACATTTTATTCCATTTTATTTCTTGTTAGCTGATTAGCTGTAACTTTTTGTTATTTTGGTGGCTGCTTCAGGATT 9406
Ma'sNightMonkey  ----------------------------TA-------------------------------------------------- 9807
VervetAGM        CATGTGCACAACGTGCAGGTTTGTTGCTTATGTATACGTGTGCCATGTTGGTGTGCTGCACCCATTAACTCGTCATTTAC 10397
Gorrilla         -------------------------------------------------------------------------------- 12181
GrabEatingMacaqueCATGTGTACAACGTGCAGGTTTGTTGCTTATGTATACATGTGCCATGTTGGTGTGCTGCACCCATTAACTTGTCATTTAC 10737
Macaque          CATGTGTACAACGTGCAGGTTTGTTGCTTATGTATACATGTGCCATGTTGGTGTGCTGCACCCATTAACTCGTCATTTAC 10781
PigTailedMacaque CATGTGCACAACGTGCAGGTTTGTTGCTTATGTATACGTGTGCCATGTTGGTGTGCTGCACCCATTAACTCGTCATTTAC 10697
Bonobo           -------------------------------------------------------------------------------- 10877
OliveBaboon      CATGTGCACAACGTGCAGGTTTGTTGCTTATGTATACGTGTGCCATGTTGGTGTGCTGCACCCATTAACTCGTCATTTAC 10151
Orangutan        -------------------------------------------------------------------------------- 11225
```

```
Majority         ATTAGGTATATCTCCTAATGCTATCCCTCCCC-TCTC------------GAC-----CTGGTGTGTAATATTCCCCTTCC

                         13690     13700     13710     13720     13730     13740     13750     13760

Human            -------------------------------------------------------------------------------- 9954
Coquerel'sSifaka TATAGCATAGTGCTGTAATCTTATCACAATCTTCCCT------------CAA-----TGGATATTTATACCACTTCCAAA 9469
Ma'sNightMonkey  -----------------------------------------------------------ATTATGTAATGTGTTCTCAGC 9828
VervetAGM        ATTAGGTATATCTCCTAATGCTATCCCTCCCC-TCTCTGCACACCCCACGACAGGCCCTGGTGTGTAATATTCCCCTTCC 10476
Gorrilla         -------------------------------------------------------------------------------- 12181
GrabEatingMacaqueATTAGGTATATCTCCTAATGCTATCCCTCCCCCTCTCTGCACACCCCACGACAGGCCCTGGTGTGTAATATTCCCCTTCC 10817
Macaque          ATTAGGTATATCTCCTAATGCTATCCCTCCCCCTCTCTGCACACCCCACGACAGGCCCTGGTGTGTAATATTCCCCTTCC 10861
PigTailedMacaque ATTAGGTATATCTCCTAATGCTATCCCTCCCCCTCTCTGCACACCCCACGACAGGCCCTGGTGTGTAATATTCCCCTTCC 10777
Bonobo           -------------------------------------------------------------------------------- 10877
OliveBaboon      ATTAGGTATATCTCCTAATGCTATCCCTCCCC-TCTCTGGACACCCCACGACAGGCCCTGGTGTGTAATATTCCCCTTCC 10230
Orangutan        -------------------------------------------------------------------------------- 11225

Majority         TGTGTCCAAGTGTTCTCATTGTT-----------------------CA------------------GT------GATAGT

                         13770     13780     13790     13800     13810     13820     13830     13840

Human            -------------------------------------------------------------------------------- 9954
Coquerel'sSifaka TATGTATATACTATCTGATTATT-------------------------------------------AT------AATAGT 9500
Ma'sNightMonkey  AGTTAGAGAGCAACCTCATTCTT-----------------------TG-------------------------------- 9853
VervetAGM        TGTGTCCAGGTGTTCTCATTGTTCATTTCCCACCTACGAGTGAGAACATGTGGTGTTTGGTTTTCTGTTCTTGTGATAGT 10556
Gorrilla         -------------------------------------------------------------------------------- 12181
GrabEatingMacaqueTGTGTCCAAGTGTTCTCATTGTTCAGTTCCCACCTATGAGTGAGAACATGCAGTGTTTGGTTTTCTGTTCTTGTGATAGT 10897
Macaque          TGTGTCCAAGTGTTCTCATTGTTCAGTTCCCACCTATGAGTGAGAACATGCAGTGTTTGGTTTTCTGTTCTTCTGATAGT 10941
PigTailedMacaque TGTGTCCAAGTGTTCTCATTGTTCAGTTCCCACTTATGAGTGAGAACATGCAGTGTTTGGTTTTCTGTTCTTGTGATAGT 10857
Bonobo           -------------------------------------------------------------------------------- 10877
OliveBaboon      TGTGTCCAAGTGTTCTCATTGTTCAGTTCCCACCTATGAGTGAGAACATGCGGTGTTTGGTTTTCTGTTCTTGTGATAGT 10310
Orangutan        -------------------------------------------------------------------------------- 11225

Majority         TTGCTGA---TGGTTTCCAGC------CAT-TCCCTACAA-------------ATCCTTTTTTATGGCTGCATAGTATTC

                         13850     13860     13870     13880     13890     13900     13910     13920

Human            -------------------------------------------------------------------------------- 9954
Coquerel'sSifaka ATGTTG----TGTTATTTACT------TCTATCCTCTTGG----------------CCTTAGTGCTGTTGTTATACATTT 9554
Ma'sNightMonkey  TTAATGA---------------------------CTATGG-------------ATACTCTTGTGTAGTTGCACCACAGTG 9893
VervetAGM        TTGCTGAGAATGGTTTCCAGCTTCATCCATGTCCCTACAAAGGACATGAACTCATCCTTTTTTATGGCTGCATAGTATTC 10636
Gorrilla         -------------------------------------------------------------------------------- 12181
GrabEatingMacaqueTTGCTGAGAATGGTTTCCAGCTTCATCCATATCCCTACAAAGGACATGAACTCATCCTTTTTTATGGCTGCATAGTATTC 10977
Macaque          TTGCTGAGAATGGTTTCCAGCTTCATCCATATCCCTACAAAGGACATGAACTCATCCTTTTTTATGGCTGCATAGTATTC 11021
PigTailedMacaque TTGCTGAGAATGGTTTCCAGCTTCATCCATATCCCTACAAAGGACATGAACTCATCCTTTTTTATGGCTGCATAGTATTC 10937
Bonobo           -------------------------------------------------------------------------------- 10877
OliveBaboon      TTGCTGAGAATGGTTTCCAGCTTCATCCATGTTCCTACAAAGGACATGAACTCATCCTTTTTTATGGCTGCATAGTATTC 10390
Orangutan        -------------------------------------------------------------------------------- 11225
```

```
Majority          CATGGTGTATATGTGCCACATTTTCTTAATCCAGTCTATCATTGATGGAGATTTGGGTTG-GTTCCAAGTCTTTGCTATT

                          13930     13940     13950     13960     13970     13980     13990     14000

Human             -------------------------------------------------------------------------------- 9954
Coquerel'sSifaka  TATT-TCTATGTATGGTATAAAGTACAAACTACATTCTTTTTGTTTGAATAACCAGTTATCTTTTAAAAAGATGTAAACA 9633
Ma'sNightMonkey   TAGTTACTGGATATACCACAATTTAGT------TTCCTACCTTGATGGAGATTTAGGTTTGAGTCCAGTTTGTAGTTATA 9967
VervetAGM         CATGGTGTATATGTGCCACATTTTCTTAATCCAGTCTNNNNNNNNNNGGCTAATTTTTTGTATTTTTAGTAGAGACAGGG 10716
Gorrilla          -------------------------------------------------------------------------------- 12181
GrabEatingMacaqueCATGGTGTATATGTGCCACATTTTCTTAATCCAGTCTATCATTGATGGAGATTTGGGTTG-GTTCCAAGTCTTTGCTATT 11056
Macaque           CATGGTGTATATGTGCCACATTTTCTTAATCCAGTCTATCATTGATGGAGATTTGGGTTG-GTTCCAAGTCTTTGCTATT 11100
PigTailedMacaque  CATGGTGTATATGTGCCACATTTTCTTAATCCAGTCTATCATTGATGGAGATTTGGGTTG-GTTCCAAGTCTTTGCTATT 11016
Bonobo            -------------------------------------------------------------------------------- 10877
OliveBaboon       CATGGTGTATATGTGCCACATTTTCTTAATCCAGTCTATCATTGATGGAGATTTGGGTTG-GTTCCAAGTCTTTGCTATT 10469
Orangutan         -------------------------------------------------------------------------------- 11225

Majority          GTGAATGGTGCCGCAATAAACATAC--G--CAT-TGTC-T-ATA----------------TT----AA----T-GGGTA-

                          14010     14020     14030     14040     14050     14060     14070     14080

Human             -------------------------------------------------------------------------------- 9954
Coquerel'sSifaka  ATAAATGTAATATTTCTACATTTCCTGTAATACAGTTACCCACA----------------TTCACTGAATACTGGGGTGA 9697
Ma'sNightMonkey   ATGTATGGAAATATTTAGAATAGA-------------------------------------------------------- 9991
VervetAGM         TTTCACCATGTTAGCCAGGATGGTCTCGATCTTCTGACCTCGTGATCCACCCATCTTGGCCTCCCAAAGTGCTGGATTAC 10796
Gorrilla          -------------------------------------------------------------------------------- 12181
GrabEatingMacaqueGTGAATGGTGCCGCAATAAACATACCTGTGCATGTGTCTTTATAG------CAGCATGATTTAT--AACCCTTTGGGTAT 11128
Macaque           GTGAATGGTGCCGCAATAAACATACCTGTGCATGTGTCTTTATAG------CAGCATGATTTAT--AACCCTTTGGGTAT 11172
PigTailedMacaque  GTGAATGGTGCCGCAATAAACATACCTGTGCATGTGTCTTTATAG------CAGCATGATTTAT--AACCCTTTGGGTAT 11088
Bonobo            -------------------------------------------------------------------------------- 10877
OliveBaboon       GTGAATGGTGCCGCAATAAACATACGTGTGCATGTGTCTTTATAG------CAGCATGATTTAT--AACCCTTTGGGTAT 10541
Orangutan         -------------------------------------------------------------------------------- 11225

Majority          A--CC--AG-AAT---AT---T---TCA--AT--TATTTCTAGT--TA--T-----AG-AATC-CCA-A------T----

                          14090     14100     14110     14120     14130     14140     14150     14160

Human             -------------------------------------------------------------------------------- 9954
Coquerel'sSifaka  TCCTCTGCAGATTTCTGGAGTTCTCTGTGCACTTCTTTTTTTCTGGTACTTTGACATGAAAACACTGGTGGGT--T---- 9771
Ma'sNightMonkey   -------------------------------------------------------------------------------- 9991
VervetAGM         AGGCGTGAGCCATCATATTCTTTTCCCAGTATTTTATGTCTAGTAACTCGTAAGAAAAAAATGATCAGATAAGTATATTT 10876
Gorrilla          -------------------------------------------------------------------------------- 12181
GrabEatingMacaqueATACCC-AGTAATGGGATCTCTGGGTCAA-ATGGTATTTCTAGTTCTAGATCCTTGAGGAATCGCCACAC-----TGTCT 11201
Macaque           ATACCC-AGTAATGGGATCTCTGGGTCAA-ATGGTATTTCTAGTTCTAGATCCTTGAGGAATCGCCACAC-----TGTCT 11245
PigTailedMacaque  ATACCC-AGTAATGGGATCTCTGGGTCAA-ATGGTATTTCTAGTTCTAGATCCTTGAGGAATCGCCACAC-----TGTCT 11161
Bonobo            -------------------------------------------------------------------------------- 10877
OliveBaboon       ATACCC-AGTAATGGGATCTCTGGGTCAA-ATGGTATTTCTAGTTCTAGATCCTTGAGGAATCGCCACAC-----TGTCT 10614
Orangutan         -------------------------------------------------------------------------------- 11225
```

```
Majority          -------------T-A-------TTT--AG----------AGTGTA--A-C-TTTCTA--T------ATC-T-TCCAGCA

                           14170     14180     14190     14200     14210     14220     14230     14240

Human             -------------------------------------------------------------------------------- 9954
Coquerel'sSifaka  -------------T-A-----GTGTTCTCG----------GACTCTTGATTCTTTCTCAGTTCAAGGAGTGTTCGAGGCT 9822
Ma'sNightMonkey   ----------------------------------------AGTAAA--------------A--------------ATGC- 10002
VervetAGM         TTAATATGCTGATTACAACATTATTTAAATATTTAGAAATAACAAATGTTCAGTGATAAGGAAATGAATCTGTTCATAAA 10956
Gorrilla          -------------------------------------------------------------------------------- 12181
GrabEatingMacaqueTCCACAA--TGGTTGAACCA--GTTTGCAGTCCCACCAGCAGTGTAAAAGCGTTTCTATTTCTCCACATCCTCTCCAGCA 11277
Macaque           TCCACAA--TGGTTGAACCA--GTTTGCAGTCCCACCAGCAGGGTAAAAGCGTTTCTATTTCTCCACATCCTCTCCAGCA 11321
PigTailedMacaque  TCCACAA--TGGTTGAACTA--GTTTGCAGTCCCACCAACAGTGTAAAAGCGTTTCTATTTCTCCACATCCTCTCCAGCA 11237
Bonobo            -------------------------------------------------------------------------------- 10877
OliveBaboon       TCCACAA--TGGTTGAACCA--GTTTGCAGTCCCACCAACAGTGTAAAAGCGTTTCTATTTCTCCACATCCTCTCCAGCA 10690
Orangutan         -------------------------------------------------------------------------------- 11225

Majority          C-TTT----TGTTT-CT-ACTTTTT--TGATCGCCAT--TAA-TGG--T-AGATG--A--T---TG--TG--T-T-ATTT

                           14250     14260     14270     14280     14290     14300     14310     14320

Human             -------------------------------------------------------------------------------- 9954
Coquerel'sSifaka  CAGCTTGATTTCCTTATAGCTTTGCTATGGTCTGTACAATCCAGGGAATAAGCTGAGGCATACATGGATTCACCTTATTT 9902
Ma'sNightMonkey   ---------------------------TATGTCAA--------------------------------------------- 10010
VervetAGM         CATTACAAATGTTTTCTAAATATTTT-TCATATGTGTGAAAAGTAGGATTAAAAATAACTTTGACAAATGCATACAATTT 11035
Gorrilla          -------------------------------------------------------------------------------- 12181
GrabEatingMacaqueCCTTT----TGTTTCCTGACTTTTTAATGATCGCCATTCTAACTGGTGTGAGATGGTATCTCATTG--TGGTTTTGATTT 11351
Macaque           CCTTT----TGTTTCCTGACTTTTTAATGATCGCCATTCTAACTGGTGTGAGATGGTATCTCATTG--TGGTTTTGATTT 11395
PigTailedMacaque  CCTTT----TGTTTCCTGACTTTTTAATGATCGCCATTCTAACTGGTGTGAGATGGTATCTCATTG--TGGTTTTGATTT 11311
Bonobo            -------------------------------------------------------------------------------- 10877
OliveBaboon       CCTTT----TGTTTCCTGACTTTTTAATGATCGCCATTCTAACTGGTGTGAGATGGTATCTCATTG--TGGTTTTGATTT 10764
Orangutan         -------------------------------------------------------------------------------- 11225

Majority          G--TTT----CTGA---CTG--GG-CAGTG---------------TTTTC------GT-TGT-GG-TGC-T--AT--C-T

                           14330     14340     14350     14360     14370     14380     14390     14400

Human             -------------------------------------------------------------------------------- 9954
Coquerel'sSifaka  GT-TTT----CTGACTTTCAGGGATCATTGT--------------TTATCATTGCCTGATGTCCAGCGTCTTGAAAACCT 9963
Ma'sNightMonkey   --------------------------AT---------------------------------------------------- 10012
VervetAGM         ATATGTGTTCCCAAAAACTGGAGGGCAGTATACCAAAATAGAGTAGTTCTCTTCAGGTTGATGGGATTCGTTTTTCATAA 11115
Gorrilla          -------------------------------------------------------------------------------- 12181
GrabEatingMacaqueGCATTTCT--CTGATGGCTGATGGCCAGTG----ATGAT-GAGCATTTTCTCACGTGTCTGTTGGCTGCATAAATGTCTT 11424
Macaque           GCATTTCT--CTGATGGCTGATGGCCAGTG----ATGAT-GAGCATTTTCTCACGTGTCTGTTGGCTGCATAAATGTCTT 11468
PigTailedMacaque  GCATTTCT--CTGATGGCTGATGGCCAGTG----ATGAT-GAGCATTTTCTCACGTGTCTGTTGGCTGCATAAATGTCTT 11384
Bonobo            -------------------------------------------------------------------------------- 10877
OliveBaboon       GCATTTCT--CTGATGGCTGATGGCCAGTG----ATGAT-GAGCATTTTCTCATGTGTCTGTTGGCTGCATAAATGTCTT 10837
Orangutan         -------------------------------------------------------------------------------- 11225
```

```
Majority          -TTTTGAGAAGTGTCTGTTCATAT-CT-CG-T-TCT----T-A-AG-G-ACAGGT--------A----------------

                          14410     14420     14430     14440     14450     14460     14470     14480

Human             -------------------------------------------------------------------------------- 9954
Coquerel'sSifaka  ATTGTTCTATATGTTTGCTTGTTTTTTCAGTTGTTT----CAACAGGGCAAATCT--------A---------------- 10015
Ma'sNightMonkey   -----GAAATTTATTTATTTA--T----------TT--------------ATTT-------------------------- 10035
VervetAGM         TTCTTTATATAT-TTTGTCTATATTCCACATTTTA-AGTATTTTATGATAAAGTTATTAATATAGGTATTAATA--TTGC 11191
Gorrilla          -------------------------------------------------------------------------------- 12181
GrabEatingMacaque CTTTTGAGAAGTGTCTGTTCATATCCTTCGCTCTCTACCATCAGAGTGAACAGGTATGGCAGAAAATTTTTGCAATCTAC 11504
Macaque           CTTTTGAGAAGTGTCTGTTCATATCCTTCGCTCTCTACCATCAGAGTGAACAGGTATGGCAGAAAATTTTTGCAATCTAC 11548
PigTailedMacaque  CTTTTGAGAAGTGTCTGTTCATATCCTTCGCTCTCTACCATCAGAGTGAACAGGTATGGCAGAAAATTTTTGCAATCTAC 11464
Bonobo            -------------------------------------------------------------------------------- 10877
OliveBaboon       CTTTTGAGAAGTGTCTGTTCATATCCTTCGCTCTCTACCATCAGAGTGAACAGGTATGGCAGAAAATTTTTGCAATCTAC 10917
Orangutan         -------------------------------------------------------------------------------- 11225

Majority          --------------------------------CT-CAAA-AACTC---C-AA-TT---AG-A-AAA-C-ACCCCATCAAA

                          14490     14500     14510     14520     14530     14540     14550     14560

Human             ----------------------------------------------------------------------A--------- 9955
Coquerel'sSifaka  --------------------------------CTTCATGTTACTCTGTCTTGACTGAAAGTAGAAGTCCACCTTTTTTTA 10063
Ma'sNightMonkey   ----------------------------------------------------------------------ATTTATTTTA 10045
VervetAGM         TTAATTGATTAAACACTGACATTTTCTTGTTTCTTTAAAAAACTTTGCATAACTT---GATATAAATCTTTTTAATTGCA 11268
Gorrilla          ----------------------------------------------------------------------A--------- 12182
GrabEatingMacaque TCATCTGACAAAGGGCTAATAT---CCAGAATCTACAAAGAACTCAAACAAATTTACAAGAAAAAAACAACCCCATCAAA 11581
Macaque           TCATCTGACAAAGGGCTAATAT---CCAGAATCTACAAAGAACTCAAACAAATTTACAAGAAAAAAACAACCCCATCAAA 11625
PigTailedMacaque  TCATCTGACAAAGGGCTAATAT---CCAGAATCTACAAAGAACTCAAACAAATTTACCAGAAAAAAACAACCCCATCAAA 11541
Bonobo            ----------------------------------------------------------------------A--------- 10878
OliveBaboon       TCATCTGACAAAGGGCTAATAT---CCAGAATCTACAAAGAACTCAAACAAATTTACAAGAAAAAAACAACCCCATCAAA 10994
Orangutan         ----------------------------------------------------------------------A--------- 11226

Majority          AAGT--GCAAAG-------------------------AATTACATAATATGGTCTCAGCAGTTATAGT-TCTACCTCATT

                          14570     14580     14590     14600     14610     14620     14630     14640

Human             --------------------------------------ATTACATAATATGGTCTCAGCAGTTGTAGA-TCTACCTCATT 9996
Coquerel'sSifaka  AAAAAAAAAAAG-------------------------AATTGCATAAAATGGTCTCAGCAGTTACAGGGTCTACTTCATA 10118
Ma'sNightMonkey   ATTT--GTACAC--------------------------AATAGAAATT-TATTTCTCACAGTTCTGGT-GGCTGGGAAGT 10095
VervetAGM         AAAAAAGCACATAAAATTTACCGTCTTAACCATTTTTAAGTGTACACT-TAAATTGTTCAGTTATTCT-TATACGTACTT 11346
Gorrilla          --------------------------------------ATTACATAATATGGTCTCAGCAGTTGTAGA-TCTACCTCATT 12223
GrabEatingMacaque AAGTGGGCAAAGGATATGAACAGACACTGTTTTTTAAAATTACATAATGTGGTCTCAGCAGTTATAGT-TCTGCCTCATT 11660
Macaque           AAGTGGGCAAAGGATATGAACAGACACTGTTTTTTAAAATTACATAATGTGGTCTCAGCAGTTATAGT-TCTGCCTCATT 11704
PigTailedMacaque  AAGTGGGCAAAGGATATGAACAGACACTGTTTTTTAAAATTACATAATGTGGTCTCAGCAGTTATAGT-TCTGCCTCATT 11620
Bonobo            --------------------------------------ATTACATAATATGGTCTCAGCAGTTGTAGA-TCTACCTCATT 10919
OliveBaboon       AAGTGGGCAAAGGATATGAACAGACACTGTTTTTTAAAATTACATAATGTGGTCTCAGCAGTTATAGT-TCTGCCTTATT 11073
Orangutan         --------------------------------------ATTACGTAATATGGTCTCAGCAGTTGCAGA-TCTACCTCATC 11267
```

```
Majority          CTTTGTTAATGACAATGGAGGATACTCT-TGTGTGGCTGCAC-CACAGTGTAGTTACTGGA----CATACCACAATTTAG
                          14650     14660     14670     14680     14690     14700     14710     14720
Human             CTTTGTTAATGACAATGGAGGATACTC---GTGTGGCTGCAC-CACAGTGTAGTTACTGGA----CATACCACAATTTAG 10068
Coquerel'sSifaka  CTTTGTTAACGACAACAGAGAATACTCT---CGTGTATGTAC-CACAGTGTAGTTACTGGA-----------CATACACA 10183
Ma'sNightMonkey   CCAAAATCAAGGTATTGGCAGATTCAGTGTCTGCTGAGGGCC-CAGTCTCTGCTTACAAGA---TGGTATCTTATTGCTA 10171
VervetAGM         AATAAT--GCCACATTGCGAAACCCATC-TCCAGAACTTTTTTCATTTTGGAGA-ACTGAAACTCTATACTGATTTTAAA 11422
Gorrilla          GTTTGTTAATGACAATGGAGGATACTCT-TGTGTGGCTGCAG-CACAGTGTAGTTACTGGA----CATACCACAATTTAG 12297
GrabEatingMacaqueCTTTGTTAATGACAATGGAGGATACTCT-TGTGTGACTGCAC-CACAGTGTAGTTACTGGA----CATACCACAATTTAG 11734
Macaque           CTTTGTTAATGACAATGGAGGATACTCT-TGTGTGACTGCAC-CACAGTGTAGTTACTGGA----CATACCACAATTTAG 11778
PigTailedMacaque  CTTTGTTAATGACAATGGAGGATACTCT-TGTGTGACTGCAC-CACAGTGTAGTTACTGGA----CATACCACAGTTTAG 11694
Bonobo            CTTTGTTAATGACAATGGAGGATACTC---ATGTGGCTGCAC-CACAGTGTAGTTACTGGA----CATACCACAATTTAG 10991
OliveBaboon       CTTTGTTAATGACAATGGAGGATACTCT-TGTGTGACTGCAC-CACAGTGTAGTTACTGGA----CATACCACAATTTAG 11147
Orangutan         CTTTTTTAATGACAATGGAGGATACTGT-TGTGTGGCTGCAC-CACAGTGCAGTTACTGGA----CATACCACAATTTAG 11341

Majority          TTTCCTACATTGATGGACATTTAGGT---TTGATTCCAGTTTGTTGT-------G-TATAACGTGTGGACATATTTAGAA
                          14730     14740     14750     14760     14770     14780     14790     14800
Human             TTTCCTACATCGATGGACATTTAGGT---TTGATTCCAGTTTGTTGT---------TATAACATGTGGACATATTTAGAA 10136
Coquerel'sSifaka  GTTCCTA---TGTTGGATATTTTGGG---TTGATTTCCATTTGTAGT---------TATAAAGTGTGGGAATATTTAGAG 10248
Ma'sNightMonkey   TGTCCTCATATGGTAGAAAGGGACAAAAAGGGAACACTGTATCCTCACAT----GGCAGAAGAGACTGAAGGACC--AGG 10245
VervetAGM         ACTCTCCCTTTTGTCCTTGCCCTGGTAACCAGCATTCTGTTTCCTGTGTCTGTGAATTTGACTACTTTAAATACCTCATA 11502
Gorrilla          TTTCCTACATTGATGGACATTTAGGT---TTGATTCCAGTTTGTCAT---------TATAATGTGTGGACATACTTAGAA 12365
GrabEatingMacaqueTTTCCTACATTGATGGACATTTAGGT---TTGATTCCAGTTTGTGGT-----TGGTTATAACGTGTGGACATATTTAGAA 11806
Macaque           TTTCCTACATTGATGGACATTTAGGT---TTGATTCCAGTTTGTGGT-----TAGTTATAACGTGTGGACATATTTAGAA 11850
PigTailedMacaque  TTTCCTACATTGATGGACATTTAGGT---TTGATTCCAGTTTGTGGT-----TAGTTATAACGTGTGGACATATTTAGAA 11766
Bonobo            TTTCCTACATTGATGGACATTTAGGT---TTGATTCCAGTTTGTTGT---------TATAACGTGTGGACATATTTAGAA 11059
OliveBaboon       TTTCCTACATTGATGGACATTTAGGT---TTGATTCCAGTTTGTGGT-----TAGTTATAACATGTGGACGTATTTAGAA 11219
Orangutan         TTTCCTACATTGCTGGACATTTAGGT---TTGATTCCAGTTTGTCAT---------TATAATGTGTGGACATATTTAGAA 11409

Majority          TAGAAGTAAAAATGCTGTGTCAA--ATGAAATTTATACTTAAAGAGTTA----CTGAAAACCACAATGCCAGAACACTCA
                          14810     14820     14830     14840     14850     14860     14870     14880
Human             TAGAAGTAAAAATGCTGTGTCAA--ATGAAATTTATACTTAAAGAGTTA----CTGAAAACCACAATGCCAGAACACTCA 10210
Coquerel'sSifaka  TAAAAGTAAATATGCTATGTCAA--ATGAAATTTATACTGAAAAAGTTA---CTTAAAAACCATAATGCCATAACACTCA 10323
Ma'sNightMonkey   CAGCTCTAGAGTCTTCTTTTTTT--TTGGAATTTATTCTTAAAGAGTGA----TTGTAAACTACAATGCCAGAATATTCA 10319
VervetAGM         TAAGTGGAATTATACAATATAGGTTAAGCATCCCTAATCTGAAAATCCAAAGTCTGAATGCTACAAAATGTGAA-ACTT- 11580
Gorrilla          TAGAAGTAAAAATGCTGTGTCAA--ATGAAATTTATACTTAAAGAGTTA----CTGAAAACCACAATGCCAGAACACTCA 12439
GrabEatingMacaqueTAGAAGTAAAAATGCTGTGTCAA--AAGAAATTTATGCTTAAAGAGTTA----CTAGAAACCACACTGCCAGAACACTCA 11880
Macaque           TAGAAGTAAAAATGCTGTGTCAA--AAGAAATTTATGCTTAAAGAGTTA----CTAGAAACCACACTGCCAGAACACTCA 11924
PigTailedMacaque  TAGAAGTAAAAATGCTGTGTCAA--AAGAAATTTATGCTTAAAGAGTTA----CTAGAAACCACACTGCCAGAACACTCA 11840
Bonobo            TAGAAGTAAAAATGCTGTGTCAA--ATGAAATTTATACTTAAAGAGTTA----CTGAAAACCACAATGCCAGAACACTCA 11133
OliveBaboon       TAGAAGTAAAAATGCTGTGTCAA--AAGAAATTTATGCTTAAAGAGTTA----CTAGAAACCACACTGCCAGAACACTCA 11293
Orangutan         TAGAAGTAAAAATGCTGTGTCAA--ATGAAATTTATACTTAAAGAGTTA----CTGAAAACCACAATGCCAGAACACTCA 11483
```

```
Majority         TTTGTTTTTTACAAGTGTGTATGGGAGTGCCTGTTTTTCCATACCCTTGGCAATTCTAGGTTTTATCAAAATTTA-GAAT

                         14890     14900     14910     14920     14930     14940     14950     14960

Human            TTTGTTTTTCACAAGTGTGTATGGGAGTGCCTATTTTTCCATACCCTTGGCAATTCTAGGTTTTATCAAAATTTAAGAAT 10290
Coquerel'sSifaka TTTGTTTTCTGTAACTGTGTATGGGAGTGCCTGTTTTTTCATACCCTTGCCAATTCTAGGTTTTATCAAAATTTA-GAAT 10402
Ma'sNightMonkey  TTTGTTTTTCACAAGTGTGTATGGGAGTGCCTGTTTTTCCATGCCCTTGGCAATTCTAGGTTTTATCAAAAT-------T 10392
VervetAGM        TTTGTTTTATTTAGTTTAGTTTAGTTTAGTTTAGTTTAGTTTAGTTTAGTTTAGTTTAGTTTTTG-----AGATG-GAGT 11654
Gorrilla         TTTGTTTTTCACAAGTGTGTATGGGAGTGCCTATTTTTCCATACCCTTGGCAATTCTAGGTTTTATCAAAATTTAAGAAT 12519
GrabEatingMacaqueTTTGTTTTTTACAGGTGTGTATGGGAGTGCCTGTTTTTCCATACCCTTGGCAATTCTAGGTTTTATCAAAATTTA-GAAT 11959
Macaque          TTTGTTTTTTACAGGTGTGTATGGGAGTGCCTGTTTTTCCATACCCTTGGCAATTCTAGGTTTTATCAAAATTTA-GAAT 12003
PigTailedMacaque TTTGTTTTTTACAGGTGTGTATGGGAGTGCCTGTTTTTCCATACCCTTGGCAATTCTAGGTTTTATCAAAATTTA-GAAT 11919
Bonobo           TTTGTTTTTCACAAGTGTGTATGGGAGTGCCTATTTTTCCATACCCTTGGCAATTCTAGGTTTTATCAAAATTTAAGAAT 11213
OliveBaboon      TTCGTTTTTTACAGGTGTGTATGGGAGTGCCTGTTTTTCCATACCCTTGGCAATTCTAGGTTTTATCAAAATTTA-GAAT 11372
Orangutan        TTTGTTTTTCACAAGTGTGTATAGGATTGCCTATTTTTCCATACCCTTGGCAATTCTAGGTTTTATCAAAATTTA-GAAT 11562

Majority         ATTAGCTTA-TTTTACAC-TAAAAATGTATTTTGTAATTTATATTTGAAATGGGTGAGGTTTTT-CAAATTTG-GCTAT

                         14970     14980     14990     15000     15010     15020     15030     15040

Human            ACTAGCTTA-TTTTACAC-TAAAAAT-TATTTTGTAATTTATATTTGAAATGGGTGAGGTTTTT-CAGATTTG-GTTAT 10365
Coquerel'sSifaka ATTAGCTAA-TTTTAGTTTTAAAAAAGTATTTTATAATTTATATTTGAAATGAGTGAGATTTTTTTAAATTTG-GCTTT 10480
Ma'sNightMonkey  ATTAGCTAAATTTTACAC--TGAAATGTATTTTATAATTTATATTTGAAATGGGTGAGGTTTTT-CAAATTTG-GCTAT 10468
VervetAGM        CTTGCTCTG-TCGCCCAGGCTGGAGTGAGTGGCGCAATCTCGGCTCACTGCAAGCTCCAACCCC--CAGGTTCATGCCAT 11731
Gorrilla         ACTAGCTTA-TTTTACAC-TAAAAAT-TATTTTGTAATTTATATTTGAAATGGGTGAGGTTTTT-CAAATTTG-GCTAT 12594
GrabEatingMacaqueATTAGATTA-TTTTACAC-TAAAAATGTATTTCGTAATTTATATTTGAAATGAGTGAAGTTTT--CAAATTTG-GCTAT 12034
Macaque          ATTAGATTA-TTTTACAC-TAAAAATGTATTTCGTAATTTATATTTGAAATGAGTGAAGTTTT--CAAATTTG-GCTAT 12078
PigTailedMacaque ATTAGATTA-TTTTACAC-TAAAAATGTATTTCGTAATTTATATTTGAAATGAGTGAAGTTTT--CAAATTTA-GCTAT 11994
Bonobo           ACTAGCTTA-TTTTACAC-TAAAAAT-TATTTTGTAATTTATATTCTGAAATGGGTGAGGTTTTT-CAAATTTG-GCTAT 11288
OliveBaboon      ATTAGATTA-TTTTACAC-TAAAAATGTATTTCGTAATTTATATTTGAAATGAGTGAAGTTTT--CAAATTTG-GCTAT 11447
Orangutan        ACTAGCTTA-TTTTACAG-TAAAAAT-TATTTTGTAATTTATATTTGAAATGGGTGAGGTTTTT-CAAATTTG-GCTAT 11637

Majority         TTATATTTC--ATTTTCTGTGAACTGCCTGTTTGTGTA-----------CTGGACCTAGTTTAATGGTCATTTTTATC--

                         15050     15060     15070     15080     15090     15100     15110     15120

Human            TTATATTTC--ATTTTCTGTGAACTGCCTGTTTGTGTA-----------CTGGACCTAGTT------------------- 10413
Coquerel'sSifaka TCATGTTTTT-ATTTTCTGTGAACTGCCTCTTTGTATA-----------CCTAACCTATTTTAATGATAATCTTTATTT- 10547
Ma'sNightMonkey  TTATATTTC--ATTTTCTGTGAACTGTCTGTTTGTCTG-----------CTGGACCTAGTTTAATGGTCATCTTTAAT-- 10533
VervetAGM        TCTCTTCTCTCAGCCTCCATCGAGTAGCTGGGACTATAAGCGGCTGCCACCACGCCCGGCTAATTTTTTTGTTTTTTT-- 11809
Gorrilla         TTATATTTC--ATTTTCTGTGAACTGCCTGTTTGTGTA-----------CTGGACCTATTTTAATGGTCATCTTTATCTT 12661
GrabEatingMacaqueCTGAATTTC--ATTTTCTGTCAACTGCTTGTTTGTGTA-----------CTGGACCTAGTTTAATGGTCATTTTTATC-- 12099
Macaque          CTGAATTTC--ATTTTCTGTCAACTGCTTGTTTGTGTA-----------CTGGACCTAGTTTAATGGTCATTTTTATC-- 12143
PigTailedMacaque CTGAATTTC--ATTTTCTGTCAACTGCTTGTTTGTGTA-----------CTGGACCTAGTTTAATGGTCATTTTTATC-- 12059
Bonobo           TTATATTTC--ATTTTCTGTGAACTGCCTGTTTGTGTA-----------CTGGACCTAGTTTAATGGTCATCTTTATCTT 11355
OliveBaboon      CTGAATTTC--ATTTTCTGTCAACTGCTTGTTTGTGTA-----------CTGGACCTAGTTTAATGGTCATTTTTATC-- 11512
Orangutan        TTATATTTC--ATTTTCTGTGAACTGCCTGTTTGTGTA-----------CTGGACCTAGTTTAATGGTCATCTTTATCTT 11704
```

```
Majority        ---ACAGTTCTTTGTATAAAAAA--ATTGATTTCATAT-----GCATTGTAGGTATTTTTT--CTTTGAGGTTTTCT---

                         15130     15140     15150     15160     15170     15180     15190     15200

Human           -----AATTC---------------------------------------------------------------------- 10418
Coquerel'sSifaka GTTGTAGCTCTTTGTATAAAAGACAGTTGATTTCATAT-----GCATTGTAGGTGTTTTT---CTTTGAGTTTTTAT--- 10616
Ma'sNightMonkey --CACAGTTCCTTGTATAAAAAA--ATTCATTTTGTAT-----GCATTGTAGATATTTTTT--CCTTGAGGTTTTTTGTT 10602
VervetAGM       ---GTTTTTCTTTTAGTAGAGAT--GGGGTTTCACTGTAGCCAGCATGGTCTCTATCTCCTGACCTCGTGATCCGCC--- 11881
Gorrilla        ATCACAGTTCTTTGTGTAAAAAA---TTGATTTCATAT-----GCCTTATAGATATTTTTT--CTTTGAGTTTTTTT--- 12728
GrabEatingMacaque---ACAGTTCTTTGTATAAAAAA--ATTGATTTCATAT-----GCATTGTAGGTATTTTTT--CTTTGAGGTTTTCT--- 12164
Macaque         ---ACAGTTCTTTGTATAAAAAA--ATTGATTTCATAT-----GCATTGTAGGTATTTTTT--CTTTGAGGTTTTCT--- 12208
PigTailedMacaque ---ACAGTTCTTTGTATAAAAAA--ATTGATTTCATAT-----GCATTGTAGGTATTTTTT--CTTTGAGGTTTTCT--- 12124
Bonobo          ATCACAGTTCTTTGTGTAAAAAA--ATTGATTTCATAT-----GCATTGTAGATATTTTTT--CTTTGAGTTTTTTT--- 11423
OliveBaboon     ---ACAGTTCTTTGTATAAAAAA--ATTGATTTCATAT-----GCATTGTGGGTATTTTTT--CTTTGAGGTTTTCT--- 11577
Orangutan       ATCACAGTTCTTTGTATAAAAAA--ATTGATTTCATAC-----GCATTGTAGATATTTTTT--ATTTGAGTTTTTTT--- 11772

Majority        --------TTTTTGCTTTTTTGATATTCTTTGT--TATAGTTTTAGGC--------ATATATATA---TTTGAATTAAGT

                         15210     15220     15230     15240     15250     15260     15270     15280

Human           --------------------------------------------------------AAATATATA-TATTTGAATTAAGT 10441
Coquerel'sSifaka --------------ATTTTTTGATGTTCTTTGT----TAATCTTGGGCATG-----ATATATACT---TCTGAATTACTA 10670
Ma'sNightMonkey TTGTTTTGTTTTTACTTTTTTGACATTCTTTG---TATAGTCTTAGAC--------ATACACATA---TTTGAATTGAGT 10668
VervetAGM       --------TGCCTCGGCCTTCCAAAGTGCTGGGATTACAGGCCCGAGCCACC----ACACCTGAAACTTTTTGAACACTT 11949
Gorrilla        --------TTTTTGCTTTTTTGATATTCTTTG---TATAGTTTTAGGCATAT----ATATATATA-TATTTGAATTAAGT 12792
GrabEatingMacaque--------TTTTTGCTTTTTTGATATTCTTTGTTGTATAGTTTTAGGC--------ATATATATA---TTTTGATTAAGT 12225
Macaque         --------TTTTTGCTTTTTTGATATTCTTTGTTGTATAGTTTTAGGC--------ATATATATA---TTTTGATTAAGT 12269
PigTailedMacaque --------TTTTTGCTTTTTTGATATTCTTTGTTGTATAGTTTTAGGC--------ATATATATA---TTTTGATTAAGT 12185
Bonobo          --------T----GCTTTTTTGATATTCTTTG---TATAGTTTTAGGCATATATATATATATATA-TATTTGAATTAAGT 11487
OliveBaboon     --------TTTTTGCTTTTTTGATATTCTTTGTTGTATAGTTTTAGGC--------ATATATATA---TTTTGATTAAGT 11638
Orangutan       --------T----GCTTTTTTGATATTCTTTG---TATAGTTTTAGGC--------ATATATATA-TATTTGAATTAAGT 11828

Majority        GATTTTGCT-TTTAAAATCCATTTGATATGTAAACATTTTTAGCCTCAGCTTCTTCATCTTTAAAAT--TAGGATTTTA-

                         15290     15300     15310     15320     15330     15340     15350     15360

Human           GATTTTATTGTTTAAAGTCCATTTGATGTGTAAACATTTTTAGCCTCAGCTTCTTCACCTTTTAAAT--TAGGATTTTA- 10518
Coquerel'sSifaka AATATTTTT--TTAAAATCTATTTGATACTTTAACATTTTTAGCTTCAGTTTCTTCATCTTTAAAATGGGGGGTTAATAA 10748
Ma'sNightMonkey GATTTCACTTTTTAAAATCCATTTGATAATTAAA-ATTTTTAACCTCAGCTTCTTCATCTTTAAAAT--TGGGATTTTAA 10745
VervetAGM       ACACGTGAT-GCTCAAAGGAAGTGCTTAGTGGAGCATTTTGGATTTTAGATT-TTCAGATTAGGAATGCTCAGGTTGTGT 12027
Gorrilla        GATTTCACTGTTTAAAATCCATTTGATGTGTAAACATTTTTAGCCTCAGCTTCTTCACCTTTAAAAT--TAGGATTTTA- 12869
GrabEatingMacaqueGATTTTGCT-TTTAAAATCCATTTGATATGTAAACATTTTTAGCCGCAGCTTCTTCATCTTTAAAAT--TAGGATTTTAT 12302
Macaque         GATTTTGCT-TTTAAAATCCATTTGATATGTAAACATTTTTAGCCGCAGCTTCTTCATCTTTAAAAT--TAGGATTTTAT 12346
PigTailedMacaque GATTTTGCT-TTTAAAATCCATTTGATATGTAAACATTTTTAGCCGCAGCTTCTTCATCTTTAAAAT--TAGGATTTTA- 12261
Bonobo          GATTTCACTGTTTAAAGTCCATTTGATGTGTAAACATTTTTAGCCTCAGCTTCTTCACCTTTAAAAT--TAGGATTTTA- 11564
OliveBaboon     GATTTTGCT-TTTAAAATCCATTTGATATGTAAACATTTTTAGCCGCAGCTTCTTCATCTTTAAAAT--TAGGATTTTA- 11714
Orangutan       GATTTCACTTTTTAAAATCCATTTGATGTGTAAACGTTTTTAGCCTCAGCTTCTTCATCTTTAAAAT--TAGGATTTTA- 11905
```

```
Majority           --------------------------------------------------------------------------------
                            15370     15380     15390     15400     15410     15420     15430     15440
Human              -------------------------------------------------------------------------------- 10518
Coquerel'sSifaka   TTAT-GA---------------------------A----------------------------------A---------- 10756
Ma'sNightMonkey    TAGTTGT------------------------------------------------------------------------- 10752
VervetAGM          TTGTCCTTTTGTGACTGGTTTACATTCACTTAACAATGTTCTCAAGGTCCATCCGTGTTGTAGCATGTGACAAGATTTCC 12107
Gorrilla           -------------------------------------------------------------------------------- 12869
GrabEatingMacaque  TTTTTATTTTATTTATTTATTTTTTTTGAGACGGAGTCTCGCTCTGTCGCCCAGGCTGGAGTGCAGTGGCCGGGTCTCAG 12382
Macaque            TTTTTATTTTATTTATTTATTTTTTTTGAGACGGAGTCTCGCTCTGTCGCCCAGGCTGGAGTGCAGTGGCCGGGTCTCAG 12426
PigTailedMacaque   -------------------------------------------------------------------------------- 12261
Bonobo             -------------------------------------------------------------------------------- 11564
OliveBaboon        -------------------------------------------------------------------------------- 11714
Orangutan          -------------------------------------------------------------------------------- 11905

Majority           --------------------------------------------------------------------------------
                            15450     15460     15470     15480     15490     15500     15510     15520
Human              -------------------------------------------------------------------------------- 10518
Coquerel'sSifaka   ---------------------------------------------------------------ATTAAA----------- 10762
Ma'sNightMonkey    -----------------------------------GAA------T-ATTAAATGTGATTGTGCATAAAAA---------- 10780
VervetAGM          TCCGTTTTTAAGGCTACATAGCATTCCATTGTGGTACACAAAAGTTGATTTGAAGCCCCCTGTGTCATCACTCATTCTTC 12187
Gorrilla           -------------------------------------------------------------------------------- 12869
GrabEatingMacaque  CTCACTGCAAGCTCCGCCTCCCGGGTTCA-----TTCTCCTGCCTCAGCCTCCCGAGTAGCTGGGACTACAGGC-CTCAC 12456
Macaque            CTCACTGCAAGCTCCGCCTCCCGGGTTCACGCCATTCTCCTGCCTCAGCCTCCGGAGTAGCTGGGACTACAGGCGCCCGC 12506
PigTailedMacaque   ----------------------------A--------------------------------------------------- 12262
Bonobo             -------------------------------------------------------------------------------- 11564
OliveBaboon        -------------------------------------------------------------------------------- 11714
Orangutan          -------------------------------------------------------------------------------- 11905

Majority           --------------------------------------------------------------------------------
                            15530     15540     15550     15560     15570     15580     15590     15600
Human              -------------------------------------------------------------------------------- 10518
Coquerel'sSifaka   -------------------T------------------------------------------------------------ 10763
Ma'sNightMonkey    -------------------------------------------------------------------------------- 10780
VervetAGM          CATAACAAAATACCATAGACTGAGTGACTTAAAAAACAGAAATTCATTTTCTCACAGTTCTGGAAACTGGGAAGTCCAAG 12267
Gorrilla           -------------------------------------------------------------------------------- 12869
GrabEatingMacaque  CACC-----CGGCTAGTTTTTTGTATTTTTTTTTTTAGTAGAGACAGGGTTTCACCATGTTAG---CCAGGATGGTCTCG 12528
Macaque            CACCTCGCCCGGCTAGTTTTTTGTATTTTTTTTTTTAGTAGAGACAGGGTTTCACCATGTTAG---CCAGGATGGTCTCG 12583
PigTailedMacaque   -------------------------------------------------------------------------------- 12262
Bonobo             -------------------------------------------------------------------------------- 11564
OliveBaboon        -------------------------------------------------------------------------------- 11714
Orangutan          -------------------------------------------------------------------------------- 11905
```

```
Majority          --------------------------------------------------------------------------------
                           15610     15620     15630     15640     15650     15660     15670     15680
Human             -------------------------------------------------------------------------------- 10518
Coquerel'sSifaka  -------------------------------------------------------------------------------- 10763
Ma'sNightMonkey   -------------------------------------------------------------------------------- 10780
VervetAGM         ATCAAGGTACCAGCAAGGCAACTTTCCTTCTGAGGCCACTTCTCTTGAAGCAAGGCCATCATTTTATTTTGTGTGCCTCA 12347
Gorrilla          -------------------------------------------------------------------------------- 12869
GrabEatingMacaque ATCTCCTGACCTTGTGATCCGCCCGTCTCGGCCTCCCAAAGTTCTGGGATTACAGGCTTGAGCCACAGCGCCTGGCTAAA 12608
Macaque           ATCTCCTGACCTTGTGATCCGCCCGTCTCGGCCTCCCAAAGTGCTGGGATTACAGGCTTGAGCCACAGCGCCCGGCTAAA 12663
PigTailedMacaque  ----------------------------------------------------------T--------------------- 12263
Bonobo            -------------------------------------------------------------------------------- 11564
OliveBaboon       -------------------------------------------------------------------------------- 11714
Orangutan         -------------------------------------------------------------------------------- 11905

Majority          ------------ATAGTTC-CACAAACTAGGTCCTT-GTTAATGTTAATGTAT-TTACATTT---------AGAATATCC
                           15690     15700     15710     15720     15730     15740     15750     15760
Human             ------------ATAGTTC-CACAAACTAGGTC------TAATGTTAATGTAT-TTACATTT---------AGAATATCC 10569
Coquerel'sSifaka  ------------GTGATTAATGCATAAAAAGTATTTTCCTAA-GTTAATGTACGCTACATAT---------AGAATATAC 10821
Ma'sNightMonkey   ------------GTATTTTCTGCAAACTAGGTCC----TCAGTGTTAATATGT-TTACACTACATTT----AGACTATCC 10839
VervetAGM         CATGACCTTTTGTTTGTCAGCACACTTTGGGGGAGTGGGTGGCACAAGCTCTC-TTGTGTTTCTTCTCATAAAGGCATTA 12426
Gorrilla          ------------ATAGTTC-CACAAACTAGGTC------TAATGTTAATGTAT-TTACATTT---------AGAATATCC 12920
GrabEatingMacaque ATTAGGATTTTAATAGTTC-CACAAACTAGGTCCTTAGTTAATGTTAATGTAT-TTGCATTT---------AGAATATCC 12677
Macaque           ATTAGGATTTTAATAGTTC-CACAAACTAGGTCCTTAGTTAATGTTAATGTAT-TTGCATTT---------AGAATATCC 12732
PigTailedMacaque  --------------AGTTC-CACAAACTAGGTCCTTAGTTAATGTTAATTTAT-TTGCATTT---------AGAATATCC 12318
Bonobo            ------------ATAGTTC-CACAAACTAGGTC------TAATGTTAATGTAT-TTACATTT---------AGAATATCC 11615
OliveBaboon       ------------ATAGTTC-CACAAACTAGGTCCTTAGTTAATGTTAATGTAT-TTGCATTT---------AGAATATCC 11771
Orangutan         ------------ATAGTTC-CACAAACTAGGT--------AG-GTTAATGTAT-TTACATTT---------AGAATATCC 11953

Majority          AA-GTTTTTATGGCTTTAGGATACAAATGAAATCTCATTTTCCA-TTATTTTATAAATTTGCTGTA----TTCAG---CT
                           15770     15780     15790     15800     15810     15820     15830     15840
Human             AA-GTTTTTATGGCTTTAGGATACAAATGAAATCTCATTTTCCA-TTATTTTATAAATTTGCTGTA----TTCAG---CT 10640
Coquerel'sSifaka  AA--ATTTTATGGCTTTAGGATAT-GTTGAAATCTCATTT---AATTTTTTAAAAATAAAGTTGTACTTATTCAA----- 10890
Ma'sNightMonkey   AA-GTTTTTATGGCTTTAGGATACAAATGAAATCTCATTTTCCA-TTATTTTATAAATTTGTTGTAGTTATTCAGAATCT 10917
VervetAGM         ATTAATATGAAGGCCCTACTCTCATGATCTCATAAAATCCTCCAGTTAACTCCTAAAGGCCCTATT----TCTAA---AT 12499
Gorrilla          AA-GTTTTTATGGCTTTAGGATACAAATGAAATCTCATTTTCCA-TTATTTTATAAATTTTCTGTA----TTCAG---CT 12991
GrabEatingMacaque AC-ATTTTTATGGCTTCAGGATACAAATGAAATCTCATTTTCCA-TTATTTTATAAATTTGCTGTA----TTCGG---CT 12748
Macaque           AC-ATTTTTATGGCTTCAGGATACAAATGAAATCTCATTTTCCA-TTATTTTATAAATTTGCTGTA----TTTGG---CT 12803
PigTailedMacaque  AC-ATTTTTATGGCTTTAGGATACAAATGAAATCTCATTTTCCA-TTATTTTATAAATTTGCTGTA----TTCGG---CT 12389
Bonobo            AA-GTTTTTATGGCTTTAGGATACAAATGAAATCTCATTTTCCA-TTATTTTATAAATTTGCTGTA----TTCAG---CT 11686
OliveBaboon       AC-ATTTTTATGGCTTTAGGATACAAATGAAATCTCATTTTCCA-TTATTTTATAAATTTTCTGTA----TTCGG---CT 11842
Orangutan         AA-GTTTTTGTGGCGTTAGGATACAAATGAAATCTCATTTTCCA-TTATTTTATAAATTTGCTATA----TTCAG---CT 12024
```

```
Majority          TTTCTTGATTTTAGACTTTTTTTT-------GTTAGCTCAGTGAGACTATAA-GAAAGAATATTTGTGAGTTTGTAATAG

                          15850     15860     15870     15880     15890     15900     15910     15920

Human             TTTCTTGATTTTAGACTTTTTTTT-------GTTAGCTCAGTGAGACTATTAAGAAAGAATATTTGTGAGTTTGTAATAG 10713
Coquerel'sSifaka  CTCATTGA-TTTAGACT-------------------CTTAGTGAGACTAAGATAAGAAAGGAAAT-TGAGTTTTTAATAT 10949
Ma'sNightMonkey   CTTCTTGATTTTAGACTTTTTTTTTGTTTTGGTTACCCCAGCGAGACTATAA-GAAAGCATATTTGTGAGTTTTTAATAG 10996
VervetAGM         TGCATCACGTTTGGGGTTAGGGCT--------TCAGCAT-GTGATTTTCGGG-GAAACATAAGCATTCAGTTCATAGTAC 12569
Gorrilla          TTTCTTGATTTTAGACTTTTTTTT-------GTTAGCTCAGTGAGACTATTAAGAAAAAAATATTTGTGAGTTTGTAATAG 13064
GrabEatingMacaqueTTTCTTGATTTTAGACTTTTTTTT-------GTTAGCTCAGTGAGACTATAA-GAAAGAATATTTGTGAGTTTGTAATAG 12820
Macaque           TTTCTTGATTTTAGACTTTTTTTT-------GTTAGCTCAGTGAGACTATAA-GAAAGAATATTTGTGAGTTTGTAATAG 12875
PigTailedMacaque  TTTCTTGATTTTAGACTTTTTTTT-------GTTAGCTCAGTGAGACTATAA-GAAAGAATATTTGTGAGTTTGTAATAG 12461
Bonobo            TTTCTTGATTTTAGACTTTTTTTT-------GTTAGCTCAGTGAGACTATTAAGAAAGAATATTTGTGAGTTTGTAATAG 11759
OliveBaboon       TTTCTTGATTTTAGACTTTTTTTT-------GTTAGCTCAGTGAGACTATAA-GAAAGAATATTTGTGAGTTTGTAATAG 11914
Orangutan         TTTCTTGAGTTTAGACTGGTTTTT-------GTTAGCTCAGTGAGACTATTA-----GAATATTTGTGAGTTTGTAATAG 12092

Majority          GAGATTTGTTGCATTTCTTTAAAATGGCTTTTTATATTCTTCGTAATTTGTAATTTCAGGGGTGTCATTT---CTGTACT

                          15930     15940     15950     15960     15970     15980     15990     16000

Human             GAGATTTGTTGCATTTCTTTAAAATGGCTTTTTATATTCTTCGTAATTTGTAATATCAGGGGTGTCATTT---CTGTACT 10790
Coquerel'sSifaka  GAGATGAGTTGCTTTTCTTTAAAATAGCTTTT-----TCTTTGTAATTTGTAAATTCAGGGGTTTTGTTT---CTGTACT 11021
Ma'sNightMonkey   GAGATTTGTTGCATTTCTTTAAAATGGCTTTTTATATTCTTTGTAGTTTGTAATTT-AGGGATGTCATTT---CTGTACT 11072
VervetAGM         CCTGCATATAGT-TGACCCATACTTCTCCCCTCCAGGTGATTACTATACTAAATTTGATGTTTACCATTCGCATCATTTT 12648
Gorrilla          GAGATTTGTTGCATTTCTTTAAAATGGCTTTTTATATTCTTCGTAATTTGTAATATCAGGAGTGTCACTT---CTGTACT 13141
GrabEatingMacaqueGAAATTTGTTAC-TTTTTTTAAAATGGCTTTTTATTTTGTTCGTAATTTGTAATTTCAGTGGTGTCATTT---CAGTACT 12896
Macaque           GAAATTTGTTAC-TTTTTTTAAAATGGCTTTTTATTTTGTTCGTAATTTGTAATTTCAGTGGTGTCATTT---CAGTACT 12951
PigTailedMacaque  GAAATTTGTTAC-TTTTTTTAAAATGGCTTTTTATTTTGTTCGTAATTTGTAATTTCAGTGGTGTCATTT---CAGTACT 12537
Bonobo            GAGATTTGTTGCATTTCTTTAAAATGGCTTTTTATATTCTTCGTAATTTGTAATATCAGGGGTGTCATTT---CTGTACT 11836
OliveBaboon       GAAATTTGTTAC-TTTTTTTAAAATGGCTTTTTATTTTGTTCGTAATTTGTAATTTCAGTGGTGTCATTT---CTGTACT 11990
Orangutan         GAGATTTGTTGCATTTCTTTAAAATGGCTTTTTATATTCTTCGTAATTTGTAATGTCAGGGGTGTCATTT---CTGTACT 12169

Majority          CTTAACCATTTTGCCAATGTGAAATCCTTGCAAAGTAATGGGCC-TATTACTTGGGAAGACAGAATTGTATGAA-ATGAC

                          16010     16020     16030     16040     16050     16060     16070     16080

Human             CTTAACCATTTTGCCAATGTGAAATCCTTGCAAAGTAATCGGCC-TATTACTTGGGAAGACAGAATTGTATGAA-ATGAC 10868
Coquerel'sSifaka  --TAATCATTTTGCTAATGTGAAATCCTTGCAAAATAATGGGCC----TA-TTGGGAAGATGGAAATGAGTGAA-ATGAT 11093
Ma'sNightMonkey   GTTAACCATTTTGCCAATGTGAAATCCATGCAGGGTAGTGGGCC-TGTTGCTTGGGAAAACAGAATTGTGTGAA-ATGAC 11150
VervetAGM         CTCATTTACTACGTTTATGT-ATTTTTATCTTAAGTAATATATGGTTTTGTTTTGCATGTTTAAACTATAGATGTACTAT 12727
Gorrilla          CTTAACCATTTTGCCAATGTGAAATCCTTGCAAAGTAATCGGCC-TATTACTTGGGAAGACAGAATTGTGTGAA-ATGAC 13219
GrabEatingMacaqueCTTAACTGTTTTGCCAATGTGAAATCCTTGCAAAGTAATGGACC-TATTACTTGGGAAGACAGAATTGTATGAA-ATGAC 12974
Macaque           CTTAACTGTTTTGCCAATGTGAAATCCTTGCAAAGTAATGGACC-TATTACTTGGGAAGACAGAATTGTATGAA-ATGAC 13029
PigTailedMacaque  CTTAACTGTTTTGCCAATGTGAAATCCTTGCAAAGTAATGGACC-TATTACTTGGGAAGACAGAATTGTATGAA-ATGAC 12615
Bonobo            CTTAACCATTTTGCCAATGTGAAATCCTTGCAAAGTAATCGGCC-TATTACTTGGGAAGACAGAATTGTATGAA-ATGAC 11914
OliveBaboon       CTTAACTGTTTTGCCAATGTGAAATCCTTGCAAAGTAATGGACC-TATTACTTGGGAAGACAGAATTGTATGAA-ATGAC 12068
Orangutan         CTTAACCATTTTGCCAATGTGAAATCCTTGCAAAGTAATGGGGC-TACTACTTGGGAAGACAGAATTGTATGAA-ATGAT 12247
```

```
Majority          TGTAGAATA-ATACAGGATGGTCC-CCAATACGGTAAAATGTTAACTTTTTAGATGTAGATTTTAAGTATGGTGAAATTT

                          16090     16100     16110     16120     16130     16140     16150     16160

Human             TGTAGAATA-ATTCAGGATGGTCC-CCAATACGGTAAAATGTTAACTTTTTAGATGTAGATTTTAAGTATGGTGAAATTT 10946
Coquerel'sSifaka  TGCAGAATA-ATACACA--GGTAC-CCAATATGGTAAAATGTTAACATTTTACATGCAGATTTTAAGTATGGTGAAATTT 11169
Ma'sNightMonkey   TATAGAATA-ATACAAGATGGTCC-CCAATATGGTAAAATGTTACCTTTTTAGATACGGATTTAAAATACGGTGAAATTT 11228
VervetAGM         CATGCTATATGTATCCTGTAATTTGCTCTTTTTGTTCAGTGTTATTTTTGTTTTTTAAGTTTATATGCATACCTCTAATT 12807
Gorrilla          TGTAGAATA-ATTCAGGATGGTCC-CCAATACGGTAAAATGTTAACTTTTTAGATGTAGATTTTAAGTATGGTGAAATTT 13297
GrabEatingMacaqueTGTAGAATA-ATAGAGGATGGTCC-CCAATACGGTAAAATGTTAACTTTTTAGATGTAGATTTTAAGTATGGTGAAAGTT 13052
Macaque           TGTAGAATA-ATAGAGGATGGTCC-CCAATACGGTAAAATGTTAACTTTTTAGATGTAGATTTTAAGTATGGTGAAAGTT 13107
PigTailedMacaque  TGTAGAATA-ATAGAGGATGGTCC-CCAATACGGTAAAATGTTAACTTTTTAGATGTAGATTTTAAGTATGGTGAAAGTT 12693
Bonobo            TGTAGAATA-ATTCAGGATGGTCC-CCAATACAGTAAAATGTTAACTTTTTAGATGTAGATTTTAAGTATGGTGAAATTT 11992
OliveBaboon       TGTAGAATA-ATAGAGGATGGTCC-CCAATACGGTAAAATGTTAACTTTTTAGATGTAGATTGTAAGTATGGTGAAAGTT 12146
Orangutan         TGTAGAATA-ATTCAGGATGGTCC-CCAATACGGTAAAATGTTAACTTTTTAGATGTAGAGTTTAAGTATGGTGAAATTT 12325

Majority          GTAGCAGTGTTCTTTAGTAAGTTAACTATTAATATATCAAGACCTA--TAACTGGACAACTAATAAATA-----ACCTAT

                          16170     16180     16190     16200     16210     16220     16230     16240

Human             GTAGCAGTGTTCTTTAGTAAGTTAACTATTAATATATCAAGACCTA--TAACTGGACAACTAATAAATA-----ACCTGT 11019
Coquerel'sSifaka  GTAGCAGTACTTTTTAGTAAGTTAAC------TAAGTTAAGACGTTA-TAACTGGACAACTAATAAAGG-----ACCTAT 11237
Ma'sNightMonkey   ATAGTAGTGTTCTTTAGTAAGTTAAC--TTAATATATCAAGATGTTA-TAACTGGATAGCTAATAA-TA-----ACCTAT 11299
VervetAGM         TT--CATTGCCGTATAGTATATGTTATATGAATATATCATTATCTTGTTGACAAATGAGTTAATATTTGTCGAGATTGAT 12885
Gorrilla          GTAGCAGTGTTCTTTAGTAAGTTAACTATTAATATATCAAGACCTA--TAACTGGACAACTAATAAATA-----ACCTAT 13370
GrabEatingMacaqueGTAGCAGTGTTCTTTAGGAAGTTAACTATTAATATCTCAAGACCTA--TAACTGGACAACTAATAAATA-----ACCTAT 13125
Macaque           GTAGCAGTGTTCTTTAGGAAGTTAACTATTAATATCTCAAGACCTA--TAACTGGACAACTAATAAATA-----ACCTAT 13180
PigTailedMacaque  GTAGCAGTGTTCTTTAGGAAGTTAACTATTAATATCTCAAGACCTA--CAACTGGACAACTAATAAATA-----ACCTAT 12766
Bonobo            GTAGCAGTGTTCTTTAGTAAGTTAACTATTAATATATCAAGACCTA--TAACTGGACAACTAATAAATA-----ACCTGT 12065
OliveBaboon       GTAGCAGTGTTCTTTAGGAAGTTAACTATTAATATCTCAAGACCTA--TAACTGGACAACTAATAAATA-----ACCTAT 12219
Orangutan         GTAGCAGTGTTCTTTAGTAAGTTAACTGTTAATATATCAAGACCTA--TAACTGGACAACTAATAAATA-----ACCTAT 12398

Majority          TTCTTAAA--ATGTTAGCTTTTTCTAA--------------CTATTCTGAAAGTACTGGTTGTAAATTGCTGAACTCATA

                          16250     16260     16270     16280     16290     16300     16310     16320

Human             TTCTTAAA--ATGTTAGCTTTTTCTAA--------------ATATTCTGAAAGTACTGGTTGTAAATTGCTGAACTCACA 11083
Coquerel'sSifaka  TTGTTAAAATAAGTTAGCTTTTTCCAAGTTGGGTTTATTAATATTTTGAAAGCAGCGATTGTAAATGGGTGAACTCATA 11317
Ma'sNightMonkey   TTCTTAAA--ATGTTAGCTTTT-CTAA--------------GTATTCTGAAGGTACTGGTTGTAAATTGCTGAACTCACA 11362
VervetAGM         TTGTCAAAT-ATTTTAAATTTTTTT----------------CCATTAC-AAACTGCT-GCTGTAAGCATTCATATGCATA 12946
Gorrilla          TTCTTAAA--ATGTTAGCTTTTTCTAA--------------GTATTCTGAAAGTACTGGTTGTAAATTGCTGAACTCACA 13434
GrabEatingMacaqueTTCTTAAA--ATGTCAGCTTTTTCTAA--------------CTATTCTGAAAGTACTAGTTGTAAATTGCTGAACTCATG 13189
Macaque           TTCTTAAA--ATGTCAGCTTTTTCTAA--------------CTATTCTGAAAGTACTAGTTGTAAATTGCTGAACTCATG 13244
PigTailedMacaque  TTCTTAAA--ATGTCAGCTTTTTCTAA--------------CTATTCTGAAAGTACTAGTTGTAAATTGCTGAACTCATG 12830
Bonobo            TTCTTAAA--ATGTTAGCTTTTTCTAA--------------ATATTCTGAAAGTACTGGTTGTAAATTGCTGAACTCACA 12129
OliveBaboon       TTCTTAAA--ATGTCAGCTTTTTCTAA--------------CTATTCTGAAAGTACTAGTTGTAAATTGCTGAACTCATG 12283
Orangutan         TTCTTAAA--ATGTTAGCTTTTTCTAA--------------ATATTCTGAAAGTACTGGTTGTAAATTGCTGAACTCATG 12462
```

```
Majority          ATTTAAATTATAATA----TGACTATTGAATTCA--CCATCATTGGCTTGCATAGAAAATTACAATCAAATAATGCTA--

                          16330     16340     16350     16360     16370     16380     16390     16400

Human             ATTTAAATTATGATA----TGACTATTGAATTCA--TCCTCATTGGCTTGCATAGAAAATTACAATCAAATAATGCTA-- 11155
Coquerel'sSifaka  ATTTAAATTGTAATATGGCTATTTATTGGATTTA--CCATCCTTGGCTTGCTTAGATAATTGCAATTAAGTATTGCTTTT 11395
Ma'sNightMonkey   ATTTAAATTATGAC-----T---------ATTCA--CCATTATTGGCTTGCACAGAAAATTACAATCAAAGAATGCTA-- 11424
VervetAGM         TTCTAAAGTGTTAGAG---TTTCTCATAGATTGTGTCTGTTTTCAAGTCG-ATTGAATATTAGCAT--ATTGCTTTCA-- 13018
Gorrilla          ATTTAAATTATGATA----TGACTATTGAATTCA--TCATCATTGGCTTGCACAGAAAATTACAATCAAATAATGCTA-- 13506
GrabEatingMacaque ATTTAAATTATAATA----TGACTGTTGAATTCA--CTATCATTGGCTTGCATAGAAAATTACAATCAAATAATGCTA-- 13261
Macaque           ATTTAAATTATAATA----TGACTGTTGAATTCA--CTATCATTGGCTTGCATAGAAAATTACAATCAAATAATGCTA-- 13316
PigTailedMacaque  ATTTAAATTATAATA----TGACTGTTGAATTCA--CTATCATTGGCTTGCATAGAAAATTACAATCAAATAATGCTA-- 12902
Bonobo            ATTTAAATTATGATA----TGACTATTGAATTCA--TCATCATTGGCTTGCATAGAAAATTACAATCAAATAATGCTA-- 12201
OliveBaboon       ATTTAAATTATAATA----TGACTGTTGAATTCA--CTGTCATTGGCTTGCATAGAAAATTACAATCAAATAATGCTA-- 12355
Orangutan         ATTTAAATTATAACA----TGACTATTGAATTCA--CCATCATTGGCTTGCATAGAAAATTACAATCAAATAATGCTA-- 12534

Majority          -------AATGTAACTTTTTTGGTAATAGT-TGAGTGTAACT----------GGTTGTTT--ATTATAG--ATGTGTTA-

                          16410     16420     16430     16440     16450     16460     16470     16480

Human             -------AATGTAACTTTTTTGGTAATAGT-TGTGTGTAACT----------GGTTGTTC--ATTATAG--ATGTGTTA- 11212
Coquerel'sSifaka  TTATACTAGAATGACTGGTT-TGTTATAATTGGTATGTTATTCAGAACTTTGTGTTGTTTGGTTTTTATTTATTTGCTTG 11474
Ma'sNightMonkey   -------AATGTAACTTTTA-GGTAATAGT-TGAGTATAATT----------GGTTGTTT--ATAATAG--ATGTGTT-- 11479
VervetAGM         -------TAGGTGATTATACTAATTATACTGTTAGTGCCGGA----------TGTAGATT--CTTGTTTCTATACATTC- 13078
Gorrilla          -------AATGTAACTTTTTTGGTAATAGT-TGTGTGTAACT----------GGTTGTTC--ATTATAG--ATGTGTTA- 13563
GrabEatingMacaque -------AATGTAACTTTTCTGGTGATAGT-TGAGTATAACT----------GGTTGTTT--ATTATGG--ATGTGTTA- 13318
Macaque           -------AATGTAACTTTTCTGGTGATAGT-TGAGTATAACT----------GGTTGTTT--ATTATGG--ATGTGTTA- 13373
PigTailedMacaque  -------AATGTAACTTTTCTGGTGATAGT-TGAGTATAACT----------GGTT------ATTATGG--ATGTGTTA- 12955
Bonobo            -------AATGTAACTTTTTTGGTAATAGT-TGTGTGTAACT----------GGTTGTTC--ATTATAG--ATGTGTTA- 12258
OliveBaboon       -------AATGTAACTTTTCTGGTGATAGT-TGAGTATAACT----------GGTTGTTT--ATTATAG--ATGTGTTA- 12412
Orangutan         -------AATGTAACTTTTTTGGTAATAGT-TGAGTGTAACT----------GGTTGTTC--ATTATAG--ATGTGTTA- 12591

Majority          ---------TTCAAAACTTTGGGTTTTTAGGTTTTGACTTTTCTTA--AAATGCTTTTCTTAAATGCTACTTAATGCTTT

                          16490     16500     16510     16520     16530     16540     16550     16560

Human             ---------TTCAAAACTTTGGGTTTTTAGGTTTTGACTTTTCTTA--AAATACTTTTCTTAAATGCTACTTAATGCTTT 11281
Coquerel'sSifaka  AACCGCCTGTCAAGAAAAATGGGTTTTTAGATTTTGATTTTTCTTA--AAATGCTTTTCTTAAATGCTACATAATGCTTG 11552
Ma'sNightMonkey   -----------CAAAACTTTGGGTGTTTAGGTTTTGACTTTCCTCA--AAATGCTTTTCTTAAATGCTACTAAATGCTTG 11546
VervetAGM         ---------TTTGAAAATCGACATTGTTGGACTT-AATTTTGCAGATCAAATGTACATGGGAA-GGGTATCTTGTGGTTT 13147
Gorrilla          ---------TTCAAAACTTTGGGTTTTTAGGTTTTGACTTTTCTTA--AAATACTTTTCTTAAATGCTACTTAATGCTTT 13632
GrabEatingMacaque ---------TTCAAAACTTTGGGTTTTTAGGTTTTGACTTTTCTTA--AAATGCTTTTCTTA--TGCTACTTAATGCTTT 13385
Macaque           ---------TTCAAAACTTTGGGTTTTTAGGTTTTGACTTTTCTTA--AAATGCTTTTCTTA--TGCTACTTAATGCTTT 13440
PigTailedMacaque  ---------TTCAAAACTTTGGGTTTTTAGGTTTTGACTTTTCTTA--AAATGCTTTTCTTA--TGCTACTTAATGCTTT 13022
Bonobo            ---------TTCAAAACTTTGGGTTTTTAGGTTTTGACTTTCCTTA--AAATACTTTCCTTAAATGCTACTTAATGCTTT 12327
OliveBaboon       ---------TTCAAAACTTTGGGTTTTTAGGTTTTGACTTTTCTTA--AAATGCTTTTCTTAAATGCTACTTAATGCTTT 12481
Orangutan         ---------TTCAAAACTTTGGGTTTTTAGGTTTTGACTTTTCTTA--AAATACTTTTCTTAAATGCTACTTAATGCTTT 12660
```

```
Majority          CTCTAGGGTATTTTTATTAGAATTTCAATTAACAGATATTTTAA--TGTCTGCGAAATGACAGTACTATGTTAGAAATTG
                          16570     16580     16590     16600     16610     16620     16630     16640
Human             CTCTAGGGTATTTTTATTAGAATTTCAATTAACAGATATTTTGA--TGTCTGCGAAATGACAGTACTATGTTAGAAATTG 11359
Coquerel'sSifaka  C--TAGGGTATTTTTATTAGAATTTCAATTTATAGACATTTTATTGTGTCTACTAAATGACAGCACTGTGTTAGAAGTTG 11630
Ma'sNightMonkey   CTCTAGGGTGTTTTTATTGGAATTTCAATTAACAGATATTTTAA--TGTCTACGAAATGACAGTACTATGTTAAAAATTG 11624
VervetAGM         TAATTTGCATTCCCTATTAATGCTTTATTTTAATGATACATTT---TATTAGTTTATTGGCCAT-TGATATTTCTTCTCT 13223
Gorrilla          CTCTAGGGTATTTTTATTAGAATTTCAATTAACAGATATTTTGA--TGTCTGTGAAATGACAGTACTATGTTAGAAATTG 13710
GrabEatingMacaqueCTCTAGGGTATTTTTATTAGAATTTCAATTAACAGATATTTTAA--TGTCTGCGAAATGACAGTACTATGTTAGATATTG 13463
Macaque           CTCTAGGGTATTTTTATTAGAATTTCAATTAACAGATATTTTAA--TGTCTGCGAAATGACAGTACTATGTTAGATATTG 13518
PigTailedMacaque  CTCTAGGGTATTTTTATCAGAATTTCAATTAACAGATATTTTAA--TGTCTGCGAAATGACAGTACTATGTTAGATATTG 13100
Bonobo            CTCTAGGGTATTTTTATTAGAATTTCAATTAACAGATATTTTGA--TGTCTGCGAAATGACAGTACTATGTTAGAAATTG 12405
OliveBaboon       CTCTAGGGTATTTTTATTAGAATTTCAATTAACAGATATTTTAA--TGTCTGCGAAATGACAGTACTATGTTAGATATTG 12559
Orangutan         CACTAGGGTATTTTTATTAGAATTTCAATTAAC--ATATTTTAA--CGTCTGCGAAATGACAGTACTATGTTAGAAATTT 12736

Majority          ATGTATTGG----------------------CTCCTTGAGGGCAGTGACCCTATTTGTTAT-----GTTTGTTCCTCCA
                          16650     16660     16670     16680     16690     16700     16710     16720
Human             ATGTATTGG----------------------CTCCTTGAGGGCAGTGACCCTATTTGTTAT-----GTTTGTTCCTCAA 11411
Coquerel'sSifaka  ATTCCAAAACA-----CTCGACAGAATATATGCTTCTGGAGGGCAGTGATCTTAATTAATAT-------------TTAA 11691
Ma'sNightMonkey   ATGTTTCAGAAATATTCAAGGCAGAATACATGTTCCTTGAGGGCAGTGACCCTATTTGTTAT-----ATTTGTTGCTCCA 11699
VervetAGM         GTGAAATGC----------------------CTGTTTATAGTTTGTTCATTTTCTTTTTGACTGTAGTTTTATCTTTTT 13280
Gorrilla          ATGTATTGG----------------------CTCCTTGAGGGCAGTGACCCTATTTGTTAT-----GTTTGTTCCTCAA 13762
GrabEatingMacaqueATGTATTGT----------------------CTCCTTGAGGGCAGTGACCCTAGTTGTTGT-----GTTTGTTCCTCCA 13515
Macaque           ATGTATTGT----------------------CTCCTTGAGGGCAGTGACCCTAGTTGTTGT-----GTTTGTTCCTCCA 13570
PigTailedMacaque  ATGTATTGT----------------------CTCCTTGAGGGCAGTGACCCTAGTTGTTGT-----GTTTGTTCCTCCA 13152
Bonobo            ATGTATTGG----------------------CTCCTTGAGGGCAGTGACCCTATTTGTTAT-----GTTTGTTCCTCAA 12457
OliveBaboon       ATGTATTGT----------------------CTCCTTGAGGGCAGTGACCCTAGTTGTTGT-----GTTTGTTCCTCCA 12611
Orangutan         ATGTATTGG----------------------CTCCTTGAGGGCAGTGACCCTATTTGTTAT-----GTTTTTTCCTCCA 12788

Majority          TCATTAATAGTACACGTGTTAAATAGTGCATACACACTTAATATTTAATAACTCTTAGAAGTGTCATAGGCTTTAATATG
                          16730     16740     16750     16760     16770     16780     16790     16800
Human             TCATTAATAGTACACATATTAAATAGTACATATACACTTAATATTTAATAACTCTTAGAAGTGTCATAGGCTTTAATATG 11491
Coquerel'sSifaka  TGACTAGAAGT------GTCATGGGCTTCAG-TATGCTTATAATCTCATGAGCCATTTAGCAACAAAAAGATAAATGCCA 11764
Ma'sNightMonkey   TCATTAATAGTACACGTATTAAGTAGTGTGTACACACATAATATTTAATAACTCTTAGAAGTATCACAGGCTTTAATATG 11779
VervetAGM         TTAAAATTGATTCCTGCTCGGCATGGTGGCT-CACGCCTATAATCC--CAACACTTGGGGAGGCCGAGGCAGGTGGATCA 13357
Gorrilla          TCATTAATAGTACACATATTAAATAGTGCATATACACTTAATATTTAATAACTCTTAGACGTGTCATAGGCTTTAATATG 13842
GrabEatingMacaqueACATTAATAGTACATGTGTTAAATAGTGCATACACACTTAATATTTAATAACTCTTAGAAGTGTCATAGGCTTTAATATG 13595
Macaque           ACATTAATAGTACATGTGTTAAATAGTGCATACACACTTAATATTTAATAACTCTTAGAAGTGTCATAGGCTTTAATATG 13650
PigTailedMacaque  ACATTAATAGTACATGTGTTAAATAGTGCATACACACTTAATATTTAATAACTCTTAGAAGTGTCATAGGCTTTAATATG 13232
Bonobo            TCATTAATAGTACACATATTAAATAGTGCATATACACTTAATACTTAATAACTCTTAGAAGTGTCATAGGCTTTAATATG 12537
OliveBaboon       ACATTAATAGTACACGTGTTAAATAGTGCATACACACTTAATATTTAATAACTCTTAGAAGTGTCATAGGCTTTAATATG 12691
Orangutan         TCATTAATAGTACACGTATTAAATAGTACATACACACTT------TAATAACTCTTAGAAGTGTCATAGGCTTTAATATG 12862
```

```
Majority          CTTATACTCTAATGAGCCATTTATCAATAAAAAGATGAATACCAAGACTATGTCTTT--TAGGACTTCATCTAGTTAGAG
                         16810     16820     16830     16840     16850     16860     16870     16880
Human             CTCATACTCTAATGAGCCATTTATCAATAAAAAGATAAATAACAAGACTATGTCTTT--TAGGACTT----TAGTTAGAG 11565
Coquerel'sSifaka  GTTTTATTGT-ACAATATTCCTTTCGCTGGAA------AT-CCAAGAGTCTGTCTTT--TAAGACCT-ATCTTGTTGGAA 11833
Ma'sNightMonkey   CTCTTAATCTTATGAGCCATTTATCAATAAAAAGATGAATACCAAGACTATGTCTTA--CAGGACCTCATCTAGTTGGAG 11857
VervetAGM         TTTGAGGTC--AGGAGTTCTAGACCAGCCTAGCCA-ACATGGCAAAACCCCGTCTCTACTAAAAGTGCAAAAAATTAGCT 13434
Gorrilla          CTCATACTCTAATGAGCCATTTATCAATAAAAAGATAAATACCAAGACTATGTCTTT--TAGGACTT----TAGTTAGAG 13916
GrabEatingMacaqueCTTATACTCTAATGAACCATTTATCAATAAAAAGGTGAATTCCAAGACTATGTCTTT--TAGGACTTCATCTAGTTAGAG 13673
Macaque           CTTATACTCTAATGAACCATTTATCAATAAAAAGGTGAATTCCAAGACTATGTCTTT--TAGGACTTCATCTAGTTAGAG 13728
PigTailedMacaque  CTTATACTCTAATGAACCATTTATCAATAAAAAGGTGAATTCCAAGACTATGTCTTT--TAGGACTTCATCTAGTTAGAG 13310
Bonobo            CTCATACTCTAATGAGCCATTTATCAATAAAAAGATAAATACCAAGACTATGTCTTT--TAGGACTT----TAGTTAGAG 12611
OliveBaboon       CTTATACTCTAATGAACCATTTATCAATAAAAAGGTGAATTCCAAGACTATGTCTTT--TAGGACTTCATCTAGTTAGAG 12769
Orangutan         CTCATACTCTAATGAGCCATTTATCAATAAAA-GATAAATACCAAGACTATGTCTTT--TAGGACTT----TAGTTAGAG 12935

Majority          AAATAAGGACCCAAGGTTTTAATTAAGTGCTATATGGCTACAGTAGGAAAGATTGGTTTGGGTTGGTAGGCTCAGAAT--
                         16890     16900     16910     16920     16930     16940     16950     16960
Human             AAATAAGGACTCAAGGTTTTAATTAAGTGCTATATGGCTACAGTAGGAAAGATTGGTTTGGGTTGGTAGGCTCAGAAT-- 11643
Coquerel'sSifaka  AGATAAGAACCCAAGATTTTAAGTA----CCTTATGTCTACAGTAGGAAAGATTAGTTTCAGTTGGTAGGATCAGAGTAG 11909
Ma'sNightMonkey   AAATACGGACCAAAGCTTTTAATTAAGTGCTATATAGCTATAGTAGAAAATACTGGTTTGGGTTGGTAGGCTCA-ACT-- 11934
VervetAGM         GGGTGTCGTGGTGGGTGTCTGCCTA---CTTGGGAGGCTGAGGCAGGAGA-ATTGCTTGAACCCAGGAGGTGGAGGTTG- 13509
Gorrilla          AAATAAGGACTCAAGGTTTTAATTAAGTGCTATATGGCTACAGTAGGAAAGATTGGTTTGGGTTGGTAGGCTCAGAAT-- 13994
GrabEatingMacaqueAAATAAGGATCCAAGGTTTTAATTAAGTGCTATGTGGCTACAGTAGGAAAGATTGATTTGGGTTGGTAGGCTCAGAAT-- 13751
Macaque           AAATAAGGATCCAAGGTTTTAATTAAGTGCTATGTGGCTACAGTAGGAAAGATTGATTTGGGTTGGTAGGCTCAGAAT-- 13806
PigTailedMacaque  AAATAAGGATCCAAGGTTTTAATTAAGTGCTATGTGGCTACAGTAGGAAAGATTGGTTTGGGTTGGTAGGCTCAGAAT-- 13388
Bonobo            AAATAAGGACTCAAGGTTTTAATTAAGTGCTATATGGCTACAGTAGGAAAGATTGGTTTGGGTTGGTAGGCTCAGAAT-- 12689
OliveBaboon       AAATAAGGATCCAAGGTTTTAATTAAGTGCTATGTGGCTACAGTAGGAAAGATTGGTTTGGGTTGGTAGGCTCAGAAT-- 12847
Orangutan         AAATAAGGACTCAGGGTTTTAATTAAGTGGTATATGGCTACAGTAGGAAAGATTGGTTTGGGTTGGTAGGCTCAGAAT-- 13013

Majority          -------------AGTGATCTGTGACCTAG-----CTAGAT---TGGGAGATA-GGGGAAGGAGTGGAGAGCAATAGGCA
                         16970     16980     16990     17000     17010     17020     17030     17040
Human             -------------AGTGGTCTGTGACCTAG-----CTAGAT---TGGGAGATA-GGAGAAAGAGTGGAGAGCAACAGGCG 11701
Coquerel'sSifaka  GTTTTATTAAAGTAGTGGGCTGTGACCTGTGCTTTATAGATAGTTGGGGGATGTGGAGAGGGAGTGGAGGGCAATACACA 11989
Ma'sNightMonkey   --------------GTGAACTGTCTGTTA-------TAGAA---TGGGTGATA-GAGGAAGGAGTGGAGAGCAACAGGCA 11989
VervetAGM         ------------CAGTGAGCCGCAATCCA------TTGCAC---TCCAACCTG-GGCGACAGAGCAAGACTCTGTTCCCC 13567
Gorrilla          -------------AGTGGTCTGTGACCTAG-----CTAGAT---TGGGAGATA-GGAGAAAGAGTGGAGACCAATAGGCA 14052
GrabEatingMacaque-------------AGTGATCTGTGACCTAG-----CCAAAT---TGGGAGATA-GGGGAAGGAGTGGAGAGCATTAGGCA 13809
Macaque           -------------AGTGATCTGTGACCTAG-----CCAAAT---TGGGAGATA-GGGGAAGGAGTGGAGAGCAATAGGCA 13864
PigTailedMacaque  -------------AGTGATCTGTGACCTAG-----CCAAAT---TGGGAGATA-GGGGAAGGAGTGGAGAGCAATAGGCA 13446
Bonobo            -------------AGTGGTCTGTGACCTAG-----CTAGAT---TGGGAGATA-GGAGAAAGAGTGGAGAGCAACAGGCG 12747
OliveBaboon       -------------AGTGATCTGTGACCTAG-----CCAAAT---TGGGAGATA-GGGGAAGGAGTGGAGAGCAATAGGCA 12905
Orangutan         -------------AGTGGTCTGTGATCTAG-----CTAGAT---TGGGAGATA-GGAGAAGGAGTGGAGAGCAATAGGCA 13071
```

```
Majority          TTGAGAGAATGACAT-AACCAAAGTTGTGTCTATAGGTTGGCATAAATTTACAGCCATTGATCAGTGAGTTGAATAATTT
                          17050     17060     17070     17080     17090     17100     17110     17120
Human             TTGAGAGAAGGACGT-AACCAAAGTTGTGTCTATAGGTTGGCATAAATTTACAGCCATTGATCAGTGAGTTGAATAGTTT 11780
Coquerel'sSifaka  TTGAGAGAATGACAT-GACCGAAGTCGTATCTATAGGCCAGTACAAATTCACAGCCATTGAGCAGTGAGTTGAATAATTT 12068
Ma'sNightMonkey   TTGAGAGAGTGACGT-AACCAAAGTTGTGTCTTTAGGATCGCATAAGTTTACAGCTATTGAGCACGGAGTTGAATACTTT 12068
VervetAGM         CGCCAAAAAAAA--------AAAATTGGATTCCTAGGATG---T---TTTATATACTCTGCACACTTAATTGTTTTGTT- 13632
Gorrilla          TTGAGAGAATGACAT-AACCAAAGTTGTGTCTATAGGTTGGCATAAATTTACAGCCATTGATCAGTGAGTTGAATCGTTT 14131
GrabEatingMacaque TTGAGAGAATGACATTAACTAAAGTTGTGTGTGTAGGTTGGCATAATTTTACAACCATTGATCAGTGAGTTGAATAATTT 13889
Macaque           TTGAGAGAATGACATTAACTAAAGTTGTGTGTGTAGGTTGGCATAATTTTACAACCATTGATCAGTGAGTTGAATAATTT 13944
PigTailedMacaque  TTGAGAGAATGACATTAACTAAAGTTGTGTGTGTAGGTTGGCATAATTTTACAACCATTGATCAGCGAGTTGAATAATTT 13526
Bonobo            TTGAGAGAATGACGT-AACCAAAGTTGTGTCTATAGGTTGGCATAAATTTACAGCCATTGATCAGTGAGTTGAATAGTTT 12826
OliveBaboon       TTGAGAGAATGACATTAACTAAAGTTGTGTGTGTAGGTTGGCATAATTTTACAACCATTGATCAGTGAATTGAATAATTT 12985
Orangutan         TTGAGAGAATGACAT-AACCAAAGTTGTGTCTATAGGTTGGCATAAATTTACAGCCATTGATCAGTGAGTTGAATAATTT 13150

Majority          GGCCTTTATGGCTAACAGTTTGTATATGGATATGAGAGAAAAGATAGAATGGCAG----GTTATATAGAAGGCCTCAATT
                          17130     17140     17150     17160     17170     17180     17190     17200
Human             GGCCTTTATGGCTAACAGCTTGTATATGGATATGAGAGAAAAGATAGAATGGCAGATAGGTTATATAGAAGACCTCAATT 11860
Coquerel'sSifaka  GGCTTTTGTGGCTAAGGGTTTGTATAAGGCTACAAGAGGAAAGATAGGATGGCAG----ATTATAG--AAGGCCTCAATT 12142
Ma'sNightMonkey   GGCTTTTATGGCTAACGATTTATATATGGATATGAGAGAAAAGATAGAATGGCAGATAGGTTATAT--AAGGCCTCAATT 12146
VervetAGM         AGTTTCTTTTGTTTTGTTTTTGTTTGAGGGAAGGGGTCTCACTACGTCACCCAGG----CTGGAGTGCAGTGGCACAGTC 13708
Gorrilla          GGCCTTTATGGCTAACAGCTTGTATATGGATATGAGAGAAAAGATAGAATGGCAGATAGGTTATATAGAAGACCTCAATT 14211
GrabEatingMacaque GGCCTTTATGGCTAAGAGTGTGTATATGGATATAAGAGAAAAGATAGAATGGCAG----GTTATATAGAAGGCCTCAATT 13965
Macaque           GGCCTTTATGGCTAAGAGTGTGTATATGGATATAAGAGAAAAGATAGAATGGCAG----GTTATATAGAAGGCCTCAATT 14020
PigTailedMacaque  GGCCTTTATGGCTAACAGTGTGTATATGGATATAAGAGAAAAGATAGAATGGCAG----GTTATATAGAAGGCCTCAATT 13602
Bonobo            GGCCTTTATGGCTAACAGCTTGTATATGGATATGAGAGAAAAGATAGAATGGCAGATAGGTTATATAGAAGACCTCAATT 12906
OliveBaboon       GGCCTTTATGGCTAACAGTGTGTTTATGGATATAAGAGAAAAGATAGAATGGCAG----GTTATATAGAAGGCCTCAATT 13061
Orangutan         GGCCTTTATGGCTAACAGCTTGTATATGGATATGAGAGAAAAGATAGAATGGCAGATAGGTTATATAGAAGGCCTCAATT 13230

Majority          ACAGGTTGTGAAATTTGTACTTCA-----TCTAGTAGGCATTAT-TCTATATACCTGTAATTTTCCA--CATAAAAGTAG
                          17210     17220     17230     17240     17250     17260     17270     17280
Human             ACAGGTAGTGAAATTTGTACTTCA-----TCTGGTAGGCATTAT-TCTATATACCTGTAATTTTCCA--CATAAAAGTAG 11932
Coquerel'sSifaka  ACAGGTTATGGCATTTGTACTTCA-----TCTAGTAGGTGCTAT-TCTGTATACCTGAAAGTTTCCATACATAAAAGTAG 12216
Ma'sNightMonkey   ACAAGTTGTGAAATTTGTACTTCA-----TCTAGTAGGCATCCT-TCTATATACCTGTAAGTTTCCA--CATAAAAGTAG 12218
VervetAGM         ACAGTCAGTGCAGTCTTGACATCCCAGGCTCTAGCAATCCTCCCACCTTTGTTTCCTGAGTAGCTGG--AACTACAGGAA 13786
Gorrilla          ACAGGTAGTGAAATTTGTACTTCA-----TCTGGTAGGCATTAT-TCTATATACTTGTAATTTTCCA--CATAAAAGTAG 14283
GrabEatingMacaque ACAGGTTGTGAAATTTGTACTTCA-----TCTAGTAGGCATTAT-TCTATATACCTGTAATTTCTCA--CATAAAAGTAG 14037
Macaque           ACAGGTTGTGAAATTTGTACTTCA-----TCTAGTAGGCATTAT-TCTATATACCTGTAATTTCTCA--CATAAAAGTAG 14092
PigTailedMacaque  ACAGGTTGTGAAATTTGTACTTCA-----TCTAGTAGGCATTAT-TCTATATACCTGTAATTTCTTG--CATAAAAGTAG 13674
Bonobo            ACAGGTAGTGAAATTTGTACTTCA-----TCTGGTAGGCATTAT-TCTATATACCTGTAATTTTCCA--CATAAAAGTAG 12978
OliveBaboon       ACAGGTTGTGAAATTTGTACTTCA-----TCTAGTAGGCATTAT-TCTATATACCTGTAATTTCTCA--CATAAAAGTAG 13133
Orangutan         ACAGGTTGTGAAATTTGTACTTCA-----TCTAGTAGGCATTAT-TCTATATATCTGTAATTTTCCA--TATAAAAGTAG 13302
```

```
Majority          CTCATTATTCTGCTTTCTTTTTTTTTTT-----------------TTGCTATA-ACAAATTTATTGTAACAACACAAAGT
                           17290     17300     17310     17320     17330     17340     17350     17360
Human             CTCATTACTCTGCTTTCTTTCTTTCTTT-------TTTTTTTTTTTTGCTATA-GCAAATTTATTGTAACAACACAAAGT 12004
Coquerel'sSifaka  GTCATTATTC---------------------------------------------------------------------- 12226
Ma'sNightMonkey   CTCATTATTC---------------------------------------------------------------------- 12228
VervetAGM         TACACCACTATGCCCAGCTAATTTTTCA-------C---------TTTTTTTGTAGAGATGGAGTCTGACTGTATTACCC 13850
Gorrilla          CTCATTATTCTGCTTTCTTTCTTTCTTT--TTT--TTTTTTTTTTTTGCTATA-GCAAATTTATTGTAACAACGCAAAGT 14358
GrabEatingMacaque CTCATTATTCTGCTTTCTTTTTTTTTTT-----------------ATGCTATA-ACAAATTTATTGTAACAACACAAAGG 14099
Macaque           CTCATTATTCTGCTTTCTTTTTTTTTTT-------T---------ATGCTATA-ACAAATTTATTGTAACAACACAAAGG 14155
PigTailedMacaque  CTCATTATTCTGCTTTCTTTTTTTTTT------------------ATGCTATA-ACAAATTTATTGTAACAACACAAAGG 13735
Bonobo            CTCATTATTCTGCTTTCTTTCTTTCTTTCTTTTTTTTTTTTTTTTTTGCTATA-GCAAATTTATTGTAACAACACAAAGT 13057
OliveBaboon       CTCATTATTCTGCTTTCCTTTTTTTTT------------------ATGCTATA-ACAAATTTATTGTAACAACACAAAGG 13194
Orangutan         CTCGTTATTC---TTTCTTTTTTTTTTT---------TTTTTTTTTTGCTATA-GCAAATTTATTGTAACAACACAAATT 13369

Majority          TATTATCTTACAGTTTTGTAGGT--GAGAAAT---CTGACACAGGTGGGCTAAAATGA---------AGGTGTTAG----
                           17370     17380     17390     17400     17410     17420     17430     17440
Human             -ATTATCTTATAGTTTTGTAGGT--GAGAAAT---CTGACACAGGTGGGCTAAAATCA---------AGGTGTTAG---- 12065
Coquerel'sSifaka  -------------------------------------------------------------------------------- 12226
Ma'sNightMonkey   ----------------T--------------------------------------------------------------- 12229
VervetAGM         AGGTTGGTTGCGAACTCCTGGGCTCAAGCAGTTCTCTCGCATTGGCCTCCTAAAATGCTGGGATTACAGGCGTGAGTCAC 13930
Gorrilla          -ATTATCTTATAGTTTTGTAGGT--GAGAAAT---CTGACACAGGTGGGCTAAAATCA---------AGGTGTTAG---- 14419
GrabEatingMacaque TATTATCTTACAGTTTTGTAGGT--GAGAAAT---CTGACACAGGTGGGCTAAAATGA---------AGGTGTTAG---- 14161
Macaque           TATTATCTTACAGTTTTGTAGGT--GAGAAAT---CTGACACAGGTGGGCTAAAATGA---------AGGTGTTAG---- 14217
PigTailedMacaque  TATTATCTTACAGTTTTGTAGGT--GAGAAAT---CTGACACAGGTGGGCTAAAATGA---------AGGTGTTAG---- 13797
Bonobo            -ATTATCTTATAGTTTTGTAGGT--GAGAAAT---CTGACACAGGTGGGCTAAAATCA---------AGGTGTTAG---- 13118
OliveBaboon       TATTATCTTACAGTTTTGTAGGT--GAGAAAT---CTGACACAGGTGGGCTAAAATGA---------AGGTGTTAG---- 13256
Orangutan         TATTATCTTATAGTTTTGTAGGT--GAGAAAT---CTGACACAGGTGGGCTAAAATCA---------AGGTGTTAG---- 13431

Majority          -----CAGGGCTGTGTTCCTTTTTGCAGGATCTGGGGGGAA-----------------TTTGTTTTCTTG-ACATATCTC
                           17450     17460     17470     17480     17490     17500     17510     17520
Human             -----CAGGGCTGTGTTCCTTTTTGCAGGATCTGGGGGGAA-----------------TTTGTTTCCTTG-ACATAACTC 12122
Coquerel'sSifaka  -------------------------------------------------------------------------------- 12226
Ma'sNightMonkey   -------------------------------------------------------------------------------- 12229
VervetAGM         TGTGCCTGGCCTGTTTTGTTAGTTATATGTCCTACAGATAACTCCTTCCTCTCTCTGACCTGTTTTTTTTTACACTGATG 14010
Gorrilla          -----CAGGGCTGTGTTCCTTTTTGCAGGATCTGGGGGGAA-----------------TTTGTTTCCTTG-ACATAACTC 14476
GrabEatingMacaque -----CAGGGCTGTGTTCCTTTTTGCAGGATCTGGGGGGGA----------------ATTTGTTTTCTTC-ACATATCTC 14219
Macaque           -----CAGGGCTGTGTTCCTTTTTGCAGGATCTGGGGGGGA----------------ATTTGTTTTCTTC-ACATATCTC 14275
PigTailedMacaque  -----CAGGGCTGTGTTCCTTTTTGCAGGATCTGGGGGGGA----------------ATTTGTTTTCTTC-ACATATCTC 13855
Bonobo            -----CAGGGCTGTGTTCCTTTTTGCAGGATCTGGGGGGAA-----------------TTTGTTTCCTTG-ACATAACTC 13175
OliveBaboon       -----CAGGGCTGTGTTCCTTTTTGCAGGATCTAGGGGGGA----------------ATTTGTTTTCTTC-ACATATCTC 13314
Orangutan         -----CAGGGCTGTGTTCCTTTTTGCAGGATCTGGGGGGAA-----------------TTTGTTTTCTTG-ACATAACTC 13488
```

```
Majority          AGCTGTTAGCTTCATCTTCAAAGAAGCAATGGC----------AAAGAAGAC----TCTCACATCA--CCTCACTGACAC
                          17530     17540     17550     17560     17570     17580     17590     17600
Human             GGCTGTTAGCTTCATCTTCAAAGAAGGAATGGC----------AAAGAAGACTCTGTCTCACATCACATCACTCCGACAC 12192
Coquerel'sSifaka  -------------------------------------------------------------------------------- 12226
Ma'sNightMonkey   ---------------------------------------------------------------------------G---- 12230
VervetAGM         AATAGAAGTTGTAAATTTTAATGTAACATTTATGTTTTCTTTAAAAAAATA----TTTAACCTAAGTCCTTACATATAG 14086
Gorrilla          GGCTGTTAGCTTCATCTTCAAAGAAGCAATGGC----------AAAGAAGACTGTGTCTCACATCACATCACTCCGACAC 14546
GrabEatingMacaque AGCTGTTAGCTTCATCTTCAAAGAAGCAATGGC----------AAAGAAGAC----TCTCACATCA--CATCACTGACAC 14283
Macaque           AGCTGTTAGCTTCATCTTCAAAGAAGCAATGGC----------AAAGAAGAC----TCTCACATCA--CATCACTGACAC 14339
PigTailedMacaque  AGCTGTTAGCTTCATCTTCAAAGAAGCAATGGC----------AAAGAAGAC----TCTCACATCA--CATCACTGACAC 13919
Bonobo            GGCTGTTAGCTTCATCTTCAAAGAAGGAATGGC----------AAAGAAGACTCTGTCTCACATCACATCACTCCGACAC 13245
OliveBaboon       AGCTGTTAGCTTCATCCTCAAAGAAGCAATGGC----------AAAGAAGAC----TCTCACATCA--CATCACTGACAC 13378
Orangutan         AGCTGTTAGCTTCATCTTCAAAGAAGCAATGGC----------AAAGAAGAC--TGTCTCACATCACATCACTCTGACAC 13556

Majority          ATTCTTGTGCCTCCCTTTCCCACTTATAGGAACCTTGTGATTACACT--GGGCCCAA--CTACAT--AATTCAGGATAAA
                          17610     17620     17630     17640     17650     17660     17670     17680
Human             ATTCTTGTGCCTCCGTTTCCCACTTACAGGGACCTTGTGATTACAAT--GGGCCCAA--CTACAT--AATAGAGGATAAA 12266
Coquerel'sSifaka  -------TGCTGTCTTC--------------------------------------------------------------- 12236
Ma'sNightMonkey   ---------TTTTCTTT-------------A------------------------------------------------- 12239
VervetAGM         GCTTGTGTATTTTATTTTGAAAGTTTTAAAATTTTGATTTTCACATTTAGGGCCTTAATCTACCTGGAATGTATTCTTTG 14166
Gorrilla          ATTCTTGTGCCTCCGTTTCCCACCTACAGGGACCTTGTGATTACAAT--GGGCCCAA--CTACAT--AATAGAGGATAAA 14620
GrabEatingMacaque ATTCTTGTGCCTCCCTTTCCCACTTATAGGAACCTTGTGATTACACT--GGGCCCAA--CTACAT--AATTCAGGATAAA 14357
Macaque           ATTCTTGTGCCTCCCTTTCCCACTTATAGGAACCTTGTGATTACACT--GGGCCCAA--CTACAT--AATTCAGGATAAA 14413
PigTailedMacaque  ATTCTTGTGCCTCCCTTTCCCACTTATAGGAACCTTGTGATTACACT--GGGCCCAA--CTACAT--AATTCAGGATAAA 13993
Bonobo            ATTCTTGTGCCTCCATTTCCCACTTACAGGGACCTTGTGATTACAGT--GGGCCCAA--CTACAT--AATAGAGGATAAA 13319
OliveBaboon       ATTCTTGTTCCTCCCTTTCCCACTTATAGGAACCTTGTGATTACACT--GGGCCCAA--CTACAT--AATTCAGGATAAA 13452
Orangutan         ATTCTTGTGCCTCCGTTTCCCACTTACAGGGACCTTGTGATTACAGT--GGGCCCAA--CTACAT--AATTCAGGATAAA 13630

Majority          TGTT---TAAATTACAAATT-AATGAAGTGGCAACT--GAA--ACAAGTTTTA-ACCTATTCACAAGAAAGG----GCTT
                          17690     17700     17710     17720     17730     17740     17750     17760
Human             TGTT---TAAATTACAAATT-AATGAAGTGGCAACT--GAA--ACAAGTTTTA-ACCTATTCTCAAGAAAGG----GCTT 12333
Coquerel'sSifaka  -------------------------------------------------------------------------------- 12236
Ma'sNightMonkey   -------------------------------------------------------------------------------- 12239
VervetAGM         TGTAGCATATGGTACAGGTTTAATTATTTTTTCTATATGGATAGCAAGTTTAGTACCTACTTGATTGAATAGTTCAGCTT 14246
Gorrilla          TGTT---TAAATTACAAATT-AATGAAGTGGCAACT--GAA--ACAAGTTTTA-ACCTATTCTCAAGAAGGG----GCTT 14687
GrabEatingMacaque TGTT---TAAATTACAAATT-AATGAAGTGGCAACTGTGAA--ACAAGTTTTA-ACCTATTCACAAGAAAGG----GCTT 14426
Macaque           TGTT---TAAATTACAAATT-AATGAAGTGGCAACT--GAA--ACAAGTTTTA-ACCTATTCACAAGAAAGG----GCTT 14480
PigTailedMacaque  TGTT---TAAATTACAAATT-AATGAAGTGGCAACT--GAA--ACAAGTTTTA-ACCTATTCACAAGAAAGG----GCTT 14060
Bonobo            TGTT---TAAATTACAAATT-AATGAAGTGGCAACT--GAA--ACAAGTTTTA-ACCTATTCTCAAGAAAGG----GCTT 13386
OliveBaboon       TGTT---TAAATTACAAATT-AATGAAGTGGCAACT--GAA--ACAAGTTTTA-ACCTATTCACAAGAAAGG----GCTT 13519
Orangutan         TGTT---TAAATTACCAATT-AATGAAGTGGCAACT--GAA--ACAAGTTTTA-ACCTATTCACAAGAAAGG----GCTT 13697
```

```
Majority          TGCTGGAGTGGTGGCTCACACCTATAATCCCAGAACTTAGGGAGGC-TGAGGCAGGCAGATCGCT-TGAGCC-CAGGAGT

                          17770     17780     17790     17800     17810     17820     17830     17840

Human             TGCTGGAATGGTGGCTCACACCTGTAATCCCAGAACTTAGGGAGGC-TGAGGCAGGCAGATCACT-TGAGCC-CAGGAGT 12410
Coquerel'sSifaka  -------------------------------------------------------------------------------- 12236
Ma'sNightMonkey   ---------------------------------------------------------GA-------------------- 12241
VervetAGM         TTATCCAGTGATCTGCTTTATTAGCCCTTCAGGTTTCCATATATTCATAGAACTGTTTCTGTGCTATGTGTGGTAGCAGT 14326
Gorrilla          TGCTGGAGTGGTGGCTCACACCTGTAATCCCAGAACTTAGGGAGGC-TGAGGCAGGCAGATCACT-TGAGCC-CAGGAGT 14764
GrabEatingMacaqueTGCTGGAGTGGTGGCTCACACCTATAATCCCAGAACTTAGGGAGGC-TGAGGCAGGCAGATCGCT-TGAGCC-CAGGAGT 14503
Macaque           TGCTGGAGTGGTGGCTCACACCTATAATCCCAGAACTTAGGGAGGC-TGAGGCAGGCAGATCGCT-TGAGCC-CAGGAGT 14557
PigTailedMacaque  TGCTGGAGTGGTGGCTCACACCTATAATCCCAGAACTTAGGGAGGC-TGAGGCAGGCAGATCGCT-TGAGCC-CAGGAGT 14137
Bonobo            TGCTGGAATGGTGGCTCACACCTGTAATCCCAGAACTTAGGGAGGC-TGAGGCAGGCAGATCACT-TGAGCC-CAGGAGT 13463
OliveBaboon       TGCTGGCGTGGTGGCTCACACCTATAATCCCAGAACTTAGGGAGGC-TGAGGCAGGCAGATCGCT-TGAGCC-CAGGAGT 13596
Orangutan         TGCTGGAGTGGTGGCTCACACCTATAATCCCAGAATTTAGGGAGGC-TGAGGCAGGCAGATCGCT-TGAGCC-CAGGAGT 13774

Majority          TTGAGACCAGCCTGGTCCTCATAGCA--AGACCCCATCTCTACAACAACTTATAAAAATTAGATAGATCTGGTGGCATGG

                          17850     17860     17870     17880     17890     17900     17910     17920

Human             TTGAGACCAGCCTGGTCCTCATAGCA--AGACCCCATCTCTACAAAAACTTTTAAAAATTAGATAGATCTGGTGGCATGG 12488
Coquerel'sSifaka  -------------------------------------------------------------------------------- 12236
Ma'sNightMonkey   --A----------------------------------------------------------------------------- 12242
VervetAGM         ACAACAGTGTCTTAATTACTATGGCATCAGCATTAAGTTTTGGAATCTTGTACAGCGAGTGTCAAAACTTTGGTTTTCAA 14406
Gorrilla          TTGAGACCAGCCTGGTCCTCATAGCA--AGACCCCATCTCTACAAAAACTTTTAAAAATCAGATAGATCTGGTGGCATGG 14842
GrabEatingMacaqueTTGAGACCAGCCTGGTCCTCATAGCA--AGACCCCATCTCTACAACAACTTAAAAAAATTAGATAGATCTGGTGGCATGG 14581
Macaque           TTGAGACCAGCCTGGTCCTCATAGCA--AGACCCCATCTCTACAACAACTTAAAAAAATTAGATAGATCTGGTGGCATGG 14635
PigTailedMacaque  TTGAGACCAGCCTGGTCCTCATAGCA--AGACCCCATCTCTACAACAACTTAAAAAAATTAGATAGATCTGGTGGCATGG 14215
Bonobo            TTGAGACCAGCCTGGTCCTCATAGCA--AGACCCCATCTCTACAAAAACTTTTAAAAATTAGATAGATCTGGTGGCATGG 13541
OliveBaboon       TTGAGACCAGCCTGGTCCTCATAGCA--AGACCCCATCTCTACAACAACTTAAAAAAATTAGATAGATCTGGTGGCATGG 13674
Orangutan         TTGAGATCAGCCTGGTCCTCGTAGCA--AGACCCCATCTCTACAAAAACT--TACAAATTAGATAGATCTGGTGGCATGG 13850

Majority          GCCTGTAGTCCCAGCTACTTGG--GAGGCTGAGACAAGAGGATC-ACCTGAGCCCAGGTGGCAGT----GAGCTGTGATT

                          17930     17940     17950     17960     17970     17980     17990     18000

Human             GCCTGTAGTCCCAGCTACTTGG--GAGGCTGAGACAAGAGGATC-ACCTGAGCCCAGGCGGCAGT----GAGCTATGATT 12561
Coquerel'sSifaka  -------------------------------------------------------------------------------- 12236
Ma'sNightMonkey   -----------------------------T--------------------------------G----------------- 12244
VervetAGM         AATTGTCTTCGTGTTTTCTTCGCTGCATTTATTTTAAGATCATTTGGCTAAGTTCTGTGAAGACTTTTTGGGTCTTATTT 14486
Gorrilla          GCCTGTAGTCCCAGCTACTTGG--GAGGCTGAGACAAGAGGATC-ACCTGAGCCCAGGCGGCAGT----GAGCTGTGATT 14915
GrabEatingMacaqueGCCTGTAGTCCCAGCTACTTGG--GAGACTGAGACAAGAGGATC-ACCTGAGCCCAGGTGGCAGT----GAGCTGTGATC 14654
Macaque           GCCTGTAGTCCCAGCTACTTGG--GAGACTGAGACAAGAGGATC-ACCTGAGCCTAGGTGGCAGT----GAGCTGTGATC 14708
PigTailedMacaque  GCCTGTAGTCCCAGCTACTTGG--GAGACTGAGACAAGAGGATC-ACCTGAGCCCAGGTGGCAGT----GAGCTGTGATC 14288
Bonobo            GCCTGTAGTCCCAGCTACTTGG--GAGGCTGAGACAAGAGGATC-ACCTGAGCCCAGGCGGCAGT----GAGCTATGATT 13614
OliveBaboon       GCCTGTAGTCCCAGCTACTTGG--GAGACTGAGACAAGAGGATC-ACCTGAGCCCAGGTGGCAGT----GAGCTGTGATC 13747
Orangutan         GCCTGTAGTCCCAGCTACTTGG--GAGGCTGAGACAAGAGGATC-ACCTGAGCCCAGGTGGCAGT----GAGCTATGATT 13923
```

```
Majority          GTATCA--CTACAGTCCAGC--CTGGGCGAGAGGATATC-CAGTCTCAAAAAAAAA-------GGAAAAGGGCTTTC--C

                         18010     18020     18030     18040     18050     18060     18070     18080

Human             GTATCA--CTACACTCCAGC--CTGGGCGAGAGGAGATC-CAGTCTCAAAAAAAAA-------GGAAAAGGGCTTTC--C 12627
Coquerel'sSifaka  -------------------------------------------------------------------------------- 12236
Ma'sNightMonkey   ---------------------------------------TAG--------------------------------------- 12247
VervetAGM         ATACTTGCCTTAAATTTAGTAGTTAATTGGGAGAACATTATAGTTGCATTATCAAAATAAAATGGCAAATTTTTTTAGC 14566
Gorrilla          GTATCA--CTACACTCCAGC--CTGGGCGAGAGGAGATC-CAGTCTCAAAAAAAAA-------GGAAAAGGGCTTTC--C 14981
GrabEatingMacaqueGTATCA--CTACAGTCCAGC--CTGGGCGAGAGGATATC-CAGTCTCAAAAAAAAA-GAAAAAGGAAAAGGGCTTTC--C 14726
Macaque           GTATCA--CTACAGTCCAGC--CTGGGCGAGAGGATATC-CAGTCTCAAAAAAAAA-GAAAAAGGAAAAGGGCTTTC--C 14780
PigTailedMacaque  ATATCA--CTACAGTCCAGC--CTGGGCGAGAGGATATC-CAGTCTCAAAAAAAAAAGAAAAAGGAAAAGGGCTTTC--C 14361
Bonobo            TTATCA--CTACACTCCAGC--CTGGGCGAGAGGAGATC-CAGTCTCAAAAAAAAA-------GGAAAAGGGCTTTC--C 13680
OliveBaboon       GTATCA--CTACAGTCCAGC--CTGGGCGAGAGGATATC-CAGTCTCAAAAAAAAAAGAAAAAGGAAAAGGGCTTTC--C 13820
Orangutan         TTATCA--CTACACTCCAGC--CTGGGTGAGAGGAGATC-CAGTCTCAAAAAAA---------GGAAAAGGGCTTTC--C 13987

Majority          TGAGGGTCAAATGAAGTACAGTTGACCCTTGAACAACATG--ATTTTGAATTACACAGGTCCACTT------ATGCATGG

                         18090     18100     18110     18120     18130     18140     18150     18160

Human             TGAGGGTCAAATGAAGTACAGTTGACCCTTGAACAACATG--ATTTTGAATTACACAGGTCCACTT------ATGCATAG 12699
Coquerel'sSifaka  -------------------------------------------------------------------------------- 12236
Ma'sNightMonkey   -------------------------------------------------------------------------------- 12247
VervetAGM         TGAAAGGCAGA-GAACTATAACATTTCTTTGAAGAATTTTCCACTTTCTAAGAGACATGCTTAATGCAGAAAATATGTTA 14645
Gorrilla          TGAGGGTCAAATGAAGTACAATTGACCCTTGAACAACATG--ATTTTGAATTACACAGGTCCACTT------ATGCATGG 15053
GrabEatingMacaqueTGAGGGTCAAATGAAGTACAGTTGACCCTTGAACAACATG--ATTTTGAACTACACAGGTCCACTT------ATGCATGG 14798
Macaque           TGAGGGTCAAATGAAGTACAGTTGACCCTTGAACAACATG--ATTTTGAACTACACAGGTCCACTT------ATGCATGG 14852
PigTailedMacaque  TGAGGGTCAAATGAAGTACAGTTGACCCTTGAACAACATG--ATTTTGAACTACACAGGTCCACTT------ATGCATGG 14433
Bonobo            TGAGGGTCAAATGAAGTACAGTTGACCCTTGAACAACATG--ATTTTGAATTACACAGGTCCACTT------ATTCATAG 13752
OliveBaboon       TGAGGGTCAAATGAAGTACAGTTGACCCTTGAACAACATG--ATTTTGAACTACACAGGTCCACTT------ATGCTTGG 13892
Orangutan         TGAGGGTCGAATGAAGTACAGTTGACCCTTGAACAGGATG--ATTTTGAATTACACAGGTCCACTT------ATGCATGG 14059

Majority          ATCCACTTATACAGAATTTTCTTCTCCCTCTTCCAC---CTAAGACAGAAAGACCAACTCTTCCTCTTCCTC---AGCCT

                         18170     18180     18190     18200     18210     18220     18230     18240

Human             ATCCACTTATACAGAATTTTCTTCTCCCTCTTCCAC---CCAAGACAGAAAGACCAACTCGTCCTCTTCCTCCTTAGCCT 12776
Coquerel'sSifaka  ------------AGAAT--------------------------------------------------------------- 12241
Ma'sNightMonkey   ------------AGAAT---------------------------------G-------------------------- 12253
VervetAGM         AAAGGATTTTAAACAGCTTATTTGTAAGGGTTGAGGGGACAGGGATACTTGGACTAATATTTCAGTTTTTCT---AAAAG 14722
Gorrilla          ATCCACTTACACAGAATTTTCTTCTCGCTCTTCCAC---CCAAGACAGA----CCAACTCGTCCTCTTCCTCCTCAGCCT 15126
GrabEatingMacaqueATCCACTTATACAGAATTTTCTTCTCCCTCTTCCAC---CTAAGACAGAAAGACCAACTCTTCCTCTTCCTC---AGCCT 14872
Macaque           ATCCACTTATACAGAATTTTCTTCTCCCTCTTCCAC---CTAAGACAGAAAGACCAACTCTTCCTCTTCCTC---AGCCT 14926
PigTailedMacaque  ATCCACTTATACAGAATTTTCTTCTCCCTCTTCCAC---CTAAGACAGAAAGACCAACTCTTCCTCTTCCTC---AGCCT 14507
Bonobo            ATCCACTTATACAGAATTTTCTTCTCCTTCTTCCAC---CCAAGACAGAAAGACCAACTCGTCCTCTTCCTCCTTAGCCT 13829
OliveBaboon       ATCCACTTATACAGAATTTTCTTCTCCCTCTTCCAC---CTAAGACAGAAAGACCAACTCTTCCTCTTCCTC---AGCCT 13966
Orangutan         ATCCACTTATACAGAATTTTCTTCTCCCTCTTCCAC---CCAAGACGGAAAGACCAACTCGTCCTCTTCCTCCTCGTCCC 14136
```

```
Majority         ACTCAGTGTAAAGACAGTG-AGGATGAAGACC--TTTATTATGA----CTGATCTCCAAAAAGATTG----GCTTCAATA

                         18250     18260     18270     18280     18290     18300     18310     18320

Human            ACTCAGCGTGAAGACAGTC-AGGATGAAGACC--TTTATTATGA----CTGATCTCCAAAAAGATTG----GCTTCAGTA 12845
Coquerel'sSifaka -----------------------ATGGAGAC-------------------AGCTCCAAAAAAGATTG----ACTTTGGTT 12275
Ma'sNightMonkey  --------------------------------------------------ATCTCCAAAAAGATTG----GCTTCAATA 12278
VervetAGM        TATTGGTTTATTGAGTACGTACCTTGAAAACTGTTTTATTGAGAGCTTCAAATTTCTCAGAATTGTATAACATTTTTGAA 14802
Gorrilla         ACTCAGCGTGAAGACAGTC-AGGATGAAGACC--TTTATTATGA----CTGATCTCCAAAAAGATTG----GCTTCAGTA 15195
GrabEatingMacaqueACTCAATGTAAAGACAGTG-AGGATGAAGACC--TTTATTATGA----CTGATCTCCAAAAAGATTG----GCTTCAATA 14941
Macaque          ACTCAATGTAAAGACAGTG-AGGATGAAGACC--TTTATTATGA----CTGATCTCCAAAAAGATTG----GCTTCAATA 14995
PigTailedMacaque ACTCAATGTAAAGACAGTG-AGGATGAAGACC--TTTATTATGA----CTGATCTCCAAAAAGATTG----GCTTCAATA 14576
Bonobo           ACTTAGCGTGAAGACAGTC-AGGATGAAGACC--TTTATTATGA----CTGATCTCCAAAAAGATTG----GCTTCAATA 13898
OliveBaboon      ACTCAATGTAAAGACAGTG-AAGATGAAGACC--TTTATTATGA----CTGATCTCCAAAAAGATTG----GCTTCAATA 14035
Orangutan        ACTCAGCGTGAAGACATTG-AGGATGAAGACC--TTTATTATGA----CCGATCTCCAAAAAGATTG----GCTTCAATA 14205

Majority         TTTCATGGAGTGCTATGAAT--TAGAATCCTGAGTCTCTATAT---TGTTAATTATTTTAGTCTCTTGGTCTGTGT----

                         18330     18340     18350     18360     18370     18380     18390     18400

Human            TTTCATGGAGTGCTATGAAT--TAGAATCCTGAGTCTCTATAT---TGTTAATTATTTTAGTCTCTTGGTCTACGT---- 12916
Coquerel'sSifaka TTACATGGGGTGCTGTGACT--TAGAATAATGAATTTCTGTAA--TTTTTAGTTATTTTAGTTTCTTACCTCGTCT---- 12347
Ma'sNightMonkey  TTTCATGGAATGCTATGAAT--TAGAATCCTGAGTCTCTGTAA--TTGTTCATTATTTTAATCTCTTGGTCTGTGT---- 12350
VervetAGM        TTCTCTCTTTTATTACTAATATCAAGCACCTATGTTTTCTTCTATTTCTTGATTTTTTTTGTCATATAATTTATTTTACT 14882
Gorrilla         TTTCATGGAGTGCTATGAAT--TAGAATCCTGAGTCTCTATAT---TGTTAATTATTTTAGTCTCTTGGTCTACGT---- 15266
GrabEatingMacaqueTTTCATGGAGTGTTATGAAT--TAGAATCCTGAGTCTCTATAT---TGTTAATTATTTTCGTCTCTTGGTCTGTGT---- 15012
Macaque          TTTCATGGAGTGTTATGAAT--TAGAATCCTGAGTCTCTATAT---TGTTAATTATTTTCGTCTCTTGGTCTGTGT---- 15066
PigTailedMacaque TTTCATGGAGTGTTATGAAT--TAGAATCCTGAGTCTCTATAT---TGTTAATTATTTTGGTCTCTTGGTCTGTGT---- 14647
Bonobo           TTTCATGGAGTGCTATGAAT--TAGAATCCTGAGTCTCTATAT---TGTTAATTATTTTAGTCTCTTGGTCTACGT---- 13969
OliveBaboon      TTTCATGGAGTGTTATGAAT--TAGAATCCTGAGTCTCTATAT---TGTTAATTATTTTAGTCTCTTGGTCTGTGT---- 14106
Orangutan        TTTCATGGAGTGCTATGAAT--TAGAATCCTCAGTCTCTATAT---TGTTAATTATTTTAGTCTCTTGGTCTATGT---- 14276

Majority         -GTGTTCCTAAACTGT-AGTAGCTTTCTTAATTTAGTTCTAGGCATACTACATTAGGGGA--------TAAGGTGGTTAA

                         18410     18420     18430     18440     18450     18460     18470     18480

Human            -GTGTTCCTAAACTGT-AGTAGCTTTCTTAATTTAGTTCTAGGCATACTACATTAGGGGA--------TAAGGTGGTTAA 12986
Coquerel'sSifaka -GTGTTGTTAAACTGT-AATAGCTTTCTTAATTTAGTTCAAGGCATACTACATTAGGGGA--------TAAAGTGTTGAA 12417
Ma'sNightMonkey  -GTGTTGCTAAATTGT-AGTAGCTTTCTTAACTTAATTCTAGGCATACCACAGTAGGGGA--------TAAGGTGGTTAA 12420
VervetAGM        GGTCTTTTCAAAGAACCAATGTTTTTATTTGTTTTCTTTTTTGTTGAGGGCAGTGAAGGGGTTCACTTTAAGTTGATCCT 14962
Gorrilla         -GTGTTCCTAAACTGT-AGTAGCTTTCTTAATTTAGTTCTAGGCATACTACATTAGGGGA--------TAAGGTGGTTAA 15336
GrabEatingMacaque-GTGTTCCTAAACTGT-AGTAGCTTTCTTAATTTAGTTCCAGGCATACTACATTAGGGGA--------TAAGGTGGTTAA 15082
Macaque          -GTGTTCCTAAACTGT-AGTAGCTTTCTTAATTTAGTTCCAGGCATACTACATTAGGGGA--------TAAGGTGGTTAA 15136
PigTailedMacaque -GTGTTCCTAAACTGT-AGTAGCTTTCTTAATTTAGTTCCAGGCATACTACATTAGGGGA--------TAAGGTGGTTAA 14717
Bonobo           -GTGTTCCTAAACTGT-AGTAGCTTTCTTAATTTAGTTCTAGGCATACTACATTAGGGGA--------TAAGGTGGTTAA 14039
OliveBaboon      -GTGTTCCCAAACTGT-AGTAGCTTTCTTAATTTAGTTCCAGGCATACTACATTAGGGGA--------TAAGGTGGTTAA 14176
Orangutan        -GTGTTTCTAAACTGT-AGTAGCTTTCTTAATTTAGTTCTAGGCATACTACATTAGGGGA--------TAAGGTGGTTAA 14346
```

```
Majority          G-------TGATATTTTCACTTTAAAAGTAATAAGGTGTCACAGAAAGTTATCACAA-GGGTTTTTTTGAGTAGTACTTC

                          18490     18500     18510     18520     18530     18540     18550     18560

Human             G-------TGATATTTTCACTTTAAAAGTAATAAGGTGTCACAGAAAGTTATCACGA--GATTTTTTTGAGCAGTACTTC 13057
Coquerel'sSifaka  G-------TAATATTTTCACTTTAAAAGTAATAAGGTATCACT-AATGTTATAAGAA-ATGTTTTTTTGAGCAAAGTT-- 12486
Ma'sNightMonkey   G-------TGATATTTCTACTTTAAAAGTAATAAGGTGTCACAGAAAGTTA----------------------------- 12464
VervetAGM         GAACTCCTTTATTTTCTTGTTTTAAAATACATTCAAGGTTAAAGGTAATCA--GCTG-ATAGCTTTGGCAGTGGTTTTTT 15039
Gorrilla          G-------TGATACTTTCACTTTAAAAGTAATAAGGTGTCACAGAAAGTTATCACGA--GATTTTTTTGAGCAGTACTTC 15407
GrabEatingMacaqueG-------TGATATTTTCACTTTAAAAGTAATAAGGTGTCACAGAAAGTTACCACAA-GAGGTTTTTTGAGTAGTACTTC 15154
Macaque           G-------TGATATTTTCACTTTAAAAGTAATAAGGTGTCACAGAAAGTTACCACAA-GAGGTTTTTTGAGTAGTACTTC 15208
PigTailedMacaque  G-------TGATATTTTCTCTTTAAAAGTAATAAGGTGTCACAGAAAGTTATCACAA-GAGGTTTTTTGAGTAGTACTTC 14789
Bonobo            G-------TGATATTTTCACTTTAAAAGTAATAAGGTGTCACAGAAAGTTATCACGA--GATTTTTTTGAGCAGTACTTC 14110
OliveBaboon       G-------TGATATTTTCACTTTAAAAGTAATAAGGTGTCACAGAAAGTTATCACAA-GAGGTTTTTTGAGTAGTACTTC 14248
Orangutan         G-------TGATATTTTCACTTTAAAAGTAGTAAGGTGTCACAGAAAGTTATCACAACAGATTTTTTTGAGCAGTACTTC 14419

Majority          TTTTTTATAACTATTGTAATGAATTTTAGA--CACAA-TAGTATCATGAAC-C-ATTGTGTGTGTATCATTCAGTCCCAA

                          18570     18580     18590     18600     18610     18620     18630     18640

Human             TTTTTTAAAACTATTATAATGAATTTTAGA--CACAA-TAGTATCATGAAC-C-ATTGTGTATCTATCATTCAGTCCCCA 13132
Coquerel'sSifaka  CTTTTTATAAGTATTGTGGTGAATTTAAACACAGCAAGTATCACAGTAGACACCATTCATTGTGTATCTATCAGTCTCAG 12566
Ma'sNightMonkey   -------------------------------------------------------------------------------- 12464
VervetAGM         TTTTTTTTTTTTTTTTTTTTTTTTTTTTAGAGTTCTCT-TTTTATTATAAAC-TTAAAGGTTTTAAGCTACACAAAAGTAG 15117
Gorrilla          TTTTTTAAAACTATTATAATGAATTTTAGA--AACAA-TAGTATCGTGAAC-C-ATTGTGTATCTATCATTCAGTCCCCA 15482
GrabEatingMacaqueTTTTTTATAACTATTGTAATGAATTTTAGA--CACAA-TAGTATCATGAAC-C-ATTGTGTGTGTATCATTCAGTCCCAA 15229
Macaque           TTTTTTATAACTATTGTAATGAATTTTAGA--CACAA-TAGTATCATGAAC-C-ATTGTGTGTGTATCATTCAGTCCCAA 15283
PigTailedMacaque  TTTTTTATAACTATTGTAATGAATTTTAGA--CACAA-TAGTATCATGAAC-C-ATTGTGTGTGTATCATTCAGTCCCAA 14864
Bonobo            TTTTTTAAAACTATTATAATGAATTTTAGA--CACAA-TAGTATCATGAAC-C-ATTGTGTATCTATCATTCAGTCCCCA 14185
OliveBaboon       TTTTTTATAACTATTGTAATGAATTTTAGA--CACAA-TAGTATCATGAAC-C-ATTGTGTGTGTATCATTCAGTCCCAA 14323
Orangutan         TTTTTTAAAACTATTATAATGAATTTTAGA--CACAA-TAGTATCATGAAC-C-ATTGTGTATCTATCATTCAGTCCCCA 14494

Majority          AAACCAT------CAGCAACCCCTAGCTA-------AAATGATGATATCCACACTCTGCTTTTCTACTTCT--GTTATTT

                          18650     18660     18670     18680     18690     18700     18710     18720

Human             AAACCAT------TAGTAACCCATAGCTA-------AAATGATGATATCCACACTCTGCTTTTCCACTTCT--GTTATTT 13197
Coquerel'sSifaka  CAATCA---------ACAGCGCACAGCTA-------AAGCTATGATATCTACACACTACTTTTCTCTTTCT--GTTATT- 12627
Ma'sNightMonkey   -------------------------------------------------------------------------------- 12464
VervetAGM         AGGGAATGGTATATAATACACTCTTTTTTTTCTGTTGGATNTCGATCTCCTGACCCTGTGATCCGCCCGCCTCGGCCTCC 15197
Gorrilla          AAACCAT------CAGCAACCCATAGCTA-------AAACGATGATATCCACACT--GCTTTTCCACTTCT--GTTATTT 15545
GrabEatingMacaqueAAACCAT------CAGCAACCCCTAGCTA-------AAATGATGATATCCAAACTCTGCTTTTCTACTTCT--GTTATTT 15294
Macaque           AAACCAT------CAGCAACCCCTAGCTA-------AAATGATGATATCCAAACTCTGCTTTTCTACTTCT--GTTATTT 15348
PigTailedMacaque  AAACCAT------CAGCAACCCCTAGCTA-------AAATGATGATATCCAAACTCTGCTTTTCTACTTCT--GTTATTT 14929
Bonobo            AAACCAT------TAGCAACCCATAGCTA-------AAATGATGATATCCACACTCTGCTTTTCCACTTCT--GTTATTT 14250
OliveBaboon       AAACCAT------CAGCAACCCCTAGCTA-------AAATGATGATATCCAAACTCTGCTTTTCTACTTCT--GTTATTT 14388
Orangutan         AAACCAT------CAGCAACCCATAGCTA-------AAACGATGATATCCACACTCTGCTTTTCCATTTCT--GTTATTT 14559
```

```
Majority          TGAGATA----AATTTCAGATAT---TCATATTGTTT----------TATCTGTAAAGGCTTCAGTGTATATCTTTAAA-

                          18730     18740     18750     18760     18770     18780     18790     18800

Human             TGAGATA----AATTTCAGATAT---TCATATTGTTT----------TATCTGTAAAGGCTTCAGTGTATATCTTTAAAG 13260
Coquerel'sSifaka  -GAAATA----AATTTCAGATAG---CCATATTGTTT----------CATCTGTAAATGTTTCA-TGTGTATCTATGAAA 12688
Ma'sNightMonkey   -----------------------------------------------------------T------------------ 12465
VervetAGM         CAAAGTGCTGGGATTACAGGCATGAGCCACCGCGCCCGGCCAGACACTGTTTTTAAAATTACATAATGTGGTCTCAGC- 15276
Gorrilla          TGAGATA----AATTTCAGATAT---TCATATTGTTT----------TATCTGTAAAGGCTTCAGTGTGTATCTTTAAAG 15608
GrabEatingMacaqueTGAGATA----AATTTCAGATAT---TCATATTGTTT----------TATCTGTAAAGGCTTCAGTGTATAACTTTAAA- 15356
Macaque           TGAGATA----AATTTCAGATAT---TCATATTGTTT----------TATCTGTAAAGGCTTCAGTGTATAACTTTAAA- 15410
PigTailedMacaque  TGAGATA----AATTTCAGATAT---TCATATTGTTT----------TATCTGTAAAGGCTTCAGTGTATAACTTTAAA- 14991
Bonobo            TGAGATA----AATTTCAGATAT---TCATATTGTTT----------TATCTGTAAAGGCTTCAGTGTATATCTTTAAAG 14313
OliveBaboon       TGAGATA----AATTTCAGATAT---TCATATTGTTT----------TATCTGTAAAGGCTTCAGTGTATAACTTTAAA- 14450
Orangutan         TGAGATA----AATATCAGATAT---TCATATTGTTT----------TATCTGTAAAGGCTTCAGTGTATATCTTTAAAG 14622

Majority          AATAAGGACTCTCTTT---------TCTTTTTAAAATATAATCAAAAG-ACTGTTAGGACAC-ACACACAAATTAAGTTA

                          18810     18820     18830     18840     18850     18860     18870     18880

Human             AATAGGGACTCTCCTCTC--------CTTTTTAAAATATAATCAAAAG-ACTGTTAGGACAC-ACACACA----AAGTTA 13326
Coquerel'sSifaka  GAAAGGGACTCTCTCT---------TCTTTTTAA--TATAACCACAAT-ACTGTTAGAACACTAAAACAATTAACAATTA 12756
Ma'sNightMonkey   ------------------------------------------CAAAAG--------------------------AG--- 12473
VervetAGM         AGTTACAGTTCTACCTCAT------TCTTTGTTAATGACAATGGAGGATACTCTTGTGTGACTGCACCACAGTGTAGTTA 15350
Gorrilla          AATAGGGACTCTCCTCTCTCTCTTTTCTTTTTAAAATATAATCAAAAG-ACTATTAGGACAC-ACACACACATTAAGTTA 15686
GrabEatingMacaqueAATAAAGACTCTCTTT---------TCTTTTTAAAATATAATCAAAAG-ACTGTTAGGACAC-ACACACAAATTAAGTTA 15425
Macaque           AATAAAGACTCTCTTT---------TCTTTTTAAAATATAATCAAAAG-ACTGTTAGGACAC-ACACACAAATTAAGTTA 15479
PigTailedMacaque  AATAAAGACTCTCTTT---------TCTTTTTAAAATATAATCAAAAG-ACTGTTAGGACAC-ACACACAAATTAAGTTA 15060
Bonobo            AATAGGGACTCTCCTCTCT----TTTCTTTTTAAAATATAATCAAAAG-ACTGTTAGGACAC-ACACACA----AAGTTA 14383
OliveBaboon       AATAAGGACTCTCTTT---------TCTTTTTAAAATATAATCAAAAG-ACTGTTAGGACAC-ACACACAAATTAAGTTA 14519
Orangutan         AATAAGGAC-----TCTC---------TTTTTAAAATATAATCAAAAG-ACTATTAGGACAC-ACACACACATTAAGTTA 14686

Majority          CTT--CATATTATAA---AATGTCAAATTTGTATTAAAATTT----CTAATT---GTCTCATAAAAGTCATAGTATTTTT

                          18890     18900     18910     18920     18930     18940     18950     18960

Human             CCT--CATATTATAA---AATGTCAAATTTGTATTAAAATTT----CTAATT---GTCTCATAAAAGTCATAGTATTTTT 13394
Coquerel'sSifaka  CTTA--ATATTATCA---AATGTTAAATTTGTGTTAAAATTT----CTAATT---GCCTCCTAAAAGTCATGGTGTTTTT 12824
Ma'sNightMonkey   ------------------------------------ATTT---------------------------------------- 12477
VervetAGM         CTGGACATACCACAATTTAGTTTCCTACATTGATGGACATTTAGGTTTGATTCCAGTTTGTGGTTAGTTATAAGGTGTGG 15430
Gorrilla          CCT--CATATTATAA---AATGTCAAATTTGTATTAAAATTT----CTAATT---GTCTCATAAAAGTCATAGTATTTTT 15754
GrabEatingMacaqueCTT--CATATTATAA---AATGTCAAATTTGTATTAAAATTT----CTAATT---GTCTCATAAAAGTCATAGTATTTTT 15493
Macaque           CTT--CATATTATAA---AATGTCAAATTTGTATTAAAATTT----CTAATT---GTCTCATAAAAGTCATAGTATTTTT 15547
PigTailedMacaque  CTT--CATATTATAA---AATGTCAAATTTGTATTAAAATTT----CTAATT---GTCTCATAAAAGTCATAGTATTTTT 15128
Bonobo            CCT--CATATTATAA---AATGTCAAATTTGTATTAAAATTT----CCAATT---GTCTCATAAAAGTCATAGTATTTTT 14451
OliveBaboon       CTT--CATATTATAA---AATGTCAAATTTGTATTAAAATTT----CTAATT---GTCTCATAAAAGTCATAGTATTTTT 14587
Orangutan         ATT--CATATTATAA---ACTGTCAAATTTGTATTAAAATTT----CTAGTT---GTCTCATAAAAGTCATAGTATTTTT 14754
```

```
Majority          ATAGG---TTTTTTTT---AGTGGGGGAAG----GAGTCATGATAAAATAAGATCAGCATATTTAACAGGT-CGTTATTA
                          18970     18980     18990     19000     19010     19020     19030     19040
Human             ATAGGGTTTTTTTTTTT--AGTGGGGGAAG----GAGTCACGATAAAATAAGATCAGCAAATTTAACAGGT-CGTTATTA 13467
Coquerel'sSifaka  ATAGGGTTTTTT-------AGTGGGGGAGG----GAATCGAAATAAGACATGATCAGCACATTTTCTAGGT-TGATATTA 12892
Ma'sNightMonkey   -------------------------------------------------------------------------------- 12477
VervetAGM         ACATA---TTTAGAAT---AGAAGTAAAAATCCTGTGTCAAAAGAAATTTATGCTTAAAGAGTTACTGGAAACCACACTG 15504
Gorrilla          ATAGGGTTTTTTTTTT---AGTGGGGGAAG----GAGTCACGATAAAATAAGATCAGCAAATTTAACAGGT-CGTTATTA 15826
GrabEatingMacaque ATAGG---TTTTTTTT---AGAGGGGGAAG----GAGTCATGATAAAATAAGATCAGCATATTTAACAGGT-CGTTATTA 15562
Macaque           ATAGG---TTTTTTTT---AGAGGGGGAAG----GAGTCATGATAAAATAAGATCAGCATATTTAACAGGT-CGTTATTA 15616
PigTailedMacaque  ATAGG---TTTTTTTT---AGAGGGGGAAG----GAGTCATGATAAAATAAGATCAGCATATTTAACAGGT-CGTTATTA 15197
Bonobo            ATAGGGTTTTTTTTTTTTTAGTGGGGGAAG----GAGTCACGATAAAATAAGATCAGCAAATTTAACAGGT-TGTTATTA 14526
OliveBaboon       ATAGG---TTTTTTTT---AGAGGGGGAAG----GAGTCATGATAAAATAAGATCAGCATATTTAACAGGT-CGTTATTA 14656
Orangutan         ATAGGGTTTTTTTTTTTT-AGTGGGGGAAG----GAGTCATGATAAAATAAGATCAGCCAATTTAACAGGT-CGTTATTA 14828

Majority          CCT-----CTTAAATATCTTTTA-----ATGTACAAGT-TCTCCGTTCCTGT-----------TTTTTG-----------
                          19050     19060     19070     19080     19090     19100     19110     19120
Human             CCT-----CTTAAATATCTTTTA-----ATGTACAAGC-TCTCCGTTCCTGT------------TTTTTG---------- 13514
Coquerel'sSifaka  TGT---CTTAAAAATATCTTTTA-----ATCTACAGGT-TCTCCCTCTATCCCTG--------TTTTTTGGGGTTTG--- 12952
Ma'sNightMonkey   -------------------------------------------------------------------------------- 12477
VervetAGM         CCAGAACACTCATTTGTTTTTTACAGGTGTGTATGGGAGTGCTTGTTTTTCCATACCCTTGGCAATTCTAGGTTTTATCA 15584
Gorrilla          CCT-----CTTAAATATCTTTTA-----ATGTACAAGT-TCTCCGTTCCTGT------------TTTTTG---------- 15873
GrabEatingMacaque CCT-----CTTAAATATATTTTA-----ATGTACAAGT-TCTCCGTTCCTGTTTTTTTTTTTTTTTTTTGGTTTTTGTTG 15631
Macaque           CCT-----CTTAAATATCTTTTA-----ATGTACAAGT-TCTCCGTTCCTGTTTTTTTGTTTTTTTTTTT-TGTTTTTTG 15684
PigTailedMacaque  CCT-----CTTAAATATCTTTTA-----ATGTACAAGT-TCTCCGTTCCTGT-----------TTTTTT----------- 15244
Bonobo            CCT-----CTTAAATATCTTTTA-----ATGTACAAGT-TCTCCGTTCCTGT------------TTTTG----------- 14572
OliveBaboon       CCT-----CTTAAATATCTTTTA-----ATGTACAAGT-TCTCCGTTCCTGT-----------TTTTTT----------- 14703
Orangutan         CCT-----CTTAAATATCTTTTA-----ATGTACAAGT-TCTCCGTTCCTGT------------TTTTTG---------- 14875

Majority          ----T-------------TTTTT---------------------------------------------------------
                          19130     19140     19150     19160     19170     19180     19190     19200
Human             ----T-------------TTTTT--------------------------------------------------------- 13520
Coquerel'sSifaka  ----TT----------TTTTTTTTTT------------------------------------------------------ 12964
Ma'sNightMonkey   -------------------------------------------------------------------------------- 12477
VervetAGM         AAATTTAGAATATTAGATTATTTTACACTAAAAATGTATTTCGTAATTTATATTTTGAAATG-AGTGAAGTTTTCAAATT 15663
Gorrilla          ----T-------------TTTTT--------------------------------------------------------- 15879
GrabEatingMacaque TTGTTGTTGTTGTTGTTGTTTTTGTTGAGACAGAGTCTCGCTTTGTCGCCCAGACTGGAGTGCAGTGGCCGGATCTCAGC 15711
Macaque           TTGTTGTTGTTGTTGTTTTTTTTGTTGAGACAGAGTCTCGCTTTGTCGCCCAGACTGGAGTGCAGTGGCCGGATCTCAGC 15764
PigTailedMacaque  ------------------TTTTT--------------------------------------------------------- 15249
Bonobo            ------------------TTTTT--------------------------------------------------------- 14577
OliveBaboon       ------------------TTT----------------------------------------------------------- 14706
Orangutan         ----TG-----------TTTTTT--------------------------------------------------------- 14883
```

```
Majority         --------------------------------------------------------------------------------

                          19210     19220     19230     19240     19250     19260     19270     19280

Human            -------------------------------------------------------------------------------- 13520
Coquerel'sSifaka -------------------------------------------------------------------------------- 12964
Ma'sNightMonkey  -------------------------------------------------------------------------------- 12477
VervetAGM        TGGCTATCTGTATTTCATTTTCTGTCAACTGCTTGTTTGTGTACTGGAC-CTAGTTTAATGGTCATTTTTATCACT--GT 15740
Gorrilla         -------------------------------------------------------------------------------- 15879
GrabEatingMacaqueTCACTGCAAGCTCCGCCTCC-GGGTTCACGCCATTCTCCTGCCTCAGCCTCCCGAGTAGCTGGGACTACAGACGCC-GCC 15789
Macaque          TCACTGCAAGCTCCGCCTCCCGGGTTCACGCCATTCTCCTGCCTCAGCCTCCCGAGTAGCTGGGACTACAGACGCCCGCC 15844
PigTailedMacaque -------------------------------------------------------------------------------- 15249
Bonobo           -------------------------------------------------------------------------------- 14577
OliveBaboon      -------------------------------------------------------------------------------- 14706
Orangutan        -------------------------------------------------------------------------------- 14883

Majority         --------------------------------------------------------------------------------

                          19290     19300     19310     19320     19330     19340     19350     19360

Human            -------------------------------------------------------------------------------- 13520
Coquerel'sSifaka -------------------------------------------------------------------------------- 12964
Ma'sNightMonkey  -------------------------------------------------------------------------------- 12477
VervetAGM        TCTTTGTATAAAAAAATTGATTTCATATGCATTGTAGATATTTTTTCTTTGAGATTTTCTT--T---TTTGCTTTTTTGA 15815
Gorrilla         -------------------------------------------------------------------------------- 15879
GrabEatingMacaqueACCTCGCCCGGCTAGGTTTT---GTATTTTAGTAGAGACGGGGTTTCACCGTGTTAGCCAGGATGGTCTCGATCTCCTGA 15866
Macaque          ACCTCGCCCGGCTAGGTTTTTGTATTTTTTAGTAGAGACGGGGTTTCACCGTGTTAGCCAGGATGGTCTCGATCTCCTGA 15924
PigTailedMacaque -------------------------------------------------------------------------------- 15249
Bonobo           -------------------------------------------------------------------------------- 14577
OliveBaboon      -------------------------------------------------------------------------------- 14706
Orangutan        -------------------------------------------------------------------------------- 14883

Majority         --------------------------------------------------------------------------------

                          19370     19380     19390     19400     19410     19420     19430     19440

Human            -------------------------------------------------------------------------------- 13520
Coquerel'sSifaka -------------------------------------------------------------------------------- 12964
Ma'sNightMonkey  -------------------------------------------------------------------------------- 12477
VervetAGM        TATTCTTTGTTGTATAGTTTTAGGCATATATGTATTCTGATTAAGTGATTTTGCTTTTAAAATCCATTTGATATGTAAAC 15895
Gorrilla         -------------------------------------------------------------------------------- 15879
GrabEatingMacaqueCCTTGTGATCCGCCCGTCTCGGCCTCCCAAAGTGCTGGGATTACAGGCTTGAGCCACCGCGCCCGGCCTGTTTTTTTTTT 15946
Macaque          CCTTGTGATCCGCCCGTCTCGGCCTCCCAGAGTGCTGGGATTACAGGCTTGAGCCACCGCACCCGGCCTGTTTTTTTTTT 16004
PigTailedMacaque -------------------------------------------------------------------------------- 15249
Bonobo           -------------------------------------------------------------------------------- 14577
OliveBaboon      -------------------------------------------------------------------------------- 14706
Orangutan        -------------------------------------------------------------------------------- 14883
```

```
Majority          -TTTTAAGTTGCATTTC-ATTTGTTGAAGAAAACTGG---TTGTTGGTCTTACACAGTTG-CC------TATAGTCTACA
                          19450     19460     19470     19480     19490     19500     19510     19520
Human             -TTTTAAGTTGCATTTC-ATTTGTTGAAGAAAACTGG---TTATTGGTCTTACACAGTTG-CC------TATAGACTACA 13588
Coquerel'sSifaka  -TTTTTGGTTGCATTTTTATTTGTTGAAGAACATTTT---TTGTTGGTCTTATAGAGTTGCCCACAGTCTGCATTCTGCA 13040
Ma'sNightMonkey   -------------------------------------------------------------------------------- 12477
VervetAGM         ATTTTTAGCCGCAGCTT-CTCCATCTTTAAAATTAGGATTTTAATAGTTCCACAAACTAGGTC------CTCAGTTAATG 15968
Gorrilla          -TTTTAAGTTGCATTTC-ATTTGTTGAAGAAAACTGG---TTATTGGTCTTACACAGTTG-CC------TATAGACTACA 15947
GrabEatingMacaqueTTTT-AAGTTGCATTTC-ATTTGTTGAAGAAAACTGG---TTGTTGGTCTTACACAGTTG-CC------TATAGTCTACA 16014
Macaque           TTTTTAAGTTGCATTTC-ATTTGTTGAAGAAAACTGG---TTGTTGGTCTTACACAGTTG-CC------TATAGTCTACA 16073
PigTailedMacaque -----AAGTTGCATTTC-ATTTGTTGAAGAAAACTGG---TTGTTGGTCTTACACAGTTG-CC------TATAGTCTACA 15313
Bonobo            -TTTTAAGTTGCATTTC-ATTTGTTGAAGAAAACTGG---TTATTGGTCTTACACAGTTG-CC------TATAGACTACA 14645
OliveBaboon       -----AAGTTGCATTTC-ATTTGTTGAAGAAAACTGG---TTGTTGGTCTTACACAGTTG-CC------TATAGTCTACA 14770
Orangutan         -TTTTAAGTTGCATTTC-GTTTGTTGAAGAAAACTGG---TTATTGGTCTTACACAGTTG-CC------TATAGACTACA 14951

Majority          TTTAC-TGATTGCGTTCTGATTTTGTAATTTAGTATGTTCTCTATGTTTCCAGTAAATTGGTAGTTG-----GATCTAGA
                          19530     19540     19550     19560     19570     19580     19590     19600
Human             TTTAC-TGATTGCGTTATGATTTTGTAATTTAGTATGTTTTCCATATTTCCAGTAAATTGGTAGTTA-----GATCTAGA 13662
Coquerel'sSifaka  TTTAC-TGGTTCTGTTCTGATGTTGTAATTTAGTATGTTCTCTATAATTCTGGTTAATTGGTTGTGG-----GATCTAGA 13114
Ma'sNightMonkey   -------------------------------------------------------------------------------- 12477
VervetAGM         TTAATGTATTTGCACTTAGAATATCCACATTTTTATGG-CTTTAGGATACAAATGAAATCTTATTTTCCATTATTTTATA 16047
Gorrilla          TTTAC-TGATTGCGTTATGATTTTGTAATTTAGTATGTTCTCCATATTTCCAGTAAATTGGTAGTTA-----GATCTAGA 16021
GrabEatingMacaqueTTTAC-TGATTGCATTCTGATTTTGTAATTTAATATGTTCTCTATGTTTCCAGTAAATTGGTAGTTG-----GATCTAAA 16088
Macaque           TTTAC-TGATTGCATTCTGATTTTGTAATTTAATATGTTCTCTATGTTTCCAGTAAATTGGTAGTTG-----GATCTAAA 16147
PigTailedMacaque TTTAC-TGATTGCATTCTGATTTTGTAATTTAATATGTTCTCTATGTTTCCAGTAAATTGGTAGTTG-----GATCTAAA 15387
Bonobo            TTTAC-TGATTGCGTTATGATTTTGTAATTTAGTATGTTCTCCATATTTCCAGTAAATTGGTAGTTA-----GATCTAGA 14719
OliveBaboon       TTTAC-TGATTGCATTCTGATTTTGTAATTTAATATGTTCTCTATGTTTCCAGTAAATTGGTAGTTG-----GATCTAAA 14844
Orangutan         TTTGC-TGATTGTGTTACGATTTTGTAATTTAGTATGTTCTCCATATTTCCAGTAAATTGGTAGTTA-----GATCTAGA 15025

Majority          GACTTGGTCAGGATCTCTGTTT--TTTTGGTAATA---TTTTTAAGTGGTGGGTTTTTATTTC--TATTAGACATGGT--
                          19610     19620     19630     19640     19650     19660     19670     19680
Human             GACTTGCTCAGGGTCTCTATTT--TTTTGGTAATA---TTTTTAAGTGGTGGGTTTTTATTTC--TGTTAGACATGGT-- 13733
Coquerel'sSifaka  GACTTCCTCAAATTCTCTTTTTGTTTTTGGTAATACTGTTTTTAAGTGGAGGTGTATTTTTTCATTAGAGGACATGGTA- 13193
Ma'sNightMonkey   -----------------------------------------------------T--------------------- 12478
VervetAGM         AATTTGCT--GTATTCGGCTTT--TCTTGATTTTAG-ACTTTTTTTTGTTAGCTCAGTGAAAC--TATAAGAAAGAGTAT 16120
Gorrilla          GACTTGCTCAGGGTCTCTATTT--TTTTGGTAATA---TTTTTAAGTGGTGGGTTTTTATTTC--TGTTAGACATGGT-- 16092
GrabEatingMacaqueGACTTGGTCAGGATCTCTGTTT--TTTTGGTAATA---TTTTTAAGTGGTGGGTTTTTATTTC--TATTAGACATGGT-- 16159
Macaque           GACTTGGTCAGGATCTCTGTTT--TTTTGGTAATA---TTTTTAAGTGGTGGGTTTTTATTTC--TATTAGACATGGT-- 16218
PigTailedMacaque GACTTGGTCAGGATCTCTGTTT--TTTTGGTAATA---TTTTTAAGTGGTGGGTTTTTATTTC--TATTAGACATGGT-- 15458
Bonobo            GACTTGCTCAGGGTCTCTATTT--TTT-GGTAATA---TTTTTAAGTGGTGGGTTTTTATTTC--TGTTAGACATGGT-- 14789
OliveBaboon       GACTTGGTCAGGATCTCCGTTT--TTTTGGTAATA---TTTTTAAGTGGTGGGTTTTTATTTC--TATTAGACATGGT-- 14915
Orangutan         GACTTGGTCAGGGTTTCTATTT--TTTTGGTAAT----TTTTTAAGTGGTGGGTTTTTATTTC--TGTTAGACATGGT-- 15095
```

```
Majority          TTCTGACTTTTCAT----GTATGTGATGTTTTTCCTAGGAACATTTCTTATACTTTACCTTGTTTGTGCTAGGCATAGTC

                          19690     19700     19710     19720     19730     19740     19750     19760

Human             TTCTGACTTTTCGT----GTATGTCATGTTTTTCCCAGGAACATTTCTTATACTTTACCTTGTTTGTGCTAGGCATAGTC 13809
Coquerel'sSifaka  -TCTGATTTTTTTGT---GTATGTGATGTTATTCCCAAGAAAATTTCTTATCCTTCACCTTGTTTGTTCTAGGCATAGTC 13269
Ma'sNightMonkey   -------------------------------------------------------------------------------- 12478
VervetAGM         TTGTGAGTTTGTAATAGGAGATTTGTTACTTTTTTTAAAATGGCTTTTTATTTTGTTCATAATTTGTAATTTCATTGGTG 16200
Gorrilla          TTCTGACTTTTCAT----GTATGTCATGTTTTTCCCAGGAACATTTCTTATACTTTACCTTGTTTGTGCTAGGCATGGTC 16168
GrabEatingMacaqueTTCTGACTTTTCAT----GTATGTGATGTTTTTCCTAGGAACATTTCTTATACTTTACCTTGTTTGTGCTAGGCATAGTC 16235
Macaque           TTCTGACTTTTCAT----GTATGTGATGTTTTTCCTAGGAACATTTCTTATACTTTACCTTGTTTGTGCTAGGCATAGTC 16294
PigTailedMacaque  TTCTGACTTTTCAT----GTATGTGATGTTTTTCCTAGGAACATTTCTTATACTTTACCTTGTTTGTGCTAGGCATAGTC 15534
Bonobo            TTCTGACTTTTCGT----GTATGTCATGTTTTTCCCAGGAACATTTCTTATACTTTACCTTGTTTGTGCTAGGCATAGTC 14865
OliveBaboon       TTCTGACTTTTCAT----GTATGTGATGTTTTTCCTAGGAACATTTCTTATACTTTACCTTGTTTGTGCTAGGCATAGTC 14991
Orangutan         TTCTGACTTTTCGT----GTATGTCATGTTTTTCCCAGGAACATTTCTTATACTTTACCTTGTTTGTGCCAGGCATAGTC 15171

Majority          TGTGCTCAATAATTATCTCTTTTTTTTTTCTTT----TTTTTG-----------------AGAGATG-GAGTC-TTGCCC

                          19770     19780     19790     19800     19810     19820     19830     19840

Human             TGTGCTCAATAATTGTCTCCTTTTTTTTTCTTTTTTTTTTTTTTTTTTTCCTGCTTTTGAGAGACAAGAGTC-TTGCCC 13888
Coquerel'sSifaka  TGTTCTCA--AATAATATTGATCAATATTCTT---------------------------AAAAGAT---A---------- 13307
Ma'sNightMonkey   ---------------------------TTTT------------------------------------------------- 12482
VervetAGM         TCATTTCTGTACTCTTAACTGTTTTGCCAATGTGAAATCCTTGC----------A----AAGTAATG-GATCTATTACTT 16265
Gorrilla          TGTGCTCAATAATTGTCTCCTTTTTTTTTTTCTTTTTTCTTTTTTT--TCCTGTTTTTTGAGAGACA-GAGTC-TTGCCC 16244
GrabEatingMacaqueTGTGCTCAATAATTATCTCTCTTTTTTTCCTGT----TTTTTG-----------------AGAGATG-GAGTC-TTGCCC 16292
Macaque           TGTGCTCAATAATTATCTCTCTTTTTTTCCTGT----TTTTTG-----------------AGAGATG-GAGTC-TTGCCC 16351
PigTailedMacaque  TGTGCTCAATAATTATCTCTCTTTTTTTCCTGT----TTTTTG-----------------AGAGATG-GAGTC-TTGCCC 15591
Bonobo            TGTGCTCAATAATTGTCTCCTTTTTTTTTTTTTTCCTGTTTT--------------TTTGAGAGACA-GAGTC-TTGCCC 14929
OliveBaboon       TGTGCTCAATAATTATCTCTCTTTTTTTCCTGT----TTTTTG-----------------AGAGATG-AAGTC-TTGCCC 15048
Orangutan         TGTGCTCAATAATTGTCTCCTTTTTTTTTTCTTTTTTTTCTGTTT-----------TTTGAGAGACG-GAGTC-TTGCCC 15238

Majority          TGTTGCCCAGGCTGGAGTGCAGTGGCCCAA----ATCTCAGTGCATCCTCTGCCTCCCGGGTTCAAGT-AATTCTCCTGC

                          19850     19860     19870     19880     19890     19900     19910     19920

Human             TGTTGCCCAGGCTGGAGTGCAGTGGCCCAATCTCAGCTCAGTGCAACCTCTGCCTCCCGGATTCAAGC-GATTCTCCTGC 13967
Coquerel'sSifaka  -----TCTACAAAGAAATG---TGTCTTAA-----------------------------------AGT----TCACTTAA 13340
Ma'sNightMonkey   -------------------------------------------------------------------------------- 12482
VervetAGM         GGGAAGACAGAATTGTATGAAATGACTAGA----ATAATAGAGGATGGTCCCCAATACGGTAAAATGTTAACTTTTTAGA 16341
Gorrilla          TGTTGCCCAGGCTGGAGTGCAGTGGCCCAATCTCAGCTCAGTGCAACCTCTGCCTCCCGGGTTCAAGC-GATTCTCCTGC 16323
GrabEatingMacaqueTGTTGCCCAGACTGGAGTGCAGTGTCCC-A----ATCTCAGCGATTCCTCTGCCTCCCGGGTTCAAAT-AATTCTCCTGC 16366
Macaque           TGTTGCCCAGACTGGAGTGCAGTGTCCC-A----ATCTCAGCGATTCCTCTGCCTCCCGGGTTCAAAT-AATTCTCCTGC 16425
PigTailedMacaque  TGTTGCCCAGACTGGAGTGCAGTGGCCT-A----ATCTCAGCGATTCCTCTGCCTCCCGGGTTCAAAT-AATTCTCCTGC 15665
Bonobo            TGTTGCCCAGGCTGGAGTGCAGTGGCCCAATCTCAGCTCAGTGCAACCTCTGCCTCCCGGGTTCAAGT-GATTCTCCTGC 15008
OliveBaboon       TATTGCCCAGGCTGGAGTGCAGTGGCCC-A----ATCTCAGCGATTCCTCTGCCACCCGGGTTCAAGT-AATTCTCCTGC 15122
Orangutan         TGTTGCCCAGGCTGGAGTGCAGTGGCCCAATCTCAGCTCAGTGCAACCTCTGCCTCCCTGGTTCAAGC-GATTCTCCTGC 15317
```

```
Majority          CTCAGCTTCCCAA-TTGCTGGAACTACAGGCGCCTGCCACCACGC--CTGGCTA-----ATTTTTGTATTTTTAGTAGGG

                          19930     19940     19950     19960     19970     19980     19990     20000

Human             CTCAGCTTCCCAAGTAGCTGGAACTACAGGCGCCTGCCACTACAC--CTGGCTA-----ATTTTTGTGTTTTTAGTAGGG 14040
Coquerel'sSifaka  TTCA-----------------------------------------------------------------TATAATAGA- 13353
Ma'sNightMonkey   -----------------------------------------------------G------------------------- 12483
VervetAGM         TGTAGATTTTAAGTATGGTGAAAGTTGTAGCAGTGTTCTTTAGGAAGTTAACTATTAATATCTCAAGACCTATAACTGGA 16421
Gorrilla          CTCAGCTTCCCAAGTAGCTGGAACTACAGGCGCCTGCCACTACAC--CTGGCTA-----ATTTTTGTGTTTTTAGTAGGG 16396
GrabEatingMacaqueCTCAGCTTCCTGA-GTGCTGGGACTACAGGCGCCTGCCACCACGC--CTGGCTA-----ATTTTTGTATTTTTAGTAGGG 16438
Macaque           CTCAGCTTCCCGA-GTGCTGGGACTACAGGCGCCTGCCACCACGC--CTGGCTA-----ATTTTTGTATTTTTAGTAGGG 16497
PigTailedMacaque  CTCAGCTTCCCAA-GTGCTGGGACTACAGGCGCCTGCCACCACGC--CTGGCTA-----ATTTTTGTATTTTTAGTAGGG 15737
Bonobo            CTCAGCTTCCCAAGTAGCTGGAACTACAGGCGCCTGCCACTACAC--CTGGCTA-----ATTTTTGTGTTTTTAGTAGGG 15081
OliveBaboon       CTCAGCTTCCCGA-GTGCTGGGACTACAGGCGCCTGCCACCACGC--CTGGCTA-----ATTTTTGTATTTTTAGTAGGG 15194
Orangutan         CTCAGCTTCCCAAGTAGTTGGAACTACAGGCGCCTGCCACCACAC--CTGGCTA-----ATTTTTGTATTTTTAGTAGGG 15390

Majority          ATAGGGTTTCACCAC--ATTGGCCAGGATGGC------TTCGAACT--CCTGACCTTAA-----GTGATCTGCCCACCTT

                          20010     20020     20030     20040     20050     20060     20070     20080

Human             ATGGGGTTTCACCAC--GTTGGCCAGGCTGAC------TTGGAACT--CCTGACCTCAA-----GTGATCTGCCCACCTT 14105
Coquerel'sSifaka  ----------A--AT----------------------------------------------------------------- 13356
Ma'sNightMonkey   --------------C----------------------------------------------------------------- 12484
VervetAGM         CAACTAATAAATAACCTATTTCTTAAAATGTCAGCTTTTTCTAACTATTCTGAAAGTACTAGTTGTAAATTGCTGAACTC 16501
Gorrilla          ATGGGGTTTCACCAC--GTTGGCCAGGCTGAC------TTCGAACT--CCTGACCTCAA-----GTGATCTGCCCACCTT 16461
GrabEatingMacaqueACAGGGTTTCACCAC--ATTGGCCAGGATGGC------TTCAAACT--CCTGACCTTAA-----GTGATCTGCCCACCTT 16503
Macaque           ACAGGGTTTCACCAC--ATTGGCCAGGATGGC------TTCAAACT--CCTGACCTTAA-----GTGATCTGCCCACCTT 16562
PigTailedMacaque  ACAGGGTTTCACCAC--ATTGGCCAGGATGGC------TTCAAACT--CCTGACCTTAA-----GTGATCTGCCCACCTT 15802
Bonobo            ATGGGGTTTCACCAC--GTTGGCCAGGCTGAC------TTCGAACT--CCTGACCTCAA-----GTGATCTGCCCACCTT 15146
OliveBaboon       ACAGGGTTTCACCAC--ATTGGCCAGGATGGC------TTCAAACT--CCTGACCTTAA-----GTGATCTGCCCACCTT 15259
Orangutan         ATGGGGTTTCACCAC--GCTGGCCAGGCTGAT------TTGGAACT--CCTGACCTCAA-----GTGATCTTCCCACCTT 15455

Majority          GGCTTCCCAA-----AGTGCTGGGATTACAGGCATGAGC---CACTGTGCCTGGCCAATAAATGTCTCTTAATG-AGTAT

                          20090     20100     20110     20120     20130     20140     20150     20160

Human             GGCTTCCCAA-----AGTGCTGGGATTATAGGCATGAGC---CACTGTGCCTGGCCAATAAGTGTCTCTTAATG-AGTAT 14176
Coquerel'sSifaka  -------------------------------------------ACTGTAC--------------------A--------- 13364
Ma'sNightMonkey   -A---------------------------------------C-------------------------------------- 12486
VervetAGM         ATGATTTAAATTATGACTGTTGAATTCACTATCATTGCCTTGCATAGAAAATTACAATCAAATAATGCTAAATGTAACTT 16581
Gorrilla          GGCTTCCCAA-----AGTGCTGGGATTATAGGCATGAGC---CACTGTGCCTGGCCAATAAGTGTCTCTTAATG-AGTAT 16532
GrabEatingMacaqueGGCTTCCCAA-----AGTGCTGGGATTACAGGCATGAGC---CACTGTGCCTGGCCAATAAATGTCTCTTAATG-AGTAT 16574
Macaque           GGCTTCCCAA-----AGTGCTGGGATTACAGGCATGAGC---CACTGTGCCTGGCCAATAAATGTCTCTTAATG-AGTAT 16633
PigTailedMacaque  GGCTTCCCAA-----AGTGCTGGGATTACAGGCATGAGC---CACTGTGCCTGGCCAATAAATGTCTCTTAATG-AGTAT 15873
Bonobo            GGCTTCCCAA-----AGTGCTGGGATTATAGGCATGAGC---CACTGTGCCTGGCCAATAAGTGTCTCTTAATG-AGTAT 15217
OliveBaboon       GGCTTCCCAA-----AGTGCTGGGATTACAGGCATGAGC---CACTGTGCCTGGCCAATAAATGTCTCTTAATG-AGTAT 15330
Orangutan         GGCTTCCCAA-----AGTGCTGGGATTACAGGCATGAGC---CACTGTGCCTGACCAATAAGTGTCTCTTAATG-AGTAT 15526
```

```
Majority        TCTTAAAGGATATCTAAAAAGAAATG-TAACTTAAAATTCACATAATAGT-AATACTATGCAGTGTTAACATTTAATGTA
                        20170     20180     20190     20200     20210     20220     20230     20240

Human           TCTTAAAGGATATCTAAAAAGAAATG-TAACTTAAAATTCACATAATAGT-AATACTATACAGTATTAACATTTAATGTA 14254
Coquerel'sSifaka ---------ATAT-TAAAATTCAATG-TAAATTGGAACTGT--TGATAGTGGATG------AGTGTTGTTTTTT--TTTT 13423
Ma'sNightMonkey ------------------------------------------------------------TA------------------ 12488
VervetAGM       TTTTTGGTGATAGTTGAGTATAACTGGTCGTTTATTATAGATGTATTATTCAAAACTTTGGGTTTTTAGGTTTTGACTTT 16661
Gorrilla        TCTTAAAGGATATCTAAAAAGAAATG-TAACTTAAAATTCACATAATAGT-AATACTATACAGTGTTAACATTTAATGTA 16610
GrabEatingMacaqueTCTTAAAGGATATCTAAAAAGAAATG-TAACTTAAAATTCACATAATAGT-AATACTATGCAATGTTAACATTTAATGTA 16652
Macaque         TCTTAAAGGATATCTAAAAAGAAATG-TAACTTAAAATTCACATAATAGT-AATACTATGCAATGTTAACATTTAATGTA 16711
PigTailedMacaque TCTTAAAGGATATCTAAAAAGAAATG-TAACTTAAAATTCACATAATAGT-AATACTATGCAATGTTAACATTTAATGTA 15951
Bonobo          TCTTAAAGGATATCTAAAAAGAAATG-TAACTTAAAATTCACATAATAGT-AATACTATACAGTGTTAACATTTAATGTA 15295
OliveBaboon     TCTTAAAGGATATCTAAAAAGAAATG-TAACTTACAATTCACATAATAGT-AATACTATGCAATGTTAACATTTAATGTA 15408
Orangutan       TCTTAAAGGATATCTAAAAAGAAATG-TAACTTAAAATTCACATAATAGT-AACACTATACAGTGTTAACATTTAATGTA 15604
```

```
Majority        AGTTGGAA--CTATTGATGGTGGACAAG-AATGTTTTGTC-AAGTTAAGAACATATGATTTAGAATGAAAAGATGAGGGT
                        20250     20260     20270     20280     20290     20300     20310     20320

Human           AGTTGGAA--CTATTGATGGTGGACAAG-AATGTTTTCTT---GTCAAGAACATACGATTTAGAATGAAAAGATGAGGGT 14328
Coquerel'sSifaka AATTGAAG------------------------GTCTTGTC-AAGTTAAGAACATGTGGTTTAGGATTAACAGACTAGAGT 13478
Ma'sNightMonkey ----------------------------------------------------A--------------------------- 12489
VervetAGM       TCTTAAAATGCTTTTCTTAAATGCTACTTAATGCTTTCTCTGGGGTATTTTTATTAGAATTTCAATTAACAGATATTTTA 16741
Gorrilla        AGCTGGAA--CTATTGATGGTGGACAAG-AATGTTTTCTT---GTCAAGAACATACGATTTAGAATGAAAAGATGAGGGT 16684
GrabEatingMacaqueAGTTGGAA--CTATTGATGGTGGACAAG-AATGTTTTGTC-AAGTTAAGAACATATGATTTGGAATGAAAAGATGAGGGT 16728
Macaque         AGTTGGAA--CTATTGATGGTGGACAAG-AATGTTTTGTC-AAGTTAAGAACATATGATTTGGAATGAAAAGATGAGGGT 16787
PigTailedMacaque AGTTGGAA--CTATTGATGGTGGACAAG-AATGTTTTGTC-AAGTTAAGAACATATGATTTGGAATGAAAAGATGAGGGT 16027
Bonobo          AGTTGGAA--CTATTGATGGTGGACAAG-AATGTTTTCTT---GTCAAGAACATACGATTTAGAATGAAAAGATGAGGGT 15369
OliveBaboon     AGTTGGAA--CTATTGATGGTGGACAAG-AATGTTTTGTC-AAGTTAGGAACATATGATTTGGAATGAAAAGATGAGGGT 15484
Orangutan       AGTTGGAA--CTATTGATGGTGGACAAG-AATGTTTTCTT---GTCAAGAACATACGATTTAGAATGAAAAGATGAGGGT 15678
```

```
Majority        TTGACTTTTATAGCATTGAAC-AGGTTAACCTCTGAGCTTCAGTTTTAT-----ACATT--TTTAAATGGTTTCAGTTGT
                        20330     20340     20350     20360     20370     20380     20390     20400

Human           TTGACTTTTATAGCATTGAAC-AGGTTAACCTCTGAGCTTCAGTTTTAT-----ACAT--TTTTAAATGGTTTCAGTTGT 14400
Coquerel'sSifaka TTGAACTCTA--ACATTGAGC-AAGTTAACTTCGGAGCCTTAGTTTTAC-----ACATT--TTTAAATGGGCTTAATTCT 13548
Ma'sNightMonkey ------------------------------------------A--TC------------------------------ 12492
VervetAGM       ATGTCTGCGAAATGACAGTACTATGTTAGAAATTGATGTGTTGTCTCCTTGAGGGCAGTGACCCTAGTTGTTGTGTTTGT 16821
Gorrilla        TTGACTTTTATAGCATTGAAC-AGGTTAACCTCTGAGCTTCAGTTTTAT-----ACAT--TTTTAAATGGTTTCAGTTGT 16756
GrabEatingMacaqueTTGACTTTTATAGCATTGAAC-AGGTTAACCTCTGAGCTTCAGTTTTAT-----ACATT--TTTAAATGGTTTAAATTGT 16800
Macaque         TTGACTTTTATAGCATTGAAC-AGGTTAACCTCTGAGCTTCAGTTTTAT-----ACATT--TTTAAATGGTTTAAGTTGT 16859
PigTailedMacaque TTGACTTTTATAGCATTGAAC-AGGTTAACCTCTGAGCTTCAGTTTTAT-----ACATT--TTTAAATGGTTTAAATTGT 16099
Bonobo          TTGACTTTTATAGCATTGAAC-AGGTTAACCTCTGAGCTTCAGTTTTAT-----ACAT--TTTTAAATGGTTTCAGTTGT 15441
OliveBaboon     TTGACTTTTATAGCATTGAAC-AGGTTAACCTCTGAGCTTCAGTTTTAT-----ACATT--TTTAAATGGTTTAAATTGT 15556
Orangutan       TTGACTTTTATAGCATTGAAC-AGGTTAACCTCTGAGCTTCAGTTTTAT-----ACATATTTTTAAATGGTTTCAGTTGT 15752
```

```
Majority          ACCTACCTCCAAAA-TTATTTTGAGTGTTAGATGAGGTAAGGAAAAGAC-----------TTATGCTGAAATGATGAGA-

                          20410     20420     20430     20440     20450     20460     20470     20480

Human             ACCTACTTCCAAAA-TTATTTTGAATGTTAGATGAGATGAGGAAAAGAC-----------TTATGCTGAAAGGATGAGA- 14467
Coquerel'sSifaka  ACCTACCTCTAAAAGTTATTTTGAAGATTAGATAATGTGTAAAAAATACAGCACAGTGTATGACTCCTAAGTGCTCAAAT 13628
Ma'sNightMonkey   -------------------------------------T------------------------------------------ 12493
VervetAGM         TCCT-CCAACATTA-ATAGTACACGTGTTAAATAGTGCATACACACTTA-----------ATATTTAATAACTCTTAGA- 16887
Gorrilla          ACCTACTTCCAAAA-TTATTTTGAATGTTAGATGAGATGAGGAAAAGAC-------------ATGCTGAAAGGATGAGA- 16821
GrabEatingMacaque ACCTACCTCCACAA-TTATTTTAAGTGTTAGATGAGGTAAGGAAAAGAC-----------TTATGCTGAAATGATGAGA- 16867
Macaque           ACCTACCTCCACAA-TTATTTTAAGTGTTAGATGAGGTAAGGAAAAGAC-----------TTATGCTGAAATGATGAGA- 16926
PigTailedMacaque  ACCTACCTCCACAA-TTATTTTAAGTGTTAGATGAGGTAAGGAAAAGAC-----------TTATGCTGAAATGATGAGA- 16166
Bonobo            ACCTACTTCCAAAA-TTATTTTGAATGTTAGATGAGATGAGGAAAAGAC-----------TTATGCTGAAAGGATGAGA- 15508
OliveBaboon       ACCTACCTCCACAA-TTATTTTAAGTGTTAGATGAGGTAAGGAAAAGAC-----------TTATGCTGAAATGATGAGA- 15623
Orangutan         ACCTACCTCCAAAA-TTATTTTGAATGTTAGATAA-----GGAAAAGAC-----------TTACGCTGAAAGGATGAGA- 15814

Majority          -ATGGTTGTTG-TTGTAGT-TCTTTAATCTGTAA------ATCTACATTTGGAAGGAACACTT--ATGGAAATCAAA--T

                          20490     20500     20510     20520     20530     20540     20550     20560

Human             -ATGGTTGTTG-TTGTAGT-TCTTTAATCTGTAA------ATCTACATTTGGAAGGAACACTTTTATGGAAATCAAA--T 14536
Coquerel'sSifaka  GATGGTTGTTA-TTGTAGT-TCTTTGTTCTGTAA------ATCCAAATTTGGAAGAAGCACTTTAATGGAAATCAAA--T 13698
Ma'sNightMonkey   -------------------------------------------------------------------------------- 12493
VervetAGM         -AGTGTCATAGGCTTTAATATGCTTATACTCTAATAAACCATTTATCAATAAAAAGGTAAATT--CCAAGACTCTGTCTT 16964
Gorrilla          -ATGGTTGTTG-TTGTAGT-TCTTTAATCTGTAA------ATCTACATTTGGAAGGAACACTTTTATGGAAATCAAA--T 16890
GrabEatingMacaque -ATGGTTGTTG-TTGTAGT-TCTTTAATCTGTAA------ATCTACATTTGGAAGGAACACTT--ATGGAAATCAAA--T 16934
Macaque           -ATGGTTGTTG-TTGTAGT-TCTTTAATCTGTAA------ATCTACATTTGGAAGGAACACTT--ATGGAAATCAAA--T 16993
PigTailedMacaque  -ATGGTTGTTG-TTGTAGT-TCTTTAATCTCTAA------ATCTACATTTGGAAGGAACACTT--ATGGAAATCAAA--T 16233
Bonobo            -ATGGTTGTTG-TTGTAGT-TCTTTAATCTGTAA------ATCTACATTTGGAAGGAACACTTTTATGGAAATCAAA--T 15577
OliveBaboon       -ATGGTTGTTG-TTGTAGT-TCTTTAATCTGTAA------ATCTACATTTGGAAGGAACACTT--ATGGTAATCAAA--T 15690
Orangutan         -ATGGTTGTTG-TTGTAGT-TCTTTAATCTGTAA------ATCTACATTTGGAAGGAACACTTTTATAGAAATCAAA--T 15883

Majority          ACAGTAAATTAATTTCTAAGAGATCCTGGAGTTCAG-GTTAAAAATAGCTGCAG-------ACAGCACTAAATATCTTTA

                          20570     20580     20590     20600     20610     20620     20630     20640

Human             ACAGTAAATTAATTTCTAAGAGGTCCTGGAGTTCAG-GTTAAAAATAGCCTCAG-------ACAGCACTAAATATCTTTA 14608
Coquerel'sSifaka  ATGATAAATTAATTTCTAAGAGATCCTGGAATTCA--GATTAAAATAGGTGCCA---G---GCAGCACTAAAGAGAATT- 13769
Ma'sNightMonkey   -------------------------------------G---------------------------CACTAAATATCTTTG 12509
VervetAGM         TTAGGACTTCATCTAGTTAGAGAAATAAGGATCCAGAGTTTTAATTAAGTGCTATGTGGCTACAGTAGGAAAGATTGGTT 17044
Gorrilla          ACAGTAAATTAATTTCTAAGAGATCCTGGAGTTCAG-GTTAAAAATAGCCTCAG-------ACAGCACTAAATATCTTTA 16962
GrabEatingMacaque ACAGTAAATTAATTTCTAAGAGATCCTGGAGTTCAG-GTTAAAAATAGCTGCAG-------ACAGGACTAAATATCTTTA 17006
Macaque           ACAGTAAATTAATTTCTAAGAGATCCTGGAGTTCAG-GTTAAAAATAGCTGCAG-------ACAGGACTAAATATCTTTA 17065
PigTailedMacaque  ACAGTAAATTAATTTCTAAGAGATCCTGGAGTTCAG-GTTAAAAATAGCTGCAG-------ACAGGACTAAATATCTTTA 16305
Bonobo            ACAGTAAATTAATTTCTAAGAGATCCTGGAGTTCAG-GTTAAAAATAGCCTCAG-------ACAGCACTAAATATCTTTA 15649
OliveBaboon       ACAGTAAATTAATTTCTAAGAGATCCTGGAGTTCAG-GTTAAAAATAGCTGCAG-------ACAGGACTAAATATCTTTA 15762
Orangutan         ACAGTAAATTAATTTCTAAGAGATCCTGGAGTTCAG-GTTAAAAATAGCTGCAG-------ACAGCACTAAATATCTTTA 15955
```

```
Majority          TT-AGTGAACAAAAAAATAAAAATGACTTAGAATTTAGC---ACAGAGAAACA-----AAGAGATGAAAATAGAAGAGGT

                          20650     20660     20670     20680     20690     20700     20710     20720

Human             TT-AGTGAACAAAAAAATAAAAATAACTTAGAATTTAGC---ACAGAGAAGCA-----AAGAGATGAAAATAGGAGAAGC 14679
Coquerel'sSifaka  ---AATGAACTAAAAAATAAAAATAACTCAGAATTTACC---ACGAAGCAATA-----GAGAGATGAAAATAGCAGAGGA 13838
Ma'sNightMonkey   TTTAATGAATAAAAAAATAAACATTACTC--AATTCAGC---ACAGAGAG---------AGAGAAGAAAATAGCAGAAGG 12575
VervetAGM         TG-GGTTGGTAGGCTCAGAATAGTGATCTGTGACCTAGCCAAACTGGGAGATAGGGGAAGGAGTGGAGAGCAATAGGCAT 17123
Gorrilla          TT-AGTGAACAAAAAAATAAAAATAACTTAGAATTTAGC---ACAGAGAAGCA-----AAGAGATGAAAATAGGAGAAGG 17033
GrabEatingMacaqueTT-AGTGAACAAAAAAATAAAAGTGACTTAGAATTTAGC---ACAGAGAAACA-----AAGAGATGAAAATAGAAGGGTT 17077
Macaque           TT-AGTGAACAAAAAAATAAAAGTGACTTAGAATTTAGC---ACAGAGAAACA-----AAGAGATGAAAATAGAAGGGTT 17136
PigTailedMacaque  TT-AGTGAACAAAAAAATAAAAATGACTTAGAATTTAGC---ACAGAGAAACA-----AAGAGATGAAAATAGAAGGGTT 16376
Bonobo            TT-AGTGAACAAAAAAATAAAAATAACTTAGAATTTAGC---ACAGAGAAGCA-----AAGAGATGAAAATAGGAGAAGG 15720
OliveBaboon       TT-AGTGAACAAAAAAATAAAAATGACTTAGAATTTAGC---ACAGAGAAACA-----AAGAGATGAAAATAGAAGAGTT 15833
Orangutan         TT-AGTGAACAAAAAAATAAAAATAACTTAGAATTTAGC---ACAGAGAAACA-----AAGAGATGAAAATAGCAGAAGA 16026

Majority          -GTTAAAAATATGTTGGAAAGGAAGAGAAGTTCTAACAAACATCTAATTAGAGTTCCAGA--AAGAAGTTAAAGATTGCA

                          20730     20740     20750     20760     20770     20780     20790     20800

Human             -GTTAAAAATGTGTTGGAAAGGAAGAGAAGTTCTAACAAACATCTAATTAGCGTTCCAGA--AA----TTAAAGATTGCA 14752
Coquerel'sSifaka  --GTTAAAAGATGA-GGAAAGGAAGAGAAGTTCTAATGAACAACTAATTAGAGTTACAGAAAGAAAAATTAAAGATAGCA 13915
Ma'sNightMonkey   -GTTAAAAATATGTTGGAAAGGAAGAGAAGTTCTAACAAACATCTAATTAGAGTTCCAGA--AAGAAATTAAACATTGCT 12652
VervetAGM         TGAGAGAAAGACATTAACTAAAGTTGTGTGTGTAGGTTGGCATAATTTTACAACCATTGATCAGTGAGTTGAATAATTTG 17203
Gorrilla          -GTTAAAAATGTGTTGGAAAGGAAGAGAAGTTCTAACAAACATCTAATTAGAGTTCCAGA--AAGAAATTAAAGATTGCA 17110
GrabEatingMacaque----AAAAATACATTGGAAAGGAAGAGAAGTTCTAACAAACATCTAATTAGAATTCCAGA--AAGAAGTTAAAGATTGCA 17151
Macaque           ----AAAAATACATTGGAAAGGAAGAGAAGTTCTAACAAACATCTAATTAGAATTCCAGA--AAGAAGTTAAAGATTGCA 17210
PigTailedMacaque  ----AAAAATACATTGGAAAGGAAGAGAAGTTCTAACAAACATCTAATTAGAATTCCAGA--AAGAAGTTAAAGATTGCA 16450
Bonobo            -GTTAAAAATGTGTTGGAAAGGAAGAGAAGTTCTAACAAACATCTAATTAGAGTTCCAGA--AAGAAATTAAAGATTGCA 15797
OliveBaboon       ----AAAAATACATTGGAAAGGAAGAGAAGTTCTAACAAACATCTAATTAGAATTCCAGA--AAGAAGTTAAAGATTGCA 15907
Orangutan         -GTTAAAAATGTGTTGGAAAGGAAGAGAAGTTCTAACAAACATCTAATTAGAGTTCCAGA--AAGAAATTAAAGATTGCA 16103

Majority          GA----AAGGCAAA--------------ATTTGAAATGCTAAA--GGCTGATAATTTTT-TAGAATTGGTGAGCTATATG

                          20810     20820     20830     20840     20850     20860     20870     20880

Human             GA----AAGGCAAA--------------ATTTGAAATGCTAAA--GGCTGATAATTTTT-CAGAATTGGTGAGCTATATG 14811
Coquerel'sSifaka  GA----GAGGCAAA--------------ATTTGAAATGCTAAA--GGCTGATAATTTTC-TAGAATTGATGAGCTTCATG 13974
Ma'sNightMonkey   GA----AAGGCAAA--------------ATTTGAAGTGCTAAA--AGCTGATAATTTTC-TAGAATTGGTGAGCTATGTG 12711
VervetAGM         GCCTTTATGGCTAACAGTGTGTATATGGATATGAGAGAAAAGATAGAATGGCAGGTTATATAGAAGGCCTCAATTACAGG 17283
Gorrilla          GA----AAGGCAAA--------------ATTTGAAATGCTAAA--GGCTGATAATTTTC-CAGAATTGGTGAGCTATATG 17169
GrabEatingMacaqueGA----AAGGCAAA--------------ATATGAAACTCTAAA--GGCTGGTAAGTTTT-TAGAATTGGTGAGCTATATG 17210
Macaque           GA----AAGGCAAA--------------ATATGAAACTCTAAA--GGCTGATAAGTTTT-TAGAATTGGTGAGCTATATG 17269
PigTailedMacaque  GA----AAGGCAAA--------------ATATGAAACGCTAAA--GGCTGATAAGTTTT-TAGAATTGGTGAGCTATATG 16509
Bonobo            GA----AAGGCAAA--------------ATTTGAAATGCTAAA--GGCTGATAATTTTT-CAGAATTGGTGGGCTATATG 15856
OliveBaboon       GA----AAGGCAAA--------------ATATGAAATGCTAAA--GGCTGATAAGTTTT-TAGAATTGGTGAGCTATATG 15966
Orangutan         GA----AAGGCAAA--------------ATTTGAAATGCTAAA--GGCTGATAATTTTT-CAGAATTGGTGAACTATATG 16162
```

```
Majority          ------AATTCTTAG---ATTTAGGAGGCACAA----GAAAACCTG-GACACGTAACAGTGAAAGAGCAGAGCACCAAAG

                          20890     20900     20910     20920     20930     20940     20950     20960

Human             ------AGTTCTTAG---ATTTAGGAGGCACAA----GAAAACCTG-GACACATAACAGTAAAAGAGCAGAGCACCAAAG 14877
Coquerel'sSifaka  ------AATTCTTAG---ATTTAGGAGGTAGAA----GAAAATCTA-GATGCGTCAGAGTGAAAGAGCAGAAAACCAAAG 14040
Ma'sNightMonkey   ------AGTTATCAG---ATTTAGGAGGCACAA----AAAAACCTA-GACACATTACAATGAAAGAGCAGAACACGAAAG 12777
VervetAGM         TTGTGAAATTTGTACTTCATCTAGTAGGCATTATTCTATATACCTGTAATTTCTCGCATAAAAGTAGCTCATTATTCTGC 17363
Gorrilla          ------AGTTCTTAG---ATTTAGGAGGCACAA----GAAAACCTG-GACACATAACAGTGAAAGAGCAGAGCACCAAAG 17235
GrabEatingMacaque------AATTCTTAG---ATTTAGGAGACACAA----GAAAACCTG-GACATGTAACAGTGAAAGAGCGGAGCACCAAAG 17276
Macaque           ------AATTCTTAG---ATTTAGGAGACACAA----GAAAACCTG-GACATGTAACAGTGAAAGAGCGGAGCACCAAAG 17335
PigTailedMacaque  ------AATTCTTAG---ATTTAGGAGACACAA----GAAAACCTG-GACATGTAACAGTGAAAGAGCAGAGCACCAAAG 16575
Bonobo            ------AGTTCTTAG---ATTTAGGAGGCACAA----GAAAACCTG-GACACATAACAGTAAAAGAGCAGAGCACCAAAG 15922
OliveBaboon       ------AATTCTTAG---ATTTAGGAGACACAA----GAAAACCTG-GACATGTAACAGTGAAAGAGCAGAGCACCAAAG 16032
Orangutan         ------AGTTCTTAG---ATTTAGGAGGCACAA----GAAAACCTG-GACACCTAACAGTGAAAGAGCAGAGCACCAAAG 16228

Majority          ACATAGAGATCTTTAAAGCAGCCAGAGAAAA-TCAGATTCCCTAAA---GAATAA-------GAATTAGAAAGTCCCAAT

                          20970     20980     20990     21000     21010     21020     21030     21040

Human             ACATAGAGATCTTTAAAGCAGCCAGAGAAAA-TCAGATTCCCTAAA---GAATAA-------GAACTAGAAAGTCCTAAT 14946
Coquerel'sSifaka  ACACA--GATCTTTAAAGAAGTCAGGGAAAAATCAGATTCCCTAAGAAGGAATAAGAACTGTTTAGTAGAAAGATACAAT 14118
Ma'sNightMonkey   ACAGAGAGATCTTTAAAGGAGCCAGAGAAAA-TGAGATTCCCTAAG---GAATAA-------GAACTAGAAAGACCCAAT 12846
VervetAGM         TTTTTTTTTTTTATGCTATAACAAATTTATT-GTAACAACACAAAG---GTATTATC-TTACAGTTTTGTAGGTGAGAAT 17438
Gorrilla          ACATAGAGATCTTTAAAGCAGCCAGAGAAAA-TCAGATTCCCTAAA---GAATAA-------GAACTAGAAAGTCCCAAT 17304
GrabEatingMacaqueACATAAAGATCTTTAAAGCAGCCAGAGAAAA-TCAGATTCCCTAAA---GAATAA-------GAATTAGAAAGCCCCAAT 17345
Macaque           ACATAAAGATCTTTAAAGCAGCCAGAGAAAA-TCAGATTCCCTAAA---GAATAA-------GAATTAGAAAGCCCCAAT 17404
PigTailedMacaque  ACATAAAGATCTTTAAAGCAGCCAGAGAAAA-TCAGATTCCCTAAA---GAATAA-------GAATTAGAAAGCCCCAAT 16644
Bonobo            ACATAGAGATCTTTAAAGCAGCCAGAGAAAA-TCAGATTCCCTAAA---GAATAA-------GAACTAGAAAGTCCCAAT 15991
OliveBaboon       ACATAAAGATCTTTAAAGCAGCCAGAGAAAA-TCAGATTCCCTGAA---GAATAA-------GAATTAGAAAGCCCCAAT 16101
Orangutan         ACATAGAGATCTTTAAAGCAGCCAGAGAAGA-TCAGATTCCCTAAA---GAATAA-------GAACTAGAAAGTCCCAAT 16297

Majority          ATGCTTCATCAGCA---TGGGCATCCAGAAGATAGTGGAATAATATTTT------CAATGTGTTGAGTGAAAATAGCTGT

                          21050     21060     21070     21080     21090     21100     21110     21120

Human             ATGCTTCATCAGCA---TGGGCATCCAGAAGATAGTGGAATAATATTTT------CAATGTGTTGAGTGAAAATAGCTGC 15017
Coquerel'sSifaka  ATGCTTCATCAGCAATATGGGGATCCAGAAGATAGTGGAATAAATTCTT------CGAAGTGTTGAGTGAAAGTAATCAT 14192
Ma'sNightMonkey   ATGCTTCATCAGCA---TAGGCATTCAGAAGATAGTGGAATAGTATTTT------CAAAGTTTTGAGTGAAAATAGCTGT 12917
VervetAGM         CTGACACAGGTGGG---CTAAAATGAAGGTGTTAGCCGGGTTGTGTTCCTTTTTGCAGGATCTGGGGGGGAGTTTGTTTT 17515
Gorrilla          ATGCTTCATCAGCA---TGGGCATCCAGAAGATAGTGGAATAATATTTT------CAACGTGTTGAGTGAAAATAGCTGC 17375
GrabEatingMacaqueATGCTTCATCAGCA---TGGGCATCCAGAAGATAGTGGAATAATATTTT------CAGTGTGTTGAGGGAAAATAGCTGT 17416
Macaque           ATGCTTCATCAGCA---TGGGCATCCAGAAGATAGTGGAATAATATTTT------CAGTATGTTGAGGGAAAATAGCTGT 17475
PigTailedMacaque  ATGCTTCATCAGCA---TGGGCATCCAGAAGATAGTGGAATAATATTTT------CAGTGTGTTGAGGGAAAATAGCTGT 16715
Bonobo            ATGCTTCATCAGCA---TGGGCATCCAGAAGATAGTGGAATAATATTTT------CAATGTGTTGAGTGAAAATAGCTGC 16062
OliveBaboon       GTGCTTCATTAGCA---TGGGCATCCAGAAGATAGTGGAATAATATTTT------CAGTGTGTTGAGGGAAAATAGCCAT 16172
Orangutan         ATGCTTCATCAGCA---TGGGCATCCAGAAGATAGTGGAATAATATTTT------CAATGTATTGAGTGAAAATAGCTGC 16368
```

```
Majority        CC-ACCTATTATTTTATAGTCAGCTAAATTATCATATAAGGAGAAGAACAAAATAAAGACACTTTTTGA----AACACTT

                        21130     21140     21150     21160     21170     21180     21190     21200

Human           CC-ACCTGTTATTTTATAGCCAGCCAAATTATCATATAAGGAGAAGAACAAAATAAAGACACTTTTTGA----AACACGT 15092
Coquerel'sSifaka CT-----ATTATTTTATAGTCAGCTAAATTATCACATAAGGATAAGAACAAAATAAAGACATTTTCTGA----AACACTC 14263
Ma'sNightMonkey CC-ACCTATTATTTTATAGTTAGCTAAATTATCAT---ATGAGAAGAACAAAATAAAGACACTTTTTGA----AACACTT 12989
VervetAGM       CT-TCACATATCTCAGCTGTTAGCTTCATCTTCAAAGAAGCAATGGCAAAGAAGACTCTCACATCACATCACTGACACAT 17594
Gorrilla        CC-ACCTGTTATTTTATAGTCAGCCAAATTATCATATAAGGAGAAGAACAAAATAAAGACACTTTTTGA----AACACGT 17450
GrabEatingMacaqueCC-ACTTATTATTTTGTAGTCAACTAAATTATCATATAAGGAGAAGAACAAAATAAAGACGCTTTTTGA----AACACTT 17491
Macaque         CC-GCTTATTATTTTGTAGTCAACTAAATTATCATATAAGGAGAAGAACAAAATAAAGACGCTTTTTGA----AACACTT 17550
PigTailedMacaque CC-ACTTATTATTTTATAGTCAACTAAATTATCATATAAGGAGAAGAACAAAATAAAGACGCTTTTTGA----AACACTT 16790
Bonobo          CC-ACCTGTTATTTTATAGCCAGCCAAATTATCATATAAGGGGAAGAACAAAATAAAGACACTTTTTGA----AACACGT 16137
OliveBaboon     CC-ACTTATTATTTTATAGTCAACTAAATTATCATATAAGGAGAAGAACAAAATAAAGACGCTTTTTGA----AACACTT 16247
Orangutan       CCCACCTATTATTTTATAGTCAGCTAACTTAT-----AAGGAGAAGAACAAAATAAAGACACTTTTTGA----AACACGT 16439

Majority        CATTCTGCCTCCAAAAAAGCAATTTAGGAAATTTAT-----CAACAGACCCACTGAAGGAATTTCTGG--AGTTTGTACT

                        21210     21220     21230     21240     21250     21260     21270     21280

Human           CATTCTGCCTCCAAAAAAGCAATTTAGGAAATGTAT-----CAACAGACCCACTGAAGGAATTTCTGG--AGTTTGTACT 15165
Coquerel'sSifaka CATTCTCCC---------GCAATTGAAGAAATTTGT-----CAACAGACCCACTGAAGGTATTACTAG--AGGTTGTACT 14327
Ma'sNightMonkey CATTCTGCCTCCAAAAAAGCAATTTAGGAAATTTAT-----CAACAGACCCACTGAAGGAGTTTCTGG--GGTTTGTATT 13062
VervetAGM       TCTTGTGCCTCCCTTTCCCACTTATAGGAACCTTGTGATTACACTGGGCCCAACTACATAATTCAGGATAAATGTTTAGA 17674
Gorrilla        CATTCTGCCTCCAAAAAAGCAGTTTAGGAAATGTAT-----CAACAGACCCACTGAAGGAATTTCTGG--AGTTTGTACT 17523
GrabEatingMacaqueCATTCTGCCTCCAAAAAAGCAATTTAGGAAATTTAT-----CAACAGATCCACTGAAGGAATTTCTGG--AGTTTGTACT 17564
Macaque         CATTCTGCCTCCAAAAAAGCAATTTAGGAAATTTAT-----CAACAGATCCACTGAAGGAATTTCTGG--AGTTTGTACT 17623
PigTailedMacaque CATTCTGTCTCCAAAAAAGCAATTTAGGAAATTTAT-----CAACAGATCCACTGAAGGAATTTCTGG--AGTTTGTACT 16863
Bonobo          CATTCTGCCTCCAAAAAAGCAATTTAGGAAATGTAT-----CAACAGACCCACTGAAGGAATTTCTGG--AGTTTGTACT 16210
OliveBaboon     CATTCTGCCTCCAAAAAAGCAATTTAGGAAATTTAT-----CAACAGATCCACTGAAGGAATTTCTGG--AGTTTGTACT 16320
Orangutan       CATTCTGCCTCCAAAAAAGCAATTTAGGAAATGTAT-----CAACAGACTCACTGAAGGAATTTCTGG--AGTTTGTACT 16512

Majority        TCAGGAAGAAAGCCAATGACACCAGAAAGGAAGTCTGACGTAGAAGTGAGGCTAATCAAGAAA-TTGGTAGGCCTGTGAG

                        21290     21300     21310     21320     21330     21340     21350     21360

Human           TCAGGAAGAAAGCCAATGACACCAGAAAGGAAGTCTGATATAGAAGTGAGGCTAATCAAGAAA-TTGGTAGGTCTGTGAG 15244
Coquerel'sSifaka TCAGGAAGAAGGCCAATGACACCAGAAAG---GTCTGAAGTAGAAGTAAGGCTAATCAAGAAA-TTGGTAAACCTGTGGG 14403
Ma'sNightMonkey TCAGGAAGAAAGCCAATGACACCAGAAAGGAGGTCTGAGGTAGAAGTTAGGCTAATTAAGAAA-TTGGTATGCCTGTGGG 13141
VervetAGM       TTACAAATTAATGAAGTGGCAACTGAAACAAGTTTTAACCTATTCACAAGA------AAGGGCTTTGCTAGAGTGGTGGC 17748
Gorrilla        TCAGGAAGAAAGCCAATGATACCAGAAAGGAAGTCTGATATAGAAGTGAAGCTAATCAAGAAA-TTGGTAGGTCTGTGAG 17602
GrabEatingMacaqueTCAGGAAGAAAGCCAATGACACCAGAAAGTAAGTCTGACGTAGAAGTGAGGCTAATCAAGAAAATTGGTAGGCCTGTGAG 17644
Macaque         TCAGGAAGAAAGCCAATGACACCAGAAAGTAAGTCTGACGTAGAAGTGAGGCTAATCAAGAAAATTGGTAGGCCTGTGAG 17703
PigTailedMacaque TCAGGAAGAAAGCCAATGACACCAGAAAGTAAGTCTGACGTAGAAGTGAGGCTAATCAAGAAAATTGGTAGGCCTGTGAG 16943
Bonobo          TCAGGAAGAAAGCCAATGACACCAGAAAGGAAGTCTGATATAGAAGTGAGGCTAATCAAGAAA-TTGGTAGGTCTGTGAG 16289
OliveBaboon     TCACGAAGAAAGCCAATGACACCAGAAAGTAAGTCTGACGTAGAAGTGAGGCTAATCAAGAAAATTGGTAGGCCTGTGAG 16400
Orangutan       TCAGGAAGAAAGCCAATGACACCAGAAAGGAAGTCTGATATAGAAGTGAGGCTAATCAAGAAA-TTGGTAGGTCTGTGAG 16591
```

```
Majority          TAAATCTAATTACTG-ACCATATAAGAATGTAATA---------------------------------GCAGT--GATTG

                          21370     21380     21390     21400     21410     21420     21430     21440

Human             TAAATCTAATTACTG-ACCATATAAGAATGTAATA---------------------------------GCAGT--GATTG 15288
Coquerel'sSifaka  TAAATCTAATTACTGAATGATATAAGAATGTAATA---------------------------------GCAGT--GATTG 14448
Ma'sNightMonkey   TAAATCTAATTACTG-ACAATATAAGAATGTAATA---------------------------------ACAGT--GTTTG 13185
VervetAGM         TCACACCTATAATCCCAGAACTTAGGGAGGCTGAG---------------------------------GCAGGCAGATCG 17795
Gorrilla          TAAATCTAATTACTG-ACCGTATAAGAATGTAATA---------------------------------GCTGT--GATTG 17646
GrabEatingMacaque TAAATCTAATTATTG-ACCATATAAGAATGTAATA---------------------------------GCAGT--GATTG 17688
Macaque           TAAATCTAATTATTG-ACCATATAAGAATGTAATA---------------------------------GCAGT--GATTG 17747
PigTailedMacaque  TAAATCTAATTATTG-ACCATATAAGAATGTAATA---------------------------------GCAGT--GATTG 16987
Bonobo            TAAATCTAATTACTG-ACCATATAAGAATGTAATA---------------------------------GCAGT--GATTG 16333
OliveBaboon       TAAACCTAATTATTG-ACCATATAAGAATGTAATA---------------------------------GCAGT--GATTG 16444
Orangutan         TAAATCTAATTACTG-ACCATATAAGAATGTAATATGGTCATTCTTACATATTACATTCTTATAATATGTAAG--AATTA 16668

Majority          ATA--------------------AGGGGTTTAAAAATAGAAT--------TAAAAATATTAAATCATAACAATA-TGTAA

                          21450     21460     21470     21480     21490     21500     21510     21520

Human             ATA--------------------AGGGGTTTAAAAATAGAAT--------TAAAAATATTAAATCATAACAATA-TGTGA 15339
Coquerel'sSifaka  ATG--------------------GGGATTTAAAAAATAGAGC--------TAAAAATGTTTAATCATGACAACA---TGT 14497
Ma'sNightMonkey   ATA--------------------AGGGGGTTAAAAATAGAAT--------TAAACATAATAAATTATAACAATA-TGTAA 13236
VervetAGM         CTTGAGCCC--------------AGGAATTTGAGACCAGCCTGGTCCTCATAGCAAGACCCCATCTCTACAACAACTTAA 17861
Gorrilla          ATA--------------------AGGGGTTTAAAAATAGAAT--------TAAAAATATTAAATCATAACAATA-TGTGA 17697
GrabEatingMacaque TTA--------------------AGGGGTTTAAAAATAGAAT--------TAAAAATACTGAATTATAACAATA-TGTAA 17739
Macaque           TTA--------------------AGGGGTTTAAAAATAGAAT--------TAAAAATACTGAATTATAACAATA-TGTAA 17798
PigTailedMacaque  TTA--------------------AGGGGTTTAAAAATAGAAT--------TAAAAATACTGAATTATAACAATA-TGTAA 17038
Bonobo            ATA--------------------AGGGGTTTAAAAATAGAAT--------TAAAAATATTAAATCATAACAATA-TGTGA 16384
OliveBaboon       TTA--------------------AGGGGTTTAAAAATAGAAT--------TAAAAATACTGAATCATAACAATA-TGTAA 16495
Orangutan         ATATTTCTTACATATTAAATATTAAAGGTTTAAAAATAGAAT--------TAAAAATATTAAATCATAACAATA-TGTAA 16739

Majority          GAGAGTGGTAGAGACACTGGAGTTTGAGTTCTGATGATCTAGTATTGATTGGAGCTGTGTTGTCAGTTACAGTAGCTACT

                          21530     21540     21550     21560     21570     21580     21590     21600

Human             GAGAGTGGTAGAGACACTGGAGTTTGAGTTCTGATGATCTAGTATTGATTGGAGCTGTGTTGTCAGTTACAGTAGCTACT 15419
Coquerel'sSifaka  AGAGTTAGTAGAGTGACTAGAGTTTTAGTTCCAATGATCTAATGTTGATTGGAGCTGCGTCATCCATTACAGTAGCTACT 14577
Ma'sNightMonkey   GAGAGCAGTAGGGTCCCTGGAG-TTAAGTTCTGATGATCTAATATTGCTTGGAGTTTTGTTGTCAGTTACAGTAGCTACC 13315
VervetAGM         AAAAATTAGATAGAT-CTGGTGGCATGGGCCTGTAGTCCCAGCTACTTGGGAGACTGAGATAAGAGGATCACCTGAGCCC 17940
Gorrilla          GAGAGTGGTATAGACACTGGAGTTTGAGTTCTGATGATCTAGTATTGATTGGAGCTGTGTTGTCAGTTACAGTAGCTACT 17777
GrabEatingMacaque GAGAGTACTATAA------------------------------------------------------------------- 17752
Macaque           GAGAGTGGTAGAGACACTGGAGTTTAAGTTCTGATGATGTAGTATTTATTGGAGCTGTGTTATCAGTTACAGTAGCTACT 17878
PigTailedMacaque  GAGAGTGGTAGAGACACTGGAGTTTAAGTTCTGATGATGTAGTATTTATTGGAGCTGTGTTATCAGTTACAGTAGCTACT 17118
Bonobo            GAGAGTGGTAGAGACACTGGAGTTTGAGTTCTGATGATCTAGTATTGATTGGAGCTGTGTTGTCAGTTACAGTAGCTACT 16464
OliveBaboon       GAGAGTGGTAGAGACATTGGAGTTTGAGTTCTGATGATGTAGTATTTATTGGAGCTGTGTTGTCAGTTACAGTAGCTACT 16575
Orangutan         GAGAGTGGTAGAGACACTGGAGTTTGAGTTCTGATGATCTAGTATTGATTGGAGCTGTGTTGTCAGTTACAGTAGCTACT 16819
```

```
Majority          AGTCAT-TTGTTAC---TGAGCACTTGACATGTATGTAGTTTAAGTTG----ATCTTCTACCTTAGTTGAAAAAT----A
                           21610     21620     21630     21640     21650     21660     21670     21680
Human             AGTCAT-TTGTTAC---TGAGCACTTGACATGTATGTAGTTTAAGTTG----ATCTTCTAC-TTAATTGAAAAAT----A 15486
Coquerel'sSifaka  AGTCATGTGGTTAT---TGAGCACTTGACATGTGTCTGGTCTAAATTG----ATCTCCT---TTAGTTGAAAAATGTAAA 14647
Ma'sNightMonkey   TGTCATCTTGTTAC---TGAGCACTTGACATGTACATAGTTTAAGCTG----ATCTTCTACCTTAGTTGAAAAAT----A 13384
VervetAGM         AGGCGG-CAGTGAGCTGTGATCGTATCACTACAGTCCAGCCTGGGCGAGAGGATATCCAGTCTCAA--AAAAAAA----A 18013
Gorrilla          AGTCAT-TTGTTAC---TGAGCACTTGACATGTATGTAGTTTAAGTTG----ATCTTCTAC-TTAATTGAAAAAT----A 17844
GrabEatingMacaque---------------------TACTTGACATGAATGTAGTTTAAGTT--------------------------------- 17778
Macaque           AGTCAT-TTGTTAC---TAAGCACTTGACATGAATGTAGTTTAAGTTG----ATTTTCTACCTTAGTTGAAAAAT----A 17946
PigTailedMacaque  AGTCAT-TTGTTAC---TAAGCACTTGACATGAATGTAGTTTAAGTTG----ATTTTCTACCTTAGTTGAAAAAT----A 17186
Bonobo            AGTCAT-TTGTTAC---TGAGCACTTGACATGTATGTAGTTTAAGTTG----ATCTTCTAC-TTAATTGAAAAAT----A 16531
OliveBaboon       AGTCAT-TTGTTAC---TGAGCACTTGACATGAATGTAGTTTAAGTTG----ATTTTCTACCTTAGTTGAAAAAT----A 16643
Orangutan         AGTGAT-TTGTTAC---TGAGCACTTGACATGTATGTAGTTTAAGTTG----ATCTTCTACCTTAGTTGAAAAAT----A 16887

Majority          CAGATTTCTAAGCCTTAGTCTGAAAGAAGAATGTAAAATATCCCAAGTTTTCATAT--TGGTTACATGTAGAACTGATAA
                           21690     21700     21710     21720     21730     21740     21750     21760
Human             CACATTTCTGAGCCTTAGTCTGAAAGAAGAATGTAAAATATCCCAAGTTTTCATAT--TGGTTACATGTACAGCGGATAA 15564
Coquerel'sSifaka  CAGATTTCTAAGTCTTAGTATAAAGGAAGAATGTAAAATATGTCAATTTTTTATAT--TGGTTACATATTGAACTGATAA 14725
Ma'sNightMonkey   CAGATTTCTAAGCCTTAGTCTGAAAGATGAATGTA------------TTTT---------TTTTTTTTTTTAAGAGATGG 13443
VervetAGM         AAAAAGGAAAAGGGCTTTCCTGAGGGTCAAATGAAGTACAGTTGACCCTTGAACAACATGATTTTGAACTACACAGGTC- 18092
Gorrilla          CACATTTCTGAGCCTTAGTCTGAAAGAAGAATGTAAAATATCCCAAGTTTTCATAT--TGGTTACATGTAGAGCTGATAA 17922
GrabEatingMacaque--------TAA---------TGAAAGAAGAATG-------------ATTTT---AT--TGGTT-CATT---AACTGATA- 17818
Macaque           CAGATTTCTAAGCCGTAGTCTGAAAGAAGAATGTAAAATATCCCACATTTTCATAT--TGGTTACATGTAGAACTGATAA 18024
PigTailedMacaque  CAGATTTCTAAGCCGTAGTCTGAAAGAAGAATGTAAAATATCCCACATTTTCATAT--TGGTTACATGTAGAACTGATAA 17264
Bonobo            CACACTTCTGAGCCTTAGTCTGGAAGAAGAATGTAAAATATCCCAAGTTTTCATAT--TGGTTACATGTACAGCTGATAA 16609
OliveBaboon       CAGATTTCTAAGCCGTAGTCTGAAAGAAGAATGTAAAATATCCCACATTTTCATAT--TGGTTACATGTAGAACTGATAA 16721
Orangutan         CACATTTCTAAGCCTTAGTCTGAAAGAAGAATGTAAAATATCCCAAGTTTTCATAT--TGGTTACATGTAGAGCTGATAA 16965

Majority          TATTTTTGTATT--TTGGGTTAAATGAAATACATTAT--GCGTTGCCTTTTCCTCTTTTTTTCTTTC-T-T-TT--T-TC
                           21770     21780     21790     21800     21810     21820     21830     21840
Human             TATTTTGGTATT--TTGGGTTAAATGAAATACATTATGTGTGTTGCCTTTTCCTCTTTTTTCCTTTC------------C 15630
Coquerel'sSifaka  TATTTT------------------GGTAT--------------------------------------------------- 14736
Ma'sNightMonkey   GGTTTCCCAT--------GTTGGCCAGGCTGGTCT-----CAAACTCCTGACCTCAGGTGATCCACC------------C 13499
VervetAGM         CACTTATGCATGGATCCACTTATACAGAATTTTCTTC--TCCCTCTTCCACCCAAGACAGAAAGACCAGCTCTTCCTCTT 18170
Gorrilla          TATTTTGGTATT--TTGGGTTAAATGAAATACATTATGTGTGTTGCCTTTTCCTCTTTTTTCCTTTC------------C 17988
GrabEatingMacaque--TTTT------------GTTAAGGAA-------------------------------TTTTCTTTCATTTCTCTCTTTC 17853
Macaque           TATTTTTGTATT--TTGGGTTAAGTGAAATGCATTAT--GCATTGCCTTTTCCTCTTTCTTTCTTTCTTTTCTTTCTTTT 18100
PigTailedMacaque  TATTTTTGTATT--TTGGGTTAAGTGAAATGCATTAT--GCATTGCCTTTTCCT----CTTTCTTTCTTTTCTTTCTCTT 17336
Bonobo            TATTTTGGCATT--TTGGGTTAAATGAAATACATTATGTGTGTTGCCTTTTCCTCTTTTTTCCTGTC------------C 16675
OliveBaboon       TATTTTTGTATT--TTGGGTTAAGTGAAATACATTAT--GCATTGCCTTTTCCTCTTTCTTTCTTTCTTTTTTTTTTTTT 16797
Orangutan         TATTTTGGTATT--TTGGGTTAAATGAAATACATTATCTGCGTTGCCTTTTCCTCTTTCTTCCTTTCTTCCTTTCTTTCC 17043
```

```
Majority        TTTTTTTA----AGA-AG-GTCT-GCT-T--C--CCAGGCTG-AGTGC-GTGGC--GATC---GCA--CT--AC------

                        21850     21860     21870     21880     21890     21900     21910     21920

Human           TTTTTTTA----AGATAGGGTCTTGCTCTGTCCACCAGGCTGGAGTGCCGTGGCATGATCATGGCAGCCTCAAC------ 15700
Coquerel'sSifaka -TTT---------------------------------------------------------------------------- 14739
Ma'sNightMonkey ACCTT--------------------------------------------------------------------------- 13504
VervetAGM       CCTCCTCAGCCTACTCAATGTAAAGACAATCAGGATGAAGACCTTTATTATGACTGACCTCCAAAAAGATTGGCTTCAAT 18250
Gorrilla        TTTTTTTA----AGATAGGGTCTTGCTCTGTCCACCAGGCTGGAGTGCCGTGGCATGATCATGGCAGCCTCAAC------ 18058
GrabEatingMacaqueCCTT---------------------------------------------------------------------------- 17857
Macaque         CTTTCTCTTT---------------------------------------------------------------------- 18110
PigTailedMacaque TCTCT--------------------------------------------------------------------------- 17341
Bonobo          TTTTTTTA----AGATAGGGTCTTGCTCTGTCCACCAGGCTGGAGTGCCGTGGCATGATCATGGCAGCCTCAAC------ 16745
OliveBaboon     TTTTTTTTTTGGAGACGGAGTCTCGCTCTGGCGCCCAGGCTGAAGTGCAGTGGCCGGATCTCAGCTCACTGCAAGCTCCG 16877
Orangutan       TTTTTTTA----AGATAGGGTCTTGCTTTTTCCACCAGGCTGGAGTGCTGTGGCATGATCATGGCAGCCTCAAC------ 17113

Majority        -CTCC-GGG-TC--GT-A--CTCC--CCTCAGCCTCCCAAGTAGCTGGGACTACAGTTGTGTGCCACCACACCTGGCTAA

                        21930     21940     21950     21960     21970     21980     21990     22000

Human           -CTCCTGGGCTCCAGTGACCCTCCCACCTCAGCCTCCCAAGTAGCTGGGACTACAGTTGTGTGCCACCACACCTGGCTAA 15779
Coquerel'sSifaka -------------------------------------------------------------------------------- 14739
Ma'sNightMonkey -------------G----------------GTCTCCCAAAGTGCTAAGATTACAGGTGTGAGCCACCACACCCAGCCAA 13554
VervetAGM       ATTTCATGGAGTGTGTTATGAATTAGAATCCTGAGTCTCTGTATTGTTAATTATTTTAGTCTCTTGGTCTGTGTGTGTTC 18330
Gorrilla        -CTCCTGGGCTCCAGTGACCCTCCCACCTCAGCCTCCCAAGTAGCTGGGACTACAGTTATGTGCCACCACACCTGACTAA 18137
GrabEatingMacaque-------------------------------------------------------------------------------- 17857
Macaque         -------------------------------------------------------------------------------- 18110
PigTailedMacaque -------------------------------------------------------------------------------- 17341
Bonobo          -CTCCTGGGCTCCAGTGACCCTCCCACCTCAGCCTCCCAAGTAGCTGGGACTACAGTTGTGTGCCACCACACCTGGCTAT 16824
OliveBaboon     CCTCCCGGGTTCACGCCATTCTCCTGCCTCAGCCTCCCGAGTAGCTGGGACTACAGGCGCCCGCCACCTCGCCCGGCTAG 16957
Orangutan       -CTCCTGGGCTCCAGTGACCCTCCCACCTCAGCCTCCCAAGTAGCTGGGACTACAGTTGTGTGCCACCACACCTGGCTAA 17192

Majority        TTT-T----AGTTTTTTT--------------------------------------GT------------------

                        22010     22020     22030     22040     22050     22060     22070     22080

Human           TTTTTG---AGTTTTTTTTT------------------------------------GT------------------ 15798
Coquerel'sSifaka -------------------------------------------------------------------------------- 14739
Ma'sNightMonkey --G------AATGTATTT-------------------------------------------------------------- 13564
VervetAGM       CTAAACTGTAGTAGCTTTCTTAATTTAGATCCAGGCATACTACGTTAGGGGATAAGGTGGTTAAGTGATATTTTCACTTT 18410
Gorrilla        TTTTTGA--GGTTTTTTT--------------------------------------GT------------------ 18155
GrabEatingMacaque-------------------------------------------------------------------------------- 17857
Macaque         -------------------------------------------------------------------------------- 18110
PigTailedMacaque -------------------------------------------------------------------------------- 17341
Bonobo          TTTGTGT--AGTTTTTTTTT------------------------------------GTTT---------------- 16846
OliveBaboon     TTTTTTTTTGTATTTTTTAGTAGAGACGGGGTTTCACCGTGTTAGCCAGGATGGTCTCGATCTCCCGACCTCGTGATCCG 17037
Orangutan       TTTTTG---AGTTTTTTTTT------------------------------------GT------------------ 17211
```

```
Majority         -----------------------AG-GA--GC-----G-T-T-T-------GCT--TCTCAAATTCCTGGGCTCAAGTGA
                         22090     22100     22110     22120     22130     22140     22150     22160
Human            -----------------------AGAGACAGCGTCTCGCTGTATCGCTTAGGCTGGTCTCAAACTCCTGGGCTCAAGTGA 15855
Coquerel'sSifaka -------------------------------------------------------------------------------- 14739
Ma'sNightMonkey  ------------------------------T--------------------A----TCCCAGGTTTTTATATTGGTTACA 13590
VervetAGM        AAAAGTAATAAGGTGTCACAGAGAGTTATCGCAAGAGGTTTTTTGAGTAGTACTTCTTTTTTATAACTATTGTAATGAAT 18490
Gorrilla         -----------------------AGAGACAGCGTCTCGCTATATTGCCTAGGCTGGTCTCAAACTCCTGGGCTCAAGTGA 18212
GrabEatingMacaque-------------------------------------------------------------------------------- 17857
Macaque          -------------------------------------------------------------------------CTCTCTC 18117
PigTailedMacaque -------------------------------------------------------------------------------- 17341
Bonobo           -----------------------AGAGACAGCGTCTCGCTATATCGCCTAGGCTGGTCTCAAATTCCTGGGCTCAAGTGA 16903
OliveBaboon      CCCGTCTCGGCCTTCCAAAGTGCTGGGATTACAGGCTTGAGCCACCGCGCCCGGCCTCTTTCTTTCTTTTCTTTCTTTTC 17117
Orangutan        -----------------------AGAGACAGCGTCTCGCTATATTGCCTAGGCTGATCTCAAACTCCTGGGCTCAAGTGA 17268

Majority         TCTTCCTGCTTTAGCCTTGCAAAGTGCTGGGATTACATGACTGAGTCTGGCTACCTTTTTCTTTTTTTCTTTCTTTTTCT
                         22170     22180     22190     22200     22210     22220     22230     22240
Human            TCCTCCTGCTTTAGCCTTGCAAAGTGCTGGGATTACAGGACTGAGTCTGGCCACCTTTTTCTTTCTTTCTTTTTTTTTT- 15934
Coquerel'sSifaka -------------------------------------------------------------------------------- 14739
Ma'sNightMonkey  TGTTGAACTGATAGCATTTTGGTATTTTGGG-TTAAATGAAATTATCT-GTTGCCTTTTCCT--TTTGCTTTCTTTTTTT 13666
VervetAGM        TTTAGACACAGTAGTATCATGAACCATTGTGTGTGTATCATTCAGTCCCAAAAACCA-------TCAGCAACCCCTAACT 18563
Gorrilla         TCCTCCCGCTTTAACCTTGCAAAGTGCTGGGATTACAGGACTGAGTCTGGCCACCTTTTTCTTTCTTTCTTTCTTTCTTT 18292
GrabEatingMacaque-------------------------------------------------------------------------------- 17857
Macaque          TCTCTCTCCCTCTCTCTCTCTCTCCCCCCCGCCTCCCTCCCTCCGTCTCCCTGTCCTCTCTCTCTCTCCCTCTCTCTCTCT 18197
PigTailedMacaque --------C-TCTCTCCCCCCCTCCCCCCGCCTGCCTCCCTCCGTCTCCCTGTCCTCTCTCTCTCCCTCTCTCTCTCT 17412
Bonobo           TCCTCCTGCTTTAGCCTTGCAAAGTGCTGGGATTACAGGACTGAGTCTGGCCACCTTTTTCTTTCTTTCTTTCTTTTTCT 16983
OliveBaboon      TTTCTCTTTCTCTCTCTCTCTCTCCCCCCCGCCTCCCTCCCTCCATCTCCCTCTCCTCTCTCTCTCT--TCTCTCTCTCT 17195
Orangutan        TACTCCCGCTTTAGCCTTGCAAAGTGCTGGGATTACAGGACTGAGTCTGGCCACCTTTTTCTTTCTTTCTTTTTTTTTTT 17348

Majority         CTTTTTTT---T-T----------T-T--AAGTCTG--------------TTT-AGATA--AGTCTTGCTCTGTCC-C
                         22250     22260     22270     22280     22290     22300     22310     22320
Human            -------------------------------AGACGG-------------------------AGTCTCCCTTTGTTG-C 15956
Coquerel'sSifaka -------------------------------------------------------------------------------- 14739
Ma'sNightMonkey  CCTTTTTT---------------------AAAGATAG-------------------------AGTCTTGCCCTGTCC-C 13698
VervetAGM        AAAATGATGATATCCAAACTCTGCTTTTCTACTTCTGTTATTTGAGATAAATTTCAGATATTCATATTGTTTTATCTGT 18643
Gorrilla         TCTTTTTTTTTTTT-----------TTT---AGACGG--------------------------AGTCTCCCTTTGTTG-C 18331
GrabEatingMacaque---------------------------------------------------TTTAAGATAGG-GTCTTGTTCTGTCC-T 17883
Macaque          CTCTTTCTTTCTCTCTCTCTCCCTGTCTGTCTCTCTCTCTCTCTTTCCTTTTTTTAAGATAGG-GTCTTGTTCTGTCC-T 18275
PigTailedMacaque CTCTCTTTCTCTCTCTCTCCCTGTCTCTCTCTCTCTCTCTCTCTTTCCTTTTTTTAAGATAGG-GTCTTGTTCTGTCC-T 17490
Bonobo           CTTTTTTTTCTTTTAGACGTTCTCCTTGTAAAGACGGTAGGT-TCT--TACCCTTTCGGTTTAAGTCTCCCTTTGTTG-C 17059
OliveBaboon      CTCTTTCTTTCTCTCTCTCTCCCTGTCTGTCTCTCTCTCTCTCTTTCCTTTTTTTAAGATAGG-GTCTTGCTCTGTCC-C 17273
Orangutan        T-TTTTT-----------------------AAGGCGG--------------------------AGTCTCCCTCTGTCA-C 17377
```

```
Majority          CCAGGCTGGAGTGCAGTGGC---AT----GATCATGGCAGCCTCTACCTCCTGGGTTCCAGTGATCCTCCTGCTTCAGCC
                           22330     22340     22350     22360     22370     22380     22390     22400
Human             CCAGGCTGGAGTACAGTGGCTCTATCTCTGCTTCCTGCAAGCTCTGCCTCCTGGGTTCACGCCATTCTCCTGCCTCAGCC 16036
Coquerel'sSifaka  ----------------------------------------------------GGGTTAAAA------------------- 14748
Ma'sNightMonkey   CCAGGCTAGAGTTCAGTGGC---AT----GATCATGGCAGCCTCAACCTCCTGGGCTCCAGTGATCCTCCCATCTCAGCC 13771
VervetAGM         AAAGGCTTCAGTGT-ATAAC---TTTAAAAATAAGGACTCTCTTTTCTTTTTAAAATATAATCAAAAGACTGTTAGGACA 18719
Gorrilla          CCAGGCTGGAGTACAGTGGCTCCATCTCTGCTTCCTGCAAGCTCTGCCTCCTGGGTTCACACCATTCTCCTGCCTCAGCC 18411
GrabEatingMacaqueCCAGGCTGGAGTGCCATGGC---AT----GATCATGGCAGCCTCAACCTCCTGTGCTCCAGTGACCCTCCCACTTCAGCC 17956
Macaque           CCAGGCTGGAGTGCCATGGC---AT----GATCATGGCAGCCTCAACCTCCTGTGCTCCAGTGACCCTCCCACTTCAGCC 18348
PigTailedMacaque  CCAGGCTGGAGTGCCATGGC---AT----GATCATGGCAGCCTCAACCTCCTGTGCTCCAGTGACCCTCCCACTTCAGCC 17563
Bonobo            CCAGGCTGGAGTACAGTGGCTCTATCTCTGCTTCCTGCAAGCTCTGCCTCCTGGGTTCACGCCATTCTCCTGCCTCAGCC 17139
OliveBaboon       CCAGGCTGGAGTGCCATGGC---AT----GATCATGGCAGCCTCAACTTCCTGTGCTCCAGTGACCCTCCCACTTCAGCC 17346
Orangutan         CCAGGCTGGAGTGCAGTGGCTCTATCTCTGCTTACTGCAAGCTCTGCCTCCCGGGTTCACGCCATTCTGCTGCCTCAGCC 17457

Majority          TCCCAAGTAGCTGGG--ACTACA-GTTGTGTGCCACCACACCTGGCT---AATTTTTGATTTTTTTGTAGAGACAGGGT
                           22410     22420     22430     22440     22450     22460     22470     22480
Human             TCCCGAGTAGCTGGG--ACTACA-GGCGCCTGCCACCACACCCGGCT--AATTTTTT-GTATTTTTAGTAGAGATGGGTT 16110
Coquerel'sSifaka  -------------------TGT--G-----TGTTACC---------------TTTTT-A----CTTT-----------T- 14770
Ma'sNightMonkey   TCCCAAGTAGCTGGG--ACTACC-GGTGTGTGCCACTACACCAGGCT---AATTTTTGAGTTTTTTTGTAGAGACAGGGT 13845
VervetAGM         CACACACACATTAAGTTACTTCATATTATAAAATGTCAAATTTGTATTAAAATTTCTAATTGTCTCATAAAAGTCATAGT 18799
Gorrilla          TCCCGAGTAGCTGGG--ACTACA-GGCGCCCGCCACCACACACGGCT--AATTTTTT-GTATTTTTAGTAGAGACGGGTT 18485
GrabEatingMacaqueTCCCAAGTAGCTGGG--ACTACA-GTTGTGTGCCACCACACCTGGCT---AATTATTGAGTTTTTTTGTAGAGACAGGGT 18030
Macaque           TCCCAAGTAGCTGGG--ACTACA-GTTGTGTGCCACCACACCTGGCT---AATTATTGAGTTTTTTTGTAGAGACAGGGT 18422
PigTailedMacaque  TCCCAAGTAGCTGGG--ACTACA-GTTGTGTGCCACCACACCTGGCT---AATTATTGAGTTTTTTTGTAGAGACAGGGT 17637
Bonobo            TCCCGAGTAGCTGGG--ACTACA-GGCGCCTGCCACCACACCCGGCT--AATTTTTT-GTATTTTTAGTAGAGACGGGTT 17213
OliveBaboon       TCCCAAGTAGCTGGG--ACTACA-GTTGTGTGCCACCACACCTGGCT---AATTATTGAGTTTTTTTGTAGAGACAGGGT 17420
Orangutan         TCTCGAGTAGCTGGG--ACTACA-GGCGCCCTCCACCAAGCCCGGCT--AATTTTTTTGTATTTTTAGTGGAGACGGTTT 17532

Majority          CTC-CTATA----TTGCCTAGGCTGGTCTGAAACTCC--TGAACTCAAGTGATCCTCCT---------GCTTTAGCCTTG
                           22490     22500     22510     22520     22530     22540     22550     22560
Human             -------------TTACC----ATGGTCTCGATCTCC--TGACCTCC--TGATCTGCCC---------ACCCTGGCCTCC 16160
Coquerel'sSifaka  --------------T--A-------------AATTACAGTGAATA-----------------------G----------- 14787
Ma'sNightMonkey   CTCACTGTG----TTGCCTAGGCTGGTCTCAAACTCT--TGGGCTCAAGTGATCCTCTT---------GCTTTAGCCTTG 13910
VervetAGM         ATTTTTATAGGTTTTTTTAGAGGGGGAAGGAGTCATGATAAAATAAGATCAGCATATTTAACAGGTCGTTATTACCTCT 18879
Gorrilla          -------------TTACC----ATGGTCTCAATCTCC--TGACCT-----GATCTGCCC---------ACCCCGGCCTCC 18532
GrabEatingMacaqueCTCGCTATA----TTGCCTAGGCTGGTCTGAAACTCC--TGGACTCAAGTGATCCTCCT---------GCTTTAGCCTTG 18095
Macaque           CTCGCTATA----TTGCCTAGGCTGGTCTGAAACTCC--TGGACTCAAGTGATCCTCCT---------GCTTTAGCCTTG 18487
PigTailedMacaque  CTCGCTATA----TTGCCTAGGCTGGTCTGAAACTCC--TGGACTCAAGTGATCCTCCT---------GCTTTAGCCTTG 17702
Bonobo            -------------TTACC----ATGGTCTCGATCTCC--TGACCT-----GATCTGCCC---------ACCCCGGCCTCC 17260
OliveBaboon       CTCGCTATA----TTGCCTAGGCTGGTCTGAAACTCC--TGGACTCAAGTGATCCTCCT---------GCTTTAGCCTTG 17485
Orangutan         CA--CTGTG----TTAGCCAGGATGGTCTCAATCTCC--TGACCTCA--TGATCTGCCC---------GCCCTGGCCTCC 17593
```

```
Majority         CAAAGTGCTGGGATTACAGGAGTGAGTCT---------GGCCACCTTTTTCTTTTTACTTTTAAAAATGTGGCTAATAGA

                         22570     22580     22590     22600     22610     22620     22630     22640

Human            CAAAGTGCTGGGATTACAGGCGTGAGCCACTGCGACCGGCCCAC-TTTTTCTTTTTACTTTTAAAAATGTGGCTAATAGA 16239
Coquerel'sSifaka -AAATTTGTAAGATTACATGTGTGGCTCA---------TGTTAC--ATTTCTTTTGGACAGTGCAAGTCTGGAGGGTAGA 14855
Ma'sNightMonkey  CAAAGTGCTGAGATTACAGGACTGAGCCT---------GGCCAGCTTTTTCTTTTTACTTTTCAAAATGTGGCTAATAGA 13981
VervetAGM        TAAATATCTTTTAATGTACAAGTTAAGTT---------CTCCGTTCCAGTTTTTTTTTTTTTTTAAGT-TGCATTTCATT 18949
Gorrilla         CAAAGTGCTGGGATTACAGGTGTGAGCCACTGCGCCCGGCCCAC-TTTTTCTTTTTACTTTTAAAAATGTGGCTAACAGA 18611
GrabEatingMacaqueCAAAGTGCTGGGATTACAGGACTGAGTCT---------GGCCACCTTTTTCTTTGTACAT----AAAT-TGACTAATAGA 18161
Macaque          CAAAGTGCTGGGATTACAGGACTGAGTCT---------GGCCACCTTTTTCTTTGTACAT----AAAT-TGGCTAATAGA 18553
PigTailedMacaque CAAAGTGCTGGGATTACAGGACTGAGTCT---------GGCCACCTTTTTCTTTGTACTTTTAAAAATGTGGCTAATAGA 17773
Bonobo           CAAAGTGCTGGGATTACAGGCGTGAGCCACTGCGACCGGCCCAC-TTTTTCTTTTTACTTTTAAAAATGTGGCTAACAGA 17339
OliveBaboon      CAAAGTGCTGGGATTACAGGACTGAATCT---------GGCCACCTTTTTCTTTGTACTTTTAAAAATGTGGCTAATAGA 17556
Orangutan        CAAAGTGCTGGGATTACAGGCATGAGCCACTGCGCCCGGCCCACCTTTTTCTTTTTACTTTTAAAAATGTGGCTAACAGA 17673

Majority         AATTTATGAGATTATATTTATGGTTCATACTACATTTCTTTTGGACAGTGC---CAGAGTGAATCAGATAAGCTTGCATT

                         22650     22660     22670     22680     22690     22700     22710     22720

Human            AATTTATGAGATTATATTTATGGTTCATACTACGTTTCTTTTGGACAGTGC---CAGAGTGAATCAGATAAGCTTGCATT 16316
Coquerel'sSifaka GATTTA-AAGTCCAAAGTGATAGATGATACGTTTGCATTTTAAAATTGTA-----AGGGTGAA------------CATA 14916
Ma'sNightMonkey  AATTTATGAGATTATATATATGGTTCATACTGCATTTCTTTTGAACTGTGC---CAGAGTGAATCAGATAAGCTTGCATT 14058
VervetAGM        TGTTGAAGAAAAC-TGGTTGTTGGTCTTACACAGTTGCCTATAGTCTGCATTTACTGATTGCATT--CTGATTTTGTAAT 19026
Gorrilla         AATTTATGAGATTATATTTATGGTTCATACTACATTTCTTTTGGACAGTGC---CAGAGTGAATCAGATAAGCTTGCATT 18688
GrabEatingMacaqueAATTTATGAGATTATATTTGTGGTTCATACTACATTTCTTTTGGACAGTGC---CAGAGTGAATCAGATAAGCTTGCGTT 18238
Macaque          AATTTATGAGATTATATTTGTGGTTCATACTACATTTCTTTTGGACAGTGC---CAGAGTGAATCAGATAAGCTTGCGTT 18630
PigTailedMacaque AATTTATGAGATTATATTTGTGGTTCATACTACATTTCTTTTGGACAGTGC---CAGAGTGAATCAGATAAGCTTGCGTT 17850
Bonobo           AATTTATGAGATTATATTTATGGTTCATACTACGTTTCTTTTGGACAGTGC---CAGAGTGAATCAGATAAGCTTGCATT 17416
OliveBaboon      AATTTATGAGATTATATTTGTGGTTCATACTACATTTCTTTTGGACAGTGC---CAGAGTGAATCAGATAAGCTTGCGTT 17633
Orangutan        AATTTATGAGATTATATTTATGGTTCATACTACATTTCTTTTGGACAGTGC---CAGAGTGAATCAGATAAGCTTGCATT 17750

Majority         TTAAAATC--CTAAGGGTG---AATACAATAGAGATAGAACCCAAAGAATTGGGGAG--------------TGGGGTTGA

                         22730     22740     22750     22760     22770     22780     22790     22800

Human            TTAAAATC--CTAAGGGTA---AATGCAATAGAGATAGAACGCAAATAATTGGGGAG--------------GGGGGTTGA 16377
Coquerel'sSifaka GTGAAAAT----AGAGATG-------GAGTATATATATAACTTCTAAAATAATGGAG--------------GGTGTGTGA 14971
Ma'sNightMonkey  TTAAAATC--CTAAGGGTG---AACACAATAGAGATAGAACACAAAAAATTGGAGAG--------------GGAGGCTGA 14119
VervetAGM        TTAATATGTACTCTATGTTTCCAGTAAATTGGTAGTTGGATCTAAAGACTTGGTCAGGATCTCTATTTTTTTGGTAATAT 19106
Gorrilla         TTAAAATC--CTAAGGGTA---AATGCAATAGAGATAGAACGCAAATAATTGGGGAG--------------AGGGGTTGA 18749
GrabEatingMacaqueTTGAAATC--CTAAGGGTG---AACACAATAGAGATAGAACCCAAAGAATTGGGGAG--------------TGGGGTTGA 18299
Macaque          TTGAAATC--CTAAGGGTG---AACACAATAGAGATAGAACCCAAAGAATTGGGGAG--------------TGGGGTTGA 18691
PigTailedMacaque TTGAAATC--CTAAGGGTGGTGAACACAATAGAGATAGAACCCAAAGAATTGGGGAG--------------TGGGGTTGA 17914
Bonobo           TTAAAATC--CTAAGGGTA---AATGCAATAGAGATAGAACGCAAATAATTGGGGAG--------------GGGGGTTGA 17477
OliveBaboon      TTGAAATC--CTACGGGTG---AACACAATAGAGATAGAACCCAAAGAATTGGGGAG--------------TGGGGTTGA 17694
Orangutan        TTAAAATC--CTAAGGGTG---AATGCAATAGAGGTAGAACACAAGTAATTGGGGAG--------------GGGAGTTGA 17811
```

```
Majority          CTGAAATTAAAGATGTATAATCCAAAAGAAGGCAAAAAAAAAAAA------GAGTAAGACAC---------AAAGTGAGC
                          22810     22820     22830     22840     22850     22860     22870     22880
Human             CTGAAATTAAAGATGTATAATCCAAAAGAAGGCAAAAAAAAA----------GGAAAGACAC---------AAAGTGAGC 16438
Coquerel'sSifaka  CTAAAATTAATGATGTGTAATCCTAAAGAAAGCAGAAAAAGAAAAGGAAA-GAATAAGACAC---------AAAGTGAGC 15041
Ma'sNightMonkey   CTGAAATTACGTATGTGTAATCCAAAAGAAGGAAGAAAAAAAGGAAA----GAATAAGACAC---------AAAGTGAGC 14186
VervetAGM         TTTTAAGTGGTGAGTTTTTATTTCTATTAGACATGGTTTCTGACTTTTCATGTCTGTGATGTTTTTCCTGGGAACATTTC 19186
Gorrilla          CTGAAATTAAAGATGTATAATCCAAAAGAAGGCAAAAAGAA-----------GGAAAGACAC---------AAAGTGAGC 18809
GrabEatingMacaqueCTGAAATTAAAGATGTATAATCCAAAAGAAGGCAAAAAAAAAAAA------GAGTAAGACAC---------AAAATTAGC 18364
Macaque           CTGAAATTAAAGATGTATAATCCAAAAGAAGGCAAAAAAAAAAAAAAA---GAGTAAGACAC---------AAAATTAGC 18759
PigTailedMacaque  CTGAAATTAAAGATGTATAATCCAAAAGAAGGCAAAAAAAAAAAAAAAA--GAGTAAGACAC---------AAAATTAGC 17983
Bonobo            CTGAAATTAAAGATGTATAATCCAAAAGAAGGCAAAAAAAA-----------GGAAAGACAC---------AAAGTGAGC 17537
OliveBaboon       CTGAAATTAAAGATGTATAATCCAAAAGAAGGCAAAAAAAAAAAAAAAAAAGAATAAGACAC---------AAAATTAGC 17765
Orangutan         CTGAAATTACAGATGTATAATCCAAAAGAAGGCAAAAAAAAAA--------GAGAAAGACAC---------AAAGTGAGC 17874

Majority          TTATATGTTAA------TAGCGTGGAAAGGTTCTGTG------AAGGGTTCCTTA----------GAACCTTTAAAGGTA
                          22890     22900     22910     22920     22930     22940     22950     22960
Human             TTATATGTTAA------TAGCGTGGAAAGGTTCTATG------AAGGGTTGTTTA----------GAGCCTTTAAGGGCA 16496
Coquerel'sSifaka  TTATATTTTAA------TGGTGTCAAAAGGCTCTATG------AATGATTCCTTT----------TAACCTTTAAAAATA 15099
Ma'sNightMonkey   TTATGTGTTAA------GAGTGTGGAAAGGTTCTGTG------AAGGGTTCCTTA----------GAACCTTTAAAGGTA 14244
VervetAGM         CTATACTTTACCTTGTTTGTGCTAGGCATAGTCTGTGCTCAATAATTGTCTCTTCTTTTTTTCCTGTTTTTTGAGAGATG 19266
Gorrilla          TTATATGTTAA------TAGCGTGGAAAGGTTCTATG------AAGGGTTGTTTA----------GAGCCTTTAAAGGTA 18867
GrabEatingMacaqueCTATATGTTAA------TAGAGTGGAAAGGTTCTGTG------GAGGGTTCCTTA----------GAACCTTTAAAGGTA 18422
Macaque           CTATATGTTAA------TAGAGTGGAAAGGTTCTGTG------AAGGGTTCCTTA----------GAACCTTTAAAGGTA 18817
PigTailedMacaque  CTATATGTTAA------TAGAGTGGAAAGGTTCTGTG------AAGGGTTCCTTA----------GAACCTTTAAAGGTA 18041
Bonobo            TTATATGTTAA------TAGCGTGGAAAGGTTCTATG------AAGGGTTGTTTA----------GAGCCTTTAAAGGTA 17595
OliveBaboon       CCATATGTTAA------TAGAGTGGAAAGGTTCTGTG------AAGGGTTCCTTA----------GAACCTTTAAAGGTA 17823
Orangutan         TTATATGTTAA------TAGCGTGGAAAGGTTCTATG------AAGAGTTCCTTA----------GAGCCTTTAAAGGTA 17932

Majority          GAATTTTCT---TGTCTGGTT---GCAGGAAAG-AGTGGCATTGTT-TTTG-------TTCACTTTTAAAGCTACAAAAA
                          22970     22980     22990     23000     23010     23020     23030     23040
Human             AAATTTTCT---TGTCTGGTT---GCAGGAAAG-AGTGGCATTGTT-TTTG-------TTCACTTTTAAAGCTACAAAAA 16561
Coquerel'sSifaka  GAATTTTCTGGTTGTCAGGTT---AGGGGCAAG-AGTGGAATCATTCTTTGTTCATTTCAGATAGAGAAAGCTACAATAA 15175
Ma'sNightMonkey   GGATTTTCT---CGTCAGGTT---GCAGGAAAG-AGTGGCATTGTT-CTTGTTCATTTTGGGTAGAGAAAGCTACAGTAA 14316
VervetAGM         GAGTCTTGC---CCTGTTGCCCAGGCTGGAGTGCAGTGGCCCAATC-TCAGCGATTCCTCTGCCTCCCGGGTTCAAGCAA 19342
Gorrilla          GAATTTTCT---TGTCTGGTT---GCAGGAAAG-AGTGGCATTGTT-TTTG-------TTCACTTTTAAAGCTACAAAAA 18932
GrabEatingMacaqueGAATTTTCT---TGTCTGGTT---GCAGGAAAG-AGTGGCATTGTT-TTTG-------TTCACTTTTGAAGCTACAAAAA 18487
Macaque           GAATTTTCT---TGTCTGGTT---GCAGGAAAG-AGTGGCATTGTT-TTTG-------TTCACTTTTGAAGCTACAAAAA 18882
PigTailedMacaque  GAATTTTCT---TGTCTGGTT---GCAGGAAAG-AGTGGCATTGTT-TTTG-------TTCACTTTTGAAGCTACAAAAA 18106
Bonobo            GAATTTTCT---TGTCTGGTT---GCAGGAAAG-AGTGGCATTGTT-TTTG-------TTCACTTTTAAAGCTACAAAAA 17660
OliveBaboon       GAATTTTCT---TGTCTGGTT---GCAGGAAAG-AGTGGCATTGTT-TTTG-------TTCACTTTTGAAGCTACAAAAA 17888
Orangutan         GAATTTTCT---TGTCTGGTT---GCAGGAAAG-AGTGGCATTGTT-TTTG-------TTCACTTTTAAAGCTACAAAAA 17997
```

```
Majority          -----------CAAGT-----AATGATGAGGAAAATTGGACATGTTGAGGAAGTAAGTAGTTT-TTTGGAATGTAGGTT
                           23050     23060     23070     23080     23090     23100     23110     23120
Human             -----------CAAGT-----AATGATGAGGAAAATTGGACATGTTGAGGAAGTAAGTAATTT-TTTGGAATGTAGGTT 16624
Coquerel'sSifaka  -----------CAA-------AATGAGAAGGAAAACTGGACATACTGAGGAAGTAAGTAGTTTGTTTGGAATGTAGGTT 15237
Ma'sNightMonkey   -----------CAAGT-----AATGATGAGGAAAATTGGGATATGTTGAGGAAGTAAGTAGGTT-GTTGGAATGTAGGTT 14379
VervetAGM         TTCTCCTGCCTCAGCTTCCCGAGTGCTG-GGACTACAGGCGCCTGCCACCACGCCTGGCTAATT-TTTGTATTTTTAGTA 19420
Gorrilla          -----------CAAGT-----AATGATGAGGAAAATTGGACATGTTGAGGAAGTAAGTAATTT-TTTGGAATGTAGGTT 18995
GrabEatingMacaque-----------CAGGT-----AATGATGAGGAAAATTGGACATGTTGAGGAAGGAAGTAGTTT-TTTGGAATGTAGGTT 18550
Macaque           -----------CAGGT-----AATGATGAGGAAAATTGGACATGTTGAGGAAGGAAGTAGTTT-TTTGGAATGTAGGTT 18945
PigTailedMacaque  -----------CAGGT-----AATGATGAGGAAAATTGGACATGTTGAGGAAGGAAGTAGTTT-TTTGGAATGTAGGTT 18169
Bonobo            -----------CAAGT-----AATGATGAGGAAAATTGGACATGTTGAGGAAGTAAGTAATTT-TTTGGAATGTAGGTT 17723
OliveBaboon       -----------CAAGT-----AATGATGAGGAAAATTGGACATGTTGAGGAAGGAAGTAGTTT-TTTGGAATGTAGGTT 17951
Orangutan         -----------CAAGT-----AATGATGAGGAAAATTGGACATGTTGAGGAAGTAAGTAATTT-TTTGGAATGTAGGTT 18060

Majority          GAGTTAGAGTAGCAGAAGACAAAATTTAAAAAGT-----AGTTTTTGAC----AGTAATATGGAGAGCTTTGAGTGCAAA
                           23130     23140     23150     23160     23170     23180     23190     23200
Human             GAGTTAGAGTAGTAGAAGACAAAATTTAAAAAGT-----AGTTTTTGAC----AGTAATATGGAGAGCTTTGAGTGCAAA 16695
Coquerel'sSifaka  GAGTTAGAGTAGTAGTAGACAAAATTTAAATAGT--------TTTTGGC----AATAATATGGAAAGCTTTGAATACAAA 15305
Ma'sNightMonkey   GAGTTAGAGTAGCTGAAGACAAAATTTAAAAAGT-----AGGTTTTGAC----AGTAATATGGAGAGTTTTGAGTGCAAA 14450
VervetAGM         GGGACAGGGTTTCACCACATTG--GCCAGGATGGCTTCAAACTCCTGACCTTAAGTGATCTGCCCACCTTGGCTTCCCAA 19498
Gorrilla          GAGTTAGAGTAGCAGAAGACAAAATTTAAAAAGT-----AGTTTTTGAC----AGTAATATGTAGAGCTTTGAGTGCAAA 19066
GrabEatingMacaqueGGGTTAGAGTAGCAGAAGACAA--TTTAAAGAGT-----AGTTTTTGAC----AGTAATATGGAGAGCTTTGAATGCAAA 18619
Macaque           GGGTTAGAGTAGCAGAAGACAA--TTTAAAGAGT-----AGTTTTTGAC----AGTAATATGGAGAGCTTTGAATGCAAA 19014
PigTailedMacaque  GGGTTAGAGTAGCAGAAGACAA--TTTAAAGAGT-----AGTTTTTGAC----AGTCATATGGAGAGCTTTGAATGCAAA 18238
Bonobo            GAGTTAGAGTAGCAGAAGACAAAATTTAAAAAGT-----AGTTTTTGAC----AGTAATATGGAGAGCTTTGAGTGCAAA 17794
OliveBaboon       GGGTTAGAGTAGCAGAAGACAA--TTTAAAGAGT-----AGTTTTTGAC----AGTAATATGGAGAGCTTTGAATGCAAA 18020
Orangutan         GAGTTAGAGTAGCAGAAGACAAAATTTAAAAAGT-----AGTTTTTGAC----AGTAATATGGAGAGCTTTGAGTGCAAA 18131

Majority          GGATTTGCAGTGAGGACCTTTGAAAATTGGACCTGCTT--------TCTCCTA-TCAGTATTT-------ATGTTTAAAT
                           23210     23220     23230     23240     23250     23260     23270     23280
Human             GGATTTGCAGTGAGGACCTTTGAAAATTGAACCTGCTT--------TCTCCTA-TCAGTATTT-------ATGTTTAAAT 16759
Coquerel'sSifaka  GTATTTGCAGTGAGGACCTTTGAAAATTTGAACTGCTTTCT----CTTTAGTA-TCAGTATTT-------ATGTTTAAAT 15373
Ma'sNightMonkey   GGATTTGCAGTGAGGACCTATGAAAATTTGATCCGTTT--------TCTCCTA-TCAGTATTT-------ATGTTTAAAT 14514
VervetAGM         AGTGCTGGGATTACAGGCATGAGCCACTGTGCCTGGCCAATAAGTGTCTCTTAATGAGTATTCTTAAAGGATATCTAAAA 19578
Gorrilla          GGATTTGCAGTGAGGACCTTTGAAAATTGAACCTGCCT--------TCTCCTA-TCAGTATTT-------ATGTTTAAAT 19130
GrabEatingMacaqueGAATTTGCAGTGAGGACCTTTGAAAATTGGACCTGCTT--------TCTCCTA-TCTGTATTT-------ATGTTTAAAT 18683
Macaque           GAATTTGCAGTGAGGACCTTTGAAAATTGGACCTGCTT--------TCTCCTA-TCTGTATTT-------ATGTTTAAAT 19078
PigTailedMacaque  GAATTTGCAGTGAGGATCTTTGAAAATTGGACCTGCTT--------TCTCCTA-TCTGTATTT-------ATGTTTAAAT 18302
Bonobo            GGATTTGCAGTGAGGACCTTTGAAAATTGAACCTGCTT--------TCTCCTA-TCAGTATTT-------ATGTTTAAAT 17858
OliveBaboon       GAATTTGCAGTGAGGACCTTTGAAAATTGGACCTGCTT--------TCTCCTA-TCTGTATTT-------ATGTTTAAAT 18084
Orangutan         GGATTTGCAGTGAGGACCTTTGAAAATTGAACCTGCTT--------TCTGCTA-TCAGTATTT-------ATGTTTAAAT 18195
```

```
Majority        A-------AACTTGATTCTT---TGTGAGTATTGTTA-TATATGTTTTCATAACTACTGTTGTAGTAGCG--TTTCTTTT

                         23290     23300     23310     23320     23330     23340     23350     23360

Human           A-------AACTTGATTCTT---TGTGAGTATTGTTA-TATATGTTTTCATAACTACTGTTGTAGTAACG--TTTCTTTT 16826
Coquerel'sSifaka A-------AACTTGATTCTT---CTTGAGTATTGTTA-CATATATTTTCATACGTA----GGTAGA---------TTTTT 15429
Ma'sNightMonkey A-------AACTTGATTCTT---TGTGAGTATTGTTAATATATATGTTTTCATAACTACTGTTATAGTAGCA-----TTTTT 14579
VervetAGM       AGAAATGTAACTTAAAATTCACATAATAGTAATACTA-TGCA-ATGTTAACATTTAATGTGGAAGTTGGAACTATTGATG 19656
Gorrilla        A-------AACTTGATTCTT---TGTGAGTATTGTTA-TATATGTTTACATAACTACTGTTGTAGTAACG--TTTCTTTT 19197
GrabEatingMacaqueA-------AACTTGACTCTC---TGTGAGTATTGTTA-TATATGTTTTCATAGCTACTGTTGTAGTAGCG--TTTCGTTT 18750
Macaque         A-------AACTTGACTCTC---TGTGAGTATTGTTA-TATATGTTTTCATAGCTACTGTTGTAGTAGCG--TTTCGTTT 19145
PigTailedMacaque A-------AACTTGACTCTC---TGTGAGTATTGTTA-TATATGTTTTCATAGCTACTGTTGTAGTAGCG--TTTCGTTT 18369
Bonobo          A-------AACTTGATTCTT---TGTGAGTATTGTTA-TATATGTTTTCATAACTACTGTTGTAGTAACG--TTTCTTTT 17925
OliveBaboon     A-------AACTTGACTCTT---TGTGAGTATTGTTA-TATATGTTTTCATAGCTACTGTTGTAGTAGCG--TTTCGTTT 18151
Orangutan       A-------AACTTGATTCTT---TGTGAGTGTTGTTA-TATATGTTTTCGTAACTACTGTTGTAAT---G--TTTCTTTT 18259

Majority        TTTT-ACTAGCTTGCTTT--CAAGCCACG--CTGTTGATTAAATATTTATGGGCCATTTTAAGGCTTCTA-ATCTTTATT

                         23370     23380     23390     23400     23410     23420     23430     23440

Human           TTTTTACTAGCTTGCTTT--CATGCCACG--CTGTTGATTAAATATTTATGGGCCATTTTAAGGCTTCTA-ATCTTCATT 16901
Coquerel'sSifaka TTTTTACTAAC---CTTT--CAATCTACC--CTGTTGATTAAATATTTATGGGCTATTTTAAGGCCTCTTAATCATCATT 15502
Ma'sNightMonkey TTTTAACTAGCTTGCTTT--CAAGCCATG--CTATTAATTAAATATTTGTGGGCCATTTTAAGGCTTCTG-TTCTTCATT 14654
VervetAGM       GTGG-ACAAGAATGTTTTGTCAAGTTAAGAACATATGATTTGGAATGAAAAGATGAGGGTTTGACTTTTATAGCATTGAA 19735
Gorrilla        TTTT-ACTAGCTTGCTTT--CATGCCACG--CTGTTGATTAAATATTTATGGGCCATTTTAAGGCTTCTA-ATCTTCATT 19271
GrabEatingMacaqueTTTT-ACTAGCTTGCTTT--CAAGCCACG--CTGTTGATTAAATATTTGTGGGCCATTTTAAGACTTCTA-ATCTTTATC 18824
Macaque         TTTT-ACTAGCTTGCTTT--CAAGCCACG--CTGTTGATTAAATATTTGTGGGCCATTTTAAGACTTCTA-ATCTTTATC 19219
PigTailedMacaque TTTT-ACTAGCTTGCTTT--CAAGCCACG--CTGTTGATTAAATATTTGTGGGCCATTTTAAGACTTCTA-ATCTTTATC 18443
Bonobo          TTTT-ACTAGCTTGCTTT--CATGCCACG--CTGTTGATTAAATATTTATGGGCCATTTTAAGGCTTCTA-ATCTTCATT 17999
OliveBaboon     TTTT-CCTAGCTTGCTTT--CAAGCCACG--CTGTTGATTAAATATTTGTGGGCCATTTTAAGACTTCTA-ATCTTTATC 18225
Orangutan       TTTT-ACTAGCTTGCTTT--CAAGCCACG--CTGTTGATTAAATATTTATGGGCCATTTTAAGGCTTCTA-ATCTTTATT 18333

Majority        TGTGATGAACTTTTGAGGAAGAGTTAGGT---TTTGTAAGTG--TTGAACT----CCAATAAACA-TAGTTCCACT----

                         23450     23460     23470     23480     23490     23500     23510     23520

Human           TGTGATGAACTTTTGAGGAAGAGTTAGGT---TTTGTAAGTG--TTGAACT----CCAATAAACA-CAGTTCCACT---- 16967
Coquerel'sSifaka TATGATGAACTTTTGCA---GAGTGAGGT---TTTATACATG--TCGGACT----TCCTTTAATA---GTTCTACT---- 15563
Ma'sNightMonkey TGTGATGAACTTCTGAG-AAGAGTTAGGT---TTTTTAAGTG--TTGAACTCCCTCTAATAAACAGTAGTTCCACT---- 14724
VervetAGM       CAGGTTAACCTCT-GAGCTTCAGTTTTATACATTTTAAATGGTTTAAATTGTA-CCTACCTCCA-CAATTATTTTAAGT 19812
Gorrilla        TGTGATGAACTTTTGAGGAAGAGTTAGGT---TTTGTAAGTG--TTGAACT----CCAATAAACA-CAGTTCCACT---- 19337
GrabEatingMacaqueTGTGATGAACTTT-GAGGAAGAGTTAGGT---TTTGTAAGTG--TTGAACT----CCAATAAACA-TAGTTCCACT---- 18889
Macaque         TGTGATGAACTTT-GAGGAACAGTTAGGT---TTTGTAAGTG--TTGAACT----CCAATAAACA-TAGTTCCACT---- 19284
PigTailedMacaque TGTGATGAACTTT-GAGGAAGAGTTAGGT---TTTGTAAGTG--TTGAACT----CCAATAAACA-TAGTTCCACT---- 18508
Bonobo          TGTGATGAACTTTTGAGGAAGAGTTAGGT---TTTGTAAGTG--TTGAACT----CCAATAAACA-CAGTTCCACT---- 18065
OliveBaboon     TGTGATGAACTTT-GAGGAAGAGTTAGGT---TTTGTAAGTG--TTGAACT----CCAATAAACA-TAGTTCCACT---- 18290
Orangutan       TGTGATGAACTTTTGAGGAAGAGTTAGGT---TTTATAAGTG--TTGAACT----CCAGTAAACA-CAGTTCCACT---- 18399
```

```
Majority          -TTCCTTAACTTCTCAAAAAGATACTTTGCTATTTGATACAGAT--------AGTTGTCTAGGCAAGCTATTA----GCA
                          23530     23540     23550     23560     23570     23580     23590     23600
Human             -TTCCTTAACTTCTCAGAAAGATACTTTGCTATTTGATACAGAT--------AGTTGTCTAGGCAAGCTATTA----GCA 17034
Coquerel'sSifaka  -----T-TTCTTCTCAGAAAGATACTTTGCAATTTGATACAGTTGTCAAACTATTTGTCTAGTCAAAGTATTT----GCA 15633
Ma'sNightMonkey   -TTCCTAAACTTCTCAAAAAGGTATTTTGCTATTTGATACAGAT--------AGTTGTGTAGGCAAGCTGTTA----CCA 14791
VervetAGM         GTTAGATGAGGTAAGGAAAAGACTTATGCTGAAATGATGAGAAT--------GGTGGTTGTTGTAGTTCTTTAATCTGTA 19884
Gorrilla          -TTCCTTAACTTCTCAGAAAGATACTTTGCTATTTGATACGGAT--------AGTTGTCTAGGCAAGCTATTA----GCA 19404
GrabEatingMacaque-TTCCTTAACTTCTCAAAAAGATTGCTGTT----TGATACAGAT--------AGTTGTCTAGGCAAGCTATTA----GCA 18952
Macaque           -TTCCTTAACTTCTCAAAAAGATTGCTGTT----TGATACAGAT--------AGTTGTCTAGGCAAGCTATTA----GCA 19347
PigTailedMacaque  -TTCCTTAACTTCTCAAAAAGATTGCTGTT----TGATACAGAT--------AGTTGTCTAGGCAAGCTATTA----GCA 18571
Bonobo            -TTCCTTAACTTCTCAGAAAGATACTTTGCTATTTGATACAGAT--------AGTTGTCTAGGCAAGCTATTA----GCA 18132
OliveBaboon       -TTCCTTAACTTCTCAAAAAGATTGCTGTT----TGATACAGAT--------AGTTGTCTAGGCAAGCTATTA----GCA 18353
Orangutan         -TTCCTTAACTTCTCAGAAAGATACTTTGCTATTTGATACAGAT--------AGTTGTCTAGGCAAGCTATTA----GCA 18466

Majority          GGGTTTCAGGTAAGACATA-GTGTT---TGCTATTTGTTTTAAGCAAACT--CTTCCCTCATTTACTCAAATTGTAGCAA
                          23610     23620     23630     23640     23650     23660     23670     23680
Human             GGGTTTCAGGTAAGACATATGTGTT---TGCTATTTGTTTTAAGCA--CT--CTTCCCTCATTTATTCAAATTGTAGCAA 17107
Coquerel'sSifaka  ATGTTTTATGTAAGAGGCAAGTATTTATTGGCTGTTTTATTAAGAGAAAT--ATCCCCTCATTTATTTCAATTGTAGCAG 15711
Ma'sNightMonkey   GGGTTTCAG-TAAGACATACGTGTT---CGCTATTTGTTTTAAGCAAACT--CTTTCCTCATTTACTCAAATTGTAGCAA 14865
VervetAGM         AATCTACATTTGGAAGGAA-CACTTA--TGGAAATCAAATACAGTAAATTAATTTCTAAGAGATCCTGGAGTTCAGGTTA 19961
Gorrilla          AGGTTTCAGGTAAGACATATGTGTT---TGCTATTTGTTTTAAGCAAACT--CTTCCCTCATTTATTCAAATTGTAGCAA 19479
GrabEatingMacaqueGGGTTTCAGGTAAGACATA-GTGTT---TGCTATTTGTTTTAAGCAAACT--CTTCCTTCACTTACTCAAATTGTAGCAA 19026
Macaque           GGGTTTCAGGTAAGACATA-GTGTT---TGCTATTTGTTTTAAGCAAACT--CTTCCTTCACTTACTCAAATTGTAGCAA 19421
PigTailedMacaque  GGGTTTCAGGTAAGACATA-GTGTT---TGCTATTTGTTTTAAGCAAACT--CTTCCTTCACTTACTCAAATTGTAGCAA 18645
Bonobo            GGGTTTCAGGTAAGACAT--GTGTT---TGCTATTTGTTTTAAGCAAACT--CTTCCCTCATTTATTCAAATTGTAGCAA 18205
OliveBaboon       GGGTTTCAGGTAAGACATA-GTGTT---TGCTATTTGTTTTAAGCAAACT--CTTCCTTCACTTACTCAAAATGTAGCAA 18427
Orangutan         GGGTTTCAGGTAAGACATATGTGTT---TGCTATTTGTTTTAAGCAAACT--CTTCCCTCATTTATTCAAATTGTAGCAA 18541

Majority          ACATCTACTACACCCATTTCCAGTTCTTTGTCTTAATAGTTCAACTATTATAAAAAG----GA---GTGGTATATATGTA
                          23690     23700     23710     23720     23730     23740     23750     23760
Human             ACATCTACTACACCCATTTCCAGTTCTTTGTCTTAATAGTTCAACTATTATAAAAA----GGA---GTGGTATATATATA 17180
Coquerel'sSifaka  ACATCTACTATACCAATTTCTAGTTCTTTATTGTAATAGTTCCACTATTATAAAAAG----GA---ATGGTATGTAA--- 15781
Ma'sNightMonkey   ACACATACTATACCCATTTCCAGTTCTTTGTCTTAATAGTTCAACTATTAAAAAAAGGAGTGATACGTAATATATATGTA 14945
VervetAGM         AAAATAGCTGCAGACAGGACTAAATATCTTTATTAGTGAACAAAAAAATAAAAATGACTTAGA---ATTTAGCACAGAGA 20038
Gorrilla          ACATCTACTACACCAATTTCCACTTCTTTGTCTTAATAGTTCAACTATTATAAAAA----GGA---GTAGTATATATATA 19552
GrabEatingMacaqueACATCTTCTACACCCATTTCCAGTTCTTTGTCTTAATAGTTCAACTATTGTAAAAAG----GA---GTGGTATATATGTA 19099
Macaque           ACATCTTCTACACCCATTTCCAGTTCTTTGTCTTAATAGTTCAACTATTGTAAAAAG----GA---GTGGTATATATGTA 19494
PigTailedMacaque  ACATCTACTACACCCATTTCCAGTTCTTTGTCTTAATAGTTCAACTATTGTAAAAAG----GA---GTGGTATATATGTA 18718
Bonobo            ACATCTACTACACCCATTTCCAGTTCTTTGTCTTAATAGTTCAACTATTATAAAAA----GGA---GTGGTATATATGTA 18278
OliveBaboon       ACATCTACTACACCCATTTCCAGTTCTTTGTCTTAATAGTTCAACTATTGTAAAAAG----GA---GTGGTATATATGTA 18500
Orangutan         ACATCTACTACACCCATTTCCAGTTCTTTGTCTTAATAGTTCCACAATTATAATAACA--GGA---GTGGTGTATATATA 18616
```

```
Majority          T----ACTCTTGGTTGGCTTTGGTTTGATTGAATTAATAAGGTG-GTTTTTTTTGTTTGT----TTTG----AGACGGAG
                           23770     23780     23790     23800     23810     23820     23830     23840

Human             TATAAACTCTTGCTTGGCTTTGGTTTGATTGAATTAATAAGGTGAGTTTTTTTTGTTTGTTTGTTTTG----AGATGGAG 17256
Coquerel'sSifaka -----ACTCTTGTGTGGCTTTTGTTTTAGTGAATTTATAAGGTAGTTTTAAAAAACACTTTG--TTTTACATTGACAGGG 15854
Ma'sNightMonkey   TATATATTCTTGCTTGGCTTTGG---G-TTGAATTTGTAAGGTGTTTTTAAAAAATACTTTAGATTGGCCGGGCGCGGTG 15021
VervetAGM         A----ACAAAGAGATGAAAATAGAAGGGTTAAA--AATACATTG-GAAAGGAAGAGAAGT----TCT------AACAAAC 20101
Gorrilla          T--AAACTCTTGCTTGGCTTTGGTTTGATTGAATTAATAAGGTGAGTTCTTTTTGTTTGT----TTTG----AGATGGAG 19622
GrabEatingMacaqueA----ACTCTTGGTAGGCTTTGGTTTGATTGAATTAATAAGGTG-GTTTTTTGTGTTTGT----TTTG----AAACGGAG 19166
Macaque           A----ACTCTTGGTAGGCTTTGGTTTGATTGAATTAATAAGGTG-GTTTTTTGTGTTTGT----TTTG----AAACGGAG 19561
PigTailedMacaque A----ACTCTTGGTAGGCTTTGGTTTGATTGAATTAATAAGGTG-GTTTTTTGTGTTTGT----TTTG----AAACGGAG 18785
Bonobo            T--AAACTCTTGCTTGGCTTTGGTTTGATTGAGTTAATAAGGTGAGTTTTTTTTGTTTGTTTGTTTTG----AGATGGAG 18352
OliveBaboon       A----ACTCTTGGTAGGCTTTGGTTTGATTGAATTAATAAGGTG-GTTTTTTGTGTTTGT----TTTG----AAACGGAG 18567
Orangutan         TATAAACTCTTGCTTGGCTTTGGTTTGATTGAATTAATAAGGTGGGTTTTTTTTGTTTGTTTGTTTTG----AGATGGAG 18692

Majority          TCTTGC-TCTGTTGCCCAGGC----TGGAGTGCAGTGGTGCA---ATCTC--GGCTCACTG--CAACCTCC----G-CTC
                           23850     23860     23870     23880     23890     23900     23910     23920

Human             TCTCGC-TCTGTTGCCCAGGC----TGGAGTGCAGTGGTGGG---ATCTC--GGCTCACTG--CAG------------- 17310
Coquerel'sSifaka TTTGAG--CCCTTGCTTTACT---TCTTCATATGATTATGAACAAATCATT-GATTCTTTG---AACCTC-------T-- 15916
Ma'sNightMonkey   GCTCACGCCTATAATCCCAGCACTTTGGGAGGCCGAGGCGGGTGGATCACGAGGCCAAGAGATTGAGACCATCCTGGTCA 15101
VervetAGM         ATCTAA-TTAGAGTTCCAGAA----AGAAATTAAAGATTGCA---GAAA---GGCAAAATA--TGAAATGCTAAAGGCTC 20168
Gorrilla          TCTCGC-TCTGTTGCCCAGGC----TGGAGTGCAGTGGTGGG---ATGTC--GGCTCACTG--CAG------------- 19676
GrabEatingMacaqueTCTTGC-TCTGTCGCCCAGGC----TGGAGTGCAGTGGTGCA---ATCTC--GGCTCACTG--CAACCTCC----GCCTC 19230
Macaque           TCTTGC-TCTGTCGCCCAGGC----TGGAGTGCAGTGGTGCA---ATCTC--GGCTCACTG--CAACCTCC----GCCTC 19625
PigTailedMacaque TCTTGC-TCTGTCGCCCAGGC----TGGAGTGCAGTGGTGCA---ATCTC--GGCTCACTG--CAACCTCC----GCCTC 18849
Bonobo            TCTCGC-TCTGTTGCCCAGGC----TGGAGTGCAGTGGTGGG---ATCTC--GGCTCACTG--CAG------------- 18406
OliveBaboon       TCTTGC-TCTGTCGCCCAGGC----TGGAGTGCAGTGGTGCA---ATCTC--CGCTCACTG--CAACCTCC----GCCTC 18631
Orangutan         TCTCAC-TCTGTTGCCCAGGC----TGGAGTGCAGTGGTGCG---ATCTC--GGCCCACTG--AAGCTCGC------CTC 18754

Majority          CCGGGTTCACGCCATTCTCCTGCCTCA-----ACCTCCCAAGTAGCTGGGACTACAGGT--GCCTGCTGCCATACCT---
                           23930     23940     23950     23960     23970     23980     23990     24000

Human             ----------GCCATTCTCCTGCCTCA-----GCCTCCCGAGTAGCTGGGACTGCAGGT--GCCTGCCACCACACCT--- 17370
Coquerel'sSifaka ----------CCTA-ATTTGTAATATAA----GAATAATAATT--CCTAACTCACATGG----TTGTTGTGAAGATT--- 15972
Ma'sNightMonkey   ACATGGTGAAACCCCGTCTCTACTAAAAATACAAAAATTAGCTAGGCAGGGCTGCAGCGTGGCCTGTAATCCCAGCTACT 15181
VervetAGM         ATAAGTTTTTAGAATTGGTGAGCTATATG---AATTCTTAGATTTAGGAGACAAAAGAA--AACC-TGGACATATAA--- 20239
Gorrilla          ----------GCCATTCTCCTGCCTCA-----GCCTCCCGAGTAGGTGGGACTGCAGGC--GCCTGCCACCACACCT--- 19736
GrabEatingMacaqueCCGGGTTCACGCCATTCTCCTGCCTCA-----ACCTCCCAAGTAGCTGGGACTACAGGT--GCCTGCTGCCATGCCT--- 19300
Macaque           CCGGGTTCACGCCATTCTCCTGCCTCA-----ACCTCCCAAGTAGCTGGGACTACAGGT--GCCTGCTGCCATGCCT--- 19695
PigTailedMacaque CCGGGTTCATGCCATTCTCCTGCCTCA-----ACCCCCCAAGTAACTGGGACTACAGGT--GCCTGCTGCCATGCCT--- 18919
Bonobo            ----------GCCATTCTCCTGCCTCA-----GCCTCCCGAGTAGCTGGGACTGCAGGT--GCCTGCCACCACACCT--- 18466
OliveBaboon       CCGGGTTCACGCCATTCTCCTGCCTCA-----ACCTCCCGAGTAGCTGGGACTACAGGT--GCCTGCTGCCATGCCT--- 18701
Orangutan         CTGGGTTCACGCCATTCTCCTGCCTCA-----GCCTCCCGAGTAGCTAGGACTGCAGGC--ACCTGCCACCACACCT--- 18824
```

```
Majority         -----GGCTAATTTTTTGTATTTTTAGTAGAGACAGGGTTTCACCGTGTTAGCCAGGATGGTCTCGAT---CTCCTGACC
                          24010     24020     24030     24040     24050     24060     24070     24080
Human            -----GGCTAATTTTTTGTATTTTTAGTAGAGATGGGGTTTCACCGTGTTAGCCAGGATGGTCTAGAT---CTCCTGACC 17442
Coquerel'sSifaka -----AAATAAGACAATAAGGTA--GGTAGAAGCAGTTTATAATCTTTCTCGT--GAGCAGTGCAAAT----ATATGAGG 16039
Ma'sNightMonkey  CAGGAGGCTGAGGCAGAGAATTGCCTGAACCAGGAGCGGAGCTTGCGGTAAGCCGAGATCGCGCCATTGCACTCCAGCCT 15261
VervetAGM        -----CAGTGAAAGAGCAGAGCACCAA-AGATATAGAGATCTTTAAAGC-AGCCAGAGAAAATCAGAT---TCCCTAAAG 20309
Gorrilla         -----GGCTAATTTTTTGTATTTTTAGTAGAGATGGGGTTTCACCGTGTTAGCCAGGATGGTCTCGAT---CTCCTGACC 19808
GrabEatingMacaque-----GGCTAATTTTTTGTATTTTTAGTAGAGACAGGATTTCACAGTGTTAGCCAGGATGGTCTTGAT---CTCCTGACC 19372
Macaque          -----GGCTAATTTTTTGTATTTTTAGTAGAGACAGGATTTCACAGTGTTAGCCAGGATGGTCTTGAT---CTCCTGACC 19767
PigTailedMacaque -----GGCTAATTTTTTGTATTTTTAGTAGAGACAGGGTTTCACCATGTTAGCCAGGATGGTCTCGAT---CTTCTGACC 18991
Bonobo           -----GGCTAATTTTTTGTACTTTTAGTAGAGATGGGGTTTCACCGTGTTAGCCAGGATGGTCTCGAT---CTCCTGACC 18538
OliveBaboon      -----GGCTAATTTTTTGTATTTTTAGTAGAGACAGGATTTCACAGTGTTAGCCAGGATGATCTTGAT---CTCCTGACC 18773
Orangutan        -----GGCTAATTTTTTGTATTTTTAGTAGAGACGGGGTTTCACCGTCTTAGCCAGGATGGTCTCAAT---CTCCTGACC 18896

Majority         TCGTG--ATCCGC------CCGCCTCGGAAA---------TACTTTGCATTGTTG-CATTGATTGGGT--TTGAGCCCC
                          24090     24100     24110     24120     24130     24140     24150     24160
Human            TTGTG--ATCCGC------CCGCCTCGGAAA---------TACTTTGCATTGTTG-GATTGATTGGGT--TTGAGCCCC 17501
Coquerel'sSifaka GTTTA--AATAGCAAAATGCCACTTTAAAA------------------AATGTGG--ACTGA--AA-----TGGATACT 16089
Ma'sNightMonkey  GGGTAACAAGAGCGAAACTCCGCCTCAAAAAAAAAAAAAAATACTTTGCATTGTTG-TATTGATTGGGT--TTCAGCCCC 15338
VervetAGM        AATAAGAATTAGA------AAGCCCCAATAT---------GCTTCATCAGCATGGGCATCCAGAAGATAGTGGAATAAT 20373
Gorrilla         TCATG--ATCTGC------CCGCCTCGGAAA---------TACTTTGCATTGTTG-GATTGATTGGGT--TTGAGCCCC 19867
GrabEatingMacaqueTCGTG--ATCCAC------CCACCTCGGAAA---------TACTTTGCATTGTTG-CATTGATTGGGT--TTGAGCCCC 19431
Macaque          TCGTG--ATCCAC------CCACCTCGGAAA---------TACTTTGCATTGTTG-CATTGATTGGGT--TTGAGCCCC 19826
PigTailedMacaque TTGTGTGATCCAC------CCATCTCGG------------CCTCCCAAAGTGCTG-GATTA--CAGGC--GTGAGC--C 19045
Bonobo           TCGTG--ATCCGC------CCGCCTCGGAAA---------TACTTTGCATTGTTG-GATTGATTGGGT--TTGAGCCCC 18597
OliveBaboon      TCGTG--ATCCGC------CCACCTCGGAAA---------TACTTTGCATTGTTG-CATTGATTGAGT--TTGAGCCCC 18832
Orangutan        TCGTG--ATCCGC------CCGCCTTGGAAA---------TACTTTGCATTGTTG-CATTGATTGG-T--TTGAGCCCC 18954

Majority         TGTTGTACTTAAGTGATTATGAACAAGTCTTTTATTCTCTGAATTTCCCTTTCCTAGTTTGTAATATTGAGGATAAT---
                          24170     24180     24190     24200     24210     24220     24230     24240
Human            TGTTGTACTTAAGTGATTATGAACAAGTCTTTTATAATCTGAATTTCCCTTTCCTAGTTTGTAATATTGAGGATAATAAT 17581
Coquerel'sSifaka TCTAATATAAGAGTAATATTTAGAAACACATAAATTATCTTATATAATGTCTTATAACTT--AATAGTATA-ATAA---- 16162
Ma'sNightMonkey  TTTTGTACTTAAGTGATGATGAACGAGTCTTTTATTCTCTTAATTTCCCTTTCCTAGTTTGTAATATTGAGGATTATTAT 15418
VervetAGM        ATTTTCAGTGTATTGAGGGAAAATA-GCTGTCCACTTATTATTTTATAGTCAACTAAATTATCATATAAGGAGAAGA--- 20449
Gorrilla         TGTTGTACTTAAGTGATTATGAACAAGTCTTTTATAATCTGAATTTCCCTTTCCTAGTTTGTAATATTGAGGATAATAAT 19947
GrabEatingMacaqueTGTTGTACTTAAGTGATAATGAACAAGCCTTTTATTCTCTGAATTTCCCTTTCTTAGTTTGTAATATTGAGGATAAT--- 19508
Macaque          TGTTGTACTTAAGTGATAATGAACAAGCCTTTTATTCTCTGAATTTCCCTTTCTTAGTTTGTAATATTGAGGATAAT--- 19903
PigTailedMacaque ------ATC--------------------------------ATATTCTTTTCCCAGTATTTTATGTCTAG--------- 19077
Bonobo           TGTTGTACTTAAGTGATTATGAACAAGTCTTTTATAATCTGAATTTCCCTTTCCTAGTTTGTAATATTGAGGATAATAAT 18677
OliveBaboon      TGTTGTACTTAAGTGATAATGAACAAGCCTTTTATTCTCTGAATTTCCCTTTCTTAGTTTGTAATATTGAGGATAAT--- 18909
Orangutan        TGTTATACTTAAGTGATTATGAACGAGTCTTTTATTCTCTGAATTTCCCTTTCCTAGTTTGTAATATTGAGGATAATAAT 19034
```

```
Majority          TCCTAACTTGCATGATTGTTGTGAAGATTAAATAAGAC--AATAATATTGGTAGGAGCTGTTTATAATCTTTCTCTCTTA
                           24250     24260     24270     24280     24290     24300     24310     24320
Human             TTCTAACTTGCATGATTGTTGTGAAGATTAAATAAGAC--AATGATATTGGTAGGAGCTCTTTATAATCTTTCTCTCTTA 17659
Coquerel'sSifaka  ----AATGCACATGGAAGTC-------TTTTATAGCTT--AGTAAGATT--TGGG---TACTTGCAAATAGTCTGGTTTA 16224
Ma'sNightMonkey   TCCTAACTTGTATGATTGTTTTGAAGATTGAATAAGAC--AATAATATTGGTAGGAGCTATTTATAATCTTTCTCTCTTA 15496
VervetAGM         ACAAAATAAAGACGCTTTTTGAAACACTTCATTCTGCCTCCAAAAAAGCAATTTAGGAAATTTATCAACAGATCCACTGA 20529
Gorrilla          TCCTAACTTGCATGATTGTTGTGAAGATTAAATAAGAC--AATGATATTGGTAGGAGCTCTTTATAATCTTTCTCTCTTA 20025
GrabEatingMacaqueTCCTAACTTGCACGATTGTTGTGAAGATTAAATAAGAC--AATAATATTGGTAGGAGCTGTTTATAATCTTTCTCTCTTA 19586
Macaque           TTCTAACTTGCACGATTGTTGTGAAGATTAAATAAGAC--AATAATATTGGTAGGAGCTGTTTATAATCTTTCTCTCTTA 19981
PigTailedMacaque  ---TAACTCAGAAAAAAAT------GATCAGATAAG----TATATTTTTAATATG--CTGATTACAACATTATTTAAATA 19142
Bonobo            TCCTAACTTGCATGATTGTTGTGAAGATTAAATAAGAC--AATGATATTGGTAGGAGCTCTTTATAATCTTTCTCTCTTA 18755
OliveBaboon       TCCTAACTTGCACGATTGTTGTGAAGATTAAATAAGAC--AATAATATTGGTAGGAGCTGTTTATAATCTTTCTCTCTTA 18987
Orangutan         TCCTAACTTGCGTGATTGTTGTGAAGATTAAATAAGAC--AATGATATTGGTAGGAGCTCTTTATAATCTTTCTCTCTTA 19112

Majority          TGTACAAT-TCAAATATATGTGGGTTTAAATACCAAAATACCACTTTAAAA-AATCTGGGC-TGAAATGGACACTTCTAA
                           24330     24340     24350     24360     24370     24380     24390     24400
Human             TGTACAAT-TCAAATATATGTCGGTTTAAATACCAAAATACCACTTTAAAA-AATCTGGGC-TGAAATGGACACTTCTAA 17736
Coquerel'sSifaka  TTTTTGATTT----TTTAT-TAGGTTTTCACAATGCATTTCTTCAGTGAAATACTCT--ATTTGAGATTGTTGTTTT--A 16295
Ma'sNightMonkey   TGTACAAT-TCAAATATATGTGGGTTTAAATACCAAAATACCTCTTTAAAA-AGTCTGAGC-TGAAATGGACACTTTTTA 15573
VervetAGM         AGGAGTTTCTGGAGTTTGTACTTCAGGAAGAAAGCCAATGACACCAGAAAGTAAGTCTGACGTAGAAGTGAGGCTAATCA 20609
Gorrilla          TGTACAAT-TCAAATATATGTCGGTTTAAATACCAAAATACCACTTTAAAA-AATCTGGGC-TGAAATGGACACTTCTAA 20102
GrabEatingMacaqueTGTACAAT-TCAAATATATGTGGGTTTAAATACCAAAATACCACTTTAAAA-AATCTAGGC-TGAAATGGACACTTCTAA 19663
Macaque           TGTACAAT-TCAAATATATGTGGGTTTAAATACCAAAATACCACTTTAAAA-AATCTAGGC-TGAAATGGACACTTCTAA 20058
PigTailedMacaque  TTTAGAAA-TAACAAATGTTCAGTGATAAGGAAATGAATCTGTTCATAAAC-ATTACAAATGTTTTCTAAATGTTTTTCA 19220
Bonobo            TGTACAAT-TCAAATATATGTCGGTTTAAATACCAAAATACCACTTTAAAA-AATCTGGGC-TGAAATGGACACTTCTAA 18832
OliveBaboon       TGTACAAT-TCAAATATATGTGGGTTTAAATACCAAAATACCACTTTAAAA-AATCTAGGC-TGAAATGGACACTTCTAA 19064
Orangutan         TGTACAAT-TCAAATATATGTCGGTTTAAATACCAAAATACCACTTTAAAA-AATCTGGGC-TGAAATGGACACTTCTAA 19189

Majority          TGTAGA--AGTA-GTGCTTTAGAAAAT---AATGCTTTGTT--------------------------A-A---A-T-T--
                           24410     24420     24430     24440     24450     24460     24470     24480
Human             TGTAGG--AGTA-GTGCTTTAGAAAAT---AATGCTTTGTT--------------------------------------- 17771
Coquerel'sSifaka  TCAAGACCTTTATGTGCTTTAGAAAAG---AATGTTTTGTT--------------------------------------- 16333
Ma'sNightMonkey   TGTAGA--AGTA-GTATTTAAGAAACATTTAAATTATGGTA-------------T-----------ATAATGTCTTATA 15625
VervetAGM         AGAAAATTGGTA-GGCCTGTGAGTAAATCTAATTATTGACCATATAAGAATGTAATAGCAGTGATTGTTAAGGAGTTTAA 20688
Gorrilla          TGTAGG--ATTA-GTGCTTTAGAAAAT---AATGCTTTGTT--------------------------------------- 20137
GrabEatingMacaqueTGTAGA--AGTA-GTGCTTTAGAAAAG---AATGCTTTGTTTCATACTCTTTTTTTTTTTTTT-TTGAGATGGAGTCTTG 19736
Macaque           TGTAGA--AGTA-GTGCTTTAGAAAAG---AATGCTTTGTTTCATACTCTTTTTTTTTTTTTT---GAGACGGAGTCTTG 20129
PigTailedMacaque  TATGTG--TGAA-AAGTAGGATTAAAA---ATAACTTTGACAAATGC--------------------ATAC--AATTTGT 19272
Bonobo            TGTAGG--AGTA-GTGCTTTAGAAAAT---AATGCTTTGTT--------------------------------------- 18867
OliveBaboon       TGTAGA--AGTA-GTGCTTTAGAAAAG---AATGCTTTGTTTCATACTCTTTTTTTTTTTTT-T-GAGACGGAGTCTTG 19136
Orangutan         TGTAGG--AGTA-GTGCTATAGAAAAT---AATGCTTTGTT--------------------------------------- 19224
```

```
Majority           --GT-------A----------C-GT---GC--T-T-------------AT------------G--------------T-

                           24490     24500     24510     24520     24530     24540     24550     24560

Human              --------------------------------------------------T----------------------------- 17772
Coquerel'sSifaka   -------------------------------------------------------------------------------- 16333
Ma'sNightMonkey    ACGTACTAGTATAATAAATGCACAGTGAAGCTTTTTATAGCTTTGTGTGTTTGCAAATTGTCTGG-------------TT 15692
VervetAGM          AAATAGAATTAAAAATACTGAATCATAACAATATGTAAGAGAGTGGTAGAGACACTGGAGTTTGAGTTCTGATGATGTAG 20768
Gorrilla           --------------------------------------------------T----------------------------- 20138
GrabEatingMacaqueCTGTGTCGCCCAGGCTGGAGTGCTGTGGCGCAATCTCTGCT--CACTACAAGCTTCGCCTCCCGGGTTC--ACGCCATTC 19812
Macaque            CTGTGTCGCCCAGGCTGGAGTGCTGTGGCGCAATCTCTGCT--CACTACAAGCTTCGCCTCCCGGGTTC--ACGCCATTC 20205
PigTailedMacaque   ATGTGTCCCCAAAA-------ACTGGAGGGCAGTAT--------ACCAAAA----------TAGAGTA--------GTTC 19319
Bonobo             --------------------------------------------------T----------------------------- 18868
OliveBaboon        CTGTGTCGCCCAGGCTGGAGTGCTGTGGCGCAATCTCTGCT--CACTGCAAGCTCCGCCTCCCGGGTTC--ACGCCATTC 19212
Orangutan          --------------------------------------------------T----------------------------- 19225

Majority           T------------------G---------------------------------------TTT--T-TAT-TTTAGT--A-

                           24570     24580     24590     24600     24610     24620     24630     24640

Human              -------------------------------------------------------------------------------- 17772
Coquerel'sSifaka   -------------------------------------------------------------------------------- 16333
Ma'sNightMonkey    T------------------G------------------------------------A--TTT--TGAATTGTTAGCAGGT 15714
VervetAGM          TATTTATTGGAGCTGTGTTGTCAGTTACAGTA--GCTACTAGTCATTTGTTACCGAGCACTTGACATGAATGTAGTTTAA 20846
Gorrilla           -------------------------------------------------------------------------------- 20138
GrabEatingMacaqueTCCTGCCTCAGCCTCCAGAGTAGCTGGGACTACAGGC--TCGCCACC------GGCTAATTTTTTGTATTTTTAGTAGAG 19884
Macaque            TCCTGCCTCAGCCTCCAGAGTAGCTGGGACTACAGGCGCCCGCCACCACGCCCGGCTAATTTTTTGTATTTTTAGTAGAG 20285
PigTailedMacaque   TC----TTCAG--------GTTGATGGGATT-----CGTTTTTCA-----------TAATTCTTTATATATTTTGTCTAT 19371
Bonobo             -------------------------------------------------------------------------------- 18868
OliveBaboon        TCCTGCCTCAGCCTCCAGAGTAGCTGGGACTACAGACGCCCGCCACCACGCCCGGCTAATTTTTTGTATTTTTAGTAGAG 19292
Orangutan          -------------------------------------------------------------------------------- 19225

Majority           -------TT----AT-TTA------AT----C---T-TT--GA-------T-AT---------T-------C--------

                           24650     24660     24670     24680     24690     24700     24710     24720

Human              -------------------------------C------------------------------------------------ 17773
Coquerel'sSifaka   -------------------------------------------------------------------------------- 16333
Ma'sNightMonkey    TTTAAAAATATGTTTATGAAGTGAAATA---CTCATTTTGAGATTGTTGTTTATCAGTGGGCCTTGAGATACT------- 15784
VervetAGM          GTTGA--TTTTCTACCTTAGTTGAAAAATACAGATTTCTAAGCCTTAGTCTGAAAGAAGAATGTAAAACATCCCACATTT 20924
Gorrilla           -------------------------------C------------------------------------------------ 20139
GrabEatingMacaqueACAGGGTTTCACCATGTTAGCCAGGATGGTCTCGATCTTCTGACCTTGTGTGATCCACCCATCTCGGCC-TCCCAAAGTG 19963
Macaque            ACAGGGTTTCACCATGTTAGCCAGGATGGTCTCGATCTTCTGACCTTGTGTGATCCACCCATCTCGGCC-TCCCAAAGTG 20364
PigTailedMacaque   A------TTCCACATTTTAA-----GTA-------TTTTATGATAGTTATTAATA--T-----TCAGTT-ATTAATATTG 19425
Bonobo             -------------------------------C------------------------------------------------ 18869
OliveBaboon        ACAGGGTTTCACTATGTTAGCCAGGATGGTCTCGATCTTCTGACCTCGGGTGATCCACCCATCTTGGCC-TCCCAAAGTG 19371
Orangutan          -------------------------------C------------------------------------------------ 19226
```

```
Majority          -----T---TT----G----A----TCATATTCTTTTCCTAGTATTTTATGTCTAGTAACTCAT--GGAAAAAAATGATCA

                          24730     24740     24750     24760     24770     24780     24790     24800

Human             ----------G-----------------TATTCTTTTCCTAGTATTTTATGTCTAGTAACTCATAAGGAAAAAAATGATCA 17826
Coquerel'sSifaka  -------------------------TCATATTCTTGTCCCAGTATTTTATGTCTTGTAA-------GGAAAAGATGATCA 16381
Ma'sNightMonkey   -----TTACAAAAGAATGCTTTGTTTCATATTCTTTTCTCAGTATTTTATGTCTAGTAACTCATA-TGAAAAAAATGATTA 15858
VervetAGM         TCATATTGGTTACATGTAGAACTGATAATATTTTTGTATTT-TGGGTTAAGTGAAATACGTTAT--GCATTGCCTTTTCC 21001
Gorrilla          ----------G-----------------TATTCTTTTCCTAGTATTTTATGTCTAGTAACTTATAAGGAAAAAAATGATCA 20192
GrabEatingMacaqueC----TGGATTACAGGCGTGAGCCATCATATTCTTTTCCCAGTATTTTATGTCTAGTAACTCA---GAAAAAAATGATCA 20036
Macaque           C----TGGATTACAGGCGTGAGCCATCATATTCTTTTCCCAGTATTTTATGTCTAGTAACTCA---GAAAAAAATGATCA 20437
PigTailedMacaque  C----TTAATT----GATTAAACACTGACATTTTCTTGT---TTCTTTA-----AAAAACTTA---GCATAACTTGAT-- 19484
Bonobo            ----------G-----------------TATTCTTTTCCTAGTATTTTATGTCTAGTAACTCATAAGGAAAAAAATGATCA 18922
OliveBaboon       C----TGGATTACAGGCGTGAGCCATCATATTCTTTTCCCAGTATTTTATGTCTAGTAACTCA---GAAAAAAATGATCA 19444
Orangutan         ----------A-----------------TATTCTTTTCCTAGTATTTTATGTCTAGTAACTCATAAGGAAAAAAATGATCA 19279

Majority          GATAAGTATATTTTTTATA--------TGCTGATTACAGCATTATTTAAATA--GTAGAAATAACAAATGTTCAGTGATA

                          24810     24820     24830     24840     24850     24860     24870     24880

Human             GATAAGTATATTTTTTAGA--------TGCTGATTACAGCATTATTTAAATA--GTAGAAATAACAACTGTTCAGTGATA 17896
Coquerel'sSifaka  GATAAATGTACTTTTTATATATAAAGATGTTGATTACAGCATTATTTACATTAAATTGAAATAACAAATGTACAGTGATA 16461
Ma'sNightMonkey   GATAAGTGTATTTTT-ATA--------TGCTGATTACAGCATTATTTAAATA--GTAGAAACAATAAATGTACAGTGATA 15927
VervetAGM         TCTTTCTTTCCTTTCTTTC--------TTTTCTTTCTCTCTCTCTCTCTCTCC-CCCTCTCTCTCTCTCTCTCTCCCCCC 21072
Gorrilla          GATAAGTATATTTTTTAGA--------TGCTGATTACAGCATTATTTAAATA--GTAGAAATAACAACTGTTCAGTGATA 20262
GrabEatingMacaqueGATAAGTATATTTTTTAATA--------TGCTGATTACAACATTATTTAAATAT-TTAGAAATAACAAATGTTCAGTGATA 20107
Macaque           GATAAGTATATTTTTTAATA--------TGCTGATTACAACATTATTTAAATAT-TTAGAAATAACAAATGTTCAGTGATA 20508
PigTailedMacaque  -ATAA--ATCTTTTTAAT---------TGC-AAAAAAAACAT----AAAATTT-ACCGTCTTAACCATTTTTAAGTGT-- 19544
Bonobo            GATAAGTATATTTTTTAGA--------TGCTGATTACAGCATTATTTAAATA--GTAGAAATAA---CTGTTCAGTGATA 18989
OliveBaboon       GATAAGTATATTTTTTAATA--------TGTTGATTACAACATTATTTAAATAT-TTAGAAATAACAAATGTTCAGTGATA 19515
Orangutan         GATAAGTATATTTTTTAGA--------TGCTGATTACAGCATTATTTAAATA--GTAGAAATAACAAATGTTCAGTGATA 19349

Majority          AGAAAATGAATAAATTCTGTTCATA-------------AACATTACAAATGTT---TTTTAAATATTTTTCATATG----

                          24890     24900     24910     24920     24930     24940     24950     24960

Human             AGACAATGAATAAATTCTGTTCATA-------------AACATTACAAATGTT---TTTTAAATATTTTTCATATG---- 17956
Coquerel'sSifaka  TGAAAATGGCTAAATTATGTTCATACAGGGAACTAATAAACATTACAAATGATATATTTTAAATATTTTTAATACA---- 16537
Ma'sNightMonkey   AGAAAATGGATAAATTCTGTTCATA-------------ACCATTACAAATGGT---GTTTCCTAAATTTTTTTGTAT--- 15988
VervetAGM         CCTCTGTCTCC-----CTCTCCTCC-------------CTCTCTCTCCTCTCT---CTCTCTTTCTTTCTCTCTTTCTCC 21131
Gorrilla          AGAAAATGAATAAATTCTGTTCATA-------------AACATTACAAATGTT---TTTAAAATATTTTTCATATG---- 20322
GrabEatingMacaqueAGGAAATGAAT-----CTGTTCATA-------------AACATTACAAATGTT---TTCTAAATGTTTTTCATATG---- 20162
Macaque           AGGAAATGAAT-----CTGTTCATA-------------AACATTACAAATGTT---TTCTAAATGTTTTTCATATG---- 20563
PigTailedMacaque  --ACACTTAAA-----TTGTTCA------------------GTTATTCTTTTT---TTTTTTTTTTTTTTTATTTA---- 19592
Bonobo            AGACAATGAATAAATTCTGTTCATA-------------AACATTACAAATGTT---TTTTAAATATTTTTCATATG---- 19049
OliveBaboon       AGGAAATGAAT-----CTGTTCGTA-------------AACATTACAAATGTT---TTCTAAATACTTTTCTTATG---- 19570
Orangutan         ACAAAA-GAATAAATTCTGTTCATA-------------AACATTACAAATGTT---TTTTAAATATTTTTCATATG---- 19408
```

```
Majority          -TGTGAAAA---GTAGGATTAAAAATAACTTTGACAAATGCATACAATTTATATGTGTCCCCAAAAGCTGGAGGGCAGTA

                          24970     24980     24990     25000     25010     25020     25030     25040

Human             -TGTGAAAA---GTAGGATTAAAAATAACTTTGACAAATGCATACAATTTATATGTGTCCCCAAAAGCTGGAGGGCAGTG 18032
Coquerel'sSifaka  -TGTGAAAAAAAGTAAGATTAAAAGTAGCTTTGACAAATATATACAGTTTATATGTGTCCCCAAAAGCTTGAGGGCAGTA 16616
Ma'sNightMonkey   ATGTGAAAA---GTAGGATTAAAAATAACTTTGACAAATACATACAATTTATATGTGTCCCCAAAAGCTGGAGGGCAGTA 16065
VervetAGM         CTGTCTGTC---TCTCTCTCTGTCTTTCCTTTTTTTAAGATAGGGTCTTGCTCTGTCCCCC---AGGCTGGAGTGCCATG 21205
Gorrilla          -TGTGAAAA---GTAGGATTAAAAATAACTTTGACAAATGCATACAATTTATGTGTGTCCCCAAAAGCTGGAGGGCAGTG 20398
GrabEatingMacaque-TGTGAAAA---GTAGGATTAAAAATAACTTTGACAAATGCATACAATTTGTATGTGTCCCCAAAAACTGGAGGGCAGTA 20238
Macaque           -TGTGAAAA---GTAGGATTAAAAATAACTTTGACAAATGCATACAATTTGTATGTGTCCCCAAAAACTGGAGGGCAGTA 20639
PigTailedMacaque  -TTT--------------------TTTTTTTTTTGAGACGGAGTCTCGCTCTGTAGCCC---AGGCTGGAGTGCAGTA 19647
Bonobo            -TGTGAAAA---GTAGGATTAAAAATAACTTTGACAAATGCATACAATTTATATGTGTCCCCAAAAGCTGGAGGGCAGTG 19125
OliveBaboon       -TGTGAAAA---GTAGGATTAAAAATAACTTTGACAAATGCATACAATTTATATGTGTCCCCAAAAACTGGAGGGCAGTA 19646
Orangutan         -TGTGAAAA---GTAGGATTAAAAATAACTTTGACAAATGCATACAATTTATATGTGTCCCCAAAAGCTGGAGGGCAGTG 19484

Majority          -TACCAAAATAGAG----TAGTTTTCTTCAGGTTGATGGGATTAGTTTTTCATAATTCTTT---ATATATTTTGTCTATA

                          25050     25060     25070     25080     25090     25100     25110     25120

Human             -TACCAAAATAGAG----TAGTTTTCTCCAGGTTGATGGGATTAGTTTTTTGTAATTCTTT---ATATATTTTCTCTATA 18104
Coquerel'sSifaka  -CACAAAAACAGAG----TAGTTTTCTTCAGGTTGATGACACTAATTCTTAAAAATTCTTTCCTATATATTTTTTATATA 16691
Ma'sNightMonkey   -TACCAAAATAGAG----TATTTTTCTTCAGGTTGATGGGAATAAGTTTTCATAATTCTTT---TCATTTTTTATAATTG 16137
VervetAGM         GCATGATCATGGCAGCCTCAACCTCCTGTGCTCCAGTGACCCTCCCACTTCA-GCCTCCCA---AGTAGCTGGGACTACA 21281
Gorrilla          -TACCAAAATAGAG----TAGTTTTCTCCAGGTTGATGGGATTAGTTTTTTGTAATTCTTT---ATATATTTTCTCTATA 20470
GrabEatingMacaque-TACCAAAATAGAG----TAGTTCTCTTCAGGTTGATGGGATTCGTTTTTCATAATTCTTT---ATATATTTTGTCTATA 20310
Macaque           -TACCAAAATAGAG----TAGTTCTCTTCAGGTTGATGGGATTCGTTTTTCATAATTCTTT---ATATATTTTGTCTATA 20711
PigTailedMacaque  -GCCGGATCT--------CAGCTCACTGCAAGCT--------CCGCCTCCCG-GGTTCACG---CCATTCTCCGGC---- 19702
Bonobo            -TACCAAAATAGAG----TAGTTTTCTCCAGGTTGATGGGATTAGTTTTTTGTAATTCTTT---ATATATTTTCTCTATA 19197
OliveBaboon       -TACCAAAATAGAG----TAGTTCTCTTCAGGTTGATGGGATTCGTTTTTCATAATTCTTT---ATATATTTTGTCTATA 19718
Orangutan         -TACCAAAATAGAG----TAGTTTTCTTCAGGTTGATGGGATTAGTTTTTTATAATTCTTT---ATATATTTTCTCTATA 19556

Majority          ----TTCCACATTTTAAGTATTTT---ATGATAAAGTTATTAATATTGATA--AGTTATTAATATTTC---TTAATTGAT

                          25130     25140     25150     25160     25170     25180     25190     25200

Human             ----TTCCACATTTTAAGTATTTT---ATGATAAAGTTATTAATATTGATA-AAGTTATTAATGTTGC---TTAATTGAT 18173
Coquerel'sSifaka  ----GTCCACATTTCTAATATTGTCTATATTTATAATATTTAATATCTA----ATCTAATGATAATTTAGCTTAATTGGT 16763
Ma'sNightMonkey   TTTTCTATTTATTTTCTCTATATTCCCATTTTTAAGTATTTTATGATTA----AGTTATTAATATTGC---TTAATTGGT 16210
VervetAGM         GTTGTGTGCCACCACACCTGGCTA---ATTATTGAGTTTTTTTTGTAGAGACAGGGTCTTGCTATATTGCGTAGGCTGGT 21358
Gorrilla          ----TTCCACATTTTAAGTATTTT---ATGATAAAGTCATTAATATTGATA-AAGTTATTAATATTGC---TTAATTGAT 20539
GrabEatingMacaque----TTCCACATTTTAAGTATTTT---ATGATAAAGTTATTAATATTCA-----GTTATTAATATTTC---TTAATTGAT 20375
Macaque           ----TTCCACATTTTAAGTATTTT---ATGATAAAGTTATTAATATTCA-----GTTATTAATATTTC---TTAATTGAT 20776
PigTailedMacaque  ----CTCAGCCTCCCAAGTAGCTG---GGGCTACAGGCGCTGCCACCTC-----GCC-CGGCTATTTT---TTGTATTTC 19766
Bonobo            ----TTCCACATTTTAAGTATTTT---ATGATAAAGTTATTAATATTGATA-AAGTTATTAATATTGC---TTAATTGAT 19266
OliveBaboon       ----TTTCACATTTTAAGTATTTT---ATGATAAAGTTATTAATATTCATA---GTTATTAATATTGC---TTAATTGAT 19785
Orangutan         ----TTCCACATTTTAAGTATTTT---ATGATAAAGTTATTAATAT-GATA-ATGTTATTAATATT-C---TTACTTGAT 19623
```

```
Majority          TAAACACT----GAC----ATTTTCTTGTTTCTTTA-------AAAAACTTTGCATAACTTGATATAAA-----------

                           25210     25220     25230     25240     25250     25260     25270     25280

Human             TAAACCCT----GAT----ATTTTCTTGTTTCTTTA-------AAAAACGTTGCATAACTTGATATAAA----------- 18227
Coquerel'sSifaka  TAAACAGC------------TTTCCTGTTTTCTTTA-------AAAAACTTAGTATAACTTGACATAAA----------- 16813
Ma'sNightMonkey   TAAACATT----GAC----TTTTTCTTGTTTCTTTA-------AAAAACTTTGCATATTATAACTTAATATA---AA--T 16270
VervetAGM         CTGAAACTCCTGGACTCAAGTGATCTTCCTGCTTTAGCCTTGCAAAGTGCTGGGATTACAGGACTGAGTCTGGCCACCTT 21438
Gorrilla          TAAACCCT----GAC----ATTTTCTTGTTTCTTTA-------AAAAA-GTTGCATAACTTGATATAAA----------- 20592
GrabEatingMacaque TAAACACT----GAC----ATTTTCTTGTTTCTTTA-------AAAAACTTAGCATAACTTGATATAAA----------- 20429
Macaque           TAAACACT----GAC----ATTTTCTTGTTTCTTTA-------AAAAACTTAGCATAACTTGATATAAA----------- 20830
PigTailedMacaque  TTAGTAGA----GAC----GGGGTTTCACCGTGTTA-------GCCAGGATGGTCTCGATCTCCTGACC----------- 19820
Bonobo            TAAACCCT----GAT----ATTTTCTTGTTTCTTTA-------AAAAACGTTGCATAACTTGATATAAA----------- 19320
OliveBaboon       TAAACACT----GAC----ATTTTCTTGTTTCTTTA-------AAAAACTTTGCATAACTTGATATAAA----------- 19839
Orangutan         TAAACACT----GAC----ATT---TTGTTTCTTTA-------AAAAACTTTGCATAACTTGATATAAA----------- 19674

Majority          --TCTTTTTAATTGTAAAAAAAAC--CACATAAAATTTACTATCTTAACCATTTTTAAGTGTACACTTAAATTGTTTAAT

                           25290     25300     25310     25320     25330     25340     25350     25360

Human             --TCTTTTTAATTGTACAAAAAAA--CACATAAAATTTACTATCTTAACCATTTTTAAGTGTACACTTAAATTGTTTAAT 18303
Coquerel'sSifaka  TCTTTCTAAAATTGTGGTAAAAAT---ACATAAAATTTACCATCTCAACTGCTTTTAAGTATACACGTAAATAGTTTAAG 16890
Ma'sNightMonkey   C-TTTTTGTGGTAAAAAAAAAAAAAAAACATAAAATTTACCATCTTAACCATTTCTAAGTGTACACTAAA---GTTTAAG 16346
VervetAGM         TTTCTTTGTACTTTCAAAAATGTGG-CTAATAGAAGTTTATGAGATTATATTTGTGGTTCATACTACATTTCTTTTGGAC 21517
Gorrilla          --TCTTTTTAATTGTACAAAAAA---CACATAAAATTTACTATCTTAACCATTTTTAAGTGTACATTTAAATTGTTTAAT 20667
GrabEatingMacaque --TCTTTTTAATTGCAAAAAAAAC-----ATAAAATTTACTGTCTTAACCATTTTTAAGTGTACACTTAAATTGTTCAGT 20502
Macaque           --TCTTTTTAATTGCAAAAAAAAC-----ATAAAATTTACTGTCTTAACCATTTTTAAGTGTACACTTAAATTGTTCAGT 20903
PigTailedMacaque  --TCGTGATCCGCCCATCTCGGCC-----TCCCAAAGTGCTGGGATTACAGGCTTGAGCCACCGCGCCCGGCTGTTCAGT 19893
Bonobo            --TATTTTTAATTGTACAAAAAA---CACATAAAATTTACTATCTTAACCATTTTTAAGTGTACACTTAAATTGTTTAAT 19395
OliveBaboon       --TCTTTTTAATTGCAAAAAAAAC-----ATAAAATTTACCATCTTAACCATTTTTAAGTGTACACTTAAATTGTTCAGT 19912
Orangutan         --TCTTTTTAATTGTACAAAAAA---CACATAAAATTTACCATC--AACCATTTTTAAGTGTACACTTCAATTGTTTAAT 19747

Majority          TATTCTTATACGTA---CTTAATAATGTTACATTGC---GAAACCCATC--TCCAGAACTTTTTTCATTTTGGA------

                           25370     25380     25390     25400     25410     25420     25430     25440

Human             TATTCTTATACGTA---CTTAATAATATTACATTGC---AAAACCCATC--TCCAGAACTTTTT-------GGA------ 18362
Coquerel'sSifaka  TACCTGTAATTCCAACCCTTTTGGATGCTGAGATGG-GAGGATCTCTTGAGGCCAGGAGTTCAAGACCTGCCTGAGCAAC 16969
Ma'sNightMonkey   TATTCTTATATGTA---CTTAATAATAATCCATTGTTGTGAAACCCATC--TCCAGAACGTTTTTCATTTTGGA------ 16415
VervetAGM         AGTGCCAGAGTGAA---TCAGATAAGCTTGGGTTTT---GAAATCCTAA--GGGTGAACACAATAGAGATAGAACCCAAA 21589
Gorrilla          TATTCTTATACGTA---CTTAATAATATTACATTGC---AAAACCCATC--TCCAGAACTTTTT-------GGA------ 20726
GrabEatingMacaque TATTCTTATACGTA---CTTAATAATGCTACATTGC---GAAACCCATC--TCCAGAACTTTTTTCATTTTGGA------ 20568
Macaque           TATTCTTATACGTA---CTTAATAATGCTACATTGC---GAAACCCATC--TCCAGAACTTTTTTCATTTTGGA------ 20969
PigTailedMacaque  TATTCTTATACGTA---CTTAATAATGCTACATTGC---GAAACCCATC--TCCAGAACTTTTTTCATTTTGGA------ 19959
Bonobo            TATTCTTATACGTA---CTTAATAATATTACATTGC---AAAACCCATC--TCCAGAACTTTTT-------GGA------ 19454
OliveBaboon       TATTCTTATACGTA---CTTAATAATGCTACATTGC---GAAACCCATC--TCCAGAACTTTTTTCATTTTGGA------ 19978
Orangutan         TATTCTTATATGTA---CTTAATAATATTACATTGT---GAAACCCATA--TCCAGAACTTTTTTCATTTTGGA------ 19813
```

```
Majority          GAACTGAGACTCTATACTGATT-------TTAAAACTCT----CCCTTTTGTCCTTGCCCTGGTAAC-------------

                           25450     25460     25470     25480     25490     25500     25510     25520

Human             GAACTGAAACTCTATACTGATT-------TTAAAACTCC----CTCTTTTGTCCTTCTGCTGGTAAC------------- 18418
Coquerel'sSifaka  ATAGTGAGACCCTGTCTCTACAAAAAAATTCAAAAAATGAGGGCGTGGTGGTACATGCCCCAGCTACTTGGGA--GGCTG 17047
Ma'sNightMonkey   GAACTGAAACCCTATGCCCATT-------TTAAAACTC-----CCCTTTTGTTCTTCCCCT------------------- 16464
VervetAGM         GAATTGGGGAGTGGGGTTGACTGAAA---TTAAAGATGT----ATAATCCAAAAGAAGGCAAAAAAAAAAAAAAAGAATA 21662
Gorrilla          GAACTGAAACTCTATACTGATT-------TTAAAACTCC----CCCTTTTGTCCTTCCGCTGGTAAC------------- 20782
GrabEatingMacaque GAACTGAGACTCTATACTGATT-------TTAAAACTCT----CCCTTTTGTCCTTGCCCTGGTAAC------------- 20624
Macaque           GAACTGAGACTCTATACTGATT-------TTAAAACTCT----CCCTTTTGTCCTTGCCCTGGTAAC------------- 21025
PigTailedMacaque  GAACTGAGACTCTATACTGATT-------TTAAAACTCT----CCCTTTTGTCCTTGCCCTGGTAAC------------- 20015
Bonobo            GAACTGAAACTCTATACTGATT-------TTAAAACTCC----CTCTTTTGTCCTTCCGCTGGTAAC------------- 19510
OliveBaboon       GAACTGAGACTCTATACTGATT-------TTAAAACTCT----CCCTTTTGTCCTTGCCCTGGTAAC------------- 20034
Orangutan         GAACTGAAGCTCTATACTGATT-------TTAAAACTCC----CCCTTTTGTCCTTCCCCTGGTAAC------------- 19869

Majority          ---CAGCATTCTATTTTCTGTGTCTATGAATTTGAC---TACTTTAAA---TACCTCA-------TATAAGTGGAATTAT

                           25530     25540     25550     25560     25570     25580     25590     25600

Human             ---CAGCATTCTATTTTCTGTGTCTATGAATTTGAC---TACTTTAAA---TACCTCA-------CATAAGTGGAATTAT 18482
Coquerel'sSifaka  AAGCAGGAGGAT--CACTTGAGTCCAGGAGTTTGAG-GTTGCAATGAGGC-AGGATGATGCTGCTGCACCCCAACCCCAG 17123
Ma'sNightMonkey   -----------T----------TC-----------------C--C--------C-TGA-------------------TAA 16476
VervetAGM         AGACACAAAATTAGCCTATAGGTTAATAGAGTGGAAAGGTTCTGTGAAGGGTTCCTTAGAACCTTTAAAGGTAGAATTTT 21742
Gorrilla          ---CAGCATTCTATTTTCTGTGTCTATGAATTTGAC---TACTTTAAA---TACCTCA-------CATAAGTGGAATTAT 20846
GrabEatingMacaque ---CAGCATTCTGTTTCCTGTGTCTATGAATTTGAC---TACTTTAAA---TACCTCA-------TATAAGTGGAATTAT 20688
Macaque           ---CAGCATTCTGTTTCCTGTGTCTATGAATTTGAC---TACTTTAAA---TACCTCA-------TATAAGTGGAATTAT 21089
PigTailedMacaque  ---CAGCTTTCTGTTTCTTGTGTCTATGAATTTGAC---TACTTTAAA---TACCTCA-------TATAAGTGGAATTAT 20079
Bonobo            ---CAGCATTCTATTTTCTGTGTCTATGAATTTGAC---TACTTTAAA---TACCTCA-------CATAAGTGGAATTAT 19574
OliveBaboon       ---CAGCATTCTGTTTCCTGTGTCTATGAATTTGAC---TACTTTAAA---TACCTCA-------TATAAGTGGAATTAT 20098
Orangutan         ---CAGCATTCTATTTTCTGTGTCTATGAATTTGAC---TACTTTAAA---TACCTCA-------CATAAGTGGAATTAT 19933

Majority          ACAATATAGGTTAAGCA-------------TCCCTAATC-----TGAAAATCCAAAGTCTG--AATGCT-ACAAAATGTG

                           25610     25620     25630     25640     25650     25660     25670     25680

Human             ACAGTATAGGTTAAGCA-------------TCCCTAATC-----TGAAAATCCAAAGTCTG--AATGCT-ACAAAATCTG 18541
Coquerel'sSifaka  ACAATAGAGAGAGATGC-----------CGTCTCAAAAGAATAGTTTAATT---ATATGTACATATGCT--TAAAATACT 17187
Ma'sNightMonkey   CCAGCACAGAAATAACA------------------------------------AATGTCCA--G-TGATAAGAAAATGTG 16517
VervetAGM         CTTGTCTGGTTGCAGGAAAGAGTGGCATTGTTTTTGTTCACTTTTGAAGCTACAAAAACAGGTAATGATGAGGAAAATTG 21822
Gorrilla          ACAATATAGGTTAAGCA-------------TCCCTAATC-----TGAAAATCCAAAGTCTG--AATGCT-ACAAAATCTG 20905
GrabEatingMacaque ACAATATAGGTTAAGCA-------------TCCCTAATC-----TGAAAATCCAAAGTCTG--AATGCT-ACAAAATGTG 20747
Macaque           ACAATATAGGTTAAGCA-------------TCCCTAATC-----TGAAAATCCAAAGTCTG--AATGCT-ACAAAATGTG 21148
PigTailedMacaque  ACAATATAGGTTAAGCA-------------TCCCTAATC-----TGAAAATCCAAAGTCTG--AATGCT-ACAAAATGTG 20138
Bonobo            ACAGTATAGGTTAAGCA-------------TCCCTAATC-----TGAAAATCCAAAGTCTG--AATGCT-ACAAAATCTG 19633
OliveBaboon       ACAATATAGGTTAAGCA-------------TCCCTAATC-----TGAAAATCCAAAGTCTG--AATGCT-ACAAAATGTG 20157
Orangutan         ACAATATAGGTTAAGCA-------------TCCCTAATC-----TGAAAATCCAAAGTCTG--AATGCT-ACAAAATCTG 19992
```

```
Majority          AAACTTTTTA------------------------TTAGTTTAGTTTAGTTTA-----------TTCA----TTAGTTTTT

                         25690     25700     25710     25720     25730     25740     25750     25760

Human             AAACTGGTGT------------------------GTGTTTTTTTTTTTTTTT-----------TTT-----TTT-TTTTT 18580
Coquerel'sSifaka  AAACTTAAGA-------------------------TGGTTTAAGTATATGTA------------------CATACACTTA 17224
Ma'sNightMonkey   -------------------------------------------------------------------------------- 16517
VervetAGM         GGACATGTTGAGGAAGGAAGTAGTTTTTTGGAATGTAGGTTGGGTTAGAGTAGCAGAAGACAATTTAAAGAGTAGTTTTT 21902
Gorrilla          AAACTGGTGT------------------------TTTTGTTTTATTTTTATT-----------TTTA----TTT-TTTTT 20945
GrabEatingMacaqueAAACTTTTTA-------------TTTTGTTTAGTTTAGTTTAGTTTAGTTCAGTTCAGTTCAGTTCA--GTTTAGTTTTT 20812
Macaque           AAACTTTTTA-------------TTTTGTTTAGTTTAGTTTAGTTTAGTTCAGTTCAGTTCAGTTCA--GTTTAGTTTTT 21213
PigTailedMacaque  AAACTTTTTA-------------TTTTGTTTAGTTTAGTTTAGTTCAGTTCAGTTCAGTTCAGTTCA--GTTTAGTTTTT 20203
Bonobo            AAACTGGTGT------------------------GTGTTTTTTTTT---------------------------------- 19655
OliveBaboon       AAACTTTTTA-------------TTTTGTTTAGTTTAGTTTAGTTCAGTTCAGTTCAGTTCAGTTCA--GTTTAGTTTTT 20222
Orangutan         AAACTTTTTT------------------------TTTTTTTTTTTT---------------------------------- 20014

Majority          GAGACGGA---GTCTTGCTCTGT-CGCCCAGG--CTGGAGTGCAG--TGGCGCAATCTCT----GCTCACTGCAAGCTCC

                         25770     25780     25790     25800     25810     25820     25830     25840

Human             GAGACGGA---GTCTTACTCTGT-CGCCCAGG--CTGGAGTGCAG--TGGCGCGATCTCT----GCTCACTGCAAGCTCC 18648
Coquerel'sSifaka  ACAATGTAACAACATTACATTTT-TGTGAAA---CAGATCTTCAGA-----ACATTTTTTTCTTGCAGAACTGAAACTCT 17295
Ma'sNightMonkey   ------------------TCTAT---------------------G--------A--ATTT-------------------- 16528
VervetAGM         GACAGTAATATGGAGAGCTTTGAGTGCAAAGAATTTGCAGTGAGGACCTTTGAAAATTGGACCTGCTTTCTTCTATCTGT 21982
Gorrilla          GAGACGGA---GTCTTACTGTGT-CGCCCAGG--CTGGAGTGCAG--TGGCGCGATCTCT----GCTCACTGCAAGCTCC 21013
GrabEatingMacaqueGAGACGGA---GTCTTGCTCTGT-CGCCCAGG--CTGGAGTGCAG--TGGTGCAATCTCG----GCTCACTGCAAGCTCC 20880
Macaque           GAGACGGA---GTCTTGCTCTGT-CGCCCAGG--CTGGAGTGCAG--TGGTGCAATCTCG----GCTCACTGCAAGCTCC 21281
PigTailedMacaque  GAGACGGA---GTCTTGCTCTGT-CGCCCAGG--CTGGAGTGCAG--TGGTGCAATCTCG----GCTCACTGCAAGCTCC 20271
Bonobo            GAGACGGA---GTCTTACTCTGT-CGCCCAGG--CTGGAGTGCAG--TGGCGCGATCTCT----GCTCACTGCAAGCTCC 19723
OliveBaboon       GAGACGGA---GTCTTGCTCTGT-CGCCCAGA--CTGGAGTGCAG--TGGCGCAATCTCG----GCTCACTGCAAGCTCC 20290
Orangutan         GAGACAGA---GTCTCACTGTGT-CGCCCAGG--CTGGAGTGCAG--TGGCGCGATCTCT----GCTCACTGCAAGCTCC 20082

Majority          ACCCCC--CAGGTTCACGCCATTTTCCTGTCTCAGCCTCC--CGAGTAGCTGGGACTATAGGCGGCTGCCACCACGCCCG

                         25850     25860     25870     25880     25890     25900     25910     25920

Human             ACCACC--CGGGTTCACGCCATCT-------------TCC--TGAGTAGCTGGGACTACAGGCGCCCGCCACCACGCCCG 18711
Coquerel'sSifaka  ATACCTATTAAAACCTCCTCTTTT------------------TGCCCAATCTCAGCCTTTGGTAACCAGCATTCTACTTT 17357
Ma'sNightMonkey   ------------------------------------------GAGTA-------CTTTAAATA-----CATCACATAGG 16553
VervetAGM         ATTTATGTTTAATTTTTTTTTTTTTTTGTNTCTCAGCCTCCATCGAGTAGCTGGGACTATAAGCGGCTGCCACCACGCCCG 22062
Gorrilla          ACCACC--CGGGTTCACGCCGTCT-------------TCC--TAAGTAGCTGGGACTACAGGCACCCGCCACCACGCCCG 21076
GrabEatingMacaqueACCCCC--CAGGTTCACGCCATTCTCCTGTCTCAGCCTCCATCGAGTAGCTGGGACTATAGGCGGCTGCCACCACGCCCG 20958
Macaque           ACCCCC--CAGGTTCACGCCATTCTCCTGTCTCAGCCTCCATCGAGTAGCTGGGACTATAGGCGGCTGCCACCACGCCCG 21359
PigTailedMacaque  ACCCCC--CAGGTTCACGCCATTCTCCTGTCTCAGCCTCCATCGAGTAGCTGGGACTATAGGCGGCTGCCACCACGCCCG 20349
Bonobo            ACCACC--CGGGTTCACGCCATCT-------------TCC--TGAGTAGCTGGGACTACAGGCGCCCGCCACCACGCCCG 19786
OliveBaboon       ACCCCC--CAGGTTCACGCCATTCTCCTGTCTCATCCTCCATCGAGTAGCTGGGACTATAGGCGGCTGCCACCATGCCCG 20368
Orangutan         ACCACC--CGGATTCACGCCATCCGCCTGCCACAGCCTCC--CGAGTAGCTGGGATTACAGGCGCCTGCCACCACGCCCA 20158
```

```
Majority          GCTAATTTTTT-TGTTTTTTTGTTTTT----CTTTTAGTAGAGATGGGGTTTCACTGTAGCCAGGATGGTCTCT-ATCTC
                          25930     25940     25950     25960     25970     25980     25990     26000
Human             GCTAATTTTTTGTATATATATATATAT----TTTTTAGTAGAGACGGGGTTTCACTGTAGCCAGGATGGACTCTTATCTC 18787
Coquerel'sSifaka  ACGAATTTGACCACTTTAGATAA-----------CTCATATAAATGGAGT----CAGTA--CAGGTTGAGCATC-----C 17415
Ma'sNightMonkey   GTGAATTATAC-------------------------AATATAGATTAAG-------------------AATC------C 16582
VervetAGM         GCTAATTTTTT-TGTTTTTTTGTTTTT----CTTTTAGTAGAGATGGGGTTTCACTGTAGCCAGCATGGTCTCT-ATCTC 22136
Gorrilla          GCTAATTTTTTGTATATATATATATATTT---TTTTTTAGTAGAGACGGGGTTTCACTGTAGCCAGGATGGACTCT-ATCTC 21152
GrabEatingMacaqueGCTAATTTTTT-TGTTTTTT-GTTTTT----CTTTTAGTAGAGATGGGGTTTCACTGTAGCCAGCATGGTCTCT-ATCTC 21031
Macaque           GCTAATTTTTT-TGTTTTTT-GTTTTT----CTTTTAGTAGAGATGGGGTTTCACTGTAGCCAGCATGGTCTCT-ATCTC 21432
PigTailedMacaque  GCTAATTTTTT-TGTTTTTT-GTTTTT----CTTTTAGTAGAGATGGGGTTTCACTGTAGCCAGCATGGTCTCT-ATCTC 20422
Bonobo            GCTAATTTTTTGTATATATATATATATAT-TTTTTAGTAGAGACGGGGTTTCACTGTAGCCAGGATGGACTCT-ATCTC 19864
OliveBaboon       GCTAATTTTTT-TGTTTTTT-GTTTTT----CTTTTAGTAGAGATGGGGTTTCACCGTAGCCAGCATGGTCTCT-ATCTC 20441
Orangutan         GCTAATTTTTTGTGTATATATATATATATTTTTTTTAGTAGAGATGGGGTTTCACCGTAGCCAGGATGGACTC--ATCTC 20236

Majority          CTGACCTCGTGATCCGCCTGCCTCGGCCTTCCAAAGTTCTGGGATTACAGGCCTGAGCCACCACACC---------TGAA
                          26010     26020     26030     26040     26050     26060     26070     26080
Human             CCGACCTCGTGATCCGCCTGCCTCGGCCTCCCAAAGTGCTGGGATTACAGGCCTGAGCCACCGCGCCTGGCCAATCTGAA 18867
Coquerel'sSifaka  CTAATATGGAAAC-----------------CCAAA--TCTGAAAT-----------GCTCCAAAATC---------TGAA 17456
Ma'sNightMonkey   CTAATCTGAAAAT-----------------CCAAAGTCT--GAAT----G-------CTACAAAATC---------TGAA 16623
VervetAGM         CTGACCTCGTGATCCGCCTGCCTCGGCCTTCCAAAGTGCTGGGATTACAGGCCCGAGCCACCACACC---------TGAA 22207
Gorrilla          CCGACCTCGTGATCCGCCTGCCTCGGCCTCCCAAAGTGCTGGGATTACAGGCCTGAGCCACCGCGCCCGGCCAGTCTGAA 21232
GrabEatingMacaqueCTGACCTCGTGATCCGCCTGCCTTGGCCTTCCAAATTTCTGGGATTACAGGCCTGAGCCACCACACC---------TGAA 21102
Macaque           CTGACCTCGTGATCCGCCTGCCTTGGCCTTCCAAATTTCTGGGATTACAGGCCTGAGCCACCACACC---------TGAA 21503
PigTailedMacaque  CTGACCTCGTGATCCGCCTGCCTTGGCCTTCCAAAGTTCTGGGATTACAGGCCTGAGCCACCACACC---------TGAA 20493
Bonobo            CCGACCTCGTGATCCGCCTGCCTCGGCCTCCCAAAGTGCTGGGATTACAGGCCTGAGCCACCGCGCCCGGCCAATCTGAA 19944
OliveBaboon       CTGACCTCGTGATCCGCCTGCCTCGGCCTTCCAAAGTTCTGGGATTACAGGCCTGAGCCACCACACC---------TGAA 20512
Orangutan         CTGACCTCGTGATCAGCCCACCTCGGCCTCCCAAAGTGCTGGGATTACAGGCCTGAGCCACCGCGCCCAGCCA-TCTGAA 20315

Majority          ACTTTTTGAGCACTTAC--TTGATGCTCAAAGGAAGTGCTTAGTGGAGCATTTTGGATTTTAGATTTTCAGATTAGGAAT
                          26090     26100     26110     26120     26130     26140     26150     26160
Human             ACTTTTTGAGCACTTAC--TTGATGCTCAAAGGAAGTGCTTAGTGGAGCATTTTGGATTTTAGATTTTCAGATTAGGAAT 18945
Coquerel'sSifaka  ACTTTTTGAGCACCAAC--ATGGTGCTCAAAGGAAATGCTCATCAGAGCATTTTGCATTTCAGATTTTCAGATTAGGGAT 17534
Ma'sNightMonkey   ACTTTTTGAGCACTTAT--GTGATGCTCAAAGGAGGTACTTAATGGAACATTTTGGATTTTAGATTTTCAGATTAGGAAT 16701
VervetAGM         ACTTTTTGAACACTTACACGTGATGCTCAAAGGAAGTGCTTAGTGGAGCATTTTGGATTTTAGATTTTCAGATTAGGAAT 22287
Gorrilla          ACTTTTTGAGCACTTAC--TTGATGCTCAAAGGAAGTGCTTAGTGGAGCATTTTGGATTTTAGATTTTCAGGTTAGGAAT 21310
GrabEatingMacaqueACTTTTTGAGCACTTACACATGATGCTCAAAGGAAGTGCTTAGTGGAGCATTTTGGATTTTAGATTTTCAGATTAGGAAT 21182
Macaque           ACTTTTTGAGCACTTACACATGATGCTCAAAGGAAGTGCTTAGTGGAGCATTTTGGATTTTAGATTTTCAGATTAGGAAT 21583
PigTailedMacaque  ACTTTTTGAGCACTTACACGTGATGCTCAAAGGAAGTGCTTAGTAGAGCATTTTGGATTTTAGATTTTCAGATTAGGAAT 20573
Bonobo            ACTTTTTGAGCACTTAC--TTGATGCTCAAAGGAAGTGCTTAGTGGAGCATTTTGGATTTTAGATTTTCAGATTAGGAAT 20022
OliveBaboon       ACTTTTTGAGCACTTACACGTGATGCTCAAAGGAAGTGCTTAGTGGAGCATTTTGGATTTTAGATTTTCAGATTAGGAAT 20592
Orangutan         ACTTTTTGAGCACTTAA--TTGATGCTCAAAGGAAGTGCTTAGTGGAGCATTTTGGATTTTAGATTTTCGGGTTAGGAAT 20393
```

```
Majority          GCTCAGGTTGTGTTTGTCCTTTTGTGACTGGCTTATATTCACTTAACATAATGTCCTCAAGGTCCATCCGTGTTGTAGCA
                          26170     26180     26190     26200     26210     26220     26230     26240
Human             GCTCAGGTTGTGTTTGTCCTTTTGTGACTGGCTTATATTCACTTAATATAATGTCCTCAGGGTCCATCCATTTTGTAGCA 19025
Coquerel'sSifaka  GCTCAGCCTATTATTGTCCTTGTGTAAGTGGCTTAT-TTCACTTAGCATAATGTCTTCAAGGTACATGCATGTTGCAGCT 17613
Ma'sNightMonkey   GCTCAGATTGTGTTTGTCCTTGTGTGACTGGCTTATTTTCACTTAATATAATGTCCTCAAGGTCCATTTGTGTTGTAGCA 16781
VervetAGM         GCTCAGGTTGTGTTTGTCCTTTTGTGACTGGTTTACATTCACTTAACA--ATGTTCTCAAGGTCCATCCGTGTTGTAGCA 22365
Gorrilla          GCTCAGGTTGTGTTTGTCCTTTTGTGACTGGCTTATATTCACTTAATATAATGTCCTCAGGGTCCATCCATTTTGTAGCA 21390
GrabEatingMacaqueGCTCAGGTTGTGTTTGTCCTTTTGTGACTGGTTTACATTCACTTAACA--ATGTTCTCAAGGTCCATCCGTGTTGTAGCA 21260
Macaque           GCTCAGGTTGTGTTTGTCCTTTTGTGACTGGTTTACATTCACTTAACA--GTGTTCTCAAGGTCCATCCGTGTTGTAGCA 21661
PigTailedMacaque  GCTCAGGTTGTGTTTGTCCTTTTGTGACTGGTTTACATTCACTTAACA--ATGTTCTCAAGGTCCATCCGTGTTGTAGCA 20651
Bonobo            GCTCAGGTCGTGTTTGTCCTTTTGTGACTGGCTTATATTCACTTAATATAATGTCCTCAGGGTCCATCCATTTTGTAGCA 20102
OliveBaboon       GCTCAGGTTGTGTTTGTCCTTTTGTGACTGGTTTACATTCACTTAACA--ATGTTCTCAAGGTCCATCCGTGTTGTAGCA 20670
Orangutan         GCTCAGGTTGTGTTTGTCCTTTTGTGACTGGCTTATATTCACTTAATATAATGTCCTCAGGGTCCATCTGTGTTGTAGCA 20473

Majority          TGTGACAAGATTTCCTCCCTTTTTAAGGCTACATAGCATTCCATTGTGGTACACAAAAGTTGATTTGAAGCCCCCTGTGT
                          26250     26260     26270     26280     26290     26300     26310     26320
Human             TGTGACAAGATTTCCTCCCTTTTTAAGGCTACATAGCATTCCATTGTGGTACACAAAAGTTCATTTGAAGCCCCCTGTGT 19105
Coquerel'sSifaka  TGTGACAGAATTTCCTTCCTTTTTAAGGCTGCATAACATTCCATCGTG-TAC--------CAATTTGAAGTCCTTTGTGT 17684
Ma'sNightMonkey   TGTAACAAGATTTCCTCCCCTTTTAAGGCCGCATAGCATTCCATTGTGGTACACGAAAGTTCGTTTGAAGCCCCCTGTGT 16861
VervetAGM         TGTGACAAGATTTCCTCCGTTTTTAAGGCTACATAGCATTCCATTGTGGTACACAAAAGTTGATTTGAAGCCCCCTGTGT 22445
Gorrilla          TGTGACAAGATTTCCTCCCTTTTTAAGGCTACATAGCATTCCATTGTGGTACACAAAAGTTCATTTGAAGCCCCCTGTGT 21470
GrabEatingMacaqueTGTGACAAGATTTCCTCCGTTTTTAAGGCTACATAGCATTCCATTGTGGTACACAAAAGTTGATTTGAAGCCCCCTGTGT 21340
Macaque           TGTGACAAGATTTCCTCCGTTTTTAAGGCTACATAGCATTCCATTGTGGTACACAAAAGTTGATTTGAAGCCCCCTGTGT 21741
PigTailedMacaque  TGTGACAAGATTTCCTCCGTTTTTAAGGCTACATAGCATTCCATTGTGGTACACAAAAGTTGATTTGAAGCCCCCTGTGT 20731
Bonobo            TGTGACAAGATTTCCTCCCTTTTTAAGGCTACATAGCATTCCATTGTGGTACACAAAAGTTCATTTGAAGCCCCCTGTGT 20182
OliveBaboon       TGTGACAAGATTTCCTCCGTTTTTAAGGCTACATAGCATTCCATTGTGGTACACAAAAGTTGATTTGAAGCCCCGTGTGT 20750
Orangutan         TGTCACAAGATTTC-TCCCTTTTTAAGGCTACATAGCATTCCATTATGGTACACAAAAGTTCATTTGAAGCCCCC-GTGT 20551

Majority          CATAACTCAGTCTTCCATAACAAAATACCATAGACTGAGTGACTTAAAAAACAGAAATTCATTTTCTCACAGTTCTGGAA
                          26330     26340     26350     26360     26370     26380     26390     26400
Human             CATAACTCAGACTTCCATAACAAAATACCATAGACTGAGTGACTTAAAAAACAGAGATTCGTTTTCTCACAGTTCTGGAA 19185
Coquerel'sSifaka  CTTAACTCAGGATACCATAATAAAACACCATAGATCAAGTGGCTTGAAAAACA--AATTTATTTTCTCACAGTTCTGAAG 17762
Ma'sNightMonkey   CATAACTCAGGTTTCCATAAGAAAATACCATAGACTGAGTGACTTAAAAAACAGAAATTTATTTTCTCCCAATTTTGGAA 16941
VervetAGM         CATCACTCATTCTTCCATAACAAAATACCATAGACTGAGTGACTTAAAAAACAGAAATTCATTTTCTCACAGTTCTGGAA 22525
Gorrilla          CATAACTCAGACTTCCATAACAAAATACCATAGACTGAGTGACTTAAAAAACAGAAATTTATTTTCTCACAGTTCTGGAA 21550
GrabEatingMacaqueCATCACTCATTCTTCCATAACAAAATACCATAGACTGAGTGACTTAAAAAAGAGAAATTCTTTTTCTCACAGTTCTGGAA 21420
Macaque           CATCACTCATTCTTCCATAACAAAATACCATAGACTGAGTGACTTAAAAAAGAGAAATTCTTTTTCTCACAGTTCTGGAA 21821
PigTailedMacaque  CATCACTCATTCTTCCATAACAAAATACCATAGACTGAGTGACTTAAAAAAGAGAAATTCTTTTTCTCACAGTTCTGGAA 20811
Bonobo            CATAACTCAGACTTCCATAACAAAATACCATAGACTGAGTGACTTAAAAAACAGAAATTTGTTTTCTCACAGTTCTGGAA 20262
OliveBaboon       CATCACTCATTCTTTCATAACAAAATACCATAGACTGAGTGACTTAAAAAAGAGAAATTCATTTTCTCACAGTTCTGGAA 20830
Orangutan         CATAACTCAGGCTTCCATAACAAAATACCATAGATTGAGTG--------GACAGAAATTTATTTTCTCACAGTTCTGGAA 20623
```

```
Majority         ACTGGGAAGTCCAAGATCAAGGTACCAGCAAGGCAACTTTCCTTCTGAGGCCACTTCTCTTGAAGTGAGG----CCACCA

                         26410     26420     26430     26440     26450     26460     26470     26480

Human            ACTGGGAAGTCCAAGATCAAGGTACCAGCAAGGCAACTTTCCTTCTGAGGCCACTTCTCTTGAAGTGAGG----CCACCA 19261
Coquerel'sSifaka ACTGGGAAGTTCAAAATCAAGGTTCTGGCAAGCTAAATTTCATTCTGAGGTCACTTCTCTTGGTTTGTAGACAGCCACCA 17842
Ma'sNightMonkey  ACTGGGAAGTCCAAGATCAAGGTATCAGCAAAGCAGTTTTCCTTCAGAGGCCACTTCTTTTGAAGTGAAG----CCACTG 17017
VervetAGM        ACTGGGAAGTCCAAGATCAAGGTACCAGCAAGGCAACTTTCCTTCTGAGGCCACTTCTCTTGAAGCAAGG----CCATCA 22601
Gorrilla         ACTGGGAAGTCCAAGATCAAGGTACCAGCAAGGCAACTTTCCTTCTGAGGCCACTTCTCTTGAAGTGAGG----CCACCA 21626
GrabEatingMacaqueACTGGGAAGTCCAAGATCAAGGTACCAGCAAGGCAACTTTCCTTCTGAGGCCACTTCTCTTGAAGCGAGG----CCATCA 21496
Macaque          ACTGGGAAGTCCAAGATCAAGGTACCAGCAAGGCAACTTTCCTTCTGAGGCCACTTCTCTTGAAGCGAGG----CCATCA 21897
PigTailedMacaque ACTGGGAAGTCCAAGATCAAGGTACCAGCAAGGCAACTTTCCTTCTGAGGCCACTTCTCTTGAAGCGAGG----CCATCA 20887
Bonobo           ACTGGGAAGTCCAAGATCAAGGTACCAGCAAGGCAACTTTCCTTCTGAGGCCACTTCTCTTGAAGTGAGG----CCACCA 20338
OliveBaboon      ACTGGGAAGTCCAAGATCAAGGTACCAGCAAGGCAGCTTTCCTTCTGAGGCCACTTCTCTTGAAGCGAGG----CCATCA 20906
Orangutan        ACTGGGAAGTCCAAGATCAAGGTACCAGCAAGGCAACTTTACTTCTGAGGCCACTTCTCTTGAAGTGAGG----CCACCA 20699

Majority         TTTTACTTTGT---GTGCCTCACATGACCTTTTCTTTGTGAGC--------------------------ACACTTTGG--

                         26490     26500     26510     26520     26530     26540     26550     26560

Human            TTTTACTTTGT---GTGCCTCACATGACCTTTTCTTTGTGAGC--------------------------ACACTTTGG-- 19310
Coquerel'sSifaka TCTTATCTTGTTTTGTGCCTCACATGACCTTTTCTTTGTGAGCCACCATCTTGCTTTGTGCCTCACATGACCTTTTGTTA 17922
Ma'sNightMonkey  TTTTACTTTGT---GTGCTTCACATGATCTTTTCTTTGTGAGC--------------------------ACACTTAGG-- 17066
VervetAGM        TTTTATTTTGT---GTGCCTCACATGACCTTTTGTTTGTCAGC--------------------------ACACTTTGG-- 22650
Gorrilla         TTTTACTTTGT---GTGCCTCACATGACCTTTTCTTTGTGAGC--------------------------ACACTTTGG-- 21675
GrabEatingMacaqueTTTTACTTTGT---GTGCCTTACATGACCTTTTCTTTGTCAGC--------------------------ACACTTTGG-- 21545
Macaque          TTTTACTTTGT---GTGCCTCACATGACCTTTTCTTTGTCAGC--------------------------ACACTTTGG-- 21946
PigTailedMacaque TTTTACTTTGT---GTGCCTCACATGACCTTTTCTTTGTCAGC--------------------------ACACTTTGG-- 20936
Bonobo           TTTTACTTTGT---GTGCCTCACATGACCTTTTCTTTGTGAGC--------------------------ACACTTTGG-- 20387
OliveBaboon      TTTTACTTTGT---GTGCCTCACATGACCTTTTCTTTGTCAGC--------------------------ACACTTTGG-- 20955
Orangutan        TTTTACTTTGT---GTGCCTCACATGACCTTTTCTTTGTGAGC--------------------------ACACTTCGG-- 20748

Majority         -------GGGAGTGGGTGGCACAAGCTCTCTGGTGTTTCTTCTCATAAAGGCATTAATCCCATCATGAAGGCCCTACTCT

                         26570     26580     26590     26600     26610     26620     26630     26640

Human            -------GGGAGTGGGTGGCAGAAGCTCTCTGGTATTTCTTCTCATAAAGGCATTAATCCCATCATGAAGGCCTTACTCT 19383
Coquerel'sSifaka GCGCGCTGAGGGTGAGGGGAACCAGCTCTCTGATGTTTCCTCTTACAA--GTGTTAATCCCGTCATGACTGCCCTACTCT 18000
Ma'sNightMonkey  -------GGGAGTGGGTGGCACAAGCTCTATGGAGTTTCTTCTCATAAAGGCATTAACCCCATCATGAAGACCCTACTTT 17139
VervetAGM        -------GGGAGTGGGTGGCACAAGCTCTCTTGTGTTTCTTCTCATAAAGGCATTAATT--AATATGAAGGCCCTACTCT 22721
Gorrilla         -------GGGAGTGGGTGGCAGAAGCTCTCTGGTGTTTCTTCTCATAAAGGCATTAATCCCATCATGAAGGCCCTACTCT 21748
GrabEatingMacaque-------GGGAGTGGGTGGCACAAGCTCTCTTGTGTTTCTTCTCATAAAGGCATTAATT--AATATGAAGGCCCTGCTCT 21616
Macaque          -------GGGAGTGGGTGGCACAAGCTCTCTTGTGTTTCTTCTCATAAAGGCATTAATT--AATATGAAGGCCCTGCTCT 22017
PigTailedMacaque -------GGGAGTGGGTGGCACAAGCTCTCTTGTGTTTCTTCTCATAAAGGCATTAATT--AATATGAAGGCCCTGCTCT 21007
Bonobo           -------GGGAGTGGGTGGCAGAAGCTCTCTGGTGTTTCTTCTCATAAAGGCATTAATCCCATCATGAAGGCCTTACTCT 20460
OliveBaboon      -------GGGAGTGGGTGGCACAAGCTCTCTTGTGTTTATTCTCATAAAGGCATTAATT--AATATGAAGGCCCTGCTCT 21026
Orangutan        -------GGGAGTGGATGGCAGAAGCTCTCTGGTGTTTCTTCTCATAAAGGCATTAATCCCATCATGAAGGCCCTACTCT 20821
```

```
Majority         CATGATCTCATGAAATCCTCCA-GTTAACTCCTAAAGGC-CCTATTTCTAAATTGCATCACATTT-GGGGTTAGGGCTTC
                         26650    26660    26670    26680    26690    26700    26710    26720
Human            CATGATCTCATGAAATCCTCCA-GTTAACTCCTAAAGGC-CCTGTTTCAAAATTGCATCACATTT-GGGGTTAGGACTTC 19460
Coquerel'sSifaka CATGACCTCATGAAACCCTA---ATTACCCTCCAAAGGCTCCCATCTCTAAATACTGTCACATTG-GGTGTTAGGGCTTC 18076
Ma'sNightMonkey  C-TGATCTCATGAAATCCTCCCCGTTAACTCCCAAAGGC-CCTATTTCTAAACTGCATCACATTTTGGGGTTAGGGTTTC 17217
VervetAGM        CATGATCTCATAAAATCCTCCA-GTTAACTCCTAAAGGC-CCTATTTCTAAATTGCATCACGTTT-GGGGTTAGGGCTTC 22798
Gorrilla         CATGATCTCATGAAATCCTCCA-GTTAACTCCTAAAGGC-CCTGTTTCAAAATTGCATCACATTT-GGGGTTAGGACTTC 21825
GrabEatingMacaqueCGTGATCTCATGAAATCCTCCA-GTTAACTCCTAAAGGC-CCTATTTCTAAATTGCATCACATTT-GGGGTTAGGGCTTC 21693
Macaque          CGTGATCTCATGAAATCCTCCA-GTTAACTCCTAAAGGC-CCTATTTCTAAATTGCATCACATTT-GGGGTTAGGGCTTC 22094
PigTailedMacaque CGTGATCTCATGAAATCCTCCA-GTTAACTCCTAAAGGC-CCTATTTCTAAATTGCATCACATTT-GGGGTTAGGGCTTC 21084
Bonobo           CATGATCTCATGAAATCCTCCA-GTTAACTCCTAAAGGC-CCTGTTTCAAAATTGCATCACATTT-GGGGTTAGGACTTC 20537
OliveBaboon      CGTGATCTCATGAAATCCTCCA-GTTAACTCCTAAAGGC-CCTATTTCTAAATTGCATCACATTT-GGGGTTAGCGCTTC 21103
Orangutan        CATGATCTTATGAAATCCTCCA-GTTAACTCTTAAAGGC-CCTATTTCTAAATTGCATTACATTT-GGGGTTAGGATTTC 20898

Majority         AGCATGTGATTTTCGGGGAAACATAAGCATTCAGTTCATAATACCCTGCATATAGTTGACCCATACTTCTCCACTCCAGG
                         26730    26740    26750    26760    26770    26780    26790    26800
Human            AGCATGTGATTTTTGGGGAAACATAAGCATTGAGTTGATAATACCCTGCATATAGTTGACCCATACTTCTCCACTCCAGG 19540
Coquerel'sSifaka A------------------ACA----CATTCAGTTCATGACACCTTGCATATACTTTACCCCTCCTT--TCACCCCAGG 18131
Ma'sNightMonkey  AGCATGTGGCTTTCGGGGAAACATAAGCATTCAGTTCATAATACTCTGCATGTAGTTGACCTGTATTT--CCACTCCAGG 17295
VervetAGM        AGCATGTGATTTTCGGGGAAACATAAGCATTCAGTTCATAGTACCCTGCATATAGTTGACCCATACTTCTCCCCTCCAGG 22878
Gorrilla         AGCATGTGATTTTTGGGGAAACATAAGCATTCAGTTGATAATACCCTGCATATAGTTGACCCATACTTCTCCACTCCAGG 21905
GrabEatingMacaqueAGCATGTGATTTTCGGGGAAACATAAGCATTCAGTTCATAATACCCTGCATATAGTTGACCCATACTTCTCTCCTCCAGG 21773
Macaque          AGCATGTGATTTTCGGGGAAACATAAGCATTCAGTTCATAATACCCTGCATATAGTTGACCCATACTTCTCTCCTCCAGG 22174
PigTailedMacaque AGCATGTGATTTTCGGGGAAACATAAGCATTCAGTTCATAATACCCTGCATATAGTTGACCCATACTTCTCTCCTCCAGG 21164
Bonobo           AGCATGTGATTTTTGGGGAAACATAAGCATTCAGTTGATAATACCCTGCATATAGTTGACCCATACTTCTCCACTCCAGG 20617
OliveBaboon      AGCATGTGATTTTCGGGGAAACATAAGCATTCAGTTCATAATACCCTGCATATAGTTGACCCATACTTCTCCCCTCCAGG 21183
Orangutan        AGCATGTGATTTTTGGGAAAACATAAGTATTCAGTTCATAATACCCTGCATATAGTTGACCCATACTTCTCCACTCCAGG 20978

Majority         TAATTACTATACTAAATTTGATGTTTACCATTCCCATCAATTTCTCATTTACTACAT-----------------------
                         26810    26820    26830    26840    26850    26860    26870    26880
Human            TAATTACTATACTAAATTTGATGTTTACCATTCCCATAAATTTCTCATTTACTACAT----------------------- 19597
Coquerel'sSifaka TAACAACCATCCGGAATTTGGTGTTTACCTTTCCTGTGAATTTTTCTTTTACTACAT----------------------- 18188
Ma'sNightMonkey  TAATTACTATCCTTAATTTGATGTTTACCATTCCTATAAATTTCTCTTTTACTACAT----------------------- 17352
VervetAGM        TGATTACTATACTAAATTTGATGTTTACCATTCGCATCATTTTCTCATTTACTACGT----------------------- 22935
Gorrilla         TAATTACTATACCAAATTTGATGTTTACCATTCCCATAAATTTCTCATTTACTACAT----------------------- 21962
GrabEatingMacaqueTGATTACTATACTAAATTTGATGTTTACCATTCGCATCATTTTCTCATTTACTACGT----------------------- 21830
Macaque          TGATTACTATACTAAATTTGATGTTTACCATTCGCATCATTTTCTCATTTACTACGT----------------------- 22231
PigTailedMacaque TGATTACTATACTAAATTTGATGTTTACCATTCGCATCATTTTCTCATTTACTACGT----------------------- 21221
Bonobo           TAATTACAATACTAAATTTGATGTTTACCATTCCCATAAATTTCTCATTTACTACAT----------------------- 20674
OliveBaboon      TGATTACTATACTAAATTTGATGTTTACCATTCGCATCATTTTCTCATTTACTACGT----------------------- 21240
Orangutan        TAATTACTATACTAAATTTGATGTTTACCATTCCCATAAATTTCTCATTTACTACATTTTTTTTTTTTTTTTTTTTTGAG 21058
```

```
Majority         --------------------------------------------------------------------------------
                         26890     26900     26910     26920     26930     26940     26950     26960
Human            -------------------------------------------------------------------------------- 19597
Coquerel'sSifaka -------------------------------------------------------------------------------- 18188
Ma'sNightMonkey  -------------------------------------------------------------------------------- 17352
VervetAGM        -------------------------------------------------------------------------------- 22935
Gorrilla         -------------------------------------------------------------------------------- 21962
GrabEatingMacaque-------------------------------------------------------------------------------- 21830
Macaque          -------------------------------------------------------------------------------- 22231
PigTailedMacaque -------------------------------------------------------------------------------- 21221
Bonobo           -------------------------------------------------------------------------------- 20674
OliveBaboon      -------------------------------------------------------------------------------- 21240
Orangutan        ACAGAGTCTCGGTCTGTTGCCCAGGCTGGAGTGCAGTGGTGCGATCTCAGCTCACTGCAAGCTCCGCCTCCCAGGTTCAC 21138

Majority         --------------------------------------------T-GTTTATGTATTTTTATCTTAAGCAATATATGGTT
                         26970     26980     26990     27000     27010     27020     27030     27040
Human            ----------------------------------------------GTTTATGTATTTTTATCTTAAGCAATATATGGTT 19631
Coquerel'sSifaka --------------------------------------------TTGTTTATGTACTTTCATCTCAAGCAGTATATGGCT 18224
Ma'sNightMonkey  ----------------------------------------------GTTTATGTATTTTTATCTTAAGCGATATATGGTT 17386
VervetAGM        --------------------------------------------T----TATGTATTTTTATCTTAAGTAATATATGGTT 22967
Gorrilla         ----------------------------------------------GTTTATGTATTTTTATCTTAAGCAATATATGGTT 21996
GrabEatingMacaque--------------------------------------------T----TATATATTTTTATCTTAAGTAATACATGGTT 21862
Macaque          --------------------------------------------T----TATATATTTTTATCTTAAGTAATACATGGTT 22263
PigTailedMacaque --------------------------------------------T----TATATATTTTTATCTTAAGTAATACATGGTT 21253
Bonobo           ----------------------------------------------GTTTATGTATTTTTATCTTAAGCAATATATGGTT 20708
OliveBaboon      --------------------------------------------T----TATATATTTTTATCTTAAGTAATACATGGTT 21272
Orangutan        GCCATTCTCCTGCCTCAGCCTCCCGAGTAGCTGGGACTTACTACATGTTTATGTATTTTTATCTTAAGCAATATATGGTT 21218

Majority         TTGTTTTGCATGTTTAAACTATA--GATGTACTATCATGCTGTATGTATTCTGT-AATTTGCTCTTTTTGTTCAGT-GTT
                         27050     27060     27070     27080     27090     27100     27110     27120
Human            TTGTTTTGCATGTTTAAACTATA--GATGTACTATCATGCTGTATGTATTCTGT-AATTTGCTTTTCTTGTTCAGT-GTT 19707
Coquerel'sSifaka TTGTTTTGCATGTTTGAACTATATAGGTGTACTGTCATGCCGAATGTATTCTGTTAACTTGCTCTTTTTGTTCATTTGCT 18304
Ma'sNightMonkey  TTGTTTTGCATGTTTAAACTATG--GAT-TACTGTGATGCCGTATGTATTCTGT-AACTTGCTCTTTTTGTTCAGT-GTT 17461
VervetAGM        TTGTTTTGCATGTTTAAACTATA--GATGTACTATCATGCTATATGTATCCTGT-AATTTGCTCTTTTTGTTCAGT-GTT 23043
Gorrilla         TTGTTTTGCATGTTTAAACTATA--GATGTACTATCATGCTGTATGTATTCTGT-AATTTGCTTTTCTTGTTCAGT-GTT 22072
GrabEatingMacaqueTTGTTTTGCATATTTAAACTATA--GATGTACTATCATGCTATATGTATTCTGT-AATTTGCTCTTTTTGTTCAGT-GTT 21938
Macaque          TTGTTTTGCATATTTAAACTATA--GATGTACTATCATGCTATATGTATTCTGT-AATTTGCTCTTTTTGTTCAGT-GTT 22339
PigTailedMacaque TTGTTTTGCATGTTTAAACTATA--GATGTACTATCATGCTATATGTATTCTGT-AATTTGCTCTTTTTGTTCAGT-GTT 21329
Bonobo           TTGTTTTGCATGTTTAAACTATA--GATGTACTATCATGCTGTATGTATTCTGT-AATTTGCTTTTCTTGTTCAGT-GTT 20784
OliveBaboon      TTGTTTTGCATGTTTAAACTATA--GATGTACTATCATGCTATATGTATTCTGT-AATTTGCTCTTTTTATTCAGT-GTT 21348
Orangutan        TGGTTTTGCATGTTTAAACTATA--GATATACTATCATGTTGTATGTATTCTGT-AATTTGCTCTTTTTGTTCAGT-GTT 21294
```

```
Majority         ATTTTTGTTTTTTAAGTTTATATGCATACCTC---TAATTTTCATTGCCATATAGTATATGAT-ATATGAATATATCATT
                          27130     27140     27150     27160     27170     27180     27190     27200
Human            ATTTTTGTT----AAGTTTATATATATA--TA---TA-TTTTCATTGCCATATAGTATATGAT-ATATGAATATATCATC 19776
Coquerel'sSifaka GTTTCTATCTTTTTAATTCATATGTAGCTCTTGATTAATTTTCATTGCCATATAATATGTGATTATATCATTACCCTACT 18384
Ma'sNightMonkey  ATTTTTGTTTTCTAAGTTTATATACATATCTC---TAATTTTCATTGCCATGTAGTACATGAT-ATATGAATATCTTGTT 17537
VervetAGM        ATTTTTGTTTTTTAAGTTTATATGCATACCTC---TAATTTTCATTGCCGTATAGTATATGTT-ATATGAATATATCATT 23119
Gorrilla         ATTTTTGTTTTTTAAGTTTGTTTATATA--TA---G--TTTTCATTGCCATATAGTATATGAT-ATATGAATATATCATC 22144
GrabEatingMacaqueATTTTTGTTTTTTAAGTTTATATGCATACCTC---TAATTTTCATTGCCATATAGTATATGTT-ATATGAATATATCATT 22014
Macaque          ATTTTTGTTTTTTAAGTTTATATGCATACCTC---TAATTTTCATTGCCATATAGTATATGTT-ATATGAATATATCATT 22415
PigTailedMacaque ATTTTTGTTTTTTAAGTTTATATGCATACCTC---TAATTTTCATTGCCATATAGTATATGTT-ATATGAATATATCATT 21405
Bonobo           ATTTTTGTTTTTTAAGTTTATATATATA--TA---TAATTTTCATTGCCATATATTATATGAT-ATATGAATATATCATC 20858
OliveBaboon      ATTTTTGTTTTTTAAGTTTATATGCATACCTC---TAATTTTCATTGCTGTATAGTATATGTT-ATATGAATATATCATT 21424
Orangutan        ATTTTTGTTTTTTAAGTTTATATATATA--TA---TAATTTTCATTGCCATATAGTATGTGAT-ATATGAATATATCATC 21368

Majority         A---------TCTTGTTGACAAAT-GAGTT----AATATTTGACGAGATTGATTTGTCAAATATTTAAATTTTTTTCCA
                          27210     27220     27230     27240     27250     27260     27270     27280
Human            ---------------TTGACAAAT-GAGTT----AATATTTGACGAGATTGATTTGTCAAATATTTAAATTTTTTTCCA 19836
Coquerel'sSifaka GACAAATATACCCTACTGACAAATTGAGGTTGTCGATATTTGACAAAGTTGATTTGCCAAATATTTCTAGTTTTTTTCTA 18464
Ma'sNightMonkey  -----------------GACACAT-GAGGC----AATATTTGACAAGATTGACTTGTCAGATATTTAAATTTTTTTCAA 17595
VervetAGM        A---------TCTTGTTGACAAAT-GAGTT----AATATTTGTCGAGATTGATTTGTCAAATATTTAAATTTTTTTCCA 23185
Gorrilla         ---------------TTGACAAAT-GAGTT----AATATTTGACGAGATTGATTTGTCAAATATTTAAATTTTTTTCCA 22204
GrabEatingMacaqueA---------TCTTGTTGACAAAT-GAGTT----AATATTTGTTGAGATTGATTTGTCAAATATTTAAACTTTTTTTCCA 22080
Macaque          A---------TCTTGTTGACAAAT-GAGTT----AATATTTGTCGAGATTGATTTGTCAAATATTTAAATTTTTTTCCA 22481
PigTailedMacaque A---------TCTTGTTGACAAAT-GAGTT----AATATTTGTCGAGATTGATTTGTCAAATATTTAAATTTTTTTCCA 21471
Bonobo           ---------------TTGACAAAT-GAGTT----AATATTTGACGAGATTGATTTGTCAAATATTTAAATTTTTTTCCA 20918
OliveBaboon      A---------TCTTGTTGACAAAT-GAGTT----AATATTTGTCGAGATTGATTTGTCAAATATTTAAATTTTTTTCCA 21490
Orangutan        ---------------TTGACAAAT-GAGT-----AATATTTGACGAGGTTGATTTGTCAAATATTTAAATTTTTTTCCA 21427

Majority         TTACAAACTGCTGCTGTAAGCATTCATATGCATATTCTAACGTGT--TAATATTAGAGTTTC------------------
                          27290     27300     27310     27320     27330     27340     27350     27360
Human            TTACAAACTGCTACTGTAAGCATTAATATGCATATTCTAACGTGT--TAATATTAGAGTTTC------------------ 19896
Coquerel'sSifaka TCACAAACTACTGCTATAAACATTCTTATGCATATTCCGACATGTATTAATATTAGAGTTTCACTAGAAGTGGAACTGCT 18544
Ma'sNightMonkey  TTACAAACTGCTGCTGTAGGCATTCATATGGATGTTCTAACATGT--TAATATTAGAGTTTC------------------ 17655
VervetAGM        TTACAAACTGCTGCTGTAAGCATTCATATGCATATTCTAAAGTGT--T------AGAGTTTC------------------ 23239
Gorrilla         TTACAAACTGCTACTGTAAGCATTAATATGCATATTCTAACATGT--TAATATTAGAGTTTC------------------ 22264
GrabEatingMacaqueTTACAAACTGCTGCTGTAAGCATTTATATGCATATTCTAACGTGT--TAATATTAGAGTTTC------------------ 22140
Macaque          TTACAAACTGCTGCTGTAAGCATTTATATGCATATTCTAACGTGT--TAATATTAGAGTTTC------------------ 22541
PigTailedMacaque TTACAAACTGCTGCTGTAAGCATTTATATGCATATTCTAACGTGT--TAATATTAGAGTTTC------------------ 21531
Bonobo           TCACAAACTGCTACTGTAAGCATTAATATGCATATTCTAACGTGT--TAATATTAGAGTTTC------------------ 20978
OliveBaboon      TTACAAACTGCTGCTGTAAGCATTCATATGCATATTCTAACGTGT--TAATATTAGAGTTTC------------------ 21550
Orangutan        TTACAAACTGCTGCTGTAAGCATCAATGTGCATATTCTAACGTGT--TAATATTAGAGTTTC------------------ 21487
```

```
Majority          ---TCATAGATTGTGTCT--TTTCAAGTCGATTGAATATTAGCATATTGCTTTCATAGGTGATTATACTAATTA---TAC
                          27370     27380     27390     27400     27410     27420     27430     27440
Human             ---TCATAGATTGTGTCT--TTTCAAATCGATTGAATATTAACATATTGCTTTCATAGGTAATTATATTAATTA---TAC 19968
Coquerel'sSifaka GGGTTAAAGGGTGTGTCTGTTTTCAAGTCAGTCAAATACTGGCATGTTGCTTTTCTAGGTGGTTGTGCTAATTTACTCTC 18624
Ma'sNightMonkey   ---TCATAGATTGTGTATGTTTTTAAATCAATTGAATATTAACATAGTGCTTTCATAGGCGATTATACCAATTA---TAC 17729
VervetAGM         ---TCATAGATTGTGTCTGTTTTCAAGTCGATTGAATATTAGCATATTGCTTTCATAGGTGATTATACTAATTA---TAC 23313
Gorrilla          ---TCATAGATTGTGTCT--TTTCAAATCGATTGAATATTAACATATTGCTTTCATAGGTAATTATACTAATTA---TAC 22336
GrabEatingMacaque---TCATAGATTGTGTCT--TTTCAAGTCGATTGAATATTAGCATATTGCTTTCATAGGTGATTATACTAATTA---CAC 22212
Macaque           ---TCATAGATTGTGTCT--TTTCAAGTCGATTGAATATTAGCATATTGCTTTCATAGGTGATTATACTAATTA---CAC 22613
PigTailedMacaque ---TCATAGATTGTGTCTGTTTTCAAGTCGATTGAATATTAGCATATTGCTTTCATAGGTGATTATACTAATTA---CAC 21605
Bonobo            ---TCATAGATTGTGTCT--TTTCAAATCGATTGAATATTAACATGTTGCTTTCATAGGTAATTATATTAATTA---TAC 21050
OliveBaboon       ---TCATAGATTGTGTCTGTTTTCAAGTCGATTGAATATTAGCATATTGCTTTCATAGGTGATTATACTAATTA---TAC 21624
Orangutan         ---TCA--GATTGTGTCT--TTTCAAATCGATTGAATGTTAACATATTGCTTTTATAAGTGATTATACTAATTA---TAC 21557

Majority          TGTTAGTGCTGGATGTAGA---TTCTTGTTTCT------ATACATTCTTTGAAAATCGGCATTGTTGGACTTTTTAATT-
                          27450     27460     27470     27480     27490     27500     27510     27520
Human             TGTTAGCGCTGCATGTAGA---TTCTTGTTTCT------ATACATTCTTTGAAAATCGGCATTGTTGGACTTTTTAATT- 20038
Coquerel'sSifaka TGTTAGTGGTAGATGTGAA---TTCCTATTTCTTACTACATATCTTTTCTGAAAATTGGCATTGTTGGACTTTTTAACTG 18701
Ma'sNightMonkey   TGTTAGTGCTGGATGTACAACATTCTTGTTTCT------ATACATTCTTTGAAAATTGGGATTGTTGGACTTTTTAATTG 17803
VervetAGM         TGTTAGTGCCGGATGTAGA---TTCTTGTTTCT------ATACATTCTTTGAAAATCGACATTGTTGGAC---TTAATT- 23380
Gorrilla          TGTTAGCGCTGCATGTAGA---TTTTTGTTTCT------ATACATTCTTTGAAAATTGGCATTGTTGGACTTTTTAATT- 22406
GrabEatingMacaqueTGTTAGTGCTGGATGTAGA---TTCTTGTTTCT------GTACATTCTTTGAAAATCGGCATTGTTGGACTTTTTAATT- 22282
Macaque           TGTTAGTGCTGGATGTAGA---TTCTTGTTTCT------GTACATTCTTTGAAAATCGGCATTGTTGGACTTTTTAATT- 22683
PigTailedMacaque TGTTAGTGCTGGATGTAGA---TTCTTGTTTCT------GTACATTCTTTGAAAATCGGCATTGTTGGACTTTTTAATT- 21675
Bonobo            TGTTAGCGCTGCATGTAGA---TTCTTGTTTCT------ATACATTCTTTGAAAATCGGCATTGTTGGACTTTTTAATT- 21120
OliveBaboon       TGTTAGTGCTGGATGTAGA---TTCTTGTTTCT------GTACATTCTTTGAAAATCGGCATTGTTGGACTTTTTAATT- 21694
Orangutan         TGTTAGTGCTGCATGTAGA---TTCTTGTTTCT------ACACGTTCTTTGAAAATCGGCATTGTTGGACTTTTTAATT- 21627

Majority          TTGCCGATCAAATGTACATGGGAAGGATATCTTGTGGTTTTAATTTGCATTCCCTATTAATGCTTTATTTTAATGATACA
                          27530     27540     27550     27560     27570     27580     27590     27600
Human             TTGCTGATCAAATGTACATGGGAAGGATATCTTGTGGTTTTAATTTGCATTCCCTATTAATGCA-------AAAAAAAAA 20111
Coquerel'sSifaka TTGCCAGTCTAATGTGCATGGGAATGATATCTTGTGAGCTTAATTTGAATTCTCAATTGATG------------------ 18763
Ma'sNightMonkey   TTGCCAATCAAATGTATATGGGAAGGATATCTTGTGGTTTT----TGTATTCCTTATTAATGCTTTCTTCTTATTATT-- 17877
VervetAGM         TTGCAGATCAAATGTACATGGGAAGGGTATCTTGTGGTTTTAATTTGCATTCCCTATTAATGCTTTATTTTAATGATACA 23460
Gorrilla          TTGCCGATCAAATGTACATGGGAAGGATATCTTGTGGTTTTAATTTGCATTCCCTATTAATGCA-------AAAAAAA-- 22477
GrabEatingMacaqueTTGCCGATCAAATGTACATGGGAAGGATATCTTGTGGTTTTAATTTGCATTCCCTATTAATGCTTTATTTTAATGATACA 22362
Macaque           TTGCCGATCAAATGTACATGGGAAGGATATCTTGTGGTTTTAATTTGCATTCCCTATTAATGCTTTATTTTAATGATACA 22763
PigTailedMacaque TTGCCGATCAAATGTACATGGGAAGGATATCTTGTGGTTTTAATTTGCATTCCCTATTAATGCTTTATTTTAATGATACA 21755
Bonobo            TTGCTGATCAAATGTACATGGGAAGGATATCTTGTGGTTTTAATTTGCATTCCCTATTAATGCA-------AAAAAAAAA 21193
OliveBaboon       TTGCCGATCAAATGTACATGGGAAGGATATCTTGTGGTTTTAATTTGCATTCCCTATTAATGCTTTATTTTAATGATACA 21774
Orangutan         TTGCCAATCAAATGTACATGGGAAGGATATCTTGTGGTTTTAATTCGCATTCCCTATTAATGCTTTATTTTAATGATATA 21707
```

```
Majority          TTTTATTAGTTTATTGGCCATTGATATTTCTTCTCTGTGAAATGC---TGTTTATAGTTTGTTCATTTTCTTTTTGACTG
                          27610     27620     27630     27640     27650     27660     27670     27680
Human             ---------------AAACATTGAT---TCCTGGGAGTT---------TTTATATACTGTGCAGACT--------AATTG 20156
Coquerel'sSifaka  -----------------------------------------A----------TACATTTTAATGATG------------- 18779
Ma'sNightMonkey   ----------TTTTGAGACGAAGTTTTGCTCTTGTTGCCCAGACT---GGAGTGCAGTGGCGTGATCTCAGTTC--ACTG 17942
VervetAGM         TTTTATTAGTTTATTGGCCATTGATATTTCTTCTCTGTGAAATGCC--TGTTTATAGTTTGTTCATTTTCTTTTTGACTG 23538
Gorrilla          ------------------CATTGAT---TCCTGAGAGTT---------TTTATATACTGTGCAGACT--------AATTG 22519
GrabEatingMacaqueTTTTATTAGTTTATTGGCCATTGATATTTCTTCTCTGTGAAATGCG--TGTTTATAGTTTGTTCATTTTCTTTTTGACTG 22440
Macaque           TTTTATTAGTTTATTGGCCATTGATATTTCTTCTCTGTGAAATGCG--TGTTTATAGTTTGTTCATTTTCTTTTTGACTG 22841
PigTailedMacaque  TTTTATTAGTTTATTGGCCATTGATATTTCTTCTCTGTGAAATGCC--TGTTTATAGTTTGTTCATTTTCTTTTTGACTG 21833
Bonobo            ---------------A--CATTGAT---TCCTGGGAGTT---------TTTATATACTGTGCAGACT--------AATTG 21236
OliveBaboon       TTTTATTAGTTTATTGGCCATTGATATTTCTTCTCTGTGATATGCC--TGTTTATAGTTTGTTCATTTTCTTTTTGACTG 21852
Orangutan         TTTTATTAGTTTATTGGCCATTAATGTTTCCTCTCTGTTAAATGCCTGTTTATATAGTTTGTTCATTTTCTTTTTGACTG 21787

Majority          TAGTTTTATCTTTTTTTAAAATTGATTCCTGCTCGGCATGGTGGCTCACGCCTATAATCCCAACACTTTGGGAG-CCGAG
                          27690     27700     27710     27720     27730     27740     27750     27760
Human             TTTTGTTATTTTTTTT-------------------------GGTTTGT------------------------------- 20179
Coquerel'sSifaka  -------------------------------------------------------------------------------- 18779
Ma'sNightMonkey   CAACTTCTCCCTCCC-A-------------------------GGTTCAAG---GTATTCTCCA-GCTT------------ 17980
VervetAGM         TAGTTTTATCTTTTTTTAAAATTGATTCCTGCTCGGCATGGTGGCTCACGCCTATAATCCCAACACTTGGGGAGGCCGAG 23618
Gorrilla          TTTTGTTAGTTTTTTT-------------------------GGTTTGT------------------------------- 22542
GrabEatingMacaqueTAGTTTTATCTTTTTTAAAAATTGATTCCTGCTCGGCATGGTGGCTCACGCCTATAATCCCAACACTTTGGGAGACCGAG 22520
Macaque           TAGTTTTATCTTTTTTAAAAATTGATTCCTGCTCGGCATGGTGGCTCACGCCTATAATCCCAACACTTTGGGAGACCGAG 22921
PigTailedMacaque  TAGTTTTATCTGTTTTTAAAATTGATTCCTGCTCGGCATGGTGGCTCACGCCTATAATCCCAACACTTTGGGAGACCGAG 21913
Bonobo            TTTTGTTATTTTTTT--------------------------GGTTGGT------------------------------- 21258
OliveBaboon       TAGTTTTATCTTTTTTTAAAATTGATTCCTGCTCGGCATGGTGGCTCACGCCTATAATCCCAACACTTTGGGAGGCCGAG 21932
Orangutan         TAGTTTTATCTTTTTTTAAAATTGATTCCTGCTCAGCGCGGTGGCTTGCGCTTATAATCTCAACACTTTGGGAGGCCGAG 21867

Majority          GC-GGTGGATCATTTGAGGTCAGGAGTTCTAGACCAGCCTAGCCAACATGGCAAAACCCCGTCTCTACTAAAAATGCA-A
                          27770     27780     27790     27800     27810     27820     27830     27840
Human             -------------------------------------------------------------------------------- 20179
Coquerel'sSifaka  -------------------------------------------------------------------------------- 18779
Ma'sNightMonkey   -----------------------------------------------------C---AGCCT------------- 17986
VervetAGM         GCAGGTGGATCATTTGAGGTCAGGAGTTCTAGACCAGCCTAGCCAACATGGCAAAACCCCGTCTCTACTAAAAGTGCAAA 23698
Gorrilla          -------------------------------------------------------------------------------- 22542
GrabEatingMacaqueGCGGGTGGATCATTTGAGGTCAGGAGTTCTAGACCAGCCTAGCCAACATGGCAAAACCCCGTCTCTACTAAAAATGCAGA 22600
Macaque           GCGGGTGGATCATTTGAGGTCAGGAGTTCTAGACCAGCCTAGCCAACATGGCAAAACCCCGTCTCTACTAAAAATGCAGA 23001
PigTailedMacaque  GCGGGTGGATCATTTGAGGTCAGGAGTTCTAGACCAGCCTAGCCAACATGGCAAAACCCCGTCTCTACTAAAAATGCAGA 21993
Bonobo            -------------------------------------------------------------------------------- 21258
OliveBaboon       GCAGGTGGATCATTTGAGGTCAGGAGTTCTAGACCAGCCTAGCCAACATGGCAAAACCCCGTCTCTACTAAAAATGCAAA 22012
Orangutan         GCAGGTGGATCACTTGAGATCAGAAGTTTGAGACCAGGCTAGCCAACATGGCAAAACCCCGTCTCTACTAAAAATACAAA 21947
```

```
Majority         A-ATTAGCTGGGTGTCGTGGTGGGTGTCTGT--T------TACTTGGGAGGCTGAGGCAGGAGAATTGCTTGAACCCAGG
                         27850     27860     27870     27880     27890     27900     27910     27920
Human            -------------------------------------------------------------------------------- 20179
Coquerel'sSifaka -------------------------------------------------------------------------------- 18779
Ma'sNightMonkey  --------------------------------------------------------------------------CCCAAG 17992
VervetAGM        AAATTAGCTGGGTGTCGTGGTGGGTGTCTGC--C------TACTTGGGAGGCTGAGGCAGGAGAATTGCTTGAACCCAGG 23770
Gorrilla         -------------------------------------------------------------------------------- 22542
GrabEatingMacaqueAGATTAGCTGGGTGTCGTGGTGGGTGTCTGTAATCCCAGTTACTTGGGAGGCTGAGGCAGGAGAATTGCTTGAACCCAGG 22680
Macaque          AGATTAGCTGGGTGTCGTGGTGGGTGTCTGTAATCCCAGTTACTTGGGAGGCTGAGGCAGGAGAATTGCTTGAACCCAGG 23081
PigTailedMacaque AGATTAGCTGGGTGTCGTGGTGGGTGTCTGTAATCCCAGTTACTTGGGAGGCTGAGGCAGGAGAATTGCTTGAACCCAGG 22073
Bonobo           -------------------------------------------------------------------------------- 21258
OliveBaboon      AAATTAGCTGGGTGTCGTGGTAGGCGTCTGTAATCCCAATTACTTGGGAGGCTGAGGCAGGAGAATTGCTTGAACCCAGG 22092
Orangutan        AAATTAGTTGGGCTTGGTGGTGGGTGTCTGTAATCTCAACTACTTGGGAGGCTGAGGCAGGAGAATTGCTTGAACCCAGG 22027

Majority         AGGTGGAGGTTGCAGTGAGC-GCAATCCATTGCA--------T----GACAGAGCAAGACTCTGT---CCCGCCAAAAAA
                         27930     27940     27950     27960     27970     27980     27990     28000
Human            -------------------------------------------------------------------------------- 20179
Coquerel'sSifaka -------------------------------------------------------------------------------- 18779
Ma'sNightMonkey  TAGC----------TGGG----AAT---------------------ACAG---------------------------- 18007
VervetAGM        AGGTGGAGGTTGCAGTGAGCCGCAATCCATTGCACTCCAACCTGGGCGACAGAGCAAGACTCTGTGCCCCCGCCAAAAAA 23850
Gorrilla         -------------------------------------------------------------------------------- 22542
GrabEatingMacaqueAGGTGGAGGTTGCAGTGAGCCGCAATCCATTGCACTCCAACCTGGGCGACAGAGCAAGACTCTGTCCCCCCGCCAAAAAA 22760
Macaque          AGGTGGAGGTTGCAGTGAGCCGCAATCCATTGCACTCCAACCTGGGCGACAGAGCAAGACTCTGTCCCCCCGCCAAAAAA 23161
PigTailedMacaque AGGTGGAGGTTGCAGTGAGCCGCAATCCATTGCACTCCAACCTGGGCGACAGAGCAAGACTCTGTCCCCCCGCCAAAAAA 22153
Bonobo           -------------------------------------------------------------------------------- 21258
OliveBaboon      AGGTGGAGGTTGCAGTGAGCTGCAATCCATTGCACTCCAACCTGGGCGACAGAGCAAGACTCTGTCCCCCCGCCAAAAAA 22172
Orangutan        AGGCGGAGGTTGCAGTGAGCTGTGATCCATTGCA--------T----GACAGAGCGAGACTCTGT---CTCAAAAAAAAA 22092

Majority         AAAAA-----TGG-TTCCTAGGAGGTTTTATATACTCTG-ACACT-AATTGTTTTGTTAGT--TTGTTTTG------TTT
                         28010     28020     28030     28040     28050     28060     28070     28080
Human            ------------------------------------------------TTGTTTTGTT-----TTGTTT-----CATTTT 20201
Coquerel'sSifaka -------------------------------------------------------------------------------- 18779
Ma'sNightMonkey  -------------------------------GCA-TGC---------ATCACCATGCC------TGGCTAA------TTT 18034
VervetAGM        AAAAAA---TTGGATTCCTAGGATGTTTTATATACTCTGCACACTTAATTGTTTTGTTAGT--TTGTTTTG------TTT 23919
Gorrilla         ------------------------------------------------TTGTTTTGTT-----TTGTTTTGTTTCGTTTT 22569
GrabEatingMacaqueAAAAA----TTGGGTTCCTAGGAGGTTTTATATACTCTGAACACTTAATTGTTTTGTTAGT--TTTTTTTG------TTT 22828
Macaque          AAAAA----TTGGGTTCCTAGGAGGTTTTATATACTCTGAACACTTAATTGTTTTGTTAGT--TTTTTTTG------TTT 23229
PigTailedMacaque AAAAA----TTGGATTCCTAGGAGGTTTTATATACTCTGCACACTTAATTGTTTTGTTAGT--TTTTTTTG------TTT 22221
Bonobo           ------------------------------------------------TTGTTTTGTT-----TTGTTT-----CGTTTT 21280
OliveBaboon      AAAAAAAAATTGGATTCCTAGGAGGTTTTATATACTCTGCACACTTAATTGTTTTGTTAGT--TTGTTTTG------TTT 22244
Orangutan        AAAAA-----TTGATTCCTAGGAGTTTTTATATACTGTGCACACT-AATTGTTTTGTTAGTTTTTGTTTTGTTTTGTTTT 22166
```

```
Majority          TGTTTTTGTTT-GAGAGACGGGGTCTCACTATGTCACCCAGGCTGGAGTGCAGTGGCTCGGTCACAG-TCACTGCAGTCT
                          28090     28100     28110     28120     28130     28140     28150     28160
Human             TGTTTTTGTTTTGAGAGACAGAGTCTCACTGTGTCATCCAGGCTGGAGTGCAGTGGCTCGGTCACAG-TCACTGCAGTCT 20280
Coquerel'sSifaka  ------------------------------TTTT------------A--------------------------------T 18785
Ma'sNightMonkey   TGTATTT-TTAGTAGAGACAGGGTTTCTCCATGTTGGTCAGGCTGG------------------------------TCT 18082
VervetAGM         TGTTTTTGTTT-GAGGGAAGGGGTCTCACTACGTCACCCAGGCTGGAGTGCAGTGGCCGGATCTCAGCTCACTGCAAGCT 23998
Gorrilla          TGTTTTTGTTTTGAGAGACAGAGTCTCACTATGTCATCCAGGCTGGAGTGCAGTGGCTCGGTCACAG-TCACTGCAGTCT 22648
GrabEatingMacaque TGTTTTTGTTT-GAGAGAAGGGATCTCACTACGTCACCCAGGCTGGAGTGCAGTGGCACAGTCACAG-TCAGTGCAGTCT 22906
Macaque           TGTTTTTGTTT-GAGAGAAGGGGTCTCACTACGTCACCCAGGCTGGAGTGCAGTGGCACAGTCACAG-TCAGTGCAGTCT 23307
PigTailedMacaque  TGTTTTTGTTT-GAGAGAAGGGGTCTCACTACGTCACCCAGGCTGGAGTGCAGTGGCACAGTCACAG-TCAGTGCAGTCT 22299
Bonobo            TGTTTTTGTTTTGAGAGACAGAGTCTCACTATGTCATCCAGGCTGGAGTGCAGTGGCTCGGTCACAG-TCACTGCAGTCT 21359
OliveBaboon       TGTTTTTGTTT-GAGAGAAGGGGTCTTACTACGTCACCCAGGCTGGAGTGCAGTGGCACAGTCACAG-TCAGTGCAGTCT 22322
Orangutan         TGTTTTTGTTTTGAGAGACAGAGTCTCACAATGTCATCCAGGCTGGAGTGCAGTGGCTCGGTCACAG-TCACTGCAGTCC 22245

Majority          TGACATCCCAGGCTCTAGCAATTCTCCCACCTTTGTTTCCTGAGTAGCTGGGACTACAGGAATACACCACTATGCCCAGC
                          28170     28180     28190     28200     28210     28220     28230     28240
Human             TGACATCCCAGGCTCTAGCAATTCTCTCACCTTTGTTTCCTGAGTAGCTGGGACTACAGGAATACACCACTATGCCCAGC 20360
Coquerel'sSifaka  TAGTTTATTGGCCATTGATGTTTCTTCTCCATTAAATGCCTGTTTA--------TATAG-----------TTTGTCCA-- 18844
Ma'sNightMonkey   TGAACTCCTGACCTCAGGTGATCCACCTGCCTCAGCCTCCCAAAGTGTTGGGATTACAGGCATGAGCCACTGCGCCCAGG 18162
VervetAGM         CCGTCTCCCGGGTTTACGCCATTCTCCTACCTCAGCCTCCCGAGCAGCTGGGACTACAGGCGCCCGCCACCTCGCCCGGC 24078
Gorrilla          TGACATCCCAGGCTCTAGCAATTCTCTCACCTTTGTTTCCTGAGTAGCTGGGACTACAGGAATACACCACTATGCCCAGC 22728
GrabEatingMacaque TGACATCCCAGGCTCTAGCAATCCTCCCACCTTTGTTTCCTGAGTAGCTGGAACTACAGGAATACACCACTATGCCCAGC 22986
Macaque           TGACATCCCAGGCTCTAGCAATCCTCCCACCTTTGTTTCCTGAGTAGCTGGAACTACAGGAATATACCACTATGCCCAGC 23387
PigTailedMacaque  TGACATCCCAGGCTCTAGCAATCCTCCCGCCTTTGTTTCCTGAGTAGCTGGAACTACAGGAATACACCACTATGCCCAGC 22379
Bonobo            TGACATCCCAGGCTCTAGCAATTCTCTCACCTTTGTTTCCTGAGTAGCTGGGACTACAGGAATACATCACTATGCCCAGC 21439
OliveBaboon       TGACATCCCAGGCTCTAGCAATCCTCCCACCTTTGTTTCCTGAGTAGCTGGAACTACAGGAATACACCACTATGCCCAGC 22402
Orangutan         TGACATCCCAGGCTCTAGCAATTCTCCCACCTTTGTTTCCTGAGTAGCTGGGACTACAGAAATACACCACTATGCCCGGC 22325

Majority          TAATTTTTCA-ATTTTTTTGTAGAGATGGAGTCTCACTGTATTACCCAGGTTGGTTGCGAACTCCTAGGCTCAAGCAGTT
                          28250     28260     28270     28280     28290     28300     28310     28320
Human             TAATTTTTCA-ATTTTTTTGTAGAGATGGAGTCTCACTGTATTACCCAGGTTTGTTGCGAACTCCCAGGCTCAAGCAGTT 20439
Coquerel'sSifaka  -----TTTTG--TTTTTTTGT---G---TGGTTTTATCTTTTTAAAAAAATTGATTG-------CTAGG-------AGAT 18897
Ma'sNightMonkey   AAATTTTTCA--ATTTTTTGTAGAGATGAAGTCTCACTATATTACCCAGGTTGGTTGTGAATGCCTAGGCTCAAACAGTT 18240
VervetAGM         TAGTTTTTTGTATTTTTTAGTAGAGACAGGGTTTCACCGGGTTAGCCAGGATGGTCTTGATCTCCTGACCTC--GTGATC 24156
Gorrilla          TAATTTTTCA-ATTTTTTTGTAGAGATGGAGTCTCACTATATTACCCAGGTTTGTTGCGAACTCCCAGGCTCAAGCAGTT 22807
GrabEatingMacaque TAATTTTTCA-ATTTTTTTGTAGAGATGGAGTCTCACTGTATTACCCAGGTTGGTTGCGAACTCCTAGGCTCAAGCAGTT 23065
Macaque           TAATTTTTCA-ATTTTTTTGTAGAGATGGAGTCTCACTGTATTACCCAGGTTGGTTGCGAACTCCTAGGCTCAAGCAGTT 23466
PigTailedMacaque  TAATTTTTCA-ATTTTTTTGTAGAGATGGAGTCTCACTGTATTACCCAGGTTGGTTGCGAACTCCTAGGCTCAAGCAGTT 22458
Bonobo            TAATTTTTCA-ATTTTTTTGTAGAGATGGAGTCTCACTGTATTACCCAGGTTTGTTGCGAACTCCCAGGCTCAAGCAGTT 21518
OliveBaboon       TAATTTTTCA-ATTTTTTTGTAGAGGTGGAGTCTCACTGTATTACCCAGGTTGGTTGCGAACTCCTAGGCTCAAGCAGTT 22481
Orangutan         TCATTTTTCA-GTTTTTTTGTAGAGATGGAGTCTCACTATATTACCCAGGTTGGTTGCGAACTCCTGGGCTCAAGCAGTT 22404
```

```
Majority         CTCCCGCATTGGCCTCCTAAAATGCTGGGATTACAGGCGTGAGTCACTGTGCCTGGCCTGTTTTGTTAGTTATATGTCCT

                         28330     28340     28350     28360     28370     28380     28390     28400

Human            CTCCCGCATTGGCCTCCTAAAGTGCTGAGATTACAGGCGTGAGTCGCTGTGCCTGGCCTGTTTTGTTAGTTACATGTCCT 20519
Coquerel'sSifaka TT------------T--ATA-TACTA----TA---------GTTACTA------------ATT----GTTATGTGTCCT 18932
Ma'sNightMonkey  CTCTCAAACTGATCTCCTGAAATGCTGGG-TTACAGGAATGAGTCACTGTGCTTGGCCTGTTTT----GTTATATGTCCT 18315
VervetAGM        CACCCGCCTCAGCCTCCCAAAGTGCTGGGATTACAGACTGGAGCCACCGCGCCCGGCCTGTATTTATGTTTAAATA---- 24232
Gorrilla         CT---GCATTGGCCTCCTAAAGTGCTGAGATTACAGGCGTGAGTCACTGTGCCTGGCCTGTTTTGTTAGTTACATGTCCT 22884
GrabEatingMacaqueCTCCCTCATTGGCCTCCTAAAATGCTGGGATTACAGGCGTGAGTCACTGTGCCTGGCCTGTTTTGTTAGTTATATGTCCT 23145
Macaque          CTCCCTCATTGGCCTCCTAAAATGCTGGGATTACAGGCGTGAGTCACTGTGCCTGGCCTGTTTTGTTAGTTATATGTCCT 23546
PigTailedMacaque CTCCCTCATTGGCCTCCTAAAATGCTGGGATTACAGGCGTGAGTCACTGTGCCTGGCCTGTTTTGTTAGTTATATGTCCT 22538
Bonobo           CTCCCGCATTGGCCTCCTAAAGTGCTGAGATTACAGGCGTGAGTCGCTGTGCCTGGCCTGTTTTGTTAGTTACATGTCCT 21598
OliveBaboon      CTCCCTCATTGGCCTCCTAAAATGCTGGGATTACAGGCGTGAGTCACTGTGCCTGGCCTGTTTTGTTAGTTATATGTCCT 22561
Orangutan        CTCCCGCATTGGCCTCCTAAAATGCTGAGATTACAGGCGTGAGTCACTGTGCCTGGCCTGTTTTGTTAGTTACGTGTCCT 22484

Majority         ACAGATAACTCCTTCCTCTCTCTGACCTGTTTTTTTTACACTGATGAATAGAAGTTGTAAATTTTAATGTAACATTTATG

                         28410     28420     28430     28440     28450     28460     28470     28480

Human            ACAGATAACTCTCTCCTCTCTCTGACCTGTTTTTTTTACACTGATGAATAG---TTGTAAATTTTAATGGGACATTTATG 20596
Coquerel'sSifaka ACAGATAATTCCTTCCTCTCTG-----TGGTTTTTTTACATTGATGAATAGAAGTTGCAAATGTTAATGTAACATTTTTC 19007
Ma'sNightMonkey  ACAGATAACTCCTTCCTCTCTGACCTGTTTTCTTTTACACTGATGAATAGAAGTTGTAAATTCTAATGTAACATTTATG 18395
VervetAGM        ------AACTTGACTCTTTGTGAGTATTGTTATATGTGTTTTCATAGCTA----CTGT----TGTA--GTAGCGTTTCTT 24296
Gorrilla         ACAGATAACTCTCTCCTCTCTCTGACCTGTTTTTTTTACACTGATGAATAG---TTGTAAATTTTAATGGGACATTTATG 22961
GrabEatingMacaqueACAGATAACTCCTTCCTCTCTCTGACCTGTTTTTTTTACACTGATGAATAGAAGTTGTAAATTTTAATGTAACATTTATG 23225
Macaque          ACAGATAACTCCTTCCTCTCTCTGACCTGTTTTTTTTACACTGATGAATAGAAGTTGTAAATTTTAATGTAACATTTATG 23626
PigTailedMacaque ACAGATAACTCCTTCCTCTCTCTGACCTGTTTTTTTTACACTGATGAATAGAAGTTGTAAATTTTAATGTAACATTTATG 22618
Bonobo           ACAGATAACTCTCTCCTCTCGCTGACCTGTTTTTTTTACACTGATGAATAG---TTGTAAATTTTAATGGGACATTTATG 21675
OliveBaboon      ACAGATAACTCCTTCCTCTCTCTGACCTGTTTTTTTTACACTGATGAATAGAAGTTGTAAATTTTAATGTAACATTTATG 22641
Orangutan        ACAGATAACTCTTTCCTCTCTCTGACCTGTTTTTTTTACACTGATGAATAG---TTGTAAATTTTAATGTGACATTTATG 22561

Majority         TTTTTCTTT-AAAAAAATATTTAACCTAAGGCCTTACATATAGGCTTGTGTATTTTATTTTGAAAGTTTTAAAATTTTGA

                         28490     28500     28510     28520     28530     28540     28550     28560

Human            TTTTTCTTTTAAAAAAACATTTAACCTAAGGCCTTACGTATAGGCTTGTGTATTTTATTTTGAAAGTTTTAAAATTTTGA 20676
Coquerel'sSifaka TTTTAAAA---AATTTTTCTTTAACTCACGGTCTTACATGTAGGCTTGTGGATTTTATTTT--A--TTTAAAAATTTTGA 19080
Ma'sNightMonkey  TTTTTCTTTTTTAAAAATATTCAACCTAAGGCCTTACATACAGGCTTGTGGATTTTATTTTGAAAGTTT----------A 18465
VervetAGM        TTTTTACT---AACTTGCTTTCAAGCCACGCTGTTGATTAAATATTTGTGGGT--CATTTTAAGACTTCTAATCTTTATC 24371
Gorrilla         TTTTTCTTTTAAAAAAACATTTAACCTAAGGCCTTACATATAGGCTTGTGTATTTTATTTTGAAAGTTTTAAAATTTTGA 23041
GrabEatingMacaqueTTTTTCTTT-AAAAAAATATTTAACCTAAGTCCTTACATACAGGCTTGTGTATTTTATTTTGAAAGTTTTAAAATTTTGA 23304
Macaque          TTTTTCTTT-AAAAAAATATTTAACCTAAGTCCTTACATACAGGCTTGTGTATTTTATTTTGAAAGTTTTAAAATTTTGA 23705
PigTailedMacaque TTTTTCTTT-AAAAAAATATTTAACCTAAGTCCTTACATACAGGCTTGTGTATTTTATTTTGAAAGTTTTAAAATTTTGA 22697
Bonobo           TTTTTCTTTTAAAAAAACATTTAACCTAAGGCCTTACGTATAGGCTTGTGTATTTTATTTTGAAAGTTTTAAAATTTTGA 21755
OliveBaboon      TTTTTCTTT-AAAAAAATATTTAACCTAAGTCCTTACATACAGGCTTGTGTATTTTATTTTGAAAGTTTTAAAATTTTGA 22720
Orangutan        TTTTTCTTTTAAAAAAATATTTAACCTAAGGCCTTACATATAGGCTTGTGTATTTTATTTTGAAAGTTTTAAAATTTTGA 22641
```

```
Majority          TTT--TCACATTTAGGGCCTTAATCTACCTGGAATTTATTCT-TTGTGTAGCATATGGTACAGGTTTAATTATTTTTTCT
                          28570     28580     28590     28600     28610     28620     28630     28640
Human             TTT--TCACATTTATGGCGTTAATCTACCTGGAATTTATTCT-TTGTGTAGCATATGTTACAGGTTTAATTATTTTTTCT 20753
Coquerel'sSifaka  TTT--TCACATTTAGGCCCTCAGTCTACCTGGAATTTAATTTTTGTGTAGTATGTGGTATAGGTTTAATTATTTTTCCT 19158
Ma'sNightMonkey   TTT---CACATTTAGGGCCATAATCTACCTGGCATTTTTTTT-TTGTGTAGCATATGGTACAGGTTTAATTATTTTTTCT 18541
VervetAGM         TGTGATGGACTTTGAGGAAGAGTTAGGTTTTGTAAGTGTTGAACTCCAATAAACATAGTTCCACTTTCCTTAACTTCTCA 24451
Gorrilla          TTT--TCACATTTATGGCCTTAATCTACCTGGAATTTATTCT-TTGTGTAGCATATGTTACAGGTTTAATTATTTTTTCT 23118
GrabEatingMacaqueTTT--TCACATTTAGGGCCTTAATCTACCTGGAATGTATTCT-TTGTGTAACATATGGTACAGGTTTAATTATTTTTTCT 23381
Macaque           TTT--TCACATTTAGGGCCTTAATCTACCTGGAATGTATTCT-TTGTGTAACATATGGTACAGGTTTAATTATTTTTTCT 23782
PigTailedMacaque  TTT--TCACATTTAGGGCCTTAATCTACCTGGAATGTATTCT-TTGTGTAACATATGGTACAGGTTTAATTATTTTTTCT 22774
Bonobo            TTT--TCACATTTATGGCGTTAATCTACCTGGAATTTATTCT-TTGTGTAGCGTATGTTACAGGTTTAATTATTTTTTCT 21832
OliveBaboon       TTT--TCACATTTAGGGTCTTAATCTACCTGGAATGTATTCT-TTGTGTAGCATATGGTACAGGTTTAATTATTTTTTCT 22797
Orangutan         TTT--TCACATTTGTGGCCTTAATCTACCTGGAATTTATTCT-TTGTGTAGCATATGGTACAGGTTTAATTTTTTTTTCT 22718

Majority          ATATGGATAGCAAGTTTAGTACCTACTTGATTGAATAGTTCAGCTTTTCTCCAGTGATCTGCTTTCTCAGCTCTTC-AGG
                          28650     28660     28670     28680     28690     28700     28710     28720
Human             ATATGGATAGCAAGCTTAGTATGTACTTGATTGAATAGCTCAGCTTTTCTCCAATGATCTGCTTTCTCAGCTCTTC-AGG 20832
Coquerel'sSifaka  GTGTGGATGATAATTTTAGTACCCACTCAATTAACTAGTTCATCTTTTCTCCAATAAGCTGCATTCTCAGCTCTTCCAAG 19238
Ma'sNightMonkey   ATATGGATAGCAAGTTTAGTACTTACTTGATTA--T---TCAGCTTTTCTCCAATGATCTGCATTCTCAGCTCTTC-AGG 18615
VervetAGM         A-AAAGATTGCT-GTTTGATACAGA--TAGTTGTCTAGGCAAGCTATTAGCAGGGTTTCAGGTAAGACATAGTGTC-TGC 24526
Gorrilla          ATATGGATAGCAAGTTTAGTACGTACTTGATTGAATAGCTCAGCTTTTCTCCAGTGATCTGCTTTCTCAGCTCTTC-AGG 23197
GrabEatingMacaqueATAAGGATAGCAAGTTTAGTACCTACTTGATTGAATAGTTCAGCTTTTATCCAGTGATCTGCTTTATTAGCCCTTC-AGG 23460
Macaque           ATAAGGATAGCAAGTTTAGTACCTACTTGATTGAATAGTTCAGCTTTTATCCAGTGATCTGCTTTATTAGCCCTTC-AGG 23861
PigTailedMacaque  ATAGGGATAGCAAGTTTAGTACCTACTTGATTGAATAGTTCAGCTTTTATCCAGTGATCTGCTTTATTAGCCCTTC-AGG 22853
Bonobo            ATATGGATAGCAAGTTTAGTATGTACTTGATTGAATAGCTCAGCTTTTCTCCAATGATCTGCTTTCTCAGCTCTTC-AGG 21911
OliveBaboon       GTAGGGATAGCAAGTTTAGTACCTACTTGATTGAATAGTTCAGCTTTTATCCAGTGATCTGCTTTATTAGCCCTTC-AGG 22876
Orangutan         ATGTGGATAGCAAGTTTAGTACCTACTTGATTGAATAGCTCAGCTTTTCTCCAATGATCTGCTTTCTCAGCTCTTC-AGG 22797

Majority          TTTCCATATATTCATAGAACTGTTTCTGTGCTATGTGTT--GTAGCAGTA-----CAA---------CAGTGTCTTAAT-
                          28730     28740     28750     28760     28770     28780     28790     28800
Human             TTTCCGTATATTCACAGAACCATTTCTGTGCTATGTCTT--GTAGCAGTA-----CAA---------CAGTGTCTTAAT- 20895
Coquerel'sSifaka  TTTCCATATATTCATGGAACTGTTTCTGGTGTATGTATTCTGTTTTATTATTTGTCAATTTCTGTACCAATATCACAGTG 19318
Ma'sNightMonkey   TTTCCATAGATTCACAGAACTGTTTCTGTGCTGCGTGTT--GTAGCGCTA-----CAA---------CAGTGTCTTAAT- 18678
VervetAGM         TATT--TGTTTTAAGCAAACTCTTCCTTCACTTATTCAA--ATTGTAGCA-----AACAT------CTACTACATCCAT- 24590
Gorrilla          TTTCCGTATATTCACAGAACCATTTCTGTGCTATGTGTT--GTAGCAGTA-----CAA---------CAGTGTCTTAAT- 23260
GrabEatingMacaqueTTTCCATATATTCATAGAACTGTTTCTGTGCTATGTGTG--GTAGCAGTA-----CAA---------CAGTGTCTTAAT- 23523
Macaque           TTTCCATATATTCATAGAACTGTTTCTGTGCTATGTGTG--GTAGCAGTA-----CAA---------CAGTGTCTTAAT- 23924
PigTailedMacaque  TTTCCATATATTCATAGAACTGTTTCTGTGCTATGTGTG--GTAGCAGTA-----CAA---------CAGTGTCTTAAT- 22916
Bonobo            TTTCCGTATATTCACAGAACCATTTCTGTGCTATGTCTT--GTAGCAGTA-----CAA---------CAGTGTCTTAAT- 21974
OliveBaboon       TTTCCATATATTCATAGAACTGTTTCTGTGCTATGTGTG--GTAGCAGTA-----CAA---------CAGTGTCTTAAT- 22939
Orangutan         TTTCCATATATTCACAGAACCATTTCTGTGCTATGTGTT--GTAGCAGTA-----CAA---------CAGTGTCTTAAT- 22860
```

```
Majority        ---TACT-ATGGCATCAGCATTAAGTTTTGGAATCTTGTACAGTG-AGTGTCAAAACTTTGTTTTTCAAAATTGTCTTGG

                        28810     28820     28830     28840     28850     28860     28870     28880

Human           ---TACTTATGGCATCAGCATTAAGTTTTGGAATCTTGTACAGTG-AGTGTCAAAACTTTGTTTTTCAAAATTGTCTTGG 20971
Coquerel'sSifaka TCTTAATTATGGTGTTAGAAT-AAATCTTGGAATCTTATAGAGTG-AATTTCAAAACTTTGTTCTTTAAAACTG----GG 19392
Ma'sNightMonkey ---TACT-ATAGCATCAGCAT-AAATCTTGGAATCTTGTACAGTG-AGTGTCAAAACTTTGTTTTTCAAAATTGTCTTGG 18752
VervetAGM       ---TTCC-A-GTTCTTTGTCTTAATAGTTCAACTGTTGTAAAAAGGAGTGGTATATATGTAAACTCTTGGTAGGCTTTGG 24665
Gorrilla        ---TACTTATGGCATCAGCATTAAGTTTTGGGATCTTGTACAGTG-AGTGTCAAAACTTTGTTTTTCAAAATTGTCTTGG 23336
GrabEatingMacaque---TACT-ATGGCATCAGCATTAAGTTTTGGAATCTTGTACAGCG-AGTGTCAAAACTTTGGTTTTCAAAATTGTCTTGG 23598
Macaque         ---TACT-ATGGCATCAGCATTAAGTTTTGGAATCTTGTACAGCG-AGTGTCAAAACTTTGGTTTTCAAAATTGTCTTGG 23999
PigTailedMacaque ---TACT-ATGGCATCAGCATTAAGTTTTGGAATCTTGTACAGCG-AGTGTCAAAACTTTGGTTTTCAAAATTGTCTTGG 22991
Bonobo          ---TACTTATGGCATCAGCATTAAGTTTTGGAATCTTGTACAGTG-AGTGTCAAAACTTTGTTTTTCAAAATTGTCTTGG 22050
OliveBaboon     ---TACT-ATGGCATCAGCATTAAGTTTTGGAATCTTGTACAGCG-AGTGTCAAAACTTTGGTTTTCAAAATTGTCTTGG 23014
Orangutan       ---TACTTATGGCATCAGCATTAAGTTTTGGAATCTTGTACAGTG-AGTGTCAAAACTTTGTTTTTCAAAATTGTCTTGG 22936

Majority        TGTTTTCTTCACTGCATTTATTTTAAGATCATTTGGCTAAGTTCTGTGAAGACTTTTTGGGTCTTATTTATACTTGCCTT

                        28890     28900     28910     28920     28930     28940     28950     28960

Human           TGTTTTCTTCACTGCATTTATTTTAAGATCATTTGGCTAAGTTCTGTGAAGGCTCTTTGGGTCTTATTTATACTTGCCTT 21051
Coquerel'sSifaka CATTTTCTTTACTACATGTATTTTAAGATCAGTTGGCTGAGTTCTGTGAGGACTTTCTGGGTTTTGTTTATAATTGTATA 19472
Ma'sNightMonkey TGTTTTCTTCACTGTATGTATTTTAAGATCATTTGGATAAGTTTTGTGAAGACTTTTTGGGTCTTATTTATACTTGCCTT 18832
VervetAGM       T------TTGATTGAATTAATAAGGTGGTTTTTTGTGTTTGTTTTGAAACGGAGTCTTG---CTCTGTCAC-CCAGGCTG 24735
Gorrilla        TGTTTTCTTCACTGCATTTATTTTAAGATCATTTGGCTAAGTTCTGTGAAGGCTCTTTGGGTCTTATTTATACTTGCCTT 23416
GrabEatingMacaqueTGTTTTCTTCGCTGCATTTATTTTAAGATCATTTGGCTAAGTTCTATGAAGACTTTTTGGGTCTTATTTATACTTGCCTT 23678
Macaque         TGTTTTCTTCGCTGCATTTATTTTAAGATCATTTGGCTAAGTTCTATGAAGACTTTTTGGGTCTTATTTATACTTGCCTT 24079
PigTailedMacaque TGTTTTCTTCGCTGCATTTATTTTAAGATCATTTGGCTAAGTTCTGTGAAGACTTTTTGGGTCTTATTTATACTTGCCTT 23071
Bonobo          TGTTTTCTTCACTGCATTTATTTTAAGATCATTTGGCTAAGTTCTGTGAAGGCTCTTTGGGTCTTATTTATACTTGCCTT 22130
OliveBaboon     TGTTTTCTTCGCTGCATTTATTTTAAGATCATTTGGCTAAGTTCTGTGAAGACTTTTTGGGTCTTATTTATACTTGCCTT 23094
Orangutan       TGTTTTCTTCGCTGCGTTTATTTTAAGATCATTTGGCTAAGTTCTGTGAAGGCTCTTTGGGTCTTATTTATACTTGCCTT 23016

Majority        AAATTTAGTAGTT---------AATTGGGAGAACATTATAGTTGCATTATCAA---AATAAAATGGCAATTTTTT--TTT

                        28970     28980     28990     29000     29010     29020     29030     29040

Human           AAATTTAGTAGTT---------AATTGGGAGAACATTATAGCTGCATTATCAA---AATAAAATGGCAATTTTTT---TT 21116
Coquerel'sSifaka AAATTTAGTAGTTCAATTAAGTAATTGGGAGAGCACTGTATTTGCATTATCAATAAAATAAAATGGCAGTGTTTA----- 19547
Ma'sNightMonkey AA-TTTAGTAGTT---------AATTGGGAGAACATTATATTTGCATTATCAA---AATAAAATGGTGATATTGT--TTT 18897
VervetAGM       GAGTGCAGTGGTG---------CAATCTCGGCTCACTGCAACCTCCACCTCCC---AGATTCATGCCATTCTCCTGCCTC 24803
Gorrilla        AAATTTAGTAGTT---------AATTGGGAGAACATTATAGCTGCATTATCAA---AATAAAATGGCAATTTTTT---TT 23481
GrabEatingMacaqueAAATTTAGTAGTT---------AATTGGGAGAACATTATAGTTGCATTATCAA---AATAAAATGGCAATTTTTT--TTT 23744
Macaque         AAATTTAGTAGTT---------AATTGGGAGAACATTATAGTTGCATTATCAA---AATAAAATGGCAATTTTTT--TTT 24145
PigTailedMacaque AAATTTAGTAGTT---------AATTGGGAGAACATTATAGTTGCATTATCAA---AATAAAATGGCAATTTTTT--TT- 23136
Bonobo          AAATTTAGTAGTT---------AATTGGGAGAACATTATAGCTGCATTATCAA---AATAAAATGGCAATTTTTT---TT 22195
OliveBaboon     AAATTTAGTAGTT---------AATTGGGAGAACATTATAGTTGCATTATCAA---AATAAAATGGCAAATTTTT--TTT 23160
Orangutan       AAATTTAGTAGTT---------AATTGGGAGAACAGTATAGCTGCATTATCAA---AATAAAATGGCAATTTTTT---TT 23081
```

```
Majority          -AGCTGAAAGGCAG--AGAACTATAA-CATTTCTTTGAAGAATTTTCCACTTTCTTCTTCATG-TATTTTC-------CT

                          29050     29060     29070     29080     29090     29100     29110     29120

Human             GAGCTGAAAGGCAG--AGAACTATAA-CATTTCTTTGAAGAATTTTCCACTTTCTTCTTCATGGTATTTTCC------CT 21187
Coquerel'sSifaka  -AGCTGAAAGGCAG--AGAATTATAA-CATTTCTTTGAAAATTTTTTCACTTCCTTCTACACAATATTTTCTGTAATGAT 19623
Ma'sNightMonkey   GAGGGGAAAGGCAG--AGAACTATAA-CATTTCTTTGAAGAATTTTCTACTTTCTTCCTCATGATATTTTCT------CT 18968
VervetAGM         AACCTCCCGAGTAGCTGGGACTACAGGTGCCTGCTGCCATGCCTGGCTAATTTTTT------------------------ 24859
Gorrilla          TAGCTGAAAGGCAG--AGAACTATAA-CATTTCTTTGAAGAATTTTCCACTTTCTTCTTCATGGTATTTTCC------CT 23552
GrabEatingMacaque-AGCTGAAAGGCAG--AGAACTATAA-CATTTCTTTGGAGAATTTTCCACTTTCTTT--------------------CT 23799
Macaque           TAGCTGAAAGGCAG--AGAACTATAA-CATTTCTTTGGAGAATTTTCCACTTTCTTT--------------------CT 24201
PigTailedMacaque  -AGCTGAAAGGCAG--AGAACTATAA-CATTTCTTTGGAGAATTTTCCACTTT------------------------CT 23187
Bonobo            GAGCTGAAAGGCAG--AGAACTATAA-CATTTCTTTGAAGAATTTTCCACTTTCTTCTTCATGGTATTTTCC------CT 22266
OliveBaboon       -AGCTGAAAGGCAG--AGAACTATAA-CATTTCTTTGAAGAATTTTCCACTTT------------------------CT 23211
Orangutan         TAGCTGAAAGGCAG--AGAATTATAA-CATTTCTTTGAAGAATATTCCACTTTCTTCTTCATGGTATTTTCC------CT 23152

Majority          AAGAGACATGCTTAATGCAGAAAATA-TGTT------AAAAGGAT-----------------------------------

                          29130     29140     29150     29160     29170     29180     29190     29200

Human             AAGAGACATGCTTAATGCAGAAAATA-TATT------AAAAGGAT----------------------------------- 21225
Coquerel'sSifaka  AAAAGACATGCTTAATGTAGAAAACTTAATTTAGGCAAAGAGGAAAATAAATCACCAATAATCCTGTCTACCACCAAAGC 19703
Ma'sNightMonkey   AAGAGACATGCTTAATGCAGAAAATAATGTT------AAAAGGAT----------------------------------- 19007
VervetAGM         -----GTATTTTTAGTAGAGACTGGA-TTTC------ACAGTGTTAG--------------------------------- 24894
Gorrilla          AAGAGACATGCTTAATGCAGAAAATA-TGTT------AAAAGGAT----------------------------------- 23590
GrabEatingMacaqueAAGAGACATGCTTAATGCAGAAAATA-TGTT------AAAAGGAT----------------------------------- 23837
Macaque           AAGAGACATGCTTAATGCAGAAAATA-TGTT------AAAAGGAT----------------------------------- 24239
PigTailedMacaque  AAGAGACATGCTTAATGCAGAAAATA-TGTT------AAAAGGAT----------------------------------- 23225
Bonobo            AAGAGACATGCTTAATGCAGAAAATA-TGTT------AAAAGGAT----------------------------------- 22304
OliveBaboon       AAGAGACATGCTTAATGCAGAAAATA-TGTT------AAAAGGAT----------------------------------- 23249
Orangutan         AAGAGACATGCTTAACGCAGAAAATA-TGTT------AAAAGGAT----------------------------------- 23190

Majority          ---------------------------------------------------TTTAAACAGCTTATATGTAAGGGATGAGG

                          29210     29220     29230     29240     29250     29260     29270     29280

Human             ---------------------------------------------------TTTAAACAGCTTATATGTAAGGGATGAGG 21254
Coquerel'sSifaka  TAGTCATCTTTGTATGTACTTGCCATCATTTTCCCTATATATTACTATGATTTTACATACCTTATATGTAAGGGAGGAGG 19783
Ma'sNightMonkey   ---------------------------------------------------TTTAAACAGCTTATATGTAAGGAGTGAGG 19036
VervetAGM         ---------------------------------------------------CCAGGATGGTCTTGATCTCCTGACCTCGT 24923
Gorrilla          ---------------------------------------------------TTTAAACAGCTTATATGTAAGGGATGAGG 23619
GrabEatingMacaque---------------------------------------------------TTTAAACAGCTTATATGTAAGGGTTGAGG 23866
Macaque           ---------------------------------------------------TTTAAACAGCTTATATGTAAGGGTTGAGG 24268
PigTailedMacaque  ---------------------------------------------------TTTAAACAGCTTATATGTAAGGGTTGAGG 23254
Bonobo            ---------------------------------------------------TTTAAACAGCTTATATGTAAGGGATGAGG 22333
OliveBaboon       ---------------------------------------------------TTTAAACAGCTTATATGTAAGGGTTGAGG 23278
Orangutan         ---------------------------------------------------TTTAAACAGCTTATATGTAAGGGATGAGG 23219
```

```
Majority         GAACAGGGATACTTGGACTAATACTTCAGTTTTTC-TAAAATTATTGGTTTATTGAGT--------ACATTCCTAGAAAA
                         29290     29300     29310     29320     29330     29340     29350     29360
Human            GAACAGGGATACTTGGACTAATATTTCAATATTTC-TAAAATTATTGGTTTATTGAGT--------ACGTTCCTAGAAAA 21325
Coquerel'sSifaka TAACAGGGATATTTTTACAAGTAGTTCAGCTTTTCCTGAATGTATTGGTTTATTCAGTTATTCAATACTATTCCAGAAAT 19863
Ma'sNightMonkey  GAACAGTCATACTTTGACAAATAGTTCAATTTTTG-TAAAACTAT--------------------ACATTCCTAAAAAA 19094
VervetAGM        GATCCACCCACCTTGGA--AATACTTT-GCATTGT-TGCATTGATTGGGT--TTGAGC--------CCCTGTTGTACTTA 24989
Gorrilla         GAACAGGGATACTTGGACTAATATTTCAATATTTC-TAAAATTATTGGTTTATTGAGT--------ACGTTCCTAGAAAA 23690
GrabEatingMacaqueGAACAGGGATACTTGGACTAATACTTCAGTTTTTC-TAAAAGTATTGGTTTATTGAGT--------ACATACCTTGAAAA 23937
Macaque          GAACAGGGATACTTGGACTAATACTTCAGTTTTTC-TAAAAGTATTGGTTTATTGAGT--------ACATACCTTGAAAA 24339
PigTailedMacaque GAACAGGGATACTTGGACTAATACTTCAGTTTTTC-TAAAAGTATTGGTTTATTGAGT--------ACATACCTTGAAAA 23325
Bonobo           GAACAGGGATACTTGGACTAATATTTCAATATTTC-TAAAATTATTGATTTATTGAGT--------ACGTTCCTAGAAAA 22404
OliveBaboon      GAACAGGGATACTTGGACTAATACTTCAGTTTTTC-TAAAAGTATTGGTTTATTGAGT--------ACATACCTTGAAAA 23349
Orangutan        GAACAGGGATACTTGGACTAATATTTCAATATTTC-TAAAATTATTGGTTTATTGAGT--------ACGTTCCTAGAAAA 23290

Majority         CT----GTTTTATTGAG--AGCTTCAAATTTCTCAGAATTGTATAGCATTTTTGAATTCTCTCTTTTGTTACTAATATCA
                         29370     29380     29390     29400     29410     29420     29430     29440
Human            CT----GTTTTATTGAA--AGCTTCAAATTTCTCAGAATTGTATAGCATTTTTGAATTCTCTCTTTTATTACTAATATCA 21399
Coquerel'sSifaka TTATCTGTTTTATTGAG--AATTTGAAATTTCTCAAAATTGTAT---AATTTTTAGTT-----TTTTATTATTAACGTCA 19933
Ma'sNightMonkey  TT----GTTTCATCGAG--AGCTTCAAATTTCTCAGAATTTTATAGCATTCTCGAATTCTCGCTTTTATTACTAATATCA 19168
VervetAGM        A-----GTGATAATGAACAAGCCTTTTATT-CTCTGAATTTC----CCTTTCCTAGTTTGTAATATTGAGGATAATTCCT 25059
Gorrilla         CT----GTTTTATTGAA--AGCTTCAAATTTCTCAGAATTGTGTAGCATTTTTGAATTCTCTCTTTTATTACTAATATCA 23764
GrabEatingMacaqueCT----GTTTTATTGAG--AGCTTCAAATTTCTCAGAATTGTATAACATTTTTGAATTCTCTCTTTTGTTACTAATATCA 24011
Macaque          CT----GTTTTATTGAG--AGCTTCAAATTTCTCAGAATTGTATAACATTTTTGAATTCTCTCTTTTGTTACTAATATCA 24413
PigTailedMacaque C------TTTTATTGAG--AGCTTCAAATTTCTCAGAATTGTATAACATTTTTGAATTCTCTCTTTTGTTACTAATATCA 23397
Bonobo           CT----GTTTTATTGAA--AGCTTCAAATTTCTCAGAATTGTATAGCATTTTTGAATTCTCTCTTTTGTTACTAATATCA 22478
OliveBaboon      CT----GTTTTATTGAG--AGCTTCAAATTTCTCAGAATTGTATAACATTTTTGAATTCTCTCTTTTGTTACTAATATCA 23423
Orangutan        CT----TTTTTATTGA----GCTTCAAATTTCTCAGAATTGTATAGCATTTTTGAATTCTCTCTTTTATTACTAATATCA 23362

Majority         AGCACCTATGTTTTCTCCTATTTCTTGATTTGTCTTGTCATAGAATTTATTTTACTGGTCTTTTCAAAGAACCAACCTTT
                         29450     29460     29470     29480     29490     29500     29510     29520
Human            AGCACCTATGTTTTCTCCTATTTCTTGATTTGTCTTGTCACAGAATTTATTTTACTGGTCTTTTCAAAGAACCAACCTTT 21479
Coquerel'sSifaka AGCATTTATGTTTTCTCCTATCTTTTGATTTGTCATGCTACAAAATTTACTTTACTGGTCTTTTCAAAGAACTGTCTTTT 20013
Ma'sNightMonkey  AGTACCTATGTTTTTTCCTGTTTCTTGATTTG-----TCATAGAATTTATTTTACTAATCTTTTCAAAGAAGCAACCTTT 19243
VervetAGM        AACTTGCAGGATTGTTGTGAAGATTAAATAAG----ACAATAATATTGGTAGGA--GCTGTTTATAATCTTTCTCTCTTA 25133
Gorrilla         AGCACCTATGTTTTCTCCTATTTTTTGATTTGTCTTGTCACAGAATTTATTTTACTGGTCTTTTCAAAGAACCAGCGTTT 23844
GrabEatingMacaqueAGCACCTATGTTTTCTTCTATTTCTTGATTTGTTTTGTCATAGAATTTATTTTACTGGTCTTTTCAAAGAACCAACCTTT 24091
Macaque          AGCACCTATGTTTTCTTCTATTTCTTGATTTGTTTTGTCATAGAATTTATTTTACTGGTCTTTTCAAAGAACCAACCTTT 24493
PigTailedMacaque AGCACCTATGTTTTCTTCTATTTCTTGATTTGTTTTGTTATAGAATTTATTTTACTGGTCTTTTCAAAGAACCAACCTTT 23477
Bonobo           AGCACCTATGTTTTCTCCTATTTCTTGATTTGTCTTGTCACAGAATTTATTTTACTGGTCTTTTCAAAGAACCAACCTTT 22558
OliveBaboon      AGCACCTATGTTTTCTTCTATTTCTTGATTTGTTTTGTCATAGAATTTATTTTATTGGTCTTTTCAAAGAACCAACCTTT 23503
Orangutan        GGCACCTATGTTTTCTCCTATTTCTTGATTTGTCTTGTCACAGAATTTATTTTACTGGTCTTTTCAAAGAACCAACCTTT 23442
```

```
Majority          TTACTTGTTTTCTTTTTTGTTGGGGGCAGTGAAGG---GAT-TCACTTTAAGTTGATCCTGAACTCCTTTATTTTCTTGT
                           29530     29540     29550     29560     29570     29580     29590     29600
Human             TTACTTGTTTTCTTTTTTGTTGGGGGCAGTGGAGG---GAT-TCACTTTAATTTGATCCTGAACTCCTTTATTTTCTT-- 21553
Coquerel'sSifaka  GTGCTTGCTTCTTTTTGTTTCATAATTCACTAATTTTTGTTTTTATCTTTTTTTGCCTTACACTAGATTTATTTTGCTGT 20093
Ma'sNightMonkey   TTACTTATTTAATTTTTCGTTGGGGGCAGGGGAGG---GAT-TTACTTTAATTTGATCCTCAACTTCTTTCTTTTCTTGT 19319
VervetAGM         TGTACAATTCAAATATATGTGGGTTTAAATACCAA---AATATCACTTTAAA--AAATCTAGGCTGAAATGGACACT--- 25205
Gorrilla          TTACTTGTTTTCTTTTTTGTTGGGGGCAGTGGAGG---GAT-TCACTTTAAGTTGATCCTGAACTCCTTTATTTTCTTGT 23920
GrabEatingMacaqueTTATTTGTTTTCTTTTTTGTTGAGGGCAGTGAAAG---GGT-TCACTTTAAGTTGATCCTGAACCCCTTTATTTTCTTGT 24167
Macaque           TTATTTGTTTTCTTTTTTGTTGAGGGCAGTGAAAG---GGT-TCACTTTAAGTTGATCCTGAACCCCTTTATTTTCTTGT 24569
PigTailedMacaque  TTATTTGTTTTCTTTTTTGTTGAGGGCAGTGAAAG---GGT-TCACTTTAAGTTGATCCTGAACCCCTTTATTTTCTTGT 23553
Bonobo            TTACTTGTTTTCTTTTTTGTTGGGGGCAGTGGAGG---GAT-TCACTTTAATTTGATCCTGAACTCCTTTATTTTCTTGT 22634
OliveBaboon       TTATTTGTTTTCTTTTTTGTTGAGGGCAGTGAAGG---GGT-TCACTTTAAGTTGATCCTGAACTCCTTTATTTTCTTGT 23579
Orangutan         TTACTTGTTTTCTTTTTGGTTGGGGG----------------TC--------------CTGAACTCCTTTATTTTCTTGT 23492

Majority          TTTAAAATGCATTCAAGGTTAAAGGTAATCAGCTGATAGCTTTGGCAGTGTTTTTTT---------------------TT
                           29610     29620     29630     29640     29650     29660     29670     29680
Human             -TTAAAATGCATTCAAGGTTAAAGGTAATCAGCTGATAGCTTTGG--CTTTTTTTTT---------------------TT 21609
Coquerel'sSifaka  TTTTTGTTTTTCGTACACTTCAGGTTGATCC--CGAACTCTTTTATTTTCTTGTTTTA--AAAATGTATTCAAGGTTAAG 20169
Ma'sNightMonkey   TTTAAAATGGATTTGAGTTTAAAGGTGATCA--T---AGCTTTGGCAATTTTTTTTT---------------------TT 19373
VervetAGM         TCTGATGTAGAAGTAGTGCTTTAGAAAAGAA--TGCTTTGTTTCATACTCTTTTTTT---------TTTTTTTTTTTTTT 25274
Gorrilla          TTTAAAATGCATTCAAGGTTAAAGGTAATCAGCTGATAGCTTTGG---C--ATTTTT---------------------TT 23974
GrabEatingMacaqueTTTAAAATACATTCAAGGTTAAAGGTAATCAGCTGATAGCTTTGGCAGTGTTTTTTT-----------TGTTTGTTTGTT 24236
Macaque           TTTAAAATACATTCAAGGTTAAAGGTAATCAGCTGATAGCTTTGGCAGTGTTTTTTT-----------TGTTTGTTTGTT 24638
PigTailedMacaque  TTTAAAATACATTCAAGGTTAAAGGTAATCAGCTGATAGCTTTGGCAGTGTTTTTTTGTTTGTTTGTTTGTTTGTTTTTT 23633
Bonobo            TTTAAAATGCATTCAAGGTTAAAGGTAATCAGCTGATAGCTTTGGTCCTTTTTTTTT---------------------TT 22693
OliveBaboon       TTTAAAATACATTCAAGGTTAAAGGTAATCAGCTGATAGCTTTGGCAGTGTTTTTTT---------------------TT 23638
Orangutan         TTTAAAATGCATTCAAGGTTAAAGGTAATCAGCTGATAGCTTTGG---CAATTTTTT---------------------TT 23548

Majority          TTTTTTACTTTTAGAGTTCTCTTTTTATTGT--AAACTTC-TT-TAAAGGTTTTAAGCTACACAAAAGTAGAGGGAATGG
                           29690     29700     29710     29720     29730     29740     29750     29760
Human             TTTTTTACTTTTAGAGTTCTCTTTTTATTGT--AAACTTCATT-TAAAGGTTTTAAGCTACACAAAAGTAGAGGGAATGG 21686
Coquerel'sSifaka  GATGGATCAGTTGGAGTTCTCTTTTCATTTTTAAAACTTCCTTATAAAAGTTTCAAAATATACAAAAGTAGAGGGAATGG 20249
Ma'sNightMonkey   ACTTTTAGAGTTCT-GTTTTTTCTTTTTTGT--AAACTTTATTATAAAGGCTTTAAGCTATACAAAAGTAGAGGGAATAG 19450
VervetAGM         TTTTTTACTTTTAGAGTTCTCTTTTTATTAT--AAACTT------AAAGGTTTTAAGCTACACAAAAGTAGAGGGAATGG 25346
Gorrilla          TTTTTTACTTTTATAGTTCTCTTTTTATTGT--AAACTTCGTTATAAAGGTTTTAAGCTACACAAAAGTAGAGGGAATGG 24052
GrabEatingMacaqueTGTTTTACTTTTAGAGTTCTCTTTTTATTAT--AAACTT------AAAGGTTTTAAGCTACACAAAAGTAGAGGGAATGG 24308
Macaque           TGTTTTACTTTTAGAGTTCTCTTTTTATTAT--AAACTT------AAAGGTTTTAAGCTACACAAAAGTAGAGGGAATGG 24710
PigTailedMacaque  TGTTTTACTTTTAGAGTTCTCTTTTTATTAT--AAACTT------AAAGGTTTTAAGCTACACAAAAGTAGAGGGAATGG 23705
Bonobo            TTTTTTACTTTTAGAGTTCTCTTTTTATTGT--AAACTTCATTATAAAGGTTTTAAGCTACACAAAAGTAGAGGGAATGG 22771
OliveBaboon       ---TTTACTTTTAGAGTTCTCTTTTTATTAT--AAACTT------AAAGGTTTTGAGCTACACAAAAGTAGAGGGAATGG 23707
Orangutan         TTTTTTACTTTTAGAGTTCTCTTTTTATTGT--AAACTTCATTATAAAGGTTTTAAGCTACACAAAAGTAGAGGGAATGG 23626
```

```
Majority          TATATAATACACTTTTTTTT----TTCTGTTGGATAATATTAATGCAAATCTCAGATCATTTCATATCATCTGCAGATAT
                          29770     29780     29790     29800     29810     29820     29830     29840
Human             TATATAATACACTTTTTTT--------CTGTTGGATAATATTAATGCAAATCTCAGATCATTTCATATAATCTGCAGATAT 21758
Coquerel'sSifaka TATAAAACCAGTTTTTTTTTCTTTTTCTCTTGGAGAATATTAAAGCAAATCTCAGATAATTTCATATCATCCCCAGATAC 20329
Ma'sNightMonkey  TATATA-----CTTTTTGT------TCTGTTGGAGAATATTAATGCAAATTGCAGA-----TCATATCATCCTCAGATAT 19514
VervetAGM         TATATAATACACTCTTTTTT----TTCTGTTGGATAGTATTTATGCAAATCTCATATCATTTCATTTCATCAGCAGCTAT 25422
Gorrilla          TATATAATACACTTTTTTT-------CTGTTGGATAATATTAGTGCAAATCTCAGATCATTTCGTATAATCTGCAGATAT 24125
GrabEatingMacaqueTATATAATACACTCTTTTTT----TTCTGTTGGATAGTATTTATGCAAATCTCATATCATTTCATTTCATCTGCAGCTAT 24384
Macaque           TATATAATACACTCTTTTTT----TTCTGTTGGATAGTATTTATGCAAATCTCATATCATTTCATTTCATCTGCAGCTAT 24786
PigTailedMacaque TATATAATACACTCTTTTTT----TTATGTTGGATAGTATTTATGCAAATCTCATA---TTTCATTTCATCTGCAGCTAT 23778
Bonobo            TATATAATACACTTTTTTT-------CTGTTGGATAATATTAATGCAAATCTCAGATCATTTCGTATAATCTGCAGATAT 22844
OliveBaboon       TATATAATACACTCTTTTTT----TTCTGTTGGGTAGTATTTATGCAAATCTCATATCATTTCATTTCATCTGCAGCTAT 23783
Orangutan         TATATAATACACTTTTTTT-------CTGTTGGATAATATTAATGCATATCTCAGATCATTTCATATAATCTGCAGATAT 23699

Majority          ATCAGAATGTGTCTCTAGCAGATATAATTTCTCTTTTAAATGTGACATTGGTACCATTA----------TAAAGTTTAAA
                          29850     29860     29870     29880     29890     29900     29910     29920
Human             ATCAGAATGTGTGTCT--------------CTCTATTAAATGTGACATTGGTACCATTA----------TAAAGTTTAAA 21814
Coquerel'sSifaka ATCAGTATGTATCTCTAACAAATATTAT--CTCTCTCAAAGGTGATATTGATACTATTAATATACCTAGTAAAATTTAAA 20407
Ma'sNightMonkey  GTCAGAATGTGTCTCTAGCAGATATAATTTCTCTTTTAAGTGTAACATTGGTACCATTA----------TAAAATTTAAA 19584
VervetAGM         AACAGAATGTGCCTCTAGCAGATACAATTTCTCTTTTAAATGTGACATTGGTACCGTTA----------TAAAGTTTAAA 25492
Gorrilla          ATCAGAATGTGTCTCT--------------CTCTATTAAATGTGACATTGGTACCATTA----------TAAAGTTTCAA 24181
GrabEatingMacaqueAACAGAATGTGCCTCTAGCAGATATAATTTCTCTTTTAAATGTGACATTGGTACTGTTA----------TAAAGTTTAAA 24454
Macaque           AACAGAATGTGCCTCTAGCAGATATAATTTCTCTTTTAAATGTGACATTGGTACTGTTA----------TAAAGTTTAAA 24856
PigTailedMacaque AAC--AATGTGCCTCTAGCAGATATAATTTCTCTTTTAAATGTGACATTGGTACTGTTA----------TAAAGTTTAAA 23846
Bonobo            ATCAGAATGTGTGTCT--------------CTCTATTAAATGTGACATTGGTACCATTA----------TAAAGTTTAAA 22900
OliveBaboon       AACAGAATGTGCCTCTAGCAGATACAATTTCTCTTTTAAATGTGACATTGGTACCGTTA----------TAAAGTTTAAA 23853
Orangutan         ATCAGAATGTGTCTCT--------------CTCTTTTAAATGTGACATTGGTACCATTA----------TAAAGTTTAAA 23755

Majority          ATAATTTCTTAATGTCATCTAATCTCCA---GTATTCAGAATACTTTTTTTTCATTATTTT----TTCTTGCCTTTTCTC
                          29930     29940     29950     29960     29970     29980     29990     30000
Human             ATAATTTCTTAATGTCGTCTAATCTCCA---GTATTCAGAATACTTTCTTTTCATTATTTTATTTTTCTTGCCTTTTCTT 21891
Coquerel'sSifaka ATAATTTCTTAATGTCATCTAATCTCCAACAGTATTCAGAATACTTTTTTTCTTTCATTTT-----CCTTGCCTTT---- 20478
Ma'sNightMonkey  ATAATTTCTTAATGTCATTTAATCTCCA---GTATTCAGAATACTTTTTTTTCATTATTTT----TTCTTGCCTTTTCTC 19657
VervetAGM         ATAATTTACTAATGTCATCTAATCCCCA---GTATTCAAAATACTTTTTTTTCATTATTTTATTTTTCTTACCTTTTCTC 25569
Gorrilla          ATAATTTCTTAATGTCGTCTAATCTCCA---GTATTCAGAATACTTTTTTTTCATTATTTTATTTTTCTTGCCTTTTCTC 24258
GrabEatingMacaqueATAATTTACTAATGTCATCTAATCTCCA---GTATTCAAAATACTTTTTTT-CATTATTTT-----TCTTACCTTTTCTC 24525
Macaque           ATAATTTACTAATGTCATCTAATCTCCA---GTATTCAAAATACTTTTTTT-CATTATTTT-----TCTTACCTTTTCTC 24927
PigTailedMacaque ATAATTTACTAATGTCATCTAATCTCCA---GTATTCAAAATACTTTTTTTTCATTATTTT-----TCTTACCTTTTCTC 23918
Bonobo            ATAATTTCTTAATGTCGTCTAATCTCCA---GTATTCAGAATACTTTTTTTTCATTATTTTATTTTTCTTGCCTTTTCTA 22977
OliveBaboon       ATAATTTACTAATGTCATCTAATCTCCA---GTATTCAAAATACTTTTTTTTCATTATTTT-----TCTTACCTTTTCTC 23925
Orangutan         ATAATTTCTTAATGTCGTCTGATCTCCA---GTATTCAGAATACTTTTTTTTCATTATTTTATTTTTCTTGCCTTTTCTC 23832
```

```
Majority          TTTTGTTGTTGTTAGATAAACTGAGTCATCTGTACAATTTCCACTTTCTGG-----------------------------
                          30010     30020     30030     30040     30050     30060     30070     30080
Human             TTTTGTTGTTGTTAGATAAAGTGAGTCATCTGTACAATTTCCACTTTCTGGCTGGGCGTGGTGGCTCACGCCTGTAATCC 21971
Coquerel'sSifaka  -------GT---TAGAGAAACTGAGTCATTTGTACAGTTTCTACTTTCTGA----------------------------- 20519
Ma'sNightMonkey   TATTGTTGTTG-TTGTTAAACTGAGTCATATGTA---TTTCCACTTTCTGG----------------------------- 19704
VervetAGM         TCTTGTTGTTGTTAGATAAACTGAGTCATCTGTACAATTTCCACTTTCTGG----------------------------- 25620
Gorrilla          TTTTGTTGT---TAGATAAACTGAGTCATCTGTACAATTTCCACTTTCTGGCTGGGCGTGGTGGCTCACGCCTGTAATCC 24335
GrabEatingMacaque TGTTGTTGTTGTTAGATAAACTGAGTCATCTGTACAATTTCCACTTTATGG----------------------------- 24576
Macaque           TGTTGTTGTTGTTAGATAAACTGAGTCATCTGTACAATTTCCACTTTATGG----------------------------- 24978
PigTailedMacaque  TGTTGTTGTTGTTAGATAAACTGAGTCATCTGTACAATTTCCACTTTATGG----------------------------- 23969
Bonobo            TTTTGTTGTTGTTAGATAAACTGAGTCATCTGTACAATTTCCACTTTCTGGCTGGGCATGGTGGCTCACGCCTGTAATCC 23057
OliveBaboon       TGTTGTTGTTGTTAGATAAACTGAGTCATCTGTACAATTTCCACTTTATGG----------------------------- 23976
Orangutan         TTTTGTTGTTGTTAGATAAACTGAGTCATCTGTACAATTTCCACTTTCTGGCCAAGCGCGGTAGCTCACGCCTGTAATCC 23912

Majority          --------------------------------------------------------------------------------
                          30090     30100     30110     30120     30130     30140     30150     30160
Human             CAGCACTTCGGGAGGCTGAGGCGGGCAGATCACGAGGTCAGGAGATCAAGACCATTTTGGCTAACACAGTGAAAACCTGT 22051
Coquerel'sSifaka  -------------------------------------------------------------------------------- 20519
Ma'sNightMonkey   -------------------------------------------------------------------------------- 19704
VervetAGM         -------------------------------------------------------------------------------- 25620
Gorrilla          CAGCACTTTGGGACGCTGAGGCGGGCAGATCACGAGGTCAGGAGATCAAAACCATCTTGGCTAACACAGTGAAAACCTGT 24415
GrabEatingMacaque -------------------------------------------------------------------------------- 24576
Macaque           -------------------------------------------------------------------------------- 24978
PigTailedMacaque  -------------------------------------------------------------------------------- 23969
Bonobo            CAGCACTTCGGGAGGCTGAGGCGGGCAGATCACGAGGTCAGGAGATCAAGACCATTTTGGCTAACACAGTGAAAACCTGT 23137
OliveBaboon       -------------------------------------------------------------------------------- 23976
Orangutan         CAGCACTTTGGGAGGCCGAGGCGG-CAGATCACGAGGTCAGGAGATCAAGACCATCTTGGCTAACACAGTGAAAACCTGT 23991

Majority          --------------------------------------------------------------------------------
                          30170     30180     30190     30200     30210     30220     30230     30240
Human             CTCTACTAAAAATACGAAAAAATTAGCCAGGCGTGGTGGGGGGCACCTGTAGTCCCAGCTACTCGGGAGGCTGAGGCAGG 22131
Coquerel'sSifaka  -------------------------------------------------------------------------------- 20519
Ma'sNightMonkey   -------------------------------------------------------------------------------- 19704
VervetAGM         -------------------------------------------------------------------------------- 25620
Gorrilla          CTCTACTAAAAATACGAAAAAATTAGCCGGGGGTGGTGGCGGGCACCTGTAGTCCCAGCTACTCGGGAGGCTGAGGCAGG 24495
GrabEatingMacaque -------------------------------------------------------------------------------- 24576
Macaque           -------------------------------------------------------------------------------- 24978
PigTailedMacaque  -------------------------------------------------------------------------------- 23969
Bonobo            CTCTACTAAAAATACGAAAAAATTAGCCAGGCGTGGTGGGGGGCACCTGTAGTCCCAGCTACTCGGAAGGCTGAGGCAGG 23217
OliveBaboon       -------------------------------------------------------------------------------- 23976
Orangutan         CTCTACTGAAAATACGAAAAAATTAGCCAGGCATGGTAGCAGGCGCCTGTAGTCCCAGCTACTCGGGAGGCTGAGGCAGG 24071
```

```
Majority        --------------------------------------------------------------------------------

                         30250     30260     30270     30280     30290     30300     30310     30320

Human           AGAATGGTGTGAACCCGGGAGGCGGAGCTTGCAGTGGCGCCACTGGACTCCAGCCTGGGTGACAGAGCAAGACTCCGTCT 22211
Coquerel'sSifaka -------------------------------------------------------------------------------- 20519
Ma'sNightMonkey -------------------------------------------------------------------------------- 19704
VervetAGM       -------------------------------------------------------------------------------- 25620
Gorrilla        AGAATGGTGTGAACCCGGGAGGCGGAGCTTGCAGTGGCGCCACTGGACTCCAGCCTGGGTGACAGAGCAAGACTCCGTCT 24575
GrabEatingMacaque-------------------------------------------------------------------------------- 24576
Macaque         -------------------------------------------------------------------------------- 24978
PigTailedMacaque -------------------------------------------------------------------------------- 23969
Bonobo          AGAATGGTGTGAACCCGGGAGGTGGAGCTTGCAGTGGCGCCACTGGACTCCAGCCTGGGTGACAGAGCAAGACTCCGTCT 23297
OliveBaboon     -------------------------------------------------------------------------------- 23976
Orangutan       AGAATGGTGTGAACCCGGGAGGCGGAGCTTGCAGTGGCGCCACTGGACTCCAGCCTGGGCAACAGAGCAAGACTCTGTCT 24151

Majority        -----------------A--------------ATTCTGCTGATTATACCTCCATGTTGTCATTTCATATGTTCCTCATG

                         30330     30340     30350     30360     30370     30380     30390     30400

Human           TAAAAAAAAAAAAAAAA---TTCCACTTTCTGGATTCTGCTGATTGTACCGCTGTGTTGTCATTTCATATGTTCTTCATG 22288
Coquerel'sSifaka --------------------------------ATTCAGCTGATTCTACCTCCGTACTGTCATTTCATACGTTCC-CATG 20565
Ma'sNightMonkey --------------------------------ATTCTGCTGATTATACCTCCATGTTGTCGTTTCATATGTTCCTCATG 19751
VervetAGM       -----------------A---------------TTCTGCTGATTATACCTCCATGTTGTCATTTCATATGTTCCTCATG 25667
Gorrilla        TAAAAAAAAAAAAAAAAAAAATTCCACTTTCTGGATTCTGCTGATTGTACCACTATGTTGTCATTTCATATGTTCTTCATG 24655
GrabEatingMacaque-----------------A---------------TTCTGCTTATTATACCTCCATGTTATCATTTCATATGTTCCTCATG 24623
Macaque         -----------------A---------------TTCTGCTTATTATACCTCCATGTTATCATTTCATATGTTCCTCATG 25025
PigTailedMacaque -----------------A---------------TTCTGCTTATTATACCTCCATGTTATCATTTCATATGTTCCTCATG 24016
Bonobo          TAAAAAAAAAAAA-------TTCCACTTTCTAGATTCTGCTGATTGTACCGCTATGTTGTCATTTCATATGTTCTTCATG 23370
OliveBaboon     -----------------A---------------TTCTGCTTATTATACCTCCATGTTGTCATTTCATGTGTTCCTCATG 24023
Orangutan       TAAAAAAAAAAAAAAAT---TCCCACTTTCTGGATTCTGCTGATTATACCGCTATGTTGTCATTTCATATGTTCCTCATG 24228

Majority        TAAACAAGTTTACCTGAAA---------TACAGGGGGT-TGATCAGACTCTGGTTTAATTTTTATCAAGAATATTTTGCA

                         30410     30420     30430     30440     30450     30460     30470     30480

Human           TAAACAAGTTTACCTGAAA---------TACAGGGGTT-TGATCAGACTCTGGTGTAATTTTTACCAAGAATATTTTGCA 22358
Coquerel'sSifaka TCCTCTGAATTTCCTGTAAACTTCTAGTTAAATGAGGTGTGATGTGATTAAGGCTTAATTTTTAACAAGAATATTTTGTA 20645
Ma'sNightMonkey TAGACAAGTTTACCTGAAA---------TACATGGGGT-TGATCAGATTCTGGTTTAATTTTTATCAAGAATATTTTGCA 19821
VervetAGM       TAAACTAGTTTACCTGAAA---------TACGGGGGGT-TGATCAGACTCTGGTTTAATTTTTATCAAGAGTATTTTGCA 25737
Gorrilla        TAAACAAGTTTACCTGAAA---------TACAGGGGGT-TGATCAGACTCTGGTGTAATTTTTACCAAGAATATTTTGCA 24725
GrabEatingMacaqueTAAACAAGTTTACCTGAAA---------TACGGGGGGT-TGATCAGACTCTGGTTTAATTTTTATCAAGAGTATTTTGCA 24693
Macaque         TAAACAAGTTTACCTGAAA---------TACGGGGGT-TGATCAGACTCTGGTTTAATTTTTATCAAGAGTATTTTGCA 25095
PigTailedMacaque TAAACAAGTTTACCTGAAA---------TACGGGGGGT-TGATCAGACTCTGGTTTAATTTTTATCAAGAGTATTTTGCA 24086
Bonobo          TAAACAAGTTTACCTGAAA---------TACAGGGGGT-TGATCAGACTCTGGTGTAATTTTTACCAAGAATATTTTGCA 23440
OliveBaboon     TAAACAAGTTTACCTGAAA---------TACGGGGGGT-TGATCAGACTCTGGTTTAATTTTTATCAAGAGTACTTTGCA 24093
Orangutan       TAAACAAGTTTACCTGAAA---------TACAGAGGGT-TGATCAGACT--GGTTTAATTTTTACCAAGAATATTTTGCA 24296
```

```
Majority          GATGTACTCTATGTAATTCCTGTTGTATCTCACTAAGAGGATCAGAACGTCATGTTACCTCTTCATAATGTTAACATTTA
                           30490     30500     30510     30520     30530     30540     30550     30560
Human             GATGTACTCTATGTAATTCCTGTTGTAT---------------------------------------------------- 22386
Coquerel'sSifaka  GATGTACTCTATGTAATGCCTATTGTATTT-ATCACACTAAGAGGAACAGAATGCTATCTCTTTGTGATGTTAACATTTA 20724
Ma'sNightMonkey   GATATACTTTGTGTAATTCCTATTGTATCTCGCTAAGGGGATCAGAATGTGTCCTTACCTCTTCCTGGTGTTAACATTTA 19901
VervetAGM         GATGTACTCTATGTAATTCCTGTTGTATCTCACTAAGACGATCAGAACCTCATGTTACCTCTTCATAATGTTAACATTTA 25817
Gorrilla          GATGTACTCTATGTAATTGCTGTTGTAG---------------------------------------------------- 24753
GrabEatingMacaqueGATGTACTCTATGTAATTCCTGTTGTATCTCACTAAGAGGATCAGAACGTCATGTTACCTCTTCATAATGTTAACATTTA 24773
Macaque           GATGTACTCTATGTAATTCCTGTTGTATCTCACTAAGAGGATCAGAACGTCATGTTACCTCTTCATAATGTTAACATTTA 25175
PigTailedMacaque  GATGTACTCTATGTAATTCCTGTTGTATCTCACTAAGAGGATCAGAACGTCATGTTACCTCTTCATAATGTTAACATTTA 24166
Bonobo            GATATACTCTATGTAATTCCTGTTGTAT---------------------------------------------------- 23468
OliveBaboon       GATGTACTCTATGTAATTCCTGTTGTATCTCACTAAGAGGATCAGAACGTCATGTTACCTCTTCATAATGTTAACATTTA 24173
Orangutan         GATGTACTCTATGTAATTCCTATTGTATCTCACTAAGAGGATCAGAATGTCTCGTTACCTCTTCATGATGTTAACATTTA 24376

Majority          TCAGTAGGTTCAGGTGTCAGCTT-GATGCATCTGTTACAGAG---TGCATCATCCTTTCACCTAATAGTTTTTAGTAGCT
                           30570     30580     30590     30600     30610     30620     30630     30640
Human             ---------------GTCAGCTT-GCTCCATCTATTACAGAG---CGCATCATCCTTTCACCTAATAGTTTTTAGTAGCT 22447
Coquerel'sSifaka  TCAATAGGTTGAAGTGTCAGCTTTGATGCATTCATTATAGAGAGCTGCATCATCCTTTCAGCTAATAGTTTTTAGTAACT 20804
Ma'sNightMonkey   TCAGTAGGTTCAGGTGTCAGCTT-GATGCATCTGTTAAAGAG---TGCATCATCCTTTCTCCTAATAGTTTTTAGTAGCT 19977
VervetAGM         TCAGTAGGTTCAGGTGTCAACTT-GATGCATCTGTTACAGAG---TGCATCATCCTTTTACCTGATAGTTTTTGGTAGCT 25893
Gorrilla          ---------------GTCAGCTT-GCTCCATCTATTACAGAG---CGCATCATCCTTTCACCTAATAGTTTTTAGTAGCT 24814
GrabEatingMacaqueTCAGTAGGTTCAGGTGTCAACTT-GATGCATCTGTTACAGAG---TGCATCATCCTTTTACCTGATAGTTTTTAGTAGCT 24849
Macaque           TCAGTAGGTTCAGGTGTCAACTT-GATGCATCTGTTACAGAG---TGCATCATCCTTTTACCTGATAGTTTTTAGTAGCT 25251
PigTailedMacaque  TCAGTAGGTTCAGGTGTCAACTT-GATGCATCTGTTACAGAG---TGCATCATCCTTTTACCTGATAGTTTTTAGTAGCT 24242
Bonobo            ---------------GTCAGCTT-GCTCCATCTATTACAGAG---TGCATCATCCTTTCACCTAATAGATTTTAGTAGCT 23529
OliveBaboon       TCAGTAGGTTCAGGTGTCAACTT-GATGCATCTGTTACAGAG---TGCATCATCCTTTTACCTGATAGTTTTTAGTAGCT 24249
Orangutan         TCGGTAGGTTCAGGTGTCAGCTT-GCTCCATCTATTGCAGAG---TGCATCATCCTTTCACCTAATAGTTTTTAGTAGCT 24452

Majority          AATGATTGCATACTTTTATTAGGTATTGCAAAAGTGGTGCTATTCTTTTTTTCTGCGTATATTAGAATTC-----TTAAG
                           30650     30660     30670     30680     30690     30700     30710     30720
Human             AATGATTGCATACTTTTATTAGGTATTGCAAAAGTGGCGCTATTCTTTTTTTCTGTGTATATTAGAATTC-----TTAAG 22522
Coquerel'sSifaka  AATGATTGCATAATTTTATTGGGTATTTCAAAAATGACAATTTTTTTTTGCATGTATTAATTTAAATTCATCTGTTAAG 20884
Ma'sNightMonkey   AGTGATTGCATAATTATATTGGGTATTGCAAAAGTGGTGCTATTCTTTTTTTGTGTGTATATTAGAATTC-----TTAAA 20052
VervetAGM         AATAATTGCATACTTTTATTAGGTATTGCGAAAGTGGTGCTATTCTTTTTTTCTGCGTATATTAGAATAC-----TTAAG 25968
Gorrilla          AATGATTGCATAATTTTATTAGGTATTGCAAAAGTGGCGCTATTCTTTTTTTCTGCATATATTAGAATTC-----TTAAG 24889
GrabEatingMacaqueAATGATTGCATACTTTTATTAGGTGTTGCGAAAGTGGTGCTATTC----------------------------------- 24894
Macaque           AATGATTGCATACTTTTATTAGGTGTTGCGAAAGTGGTGCTATTCTTTTTTTCTGCGTATATTAGAATTC-----TTAAG 25326
PigTailedMacaque  AATGATTGCATACTTTTATTAGGTGTTGCGAAAGTGGTGCTATTCTTTTTTTCTGCGTATATTAGAATTC-----TTAAG 24317
Bonobo            AATGATTGCATAATTTTATTAGGTATTGCAAAAGTGGCGCTATTCTTTTTTTCTGCGTATATTAGAATTC-----TTAAG 23604
OliveBaboon       AATGATTGCATACTTTTATTAGGTGTTGCAAAAGTGGTGCTATTCTTTTTTTCTGCGTATATTAGAATTC-----TTAAG 24324
Orangutan         AATGATTGCATAATTTTATTAGGTATTGCAAAAGTGGCGCTATTCTTTTTTTCTGCATATATTAGAATTC-----TTAAG 24527
```

```
Majority          AATTTTATCCATCAACTCTTTGGTCATATGTAAGTACCC-TTTTTATAAGAAAAGCAGGAGAGATTTT-GACTTTTTAAG
                          30730     30740     30750     30760     30770     30780     30790     30800
Human             AATTATATCCATCAACTGTTTGGTCATACGTAAGTACC--TTTTTATAAGAAAAGCAGGAGAGAATTT-GACTTCTTAAG 22599
Coquerel'sSifaka  AACTTTATCCATCAACTATTTGGTCACATTGAAGTACAC-TTTTTATAAGAAAAGCAGGACAGATTTTTGACTTTTTAAA 20963
Ma'sNightMonkey   AATTTTATCCATCAACTATTTGGTCATACTTAAGTACCCTTTTTTATAAGAAAAACAGGAGAGATTTT-AACTTTCTAAG 20131
VervetAGM         AATTTTATCCATCAACTCTTTGGTCATATGTAAGTACCC-TTTTTATAAGGAAAGCAGGAGAGATTTT-GACTTTTTAAG 26046
Gorrilla          AATTATATCCATCAACTGTTTGGTCATACGTAAGTACCC-TTTTTATAAGAAAAGCAGGAGAGAATTT-GACTTCTTAAG 24967
GrabEatingMacaque---------------------------------------------------------------------------AAG 24897
Macaque           AATTTTATCCATCAACTCTTTGGTCATATGTAAGTACCC-TTTTTATAAGGAAAGCAGGAGAGATTTT-GACTTTTTAAG 25404
PigTailedMacaque  AATTTTACCCATCAACTCTTTGGTCATATGTAAGTACCC-TTTTTATAAGGAAAGCAGGAGAGATTTT-GACTTTTTAAG 24395
Bonobo            AATTATATCCATCAACTGTTTGGTCATACGTAAGTACCC-TTTTTATACGAAAAGCAGGAGAGAATTT-GACTTCTTAAG 23682
OliveBaboon       AATTTTATCCATCAACTCTTTGGTCATATGTAAGTACCC-TTTTTATAAGGAAAGCAGGAGAGATTTT-GGCTTTTTAAG 24402
Orangutan         AATTATATCCATCAACTATTTGGTCATACATAAGTACCC-TTTTTATAAGAAAAGCAGGAGAGATTTT-GACTTTTTAAG 24605

Majority          ----TTTTCTGATGAGTTGGTACCCTAA-----AACCTCCAGTATAACCAATATGAGTTCCTTTTTGTTGTTGTTAGTAT
                          30810     30820     30830     30840     30850     30860     30870     30880
Human             ----TTTTCTGATGAGTTGTTACCCTAA-----AACCTCCAGTATAATCAATATGAGTTCCTTTTTGTTGCTGTTAGTAT 22670
Coquerel'sSifaka  AACTTTTCCTGAGGAGTTGGTACTCCAGC----AACCTCCA-CATAACCAATGTGAGTTCCTTTTTGTTGTT---AGTAT 21035
Ma'sNightMonkey   ----TTTTCTGATGAGTTAGTACCCTAACAAACAACCTCCAGTATAACCAATATGAGTTCCTTTTTGTTGCTGTTAGTAC 20207
VervetAGM         ----TTTTCTGATGAGTTGGTACCCTAA-----AACCTCCAATATAACCAGTATGAGTTCCTTTTTGTTGTTGTTAGTAT 26117
Gorrilla          ----TTTTCTGATGAGTTGTTACCCTAA-----AACCTCCAGTATAATCAATATGAGTTCCTTTTTGTTGCTGTTAGTAT 25038
GrabEatingMacaque----TTTTCTGATGAGTTGGTACCCTAA-----AACCTCCAATATAACCAGTATGAGTTCCTTTTTGTTGTT---AGTAT 24965
Macaque           ----TTTTCTGATGAGTTGGTACCCTAA-----AACCTCCAATATAACCAGTATGAGTTCCTTTTTGTTGTT---AGTAT 25472
PigTailedMacaque  ----TTTTCTGATGAGTTGGTACCCTAA-----AACCTCCAATATAACCAGTATGAGTTCCTTTTTGTTGTT---AGTAT 24463
Bonobo            ----TTTTCTGATGAGTTGTTACCCTAA-----AACCTCCAGTATAATCAATATGAGTTCCTTTTTGTTGCTGTTAGTAT 23753
OliveBaboon       ----TTTTCTGATGAGTTGGTACCCTAA-----AACCTCCAATATAACCAGTATGAGTTCCTTTTTGTTGTT---AGTAT 24470
Orangutan         ----TTTTCTGATGAGTTGGTACCCTAA-----CACCTCCAGTATAATCAAT--GAGTTCCTTTTTGTTGCTGTTAGTAT 24674

Majority          TGTCGCAAAAGCAAAGAGTTTAATAACATTTATTTTCTAGTGTATTGTAGTAATCATTCTTTTTTTTT---AATTTCTAA
                          30890     30900     30910     30920     30930     30940     30950     30960
Human             CGTCGCAACAGCAAAGAGTTTAATAACATTTATTTTCTAGTGTATTGCAGTAATCATTCTTCTTTTTTTTAAATTTCTAA 22750
Coquerel'sSifaka  CATTATGAAAGCAAAGATTTTAA---CATTTATTTTCATTCTATTATAGTGATCATTCTTTTTT-----AAATGCTTAA 21107
Ma'sNightMonkey   TGTCACAAAAGCAAAGATTTTAATAACATTTATTTTCTAGTGTATTGCCGTGATCATTCCTAA--------AATTTCTAA 20279
VervetAGM         TGTTGCAAAAGCAAAGAGTTTAATAATATTTATTTGCTAATGTATTGTAGTAATCATTCTTTTTTTTTT--AATCTCTGA 26195
Gorrilla          TGTCGCAACAGCAAAGAGTTTAATAACATTTATTTTCTAGTGTATTGCAGTAATCATTCTTCTTTTTTTT-AATTTCTAA 25117
GrabEatingMacaqueTGTCTCAAAAGCAAAGAGTTTAATAACATTTATTTGCTAATGTATTGTAGTAATCATTCTTTTTTTTA---AATCTCTGA 25042
Macaque           TGTCGCAAAAGCAAAGAGTTTAATAACATTTATTTGCTAATGTATTGTAGTAATCATTCTTTTTTTTA---AATCTCTGA 25549
PigTailedMacaque  TGTCGCAAAAGCAAAGAGTTTAATAACATTTATTTGCTAATGTATTGTAGTAATCATTCTTTTTTTTA---AATCTCTGA 24540
Bonobo            CGTCGCGACAGCAAAGAGTTTAATAACATTTATTTTCTAGTGTATTGCAGTAATCATTCTTCTTTTTTT--AATTTCTAA 23831
OliveBaboon       TGTCGCAAAAGCAAAGAGTTTAATAACATTTATTTGCTAATGTATTGTAGTAATCATTCTTTTTTTAA---AATCTCTGA 24547
Orangutan         CGTCGCAACAGCAAAGAGTTTAATAACGTTTATTTTCTGGTGTATTGCAGTAATCATTCTTTTTTTT----AATTTCTAA 24750
```

```
Majority          GCTGTTT-ATT----AAATGAAAAG----AGAACAGTGCTAAGCA----GCTTGTAT-GTGTGTGGGT------------

                          30970     30980     30990     31000     31010     31020     31030     31040

Human             GCTGTTTTATT----AAATGAAAAG----AGAACAATGCTAAGCA----GCTTGTATGGTGTGTGTGT------------ 22806
Coquerel'sSifaka  ATTGTTTCATCTTGGGTCTGCAAGATTTCAGAACACCAATAGGTTTGATACTTGCTTAAAGTATCATTTTTTGCTCCAGA 21187
Ma'sNightMonkey   GCTATTTTATT----AAATGAAAAG----AGAACAATGCTAAGCA----GCTTGTAT-------G--G------------ 20326
VervetAGM         GCTGTT---------AAATGAAAAG----AGAACAGTGCTAAGCA----GCTTGTAT--------GGT------------ 26238
Gorilla           GCTGTTTCATT----AAATGAAAAG----AGAACAATGCTAAGCA----GCTTGTATGGTGTGTGTGTG----------- 25174
GrabEatingMacaqueGCTGTT---------AAATGAAAAG----AGAACAGTGCTAAGCA----GCTTGTAT--GGGGGGGGT------------ 25091
Macaque           GCTGTT---------AAATGAAAAG----AGAACAGTGCTAAGCA----GCTTGTAT--GGGGGGGGT------------ 25598
PigTailedMacaque  GCTGTC---------AAATGAAAAG----AGAACAGTGCTAAGCA----GCTTGTATTGGGGGGGGGG------------ 24591
Bonobo            GCTGTTTTATT----AAATGAAAAG----AGAACAATGCTAAGCA----GCTTGTATGGTGTGTGTGTGTGTGT-----G 23894
OliveBaboon       GCTGTT---------AAATGAAAAG----AGAACAGTGCTAAGCA----GCTTGTAT--GGGGGGGGT------------ 24596
Orangutan         GCTATTTTATT----AAAAGAAAAG----AGAACAGTGCTAAGCA----GCTTGTATGGTGTGTGTGTG----------- 24807

Majority          -GTGTGTGTTTGTGTTTG---------TATGAATTGTTAA--AAACGCGGTGGCTCAAGCCTGTAATTCCAGCACTTTGG

                          31050     31060     31070     31080     31090     31100     31110     31120

Human             TGTGTGGGTTTTTATTTT--------GTATGAAT-GTTAA--AAACACGGTGGCTAAAGCCTGTAATCCCAGCATTTTGG 22875
Coquerel'sSifaka  CCTTTGATCAGATATTTCTTTAAAAAGTCCTGGTTTCTGGCCGGGCGCGGTGGCTCACGCCTGTAATCCTAGCACTCTGG 21267
Ma'sNightMonkey   --TATGG--TTTTTTTTG---------TGTGAAT---------------------------------------------- 20347
VervetAGM         -GTGTGT----GTGTGTG---------TATGAATTGTGAA--AAACCCGGTGACCCAAGCCTGTAATTCCAGCACTTTGG 26302
Gorilla           TGTGTGGTTTTTTTTTTTTTTTTTTTGTATGAAT-GTTAA--AAACATGGTGGCTCAAGCCTGTAATCCCAGCATTTTGG 25251
GrabEatingMacaque-GTGTGTGTCTGTGTGTG---------TATGAATTGTGAA--AAACGCGGTGACTCAAGCCTGTAATTCCAGCACTTTGG 25159
Macaque           -GTGTGTGTCTGTGTGTG---------TATGAATTGTGAA--AAACGCGGTGACTCAAGCCTGTAATTCCAGCACTTTGG 25666
PigTailedMacaque  -GTGTGTGTCTGTGTGTG---------TATGAATTGTGAA--AAACGCGGTGACTCAAGCCTGTAATTCCAGCACTTTGG 24659
Bonobo            TGTGTGGGTTTTTTTTTTTTTTT---GTATGAAT-GTTAA--AAACACGGTGGCTCAAGCCTGTAATCCCAGCATTTTGG 23968
OliveBaboon       -GTGTGTGTCTGTGTGTG---------TATGAATTGTGAA--AAACGCGGTGACTCAAGCCTGTAATTCCAGCACTTTGG 24664
Orangutan         TGTGTGTGTGTGTGTTTA---------TATGAAT-GTTAA--AAACACGGTGGCTCAAGCCTGTAATCCCAGCATTTTGG 24875

Majority          GAGGCCAAGGTGGGCGGATAAC--AAGATCAGGAGCTTGAGACCTGCCTGG--------------CC-------------

                          31130     31140     31150     31160     31170     31180     31190     31200

Human             GAGGCCAAGGTGGTTGGATCAC--AAGGTCAGGAGCTTGAGACCAGCCTGG--------------CTCTACTAAAAATAA 22939
Coquerel'sSifaka  GAGGCTGAGGCGGGAGGATTGCTTGAGCTCAGGAGTTCGAGACCAGCCTGAGCAAGAGTGAGACCCTGTCTCTACTATGA 21347
Ma'sNightMonkey   ----------------------------------------G--------------------------------------- 20348
VervetAGM         GAGGCCAAGGTGGGCAGATAAC--AAGATCAGGAGCTTGAGACCTGCCTGG--------------CC------------- 26353
Gorilla           GAGGCCAAGGTGGTTGGATCAC--AAGGTCAGGAGCTTGAGACCAGCCTGG--------------CTCTGCTAAAAATGA 25315
GrabEatingMacaqueGAGGCCAAGGTGGGCAGATAAC--AAGATCAGGAGCTTGAGACCTGCCTGG--------------CC------------- 25210
Macaque           GAGGCCAAGGTGGGCAGATAAC--AAGATCAGGAGCTTGAGACCTGCCTGG--------------CC------------- 25717
PigTailedMacaque  GAGGCCAAGGTGGGCAGATAAC--AAGATCAGGAGCTTGAGACCTGCCTGG--------------CC------------- 24710
Bonobo            GAGGCCAAGGTGGTTGGATCAC--AAGGTCAGGAGCTTGAGACCAGCCTGG--------------CTCTACTAAAAATAA 24032
OliveBaboon       GAGGCCAAGGTGGGCAGATAAC--ATGATCAGGAGCTTGAGACCTGCCTGG--------------CC------------- 24715
Orangutan         GAGGCCAAGGTGGTTGGATCAC--AAGGTCAGGAGCTTGAGACCAGCCTGG--------------C----C--------- 24926
```

```
Majority          AATGTGGTGAAACCC-CGTCTCTACTAAAAATACAGAAA-TTAGCCCTTTGTGGT-GCGCGCGCCTATAGTCCCAGCTAC

                          31210     31220     31230     31240     31250     31260     31270     31280

Human             AATGTGGTGAAACCC-CGTCTCTACTAAAAATACAAAAA-TTAGCCCTTTGTTGTTGCACGCGCCTATAGTCCCAGCTAC 23017
Coquerel'sSifaka  AATAGAAAGAAATTAGCAAAACAACTAAAAATAGAAAAAATTAGCCGGGCACAGTGGGGCGTGCCTGTATTCCCAGCTAC 21427
Ma'sNightMonkey   ---------------------------TT--------------------------------------------------- 20350
VervetAGM         AATGTGGTGAAACCC-CGTCTCTACTAAAAATACAGAAA-TTAGCCCTTTGTGGG-GCGCGCACCTGTAGTCCCAGCTAC 26430
Gorrilla          AATGTGGTGAAACCC-CGTCTCTACGAAAAATACAAAAA-TTAGCCCTTTGTTGTTGCACGCGCCTATAGTCCCAGCTAC 25393
GrabEatingMacaque AATGTGGTGAAACCC-CGTCTCTACTAAAAATACAGAAA-TTAGCCCTTTGTGGG-GCGCGCGCCTATAGTCCCAGCTAC 25287
Macaque           AATGTGGTGAAACCC-CGTCTCTACTAAAAATACAGAAA-TTAGCCCTTTGTGGG-GCGCGCGCCTATAGTCCCAGCTAC 25794
PigTailedMacaque  AATGTGGTGAAACCC-CGTCTCTACTAAAAATACAGAAA-TTAGCCCTTTGTGGG-GCGCGCGCCTGTAGTCCCAGCTAC 24787
Bonobo            AATGTGGTGAGACCC-CGTCTCTACTAAAAATACAAAAA-TTAGCCCTTTGTTGTTGCACGCGCCTATAGTCCCAGCTAC 24110
OliveBaboon       AATGTGGTGAAACCC-CGTCTCTACTAAAAATACAGAAA-TTAGCCCTTTGTGGG-GCGCGCGCCTGTAGTCCCAGCTAC 24792
Orangutan         AATGTGGTGAAACCC-CGTCTCTACTAAAAATACAAAAA-TTAAC--TTTGTGGTTGCACGTGCCTATAGTCCCAGCTAC 25002

Majority          TC-GGGAGGCTGAG-----GCGGAAAAATCGCTTGAACCTGGG---------------------------------AGAT

                          31290     31300     31310     31320     31330     31340     31350     31360

Human             TC-GGGAGGCTGAG-----GCAGAAAAATTGCTTGAACCTGGGGAGGCTGAGGCAGAAAAATTACTTGAACCCGGGAGAC 23091
Coquerel'sSifaka  CCCGGGAGGCTGAGCTGAGGCAGGAGGATCGCTTGAGCCCAGA---------------------------------AGTC 21474
Ma'sNightMonkey   -----------------------AAAAA-----------------T---------------------------------- 20356
VervetAGM         TC-GGGAGGCTGAG-----GCGGAAAAATCGCTTGAACCTGGG---------------------------------AGAT 26471
Gorrilla          TT-GGGAGGCTGAG-----GCAGAAAAATTGCTTGAACCTGGGGAGGCTGAGGCAGCAAAATTACTTGAACCCGGGAGAC 25467
GrabEatingMacaque TC-GGGAGGCTGAG-----GCGGAAAAATCGCTTGAACCTGGG---------------------------------AGAT 25328
Macaque           TC-GGGAGGCTGAG-----GCGGAAAAATCGCTTGAACCTGGG---------------------------------AGAT 25835
PigTailedMacaque  TC-GGGAGGCTGAG-----GCGGAAAAATCGCTTGAACCTGGG---------------------------------AGAT 24828
Bonobo            TC-GGGAGGCTGAG-----GCAGAAAAATTGCTTGAACCTGGGGAGGCTGAGGCAGAAAAATTACTTGAACCCGGGAGAC 24184
OliveBaboon       TC-GGGAGGCTGAG-----GCGGAAAAATCGCTTGAACCTGGG---------------------------------AGAT 24833
Orangutan         TT-GGGAGGCTGAG-----GCAGAAAAATCGCTTGAACCTGGGGAGGCTGAAGCAGAAAAATCGCTTGAACCCGGGAGAC 25076

Majority          GGAGGTTGCAGTGAGCCGAGATCGTGCCACTGCACTCCAGCCTGGGCAACAGACTGAGATTCC-ATCTC-AAAGACAAAA

                          31370     31380     31390     31400     31410     31420     31430     31440

Human             AGAGGTTGCAGCGAGCCGAGATTGCGCCACTGCACTCCAGCCTGGGCAACAGACTGAGATTCC-ATCT--AAAAACAAAA 23168
Coquerel'sSifaka  TGAGGTTGCTGTGAGCTAGGCTGATGCCATGGCACTCTAGCTCAGGCAAGAGAGTGAGACTCTTGTCTCAAAAAAAAAAA 21554
Ma'sNightMonkey   -------------------------------------------------------------------------------- 20356
VervetAGM         GGAGGTTGCAGTGAGCCGAGATCGTACCACTGCACTCCAGCCTGGGCAACAGACTGAGATTCC-ATCTC-AAAGACAAAA 26549
Gorrilla          AGAGGTTGCAGCGAGCCGAGATTGCGCCACTGCACTCCAGCCTGGGCAGCAGACTGAGATTCC-ATCTC-AAAAACAAAA 25545
GrabEatingMacaque GGAGGTTGCAGTGAGCCGAGATCGTACCACTGCACTCCAGCCTGGGCAACAGACTGAGATTCC-ATCTC-AAAGACAAAA 25406
Macaque           GGAGGTTGCAGTGAGCCGAGATCGTACCACTGCACTCCAGCCTGGGCAACAGACTGAGATTCC-ATCTC-AAAGACAAAA 25913
PigTailedMacaque  GGAGGTTGCAGTGAGCCGAGATCGTACCACTGCACTCCAGCCTGGGCAACAGACTGAGATTCC-ATCTC-AAAGACAAAA 24906
Bonobo            AGAGGTTGCAGCGAGCCGAGATTGCGCCACTGCACTCCAGCCTGGGCAACAGACTGAGATTCC-ATCTC-AAAAACAAAA 24262
OliveBaboon       GGAGGTTGCAGTGAGCCGAGATCGTACCACTGCACTCCAGCCTGGGCAACAGACTGAGATTCC-ATCTC-AAAGACAAAA 24911
Orangutan         GGAGGTTGCAGCAAGCCGAGATCGCGCCACTGCACTCCAGCCTGGGTGACAGACTGAGATTCC-ATCTC-AAAAACAAAA 25154
```

```
Majority          CCATGTATATATGTAAGTTTGAGGAAAAGAATTGTATGAAAATTCAGGATAATTAATGAAAGTAAATGAGAACTTCTTTA
                          31450     31460     31470     31480     31490     31500     31510     31520
Human             CCATGTATATATGTAAGTTTGAGGAAAAGAGTTGTATGAAAATTCAGGATAATTAATAAAAGTAAACGAGAACTTCTTTA 23248
Coquerel'sSifaka  AAAAGTCCTGATTTCTATGGGTAGGAAATGGTCCTTAGAAA--CCACAATAATACGAGTACCAAGATGCTAATTGCTTTT 21632
Ma'sNightMonkey   ----GTATATATGTAAGTTTGAGGAAAATAATTGTATGAAAATTCAGAATAA---ACGAAAGTAAACAAGAACATCTTTA 20429
VervetAGM         CCATGTATATATGTAAGTTTGAGGAAAAGAATTGTATGAAAATTCAGGATAATTAATGAAAGTAAATGAGAACCTCTTTA 26629
Gorrilla          CCATGTATGTATGTAAGTTTGAGGAAAAGAGTTGTATGAAAATTCAGGATAATTAATAAAAGTAAATGAGAACTTCTTTA 25625
GrabEatingMacaqueCCATGTATATATGTAAGTTTGAGGAAAAGAATTGTATGAAAATTCAGGATAATTAATGAAAGTAAATGAGAACTTCTTTA 25486
Macaque           CCATGTATATATGTAAGTTTGAGGAAAAGAATTGTATGAAAATTCAGGATAA----TGAAAGTAAATGAGAACTTCTTTA 25989
PigTailedMacaque  CCATGTATATATGTAAGTTTGAGGAAAAGAATTGTATGAAAATTCAGGATAATTAATGAAAGTAAATGAGAACTTCTTTA 24986
Bonobo            CCGTGTATATATGTAAGTTTGAGGAAAAGAGTTGTATGAAAATTCAGGATAATTAATAAAAGTAAACGAGAACTTCTTTA 24342
OliveBaboon       CCATGTATATATGTAAGTTTGAGGAAAAGAATTGTATGAAAATTCAGGATAATTAATGAAAGTAAATGAGAACTTCTTTA 24991
Orangutan         CCATGTATATATGTAAGTTTGAGGAAAAGAATTGTATGAAAATTCAGGATAATTGATAAAAGCAAACGAGAACTTCTTTA 25234

Majority          TATTTTCTG---------TTTTTTTTTT-------AATTTTCTCAGTAAATGTTTGAATTTTTTTAATGTAGAAAATGT
                          31530     31540     31550     31560     31570     31580     31590     31600
Human             CATATATAT---------ATATTTTTT---------AATTTTCTCAGTTAATGTTTGAATTTTTTTAACGTAGAAAATGT 23310
Coquerel'sSifaka  AGGTGGTTGTTATTTCAAGGCTCTTTTAGTGGACAGTATTTTTTAAAGATAGAAATACATTATGAGCTCGTTTGTAATTT 21712
Ma'sNightMonkey   TATTTTCTG----------GGTTGTTAAA-------AATTTTCTCAGTTAATGGTTGCATTTTTTTAATGTAAAAAATGT 20492
VervetAGM         TATTTTCTGGGTTTTTTTTTTTTTTTTTTTTTTTTTGAATTTTCTCAGTAAATGTTTGAATGTTTTTAATGTAGAAAATGT 26709
Gorrilla          TATTTTCTG---------GTTTTTTTTA-------AATTTTCTCAGTTAATGTTTGAATTTCTTTAATGTAGAAAATGT 25689
GrabEatingMacaqueTATTTTCTG---------TTTTTTTTTTTTTTTTTAAATTTTCTCAGTAAATGTTTGAATGTTTTTAATGTAGAAAATGT 25557
Macaque           TATTTTCTG---------TTTTTTTT----------AATTTTCTCAGTAAATGTTTGAATGTTTTTAATGTAGAAAATGT 26050
PigTailedMacaque  TATTTTGTT---------TTGTTTTTTTTT-----AATTTTCTCAGTAAATGTTTGAATGTTTTTAATGTAGAAAATGT 25052
Bonobo            TATATATAT---------ATATTTTT----------AATTTTCTCAGTTAATGTTTGAATTTTTTTAACGTAGAAAATGT 24403
OliveBaboon       TATTTTCTG---------TTGTTTTTTTTTT----AATTTTCTCAGTAAATGTTTGAATGTTTTTAATGTAGAAAATGT 25058
Orangutan         TGGTTTTTT---------TTTTTTTTTT-------AATTTTCTCAGTTAATGTTTGAATTTTTTTAATGTAGAAAATGT 25298

Majority          CAGTGGGGTTCTCTGGGGAGAAAGATTATAGTCCATGATTCCTCCTATTGTATAT---ATACCTGTATTAAAATATGC-A
                          31610     31620     31630     31640     31650     31660     31670     31680
Human             CAATGGGGTTATCTGGGGAGAAAGATTATAGTCCATGAGTCCCCCTGTTGTATAT---ATACCTGTATTAAAATATGC-A 23386
Coquerel'sSifaka  CTATTCATATTTAAAGTTACACAGCTTTTACTTCTATCTTTAGAAGATTTTCTATTTTATATTTCTATCTCTGTAAACTG 21792
Ma'sNightMonkey   CAGTGGGGTTATCTGGGCAGAAAGATTATAGTCCATGATGCCTCTGATTGTGTAT---ATGCCTGTATTAAAATATGC-G 20568
VervetAGM         CAGTGGGGTTCTCTGGAGAGAAAGATTATAGTATATGATTCCTCCTATTGTATAT---ATGCCTGTATTAAAATATGC-A 26785
Gorrilla          CAATGGGGTTATCTGGGGAGAAAGATTATAGTCCATGATTCCTCCTGTTGTATAT---ATACCTGTATTAAAATATGC-A 25765
GrabEatingMacaqueCAGTGGGGTTCTCTGGAGAGAAAGATTATAGTACATGATTCCTCCTATTGTGTAT---ATACCTGTATTAAAATATGC-A 25633
Macaque           CAGTGGGGTTCTCTGGAGAGAAAGATTATAGTACATGATTCCTCCTATTGTGTAT---ATACCTGTATTAAAATATGC-A 26126
PigTailedMacaque  CAGTGGGGTTCTCTGGAGAGAAAGATTATAGTACATGATTCCTCCTATGGTATAT---ATGCCTGTATTAAAATATGC-A 25128
Bonobo            CAATGGGGTTATCTGGGGAGAAAGATTGTAGTCCATGAGTCCCCCTGTTGTATAT---ATACCTGTATTAAAATATGC-A 24479
OliveBaboon       CAGTGGGGTTCTCTGGAGAGAAAGATTATAGTACATGATTCCTCCTATTGTATAT---ATGCCTGTATTAAAATATGC-A 25134
Orangutan         CAATGGGGTTATCTGGGGAGAAAGATTATAGTCCATGATTCCTCCTATTATATAT---ATACCTGTATTAAAATATGC-A 25374
```

```
Majority          ATAAATGCTATTTTTAAAAATGTCTTATTACATAGAAAAGAA-GACCAGAATACTAGCTCTCCACGTC-TATGTCTTGAT
                          31690     31700     31710     31720     31730     31740     31750     31760
Human             ATAAATGCTATTTTAAAAAATGTCTTATTACATAGAACAGAA-GACCAGAATACTAGCTCTCCACGTCTTATGTCTTGAT 23465
Coquerel'sSifaka  AAAAATCTTACTGGCATTGACATTATTACATATATAAACGAATAACTAGTTTTTTAAAAAGCAACGTC-AATATTATACT 21871
Ma'sNightMonkey   ATAAATGCTATTTTAAAAAATGTCTTATTACATAGAAAAGAA-GACCAGAATACTAGCTCTCCAGGTCTCATATCTTGAT 20647
VervetAGM         ATAAATGTGATTTTTAAAAATGTCTTATTACATAGAAAAGAA-GACCAGAATACTAGCTCTACACGT--TATGTCTTGAT 26862
Gorrilla          ATAAATGCTATTTTAAAAAATGTCTTATTACATAGAAAAGAA-GACCACAATACTAGCTCTCCACGTCTTATGTCTTGAT 25844
GrabEatingMacaque ATAAATGTGATTTTTAAAAATGTCTTATTACATAGAAAAGAA-GACCAGAATACTAGCTCTACACGT--TATGTCTTGAT 25710
Macaque           ATAAATGCGATTTTTAAAAATGTCTTATTACATAGAAAAGAA-GACCAGAATACTAGCTCTACACGT--TATGTCTTGAT 26203
PigTailedMacaque  ATAAATGCGATTTTTAAAAATGTCTTATTACATAGAAAAGAA-GACCAGAATACTAGCTCTACACGT--TATGTCTTGAT 25205
Bonobo            ATAAATGCTATTTTTAAAAATGTCTTATTACATAGAAAAGAA-GACCAGAATACTAGCTCTCCACGTCTTACGTCTTGAT 24558
OliveBaboon       ATAAATGCGATTTTTAAAAATGTCTTATTACATAGAAAAGAA-GACCAGAATACTAGCTCTACACGT--TATGTCTTGAT 25211
Orangutan         ATAAATGCTATTTTTAAAAATGTCTTATTGCATAGAAAAGAA-GACTGCAATACTAGCTCTCCACGTCTCATGTCTTGAT 25453

Majority          CA-GTGAAGATTAGCTAGCAAAAC--AACCATAACTTAGGGATAGCTTTATTTGTGGGAAGTGTCATTTAGTATGCCATG
                          31770     31780     31790     31800     31810     31820     31830     31840
Human             CA-GTGAAGATTAGCTAGCAAAAC--AACCATAACTTAGGGATAGCTTTATTTGTGGGAAGTGTCATTTAGTATGTCATG 23542
Coquerel'sSifaka  AACATTGTAATTATTGAATATAGTTTAAAGTTTTTTTGCAGTCACTTTTGTCCTTAAATTATATTCTACAAAGAGTAGAC 21951
Ma'sNightMonkey   CA-GAGAAAATTAGCTAGCGAAAC--AACCGTAACTTAGGGATAGCTTTATTTGTAGGAAGTGTTATTTAGTATGCCATG 20724
VervetAGM         CA-GTGAATATTAGCTAGCAAAAC--AACCATAACTTAGGGATAGCTTTATTTGTGGGAAGTGTCATTTAGTATGCCATG 26939
Gorrilla          CA-GTGAAGATTAGCTAGCAAAAC--AACCATAACTTAGGGATAGCTTTATTTGTGGGAAGTGTCATTTAGTATGTCATG 25921
GrabEatingMacaque CA-GTGAAGATTAGCTAGCAAAAC--AACCATAACTTAGGGATAGCTCTATTTGTGGGAAGTGTAATTCAGTATGCCATG 25787
Macaque           CA-GTGAAGATTAGCTAGCAAAAC--AACCATAACTTAGGGATAGCTTTATTTGTGGGAAGTGTAATTCAGTATGCCATG 26280
PigTailedMacaque  CA-GTGAAGATTAGCTAGCAAAAC--AACCATAACTTAGGGATAGCTTTATTTGTGGGAAGTGTAATTCAGTATGCCATG 25282
Bonobo            CA-GTGAAGATTAGCTAGCAAAAC--AACCATAACTTAGGGATAGCTTTATTTGTGGGAGGTGTCATTTAGTATGTCATG 24635
OliveBaboon       CA-GTGAAGATTAGCTAGCAAAAC--AACCATAACTTAGGGATAGCTTTATTTGTGGGAAGTGTCATTTAGTATGCCATG 25288
Orangutan         CA-GTGAAGATTAGCTAGCAAAAC--AACCATAACTTAGGGATAGCTTTATTTGTGGGAAGTGTCATTTAGTATGTCATG 25530

Majority          T---------ATTAAAAATCATTTGTAAAGTCTTTAAAAGAGCATCTTATCTTTTTT-GT-----AGGTGATAATATTG
                          31850     31860     31870     31880     31890     31900     31910     31920
Human             T---------ATTAAAAATCATTTGT--AGTCTTTCAGAGAGCATCTTATCTTTCTT-GT-----AGGTGATAGTATTG 23604
Coquerel'sSifaka  AGTATAGTACAATTACAGA--ATTTTAAAATTTATTGAAATAATTCTCTGTGTGGTTCTACTGCCAAGTTAGTAGTTAGG 22029
Ma'sNightMonkey   TTCCAGTAAATATTAAAAGTCATT-GCAAAGTGTTTAAAAGAACATCTTATCTTTTTTTGT-----AGGTAATAATATTG 20798
VervetAGM         T---------ATTAAAAATCATTTGTAAAGTCTTTAAAA--GCATCTTATCTTTTTT-GT-----AGGTGATAATATTG 27001
Gorrilla          T---------ATTAAAAATCATTTGT--AGTCTTTCAGAGAGCATCTTATCTTTCTT-GT-----AGGTGATAGTATTG 25983
GrabEatingMacaque T---------ATTAAAAATCATTTGTAAAGTCTTTAAAAGAGCATCTTATCTTTTTT-GT-----AGGTGATAATATTG 25851
Macaque           T---------ATTAAAAATCATTTGTAAAGTCTTTAAAAGAGCATCTTATCTTTTTT-GT-----AGGTGATAATATTG 26344
PigTailedMacaque  T---------ATTAAAAATCATTTGTAAAGTCTTTAAAAGAGCATCTTATCTTTTTT-GT-----AGGTGATAATATTG 25346
Bonobo            T---------ATTAAAAATCATTTGT--AGTCTTTCAGAGAGCATCTTATCTTTCTT-GT-----AGGTGATAGTATTG 24697
OliveBaboon       T---------ATTAAAAATCATTTGTAAAGTCTTTAAAAGAGCATCTTATCTTTTTT-GT-----AGGTGATAATATTG 25352
Orangutan         T---------ATTAAAAATCATTTGT--AGTCTTTCAGAGAGCATCTTATCTTTCTT-GT-----AGGTGATAGTATTG 25592
```

```
Majority          GCTATCTATGTAATGTCTTAAGTTCTTTAAATTATCCTTTTTCTTATTTTTTGTGTACATTGTTTATAAAATA----ATG
                           31930     31940     31950     31960     31970     31980     31990     32000

Human             GCTATCTATGTAGTGTCTTAAGTTCTTTAAATTATCCTTATTCTTACTTTTTGTGTACATTGTTTATAAAATA----ATG 23680
Coquerel'sSifaka  TTTGTTTTCATTTTGTGTTTAATTTTTAGGGATTTTTTTTTTTTTTTTACTTTAAAATAAATTTTCCAAAATC---AAAA 22106
Ma'sNightMonkey   GCTATCTGTGTAATGTCTTAAGTTAATTAAATTATCCTCATCTTATTTTTTTGTGTACATTGTTTATAAAATAGTTAATG 20878
VervetAGM         GCTATCTATGTAATGTCTTAAGTTCCTTAAATTATCCTTTTTCTTATTTTTTGTGTACATTGTTTATAAAATA----ATG 27077
Gorrilla          GCTATCTACGTAGTGTCTTAAGTTCTTTAAATTATCCTTATTCTTACTTTTTGTGTATATTGTTTATAAAATA----ATG 26059
GrabEatingMacaqueGCTATCTATGTAATGTCTTAAGTTCCTTAAATTATCCTTTTTCTTATTTTTTGTGTACATTGTTTATAAAATA----ATG 25927
Macaque           GCTATCTATGTAATGTCTTAAGTTCCTTAAATTATCCTTTTTCTTATTTTTTGTGTACATTGTTTATAAAATA----ATG 26420
PigTailedMacaque  GCTATCTATGTAATGTCTTAAGTTCCTTAAATTATCCTTTTTCTTATTTTTTGTGTACATTGTTTATAAAATA----ATG 25422
Bonobo            GCTATCTATGTAGTGTCTTAAGTTCTTTAAATTATCCTTATTCTTACTTTTTGTGTACATTGTTTATAAAATA----ATG 24773
OliveBaboon       GCTATCTATGTAATGTCTTAAGTTCCTTAAATTATCCTTTTTCTTATTTTTTGTGTACATTGTTTATAAAATA----ATA 25428
Orangutan         GCTATCTATGTAGTGTCTTAAGTTCTTTAAATTATCCTTATTCTTACTTTTTGTGTACATTGTTTATAAAATA----ATG 25668

Majority          TGATCATCTTAATCTACCATTTT---GA------TTATGGTTTGATCATTCTTTT--------ACTA--TTTCCACTTGT
                           32010     32020     32030     32040     32050     32060     32070     32080

Human             TGATCATCTTAATCTACCATTTT---GA------TTATGGTTTGATCATTCTTTT--------ACTA--TTTCCACTTGT 23741
Coquerel'sSifaka  CTATCATACAAGCTTATAGTTTTTCTGACAAGCTTCATAATATGCTACTTTTCTACAGAGAAATCTAGCCTTCCACTCCT 22186
Ma'sNightMonkey   TCATCATCTTAATCTACCATTTC---GA------TTGTGGTTTGATCATTCTTTT--------ACTA--TTTTTACTTGT 20939
VervetAGM         TGATCATCTTAATCTACCATTTT---GA------TTATGGTTTGATCATTCTTTT--------ACTA--TTTCCACTTGT 27138
Gorrilla          TGATCATCTTAATCTACCATTTT---GA------TTATGGTTTGATCATTCTTTT--------ACTA--TTTCCACTTGT 26120
GrabEatingMacaqueTGATCATCTTAATCTACCATTTT---GA------TTATGGTTTGATCATTCTTT---------ACTA--TTTCCATTTGT 25987
Macaque           TGATCATCTTAATCTACCATTTT---GA------TTATGGTTTGATCATTCTTT---------ACTA--TTTCCACTTGC 26480
PigTailedMacaque  TGATCATCTTAATCTACCATTTT---GA------TTATGGTTTGATCATTCTTT---------ACTA--TTTCCACTTGT 25482
Bonobo            TGATCATCTTAATCTATCATTTT---GA------TTATGGTTTGATCATTCTTTT--------ACTA--TTTCCGCTTGT 24834
OliveBaboon       TGATCATCTTAATCTACCATTTT---GA------TTATGGTTTGATCATTCTTT---------ACTA--TTTCCACTTGT 25488
Orangutan         TGATCATCTTAATCTACCATTTT---GA------TTATGGTTTGATCATTCTTTT--------ACTA--TTTCCACTTGT 25729

Majority          TAGTAAAGCCTTTGTACTTATTAAATTTTGATAAAAATGATTATTATAACATGCCTGGTTT-----TACTTAAGT--GTC
                           32090     32100     32110     32120     32130     32140     32150     32160

Human             TAGCAAAGCCTTTATACTTACTAAATTATGATAAAAATGATTATTATAACATGCCCGGTTT-----TACTTAAAT--GTC 23814
Coquerel'sSifaka  GTCTAGTCTACTTATTTTCTCTCTGCCTTTATCAGTATCTGTTTTCATAAATTTTTGGTTTATCCCTCTTTTATTCAATA 22266
Ma'sNightMonkey   TAGTAAAGCCTTTGTACCTATTACATTTTGATAAAGATGAATTTTATAACATGCCTAGTTT-----TACTTAAGT--GTC 21012
VervetAGM         TAATAAAGACTTTGTACTTATTAAATTTTGATGAAAATGATTATTATAACATGCCTGGTTT-----TACTTAAGT--GCC 27211
Gorrilla          TAGTAAAGCCTTTATACTTATTAAATTATGATAAAAATGATTATTATAACATGCCCGGTTT-----TACTTAAAT--GTC 26193
GrabEatingMacaqueTAATAAAGACTTTGTACTTATTAAATTTTGATAAAAAGGATTATTATAACATGCCTGGTTT-----TACTTAAGT--GCC 26060
Macaque           TAATAAAGACTTTGTACTTATTAAATTTTGATGAAAAGGATTATTATAACATGCCTGGTTT-----TACTTAAGT--GCC 26553
PigTailedMacaque  TAATAAAGACTTTGTACTTATTAAATTTTGGTGAAAAGGATTATTATAACATGCCTGGTTT-----TACTTAAGT--GCC 25555
Bonobo            TAGTAAAGCCTTTATACTTATTAAATTATGATAAAAATGATTATTATAACATGCCTGGTTT-----TACTTAAAT--ATC 24907
OliveBaboon       TAATAAAGACTTTGTACTTATTAAATTTTGATGA---GGATTATTATAACATGCCTGGTTT-----TACTTAAGT--GCC 25558
Orangutan         TAGTAAAGCCTTTATACTTACTAAATTTTGATAAAAATGATTATTATAACATGCCTGGTTT-----TACTTAAGT--GTC 25802
```

```
Majority          TTATTTAAACTTTCTGATTGTTATTCTTTCTTAT----TCAGGTTTCTTATTGTCATGGTTTACATC-TGTTTTCAGGTG

                          32170     32180     32190     32200     32210     32220     32230     32240

Human             TTATTTAAACGTTCTGATTGTTATTCTTTCTTAT----TCAGGTTTCTTATTGTCATGTTTTATGTC-TGTTTTCAGGTG 23889
Coquerel'sSifaka  TAATGTGAATTTCTTCCTACCCTCTGCCTCCCACAACATTAGGTGAAACGT-GGCACAATATACAGAATGTTTTATAGCT 22345
Ma'sNightMonkey   TTATTTAAATTTTCTCATTGTTATTCTTTCTTAC----TTAGGTTTCTTATTGTCATGGTTTATATC-TGTTTTCAGGTG 21087
VervetAGM         TTATTTAAACTTTCTGATTGTAATTCTT------------AGGTTTCTTATTGTCGTGGTTTACATC-TGTTTTCAGGTG 27278
Gorrilla          TTATTTAAACGTTCTGATTATTATTCTT----AT----TCAGGTTTCTTATTGTCATGGTTTATATC-TGTTTTCAGGTG 26264
GrabEatingMacaqueTTATTTAAACTTTCTGATTGTAATTCTTTCTTTG----TCAGGTTTCTTATTGTTGTGGTTTACATC-TGTTTTCAGGTG 26135
Macaque           TTATTTAAACTTTCTGATTGTAATTCTTTCTTTG----TCAGGTTTCTTATTGTTGTGGTTTACATC-TGTTTTCAGGTG 26628
PigTailedMacaque  TTATTTAAACTTTCTGATTGTAATTCTTTCTTTG----TCAGGTTTCTTATTGTTGTGGTTTACATC-TGTTTTCAGGTG 25630
Bonobo            TTATTTAAACGTTCTGATTGTTATTCTTTCTTAT----TCAGGTTTCTTATTGTCATGTTTTATGTC-TGTTTTCAGGTG 24982
OliveBaboon       TTATTTAAACTTTCTGATTGTAATTCTTTCTTTG----TCAGGTTTCTTATTGTCGTGGTTTACATC-TGTTTTCAGGTG 25633
Orangutan         TTATTTAAACTTTCTGATTGTTATTCTTTCTTAT----TCAGGTTTCTTATTATCATGGTTTACATC-TGTTTTCAGGTG 25877

Majority          TAATTGTCCTCTC-----ACAAATGTATCCCA-----TTTTGGGTGATAAAATACCTGA--TCACCTTATCCTTATAGTT

                          32250     32260     32270     32280     32290     32300     32310     32320

Human             TAATTGTCCTCTC-----ACAAATGTATCCCA-----TTTTGGGTGACAAAATACCTGA--TCACCTTATCCTTATAGTT 23957
Coquerel'sSifaka  TGGCTTTTTTACTTAATGATAAATGTATCCTGGAAATCACTACATGGCAGTATATGGAAAATTTTCTCATTTCTTCCTGC 22425
Ma'sNightMonkey   TAATAGTCCTTCC-----ACAAATGTATCCCA-----TTTTGGATGATAAAATATCTGA--TCACCTTATCCTTATAGTT 21155
VervetAGM         TAATTGTCCTCTC-----ACAAATGTATCCCA-----TTTTGGGTGATAAAATACTTGA--TCACCTTATCCTTATAGTT 27346
Gorrilla          TAATTGTCCTCTC-----ACAAATGTATCCCA-----TTTTGGGTGACAAAATACCTGA--TCACCTTATCCTTATAGTT 26332
GrabEatingMacaqueTAATTGTCCTCTC-----ACAAATGTATCCCA-----TTTTGGGTGATAAAATACCTGA--TCACCTTATCCTTATAGTT 26203
Macaque           TAATTGTCCTCTC-----ACAAATGTATCCCA-----TTTTGGGTGATAAAATACCTGA--TCACCTTATCCTTATAGTT 26696
PigTailedMacaque  TAATTGTCCTCTC-----ACAAATGTATCCCA-----TTTTGGGTGATAAAATACCTGA--TCACCTTATCCTTATAGTT 25698
Bonobo            TAATTGTCCTCTC-----ACAAATGTATCCCA-----TTTTGGGTGACAAAATACCTGA--TCACCTTATCCTTATAGTT 25050
OliveBaboon       TAATTGTCCTCTC-----ACAAATGTATCCCA-----TTTTGGGTGATAAAATACCTGA--TCACCTTATCCTTATAGTT 25701
Orangutan         TAATTGTCCTCTC-----ACAAATGTATCCCA-----TTTTGGGTGACAAAATACCTGA--TCACCTTATCCTTACAGTT 25945

Majority          TTCTCCCTAGAATCTTTTTTGTTATATTAATCAAAGTTTATT--TGTGAGAA----TGTTATGAAATATAAGAAAACGAA

                          32330     32340     32350     32360     32370     32380     32390     32400

Human             TTCTCCCTAGAATCTTTTTTGTTATATTAATCAAAGTTTATT--TGTGAGAA----TGTTATGAAATATAAGAAAATGAA 24031
Coquerel'sSifaka  TGCTGCATAGTTCTTTTAGACTGTTTTTTTCCCAGTCTCTTACTATTACAAATTATGTTATGGTGAATAGCTTTGTGCA 22505
Ma'sNightMonkey   TTCTCCCTAGAATCTTCTTTGTTATATTAATTAAAGTTTATA--TGTGAGAA----TGTTATGAAATACAAGAAAACAAA 21229
VervetAGM         TTCTCCCTAGAATCTTTTTTGTTATATTAATCGAAGTTTATT--TGTGAGAA----TGTTATGAAATGTAAGAAAACGAA 27420
Gorrilla          TTCTCTCTAGAATCTTTTTTGTTATATCAATCAAAGTTTATT--TGTGAGAA----TATTATGAAATATAAAAAAATGAA 26406
GrabEatingMacaqueTTCTCCCTAGAATCTTTTTTGTTATATTAATCAAAGTTTATT--TGTGAGAA----TGTTGTGAAATGTAAGAAAACGAA 26277
Macaque           TTCTCCCTAGAATCTTTTTTGTTATATTAATCAAAGTTTATT--TGTGAGAA----TGTTGTGAAATGTAAGAAAACGAA 26770
PigTailedMacaque  TTCTCCCTAGAATCTTTTTTGTCATATTAATCAAAGTTTATT--TGTGAGAA----TGTTGTGAAATGTAAGAAAACGAA 25772
Bonobo            TTCTCCCTAGAATCTTTTTTGTTATATTAATCAAAGTTTATT--TGTGAGAA----TGTTATGAAATATAAGAAAATGAA 25124
OliveBaboon       TTCTCCCTAGAATCTTTTTTGTTATATTAATCAAAGTTTATT--TGTGAGAA----TGTTGTGAAATGTAAGAAAACGAA 25775
Orangutan         TTCTCCCTAGAATCTTTTTTGTTATATTAATCAAAGTTTATT--TGTGAGAA----TGTTATGAAATATAAGAAAATGAA 26019
```

```
Majority          CATCATGAACCTGCCACTCAAATAAAAAA---------CTT-------GAATGCTACCAATACTGTTGTATATTCCATAT
                           32410     32420     32430     32440     32450     32460     32470     32480
Human             CATCATGAACCTGTCACTCAAATAAAAAAA--------CTT-------GAATGTTACCAATACTATTGTATATTCCATAT 24096
Coquerel'sSifaka  TAT-GTAATTTTGCTGGTATAAAAAAATACCAGTATATCTTTCAGATAGAGTCCTAAAAGTGAAATTGCTGGGTCAGTGT 22584
Ma'sNightMonkey   CATCGTGAACCTACTACTCAAATAAAAAA---------CTT-------GAATGTTACCAATATTGTTGTGTATTCCATGT 21293
VervetAGM         CATCATGAACCTGCCACTCAAATAAAAAA---------CTT-------GAATGCTCCCAATACTTTTGTATATTCCATAT 27484
Gorrilla          CATCATGAACCTGCCACTCAAATAAAAAAAA-------CTT-------GAATGTTACCAATACTATTGTATATTCCATAT 26472
GrabEatingMacaqueCATCATGAACCTGCCACTCAAATAAAAAA---------CTT-------GAATGCTACCAATACTGTTGTATATTCCATAT 26341
Macaque           CATCATGAACCTGCCACTCAAATAAAAAA---------CTT-------GAATGCTACCAATACTGTTGTATATTCCATAT 26834
PigTailedMacaque  CATCATGAACCTGCCACTCAAATAAAAAA---------CTT-------GAATGCTACCAATACTGTTGTATATTCCATAT 25836
Bonobo            CATCATGAACCTACCACTCAAATAAAAAAA--------CTT-------GAATGTTACCAATACTATTGTATATTCCATAT 25189
OliveBaboon       CATCACGAACCTGCCACTCAAATAAAAAA---------CTT-------GAATGCTACCAATACTGTTGTATATTCCATAT 25839
Orangutan         CATCATGAACCTGCCATTCAAATAAAAAAG--------CTT-------GAATGTTACCAATACTGTTGTATATTCCATAT 26084

Majority          GCAG-----CAATATTGCTTTTTTTTTTTTTTTTTTT---GAGAAAGAGTCTAGTTCTGTCACCCAGGCTGG--AGTGCA
                           32490     32500     32510     32520     32530     32540     32550     32560
Human             GCAG-----CAATATTGCTTTTTTTTTTTT--------------AGAGTCTTGCTCTGATACCCACGCTGG--AGTGCA 24154
Coquerel'sSifaka  GTAAATACATAATTTTACTAGCTATTGCTGAGTTCTTTTTGTTGTAGGAATTGTATTTGCTATCATAACAGGTAAGTTTG 22664
Ma'sNightMonkey   GCAA-----CAATATTGCTTTTT--------------------------------------------------------- 21311
VervetAGM         GCAG-----CAATATTGCTTTTTTTTTTTTTTTTTTTTTG-GAGAAAGAGTCTAGTTCTGTCACCCAGGCTGG--AGTGCA 27556
Gorrilla          GCAG-----CAATATTGCTTTTTTNNNNNNNNNNNNNN---NN---NNNNNNNNNNNNNNNNNNNNNNNNNNN--NNNNNN 26539
GrabEatingMacaqueGCAG-----CAGTATTGCTTTTTTTTTTTTTTTTT-----GGGAAAGAGTCTAGTTCTGTCACCCAGGCTGG--AGTGCA 26409
Macaque           GCAG-----CAGTATTGCTTTTTTTTTTTTTTTTTTTTT--GGGAAAGAGTCTAGTTCTGTCACCCAGGCTGG--AGTGCA 26905
PigTailedMacaque  GCAG-----CAATATTGCTTTTTTTTTTTTTTTTTTT---GAGAAAGAGTCTAGTTCTGTCACCCAGGCTGG--AGTGCA 25906
Bonobo            GCAG-----CAATATTGCTTTTTTTTTTTTTTTTT----------AGAGTCTTGCTGTGATACCCACGCTGG--AGTGCA 25252
OliveBaboon       GCAG-----CAATATTGCTTTTTTTTTTTTTTTTTTTTTTTGAGAAAGAGTCTAGTTCTGTCACCCAGGCTGG--AGTGCA 25912
Orangutan         GCAA-----CAATATTGCTTTTTTTTTTTTTTTTTTT---TAAGAAGAGTCTTGCTTTGATACCCACGCTGG--AGTGCA 26154

Majority          GTGA--TGCTATCTCAGCTCATTGCAAGCTCCGCCTCC-------CTGGTTCACACCATTCTCCTGCCTCAGCCTCC-TG
                           32570     32580     32590     32600     32610     32620     32630     32640
Human             GTGG--TGTCATCTCGGCTCACAGCAAGCTTCGCCTCC-------CTGGTTCACCCCATTCTCCTGCCTCAGCCTCC-CG 24224
Coquerel'sSifaka  GTTGCCTATTTCCCCATAGTCTATCAGGGTATATTGTCAAAAGTTTGGGGTTTTGCCAGGCAGATAGGTGAGACAGCATC 22744
Ma'sNightMonkey   -------------------------------------------------------------------------------- 21311
VervetAGM         GTGA--TGCTATCTCAGCTCATTGCAAGCTCCGCCTCC-------CTGGTTCACACCATTCTCCTGCCTCAGCCTCC-TG 27626
Gorrilla          NNNN--NNNNNNNNNNNNNNNNNNNNNNNNNNNNNNNN-------NNNNNNNNNNNNNNNNNNNNNNNNNNNNNNN-NN 26609
GrabEatingMacaqueGTGA--TGCTATCTCAGCTCATTGCAAGCTCCGCCTCC-------CTGGTTCACACCATTCTCCTGCCTCAGCCTCC-TG 26479
Macaque           GTGA--TGCTATCTCAGCTCATTGCAAGCTCCGCCTCC-------CTGGTTCACACCATTCTCCTGCCTCAGCCTCC-TG 26975
PigTailedMacaque  GTGA--TGCTATCTCAGCTCATTGCAAGCTCCGCCTCC-------CTGGTTCACACCATTCTCCTGCCTCAGCCTCC-TG 25976
Bonobo            GTGG--CGTCATCTTGGCTCACTGCAAGCTCCGCCTCC-------CTGGTTCACCCCATTCTCCTGCCTCAGCCTCC-CG 25322
OliveBaboon       GTGA--TGCTATCTCAGCTCATTGCAAGCTCCGCCTCC-------CTGGTTCACACCATTCTCCTGCCTCAGCCTCC-TG 25982
Orangutan         GTGG--CGTGATCTTGGCTCACTGCAAGATCCGCCTCC-------CTGGTTCACCCCATTCTCCTGCCTCAGCCTCC-CG 26224
```

```
Majority         CATAGCTGGGACTACAGGCGCCCACCACCACACCCGCCTAATTTTTTGTATATTTAGTAGAGGCGGGGTTTCAGCATGTT

                         32650     32660     32670     32680     32690     32700     32710     32720

Human            AGTAGCTGGGACTTCAGGCACCCGCCACCACACCCGGCTAATTTTTTGAATTTTTAGTAGAGACGGGGTTTCACCCTGTT 24304
Coquerel'sSifaka TAAATGTAGTATTAATTTTCATTGTCTTAATGTGAGCAAGGTGAAACAACTTTTCAGTTTAAAAATAATTTCTCTTACCT 22824
Ma'sNightMonkey  -------------------------------------------------------------------------------- 21311
VervetAGM        CATAGCTGGGACTACAGGCGCCCACCACCACACCCGCCTAATTTTTTGTATATT-AGTAGAGGTGGGGTTTCAGCATGTT 27705
Gorrilla         NNNNNNNNNNNNNNNNNNNNNNNNNNNNNNNNNNNNNNNNNNNNNNNNNNNNNNNNNNNNNNNNNNNNNNNNNNNNNNNN 26689
GrabEatingMacaqueCATAGCTGGGACTACAGGCGCCCACCACCACACCCGCCTAATTTTTTGTATATTTAGTAGAGGCGGGGTTTCAGCATGTT 26559
Macaque          CATAGCTGGGACTACAGGCGCCCACCACCACACCCGCCTAATTTTTTGTATATTTAGTAGAGGCGGGGTTTCAGCATGTT 27055
PigTailedMacaque CATAGCTGGGACTACAGGCGCCCACCACCACACCCGCCTAATTTTTTGTATATTTAGTAGAGGCGGGGTTTCAGCATGTT 26056
Bonobo           AGTAGCTGGGACTACAGGCACCCGCCACCACACCCGGCTAATTTTTTGAATTTTTAGTAGAGACGGGGTTTCACCCTGTT 25402
OliveBaboon      CATAGCTGGGACTACAGGCGCCCACCACCACACCCACCTAATTTTTTGTATATTTAGTAGAGGCGGGGTTTCAGCATGTT 26062
Orangutan        AGTAGCTGGGACTACAGGTGCCCACCACCACACCCGGCTAATTTTTTGAATTTTTAGTAGAGACGGGGTTTCACCCTGTT 26304

Majority         A-GCCAGGATGGTTTCT-ATCTCCTGACCTCGTTATCCGTCCGC--GTCGGCC------TCCCAAAGTGCTGGGATTATA

                         32730     32740     32750     32760     32770     32780     32790     32800

Human            A-GCCAGGATGGTTTTG-ATCTCATGACCTCGTGATCCGTCTGC--CTCGGCC------TCCCAAAGTGCTGGGATTATA 24374
Coquerel'sSifaka ATACTGGGAACTATTCTTATCTCTTG-CCTGGTTCTCTGTAGGATAACTGGTCATCTTTTTCTAAATTTTTGTAAGTCTG 22903
Ma'sNightMonkey  -------------------------------------------------------------------------------- 21311
VervetAGM        A-GCTAAGATGGTCTCT-ATCTCCTGACCTCATTATATGCCCGC--ATCGGCC------TCCCAAAGTGCTGCGATTACA 27775
Gorrilla         N-NNNNNNNTGGTTTTG-ATCTCATGACCTCGTGATCCGTCCGC--CTTGGCC------TCCCAAAGTGCTGGGATTATA 26759
GrabEatingMacaqueA-GCCAGGATGGTCTCT-ATCTCCTGACCTCATTATACGCCCGC--GTCGGCC------TCCCAGAGTGCTGGGATTACA 26629
Macaque          A-GCCAGGATGGTCTCT-ATCTCCTGACCTCATTATACGCCCGC--GTCGGCC------TCCCAGAGTGCTGGGATTACA 27125
PigTailedMacaque A-GCCAGGATGGTCTCT-ATCTCCTGACCTCATTATACGCCCGC--GTCGGCC------TCCCAAAGTGCTGGGATTACA 26126
Bonobo           A-GCCAGGATGGTTTTG-ATCTCATGACCTCGTGATCTGTCCGC--CTCGGCC------TCCCAAAGTGCTGGGATTATA 25472
OliveBaboon      A-GCCAGGATGGTCTCTTATCTCCTGACCTCATTATACGCCTGC--GTCGGCC------TCCCAAAGTGCTGGGATTACA 26133
Orangutan        A-GCCAGGATGGTTTTG-ATCTCATGACCTCGTGATCCATCCAC--CTCGGCC------TCCCAAAGTGCTGGGATTATA 26374

Majority         -GGTGTGAGCTACTGTGCCCAGCCCAATATTGCTTTTT---AATCCCCCT----TCTTACCCTGTCATGGGTAGGT----

                         32810     32820     32830     32840     32850     32860     32870     32880

Human            -GGTGTGAGCTACTGCGCCCAGCCCAATATTGCTTTTT---AATCCCCCT----TCTTACCCTGCCATGGGTAGGT---- 24442
Coquerel'sSifaka TGTTGGAAGTTAAGGTTCCCTTTTTGATCCAGTTATTTGGGAATTATTTCAAAATTTTATCTCATTAAATTAAAACACTT 22983
Ma'sNightMonkey  ----------------------------A-------------ATCTCCCT----TTTTACCCTATCATGGGTAGGT---- 21342
VervetAGM        -GGCGTGAGCCACTGCGCCCAGCCCAATATTGCTTTTT---AATTCCCCT----TCTTACCCTGTCATGGGTAGGT---- 27843
Gorrilla         -GGTGTGAGCTACTGCGCCCAGCCCAATACTGCTTTTT---AATCCCCCT----TCTTACCCTGTCATCGGTAGGT---- 26827
GrabEatingMacaque-GGCGTGAGCCACTGTGCCCAGCCCAATATTGCTTTTT---AATCCCCCT----TCTTACCCTGTCATGGGTAGGT---- 26697
Macaque          -GGCGTGAGCCACTGTGCCCAGCCCAATATTGCTTTTT---AATCCCCCT----TCTTACCCTGTCATGGGTAGGT---- 27193
PigTailedMacaque -GGTGTGAGCCACTGTGCCCAGCCCAATATTGCTTTTT---AATCCCCCT----TCTTACCCTGTCATGGGTAGGT---- 26194
Bonobo           -GGTGTGAGCTACTGCGCCCAGCCCAATATTGCTTTTT---AATCCCCCT----TCTTACCCTGTCATGGGTAGGT---- 25540
OliveBaboon      -GACGTGAGCCACTGTGCCCAGCCCAGTATTGCTTTTT---AATCCCCCT----TCTTACCCTGTCATGGGTAGGT---- 26201
Orangutan        -GGTGTGAGCTACTGCGCCCAGCCCAATATTGCTTTTT---AATCCCCCT----TCTTACCCTGTCATGGGTAGGT---- 26442
```

```
Majority         ---GGCTGCCACAACTTTTGCCTTATTTTTTCAATATGGATTTTTCATGTAAATGACAAAAATATATT-----TTTGTTT
                          32890     32900     32910     32920     32930     32940     32950     32960
Human            ---GGCTGCCACAACTTTTGCCTTATTTTTTCAATATGGATTTTTCATGTAAATGACAAAAATATATT------TAGTTT 24513
Coquerel'sSifaka TACGATTGTTCCATTTTTTTCTCTATTCTTCTCATTAAAATGTCTTATTGTAGTTGCCCAAATATTAT-AGTCTATACTT 23062
Ma'sNightMonkey  ---GACTGCCACAACTTTTCCCTTAATTTTTCAATATGGATTTTTCATGTAAATGACAAAAATATATTTAGTTTAGGGTT 21419
VervetAGM        ---GGCTGCCACAACTTTTGCCTTATTTTTTCAATATGGATTTTTCATGTAAATGACAAAAATATATT------TTGTTT 27914
Gorrilla         ---GGCTGCCACAACTTTTGCCTTATTTTTTCAATATGGATTTTTCATGTAAATGAAAAAAATATATT------TAGTTT 26898
GrabEatingMacaque---GGCTGCCACAACTTTTGCCTTATTTTTTCAATATGGATTTTTCATGTAAATGACAAAAATATATTTTGTTTTTTTT 26774
Macaque          ---GGCTGCCACAACTTTTGCCTTATTTTTTCAATATGGATTTTTCATGTAAATGACAAAAATATATTTTGTTTTTTTT 27270
PigTailedMacaque ---GGCTGCCACAACTTTTGCCTTATTTTTTCAATATGGATTTTTCATGTAAATGACAAAAATATATA-----TATATAT 26266
Bonobo           ---GGCTGCCACAACTTTTGCCTTATTTTTTCAATATGGATTATTCATGTAAATGACAAAAATATATT------TAGTTT 25611
OliveBaboon      ---GGCTGCCACAACTTTTGCCTTATTTTTTCAATATGGATTTTTCATGTAAATGACAAAAATATATTTTGT-TTTTTTT 26277
Orangutan        ---GGCTGCCACAACTTTTGCCTTATTTTTTCAATATGAATTTTTCATGTCAATGACAAAAATATATT------TAGGTT 26513

Majority         TTTTTT--AGTTTATGGAAATG-GTATGCC----CATATAATGTTTTCC----ATGTCTTGTGT---TTT-AATTCAATA
                          32970     32980     32990     33000     33010     33020     33030     33040
Human            TTTTTT--AGTTTATGGAAACG-GTATGCC----TATATAATGTTTTCC----ATGTCTTGTGT---TTT-AATTCAATA 24578
Coquerel'sSifaka CTTTATGGAATTCATTGACATATGCATCATATAGTATATGATTTTCTTTTGTGATTTCTCACGGACATTTAAATAGAATG 23142
Ma'sNightMonkey  TTTTTTTTCAGTTATGGAAATG-GTATGCC----CATATAATGTTTTCT----GTGTTTTGTGT---TTT-CATTCAATA 21486
VervetAGM        TTGTTT--AGTTTATGGAAATG-GTATGCC----CATATGATGTTTTCC----ATGTCTTGTGT---TTT-AATTCAATA 27979
Gorrilla         TTTTTT--AGTTTATGGAAATG-GTATGCC----CATATAATGTTTTCC----ATGTCTTGTGT---TTTTAATTCAATA 26964
GrabEatingMacaqueTTTTTT--AGTTTATGGAAATG-GTATGCC----CATATAATGTTTTCC----ATGTCTTGTGT---TTT-AATTCAATA 26839
Macaque          TTTTTTTTAGTTTATGGAAATG-GTATGCC----CATATAATGTTTTCC----ATGTCTTGTGT---TTT-AATTCAATA 27337
PigTailedMacaque ATTTTT--AGTTTATGGAAATG-GTATGCC----CATATAATGTTTTCC----ATGTCTTGTGT---TTT-AATTCAATA 26331
Bonobo           TTTTTT--AGTTTATGGAAATG-GTATGCC----CATATAATGTTTTCC----ATGTCTTGTGT---TTT-AATTCAATA 25676
OliveBaboon      TTTTTT--AGTTTATGGAAATG-GTATGCC----CATATAATGTTTTCC----ATGTCTTGTGT---TTT-AATTCAATA 26342
Orangutan        TTTTTT--AGTTTATGGAAATG-GTATGCC----CATATAATGTTTTCC----GTGTCTTGTGT---TTT-AATTCAATA 26578

Majority         ATTATTCTA---ATAAAT---AGCTGTATTTTTTGGATAGTTT-----TATAATA-ATCATTGCTGTGGTATACTCTTT-
                          33050     33060     33070     33080     33090     33100     33110     33120
Human            ATGATTCTA---ATAAAT---AGCCGTATTTTTTGGATAGTTT-----TATAATA-CTCATTGCTGTGATATACTCTTT- 24645
Coquerel'sSifaka GGTAAACTGCACATAACTTAAATGTGCATCAATAGGGGATTTAAAACTATGATATACCAACATAGTAGAATGCTGTGCG 23222
Ma'sNightMonkey  ATGATTCTA---ATAAAT---AGCCATATTTTGTGAGTAGTTA-----TATAATA-ATCATT---GTGGTATACTTTTT- 21550
VervetAGM        ATTATTCTA---ATAAAT---AGCTGTATTTTTTGGGTAGTTT-----TATAATA-ATCATTGCTGTGGTATACTCTTT- 28046
Gorrilla         ATGATTCTA---ATAAAT---AGCCGTATTTTTTGGATAGTTT-----TGTAATA-CTCATTGCTGTGATATACTCTT-- 27030
GrabEatingMacaqueATTATTCTA---ATAAAT---AGCTGTATTTTTTGGATAGTTT-----TATAATA-ATCATTGCTGTGGTATACTCTTT- 26906
Macaque          ATTATTCTA---ATAAAT---AGCTGTATTTTTTGGATAGTTT-----TATAATA-ATCATTGCTGTGGTATACTCTTT- 27404
PigTailedMacaque ATTATTCTA---ATAAAT---AGCTGTATTTTTTGGATAGTTT-----TATAATA-ATCATTGCTGTGGTATACTCTTT- 26398
Bonobo           ATGATTCTA---ATAA------GCCGTATTTTTTGGATAGTTT-----TATAATA-CTCATTGCTGTGATATACTCTTT- 25740
OliveBaboon      ATTATTCTA---ATAAAT---AGCTGTATTTTTTGGATGGTTT-----TATAATA-ATCATTGCTGTGGTATACTCTTT- 26409
Orangutan        ATGATTCTA---ATAAAT---AGCCGTATTTTTTGGGTAGTTT-----TATAATA-CTCATTGCTGTGATACACTCTTT- 26645
```

```
Majority          ----T-GTGAAAAT---ACCCATTTTATCTCT---TTTATTGGACATT-TGGGTTGATGGAC--ATTTGAATATTTAAAG

                          33130     33140     33150     33160     33170     33180     33190     33200

Human             ----T-GTGAAAAT---ACCCATTTTATCT-----TTTATTGGACATT-TGGGTTGATGGAC--ATTTGAATATTTAAAG 24709
Coquerel'sSifaka  GCTGTTGAAAGAATGAAACAGATTTTATATGATGATTTAAAAAAAATTCTAAGCTGTTTTCTTAATTGAAAAGAATAATG 23302
Ma'sNightMonkey   ----TTGTGGAAAT---ACTCATTTTATCTCT---TTTACTGGAAATG-TGGGTTGATGAAC--ATTTCAATATTTAAAG 21617
VervetAGM         ----T-GTGAAAAT---ACCCATTTTATCTCT---TTTATTGGACATT-TGGGTGGATGGAC--ATTTGAATATTTAAAG 28112
Gorrilla          ------GTGAAAAT---ACCCATTTTATCT-----TTTATTGGACATT-TGGGTTGATGGAC--ATTTGAATATTTAAAG 27093
GrabEatingMacaque----T-GTGAAAAT---ACCCATTTTATCTCT---TTTATTGGACATT-TGGGTTGATGGAC--ATTTGAATATTTAAAG 26972
Macaque           ----T-GTGAAAAT---ACCCATTTTATCTCT---TTTATTGGACATT-TGGGTTGATGGAC--ATTTGAATATTTAAAG 27470
PigTailedMacaque  ----T-GTGAAAAT---ACCCATTTTATCTCT---TTTATTGGACATT-TGGGTTGATGGAC--ATTTGAATATTTAAAG 26464
Bonobo            ----T-GTGAAAAT---ACCCATTTTATCT-----TTTATTGGACATT-TGGGTTGATGGAC--ATTTGAATATTTAAAG 25804
OliveBaboon       ----T-GTGAAAAT---ACCCATTTTATCTCT---TTTATTGGACATT-TGGGTTGATGGAC--ATTTGAATATTTAAAG 26475
Orangutan         ----T-GTGAAAAT---ACCCATTTTATCTCT---TTTATTGGACATT-TGGGTTGATGGAC--ATTTGAATATTTAAAG 26711

Majority          ATTTTGACTTAGGCCAGGCATGGTGGCTCACGCCTGT----AATCACAGCACTTTGGGAGGCC-AGGCGGGTGGATCAC

                          33210     33220     33230     33240     33250     33260     33270     33280

Human             ATTTTGACTTAGGCCGGGCGTGGTGGCTCAAGCTTGT----AATCCCAGCACTTTGGGAGGCC-AGGCGGGCAGATCAC 24784
Coquerel'sSifaka  CTTTTTTTGTATGACAAAAACATATACATATATCAGTTTCGGAGGAAAGAATTGTATGAAACTTAAGGATAATTAATTTT 23382
Ma'sNightMonkey   ATTTTGACTTCGGCTGGGCATGGTGGGTCACACCTGT----AATCTCAGCACTTTGGGAGGCC-AAGGAGGGTAGATCAC 21692
VervetAGM         ATTTTGACTTACGCCAGGCATGGTGGCTCATGCCTGT----AATCACAGCACTTTGGGAGACC-AGGCGGGTGGATCAC 28187
Gorrilla          ATTTTGACTTAGGCCGGGCGTGGTGGCTCAAGCTTGT----AATCCCAGCACTTTGGGAGGCC-AGGCGGGCAGATCAC 27168
GrabEatingMacaqueATTTTGACTTACGCCAGGCATGGTGGCTCACGCCTGT----AATCACAGCACTTTGGGAGGCC-AGGCGGGTGGATCAC 27047
Macaque           ATTTTGACTTACGCCAGGCATGGTGGCTCACGCCTGT----AATCACAGCACTTTGGGAGGCC-AGGCGGGTGGATCAC 27545
PigTailedMacaque  ATTTTGACTTACGCCAGGCATGGTGGCTCACGCCTGT----AATCACAGCACTTTGGGAGACC-AGGCGGGTGGATCAC 26539
Bonobo            ATTTTGACTTAGGCCGGGCGTGGTGGCTCAAGCTTGT----AATCCCAGCACTTTGGGAGGCC-AGGCGGGCAGATCAC 25879
OliveBaboon       ATTTTGACTTACGCCAGGCATGGTGGCTCACGCCTGT----AATCACAGCACTTTGGGAGGCC-AGGCGGGTGGATCAC 26550
Orangutan         ATTTTGACTTAGGCCGGGCGTGGTGGCTCAAGCCTGT----AATTCCAGCACTTTGGGAGGCC-AGGCAGGCAGATCAC 26786

Majority          AAGGTC----AGGAGTTCGAGACCAGTGTGGCCAACATAGTGAAACCCC---ATCTTTACTAAAAAT-ACAAAAATTAGC

                          33290     33300     33310     33320     33330     33340     33350     33360

Human             AAGGTC----AGGAGTTTGAGACCAGCCTGGCCAACATAGTGAAACCCCC--ATCTCTACTAAAAAT-ACCATAATTAGC 24857
Coquerel'sSifaka  GGGGTGGAAGAGAACTTCCATTTTTCATTTTTTACTTTTTTGAATTTTCTAAATTAATACTTGAATTTGTTTTAATTTTT 23462
Ma'sNightMonkey   GAGGTT----GGAAGTTCGAGACCAGCTTGGCTAACATAGTGAAACCCCT--GTCTCTACTAAAAAT-ACAAAAATTAGC 21765
VervetAGM         AAGATC----AAGAGTTCGAGACCATTGTGGCCAACATAGTGAAACCCC---ATCTTTACTAAAAAT-ACAAAAATTAGC 28259
Gorrilla          AAGGTC----AGGAGTTCGAGACCAGCCTGGCCAACATAGTGAAACCCCC--ATCTCTACTAAAAAT-ACCATAATTAGC 27241
GrabEatingMacaqueAAGGTC----AGGAGTTCGAGACCAGTGTGGCCAACATAGTGAAACCCC---ATCTTTACTAAAAAT-ACAAAAATTAGC 27119
Macaque           AAGGTC----AGGAGTTCGAGACCAGTGTGGCCAACATAGTGAAACCCC---ATCTTTACTAAAAAT-ACAAAAATTAGC 27617
PigTailedMacaque  AAGGTC----AGGAGTTCGAGACCAGTGTGGCCAACATAGTGAAACCCC---ATCTTTACTAAAAAT-ACAAAAATTAGC 26611
Bonobo            AAGGTC----AGGAGTTCGAGACCAGCCTGGCCAACATAGTGAAACCCCC--ATCTCTACTAAAAAT-ACCATAATTAGC 25952
OliveBaboon       AAGGTC----AGGAGTTCGAGACCAGTGTGGCCTACATAGTGAAACCCC---ATCTTTACTAAAAAT-ACAAAAATTAGC 26622
Orangutan         A--G---------AGTTCGAGACCAGCCTGGCCAACATAGCGAAGCCCC---GTCTCTACTAAAAAT-ACCATAATTAGC 26851
```

```
Majority          CAGGTGT-------GGTGGTGTGTGCCTGTAGTCCCAGCTACTCAGGAGGCTGAGGCAGGAGAATCGCTTGAACCTGGGA

                           33370     33380     33390     33400     33410     33420     33430     33440

Human             GGGGTAT-------GGTGGTGGGTGCCTGTAGTCCCAGCTACTCAGGAGGCTGAGACAGGAGAATCGCTTGAACCCAGGA 24930
Coquerel'sSifaka  AAAATGTCAGCAGGGGTTATATGGACAGAAAGATTATGGTCCATGGTTCTTCTTTTTTGCGTATATGCCTGTATTTAAAA 23542
Ma'sNightMonkey   TGGGTGT-------GATGGAGGGTGCCTGTATTCTCAGCTACTTGAGAGGCTGAGGCAGGAGAATTGCTTGAACCTGGGA 21838
VervetAGM         CAGGTGT-------GGTGGTGTGCGCCTGTAGTCCCAGCTACTCAGGAAACTGAGGCAGGAGAATCGCTTGAACCTGGGA 28332
Gorrilla          GGGGTAT-------GGTGGTGGGTGCCCGTAGTCCCAGCTACTCAGGAGGCTGAGACAGGAGAATCGCTTGAACCCAGGA 27314
GrabEatingMacaqueCAGGTGT-------GGTGGTGTGCGCCTGTAGTCCCAGCTACTCAGGAGACTGAGGCAGGAGAATCGCTTGAACCTGGGA 27192
Macaque           CAGGTGT-------GGTGGTGTGCGCCTGTAGTCCCAGCTACTCAGGAGACTGAGGCAGGAGAATCGCTTGAACCTGGGA 27690
PigTailedMacaque  CAGGTGT-------GGTGGTGTGCGCCTGTAGTCCCAGCTACTCAGGAGACTGAGGCCGGAGAATCGCTTGAACCTGGGA 26684
Bonobo            GGGGTAT-------GGTGGTGGGTGCCTGTAGTCCCAGCTACTCAGGAGGCTGAGACAGGAGAATCGCTTGAACCCAGGA 26025
OliveBaboon       CAGGTGT-------GGTGGTGTGCGCCTGTAGTCCCAGCTACTCAGGAGACTGAGGCAGGAGAATCGCTTGAACCTGGGA 26695
Orangutan         CGGGTAT-------GGTGGTGGGTGCCTGTAGCCCCAGCTACTCAGGAGGCTGAGACAGGAGAATCACTTGAACCCAGGA 26924

Majority          GGCGGAG-GTTGCAGTGAGCTGAGACTGCGCCATTGCACT---------CCAGCCTGGGTGACAGA---CTGAGACTCCA

                           33450     33460     33470     33480     33490     33500     33510     33520

Human             GGCGGAG-GTTGCAGTGAGCTGAGACCGCGCCATTACACT---------CCAGCCTGTGTGACAGA---CTGAGACTCCA 24997
Coquerel'sSifaka  TACACAATATTATTTTTTAAAAGTCTTTTTCCATAACTAGAAGAGATGATCAGAGTATGAGACAGAATTCTTTGATTACC 23622
Ma'sNightMonkey   GGCAGAG-GTTGCGGGGAGCTGAGGTTGCCCCATTGCACT---------CCAGCCTGGGTGACAGA---ATGAGACTACG 21905
VervetAGM         GGTGGAG-GTTGCAGTGAGCTGAGACTGCGCCTTTGCACT---------CCAGCCTGGGCGACAGA---CCGAGACTCCA 28399
Gorrilla          GGCGGAG-GTTGCAGTGAGCTGAGACTGCGCCATTACACT---------CCAGCCTGTGTGACAGA---CTGAGACTCCA 27381
GrabEatingMacaqueGGTGGAG-GTTGCAGTGAGCTGAGACTGCGCCATTGCACT---------GCAGCCTGGGCGACAGA---CTGAGACTCCA 27259
Macaque           GGTGGAG-GTTGCAGTGAGCTGAGACTGCGCCATTGCACT---------CCAGCCTGGGCGACAGA---CTGAGACTCCA 27757
PigTailedMacaque  GGTGGAG-GTTGCAGTGAGCTGAGACTGCGCCATTGCACT---------CCAGCCTGGGCGACAGA---CTGAGACTCCA 26751
Bonobo            GGCGGAG-GTTGCAGTGAGCTGAGACCATGCCATTACACT---------CCAGCCTGTGTGACAGA---CTGAGACTCCA 26092
OliveBaboon       GGTGGAG-GTTGCAGTGAGCTGAGACTGCACCATTGCACT---------CCAGCCTGGGCGACAGA---CTGAGACTCCA 26762
Orangutan         GGCGGAG-GTTGCAGTGAGCTGAGACCGCGCCATTACTCT---------CCAGCCTGTGTGACAGA---CCAAGACTCCA 26991

Majority          TCTCAAAAAAAAA-A-A-AA------------AAAAAA-AGATT--TTGACTTTAAGAACAATACTGCTGTGAACTT--T

                           33530     33540     33550     33560     33570     33580     33590     33600

Human             TCTCAAAAACAAACAAAAAAAAAC--AACAACAAAAAA-AGATT--TTGACTTTAAGAACAATACTGCTGTGAACTT--C 25070
Coquerel'sSifaka  TCTTCAGGTCTCATATCTTGTCACTGAAGATTAGAAAGCAAAATAACTAACCCTAGGGATAGCTTTGTGGGAAATGTCAT 23702
Ma'sNightMonkey   TCTCAAAAAAGAAAAAAGAA------------AAAAATAAGATT--TTGTCTTTAAGAACAATACTGCTGTGAACTT--T 21969
VervetAGM         TCTCAAAAAAAAA-------------------A--AAA--GATT--TTGACTTCAAGAACAATACTGCTGTGAACTT--T 28452
Gorrilla          TCTCAAAAACAAAAACAAAAAAAC--AACAA-AAAAAA-AGATT--TTGACTTTAAGAACAATACTGCTGTGAACTT--C 27453
GrabEatingMacaqueTCTCAAAAAAA---------------------A--AAA--GATT--TTGACTTTAAGAACAATACTGCTGTGAACTT--T 27310
Macaque           TCTCAAAAAAATA-------------------A--AAA--GATT--TTGACTTTAAGAACAATACTGCTGTGAACTT--T 27810
PigTailedMacaque  TCTCAAAAAAA-A-------------------A--AAA--GATT--TTGACTTTAAGAACAATACTGCTGTGAACTT--T 26803
Bonobo            TCTCAAAAACAAAACAAAAACAAC--AACAACAAAAAA-AGATT--TTGACTTTAAGAACAATACTGCTGTGAACTT--C 26165
OliveBaboon       TCTCAAAAAAA---------------------A--AAA--GATT--TTGACTTTGAGAACAATACTGCTGTGAACTT--T 26813
Orangutan         TCTCAAAAAAAAAAAAAAAAAAAA--AAAAAAAAAAAACAGATT--TTGACTTTAAGAACAATACTGCTGTGAACTT--T 27065
```

```
Majority          TTTGTAC-TTGTTTCCTGGTACAT------TTATGCAAGCGTTTTTGAGAGTGTATAT--------TTAAAGGTA-----
                           33610     33620     33630     33640     33650     33660     33670     33680
Human             TTTGTAC-TTGTTTCCTGGTGCAT------TTATGCAAGCGTTTTTGAGAGGGTATAT--------TTAAAGGTA----- 25130
Coquerel'sSifaka  GTAGTATGTTATGTACCAATAAATATTAAGTCATTGTAAAGTCTTTAAAAGAGCATATCATCTTCTTTGTAGGTAATAAC 23782
Ma'sNightMonkey   TTTGTAC-TTGTTTCCTGGTGCAT------TTATGCAAGTGTTTTTGAGAGTATATGT--------CTAAAGGTA----- 22029
VervetAGM         TTTGTAC-TTGTTTCCTGGTACAT------TTATGCAAGCGTTTTTGAGAGTGTATAT--------TTAAAGGTA----- 28512
Gorrilla          TTTGTAC-TTGTTTCCTGGTGCAT------TTATGCAAGCGTTTTTGAGAGGGTATAT--------TTAAAGGTA----- 27513
GrabEatingMacaqueTTTGTAC-TTGTTTCCTGGTACAT------TTATGCAAGCGTTTATGAGAGTGTATAT--------TTAAAGGTA----- 27370
Macaque           TTTGTAC-TTGTTTCCTGGTACAT------TTATGCAAGCGTTTATGAGAGTGTATAT--------TTAAAGGTA----- 27870
PigTailedMacaque  TTTGTAC-TTGTTTCCTGGTACAT------TTATGCAAGCGTTTATGAGAGTGTATAT--------TTAAAGGTA----- 26863
Bonobo            TTTGTAC-TTGTTTCCTGGTGCAT------TTATGCAAGCGTTTTTGAGAGGGTATAT--------TTAAAGGTA----- 26225
OliveBaboon       TTTGTAC-TTGTTTCCTGGTACAT------TTATGCAAGCGTTTTTGAGAGTGTATAT--------TTAAAGGTA----- 26873
Orangutan         TTTGTAC-TTGTTTCCTGGTGCAT------TTATGCAAGCGTTTTTGAGAAGGTATAT--------TTAAAGGTA----- 27125

Majority          --TAGGTATATATTGCT--AGTGTGTAAAATAAACTTTACGAGATAGCACTAGATTATTTTCCAGTGTATTGTTCCTATT
                           33690     33700     33710     33720     33730     33740     33750     33760
Human             --TAGGTATATATTACT--AGTGTGTAAAATAAACTTTATGAGACAGTACTAGATTATTTTCCAGTGTATTGTTCCTATT 25206
Coquerel'sSifaka  ATTGGCTGTCTATTTCTTAAGTTTATTAAGTTATTCTTTTTTGTATACATT-GCTTATGAAATAATGTTATATCTTAATC 23861
Ma'sNightMonkey   --TAGGTATGTATTGCT--AGTATGTAGAGTAAACTTCACGAGACAGTACTAGATTATTTTCCAGTGTATTGTCCCTATT 22105
VervetAGM         --TAGATACGTATTGCT--AGTGTGTAAAATAAACTTTACGAGATAGCACTAGATTATTTTCCAGTATATTGTTCCTATT 28588
Gorrilla          --TTGGTATATATTACT--AGTGTGTAAAATAAACTTTACGAGACAGTACTAGATTATTTTCCAGTGTATTGTTCCTATT 27589
GrabEatingMacaque--TAGATATATATTGCT--AGTGTGTAAAATAAACTTTACGAGATAGCACTAGATTATTTTCCAGTGTATTGTTCCTATT 27446
Macaque           --TAGATACATATTGCT--AGTGTGTAAAATAAACTTTACGAGATAGCACTAGATTATTTTCCAGTGTATTGTTCCTATT 27946
PigTailedMacaque  --TAGATACATATTGCT--AGTGTGTAAAATAAACTTTACGAGATAGCACTAGATTATTTTCCAGTGTATTGTTCCTATT 26939
Bonobo            --TAGGTATATATTACT--AGTGTGTAAAATAAACTTTATGAGACAGTACTAGATTATTTTCCAGTGTATTGTTCCTATT 26301
OliveBaboon       --TGGATACATATTGCT--AGTGTGTAAAATAAACTTTACGAGATAGCACTAGATTATTTTCCAGTGTTTTGTTCCTATT 26949
Orangutan         --TAGGTATATATTACT--AATGTGTAAAATAAACTTTATGAGACAGTAGTAGATTATTTTCCAGTGTGTTGTTCCTATT 27201

Majority          TATGTTCTCACCAGTAGTGCGTAGGAGTTCTTG---CTGTTTCA------GATCCCCACCAGC---ACTTGGCTAAGTAT
                           33770     33780     33790     33800     33810     33820     33830     33840
Human             TATGTTCTCACCAGTAGTGCGTAGGAGTTCTTG---CTGTTTCA------GATCCCCACCAGC---ACTTGGCTAGGTAT 25274
Coquerel'sSifaka  TACCATTTTGAGTATGGTTTGATTAAATTACTGTTTCTGCTTGATAGTGGAACCTCTACTTACTAAATTTTGGTAAATAT 23941
Ma'sNightMonkey   TATGTTCTCACCAGTAGTGCATACTAGTTCTTG---CTGTTTCA------GATCCCTACCAGC---ACTTGGCTAAGTAT 22173
VervetAGM         TATGTTCTCACCAGTAGTGCGTAGGAGTTCTTG---CTGTTTCA------GATCCCCACCAGC---ACTTGGCTAAGTA- 28655
Gorrilla          TATGTTCTCACCAGTAGTGCGTAGGAGTTCTTG---CTGTTTCA------GATCCCCACCAGC---ACTTGGCTAGGTAT 27657
GrabEatingMacaqueTATGTTCTCACCAGTAGTGCGTAGGAGTTCTTG---CTTTTTCA------GATCCCCACCAGC---ACTTGGCTAAGTA- 27513
Macaque           TATGTTCTCACCAGTAGTGCGTAGGAGTTCTTG---CTTTTTCA------GATCCCCACCAGC---ACTTGGCTAAGTA- 28013
PigTailedMacaque  TATGTTCTCACCAGTAGTGCGTAGGAGTTCTTG---CTGTTTCA------GATCCCCACCAGC---ACTTGGCTAAGTA- 27006
Bonobo            TATGTTCTCACCAGTAGTGCGTAGGAGTTCTTG---CTGTTTCA------GATCCCCACCAGC---ACTTGGCTAGGTAT 26369
OliveBaboon       TATGTTCTCACCAGTAGTGCGTAGGAGTTCTTG---CTGTTTCA------GATCCCCACCAGC---ACTTGGCTAAGTA- 27016
Orangutan         TGTGTTCTCACCAGTAGTGCGTAGGAGGTCTTG---CTGTTTCA------TATCCCCACGAGC---ACTTGGCTAGGTAT 27269
```

```
Majority         T-AATTTCTTAATTTTTACC-AATTTGGTGTGTAGTATTTTCTCTGGTCTTCCTTTGTCTTTCCCTGATTATTAATGA-G
                         33850     33860     33870     33880     33890     33900     33910     33920
Human            TTAATTTCTTAATTTTTAAC-AATTTGGTGTGTAGTATATTCTCTGGTCTTCCTTTGTCTTACCCTGATTATTAATGA-G 25352
Coquerel'sSifaka G-AATATTATAACGCATGTCTGGTTTTATTTG-AGTGTCTTATTTAAACTTATTGCCATTTCTTAGGGTTCTTATTGTCA 24019
Ma'sNightMonkey  TTAATTTCTTAATTTTTAAC-AATGTGGTGTGTTGTATCTTCTCTGGT----CTTTGTCTTTGCCTGATTATAAATGA-G 22247
VervetAGM        ---ATTTCTTAATTTTTACC-AATTTGGTGTGTAGTATTTTCTCTGGTCTTCCTTTGTCTTTCCCTGATTATTAATGA-G 28730
Gorrilla         TTAATTTCTTAATTTTTAAC-AATTTGGTGTGTAGTATATTCTCTGGTCTTCCTTTGTCTTTCCCTGATTATTAATGA-G 27735
GrabEatingMacaque---ATTTCTTAATTTTTACC-AATTTGGTGTGTAGTATTTTCTCTGGTCTTCCTTTGTCTTTCCCTGATTATTAATGA-G 27588
Macaque          ---ATTTCTTAATTTTTACC-AATTTGGTGTGTAGTATTTTCTCTGGTCTTCCTTTGTCTTTCCCTGATTATTAATGA-G 28088
PigTailedMacaque ---ATTTCTTAATTTTTACC-AATTTGGTGTGTAGTATTTTCTCTGGTCTTCCTTTGTCTTTCCCTGATTATTTATGA-G 27081
Bonobo           TTAATTTCTTAATTTTTAAC-AATTTGGTGTGTAGTATATTCTCTGGTCTTCCTTTGTCTTTCCCTGATTATTAATGA-G 26447
OliveBaboon      ---ATTTCTTAATTTTTACC-GGTTTGGTGTGTAGTATTTTCTCTGGTCTTCCTTTGTCTTTCCCTGATTATTAATGA-G 27091
Orangutan        TTAATTTCTTAATTTTTAAC-AATTTGGTGTGTAGTGTATTCTCTGGTCTTCCTTTGTCTTTCCCTGATTATTAATAA-G 27347

Majority         GTCTTACATCTTTTTTTCCCCCACATTTATGGCCAT-GGATGTTTCTCTTTTGTGAAATTCTGTGAAATGCCTATTTGTG
                         33930     33940     33950     33960     33970     33980     33990     34000
Human            GTCTTACATCTTTTTTCCCCCCACATTTATGGCCAT-GGATGTTTCTCTTTTGTGAAATTCTGTTAAATGCCTATTTGTG 25431
Coquerel'sSifaka GGTTTACATCTATTTTTAGGTGTAATTGTTCTTCACAGAAGTGTCCCATTTTGGGTGATTAAAGAGTCACCTTATCCTTA 24099
Ma'sNightMonkey  GTCTTACATCTTTTTTTCCCC-ACCTTTATGGCCTTTGGATGTTTCTGTTTCGTGAAATTCTGTGAAATGCCTATTCGTG 22326
VervetAGM        GTCTTACGTCTTTTTTTCCCCCACATTTATGGCCAT-GGATGTTTCTCTTTTGTGAAATTCTGTGAAATGCTTATTTGTG 28809
Gorrilla         GTCTTACATCTTTTTTCCCCCCGCATTTATGGCCAT-GGATGTTTCTCTTTTGTGAAATTCTGTTAAATGCCTATTTGTG 27814
GrabEatingMacaqueGTCTTACGTCTTTTTTTCCCCCACATTTATGGCCGT-GGATGTTTCTCTTTTGTGAAATTCTGTGAAATGCCTATTTGTG 27667
Macaque          GTCTTACGTCTTTTTTTCCCCCACATTTATGGCCAT-GGATGTTTCTCTTTTGTGAAATTCTGTGAAATGCCTATTTGTG 28167
PigTailedMacaque GTCTTACGTCTTTTTTTCCCCCACATTTATGGCCGT-GGATGTTTCTCTTTTGTGAAATTCTGTGAAATGCCTATTTGTG 27160
Bonobo           GTCTTACATCTTTTTTCCCCCCATATTTATGGCCAT-GGATGTTTCTCTTTTGTGAAATTCTGTTAAATGCGTATTTGTG 26526
OliveBaboon      GTCTTACGTCTTTTTTTCCCCCACATTTATGGCCAT-GGATGTTTCTCTTTTGTGAAATTCTGTGAAATGCCTATTTGTG 27170
Orangutan        GTCTTACATCTTTTTTTCCCCCACATTTATGGACAT-GGGTGTTTCTCTTCTGTGAAATTCTGTTAAATCCCTATTGGTG 27426

Majority         TTTTTTGCTTGATCAAAATATGAGTTTATTATT-TAACTGATTTTGTAGTTTTAAAAAATTGCTGGGGGCAG--TGGCTT
                         34010     34020     34030     34040     34050     34060     34070     34080
Human            TTTTTTGCTTGAT-AAAATGTGAGCTTATTATT-TAACTGATTTTGTAGTTTTAAAAAATTGCTGGGGGCG--GTGGCTT 25507
Coquerel'sSifaka TTTATTGTTTTCT---TCCTAGAATCTTTTGTTATAGCCCGCAAGATATATTTGTGAGGATACTGTGAAATATAAAATGA 24176
Ma'sNightMonkey  TTTTTTGCCTGATAAAAATTTGAGTTTATTATT-TAACTGATTTTGTAGTTTTAAAAAATTGCTGGGGGCAG------TG 22399
VervetAGM        TTTTTTGCTTGATCAAAATATGAGTTTATTATT-TAACTGATTTTGTAGTTTTAAAACATTGCTGGAGGCAG--TGGCTT 28886
Gorrilla         TTTTTTGCTTGAT-AAAATGTGAGCTTATTATT-TAACTGATTTTGTAGTTTTAAAAAATTGCTGGGGGCG--GTGGCTT 27890
GrabEatingMacaqueTTTTTTGCTTGATCAAAATATGAGTTTATTATT-TAAATGATTTTGTAGTTTTAAAAAATTGCCGGAGGCAG--TGGCTT 27744
Macaque          TTTTTTGCTTGATCAAAATATGAGTTTATTATT-TAAATGATTTTGTAGTTTTAAAAAATTGCCGGAGGCAG--TGGCTT 28244
PigTailedMacaque TTTTTTGCTTGATCAAAATATGAGTTTATTATT-TAAATGATTTTGTAGTTTTAAAAAATTGCCGGAGGCAG--TGGCTT 27237
Bonobo           TTTTTTGCTTGAT-AAAATGTGAGCTTATTATT-TAACTGATTTTGTAGTTTTAAAAAATTGCTGGGGGCG--ATGGCTT 26602
OliveBaboon      TTTTTTGCTTGATCAAAATATGAGTTTATTATT-TAACTGATTTTGTAGTTTTAAA--ATTGCCG-AGGTA------TTT 27240
Orangutan        TTTTTTGCTTGAT-AAAGTGTGAGTTTATTATT-TAACTAATTTTGTAGTTTTAAAAAATGGCTGGGGGCGCGGTGGCTT 27504
```

```
Majority          GCGTCTGTAATCTCAGCTATTTGGGAGGCTGAGGGAGGAGGATTGCTTGTGGGGCCCAGGAGTT-TGAGGCTGCAGTGAG

                           34090     34100     34110     34120     34130     34140     34150     34160

Human             GCGTCTGTAATCTCAGCTTTTTGGGAGGCTGAAGGAGGAGGATTGCTTGTGGGGCCCAGGAGTT-TGAGGCTGCAGTGAG 25586
Coquerel'sSifaka  ACACCATGAATCT--GCTACCCA--AATAAAAAACTTGAATGTTACCAGTATTATGTATATATCATATGGACCTTTTTAT 24252
Ma'sNightMonkey   CCCTCTGTAATCTTAGCTATTTGGGAGGCTGAGGCAGGAGGATTGCTTGTGGGGCCCAGGAGTT-TGAGGCTGCAGTGAG 22478
VervetAGM         GCGTCTGTAATCTCAGCTATTTGGGAGGCTGAGGGAGGAGGATTGCTTGTGGGGCCCAGGAGTT-TGAGGCTGCAGTGAG 28965
Gorrilla          GCGTCTGTAATCTCAGCTTTTTGGGAGGCTGAAGGAGGAGGATTGCTTGTGGGGCCCAGGAGTT-TGAGGCTGCAGTGAG 27969
GrabEatingMacaqueGCGTCTGTAATCTCAGCTATTTGGGAGGCTGAGGGAGGAGGATTGCTTGTGGGGCCCAGGAGTT-TGAGGCTGCAGTGAA 27823
Macaque           GCGTCTGTAATCTCAGCTATTTGGGAGGCTGAGGGAGGAGGATTGCTTGTGGGGCCCAGGAGTT-TGAGGCTGCAGTGAA 28323
PigTailedMacaque  GCGTCTGTAATCTCAGCTATTTGGGAGGCTGAGGGAGGAGGATTGCTTGTGGGGCCCAGGAGTT-TGAGGCTGCAGTGAA 27316
Bonobo            GCGTCTGTAATCTCAGCTTTTTGGGAGGCTGAAGGAGGAGGATTGCTTGTGGGGCCCAGGAGTT-TGAGGCTGCAGTGAG 26681
OliveBaboon       ATGCCTGTAATTT-AGCTATTTG--AGGCTGAGG-AGGAGGATTGCTTG--GGGCCCAGGAGTT-TGAGGCCGCAGTGA- 27312
Orangutan         GCGTCTGTAATCTCAGCTTTTTGGGAGGCTGAAGGAGGAGGATTGCTTGTGGGGCCCAGGAGTT-TGAGGCTGCAGTGAG 27583

Majority          CTATGATTGT--TCCATTGCACTCTAGCTTGGTGACAGAGCAAGACCTTGTCTGA-------AAAAA-------AAAAAA

                           34170     34180     34190     34200     34210     34220     34230     34240

Human             CTATGATCGT--TCCATTGCACTCTAGCTTGGTGACAGAGCAAGACTTTGTCT--------GAAAAA-------AAAAAA 25649
Coquerel'sSifaka  CCTCCCCCCCTTCTTACCCGACCATGGGTAGATGACTGCCATAAATTTCTTATTCTTTCCAGTATGGTTTTGTTACGCAA 24332
Ma'sNightMonkey   CTATGATCAT--TCCACTGCAGTCTAGTTTGGTGGCAGAGCAAGACCTTGTCTC--------AAAAA-----------CA 22537
VervetAGM         CTATCATTGC--TCCATTGCACTCTAGCTTGGTGACAGAGCAAGACCTTGTCTGGGGGGGAGAAAAAA------AAAAAA 29037
Gorrilla          CTATGATCGT--TCCATTGCACTCTAGCTTGGTGACAGAGCAAGACTTTGTCTGAAA---AAAAAAA-------AAAAAA 28037
GrabEatingMacaqueCTATGATTGT--TCCATTGCACTCTAGCTTGGTGACAGAGCAAGACCTTGTCTGA-------AAAAA-----------AA 27883
Macaque           CTATGATTGT--TCCATTGCACTCTAGCTTGGTGACAGAGCAAGACCTTGTCTGA-------AAAAA-----------AA 28383
PigTailedMacaque  CTATGATTGT--TCCATTGCACTCTAGCTTGGTGACAGAGCAAGACCTTGTCTGA-------AAAAA-----------AA 27376
Bonobo            CTATGATCGT--TCCATTGCACTCTAGCTTGGTGACAGAGCAAGACTTTGTCTAG------GAAAAA-------AAAAAA 26746
OliveBaboon       TGGTGATTGAAACCGATTGTGACCCCATTT----ATGGAGCAAGACCTTGTCTCA-------GCAGGT------CTTTAC 27375
Orangutan         CTATGATTGT--TCCATTGCACTCTAGCTTGGTGACAGAGCAAGACCTTGTCT---------------------GAAAAA 27640

Majority          AAAAAAGGTGTGTGTTTT--------ACGGTTAC-TGATT---GTTTTTTG-----GTTATTTGTG-TTGCTGG--TG--

                           34250     34260     34270     34280     34290     34300     34310     34320

Human             AAAAAAAGTGTGTGTTTT--------ACAGTTAC-TGATT---GTCTTTTG-----GTTATTTGTG-TTGCTGG--TG-- 25707
Coquerel'sSifaka  ATGACAAAAATGTATTTAGTTTTTTTTGAGTTGCATGAAAATGGTATACTCA-TATGTTTTCTGTGACTTGTGTTTTAAT 24411
Ma'sNightMonkey   AAAAAAAGTGTGTGTTTT--------ACAGCTAC-TGGTT---GTCTTTTG-----GTTATTTGTG-TTGCTGG--TG-- 22595
VervetAGM         AAAAAAGGTGTGTCTTTT--------AGGGTTAC-TGATT---GTTTTTTT-----GTTATTTGTG-TTGCTGG--TG-- 29095
Gorrilla          AAAAAAAGTGTGTGTTTT--------ACAGTTAC-TGATT---GTCTTTTG-----GTTATTTGTG-TTGCTGG--TG-- 28095
GrabEatingMacaqueAAAAAAGGTGTGTCTTTT--------ACGGTTAC-CG-------TTTTCTC-----ATT--------TTGCT-------- 27926
Macaque           AAAAAAGGTGTGTCTTTT--------ACGGTTAC-CGATT---GTTTTTTG-----GTTATTTGTG-TTGCTGG--TG-- 28441
PigTailedMacaque  AAAAAAGGTGTGTCTTTT--------ACGGTTAC-CGATT---GTTTTTTG-----GTTATTTGTG-TTGCTGG--TG-- 27434
Bonobo            AAAAAAAGTGTGTGTTTT--------ACAGTTAC-TGACT---GTCTTTTG-----GTTATTTGTG-TTGCTGG--TG-- 26804
OliveBaboon       CGGATTGTTTTGTTATTTTGTTGCTGGTGTTCAC-CAGTTTAGCTTTCTGTCTTTGTCACTCTTT-ATGCTGTCTTG-- 27451
Orangutan         AAAAA--GTGTGTGTTTT--------ACGGTTAC-TGATT---GTCTTTTG-----GTTATTTGTG-TTGCTAA--TG-- 27696
```

```
Majority          TCTTTTCCCAGTTTG-TAGCTTGCCTTGTCAC-TTCTTTATGCTGTCTTGTC-TGTCTGTCTCT-----TTCTCTCCTTC

                           34330     34340     34350     34360     34370     34380     34390     34400

Human             TCTTTTCCCAGTTTG-TAGCTTGCCTTGTCAC-TTCTTTATGCTGTCTTGTC-TGTCTGTCTGT-----CTGTCTCCCTC 25779
Coquerel'sSifaka  TCAATAATGATTCTAATAGCTAACCATATTTTTTGTGTGTAATTGTGTAATAATACTTTTCTATGAAAATACCCAATTTA 24491
Ma'sNightMonkey   TCTTTTCCCAGTCTT-TATGCTGTCCTGTCTTGTTCGATCGATTGATCGATC-TTCCTACCTAC-----CT--------- 22659
VervetAGM         TCTTTTCCCAGTTTG-TAGCTTGCCTTGTCAC-TTCTTTATGCTGTCTTGTCTTGACTCACTCT-----TTCTCTCTCTC 29168
Gorrilla          TCTTTTCCCAGTTTG-TAGCTTGCCTTGTCAC-TTCTTTATGCTGTCTTGTC-TGTCTGTCTGT-----CTCCCTCCCTC 28167
GrabEatingMacaque---TTTCCCAGTT-----ACTTGCCTCTTGA-----------------TGTCTTGA------------------------ 27957
Macaque           TCTTTTCCCAGTTTG-TAGCTTGCCTTGTCAC-TTCTTTATGCTGTCTTGTCTTGACTCACTCT-----TTCTCTCCTTC 28514
PigTailedMacaque  TCTTTTCCCAGTTTG-TAGCTTGCCTTGTCAC-TTCTTTATGCTGTCTTGTCTTGACTCACTCT-----TTCTCTCCTTC 27507
Bonobo            TCTTTTCCCAGTTTG-TAGCTTGCCTTGTCAC-TTCTTTATGCTGTCTTGTC-TGTCTGTCT------------------ 26863
OliveBaboon       TCTTGACTCACTTTT-CTCCTTCCTTCCTTCC-TTCCTTCCTTCCTTCCTTCCTTCCTTCCTTC-----CCTCTTCCTTC 27524
Orangutan         TCTTTTCCCAGTTGG-TAGCTTGCCTTGTCAC-TTCTTTATGCTGTCTTGTC-TGTCTGTCT------------------ 27755

Majority          CTTCCTTCCT-------------------------------------------------------------------TCC

                           34410     34420     34430     34440     34450     34460     34470     34480

Human             CCTCCCTCCCC------------------------------------------------------------------CTC 25793
Coquerel'sSifaka  TTTCTTTTCCTGGACCTTTGGGTTGGTGGACATTTGGATATTTAAAGTTTTTGACTTTAAGAACAATACTGCAGTTAACA 24571
Ma'sNightMonkey   -----------------------------------------------------------------------------TCA 22662
VervetAGM         CTTCCTTCCTC------------------------------------------------------------------NNN 29182
Gorrilla          CCTCCCNNNNN------------------------------------------------------------------NNN 28181
GrabEatingMacaque-----------------------------------------------------------------------------TCA 27960
Macaque           CTTCCTTCCTTCCTTCTTTCCTTCCTTCCTTCCTTCCTTCCTTCCTTCCTTCCTTCCTTCCTTCCTTCCT-------TCC 28587
PigTailedMacaque  CTTCCTTCCTT-------------------------------------------------CCTTCCTTCCT-------TCC 27532
Bonobo            -------------------------------------------------------------------------------- 26863
OliveBaboon       CTCCCTCCCTCCCTCCCTCCTCCCTCCCTCCCTCTCTCTTTTCTCTCTCTCTCTCTGTTTCTTTCTTTTTAGATAATTCT 27604
Orangutan         -------------------------------------------------------------------------------- 27755

Majority          TTTCTCTCTTT----------------CTCTCTCTCTTTCT--------------------------CTCTCT-----CC

                           34490     34500     34510     34520     34530     34540     34550     34560

Human             CCTCCGTCCGT----------------CTGTCCCTCCCTCC--------------------------CTCCCT-----CC 25826
Coquerel'sSifaka  TTTTTGTGTTTGTTTCCTGGTGCATTTATGCAAGAGTTTTTTTAGCTTACATATCATAAGTGTATATATTGCTAGGGTG 24651
Ma'sNightMonkey   TACCTATCTAT----------------CTATCTACCTACG---------------------------TACCT-----AC 22693
VervetAGM         NNNNNNNCTCT----------------CTCTCTCTCTCTCT--------------------------CTCTCT-----CT 29215
Gorrilla          NNNNNNNNNNN----------------NNNNNNNNNNN-NN--------------------------NNNNNN-----NN 28213
GrabEatingMacaqueTTTCTCTCTTT----------------CTCT------------------------------------------------- 27975
Macaque           TTTCTCTCTCT----------------CTCTTTCTCTCTCT--------------------------CTCTCT-----CT 28620
PigTailedMacaque  TTCCTCTCTCT----------------CTCTCTTTCTTTCT--------------------------CTCTCT-----CT 27565
Bonobo            --------------------------------------CC--------------------------CTCCCT-----CC 26873
OliveBaboon       TTTTTTTTTTTGAGACGGGAGTCTCGCTTCTGTCGCTTCCAGGCTGGAGTGCAGGGCCAGATCTCAGCTCACTGCAAGCC 27684
Orangutan         -------------------------------------------------------------------------------- 27755
```

```
Majority         CTCCCTTCCTCCCTCCCTCCCTCCCTC-----------------------------------------------------

                         34570     34580     34590     34600     34610     34620     34630     34640

Human            CTCCCTCCCTCCCTCCCTCCCTCCCTT----------------------------------------------------- 25853
Coquerel'sSifaka TGGAGTATGTTAAACTGTACAAGACAGATTGTTTTCCAGTGTATAATTCCAATTTATGTTTTCACCAGTGATGCTTAAGA 24731
Ma'sNightMonkey  CTACCTACCTACCTACCTACCTACCTA----------------------------------------------------- 22720
VervetAGM        CTCTCTT-CTTCTTCTCTCTCTCTCTC----------------------------------------------------- 29241
Gorrilla         NNNNNTCCATCCGTCCCTCCCTCCCTC----------------------------------------------------- 28240
GrabEatingMacaque-------------------------------------------------------------------------------- 27975
Macaque          CTTTCTTTCTCTCTCTCTCTCTCTCTCTTTCTTTCTCTC----------------------------------------- 28659
PigTailedMacaque CTCTCTTTCTTTCTCTCTCTCTCTCTCT------CTCTC----------------------------------------- 27598
Bonobo           CTCCCTCCCTCCCTCCCTTCCTTCCTT----------------------------------------------------- 26900
OliveBaboon      CGCCCCTGGTTTACGCCATTCTCCCTGCCTCAGCCTCCCCGAAAGTAGCTGGGATTACAGCGCCGCCACCGCCACTCGCT 27764
Orangutan        C--CCTCCCTCCCTCCCT--CTCCTTC----------------------------------------------------- 27778

Majority         --TCTCCCTTTCTTT-----------------------------------------------------------------

                         34650     34660     34670     34680     34690     34700     34710     34720

Human            --CCTTCCTTCCTTC----------------------------------------------------------------- 25866
Coquerel'sSifaka GTTCTTCCTATCCTCACTAGCACTTGGTTAAATATTTAATTTCTTAATTTTTGGCAGTCTGGTATGTAGTATCTTCTTTG 24811
Ma'sNightMonkey  --CCTACCTACATGTG---------------------------------------------------------------- 22734
VervetAGM        --TCTCTCTCTCTCT----------------------------------------------------------------- 29254
Gorrilla         --CCTCCCTCCCTTC----------------------------------------------------------------- 28253
GrabEatingMacaque---CTCTGTTTCTTT----------------------------------------------------------------- 27987
Macaque          --TCTCTGTTTCTTT----------------------------------------------------------------- 28672
PigTailedMacaque --TCTCTCTCTCTCT----------------------------------------------------------------- 27611
Bonobo           --CCTTCCTTCCTTC----------------------------------------------------------------- 26913
OliveBaboon      AGTTTTTGCATTTTTAGTAGAGACGGTTTCACCCGCTAGCCAGGATGGTCTCGATCTCCTGACCTCGTGATCCGCCCGTC 27844
Orangutan        --CCTCCCTTCCTCC----------------------------------------------------------------- 27791

Majority         ------------------------------------------------CTTTCTTCC------TTTCTACT--TTACTTT

                         34730     34740     34750     34760     34770     34780     34790     34800

Human            ------------------------------------------------CTTCCTTCC------TTCCTACT--TTACTTT 25890
Coquerel'sSifaka GTCTTTTCTTGTCTTTCCCTGATTA-----TTAAGGAGGTGTTTCATCTTTTTCCCCCACATTTGTGACCACTAGTGTT 24886
Ma'sNightMonkey  ---------------------------------------------CAGGTTTGTTACG-------TGGGTA--TGGTATG 22760
VervetAGM        ------------------------------------------------CTTTCTTTTAAATAATTTCAACT--TTACTTT 29284
Gorrilla         ------------------------------------------------CTTCCTTCC------TTCCTACT--TTACTTT 28277
GrabEatingMacaque------------------------------------------------CTTTCT---------------------GCTCT 27998
Macaque          ------------------------------------------------CTTTCTTTTAAATAATTTCAACT--TTACTTT 28702
PigTailedMacaque ------------------------------------------------CTTTCTTTTAAATAATTTCAACT--TTACTTT 27641
Bonobo           ------------------------------------------------CTTCCTTCC------TTCCTACT--TTACTTT 26937
OliveBaboon      TGGGCCTCCCAAAGTGCTGGGATTACAGGCTTGAGCCACCGTGCCCGGCCCTCTTTTAGATAATTTCAACT--TTACTTT 27922
Orangutan        ------------------------------------------------CTTCCTTCC------TTCCTACT--TTACTTT 27815
```

```
Majority          AGATTC----AGGGTTACATGTGCAGGTTTGTTACATGGGTATATGGCATGAAGCTGAAGTTTGCAATACAAGTGATCCT
                          34810     34820     34830     34840     34850     34860     34870     34880
Human             AGATTC----AGGGTTACATGTGCAGGTTTGTTACATGGGTATATGGCATGAAGCTGAGGTTTGCAATACAAGTGATCCT 25966
Coquerel'sSifaka  ACTCTTTTGTGAAATTCCATGAAATGTCTTTTTGTGTCTTTAGCTTCATAAAAATTTCAATTTATTATTTAACTGATTTT 24966
Ma'sNightMonkey   ATGTT------GAGTTACATGTGCAGGTTTGTTACATGGATATATGGTATGATGTTGAGATTTGCAATACAAGTGATCCT 22834
VervetAGM         AGATTC----AGGGTTACATGTGCAGGTTTATTATATGGGTATATGGCATGAAACTGAAGTTTGCAATACAAGTGATCCT 29360
Gorrilla          AGATTC----AGGGTTACATGTGCAGGTTTGTTACATGGGTATATGGCATGAAGCTGAGGTTTGCAATACAAGTGATCCT 28353
GrabEatingMacaqueGGGT---------------------------------------ATGATCTGAAGCTGAAGTTTT-------AGTGATC-- 28030
Macaque           AGATTC----AGGGTTACATGTGCAGGTTTATTACATGGGTATATGGCATGAAGCTGAAGTTTGCAATACAAGTGATCCT 28778
PigTailedMacaque  AGATTC----AGGGTTACATGTGCAGGTTTATTACATGGGTATATGGCATGAAGCTGAAGTTTGCAATACAAGTGATCCT 27717
Bonobo            AGATTC----AGGGTTACATGTGCAGGTTTGTTACATGGGTATATGGCATGAAGCTGAGGTTTGCAATACAAGTGATCCT 27013
OliveBaboon       AGATTC----AGGGTTACATGTGCAGGTTTATTACATGGGTATATGGTATGAAGCTGAAGTTTGCAATACAAGTGATCCT 27998
Orangutan         AGATTC----AGGGTTACATGTGCAGGTTTGTTACATGGGTATATGGCATGAAGCTGAGGTTTGCAATACAAGTGATCCT 27891

Majority          GTCAGCCAGATAGTGAGCTCAGTACCCAGCAGTTA---GATTTACAATC-CTTTCTCCCTTCCC----CTGCCACT---A
                          34890     34900     34910     34920     34930     34940     34950     34960
Human             GTCAGCCAGAGAGTGAGCTCAGTACCCAACAGTTA---GATTTACAATC-CTTTCTCCCTCCCC----CCACCACT---A 26035
Coquerel'sSifaka  GT-AGTTGTATTATGA--TTATTTTCTGTTGATTATATGTTTTGCTGTTACCTTCTCCCAGCTTGTTGCTTGCATTGTCA 25043
Ma'sNightMonkey   GTCATCCAGATAGTGAGCACAGTACCCAACAGTTA---GGTTTACAATA-CTTTCCCCCCACCCAACCCCGCCACT---A 22907
VervetAGM         GTCAGCCAGATAGTGAGCTCAGTACCCAGCAGTTA---GATTTACAATC-CTTTCTCCCTTCCC----CTGCCACT---A 29429
Gorrilla          GTCAGCCAGAGAGTGAGCTCAGTACCCAACAGTTA---GATTTACAATC-CTTTCTCCCTCCCC----CCACCACT---A 28422
GrabEatingMacaque---AGCCTGA-----------GCATCGAGCAGTTA---GATTTACAATC-CTTTCTCCCTTCCC----CTGCCACT---A 28085
Macaque           GTCAGCCAGATAGTGAGCTCAGTACCGAGCAGTTA---GATTTACAATC-CTTTCTCCCTTCCC----CTGCCACT---A 28847
PigTailedMacaque  GTCAGCCAGATAGTGAGCTCAGTACCCAGCAGTTA---GATTTACAATC-CTTTCTCCCTTCCC----CTGCCACT---A 27786
Bonobo            GTCAGCCAGAGAGTGAGCTCAGTACCCAACAGTTA---GATTTACAATC-CTTTCTCCCTCCCC----CCACCACT---A 27082
OliveBaboon       GTCAGCCAGATAGTGAGCTCAGTACCCAGCAGTTA---GATTTACAATC-CTTTCTCCCTTCCC----CTACCACT---A 28067
Orangutan         GTCAGCCAGAGAGTGAGCTCAGTACTCAACAGTTA---GATTTACAATC-CTTTCTGCCTCCCC----CCGCCTCT---A 27960

Majority          GTTCCCAGTGCT-TATTGTTGCGGATT--AATACAAATTCTTAATGTATAGAGTTTTAATGTGACCACAGATTCCCTTTG
                          34970     34980     34990     35000     35010     35020     35030     35040
Human             GTTCCCAGTGCA-TATTGTTGCGGATT--AATACAGATTCTTAATGTATAG--TTTTAATGTGGCCACAAATTCCCTTTG 26110
Coquerel'sSifaka  CTTCTCTATGCTATCTTTTGATGAATAGAAGTTATTAATTTTAATGTATAGAGTTTTAATATGGCTGCAGATTCCCTTTG 25123
Ma'sNightMonkey   GTTCCCAGTGTTATATTGTTGCGAATT--AATACACATTCTTAATGTATAGAGTGTTAATATGGCCACAAATTCCCATTG 22985
VervetAGM         GTTCCCAGTGCT-TATTGTTGCAGATT--AATACAAATTCTTAATGCATAGAGTTTTAATGTGACCACAGATTCCCTTTG 29506
Gorrilla          GTTCCCAGTGCT-TATTGTTGCGGATT--AATACAGATTCTTGATGTATAGAGTTTTAATGTGGCCACAAATTCCCTTTG 28499
GrabEatingMacaqueGTTCCCAGTGCT-TATTGTTGCAGATT--AATACAAATTCTTAATGCATAGAGTTTTAATGTGACCACAGATTCCCTTTG 28162
Macaque           GTTCCCAGTGCT-TATTGTTGCAGAT---AATACAAATTCTTAATGCATAGAGTTTTAATGTGACCACAGATTCCCTTTG 28923
PigTailedMacaque  GTTCCCAGTGCT-TATTGTTGCAGATT--AATACAAATTCTTAATGCATAGAGTTTTAATGTGACCACAGATTCCCTTTG 27863
Bonobo            GTTCCCAGTGCA-TATTGTTGCGGATT--AATACAGATTCTTAATGTATAGAGTTTTAATGTGACCACAAATTCCCTTTG 27159
OliveBaboon       GTTCCCAGTGCT-TATTGTTGCAGATT--AATACAAATTCTTAATGCATAGAGTTTTAATGTGACCACAGATTCCCTTTG 28144
Orangutan         GTTCCCAGTGCA-TATTGCTGCGGATT--AATACAGATTCTTAATGTATAGAGTTTTAATGTGGCCACAAATTCCCTTTG 28037
```

```
Majority        CATCTTAGATAAAAATCATTTATATGATCCCATTCTTTACACATTCCAAATTTTCCTTTGCTCTTGT-GGACATCTTCCA

                        35050     35060     35070     35080     35090     35100     35110     35120

Human           CATCTTAGATAAAAATAATTTATATGATCCCATTCTTTACACATTCCAAATTTTCCTTTGCTCTTCT-GGACATCTTCCA 26189
Coquerel'sSifaka CATCTTGGATAAAAATCATTTATATGATCCCATTCTTTATCTATGCTTAATTTTCCCTAGCTCCCT--GGGTATCTTC-A 25200
Ma'sNightMonkey CATCTTAGACAAAAATCATTTATATGATCCCATTCTTTATACATTCCAAATTTTCCTTTGCTCTTCT-GGACATCTTCCA 23064
VervetAGM       CATCTTAGATAAAAATCATTTATATGATCCCATTCTTTACACATTCCAAATTTTCCTTTGCTCTTGTTGGACGTCTTCCA 29586
Gorrilla        CATCTTAGATAAAAATAATTTATATGATCCCATTCTTTACACATTCCAAATTTTCCTTTGCTCTTCT-GGACATCTTCCA 28578
GrabEatingMacaqueCATCTTAGATAAAAATCATTTATATGATCCCATTCTTTACACATTCCAAATTTTCCTTTGCTCTTGTCGGACATCTTCCA 28242
Macaque         CATCTTAGATAAAAATCATTTATATGATCCCATTCTTTACACATTCCAAATTTTCCTTTGCTCTTGTCGGACATCTTCCA 29003
PigTailedMacaque CATCTTAGATAAAAATCATTTATATGATCCCATTCTTTACACATTCCAAATTTTCCTTTGCTCTTGTCGGACATCTTCCA 27943
Bonobo          CATCTTAGATAAAAATAATTTATATGATCCCATTCTTTACACATTCCAAATTTTCCTTTGCTCTTTT-GGACATCTTCCA 27238
OliveBaboon     CATCTTAGGTAAAAATCATTTATATGATCCCATTCTTTACACATTCCAAATTTTCCTTTGCTCTTGTCGGACATCTTCCA 28224
Orangutan       CATCTTAGATAAAAATAATTTATATGATCCCATTCTTTACACATTCCAAATTTTCTCTTGCTCTTGT-GGACATCTTCCA 28116

Majority        TGTTGATTGTTGGAAGTCTTGAAGGCAATCTCTAATAACAGTTTTTAGCTTTTCAACTGTTGCTGTGATACTGTTTCTTA

                        35130     35140     35150     35160     35170     35180     35190     35200

Human           TGTTGATTGCTGGAAGTCTTGAAGGCAATCCCAAATAACAGTTTTTAGCTTTTCAACTGTTGCTGTGATACGGTTTCTTA 26269
Coquerel'sSifaka TGTTAATTGTTGGAAGTCTT--------TGTCTAATAACAGTTTTTAGGTTTTCAACTGTTGTCGCAATACAGTTTCTTA 25272
Ma'sNightMonkey TGTTGATTGTTGTAAGTCTTGAAAGAAATCCCTACTAACAGTTTTTAGCTTTTCAGCTGTTGCTGTGATA--GTTTATTA 23142
VervetAGM       TGTTGATTGTTGGAATTCTTGAAGGCAATCTCTAATAACAGTTTTTAGCTCCTCAACTGTTGCTATGATACTGTTTCTTA 29666
Gorrilla        TATTTATTGCTGGAAGTCTTGAAGGCAATCCCTAATAACGGTTTTTAGCTTTTCAACTGTTGCTGTGATACGGTTTCTTA 28658
GrabEatingMacaqueTGTTGATTGTTGGAAGTCTTGAAGGCAATCTCTAATAACAGTTTTTAGCTCCTCAACTGTTGCTATGATACTGTTTCTTA 28322
Macaque         TGTTGATTGTTGGAAGTCTTGAAGGCAATCTCTAATAACAGTTTTTAGCTCCTCAACTGTTGCTATGATACTGTTTCTTA 29083
PigTailedMacaque TGTTGATTGTTGGAAGTCTTGAAGGCAATCTCTAATAACAGTTTTTAGCTCCTCAACTGTTGCTATGATACTGTTCCTTA 28023
Bonobo          TGTTGATTGCTGGAAGTCTTGAAGGCAATCCCTAATAACAGTTTTTAGCTTTTCAACTGTTGCTGTGATATGGTTTCTTA 27318
OliveBaboon     TGTTGATTGTTGGAAGTCTTGAAGGCAATCTCTAATAACAGTTTTTAGCTCCTCAACTGTTGCTATGATACTGTTTCTTA 28304
Orangutan       TGTTGATTACTGGAAGTCTTGAAGGCAATCCCTAATAACGGTTTTTAGCTTTTCAACTGTTGCTGTGATACGGTTTCTTA 28196

Majority        TAAAGAGATT----------TTTG-AGTAGAATTTATCAGTATTTTCTTTCATATTCAGTTTTCTTGTGTCTTAATTTAA

                        35210     35220     35230     35240     35250     35260     35270     35280

Human           TA--GAGATT----------TTTGTAGTAGAATTTATCAGTATTTTCTTTCATGTTCAGTTTTCTTGTGTCTTAATTTAA 26337
Coquerel'sSifaka CATGGAGATTATGTAGCTATTTTGTAGTATAATTTATCCATATTTTCTTTCATGTTTAATTTTCTCGTGTCTTAATATAA 25352
Ma'sNightMonkey TAAAGAAATT----------TTGT-AGTAGAATTTATCAGTTTTTTTCTTCATACTCAGTTTCCTTGTGTCTTAATTTAA 23211
VervetAGM       TAAAGAGATT----------TTAA-GGTAGAATTTATCAGTATGTTCTTTCATATTCAGTTTTCTTGTATCTTAATTTAA 29735
Gorrilla        TA--GAGATT----------TTTGTAGTAGAATTTATCAGTATTTTCTTTCATGTTCAGTTTTCTTGTGTCTTAATTTAA 28726
GrabEatingMacaqueTAAAGAGATT----------TTAA-GGTAGAATTTATCAGTATGTTCTTTCATATTCAGTTTTCTTGTATCTTAATTTAA 28391
Macaque         TAAAGAGATT----------TTAA-GGTAGAATTTATCAGTATGTTCTTTCATATTCAGTTTTCTTGTATCTTAATTTAA 29152
PigTailedMacaque TAAAGAGATT----------TTAA-GGTAGAATTTATCAGTATGTTCTTTCATATTCAGTTTTCTTGTATCTTAATTTAA 28092
Bonobo          TA--GAGATT----------TTTGTAGTAGAATTTATCAGTATTTTCTTTCATGTTCAGTTTTCTTGTGTCTTAATTTAA 27386
OliveBaboon     TAAAGAGATT----------TTAA-AGTAGAATTTATCAGTATGTTCTTTCATATTCAGTTTTCTTGTATCTTAATTTAA 28373
Orangutan       TAAAGAGATT----------TTTGTAGTAGAATTTATCAGTATTTTCTTTCATGTTCAGTTTTCTTGTGTCTTAATTTGA 28266
```

```
Majority          AAATCCTTACATACTCAGAGTTCATAAAGGTA--------TATTTTCTTCTGAAATTTTTAAAAATTTTGCTTTTTATGT
                          35290     35300     35310     35320     35330     35340     35350     35360
Human             AAATCCTTACATACTCAGAGTTCATAAAGATACT-----ATATTTTCTTCTGAAATTTTAAAAAATTTTGCTTTTTATAT 26412
Coquerel'sSifaka  AAAGTC-------CTTAGTGTTCATAAAGATATTTTCATATATTTTCATCTAAAATTTTTTAAAATTTTGCTTTTTACAT 25425
Ma'sNightMonkey   AAATTCTTACATACTCAGAGTTCATAAAGGTATT-----ATATTTTCTTCTGAAATTTTAAAACATTTTGCTTTTTATGT 23286
VervetAGM         AAATCCTTACATACTAAGAGTTCATAAAGGTA----------TTTTCTTCTGAAATTTTTTAAAAATTTGCTTTTTATGT 29805
Gorrilla          AAATCCTTACATACTCAGAGTTCATAAAGATACT-----ATATTTTCTTCAGAAATTTTAAAAAATTTTGCTTTTTATAT 28801
GrabEatingMacaqueAAATCCTTACATACTAAGAGTTCATAAAGGTA----------TTTTCTTCTGAAATTTTTCAAAAATTTGCCTTTTATGT 28461
Macaque           AAATCCTTACATACTAAGAGTTCATAAAGGTA----------TTTTCTTCTGAAATTTTTCAAAAATTTGCCTTTTATGT 29222
PigTailedMacaque  AAATCCTTACATAGTAAGAGTTCATAAAGGTA----------TTTTCTTCTGAAATTTTTCAAAAATTTGCTTTTTATGT 28162
Bonobo            AAATCCTTACATACTCAGAGTTCATAAAGATACT-----ATATT-----CTGAAATTTTAAAAAATTTTGCTTTTTATAT 27456
OliveBaboon       AAATCCTTACATACTAAGAGTTCATAAAGGTA----------TTTTCTTCTGAAATTTTTTAAAAATTTGCTTTTTATGT 28443
Orangutan         AAATCCTTATATACTCAGAGTTCATAAAGATA--------TATTTTCTTCTGAAATTTTAAAAAATTTTGCTTTTTATGT 28338

Majority          TTATCTCTATCAGAAAATTATTTTTGTGTATTGTATTTGGTAGGGATCTGGTTTCATTTTTTT-GCCCTTATTAAGATAG
                          35370     35380     35390     35400     35410     35420     35430     35440
Human             TTATC------AGAAAATTATTTTTGTGTATTGTATTTAGTAGGGATCTGGTTTCATTTTTTT-GCCCTTATTAAGATAG 26485
Coquerel'sSifaka  TTGTCTCT---ATCAGTTATTTTTTGTGTTTTGTATTAGGTAGGGATCCAGTTTTATTTTTTTTGCCCTTGTAGAGATAG 25502
Ma'sNightMonkey   TTATCTCTGTCAGAAAATTATTTTTGTGTATTGTATTTGGTAGGGATCCGGCTTTATTTTTT--GCCTTTATTAAAATAG 23364
VervetAGM         TTATCTCTATCAGAAAATTATTTTTGTATATTGTATTTGGTAGGGATCTGGTTTCATTTTTTT-GCCCTTATTAAGATAG 29884
Gorrilla          TTATC------AGAAAATTATTTTTGTGTATTGTATTTAGTAGGGATCTGGTTTCATTTTTTT-GCCCTTATTAAGATAG 28874
GrabEatingMacaqueTTATCTCTATCA--AAATTACTTTTGTGTATTGTATTTGGTAGGGATCTGGTTTCATTTTTTT-GCCCTTATTAAGATAG 28538
Macaque           TTATCTCTATCA--AAATTACTTTTGTGTATTGTATTTGGTAGGGATCTGGTTTCATTTTTTT-GCCCTTATTAAGATAG 29299
PigTailedMacaque  TTATCTCTATCA--AAATTATTTTTGTGTATTGTATTTGGTAGGGATCTGGTTTCATTTTTTT-GCCCTTATTAAGATAG 28239
Bonobo            TTATC------AGAAAATTATTTTTGTGTATTGTATTTAGTAGGGATCTGGTTTCATTTTTTT-GCCCTTATTAAGATAG 27529
OliveBaboon       TTATCTCTATCA--AAATTATTTTTGTGTATTGTATTTGGTAGGGATCTGGTTTCATTTTTTT-GCCCTTATTAAGATAG 28520
Orangutan         TTATC------AGAAAATTATTTTTGTGT-----ATTTAGTAGGGATCTGGTTTCATTTTTTT-GCCCTTATTAAGATAG 28406

Majority          TCAATTAAATTGAAATCTCTGTAGTGACTTTTT-------CATATTACAGGTGTATATATCATAAGGTCAAGGCTGAAAA
                          35450     35460     35470     35480     35490     35500     35510     35520
Human             TCAATTAAATTGAAATCTCTGTAATGACTTTTT-------CATATTACAGGTATATATATCGTAAGGCAAAGGCTGAAAA 26558
Coquerel'sSifaka  GCAATTAAACTGAAATGTGCAGTGCCATTTTTTTCCCCTCCACATTTCAGGTGTATATATCTTAGGGTAAAGGCTAAGCT 25582
Ma'sNightMonkey   TCAATTAAATGGAAATCTCTGTAGTGACTTTTT-------CGTAGTACAGGTGTATATATCATAAGGTGAAGGCTGCGAA 23437
VervetAGM         TCAA----ATTGAAATCTCTGTAGTGACTTTTT-------CATATTACAGGTGTGTATATCGAAAGGTCAAGGCTGAAAA 29953
Gorrilla          TCAATTAAATTGAAATCTCTGTAATGACTTTTT-------CATATTACAGGTATATATATCGTAAGGCAAAGGCTGAAAA 28947
GrabEatingMacaqueTCAA----ATTGAAATCTCTGTAGTGACTTTTT-------CATATTACAGGTGTGTATATCAAAAAGTCAAGGCTGAAAA 28607
Macaque           TCAA----ATTGAAATCTCTGTAGTGACTTTTT-------CATATTACAGGTGTGTATATCAAAAAGTCAAGGCTGAAAA 29368
PigTailedMacaque  TCAA----ATTGAAATCTCTGTAGTGACTTTTT-------CATATTACAGGTGTGTATATCAAAAAGTCAAGGCTGAAAA 28308
Bonobo            TCAATTAAATTGAAATCTCTGTAATGACTTTTT-------CATATTACAGGTATATATATCGTAAGGCAAAGGCTGAAAA 27602
OliveBaboon       TCAA----ATTGAAATCTCTGTAGTGACTTTTT-------CATATTACAGGTGTGTATATCAAAAGGTCAAGGCTGAAAA 28589
Orangutan         TCAATTAAATTGAAATCTCTGTAGTGACTTTTT-------CATATTACAGGTGTATATATCATAAGGTGAAGGCTGAAAA 28479
```

```
Majority          A--ACTATCATAACAAAATGCAGAACATAGTGGCATAAATAGACTAGAAGGTTTTTTT---------TGTTTCTTTTTCT

                          35530     35540     35550     35560     35570     35580     35590     35600

Human             A--ACTATCATAACAAAATGCAGAATATGGTGGCATAAATAAA--AGAGTTTTTT------------TGTTTCTTTTTCT 26622
Coquerel'sSifaka  ACTGTAACTATTAATAAATGCAGAGCATAGTAGCATAAACAGATTAGAAGCTTAT-------------------TTCTTT 25643
Ma'sNightMonkey   A--ACTACCATAACAAAATGCAGAACATAGTGGCATAAATAGACGAGAAGGTTTTTTTTTGTTTATTTCTTTCTTTTTCT 23515
VervetAGM         A--TCTATCATAACAAAATACAGAACATAGTGGCATAAATAGACTAGAAGGTTTTTTT---------TGTTTCTTTTTCT 30022
Gorrilla          A--ACTATCATAACAAAATGCAGAATATGGTGGCATAAATAA----GAGTTTTTT------------TGTTTCTTTTTCT 29009
GrabEatingMacaqueA--ACTATCATAACAAAATGCAGAACATAGTGGCATAAATACACTAGAAGGTTTTTTT---------TGTTTCTTTTTCT 28676
Macaque           A--ACTATCATAACAAAATGCAGAACATAGTGGCATAAATACACTAGAAGGTTTTTTT---------TGTTTCTTTTTCT 29437
PigTailedMacaque  A--ACTATCATAACAAAATGCAGAACATAGTGGCATAAATACACTAGAAGGTTTTTTT---------TGTTTCTTTTTCT 28377
Bonobo            A--ACTATCATAACAAGATGCAGAATATGGTGGCATAAATAAA--AGAGTATTTT------------TGTTTCTTTTTCT 27666
OliveBaboon       A--ACTATCATAACAAAATGCAGAACATAGTGGCATAAATAGACTAGAAGGTTTTTTT---------TGTTTCTTTTTCT 28658
Orangutan         A--ACTATCATAACAAAATGCAGAACATAGTGGCATAAATAGA--GGGTTTTTTTGTT---------TGTTTCTTTTTCT 28546

Majority          GTCATAGCAAAAGTCCTGCT---GGTATAAGAAAGTGGTATCAGTCTGCTCCATAAGG-TTATTCACTGGGCCAAGTTTC

                          35610     35620     35630     35640     35650     35660     35670     35680

Human             GTCATAGCAGAAGTCCTGTT---GGTATAAGAAAGTGGTATCAGTCTGCTCCATAAGG-TTATTCACTGGGCCAAGTTTC 26698
Coquerel'sSifaka  CTCATAGCAAAAATCTTTCTCGTGGTTTAAGAAAATGTTATCAATCTGCTCCATAAGGGTTATTCACTGGGCCAGGTTTC 25723
Ma'sNightMonkey   CTCATAGCAAAAATCCTTCT---G--ATAAGAAAGTGGTATCAATCTGCTTCATAAGG-TTATTCACTGGGCCAAGTTTC 23589
VervetAGM         GTCATAGCAAAAGTCCTGCT---GGTATAAGAAAGTGATATGAGTCTGCTCCATAAGA-TTATTCACTGGGCCAGGTTTC 30098
Gorrilla          GTCATAGCAAAAGTCCTGTT---GGTATAAGAAAGTGGTATCAGTCTGCTCCATAAGG-TTATTTACTGGGCCAAGTTTC 29085
GrabEatingMacaqueGTCATAGCAAAAGTCCTGCT---GGTATAAGAAAGTGATATGAGTCTGCTCCATAAGA-TTATTCACTGGGCCAAGTTTC 28752
Macaque           GTCATAGCAAAAGTCCTGCT---GGTATAAGAAAGTGATATGAGTCTGCTCCATAAGA-TTATTCACTGGGCCAAGTTTC 29513
PigTailedMacaque  GTCATAGCAAAAGTCCTGCT---GGTATAAGAAAGTGATATGAGTCTGCTCCATAAGA-TTATTCACTGGGCCAAGTTTC 28453
Bonobo            GTCATAGCAAAAGTCCTGTT---GGTGTAAGAAAGTGGTATCAGTCTGCTCCATAAGG-TTATTCACTGGGCCAAGTTTC 27742
OliveBaboon       GTCATAGCAAAAGTCCTGCT---GGTGTAAGAAAGTGATATGAGTCTGCTCCATAAGA-TTATTCACTGGGCCAAGTTTC 28734
Orangutan         GTCATAGCAAAAGTCCTGCT---GGTACAAGAAAGTGGTATCAATCTGCTCCATAAGG-TTATTCACTGGGCCAAGTTTC 28622

Majority          TTTCATATTGTTGCTTCACTAATCCCTAGGCTTTTGTTCTTGGCTATGTGGTCTGTTGATATGTGATTGTGCTCT---CC

                          35690     35700     35710     35720     35730     35740     35750     35760

Human             TTTCATATTGTTGCTTCACTGATCCCTAGGCTTTTGTTCTTGGCTATGTGGTCTGTTGATGTGTGATTGTGCTCTTCTCC 26778
Coquerel'sSifaka  TTTCATCTTGTTGCTTCACTAATCCCTAGGCTTTTGT-CTTTGCTGTGTGATCTGTTTCTATATAATTGTGCTGT---CC 25799
Ma'sNightMonkey   TTTCATATTGTTACTTCACTAATCCCTAGGCTTTTGTTCTTGGCTTCGTAGTCTGTTGATGTGTAATTGTGCTCT---CC 23666
VervetAGM         TTTCATATTGTTGCTTCACTAATCCCTAGGTTTTTGTTCTTGGCTAAGTGGTCTGTTGATACGTGATTGTGCTCT---CC 30175
Gorrilla          TTTCATATTGTTGCTTCACTGATCCCTAGGCTTTTGTTCTTGGCTATGTGGTCTGTTGATGTGTGATTGTGCTCTTCTCC 29165
GrabEatingMacaqueTTTGATATTGTTGCTTCACTAATCCCTAGGCTTTTGTTCTTGGCTATGTGATCTGTTGATACGTGATTGTGCTCT---CC 28829
Macaque           TTTGATATTGTTGCTTCACTAATCCCTAGGCTTTTGTTCTTGGCTATGTGATCTGTTGATACGTGATTGTGCTCT---CC 29590
PigTailedMacaque  TTTGATATTGTTGCTTCACTAATCCCTAGGCTTTTGTTCTTGGCTATGTGATCTGTTGATACGTGATTGTGCTCT---CC 28530
Bonobo            TTTCATATTGTTGCTTCACTGATCCCTAGGCTTTTGTTCTTGGCTATGTGGTCTGTTGATGTGTGATTGTGCTCTTCTCC 27822
OliveBaboon       TTTGATATTGTTGCTTCACTAATCCCTAGGCTTTTGTTCTTGGCTCTGTGGTCTGTTGATACGTGATTGTGCTCT---CC 28811
Orangutan         TTTCATATTGTTGCTTCACTGATCACTAGGCTTTTGTTCTTGGCTATGTGGTCTGTTGATGTGTGATTGTGCTCT---CC 28699
```

```
Majority          TTGTTTTTAAAAAATGTTTTCCCTGTAGTTGTCT-ATTTCGTTGTCTTTTTTT--AAAAAAAAGTTAGCTTTTGATTT-T

                          35770     35780     35790     35800     35810     35820     35830     35840

Human             TTGTTTAAAAAAAAAGTTTTCCCTGTATTTGTGT-ATTTCATTGTCTTTTTTT----TTTAAAGTCAGCTTTTGATTT-T 26852
Coquerel'sSifaka  TAATTTTTTTTTTTATCTCCCTGTAGGTTTGTCT-ATTTATTGTCTTTTTTTTTCTTTTAAAGTTAGCTTTTGGTTTAT 25878
Ma'sNightMonkey   TTGTTTAAAAACAAGTTTTCCCTGTAGTTGTCTTATTTTGTCTTTTTTTTTTTAAAAAAAAGTCAGCTTTTGACTT-T 23745
VervetAGM         TTGTTTTTAAAAAATGTTTTCCCTGTAGTTTTCT-ATTTCGTTGTCTTTTTTT--AAAAAAAAGTTAGCTTTTGATTT-T 30251
Gorrilla          TTGTTTAAAAAAAAAGTTTTCCCTGTAGTTGTCT-ATTTCGTTGTCTTTTTTT----TTAAAAGTCAGCTTTTGATTT-T 29239
GrabEatingMacaqueTTGTTTTTAAAAAATGTTTTCCCTGTAGTTTTCT-ATTTCGTTGTCTTTTTTAA-AAAAAAAAGTTAGCTTTTGATTT-T 28906
Macaque           TTGTTTTTAAAAAATGTTTTCCCTGTAGTTTTCT-ATTTCGTTGTCTTTTTTAA-AAAAAAAAGTTAGCTTTTGATTT-T 29667
PigTailedMacaque  TTGTTTTTAAAAAATGTTTTCCCTGTAGTTTTCT-ATTTCGTTGTCTTTTTTTT-AAAAAAAAGTTAGCTTTTGATTT-T 28607
Bonobo            TTGTTTAAAAAAAAAGTTTTCCCTGTATTTGTGT-ATTTCATTGTCTTTTTTA----AAAAAAGTCAGCTTTTGATTT-T 27896
OliveBaboon       TTGTTTTTAAAAAATGTTTTCCCTGTAGTTTTCT-ATTTCATTGTCTTTTTTTA-AAAAAAAAGTTAGCTTTTGATTT-T 28888
Orangutan         TTGTTTAAAAAAAGTGTTTTCCCTGTAGTTGTCT-ATTTTGTTGTCTTTTTTG----AAAAAAGTCAGCTTTTGATTT-T 28773

Majority          ACTGATC--------CTCTTCATTGTAC----CATTGTTTTCTCTATTTTTATTGGTTGCATGAGTCAGGGCTTTTGCAA

                          35850     35860     35870     35880     35890     35900     35910     35920

Human             ACTGATC--------CTCTTCATTGTAC----CATTGTTTTCTCTATTTTTATTGGTTGCGTGAGTCAGGGCTTTTGCAA 26920
Coquerel'sSifaka  ACTAAGCAGACAAGACATGGTATTCTTCAGTGTATTACTGTTTCTGTTTTTATTGGTTGTGTGAGTTAGTGCTTTC-CCA 25957
Ma'sNightMonkey   TTTGATC--------GTCTTCATTGTCC----CATTGTTTTCTCTATTTTTATTGGGTGTGTGAATCAGTGCTTTTGCAA 23813
VervetAGM         ACAGATC--------CTCTTCATTATAC----CATTGTCTTCTCTATTTTTATTTGTTGCATGAGTCAGGGCTTTTGCAA 30319
Gorrilla          ACTGATC--------CTCTTCATTGTAC----CATTGTTTTCTCTATTTTTATTGGTTGCGTGAGTCAGGACTTTTGCAA 29307
GrabEatingMacaqueACAGATC--------CTCTTCATTAT---------TGTCTTCTCTATTTTTATTTGTTGCATGAGTCAGGGCTTTTGCAA 28969
Macaque           ACAGATC--------CTCTTCATTAT---------TGTCTTCTCTATTTTTATTTGTTGCATGAGTCAGGGCTTTTGCAA 29730
PigTailedMacaque  ACAGATC--------CTCTTCATTATAC----CATTGTCTTCTCTATTTTTATTTGTTGCATGAGTCAGGGCTTTTGCAA 28675
Bonobo            ACTGATC--------CTCTTCATTGTAC----CATTGTTTTCTCTATTTTTATTGGTTGCGTGAGTCAGGGCTTTTGCAA 27964
OliveBaboon       ACAGATC--------CTCTTCATTATACA-TACATTGTCTTCTCTATTTTTATTTGTTGCATGAGTCAGGGCTTTTGCAA 28959
Orangutan         ACTGATC--------CTCTTCATTGTAC----CATTGTTTTCTCTATTTTTATTGGTTGCATGAGTCAGGGCTTTTGCAA 28841

Majority          GCCTTTAATAGGTAGGTGGTATTCA------------------------------------------------GTCCACC

                          35930     35940     35950     35960     35970     35980     35990     36000

Human             GTCTTTAATAGGTAGGTGGTATTCA------------------------------------------------GTCCACT 26952
Coquerel'sSifaka  GCCTTTAATAGGTAGGTAGCACTCA------------------------------------------------GCCTACC 25989
Ma'sNightMonkey   GCCTTTAATAGGTAGGTAGCATTTACACTTGGTCTTAACCAAAAGGCCAAGAAGCGATGGTAGGTAGCATTTAGTCCACC 23893
VervetAGM         GCCTTTAATAGGTAGGTGGTATTCA------------------------------------------------GTCCACC 30351
Gorrilla          GTCTTTAATAGGTAGGTGGTATTCA------------------------------------------------GTCCACT 29339
GrabEatingMacaqueGCCTTTAATAGGTAGGTGGTATTCA------------------------------------------------GTCCACC 29001
Macaque           GCCTTTAATAGGTAGGTGGTATTCA------------------------------------------------GTCCACC 29762
PigTailedMacaque  GCCTTTAATAGGTAGGTGGTATTCA------------------------------------------------GTCCACC 28707
Bonobo            GTCTTTAATAGGTAGGTGGTATTCA------------------------------------------------GTCCACT 27996
OliveBaboon       GCCTTTAATAGGTAGGTGGTATTCA------------------------------------------------GTCCACC 28991
Orangutan         GCCTTTAATAGGTAGGTGGTATGCA------------------------------------------------CCCCACG 28873
```

```
Majority          TCTTCATCATCTTTGTGGCACCTCTGT--TTAAGGAGACCAGGAATATTCACCTGGGTACGTACCTTTCTTCCTCTCTGA

                          36010     36020     36030     36040     36050     36060     36070     36080

Human             TCTTCATCATCTTGGTGGCACCTCTGT--TTAAGGAGACCAGGAATACTCACCTGGGTACGTACCTTTCTTCCTCTCTGA 27030
Coquerel'sSifaka  TCTTCATTTCCTTGGTAGCACCTCTGTGTTTAAGGAGTCCAGGA-TAATCATGTCGGTGCATACCTTCCTCCATCTTGGA 26068
Ma'sNightMonkey   TCTTCATCACCTTCGTTGCACCTCTGTGTTTAAGGAGAACAGAAATACCCACCTGGGTACGTATATTTCTTCCTCTCTGA 23973
VervetAGM         TCTTCATCATCTTTGTGGCACCTCTGT--TTAAGGAGACCAGGAATATTCACCTAGGTACGTACCTTTCTTCCTCTCTGA 30429
Gorrilla          TCTTCATCATCTTGGTGGCACCTCTGT--TTAAGGAGACCAGGAATACTCACCTGGGTACGTACCTTTCTTCCTCTCTGA 29417
GrabEatingMacaqueTCTTCATCATCTTTGTGGCACCTCTGT--TTAAGGAGACCAGGAATATTCACCTAGGTACGTACCTTTCTTCCTCTCTGA 29079
Macaque           TCTTCATCATCTTTGTGGCACCTCTGT--TTAAGGAGACCAGGAATATTCACCTAGGTACGTACCTTTCTTCCTCTCTGA 29840
PigTailedMacaque  TCTTCATCATCTTTGTGGCACCTCTGT--TTAAGGAGACCAGGAATATTCACCTAGGTACGTACCTTTCTTCCTCTCTGA 28785
Bonobo            TCTTCATCATCTTGGTGGCACCTCTGT--TTAAGGAGACCAGGAATACTCACCTGGGTACGTACCTTTCTTCCTCTCTGA 28074
OliveBaboon       TCTTCATCATCTTTGTGGCACCTCTGT--TTAAGGAGACCAGGAATATTCACCTAGGTATGTACCTTTCTTCCTCTCTGA 29069
Orangutan         TCTTCATCATCTTGGTGGCACCTCTGT--TTAAGGAGACCAGGAATACTCACCTGGGTACGCACCTTTCTTCCTCTCTGA 28951

Majority          GAATCTGTTTTTCCTCAGGAACTTATTATTTGTGTCCTTGCCCTGTGTCTTTCTGTCATGGTTCCCTAGGCTGACATCTC

                          36090     36100     36110     36120     36130     36140     36150     36160

Human             TAATCTATTTTTCCTCAGGAACTTAATATTTGTGTCCTTGCCCGGTGTCTTTCTGTCATGGTTCCCTAGGCTGCCATCTC 27110
Coquerel'sSifaka  GAATATGGTTTTCCTTAGGAACTTGATACTCATGTCCTTG-GCCATGTTTTTCGGTCGTGGTTCCCTAGGTTGACATCTC 26147
Ma'sNightMonkey   AAATCTACTTTTCCTCAGGAACTTACTATTCATGTCCTTGCGCTGTGTCTTTCTGTCATGGTTCCCTAGGCTGACATCTC 24053
VervetAGM         GAATGTGTTTTTCCTCAGGAACTTATTATTTGTGTCCTTGCCCTGTGTCTTCCTCTCATGGTTCCCTAGGCTGACATCTC 30509
Gorrilla          TAATCTATTTTTCCTCAGGAACTTAATATTTGTGTCCTTGCCCTGTGTCTTTCTGTCATGGTTCCCTAGGCTGCCATCTC 29497
GrabEatingMacaqueGAATCTGTTTTTCCTCAGGAACTTATTATTTGTGTCCTTGCCCTGTGTCTTTCTCTCATGGTTCCCCAGGCTGACATCTC 29159
Macaque           GAATCTGTTTTTCCTCAGGAACTTATTATTTGTGTCCTTGCCCTGTGTCTTTCTCTCATGGTTCCCCAGGCTGACATCTC 29920
PigTailedMacaque  GAATCTGTTTTTCCTCAGGAACTTATTATTTGTGTCCTTGCCCTGTGTCTTTCTCTCATGGTTCCCCAGGCTGACATCTC 28865
Bonobo            TAATCTATTTTTCCTCAGGAACTTAATATTTGTGTCCTTGCCCTGTGTCTTTCTGTCATGGTTCCCTAGGCTGCCATCTC 28154
OliveBaboon       GAATCTGTTTTTCCTCAGGAACTTATTATTTGTGTCCTTGCCCTGTGTCTTTCTCTCATGGTTCCCTAGGCTGACATCTC 29149
Orangutan         TAATCTACTTTTCCTCGGGAACTTAATATTTGTGTCCTTGCCCTGTGTCTTTCTGTCATGGTTCCCTAGGCTGCCATCTC 29031

Majority          CAGAGGAGAAAATGTTTGGTTAGCTCTCAGTCCCAAACCATAGCAGAGCCCAGAGACTCTTGTCACCCTCAGCCTTCAGT

                          36170     36180     36190     36200     36210     36220     36230     36240

Human             CACAGGAGAAAATGTTTGGTTAGTTCTCAGTCCCAAACCATAGCAGAGCCCAGAGACTCTTGTCACCCTCAGCCTTCAGT 27190
Coquerel'sSifaka  CAGAGGAGAACGTGTTTGGTCAGCTCTCAGTGCTGAACCCTAGCAGAGCCCGGGGACTCTTGTCCACCGCAACCATCAGT 26227
Ma'sNightMonkey   CAGAGGAAAAAATGTTTGGTTAGCTCTCAGTTCCAAATCATAGCAGAGCCCAGAGACTCTTGTCAACTTGAGCCTTCAGT 24133
VervetAGM         CAGAGAAGAAAATGTTTGGTTAGCTCTCAGTCCCAAACTATAGCAGAGCCCAGAGACTGTTGTCACCCTCAGCCTTCAGT 30589
Gorrilla          CACAGGAGAAAATGTTTGGTTAGTTCTCAGTCCCAAACCATAGCAGAGCCCAGAGACTCTTGTCACCCTCAGCCTTCAGT 29577
GrabEatingMacaqueCAGAGGAGAAAATGTTTGGTTAGCTCTCAGTCCCAAACCATAGCAGAGCCCAGAGACTGTTGTCACTCTCAGCCTTCAGT 29239
Macaque           CAGAGGAGAAAATGTTTGGTTAGCTCTCAGTCCCAAACCATAGCAGAGCCCAGAGACTGTTGTCACTCTCAGCCTTCAGT 30000
PigTailedMacaque  CAGAGGAGAAAATGTTTGGTTAGCTCTCAGTCCCAAACCATAGCAGAGCCCAGAGACTGTTGTCACTCTCAGCCTTCAGT 28945
Bonobo            CACAGGAGAAAATGTTTGGTTAGTTCTCAGTCCCAAACCATAGCAGAGCCCAGAGACTCTTGTCACCCTCAGCCTTCAGT 28234
OliveBaboon       CAGAGGAGAAAATGTTTGGTTAGCTCTCAGTCCCAAACCATAGCAGAGCCCAGAGACTGTTGTCACTCTCAGCCTTCAGT 29229
Orangutan         CAGAGGAGAAAATGTTTGGTTAGTTCTCAGTCCCAAACCATAGCAGAGCCCAGAGACCCTTGTCACCTTCAGCCTTCAGT 29111
```

```
Majority          CCTTGGTTGCCTTCTTGGCAGACC-TGGCAGCTCTTCCACCTGGAAGTGATACATGGTATTAATAGTTGGACTGGACTAT

                          36250     36260     36270     36280     36290     36300     36310     36320

Human             CCTTGGTTGCCTTCTTCGCAGACC-TGGCAGCTCTTCCACCTGAAAGTGATACATGGTATTAATAGTTGGACTAGACTAT 27269
Coquerel'sSifaka  TCTTGTTTACCTTCCTGGCAGCCCCTAGCAGCTTTTCCACCTGGAAATGATAGCTGGT------AGTTGCATTAGATTAT 26301
Ma'sNightMonkey   TCTTGGTTGCCTTCTTGGCAGCCC-TAGCAGCTCTTCCACCTGGAAGTGATACATGGTCTTAATAGTTGGACTGTACTAT 24212
VervetAGM         CCTTGGTTGCCTTCTTGGCAAACC-TGGCAGCTCTTCCACCTGGAAGTGATACATGGTATTAATAGTTGGACTGGACTAT 30668
Gorrilla          CCTTGGTTGCCTTCTTGGCAGACC-TGGCAGCTCTTCCACCTGAAAGTGATACATGGTATTAATAGTTGGACTAGACTAT 29656
GrabEatingMacaqueCCTTGGTTGCCTTCTTGGCAGACC-TGGCATCTCTTCCACCTGGAAGTGATACATGGTATTAATAGTTGGACTGGACTAT 29318
Macaque           CCTTGGTTGCCTTCTTGGCAGACC-TGGCATCTCTTCCACCGGGAAGTGATACATGGTATTAATAGTTGGACTGGACTAT 30079
PigTailedMacaque  CCTTGGTTGCCTTCTTGGCAGACC-TGGCAGCTCTTCCACCTGGAAGTGATACATGGTATTAATAGTTGGACTGGACTAT 29024
Bonobo            CCTTGGTTGCCTTCTTCGCAGACC-TGGCAGCTCTTCCACCTGAAAGTGATACATGGTATTAATAGTTGGACTAGACTGT 28313
OliveBaboon       CCTTGGTTGCCTTCTTGGCAGACC-TGGCAGCTCTTCCACCTGGAAGTGATACATGGTATTAATAGTTGGACTGGACTAT 29308
Orangutan         CCTTGGTTGTCTTCTTGGCAGACC-TGGCAGCTCTTCCACCTGAAAGTGATACATGGTATTAATAGTTGGACTAGACTAT 29190

Majority          TATGATAAACAGAGTATCTAATAACCATGAGAAGAAAAAACAAGAAATTTGTAACATTGAAATGAGTGC-AAATGGCCTT

                          36330     36340     36350     36360     36370     36380     36390     36400

Human             TATGATAAACAGAGTATCTAATAACCATGAGAATAAAAAACAAGAAATTTGTAACATTGAAATGAGTGCTAAATGGCCTT 27349
Coquerel'sSifaka  TATGATGAAAAGAATATCTAATAACCATGAGAAGAAAAAATAAGACATTTATATCATTGAAATGAATGCCAAATGGCCTT 26381
Ma'sNightMonkey   TATGA----CAGAATATCTAACAACCTTGAGAAGAAAAAACAAGAAATTTGTAACATTGAAATGAGTGCCAAATGGCATT 24288
VervetAGM         TATGATAAACAGAGTATCTAATAACCATGAAAAGAAAAAACAAGAAATTTGTAACGTTGAAATGAGTG---AATGGCCTT 30745
Gorrilla          TATGATAAACAGAGTATCTAATAACCATGAGAATAAAAAACAAGAAATTTGTAACATTTAAATGAGTGCTAAATGGCCTT 29736
GrabEatingMacaqueTATGATAAACAGAGTATCTAATAACCATGAAAAGAAAAAACAAGAAATTTGTAACATTGAAATGAGTG---AATGGCCTT 29395
Macaque           TATGATAAACAGAGTATCTAATAACCATGAAAAGAAAAAACAAGAAATTTGTAACATTGAAATGAGTG---AATGGCCTT 30156
PigTailedMacaque  TATGATAAACAGAGTATCTAATAACCATGAAAAGAAAAAACAAGAAATTTGTAACATTGAAATGAGTG---AATGGCCTT 29101
Bonobo            TATGATAAACAGAGTATCTAATAACCATGAGAATAAAAAACAAGAAATTTGTAACATTGAAATGAGTGCTAAATGGCCTT 28393
OliveBaboon       TATGATAAACAGAGTATCTAATAACCATGAAAAGAAAAAACAAGAAATTTGTAACATTGAAATGAGTG---AATGGCCTT 29385
Orangutan         TAT-------AGAGTATCTAATAAACATAAGAATAAAAAACAAGAAATTTGTAACATTGAAATGAGTGCTAAATGGCCTT 29263

Majority          TAATCTGTACTTTCTTTTGCATTTTTTTTTTT--AAGTCATTTCTTTCTCTGA----CGTGTAGAGTGTATCTTCCTATT

                          36410     36420     36430     36440     36450     36460     36470     36480

Human             TAATCTGTACTTGCTTTTGCACATTTTTTTTT--AAGTGGTTTCTTTCTCTGA----CGTGTAGAGTGTATCTTCCTATT 27423
Coquerel'sSifaka  TAATCTGAACTTACTTTTGCACTTTTTTTA----AAGTCATTTCTTTCACTGATTGACTTACAACTTGTATCTTCGCGTA 26457
Ma'sNightMonkey   TAATCTGTACTTACTTTTGCACTTTTTTAAA---AAGTCATTTCTTTCTCTGA----CATGTAGAGTGTGTCTTCCTGTT 24361
VervetAGM         TAATCTGTACTTTTGCATTTTTTTTTTTTT-----AGTCATTTCTTTCTCTGA----CGTGTAGAGTGTATCTTCCTATT 30816
Gorrilla          TAATCTGTACTTGCTTTTGCACATTTTTTTTTTTAAGTGGTTTCTTTCTCTGA----CGTGTAGAGTGTATCTTCGTATT 29812
GrabEatingMacaqueTAATCTGTACTTTTGCATTTTTTTTTTTTTTTTT--AGTCATTTCTTTCTCTGA----CGTGTAGAGTGTATCTTCCTATT 29469
Macaque           TAATCTGTACTTTTGCATTTTTTTTTTTTTTTTTTAGTCATTTCTTTCTCTGA----CGTGTAGAGTGTATCTTCCTATT 30232
PigTailedMacaque  TAATCTGTACTTTTGCATTTTTTTTTTTTTTTTT-AGTCATTTCTTTCTCTGA----CGTGTAGAGTGTATCTTCCTATT 29176
Bonobo            TAATCTGTACTTGCTTTTGCACATTTTTTTTT--AAGTGGTTTCTTTCTCTGA----CGTGTAGAGTGTATCTTCTTATT 28467
OliveBaboon       TAATCTGTACTTTTGGATTTTTTTTTTTTTTT---AGTCATTTCTTTCTCTGA----CGTGTAGAGTGTATCTTCCTATT 29458
Orangutan         TAATCTGTACTTACTTTTGCATTTTTTTTTTT--AAGTGGTTTCTTTCTCTGA----CGTGTAGAGTGTATCTTCCTATT 29337
```

```
Majority           TCTGCATTTTC----TTGTTTCTGTGGGATGTTAGTTTCTCTTGAAATGGTCATTGATATTACACAGGAAGAAAAGTTTC
                           36490     36500     36510     36520     36530     36540     36550     36560
Human              TCTGAATTTTC----TTGTTTCTGTGGGATGTTAGTTTCTCTTGAAATGGTCATTGATATTACTCAGGAAGAAAAGTTTC 27499
Coquerel'sSifaka   TCAGCATTTTCCTAATTGTTTCTCTGGGATGCTGACT--TCTTCAGATGGTCATTGATATTATACAGGAAGAAAAGTTTC 26535
Ma'sNightMonkey    TCTGCATTTTC----TTGTTTCTGTGGGATGTTAGTGACTCTTGAAATAGTCATTGGTATTACACTGAAAGAAAAGTTTC 24437
VervetAGM          TTTGCATTTTC----TTATTTCTGTGGGATGTTAGTTTCTCTTGAAATGATCATTGATATTACACAGGAAGAAAAGTTTC 30892
Gorrilla           TCTGAATTTTC----TTGTTTCTGTGGGATGTTAGTTTCTCTTGAAATGGTCATTGATATTACTCAGGAAGAAAAGTTTC 29888
GrabEatingMacaque  TTTGCATTTTC----TTATTTCTGTGGGATGTTAGTTTCTCTTGAAAT-ACCATTGATATTACACAGGAAGAAAAGTTTC 29544
Macaque            TTTGCATTTTC----TTATTTCTGTGGGATGTTAGTTTCTCTTGAAAT-ATCATTGATATTACACAGGAAGAAAAGTTTC 30307
PigTailedMacaque   TTTGCATTTTC----TTATTTCTGTGGGATGTTAGTTTCTCTTGAAATGATCATTGATATTACACAGGAAGAAAAGTTTC 29252
Bonobo             TCTGAATTTTC----TTGTTTCTATGGGATGTTAGTTTCTCTTGAAATGGTCATTGATATTACTCAGGAAGAAAAGTTTC 28543
OliveBaboon        TTTGCATTTTC----TTATTTCTGTGGGATGTTAGTTTCTCTTGAAATGATCATTGATATTACACAGGAAGAAAAGTTTC 29534
Orangutan          TCTGCATTTTC----TTGTTTCTGTGGGATGTTAGTTTCTCTTGAAATGGTCATTGATATTACTCAGGAAGAAAAGTTTC 29413

Majority           TGTGGTCAGATTTAATAAGAGATATCTATGCTAGTCTTAACATTTAATTTCTTTT----AGTTGAATCTAA----AATTG
                           36570     36580     36590     36600     36610     36620     36630     36640
Human              TGTGGTCAGATTTAATAAGAGATATCTATGCTAGTCTTAACATTTAATTTCTTTT----AGTTGAATCTAA----AATTG 27571
Coquerel'sSifaka   TTTGGTTAGATTTAACAAGAGACATCAATGCCAATCTTAAAATTTAATTTCTTCTTTTAGGTTGAATCCAAGATCAATTG 26615
Ma'sNightMonkey    TGTGGTCAGATTTAATAAGAGACATCAATGCTAGTCTTAACATTTAATTTCTTTT----AGTTGAATCTGA----GATTG 24509
VervetAGM          TATGGTCAGATTTAATAAGA-GTAT-TACGCTAGTCTTAACATTTAATTTCTTTT----AGTTGAATCTAA----AATTG 30962
Gorrilla           TGTGGTCAGATTTAATAAGAGATATCTGTGCTAGTCTTAACATTTAATTTCTTTT----AGTTGAATCTAA----AATTG 29960
GrabEatingMacaque  TATGGTCAGATTTAATAAGA-GTAT-TACGCTAGTCTTAACATTTAATTTCTTTT----AGTTGAATCTAA----AATTG 29614
Macaque            TATGGTCAGATTTAATAAGA-GTAT-TACGCTAGTCTTAACATTTAATTTCTTTT----AGTTGAATCTAA----AATTG 30377
PigTailedMacaque   TGTGGTCAGATTTAATAA---GTAT-TATGCTAGTCTTAACATTTAATTTCTTTT----AGTTGAATCTAA----AATTG 29320
Bonobo             TGTGGTCAGATTTAATAAGAGATATCTATGCTAGTCTTAACATTTAATTTCTTTT----AGTTGAATCTAA----AATTG 28615
OliveBaboon        TGTGGTCAGATTTAATAAGA-GTAT-TACGCTAGTCTTAACATTTAATTTCTTTT----AGTTGAATCTAA----AATTG 29604
Orangutan          TGTGGTCAGATTTAATAAGAGATATCTGTGCTAGTCTTAACATTTAATTTCTTTT----AGTTGAATCTAA----AATTT 29485

Majority           ACATCATTCTTAGATGAATTAAAAATTTT--AAAGAGCATATATTCTTTAGTAGGTATTTTGATTCT------AAGTACC
                           36650     36660     36670     36680     36690     36700     36710     36720
Human              ATATCATTCTTAGATTAATTAAAAATTTTTTAAAGAGCATGTATTCTTTAGTA----TTTTGATTCT------AAGTACC 27641
Coquerel'sSifaka   ACATCTATCTTAGATGAATTAAAATGTTT--AAAAAGTGTATATTCCT-AGTTCAAATTTGATTCTGAGTTTAAGTACC 26692
Ma'sNightMonkey    ACAT---TCTTAGGTGAATTAAAAATTTT--AAAAAGCATGTATTCTTTAGTAGATATTTTGATTCT------AAGTACC 24578
VervetAGM          ACATCATTCTTAGATGAATTAAAAAATTT--AAAGAGCACATATTCTTTCGTAGGTATTTTGATTCT------AAGTACC 31034
Gorrilla           ATAT---TCTTAGATGAATTAAAAATTTTTTAAAGAGCATGTATTCTTTAGTA----TTTTGATTCC------AAGTACC 30027
GrabEatingMacaque  ACATCATTCTTAGATGAATTTAAAAATTT--AAAGAGCACATATTCTTTCGTAGGTATTTTGATTCT------AAGAACC 29686
Macaque            ACATCATTCTTAGATGAATTAAAAAATTT--AAAGAGCACATATTCTTTCGTAGGTATTTTGATTCT------AAGAACC 30449
PigTailedMacaque   ACATCATTCTTAGATGAATTAAAAAATTT--AAAGAGCACATATTCTTTCGTAGGTATTTTGATTCT------AAGAACC 29392
Bonobo             ATATCATTCTTAGATGAATTAAAAATTTTTTAAAGAGCATGTATTCTTTAGTA----TTTTGATTCT------AAGTACC 28685
OliveBaboon        ACATCATTCTTAGATGAATTAAAAAATTT--AAAGAGCACATATTCTTTCGTAGGTATTTTGATTCT------AAGAACC 29676
Orangutan          CTATCGTTCTTAGATGAACTAAAAATTTTTTAAAGAGCATGTATTCTTTAGTAAGTATTTTGATTCT------AATTACC 29559
```

```
Majority        ACTAATTTGATTTGTTTCAGCAGTCATTAATGTGGGGCAGTCATCAATCACTACC-ATTGATCATTTATTTCATATACAT

                        36730     36740     36750     36760     36770     36780     36790     36800

Human           ACTAATTTGATTTGTTTCAACAGTCATTAATGTGGGGCAGTCATCAATCACTACC-ATTGATCATTTATTTCATATACAT 27720
Coquerel'sSifaka ATTAATTTGATTTGTTTCAATAGTCACTAATGTGGAATAGCCATCAATCACTACT-ATGGATCATTCATTTTATATATAT 26771
Ma'sNightMonkey ATTAATTTGATTTGCTTCAGCAGTCATTAACGTAGGGCAGTCATTAATCACTACC-ATTGATCATTTATTTCATATACAG 24657
VervetAGM       ACTAATTTGATTTGTTTCAGCAGTCATTAATGTGGGGCAGTCGTCAGTCACTACCCATTGATCATTTATTTCATATACAT 31114
Gorrilla        ACTAATGTGATTTGTTTCAACAGTCATTAATGTGGGGCAGTCATCAATCACTACC-ATTGATCATTTATTTCATATACAT 30106
GrabEatingMacaqueACTAATTTGATTTGTTTCAGCAGTCATTAATGTGGGGCAGTCGTCAGTCACTACCCATTGATCATTTATTCCATATACAT 29766
Macaque         ACTAATTTGATTTGTTTCAGCAGTCATTAATGTGGGGCAGTCGTCAGTCACTACCCATTGATCATTTATTCCATATACAT 30529
PigTailedMacaque ACTAATTTGATTTGTTTCAGCAGTCATTAATGTGGGGCAGTCGTCAGTCACTACCCATTGATCATTTATTCCATATACAT 29472
Bonobo          ACTAATTTGACTTGTTTCAACAGTCATTAATGTGGGGCAGTCATCAATCACTACC-ATTGATCATTTATTTCATATACAT 28764
OliveBaboon     ACTAATTTGATTTGTTTTAGCAGTCATTAATGTGGGGCAGTCGTCAGTCACTACCCATTGATCATTTATTTCATATATAT 29756
Orangutan       ACTAATTTGATTTGCTTCAGCAGTCATTAATGTGGGGCAGTCATCAATCACTACC-ATTGATCATTTATTTCATATACAT 29638

Majority        TCTAAGTTAAATGACCAGGCTGAAATAATTCAGGTGAAATTATTCTGTCAGTTAATGTGGTAATGTTAACATTTATCAGT

                        36810     36820     36830     36840     36850     36860     36870     36880

Human           TGTAAGTTAAATGACCAGGCTGAAATAATTCAGATGAAATTATTCTGTCAGTTAATGTGGTAATGTTAACATTTATCAGT 27800
Coquerel'sSifaka TCCAAGTTAAACAACTAGGCTTAGATAATTCAAGTGAAACTGTTCCATCAGTCAACGTGGTAATGTTAACATTTATCAAT 26851
Ma'sNightMonkey TCTAAGTTAAATGACCAATCTGAAATAGTTCAAGTGAAATTATTCTGTCAGTTAATGTGGTAATGTTAACATATATCAAT 24737
VervetAGM       TCTAAGTTAAATGATCAGACTGAAATAATTCAGGTGAAATTATTCTGTCAGTTAATGTGGTAATGTTAACATTTATCAGT 31194
Gorrilla        TGTAAGTTAAATGACCAGGCTGAAATAATTCAGATGAAATTATTCTGTCAGTTAATGTGGTAATGTTAACATTTATCAGT 30186
GrabEatingMacaqueTCTAAGTTAAATGATCAGACTGAAATAATTCAGGTGAAATTATTCTATCAGTTAATGTGGTAATGTTAACATTTATCAGT 29846
Macaque         TCTAAGTTAAATGATCAAACTGAAATAATTCAGGTGAAATTATTCTATCAGTTAATGTGGTAATGTTAACATTTATCAGT 30609
PigTailedMacaque TCTAAGTTAAATGATCAGACTGAAATAATTCAGGTGAAATTATTCTATCAGTTAATGTGGTAATGTTAACATTTATCAGT 29552
Bonobo          TGTAAGTTAAATGACCAGGCTGAAATAATTCAGATGAAATTATTCTGTCAGTTAATGTGGTAATGTTAACATTTATCAGT 28844
OliveBaboon     TCTAAGTTAAATGATCAGACTGAAATAATTCAGGTGAAATTATTCTATCAGTTAATGTGGTAATGTTAACATTTATCAGT 29836
Orangutan       TGTAAGTTAAATGACCAGGCTGAAATAATTCAGGTGAAATTATTCTGTCAGTTAATGTGGTAATGTTAACATTTATCAGT 29718

Majority        AGTGCTGAATCACTTCTTTTGGGCATAATAGTGGTAAGCTTTTT-GCTCATTGGCAAAGACAGGCATAATTGCTTTTTT-

                        36890     36900     36910     36920     36930     36940     36950     36960

Human           AGTGCTGAATCACTTCTTTTGGGCATAATAGTGGTAAGCTTTTT-GCTCAATG-CAAAGACAGGCATAATTGCTTTTTTC 27878
Coquerel'sSifaka AGGGCAGAATCACTTCTTTTGGGCATAATAATGGTAGACTTTTT-ACTCAGTGGAAAAGCAAAACACAGTTGGTTTTGT- 26929
Ma'sNightMonkey AGTGCTGAATCACTTCTTTTGGGCATAATAGTGGTAAGCTTTTT-GCTCATTG-GAAAGACAGGCATAATTGCTTTTTTA 24815
VervetAGM       AGTGCTGAATCACTTCTTTTGGGCATAATAGTGGTAAGCTTTTTTGCTTATTGGCAAAGACAGGCATAATTGCTTTTTT- 31273
Gorrilla        AGTGCTGAATCACTTCTTTTGGGCATAATAGTGGTAAGCTTTTT-GCTCAATG-CAAAGGCAGGCATAATTGCTTTTTTT 30264
GrabEatingMacaqueAGTGCTGAATCACTTCTTTTGGGCATAATAGTGGTAAGCTTTTTTGCTTATTGGCAAAGACAGGCATAATTGCTTTTTT- 29925
Macaque         AGTGCTGAATCACTTCTTTTGGGCATAATAGTGGTAAGCTTTTTTGCTTATTGGCAAAGACAGGCATAATTGCTTTTTT- 30688
PigTailedMacaque AGTGCTGAATCACTACTTTTGGGCATAATAGTGGTAAGCTTTTTTGCTTATTGGCAAAGACAGGCATAATTGCTTTTTT- 29631
Bonobo          AGTGCTGAATCACTTCTTTTGGGCATAATAGTGGTAAGCTTTTT-GCTCAATG-CAAAGACAGGCATAATTGCTTTTTT- 28921
OliveBaboon     AGTGCTGAATCACTTCTTTTGGGCATAATAGTGGTAAGCTTTTTTGCTTATTGGCAAAGACAGGCATAATTGCTTTTTT- 29915
Orangutan       AGTGCTGAATCACTTCTTTTGGGCATAATAGTGGTAAGCTTTTT-GCTCAATG-CAAAGACAGGCATAATTGCTTTTTT- 29795
```

```
Majority          AAATAAAAATGTAATTTGTAAAAATACTCC--AGTAATTTTTGTAAAATCTCAAAATTGCAAATAATTTGACATTATTCT
                          36970     36980     36990     37000     37010     37020     37030     37040
Human             AAATAAAAATGTAATTTGTAAAAATACTCC--AGTAATTTTTGTAAAATCTCAAAATTGCAAATAATTTGACGTTATTCT 27956
Coquerel'sSifaka  ----ATTAATGTAATTTATAAAAAAATTCTTTAGTAATTTTGATGAAATCTCAAAATTGCAGATAATTTGATATTCTTCT 27005
Ma'sNightMonkey   AAATAAAAATGTAATTTGTAAAAATACTCC--AGTAATTTTTGTAACATCTCAAAATTGCAAATAATTTGACATTATTCT 24893
VervetAGM         AAATAAAAATGTAATTTGTAAAAATACTCC--AGTAATTTTTGTAAAATCTCAAAATTGCAAATAATTTGACATTATTCT 31351
Gorrilla          AAATAAAAATGTAATTTGTAAAAATATTCC--AGTAATTTTTGTAAAATCTCAAAATTGCAAATAATTTGACGTTATTCT 30342
GrabEatingMacaqueAAATAAAAATGTAATTTGTAAAAATACTCC--AGTAATTTTTGTAAAATCTCAAAATTGCAAATAATTTGACATTATTCT 30003
Macaque           AAATAAAAATGTAATTTGTAAAAATACTCC--AGTAATTTTTGTAAAATCTCAAAATTGCAAATAATTTGACATTATTCT 30766
PigTailedMacaque  AAATAAAAATGTAATTTGTAAAAATACTCC--AGTAATTTTTGTAAAATCTCAAAATTGCAAATAATTTGACATTATTCT 29709
Bonobo            AAATAAAAATGTAATTTGTAAAAATACTCC--AGTAATTTTTGTAAAATCTCAAAATTGCAAATAATTTGACGTTATTCT 28999
OliveBaboon       AAATAAAAATGTAATTTGTAAAAATACTCC--AGTAATTTTTGTAAAATCTCAAAATTGCAAATAATTTGACATTATTCT 29993
Orangutan         AAATAAAAATGTGATTTGTAAAAATACTTC--AGTAATTTTTGTAAAATCTCAAAATTGCAAATAATTTGACATTATTCT 29873

Majority          ----CAGTAATGAAGGCTGTTAAATCTGCTCTCTTAACAGCAGGTATGAAATCATTTGAGGAAGAAAAAAGGTCATAAAT
                          37050     37060     37070     37080     37090     37100     37110     37120
Human             ----CAGTAATGAAGGCTGTTAAATCTCCTCTCTTAACGGCAGGTATGAAATCATTTGAGGAAGAAAAAAGTTCATAAAT 28032
Coquerel'sSifaka  GACACACTTATGAAGGCTATTAGATCTACTCT--TAAGAGCAGGTATGAAATCACTTCAAGAAGAAAAAAGACCACAAAT 27083
Ma'sNightMonkey   ----CAGTAATGAAGATTGTTAAATCTGCTCTCCTAACAGCAAGTATGAAATCATTTGAGTAAGAAAAAAGATTATAAAT 24969
VervetAGM         ----CAGTAATGAAGGCTGTTAAATCTGCTCTCCTAACAGCAGGTATGAAACCATTTGAGGAAGAAAAAAGGGCATAAAT 31427
Gorrilla          ----CAGTAATGAAGGCTGTTAAATCTCCTCTCCTAACGGCAGGTATGAAATCATTTGAGGAAGAAAAAAGTTCATAAAT 30418
GrabEatingMacaque----CAGTAATGAAGGCTGTTAAATCTGCTCTCTTAACAGCAGGTATGAAACCATTTGAGCAAGAAAAA-GGTCATAAAT 30078
Macaque           ----CAGTAATGAAGGCTGTTAAATCTGCTCTCTTAACAGCAGGTATGAAACCATTTGAGGAAGAAAAA-GGTCATAAAT 30841
PigTailedMacaque  ----CAGTAATGAAGGCTGTTAAATCTGCTCTCTTAACAGCAGGTATGAAACCATTTGAGGAAGAAAAAAGGTCATAAAT 29785
Bonobo            ----CAGTAATGAAGGCTGTTAAATCTCCTCTCTTAACGGCAGGTATGAAATCATTTGAGGAAGAAAAAAGTTCATAAAT 29075
OliveBaboon       ----CAGTAATGAAGGCTGTTAAATCTGCTCTCTTAACAGCAGGTATGAAACCATTTGAGGAAGAAGAAAGGTCATAAAT 30069
Orangutan         ----CAGTAATGAAGGCCGTTAAATCTGCTCTCTTAACGGCAGGTGTGAAATCATTTGAGGAAGAAAAAAGTTCATAAAT 29949

Majority          ACAAACTTAAAATAAAA-------AGACTTTTCCATGCAAAAAGATGCATTGCAGGAAGGAGGAAGAGTGTCCTTCATAG
                          37130     37140     37150     37160     37170     37180     37190     37200
Human             ACAAACTTAAAAAAAAA--TGGAAAGACTTTTCCATGCAAAAAGATGCATTGCAGGAAGGAGGAAGAGTGTCCTTCATAG 28110
Coquerel'sSifaka  ACAAAATTCAAAGAAAAAATT-AAAGACTTTGCCATGCATAAAGATGCATTGTAGGAAGGAAGAAGAGAGCATCTCC--G 27160
Ma'sNightMonkey   ATAAACTTAAAAAAAAA--TGGAAATACTTTTCCATGCAAAAAGATGCATTGCAGGAACAAGGAAGAGTGTCATGCACAG 25047
VervetAGM         ACAAACTTAAAATGGAA-------AGACTTTTCCATGCAAAAAGATGCATTGCAGGAAGGAGGAAGAGTATCCTTCATAG 31500
Gorrilla          ACAAACTTAAAAAAAAAAATGGAAAGACTTTTCCATGCAAAAAGATGCATTGCAGGAAGGAGGAAGAGTGTCCTTCATAG 30498
GrabEatingMacaqueACAAGCTTAAAATGGAA-------AGACTTTTCCATGCAAAAAGATGCATTGCAGGAAGGAGGAAGAGTATCCTTCATAG 30151
Macaque           ACAAGCTTAAAATGGAA-------AGACTTTTCCATGCAAAAAGATGCATTGCAGGAAGGAGGAAGAGTATCCTTCATAG 30914
PigTailedMacaque  ACAAACTTAAAATGGAA-------AGACTTTTCCATGCAAAAAGATGCATTGCAGGAAGGAGGAAGAGTATCCTTCATAG 29858
Bonobo            ACAAACTTAAAAAAAAA--TGGAAAGACTTTTCCATGCAAAAAGATGCATTGCAGGAAGGAGGAAGAGTGTCCTTCATAG 29153
OliveBaboon       ACAAACTTAAAATGGAA-------AGACTTTTCCATGCAAAAAGATGCATTGCA-------------------------- 30116
Orangutan         ACAAACTTAAAAAAA---------------T-----GCAAAAAGGTGCATTGCAGGAAGGAGGAAGAGTGTCTTTCATAG 30009
```

```
Majority          CACACCACAAGTCAGTATTAGAGGCTTTCTTTTATACTTGAGTAGACCCAAGATTCCCCACCTATCATCT--TCCAAAA-
                           37210     37220     37230     37240     37250     37260     37270     37280
Human             CACACCACAAGTCAGTATTAGAGGCTTGCTTTTCTACTTGAGTAGACCCAAGATTCCCCACCTATCGTCT--TCCAAAG- 28187
Coquerel'sSifaka  CACACCACAAGTCAGAATTAGAGGTTTTGTTTTGTACTTGAGCAGATCCAAGATTCCCCACCTATCATCT--TCCAGAA- 27237
Ma'sNightMonkey   CACACCACAAGTCAGAATTAGAGGCTTTCTTTTGTATTTGAATAGACTCAAGATTCCCTACCCATCATCTCTTCCAGAA- 25126
VervetAGM         CACACCACAAGTCAGTGTTAGAGGCTTTCTTTTATACTTGAGTAGGCCCAAGATTCCCCACCTATCATCT--TCCAAAA- 31577
Gorrilla          CACACCACAAGTCAGTATTAGAGGCTTGCTTTTCTACTTGAGTAGACCCAAGATTCCCCACCTATCGTCT--TCCAAAG- 30575
GrabEatingMacaqueCACACCACAAGTCAGTGTTAGAGGCTTTCTTTTATACTTGAGTAGGCCCAAGATTCCCCACCTATCATCT--TCCAAAA- 30228
Macaque           CACACCACAAGTCAGTGTTAGAGGCTTTCTTTTATACTTGAGTAGGCCCAAGATTCCCCACCTATCATCT--TCCAAAA- 30991
PigTailedMacaque  CACACCACAAGTCAATGTTAGAGGCTTTCTTTTATACTTGAGTAGGCCCAAGATTCCCCACCTATCATCT--TCCAAAA- 29935
Bonobo            CACACCACAAGTCAGTATTAGAGGCTTGCTTTTCTACTTGAGTAGACCCAAGATTCCCCACCTATCGTCT--TCCAAAG- 29230
OliveBaboon       --CACCACAAGTCAGTGTTAGAGGCTTTCTTTTATACTTGAGTAGGCCCAAGATTCCCCACCTATCATCT--TCCAAAA- 30191
Orangutan         CACACCACAAGTTAGTATTAGAGGCTTGCTTTTCTACTTGAGTAGACCCAAGATTCCCCACCTATCGTCT--TCCAAAGA 30087

Majority          --TTACCTGTGATCCACTGTAATCACAAGTTTTACAGTGG----TATACTGTAGGTGTAT---GGAAGGATTTGAATTCA
                           37290     37300     37310     37320     37330     37340     37350     37360
Human             --TTACTTGTGATCCACTGCAATCACAAGTTTTACAGTGTG---TATACTCTAGGTGTAT---GGAAGGATTTGAATTCA 28259
Coquerel'sSifaka  --TTACGTGTGATCCACTGTAATCACAAGTATTACAGTGAAGTATGTATTACAGTAGAAGTATGGAAGGATTTTGATTCT 27315
Ma'sNightMonkey   --TTACCTGTGACCCACTGTAATCACAGGTTTTACGGTGG----GATACTGTAGATTTATTTCGGAAGGATTTGAATTCA 25200
VervetAGM         --TTACCTGTGATCCAGTGTAATCACAGGTTTTGCAGTGG----TATACTGTAGGTGTAT---GGAAGGATTTGAATTCA 31648
Gorrilla          --TTACTTGTGATCCACTGCAATCACAAGTTTTACAGTGT----TATACTCTAGGTGTAT---GGAAGGATTTGAATTCA 30646
GrabEatingMacaque--TTACCTGTGATCCACTGTAATCACAAGTTTTGCAGTGG----TATACTGTAGGTGTAT---GGAAGGATTTGAATTCA 30299
Macaque           --TTACCTGTGATCCACTGTAATCACAAGTTTTGCAGTGG----TATACTGTAGGTGTAT---GGAAGGATTTGAATTCA 31062
PigTailedMacaque  --TTACCTGTGATCCACTATAATCACAAGTTTTGCAGTGG----TATACTGTAGGTGTAT---GGAAGGATTTGAATTCA 30006
Bonobo            --TTACTTGTGATCCACTGCAATCACAAGTTTTACAGTGT----TATACTCTAGGTGTAT---GGAAGGATTTGAATTCA 29301
OliveBaboon       --TTACCTGTGATCCACTGTAATCACCAGTTTTGCAGTGG----TATACTGTAGGTGTAT---GGAAGGATTTGAATTCA 30262
Orangutan         AGTTACCTGTGATCCACTGCAATCACAAGTTTTACAGTGT----TATACTCCAGGTGTAT---GGAAGGATTTGAATTCA 30160

Majority          CAAATAGTCAAACATCTAAAGAATAAGCTGATAA-------TAATACATTTGAAAAGAGGTCAGGT---ATATTTTTGAA
                           37370     37380     37390     37400     37410     37420     37430     37440
Human             CAAATAGTCAAACATCAGAAGAATAAGCTGATAA-------TAATACATTTGAAAAGAGGTCAAGT---ATATTTTTGAA 28329
Coquerel'sSifaka  GCAA-------ACATCTAGAAAATAAACTGATAG--------TACACATTTGAAAAGAGGTCAGAT---GTACTTTTGAA 27377
Ma'sNightMonkey   TAAATAGTTAAACATATAAAGAATAAGCTGATAACTGATAATAATACATTTGAAAAGAGGTCAGGTGGTATACTTTTGAA 25280
VervetAGM         CAAATAGTCAGACATCTAAAGAATACGCTGCTGA-------TAATGCATTTGAAAAGAGGTCAGGT---ATATTTTTGAA 31718
Gorrilla          CAAATAGTCAAACATCAGAAGAATAAGCTGATAA-------TAATACATTTGAAAAGAGGTCAAGT---ATATTTTTGAA 30716
GrabEatingMacaqueCAAATAG----ACATCTAAAGAATAAGCTGATAA-------TAATGCATTTGAAAAGAGGTCAGGT---ATATTTTTGAA 30365
Macaque           CAAATAG----ACATCTAAAGAATAAGCTGATAA-------TAATGCATTTGAAAAGAGGTCAGGT---ATATTTTTGAA 31128
PigTailedMacaque  CAAATAG----ACATCTAAAGAATAAGCTGATAA-------TAATGCATTTGAAAAGAGGTCAGGT---ATATTTTTGAA 30072
Bonobo            CAAATAGTCAAACATCAGAAGAATAAGCTGATAA-------TAATACATTTGAAAAGAGGTCAAGT---ATATTTTTGAA 29371
OliveBaboon       CAAATAG----ATATCTAAAGAATAAGCTGATAA-------TAATGCATTTGAAAAGAGGTCAGGT---ATATTTTTGAA 30328
Orangutan         CAAATAGTCAAACATCAAAAGAATAAGCTGATAA-------TAATACATTTGAAAAGAGGTCAGGT---ATATTTTTGAA 30230
```

```
Majority          AACTAGGAGAGTTAAAGTGTTTTT-GTGAACTAAGGAACAAATAAGGCATTGACAATATTTTAATTGATTAGGAATTTTG
                          37450    37460    37470    37480    37490    37500    37510    37520
Human             AACTAGGAGAGTTAAAGTGTTTTT-GTGAACTAAGGAACAAATAAGGCATTGACAATATTTTACTTGATTAGGAATTTTG 28408
Coquerel'sSifaka  AACTAGGGGAATTAAAGTGTTTTTTGAAAACTAAGGAG---ATAAGACATTGAAAATATTTTAAGGGGTTAGAAATTTTG 27454
Ma'sNightMonkey   AACTTGGAGAGTTAAAGTGTTTTT-GTGAAATAAGGAAAATACATGACACTGACAATATTTTAAGGGACTAGGAATTTTG 25359
VervetAGM         AACTAGGAGAGT-----TATTTTT-GTGAACTAAGGAACAAATAACGCATTGACAATATTTTAAATGATTAGGAATTTTG 31792
Gorrilla          AACTAGGAGAGTTAAAGTGTTTTT-GTGAACTAAGGAACAAATAAGGCATTGACAATATTTTACTTGATTAGGAATTTTG 30795
GrabEatingMacaqueAACTAGGAGAGTTAAAGTATTTTT-GTGAACTAAGGAACAAATAAGGCATTGACAATATTTTAATTGATTAGGAATTTTG 30444
Macaque           AACTAGGAGAGTTAAAGTATTTTT-GTGAACTAAGGAACAAATAAGGCATTGACAATATTTTAATTGATTAGGAATTTTG 31207
PigTailedMacaque  AACTAGGAGAGTTAAAGTATTTTT-GTGAACTAAGGAACAAATAAGGCATTGACAATATTTTAATTGATTAGGAATTTTG 30151
Bonobo            AACTAGGAGAGTTAAAGTGTTTTT-GTGAACTAAGGAACAAATAAGGCATTGACAATATTTTACTTGATTAGGAATTTTG 29450
OliveBaboon       AACTAGGAGAGTTAAAGTATTTTT-GTGAACTAAGGAACAAATAAGGCATTGACAATATTTTAATTGATTAGGAATTTTG 30407
Orangutan         AACTAGGAGAGTTAAAGTGTTTTT-GTGAACTAAGGAACAAATAAGGCATTGACAATATTTTACTTGATTAGGAATTTTG 30309

Majority          TTCAGGTGAGTAAAACATTAAGGATCGTTTCAGTTTTGTTTTTGTTTGTTTTTTACTTTTAGAATATCTGATCTGGACTT
                          37530    37540    37550    37560    37570    37580    37590    37600
Human             TTCAGGTGAGTAAAACTTTAAGCATCACTTAAGTTTTGTTTTTGTTTC----TTACTTTTAGAATATCTGATCTGGACTT 28484
Coquerel'sSifaka  TTCAGGTGACTAAAACATTAAGGACCTCTTTACAGTTTTATTTGGTT----TTTACTTTTAGCATGTCTGATGTGGAGTT 27530
Ma'sNightMonkey   TTCAGGTGAGTAAAACATCAAGGATCGCTTCAGTTTTGTTTTTGTTTGTTTTTTACTTTTAGAGTATCTGATCTGGACTT 25439
VervetAGM         TTCAGGTTAGTAAA-CATTAAGGATCGTTTCAGTTTTGTTTTTGTTTGTTTTTTACTTTTAGAATATCTAATCTGGACTT 31871
Gorrilla          TTCAGGTGAGTAAAACTTTAAGCATCGCTTAAGTTTTGTTTTTGTT--------ACTTTTAGAATATCTGATCTGGACTT 30867
GrabEatingMacaqueTTCAGGTTGGTAAA-CATTAAGGATCGTTTCAGTTTTGTTTTTGTTTGTTTTTTACTTTTAAAATATCTAATCTGGACTT 30523
Macaque           TTCAGGTTGGTAAA-CATTAAGGATCGTTTCAGTTTTGTTTTTGTTTGTTTTTTACTTTTAAAATATCTAATCTGGACTT 31286
PigTailedMacaque  TTCAGGTTAGTAAA-CATTAAGGATCGTTTCAGTTTTGTTTTTGTTTGTTTTTTACTTTTAAAATATCTAATCTGGACTT 30230
Bonobo            TTCAGGTGAGTAAAACTTTAAGC-----TTAAGTTTTGTTTTTGTTTG----TTACTGTTAGAATATCTGATCTGGACTT 29521
OliveBaboon       TTCAGGTTAGTAAA-CATTAAGGATCGTTTCAGTTTTGTTTTTGTTTGTTTTTTACTTTTAAAATATCTAATCTGGACTT 30486
Orangutan         TTCAGGTGAGTAAAACTTCAAGCATCGCTTAAGTTTTGTTTTTGTTTG----TTACTTTTAGAATATCTGATCTGGACTT 30385

Majority          CTGTGGATTTATGGAAGGGCTGGCTTTATTT----CTCTATCCTGTGGTGGGGGGCCAACTTTGATTTT-GTATACGGAG
                          37610    37620    37630    37640    37650    37660    37670    37680
Human             CTGTGGATTTATGGAAGAGCTGGCTTTATTT----CTCTATCCTGTGG-GAGGGGCAAACTTTGATTTT-GTATGTGGAG 28558
Coquerel'sSifaka  CTGTTAACTTATAGAAGGGCTGATTTTATTTACTTCCTCATCCTATGG-GGGGGGTTGACTTTGCTTTTTGTATGTGGAG 27609
Ma'sNightMonkey   CTGTGAATTTATGGAAGGGCTGGCTTTATTT----CCGTATCCTGTGGTGGGGGGGCAACTTTGATTTT-ATATATGGAG 25514
VervetAGM         CTGTGGGTTTATGGAAGGGCTGGCTTTATCT----CTCTATCCTGTGGTGGCGGGCCAACCTTGAGTTT-GTATACAGAG 31946
Gorrilla          CTGTGGATTTATGGAAGGGCTGGCTTTATTT----CTCTATCCTGTGG-GAGGGGCAAACTTTGATTTT-GTATGCGGAG 30941
GrabEatingMacaqueCTGTGGGTTTATGGAAGGGCTGGCTTTATCT----CTCTATCCTGTGGTGGCGGGCCAACCTTGATTTT-GTATACGGAG 30598
Macaque           CTGTGGGTTTATGGAAGGGCTGGCTTTATCT----CTCTATCCTGTGGTGGCGGGCCAACCTTGATTTT-GTATACGGAG 31361
PigTailedMacaque  CTGTGGGTTTATGGAAGGGCTGGCTTTATCT----CTCTATCCTGTGGTGGCGGGCCAACCTTGATTTT-GTATACGGAG 30305
Bonobo            CTGTGGATTTATGGAAGGGCTGGCTTTATTT----CTCTATCCTGTGG-GAGGGGCAAACTTTGATTTT-GTATGCGGAG 29595
OliveBaboon       CTGTGGGTTTATGGAAGGGCTGGCTTTATCT----CTCTATCCTGTGGTGGCGGGCCAACCTTGATTTT-GTATACGGAG 30561
Orangutan         CTGTGGATTTATGGAAGGGCTGGCTTTATTT----CTGTATCCTGTGGTGAGGGGCCAACTTTGATTTT-GTATGCAGAG 30460
```

```
Majority         TAACTTGACCTTCAGTTTTTTT--GGTCTAAGAACAAAGGCCAAAAAATATGTAACAGTTAATAATAA----CGTTAATT

                         37690     37700     37710     37720     37730     37740     37750     37760

Human            TAACTTGACCTTCAGTTTTTTT--GGTCTGAGAACAAAGGCCAAAAAATATGTAACAGTTAATAACG--------TAATT 28628
Coquerel'sSifaka TAACTTGATTCACAGTTTTTTC---ATCAAAGAACAAAGGCCAAAAAATATGTAGCAGTTGTTATTAATCAGCATTTGTT 27686
Ma'sNightMonkey  TAACTTGACCTTCAGTTTTTTT--GATTTAAGAACAAAGGCCAAAAAATATGTAACAGTTGTTAATAA-------TAG-- 25583
VervetAGM        TAACTTGACCTTCAGTTTTTTTTTGGTCTAAGAACAAAGGCCAAAAAATATGTAACTGTTAATAATAA----CGTTAATT 32022
Gorrilla         TAGCTTGACCTTCAGTTTTTTT--GGTCTGAGGACAAAGGCCAAAAAATATGTAACAGTTAATAACG--------TAATT 31011
GrabEatingMacaqueTAACTTGACCTTCAGTTTTTTTT-GGTCTAAGAACAAAGGCCAAAAAATATGTAACTGTTAATAATAA----CGTTAATT 30673
Macaque          TAACTTGACCTTCAGTTTTTTTT-GGTCTAAGAACAAAGGCCAAAAAATATGTAACTGTTAATAATAA----CGTTAATT 31436
PigTailedMacaque TAACTTGACCTTCAGTTTTTTTT-GGTCTAAGAACAAAGGCCAAAAAATATGTAACTGTTAATAATAA----CGTTAATT 30380
Bonobo           TAACTTGACCTTCAGTTTTTTT--GGTCTGAGAACAAAGGCCAAAAAATATGTAACAGTTAATAACG--------TAATT 29665
OliveBaboon      TAACTTGACTTTCAGTTTTTTTT-GGTCTAAGAACAAAGGCCAAAAAATATGTAACTGATAATAATAA----CGTTAATT 30636
Orangutan        TAACTTGACCTTCAGTTTTTTT--GGTCTGAGAACAAAGGCCAAAAAATATGTAACAGTTAATAATAA----CGTTAATT 30534

Majority         CACTTACTTAGTCAGTAAATATGTGGGGACATTGTACTTGGTTGACACTTGGGAATATTATAGTGAGCACTACTGATGTG

                         37770     37780     37790     37800     37810     37820     37830     37840

Human            CACTTACTTAGTCAATAAATATGTGGGGACATTGTACTTGGTTGACACTTGGGAATATTGTAGTGAGCACTACTGATGTG 28708
Coquerel'sSifaka AACTTATTTAGTCAATAAATATGTATGGACACTGTATTTGGTTGACACTTGGGAATATTATAGTGAACACTA-TGATGTG 27765
Ma'sNightMonkey  CATTAACT----------------------ATT-----TGGTTGACACTTGGGAATATTATAGTGAGCACTACTGATGTG 25636
VervetAGM        CACTTACTGAGTCAGTAAATATGTGGGAACGTTGTACTTGGTTGACACTTGGGAATATTATAGTGAGCACTACTGATGTG 32102
Gorrilla         CACTTACTTAGTCAATAAATATGTGGGGACATTGTACTTGGTTGACACTTGGGAATATTATAGTGAGCACTACTGATGTG 31091
GrabEatingMacaqueCACTTACTGAGTCAGTAAATATGTGGGAACGTTGTACTTGGTTGACACTTGGGAATATTATAGTGAGCACTACTGATGTG 30753
Macaque          CACTTACTGAGTCAGTAAATATGTGGGAACGTTGTACTTGGTTGACACTTGGGAATATTATAGTGAGCACTACTGATGTG 31516
PigTailedMacaque CACTTACTGAGTCAGTAAATATGTGGGAACGTTGTACTTGGTTGACACTTGGGAATATTATAGTGAGCACTACTGATGTG 30460
Bonobo           CACTTACTTAGTCAATAAATATGTGGGGACATTGTACTTGGTTGACACTTGGGAATATTCTAGTGAGCACTACTGATGTG 29745
OliveBaboon      CACTTACTGAGTCAGTAAATATGTGGGAACGTTGTACTTGGTTGACACTTGGGAATATTATAGTGAGCACTACTGATGTG 30716
Orangutan        CATTTACTTAGTCAATAAATATGTGGGGACATTGTACTTGGTTGACACTTGGGAATATTATAGTGAGCACTACTGATGTG 30614

Majority         GGCCAGGCACTTTGCCTGGTAATCACTGTTCATGTATTATCACATTTAATCCTAAGGGCTGTGATGTAGATGT-TAT--C

                         37850     37860     37870     37880     37890     37900     37910     37920

Human            AGCCAGGCACTTTGCCTGGTAATCACTGTTCGTGTAT-----AATTTAATCCTAAGGGCTGTGATGTAGATGT-TAT--C 28780
Coquerel'sSifaka GGCCATGTACTTTGGCAGGCAATCCCTATTCATGTATTATCACATTTAATCCTAAGGGCTGTGATGTAGGTGGATATTAC 27845
Ma'sNightMonkey  GGCCATGCACTTTGCCTGGTAATCACTGTTCATGTATTACCATATTTAATCCTATGGGCTGTGATGTAGATGT-TAT--C 25713
VervetAGM        GGCCAGGCACTTTGCCTGGTAATCACTGTTCATGTATTACCACATTTCATCCTAAGGGCTGTGATGTGGATGT-TAT--C 32179
Gorrilla         AGCCAGGCACTTTGCCTGGTAATCACTGTTCGTGTAT-----AATTTAATCCTAAGGGCTGTGATGTAGATGT-TAT--C 31163
GrabEatingMacaqueGGCCAGGCACTTTGCCTGGTAATCACTGTTCATGTATTATCACATTTAATCCTAAGGGCTGTGATGTGGATGT-TAT--C 30830
Macaque          GGCCAGGCACTTTGCCTGGTAATCACTGTTCATGTATTATCACATTTAATCCTAAGGGCTGTGATGTGGATGT-TAT--C 31593
PigTailedMacaque GGCCAGGCACTTTGCCTGGTAATCACTGTTCATGTATTATCACATTTAATCCTAAGGGCTGTGATGTGGATGT-TAT--C 30537
Bonobo           AGCCAGGCACTTTGCCTGGTAATCACTGTTCGTGTAT-----AATTTAATCCTAAGGGCTGTGATGTAGATGT-TAT--C 29817
OliveBaboon      GGCCAGGCACTTTGCCTGGTAATCACTGTTCATATATTATCACATTTAATCCTAAGGGCTGTGATGTGGATGT-TAT--C 30793
Orangutan        AGCCAGGCACTTTGCCTGGTAATCACTGTTCGTGTATTACTTAATTTAATCCTAAGGGCTGTGATGTAGATGT-TAT--C 30691
```

```
Majority        CCTATTTTACAAATGAAGAACTTGAGGCTTAGAATGATTAAGTACTGTTA-CCTAACTGATAAGTGGAAGCTGAGCTTCA

                        37930    37940    37950    37960    37970    37980    37990    38000

Human           CCTGTTTTACAAATAAAGAACTTCAGGCTTAGAATGATTAAGTACTGTTA-CCTAACTGATAAGTGGAAGCTGAGCTCCA 28859
Coquerel'sSifaka CCTGTATTACAAATGAAGAATGTTAGGTTTAGAATGGTTAAGTACAGTTA-AATAGCTGATA-GTGGAAGCTGAACTTCA 27923
Ma'sNightMonkey CCTGTCTTACAAATGAAGAACTTTAGGCTTAGAATGATTAAGTACAGTTA-CCTAACTGATAAGTTGAAGCTGAGCTCCA 25792
VervetAGM       CCTATTTTACAAATGAAGAACTTGAGGCTTAGAGTGATTAAGTACTGTTAACCTAACTGGTAAGTGGAAGTTGAGCTTCA 32259
Gorrilla        CCTGTTTTACAAATAAAGAACTTCAGGCTTAGAATGATTAAGTACTGTTA-CCTAACTGATAAGTGGAAGCTGAGCTCCA 31242
GrabEatingMacaqueCCTATTTTACAAATGAAGAACTTGAGGCTTAGAGTGATTAAATACTGTTAACCTAACTGGTAAGTGGAAGTTGAGCTTCA 30910
Macaque         CCTATTTTACAAATGAAGAACTTGAGGCTTAGAGTGATTAAATACTGTTAACCTAACTGGTAAGTGGAAGTTGAGCTTCA 31673
PigTailedMacaque CCTATTTTACAAATGAAGAACTTGAGGCTTAGAGTGATTAAATACTGTTAACCTAACTGGTAAGTGGAAGTTGAGCTTCA 30617
Bonobo          CCTGTGTTACAAATAAAGAACTTCAGGCTTAGAATGATTAAGTACTGTTA-CCTAACTGATAAGTGGAAGCTGAGCTCCA 29896
OliveBaboon     CCTATTTTACAAATGAAGAACTTGAGGCTTAGAGTGATTAAGTACTGTTAACCTAACTGGTAAGTGGAAGTTGAGCTTCA 30873
Orangutan       CCTATTTTACAAATAAAGAACTTCAGGCTTGGAATGATTAAGTACTGTTA-CCTAACTGATAAGTGGAAGCTCAGCTCCA 30770

Majority        GAGCATCCTTTCTTAATACCATATTTTCTACTTGTCATGAAAACATGAAGAAAGCTAGGAAACATTTTCTGT-GTTGATA

                        38010    38020    38030    38040    38050    38060    38070    38080

Human           GAGCACCCTTTCTTAATACAGTATTTTCTACTTGTCATGAAAACATGAAGAAAGCTAGGAAACATTTTCTGT-GTTGATA 28938
Coquerel'sSifaka GAGTAGTTTTATTTAAAACCATATTATATA----TTGTGAAAACAT----AGAACTAGGAAACATTTTCTGTTGTATATA 27995
Ma'sNightMonkey AAGCCCCCTTTCTTAATACCATATTGTCTACTTGTCATGAAAACATGAAGAAAGCTAGGAAAGATTTTCTTTGTTGATA 25872
VervetAGM       GAGCATCCTTTCTTAATACCATATTTTCTACTTGTCATGAAAACTTGAAGAAAGCTAGGAAACATCTATTGT---TGATA 32336
Gorrilla        GAGCACCCTTTCTTAATACAGTATTTTCTACTTGTCATGAAAACATGAAGAAAGCTAGGAAACATTTTCTTT-GTTGTTA 31321
GrabEatingMacaqueGAGCATCCTTTCTTAATACCATATTTTCTACTTGTCATGACAACTTGAAGAAAGCTAGGAAACATCTATTGT---TGATA 30987
Macaque         GAGCATCCTTTCTTAATACCATATTTTCTACTTGTCATGACAACTTGAAGAAAGCTAGGAAACATCTATTGT---TGATA 31750
PigTailedMacaque GAGCATCCTTTCTTAATACCATATTTTCTACTTGTCATGAAAACTTGAAGAAAGCTAGGAAACATCTATTGT---TGATA 30694
Bonobo          GAGCACCCTTTCTTAATACAGTATTTTCTACTTGTCATGAAAACATGAAGAAAGCTAGGAAACATTTTCTGT-GTTGATA 29975
OliveBaboon     GAGCATCCTTTCTTAATACCATATTTTCTACTTGTCATGAAAACTTGAAGAAAGCTAGGAAACATCTATTGT---TGATA 30950
Orangutan       GAGCACCCTTTCTTAATACCGTATTTTCTACTTGTCCTGAAAACACGAAGAAAGCTAGGAAACATTTTCTGTTGTTGATA 30850

Majority        ATGAGCCAATA--ATGTGTATCAGTGGTTCTTAAACTTTAGCATGCATCAGAAACACTTGAGGATTTTTTTTTA--AACC

                        38090    38100    38110    38120    38130    38140    38150    38160

Human           ATGAGCCAATA--ATGTGTATCAGTGGTTCTTAAACTTTAGCATGCATCAGAAACACTTGGGGATTTTTTTTTTTTAAACC 29016
Coquerel'sSifaka GTGGACCAATATAATGTATATCAGTGG-TCTTAAATGTTAGCACACATCAGAAACAGCAGGAGGATTTGTTAAA--A-TT 28071
Ma'sNightMonkey TGGAGCCAGTA--AAGTATATCACTGATTCTTAAACTTTAGCATGCGTCAGAAACACTTGAGG-TTTTTTTTAA--AACC 25947
VervetAGM       ATGAGCCAATA--ATGTGTATCAGTGGCTCTTAAACTTTTGCATGCATGAGAAACACTTGAGGATTTTTTTTTA--AACC 32412
Gorrilla        ATGAGCCAATA--ATGTGTATCAGTGGTTCTTAAACTTTAGCATGCATCAGAAACACTTGGGGATTTTTTTTTA-AAACC 31398
GrabEatingMacaqueATGAGCCAATA--ATGTGTATCAGTGGCTCTTAAACTTTTGCATGCATGAGAAACACTTGAGGATTTTTTTTTA--AACC 31063
Macaque         ATGAGCCAATA--ATGTGTATCAGTGGCTCTTAAACTTTTGCATGCATGAGAAACACTTGAGGATTTTTTTTTA--AACC 31826
PigTailedMacaque ATGAACCAATA--ATGTGTATCAGTGGCTCTTAAACTTTTGCATGCATGAGAAACACTTGAGGATTTTTTTTTA--AACC 30770
Bonobo          ATGAGCCAATA--ATGTGTATCAGTGGTTCTTAAACTTTAGCATGCATCAGAAACACTTGGGGATTTTTTTTTTTTAACC 30053
OliveBaboon     ATGAGCCAATA--ATGTGTATCAGTGGCTCTTAAACTTTTGCATGCATGAGAAACACTTGAGGATTTTTTTTTA--AACC 31026
Orangutan       ATGAGCCAATA--ATGTGTATCAGTGGTTCTTAAACTTTAGCATGCATCAGAAACACTTGAGGATTTTTTTTTTTTAAACC 30928
```

```
Majority         TGGG----------------------------------------------------------------------------
                         38170     38180     38190     38200     38210     38220     38230     38240

Human            TGGGG--------------------------------------------------------------------------- 29021
Coquerel'sSifaka TAAG---------------------------------------------------------------------------- 28075
Ma'sNightMonkey  TGGG---------------------------------------------------------------------------- 25951
VervetAGM        TGGC---------------------------------------------------------------------------- 32416
Gorrilla         TGGG---------------------------------------------------------------------------- 31402
GrabEatingMacaqueTGGC---------------------------------------------------------------------------- 31067
Macaque          TGGC---------------------------------------------------------------------------- 31830
PigTailedMacaque TGGC---------------------------------------------------------------------------- 30774
Bonobo           TGGG---------------------------------------------------------------------------- 30057
OliveBaboon      TGGC---------------------------------------------------------------------------- 31030
Orangutan        TGGGTTTTTAGATTCCACCCTCAGAGTTTCTTATTCAGTAGGTCTGGGATTGGACCTGAGAACATGTACTGTCAGTAAGT 31008

Majority         --------------------------------------TTTTTGGACCCCACCCTCAGAGTTTCTTATTCAGTAGGTCTG
                         38250     38260     38270     38280     38290     38300     38310     38320

Human            --------------------------------------TTTTTAGATCCCACCCTCAGAGTTTCTTATTCAGTAGGTCTG 29063
Coquerel'sSifaka --------------------------------------TTGTTGGACCCCACCCTAAGTTTCTTATTCTCAGTAGGTCTA 28117
Ma'sNightMonkey  --------------------------------------GTTTTGGACCATACTCTC----TTTCTTATTCAGTAGGTCTG 25989
VervetAGM        --------------------------------------GTTTTGGACCCCACCCTCAGAGTTTCTTATTCAGTAGGTCTG 32458
Gorrilla         --------------------------------------TTTTTAGATCCCACCCTCAGAGTTTCTTATTCAGTAGGTCTG 31444
GrabEatingMacaque--------------------------------------TTTTTGGACCGCACCCTCAGAATTTCTTATTCAGTAGGTCTG 31109
Macaque          --------------------------------------TTTTTGGACCGCACCCTCAGAATTTCTTATTCAGTAGGTCTG 31872
PigTailedMacaque --------------------------------------TTTTTGGACCCCACCCTCAGAATTTCTTATTCAGTAGGTCTG 30816
Bonobo           --------------------------------------TTTTTAGATCCCACCCTCAGAGTTTCTTATTCAGTAGGTCTG 30099
OliveBaboon      --------------------------------------TTTTTGGACCCCACCCTCAGAATTTCTTATTCAGTAGGTCTG 31072
Orangutan        TTCCAGGTCTATTGAGGATTTTTTTTTTAAAACCTGGGTTTTTAGATCCCACCCTCAGAGTTTCTTATTCAGTAGGTCTG 31088

Majority         GGATTGGACCTGAGAACATGTATTGTCAGTAAGTTTCCAGGTCTAATCTTAGGATTAGGGACCACACTTTGAGAATCGCT
                         38330     38340     38350     38360     38370     38380     38390     38400

Human            GGATTGGACCTGAGAACATATACTGTCAGTAAGCTTCCAGGTCTAATCTTAGGATTAGGGACCACACTTTGAGAATGATT 29143
Coquerel'sSifaka AGAT-GGACCTGAGAACATGTATTGCCAACAGGTTTCCAGGTCTG--------AT----------------------GTT 28166
Ma'sNightMonkey  GGATTAGACCTGAGAACACGTATTGTCAGTAAGTTTCCAGGTCTGATCTTTGGATTAGGGACCACACTTTG-GAATCACT 26068
VervetAGM        GGATTGGACCTGAGAACGTGTATTATCAGTAATTTTCCAGGTCTAATCTTAGGATTAGGCACCTCACTTTGAGAATCGCT 32538
Gorrilla         GAATTGGACCTGAGAACATATACTGTCAGTCAGTTTCCAGGTCTAATCTTAGGATTAGGGACCACACTTTGAGAATCATT 31524
GrabEatingMacaqueGGATTGGACCTGAGAACATGTATTATCAGTAAGTTTCCAGGTCTAATCTTAGGATTAGGCACCACACTTTGAGAATCGCT 31189
Macaque          GGATTGGACCTGAGAACATGTATTATCAGTAAGTTTCCAGGTCTAATCTTAGGATTAGGCACCACACTTTGAGAATCGCT 31952
PigTailedMacaque GGATTGGACCTGAGAACATGTATTATCAGTAAGTTTCCAGGTCTAATCTTAGGATTAGGCACCACACTTTGAGAATCGCT 30896
Bonobo           GGATTGGACCTGAGAACATATACTGTCAGTAAGCTTCCAGGTCTAATCTTAGGATTAGGGACCACACTTTGAGAATCATT 30179
OliveBaboon      GGATTGGATCTGAGAACATGTATTATCAGTAAGTTTCCAGGTCTAATCTTAGGATTAGGCACCACACTTTGAGAATCGCT 31152
Orangutan        GGATTGGACCTGAGAACATGTACTGTCAGTAAGTTTCCAGGTCTAATCTTAGGATTAGGGACCACACTTTGAGAATCACT 31168
```

```
Majority          AGAGTATTCAATTAGATCTTAGGGACTACACTGTGAGAATCACTAATGTATACAATTCTGCTCATCATGTTCTTATAAAA
                           38410     38420     38430     38440     38450     38460     38470     38480
Human             AGAGTATTCAATTAGATCTTAGGGACTACACTGTGAGAATCAGTAATGTATACAATTCTGCTCATCATGTTCTTATAAAA 29223
Coquerel'sSifaka  --------------GATTTTAGGGACTACACTTTGAGAATCACTAAAATATCTAATTCTGCTCATTATA-CCTTATACAA 28231
Ma'sNightMonkey   AAAGTATACAATTAGATCTTAGGGACTACACTATGAGAATCACTAGAGTATACAGTTCTCCTCATCATGTCCTTATAAAA 26148
VervetAGM         AGAGTATTCAATTAGATCTTAGGGACTACACTGTGAGAATCACTAATGTATACACTTCTGCTCATCATGTTCTTATAAAA 32618
Gorrilla          AGAGTATTCAATTAGATCTTAGGGACTACACTGTGAGAATCAGTAATGTATACAATTCTGCTCATCATGTTCTTATAAA- 31603
GrabEatingMacaqueAGAGTATTCAATTAGATCTTAGGGACTACACTGTGAGAATCACTAATGTATACAATTCTGCTCATCATGTTCTTATAAAA 31269
Macaque           AGAGTATTCAATTAGATCTTAGGGACTACACTGTGAGAATCACTAATGTATACAATTCTGCTCATCATGTTCTTATAAAA 32032
PigTailedMacaque  AGAGTATTCAATTAGATCTTAGGGACTACACTGTGAGAATCACTAATGTATACAATTCTGCTCATCATGTTCTTATAAAA 30976
Bonobo            AGAGTATTCAATTAGATCTTAGGGACTACACTGTGAGAATCAGTAATGTATACAATTCTGCTCATCATGTTCTTATAAAA 30259
OliveBaboon       AGAATATTCAATTAGATCTTAGGGACTACACTGTGAGAATCACTAATGTATACAATTCTGCTCATCATGTTCTTACAAAA 31232
Orangutan         AGAGTATTCAATTAGATCTTAGGGACTACACTGTGAGAATCAGTAACGTATACAATTCTGCTCATCATGTTCTTATAAAA 31248

Majority          CTA----TGCCATGCCAAATCTTCCATATCTTGATTAGAAATTGGTTAAGAAATTAGGCTTGGTGTAAATACGGTATCAA
                           38490     38500     38510     38520     38530     38540     38550     38560
Human             CTA----TGCCATGCCAGATCTTCCATATCTTGATTAGAAATTGGTTAAGAAATTAGGCTTGGTGTAAATACAGTATCAA 29299
Coquerel'sSifaka  CTAAGCATGCCATGCCAAATCTTCCATATCTTGATTAGAAATT------------AGACTTAGTGCAAATACGGTATTAA 28299
Ma'sNightMonkey   CTA----TGCCATTCCAAATCTTCCATATCTTGATTAGAAATTGGTTTATAAATTAGACTTGGTGTAAATATAGTATTAA 26224
VervetAGM         TTA----TACCATGCCAAATCTTCCATATCTAGTTTAGAAATTGGTTAAGAACTTAAGCCTGGTGTAAATACGGTATCAG 32694
Gorrilla          CTA----TGCCATGCCAGATCTTCCATATCTTGATTAGAAATTGGTTAAGAAATTAGGCTTGGTGTAAATACAGTATCAA 31679
GrabEatingMacaqueTTA----TGCCATGCCAAATCTTCCATATCTAGTTTAGAAATTGGTTAAGAAATTAAGCCTGGTGTAAATACGGTATCAA 31345
Macaque           TTA----TGCCATGCCAAATCTTCCATATCTAGTTTAGAAATTGGTTAAGAAATTAAGCCTGGTGTAAATACGGTATCAA 32108
PigTailedMacaque  TTA----TGCCATGCCAAATCTTCCATATCTAGTTTAGAAATTGGTTAAGAAATTAAGCCTGGTGTAAATATGGTATCAA 31052
Bonobo            CTA----TGCCATGCCAGATCTTCCATATCTTGATTAGAAATTGGTTAAGAAATTAGGCTTGGTGTAAATACAGTATCAA 30335
OliveBaboon       TTA----TGCCATGCCAAATCTTCCATATCTAGTTTAGAAATTGGTTAAGAAATTAAGCCTGGTGTAAATACGGTATCAA 31308
Orangutan         CTA----TGCCATGCCAAATCTTCCATATCTTGATTAGAAATTGGTTAAGAAATTAGGCTTGGTGTAAATACAGTATCAA 31324

Majority          TGTGGCTTGAAACT--GGTTGTATTTTTCTG-TTTTTTTCTC----TGTGCATTTGATGCTAATAAGTAGAAAGCTCTTA
                           38570     38580     38590     38600     38610     38620     38630     38640
Human             TGTGGCTTGAAACT--GGTTGTATTTTTCTG-TTTTTTTTTTTCTTTGTGCATTTGATGCTAATAAGTAGAAAGCTCTTA 29376
Coquerel'sSifaka  TGTGGCATGAAACCATGCTTTAATTTTTCCGATTCCAGTATTTCTCTGAGCATTTGATGCTGATGAGCAGAAAGCTTTTA 28379
Ma'sNightMonkey   TATGGCTTGAAACTATGGTTGTATTTTTCTG-TTTTGTTTTCCCTCTGTGCATTTGGTGCTAATAAGTAGAAAGCTCTTA 26303
VervetAGM         TGTGACTTGAAACT--GGTTGTATTTTTTGG-TTTTTTTCTC----TGCGCATTTGATGTCAATAAGTAGAAAGC----- 32762
Gorrilla          TGTGGCTTGAAACT--GGTTGTATTTTTCTG-TTTTTTTTTT-CTCTGTGCATTTGATGCTAATAAGTAGAAAGCTCTTA 31755
GrabEatingMacaqueTGTGACTTGAAACT--GGTTGTATTTTTTGG-TTTTTTTCTC----TGCGCATTTGATGCCAATAAGTAGAAAGCTCTTA 31418
Macaque           TGTGACTTGAAACT--GGTTGTATTTTTTGG-TTTTTTTCTC----TGCGCATTTGATGCCAATAAGTAGAAAGCTCTTA 32181
PigTailedMacaque  TGTGACTTGAAACT--GGTTGTATTTTTTGG-TTTTTTTCTC----TGCGCATTTGATGCCAATAAGTAGAAAGCTCTTA 31125
Bonobo            TGTGGCTTGAAACT--GGTTGTATTTTTCTG-TTTTTTTTTT-CTTTGTGCATTTGATGCTAATAAGTAGAAAGCTCTTA 30411
OliveBaboon       TGTGACTTGAAACT--GGTTGTATTTTTTGG-TTTTTTTCTC----TGCGCATTTGATGCCAATAAGTAGAAAGCTCTTA 31381
Orangutan         CGTGGCTTGAAATT--GGTTGTATTTTTCTG-TTTTTTTCTC----TGTGCATTTGATGCTAATAAGTAGAAAGCTCTTA 31397
```

```
Majority          AAATATTTAAGGGAGATGACCTATGGATATCTGTTTACTGTTCTCATATTTGTTGTAATAC---AGTTTATTCATTATGA

                          38650     38660     38670     38680     38690     38700     38710     38720

Human             AAATATTTAAGGGAGACAACCTATGGATATCTGTTTACTCTTCTCATATTTGTTGTAATAC---AGTTTATTCATTATGA 29453
Coquerel'sSifaka  AAACATTTAAGGGAGATGACCTA-GAATATCTGTTTACCATTTTCACATGTGTTGTAATATGAAAGCTTATTAATTATGA 28458
Ma'sNightMonkey   AAATATCGAGGAGATACGACCTATGAATATCTGTTTGCTGTTCTCACACTTGTTGTAATAC---AATTTATTCATTATGA 26380
VervetAGM         --------AAGGGAGATGACCTATGGATATCTGTTTACTGTTCTCATATTTGTTGTAATAC---AGTTTATTCATTATGA 32831
Gorrilla          AAATATTTAAGGGAGACAACTTATGGATATCTGTTTACTGTTCTCCTATTTGTTGTAATAC---AGTTTATTCATTATGA 31832
GrabEatingMacaqueAAATATTTAAGGGAGATGACCTATGGATATCTGTTTACCGTTCTCATATTTGTTGTAATAC---AGTTTATTCATTATGA 31495
Macaque           AAATATTTAAGGGAGATGACCTATGGATATCTGTTTACCGTTCTCATATTTGTTGTAATAC---AGTTTATTCATTATGA 32258
PigTailedMacaque  AAATATTTAAGGGAGATGACCTATGGATATCTGTTTACCGTTCTCATATTTGTTGTAATAC---AGTTTATTCATTATGA 31202
Bonobo            AAATATTTAAGGGAGACAACCTATGGATATCTGTTTACTGTTCTCATATTTGTTGTAATAC---AGTTTATTCATTATGA 30488
OliveBaboon       AAATATTTAAGGGAGATGACCTATGGATATCTGTTTACCGTTCTCATATTTGTTGTAATAC---AGTTTATTCATTATGA 31458
Orangutan         AAATATTTAAGGGAGACAACCTATTGATATCTGTTTACTGTTTTCATGTTTGTTGTAATAC---AGTTTATTCATTATGA 31474

Majority          GTTTAAATACAAATTAGCATAAAGCTTATGGTTAA-TTTGGATTTT-TTGGACTTTCTGAAATGCTTTGATAATTCATTA

                          38730     38740     38750     38760     38770     38780     38790     38800

Human             GTTTAAATACAAATTAGCGTAAAGCTTATGGTTAA-TTTGGATTTT-TTGGACTTTCTGAAATGCTTTGATAATTCATTG 29531
Coquerel'sSifaka  GTTTAA-CACAAATCAGCATAAGGCTTATGGTTAA-TTTGGATTTTCTTTGACTTTCTGAAATGCTTTGATAA---ATTA 28533
Ma'sNightMonkey   GTTTAAATATAAATTAGCATAAAGCTTATGGTTAA-TTTGGATTTT-TTGGACTTTCTGAAATGCTTTGATAATTCATTA 26458
VervetAGM         ATTTAAATACAAATTAGCATAAAGCTTATGGTTAAATTTGGATTTT-TTGGACTTTCTGAAATGCTTTGATAATTCATTA 32910
Gorrilla          GTTTAAATACAAATTAGCGTAAAGCTTATGGTTAA-TTTGGATTTT-TTGGACTTTCTGAAATGCTTTGATAATTCATTG 31910
GrabEatingMacaqueGTTTAAATACAAATTAGCATAAAGCTTATGGTTAAATTTGGATTTT-TTGGACTTTCTGAAATGCTTTGATAATTCATTA 31574
Macaque           GTTTAAATACAAATTAGCATAAAGCTTATGGTTAAATTTGGATTTT-TTGGACTTTCTGAAATGCTTTGATAATTCATTA 32337
PigTailedMacaque  GTTTAAATACAAATTAGCATAAAGCTTATGGTTAAATTTGGATTTT-TTGGACTTTCTGAAATGCTTTGATAATTCATTA 31281
Bonobo            GTTTAAATACAAATTAGCGTAAAGCTTATGGTTAA-TTTGGATTTT-TTGGACTTTCTGAAATGCTTTGATAATTCATTG 30566
OliveBaboon       GTTGAAATACAAATTAGCATAAAGCTTATGGTTAAATTTGGATTTT-TTGGACTTTCTGAAATGCTTTGATAATTCATTA 31537
Orangutan         GTTTAAATACTAATTAGCCTGAAGCTTATGGTTAA-TTTGGATTTT-TTGTACTTTCTGAAATGCTTTGATAATTCATTA 31552

Majority          TCTCCTTTGCACATTTTTTT--CATTTTGATGGTTGCATTTAACTTCTTTGTACTGATTGGTT-TTTTAAATC-AGCCAG

                          38810     38820     38830     38840     38850     38860     38870     38880

Human             CCTCCTTTGCACATTTTTTT--CATTTTGATGGTTGCATTTAACTTCTTTGTACTGATT-----TTTTAAATC-AGCCAG 29603
Coquerel'sSifaka  TGTCCTTTATACATTTTTTTT-CATTTTGATGGTTGCATTCCACTTTTTTGTACTGATTGGTTGTTTTAGATCCAGCCAG 28612
Ma'sNightMonkey   TCTCCTTTGCACATTTTTTTTTCATTTTGATGGTTGCATTTAACTTTTTTGTATTGATTGGTT-GTTTAAATC-AGCCAG 26536
VervetAGM         TCTCCTTTGCACATTTTTTT--CATTTTGGTGGTTGCATTTAACTTCTTTGTACTGATTGGTT-ATTTAAATC-AGCCAG 32986
Gorrilla          CCTCCTTTGCACATTTTTTT--CATTTTGATGGTTGCATTTAACTTCTTTGTACTGATT-----TTTTAAATC-AGCCAG 31982
GrabEatingMacaqueTCTCCTTTGCACATTTTTTT--CATTTTGATGGTTGCATTTAACTTCTTTGTACTCATTGGTT-GTTTAAATC-AGCCAG 31650
Macaque           TCTCCTTTGCACATTTTTTT--CATTTTGATGGTTGCATTTAACTTCTTTGTACTCATTGGTT-GTTTAAATC-AGCCAG 32413
PigTailedMacaque  TCTCCTTTGCACATTTTTTT--CATTTTGATGGTTGCATTTAACTTCTTTGTACTGATTGGTT-GTTTAAATC-AGCCAG 31357
Bonobo            CCTCCTTTGCACATTTTTTT--CATTTTGATGGTTGCATTTAACTTCTTTGTACTGATT-----TTTTAAATC-AGCCAG 30638
OliveBaboon       TCTCCTTTGCACATTTTTTT--CATTTTGATGGTTGCATTTAACTTCTTTGTACTGATTGGTT-GTTTAAATC-AGCCAG 31613
Orangutan         TCTCCTTTGCACATTTTTTT--CATTTTGATGGTTGCATTTAACTTTTTTGTACTGATT-----TTTTAAATC-AGCCGG 31624
```

```
Majority         TATTGATTTAACCTATTTTAAATACTAATATCCATGTATGTAAATATA--CATAACCAAGGCAAAACAGTTGATTTTAGC

                         38890     38900     38910     38920     38930     38940     38950     38960

Human            TATTAATTTAACCTATTTCAATACTAATATCCATGTATGTAAATATC--CATAACTAAGGCAAAACAGTTGATTTTAGC 29681
Coquerel'sSifaka TATTGAGTTAGC-TATTTTAAATATTAATACCCATGTATGTAAATATATACATAAAGTAGGCAAAAGAGTTGATTT-AGC 28690
Ma'sNightMonkey  TATTGATTGAACCTATTTTAAATACTAATACCCATGTATGTAAATATA--CATAACAAAGGCAAAACAGTTGATTTTAGC 26614
VervetAGM        TATTGATTTAACCTATTT-AAATACTAATATCCATGTATGTAAATATA--CATAGCCAAGGCAAAACAGTTGATTTTAGT 33063
Gorrilla         TATTAATTTAACCTATTTTAAATACTAATATCCGTGTATGTAAATATC--CATAACTAAGGCAAAACAGTTGATTTTAGC 32060
GrabEatingMacaqueTATTGATTTAACCTATTT-AAATACTAATATCCATGTATGTAAATATA--CATAGCCAAGGCAAAACAGTTGATTTTAGT 31727
Macaque          TATTGATTTAACCTATTT-AAATACTAATATCCATGTATGTAAATATA--CATAGCCAAGGCAAAACAGTTGATTTTAGT 32490
PigTailedMacaque TATTGATTTAACCTATTT-AAATACTAATATCCATGTATGTAAATATA--CATAGCCAAGGCAAAACAGTTGATTTTAGT 31434
Bonobo           TATTAATTTAACCTATTTTAAATACTAATATCCATGTATGTAAATATC--CATAACTAAGTCAAAACAGTTGATTTTAGC 30716
OliveBaboon      TATTGATTTAACCTATTT-AAATACTAATAACCATGTATGTAAATATA--CATAGCCAAGGCAAAACAGTTGATTTTAGT 31690
Orangutan        TGTTGATTTAACCTATTTTAAATACTAATATCCATGTATGTAAATACA--CATAACCAAGGCAAAACAGTTGATTTTAGC 31702

Majority         TAGTTTAAATATTAGTCCATT----CATATTTTTGTAAATACATAACATTTTCAAAATAGCTAAAATGTCACTGGCTGGT

                         38970     38980     38990     39000     39010     39020     39030     39040

Human            TAGTTTAAATATTAGTCCATT----CATATTTTTGTAAGTACATAACATTTTCAAAATAGCTAAAATGTCACTGGCTGGT 29757
Coquerel'sSifaka TAGTTTAAATAGTAGTCTACTCACATATATTTCTGTAAATACAT-----TTGCAAAATAACTAAAACATCACTAACTGGT 28765
Ma'sNightMonkey  TAGTTTAAATACTAGTCTATTGTTCATATTTTTGTAAATACATAG-ATTATCAAAACAGCTAAAATGTCACTGGCTAGT 26693
VervetAGM        TAGTTTAAATATTAGTCCATT----CATATTTTTGTAAATACATAACATTTTCAAAATAGCTAAAATGTCACTGGCTGGT 33139
Gorrilla         TAGTTTAAATATTAGTCCATT----CATATTTTTGTAAATACATAACATTTTCAAAATAGCTAAAATGTCACTGGCTGGT 32136
GrabEatingMacaqueTAGTTTAAATATTAGTCCATT----CATATTTTTGTAAATACATAACATTTTCAAAATAGCTAAAATGTCACTGGCTGGT 31803
Macaque          TAGTTTAAATATTAGTCCATT----CATATTTTTGTAAATACATAACATTTTCAAAATAGCTAAAATGTCACTGGCTGGT 32566
PigTailedMacaque TAGTTTAAATATTAGTCCATT----CATATTTTTGTAAATACATAACATTTTCAAAATAGCTAAAATGTCACTGGCTGGT 31510
Bonobo           TAGTTTAAATATTAGTCCATT----CATATTTTTGTAAATACATAACATTTTCAAAATAGCTAAAATGTCACTGGCTGGT 30792
OliveBaboon      TAGTTTAAATATTAGTCCATT----CATATTTTTGTAAATACATAACATTTTCAAAATAGCTAAAATGTCACTGGCTGGT 31766
Orangutan        TAGTTTAAATATTAGTCCATT----CATATTTTTGTAAATACATAACATTTTCAAAATAGCTAAAATGTCACTGGCTGGT 31778

Majority         TTGAACAACTGTGCAGTTCCCAAACAGATGAATGTATAACCTTTTCTCTAAAGAATTTTAAGACATGACCTTATTTTTTT

                         39050     39060     39070     39080     39090     39100     39110     39120

Human            TTGAACAACTGTGCAGTTACCGAAGAGATGAATGTATAACCTTTTCTGTAAAGAATTTTAAGACATGACCTTATTTTTTT 29837
Coquerel'sSifaka TTAAACAACTGTGCAGTTCTAAAACATGTACATTTTTAACCTTTTCTTTAAAAGATGTTAGGGTATGACGCTATCTGTTT 28845
Ma'sNightMonkey  ATGAAGAACTGTGCGGTTCCCAAACAGATGAATGTATAACCTTTTCTCTAAAGAATTTTAAGACATGACCTTATTTTT-- 26771
VervetAGM        TTGAACAACTGTGCAGTTCCCAAACAGATGAATGTATAACCTTTTCTCTAAAGAATTTTAAGACATAACCTTATTTTTTT 33219
Gorrilla         TTGAACAACTGTGCAGTTCCCAAAGAGATGAATGTATAACCTTTTCTGTAAAGAATTTTAAGACATGACCTTATTTTTTT 32216
GrabEatingMacaqueTTAAACAACTGTGCAGTTCCCAAACAGATGAATGTATAACCTTTTCTCTA-----TTTTAAGACATAACCTTATTTTTTT 31878
Macaque          TTAAACAACTGTGCAGTTCCCAAACAGATGAATGTATAACCTTTTCTCTA-----TTTTAAGACATAACCTTATTTTTTT 32641
PigTailedMacaque TTAAACAACTGTGCAGTTCCCAAACAGATGAATGTATAACCTTTTCTCTA-----TTTTAAGACATAACCTTATTTTTTT 31585
Bonobo           TTGAACAACTGTGCAGTTACCGAAGAGATGAATGTATAACCTTTTCTGTAAAGAATTTTAAGACATGACCTTATTTTTTT 30872
OliveBaboon      TTAAACAACTGTGCAGTTCCCAAACAGTTGAATGTATAACCTTTTCTCTA-----TTTTAAGACATAACCTTATTTTTTT 31841
Orangutan        TTGAACAACTGTGCAGTTCCCAAAGAAATGAATGTATAACCTTTTCTGTAAAGAATTTTAAGACATGACCTTATTTTTTT 31858
```

```
Majority         --------GCAGAAAGAAATCTC-TCATTCCAAAGATTTTGCCTTTGCTTTCAGTGTTCTAGCCACTGAAGTATATACCT

                         39130     39140     39150     39160     39170     39180     39190     39200

Human            --------GCAGAAAGAAATCTCTTCATTCCAAAGATTTTGCCTTTGCTTTCGGTGTTCTAGCCACTGAAGTATATACCT 29909
Coquerel'sSifaka TTATTCTAACAGAAAGAAATTTC-TCATTCCATA--TTTTGCTTTTGCTTTCAATGTTCTACCCC------TATATGCCC 28916
Ma'sNightMonkey  --------GCAGAAAAAAATTTTCTCATTCCATAGATTTTGCCTTTGCTTTCAACGTTCTAGCTACTGAAGTATATACCT 26843
VervetAGM        --------GCAGAAAGAAATCTC-TCATTCCAAAGATTTTGCCTTTGCTTTCAGTGTTCTAGCCACTGAAGTATATACCT 33290
Gorrilla         --------GCAGAAAGAAATCTCTTCATTCCAAAGATTTTGCCTTTGCTTTCAGTGTTCTAGCCACTGAAGTATATACCT 32288
GrabEatingMacaque--------GCAGAAAGAAATCTC-TCATTCCAAAGATTTTGCCTTTGCTTTCAGTGTTCTAGCCACTGAAGTATATACCT 31949
Macaque          --------GCAGAAAGAAATCTC-TCATTCCAAAGATTTTGCCTTTGCTTTCAGTGTTCTAGCCACTGAAGTATATACCT 32712
PigTailedMacaque --------GCAGAAAGAAATCTC-TCATTCCAAAGATTTTGCCTTTGCTTTCAGTGTTCTAGCCACTGAAGTATATACCT 31656
Bonobo           --------GCAGAAAGAAATCTCTTCATTCCAAAGATTTTGCCTTTGCTTTCAGTGTTCTAGCCACTGAAGTATATACCT 30944
OliveBaboon      --------GCAGAAAGAAATCTC-TCATTCCAAAGATTTTGTCTTTGCTTTCAGTGTTCTAGCCTCTGAAGTATATACCT 31912
Orangutan        --------GCAGAAAA----TTTCTCATTCCAAAGATTTTGCCTTTGCTTTCAGTGTTCTAGCCACTGAAATATATACCT 31926

Majority         GTTTAATTTTAATAATGATGATGATATTGATATCTAACTCACCAGAATGAAGTTCTAATTTTCTAAAGACCTGAGTTATC

                         39210     39220     39230     39240     39250     39260     39270     39280

Human            GTTTAATTTTAATAATGATGAAGATATTGATATCTAACTCACCAGAATGAAGTTCTAATTTTCTAAAGACCTGAATTATC 29989
Coquerel'sSifaka GTTTAATTTTAATGATGATGATGA---TGATGTCCAACTCACCAATATGAAATTCTCATTTTCCTAAGACTTGAGTTATC 28993
Ma'sNightMonkey  GTTTAATTTTAATAATGATGAT-ACGTTGATATCTAACTCACCAGAATGAAGTTCTAATTTTCTAAAGACCTGAGTTATC 26922
VervetAGM        GCTTAATTTTAATAATGATGATGATACTCATATCTGACTCACCAGAATGAAGTTCTAATTTTATAAAGATCTGAGTTATC 33370
Gorrilla         GTTTAATTTTAATAATGATGATGATATTGATATCTAACTCACCAGAATGAAGTTCTAATTTTCTAAAGACCTGAATTATC 32368
GrabEatingMacaqueGCTTAATTTTAATAATGATGATGATACTGATATCTAACTCACCAGAATGAAGTTCTAATTTTATAAAAATCTGAGTTATC 32029
Macaque          GCTTAATTTTAATAATGATGATGATACTGATATCTAACTCACCAGAATGAAGTTCTAATTTTATAAAAATCTGAGTTATC 32792
PigTailedMacaque GCTTAATTTTAATAATGATGATGATACTGATATCTAACTCACCAGAATGAAGTTCTAATTTTATAAAAATCTGAGTTATC 31736
Bonobo           GTTTAATTTTAATAATGATGATGATATTGATATCTAACTCACCAGAATGAAGTTCTAATTTTCTAAAGACCTGAATTATC 31024
OliveBaboon      GCTTAATTTTAATAATGATGATGATACTGATATCTAACTCACCAGAATGAAGTTCTAATTTTATAAAAATCTGAGTTATC 31992
Orangutan        GTTTAATTTTAATAATGATGATGATATTGATATCTAACTCACCAGAATGAAGTTCTAATTTTCTAAAGACGTGAATTATC 32006

Majority         TGGATGAATAAAGGACCATTGG-----GATGGAGGATTTATA------GAGAACGTTTCTGAGA---AAGTCTAGCTGG-

                         39290     39300     39310     39320     39330     39340     39350     39360

Human            TGGATGAATAAAGGACCATTGG-----GATGGAGGATTTATA------GA--ACGTTTCTGAGA---AAGTCTAGCTGT- 30052
Coquerel'sSifaka TGGATGAAGAAAGGGCCCTTGGTAAAAGGCAAGAGATTTGTATGATCTGAAGAGGCCCTTGGGATGGAGGATTATATGGC 29073
Ma'sNightMonkey  TGGATGAATAAAGGGCCATTGG-----GATGGAGGATTTATA------GA--ACATTTCTGAGA---AAGTCTAGCTAG- 26985
VervetAGM        TGGATGAATAAAGGACCATTGG-----GATGGAGGATGTAGA------GAGAACGTTTCTGAGA---AAGTCTAGCTGG- 33435
Gorrilla         TGGATGAATAAAGGACCATTGG-----GATGGAGGATTTATA------GA--ACGTTTCTGAGA---AAGTCTAGCTGT- 32431
GrabEatingMacaqueTGCATGAATAAAGGACCATTGG-----GATGGAGGATGTAGA------GAGAACGTTTCTGAGA---AAGTCTAGCTGG- 32094
Macaque          TGCATGAATAAAGGACCATTGG-----GATGGAGGATGTAGA------GAGAACGTTTCTGAGA---AAGTCTAGCTGG- 32857
PigTailedMacaque TGCATGAATAAAGGACCATTGG-----GATGGAGGATGTAGA------GAGAACGTTTCTGAGA---AAGTCTAGCTGG- 31801
Bonobo           TGGATGAATAAAGGACCATTGG-----GATGGAGGATTTATA------GA--ACGTTTCTGAGA---AAGTCTAGCTGT- 31087
OliveBaboon      TGCATGAATAAAGGACCATTGG-----GATGGAGGATGTAGA------GAGAACGTTTCTGAGA---AAGTCTAGCTAG- 32057
Orangutan        TGGATGAATAAACGACCATTGG-----GATGGAGGATTTATA------GA--ACATTTCTGAGA---AAGTCTAGCTGG- 32069
```

```
Majority          ------TGTGGCTTTGGTTTGTTTGTAAACAATTTGAGATAGCTTTCCAAAGCTTTTGCTTATAGAATCTAATCACTTTT

                          39370     39380     39390     39400     39410     39420     39430     39440

Human             ------TGTGGCTTTGGTTTGTTTGTAAACAATT-GAGATAGCTTTCCAAAGCTTTTGCTTATAGAATCTAATCACTTTT 30125
Coquerel'sSifaka  ATTAGTTTTTGCTTTTTTTTTTTTGATAAAAAATCAAGGTAGCTTTCCAAAGGTTTTAATTATGGAAGCTAATGATCTTT 29153
Ma'sNightMonkey   ------TTTGGCTTTGGTTTGTTTGTAAAAAATT-GAGATAGCTTTCCAAAGCTTTTGCTTATAGAATCTAATCAGTTTT 27058
VervetAGM         ------TGTGGCTTTGGTTTGTTTGTAAACAATTTGAGATAGCGTTCCAAAGCTTTTGCTTATAGAATCTAATCACTTTT 33509
Gorrilla          ------TGTGGCTTTGGTTTGTTTGTAAACAATT-GAGATAGCTTTCCAAAGCTTTTGCTTATAGAATCTAATCACTTTT 32504
GrabEatingMacaque------TGTGGCTTTGGTTTGTTTGTAAACAATTTGAGATAGCTTTCCAAAGCTTTTGCTTATAGAATCTAATCACTTTT 32168
Macaque           ------TGTGGCTTTGGTTTGTTTGTAAACAATTTGAGATAGCTTTCCAAAGCTTTTGCTTATAGAATCTAATCACTTTT 32931
PigTailedMacaque  ------TGTGGCTTTGGTTTGTTTGTAAACAATTTGAGATAGCTTTCCAAAGCTTTTGCTTATAGAATCTAATCACTTTT 31875
Bonobo            ------TGTGGCTTTGGTTTGTTTGTAAACAATT-GAGATAGCTTTCCAAAGCTTTTGCTTATAAAATCTAATCACTTTT 31160
OliveBaboon       ------TGTGGCTTTGGTTTGTTTGTAAACAATTTGAGGTAGCTTTCCAAAGCTTTTGCTTATAGAATCTAATCACTTTT 32131
Orangutan         ------TGTGGCTTTGGTTTGTTTGTAAACAATT-GAGATAGCTTTCCAAAGCTTTTGCTTATAGAATCTAATCACTTTT 32142

Majority          TCTATTTATTGTCTTTTGGACTTA----AAAA--AAGGAAAGGGAACACTGTTGCCTTTGTGAGAATGGGAGAGGGATAG

                          39450     39460     39470     39480     39490     39500     39510     39520

Human             TCTATTTATTGTCTTTTGGGCTTAGAGTAAAG--AAGGGAAGGGAACACTGTTGCCTTTGTGAGAATGGGAGAGGGATAG 30203
Coquerel'sSifaka  CCTATTTATTGTCTTTTGGACTTGAGTGACAG--GAGGAAAGGGAACACT--TGCTTTTGTGGGAAAGGGAGACAGATAG 29229
Ma'sNightMonkey   TCTGTGTATTATCTTTTGGATTTGGATTAAAATGAAGGGAAAGGAACACTGTTGCGTTTGTGAGAATGGGAGAGGGATAC 27138
VervetAGM         TCTATTTATTGCCTTTTGGACTTA----AAGA--AGGGAAGGGGAACATTGTTGCCTTTGTGAGAATGGGGGAGGGATAG 33583
Gorrilla          TCTATTTATTGTCTTTTGGGCTTAGAGTAAAG--AAGGGAAGGGAACACTGTTGCCTTTTTGAGAATGGGAGAGGGATAG 32582
GrabEatingMacaqueTCTATTTATTGCCTTTTGGACTTA----AAGA--AGGGAGGGGGAACATTGTTGCCTTTGTGAGAATGGGGGAGGGATAG 32242
Macaque           TCTATTTATTGCCTTTTGGACTTA----AAGA--AGGGAGGGGGAACATTGTTGCCTTTGTGAGAATGGGGGAGGGATAG 33005
PigTailedMacaque  TCTATTTATTGCCTTTTGGACTTA----AAGA--AGGGAGGGGGAACATTGTTGCCTTTGTGAGAATGGGGGAGGGATAG 31949
Bonobo            TCTATTTATTGTCTTTTGGGCTT-----AAAG--AAGGGAAGGGAACACTGTTGCCTTTGTGAGAATGGGAGAGGGATAG 31233
OliveBaboon       TCTATTTATTGCCTTTTGGACTTA----AAGA--AGGGAAGGGGAACATTGTTGCCTTTGTGAGAATGGGAGAGGGATAG 32205
Orangutan         TCTATTTATTGTCTTTTGGACTTAGATTAAAG--AAGGGAAGGGAACACTGTTGCCTTTCTGAGAATGGGAGAGGGATAG 32220

Majority          ATGGGAGAGTAGAGGGATGAAGTTAATTTTTTATTATAGGGATTAACATTTTTCACTATTCCCAATAGTGTAACATTTGA

                          39530     39540     39550     39560     39570     39580     39590     39600

Human             ATGGGAGAGTAGAGGGATGAAGTTAATTTTTTATTATAGGGATTAACATTTTTAACTATTCCCAATAGTGTAACATTTGA 30283
Coquerel'sSifaka  ATGAGAGAGTAGAGGGGTGAAGTTAATTTTTT---A-----------AT--TT----ATTCTCAATAATGTAACATATGA 29289
Ma'sNightMonkey   ATGGGAGAGTAGAGGGATTAAGTTAATTTTTTATTATAGAGATTGACATTTTTATCTATTCCCAGGAGTGTAACATTTGA 27218
VervetAGM         ATGGGAGAGTAGAGGGATGAAGTTAATTTTTTATTATAGGGATTAACATTTTTCACTGTTTCCAATAGTGTAACATTTGA 33663
Gorrilla          ATGGGAGAGTAGAGGGATGAAGTTAATTTTTTATTATTGGGATTAACATTTTTAACTATTCCCAATAGTGTAACATTTGG 32662
GrabEatingMacaqueATGGGAGAGTAGAGGGATGAAGTTAATTTTTTATTATAGGGATTAACATTTTTCACTGTTCCCAATAGTGTAACATTTGA 32322
Macaque           ATGGGAGAGTAGAGGGATGAAGTTAATTTTTTATTATAGGGATTAACATTTTTCACTGTTCCCAATAGTGTAACATTTGA 33085
PigTailedMacaque  ATGGGAGAGTAGAGGGATGAAGTTAATTTTTTATTATAGGGATTAACATTTTTCACTGTTCCCAGTAGTGTAACATTTGA 32029
Bonobo            ATGGGAGAGTAGAAGGATGAAGTTAATTTTTTATTATAGGGATTAACATTTTTAACTATTCCCAATAGTGTAACATTTGA 31313
OliveBaboon       ATGGGAGAGTAGAGGGATGAAGTTAATTTTTTATTATAGGGATTAACATTTTTCACTGTTTCCAATAGTGTGACATTTGA 32285
Orangutan         ATGGGAGAGTAGAGGGATGAAGTTAATTTTTTATTATAGGGATTAACATTTTTAACTATTCCCAATAGTGTAACATTTGA 32300
```

```
Majority          CATTTGTTT----AAGTGAAAGGCTTTTAGATTAATATTTTTAAATTTAAAGATGTCTTATGCTAATTTGTTTTTGTTTT
                          39610     39620     39630     39640     39650     39660     39670     39680
Human             CATTT--------AAGTGAAAGGCTTTTAGATTAATATTTTTAAATTTAAAGATGTCTTATGCTAATTTGTTTTTGTTTT 30355
Coquerel'sSifaka  TATTTGCTTGCATGCATGACAGGCTTTCAGATTAATATTTTAAAATTTTAATATAACTTATGCTAGTTTGTTTTTGTTTT 29369
Ma'sNightMonkey   CATTTGTTT----AAGTGAAAGGCTTT-AGGTTAATATTTTGAAATTTAAAGATGTCTTATGCTAATTTGTTTTTGTTTT 27293
VervetAGM         CATTTGTTT----AAGTGAAAGACTTTTAGATTAATATTTTTAAATTTAAAGATGTCTTATGCTAACTTGTTTTTGTTTT 33739
Gorrilla          CATTT--------AAGTGAAAGGCTTTTAGATTAATATTTTTAAATTTAAAGATGTCTTATGCTAATTTGTTTTTGTTTT 32734
GrabEatingMacaqueCATTT--------AAGTGAAAGACTTTTAGATTAATATTTTTAAATTTAAAGATATCTTATGCTAACTTGTTTTTGTTTT 32394
Macaque           CATTTGTTT----AAGTGAAAGACTTTTAGATTAATATTTTTAAATTTAAAGATATCTTATGCTAACTTGTTTTTGTTTT 33161
PigTailedMacaque  CATTTGTTT----AAGTGAAAGACTTTTAGATTAATATTTTTAAATTTAAAGATATCTTATGCTAACTTGTTTTTGTTTT 32105
Bonobo            CATTT--------AAGTGAAAGGCTTTTAGATTAATATTTTTAAATTTAAAGATGTCTTATGCTAATTTGTTTTTGTTTT 31385
OliveBaboon       CATTTGTTT----AAGTGAAAGACTTTTAGATTAATATTTTTAAATTTAAAGATATCTTATGCTAACTTGTTTTTGTTTT 32361
Orangutan         CATTT--------AAGTGAAAGGCTTTTAGATTAATATTTTTAAATTTAAAGATGTCTTATGCTAATTTGTTTTTGTTTT 32372

Majority          TATAGAGAGGATCTGACGAACTACTCTCAGGCAGTGTTCTCAGTAGTCCAAACTCTAATATGAGCAGCATGGTAGTTACA
                          39690     39700     39710     39720     39730     39740     39750     39760
Human             CATAGAGAGGATCTGACGAACTACTCTCAGGCAGTGTTCTCAGTAGTCCGAACTCTAATATGAGCAGCATGGTAGTTACA 30435
Coquerel'sSifaka  TATAGAGAGGATCTGACGAACTACTCTCAGGCAGTGTTCTCAGTAGTCCAAACTCTAATATGAGCAGCATGGTAGTTACA 29449
Ma'sNightMonkey   TATAGAGAGGATCTGACGAACTACTCTCAGGCAGTGTTCTCAGTAGTCCAAACTCTAATATGAGCAGCATGGTAGTTACA 27373
VervetAGM         TATAGAGAGGATCTGACGAACTGCTCTCAGGCAGTGTTCTCAGTAGTCCAAACTCTAATATGAGCAGCATGGTAGTTACA 33819
Gorrilla          TATAGAGAGGATCTGACGAACTACTCTCAGGCAGTGTTCTCAGTAGTCCGAACTCTAATATGAGCAGCATGGTAGTTACA 32814
GrabEatingMacaqueTATAGAGAGGATCTGACGAACTGCTCTCAGGCAGTGTTCTCAGTAGTCCAAACTCTAATATGAGCAGCATGGTAGTTACA 32474
Macaque           TATAGAGAGGATCTGACGAACTGCTCTCAGGCAGTGTTCTCAGTAGTCCAAACTCTAATATGAGCAGCATGGTAGTTACA 33241
PigTailedMacaque  TATAGAGAGGATCTGACGAACTGCTCTCAGGCAGTGTTCTCAGTAGTCCAAACTCTAATATGAGCAGCATGGTAGTTACA 32185
Bonobo            CATAGAGAGGATCTGACGAACTACTCTCAGGCAGTGTTCTCAGTAGTCCGAACTCTAATATGAGCAGCATGGTAGTTACA 31465
OliveBaboon       TATAGAGAGGATCTGACGAACTGCTCTCAGGCAGTGTTCTCAGTAGTCCAAACTCTAATATGAGCAGCATGGTAGTTACA 32441
Orangutan         TATAGAGAGGATCTGACGAACTACTCTCAGGCAGTGTTCTCAGTAGTCCAAACTCTAATATGAGCAGCATGGTAGTTACA 32452

Majority          GGTAAGTGTTACTCTCTTAGCAGTCTGTCATT---TGCCATTTTAGGGGGCAGAATGTTAAAACTCAAATTTAACTCTGT
                          39770     39780     39790     39800     39810     39820     39830     39840
Human             GGTAAGTGTTACTCTCTTAGCAGTCTGTCATT---TGCCATTTTAGGGGGCAGAATGTTAAAACTCAAATTTAACCCTGT 30512
Coquerel'sSifaka  GGTAAGTATTACTCTC--AGCAGTTTGTCATTATTTGCCATTTTATGGGGCAGAATGTTAAAGCAAAAATTTA-TTCTGT 29526
Ma'sNightMonkey   GGTAAGTATTACTCTCTTAGCAGTCTGTCATT---TGCCATTTTAGGGAGCAGAATGTTAAAACTCAAATTGAACTCTGT 27450
VervetAGM         GGTAAGTGTTACTCTCTTAGCAGTCTGTCATT---TACCATTTTAGGGGGCAGAGTGTTAAAACTCAAATTTAACTGTGT 33896
Gorrilla          GGTAAGTGTTACTCTCTTAGCAGTCTGTCATT---TGCCATTTTAGGGGGCAGAATGTTAAAACTCAAATTTAACCCTGT 32891
GrabEatingMacaqueGGTAAGTGTTACTCTCTTAGCAGTCTGTCATT---TACCATTTTAGGGGGCAGAATGTTAAAACTCAAATTTAACTCTGT 32551
Macaque           GGTAAGTGTTACTCTCTTAGCAGTCTGTCATT---TACCATTTTAGGGGGCAGAATGTTAAAACTCAAATTTAACTCTGT 33318
PigTailedMacaque  GGTAAGTGTTACTCTATTAGCAGTCTGTCATT---TACCATTTTAGGGGGCAGAATGTTAAAACTCAAATTTAACTCTGT 32262
Bonobo            GGTAAGTGTTACTCTCTTAGCAGTCTGTCATT---TGCCATTTTAGGGGGCAGAATGTTAAAACTCAAATTTAACCCTGT 31542
OliveBaboon       GGTAAGTGTTACTCTCTTAGCAGTCTGTCATT---TACCATTTTAGGGGGCAGAATGTTAAAACTCAAATTTAACTCTGT 32518
Orangutan         GGTAAGTGTTACTCTGTTAGCAGTCTGTCATT---TGCCATTTTAGGGGGCAGAATGTTAAAACTCAAATTTAGCTCTGT 32529
```

```
Majority          TATACCAGGAAAGCCTTT-------------------------CTTTTTAAATGCTGTGTTGAAACATTTTAGAGTAAAT
                          39850     39860     39870     39880     39890     39900     39910     39920
Human             TATACCAGGAAAGCCTTT-------------------------CTTTTTAAATGC-GTGTTGAAACATTTTAGAGTAAAT 30566
Coquerel'sSifaka  TACATCAGAGTAGCCTTT---------------------------CTTTAAATGCTATGTTAAAACATTTTAGAGGAAAT 29579
Ma'sNightMonkey   TTTACCAGAAAAGCCTTT-------------------------CTTTTTAAATGCTGTGTTGAAACATTTTAGAGTAAAT 27505
VervetAGM         TATACCAGGAAAGCCTTTTGTTTTGTTTTGTTTTTAAGCCTTTCTTTTTAAATGCTGTGTTGAAACATTTTAGAGTAAAT 33976
Gorrilla          TATACCAGGAAAGCCTTT-------------------------CTTTTTAAATGCTGTGTTGAAACATTTTAGAGTAAAT 32946
GrabEatingMacaqueTATACTAGGAAAGCCTTTTTTTTT-TT-------AAAGCCTTTCTTTTTAAATGCTGTGTTGAAACATTTTAGAGTAAAT 32623
Macaque           TATACTAGGAAAGCCTTTTTTTTT-TTT------AAAGCCTTTCTTTTTAAATGCTGTGTTGAAACATTTTAGAGTAAAT 33391
PigTailedMacaque  TATACTAGGAAAGCCTTTTTTTTT-TTTTTTTTTTAAGCCTTTCTTTTTAAATGCTGTGTTGAAACATTTTAGAGTAAAT 32341
Bonobo            TATACCAGGAAAGCCTTT-------------------------CTTTTTAAATGC-GTATTGAAACATTTTAGAGTAAAT 31596
OliveBaboon       TATACTAGGAAAGCCTTTTTTTTT-TTT----TTCAAGCCTTTCTTTTTAAATGCTGTGTTGAAACATTTTAGAGTAAAT 32593
Orangutan         TATACCAGGAAAGCCTTT-------------------------CTTTTTAAATGCTGTGTTGAAACATTTTAGAGTAAAT 32584

Majority          TTT-ACTAGAGTATATATGAGCTGAGTGAAAACAAAC----AAAAAAACCTGAAG-TGGTCACTTGG--GAGCAAGTTAC
                          39930     39940     39950     39960     39970     39980     39990     40000
Human             TTT-ACTAAAGTATATATGAGCCGAGTGGAAAAAAA-----AAAAAAACCTGAAGCTGGTAACTTTGGTGAACAAGTTAC 30640
Coquerel'sSifaka  TTTTACTAGTATATAAATGAGTCCAGTG-------------GGAAAATCTTGAAGTTGGTGACTTGT--GGGCAAGTAAC 29644
Ma'sNightMonkey   TTT-ACTAGAGTATATATGAGCTGAGTGAAAAAAA------AAAAACACCTTAAGATGGTGACTTTGATGAACAAGTTAC 27578
VervetAGM         TTT-ACTAGAGTATATATGAGCTGAGTGAAAACAAAC----AAACAAA--AAAAA--AAACACCTGA---AGTTGGTGAC 34044
Gorrilla          TTT-ACTAAAGTATATATGAGCTGAGTGAAAAAAAAAAAAAAAAAAAAACCTGAAGCTGGTAACTTTGGTGAACAAGTTAC 33025
GrabEatingMacaqueTTT-ACTAGAGTATATATGAGCTGAGTGAAAACAAAC----AAACAAA----A------CCACCTGA---AGTTGGTGAG 32685
Macaque           TTT-ACTAGAGTATATATGAGCTGAGTGAAAACAAAC----AAACAAA----A------CCACCTGA---AGTTGGTGAG 33453
PigTailedMacaque  TTT-ACTAGAGTATGTATGAGCTGAGTGAAAACAAAC----AAACAAA----AAA--AACCACCTGA---AGTTGGTGAC 32407
Bonobo            TTT-ACTAAAGTATATATGAGCCGAGTG--AAAAA------AAAAAAACCTGAAGCTGGTAACTTTGGTGAACAAGTTAC 31667
OliveBaboon       TTT-ACTAGAGTGTATATGAGCTGAGTGAAAACAAAC----AAACAAACAAAAAA--AACCACCTGA---AGTTGGTGAC 32663
Orangutan         TTT-ACTAGAGTATATATGAGCTGAGTGAAAAAAAAAAAAAAAAAACACCTGAAGCTGGTAACTTTGGTGAACAAGTTAC 32663

Majority          TCTTAAGTTTGATTGCTTTTCATTTTTGAACCACAAAT--GTAATTCT-TCTCACAT-TATAGTCCCCTCATGTGCCCCT
                          40010     40020     40030     40040     40050     40060     40070     40080
Human             TCTTAAATTTGATTGCTTTTCATTTTTGAACCACAAAT--GTAATTCTGTCTCACAT-TATAGTCCCTTCATCTGCCCCT 30717
Coquerel'sSifaka  TCATAAGTCTGATTGCCTTT--TTTTTGGATCACAAAGATATAATCCTTTCTCTTTCATATAATCCCCTCA----CCCCT 29718
Ma'sNightMonkey   TCTTGAGTTTGATTGCTTTTCATTTTTGAATCACAAATATGTAATTCTTTCTCACAT-TATAGTCCCCTCAAGTGCCTCT 27657
VervetAGM         TCTTAAGTTTGATTGCTTTTCATTTTTGAACCACAAAT--GTAATTCT----CACAT-TATAGTCCCCTCATGTGCCCCT 34117
Gorrilla          TCTTAAATTTGATTGCTTTTCATTTTTGAACCACAAAT--GTAATACTGTCTCACAT-TATAGTCCCTTCATGTGCCCCT 33102
GrabEatingMacaqueTCTTAAGTTTGATTGCTTTTCATTTTTGAACCACAAAT--GTAATTCT----CACAT-TATAGTCCCGTCATGTGCCCCT 32758
Macaque           TCTTAAGTTTGATTGCTTTTCATTTTTGAACCACAAAT--GTAATTCT----CACAT-TATAGTCCCCTCATGTGCCCCT 33526
PigTailedMacaque  TCTTAAGTTTGATTGCTTTTCATTTTTGAACCACAAAT--GTAATTCT----CACAT-TATAGTCCCCTCATGTGCCCCT 32480
Bonobo            TCTTAAATTTGATTGCTTTTCATTTTTGAACCACAAAT--GTAATTCTGTCTCACAT-TATAGCCCCTTCATCTGCCCCT 31744
OliveBaboon       TCTTAAGTTTGATTGCTTTTCATTTTTGAACCACAAAT--GTAATTCT----CACAT-TATAGTCCCCTCATGTGCCCCT 32736
Orangutan         -CTTAAGTTTGATTGCTTTTCATTTTTAAACCACAAAT--GTAATTCTTTATCACAT-TATAGTCCCTTCATGTGCCCCT 32739
```

```
Majority          CTGGTATTTTTATTTTGTTTTTATTTTTTGTGTTTGTGTGTCATATAATATTTTT-ACTTTAAGTATTT----ATGCCAC

                          40090     40100     40110     40120     40130     40140     40150     40160

Human             CTGGTATTTTTATTTTGTTTTTATTTTTTGTGTTTGTGTGTCATATAATATTTTT-ACTTTAAGTATTT----ATGCCAC 30792
Coquerel'sSifaka  TTTGTATT------TTTTTTTTAAT-------TTTTTAATCATAGAATATTTTT-ACTTTAAGTATTTCTTTATGCCAT 29784
Ma'sNightMonkey   CTGTTAT-------TTGTTTTT--------TGTTTGTGTGGCATAGAATGTTTTTTACTTTAAGTATTTGTTTATACCAC 27722
VervetAGM         CTGGTTTTTTTATTTTGTTTTTATTTTTTGTGTTTGTGTGTCATATAATATTTTT-ACT----GTATTT----ATGCCAC 34188
Gorrilla          CTGGTATTTATATTTTGTTTTTATTTTTTGTGTTTGTGTGTCATATAATATTTTC-ACTTTAAGTATTT----ATGCCAC 33177
GrabEatingMacaqueCTGGTATTTTTATTTTGTTTTTATTTTTTGTGTTTGTGTGTCATATAATATTTTT-ACTTTAAGTATTT----ATGCCAC 32833
Macaque           CTGGTATTTTTATTTTGTTTTTATTTTTTGTGTTTGTGTGTCATATAATATTTTT-ACTTTAAGTATTT----ATGCCAC 33601
PigTailedMacaque  CTGGTATTTTTATTTTGTTTTTATTTTTTGTGTTTGTGTGTCATATAATATTTTT-ACTTTAAGTATTT----ATGCCAC 32555
Bonobo            CTGGTATTTTTATTTTGTTTTTATTTTTTGTGTTTGTGTGTCATATAATATTTTT-ACTTTAAGTATTT----ATGCCAC 31819
OliveBaboon       CTGGTATTTTTATTTTGTTTTCATTTTTTGTGTTTGTGTGTCATATAATATTTTT-ACTTTAAGTATTT----ATGCCAC 32811
Orangutan         CTGGTATTTTTATTTTGTTTTTATTTTTTGTGTTTGTGTGTCATA--ATATTTTT-ACTTTAAGTATTT----ATGCCAC 32812

Majority          AAACCCTAAAACCCAAATTTTAAGTAAAAACCAAAGAATTCAGCTACATAGTTG-------------------------C

                          40170     40180     40190     40200     40210     40220     40230     40240

Human             AAACCCTAAAACCCAAATTTTAAGTAAAAACCGAAGAATTCAGCTACATAGTTGTTTAAAACCATTGCAACCATAGTTGC 30872
Coquerel'sSifaka  AAACCCTAAAACCCAAACTTTAAATAAAACCCAAAAAATTCTGCTACATAACTATTT------T-----------CTTTC 29847
Ma'sNightMonkey   AAACCCTAAAACCCAAATTTTTGGTAAAAACCAAAGAATTCAGCAACATAGTTG-------------------------- 27776
VervetAGM         AA-CCCTAAAACCCACATTGTAAGTAAAAACCAAAGAATTCAGCTACATAGTTG-------------------------C 34242
Gorrilla          AAACCGTAAAACCCAAATTTCAAGTAAAAACCAAAGAATTCAGCTACATAGTTGTTTAAAACCATTGCAACCATAGTTGC 33257
GrabEatingMacaqueAA-CCCTAAAACCCACATTGTAAGTAAAAACCAAAGAATTCAGCTACATAGTTG-------------------------C 32887
Macaque           AA-CCCTAAAACCCACATTGTAAGTAAAAACCAAAGAATTCAGCTACATAGTTG-------------------------C 33655
PigTailedMacaque  AA-CCCTAAAACCCACATTGTAAGTAAAAACCAAAGAATTCAGCTACATAGTTG-------------------------C 32609
Bonobo            AAACCCTAAAACCCAAATTTTAAGTAAAAACCGAAGAATTCAGCTACATAGTTGTTTAAAACCATTGCAACCATAGTTGC 31899
OliveBaboon       AA-CCCTAAAACCCACATTGTAAGTAAAAACCAAAGAATTCAGCTACATAGTTG-------------------------C 32865
Orangutan         AAACCCTAAAACCCAAATTTTAAGTAAAAACCAAAGAATTCAGCTACATAGTTGTTTAAAACCATTGCAACCATAGTTGC 32892

Majority          TATGGTTTTAAC-TAATTTTTTGCTGAAACAGTATTTTAATTCTTAGA-GTTGATCCTTTTATGTTGTCTACCTGCATAT

                          40250     40260     40270     40280     40290     40300     40310     40320

Human             TATGGTTTTAAC-TAATTTTTTGCTGAAACAGTATTTTAATTCT--GA-GTTGATCCTTTTATGTTGTCTACCTGCATAT 30948
Coquerel'sSifaka  TGCGGTTTGAACCTTTTTCTTTGCTGTAACAATTCTTTGAAT-T--GA-TTTTTTTTTTTTATGTTGTTGACTTGCAAAT 29923
Ma'sNightMonkey   -AAAACTC------ATTTTTTTGCTGAAACAATATGTTAATTCTTTGA-ATTGATCCTTTTATGTTGTCTACCTGCATAT 27848
VervetAGM         AATGGTTTTAAC-TAATTTTCTGCTGAAACAGTATTTTAATTCTTAGAAGTTGATCCTTTTATGTTGTCTACCTGCATAT 34321
Gorrilla          TATGGTTTTAAC-TAATTTTTTGCTGAAACAGTATTTTAATTCT--GA-GTTGATCCTTTTATGTTGTCTACCTGCATAT 33333
GrabEatingMacaqueAATGGTTTTAAC-TAATTTTTTGCTGAAACAGTATTTTAATTCTTAGA-GTTGATCCTTTTATGTTGTCTACCTGCATAT 32965
Macaque           AATGGTTTTAAC-TAATTTTTTGCTGAAACAGTATTTTAATTCTTAGA-GTTGATCCTTTTATGTTGTCTACCTGCATAT 33733
PigTailedMacaque  AATGGTTTTAAC-TAATTTTTTGCTGAAACAGTATTTTAATTCTTAGA-GTTGATCCTTTTATGTTGTCTACCTGCATAT 32687
Bonobo            TATGGTTTTAAC-TAATTTTTTGCTGAAACAGTATTTTAATTCT--GA-GTTGATCCTTTTATGTTGTCTACCTGCATAT 31975
OliveBaboon       AATGGTTTTAAC-TAATTTTTTGCTGAAACAGTATTTTAATTCTTAGA-GTTGATCCTTTTATGTTGTCTACCTGCATAT 32943
Orangutan         TATGGTTTTAAC-TAATTTTTTGCTGAAACAGTATTTTA---------------TCCTTTTATGTTGTCTACCTGCATAT 32956
```

```
Majority          TTAAGATGATTTGTTTTAATCTTCAAAGTTAGAGTTA-AAAAAAATTGTATATGTCTGAAATAATGTTGGTTGGTTGTAG

                          40330     40340     40350     40360     40370     40380     40390     40400

Human             TTAAGATGATTTGTTTTAATCTTCAAAGTTAGAGTTA-AAAAAAATTGTATATGTCTGAAATAATGTTGTTT----GTAG 31023
Coquerel'sSifaka  TTAAGATGATTTGTTT-AACTTTCAGAGTTAGAGTTTTTCTTAAATTGTATATGTCTGAAATAACGTTGGTTGGTTGTAG 30002
Ma'sNightMonkey   TTAAGATGATTTGTTT-AATCTTCAAACTTAGAGTTT-TAAAAAGTCGTATATGCCTGAAATAATGTTGGTTGTTTGTAG 27926
VervetAGM         TTAAGATGATTTGTTTTAATCTTCAAAGTTACAGTTA-AAAAAAATT-TGTGTATCTGAAATAATGTTGGTTTGTTGTAG 34399
Gorrilla          TTAAGATGATTTGTTTTAATCTTCAAAGTTAGAGTTA-AAAAAAATTGTATATGTCTGAAATAATGTTGTTT----GTAG 33408
GrabEatingMacaqueTTAAGATGATTTGTTTTAATCTTCAAAGTTAGAGTTA-AAAAAAATT-TGTTTATCTGAAATAATGTTGGTTGGTTGTAG 33043
Macaque           TTAAGATGATTTGTTTTAATCTTCAAAGTTAGAGTTA-AAAAAAATT-TGTTTATCTGAAATAATGTTGGTTGGTTGTAG 33811
PigTailedMacaque  TTAAGATGATTTGTTTTAATCTTCAAAGTTAGAGTTA-AAAAAAATT-TGTTTATCTGAAATAATGTTGGTTGGTTGTAG 32765
Bonobo            TTAAGATGATTTGTTTTAATCTTCAAAGTTAGAGTTT-AAAAAAATTGTATATGTCTGAAATAATGTTGTTT----GTAG 32050
OliveBaboon       TTAAGATGATTTGTTTTAATCTTCAAAGTTAGAGTTA-AAAAAAATT-TGTTTATCTGAAATAATGTTGGTTGGTTGTAG 33021
Orangutan         TTACGATGATTTGTTTTAATCTTCAGAGTTAAA-----AAAAAAATTGTATATGTCTGAAACAATGTTGTTT----GTAG 33027

Majority          GATCTGTCAGTAAACCAATATAGACCAAATTTTAGACCTTAGTTTTATGGCTGATGGGCCAAATTTTTTTTTTTTTTT-T

                          40410     40420     40430     40440     40450     40460     40470     40480

Human             CATCTGTCAGTAAAACAATATAGACCAAATTTTAGACCTTAGTTTTATGGCTGATGGGCCAAATTTTTTTTT-------- 31095
Coquerel'sSifaka  ----TGCCTAGAAACAAATATAGACTAA-TTTTATACCTTAATTTTATGGCTGATGAACCAA------------------ 30059
Ma'sNightMonkey   ----TATCAGTAAACAAATATAGACCAAATTTTAGACCTTAGTTTTATGGCTGATGGACCAATTTTTTTTTCCTTAGTAT 28002
VervetAGM         GATCTGTTAGTAAACCAATATAGACCAAATTTTAGACCTTAGTTTTATGGCTGTTGGGCCAAATTTTTTTTTTTTTTT-T 34478
Gorrilla          CATCTGTCAGTAAAACAATATAGACCAAATTTTAGACCTTAGTTTTATGGCTGATGGGCCAAATTTTTTTTTT------- 33481
GrabEatingMacaqueGATCTGTTAGTAAACCAATATAGACCAAATTTTAGACCTTAGTTTTATGGCTGTTGGGCCAAATTTTTTTTTTTTTTT-T 33122
Macaque           GATCTGTTAGTAAACCAATATAGACCAAATTTTAGACCTTAGTTTTATGGCTGTTGGGCCAAATTTTTTTTTTTTTTT-T 33890
PigTailedMacaque  GATCTGTTAGTAAACCAATATAGACCAAATTTTAGACCTTAGTTTTATGGCTGTTGGGCCAAATTTTTTTTTTTTTTT-T 32844
Bonobo            CATCTGTCAGTAAAACAATATAGACCAAATTTTAGACCTTAGTTTTATGGCTGATGGGCCAAATTTTTTTTT-------- 32122
OliveBaboon       GATCTGTTAGTAAACCAATATAGACCAAATTTTAGACCTTAGTTTTATGGCTGTTGGGCCAAATTTTTTTTTTTTTTT-- 33099
Orangutan         CATCTGTCAGTAAAACAATATAGACCAAATTTTAGAGCTTAGTTTTATGGCTGATGGGCCAAATTTTTTTTTTTTTTT-T 33106

Majority          TT-GTATTTTGTTTTGTTTTGCTTGAATTAACGTGTTCTCATCTGAATGGGCCAAATTTTGAATTTGATGATTATTATTA

                          40490     40500     40510     40520     40530     40540     40550     40560

Human             ---GTATTTTGTTTAGTTTTGCTTGAATTGACGTGTTCTCATCTGAATGGGCCAAATTTTGAATTTGATTATTATTATTA 31172
Coquerel'sSifaka  ----------------------------------------------------ATTTTGAATCTG---ATTATTATTG 30081
Ma'sNightMonkey   TTTGTTTTTTTTTTTGTTT-GTTTGAACTTACGTGTTCTCATCTGAATGGAACAAATTTTGAATCTGAGTATTATTACTA 28081
VervetAGM         TT-GTATTTTGTTTTGTTTTGCTTGAATTAACGTGTTCTCATCTGAATGGGCCAAATTTTGAATTTGATGATTATTATTA 34557
Gorrilla          ---GTATTTTGTTTAGTTTTGCTTGAATTGATGTGTTCTCATCTGAATGGGCCAAATTTTGAATTTGATTATTATTATTA 33558
GrabEatingMacaqueTTTGTATTTTGTTTTGTTTTGCTTGAATTAACGTGTTCTCATCTGAATGGGCCAAATTTTGAATTTGATGATTATTATTA 33202
Macaque           TTTGTATTTTGTTTTGTTTTGCTTGAATTAACGTGTTCTCATCTGAATGGGCCAAATTTTGAATTTGATGATTATTATTA 33970
PigTailedMacaque  TT-GTATTTTGTTTTGTTTTGCTTGAATTAACGTGTTCTCATCTGAATGGGCAAAATTTTGAATTTGATGATGATTATTA 32923
Bonobo            ---GTATTTTGTTTAGTTTTGCTTGAATTGACGTGTTCTCATCTGAATGGGCCAAATTTTGAATTTGACTATTATTATTA 32199
OliveBaboon       ---GTATTTTGTTTTGTTTTGCTTGAATTAACGTGTTCTCATCTGAATGGGCCAAATTTTGAATTTGATGATTATTATTA 33176
Orangutan         TT-GTATTTTGTTTAGTTTTTCTTGAATTGACGTGTTCTCATCTGAATGGGCCAAATTTTGAATTTGATAATTATTATCA 33185
```

```
Majority         TCAAATGAATTTACCTCACAACTCAACATTTTACATTATTTACCTGAAACCAAAGTTCCTTTTT-CTGTATTTTAACTTT
                         40570     40580     40590     40600     40610     40620     40630     40640
Human            TCAAATGAATTTACCTCACAACTCCACATTTTACATTATTTACCTGAAACCAAAGTTCCTTTTT-CTGTATTTTAACTTT 31251
Coquerel'sSifaka TTGAATGAATT-ACCTTATAACCCTGCATTTCACATTATTTACTTCAAACCGAAGTTCCTTTTTTCTGTTATTTTTTCTT 30160
Ma'sNightMonkey  TCGAAGGAATTTACCTCACAACTCAACATTTTACATTATTTACCTGAAACCAAAGTTCCTTTTT-CTGTATTTTAACTTT 28160
VervetAGM        TCAAATGAATTTACCTCACAGCTCAACATTTTACATTATTTACCTGAAACTAAAGTTCCTTTTT-CTGTATTTTAACTTT 34636
Gorrilla         TCAAATGAATTTACCTCACAACTCCACATTTTACATTATTTACCTGAAACCAAAGTTCCTTTTT-CTGTATTTTAACTTT 33637
GrabEatingMacaqueTCAAATGAATTTACCTCACAGCTCAACATTTTACATTATTTACCTGAAACTAAAGTTCCTTTTT-CTGTATTTTAACTTT 33281
Macaque          TCAAATGAATTTACCTCACAGCTCAACATTTTACATTATTTACCTGAAACTAAAGTTCCTTTTT-CTGTATTTTAACTTT 34049
PigTailedMacaque TCAAATGAATTTACCTCACAGCTCAACATTTTACATTATTTACCTGAAACTAAAGTTCCTTTTT-CTGTATTTTAACTTT 33002
Bonobo           TCAAATGAATTTACCTCACAACTCCACATTTTACATTATTTACCTGAAACCAAAGTTCCTTTTT-CTGTATTTTAACTTT 32278
OliveBaboon      TCAAATGAATTTACCTCACAGCTCAACATTTTACATTATTTACCTGAAACCAAAGTTCCTTTTT-CTGTATTTTAACTTT 33255
Orangutan        TCAAATGAATTTACCTTACAACTCCACATTTTACATTATTTACCTGAAACCAAGGTTCCTTTTT-CTGTATTTTAACTTT 33264

Majority         GAATTATTTGCTTGAGGAACGAATTATATTTGGCAATAAAAGGATCATAAAAGACTAGAAGACATTTTGATTTCCCTTAG
                         40650     40660     40670     40680     40690     40700     40710     40720
Human            GAATTATTTGCTTGAAGAACAAATTATATTTGGCAATAAAAGGATCATGAAAGACTAGAAGACATTTTGATTTCCCTTAG 31331
Coquerel'sSifaka GAATTACTTGCTTGAGGAATTAATTATATTGGGCAATAAAGGGATCTTAAAAGACTAGAAGACATTTTGATTTCCTTCAT 30240
Ma'sNightMonkey  GAATTATTTGCTTGAGGAACAAATT-TATTTGCATTAAAAGGATTGTAAAAGACTAGAAGACATTTTGATTTCCCTTAG 28239
VervetAGM        GAATTATTTGCTTGAGGAACGAATTATATTTGGCAATAAAAGGATCGTAAAAGACTAGAAGACATTTCGATTTCCCTTAG 34716
Gorrilla         GAATTATTTGCTTGAAGAACAAATTATATCTGGCAATAAAAGGATCATGAAAGACTAGAAGACATTTTGATTTACCTTAG 33717
GrabEatingMacaqueGAATTATTTGCTTGAGGAACGAATTATATTTGGCAATAAAAGGATCATAAAAGACTAGAAGACATTTTGATTTCCCTTAG 33361
Macaque          GAATTATTTGCTTGAGGAACGAATTATATTTGGCAATAAAAGGATCATAAAAGACTAGAAGACATTTTGATTTCCCTTAG 34129
PigTailedMacaque GAATTATTTGCTTGAGGAACGAATTATATTTGGCAATAAAAGGATCATAAAAGACTAGAAGACATTTTGATTTCCCTTAG 33082
Bonobo           GAATTATTTGCTTGAAGAACAAATTATATTTGGCAATAAAAGGATCATGAAAGACTAGAAGACATTTTGATTTCCCTTAG 32358
OliveBaboon      GAATTATTTGCTTGAGGAACGAATTATATTTGGCAATAAAAGGATCATAAAAGACTAGAAGACATTTTGATTTCCCTTAG 33335
Orangutan        GAATTATTTGCTTGAGGAACAAATTATATTTGGCAATAAAAGGATCATGAAAGACTAGAAGAGATTTTGATTTCCCTTAG 33344

Majority         GTAGCTGTGCTTA-TGTATACATACATCTTGACACACATA--AACACAAA-CACACATCTCTGACAATTCAGTTAACTAA
                         40730     40740     40750     40760     40770     40780     40790     40800
Human            GTAGCTGTGCTTAATGTATACATACATCTTGACACACACA--AACACAAA-CACACATCTCTGACAATTCAGTTAACTAA 31408
Coquerel'sSifaka AGAGCCAT---TA-CTTGTACATACAT-----------------A-------CGCATCCCTTATGGTTCAGTTAACTAA 30291
Ma'sNightMonkey  GTATCCGTGCTTA-TGTATACATACATCTTGACACA------AACACA---CACACATCTCTGACAGTTCAGTTAACTAA 28309
VervetAGM        GTAGCTGTGCTTA-TGTATACATACATCTTGACACACATA--AACACAAAACACACATCTCTGACAATTCAGTTAACTAA 34793
Gorrilla         GTAGCTGTGCTTAATGTATACATAAATCTTGACACACACACAAACACAAA-CACACATCTCTGACAATTCAGTTAACTAA 33796
GrabEatingMacaqueGTATCTGTGCTTA-TGTATACATACATCTTGACACACATA--AACACAAAACACACATCTCTGACAATTCAGTTAACTAA 33438
Macaque          GTATCTGTGCTTA-TGTATACATACATCTTGACACACATA--AACACAAAACACACATCTCTGACAATTCAGTTAACTAA 34206
PigTailedMacaque GTAGCTGTGCTTA-TGTATACATACATCTTGACACACATA--AACACAAAACACACATCTCTGACAATTCAGTTAACTAA 33159
Bonobo           GTAGCTGTGCTTAATGTATACATACATCTTGACACACACA--AACACAAA-CACACATCTCTGACAATTCAGTTAACTAA 32435
OliveBaboon      GTAGCTGTGCTTA-TGTATACATACATCTTGACACACATA--AACACAAAACACACATCTCTGACAATTCAGTTAACTAA 33412
Orangutan        GTAGCTGTGCTTA-TGTATACATATATCTTGACACACACA--AACACAAA-CACACATC--TGACAATTCAGTTAACTAA 33418
```

```
Majority          AAATTATAAGGAC----TTAACAGATAA--TAGTCATGATGTGAAGATAAATTTGTCAGCAGGTCATTATCATTGATTTT
                          40810     40820     40830     40840     40850     40860     40870     40880
Human             AAATTATAAGGAC----TTAACAGATAA--TAGTCATGATGTGAAGATAAATTTGTCAACAGGTCATTATCATTGATTTT 31482
Coquerel'sSifaka  AAATTACAAGGACGTATTTACCAAATACAGTCATAATGATGTGAAGAGGAATTTGCCAACACATCATTATTACTGATTTT 30371
Ma'sNightMonkey   AAATTATGAGGAC----TTAACAGATA---TAGTCATGATGTGAAGATAAATTTGTCAGTAGGTCATCATCACTGATTTT 28382
VervetAGM         AAATTATAAGGAC----TTAACAGATAA--TAGTCACGATTTGAAGATAAAGTTGTCAGCAGGTCATTATCATTGATTTT 34867
Gorrilla          AAATTATAAGGAC----TTAACAGATAA--TAGTCATGATGTGAAGATAAATTTGTCAACAGGTCATTATCATTGATTTT 33870
GrabEatingMacaqueAAATTGTAAGGAC----TTAACAGATAA--TAGTCACGATTTGAAGATAAATTTGTCAGCAGGTCATTATCATTGATTTT 33512
Macaque           AAATTGTAAGGAC----TTAACAGATAA--TAGTCACGATTTGAAGATAAATTTGTCAGCAGGTCATTATCATTGATTTT 34280
PigTailedMacaque  AAATTGTAAGGAC----TTAACAGATAA--TAGTCACGATTTGAAGATAAATTTGTCAGCAGGTCATTATCATTGATTTT 33233
Bonobo            AAATTATAAGGAC----TTAACAGATAA--TAGTCATGATGTGAAGATAAATTTGTCAACAGGTCATTATCATTGATTTT 32509
OliveBaboon       AAATTATAAGGAC----TTAACAGATAA--TAGTCACGATTTGAAGATAAATTTGTCAGCAGGTCATTATCATTGATTTT 33486
Orangutan         AAATTATAAGGAC----TTAACAGATAA--TAGTCATGATGTGAAGATAAATTTGTCAACAGGTCATTATCATTGATTTT 33492

Majority          GCTATGTAGTGATTACAAAGCAAAATCACTGGGCCATGATTTTGATAGTCAAAGTACCATGTACACATAATTGAACAAAT
                          40890     40900     40910     40920     40930     40940     40950     40960
Human             GCTATGTAATGATTACAAAGCAAAATCACTGGGCCTTGATTTTGATAGTCAAAGTACCATGTACACATAATTGAACAAAT 31562
Coquerel'sSifaka  TCTATGTAGTGATTATAAAGTAAAATCACAAGGCAGTGATTTTGATAGTCAAAGTACCATGTGCAAATAATTCACCCAAT 30451
Ma'sNightMonkey   GCTATGTAATAATTATAAAGCAAAATCACTGGACCATGATTTTGATAGTTAAAGTACCATGTACAGCTAACTGAATGAAT 28462
VervetAGM         GCTATGTAGTGATTACAAAGCAAAATCACTGGGCCGTGATTTTGATAGTCAAAGTACCATGTACACATACTTGAACAAAT 34947
Gorrilla          GCTATGTAATGATTACAAAGCAAAATCACTGGGCCTTGATTTTGATAGTCAAAGTACGATGTACACATAATTGAACAAAT 33950
GrabEatingMacaqueGCTATGTAGTGATTACAAAGCAAAATCACTGGGCCATGATTTTGATAGTCAAAGTACCATGTACACGTAATTGAACAAAT 33592
Macaque           GCTATGTAGTGATTACAAAGCAAAATCACTGGGCCATGATTTTGATAGTCAAAGTACCATGTACACGTAATTGAACAGAT 34360
PigTailedMacaque  GCTATGTAGTGATTACAAAGCAAAATCACTGGGCCATGATTTTGATAGTCAAAGTACCATGTACACGTAATTGAACAAAT 33313
Bonobo            GCTATGTAATGATTACAAAGCAAAATCACTGGGCCTTGATTTTGATAGTCAAAGTACCATGTACACATAATTGAACAAAT 32589
OliveBaboon       GCTACGTAGTGATTACAAAGCAAAATCACTGGGCCATGATTTTGATAGTCAGAGTACCATGTACACGTAATTGAACAAAT 33566
Orangutan         GCTATGTAATGATTACAAAGCAAAATCACTGGGCCTTGATTTTGATAGTCAAAGTACCATGTACACATAGTTGAACAAAT 33572

Majority          AGGCTTAAGTGTGT--AACT--GGTTTGTCGGTTTCTCTTTTTGTTT----TTACAATGCATACTGTGATTGATAATGGG
                          40970     40980     40990     41000     41010     41020     41030     41040
Human             AGGCAGAAGTGTGT--AACTCTGGTTTGTCGGTTTCTCTTTTTGTTT----TTACAATGCATACTGTGATCGATAATGGG 31636
Coquerel'sSifaka  AGGCTCAAGTGTCTTTAACTCTGCTTTGTCAGTTTCTCTTTTTA---------ATAATATATATTGTGATTGATAACGGA 30522
Ma'sNightMonkey   AGGCATAAGTGTGT--AACT--GGTTTGTCAGTTTCTCTTTTTGTTTGTTGTTCCAATGCATATTGTGATTGTTAATGGG 28538
VervetAGM         AGGCTTAAGTGTGT--AACT------TGTCGGTTTCTCTTCTTGTTT----TTACAATGCATACTGTGATTGATAATGGG 35015
Gorrilla          AGGTAGAAGTGTGT--AACTCTGGTTTGTCGGTTTGTCTTTTTGTTT----TTACAATGCATACTGTGATCGATAATGGG 34024
GrabEatingMacaqueAGGCTTAAGTGTGT--AACT------TGTCGGTTTCTCTTTTTGTTT----TTCCAATGCATACTGTGATTGATAATGGG 33660
Macaque           AGGCTTAAGTGTGT--AACT------TGTCGGTTTCTCTTTTTGTTT----TTCCAATGCATACTGTGATTGATAATGGG 34428
PigTailedMacaque  AGGCTTAAGTGTGT--AACT------TGTCGGTTTCTCTTTTTGTTT----TTACAATGCATACTGTGATTGATAATGGG 33381
Bonobo            AGGCAGAAGTGTGT--AACTCTGGTTTGTCGGTTTCTCTCTTTGTTT----TTACAATGCATACTGTGATCGATAATGGG 32663
OliveBaboon       AGGCTTAAGTGTGT--ACCT------TGTCGGTTTCTCTTTTTGTTT----TTACAATGCATACTGTGATTGATAATGGG 33634
Orangutan         AGGCAGAAGTGTGT--AACTCTGGTTTGTCGATTTCTCTTTTTGTTT----TTACAATGCATACTGTGATCGATAATGGG 33646
```

```
Majority          AAATTAA-TTCCAAATTAATTCCTA-ATATTTAGGGCTCATGACAGAGTAACTAGTAGCAGCCTTGACCCCTTTTTCAGT
                          41050     41060     41070     41080     41090     41100     41110     41120
Human             AAATTAA-TTTGGAATTAATTTCCAAATATTTAGGGCTAGTGACAGAGTAACTAGTAGCAGCCTTGGCCCCTTTTTCAGT 31715
Coquerel'sSifaka  AATGTTTTTTTAAAATTAAT--------ATTTAGGGCT-ATGGCAGAGTAATTGGTAGCAGCTTTGCCCTCATTTCCAAT 30593
Ma'sNightMonkey   AATGTT--TTCCAAATTAAT------AT--TTAGGGCTAATGACAGAG----TAGTAGCAGCCTTGACCCCTTTTTCAGT 28604
VervetAGM         AAATTAA-TTCCAAATTAATTCCTA-ATATTTAGGGCTCATGACAAAATAACTAGTAGTAGCCTTGACCCGTTTTTCAGT 35093
Gorrilla          AAATTAA-TTTGGAATTAATTTCCAAATGTTTAAGGCTAGTGACAGAGTAACTAGTAGCAGCCTTGGCACCTTTTTCAGT 34103
GrabEatingMacaqueAAATTAA-TTCCAAATTAATTCCTA-ATATTTAGGGCTCATGACAGAATAACTAGTAGTAGCCTTGACCCCTTTTTCGGT 33738
Macaque           AAATTAA-TTCCAAATTAATTCCTA-ATATTTAGGGCTCATGACAGAATAACTAGTAGTAGCCTTGACCCCTTTTTCGGT 34506
PigTailedMacaque  AAATTAA-TTCCAAATTAATTCCTA-ATATTTAGGGCTCATGACAGAATAACTAGTAGTAGCCTTGACCCCTTTTTCGGT 33459
Bonobo            AAATTAA-TTTGGAATTAATTTCCAAATATTTAGGGCTAGTGACAGAGTAACTAGTAGCAGCCTTGGCCCCTTTTTCAGT 32742
OliveBaboon       AAATTAA-TTCCAAATTAATTCCTA-ATATTTAGGGCTCATGACAGAATAACTAGTAGTAGCCTTGACCCCTTTTTCGGT 33712
Orangutan         AAATTAA-TTTGGAATTAATTTCCACATATTTAGGGCTAGTGACAGAGTAACTAGTAGCAGCTTTGGCCCCTTTTTCAGT 33725

Majority          CTCCCCCCAAAACCAAAAACTATGTAACTGAATAAAATATGTGGAACAACTGTTGTTTGGAAGTGATGAGCAGACGGTAT
                          41130     41140     41150     41160     41170     41180     41190     41200
Human             CTCCCCCCAAAACCAAAAAGTATGTAACTGAATAAAATACGTGGAACAACTGTTGTTTGGAAGTGATGAGCAGACGGTAT 31795
Coquerel'sSifaka  CTCCTCCCCGA-TCAAAAACTGTATAAATGAATGAAACA--TGAAACAACTGTTGTCTGGAATTGATGAGGAAACAATAC 30670
Ma'sNightMonkey   CTCCTCCCAAAACCAAAAACTATGTAACTGAATAAAATATGTGGAACAACTGTTGTTTGGAAGTGATGAGCAGACAGTAT 28684
VervetAGM         CTCCCCCCAAAACCAAAAACAATGTAACTGAATAAAATATGTGGAACAACTGTTGTTTGGAAGTGATGAGCAGACGGTAT 35173
Gorrilla          CTCCCCCCAAAACCAAAAAGTATGTAACTGAATAAAATACGTGGAACAACTGTTGTTTGGAAGTGATGAGCAGACGGTAT 34183
GrabEatingMacaqueCTCCCCACAAAACCAAAAACTGTGTAACTGAATAAAATATGTGGAACAACTGTTGTTTGGAAGTGATGAGCAGACGGTAT 33818
Macaque           CTCCCCACAAAACCAAAAACTGTGTAACTGAATAAAATATGTGGAACAACTGTTGTTTGGAAGTGATGAGCAGACGGTAT 34586
PigTailedMacaque  CTCCCCACAAAACCAAAAACTGTGTAACTGAATAAAATATGTGGAACAACTGTTGTTTGGAAGTGATGAGCAGACGGTGT 33539
Bonobo            CTCCCCCCAAAACCAAAAAGTATGTAACTGAATAAGATACGTGAAACAACTGTTGTTTGGAAGTGATGAGCAGACAGTAT 32822
OliveBaboon       CTCCCCGCAAAACCAAAAACTATGTAACTGAATAAAATATGTGGAACAACTGTTGTTTGGAAGTGATGAGCAGACGGTAT 33792
Orangutan         CTCCCCCCAAAACCAAAAACTATGTAACTGAATAAAATACGTGGAACAACTGTTGTTTGGAAGTGATGAGCAGACAGTAT 33805

Majority          AAGATCAT---CCTTGAGAGAAGG---------------GTAGCATGTGAGGTA----------------------AG
                          41210     41220     41230     41240     41250     41260     41270     41280
Human             AAGATCAT---CCTTGAGAGAAGG---------------GGTACACGTGAGGTA----------------------AG 31833
Coquerel'sSifaka  AAGATCATGATCTTTGAGAGAAGGTAAACCTTCTCTCAAAATAACCTGTGAACTACAAAGGGAAGGCTCATCTCCCTGAG 30750
Ma'sNightMonkey   AAGATCAT---CCTTGAGGGAAGG---------------GTAATGCCTGAGCTA----------------------AG 28722
VervetAGM         AAGATCAT---CCTTGAGAGAAGG---------------GTAGCATGTGAGGTA----------------------AG 35211
Gorrilla          AAGATCAT---CCTTGAGAGAAGG---------------GGTACACGTGAGGTA----------------------AG 34221
GrabEatingMacaqueAAGATCAT---CCTTGAGAGAAGG---------------GTAGCATGTGAGGTA----------------------AG 33856
Macaque           AAGATCAT---CCTTGAGAGAAGG---------------GTAGCATGTGAGGTA----------------------AG 34624
PigTailedMacaque  AAGATCAT---CCTTGAGAGAAGG---------------GTAGCATGTGAGGTA----------------------AG 33577
Bonobo            AAGATCAT---CCTTGAGAGAAGG---------------GGTACAGGTGAGGTA----------------------AG 32860
OliveBaboon       AAGATCAT---GTTTGAGAGAAGG---------------GTAGCATGTGAGGTA----------------------AG 33830
Orangutan         AAGATCAT---CCTTGAGAGAAGG---------------GGTGCACGTGAG--------------------------- 33838
```

```
Majority          TATCATGAACACTCTGGCTTTCTGCCTTGGACTGTTTTACTGCTGTAAACCACAGAGAAGTGGAGAGCTCAGAGTATACA

                          41290     41300     41310     41320     41330     41340     41350     41360

Human             TATCATGAACACTCTGGCTTTCTGTCTTGGACTATTTTACTGCTCTAAACCACAGAGAAGTGGAGAGCCCAGAGTATACA 31913
Coquerel'sSifaka  CGTCATGATGACTCTGGCTTTCTGCTTTGGGCTACCTTACTCCTATAGCACAGGGAAAGAGAGCTCAAGCAGAGTATACC 30830
Ma'sNightMonkey   TATCGTAAACACTCTGGCGTTTTGCCTTAGACTACTTTACTGCTGTAAACCACA-AGAAGTGGAGAACCCAGAGTATACA 28801
VervetAGM         TATCATGAACACTCTGGCTTTCTGCCTTGGACCGCTTTACTGCTGTAAACCACAGAGAAGTGGAGAGCTCAG--TATACA 35289
Gorrilla          TATCATGAACGCTCTGGCTTTCTGTCTTGGACTGTTTTACTGCTCTAAACCACAGAGAAGTGGAGAGCCCAGAGTATACA 34301
GrabEatingMacaqueTATCATGAACACTCTGGCTTTCTGCCTTGGACCGTTTCACTGCTGTAAACCACAGAGAAGTGGAGAGCTCAG--TATACA 33934
Macaque           TATCATGAACACTCTGGCTTTCTGCCTTGGACCGTTTCACTGCTGTAAACCACAGAGAAGTGGAGAGCTCAG--TATACA 34702
PigTailedMacaque  TATCATGAACACTCTGGCTTTCTGCCTTGGACCGTTTCACTGCTGTAAACCACAGAGAAGTGGAGAGCTCAG--TATACA 33655
Bonobo            TATCATGAACACTCTGGCTTTCTGTCTTGGACTATTTTACTGCTCTAAACCACAGAGAAGTGGAGAGCCCAGAGTATACA 32940
OliveBaboon       TATCATGAACACTCTGGCTTTCTGCCTTGGACTGTTTTACTGTTGTAAACCACAGAGAAGTGGAGAGATCAG--TATACA 33908
Orangutan         TATCATGAACACTCTGGCTTTCTGCCTTGGACTATTTTACTGTTCTAAACCACAGAGAATTGGAGAGCTCAGAGTATACA 33918

Majority          GGTCTCACTGAGCTGAAGAGACTAAGAGTGGGTGTCAGAGCTGCTCAGTCATTTGGAATTTATGGGGAAG--TCCTGGAG

                          41370     41380     41390     41400     41410     41420     41430     41440

Human             GGTCTCACTGAGCTGAAGAGACCAAGAGTGGGTGTCAGGGCTGCCCAATCATTTGGAATTTATGGAGAAG--TCCTGGAG 31991
Coquerel'sSifaka  GGTGTTACTGAGCTGAAGAGACTAAGAGTGTAATTCAAAGCTGTTCAAACAT--AAAATTTGTGGGGAAGGGCCCTGGAG 30908
Ma'sNightMonkey   GGTCTCACTGAGCTGAAGAGACCAAGACTGGGAATCAGAGCTGTTCAGTCATTTGGAATTTGTGGGGAAG--TCTTGAGG 28879
VervetAGM         GGTCTCACTGAGCTGAAGAGACTAAGAGTGGGTGTCAGAACTGCTCAGTCATTTGGAATTTATGGGGAAG--TCCTGGAG 35367
Gorrilla          GGTCTCACTGAGCTGAAGAGACCAAGAGTGGGTGTCAGGGCTGCCCAATCATTTGGAATTTATGGAGAAG--TCCTGGAG 34379
GrabEatingMacaqueGGTCTCACTGAGCTGAAGAGACTAAGAGTGGGTGTCAGAACTGCTCAGTCATTTGGAATTTATGGGGAAG--TCCTGGAG 34012
Macaque           GGTCTCACTGAGCTGAAGAGACTAAGAGTGGGTGTCAGAACTGCTCAGTCATTTGGAATTTATGGGGAAG--TCCTGGAG 34780
PigTailedMacaque  GGTCTCACTGAGCTGAAGAGACTAAGAGTGGGTGTCAGAACTGCTCAGTCATTTGGAATTTATGGGGAAG--TCCTGGAG 33733
Bonobo            GGTCTCACTGAGCTGAAGAGACCAAGAGTGGGTGTCAGGGCTGCCCAATCATTTGGAATTTATGGAGAAG--TCCTGGAG 33018
OliveBaboon       GGTCTCACTGAGCTGAAGAGACTAAGAGTGGGTGTCAGAACTGCTCAGTCGTTTGGAATTTATGGGGAAG--TCCTGGAG 33986
Orangutan         GGTCTCACTGAGCTGAAGAGACCGAGAGTGGGTGTCAGAGCTGCTCAATCATTTGGAATTTATGGAGAAG--TCCTGGAG 33996

Majority          AAAAGAGAGCTGTCTAGGGGTTTAGGAATAGAGCAGGGGGCAGAATTTAGTATGGTCATT-CACTTCATTCAGAGTTTTG

                          41450     41460     41470     41480     41490     41500     41510     41520

Human             AAAAGAGAGCTGTCTAGGGGTTTAGGAATAGAGCAGGGGGCAGAATTTAGTATGGTCATTTCACTTCATTCACAGTTTTG 32071
Coquerel'sSifaka  ATAAGGAAATTGTCTGGGGGTTTATACATAGGGTAGGGGACAGAATTTGTTGTGGGGATT-CACTTCATTCAGAATTTTG 30987
Ma'sNightMonkey   AAAATAAAGCTGTCTAGGGGTTTAGGAATACAGCAAGGGGCAGAATTTAGTGTGGTCATT-CACTTCATTCAGAGTTTTG 28958
VervetAGM         AAAAGAGAGCTGTCTAGGGGTTTAGGAGTAGAACAGGGGGCAGAATTTAGTATGGTCATT-CACTTCATTCAGAGTTTTG 35446
Gorrilla          AAAAGAGAGCTGTCTAGGGGTTTAGGAATAGAGCAGGGGGCAGAATTTAGTATGGTCATTTCACTTCATTCAGAGTTTTG 34459
GrabEatingMacaqueAAAAGAGAGCTGTCTAGGGGTTTAGGGGTAGAACAGAGGGCAGAATTTAGTATGGTCATT-CACTTCATTCAGAGTTTTG 34091
Macaque           AAAAGAGAGCTGTCTAGGGGTTTAGGGGTAGAACAGGGGGCAGAATTTAGTATGGTCATT-CACTTCATTCAGAGTTTTG 34859
PigTailedMacaque  AAAAGAGAGCTGTCTAGGGGTTTAGGGGTAGAACAGGGGGCAGAATTTAGTATGGTCATT-CACTTCATTCAGAGTTTTG 33812
Bonobo            AAAAGAGAGCTGTCTAGGGGTTTAGGAATAGAGCAGGGGGCAGAATTTAGTATGGTCATTTCACTTCATTCACAGTTTTG 33098
OliveBaboon       AAAAGAGAGCTGTCTAGGGGTTTAGGAGTAGAACAGGGGGCAGAATTTAGTATGGTCATT-CACTTCATTCAGAGTTTTG 34065
Orangutan         AAAAGAGAGCTGTCTAGAGGTTTAGGAATAGAGCAGGGGGCAGAATTTAGTATGGTCATT-CACTTCATTCAGAGTTTTG 34075
```

```
Majority          GATAATGACTGGGTTACATGTGTGCAGGTTGAGACTTT-GCCAGGTCTGTCAGAAATTGTCTGCTCTGGGGCTAA-----

                           41530     41540     41550     41560     41570     41580     41590     41600

Human             GATAATGACTTGGTTACATGTGTGCAGGTTGAGGCTTT-GCCAGGTCTGTCAGAAATTGTCTGCTCTGGGGCTAA----- 32145
Coquerel'sSifaka  GATAATGGCTGGTCTGTGTGTGTGCAGACTGAGACTTTTGCAAGGTCTGTCAGAAATCACCTGCTCTGGGGCTAAGAGCT 31067
Ma'sNightMonkey   GATAATGACTGGGTTACATGTGTGCAGGT-----------------CTATCAGAAGTTGTCTGCTCTGGGGCTAA----- 29016
VervetAGM         GATAATGACTGGGTTACATGTGTGCAGGTTGAGACTTT-GCCAGGTCTATCAGAAATTGTCTGCTCTGGGGCTAA----- 35520
Gorrilla          GATAATGACTGGGTTACATGTGTGCAGGTTGAGGCTTT-GCCAGGTCTATCAGAAGTTGTCTGCTCCGGGGCTAA----- 34533
GrabEatingMacaqueGATAATGACTGGGTTACATGTGTGCAGGTTGAGACTTT-GCCAGGTCTGTCAGAAATTGTCTGCTCTGGGGCTAA----- 34165
Macaque           GATAATGACTGGGTTACATGTGTGCAGGTTGAGACTTT-GCCAGGTCTGTCAGAAATTGTCTGCTCTGGGGCTAA----- 34933
PigTailedMacaque  GATGATGACTGGGTTACATGTGTGCAGGTTGAGACTTT-GCCAGGTCTGTCAGAAATTGTCTGCTCTGGGGCTAA----- 33886
Bonobo            GATAATGACTGGGTTACATGTGTGCAGGTTGAGGCTTT-GCCAGGTCTATCAGAAATTGTCTGCTCTGGGGCTAA----- 33172
OliveBaboon       GATAATGACTGGGTTACATGTGTGCAGGTTGAGAGTTT-GCCAGGTCTGTCAGAAATTGTCTGCTCTGGGGCTAA----- 34139
Orangutan         GATAATGACTGGGTTACATGTGTGCAGGTTGAGGTTTT-GCCAGGTCTATCAGAAATTGTTTGCTCTGGGGCTAA----- 34149

Majority          --ATGGTGATTCCATAGGTTGCATAGTACTCTGAAACATTG------------------------GAGTTCTGAGAAGTC

                           41610     41620     41630     41640     41650     41660     41670     41680

Human             --ATGGTGATTCCATAGGTTGCACGGTACTCTGAAACATTG------------------------GAGTTCTGAGAAGTC 32199
Coquerel'sSifaka  AAATGGTAGTTCCGTAGGTTGAGCAGTTCTCTAAGACATTGCAGTTGTGGGAAGTCAGACATTAAGAGTTCTGGAAAGTC 31147
Ma'sNightMonkey   --ATGGTGATTCCATAAGTTGCATAGTACTCTGCAACATTG------------------------GAGTTCTGAGAAGTC 29070
VervetAGM         --ATAGTGATTCCATAGGTTGCATAGTACTCTGAAACATTG------------------------GAGCTCTGAGAAGTC 35574
Gorrilla          --ATGGTGATTCCATAGGTTGCACAGTACTCTGAAACATTG------------------------GAGTTCTGAGAAGTC 34587
GrabEatingMacaque--ACAGTGATTCCATAGGTTGCATAGTACTCTGAAACATTG------------------------GAGTTCTGAGAAGTC 34219
Macaque           --ACAGTGATTCCATAGGTTGCATAGTACTCTGAAACATTG------------------------GAGTTCTGAGAAGTC 34987
PigTailedMacaque  --ACAGTGATTCCATAGGTTGCATAGTACTCTGAAACATTG------------------------GAGTTCTGAGAAGTC 33940
Bonobo            --ATGGTGATTCCATAGGTTGCACGGTACTCTGAAACATTG------------------------GAGTTCTGAGAAGTC 33226
OliveBaboon       --ACAGTGATTCCATAGATTGCATAGTACTCTGAAACATTG------------------------GAGTTCTGAGAAGTC 34193
Orangutan         --ATGGTGATTCCATAGGTTGCAGAGTACTCTGAAACATTG------------------------GAGTTCTGAGAAGTC 34203

Majority          AGAATGCAGACATCTTGCAGGGTACTTCAAACACTCCATTTAGACTACAGAAAGCCTGGACCTGAATAGAAAGGATCACA

                           41690     41700     41710     41720     41730     41740     41750     41760

Human             AGAATGCAGACATATTGCAGGGTACTTCAAACACTCCATTTAGACTACAGAAAGCCTGGACCTGAATAGAAAGGATCACA 32279
Coquerel'sSifaka  AGAATGCAGACATCTTGCAGAGTACCTCAGACATTCCATTTAGACTAC-GAAAGCTCAGTCCTTAA-----AGGATCA-- 31219
Ma'sNightMonkey   AGAATGCAGACATCTTGTAGGGTACTTCAGACACTCCATTTAGACTACAGAAAGCCCAGGCCTGAATAAATAGGATCACA 29150
VervetAGM         AGAATGCAGACATCTTGCAGGGTACTTCAAACACTCCATTTAGACTACAGAAAGCCTGGACCTGAATAGAAAGGATCATA 35654
Gorrilla          AGAATGCAGACATATTGCAGGGTACTTCAAACACTCCATTTAGACTACAGAAAGCCTGGACCTGAATAGAAAGGATCACA 34667
GrabEatingMacaqueAGAATGCAGACACCTTGCAGGGTACTTCAAACACTCCATTTAGACTACAGAAAGCCTGGACCTGAATAGAAAGGATCACA 34299
Macaque           AGAATGCAGACACCTTGCAGGGTACTTCAAACACTCCATTTAGACTACAGAAAGCCTGGACCTGAATAGAAAGGATCACA 35067
PigTailedMacaque  AGAATGCAGACACCTTGCAGGGTACTTCAAACACTCCATTTAGACTACAGAAAGCCTGGACCTGAATAGAAAGGATCACA 34020
Bonobo            AGAATGCAGACATCTTGCAGGGTACTTCAAACACTCCATTTAGACTACAGAAAGCCTGGACCTGAATAGAAAGGATCACA 33306
OliveBaboon       AGAATGCAGACATCTTGCAGGGTACTTCAAACACTCCATTTAGACTACAGAAAGCCTGGACCTGAATAGAAAGGATCACA 34273
Orangutan         AGAATGCAGACATCTTGCAGGGTACTTCAAACACTCCATTTGGACTACAGAAAGCCTGGACTTGAATAGAAAGGATCACA 34283
```

```
Majority          CCCTATAGGAAGTGCCATCTATAAGACTGAGATAAAAACCAAAAACCTAACCCGATAGGAA-GTGAAGGATTCACCAGTT

                          41770     41780     41790     41800     41810     41820     41830     41840

Human             CCCTATAGGAAGTGCCATCTATAAGACTGAGATAAAAACCAGAAACCTAGCCCGATAGGAA-GTGAAGGATTCACCAGTT 32358
Coquerel'sSifaka  CTCTATAGTAAGAGCCATCCATAAGACT-AGTTAA--ACTGAAAATCTAATCTGACAGAAATGTAAAGGATTCACCAGT- 31295
Ma'sNightMonkey   CCCTATGGGAAGTGCCATCTATAAGACTAAGATAAAAACCAAAAACCTAACCCAATAGGAA-GTGAAGGATTCACCAGTT 29229
VervetAGM         CCCCATAGGAAGTGCCATCTATAAGACTGAGATAAAAACAAAAAACCAAACCCAATAGGAA-GTGAAGGATTCACCAGTT 35733
Gorrilla          CCCTATAGGAAGTGCCATCTATAAGACTGAGATAAAAACCAGAAACCTAGCCCGATAGGAA-ATGAAGGACTCACCAGTT 34746
GrabEatingMacaqueCCCCATAGGAAGTGCCATCTATAAGACTGAGATAAAAACAAAAAACCAAACCCGATAGGAA-GTGAAGGATTCACCAGTT 34378
Macaque           CCCCATAGGAAGTGCCATCTATAAGACTGAGATAAAAACAAAAAACCAAACCCGATAGGAA-GTGAAGGATTCACCAGTT 35146
PigTailedMacaque  CCCCATAGGAAGTGCCATCTATAAGACTGAGATAAAAACAAAAAACCAAACCCGATAGGAA-GTGAAGGATTCACCAGTT 34099
Bonobo            CCCTATAGGAAGTGCCATCTATAAGACTGAGATAAAAACCAGAAACCTAGCCCGATAGGAA-GTGAAGGATTCACCAGTT 33385
OliveBaboon       CCCCATAGGAAGTGCCATCTATAAGACTGAGATAAAAACAAAAAACCAAACCCGATAGGAA-GTGAAGGATTCACCAGTT 34352
Orangutan         CCCTGTAGGAAGTGCCATCTGTAAGACTGAGATAAAAACCAGAAACCTAACCCGATAGGAA-GTGAAGGATTCACCAGTT 34362

Majority          AATCTAACCAGTAACTGATGATGGATTAACCAATAATTTAGTTGCCTTCCAAAACA-AAACAACACCCCC-AAGAAAGAC

                          41850     41860     41870     41880     41890     41900     41910     41920

Human             AATCTAACCAGTAACTGATGAAGGATTAATCAGTAATTTAGTTGCCTTCCAAAACA-AAACAACACCCCC-AAGAAAGA- 32435
Coquerel'sSifaka  AATATAA-----------------------------------TTGCCTGCCAAAACAGAAACACTACCCCCTAAGGCAGAC 31341
Ma'sNightMonkey   AATCTAA-----------------------------TTTAGTTGCCTTCCAAAACA-AAACAACACCCTC-AAGGAAGAC 29278
VervetAGM         AATCTAACCAGTAACTGATGATGGATTAACCAATAATTTAGTTGCCTTTCAAAACA-AAATAACACCCCC-AAGAAAGAC 35811
Gorrilla          AATCTAACCAGTAACTGATGAAGGATTAACCAGTAATTTAGTTGCCTTCCAAAACA-AAACAACACCCCC-AAGAAAGA- 34823
GrabEatingMacaqueAATCTAACCAGTAACTGATGATGGATTAACCAATAATTTAGTTGCCTTTCAAAACA-AAATAACACCCCC-AAGAAAGAC 34456
Macaque           AATCTAACCAGTAACTGATGATGGATTAACCAATAATTTAGTTGCCTTTCAAAACA-AAATAACACCCCC-AAGAAAGAC 35224
PigTailedMacaque  AATCTAACCAGTAACTGATGATGGATTAACCAATAATTTAGTTGCCTTTCAAAACA-AAATAACACCCCC-AAGAAAGAC 34177
Bonobo            AATCTAACCAGTAACTGATGAAGGATTAATCAGTAATTTAGTTGCCTTCCAAAACA-AAACAACACCCCC-AAGAAAGA- 33462
OliveBaboon       AATCTAACCAGTAACTGATGATGGATTAACCAATAATTTAGTTGCCTTTCAAAACA-AAATAACACCCCC-AAGAAAGAC 34430
Orangutan         AATCTAACCAGTAACTGATGAAGGATTAACCAGTAATTTAGTTGCCCTCCAAAACA-AAACAACACCCCC-AAGAAAGA- 34439

Majority          AACATGATATAGTTTTCCTGCAACTATCTTCCAT-AATATCTAGCATACCATCAAAAATTACAAGTAATGTGAAGAAGCA

                          41930     41940     41950     41960     41970     41980     41990     42000

Human             --CATGATATAGTTTTCCTGCAACTATCTTCCAT-AATATCTAGTATACCATCAAAAATTACAAGTGATGTGCAGAAGCA 32512
Coquerel'sSifaka  AGCATGATTCAGTGTTCCTGTAC--TTCT-CCAT-AATATCTAGCATACCATCAAAAATTACAGGTCATGTGAAGAAGCA 31417
Ma'sNightMonkey   AACATGATACAATTTTCCTGCAACTGTCTTCCGTTAATATCTAGCATATCATCAAAAATTACAAGTAATGTGAAGAAGCA 29358
VervetAGM         AACATGATATAGTTTTCCTGCTACTATCTTCCAT-AATATCTACCATACCATCAGAAATTACAAGTAATGTGAAGAAGCA 35890
Gorrilla          --CATGATATAGTTTTCCTGCAACTATCTTCCAT-AATATCTAGCATACCATCAAAAATTACAGGTGATGTGCAGAAGCA 34900
GrabEatingMacaqueAACATGATATAGTTTTCTTGCTACTATCTTCCAT-AATATCTACCATACCATCAGAAATTATAAGTAATGTGAAGAAGCA 34535
Macaque           AACATGATATAGTTTTCTTGCTACTATCTTCCAT-AATATCTACCATACCATCAGAAATTATAAGTAATGTGAAGAAGCA 35303
PigTailedMacaque  AACATGATATAGTTTTCTTGCTACTATCTTCCAT-AATATCTACCATACCATCAGAAATTACAAGAAATGTGAAGAAGCA 34256
Bonobo            --CATGATATAGTTGTCCTGCAACTATCTTCCAT-AATATCTAGTATACCATCAAAAATTACAAGTGATGTGCAGAAGCA 33539
OliveBaboon       AACATGATATAGTTTTCTTGCTACTATCTTCCAT-AATATCTACCGTACCATCAGAAATTACAAGTAATGTGAAGAAGCA 34509
Orangutan         --CATGATACAGTTTTCCTGCAACCATCTTCCAT-AATATCTAGCATACCATCAAAAATTTCAAGTGATGTGAAGAAGCA 34516
```

```
Majority          GGAAAGTAACCCATGAAATATAAAGA-CACATTTAATAGGAACAGATTCCAAAATGACCTAGA-GTTAGAATTAGCAGAC

                          42010     42020     42030     42040     42050     42060     42070     42080

Human             GGAAAGTAACCCATGAAATATAAAGAACACATTTAATAGGAACAGATTCCAAAATGACCTAGA-GTTAGAATTAGCAGAC 32591
Coquerel'sSifaka  GGAAAATAAAATGTAAACTACAAAGA-GGCATTCAATAGGAGCGGACTTGAAAATTATCTAGATAACAGAATTAGCA--- 31493
Ma'sNightMonkey   GGAAAATAAAACATTAACCATAAGGA-AACATTTAATAGGAACAGATTCCAAAATGACCTAGA-GTTAGAATTGGCAGAC 29436
VervetAGM         GAAAAGTAACCCATGAAATATAAAGA-CACATTGAATAGGAACAGATTCCAAAATGACCTAGA-GTTAGAATTAGCAGAC 35968
Gorrilla          GGAAAGTAACCCATGAAATACAAAGAACACATTTAATAGGAACAGATTCCAAAATGACCTAGA-GTTAGAATTAGCAGAC 34979
GrabEatingMacaqueGGAAAGTAACCCATGAAATATAAAGA-CACATTGAATAGGAACAGATTCCAAAATGACCTAGA-GTTAGAATTAGCAGAC 34613
Macaque           GGAAAGTAACCCATGAAATATAAAGA-CACATTGAATAGGAACAGATTCCAAAATGACCTAGA-GTTAGAATTAGCAGAC 35381
PigTailedMacaque  GGAAAGTAACCCATGAAATATAAAGA-CACATTGAATAGGAACAGATTCCAAAATGACCTAGA-GTTAGAATTAGCAGAC 34334
Bonobo            GGAAAGTAACCCATGAAATATAAAGAACACATTTAATAGGAACAGATTCCAAAATGACCTAGA-GTTAGAATTAGCAGAC 33618
OliveBaboon       GGAAAGTAACCCATGAAATATAAAGA-CACATTGAATAGGAACAGATTCCAAAATGACCTAGA-GTTAGAATTAGCAGAC 34587
Orangutan         GGAAAGTAACCCATGAAATATAAAGA-CACATTTAATAGGAACAGATTCCAAAATGACCTAGA-GTTAGAATTAGCAGAC 34594

Majority          AAGGACGATAAGTATTTTAAGCTGTAAAGGAAAAGATTGAAATAATGAGTGA-----TGGAGGAGTGTG-TTAAGAGATA

                          42090     42100     42110     42120     42130     42140     42150     42160

Human             AAGGACGATAAGTATTTTAAGCTGTAAAGGAAAAGATTGAAATAATGAGTGA-----TGGAGGAGTGTGTTGAAGAGATA 32666
Coquerel'sSifaka  -AAGATGATACATATGTTAAGCTATAAAGGAGAAGATTGACATAATGATTGAACACATGGAGGAATATG-TTAAGAGATA 31571
Ma'sNightMonkey   AAGGACGGTAAGTATTTTAAGCTGTAAAGAAAAAGATTGAAATAATGAGTGA-----TGGAGGAGTGTGTTTAAGAGATA 29511
VervetAGM         AAGGACC-TAAGTATTTTAAGTTGTAAAGGAAAAGATTGAAATAATGAATGA-----TGGAGGAGTGT--TTAAGAGATA 36040
Gorrilla          AAGGACGATAAGTATTTTAAGCTGTAAAGGAAAAGATTGAAATAATGAGTGA-----TGGAGGAGTGTGTTGAAGAGATA 35054
GrabEatingMacaqueAAGGACG-TA----TTTTAAGTTGTAAAGGAAAAGATTGAAATAATGAATGA-----TGGAGGAGTGC--TTAAGAGATA 34681
Macaque           AAGGACG-TA----TTTTAAGTTGTAAAGGAAAAGATTGAAATAATGAATGA-----TGGAGGAGTGC--TTAAGAGATA 35449
PigTailedMacaque  AAGGACG-TA----TTTTAAGTTGTAAAGGAAAAGATTGAAATAATGAATGA-----TGGAGGAGTGT--TTAAGAGATA 34402
Bonobo            AAGGACAATAAGTATTTTAAGCTGTAAAGGAAAAGATTGAAATAATGAGTGA-----TGGAGGAGTGTGTTGAAGAGATA 33693
OliveBaboon       AAGGACG-TAAGTATTTTAAGTTGTAAAGGAAAAGATTGAAATAATGAATGA-----TGGAGGAGTGT--TTAAGAGAGA 34659
Orangutan         AAGGACGATAAGTATTTTAAGCTGTACAGGAAAAGATTGAAATAATGAGTGA-----TGGAGGAGTGTGGTTAAGAGATA 34669

Majority          AAAAA--CTATAAAGAAAGCATGTAGAAATATTCTAATTGAAAAATACAGTATTTGAAATAGTTGGGACTAATTCACTGG

                          42170     42180     42190     42200     42210     42220     42230     42240

Human             AAAAA--CTATAAAGAAAGCATGTAGAAATATTCTAATTGAAAAATACAGTTTTTGAAATAGTTGGGACTGATTCACTGG 32744
Coquerel'sSifaka  A------CTGTAAAGAC--TAACAACAAATTCTATACTTAATGG----------GGAAATACT--GAAT--ATTTTCTCC 31629
Ma'sNightMonkey   AAAAG--CTATAAATAAAGCGTGTAGATATATTCTAATTAAAAA---TAATATTTGAAATGGTTGGGGCGAATTCACTGG 29586
VervetAGM         AAAAC--CTATAAAGAAAGCATGTAGAAATATTCTAATTGAAAAATGTAGTATTTGAAATAATTGGGACTAATTCACTGG 36118
Gorrilla          AAAAA--CTATAAAGAAAGCATGTAGAAATATTCTAATTGAAAAATACAGTTTTTGAAATAGTTGAGACTAATTCACTGG 35132
GrabEatingMacaqueAAAAA--CTATGAAGAAAGCATGTAGAAATATTCTAATTGAAAAATATAGTATTTGAAATAGTTGGGACTAATTCACTGG 34759
Macaque           AAAAA--CTATGAAGAAAGCATGTAGAAATATTCTAATTGAAAAATATAGTATTTGAAATAGTTGGGACTAATTCACTGG 35527
PigTailedMacaque  AAAAA--CTATGAAGAAAGCGTGTAGAAATATTCTAATTGAAAAATACAGTATTTGAAATAGTTGGGACTAATTCACTGG 34480
Bonobo            AAAAA--CTATAAAGAAAGCATGTAGAAATATTCTAATTGAAAAATACAGTTTTTGAAATAGTTGGGACTAATTCACTGG 33771
OliveBaboon       AAAAA--CTATAAAGAAAGCATGTAGAAATATTCTAATTGAAAAATACAGTATTTGAAATAGTTGGGACTAATTCACTGA 34737
Orangutan         AAAAAAACTATAAAGAAAGCATGTAGAAATATTCTAATTGAAAAATACAGTTTTTGAAATAGTTGGGACTAATTCACTGG 34749
```

```
Majority          TTGGAACTAATAGCAGATTGGAGACAA--GAAGAAATGTTAGTGAACT---TGAATACAGTTGAATACAGTGATTAAATC

                           42250     42260     42270     42280     42290     42300     42310     42320

Human             TTGGAACTAGTAGCAGATTGGAGACAATAGAAGAAATGTTAGTGAACT---TGAATACTGTTGAATACAGTGATTAAATC 32821
Coquerel'sSifaka  TTAAAAACAG----GAATT--AGGCAA--GGGTGTCTATTTCACAATT-------TTTTTCTAGCCTTCTGTTTATATC 31694
Ma'sNightMonkey   TTGGAACTAATAGCAGATTGGACACAGTAGAAGAAATGTTAGTGAATT---TGAATGTAGTTGGATACAGTGATTAAATC 29663
VervetAGM         TTGGAACTAATAGCAGATTGGAGACAA--GAAGATATGTTAGTGAACT---TGAATACACGTGAATACAATGATTAAATC 36193
Gorrilla          TTGGAACTAATAGCAGATTGGAGACAATAGAAGAAATGTTAGTGAATTCATTGAATACTGTTGAATACAGTGATTAAATC 35212
GrabEatingMacaqueTTGGAACTAATAGCAGATTGGAGACAA--GAAGAAATGTTAGTGAACT---TGAATACACATGAATACAATGATTAAATC 34834
Macaque           TTGGAACTAATAGCAGATTGGAGACAA--GAAGAAATGTTAGTGAACT---TGAATACACATGAATACAATGATTAAATC 35602
PigTailedMacaque  TTGGAACTAATAGCAGATTGGAGACAA--GAAGAAATGTTAGTGAACT---TGAATACACATGAATACAATGATTAAATC 34555
Bonobo            TTGGAACTAATAGCAGATTGGAGACAATAGAAGAAATGTTAGTGAACT---TGAATACTGTTGAATACAGTGATTAAATC 33848
OliveBaboon       TTGGAACTAATAGCAGATTGGAGACAA--GAAGAAATGTTAGTGAACT---TGAATACACATGAATACAATGATTAAATC 34812
Orangutan         TCGGAACTAATAGCAGATTGGAGACAATAGAAGAAATGTTAGTGAACT---TGAATACAGATGAATACAGTGATTAAATC 34826

Majority          TGTCAGAGAGGAGAAAGTATACCTAATGGACAATGAAGCAGTCTGACATGTAATTTGAGTTTCAGTGTGAGAATGTGGGG

                           42330     42340     42350     42360     42370     42380     42390     42400

Human             TGTCAGAGAGGAGAAAGTATATCTAATGGACAATGAAGCAGTCTGACATGTAAGTTGAATTTCAGAGTGAGAATGTGGGG 32901
Coquerel'sSifaka  AGGCAAGTAAAATAAAAGGTATAAAGATTGCAAAGGAGAAGTAAAACTCTTA--------TTCAGTATCAACATGTT--- 31763
Ma'sNightMonkey   TGCCAGAGAGGAGAAAATACACCTCATGGGCAATGATGCAGTCTGACATGTAATTTGAGTTTCAGAGTGAGAATGTGGGG 29743
VervetAGM         TTTCAGAGAGGAGAAAGTATACCTAATGGACAGTGAAGCAGTCTGACATATAATTTGAGTTTCAGTGTGAGAATGTGGAG 36273
Gorrilla          TGTCAGAGAGGAGAAAGTATACCTAATGGACAATGAAGCAGTCTGACATGTAAGTTGAATTTCAGAGTGAGAATGTGGGG 35292
GrabEatingMacaqueTTTCAGAGAGGAGAAAGTATACCTAATGGACAATGAAGCAGTCTGACATATAATTTGAGCTTCAGTGTGAGAATGTGGAG 34914
Macaque           TTTCAGAGAGGAGAAAGTATACCTAATGGACAATGAAGCAGTCTGACATATAATTTGAGCTTCAGTGTGAGAATGTGGAG 35682
PigTailedMacaque  TTTCAGAGAGGAGAAAGTATACCTAATGGACAATGAAGCAGTCTGACATATAATTTGAGCTTCAGTGTGAGAATGTGGAG 34635
Bonobo            TGTCAGAGAGGAGAAAGTATACCTAATGGACAATGAAGCAGTCTGACATGTAAGTTGAATTTCAGAGTGAGAATGTGGGG 33928
OliveBaboon       TTTCAGAGAGGAGAAAGTATACCTAATGGACAATGAAGCAGTCTGACATATAATTTGAGCTTCAGTGTGAGAATGTGGAG 34892
Orangutan         TGTCAGAGAGGAGAAAGTATACCTAATGGACAATGAAGCTGTCTGACATGTAATTTGAATTTCAGAGTGAGAATGTGGGG 34906

Majority          GGAAAAAACATATATCTGTTTGAGGTAGTAATGACTGAGAACTTCCAAAATTTGAATAAAATATATCAACTTACATTCCT

                           42410     42420     42430     42440     42450     42460     42470     42480

Human             GAATAAAACATCTATCTGTTTGAGATAGTAATGACTGAGAACTTCTAAAATTTGAATAAAATATATCAACTTACATTCCT 32981
Coquerel'sSifaka  ----------TAT-----TTTAAAATAGAAAACCCTGAAGGGATCCAC-----------AAAAGATCATCTGGTAGAACT 31817
Ma'sNightMonkey   AAG-AAAGCATGTATCTCTTTGAGGTAGTCATGACAGAGAACTTCTACAATTTGAATAAAATATATCAATTTGCAGTCTT 29822
VervetAGM         GGAAAAAACATATATTTGTTTGAGGTAGTAATGACTGAGAACTTACAAAATTTGAATAAAATATATCAACTTACATTCCT 36353
Gorrilla          GAATAAAACATGTATCTGTTTGAGATAGTAATGACTGAGAACTTCTAAAATTTGAATAAAATATATCAACTTACATTCCT 35372
GrabEatingMacaqueGGAAAAAACATAT--CTGTTTGAGGTAGTAATGACTGAGAACTTACAAAATTTGAATAAAATATATCAACTTACATTCCT 34992
Macaque           GGAAAAAACATATATCTGTTTGAGGTAGTAATGACTGAGAACTTACAAAATTTGAATAAAATATATCAACTTACATTCCT 35762
PigTailedMacaque  GGAAAAAACATATATCTGTTTGAGGTAGTAATGACTGAGAACTTACAAAATTTGAATAAAATATATCAACTTACATTCCT 34715
Bonobo            GAATAAAACATGTATCTGTTTGAGATAGTAATGACTGAGAACTTCTAAAATTTGAATAAAATATATCAACTTACATTCCT 34008
OliveBaboon       GGAAAAAACATATATCTGTTTGAGGTAGTAATGACTGAGAACTTACAAAATTTGAATAAAATATATCAACTTACATTCCT 34972
Orangutan         GAA-AAAACATGTATCTGTTTGAGATAGTAATGACTGAGAACTTCTAAAATTTAAATAAAATATATCAACTTA-ATTCCT 34984
```

```
Majority         AAGAAGCTCAGGTAAACTCCTCATGTAGGCTAAATAAAAAGAACCATACTTAGACACATAGTAGTCAAAATATTGAAAAC

                         42490     42500     42510     42520     42530     42540     42550     42560

Human            AAGAAGCTCAGGTAAACTCCTCATGTAGGCTAAATAAAAAGAACCATACTTAGACACATAGTAGTCAAAATATTGAAAAC 33061
Coquerel'sSifaka AATAAGT-------AACTT------TAGAATGATTACAGGATACAAGTTCTATATATAAA------AGTGTATTGTTTCC 31878
Ma'sNightMonkey  AAGAAGTTCAGGTAAACTCCCCATGTAGGCTAAATGAAAAGAACCATACTTAGACACACAGT---CAAAATACTGAAAAC 29899
VervetAGM        AAGAAGCTCAGGTAAACTCCTCATGTAGGCTAAATAAAAAGAACCATACTTAGACACATAGTAGTCAAAATATTGAAAAC 36433
Gorrilla         AAGAAGCTCAGGTAAACTC---ATGTAGGCTAAATAAAAAGAACCACACTTAGACACATAGTAGTCAAAATATTGAAAAC 35449
GrabEatingMacaqueAAGAAACTCAGGTAAACTCCTCATGTAGGCTAAATAAAAAGAACCATACTTAGACACATAGTGGTCAAAATATTGAAAAC 35072
Macaque          AAGAAGCTCAGGTAAACTCCTCATGTAGGCTAAATAAAAAGAACCATACTTAGACACATAGTGGTCAAAATATTGAAAAC 35842
PigTailedMacaque AAGAAGCTCAGGTAAACTCCTCATGTAGGCTAAATAAAAAGAACCATACTTAGACACATAGTGGTCAAAATATTGAAAAC 34795
Bonobo           AAGAAGCTCAGGTAAACTCCTCATGTAGGCTAAATAAAAAGAACCACACTTAGACACATAGTAGTCAAAATATTGAAAAC 34088
OliveBaboon      AAGAAGCTCAGGTAAACTCCTCATGTAGGCTAAATAAAAAGAACCATACTTAGACACATAGTGGTCAAAATATTGAAAAC 35052
Orangutan        CAGAAGCTCAGGTAAACTCCTCAGGTAGGCTAAATAAAAAGAACCATACTTAGACACATAGTAGTCAAAATATTGAAAAC 35064

Majority         TAGAAATGAAGAGCAAATCTAAACAGCCGGGAAAAAGACATCTAACAGGAGACCAATACCTTGTATTAAAGGGAAAAAAA

                         42570     42580     42590     42600     42610     42620     42630     42640

Human            TAGAAATGAAGAGCAAATCTAAACAGCTGCGAAAAAAACGTCTAACAGGAGACCAACACCTCGTATTAAAGGGGGAAAAA 33141
Coquerel'sSifaka TTATACTAGC-AGCAAAT-T-----GTTGGAAAATGAAATTTTAA---GAAATCCA-----TTTACAAAAGCATCAGAAA 31943
Ma'sNightMonkey  TAGCAGTGAAGAGCAAATCTAAACAGCTGGGAAAAAGACATCTAACAGGAGACCAACACCTTGTACAAAAGGGAAAAAAA 29979
VervetAGM        TAGAAATGAAGAGCAAATCTAAACAGCCAGGAAAAAGACATCTAACAGGAGACCAATACGTTGTATTAAAGAGAAAAAAA 36513
Gorrilla         TAGAAACGAAGAGCAAATCTAAACAGCTGCGAAAAAAACGTCTAACAGGAGACCAACACCTTGTATTAAAGGGGAAAAAA 35529
GrabEatingMacaqueTAGAAATGAAGAGCAAATCTAAACAGCCAGGAAAAAGACATCTAACAGGAGACCAATACCTTGTATTAAAGAGAAAAAAA 35152
Macaque          TAGAAATGAAGAGCAAATCTAAACAGCCAGGAAAAAGACATCTAACAGGAGACCAATACCTTGTATTAAAGAGAAAAAAA 35922
PigTailedMacaque TAGAAATGAAGAGCAAATCTAAACAGCCAGGAAAAAGACATTTAACAGGAGACCAATACCTTGTATTAAAGAGAAAAAAA 34875
Bonobo           TAGAAATGAAGAGCAAAGCTAAACAGCTGCGAAAAAAACGTCTAACAGGAGACCAACACCTTGTATTAAAGGGGGAAAAA 34168
OliveBaboon      TAGAAATGAAGAGCAAATCTAAACAGCCAGGAAGAAGACATCTAACAGGAGACCAATACCTTGTATTAAAGAGAAAAAAA 35132
Orangutan        TGGAAATGA---GCAAATCTAAACAGCCGCGAAAAAAACGTCTAACAGGAGACCAACACCTTGTATTAAAGGGGAAAAAA 35141

Majority         AAA-----GCGAATTTCTTTAGACTGAATGGAATGACATTAGATAACTTGGCTCTGCAGGAGATAAAGAGGAGTACTGGG

                         42650     42660     42670     42680     42690     42700     42710     42720

Human            AA------GCGAATTTCTTTAGACTGAATGGCAT----------AACTTGGCTCTACAGGGGATAA-GAGGAGTACTGGG 33204
Coquerel'sSifaka GA--------CAAAATATCAGGAACAAATTTAAC-A--------AA---AGATGTATAAGACATCT------ATACCAAT 31997
Ma'sNightMonkey  AG-------CAAATTTCTTTAGAGTGAATGGGATGACATTAGATAACTTGGATCTACAGGAGATGAGGAGTAGTACTGGG 30052
VervetAGM        AAAAAA--GAGAATTTCTTTAGACTGAATGGAATGACATTAGGTAACTTGGCTCTGCAGGAGAAAAAGAGGAGTACTGGG 36591
Gorrilla         AAA-----GCGAATTTGTTTAGACTGAATGGCAT----------AACTTGGCTCTACAGGGGATAA-GAGGAGTACTGGG 35593
GrabEatingMacaqueAAAAAAAAGAGAATTTCTTTAGACTGAATGGAATGACATTAGATAACTTGGCTCTGCAGGAGATAAAGAGGAGTACTGGG 35232
Macaque          AAAAAAA-GAGAATTTCTTTAGACTGAATGGAATGACATTAGATAACTTGGCTCTGCAGGAGATAAAGAGGAGTACTGGG 36001
PigTailedMacaque AAAAAAA-GAGAATTTCTTTAGACTGAATGGAATGACATTAGATAACTTGGCTCTGCAGGAGATAAAGAGGAGTACTGGG 34954
Bonobo           AA------GCGAATTTCTTTAGACTGAATGGCAT----------AACTTGGCTCTACAGGGGATAA-GAGGAGTACTGGG 34231
OliveBaboon      AAAA----GAGAATTTCTTTAGACTGAATGGAATGACATTAGATAACTTGGCTCTGCAGGAGATAAAGAAGAGTACTGGG 35208
Orangutan        AAA-----GCGGATTTCTTTAGTCTGAATGGGATGACATTAGATAACTTGGCTCTGCAGGGGATAA-GAGGAGTACTGGG 35215
```

```
Majority          AATGGTTAATATGTGTATAAATTTTAAAAGGGCTCTTTCTTTTCCTCTCAATTTCTTTGAAAGACTTTATGTAAACAACA
                          42730     42740     42750     42760     42770     42780     42790     42800
Human             AATGGTTAATATGTGTATAAATTTTAAAAGGGCTCTCTCTTTTCCTATCAGTTTCTTTGAAAGCCTTTATGTAAACAACA 33284
Coquerel'sSifaka  AACTTTAAGATGCTGCCAGAAGTTTAAACAGACC--------------TAAATAAAGGGAGAGA-------TAGACCATG 32056
Ma'sNightMonkey   AATGGTAAATACATGTATACATTTTAAAAAGTCTCTGTCTTTTCCTCTCAATTTCTTTGAAAGACTTTGTATAAACGACA 30132
VervetAGM         AGTGGTTAATATGTGTATAAATTTTAAAAGGGCTCTTTCTTTTCCTCTCAATTTCTTTGAAAGACTTTATGTAAACAACA 36671
Gorrilla          AATGGTTAATATGTGTATAAATTTTAAAAGGGCTCTCTCTTTTCCTATCAGTTTCTTTGAAAGCCTTTATGTAAACAACA 35673
GrabEatingMacaque AGTGGTTAATATGTGTATAAATTTTAAAAGGGCTCTTTCTTTTCCTCTCAATTTCTTTGAAAGACTTTATGTAAACAACA 35312
Macaque           AGTGGTTAATATGTGTATAAATTTTAAAAGGGCTCTTTCTTTTCCTCTCAATTTCTTTGAAAGACTTTATGTAAACAACA 36081
PigTailedMacaque  AGTGGTTAATATGTGTATAAATTTTAAAAGGGCTCTTTCTTTTCCTCTCAATTTCTTTGAAAGACTTTATGTAAACAACA 35034
Bonobo            AATGGTTAATATGTGTATAAATTTTAAAAGGGCTCTCTCTTTTCCTATCAGTTTCTTTGAAAGCCTTTATGTAAACAACA 34311
OliveBaboon       AGTGGTTAATATGTGTATAAATTTTAAAAGGGCTCTTTCTTTTCCTCTCAATTTCTTTGAAAGACTTTATGTAAACAACA 35288
Orangutan         AATGGTTAATATGTGTATAAATTTTAAAAGGGCTCTCTCTTTTCCTGTCAGTTTCTTTGAAAGCTTTTATGTAAACAGCA 35295

Majority          ACTTTGTATTATGGGGTTTATTATGTAAATGGAT-TTTATATGACAACAGTTAGTACAAAGTGAAGGTAAATGGAATTTT
                          42810     42820     42830     42840     42850     42860     42870     42880
Human             ACTTTGTATTATGGGGTTTATTATGTAAATGGATGTTTATATGACAACAGTTAGTACAAAGTGAAGATAAATGGAATTCT 33364
Coquerel'sSifaka  TTCATGAATTAGAAAGCTCAGTATATTAACA-----T-GT-------CAGTTCTTTCCAAAT-----TGGATGGCATTAA 32118
Ma'sNightMonkey   ACTTTGTATTACGGGGTTTAGTATGTAAATGGATGTTTATATGACAACAATTAGTACAAAGTGAAGATAAATGGAATTTT 30212
VervetAGM         ACTTTGTATTATGGGGTTTATTATGTAAATGGAT-TTTATATGACAACAGTTAGTACAAAGTGAAGGTAAGTGGAATTTT 36750
Gorrilla          ACTTTGTATTATGGGGTTTATTATGTAAATAGATGTTTATATGACAACAGTTAGTACAAAGTGAAGATAAATGGAATTCT 35753
GrabEatingMacaque ACTTTGTATTATGGGGTTTATTATGTAAATGGAT-TTTATATGACAACTGTTAGTACAAAGTGAAGGTAAATGGAATTTT 35391
Macaque           ACTTTGTATTATGGGGTTTATTATGTAAATGGAT-TTTATATGACAGCTGTTAGTACAAAGTGAAGGTAAATGGAATTTT 36160
PigTailedMacaque  ACTTTGTATTATGGGGTTTATTATGTAAATGGAT-TTTATATGACAACTGTTAGTACAAAGTGAAGGTAAATGGAATTTT 35113
Bonobo            ACTTTGTATTATGGGGTTTATTATGTAAATGGATGTTTATATGACAACAGTTAGTACAAAGTGAAGATAAATGGAATTCT 34391
OliveBaboon       ACTTTGTATTATGGGGTTTATTATGTAAATGGAT-TTTATATGACAACAGTTAGTACAAAGTGAAGGTAAATGGAATTTT 35367
Orangutan         ACTTTGTATTATGGGGTTTATTATGTAAATGGATGTTTATATGACAACAGTTAGTACAAAGTGAAGATAAATGGAATTCT 35375

Majority          TCAGTGGTAAGGTTCTTATATTTTACCTGAAGTGATG-CAGTGTTAACTTT-AATATGAAAAGATTCTG-----TTAAGG
                          42890     42900     42910     42920     42930     42940     42950     42960
Human             TCAGTGGTAAGGTTCTTATATTTTACCTGAAGTGATA-CAGTGTTAACTCT-AACATAAAAAGATTCTG-----TTAAGG 33437
Coquerel'sSifaka  TCTGTAATTA---------ATCAAGCCCAGTAGGCTT-TAAAGTTGAAATT---AGTGAGTAGATTCTA----------- 32174
Ma'sNightMonkey   TCAGTGGTAAGGTTCTTGTATTTTACATAAAATGTTA-CAGTGTTAACGCT-AACATAAAAATATTCTGATAAGTTAAAG 30290
VervetAGM         TCAGTGGTAAGATTCTTATATTTTACCTGAAGTGATG-CAGTGTTAACTTT-AATATGAAAAGATTCTG-----TTAAGG 36823
Gorrilla          TTAGTGGTAAGGTTCTTATATTTTACCTGAAGTGATA-CAGTGTTAACTCT-AACATAAAAAGATTCTG-----TTAAGG 35826
GrabEatingMacaque TCAGTGGTAAGATTCTTATATTTTACCTGAAGTGATG-CAGTGTTAACTTT-AATATGAAAAGATTCTG-----TTAAGG 35464
Macaque           TCAGTGGTAAGATTCTTATATTTTACCTGAAGTGATG-CAGTGTTAACTTT-AATATGAAAAGATTCTG-----TTAAGG 36233
PigTailedMacaque  TCAGTGGTAAGATTCTTATATTTTACCTGAAGTGATG-CAGTGTTAACTTT-AATATGAAAAGATTCTG-----TTAAGG 35186
Bonobo            TCAGTGGTAAGGTTCTTATATTTTACCTGAAGTGATA-CAGTGTTAACTCT-AACATAAAAAGATTCCG-----TTAAGG 34464
OliveBaboon       TCAGTGGTAAGATTCTTATATTTTACCTGAAGTGATG-CAGTGTTAACTTTTAATATGAGAAGATTCTG-----TTAAGG 35441
Orangutan         TCAGTGGTAAGGTTCTTATATTTTACCTGAAGTGGTAACAGTGTTAACTCT-AACGTAAAAAGATTCTG-----TTAAGG 35449
```

```
Majority          TTGCATATCAAAATTCTAAGTGTAATCTCATTTAAAAAGATTTAAAGAA-GTATAGCTAAAAGCCAATAGAGGAAAAATT

                           42970     42980     42990     43000     43010     43020     43030     43040

Human             TTGCATATCAAAATTCTAAGTGTAATCTCATTTAAAAAGATAAAAAGAA-GTATAACGAAAAGCCAATAGAGGAAAAATT 33516
Coquerel'sSifaka -----------------------AAATTAATTTGGAAATA---AAATGAGCTAAAACCATT--TTGAAAACGGAAAAGTT 32226
Ma'sNightMonkey  TTGCATATCAAAATTCTAAGTGTAATCACATTTAAAAAGATTTAAAGACTGTATAGCTAAAAGCCAGTAGAGGAAGAATT 30370
VervetAGM        TTGCATATCAAAATTCTAAGTGTAATCTCATTTAAAAAGATTTAAAGAA-GTATAGCTAAAAGCCAATAGAGGAAAAATT 36902
Gorrilla         TTGCATATCAAAATTCTAAGTGTAATCTCATTTAAAAAGATATAAAGAA-GTATAACGAAAAGCCAATAGAGGAAAAATT 35905
GrabEatingMacaqueTTGCATATCAAAATTCTAAGTGTAATCTCATTTAAAAAGATTTAAAGAA-GTATAGCTAAAAGCCAATAGAGGAAAAATT 35543
Macaque          TTGCATATCAAAATTCTAAGTGTAATCTCATTTAAAAAGATTTAAAGAA-GTATAGCTAAAAGCCAATAGAGGAAAAATT 36312
PigTailedMacaque TTGCATTTCAAAATTCTAAGTGTAATCTCATTTAAAAAGATTTAAAGAA-GTATAGCTAAAAGCCAATAGAGGAAAAATT 35265
Bonobo           TTGCATATCAAAATTCTAAGTGTAATCTCATTTAAAAAGATATAAAGAA-GTATAACGAAAAGCCAATAGAGGAAAAATT 34543
OliveBaboon      TTGCATATCAAAATTCTAAGTGTAATCTCATTTAAAAAGATTTAAAGAA-GTATAGCTAAAAGCCAATAGAGGAAAAATT 35520
Orangutan        TTGCATATCAAAATTCTAAGTGTAATCTCATTTAAAAAGATACAAAGAA-GTATAACGAAAAGCCAATAGAGGAAAAATT 35528

Majority          GGAGCATTGA---CTACCTGTTTTCAAGAATTACTGCAAAGCTACAGTAATTA--AAAATAGCATCATAATGACATAAAG

                           43050     43060     43070     43080     43090     43100     43110     43120

Human            GGAGCATTGA---CTACCAGTTTTCAAGAATTACTGCAAAGCTACGGTAATTA--AAAATAGCATCATAATAACATAAAG 33591
Coquerel'sSifaka GAAACAC--A-------CTGACTTCAAGATTCACTGTAAACCTATAGTTAATT-----AAAGCA--ATATTAGCAT---G 32287
Ma'sNightMonkey  GAAGCATTTA---CTACCTGTTTTCAAGAATTGCTGCAAAGCTACAGTAATTA--AAAATAGCATCAGATTGACATAAAG 30445
VervetAGM        GGAGCATTGATGACTACCTGTTTTCAAGAATTACTGCAAAGCTACAGTAATTA--AAAATAGCATCATAATGACATAAAG 36980
Gorrilla         GGAGCATTGA---CCACCAGTTTTCAAGAATTACTGCAAAGCTACGGTAATTATAAAAATAGCATCATAATGACATAAAG 35982
GrabEatingMacaqueGGAGCATTGATGACTACCTGTTTTCAAGAATTACTGCAAAGCTACAGTAATTA--AAAATAGCATCATAATGACATAAAG 35621
Macaque          GGAGCATTGATGACTACCTGTTTTCAAGAATTACTGCAAAGCTACAGTAATTA--AAAATAGCATCATAATGACATAAAG 36390
PigTailedMacaque GGAGCATTGATGACTACCTGTTTTCAAGAATTACTGCAAAGCTACAGTAATTA--AAAATAGCATCATAATGACATAAAG 35343
Bonobo           GGAGCATTGA---CTACCAGTTTTCAAGAATTACTGCAAAGCTACGGTAATTA--AAAATAGCATCATAATAACATAAAG 34618
OliveBaboon      GGAGCATTGATGACTACCTGTTTTCAAGAATTACTGCAAAGCTACAGTAATTA--AAAATAGCATCATAATGACATAAAG 35598
Orangutan        GGAACATTGA---CTACCAGTTTTCAAGAATTACTGCAAAGCTACAGTAATTA--CAAATAGCATCATAACGACATAAAG 35603

Majority          A----TAAATAGGTCAGTGAGACAGAGCAGAGAGTTCAGAAATTAACTCACACAATTATGGATAACTGATTTTTGACAAA

                           43130     43140     43150     43160     43170     43180     43190     43200

Human            A----TAAATAGGTCAGTGAGACAGAGCAGAGAGTTCAGAAGTTAACTCACACAATTATGGATAACTGATTTTTGACAAA 33667
Coquerel'sSifaka ------------GTTAGTGCCAC--AGAATAGCGTTCAGAAATAAGCTCACAT--TTAAGGTCAGTTGATTATTTACAAA 32351
Ma'sNightMonkey  ATAAATAGGTAGGTCAGTGAGACAGAGCAGAGAATTCAGAAATTAACTTACATAATTATGGATGACTGATTTTTGACAAA 30525
VervetAGM        A----TAAATAGGTCAGTGAGA----GCAGAGAGTTCGGAAATTAACTCACACAATTATGGATAACTGGTTTTTGACAGA 37052
Gorrilla         A----TAAATAGGTCAGTGAGACAGAGCAGAGAGTTCAGAAGTTAACTCACACAATTATGGATAACTGATTTTTGACAAA 36058
GrabEatingMacaqueA----TAAATAGGTCAGTGAGACAGAGCAGAGAGTTCAGAAATTAACTCACACAATTATGGATAACTGGTTTTTGACAGA 35697
Macaque          A----TAAATAGGTCAGTGAGACAGAGCAGAGAGTTCAGAAATTAACTCACACAATTATGGATAACTGGTTTTTGACAGA 36466
PigTailedMacaque A----TAAATAGGTCAGTGAGACAGAGCAGAGAGTTCAGAAATTAACTCACACAATTATGGATAACTGGTTTTTGACAGA 35419
Bonobo           A----TAAATAGGTCAGTGAGACAGAGCAGAGAGTTCAGAAGTTAACTCACACAATTATGGATAACTGATTTTTGACAAA 34694
OliveBaboon      A----TAAATAGGTCAGTGAGACAGAGCAGAGAGTTCAGAAATTAACTCACACAATTACGGATAACTGGTTTTTGACAGA 35674
Orangutan        AAAGATAAATAGGTCAGTGAGATAGAGCAGAGAGTTCAGAAGTTAACTCACACAATTATGGATAACTGATTTTTGACAAA 35683
```

```
Majority          GGTGCAAAGTCAATTCAGTGG-AGAAAGAGTAGACTTC--AACAAAGGGTACTATAGCAGTTGGATCTAT-CCATCTGCA
                          43210     43220     43230     43240     43250     43260     43270     43280
Human             GGTGCAAAGTCAATTCAGTGG-AGAAAAAGTAGACTTCC-AACAAAGGGTGCTACAGCAGTTGGAGCTAT-CCGTATGCA 33744
Coquerel'sSifaka  GTTGCCTTGGTAACTCAAGGG--GAAAGAGAAGTCTTTTCAACAAATGGTGCCAGAACAGCTTGAAACACATTAAAAATA 32429
Ma'sNightMonkey   GGTGCAAAGTCAGTTCAGTGG-AGAAAGAGTAGACTTC--CACAAACGGTGCTGTAGCAGTTGGATCTAT-CCATATGCA 30601
VervetAGM         GGTGCAAAGTCAATTCAGTGGGAAAAAGAGTAGACTTC--AACAAAGGGTACTATAGCAGTTGGATCTAT-CCATCTGCA 37129
Gorrilla          GGTGCAAAGTCAATTCAGTGG-AGAAAAAGTAGACTTCC-AACAAAGGGTACTATAGCAGTTGGAGCTAT-CCGTATGCA 36135
GrabEatingMacaqueGGTGCAAAGTCAATTCAGTGGGAAAAAGAGTAGACTTC--AACAAAGGTTACTATAGCAGTTGGATCTAT-CCATCTGCA 35774
Macaque           GGTGCAAAGTCAATTCAGTGGGAAAAAGAGTAGACTTC--AACAAAGGTTACTATAGCAGTTGGATCTAT-CCATCTGCA 36543
PigTailedMacaque  GGTGCAAAGTCAATTCAGTGGGAAAAAGAGTAGACTTC--AACAAAGGTTACTATAGCAGTTGGATCTAT-CCATCTGCA 35496
Bonobo            GGTGCAAAGTCAGTTCAGTGG-AGAAAAAGTAGACTTCC-AGCAAAGGGTGCTATAGCAGTTGGAGCTAT-CCGTATGCA 34771
OliveBaboon       GGTGCAAAGTCAATTCAGTGGGAAAAAGAGTAGACTTC--AACAAAGGTTACTATAGCAGTTGGATCTAT-CCATCTGCA 35751
Orangutan         GGTGCAAAGTCAATTCAGTGG-AGAAAAAGTAGACTTC--AACAAAGGGTGCTATAGCAGTTGGATCTAT-CCGTGTACA 35759

Majority          AAAAAA-TGATCTTGGATCTGTATCTCATACTACATATGAAAATTAACTCCAGATGGATCATGGCCTAAA-TGTAAAACC
                          43290     43300     43310     43320     43330     43340     43350     43360
Human             AAAAAA-TGATCTTGGATCTGTATCTCACACTGCATATGAAAATTAACTCCAAATGGATCATGGCCTAA--TGTAAAACC 33821
Coquerel'sSifaka  GAAAAA--GAAACTTAAACTCTATGTCATACCATACAAAAAAT------GAGATTAATTATATACTAAACCGTAAAAAC 32501
Ma'sNightMonkey   AAAAGT--GATTTTGGATCTGTATCTCACACTACATATGAAAATTAACTCCAAATGGATCGTGGCCTAAA-TGTAAAACC 30678
VervetAGM         AAAAAAATGATCTTGGATATGTATCTCATACTACATATGAAAATTAACTCCAGATGGATCATGACCTAAA-TGTAAAACC 37208
Gorrilla          AAAAAA-TGATCTTGGATCTGTATCTCACACTGCATATGAAAATTAACTCCAAATGGATCATGGCCTAAA-TGTAAAACC 36213
GrabEatingMacaqueAAAAAAAAGATCTTGGATATGTATCTCATACTACATATGAAAATTAACTCCAGATGGATCATGACCTAAA-TGTAAAACC 35853
Macaque           AAAAAAAAGATCTTGGATATGTATCTCATACTACATATGAAAATTAACTCCAGATGGATCATGACCTAAA-TGTAAAACC 36622
PigTailedMacaque  AAAAAAAAGATCTTGGATATGTATCTCATACTACATATGAAAATTAACTCCAGATGGATCATGACCTAAA-TGTAAAACC 35575
Bonobo            AAAAAA-TGATCTTGGATCTGTATCTCACACTGCATATGAAAATTAACTCCAAATGGATCATGGCCTAA--TGTAAAACC 34848
OliveBaboon       AAAAAAATGATCTTGGATATGTATCTCATACTACATATGAAAATTAACTCCAGATGGATCATGACCTAAA-TGTAAAACC 35830
Orangutan         AAAAAA-TGATCTTGGATCTGTATCTCACACTACCTATGCAAATTAACTCGAAATGGATCATGGCCTAAA-TGTAAAACC 35837

Majority          AAAAATTATAAAACCTCTAGAAAAAAACAC---AGAAAATCTTTGTGACTTTGGATTAAGCAGAAATGTATTTGATATGA
                          43370     43380     43390     43400     43410     43420     43430     43440
Human             AAAAATTATAAAATCTCTAGAAAAAAATAC---AGAAAATCTTCGTGACTTTGGATTAAGCAGGAATGTATTTGATATGA 33898
Coquerel'sSifaka  TA----TATAAAGCTTTTAAGAAAAATCACA--GGATAAACTCATAAACCTTGGGGTAAATG---ATTTCTTAGCAAAGA 32572
Ma'sNightMonkey   AAAAATTATAAAACCTCTAGAAGAAAACATGGTAGAAAATCTTTGTGACCTTGGATTAAGCAGAAATGTATTTGATATGA 30758
VervetAGM         AAAAATTATAAAACCTCTAGAAAAAGACAC---AGAAAATCCTTGTGACTTTGGATTAAGCAGAAATGTATTTGATATAA 37285
Gorrilla          AAAAATTATAAAATCTCTAGAAAAAAATAC---AGAAAATCTTTGTCACTTTGGATTAAGCAGAAATGTATTTGATGTGA 36290
GrabEatingMacaqueAAAAATTATAAAACCTCTAGAAAAAAACAC---AGAAAATCTTTGTGACTTTGGATTAAGCAGAAATGTGTTTGATATAA 35930
Macaque           AAAAATTATAAAACCTCTAGAAAAAAACAC---AGAAAATCTTTGTGACTTTGGATTAAGCAGAAGTGTATTTGATATAA 36699
PigTailedMacaque  AAAAATTATAAAACCTCTAGAAAAAAACAC---AGAAAATCTTTGTGACTTTGGATTAAGCAGAAATGTATTTGATATAA 35652
Bonobo            AAAAATTATAAAATCTCTAGAAAAAAATAC---AGAAAATCTTTGTGACTTTGGATTAAGCAGAAATGTATTTGATATGA 34925
OliveBaboon       AAAAATTATAAAACCTCTAGAAAAAAACAC---AGAAAATCTTTGTGACTTTGGATTAAGCAGAAATGTATTTGATATAA 35907
Orangutan         AAAAATTATAAAACCTCTAGAAAAAAATAC---AGAAAATCTTTGTGACTTTGGATTAAGCAGAAATGTATTTGATATGA 35914
```

```
Majority          CCCTAAAAGCACAATCTATTAA-AAAATGGAATTTGACATCAGAATTAAAATCTTGTGCCCTTCAAAAGAAACTGTTA-G
                          43450     43460     43470     43480     43490     43500     43510     43520
Human             CCCTAAAAGCACAATCTATTAA-AAAATGGAATTTGACATCAGAATTAAAATCTTCTGCCATTCAAAAGAAACTGCTA-G 33976
Coquerel'sSifaka  TACAAAAATCACTAATCATAAAGTAATTATAAACTGG-ATCAAAAGGGGAAATTT--GCTCATCAAAAGACA---TTAAG 32646
Ma'sNightMonkey   TCCTAAAAGCATAATCTATTAAGAAAATGGATTTTGACATTAGAATTAAAAACTTCTGCCCTTTGAACCAAACTGTTAAG 30838
VervetAGM         CCCTAAAAGTATAATCTGTTAA-AAAATGGAATTTGACATCAGAATTAAAATCTTGTACCGTTCAAAAGAAACTGTTA-G 37363
Gorrilla          CCCTAAAAGCACAATCTATTAA-AAAATGGAATTTGACATCAGAATTAAAATCTTCTGCCATTCAAAAGAAACTCTTA-G 36368
GrabEatingMacaqueCCCTAAAAGTACAATCTGTTAA-AAAATGGAATTTGACATCAGAATTAAAATCTTGTACCCTTCAAAAGAAACTGTTA-G 36008
Macaque           CCCTAAAAGTACAATCTGTTAA-AAAATGGAATTTGACATCAGAATTAAAATCTTGTACCCTTCAAAAGAAACTGTTA-G 36777
PigTailedMacaque  CCCTAAAAGTACAATCTGTTAA-AAAATGGAATTTGACATCAGAATTAAAATCTTGTACCCTTCAAAAGAAACTGTTA-G 35730
Bonobo            CCCTAAAAGCACAATCTATTAA-AAAATGGAATTTGACATCAGAATTAAAATCTTCTGCCATTCAAAAGAAACTGCTA-G 35003
OliveBaboon       CCCTAAAAGTACAATCTGTTAA-AAAATGGAATTTGACATCAGAATTAAAATCTTGTACCCTTCAAAAGAAACTGTTA-G 35985
Orangutan         CCCTAAAAGCACAATCTATTAA-AAAATGGAATTTGACATTAGAATTAAAATCTTCAGCCATTCAAAAGAAACTGTTA-G 35992

Majority          GGAATGAAAAGACAAGCCATA--GACTGGAAGAACAT---CATAACAAAGCATATAGCTGATAAAGGACTTCTATCCAGA
                          43530     43540     43550     43560     43570     43580     43590     43600
Human             GGAATGAAAAGACAAGCCATA--AACTGGAAGAACAT---CATAACAAAGCATATAGCTGATAAAGGACTTCTATCCAGA 34051
Coquerel'sSifaka  ACAATGAAAAAGGAAGGCAAAACGAACAGAATGATATTCACATATGAATGCATATATATGACAAAGGGCTTGTAT----- 32721
Ma'sNightMonkey   GGAGTGAAAAGACAAGCCGTA--GACTGGCAGAGCAT---CATAACAAA--ACAGAGCTGATACAGGACTTCTAT-TAGA 30910
VervetAGM         GGAATGAAAAGACAAGCCATA--GACTGGAAGAACAT---CATAACAAAGCATATAGCTGATAAAGGACTTCTATCCAGA 37438
Gorrilla          GGAATGAAAAGACAAGCCATA--AACTGGAAGAACAT---CATAACAAAGCATATAGCTGATAAAGGACTTCTATCCAGA 36443
GrabEatingMacaqueGGAATGAAAAGACAAGCCATA--GACTGAAAGAACAT---CATAACAAATCATATAGCTGATAAAGGACTTCTATCCAGA 36083
Macaque           GGAATGAAAAGACAAGCCATA--GACTGAAAGAACAT---CATAACAAAGCATATAGCTGATAAAGGACTTCTATCCAGA 36852
PigTailedMacaque  GGAATGAAAAGACAAGCCATA--GACTGGAAGAACAT---CATAACAAAGCATATAGCTGATAAAGGACTTCTATCTAGA 35805
Bonobo            GGAATGAAAAGACAAACCATA--AACTGGAAGAACAT---CATAACAAAGCATATAGCTGATAAAGGACTTCTATCCAGA 35078
OliveBaboon       GGAATGAAAAGACAAGCCATA--GACTGGAAGAACAT---CATAACAAAGCATATAGCTGATAAAGGACTTCTATCCAGA 36060
Orangutan         GGAATGAAAAGACAAGCCATA--AACTGGAAGAACAT---CATAACAAAGCATATAGCTGATAAAGGACTTCTATCCAGA 36067

Majority          ATATTAAAAGATTCAGTATATGTAAACTCAGCAGTCAGGCAACAAACTACTCAATGAATTGGCCAAAATATTTGAACAGG
                          43610     43620     43630     43640     43650     43660     43670     43680
Human             ATATTAAAAGATTCAGTATATGTAAAATCAGCAGTCAAGCAACAAACTACTCAATGAATTGGCCAAAATATTTGAACAGG 34131
Coquerel'sSifaka  ---------CCTCAAGTATTTGTAAAG--GACTCTTACACTGTCATCAATATG----AACAGCCCAATT--TTAAAAAAG 32784
Ma'sNightMonkey   ATATTAAAAATTTTAATATATGTAAACTCAACAGTAAGGCAACAAACAACTCG----ATTGGCTAAAATATTTGAACAGA 30986
VervetAGM         ATATTAAAAGATTCAGTATATGTAAACTCAGCAGTCAGGCAACAGACTACTCAATGAATTGGCCAAAATATTTGAACAGG 37518
Gorrilla          ATATTAAAAGATTCAGTATATGTAAAATCAGCAGTCAAGCAACAAACTACTCAATGAATTGGCCAAAATATTTGAACAGG 36523
GrabEatingMacaqueATATTAAAAGATGCAGTATATGTAAACTCAGCAGTCAGGCAACAGACTACTCAATGAATTGGCCAAAATATTTGAACAGG 36163
Macaque           ATATTAAAAGATGCAGTATATGTAAACTCAGCAGTCAGGCAACAGACTACTCAATGAATTGGCCAAAATATTTGAACAGG 36932
PigTailedMacaque  ATATTAAAAGATGCAGTATATGTAAACTCAGCAGTCAGGCAACAGACTACTCAGTGAATTGGCCAAAATATTTGAACAGG 35885
Bonobo            ATATTAAAAGATTCAGTATATGTAAAATCAGCAGTCAAGCAACAAACTACTCAATGAATTGGCCAAAATATTTGAACAGG 35158
OliveBaboon       ATATTAAAAGATGTAGTATATGTAAACTCAGCAGTCAGGCAACAGACTACTCAATGAATTGGCCAAAATATTTGACCAGG 36140
Orangutan         ATATTAAAAGATTCAGTAAATGTAAAATCAACAATCAAGCAACAAACTACTCAATGAATTGGCCAAAATATTTGAACAGG 36147
```

```
Majority          TAGTACAGCACAAAAGATACACAGATGGTAAGTAAGCACATGAAAAAGCTGATCAGCATCATTAGTTATTAGGGAAATGA

                          43690     43700     43710     43720     43730     43740     43750     43760

Human             TAGTACAGCACAAAAGATACACAGATGGTAAGTAAGCACGTGAAAAAGCTGGTCAGCATCATTAGTTATTAGGGAAATGA 34211
Coquerel'sSifaka  GGG--C--CAAACAAGTTATAAGAATGGGTAATAAGTACAT-AAAAAGAT-A-------TATTAGTTATGAAGTAAACAC 32851
Ma'sNightMonkey   TAGTTCAGCACAAAAGGTATACAGATGGCAAATAAGCACATGAAAAAGACGATCAGCATCATTAGTCATTACGGAAATGA 31066
VervetAGM         TAGTATTGCACAAGAGATACACAGATGGTAAGTAAGCACATGGAAAAGCTGATCAGCATCATTAGTTATTAGGGAAATGA 37598
Gorrilla          TAGTACAGCACAAAAGATATACAGATGGTAAGTAAGCACGTGAAAAAGCTGGTCAGCATCATTAGTTATTAGGGAAATGA 36603
GrabEatingMacaqueTAATATTGCACAAGAGATACACAGATGGTAAGTAAGCACATGGAAAAGCTGATCAGCATCATTAGTTATTAGGGAAATGA 36243
Macaque           TAATATTGCACAAGAGATACACAGATGGTAAGTAAGCACATGGAAAAGCTGATCAGCATCATTAGTTATTAGGGAAATGA 37012
PigTailedMacaque  TAATAT-GCACAAGAGATACACAGATGGTAAGTAAGCACATGGAAAAGCTGATCAGCATCATTAGTTATTAGGGAAATGA 35964
Bonobo            TAGTACAGCACAAAAGATATACAGATGGTAAGTAAGCACGTGAAAAAGCTGGTCAGCATCATTAGTTATTAGGGAAATGA 35238
OliveBaboon       TAGTATTGCACAAGAGATACACAGATGGTAAGTAAGCACATGGAAAAGCTGATCAGCATCATTAGTTATTAGGGAAATGA 36220
Orangutan         TAGTACAGCACAAAAGACATACAGATAGTAAGTAAGCACATGAAAAAGCTGATCAGCATCGTTAGTCATTAGGGAAATGA 36227

Majority          AAATTAAAACTACAGTGAGATAACATTACAAACCTATTAATAGTGACTAAAATTAAAAAGAGTGCCATCTTGGAAAGCAT

                          43770     43780     43790     43800     43810     43820     43830     43840

Human             AAATTAAAACTACAGTGAGATAATATTACAAACCTATTAATAGTGACTAAAATTAAAAAGAGTGCCATCTTGGAAAGCAT 34291
Coquerel'sSifaka  AAGTTAAAACCATTATGAGATAGCCTT-CACACCAACTAG-AACAGCTAAAATTAAACAGATTGGCAACACCAAATG--- 32926
Ma'sNightMonkey   AAATTAAAACTACAGTGAGATAACATTACAAACCTATTAATGGTGACTAAAATTAAA--GAGTGCCATCTTGGAAAGCAT 31144
VervetAGM         AAATTAAAACTACAGTGAAATAACATTACAAACCTATTAATAGTGACTAAGATTAAAAAGAATGCCATCTTGGAAAGCAT 37678
Gorrilla          AAATTAAAACTACAATGAGATAATATTACAAACCTATTAATAGTGACTAAAATTAAAAAGAGTGCCATCGTGGAAAGCAT 36683
GrabEatingMacaqueAAATTAAAACTACAGTGAAATAACATTACAAACTTATTAATAGTGACTAAGATTAAAAAGAGTGCCATCTTGGAAAGCAT 36323
Macaque           AAATTAAAACTACAGTGAAATAACATTACAAACCTATTAATAGTGACTAAGATTAAAAAGAGTGCCATCTTGGAAAGCAT 37092
PigTailedMacaque  AAATTAAAACTACAGTGAAATAACATTACAAACCTATTAATAGTGACTAAGATTAAAAAGAGTGCCATCTTGGAAAGCAT 36044
Bonobo            AAATTAAAACTACAGTGAGATAATATTACAAACCTATTAATAGTGACTAAAATTAAAAAGAGTGCCATCTTGGAAAGCAT 35318
OliveBaboon       AAATTAAAACTACAGTGAAATAACATTACAAACCTATTAATAGTGACTAAGATTAAAAAGAGTGCCATCTTGGAAAGCAT 36300
Orangutan         AAATTAAAACTACAGTGAGATAATATTACAAACCTATTAATAGTGACTAAAATTAAAAAGAGTGCCATCTTGGAAAGCGT 36307

Majority          GATTTGGGAAGAATGTGGAAGAATCATAATATTCACAATACTGCTGATGGGAATGTAAAATCATAGAACTACTTTGGAAA

                          43850     43860     43870     43880     43890     43900     43910     43920

Human             GAGTTGGGAAGAATGTGGAAGAATGTTAATATTCACAATACTGCTGATGGGAATGTAAAATCGTAG---TACTTTGGAAA 34368
Coquerel'sSifaka  ---TTGATGAAGATACGAAACTATCTTA----------T--TGCTGCTGAGGGTATAGAATAGTTG---------GGAAG 32982
Ma'sNightMonkey   GAATAGGAAAGAATGTGGAAGAATCATAATATTCACAATACCGTTGATGGGAATGTAAAATCATAGAACTACTTTGGGAA 31224
VervetAGM         GATTTGGGAAGAATGAGGAAGAATCATAATATTCACAATACTGCTGATGGGAATGTAAAATCGTAGAACTACTTTGGAAA 37758
Gorrilla          GAGTTGGGAAGAATGTGGAAGAATCATAATATTCACAATACTGCTGATGGGAATGTAAAATCGTAG---TACTTTGGAAA 36760
GrabEatingMacaqueGATTTGGGAAGAATGAGGAAGAATCATAATATTCACAATACTGCTGATAGGAATGTAAAATCATAGAACTACTTTGGAAA 36403
Macaque           GATTTGGGAAGAATGAGGAAGAATCATAATATTCACAATACTGCTGATGGGAGTGTAAAATCATAGAACTACTTTGGAAA 37172
PigTailedMacaque  GATTTGGGAAGAATGAGGAAGAATCATAATATTCACAATACTGCTGATGGGAATGTAAAATCATAGAACTACTTTGGAAA 36124
Bonobo            GAGTTGGGAAGAATGTGGAAGAATGTTAATATTCACAATACTGCTGATGGGAATGTAAAATCGTAG---TACTTTGGAAA 35395
OliveBaboon       GATTTGGGAAGAATGAGGAAGAATCATAATATTCACAATACTGCTGATGGGAATGTAAAATCATAGAACTACTTTGGAAA 36380
Orangutan         GAGTTGGGAAGAATGTGGAAGAATCATAATATTCACAATACTGCTGATGGGAATGTAAAATCATAG---TACTTTGGAAA 36384
```

```
Majority          ACAATTTGGTAGTTTCTCCATGATCCAGATTATTTGTCTCCTAAGTATTTA----CCCAAGAGAAATGAAGGCATCTGTT
                          43930     43940     43950     43960     43970     43980     43990     44000
Human             ACAATCTGGTAGTTTCTCCATGATCCAGGTTATTTGTCTCCTAAGCATTTA----CCCAAGAGAAAAGAAGGCATCTATT 34444
Coquerel'sSifaka  ATAGTCTGGCATTTTCTT-ATG--CCAAACTA--------------ACTTA----------------------------- 33016
Ma'sNightMonkey   ACAATTTGGCAGTTTCTCCATGATCCAGGTTATTTGTCTCCTAAGCATTTA----CCCAAGAGAAACGAAGCCATCT-TT 31299
VervetAGM         ACAATTTGGTAGTTTCTCCATGATCCAGATTATTTGTCTCCTAAGTATTTATTTACCCAAGAGAAATGAAGGCATCTGTT 37838
Gorilla           ACAATCCGGTAGTTTCTCCATGATCCAGGTTATTTGTCTCCTAAGCATTTA----CCCAAGAGAAAATAAGGCATCTATT 36836
GrabEatingMacaque ACAATTTGATAGTTTCTTCATGATCCAGATTATTTGTCTCCTAAGTATTTA----CCCAAGAGAAGTGAAGGCATCTGTT 36479
Macaque           ACAATTTGATAGTTTCTTCATGATCCAGATTATTTGTCTCCTAAGTATTTA----CCCAAGAGAAGTGAAGGCATCTGTT 37248
PigTailedMacaque  ACAATTTGGTAGTTTCTTCATGATCCAGATTATTTGTCTCCTAAGTATTTA----CCCAAGAGAAATGAAGGCATCTGTT 36200
Bonobo            ACAATCTGGTAGTTTCTCCATGATCCAGGTTATTTGTCTCCTAAGCATTTA----CCCAAGAGAAAAGAAGGCATCTATT 35471
OliveBaboon       ACAATTTGGTAGTTTCTTCATGATCCAGATTATTTGTCTCCTAAGTATTTA----CCCAAGAGAAATGAAGGCATCTGTT 36456
Orangutan         ACAATTTGGTAGTTTCTCTATGATCCAGGTTATTTGTCTCCTAAGCATTTA----CCGAAGAGAAATGAAGGCATCTGTT 36460

Majority          CATTAAAAAACTCTCATGTTAATGTTCATAGCCATTTTATTTGTAATCACCAAAATGTGAAACAAA-CATTCATCAACAG
                          44010     44020     44030     44040     44050     44060     44070     44080
Human             CATTAAAAAACTCTCATGTTAATGTTCATAGCCATTTTATTTGTAATCACCAAAATGTGAAACAAA-CATTCATCAACAG 34523
Coquerel'sSifaka  -----------------------------------------TGTAGT--------------------------------- 33022
Ma'sNightMonkey   CATTAAAAAACTCTCATGTTAATGTTCCTAGTCATTTTATTTGTAATCACCAAAATGTGAAACAAA-CATTCATCAACAG 31378
VervetAGM         CATTAAAAAGCTCTCATGTTAATATTCATAGCCATTTTATTTGTAATCACCAAAATGTGAAACAAA-CAAACATCAGCAG 37917
Gorilla           CATTAAAAAACTCTCATGTTAATGTTCATAGCCATTTTATTTGTAATCACCAAAATGTGAAACAAAACATTCATCAACAG 36916
GrabEatingMacaque CATTAAAAAACTCTCATGTTAATGTTCATAGCCATTTTATTTGTAATCACCAAAATGTGAAACATT-CATTCATCAACAG 36558
Macaque           CATTAAAAAACTCTCATGTTAATGTTCATAGCCATTTTATTTGTAATCACCAAAATGTGAAACAAA-CATTCATCAACAG 37327
PigTailedMacaque  CATTAAAAAACTCTCATGTTAATGTTCATAGCCATTTTTTTTGTAATCACCAAAATGTGAAACA---CATTCATCAACAG 36277
Bonobo            CATTAAAAAACTCTCATGTTAATGTTCATAGCCATTTTATTTGTAATCACCAAAATGTGAAACAAA-CATTCATCAACAG 35550
OliveBaboon       CATTAAAAAACTCTCATGTTAATGTTCATAGCCATTTTATTTGTAATCACCAAAATGTGAAACAAA-CATTCATCAACAG 36535
Orangutan         CATTAAAAAGCTCTCATGTTAATGTTCATAGCCATTTTATTTGTAGTCACCAAAATGTGAAACAAA-CATTCATCAATGG 36539

Majority          ATGAACTAATAAGTAGATTCCTGTTTT------------------GGCATCATATGCTTTTCTGTACAGTGGAATATAAA
                          44090     44100     44110     44120     44130     44140     44150     44160
Human             ATGAACTAATAAGTAGATTCCTGTTTT------------------GGCATCATATGCTTTTCCATACAGTGGAATACAA- 34584
Coquerel'sSifaka  -------AC---------TTCTACTTT----------------------------------------------------- 33033
Ma'sNightMonkey   ATGAACTAATAAATCGATTCCTGTTTTTATTAGGGAATAAAAACAGGAATCATATGCTTTTCCATACAGTAGAATACAAC 31458
VervetAGM         ATGAACTAATAAGTAGATTCCTGTTTT------------------GGCATCATATGCTTTTCTGTACAGTGGAATATAAA 37979
Gorilla           ATGAACTAATAAGTAGATTCCTGTTTT------------------GGCATCATATGCTTTTCCATACAGTGGAATACAA- 36977
GrabEatingMacaque ATGAACTAATAAGTAGATTCCTGTTTT------------------GGCATCATATGCTTTTCTGTACAGTGGAATATAAA 36620
Macaque           ATGAACTAATAAGTAGATTCCTGTTTT------------------GGCATCATATGCTTTTCTGTACAGTGGAATATAAA 37389
PigTailedMacaque  ATGAACTAATAAGTAGATTCCTGTTTT------------------GGCATCATATGCTTTTCTGTACAGTGGAATATAAA 36339
Bonobo            ATGAACTAATAAGTAGATTCCTGTTTT------------------GGCATCATATGCTTTTCCATACAGTGGAATAAAA- 35611
OliveBaboon       ATGAACTAATAAGTAGATTCCTGTTTT------------------GGCATCATATGCTTTTCTGTACAGTGGAATATAAA 36597
Orangutan         ATGAACTAA---GTAGATTCCTGTTTT------------------GGCATCATATGCTTTTCCGTACAGTGGAATACAA- 36597
```

```
Majority          AACAGCCATATAGAATGAGCTATTGATAGACACAATAGCAAGAATGAATCTCCAAACAATTATGTTGTGTAAAAGATGAG
                           44170     44180     44190     44200     44210     44220     44230     44240
Human             --CAGCCATGTAGAATGAGCTATTGATAGACACAACAGCAAGAATGAATCTCCAAACAATTATGTTATGTAAAAGATGGG 34662
Coquerel'sSifaka  --------------------------------------------TAGTTTTTTATCCAATGACAATGTACAAAA------ 33063
Ma'sNightMonkey   AACAGCCATATAGAGTGAACTGTTGATAGACACAACAGCAAGAATGAATCTCCAAACAATTATGTTGTGTAAAAGAT-AC 31537
VervetAGM         AACAGCCATATAGAATGAGCTGTTGATACACACAATAGCAAGAATGAATCTCCAAACAGTTATGTTGTGTAAAAGATGAG 38059
Gorrilla          --CAGCCATGTAGAATGAGCTATTGATAGACACAACAGCAAGAATGAATCTCCAAACAATTATGTTATGTAAAAGATGGG 37055
GrabEatingMacaqueAACAGCCATATAGAATGAGCTGTTGATAAACACAATAGCAAGAATGAATCTCCAAACAGTTATGTTGTGTAAAAGATGAG 36700
Macaque           AACAGCCATATAGAATGAGCTATTGATAAACACAATAGCAAGAATGAATCTCCAAACAGTTATGTTGTGTAAAAGATGAG 37469
PigTailedMacaque  AACAGCCATATAGAATGAGCTATTGATAAACACAATAGCAAGAATGAATCTCCAAACAGTTATGTTGTGTAAAAGATGAG 36419
Bonobo            --CAGCCATGTAGAATGAGCTATTGATAGACACAACAGCAATAATGAATCTCCAAACAATTATGTTATGTAAAAGATGGG 35689
OliveBaboon       AACAGCCATATAGAATGAGCTATTGATAAACACAATAGCAAGAATGAATCTCCAAACAGTTATGTTGTGTAAAAGATGAG 36677
Orangutan         --CAGCCATATAGAATGAGCTGTTGATACACACAGCAGCAAGAATGAATCTCCAAACAATTATGTTATGTAAAAGATGGG 36675

Majority          AGAGAAAAAGAGTACATCCTGTATGATTCCATTTATATAAAACTCTGGGTAATGCAA-ACTAAT--CTAAAGCAAATTCG
                           44250     44260     44270     44280     44290     44300     44310     44320
Human             AGAAAAAAAGACTACATCCTGTATGATTCCATTTATATAAAACTCTGGGAAATGCAA-ACTAAT--CTAAAGCAAATTCA 34739
Coquerel'sSifaka  -------------------------CTT--------------------G---TACAA----------------ATATGTG 33079
Ma'sNightMonkey   TGGGAATCAAAGTAGAAATAACGACTTTCAAAGAAAGTGGAAATATATGGAGTATAATAGTATAATGTAAATAATATAAA 31617
VervetAGM         AGAGAAAAAGAGTACATCCTGTATGATTCCATTTATATAAAACTCTGGGTAATGCAA-ACTAATATCTAAAGCAAATTCG 38138
Gorrilla          AGAAAAAAAGACTACATCCTGTATGATTCCATTTATATAAAACTGTGGGAAATGCAA-ACTAAT--CTAAAGCAAATTCA 37132
GrabEatingMacaqueAGAGAAAAAGAGTACATCCTGTATGATTCCATTTATATAAAACTCTGGGTAATGCAA-ACTAAT--CTAAAGCAAATTCG 36777
Macaque           AGAGAAAAAGAGTACATCCTGTATGATTCCATTTATATAAAACTCTGGGTAATGCAA-ACTAAT--CTAAAGCAAATTCG 37546
PigTailedMacaque  AGAGAAAAAGAGTACATCCTGTATGATTCCATTTATATAAAACTCTGGGTAATGCAA-AGTAAT--CTAAAGCAAATTCG 36496
Bonobo            AGAAAAAAAGACTACATCCTGTATGATTCCATTTATATAAAACTCTGGGAAATGCAA-ACTAAT--CTAAAGCAAATTCA 35766
OliveBaboon       AGAGAAAAAGAGTACATCCTGTATGATTCCATTTATATAAAACTCTGGGTAATGCAA-ACTAAT--CTAAAGCAAATTCG 36754
Orangutan         AGAAAAAAAGAGTACATCCTGTATGATTCCATTTACATAAAACTCTGGGAAATGCAA-ACTAAT--CTAAAGCAAATTCG 36752

Majority          TGGTTCCTAGGAGTTTAAGAGTGGGAAAGGACAGGCTGGAGG------GGTGACAGAGGGACACAAGAAAACTCAAGGGT
                           44330     44340     44350     44360     44370     44380     44390     44400
Human             TGGTTCTTAGGAGTTTAAGAGTGGGAAAGGACAGGCAGGAGG------GGTGGCAAAGGGACACAGGAAAACTCGGGAGT 34813
Coquerel'sSifaka  T-------------------A---------------------------------------------------TCAAG--- 33086
Ma'sNightMonkey   GTGGAGATAGCGCTTTCAAAAAAAGTGGAGATATAGAGAATATAATAGGAAAATAAGGCAATGTTACAAATGTTAAGGCT 31697
VervetAGM         TGGTTCCTAGGAGTTTAAGAGTGGGAAAGGACAGGCTGGAGG------GGTGACAGAGGGACACAAGAAAACTCAAGGGT 38212
Gorrilla          TGGTTCTTAGGAGTTTAAGAGTGGGAAAGGACAGGCAGGAGGAG---GGGTGGCAAAGGGACGCAGGAAAACTCGGGGGT 37209
GrabEatingMacaqueTGGTTCCTAGGAGTTTAAGAGTGGGAAAGGACAGGCTGGAGG------GGTGACAGAGGGACACAAGAAAACTCAAGGGT 36851
Macaque           TGGTTCCTAGGAGTTTAAGAGTGGGAAAGGACAGGCTGGAGG------GGTGACAGAGGGACACAAGAAAACTCAAGGGT 37620
PigTailedMacaque  TGGTTCCTAGGAGTTTAAGAGTGGGAAAGGACAGGCTGGAGG------GGTGACAGAGGGACACAAGAAAACTCAAGGGT 36570
Bonobo            TGGTTCTTAGGAGTTTAAGAGTGGGAAAGGACAGGCAGGAGG------GGTGGCAAAGGGACACAGGAAAACTCGGGGGT 35840
OliveBaboon       TGGTTCCTAGGAGTTTAAGAGTGGGAAAGGACAGGCTGGAGG------GGTGACAGAGGGACACAAGAAAACTCAAGGGT 36828
Orangutan         TGGTTCCTAGGAGTTTAAGAGTGGGAAAGGACAGGCAGGAGG------GGTGGCAAAGGGACACAAGAAAACTCGGGGGT 36826
```

```
Majority          GATGGACATATAAATTATTGTGATGGTGGTTTTATGGATGTATAT-GTATGTCAAAACTTGTCAAATTTTATAATTTAAA
                          44410     44420     44430     44440     44450     44460     44470     44480
Human             GATGGACATATAAATTATTGTGATGGTGGTTTTATGGATGTATGC-ATATGTCAAAACTTGTCAAATTTTATAATTTAAA 34892
Coquerel'sSifaka  ------------AACTTTATTGATAATGG-T-------------------CCCAAACTT----GAA----ATAACCCGAA 33126
Ma'sNightMonkey   AACCAATTCAGCAACTTAAATGAGATTAAGTTTCTTTACGAATGCAGTTTACCAAAACT----GAATCTGATTAGCTGTA 31773
VervetAGM         GATGGACATATAAATTATTGTGATGGTGGTTTTATGGATGTATAT-GTATGTCAAAACTTGTCAAATATTATAATTTAAA 38291
Gorrilla          GATGGACATATAAATTATTGTGATGGTGGTTTTATGGATGTATGT-ATATGTCAAAACTTGTCAAATTTTATAATTTAAA 37288
GrabEatingMacaqueGATGGACATATAAATTATTGTGATGGTGGTTTTATGGATGTATAT-GTATGTCAAAACTTGTCAAATTTTATAATTTAAA 36930
Macaque           GATGGACATATAAATTATTGTGATGGTGGTTTTATGGATGTATAT-GTATGTCAAAACTTGTCAAATTTTATAATTTAAA 37699
PigTailedMacaque  GATGGACATATAAATTATTGTGATGGTGGTTTTATGGATGTATAT-GTATGTCAAAACTTGTCAAATTTTATAATTTAAA 36649
Bonobo            GATGGACATATAAATTATTGTGATGGTGGTTTTATGGATGTATGT-AAATGTCAAAACTTGTCAAATTTTATAATTTAAA 35919
OliveBaboon       GATGGACATATAAATTATTGTGATGGTGGTTTTATGGATGTATAT-GTATGTCAAAACTTGTCAAATTTTATAATTTAAA 36907
Orangutan         GATGGACATATAAATTATTGTGATGGTGGTTTTATGGATGTATAT-ATATGTCAAAACTTGTCAAATTTTATAATTTAAA 36905

Majority          TACATGTACACATACTAGAATGGCCGAACTTCAGAACATTGATAACACCAAATGCTGGCAAGGATGTGGAGCAGCAGGAA
                          44490     44500     44510     44520     44530     44540     44550     44560
Human             TACATATACACATCCTAGAATGGCCGAACTTCAGAACATTGATAACACCAAATGCTGGCAAGGATGTGGAGCAACAGGAA 34972
Coquerel'sSifaka  TGT-----------CTA-----G--------CAGGACAATG-----------------------------------GA- 33145
Ma'sNightMonkey   TAC---TAC-TGTATTATACAAATTGGAATTTATAATTAAGAGATTAGGCATAGGTGGTTTCACTATTGAACTTTGTGAA 31849
VervetAGM         TACATGTACACATACTAGAATGGCCGAACTTCAGAACATTGATAACACCAAATGCTGGCAAGGATGTGGAGCAGCAGGAA 38371
Gorrilla          TACATGTACACATCCTAGAATGGCCGAACTTCAGAACATTGATAACACCAAATGCTGGCAAGGATGTGGAGCAACAGGAA 37368
GrabEatingMacaqueTACATGTACACATACTAGAATGGCCGAACTTCAGAACATTGATAACACCAAATGCTGGCAAGGATGTGGAGCAGCAGGAA 37010
Macaque           TACATGTACACATACTAGAATGGCCGAACTTCAGAACATTGATAACACCAAATGCTGGCAAGGATGTGGAGCAGCAGGAA 37779
PigTailedMacaque  TACATGTACACATACTAGAATGGCCGAACTTCAGAACATTGATAACACCAAATGCTGGCAAGGATGTGGAGCAGCAGGAA 36729
Bonobo            TACATGTACACATCCTAGAATGGCCGAACTTCAGAACATTGATAACACCAAATGCTGGCAAGGATGTGGAGCAACAGGAA 35999
OliveBaboon       TACATGTACACATACTAGAATGGCCGAACTTCAGAACATTGATAACACCAAATGCTGGCAAGGATGTGGAGCAGCAGGAA 36987
Orangutan         TACATGTACACATACTAGAATGGCCGAACTTCAGAACATAGATAACACCAAATGATGGCAAGGATGTGGAGCAGCAGGAA 36985

Majority          CTCTCACTTACTGCTTGGTGGAATGCAGAGTGGTACAGCCACTTTGGAAGACAATTTGGCAGTTTCTTAGAAAACTATAT
                          44570     44580     44590     44600     44610     44620     44630     44640
Human             CTCTCACTTACTGCTCAGTGGAATGCAGAGTGGTACAGCCACTTTGGAAGACAATTTGGCAATTTCTTAGAAAACTATAC 35052
Coquerel'sSifaka  -------------------------------------------------------------------------------- 33145
Ma'sNightMonkey   A-CTAAGAAAATAATAGCTGTCTTTGAAAATGGAGGAG-------GGAATACTACCCTGTTCACTTTATGAAGCCATTGT 31921
VervetAGM         CTCTCACTTATTGCTTGGTGGAATGCAGAGTGGTACAGCCACTTTGGAAGACAATTTGGCAGTTTCTTAGAAAACTATAT 38451
Gorrilla          CTCTCACTTACTGCTCAGTGGAATGCAGAGTGGTACAGCCACTTTGGAAGACAATTTGGCAATTTCTTAGAAAACTATAC 37448
GrabEatingMacaqueCTCTCACTTACTGCTTGGTGGAATGCAGAGTGGTACAGCCACTTTGGAAGACAATTTGGCAGTTTCTTAGAAAACCATAT 37090
Macaque           CTCTCACTTACTGCTTGGTGGAATGCAGAGTGGTACAGCCACTTTGGAAGACAATTTGGCAGTTTCTTAGAAAACTATAT 37859
PigTailedMacaque  CTCTCACTTACTGCTTGGTGGAATGCAGAGTGGTACAGCCACTTTGGAAGACAATTTGGCAGTTTCTTAGAAAACTATAT 36809
Bonobo            CTCTCACTTACTGCTCAGTGGAATGCAGAGTGGTACAGCCACTTTGGAAGATAATTTGGCAATTTCTTAGAAAACTATAC 36079
OliveBaboon       CTCTCACTTATTGCTTGGTGGAATGCAGAGTAGTACAGCCACTTTGGAAGACAATTTGGCAGTTTCTTAGAAAACTATAT 37067
Orangutan         CTCT--CTTATTGCTCGGTGGAATGCAGAGTGGTACAGCCACTTTGGAAGACAATTTGGCAATTTCTTAGAAAACTATAC 37063
```

```
Majority          GGCCGGGCGCAGTGGCTCATGCCGGTAATCCCA-CAGTTTGGGAAGCCGAGGCAGGCGGATCACCTGAGGTCGGGAGTTC

                          44650     44660     44670     44680     44690     44700     44710     44720

Human             GGCCGGGCACAGTGGCTTACGCCTGTAATCCCAGCGCTTTGGGAAGCCAAGGCAGGCGGATCACCTGAGGTCAGAAGTTC 35132
Coquerel'sSifaka  ------------------------------------------------------------TAA----------AT 33150
Ma'sNightMonkey   AACTGACAGTAAAACCTAACAAGGATAAT------AGATTAAAGATTTCAGACTAGTATTTCTCATTACCT----AATGC 31991
VervetAGM         GGCCGGGCGCAGTGGCTCATGCTGGTAATTCCA-CGGTTTGGGAAGCCGAGGCAGGCGGATCACCTGAGGTCGGGAGTTC 38530
Gorrilla          GGCCGGGCACAGTGGCTCACGCCTGTAATCCCAGCACTTTGGGAAGCCAAGGCAGGCAGATCACCTGAGGTCGGAAGTTC 37528
GrabEatingMacaque GGCCGGGCGCAGTGGCTCATGCCGGTAATCCCA-CAGTTTGGGAAGCCGAGGCAGGCAGATCACCTGAGGTCGGGAGTTC 37169
Macaque           GGCCGGGCGCAGTGGCTCATGCCGGTAATCCCA-CAGTTTGGGAAGCCGAGGCAGGCGGATCATCTGAGGTCGGGAGTTC 37938
PigTailedMacaque  GGCCGGGCGCAGTGGCTCATGCCGGTAATCCCA-CAGTTTGGGAAGCCGAGGCAGGCGGATCACCTGAGGTCGGGAGTTC 36888
Bonobo            AGCCGGGCACAGTGGCTCACGCCTGTAATCCCAGCACTTTGGGAAGCCAAGGCAGGCGGATCACCTGAGGTCGGAAGTTC 36159
OliveBaboon       GGCCAGGCGCAGTGGCTCATGCCGGTAATCCCA-CAGTTTGGGAAGCCGAGGCAGGCGGATCACCTGAGGTCGGGAGGTC 37146
Orangutan         GGCCGGGCACAGTGGCTCACACCTGTAATCCCAGCACTTTGGGAAGCCAAGGCAGGCAGATCACCTGAGGTCGGGAGTTC 37143
```

```
Majority          AAAACCAGCCTGGCCAACATGGAGAAACCCTGTCTCTACTAAAACATACAAAATTAGCCAGGCGTGGTGGTGCATACCTG

                          44730     44740     44750     44760     44770     44780     44790     44800

Human             AAAACCAGCCTGGCCAACATGGAGAAACCCGATCTCTACTAAAAAATACAAAATTAGCTAGGCGTGGTGGTGCATGCCTG 35212
Coquerel'sSifaka  T--GT-GGTATAGTCAGACAGTGGATTGCTACTCATTACTA-----TTAAAAAGAA--T------GGTTACTAATACATG 33214
Ma'sNightMonkey   A-AATATTCTTAAAGAAAATGTAGATAAATCACCTCCAGCAGTATATGAGAGACCGTTCCAAC--TGAGACTTATCGCAG 32068
VervetAGM         AAAACCAGCCTGGCCAACATGGAGAAACCCTGTCTCTACTAAAACATACAAAATTAGCCAGGCGTGGTAGTGCATACCTG 38610
Gorrilla          AAAACCAGCCTGGCCAACATGGAGAAACCCAATCTCTACTAAAAAATACAAAATTAGCCAGGCGTGGTGGTGCATGCCTG 37608
GrabEatingMacaque AAAACCAGCCTGGCCAACATGGAGAAACCCTGTCTCTACTAAAACATGCAAAATTAGCCAGGCGTGGTGGTGCATACCTG 37249
Macaque           AAAACCAGCCTGGCCAACATGGAGAAACCCTGTCTCTACTAAAACATGCAAAATTAGCCAGGCGTGGTGGTGCATACCTG 38018
PigTailedMacaque  AAAACCAGCCTGGCCAACATGGAGAAACCCTGTCTCTACTAAAACATGCAAAATTAGCCAGGCGTGGTGGTGCATACCTG 36968
Bonobo            AAAACCAGCCTGGCCAACATGGAGAAACCCGATCTCTACTAAAAAATACAAAATTAGCCAGGCGTGGTGGTGCATGCCTG 36239
OliveBaboon       AGAACCAGCCTGGCCAACATGGAGAAACCCTGTCTCTACTAAAACATACAAAATTAGCCAGGCGTGGTGGTGCATACCTG 37226
Orangutan         AAAACCAGCCTGGCCAACATGGAGAAACCTGATCGCTACTAAAAAATACAAAATTAGCCAGGCGTGGTGGTGCATGCCTG 37223
```

```
Majority          TAATCCCAGCTACTCAGGAGCCTGAGGCAGGAGAATTGCTTGAACCCAGGAGGTGGAGGTTGCGGTGAGCCAAGATTGCG

                          44810     44820     44830     44840     44850     44860     44870     44880

Human             TAATCCCAGCCACTCAGGAGGCTGAAGCAGGAGAATTACTTGAACCCAGGAGGTGGAGGTTGCAGTGAGCCAGGATCACG 35292
Coquerel'sSifaka  CAAT---------------------------AACA---TGAGTG-AAT-----------------A------------ 33232
Ma'sNightMonkey   GATTATAAGATTT------GTTTAACATTTGGAAATGAACTCAATGTAATTTGTCACAACAAAAG-AATGAAGGAGGAAA 32141
VervetAGM         TAATCCCAGCTACCCAGGAGCCTGAGGCAGGAGAATCGCTTGAACCCAGGAGGTGGAGGTTGCGGTGAGCCAAGATTGCG 38690
Gorrilla          TAATCCCAGCCACTCAGGAGGCTGAAGCAGGAGAATTATTTGAACCCAGGAGGTGGAGGTTGCAGTGAGCCAGGATCACG 37688
GrabEatingMacaque TAATCCCAGCTACCCAGGAGCCTGAGGCAGGAGAATTGCTTGAACCCAGGAGGTGGAGGTTGTGGTGAGCCAAGATTGTG 37329
Macaque           TAATCCCAGCTACCCAGGAGCCTGAGGCAGGAGAATTGCTTGAACCCAGGAGGTGGAGGTTGTGGTGAGCCAAGATTGTG 38098
PigTailedMacaque  TAATCCCAGCTACCCAGGAGCCTGAGGCAGGAGTATTGCTTGAACCCAGGAGGTGGAGGTTGTGGTGAGCCAAGATTGCG 37048
Bonobo            TAATCCCAGCCACTCAGGAGGCTGAAGCGGGAGAATTACTTGAACCCAGGAGGTGGAGGTTGCAGTGAGCCAGGATCACG 36319
OliveBaboon       TAATCCCAGCTACTCAGGAGCCTGAGGCAGGAGAATCGCTTGAACCCAGGAGGTGGAGGTTGCGGTGAGCCAAGATTGCG 37306
Orangutan         TAATCCCAGGTACTCAGGAGGCTGAAGCAGGAGAATCGCTTGAACTCAGGAGGTGGAGGTTGTGGTGAGCCAAGATCGTG 37303
```

```
Majority          CC-ATTTCACTCCAACCTGGGCAACGAGAGTGAGACTCCATCTCAAATATACTTTTATTG-TATGATCCAGCAGTTGTGC

                          44890     44900     44910     44920     44930     44940     44950     44960

Human             CCCATTTCACTCCAACCTGGGCAACAAGAGCGAGACTCCATCTCAAATATACTTTCATTG-TATGATCCAGCAGTTGTGC 35371
Coquerel'sSifaka  -------C-------------------------------------TAAACATTAAATG-------------------- 33246
Ma'sNightMonkey   ACTATCTCTCAGAAAACTAGTAAGAGAAGGTCCTTTTACAAAGTAAATAAGGGGTATCTACTGAGAGCCTACAACAAATT 32221
VervetAGM         CC-ATTGCACTCCAACCTGGGCAACGAGAGTGAGACTCCATCTCAAATATACTTTTATTG-TATGATCCAGCAGTTGTGC 38768
Gorrilla          CC-ATTTCACTCCAACCTGGGCAACAAGAGCGAGACTCCATCTCAAATATACTTTCATTG-TATGATCCAGCAGTTGTGC 37766
GrabEatingMacaqueCC-ATTGCACTCCAACCTGGGCAACGAGAGTGAGACTCCATCTCAAATATACTTTTATTG-TATGATCCAGCAGTTGTGC 37407
Macaque           CC-ATTGCACTCCAACCTGGGCAACGAGAGTGAGACTCCATCTCAAATATACTTTTATTG-TATGATCCAGCAGTTGTGC 38176
PigTailedMacaque  CC-ATTGCACTCCAACCTGGGCAATGAGAGTGAGACTCCATCTCAAATATACTTTTATTG-TATGATCCAGCAGTTGTGC 37126
Bonobo            CC-ATTTCACTCCAACCTGGGCAACAAGAGCGAGACTCCATCTCAAATATACTTTCATTG-TATGATCCAGCAGTTGTGC 36397
OliveBaboon       CC-ATTGCACTCCAACCTGGGCAACGAGAGTGAGACTCCATCTCAAATATACTTTTATTG-TATGATCCAGCAGTTGTGC 37384
Orangutan         CC-ATTTCACTCCAACCTGGGCAACAAGAGCGAGACTCCATCTCAAATATACTTTCATTG-TGTGATCCAGCAGTTGTGC 37381

Majority          TCCCTGGTATTTGCCTAA-AGGAGTTGAAAACTTAGTCTGCACAGAAAGCAGCTC--ATGGATGTTTATA-GCACTTTTC

                          44970     44980     44990     45000     45010     45020     45030     45040

Human             TCCCTGGTATTTGCCTAA-AGGAGTTGAAAACTTAGTCTGCGCAGAAAGTAGCAC--ATGGATGTTTATA-GCACCTTTC 35447
Coquerel'sSifaka  ------------GAGTAA---------------------------------------------------A---A------ 33254
Ma'sNightMonkey   TCATACTTACTGGGGAAATAGTGAATGTTTCCTTTTTAAAAACAGAAAGAAGCAAGGATGTCTGTTTTCACAAATTTTTC 32301
VervetAGM         TCCCTGGTATTTGCCTAA-AGGAGTTGAAAACTTAGTCTGCACAGAAAGCAGCTC--ATGGATGTTTATA-GCACTTTTC 38844
Gorrilla          TCCCTGGTATTTGCCTAA-AGGAGTTGAAAACTTAGTCTGCGCAGAAAGTAGCAC--ATGGATGTTTATA-GCACCTTTC 37842
GrabEatingMacaqueTCCCTGGTATTTGCCTAA-AGGAGTTGAAAACTTAGTCTGCACAGAAAGCAGCTC--ATGGATGTTTATA-GCACTTTTC 37483
Macaque           TCCCTGGTATTTGCCTAA-AGGAGTTGAAAACTTAGTCTGCACAGAAAGCAGCTC--ATGGATGTTTATA-GCACTTTTC 38252
PigTailedMacaque  TCCCTGGTACTTGCCTAA-AGGAGTTGAAAACTTAGTCTGCACAGAAAGCAGCTC--ATGGATGTTTATA-GCACTTTTC 37202
Bonobo            TCACTGGTATTTGCCTAA-AGGAGTTGAAAACTTAGTCTGCGCAGAAAGTAGCAC--ATGGATGTTTATA-GCACCTTTC 36473
OliveBaboon       TCCCTGGTATTTGCCTAA-AGGAGTTGAAAACTTAGTCTGCACAGAAAGCAGCTC--ATGGATGTTTATA-GCACTTTTC 37460
Orangutan         TCCCTGGTATTTGCCTAA-AGGAGTTGAAAACTTAGTCTGCGCAGCAAGTAGCAC--GTGGATGTTTATA-GCACCTTTC 37457

Majority          TTTATAGTTGCCAGA--ACTTCATAGCAACCAAGAAGTCCTTTAGTAGGTAAATGGCCAAATAAACTGTGGTGTATATCC

                          45050     45060     45070     45080     45090     45100     45110     45120

Human             TTTATAGTTGCCAGA--ACTTCATAGCAACCAAGAAGTCCTTTAGGAGGTGAATGGCCAAATAAACTGTGGTGTATATTC 35525
Coquerel'sSifaka  ------GATGCCAGA------CAT--------AAGAGTACAT--------------------AACTCTGTGATTT---- 33289
Ma'sNightMonkey   CCCCTAGCTTTCTGCTTACATTAAGGCAGGTGAAAGACATAAAGATTGGGAAGGAAGAAAGAAAACTGTCGTATTCA-CC 32380
VervetAGM         TTTATAATTGCCAGA--ACTTCATAGCAACCAAGAAGTCCTTTAGTAGGTAAATGGCCAAATAAATTGTGGTGTATATCC 38922
Gorrilla          TTTATAGTTGCCAGA--ACTTCATAGCAACCAAGAAGTCCTTTAGGAGGCGAATGGCCAAATAAACTGTGGTGTATATTC 37920
GrabEatingMacaqueTTTATAATTGCCAGA--ACTTCATAGCAACCAAGAAGTCCTTTAGTAGGTAAATGGCCAAATAAATTGTGGTGTATATCC 37561
Macaque           TTTATAATTGCCAGA--ACTTCATAGCAACCAAGAAGTCCTTTAGTAGGTAAATGGCCAAATAAATTGTGGTGTATATCC 38330
PigTailedMacaque  TTTATAATTGCCAGA--ACTTCATAGCAACCAAGAAGTCCTTTAGTAGGTAAATGGCCAAATAAATTGTGGTGTATATCC 37280
Bonobo            TTTATAGTTGCCAGA--ACTTCATAGCAACCAAGAAGTCCTTTAGGAGGTGAATGGCCAAATAAACTGTGGTGTATATTC 36551
OliveBaboon       TTTATAATTGCTAGA--ACTTCATAGCAACTAAGAAGTCCTT----AG-TAAATGGCCAAATAAATTGTGGTATATATCC 37533
Orangutan         TTTATAGTTGCCAGA--ACTTCATAGCAACCAAGAAGTCCTTTAGTAGGTGAATGGCCAAATAAACTGTGGTGTATATTC 37535
```

```
Majority        AAACAGTATAATGTTAATCAGCACTAAATGGAAATGAGCTATCA-AGCCATGGAGAACCTTCAGTGCGTATT---ACTAA

                        45130     45140     45150     45160     45170     45180     45190     45200

Human           AGACAGTATAATGTTAATCAGCACTAAATGGAAATGAGCTATCA-AGCCATGGAGAACCTTCAGTGAACATT---ACTAA 35601
Coquerel'sSifaka ------TATAGTATTT---------------------CTATGA-----------------------------------A 33306
Ma'sNightMonkey ATTCAACATGATTTTTAAAATAGAATACCTAAAGTAATCTACAACAGATTTGGTAGAACTAATAAGCGAATTTAGAATAA 32460
VervetAGM       AAACAGTATAATGTTAATCAGCACTAAATGGAAATGAGCTATCA-AGCCATGGAGAACCTTCAGTGCGTATT---ACTAA 38998
Gorrilla        AGACAGTATAATGTTAATCAGCACTAAATGGAAATGAGCTATCA-AGCCATGGAGAACCTTCAGTGTACATT---ACTAA 37996
GrabEatingMacaqueAAACAGTATAGTGTTAATCAGCACTAAATGGAAATGAGCTATCA-AGCCATGGAGAACCTTCAGTGCGTATT---ACTAA 37637
Macaque         AAACAGTATAGTGTTAATCAGCACTAAATGGAAATGAGCTATCA-AGCCATGGAGAACCTTCAGTGCGTATT---ACTAA 38406
PigTailedMacaque AAACAGTATAGTGTTAATCAGCACTAAATGGAAATGAGCTATCA-AGCCATGGAGAACCTTCAGTGCGTATT---ACTAA 37356
Bonobo          AGACAGTATAATGTTAATCAGCACTAAATGGAAATGAGCTATCA-AGCCATGGAGAACCTTCAGTGTACATT---ACTAA 36627
OliveBaboon     AAACAGTATAGTGTTAATCAGCACTAAATGGAAATGAGCTATCA-AGCCATGGAGAACCTTCGGTGCGTATT---ACTAA 37609
Orangutan       AGACAGTATAATGTTAATCAGCACTAAATGGAAATGAGCTATCA-AGCCATGGAGAACCTTCAGTGTATATT---ACTAA 37611

Majority        GTAAAAGAAGCCAGTTTGAAAATTCTACATACCATGTGATTCCAACTATATGACATTCTGGAAAAAGCAAAATTATGGAA

                        45210     45220     45230     45240     45250     45260     45270     45280

Human           ATAAAAGAAGCCACTCTGAAAATTCTGCATACCATATGATTCCAACTATATGACATTCTGGAAAAAGCAAAATTATGGAG 35681
Coquerel'sSifaka GTTAAAGA--------GCAGA----------TAATTGGCTTTTAGTT-------------TAGAAACTAGAATAATGGA- 33354
Ma'sNightMonkey TTATAGGATATAAGTTCTATG--TACAAATATAGTCTCCTTATACTAAAAGCAAATTCTTGGAAAATGAAATTTTTAAAA 32538
VervetAGM       GTAAAAGAAGCCAGTTTGAAAATCCTACATACCATGTGATTCCAACTATATGACATTCTGTAAAAAGCAAAATTATGGAA 39078
Gorrilla        ATAAAAGAAGCCACTCTGAAAATTCTGCATACCATATGATTCCAACTATATGACATTCTGGAAAAAGCAAAATTATGGAG 38076
GrabEatingMacaqueGTAAAAGAAGCCAGTTTGAAAATCCTATATACCATGTGATTCCAACTATATGAGATTCTGGAAAAAGCAAAATTATGGAA 37717
Macaque         GTAAAAGAAGCCAGTTTGAAAATCCTATATACCATGTGATTCCAACTATATGAGATTCTGGAAAAAGCAAAATTATGGAA 38486
PigTailedMacaque GTAAAAGAAGCCAGTTTGAAAATCCTACATACCATGTGATTCCAACTATATGACATTCTGGAAAAAGCAAAATTATGGAA 37436
Bonobo          ATAAAAGAAGCCACTCTGAAAATTCTGCATACCATGTGATTCCAACTATATGACATTCTGGAAAAAGCAAAATTATGGAG 36707
OliveBaboon     GTAAAAGAAGCCAGTTTGAAAATCCTACATACCATGTGATTCTAACTATATGACATTCTGGAAAAAGCAAAATTATGGAA 37689
Orangutan       GTGAAAGAAGCCACTCTGAAAATTCTACATACCATATGATTCCAACTATTTGACATTCTGGAAAAAGCAAAATTATGGAG 37691

Majority        ACA-GTAAAAATCAGTGGTTGCTAGGATTTGCGAGTTGAGGGAGGGATGAATTGGTGGAGTACAGAGGATTT-TAGGGCA

                        45290     45300     45310     45320     45330     45340     45350     45360

Human           ACA-GTAAAAATCAGTGGTTGCTAGGGTTTAGGGGTGGAGGGAGGGATGAACTGGTGGAGTACAGAGGATTT-TAGGGTA 35759
Coquerel'sSifaka ------------------TGGCTGTGATGT-------T---GGGAGCTTAACAGGAAGTAGAAAGGAATTTTCTGGGGTG 33406
Ma'sNightMonkey ATTCATTTGTAAAAGTATCAGACAAAATAT-----CAGGAAGAAAATTTAACAAAAGATGTGTAAGACATCTGCACCAAT 32613
VervetAGM       ACA-GTAAAAATCAGTGGTTGCTAGGATTTGCGAGTTGAGGGAGGGATGAATTGGTGGAGTACAGAGGATTT-TAGGGCA 39156
Gorrilla        ACA-GTAAAAATCAGTGGTTGCTAGGGTTTAGGGGTTGAGGGAGGGATGAATTGGTGGAGTACAGAGGATTT-TACGGCA 38154
GrabEatingMacaqueACA-GTAAAAATCAGTGGTTGCAAGGATTTGCGAGTTGAGGGAGGGATGAATTGGTGGAGTACAGAGGATTT-TAGGGCA 37795
Macaque         ACA-GTAAAAATCAGTGGTTGCAAGGATTTGCGAGTTGAGGGAGGGATGAATTGGTGGAGTACAGAGGATTT-TAGGGCA 38564
PigTailedMacaque ACA-GTAAAAATCAGTGGTTGCAAGGATTTGCGAGTTGAGGGAGGGATGAATTGGTGGAGTACAGAGGATTT-TAGGGCA 37514
Bonobo          ACA-GTAAAAATCAGTGGTTGCTAGGGTTTAGGGGTTGAGGGAGGGATGAATTGGTGGAGTACAGAGGATTT-TAGGGCA 36785
OliveBaboon     ACA-GTAAAAATCAGTGGTTGCTAGGATTTGCGAGTTGAGGGAGGGATGAATTGGTGGAGTACAGAGGATTT-TAGGGTA 37767
Orangutan       ACA-GTAAAAATCTGTGGTTGCTAGGGTTTGGGGGTTGAGGGAGGGATGAATTGGTGGAGTACAGAGGATTT-TAGGGCA 37769
```

```
Majority          ATGAAAATATTCTGCATG----CTATAATGATAGATACATATCATAAATTTTTCCAAACCCATAGAATGTAAGACACTTG

                           45370     45380     45390     45400     45410     45420     45430     45440

Human             CTGAAAATATTCTGCATGATA-CTATAATGATAGATACATGTCATACATTTTTCCAAACCCATAGAATGTAAGACACTTG 35838
Coquerel'sSifaka  ATGGAAATGTTCTATATC---------------AAT-------------------------TAAGTTGTGAAA--C--- 33440
Ma'sNightMonkey   ATGAAACACTGCTAAGAGAAGTTTAAAAAGACTTAAATAAATGAAGACATAGACCATGTTCATGAATTAGAAGGCTCAAT 32693
VervetAGM         ATGAAAATATTCTGCATG----CTATAATGATAAATACATATCATAAATTTTTCCAAACCCATAGAATGTAAGACACTTG 39232
Gorrilla          ATGAAAATATTCTGCATGATA-CTATAATGATAGATACATGTCATACATTTTCCCAAACCCATAGAATGTAAGACACTTG 38233
GrabEatingMacaqueATGAAAATATTCTGCATG----CTATAATGATAGATACATATCATAAATTTTTCCAAACCCATAGAATGTAAGACACTTG 37871
Macaque           ATGAAAATATTCTGCATG----CTATAATGATAGATACATATCATAAATTTTTCCAAACCCATAGAATGTAAGACACTTG 38640
PigTailedMacaque  ATGAAAATATTCTGCATG----CTATAATGATAGATACATATCATAAATTTTTCCAAACCCATAGAATGTAAGACACTTG 37590
Bonobo            CTGAAAATATTCTGCATGATA-CTATAATGATAGATACATGTCATACATTTTTCCAAACCCATAGAATGTAAGACACTTG 36864
OliveBaboon       ATGAAAATATTCTGCATG----CTATAATGATAGATACATACCATAAATTTTTCCAAACCCATAGAATGTAAGACACTTG 37843
Orangutan         ATGAAAATATTCTGCATGATA-CTATAATGATAGATACATGTGATACATTTTTCCAAACCCGTAGAATGTAAGACACTTG 37848

Majority          GAGTGAACCCCAAGGTAACTACAGACTTTGAGTGATTATAATGTGTCAGTGTAGGTTCATCAGTTGT-AACAATGTGCCA

                           45450     45460     45470     45480     45490     45500     45510     45520

Human             GAGTGAACCTGGAGGTAACTACAGACTTTGAGTGATTATAATGTGTCAGTGTAGGTTCATCAGTTGT-AACAATGTGCCA 35917
Coquerel'sSifaka  --------A----------TCCAAAAT----------T------------------------TGT------------ 33453
Ma'sNightMonkey   ATGTTAACATTCCAGTTCTCCCTAAATTGGAATGGC-AT----TG--AATCTAAATTCATTAAGTCTCACAGACTTTTAA 32766
VervetAGM         GAGTGAACCCCAAGGTAACTGCAGACTTTGAGTGATTATAATGTGTCAGTGTAGGTTCATCAGTTGT-AACAATGTGCCA 39311
Gorrilla          GAGTGAACCTGGAGGTAACTACAGACTTTGAGTGATTATAATGTGTCAGTGTAGGTTCATCAGTTGT-AACAATGTGCCA 38312
GrabEatingMacaqueGAGTGAACCCCAAGGTAACTACAGACTTTGAGTGATTATAATGTGTCAGTGTAGGTTCATCAGTTGT-AACAATGTGCCA 37950
Macaque           GAGTGAACCCCAAGGTAACTACAGACTTTGAGTGATTATAATGTGTCAGTGTAGGTTCATCAGTTGT-AACAATGTGCCA 38719
PigTailedMacaque  GAGTGAACCCCAAGGTAACTACAGACTTTGAGTGATTATAATGTGTCAGTGTAGGTTCATCAGTTGTTAACAATGTGCCA 37670
Bonobo            GAGTGAACCTGGAGGTAACTACAGACTTTGAGTGATTATAATGTGTCAGTGTAGGTTCATCAGTTGT-AACAATGTGCCA 36943
OliveBaboon       GAGTGAACCCCAAGGTAACTACAGACTTTGAGTGATTATAGTGTGTCAGTGTAGGTTCATCAGTTGT-AACAATGTGCCA 37922
Orangutan         GAGTGAACCCGGAGGTAACTACAGACT--GAGTGATTATAATGTGTCAGTGTAGGTTCATCAGTTGT-AACAATGTGCCA 37925

Majority          TCTGG---TGGGAGATGTTGATAAAGGGGAGGCTATTTGGG-GCAGGAGGCAGGGA-AATCTCTCTGCTTTCCTCTCCAT

                           45530     45540     45550     45560     45570     45580     45590     45600

Human             TCTGG---TGGGAGATGTTGATAAAGGGGAGGCTATTTGGG-GCAGGAGGCAGGGA-AATCTCTCTGCTTTCCTCTCCAT 35992
Coquerel'sSifaka  ----T----GAAAGCAGTAGATTA---------TCTTTGGG-------AACAAGTA----------------------GT 33487
Ma'sNightMonkey   ATTGAAATTGAGAGTAGTTTCTAAAATA-----TATGTGGAAATGAAAAGGAGCTAGAATAAAACTATTTTGAAGTAAAT 32841
VervetAGM         TCTGG---TGGGAGATGTTGATAAAGGGGAGGCTATTTGGGAGCAGGAGGCAGGGA-AATCTCTCTGCTTTCCTCTCCAT 39387
Gorrilla          TCTGG---TGGGAGATGTTGATAAAGGGGAGGCTGTTTGGG-GCAGGAGGCAGGGA-AATCTCTCTGCTTTCCTCTCCAT 38387
GrabEatingMacaqueTCTGG---TGGGAGATGTTGATAAAGGGGAGGCTATTTGGGGGCAGGAGGCAAGGA-AATCTCTCTGCTTTCCTCTCCAC 38026
Macaque           TCTGG---TGGGAGATGTTGATAAAGGGGAGGCTATTTGGGGGCAGGAGGCAAGGA-AATCTCTCTGCTTTCCTCTCCAC 38795
PigTailedMacaque  TCTGG---TGGGAGATGTTGATAAAGGGGAGGCTATTTGGGGGCAGGAGGCAAGGA-AATCTCTCTGCTTTCCTCTCCAC 37746
Bonobo            TCTGG---TGGGAGATGTTGATAAAGGGGAGGCTATTTGGG-GCAGGAGGCAGGGA-AATCTCTCTGCTTTCCTCTCCAT 37018
OliveBaboon       TCTGG---TGGGAGATGTTGATAAAGGGGAGGCTATTTGGGGGCAGGAGGCAAGGA-AATCTCTCTGCTTTCCTCTCCGC 37998
Orangutan         TCTGG---TGGGAGATGTTGATAAAGGGGAGGCTATTTGGG-GCAGGAGGCAGGGA-AATCTCTCTGCTTTCCTCTCCAT 38000
```

```
Majority          TTTGCTG-TGTACCTAA-ACTTACTCTGAAGAATGTCTTTGA--AAATTTATTCATGTGCTTGCATTGC-ATTTCTTTTG
                           45610     45620     45630     45640     45650     45660     45670     45680
Human             TTTGCTG-TGTACCTAA-ACTTACTCTGAAAAATGTCTTTGA--AAATTTACTCATGCGCTTGCATTGC-ATTTCTTTTG 36067
Coquerel'sSifaka  TTTGTTG------------------------------------------------------------------------G 33495
Ma'sNightMonkey   TGTGCCACTGTTTTCAAGACTTAACCACAAACCTGTAGTTAATCAAAACAATTTA-GTACTAGCATAGTTAGTGACATAG 32920
VervetAGM         TTTGCTG-TGTACCTAA-ACTTACTCTGAAGAATATCTTTGA--AAATTTATTCATGTGCTTGCATTGC-ATTTCTTTTG 39462
Gorrilla          TTTGCTG-TGTACCTAA-ACTTACTCTGAAAAATGTCTTTGA--AAATTTACTCATGTGCTTGCATTGC-ATTTCTTTTG 38462
GrabEatingMacaqueTTTGCTG-TGTACCTAA-ACTTACTCTGAAGAATATCTTTGA--AAATTTATTCATGTGCTTGCATTGC-ATTTCTTTTG 38101
Macaque           TTTGCTG-TGTACCTAA-ACTTACTCTGAAGAATATCTTTGA--AAATTTATTCATGTGCTTGCATTGC-ATTTCTTTTG 38870
PigTailedMacaque  TTTGCTG-TGTACCTAA-ACTTACTCTGAAGAATATCTTTGA--AAATTTATTCATGTGCTTGCATTGC-ATTTCTTTTG 37821
Bonobo            TTTGCTG-TGTACCTAA-ACTTACTCTGAAAAATGTCATTGA--AAATTTACTCATGCGCTTGCATTGC-ATTTCTTTTG 37093
OliveBaboon       TTTGCTG-TGTACCTAA-ACTTACTCTGAAGAATATCTTTGA--AAATTTATTCATGTGCTTGCATTGC-ATTTCTTTTG 38073
Orangutan         TTTGCTG-TATACGTAA-ACTTACTCTGAAAAATGTCTTTGA--AAATTTACTCATTTGCTTGCATTGC-ATTTCTTTTG 38075

Majority          GATATTACTGGTCTAAGTAGTCCAATTAAAAGACAAAGATT-------G-------------------------------
                           45690     45700     45710     45720     45730     45740     45750     45760
Human             GATATTACTGGTCTAAGTAGTCCAGTTAAAAGACAAAGATT-------G------------------------------- 36109
Coquerel'sSifaka  AATAT--------------------------------------------------------------------------- 33500
Ma'sNightMonkey   AATAGGAATAAACTCATGTGTTTA---AAGATGTCTTGGTTA-----------------------C-------------- 32960
VervetAGM         GATATTACTGGTCTAAGTAGTCCAATTAAAAGACAAAGATT--------------------G------------------ 39504
Gorrilla          GGTATTACTGGTCTAAGTAGTCCAGTTAAAAGACAAAGATT-------G------------------------------- 38504
GrabEatingMacaqueGATATTACTGGTCTAAGTAGTCCAATTAAAAGACAAAGATTGGGCCGGGCGCGGTGGCTCAAGCCTGTAATCCCAGCACT 38181
Macaque           GATATTACTGGTCTAAGTAGTCCAATTAAAAGACAAAGATTGGGCCGGGCGCGGTGGCTCAAGCCTGTAATCCCAGCACT 38950
PigTailedMacaque  GATATTACTGGTCTAAGTAGTCCAATTAAAAGACAAAGATTGGGCCGGGCGCGGTGGCTCAAGCCTGTAATCCCAACACT 37901
Bonobo            GATATTACTGGTCTAAGTAGTCCAGTTAAAAGACAAAGATT-------G------------------------------- 37135
OliveBaboon       GATATTACTGGTCTAAGTAGTCCAATTAAAAGACAAAGATTGGGCCGGGCGCGGTGGCTCAAGCCTGCAATCCCAGCACT 38153
Orangutan         GATATTACTGGTCTAAGTAGTCCAGTTAAAAGACAAAGATT-------G------------------------------- 38117

Majority          --------------------------------------------------------------------------------
                           45770     45780     45790     45800     45810     45820     45830     45840
Human             -------------------------------------------------------------------------------- 36109
Coquerel'sSifaka  -------------------------------------------------------------------------------- 33500
Ma'sNightMonkey   -------------------------------------------------------------------------------- 32960
VervetAGM         -------------------------------------------------------------------------------- 39504
Gorrilla          -------------------------------------------------------------------------------- 38504
GrabEatingMacaqueTTGGGAGGCCGAGACGGGCGGATCACGAGGTCAGGAGATCGAGACCATCCCGGCTAACACGGTGAAACCCCTTCTCTACT 38261
Macaque           TTGGGAGGCCGAGACGGGCGGATCACGAGGTCAGGAGATCGAGACCATCCCGGCTAACACGGTGAAACCTCGTCTCTACT 39030
PigTailedMacaque  TTGGGAGGCCGAGACGGGCGGATCACGAGGTCAGGAGATGGAGACCATCCCGGCTAACACGGTGAAACCCCTTCTCTACT 37981
Bonobo            -------------------------------------------------------------------------------- 37135
OliveBaboon       TTGGGAGGCCGAGACGGGCGGATCACGAGGTCAGGAGATCGAGACTATCCTGGCTAACACGGTGAAACCCCGTCTCTACT 38233
Orangutan         -------------------------------------------------------------------------------- 38117
```

```
Majority          --------------------------------------------------------------------------------

                          45850     45860     45870     45880     45890     45900     45910     45920

Human             -------------------------------------------------------------------------------- 36109
Coquerel'sSifaka  -------------------------------------------------------------------------------- 33500
Ma'sNightMonkey   -------------------------------------------------------------------------------- 32960
VervetAGM         -------------------------------------------------------------------------------- 39504
Gorrilla          -------------------------------------------------------------------------------- 38504
GrabEatingMacaque AAAAAAAAATACAAAAAAACTAGCCGGGGGAGATGGCGGGTGCCTGTAGTCCCAGCTACTCGGGAGGCTAAGGCAGGAGA 38341
Macaque           AAAAAAAAATACAAAAAAACTAGCCGGGGGAGATGGCGGGTGCCTGTAGTCCCAGCTACTCGGGAGGCTAAGGCAGGAGA 39110
PigTailedMacaque  AAAAAAAAATACAAAAAAACTAGCCGGGGGAGATGGCGGGCGCCTGTAGTCCCAGCTACTCGGGAGGCTAAGGCAGGAGA 38061
Bonobo            -------------------------------------------------------------------------------- 37135
OliveBaboon       AAAAAAAAATACAAAAAAACTAGCCGGGGGAGATGGCGGGCGCCTGTAGTCCCAGCTACTCGGGAGGCTAAGGCAGGAGA 38313
Orangutan         -------------------------------------------------------------------------------- 38117

Majority          --------------------------------------------------------------------------------

                          45930     45940     45950     45960     45970     45980     45990     46000

Human             -------------------------------------------------------------------------------- 36109
Coquerel'sSifaka  -------------------------------------------------------------------------------- 33500
Ma'sNightMonkey   -------------------------------------------------------------------------------- 32960
VervetAGM         -------------------------------------------------------------------------------- 39504
Gorrilla          -------------------------------------------------------------------------------- 38504
GrabEatingMacaque ATGGCGTGAACCCGGGAGGCGGAGCTTGCAGTGAGCCGAGAT--CGCCACTGCACTCCAGCCTGGGTGACAGAGCAAGAC 38419
Macaque           ATGGCGTGAACCCGGGAGGCGGAGCTTGCAGTGAGCCGAGATTGCGCCACTGCACTCCAGCCTGGGTGACAGAGCGAGAC 39190
PigTailedMacaque  ATGGCGTGAACCCAGGAGGCGGAGCTTGCAGTGAGCCGAGATTGCGCCACTGCACTCCAGCCTGGGTGACAGAGCGAGAC 38141
Bonobo            -------------------------------------------------------------------------------- 37135
OliveBaboon       ATGGCGTGAACCCGGGAGGCGGAGCTTGCAGTGAGCCGAGATTGCGCCACTGCACTCCAGCCTGGGTGACAGAGCGAGAC 38393
Orangutan         -------------------------------------------------------------------------------- 38117

Majority          --------------------------------------------TCAGACTTGATTAAAAAGCAAGGCCTAACTACACTG

                          46010     46020     46030     46040     46050     46060     46070     46080

Human             --------------------------------------------TCAGACTTGATTAAAAAGCAAGGCCTAACTACACTG 36145
Coquerel'sSifaka  -------------------------------------------------------------------------------- 33500
Ma'sNightMonkey   --------------------------------------------TCA---TTGAGGAAAGAG-AAGGCTTTTCAATAAAC 32992
VervetAGM         --------------------------------------------TCAGACTTGATTAAAAAGCAAGGCCTAACTACACTG 39540
Gorrilla          --------------------------------------------TCAGACTTGATTAAAAAGCAAGGCCTAACTACACTG 38540
GrabEatingMacaque TCCGTCTCAAAAAAAAAAAAAAAAAAAAAAAAGACAAAAGATTGTCAGACTTGATTAAAAAGCAAGGCCTAACTACACTG 38499
Macaque           TCCGTCTCAAAAAAAAAAAAAAGACAAA-----------GATTGTCAGACTTGATTAAAAAGCAAGGCCTAACTACACTG 39259
PigTailedMacaque  TCCGTCTCAAAAAAAAAAAAAAAAGACA---------AAGATTGTCGGACTTGATTAAAAAGCAAGGCCTAACTACACTG 38212
Bonobo            --------------------------------------------TCAGACTTGATTAAAAAGCAAGGCCTAACTACACTG 37171
OliveBaboon       TCCATCTCAAAAAAAAAAAAAAAGACAA----------AGATTGTCAGACTTGATTAAAAAGCAAGGCCTAACTACACTG 38463
Orangutan         --------------------------------------------TCAGACTTGATTAAAAAGCAAGGCCTAACTACAC-- 38151
```

```
Majority          TCTATACGAGGGTCAGGTGGAAATACCAAGATGCAGGTTGAAAGTAAAAAGATGGAAAGTGATATTCCATGCAAACAGTA

                          46090     46100     46110     46120     46130     46140     46150     46160

Human             TCAATACGAGGGTCAGGTAGAAATATCAAGATGCAGGTTGAAAGTAAAAAGATGGAAAATGATATTCCGTGCAAACAGTA 36225
Coquerel'sSifaka  -------------------------------------------------------------------------------- 33500
Ma'sNightMonkey   GTGCCAGAACAGCTGAATATACATGCAAAAAAGCAAA--AGAAACTCAACCCTACATTAC-ATATAGAACACTGGGATTA 33069
VervetAGM         TCTATACAAGGGTCAGGTGGAAATACCAAGGTGCAGGTTGAAAGTAAAAAGATGGAAAGTGATACTCCATGCAAACAGTA 39620
Gorrilla          TCAATACGAGGGTCAGGTAGAAATATCAAGATGCAGGTTGAAAGTAAAAAGATGGAAAATGATATTCCGTGCAAACAGTA 38620
GrabEatingMacaqueCCTATAGGAGGGTCAGGTGGAAATACCAAGATGCAGGTTGAAAGTAAAAAGATGGAAAGTGATATTCCATGCAAACAGTA 38579
Macaque           CCTATACGAGGGTCAGGTGGAAATACCAAGATGCAGGTTGAAAGTAAAAAGATGGAAAGTGATATTCCATGCAAACAGTA 39339
PigTailedMacaque  CCTATACGAGGGTCAGGTGGAAATACCAAGATGCAGGTTGAAAGTAAAAAGATGGAAAGTGATATTCCATGCAAACAGTA 38292
Bonobo            TCAATACGAGGGTCAGGTAGAAATATCAAGATGCAGGTTGAAAGTAAAAAGATGGAAAATGATACTTTGTGCAAACAGTA 37251
OliveBaboon       TCTATACGAGGATCAGGTGGAAATACCAAGATGCAGGTTGAAAGTAAAAAGATGGAAAGTGATATTCCATGCAAACAGTA 38543
Orangutan         TCTATACGAGGGTCAGGTGGAAATATCAAGATGCGGGTTGAAAGTAAAAAGATGGAAAATGATACTCCATGCAAACAGTA 38231

Majority          AGCGCGAGAAAGTTGATATGATTATATTACTAGACAGA--AAAATTTAAGACAAGAAGTATTACCAGATATAAAAAAGCA

                          46170     46180     46190     46200     46210     46220     46230     46240

Human             AGCACAAGAAAGTTGATATGATTATATTACTAGACAGA--AAAATTTAAGACAAA---TATTACCAGATATAAAAAAGCA 36300
Coquerel'sSifaka  -------------------------------------------------------------------------------- 33500
Ma'sNightMonkey   ATTATATAAAAACTTACCTAA--AAACTAATATATAGA---GCTTTTAGGAAAAA------TCCTAGGC-TATACTTGCA 33137
VervetAGM         AGCGTGAGAAAGTTGATATGATTATATTACTAGACAGA--AAAATTTAAGACAAGAAGTATTACCAAATATAAAAAAGCA 39698
Gorrilla          AGCACAAGAAAGTTGATATGATTATATTACTAGACAGAGAAAAATTTAAGACAAG---TATTACCAGATATAAAAAAGCA 38697
GrabEatingMacaqueAGCGCGAGAAAGTTGATATGATTATATTACTAGACAGA--AAAATTTAAGACAAGAAGTATTACCAAATATAAAAAAGCA 38657
Macaque           AGCGCGAGAAAGTTGATATGATTATATTACTAGACAGA--AAAATTTAAGACAAGAAGTATTACCAAATATAAAAAAGCA 39417
PigTailedMacaque  AGCGCGAGAAAGTTGATATGATTATATTACTAGACAGA--AAACTTTAAGACAAGAAGTATTACCAAATATAAAAAAGCA 38370
Bonobo            AGCACAAGAAAGTTGATATGATTATATTACTAGACAGAGAAAAATTTAAGACAAA---TATTACCAGATATAAAAAAGCA 37328
OliveBaboon       AGCGTGAGCAAGTTGATATGATTATATTACTAGACAGA--AAAATTTAAGACAAGAAGTATTACCAAATATAAAAAAGCA 38621
Orangutan         AGCACAAGAAAGTTGATATGATTATATTACTAGACAGAGAAAAATTTGAGACAAGAAGTATTACCAGATATAAACAAGCA 38311

Majority          TATTTCATAATGCTGAAGGGTCAGTTTATCAGAAAACATTTCTTAATTTGTATGCCA--ATAATAACAA-----AGTATA

                          46250     46260     46270     46280     46290     46300     46310     46320

Human             TATTTCACAATGTTGAAGGGTCAGTTTATCAGAAAACATTTCTTAATTTGTATGCCA--ATAATAACAAATCAAAGTATA 36378
Coquerel'sSifaka  -----------------------------------------------------------------GAA--------ATG 33506
Ma'sNightMonkey   ATTTTGGGATAAATGAAGATTTCTTAAAACATAAAGGTATAGATAAACTTGACTCCATCAAAATGGGAAATTTCTGCTTA 33217
VervetAGM         TATTTCATAATGCTGAAGGGTCAGTTTATCAGAAAACATTTCTTAATTTGTATGCCA--ATAATAACAA-----AGTATA 39771
Gorrilla          TATTTCACAATGTTGAAGGGTCAGTTTATCAGAAAACATTTCTTAATTTGTATGCCA--ATAATAACAAATTAAAGTATA 38775
GrabEatingMacaqueTATTTCATAATGCTGAAGGGTCAGTTTATCAGAAAACATTTCTTAATTTGTATGCCA--ATAATAACAA-----AGTATG 38730
Macaque           TATTTCATAATGCTGAAGGGTCAGTTTATCAGAAAACATTTCTTAATTTGTATGCCA--ATAATAACAA-----AGTATG 39490
PigTailedMacaque  TATTTCATAATGCTGAAGGGTCAGTTTATCAGAAAACATTTCTTAATTTGTATGCCA--ATAATAACAA-----AGTATG 38443
Bonobo            TATTTCACAATGTTGAAGGGTCAGTTTATCAGAAAACATTTCTTAATTTGTATGCCA--ATAATAACAACTTAAAGTATA 37406
OliveBaboon       TATTTCATAATGCTGAAGAGTCAGTTTATCAGAAAACATTTCTTAATTTGTATGCCA--ATAATAACAA-----AGTATA 38694
Orangutan         TATTTCACAATGTTGAAGGGTCAGTTTATCAGAAAACGTTTCTTAATTTGTATGCCA--ATAATAAGAAATTAAAGTATA 38389
```

```
Majority        TTAAGAAAAAGATCACAGAGCTAAGTAGAGAAACAGAAATCCACAATCATAGCCTGATTTTTT-AAACTATAATTTATA-
                        46330     46340     46350     46360     46370     46380     46390     46400
Human           TTAAGAAAAACATCACAGAACTAAGTAGAGAAACAAAAATCCACAATCATAGCCTGGTTTTTT-AAAACATAATTTATG- 36456
Coquerel'sSifaka TTAAGAGAGAG-----AGAGAGAGAGAGAGAGA--------------GAGAGCGTG---T------------------- 33544
Ma'sNightMonkey ATCAAAAGACTTTGTTAAGACAATGAAAAGGCA-AGGCACAGACTTGGAGAATATAATATTTACATATCAATACTTATAT 33296
VervetAGM       TTAAGAAAAACATCACAGAGCTAAGTAGAGAAACAGAAATCCACAATCATAGCCTGATTTTTT-AAACTATAATTTGGA- 39849
Gorrilla        TTAAGAAAAACATCACAGAACTAAGTAGAGAAACAGAAATCCACAATCATAGCCTGGTTTTTT-AAAACATAATTTATG- 38853
GrabEatingMacaqueTTAAGAAAAAGATCACAGAGCTAAGTAGAGAAACAGAAATCCACAATCACAGCCTGATTTTTT-AAACTATAATTTAGA- 38808
Macaque         TTAAGAAAAAGATCACAGAGCTAAGTAGAGAAACAGAAATCCACAATCATAGCCTGATTTTTT-AAACTATAATTTAGA- 39568
PigTailedMacaque TTAAGAAAAAGATCACAGAGCTAAGTAGAGAAACAGAAATCCACAATCATAGCCTGATTTTTT-TTTTTTTTTTTTTGA- 38521
Bonobo          TTAAGAAAAACATCACAGAACTAAGTAGAGAAACAGAAATCCACAATCATAGCCTGGTTTTTT-AAAACATAATTTATG- 37484
OliveBaboon     TTAAGAAAAAGATCACAGAGCTAAGTAGAGAAACAGAAATCCACAATCATAGCCTGATTTTTT-AAACTATAATTTAGA- 38772
Orangutan       TTAAGAAAAGCATCACAGAACTAAGTAGAGAAACAGAAATCCACAATCATAGCCTGATTTTTTTAAAACATAATTTATG- 38468

Majority        GTCATTGATAGGACAGTTACAGTCCTATCCCCCAAATAAAAA---GATACATAATTTGAAAAGTGCTATCATGTTGGCTT
                        46410     46420     46430     46440     46450     46460     46470     46480
Human           GCAATTGATAGGATAGT-ACAGTCCTAGCCCCCAAATAAAAA---GATACATAATTTGAAAAGTGCTATCATGTTGGCTT 36532
Coquerel'sSifaka GT-G---------------------------------------------------------------------------- 33547
Ma'sNightMonkey GTGACTG-TA--TTAGT-CCATTTTCAGGCTGCTGATAAAGACA-TACCCGAGACTGGGCAATTTACAAAAGAAAGAGAT 33371
VervetAGM       GTCATTGATAGGACAGTTACAGTCCTATCCCCCAAATAAAAA---GATACATAATTTGAAAAATGCTATCATGTTGGCTT 39926
Gorrilla        GTAATTGATAGGACAGT-ACAGTCCTAGCCCCCAAATAAAAA---GAAACATAATTTGAAAAGTGCTATCATGTTGGCTT 38929
GrabEatingMacaqueGTCATTGATAGGACAGTTACAGTCCTATCCCCCAAATAAAAA---GATACATAATTTGAAAAATGCTGTCATGTTGGCTT 38885
Macaque         GTCATTGATAGGACAGTTACAGTCCTATCCCCCAAATAAAAA---GATACATAATTTGAAAAATGCTGTCATGTTGGCTT 39645
PigTailedMacaque GACG---------GAGTCTC-GCTCTGTCGCCCAGGCTGGAGTGCAGTGGCCGGATCTCAGCTCACTGCAAGCTCCGCCT 38591
Bonobo          GTAATTGATAGGATAGT-ACCGTCCTAGCCCCCAAATAAAAA---GATACATAATTTGAAAAGTGCTATCATGTTGGCTT 37560
OliveBaboon     GTCATTGATAGGACAGTTACAGTCCTATCCCCCAAATAAAAA---GATACATAATTTGAAAAATGCTGTCATGTTGGCTT 38849
Orangutan       GTAATTGATAGTACAGTTACAGTCCTATCCCCCAAATAAAAA---GATATATAATTTGAAAAGTGCTATCATGTTGGCTT 38545

Majority        AACTCACATTTATAAAACTATACCCAGTAACTGTAAAATAACTTCAAGTGTACATAGAACATTCACAATGATAGACCA-T
                        46490     46500     46510     46520     46530     46540     46550     46560
Human           AACTCACATTTATAAAACTATACCCAGTAACTGTAAAATAACTTCAAGTGTACATAGAACATTCACAATGATAGACCT-T 36611
Coquerel'sSifaka ----TGTGTGTGTGTG--TGTGTG------------------T--GTGTGTGTGNNNNNNNNNN--N------------- 33586
Ma'sNightMonkey TTAGTGGATTTACAGGTTCATGTGAAGCCTCAGCATAATGGTGGAAGGTAAGGAGGAACAACTCACA-TCTTATATGG-- 33448
VervetAGM       AACTCACATTTATAAAACTATACCCAGTAACTGTAAAATAACTTCAAGTGTACATAGAACATTCACAGTGATAGACTA-T 40005
Gorrilla        AACTCACATTTATAAAACTATACCCAGTAACTGTAAAATAACTTCAAGTGTGCATAGAATATTCACAATGATAGGCCT-T 39008
GrabEatingMacaqueAACTCACATTTATAAAACTATACCCAGTAACTGTAAAATAACTTCAAGTGTACATAGAACATTCACAATGGTAGACCA-T 38964
Macaque         AACTCACATTTATAAAACTATACCCAGTAACTGTAAAATAACTTCAAGTGTACATAGAACATTCACAATGGTAGACCA-T 39724
PigTailedMacaque CCCGGGTTCACGCCATTCTCCGGCCTCAGCCTCCCGAGTAGCT--GGGACTACAGGCGCCCGCCACCTCGCCCGGCTAGT 38669
Bonobo          AACTAACATTTATAAAACTATACCCAGTAACTGTAAAATAACTTCAGGTGTACATAGAACATTCACAATGGTAGACCT-T 37639
OliveBaboon     AACTCACATGTATAAAACTATACCCAGTAACTGTAAAATAACTTCAAGTGTACATAGAGCATTCACAATGATAGACCA-T 38928
Orangutan       AACTTACATTTATAAAACTATACCCAGTAACTGTAAAATAACTTCAAGTGTACATAGAACATTCACAATGATAGACCT-T 38624
```

```
Majority         ATATTGGATTACAAAATAGCTCAATAAATTTCAAGTGATTGACAGTTCATGTAG----TATCTGACTATAGTGGAATTAA
                         46570     46580     46590     46600     46610     46620     46630     46640
Human            ATATTGGATTATAAAATAACTCAATAAATTTCAAATGATTGACAGCTAATGTA---TCTGACTGACTATAGTGGAATTAA 36688
Coquerel'sSifaka --NNNNNNNNNNNNNN---NNNNNNNNNNNNNNNNNNNNNNNNNNNGTGTGTG--------------------------- 33634
Ma'sNightMonkey  --ATGGCAGCAGGCAAAGAGAAGAGAGCTTTTGTAGGGAAACTCCCCTTTTTAA-AACCATCAGATCTTGTGAGACGTAT 33525
VervetAGM        ATATTGGATTACAAAATAGCTCAATAAATTTCAAGTTATTGACAGTTCATATAG----TATATGACTATAGTGGAATTAA 40081
Gorrilla         ATATTGGATTATAAAATAGCTCAATAAATTTCAAATGATTGACAGCTAATGTAGTATCTGACTGACTATAGTGGAATTAA 39088
GrabEatingMacaqueGTATTGGATTACAAAATAGCTCAATAAATTTCAAGTGATTGACAGTTCATATAG----TATATGACTATAGTGGAATTAA 39040
Macaque          GTATTGGATTACAAAATAGCTCAATAAATTTCAAGTGATTGACAGTTCATATAG----TATATGACTATAGTGGAATTAA 39800
PigTailedMacaque TTTTTGTATTTCTTAATAGAGACGGGGTTTCACCGTGTTAGCCAGGATGGTTTC----GATCTCCTGACCTCGTGATCCG 38745
Bonobo           ATATTGGATTATAAAATAACTCAATAAATTTCAAATGATTGACAGCTAATGTACTATCTGACTGACTATAGTGGAATTAA 37719
OliveBaboon      GTATTGGATTACAAAATAGCTCAATAAATTTCAAGTGATTGACAGTTCATATAG----TATATGACTATAGTGGAATTAA 39004
Orangutan        ATATTGGATTATAAAATAACTCAATAAATTTCAAATGATTGACAGCTCATGTAGTATCTGACTGACTGTAGTGGAATTAA 38704

Majority         ATTA----GAAATCAAAAATAGACATCTAGGAAAGTATGAAATATTAGAAATTATACAATTGTGAACAATACATGGATTA
                         46650     46660     46670     46680     46690     46700     46710     46720
Human            ATTA----GAAATCAAAAATAGACATCTAGGAAAGTACTAAATATTAGAAATTACACAATCGTGAACAATACATGGATTA 36764
Coquerel'sSifaka ----------------------------------------------------TGTGTGTGTGTG--------------TG 33648
Ma'sNightMonkey  TTCA-----CTATCACGAG--AACAGCATGGGAAAGACCAACCTTCATGATTTGGTTACTTCCCACCAGGTCCCTCACAC 33598
VervetAGM        ---------AAATCAAAAATAGACATCTAGGAAAGTATGAAATATTAGAAATTATACAATTGTGAACAATACATGGATTA 40152
Gorrilla         ATTA----GAAATCAAAAATAGACATCTAGGAAAGTACCAAATATTAGAAATTACACAATCGTGAACAATACATGGATTA 39164
GrabEatingMacaqueATTA----GAAATCAAAAATAGACATCTAGGAAAGTATGAAATATTAGAAATTATACAATTGTGAACAGCACATGGATTA 39116
Macaque          ATTA----GAAATCAAAAATAGACATCTAGGAAAGTATGAAATATTAGAAATTATACAATTGTGAACAGCACATGGATTA 39876
PigTailedMacaque CCCGTCTCGGCCTCCCAAAGTGCTGGGATTACAGGCTTGAGCCACCGCGCCCGGCCAGCCTGATTTTTTAAACTATAATT 38825
Bonobo           ATTA----GAAATCAAAAATAGACATCTAGGAAAGTACCAAATATTAGAAATTACACAATCGTGAACAATACATGGATTA 37795
OliveBaboon      ATTA----GAAATCAAAAATAGACATCTAGGAAAGTATGAAATATTAGAAATTATACAATTGTGAACAACACATGGATTA 39080
Orangutan        ATTA----GAAATCAAAAATAGACACCCAGGAAAGTACCAAATATTAGAAATTACACAATTGTGAACAATACATGGATTA 38780

Majority         TAGAAGTGTTA---------------------------------------------------------------------
                         46730     46740     46750     46760     46770     46780     46790     46800
Human            TAGAAGTGTTA--------------------------------------------------------------------- 36775
Coquerel'sSifaka TGTGTGTGTG---------------------------------------------------------------------- 33658
Ma'sNightMonkey  AACACTTGGAA-------------------A------------------------------------------------- 33610
VervetAGM        TAGAAGTGTTA--------------------------------------------------------------------- 40163
Gorrilla         TAGACGTGTTA--------------------------------------------------------------------- 39175
GrabEatingMacaqueTAGAAGTGTTACCACAA-GGACATATTTTTTTTTTTTTTTTT----GAGACGGAGTCTCG----CTCTGTCGC-CAGGCT 39186
Macaque          TAGAAGTGTTACCACAA-GGACATATTTTTTTTTTTTTTTTTTTTTGAGACGGAGTCTCG----CTCTGTCGCGCAGGCT 39951
PigTailedMacaque TAGAGTCATTGATAGGACAGTTACAGTCCTATCCCCCAAATAAAAAGATACATAATTTGAAAAATGCTGTCATGTTGGCT 38905
Bonobo           TAGAAGTGTTA--------------------------------------------------------------------- 37806
OliveBaboon      TAGAAGTGTT--------------------A------------------------------------------------- 39091
Orangutan        TAGAAGTGTTA--------------------------------------------------------------------- 38791
```

```
Majority           --------------------------------------------------------------------------------

                            46810     46820     46830     46840     46850     46860     46870     46880

Human              -------------------------------------------------------------------------------- 36775
Coquerel'sSifaka   -------------------------------------------------------------------------------- 33658
Ma'sNightMonkey    -------------------------------------------------------------------------------- 33610
VervetAGM          -------------------------------------------------------------------------------- 40163
Gorrilla           -------------------------------------------------------------------------------- 39175
GrabEatingMacaque  GGAGT-GCA---GTGGCGCGATCTCGGCTCACTGCAAGCTCCGCCTCCCAGGTTCACGCCATTCTCCTGCCTCAGCCTCC 39262
Macaque            GGAGT-GCA---GTGGTGCGATCTCGGCTCACTGCAAGCTCCGCCTCCCGGGTTCACGCCATTCGCCTGCCTCAGCCTCC 40027
PigTailedMacaque   TAACTCACATTTATAAAACTATACCCAGTAACTGTAAAATA---ACTTCAAGTGTACATAGAACATTCACAATGGTAGAC 38982
Bonobo             -------------------------------------------------------------------------------- 37806
OliveBaboon        -------------------------------------------------------------------------------- 39091
Orangutan          -------------------------------------------------------------------------------- 38791

Majority           --------------------------------------------------------------------------------

                            46890     46900     46910     46920     46930     46940     46950     46960

Human              -------------------------------------------------------------------------------- 36775
Coquerel'sSifaka   -------------------------------------------------------------------------------- 33658
Ma'sNightMonkey    --------------T----------------------------------------------------------------- 33611
VervetAGM          -------------------------------------------------------------------------------- 40163
Gorrilla           -------------------------------------------------------------------------------- 39175
GrabEatingMacaque  CGAGTAGCTGGGACTACAGGCGCCCACAACCGCGCTCGGCTAATTTTTTTGTATTTTTAGTAGA-GACGGGGTTTCACCG 39341
Macaque            CGAGTAGCTGGGACTACAGGCGCCCACAACCGCGCCCGGCTAATTTTTT-GTATTTTTAGTAGA-GACGGGGTTTCACCG 40105
PigTailedMacaque   CATGTA--TTGGATTACAAAATAGCTCAATAAATTTCAAGTGATTGACA-GTTCATATAGTATATGACTATAGTGGAAT- 39058
Bonobo             -------------------------------------------------------------------------------- 37806
OliveBaboon        -------------------------------------------------------------------------------- 39091
Orangutan          -------------------------------------------------------------------------------- 38791

Majority           --------------------------------------------------------------------------------

                            46970     46980     46990     47000     47010     47020     47030     47040

Human              -------------------------------------------------------------------------------- 36775
Coquerel'sSifaka   -------------------------------------------------------------------------------- 33658
Ma'sNightMonkey    -------------------------------------------------------------------------------- 33611
VervetAGM          -------------------------------------------------------------------------------- 40163
Gorrilla           -------------------------------------------------------------------------------- 39175
GrabEatingMacaque  TGGTCTCGATCTCCTGACCTTGTGATCCGCCCGCCTCGGCCTCCCA-AAGTGCTGAGATT----ACAG-GCGTGAGCCAC 39415
Macaque            TGGTCTCGATCTCCTGACCTTGTGATCCGCCCGCCTCGGCCTCCCA-AAGTGCTGAGATT----ACAG-GCGTGAGCCAC 40179
PigTailedMacaque   TAAATTAGAAATCAAAAATAGACATCTAGGAAAGTATGAAATATTAGAAATTATACAATTGTGAACAGCACATGGATTAT 39138
Bonobo             -------------------------------------------------------------------------------- 37806
OliveBaboon        -------------------------------------------------------------------------------- 39091
Orangutan          -------------------------------------------------------------------------------- 38791
```

```
Majority          ----------CCACAAGGACATATTTTTG---ACTGAATGAAAATATATCAAAATTTGTTGGATACGACCCAAGGGTGTG
                          47050     47060     47070     47080     47090     47100     47110     47120
Human             ----------CCACAAGGACATATTTTTG---ACTGAATGAAAATATATCAAAATTTGTTGGATACTACCCAAGGGTGTG 36842
Coquerel'sSifaka  ---------------------------TG-------------------------------------TGTGTGTGTGTGTG 33674
Ma'sNightMonkey   ----------TCAGGAGGAGATTTGGATGGGGACACAGCCAAACCATATCAATGACAAAGGACTTGTATTCCAAAGGACT 33681
VervetAGM         ----------CCACAAGGACATATTTTTG---ACTGAATGAAAATATATCAAAATTTGTTGGATACGACCCAAGGGCGTG 40230
Gorrilla          ----------CCACAAGGACATATTTTTG---ACTGAATGAAAATATATCAAAATTTGTTGGATACTACCCAAGGGTGTG 39242
GrabEatingMacaqueCGCGCCCGGCCCACAAGGACATATTTTTG---ACTGAATGAAAATATATCAAAATTTGTTGGATACGACCCAAGGGAGTG 39492
Macaque           CGCGCCCGGCCCACAAGGACATATTTTTG---ACTGAATGAAAATATATCAAAATTTGTTGGATACGACCCAAGGGAGTG 40256
PigTailedMacaque  AGAAGTGTTACCACAAGGACATATTTTTG---ACTGAATGAAAATATATCAAAATTTGTTGGATACGACCCAAGGGCGTG 39215
Bonobo            ----------CCACAAGGACATATTTTTG---ACTGAATGAAAATATATCAAAATTTGTTGGATACTACCCAAGGGTATG 37873
OliveBaboon       ----------CCACAAGGACATATTTTTG---ACTGAATGAAAATATATCAAAATTTGTTGGATACGACCCAAGGGCGTG 39158
Orangutan         ----------CCACAAGGACATATTTTTG---ACTGAATGAAAATATATCAAAATTTGTTGGATACGACCCAAGGGTGTG 38858

Majority          TATGGTGTA--AAGTATGTCTTCAAATACCTATATTATAAAAAAAAGGTTTAAAACTCCCTATTTAAG-ACTGACCAAG-
                          47130     47140     47150     47160     47170     47180     47190     47200
Human             TATGGTGAA--AAGTATGTCTTCAAATACCTATATTACAAAAAAA-GGTTTAAAACTCCCAATTTAAG-ACTGACCAAGG 36918
Coquerel'sSifaka  TGTATTGCTT---GAACTTCACCAGAT--TTACATTATAGAAGAG---TTTTGTTCCATTTGTATT--------TCA--- 33735
Ma'sNightMonkey   TGTAAAGGA--------CTCTTCCAATCACTATATATAAGCAGCCCAATTTTTTTTATAAGAATGGTAACAAGCACGT 33753
VervetAGM         TATGGTGTGTAAAGTATGTCTTCAAATACCTATATTATAAAAAAAAGGTTTAGAACTCCCTATTTAAG-ACTGACCAAG- 40308
Gorrilla          TATGGTGAA--AAGTATGTCTTCAAATACCTATATTACAAAAAAA-GGTTTAAAACTCCCTATTTAAG-ACTGACCAAGG 39318
GrabEatingMacaqueTATGGTGTG--AAGTATGTCTTCAAATACCTATATTATAAAAAAAAGGTTTAAAACTCCCTATTTAAG-ACTGACCAAG- 39568
Macaque           TATGGTGTG--AAGTATGTCTTCAAATACCTATATTATAAAAAAAAGGTTTAAAACTCCCTATTTAAG-ACTGACCAAG- 40332
PigTailedMacaque  TATGGTGTG--AAGTATGTCTTCAAATACCTATATTATAAAAAAAAGGTTTAAAACTCCCTATTTAAG-ACTGACCAAG- 39291
Bonobo            TATGGTGAA--AAGTATGTCTTCAAATACCTATATTACAAAAAAA-GGTTTAAAACTCCCAATTTAAG-AGTGACCAAGG 37949
OliveBaboon       TATGGTGTA--AAGTATGTCTTCAAATACCTATATTATAAAAAAAAGGTTGAAAACTCCCTATTTAAG-ACTGACCAAG- 39234
Orangutan         TATGGTGAA--AAGCATGTCTTCAAATACCTATATTACAAAAAAA-GGTTTAAAAGTCCCTATTTAAG-ACTGACCAAGA 38934

Majority          -AAAAGACATA--AATTACCAGTG---TGGGAATCAAAGTCGAGATAA--TCACTTTCACAGAAAGTGGAAATGTACAGA
                          47210     47220     47230     47240     47250     47260     47270     47280
Human             AAAAAGACATA--AATTACCAATAC--TGGAAATCAAGGTCGAGATAA--TCACTTTCAGAGAAAGTGGATATATGCAGA 36992
Coquerel'sSifaka  ----------------------------GGGGGCCAGGGTCAGTA-------------A---------T--GTATATAGG 33763
Ma'sNightMonkey   GAAAAGATACATTAGTCATCAGGTGATCACAAGTCAAAACCATTATGAGGTAGAATTTACACCAACTAGA-ATGGCTAAA 33832
VervetAGM         -AAAAGACATA--AATTACCAGTC---TGGGAATCAAAGTCGAGATAA--TCACTTTCACAGAAAGTGGAAATGTACAGA 40380
Gorrilla          AAAAAGACATA--AATTACCAATAC--TGGAAATCAAAGTCGAGATAA--TCACTTTCGGAGAAAGTGGAAATATGCAGA 39392
GrabEatingMacaque-AAAAGACATA--ATTTACCAGTG---TGGGAATCAAAGTCGAGATAA--TCACTTTCACAGAAAGTGGAAATGTACAGA 39640
Macaque           -AAAAGACATA--ATTTACCAGTG---TGGGAATCAAAGTCGAGATAA--TCACTTTCACAGAAAGTGGAAATGTACAGA 40404
PigTailedMacaque  -AAAAGACATA--AATTACCAGTG---TGGGAATCAAAGTCGAGATAA--TCACTTTCACAGAAAGTGGAAATTTACAGA 39363
Bonobo            AAAAAGACATA--AATTACCAATAC--TGGAAATCAAGGTCGAGATAA--TCACTTTCAGAGAAAGTGGATATATGCAGA 38023
OliveBaboon       -AAAAGACATA--AATTACCAGTG---TGGGAATCAAAGTCGAGATAA--TCACTTTCACAGAAAGTGGAAATGTACAGA 39306
Orangutan         AAAAAGACATA--AATTACCAATAC--TGGAAATCAAAGTCGAGATAA--TCACTTTCAGAGGAAGTGGAAATATACAGA 39008
```

```
Majority          GTATAATAGTATAACATTAAT-AATATAAA-GTGGAGCTAGCACTTTCAATAAAATCAGAGATATATAGAATATAATAGG
                          47290     47300     47310     47320     47330     47340     47350     47360
Human             GTATAATAGTATAACATAAAT-AATATAAA-GTGGAGATAGCACTTTCAATAAAAGCAGAGATATATAGAATATAATAGG 37070
Coquerel'sSifaka  GGAGAGTAGTCGAAAA---------------------------CTTT----------------ATA-ATAATCTGCTCTA 33799
Ma'sNightMonkey   CTAAAATAGGTTGGCAACACTGAATGTTGATGAAGATGTGGAACTGTCATTCATTACTGCTGAGTATATAACATAGTTGG 33912
VervetAGM         GTATAATAGTATAACATTAAT-AATATAAA-ATGGAGCTAGCACTTTCAATAAAATCAGAGATACATAGAATATAATAGG 40458
Gorrilla          GTATAATAGTATAACATAAAT-AATATAAA-GTGGAGATAGCACTTTCAATAAAAGCAAAGATATATAGAATATAATAGG 39470
GrabEatingMacaque GTATAATAGTATAACATTAAT-AATATAAA-GTGGAGCTAGCACTTTCAATAAAATCAGAGATATATAGAATATAATAGG 39718
Macaque           GTATAATCGTATAACATTAAT-AATATAAA-GTGGAGCTAGCACTTTCAATAAAATCAGAGATATATAGAATATAATAGG 40482
PigTailedMacaque  GTATAATAGTATAACA---AT-AACATAAA-GTGGAGCTAGCACTTTCAATAAAATCAGAGATATATAGAATATAATAGG 39438
Bonobo            GTATAATAGTATAACATAAAT-AATATAAA-GTGGAGATAGCACTTTCAATAAAAGCAGAGATATATAGAATATAATAGG 38101
OliveBaboon       GTATAATAGTATAACATTAAT-AATATAAA-GTGGAGCTAGCACTCTCAATAAAATCAGAGATATGTAGAATATAATAGG 39384
Orangutan         GTATAATAGTATAGCATAAAT-AATTTAAA-GTAGAGATAGCACTTTCAATAAAAGCAGAGGTATATAGAATATAGTAGG 39086

Majority          AAAATAA-----TATTACTAATGTTGTTAAGGCTAACAAATTCAACAACTTAGATGAAGTAAGTTTCTTGACAAATGCAA
                          47370     47380     47390     47400     47410     47420     47430     47440
Human             AAAATAAGTCAATATTAGTAATGTTGTTAAGGCTAACAAATTCAGCAACTTAGATGAAA--AGTTTCTTGACAAATGCAA 37148
Coquerel'sSifaka  GAGATAG--------------------CAAGGTCATGAATTTTAGGGTCTTGGAAGTAG------------AATATGCAG 33847
Ma'sNightMonkey   GAAAGTAGTTTTTCCTTTTTTTTTTTTTTTTTTTTTNNNNNNNNNNNNNNNNNNNNNNNNNNNNNNNNNNNNNNNNNNNNNN 33992
VervetAGM         AAAATAT-----TATTACCAGTGTTGTTAAGGCTAACACATTCAACAACTTAGATGAAGTAAGTTTCTTGACAAATGCAA 40533
Gorrilla          AAAATAAGTCGATATTACTAATGTTGTTAAGGCTAACAAATTCAGCAACTTAGATGAAATAAGTTTCTTGACAAATGCAA 39550
GrabEatingMacaque AAAATAA-----TATTACCAATGTTGTTAAGGCTAACAAATTCAACAACTTAGATGAAGTAAGTTTCTTGACAAATGCAA 39793
Macaque           AAAATAA-----TATTACCAATGTTGTTAAGGCTAACAAATTCAACAACTTAGATGAAGTAAGTTTCTTGACAAATGCAA 40557
PigTailedMacaque  AAAATAA-----TATTACCAATGTTGTTAAGGCTAACAAATTCAACAACTTAGATGAAGTAAGTTTCTTGACAAATGCAA 39513
Bonobo            AAAATAAGTCAATATTACTAATGTTGTTAAGGCTAACAAATTCAGCAACTTAGATGAAA--AGTTTCTTGACAAATGCAA 38179
OliveBaboon       AAAATAA-----TATTACCAATGTTGTTAAGGCTAACAAATTCAACAACTTAGATGAAGTAAGTTTCTTGACAAATGCAA 39459
Orangutan         AAAATAAGT-AATATTACTAATGT---TAAGGCTAACAGATTCAACAACTTAGATGAAATAAGTTTCTTGACAAATGCAA 39162

Majority          AACTGACTTAAGTATATAGCTTAACAGCTATATACTACTGTATTATACAAATCGGAATTTATAATTAAAAAAATATTTCC
                          47450     47460     47470     47480     47490     47500     47510     47520
Human             AACTGACTTAAGTATATAGCTTAACAGCTATATACTACTGTATTATACAAATTGGAATTTATAATTAAAAAAATCTTTCC 37228
Coquerel'sSifaka  GAATGAT-------------GTATCAAGAAATTACAACTCAGTTATTTGGAAGAAGATGAGTTTGGGGAAAAATACTAGT 33914
Ma'sNightMonkey   NNNNNNNNNNNNNNNNNNNNNNNNNNNNNNNNNNNNNNNNNNNNNNNNNNNNNNNNNNNNNNNNNNNNNNNNNNNNNNNN 34072
VervetAGM         AACTGACTTAAGTATATAGCTTAACAGCTATATACTACTGTATTATGCAAATCGGAATTTATAATTAAAAAAATATTTCC 40613
Gorrilla          AACTGACTTAAGTATATAGCTTAACAGCTATATACTACTGTATTATACAAATTGGAATTTATAATTAAAAAAATCTTTCC 39630
GrabEatingMacaque AACTGACTTAAGTATATAGCTTAACAGCTATATACTACTGTATTATACAAATCGGAATTTATAATTAAAAAAATATTTCC 39873
Macaque           AACTGACTTAAGTATATAGCTTAACAGCTATATACTACTGTATTATACAAATCGGAATTTATAATTAAAAAAATATTTCC 40637
PigTailedMacaque  AACTGACTTAAGTATATAGCTTAACAGCTATATACTACTGTATTATACAAATCGGAATTTATAATTAAAAAAATATTTCC 39593
Bonobo            AACTGAC--AAGTATATAGCTTAACAGCTATATACTACTGTATTATACAAATTGGAATTTATAATTAAAAAAATCTTTCC 38257
OliveBaboon       AACTGACTTAAGTATATAGCTTAACAGCTATATA---CTGTATTATACAAATCGGAATTTATAATTAAAAAAATATTTCC 39536
Orangutan         AAGTGACT------TATAGCTTAACAGCTGTATACTACTGTATTATACAGATTGGAATTTATAATTAAAAAAATCTTTCC 39236
```

```
Majority          ACAAAGATTACTCTAGGCATAGGTTGTTTTACTATTGAATTTTATGAAATTTTAAAGAAATAATAGCTGTCTTTGAAAAT
                          47530     47540     47550     47560     47570     47580     47590     47600
Human             ACAAAGATTACTCTAGTCATAGATGGTTTCACTATTGAATTTTATGAAATTTTAAAGGAATACTAGCTGTCTTTGAAAAT 37308
Coquerel'sSifaka  TTTAGAATCACT--GG--GTGGATGATGGTACCATT-AA-----CAAAGGGGGAAAAGTGGAAAAGAAGATTTTTGGTAG 33984
Ma'sNightMonkey   NNNNNNNNNNNNNNNNNNNNNNNNNNNNNNNNNNNNNNNNNNNNNNNNNNNNNNNNNNNNNNNNNNNNNNNNNNNNNNNN 34152
VervetAGM         ACAAAGATTACTCTAGGCATAGGTTGTTTTACTATTGAATTTTATGAAATTTTAAAGAAATAATAGCTGTCTTTGAAAAT 40693
Gorrilla          ACAAAGATTACTCTAGGCATAGATGGTTTCACTATTGAATTTTATGAAATTTTAAAGGAATACTAGCTGTCTTTGAAAAT 39710
GrabEatingMacaqueACAAAGATTACTCTAGGCATAGGTTGTTTTACTATTGAATTTTATGAAATTTTAAAGAAATAATAGCTGTCTTTGAAAAT 39953
Macaque           ACAAAGATTACTCTAGGCATAGGTTGTTTTACTATTGAATTTTATGAAATTTTAAAGAAATAATAGCTGTCTTTGAAAAT 40717
PigTailedMacaque  ACAAAGATTACTCTAGGCATAGGTTGTTTTACTATTGAATTTCATGAAATTTTAAAGAAATAATAGCTGTCTTTGAAAAT 39673
Bonobo            ACAAAGATTACTCTAGTCATAGATGGTTTCACTATTGAATTTTATGAAATTTTAAAGGAATACTAGCTGTCTTTGAAAAT 38337
OliveBaboon       ACAAAGATTACTCTAGGCATAGGTTGTTTTACTATTGAATTTTACGAAATTTTAAAGAAATAATAGCTGTCTTTGAAAAT 39616
Orangutan         ACAAAGATTATTCTAGGCATAGATGGTTTCACTATTGAATTTTATGAAATTTTAAAGAAATAATAGCTGTCTTTGAAAAT 39316

Majority          GGAGGAGGGAATACTGCCCTGTTCACTTTATGAAGCCACTATAACTGACAGCAAAACCTGACAAGGGTAATATATCAAGA
                          47610     47620     47630     47640     47650     47660     47670     47680
Human             GGAGGAGGGAATACTACCCTGTTCACTTTATGAAACCACTATAACTGACAGCAAAACCTGACAAGGGTAATATATCAAGA 37388
Coquerel'sSifaka  AAAGAATGAATCTATGACTCTTCCGTTTTTTAAAG----TATGTTTAGGAATGATACATAATGTGCTTTATGACCGAAGA 34060
Ma'sNightMonkey   NNNNNNNNNNNNNNNNNNNNNNNNNNNNNNNNNNNNNNNNNNNNNNNNNNNNNNNNNNNNNNNNNNNNNNNNNNNNNNNN 34232
VervetAGM         GGAGGAGGAAATACTGCCCTGTTCACTTTATGAAGCCACTATAACTGACAGCAAAACCTGACAAGGGTAATATATCAAGA 40773
Gorrilla          GGAGGAGGGAATACTACCCTGTTCACTTTATGAAACCACTATAACTGACAGCAAAACCTGACAAGGGTAATATATCAAGA 39790
GrabEatingMacaqueGGAGGAGGGAATACTGCCCTGTTCACTTTATGAAGCCACTATAACTGACAGCAAAACCTGACAAGGGTAATATATCAAGA 40033
Macaque           GGAGGAGGGAATACTGCCCTGTTCACTTTATGAAGCCACTATAACTGACAGCAAAACCTGACAAGGGTAATATATCAAGA 40797
PigTailedMacaque  GGAGGAGGGAATACTGCCCTGTTCACTTTATGAAGCCACTATAACTGACAGCAAAACCTGACAAGGGTAATATATCAAGA 39753
Bonobo            GGAGGAGGGAATACTACCCTGTTCACTTTATGAAACCACTATAACTGACAGCAAAACCTGACAAGGGTAATATATCAAGA 38417
OliveBaboon       GGAGGAGGGAATACTGCCCTGTTCACTTTATGAAGCCACTATAACTGACAGCAAAACCTGACAAGGGTAATATATCAAGA 39696
Orangutan         GGAGGAGGGAATACTACCCTGTTCACTTTATGAAACCACTATAACTGACAGCAAAACCTGACAAGGGTAATATATCAAGA 39396

Majority          TTTCAGACTAGTATTTCTCATTAACCTAAATGCATATATTCTTAAGAAAATGTTGGTAAATCACCTCCAGTAGAATATGA
                          47690     47700     47710     47720     47730     47740     47750     47760
Human             TTTCAGACTAGTATTTCTCATTAACCTAAATGCATATATTCTTAAGAAAATGTTGGTAAATCACCTCCGGTAGAATATGA 37468
Coquerel'sSifaka  ATCCGTGTAATTATTTTGTAGTGATGGAATGAAATTC---CTTAAAGCCTTGTTTTAACATA---------AGAAATTTA 34128
Ma'sNightMonkey   NNNNNNNNNNNNNNNNNNNNNNNNNNNNNNNNNNNNNNNNNNNNNNNNNNNNNNNNNNNNNNNNNNNNNNNNNNNNNNNN 34312
VervetAGM         TTTCAGACTAGTATTTCTCATTAACCTAAATGCATATATTCTTAAGAAAATGTTGGTAAATC-CCTCCAGTAGAATATGA 40852
Gorrilla          TTTCAGACTAGTATTTCTTATTAACCTAAATGCATATATTCTTAAGAAAATGTTGGTAAATCACCTCCAGTAGAATATGA 39870
GrabEatingMacaqueTTTCAGACTAGTATTTCTCATTACCCTAAATGCATATATTCTTAAGAAAATGTTGGTAAATCACCTCCAGTAGAATATGA 40113
Macaque           TTTCAGACTAGTATTTCTCATTACCCTAAATGCATATATTCTTAAGAAAATGTTGGTAAATCACCTCCAGTAGAATATGA 40877
PigTailedMacaque  TTTCAGACTAGTATTTCTCATTAACCTAAATGCATATATTCTTAAGAAAATGTTGGTAAATCACCTCCAGTAGAATATGA 39833
Bonobo            TTTCAGACTAGTATTTCTCATTAACCTCAATGCATATATTCTTAAGAAAATGTTGGTAAATCACCTCCGGTAGAATATGA 38497
OliveBaboon       TTTCAGACTAGTATTTCTCATTAACCTAAATGCATAT--TCTTAAGAAAATGTTGATAAATCACCTCCAGTAGAATATGA 39774
Orangutan         TTTCAGACTAGTATTTCTCATTAACCTAAATGCATATATTCTTAAGAAAATGTTGGTAAATCACCTCCAGTAGAATATGA 39476
```

```
Majority          GAGGCAATTCTGAGACTTA-C----ACAGGATTGTAAGGTTTGTTTAACATTTGAAAATGAACTCAGTGTAATTTGTCAC

                         47770     47780     47790     47800     47810     47820     47830     47840

Human             GAGACAATTCTGAGACTTATC----GCAGGATTGTAAGGTTTGTTTAACATTTGAAAATGAACTCAGTGTAATTTGTCAC 37544
Coquerel'sSifaka  CAGGCAATCAATTTATTTAAC----CTCTATGTACAGAGAAAACTTTAAGTGTTAAGATTT-TTCTGTTTTTTTTCTAAC 34203
Ma'sNightMonkey   NNNNNNNNNNNNNNNNNNN------NNNNNNNNNNNNNNNNNNNNNNNNNNNNNNNNNNNNNNNNNNNNNNNNNNNNNNN 34386
VervetAGM         GAGGCAATTCTGAGACTTA-TC---ACAGGGTTGTAAGGTTTGTTTAACATTTGAAAATGAACTCAGTGTAATTTGTCAC 40928
Gorrilla          GAGACAATTCTGAGACTTACC----GCAGGATTGTAAGGTTTGTTTAACATTTGAAAATGAACTCAGTGTAATTTGTCAC 39946
GrabEatingMacaqueGAGGCAATTCTGAGACTTA-C----ACAGGATTGTAAGGTTTGTTTAACATTTGAAAATGAACTCAGTGTAATTTGTCAC 40188
Macaque           GAGGCAATTCTGAGACTTA-CC---ACAGGATTGTAAGGTTTGTTTAACATTTGAAAATGAACTCAGTGTAATTTGTCAC 40953
PigTailedMacaque  GAGGCAATTCTGAGACTTAACC---ACAGGATTGTAAGGTTTGTTTAACATTTGAAAATGAACTCAGTGTAATTTGTCAC 39910
Bonobo            GAGACAATTCTGAGACTTATCATAAGCAGGATTGTAAGGTTTGTTTAACATTTGAAAATGAACTCAGTGTAATTTGTCAC 38577
OliveBaboon       GAGGCAATTCTGAGACTTA-CC---ACAGGATTGTAAGGTTTGTTTAACATTTGAAAATGAACTCAGTGTAATTTGTCAC 39850
Orangutan         GAGACAATTGTGAGACTTACT----GCAGGTTTGTAAGGTTTGTTTAGCATTTGAAAATGAATTCAGTGTAATTTGTCAC 39552

Majority          AGTGATAGAATGAAGAAGGAAAAATATCTCTCAGAAAACTAGTAAGAGAAGGAGCTTTTTACAATGTAAATAAGGGGTAT

                         47850     47860     47870     47880     47890     47900     47910     47920

Human             AATGATAGAATGAAGAAAGAAGAATTTCTCTCAGAAAACTAGGAAGAGAAGGAGCTTTT-ACAATGTAAATAAGGGGTAT 37623
Coquerel'sSifaka  AAAGAAAGATCAAAGTCTGCA---TATGTTCTAACACACCAATTTGAATA----CTGTTACTCATAAGAACAGATAGCAT 34276
Ma'sNightMonkey   NNNNNNNNNNNNNNNNNNNNNNNNNNNNNNNNNNNNNNNNNNNNNNNNNNNNNNNNNNNNNNNNNNNNNNNNNNNNNNNN 34466
VervetAGM         AGTGATAGAATGAAGAAGGAAAAATATCTCTCAGAAAACTAGTAAGAGAAGGAGCTTTTTACACTGTAAATAAGGGGTAT 41008
Gorrilla          AATGATAGAATGAAGAAAGAAGAATTTCTCTCAGAAAACTAGGAAGAGAAGGAGCTTTT-ACAATGTAAATAAGGGGTAT 40025
GrabEatingMacaqueAGTGATAGAATGAAGAAGGAAAAATATCTCTCAGAAAACTAGTAAGAGAAGGAGCTTTTTACAATGTAAATAAGGGGTAT 40268
Macaque           AGTGATAGAATGAAGAAGGAAAAATATCTCTCAGAAAACTAGTAAGAGAAGGAGCTTTTTACAATGTAAATAAGGGGTAT 41033
PigTailedMacaque  AGTGATAGAATGAAGAAGGAAAAATATCTCTCAGAAAACTAGTAAGAGAAGGAGCTTTTTACAATGTAAATAAGGGGTAT 39990
Bonobo            AATGATAGAATGAAGAAAGAAGAATTTCTCTCAGAAAACTAGGAAGAGAAGGAGCTTTT-ACAATGTAAATAAGGGGTAT 38656
OliveBaboon       AGTGATAGAATGAAGAAGGAAAAATATCTCTCAGAAAACTAGTAAGAGAAGGAGCTTTTTACAATGTAAATAAGGGGTAT 39930
Orangutan         AATGATAGAATGAAGAAGGAAGAATCTCTCTCAGAAAGCTAGGAAGAGAAGGAGCTTTT-ACAATGTAAATAAGGGGTAT 39631

Majority          CTA---AGAACCTACAACAAATTTCATACTTAATGGAGAAATATTGAATGTTTTCTTCTTAAAATCAGAAATGGAGCAAG

                         47930     47940     47950     47960     47970     47980     47990     48000

Human             CTACTAAGAACCTACAACAAATTTCATACTTAATGGAGAAATATTGAATGTTTTCTTCTTAAAAACAGAAATGAAGCAAG 37703
Coquerel'sSifaka  AAA----AGACCTTTAAAAAAC--------T----------GGT-GATTATATTTGCCT-------GGAAA------AAT 34320
Ma'sNightMonkey   NNN---NNNNNNNNNNNNNNNNNNNNNNNNNNNNNNNNNNNNNNNNNNNNNNNNNNNNNNNNNNNNNNNNNNNNNNNNNN 34543
VervetAGM         CTA---AGAACCTACAACAAATTTCATACTTAATGGAGAAATATTGAATATTTTCTTCTTAAAATCAGAAATGGAGCAAG 41085
Gorrilla          CTACTAAGAACCTACAACAAATTTCATACTTAATGGAGAAATATTGAATGTTTTCTTCTTAAAAACAGAAATGAAGCCAG 40105
GrabEatingMacaqueCTA---AGAACCTACAAAAAATTTCATACTTAATGGGGAAATATTGAATGTTTTCTTCTTAAAATCAGAAATGGAGCAAG 40345
Macaque           CTA---AGAACCTACAACAAATTTCATACTTAATGGGGAAATATTGAATGTTTTCTTCTTAAAATCAGAAATGGAGCAAG 41110
PigTailedMacaque  CTA---AGAACCTACAACAAATTTCATACTTAATGGGGAAATATTGAATGTTTTCTTCTTAAAATCAGAAATGGAGCAAG 40067
Bonobo            CTACTAAGAACCTACAACAAATTTCATACTTAATGGAGAAATATTGAATGTTTTCTTCTTAAAAACAGAAATGAAGCAAG 38736
OliveBaboon       CTA---AGAACCTACAACAAATTTCATACTTAATGGGGAAATATTGAATGTTTTCTTCTTAAAATCAGAAATGGAGCAAG 40007
Orangutan         CTACTAAGAACCTACAACAAATTTCATACTTAATGGAGAAATATTGAATGTTTTCTTCTTAAAAACAGAAATGAAGCAAG 39711
```

```
Majority         GGTGTCTGTTTTCTCAATTTTTT--CCCCTAGCTTTCTGCTTACATTAAAGCAGGTGAAATAAAAGACATAAAGATTGTA
                        48010     48020     48030     48040     48050     48060     48070     48080
Human            GATGTCTGTTTTCTCAATTTTTC--CCCTTAGCTTTCTGCTTACATTAAGGCAGGTGAAATAAAAGACATAAAGATTGTA 37781
Coquerel'sSifaka ATTCCCTATGGTTCAGATGACTC---AGAAGGACTTTTGCTGACAATGTTACAAG--------AACACTTCCTGGTTATC 34389
Ma'sNightMonkey  NNNNNNNNNNNNNNNNNNNNNNN--NNNNNNNNNNNNNNNNNNNNNNNNNNNNNNNNNNNNNNNNNNNNNNNNNNNNNNN 34621
VervetAGM        GGTGTCTGTTTTCTCAATTTTTTCCCCCTTAACTTTCTGCTTACATTAAAGCAGGTGAAATAAAAGACATAAAGGTTGTA 41165
Gorrilla         GATGTCTGTTTTCTCAATTTTTC--CCCTTAGCTTTCTGCTTACATTAAGGCAGGTGAAATAAAAGACATAAAGATTGTA 40183
GrabEatingMacaqueGGTGTCTGTTTTCTCAATTTTTT-CCCCCTAACTTTCTGCTTACATTAAAGCAGGTGAAATAAAAGACATAAAGATTGTA 40424
Macaque          GGTGTCTGTTTTCTCAATTTTTT-CCCCCTAACTTTCTGCTTACATTAAAGCAGGTGAAATAAAAGACATAAAGATTGTA 41189
PigTailedMacaque GGTGTCTGTTTTCTCAATTTTTTTCCCCCTAACTTTCTGCTTACATTAAAGCAGGTGAAATCAAAGACATAAAGATTGTA 40147
Bonobo           GATGTCTGTTTTCTCAATTTTTC--CCCTTAGCTTTCTGCTTACATTAAGGCAGGTGAAATAAAAGACATAAAGATTGTA 38814
OliveBaboon      GGTGTCTGTTTTCTCAATTTTTTTCCCCCTAACTTTCTGCTTACATTAAAGCAGGTGAAATAAAAGACATAAAGATTGTA 40087
Orangutan        GATGTCTGTTTTCTCAATTTTTC--CCCCTAGCTTTTGCTTACATTAAGGCAGGTGAAATAAAAGACATAAAGATTGTA 39789

Majority         AAGGAAGAAAGAAAACTGTCTTATTCACTGTTCAACATGCTTTTTAAAATAGAAGACCTAAAGGAATCTACAACAGATTT
                        48090     48100     48110     48120     48130     48140     48150     48160
Human            AAGGAAGAAAGAAAACTGTCTTATTCACCATTCAACATGCTTTTTAAAGTAGAAGACCTGAAGGACTCTACAACAGATTT 37861
Coquerel'sSifaka AAATATACAACAGGATT-TCATTTTTATGTTTCAATGGCCAGCTTTATCCAAAATGTTT---C----TTCTAATTGATTT 34461
Ma'sNightMonkey  NNNNNNNNNNNNNNNNNNNNNNNNNNNNNNNNNNNNNNNNNNNNNNNNNNNNNNNNNNNNNNNNNNNNNNNNNNNNNNNN 34701
VervetAGM        AAGGAAGAAAGAAAACTGTCTTATTCACTGTTCAACATGCTTTTTAAAATAGAAGACCTAAAGGAATCTACAACAGATTT 41245
Gorrilla         AAGGAAGAAAGAAAACTGTCTTATTCACCATTCAACATGCTTTTTAAAGTAGAAGACCTAAAGGAATCTACAACAGATTT 40263
GrabEatingMacaqueAAGGAAGAAAGAAAACTGTCTTATTCACTGTTCAACATGCTTTTTAAGATAGAAGACCTAAAGGAATCTACAACAGATTT 40504
Macaque          AAGGAAGAAAGAAAACTGTCTTATTCACTGTTCAACATGCTTTTTAAGATAGAAGACCTAAAGGAATCTACAACAGATTT 41269
PigTailedMacaque AAGGAAGAAAGAAAACTGTCTTATTCACTGTTCAACATGCTTTTTAAAATAGAAGACCTAAAGGAATCTACAACAGATTT 40227
Bonobo           AAGGAAGAAAGAAAACTGTCTTATTCACCATTCAACATGCTTTTTAAAGTAGAAGACCTGAAG-ACTCTACAACAGATTT 38893
OliveBaboon      AAGGAAGAAAGAAAACTGTCTTATTCACTGTTCAACATGCTTTTTAAAATAGAAGACCTAAAGGAATCTACAACAGATTT 40167
Orangutan        AAGGAAGAAAGAAAACTGTCTTATTCACCATTCAACATGCTTTTTAAAATAGAAGACCTAAAGGTATCTACAACAGATTT 39869

Majority         GGTAGAACTAATAAGTGAATTTAGAATAATTATAGGATACAAGTTCTGTATACAAAAATAGTCTCCTTATACTTG-CAGC
                        48170     48180     48190     48200     48210     48220     48230     48240
Human            GGTAGAACTAATAAGTGAATTTAGAATAATTATAGGATACAAGTTCTATGTACAGAAATAGTCTCCTTATACTTGGCAGC 37941
Coquerel'sSifaka --------TACTATAGAATTC------------TGTTTCCAAAACTATCTCTCAACAGCCTTTCCCTTATCTGTG----T 34517
Ma'sNightMonkey  NNNNNNNNNNNNNNNNNNNNN------------NNNNNNNNNNNNNNNNNNNNNNNNNNNNNNNNNNNNNNNNNNN-NNNN 34768
VervetAGM        GGTAGAACTAATAAGTGAATTTAGAATAATTATAGGATACAAGTTCTGTGTACAAAAATAGTCTCCTTATACTAG-CAGC 41324
Gorrilla         GGTAGAACTAATAAGTGAATTTAGAATAATTATAGGATACAAGTTCTATGTACAGAAATAGTCTCCTTATACTTG-CAGC 40342
GrabEatingMacaqueGGTAGAACTAATAAGTGAATTTAGAATAATTATAGGATACAAGTTCTGTATACAAAAATAGTCTCCTTATACTAG-CAGC 40583
Macaque          GGTAGAACTAATAAGTGAATTTAGAATAATTATAGGATACAAGTTCTGTATACAAAAATAGTCTCCTTATACTAG-CAGC 41348
PigTailedMacaque GGTAGAACTAATAAGTGAATTTAGAATAATTATAGGATACAAGTTCTGTATACAAAAATAGTCTCCTTATACTAG-CAGC 40306
Bonobo           GGTAGAACTAATAAGTGAATTTAGAATAATTATAGGATACAAGTTCTATGTACAGAAATAGTCTCCTTATACTTG-CAGC 38972
OliveBaboon      GGTAGAACTAATAAGTGAATTTAGAATAATTATAGGATACAAGTTCTGTATACAAAAATAGTCTCCTTATACTAG-CAGC 40246
Orangutan        GGTAGAACTAATAAGTGAATT------------AT-ATACAAGTTCTATATACAGAAATAGTCTCCTTATACTTG-CAGC 39935
```

```
Majority        AAATTCTTAAAAAATGAAATTTTAAAAAATCTATTTATTAAAAGTATCAGACAAA--ATATCAGGAAAAAAA-CTTGACA
                         48250     48260     48270     48280     48290     48300     48310     48320
Human           AAATTCTTAGAAAATGAAATTTTAAAAAATCTGTTTAT-AAAAGTATCAGACAAA--ATATCAGGAAAAAAA-TTTGACA 38017
Coquerel'sSifaka AGACTTTCCTTCTCTGTACCTATTGAACAGATACTTTG------TGTCAAAGAAA--AAATTGACTTTAGAA----GTGA 34585
Ma'sNightMonkey NNNNNNNNNNNNNNNNNNNNNNNNNNNNNNNNNNNNNNNNNNNNNNNNNNNNNNN--NNNNNNNNNNNNNNNNNNNNNNN 34846
VervetAGM       AAATTCTTAAAAAATGAAATTTTAAAAAATCCATTTATTAAAAGTATCAGACAAAAAAATATCAGGAAAAAAA-CTTGACA 41403
Gorrilla        AAATTCTTAGAAAATGAAATTTTAAAAAATCTGTTTAT-AAAAGTATCAGACAAA--ATATCAGGAAAAAAAAATTTGACA 40419
GrabEatingMacaqueAAATTCTTAAAAAATGAAATTTTAAAAAATCCATTTATTAAAAGTAGCAGACAAAAAAATATCAGGAAAAAAA-CTTGACA 40662
Macaque         AAATTCTTAAAAAATGAAATTTTAAAAAATCCATTTATTAAAAGTAGCAGACAAAAAAATATCAGGAAAAAAA-CTTGACA 41427
PigTailedMacaque AAATTCTTAAAAAATGAAATTTTAAAAAATCCATTTATTAAAAGTAGCAGACAAAAAAATATCAGGAAAAAAA-CTTGACA 40385
Bonobo          AAATTCTTAGAAAATGAAATTTTAAAAAATCTGTTTAT-AAAAGTATCAGACAAA--ATATCAGGAAAAAAA-TTTGACA 39048
OliveBaboon     AAATTCTTAAAAAATGAAATTTTAGAAAATCCATTTATTAAAAGTAGCAGACAAAAAAATATCAGGAAAAAAA-CTTGACA 40325
Orangutan       AAATTCTTAGAAAATGAAATTAAAAAAAATCTGTTTGT-AAAAGTATCAGACAGA--ATATCAGGAAAAAAAAATTTGACA 40012

Majority        AAAGATGTGTAAGACATCTGCACCAATATGAAACACTGCTAAGAGAAGTTTTAAAAAGACTT-ACATGAAGACATAGACC
                         48330     48340     48350     48360     48370     48380     48390     48400
Human           AAAGATATGTAAGACATCTACACCAATATGAAACACTGCTAAGAGAAGTTTTAAAAAGACTT-ACATGAAGACGTAGACC 38096
Coquerel'sSifaka GAAATATTATCAGAAACTTAATCTGAGGTAAATAGCTCATAAGTTGA--TATAATAAGA----ATATTTTGTAATAAATT 34659
Ma'sNightMonkey NNNNNNNNNNNNNNNNNNNNNNNNNNNNNNNNNNNNNNNNNNNNNNNNNNNNNNNNNNNNNNNNNNNNNNNNNNNNNNNN 34926
VervetAGM       AAAGATGTGTAAGACATCTGCACCAATATGAAACACTGCTAAGAGAAGTTTTAAAAAGACTTTACATGAAGACATAGACC 41483
Gorrilla        AAAGATATGTAAGACATCTACACCAATATGAAACACTGCTAAGAGAAGTTTTAAAAAGACTT-ACATGAAGACGTAGACC 40498
GrabEatingMacaqueAAAGATGTGTAAGACATCTGCACCAATATGAAACACTGCTAAGAGAAGTTTTAAAAAGACTT-ACATGAAGACATAGACC 40741
Macaque         AAAGATGTGTAAGACATCTGCACCAATATGAAACACTGCTAAGAGAAGTTTTAAAAAGACTT-ACATGAAGACATAGACC 41506
PigTailedMacaque AAAGATGTGTAAGACATCTGTACCAATATGAAACACTGCTAAGAGAAGTTTTAAAAAGACTT-ACATGAAGACATAGACC 40464
Bonobo          AAAGATATGTAAGACATCTACACCAATATGAAACACTGCTAAGAGAAGTTTTAAAAAGACTT-ACATGAAGATGTAGACC 39127
OliveBaboon     AAAGATGTGTAAGACATCTGCACCAATATGAAACACTGCTAAGAGAAGTTTTAAAAAGACTT-ACATGAAGACATAGACC 40404
Orangutan       AAAGATGTGTAAGACATCTACACCAATATGAAACACTGCTAAGAGAAGTTTTAAAAAGACTT-ACATGAAGACGTAGACC 40091

Majority        ATGTTCATGAATTAGAAGGCTCAGTATGTTAACATGCCAGTTCTCCCTAAATTGGGATGGCATTGAATCTATAAGGACTT
                         48410     48420     48430     48440     48450     48460     48470     48480
Human           ATGTTCATGAATTAGAAGGCTCAATATGTTAACATGCCAGTTCTCCCTAAATTGGGATGGCATTGAATCTATAAAGACTT 38176
Coquerel'sSifaka CCTTCAGGAGAAATAGAAGTAAAATATTTAAAAAATACAAGTAGCCCCATCTAGCATTGGAATTAGATTT-T-------- 34730
Ma'sNightMonkey NNNNNNNNNNNNNNNNNNNNNNNNNNNNNNNNNNNNNNNNNNNNNNNNNNNNNNNNNNNNNNNNNNNNNNNNNNNNNNNN 35006
VervetAGM       ATGTTCATGAATTAGAAGGCTCAGTATGTTAACATGCCAGTTCTCCCTAAATTGGGATGACATTGAATCTATAAGGACTT 41563
Gorrilla        ATGTTCATGAATTAGAAGGCTCAATATGTTAACATGCCAGTTCTCCCTAAACTGGGATGGCATTGAATCTATAAAGACTT 40578
GrabEatingMacaqueATGTTCATGAATTAGAAGGCTCAGTATGTTAACATGCCAGTTCTCCCTCAATTGGGATGGCATTGAATCTATAAGGACTT 40821
Macaque         ATGTTCATGAATTAGAAGGCTCAGTATGTTAACATGCCAGTTCTCCCTAAATTGGGATGGCATTGAATCTATAAGGACTT 41586
PigTailedMacaque ATGTTCATGAATTAGAAGGCTCAGTATGTTAACATGCCAGTTCTCCCTAAATTGGGATGGCATTGAATCTATAAGGACTT 40544
Bonobo          ATGTTCATGAATTAGAAGGCTCAATATGTTAACATGCCAGTTCTCCCTAAATTGGGATGGCATTGAATCTATAAAGACTT 39207
OliveBaboon     ATGTTCATGAATTAGAAGGCTCAGTATGTTAACATGCCAGTTCTCCCTAAATTGGGATGGCATTGAATCTGTAAGGACTT 40484
Orangutan       ATGTTCATGAATTAGAAGGCTCAATATGTTAACATGCCAGTTCTCCCTAAATTGGGATGGCATTGAATCTGTAAAGACTT 40171
```

```
Majority          CTAAATTGAGAGTAGATTCTAAAATTTATGTGGAAATAAAAAGGAGCTAGAATAATTAAAACCATTTTGAAAAAGTAAAT
                          48490     48500     48510     48520     48530     48540     48550     48560
Human             ATAAATTGAGAGTACATTCTAAAATTTATGTGGAAATAAAAAGGAGCTAGGACAATTAAAACCATTTTGAAAAAGTAAAT 38256
Coquerel'sSifaka  ---A-TTGAAAGGAATTTGTAA---CTCTTTGGCAGTGGGAGGAGGCTTGA-----------CAATTGGAAGGTATAGG- 34791
Ma'sNightMonkey   NNNNNNNNNNNNNNNNNNNNNNNNNNNNNNNNNNNNNNNNNNNNNNNNNNNNNNNNNNNNNNNNNNNNNNNNNNNNNNNN 35086
VervetAGM         CTAAATTGAGAGTAGATTCTAAAATTTATGTGGAAATAAAAAGGAGCTAAAATAATTAAAACCATTTTGAAAAAGTAAAT 41643
Gorrilla          ATAAATTGAGAGTAGATTCTAAAATTTACGTGGAAATAAAAAGGAGCTAGGACAATTAAAACCATTTTGAAAAAGTAAAT 40658
GrabEatingMacaqueCTAAATTGAGAGTAGATTCTAAAATTTATGTGGAAATAAAAAGGAGCTAAAATAATTAAAACAATTTTGAAAAAGTAAAT 40901
Macaque           CTAAATTGAGAGTAGATTCTAAAATTTATGTGGAAATAAAAAGGAGCTAAAATAATTAAAACAATTTTGAAAAAGTAAAT 41666
PigTailedMacaque  CTAAATTGAGAGTAGATTCTAAAATTTATGTGGAAATAAAAAGGAGCTAAAATAATTAAAACAATTTTGAAAAAGTAAAT 40624
Bonobo            ATAAATTGAGAGTACATTCTAAAATTTATGTGGAAATAAAAAGGAGCTAGGACAATTAAAACCATTTTGAAAAAGTAAAT 39287
OliveBaboon       CTAAATTGAGAGTAGATTCTAAAATTTATGTGGAAATAAAAAGGAGCTAAAATAATTAAAACCATTTTGAAAAAGTAAAT 40564
Orangutan         ATAAATTGAGAGTAGATTCTAAAATTTATTTGGAAATAAAAAGGAGCTAGGACAATTAAAATCATTTTGAAAAAGTAAAT 40251

Majority          TTTGCCACTGTTCTCAGGACTCACCACAAACCTGTAGTTAATCCAAAACAATTTAGTACTAGCATAGTTAGTGACA----
                          48570     48580     48590     48600     48610     48620     48630     48640
Human             TTTGCCACAGTTCTCAAGACTCACTACAAACCTGTAGTTAATCCAAAACAGTTTACTACTAGCATAGTTAGTGACACAGA 38336
Coquerel'sSifaka  --------------CT-------CTTTAATTTCTTAGTCATTTC-------TTTATTACT----------GT--GG---- 34827
Ma'sNightMonkey   NNNNNNNNNNNNNNNNNNNNNNNNNNNNNNNNNNNNNNNNNNNNNNNNNNNNNNNNNNNNNNNNNNNNNNNNNNNN---- 35162
VervetAGM         TTTGCCACTGTTCTCAGGACTCACCACAAACCTGTAGTTAATCCAAAACAATTTAGTACTAGCATAGTTAGTGACA---- 41719
Gorrilla          TTTGCCACAGTTCTCAAGACTCACTACAAACCTGTAGTTAATCCAAAATAGTCTACTACTAGCATAGTTAGTGACACAGA 40738
GrabEatingMacaqueTTTGCCACTGTTCTCAGGACTCACCACAAACCTGTAGTTAATCCAAAACAATTTAGTGCTAGCATAGTTAGTGACA---- 40977
Macaque           TTTGCCACTGTTCTCAGGACTCACCACAAACCTGTAGTTAATCCAAAACAATTTAGTGCTAGCATAGTTAGTGACA---- 41742
PigTailedMacaque  TTTGCCACTGTTCTCAGGACTCACCACAAACCTGTAGTTAATCCAAAACAATTTAGTGCTAGCATAGTTAGTGACA---- 40700
Bonobo            TTTGCCACAGTTCTCAAGACTCACTACAAACCTGTAGTTAATCCAAAACAGTTTACTACTAGCATAGTTAGTGACACAGA 39367
OliveBaboon       TTTGCCACTTTTCTCAGGACTCACCACAAACCTGCAGTTAATCCAAAACAATTTAGTGCTAGCATAGTTAGTGACA---- 40640
Orangutan         TTTGCCACAGTTCTCAAGACTCACCACAAACCTGTAGTTAATCCAAAACAGTTTACCACTAGCATAGTTAGTGACATACA 40331

Majority          -TAGTGATCAGGGAATAACTCATGCATTAAAGTTGCCTTGGTCACTCAGTGAAGAAAGAGAAGGCTTTTA---AACAAAT
                          48650     48660     48670     48680     48690     48700     48710     48720
Human             ATAGCGTTCAGGGAATAACTCATGCATTAAAGTTGCCTTGATCACTCAGTGAAGAAAGAGAAGGCCTTTC---AACAGAT 38413
Coquerel'sSifaka  ------------AAATGGACTATCCCTTTAGGATAGTTTTGTTACTCTGT----AATAGCAAGATTTATT-----TACTA 34886
Ma'sNightMonkey   -NNNNNNNNNNNNNNNNNNNNNNNNNNNNNNNNNNNNNNNNNNNNNNNNNNNNNNNNNNNNNNNNNNNN---NNNNNNN 35238
VervetAGM         -TAGTGATCAGGGAATAACTCATGCATTAAAGTTACCTTGGTCACTCAATGAAGAAAGAGAAGGCTTTTA---AACAAAT 41795
Gorrilla          ATAGTGTTCAGGGAATCACTCATGCATTAAAGTTGCCTTGATCATTCAGTGAAGAAAGAGAAGGCCTTTC---AACAGAT 40815
GrabEatingMacaque-TAGTGATCAGGGAATAACTCATGCATTAAAGTTGCCTTGGTCACTCAATGAAGAAAGAGAAGGCTTTTA---AACAAAT 41053
Macaque           -TAGTGATCAGGGAATAACTCATGCATTAAAGTTGCCTTGGTCACTCAATGAAGAAAGAGAAGGCTTTTA---AACAAAT 41818
PigTailedMacaque  -TAGTGATCAGGGAATAACTCATGCATTAAAGTTGCCTTGGTCACTCAATGAAGAAAGAGAAGGCTTTTA---AACAAAT 40776
Bonobo            ATAGCGTTCAGGGAATAACTCATGCATTAAAGTTGCCTTGATCACTCAGTGAAGAAAGAGAAGGGTGTGCTAGAACAGAT 39447
OliveBaboon       -TAGTGATCAGGGAATAACTCATGCATTAAAGTTGCCTTGGTCACTCAATGAAGAAAGAGAAGGCTTTTA---AACAAAT 40716
Orangutan         ATAGTGTTCAGGGAATAACTCATGCATTAAAGTTGCCTTGATCACTCAGTGAAGAAAGAGAAGGCCTTTC---AACAGAT 40408
```

```
Majority         GTGCTAGAACAGCTGAATATACATACAAAAAAG-CAAAAGAAACTCAAACCCTACCTTATTGCATATAAAACAATGAGAT

                         48730     48740     48750     48760     48770     48780     48790     48800

Human            GTGCTAGAACAGCTGAATATACATACAAAAAAG-CAAAAGAAACTTAAACCCTACATCATTCTGTATAAAACAATGAGAT 38492
Coquerel'sSifaka CTGCTAAAAGTGTGACTTTTGCTTTAAGTGAAG--GCACTGAAATCATCCCATACATTTTGGTACGTGTTACTCAGAACT 34964
Ma'sNightMonkey  NNNNNNNNNNNNNNNNNNNNNNNNNNNNNNNNN-NNNNNNNNNNNNNNNNNNNNNNNNNNNNNNNNNNNNNNNNNNNNNN 35317
VervetAGM        GTGCTAGAACAGCTGAATATACATACAAAAAAA-CAAAAGAAACTCAAACCCTACCTTATTGCATATAAAACAATGAGAT 41874
Gorrilla         GTGCTAGAACAGCTGAATATACATACAAAAAAG-CAAAAGAAACTTAAACCCTACATCATTCTGTATAAAACAATGAGAT 40894
GrabEatingMacaqueGTGCTAGAACAGCTGAATATACGTACAAAAAAA-CAAAAGAAACTCAAACCCTACCTTATTGCATATAAAACAATGAGAT 41132
Macaque          GTGCTAGAACAGCTGAATATACGTACAAAAAAA-CAAAAGAAACTCAAACCCTACCTTATTGCATATAAAACAATGAGAT 41897
PigTailedMacaque GTGCTAGAACAGCTGAATATACGTACAAAAAAA-CAAAAGAAACTCAAACCCTACCTTATTGCATATAAAACAATGAGAT 40855
Bonobo           GTGCTAGAACAGCTGAATATACATACAAAAAAG-CAAAAGAAACTTAAACCCTACATCATTCTGTATAAAACAATGAGAT 39526
OliveBaboon      GTGCTAGAACAGCTGAATATACATACAAAAAAAACAAAAGAAACTCAAACCCTACCTTATTGCATATAAAACAATGAGAT 40796
Orangutan        GTGCTAGAACAGCTGAATATACGTACAAAAAAG-TAAAAGAAACTCAAACCCGACATCATTCCATATAAAACAATGAGAT 40487

Majority         TA----TATAAAAACTAATGTATAGAGTTTTTAGGAAAAAGCATAGGATATACTTGCCACTTTGGGATAAATGAAGATTT

                         48810     48820     48830     48840     48850     48860     48870     48880

Human            TAATTATATAAAAACTAATATATAGAGTTTTTAGGAAAAAGCATAGGATATACTTGCAACTTTGGGATAAATGAAGATTT 38572
Coquerel'sSifaka CT-----G---AGATTTTCATTTTAATTTTTACAGACATTTTTGATGTGATGCCCTTTCATTTTGTTATAATGC---TTT 35033
Ma'sNightMonkey  NN----NNNNNNNNNNNNNNNNNNNNNNNNNNNNNNNNNNNNNNNNNNNNNNNNNNNNNNNNNNNNNNNNNNNNNNNNNN 35393
VervetAGM        TAATTATATAAAAACTAATGTATAGAGTTTTTAGGAAAAAGCATAGGATATACTTGCCACTTTGGGATAAATGAAGATT- 41953
Gorrilla         TAATTATATAAAAACTAATATATAGAGTTTTTAGGAAAAAGCATAGGATATACTTGCAACTTTGGGATAAATGAAGATTT 40974
GrabEatingMacaqueTA----TATAAAAACTAATGTATAGAGTTTTTAGGAAAAAGCATAGGGTATACTTGCCACTTTGGGATAAATGAAGATTT 41208
Macaque          TA----TATAAAAACTAATGTGTAGCGTTTTTAGGAAAAAGCATAGGGTATACTTGCCACTTTGGGATAAATGAAGATTT 41973
PigTailedMacaque TA----TATAAAAACTAATGTATAGAGTTTTTAGGAAAAAGCATAGGGTATACTTGCCACTTTGGGATAAATGAAGATTT 40931
Bonobo           TAATTATATAAAAACTAATATATAGAGTTTTTAGGAAAAAGCATAGGATATACTTGCAACTTTGGGATAAATGAAGATTT 39606
OliveBaboon      TA----TATAAAAACTAATGTATAGAGTTTTTAGGAAAAAGCATAGGGTATACTTGCCACTTTGGGATAAATGAAGATTT 40872
Orangutan        TAATTATATAAAAACTAATATATAGAGTTTTTAGGAAAAAGCCTAGGATATACTTGCAACTTTGGGATAAATGAAGATTT 40567

Majority         CTTAAAACA--AAAATTACTCATCTTAAAGGAATATATAAACTTGACTCCATCAACATGGGAAATTTCAAA-----TTGT

                         48890     48900     48910     48920     48930     48940     48950     48960

Human            CTTAAAATAGAAAAAT-------CTTAAAGGAATATATAAACTTGACTCCATCAAAATGGGAAATTTCTGC-----TTCA 38640
Coquerel'sSifaka T-------G------------------A--GAGTATTCCAAAGAGATTATTTCACAGTTCGAA--------------TGT 35072
Ma'sNightMonkey  NNNNNNNNN--NNNN-----------NNNNNNNNNNNNNNNNNNNNNNNNNNNNNNNNNNNNNNNNNNNNN-----NNNN 35455
VervetAGM        --TAAAACA--AAAATTACTCATCCTAAAGGAATATATAAACTTGACTCCATCAACATGGGAAATTTCAAAAGACATTGT 42029
Gorrilla         CTTAAAATAGAAAAA-----------AAAGGAATATATAAACTTGACTACTTCAAAATGGGAAATTTCTGC-----TTAA 41038
GrabEatingMacaqueCTTAAAACA--AAAATTACTCATCTTAAAGGAATATATAAACTTGACTCCATCAGCATGGGAAATTTCAAAAGACATTGT 41286
Macaque          CTTAAAACA--AAAATTACTCATCTTAAAGGAATATATAAACTTGACTCCATCAGCATGGGAAATTTCAAAAGACATTGT 42051
PigTailedMacaque CTTAAAACA--AAAATTACTCATCTTAAAGGAATATATAAACTTGACTCCATCAGCATGGGAAATTTCAAAAGACATTGT 41009
Bonobo           CTTAAAATAGAAAAAT-------CTTAAAGGAATATATAAACTTGACTCCATCAAAATGGGAAATTTCTGC-----TTAA 39674
OliveBaboon      CTTAAAACA--AAAATTACTCATCTTAAAGGAATATATAAACTTGACTCCATCAGCATGGGAAATTTCAAAAGACATTGT 40950
Orangutan        CTTAAAATAGAAAAATTACTCATCTTAAAGGAATATATAAACTTGACTCCATCAAAATGGGAAATTTCTGC-----TTAC 40642
```

```
Majority          TCAAAAGACATTGTTAAGACAATGAAAAGGCAAGGCACAGACTTGGAGAATATAATATTTACATATCAGTACATATATAT
                          48970     48980     48990     49000     49010     49020     49030     49040
Human             TCAAAAGACATTGTTAAGACATTGAAAAGACAAGGCACAGACTTGGAGAATATAATATTTGTGTATCAATACATGCATAT 38720
Coquerel'sSifaka  TTATGAGA----GTTCTAACAATGT--------GTCTTGCATATAGAGATAATGAGAGCCTTCCTTTAGGTGCCGTCTGT 35140
Ma'sNightMonkey   NNNNNNNNNNNNNNNNNNNNNNNNNNNNNNNNNNNNNNNNNNNNNNNNNNNNNNNNNNNNNNNNNNNNNNNNNNNNNNNN 35535
VervetAGM         TCAAAAGACATTGTTAAGACAATGAAAAGGCAAGGCACAGACTTGGAGAATATAATATTTACATATCAGTACATATATAT 42109
Gorrilla          TCAAAAGACATTGTTAAGACACTGAAAAGACAAGGCACAGACTTGGAGAATATAATATTTGCGTGTCAATACATACATAT 41118
GrabEatingMacaqueTCAAAAGACATTGTTAAGACAATGAAAAGGCAAGGCACAGACTTGGAGAATATAATATTTACATATCAGTACATATATAT 41366
Macaque           TCAAAAGACATTGTTAAGACAATGAAAAGGCAAGGCACAGACTTGGAGAATATAATATTTACATATCAGTACATATATAT 42131
PigTailedMacaque  TCAAAAGACATTGTTAAGACAATGAAAAGGCAAGGCACAGACTTGGAGAATATAATATTTACATATCAGTACATATATAT 41089
Bonobo            TCAGAAGACATTGTTAAGACACTGAAAAGACAAGGCACAGACTTGGAGAATATAATATTTGTGTATCAATACATACATAT 39754
OliveBaboon       TCAAACGACATTGTTAAGACAATGAAAAGGCAAGGCACAGACTTGGAGAATATAATATTTACATATCAGTACATATATAT 41030
Orangutan         TCAAAAGACATTGTTAAGACACTGAAAAGACAAGGCACAGACTTGGAGAATATAATATTTGCATATCAATACATATATAT 40722

Majority          GACTGTATTAGTCCATTTTTATGCTGCTGATAAAGACGTACCTGAGACTGGG--------CAATTTACAAAAGAAACCAG
                          49050     49060     49070     49080     49090     49100     49110     49120
Human             GACTGTATTAGTCCATTTTCATGCTGCTGATAAAGACGTACCCGAGACTGGG----CGGGCAATTTATGAAAGAAACCAG 38796
Coquerel'sSifaka  -ATTTTTCTAGCTCATGCCTAAGGTACTTTTG-----GCATCTGGGCTTTGC-----AG-CCACTTGGAAATGCAGAGAG 35208
Ma'sNightMonkey   NNNNNNNNNNNNNNNNNNNNNNNNNNNNNNNNNNNNNNNNNNNNNNNN--------NNNNNNNNNNNNNNNNNNNN 35607
VervetAGM         GACTGTATTAGTCCATTTTTATGCTGCTGATAAAGACATACCTGAGACCGGG--------CAATTTACAAAAGAAACCAG 42181
Gorrilla          GACTGTATTAGTCCATTTTCATGCTGCTGATAAAGACGTACCTGAGACTGGG----CAGGCAATTTATGAAAGAAACCAG 41194
GrabEatingMacaqueGACTGTATTAGTCCATTTTTATGCTGCTGATAAAGACATACCTGAGACCGGG--------CAATTTACAAAAGAAACCAG 41438
Macaque           GACTGTATTAGTCCATTTTTATGCTGCTGATAAAGACATACCTGAGACTGGG--------CAATTTACAAAAGAAACCAG 42203
PigTailedMacaque  GACTGTATTAGTCCATTTTTATGCTGCTGATAAAGACATACCTGAGACCGGG--------CAATTTACAAAAGAAACCAG 41161
Bonobo            GACTGTATTAGTCCATTTTCATGCTGCTGATAAAGACGTACCCGAGACTGGG----CGGGCAATTTATGAAAGAAACCAG 39830
OliveBaboon       GACTGTATTAGTCCATTTTTATGCTGCTGATAAAGACATACTTGAGACCGGG--------CAATTTACAAAAGAAACCAG 41102
Orangutan         GACTGTATTAGTCCATTTTCATGCTGCTGATAAAGACGTACCTGAGACTGGGTGGGCGGGCAATTTGTGAAAGAAACCAG 40802

Majority          TTTAATGGATTTACAGTTCCATGTGACTGGGGAAGCCTCATAATCATGGTGGAAGGCAAGGAGGAGCAAATCTACATCTT
                          49130     49140     49150     49160     49170     49180     49190     49200
Human             TTTAATGAATTTACAATTCTATGTGACTGGGGAAGCCTCATAATCATGGTGGAAGGCAAGGAGGAGCAAGTC-ACATCTT 38875
Coquerel'sSifaka  TTACGTCTTTTTACAGT-----AAGTCTAGTGAGGCTTTTTA--TGTGGTGGGTGTTGGAGTTAACTAAACA-----GCC 35276
Ma'sNightMonkey   NNNNNNNNNNNNNNNNNNNNNNNNNNNNNNNNNNNNNNNNNNNNNNNNNNNNNNNNNNNNNNNNNNNNNNNNNNNNNNNN 35687
VervetAGM         TTTAATGGATTTACAGTTCCATGTGACTGGGGAAACCTCACAATCATGGTGGAAGGCAAGGAGGAGCAAATCTGCATCTT 42261
Gorrilla          TTTAATGAATTTACAATTCTGTGTGACTGGGGAAGCCTCATAATCATGGTGGAAGGCAAGGAGGAGCAAGTC-ACATCTT 41273
GrabEatingMacaqueTTTAATGGATTTACAGTTCCATGTGACTGGGGAAACCTCACAATCATGGTGGAAGGCAAGGAGGAGCAAATCTACATCTT 41518
Macaque           TTTAATGGATTTACAGTTCCATGTGACTGGGGAAACCTCACAATCATGGTGGAAGGCAAGGAGGAGCAAATCTACATCTT 42283
PigTailedMacaque  TTTAATGGATTTACAGTTCCATGTGACTGGGGAAACCTCACAATCATGGTGGAAGGCAAGGAGGAGCAAATCTACATCTT 41241
Bonobo            TTTAATGAATTTACAATTCTATGTGACTGGGGAAGCCTCATAATCATGGTGGAAGGCAAGGAGGAGCAAGTC-ACATCTT 39909
OliveBaboon       TTTAATGGATTTACAGTTCCATGTGACTGGGGAAACCTCACAATCATGGTGGAAGGCAAGGAGGAGCAAATCTACATCTT 41182
Orangutan         TTTAATGAATTTACAGTTCTGTGTGACTGGGGAAGCCTCATAATCATGGTGGAAGGCAAGGAGGAGTAAGTC-ACATCTT 40881
```

```
Majority         ACATGGATGGCAACAGGCAAAGAAAAGAGAGCTTGTGCAGGGAAACTCTCCCCTTTTTAAAACCGTGAGACTTACTTCAT

                          49210     49220     49230     49240     49250     49260     49270     49280

Human            ACGTAGATGGCAACAGGCAAAGAAAAGAGAGCTTGTGCGGGGAAACTCTCCCCTTTTTAAAACCATGAGACTTACTTCAC 38955
Coquerel'sSifaka AGTGGAATGACAGAAGTACCA------TCTATCTGTATGTATCTGCTCTTTTTTTTTTTTTTTTTGGAATTTCTGTTTC 35350
Ma'sNightMonkey  NNNNNNNNNNNNNNNNNNNNNNNNNNNNNNNNNNNNNNNNNNNNNNNNNNNNNNNNNNNNNNNNNNNNNNNNNNNNNNNN 35767
VervetAGM        ACATGGATGGCAACAGGCAAA-----GAGAGCTTGTGCAGGGAAACTCTCCCCTTTTTAAAACTGAGAGACATACTTCAT 42336
Gorrilla         ACATAGATGGCAGCAGGCAAAGAAAAGAGAGCTTGTGCAGGGAAACTCTCCCCTTTTTAAAACTGTGAGACTTACTTCAC 41353
GrabEatingMacaqueACATGGATGGCAACAGGCAAAGAAAAGAGAGCTTGTGCAGGGAAACTCTTCCCTTTTTAAAACCGAGAGACATACTTCAT 41598
Macaque          ACATGGATGGCAACAGGCAAAGAAAAGAGAGCTTGTGCAGGGAAACTCTTCCCTTTTTAAAACCGAGAGACATACTTCAT 42363
PigTailedMacaque ACATGGATGGCAACAGGCAAAGAAAAGAGAGCTTGTGCAGGGAAACTCTCCCCTTTTTAAAACCGAGAGACATACTTCAT 41321
Bonobo           ACGTAGATGGCAACAGGCAAAGAAAAGAGAGCTTGTGCAGGGAAACTCTCCCCTTTTTAAAACCGTGAGACTTACTTCAC 39989
OliveBaboon      ACATGGATGGCAACAGGCAAAGAAAAGAGAGCTTGTGCAGGGAAACTCTCCCCTTTTTAAAACCGAGAGACATACTTCAT 41262
Orangutan        ACATAGATGGCAACAGGCAAAGAAAAGAGAGTTTGTGCAGGGAAACTCTCCCCTTTTTAAAACCGTGAGACTTACTTCAC 40961

Majority         TATCACGAGAACAGCATGGGAAAGACTTGCCCCCATGATTCAGTTACCTCCCACCAGGTCCCTCCCACAACACATGGGAA

                          49290     49300     49310     49320     49330     49340     49350     49360

Human            TGTCACGAGAACAGCATTGGAAAGACCTGCCCCCATGATTCAGTTAACTCCCACCAGGTCCCTCCCACAACACATGGGAA 39035
Coquerel'sSifaka TTGTGTAAGAAT-G-------AATACTGGCATTCATTTTTC-------T-----TAGAGTCTTTTTGTTTTATTTTTTTT 35410
Ma'sNightMonkey  NNNNNNNNNNNNNNNNNNNNNNNNNNNNNNNNNNNNNNNNNNNNNNNNNNNNNNNNNNNNNNNNNNNNNNNNNNNNNNNN 35847
VervetAGM        TATCACGAGAACAACATGGGAAAGACTTGCCCCCATGATTCAGTTACCTCCCACCAGGTCCTTCCCACAACACATGGGAA 42416
Gorrilla         TGTCACGAGAACAGCATTGTAAAGACCTGCCCCCATGATTCAGTTAACTCCCACCAGGTCCCTCCCACAACACATGGGAA 41433
GrabEatingMacaqueTATCACGAGAACAACATGGGAAAGACTTGCCCCCATGATTCAGTTACCTCCCACCAGGTCCCTCCCACAACACATGGGAA 41678
Macaque          TATCACGAGAACAACATGGGAAAGACTTGCCCCCATGATTCAGTTACCTCCCACCAGGTCCCTCCCACAACACATGGGAA 42443
PigTailedMacaque CATCACGAGAACAACATGGGAAAGACTTGCCCCCATGATTCAGTTACCTCCCACCAGGTCCCTCCCACAACACATGGGAA 41401
Bonobo           TGTCACGAGAACAGCATTGGAAAGACCTGCCCCCATGATTCAGTTAACTCCCACCAGGTCCCTCCCACAACACATGGCAA 40069
OliveBaboon      TATCACGAGAACAACATGGGAAAGACTTGCCCCCATGATTCAGTTACCTCCCACCAGGTCCCTCCCACAACACATGAGAA 41342
Orangutan        TGTCACGGGAACAGCATTGGAAAGACCTGCCCCCGTGATTCAGTTACCTCCCACCAGGTCCCTCCCACAACACATGGGAA 41041

Majority         TTCAAGAGGAGATTTGGGTGGGGGACACAGCCAAACCGTATCAATGATAAAGGACTTGTATCCCAAAAGACTTGTAAAAA

                          49370     49380     49390     49400     49410     49420     49430     49440

Human            TTCAAGAGGAGATGTGGGTGGGGA-CACAGCCAAACCATATCAATGACAAAGGACTTGTATCCCAAAGGACTTGTAAAAA 39114
Coquerel'sSifaka TTTCCTAGA-------G--------------CCAGCAGCTTCTGTAACACCGTGCTTTGCCTTTAAG-----TCTAATTA 35464
Ma'sNightMonkey  NNNNNNNNNNNNNNNNNNNNNNNNNNNNNNNNNNNNNNNNNNNNNNNNNNNNNNNNNNNNNNNNNNNNNNNNNNNNNNNN 35927
VervetAGM        TTCAAGAGGAGATTTGGGTGGGGGACACAGCCAAACCGTATCAATGATAAAGGACTTGTATCCCAAAGGACTTGTAAAAA 42496
Gorrilla         TTCAAGAGGAGATGTGGGTGGGG--CACAGCCAAACCATATCAATGACAAAGGACTTGTATCCCAAAGGACTTGTAAAAA 41511
GrabEatingMacaqueTTCAGGAGGAGATTTGGGTGGGGGACACAGCCAAACCGTATCAATGATAAAGGACTTGTATCCCAAAAGACTTGTAAAAA 41758
Macaque          TTCAGGAGGAGATTTGGGTGGGGGACACAGCCAAACCGTATCAATGATAAAGGACTTGTATCCCAAAAGACTTGTAAAAA 42523
PigTailedMacaque TTCAGGAGGAGATTTGGGTGGGGGACACAGCCAAACCGTATCAATGATAAAGGACTTGTATCCCAAAAGACTTGTAAAAA 41481
Bonobo           TTCAAGAGGAGATGTGGGTGGGGA-CACAGCCAAACCATATCAACGACAAAGGACTTGTATCCCAAAGGACTTGTAAAAA 40148
OliveBaboon      TTCAAGAGGAGATTTGGGTGGGGGACACAGCCAAACCGTATCAATGATAAAGGACTTGTATCCCAAAAGACTTGTAAAAA 41422
Orangutan        TTCAAGAGGAGATGTGGGTGGGGA-CACAGCCAAACCATATCAATGACAAAGGACTTGTATCCCAGAAGACTTGTAAAAA 41120
```

```
Majority          AA-----------A---CTCCAATCAATAAATATGAGCAGCCCAATTTTTTT--AAGTGGCAAAAGAAGTGACATTATAA
                          49450     49460     49470     49480     49490     49500     49510     49520
Human             AAAA---------ACTCCTCCAATCAATAAATATGAGCAGCCCAATTTTTTTTTAAGTGGCAAAAGAAGTGACATTATAA 39185
Coquerel'sSifaka  AA-------------------------------------------ACTGCTTT--------------GCCTCTCAGTATCA 35488
Ma'sNightMonkey   NN---------------NNNNN------------------NNNNNNNNNNN--NNNNNNNNNNNNNNNNNNNNNNNNNN 35971
VervetAGM         AA-----------G---CTCCAA------------------TCAATTTTTTT--AAGTGGCAAAAGAAGTGACATTATAA 42542
Gorrilla          AAAAGCTCCTCCAACTCCTCCAATCAATACATATGAGCAGCCCAATTTTTTTTTAAGTGGCAAAAGAAGTGACATTATAA 41591
GrabEatingMacaqueAA-----------A---CTCCAATCAATAAATATGAGCAGCCCAATTTTTTT--AAGTGGCAAAAGAAGTGACATTATAA 41822
Macaque           AA-----------G---CTCCAATCAATAAATATGAGCAGCCCAATTTTTTT--AAGTGGCAAAAGAAGTGACATTATAA 42587
PigTailedMacaque  AA-----------A---CTCCA----ATAAATATGAGCAGCCCAATTTTTTT--AAGTGGCAAAAGAAGTGACATTATAA 41541
Bonobo            AAAA---------ACTCCTCCAATCAATAAATATGAGCAGCCCAATTTTTTTTTAAGTGGCAAAAGAAGTGACATTATAA 40219
OliveBaboon       AA-----------A---CTCCAATCAATAAATATGAGCAGCCCAATTTTTTT--AAGTGGCAAAAGAAGTGACATTATAA 41486
Orangutan         AA-----------ACTCCTCCAATCAATAAATATGAGCAGCCCAATTTTTTT-AAGTGACAAAAGAAGTGACATTATAA 41188

Majority          GAATGGGTAACAAGCACACGGAAAGATTACACTAGCCATCAGGTAATCACAAGTCAAAACCATTATGAGACA--ATTTAC
                          49530     49540     49550     49560     49570     49580     49590     49600
Human             GGATGGGTAACAAGCACATGAAAAGATAATGTTAGTCATCAGGTGATCACAAGTCAAAACCATTA-GAGATAGAATTTAC 39264
Coquerel'sSifaka  TATCTGATAATAA---------------------------AAGGAGTTAC----CAAAACTTTCAT--------A----- 35524
Ma'sNightMonkey   NNNNNNNNNNNNNNNNNNNNNNNNNNNNNNNNNNNNNNNNNNNNNNNNNNNNNNNNNNNNNNNNNNNNNN--NNNNNN 36049
VervetAGM         GAATGGGTAACAAGCACACGGAAAGATTACACTAGCCATCAGGTAATCACAAGTCAAAACCATTATGAGACA--ATTTAC 42620
Gorrilla          GGATGGGTAACAAGCACATGAAAAGATAATGTTAGTCATCAGTTGATCACAAGTCAAAACCATTATGAGATAGAATTTAC 41671
GrabEatingMacaqueGAATGGGTAACAAGCACACGGAAAGATTACACTAGCCATCAGGTAATCACAAGTCAAAACCATTATGAGACA--ATTGAC 41900
Macaque           GAATGGGTAACAAGCACACGGAAAGATTACACTAGCCATCAGGTAATCACAAGTCAAAACCATTATGAGACA--ATTGAC 42665
PigTailedMacaque  GAATGGGTAACAAGCACACGGAAAGATTACACTAGCCATCAGGTAATCACAAGTCAAAACCATTATGAGACA--ATTTAC 41619
Bonobo            GGATGGGTAACAAGCACATGAAAAGATAATGTTAGTCATCAGGTGATCACAAGTCAAAACCATTA-GAGATAGAATTTAC 40298
OliveBaboon       GAATGAGTAACAAGCACACGGAAAGATTACACTAGCCATCAGGTAATCACAAGTCAAAACCATTATGAGACA--ATTTAC 41564
Orangutan         GGATGGGTAACAAGCACATGAAAAGATAACATTAGTCATCACGTAATCACGAGTCAAAACCATTATGAGATAGAATTTAC 41268

Majority          ACCAACTAGAATGGCTAAAATTAAATAGGTTGGCAATACCTAATGTTGATGAAGATGTGGAACTGCCTTTGATTACTGCT
                          49610     49620     49630     49640     49650     49660     49670     49680
Human             ACCAACTAGAATGGCTAAAATTAAATAGATTGGCAATACCGAATGTTGATGAAGATGTGGAACTGTCTTTAATTACTGCT 39344
Coquerel'sSifaka  ---------AATGCATGAGTCTGAAAGTCTTGGAG------AATGAAGATGTACTTATAATTTGCCAAATAGTTTTCTCT 35589
Ma'sNightMonkey   NNNNNNNNNNNNNNNNNNNNNNNNNNNNNNNNNNNNNNNNNNNNNNNNNNNNNNNNNNNNNNNNNNNNNNNNNNNNNNNN 36129
VervetAGM         ACCAACTAGAATGGCTAAAATCAAATAGGTTGGCAATACCTAATGTTGATGAAGATGTGGAACTGCCTTTGATTACTGCT 42700
Gorrilla          ACCAACTAGAATGGCTAAAATTAAATAGATTGGCAACACCGAATGTTGATGAAGATGTGGAACTGTCTTTAATTACTGCT 41751
GrabEatingMacaqueACCAACTAGAATGGCTAAAATCAAATAGGTTGGCAATACCTAATGTTGATGAAGATGTGGAACTGCCTTTGATTACTGCT 41980
Macaque           ACCAACTAGAATGGCTAAAATCAAATAGGTTGGCAATACCTAATGTTGATGAAAATGTGGAACTGCCTTTGATTACTGCT 42745
PigTailedMacaque  ACCAACTAGAATGGCTAAAATCAAATAGGTTGGCAATACCTAATGTTGATGAAGATGTGGAACTGCCTTTGATTACTGCT 41699
Bonobo            ACCAACTAGAATGGCTAAAATTAAATAGATTGGCAACACCGAATGTTGATGAAGATGTGGAACTGTCTTTAATTACTGCT 40378
OliveBaboon       ACCAACTAGAATGGCTAAAATCAAATAGGTTGGCAATACCTAATGTTGATGAAGATGTGGAACTGCCTTTGATTACTGCT 41644
Orangutan         ACCAACTAGAATGGCTAAAATTAAATAGATTGGCAACACCGAATGTTGATGAAGATGTGGAACTGTCTTTAATTACTGCT 41348
```

```
Majority         GAGTATGTAACATAGTTGGGAAAATAGTTTGC-TTTTTTTTTTTTTTTT------------G-GACAGAGTCTTGTTCTG

                         49690     49700     49710     49720     49730     49740     49750     49760

Human            GAGTATATAACATAGTTGGGAAAATAGTTTGCTTTTTTTTTTTTTTTTTTTTTTTT-----GAGACAGAGTCTTGCTCTG 39419
Coquerel'sSifaka GGACAGATGAAGTAT-----AAAGT----------TTTTTTTTTTTTTT------------GACACAGAGTCTTGCTCTG 35642
Ma'sNightMonkey  NNNNNNNNNNNNNNNNNNNNNNNNN-----------NNNNNNNNNNNNN--------------NNNNNNNNNNNNNNNNN 36184
VervetAGM        GAGTATGTAACATAGTTGGGAAAATAGTTTGC----CTTTTTTTTTTTG------------A-GACAGAGTCTTGTTCTG 42763
Gorilla          GAGTATATAACATAGTTGGGAAAATAGTTTGCTTTTTTTTTTTTTTTTTTTTTTTTTTTTTGAGACAGAGTCTTGCTCTG 41831
GrabEatingMacaqueGAGTATGTAACATAGTTGGGAAAAT-----------TTTTTTTTTTTTG------------A-GACAGAGTCTTGTTCTG 42036
Macaque          GAGTATGTAACATAGTTGGGAAAAT-----------TTTTTTTTTTTTG------------A-GACAGAGTCTTGTTCTG 42801
PigTailedMacaque GAGTATGTAACATAGTTGGGAAAATAGTTTGCCTTTTTCTTTTTTTTTG------------A-GACAGAGTCTTGTTCTG 41766
Bonobo           GAGTATATAACATAGTTGGGAAAATAGTTTGCTTTTTTTTTTTTTTTTT------------GAGACAGAGTCTTGCTCTG 40446
OliveBaboon      GAGTATGTAACATAGTTGGGAAAACAGTTTGCCTTTTTTTTTTTTTTTG------------A-GACAGAGTCTTGTTCTG 41711
Orangutan        GAGTATATAACATAGTTGGGAAAATAGTTTGCTTTTTTTTTTTTTTTTTTTTTTT------GAGACAGAGTCTTGCTCTG 41422

Majority         TCACC-AGGCTGGAATG--CAGTGTGGCATGATCTTGGCTT--ACTGCAACCTCCGCCTCTTGGGTTCAGGTGATTCTCC

                         49770     49780     49790     49800     49810     49820     49830     49840

Human            TCACC-AGGCTGGAATG--CAGT--GGCATGATCTCAGCTT--ACTGCAACCTCCGCCTCCTGGGTTCAAGCGATTCTCC 39492
Coquerel'sSifaka TTGCCCGGGCTAGAGTGAGTGCCGTGGCGTCAGCCTAGCTC--ACAGCAACCTCAAACTCCTGGGCTCAAGCGATCCTTC 35720
Ma'sNightMonkey  NNNNN-NNNNNNNNNNN--NNNN--NNNNNNNNNNNNNNNN--NNNNNNNNNNNNNNNNNNNNNNNNNNNNNNNNNNNNN 36257
VervetAGM        TCACC-AGGCTAGAATG--CAGTGTGGCATGATCTTGGCTT--ACTGCAACCTCCGCCTCTTGGGTTCAGGTGATTCTCC 42838
Gorilla          TCACC-AGGCTGGAATG--CAGT--GGCATGATCTCAGCTT--ACTGCAACCTCTGCCTCCTGGGTTCAAGCGATTCTCC 41904
GrabEatingMacaqueTCACC-AGGCTGGAATG--CAGTGTGGCATGATCTTGGCTTAAACTGCAACCTCCGCCTCTTGGGTTCAGGTGATTCTCC 42113
Macaque          TCACC-AGGCTGGAATG--CAGTGTGGCATGATCTTGGCTTAAACTGCAACCTCCGCCTCTTGGGTTCAGGTGATTCTCC 42878
PigTailedMacaque TCACC-AGGCTGGAATG--CAGTGTGGCATGATCTTGGCTTAAACTGCAACCTCCGCCTCTTGGGTTCAGGTGATTCTCC 41843
Bonobo           TCACC-AGGCTGGAATG--CAGT--GGCATGATCTCAGCTT--ACTGCAACCTCCGCCTCCTGGGTTCAAGTGATTCTCC 40519
OliveBaboon      TCACC-AGGCTGGAATG--CAGTGTGGCATGATCTTGGCTT--ACTGCAACCTCCGCCTCTTGGGTTCAGGTGATTCTCC 41786
Orangutan        TCACC-AGGCTGGAATG--CAGT--GGCATGATCTCAGCTT--ACTGCAACCTCCACCTCCTGGGTTCAAGCAATTCTCC 41495

Majority         TGCCTCAGGCTCTTGAGTAGCTGGCACTACAGGCGCACGCCACCACACCCAGCTAATTTTTTGTATTTTTAGTAGAGACT

                         49850     49860     49870     49880     49890     49900     49910     49920

Human            TGCCTCAGCCTCCTGAGTAGCTGGAACTACAGGTGCACGCCACCACACCCGGCTAATTTTTTGTATTTTTAGTAGAGACT 39572
Coquerel'sSifaka TGCCTCAGCCTCCCGAGTAGCTGGGACTACAGGCATGCACCACCATGCCCTGCTAATTTTTTCTAT-----ATAGATATT 35795
Ma'sNightMonkey  NNNNNNNNNNNNNNNNNNNNNNNNNNNNNNNNNNNNNNNNNNNNNNNNNNNNNNNNNNNNNNNNNNNNNNNNNNNNNNNN 36337
VervetAGM        TGCCTCAGGCTCTTGAGTAGCTGGCACTACACGCGCACGCCACGACACCCAGCTAATTTTTTGTATTTTTAGTAGAGACT 42918
Gorilla          TGCCTCAGCCTCCTGAGTAGCTGGAACTACAGGTGCACGCCACCACACCCAGCTAATTTTTTGTATTTTTAGTAGAGACT 41984
GrabEatingMacaqueTGCCTCAGGCTCTTGAGTAGCTGGCACTACACGCGCACGCCACGACACCCAGCTAATTTTTTGTATTTTTAGTAGAGACT 42193
Macaque          TGCCTCAGGCTCTTGAGTAGCTGGCACTACACGCGCACGCCACAACACCCAGCTAATTTTTTGTATTTTTAGTAGAGACT 42958
PigTailedMacaque TGCCTCAGGCTCTTGAGTAGCTGGCACTACATGCGCACGCCACGACACCCAGCTAATTTTTTGTATTTTTAGTAGAGACT 41923
Bonobo           TGCCTCAGCCTCCCGAGTAGCTGGAAGTACAGGTGCACGCCACCACACCCGGCTAATTTTTTGTATTTTTAGTAGAGACT 40599
OliveBaboon      TGCCTCAGGCTCTTGAGTAGCTGGCACTACACGCGCACGCCACGACACCCAGCTAATTTTTTGTATTTTTAGTAGAGACT 41866
Orangutan        TGCCTCAGCCTCCCGAGTAGCTGGAACTACAGGTGCACACCACCACACCCAGCTAATTTTTTGTATTTTTGGTAGAGACT 41575
```

```
Majority          GGGTTTCGCCATGTTGGCCAGGATGGTCTTGCTCTTTTGACCTTGTGATCCACCCACCTCGGCCTCCCAAAGTGCTGGGA
                          49930     49940     49950     49960     49970     49980     49990     50000
Human             GGGTTTCGCCATGTTGGCCAGGATGGTCTTGCTCTCTTGACGTTGTGATCTGCCCACGTCGGCCTCCCAGAGTGCTGGGA 39652
Coquerel'sSifaka  ---TTTAGTTGTCCAGATCA------TTTCTTTCTAT-GTTTTAGTAGAGACGGGGTCTCGCTCTTGCTCAG-GCTGGTC 35864
Ma'sNightMonkey   NNNNNNNNNNNNNNNNNNNNNNNNNNNNNNNNNNNNNNNNNNNNNNNNNNNNNNNNNNNNNNNNNNNNNNNNNNNNNNNN 36417
VervetAGM         GGGTTTCCCCATGTTGGCCAGGATGGTACTGCTCTTTTGACCTTGTGATCCACCCACCTTGGCCTCCCAAAGTGCTGGGA 42998
Gorrilla          GGGTTTCACCATGTTGGCCAGGATGGTCTTGCTCTCTTGACGTTGTGATCCACCCACGTCGGCCTCCCAGAGTGCTGGGA 42064
GrabEatingMacaqueGGGTTTCGCCATGTTGGCCAGGATGGTCTTGCTCTTTTGACCTTGTGATTCACCCACCTCGGCCTCCCAAAGCGCTGGGA 42273
Macaque           GGGTTTCGCCATGTTGGCCAGGATGGTCTTGCTCTTTTGACCTTGTGATTCACCCACCTCGGCCTCCCAAAGCGCTGGGA 43038
PigTailedMacaque  GGGTTTCGCCATGTTGGCCAGGATGGTCTTGCTCTTTTGACCTTGTGATCCACCCACCTCGGCCTCCCAAAGCGCTGGGA 42003
Bonobo            GGGTTTCGCCATGTTGGCCAGGATGGTCTTGCTCTCTTGACGTTGTGATCCGCCCACGTCGGCCTCCCAGAGTGCTGGGA 40679
OliveBaboon       GGGTTTCGCCATGTTGGCCAGGATGGTCTTGCTCTTTTGACCTTGTGATCCACCCACCTCGGCCTCCCAAAGTGCTGGGA 41946
Orangutan         GGGTTTCGCCATGTTGGCCAGGATGGTCTTGCTCTCTTGACCTTGTGATCCGCCCACTTCGGCCTCCCAGAGTGCTGGGA 41655

Majority          TTACTGGCGTGAGCCACTGTGCCTGACCTAGTTTGCCATTTTCTTACGGAGTTAAACATAAACGTACTTTATGGTCCATA
                          50010     50020     50030     50040     50050     50060     50070     50080
Human             TTACAGGCCTGAGCCACTGCGTCTGACCTAGTTTACTATTTTCTTACGGAGTTAAACATTAACCTATTTTATGGTCCATA 39732
Coquerel'sSifaka  TTGAATTCCTGAGCTCAAACGATCCACCCGCCTCGGC---CTCCCAGAGTGCTAGG-A----------TTACAGGCGTGA 35930
Ma'sNightMonkey   NNNNNNNNNNNNNNNNNNNNNNNNNNNNNNNNNNNNNNNNNNNNNNNNNNNNNNNNNNNNNNNNNNNNNNNNNNNNNNNN 36497
VervetAGM         TTACTGGCGTGAGCCACTGTGCCTGACCTAGTTTGCCATTTTCTTACGGCGTTAAACATAAACGTACTTTATGGTCCATA 43078
Gorrilla          TTACAAGCCTGAGCCACTGCATCTGGCCTAGTTTGCTATTTTCTTACGGAGTTAAACATTAACCTACTTTATGGTCCATA 42144
GrabEatingMacaqueTTACTGGCGTGAGCCACTGTGCCTGACCTAGTTTGCCATTTTCTTACGGAGTTAAACATAAACGTACTTTATGGTCCATA 42353
Macaque           TTACTGGCGTGAGCCACTGTGCCTGACCTAGTTTGCCATTTTCTTACGGAGTTAAACATAAATGTACTTTATGGTCCATA 43118
PigTailedMacaque  TTACTGGCGTGAGCCACTGTGCCTGACCTAGTTTGCCATTTTCTTCCGGAGTTAAACATAAACGTACTTTATGGTCCATA 42083
Bonobo            TTACAGGCCTGAGCCACTGCGTCTGACCTAGTTTGCTATTTTCTTACGGAGTTAAACATTAACCTACTTTATGGTCCATA 40759
OliveBaboon       TTACTGGCGTGAGCCACTGTGCCTGACCTAGTTTGCCATTTTCTTACGGAGTTAAACATAAACGTACTTTATGGTCCGTA 42026
Orangutan         TTACAGGCGTGAGCCACTGCGCCTGGCCTAGTTTGCTGTTTTCTTACAGAGTTAAACATTAACCTACTTTATGGTCCATA 41735

Majority          ACTTCTACTTTTAGTTTTTTACCCAGTAATAACATGTTTGCTGTAAAACTTGTATGTGTATCAAAAACTTGACTGATAAT
                          50090     50100     50110     50120     50130     50140     50150     50160
Human             ACTTCTACTTTTAGTTTTTTACCCAGTAAGAACATGTTTGCTGTAAAACTTGTATGTGTATCAAAAACTTTACTGATAAT 39812
Coquerel'sSifaka  ACCACCGCGCCCGGCCTG-----AAGTATAAAGTTTTGAATTGTTACACTTTTATGTGGGTATGCAATTGAAAGTATGTT 36005
Ma'sNightMonkey   NNNNNNNNNNNNNNNNNNNNNNNNNNNNNNNNNNNNNNNNNNNNNNNNNNNNNNNNNNNNNNNNNNNNNNNNNNNNNNNN 36577
VervetAGM         ACTTCTACTTTTAGTTTCTTACCCAGTAATAACATGTTTGCTGTAAAACTTGTATGTGTATCAAAAACTTGACTGATAAT 43158
Gorrilla          ACTTCTACTTTTAGTTTTTTACCGAGTAAGAACATGTTTGCTGTAAAACTTGTATGTGTATCAAAAACTTTACTGATAAT 42224
GrabEatingMacaqueACTTCTACTTTTAGTTTTTTACCCAGTAATAACATGTTTGCTGTAAAACTTGTATGTGTATCAAAAACTTGACTGATAAT 42433
Macaque           ACTTCTACTTTTAGTTTTTTACCCAGTAATAACATGTTTGCTGTAAAACTTGTATGTGTATCAAAAACTTGACTGATAAT 43198
PigTailedMacaque  ACTTCTACTTTTAGTTTTTTACCCAGTAATAACATGTTTGCTGTAAAACTTGTATGTGTATCAAAAACTTGACTGATAAT 42163
Bonobo            ACTTCTACTTTTAGTTTTTTACCCAGTAAGAACCTGTTTGCTGTAAAACTTGTATGTGTATCAAAAACTTTACTGATAAT 40839
OliveBaboon       ACTTCTACTTTTAGTTTTTTACCCAGTAATAACATGTTTGCTGTAAAACTTGTATGTGTATCAAAAACTTGACTGATAAT 42106
Orangutan         ACTTCTACTTTTAGTTTTTTACCCAGTAAAAACATGTTTGCTGTAAAACTTGTATGTGTATCAAAAACTTTACTGATAAT 41815
```

```
Majority          GGTCCCAAACTTGAAATAACACAAATGTTTATCAACAGGAAAATGGATAAATTGTGGTATGATCATACAGTATATTACTA
                          50170     50180     50190     50200     50210     50220     50230     50240
Human             GGTCCCAAACTTGAAATAACCCAAATGTTTATCAACAGGAAAATGGATAAATTGTGGTATAATCATATAGTATATTACTA 39892
Coquerel'sSifaka  AGTGAACGCGTGGGACTAAAATAATAGATTTACA--AATTAAATAAGAAGTTGATTGTAAAACCCCAGTGCTGGAGTGTT 36083
Ma'sNightMonkey   NNNNNNNNNNNNNNNNNNNNNNNNNNNNNNNNNNNNNNNNNNNNNNNNNNNNNNNNNNNNNNNNNNNNNNNNNNNNNNNN 36657
VervetAGM         GGTCCCAAACTTGAAATAACACAAATGTTTATCAACAGGAAAATTGATAAATTGTGGTATGATCATACAATATATTACTA 43238
Gorrilla          GGTCCCAAACTTGAAATAACCCAAATGTTTATCAACAGGAAAATGGATAAATTGTGGTATAATCATATAGTATATTACTA 42304
GrabEatingMacaque GGTCCCAAACTTGAAATAACACAAATGTTTATCAACAGGAAAATGGATAAATTGTGGTATGATCATACAATATATTACTA 42513
Macaque           GGTCCCAAACTTGAAATAACACAAATGTTTATCAGCAGGAAAATGGATAAATTGTGGTATGATCATACAATATATTACTA 43278
PigTailedMacaque  GGTCCCAAACTTGAAATAACACAAATGTTTATCAACAGGAAAATGGATAAATTGTGGTATGATCATACAATATATTACTA 42243
Bonobo            GGTCCCAAACTTGAAATAACCCAAATGTTTATCAACAGGAAAATGGATAAATTGTGGTATAATCATATAGTATATTACTA 40919
OliveBaboon       GGTCCCAAACTTGAAATAACACAAATGTTTATCAACAGGAAAATGGATAAATTGTGGTATGATCATACAATATATTACTA 42186
Orangutan         GGTCCCAAACTTGAAATAACCCAAATGTTTATCAACAGGAAAATGGATAAATTGTGGTATAATCATACAGTATATTACTA 41895

Majority          CTTACTAATAACAAAAGAGTGGTTAGTAGTACATGCGAAAACATGGGTGAATATTAAGCATTGTATGAAGCAAAAGAAAC
                          50250     50260     50270     50280     50290     50300     50310     50320
Human             CTTACTAATAACAAAAGAATGGTTAGTAGTACATGCGAAAACATGGGTGAATAGTAAGCATTGTATGAAGCAAAAGAAAC 39972
Coquerel'sSifaka  TATAATGATCAGGGAAAGATAGGAAGGACCTGGGGCCAAAACAGCTTGGGAAATTAATAATTTTGGGGAATGAATGAGTA 36163
Ma'sNightMonkey   NNNNNNNNNNNNNNNNNNNNNNNNNNNNNNNNNNNNNNNNNNNNNNNNNNNNNNNNNNNNNNNNNNNNNNNNNNNNNNNN 36737
VervetAGM         CTTACTAATAACAAAAGAGTGGTTAGTAATACATGCGAAAACATGGGTGAATATTAAGCATTGTATGAAGCAAAAGAAAC 43318
Gorrilla          CTTACTAATAACAAAAGAATGGTTAGTAGTACATGCGAAAACATGGGTGAGTAGTAAGCATTGTATGAAGCAAAAGAAAC 42384
GrabEatingMacaque CTTACTGATAACAGAAGAGTGGTTAGTAATACATGCGAAAACATGGGTGAATATTAAGCATTGTATGAAGCAAAATAAAC 42593
Macaque           CTTACTGATAACAGAAGAGTGGTTAGTAATACATGCGAAAACATGGGTGAATATTAAGCATTGTATGAAGCAAAATAAAC 43358
PigTailedMacaque  CTTACTAATAACAGAAGAGTGGTTAGTAATACATGCGAAAACATGGGTGAATATTAAGCATTGTATGAAGCAAAAGAAAC 42323
Bonobo            CTTACTAATAACAAAAGAATGGTTAGTAGTACATGCGAAAACATGGGTGAATAGTAAGCATTGTATGAAGCAAAAGAAAC 40999
OliveBaboon       CTTACTAATAACAAAAGAGTGGTTAGTAGTACATGCGAAAACATGGGTGAATATTAAGCATTGTATGAAGCAAAAGAAAC 42266
Orangutan         CTTACTAATAACAAAAGAATGGTTAGTAGTACATGTGAAAACATGGGTGAATATCAAGCATTGTATGAAGCAAAAGAAAC 41975

Majority          CAGACAGACCTGAAAGAGTACATAGAGTGATTCAATTTCTTGAAAGTTTAAGAGCAGATCATTAGTTTTTTGTTTAGAAA
                          50330     50340     50350     50360     50370     50380     50390     50400
Human             CAGACAGACCTGAAAGAGTACATAGTGTGATTCCATTTCTTGAAAGTTTAAGAGCAGATCATTAATTTTTTGTTTAGAAA 40052
Coquerel'sSifaka  AGCAGAAGGTTCAAAGCACGTATCAAATGAGTCAGGG----GAAGTAAGAAAAGTAGA--AGTAGAAATTGTTAGGGGTG 36237
Ma'sNightMonkey   NNNNNNNNNNNNNNNNNNNNNNNNNNNNNNNNNNNNNNNNNNNNNNNNNNNNNNNNNNNNNNNNNNNNNNNNNNNNNNNN 36817
VervetAGM         CAGACAGACCTGAAAGAGTACATAGAGTGATTCAATT-CTTGAAAGTTTAAGAGCAGATCATTAGTTTTTTGTTTAGAAA 43397
Gorrilla          CAGACAGACCTGAAAGAGTACGTAGTGTGATTCCATTTCTTGAAAGTTTAAGAGCAGATCATTAATTTTTTGTTTAGAAA 42464
GrabEatingMacaque CAGACAGACCTGAAAGAGTACATAGAGTGATTCAATTTCTTGAAAGTTTAAGAGCAGATCATTAGTTTTTTGTTTAGAAA 42673
Macaque           CAGACAGACCTGAAAGAGTACATAGAGTGATTCAATTTCTTGAAAGTTTAAGAGCAGATCATTAGTTTTTTGTTTAGAAA 43438
PigTailedMacaque  CAGACAGACCTGAAAGAGTACATAGAGTGATTCAATTTCTTGAAAGTTTAAGAGCAGATCATTAGTTTTTTGTTTAGAAA 42403
Bonobo            CAGACAGACCTGAAAGAGTACATAGTGTGATTCCATTTCTTGAAAGTTTAAGAGCAGATCATTAATTTTTTGTTTAGAAA 41079
OliveBaboon       CAGACAGACCTGAAAGAGTACATAGAGTGATTCAATTTCTTGAAAGTTTAAGAGCAGATCATTAGTTTTTTGTTTAGAAA 42346
Orangutan         CAGACAGACCTGAAAGAGTACATAGTGTGATTCCATTTCTTGAAAGTTTAAGAGCAGATTATTAATTTTTTGTTTAGTAA 42055
```

```
Majority          CCAGAATGTTGGAGATTAACAGAAAGGGAATATGGTAGCATTTTCTGGTGTGATGGAAACGTCCTATGTTAATAAACTAA
                           50410     50420     50430     50440     50450     50460     50470     50480
Human             CCAGAATGTTGGAGATTAACAGAAAGGGAATATGGTGGCATTTTCTGGTGTGATGGAAACGTCCTATGTTAATAAACTAA 40132
Coquerel'sSifaka  CTGTGAGAACAGAGAAATTCATTAAGGAAGAGAGTTCACAGTTCCT----TG--G------TCACTGGTGGATATGGTGA 36305
Ma'sNightMonkey   NNNNNNNNNNNNNNNNNNNNNNNNNNNNNNNNNNNNNNNNNNNNNNNNNNNNNNNNNNNNNNNNNNNNNNNNNNNNNNNN 36897
VervetAGM         CCAGAATGTTGGAGATTAACAGAAAGGGAATATGGTAGCATTTTCTGGTGTGATGGAAACGTCCTGTGTTAATAAACTAA 43477
Gorrilla          CCAGAATGTTGGAGATTAACAGAAAGGGAATACGGTGGCATTTTCTGGTGTGATGGAAACGTCCTATGTTAATAAACTAA 42544
GrabEatingMacaqueCCAGAATGTTGGAGATTAACAGAAAGGGAATATGGTAGCATTTTCTGGTGTGATGGAAACGTCCTATGTTAATAAACTAA 42753
Macaque           CCAGAATGTTGGAGATTAACAGAAAGGGAATATGGTAGCATTTTCTGGTGTGATGGAAACGTCCTATGTTAATAAACTAA 43518
PigTailedMacaque  CCAGAATGTTGGAGATTAACAGAAAGGGAATATGGTAGCATTTTCTGGTGTGATGGAAACGTCCTATGTTAATAAACTAA 42483
Bonobo            CCAGAATGTTGGAGATTAACAGAAAGGGAAAACGGTGGCATTTTCTGGTGTGATGGAAACGTCCTATGTTAATAAACTAA 41159
OliveBaboon       CCAGAATGTTGGAGATTAACAGAAAGGGAATATGGTAGCATTTTCTGGTGTGATGGAAACGTCCTATGTTAGTAAACTAA 42426
Orangutan         CCAGAATTTTGGAGATTAACAGAAAGGGAACATGGTGGCATTTTCTGGTGTGATGGAAACGTCCTATGTTAATAAACTAA 42135

Majority          GTTGCAAAACAAATCCAAAATTTGTTTGAAGCAGTAGATTATCTCTGAGAACTCTGAATTACCAGTAGTTGTGTCTTGCT
                           50490     50500     50510     50520     50530     50540     50550     50560
Human             GTTGCAAAACAAATCCAAAAGTTGTTTGAAGCAGTAGATTATCTCTGAGAACTCTGAATTTCCAGTAGTTGTGTCTTGCT 40212
Coquerel'sSifaka  TTTTAAAAATTACCCATGATCTTGATAAGACCCATTTATT-----TGAAAATTGCGGGTAAAAGCGAATGGAGAACAAAG 36380
Ma'sNightMonkey   NNNNNNNNNNNNNNNNNNNNNNNNNNNNNNNNNNNNNNNNNNNNNNNNNNNNNNNNNNNNNNNNNNNNNNNNNNNNNNNN 36977
VervetAGM         GTTGCAAAACAAATCCAAAATTTGTTTGAAGCAGTAGATTATCTCTGAGAACTCTGAATTACCAGTAGTTGTGTCTTGCT 43557
Gorrilla          GTTGCAAAACAAATCCAAAATTTGTTTGAAGCAGTAGATTATCTCTGAGAACTCTGAATTTCCAGTAGTGGTGTCTTGCT 42624
GrabEatingMacaqueGTTGCAGAACAAATCCAAAATTTGTTTGAAGCAGTAGATTATCTCTGAGAACTCTGAATTACCAGTAGTTGTGTCTTGCT 42833
Macaque           GTTGCAAAACAAATCCAAAATTTGTTTGAAGTAGTAGATTATCTCTGAGAACTCTGAATTACCAGTAGTTGTGTCTTGCT 43598
PigTailedMacaque  GTTGCAAAACAAATCCAAAATTTGTTTGAAGCAGTAGATTATCTCTGAGAACTCTGAATTACCAGTAGTTGTGTCTTGCT 42563
Bonobo            GTTGCAAAACAAATCCAAAATTTGTTTGAAGCAGTAGATTATCTCTGAGAACTCTGAATTTCCAGTAGTTGTGTCATGCT 41239
OliveBaboon       GTTGCAAAACAAATCCAAAATTTGTTTGAAGCAGTAGATTATCTCTGAGAACTCTGAATTACCAGTAGTTGTGTCTTGCT 42506
Orangutan         GTTGCAAAACAAATCCAAAATTTGTTTGAAGCAGTAGATTATCTCTGAGAACTCTGAATTACCAGTAGTTGTGTCTTGCT 42215

Majority          GTAATGCGAAATATGAGATTGGGAATGCCTAGGTGAGTCCATGTAAGCAGGGGCCTAGATATGGGATTTGGGGTGTGTGT
                           50570     50580     50590     50600     50610     50620     50630     50640
Human             GTAATGAGAAATATGAGATTGGGAATGACTAGGTGAGTCCATGTAG-CAGGGGCCTAGATGTGGGATTTGATGTGTGTGT 40291
Coquerel'sSifaka  ATAAGTCAGGAGGAGAAA-----AGTGATGGACTGAT--C----------------AGAGGTGAAGATGG---------- 36427
Ma'sNightMonkey   NNNNNNNNNNNNNNNNNNNNNNNNNNNNNNNNGTGTGTGTGTGTGTGTGTGTGTGTGTGTGGTGGTGGTGGT------ 37051
VervetAGM         ATAATGCGAAATATGAGATTGGGAATGCCTAGGTGAGTCCATGTAAGCAGGGGCCTAGATACGGGATTTGGGGTGGGGGT 43637
Gorrilla          GTAATGAGAAATATGAGATTGGGAATGACTAGGTGAGTCCATGTAG-CAGGGGCCTAGATGTGGGATTTG------ATGT 42697
GrabEatingMacaqueATAATGCGAAATATGAGATTGGGAATGCCTAGGTGAGTCCATGTAAGCAGGGGCCTAGATATGGGATTTGGGGTGTGTGT 42913
Macaque           ATAATGCGAAATACGAGATTGGGAATGCCTAGGTGAGTCCATGTAAGCAGGGGCCTAGATATGGGATTTGGGGTGTGTGT 43678
PigTailedMacaque  GTAATGCGAAATATGAGATTGGGAATGCCTAGGTGAGTCCATGTAAGCAGGGGCCTAGATATGGGATTTGGGGTGTGTGT 42643
Bonobo            GTAATGAGAAATATGAGATTGGGAATGACTAGGTGAGTCCATGTAG-CAGGGGCCTAGATGTGGGATTTG--GTGTGTGT 41316
OliveBaboon       ATAATGCGAAATATGATATCGGGAATGCCTAGGTGAGTCCATGTAAGCAGGGGCCTAGATATGGGATTTGGGGTGTGTGT 42586
Orangutan         GTAATGCGAAATATGAGATTGGGAATGACTAGGTGAGTCCATGTAGGCAGGGGCCTAGATATGGGATTTG---------- 42285
```

```
Majority         GTGTGTGTGTGTGTGTGTGTGTGTGTGT----------------------------------------------------

                         50650     50660     50670     50680     50690     50700     50710     50720

Human            GTGTGTGTGTGTGTGTGTGTGTGTGTGT---------------------------------------------------- 40319
Coquerel'sSifaka -------------------------------------------------------------------------------- 36427
Ma'sNightMonkey  G---GTGTGTGTGTGGTGGTGTGTGTGT---------------------------------------------------- 37076
VervetAGM        GTGTGTGTGTGTGTGTGTGTGTGTGTGT---------------------------------------------------- 43665
Gorrilla         GTGTGTGTGTGTGTGTGTGTGTGTGTGT---------------------------------------------------- 42725
GrabEatingMacaqueGTGTGTGTGTGTGTGTGTGTGTGTGTGT----------------------AGCTTGGACTTTGTTGTGTGTGTGTGTGTG 42971
Macaque          GTGTGTGTGTGTGTGTGTGTGTGTGTGTGTGTGTGTGTGTGTGTGTGTGTAGCTTGGACTTTGTTGTGTGTGTGTGTGTG 43758
PigTailedMacaque GTGTGTGTGTGTGTGTGTGTGTGTGTGT---------------------------------------------------- 42671
Bonobo           GTGTGTGTGTGTGTGTGTGCGTGTGTGT---------------------------------------------------- 41344
OliveBaboon      GTGTGTGTTTGTGTGTGTGT----GT------------------------------------------------------ 42608
Orangutan        ----ATGTGTGTGTGTGTGTGTGTGTGT---------------------------------------------------- 42309

Majority         ------------------------GTGTGT--------------------------------------------------

                         50730     50740     50750     50760     50770     50780     50790     50800

Human            ------------------------GTGTGT-------------------------------------------------- 40325
Coquerel'sSifaka -------------------------------------------------------------------------------- 36427
Ma'sNightMonkey  -------------------------------------------------------------------------------- 37076
VervetAGM        --------------------------GT---------------------------------------------------- 43667
Gorrilla         ------------------------GTGTAT-------------------------------------------------- 42731
GrabEatingMacaqueTGTAGCTTGGACTTTGTTGTGTGTGTGTGTGTGT------AGCTTGGACTTTGTTTTGTGTGTGTGTGTGTGTGTGTG 43045
Macaque          TGTAGCTTGGACTTTGTTGTGTGTGTGTGTGTGTGTGTGTAGCTTGGACTTTGTTTTGTGTGTGTGTGTGTGTGTGTG 43838
PigTailedMacaque -G----T-----------------GTGTGTGTGT---------------------------------------------- 42683
Bonobo           ------------------------GCGTAT-------------------------------------------------- 41350
OliveBaboon      -------------------------------------------------------------------------------- 42608
Orangutan        ------------------------GTGTGT-------------------------------------------------- 42315

Majority         ---------AGCTTGGACTTTACCAGATTTACATTTTAGAAAGAATTTTTGTTCCATTTGTATTTCAGAGG------CAG

                         50810     50820     50830     50840     50850     50860     50870     50880

Human            ---------AGCTTGGACTTTAACAGATTTACATTTTAGAAAGAATTTTTGTTCCATTTGTATTTCACAGGTAGGATCAG 40396
Coquerel'sSifaka -------------------TTGAC----------TTTAGAAT---------TG--------------------------- 36442
Ma'sNightMonkey  -----------CTTGGACTTTAACAGATTTACATTTTAGAAA---T-TTTGTTCCAATTTTATTTCAGAGGCAGGATCAG 37141
VervetAGM        ---------AGCTTGGACTTTACCAGATTTACATTTTAGAAAGAATTTTTGTTCCATTTGTATTTCAGAGG------CAG 43732
Gorrilla         ---------AGCTTGGACTTTAACAGATTTACATTTTAGAAAGAATTTTTGTTCCATTTGTATTTCACAGGTAGGATCAG 42802
GrabEatingMacaqueTGTGTGTGTAGCTTGGACTTTACCAGATTTACATTTTAGAAAGAATTTTTGTTCCATTTGTATTTCAGAGG------CAG 43119
Macaque          TGTGTGT--AGCTTGGACTTTACCAGATTTACATTTTAGAAAGAATTTTTGTTCCATTTGTATTTCAGAGG------CAG 43910
PigTailedMacaque ---------AGCTTGGACTTTACCAGATTTACATTTTAGAAAGAATTTTTGTTCCATTTGTATTTCAGAGG------CAG 42748
Bonobo           ---------AGCTTGGACTTTAACAGATTTACATTTTAGAAAGA-TTTTTGTTCCATTTGTATTTCACAGGTAGGATCAG 41420
OliveBaboon      ---------AGCTTGGACTTTACCAGATTTACATTTTAGAAAGAATTTTTGTTCCATTTGTATTTCAGAGG------CAG 42673
Orangutan        ---------AGCTTGGACTTTACCAGATTTACATTTGAGAAAGAATTTTTGTTCCATTTGTATTTCAGAAGCAGGATCAG 42386
```

```
Majority          TATTGTATAGAGGGGAGAATAGTTTTAAAACATTACCGTAGTTCATTCCAAAGATACCAAGGTCCTGAATTTTAGGGTCT
                          50890     50900     50910     50920     50930     50940     50950     50960
Human             TATTGTATAGAGGGGAGAATAGTTTTAAAACATTACGGTAGTTCATTCCAAAGATACCAAGGTCCTGAATTTTAGGGTCT 40476
Coquerel'sSifaka  --------AT---------------------------------------------------------------------- 36444
Ma'sNightMonkey   TATTGTATAGAGGGGAGAATAGTTA-AAAACATTACAGTAGTTTATTCCAAAGATATCAAGGTCCTGAATTTTAGGATCT 37220
VervetAGM         TATTGTATAGAGGGGAGAATAGTTTTAAAACATTACCGTAGTTCATTCCAGAGATACCAAGGTCCTGAATTTTAGGGTCT 43812
Gorrilla          TATTGTATAGAGGGGAGAATAGTTTTAAAACATTACGGTAGTTCATTCCAAAGATACCAAGGTCCTGAATTTTAGGGTTT 42882
GrabEatingMacaqueTATTGTATACAGGGGAGAATAGTTTTAAAACATTACCGTAGTTCATTCCAAAGATACCAAGGTCCTGAATTTTAGGGTCT 43199
Macaque           TATTGTATACAGGGGAGAATAGTTTTAAAACATTACCGTAGTTCATTCCAAAGATACCAAGTTCCTGAATTTTAGGGTCT 43990
PigTailedMacaque  TATTGTATAGAGGGGAGAATAGTTTTAAAACATTACCGTAGTTCATTCCAAAGATACCAAGGTCCTGAATTTTAGGGTCT 42828
Bonobo            TATTGTATAGCGGGGAGAATAGTTTTAAAACATTACGGTAGTTCATTCCAAAGATACCAAGGTCCTGAATTTTAGGGTCT 41500
OliveBaboon       TATTGTATAGAGGGGAGAATAGTTTTAAAACATTACCGTAGTTCATTCCAAAGATACCAAGGTCCTGAATTTTAGGGTCT 42753
Orangutan         TATTGTATAGAGAGGAGAATAGTTTTAAAACATTACAGTAGTTCATTCCAAAGATACCAAGGTCCTGAATTTTAGGGTCT 42466

Majority          TG-GAAGGAGAATATGCAGGAAATGTTA---------AAACGAG----------TTATCTGGAAGAAGACAAGTTTGGAA
                          50970     50980     50990     51000     51010     51020     51030     51040
Human             TG-GAAGGAGAAT--GCAGGAAATGATA---------AAACGAG----------TTATCTGGAAGAAGACAAGTTTGGAA 40534
Coquerel'sSifaka  ---GTAGAAGTTCG-GTTTTGAGTTTCG---------AAGGAAG-----------TGTTTAAGTAGAAACAGTTTTAAAA 36500
Ma'sNightMonkey   TG-GAAGGAGAATATGCAGGAAATGATATATGTCAAGAAATGAGATGTTTGAGTTATTTGGAATAAGACAAGTTTGGAA 37299
VervetAGM         TG-GAAGGAGAATATGCAGGAGATGTTA---------AAACGAG----------TTATCCGGAAGAAGACAAGTTTGGAA 43872
Gorrilla          TTCGAAGGAGAATATGCAGGAAATGATA---------AAACGAG----------TTATCTGGAGGAAAACAAGTTTGGAA 42943
GrabEatingMacaqueTG-GAAGGAGAATATGCAGGAGATGTTA---------AAACGAG----------TTATCTGGAAGAAGACAAGTTTGGAA 43259
Macaque           TG-GAAGGAGAATATGCAGGAGATGTTA---------AAACGAG----------TTATCTGGAAGAAGACAAGTTTGGAA 44050
PigTailedMacaque  TG-GAAGGAGAATATGCAGGAGATGTTA---------AAACGAG----------TTATCTGGAAGAAGACAAGTTTGGAA 42888
Bonobo            TG-GAAGGATAATATGCAGGAAATGATA---------AAACGAG----------TTATCTGGAAGAAGACAAGTTTGGAA 41560
OliveBaboon       TG-GAAGGAGAATATGCAGGAGATGTTA---------AAACGAG----------TTATCTGGAAGAAGACAAGTTTGGAA 42813
Orangutan         TG-GAAGGAGAAGATGCAGGAAATGATA---------AAATGAG----------TTACCTGGAAGAAGACAAGTTTGGAA 42526

Majority          TAATATTAAGGAGTTTTGTCTTAGAAT--CACTGGGTTGGTGGAAGAT-GATGCCATTAACAAAGG-GGGAAAAGTAGGA
                          51050     51060     51070     51080     51090     51100     51110     51120
Human             TAATATTAAAGAGTTTTGTCTTAGAAT--CACTGGGTTGGTAGAAGAT-GATGCCATTAACAAAGG-GGGAAAAGTAGGA 40610
Coquerel'sSifaka  TTTTGTTTT-GAATTT------A---T------GAGTAGAT-GAACAT--CCTCCCTTAGCAAAG-----------G-- 36548
Ma'sNightMonkey   GAATATTA--GAGTTTTGTCTTAGAAT--CACTGGGTTGGTGGAAGAT-GATGCCGTTAACAAAGGAGGGAAAAGTAGGA 37374
VervetAGM         TAATATGAAGGAGTTTTGTCTTAGAAT--CACTCGGTTGGTGGAAGAT-GATGCCATTAACAAAGG-GGGAAAAGTAGGA 43948
Gorrilla          TAATATTAAAGAGTTTTGTCTTAGAAT--CACTGGGTTGGTAGAAGATTGATGCCATTAACAAAGG-GGAAAAAGTAGGA 43020
GrabEatingMacaqueTAATATGAAGGAGTTTTGTCTTAGAATTCCACTGGGTTGGTGGAAGAT-GATGCCATTAACAAAGG-GGGAAAAGTAGGA 43337
Macaque           TAATATGAAGGAGTTTTGTCTTAGAATTCCACTGGGTTGGTGGAAGAT-GATGCCATTAACAAAGG-GGGAAAAGTAGGA 44128
PigTailedMacaque  TAATATGAAGGAGTTTTGTCTTAGAAT--CACTGGGTTGGTGGAAGAT-GATGCCATTAACAAAGG-GGGAAAAGTAGGA 42964
Bonobo            TAATATTAAAGAG-----------------------------AAGAT-GATGCCATTAACAAAGG-GGGGAAAGTAGGA 41608
OliveBaboon       TAATATGAAGGAGTTTTGTCTTAGAAT--CACTGGGTTGGTGGAAGAT-GATGCCATTAACAAAGG-GGGAAAAGTAGGA 42889
Orangutan         TAATATTAAAGAGTTTTGTCTTAGAAT--CACTGGGTTTGTCGAAGAT-GATGCCATTAACAAAGG-GGGAAAAGTAGGA 42602
```

```
Majority         ----AAAGGAGACTCGTTTTTGGTGGAAAGAAAGAATAATCTGCTACTGTTCTATTTTTCT-------------------

                        51130     51140     51150     51160     51170     51180     51190     51200

Human            ----AAAGGAGACTCGTTTTTGGTGGGAAAAAAGAATAATCCGCTACTGTTCTGTTTTTTTTTGTTTGTTTTTTGTTTTT 40686
Coquerel'sSifaka ----------------CTTTT----AAAAGA-----------G---CT-------------------------------- 36562
Ma'sNightMonkey  ----AAAGGAGACTTGTTTTTGGTGGAAAGAAAGAATAATTTGCTACTGTTCTATTTTTTT------------------- 37431
VervetAGM        GAAAAAAGGAGACTCGTTTTTGGTGGAAAGAAAGAATAATCTGCTACTGTTATATTTTTCT------------------- 44009
Gorrilla         ----AAAGGAGACTCGTTTTTGGTGGAAAAAAAGAATAATCTGCTACTGTTCTGTTTTT-----T--------------- 43076
GrabEatingMacaqueGAACAAAGGAGACTCGTTTTTGGTGGAAAGAAAGAATAATCTGCTACTGCTCTATTTTTCT------------------- 43398
Macaque          GAACAAAGGAGACTCGTTTTTGGTGGAAAGAAAGAATAATCTGCTACTGCTCTATTTTTCT------------------- 44189
PigTailedMacaque GAACAAAGGAGACTCGTTTTTGGTGGAAAGAAAGAATAATCTGCTACTGCTCTATTTTTCT------------------- 43025
Bonobo           ----AAAGGAGACTCGTTTTTGGTGGGAAAAAAGAATAATCTGCTACTGTTCTGTTTTT-----T--------------- 41664
OliveBaboon      GAACAAAGGAGACTCGTTTTTGGTGGAAAGAAAGAATAATCTGCTACTGCTCTATTTTTCT------------------- 42950
Orangutan        ----AAAGGAAACTCGTTTTTGGTGGAAAAAAAGAATAATCTGCTACTGTTCTATTTTT-----T--------------- 42658

Majority         --------------------------------------------------------------------------------

                        51210     51220     51230     51240     51250     51260     51270     51280

Human            TATTTTTTATTGATCATTCTTGGGTGTTTCTCGCAGAGGGGGATTTGGCAGGGTCATAGGACAATAGTGGAGGGAAGGTC 40766
Coquerel'sSifaka -------------------------------------------------------------------------------- 36562
Ma'sNightMonkey  -------------------------------------------------------------------------------- 37431
VervetAGM        -------------------------------------------------------------------------------- 44009
Gorrilla         -------------------------------------------------------------------------------- 43076
GrabEatingMacaque-------------------------------------------------------------------------------- 43398
Macaque          -------------------------------------------------------------------------------- 44189
PigTailedMacaque -------------------------------------------------------------------------------- 43025
Bonobo           --------------------------------------------------------T----------------------- 41665
OliveBaboon      -------------------------------------------------------------------------------- 42950
Orangutan        -------------------------------------------------------------------------------- 42658

Majority         --------------------------------------------------------------------------------

                        51290     51300     51310     51320     51330     51340     51350     51360

Human            AGCAGATAAACAAGTGAACAAAGGTCTCTGGTTTTCCTAGGCAGAGGACCCTGCGGACTTCCGCAGTGTTTGTGTCCCTG 40846
Coquerel'sSifaka -------------------------------------------------------------------------------- 36562
Ma'sNightMonkey  -------------------------------------------------------------------------------- 37431
VervetAGM        -------------------------------------------------------------------------------- 44009
Gorrilla         --------------------------------T----------------------------------------------- 43077
GrabEatingMacaque-------------------------------------------------------------------------------- 43398
Macaque          -------------------------------------------------------------------------------- 44189
PigTailedMacaque -------------------------------------------------------------------------------- 43025
Bonobo           -------------------------------------------------------------------------------- 41665
OliveBaboon      -------------------------------------------------------------------------------- 42950
Orangutan        ---------------------------------T---------------------------------------------- 42659
```

```
Majority         --------------------------------------------------------------------------------

                          51370     51380     51390     51400     51410     51420     51430     51440

Human            GGTCCTTGAGATTAGGGAGTGGTGATGACTCTTAACGAGCATGCTGCCTTCAAGCATCTGTTTAACAAAGCACATCTTGC 40926
Coquerel'sSifaka -------------------------------------------------------------------------------- 36562
Ma'sNightMonkey  -------------------------------------------------------------------------------- 37431
VervetAGM        -------------------------------------------------------------------------------- 44009
Gorrilla         -------------------------------------------------------------------------------- 43077
GrabEatingMacaque-------------------------------------------------------------------------------- 43398
Macaque          -------------------------------------------------------------------------------- 44189
PigTailedMacaque -------------------------------------------------------------------------------- 43025
Bonobo           -------------------------------------------------------------------------------- 41665
OliveBaboon      -------------------------------------------------------------------------------- 42950
Orangutan        -------------------------------------------------------------------------------- 42659

Majority         --------------------------------------------------------------------------------

                          51450     51460     51470     51480     51490     51500     51510     51520

Human            ACCGCCCTTAATCCATTTAACCCTGAGTGGACACAGCACATGTTTCAGAGAGCACAGGGTTGGGGGTAAGGTCACAGATC 41006
Coquerel'sSifaka -------------------------------------------------------------------------------- 36562
Ma'sNightMonkey  -------------------------------------------------------------------------------- 37431
VervetAGM        -------------------------------------------------------------------------------- 44009
Gorrilla         -------------------------------------------------------------------------------- 43077
GrabEatingMacaque-------------------------------------------------------------------------------- 43398
Macaque          -------------------------------------------------------------------------------- 44189
PigTailedMacaque -------------------------------------------------------------------------------- 43025
Bonobo           -------------------------------------------------------------------------------- 41665
OliveBaboon      -------------------------------------------------------------------------------- 42950
Orangutan        -------------------------------------------------------------------------------- 42659

Majority         --------------------------------------------------------------------------------

                          51530     51540     51550     51560     51570     51580     51590     51600

Human            AACAGGATAAGAATTTTTCTTAGTACAGAACAAAATGAAAAGTCTCCCATGTCTACCTCTTTCTACACAGACACGGCAAC 41086
Coquerel'sSifaka -------------------------------------------------------------------------------- 36562
Ma'sNightMonkey  -------------------------------------------------------------------------------- 37431
VervetAGM        -------------------------------------------------------------------------------- 44009
Gorrilla         -------------------------------------------------------------------------------- 43077
GrabEatingMacaque-------------------------------------------------------------------------------- 43398
Macaque          -------------------------------------------------------------------------------- 44189
PigTailedMacaque -------------------------------------------------------------------------------- 43025
Bonobo           -------------------------------------------------------------------------------- 41665
OliveBaboon      -------------------------------------------------------------------------------- 42950
Orangutan        -------------------------------------------------------------------------------- 42659
```

```
Majority         --------------------------------------------------------------------------------

                         51610     51620     51630     51640     51650     51660     51670     51680

Human            CATCCGATTTCTCAATCTTTTCCCCACCTTTCCCCCCTTTCTATTCCACAAAACCGCCATTGTCATCATGGCCCGTTCTC 41166
Coquerel'sSifaka -------------------------------------------------------------------------------- 36562
Ma'sNightMonkey  -------------------------------------------------------------------------------- 37431
VervetAGM        -------------------------------------------------------------------------------- 44009
Gorrilla         -------------------------------------------------------------------------------- 43077
GrabEatingMacaque-------------------------------------------------------------------------------- 43398
Macaque          -------------------------------------------------------------------------------- 44189
PigTailedMacaque -------------------------------------------------------------------------------- 43025
Bonobo           -------------------------------------------------------------------------------- 41665
OliveBaboon      -------------------------------------------------------------------------------- 42950
Orangutan        -------------------------------------------------------------------------------- 42659

Majority         --------------------------------------------------------------------------------

                         51690     51700     51710     51720     51730     51740     51750     51760

Human            AATGAGCTGTTGGGTACACCTCCCAGACGGGGTGGTGGCCGGGCAGAGGGGCTCCTCACTTCCCAGTAGGGGCGGCCGGG 41246
Coquerel'sSifaka -------------------------------------------------------------------------------- 36562
Ma'sNightMonkey  -------------------------------------------------------------------------------- 37431
VervetAGM        -------------------------------------------------------------------------------- 44009
Gorrilla         -------------------------------------------------------------------------------- 43077
GrabEatingMacaque-------------------------------------------------------------------------------- 43398
Macaque          -------------------------------------------------------------------------------- 44189
PigTailedMacaque -------------------------------------------------------------------------------- 43025
Bonobo           -------------------------------------------------------------------------------- 41665
OliveBaboon      -------------------------------------------------------------------------------- 42950
Orangutan        -------------------------------------------------------------------------------- 42659

Majority         --------------------------------------------------------------------------------

                         51770     51780     51790     51800     51810     51820     51830     51840

Human            CAGAGGCGCCCCTCACTCCCCAGTAGGGGCAGCCGGGCAGAGGCGCCCCTCACCTCCCGGATGGGGCGGCTGGCCGGGCG 41326
Coquerel'sSifaka -------------------------------------------------------------------------------- 36562
Ma'sNightMonkey  -------------------------------------------------------------------------------- 37431
VervetAGM        -------------------------------------------------------------------------------- 44009
Gorrilla         -------------------------------------------------------------------------------- 43077
GrabEatingMacaque-------------------------------------------------------------------------------- 43398
Macaque          -------------------------------------------------------------------------------- 44189
PigTailedMacaque -------------------------------------------------------------------------------- 43025
Bonobo           -------------------------------------------------------------------------------- 41665
OliveBaboon      -------------------------------------------------------------------------------- 42950
Orangutan        -------------------------------------------------------------------------------- 42659
```

```
Majority         --------------------------------------------------------------------------------

                         51850     51860     51870     51880     51890     51900     51910     51920

Human            GGGGGCTGACCCCCCCACCTCCCTCCCGGATGGGGCAGCTGGCCGGGCGGGGGGCTGACCCCCCCACCTCCCTCCCGGAC 41406
Coquerel'sSifaka -------------------------------------------------------------------------------- 36562
Ma'sNightMonkey  -------------------------------------------------------------------------------- 37431
VervetAGM        -------------------------------------------------------------------------------- 44009
Gorrilla         -------------------------------------------------------------------------------- 43077
GrabEatingMacaque-------------------------------------------------------------------------------- 43398
Macaque          -------------------------------------------------------------------------------- 44189
PigTailedMacaque -------------------------------------------------------------------------------- 43025
Bonobo           -------------------------------------------------------------------------------- 41665
OliveBaboon      -------------------------------------------------------------------------------- 42950
Orangutan        -------------------------------------------------------------------------------- 42659

Majority         --------------------------------------------------------------------------------

                         51930     51940     51950     51960     51970     51980     51990     52000

Human            GGGGCGGCTGGCCGGGCTGGGGGCTGACCCCCGCCACCTCCCTCCCGGACGGGGCGGCTGGCCGGGCTGGGGGCTGACCC 41486
Coquerel'sSifaka -------------------------------------------------------------------------------- 36562
Ma'sNightMonkey  -------------------------------------------------------------------------------- 37431
VervetAGM        -------------------------------------------------------------------------------- 44009
Gorrilla         -------------------------------------------------------------------------------- 43077
GrabEatingMacaque-------------------------------------------------------------------------------- 43398
Macaque          -------------------------------------------------------------------------------- 44189
PigTailedMacaque -------------------------------------------------------------------------------- 43025
Bonobo           -------------------------------------------------------------------------------- 41665
OliveBaboon      -------------------------------------------------------------------------------- 42950
Orangutan        -------------------------------------------------------------------------------- 42659

Majority         --------------------------------------------------------------------------------

                         52010     52020     52030     52040     52050     52060     52070     52080

Human            CCCGACCTCCTTCCCGGACGGGGCGGCTGGCCGGGCAGAGGGGCTCCTCACTTCCCAGTAGGGGCGGCCGGGCAGAGGCG 41566
Coquerel'sSifaka -------------------------------------------------------------------------------- 36562
Ma'sNightMonkey  -------------------------------------------------------------------------------- 37431
VervetAGM        -------------------------------------------------------------------------------- 44009
Gorrilla         -------------------------------------------------------------------------------- 43077
GrabEatingMacaque-------------------------------------------------------------------------------- 43398
Macaque          -------------------------------------------------------------------------------- 44189
PigTailedMacaque -------------------------------------------------------------------------------- 43025
Bonobo           -------------------------------------------------------------------------------- 41665
OliveBaboon      -------------------------------------------------------------------------------- 42950
Orangutan        -------------------------------------------------------------------------------- 42659
```

```
Majority         --------------------------------------------------------------------------------

                         52090     52100     52110     52120     52130     52140     52150     52160

Human            CCCCTCACCTCCCGGAAGGGGCGGCTGGCCGGGCGGGGGTCTGACCCCCCCACCTCCCTCCTGGATGGGGCGGCTGGCCT 41646
Coquerel'sSifaka -------------------------------------------------------------------------------- 36562
Ma'sNightMonkey  ------A------------------------------------------------------------------------- 37432
VervetAGM        -------------------------------------------------------------------------------- 44009
Gorrilla         -------------------------------------------------------------------------------- 43077
GrabEatingMacaque-------------------------------------------------------------------------------- 43398
Macaque          -------------------------------------------------------------------------------- 44189
PigTailedMacaque -------------------------------------------------------------------------------- 43025
Bonobo           -------------------------------------------------------------------------------- 41665
OliveBaboon      -------------------------------------------------------------------------------- 42950
Orangutan        -------------------------------------------------------------------------------- 42659

Majority         --------------------------------------------------------------------------------

                         52170     52180     52190     52200     52210     52220     52230     52240

Human            GGCGGGGGCTGACCCCCACCTCCCTCCCAGACGGGGTGACTGCCGGGCGGAGACGCTCCTCACTTCCCAGACGGCGTGGC 41726
Coquerel'sSifaka -------------------------------------------------------------------------------- 36562
Ma'sNightMonkey  -------------------------------------------------------------------------------- 37432
VervetAGM        -------------------------------------------------------------------------------- 44009
Gorrilla         -------------------------------------------------------------------------------- 43077
GrabEatingMacaque-------------------------------------------------------------------------------- 43398
Macaque          -------------------------------------------------------------------------------- 44189
PigTailedMacaque -------------------------------------------------------------------------------- 43025
Bonobo           -------------------------------------------------------------------------------- 41665
OliveBaboon      -------------------------------------------------------------------------------- 42950
Orangutan        -------------------------------------------------------------------------------- 42659

Majority         --------------------------------------------------------------------------------

                         52250     52260     52270     52280     52290     52300     52310     52320

Human            TGCCGGGCGGAGGGGCTCCTCACTTCTCAGATGGGGCGGCTGCTGGGCGGAGGGACTCCTCGCTTCTCAGATGGGGCGGC 41806
Coquerel'sSifaka -------------------------------------------------------------------------------- 36562
Ma'sNightMonkey  -------------------------------------------------------------------------------- 37432
VervetAGM        -------------------------------------------------------------------------------- 44009
Gorrilla         -------------------------------------------------------------------------------- 43077
GrabEatingMacaque-------------------------------------------------------------------------------- 43398
Macaque          -------------------------------------------------------------------------------- 44189
PigTailedMacaque -------------------------------------------------------------------------------- 43025
Bonobo           -------------------------------------------------------------------------------- 41665
OliveBaboon      -------------------------------------------------------------------------------- 42950
Orangutan        -------------------------------------------------------------------------------- 42659
```

```
Majority        --------------------------------------------------------------------------------
                         52330     52340     52350     52360     52370     52380     52390     52400
Human           CGGGCCGAGACGCTCCTCACCTCCCAGACGGGGTCGCGGCCGGGTAGAGGCGCTCCTCACATCCCAGACGGGGCGGCGGG 41886
Coquerel'sSifaka -------------------------------------------------------------------------------- 36562
Ma'sNightMonkey --------------------------------------------------------------------------------- 37432
VervetAGM       -------------------------------------------------------------------------------- 44009
Gorrilla        -------------------------------------------------------------------------------- 43077
GrabEatingMacaque-------------------------------------------------------------------------------- 43398
Macaque         -------------------------------------------------------------------------------- 44189
PigTailedMacaque -------------------------------------------------------------------------------- 43025
Bonobo          -------------------------------------------------------------------------------- 41665
OliveBaboon     -------------------------------------------------------------------------------- 42950
Orangutan       -------------------------------------------------------------------------------- 42659

Majority        --------------------------------------------------------------------------------
                         52410     52420     52430     52440     52450     52460     52470     52480
Human           GCAGCGGCGCTCCCCACATCTCAGACGATGGGCGGCCGGGCAGAGACGCTCCTCACTTCCTAGATGGGATGGCGGCCGGG 41966
Coquerel'sSifaka -------------------------------------------------------------------------------- 36562
Ma'sNightMonkey -------------------------------------------------------------------------------- 37432
VervetAGM       -------------------------------------------------------------------------------- 44009
Gorrilla        -------------------------------------------------------------------------------- 43077
GrabEatingMacaque-------------------------------------------------------------------------------- 43398
Macaque         -------------------------------------------------------------------------------- 44189
PigTailedMacaque -------------------------------------------------------------------------------- 43025
Bonobo          -------------------------------------------------------------------------------- 41665
OliveBaboon     -------------------------------------------------------------------------------- 42950
Orangutan       -------------------------------------------------------------------------------- 42659

Majority        --------------------------------------------------------------------------------
                         52490     52500     52510     52520     52530     52540     52550     52560
Human           AAGAGGCGCTCCTCACTTCCTAGATGGGATGGCGGCCGGGCAGAGACCTCCTTTCTTTCCAGACTGGGCAGCCAGGCAGA 42046
Coquerel'sSifaka -------------------------------------------------------------------------------- 36562
Ma'sNightMonkey -------------------------------------------------------------------------------- 37432
VervetAGM       -------------------------------------------------------------------------------- 44009
Gorrilla        -------------------------------------------------------------------------------- 43077
GrabEatingMacaque-------------------------------------------------------------------------------- 43398
Macaque         -------------------------------------------------------------------------------- 44189
PigTailedMacaque -------------------------------------------------------------------------------- 43025
Bonobo          -------------------------------------------------------------------------------- 41665
OliveBaboon     -------------------------------------------------------------------------------- 42950
Orangutan       -------------------------------------------------------------------------------- 42659
```

```
Majority         --------------------------------------------------------------------------------

                         52570     52580     52590     52600     52610     52620     52630     52640

Human            GGGGCTCCTCACGTTCCAGACGATGGGCGGCCAGGCAGAGACGCTCCTCACTTCCCAAACGGGGTGGCGGCTGGGCAGAG 42126
Coquerel'sSifaka -------------------------------------------------------------------------------- 36562
Ma'sNightMonkey  -------------------------------------------------------------------------------- 37432
VervetAGM        -------------------------------------------------------------------------------- 44009
Gorrilla         -------------------------------------------------------------------------------- 43077
GrabEatingMacaque-------------------------------------------------------------------------------- 43398
Macaque          -------------------------------------------------------------------------------- 44189
PigTailedMacaque -------------------------------------------------------------------------------- 43025
Bonobo           -------------------------------------------------------------------------------- 41665
OliveBaboon      -------------------------------------------------------------------------------- 42950
Orangutan        -------------------------------------------------------------------------------- 42659

Majority         --------------------------------------------------------------------------------

                         52650     52660     52670     52680     52690     52700     52710     52720

Human            GCTGCAATCTCGGCACTTTGGGAGGCCAAGGCAGGCGGCTGGGAGGTGGAGGTTGTAACGAGCCGAGATCACGCCACTGC 42206
Coquerel'sSifaka -------------------------------------------------------------------------------- 36562
Ma'sNightMonkey  -------------------------------------------------------------------------------- 37432
VervetAGM        -------------------------------------------------------------------------------- 44009
Gorrilla         -------------------------------------------------------------------------------- 43077
GrabEatingMacaque-------------------------------------------------------------------------------- 43398
Macaque          -------------------------------------------------------------------------------- 44189
PigTailedMacaque -------------------------------------------------------------------------------- 43025
Bonobo           -------------------------------------------------------------------------------- 41665
OliveBaboon      -------------------------------------------------------------------------------- 42950
Orangutan        -------------------------------------------------------------------------------- 42659

Majority         --------------------------------------------------------------------------------

                         52730     52740     52750     52760     52770     52780     52790     52800

Human            ACTCCAGCCTGGGCACCATTGAGCACTGAGTGAACCAGACTCCGTCTGCAATTCCGGCACCTCGGGAGGCCGAGGCTGGC 42286
Coquerel'sSifaka -------------------------------------------------------------------------------- 36562
Ma'sNightMonkey  -------------------------------------------------------------------------------- 37432
VervetAGM        -------------------------------------------------------------------------------- 44009
Gorrilla         -------------------------------------------------------------------------------- 43077
GrabEatingMacaque-------------------------------------------------------------------------------- 43398
Macaque          -------------------------------------------------------------------------------- 44189
PigTailedMacaque -------------------------------------------------------------------------------- 43025
Bonobo           -------------------------------------------------------------------------------- 41665
OliveBaboon      -------------------------------------------------------------------------------- 42950
Orangutan        -------------------------------------------------------------------------------- 42659
```

```
Majority          --------------------------------------------------------------------------------

                           52810     52820     52830     52840     52850     52860     52870     52880

Human             GGATCACTCGCGGTTAGGAGCTGCAGACCAGCCCGGCCAACACAGCGAAACCCCGTCTCCACCAAAAAAATACGAAAACC 42366
Coquerel'sSifaka  -------------------------------------------------------------------------------- 36562
Ma'sNightMonkey   -------------------------------------------------------------------------------- 37432
VervetAGM         -------------------------------------------------------------------------------- 44009
Gorrilla          -------------------------------------------------------------------------------- 43077
GrabEatingMacaque -------------------------------------------------------------------------------- 43398
Macaque           -------------------------------------------------------------------------------- 44189
PigTailedMacaque  -------------------------------------------------------------------------------- 43025
Bonobo            -------------------------------------------------------------------------------- 41665
OliveBaboon       -------------------------------------------------------------------------------- 42950
Orangutan         -------------------------------------------------------------------------------- 42659

Majority          --------------------------------------------------------------------------------

                           52890     52900     52910     52920     52930     52940     52950     52960

Human             AGTCAGGCGTGGCGGCGCGTGCCTGCAATAGCAGGCACTCGGCAGGCTGAGGCAGGAGAATCAGGCAGGGAGGTTGCAGT 42446
Coquerel'sSifaka  -------------------------------------------------------------------------------- 36562
Ma'sNightMonkey   -------------------------------------------------------------------------------- 37432
VervetAGM         -------------------------------------------------------------------------------- 44009
Gorrilla          -------------------------------------------------------------------------------- 43077
GrabEatingMacaque -------------------------------------------------------------------------------- 43398
Macaque           -------------------------------------------------------------------------------- 44189
PigTailedMacaque  -------------------------------------------------------------------------------- 43025
Bonobo            -------------------------------------------------------------------------------- 41665
OliveBaboon       -------------------------------------------------------------------------------- 42950
Orangutan         -------------------------------------------------------------------------------- 42659

Majority          --------------------------------------------------------------------------------

                           52970     52980     52990     53000     53010     53020     53030     53040

Human             GAGTCGAGATGGCAGCAGTACAGTCCAGCTTCGGCTCGTCATCAGAGGGAGACTGTGGAAAGAGAGGGAGAGGGAGACCG 42526
Coquerel'sSifaka  -------------------------------------------------------------------------------- 36562
Ma'sNightMonkey   -------------------------------------------------------------------------------- 37432
VervetAGM         -------------------------------------------------------------------------------- 44009
Gorrilla          -------------------------------------------------------------------------------- 43077
GrabEatingMacaque -------------------------------------------------------------------------------- 43398
Macaque           -------------------------------------------------------------------------------- 44189
PigTailedMacaque  -------------------------------------------------------------------------------- 43025
Bonobo            -------------------------------------------------------------------------------- 41665
OliveBaboon       -------------------------------------------------------------------------------- 42950
Orangutan         -------------------------------------------------------------------------------- 42659
```

```
Majority          ----------------------------------------------------------AACTATGCTTTAGGATGATATA
                          53050     53060     53070     53080     53090     53100     53110     53120
Human             TGGGGAGAGGAGGGAGGGGGAGGGGGAGGGGGAGGGGGAGGGCTGTTCTGTTTTTTTTAACTATGCTTTAGGATGATATA 42606
Coquerel'sSifaka  -------------------------------------------------------------------------------- 36562
Ma'sNightMonkey   ----------------------------------------------------------AACTATGCTTTAGAGATGATATG 37455
VervetAGM         ----------------------------------------------------------AACTATGCATTAGGGATGATATA 44032
Gorrilla          ----------------------------------------------------------AACTATGCTTTAGGATAATATA 43100
GrabEatingMacaque ----------------------------------------------------------AACTATGCATTAGGGATGATATA 43421
Macaque           ----------------------------------------------------------AACTATGCATTAGGGATGATATA 44212
PigTailedMacaque  ----------------------------------------------------------AACTATGCATTAGGGATGATATA 43048
Bonobo            ----------------------------------------------------------AACTGTGCTTTAGAGATGATATA 41688
OliveBaboon       ----------------------------------------------------------AACTGTGCATTAGGGATGATATA 42973
Orangutan         ----------------------------------------------------------AACTATGCTTTAGGGATGATATG 42682

Majority          TGGTGTACTTCATGACCAAAGAATCAATCTAATCTTTTTGTAATGATGGCATAAAATTCCTTTAAGCCTTGTTTTAACAC
                          53130     53140     53150     53160     53170     53180     53190     53200
Human             TAATGTACTTCATGACCAAGGAATCAATCTAATCTTTTTGTAATGATGGCATAAAATTCCTTTAAGCCTTGTTTTAATAC 42686
Coquerel'sSifaka  -------------------------------------------------------------------------------- 36562
Ma'sNightMonkey   TAATGTATTTCATGACCAAATAATCAATCTAATCTTTTTGTAGTGGTGGCATAAAATTCCTTAAAGCCTTGTTTTAACAT 37535
VervetAGM         TGGTGTACTTCATGACCAAAGAATCAATCTAATCTTTTTGTAATGATGGCATCAAATTCCTTTAAGGCTTGTTTTAACAC 44112
Gorrilla          TAATGTACTTCATGACCAAGGAATCAATCTAATCTTTTTGTAATGATGGCATAAAATTCCTTTAAGCCTTGTTTTAATAC 43180
GrabEatingMacaque TGGTGTACTTCATGACCAAAGAATCAATCTAATCTTTTTGTAATGATGGCATAAAATTCCTTTAAG-CTTGTTTTAACAC 43500
Macaque           TGGTGTACTTCATGACCAAAGAATCAATCTAATCTTTTTGTAATGATGGCATAAAATTCCTTTAAG-CTTGTTTTAACAC 44291
PigTailedMacaque  TGGTGTACTTCATGACCAAAGAATCAATCTAATCTTTTTGTAATGATGGCATAAAATTCCTTTAAG-CTTGTTTTAACAC 43127
Bonobo            TAATGTACTTCATGACCAAGGAATCAATCTAATCTTTTTGTAATGATGGCATAAAATTCCTTTAAGCCTTGTTTTAATAC 41768
OliveBaboon       TGGTGTACTTCATGACCAAAGAATCAATCTAATCTTTTTGTAATGATGGCATAAAATTCCTTTAAG-CTTGTTTTAACAC 43052
Orangutan         TAATGTACTTCATGACCAAAGAATCAATCTAATCTTTCTGTAGTGATGCCATAAAATTCCTTTAAGCCTTGTTTTAATAC 42762

Majority          AAGAAGTCAGTTTATTTAACCTCTGTGTACAGAGGAAATGCTGAGTTTTAAGATTTTTCTATTTTTTT---------CTG
                          53210     53220     53230     53240     53250     53260     53270     53280
Human             AAGAAGTCAGTTTATTTAACCTCCATGTACAGAGGAAATACTGAGTTTTAAGATTTTTCTATTTTTTTT--------CTG 42758
Coquerel'sSifaka  -------------------------------------------------------------------------------- 36562
Ma'sNightMonkey   AAGAAGTCAGTTTATTTAACCTTTGTGTACAGAGGAAATACTAAGTTTTAAGATTTTTCTGTTTTTTTTTTTTTTTTCTG 37615
VervetAGM         AAGAAGTCAGTTTATTTAACCTCTGCGTACAGAGGAAATGCTGAGTTTTAAGATTTTTCTATTTTTTT---------CTG 44183
Gorrilla          AAGAAGTCAGTTTATTTAACCTCCATGTAGAGAGGAAATACTGAGTTTTAAGATTTTTCTATTTTTTTT--------CTG 43252
GrabEatingMacaque AAGAAGTCAGTTTATTTAACCTCTGCGTACAGAGGAAATGCTGAGTTTTAAGATTTTTCTATTTTTTT---------CTG 43571
Macaque           AAGAAGTCAGTTTATTTAACTTCTGCGTACAGAGGAAATGCTGAGTTTTAAGATTTTTCTATTTTTTT---------CTG 44362
PigTailedMacaque  AAGAAGTCAGTTTATTTAACCTCTGCGTACAGAGGAAATGCTGAGTTTTAAGATTTTTCTATTTTTTT---------CTG 43198
Bonobo            AAGAAGTCAGTTTATTTAACCTCCATGTACAGAGGAAATACTGAGTTTTAAGATTTTTCTATTTTTTTT--------CTG 41840
OliveBaboon       AAGAAGTCAGTTTATTTAACCTCTGCGTACAGAGGAAATGCTGAGTTTTAAGATTTTTCTATTTTTTT---------CTG 43123
Orangutan         AAGAAGTCAATTTATTTAACCTCCATGTACAGAGGAAATACTGAGTTTTAAGATTTTTCTATTTTTTTT--------CTG 42834
```

```
Majority          ACAAAGA----TGAAAGTCTGCATATATTC--------------------------------------------------

                           53290     53300     53310     53320     53330     53340     53350     53360

Human             ACAAAAA----TGAAAGTCTGCATATATTC-------------------------------------------------- 42784
Coquerel'sSifaka  -------------------------------------------------------------------------------- 36562
Ma'sNightMonkey   ACAAAGAAAGATGAAAGTCTGCATATATTC-------------------------------------------------- 37645
VervetAGM         ACAAAGA----TGAAAGTCTGCATATATTC-------------------------------------------------- 44209
Gorrilla          ACAAAGA----TGAAAGTCTGCATATATTC-------------------------------------------------- 43278
GrabEatingMacaqueACAAAGA----TGAAAGTCTGCATATATTC-------------------------------------------------- 43597
Macaque           ACAAAGA----TGAAAGTCTGCATATATTC-------------------------------------------------- 44388
PigTailedMacaque  ACAAAGA----TGAAAGTCTGCATATATTCGGCCGGGCGCGGTGGCTCACGCCTGTAATCCCAGCACTTTGGGAGGCCGA 43274
Bonobo            ACAAAGA----TGAAAGTCTGCATATATTC-------------------------------------------------- 41866
OliveBaboon       ACAAAGA----TGAAAGTCTGCATATATTC-------------------------------------------------- 43149
Orangutan         ACAAAGA----TGAAAGTCTGCATATATTC-------------------------------------------------- 42860

Majority          --------------------------------------------------------------------------------

                           53370     53380     53390     53400     53410     53420     53430     53440

Human             -------------------------------------------------------------------------------- 42784
Coquerel'sSifaka  -------------------------------------------------------------------------------- 36562
Ma'sNightMonkey   -------------------------------------------------------------------------------- 37645
VervetAGM         -------------------------------------------------------------------------------- 44209
Gorrilla          -------------------------------------------------------------------------------- 43278
GrabEatingMacaque-------------------------------------------------------------------------------- 43597
Macaque           -------------------------------------------------------------------------------- 44388
PigTailedMacaque  GGCGGGCGGATCACAAGGTCAGGAGATCGAGACCACGGTGAAACCCCGTCTCTACTAAAAATACAAAAAATTAGCCGGGC 43354
Bonobo            -------------------------------------------------------------------------------- 41866
OliveBaboon       -------------------------------------------------------------------------------- 43149
Orangutan         -------------------------------------------------------------------------------- 42860

Majority          --------------------------------------------------------------------------------

                           53450     53460     53470     53480     53490     53500     53510     53520

Human             -------------------------------------------------------------------------------- 42784
Coquerel'sSifaka  -------------------------------------------------------------------------------- 36562
Ma'sNightMonkey   -------------------------------------------------------------------------------- 37645
VervetAGM         -------------------------------------------------------------------------------- 44209
Gorrilla          -------------------------------------------------------------------------------- 43278
GrabEatingMacaque-------------------------------------------------------------------------------- 43597
Macaque           -------------------------------------------------------------------------------- 44388
PigTailedMacaque  GCGGTTGTGGCGCCTGTAGTCCCAGCTACTCGGGAGGCTGAGGCAGGAGAATGGCGTGAACCCGGGAGGCGGAGCTTGC 43434
Bonobo            -------------------------------------------------------------------------------- 41866
OliveBaboon       -------------------------------------------------------------------------------- 43149
Orangutan         -------------------------------------------------------------------------------- 42860
```

```
Majority        --------------------------------------------------------------------------------

                        53530     53540     53550     53560     53570     53580     53590     53600

Human           -------------------------------------------------------------------------------- 42784
Coquerel'sSifaka -------------------------------------------------------------------------------- 36562
Ma'sNightMonkey -------------------------------------------------------------------------------- 37645
VervetAGM       -------------------------------------------------------------------------------- 44209
Gorrilla        -------------------------------------------------------------------------------- 43278
GrabEatingMacaque-------------------------------------------------------------------------------- 43597
Macaque         -------------------------------------------------------------------------------- 44388
PigTailedMacaque AGTGAGCCGAGATCGCGCCACTGCACTCCAGCCTGGGCGACAGAGCGAGACTCCGTCTCAAAAAAAAAAAAAAAAAGAAA 43514
Bonobo          -------------------------------------------------------------------------------- 41866
OliveBaboon     -------------------------------------------------------------------------------- 43149
Orangutan       -------------------------------------------------------------------------------- 42860

Majority        --------------TAAGAGACACCAATTTGAATACTGTTGCTTATAAGAGCAAATAGCATAAAATACCTTTAAAAATTG

                        53610     53620     53630     53640     53650     53660     53670     53680

Human           --------------TAAGAGACACCAATTTGAATACTGTTGCTTATAAGAGCAAATAGCATAAAAGACCTTTAAAAATTG 42850
Coquerel'sSifaka -------------------------------------------------------------------------------- 36562
Ma'sNightMonkey --------------TAAGAGACAGCAATTTGAATACTGTTGCTTGTAAGAGCACATAGCATAAAAGACCTTTAAAAACTG 37711
VervetAGM       --------------TAAGAGACACTAGTTTGAATACTGTCGCTTATAAGAGCAAATAGCATAAAATACGTTTAAAAATTG 44275
Gorrilla        --------------TAAGAGACACCCATTTGAATACTGTTGCTTATAAGAGCAAATAGCATAAAAGACCTTTAAAAATTG 43344
GrabEatingMacaque--------------TAAGAGACACCAGTTTGAATACTGTCGCTTATAAGAGCAAATAGCATAAAATACCTTTAAAAATTG 43663
Macaque         --------------TAAGAGACACCAGTTTGAATACTGTCGCTTATAAGAGCAAATAGCATAAAATACCTTTAAAAATTG 44454
PigTailedMacaque GTCTGCATATATTCTAAGAGACACCAATTTGAATACTGTCGCTTATAAGAGCAAATAGCATAAAATACCTTTAAAAATTG 43594
Bonobo          --------------TAAGAGACACCAATTTGAATACTGTTGCTTATAAGAGCAAATAGCATAAAAGACCTTTAAAAATTG 41932
OliveBaboon     --------------TAAGAGACACCAGTTTGAATACTGTCGCTTAGAAGAGCAAATAGCATAAAATACCTTTAAAAATTG 43215
Orangutan       --------------TAAGAGACACCAATTTGAATACTGTTGCTTATAAGAGCAAATAGCATAACAGACCTTTAAAAATTG 42926

Majority        GTGATTATATTTGCCTGGAAAAATACTCCATGTAATTCAGATGACTCAAAAGGACATTGGTAGACAATGTTACAAGGACA

                        53690     53700     53710     53720     53730     53740     53750     53760

Human           GTGATTATATTTGCCTGGAAAAATACTCCATGTAATTCAGATGACTCAAAAGGACGTTGGTAGACAATGTTACAAGGACA 42930
Coquerel'sSifaka -------------------------------------------------------------------------------- 36562
Ma'sNightMonkey GTGATTATATTTGCCTGGAAAAATACTCCATGTGATTCAGGTGACTCAAAAGGACTTTGGTAGACAGTGTTACAAGGACA 37791
VervetAGM       GTGATTATATTTGCCTGGAAAAATAATCCATGTAATTCAGATGACTCAAAAGGACATTGGTAGACAATGTTACAAGGACA 44355
Gorrilla        GTGATTATATTTGCCTGGAAAAATACTCCATGTAATTCAGATGACTCAAAAGGACATTGGTAGACAATGTTACAAGGACA 43424
GrabEatingMacaqueGTGATTATATTTGCCTGGAAAAATAATCCATGTAATTCAGATGACTCAAAAGGACATTGGTAGACAATGTTACAAGGACA 43743
Macaque         GTGATTATATTTGCCTGGAAAAATAATCCATGAAATTCAGATGATTCAAAAGGACATTGGTAGACAATGTTACAAGGACA 44534
PigTailedMacaque GTGATTATATTTGCCTGGAAAAATAATCCATGTAATTCAGATGACTCAAAAGGACATTGGTAGACAATGTTACAAGGACA 43674
Bonobo          GTGATTATATTTGCCTGGAAAAATACTCCATGTAATTCAGATGACTCAAAAGGACGTTGGTAGACAATGTTACAAGGACA 42012
OliveBaboon     GTGATTATATTTACCTGGAAAAATAATCCATGTAATTCAGATGACTCAAAAGGACATTGGTAGACAATGTTACAAGGACA 43295
Orangutan       GTGATTATATTTGCCTGGAAAAATACTCCATGTAATTCAGATGACTCAAAAGGACGTTGGTAGACAATGTTACAAGGACA 43006
```

```
Majority         CTTTT-ATGGTTATCAGATATAGTACAGGATTTCATTTTTATGTTCCAGCTTCCAGCTTTATCTAAAATATTTCTTGTAA

                         53770     53780     53790     53800     53810     53820     53830     53840

Human            CTTTT-ATGGTTATCAGATATAGTACAGGATTTCATTTTTATGTTCCAGCTTCCAGCTTTATCTAAAATATTTCTTGTAA 43009
Coquerel'sSifaka -------------------------------------------------------------------------------- 36562
Ma'sNightMonkey  CTTTTTATGGTTATCAGATATAGTACAGGATTTCATTTTTATGTTCCAGCTTCCAGCTTTATCTAAAATATTTCTTCCAA 37871
VervetAGM        CTTTT-ATGGTTATTAGATATAGTACAGGATTTCATTTTTATGTTCCAGCTTCCAGCTTTATCTAAAATATTTCTTGTAA 44434
Gorrilla         CTTTT-ATGGTTATCAGATATAGTACAGGATTTCATTTTTATGTTCCAGCTTCCAGCTTTATCTAAAATATTTCTTGTAA 43503
GrabEatingMacaqueCTTTT-ATGGTTATCAGATATAGTACAGGATTTCATTTTTATGTTCCAGCTTCCAGCTTTATCTAAAATATTTCTTGTAA 43822
Macaque          CTTTT-ATGGTTATCAGATATAGTACAGGATTTCATTTTTATGTTCCAGCTTCCAGCTTTATCTAAAATATTTCTTGTAA 44613
PigTailedMacaque CTTTT-ATGGTTATCAGATATAGTACAGGATTTCATTTTTATGTTCCAGCTTCCAGCTTTATCTAAAATATTTCTTGTAA 43753
Bonobo           CTTTT-ATGGTTATCAGATATAGTACAGGATTTCATTTTTATGTTGCAGCTTCCAGCTTTATCTAAAATATTTCTTGTAA 42091
OliveBaboon      CTTTT-ATGATTATCAGATATAGTACAGGATTTCATTTTTATGTTCCAGCTTCCAGCTTTATCTAAAATATTTCTTGTAA 43374
Orangutan        CTTTT-ATGGTTATCAGATATAGCACAGGATTTCATTTTTATGTTCCAGCTTCCAGCTTTATCTAAAATATTTCTTGTAA 43085

Majority         TAGATTTTACTGTAGAATTGTTTCCTAAGGCATCTGTCTCAACAGCCTTTCTCTTATCTGTGCAGACTTTTCTTTCTCTG

                         53850     53860     53870     53880     53890     53900     53910     53920

Human            TAGATTTTACTGTAGAATTGTTTCCTAAGGCATCTGTCTCAACAGCCTTTCCCTTATCTGTGCAGACTTTTCTTTCTCTG 43089
Coquerel'sSifaka -------------------------------------------------------------------------------- 36562
Ma'sNightMonkey  TATGTTTTACTGTAGAATTGTTTCCAAAAGCATCTGTCTCAGCAGTTGTTCCCTTACCTGTGCAGACTTTTCTTTCTCTG 37951
VervetAGM        TAGATTTTACTGTAGAATTGTTTCCTAAGGCATCTGTCTCAACAGCCTTTCTCTTATCTATGCAGACTTTTCTTTCTCTG 44514
Gorrilla         TAGATTTTACTGTAGAATTGTTTCCTAAGGCATCTGTCTCAACAGCCTTTCCCTTATCTGTGCAGACTTTTCTTTCTCTG 43583
GrabEatingMacaqueTAGATTTTACTGTAGAATTGTTTCCTAAGGCATCTGTCTCAACAGCCTTTCTCTTACCTATGCAGACTTTTCTTTCTCTG 43902
Macaque          TAGATTTTACTGTAGAATTGTTTCCTAAGGCATCTGTCTCAACAGCCTTTCTCTTACCTATGCAGACTTTTCTTTCTCTG 44693
PigTailedMacaque TAGATTTTACTGTAGAATTGTTTCCTAAGGCATCTGTCTCAACAGCCTTTCTCTTACCTATGCAGACTTTTCTTTCTCTG 43833
Bonobo           TAGATTTTACTGTAGAATTGTTTCCTAAGGCATCTGTCTCAACAGCCTTTCCCTTATCTGTGCAGACTTTTCTTTCTCTG 42171
OliveBaboon      TAGATTTTACTGTAGAATTGTTTCCTAAGGCATCTGTCTCAACAGCCTTTCTCTTATCTATACAGACTTTTCTTTCTCTG 43454
Orangutan        TAGATTTTACTGTAGAATTGTTTCCTAAGGCATCTGTCGCAACAGCCTTTCCCTTATCTGTGCAGACTTTTCTTTCTCTG 43165

Majority         TACCTGTTGAAT-AGGTGCTTTTTGTCAAAGAAAAAA-TTAATTTTACAGGTTAGAAAATTTCATTTTATTAGAAACTTA

                         53930     53940     53950     53960     53970     53980     53990     54000

Human            TACTTGTTGAAT-AGGTGCTTTTTGTCAAAGAAAAAA-ATAATTTTACAGGTGAAAAAATTTCATGTTATTAGAAACTTA 43167
Coquerel'sSifaka -------------------------------------------------------------------------------- 36562
Ma'sNightMonkey  TGCCTATTGAAT-AGGTGCTTTTTATCAAAGAAAAAAATTAATTTTATAGATGAGAAAAATTCGTGTTATTAGAAACTTA 38030
VervetAGM        TACCAGTTGAAT-AGGTGCTTTTTGTCAAAGAAAAAA-TTAATTTTACAGGTTAGAAAATTTCATTTTATTAGAAAC--A 44590
Gorrilla         TACTTGTTGAAT-AGGTGCTTTTTGTCAAAGAAAAAA-TTAATTTTACAAGTGAAAAAATTTCATGTTATTAGAAACTTA 43661
GrabEatingMacaqueTACCTGTTGAAT-AGGTGCTTTTTGTCAAAGAAAAAA-TTAATTTTACAGGTTAGAAAATTTCATTTTATTAGAAACTTA 43980
Macaque          TACCTGTTGAAT-AGGTGCTTTTTGTCAAAGAAAAAA-TTAATTTTACAGGTTAGAAAATTTCATTTTATTAGAAACTTA 44771
PigTailedMacaque TACCTGTTGAAT-AGGTGCTTTTTGTCAAAGAAAAAA-TTAATTTTACAGGTTAGAAAATTTCATTTTATTAGAAACTTA 43911
Bonobo           TACTTGTTGAATTAGGTGCTTTTTGTTGAAGAAAAAA-TTAATTTTACAGGTGAAAAAATTTCATGTTATTAGAAACTTA 42250
OliveBaboon      TACCTGTTGAAT-AGGTGCTTTTTGTCAAAGAAAAAA-TTAATTTTACAGGTTAGAAAATTTCTTTTTATTAGAAACTTA 43532
Orangutan        TACCTGTTGAAT-AGGTGCTTTTTGTCAAAGAAAAAA-TTAATTTTACAGGTGAGAAAATTTCATGTTATTAGAAACTTA 43243
```

```
Majority          ATCTCAGGTAAATAAATGTTTAATAAATTGATACAGTAAGGACATTTTCTAATGAATTGGTTCCTTCGAGAGATATAAAA

                          54010     54020     54030     54040     54050     54060     54070     54080

Human             ATCTCAGGTAAATAAATGTTTAATAAATTGATACAGTAAGGACATTTTCTAATGAATTGGTTCCTTCAAGAGATATAAAA 43247
Coquerel'sSifaka  -------------------------------------------------------------------------------- 36562
Ma'sNightMonkey   ATCTCAGGTAAATAA--ATCTAATAAATTAATATAGTAAGGACATTTTCTAATAAATTGGTTCCTTCAGGAGATATAAAA 38108
VervetAGM         ATCTCAGGTAAATAAATGTTTAATAAATTGATACAGTAAGGACATTTTCTAATGAATTGGTTCCTTCGAGAGATATATAA 44670
Gorrilla          ATCTCAGGTAAATAAATGTTTAATAAATTGATACAGTAAGGACATTTTCTAATGAATTGGTTCCTTCAAGAGATATAGAA 43741
GrabEatingMacaqueATCTCAGGTAAATAAATGTTTAATAAATTGATACAGTAAGGACATTTTCTAATGAATTGGTTCCTTCGAGAGATATAAAA 44060
Macaque           ATCTCAGGTAAATAAATGTTTAATAAATTGATACAGTAAGGACATTTTCTAATGAATTGGTTCCTTCGAGAGATATAAAA 44851
PigTailedMacaque  ATCTCAGGTAAATAAATGTTTAATAAATTGATACAGTAAGGACATTTTCTAATGAATTGGTTCCTTCGAGAGATATAAAA 43991
Bonobo            ATCTCAGGTAAATAAATGTTTAATAAATTGATACAGTAAGGACATTTTCTAATGAATTGGTTCCTTCAAGAGATATTAAA 42330
OliveBaboon       ATCTCAGGTAAA----TGTTTAATAAATTGATACAGTAAGGACATTTTCTAATGAATTGGTTCCTTCGAGAGATATAAAA 43608
Orangutan         ATCTCAGGTAAATAAATGTTTAATAAATTGATACAGTAAGAACATTTTCTAATGAATTGGTTCCTTCAAGAGATATAAAA 43323

Majority          GTGAAATATTTC--------------------------------------------------------------------

                          54090     54100     54110     54120     54130     54140     54150     54160

Human             GTGAAATATTTC-------------------------------------------------------------------- 43259
Coquerel'sSifaka  -------------------------------------------------------------------------------- 36562
Ma'sNightMonkey   GTGAAATATTTCGGGCCGGGCACGGTGGCTCACGCCTGTAATCCCAGCACTTTGGGAGGCAGAGGCGGGTGGATCACGAG 38188
VervetAGM         GTGAAATATTTC-------------------------------------------------------------------- 44682
Gorrilla          GTGAAATATTTC-------------------------------------------------------------------- 43753
GrabEatingMacaqueGTGAAATATTTC-------------------------------------------------------------------- 44072
Macaque           GTGAAATATTTC-------------------------------------------------------------------- 44863
PigTailedMacaque  GTGAAATATTTC-------------------------------------------------------------------- 44003
Bonobo            GTGAAATATTTC-------------------------------------------------------------------- 42342
OliveBaboon       GTGAAATATTTC-------------------------------------------------------------------- 43620
Orangutan         GTGAAATATTTC-------------------------------------------------------------------- 43335

Majority          --------------------------------------------------------------------------------

                          54170     54180     54190     54200     54210     54220     54230     54240

Human             -------------------------------------------------------------------------------- 43259
Coquerel'sSifaka  -------------------------------------------------------------------------------- 36562
Ma'sNightMonkey   GTTAAGAGATCGAGACCATCCTAGTCAACATGGTGAAACCCTGTCTCTTCTAAAAATACAAAAAATTAGCTGGGCATGGT 38268
VervetAGM         -------------------------------------------------------------------------------- 44682
Gorrilla          -------------------------------------------------------------------------------- 43753
GrabEatingMacaque-------------------------------------------------------------------------------- 44072
Macaque           -------------------------------------------------------------------------------- 44863
PigTailedMacaque  -------------------------------------------------------------------------------- 44003
Bonobo            -------------------------------------------------------------------------------- 42342
OliveBaboon       -------------------------------------------------------------------------------- 43620
Orangutan         -------------------------------------------------------------------------------- 43335
```

```
Majority          --------------------------------------------------------------------------------

                           54250     54260     54270     54280     54290     54300     54310     54320

Human             -------------------------------------------------------------------------------- 43259
Coquerel'sSifaka  -------------------------------------------------------------------------------- 36562
Ma'sNightMonkey   GGCGCATGCCTGTAATCCCAGCTCCTCAGGAGGCTGAGGCAGGAGAATTGCCTGAACCCAGGAGGCGGAGGTTGCGGTGA 38348
VervetAGM         -------------------------------------------------------------------------------- 44682
Gorrilla          -------------------------------------------------------------------------------- 43753
GrabEatingMacaque-------------------------------------------------------------------------------- 44072
Macaque           -------------------------------------------------------------------------------- 44863
PigTailedMacaque  -------------------------------------------------------------------------------- 44003
Bonobo            -------------------------------------------------------------------------------- 42342
OliveBaboon       -------------------------------------------------------------------------------- 43620
Orangutan         -------------------------------------------------------------------------------- 43335

Majority          --------------------------------------------------------------------------------

                           54330     54340     54350     54360     54370     54380     54390     54400

Human             -------------------------------------------------------------------------------- 43259
Coquerel'sSifaka  -------------------------------------------------------------------------------- 36562
Ma'sNightMonkey   GCCGAGATCGTGCCATTGCACTCCAGCCTGAGTAACAAGAGTGAAACTCCGTCTCAAAAATAAAAATAAAAATAAAAATA 38428
VervetAGM         -------------------------------------------------------------------------------- 44682
Gorrilla          -------------------------------------------------------------------------------- 43753
GrabEatingMacaque-------------------------------------------------------------------------------- 44072
Macaque           -------------------------------------------------------------------------------- 44863
PigTailedMacaque  -------------------------------------------------------------------------------- 44003
Bonobo            -------------------------------------------------------------------------------- 42342
OliveBaboon       -------------------------------------------------------------------------------- 43620
Orangutan         -------------------------------------------------------------------------------- 43335

Majority          ---------------------------AAAAATACAACTGTTCCCAAGTAGCATTAGAATTTGATTTTGTTAAATTTGTA

                           54410     54420     54430     54440     54450     54460     54470     54480

Human             ---------------------------AAAAATACAACTGTTCCTAAGTAGCATTAGAATTTGATTTTGTTAAATTTGTA 43312
Coquerel'sSifaka  -------------------------------------------------------------------------------- 36562
Ma'sNightMonkey   AAAAAATAAAAAAAAGTGAAATATTTCAAAAATACAACTGTTCCCAGGTAGCATTAGAATTTGATTTTGTTAAATCTGTA 38508
VervetAGM         ---------------------------AAAAATACAACTGTTCCCAAGTAGCATTAGAATTTGATTTTGTTAAATTTGTA 44735
Gorrilla          ---------------------------AAAAATACAACTGTTCCTAAGTAGCATTAGAATTTGATTTTGTTAAATTTGTA 43806
GrabEatingMacaque---------------------------AAAAATACAACTGTTCCCAAGTAGCATTAGAATTTGATTTTGTTAAATTTGTA 44125
Macaque           ---------------------------AAAAATACAACTGTTCCCAAGTAGCATTAGAATTTGATTTTGTTAAATTTGTA 44916
PigTailedMacaque  ---------------------------AAAAATACAACTGTTCCCAAGTAGCATTAGAATTTGATTTTGTTAAATTTGTA 44056
Bonobo            ---------------------------AAAAATACAACTGTTCCTAAGTAGCATTAGAATTTGATTTTGTTAAATTTGTA 42395
OliveBaboon       ---------------------------AAAAATACAACTGTTCCCAAGTAGCATTAGAATTTGATTTTGTTAAATTTGTA 43673
Orangutan         ---------------------------AAAAATATGACTGTTCCCAAGTAGCATTAGAATTTGATTTTGTTAAGTTTGTA 43388
```

```
Majority          ACTATTTAGCAGTGACTAGGAGGCTTGACAATTCTAAGGTATAGTCTCTTTAATTTTTT-AGTGATTTCTTTGTAGCTAT
                          54490     54500     54510     54520     54530     54540     54550     54560
Human             ACTATTTAGCAGTGACTATGAGGCTTGACAATTCTAAGGTACAGTCTCTTTAATTTTCT-AGTGATTTCTTTGTAGCTAC 43391
Coquerel'sSifaka  --------------------G-------GA----TAAGGTATAG-------AA--------------------------- 36577
Ma'sNightMonkey   ACTATTTAGCAGTGAGTAGGAGGCTTGACAATTGTAAGGCATGGTCTCTTTAATTTTTTTAGTGATTTTGTTGTAGCCAT 38588
VervetAGM         ACTATTTAGCAGTGACTAGGAGACTTGACAATTCTAAGGTATAGTCTCTTTAATTTTTT-AGTGATTTCTTTGTAGATAT 44814
Gorrilla          ACTATTTAGCAGTGACTATGAGGCTTGGCAATTCTAAGGTACAGTCTCTTTAATTTTCT-AGTGATTTCTTTGTAGCTAC 43885
GrabEatingMacaqueACTATTTAGCAGTGACTAGGAGACTTGACAATTCTAAGATATAGTCTCTTTAATTTTTT-AGTGATTTCTTTGTAGATAT 44204
Macaque           ACTATTTAGCAGTGACTAGGAGACTTGACAATTCTAAGATATAGTCTCTTTAATTTTTT-AGTGATTTCTTTGTAGATAT 44995
PigTailedMacaque  ACTATTTAGCAGTGACTAGGAGACTTGACAATTCTAAGGTATAGTCTCTTTAATTTTTT-AGTGATTTCTTTGTAGATAT 44135
Bonobo            ACTATTTAGCAGTGACTGTGAGGCTTGACAATTCTAAGGTACAGTCTCTATAATTTTCT-AGTGATTTCTTTGTAGCTAC 42474
OliveBaboon       ACTATTTAGCAGTGACTAGGAGACTTGACAATTCTAAGGTATAGTCTCTTTAATTTTTT-AGTGATTTCTTTGTAGATAT 43752
Orangutan         ACTATTTAGCAGTGACTACGAGGCTTGACAATTCTAAGGTACAGTCTCTTTAATTTTCT-AGTGATTTCTTTGTAGCTAC 43467

Majority          GGAACTGGAATATCCCTTTAGGTA----TACGTATTTCTGCCAGTGCTGATTGTTACTCTGTAATAGCAGGATTTTTTGA
                          54570     54580     54590     54600     54610     54620     54630     54640
Human             GGAACTGGAATATCCCTTTAGGTA----TACATATTTCTGCCAGTGGTGATTGTTACTCTGTAATAGCAGGATTTTCTGA 43467
Coquerel'sSifaka  ---AATTGTATGTACTTAAAG------------ATTTCTACCA------------------------------------- 36605
Ma'sNightMonkey   GGAAATGGAATATCCCTTTAGGTA----TACATATTTCTGCCAGTGCTGATTGTTACTCTGTAA-AGCAGGACTTAAAAA 38663
VervetAGM         GGAACTGGAATATCCCTTTAGGTA----TACGTATTTCTGCCAGTGCTGATTGTTACTCTGTAATAGCAGGATATTTTGA 44890
Gorrilla          GGAACTGGAATATCCCTTTAGGTA----TACATATTTCTGCCAATGCTGATTGTTACTCTGTAATAGCAGGATTTTTTGA 43961
GrabEatingMacaqueGGAACTGGAATATCCCTTTAGGTA----TACGTATTTCTGCCAGTGCTGATTGTTACTCTATAATAGCAGGATTTTTTGA 44280
Macaque           GGAACTGGAATATCCCTTTAGGTA----TACGTATTTCTGCCAGTGCTGATTGTTACTCTATAATAGCAGGATTTTTTGA 45071
PigTailedMacaque  GGAACTGGAATATCCCTTTAGGTA----TACGTATTTCTGCCAGTGCTGATTGTTACTCTATAATAGCAGGATTTTTTGA 44211
Bonobo            GGAACTGGAATATCCCTTTAGGTA----TACATATTTCTGCCAATGCTGATTGTTACTCTGTAATAGCAGGATTTTCTGA 42550
OliveBaboon       GGAACTGGAATATCCCTTTAGGTA----TATGTATTTCTGCCAGTGCTGATTGTTACTCTGTAATAGCAGGATTTTTTGA 43828
Orangutan         GGAACCGGAATATCCCTTTAGGTAGGTATACATATTTCTGCCATTGCCGATTGTTACTCTGTAATAGCAGGATTTTTTGA 43547

Majority          AAA-TTATTGCTAAACATGACATTTTGCTCTACCTGAAAGCAGTTAAACCATTCCAAAGAGTCTGGAACATTTTACTTAG
                          54650     54660     54670     54680     54690     54700     54710     54720
Human             AAA-TTATTGCTAAACATGACATTTTGCTCTACCTGAAAGCAGTTAAACCATTCCATAGAGTCTGGAACATTTTACTTAG 43546
Coquerel'sSifaka  --------------------------------------------------------------------------T-T---- 36607
Ma'sNightMonkey   AAAATTATTGCTAAAAATGACATTTTGCTCTACTGGAAAGCACTTAAACCATTCCATAGAGTCTGGAACATTTTACTTAG 38743
VervetAGM         AAA-TTATTGCTAAACATGACATTTTGCTCTACCTGAAAGCAGTTAAACGATTCCAAAGAGTCTGGAACATTTTACTTAG 44969
Gorrilla          AAA-TTATTGCTAAACATGACATTTTGCTCTACCTGAAAGCAGTTAAACCATTCCATAGAGTCTGGAACATTTTACTTAG 44040
GrabEatingMacaqueAAA-TTATTGCTAAACATGACATTTTGCTCTACCTGAAAGCAGTTAAACCATTCCAAAGAGTCTGGAACATTTTACTTAG 44359
Macaque           AAA-TTATTGGTAAACATGACATTTTGCTCTACCTGAAAGCAGTTAAACCATTCCAAAGAGTCTGGAACATTTTACTTAG 45150
PigTailedMacaque  AAA-TTATTGCTAAACATGACATTTTGCTCTACCTGAAAGCAGTTAAACCATTCCAAAGAGTCTGGAACATTTTACTTAG 44290
Bonobo            AAA-TTATTGCTAAACATGACATTTTGCTCTACCTGAAAGCAGTTAAACCATTCCATAGAGTCTGGAACATTTTACTTAG 42629
OliveBaboon       AAA-TTATTGCTAAACATGACATTTTGCTCCACCTGAAAGCAGTTAAACCATTCCAAAGAGTCTGGAACATTTTACTTAG 43907
Orangutan         AAA-TTATTGCTAAACATGACAGTTTGCTCTACCTGAAAGTAGTTAAACCATTCCACAGAGTCTGGAACATTTTACTTAG 43626
```

```
Majority          AACTCTTTTAAGATTTTCATTTTGACTTTTACAGAC--ATATTTTTGAGGTGATGTCATTTTGTTTTGCTATAATGTTTT
                          54730     54740     54750     54760     54770     54780     54790     54800
Human             AACTCTTTTAAGATTTTCATTTTAACTTTTACAGAC--ATATTTTTGAGGTGATGTCATTCT-TTTTGCTATAATGTTTT 43623
Coquerel'sSifaka  AG------------------------T--AC--A------TTACTAC----ATGCCTTGTTAATTGAACATAAAGTCCT 36648
Ma'sNightMonkey   AACTCTTTTAAGATTTTCATTTTAACTTTTATAGACTTACATTTTTGCGGCAATGTCATTTTGTTTTGTTACAGTGTTTT 38823
VervetAGM         AACTCTTTCAAGATTTTCATTTTGACTTTTACAGAC--ATATTTTTGAGGTGATGTCATTTCGTTTTGCTATAATGTTTT 45047
Gorrilla          AACTCTTTTAAGATTTTCATTTTAACTTTTACAGAC--ATATTTTTGAGGTGATGTCATTCT-TTTTGCTATAATGTTTT 44117
GrabEatingMacaqueAACTCTTTTAAGATTTTCATTTTGACTTTTACAGAC--ATATTTTTGAGGTGATGTCATTTCGTTTTGCTATAATGTTTT 44437
Macaque           AACTCTTTTAAGATTTTCATTTTGACTTTTACAGAC--ATATTTTTGAGGTGATGTCATTTCGTTTTGCTATAATGTTTT 45228
PigTailedMacaque  AACTCTTTTAAGATTTTCATTTTGACTTTTACAGAC--ATATTTTTGAGGTGATGTCATTTCGTTTTGCTATAATGTTTT 44368
Bonobo            AACTCTTTTAAGATTTTCATTTTAACTTTTACAGAC--ATATTTTTGAGGTGATGTCATTCT-TTTTGCTATAATGTTTT 42706
OliveBaboon       AACTCTTTTAAGATTTTCATTTTGACTTTTACAGAC--ATATTTTTGAGGTGATGTCATTTCGTTTTGCTATAATGTTTT 43985
Orangutan         AACTCTTTTAAGATTTTCATTTTAACTTTTACAGAC--ATATTTTTGAGGTGATGTCATTTTGTTTTGCTATAATGTTTT 43704

Majority          GAGAGTATTCCAGTAGATTATTTCATAGTTTTACTATTTATGAAGGTTTTAACAACTTA-TCTTCTTGC-----------
                          54810     54820     54830     54840     54850     54860     54870     54880
Human             GAAAGTATTCCAGTAGATTATTTCATAGTTTTACTATTTATAAAGGTTTTAACAACTCA-TCTTCTTGCATATAAAGGTA 43702
Coquerel'sSifaka  AATATTATT----TGAATTATTAC-TAGTTTTAC----------------A-TACCCAA--------------------- 36685
Ma'sNightMonkey   GAGAGTATTCCAGTAGATTATTTCAGAGTTTTAAGATTTATGAGCATTTTAACAACTTAACCTTCTTGC----------- 38892
VervetAGM         GAGAGTATTCCAGTAGATTATTTCATGGTTTTACTATTTATGAAGGCTTTAACAAGTTA-TCTTCTTGC----------- 45115
Gorrilla          GAAAGTATTCCAGTAGATTACTTCATAGTTTTACTATTTATGAAGGTTTTAACAACTCA-TCTTCTTGCATATAAAGGTA 44196
GrabEatingMacaqueGAGAGTATTCCAGTAGATTATTTCATAGTTTTACTATTTATGAAGGCTTTAACAAGTTA-TCTTCTTGC----------- 44505
Macaque           GAGAGTATTCCAGTAGATTATTTCATAGTTTTACTATTTATGAAGGCTTTAACAAGTTA-TCTTCTTGC----------- 45296
PigTailedMacaque  GAGAGTATTCCAGTAGATTATTTCATAGTTTTACTATTTATGAAGGCTTTAACAAGTTA-TCTTCTTGC----------- 44436
Bonobo            GAAAGTATTCCAGTAGATTATTTCATAGTTTTACTATTTGTAAAGGTTTTAACAACTCA-TCTTCTTGCATATAAAGGTA 42785
OliveBaboon       GAGAGTATTCCAGTAGATTATTTCATAGTTTTACTATTTATGAAGGCTTTAACAAGTTA-TCTTCTTGC----------- 44053
Orangutan         GAAAGTATTCCAGTATATTATTTCATAGTTTTACTATTTATGAGGGTTTTAACAACTTA-TCTTCTT------------- 43770

Majority          ------------------------------------------------------------ATATAAAGGTAGTGAGAGCC
                          54890     54900     54910     54920     54930     54940     54950     54960
Human             GTGAGAGCCTTATTTCATAGTGTTACTATTTATGAGGGTTTTAACAACTTATCTTCTTGTATATAAAGGTAGTGAGAGCC 43782
Coquerel'sSifaka  -------------------------------------------------------------------------------- 36685
Ma'sNightMonkey   ------------------------------------------------------------ATATAAAGGTAGTGAGAGCC 38912
VervetAGM         ------------------------------------------------------------ATATAAAGGTAGTGAGAGCC 45135
Gorrilla          GTGAGAGCCTTATTTCATAGTGTTA---TTTATGAGGGTTTTAACAACTTACCTTCTTGTATATAAAGGTAGTGAGAGCC 44273
GrabEatingMacaque------------------------------------------------------------ATATAAAGGTAGTGAGAGCA 44525
Macaque           ------------------------------------------------------------ATATAAAGGTAGTGAGAGCA 45316
PigTailedMacaque  ------------------------------------------------------------ATATAAAGGTAGTGAGAGCA 44456
Bonobo            GTGAGAGCCTTATTTCATAGTGTTACTATTTATGAGGGTTTTAACAACTTATCTTCTTGTATATAAAGGTAGTGAGAGCC 42865
OliveBaboon       ------------------------------------------------------------ATATAAAGGTAGTGAGAGCA 44073
Orangutan         ------------------------------C-----------------------------ATATAAGGGTAGTGAGAGCC 43791
```

```
Majority         TTCTCTAGGTGCCTTCTGTGTTTTTCCAGCTCATGCCTAATATACTTTTGACAACTAGGCCACACAACTACTTGGAAATA
                          54970     54980     54990     55000     55010     55020     55030     55040
Human            TTCTCTAGGTGCCTTCTGTGTTTTTCCAGCTCATGCCTAATATGCTTTTGACAACTACACCACACAACTACTTGGAAATA 43862
Coquerel'sSifaka ----ACAAAAG-TTTCTGGGTTT------------------------------AATGGG---------------GGAATG 36715
Ma'sNightMonkey  TTCTCTAGGTGCTTTCTGTGTTTTTCCAGCTCATGCCTGATATACTTTTGGCAACTAGGCCACACAACTACTTACAAATA 38992
VervetAGM        TTCTCTAGGTGCCTTCTGTGTTTTTCCAGCTCATGCCTAACATACTTTGGACAACTAGGCCATACAACTACTTGGAAATA 45215
Gorrilla         TTCTCTAGGTGCCTTCTGTGTTTTTCCAGCTCATGCCTAATATGCTTTTGACAACTATGCCACACAACTACTTGGAAGTA 44353
GrabEatingMacaqueTTCTCTAGGTGCCTTCTGTGTTTTTCCAGCTCATGCCTAATATACTTTGGACAACTAGGCCACACAACTACTTGGAAATA 44605
Macaque          TTCTCTAGGTGCCTTCTGTGTTTTTCCAGCTCATGCCTAATATACTTTGGACAACTAGGCCACACAACTACTTGGAAATA 45396
PigTailedMacaque TTCTCTAGGTGCCTTCTGTGTTTTTCCAGCTCATGCCTAATATACTTTGGACAACTAGGCCACACAACTACTTGGAAATA 44536
Bonobo           TTCTCTAGGTGCCTTCTGTGTTTTTCCAGCTCATGCCTAATATGCTTTTGACAACTACGCCACACAACTACTTGGAAATA 42945
OliveBaboon      TTCTCTAGGTGCCTTCTTTGTTTTTCCAGCTCATGCCTAATATACTTTGGACAACTAGGCCACACAACTACTTGGAAATA 44153
Orangutan        TTCTCTAGGTGCCTTCTGTGTTTTTCCAGCTCATGCCTAATATACTTTTGACAACTAGGCCACACAACTACTTGGAAATA 43871

Majority         CAAGAGTTAATAG-TTTCCTTTTACGGAAACCTCGTGAGGCTTTTTGTGTGGTGGGTGTTGCAGA-AACAGCCAACAGAA
                          55050     55060     55070     55080     55090     55100     55110     55120
Human            CAAGAGTTAATAG-TTACCTTTTACGGAAACCTGGTGAGGCTTTTTGCGTGGTGGCTGTTGCAGA-AACAGCCAACAGAA 43940
Coquerel'sSifaka GAAGA---AAAGG-----AT--CATGGGAAGAAATTCAGA-TTATTCTGTAGT-----CTTCTGA--------A---GAA 36768
Ma'sNightMonkey  CAAGATTTAATAG-TTGCCTTTTACGGAAACCTAGTGAGGCTTTTTGTGTGGTGGCTGTTGCAGTTAACAACCAGCAGAA 39071
VervetAGM        CAAGAGTTAATAGGTTTCCTTTTATGGAAACCTCGTGAGGCTTTTTGTGTGGTGGGTGTTGCAGA-AACAGCCAACAGAA 45294
Gorrilla         CAAGAGTTAATAG-TTGCCTTTTACGGAAACCTAGTGAGGCTTTTTGTGTGGTGGCTGTTGCAGA-AACAGCCAACAGAA 44431
GrabEatingMacaqueCAAGAGTTAATAGGTTTCCTTTTACGGAAACCTCGTGAGGCTTTTTGTGTGGTGGGTGTTGCAGA-AACAGCCAACAGAA 44684
Macaque          CAAGAGTTAATAGGTTTCCTTTTACGGAAACCTCGTGAGGCTTTTTGTGTGGTGGGTGTTGCAGA-AACAGCCAACAGAA 45475
PigTailedMacaque CAAGAGTTAATAGGTTTCCTTTTACGGAAACCTCGTGAGGCTTTTTGTGTGGTGGGTGTTGCAGA-AACAGCCAACAGAA 44615
Bonobo           TAAGAGTTAATAG-TTACCTTTTACGGAAACCTAGTGAGGCTTTTTGTGTGGTGGCTGTTGCAGA-AACAGCCAACAGAA 43023
OliveBaboon      CAAGAGTTAATAGGTTTCCTTTTACGGAAACCTCGTGAGGCTTTTTGTGTGGTGGGTGTTGCAGA-AACAGCCAACAGAA 44232
Orangutan        CAAGAGTTAATAA-TTACCTTTTACGGAAACCTAGTGAGGCTTTTTGTGTGGTGGCTGTTGCAGA-AACAGCCAACAGAA 43949

Majority         TAACAGAAGTACCACCTATCTGCATCTACCTGCTTTTTTTCTT--GTTAAGTCTTCTTTGGAATTTGTTTCTCATACAGG
                          55130     55140     55150     55160     55170     55180     55190     55200
Human            TAGCAGAAGTACCACCTATCTGCACCTACCTGCTTTTTTTCTC--TTTAACTCTTCTTTGGAATTTGTTTCTCATACAGG 44018
Coquerel'sSifaka TCATAGAACTGTAACC---CTG-ATAGACCCAATTCT-------------------------ACTTACTTCTCTGATAGG 36819
Ma'sNightMonkey  AAACAGAAGTACCACCAATCTGCATCTACCTGCTTTTTTTCTCTCGTTAAATCTTCCTTGGAATTTGTTTCTCATATAGG 39151
VervetAGM        TAACAGAAGTACCACCTATCTGCATCTACCTGCTTTTTTTCTT--GTTAAGTCTTCTTTGGAATTTGTTTCTTTTACAGG 45372
Gorrilla         TAGCAGAAGTACCACCTATCTGCATCTACCTGCTTTTTTTCTC--GTTAACTCTTTTTTGGAATTTGTTTCTCATACAGG 44509
GrabEatingMacaqueTAACAGAAGTACCACCTATCTGCATCTACCTGCTTTTTTTCTT--GTTAAGTCTTCTTTGGAATTTGTTTCTCATACAGG 44762
Macaque          TAACAGAAGTACCACCTATCTGCATCTACCTGCTTTTTTTCTT--GTTAAGTCTTCTTTGGAATTTGTTTCTCATACAGG 45553
PigTailedMacaque TAACAGAAGTACCACCTATCTGAATCTACCTGCTTTTTTTCTT--GTTAAGTCTTCTTTGGAATTTGTTTCTCATACAGG 44693
Bonobo           TAGCAGAAGTACCACCTATCTGCATCTACCTGCTTTTTTTCTC--TTTAACTCTTCTTTGGAATTTGTTTCTCATACAGG 43101
OliveBaboon      TAACAGAAATACCACCTATCTGCATCTACCTGCTTTTTTTCTT--GTTAAGTCTTCTTTGGAATTTGTTTCTCATACAGG 44310
Orangutan        TAACAGAAGTACCACCTATCTGCATCTACCTGCTTTTTTTCTC--GTTAACTCTTCTTTAGAATTTGTTTCTCATACAGG 44027
```

```
Majority          AAAGAGTAGCAGCATTCATTTTTTTTT-CTTAGCTTAGAGTCTTTTT-AT---TTTGTTTTTTGTTTTTCTAAAGCTGGA

                          55210     55220     55230     55240     55250     55260     55270     55280

Human             AAAGAGTAGCAGCATTCATTTTTTTTTTCTTAGCTTAGAGTCTTTTT-AT---TTTGTTTTTTGTTTTTCTAAAGCTAGA 44094
Coquerel'sSifaka  -----GTGATAA-ATGAATGGCCT--------GC----------------------------ATGTTTCCTAAGCTGAG 36856
Ma'sNightMonkey   AAAGAGTAGCAGCATTCATTGTTTTT--CTTAGCTTAGAGTTTTTTTTAT---TTTGTTTTTTGTTTTTCTAAAGCTAGA 39226
VervetAGM         AAAGAGTAGCAGCATTCATTTTTTTTTTCTTAGCTTAGAGTCTTTT--ATTTATTTATTTATTGTTTTTCTAAAGCTGGA 45450
Gorrilla          AAAGAGTAGCAGCATTCATTTTTTTTTC--TTAGCTTAGAGTCTTTTT-AT---TTTGTTTTTTGTTTTTCTAAAGCTAGA 44583
GrabEatingMacaqueAAAGAGTAGCAGCATTCATTTTTTTTT-CTTAGG----AGTCTTTTT-ATTTATTTATTTTTTGTTTTTCTAAAGCTGGA 44836
Macaque           AAAGAGTAGCAGCATTCATTTTTTTTT-CTTAG-----AGTCTTTTT-ATTTATTTATTTTTTGTTTTTCTAAAGCTGGA 45626
PigTailedMacaque  AAAGAGTAGCAGCATTCATTTTTTTT--CTTAG-----AGTCTTTTT-ATTTATTTATTTTTTGTTTTTCTAAAGCTGGA 44765
Bonobo            AAAGAGTAGCAGCATTCATTTTTTTTT-CTTAGCTTAGAGTCTTTTT-AT---TTTGTTTTTTGTTTTTCTAAAGCTAGA 43176
OliveBaboon       AAAGAGTAGCAGCATTCATTTTTTTTT-CTTAGCTTAGAGTCTTTTT-ATTTATTTATTTTTTGTTTTTCTAAAGCTGGA 44388
Orangutan         AAACAGTAGCAGCATTCGTTTTTTTTC--TTAGCTTAGAGTCTTTTT-AT---TTTGTTTTTTGTTTTTCTAAAGCTAGA 44101

Majority          AAACACTGCTTTGCCTTGAAATCTAAAACTGCTTAGAATTAAAACTGCTTTGCCTCTCAGTTTCGTATTTGATAAAGAGA

                          55290     55300     55310     55320     55330     55340     55350     55360

Human             AAACACTGCTTTGCCTTGAAGTCTAAAACTGCTTAGAATTAAAACTTCTTTGCCTCTCAGTTTCATATTTGATAAAGAGA 44174
Coquerel'sSifaka  GGCCAC--CCTTTC--------------------------------TTTTGCCCCCACCCCTTTTTTTTACTA----GG 36897
Ma'sNightMonkey   AAGCACTGCTTTGCCTTTAAATCTAAAACTGCTTAGAATTAAAACTGCTTTGCCTCTCAGTTTCATATTTGATA----GA 39302
VervetAGM         AAACACTGCTTTGCCTTGAAATCTAAAACTGCTTAGAATTAAAACTGTTTTGCCTCTCAGGTTCGTATTTGATAAAGAGA 45530
Gorrilla          AAACACTGCTTTGCCTTGAAGTCTAAAACTGCTTAGAATTAAAACTGCTTTGCCTCTCAGTTTCATATTTGATAAAGAGA 44663
GrabEatingMacaqueAAACACTGCTTTGCTTTGAAATCTAAAACTGCTTAGAATTAAAACTGCTTTGCCTCTCAGTTTCGTATTTGATAAAGAGA 44916
Macaque           AAACACTGCTTTGCTTTGAAATCTAAAACTGCTTAGAATTAAAACTGCTTTGCCTCTCAGTTTCGTATTTGATAAAGAGA 45706
PigTailedMacaque  AAACACTGCTTTGCTTTGAAATCTAAAACTGCTTAGAATTAAAACTGCTTTGCCTCTCAGTTTCGTATTTGATAAAGAGA 44845
Bonobo            AAACACTGCTTTGCCTTGAAGTCTAAAACTGCTTAGAATTAAAACTGCTTTGCCTCTCAGTTTCATATTTGATAAAGAGA 43256
OliveBaboon       AAACACTGCTTTGCCTTGAAATCTAAAACTGCTTAGAATTAAAACTGCTTTGCCTCTCAGTTTCGTATTTGATAAAGAGA 44468
Orangutan         AAACACTGCTTTGCCTTGAAGTCTAAAACTGTTTAGAATTAAAACTGCTTTGCCTTTCAGTTTCATATTTGATAAAGAGA 44181

Majority          GTTAGGAAAACTTTTATAAATGTGTG--TCTCTGAAAGACTTGGAGAATGAAGATGTACTTAAGTTTTGTCACTAGTTTT

                          55370     55380     55390     55400     55410     55420     55430     55440

Human             GTTAGGAAAACTTTTATAAATGTGTG--TCTCTGAAAGACTTGGAGAATGAAGATGTACCTAAGTTTTGTCACTGATTTT 44252
Coquerel'sSifaka  CTTGGG----TTTTTATATATGAAAA--------TA-GGCTTGGT---------TATGATTAAA---------------- 36939
Ma'sNightMonkey   GTTAGGAAAGCTTTTATAAATGTATGAGTCTCTGAAAGACTTGGAGAATGAAGATGTACTTAAGTTTTGTCACTAGTGTT 39382
VervetAGM         GTTAGGAAAACTTTTATAAATGTGTG--CCTCTGAAAGACTTGGAGAATTAAGATGTACTTAAGTTTTGTCGCTAGTTTT 45608
Gorrilla          GTTAGGAAAACTTTTATAAATGTGTG--TCTCTGAAAGACTTGGAGAATGAAGATGTACTTAAGTTTTGTCACTGGTTTT 44741
GrabEatingMacaqueGTTAGGAAAACTTTTATAAATGTGTG--CCTCTGAAAGACTTGGAGAATGAAGATGTACTTAAGTTTTGTCACTAGTTTT 44994
Macaque           GTTAGGAAAACTTTTATAAATGTGTG--CCTCTGAAAGACTTGGAGAATGAAGATGTACTTAAGTTTTGTCACTAGTTTT 45784
PigTailedMacaque  GTTAGGAAAACTTTTATAAATGTGTG--CCTCTGAAAGACTTGGAGAATGAAGATGTACTTAAGTTTTGTCACTAGTTTT 44923
Bonobo            GTTAGGAAAACTTTCATAAATGTGTG--TCTCTGAAAGACTTGGAGAATGAAGATGTACCTAAGTTTTGTCACTGGTTTT 43334
OliveBaboon       GTTAGGAAAACTTTTATAAATGTGTG--CCTCTGAAAGACTTGGAGAATGAAGATGTACTTAAGTTTTGTCACTAGTTTT 44546
Orangutan         GTTAGGGAAACTTTTATAAATGTGTG--TCTCTGAAAGACTTGGAGAATGAAGATGTACTTAAGTTTTGTCACTGGTTTT 44259
```

```
Majority          CATTGGACGGAAG-------------------------CATAAGGTTTTGCAGTGTTGTATTTTATATTGGTACACAGTT
                           55450     55460     55470     55480     55490     55500     55510     55520
Human             CATTGGACAGAAG-------------------------TATAAGGTTTTGCCGTGTCGTATTTTATATTTGTACACAGTT 44307
Coquerel'sSifaka  ----------------------------------------------------GGAATCCCGTTTCCTAGTTGCACATT-CT 36967
Ma'sNightMonkey   TGTTGGACAGAAGTTTAAGACGTTGTATAAGTATAAGACAGAAGTATTTACAACGTTGTATTTTATACTGGTACACAGTT 39462
VervetAGM         CATTGGACGGAAG-------------------------CATAAGTTTTTGCAGTGTTGTATTTTATATTGGTACACAGTT 45663
Gorrilla          CATTGGACAGAAG-------------------------TATAAGGTTTTGCCGTGTTGTATTTTATATTTGTACACAGTT 44796
GrabEatingMacaqueCACTGGACGGAAG-------------------------CATAAGGTTTTGCAGTGTTGTATTTTATATTGGTACACAGTT 45049
Macaque           CACTGGACGGAAG-------------------------CATAAGGTTTTGCAGTGTTGTATTTTATATTGGTACACAGTT 45839
PigTailedMacaque  CACTGGATGGAAG-------------------------CATAAGGTTTTGCAGTGTTGTATTTTATATTGGTACACAGTT 44978
Bonobo            CATTGGACAGAAG-------------------------TATAAGGTTTTGCCGTGTTGTATTTTATATTTGTACACAGTT 43389
OliveBaboon       CATTGGAAGGAAG-------------------------CATAAGGTTTTGCAGTGTTGTATTTTATATTGGTACACAGTT 44601
Orangutan         CATT------------------------------------A-GGTTTTGCAGTGTTGTATTTTATATTTGTACACAGTT 44301

Majority          GAAAGTATAATGGTGAATGAGTAGGACTAAA-TAATTTACACATTATATGAGAAAGTAGGCCAGGTGCTGTGGCTCATGC
                           55530     55540     55550     55560     55570     55580     55590     55600
Human             GAAAGTATAACGGTGAATGAGTAGGACTAAAATAATTTACAAATTATATGAGAAAGTAGGCCAGGTGCTGTGGCTCATGC 44387
Coquerel'sSifaka  CATAGTATCA-----A-TTATTTGAAGTGAA--GACTTTCATAATATTT-----AGTA--CTACG--------------- 37017
Ma'sNightMonkey   GAAAGTATGA---TGAATGAGTAGGACTAAGATAATTTACAAATTATATGAGAAAGTATGCCAGGTGCGGTGGCTCACGC 39539
VervetAGM         GAAAGTATAATGGTGAATGAGTAGGACTAAA-TAATTTACACATTATATGAGAAAGTAGGCCAGGTGCTGTTGCTCACGC 45742
Gorrilla          GAAAGTATAACGGTGAATGAGTAGGACTAAAATAATTTACAAATTATCTGAGAAAGTAGGCCAGGTGCTGTGGCTCATGC 44876
GrabEatingMacaqueGAAAGTATAATGGTGAATGAGTAGGACTAAA-TAATTTACACATTATATGAGAAAGTAGGCCAGGTTCTGTTGCTCATGC 45128
Macaque           GAAAGTATAATGGTGAATGAGTAGGACTAAA-TAATTTACACATTATATGAGAAAGTAGGCCAGGTTCTGTTGCTCATGC 45918
PigTailedMacaque  GAAAGTATAATGGTGAATGAGTAGGACTAAA-TAATTTACACATTATATGAGAAAGTAGGCCAGG---T----------- 45043
Bonobo            GAAAGTATAACGGTGAATGAGTAGGACTAAAATAATTTACAAATTATATGAGAAAGTAGGCCAGGTGCTGTGGCTCATGC 43469
OliveBaboon       GAAAGTATAATGGTGAATGAGTAGGACTAAA-TAATTTACACATTATATGAGAAAGTAGGCCAGGTTCTGTTGCTCATGC 44680
Orangutan         GAAAGCATAATGGTGAATGAGTAGGACTAAAATAATTTACAAATTACATGAGAAAGTAGGCCAGGTGCTGTGGCTCATGC 44381

Majority          CTGTATTTCAGCACTTTGGGAGGCTGAGGCAGGTGGATCACTTGAGGCCAGGAGTTGGAGACCAGCCT---GGCCAACAT
                           55610     55620     55630     55640     55650     55660     55670     55680
Human             CTGTATTTCAGCACTTTGGGAGGCTGAGGCAGGTGGATCACTTGAGGCCAGGAGTTCGAGACCAGCCT---GGCCAACAT 44464
Coquerel'sSifaka  -------------------------------------------------------------------------------- 37017
Ma'sNightMonkey   CTGTATTTCAGCACTTTAGGAGGCTGAGGTGGGCAAATCACTTGAGGCCAGGAGTTTGAGACCAGTCT---GGCCAACAT 39616
VervetAGM         CTGTATTTCAGCACTTTGGGAGGCTGAGGCAGGTGGATCACTTGAGGCCAGGAGTTGAAGACCAGCCTCCTGGCCAACAT 45822
Gorrilla          CTGTATTTCAGCACTTTGGGAGGCTGAGGCAGGTGGATCACTTGAGGCCAGGAGTTCGAGACCAGCCT---GGCCAACAT 44953
GrabEatingMacaqueCTGTATTTCAGCACTTTGGGAGGCTGAGGCAGGTGGATCACTTGAGGCCAGGAGTTGAAGACCAGCCT---GGCCAACAT 45205
Macaque           CTGTATTTCAGCACTTTGGGAGGCTGAGGCAGGTGGATCACTTGAGGCCAGGAGTTGAAGACCAGCCT---GGCCAACAT 45995
PigTailedMacaque  ---------AG----------------A-TAGATAGAT------------------------------------------ 45055
Bonobo            CTGTATTTCAGCACTTTGGGAGGCTGAGGCAGGTGGATCACTTGAGGCCAGGAGTTCGAGACCAGCCT---GGCCAACAT 43546
OliveBaboon       CTGTATTTCAGCACTTTGGGAGGCTGAGGCAGGTGGATCACTTGAGGCCAGGAGTTGAAGACCAGCCT---GGCCAACAT 44757
Orangutan         CTGTATTTCAGCACTTTGGGAGGCTGAGGCAGGTGGATCACTTGAGGCCAGGAGTTAGAGACCAGCCT---GGCCGACAT 44458
```

```
Majority         GGTGAAACCCCATCTCTACTGAAAATACAAAAATTAGCTGGGTGTGGTGGTGCACGCCTGTATTCCCAGCTACATGGGAG

                          55690     55700     55710     55720     55730     55740     55750     55760

Human            GGTGAAACCCCATCTCTACTGAAAATAGAAAAATTAGCTGGGTGTGGTGGTGCACACCTGAATTCCCAGCTACATGGGAG 44544
Coquerel'sSifaka -----------AT-------AAAAATA----------CTTAGT------------------------AGCCACCT----- 37040
Ma'sNightMonkey  GATAAA--CCTGTGTCTACTAAAAATACAAAAATTAGCTGGATGTGGTGGTGCACACCTGTAATCCCAGCTACTTGGGCG 39694
VervetAGM        GGTGAAACCCCATCTCTACTGAAAATACAAAAATTAGCTGGGTGTGGTGGTGCACGCCTATATTCCCAGCTACATGGGAG 45902
Gorrilla         GGTGAAACCCCATCTCTACTGAAAATACAAAAATTAGCTGGGTGTGGTGGTGCACACCTGAATTCCCAGCTACATGGGAG 45033
GrabEatingMacaqueGGTGAAACCCCATCTCTACTGAAAATACAAAAATTAGCTGGGTGTGGTGGTGCACGCCTATATTCCCAGCTACATGGGAG 45285
Macaque          GGTGAAACCCCATCTCTACTGAAAATACAAAAATTAGCTGGGTGTGGTGGTGCACGCCTATATTCCCAGCTACATGGGAG 46075
PigTailedMacaque ----------------------AGAT--A-------GATAGAT------------------------AGATAGAT----- 45075
Bonobo           GATGAAACCCCATCTCTACTGAAAATACAAAAATTAGCTGGGTGTGGTGGTGCACACCTGAATTCCCAGCTACATGGGAG 43626
OliveBaboon      GGTGAAACCCCATCTCTACTGAAAATACAAAAATTAGCGGGGTGTGGTGGTGCACGCCTATATTCCCAGCTACATGGGAG 44837
Orangutan        GGTGAAACCCCATCTCTACTGAAAATACAAAAATTAGCTGGGTATGGTGGTGCACGCCTGAATTCCCAGCTACATGGGAG 44538

Majority         GCTGTGGCACGAGAATCACCTAAACCTGGGAAGTGGAGGTTGTAGTCAACCAAGATCACGCCACTGCACTCCAGCCTGGC

                          55770     55780     55790     55800     55810     55820     55830     55840

Human            GCTGTGGCACGAGAATCACTTAAACCTGGGAAGTGGAGGTTGTAGTCAACCAAGATCATGCCACTGCACTCCAGCCTGGC 44624
Coquerel'sSifaka ---------------TCTC---------------GAAGCTAGT--------A--------------------------- 37054
Ma'sNightMonkey  GCTGTGGCATGAGAATCACCTAAACCTGGGAAGTGGAGGTTGTAGTGAAGTAAGAGCACGCCACTGCACTCCAGC----- 39769
VervetAGM        GCTGTGGCACGAGAATCACCTAAACCTGGGAAGTGGAGATTATAGTCAACCAAGATCACGCCACTGCACTCCAGCCTGGC 45982
Gorrilla         GCTGTGGCACGAGAATCACTTAAACCTGGGAAGTGGAGGTTGTAGTCAACCAAGATCACGCCACTGCACTCCAGCCTGGC 45113
GrabEatingMacaqueGCTGTGGCACGAGAATCACCTAAACCTGGGAAGTGGAGGTTGTAGTCAACCAAGATCACGCCACTGCACTCCAGCT---- 45361
Macaque          GCTGTGGCACGAGAATCACCTAAACCTCGGAAGTGGAGGTTGTAGTCAACCAAGATCACGCCACTGCACTCCAGCT---- 46151
PigTailedMacaque ------------------------------------AG------------------------------------------ 45077
Bonobo           GCTGTGGCACGAGAATCACTTAAACCTGGGAAGTGGAGGTTGTAGTCAACCAAGATCATGCCACTGCACTCCAGCCTGGC 43706
OliveBaboon      GCTGTGGCACGAGAATCACCTGAACCTGGGAAGTGGAGGTTGTAGTCAACCAAGATCACGCCACTGCACTCCAGCCTGGC 44917
Orangutan        GCTGTGGCACAAGAATCACTTAAACCTGGGAAGTGGAGGTTGTAGTCAACCAAGATCACGCCACTGTACTCCAGCCTGGC 44618

Majority         AATACAGCAAGACTCTGTCTC--ATAGAT-AGATAGATAGATAGATAGATAGATAGAT-GAT-GATA----GATAGATAG

                          55850     55860     55870     55880     55890     55900     55910     55920

Human            AATACAGCAAGACTCTGTCT-------------------------TAGATAGATAGATAGATAGATA----GATAGATAG 44675
Coquerel'sSifaka ---------------------------AC-----------------TAACTGAAACAGT--------------------- 37069
Ma'sNightMonkey  --------AAGACT--GTCTC----AGAT-AGATAGATAGATAGATAGATAGATAGAT-------------A------- 39813
VervetAGM        AATACAGCAAGACTCTGTCTC--ATAGATTAGATAGATAGATAGATGGATGGATGGATGGATGGATGGATGGATGGATGG 46060
Gorrilla         AATACAGCAAGACTCTGTCA----TAGAT-AGATAGATAGATAGATAGATAGATAGATAGATAGATA----GATAGATAG 45184
GrabEatingMacaqueAGTACAGCAAGACTCTGTCTC--ATAGATTAGATGGATGGATGGATGGATGGATGGAT---------------------- 45417
Macaque          AGTACAGCAAGACTCTGTCTC--ATAGATTAGATGGATGGATGGATGGATGGATGGATGGATGGATAGATAGATAGATAG 46229
PigTailedMacaque -----------A------------TAGAT-AGATAGAGAGAGAGAGAGAGAGAGAGAG---------------------- 45111
Bonobo           AATACAGCAAGACTCTGTCTCAGATAGAT-AGATAGATAGATAGATAGATAGATAGATAGATAGATA----GATAGATAG 43781
OliveBaboon      AATACAGCAAGACTCTGTCTC--ATAGATTAGATGGATGGATGGATGGATGAATGGAT---------------------- 44973
Orangutan        AATACAGCAAGACTCTGTCATTGATAGAT-AGATAGATAGATAGATAGATAGATAGATAGATAGATAATAAGATAGATAG 44697
```

```
Majority          ATA-----------------------------------------------------------------------------
                           55930     55940     55950     55960     55970     55980     55990     56000
Human             ATA----------------------------------------------------------------------------- 44678
Coquerel'sSifaka  -------------------------------------------------------------------------------- 37069
Ma'sNightMonkey   -------------------------------------------------------------------------------- 39813
VervetAGM         ATGGATGGATGGATTATAGTCAACCAAGATCACGCCACTGCACTCCAGCCTGGCAATACAGCAAGACTCTGTCTCATAGA 46140
Gorrilla          ATA----------------------------------------------------------------------------- 45187
GrabEatingMacaque -------------------------------------------------------------------------------- 45417
Macaque           ATAGATAGATAGAT------------------------------------------------------------------ 46243
PigTailedMacaque  -------------------------------------------------------------------------------- 45111
Bonobo            ATA----------------------------------------------------------------------------- 43784
OliveBaboon       -------------------------------------------------------------------------------- 44973
Orangutan         ATAATAAGATAAGATA-----------GA-----------------------------------------------TAAG 44719

Majority          -------------------------------------------------------GAT-GATAGATAGATAGATACTGTG
                           56010     56020     56030     56040     56050     56060     56070     56080
Human             -------------------------------------------------------------------GATAGATACTGTG 44691
Coquerel'sSifaka  -------------------------------------------------------------------------------- 37069
Ma'sNightMonkey   -------------------------------------------------------------------GATAGATATTGTG 39826
VervetAGM         TTAGATAGATAGATGGATGGATGGATGGATGGATGGATGGATGGATGGATGGATAGATAGATAGATAGATAGATACTGTG 46220
Gorrilla          -------------------------------------------------------------------GATAGATACTGTG 45200
GrabEatingMacaque ----------------------------------------------AGATAGATAGATAGATAGATAGATAGATACTGTG 45451
Macaque           ----------------------------------------------AGATAGATAGATAGATAGATAGATAGATACTGTG 46277
PigTailedMacaque  ----------------------------------------------AGAGAGATAGATAGATAGATAGATAGATACTGTG 45145
Bonobo            -------------------------------------------------------------------GATAGATACTGTG 43797
OliveBaboon       ------------------------------------------------------GGATGGATAGATAGATAGATACTGTG 44999
Orangutan         ATAGATAATAAGATAGATAATAAGATAGATA---AGATAGATAGATAGATAG--GATAGATAAGATAGATAGATATTGTG 44794

Majority          AAACCCAG-TGCTAGAGTGTTTATA-TT-AATAAGTAAAGATAGGTAGAGTATCGGAACAAAAACAGCATGGGAAACT-A
                           56090     56100     56110     56120     56130     56140     56150     56160
Human             AAACCCAG-TGCTAGAGTGTTTATA-TTTAATAAGTAAAGATAGGTAGAGTATCGGAACAAAAACAGCATGGGAAACT-A 44768
Coquerel'sSifaka  -------------------------------------------------------------------------------- 37069
Ma'sNightMonkey   AAACCCAGGTGCTAGAGTGTTTATAATTTAATAAGTAAAGATAGGTAGAACATTGGAACAAAAATAGCATGGGAAACT-A 39905
VervetAGM         AAACCCAG-TGCTAGAGTGTTTATG-TT-AATCAGTAAAGATAGGTAGAGTATCGGAACAAAAACAGCATGGGAAACTTA 46297
Gorrilla          AAACCCAG-TGCTAGAGTGTTTATA-TTTAATAAGTAAAGATAGGTAGAGTATCGGAACAAAAACAGCATGGGAAACT-A 45277
GrabEatingMacaque AAACCCAG-TGCTAGAGTGTTTATA-TT-AATAAGTAAAGATAGGTAGAGTATCGGAACAAAAACGGCATGGGAAACTTA 45528
Macaque           AAACCCAG-TGCTAGAGTGTTTATA-TT-AATAAGTAAAGATAGGTAGAGTATCGGAACAAAAACAGCATGGGAAACTTA 46354
PigTailedMacaque  AAACCCAG-TGCTAGAGTGTTTATA-TT-AATAAGTAAAGATAGGTAGAGTATCGGAACAAAAACAGCATGGGAAACTTA 45222
Bonobo            AAACCCAG-TGCTAGAGTGTTTATA-TTTAATAAGTAAAGATAGGTAGAGTATCGGAACAAAAACAGCATGGGAAACT-A 43874
OliveBaboon       AAACCCAG-TGCTAGAGTGTTTATA-TT-AATAAGTAAAGATAGGTAGAGTATCGGAACAAAAACAGCATGGGAAACTTA 45076
Orangutan         AAACCCAG-TGCTAGAGTGTTTATA-TTTAATAAGTACAGATAGGTAGAGTATTGGAACAAAAACAGCATGGGAAACT-A 44871
```

```
Majority          ACAGTTTTGGGGAATTAATGAGTAATCGGGATTCAAAGCAAGTATCAGATGAGTCAGGGGAAATGAGAAAAGTAGAAGTG
                          56170     56180     56190     56200     56210     56220     56230     56240
Human             ACAATTTTGGGGAATGAATGAGTAATCAGGATTCAAAGCAAGTATCAAATGAGTC--GGGAAATTAGAAAAGTAGAAATT 44846
Coquerel'sSifaka  ----------------------------------------ATCAGGT-AGT---G-TAAATTTCGGTAGTT-ACACA 37100
Ma'sNightMonkey   ACAATTTTGGGGAATGAGTGAGTAATCAGGATTCAAAGCAAGTATCAAATGAGTCAGGGGAAATGAGAAAAGTAGAAGTG 39985
VervetAGM         ACAGTTTTGGGGAATTAATGAGTAATCGGGATTCAAAGCAAGTGTCAGATGAGTCAGGGGAAATGAGAAAAGTAGAAGTG 46377
Gorrilla          ACAATTTTGGGGAATGAATGAGTAATCAGGATTCAAAGCAAGTATCAAATGAGTCAGGGGAAATTAGAAAAGTAGAAATT 45357
GrabEatingMacaqueACAGTTTTGGGGAATTAATGAGTAATCGGGATTCAAAGCAAGTGTCAGATGAGTC-GGGGAAATGAGAAAAGTAGAAGTG 45607
Macaque           ACAGTTTTGGGGAATTAATGAGTAATCGGGATTCAAAGCAAGTGTCAGATGAGTC-GGGGAAATGAGAAAAGTAGAAGTG 46433
PigTailedMacaque  ACAGTTTTGGGGAATTAATGAGTAATCGGGATTCAAAGCAAGTGTCAGATGAGTC-GGGGAAATGAGAAAAGTAGAAGTG 45301
Bonobo            ACAGTTTTGGGGAATGAATGAGTAATCAGGATTCAAAGCAAGTATCAGATGAGTCAGGGGAAATTAGAAAAGTAGAAATT 43954
OliveBaboon       ACAGTTTTGGGGAATTAATGAGTAATCGGGATTCAAAGCAAGTGTCAGATGAGTCAGGGGAAATGAGAAAAGTAGAAGTG 45156
Orangutan         ACAATTTTGGGGAATGAATGAGTAATCAGGATTCAAAGCAAGTATCAAATGAGTCAGGGGAAATTAGAAAAGTAGAAATT 44951
```

```
Majority          GAAATTTT-AGGGGTGCTATTAGA-----GAAATTCAGTGAGGAAGAGAGGGTTCACAGTTCCTTGTTGACTGATGGATG
                          56250     56260     56270     56280     56290     56300     56310     56320
Human             GAAGTTTTTAGGGGTGCTATTAGA-----GAAATTCAGTGAGGAAGAGAGGGTTCACAGTTCCTTGTTGACTGATGGATG 44921
Coquerel'sSifaka  GTAGCT----GGCTTGGTCTTTGG-------------GAGCAGAAG---------ACAGATCTTTTTTAGGCCATCCATG 37154
Ma'sNightMonkey   GAAATTTTTAGGGGTGCTATTAGAACAGAGAAATTCAGTGAGGAGGTGAGGGTTCCAAGTTCCTTGTTGACTGATGGATG 40065
VervetAGM         GAAATTTT-AGGGGTGCTATTAGA-----GAAATTCAGTGAGGAAGAAAGGGTTCACAGTTCCTTGTTGACTGATGGATG 46451
Gorrilla          GAAGTTTTTAGGGGTGCTATTAGAACAGAGAAATTCAGTGAGGAAGAGAGGGTTCACAGTTCCTTGTTGACTGATGGATG 45437
GrabEatingMacaqueGAAATTTT-AGGGGTGCTATTAGA-----GAAATTCAGTGAGGAAGAAAGGGTTCACAGTTCCTTGTCGACTGATGGATG 45681
Macaque           GAAATTTT-AGGGGTGCTATTAGA-----GAAATTCAGTGAGGAAGAAAGGGTTCACAGTTCCTTGTCGACTGATGGATG 46507
PigTailedMacaque  GAAATTTT-AGGGGTGCTATTAGA-----GAAATTCAGTGAGGAAGAAAGGGTTCACAGTTCCTTGTCGACTGATGGATG 45375
Bonobo            GAAGTTTTTAGGGGTGCTATTAGA-----GAAATTCAGTGAGGAAGAGAAGGTTCACAGTTCCTTGTTGACTGATGGATG 44029
OliveBaboon       GAAATTTT-AGGGGTGCTATTAGA-----GAAATTCAGTGAGGAAGAAAGGGTTCACAGTTCCTTGTCGACTGATGGATG 45230
Orangutan         GAAATTTTTAGGGGTGCTGTTAGA-----GAAATTCAGTGAGGAAGAGAGGGTTCACAGTTTCTTGTTGACTGATGGATG 45026
```

```
Majority          TGGTGATTTTACAAATTACCCATGATC-TTGC---------------------CGACACCCATTTATGTAAAAATTGCA
                          56330     56340     56350     56360     56370     56380     56390     56400
Human             TGGTGATTTAACAAATTACCCATGATCCTTGC---------------------CGACACCCATTTATGTAAAAATTGCA 44979
Coquerel'sSifaka  TG-TAATGTGA-AGTTTGTCT-------TTGG----------------------------GATTC---TT----TTGCA 37189
Ma'sNightMonkey   TGGTGATTTCACAGATTACCCACGATC-TTGG---------------------CGACATCCATTTATGTAAAAATCGCA 40122
VervetAGM         TGGTGATTTTAAAAATTACCCATGAGC-TTGCTGACACCCATTTATGTAAAATTCGACACCCATTTATGTAAAAATTGCA 46530
Gorrilla          TGGTGATTTAACAAATTACCCATGATC-TTGC---------------------CGACACCCATTTATGTAAAAATTGCA 45494
GrabEatingMacaqueTGGTGATTTTAAAAATTACCCATGATC-TTGCCGACACCCATTTATGTAAAATTCGACACCCATTTATGTAAAAATTGCA 45760
Macaque           TGGTGATTTTAAAAATTACCCATGATC-TTGCCGACACCCATTTATGTAAAATTCGACACCCATTTATGTAAAAATTGCA 46586
PigTailedMacaque  TGGTGATTTTAAAAATTACCCATGATC-TTGCCGACACCCATTTATGTAAAATTCGACACCCATTTATGTAAAAATTGCA 45454
Bonobo            TGGTGATTTAACAAATTACCCATGATC-TTGC---------------------CGACACCCATTTATGTAAAAATTGCA 44086
OliveBaboon       TGGTGATTTTAAAAATTACCCATGATC-TTGCCGACACCCATTTATGTAAAATTCGACACCCATTTATGTAAAAATTGCA 45309
Orangutan         TGGTGATTTAACAGATTACCCATGATC-TTGC---------------------CGACACCCATTTATGTAAAAATTGCA 45083
```

```
Majority          GGTAAAAGCTAGTGGAGAACAAAGGTCAGTCAGCAGGAGAAAAGTGATGTACTGAT-AGAGGTGAAGATGCTGGCTTTAG
                          56410     56420     56430     56440     56450     56460     56470     56480
Human             GGTAAAAGCTAGTGGAGAACAAAGGTCAGTCAGCAGGAGAAAAGTGATGTACTGATCAGAGGTGAAGATGCTGGCTTTAG 45059
Coquerel'sSifaka  G--TGTAGCGGTTTAAGAGCACAGGCCAG--------------GTGGGGCA-TGGT----GGCTAACAC-------CTGT 37241
Ma'sNightMonkey   GGTAAAAGCTAGTGGAGAACAAAGGTCAGTCAGTATGAGAAAAGTGACGTACTGATCACAGGCGAAGATGCTGGCTTTAG 40202
VervetAGM         GGTAAAAGCTAGTGGAGAACAAAGGTCAGTCAGCAGGAGAAAAGTGATGTACTGAT-AGAGGTGAAGATGCTGGCTTTAG 46609
Gorrilla          GGTAAAAGCTAGTGGAGAACAAAGGTCAGTCAGCAGGAGAAAAGTGATGTACTGATCAGAGGTGAAGATGCTGGCTTTAG 45574
GrabEatingMacaqueGGTAAAAGCTAGTGGAGAACAAAGGTCAGTCAGAAGGAGAAAAGTGATGTACTGAT-AGAGGTGAAGATGCTGGCTTTAG 45839
Macaque           GGTAAAAGCTAGTGGAGAACAAAGGTCAGTCAGAAGGAGAAAAGTGATGTACTGAT-AGAGGTGAAGATGCTGGCTTTAG 46665
PigTailedMacaque  GGTAAAAGCTAGTGGAGAACAAAGGTCAGTCAGCAGGAGAAAAGTGATGTACTGAT-AGAGGTGAAGATGCTGGCTTTAG 45533
Bonobo            GGTAAAAGCTAGTGGAGAACAAAGGTCAGTCAGCAGGAGAAAAGTGATGTACTGATCAGAGGTGAAGATGCTGGCTTTAG 44166
OliveBaboon       GGTAAAAGCTAGTGGAGAACAAAGGTCAGTCAGCAGGAGAAAAGTGATGTACTGAT-AGAGGTGAAGATGCTGACTTTAG 45388
Orangutan         G-TAAAAGCTAGTGGAGAACAAAGGTCAGTCAGCAGGAGAAAAGTGATGTACTGATCAGAGGTGAAGATGCTGGCTTTAG 45162

Majority          AATTGAGGTAGAAAA-CTGGTTTTGAGTTTGGAAGAGAAGTATTCAAGTAGAAACCATTTTAAAAT-------TTTATTT
                          56490     56500     56510     56520     56530     56540     56550     56560
Human             AATTGAGGTGAAAAA-CTGGTTTTGAGTTTGGAAGAGAAGTATTCAAGTAGAAACCATTTTAAAAT-------TTTATTT 45131
Coquerel'sSifaka  AATCCCAGC----A--CT--TTTGGAAGCCGCAGAAGGAGGATC--GCTTGAGGCCA----GGAGT-------------- 37293
Ma'sNightMonkey   AATTGAAGTAGAAAA-CCGATTTTGAGTTTGGAAAAGAAGTGTTCAAGTAGAAAGCATTTTAAAATGGTTTATTTTATTT 40281
VervetAGM         AATTGAGGTAGAAAA-CTGGTTTTGAGTTTGAAAGAGAAGTATTCAAGTAGAAACCATTTTAAAAT-------TTTATTT 46681
Gorrilla          AATTGAGGTAAAAAA-CTGGTTTTGAGTTTGGAAGAGAAGTATTCAAGTAGAAACTATTTTAAAAT-------TTTATTT 45646
GrabEatingMacaqueAATTGAGGTAGAAAA-CTGGTTTTGAGGTTGAAAGAGAAATATTCAAGTAGAAACCATTTTAAAAT-------TTTATTT 45911
Macaque           AATTGAGGTAGAAAA-CTGGTTTTGAGGTTGAAAGAGAAATATTCAAGTAGAAACCATTTTAAAAT-------TTTATTT 46737
PigTailedMacaque  AATTGAGGTAGAAAA-CTGGTTTTGAGGTTGAAAGAGAAATATTCAAGTAGAAACCATTTTAAAAT-------TTTATTT 45605
Bonobo            AATTGAGGTGAAAAA-CTGGTTTTGAGTTTAGAAGAGAAGTATTCAAGTAGAAACCATTTTAAAAT-------TTTATTT 44238
OliveBaboon       AATTGAGGTAGAAAA-CTGGTTTTGAGTTTGAAAGAGAAATATTCAAGTAGAAACCATTTTAAAAT-------TTTATTT 45460
Orangutan         AATTGAGGTAAAAAAACTGGTTTTGAGTTTGGAAGAGAAGTATTCAAGTAGAAACCATTTAAAAAT-------TTTATTT 45235

Majority          GCTTTGAATCTTTGAGTACATGAACATCTTCCCTAAGCAAGAGGCTTTA-AAAGAGTTGAATAATATATGGAAAATGGTG
                          56570     56580     56590     56600     56610     56620     56630     56640
Human             GCTTTGAATCCGTGATTACATGAACATCTTCCCTAAGCAAGAGGCTTTG-AAAGAGTTGAATAATGTATGGAAAATGGTG 45210
Coquerel'sSifaka  ---TTGAGACC-----AGCCTGAACAACATGGGAGAGCC--TGTCTCTACAAAAAATAGAAAAATTAGCTGGGTGTGGTG 37363
Ma'sNightMonkey   GCTTTGAATC--TGAATACCTGAACATCTTCCCTTAGCA-GAGGCTTTA-AAAGAGTTGAGTAATATATGGAAAATGGTG 40357
VervetAGM         GCTTTGAATCTTTGAGTACATGAACATCTTCCCTAAGCAAGAGGCTTTA-AAAGAGTTGAATAATATATGGAAAATGGTG 46760
Gorrilla          GCTTTGAATCCGTGATTACATGAACATCTTCCCTAAGCAAGAGGCTTTG-AAAGAGTTGAATAATATATGGAAAATGGTG 45725
GrabEatingMacaqueGCTTTGAATCTTTGAGTACATGAACATCTTCCCTAAGCAAGAGGCTTTA-AAAGAGTTGAATAATATATGGAAAATGGTG 45990
Macaque           GCTTTGAATCTTTGAGTACATGAACATCTTCCCTAAGCAAGAGGCTTTA-AAAGAGTTGAATAATATATGGAAAATGGTG 46816
PigTailedMacaque  GCTTTGAATCTTTGAGTACATGAACATCTTCCCTAAGCAAGAGGCTTTA-AAAGAGTTGAATAATATATGGAAAATGGTG 45684
Bonobo            GCTTTGAATCCGTGATTACATGAATATCTTCCCTAAGCAAGAGGCTTTG-AAAGAGTT---TAATATATGGAAAATGGTG 44314
OliveBaboon       GCTTTGAATCTTTGAGTACATGAACATCTTCCCTAAGCAAGAGGCTTTA-AAAGAGTTGAATAATATATGGAAAATGGTG 45539
Orangutan         GCTTTGAATCCATGATTACATGAACATCTTCCCTAAACAAGAGGCTTTA-AAAGAGTTGAATAATATATGGAAAATGGTG 45314
```

```
Majority          TGCTTAAAGATTTTT-ACATTCATATGCAATGAATAAAATTTATATGCATAAATAAAAGCATTACCTCATGCCTTCTTAA

                           56650     56660     56670     56680     56690     56700     56710     56720

Human             TGCTTAAAGATTTTTTACATTCATATACAATGAATAAAATTTATATGCATAAATAAAAGCATTACCTCATGCCTTCTTAA 45290
Coquerel'sSifaka  CGC----ACATCTAT---AGTCCCAGCTACTCAGGAA---------GCTGAGGCAGGAGGATT--------CC------- 37412
Ma'sNightMonkey   TGCTTAAAGATTTCT-ACATTCATATGCAATGAATAAAATTTATATGCATAAATAAAAGCATTACCTCATGCAT---TAG 40433
VervetAGM         TACTTAAAGATTTTT-ACATTCATATGCAATGAATAAAATTTATATGCATAAATAAAAGCATTACCTCATACCTTCTTAA 46839
Gorrilla          TGCTTAAAGATTTTT-ACATTCATATGCAATGAATAAAATTTATATGCATAAATAAAAGCATTACCTCATGCCTTCTTAA 45804
GrabEatingMacaqueTGCTTAAAGATTTTT-ACATTCATATGCAATGAATAAAATTTATATGCATAAATAAAAGCATTACCTCATACCTTCTTAA 46069
Macaque           TGCTTAAAGATTTTT-ACATTCATATGCAATGAATAAAATTTATATGCATAAATAAAAGCATTACCTCATACCTTCTTAA 46895
PigTailedMacaque  TGCTTAAAGATTTTT-ACATTCATATGCAATGAATAAAATTTATATGCATAAATAAAAGCATTACCTCATACCTTCTTAA 45763
Bonobo            TGCTTAAAGATTTTT-ACATTCATATACAATGAATAAAATTTATATGCATAAATAAAAGCATTACCTCATGCCTTCTTAA 44393
OliveBaboon       TGCTTAAAGATTTTT-ACATTCATATGCAATGAATGAAATTTATATACATAAATAAAAGCATTACCTCATACCTTCTTAA 45618
Orangutan         TGCTTAAAGATTTTT------CATATGCAATGAATAAAATTTATATGCATAAATAAAAGCATTACCTCATGCCTTCTTAA 45388

Majority          TTGAACATAAACATTTGAATTATTACTGGTTTT-ACACACCCAAACAAAAGTTCTGGGTTTACTGGAGGAATGGAAGGGA

                           56730     56740     56750     56760     56770     56780     56790     56800

Human             CTGAACATAAACATTTGAATTATTACTGGTTTT-ACACGCCCAAACAAAAGTTCTGGGTTTACTGGAGGAATGGAAGGGA 45369
Coquerel'sSifaka  TTGAGCCCAAGAGTTTGAG--GTTGCTG----TGACCCGTGATGACACCA-CTGCACGCTAGCCCAGGCAATAGAGCG-- 37483
Ma'sNightMonkey   TTGAACGTAAACATTTGAATTATTACTAGTTTT-ACACACCCAAACAAAAGTTCTAGGTTTACTATAGGAATGGAAGG-A 40511
VervetAGM         TTGAACATAAACATTTGAATTATTACTGGTTTT-ACACACCCAAACAAAAGTTCTGGGTTTACTGGAGGAATGGAAGGGA 46918
Gorrilla          CTGAACATAAACATTTGAATTATTACTGGTTTT-ACACGCCCAAACAAAAGTTCTGGGTTTACTGGAGGAATGGAAGGGA 45883
GrabEatingMacaqueTTGAACA--AACATTTGAATTATTACTGGTTTTTACACACCCAAACAAAAGTTCTGGGTTTCCTGGAGGAATGGAAGGGA 46147
Macaque           TTGAACA--AACATTTGAATTATTACTGGTTTTTACACACCCAAACAAAAGTTCTGGGTTTCCTGGAGGAATGGAAGGGA 46973
PigTailedMacaque  TTGAACATAAACATTTGAATTATTACTGGTTTTTACACACCCAAACAAAAGTTCTGGGTTTCCTGGAGGAATGGAAGGGA 45843
Bonobo            CTGAACATAAACATTTGAATTATTACTGGTTTT-ACACGCCCAAACAAAAGTTCTGGGTTTACTGGAGGAATGGAAGGGA 44472
OliveBaboon       TTGAACATAAACATTTGAATTATTACTGGTTTT-ACACACCCAAACAAAAGTTCTGGGTTTCCTGGAGGAATGGAAGGGA 45697
Orangutan         CTGAACATAAACATTTGAATTATTACTGGTTTT-ACACACCCAAACAAAAGTTCTGGGTTTACTGGAGGAATGGAAGGGA 45467

Majority          AAGATCTCTTTCATTCCCTCAGTAGACCTTTCCCATTCAGGATCATGGGAAGATGCTGAAGAATTGTAGAACTGTAACAC

                           56810     56820     56830     56840     56850     56860     56870     56880

Human             AAGATCTCTTTCATTCCCCCAGTAAACCTTTCCCATTCAGGATCATGGGAAGATGCTGAAGAATTGTAGAACTGTAACAC 45449
Coquerel'sSifaka  -----------------------AGACCCTGTCTCT-------------AAAACGTGAAAGAA------AACCTCCACAG 37521
Ma'sNightMonkey   AAGATTTCTTTCATTCCCTCAGTAAACCTTTCCCATTTAGGATCTTGGGAAGATGATGAAGAATCATAGAACTGTAGCAC 40591
VervetAGM         AAGATCTCTTTCATTCCCTCAGTAGACCTTTCCCATTCAGGATCATGGGAAGATGCTGAAGAACCGTAGAACTGTAACAC 46998
Gorrilla          AAGATCTCTTTCATTCCCCCAGTAAACCTTTCCCATTCGGGATCATGGGAAGATGCTGAAGAATTGTAGAACTGTAACAC 45963
GrabEatingMacaqueAAGATCTCTTTCATTCCCTCAGTAGACCTTTCCTATTCAGGATCGTGGGAAGATGCTGAAGAACTGTAGAACTGTAACAC 46227
Macaque           AAGATCTCTTTCATTCCCTCAGTAGACCTTTCCTATTCAGGATCGTGGGAAGATGCTGAAGAACTGTAGAACTGTAACAC 47053
PigTailedMacaque  AAGATCTCTTTCATTCCCTCAGTAGACCTTTCCTATTCAGGGTCGTGGGAAGATGCTGAAGAACTGTAGAACTGTAACAC 45923
Bonobo            AAGATCTCTTTCATTCCCCCAGTAAACCTTTCCCATTCAGGATCATGGGAAGATGCTGAAGAATTGTAGAACTGTAACAC 44552
OliveBaboon       AAGATCTCTTTCATTCCCTCAGTAGACCTTTCCCATTCAGGATCATGGGAAGATGCTGAAGAACTGTAGAACTGTAACAC 45777
Orangutan         AAGATCTCTTTCATTCTCCCAGTAAACCTTTTCCATTCAGGATCATGGGAAGATGCTGAAGAATTGTAGAACTGTAACAC 45547
```

```
Majority         AGGCAGACCTAATGCTTCGTAAGTTGTGAAC------CACACATTTTTGGGCCCTTTTTCGGTGTTGCTGGAGTGTTTTT
                         56890     56900     56910     56920     56930     56940     56950     56960

Human            AGGCAGACCTAATGCTTCATAAGCTA--ATG------TTTCCATTTTTGGACCCTTTTTCGGTGTTGCTGGAGTGTGTTT 45521
Coquerel'sSifaka ACTGTGGAGTCACACTCCCTGAGTTTTAATC-----------CTTTTCCTACTCCTCTCTCTCTTTCTTGCCTTGCCTTT 37590
Ma'sNightMonkey  AGTCAGACCTAATGTTTCATAAGCTGTGAAC------CACACATTTTTGGGCCCCTTTTTGGTGTTGCTGGAGTGTTTTT 40665
VervetAGM        AGGCAGACCTAATGCTTTGTAAGTTCACAACTTTTTCACAAAATTTTGGGCCCTTTTTCAGTGTTGCTGGAGTGTTTTT 47078
Gorrilla         AGGCAGACCTAATGTTTCATAAGCTA--ATG------TTTCCATTTTTGGACCCTTTTTCGGTGTTGCTGGAGTGTTTTT 46035
GrabEatingMacaqueAGGCAGACCTAATGCTTTGTAAGTTGTGAAC------CACAAAATTTTGGGCCCTTTTTCAGTGTTGCTGGAGTGTTTTT 46301
Macaque          AGGCAGACCTAATGCTTTGTAAGTTGTGAAC------CACAAAATTTTGGGCCCTTTTTCAGTGTTGCTGGAGTGTTTTT 47127
PigTailedMacaque AGGCAGACCTAATGCTTTGTAAGTTGTGAAC------CACAAAATTTTGGGCCCTTTTTCAGTGTTGCTGGAGTGTTTTT 45997
Bonobo           AGGCAGACCTAATGCTTCATAAGCTA--ATG------TTTCCATTTTTGGACCCTTTTTCGATGTTGCTGGAGTGTTTTT 44624
OliveBaboon      AGGCAGACCTAATGCTTTGTAAGTTGTGAAC------CACAAAATTTTGGGCCCTTTTTCAGTGTTGCTGGAGTGTTTTT 45851
Orangutan        AGGCAGACCTAGTGTTTCATAAGCTGTGAAC------CACACATTTTTGGACCCTTTTTCGGTGTTGCTGGAGTGTTTTT 45621

Majority         ATATTTGAAAATAGGCTTTGTTATATTATTAAAGGAGTCCCCATTCCCA------CATTCTCATAGTATCATTTATTTAA
                         56970     56980     56990     57000     57010     57020     57030     57040

Human            ATATTTGAAAATAGGCTTTGTTATATTATTAAAGGAGCCCCCATTCCCAGTTTCACATTCTCATAGTATCATTTATTTAA 45601
Coquerel'sSifaka CCTTT-----------TTCCTTTTCCCCTT---------CCTTTTCCCC--------TTTTCCT----TCCTTTCTTT-- 37636
Ma'sNightMonkey  GTGTTTGAAAATAGACTTTGTTGTATTATTAATGGAGTCCCCATTCCCAGTTTCACATTCTCATAGTATCGTTTATTTAA 40745
VervetAGM        ATATTTGAAAATAGGCTTTGTTATATTATTAAAGGAGTCCCCATTCCCA------CATTCTCATAGTATCATTTATTTAA 47152
Gorrilla         GTATTTGAAAATAGGCTTTGTTATATTATTAAAGGAGCCCCCATTCCCAGTTTCACATTCTCATAGTATCATTTATTTAA 46115
GrabEatingMacaqueATATTAGAAAATAGGCTTTGTTATATTATTAAAGGAGTCCCCATTCCCA------CATTCTCATAGTATCATTTATTTAA 46375
Macaque          ATATTAGAAAATAGGCTTTGTTATATTATTAAAGGAGTCCCCATTCCCA------CATTCTCATAGTATCATTTATTTAA 47201
PigTailedMacaque ATATTAGAAAATAAGCTTTGTTATATTATTAAAGGAGTCCCCATTCCCA------CATTCTCATAGTATCATTTATTTAA 46071
Bonobo           ATATTTGAAAATAGGCTTTGTTATATTATTAAAGGAGCCCCCATTCCCAGTTTCACATTCTCATAGTATCATTTATTTAA 44704
OliveBaboon      ATATTTGAAAATAGGCTTTGTTATATTATTAAAGGAGTCCCCATTCCCA------CATTCTCATAGTATCATTTATTTAA 45925
Orangutan        ATATTTGAAAATAGGCTTTGTTATATTATTAAAGGAGCCCCCATTCCCAGTTTCACATTCTCGTAGTATCATTTATTTAA 45701

Majority         AATGAAGACTTTTA--TAATATTTGGCACTAAGATATGAATACTTAGT-AGTCACCCTCTGGAAGCTATTAACTAACTGA
                         57050     57060     57070     57080     57090     57100     57110     57120

Human            AATGAAGACTTTTA--TAATATTTGGCACTAAGATATGAATACTTAGT-AGTCACCTCATGGAAGCTATTAACT----GA 45674
Coquerel'sSifaka --------CTT----------TTCTTCCCT----------TCCTC---------CCCTTT------------C------ 37660
Ma'sNightMonkey  AATGAAGACTT-TA--TAGTATTTTGCACTAAGATACGAACACTTAGT-AGTCACCCTCTTGAAGCTTTTAGCTAACTAA 40821
VervetAGM        AATGAAGACTTAAAAATAATATTTGGCACTAAGATATGAATACTTAGT-AGTCACCCTCTGGAAGCTATTAACTAACTGA 47231
Gorrilla         AATGAAGACT--TA--TAATATTTGGCACCAAGATATGAATACTTAGT-AGTCACCCCATGGAAGCTATTAACT----GA 46186
GrabEatingMacaqueAATGAAGACTTTTA--TAATATTTGGCACTAAGATGTGAATACTTAGT-AGTCACCCTCTGGAAGCTATTAACTAACTGA 46452
Macaque          AATGAAGACTTTTA--TAATATTTGGCACTAAGATATGAATACTTAGT-AGTCACCCTCTGGAAGCTATTAACTAACTGA 47278
PigTailedMacaque AATGAAGACTTTTA--TAACATTTGGCATTAAGATATGAATACTTAGT-AGTCACCCTCTGGAAGCTATTAACTAACTGA 46148
Bonobo           AATGAAGACTTTTA--TAATATTTGGCACTAAGATATGAATACTTAGT-AGTCACCCCATGGAAGCTATTAACT----GA 44777
OliveBaboon      AATGAAGACTTTAA--TAATATTTGGCACTAAGATATGAATACTTAGT-AGAAACCCTCTGGAAGCTATTAACTAACTGA 46002
Orangutan        AATGAAGACTTTTA--TAATATTTGGCACTAAGATATGAATACTTAGTTAGTCACCCCATGGAAGCTATTAACTAACTGA 45779
```

```
Majority         GGGAGTGTCAGGTGGTGTAAATGTTGATAGTTTACTACAGTAACTGGCTTGGTCTTTGGGAGAAAAGGACAGATCTTTTT

                         57130     57140     57150     57160     57170     57180     57190     57200

Human            GGGAGTGTCAGGTAGTGTAAATGTTGATAGTTTACTACAGTAACTGGCTTGGTCTTTGGGAGAAGAGGACAGATCTTTTT 45754
Coquerel'sSifaka ---------------------------TTTT------------CTTCCTTTTTCCTT------------C----CTTCCT 37685
Ma'sNightMonkey  GGAAGTGGCAGGTAATGTAGATGTTATTGGTTTAACTATATAACTGGCTTGGTCTTTGTAAGAAAAGGACAGATC-TTTT 40900
VervetAGM        GGGAGTGTCAGGTGGTGTAAATGTTGATAGTTTACTACAGTAACTGGCTTGGTCTTTGGGAGAAAAGGACAGATCTTTTT 47311
Gorrilla         GGGAGTGTCAGGTAGTGTAAATGTTGATAGTTTACTACAGTAACTGGCTTGGTCTTTGGGAGAAGAGGACAGATCTTTTT 46266
GrabEatingMacaqueGGGAGTGTCAGGTGGTGCAAATGTTGATCGTTTACTACAGTAACTGGCTTGGTCTTTGGGAGAAAAGGACAGATCTTTTT 46532
Macaque          GGGAGTGTCAGGTGGTGCAAATGTTGATCGTTTACTACAGTAACTGGCTTGGTCTTTGGGAGAAAAGGACAGATCTTTTT 47358
PigTailedMacaque GGGAGTGTCAGGTGGTGCAAATGTTGATCGTTTACTACAGTAACTGGCTTGGTCTTTGGGAGAAAAGGACAGATCGTTTT 46228
Bonobo           GGGAGTGTCAGGTAGTGTAAATGTTGATAGTTTACTACAGTAACTGGCTTGGTCTTTGGGAGAAGAGGACAGATCTTTTT 44857
OliveBaboon      GGGAGTGTCAGGTGGTGTAAATGTTGATCGTTTACTACAGTAACTGGCTTGGTCTTTGGGAGAAAAGGACAGATCTTTTT 46082
Orangutan        GGGAGTGTCAGATAGTGTAAATGTTGATAGTTTACTACAGTAACTGGCTTGGTCTTTGGGAGAAAAGGACAGATCTTTTT 45859

Majority         TAGGCAACCTGTATGTAATGTAAAGTTTGTATTTGAGTTCCTTCTATAGTGTAGTTGTTTAAGAGCACAGACTA-GGAA-

                         57210     57220     57230     57240     57250     57260     57270     57280

Human            TAGGCAACCTGTATGTAATGTAATGTTTGTTTTTGAGATCCTACTATAGTGTAGTTGTTGAAGAGCACAGACTATGGAA- 45833
Coquerel'sSifaka TTCGCTACCT----------------TCCCTTTC---TTTCTTTT----------------------------------- 37711
Ma'sNightMonkey  TAGGCAATCTGTATGTAATGTAAAGTTTGTATTTGAGATCCTTCTATAGTGTAGTTGTTAAAGAGCACAGACTATGGAAA 40980
VervetAGM        TAGGCAACCTGTATGTAATGTAAAGTTTGTATCTGAGTTCCTTCTATAGTGTAGTTGTTTAAGAGCACAGACTA-GGAA- 47389
Gorrilla         TAGGCAACCTGTATGTAATGTAAAGTTTGTTTTTGAGATCCTACTATAGTGTAGTTGTTGAAGAGCACAGACTATGGAA- 46345
GrabEatingMacaqueTAGGCAACCTGTATGTAATGTAAAGTTTGTATTTGAGTTCCTTCTATAGTGTAGTTGTTTAAGAGCACAGACTA-GGAA- 46610
Macaque          TAGGCAACCTGTATGTAATGTAAAGTTTGTATTTGAGTTCCTTCTATAGTGTAGTTGTTTAAGAGCACAGACTA-GGAA- 47436
PigTailedMacaque TAGGCAACCTGTATGTAACGTAAAGTTTGTATTTGAGTTCCTTCTATAGTGTAGTTGTTTAAGAGCACAGACTA-GGAA- 46306
Bonobo           TAGGCAACCTGTATGTAATGTAATGTTTGTTTCTGAGATCCTACTATGGTGTAGTTGTTGAAGAGCACAGACTATGGAA- 44936
OliveBaboon      TAGGCAACCTGTATGTAATGTAAAGTTTGTATTTGAGTTCCTTCTATAGTGTAGTTGTTTAAGAGCACAGACTA-GGAA- 46160
Orangutan        TAGGCAACCTGTATGTAATGTAAAGTTTGTTTTTGAGATCCTTCTATAGTGTAGTTGTTGAAGAGCACAGACTATGGAA- 45938

Majority         -TCATACTCCCTGAGTTTAATCCTCTCTCTACTACTTCTT--GTCTGTACCACTTAGTAACTATCTAATCTTGGGCATTT

                         57290     57300     57310     57320     57330     57340     57350     57360

Human            -TCATGCTCCCTGAGTTTAATCCTTTCTCTGCTACTTCCT--GTCTATACCACTTAGTAACTATCTAATCTTGGGCATTT 45910
Coquerel'sSifaka ------CTTTCTTCTTTT--CTTTCTTTCTACCACTTAGT------A-ACTATCTGAC--CT----GACTTTGGGCGTTT 37770
Ma'sNightMonkey  ACCATACTCCCTGAATTTAATCCTTTCTCTACTGCTTCCT--GTATGTACCATTTAGTAACT----AATCTTGGACATTT 41054
VervetAGM        -TCATACTCCCTGAGTTTAATCCTCTCTCTACTACCTCTTTTGTCTGTACCACTTAGTAACTATCTAATCTTGGGCATTT 47468
Gorrilla         -TCATGCTCCCTGAGTTTAATCCTTTCTCTACTACTTCCT--GTCTATACCACTTAGTAACTATCTAATCTTGGGCATTT 46422
GrabEatingMacaque-TCATACTCCCTGAGTTTAATCCTCTCTCTACTACCTCTT--GTCTGTACCACTTAGTAACTATCTAATCTTGGGCATTT 46687
Macaque          -TCATACTCCCTGAGTTTAATCCTCTCTCTACTACCTCTT--GTCTGTACCACTTAGTAACTATCTAATCTTGGGCATTT 47513
PigTailedMacaque -TCATACTCCCTGAGTTTAATCCTCTCTCTACTACCTCTT--GTCTGTACCACTTAGTAACTATCTAATCTTGGGCATTT 46383
Bonobo           -TCATGCTCCCTGAGTTTAATCCTTTCTCTACTACTTCCT--GTCTATACCACTTAGTAACTATCTAATCTTGGGCGTTT 45013
OliveBaboon      -TCATACTCCCTGAGTTTAATCCTCTCTCTACTACCTCTT--GTCTGTACCACTTAGTAACTATCTAATCTTGGGCATTT 46237
Orangutan        -TCATGCTCCCTGAGTTTAATCCTTTCTCTAGTACTTCCT--ATCTATACCACTTAGTAACTATTTAATCTTGGGCATTT 46015
```

```
Majority          AACTAATTGTTACCT-GTAAATGGGGTTGATAGGTCATGATGATAGCAATCTGAGAGTTGTAAATTGCCTGGCATGTAGT

                          57370     57380     57390     57400     57410     57420     57430     57440

Human             AACTAATTGTTACCT-GTAAATGGGGTTGATAGGTCATAATGATAGCAATCTGAGAGTTGTAAATTGCCCGGCATGTAGT 45989
Coquerel'sSifaka  AACTAGCTCTTACCG-ATAAATGA---CAATTGGTCATGATAATAGCGATCTTAGAGTTGTCAGTTGCCTGGCATGTAGT 37846
Ma'sNightMonkey   AACTAATTGCTACCT-GTAAATGGGGTTGATAGGTCATGATGATAGCAATCTGAGAGTTGTAAATTGCCTGGCATGCGTT 41133
VervetAGM         AACTAATTGTTACCT-GTAGATGGGGTTGATAGGTTGTGATGATAGCAATCTGAGAGTTATAAATTGCTTGGCATGTAGT 47547
Gorrilla          AACTAATTGTTACCT-GTAAATGGGGTTGATAGGTCATGATGATAGCAATCTGAGAGTTGTAAATTGCCTGGCATGTAGT 46501
GrabEatingMacaqueAACTAATTGTTACCT-GTAAATGGGGTTGATAGGTCGTGATGATAGCAATCTGAGAGTTATAAATTGCTTGGCATGTAGT 46766
Macaque           AACTAATTGTTACCT-GTAAATGGGGTTGATAGGTCGTGATGATAGCAATCTGAGAGTTATAAATTGCTTGGCATGTAGT 47592
PigTailedMacaque  AACTAATTGTTACCT-GTAAATGGGGTTGATAGGTCGTAATGATAGCAATCTGAGAGTTATAAATTGCTTGGCATGTAGT 46462
Bonobo            AACTAATTGTTACCT-GTAAATGGGGTTGATAGGTCATAATGATAGCAATCTGAGAGTTGTAAATTGCCTGGCATGTAGT 45092
OliveBaboon       AACTAATTGTTACCT-GTAAATGGGGTTGACAGGTCGTGATGATAGCAATCTGAGAGTTATAAATTGCTTGGCATGTAGT 46316
Orangutan         AACTAATTGTTACCTTGTAAATGGGGTTGATAGGTCATGATGATAGCAATCTGAGAGTTTTAAATTGCCTGGCATGTAGT 46095

Majority          TAATTAGCAGTGAATGTTAAATATTGATGCAAAATAAAATTATATCTTTTCTGTATTTTT-CTAAGAAGTCAAGATCTTT

                          57450     57460     57470     57480     57490     57500     57510     57520

Human             TAATTAGCAGTGAATATTAAATATTGATGCAAAATAAAATTACATCTTTTCTATATTTTTTCCAAGAAGTCAAGATCTTT 46069
Coquerel'sSifaka  TGACTTTCAGTGAATGTTAAATACTGCTGCAAAATAAAATTATAT--TTTCTGTATTTTT-CTAAGAAACCCAGATGTTT 37923
Ma'sNightMonkey   TAATTTGCAGTGAATATTAAATATTGATGCAAAACAAAATTACATCTTTTCTATATTTTT-CCAAGAAGTCAAGATCTTT 41212
VervetAGM         TAATTAGCAGTGAATGTTAAATATTGATGCAAAATAAAATTATGTCTTTTCTGTATTTTT-CTAAGACGTCAAGGTCTTT 47626
Gorrilla          TAATTAGCAGTGAATGTTAAATATTGATGCAAAATAAAATTACATTTTTTCTATATTTTTTCCAAGAAGTCAAGATCTTT 46581
GrabEatingMacaqueTAATTAGCAGTGAATGTTAAATATTGATGCAAAATAAAATTATGTCTTTTCTGTATTTTT-CTAAGACGTCAAGATCTTT 46845
Macaque           TAATTAGCAGTGAATGTTAAATATTGATGCAAAATAAAATTATGTCTTTTCTGTATTTTT-CTAAGACGTCAAGATCTTT 47671
PigTailedMacaque  TAATTAGCAGTGAATGTTAAATATTGATGCAAAATAAAATTATGTCTTTTCTGTATTTTT-CTAAGACGTCAAGATCTTT 46541
Bonobo            TAATTAGCAGTGAATATTAAATATTGATGCAAAATAAAATTACATCTTTTCTATATTTTTTCCAAGAAGTCAAGATCTTT 45172
OliveBaboon       TAATTAGCAGTGAATGTTAAATATTGATGCAAAATAAAATTATGTCTTTTCTGTATTTTT-CTAAGACGTCAAGATCTTT 46395
Orangutan         TAATTAGCAGTGAATGTTAAATATTGATGCAAAATAAAATTATATCTTTTCTATATTTTTTCCAAGAAGTCAAGATCTTT 46175

Majority          GTCTTATCCTTTGATTCTCAAAAGTTTCCTACCTTCTTTCTGTGTTATTCAGCTGTTTTACTTCTGCTCAACATCCACTG

                          57530     57540     57550     57560     57570     57580     57590     57600

Human             GTCTTATCCTTTGATTCTCAGAAGTTTCCTACGTTCTTTCTGTGTTATTCAGCTATTTTACTTCTGCTTAATATCCACTA 46149
Coquerel'sSifaka  ATCTTAATCCTTGATTGTCAAAAGTATACCACCACCTTTCCATGTTATTTAGCTGTTTGATTTCTGCCCGCCATGCACTG 38003
Ma'sNightMonkey   GTCTTATCCTTCAATTCTTAAAGGTCTCCTACCTTCTTTCTGTATTATTCAGCTGTTTTACTTCTGTTCAACATCCACTG 41292
VervetAGM         GTCTTATCCTTTGATTCTCAAAAGTTTCCTACCTTCTTTCTGTGTTATTCAGCTGTTGTACTTCTGCTCAACATCCGCTG 47706
Gorrilla          GTCTTATCCTTTGATTCTCAGAAGTTTCCTACCTTCTTTCTGTGTTATTCAGCTATTTTACTTCTGCTTAATATCCACTA 46661
GrabEatingMacaqueGTCTT-----TTGATTCTCAAAAGTTTCCTACCTTCTTTCTGTGTTATTCAGCTGTTGTACTTCTGCTCAACATCCGCTG 46920
Macaque           GTCTT-----TTGATTCTCAAAAGTGTCCTACCTTCTTTCTGTGTTATTCAGCTGTTGTACTTCTGCTCAACATCCGCTG 47746
PigTailedMacaque  GTCTT-----TTGATTCTCAAAAGTTTCCTACCTTCTTTCTGTGTTATTCAGCTGTTGTACTTCTGCTCAACATCCGCTG 46616
Bonobo            GTCTTATCCTTTGATTCTCAGAAGTTTCCTACGTTCTTTCTGTGTTATTCAGCTATTTTACTTTTGCTTAATATCCACTA 45252
OliveBaboon       GTCTTATCCTTTGATTCTCAAAAGTTTCCTACCTTCTTTCTGTGTTACTCAGCTGTTGTACTTCTGCTCAACATCCGCTG 46475
Orangutan         GTCCTATCCTTTAATTCTCAAAAGTTTCCTATCTTCTTTCTGTGTTATTCAGCTGTTTTACTTCTGCTCAACATCCACTA 46255
```

```
Majority          TCCAGAAAACATCCAATACAAATCCAAAGTAA---ATTATTTAATTTCCTTAATAATAGGTTTAGGCAGGCTTTTGCCTT

                          57610     57620     57630     57640     57650     57660     57670     57680

Human             TCCAGAAAACATCCAACACAAATCCAAAATAA---ATTATTTAATTTCCTTAATAATAGGTTTAGGCAGGCTTTTGCCTT 46226
Coquerel'sSifaka  TCCAGAAAGTATCCAGTACCAATCCAAAACAA-----TAAACTATTCCCTTAATAACAAGTTTAGGTAGGTTTTTGCATT 38078
Ma'sNightMonkey   TCCAGAAAACATCCAGCACAAATCCAAAATAATAAATTATTTGATTTCCTTAATAATAGGTTTAGGCAGGCTTTTGCCTT 41372
VervetAGM         TCCAGAAAACATCCAATACAAATCCAAAGTAA---ATTATTTAATTTCCGTAATAATAGGTTTAGGCAGGCTTTTGCCTT 47783
Gorrilla          TCCAGAAAACATCCAACACAAATCCAAAGTAA---ATTATTTAATTTCCTTAATAATAGGTTTAGGCAGGCGTTTGCCTT 46738
GrabEatingMacaqueTCCAGAAAACATCCAATACAAATCCAAAGTAA---ATTATTTAATTTCCGTAATAATAGGTTTAGGCAGGCTTTTGCCTT 46997
Macaque           TCCAGAAAACATCCAATACAAATCCAAAGTAA---ATTATTTAATTTCCGTAATAATAGGTTTAGGCAGGCTTTTGCCTT 47823
PigTailedMacaque  TCCAGAAAACATCCAATACAAATCCAAAGTAA---ATTATTTAATTTCCGTAATAATAGGTTTAGGCAGGCTTTTGCCTT 46693
Bonobo            TCCAGAAAACATCCAACACAAATCCAAAGTAA---ATTATTTAATTTCCTTAATAATAGATTTAGGCAGGCTTTTGCCTT 45329
OliveBaboon       TCCAGAAAACATCCAATACAAATCCAAAGTAA---ATTATTTAATTTCCGTAATAATAGGTTTAGGCAGTCTTTTGCCTT 46552
Orangutan         TCCAGAAAACATCCAACACAAATCCAAAGTAA---ATTATTTAATTTCCTTAATAATAGGTTTAGGCAGGCTTTTGCCTT 46332

Majority          TTGGGAATTATTTTTGGAAAATCATTTGAAAGGGGAAGCCTTTTTTCTCCTTATGGGTACAGCGATAGGTAGTATCTTTA

                          57690     57700     57710     57720     57730     57740     57750     57760

Human             TTGGGAATTATTTTTGGAAAATCATTTGAAAGGGGAAGCCTTTTTTCTCCTTATGGGTACAGTGATAGGTAGTATCTTTA 46306
Coquerel'sSifaka  TTGGATATTATAATCTGAAAATTGCATGAAAAAG-AAGCCTTTTTTCCCCTTACAGGTACAGTGATAAGTAGTATCTTTA 38157
Ma'sNightMonkey   TTGGGAAGTATTTTCTGAAAATCATTTGAAAGAGGAAGCCTTGTTTCTTCTTATGGGTACAGTGATAT----TATCTTTA 41448
VervetAGM         TTGGGAATTATTTTTGGAAAATCATTTGAAAGGGGAAGCCTTTTTTCTCCTTAAGGGTACAGCGATAGGTAGTAGCTTTA 47863
Gorrilla          TTGGGAATTATTTTTAGAAAATCATTTGAAAGGGGAAGCCTTTTTTCTCCTTATGGGTACAGCGATAGGTAGTATCTTTT 46818
GrabEatingMacaqueTTGGGAATTATTTTTGGAAAATCATTTGAAAGGGGAAGCCTTTTTTCTCCTTATGGGTACAGCGATAGGTAGTATCTTTA 47077
Macaque           TTGGGAATTATTTTTGGAAAATCATTTGAAAGGGGAAGCCTTTTTTCTCCTTATGGGTACAGCGATAGGTAGTATCTTTA 47903
PigTailedMacaque  TTGGGAATTATTTTTGGAAAATCATTTGAAAGGGGAAGCCTTTTTTCTCCTTATGGGTACAGCGATAGGTAGTATCTTTA 46773
Bonobo            TTGGGAATTATTTTTGGAAAATCATTTGAAAGGGGAAGCCTTTTTTCTCCTTATGGGTACAGTGATAGGTAGTATCTTTA 45409
OliveBaboon       TTGGGAATTATTTTTGGAAAATCATTTGAAAAGGGAAGCCTTTTTTCTCCTTATGGGTACAGCGATAGGTAGTATCTTTA 46632
Orangutan         TTGGGAATTATTTTCGGAAAATCATTTGAAAGGGGAAGCCTTTTTTCTCCTTATGGGTACAGCGATAGGTGGTATCTTTA 46412

Majority          TTGGTGTTTTTTTTTT----CCTTGTCTTTCTCCCAGAAGGAAAAGGACAGACCAAATTAGGAGAAACTGAAATTTGCTT

                          57770     57780     57790     57800     57810     57820     57830     57840

Human             TTGGTCTTTTTTTTTTCC-----TTGTCTTTCTCCCAGAAGGAAAAGGACAGACCAAATTAGGAGAAACTGAAATTTGCTT 46381
Coquerel'sSifaka  TTTTGGTGGGTTTTTT----C-TTACCTTTCTCCAAGAAGGAA--GGACAGATCATATTTGAAGAG-CTGAAATTTGCTG 38229
Ma'sNightMonkey   TTCGTGTTTGTTTTTT----CCTTGTCTTTGTTCCAGAAGGAAGTGGACAGACCAAATTAGGAGAAACTGAAATTTGCGT 41524
VervetAGM         TTGGTGGTTTTTTTTT----CCTTGTCTTTCTCCCAGAAGGAAAAGGACAGACCAAATTAGGAGAAACTGAAATTTACTT 47939
Gorrilla          TTGGTCTTTTTTTTTTTTTTCCTTGTCCTTCTCCCAGAAGGAAAAGGACAGACCAAATTAGGAGAAACTAAAATTTGCTT 46898
GrabEatingMacaqueTTGGTGTTTTTTTTT-----CCTTGTCTTTCTCCCAGAAGGAAAAGGACAGACCAAATTAGGAGAAACTGAAATTTACTT 47152
Macaque           TTGGTGTTTTTTTTT-----CCTTGTCTTTCTCCCAGAAGGAAAAGGACAGACCAAATTAGGAGAAACTGAAATTTACTT 47978
PigTailedMacaque  TTGGTGTTTTTTTTTTTT--CCTTGTCTTTCTCCCAGAAGGAAAAGGACAGACCAAATTAGGAGAAACTGAAATTTACTT 46851
Bonobo            TTGGTCTTTTTTTTTCCC----TTGTCTTTCTCCCAGAAGGAAAAGGACAGACCAAATTAGGAGAAACTGAAATTTGCTT 45485
OliveBaboon       TTGGTGTTTTTTTTTTTC--CCTTGTCTTTCTCCCAGAAGGAAAAGGACAGACCAAATTAGGAGAAACTGAAATTTACTT 46710
Orangutan         TTGGTCTTTTTTTTTTCC----TTGTCTTTCTCCCAGAAGGAAAAGGACAGACCAAATTAGGAGAAACTGAAATTTGCTT 46488
```

```
Majority         TTTTGAGTATCGTGATACCATAACCTATAACACATGGGGCTTTAGGTTGGATTGTGAAGTATTTGTTCTTGTTATTCAAA
                         57850     57860     57870     57880     57890     57900     57910     57920
Human            TTTTGAGTATCCTGATACCATAAC-TATAACACATGGGGCTTTAGGTTGGATTGTGAAGTATTTGTTCTCGTTATTCAAA 46460
Coquerel'sSifaka TTTTGAGTATCCTAATACTGTAAC-TATAACACATGGGGCTCTAGGTTGGATTATGAAATATTTGTCCTTGTTATTCAAA 38308
Ma'sNightMonkey  TTTTGAATATCTTGATACCATAACCTATAACACATGGGGCTTTAGTTTGCATTGTGAA-TATTTGTTGTTGTTATTCAAA 41603
VervetAGM        TTTTGAGTATCGTGATGCTATAACGTGTAACACATGGGGCTTTAGGTTGGATTATGAAGTAT----TCTTGTTATTCAAA 48015
Gorrilla         TTTTGAGTATCCTGATACCGTAAC-TATAACACATGGGGCTTTAGGTTGGATTGTGAAGTATTCGTTCTCGTTATTCAAA 46977
GrabEatingMacaqueTTTTGAGTATCGTGATACCATAACCTATAACACATGGGGCTTTAGGTTGTACTGTGTAGTATTTGTTCTTGTTATTCAAA 47232
Macaque          TTTTGAGTATCGTGATACCATAACCTATAACACATGGGGCTTTAGGTTGAACTGTGTAGTATTTGTTCTTGTTACTCAAA 48058
PigTailedMacaque TTTTGAGTATCGTGATACCATAACCTATAACACATGGGGCTTTAGGTTGGACTGTGAAGTATTTGTTCTTGTTATTCAAA 46931
Bonobo           TTTTGAGTATCCTGATACCATAAC-TATAACACATGGGGCTTTAGGTTGGATTGTGAAGTATTTGTTCTCGTTATTCAAA 45564
OliveBaboon      TT--GAGTATCGTGATACCATAACCTGTAACACATGGGGCTTTAGGTTGGATTGTGAAGTATTTGTTCTTGTTATTCAAA 46788
Orangutan        TTTTGAGTATCCTGATACCATAACCTATAACACATGGGACTTTAGGTTGGATTGTGAAGTATTTGTTCTTGTTATTCAAA 46568

Majority         CAAACTAATGAACAAGAACAAATTATCAAGTTTCTGTACTCAGACTGCTTTG-TCAAAAAAAGGTCTTAGTCCTGTATCA
                         57930     57940     57950     57960     57970     57980     57990     58000
Human            CAAACTAATGAACAAGAACAAATTATCAAGTTTCTGTACTCAGACTGCTTTG-TCAAAAAAAGGTCTTAGTCCTGTATCA 46539
Coquerel'sSifaka C----TAATGAGTAAGAACAAACTATCAGGTTTCTGAACTAAGACGGTTTTGGCCAAAAAAAGGTCTTAGTCCTACATTG 38384
Ma'sNightMonkey  CAAAATAATGAACAAGAACAAACTATCAAGTTTCTATACTCAGTCTACTTTG-TCAAAAAA-GATCTTAGTCTTACATCA 41681
VervetAGM        CAAACTAATGAACGAGAACAGATTATCAAGTTTCAGTATTCAGACTGCTTTG-TCAAAAAAAGGTCTTAGTCCTGTATCA 48094
Gorrilla         CAAACTAATGAACAAGAACAAATTATCAAGTTTCTGTACTCAGACTGCTTTG-TCAAAAAAAGGTCTTAGTCCTGTATCA 47056
GrabEatingMacaqueCAAACTAATGAACGAGAACAGATTATCAAGTTTCAGTATTCACACTGCTTTG-TCAAAAAAAGGTCTTAGTCCTGTATCA 47311
Macaque          CAAACTAATGAATGAGAACAGATTATCAAGTTTCAGTATTCACACTGCTTTG-TCAAAAAAAGGTCTTAGTCCTGTATCA 48137
PigTailedMacaque CAAACTAATGAACGAGAACAGATTATCAAGTTTCAGTATTCAGACTGCTTTG-TCAAAAAAAGGTCTTAGTCCTGTATCA 47010
Bonobo           CAAACTAATGAACAAGAACAAATTATCAAGTTTCTGTACTCAGACTGCTTTG-TCAAAAAAAGGTCTTAGTCCTGTATCA 45643
OliveBaboon      CAAACTAATGAACGAGAACAGATTATCAAGTTTCAGTATTCAGACTGCTTTG-TCAAAAAAAGGTCTTAGTCCTGTATCA 46867
Orangutan        CAAACTAATGAACAAGAACAAATTATCAAGTTTCTGTACTCAGACTGCTTTG-TCAAAAAAAGGTCTTAGTCCTGTATCA 46647

Majority         GAAGAA-TATGTGTTCCTTGTTGACTGATGCCAGTTGTTGTGTGTGCCAATTACATGTTTTAAAGTATATGTGTATTTTT
                         58010     58020     58030     58040     58050     58060     58070     58080
Human            GAATAA-TATGTGTTCCTTGTTGACTGATGCCGATTGTTGTGTGTGCCCATTATGTGTTTTAAAGTATATGTGTATTTTT 46618
Coquerel'sSifaka GAAGAA----------TATGC------------------ATGTATGCCAGTTACATGTTTTAAGTTTTGTATGTATTTTT 38436
Ma'sNightMonkey  GAAGAA-TATGTGTATTATGT-------G---------TGCGTGTGCCAATTATATGTTTTAAAGTATATGTGTATTTTT 41744
VervetAGM        GAAGAAATATGTGTTCCTTGTTGACTGATGCCAGTTGTTGTGTGTGCCAATTACATGTTTTAAAGTATATGCATATTTTT 48174
Gorrilla         GAATAA-TATGTGTTCCTTGTTGACTGATGCCGATTGTTGTGTGTGCCCATTACGTGTTTTAAAGTATATGTGTATTTTT 47135
GrabEatingMacaqueGAAGAA-TATGTGTTCCTTGTTGACTGATGCCAGTTGTTGTGTGTGCCAATTACATGTTTTAAAGTATATGCATATTTTT 47390
Macaque          GAAGAA-TATGTGTTCCTTGTTGACTGATGCCAGTTGTTGTGTGTGCCAATTACATGTTTTAAAGTATATGCATATTTTT 48216
PigTailedMacaque GAAGAA-TATGTGTTCCTTGTTGACTGATGCCAGTTGTTGTGTGTGCCAATTACATGTTTTAAAGTATATGCATATTTTT 47089
Bonobo           GAATAA-TAAGTGTTCCTTGTTGACTGATGCCGGTTGTTGTGTGTGCCCATTATGTGTTTTAAAGCATATGTGTATTTTT 45722
OliveBaboon      GAAGAA-TATGTGTTCCTTGTTGACTGATGCCAGTTGTTGTGTGTGCCAATTACATGTTTTAAAGTATATGCATATTTTT 46946
Orangutan        GAAGAA-T--GTGTTCCTTATTGACTGATGCTGATTGTTGTGTGTGCCAATTACATGTTTTAAAG------TGTATTTTT 46718
```

```
Majority          TGGATTCTGTGCTTTAGCTTACATTTTCAGTTAACTGGCCTAGAGATGATTTTGTTAGATAAGATACTACAAAATGCAAA
                           58090     58100     58110     58120     58130     58140     58150     58160
Human             TGGATTCTGTGCTTTAGCTTACATTTTCAATTAACGGGCCTAGAGATGATTTTGTTAGATAAGATACTACAAAATGCAAA 46698
Coquerel'sSifaka  TTCATTCCCTGTTTTAACTTACATTT-CAGTTAACAGACCCAATGATGATTTTGTTAAATAAGATACCACAAAATAGAAA 38515
Ma'sNightMonkey   TGAATTCTGTGCTTTAGCTTACATTTTCAATTAACGGGCCTAGAGATGATTTTGTTAGATAAGATACGACAAATTAGAAA 41824
VervetAGM         TGGATTCTGTGCTTTAGCTTACATTTTCTGTTAACTGGCCTCGAGATGATTTTGTTAGATAAGATACTACAAAATGCAAA 48254
Gorrilla          TGGATTCTGTGCTTTAGCTTACATTTTCAATTAACGGGCCTAGAGATGATTTTGTTAGATAAGATACTACAAAATGCAAA 47215
GrabEatingMacaqueTGGATTCTGTGCTTTAGCTTACATTTTCTGTTAACTGGCCTCGAGATGATTTTGTTAGATAAGATACTACAAAATGCAAA 47470
Macaque           TGGATTCTGTGCTTTAGCTTACATTTTCTGTTAACTGGCCTCGAGATGATTTTGTTAGATAAGATACTACAAAATGCAAA 48296
PigTailedMacaque  TGGATTCTGTGCTTTAGCTTACATTTTCTGTTAACTGGCCTCGAGATGATTTTGTTAGATAAGATACTACAAAATGCAAA 47169
Bonobo            TGGATTCTGTGCTTTAGCTTACATTTTCAATTAATGGGCCTAGAGATGATTTTGTTAGATAAGATACTACAAAATGCAAA 45802
OliveBaboon       TGGATTCTGTGCTTTAGCTTACATTTTCTGTTAACTGGCCTCGAGATGATTTTGTTAGATAAGATACTACAAAATGCAAA 47026
Orangutan         TGGATTCTGTGCTTTGGCTTACATTTTCAATTAACGGGCCTAGAGATGATTTTGTTAGATAACATACTACAAAATGCAAA 46798

Majority          GTTCAGTCT----CTTCCTTTTGACAAATTCAGGTGCCCTGTGCACACACAAATCTTTGTCAGTAGTGAAAGGACATGTG
                           58170     58180     58190     58200     58210     58220     58230     58240
Human             GTTCAGTCT----CTTCCTTTTGACAAATTCAGGTGCCCTGTGCGCACACAAATCTTTGTCAGTAGTGAAAGGACATGTG 46774
Coquerel'sSifaka  GTTTAGCCTCTTTCTTCTTTTTGACAAGTTCAGGTGCCT-GTGCATGCACAAACCTTCCTCGGTAATGAAAGGAGGTATG 38594
Ma'sNightMonkey   GTTCAGTCTT---CTTCCTTTTGACAA-----GGTGCCA-GCGCACACACAAATCTTTGTTAGTAGTGAAAGGACATGTG 41895
VervetAGM         GTTCAGTCT----CTTCCTTTTGACAAATTCAGGTGCCCTGTGCACACACAAATCTTTGCCAGTAGTGAAAGGACATGGG 48330
Gorrilla          GTTCAGTCT----CTTCCTTTTGACAAATTCAGGTGCCCTGTGCGCACACAAATCTTTGTCAGTAGTGAAAGGACATGTG 47291
GrabEatingMacaqueGTTCAGTCT----CTTCCTTTTGACAAATTCAGGTGCCCTGTGCACACACAAATCTTTGTCAGTAGTGAAAGGACATGGG 47546
Macaque           GTTCAGTCT----CTTCCTTTTGACAAATTCAGGTGCCCTGTGCACACACAAATCTTTGTCAGTAGTGAAAGGACATGGG 48372
PigTailedMacaque  GTTCAGTCT----CTTCCTTTTGACAAATTCAGGTGCCCTGTGCCCACATAAATCTTTGTCAGTAGTGAAAGGACATGGG 47245
Bonobo            GTTCAGCCT----CTTCCTTTTGACAAATTCAGGTGCCCTGTGCGCACACAAATCTTTGTCAGTAGTGAAAGGACATGTG 45878
OliveBaboon       GTTTAGTCT----CTTCCTTTTGACAAATTCAGGTGCCCTGTGCACACACAAATCTTTGTCAGTAGTGAAAGGACATGGG 47102
Orangutan         GTTCAGTCC----CTTCCTTTTGACAAATTCAGATGCCCTGTGCACACACAAATCTTTGTCAGTAGTGAAAGGACATGTG 46874

Majority          -ATAGCTTCCTGTAGTTTTTT-----------------------------------------------------------
                           58250     58260     58270     58280     58290     58300     58310     58320
Human             -ATAGCTTCCTGTAGTTTTTT----------------------------------------------------------- 46794
Coquerel'sSifaka  GATAGCTTCCTATAGTTTTTC----------------------------------------------------------- 38615
Ma'sNightMonkey   -ATAGCTTGCTGTAGTTTTTT----------------------------------------------------------- 41915
VervetAGM         -ATAGCTTCCTGTAGTTTTTTTTTTTTT-------GAGACGTAGTCTCGCTCTATTGCCTAGGCTGTAGTGCGGTGGCGCG 48402
Gorrilla          -ATAGCTTCCTGTAGTTTTTT----------------------------------------------------------- 47311
GrabEatingMacaque-ATAGCTTCCTGTAGTTTTTTTTTTTTTTTTTTTTTGAGACGTAGTCTCGCTCTGTTGCCCAGGCTGGAGTGCGGTGGCGCG 47625
Macaque           -ATAGCTTCCTGTAGTTTTTTTTTTTTTTTTTT---GAGACGTAGTCTCGCTCTGTTGCCCAGGCTGGAGTGCGGTGGCGCG 48448
PigTailedMacaque  -ATAGCTTCCTGTAGTTTTTTTTTTTTTTTTT----GAGACGTAGTCTCGCTCTGTTGCCCAGGCTGGAGTGCGGTGGCGCG 47320
Bonobo            -ATAGCTTCCTGTAGTTTTTT----------------------------------------------------------- 45898
OliveBaboon       -ATAGCTTCCTGTAGTTTTTTTTTCTTTT------GAGACGGAGTCTCGCTCTGTTGCCCACGCTGGAGTGCGGTGGCGCG 47175
Orangutan         -ATAGCTTCCTGTAGTTTTTT----------------------------------------------------------- 46894
```

```
Majority         --------------------------------------------------------------------------T-----

                         58330     58340     58350     58360     58370     58380     58390     58400

Human            -------------------------------------------------------------------------------- 46794
Coquerel'sSifaka --------------------------------------------------------------------------T----- 38616
Ma'sNightMonkey  -------------------------------------------------------------------------------- 41915
VervetAGM        ACCTCCGCTCACTGCAAGCTCTGCCTCCCAGGTTCACGCCATTCTCCTGCCTCAGCCTCCGGAGTAGCTGGGACTACAGG 48482
Gorrilla         -------------------------------------------------------------------------------- 47311
GrabEatingMacaqueACCTCCGCTCACTGCAAGCTCTGCCTCCCAGGTTCACGCCATTCTCCTGCCTCAGCCTCCCGAGTAGCTGGGACTACAGG 47705
Macaque          ACCTCCGCTCACTGCAAGCTCTGCCTCCCAGGTTCACGCCATTCTCCTGCCTCAGCCTCCCGAGTAGCTGGGACTACAGG 48528
PigTailedMacaque ACCTCCGCTCACTGCAAGCTCTGTCTCCCAGGTTCACGCCATTCTCCTGCCTCAGCCTCCCGAGTAGCTGGGACTACAGG 47400
Bonobo           -------------------------------------------------------------------------------- 45898
OliveBaboon      ACCTCCGCTCACTGCAAGCTCTGCCTCCCAGGTTCACGCCATTCTCCTGCCTCAGCCTCCCGAGTAGCTGGGACTACAGG 47255
Orangutan        -------------------------------------------------------------------------------- 46894

Majority         --------------------------------------------------------------------------------

                         58410     58420     58430     58440     58450     58460     58470     58480

Human            -------------------------------------------------------------------------------- 46794
Coquerel'sSifaka -------------------------------------------------------------------------------- 38616
Ma'sNightMonkey  -------------------------------------------------------------------------------- 41915
VervetAGM        CGCCTGCCACCACGCCCGGCTAATTTTTTGTATTTTTTTTTAGTAGAGACGGGGTTTCACTGTGTTAGCCAAGATGGTG 48562
Gorrilla         -------------------------------------------------------------------------------- 47311
GrabEatingMacaqueCGCCTGCCACCACGCCCAGCTAATTTTTTGTATTTTTTTTTAGTAGAGACGGGGATTTCACTGTGTTAGCCAGGATGGTG 47785
Macaque          CGCCTGCCACCACGCCCAGCTAATTTTTTGTATTTTTTTTTAGTAGAGACGGGGATTTCACTGTGTTAGCCAGGATGGTG 48608
PigTailedMacaque CGCCTGCCACCACGCCCAGCTCATTTTTTGTATTTTTTTTTAGTAGAGACGGGGGTTTCACTGTGTTAGCCAGGATGGTG 47480
Bonobo           -------------------------------------------------------------------------------- 45898
OliveBaboon      CACCTGCCACCACACCCAGCTAATTTTTTGTATTTTTTTTTAGTAGAGACGGGGGTTTCACTGTGTTAGCCAGGATGGTG 47335
Orangutan        -------------------------------------------------------------------------------- 46894

Majority         --------------------------------------------------------------------------------

                         58490     58500     58510     58520     58530     58540     58550     58560

Human            -------------------------------------------------------------------------------- 46794
Coquerel'sSifaka -------------------------------------------------------------------------------- 38616
Ma'sNightMonkey  -------------------------------------------------------------------------------- 41915
VervetAGM        TCGATCGCCAGACCTTGTGATCCCCCTCTGCCTCCCAAAGTGCTGGGATTACAGGCGTGAGCCACCGCACCCCGGCCAGC 48642
Gorrilla         -------------------------------------------------------------------------------- 47311
GrabEatingMacaqueTTGATCGCCTGACCTCGTGATCCCCCTCCGCCTCCCAAGGTGCTGGGATTACAGGCGTGAGCCACCGCACCCCGGCCAGC 47865
Macaque          TTGATCGCCTGACCTCGTGATCCCCCTCCGCCTCCCAAGGTGCTGGGATTACAGGCGTGAGCCACCGCACCCCGGCCAGC 48688
PigTailedMacaque TTGATCGCCTGACCTCGTGATCCCCCTCTGCCTCCCAAGGTGCTGGGATTACAGGCGTGAGCCACCGCACCCCGGCCAGC 47560
Bonobo           -------------------------------------------------------------------------------- 45898
OliveBaboon      TCAATCGCCTGACCTCGTGATCCCCCTCCGCCTCCCAAAGTGCTGGGATTACAGGCGTGAGCCACCGCACCCCGGCCAGC 47415
Orangutan        -------------------------------------------------------------------------------- 46894
```

```
Majority          ---------------CATATTTCTGGAGTGGTGAAGAGCTAGCCAGCCAAGATTTTCAGGGTAATATATTCAGAAGCCAG

                         58570     58580     58590     58600     58610     58620     58630     58640

Human             ---------------CATATTTCTGGAGTGGTGAAGAGCTACCCAGCCAAGATTTTCAGGGCAATATATTCAGAAGCCAG 46859
Coquerel'sSifaka  -----------------TATTTTTGGAGTTATGCAGAGCTAGCCAGCCAAGATTTTCAGGGTAGTATGTTCAGAAGCCAT 38679
Ma'sNightMonkey   ---------------CATATTTCTGGAGTGGTGAAGAGCTAGCCAGCCAAGATTTACAGGGTAATGTATTCAGAAGCCAG 41980
VervetAGM         TTCCTGTAGTTTTTTCATATTTCTGGAGTGGTGAAGAGCTAGCCAGCCAAGATTTTCAGGGTAATATATTCAGAAGCCAG 48722
Gorrilla          ---------------CATATTTGTGGAGTGGTGAAGAGCTACCCAGCCAAGATTTTCAGGGTAATATATTCAGAAGCCAG 47376
GrabEatingMacaqueTTCCTGTAGTTTTTTCATATTTCTGGAGTGGTGAAGAGCTAGCCAGCCAAGATTTTCAGGGTAATATATTCAGAAGCCAG 47945
Macaque           TTCCTGTAGTTTTTTCATATTTCTGGAGTGGTGAAGAGCTAGCCAGCCAAGATTTTCAGGGTAATATATTCAGAAGCCAG 48768
PigTailedMacaque  TTCCTGTAGTTTTTTCATATTTCTGGAGTGGTGAAGTGCTAGCCAGCCAAGATTTTCAGGGTAATATATTCAGAAGCCAG 47640
Bonobo            ---------------CATATTTCTGGAGTGGTGAAGAGCTACCCAGACAAGATTTTCAGGGTAATATATTCAGAAGCCAG 45963
OliveBaboon       TTCCTGTAGTTTTTTCATATTTCTGGAGTGGTGAAGAGCTAGCCAGCCAAGATTTTCAGGGTAATATATTCAGAAGCCAG 47495
Orangutan         ---------------CATATTTCTGGAGTGGTGAAGAGCTAGCTAGCCAAGATTTTCAGGGTAGTATATTCAGAAGCCAG 46959

Majority          AGCTTACT---CTTCTGTACAAAGACAACCTAAGGGGATTTTTAAATGCATTGGCTAGCTGTTCAAGTATATAAACCTAA

                         58650     58660     58670     58680     58690     58700     58710     58720

Human             AGCTTACT---CTTCTGTACAAAGACAACCTAAGGGGATTTTTAAATGCATTGGCTTGCCGTTCAAGTATATAAACCTAA 46936
Coquerel'sSifaka  AGTTTACTTCTCTTTTGTACAAAGGCAACCTAAGGAGATTTTTAAATGCATTGGCTGGCCATTCAAGTATACAAGCCTAA 38759
Ma'sNightMonkey   AGCTCACT---CTTCTGTACAAAGACAACCTAAAGGGATTTTTAATTGCATTGGTTAGCCATTCAAGTATATAAACCTAA 42057
VervetAGM         AGCTTACT---CTTCTGTACAAAGACAACCTAAGGGAATTTTTAAATGCATTGGCTGGCTGTTCAAGTATATAAACCTAA 48799
Gorrilla          AGCTTACT---CTTCTGTACAAAGACAACCTAAGGGGATTTTTAAATGCATTGGCTTGCTGTTCAAGTATATAAACCTAA 47453
GrabEatingMacaqueAGCTTACT---CTTCTGTACAAAGACAACCTAAGGGAATTTTTAAATGCATTGGCTAGCTGTTCAAGTATATAAACCTAA 48022
Macaque           AGCTTACT---CTTCTGTACAAAGACAACCTAAGGGAATTTTTAAATGCATTGGCTAGCTGTTCAAGTATATAAACCTAA 48845
PigTailedMacaque  AGCTTACT---CTTCTGTACAAAGACAACCTAAGGGAATTTTTAAATGCATTGGCTAGCTGTTCAAGTATATAAACCTAA 47717
Bonobo            AGCCTACT---CTTCTGTACAAAGACAACCTAAGGGGATTTTTAAATGCATTGGCTTGCCGTTCAAGTATATAAACCTAA 46040
OliveBaboon       AGCTTACT---CTTCTGTACAAAGACAACCTAAGGGAATTTTTAAATGCATTGGCTAGCTGTTCAAGTATATAAACCTAA 47572
Orangutan         AGCTTACT---CTTCTATACAAAGACAACCTAAGGGGATTTTTAAATGCATTGGCTGGCCATTGAAGTATATAAACCTAA 47036

Majority          GTGAAACTCCTAGAGTAAAATATGACTTAAGTATAGCTTGA-------------------GTCCATATATTATCTCTTA-

                         58730     58740     58750     58760     58770     58780     58790     58800

Human             GTGAAACTCCTAGAGTAAAATATGACTTAAGTATAGCTTGA-------------------GTCCATATATTATCACTTA- 46996
Coquerel'sSifaka  GTGAAACTTTTAGAGTAGAATATGACTTAAGTATAGCTTGACAGGTAAATTATCGAGTGAGTGCACATACTATCTCCTA- 38838
Ma'sNightMonkey   GTGAAACTCCTAGGGTAAAATATGACTTAAGTATAGCTTGA-------------------GTTCATATATTATCTCTTA- 42117
VervetAGM         GTGAAACTCCTACAGTAAAATATGACTTAAGTATAGCTTGA-------------------GTCCATATATTATCTCTTA- 48859
Gorrilla          GTGAAACTCCTAGAGTAAAATATGACTTAAGTATAGCTTGA-------------------GTCCATATATTATCTCTTAA 47514
GrabEatingMacaqueGTGAAACTCCTACAGTAAAATATGACTTAAGTATAGCTTGA-------------------GTCCATATATTATCTCTTA- 48082
Macaque           GTGAAACTCCTACAGTAAAATATGACTTAAGTATAGCTTGA-------------------GTCCATATATTATCTCTTA- 48905
PigTailedMacaque  GTGAAACTCCTACAGTAAAATATGACTTAAGTATAGCTTGA-------------------GTCCATATATTATCTCTTA- 47777
Bonobo            GTGAAACTCCTAGAGTAAAATATGACTTAAGTATAGCTTGA-------------------GTCCATATATTATCTCTTA- 46100
OliveBaboon       GTGAAACTCCTACAGTAAAATATGACTTAAGTATAGCTTGA-------------------GTCCATATATTGTCTCTTA- 47632
Orangutan         GTGAAACTCCTGGAGTAAAATATGACTTAAGTATAGCTTGA-------------------GTCCATATATTATCTCTTA- 47096
```

```
Majority          GAGATACCTTCTCTGGTCATCCAATAAAGTCATTACTACCATCA-TCTTCCAGTCCTAGACAAAGTCACTTTTAAGCACA
                          58810     58820     58830     58840     58850     58860     58870     58880
Human             GAGATACCTTCTCTGATCATCCAATAAAGTCATTACTACTATCAATCTTCCAGTCCTAGACAAAGTAACTTTTAAGCACA 47076
Coquerel'sSifaka  GAGATAACTTCTGTGATTATACAATAAAGTCATTACCACCACTC-TATCCCGGTCATTGGCAAATTCACTTTTAATCACA 38917
Ma'sNightMonkey   GAGATACCTTCTCTGGTCATCAAATAAAGTCATTACTGCTAATCACCTTCCTGTCCTAGACAAAGTCACTTTTAATCACA 42197
VervetAGM         GAGATACCTTCTCTGGTCATCCAGTAAAATCATTACTACCATCA-TCTTCCAGTCCTAGACAAAGTCACTTTTAAGCACA 48938
Gorrilla          GAGATACCTTCTCTGATCATCCAATAAAGTCATTACTACTATCAATCTTCCAGTCCTAGACAAAGTCACTTTTAAGCACA 47594
GrabEatingMacaqueGAGATACCTTCTCTGGTCATCCAGTAAAATCATTACTACCATCA-TCTTCAAGTCCTAGACAAAGTCACTTTTAAGCACA 48161
Macaque           GAGATACCTTCTCTGGTCATCCAGTAAAATCATTACTACCATCA-TCTTCCAGTCCTAGACAAAGTCACTTTTAAGCACA 48984
PigTailedMacaque  GAGATACCTTCTCTGGTCATCCAGTAAAATCATTACTACCATCA-TCTTCCAGTCCTAGACAAAGTCACTTTTAAGCACA 47856
Bonobo            GAGATACCTTCTCTGATCATCCAATAAAGTCATTACTACTATCAATCTTCCAGTCCTAGACAAAGTAACTTTTAAGCACA 46180
OliveBaboon       GAGATACCTTGTCTGGTCATCCAGTAAAATCATTACTACCATCA-TCTTCCAGTCCTAGACAAAGTCACTTTTAAGCACA 47711
Orangutan         GAGATACCTTCTCTGATCATCCAATAAAGCCATTACTACTATCA-TCTTCCAGTCCCAGACAAAGTCACTTTTAAGCACA 47175

Majority          TCACT--GTTTTACTT----CATAGCACATACC-AT----AGAAAGTATCTGGTTCAGGTACTAGTTAGTCCAGCTC-CC
                          58890     58900     58910     58920     58930     58940     58950     58960
Human             TCACT--GTTTTACCT----CACAGCATATACC-GT----AGAAAGTATCTGGTTCAGGTACTAGTTAGTCCAGCTC-CC 47144
Coquerel'sSifaka  TCACTCCGTTTTACTTTCTTCATAGTACTTACCCACTATAAGACATTATCTGGTTCATGTAGT--CCACCTCTCCCACTA 38995
Ma'sNightMonkey   CCACT--GTTTTACTTTATTCATAGCACTTTTC-ACTATAAGAAAGTATCTGGTTCATGTACTAGTTAGTCCAGCTCTCC 42274
VervetAGM         TCACT--GTTTTACTT----CATAGCACGTACC-AC----AGAAAGTATCTGGTTCAGGTACTAGTTAGTCTAGCTC-CC 49006
Gorrilla          TCACT--GTTTTACCT----CACAGCACATACC-GT----AGAAAGTATCTGGTTCAGGTACTAGTTAGTCCAGCTC-CC 47662
GrabEatingMacaqueTCACT--GTTTTACTT----CATAACACATACC-AT----AGAAAGTATCTGGTTCAGGTACTAGTTAGTCCAGCTC-CC 48229
Macaque           TCACT--GTTTTACTT----CATAACACATACC-AT----AGAAAGTATCTGGTTCAGGTACTAGTTAGTCCAGCTC-CC 49052
PigTailedMacaque  TCACT--GTTTTACTT----CATAGCACATACC-AT----AGAAAGTATCTGGTTCAGGTACTAGTTAGTCCAGCTC-CC 47924
Bonobo            TCACT--GTTTTACCT----CACAGCACATACC-GT----AGAAAGTATCTGGTTCAGGTACTAGTTAGTCCAGCTC-CC 46248
OliveBaboon       TCACT--GTTTTACTT----CATAGCACATACC-AT----AGAAAGTATCTGGTTCAGGTACTAGTTAGTCCAGCTC-CC 47779
Orangutan         TCACT--GTTTTACCT----CACAGCACGTACC-AT----AGAAAGTATCTGGTTCAAGTACTAGTTAGTCCAGCTC-CC 47243

Majority          CACTATAAGGTATCAGGAATCATA--ATCATCTTGTTCACTCTGCTGTACCCTCAGAACGTAAAACATTGCTAGACACAT
                          58970     58980     58990     59000     59010     59020     59030     59040
Human             TACTATAAGGTATCAGGAATCATATAATCATCTTGTTCACTCT-CTGTACCCTCAGAACGTAAAACATTGCTAGACACAT 47223
Coquerel'sSifaka  GACTATAGAGCATCAGGAATCATA--TTTATCTTGTTCATTGT--CTCATCTCCAGAGCCTAAAACAGTGCTAGGCACAT 39071
Ma'sNightMonkey   CACTATAGGGTATCAGGAATCATA--ATCATCTTGTTCACGCTGCTGTACCCTCGGAACGTAAAACATTGCTAGATGCAT 42352
VervetAGM         CACTATAAGGTATCAGGAATCATA--ATCGTTTTGTTCACTCTGCTGTACCCTCAGAACGTAAAACGTTGCTAGACACAT 49084
Gorrilla          TACTATAAGGTATCAGGAATCATATAATCATCTTGTTCACTCT-CTGTACCCTCAGAACATAAAACATTGCTAGACACAT 47741
GrabEatingMacaqueCACTATAAGGTATCAGGAATCATA--ATCGTTTTGTTCACTCTGCTGTACCCTCAGAACGTAAAACGTTGCTAGACACAT 48307
Macaque           CACTATAAGGTATCAGGAATCATA--ATCGTTTTGTTCACTCTGCTGTACCCTCAGAACGTAAAACGTTGCTAGACACAT 49130
PigTailedMacaque  CACTATAAGGTATCAGGAATCATA--ATCGTTTTGTTCACTCTGCTGTACCCTCAGAACGTAAAACGTTGCTAGACACAT 48002
Bonobo            TACTATAAGGTATCAGGAATCATATAATCATCTTGTTCACTCT-CTGTACCCTCAGAACGTAAAACATTGCTAGACACAT 46327
OliveBaboon       CACTATAAGGTATCAGGAATCATA--ATCGTTTTGTTCACTCTGCTCTACCCTCAGAACGTAAAACGTTGCTAGACACAT 47857
Orangutan         CACTATAAGGTATCAGGAATCATA--ATCATCTTGTTCACTCTGCTGTACCCTCAGAATGTAAAACATTGCTAGACACAT 47321
```

```
Majority         CCCTGAATACCCAGTAAATGTTTAAGGAATTAATGAGTGAATTAATTTTGAAGACACTTT--GGAGACCAAGATAATCTT
                         59050     59060     59070     59080     59090     59100     59110     59120
Human            CCCTGAATACCCAGTAAATGTTTAAGGAATTAATGAGTGAATTAATTTTGAAGATACGTT--GGAGACCAAGATAATCTG 47301
Coquerel'sSifaka CCTCGA-TACTCAGTGAATGTTTAAAAAATTCATGAGTGAATGCATTTTGAAAAAACTTAAAGAAGATCAAGACGATCTT 39150
Ma'sNightMonkey  CCCTGAATACTCAGTAAATGTTTAAG-AATTAATGAGTGAATTGATTTTAAAGACACTT---AGAGACCAAGATAATCTT 42428
VervetAGM        CCCTGAATACCCAGTAAATGTTTAAGGAATTAATGAGTGAATTAATTTTGAAGACACTTT--GGAGACCAAGATAATCTT 49162
Gorrilla         CCCTGAATACCCAGTAAATGTTTAAGGAATTAATGAGTGAATTAATTTTGAAGATACTTG--GGAGACCAAGATAATCTG 47819
GrabEatingMacaqueCCCTGAATACCCAGTAAATGTTTAAGGAATTAATGAGTGAATTAATTTTGAAGACACTTT--GGAGACCAAGATAATCTT 48385
Macaque          CCCTGAATACCCAGTAAATGTTTAAGGAATTAATGAGTGAATTAATTTTGAAGACACTTT--GGAGACCAAGATAATCTT 49208
PigTailedMacaque CCCTGAATACCCAGTAAATGTTTAAGGAATTAATGAGTGAATTAATTTTGAAGACACTTT--GGAGACCAAGATAATCTT 48080
Bonobo           CCCTGAATACCCAGTAGATGTTTAAGGAATTAATGAGTGAATTAATTTTGAAGATACTTT--GGAGACCAAGATAATCTG 46405
OliveBaboon      CCCTGAATACCCAGTAAATGTTTAAGGAATTAATGAGTGAATTAATTTTGAAGACACTTT--GGAGACCAAGATAATCTT 47935
Orangutan        CCCTGAATACCCAGTAAATGTTTAAGGAATTAATGAGTGAATTAATTTTGAAGATACTTT--GGAGACCAAGATAATCTG 47399

Majority         ACAGTCTTACAGATATTGTTGAACATACACAAACACATTATATTGTTTATAGGTTGTGTTGAATGTTTTGGGAAATACCA
                         59130     59140     59150     59160     59170     59180     59190     59200
Human            ACAGTCTTATAGATGTTGTTGAACATACACAAACACATTATATAGTTTATAGGTTGTGTTGAATGTTTTAGGAAATACCA 47381
Coquerel'sSifaka AGAGTTTTGTAGATATTGTTGAGTATACACA----CACGATATTGTTTATAGATTGTGTTGAATGTTTTGGCAAATACTA 39226
Ma'sNightMonkey  A--GTCTTACAGATATTGTTGAACATACACAAATACATTATATTGTTTATAGGTTGTGTTGAATGTATTGGGGAATACCA 42506
VervetAGM        ACAGTCTTACAGATATTGTTGAACATATACAAACACATTATATTGTTTATAGGTTGTGTTGAATGTTTTGGGAAATACCA 49242
Gorrilla         ACAGTCTTATAGATGTTGTTGAACATACACAAACACATTATATAGTTTATAGGTTGTGTTTAATGTTTTAGGAAATACCA 47899
GrabEatingMacaqueACAGTCTTACAGATATTGTTGAACATATACAAACACATTATATTGTTTATAGGTTGTGTTGAATGTTTTGGGAAATACCA 48465
Macaque          ACAGTCTTACAGATATTGTTGAACATACACAAACACATTATATTGTTTATAGGTTGTGTTGAATGTTTTGGGAAATACCA 49288
PigTailedMacaque ACAGTCTTACAGATATTGTTGAACATATACAAACACATTATATTGTTTATAGGTTGTGTTGAATGTTTTGGGAAATACCA 48160
Bonobo           ACAGTCTTATAGATGTTGTTGAACATACACAAACACATTATATAGTTTATAGGTTGTGTTGAATGTTTTAGGAAATACCA 46485
OliveBaboon      ACAGTCTTACAGATATTGTTGAACATATACAAACACATTATATTGTTTATAGGTTGTGTTGAATGTTTTGGGAAATACCA 48015
Orangutan        ACAGGCTTATAGATATTGTT-AACATACACAAACACATTATATTGTTTATAGGTTGTATTGAATGTTTTGGGAAATACCA 47478

Majority         TAAATTACCTAAAGTTTTATACAAAATGG-AGTATACATGTATATTTCTGAATGTGTCAAGATCCTTCATTTTAGCTACA
                         59210     59220     59230     59240     59250     59260     59270     59280
Human            TAAATTACCTAAAGTTTTATACAAAATGG-AGTATACATGTATATTTCTGAATGTGTCAAGATCCTTCATTTTAGCTA-A 47459
Coquerel'sSifaka TAAAGTACCTACCATTTTATTCAAAATGG-ACTATCTATGTATATTTCTGAATGTGTAAGGATCCTTCAATTTAGTTAGA 39305
Ma'sNightMonkey  TAAAGTACCTAAAATTTTACGCAAAGTGG-AGTATGCATGTATATTTCTGAATGTGTCGAGATCCTTCATTTTAACTACA 42585
VervetAGM        TAAATTACCTAAAGGTTTATACAAAATGG-AGTATACATGTATATTTCTGAATGTGTCAAGATCCTTCATTTTAGCTACA 49321
Gorrilla         TAAATTACCTAAAGTTTTATACAAAATGG-AGTATACATGTATATTTCTGAATGTGTCAAGATCCTTCATTTTAGCTA-A 47977
GrabEatingMacaqueTAAATTACCTAAAGGTTTATACAAAATGG-AGTATACATGTATATTTCTGAATGTGTCAAGATCCTTCATTTTAGCTACA 48544
Macaque          TAAATTACCTAAAGGTTTATACAAAATGGGAGTATACATGTATATTTCTGAATGTGTCAAGATCCTTCATTTTAGCTACA 49368
PigTailedMacaque TAAATTACCTAAAGGTTTATACAAAATGG-AGTATACATGTATATTTCTGAATGTGTCAAGATCCTTCATTTTAGCTACA 48239
Bonobo           TAAATTACCTAAAGTTTTATACAAAATGG-AGTATACATGTATATTTCTGAATGTGTCAAGATCCTTCATTTTAGCTA-A 46563
OliveBaboon      TAAATTACCTAAAGGTTTATACAAAATGG-AGTATATATGTATATTTCTGAATGTGTCAAGATCCTTCATTTTAGCTACA 48094
Orangutan        TAAATTACCTAAAGTTTTATACAAAATGG-AGTATACATGTATATTTCTGAATGTGTCAAGATCCTTCATTTTAGCTACA 47557
```

```
Majority          GTCTCAGAAATATCCTTGAGATCCTAAAAGATTAAGAACCATTGATTTGGCATAGTAGATA-ACTGCAAGTCCTTCAAAC

                          59290     59300     59310     59320     59330     59340     59350     59360

Human             GTCTCAGAAATATCCTTGAGATCCTAAAAGACTAAGAACCATTGATCTGGCATAGTAGATATACTGCAAGTCCTTCAAAC 47539
Coquerel'sSifaka  GTCTCAGAAATATCCTTGAGTCCATAGAAGGTTAAGAAACGTCAGTATGGCATAGTGAATAGACTACAAATTCTTTAAAC 39385
Ma'sNightMonkey   GTCTCAGAAAT---CTTGAGTTGCTGAAAGATTAAGAACCATTTATCTGGCATAGTAGATAGACTGCAACTCCTTTAAAC 42662
VervetAGM         GTCTCAGAAATATCCTTGAGATCCTAAAAGATTGAGAACCATTGATTTGGCATAGTAGAT----TGCAAGTCCTTCAAAC 49397
Gorrilla          GTCTCAGAAATATCCTTGAGATCCTAAAAGACTAAGAACCATTGATCTGGCATAGTAGATATGCTGCAAGTCCTTCAAAC 48057
GrabEatingMacaqueGTCTCAGAAATATCCTTGAGATCCTAAAAGATTGAGAACCATTGATTTGGCATAGTAGAT----TGCAAGTCCTTCAAAC 48620
Macaque           GTCTCAGAAATATCCTTGAGATCCTAAAAGATTGAGAACCATTGATTTGGCATAGTAGAT----TGCAAGTCCTTCAGAC 49444
PigTailedMacaque  GTCTCAGAAATATCCTTGAGATCCTAAAAGATTGAGAACCATTGATTTGGCATAGTAGAT----TGCAAGTCCTTCAAAC 48315
Bonobo            GTCTCAGAAATATCCTTGAGATCCTAAAAGACTAAGAACCATTGATCTGGCGTAGTAGATATACTGCAAGTCCTTCAAAC 46643
OliveBaboon       GTCTCAGAAATATCCTTGAGATCCTAAAAGATTGAGAACCATTGATTTGGCATAGTAGAT----TGCAAGTCCTTCAAAC 48170
Orangutan         GTCTCAGAAATATCCTTGAGATCCTAAAAGACTAAGAACTATTGATCTGGCATAGTAGATATACTGCAAGTCCTTCAGAC 47637

Majority          AACTCTATTTAAA-TATTACCTATTTTATAGGCATTTTGAATAATGTTTTTATTTTTTGGTGAAGATTTGGAATGAAGGC

                          59370     59380     59390     59400     59410     59420     59430     59440

Human             AACTGTATTTAAA-TATTACCTATTTTATAGGCATTTTGAATAATATTTTTATTTTTTGGTGAAGTTTTGGAATGAAGAC 47618
Coquerel'sSifaka  AATTCTACATAAAACACCACCTATTTTATAGCCATCTTTAACA---TTTTTGCTTTT-GGTGTAGCTTTGGAATGAAGGC 39461
Ma'sNightMonkey   AACTCCATTTAAA-TATTACCTATTTTATAGGCATTTTGAATA---TTTTTATTTTT-GGTGAATATTTGAAATGAAGAC 42737
VervetAGM         AACTCTATTTAAA-TATTACCTGTTTTATAGGCATCTTGAATAATGTTTTTATTTTTTGGTGAAGATTTGGAATGAAGGC 49476
Gorrilla          AACTGTATTTAAA-TATTACCTATTTTATAGGCATTTTGAATAATATTTTTATTTTTTGGTGAAGTTTTGGAATGAAGAC 48136
GrabEatingMacaqueAACTCTATTTAAA-TATTACCTATTTTATAGGCATTTTGAATAATGTTTTTATTTTTTGGTGAAGATTTGGAATGAAGGC 48699
Macaque           AACTCTATTTAAA-TATTACCTATTTTATAGGCATTTTGAATAATGTTTTTATTTTTTGGTGAAGATTTGGAATGAAGGC 49523
PigTailedMacaque  AACTCTATTTAAA-TATTACCTATTTTATAGGCATTTTGAATAATGTTTTTATTTTTTGGTGAAGATTTGGAATGAAGGC 48394
Bonobo            AACTGTATTTAAA-TATTACCTATTTTATAGGCATTTTGAATAATATTTTTATTTTTTGGTGAAGTTTTGGAATGAAGAC 46722
OliveBaboon       AACTCTATTTAAA-TATTACCTATTTTATAGGCATTTTGAATAATGTTTTTATTTTTTGGTGAAGATTTGGAATGAAGGC 48249
Orangutan         AACTATATTTAAA-TATTATCTATTTTATAGGCATTTTGAATAATATTTTTATTTTTTGGTGAAGATTTGGAATGAAGAC 47716

Majority          AGTTAACTAATTTGTTGATTGAATTATATTACTTT-----AAATCTCATGGTAACAGAATAAGGTAATTAAGTTCATGAG

                          59450     59460     59470     59480     59490     59500     59510     59520

Human             AGTTAACTAATTTGTTGATTGAATTATGTTACTTTACTTTAAATCTCACGGTAACAGAATAAGGTAATTAAGTTCATGAG 47698
Coquerel'sSifaka  AGGTAACTAATTTGTTGATTGAAATATATTAGTTTACTTTAACGTTCAAGGTAACAGAATAAAGTAATTAATTTCTTGAG 39541
Ma'sNightMonkey   AGTTAACTAA---------TGAAGTATATTAGTTT-----AAATCTCACAGTAACAGAATAAGGTAATTAAGTTCATGAG 42803
VervetAGM         CGTTAACTAATTTGTTGATTGAATTATATTACTTT-----AAATCTCATGCTAATAGAATAAGATAATTAAGTTCATGAG 49551
Gorrilla          AGTTAACTAATTTGTTGATTGAGTTATGTTACTTTACTTTAAATCTCATGGTAACAGAATAAGGTAATTAAGTTCATGAG 48216
GrabEatingMacaqueCATTAACTAATTTGTTGATTAAATTATATTACTTT-----AAATCTCATGGTAATAGAATAAGATAATTAAGTTCATGAG 48774
Macaque           CATTAACTAATTTGTTGATTAAATTATATTACTTT-----AAATCTCATGGTAATAGAATAAGATAATTAAGTTCATGAG 49598
PigTailedMacaque  CGTTAACTAATTTGTTGATTAAATTATATTACTTT-----AAATCTCATGGTAATAGAATAAGATAATTAAGTTCATGAG 48469
Bonobo            AGTTAACTAATTTGTTGATTGAATTATGTTACTTT-----AAATCTCACGATAACAGAATAAGGTAACTAAGTTCATGAG 46797
OliveBaboon       CGTTAACTAATTTGTTGATTAAATTATATTACTTT-----AAATCTCATGGTAATAGAATAAGATAATTAAGTTCATGAG 48324
Orangutan         AGTTAACTAATTTGTTGATTGAATTATGTTAATTTACTTTAAATCTCACGGTAACAGAATAAGGTAATTAAGTTCATGAG 47796
```

```
Majority          TGATTTAGAGTATATT-GATGTTAATAGTCACAAAATATT-----AGGTAGTTTAGATGGTAATAGTAATATATTAGGAG
                          59530     59540     59550     59560     59570     59580     59590     59600
Human             TGATTTAGAGTATATT-GATGTTAATAGTCACAAAATATT-----AGGTAGTTTAGACGGCAATAGTAATATATCAGGAG 47772
Coquerel'sSifaka  TGATTCATAGTATATT-GATGTTAATAGC-ACAAAATATTTAAGTAGATAGATTAGGTGGTAATATTAACATATTAGGAA 39619
Ma'sNightMonkey   AGATTTAGAGTATATT----------GAT-----------------GTAGTTTAGATGGTAATAGTAATATATTAGGAG 42855
VervetAGM         TAATTTAGAGTATATA-GATCTTAATAGTCACAAAATATT-----AGGTAGTTTAGATGGTAATAGTAATATATTAGAAG 49625
Gorrilla          TGATTTAGAGTATATT-GATGTTAATAGTCACAAAATATT-----AGGTAGTTTAGACGGTAATAGTAATATATCAGGAG 48290
GrabEatingMacaqueTGATTTAGAATATATTTGATCTTAATAGTCACAAAATATT-----AGGTAGTTTAGATGGTAATAGTAATATATTAGAAG 48849
Macaque           TGATTTAGAGTATATT-GATCTTAATAGTCACAAAATATT-----AGGTAGTTTAGATGGTAATAGTAATATATTAGAAG 49672
PigTailedMacaque  TGATTTAGAGTATATT-GATCTTAATAGTCACAAAATATT-----AGGTAGTTTAGATGGTAATAGTAATATATTAGAAG 48543
Bonobo            TGATTTAGAGTATATT-GATGTTAATAGTCACAAAATATT-----AGGTAGTTTAGACGGTAATAGTAATATATCAGGAG 46871
OliveBaboon       TGATTTAGAGTATATT-GATCTTAATAGTCACAAAATATT-----AGGTAGTTTAGATGGTAATAGTAATATATTAGAAG 48398
Orangutan         TGATTTAGAGTATATT-GATGTTAATAGTCACAAAATATT-----AGGTACTTTAGATGGTAATAGTAATATATCAGGAG 47870

Majority          AGGCTCATTGTAGATAAGCCATAATATGGATTGACTGCATTATCATTTTTATAAACTTTACATTCTTTGATCCCACCTTC
                          59610     59620     59630     59640     59650     59660     59670     59680
Human             AGGCTCATTGTAGATAAGCCATAATATGGATTGACTGCATTATCATTTTTATAAACTTTACATTCTTTGATCCCACCTTC 47852
Coquerel'sSifaka  AGGCTCAGTGTAGATAAGCCATAATGTGGATTGACTACATAGTCATTTTT-TTAGCATTGCCCTCTTTGATCCCACTTTT 39698
Ma'sNightMonkey   AGGCTCATTGTAGGTAAGCCATAATATGGATTGACTGCATAATCATTTTTATAAGCTTTACATTCTTCGATCCCACCTTC 42935
VervetAGM         AAGCTCATTGTAGATAAGCCATAAT-TGGATTGACTGCATTATCAGTTTTATAAACTTTACATTCTTTGATCCCACATTC 49704
Gorrilla          AGGCTCATTGTAGATAAGCCATAATATGGATTGACTGCATTATCATTTTTATAAACTTTACATTCTTTGATCCCACCTTC 48370
GrabEatingMacaqueAAGCTCATTGTAGATAAGCCATAAT-TGGATTGACTGCATTATCAGTTTTGTAAACTTTACATTCTTTGATCCCACATTC 48928
Macaque           AAGCTCATTGTAGATAAGCCATAAT-TGGATTGACTGCATTATCAGTTTTGTAAACTTTACATTCTTTGATCCCACATTC 49751
PigTailedMacaque  AAGCTCATTGTAGATAAGCCATAAT-TGGATTGACTGCATTATCAGTTTTGTAAACTTTACATTCTTTGATCCCACATTC 48622
Bonobo            AGGCTCATTGTAGATAAGCCATAATATGGATTGACTGCATTATCATTTTTATAAACTTTACATTCTTTGATCCCACCTTC 46951
OliveBaboon       AAGCTCATTGTAGATAAGCCATAAT-TGGATTGACTGCATTATCAGTTTTGTAAACTTTACATTCTTTGATCCCACATTC 48477
Orangutan         AGGCTCATTGTAGATAAGCCATAATATGGATTGACTGCATTATCATTTTTATAAACTTTACATTCTTTGATCCCACCTTC 47950

Majority          AAGTACA---TTTTTCAAATCAAACAATTTATAATTTCTCAGGTAAAATTATAATCTTCCTAATTTTTGTCAACCCAGTT
                          59690     59700     59710     59720     59730     59740     59750     59760
Human             AAGTACA---TTTTTCAAATCAAACAATTTATAATTTCTCAGGTAAAATTATAATCTTCCTAATTTTGGTCAAACCAGTT 47929
Coquerel'sSifaka  GAGTACAACATTTTTGAAATTGAACAATTTATAATTTTTCAGGTAAAATTATAATCTTCATAATTTTTGCTAACTCAGTT 39778
Ma'sNightMonkey   AAGTACA---TTTTCCAAATTGAACAATTTATAATTTCTCAGTTAAA-TTATAATCTCCCTAATTTTTGTCAGCCCAGTT 43011
VervetAGM         AAGTACA---TTTTTCATATCGAACAATTTATCATTTCTCAGGTAAAATTAT---CTTCCTAATTTTTGTCAACCTAGTT 49778
Gorrilla          AAGTACA---TTTTTCAAATCAAACAATTTATAATTTCTCAGGTAAAATTATAATATTCCTAATTTTGGTCAAACCAGTT 48447
GrabEatingMacaqueAAGTACG---TTTTTCAAATCAAACAATTTATCATTTCTCAGGTAAAATTAT---CTTCCTAATTTTTGTCAACCTAGTT 49002
Macaque           AAGTACG---TTTTTCAAATCAAACAATTTATCATTTCTCAGGTAAAATTAT---CTTCCTAATTTTTGTCAACCTAGTT 49825
PigTailedMacaque  AAGTACG---TTTTTCAAATCAAACAATTTATCATTTCTCAGGTAAAATTAT---CTTCCTAATTTTTGTCAACCTAGTT 48696
Bonobo            AAGTACA---TTTTTCAAATCAAACAATTTATAATTTCTCAGGTAAAATTATAATCTTCCTAATTTTGGTCAAACCAGTT 47028
OliveBaboon       AAGTACG---TTTTTCAAATTGAACAATTTATCATTTCTCAGGTAAAATTAT---CTTCCTAATTTTTGTCAACCTAGTT 48551
Orangutan         AAGTACA---TTTTTCAAATCAAACAATTTATAATTTCTCAGGTAAAATTATAATCTTCCTAATTTTTGTCAACCCAGTT 48027
```

```
Majority        TTTTCACTTTTTAAAAATATTCATATTAGTTCATTATAAGATGATTAAATTACAAGATACAGTCATAAATCTTGGAGATA

                         59770     59780     59790     59800     59810     59820     59830     59840

Human           TTTTCACTTTTTAAAAATGTTCATATTAGTTCATTATAAGATGATTAAATTACAAGATACAGTCATAAATCTTGGGGATA 48009
Coquerel'sSifaka TTTTCACTTTTTTTTGGTATTTCTGTTAGTTTGTCATAAAATGATTAAATTGCAAGATACAGTCATGCCTC----ACTTA 39854
Ma'sNightMonkey TTA-CACTTTTTAGAAATATT--TCTTAGTCCATTAAAAGATGATT-----ACAGTATACAGTCATAATTCTTGGAGATC 43083
VervetAGM       TTTTCACTTTT-AAAAATATTCATATTAGTTCATTATAAGATGATTCAGTTACAAGATACAGTCATAAATCTTGGAGATA 49857
Gorrilla        TTTTCACTTTTTAAAAATGTTCATATTAGTTCATTATAAGATGATTAAATTACAAGATACAGTCATAAATCTTGGGGATA 48527
GrabEatingMacaqueTTTTCACTTTT-AAAAATATTCATATTAGTTCATTATAAGATGATTAAATTACAAGATACAGTCATAAATCTTGGAGATA 49081
Macaque         TTTTCACTTTT-AAAAATATTCATATTAGTTCATTATAAGATGATTAAATTACAAGATACAGTCATAAATCTTGGAGATA 49904
PigTailedMacaque TTTTCACTTTT-AAAAATATTCATATTAGTTCATTATAAGATGATTAAATTACAAGATACAGTCATAAATCTTGGAGATA 48775
Bonobo          TTTTCACTTTTTAAAAATGTTCATATTAGTTCATTATAAGATGATTAAATTACAAGATACAGTCATAAATCTTGGGGATA 47108
OliveBaboon     TTTTCACTTTT-AAAAATATTCATATTAGTTCATTATAAGATGATTAAATTACAAGATACAGTCATAAATCTTGGAGATA 48630
Orangutan       TTTTCACTTTTTAAAAATGTTCATATTAGTTCATTATAAGATGATTAAATTACAAGATACAGTCATAAATCTTGGGGATA 48107

Majority        GTGATGGGGATACCCTCCGAGAAATGCATTGTTAGGTAATTTTGTCACTGCATAAATGTCATAGAGT-ACATTTACACAA

                         59850     59860     59870     59880     59890     59900     59910     59920

Human           GTGATGAGGATACTCTCGGAGAAATGCATTGTTAGGTAATTTTGTCACTGCATAAATGTCATAGAGT-ACATTTGCACAA 48088
Coquerel'sSifaka ATGATGAGGTTATATTCTGAGAAATGCTTTGTTAGGTGATTTTGTCATTGTACAAA---CATAGAATTGTACTTACACAA 39931
Ma'sNightMonkey ATGGTGGGGATACCCTTTAAGAAACGCATTGTTAGGTAATTTTGTCATTGCGCAAATGTCATAGAGT-ACATTTACACAA 43162
VervetAGM       GTGATGGGGATACCCTCCGGGAAATGCATTGTTAGGTAATTTTGTCACTGCATAAATGTCATAGAGT-ACATTTACACAA 49936
Gorrilla        GTGATGAGGATACTCTTGGAGAAATGCATTGTTAGGTAATTTTGTCACTGCATAAATGTCATAGAGT-ACATTTGCACAA 48606
GrabEatingMacaqueGTGATGGGGATACCCTCCGGGAAGTGCATTGTTAGGTAATTTTGTCACTGCATAAATGTCATAGAGT-ACATTTACACAA 49160
Macaque         GTGATGGGGATACCCTCCGGGAAATGCATTGTTAGGTAATTTTGTCACTGCATAAATGTCATAGAGT-ACATTTACACAA 49983
PigTailedMacaque GTGATGGGGATACCCTCCGGGAAATGCATTGTTAGGTAATTTTGTCACTGCATAAATGTCATAGAGT-ACATTTACACAA 48854
Bonobo          GTGATGAGGATACTCTCGCAGAAATGCATTGTTAGGTAATTTTGTCACTGCATAAATGTCATAGAGT-ACATTTGCACAA 47187
OliveBaboon     GTGATGGGGATACCCTCCGGGAAATGCATTGTTAGGTAATTTTGTCACTGCATAAATGTCATAGAGT-ACATTTACACAA 48709
Orangutan       GTGATGAGGATACCCTCGGAGAAATGCATTGTTAGGTAATTTTGTCATTGCATAAATGTCATAGAGT-ACATTTACACAA 48186

Majority        ACCT----GCTACACATGTAGGC-TATATGGTATATGTAG-----CCTTTTGCTCCTAGGCA----ACAAACTTGTATAG

                         59930     59940     59950     59960     59970     59980     59990     60000

Human           ACCT----GCTACACATGTAGGC-TATATGGTATATGTAG-----CCTTTTGCTCCTAGGCA----ACAAATTTG-ACAG 48153
Coquerel'sSifaka ACCTAGATGGTATAGCTTAGTGCACACCTAGGCAATGTGGTGTAGTCTGTTGTGCCTACGCT----ACAAATCTGTGTGG 40007
Ma'sNightMonkey ACCT----GCTACACATGCAGGC-TATATGGTATATGTAGG-TAGCCTTTTGCTCCTAGGCAGGCAACAAACTTGCACAG 43236
VervetAGM       ACAT----GCTACCCATGTAGGC-TATATGGTATATGTAG-----CCTTTTGCTCCTAGGCA----ACCAACTTGTATAG 50002
Gorrilla        ACCT----GCTACACGTGTAGGC-TATATGGTATATGTAG-----CCTTTTGCTCCTAGGCA----ACAAACTTG-ACAG 48671
GrabEatingMacaqueACGT----GCTACCCATGTAGGC-TATATGGTATATGTAG-----CCTTTTGCTCCTAGGCA----ACCAACTTGTATAG 49226
Macaque         ACGT----GCTACCCATGTAGGC-TATATGGTATATGTAG-----CCTTTTGCTCCTAGGCA----ACCAACTTGTATAG 50049
PigTailedMacaque ACAT----GCTACCCATGTAGGC-TATATGGTATATGTAG-----CCTTTTGCTCCTAGGCA----ACCAACTTGTATAG 48920
Bonobo          ACCT----GCTACACATGTAGGC-TATATGGTATATGTAG-----CCTTTTGCTCCTAGGCA----ACAAACTTG-ACAG 47252
OliveBaboon     ACAT----GCTACCCATGTAGGC-TATATGGTATATGTAG-----CCTTTTGCTCCTAGGCA----ACCAACTTGTATAG 48775
Orangutan       ACCT----GCCACACATGTAGGC-AATGTGGTGTATGTAG-----CCTTTTGCTCCTAGGCA----ACAAACTTG-ACAG 48251
```

```
Majority          CATGTTA-CTGTTCTGAATACTGGAGGCAGTTGTAACACAGTAGTAAGTATTGATGTATCTAAACAGAAA-AGGTACAAT

                        60010     60020     60030     60040     60050     60060     60070     60080

Human             CATGTTA-CTGTTCTGAATACTGGAGGCAGTTGCAACACAGTAATAAGTATTGATGTATCTAAACAGAAA-AGGTACAAT 48231
Coquerel'sSifaka  CATGTTAACTGTACTGAATACTGAAGGCGGTTATAACACGTTAAT---TATATATGTATTTAAACATATCTAGATATAGA 40084
Ma'sNightMonkey   CATGTTA-CTGTTCTGAATACTGGAGGCAGTTGTAACACAATAGGAAGCATTGGTATATCTAAACAGAAA-AGGTACAAT 43314
VervetAGM         CATGTTA-CTGTTCTGAATACTGGAGGCAGTTGTAACACAGTAGTAAGTATTGATGTTTCTAAACAGAAA-AGGTACAAT 50080
Gorrilla          CATGTTA-CTGTTCTGAATACTGGAGGCAGTTGTAACACAGTAGTAAGTATTGATGTATCTAAACAGAAA-AGGTACAAT 48749
GrabEatingMacaqueCATGTTA-CTGTTCTGAATACTGGAGGCAGTTGTAACACAGTAGTAAGTATTGATGTTTCTAAACAGAAA-AGGTACAAT 49304
Macaque           CATGTTA-CTGTTCTGAATACTGGAGGCAGTTGTAACACAGTAGTAAGTATTGATGTTTCTAAACAGAAA-AGGTACAAT 50127
PigTailedMacaque  CATGTTA-CTGTTCTGAATACTGGAGGCAGTTGTAACACAGTAGTAAGTATTGATGTTTCTAAACAGAAA-AGGTACAAT 48998
Bonobo            CATGTTA-CTGTTCTGAATACTGGAGGCAGTTGTAACACAGTAATAAGTATTGATGTATCTAAACAGAAA-AGGTACAAT 47330
OliveBaboon       CATGTTA-CTGTTCTGAATACTGGAGGCAGTTGTAACACAGTAGTAAGTATTGATGTTTCTAAACAGAAA-AGGTACAAT 48853
Orangutan         CATGTTA-CTCTTCTGAATACTGGAGGCAGTTGTAACACAGTAGTAAGTATTGATGTATCTAAACAGAAA-AGGTACAAT 48329

Majority          AAAAATATGGTAT---TATAATCTTATGGGACCACCATTGTATATGTGGCCTGTCAATGACCAAAATGTAGTTA-TGTGG

                        60090     60100     60110     60120     60130     60140     60150     60160

Human             AAAAATATGGTAT---TATAATCTTATGGGACCACCATTGTATATGTGGCCTGTCAATGACCAAAATGTAGTTA-TGTGG 48307
Coquerel'sSifaka  AAAGGTACAGTAAAAACATAATCCTGTGGCACCACCATCATATATGTGGCCCATTGTTGACCAAAATATAATTG-TGTGG 40163
Ma'sNightMonkey   AAAAATATTGATT---TATAATCTTATGGGACTACCATTGTATATGTGGCCTGTCAGTGACCAAAATGTAGTCA-TGTGT 43390
VervetAGM         AAAAATACGGTAT---TATAATCTTATGGGACCACCATTGTATATGTGGCCTCTCAGTGACCAAAATGTAGTTAATGTGG 50157
Gorrilla          AAAAATATGGTAT---TATAATCTTATGGGACCACCATTGTATATGCGGCCTGTCAATGACCAAAATGTAGTTA-TGTGG 48825
GrabEatingMacaqueAAAAATATGGTAT---TATAATCTTATGGGACCACCATTGTATATGTGGCCTGTCAATGACCAAAATGTAGTTAATGTGG 49381
Macaque           AAAAATATGGTAT---TATAATCTTATGGGACCACCATTGTATATGTGGCCTGTCAATGACCAAAATGTAGTTAATGTGG 50204
PigTailedMacaque  AAAAATATGGTAT---TATAATCTTATGGGACCACCATTGTATATGTGGCCTGTCAATGACCAAAATGTAGTTAATGTGG 49075
Bonobo            AAAAATATGGTAT---TATAATCTTATGGGACCACCATTGTATATGTGGCCTGTCAATGACCAAAATGTAGTTA-TGTGG 47406
OliveBaboon       AAAAATACGGTAT---TATAATCTTATGGGACCACCATTGTATATGTGGCCTGTCAGTGACCAAAATGTAGTTAATGTGG 48930
Orangutan         AAAAATATGGTAT---TATAATCTTATGGGACCACCATTGTAGATGCGGCCTGTCAGTGACCAAAATGTAGTTA-TGTGG 48405

Majority          CACGTGACTGTGATTGTATTCTTTAGTC-----ATTGGACAGTTTCTTTATGAGATTACATTTTAATGTTGTTGCTTATT

                        60170     60180     60190     60200     60210     60220     60230     60240

Human             CACATGACTGTGATTGTATTCTTTAGTC-----ATTGGACAGTTTCTTTATGAGATTACATTTTAATGTTGTTGCTTATT 48382
Coquerel'sSifaka  TACATGGCTGTAATTGTATTCTTTAGTC-----ATTGTGTAGTTTCTT--TGAGATTGCATTTTAATGTTTTTGTTTATC 40236
Ma'sNightMonkey   TACATGACTGTGATAGTATTTTTCAGTC-----ATTGGACAGTTTCTTTATGAGATTACATTTTAATGTTGTTGCTTATT 43465
VervetAGM         CACGTGACTGTGATTGTATTCTTTAGTCTAGTCATTGGACAGTTTCTTTATGAGATTACATTTTAATGTTATT------- 50230
Gorrilla          CACGTGACTGTGATTGTATTCTTTAGTC-----ATTGGACAGTTTCTTTATGAGATTACATTTTAATGTTGTTGCTTATT 48900
GrabEatingMacaqueCACGTGACTGTGATTGTATTCTTTAGTC-----ATTGGACAGTTTCTTTATGAGATTACATTTTAATGTTGTT----ATT 49452
Macaque           CACGTGACTGTGATTGTATTCTTTAGTC-----ATTGGACAGTTTCTTTATGAGATTACATTTTAATGTTGTT----ATT 50275
PigTailedMacaque  CACGTGACTGTGATTGTATTCTTTAGTC-----ATTGGACAGTTTCTTTATGAGATTACATTTTAATGTTGTT----ATT 49146
Bonobo            CACATGACTGTGATTGTATTCTTTAGTC-----ATTGGACAGTTTCTTTATGAGATTACATTTTAATGTTGTTGCTTATT 47481
OliveBaboon       CACGTGACTGTGATTGTATTCTTTAGTC-----ATTGGACAGTTTCTTTATGAGATTACATTTTAATGTTGTTG-TTATT 49004
Orangutan         CACATGACTGTGATTGTATTCTTTAGTC-----ATTGGACAGTTTCTTTATGAGATTACATTTTAATGTTGTTGCTTATT 48480
```

```
Majority          AATAATTACTCTTTATA-ATACTGTTGAGAAACACAT----TAACAGCAATCTTAACTAATTGATAATTTCATTTAATAA

                           60250     60260     60270     60280     60290     60300     60310     60320

Human             AATAATTACTCTTTATA-ATACTGTTAAGAAAC--AT----TAACAGCAATCTTAAATAGTTGATAATTTCATTTAATAA 48455
Coquerel'sSifaka  ACTTACTACTCTTTACATATACTGTTCGTAAATATATCGTTTAACAGCAATCCTAACTAATTGATA--CTCATTTAGTAA 40314
Ma'sNightMonkey   AATA-TTACTCTTTACA-ATGTTGTTGAGAAACATAT----AAACAGCAATCTTAACTAATTGATAATTTCATTATGGAA 43539
VervetAGM         AATAATTACTCTTTATA-ATACTGTTGAGAAACACAT----TAACAGCAATCTTAACTAATTGATAATTTCATTTAATAA 50305
Gorrilla          AATAATTACTCTTTATA-ATACTGTTAAGAAAC--AT----TAACAGCAATCTTAAATAATTGATAATTTCATTTAATAA 48973
GrabEatingMacaqueAATAATTACTCTTTATA-ATACTATTGAGAAACACAT----TAACAGCAATCTTAACTAATTGATAATTTCATTTAATAA 49527
Macaque           AATAATTACTCTTTATA-ATACTATTGAGAAACACAT----TAACAGCAATCTTAACTAATTGATAATTTCATTTAATAA 50350
PigTailedMacaque  AATAATTACTCTTTATA-ATACTATTGAGAAACACAT----TAACAGCAATCTTAACTAATTGATAATTTCATTTAATAA 49221
Bonobo            AATAATTACTCTTTATA-ATACTGTTAAGAAAC--AT----TAACAGCAATCTTAAATAGTTGATAATTTCATTTAATAA 47554
OliveBaboon       AATAATTACTCTTTATA-ATACTGTTGAGAAACACAT----TAACAGCAATCTTAACTAATTGATAATTTCATTTAATAA 49079
Orangutan         AATAATTACTCTTTATA-ATGCTGTTGAGAAAC--AT----TAACAGCAATCTTAACTAATTGATAATTTCATTTAATAA 48553

Majority          TA---GGTGGATATGATTTT----ATTCCTTTGAAAGCCTAGCATTTG-AATTTTTAAGAATTATTTAAGTATTCTCAGC

                           60330     60340     60350     60360     60370     60380     60390     60400

Human             TAATAGGTGGATATGATTTT----ATTCCTTTGAAAGCCTGGCATTTG-AATTTTCAAGAATTATTTAAGTATTCTCAGC 48530
Coquerel'sSifaka  TAAGCGATGGATATGATTTTCTCTATTCCTTTGAAAGCTCAGTTTTTTTAATTGTTAGGAATTATTCAGGTATACCCAGG 40394
Ma'sNightMonkey   TA---GGTAGATATGATTTT----ATTCCTTTGAAAGTCTAGTTTTTG-AATT------------TTAAGTATTACCAGC 43599
VervetAGM         TA---GGTGGATGGGATTTT----ATTCCTTTGAAAGCCTAGCATTTG-AATTTTTAAGAATTATTTAAGTATTCTCAGA 50377
Gorrilla          TAATAGGTGGATATGATTTT----ATTCCTTTGAAAACCTGGCATTTG-AATTATCAAGAATTATTTAAGTATTCTCAGC 49048
GrabEatingMacaqueTA---GGTGGATGGGATTTT----ATTCCTTTGAAAGCCTAGCATTTG-AATTTTTAAGAATTATTTAAGTATTCTCAGA 49599
Macaque           TA---GGTGGATGGGATTTT----ATTCCTTTGAAAGCCTAGCATTTG-AATTTTTAAGAATTATTTAGGTATTCTCAGA 50422
PigTailedMacaque  TA---GGTGGATGGGATTTT----ATTCCTTTGAAAGCCTAGCATTTG-AATTTTTAAGAATTATTTAAGTATTCTCAGA 49293
Bonobo            TAATAGGTGGATATGATTTT----ATTCCTTTGAAAGGCTGGCATTTG-AATTTTCAAGAATTATTTAAGTAGTCTCAGC 47629
OliveBaboon       TA---GGTGGATGGGATTTT----ATTCCTTTGAAAGCCTAAAATTTG-AATTTTTAAGAATTATTTAAGTATTCTCAGA 49151
Orangutan         TAATAGGTGGATATGATTTT----ATTCCTTTGAAAGCCTAGCGTTTG-AATTTTCAAGAATTATTTAAGTATTCTCAGC 48628

Majority          TATGTTTTTAAGTTCTTTT-------------------------------------------------------------

                           60410     60420     60430     60440     60450     60460     60470     60480

Human             TATGTTTTTAAGTTCTTTT------------------------------------------------------------- 48549
Coquerel'sSifaka  TATGTTTTTAAGGTTTTTTAAAATTTGTTTTGTAACATAATGAGTATGATGTACACCATCTGGGGGATGGACATGCTTGA 40474
Ma'sNightMonkey   TTTGTTTTTAAGTTCTTTT------------------------------------------------------------- 43618
VervetAGM         TACGTTTTTAAGTTCTTTT------------------------------------------------------------- 50396
Gorrilla          TATGTTTTTAAGTTCTTTT------------------------------------------------------------- 49067
GrabEatingMacaqueTACGTTTTTAAGTTCTTTT------------------------------------------------------------- 49618
Macaque           TACGTTTTTAAGTTCTTTT------------------------------------------------------------- 50441
PigTailedMacaque  TACGTTTTTAAGTTCTTTT------------------------------------------------------------- 49312
Bonobo            TATGTTTT-AAGTTCCTTT------------------------------------------------------------- 47647
OliveBaboon       TACGTTTTTAAGTTCTTTT------------------------------------------------------------- 49170
Orangutan         TATGTTTTCAAGTTCTTTT------------------------------------------------------------- 48647
```

```
Majority          ----------------------------------------------------------------------TGGAAATTTG
                          60490     60500     60510     60520     60530     60540     60550     60560
Human             ----------------------------------------------------------------------TGGAAATTTG 48559
Coquerel'sSifaka  AGCTCTGCCTTGGGGTGGGGGGACAAGGACAATGTAACCTAAATATTTGTACCCCCATAATATGCAGAAATAAAAATTTG 40554
Ma'sNightMonkey   ----------------------------------------------------------------------TGGAAATTTG 43628
VervetAGM         ----------------------------------------------------------------------TGGAAATTTG 50406
Gorrilla          ----------------------------------------------------------------------TGGAAATTTG 49077
GrabEatingMacaque ----------------------------------------------------------------------TGGAAATTTG 49628
Macaque           ----------------------------------------------------------------------TGGAAATTTG 50451
PigTailedMacaque  ----------------------------------------------------------------------TGGAAATTTG 49322
Bonobo            ----------------------------------------------------------------------TGGAAATTTG 47657
OliveBaboon       ----------------------------------------------------------------------TGGAAATTTG 49180
Orangutan         ----------------------------------------------------------------------TGGAAATTTG 48657

Majority          T-----AGTTTTTT-ACACAAATA-GGAAAATTCATACATTCTTTTATTGTTGATATATAAAGACTATTTTTT-AATGTC
                          60570     60580     60590     60600     60610     60620     60630     60640
Human             T-----AGTTTTTTTACACAAATA-GGAAAATTCATACATTCTTTTATTGTTGATATATAAGGACTGTTTTTT-AATGTC 48632
Coquerel'sSifaka  TTTTGTAGTTTTTT-GCACGAATAAGGAAAATTACTATATTCTTTTATTGTTAATATATAGAGACTATTTTTTTAATGTC 40633
Ma'sNightMonkey   TTTTGTAGGTTTTT-ACACAAATA-GGAAAATTCATACATTCTTTTATTGTTGCTATATAAAGATTATTTTTT-AATGTC 43705
VervetAGM         T-----AGTTTTTT-ACATAAATA-GGAAGATTCATACATTCTTTTATTGTTGATATATAAAGACTATTTTTT-AATGTC 50478
Gorrilla          T-----AGTTTTTTTACACAAATA-GGAAAATTCATACATTCTTTTATTGTTGATATATAAGGACTGTTTGTT-AATGTC 49150
GrabEatingMacaque T-----AGTTTTTT-ACATAAATA-GGAAGATTCATACATTCTTTTATTGTTGATATATAAAGACTATTTTTT-AATGTC 49700
Macaque           T-----AGTTTTTT-ACATAAAGA-GGAAGATTCATACATTCTTTTATTGTTGATATATAAAGACTATTTTTT-AATGTC 50523
PigTailedMacaque  T-----AGTTTTTT-ACATAAATA-GGAAGATTCATACATTCTTTTATTGTTGATATATAAAGACTATTTTTT-AATGTC 49394
Bonobo            T-----AGTTTTTT-ACACAAATA-GGAAAATTCATACATTCTTTTATTGTTGATATATAAGGACTGTTTTTT-AATGTC 47729
OliveBaboon       T-----AGTTTTTT-ACATAAATA-GGAAGATTCATACATTCTTTTATTGTTGATATATAAAGACTATTTTTT-AATGTC 49252
Orangutan         T-----AGTTTTTTTACACAAATA-GGAAAATTCATACATTCTTTTATTGTTGATGTATAAAGACTGTTTTTT-AATGTC 48730

Majority          TTTCAGCCAATGGTAATGATAGTAAAAAATTTAAAGGAGAAGATAAAATGGATGGTGCTCCTTCTCGTGTACTTCATATT
                          60650     60660     60670     60680     60690     60700     60710     60720
Human             TTTCAGCCAATGGTAATGATAGTAAAAAATTTAAAGGAGAAGATAAAATGGATGGTGCTCCTTCTCGTGTACTTCATATT 48712
Coquerel'sSifaka  TTTCAGCCAATGGTAATGATAGTAAAAAATTTAAAGGAGAAGATAAAATGGATGGTGCACCTTCTCGTGTACTTCATATT 40713
Ma'sNightMonkey   TTTCAGCCAATGGTAATGATAGTAAAAAATTTAAAGGAGAAGATAAAATGGATGGTGCTCCTTCTCGTGTACTTCATATT 43785
VervetAGM         TTTCAGCCAATGGTAATGATAGCAAAAAATTTAAAGGAGAAGATAAAATGGATGGTGCTCCTTCTCGTGTACTTCATATT 50558
Gorrilla          TTTCAGCCAATGGTAATGATAGTAAAAAATTTAAAGGAGAAGATAAAATGGATGGTGCTCCTTCTCGTGTACTTCATATT 49230
GrabEatingMacaque TTTCAGCCAATGGTAATGATAGTAAAAAATTTAAAGGAGAAGATAAAATGGATGGTGCTCCTTCTCGTGTACTTCATATT 49780
Macaque           TTTCAGCCAATGGTAATGATAGTAAAAAATTTAAAGGAGAAGATAAAATGGATGGTGCTCCTTCTCGTGTACTTCATATT 50603
PigTailedMacaque  TTTCAGCCAATGGTAATGATAGTAAAAAATTTAAAGGAGAAGATAAAATGGATGGTGCGCCTTCTCGTGTACTTCATATT 49474
Bonobo            TTTCAGCCAATGGTAATGATAGTAAAAAATTTAAAGGAGAAGATAAAATGGATGGTGCTCCTTCTCGTGTACTTCATATT 47809
OliveBaboon       TTTCAGCCAATGGTAATGATAGTAAAAAATTTAAAGGAGAAGATAAAATGGATGGTGCTCCTTCTCGTGTACTTCATATT 49332
Orangutan         TTTCAGCCAATGGTAACGATAGTAAAAAATTTAAAGGAGAAGATAAAATGGATGGTGCTCCTTCTCGTGTACTTCATATT 48810
```

```
Majority         CGAAAATTACCTGGGGAAGTAACAGAAACTGAAGTTATTGCTTTAGGCTTACCTTTTGGTAAGGTGACCAACATCCTTAT

                         60730     60740     60750     60760     60770     60780     60790     60800

Human            CGAAAATTACCTGGGGAAGTAACAGAAACTGAAGTTATTGCTTTAGGCTTACCTTTTGGTAAGGTGACCAACATCCTTAT 48792
Coquerel'sSifaka CGGAAATTACCTGGAGAAGTGACAGAAACTGAAGTTATTGCTTTAGGCTTACCTTTTGGTAAGGTGACCAACATCCTTAT 40793
Ma'sNightMonkey  CGAAAATTACCTGGGGAAGTAACAGAAACTGAAGTTATTGCTTTAGGCTTACCTTTTGGCAAGGTGACCAACATCCTTAT 43865
VervetAGM        CGAAAATTACCTGGGGAAGTAACAGAAACTGAAGTTATTGCTTTAGGCTTACCTTTTGGTAAGGTGACCAACATCCTTAT 50638
Gorrilla         CGAAAATTACCTGGGGAAGTAACAGAAACTGAAGTTATTGCTTTAGGCTTACCTTTTGGTAAGGTGACCAACATCCTTAT 49310
GrabEatingMacaqueCGAAAATTACCTGGGGAAGTAACAGAAACTGAAGTTATTGCTTTAGGCTTACCTTTTGGTAAGGTGACCAACATCCTTAT 49860
Macaque          CGAAAATTACCTGGGGAAGTAACAGAAACTGAAGTTATTGCTTTAGGCTTACCTTTTGGTAAGGTGACCAACATCCTTAT 50683
PigTailedMacaque CGAAAATTACCTGGGGAAGTAACAGAAACTGAAGTTATTGCTTTAGGCTTACCTTTTGGTAAGGTGACCAACATCCTTAT 49554
Bonobo           CGAAAATTACCTGGGGAAGTAACAGAAACTGAAGTTATTGCTTTAGGCTTACCTTTTGGTAAGGTGACCAACATCCTTAT 47889
OliveBaboon      CGAAAATTACCTGGGGAAGTAACAGAAACTGAAGTTATTGCTTTAGGCTTACCTTTTGGTAAGGTGACCAACATCCTTAT 49412
Orangutan        CGAAAATTACCTGGGGAAGTAACAGAAACTGAAGTTATTGCTTTAGGCTTACCTTTTGGTAAGGTGACCAACATCCTTAT 48890

Majority         GCTGAAAGGAAAAAATCAGGTACACTTCTTTTGGGGTTTATGAAATGTTAAACCCCAAACT--ATCCAGCAGGTGTGAAT

                         60810     60820     60830     60840     60850     60860     60870     60880

Human            GCTGAAAGGAAAAAATCAGGTACACTTCTTTCAGGGTTTATGAAATGTTAAACCCCAAACT--ATCCAGCAGGTGTGAAT 48870
Coquerel'sSifaka GCTGAAAGGAAAAAATCAGGTACAGTTCTTTTAGGGTTTATGACATGTTAAACTCCAAACT--ACCCAACAGATATGAAT 40871
Ma'sNightMonkey  GCTGAAAGGAAAAAATCAGGTACACTTCTTTCGGGGGTTATAAAATGTTAAACCCCAAACTGTATCCAGCAG-TGTGAAT 43944
VervetAGM        GCTGAAAGGAAAAAATCAGGTACACTTTTTTTGGGGTTTATGAAATGTTAAACCCCAAACT--ATCCAGCAGATGTGAAT 50716
Gorrilla         GCTGAAAGGAAAAAATCAGGTACACTTCTTTCAGGGTATATGAAATGTTAAACCCCAAACT--GTCCAGCAGGTGTGAAT 49388
GrabEatingMacaqueGCTGAAAGGAAAAAATCAGGTACACTTTTTTTGGGGTTTATGAAATGTTAAACCCCAAACT--ATCCAGCAGGTGTGAAT 49938
Macaque          GCTGAAAGGAAAAAATCAGGTACACTTTTTTTGGGGTTTATGAAATGTTAAACCCCAAACT--ATCCAGCAGGTGTGAAT 50761
PigTailedMacaque GCTGAAAGGAAAAAATCAGGTACACTTTTTTTGGGGTTTATGAAATGTTAAACCCCAAACT--ATCCAGCAGGTGTGAAT 49632
Bonobo           GCTGAAAGGAAAAAATCAGGTACACTTCTTTCAGGGTTTATGAAATGTTAAACCCCAAACT--ACCCAGCAGGTGTGAAT 47967
OliveBaboon      GCTGAAAGGAAAAAATCAGGTACACTTTTTTTGGGGTTTATGAAATGTTAAACCCCAAACT--ATCCAGCAGGTGTGAAT 49490
Orangutan        GCTGAAAGGAAAAAATCAGGTATACTTCTTTCGGGGTTTATGAAATGTTAAACCCCAAACT--ATCCAGCAGGTGTGAAT 48968

Majority         TAA-T----TGTAA-AAGGGAGAAAATAGTAGGTTATTTTTCTTAAAGAAC-----AGAAGTCTTATTCTCACTTTTTCG

                         60890     60900     60910     60920     60930     60940     60950     60960

Human            TAA-T----TGTAA-AAGGGAGAAAATAGTAGGTTATTTTTCTTAAAGAAC-----AGAAGTCTTATTCTCACTTTTTCG 48939
Coquerel'sSifaka TAACTGCCACCTAATAAAAGGGTAGAAAATAGGTTAATTTTCTGGAAGAACGTAACAGAAGTCTTACTTTAACTTTTCTT 40951
Ma'sNightMonkey  TAA-T----TGTAA-AAGGGAGAAAATAGTAGGTTATTTTTCTT---GAAC-----AGAAGTCTTAGTCTCACTTTTTCG 44010
VervetAGM        TAA-T----TGTAA-AAGGGAGAAAATAGTAGGTTATTTTTCTTAAAGAAC-----AGAAGTCATATTCTCAGTTTTTCA 50785
Gorrilla         TAAAT----TGTAA-AAGGGAGAAAATAGTAGGTTATTTTTCTTAAAGAAC-----AGAAGTCTTACTCTCACTTTTTCG 49458
GrabEatingMacaqueTAA-T----TGTAA-AAGGGAGAAAATAGGAGGTTATTTTTCTTAAAGAAC-----AGAAGTTGTATTCTCAGTTTTTCA 50007
Macaque          TAA-T----TGTAA-AAGGGAGAAAATAGGAGGTTATTTTTCTTAAAGAAC-----AGAAGTTGTATTCTCAGTTTTTCA 50830
PigTailedMacaque TAA-T----TGTAA-AAGGGAGAAAATAGTAGGTTATTTTTCTTAAAGAAC-----AGAAGTTGTATTCTCAGTTTTTCA 49701
Bonobo           TAA-T----TGTAA-AAGGGAGAAAATAGTAGGTTATTTTTCTTAAAGAAC-----AGAAGTCTTATTCTCACTTTTTCG 48036
OliveBaboon      TAA-T----TGTAA-AAGGGAGAAAATAGTAGGTTATTTTTCTTAAAGAAC-----AGAAGTCGTATTCTCAGTTTTTCA 49559
Orangutan        TAA-T----TGTAA-AAGGGAGAAAATAGTAGGTTATTTTTCTTAAAGAAC-----AGAAGTCTTATTCTCACTTTTTCG 49037
```

```
Majority          AGTAGATATCTGTGGGAAACTAATGCTAGGTAGCCAAAGTGAATGAATCACTATAGTTTTTAAACTTTGCCTTTTGAATT

                          60970    60980    60990    61000    61010    61020    61030    61040

Human             AGTAGATGTCTGTGGGAAACTAATGCTAGGTAGCCAAAGTGAGTGAATCACTATAGTTTTTAAACTTTGTCTTTTGAATT 49019
Coquerel'sSifaka  GGTAGATATCGGTGGGAAACTAATGCTAGATAGCCAAAATGAATGAGTCACCGTAGTGTTGAGATTTTGCCTTTTGAATT 41031
Ma'sNightMonkey   AGTAGATGTCTGTGGGAAACAAATGCTAGGTAGCCAAAGTGAATGAGTCACCATAGTTTTTAAACTTTGCCTTTTGTATT 44090
VervetAGM         AGTAGGTATCTGTGGGAAACTAATGCTAGGTAGCCAAATTGAATGAATCACTATAGTTTTTAAACTTTGCCTTTTGAATT 50865
Gorrilla          AGTAGATGTCTGTGGGAAACTAATGCTAGGTAGCCAAAGTGAATGAATCACTATAGTTTTTAAACTTTGTCTTTTGAATT 49538
GrabEatingMacaqueAGTAGGTATCTGTGGGAAACTAATGCTAGGTAGCCAAATTGAAT----CACTATAGTGTTTAAACTTTGCCTTTTGAATT 50083
Macaque           AGTAGGTATCTGTGGGAAACTAATGCTAGGTAGCCAAATTGAAT----CACTATAGTTTTTAAACTTTGCCTTTTGAATT 50906
PigTailedMacaque  AGTAGGTATCTGTGGGAAACTAATGCTAGGTAGCCAAATTGAAT----CACTATAGTTTTTAAACTTTGCCTTTTGAATT 49777
Bonobo            AGTAGATGTCTGTGGGAAACTAATGCTAGGTAGCCAAAGTGAATGAATCACTATAGTTTTTAAACTTTGTCTTTTGAATT 48116
OliveBaboon       AGTAGGTATCTGTGGGAAACTAATGCTAGGTAGCCAAACTGAATGAATCACTGTAGTTTTTAAACTTTGCCTTTTGAATT 49639
Orangutan         AGTAGATGTCTGTGAGAAACTAATGCTAGGTAGCCAAAGTGAATGAATCACTATAGTTTTTAAACTTTGCCTTTTGAATT 49117

Majority          CACTTTA-----------GGGCTGTAA-----------------TTGAGTAATTGTTATGAAATTGTGGAAAATACAAAT

                          61050    61060    61070    61080    61090    61100    61110    61120

Human             GACTTTA-----------GGGCTCTAA-----------------TTGAATAATTGTTATGAAATTGTGGAAAATACAAAT 49071
Coquerel'sSifaka  TACTTTATGACTCGAACAGGGTTCTAAATTTAACTCTTTAGGACTTTACTTGTTATAATGAAACTGTGGGAAATACGAAT 41111
Ma'sNightMonkey   CACTTTA-----------AGGCTCTAA-----------------TTGAATAATTGTTATGAAATTGTGGAAAATACAAAT 44142
VervetAGM         CACTTAA-----------GGGCTGTGA-----------------TTGAGTAATTGTTATGAAATTGTGGAAAATACAAAT 50917
Gorrilla          GACTTTA-----------GGGCTTTAA-----------------GTGAATAATTGTTATGAAATTGTGGAAAATACAAAT 49590
GrabEatingMacaqueCACTTTA-----------GGGCTGTAA-----------------TTGAGTAATTGTTATGAAATTGTGGAAAATACAAAT 50135
Macaque           CACTTTA-----------GGGCTGTAA-----------------TTGAGTAATTGTTATGAAATTGTGGAAAATACAAAT 50958
PigTailedMacaque  CACTTTA-----------GGGCTGTAA-----------------TTGAGTAATTGTTATGAAATTGTGGAAAATACAAAT 49829
Bonobo            GACTTTA-----------GGGCTCTAA-----------------TTGAATAATTGTTATGAAATTGTGGAAAATACAAAT 48168
OliveBaboon       CACTTTA-----------GGGCTGTAA-----------------TTGAGTAATTGTTATGAAATTGTGGAAAATACAAAT 49691
Orangutan         CACTTTA-----------GGGCTCTAA-----------------TTGAATAATTGTTATGAAATTGTGGAAAATACAAAG 49169

Majority          GAAGTATTTCTTTTACTGTGGCTCTGTTCTTTG--ATATCAGAAATCAGTAGAGGTA-CTAGTCGCAT------------

                          61130    61140    61150    61160    61170    61180    61190    61200

Human             GAAGTATTTCTTTTACTGTGGCTCTGTTCTTTG--GTATCAGAAATCAGCAGGGGTA-CTAGTCGCAT------------ 49136
Coquerel'sSifaka  GAAGTATTTCTTTTATTGTGGCTGTGTTCTTTGTGATATCAGAAATCAGTAATGATATCTGGTAGCATTAGCAGAAATTA 41191
Ma'sNightMonkey   GAAGTATATCTTTTACTGTGGCTCTGCTCTTTGTGATATCAGAAGTCAGTAGAGGTA-CTAGTCGCAT------------ 44209
VervetAGM         GAAGTATTTCTTTTACTGTGGCTCTGT-CTTGG--ATATCAGAAATCAGTAGAAGTA-CTAGTCGCAT------------ 50981
Gorrilla          GAAGTGTTTCTTTTACTGTGGCTCTGTTCTTTG--ATATCAGAAATCAGTAGGGGTA-CTAGTCGCAT------------ 49655
GrabEatingMacaqueGAAGTATTTCTTTTACTGTGGCTCTGT-CTTGG--ATATCAGAAATCAGTAGAAGTA-CTAGTCGCAT------------ 50199
Macaque           GAAGTATTTCTTTTACTGTGGCTCTGT-CTTGG--ATATCAGAAATCAGTAGAAGTA-CTAGTCGCAT------------ 51022
PigTailedMacaque  GAAGTATTTCTTTTACTGTGGCTCTGT-CTTGG--ATACCAGAAATCAGTAGAAGTA-CTAGTCGCAT------------ 49893
Bonobo            GAAGTATTTCTTTTACTGTGGCTCTGTTCTTTG--ATATCAGAAATCAGTAGGGGTA-CTAGTCGCAT------------ 48233
OliveBaboon       GAAGTATTTCTTTTACTGTGGCTCTGT-CTTGG--ATATCAGAAATCAGTAGAAGTA-CTAGTCGCAT------------ 49755
Orangutan         GAAGTATTTCTTTTACTGTGGCTCTGTTCTTTG--ATACTGGAAATCAGTAGGGCTA-CTAGTCGCAT------------ 49234
```

```
Majority          -------------------CTTTCCTTAA-------GCTTACTTTATGAG---------------AATATGAAATTTTAT
                          61210     61220     61230     61240     61250     61260     61270     61280
Human             -------------------CTTTCCTTAA-------GCTT----TATGAG---------------AATATGAAATTTTAT 49171
Coquerel'sSifaka  GGGAGTAATTCTGGATATTCTTCTTTTAATAGATGTAACTATATCATTAGGGCCAAGGTATATTAAATATGAAATTTTAT 41271
Ma'sNightMonkey   -------------------CTTTCCTTAA-------GCTTACTTTATGAG---------------AATATGAAATTTTAT 44248
VervetAGM         -------------------CTTTCCTTAA-------GCTTACTTTATGAG---------------AATATGCAATTTTGT 51020
Gorrilla          -------------------CTTTCCTTAA-------GCTT----TATGAG---------------AATATGAAATTTTAT 49690
GrabEatingMacaque -------------------CTTTCCTTAA-------GCTTACTTTATGAG---------------AATATGCAATTTTAT 50238
Macaque           -------------------CTTTCCTTAA-------GCTTACTTTATGAG---------------AATATGCAATTTTAT 51061
PigTailedMacaque  -------------------CTTTCCTGAA-------GCTTACTTTATGAG---------------AATATGCAATTTTAT 49932
Bonobo            -------------------CTTTCCTTAA-------GCTT----TATGAG---------------AATATGAAATTTTAT 48268
OliveBaboon       -------------------CTTTCCTTAA-------GCTTACTTTATGAG---------------AATATGCAATTTTAT 49794
Orangutan         -------------------CTTTCCTTAA-------GCTTACTTTATGAG---------------AATATGAAATTTTAT 49273

Majority          TTAAAAATATTAAATGATACCATTTAGAAGAATTTATGAAATTTAAC-ACTCCCTTTTATATACTTAATGTTAATCTGAC
                          61290     61300     61310     61320     61330     61340     61350     61360
Human             TTAAAAATGTTAAATGATACCATTTAGAAGAATTTATGAAATTTAAC-ACTCCCTTTTATATACTTGAGGTTAATCTGAC 49250
Coquerel'sSifaka  TTTAAAATTTTTAATGGTATCATTTAAAAGAATTTATAAAGTTTAATTAATAACATTCCTGTGATTATAGTTAATCTGAT 41351
Ma'sNightMonkey   TTAAAATTTTTAAATGATACCATTTAGAAGAATGTATGAAATTTAAC-ACTTCCTTTTATATAGTTAATGTTAATCTGAC 44327
VervetAGM         TTAAAAATATTAAATGATGCCATTTAGAGTAATTTATGAAATTTAAC-ACTCCCTTTTATATACTTAATGTTAATCTGAC 51099
Gorrilla          TTAAAAATGTTAAATGATACCATTTAGAAGAATTTATGAAATTTAAC-ACTTCCTTTTATATACTTGAGGTTAATCTGAC 49769
GrabEatingMacaque TTAAAAATATTAAATGATGCCATTTAGAATAATTTATGAAATTTAAC-ACTCCCTTTTATATACTTAATGTTAATCTGAC 50317
Macaque           TTAAAAATATTAAATGATGCCATTTAGAATAATTTATGAAATTTAAC-ACTCCCTTTTATATACTTAATGTTAATCTGAC 51140
PigTailedMacaque  TTAAAAATATTAAATGATGCCATTTAGAATAATTTATGAAATTTAAC-ACTCCCTTTTATATACTTAATGTTAATCTGAC 50011
Bonobo            TTAAAAATGTTAAATGATACCATTTAGAAGAGTTTATGAAATTTAAC-ACTCCCTTTTATATACTTGAGGTTAATCTGAC 48347
OliveBaboon       TTAAAAATATTAAATGATGCCATTTAGAATAATTTATGAAATTTAAC-ACTCCCTTTTATATACTTAATGTTAATCTGAC 49873
Orangutan         TTAAAAATGTTAAATGATACCATTTAGAAGAATTTATGAAATTTAAC-ACTCCCTTTTATATACTTAAGGTTAATCTGAC 49352

Majority          ATTTAATTTGTAATTTTTTTCTTTGTCTCTGTATAATTATACTGCTTTTTAAATGACTTG-GAAATCTTATATTCTCTAG
                          61370     61380     61390     61400     61410     61420     61430     61440
Human             ATTTAATTTGTAATTTTGTTCTTTGTCTCTATATAATTATACTGCTTTTAAAATGACTTG-GAAATCTTACATTCTCTAG 49329
Coquerel'sSifaka  GTTT--T-TTTAATTTTTTTCTTTGTATA---ATAATTATACTGCTTATCAAACGACTTTTGAGATCTCATATTCTTTAG 41425
Ma'sNightMonkey   ATTTAATTTGTAATTGTTTGCTTTGTCTCTATAAAATTATATTGCTTTTGAAATGACTTT-GAAATCTTATATTCTCTAG 44406
VervetAGM         ATTTAATTTGTAATGTTTTTCTTTGTCTCTGTATAATTATACTGCTTTTTAAATGACTTG-GAAATCTTATATTCTCTAG 51178
Gorrilla          ATTTAATTTGTAATTTTGTTCTTTGTCTCTATATAATTATACTGCTTTTAAAATGACTTG-GAAATCTTACATTCTCTAC 49848
GrabEatingMacaque ATTTAATTTGTAATGTTTTTCTTTGTCTCTGTATAATTATACTGCTTTGTAAATGATTTG-GAAATCATATATTCTCTAG 50396
Macaque           ATTTAATTTGTAATGTTTTTCTTTGTCTCTGTATAATTATACTGCTTTGTAAATGACTTG-GAAATCATATATTCTCTAG 51219
PigTailedMacaque  ATTTAATTTGTAATGTTTTTCTTTGTCTCTGTATAATTATACTGCTTTTTAAATGACTTG-GAAATCATATATTCTCTAG 50090
Bonobo            ATTTAATTTGTAATTTTGTTCTTTGTCTCTATATAATTATACTGCTTTTAAAATGACTTG-GAAATCTTACATTCTCTAG 48426
OliveBaboon       ATTTAATTTGTAATGTTTTTCTTTGTCTCTGTATAATTATACTGCTTTTTAAATGACTTG-GAAATCATATATTCTCTAG 49952
Orangutan         ATTTAATTTGTAATTTTGTTCTTTGTCTCTATATAATTATACTGCTTTTTAAATGACTTG-GAAATCTTATATTCTTTAG 49431
```

```
Majority          GGTAGAAGTGGTAAAATTCTGATGCTAGAAAATGTTTACATTTTCTCTAGCATTTGTTGTGGGAA-TAGATTTGT-GGGT

                          61450     61460     61470     61480     61490     61500     61510     61520

Human             GGTAGAAGTGGTAAAATTCTGATGCTAGAAAATGTTTACATTTTCTCTAGCATTTGTTGCAAGAA-TAGATTTGT-GGGT 49407
Coquerel'sSifaka  GGTAGAAATAGTAAAATTCTGATGCTG-----------CATGTCCTCTAGCTTTTGTTGGGGAAAATAGATTTGTAGGGT 41494
Ma'sNightMonkey   GGTAGAAGTGGTAAAATTCTGATGCTAGAAAATGTTTACATTTTCTCTAGCATTTGTTATGGGAA-TAGATTTGT-GAGT 44484
VervetAGM         GGTAGAAGTGGTAAAATTCTGATGCTAGAAAATATTTACATTTTCTCTAGCATTTGCTGTGGGAA-TAGATTTGT-GGGT 51256
Gorrilla          AGTAGAAGTGGTAAAATTCTGATGCTAGAAAATGTTTACATTTTCTCTAGCATTTGTTGCAAGAA-TAGATTTGT-GGGT 49926
GrabEatingMacaqueGGTAGAAGTGGTAAAATTCTGATGCTAGAAAATATTTACATTTTCTCTAGCATTTGCTGTGGGAA-TAGATTTGT-GGGT 50474
Macaque           GGTAGAAGTGGTAAAATTCTGATGCTAGAAAATATTTACATTTTCTCTAGCATTTGCTGTGGGAA-TAGATTTGT-GGGT 51297
PigTailedMacaque  GGTAGAAGTGGTAAAATTCTGATGCTAGAAAATATTTACATTTTCTCTAGCATTTGCTGTGGGAA-TAGATTTGT-GGGT 50168
Bonobo            GGTAGAAGTGGTAAAATTCTGATGCTAGAAAATGTTTACATTTTCTCTAGCATTTGTTGCAAGAA-TAGATTTGT-GGGT 48504
OliveBaboon       GGTAGAAGTGGTAAAATTCTGATGCTAGAAAATATTTACATTTTCTGTAGCATTTGCTGTGGGAA-TAGATTTGT-GGGT 50030
Orangutan         GGTAGAAGTGGTAAAATTCTGATGCTAGAAAATGTTTACATTTTCTCTAGCATTTGTTGCGGGAA-TA-ATTTGT-GGGT 49508

Majority          GTGAAAAATCATAGGTGGAAGGCAGAAGCAGCATGTACTCAGTT------TTCTTCTGAGGTTAGCTTTAACTGTATACC

                          61530     61540     61550     61560     61570     61580     61590     61600

Human             GTGAAAAATCATAGGTGGAAGGCAGAAGCAGCGTGTACTCGGTT------TTCTTCTGAGGTTAGCTTTAACTGTATACC 49481
Coquerel'sSifaka  GTAAAAAATCGTAGGTGGAAGGCAGAAGCACCAGGTACTCAGCT------TTCTTCTGAGATTAGGTTTAATTGTATACC 41568
Ma'sNightMonkey   GTGAAAA-TCGTAAGTGGAAAGCAGAAACGGCATGTACTTAGTT------TTCTTCTAAGGTTATTTTTAACTGTATACC 44557
VervetAGM         GTGAAAAATCATAGGTGGAAGGTAGAAGCAGCATGTACTCAGTTCTAACTTTCTTCTGAGGTTAGCTTTAACTGTATACC 51336
Gorrilla          GTGAAAAATCATAGGTGGAAGGCAGAAGCAGCATGTACTCGGTT------TTCTTCTGAGGTTAGCTTTAACTGTATACC 50000
GrabEatingMacaqueGTGAAAAATCATAGGTGGAAG-----------GTGTACTCAGTTCTAACTTTCTTCTGAGGTTAGCTTTAACTGTATACC 50543
Macaque           GTGAAAAATCATAGGTGGAAG-----------ATGTACTCAGTTCTAACTTTCTTCTGAGGTTAGCTTTAACTGTATACC 51366
PigTailedMacaque  GTGAAAAATCATAGGTGGAAGGTAGAAGCAGCATGTACTCAGTTCTAACTTTCTTCTGAGGTTAGCTTTAACTGTATACC 50248
Bonobo            GTGAAAAATCATAGGTGGAAGGCAGAAGCAGCGTGTACTCGGTT------TTCTTCTGAGGTTAGCTTTAACTGTATACC 48578
OliveBaboon       GTGAAAAATCATAGGTGGAAGGTAGAAGCAGCATGTACTCAGTTCTAACTTTCTTCTGAGGTTAGCTTTAACTGTATACC 50110
Orangutan         GTGAAAAATCATAGGTGGAAGGCAGAAGCAGCATGTACTCAGTT------TTCTTCTGAGGTTAGCTTTAACTGTATACC 49582

Majority          TAAACTTCAACAAGAGATTTCTAATTCTACTTAT-AGAACATTTAAAAGTAACTTGAAAGATTATATTCTAGATAATTGA

                          61610     61620     61630     61640     61650     61660     61670     61680

Human             TAAACTTCAACAAGAGATTTCTAATTCTGCTTATCAGAACATTTAAAAGTAACCTGAAGGATTATATTCTAGATAATTGA 49561
Coquerel'sSifaka  TAAACTTCAATAAGAGATTTTCAGTTCTACTTATCAGAACATTTAAATGTAACTTGAAAGATTATATTCTAGATAATTGA 41648
Ma'sNightMonkey   TAAACTTCAATAAGAGATTTCTAATTCTACTTATTAGAACATTTAAAAGTAACTTGAAAAAT---ATTTTAGATAATTGA 44634
VervetAGM         TAAACTTCAACAAGAGATTTCTAATTCTACTTAT--------TTAAAAGTAACTTGAAAGATTATATTCTAGATAATTGA 51408
Gorrilla          TAAACTTCAACAAGAGATTTCTAATTCTACTTATTAGAACACTTAAAAGTAACCTGAAGGATTATATTCTAGATAATTGA 50080
GrabEatingMacaqueTAAACTTCAACAAGAGATTTCTAATTCTACTTAT--------TTAAAAGTAACTTGAAAGATTATATTCTAGATAATTGA 50615
Macaque           TAAACTTCAACAAGAGATTTCTAATTCTACTTAT--------TTAAAAGTAACTTGAAAGATTATATTCTAGATAATTGA 51438
PigTailedMacaque  TAAACTTCAACAAGAGATTTCTAATTCTACTTAT--------TTAAAAGTAACTTGAAAGATTATATTCTAGATAATTGA 50320
Bonobo            TAAACTTCAACAAGAGATTTCTAATTCTACTTATTAGAACATTTAAAAGTAACCTGAAGGATTATATTCTAGATAATTGA 48658
OliveBaboon       TAAACTTC--CAAGAGATTTCTAATTCTACTTAT--------TTAAAAGTAACTTGAAAGATTATATTCTAGATAATTGA 50180
Orangutan         TAAACTTCAACAAGAGATTTCTAAATCTACTTATTAGAACATTTAAAAGTAACTTGAAAGATTATATTCTAGATAATTGA 49662
```

```
Majority         TTA----AAATCAGCTGAATAGATTGCTCAGATA------TTAATTTTATTTGAATTTATAGTATTAAAAATTGTGCTAC

                         61690     61700     61710     61720     61730     61740     61750     61760

Human            TTA----AAATCATCTGAATAGATTGCTTAGATA------TTAATTTTATTTGAATTTATAGTATTAAAAATTGTGCTAC 49631
Coquerel'sSifaka TTTTTCTAAGTCTGCTGAATAGATTACACAGATATTGAAATTTATTTGAATTTAATGTATATTATTATAATTTTTGTTAT 41728
Ma'sNightMonkey  TTA----AAATCAGCTGAATAGATTGCACAGATACT----TTTATTTGAATTTAATTTATAGTATTAAAATTTTTGCTAC 44706
VervetAGM        TTA----AAATCAGCTGAATAGATTGCTCAGATA------TTAATTTTATTTGAATTTATAGTATTAAAAATTGTGCTAC 51478
Gorrilla         TTA----AAATCATCTGAATAGATTGCTTAGATA------TTAATTTTATTTGAATGTATAGTATTAAAAATTGTGCTAC 50150
GrabEatingMacaqueTTA----AAATCAACTGAATAGATTGCTCAGATA------TTAATTTTATTTGAATTTATAGTATTAAAAATTGTGCTAC 50685
Macaque          TTA----AAATCAACTGAATAGATTGCTCAGATA------TTAATTTTATTTGAATTTATAGTATTAAAAATTGTGCTAC 51508
PigTailedMacaque TTA----AAATCAACTGAATAGATTGCTCAGATA------TTAATTTTATTTGAATTTATAGTATTAAAAATTGTGCTAC 50390
Bonobo           TTA----AAATCATCTGAATAGATTGCTTAGATA------TTAATTTTATTTGAATTTATAGTATTAAAAATTGTGCTAC 48728
OliveBaboon      TTA----AAATCAGCTGAATAGATTGCTCAGATA------TTAATTTTATTTGAATTTATAGTATTAAAAATTGTGCTAC 50250
Orangutan        TTA----AAATCAGCAGAATAGATTGCTTAGCTA------TTAATTTTATTTGAATTTATAGTATTAAAAATTGTGTTAC 49732

Majority         TTAAATTTTCAGGCATTTTTGGAACTAGCAACCGAGGAAGCAGCTATTACTATGGTTAATTACTATTCTGCTGTGACACC

                         61770     61780     61790     61800     61810     61820     61830     61840

Human            TTAAATTTTCAGGCATTTTTGGAACTAGCAACCGAGGAAGCAGCTATTACTATGGTTAATTACTATTCTGCTGTGACACC 49711
Coquerel'sSifaka TT-----TTCAGGCATTTTTGGAACTAGCAACAGAGGAAGCAGCTATTACTATGGTTAATTACTATTCTGCTGTGACACC 41803
Ma'sNightMonkey  TTAAATTTTCAGGCATTTTTGGAGCTAGCAACTGAGGAAGCAGCTATTACTATGGTTAATTACTATTCTGCTGTGACACC 44786
VervetAGM        TTAAATTTTCAGGCATTTTTGGAACTAGCAACTGAGGAAGCAGCTATTACTATGGTTAATTACTATTCTGCCGTGACACC 51558
Gorrilla         TTAAATTTTCAGGCATTTTTGGAACTAGCAACCGAGGAAGCAGCTATTACTATGGTTAATTACTATTCTGCTGTGACACC 50230
GrabEatingMacaqueTTAAATTTTCAGGCATTTTTGGAACTAGCAACCGAGGAAGCAGCTATTACTATGGTTAATTACTATTCTGCCGTGACACC 50765
Macaque          TTAAATTTTCAGGCATTTTTGGAACTAGCAACCGAGGAAGCAGCTATTACTATGGTTAATTACTATTCTGCCGTGACACC 51588
PigTailedMacaque TTAAATTTTCAGGCATTTTTGGAACTAGCAACCGAGGAAGCAGCTATTACTATGGTTAATTACTATTCTGCCGTGACACC 50470
Bonobo           TTAAATTTTCAGGCATTTTTGGAACTAGCAACCGAGGAAGCAGCTATTACCATGGTTAATTACTATTCTGCTGTGACACC 48808
OliveBaboon      TTAAATTTTCAGGCATTTTTGGAACTAGCAACCGAGGAAGCAGCTATTACTATGGTTAATTACTATTCTGCCGTGACACC 50330
Orangutan        TTAAATTTTCAGGCATTTTTGGAACTAGCAACCGAGGAAGCAGCTATTACTATGGTTAATTACTATTCTGCTGTGACACC 49812

Majority         TCATCTTCGTAACCAACCGATATATATCCAGTACTCGAATCACAAAGAACTAAAGACAGATAATACATTAAACCAAGTAA

                         61850     61860     61870     61880     61890     61900     61910     61920

Human            TCATCTTCGTAACCAACCAATATATATCCAGTACTCGAATCACAAAGAACTAAAGACAGATAATACATTAAACCAAGTAA 49791
Coquerel'sSifaka TCATCTTCGTAACCAACCAATTTATATCCAGTATTCCAATCACAAAGAGCTAAAGACAGATAATACATTAAACCAAGTGA 41883
Ma'sNightMonkey  TCATCTTCGTAACCAACCGATATATATCCAGTACTCCAATCACAAAGAACTAAAGACAGATAACACATTAAACCAAGTAA 44866
VervetAGM        TCATCTTCGTAACCAACCGATATATATCCAGTACTCGAATCACAAAGAACTAAAGACAGATAATACATTAAACCAAGTAA 51638
Gorrilla         TCATCTTCGTAACCAACCAATATATATCCAGTACTCGAATCACAAAGAACTAAAGACAGATAATACATTAAACCAAGTAA 50310
GrabEatingMacaqueTCACCTTCGTAACCAACCGATATATATCCAGTACTCGAATCACAAAGAACTAAAGACAGATAATACATTAAACCAAGTAA 50845
Macaque          TCACCTTCGTAACCAACCGATATATATCCAGTACTCGAATCACAAAGAACTAAAGACAGATAATACATTAAACCAAGTAA 51668
PigTailedMacaque TCACCTTCGTAACCAACCGATATATATCCAGTACTCGAATCACAAAGAACTAAAGACAGATAATACATTAAACCAAGTAA 50550
Bonobo           TCATCTTCGTAACCAACCAATATATATCCAGTACTCGAATCACAAAGAACTGAAGACAGATAATACATTAAACCAAGTAA 48888
OliveBaboon      TCATCTTCGTAACCAACCGATATATATCCAGTACTCGAATCACAAAGAACTAAAGACAGATAATACATTAAACCAAGTAA 50410
Orangutan        TCATCTTCGTAACCAACCAATATATATCCAGTACTCGAATCACAAAGAACTAAAGACAGATAATACATTAAACCAAGTAA 49892
```

```
Majority          GTATGTGTAGGTACATAAATAAAATGGCCTAGAACATATTATGAATCTCATAA-ACATTAATAGGAAG-GAAATGTTTAC

                          61930     61940     61950     61960     61970     61980     61990     62000

Human             GTATGTGTAGGTACATAAATAAAATGGCCTAGAACATATTATGAATCTCATAA-ACATTAATAGGAAG-GAAATGTTTAC 49869
Coquerel'sSifaka GTATGTGTAGGTACATAATTAAAATGGCCTAGAACATATTAAGAATCTTATAACATAGTAACGGGAAATAAAATATTTAA 41963
Ma'sNightMonkey   GTATGTGTAGGTACATAAATAAAATGGCCTAGAACGTATTATGAGTCTCATAA-ACATTAACAGGAAG-GAAATGTTAAC 44944
VervetAGM         GTATGTGTAGGTGCATAAATAAAATGGCCTAGAACATATTATGAATCTCATAA-ACTTTAATAGGAAG-GAAATGTTTAC 51716
Gorrilla          GTATGTGTAGGTACATAAATAAAATGGCCTAGAACATATTATGAATCTCATAA-ACATTAATAGGAAG-GAAATGTTTAC 50388
GrabEatingMacaqueGTATGTGTAGATGCATAAATAAAATGGCCTAGAACATATTATGAATCTCATAA-ACATTAATAGGAAG-GAAATGTTTAC 50923
Macaque           GTATGTGTAGATGCATAAATAAAATGGCCTAGAACATATTATGAATCTCATAA-ACATTAATAGGAAG-GAAATGTTTAC 51746
PigTailedMacaque GTATGTGTAGGTGCATAAATAAAATGGCCTAGAACATATTATGAATCTCATAA-ACATTAATAGGAAG-GAAATGTTTAC 50628
Bonobo            GTATGTGTAGGTACATAAATAAAATGGCCTAGAACATATTGTGAATCTCATAA-ACATTAATAGGAAG-GAAATGTTTAC 48966
OliveBaboon       GTATGTGTAGGTGCATAAATAAAATGGCCTAGAACATATTATAAATCTCATAA-ACATTAATAGGAAG-GAAATGTTTAC 50488
Orangutan         GTGTGTGTAGGTACATAAATAAAATGGCCTAGAACATATTATGAATCTCATAA-ACATTAATAGGAAG-GAAATGTTTAC 49970

Majority          CTGTTCAGA---TGTTCCTTATAGGCATTTTCTTGTTATTTTT-------------------------------------

                          62010     62020     62030     62040     62050     62060     62070     62080

Human             CTGTTCAGA---TGTTCCTTATAGGCATTTTCTTGTTATTTTT------------------------------------- 49909
Coquerel'sSifaka CCACTGAGAAAATGTTTCT-ATAGACATTTTCTTATTATCTTT------------------------------------- 42005
Ma'sNightMonkey   CCATTCAGA---TGTTTCTTACAGACATTTTCTTGTAATTTTT------------------------------------- 44984
VervetAGM         CTGTTCAGA---TGTTCCTTATAGGCATTTTCTTGTTATTTTT------------------------------------- 51756
Gorrilla          CTGTTCAGA---TGTTCCTTATAGGCATTTTCTTGTTATTTTT------------------------------------- 50428
GrabEatingMacaqueCTGTTCAGA---TGTTCCTTATAGGCATCTTCTTGTTATTTTT------------------------------------- 50963
Macaque           CTGTTCAGA---TGTTCCTTATAGGCATCTTCTTGTTATTTTT------------------------------------- 51786
PigTailedMacaque CTGTTCAGA---TGTTGCTTATAGGCATCTTCTTGTTATTTTT------------------------------------- 50668
Bonobo            CTGTTCAGA---TGTTCCTTATAGGCATTTTCTTGTTATTTTT------------------------------------- 49006
OliveBaboon       CTGTTCAGA---TGTTCCTTATAGGCATTTTCTTGTTATTTTT------------------------------------- 50528
Orangutan         CTGTTCAAA---TGTTCCTTATAGGCATTTTCTTGTTATTTTTTTATTTTTTTTATTTTTTTATTTTTTTAGATTAACCC 50047

Majority          --------------------------------------------------------------------------------

                          62090     62100     62110     62120     62130     62140     62150     62160

Human             -------------------------------------------------------------------------------- 49909
Coquerel'sSifaka -------------------------------------------------------------------------------- 42005
Ma'sNightMonkey   -------------------------------------------------AAGGTATTTT----------T---------- 44995
VervetAGM         -------------------------------------------------------------------------------- 51756
Gorrilla          -------------------------------------------------------------------------------- 50428
GrabEatingMacaque-------------------------------------------------------------------------------- 50963
Macaque           -------------------------------------------------------------------------------- 51786
PigTailedMacaque -------------------------------------------------------------------------------- 50668
Bonobo            -------------------------------------------------------------------------------- 49006
OliveBaboon       -------------------------------------------------------------------------------- 50528
Orangutan         AGTCTTGGGTATGTCTTTATTAGCAGCATGAGAACGCACTAATACAAATAGAGTATTTTTTTTTTCTGTTACCATGGGTA 50127
```

```
Majority          --------------------------------------------------------------------------------

                           62170     62180     62190     62200     62210     62220     62230     62240

Human             -------------------------------------------------------------------------------- 49909
Coquerel'sSifaka  -------------------------------------------------------------------------------- 42005
Ma'sNightMonkey   ----------------------------------------GT-------------------------------------- 44997
VervetAGM         -------------------------------------------------------------------------------- 51756
Gorrilla          -------------------------------------------------------------------------------- 50428
GrabEatingMacaque -------------------------------------------------------------------------------- 50963
Macaque           -------------------------------------------------------------------------------- 51786
PigTailedMacaque  -------------------------------------------------------------------------------- 50668
Bonobo            -------------------------------------------------------------------------------- 49006
OliveBaboon       -------------------------------------------------------------------------------- 50528
Orangutan         AGTTGCAATAGAGTATTATTAACTACAGTTACTGTGTTGTACATTAGATCTCCAGAACCTATTCATCCTGCATAACTGAA 50207

Majority          --------------------------------------------------------------------------------

                           62250     62260     62270     62280     62290     62300     62310     62320

Human             -------------------------------------------------------------------------------- 49909
Coquerel'sSifaka  -------------------------------------------------------------------------------- 42005
Ma'sNightMonkey   -------------------------------------------------------------------------------- 44997
VervetAGM         -------------------------------------------------------------------------------- 51756
Gorrilla          -------------------------------------------------------------------------------- 50428
GrabEatingMacaque -------------------------------------------------------------------------------- 50963
Macaque           -------------------------------------------------------------------------------- 51786
PigTailedMacaque  -------------------------------------------------------------------------------- 50668
Bonobo            -------------------------------------------------------------------------------- 49006
OliveBaboon       -------------------------------------------------------------------------------- 50528
Orangutan         CTTTGCAGACTTTGACCAACATCTCCCTATTTCCTGCAACCTTTATCCCCTGAAAACCACCACTCCGCTTTCTGTTTCTG 50287

Majority          --------------------------------------------------------------------------------

                           62330     62340     62350     62360     62370     62380     62390     62400

Human             -------------------------------------------------------------------------------- 49909
Coquerel'sSifaka  -------------------------------------------------------------------------------- 42005
Ma'sNightMonkey   --------------------------G----------------------------------------------------- 44998
VervetAGM         -------------------------------------------------------------------------------- 51756
Gorrilla          -------------------------------------------------------------------------------- 50428
GrabEatingMacaque -------------------------------------------------------------------------------- 50963
Macaque           -------------------------------------------------------------------------------- 51786
PigTailedMacaque  -------------------------------------------------------------------------------- 50668
Bonobo            -------------------------------------------------------------------------------- 49006
OliveBaboon       -------------------------------------------------------------------------------- 50528
Orangutan         AATTCAACTTTTTACGTTATATAAATGAGATAATGCAGTATTTATCTTTCTGTGCTTGGCTGATGTTTTTTTTTCTTTTT 50367
```

```
Majority         --------------------------------------------------------------------------------

                          62410     62420     62430     62440     62450     62460     62470     62480

Human            -------------------------------------------------------------------------------- 49909
Coquerel'sSifaka -------------------------------------------------------------------------------- 42005
Ma'sNightMonkey  ------------C--AGAATGTTTCTAAGTATTAAATACTATGTAAGTTTGAATTCCTTTAAAGCCCTGGTAAATGTAGT 45064
VervetAGM        -------------------------------------------------------------------------------- 51756
Gorrilla         -------------------------------------------------------------------------------- 50428
GrabEatingMacaque-------------------------------------------------------------------------------- 50963
Macaque          -------------------------------------------------------------------------------- 51786
PigTailedMacaque -------------------------------------------------------------------------------- 50668
Bonobo           -------------------------------------------------------------------------------- 49006
OliveBaboon      -------------------------------------------------------------------------------- 50528
Orangutan        TTTCTTTTAATTTCTGAAGTATTTATTGATCATTCTTGGGTGTTTCTCGGAGAGGGGGATATGGCAGGGTCATAGGATAA 50447

Majority         --------------------------------------------------------------------------------

                          62490     62500     62510     62520     62530     62540     62550     62560

Human            -------------------------------------------------------------------------------- 49909
Coquerel'sSifaka -------------------------------------------------------------------------------- 42005
Ma'sNightMonkey  TTTTCTTGTTGCAAGTTAAAATTTTTGTGTAGAAACTATCAGGTGGGATTTATTAGTAGCATAAACTATTTATACAGTTC 45144
VervetAGM        -------------------------------------------------------------------------------- 51756
Gorrilla         -------------------------------------------------------------------------------- 50428
GrabEatingMacaque-------------------------------------------------------------------------------- 50963
Macaque          -------------------------------------------------------------------------------- 51786
PigTailedMacaque -------------------------------------------------------------------------------- 50668
Bonobo           -------------------------------------------------------------------------------- 49006
OliveBaboon      -------------------------------------------------------------------------------- 50528
Orangutan        TAGTGGAGAGAAAGTCAGGAGATAAACACATGAACAAAGGTCTCTGGTTTTCCTAGGCAGAGGACCCTGCGGCCTTCTGC 50527

Majority         --------------------------------------------------------------------------------

                          62570     62580     62590     62600     62610     62620     62630     62640

Human            -------------------------------------------------------------------------------- 49909
Coquerel'sSifaka -------------------------------------------------------------------------------- 42005
Ma'sNightMonkey  TC------------------------------------------------------------------------------ 45146
VervetAGM        -------------------------------------------------------------------------------- 51756
Gorrilla         -------------------------------------------------------------------------------- 50428
GrabEatingMacaque-------------------------------------------------------------------------------- 50963
Macaque          -------------------------------------------------------------------------------- 51786
PigTailedMacaque -------------------------------------------------------------------------------- 50668
Bonobo           -------------------------------------------------------------------------------- 49006
OliveBaboon      -------------------------------------------------------------------------------- 50528
Orangutan        AGTGTTTGTGCCCCTGAGTACTTGAGATTAGGGAGTGGTGATGACTCTTAAAGAGCATGCTGCCTTCAAGCATCTGTTTA 50607
```

```
Majority         --------------------------------------------------------------------------------

                         62650     62660     62670     62680     62690     62700     62710     62720

Human            -------------------------------------------------------------------------------- 49909
Coquerel'sSifaka -------------------------------------------------------------------------------- 42005
Ma'sNightMonkey  -------------------------------------------------------------------------------- 45146
VervetAGM        -------------------------------------------------------------------------------- 51756
Gorrilla         -------------------------------------------------------------------------------- 50428
GrabEatingMacaque-------------------------------------------------------------------------------- 50963
Macaque          -------------------------------------------------------------------------------- 51786
PigTailedMacaque -------------------------------------------------------------------------------- 50668
Bonobo           -------------------------------------------------------------------------------- 49006
OliveBaboon      -------------------------------------------------------------------------------- 50528
Orangutan        ACAAAGCACATCTTGCACCACCCTTAATCCATTTAATCCTGAGTTGACACAGCACATGTTTCAGAGAGCAGGGGGCTGGG 50687

Majority         --------------------------------------------------------------------------------

                         62730     62740     62750     62760     62770     62780     62790     62800

Human            -------------------------------------------------------------------------------- 49909
Coquerel'sSifaka -------------------------------------------------------------------------------- 42005
Ma'sNightMonkey  -------------------------------------------------------------------------------- 45146
VervetAGM        -------------------------------------------------------------------------------- 51756
Gorrilla         -------------------------------------------------------------------------------- 50428
GrabEatingMacaque-------------------------------------------------------------------------------- 50963
Macaque          -------------------------------------------------------------------------------- 51786
PigTailedMacaque -------------------------------------------------------------------------------- 50668
Bonobo           -------------------------------------------------------------------------------- 49006
OliveBaboon      -------------------------------------------------------------------------------- 50528
Orangutan        GGAAAGGCCATAGATCAACAGCATCCCAAGGCAGAAGAATTTCTCCTAGTCAGAACAAAATGGAGTCTCCTATGCCCACC 50767

Majority         --------------------------------------------------------------------------------

                         62810     62820     62830     62840     62850     62860     62870     62880

Human            -------------------------------------------------------------------------------- 49909
Coquerel'sSifaka -------------------------------------------------------------------------------- 42005
Ma'sNightMonkey  -------------------------------------------------------------------------------- 45146
VervetAGM        -------------------------------------------------------------------------------- 51756
Gorrilla         -------------------------------------------------------------------------------- 50428
GrabEatingMacaque-------------------------------------------------------------------------------- 50963
Macaque          -------------------------------------------------------------------------------- 51786
PigTailedMacaque -------------------------------------------------------------------------------- 50668
Bonobo           -------------------------------------------------------------------------------- 49006
OliveBaboon      -------------------------------------------------------------------------------- 50528
Orangutan        CCTTTCTACACAGACACAGCAACAATCTGAACTCTCCTTCCTTTCCCCACACTTCCTCCCCTTCTCTTCAACAAAACCGC 50847
```

```
Majority         --------------------------------------------------------------------------------

                          62890     62900     62910     62920     62930     62940     62950     62960

Human            -------------------------------------------------------------------------------- 49909
Coquerel'sSifaka -------------------------------------------------------------------------------- 42005
Ma'sNightMonkey  -------------------------------------------------------------------------------- 45146
VervetAGM        -------------------------------------------------------------------------------- 51756
Gorrilla         -------------------------------------------------------------------------------- 50428
GrabEatingMacaque-------------------------------------------------------------------------------- 50963
Macaque          -------------------------------------------------------------------------------- 51786
PigTailedMacaque -------------------------------------------------------------------------------- 50668
Bonobo           -------------------------------------------------------------------------------- 49006
OliveBaboon      -------------------------------------------------------------------------------- 50528
Orangutan        CATCATCCTCATGGCCCGCTCCCGATGGTCGCTGTCTCTTCGGAGCTGTTGGGTACACCTCCCAGACAGGGCGGCTGGGC 50927

Majority         --------------------------------------------------------------------------------

                          62970     62980     62990     63000     63010     63020     63030     63040

Human            -------------------------------------------------------------------------------- 49909
Coquerel'sSifaka -------------------------------------------------------------------------------- 42005
Ma'sNightMonkey  ----------------------------------CTGCTTAGA-A----TT---T------------------------- 45159
VervetAGM        -------------------------------------------------------------------------------- 51756
Gorrilla         -------------------------------------------------------------------------------- 50428
GrabEatingMacaque-------------------------------------------------------------------------------- 50963
Macaque          -------------------------------------------------------------------------------- 51786
PigTailedMacaque -------------------------------------------------------------------------------- 50668
Bonobo           -------------------------------------------------------------------------------- 49006
OliveBaboon      -------------------------------------------------------------------------------- 50528
Orangutan        AGAGGCGCCCCTCGCTTCCCAGATGGGGCCGCCCGGGCAGAGGCGCTCCTCTCCTCCCAGACGGGGCGGCCGGGCAGAGG 51007

Majority         --------------------------------------------------------------------------------

                          63050     63060     63070     63080     63090     63100     63110     63120

Human            -------------------------------------------------------------------------------- 49909
Coquerel'sSifaka -------------------------------------------------------------------------------- 42005
Ma'sNightMonkey  ----------------------AAGGTATGAAGTTGGGTGCCTTTGGTTATTTGACTTTTTATAGGTCTTAATTTCTTAT 45217
VervetAGM        -------------------------------------------------------------------------------- 51756
Gorrilla         -------------------------------------------------------------------------------- 50428
GrabEatingMacaque-------------------------------------------------------------------------------- 50963
Macaque          -------------------------------------------------------------------------------- 51786
PigTailedMacaque -------------------------------------------------------------------------------- 50668
Bonobo           -------------------------------------------------------------------------------- 49006
OliveBaboon      -------------------------------------------------------------------------------- 50528
Orangutan        CGCTCCTCACTTCCCCGACGGGGCCGCCCGGGCAGAGGCGCTCCTCGCTTCCCAGACGGGGCCGCCCGGGCAGAGGCGCT 51087
```

```
Majority          ----------------------------------------------------------------------AAGGTATTTT

                         63130     63140     63150     63160     63170     63180     63190     63200

Human             ----------------------------------------------------------------------AAGGTATTTT 49919
Coquerel'sSifaka  ----------------------------------------------------------------------ACGATATTTT 42015
Ma'sNightMonkey   GGAAAAATTTTTTTGAAAATGTGTTAATGGTTTTCCTGGAAAAGAAGTCTTAAGGTTGAAGAATATATGAATGTATTGA 45297
VervetAGM         ----------------------------------------------------------------------AAGGTATTTT 51766
Gorrilla          ----------------------------------------------------------------------AAGGTATTTT 50438
GrabEatingMacaque ----------------------------------------------------------------------AAGGTATTTT 50973
Macaque           ----------------------------------------------------------------------AAGGTATTTT 51796
PigTailedMacaque  ----------------------------------------------------------------------AAGGTATTTT 50678
Bonobo            ----------------------------------------------------------------------AAGGTATTTT 49016
OliveBaboon       ----------------------------------------------------------------------AAGGTATTTT 50538
Orangutan         CCCAGACCGGGTGGCAGCCGGGCAGAGGCGCTCCTCACCTCCCAGACGGGGCGGCCTTCTTGTTATTTTTAAGGTATTTT 51167

Majority          CATGCAGAATGTTTCTAAGTATTAAATACTATGTCAT-TTTGAATTCCTTTAAACCCCTGATCAAA--TGTTCTTTTTCT

                         63210     63220     63230     63240     63250     63260     63270     63280

Human             CATGCAGAATGTTTCTAAGTATTAAATACTATGTCAT-TTTGAATTCCTTTAATCCCCTGGTCAAA--TGTTCTTTTTCT 49996
Coquerel'sSifaka  CATGCAGAATGTCCT-AAGTATTACATGTCATAGAGT-TTTGAATTCCTTTAAAGCCCTGATCAAAAATGTTCTTTTTCT 42093
Ma'sNightMonkey   TGTTTTAAAAATATA-ATGTAGAAGATACTGTTTAAAATTCATATATCTAAAAATTAGTATTAGAA--CGCTTTTGTTTT 45374
VervetAGM         CATGCAGAATGTTTCTGAGTATTAAATACTATGTCAT-TTTGAATTCCTTTAAACCCCTGATCAAA--TGTTCTTTTTCT 51843
Gorrilla          CATGCAGAATGTTTCTAAGTATTAAATACTATGTCAT-TTTGAATTCCTTTAACCCCCTGGTCAAA--TGTTCTTTTTCT 50515
GrabEatingMacaque CATGCAGAATGTTTCTGAGTATTAAATACTATGTCAT-TTTAAATTCCTTTAAACCCCTGATCAAA--TGTTCTTTTTCT 51050
Macaque           CATGCAGAATGTTTCTGAGTATTAAATACTATGTCAT-TTTAAATTCCTTTAAACCCCTGATCAAA--TGTTCTTTTTCT 51873
PigTailedMacaque  CATGCAGAATGTTTCTGAGTATTAAATACTATGTCAT-TTTAAATTCCTTTAAACCCCTGATCAAA--TGTTCTTTTTCT 50755
Bonobo            CATGCAGAATGTTTCTAAGTATTAAATACTATGTCAT-TTTGAATTCCTTTAACCCCCTGGTCAAA--TGTTCTTTTTCT 49093
OliveBaboon       CATGCAGAATGTTTCTGAGTATTAAATACTATGTCAT-TTTGAATTCCTTTAAACCCCTGATCAAA--TGTTCTTTTTCT 50615
Orangutan         CATGCAGAATGTTTCTAAGTATTAAATACTATGTCAT-TTTGAATTCCTTTAAACCCCTGGTCAAA--TGTTCTTTTTCT 51244

Majority          TGTTGCAAGTTAAAATTTTTGTGTAGAAGCTTATCAGGTAGGATTTGTTATTAGCATAAACTATTTATACGACTCTCCTG

                         63290     63300     63310     63320     63330     63340     63350     63360

Human             TGTTGCAAGTTAAAATTTTTGTGTAGAAACTTATCAGGTAGGATTTATTATTAGCATAAACTGTTTATACAACTCTCCTG 50076
Coquerel'sSifaka  TATTGCAAGTTAAAATTTTTATGTAAAAACTATTTAAGAGGTAGGACTTATTAACACAAATGATTTATACAGCTCTTCTA 42173
Ma'sNightMonkey   TATTTTTTGTTTTTTCTTTTGAGATAGAGTTTCACTCGTGTTGCCCAG-GCTGGAGTGCAGTGTTGCAATTGCGG-CTCA 45452
VervetAGM         TGTTGCAAGTTAAAATTTTTGTGGAGAAGCTTATCAGGTAGGATTTGTTATTAGCATAAACTATTTATACGACTCTCCTG 51923
Gorrilla          TGTTGCAAGTTAAAATTTTTGTGTAGAAACCTATCAGGTAGGATTTATTATTAGCATAAACTATTTATACAACTCTCCTG 50595
GrabEatingMacaque TGTTGCAAGTTAAAATTTTTGTGGAGAAGCTTATCAGGTAGGATTTGTTATTAGCATAAACTATTTATACGACTCTCCTG 51130
Macaque           TGTTGCAAGTTAAAATTTTTGTGGAGAAGCTTATCAGGTAGGATTTGTTATTAGCATAAACTATTTATACGACTCTCCTG 51953
PigTailedMacaque  TGTTGCAAGTTAAAATTTTTGTGGAGAAGCTTATCAGGTAGGATTTGTTATTAGCATAAACTATTTATACGACTCTCCTG 50835
Bonobo            TGTTGCAAGTTAAAATTTTTGTGTAGAAACTTATCGGGTAGGATTTATTATTAGCATAAACTGTTTATACAACTCTCCTG 49173
OliveBaboon       TGTTGCAAGTTAAAATTTTTGTGGAGAAGCTTATCAGGTAGGATTTGTTATTAGCATAAACTATTTGTACGACTCTCCTG 50695
Orangutan         TGTTGCAAGTTAAAATTTTTGTGTAGAAACTTATCAGGTAGGATTTATTATTAGCATAAACTATTTATACAACTCTCCTG 51324
```

```
Majority         CTTAGAATTTAAGGTATGAAGTTCAGTGCCTTTGGTTATTTGACTTTTTTTATAAGTTTTAATTTCTTATGGAAAAAATT

                         63370     63380     63390     63400     63410     63420     63430     63440

Human            CTTAGAATTTAAGGTAAGAAGTTCAGTGCCTTTGGTTATTTGATTTTTTTTATAGGTCTTAATTTCTTACGGAAAATTTT 50156
Coquerel'sSifaka CTTGGACTTTAAGGTATGAAGTTCAGTGGCTTTGGTTATTTGACTTTTT--ATAGGTCTTAATTTCTTGTGGAAAAATTC 42251
Ma'sNightMonkey  CTGCAACCTCTGCCTCCTGGGTTCAAGCGATTCTCCCACCTCAGCCTCCCAAGTAGCTGGGATTACAGGCGTATACCACC 45532
VervetAGM        CTTAGAATTTAAGGTATGAAGTTCAGTGCCTTTGGTTATTTGACTTTTTTTATAAGTTTTAATTTCTTATGGAAAAAAAA 52003
Gorrilla         CTTAGAATTTAAGGTAAGAAGCTCAGTGCCTTTGGTTATTTGATTTTTTTTATAGGTCTTAATTTCTTACGGAAAATTTT 50675
GrabEatingMacaqueCTTAGAATTTAAGGTATGAAGTTCAGTGCCTTTGGTTATTTGACTTTTTTTATAAGTTTTAATTTCTTATGGAAAAAAAT 51210
Macaque          CTTAGAATTTAAGGTATGAAGTTCAGTGCCTTTGGTTATTTGACTTTTTTTATAAGTTTTAATTTCTTATGGAAAAAAAT 52033
PigTailedMacaque CTTAGAATTTAAGGTATGAAGTTCAGTGCCTTTGGTTATTTGACTTTTTTTATAAGTTTTAATTTCTTATGGAAAAAAAT 50915
Bonobo           CTTAGAATTTAAGGTAAGAAGTTCAGTGCCTTTGGTTATTTGATTTTTTTTATAGGTCTTAATTTCTTACGGAAAATTTT 49253
OliveBaboon      CTTAGAATTTAAGGTATGAAGTTCAGTGCCTTTGGTTATTTGACTTTTTTTATAAGTTTTAATTTCTTATGGAAAAAAAT 50775
Orangutan        CTTAGAATTTAAGGTATGAAGTTCAGTGCCTTTGGTTATTTGACTTTTTTTATAGGTCTTAATTTCTTATGGAAAATTTT 51404

Majority         TTTTTGAAGATGTGTTAAT-GGTTTTCCTGGAAAAGAAAT--CTTAAGGATGA--AGAATATATGAATGTATTGATGTTT

                         63450     63460     63470     63480     63490     63500     63510     63520

Human            TTTTTGAAGATGTGTTAAT-GGTTTTCCTGGAAAAGAAAT--CTTAAGGATGA--AGAATATGTGAATGTATTGATGTTT 50231
Coquerel'sSifaka CTTGTGAAGATGTGTTAATTGATTTTCCTGGAAAAAACAT--CTTATGGATNN--NNNNNNNNNNNNNNNNNNNNNNNNN 42327
Ma'sNightMonkey  ACACC-TGGCTAATTTTGT-ATTTTCAATAGAGATGGGGTTTCTCCATGTTGGTCGGCTGGTCTCGAACTCCTGACCTCA 45610
VervetAGM        TTTTGGAAGATGTGTTAAT-GGTTTTCCTGGAAAACAAAT--CTTCAGAATGA--AGAATATATGAATGTATTGATGTTT 52078
Gorrilla         TTTTTGAAGATGTGTTAAT-GGTTTTCCTGGAAAAGAAAT--CTTAAGGATGA--AGAATATGTGAATGTATTGATGTTT 50750
GrabEatingMacaqueTTTTG-AAGATGTGTTAAT-GGTTTTCCTGGAAAAGAAAT--CTTAAGGATGA--GGAATATATGAATGTATTGATGTTT 51284
Macaque          TTTTG-AAGATGTGTTAAT-GGTTTTCCTGGAAAAGAAAT--CTTAAGGATGA--GGAATATATGAATGTATTGATGTTT 52107
PigTailedMacaque TTTTG-AAGATGTGTTAAT-GGTTTTCCTGGAAAAGAAAT--CTTAAGGATGA--GGAATATATGAATGTATTGATGTTT 50989
Bonobo           TTTTTGAAGATGTGTTAAT-GGTTTTCCTGGAAAAGAAAT--CTTAAGGATGA--AGAATATGTGAATGTATTGATGTTT 49328
OliveBaboon      TTTTG-AAGATGTGTTAAT-GGTTTTCCTGGAAAAGAAAT--CTTAAGGATGA--AGAATATATGAATGTATTGATGTTT 50849
Orangutan        TTTTTGAAGATGTGTTCAT-GGTTTTCCTGGAAAAGAAAT--CTTAAGGATGA--AGAATATATGAATGTATTGATGTTT 51479

Majority         TAAAAATGTAATGTAGAAGATATT----GT-TTAAAATTAATGTATTTAAAAATAGCAT------TAGAATGTTTTCT-G

                         63530     63540     63550     63560     63570     63580     63590     63600

Human            TAAAAATGTAATATAGAAGATATT----GT-TTAAAATTAATGTATTCAAAAATAGCAT------TAGAATGTTTTCC-G 50299
Coquerel'sSifaka NNNNNNNNNNNNNNNNNNNNNNNN-------NNNNNNNNNNNNNNNNNNNNNNNNNNNNN--------------------- 42379
Ma'sNightMonkey  GGTGATCCGACCACCTCAGCCTCCCGGAGTGTTGGGATTACAGGGGTGAGCCACCATGCCTGGCCTAGAATATTTTTTTG 45690
VervetAGM        TAAAAATGTAATGTAGAAGATATT----AT-TTAAAATTAATGTATTTAAAAATAGCAT------TAGAATGTTTTCT-G 52146
Gorrilla         TAAAAATGTAATATAGAAGATATT----GT-TTAAAATTAATGTATTTAAAAATAGCAT------TAGAATGTTTTCT-G 50818
GrabEatingMacaqueTAAAAATGTAATGTAGAGGATATT----AT-TTAAAATTAATGTATTTAAAAATAGCAT------TAGAATGTTTTCT-G 51352
Macaque          TAAAAATGTAATGTAGAAGATATT----AT-TTAAAATTAATGTATTTAAAAATAGCAT------TAGAATGTTTTCT-G 52175
PigTailedMacaque TAAAAATGTAATGTAGAAGATATT----AT-TTAAAATTAATGTATTTAAAAATAGCAT------TAGAATGTTTTCT-G 51057
Bonobo           TAAAAATGTAATATAGAAGATATT----GT-TTAAAATTAATGTATTCAAAAATAGCAT------TAGAATGTGTTCT-G 49396
OliveBaboon      TAAAAATGTAATGTAGAAGATATT----AT-TTAAAATTAATGTATTTAAAAATAGCAT------TAGAATGTTTTCT-G 50917
Orangutan        TAAAAATGTAATATAGAAGATATT----GT-TTAAAATTAATGTATTTAAAAATAGCAT------TAGAATGTTTTCT-G 51547
```

```
Majority          ACCTACATTATAA--GACTGCTGTAATTATCTAAAACTTT-CGCTGTTTTAATATAGTTTTAAAACTAATGATATCTTTC
                          63610     63620     63630     63640     63650     63660     63670     63680
Human             ACCTACATTATAA--GACTGCTGTAATGATCTAAAACTTT-AGCTGTTTTAATATAGTTTTAAAACTAATGATATCTTTC 50376
Coquerel'sSifaka  -----------------------------------------NNNNNNNNNNNNNNNNNNNNNNNNNNNNNNNNNNNNNNN 42418
Ma'sNightMonkey   AGCTACATTGTAAAAGACTGCTGTAATAATCTAAAATTTT-AGTTCTTTTAGTGTAGTTTTAAAAGTAATTATATCTTTC 45769
VervetAGM         ACCTACATTATAA--GACTGCTGTAATTATCTAAAACTTTTCGCTGTTTTAATATAGTTTTAAAACTAATGATGTCTTTC 52224
Gorrilla          ACCTACATTATAA--GACTGCTGTAATGATCTAAAACTTT-AGCTGTTTTAATATAGTTTTAAAACTAATGATATCTTTC 50895
GrabEatingMacaqueACCTACATTATAA--GACTGCTGTAATTATCTAAAACTTT-CGCTGTTTTAATATAGTTTTAAAACTAATGATATCTTTC 51429
Macaque           ACCTACATTATAA--GACTGCTGTAATTATCTAAAACTTT-CGCTGTTTTAATATAGTTTTAAAACTAATGATATCTTTC 52252
PigTailedMacaque  ACCTACATTATAA--GACTGCTGTAATTATCTAAAACTTT-CGCTGTTTTAATATAGTTTTAAAACTAATGATATCTTTC 51134
Bonobo            ACCTACATTATAA--GACTGCTGTAATGATCTAAAACTTT-AGCTGTTTTAATATAGTTTTAAAACTAATGATATCTTTC 49473
OliveBaboon       ACCTACATTATAA--GACTGCTGTAATTATCTAAAACTTT-CGCTGTTTTAACATAGTTTTAAAACTAATGATATCTTTC 50994
Orangutan         ACCTACATTATAA--GACTGCTGTAATGATCTAAAACTTT-AGCTGTTTTAATATAGTTTTAAAACTAATGGTATCTTTC 51624

Majority          TCTGTCAGTAAAATACAGACTTTTTCTTAA------------TAAAAATGTAATGGAAAACTATTCCTCATAGATTTTTG
                          63690     63700     63710     63720     63730     63740     63750     63760
Human             TCTGTCAGTAAAATACAAACTTTTTCTTAA------------TAAAAATGTAATGGAAAACTGTTCCTCATAGATTTTTG 50444
Coquerel'sSifaka  NNNNNNNNNNNNNNNNNNNNNNNNNNNNNN------------NNNNNNNNNNNNNNNNNNNNNNNNNNNNNNNNNNNNNNNN 42486
Ma'sNightMonkey   TCTTTGAGTACAAAACAAACTTTTTCTTAAATATACATTTCTTAAAAATGTAATGGAAAACTATGCCTCATAGATTTTTG 45849
VervetAGM         TCTGTCAGTAAAATACAGACTTTTTCTTAA------------TAAAAATGTAACGGAAAACTATTCCTCATAGATTTTTG 52292
Gorrilla          TCTGTCAGTAAAATACAAACTTTTTCTTAA------------TAAAAATGTAATGGAAAACTGTTCCTCATAGATTTTTG 50963
GrabEatingMacaqueTCTGTCAGTAAAATACAGACTTTTTCTTAA------------TAAAAATGTAACGGAAAACTATTCCTTATAGATTTTTG 51497
Macaque           TCTGTCAGTAAAATACAGACTTTTTCTTAA------------TAAAAATGTAACGGAAAACTATTCCTTATAGATTTTTG 52320
PigTailedMacaque  TCTGTCAGTAAAATACAGACTTTTTCTTAA------------TAAAAATGTAACGGAAAACTATTCCTCATAGATTTTTG 51202
Bonobo            TCTGTCAGTAAAATACAAACTTTTTCTTAA------------TAAAAATGTAATGGAAAACTGTTCCTCATAGATTTTTG 49541
OliveBaboon       TCTGTCAGTAAAGTACAGACTTTTTCTTAA------------TAAAAATGTAACGGAAAACTATTCCTCATAGATTTTTG 51062
Orangutan         TCTGTCAGTAAAATACAAACTTTTTCTTAA------------TAAAAATGTAATGGAAAACTATTCCTCATAGATTTTTG 51692

Majority          TCACTTTACAAAGTGACAAAATGGTTTTGATAGTTTATGAAAAATAAGCTTGTAAAACTTTTTACCTAAAAGATAGGACT
                          63770     63780     63790     63800     63810     63820     63830     63840
Human             TCACTTTACAAAGTGACAAAATCATTTTGTTAGTTTATGGAAAATAAGCTTGTAAAACTTTTTACCTAAAAGATAGGACT 50524
Coquerel'sSifaka  NNNNNNNNNNNNNNNNNNNNNNNNNNNN------------NN--NNNNNNNNNNNNNNNNNNNNNNNNNNNNNNNNNNNNNN 42552
Ma'sNightMonkey   TCGCTTTTTGCAGTGACAAAGTCGTGTTGATAGTTTATGAAAAACAAGCTTGTAAAACTTTTGACCTGAAAGATAAGGCT 45929
VervetAGM         TCACTTTACAAAGTGACAAAATGGTTTAG----TTTATGAAAAATAAGCTTGTAAAACTTTTTACCTAAAAAATAGGACT 52368
Gorrilla          TCACTTTACAAAGTGACAAAATCATTTTGTTAGTTTATGGAAAATAAGCTTGTAAAACTTTTTACCTAAAAGATAGGACT 51043
GrabEatingMacaqueTCACTTTACAAAGTGACAAAATGGTTTAGATAGTTTATGAAAAATAAGCTTGTAAAACTTTTTACCTAAAAGATAGGACT 51577
Macaque           TCACTTTACAAAGTGACAAAATGGTTTAGATAGTTTATGAAAAATAAGCTTGTAAAACTTTTTACCTAAAAGATAGGACT 52400
PigTailedMacaque  TCACTTTACAAAGTGACAAAATGGTTTAGATAGTTTATGAAAAATAAGCTTGTAAAACTTTTTACCTAAAAGATAGGACT 51282
Bonobo            TCACTTTACAAAGTGACAAAATCATTTTGTTAGTTTATGGAAAATAAGCTTGTAAAACTTTTTACCTAAAAGATAGGACT 49621
OliveBaboon       TCACTTTACAAAGTGACAAAATGGTTTAGATAGTTTATGAAAAATAAGCTTGTAAAACTTTTTACCTAAAAGATAGGACT 51142
Orangutan         TCACTTCACAAAGTGACAAAATCATTTTGATAGTTTATGGAAAATAAGCTTGTAAAACTTTTTACCTAAAAGATAGGACT 51772
```

```
Majority          GAAATTTCAGCTGTT---TTTTTAATTTGATGATGAATTTTTAATTTCTTTTGAAAAAGAATGTATGCTTCTAATAATTT
                          63850     63860     63870     63880     63890     63900     63910     63920
Human             GAAATTTCAGCT------TTTTTAATTTGATGATGAGTTTTTAATTTCTTTTGAAAAAGAATGTATGCTTCTAATAATTT 50598
Coquerel'sSifaka  NNNNNNNNNNNN------NNNNNNNNNNNNNNNNNNNNNNNNNNNNNNNNNNNNNNNNNNNNNNNNNNNNNNNNNNNNNN 42626
Ma'sNightMonkey   CAAATTTCAGCTGTTTTTTTTTTTAATGTGTTGATAAATTTTTGATTT-TTTTGAAAAATGATGTACACTTGTAATAATTT 46008
VervetAGM         GAAATTTCAGCTGTT---TTTTTAATTTGATGATGAATTTTTAATTTCTTTTGAAAAAGAATGTATGCTTCTAATAATT- 52444
Gorrilla          GAAATTTCAGCTGTT---TTTTTAATTTAATGATGAGTTTTTAATTTCTTTTGAAAAAGAATGTATGCTTCTAATAATTT 51120
GrabEatingMacaqueGAAATTTCAGCTGGT---TTTTTAATTTGATCATGAATTTTTAATTTCTTTTGAAAAAGAATGTACGCTTCTAATAATT- 51653
Macaque           GAAATTTCAGCTGGT---TTTTTAATTTGATGATGAATTTTTAATTTCTTTTGAAAAAGAATGTACGCTTCTAATAATT- 52476
PigTailedMacaque  GAAATTTCAGCTGTT---TTTTTAATTTGATGATGAATTTTTAATTTCTTTTGAAAAAGAATGTACGCTTCTAATAATT- 51358
Bonobo            GAAATTTCAGCTGTT---TTTTTAATTTGATGATGAGTTTTTAATTTCTTTTGAAAAAGAATGTATGCTTCTAATAATTT 49698
OliveBaboon       GAAATTTCAGCTGTT---TTTTTAATTTGATGATGAATTCTTAATTTCTTTTGAAAAAGAATGCACGCTTCTAATAATT- 51218
Orangutan         GAAATTGCAGCTGTT---TTTTTAATTTGATGATGAGTTTTTAATTTCTTTTGAAAAAGAATGTATACTTCCAATAATTT 51849
```

```
Majority          ATCAAGAGGAAGAATACCAAAAGAAAATATCTGCTGTTCTTTCTTTTTACTTAGATTTTTTTTT-----GCATTTTTAAC
                          63930     63940     63950     63960     63970     63980     63990     64000
Human             ATCAAGAGGAAGAATACCAAAAGAAAATATCTGCTCTTCTTTCTTTTTACTTAGATTTTTTT-------GCATTTTTAAC 50671
Coquerel'sSifaka  NNNNNNNNNNNNNNNNNNNNNNNNNNNNNNNNNNNNNNNNNNNNNNNNNNNNNNNNNNNNNN-------NNNNNNNNNNN 42699
Ma'sNightMonkey   ATCAAGACTAAGTATACCAAAAGAAAGTATCTGCTCTTCTTTCTTTTTACTTAGATTTCTTTTTTTGTGGCATTTTTAAC 46088
VervetAGM         ATCAAGAGGAAGAATACCAAAAGAAAATATCTGCTGTTCTTTCTTTTTACTTAGATTTTTTTTT-----GCATTTTTAAC 52519
Gorrilla          ATCTAGAGGAAGAATACCAAAAGAAAATATCTGCTCTTCTTTCTTTTTACTTAGATTTTTTT-------GCATTTTTAAC 51193
GrabEatingMacaqueATCAAGAGGAAGAATACCAAAAGAAAATATCTGCTGTTCTTTCTTTTTACTTAGATTTTTTTTT-----GCATTTTTAAC 51728
Macaque           ATCAAGAGGAAGAATACCAAAAGAAAATATCTGCTGTTCTTTCTTTTTACTTAGATTTTTTTTT-----GCATTTTTAAC 52551
PigTailedMacaque  ATCAAGAGGAAGAATACCAAAAGAAAATATCTGCTGTTCTTTCTTTTTACTTAGATTTTTTTTT-----GCATTTTTAAC 51433
Bonobo            ATCAAGAGGAAGAATACCAAAAGAAAATATCTGCTCTTCTTTCTTTTTACTTAGATTTTTTT-------GCATTTTTAAC 49771
OliveBaboon       ATCAAGAGGAAGAATACCAAAAGAAAATATCTGCTGTTCTTTCTTTTTACTTAGATTTTTTTTTTT---GCATTTTTAAC 51295
Orangutan         ATCAAGAGGAAGAATACCAAAAGAAAATATCTGCTCTTCTTTCTTTTTACTTAGATTTTTTT-------GCATTTTTAAC 51922
```

```
Majority          TTATTTTTAACAAATAATTGTATATACTTAATGGTGTACAATGTGATGTTTTAATACATATATA--TTGTGGAATGATCA
                          64010     64020     64030     64040     64050     64060     64070     64080
Human             TTATTTTTAACAAATAATTGTTTATACTTA-TGGTGTACAATGTGATGTTTTAATACATATATA--TTATGGAATGATCA 50748
Coquerel'sSifaka  NNNNNNNNNNNNNNNNNNNNNNNNNNNNNNNNTGGGGTACAGTGTGATGTCTTGATACATATATACCTTGTGGAATGATCA 42779
Ma'sNightMonkey   TTGTTTTTAACAAACAATTGTATATACTTA-TGGTTTACAATGTGATGTTCTGATACATATATA--TTGCGGGATGATCA 46165
VervetAGM         TTATTTTTAACAAATAATGGTATATACTTAACGGTGTTCAAGGTGATGTTTTAATACATATATA--TAGTGGAATGATCA 52597
Gorrilla          TTATTTTTAACAAATAATTGTTTATACTTA-TGGTGTACAATGTGATGTTTTAATACATATATA--TTATGGAATGATCA 51270
GrabEatingMacaqueTTATTTTTAACAAATAATGGTATATACTTAACGGTGTTCAAGGTGATGTTTTAATACATATATA--TAGTGGAATGACCA 51806
Macaque           TTATTTTTAACAAATAATGGTATATACTTAACGGTGTTCAAGGTGATGTTTTAATACATATATA--TAGTGGAATGACCA 52629
PigTailedMacaque  TTATTTTTAACAAATAATGGTATATACTTAACGGTGTTCAAAGTGATGTTTTAATACATATATA--TAGTGGAATGACCA 51511
Bonobo            TTATTTTTAACAAATAATTGTTTATACTTA-TGGTGTACAATGTGATGTTTTAATACATATATA--TTATGGAATGATCA 49848
OliveBaboon       TTATTTTTAACAAATAATAATATATACTTAACGGTGTTCAAGGTGATGTTTTAATACATATATA--TAGTGGAATGACCA 51373
Orangutan         TTATTTTTAACAAATAATTGTATGTACTTA-TGGTGTACAATGCAATGTTTTAATACATATATA--TTATGGAATGATCA 51999
```

```
Majority          AATCTGGCTAGTTAACATATTCATCACTTCCAATACTTACAATTTCTTTATGGTAAGAACATTTAAAAT---TCCTTTAG

                          64090     64100     64110     64120     64130     64140     64150     64160

Human             AATCTGGCTGGTTAACATATTCATCTCTTC-AGTACTTACAATTTCTTTATGGTAAGAACATTTAAAAT---TCCTTTAG 50824
Coquerel'sSifaka  AATTAGGCC----AACATATCCATCACCTA-AATAGTTA---TTTCTATGTGGTAAAGACATGTAAAATCCTTCTTTTAG 42851
Ma'sNightMonkey   AATCTGGTTAGTTAACATATTCATCACTTCAAATACTTACAATTTCTTTATGGTAAGAACATTTAAAAT---TCCTTTAG 46242
VervetAGM         AATATGGCTAGTTAACATGCTCATCATTTCAGTACTTACAATTTCTTTATGGTAAGAACATTTAAAAT---TCCTTTAG 52674
Gorrilla          AATCTGGCTGGTTAACGTATTCATCTCTTCCAATACTTAGAATTTCTTTATGGTAAGAACATTTAAAAT---TCCTTTAG 51347
GrabEatingMacaqueAATATGGCTAGTTAACATGCTCATCATTTCCAATACTTACAATTTCTTTATGGTAAGAACATTTAAAAT---TCCTTTAG 51883
Macaque           AATATGGCTAGTTAACATGCTCATCATTTCCAATACTTACAATTTCTTTATGGTAAGAACATTTAAAAT---TCCTTTAG 52706
PigTailedMacaque  AATATGGCTAGTTAACATGCTCATCATTTCCAATACTTACAATTTCTTTATGGTAAGAACATTTAAAAT---TCCTTTAG 51588
Bonobo            AATCTGGCTGGTTAACATATTCATCTCTTCCAGTACTTACAATTTCTTTATGGTAAGAACATTTAAAAT---TCCTTTAG 49925
OliveBaboon       AATATGGCTAGTTAACATGCTCATCATTTCCAATACTTACAATTTCTTTATGGTAAGAACATTTAAAAT---TCCTTTAG 51450
Orangutan         AATCTGGCTAGT-AACATATTCATCACTTCCAATACTTACAATTTCTTTATGGTAAGAACATTTAAAAT---TCCTTTAG 52075

Majority          CTATTTTG-A-GTATACAGTGGAATGTGGAAAAAC--------TGTTTTTCCTCTGCTCACACCACAACAATCAACACAG

                          64170     64180     64190     64200     64210     64220     64230     64240

Human             CTATTTTGGAAGTATACAGTGGAATGTGGAAAAAC--------TGTTTTTCCTCTGCTCACACCACAACAATCAACACAG 50896
Coquerel'sSifaka  CTATTTTG-AAATACGCAGTTGAATGTGGAAAGACCCAAGCTATTTCTCTTCTGCTCTCACACCACGACAATTAACATGG 42930
Ma'sNightMonkey   CTATTTGG-AGGTGTACAGCGGAATGTGGAAAAAC--------TTTTTCCTCTGCTCTCATACCACAACAATCAGCACAG 46313
VervetAGM         ------------TATACAGTGGAATGTGGAAAAAC--------TGTTTTTCCTCTGCTCACACCACAACAATCAACATAG 52734
Gorrilla          CTATTTTG-AAGTATACAGTGGAATGTGGAAAAAC--------TGTTTTTCCTCTGCTCACACCACAACAATCAACACAG 51418
GrabEatingMacaque------------TACACAGTGGAATGTGGAAAAAC--------TGTTTTTCCTCTGCTCACACCACAACAATCAACACAG 51943
Macaque           ------------TACACAGTGGAATGTGGAAAAAC--------TGTTTTTCCTCTGCTCACACCACAGCAATCAACACAG 52766
PigTailedMacaque  ------------TACACAGTGGAATGTGGAAAAAC--------TGTTTTTCCTCTGCTCACACCACAACAATCAACACAG 51648
Bonobo            CTATTTTG-AAGTATACAGTGGAATGTGGAAAAAC--------TGTTTTTCCTCTGCTCACACCACAACAATCAACACAG 49996
OliveBaboon       ------------TATACAGTGGAATGTGGAAAAAC--------TGTTTTTCCTGTGCTCACACCACAACAATCAACACAG 51510
Orangutan         CTATTTTG-AGGTATACAGTGGAATGTGGAAAAAC--------TGTTTTTCCTCTGCTCACACCACAACAATCAACACAG 52146

Majority          AAGGCTTCTGTGGCCAGAAATGTGGGGGAATTTTTTTCC-ACATGCCAAGCAAGCAGTCAGGTCTACAGTG---------

                          64250     64260     64270     64280     64290     64300     64310     64320

Human             AAGGCTTCTGTGGCCTCAAATGTGGGGGAATTTTTTTCC---ACGCCAAGCAAGCAGTCAGGTCTGCAGTG--------- 50964
Coquerel'sSifaka  AAAACTTC-GTGACC--AAATATGGAGGAATTTTTCCCG-ACATACCAAGCAAGCAA---A---------A--------- 42985
Ma'sNightMonkey   AAGACTTCTGTGGCCACAAATGTGGGGGAGTTTTTTTCC-ACATGCCAAGCAAGCAGTCAGGACTACAGTGGACACTAGC 46392
VervetAGM         AAGGCTTCTGCGTCCAGAAATGTGGGGGAATTTTTTTTCCACATGGCAAGCAAGCAGTCAGGTCTACAGTG--------- 52805
Gorrilla          AAGGCTTCTGTGGCCTCACATGTGGGGGAATTTTTTTCT---ACGCCAAGCAAGCAGTCAGGTCTGCAGTG--------- 51486
GrabEatingMacaqueAAGGCTTCTGCGGCCAGAAATGTGAGGGAATTTTTTTTCCACATGGCAAGCAAGCAGTCAGGTCTACAGTG--------- 52014
Macaque           AAGGCTTCTGCGGCCAGAAATGTGAGGGAATTTTTTTTCCACATGGCAAGCAAGCAGTCAGGTCTACAGTG--------- 52837
PigTailedMacaque  AAGGCTTCTGCGGCCAGAAATGTGAGGGAATTTTTTTTCCACATGGCAAGCAAGCAGTCAGGTCTACAGTG--------- 51719
Bonobo            AAGGCTTCTGTGGCCTCAAATGTGGGGGAATTTTTTTCC---ACGCCAAGCAAGCAGTCAGGTCTGCAGTG--------- 50064
OliveBaboon       AAGGCTTCTGCGGCCAGAAATGTGAGGGAATTTTTTTTCCACATGGCAAGCAAGCAGTCAGGTCTACAGTG--------- 51581
Orangutan         AAGGCTTCTGTGGCCACAAATGTGGGGGAATTTTTTTCC---ACGCCAAGCAAGCAGTCAGGTCTGCAGTG--------- 52214
```

```
Majority        -----TTCTCCAACTTAGCTCAGTTTCAACACTATCTGCCAGGAGATAGCATCAGATCCCACAGGTTGAGGGCTCAGTCC

                        64330     64340     64350     64360     64370     64380     64390     64400

Human           -----TTCTCCAACTTAGCTCAGTTTCAACACTGTCTGCCGGGAGATAGCATCAGATCCCACAGGTTGAGGGCTCAGTCC 51039
Coquerel'sSifaka ------CCTCCAACCCAGTTCATTTCCAACACCACCTACTTGGATATAACATCAGATCCCACAGGTTGAGGGCTTAATCC 43059
Ma'sNightMonkey CTGTGTTCTCCAACTTAGCTCAGTTCCAATGCTTTCTGCCAGGAGATAGCATCAGATCCCACAGGTCGAGGGCTCAGTCC 46472
VervetAGM       -----TTCTCCAACTTAGCTCAGTTTCAACACTATCTGCCAGGAGATAGCATCAGATCCCACAGGTTGAGGGCTCAGTCC 52880
Gorrilla        -----TTCTCCAACTTAGCTCAGTTTGAACACTGTCTGCCGGGAGATAGCATCAGATCCCACAGGTTGAGGGCTCAGTCC 51561
GrabEatingMacaque-----TTCTCCATCTTAGCTCAGTTTCAACACTATCTGCCAGGAGATAGCATCAGATCCCACAGGTTGAGGGCTCAGTCC 52089
Macaque         -----TTCTCCAACTTAGCTCAGTTTCAACACTATCTGCCAGGAGATAGCATCAGATCCCACAGGTTGAGGGCTCAGTCC 52912
PigTailedMacaque -----TTCTCCAACTTAGCTCAGTTTCAACACTATCTGCCAGGAGATAGCATCAGATCCCACAGGTTGAGGGCTCAGTCC 51794
Bonobo          -----TTCTCCAACTTAGCTCAGTTTCAATACTGTCTGCCGGGAGATAGCATCAGATCCCACAGGTTGAGGGCTCAGTCC 50139
OliveBaboon     -----TTCTCCAACTTAGCTCAGTTTCAACACTATCTGCCAGGAGATAGCATCAGATCCCACAGGTTGAGGGCTCAGTCC 51656
Orangutan       -----TTCTCCAACTTAGGTCAGTTTCAACACTGTCTGCCGGGAGATAGCATCAGATCCCACAGGTTGAGGGCTCAGTCC 52289

Majority        CACAAGAT---CTCCTCAGACACCAGCAAGTCAGGCCTCTGGAACTTCTGATTGACCAGCTTCAAGTTGGGGTTCCCACA

                        64410     64420     64430     64440     64450     64460     64470     64480

Human           CACAAGAT---CTCCTGAGACACCAGCAAGTCAGGCCTCTGGAACTTCTGATTGACCAGCTTCAAGTTGGGGTTCCCACA 51116
Coquerel'sSifaka CACAAAACAGACCCCTCCCCTTCTGACCCGTTGCAGGTCAGGCCTCTGGAACCAACCGACTTCAATTGGTGGTTCCCGTG 43139
Ma'sNightMonkey CACAAGAC---CTCCTCAGACACCAGCAAGTCAGACCTCCGGAACTTCTCATTGACCAACTTCAAGTTGGGATTTCCACA 46549
VervetAGM       CACAAGAT---GTCCTCAGACACCAGCAAGTCAGGCCTGTGGAACTTCTGATTGACCAGCTTTAAGTTAGGGTTCCCACA 52957
Gorrilla        CACAAGAT---CTCCTGAGACACCAGCAAGTCAGGCCTCTGGAACTTCTGATTGACCAGCTTCAAGTTGGGGTTCCCACA 51638
GrabEatingMacaqueCACAAGAT---GTCCTCAGACACCAGCAAGTCAGGCCTCTGGAACTTCTGATTGACCAGCTTTAAGTTAGGGTTCCTACA 52166
Macaque         CACAAGAT---GTCCTCAGACACCAGCAAGTCAGGCCTCTGGAACTTCTGATTGACCAGCTTTAAGTTAGGGTTCCCACA 52989
PigTailedMacaque CACAAGAT---GTCCTCAGACACCAGCAAGTCAGGCCTCTGGAACTTCTGATTGACCAGCTTTAAGTTAGGGTTCCCACA 51871
Bonobo          CACAAGAT---CTCCTGAGACACCAGCAAGTCAGGCCTCTGGAACTTCTGATTGACCAGCTTCAAGTTGGGGTTCCCACA 50216
OliveBaboon     CACAAGAT---GTCCTCAGACACCAGCAAGTCAGGCCTCTGGAACTTCTGATTGACCAGCTTTAAGTTAGGGTTCCCACA 51733
Orangutan       CACAAGAT---CTCCTCAGACACCAGCAAGTCAGGCCTCTGGAACTTCTGATTGACCAGCTTCAAGTTGGGGTTCCCACA 52366

Majority        ACCCCCT-------------TTTTGGATTTCGTTAATTTGCTAGAGCAGCACAGAGAAC-TCAAGGAAACATTTACTGA

                        64490     64500     64510     64520     64530     64540     64550     64560

Human           ACCCCCT-------------TTTTGGATTTCATTAATTTGCTAGAGCAGCACACAGAAC-TCAAGGAAACATTTACTGA 51181
Coquerel'sSifaka ATCCCCT-------------CTTTGTTTTACAGATGTTTACCAGTTTATTATAAACAATATTACAAAGATGTAGATGAA 43205
Ma'sNightMonkey ACCCCCT-------------TTTTGGATTTTGTTAATTAGCTAGACCAGCGCACAGAAC-TCAAGGAAACATTTACT-- 46612
VervetAGM       GCCCCCT-------------TATTGGATTTCGTTAATTTGCTAGAGCAGCACAGAGAAC-TCAAGGAAACATTTACTGA 53022
Gorrilla        ACCCCCT-------------TTTTGGATTTCATTAATTTGCTAGAGCAGCACACAGAAC-TCAAGGAAACATTTACTGA 51703
GrabEatingMacaqueGCCCCCT-------------TTTTGGATTTCGTTAATTTGCTAGAGCAGCACAGAGAAC-TCAAGGAAACATTTACTGA 52231
Macaque         GCCCCCT-------------TTTTGGATTTCGTTAATTTGCTAGAGCAGCACAGAGAAC-TCAAGGAAACATTTACTGA 53054
PigTailedMacaque GCCCCCT-------------TTTTGGATTTCGTTAATTTGCTAGAGCAGCACAGAGAAC-TCAAGGAAACATTTACTGA 51936
Bonobo          ACCCCCT-------------TTTTGGATTTCATTAATTTGCTAGAGCAGCACACAGAAC-TCAAGGAAACATTTACTGA 50281
OliveBaboon     GCCCCCT-------------TTTTGGATTTCGTTAATTTGCTAGAGCAGCACAGAGAAC-TCAAGGAAACATTTACTGA 51798
Orangutan       ACCCCCCCCCCCCGCCCCCGCTTTTGGATTTCATTAATTTGCTAGAGCAGCACACAGAAC-TCAAGGAAACATTTACTGA 52445
```

```
Majority        GATTTACTAGTTTATTGTATAGGATATTACAAAGGATAATAAGCCTCTA-ATCATGCCTTGGTCTTTCAGGTGACCAGTC

                        64570     64580     64590     64600     64610     64620     64630     64640

Human           GATTTACTAGTTTATTATATAGGATATTAAAAAGGATAATAAGCCTCTA-ATCATGCCTTGATCTTTCAGGTGACCAGTC 51260
Coquerel'sSifaka AA------AATGCATAGGGCAAGATATGGGAAAGGGCAGGGAGCTTCCATGCCCTCTTTGGGTACGCTACCCCTCCAGGA 43279
Ma'sNightMonkey --------AGTTTATTGTATAGGATATTATAGAGGATGATATGCCTCTA-ATTATGCCTAGGTCTTTCAGGTGACCAGCC 46683
VervetAGM       GATTTACTAGTTTATTGTATAGGATAGTACAAAGAATAATAAGCCTCTA-ATCATGCCTTGGTCTTTCAGGTGACCAGTC 53101
Gorrilla        GATTTACTAGTTTATTATATAGGATATTAAAAAGGATAATAAGCCTCTA-ATCATGCCTTGATCTTTCAGGTGACCAGTC 51782
GrabEatingMacaqueGATTTACTAGTTTATTGTATAGGATAGTACAAAGGATAATAAGCCTCTA-ATCATGCCTTGGTCTTTCAGGTGACCAGTC 52310
Macaque         GATTTACTAGTTTATTGTATAGGATAGTACAAAGGATAATAAGCCTCTA-ATCATGCCTTGGTCTTTCAGGTGACCAGTC 53133
PigTailedMacaque GATTTACTAGTTTATTGTATAGGATAGTACAAAGGATAATAAGCCTCTA-ATCATGCCTTGGTCTTTCAGGTGACCAGTC 52015
Bonobo          GATTTACTAGTTTATTATATAGGATATTAAAAAGGATCATAAGCCTCTA-ATCATGCCTTGATCTTTCAGGTGACCAGTC 50360
OliveBaboon     GATTTACTAGTTTATTGTATAGGATAGTACAAAGGATAATAAGCCTCTA-ATCATGCCTTGGTCTTTCAGGTGACCAGTC 51877
Orangutan       GATTTACTAGTTTATTATATAGGATATTACAAAGGATAATAAGCCTCTA-ATCATGCCTTGGTCTTTCAGGTGACCAGTC 52524

Majority        CCCATCCTGAAGTGGGGCTGCCAGCCATCAGTCAACTCATTAGCATACAA--------------TTTGGAGATTTATTAG

                        64650     64660     64670     64680     64690     64700     64710     64720

Human           CCCATCTTAAAGTGGGGCTGCCAGCCATCAGTCAACTCATTAGCATACAAAATATATCA----TTTTGGAGATTTATTAG 51336
Coquerel'sSifaka ACC-TCCACATGTTTAGCTATC--CTAGAAGCT--CTCCTAACCCTGTCT-------------TTTTGGGGTTTTATGGA 43341
Ma'sNightMonkey CCCATCCTGAAGTGGGGCTGTCAGCCATAAGTCAAGTCATTAGCATACGAAATATATCAATCACTTTGGAGATTTATTAG 46763
VervetAGM       CCCATCCTGAAGTGGGGCTGCCAGCCATCAGTCAACTCATTAGCATACAA------------------------------ 53151
Gorrilla        CGCATCTTAAAGTGGGGCTGCCAGCCATCAGTCAACTCATTAGCATACAAAATATATCA----TTTTGGAGATTTATTAG 51858
GrabEatingMacaqueCCCATCCTGAAGTGGGGCTGCCAGCCATCAGTCAACTCATTAGCATACAA------------------------------ 52360
Macaque         CCCATCCTGAAGTGGGGCTGCCAGCCATCAGTCAACTCATTAGCATACAA------------------------------ 53183
PigTailedMacaque CCCATCCTGAAGTGGGGCTGCCAGCCATCAGTGAACTCATTAGCATACAA------------------------------ 52065
Bonobo          CCCATCTTAAAGTGGGGCTGCCAGCCATCAGTCAACTCATTAGCATACAAAATATATCA----TTTTGGAGATTTATTAG 50436
OliveBaboon     CCCATCCTGAAGTGGGGCTGCCAGCCATCAGTCAACTCATTAGCATACAA------------------------------ 51927
Orangutan       CCCATCCTAAAGTGGGGCTGCCAGCCATCAGTCAACTTATTAGCATACAAAATATATCA----CTTTGGAGACTTATTAG 52600

Majority        GAATTTA---TTGTAT-----G-----------G------------------------------ATATTATGCATT

                        64730     64740     64750     64760     64770     64780     64790     64800

Human           GAATTTAGAGTTGTATGCCAGGAAATGGGGTTGAGGACCAAATGTCTATTTTACAATGTCACAATACAGTATTATGCATT 51416
Coquerel'sSifaka GGCTTCA---TTACAT-----A-----------G----------------------------------ACATGATTGATT 43368
Ma'sNightMonkey GAATTTAGAGTTGTATGCCAGGAAATGGGTTTGAGGACTAAATGTTTATTTTACAGTGTCACAACAAAACAATATGCATT 46843
VervetAGM       --------------------------------------------------------------------TATTATGCATT 53162
Gorrilla        GAATTTAGAGTTGTATGCCAGGAAATGGGGTTGAGGACCAAATGTCTATTTTACAATGTCACAATACAATATTATGCATT 51938
GrabEatingMacaque--------------------------------------------------------------------TATTATGCATT 52371
Macaque         --------------------------------------------------------------------TATTATGCATT 53194
PigTailedMacaque --------------------------------------------------------------------TATTATGCATT 52076
Bonobo          GAATTTAGAGTTGTATGCCAGGAAATGGGGTTGAGGACCAAATGTCTATTTTACAATGTCACAATACAATATTATGCATT 50516
OliveBaboon     --------------------------------------------------------------------TATTATGCATT 51938
Orangutan       GAATTTGGAGTTGTATGACAGGAAATGGGGTTGAGGACCAAATGTCTATTTTACAATGTCACAATACAATATTATGTATT 52680
```

```
Majority          ACAGTAGTTGCCAT-GCTGTGCAGTAGATCACCAGAACC-ATTCTTCCTGTCTGAAACTT-TGTACTTTGACCATCATCT
                          64810     64820     64830     64840     64850     64860     64870     64880
Human             ACAGTAGTTGCCAT-GCTGTGCGGTAGATCACCAGAACCTATTCTTCCTGTCTGAAACTT-TGTACTTTGACCATCATCA 51494
Coquerel'sSifaka  AAACCATTGGCCATTGGTGATCAACTTAACATTCAGCTC-TTTTTTCCTCCCTGGAGGTTGTGGGGGTGGGACTGAAAGT 43447
Ma'sNightMonkey   ACAATAGTTGCCAT-GCTGTGCAGTAGATCACGGGAACCTATTCTTCCTCTCTGAAACTT-TGTACTTTGACCATCATCT 46921
VervetAGM         ACAGTAGTTGCCAT-GCTGTACAGCAGATCACCAGAACC-ATTCTTCCTGTCTGAAACTT-TGTACTTTGACCATCATCT 53239
Gorrilla          ACAGTAGTTGCCAT-GCTGTGCGGTAGATCACCAGAACCTATTCTTCCTGGCTGAAACTT-TGTACTTTGACCATCATCA 52016
GrabEatingMacaqueACAGTAGTTGCCAT-GCTGTACAGTAGATCACCAGAACC-ATTCTTCCTGTCTGAAACTT-TGTACTTTGACCATCATCT 52448
Macaque           ACAGTAGTTGCCAT-GCTGTACAGTAGATCACCAGAACC-ATTCTTCCTGTCTGAAACTT-TGTACTTTGACCATCATCT 53271
PigTailedMacaque  ACAGTAGTTGCCAT-GCTGTACAGTAGATCACCAGAACC-ATTCTTCCTGTCTGAAACTT-TGTACTTTGACCATCATCT 52153
Bonobo            ACAGTAGTTGCCAT-GCTGTGCGGTAGATCACCAGAACCTATTCTTCCTGTCTGAAACTT-TGTACTTTGACCATCATCA 50594
OliveBaboon       ACAGTAGTTGCCAT-GCTGTACAGTAGATCACCAGAACC-ATTCTTCCTGTCTGAAACTT-TGTACTTTGACCATCATCT 52015
Orangutan         ACAGTAGTTGTCAT-GCTGTGCAGTAGATCACCAGAACCTATTCTTCCTGTCTGAAACTT-TGTACTTTGACCATCATCT 52758

Majority          CCCCTTTCCCTGTTCATTCCTCCCTCCCCCA-GCCTCTGGTGACTATCTTTCTACTCTCTACTAATGTGAGTTCAGCTTT
                          64890     64900     64910     64920     64930     64940     64950     64960
Human             CCCCTT-CCCTGTTCACTCCTCCCTCCCCCA-GCCTCTGGTGACTATTTTTCTACTCTCTACTAATGTGAGTTCAGCTTT 51572
Coquerel'sSifaka  CCCTACCTTTTAATCATGTCTTAGTCTTTCAGGCCACCAGCCCCTATCCT--AAACCGGGGCTGCCA-GCCATCAATTAA 43524
Ma'sNightMonkey   CCCCTTTCCCTGTTCACTCCTTCCTTCCTCAAGTCTCTGGTGACTATCTTTCTACTCTCTACTAAAATGAGTTCAACTTT 47001
VervetAGM         CCCCTTTCCCTGTTCATTCCTCCCTCCCCCA-GCTTCTGGTGACTGTCTTTCTACTCTCTACTAATGTGAGTTCTGCTTT 53318
Gorrilla          CCCCTTTCCCTGTTCACTCCTCCCTCCCCCA-GCCTCTGGTGACTATTTTTCTGCTCTCTACTAATGTGAGTTCAGCTTT 52095
GrabEatingMacaqueCCCCTTTCCCTGTTCATTCCTCCCTCCCCCA-GCTTCTGGTGACTATCTTTCTACTCT--ACTGATGTGAGTTCTGCTTT 52525
Macaque           CCCCTTTCCCTGTTCATTCCTCCCTCCCCCA-GCTTCTGGTGACTATCTTTCTACTCT--ACTGATGTGAGTTCTGCTTT 53348
PigTailedMacaque  CCCCTTTCCCTGTTCATTCCTCCCTCCCCCA-GCTTCTGGTGACTATCTTTCTACTCT--ACTGATGTGAGTTCTGCTTT 52230
Bonobo            CCCCTT-CCCTGTTCACTCCTCCCTCCCCCA-GCCTCTGGTGACTATTTTTCTACTCTCTACTAATGTGAGTTCAGCTTT 50672
OliveBaboon       CCCCTTTCCCTGTTCATTCCTCCCTCCCCCA-GCTTCTGGTGACTATCTTTCTACTCTCTACTGATGTGAGTTCTGCTTT 52094
Orangutan         GCCCTTTCCCTGTTCACTCCTCCCTCCCCCA-GCCTCTGGTGACTATTTTTCTACTCTCTACTACTGTGAGTTCAGCTTT 52837

Majority          TTTAGATTCCATATGTAAGTGAGATAATGTGTCATTTGTCTTTCTTTCTTTGGCTTAT-------TCCCTTAGCACACAC
                          64970     64980     64990     65000     65010     65020     65030     65040
Human             TTTAGATTACATATGTAAGTGAGATAATGTGTTATTTGTCTTTCTTTGTTTGACTTAT-------TCACTTAGCACACAC 51645
Coquerel'sSifaka  CTCAT-TAGCATACAAAAAGACATTACTTTGGCAATT-CCAAGGATTTTAGGAGTTGT--------GTACCAGGAAATAG 43594
Ma'sNightMonkey   TTTAGATTCCACATATAAATGAGATAATGTGGCATTTGTCTTTATTTGCTTGGCTTAT-------TCACTTAGCATACAC 47074
VervetAGM         TTTAGATTCCATATGTAAGTGAGATAATGTGTCGTTTGTCTTTCTTTCTTTGGCTTAT-------TCCCTTAGCGCACAC 53391
Gorrilla          TTTAGATTACATATGTAAGTGAGATAATGTGGTATTTGTCTTTCTTTGTTTGACTTAT-------TCACTTAGCACACAC 52168
GrabEatingMacaqueTTTAGATTCCATATGTAAGTGAGATAATGTGTCCTTTGTCTTTCTTTCTTTGGCTTATTCCTTATTCCCTTAGCGCACAC 52605
Macaque           TTTAGATTCCATATGTAAGTGAGATAATGTGTCCTTTGTCTTTCTTTCTTTGGCTTATTCCTTATTCCCTTAGCGCACAC 53428
PigTailedMacaque  TTTAGATTCCATATGTAAGTGAGATAATGTGTCGTTTGTCTTTCTTTCTTTGGCTTATTCCTTATTCCCTTAGCGCACAC 52310
Bonobo            TTTAGATTACATATGTAAGTGAGATAATGTGGTATTTGTCTTTCTTTGTTTGACTTAT-------TCACTTAGCACACAC 50745
OliveBaboon       TTTAGATTCCATATGTAAGTGAGATAATGTGTCGTTTGTCTTTCTTTCTTTGGCTTATTCCTTATTCCCTTAGCGCACAC 52174
Orangutan         TTTAGATTACATATGTAAGTGAGATAATGTGGTATTTGTCTTTCTTTCTTTGGCTTAT-------TCACTTAGCACACAC 52910
```

```
Majority          AGTGTCCTCTTAAAATGGCATTTAATATCCCATTTCTATTTCATTATTATTTAAGTAATTTGCAAGCACATTCTCATTTT
                          65050     65060     65070     65080     65090     65100     65110     65120
Human             AGTGTCCTCTTAAAATGACATTTAATATCCCATTTCTAGTTCGTTATTATTTAAGTAATTTGCAAGTACATTCTCATTTT 51725
Coquerel'sSifaka  GTTGAAGACCAAATATA-TATTTTACAGTATCACAACAATACATTATTATT-----A-ACTGTAGTTGCCATGCTATTT- 43666
Ma'sNightMonkey   AGTGTCTTCTTAAAATG--CTGTAATATCCTATTTGTATTTCATTATTATTCAAGTAATTTGCGAACACATTATCATTTT 47152
VervetAGM         AGTGTCCTCTTAAAATGGCATTTAATGTCCCATTTCTATTTCATTATTATTT----AATTTGCAAGCACATTCTCATGTT 53467
Gorrilla          AGTGTCCTCTTAAAATGACATTTAATATCCCATTTCTAGTTCGTTATTATTTAAGTAATTTCCAAGTACATTCTCATTTT 52248
GrabEatingMacaqueAGTGTCCTCTTAAAATGGCATTTAATGTCCCATTTCTATTTCATTATTATTTAAGTAATTTGCAAGCACATTCTCATGTT 52685
Macaque           AGTGTCCTCTTAAAATGGCATTTAATGTCCCATTTCTATTTCATTATTATTTAAGTAATTTGCAAGCACATTCTCATGTT 53508
PigTailedMacaque  AGTGTCCTCTTAAAATGGCATTTAATGTCCTATTTCTATTTCATTATTATTTAAGTAATTTGCAAGCACATTCTCATGTT 52390
Bonobo            AGTGTCCTCTTAAAATGACATTTAATATCCCATTTCTAGTTCGTTATTATTTAAGTAATTTGCAAGTACATTCTCATTTT 50825
OliveBaboon       AGTGTCCTCTTAAAATGGCATTTAATGTCCCATTTCTATTTCATTATTATT-----AATTTGCAAGCACATTCTCATGTT 52249
Orangutan         AGTGTCCTCTTAAAATGACATTTAATATCCCATTTCTAGTTCATTATTATTTAAGTAATTTGCAAGTACGTTCTCATTTT 52990

Majority          AAAAGATTAAACGAAAGGAACATGTAAAGAATAAAGCTGGGGACTAGGCTTGGTGGCTCACACCAGTAATCCCAGCACTT
                          65130     65140     65150     65160     65170     65180     65190     65200
Human             AAAAGATTAAACGAAAGGAACATGTAAAGAATAAAGCTGGGGGCTGGGCTTGGTGGCTTATACTGGTAATCCCAGCACTT 51805
Coquerel'sSifaka  -----AGCAGAGCACCAGAACCTATTCTTCCTAA--CT-GAAACTGTACCTTTTGACCAACATC-----TCCCTTTTCCC 43733
Ma'sNightMonkey   AAAAGATTAAACAAAAGGAACATGTAAAGAATAAAGCTTGGGGTCAGGTGTGGTGGCTCACGCCTGTAATCCCAGCACTT 47232
VervetAGM         AAAAGATTAAACGAAAGGAACACGTAAAGAATAAAGCTGGGGACTAGGCTTGGTGGCTCACACCAGTAATCGCAGCACTT 53547
Gorrilla          AAAAGATTAAACGAAAGGAACATGTAAAGAATAAAGCTGGGGGCTGGGCTTGGTGGCTTATACTGGTAATCCCAGCACTT 52328
GrabEatingMacaqueAAAAGATTAAACAAAAGGAACACGTAAAGAATAAAGCTGGGGACTAGGCTTGGTGGCTCACACCAGTAATCCCAGCACTT 52765
Macaque           AAAAGATTAAACAAAAGGAACACGTAAAGAATAAAGCTGGGGACTAGGCTTGGTGGCTCACACCAGTAATCCCAGCACTT 53588
PigTailedMacaque  AAAAGATTAAACAAAAGGAACACGTAAAAAATAAAGCTGGGGACTAGGCTTGGTGGCTCACACCAGTAATCCCAGCACTT 52470
Bonobo            AAAAGATTAAACGAAAGGAACATGTAAAGAATAAAGTTGGGGGCTGGGCTTAGGGGCTTATACTGGTAATCCCAGCACTT 50905
OliveBaboon       AAAAGATTAAACAAAAGGAACATGTAAAGAATAAAGCTGGGGACTAGGCTTGGTGGCTCACACCAGTAATCCCAGCACTT 52329
Orangutan         AAAAGATTAAACGAAAGGAACATGTAAAGAATAAAGCTGGGGGCTGGGCTTGGTGGCTCATACTGGTAATCCCAGCACTT 53070

Majority          TGGGAGGCTGAGGCGGACGGATTACCTGAGGTCAGGAGTTCAAGACCCCCCT-----------GACCAACATGG-CGAAA
                          65210     65220     65230     65240     65250     65260     65270     65280
Human             TGGGAGGCTGAGGCGGGTGGATTACCTGAGGTCAGGAGTTCAAGACCCGCCT-----------GACCAACATGG-TGAAA 51873
Coquerel'sSifaka  TGTCTACTTCTCCCTCCCTGTT--CCCAGCCTCTAGTAACCAGAACAAATTA------------ATTTATATGAATTCAA 43799
Ma'sNightMonkey   TGGGAGGCTGAGGCAGATGGATCACCTGAGGTCAGGAGTTTGAGACCAGCCT-----------GGCCAACATGG-CAAAA 47300
VervetAGM         TGGGAGGCTGAGGCGGACGGATTACCTGAGGTCAGGAGTTCAAGACCCCCCCCACC-------GACCAACATGG-CAAAA 53619
Gorrilla          TGGGAGGCTGAGACGGGCGGATTACCTGAGGTCAGGAGTTCAAGACCTGCCT-----------GACCAACATGG-TGAAA 52396
GrabEatingMacaqueTGGGAGGCTGAGGCGGACGGATTACCTGAGGTCAGGAGTTCAAGACCCCCCCCCCC--------GACCAACATGG-CGAAA 52836
Macaque           TGGGAGGCTGAGGCGGACGGATTACCTGAGGTCAGGAGTTCAAGACCCCCCCCCCCCCC-----GACCAACATGG-CGAAA 53662
PigTailedMacaque  TGGGAGGCTGAGGCGGACGGATTACCTGAGGTCAGGAGTTCAAGACCCCCCCCCCC-------GACCAACATGG-CGAAA 52542
Bonobo            TGGGAGGCTGAGGCGGGTGGATTACCTGAGGTCAGGAGTTCAAGACCCGCCT-----------GACCAACATGG-TGAAA 50973
OliveBaboon       TGGGAGGCTGAGGCGGACGGATTACCTGAGGTCAGGAGTTCAAGACCCCCCCCCCCCCAGCCCGACCAACATGG-CGAAA 52408
Orangutan         TGGGAGGCTGAGGTGGGTGGATTACCTGAGGTCAGGAGTTCAAGACCCTCCT-----------GACCAACATGG-TGAAA 53138
```

```
Majority          CCCTGTCTCTACTAAAAATACAAAAAGGAAAGAAAAA----A-------AATTTAGCTGGGCATGG-TGCCAGATGTCTG

                          65290     65300     65310     65320     65330     65340     65350     65360

Human             CCCTGAGTCTACTAAAAATACAAAAAAAAAAAA----------------AAATTAGCCAGGCATGG-TGCCAGGTGCCTG 51936
Coquerel'sSifaka  CTTTTTTTAGGTTCCACATATAAGTGAGATCATGTAG------------TATTTGTCTCGCTCTGCCTGGCTTATTTCAC 43867
Ma'sNightMonkey   CCCTGTCTCTACTAAAAATACAAAAAGAAAAAGAAAATTAAAAAAAAGAAAATTAGCTGATCATGG-TGGCAGGTGCCTG 47379
VervetAGM         CCCTGTCTCTACTAAAAATACAAAAAGGAAAGAAAAA----A-------ACTTTAGCTGGGCATGG-TGCCAGATGTCTG 53687
Gorrilla          CCCTGAGTCTACTAAAAATGCAAAAAAAAAAAAAAAA----AA------AAATTAGCCAGGCATGG-TGCCAGGTGCCTG 52465
GrabEatingMacaqueCCCTGTCTCTACTAAAAATACAAAAAGGAAAGAAAAA----A-------A-TTTAGCTGGGCATGG-TGCCAGATGTCTG 52903
Macaque           CCCTGTCTCTACTAAAAATACAAAAAGGAAAGAAAAA----A-------AATTTAGCTGGGCATGG-TGCCAGATGTCTG 53730
PigTailedMacaque  CCCTGTCTCTACTAAAAATACGAAAAGGAAAGAAAAA----A-------AATTTAGCAGGGCATGG-TGCCAGATGTCTG 52610
Bonobo            CCCTGAGTCTACTAAAAATACAAAAAAA---------------------AAATTAGCCAGGCATGG-TGCCAGGTGCCTG 51031
OliveBaboon       CCCTGTCTCTACTAAAAATACAAAAAGGAAAGAAAAA----A-------AATTTAGCTGGGCATGG-TGCCAGATGTCTG 52476
Orangutan         CCCTGAGTCTACTAAAAATACAAAAAAAAAAAAAAAA----AAAAAAAAAAATCAGCCAGGCATGG-TGCCAGGTGCCTG 53213

Majority          TAATCCCAGA-TACTCGGAGGCTGAGGCA-GAGAGAGTTGCTTGAACCCGGGAGG-CGGAGCTTGCAGTGATCCCAGAT-

                          65370     65380     65390     65400     65410     65420     65430     65440

Human             TAATCCCAGC-TACTTGGAGGCTGAGGCAAGAGAGAATTGCTTGAACCCGGGGGGGTGGAGCTTGCGGTGATCCCAGAT- 52014
Coquerel'sSifaka  TTAGCACAATGTCCTTTAAAAATGGCGTTTGATATCCCAGTTTTTTTTTATTAAT--GTAATTTA---TGAACACATTT- 43941
Ma'sNightMonkey   TAATCGCAGC-TACTTGGAGGCTGAGGCA-AAGAGAATTGCTTGAACCGGGGAGG-CAGAGCTTGCAGTGAACCCAGATC 47456
VervetAGM         TAATCCCAGA-TACTAGGAGGCTGAGGCA-GAGAGAGTTGCTTGAACCCGGGAGG-CGGAGCTTGCGGTGATCCCAGAT- 53763
Gorrilla          TAATCCCAGG-TACTCGGAGGCTGAGGCAAGAGAGAATTGCTTGAACCCGGGGGG-TGGAGCTTGCGGTGATCCCAGAT- 52542
GrabEatingMacaqueTAATCCCAGA-TACTTGGAGGCTGAGGCA-GAGAGAGTTGCTTGAACCTGGGAGG-CGGAGCTTGCAGTGATCCCAGAT- 52979
Macaque           TAATCCCAGA-TACTCGGAGGCTGAGGCA-GAGAGAGTTGCTTGAACCCGGGAGG-CGGAGCTTGCAGTGATCCCAGAT- 53806
PigTailedMacaque  TAATCCCAGA-TACTCGGAGGCTGAGGCA-GAGAGAGTTGCTTGAACCCGGGAGG-CGGAGCTTGCAGTGATCCCAGAT- 52686
Bonobo            TAATCCCAGC-TACTCAGAGGCTGAGGCAAGAGAGAATTGCTTGAACCCGGGGGGGTGGAGCTTGCGGTGATCCCAGAT- 51109
OliveBaboon       TAATCCCAGA-TACTCGGAGGCTGAGGCA-GAGAGAGTTGCTTGAACCCGGGAGG-CGGAGCTTGCAGTGATCCCAGAT- 52552
Orangutan         TAATCCCAGC-TACTCGGAGGCTGAGGCA-GAGAGAATTGCTTGAACCCGGGAGG-TGGACCTTGCAGTGATCCCAGAT- 53289

Majority          GCGCCACTGCACTCCGGCCTGGGTGACA----GAGCGAGACTCTGCC----------------AAAAAAAAGAAC----A

                          65450     65460     65470     65480     65490     65500     65510     65520

Human             GCGTCACTGCACTCCAGCCTGGGTGACA----GAGCGAGACTCTATCTCAAAAAAAAAAA---AAAAAAAAAAAAGAGTA 52087
Coquerel'sSifaka  -T--CATTTTAA-AAGGTTTAAGTGAAA---------GGAACATGTA----------------AAGAATAAAGAT----- 43987
Ma'sNightMonkey   GTGCCTGTGCACTCCAGCCTGGGTGACATCTAAAAAAAAAAAAAGAATAAAGAGACCCCATCTAAAAAAAAAAAAAGAATA 47536
VervetAGM         GCGCCACTGCACTCCGGCCTGGGTGACA----GAGCGAGACTCTGCC----------------AAAAAAAAGAAC----A 53819
Gorrilla          GCGTCACTGCACTCCAGCCTGGGTGACA----GAGCGAGACTCTGGCTCAAAAAAAAAAA---AAAAAAAAGAAT----A 52611
GrabEatingMacaqueGCGCCACTGCACTCCGGCCTGGGTGACA----GAGCGAGACTCTGCC----------------AAAAAAAAGAAC----A 53035
Macaque           GCGCCACTGCACTCCGGCCTGGGTGACA----GAGCGAGACTCTGCC----------------ATAAAAAAGAAC----A 53862
PigTailedMacaque  GCGCCACTGCACTCCGGCCTGGGTGACA----GAGCGAGACTCTGCC----------------AAAAAAAAGAAC----A 52742
Bonobo            GCGTCACTGCACTCCAGCCTGGGTGACA----GAGCGAGACTCTTAATCTACAAAAAAAA---AAAAAAAAAAGAA-ATAA 51181
OliveBaboon       GCTCCACTGCACTCCGGCCTGGGTGACA----GAGCGAGACTCTGCC----------------AAAAAAAAGAAC----A 52608
Orangutan         GCGCCACTGCACTACAGCCTGGGTGACA----GAGCGAGACTCTGTCTC-----AAAAAA---AAAAAAAAGAAT----C 53353
```

```
Majority          AAGCTGGCGCCTACACATGGATG--TGTGGAGG-AGGGGGACTT------------AGGATGACCGGTGGAAGCCCCATT

                          65530     65540     65550     65560     65570     65580     65590     65600

Human             AAGCTGAGGCCTACACATGGATGGATGTGGAGG-AGGGGCACTT------------AGGATGACTAGTGGAAGCCCCATT 52154
Coquerel'sSifaka  -AGT--GCGTCTACAGATAGATGTCTGTGTGAG-AGGAGGACTTGTAATTTTAAATAGGATGACCAGTGCAAGTCCCATT 44063
Ma'sNightMonkey   AAGCTGGAGCACCTACATAGATGTTTGTGGAAG-AGGGGGACTTGTAATTTTAAATAGGATGACTGGTAGATGCCGCATT 47615
VervetAGM         AAGCTGGCGCCTACACATGGAT----GTGGAGG-AGGGGGACTT------------AGGATGACCGTTGGAAGCCCCATT 53882
Gorrilla          AAGCTGAGGCCTACCCATGGATGGATGTGGAGG-AGGGGCACTT------------AGGATGACTAGTGGAAGCCCCATT 52678
GrabEatingMacaqueAAGCTGGCGCCTACGCATGGAT----GTGGAGG-AGGGGGACTT------------AGGATGACCGTTGGAAGCCCCATT 53098
Macaque           AAGCTGGCGCCTACGCATGGAT----GTGGAGG-AGGGGGACTT------------AGGATGACCGTTGGAAGCCCCATT 53925
PigTailedMacaque  AAGCTGGCGCCTACGCATGGAT----GTGGAGG-AGGGGGACTT------------AGGATGACCGTTGGAAGCCCCATT 52805
Bonobo            AGACTGAGGCCTACACATGGATGGATGTGGAGGGAGGGGCACTT------------AGGATGACTAGTGGAAACCCCATT 51249
OliveBaboon       AAGCTGGCGCCTACGCATGGAT----GTGGAGG-AGGGGGACTT------------AGGATGACCGTTGGAAGCCCCATT 52671
Orangutan         AAGCTAGGGCCTACACATGGATAGATGTGGAGG-AGGGGCACTT------------AGGATGACCAGTGGACGCCCCATT 53420

Majority          GAGAGGGGATATTTGAATAATGATCTTCAAAGTGGTTAGGGAATAAGCCACTTATTCCAGACAGAAGGAATAATCAATGC

                          65610     65620     65630     65640     65650     65660     65670     65680

Human             GAGAGGGGATATTTGAATAATGAACTTCAAAGTGGTTAGGGAATAAGCCACTTATTCCAGACAGAAGGAATAATCAATGC 52234
Coquerel'sSifaka  GAGAAGGGATATTTGAATAATGACCTTCAAAGTGATGAAGGAATAAGCCACTTATTCCAGACAGAAGGAATAATCAATGT 44143
Ma'sNightMonkey   GAGAGGGGATATTTGAATAATGAACTTCAAGGTGGTTAGGGAATAAGCCACTTATTCCAGACAGAAGGAATAATCAAGGC 47695
VervetAGM         GAGAGGGGATATTTGAATAATGATCTTCAAAGTGGTTAGGGAATAAGCCACTTACTTGAGACAGAAGGAATAATCAGTGC 53962
Gorrilla          GAG--GGGATATTTGAATAATGAACTTCAAAGTGGTTAGGGAATAAGCCACTTATTCCAGACAGAAGGAATAATCAATGC 52756
GrabEatingMacaqueGAGAGGGGATATTTGAATAATGATCTTCAAAGTGGTTAGGGAATAAGCCACTTACTTCAGACAGAAGGAATAATCAGTGC 53178
Macaque           GAGAGGGGATATTTGAATAATGATCTTCAAAGTGGTTAGGGAATAAGCCACTTACTTCAGACAGAAGGAATAATCAGTGC 54005
PigTailedMacaque  GAGAGGGGATATTTGAATAATGATCTTCAAAGTGGTTAGGGAATAAGCCACTTACTTCAGACAGAAGGAATAATCAGTGC 52885
Bonobo            GAGAGGGGATATTTGAATAACGAACTTCAAAGTGGTTAGGGAATAAGCCACTTATTCCAGACAGAAGGAATAATCAATGC 51329
OliveBaboon       GAGAGGGGATATTTGAATAATGATCTTCAAAGTGGTTAGGGAATAAGCCACTTACTTCAGACAGAAGGAATAATCAGTGC 52751
Orangutan         GAGAGGGGATATTTGAATAATGAACTTCAAAGTGGTTAGGTAATAAGCCACTTATTCCAGACCGAAGGAATAATCAATGC 53500

Majority          AAAGTTCCTTCTGTTG----AAAAAAATGCTTTTATAATTTTATTTTTCATTTTCTAAAGCCCTTCAATCTCCTT-----

                          65690     65700     65710     65720     65730     65740     65750     65760

Human             AAAGTTCCTTCTGTTG----AAAAAAATGCTTTTATAATTTTATTTTTCATCTTCTAAAGCCCTTCAGTCTCCTT----- 52305
Coquerel'sSifaka  AAAGTTCCTTCTGTTG----AAGAAAAAGTTTTGTAATCATATTTTTTATTTTCAAAAGCCCTTCAGTCTTCTT----- 44214
Ma'sNightMonkey   AAAGTTCCTTGTGTTGGAAAAAAAAAAATGCTTTTATAATTATATTTTTCCTTTTCTAAAGCCTTTCAATCTCCTT----- 47770
VervetAGM         AAAGTTC-TTCTGTTG----AAAAAAATGCTTTTACAATTTTATTTTTAATTTTCTAAAGCCCTTCAATCTCCTT----- 54032
Gorrilla          AAAGTTCCTTCTGTTG----AAAAAAATGCTTTTATAATTTTATTTTTCATTTTCTAAAGCCCTTCAGTCTCCTT----- 52827
GrabEatingMacaqueAAAGTTC-TTCTGTTG----AAAAAAATGCTTT-ACAATTTTATTTTTCATTTTCTAAAGCCCTTCAATCTCCTT----- 53247
Macaque           AAAGTTC-TTCTGTTG----AAAAAAATGCTTT-ACAATTTTATTTTTCATTTTCTAAAGCCCTTCAATCTCCTT----- 54074
PigTailedMacaque  AAAGTTC-TTCTGTTG----AAAAAAATGCTTT-ACAATTTTATTTTTCATTTTCTAAAGCCCTTCAATCTCCTT----- 52954
Bonobo            AAAGTTCCTTCTGTTG----AAAAAAATGCTTTTATAATTTTATTTTTCATTTTCTAAAGCCCTTCAGTCTCCTT----- 51400
OliveBaboon       AAAGTTC-TTCTGTTG----AAAAAAATGCTTTTACAATTTTATTTTTCATTTTCTAAAGCCCTTCAATCTCCTT----- 52821
Orangutan         AAAGTTCCTTCTGTTG----AAAAAAATGCTTTTATAATTTTATTTTTCATTTTCTAAAGCCCTTCAATCTCCTTGATTT 53576
```

```
Majority          -------------TGGTCATCT---------------------TTTTTTTTTGTAGTTGGTTATGTCTTATG-A--CAAC

                         65770     65780     65790     65800     65810     65820     65830     65840

Human             -------------TGGTCAT-----------------------CTTTTTTTTGTAGTTGGTTATGCCTTAA-----CAAC 52344
Coquerel'sSifaka  -------------TGATTATCC-T-------------------TTTTGCATTGTATTTGTTCGTATCTTAGGGACTTTAC 44261
Ma'sNightMonkey   -------------TGGTCAT-----------------------CTTTTTTTTGTAGTTGGTTATGTCTTACG-ACTTAAC 47813
VervetAGM         -------------TGGTCATCT---------------------TTTTTTTTTGTAGTCAGTTATGTCTTGTG-A--CAGC 54075
Gorrilla          -------------TGGTCAC-----------------------CTTTTTTTTGTAGTTGGTTATGCCTTAAG-A--CAAC 52868
GrabEatingMacaque-------------TGGTCATCT---------------------TTTTTTT--GTAGTCAGTTATGTCTTGTG-A--CAGC 53288
Macaque           -------------TGGTCATCT---------------------TTTTTTTT-GTAGTCAGTTATGTCTTGTG-A--CAGC 54116
PigTailedMacaque  -------------TGGTCATCT---------------------TTTTTTTT-GTAGTCAGTTATGTCTTGTG-A--CAGC 52996
Bonobo            -------------TGGTCAT-----------------------CTTTTTTTTGTAGTTGGTTATGCCTTAAG-A--CAAC 51441
OliveBaboon       -------------TGGTCATCT---------------------TTTTTTTT-GTAGTCAGTTATGTCTTGTG-A--CAGC 52863
Orangutan         TTTTTTTTGTAGTTGGTTATGCCTTAAGACAACAGTATTTATATCTTTTTTTGTAGTTGGTTTTGCCTTACG-A--CAAC 53653

Majority          AGTACTTTGAGGATACTAATTACAGTATTTAAAAAATACATCACTCTATGCTGAGTTAT---TTTTTCAGTGTTAGCATT

                         65850     65860     65870     65880     65890     65900     65910     65920

Human             AGTACTTTGAGGATACTAGTTACAGTATTTAAAAAATACGTCACTATATGCTGAGTTAT---TTTTTCAGTGTTAGCATT 52421
Coquerel'sSifaka  AGTTCTTTGAGGATACTAATTATAGTATTTTAAAAATACTTCAC-----GATGAATTATCTTTTCTTCAGCATTAGGTTT 44336
Ma'sNightMonkey   GGTACTTTGAGGATACTAATTGGCGTATTTAAATAATACGTCAGTATATACTGAGTTATCATTTTTTCAGTGTTAGCATT 47893
VervetAGM         AGTACTTTGAGGATACTAATTACAGTATTTAAAAAATATATCACTCTATGCTGAGTTAT---TTTTTCAGTGTTAGCATT 54152
Gorrilla          AGTACTTTGAGGATACTAGTTACAGTATTTAAAAAATACATCACTATATGCTGAGTTAT---TTTTTCAGTGTTAGCATT 52945
GrabEatingMacaqueAGTACTTTGAGGATACTAATTACAGTATTTAAAAAATATATCACTCTATGCTGAGTTAT---TTTTTCAGTGTTAGCATT 53365
Macaque           AGTACTTTGAGGATACTAATTACAGTATTTAAAAAATATATCACTCTATGCTGAGTTAT---TTTTTCAGTGTTAGCATT 54193
PigTailedMacaque  AGTACTTTGAGGATACTAATTACAGTATTTAAAAAATATATCACTCTATGCTGAGTTAT---TTTTTCAGTGTTAGCATT 53073
Bonobo            AGTACTTTGAGGATACTAGTTACAGTATTTAAAAAATACGTCACTATATGCTGAGTTAT---TTTTTCAGTGTTAGCATT 51518
OliveBaboon       AGTACTTTGAGGATACTAATTACAGTATTTAAAAAATATATCACTCTATGCTGAGTTAT---TTTTTCAGTGTTAGCATT 52940
Orangutan         AGTATTTTGAGGATACTAATTACAGTATTTAAAAAATACATCACTATATGCTGAGTTAT---TTTTTCAGTGTTAGCATT 53730

Majority          TATGTTTATCTTGTTCTCACTCTCTT-CATCCCCCGTTTGTTGTCTTTTCATATTTCAGAATGGACAATTAGATTTTGTT

                         65930     65940     65950     65960     65970     65980     65990     66000

Human             TATGTTTATCTTGTTCTCATTCTCTT-CATCCCCCCTTTGTTGTCTTTTCGTGTTTCAGAATGGACAATTAGATTTTGTT 52500
Coquerel'sSifaka  TATGTTTATCTTGTTCTCAGTCTCCT---TTCCCCCTTTTTATCTGTTCATATTTTATAATGGACAATTAGGGTTTACT 44413
Ma'sNightMonkey   TCTGTTTATCTTGTTCTTACTCTCTTTCATCCCCCCTCTGTTGTCTTTTCATATTTCAGAATGGACAATTAGATTTTGTT 47973
VervetAGM         TATGTTTATCTTGTTCTCACTCTCTT-CATCCCCCGTTTGTTGTCTCTTCATATTTCAGAATGGACAATTATATTTTGTT 54231
Gorrilla          TATGTTTATCTTGTTCTCATTCTCTT-CATCCCCCCTTTGTTGTCTTTTCGTGTTTCAGAATGGACAATTGGATTTTGTT 53024
GrabEatingMacaqueTATGTTTATCTTGTTCTCACTCTCTT-CATCCCCTGTTTGTTGTCTCTTCATATTTCAGAATGGACAATTATATTTTGTT 53444
Macaque           TATGTTTATCTTGTTCTCACTCTCTT-CATCCCCTGTTTGTTGTCTCTTCATATTTCAGAATGGACAATTATATTTTGTT 54272
PigTailedMacaque  TATGTTTATCTTGTTCTCACTCTCTT-CATCCCCTGTTTGTTGTCTCTTCATATTTCAGAATGGACAATTATATTTTGTT 53152
Bonobo            TATGTTTATCTTGTTCTCATTCTCTT-CATCCCCCCTTTGTTGTCTTTTCGTGTTTCAGAATGGACAATTAGATTTTGTT 51597
OliveBaboon       TATGTGTATCTTGTTCTCACTCTCTT-CATCCCCCGTTTGTTGTCTCTTCATATTTCAGAATGGACAATTATATTTTGTT 53019
Orangutan         TATGTTTATCTTGTTCTCATTCTCTT-CATCCCCC-TTTGTTGTCTTTTCGTGTTTCAGAATGGACAATTAGATTTTGTT 53808
```

```
Majority         AAAGTTGTCTGCTTTCCTCTAGGATAAAGTAATGAAGAGTGTCTACTAAGCTGTTA------CTCCCATTCCCCCCAAAT
                         66010     66020     66030     66040     66050     66060     66070     66080
Human            AAAGTTGTATGCTTTCCTCTAGGATAAAGTAATGAAGAGCGTTTACTAAGCTGTTA------CCCCCATTCCCCCCAAAT 52574
Coquerel'sSifaka GGACTTGCGTGCTTTTCTCTCAGGTAAAGTAATGAGT--TGGATCCTAAGCTGGNN------NNNNNNNNNNNNNNNNNN 44485
Ma'sNightMonkey  AGAGTTGTGTGCTTTCCTCTAGGATAAAATACTGAAGAATGTATACTAAGCTGTTATCCCCACCCCCATTCTCCCTAAAT 48053
VervetAGM        AAAGTTGTCTGCTTTCCTCTAGGATAAAGTAATGAAGAGTGTCTACCAAGCTGTTA------CTCCCATCCCCCCCAAAT 54305
Gorrilla         AAAGTTGTATGCTTTCCTCTAGGATAAAGTAATGAAGAGTGTCTACTAAGCTGTTA------CCCCCATTCCCCCCAGAT 53098
GrabEatingMacaqueAAAGTTGTCTGCTTTCCTCTAGGATAAAGTAATGAAGAGTGTCTACCAAGCTGTTA------CTCCCATCCCCCCCAAAT 53518
Macaque          AAAGTTGTCTGCTTTCCTCTAGGATAAAGTAATGAAGAGTGTCTACCAAGCTGTTA------CTCCCATCCCCCCCAAAT 54346
PigTailedMacaque AAAGTTGTCTGCTTTCCTCTAGGATAAAGTAATGAAGAGTGTCTACCAAGCTGTTA------CTCCCATCCCCCCCAAAT 53226
Bonobo           AAAGTTGTATGCTTTCCTCTAGGATAAAGTAATGAAGAGTGTCTACTAAGCTGTTA------CCCCCATTCCCCCCAAAT 51671
OliveBaboon      AAAGTTGTCTGCTTTCCTCTAGGATAAAGTAATGAAGAGTGTCTACCAAGCTGTTA------CTCCCATCCCCCCCAAAT 53093
Orangutan        AAAGTTGTATGCTTTTCTCTAGGATAAAGTAATGAAGAGCGTCTACTAAGCTGTTA------CCCCCATTCCCCCCAAAT 53882

Majority         ACCAAAGAG-CT-CTTATTCTGGGTCA-CAAAACTCCCTTTAGAATTTCTGGTACCA-----------------------
                         66090     66100     66110     66120     66130     66140     66150     66160
Human            ACCAAAGAGGCT-CTTATTCTGGGTCAACAAAACTCCCTTTAGAATTTCTGGTACCAGGCTGTGCGTGGTGGCTCACACT 52653
Coquerel'sSifaka NNNNNNNNN----NNNNNNNNNNNNNN-NNNNNNNNNNNNNNNNNNNNNNNNNNNNNN----------------------- 44537
Ma'sNightMonkey  ACCAAAGAGGCC-TTTATTCTGGGCCAACAAAACTCCCTTTAGAATATCTGGTACCA----------------------- 48109
VervetAGM        ACCAAAGAG-CT-CTTATTCTGGGTCA-CAAAACTCCCTTTAGAATTTCTGGTACCA----------------------- 54359
Gorrilla         ACCAAAGAGGCTGTTTATTCTGGGTCAACAAAACTCCCTTTAGAATTTCTGGTACCATGCTGGGCGTGGTGGCTCACACT 53178
GrabEatingMacaqueACCAAAGAG-CT-CTTATTCTGGGTCA-CAAAACTCCCTTTAGAATTTCTGGTACCA----------------------- 53572
Macaque          ACCAAAGAG-CT-CTTATTCTGGGTCA-CAAAACTCCCTTTAGAATTTCTGGTACCA----------------------- 54400
PigTailedMacaque ACCAAAGAG-CT-CTTATTCTGGGTCA-CAAAACTCCCTTTAGAATTTCTGGTACCA----------------------- 53280
Bonobo           ACCAAAGAGGCT-CTTATTCTGGGTCAACAAAACTCCCTTTAGAATTTCTGGTACCAGGCTGGGCGTGGTGGCTCACACT 51750
OliveBaboon      ACCAAAGAG-CT-CTTATTCTGGGTCA-CAAAACTCCCTTTAGAATTTCTGGTACCA----------------------- 53147
Orangutan        ATCAAAGAGGCT-CTTATTCTGGGTCAACAAAACTCCCTTTAGAATTTCTGGTACCAGGCTGGGCGTGGTGGCTCACACT 53961

Majority         --------------------------------------------------------------------------------
                         66170     66180     66190     66200     66210     66220     66230     66240
Human            TGTAATCCCAGCACTTTGGGAGGCTGAGGTGGGCGGATCACGAGGTCAGGAGATCGAGACCATGGTGAAACCCCGTCTGT 52733
Coquerel'sSifaka -------------------------------------------------------------------------------- 44537
Ma'sNightMonkey  -------------------------------------------------------------------------------- 48109
VervetAGM        -------------------------------------------------------------------------------- 54359
Gorrilla         TGTAATCCCAGCACTTTGGGAGGCTGAGGTGGGCGGATCATGAGGTCAGGAGATCGAGACCATGGTGAAACCCCGTCTCT 53258
GrabEatingMacaque-------------------------------------------------------------------------------- 53572
Macaque          -------------------------------------------------------------------------------- 54400
PigTailedMacaque -------------------------------------------------------------------------------- 53280
Bonobo           TGTAATCCCAGCACTTTGGGAGGCTGAGGTGGGCGGATCACGAGGTCAGGAGATCGAGACCATGGTGAAAACCCGTCTCT 51830
OliveBaboon      -------------------------------------------------------------------------------- 53147
Orangutan        TGTAATTCCAGCACTTTGGGAGGCCAAGGTGGGCGGATCACGAGGTCAGGAGATCAAGACCATGGTGAAACCCCGTCTCT 54041
```

```
Majority        --------------------------------------------------------------------------------

                         66250     66260     66270     66280     66290     66300     66310     66320

Human           ACTAAAAATACGAAAATTAGCTGGGCGTGGTGGCGGGCGCCTGTAGTCCCAGCTACTCGGGAGGCTGAGGCAGGAGAATG 52813
Coquerel'sSifaka -------------------------------------------------------------------------------- 44537
Ma'sNightMonkey -------------------------------------------------------------------------------- 48109
VervetAGM       -------------------------------------------------------------------------------- 54359
Gorrilla        ACTAAAAATACGAAAATTAGCTGGGCGTGGTGGTGGGCGCCTGTAGTCCCAGCTACTCGGAAGGCTGAGGCAGGAGAATG 53338
GrabEatingMacaque-------------------------------------------------------------------------------- 53572
Macaque         -------------------------------------------------------------------------------- 54400
PigTailedMacaque -------------------------------------------------------------------------------- 53280
Bonobo          ACTAAAAATACGAAAATTAGCTGGGCGTGGTGGTGGGCGCCTGTAGTCCCAGCTACTCGGGAGGCTGAGGCAGGAGAATG 51910
OliveBaboon     -------------------------------------------------------------------------------- 53147
Orangutan       ACTAAAAATACAAAAGTTAGCCGGACGTGGTGGCAGGCGCCTGTAGTCCCAGCTACTCGGGAGGCTGAGGCAGGAGAATG 54121

Majority        --------------------------------------------------------------------------------

                         66330     66340     66350     66360     66370     66380     66390     66400

Human           GTGCAGACCCAGGAGGCGGAGCTTGCAGTGAGCCGAGATCGCGCCACTGCACTTCAGCCTGGGCGACAGAGCGAGACTCT 52893
Coquerel'sSifaka -------------------------------------------------------------------------------- 44537
Ma'sNightMonkey -------------------------------------------------------------------------------- 48109
VervetAGM       -------------------------------------------------------------------------------- 54359
Gorrilla        GTGCAGACCCGGGAGGCAGAGCTTGCAGTGAGCCGAGATCGCGCTGCTGCATTCCAGCCTGGGCGACAGAGCGAGACTCT 53418
GrabEatingMacaque-------------------------------------------------------------------------------- 53572
Macaque         -------------------------------------------------------------------------------- 54400
PigTailedMacaque -------------------------------------------------------------------------------- 53280
Bonobo          GTGCAGACCCAGGAGGCAGAGCTTGCAGTGAGCCGAGATCGCGCCACTGCACTTCAGCCTGGGCGACAGAACGAGACTCT 51990
OliveBaboon     -------------------------------------------------------------------------------- 53147
Orangutan       GCGTGAACCCAGGAGGCGGAGCTTGCAGTGAGCTGAGATTGCGCCACTGCACTCCAGCCTGGGTGACAGAGCGAGACTCT 54201

Majority        --------------------------------------------------------GCAGGGCACTTTGGCTCGTGCCTG

                         66410     66420     66430     66440     66450     66460     66470     66480

Human           GTCTCA----AAAAAAAAAAAAAAAAAGAATTCCTGGTACCA--------------GCAGGGCACTTTGGCTCATGCCTG 52955
Coquerel'sSifaka -------------------------------------------------------------------------------- 44537
Ma'sNightMonkey --------------------------------------------------------GCAGAACACTTTGGCTCATGCCCA 48133
VervetAGM       --------------------------------------------------------GCAGGGCACTTTGGCTCGTGCCTG 54383
Gorrilla        GTCTCAAAAAAAAAAAAAAAAAAAAAAGAATTTCTGGTACCA--------------GTGGGGCACTTTGGCTCATGCCTG 53484
GrabEatingMacaque--------------------------------------------------------GCAGGGCACTTTGGCTCGTGCGTG 53596
Macaque         --------------------------------------------------------GCAGGGCACTTTGGCTCGTGCGTG 54424
PigTailedMacaque --------------------------------------------------------GCAGGGCACTTTGGCTCGTGCGTG 53304
Bonobo          GTCT------ACAAAAAAAAAAAAAAAAGAATTCCTGGTACCAAAGCAGGTACCAAAGCAGGGCACTTTGGCTCATGCCTG 52064
OliveBaboon     --------------------------------------------------------GCAGGGCACTTTGGCTCGTGCGTG 53171
Orangutan       GTATCA---AAAAAAAAAAAAAAAAAAGAATTTCTGGTACCA--------------GCAGGGCACTTTGGCTCATGCCTG 54264
```

```
Majority          TAATCCCAGCACTTTGAGAGGCTGAGGTAAGTTCGGGGCAGCAGTGAACTG--GTCACACCACTGCACTCCAGCCTCAGC
                          66490     66500     66510     66520     66530     66540     66550     66560
Human             TAATCCCAGCACTTTGAGAAGCTGAGGTAAATTCAGGGCAGCAGTGAACTG--GTCACACCACTGCACTCCAGCCTCAGC 53033
Coquerel'sSifaka  -------------------------NNNNNNNNNNNNNNNNNNNNNNNNN------------NNNNNNN---------- 44569
Ma'sNightMonkey   TAATCCCAGCACTTTGAAAGGCTGATGCAAGTTCAGGGCAGCAGTGAACTGTAATCACACAACTGCACCCCAGCCTTGTC 48213
VervetAGM         TAATCCCGGCACTTTGAGAGACTGAGGTAAGTTCGGGGCAGCAGTGAACTG--GTCACACCACTGCACTCCAGCCTCGGC 54461
Gorrilla          TAATCCCAGCACTTTGAGAAGCTTAGGTAAATTCAGGGCAGCAGTGAACTG--GTCACACCAGTGCACTCCAGCCTCAGC 53562
GrabEatingMacaqueTAATCCCAGCACTTTGAGAGACTGAGGTAAGTTCGGGGCAGCAGTGAACTG--GTCACACCACTGCACTCCAGCCTCAGC 53674
Macaque           TAATCCCAGCACTTTGAGAGACTGAGGTAAGTTCGGGGCAGCAGTGAACTG--GTCACACCACTGCACTCCAGCCTCAGC 54502
PigTailedMacaque  TAATCCCAGCACTTTGAGAGACTGAGGTAAGTTCGGGGCAGCAGTGAACTG--GTCACACCACTGCACTCCAGCCTCAGC 53382
Bonobo            TAATCCCAGCACTTTGAGAAGCTGAGGTAAATTCAGGGCAGCAGTGAACTG--GTCACACCACTGCACTCCAGCCTCAGC 52142
OliveBaboon       TAATCCCAGCACTTTGAGAGACTGAGGTAAGTTCGGGGCAGCAGTGAACTG--GTCACACCACTGCACTCCAGCCTCGGC 53249
Orangutan         TAATCCCAACACTTTGAGAAGCTGAGGTAAATTCGGGGCAGCAGTGAACTG--GTCACACCACTGCACTCCAGCCTCAGC 54342

Majority          ATCAAAGTGAGACCCAGTCTCTTAAAAAAAAAAAA-------AAGAAAGAAAGAAATTCTGGTTCCTTTAAGGGAGAGGT
                          66570     66580     66590     66600     66610     66620     66630     66640
Human             AACAAAGCAAGACACAGTCTCTTAAAAAAAAAAAAA------------GGAAGAAATTCTGGTTCCTTTAAGGGAGAT-T 53100
Coquerel'sSifaka  -NCAAA--GAGGCCTTTCTTCCTGGCCAAGAAACT---C-------ACTGTAGAATTTCTGGTTCCTTTGAGGCCAGGCT 44636
Ma'sNightMonkey   AACAAAGTGAGACCCAGTCTCTTAAAAAAAAAACA-------GAGAAGAAAATAAATTCTGGTTTCTTTAAGGAAGAGTT 48286
VervetAGM         ATCAAAATGAGACCCAGTGTCTTAAAAAAAACAAA-------AAGAAAGAAAGAAATTATGGTGCCTTTAAGAGAGAGGT 54534
Gorrilla          AACAAAGTGAGACACAGTCTCTTAAAAAAAAAAAG-------------GAAGAAATTCTGGTTCCTTTAAGGGAGAT-T 53627
GrabEatingMacaqueATCAAAATGAGACCCAGTCTCTTAAAAAAAAAAAA-GAAATAAAGAAAGAAGGAAATTCTGGTGCCTTTAAGAGAGAGGT 53753
Macaque           ATCAAAATGAGACCCAGTCTCTTAAAAAAAAAAAA-GAAATAAAGAAAGAAGGAAATTCTGGTGCCTTTAAGAGAGAGGT 54581
PigTailedMacaque  ATCAAAATGAGACCCAGTCTCTTAAAAAAAAAAAA-GAAATAAAGAAAGAAGGAAATTCTGGTGCCTTTAAGAGAGAGGT 53461
Bonobo            AACAAAGCGAGAAACAGTCTCTTAAAAAAAAAAAA-------------GGAAGAAATTCTGGTTCCTTTAAGGGAGAT-T 52208
OliveBaboon       ATCAAAATGAGACCCAGTCTCTTAAAAAAAAAAAAAGAAATAAAGAAAGAAGGAAATTCTGGTGCCTTTAAGAGAGAGGT 53329
Orangutan         AACAAAGTGAGACACAGTCTCTTAAAAAAAAAAAG-------------AAAGAAATTCTGGATCCTTTAAGGTAGAT-T 54407

Majority          TTTT----GTTGTTTTTGTTTT-TTAATGGTGAGGGTACAAGTTGGTCA---GAGGAGGGAGAAGAGAAATTTAGCTCT-
                          66650     66660     66670     66680     66690     66700     66710     66720
Human             TGTT----GTTGTTTTCTTCTT-TTAATGGTGAGGGTACAAGTTGGTCA---CAGGAGGGAGAAGAGAAATTTAGCTCT- 53171
Coquerel'sSifaka  TTTTAATTGTTGTTTTTGTTTTTTTAATGGTGAAGGGGCAAGTTGATCAAGAGGAGAGGGAAAAGAGGTGTTTCATTGTG 44716
Ma'sNightMonkey   TTCT----GTTATT-TT------TTAATGGTGAGGGGACAAGTTGGTCA---AAAGAGGGAGAAGAGAAATTTAGCTCT- 48351
VervetAGM         TTTT----TTTGTTATTGTTGT-TCGATGGTGAGGGTACAAGTTGGTCA---GAGGAGGGAGAAGAGAAATTTAGCTCT- 54605
Gorrilla          TGTT----GTTGTTTTCTTCTT-TTAATGGTGAGGGTACAAGTTGGTCG---CAGGAGGGAGAAGAGAAATTTAGCTCT- 53698
GrabEatingMacaqueTTTT----GTTGTTGTTGTTGT-TTGATGGTGAGGGTACAAGTTGGTCA---GAGGAGGGAGAAGAGAAATTTAGCTCT- 53824
Macaque           TTTT----GTTGTTGTTGTTGT-TTGATGGTGAGGGTACAAGTTGGTCA---GAGGAGGGAGAAGAGAAATTTAGCTCT- 54652
PigTailedMacaque  TTTT----TTTGTTGTTGTTGT-TTGATGGTGAGGGTACAAGTTGGTCA---GAGGAGGGAGAAGAGAAATTTAGCTCT- 53532
Bonobo            TGTT----GTTGTTTTCTTCTT-TTAATGGTGAGGGTACAAGTTGGTCA---CAGGAGGGAGAAGAGAAATTTAGCTCT- 52279
OliveBaboon       TTTT----GTTGTTGTTGTTGT-TTGATGGTGAGGGTACAAGTTGGTCA---GAGGAGGGAGAAGAGAAATTTAGCTCT- 53400
Orangutan         TGTT----GTTGTTTTCTTCTT-TTAATGGTGAGGGTACAAGTTGGTCA---GAGGAGGGAGAAGAGAAATTTAGCTCT- 54478
```

```
Majority         TT-GTTAATGAACTAGACTTTCTGTTCATCCATTTTTCACGCACTCC-CTCCTCCTGTGAACCTGCCAATTTCTAATTTG

                         66730     66740     66750     66760     66770     66780     66790     66800

Human            TTTGTTAATGAACTAGACTTTCTGTTCGTCTGTTTTTCACCCACTCC-CTCCTCCTGTGAACCTGCCAATTTCTGATTTG 53250
Coquerel'sSifaka TTTGTTCATTAGCTAGACTTTCCATTTATCCTTTTGTTTTGTCCCTC-TCCCGTCTGTGAACCTGCTAATTTCTGATCTG 44795
Ma'sNightMonkey  TT-GTTAATGAGCTAGACTTTCGGTTCATCCTTTTTTCACCCACTCTTCTCCTCCTGTGAACCTGCCAATT--------- 48421
VervetAGM        TT-GTTAATGAACTAGACTTTCTGTTCATCCATTTTTCACGCACTCC-CTCCTCCTGTGAACCTCCCAATTTCTAATTTG 54683
Gorrilla         TTTGTTAATGAGCTAGACTTTCTGTTCGTCCGTTTTTCACCCACTCC-CTCCTCCTGTGAACCTGCCAATTTCTGATTTG 53777
GrabEatingMacaqueTT-GTTAATGAACTAGACTTTCTGTTCATCCATTTTTCACGCACTCC-CTCCTCCTGTGAACCTGCCAATTTCTAATTTG 53902
Macaque          TT-GTTAATGAACTAGACTTTCTGTTCATCCATTTTTCACGCACTCC-CTCCTCCTGTGAACCTGCCAATTTCTAATTTG 54730
PigTailedMacaque TT-GTTAATGAACTAGACTTTCTGTTCATCCATTTTTCACGCACTCC-CTCCTCCTGTGAACCTGCCAATTTCTAATTTG 53610
Bonobo           TTTGTTAATGAACTAGACTTTCTGTTCGTCTGTTTTTCACCCACTCC-CTCCTCCTGTGAACCTGCCAATTTCTGATTTG 52358
OliveBaboon      TT-GTTAATGAACTAGACTTTCTGTTCATCCATTTTTCACGCACTCC-CTCCTCCTGTGAACCTGCCAATTTCTAATTTG 53478
Orangutan        TTTGTTAATGAACTAGACTTTCTGTTCATCCGTTTTTCACCCACTCC-CTCCTCCTGTGAACCTGCCAATTTCTAATTTG 54557

Majority         GTTCCTGTGGCTGCTACCAGTATAACTCCTGTTTGCTGTGTGTTCACTGTGTCTCCAGTCTATTTGGTTCCATTCATTTT

                         66810     66820     66830     66840     66850     66860     66870     66880

Human            GTTCCTGTGGCTGCTACCAGTATAACACCTGTTTGCTGTGTGTTCACTGTGTCTCCAGTCCATTTGGTTCCATTCATTTT 53330
Coquerel'sSifaka GTTCCTGTGGCAA--ACCAGTGTAACCCCTGTTTGCTATGTATTCACTGTGCCTTCTGTCT-----CTTTGGTTCATTTT 44868
Ma'sNightMonkey  ---------------ACCA-----------GT---------------------T----------TGGTTCCATTCATTTT 48444
VervetAGM        GTTCCTGTGACTGCTACCAATATAACTCCTGTTTGCTGTGTGTTCACTGTGTCTCCAGTCTGTTTGGTTCCATTCATTTT 54763
Gorrilla         GTTCCTGTGGCTGCTACCAGTATAACACCTGTTTGCTGTGTGTTCACTGTGTCTCCAGTCCATTTGGTTCCATTCATTTT 53857
GrabEatingMacaqueGTTCCTGTGACTGCTACCAATATAACTCCTGTTTGCTGTGTGTTCACTGTGTCTCCAGTCTATTTGGTTCCATTCATTTT 53982
Macaque          GTTCCTGTGACTGCTACCAATATAACTCCTGTTTGCTGTGTGTTCACTGTGTCTCCAGTCTATTTGGTTCCATTCATTTT 54810
PigTailedMacaque GTTCCTGTGACTGCTACCAATATAACTCCTGTTTGCTGTGTGTTCACTGTGTCTCCAGTCTATTTGGTTCCATTCATTTT 53690
Bonobo           GTTCCTGTGGCTGCTACCAGTATAACACCTGTTTGCTCTGTGTTCACTGTGTCTCCAGTCCATTTAGTTCCATTCATTTT 52438
OliveBaboon      GTTCCTGTGACTGCTACCAATATAACTCCTATTTGCTGTGTGTTCACTGTGTCTCCAGTCTATTTGGTTCCATTCATTTT 53558
Orangutan        GTTCCTGTGGCTGCTACCAGTATAACACCTGTTTGCTGTGTGTTCACTGTGTCTCCAGTCCGTTTGGTTCCATTCATTTT 54637

Majority         CTGTGTTCCAGGAATTTGATAAAATTATTCTCAGCTAATGATGACCTTCCCTCCCTTCAGT------GTTTGTTT-ATTT

                         66890     66900     66910     66920     66930     66940     66950     66960

Human            CTGTGTTCCAGGAATTTGATAAAATTATTCTCAGCTAATGATAACCTTCCCTCCCTTCAGT------GTTTGTTT-ATTT 53403
Coquerel'sSifaka CTGTGTTCTAAAAATTTAATAAAATTATTTTCAGCTAATGGTGACCTTTGCTCTGTTCAGTGTTTTTGTTTATTTCATTT 44948
Ma'sNightMonkey  CTGTGTTCCAGGAATTTGATAAAATTATTCTTAGCTAATGATGACCTTAACTCCCTTCAGT------GTTTGTTT-ATTT 48517
VervetAGM        CTGTGTTCCAGGAATTTGATAAAATTATTCTCAGCTAATGATGACCTTCCCTCCCTT----------GTTTGTTT-ATTT 54832
Gorrilla         CTGTGTTCCAGGAATTTGATAAAATTATTCTCAGCTAATGATAACCTTCCCTCCCTTCAGT------GTTTGTTT-ATTT 53930
GrabEatingMacaqueCTGTGTTCCAGGAATTTGATAAAATTATTCTCAGCTAATGATGACCTTCCCTCCCTTCAGT------GTTTGTTT-ATTT 54055
Macaque          CTGTGTTCCAGGAATTTGATAAAATTATTCTCAGCTAATGATGACCTTCCCTCCCTTCAGT------GTTTGTTT-ATTT 54883
PigTailedMacaque CTGTGTTCCAGGAATTTGATAAAATTATTCTCAGCTAATGATGACCTTCCCTCCCTTCAGT------GTTTGTTT-ATTT 53763
Bonobo           CTGTGTTCCAGGAATTTGATAAAATTATTCTCAGCTAATGATAACCTTCCCTCCCTTCAGT------GTTTGTTT-ATTT 52511
OliveBaboon      CTGTGTTCCAGGAATTTGATAAAATTATTCTCAGCTAATGATGACCTTCCCTCCCTTCAGT------GTTTGTTT-ATTT 53631
Orangutan        CTGTATTCCAGGAATTTGATAAAATTATTCTCAGCTAATGATAACCTTCCCTCCCTTCAGT------GTTTGTTT-ATTT 54710
```

```
Majority         TATATTCATTCACTTCTAGGCATTTTAATAGGGTTTCATAATAGAAGACATGCAAATGAGTATGCATTAACCATGAATTC

                         66970     66980     66990     67000     67010     67020     67030     67040

Human            TATATTCATTCACTTCTAGGCATTTTAATAGAGTTTCATAATAGAAGACATGCAAATGAGTATGCATTAACCATGAATTC 53483
Coquerel'sSifaka TATATTCATTCACCTATAGGCATTTTAATAGAATTTCATAACCAAAGAGTGGCAAATGAATATGTGTTAACCATGAATTC 45028
Ma'sNightMonkey  TATATTCATTTATTTCTAGGCATTTTAATAGGGTTTCATAATAGAAAACATGCACATGAGTATGCATTAACTATGAATTC 48597
VervetAGM        TATATTCATTCACTTCTAGGCATTTTAATAGGGTTTCATAATAGAAGACATGCAAATGAGTATGCATTAACCATGAATTC 54912
Gorrilla         TATATTCATTCACTTCTAGGCATTTTAATAGAGTTTCATAATAGAAGACATGCAAATGAGTATGCATTAACCATGAATTC 54010
GrabEatingMacaqueTATATTCATTCACTTCTAGGCATTTTAATAGGGTTTCATAATAGAAGACATGCAAATGGGTATGCATTAACCATGAATTC 54135
Macaque          TATATTCATTCACTTCTAGGCATTTTAATAGGGTTTCATAATAGAAGACATGCAAATGGGTATGCATTAACCATGAATTC 54963
PigTailedMacaque TATATTCATTCACTTCTAGGCATTTTAATAGGGTTTCATAATAGAAGACATGCAAATGGGTATGCATTAACCATGAATTC 53843
Bonobo           TATATTCATTCACTTCTAGGCATTTTAATAGAGTTTCATAATAGAAGACATGCAAATGAGTATACATTAACCATGAATTC 52591
OliveBaboon      TATATTCATTCACTTCTAGGCATTTTAATAGGGTTTCATAATAGAAGACATGCAAATGGGTATGCATTAACCATGAATTC 53711
Orangutan        TATATTCATTCACTTCTAGGCATTTTAATAGAGTTTCATAATAGAAGACATGCAAATGAGTATGCATTAACCATGAATTC 54790

Majority         TTCCACATCCCTTCATGAAATCACCAGGTCCTATTAGTTCACACTTTTTAATTATCTGCCTGGTTTTCTCCATCTTTCCT

                         67050     67060     67070     67080     67090     67100     67110     67120

Human            TTTCACATCCGTTCATGAAATCACCAGGTCCTATTAGTTCACACTTTTTAATTATCTGCCTGGTTTTCTCCATCTTTCCT 53563
Coquerel'sSifaka TTCCACAACCCTTCATAAAATCACCAAGACCTATCAGTTTACTCACCTTAATTAACTGCCTAGTTGTCTTCATCTCTCC- 45107
Ma'sNightMonkey  TTCCACATCTCTTCATGAAATCATCAGGTCCTATCAGTTCACACTTTTTAATTATCTGCGTAGTTTCTCCATCTTTCC- 48676
VervetAGM        TTCCACATCCCTTCTTGAAATCACCAGGTCCTATTAGTTCACACTTTTTAATTATCTGCCTGGTTTTCTCCATCTTTCCT 54992
Gorrilla         TTCCACATCCCTTCATGAAATCACCAGGTCCTATTAGTTCACACTTTTTAATTATCTGCCTGGTTTTCTCCATCTTTCCT 54090
GrabEatingMacaqueTTCCACATCCCTTCTTGAAATCACCAGGTCCTATTAGTTCACACTTTTTAATTATCTGCCTTGTTTTCTCCATCTTTCCT 54215
Macaque          TTCCACATCCCTTCTTGAAATCACCAGGTCCTATTAGTTCACACTTTTTAATTATCTGCCTTGTTTTCTCCATCTTTCCT 55043
PigTailedMacaque TTCCACATCCCTTCTTGAAATCACCAGGTCCTATTAGTTCACACTTTTTAATTATCTGCCTTGTTTTCTCCATCTTTCCT 53923
Bonobo           TTCCACATCCCTTCATGAAATCACCAGGTCCTATTAGTTCACACTTTTTAATTATCTGCCTGGTTTTCTCCATCTTTCCT 52671
OliveBaboon      TTCCACATCCCTTCTTGAAATCACCAGGTCCTATTAGTTCATACTTTTTAATTATCTGCCTGGTTTTCTCCATCTTTCCT 53791
Orangutan        TTCCACATCCCTTCATGAAATCACCAGGTCCTGTTAGTTCACACTTTTTAATTACCTGCCTGGTTTTCTCCATCTTTCCT 54870

Majority         GCTACTTGTATTGCTCAAACACACTCATCTGTTGGCTATATTACTACAACAGTTCTCTTTATAAACTACTTTCTTTTTCT

                         67130     67140     67150     67160     67170     67180     67190     67200

Human            GCTACTTGTATTGCTCAAACACACTCACCTGTTGGCTATATTACTACGACAGTTCTCTTTATAAACTACTTTCTTTTTCT 53643
Coquerel'sSifaka --CACT-GTATTTCTCAGAC-TAGTCACCTCTTGGCTCTGTTATTACAACAGTTCTCTTACTAATCTGCTTTCTT--ACT 45181
Ma'sNightMonkey  --TACTTGTATTACTCAAACACACTCATCTCTTGACTATGTTACTACAACATTTCTCTTTATAAACTACTTTCTTTTCCT 48754
VervetAGM        GCTACTTGTATTGCTCAGACACACTCATCTGTTGGCTATATTACTACAACAGTTATCTTTATAAACTACTTTCTTTTTCT 55072
Gorrilla         GCTACTTGTATTGCTCAAACACACTCACCTGTTGGCTATATTACTACGACAGTTCTCTTTATAAACTACTTTCTTTTTCT 54170
GrabEatingMacaqueGCTACTTGTATTGCTCAAACACACTCATCTGTTGGCTATATTACTACAACAGTTATCTTTATAAACTACTTTCTTTTTCT 54295
Macaque          GCTACTTGTATTGCTCAAACACACTCATCTGTTGGCTATATTACTACAACAGTTATCTTTATAAACTACTTTCTTTTTCT 55123
PigTailedMacaque GCTACTTGTATTGCTCAAACACACTCATCTGTTGGCTATATTACTACAACAGTTATCTTTATAAACTACTTTCTTTTTCT 54003
Bonobo           GCTACTTGTATTGCTCAAACACACTCACCTGTTGGCTATATTACTACGACAGTTCTCTTTATAAACTACTTTCTTTTTCT 52751
OliveBaboon      GCTACTTGTATTGCTCAAACACACTCATCTGTTGGCTATATTACTACAACAGTTATCTTTATAAACTACTTTCTTTTTCT 53871
Orangutan        GCTACTTGTATTGCTCAAACACACTCACCTGTTGGCTATATTACTACGACAGTTCTCTTTATAAACTACTTTCTTTTTCT 54950
```

```
Majority          TTTCCCTCTTGGAATTTTTT-CTCCCTAGTACAGCCAGTGCGTATCTTTCTCAAATGCACATATCTAAGTTACTCCCATG

                          67210     67220     67230     67240     67250     67260     67270     67280

Human             TTTCCCTTCTGGAATTTTTTTCTCGCTAGTACAGCCAGTACGTATCTTTCTCAAATGCACATATCTAAGTTACTCCCATG 53723
Coquerel'sSifaka  TTTCCCTCTTGAATTCTTTT--TCCCTAGTGCAGCTGGTGTATATCCTTCTGAAAT--------CTAAGTTACCTCCCTG 45251
Ma'sNightMonkey   TTTCCCTCTTGGAGTTTTTT-CTCTCTAGTACAGCCAGTTAATATCTTTCTCAAATTCACCTATCTAAGTT-CTCCCATG 48832
VervetAGM         TTTCCCTCTTGGAATTTTTT-CTGCCTAGTACAGCCAGTGCGTATCTTTCTCAAATGCACATATCTAAGTTACTCCCATG 55151
Gorrilla          TTTCCCTTCTGGAATTTTTTTCTCGCTAGTACAGCCAGTACGTATCTTTCTCAAATGCACATATCTAAGTTACTCCCATG 54250
GrabEatingMacaqueTTTCCCTCTTGGAATTTTTT-CTCCCTAGTACAGCCAGTGCGTATCTTTCTCAAATGCACATATCTAAGTTACGCCCATG 54374
Macaque           TTTCCCTCTTGGAATTTTTT-CTGCCTAGTACAGCCAGTGCGTATCTTTCTCAAATGCACATATCTAAGTTACGCCCATG 55202
PigTailedMacaque  TTTCCCTCTTGGAATTTTTT-CTGCCTAGTACAGCCAGTGCATATCTTTCTCAAATGCACATATCTAAGTTACGCCCATG 54082
Bonobo            TTTCCCTTCTGGAATTTTTTTCTCGCTAGTACAGCCAGTACGTATCTTTCTCAAATGCACATATCTAAGTTACTCCCATG 52831
OliveBaboon       TTTCCCTCTTGGAATTTTTT-CTGCCTAGTACAGCCAGTGCGTATCTTTCTCAAATGCACATATCTAAGTTACTCCCATG 53950
Orangutan         TTTCCCTCCTGGAATTTTTTTCTCCCTAGTACAGCCAGTACATATCTTTCTCAAATGCACATATCTAAGTTACTCCCATG 55030

Majority          CAGTAGGTCCTGTTCATGCTCAGGGTAAAGGCCTTTTCTGAGATTACTGCT--TTTTGTCCTCTGTT-T--GACTACTCC

                          67290     67300     67310     67320     67330     67340     67350     67360

Human             CAGTAGGTCCTGTTCATGCTCAGGCTAAAGGCCCTTTCTGAGCTTACTACT--TTTTGTCTTGTCTTAT--GACTACTCC 53799
Coquerel'sSifaka  CAGTAGGTCCTTTTCATGTTCAGGATAAAGGCCCTTTCTCAGATCACTGCTATTTTTGTTCTCTGTCTTATGGCTGGTCC 45331
Ma'sNightMonkey   CAGTATATCCTGTTCATGCTTAGGGTAAAGGCCATTTCTGAGATCACTACT--TTTTGTCTTCTGTCTTATGACTACTCC 48910
VervetAGM         CAGTAGGTCCTGTTCATGCTCAGGGTAAAGGCCTTTTCTGAGATTACTGCT--CTTTGTCCTCTGTT------CTACTTC 55223
Gorrilla          CAGTAGGTCCTGTTCATGCTCAGGCTAAAGGCCCTTTCTGAGCTTACTACT--TTTTGTCTTGTCTTAT--GATTACTCC 54326
GrabEatingMacaqueCAGTAGGTCCTGTTCATGCTCAGGGTAAAGGCCTTTTCTGAGATTACTGCT--CTTTGTCCTCTGTT------CTACTTC 54446
Macaque           CAGTAGGTCCTGTTCATGCTCAGGGTAAAGGCCTTTTCTGAGATTACTGCT--CTTTGTCCTCTGTT------CTACTTC 55274
PigTailedMacaque  CAGTAGGTCCTGTTCATGCTCAGGGTAAAGGCCTTTTCTGAGATTACTGCT--CTTTGTCCTCTGTT------CTACTTC 54154
Bonobo            CAGTAGGTCCTGTTCATGCTCAGGCTAAAGGCCCTTTCTGAGCTTACTACT--TTTTGTCTTGTCTTAT--GACTACTCC 52907
OliveBaboon       CAGTAGGTCCTGTTCATGCTCAGGGTAAAGGCCTTTTCTGAGATTACTGCT--CTTTGTCCTCTGTT------CTACTTC 54022
Orangutan         CAGTAGGTCCTGTTCATACTCAGGGTAAAGGCCCTTTCTGAGATTACTACT--TTTTGTCTTGTCTTAT--GACTACTCC 55106

Majority          CACCCTATTTGCTTATGCTTCTGTCCCAAAAGTAATGGTGTATCCTGTAACCTGGCATGCTTTCTCCTGATGTCAGTTGT

                          67370     67380     67390     67400     67410     67420     67430     67440

Human             CACCCTATTTGCTTATGCTTCTGTCCCAAAAGTAATGGTGCATCCTGTAACCTGGCATGTTTTCTCTTGATGTCAGTCGT 53879
Coquerel'sSifaka  TGTCCTATTTGCTTGTGCTTCTGTCCCAAAAGTAATGCTAGATCCTCAAACTTGGCATGCTTTCTGTTGATTTCAGTCTT 45411
Ma'sNightMonkey   CACCCTATTTGCTTATGCTTCTGTCCCAAAAGTAATGGTGCATCCTATAACCTGGAATGCTTTCTTCTGATGTCAGTTAT 48990
VervetAGM         CACCCTATTTGCTTATGCTTCTGTCCCAAAAGTAATGGTGTATCCTGTAACCTGGCATGCTTTCTCCTGATGTTAGTTGT 55303
Gorrilla          CACCCTATTTGCTTATGCTTCTGTCCCAAAAGTAATGGTGCATCCTGTAACCTGGCATGTTTTCTCTTGATGTCAGTCGT 54406
GrabEatingMacaqueCACCCTATTTGCTTATGCTTCTGTCCCAAAAGTAATGGTGTATCCTGTAACCTGGCATGCTTTCTCCTGATGTTAGTTGT 54526
Macaque           CACCCTATTTGCTTATGCTTCTGTCCCAAAAGTAATGGTGTATCCTGTAACCTGGCATGCTTTCTCCTGATGTTAGTTGT 55354
PigTailedMacaque  CACCCTATTTGCTTATGCTTCTGTCCCAAAAGTAATGGTGTATCCTGTAACCTGGCATGCTTTCTCCTGATGTTAGTTGT 54234
Bonobo            CACCCTATTTGCTTATGCTTCTGTCCCAAAAGTAATGGTGCATCCTGTAACCTGGCATGTTTTCTCTTGATGTCAGTCGT 52987
OliveBaboon       CACCCTATTTGCTTATGCTTCTGTCCCAAAAGTAATGGTGTATCCTGTAACCTGGCATGCTTTCTCCTGATGTTAGTTGT 54102
Orangutan         CACCCTATTT-CTTATGCTTCTGCCCCAAAAATAATGGTGCATCCTGTAACCTGGCATGCTTTCTCCTGATGTCAGTTGT 55185
```

```
Majority        TTGCTGCCATTATGTCACTG------CTCTCATGCATGGAATATTGTGGGTTG---GCCTGCCTTTTGCACCACCCTTCC

                        67450     67460     67470     67480     67490     67500     67510     67520

Human           TTGCTGCCATTATGTCACTG------CTCTCATGCATGGAATATTGTGGGTTG---GCCTGCCTTTTGCACCACCCTTCC 53950
Coquerel'sSifaka TTGCTGCCATTATGTCACTGTGTTCTCACTTACATGTGGAATATTGTGGCTTGTCTGCCTGCCTT-TGCGCCACCCTTCC 45490
Ma'sNightMonkey TT-CTGCTGTTATGTCACTG------CTCTCATGCATGGAATATTGTGGGTTG---GCCTGCCTTGTGCACCACACTTCC 49060
VervetAGM       TTGCTGCCATTATGTCACTG------GTCTCATGCATGGAATATTGTGGGTTG---GCCTGCCTTTTGCACCAACCTTCC 55374
Gorrilla        TTGCTGCCATTATGTCACTG------CTCTCATGCATGGAATATTGTGGGTTG---GCCTGCCTTTTGCACCACCCTTCC 54477
GrabEatingMacaqueTTGCTGCCATTATGTCACTG------CTCTCATGCATGGAATATTGTGGGTTG---GCCTGCCTTTTGCACCAACCTTCC 54597
Macaque         TTGCTGCCATTATGTCACTG------CTCTCATGCATGGAATATTGTGGGTTG---GCCTGCCTTTTGCACCAACCTTCC 55425
PigTailedMacaque TTGCTGCCATTATGTCACTG------CTCTCATGCATGGAATATTGTGGGTTG---GCCTGCCTTTTGCACCAACCTTCC 54305
Bonobo          TTGCTGCCATTATGTCACTG------CTCTCATGCATGGAATATTGTGGGTTG---GCCTGCCTTTTGCACCACCCTTCC 53058
OliveBaboon     TTGCTGCCATTATGTCACTG------CCCTCATGCATGGAATATTGTGGATTG---GCCTGCCTTTTGTACCAACCTTCC 54173
Orangutan       TTGCTGCCATTATGTCACTG------CTCTCATGCATGGAATATTGTGGGTTG---GCCTGCCTTTTGCACCGCCCTTCC 55256

Majority        TCCACCTCACACTTCACATGGCCAGT-GTTAAATATCTTGAGGTCTCCCTTTAGCTATTTCTTCCCCTA-AAAGCTATCC

                        67530     67540     67550     67560     67570     67580     67590     67600

Human           TCCACCTCACACTTCACATGGCCAGT-GTTAAATATCTTGAGGTCTCCCTTTAGCTATTTCTTCTCCTA-AAAGCTATCC 54028
Coquerel'sSifaka ---ACCCCACACTTTATGTGGCCATTTGCTAAATATCTTTAGGTCTCCCTTTAGCTATTTTTTCCTTTAGAAAGCCATCC 45567
Ma'sNightMonkey ---ACCTCACACTTCACATGGCCAGT-GTTAAATATCTTGAGGTTTCCCTTTAGCTATTTCTTCTCCTA-AAAGCTATCC 49135
VervetAGM       TCCACCTCACATTTCACATGACCAGT-GTTAAATATCTTGAGGTCTCCCTTTAGCTATTACTTCCCCTG-AAAGCTATCC 55452
Gorrilla        TCCACCTCACACTTCACATGGCCAGT-GTTAAATATCTTGAGGTCTCCCTTTAGCTATTTCTTCTCCTA-AAAGTTATCC 54555
GrabEatingMacaqueTCCACCTCACACTTCACATGACCAGT-GTTAAATATCTTGAGGTCTCCCTTTAGCTATTACTTCCCCTA-AAAGCTATCC 54675
Macaque         TCCACCTCACACTTCACATGACCAGT-GTTAAATATCTTGAGGTCTCCCTTTAGCTATTACTTCCCCTA-AAAGCTATCC 55503
PigTailedMacaque TCCACCTCACACTTCACATGACCAGT-GTTAAGTATCTTGAGGTCTCCCTTTAGCTATTACTTCCCCTA-AAAGCTATCC 54383
Bonobo          TCCACCTCACACTTCACATGGCCAGT-GTTAAATATCTTGAGGTCTCCCTTTAGCTATTTCTTCTCCTA-AAAGCTATCC 53136
OliveBaboon     TCCACCTCACACTTCACATGACCAGT-GTTAAATATCTTGAGGTCTCCCTTTAGCTATTACTTCCCCTA-AAAGCTATCC 54251
Orangutan       TCCATTTCACACTTCACATGGCCAGT-GTTAAATATCTTGAGGTCTCCCTTTAGATATTTCGTCCCCTA-AAAGCTATCC 55334

Majority        CCTGATAAGCAAAGAGTAGATTAGGTTTGGATTGAGAGCCTTAACTCCAAATGAGATACTGCTATGCACATAACCAGAGT

                        67610     67620     67630     67640     67650     67660     67670     67680

Human           CCTGATAAGCAAAGAGTAGATTAGGTTTGGATTGAGAGTGTTAACTCCGAATGAGATACTGCTATGCACATAACCAGAGT 54108
Coquerel'sSifaka --TGATA--CACAGAGTGGATTAGATTTGGATTGAGGGCCTTAAATGCAAAT---------------------------- 45615
Ma'sNightMonkey CATGATAAGCAAAGAATAGATTAGGTTTGGATTGAGAGCCTTAACTCCAAATGAGATACTGCTATGCACATAACCAGAGT 49215
VervetAGM       CCTGATAAGCAAAGAGTAGATTAGGTTTGGATTGAGAGCCTTAACTCCAAATGAGATACTGCTATGCACATAACCAGAGT 55532
Gorrilla        CCTGATAAGCAAAGAGTAGATTAGGTTTGGATTGAGAGTGTTAACTCCGAATGAGATATTGCTATGCACATAACCAGAGT 54635
GrabEatingMacaqueCCTGATAAGCAAAGAGTAGATTAGGTTTGGATTGAGAGCCTTAACTCCAAATGAGATACCGCTATGCACATAACCAGAGT 54755
Macaque         CCTGATAAGCAAAGAGTAGATTAGGTTTGGATTGAGAGCCTTAACTCCAAATGAGATACCGCTATGCACATAACCAGAGT 55583
PigTailedMacaque CCTGATAAGCAAAGAGTAGATTAGGTTTGGATTGAGAGCCTTAACTCCAAATGAGATACCGCTATGCACATAACCAGAGT 54463
Bonobo          CCTGATAAGCAAAGAGTAGATTAGGTTTGGATTGAGAGTGTTAACTCCGAATGAGATACTGCTATGCACATAACCAGAGT 53216
OliveBaboon     CCTGATAAGCAAAGAGTAGATTAGGTTTGGATTGAGAGCCTTAACTCCAAATGAGATACCGCTATGCACATAACCAGAGT 54331
Orangutan       CCTGATAAGCAAAGAGTAGATTAGGTTTGGATTGAGAGCATTAACTCCGAATGAGATACTGCTATGCACATAACCAGAGT 55414
```

```
Majority         GGCTAAAATTAAAAGGATAGGTAATAAAATATTGGTGACAG---------------------------------------

                         67690     67700     67710     67720     67730     67740     67750     67760

Human            GGCTAAAATTAAAAGGATAGGTAATAAAATACTGGTGACAA--------------------------------------- 54149
Coquerel'sSifaka ---TAAAATCACAGGGG---------AA-TACTA---A------------------------------------------ 45637
Ma'sNightMonkey  GGTCAAAATTAAAAGGATAGGTAATAAAATGTTGGTGACAATGTAGAAAAGCTTGAACTCTCATATATATGATGAAAGGA 49295
VervetAGM        GGCTAAAATTAAAAGGATGGGTAATAAAATATTGGTGACAG--------------------------------------- 55573
Gorrilla         GGCTAAAATTAAAAGGATAGGTAATAAAATACTGGTGACAA--------------------------------------- 54676
GrabEatingMacaqueGGCTAAAATTAAAAGGATAGGTAGTAAAATATTGGTGACAG--------------------------------------- 54796
Macaque          GGCTAAAATTAAAAGGATAGGTAATAAAATATTGGTGACAG--------------------------------------- 55624
PigTailedMacaque GGCTAAAATTAAAAGGATAGGTAATAAAATATTGGTGACAG--------------------------------------- 54504
Bonobo           GGCTAAAATTAAAAGGATAGGTAATAAAATACTGGTGACAA--------------------------------------- 53257
OliveBaboon      GGCTAAAATTAAAAGGATAGGTAATAAAATATTGGTGACAG--------------------------------------- 54372
Orangutan        GGCTAAAATTAAAAGGATAGGTAATAAAATACTGGTGACAA--------------------------------------- 55455

Majority         CCATTTTTGAAAACTTGAAATATCTACTAAAGTTGAGCATATA-CTATTCTTTGACATA--------GT-----------

                         67770     67780     67790     67800     67810     67820     67830     67840

Human            CCATTTTTGAAAACTCGAAATATCTACTAAAGTTGAGCATATAGCTATTCTTTGATGTA--------GT----------- 54210
Coquerel'sSifaka CCACGCGTATATACCAGA----CTGACTAAAATTAAAAGAGA---AGT----AACATG---------------------- 45685
Ma'sNightMonkey  CCATTTTTGAAAACTTGAAATTTCTACTAAAGTTGAGCATGTATCTATT-TTTGACATA--------GT----------- 49355
VervetAGM        CCAGTTTTGAAAACTTGGAATATCTGCTAAAGTTGAGCATATA-CTATTCTTTGACATAAACTCAAAGTTTACTCCTAGG 55652
Gorrilla         CCATTTTTGAAAACTCGAAATATCTACTAAAGTTGAGCATATACCTATTCTTTGACGTA--------GT----------- 54737
GrabEatingMacaqueCCAGTTTTGAAAACTTGGAATATCTGCTAAAGTTGAGCATATA-CTATTCTTTGACATAAACTCAAAGT----------- 54864
Macaque          CCAGTTTTGAAAACTTGGAATATCTGCTAAAGTTGAGCATATA-CTATTCTTTGACATAAACTCAAAGT----------- 55692
PigTailedMacaque CCAGTTTTGAAAACTTGGAATATCTGCTGAAGTTGAGCATATA-CTATTCTTTGACATAAACTCAAAGTTTACTCCTAGG 54583
Bonobo           CCATTTTTGAAAACTCGAAATATCTACTAAAGTTGAGCATATAGCTATTCTTTGACGTA--------GT----------- 53318
OliveBaboon      CCAGTTTTGAAAACTTGGAATATCTGCTAAAGTTGAGCATATA-CTATTCTTTGACATAAACTCAAAGTTTACTCCTAGG 54451
Orangutan        CCATTTTTGAAAACTTGAAATATCTATTGAAGTTGAGCATATACCTATTCTTTGATGTA--------GT----------- 55516

Majority         ------AAACTCGCTCCTAGTTAACATACCTGAGAATATATACATATGTATGTTAAAAGATATGTCCAAGAAAATTCATG

                         67850     67860     67870     67880     67890     67900     67910     67920

Human            ------AAACTCGCTCCTAGTTAACATACCTGAGAACATGTACATAGGTATGTTAAAAGATATGTACAAGAAAGTTCATG 54284
Coquerel'sSifaka --------------ACAATGTAAAGAAACTTGA----ATTTTCATATATAAGTGATGGGA-ATGT-------AATTGATA 45739
Ma'sNightMonkey  ------AAACTCGCTCCTAGTTA-CATACCTGAGAACATATACATAGGTATGTTAAAAGAGATGTACTAGAAAGTGAATG 49428
VervetAGM        AGGAGTAAACTCGCTCCTAGTTAACATACCTGAGAATATATACATATGTATGTTAAAAGATATGTCCAAGAAAATTCATG 55732
Gorrilla         ------AAACTCGCTCCTAGTTAACATACCTGAGAACATGTACATAGGTATGTTAAAAGATATGTACAAGAAAGTTCATG 54811
GrabEatingMacaque------AAACTCGCTCCTAGTTAACATACCTGAGAATATATACATATGTATGTTAAAAGATATGTCCAAGAAAATTCATG 54938
Macaque          ------AAACTCGCTCCTAGTTAACATACCTGAGAATATATACATATGTATGTTAAAAGATATGTCCAAGAAAATTCATG 55766
PigTailedMacaque AGGAGTAAACTCGCTCCTAGTTAACATACCTGAGAATATATACATATGTATGTTAAAAGATATGTCCAAGAAAATTCATG 54663
Bonobo           ------AAACTCGCTCCTAGTTAACATACCTGAGAACATGTACATAGGTATGTTAAAAGATATGTACAAGAAAGTTCATG 53392
OliveBaboon      AGGAGTAAACTCGCTCCTAGTTAACATACCTGAGAATATATACATATGTATGTTAAAAGATATGTCCAAGAAAATTCATG 54531
Orangutan        ------AAACTCGCTCCTAGTTAACATACCTGAGAACGTATACATATGTATATTAAAAGATACGTACAAGAAAGTTCATG 55590
```

```
Majority          GAACACTGTATGTAATAGCCCAAAAAACTATAAATGATCCAAATGTTCAACAGCAGCAGAATATGTTGTGGTGTATTCAT
                          67930     67940     67950     67960     67970     67980     67990     68000
Human             GAACACTGTATGTAATAGCCCAAAAAACTATAAATCATCCAAATGTTCAACAGCAGCAGAATATGTTGTGTTGTATTCAT 54364
Coquerel'sSifaka  TAGAACCATTTTTAAAAACTTGAA-------ATATCTACTAAA-GTTGAGCAT-----GTATGTGTTCT------TTGAC 45800
Ma'sNightMonkey   GAACACTGTATGTAATAGCCCAAA--GCCACAAATTATCCAAATGTTCAACAGCAGCAGAATTTGTTGTGGTGTATTCAT 49506
VervetAGM         GAACACTGTATGTAGTAGTCCAAAAAACTATAAATGATCCAAATGTTCAACAGCAGCAGAATATGTTATGGTGTATTCAT 55812
Gorrilla          GAACACTGTATGTAATAGCCCAAAAAACTATAAATCATCCAAATGTACAACAGCAGCAGAATATGTTGTGGTGTATTCAT 54891
GrabEatingMacaqueGAACACTGTATGTAGTAGTCCAAAAAACTATAAATGATCCAAATGTTCAACAGCAGCAGAATATGTTGTGGTGTATTCAT 55018
Macaque           GAACACTGTATGTAGTAGTCCAAAAAACTATAAATGATCCAAATGTTCAACAGCAGCAGAATATGTTGTGGTGTATTCAT 55846
PigTailedMacaque  GAACACTGTA----GTAGTCCAAAAAACTATAAATGATCCAAATGTTCAACAGCAGCAGAATATGTTGTGGTGTATTCAT 54739
Bonobo            GAACACTGTATGTAATAGCCCAAAAAACTATAAATCATCCAAATGTTCAACAGCAGCAGAATATGTTGTGGTGTATTCAT 53472
OliveBaboon       CAACACTGTATGTAGTAGTCCAAAAAACTGTAAGTGATCCAAATGTTCATCAGCAGCAGAATATGTTGTGGTGTATTCAT 54611
Orangutan         GAACACTGTATGTAATAGCCCAAAAAATTATAAATCATCCAAATGTTCAACAGCAGCAGAATATGTTGTGGTGTGTTCAT 55670

Majority          TTAGTGGAATA----------TT--------------------------------------------G----------
                          68010     68020     68030     68040     68050     68060     68070     68080
Human             TTAGTGGAATA----------TT--------------------------------------------G---------- 54378
Coquerel'sSifaka  TTAGTA-ATTA-------------------------------------------------------------------- 45810
Ma'sNightMonkey   TTAGCAGAATA----------CT-------------------------------------------------------- 49519
VervetAGM         TTAGTGGAATATTGCTTTTTTTTT--------------------GAGATGGAGTTTCAAGCCTAGGTTGGAGTGCAATGG 55872
Gorrilla          TTAGTGGAATA----------TT--------------------------------------------G---------- 54905
GrabEatingMacaqueTTAGTGGAATATTGCTTTTTTTTTTTTTTTTTTTTT--------GAGATGGAGTTTCAAGCCTAGGTTGGAGTGCAATGG 55090
Macaque           TTAGTGGAATATTGCTTTTTTTTTTTTTTTTTTTTTTTTTTTTTTGAGATGGAGTTTCAAGCCTAGGTTGGAGTGCAATGG 55926
PigTailedMacaque  TTAGTGGAATATTGCTTTTTTTTTTTTTTTTTTTTTTTT-----GAGATGGAGTTTCAAGCCTAGGTTGGAGTGCAATGG 54814
Bonobo            TTAGTGGAATA----------TT--------------------------------------------G---------- 53486
OliveBaboon       TTAGTGGAATATTGCCTTTTTTTTTTTTTTTTTTTTTTT-----GAGATGGAGTTTCAAGCCTAGGTTGGAGTGCAATGG 54686
Orangutan         TTAGTGGAGTA----------TT--------------------------------------------G---------- 55684

Majority          --------------------------------------------------------------------------------
                          68090     68100     68110     68120     68130     68140     68150     68160
Human             -------------------------------------------------------------------------------- 54378
Coquerel'sSifaka  -------------------------------------------------------------------------------- 45810
Ma'sNightMonkey   -------------------------------------------------------------------------------- 49519
VervetAGM         CGTGATCTCAGCTCACCGCAACCTCTGCCTCCCAGGTGCAAGCAATTCTCCTGCCTCAGCCTCCTGAGTAGCTGGGATTA 55952
Gorrilla          -------------------------------------------------------------------------------- 54905
GrabEatingMacaqueCGTGATCTCAGCTCACCGCAACCTCTGCCTCCCAGGTGCAAGCGATTCTCCTGCCTCAGCCTCCCGAGTAGCTGGGATTA 55170
Macaque           CGTGATCTCAGCTCACCGCAACCTCTGCCTCCCAGGTGCAAGCGATTCTCCTGCCTCAGCCTCCCGAGTAGCTGGGATTA 56006
PigTailedMacaque  CGTGATCTCAGCTCACCGCAACCTCTGCCTCCCAGGTGCAAGTGATTCTCCTGCCTCAGCCTCCCAAGTAGCTGGGATTA 54894
Bonobo            -------------------------------------------------------------------------------- 53486
OliveBaboon       CGTGATCTCAGCTCACCGCAACCTCTGCCTCCCAGGTGCAAGCGATTCTCCTGCCTCAGCCTCCTGAGTAGCTGGGATTA 54766
Orangutan         -------------------------------------------------------------------------------- 55684
```

```
Majority          ---------------------------------------G----------------------------------------

                          68170     68180     68190     68200     68210     68220     68230     68240

Human             -------------------------------------------------------------------------------- 54378
Coquerel'sSifaka  -------------------------------------------------------------------------------- 45810
Ma'sNightMonkey   ---------------------------------------G---------------------------------------- 49520
VervetAGM         CAGGCATGCACCACCATGCCAGGCTAATTTTGTATTTTTAGTAAAGATGGGGTTTCTCTATGTTGGTCAGGCTGGTCTCG 56032
Gorrilla          -------------------------------------------------------------------------------- 54905
GrabEatingMacaque CAGGCATGCACCACCATGCCAGGCTAATTTTGTATTTTTAGTAAAGACGGTGTTTCTCTATGTTGGTCAGGCTGGTCTCG 55250
Macaque           CAGGCATGCACCACCATGCCAGGCTAATTTTGTATTTTTAGTAAAGACGGTGTTTCTCTATGTTGGTCAGGCTGGTCTCG 56086
PigTailedMacaque  CAGGCATGCACCACCATGCCAGGCTAATTTTGTATTTTTAGTAAAGACGGTGTTTCTCTATGTTGGTCAGGCTGGTCTCG 54974
Bonobo            -------------------------------------------------------------------------------- 53486
OliveBaboon       CAGGCATGCACCACCATGCCAGGCTAATTTTGTATTTTTAGTAAAGACGGGGTTTCTCTCTGTTGGTCAGGCTGGTCTCG 54846
Orangutan         -------------------------------------------------------------------------------- 55684

Majority          --------------------------------------------------------------------------------

                          68250     68260     68270     68280     68290     68300     68310     68320

Human             -------------------------------------------------------------------------------- 54378
Coquerel'sSifaka  -------------------------------------------------------------------------------- 45810
Ma'sNightMonkey   -------------------------------------------------------------------------------- 49520
VervetAGM         AACTCCTGACCTCAGGTGATCCACCCACCTCGGCCTCCCAGAGTGCTGGGATTACAGGCGTGAGCCACCATGCCCGGTCG 56112
Gorrilla          -------------------------------------------------------------------------------- 54905
GrabEatingMacaque AACTCCTGACCTCAGGTGATCCACCCACCTCGGCCTCCCAGAGTGCTGGGATTACAGGCGTGAGCCACCATGCCCGGTCG 55330
Macaque           AACTCCTGACCTCAGGTGATCCACCCACCTCGGCCTCCCAGAGTGCTGGGATTACAGGCGTGAGCCACCATGCCCGGTCG 56166
PigTailedMacaque  AACTCCTGACCTCAGGTGATCCACCCACCTCGGCCTCCCAGAGTGCTGGGATTACAGGCGTGAGCCACCATGCCCGGTCG 55054
Bonobo            -------------------------------------------------------------------------------- 53486
OliveBaboon       AACTCCTGACCTCAGGTGATCCACCCACCTCGGCCTCCCAGAGTGCTGGGATTACAGGCGTGAGCCACCATGCGCGGTCG 54926
Orangutan         -------------------------------------------------------------------------------- 55684

Majority          --------TATGGTAATTAAAATGGACAAGCTACTGCT----ACCTGCAGAACATGGGTAGTGTACATCATAA--TTACT

                          68330     68340     68350     68360     68370     68380     68390     68400

Human             --------TATAGTAATAAAAATGAACAAGCTACTGCT----GCCTGCAAAACATGGGTAGTGTACATCATAA--TTATT 54444
Coquerel'sSifaka  -----------------------------TCTACTCCT---TAGTTAGATACCGGAGAAAACGTGTGTGTGTG--TATCT 45856
Ma'sNightMonkey   --------TATGATAATAAAAATGAACAAGCTACTGCTTGCTACCTGCAAAACATGGGTAGTGTACATCATAAATTTATT 49592
VervetAGM         GAATATTGTATGGTAAATAAAGTGGACAAGCTACTGCT----ACATGCAGAACATGGATAGTGTACATCATAA--TTACT 56186
Gorrilla          --------TATAGTAATAAAAATGAACAAGCTACTGCT----GCCTGCAAAACATGGGTAGTGTACATCATAA--TTATT 54971
GrabEatingMacaque GAATATTGTATGGTAAATAAAATGGACAAGCTACTGCT----ACGTGCAGAACATGGGTAGTGTACATCATAA--TTACT 55404
Macaque           GAATATTGTATGGTAAATAAAATGGACAAGCTACTGCT----ACGTGCAGAACATGGGTAGTGTACATCATAA--TTACT 56240
PigTailedMacaque  GAATATTGTATGGTAAATAAAATGGACAAGCTACTGCT----ACGTGCAGAACATGGGTAGTGTACATCATAA--TTACT 55128
Bonobo            --------TATAGTAATAAAAATGAACAAGCTACTGCT----GCCTGCAAAACATGGGTAGTGTACATCATAA--TTATT 53552
OliveBaboon       GAATATTGTATGGTAAATAAAATGGACAAGCTACTGCT----ACGTGCAGAACATGGGTAGTGTACATCATAA--TTACT 55000
Orangutan         --------TATAGTAATAAAAATGAACAAGCTACTGCT----GCCTGCAAAACATGGGTAGTGTACATCATAA--TTATT 55750
```

```
Majority          GAAAGAAGCCAAACCTAAGTAGAATACATCTGATACGATTCCATTTTTGTAACTTGGAGGAAGCATGAGTATGGCTTCCA

                          68410     68420     68430     68440     68450     68460     68470     68480

Human             GGAAGAAGCCAAACCAAAGTAGAATACATCTGATATGATTCCATTTATGTAACTTGGAGGAAGCGTGAGTATGGCTTCTA 54524
Coquerel'sSifaka  ATGCATATCAAAACATATATATGAGGTAGCTCATGAAACACTGTAGTAGTAGCTCAAAGC-----TGGAAATGA--TCCA 45929
Ma'sNightMonkey   GAAAGAAGCCAAACCAAACTAGAACACATCTGATAAGATTCCATTTATGTAACTTGGAGGAAGCATGAGTATGGCTTCTA 49672
VervetAGM         GAAAGAAGCCAAACCTAAGTAGAATACATCTGATACGATTCCATTTTTGTAACATGCAGGAAGCATGAATATGGCTTACA 56266
Gorrilla          GGAAGAAGCCAAACCAAAGTAGAATACATCTGATATGATTCCATTTATGTAACTTGGAGGAAGCGTGAGTATGGCTTCTA 55051
GrabEatingMacaqueGAAAGAAGCCAAACCTAAGTAGAATACATCTGATACGATTCCATTTTTGTAACATGGAGGAAGCATGAGTATGGCTTACA 55484
Macaque           GAAAGAAGCCAAACCTAAGTAGAATACATCTGATACGATTCCATTTTTGTAACATGGAGGAAGCATGAGTATGGCTTACA 56320
PigTailedMacaque  GAAAGAAGCCAAACCTAAGTAGAATACATCTGATACGATTCCATTTTTGTAACATGGAGGAAGCATGAGTATGGCTTACA 55208
Bonobo            GGAAGAAGCCAAACCAAAGTAGAATACATCTGATATGATTCCATTTATGTAACTTGGAGGAAGCGTGAGTATGGCTTCTA 53632
OliveBaboon       GAAAGAAGCCAAACCTAAGTAGAATACATCTGATACGATTCCATTTTTGTAACATGGAGGAAGCATGATTATGGCTTACA 55080
Orangutan         GAAAGAAGCCAAACCAAAGTAGAATACATCTGATACGATTCCATTTATGTAACTTGGAGGAAGCATGAGTATGGCTTCTA 55830

Majority          GAGTGTTGATAATATGTTGTTTTTT-ATCTGGGTAGTTATTTCACAGAGATGTCCAATTTGTGGTATTTTATGGAGCTTT

                          68490     68500     68510     68520     68530     68540     68550     68560

Human             GAGTGTTGATAATATACTGTTTTTT-ATCTGGGTAATTATTTCACAGAGATGTCCAATTTGTGGTATTTTATGGAGCTTT 54603
Coquerel'sSifaka  AATGTTTAGGAGCA------------------GTAG----------AA--TAT----TTTGTGGTAT---AT------TC 45966
Ma'sNightMonkey   GAGGGTTGATAATATGCTGTTTTTT-ATCTGGGTAGTTACTTCACAAAGATGCCCAATTTGTGATATTTTATGGAGTTTT 49751
VervetAGM         GAGTGTTGATAATATGTCGTTTTTTTATCTGGGTAGTTATTTCACAGAAATGTCCAATTTGTGGTATTTTATGGAGCTTT 56346
Gorrilla          GAATGTTGATAATATGCTGTTTTTT-ATCTGGGTAATTATTTCACAGAGATGTCCAATTTGTGGTATTTTATGGAGCTTT 55130
GrabEatingMacaqueGAGTGTTGATAATATGTCGTTTTTT-ATCTGGGTAGTTATTTCACAGAGATGTCCAATTTGTGGTATTTTATGGAGCTTT 55563
Macaque           GAGTGTTGATAATATGTCGTTTTTT-ATCTGGGTAGTTATTTCACAGAGATGTCCAATTTGTGGTATTTTATGGAGCTTT 56399
PigTailedMacaque  GAGTGTTGATAATATGTCGTTTTTT-ATCTGGGTAGTTATTTCACAGAGATGTCCAATTTGTGGTATTTTATGGAGCTTT 55287
Bonobo            GAGTGTTGATAATATGCTGTTTTTT-ATCTGGGTAATTATTTCACAGAGATGTCCAATTTGTGGTATTTTATGGAGCTTT 53711
OliveBaboon       GAGTGTTGATAATACGTTGTTTTTT-ATCTGGGTAGTTATTTCACAGAGATGTCCAATTTGTGGTATTTTATGGAGCTTT 55159
Orangutan         GGGTGTTGATAATA-------T-----------------------------------------------------------TT 55847

Majority          ATAC--TTAGGTTTTGTGTCCTTTTCT--GTGTGTGTGTAATATTTTGATAAGAAGTTTTATTAAAATCTAGGTTGGCAA

                          68570     68580     68590     68600     68610     68620     68630     68640

Human             ATAC--TTAGGTTTTGTGTCCTTTTGT--GTGTGTGTGTAATATTTTGATAAGAAGTTTTATTAAAATCTAGGTTTGCAA 54679
Coquerel'sSifaka  ATCCACTTAGGATTTGTGTCCTTTACT--GTGTATGTGCAATATTTTGATT---A-----ATTGAAATTTAGCTTGGCAA 46036
Ma'sNightMonkey   ATACACTTAGGATTTGTATCCTTTTCTGAGTGTGTGTGTAATATTTTGATAAGGAGTTTTATTAAAATACAGATTGGCAA 49831
VervetAGM         ATAC--TTAGGTTTTGTGTCCTTTTCT--CTCTGTGTGTAATATTTTGGTAAGAAGTTTTATTAAAGTCTATGTTGGCAA 56422
Gorrilla          ATAC--TTAGGTTTTGTGTCCTTTTGT--GTGTGTGTGTAATATTTTGATA---AGTTTTATTAAAATCTAGGTTTGCAA 55203
GrabEatingMacaqueATAC--TTAGGTTTTGTGTCCTTTTCT--GTGTGTGTGTAATATTTTGGTAAGAAGTTTTATTAAAATCTATGTTGGCAA 55639
Macaque           ATAC--TTAGGTTTTGTGTCCTTTTCT--GTGTGTGTGTAATATTTTGGTAAGAAGTTTTATTAAAATCTATGTTGGCAA 56475
PigTailedMacaque  ATAC--TTAGGTTTTGTGTCCTTTTCT--GTGTGTGTGTAATATTTTGGTAAGAAGTTTTATTAAAATCTATGTTGGCAA 55363
Bonobo            ATAC--TTAGGTTTTGTGTCCTTTTGT--GTGTGTGTGTAATATTTTGATAAGAAGTTTTATTAAAATCTAGGTTTGCAA 53787
OliveBaboon       ATAC--TTAGGTTTTGTGTCCTTTTCT--GTGTGTGTGTAATATTTTGGTAAGAAGTTTTATTAAAATCTACGTTGGCAA 55235
Orangutan         ATAT--ATA------------T------ATATGCTTGTA-------GATG-------TAGCTAATATCT---------- 55882
```

```
Majority          GCCAACTCTGCTCCATGAATTATAAGAAAAAA-TATATGAAGTCTTTAAAAAAATGAAATCATAAAGAAATGTATGAAGT
                          68650     68660     68670     68680     68690     68700     68710     68720
Human             GCCAACACTGCTCCATGAATTATAAGAAAAAA-TATATGAAGTCATTAAAAA--TGAAATCATAAAGAAATGTATGAAGT 54756
Coquerel'sSifaka  GCCAACTGCAATCCATGAATTA---GAAAAAG--ACATG---------------------------------TGTGAAGT 46078
Ma'sNightMonkey   GCCAACTCTGCTCCATGAATTA-GAGAAAAAA-TATATGAACTCATTAAAAAAATGAAATAATAAA--AATATATGAAGT 49907
VervetAGM         GCCAACTCTGCTCCATGAATTATAAGAAAAAAATATATGAAGTCTTTAAAAAAATGAAATCATAAAGAAATGTATGAAGT 56502
Gorrilla          GCCAACGCTGCTCCATGAATTATAAGAAAAAA-TATATGAAGTCATTAAAAACATGAAATCATAAAGAAATGTATGAAGT 55282
GrabEatingMacaqueGCCAACGCTGCTCCATGAATTATAAGAAAAAA-TATATGAAGTCTTTAAAAAAATGAAATCATAAAGAAATGTATGAAGT 55718
Macaque           GCCAACTCTGCTCCATGAATTATAAGAAAAAA-TATATGAAGTCTTTAAAAAAATGAAATCATAAAGAAATGTATGAAGT 56554
PigTailedMacaque  GCCAACTCTGCTCCATGAATTATAAGAAAAAA-TATATGAAGTCTTTAAAAAAATGAAATCATAAAGAAATGTATGAAGT 55442
Bonobo            GCCAACACTGCTCCATGAATTATAAGAAAAAA-TATATGAAGTCATTAAAAA--TGAAATCATAAAGCAATGTATGAAGT 53864
OliveBaboon       GCCAACTCTGCTCCATGAATTATAAGAAAAAA-TATATGAAGTCTTTAAAAAAATGAAATCATAAAGAAATGTATGAAGT 55314
Orangutan         ------------------C------------------------------------------------------------- 55883

Majority          AATTTGTTACTGGTGTGGGAGAACATGATTGTTAAGCCATAAAAGGAAGATAGAGAACAATGAATAGTGTTAAATAAAAG
                          68730     68740     68750     68760     68770     68780     68790     68800
Human             AATTTGTTACTGATGTGAGAGAACATGATTGTTAAGCCATAAAAGGAAGATAGAGAACAATGAATAGTATTAAATAAAAG 54836
Coquerel'sSifaka  CATTTGTTACTGATATGGAACA-CATAA---TTAAGCTAAAAAAGGAAGATGGAGAACAATAA----------------- 46137
Ma'sNightMonkey   AATTTGTTACTGATGTGGGAAAACATGATTGTTAAGCCATAAAAGGAAAATAGAGAACAGTGAATAGTATTAAATAAAAG 49987
VervetAGM         AATTTGTTACTGGTGTGGGAGAACATGATTGTTAAGCCATAAAAGGAAGATAGAGAACAATAAATAGTGTTAAATAAAAG 56582
Gorrilla          AATTTGTTACTGATGTGAGAGAACATGATTGTTAAGCCATAAAAGGAAGATAGAGAACAATGAGTAGTATTAAATAAAAG 55362
GrabEatingMacaqueAATTTGTTACTGGTGTGGGAGAACATGATTGTTAAGCCATAAAAGGAAGATAGAGAACAATGAATAGTGTTAAATAAAAG 55798
Macaque           AATTTGTTACTGGTGTGGGAGAACATGATTGTTAAGCCATAAAAGGAAGATAGAGAACAATGAATAGTGTTAAATAAAAG 56634
PigTailedMacaque  AATTTGTTACTGGTGTGGTAGAACATGATTGTTAAGCCATAAAAGGAAGATAGAGAACAATGAATAGTGTTAAATAAAAG 55522
Bonobo            AATTTGTTACTGATGTGAGAGAACATGATTGTTAAGCCATAAAAGGAAGATAGAGAACAATGAATAGTATT----AAAAG 53940
OliveBaboon       AATTTGTTACTGGTGTGGGAGAACATGATTGTTAAGCCATAAAAGGAAGATAGAGAACAATGAATAGTGTTAAATAAAAG 55394
Orangutan         ---------------TGGAAG-----GA---------------------------------------------------- 55891

Majority          TATGAATAGTATTGAATTAAAA-GTATGAATAGTATTGAATAAAA-TATGAATAGTATTGGGAAAAAA--TATGGAATAA
                          68810     68820     68830     68840     68850     68860     68870     68880
Human             TATGAATAGTATTGAATAAAA--------A----------------TATGAATAGTATTG-AAAAAAA--TACAGACTAA 54889
Coquerel'sSifaka  ---------------------------------------------------ATCGTACTAAAAAAAAG--TACATAATAG 46164
Ma'sNightMonkey   TATGAATAGTATTGAATTAAAA-GTATGAATAGTATTGAATAAAAATAAGAATAGTATTGGGAAAAAG--TATGGAATAA 50064
VervetAGM         TATGAATAGTATTGAATTAAAAAGTATGAATAGTATTGAATAAAA-TATGAATAGTATTGGAGAAAAAAGTATGGAATAA 56661
Gorrilla          TATGAATACTATTGAATAAAA--------A----------------TATGAATAGTATTGGAAAAAAA--TACGGAATAA 55416
GrabEatingMacaqueTATGAATAGTATTGAATTAAAAAGTATGAATAGTATTGAATAAAA-TATGAATAGTATTGGGAAAAAA-GTATGGAATAA 55876
Macaque           TATGAATAGTATTGAATTAAAAAGTATGAATAGTATTGAATAAAA-TATGAATAGTATTGGGAAAAAA-GTATGGAATAA 56712
PigTailedMacaque  TATGAATAGTATTGAATTAAAAAGTATGAATAGTATTGAATAAAA-TATGAATAGTATTGGGAAAAAA-GTATGGAATAA 55600
Bonobo            TATGAATAGTATTGAATAAAA--------A----------------TATGAATAGTATTG-AAAAAAA--TACGGACTAA 53993
OliveBaboon       TATGAATAGTATTGAATTAAAAAGTATGAATAGTATTGAATAAAA-TATGAATAGTATTGGGAAAAAAAGTATGGAATAA 55473
Orangutan         -------------------------------------------------------TACCG-------------------- 55896
```

```
Majority          GTATAATATTTGTGT-----------------TATATATATGCTT-GTAGATGTAGCTAGTATCTCTGGAAGGATACCTA

                           68890     68900     68910     68920     68930     68940     68950     68960

Human             GTATAATATTTATAT------------------ATATAT--GCTT-GTAGATGTAGCTAGTATCTCTGGAAGGATATCGA 54948
Coquerel'sSifaka  ATATA---------------------------TACATGTATGCTTTATATATGTAGATGATATCTCTGGAAGAATACCCA 46217
Ma'sNightMonkey   GTATAAGATTAAT--------------------ACATATATACTT-GTAGATGTAGATAGTATCTTTGGAAGGATGCCTA 50123
VervetAGM         GTATAATATTTGTGTGTGTGTGTGTGTCTATATATATATATGCTT-GTAGATGTAGCTAGTGTCTCTGGAAGGATACCTA 56740
Gorrilla          GTATAATATTTATAT------------------ATATAT--GCTT-GTAGATGTAGCTAGTATCTCTGGAAGGATACGGA 55475
GrabEatingMacaqueGTATAATATTTGTGT----------------GTATATATATGCTT-GTAGATGTAGCTAGTATCTCTGGAAGGATACCTA 55939
Macaque           GTATAATATTTGTGT----------------ATATATATATGCTT-GTAGATGTAGCTAGTATCTCTGGAAGGATACCTA 56775
PigTailedMacaque  GTATAATATTTGTGT----------------ATATATATATGCTT-GTAGATGTAGCTAGTATCTCTGGAAGGATACCTA 55663
Bonobo            GTATAATATTTATAT------------------ATATATATGCTT-GTAGATGTAGCTAGTATCTCTGGAAGGATATCAA 54054
OliveBaboon       GTATAATATTTGTGT----------------GTATATATATGCTT-GTAGATGTAGCTAGTATCTCTGGAAGGATACCTA 55536
Orangutan         -------------------------------------A------------A--------------------G-ATACTGA 55906

Majority          AGATAATACTG-TGAGGGGTTGGTAGGGGAAAATAGACTGGGTGACAGAGTTGCAGGGGAATTTTTAATCTTTTACACCA

                           68970     68980     68990     69000     69010     69020     69030     69040

Human             AGATAATACTG-TGAGGGGTTGGTAGGGGAAAATAGACTGGGTGACAGAGTTGCAGGGGAATTTTTAATCTTTAATACCA 55027
Coquerel'sSifaka  AGATAATACTGGTGAGGGTTTGGTAAGGAAAAGCAGACTGAGTAACAGAGTTGCCATG-A--------TC----AGGTGA 46284
Ma'sNightMonkey   AGATAATACTG-TGAGGGGTTGGTAGGGGTAAGTAGACTAGGTGACAGCGTTGCAGGGGAATTTTCAATCTTTTACACCA 50202
VervetAGM         AGATAATACTG-TAAGGGGTTGGTAGGGGAAAATAGAATGGATGACAGAGTTACAGGGGAATTTTTAATCTTTTACACCA 56819
Gorrilla          AGATAATACTG-CGAGGGGTTGGTAGGGGAAAATAGACTGGGTGACAGAGTTGCAGGGGAATTTTTAATCTTTAATACCA 55554
GrabEatingMacaqueAGATAATACTG-TAAGGGGTTGGTAGGGGAAAATAGAATGGATGACAGAGTTGCAGAGGAATTTTTAATCTTTTACACCA 56018
Macaque           AGATAATACTG-TAAGGGGTTGGTAGGGGAAAATAGAATGGATGACAGAGTTGCAGAGGAATTTTTAATCTTTTACACCA 56854
PigTailedMacaque  AGATAATACTG-TAAGGGGTTGGTAGGGGAAAATAGAATGGATGACAGAGTTGCAGAGGAATTTTTAATCTTTTACACCA 55742
Bonobo            AGATAATGCTG-TGAGGGGTTGGTAGGGGAAAATAGACTGGGTGACAGAGTTGCAGGGGAATTTTTAATCTTTAATACCA 54133
OliveBaboon       AGATAATACTG-TAAGGGGTTGGTAGGGGAAAATAGAATGGATGACAGAGTTGCAGGGGAATTTTTAATCTTTTACACCA 55615
Orangutan         AGATAATACTG-TGAGGGATTGGTAGGGGAAAATGGACTAGGTGACAGAGTTGCAGGAGAATTTTTAATCTTTTATACCA 55985

Majority          CTTTTATACCATGATCATGGATTCCTATTTTAAAAAACACATTTTATTATTTGAAGCAGTAGATGTTAGTGTTTTTCTAA

                           69050     69060     69070     69080     69090     69100     69110     69120

Human             CTTTTATACCATGATCATGGATTCCTATTTTAGAAAACA--TTTTATTATTTGAAGCAG---ATGTTAGTGTTTTTCTAA 55102
Coquerel'sSifaka  CTGCTATTTTAAAAAC----AC--ATTTTT-----------TTTTGTTATTTGAAACAGTAGATGTTAGTGTTTTTCTAC 46347
Ma'sNightMonkey   CTGTCGTATCATTATCATAGATTCCTATTTTTAAAAACACATTTTATTATTTGAAGCAGCAGATGTTAGTGTTTTTCTAA 50282
VervetAGM         CTTTTATACCATGATCCTGGATTCCTATTTTAAAAAACACATTTTATTATTTGAAGCAGTAGATGTTAGTGTTTTTCTAA 56899
Gorrilla          CTTTTATACCATGATCATGGATTCCTATTTTAGAAAACA--TTTTATGATTTGAAGCAGCAGATGTTAGTGTTTTTCTAA 55632
GrabEatingMacaqueCTTTTATACCATGATCATGGATTCCTATTTTAAAAAACACATTTTATTATTTGAAGCAGTAGATGTTAGTGTTTTTCTAA 56098
Macaque           CTTTTATACCATGATCATGGATTCCTATTTTAAAAAACACATTTTATTATTTGAAGCAGTAGATGTTAGTGTTTTTCTAA 56934
PigTailedMacaque  CTTTTATACCATGATCATGGATTCCTATTTTAAAAAACACATTTTATTATTTGAAGCAGTAGATGTTAGTGTTTTTCTAA 55822
Bonobo            CTTTTATACCATGATCATGGATTCCTATTTTAGAAAACA--TTTTATTATTTGAAGCAG---ATGTTAGTGTTTTTCTAA 54208
OliveBaboon       CTTTTATACCATGATCATGGATTCCTATTTTAAAAAACACATTTTATTATTTGAAGCAGTAGATGTTAGTGTTTTTCTAA 55695
Orangutan         CTTTTATATCATGATCATGGATTCCTGTTTTAAAAAACACATTTTATTATTTGAAGCAGCAGATGTTAGTGTTTTTCTAA 56065
```

```
Majority         GTTGTGGTAGTCT-ATGCTTGTTATTATAGATAAATACC-GTAAGAACATGTTCACTAATGAAGA---TAATATTGCAAT

                         69130     69140     69150     69160     69170     69180     69190     69200

Human            GTTGTGGTAGTCT-ATGCTTGTTATTATAGATAAATACA-GTAAGAACATGTTCACTAATGAAGAAGATAATATTGCAAT 55180
Coquerel'sSifaka TTTGTGGTAGTCC-ATAGTTCTTGTTATAGATATATATTTGTAAGAACATGTTCACTAACGAAGAA-ATAAAATTGCCAT 46425
Ma'sNightMonkey  GTTGTGGTAGTCTTATGCTTGTTATTATAGATAAATATA-GTAAGAACATGTTCACTAATGAAGA---TAATATTGCAGT 50358
VervetAGM        GTTGTGGTAGTCT-ATGCTTGTTATTATAGATAAATACC-GTAAGAACATGTTCACTAATGAAGA---TAATATTGCAAT 56974
Gorrilla         GTTGTCGTAGTCT-ATGCTTGTTACTATAGATAAATACA-GTAAGAACATGTTCACTAATGAAGAAGATAATATTGCAAT 55710
GrabEatingMacaqueGTTGTGGTAGTCT-ATGCTTGTTATTATAGGTAAATACC-ATAAGAACATGTTCACTAATGAAGA---TAATATTGCAAT 56173
Macaque          GTTGTGGTAGTCT-ATGCTTGTTATTATAGGTAAATACC-ATAAGAACATGTTCACTAATGAAGA---TAATATTGCAAT 57009
PigTailedMacaque GTTGTGGTAGTCT-ATGCTTGTTATTATAGGTAAATACC-ATAAGAACATGTTCACTAATGAAGA---TAATATTGCAAT 55897
Bonobo           GTTGTGGTAGTCT-ATGCTTGTTATTATAGATAAATACA-GTAAGAACATGTTCACTAATGAAGAAGATAATATTGCAAT 54286
OliveBaboon      GTTGTGGTAGTCT-GTGCTTGTTATTATAGGTAAATACC-ATAAGAACATGTTCACTAATGAAGA---TAATATTGCAAT 55770
Orangutan        GTTGTGGTAGTCT-ATGCTTGTTATTATAGATAAATACA-GTAAGAACATGTTCACTAATGAAGAAGATAATATTGCAAT 56143

Majority         GAGCTACTTAAAATATTTAAA-TATATTTAAACATTTGAATAGATA----CTGCTCTATAAATCTAATTTTACCAATTTA

                         69210     69220     69230     69240     69250     69260     69270     69280

Human            GAGCTACTTAAAATATTTAAA-TACATTTAAACATTTGAATAGATA----CTGCTCTATAAAACTAATTTTACCAATTTA 55255
Coquerel'sSifaka GAGCTACTTAAAATATTTGAAGTATATTCAAACATTTAAATAGATAAATGTTGCTCTAGAAATCTAATTTTACCTGTT-- 46503
Ma'sNightMonkey  GAGCTACTTACAATATTTAAA-TATGTTTAAACATTTGAATAGATA----TTGCTCTCTAAATCTGATTTTACCAATTTA 50433
VervetAGM        GAGCTACTTAAAATATTTAAA-TATATTTAAACATTTGAATAGCTA----CTGCTCTATAAATCTAATTTTACCAATTTA 57049
Gorrilla         GCGCTACTTAAAATATTTAAA-TATATTTAAACATTTGAATAGATA----CAGCTCTATAAATCTAATTTTACCAATTTA 55785
GrabEatingMacaqueGAGCTACTTAAAATATTTAAA-TATATTTAAACATTTGAATAGCTA----CTGCTCTATAAATCTAATTTTACCAATTTA 56248
Macaque          GAGCTACTTAAAATATTTAAA-TATATTTAAACATTTGAATAGCTA----CTGCTCTATAAATCTAATTTTACCAATTTA 57084
PigTailedMacaque GAGCTACTTAAAATATTTAAA-TATATTTAAACATTTGAATAGCTA----CTGCTCTATAAATCTAATTTTACCAATTTA 55972
Bonobo           GAGCTACTT--------TAAA-TATATTTAAACATTTGAATAGATA----CTGCTCTATAAATCTAATTTTACCAATTTA 54353
OliveBaboon      GAGCTACTTAAAATATTTAAA-TATATTTAAACATTTGAATAGGTA----CTGCTCTATAAATCTAAATTTACCAATTTA 55845
Orangutan        GAGCTACTTAAAATATTTAAA-TATATTTAAACATTTGAATAGATA----CTGGTCTATAAATCTAATTTTACCAATTTA 56218

Majority         CAATTATTTTTTCAATAATAGTATCAAGATGTCTACTAAAATAAATAATCTATCACATTTCAAAATGTTGGGTTTTAATT

                         69290     69300     69310     69320     69330     69340     69350     69360

Human            CAATTATTTTTTCAATAATAGTATCAAGATGTCTACTAAAATAA----TCTATCACATTTCAAAATGTTGAGTTTTAATT 55331
Coquerel'sSifaka -AATAACTTCTT----AGTAATATAAAAGTGTCTGTTAAC-TA-----TCTGTCATGTTTCAAAATGTTGGGTTTAAATT 46572
Ma'sNightMonkey  CAGTTATTTTTC----AATAATATCAAGATGTCTACTAAAATAAACAATA-ATCGTGTTTCAAATTGTTCTGTTTTAATT 50508
VervetAGM        CAATTAGTTTTTCAATAGTAGTATCAAGATGTCTACTAAAATAAATAATCTATTACATTTCAAAATGTCGGGTTTTAGTA 57129
Gorrilla         CAATTATTTTTTCAATAATAGTATCAAGATGTCTACTAAAAT------T--ATCACATTTCAAAATGTTGAGTTTTAATT 55857
GrabEatingMacaqueCAATTAGTTTTTCAATAATAGTATCAAGATGTCTACTAAAATAAATAATCTATTACATTTCAAAATGTTGGGTTTTAGTA 56328
Macaque          CGATTAGTTTTTCAATAATAGTATCAAGATGTCTACTAAAATAAATAATCTATTACATTTCAAAATGTTGGGTTTTAGTA 57164
PigTailedMacaque CAATTAGTTTTTCAATAATAGTATCAAGATGTCTACTAAAATAAATAATCTATTACATTTCAAAATGTTGGGTTTTAGTA 56052
Bonobo           CAATTATTTTTTCAATAATAGTATCAAGATGTCTACTAAAATAA----TCTATCACATTTCAAAATGTTGAGTTTTAATT 54429
OliveBaboon      CAATTAGTTTTTCAATAATAGTATCAAGATGTCTACTAAAATAAATAATCTATTACATTTCAAAATGTTGGGTTTTAGTA 55925
Orangutan        CAATTATTTTTTCAATAATAGTATCAAGATGTCTACTAAAATAA----TCTATCACATTTCAAAATGTTGAGTTTTAATT 56294
```

```
Majority          AG----TCATTTTGGCTAATACATAAATTTATAGATAAAATTA---AGTAACATTTAAATGATTGTTTAGATATGTATCT

                          69370     69380     69390     69400     69410     69420     69430     69440

Human             AGTAAGTCATTTTGACTAATACATAAATTTATAGATAAAATTA---AGTAGCATTTAAATGATTGTTTAGATATATATCT 55408
Coquerel'sSifaka  AA----TGATTC--ACAGTTACTTCAGTTTATTATTTAAATAGC--ATTTAAATGATGTAGTTGGTCTAGATGAATATGT 46644
Ma'sNightMonkey   AAGAAGTCATTTTGGCTAATATATACATTTATACATAAAATTATTAAGTAGCATTTAAATGCTTGTTTAGATATGTATGT 50588
VervetAGM         AG----TCATTTTGGCTAATACATAAATTTATAGATAAAATTA---AGTAACATTTAAATGATTGTGTAGATATGTATCT 57202
Gorrilla          AGTAAGTCATTTTGGCTAATACATAAATTTATAGATAAAATTA---AGTAGCATTTAAATGATTGTTTAGATATGTATCT 55934
GrabEatingMacaqueAG----TCATTTTGGCTAATATATAAATTTATAGATAAAATTA---AGTAACATTTAAATGATTGTGTAGATATGTATCT 56401
Macaque           AG----TCATTTTGGCTAATATATAAATTTATAGATAAAATTA---AGTAACATTTAAATGATTGTGTAGATATGTATCT 57237
PigTailedMacaque  AG----TCATTTTGGCTAATATATAAATTTATAGATAAAATTA---AGTAACATTTAAATGATTGTGTAGATATGTATCT 56125
Bonobo            AGTAAGTCATTTTGGCTAATACATAAATTTATAGATAAAATTA---AGTAGCATTTAAATGATTGTTTAGATATATATCT 54506
OliveBaboon       AG----TCATTTTGGCTAATATATAAATTTATAGATAAAATTA---AGTAACATTTAAATGATTGTGTAGATATGTATCT 55998
Orangutan         AG----TCATTTTGGCTAATACATAAATTTATAGATAAAATTA---AGTAGCATTTAAATGATCGTTTAGATATGTATCT 56367

Majority          GGACTCT--------CAAGCCATGTGATCTGTGATTATATAATATTTTAGTAAAATGACTGTTCTTGTTTATTCTCCTAA

                          69450     69460     69470     69480     69490     69500     69510     69520

Human             GGACTCT--------CAAGCCGTGTGATCTGTGGTTATATAATATTTTAGTAAAATGACTTTTCTTGTTTATTCTCCTGA 55480
Coquerel'sSifaka  GGACTCTTTGAGTCTCAAGCTCTATGATCCCTGGTTATATAATATTTCAGCAAAATGGCTGTTATTATTTATTCCCATAA 46724
Ma'sNightMonkey   GGACTCTTTCATTCTCAAGCCATGTGATCTGTGATTTTATAATATTTTAGTAAAATGACTGTTCTTGTTTTTTCTCCCGA 50668
VervetAGM         GGACTCT--------CAAGCCATGTGATCTGTGATTATATAATGTTTTAGTAAAATGACTGTTCTTGTTTATTCTCCTAA 57274
Gorrilla          GGACTCT--------CAAGCTATGTGATCTGTGGTTATATAATATTTTAGTAAAATGACTTTTCTTGTTTATTCTCCTGA 56006
GrabEatingMacaqueGGACTCT--------CAAGCCATGTGATCTGTGATTATATAATATTTTAGTAAAATGACTGTTCTTGTTTATTCTCCTAA 56473
Macaque           GGACTCT--------CAAGCCATGTGATCTGTGATTATATAATATTTTAGTAAAATGACTGTTCTTGTTTATTCTCCTAA 57309
PigTailedMacaque  GGACTCT--------CAAGCCATGTGATCTGTGATTATATAATATTTTAGTAAAATGACTGTTCTTGTTTATTCTCCTAA 56197
Bonobo            GGACTCT--------CAAGCCGTGTGATCTGTGGTTATATAATATTTTAGTAAAATGACTTTTCTTGTTTATTCTCCTGA 54578
OliveBaboon       GGACTCT--------CAAGCCATGTGATCTGTGATTATATAATATTTTAGTAAAATGACTGTTCTTGTTTATTCTCCTAA 56070
Orangutan         GAACTCT--------CAAGCCGTGTGATCTGTGGTTATATA---TTTTAGTAAAATGACTGTTCTTGTTTATTCTCCTGA 56436

Majority          GTGTCCTAATTCTTTTGTATTCTCTTT------CATTAACCTGTTAATGTAGATTTTTCAAACT--GTAAAGCTATGTAT

                          69530     69540     69550     69560     69570     69580     69590     69600

Human             GTGTCCTAATTCTTTTGTATTCTCTTT------CATTAACCTGTTGATATAGATTTTTCAAACT--GTAAACCTATGTAT 55552
Coquerel'sSifaka  ATGCCCTAAATCTTTTGTGTTCTCTTT-CC---CTTTTATTCATCACTGTGCAGTTTGTATTCCT-ATTTATCTGCTAAT 46799
Ma'sNightMonkey   GTGTCCTAATTCTTTTGTATTTTCTTTTCCTTTCTTTAACCTGTTAATATAGATTTTTCAAACTTTGTAAAGCTATGTGT 50748
VervetAGM         GTATCTTAATTCTTTTGTATTCTCTTT------CATTAAC-TGTTAATGTAGATTTTTCAAACT--GTAAAGCTATGTAT 57345
Gorrilla          GTGTCCTAATTCTTTTGTATTCTCTTT------CATTAACCTGTTAATATAGATTTTTCAAACT--ATAAACCTATGTAT 56078
GrabEatingMacaqueGTATCTTAATTCTTTTGTATTCTCTTT------CATTAAC-TGTTAATGTAGATTTTTCAAACT--GTAAAGCTATGTAT 56544
Macaque           GTATCTTAATTCTTTTGTATTCTCTTT------CATTAAC-TGTTAATGTAGATTTTTCAAACT--GTAAAGCTATGTAT 57380
PigTailedMacaque  GTATCTTAATTCTTTTGTATTCTCTTT------CATTAAC-TGTTAATGTAGATTTTTCAAACT--GTAAAGCTATGTAT 56268
Bonobo            GTGTCCTAATTCTTTTATATTCTCTTT------CATTAACCTGTTGATATAGATTTTTCAAACT--GTAAACCTATGTAT 54650
OliveBaboon       GTATCTTAATTCTTTTGTATTCTCTTT------CATTAAC-TGTTAATGTAGATTTTTCAAACT--GTAAAGCTATGTAT 56141
Orangutan         GTGTCCTAATTCTTTTGTATTCTCTTT------CATTAACCTGTTAATATAGATTTTTCAAACT--GTAAACCTATGTAT 56508
```

```
Majority          AGGTATTTGTGGTATTTGTCATTGTTATATTTTAGAACTATTTGCCTTTGTA-----TGTTTTTTGACCACAGATAAGGT
                          69610     69620     69630     69640     69650     69660     69670     69680
Human             AGGTATTTGTGGTATTTGTCATTGTTATATTTTAGAACTATTTGCCTTTGTA-----TGTTTTTTGACCACAGATAAGGT 55627
Coquerel'sSifaka  ATAGATTTTTCAAATT-A----TAAGGCAATGTAGAGGTATTTGTCATTGTTATATATGTTTTCTGACCCCAGATAAGGT 46874
Ma'sNightMonkey   AGGTATTTGTGGTATTTGTCGTTGTTATATTTTAGAACTATTTGCCTTTGTA-----TATTTTTTGGCCACAGATAAGGT 50823
VervetAGM         AGGTATTTGTGGTATTTGTCATTGTTACACTTTAGAACTATTTGCCTTTGT------TGTTTTTTGACCACAGATAAGGT 57419
Gorrilla          AGGTATTTGTGGTATTTGTCATTGTTATATTTTAGAACTATTTGCCTTTGTA-----TGTTTTTTGACCACAGATAAGGT 56153
GrabEatingMacaqueAGGTATTTGTGGTATTTGTCATTGTTATACTTTAGAACTATTTGCCTTTGT------TGTTTTTTGACCACAGATAAGGT 56618
Macaque           AGGTATTTGTGGTATTTGTCATTGTTATACTTTAGAACTATTTGCCTTTGT------TGTTTTTTGACCACAGATAAGGT 57454
PigTailedMacaque  AGGTATTTGTGGTATTTGTCATTGTTATACTTTAGAACTATTTGCCTTTGT------TGTTTTTTGACCACAGATAAGGT 56342
Bonobo            AGGTATTTGTGGTATTTGTCATTGTTATATTTTAGAACTATTTGCCTTTGTA-----TGTTTTTTGACCACAGATAAGGT 54725
OliveBaboon       AGGTATTTGTGGTATTTGTCATTGTTATACTTTAGAACTATTTGCCTTTGT------TGTTTTTTGACCACAGATAAGGT 56215
Orangutan         AGGTATTTGTGGTATTTGTCATTGTTATATTTTAGAACTATTTGCCTTAGTA-----TGTTTTTTGACCACAGATAAGGT 56583

Majority          AGAAAGAAATTATTTCCTTATCATAGACTGTGTCATAATAATAA---TAGGAAAGGGATAATAGTAATAAAGGCAGTCTT
                          69690     69700     69710     69720     69730     69740     69750     69760
Human             AGAAAGAAATTATTTCCTTATCATAGACTG--TCATCATAATAA---CAGGAAAGGGATAATAGTAATAAAGGCAGTCTT 55702
Coquerel'sSifaka  ----AGGAATTATTTCCTTATCAGAGCATGTGTCACAATGATAA---TAAGAAAGGCATAATAGTAATAAAAGCTTTCTT 46947
Ma'sNightMonkey   AAAAAGAAATTATTTCCTTATAGAATCTGTCATAATAATAATAATAATAGGAAAGGGATAATAGTAATAAAGGCAGTCTT 50903
VervetAGM         AGAAAGAAATTATGTCCTTATCATAGACTCTGTCATAATAATAA---TAGGAAAGGGATAATAGTAATAAAGGCAGTCTT 57496
Gorrilla          GGAAAGAAATTATTTCCTTATCATAGACTG--TCATAATAATAA---CAGGAAAGGGATAATAGTAATAAAGGCAGTCTT 56228
GrabEatingMacaqueAGAAAGAAATTATATCCTTATCATAGACTCTGTCATAATAATAA---TAGGAAAGGGATAATAGTAATAAAGGCAGCCTT 56695
Macaque           AGAAAGAAATTATGTCCTTATCATAGACTCTGTCATAATAATAA---TAGGAAAGGGATAATAGTAATAAAGGCAGCCTT 57531
PigTailedMacaque  AGAAAGAAATTATGTCCTTATCATAGACTC--TCATAATAATAA---TAGGAAAGGGATAATAGTAATAAAGGCAGTCTT 56417
Bonobo            AGAAAGAAATTATTTCCTTATCATAGACTG--TCATAATAATAA---CAGGAAAGGGATAATAGTAATAAAGGCAGTCTT 54800
OliveBaboon       AGAAAGAAATTATGTCCGTATCATAGACTCTGTCATAATAATAA---TAGGAAAGGGATAATAGTAATAAAGGCAGTCTT 56292
Orangutan         AGAAAGAAATTATTTCCTTATCATAGACTG--TCATAATAATAATAACAGGAAAGGGATAATAGTAATAAAGGCAGTCTT 56661

Majority          TTGG----AGCATTTAACATAATATTTGATTGACAGATTTC-CTACAAATACTGTGTTTATTCAATTTTATGGTTATATT
                          69770     69780     69790     69800     69810     69820     69830     69840
Human             TTGG----AGCATTTAACATAATATTTGATTGACAGATTTC-CTACAAATACTGTGTTTATTCAATTTTATGGGTATATT 55777
Coquerel'sSifaka  TTGA----AGCATTTAACATTTGATTTGATTGACAGATTTC-CTAAAAATACTGTATTTATTCAATTATATA--TATTTT 47020
Ma'sNightMonkey   TTGG----AGCATTTAACATATTATTTGATCGACAGATTTTTCTACAAATACTGTGTTTATTCAGTTTTATGGGTATATT 50979
VervetAGM         TTGG----AGCATTTAACATAATATTTGATTGGAAGATTTC-CTACAAATACTGTGTTTATTCAATTTTATGGTTATATT 57571
Gorrilla          TTGG----AGCATTTAACATAATATTTGATTGACAGATTTC-CTACAAATACTGTGTTTATTCAATTTTATGGGTATATT 56303
GrabEatingMacaqueTTGGAGGGAGCATTTAACATAATATTTGATTGGAAGATTTC-CTACAAATACTATGTTTATTCAATTTTATGGTTATATT 56774
Macaque           TTGGAGGGAGCATTTAACATAATATTTGATTGGAAGATTTC-CTACAAATACTATGTTTATTCAATTTTATGGTTATATT 57610
PigTailedMacaque  TTGGAGGGAGCATTTAACATAATATTTGATTGGAAGATTTC-CTACAAATACTATGTTTATTCAATTTTATGGTTATATT 56496
Bonobo            TTGG----AGCATTTAACATAATATTTGATTGACAGATTTC-CTACAAATACTGTGTTTATTCAATTTTATGGGTATATT 54875
OliveBaboon       TTGGAGGGAGCATTTAACATAATATTTGATTGGAAGATTTC-CTACAAATACTATGTTTATTCAATTTTATGGTTATATT 56371
Orangutan         TTGG----AGCATTTAACATAATATTTGATTGACGGATTTC-CTACAAATACTGTGTTTATTCAATTTTATGGGTATATT 56736
```

```
Majority          TTTTCACATTTTAAAATCTCAGAAATTTGATGTGTCCTACAGTAGTCTATCTTCCATAGTTCAATTGGCAACATCT-ATT

                          69850     69860     69870     69880     69890     69900     69910     69920

Human             TTTTCGCATTTTAAAATCTCAGAAATTTGATGTGTCCTGCAATAGTCTATCTTCCATAGTTCAATTGGCAACATCTTATT 55857
Coquerel'sSifaka  TTT-CACATTTTA----T-------TT-----------------TTTAT------------------------------- 47040
Ma'sNightMonkey   TTT-CACATTTTAAAATC--AGAAATTTGATGTGTTCTACAATAGTCTAT---------TTCAGTTGACAACATCTTATT 51047
VervetAGM         TTTTCACATTTTAAAATCTCAGAAATTTGATGTGTCCTACAGTAGTCTATCTTCCGTAGTTCAATTGGCAACATCT-ATT 57650
Gorrilla          TTTTCGCATTTTAGAATCTCAGAAATTTGATGTGTCCTACAATAGTCTATCTTCCATAGTTCAGTTGGCAACATCTTATT 56383
GrabEatingMacaqueTTTTCACATTTTAAAATCTCAGAAATTTGATGTGTCCTACAGTAGTCTATCTTCCATAGTTCAATTGGCAACATCT-ATT 56853
Macaque           TTTTCACATTTTAAAATCTCAGAAATTTGATGTGTCCTACAGTAGTCTATCTTCCATAGTTCAATTGGCAACATCT-ATT 57689
PigTailedMacaque  TTTTCACATTTTAAAATCTCAGAAATTTGATGTGTCCTACAGTAGTCTATCTTCCATAGTTCAATTGGCAACATCT-ATT 56575
Bonobo            TTTTCGCATTTTAAAATCTCAGAAATTTGATGTGTCCTGCAATAGTCTATCTTCCATAGTTCAATTGGCAACATCTTATT 54955
OliveBaboon       TTTTCACATTTTAAAATCTCAGAAATTTGATGTGTCCTACAGTAGTCTATCTTCCATAGTTCAATTGGCAACATCT-ATT 56450
Orangutan         TTTTCACATTTTAAAGTCTCAGAAATTTGATGTGTCCTACAATAGTCTATCTTCCATAGTTCAATTGGCAAAATCTTATT 56816
```

```
Majority          AGTCATACTTAAAACAATAGTGCATTTTATAATTGATGGTGTATTTGATACCGTAAAATGTATGGGAAATATCCTTCATA

                          69930     69940     69950     69960     69970     69980     69990     70000

Human             AGTCATACATAAAACAATAGTGCATTTTATAATTAATGGTGTATTTGATACCATAAAATGTATGGGAAATATTCTTCATA 55937
Coquerel'sSifaka  -----------------T------TTTTTTAATT----TT-TTTTTGACAC---AGAG----T-----------CTCACT 47074
Ma'sNightMonkey   AGTTGTACATAAAACAATAGTGCATTTTATAATTGATGGTGTATGTGATACCATAAAACGCATGAGAAATAGCCTTCATA 51127
VervetAGM         AGTCATACTTAAAACAATAGTGCATTTTATAATTGATGGTGTATTTGATACCGTAAAATGTATGGGAAATATCCTTCATA 57730
Gorrilla          AGTCATACATAAAACAATAGTGCATTTTATAATTGATGGTGTATTTGATACCATAAAATGTATGGGAAATATTCTTCATA 56463
GrabEatingMacaqueAGTCATACTTAAAACAATAGTGCATTTTATAATTGATGGTGTATTTGATACCGTAAAATGTATGGGAAATATCCTTCATA 56933
Macaque           AGTCATACTTAAAACAATAGTGCATTTTATAATTGATGGTGTATTTGATACCGTAAAATGTATGGGAAATATCCTTCATA 57769
PigTailedMacaque  AGTTATACTTAAAACAATAGTGCATTTTATAATTGATGGTGTATTTGATACCGTAAAATGTATGGGAAATATCCTTCATA 56655
Bonobo            AGTCATACATAAAACAATAGTGCATTTTATAATTAATGGTGTATTTGATACCATAAAATGTATGGGAAATATTCTTCATA 55035
OliveBaboon       AGTCATACTTAAAACAATAGTGCATTTTATAATTGATGGTGTATTTGATACTGTAAAATGTATGGGAAATATCCTTCATA 56530
Orangutan         AGTCATACGTAAAACAATAGTGCATTTTATAATTGATGGTATATTTGATACCATAAAATGTATGGGAAATATCCTTCATA 56896
```

```
Majority          TTATATCTTGAAGTTTACCTGAGAATTTTTTGGTTTTGGACTGGACTACACATCG-GTTTCTATTCTAAAAATTTTGGTG

                          70010     70020     70030     70040     70050     70060     70070     70080

Human             TTATATCTTGAAGTTTACCTGAGAATTTTGTGGTTTTGGACTAGACTACACGTTG-GTTTCTATTCTAAAAATTTTGGTG 56016
Coquerel'sSifaka  CTGTTGCCTAGA-----CT--AGAGTGCCATGGCATCAGCCTAG--CTCACAGCA-------ACCTCAAACTCCTGGGCT 47138
Ma'sNightMonkey   TTATAACTTAAAGTTTACCTGAAAATTTTGT--TTTTGGACTGGAGTACACATCA-GTTTCTATTCTAAAAATTTTAGTC 51204
VervetAGM         TAATATCTTGAAGTTTACCTGAGAATTTTTTGATTTTGGACTGGACTACACATCG-ATTTCTATTCTAAAAATTTTGGTA 57809
Gorrilla          TTATATCTTGAAGTTTACCTGAGAATTTTGTGGTTTTGGACTAGACTACACGTCAAGTTTCTTTTCCAAAAATTTTGGTG 56543
GrabEatingMacaqueTAATATCTTGAAGTTTACCTGAGAATTTTTTGGTTTTGGACTGGACTACACATCG-ATTTCTAATCTAAAAATTTTGGTG 57012
Macaque           TAATATCTTGAAGTTTACCTGAGAATTTTTTGGTTTTGGACTGGACTACACATCG-ATTTCTAATCTAAAAATTTTGGTG 57848
PigTailedMacaque  TAATATCTTGAAGTTTACCTGAGAATTTTTTGGTTTTGGACTGGACTACACATCG-ATTTCTATTCTAAAAATTTTGGTG 56734
Bonobo            TTATATCTTGAAGTTTACCTGAGAATTTTGTGGTTTTGGACTAGACTACACGTTG-GTTTCTATTCTAAAAATTTTGGTG 55114
OliveBaboon       TAATATCTTGAAGTTTACCTGAGAATTTTTTGGTTTTGGACTGGACTACACATCG-ATTTCTATTCTAAAAATTTTGGTA 56609
Orangutan         TTGTATCTTGAAGTTTACCTGAGAATTTTGTGGTTTTGGACTAGACTACACGTTG-GTTTCTATTCTAAAAATTTTGGTG 56975
```

```
Majority          TCAGAAGTTAAGTGTAGTATGAAAAATGTATTTTCTGAACGTTCCTATTTTAAGATTGTATTAGAAATAATAAGCTTTAT

                          70090     70100     70110     70120     70130     70140     70150     70160

Human             TCAGAAGTTAAGTGTAGTATGAAAAATGTATTTTCTGAATGTTCCTATTTTAAGATTGTATTAGAAATAATAAGCTTTAT 56096
Coquerel'sSifaka  CCAGCAATCC------------------TACTGCCTTAGCCTCCC-----AA-----GTAGCTGGGACTACAGGCAT--- 47187
Ma'sNightMonkey   TCAGAAGTTTAGTGTAGTATGAAAAATTTATATTCTAATTGCTCCTGTTTTAAGATTGTATTAGAAATAATAAGCTTTCT 51284
VervetAGM         TCAGAAGTTAAGTGTAGTATGAAAAATGTATTTTCTGCACGTTCCTATTTTAAGATTGTATTAGAAATAATAAGCTTTAT 57889
Gorrilla          TCAGAAGTTAAGTGTAGTATGAAAAATGTATTTTCTGAATGTTCCTATTTTAAGATTGTATTAGAAATAATAAGCTTTAT 56623
GrabEatingMacaqueTCAGAAGTTAAGTGTAGTATGAAAAATGTATTTTCTGCACGTTCCTATTTTAAGATTGTATTAGAAATAATAAGCTTTAT 57092
Macaque           TCAGAAGTTAAGTGTAGTATGAAAAATGTATTTTCTGCACGTTCCTATTTTAAGATTGTATTAGAAATAATAAGCTTTAT 57928
PigTailedMacaque  TCAGAAGTTAAGTGTAGTATGAAAAATGTATTTTCTGCACGTTCCTATTTTAAGATTGTATTAGAAATAATAAGCTTTAT 56814
Bonobo            TCAGAAGTTAAGTGTAGTATGAAAAATGTATTTTCTGAATGTTCCTATTTTAAGATTGTATTAGAAATAATAAGCTTTAT 55194
OliveBaboon       TCAGAAGTTAAGTGTAGTATGAAAAATGTATTTTCTGCACATAC--ATTT---------ATTAGAAATAATAAGCTTT-- 56676
Orangutan         TCAGAAGTTAAGTGTAGTATGAAAAATGTATTTTCTGAATGTTCCTATTTTAAGATTATATTAGAAATAATAAGCTTTAT 57055

Majority          GGGAGTTCTTTTGGATAATTCAGTAACCCCCTTGTTGAGATTTACAATGTAACTGTTTTATGGAGTGATTAATGTTAAAG

                          70170     70180     70190     70200     70210     70220     70230     70240

Human             GGGAGTTCTTTTGGATAATTCAGTAACCCCCTTGTTGAGATTTACAACGTACCTGTTTTATGGAGTGATTAATGTTAAAG 56176
Coquerel'sSifaka  ------------GCGCCACCATG--CCCGGCTAATTT---TTTAT-ATATATATATATT----------T---------- 47229
Ma'sNightMonkey   GGGAGTTATTTTGGATAATTTAGTAACCCCCTCGTTGAGATTTATGATGTATCTGTTATATGGAGTGATTAA------AG 51358
VervetAGM         GGGAGTTCTTTTGGATAATTCAGTAACCCCCTTGTTGAGATTTACAATGTAACTGTTTTATGGAGTGATTAATGTTAAAG 57969
Gorrilla          GGGAGTTCTTTTGGATAATTCAGTAACCCCCTTGTTGAGATTTACAATGTACCTGTTTTATGGAGTGATTAATGTTAAAG 56703
GrabEatingMacaqueGGGAATTCTTTTGGATAATTCAGTAACCCCCTTGTTGAGATTTACAACGTCACTGTTTTTTGGAGTGATTAATGTTAAAG 57172
Macaque           GGGAATTCTTTTGGATAATTCAGTAACCCCCTTGTTGAGATTTACAACGTCACTGTTTTTTGGAGTGATTAATGTTAAAG 58008
PigTailedMacaque  GGGAATTCTTTTGGATAATTCAGTAACCCCCTTGTTGAGATTTACAACGTCACTGTTTTTTGGAGTGATTTATGTTAAAG 56894
Bonobo            GGGAGTTCTTTTGGATAATTCAGTAACCCCCTTGTTGAGATTTACAATGTACCTGTTTTATGGAGTGATTAATGTTAAAG 55274
OliveBaboon       ----ATTCTTTTGGATAATTCAGTAACCCCCTTGTTGAGATTTACAATGTAACTGTTTTATGGAGTGATTAATGTTAAAG 56752
Orangutan         GGGAGTTCTTTTGGATAATTCAGTAACCCCCTTGTTGAGATTTACAATGTACCTGTTTTATGGAGTGATTAATGTTACAG 57135

Majority          TTTTATTGTTCTCTAAATTTCTGTTTAAAACATTTTCTGATGATACATGTGTCTGTAAAACTACATCTTGTT-TTTTGAG

                          70250     70260     70270     70280     70290     70300     70310     70320

Human             TTGTATT---CTCTAAATTTCTGTTTAAAACATTTTCTGATGGTACATGTATTTATAAAACTACATCTTGTTCTTTTGAG 56253
Coquerel'sSifaka  TTTTTTT----TTTTAATTTC------AG-CATATTATGGTAGTACAGATGTT--------T------------------ 47272
Ma'sNightMonkey   TGTTATT---CTCTAAATTTCTGTTTAAAACATTTTTGAGTATAGAGGTGTCTGTAAAACTCTATCTTGTTCTTTTGAG 51435
VervetAGM         TTTTATTGTTCTCTAAATTTCTGTTTAAAACATTTTCTGATGATACATGTGTCTGTAAAACTACATCTTGTT-TTTTGAG 58048
Gorrilla          TTTTATTGTTCTCTAAATTTCTGTTTAAAACATTTTCTGATGGTACATGTGTCTATAAAACTACATCTTGTTCTTTTGAG 56783
GrabEatingMacaqueTTTTATTGTTCTCTAGATTTCTGTTTAAAACATTTTCTGATGATACATGTGTCTGTAAAACTACATCTTGTT-TTTTGAG 57251
Macaque           TTTTATTGTTCTCTAGATTTCTGTTTAAAACATTTTCTGATGATACATGTGTCTGTAAAACTACATCTTGTT-TTTTGAG 58087
PigTailedMacaque  TTTTATTGTTCTCTAGATTTCTGTTTAAAACATTTTCTGATGATACATGTGTCTGTAAAACTACATCTTGTT-TTTTGAG 56973
Bonobo            TTTTATTGTTCTCTAAATTTCTGTTTAAAACATTTTCTGATGGTACATGTGTTTCTAAAACTACATCATGTTCTTTTGAG 55354
OliveBaboon       TTTTTTTGTTCTCTAGATTTCTGTTTAAAACATTTTCTGATGATACATGTGTCTGTAAAACTACATCTTGTT-TTTTGAG 56831
Orangutan         TTTTATTGTTTTCCAAATTTCTGTTTAAAACATTTTCTGATGGTATATGTGTCTGTAAAACTACATCTTGTTCTTTTGAG 57215
```

```
Majority         AATAAATTACATATCGTATCTTTG--CTTGTTCAACATTGATCCCAAAGCCTGGATAATTTTTTTGAATGAAATACTAGT

                         70330     70340     70350     70360     70370     70380     70390     70400

Human            AATAAATTACATATCATACCTTTG--CATGTTCAACATTGATCCCAAAGCCTGGATAATTTTTTTGAATGAAATACTAGT 56331
Coquerel'sSifaka -A--GGTTACGTATGTTGCCCTTG----C---CCACCCTCCCCCCAAGTCCG---AGTTTC--------AAATAT--GT 47329
Ma'sNightMonkey  AATGAATTACATATCCTATCTTTTTCCTTGTTTAACATTGATCCCAAAGCCTGGATACTTTTTTTGAACGAAATACTAGT 51515
VervetAGM        AATAAATTATATATCGTATCTTTG--CTTGTTCAACATTGATTC-AAAGCCTGGATAATTTTTTTGAATGAAATACTAGT 58125
Gorrilla         AATAAATTACATATCATACCTTTG--CTTGTTCAACATTGATCCCAAAGCCTGGATAATTTTTTTGACTGAAATACTAGT 56861
GrabEatingMacaqueAATAAATTATATATCGTATCTTTG--CTTGTTCAACATTGATTC-AAAGCCTGGATAATTTTTTTGAATGAAATACTAGT 57328
Macaque          AATAAATTATATATCGTATCTTTG--CTTGTTCAACATTGATTC-AAAGCCTGGATAATTTTTTTGAATGAAATACTAGT 58164
PigTailedMacaque AATAAATTATATATCGTATCTTTG--CTTGTTCAACATTGATTC-AAAGCCTGGATAATTTTTTTGAATGAAATACTAGT 57050
Bonobo           AATAAATTACATATCATACCTTTG--CTTGTTCAACATTGATCCCAAAGCCTGGATAATTTTTTTGAATGAAATACTAGT 55432
OliveBaboon      AATAAATTATATATCGTGTCTTTG--CTTGTTCAACATTGATTC-AAAGCCTGGATAATTTTTTTGAATGAAATACTAGT 56908
Orangutan        AATAAATTACATATCATACCTTTG--CTTGTTCAACATTGATCCCAAAGCCTGGATAATTTTTTTGAATGAAATACTAGT 57293

Majority         AGAATATTTGATTTATTAAACAGGTAGTACCTGGCTCATGG-----------CAGGTGCTTAAAAATATTTGTTGAGTGG

                         70410     70420     70430     70440     70450     70460     70470     70480

Human            AGAATATTTGATTTATTAAACAGGTAGTACCTGGCTCATGG-----------CAGGTGCTTAAAAATATCTGTTGAGGGG 56400
Coquerel'sSifaka GCATTCTTTG------------GGTGGTGCACATTGCACAC-----------TTTGTGTATGCATATACCCGT------- 47379
Ma'sNightMonkey  AGAATATTTGACATATTAAACAGCTAATACCTGGCTCATATACTCATAATTCCAGGTGCTTAAAAATAATTGTCGAGTGG 51595
VervetAGM        AGAATATTTGATTTATTAAACAGGTAGTACCTGGCTCATGG-----------CAGGTGCTTAAAAATATTTGTTGAGTGG 58194
Gorrilla         AGAATATTTGATTTATTAAACAGGTAGTACCTGGCTCATGG-----------CAGGTGCTTAAAAATATTTGTTGAGTGG 56930
GrabEatingMacaqueAGAATATTTGATTTATTAAGCAGGTAGTACCTGGCTCATGG-----------CAGGTGCTTAAAAATATTTGTTGGGTGG 57397
Macaque          AGAATATTTGATTTATTAAGCAGGTAGTACCTGGCTCATGG-----------CAGGTGCTTAAAAATATTTGTTGGGTGG 58233
PigTailedMacaque AGAATATTTGATTTATTAAACAGGTAGTACCTGGCTCATGG-----------CAGGTGCTTAAAAATATTTGTTGGGTGG 57119
Bonobo           AGAATATTTGATTTATTAAACAGGTAGTACCTGGCTCATGG-----------CAGGTGCTTAAAAATATTTGTTGAGTGG 55501
OliveBaboon      AGAATATTTGATTTATTAAACAGGTAGTACCTGGCTCATGG-----------CAGGTGCTTAAAAATATTTGTTGGGTGG 56977
Orangutan        AGAATATTTGATTTATTAAACAGGTAGTACCTGGCTCATGG-----------CAGGTGCTTAAAAATATTTGTTGAGTGG 57362

Majority         CCATTAAATTTTGTTTAATCTGTGTAAGTTCTAGGAACAAAGTGAACTAGTAGTGT---AACAGGTAGCAAAGTATGTGA

                         70490     70500     70510     70520     70530     70540     70550     70560

Human            CCATTTAATTTTGTTTAATCTGTGTAAGTTCTAGGAACAAAGTGAACTAGTAGTGT---AACAGGTTGCAAAGTATGTGA 56477
Coquerel'sSifaka -C-----------------CCTCCCCAGCCCCCCGCCCAACAT---CCAATAG---------------------ATGTTA 47417
Ma'sNightMonkey  GCATTAAATTTTGTTTAATCTGTGTAAGTTCTAGGAACAAAGTGAACTAGTAGTGT---AACAGGTAGCAAAATATATGA 51672
VervetAGM        CCATTAAATGTTGTTTAATCTGTGTAAGTTCTAGGAACAAAGTGAACTAGTAGTGT---AACAGGTAGCAAAGTATGTGA 58271
Gorrilla         CCATTTAATTTTGTTTAATCTGTGTAAGTTCTAGGAACAAAGTGAACTAGTAGTGT---AACAGGTAGCAAAGTATGTGA 57007
GrabEatingMacaqueCCATTAAATGTTGTTTAATCTGTGTAAGTTCTAGGAACAAAGTGAACTAGTAGTGT---AACAGGTAGCAAAGTATGTGA 57474
Macaque          CCATTAAATGTTGTTTAATCTGTGTAAGTTCTAGGAACAAAGTGAACTAGTAGTGT---AACAGGTAGCAAAGTATGTGA 58310
PigTailedMacaque CCATTAAATGTTGTTTAATCTGTGTAAGTTCTAGGAACAAAGTGAACTAGTAGTGT---AACAGGTAGCAAAGTATGTGA 57196
Bonobo           CCATTTAATTTTGTTTAATCTGTGTAAGTTCTAGGAACAAAGTGAACTAGTAGTGT---AACAGGTAGCAAAGTATGTGA 55578
OliveBaboon      CCATTAAATGTTGTTTAATCTGTGTAAGTTCTAGGAACAAAGTGAACTAGTAGTGT---AACAGGTAGCAAAGTATGTGA 57054
Orangutan        CCATTTAATTTTGTTTAATCTGTGTAAGTTCTAAGAACAAAGTGAACTAGTAGTGTTGTAACAGGTAGCAAGGTATGTGA 57442
```

```
Majority          CAATAATGGGTATGTTTCTAAACTACATAATATTAATTTCCAGCGTGCTCAAGCAGTTCTTCAAGCTGTGACAGCTGTCC

                          70570     70580     70590     70600     70610     70620     70630     70640

Human             CAATAATGGGTATGTTTCTAAACTACATAATCTTAATTTCCAGCGTGCTCAGGCAGTTCTTCAAGCTGTGACAGCTGTCC 56557
Coquerel'sSifaka  TTCTCATATGTGCACTTATGTGTTGTATA-TATATATTTTTAGT-TGTCCAGCTAATTTCT------T---------TCT 47480
Ma'sNightMonkey   CATTAATAGATGTGTTTCTAAACTACTTAATCTTAATTTCCAGCGTGCTCAAGCAGTTCTTCAAGCTGTGACAGCTGTCC 51752
VervetAGM         CAATAGTGGGTATGTTTCTAAACTACATAATATTAATTTCCAGCGTGCTCAAGCAGTTCTTCAAGCTGTGACAGCTGTCC 58351
Gorrilla          CAATAATGGGTATGTTTCTAAACTACATAATCTTAATTTCCAGCGTGCTCAAGCAGTTCTTCAAGCTGTGACAGCTGTCC 57087
GrabEatingMacaqueCGATAGTGGGTATGTTTCTAAACTACATAATATTAATTTCCAGCGTGCTCAAGCAGTTCTTCAAGCTGTGACAGCTGTCC 57554
Macaque           CGATAGTGGGTATGTTTCTAAACTACATAATATTAATTTCCAGCGTGCTCAAGCAGTTCTTCAAGCTGTGACAGCTGTCC 58390
PigTailedMacaque  CGATAGTGGGTATGTTTCTAAACTACATAATATTAATTTCCAGCGTGCTCAAGCAGTTCTTCAAGCTGTGACAGCTGTCC 57276
Bonobo            CAATAATGGGTATGTTTCTAAACTACATAATCTTAATTTCCAGCGTGCTCAAGCAGTTCTTCAAGCTGTGACAGCTGTCC 55658
OliveBaboon       CAATAGTGGGTATGTTTCTAAACTACATAATATTAATTTCCAGCGTGCTCAAGCAGTTCTTCAAGCTGTGACAGCTGTCC 57134
Orangutan         CAATAATGGGTATGTTTCTAAACTACATAATCTTAATTTCCAGCGTGCTCAAGCAGTTCTTCAAGCTGTGACAGCTGTCC 57522

Majority          AGACAGCAAATACTCCTCTTAGTGGCACCACAGTTAGCGAGAGTGCAGTGACTCCAGCCCAGAGTCCAGTACTTAGAATA

                          70650     70660     70670     70680     70690     70700     70710     70720

Human             AGACAGCAAATACTCCTCTTAGTGGCACCACAGTTAGCGAGAGTGCAGTGACTCCAGCCCAGAGTCCAGTACTTAGAATA 56637
Coquerel'sSifaka  --A----------TTTTTTTAGTGGAGACGGGGT---C----------TCGCTCTAGCTCAG------------------ 47517
Ma'sNightMonkey   AGACAGCAAATACTCCTCTTAGTGGCACCACAGTTAGCGAGAGTGCAGTGACTCCAGCCCAGAGTCCAGTACTTAGAATA 51832
VervetAGM         AGACAGCAAATACTCCTCTTAGTGGCACCACAGTTAGCGAGAGTGCAGTGACTCCAGCCCAGAGTCCAGTACTTAGAATA 58431
Gorrilla          AGACAGCAAATACTCCTCTTAGTGGCACCACAGTTAGCGAGAGTGCAGTGACTCCAGCCCAGAGTCCAGTACTTAGAATA 57167
GrabEatingMacaqueAGACAGCAAATACTCCTCTTAGTGGCACCACAGTTAGCGAGAGTGCAGTGACTCCAGCCCAGAGTCCAGTACTTAGAATA 57634
Macaque           AGACAGCAAATACTCCTCTTAGTGGCACCACAGTTAGCGAGAGTGCAGTGACTCCAGCCCAGAGTCCAGTACTTAGAATA 58470
PigTailedMacaque  AGACAGCAAATACTCCTCTTAGTGGCACCACAGTTAGCGAGAGTGCAGTGACTCCAGCCCAGAGTCCAGTACTTAGAATA 57356
Bonobo            AGACAGCAAATACTCCTCTTAGTGGCACCACAGTTAGCGAGAGTGCAGTGACTCCAGCCCAGAGTCCAGTACTTAGAATA 55738
OliveBaboon       AGACAGCAAATACTCCTCTTAGTGGCACCACAGTTAGCGAGAGTGCAGTGACTCCAGCCCAGAGTCCAGTACTTAGAATA 57214
Orangutan         AGACAGCAAATACTCCTCTTAGTGGCACCACAGTTAGCGAGAGTGCAGTGACTCCAGCCCAGAGTCCAGTACTTAGAATA 57602

Majority          ATTATTGACAACATGTACTACCCTGTAACACTTGATGTTCTTCACCAAGTAAGTTTAATCTGCATAATTACCTATAAATT

                          70730     70740     70750     70760     70770     70780     70790     70800

Human             ATTATTGACAACATGTACTACCCTGTAACACTTGATGTTCTTCACCAAGTAAGTTTAATCTGCATAATTACCTATAAATT 56717
Coquerel'sSifaka  -------G---------CTGGTCTCAAACTCCTGA-GTT------CAAACAA--TCCACCCGCCTCAG--CCTCCCA--- 47567
Ma'sNightMonkey   ATTATTGACAACATGTACTACCCTGTAACACTTGATGTTCTTCACCAAGTAAGTTTAATCTGCTTAATTACCTATAA--- 51909
VervetAGM         ATTATTGACAACATGTACTACCCTGTAACACTTGATGTTCTTCACCAAGTAAGTTTAATCTGCATAATTACCTATAAATT 58511
Gorrilla          ATTATTGACAACATGTACTACCCTGTAACACTTGATGTTCTTCACCAAGTAAGTTTAATCTGCATAATTACCTATAAATT 57247
GrabEatingMacaqueATTATTGACAACATGTACTACCCTGTAACACTTGATGTTCTTCACCAAGTAAGTTTAATCTTCATAATTACCTATAAATT 57714
Macaque           ATTATTGACAACATGTACTACCCTGTAACACTTGATGTTCTTCACCAAGTAAGTTTAATCTTCATAATTACCTATAAATT 58550
PigTailedMacaque  ATTATTGACAACATGTACTACCCTGTAACACTTGATGTTCTTCACCAAGTAAGTTTAATCTTCATAATTACCTATAAGTT 57436
Bonobo            ATTATTGACAACATGTACTACCCTGTAACACTTGATGTTCTTCACCAAGTAAGTTTAATCTGCATAATTACCTATAAATT 55818
OliveBaboon       ATTATTGACAACATGTACTACCCTGTAACACTTGATGTTCTTCACCAAGTAAGTTTAGTCTGCATAATTACCTATAAATT 57294
Orangutan         ATTATTGACAACATGTACTACCCTGTAACACTTGATGTTCTTCACCAAGTAAGTTTAATCTGCATAAT-ACCTATAAATT 57681
```

```
Majority          AGAGAAATAATAATATAGTAAATAAGTAATGTTTTGTTTTTAATGTTTTAACAGATATTTTCTAAGTTTGGTGCTGTTTT
                          70810     70820     70830     70840     70850     70860     70870     70880
Human             AGAGAAATAATAATATAGTAAATAAGTAATGTTTTGATTTTAATGTTTTAACAGATATTTTCTAAGTTTGGTGCTGTATT 56797
Coquerel'sSifaka  -GAGTGCTAGGATTACAG-GAGTGAGCAA-----------CCACACCCGGCC---TATTTTTCACATTT--TAAAATCTT 47629
Ma'sNightMonkey   -GATAAATAATAATATACTCAGTAAATAATGTTTTGATTTTAATGTTTTAACAGATATTTTCTAAATTTGGTGCTGTATT 51988
VervetAGM         AGAGAAATAATAATATAGTAAATAAGTAACGTTTTATTTTTAATGTTTTAACAGATATTTTCTAAGTTTGGTGCTGTTTT 58591
Gorrilla          AGAGAAATAATAATATAGTAAATAAGTAATGTTTTGATTTTAATGTTTTAACAGATATTTTCTAAGTTTGGTGCTGTATT 57327
GrabEatingMacaqueAGAGAAATAATAATATAGTAAATAAGTAACGTTTTATTTTTAATGTTTTAACAGATATTTTCTAAGTTTGGTGCTGTTTT 57794
Macaque           AGAGAAATAATAATATAGTAAATAAGTAACGTTTTATTTTTAATGTTTTAACAGATATTTTCTAAGTTTGGTGCTGTTTT 58630
PigTailedMacaque  AGAGAAATAATAATATAGTAAATAAGTAATGTTTTATTTTTAATGTTTTAACAGATATTTTCTAAGTTTGGTGCTGTTTT 57516
Bonobo            AGAGAAATAATAATATAGTAAATAAGTAATGTTTTGATTTTAATGTTTTAACAGATATTTTCTAAGTTTGGTGCTGTATT 55898
OliveBaboon       AGAGAAATAATAATATAGTAAATAAGTAACGTTTTATTTTTAATGTTTTAACAGATATTTTCTAAGTTTGGTGCTGTTTT 57374
Orangutan         CGAGAAATAATAATATAGTAAATAAATAATGTTTTGATTTTAATGTTTTAACAGATATTTTCTAAGTTTGGTGCTGTATT 57761

Majority          GAAGATAATCACATTTACAAAAAATAACCAGTTTCAAGCTTTGCTCCAGTATGGTGATCCAGTAAATGCTCAACAAGCAA
                          70890     70900     70910     70920     70930     70940     70950     70960
Human             GAAGATAATCACATTTACAAAAAATAACCAGTTTCAAGCTTTGCTCCAGTATGGTGATCCAGTAAATGCTCAACAAGCAA 56877
Coquerel'sSifaka  GTAAAT-----------CAAGATGTATCTTG---------------CAGTG-GTTGATCCATCAGA-GTTTAATTGGCAA 47681
Ma'sNightMonkey   GAAGATAATCACATTTACAAAAAATAACCAGTTTCAAGCTTTGCTCCAGTATGGTGATCCAGTAAATGCTCAACAAGCAA 52068
VervetAGM         GAAGATAATCACATTTACAAAAAACAACCAGTTTCAAGCTTTGCTCCAGTATGGTGATCCAGTAAATGCTCAACAAGCAA 58671
Gorrilla          GAAGATAATCACATTTACAAAAAATAACCAGTTTCAAGCTTTGCTCCAGTATGGTGATCCAGTAAATGCTCAACAAGCAA 57407
GrabEatingMacaqueGAAGATAATCACATTTACAAAAAACAACCAGTTTCAAGCTTTGCTCCAGTATGGTGATCCAGTAAATGCTCAACAAGCAA 57874
Macaque           GAAGATAATCACATTTACAAAAAACAACCAGTTTCAAGCTTTGCTCCAGTATGGTGATCCAGTAAATGCTCAACAAGCAA 58710
PigTailedMacaque  GAAGATAATCACATTTACAAAAAACAACCAGTTTCAAGCTTTGCTCCAGTATGGTGATCCAGTAAATGCTCAACAAGCAA 57596
Bonobo            GAAGATAATCACATTTACAAAAAATAACCAGTTTCAAGCTTTGCTCCAGTATGGTGATCCAGTAAATGCTCAACAAGCAA 55978
OliveBaboon       GAAGATAATCACATTTACAAAAAACAACCAGTTTCAAGCTTTGCTCCAGTATGGTGATCCAGTAAATGCTCAACAAGCAA 57454
Orangutan         GAAGATAATCACATTTACAAAAAATAACCAGTTTCAAGCTTTGCTCCAGTATGGTGATCCAGTAAATGCTCAACAGGCAA 57841

Majority          AACTAGTAAGTCTTTCTTTTGAGATGGTGATTTTTTTTAATTGAAATGTATATGTAAAAATATATATATA----------
                          70970     70980     70990     71000     71010     71020     71030     71040
Human             AACTAGTAAGTCTTTCTTTTGAGATGGTGATTTTTTTTTATTGAAATGTATATGTAGAAAAATATATATATATGTATGTA 56957
Coquerel'sSifaka  ---CA---------TCTTATTAGT--------C----------A------CACATAAAAT-------------------- 47705
Ma'sNightMonkey   AACTAGTAAGTCTTTCTTTTGAGCTACTGATGTTTTTCATTGAGATGTATATGTAAAAT-ATATATGTG----TATATA 52143
VervetAGM         AACTAGTAAGTCTTTCTTTTGAGATGGTGATTTTTTTTAATTGAAATGTATATGTAAAAA-ATATATATA---------- 58740
Gorrilla          AACTAGTAAGTCTTTCTTTTGAGATGGTGATTTTTTTTT-ATTGAAGTGTATATATAGAAAAATATATATATATGTATGTA 57486
GrabEatingMacaqueAACTAGTAAGTCTTTCTTTTGAGATAGTGATTTTTTTTAATTGAAATGTATATGTAAAAATATATACATA---------- 57944
Macaque           AACTAGTAAGTCTTTCTTTTGAGATAGTGATTTTTTTTAATTGAAATGTATATGTAAAAATATATACATA---------- 58780
PigTailedMacaque  AACTAGTAAGTCTTTCTTTTGAGATAGTGATTTTTTTTAATTGAAATGTATATGTAAAAATATATACATA---------- 57666
Bonobo            AACTAGTAAGTCTTTCTTTTGAGATGGTGATTTTTTTTTATTGAAATGTATATGTAGAAAAATATATATATATGTATGTA 56058
OliveBaboon       AACTAGTAAGTCTTTCTTTTGAGATAGTGATTTTTTT-AATTGAAATGTATATGTAAAAATATATATATA---------- 57523
Orangutan         AACTAGTAAGTCTTTCTTTTGAGATGGTGATTTTTTTT-ATTGAAATGTATATGTAGAAAAATATATATA--TGTATGTA 57918
```

```
Majority         TACTTAATGATATCTTGCAGTAT---AGATAGTAGAAATTGATAGCATTAAAATAATATTAAGAAACCTTAATATTTGTC

                         71050     71060     71070     71080     71090     71100     71110     71120

Human            TACTTAATGATATCTTGTAGCATTACAGATAGTAGAAATTGATGGCATTAA-----TATTAAGAAACCTTAATATTTGTC 57032
Coquerel'sSifaka --AATAGCG-CATTTTACAATT----A-ATCGTA-TATTCGATACCATAAAATGTATGGGAAATATCCTTCATATTA--T 47774
Ma'sNightMonkey  TACTTAATGATACCTTGTAGCATTATAGATAGTAGTAATTGATGGCATTAAAA---TATTAAGAAACCTTAATATTTGTC 52220
VervetAGM        TACTTAATGATATCTTGCAGTA----AGATAGTAGAAACTGATAGCATTAAAATAATATTAAGAAACCTTAATATTTGTC 58816
Gorrilla         TACTTAATGATATCTTGTAGCATTACAGATAGTAGAAATTGATGGCATTAA-----TATTAAGAAACCTTAATATTTATC 57561
GrabEatingMacaqueTACTTAATGATATCTTGCAGTAT---AGATAGTAGAAATTGATAGCATTAAAATAATATTAAGAAACCTTAATATTTGTC 58021
Macaque          TACTTAATGATATCTTGCAGTAT---AGATAGTAGAAATTGATAGCATTAAAATAATATTAAGAAACCTTAATATTTGTC 58857
PigTailedMacaque TACTTAATGATATCTTGCAGTAT---AGATAGTAGAAATTGATAGCATTAAAATAATATTAAGAAACCTTAATATTTGTC 57743
Bonobo           TACTTAATGATATCTTGTAGCATTACAGATAGTAGAAATTGATGGCATTAA-----TATTAAGAAACCTTAATATTTGTC 56133
OliveBaboon      TACTTAATGATATCTTGCAGTAT---AGATAGTAGAAATTGATAGCATTAAAATAATATTAAGAAACCTTAATATTTGTC 57600
Orangutan        TACTTAATGATATCTTGTAGCATTATAGATAGTAGAAATTGATGGCATTAAAATAACATTAAGAAACCTTAATATTTGTC 57998

Majority         ATAAAAAAGAAATATTTATTTTTAGGAAAATCATAGTTGTAGTTAACCATAATGTAGGCTTCTATTATTTTACTTTTTTT

                         71130     71140     71150     71160     71170     71180     71190     71200

Human            ATAAAAGA--AATATTTATTTTTAGGAAAATCATAGTTGTAGTTAACCATGATGTGGGTTTCTATTATTTTACTTTTTTT 57110
Coquerel'sSifaka ATCTTAAAG---------TTTTCCTGAAAAT----GTTCTGGTTTTGTACAGAGTACACATCAGT-------CTTTGTTC 47834
Ma'sNightMonkey  ATTAAAAA--AATGTTTATTTTTAGGAAAATCATAGTTACAGTTAATCATAATGTAGGCTTCTTTTTTTTCACGTTTTTG 52298
VervetAGM        ATAAAAAAGAAATACTTATTTTTAGGAAAATCATAGTTGTAGTTAACCATAATGTAGGCTTCTATTATTTTGCTTTTTTT 58896
Gorrilla         ATAAAAGAGAAATATTTATTTTTAGGAAAATCATAGTTGTAGTTAACCATAATGTGGGTTTCTATTATTTTACTTTTTTT 57641
GrabEatingMacaqueATAAAAAAGAAATACTTATTTTTAGGAAAATCATAGTTGTAGTTAACCATAATGTAGGCTTCTATTATTTTACTTTTTTT 58101
Macaque          ATAAAAAAGAAAT----ATTTTTAGGAAAATCATAGTTGTAGTTAACCATAATGTAGGCTTCTATTATTTTACTTTTTTT 58933
PigTailedMacaque ATAAAAAAGAAATACTTATTTTTAGGAAAATCATAGTTGTAGTTAACCATAATGTAGGCTTCTATTATTTTACTTTTTTT 57823
Bonobo           ATAAAAGA--GAAATTTATTTTTAGGAAAATCATAGTTGTAGTTAACCATAATGTGGGTTTCTATTATTTTACTTTTTTT 56211
OliveBaboon      ATAAAAAAGAAATACTTATTTTTAGGAAAA------TTGTAGTTAACCATAATGTAGGCTTCTATTATTTTACTTTTTTT 57674
Orangutan        ATAAAAGAGAAATATTTATTTTTAGGAAAATCATAGTTGTAATTAACCATAATATAGGCTTCTATTATTTTACTTTTTTT 58078

Majority         TTTTTTT--TAATTTAAGGACACAAGTTTGTACTCTTAAGAAAAAAGCAGGACTATATTGTGGATGTGCACCTGCATAAG

                         71210     71220     71230     71240     71250     71260     71270     71280

Human            TTTTTTTTTTAATTTAAGGACAAAAGTTTGTACTCTTAAGAAAAAAGCAGGACTATATTGTGAATGTGCACCAGCATAAG 57190
Coquerel'sSifaka TAAA-----AATTTTGGTATCAGAAGTTAATAGTATGAAAAAA------G-ACT-TATT-------TGTATCC---TAAG 47891
Ma'sNightMonkey  TTTT-----AAATTTAAGGACAAAAGTCTGTGCTGTTAAGAAAAAAGCAGGACTACATTGTGGATGCACATCTGCGTAAG 52373
VervetAGM        TTTTTTT---AATTAAAGGACACAAGTTTGTACTCTTAAGAAAAAAGCAGGACTATGTTGGGGATGTGCACCTGCATAAG 58973
Gorrilla         TTTTTT--TTAATTTAAGGACAAAAGTTTGTACTCTTAAGAAAAAAGCAGGACTGTATTGTGAATGTGCACCAGCAGAAG 57719
GrabEatingMacaqueTTTTTTTT-AAATTTAAGGACACAAGTTTGTACTCTTAAGAAAAAAGCAGGACTATGTTGGGGATGTGCACCTGCATAAA 58180
Macaque          TTTTTTTT-AAATTTAAGGACACAAGTTTGTACTCTTAAGAAAAAAGCAGGACTATGTTGGGGATGTGCACCTGCATAAA 59012
PigTailedMacaque TTTTTTTT-TAATTTAAGGACACAAGTTTGTACTCTTAAGAAAAAAGCAGGACTATGTTGGGGATGTGCACCTGCATAAA 57902
Bonobo           TTT------TAATTTAAGGACAAAAGTTTGTACTCTTAAGAAAAAAGCAGGACTATATTGTGAATGTGCACCAGCATAAG 56285
OliveBaboon      TTTTTTTT-TAATTTAAGGACACAAGTTTGTACTCTCAAGAAAAAAGCAGGACTATGTTGGGGATGTGCACCTGCATAAA 57753
Orangutan        TTTTTT--TTAATTTAAGGACGAAAGTTTGTACTCTTAAGAAAAAAGCAGGACTATATTGTGGATGTGCACCAGCATAAG 58156
```

```
Majority          ATTTGAAGTGGCTTTTAGTGGGAATTTCAAAGTAATTAGTTTTCAAGAGAGTTTGGCTGAAATACT------CAGATGTG

                          71290     71300     71310     71320     71330     71340     71350     71360

Human             ATTTGAAGTGGCTTTTGCTGGGAATTTCAAAGTAATTAGTTTTCAAAAGAGTTTGGCTGAAATATA------CAGATGTG 57264
Coquerel'sSifaka  ATT--------------------------C--C-------TTT------------------------------------- 47899
Ma'sNightMonkey   ATTTGAAGTGGCTTTTAGTGGGAGTTTCAAAGTAA----TTTTCAAGACAGTTTGGCTGAAATACATCAATACAGATGTG 52449
VervetAGM         ATTTGAAGTGGCTTTTAGTGGGAATTTCAAAGTAATTAGTTTTCAAGAGAGTTTGGCTGAAATACT------CAGATGCG 59047
Gorrilla          ATTTGAAGTGGCTTTTGTTGGGAATTTCAAAGTAATTAATTTTCAAAAGAGTTTGGCTGAAATATA------CAGATGTG 57793
GrabEatingMacaqueATTTGAAGTGGCTTTCAGTGGGAATTTCAAAGTAATTAGTTTTCAAGAGAGTTTGGCTGAAATACT------CAGATGTG 58254
Macaque           ATTTGAAGTGGCTTTCAGTGGGAATTTCAAAGTAATTAGTTTTCAAGAGAGTTTGGCTGAAATACT------CAGATGTG 59086
PigTailedMacaque  ATTTGAAGTGGCTTTTAGTGGGAATTTCAAAGTAATTAGTTTTCAAGAGAGTTTGGCTGAAATACT------CAGATGTG 57976
Bonobo            ATTTGAAGTGGCTTTTGCTGGGAATTTCAAAGTAATTAGTTTTCAAAAGAGTTTGGCTGAAATATA------CAGATGTG 56359
OliveBaboon       ATTTGAAGTGACTTTTAGTGGGAATTTCAAAGTAACTAGTTTTCAAGAGAGTTTGGCTGAAATACT------CAGATGTG 57827
Orangutan         ATTTGAAGTGGCTTTTACTGGGAATTTCAAAGTAATTAGTTTTCAAGAGAGTTTGGCTGAAATACA------CAGATGTG 58230

Majority          GCTCAAATGCTATGATAGGCTCTCAGTTTATTTAAAACTATATATTCTCT-TAATGTCTTTTTTTT--AAAGGTTTTATG

                          71370     71380     71390     71400     71410     71420     71430     71440

Human             GCTCAAATGCTATGATAGGCTCTCAGTTTATTTAAAACTATATATTCTCTTTAATGTCTTTTTTTT--AAAGGTTTTATG 57342
Coquerel'sSifaka  --TTAAAGATTATATTAG------AATTAAT---AAGCTTTATA-----------------------GGAGGTCTTTTG 47944
Ma'sNightMonkey   GCTCAAATGCTATGATAGGTTCTCAGTTTATTTAAAACTCTATATTCTCT-TAATGTCTTTTTTAAAAAAAGTTTTATG 52528
VervetAGM         GCTCAAATGCTATGATAGGCTCTCAGTTTATTTAAAATTATGTATTATCTTTAATGTCTTTTTTTA---AAGATTTTATG 59124
Gorrilla          GCTCAAATGCTATGATAGGCTCTCAGTTTATTTAAAACTATATATTCTCTTTAATGTCTTTTTTTTT-AAAGGTTTTATG 57872
GrabEatingMacaqueGCTCAAATGCTATGATAGGCTCTCAGTTTATTTAAAATTATATATT-----TAATGTCTTTTTTTT--AAAGATTTTATG 58327
Macaque           GCTCAAATGCTATGATAGGCTCTCAGTTTATTTAAAATTATATATT-----TAATGTCTTTTTTTA--AAAGATTTTATG 59159
PigTailedMacaque  GCTCAAATGCTATGATAGGCTCTCAGTTTATTTAAAATTATATATT-----TAATGTCTTTTTTTT--AAAGATTTTATG 58049
Bonobo            GCTCAAATGCTATGATAGGCTCTCAGTTTATTTAAAACTATATATTCTCTTTAATGTCTTTTTTTTT-AAAGGTTTTATG 56438
OliveBaboon       GCTCAAATGCTATGATAGGCTCTCAGTTTATTTAAAATTATATATT-----TAATGTCTTTTTTTTTTAAAGATTTTATG 57902
Orangutan         GCTCAAATGCTATGATAGGCTCTCAGTTTATTTAAAACTATATATTCTCTTTAATGTCTTTTTTTT--AAAGGTTTTATG 58308

Majority          AATAGGTTTCTAGTCA-CTTTTTATTTATATCATTTATCTTTTTT-GTCATTGTTATTTATGGAGGATGTGAGGTCATTG

                          71450     71460     71470     71480     71490     71500     71510     71520

Human             AATAGGTTTCTAGTCA-CTTTTTATTCATATCATTTATCTTTTTT-GTCATTGTTACATATGGAGGATGTAAGGTCATTG 57420
Coquerel'sSifaka  AATAA---TCCAGTAATCTCCTTGTTGAGATTTACCATGTATCT--GTTTTATTTA---GTGAATAATGTTAGATTA--- 48013
Ma'sNightMonkey   AATATGTTTCTAGTCAACTTTTTATTCATATCATTTATCTTTTTTTGTTGTTGTCA--TATGGAGGATATGAGGTCATTA 52606
VervetAGM         AATAGGTTTCTAGTCA-CTTTTTATTTATATCATTTATCTTTTTT-GTCATTGTTATTTATGGAGGATGTGAGGTCATTG 59202
Gorrilla          AATAGGTTTCTAGTCA-CTTTTTATTCATATCATTTATCTTTTTT-GTCATTGTTACATATGGAGGATGTAAGGTCATTG 57950
GrabEatingMacaqueAATAGGTTTCTAGACA-CATTTTATTTATATCATTTATCTTTTTT-GTCATTGTTATTTATGGAGGATGTGAGGTCATTG 58405
Macaque           AATAGGTTTCTAGACA-CATTTTATTTATATCATTTATCTTTTTT-GTCATTGTTATTTATGGAGGATGTGAGGTCATTG 59237
PigTailedMacaque  AGTAGGTTTCTAGTCA-CATTTTATTTATATCATTTATCTTTTTT-GTCATTGTTATTTATGGAGGATGTGAGGTCATTG 58127
Bonobo            AATAGGTTTCTAGTCA-CTTTTTATTCATATCATTTATCTTTTTT-GTCATTGTTACATATGGAGGATGTAAGGTCATTG 56516
OliveBaboon       AATAGGTTTCTAGTCA-CTTTTTATTTATATCATTTATCTTTTTT-GTCATTGTTATTTATGGAGGATGTGAGGTCATTG 57980
Orangutan         AATAGGTTTCTAGTCA-CTTTTTATTCATATCATTTATCTTTTTT-GTCATTGTTATATATGGAGGATGTAAGGTCATTG 58386
```

```
Majority          AGAGATGTAAAATTTCC--TGTCAAGGCTGCCACTGAAAACTGCAAACCACATCTTTGCTTCCAAGTACCTTAATCTTCT
                          71530     71540     71550     71560     71570     71580     71590     71600
Human             AGAGATGTAAAATTTCC--CATCAAGGCTGCCACTGAAAACTGCGCACCACATCTTTGCTTCCAAGTACCTTAATCTTCT 57498
Coquerel'sSifaka  -AAGTTTTATTGTTCTC----TAAAAGTTTCTGTTTAAAAC--------ATTTTCTGGTT---ATGTACAT----C---T 48070
Ma'sNightMonkey   AGACATGTAAAATTTCCCCTGTCAGGGCTGCTGCTGAAAACGGCATACCACATCTTTGCTTCCAAGTACCTTAATCTTCT 52686
VervetAGM         AGAAATGTAAAATTTCC--TGTCAAGGCTGCCACTGAAAACTGCAAACCACATCTTTGCTTCCAAGTACCTTAATCTTCT 59280
Gorrilla          AGAGATGTAAAATTTCC--CGTCAAGGCTGCCACTGAAAACTGCATACCACATCTTTGCTTCCAAGTACCTTAATCTTCT 58028
GrabEatingMacaqueAGAAATGTAAAATTTCC--TGTCAAGGCTGCCACTGAAAACTGCAAACCACATCTTTGCTTCCAAGTACCTTAATCTTCT 58483
Macaque           AGAAATGTAAAATTTCC--TGTCAAGGCTGCCACTGAAAACTGCAAACCACATCTTTGCTTCCAAGTACCTTAATCTTCT 59315
PigTailedMacaque  AGAAATGTAAAATTTCC--TGTCAAGGCTGCCACTGAAAACTGCAAACCACATCTTTGCTTCCAAGTACCTTAATCTTCT 58205
Bonobo            AGAGATGTAAAATTTCC--CGTCAAGGCTGCCACTGAAAACTGCATACCACATCTTTGCTTCCAAGTACCTTAATCTTCT 56594
OliveBaboon       AGAAATGTAAAATTTCC--TGTCAAGGCTGCCACTGAAAACTGCAAACCACATCTTTGCTTCCAAGCACCTTAATCTTCT 58058
Orangutan         AGAGATGTAAAATTTTC--TGTCAAGGCTGCCACTGAAAACTGCATACCACGTCTTTGCTTCCAAGTACCTTAATCTTCT 58464

Majority          GTTTGTATATCTGAAATCTACTTTTGCTCTGTCTCTTTAACTTTTTGTGCCATAGTATCTCATTATTATTACTATTCCAT
                          71610     71620     71630     71640     71650     71660     71670     71680
Human             GTTTGTATATCTTAAATCTACTTTTGCTCTGTCTCTTTAACTTTTTGTGCCATAGTATCTCATTATTATTACTATTCCAT 57578
Coquerel'sSifaka  GCC----------AAACTAAATTTTGTTCT------------TTTGAGAATAAATTA---CATTGC-----CTATT--TT 48118
Ma'sNightMonkey   GTTTGTATGTCTTAAATCTACTTTTGCTTTGTCTCTCTAACTTTTTGTGCCATAGTATCTCATTATTATTACTATTCCAT 52766
VervetAGM         GTTTGTATATCTGAAATCTACTTTTGTTCTGTCTCTTTAACTTTTTGTGCCATAGTATCTCATTATTATTACTATTCCAT 59360
Gorrilla          GTTTGTATATCTGAAATCTACTTTTGCTCTGTCTCTTTAACTTTTTGTGCCATAGTATCTTATTATTATTACTATTCCAT 58108
GrabEatingMacaqueGTTTGTATATCTGAAATCTACTTTTGCTCTGTCTCTTTAACTTTTTGTGCTATAGTATCTCATTATTATTACTATTCCAT 58563
Macaque           GTTTGTATATCTGAAATCTACTTTTGCTCTGTCTCTTTAACTTTTTGTGCTATAGTATCTCATTATTATTACTATTCCAT 59395
PigTailedMacaque  GTTTGTATATCTGAAATCTACTTTTGCTCTGTCTCTTTAACTTTTTGTGCCATAGTATCTTATTATTATTACTATTCCAT 58285
Bonobo            GTTTGTATATCTTAAATCTACTTTTGCTCTGTCTCTTTAACTTTTTGTGCCATAGTATCTCATTATTATTACTATTCCAT 56674
OliveBaboon       GTTTGTATATCTGAAATCTACTTTTGCTCTGTCTCTTTAACTTTTTGTGCCATAGTATCTCATTATTATTACTATTCCGT 58138
Orangutan         GTTTGTATATCTTAAATCTACTTTTGCTCTGTCTCTTTAACTTTTTGTGCCATAGTATCTCATTATTATTACTATTCCAT 58544

Majority          TATTAAGCCATTA----AAAATTCATTATTAAGCC---------------------------------------------
                          71690     71700     71710     71720     71730     71740     71750     71760
Human             TATTAAGCCATTA----AAAATTCATTATTAAGCC--------------------------------------------- 57609
Coquerel'sSifaka  TGC----CTGTTG----AATGTTGATCCCAAAACC--------------------------------------------- 48145
Ma'sNightMonkey   TATTAAGACATTATTATGAAACCATTTGATAAACTTACCTTTGTCTTAACTGGTTTTCTTTCTTTCTTTTTTTTTTTTTT 52846
VervetAGM         TATTAAGCCATTA----AAAATTCATTATTAAGCC--------------------------------------------- 59391
Gorrilla          TATTAAGCCATTA----AAAATTCATTATTAAGCC--------------------------------------------- 58139
GrabEatingMacaqueTATTAAGCCATTA----AAAATTCGTTATTAAGCC--------------------------------------------- 58594
Macaque           TATTAAGCCATTA----AAAATTCGTTATTAAGCC--------------------------------------------- 59426
PigTailedMacaque  TATTAAGCCATTA----AAAATTCGTTATTAAGCC--------------------------------------------- 58316
Bonobo            TATTAAGCCATTA----AAAATTCATTATTAAGCC--------------------------------------------- 56705
OliveBaboon       TATTAAGCCATTA----AAAATTCATTATTAAGCC--------------------------------------------- 58169
Orangutan         TATTAAGCCATTA----AAAATTCATTATTAAGCC--------------------------------------------- 58575
```

```
Majority          --------------------------------------------------------------------------------
                          71770     71780     71790     71800     71810     71820     71830     71840
Human             -------------------------------------------------------------------------------- 57609
Coquerel'sSifaka  -------------------------------------------------------------------------------- 48145
Ma'sNightMonkey   AATAGAGATGGGGTTTCTCCATGTTGGTCAGGCTGGTCTCGAACTCCCAACCTCAGGTGATCCGCCCATCTCAGCCTCCC 52926
VervetAGM         -------------------------------------------------------------------------------- 59391
Gorrilla          -------------------------------------------------------------------------------- 58139
GrabEatingMacaque -------------------------------------------------------------------------------- 58594
Macaque           -------------------------------------------------------------------------------- 59426
PigTailedMacaque  -------------------------------------------------------------------------------- 58316
Bonobo            -------------------------------------------------------------------------------- 56705
OliveBaboon       -------------------------------------------------------------------------------- 58169
Orangutan         -------------------------------------------------------------------------------- 58575

Majority          ------------------------------------------ATTATTA-----TGAAACCATTT---------------
                          71850     71860     71870     71880     71890     71900     71910     71920
Human             ------------------------------------------ATTATTA-----TGAAACCATTT--------------- 57627
Coquerel'sSifaka  -------------------------------------------------------T-A---------------------- 48147
Ma'sNightMonkey   AAAGTGCTGGGATTGCAGGCGTGAGCCACCGCGCCCGGCTAAATTTTTAGTTGTTTGAACTATTTTCTTTCTTTTCTTTT 53006
VervetAGM         ------------------------------------------ATTATTA-----TGAAACCATTT--------------- 59409
Gorrilla          ------------------------------------------ATTATTA-----TGAAACCATTT--------------- 58157
GrabEatingMacaque ------------------------------------------ATTATTA-----TGAAACCATTT--------------- 58612
Macaque           ------------------------------------------ATTATTA-----TGAAACCATTT--------------- 59444
PigTailedMacaque  ------------------------------------------ATTATTA-----TGAAACCATTT--------------- 58334
Bonobo            ------------------------------------------ATTATTA-----TGAAACCGTTT--------------- 56723
OliveBaboon       ------------------------------------------ATTATTA-----TGAAACCATTT--------------- 58187
Orangutan         ------------------------------------------ATTATTA-----TGAAACCATTT--------------- 58593

Majority          -----------------------------------------AATAA----------------------------------
                          71930     71940     71950     71960     71970     71980     71990     72000
Human             -----------------------------------------AATAA---------------------------------- 57632
Coquerel'sSifaka  -----------------------------------------GATAA---------------------------------- 48152
Ma'sNightMonkey   CTTTTTCTTTTTCCTTCTTTTTTTGTTTTTTTTTTTTGGTTAACAGAATCTTGCTCTGTCACCCAGGCTGGAGTGCAGTG 53086
VervetAGM         -----------------------------------------AATAA---------------------------------- 59414
Gorrilla          -----------------------------------------AATAA---------------------------------- 58162
GrabEatingMacaque -----------------------------------------AATAA---------------------------------- 58617
Macaque           -----------------------------------------AATAA---------------------------------- 59449
PigTailedMacaque  -----------------------------------------AATAA---------------------------------- 58339
Bonobo            -----------------------------------------AATAA---------------------------------- 56728
OliveBaboon       -----------------------------------------AATAA---------------------------------- 58192
Orangutan         -----------------------------------------AATAA---------------------------------- 58598
```

```
Majority          -----------GCTTAC-------------------ATTTGTCTTAACTGGTTTTCTTTCTTTCGTTCGTTCTTTTACTT
                           72010     72020     72030     72040     72050     72060     72070     72080
Human             -----------GCTTAC-------------------ATTTATCTTAACTGGTTTTCTTCCTTTCATTCATTCTTTTACTT 57682
Coquerel'sSifaka  -------------------------------------------------------------------------------- 48152
Ma'sNightMonkey   GTGCAGTCTTGGCTCACTGCAACCTTCACCTCCTGGGTTCGTCTTAACTAGTTTTCTTCCTTTCATTCTTTGTTTTACTT 53166
VervetAGM         -----------GCTTAC-------------------ATTTGTCTTAACTGGTTTTCTTTCTTTCGTTCCTTCTTTTACTT 59464
Gorrilla          -----------GCTTAC-------------------ATTTGTCTTAACTGGTTTTCTTCCTTTCATTCATTCTTTTACTT 58212
GrabEatingMacaque-----------GCTTAC-------------------ATTTGTCGTAACTGGTTTTCTTTCGTTCGTTCGTTCTTTTACTT 58667
Macaque           -----------GCTTAC-------------------ATTTGTCGTAACTGGTTTTCTTTCGTTCGTTCGTTCTTTTACTT 59499
PigTailedMacaque  -----------GCTTAC-------------------ATTTGTCGTAACTGGTTTTCTTTCGTTCGTTCGTTCTTTTACTT 58389
Bonobo            -----------GCTTAC-------------------ATTTATCTTAACTGGTTTTCTTCCTTTCATTCATTCTTTTACTT 56778
OliveBaboon       -----------GCTTAC-------------------ATTTGTCTTAACTGGTTTTCTTTCGTTCGTTCGTTCTTTTACTT 58242
Orangutan         -----------GCTTAC-------------------ATTTGTCTTAACTGGTTTTCTTCCTTTCATTCTTTCTTTTACTT 58648

Majority          TCAGATTAATTATTGTCTCTAATACTTAAATGCTTCCTCCAGATCAAGATTACTTCTGTGGAGCCCAGACCATTTTTTAT
                           72090     72100     72110     72120     72130     72140     72150     72160
Human             TCAGATTAATTATTGTCTCTAATACTTAAATGCTTCCTCCAGATCAAGATTACTTCTGTGGAGCCCAGACCATTTTTTAC 57762
Coquerel'sSifaka  -----------------------------------------------------------------------TTTTTTTT 48161
Ma'sNightMonkey   TCAGACTAATTATTGTTTCTAATACTTAAATGCCTCCTCCAGATCAAGATCACTTCTGTGGAGCCCAGACCATTTTTAAT 53246
VervetAGM         TCAGATTAATTATTGTCTCTAATACTTAAATGCTTCCTCCAGATCAAGATTACTTCTGTGGAGCCCAGACCATTTTTTAT 59544
Gorrilla          TCAGATTAATTATTGTCTCTAATACTTAAATGCTTCCTCCAGATCAAGATTACTTCTGTGGAACCCAGACCATTTTTTAC 58292
GrabEatingMacaqueTCAGATTAATTATTCT----AATACTTAAATGCTTCCTCCAGATCAAGATTACTTCTGTGGAGCCCAGACCATTTTTTAT 58743
Macaque           TCAGATTAATTATTCT----AATACTTAAATGCTTCCTCCAGATCAAGATTACTTGTGTGGAGCCCAGACCATTTTTTAT 59575
PigTailedMacaque  TCAGATTAATTATTCT----AATACTTAAATGCTTCCTCCAGATCAAGATTACTTCTGTGGGGCCCAGACCATTTTTTAT 58465
Bonobo            TCAGATTAATTATTGTCACTAATACTTAAATGCTTCCTCCAGATCAAGATTACTTCTGTGGAGCCCAGACCATTTTTTAC 56858
OliveBaboon       TCAGATTAATTATTCTCTCTAATACTTAAATGCTTCCTCCAGATCAAGATTACTTCTGTGGAGCCCAGACCATTTTTTAT 58322
Orangutan         TCAGATTAATTATTGTCTCTAATACTTAAATGCTTCCTCCAGATCAAGATTACTTCTGTGGAGCCCAGACCATTTTTTAT 58728

Majority          AATTGTTTCATTGCATTTGCATCCTCACAACCTCATTTCTGGAGCAAAATTAGTTGTAGGTAGCAGAAAGGCTATACAAG
                           72170     72180     72190     72200     72210     72220     72230     72240
Human             AATTGTTTCATTGCATTTCCATCCACACAACCTCATTTTGGGAGCAAAATTAGTTGTAGGTAGCAGAAAGGCTATACAAG 57842
Coquerel'sSifaka  AACT--------------------A--AACACT---------AGTAGAAT--ATTTAATTA--TGAA--------CAGA 48198
Ma'sNightMonkey   AATTGTTTCATTGCATTTCCATCCTCACAACCTCATTTCGGGAACAAAATTAGTTTTAGGTAGCAGAAAGGCTGTACAAG 53326
VervetAGM         AATTGTTTCGTTGCGTTTGCATCCTCACAACCTCATTTCTGGAGCAAAATTAGTTGTAGGTAGCAGAAAGGCTATACAAG 59624
Gorrilla          AATTGTTTCATTGCATTTCCATCCACACAACCTCATTTTGGGAGCAAAATTAGTTGTAGGTAGCAGAAAGGCTATACAAG 58372
GrabEatingMacaqueAATTGTTTCATTGCATTTGCATCCTCACAACCTCATTTCTGGAGCAAAATTAGTTGTAGGTAGCAGAAAGGCTATACAAG 58823
Macaque           AATTGTTTCATTGCATTTGCATCCTCACAACCTCATTTCTGGAGCAAAATTAGTTGTAGGTAGCAGAAAGGCTATACAAG 59655
PigTailedMacaque  AATTGTTTCATTGCATTTGCATCCTCACAACCTCATTTCTGGAGCAAAATTAGTTGTAGGTAGCAGAAAGGCTATACAAG 58545
Bonobo            AATTGTTTCATTGCATTTCCATCCACACAACCTCATTTTGGGAGCAAAATTAGTTGTAGGTAGCAGAAAGGCTATACAAG 56938
OliveBaboon       AATTGTTTCATTGCATTTGCATCCTCACAACCTCATTTCTGGAGCAAAATTAGTTGTAGGTAGCAGAAAGGCTATACAAG 58402
Orangutan         AATTGTTTCATTGCATTTCCATCCTCACAACCTCATTTTGGGAGCAAAATTAGTTGTAGGTAGCAGAAATGCTATACAAG 58808
```

```
Majority          TAATAATAGCATAATTGTAATAGTTTACCAAGTGCTATAGTCTCATTTTTTCTGACCACTACTCTATGTTGTCAACAAAA
                          72250     72260     72270     72280     72290     72300     72310     72320
Human             TAATAATAGCATAATTGTAATAGTTTACCAAGTGCCGTAGTCTCATTTTTTCTGACCACTACTCTATATTGTCAACAAAA 57922
Coquerel'sSifaka  TAATACCTGCCTCATAGCAGATGCTCA-AAAATAC-ATAGT-------------GAGCATTAA---ATTTGTTTATGTA- 48260
Ma'sNightMonkey   TAATAGTAGCATAATTGTAATAGTTTACCAAGTGCTATAGTCAT-TTTTTTCTGACTACTACTCTGTATTGTCTACAAAA 53405
VervetAGM         TAATAATAGCATAATTGTAATAGTTTACAAAGTGCTATAGTCTCATTTTTTCTGACCACTACTCTATGTTATCAGCAAAA 59704
Gorrilla          TAATAATAGCATAATTGTAATAATTTACCAAGTGCCGTAGTCTCATTTTTTCTGACCACTACTCTATATTGTCAACAAAA 58452
GrabEatingMacaqueTAATAATAGCATAATTGTAATAGTTTACCAAGTGCTATAGTCTCATTTTTTCTGACCACTACTCTATGTTATCAGCAAAA 58903
Macaque           TAATAATAGCATAATTGTAATAGTTTACCAAGTGCTATAGTCTCATTTTTTCTGACCACTACTCTATGTTATCAGCAAAA 59735
PigTailedMacaque  TAATAATAGCATAATTGTAATAGTTTACCAAGTGCTATAGTCTCATTTTTTCTGACCACTACTCTATGTTATCAGCAAAA 58625
Bonobo            TAATAATAGCATAATTGTAATAGTTTACCAAGTGCTGTAGTCTCATTTTTTCTGACCACTACTCTATATTGTCAACAAAA 57018
OliveBaboon       TAATAATAGCATAATTGTAATAGTTTACCAAGTGCTATAGTCTCATTTTTTCTGACCACTACTCTATGTTATCAGCAAAA 58482
Orangutan         TAATAATAGCATAATTGTAATAGTTTACCAAGTGCTGTAGTGTCATTTTTTCTGACCACTACTCTATATTGTCAACAAAA 58888

Majority          CAGGGGTTTCCCCTGTGTTACAAGAGGATGCTAAGATTTAAAG--GTGAATTACTTATAATGAACACTACCTATAAGGTA
                          72330     72340     72350     72360     72370     72380     72390     72400
Human             CAGGGTTTTCCCCTGCGTTACAAGAGGATGCTAAGATTTAAAGAGGTGAATTACTTACAGTGAACACCACCTATAAGGTA 58002
Coquerel'sSifaka  -------------TGTTCTAGAAAAGAA--ATGAA--CTAATA----GTATAGTATGTAGCAAAGTATATGACAATGATG 48319
Ma'sNightMonkey   GAGGGGTTTTTCCTGTGTTACAAGGGGATGCTAAGATTTAAAGATGTGCATTACTTATAATGAACATTGCCTGTAAGGTC 53485
VervetAGM         CAGGG-TTTCCCCTTTGTTACAAGATGATGCTAAGATTTAAAG--GTGAATTACTTATAATGAACACTACCTATAAGGTA 59781
Gorrilla          CAGGGTTTTCCCCTGCGTTACAAGAGGATGCTAAGATTTAAAGAGGTGAATTACTTACAGTGAACACCACCTATAAGGTA 58532
GrabEatingMacaqueCAGGGGTTTCCCCTTTGTTACAAGAGGATGCTAAGATTTAAAG--GTGAATTACTTATAATGAACACTACCTATAAGGTA 58981
Macaque           CAGGGGTTTCCCCTTTGTTACAAGAGGATGCTAAGATTTAAAG--GTGAATTACTTATAATGAACACTACCTATAAGGTA 59813
PigTailedMacaque  CAGGGGTTTCCCCTTTGTTACAAGAGGATGCTAAGATTTAAAG--GTGAATTACTTATAATGAACACTACCTATAAGGTA 58703
Bonobo            CAGGGTTTTCCCCTGCGTTACAAGAGGATGCTAAGATTTAAAGAGGTGAATTACTTACAGTGAACACCACCTATAAGGTA 57098
OliveBaboon       CAGGGGTTTCTCCTTTGTTACAAGAGGATGCTAAGTTTTAAAG--GTGAATTACTTATAATGAACACTACCTATAAGGTA 58560
Orangutan         CAGGGTTTTCCCCTGCGTTACAGGAGGATGCTAAGATTTAAAGAGGTGAATTACTTACAGTGAACACCACCTATAAGGTA 58968

Majority          GACATTTTTTCAGTCTT---------AGCCTTTCTCTC-AGTATGCTGCTCCTCACTGGTTCTCAGGTATAATCTAATCT
                          72410     72420     72430     72440     72450     72460     72470     72480
Human             GACATTTTTTCAGTCTT---------AGCCTTTCTCTCCAGTATGCTGCTTCTCACTGGTTCTCGGGTATAATCTAATCT 58073
Coquerel'sSifaka  GGTGTGTTTCTAAACTA------------CTTAATCTT-AATTTTCAGC---------GTGCTCAAGCAG-------T-- 48368
Ma'sNightMonkey   AACATTTTTTCAGTCTTCCTTTCAGTACCCTTTCTCTCCAGTATGCTGCTCCTCACTGGCTCTCAGGTATAATCTAATCT 53565
VervetAGM         GACATTTTTTCAGTCTC---------AGCCTTTCTTTC-AGTATGCTGCTCCTCACTGGTTCTCAGGTATAATCTAATCT 59851
Gorrilla          GACATTTTTTCAGTCTT---------AGCTTTTCTCTCCAGTATGCTGCTTCTCACTGGTTCTCGGGTATAATCTAATCT 58603
GrabEatingMacaqueGACGTTTTTTCAGTCTC---------AGACTTTCTTTC-AGTATGCTGCTCCTCACTGGTTCTCAGGTATAATCTAATCT 59051
Macaque           GACGTTTTTTCAGTCTC---------AGACTTTCTTTC-AGTATGCTGCTCCTCACTGGTTCTCAGGTATAATCTAATCT 59883
PigTailedMacaque  GACGTTTTTTCAGTCTC---------AGACTTTCTTTC-AGTATGCTGCTCCTCACTGGTTCTCAGGTATAATCTAATCT 58773
Bonobo            GACATTTTTTCAGTCTT---------AGCCTTTCTCTCCAGTATGCTGCTTCTCACTGGTTCTCGGGTATAATCTAATCT 57169
OliveBaboon       GACGTTTTTTCAGTCTC---------AGCCTTTCTTTC-AGTATGCTGCTCCTCACTGGTTCTCAGGTATAATCTAATCT 58630
Orangutan         GACATTTTTTCAGTCTT---------AGCCTTTCTCTCCAGTACGCTGCTTCTCACTGGTTCTCGGGTATAATCTAATCT 59039
```

```
Majority          TATTATGCTTTCCTCTTCAGGCATAGGTTGACAATTAGAATTCTTTTTTTTC-------CAAAAAATGATTATCCTCCTT

                          72490     72500     72510     72520     72530     72540     72550     72560

Human             TACTGTGCTTTCCGCTTCAGGCATAGGTTAATAATTAGAATTCTTTTTTTTTGT----CCAAAAAATGATTATCCTCCTT 58149
Coquerel'sSifaka -------------TCTTCAAGCTG----TGACAGCTG------------TCC--------AGACAGCAAATACTC--CTC 48409
Ma'sNightMonkey   TATTAGACTTTCCTTTTCAGGCATAGGTTAACAATTAGAATTCTTTTTTTCC---------AAAAATAAGTAACC-CTTT 53635
VervetAGM         TATTATGCTTTCCTCTTCAGGCATAGGTTGACAATTAGAATTCTTTTTTTTC-------CAAAAAATGATTATCCTCCTT 59924
Gorrilla          TACTGAGCTTTCCGCTTCAGGCATAGGTTAACAATTAGAATTCTTTTTTTTTTTTT-CCAAAAAGTGATTATCCTCCTT 58682
GrabEatingMacaqueTATTATGCTTTCCTCTTCAGGCATAGGTTGACAATTAGAATTCTTTTTTTTC-------CAAAAAATGAGTATCCTCCTT 59124
Macaque           TATTATGCTTTCCTCTTCAGGCATAGGTTGACAATTAGAATTCTTTTTTTTC-------CAAAAAATGAGTATCCTCCTT 59956
PigTailedMacaque TATTATGCTTTCCTCTTCAGGCATAGGTTGACAATTAGAATTCTTTTTTTTC-------CAAAAAATGAGTATCCTCCTT 58846
Bonobo            TACTGTGCTTTCCGCTTCAGGCATAGGTTAATAATTAGAATTCTTTTTTTTTT----CCAAAAAATGATTATCCTCCTT 57245
OliveBaboon       TATTATGCTTTCCTCTTCAGGCATAGGTTGACAATTAGAATTCTTTTTTTTC-------CAAAAAATGATTATCCTCCTT 58703
Orangutan         TATTATGCTTTCTGCTTCAGGCATAGGTTAACAATTAGAATTCTTTCTTTTTTTTTTTCCAAAAAATGATTATCCTCCTT 59119

Majority          TCACATGCCCTGGCTACCAAAATCAATGTGGTATGAGGTTTTATGATTAGGAGACAATCTAGAAGTCATGGTTTCTGTGA

                          72570     72580     72590     72600     72610     72620     72630     72640

Human             TCACATGCCCTGGCTACCAAAATCAATGTGGTATGAGGTTTTATGAATAGGAGACAATCTAGAAGTCATGGTTTCTGTGA 58229
Coquerel'sSifaka TTA------GTGGC-ACCACAGTGAGTG------AGAGTGCAGTGACT-----CCAGCCCAGA------G---------- 48455
Ma'sNightMonkey   TCACATGCCCTGGCTACCAAAATCAATGTGGTATGAGGTTTTATCAGTAGGAGACAATCTAGAAGTCATGGTTTCTGTGG 53715
VervetAGM         TCACATGCCCTGGCTACCAAAATCAATGTGGTATGAGGTTTTATGATTAGGAGACAATCTAGAAATCATGGTTTCTGTGA 60004
Gorrilla          TCACATGCCCTGGCTACCAAAATCAATGTGGTATGAGGTTTTATGAATAGGAGACAATCTAGAAGTCATGGTTTCTGTGA 58762
GrabEatingMacaqueTCACATGCTGTGGCTACCAAAATCAATGTGGTATGAGGTTTTATGATTAGGAGACAATCTAGAAATCATGGTTTCTGTGA 59204
Macaque           TCACATGCTGTGGCTACCAAAATCAATGTGGTATGAGGTTTTATGATTAGGAGACAATCTAGAAATCATGGTTTCTGTGA 60036
PigTailedMacaque TCACATGCCGTGGCTACCAAAATCAATGTGGTATGAGGTTTTATGATTAGGAGACAATCTAGAAATCATGGTTTCTGTGA 58926
Bonobo            TCACATGCCCTGGCTACCAAAATCAATGTGGTATGAGGTTTTATGAATAGGAGACAATCTAGAAGTCATGGTTTCTGTGA 57325
OliveBaboon       TCACATGCCCTGGCTACCAAAATCAATGTGGTATGAGGTTTTATGATTAGGAGACAATCTAGAAATCATGGTTTCTGTGA 58783
Orangutan         TCACATGCCCTGGCTACCAAAATCAGTGTGGTATGAGGTTTTATAAATAGGAGACAGTCTAGAAGTCGTGGTTTCTGTGA 59199

Majority          CATTTTTCACAACTTCAAACATTTAAATATAGTCTACATTTTTCTTGGTGTGGAATATTTTCCTTGCTCTTCTGACCTCA

                          72650     72660     72670     72680     72690     72700     72710     72720

Human             CATTTTTCACAACTTCAAACGTTTAAATATAGTCTACATTTTTCTTGGTGTGGAATATTTTCCTTGCTCTTCTGACCCCA 58309
Coquerel'sSifaka -----TCCAGTACTTAGAATAATTATTGACAATATGTACTACCCT------GTAACACTTG--ATGTTCTTC-------- 48514
Ma'sNightMonkey   CATTTTTCACAACTTCAGACATTGAAATATAGTGTACATTTTTCTTGATGTGGAATATTTTCCTTGCTCTTCTG-CCCTG 53794
VervetAGM         CATTTTTCACAACTTCAAACATTTAAATATAGTCTACATTTTTCTTGGTGTGGAATATTTTCCTTGCTCTTCTGACCTCA 60084
Gorrilla          CATTTTTCACAACTTCAAACATTTAAATATAGTCTACATTTTTCTTGATGTGGAATATTTTCCTTGCTCTTCTGACCCCA 58842
GrabEatingMacaqueCATTTTTCACAACTTCAAACATTTAAATATAGTCTACATTTTTCTTGGTGTGGAATATTTTCCTTGCTCTTCTGACCTCA 59284
Macaque           CATTTTTCACAACTTCAAACATTTAAATATAGTCTACATTTTTCTTGGTGTGGAATATTTTCCTTGCTCTTCTGACCTCA 60116
PigTailedMacaque CATTTTTCACAACTTCAAACATTTAAATATAGTCTACATTTTTCTTGGTGTGGAATATTTTCCTTGCTCTTCTGACCTCA 59006
Bonobo            CATTTTTCACAACTTCAAACATTTAAATATAGTCTACATTTTTCTTGGTGTGGAATATTTTCCTTGCTCTTCTGACCCCA 57405
OliveBaboon       CATTTTTCACAACTTCAAACATTTAAATATAGTCTACATTTTTCTTGGTGTGGAATATTTTCCTTGCTCTTCTGACCTCA 58863
Orangutan         CATTTTTCACAACTTCAAACATTTAAATATAGTTTACATTTTTCTTGGTGTGGAATATTTTCCTTGCTCTTCTGACCCCA 59279
```

```
Majority          AAGAAAAGTATTTGTTAGAGGACCAATTAAATGAAATTTG----GGGATTATACTGTATTTGAGCTATAATTCTTATGGG

                          72730     72740     72750     72760     72770     72780     72790     72800

Human             AAGAAAAGTATTTGTTAGAGGACCAATTAAATGAAATTTGAGTGGGGATTATACTGTGTTTGAGCTATAATTCTTAAGGG 58389
Coquerel'sSifaka  ---------------------ACCAAGTAAGTTTAACTTA-------ATTTGCCTGC---TTACCTATAA-----ATTAG 48558
Ma'sNightMonkey   AGGAAAAGTATTTGTTAAAGGACCAATTAAATGAAATATGACTGGGAATTATACTGTATTTGGGCTATAATTCTTATGGG 53874
VervetAGM         AAGAAAAGTATTTGTTAGAGGACCAATTAAATGAAATTTG----GGGATTATACTGTATTTGAGCTATAATTCTTATGGG 60160
Gorrilla          AAGAAAAGTATTTGTTAGAGGACCAATTAAATGAAATTTGAGTGGGAATTATACTGTGTTTGAGCTATAATTCGTATGGG 58922
GrabEatingMacaqueAAGAAAAGTATTTGTTAGAGGACCAATTAAATGAAATTTG----GGGATTGTACTGCATTTGAGCTATAATTCTTATGGG 59360
Macaque           AAGAAAAGTATTTGTTAGAGGACCAATTAAATGAAATTTG----GGGATTGTACTGCATTTGAGCTATAATTCTTATGGG 60192
PigTailedMacaque  AAGAAAAGTATTTGTTAGAGGACCAATTAAATGAAATTTG----GGGATTGTACTGCATTTGAGCTATAATTCTTATGGG 59082
Bonobo            AAGAAAAGTATTTGTTAGAGGACCAATTAAATGAAATTTGAGTGGGGATTATACTGTGTTTGAGCTATAATTCTTATGGG 57485
OliveBaboon       AAGAAAAGTATTTGTTAGAGGACCAATTAAATGAAATTTG----GGGATTATACTGTATTTAAGCTATAATTCTTACGGG 58939
Orangutan         AAGAAAAGTATTTGTTAGAGGACCAATTAAATGAAATTTGAATGGGGATTATACTGTGTTTGAGCTATAATTCTTATGGG 59359

Majority          GAAAATAGTTTATATGTGTATCTTGTCTGAAGTAATACATAGTTA-CATTTAATCTCAT---AAAAG----TACCTCAAT

                          72810     72820     72830     72840     72850     72860     72870     72880

Human             GAAAATTGTTTATATGTGTATCTTGTCTGAAGTAATACATAGTTA-CATTAAATCTCAT---AAAAG----TACCTCAAT 58461
Coquerel'sSifaka  AGAAATA----A---------------TAATATAGTAGATAAATA------------AT------------GTTTTGAT 48594
Ma'sNightMonkey   GAAAAT-----AT--GTGTATCTTGTCTGAAGTAATACATAGTTA-CATTTAATCTCATCATAAAAGATAGTACCTCAAT 53946
VervetAGM         GAAAATAGTTTATATGTGTATCTTGTCTGAAGTAATACATGGTTA-CATTTAATCTCAT---AAAAG----TACCTCAAT 60232
Gorrilla          GAAAATTGTTTATATGTGTATCTTGTCTGAAGTAATACATAGTTAACGTTAAATCTCGT---AAAAG----TACCTCAGT 58995
GrabEatingMacaqueGAAAATAGTTTATATGTGTATCTTGTCTGAAATAATACATGATTA-CATTTAATCTCAT---AAAAG----TACCTCAAT 59432
Macaque           GAAAATAGTTTATATGTGTATCTTGTCTGAAATAATACATGATTA-CATTTAATCTCAT---AAAAG----TACCTCAAT 60264
PigTailedMacaque  GAAAATAGTTTATATGTGTATCTTGTCTGAAATAATACATGATTA-CATTTAATCTCAT---AAAAG----TACCTCAAT 59154
Bonobo            GAAAATTGTTTATATGTGTATCTTGTCTGAAGTAATACATAGTTA-CATTAAATCTCAT---AAAAG----TACCTCAAT 57557
OliveBaboon       GAAAATAGTTTATATGTGTATCTTGTCTGAAGTAATACATGATTA-CATTTAATCTCAT---AAAAG----TACCTCAAT 59011
Orangutan         GAAAATAGTTTATATGTGTATCTTGTCTGAAGTAATACATAGTTA-CATTTAATCTCATCATAAAAG----TACCTCAAT 59434

Majority          TA-TGTCAACTG----TTTGCAATTTTCGTTTAATCTTCTGTGAACAGATACTAAGCATCCAACTATAATATTGATGTTT

                          72890     72900     72910     72920     72930     72940     72950     72960

Human             TG-TGTCAACTG----TTTGACATTTTTGTTTAATCTTCTGTGAACAGATACTAAGCATCCAACTATGATATTGATGTTT 58536
Coquerel'sSifaka  ----------------------------TTTAATGTTTT---AACAGATAT----TTTCTAAGT------TTGGTGCT- 48631
Ma'sNightMonkey   TA-TGTCAACTGAAATTTTGAAGTTTTCATTTAATCTTCTGTGAACAGATAATAAGCATCCAACTATAATATTGATGTTT 54025
VervetAGM         TAAAGTCAACTG----TTTGCAATTTTCATTTAAACTTCTGTGAACAGATAC-AAGCATCCAACTATAATATTGATGTTT 60307
Gorrilla          TG-TGTCAACTG----TTTGAAATTTTTGTTTAATCTTCTGTGAACAGATACTAAGCATCCAACTATGATATTGATGTTT 59070
GrabEatingMacaqueTAATGTCAACTG----TTTGCAATTTTCGTTTAATCTTCTGTGAACAGATACTAAGCATCCAACTATAATATTGATGTTT 59508
Macaque           TAATGTCAACTG----TTTGCAATTTTCGTTTAATCTTCTGTGAACAGATACTAAGCATCCAACTATAATATTGATGTTT 60340
PigTailedMacaque  TAATGTCAACTG----TTTGCAATTTTCGTTTAATCTTCTGTGAACAGATACTAAGCATCCAACTATAATATTGATGTTT 59230
Bonobo            TG-TGTCAACTG----TTTGAAATTTTTGTTTAATCTTCTGTGAACAGATACTAAGCATCCAACTATGATATTGATGTTT 57632
OliveBaboon       ----GTCAACTG----TTTGCAATTTTCGTTTAATCTTCTGTGAACAGATACTAAGCATCCAACTATAATATTGATGTTT 59083
Orangutan         TA-TGTCAACTG----TTTGAAATTTTTGTTTAATCTTCTGTGAACAGATACTAAGCATCCAACTATAATATTGATGTTT 59509
```

```
Majority          GTGCACAGGTATGCACTCAGGTCTTCTTGACAAAGATCTAAAGTTATAATTCTAAGAGAGTAGATAATTGTTTTATTAAG

                          72970     72980     72990     73000     73010     73020     73030     73040

Human             GTGCACAGGTATGCACTCAGGTCTTCTTGACAAAGATCTAAAGTTATAATTCTAAGAGAGTAGATAATTGTTTTACTAAG 58616
Coquerel'sSifaka  --------GTAT---------------TG---AAGAT---A-ATCACATTTACAAAA-----AATAACCAGTTT--CAAG 48674
Ma'sNightMonkey   GTCCACAAGTATATACTCAGGTCTTTTTGACAAACATCTTAAGTTATAATTCTAAGAGAGTAGATCATTGTTTTATTAAG 54105
VervetAGM         GTGCACAGGTATGCACTCAGGTCTTCTTGACAAAGATTTAAAGTTATAATTCTAAGACAGTAGATAATTGTTTTATTAAG 60387
Gorrilla          GTGCACAGGTATGCACTCAGGTCTTCTTGACAAATATCTAAAGTTATAATTCTAAGAGAGTAGATAATTGTTTTACTAAG 59150
GrabEatingMacaqueGTGCACAGGTATGCACTCAGGTCTTCTTGGCAAAGATCTAAAGTTATAATTCTAAGATAGTAGATAATTGTTTTATTAAG 59588
Macaque           GTGCACAGGTATGCACTCAGGTCTTCTTGGCAAAGATCTAAAGTTATAATTCTAAGATAGTAGATAATTGTTTTATTAAG 60420
PigTailedMacaque  GTGCACAGGTATGCACTCAGGTCTTCTTGGCAAAGATCTAAAGTTTTAATTCTAAGACAGTAGATAATTGTTTTATTAAG 59310
Bonobo            GTGCACAGGTATGCACTCAGGTCTTCTTGACAAAGATCTAAAGTTATAATTCTAAGAGAGTAGATAATTGTTTTACTAAG 57712
OliveBaboon       GTGCACAGGTATGCACTCAGGTCTTCTTGACAAAGATCTAAAGTTATAATTCGAAGACAGTAGATAATTGTTTTATTAAG 59163
Orangutan         GTGCACAGGTATGCACTCAGGTCTTCTTGACAAAGATCTAAAGTTAAAATTCTAAGAGAGTAGATAATTGTTTTACTAAG 59589

Majority          TACTTATTCTGGTATCAGAACTCACACTTCACTTATTAGAGAAGGAAAAATAAATTTGTTTCCTAAACTTGCTTTGCCCC

                          73050     73060     73070     73080     73090     73100     73110     73120

Human             TACTTATTCTGGTATCAGAACTCACACTTCACTTATTACAGAAGGAAAAAGAAATTTGTTTCCTAAACTTGCTTTGCCCC 58696
Coquerel'sSifaka  CTTTGCTTC-AGTATGG----TGATCCAGTAAATGCTCAACAAGCAAAATTAG---TAAGTCTAGAGCTTTTTTTGCCAC 48746
Ma'sNightMonkey   TACTTATTC-GGTATCA--ATTCACACTTCACATACTAGAGAAGGAAAAAGAAATTTGTTTCCTAAACTTGCTTTGCCCC 54182
VervetAGM         TACTTATTCTGGTATCAGAACTCACACTTCACTTATTAG-GAAGGAAAAATAAATTTGTTTCCTAAACTTGCTTTGCCCC 60466
Gorrilla          TACTTATTCTGGTATCAGAACTCACACTTCACTTATTACAGAAGCAAAAAGAAATTTGTTTCCTAAACTTGCTTTGCCCC 59230
GrabEatingMacaqueTACTTATTCTGGTATCAGAACTCACACTTCACTTATTAGAGAAGGAAAAATAAATTTGTTTCCTAAACTTGCTTTGCCCC 59668
Macaque           TACTTATTCTGGTATCAGAACTCACACTTCACTTATTAGAGAAGGAAAAATAAATTTGTTTCCTAAACTTGCTTTGCCCC 60500
PigTailedMacaque  TACTTATTCTGGTATCAGAACTCACACTTCACTTATTAGAGAAGGAAAAATAAATTTGTTTCCTAAACTTGCTTTGCCCC 59390
Bonobo            TACTTATTCTGGTATCAGAACTCACACTTCACTTATTACAGAAGGAAAAAGAAATTTGTTTCCTAAACTTGCTTTGCCCC 57792
OliveBaboon       TACTTATTCTGGTATCAGAACTCACACTTCACTTATTAGAGAAGGAAAAATAAATTTGTTTCCTAAACTTGCTTTGCCCC 59243
Orangutan         TACTTATTCTGGTATCAGAACTCACACTTCACTTATTAGAGAAGGAAAAAGAAATTTGTTTCCTAAACTTGCTTTGCCCC 59669

Majority          ATTACCTTGTAATCTAGACCCTTTGGTAAAATTTACATTTTTGATAACTACAGTAATGT-TCATTTTACATGCCTGCATC

                          73130     73140     73150     73160     73170     73180     73190     73200

Human             ATTACCTTGTAATCTAGACCCTTTGGTAAAATTTGCATTTTTGATAACTACAGTAATGT-TCATTTTACTTGCCTGCATC 58775
Coquerel'sSifaka  GGTGGTTTTTTTTTT-----CATTGATATA-TATATATTTTG-----TATATTAATATGTCAATATAGCTAATTTTATT 48815
Ma'sNightMonkey   TTTACCTTGTAATCTAGACCCTTTGTTAAAATTTACATTTTTGATAACTAGAGTAATGT-TCATTTTACATGCCTGCATC 54261
VervetAGM         ATTACCTTGTAATCTAGACCCTTTGGTAAAATTTACATTTTTGATAACTAAGGTAATGT-TCATTTTACATGCTTGCATC 60545
Gorrilla          ATTACCTTGTAATCTAGACCCTTTGGTAAAATTTGCATTTTTGATAACTACAGTAATGT-TCATTTTACATGCCTGCATC 59309
GrabEatingMacaqueATTACCTTGTAATCTAGACCCTTTGGTAAAATTTACATTTTTGATAACTGCAGTAATGT-TCATTTTACATGCCTGCATC 59747
Macaque           ATTACCTTGTAATCTAGACCCTTTGGTAAAATTTACATTTTTGATAACTGCAGTAATGT-TCATTTTACATGCCTGCATC 60579
PigTailedMacaque  ATTACCTTGTAATCTAGACCCTTTGGTAAAATTTACATTTTTGATAACTACAGTAATGT-TCATTTTACATGCCTGCATC 59469
Bonobo            AGTACCTTGTAATCTAGACCCTTTGGTAAAATTTGCATTTTTGATAACTACAGTAATGT-TCATTTTACTTGCCTGCATC 57871
OliveBaboon       ATTACCTTGTAATCTAGACCCTTTGGTAAAATTTACATTTTTGATAACTACAGTAATGT-TCGTTTTACATGCCTGCATC 59322
Orangutan         ATTACCTTGTAATCTAGACCCTTTGGTAAAATTTACATTTTTGATAACTACAGTAATGT-TGATTTTACATGCCTGCATC 59748
```

```
Majority          TGGGCTCTACCTAGAGAATATCACATAATA--ACATAATCTGACAATTGGTACCAGTTTAAATCCATCAGCATCAACTAC
                           73210     73220     73230     73240     73250     73260     73270     73280
Human             TGGGCTCTACTTAGAGAATATCACATAATATTACATAATCTGACAATTGGTACCAGTTTAAATCCATCAACGTCAACTAC 58855
Coquerel'sSifaka  --AATCCTGTC---ATAATTTAGCATACCA---TGTAGCATTATAGATGGTA-------GAAATTGGTAGCATTAA--A- 48877
Ma'sNightMonkey   TGGGCTCTACCTAGAGAATATCACATAATATTATATAATCTGACAGTTGGTACCAGTTTAAATCCATCAGCATCAACTAC 54341
VervetAGM         TGGGCTTTACCTAGAGAATATCACATAATA--ACATAATCTGACAATTGGTACCAGTTTAAATCCATCAGCATCAACTAC 60623
Gorrilla          TGGGCTCTACTTAGAGAATATCACATAATATTACATAATCTGACAATTGGTACCGGTTTAAATCCATCAACATCAACTAC 59389
GrabEatingMacaqueTGGGCTTTACCTAGAGAATATCACATAATA--ACATAATTTGACAATTGGTACCAGTTTAAATCCATCAGCATCAACTAC 59825
Macaque           TGGGCTTTACCTAGAGAATATCACATAATA--ACATAATTTGACAATTGGTACCAGTTTAAATCCATCAGCATCAACTAC 60657
PigTailedMacaque  TGGGCTTTACCTAGAGAATATCACATAATA--ACATAATTTGACAATTGGTACCAGTTTAAATCCACCAGCATCAACTAC 59547
Bonobo            TGGGCTCTACTTAGAGAATATCACACAATATTACATAATCTGACAATTGGTACCGGTTTAAATCCATCAACATCAACTAC 57951
OliveBaboon       TGGGTTTTACCTAGAGAATATCACATAATA--ACATAATCTGACAATTAGTACCAGTTTAAATCCATCAGCATCAACTAC 59400
Orangutan         TGGGTTCTACTTAGAGAATATCACATAATATTACGTAATCTGACAATTGGTACCAGTTTAAATCCATCAGCATCAACTAC 59828

Majority          AAATAAGCCCTCTACTCTAGTTTAAAAATCTCTTCACATTTCTTCAGTCAACAGTTTTTCTGTTTTTCACAATGCCTCTA
                           73290     73300     73310     73320     73330     73340     73350     73360
Human             AAGTAAGCCCTCTACTCTACTTTAAAAATCTCTTCCCATTTCTTCAGTCAACAGTTTTTCTGTTTTTCACAATGCCTATG 58935
Coquerel'sSifaka  --ATAA-----------TATTAGGAAA-------------CCTTAGT-------------ATTTGTCATA--------- 48908
Ma'sNightMonkey   AAATAAGCCCTC-----TACTTTTAAAATCTCTTCACATTTCTTCAGTCAACA-TGTTTTTGTTTTTCATAATGCCTATA 54415
VervetAGM         AAAGAAGCCCCC-ACTCTAGTTTAAAAATCTCCTCACATTTCTTCAGTCAACAGTTTTTCTGTTTTTCACAATGCCTCTA 60702
Gorrilla          AAATAAGCCCTCTACTCTACTTTAAAAATCTCTTCCCATTTCTTCAGTCAACAGTTTTTCTGTTTTTCACAATGCCTACG 59469
GrabEatingMacaqueAAAGAAGCCCCCTACTCTAGTTTAAAAATCTCCTCACATTTCTTCAGTCAACAGTTTCTCTGTTTTTCACAGTGCCTCTA 59905
Macaque           AAAGAAGCCCCCTACTCTAGTTTAAAAATCTCCTCACATTTCTTCAGTCAACAGTTTCTCTGTTTTTCACAGTGCCTCTA 60737
PigTailedMacaque  AAAGAAGCCCCCTACTCTAGTTTAAAAATCTCCTCACATTTCTTCAGTCAACAGTTTCTCTGTTTTTCACAGTGCCTCTA 59627
Bonobo            AAATAAGCCCTCTACTCTACTTTAAAAATCTCTTCCCATTTATTCAGTCAACAGTTTTTCTGTTTTTCACAATGCCTATG 58031
OliveBaboon       AAAGAAGCCCCCTACTCTAGTTTAAAAATCTCCTCACATTTCTTCAGTCAACAGTTTTTCTGTTTTTCACAATGCCTCTA 59480
Orangutan         AAATAAGCCCTCTACTCTACTTTAAAAATCTCTTCACATTTCTTCAGTCAACAGTTTTTCTGTTTTTCACAATGCCTATA 59908

Majority          TCAGATAGAAATTTTCCCTTCTACCATATCTCTCGTCACTCTCACTTGCTTGAAGAAGAAAATCACATGGAAGCTTCATC
                           73370     73380     73390     73400     73410     73420     73430     73440
Human             TCAGATAGAAATTTTCCCTTCTACCATATCTCTCCTCACTCTCACTTGCTTGAAGAGGAAAATCACATGGAAGCTTCATC 59015
Coquerel'sSifaka  --AAAAAGAAATGTTTATTTTTAGACT----------------A----------------------ATGATAGCTGTG-- 48946
Ma'sNightMonkey   TCAGATAGAAATTTTCCCTTCTACCATATCTCTTCTCAATC--ACTTGCTTGAAGAAGAAAATCACATGAAAGCTTCATC 54493
VervetAGM         TCAGATAGAAATTTTCCCTTCTACCATATCTCTCGTCACTCTCACTTGCTTGAAGAAGAAAATCACATGGAAGCTTCATC 60782
Gorrilla          TCAGATAGAAATTTTCCCTTCTACCATATGTCTCCTCACTCTCACTTGCTTGAAGAGGAAAATCACATGGAAGCTTCATC 59549
GrabEatingMacaqueTCAGATAGAAATTTTCCCTTCTACCATATCTCTCGTCACTCTCACTTGCTTGAAGAAGAAAATCACATGGAAGCCTCATC 59985
Macaque           TCAGATAGAAATTTTCCCTTCTACCATATCTCTCGTCACTCTCACTTGCTTGAAGAAGAAAATCACATGGAAGCCTCATC 60817
PigTailedMacaque  TCAGATAGAAATTTTCCCTTCTACCATATCTCTCGTCACTCTCACTTGCTTGAAGAAGAAAATCACATGGAAGCCTCATC 59707
Bonobo            TCAGATAGAAATTTTCCCTTCTACCATGTCTCTTCTCACTCTCACTTGCTTGAAGAGGAAAATCACATGGAAGCTTCATC 58111
OliveBaboon       TCAGATAGAAATTTTCCCTTCTACCATATCTCTCGTCACTCTCACTTGCTTGAAGAAGAAAATCACATGGAAGCCTCATC 59560
Orangutan         TCAGATAGAAATTTTCCCTTCTACCATATCTCTCCTCACTCTCACTTGCTTGAAGAGGAAAATCACATGGAAGCTTCATC 59988
```

```
Majority          AGTTTTTCTTGCAAAATCTGCTAATACTCACACTTGTCTTTCTCTTCTAATAAAAGGCATAGATGTTTAGTTGTCTAAGA

                          73450     73460     73470     73480     73490     73500     73510     73520

Human             AGCTTTTCTTGCAAAATCTGCTAATACTCACACTTGTCTTTCTCTTTTAATAAAAGGCATAGATGTTTAGTTATCTAAGA 59095
Coquerel'sSifaka  ------------A---TTATCCATAACATAGACTTGCATTATTTT-----------ACATTTCTGTTTGATT---TAGGT 48997
Ma'sNightMonkey   AGCTTTTCTTGCAAAATCTGCTAATACTCA-ACTTGTCTTTCTCTTCTAATAAAAAGCATGCATGTTTAGTTGTCTAAGA 54572
VervetAGM         AGTTTTTCTTGCAAAATCTGCTAATACTCACACTTGTCTTTCTCTTCTAATAAAAGGCATAGATGTTTAGTTATCTAAGA 60862
Gorrilla          AGCTTTTCTTGAAAAATCTGCTAATACTCACACTTGTCTTTCTCTTTTAATAAAAGGCATAGATGTTTAGTTATCTAAGA 59629
GrabEatingMacaqueAGTTTTTCTTGCAAAATCTGCTAATACTCACACTTGTCTTTCTCTTCTAATAAAAGGCATAGATGTTTAGTTGTGTAAGA 60065
Macaque           AGTTTTTCTTGCAAAATCTGCTAATACTCACACTTGTCTTTCTCTTCTAATAAAAGGCATAGATGTTTAGTTGTGTAAGA 60897
PigTailedMacaque  AGTTTTTCTTGCAAAATCTGCTAATACTCACACTTGTCTTTCTCTTCTAATAAAAGGCATAGATGTTTAGTTGTGTAAGA 59787
Bonobo            AGCTTTTCTTGCAAAATCTGCTAATACTCACACTTGTCTTTCTCTTTTAATAAAAGGCATAGATGTTTAGTTATCTAAGA 58191
OliveBaboon       AGTTTTTCTTGCAAAATCTGCTAATACTCACACTTGTCTTTCTCTTCTAATAAAAGGCATAGATGTTTAGTTGTCTAAGA 59640
Orangutan         AGCTTTTCTTGCAAAATCTGCTAATACTCACACTTGTCTTTCTCTTTTAATAAAAGGCATAGATGTTTAGTTATCTAAGA 60068

Majority          CCAATAAACACCAAAGCCTATCGTCTTCTGCCTCTCAAGAGACTGCACTATCTGTCATCCCCTCTTTCTCATTTGTTTCT

                          73530     73540     73550     73560     73570     73580     73590     73600

Human             CCAATAAACACCAAAGCCTATCGTCTTCTGCCTGTCAAGAGACTGCACTATCTGTCATCCCCT--TTCTCATTTATTTCT 59173
Coquerel'sSifaka  ACAACAAAA-------------GTTTTGTGCTCTTGAGAAGATAGCAGGA-------T---------------------- 49035
Ma'sNightMonkey   CCAATAAACACCAAAGCCTATCATCTTCTGCCTTTCAGGAGACTGCACTATCAGTCATCCCCTCTTTCTCGTTTATTTAT 54652
VervetAGM         CCAATAAACACCAAAGCCTATCGTCTTCTGCCTCTCAAGAGACTGCACTATCTGTCATCCCCTCTTTCTCATTTGTTTCT 60942
Gorrilla          CCAATAAACACCAAAGCCTATCGTCTTCTGCCTCTCAAGAGACTGCACTATCTGTCCTCCCCT--TTCTCATTTATTTCT 59707
GrabEatingMacaqueCCAATAAACACCAAAGCCTATCGTCTTCTGCCTCTCAAGAGACTGCACTATCTGTCATCCCCTCTTTCTCATTTGTTTCT 60145
Macaque           CCAATAAACACCAAAGCCTATTGTCTTCTGCCTCTCAAGAGACTGCACTATCTGTCATCCCCTCTTTCTCATTTGTTTCT 60977
PigTailedMacaque  CCAATAAACACCAAAGCCTATCGTCTTCTGCCTCTCGAGAGACTGCACTATTTGTCATCCCCTCTTTCTCATTTGTTTCT 59867
Bonobo            CCAATAAACACCAAAGCCTATCGTCTTCTGCCTCTCAAGAGACTGCACTATCTGTCGTCCCCT--TTCTCATTTATTTCT 58269
OliveBaboon       CCAATAAACACCAAAGCCTATCGTCTTCTGCCTCTCAAGAGACTGCACTATCTGTCATCCCCTCTTTCTCATTTGTTTCT 59720
Orangutan         CCAGTAAACACCAAAGCCTATCGTCTTCTGCCTCTCAAGAGACTGCACTATCTGTCATCCCCTCTTTCTCATTTATTTTT 60148

Majority          CTCTATATTGGCCACTTTTCACCAGTTTCAAGCATTCTCAGCCTCTTTCATTTTAAATGAATTTACCTTCAACTTTCAAT

                          73610     73620     73630     73640     73650     73660     73670     73680

Human             CTCTATATTGGCCACTTTTCACCAGTTTCAAGCATTCTCAGCTTCTTTCATTTTAAATGAATTTACCTTCAACTTTCAAT 59253
Coquerel'sSifaka  ---TGTATTG----------------TGCATGCATACTTG---TAAAGGATTTTAAGTGG--------------TTT--C 49077
Ma'sNightMonkey   CTTTATACTGGCCACTTTTTGCCAGTTTCAAGCATTCTCAGCCTCTTTGATTTTAAATGAATTTACCCTCAACTTGGAAT 54732
VervetAGM         CTCTATGTTGGCCACATTTCACCAGTTTCAAGTATTCTCAGCCTCTTTCATTTTAAATGAATTTACCTTCAACTTTCAAT 61022
Gorrilla          CTCTATATTGGCCACTTTTCACCAGTTTCAAGCATTCTCAGCTTCTTTCATTTTAAATGAATTTACCTTCAACTTTCAAT 59787
GrabEatingMacaqueCTCTATGTTGGCCACTTTTCACCAGTTTCAAGTATTCTCAGCCTCTTACATTTTAAATGAATTTACCTTCAACTTTCAAT 60225
Macaque           CTCTATGTTGGCCACTTTTCACCAGTTTCAAGTATTCTCAGCCTCTTACATTTTAAATGAATTTACCTTCAACTTTCAAT 61057
PigTailedMacaque  CTCTATGTTGGCCACTTTTCACCAGTTTCAAGTATTCTCAGCCTCTTACATTTTAAATGAATTTACCTTCAACTTTCAAT 59947
Bonobo            CTCTATATTGGCCACTTTTCACCAGTTTCAAGCATTCTCAGCTTCTTTCATTTTAAATGAATTTACCTTCAACTTTCAAT 58349
OliveBaboon       CTCTATGTTGGCCACTTTTCACCAGTTTCAAGTATTCTCAGCCTCTTTCATTTTAAATGAATTTACCTTCAACTTTCAAT 59800
Orangutan         CACTATATTGGCCACTTTTCACCAGTTTCAAGCATTCTCAGCTTCTTTCATTTTAAATGAATTTATCTTCAACTTTCAAT 60228
```

```
Majority          CCATCCATCTATAACTCTACATACTTTCTATCACCCTGCCTCCCCTCTCCCTTTTCACTCAT-----GACTTCTTAAGTA

                          73690     73700     73710     73720     73730     73740     73750     73760

Human             CCATCCATCTATAACTCTACGTACTTTCTATCACCCTGCCTCCCCTCTCCCTTTTCACTCATAACCAGACTTCTTAAATA 59333
Coquerel'sSifaka  CAGAG-----GGAGTTTTA------------------------------------------------------------- 49091
Ma'sNightMonkey   CCATCCATCTGTAACTCTACATACTTTCTATCACCCTGCCTCTCTTCTCCCTTTTCATTCATAACCAGACTTCTTAAATA 54812
VervetAGM         CCATCCATCAATAACTCTACATACTTTCTGTCACCCTGCCTCCCCTCTCCCTTTTCACTCAT-----GACTT---AAGTA 61094
Gorrilla          CCATCCATCTATAACTCTACATACTTTCTATCACCCTGCCTCCCTTCTCCCTTTTCACTCATAACCAGACTTCTTAAACA 59867
GrabEatingMacaqueCCATCCATCTATAACTCTAC----TTTCTATCACCCTTCCTCCCCTCTCCCTTTTCACTCAT-----GACTTCTTAAGTA 60296
Macaque           CCATCCATCTATAACTCTAC----TTTCTATCACCCTTCCTCCCCTCTCCCTTTTCACTCAT-----GACTTCTTAAGTA 61128
PigTailedMacaque  CCATCCGTCTATAACTCTAC----TTTCTATCACCCTTCCTCCCCTCTCCCTTTTCACTCAT-----GACTTCTTAAGTA 60018
Bonobo            CCATCCATCTATAACTCTACATACTTTCTATCACCCTGCCTCCCCTCTCCCTTTTCACTCATAACCAGACTTCTTAAATA 58429
OliveBaboon       CCATCCATCTATAACTCTAC----TTTCTATCACCCTGCCTCCCCTCTCCCTTTTCACTCAT-----GACTTCATAAGTA 59871
Orangutan         CCATC-ATCTGTAACTCTACATACTTTCTATCACCCTGCCTCCCCTCTCCCTTTTCACTCATAACCAGACTTCTTAAATA 60307

Majority          GGCTATCTTTGTTGTTTATT--CTC---ATTT-CTCTACTTACTATAATTTGGTTTTAGCACTGTTGACCCAAATTGCTC

                          73770     73780     73790     73800     73810     73820     73830     73840

Human             GTCTATCTTTGTTGTTTATTATCTC---ATTT-CTCTACTTACTATTATTTGGTTTTAGCACTGTTGACCCAAATTGCTC 59409
Coquerel'sSifaka  -------------------------------------------------------------------------------- 49091
Ma'sNightMonkey   GGCTATCTTTGTTGTTTATTATCTCCTCATTTTCTCTACTTATTATAATTTGGTTTTAGCACTGTTTACCCAAATTGCTC 54892
VervetAGM         GGCTATCCTTGTTGTTTATT--CTC---ATTT-CTCTACTTACTATAATTTGGTTTTAGCACTGTTGACCCAAGTTACTC 61168
Gorrilla          GGCTATCTTTGTTGTTTATTATCTCCTCATTT-CTCTACTTACTATTATTTGGTTTTAGCACTGTTGACCCAAATTGCTC 59946
GrabEatingMacaqueGGCTATCCTTGTTGTTTATT--CTC---ATTT-CTCTACTTACTATAATTTGGTTTTAGCACTGTTGACCCAAATTACTC 60370
Macaque           GGCTATCCTTGTTGTTTATT--CTC---ATTT-CTCTACTTACTATAATTTGGTTTTAGCACTGTTGACCCAAATTACTC 61202
PigTailedMacaque  GGCTATCCTTGTTGTTTATT--CTC---ATTT-CTCTACTTACTATAATTTGGTTTTAGCACTGTTGACCCAAATTACTC 60092
Bonobo            GGCTATCTTTGTTGTTTATTATCTCCTCATTT-CTCTACTTACTATTATTTGGTTTTAGCACTGTTGACCCAAATTGCTC 58508
OliveBaboon       GGCTATCCTTGTTGTTTATT--CTC---ATTT-CTCTACTTACTATAATTTGGTTTTAGCACTGTTGACCCAAATTACTC 59945
Orangutan         GGCTATCTTTGTTGTTTATTATCTCCTCATTT-CTCTACTTACTATAATTTGGTTTCAGCACTGTTGACCCAAATTGCTC 60386

Majority          TTCCCCAGTGTCACCAGTGATT-ATTAGAGTAGCTAAATGCTAGTGGTATGATTCAGTCCTTGTCTTACTTAATATCTAG

                          73850     73860     73870     73880     73890     73900     73910     73920

Human             TTCCTCAGCGTCACCAGCGATT-AGTAGAGTAGCTAAATGCTAGTGATATGATTTAGTCCTTGTCTTACTTAATACCTAG 59488
Coquerel'sSifaka  -------------------------------------------------------------------------------- 49091
Ma'sNightMonkey   TTCC-CAGTGTTACCAGTGATT-ATTAGAGTAGCTAAATGCCAGTGATACGATTCAGTCCATACCTTACTTAATATTTAG 54970
VervetAGM         TTCCCCAGTGCCACCAGTGATT-ATTAGAGTAGCTAAATGCTAGTGGTATGATTCAGTCCTTGTCTTACTTAATATCTAG 61247
Gorrilla          TTCCTCAGCGTCACCAGTGATT-AGTAGAGTAGCTAAATGCTAGTGATATGATTTAGTCCTTGTCTTACTTAATACCTAG 60025
GrabEatingMacaqueTTCCCCAGTGCCACCAGTGATT-ATTAGAGTAGCTAAATGCTAGTGGTATGATTCAGTCCTTGTCTTACTTAATATCTAG 60449
Macaque           TTCCCCAGTGCCACCAGTGATT-ATTAGAGTAGCTAAATGCTAGTGGTATGATTCAGTCCTTGTCTTACTTAATATCTAG 61281
PigTailedMacaque  TTCCCCAGTGCCACCAGTGATT-ATTAGAGTAGCTAAATGCTAGTGGTATGATTCAGTCCTTGTCTTACTTAATATCTAG 60171
Bonobo            TTCCTCAGCATCACCAGTGATT-AGTAGAGTAGCTAAATGCTAGTGATATGATTTAGTCCTTGTCTTACTTAATACCTAG 58587
OliveBaboon       TTCCCCAGTGCCACCAGTGATT-ATTAGAGTAGCTAAATGCTAGTGGTATGATTCAGTCCTTGTCTTACTTAATATCTAG 60024
Orangutan         TTTCCCAGTGTCACCAGTGATTTATTAGAGTAGCTAAATGCTAGTGATATGATTTAGTCCTTGTCTTACTTAATATCTAG 60466
```

```
Majority          ATAATATTTGGCACTGTGCACCATTGTCTTAAAATTCTCTTTCTTCCTGGTCTCTGATACTTCTCACCTAGATTTATCCT
                          73930     73940     73950     73960     73970     73980     73990     74000
Human             ATAATATTTGGCACTGTGCACCATTGTCTTAAAATTCTCTTTCTTCCTGGTCTCTGATACTTCTCACCTGGATTTATCCT 59568
Coquerel'sSifaka  -------------------------------------------------------------------------------- 49091
Ma'sNightMonkey   ATAATAATTGGTGCTGTATACCATTGTCTTAAAATTCTTTCTCTTCCTGGTCTCTGATACTTCTCACCTAGATTTACCCT 55050
VervetAGM         ATAATATTTGACACTGTGCACCATTATCTTAAAATTCTCTTTCTTCCTGGTCTCTGATACTTCTCACGTAGATTTATCCT 61327
Gorrilla          ATAATATTTGGCACTGTGCACCATTGTCTTAAAATTCTCTTTCTTCCTGGTCTCTGATACTTCTCACCTGGATTTATCCT 60105
GrabEatingMacaqueATAATATTTGACACTGTGCACCATTATCTTAAAATTCTCTTTCTTCCTGGTCTCTGATACTTCTCACCTAGATTTATCCT 60529
Macaque           ATAATATTTGACACTGTGCACCATTATCTTAAAATTCTCTTTCTTCCTGGTCTCTGATACTTCTCACCTAGATTTATCCT 61361
PigTailedMacaque  ATAATATTTGACACTGTGCACCCTTATCTTAAAATTCTCTTTCTTCCTGGTCTCTGATACTTCTCACCTAGATTTATCCT 60251
Bonobo            ATAATATTTGGCACTGTGCACCATTGTCTTAAAATTCTCTTTCTTCCTGGTCTCTGATACTTCTCACCTGGATTTATCCT 58667
OliveBaboon       ATAATATTTGACACTGTGCACCATTATCTTAAAATTCTCTTTCTTCCTGGTCTCTGATACTTCTCACCTAGATTTATCCT 60104
Orangutan         ATAATATTTAGCACTGTGCACCATTGTCTTAAAATTCTCTTTCTTCCTGGTCTCTGATACTTCTCACGTAGGTTTATCCT 60546

Majority          ACCTCTCTGAC--TTTCTTCCTTGTCATGTCACTCCCCTTCTACCTGGGAGGCCTCACCTGCTCTCACAGCTCCGTTAAA
                          74010     74020     74030     74040     74050     74060     74070     74080
Human             ACCTCTCTA----TTTCTTCCTTGTCTTGTAACTCCCCTTCTACCTGGGAGGCCTCACCTGCTGTCACAGCTCCATTAAA 59644
Coquerel'sSifaka  -------------------------------------------------------------------------------- 49091
Ma'sNightMonkey   ACCTCTCTGGC--TATCTTCCTTGTCAGGTT-CTCCCCTTCTGCCTGGGAGGCCTCACCTGCTCTCACAGGTCAGTTAAA 55127
VervetAGM         ACCCCTCTGACTGTTTCTTCCTTGTCATGTCACTCCCCTTCTACCTGGGAGGCCTCACCTGCTCTCACACCTCTGTTAGA 61407
Gorrilla          ACCTCTCTA----TTTCTTCCTTGTCTTGTAACTCCCCTTCTACCTGGGAGGCCTCACCTGCTGTCACAGCTCCATTAAA 60181
GrabEatingMacaqueACCCCTCTGACTGTTTCTTCCTTGTCATGTCACTCCCCTTCTACCTGGGAGGCCTCACCTGCTCTCACAGCTCCGTTAAA 60609
Macaque           ACCCCTCTGACTGTTTCTTCCTTGTCATGTCACTCCCCTTCTACCTGGGAGGCCTCACCTGCTCTCACAGCTCCGTTAAA 61441
PigTailedMacaque  ACCCCTCTGACTGTTTCTTCCTTGTCATGTCACTCCCCTTCTACCTGGGAGGCCTCACCTGCTCTCACAGCTCCGTTAAA 60331
Bonobo            ACCTCTCTA----TTTCTTCCTTGTCTTGTAACTCCCCTTCTACCTGGGAGGCCTCACCTGCTGTCACAGCTCCATTAAA 58743
OliveBaboon       ACCCCTCTGACTGTTTCTTCCTTGTCATGTCACTCCCCTTCTACCTGGGAGGCCTCACCTGCTCTCACAGCTCCGTTAAA 60184
Orangutan         ACCTCTCTA----TTTCTTCCTTGTCTTGTAACTCCCCTTCTACCTGGGAGGCCTCACCTGCTCTCACAGCTCCATTAAA 60622

Majority          CCTCT-GTGATTCAGACAGTGTTCACATCACATTTTCTAGCTCAGACCTTTCCTAAGAGACCCACACTAAGAACCTGGCA
                          74090     74100     74110     74120     74130     74140     74150     74160
Human             CCTCTTATGATTCAGACAGAGTTCACATCACATTTTCTAGCTCAGACCTCTCCTAAGAGACCCACACTAAGAACCTGGCG 59724
Coquerel'sSifaka  -------------------------------------------------------------------------------- 49091
Ma'sNightMonkey   CCTCT-GTGATTCA----GTGTTCACATCACATTTTCTAGCTCAGACCTCTCTTAAGAGACCCACGCTAGGAACCTGGCA 55202
VervetAGM         CCTCT-ATGATTCAGACAGTGTTCACATCACATTTTGTAGCTCAGACCTTTCCTAAGAGACCCACACTAAGAACCTGGCA 61486
Gorrilla          CCTCT-GTGATTCAGACAGAGTTCACGTCACATTTTCTAGCTCAGACCTCTCCTAAGAGACCCACACTAAGAACCTGGTG 60260
GrabEatingMacaqueCCTCT-GTGATTCAGACAGTGTTCACATCACATTTTCTAGCTCAGACCTTTCCTAAGAGACCCACACTAAGAACCTGGCA 60688
Macaque           CCTCT-GTGATTCAGACAGTGTTCACATCACATTTTCTAGCTCAGACCTTTCCTAAGAGACCCACACTAAGAACCTGGCA 61520
PigTailedMacaque  CCTCT-GTGATTCAGACAGTGTTCACATCACATTTTCTAGCTCAGACCTTTCCTAAGAGACCCACACTAAGAACCTGGCA 60410
Bonobo            CCTCT-GTGATTCAGACAGAGTTCACATCACATTTTCTAGCTCAGACCTCTCCTAAGAGACCCACACTAAGAACCTGGTG 58822
OliveBaboon       CCTCT-GTGATTCAGACAGTGTTCACATCACATTTTCTAGCTCAGACCTTTCCTAAGAGACCCACACTAAGAACCTGGCA 60263
Orangutan         CCTCT-GTGATTCAAACAGAGTTCACATCACATTTTCTAGCTCAGACCTCTCCTAAGAGACTCACACTAAGAACCTGGCG 60701
```

```
Majority          GTCTAATGATTATCTTCAAACTTAGTGTGTTCAATCCAGCTCCTGTTTTTGGTGTCTACTGT--TAGTAAAGTGCAAGAT
                          74170     74180     74190     74200     74210     74220     74230     74240
Human             GACTAATGAATATCTTCAAAC--AGTGTGTTCAATCTAGCTCCTGTTTTTGGTGTCCGCTGTGTTAGGAAAGTGCAAGAT 59802
Coquerel'sSifaka  -------------------------------------------------------------------------------- 49091
Ma'sNightMonkey   GACTAATGAATATCTTCAAACTTAGTGTGTTCAATCCAACTCCTGTTTTTGATGTCCCCTGTGTTAGTAAAGTGCAAGAT 55282
VervetAGM         GTCTAATGATTATCTTCAAACTTAGTGTGTTCAATCCAGCTCCTGTTTTTGGTGTCTACTGT--TAGTAAAGTGCAAGAT 61564
Gorrilla          GACTAATGAATATCTTCAAACTTAGTGTGTTCAATCTAGCTCCTGTTTTTGGTGTCCGCTGTGTTAGTAAAGTGCAAGAT 60340
GrabEatingMacaqueGTCTAATGATTATCTTCAAACTTAGTGTGTTCAATCCAGCTCCTGTTTTTGGTGTCTACTGT--TAGTAAAGTGCAAGAT 60766
Macaque           GTCTAATGATTATCTTCAAACTTAGTGTGTTCAATCCAGCTCCTGTTTTTGGTGTCTACTGT--TAGTAAAGTGCAAGAT 61598
PigTailedMacaque  GTCTAATGATTATCTTCAAACTTAGTGTGTTCAATCCAGCTCCTGTTTTTGGTGTCTACTGT--TAGTAAAGTGCAAGAT 60488
Bonobo            GACTAATGAATATCTTCAAAC--AGTGTGTTCAATCTAGCTCCTGTTTTTGGTGTCCGCTGTGTTAGTAAAGTGCAAGAT 58900
OliveBaboon       GTCTAATGATTATCTTCAAACTTAGTGTGTTCAATCCAGCTCCTGTTTTTGGTGTCTACTGT--TAGTAAAGTGCAAGAT 60341
Orangutan         GACTAATGAATATCTTCAAACTTAGTGTGTTCAATCCAGCTCCTGTTTTTGGTGTCCACTGTGTTAGTAAAGTGCAAGAT 60781

Majority          ACCAACTTCAGTCTCATTAACAAGCCAGAAAACATGGTATTTTTCCCCTTCCTTCCAGCCTATCATTAGATATTATTGAT
                          74250     74260     74270     74280     74290     74300     74310     74320
Human             ACCAACTTCAGTCTCATTAACAAGCCAGTAAACATGGTATTTTTCCCTTTCCTTCCAACCTGTCATCAGATATTATTGAT 59882
Coquerel'sSifaka  -------------------------------------------------------------------------------- 49091
Ma'sNightMonkey   ACCAACTTCAGACTCATTAACAAGCCAGAAAGCAT---ATTTTTCCCTTTCCTTCCAGTCTATCTTCAGATACTATTGAT 55359
VervetAGM         ACCAACTTCAGTCTCATTAACAAGCCAGAAAACATGGTATTTTTCCCCTTCCTTCCAGCCTATCATTAGATATTATTGAT 61644
Gorrilla          ACCAACTTCAGTCTCATTAACAAGCCAGTAAACATGGTATTTTTCCCCTTCCTTCCAACCTGTCATCAGATATTATTGAT 60420
GrabEatingMacaqueACCAACGTCAGTCTCATTAACAAGCCAGAAAACATGGTATTTTTCCCCTTCCTTCCAGCCTATCATTAGATATTATTGAT 60846
Macaque           ACCAACGTCAGTCTCATTAACAAGCCAGAAAACATGGTATTTTTCCCCTTCCTTCCAGCCTATCATTAGATATTATTGAT 61678
PigTailedMacaque  ACCAACGTCAGTCTCATTAACAAGCCAGAAAACATGGTATTTTTCCCCTTCCTTCCAGCCTATCATTAGATATTATTGAT 60568
Bonobo            ACCAACTTCAGTCTCATTAACAAGCCAGTAAACATGGTATTTTTCCCCTTCCTTCCAACCTGTCATCAGATATTATTGAT 58980
OliveBaboon       ACCAACTTCAGTCTCATTAACAAGCCAGAAAACATGGTATTTTTCCCCTTCCTTCCAGCCTATCATTAGATATTATTGAT 60421
Orangutan         ACCAACTTCAGTCTCATTAACAAGCCAGTAAGCATGGTATTTTTCCCATTCTTGCCAGCCTTTCGTCAGATATTATTGAT 60861

Majority          TATACCTCC-TAAATTTTTGTTGAATCTGGCCTTTTTTCTCC-AACTCTTTTGCTGCCACTGCAGTCTAAGCC--AGTGT
                          74330     74340     74350     74360     74370     74380     74390     74400
Human             TATACCTCC-TAAATTTTTGTTGAATCTGGCCTTTTTTCTCC-AACTCTT--GCTACCATTGCAGTCTAAGCC--AGTGT 59956
Coquerel'sSifaka  -------------------------------------------------------------------------------- 49091
Ma'sNightMonkey   TATACCTCCCTAAATTTTTGTTGAATCTGGCCTTTTTTCTCTTAACTCTTTGGTTACCATTCCAGTATAAGCC--AGTGT 55437
VervetAGM         TATACCTCC-TAAATTTTTGTTGAATCTGGCCTTTTTTCTCC-AACTCTTTTGCTGCCACTACAGTCTAAGCC--AGTGT 61720
Gorrilla          TATACCTCC-TAAATTTTTGTTGAATCTGGCCTTTTTTCTCC-AACTCTT--GCTACCACTGCAGTCTAAGCG--AGTGT 60494
GrabEatingMacaqueTATACCTCC-TAAATTTTTGTTGAATCTGGCCTTTTTTCTCC-AACTCTTTTGCTGTCACTGCAGTCTAAGCC--AGTGT 60922
Macaque           TATACCTCC-TAAATTTTTGTTGAATCTGGCCTTTTTTCTCC-AACTCTTTTGCTGTCACTGCAGTCTAAGCC--AGTGT 61754
PigTailedMacaque  TATACCTCC-TAAATTTTTGTTGAATCTGGCCTTTTTTCTCC-AACTCTTTTGCTGCCACTGCAGTCTAAGCC--AGTGT 60644
Bonobo            TATACCTCC-TAAATTTTTGTTGAATCTGGCCTTTTTTCTCC-AACTCTT--GCTACCACTGCAGTCTAAGCC--AGTGT 59054
OliveBaboon       TATACCTCC-TAAATTTTTGTTGAATCTGGCCTTTTTTCTCC-AACTCTTTTGCTGCCACTGCAGTCTAAGCCCCAGTGT 60499
Orangutan         TATACCTCC-TAAATTTTTGTTGAATCTGGCCTTTTTTCTCC-AACTCTT--GCTACTACTGCAGTCTAAGCT--AGTGT 60935
```

```
Majority          TATTCAAGATGCTGGTCTCTCAAGGTAGT-AGTGTGTGTCACACTGAAATCAGCATACTTCTAGCAA-CAGTATGCTTAA
                          74410     74420     74430     74440     74450     74460     74470     74480
Human             TATTCAAGATGCTGGTCTCTCAAGGTAGT-AGTGTGTGTCAAACTGAAATCAGCATACTTCTAGCAA-CAGTATGCTTAA 60034
Coquerel'sSifaka  -------------------------------------------------------------------------------- 49091
Ma'sNightMonkey   TACTCAAGATGCTGGTTTCTCAAGGTAGG-AGTGTGTGTCAAACTGAAATCAGCATACTTCTAGCAA-CAGTATGCTTAA 55515
VervetAGM         TATTCAAGATGCTGGTCTCTCAAGGTAGTTAGTGTATGTCACACTGAAATCAGCATACTTCTAGCAAACAGTATACTTAA 61800
Gorrilla          TATTCAAGATGCTGGTCTCTCAAGGTAGT-AGTGTGTGTCCAACTGAAATCAGCATACTTCTAGCAA-CAGTATGCTTAA 60572
GrabEatingMacaqueTATTCAAGATGCTGGTCTCTCAAGGTAGTTAGTGTATGTCACACTGAAATCAGCATACTTCTAGCAAACAGTATACTTAA 61002
Macaque           TATTCAAGATGCTGGTCTCTCAAGGTAGTTAGTGTATGTCACACTGAAATCAGCATACTTCTAGCAAACAGTATACTTAA 61834
PigTailedMacaque  TATTCAAGATGCTGGTCTCTCAAGGTAGTTAGTGTATGTCACACTGAAATCAGCATACTTCTAGCAAACAGTATACTTAA 60724
Bonobo            TATTCAAGATGCTGATCTCTCAAGGTAGT-AGTGTGTGTCAAACTGAAATCAGCATACTTCTAGCAA-CAGTATGCTTAA 59132
OliveBaboon       TATTCAAGATGCTGGTCTCTCAAGGTAGTTAGTGTATGTCACACTGAAATCAGCATACTTCTAGCAAACAGTATACTTAA 60579
Orangutan         TATTCAAGATGCTGGTCTCTCAAGGTAGT-AGTGTGTGTCAAGCTGAAATCAGCATACCTCTAGCAA-CAGTATGCTTAA 61013

Majority          TGTCGATATTGACGTAAATTTTGACACAGACTT---ATATTCATGCAGAGTGGCCTGGGCTGCTGGACCGTGAATCATGA
                          74490     74500     74510     74520     74530     74540     74550     74560
Human             TGTCAATATTGGCATAAATTTTGACACAGACTTCTTATATTCATGCAGAGTAGCCTGGGCTTCTGGACCGTCAATCATAA 60114
Coquerel'sSifaka  -------------------------------------------------------------------------------- 49091
Ma'sNightMonkey   TGTCAATATTGACATAAATTTTGACACAAACTTCTTATATTCGTGCAGAGTAACCTGGATTGCTGAACCATGCATCATAA 55595
VervetAGM         TGTTGATATTGACGTAAATTTTGACACAGACTT---ATATTCATGCAGAGTGGCCTGGGCTGCTGGACCGTGAATCATGA 61877
Gorrilla          TGTCAATATTGGCATAAATTTTGACACAGACTTCTTACATTCATGCAGAGTAGCCTGGGCTTCTGGACCGTCAATCATAA 60652
GrabEatingMacaqueTGTCGATATTGACGTAAATTTTGACACAGACTT---ATATTCATGCAGAGTGGCCTGGGCTGCTGGACCGTGAATCATGA 61079
Macaque           TGTCGATATTGACGTAAATTTTGACACAGACTT---ATATTCATGCAGAGTGGCCTGGGCTGCTGGACCGTGAATCATGA 61911
PigTailedMacaque  TGTCGATATTGACGTAAATTTTGACACAGACTT---ATATTCATGCAGAGTGGCCTGGGCTGCTGGACCGTGAATCATGA 60801
Bonobo            TGTCAATATTGGCATAAATTTTGACACAGACTTCTTATATTCATGCAGAGTAGCCTGGGCTTCTGGACCGTCAATCATAA 59212
OliveBaboon       TGTCGATATTGACGTAAATTTTGACACAGACTT---ATATTCATGCAGAGTGGCCTGGGCTGCTGGACCGTGAATCATGA 60656
Orangutan         TGTTGATATTGGCATAAATTTTGACACAGACTTCTTATATTCATGCAGAGTAGCTTGGGCTTCTGGACCGTCAATCATAA 61093

Majority          TTTGAGTAGCATTGATGTAGTAATAGTTCTCTTTCATGTCTCTACTTCCTTGGCCTTCTGACTGAACTCACCATTTTGAC
                          74570     74580     74590     74600     74610     74620     74630     74640
Human             TTTAAGTAGCATTGATGTAGTAATAATTCTCTTTCATATCTCCACTTCCTTGGCCTTCTGACTGAACTCACCATTTTGAC 60194
Coquerel'sSifaka  -------------------------------------------------------------------------------- 49091
Ma'sNightMonkey   TTTTAGTAGCACTGATGTAGTCATCATTCTCTTTCATAGCTCTACTTCACTGGCCTTCTGACTGAACTCACCATTTTGAC 55675
VervetAGM         TTTGAGTAGCATTGATGTAGTAATAGTTCTCTTTCATGTCTCTACTTCCTTGGCCTTCTGACTGAACTCACCATTTTGAC 61957
Gorrilla          TTTAAGTAGCATTGATGTAGTAATAATTCTCTTTCATATCTCCACTTCCTTGGCCTTCTGACTGAACTCACCATTTTGAC 60732
GrabEatingMacaqueTTTGAGTAGCATTGATGTAGTAATAGTTCTCTTTCATGTCTCTCCTTCCTTGGCCTTCTGACTGAACTCACCATTTTGAC 61159
Macaque           TTTGAGTACCATTGATGTAGTAATAGTTCTCTTTCATGTCTCTCCTTCCTTGGCCTTCTGACTGAACTCACCATTTTGAC 61991
PigTailedMacaque  TTTGAGTAGCATTGATGTAGTAATAGTTCTCTTTCATGTCTCTCCTTCCTTGGCCTTCTGACTGAACTCACCATTTTGAC 60881
Bonobo            TTTAAGTAGCATTGATGTAGTAATAATTCTCTTTCATATCTCCACTTCCTTGGCCTTCTGACTGAACTCACCATTTTGAC 59292
OliveBaboon       TTTGAGTAACATTGATGTAGTAATAGTTCTCTTTCATGTCTCTACTTCCTTGGCCTTCTGACTGAACTCACCATTTTGAC 60736
Orangutan         TTTAAGTAGCATTGATGTAGTAATAATTCTCTTTCATATCTCTACTTCCTTGGCCTTCTGACTGAACTCACCATTTTGAC 61173
```

```
Majority          ATCTTCCTCTGCCTCCATTATTTTTCCACAGAGTACTAAGAATGATCTCATCCTC-AAGTCATATTGCTTCTAATCACAA

                          74650     74660     74670     74680     74690     74700     74710     74720

Human             ATCTTCCCCTACCTCCATTATTTTTCCACAGAGTACTAGGAATCATCTCAACGTC-AAGTCATATTGCTTCTAATCACAA 60273
Coquerel'sSifaka  -------------------------------------------------------------------------------- 49091
Ma'sNightMonkey   ATCTTCCTCTACCTCCATTGTTTTTCCACAGAGGACCAAAAATGATCTCAACTTCCAAGTCATATTGCTTTCAATCACAA 55755
VervetAGM         ATCTTCCTCTGCCTCCATTATTTTTCCACAGAGTACTAAGAATTATATCATCCTC-AAGTCATATTGCTTCTAATCACAA 62036
Gorrilla          ATCTTCCCCTACCTCCATTATTTTTCCACAGAGTACTAAGAATCATCTCAACGTC-AAGTCATATTTCTTCTAATCACAA 60811
GrabEatingMacaqueATCTTCCTCTGCCTCCATTATTTTTCCACAGAGTACTAAGAATGATATCATCCTC-AAGTCATATTGCTTCTAATCACAA 61238
Macaque           ATCTTCCTCTGCCTCCATTATTTTTCCACAGAGTACTAAGAATGATATCATCCTC-AAGTCATATTGCTTCTAATCACAA 62070
PigTailedMacaque  ATCTTCCTCTGCCTCCATTATTTTTCCACAGAGTACTAAGAATGATATCATCCTC-AAGTCATATTGCTTCTAATCACAA 60960
Bonobo            ATCTTCCCCTACCTCCATTATTTTTCCACAGAGTACTAAGAATCATCTCAACGTC-AAGTCATATTGCTTCTAATCACAA 59371
OliveBaboon       ATCTTCCTCTGCCTCCATTATTTTTCCACAGAGTACTAAGAATGATATCATCCTC-AAGTCATATTGCTTCTAATCACAA 60815
Orangutan         ATCTTCCCCTACCTCCATTATTTTTCCA--GAGTACTAAGAATCATCTCAACCTC-AAGTCATATTGCTTCTAATCACAA 61250

Majority          TACCCTTTAGTGACTTTTTTGTCCTTGTGATGAAGTCCACAGTTCTTATCATGG-------CTTATAAAACTCTAAATCA

                          74730     74740     74750     74760     74770     74780     74790     74800

Human             TACCCTTTAGTGACTTTTTTGTCCTTGCAATAAAGTCCACAGTCCTTATCATGG-------CTTATAAGACTCTAAATCA 60346
Coquerel'sSifaka  -------------------------------------------------------------------------------- 49091
Ma'sNightMonkey   TGCCCTTTAGTGACTTTGT-GTCCTTGTGATGAAGTCCACAGTCCTTATCATGG-------CTTACAAAACTCTAAATCA 55827
VervetAGM         TACCCTTTAGTGACTTTTTTGTCCTTGTGATGAAGTCCACAGTTCTTATCATGGATCGTGGCTTATAAAACTCTAAATCA 62116
Gorrilla          TACCCTTTAGTGACTTTTTTGTCCTTGCAATAAAGTCCACAGTCCTTATCATGG-------CTTATAAGACTCTAAGTCA 60884
GrabEatingMacaqueTACCCTTTAGTGACTTTTTTGTCCTTGTGATGAAGTCCACAGTTCTTATCATGGATCATGGCTTATAAAACTCTAAATCA 61318
Macaque           TACCCTTTAGTGACTTTTTTGTCCTTGTGATGAAGTCCACAGTTCTTATCATGGATCATGGCTTATAAAACTCTAAATCA 62150
PigTailedMacaque  TACCCTTTAGTGACTTTTTTGTCCTTGTGATGAAGTCCACAGTTCTTATCATGGATCATGGCTTATAAAACTCTAAATCA 61040
Bonobo            TACCCTTTAGTGACTTTTTTGTCCTTGCAATAAAGTCCACAGTCCTTATCATGG-------CTTATAAGACTCTAAATCA 59444
OliveBaboon       TACCCTTTAGTGACTTTTTTGTCCTTGTGATGAAGTCCACAGTTCTTATCATGGATCATGGCTTATAAAACTCTAAATCA 60895
Orangutan         TACCCTTTAGTGACTTTTTTGTCCTTGCAATAAAGTCCACAGTCCTTATCATGG-------CTTATAAGACTCTAAATCA 61323

Majority          GTCCTCAGCTTGCCTCTCCATTCTCCTCTCATGCCTTTTT-AGCTATATTGGAGTTCTTTAAATAGCTTGCATATACTGT

                          74810     74820     74830     74840     74850     74860     74870     74880

Human             GTCCTCAGCTTGCCTCTCCATTCTCCTCTCATGCCTTTTT-AGCTATATTGGACTTCTTTAAATAGCTTGCATATACTGT 60425
Coquerel'sSifaka  -------------------------------------------------------------------------------- 49091
Ma'sNightMonkey   GTCCTCAGCTTGCCTCTCCAGTCTCTTCTCATGACTTTTT-AGCTATTTTGGACTTCTTTAAATAGCTTCCATATACTGT 55906
VervetAGM         GTCCTCAGCTTGCCTCTCCATTCTCCTCTCATGCCTTTTT-AGCTATATTGGAGTTCTTTAAATAGCTTGCATACACTGT 62195
Gorrilla          GTCCTCAGCTTGCCTCTCCATTCTCCTCTCATGCCTTTTTTAGCTATATTGGACTTCTTTAAATAGCTTGCATATACTGT 60964
GrabEatingMacaqueGTCCTCAGCTTGCCTCTCCATTCTCCTCTCATGCCTTTTT-AGCTATATTGGAGTTCTTTAAATAGCTTGCATACACTGT 61397
Macaque           GTCCTCAGCTTGCCTCTCCATTCTCCTCTCATGCCTTTTT-AGCTATATTGGAGTTCTTTAAATAGCTTGCATACACTGT 62229
PigTailedMacaque  GTCCTCAGCTTGCCTCTCCATTCTCCTCTCATGCCTTTTT-AGCTATATTGGAGTTCTTTAAATAGCTTGCATACACTGT 61119
Bonobo            GTCCTCAGCTTGCCTCTCCATTCTCCTCTCATGCCTTTTT-AGCTATATTGGACTTCTTTAAATAGCTTGCGTATACTGT 59523
OliveBaboon       GTCCTCAGCTTGCCTCTCCATTCTCCTCTCATGCCTTTTT-AGCTATGTTGGAGTTCTTTAAATAGCTTGCATACACTGT 60974
Orangutan         GTCCTCAGCTTGCCTCTCCATTCTCCTCTCATGCCTTTTT-AGCTATATTGGACTTCTTTAAATAGCTTGCATATACTGT 61402
```

```
Majority          ATTTTCTCTGATGTCT-------------------TGATCTTACAGATTGCTGTTCCATTTGCTAATACTATTTTTT-CT
                          74890     74900     74910     74920     74930     74940     74950     74960
Human             ATTTTCTCTGATGTCT-------------------CGATCTTACAGATTGCTGTTCCATTTGCTAATACTATTTTTT-CT 60485
Coquerel'sSifaka  -------------------------------------AAATAATAAATT--TGGTTTTTGAGATAAT------TTGG--- 49123
Ma'sNightMonkey   ATTTTCTCTGATGTCTGGTAAAATCTCTGATGTCTCAGTCTTACAGATTGCTGTTCCATTTGCTAATACTATTTTTT-CT 55985
VervetAGM         ATTTTCTCTGATGTCT-------------------TGATCTTACAGATTGCTGTTCCATTTGCTAATACTATTTTTTTCT 62256
Gorrilla          ATTTTCTCTGATGTCT-------------------CGATCTTACAGATTGCTGTTCCATTTGCTAATACTATTTTTT-CT 61024
GrabEatingMacaqueATTTTCTCTGATGTCT-------------------TGATCTTACAGATTGCTGTTCCATTTGCTAATACTATTTTTT-CT 61457
Macaque           ATTTTCTCTGATGTCT-------------------TGATCTTACAGATTGCTGTTCCATTTGCTAATACTATTTTTT-CT 62289
PigTailedMacaque  ATTTTCTCTGATGTCT-------------------TGATCTTACAGATTGCTGTTCCATTTGCTAATACTATTTTTT-CT 61179
Bonobo            ATTTTCTCTGATGTCT-------------------CGATCTTACAGATTGCTGTTCCATTTGCTAATACTATTTTTT-CT 59583
OliveBaboon       ATTTTCTCTGATGTCT-------------------TGATCTTACAGATTGCTGTTCCATTTGCTAATACTATTTTTTTCT 61035
Orangutan         ATTTTCTCTGATGTCT-------------------CGATCTTACAGATTGCTGTTCCATTTGCTAATACTATTTTTT-CT 61462

Majority          CTTCATCTCTTTATGCCCAACTTCTGCTTCTCTTTGTCTTTCAAGTATCAGTTTAGAAGTCTCTTTCTGAGAGAAGTCTT
                          74970     74980     74990     75000     75010     75020     75030     75040
Human             CTTCATCTCTTTATGCCCAACTTCTGCTTCTCTTTGTCTTTCAAGTATCAGTTTAGAAGCCTCTTCCTGAGATAAGTCTT 60565
Coquerel'sSifaka  CTGAA-----ATATATCAA--------------------TACAGATGT-GGTTCAGATG-------CTATGATAGGCTCT 49170
Ma'sNightMonkey   CTTCATCTCTTTATGCCCAACTTCTGCTTATCTTCGTCTTTCAGGTATCAGTTTAGAAGTCTCTTCTTGAGAGAAGTCTT 56065
VervetAGM         CTTCATCTCTTTATGCCCAACTTCTGCTCCTCTTTGTCTTTCAAGCATTAGTTTAGAAGTCTCTTTCTGAGAGAAGTCGT 62336
Gorrilla          ATTCATCTCTTTATGCCCAACTTCTGCTTCTCTTTGTCTTTCAAGTATCAGTTTAGAAGCCTCTTCCTGAGATAAGTCTT 61104
GrabEatingMacaqueCTTCATCTCTTTATGCCCAACTTCTGCTTCTCTTTGTCTTTCAAGTATCGGTTTAGAAGTCTCTTTCTGAGAGAAGTCTT 61537
Macaque           CTTCATCTCTTTATGCCCAACTTCTGCTTCTCTTTGTCTTTCAAGTATCGGTTTAGAAGTCTCTTTCTGAGAGAAGTCTT 62369
PigTailedMacaque  CTTCATCTCTTTATGCCCAACTTCTGCTTCTCTTTGTCTTTCAAGTATCGGTTTAGAAGTCTCTTTCTGAGAGAAGTCTT 61259
Bonobo            CTTCATCTCTTTATGCCCAACTTCTGCTTCTCTTTGTCTTTCAAGTATCAGTTTAGAAGCCTCTTCCTGAGATAAGTCTT 59663
OliveBaboon       CTTCATCTCTTTATGCCCAACTTCTGCTTCTCTTTGTCTTTCAAGTATCAGTTTAGAAGTCTCTTTCTGAGAGAAGTCTT 61115
Orangutan         CTTCATCTCTTTATGCCCAACTTCTGCTTCTCTTTGTCTTTCAAGTATCAGTTTAGAAGCCTCTTCCTGAGATAAGTCTT 61542

Majority          CTGTAACATTTCAACTAGATTAGATCCCTTCCTTTCTTAGTTCTCAGTGCCACACATAAAATTTTTAATTTGTTAAATGT
                          75050     75060     75070     75080     75090     75100     75110     75120
Human             CTGTAACATTTCAACTAGATTAGATCCCTTCCTTTCTTAGTTCTCAGTATCACACATAAAATTTATAATTTGTTAAATGT 60645
Coquerel'sSifaka  CAGTTT-----------ATTTAGGTCTATATCCTCTATTCTTTTAAAGATATTTAGAAAACA---GATTTTCTGAGTAG 49236
Ma'sNightMonkey   CCGTAA------GACTAGATTAGATCCCTTCCTTTCTTAGTTCTCAGTAACACACATAAAATTTATAATTTGTTAAATGT 56139
VervetAGM         CTGTAACATTTCAACTAGATTAGATCCCTTCCTTTCTTAGTTATCAGTGCCACACATACAATTTTTAATTTGTTAAATGT 62416
Gorrilla          CTGTAACATTTCAACTAGATTAGATCCCTTCCTTTCTTAGTTCTCAGTATCACACATAAAATTTATAATTTGTTAAATGT 61184
GrabEatingMacaqueCTGTAACATTTCAACTAGATTAGATCCCTTCCTTTCTTAGTTATCAGTGCCACACATACAATTTTTAATTTGTTAAATGT 61617
Macaque           CTGTAACATTTCAACTAGATTAGATCCCTTCCTTTCTTAGTTATCAGTGCCACACATACAATTTTTAATTTGTTAAATGT 62449
PigTailedMacaque  CTGTAACATTTCAACTAGATTAGATCCCTTCCTTTCCCAGTTATCAGTGCCACACATACAATTTTTAATTTGTTAAATGT 61339
Bonobo            CTGTAACATTTCAACTAGATTAGATCCCTTCCTTTCTTAGTTCTCAGTATCACACATAAAATTTATAATTTGTTAAATGT 59743
OliveBaboon       CTGTAACATTTCAACTAGATTAGATCCCTTCCTTTCTTAGTTATCAGTGCCACACATACAATTTTTAATTTGTTAAATGT 61195
Orangutan         CTGTAACATTTCAACTGGATTAGATCCCTTCCTTTCTTAGTTCTCAGTATCACACATAAAATTTATAATTTGTTAAATGT 61622
```

```
Majority          GTATATTTTCTGTTGGACTGTAAGTTCTGAGTACCATGTCTGCTTTGTTCTCTGCTGTATTT---AAGCATTGACATTTA
                          75130     75140     75150     75160     75170     75180     75190     75200
Human             GTATATTTTCTGTCGGACTGTAAGTTCTGAGTACCATGTCTGCTTTGCTTTCTGCTGTATTTATTAAACATTGACATTCA 60725
Coquerel'sSifaka  --AT---TCCTAGTCTACTTTTTATTCCCATCATT-TGTCTTTTTTGTTGT-TGTTATGTAT---GGAGGGTGAGAGACA 49306
Ma'sNightMonkey   GTGTATTTTCTGTTGGACTGTAAGTTCTGAGTACCATGTCTGCTTTGTACTCTGCTATATTTCTTAAGCATTGACATTTA 56219
VervetAGM         GTATATTTTCTGTTGGACTGTAAGTTCTGAGTACCATGTCTGCTTTATTCTCTGCTGTATT----AAGCATTGACATTTA 62492
Gorrilla          GTATATTTTCTGTCGCACTGTAAGTTCTGAGTACCATGTCTGCTTTGCTTTCTGCTGTATTTATTAAACATTGACATTCA 61264
GrabEatingMacaqueGTATATTTTCTGTTGGACTGTAAGTTCTGAGTACCATGTCTGCTTTATTCTCTGCTGTATT----AAGCATTGACATTTA 61693
Macaque           GTATATTTTCTGTTGGACTGTAAGTTCTGAGTACCATGTCTGCTTTATTCTCTGCTGTATT----AAGCATTGACATTTA 62525
PigTailedMacaque  GTATATATTCTGTTGGACTGTAAGTTCTGAGTACCATGTCTGCTTTATTCTCTGCTGTATT----AAGCGTTGACATTTA 61415
Bonobo            GTATATTTTCTGTCGGACTGTAAGTTCTGAGTACCATGTCTGCTTTGCTTTCTGCTGTATTTATTAAACATTGACATTCA 59823
OliveBaboon       GTATATTTTCTATTGGACTGTAAGTTCTGAGTACCATGTCTGCTTTATTCTCTGCTGTATT----AAGCATTGACATTTA 61271
Orangutan         GTATATTTTCTGTTGGACTGTAAGTTCTGAGTACCATGTCTGCTTTGTTCTCTGCTGTATTTATTAAGCATTGACATTTA 61702

Majority          GGGTGAAAATCAGAAGTCATAGTAACAATCCTAATTATCAATAAAAAGTATTTTATTCGATCCTCATATTTATTAAAGGA
                          75210     75220     75230     75240     75250     75260     75270     75280
Human             GGGTGAAAATCAGAAGTCATAGTAACAATCTTAATTATCAATAAAAAGTATTTTATGCGATCCTCATATTTATTAAAGAA 60805
Coquerel'sSifaka  --ATTAAAAGAAGTAAAACTTGCCCATGTCATGACTACCATTGAAAACTGCGTAGCA-AATCTTTACTTCC--------A 49375
Ma'sNightMonkey   GGGTGATAATCAGAAATCATAGTGAGAACCCTAATTATCAATAAAAAGTATTTTATATGATCCTCATATTTATTAAAGAA 56299
VervetAGM         GGGTGAAAATCAGAAGTCATAGTAACAATCCTAATTATCTATAAAAAGTATTTTATTCGATCCTCATATTTATTAAAGGA 62572
Gorrilla          GGGTGAAAATCAGAAGTCATAGTAACAATCTTAATTATCAATAAAAAGTATTTTATGCGATCTTCATATTGATTAAAGAA 61344
GrabEatingMacaqueGGGTGAAAATCAGAAGTCATAGTAACAATCCTAATTATCTATAAAAAGTATTTTATTCGATCCTCATATTTATTAAAGGA 61773
Macaque           GGGTGAAAATCAGAAGTCATAGTAACAATCCTAATTATCTATAAAAAGTATTTTATTCGATCCTCATATTTATTAAAGGA 62605
PigTailedMacaque  GGGTGAAAATCAGAAGTCATAGTAACAATCCTAATTATCTATAAAAAGTATTTTATTCGATCCTCATATTTATTAAAGGA 61495
Bonobo            GGGTGAAAATCAGAAGTCATAGTAACAATCTTAATGATCAATAAAAAGTATTTTATGCGATCCTCATATTTATTAAAGAA 59903
OliveBaboon       GGGTGAAAATCAGAAGTCATAGTAACAATCGTAATTATCTATAAAAAGTATTTTATTCGATCCTCATATTTATTAAAGGA 61351
Orangutan         GGGTGAAAATCAGAAGTCATAGTAACAATCCTAATTATCAATAAAAAGTATTTTATGTGATCCTCATATTTATTAAAGAA 61782

Majority          AACACCTTAATACCTT-TTAGAAAATCTTGTTTATTTATTGGAAAGTAGTGTATTAGGCAAAGATGTGTCTTCT--TGGC
                          75290     75300     75310     75320     75330     75340     75350     75360
Human             AACACCTTAATACCTT-TCAGAAAATCTTGTTTATTTATTGGAAAGTAGTGTATTAGGCAAAGATGCGTCTTCTCTTGGC 60884
Coquerel'sSifaka  AATGCCTTAATCTTCTGTTTGTATATCTT--------ACTTAAATCTACTTT----G-C-----TCTATCTTTT--TAAC 49435
Ma'sNightMonkey   CACATCTTAATACCTT-TTAGAAAATCTTGTTTATTTTTGGAAAACAAT-----AGGCAAACATGTGGCTTCTCGTGGC 56373
VervetAGM         AACACCTTAATACCTT-TTAGAAAGTCTTGTTTATTTATTGGAAAGTAGTGTATTAGGCAAAGATGTGCCTTCT--TGGC 62649
Gorrilla          AACACCTTAATACCTT-TCAGAAAATCTTGTTTATTTATTGGAAAGTAGTGTATTAGGCAAAGATGCGTCTTCTCTTGGC 61423
GrabEatingMacaqueAACACCTTAATACCTT-TTAGAAAATCTT--------ATTGGAAAGTAGTGTATTAGGCAAAGATGTGCCTTCT--TGGC 61842
Macaque           AACACCTTAATACCTT-TTAGAAAATCTTGTTTATTTATTGGAAAGTAGTGTATTAGGCAAAGATGTGCCTTCT--TGGC 62682
PigTailedMacaque  AACACCTTAATACCTT-CTAGAAAATCTTGTTTATTTATTGGAAAGTAGTGTATTAGGCAAAGATGTGCCTTCT--TGGC 61572
Bonobo            AACACCTTAATACCTT-TCAGAAAATCTTGTTTATGTATTGGAAAGTAGTGTATTAGGCAAAGATGCGTCTTCTCTTGGC 59982
OliveBaboon       AACACCTTAATACCTT-TTAGAAAATCTTGTTTATTTATTGGAAAGTAGTGTATTAGGCAAAGATGTGCCTTCT--TGGC 61428
Orangutan         AACACCTTAATACCTT-TTAGAAAATCTTGTTTATTTATTGGAAAGTAGTGTATTAGGCAAAGATGTGTCTTCTCTTAGC 61861
```

```
Majority          ATTCTAATTCAGCTTTTGTAATGTTGAGGAATATAATTACTTGATGCATAAAAATATCAGTCTTTTTAA-CAGTATAATA
                          75370     75380     75390     75400     75410     75420     75430     75440
Human             TTTCTAATTCAGCTTTTATAATGTTGAGGAATATAATTACTTGATGCATAAAAATATCAATCTTTTTAA-TAGTATAATA 60963
Coquerel'sSifaka  TTTTTGTGCCATATATCTCGTTATTCCG--TT-------CTTAAGCCATTAAAAATTCA--TTATTAAACCATTATTAGG 49504
Ma'sNightMonkey   TTTCTAATTCAGCTTTTACAGTGTTGAGGAATGTAATTAGTTGATACATAAAAAAA-CAGTCTTTTTAA-TAGTATAATA 56451
VervetAGM         ATTCTAATTCAGCTTTTGTAATGTTGAGGACTATAATTACTTGATGCATAAAAATACCAGTCTTTTTAA-CAGTATAATA 62728
Gorrilla          TTTCTAATTCAGCTTTTATAATGTTGAGGAATATAATTACTTGATGCATAAAAATATCAATCTTTTTAA-TAGTATAATA 61502
GrabEatingMacaqueATTCTAATTCAGCTTTTGTAATGTTGAGGAATATAATTACTTGATGCATAAAAATACCAGTCTTTTTTA-CAGTATAATA 61921
Macaque           ATTCTAATTCAGCTTTTGTAATGTTGAGGAATATAATTACTTGATGCATAAAAATACCAGTCTTTTTTAACAGTATAATA 62762
PigTailedMacaque  ATTCTAATTCAGCTTTTGTAATGTTGAGGAATATAATTACTTGATGCATAAAAATACCAGTCTTTTTTA-CAGTATAATA 61651
Bonobo            TTTCTAATTCAGCTTTTATAATGTTGAGGAATATAATTACTTGATGCATAAAAATATCAATCTTTTTAA-TAGTATAATA 60061
OliveBaboon       ATTCTAATTCAGCTTTTGTAATGTTGAGGAATATAATTACTTGACGCATAAAAATACCAGTCTTTTTAA-CAGTATAATA 61507
Orangutan         ATTCTAATTCAGCTTTTAAAATGTTGAGGAATATAATTACTTGATGCATAAAAATATCAATCTTTTTAA-TAGTATAATA 61940

Majority          AGACAGCTAATGTAATCTCCTTTTCACCTGGGATT-TAAAAATTGTTTT-ACGTAGTCCTTACATTATAACACACATGAG
                          75450     75460     75470     75480     75490     75500     75510     75520
Human             AGACATATAATGTAATCTCCTTTTCACCTGGGATT-TAAAAATTGTTTTTACATAGTCCTTACATTATAACACACATAAG 61042
Coquerel'sSifaka  AAACCATTAAATCAACTGTCTTT--ACTTACATTTGTCTTACTTGTTTT----TCTCCCTTTCACTCTTTCTTTTACTTT 49578
Ma'sNightMonkey   AGACA--CAGTGTAATCTCCTTTTCACTTGGAATT-TAAAAATTATTTT-ATGTAGTCCTTAAATTACAACACACGTGAG 56527
VervetAGM         AGACAGCTAATGTAATCTCCTTTTCACCTGGGATT-TAAAAATTGTTTT-ACGTAGTCCTTACATTATAACACACATGAG 62806
Gorrilla          AGACATATAATGTAATCTCCTTTTCACCTGGGATT-TAAAAATTGTTTTTACATAGTCCTTACGTTATAACACACATAAG 61581
GrabEatingMacaqueAGACAGCTAATGTAATCTCCTTTTCACCTGGGATT-TAAAAATTGTTTT-ATGTAGTCCTTACATTATAACACACATGAG 61999
Macaque           AGACAGCTAATGTAATCTCCTTTTCACCTGGGATT-TAAAAATTGTTTT-ACGTAGTCCTTACATTATAACACACATGAG 62840
PigTailedMacaque  AGACAGCTAATGTAATCTCCTTTTCACCTGGGATT-TAAAAATTGTTTT-ACGTAGTCCTTACATTATAACACACATGAG 61729
Bonobo            AGACATATAATGTAATCTCCTTTTCACCTGGGATT-TAAAAATTGTTTTTACATAGTCCTTACATTATAACACACATAAG 60140
OliveBaboon       AGACAGCTAATGTAATCTCCTTTTCACCTGGGATT-TAAAAATTGTTTT-ACGTAGTCCTTACATTATAACACACATGAG 61585
Orangutan         AGACAGATAATGTAATCTCCTTTTCACCTGGGATT-TAAAAATTGTTTT-ACATAGTCCTTACATTATAACACACATAAG 62018

Majority          TAGGAAATGGTTCTTTTT-GGTTTTTGTTATTAGAAACAGCTTTTCAGAAACAT---GTGTTTATTAGGAATATCTTTAG
                          75530     75540     75550     75560     75570     75580     75590     75600
Human             TAGGAAATGGTTCTTTTT-GGTTTTTGTTATTAGAAACAGCTTTTCAGAAACAT---GTCTTTATTAGGAATATCTTTAG 61118
Coquerel'sSifaka  CAG--ACTAATTAT--GA---TCTCTAATACTTCAGT--GCTTCCTTCAGA--T-----------CAAGATTACTTCCAT 49636
Ma'sNightMonkey   TAGGAAATGGTTGTTTTTGTTTTTTTGTTAATAGAAACAGCTTTTCAGAAGCAT---GTCTTTATTAGGAATATCTTTAG 56604
VervetAGM         TAGGAAATGGTTCTTTTT-AGTTTTTGTTATTAGAAACAGCTTTTCAGAAACATTATGTGTTTATTAGGAATATCT--AG 62883
Gorrilla          TAGGAAATGGTTCTTTTT-GGTTTTTGTTATTAGAAACATCTTTTCAGAAACAT---GTCTTTATTAGGAATATCTTTAG 61657
GrabEatingMacaqueTAGGAAATGGTTCTTTTT-GGTTTTTGTTATTAGAAACAGCTTTTCAGAAACATTATGTGTTTTTTAGGAATATCT--AG 62076
Macaque           TAGGAAATGGTTCTTTTT-GGTTTTTGTTATTAGAAACAGCTTTTCAGAAACATTATGTGTTTTTTAGGAATATCT--AG 62917
PigTailedMacaque  TAGGAAATGGTTCTTTTTTGGTTTTTGTTATTAGAAACAGCTTTTCAGAAACATTATGTGTTTCTTAGGAATATCT--AG 61807
Bonobo            TAGGAAATGGTTCTTTTT-GGTTTTTGTTATTAGAAACAGCTTTTCAGAAACAT---GTCTTTATTAGGAATATCTTTAG 60216
OliveBaboon       TAGGAAATGGTTCTTCTT-GGTTTTTGTTATTAGAAACAGCTTTTCGGAAACATTATGTGTTTATTAGGAATATCT--AG 61662
Orangutan         TAGGAAATGGTTCTTTTC-AGTTTTTGTTATTAGAAACAGCTTTTCAGAAACAT---GTCTTTATTAGGAATATCTTTAG 62094
```

```
Majority          TTGACACTGGACAGTGATATTGTGTCTTCTGGAAGTCCTGTTTATATGATCATTTTATTTTAACATAT-AAGTGGGTTGT

                          75610     75620     75630     75640     75650     75660     75670     75680

Human             TTGACACTGGACAGT-ATATTGTGTCTTCTGGAAGTCCTGTTTATATGATCATTTTATTTTAACATAT-AAGTGGGTTGT 61196
Coquerel'sSifaka  GGAACCCAGAACATT--TTTTGTTTCTGTTTCA--TTGCATTTCTATGCTCAG---AATCTCATTTG----GGAGGCAAA 49705
Ma'sNightMonkey   TTGACACTGGACATTGATACTGTGTCTTTTGGAAGTCCTGTTTATATGATCATTTTATTTTAACATAT-AAGTGGGTTGT 56683
VervetAGM         TTGACACTGGACAGTGATATTGTGTCTTCTGGAAGTCCTGTTTATATGATCATTTTATTTTAACATAT-AAGTGGGTTGT 62962
Gorrilla          TTGACACTGGACAGT-ATATTGTGTCTTCTGGAAGTTCTGTTTATATGATCATTTTATTTTAACATATTAAGTGAGTTGT 61736
GrabEatingMacaqueTTGACACTGGACAGTGATATTGTGTCTTCTGGAAGTCCTGTTTATATGATCATTTTATTTTAACGTAT-AAGTGGGTTGT 62155
Macaque           TTGACACTGGACAGTGATATTGTGTCTTCTGGAAGTCCTGTTTATATGATCATTTTATTTTAACGTAT-AAGTGGGTTGT 62996
PigTailedMacaque  TTGACACTGGACAGTGATATTGTGTCTTCTGGAAGTCCTGTTTATATGATCATTTTATTTTAACGTAT-AAGTGGGTTGT 61886
Bonobo            TTGACACTGGACAGT-ATATTGTGTCTTCTGGAAGTCCTGTTTATATGATCATTTTATTTTAACATAT-AAGTGGGTTGT 60294
OliveBaboon       TTGACACTGGACAGTGATATTGTGTCTTCTGGAAGTCCTGTTTATATGATCATTTTATTTTAACGTAT-AAGTGGGTTGT 61741
Orangutan         TTGACACTGGACAGT-ATATTGTGTCTTTTGGAAGTCCTGTTCATATGATCATTTTATTTTAACATAT-AAGTGGGTTGA 62172

Majority          TTCTTATTATTTATTTCTTCCAGGAGATTCCATTTCATCACTAA----TAATAAAAATAAATATGAGGTAAAACATTTGT

                          75690     75700     75710     75720     75730     75740     75750     75760

Human             TTCTTATTCTTTATTTCTTCCAGGAGATTCCATTTCATCACTAACTGATAATAAAAATAAATATGAGATAAAACATTTGC 61276
Coquerel'sSifaka  -----AATAGCTTTAGGTAGCAGAA-AG---GCTGTATAAGTAA----TAATACAATTGTA-ATAGTTTACCAA----GT 49767
Ma'sNightMonkey   TTCTTATTATTTATTTCTTCTAGGAGATTGTATTTCATCACTAAC--ATAATAAAAATAAATATGAGGTAAAAA------ 56755
VervetAGM         TTCTTATTATTTATTTCTTCCAGGAGATTCCATTTCATCACTAA----TAATAAAAATAAATATGAGGTAAAACATTTGT 63038
Gorrilla          TTCTTATTCTTTATTTCTTCCAGGAGATTCCATTTCATCACTAACTGATAATAAAAATAAATATGAGATAAAACATTTGC 61816
GrabEatingMacaqueTTCTTATTATTTATTTCTTCCAGGAGATTCCATTTCATCATTAA----TAATAAAAATAAATATGAGGTAAAACATTTGT 62231
Macaque           TTCTTATTATTTATTTCTTCCAGGAGATTCCATTTCATCACTAA----TAATAAAAATAAATATGAGGTAAAACATTTGT 63072
PigTailedMacaque  TTCTTATTATTTATTTCTTCCAGGAGATTCCATTTCATCACTAA----TAATAAAAATAAATATGAGGTAAAACATTTGT 61962
Bonobo            TTCCTATTCTTTATTTCTTCCAGGAGATTCCATTTCATCACTAACTGATAATAAAAATAAATATGAGATAAAACATTTGC 60374
OliveBaboon       TTCTTATTATTTATTTCTTCCAGGAGATTCCATTTCATCACTAA----TAATAAAAATAAATATGAGGTAAAACATTTGT 61817
Orangutan         TTCTTATTATTTATTTCTTCCAGGAGATTCCATTTCATCACTAACTGATAATAAAAATAAATATGAGATAAAACATTTGC 62252

Majority          GAAAGTAAGATTATGTCATTCATTTCCTTCTCGCAGAGTACACATTGCTTTATGCAGATTCTTTGAGAGACCTAATGGAA

                          75770     75780     75790     75800     75810     75820     75830     75840

Human             AGCAGTAAGATTATGTCATTCATTTCCTTCTCACAGAGTACACATTGCTTTATGCAGATTCTTTGAGAGACCTAATGGAA 61356
Coquerel'sSifaka  ---------GCTATAATCTCATTTTTTTTCT----GA-C---CATTACTTTATA-----TTCTAAACAGAACAGGTTTT- 49824
Ma'sNightMonkey   -----------TATATCATTCATTTTCTTCTCACAGAGTAAGCATTGTTTTATGCAGAGTCTTTGAGACAACTAATGGAA 56824
VervetAGM         GAAAGTAAGATTATGTCATTCATTTCCTTCTCGCAGAGTACACATTGCTTTATGCAGATTCTTTGAGAGACCTAATGGAA 63118
Gorrilla          AGCAGTAAGATTATGTCATTCATTTCCTTCTCACAGAGTACACATTGCTTTATGCAGATTCTTTGAGAGACCTAATGGAA 61896
GrabEatingMacaqueGAAAGTAAGATTGTGTCATTCATTTCCTTCTCGCAGAGTACACATTGCTTTATGCAGATTCTTTGAGAGACCTAATGGA- 62310
Macaque           GAAAGTAAGATTGTGTCATTCATTTCCTTCTCGCAGAGTACACATTGCTTTATGCAGATTCTTTGAGAGACCTAATGGA- 63151
PigTailedMacaque  GAAAGTAAGATTGTGTCATTCATTTCCTTCTCGCAGAGTACACATTGCTTTATGCAGATTCTTTGAGAGACCTAATGGA- 62041
Bonobo            AGCAGTAAGATTATGTCATTCATTTCCTTCTCACAGAGTACACATTGCTTTATGCAGATTCTTTGAGAGACCTAATGGAA 60454
OliveBaboon       GAAAGTAAGATTGTGTCATTCATTTCCTTCTCGCAGAGTACACATTGCTTTATGCAGATTCTTTGAGAGACCTAATGGA- 61896
Orangutan         GGCAGTAAGATTATGTCATTCATTTTCTTCTCACAGAGTACACATTGCTTTATGCACATTCTTTGAGAGACCTAGTGGAA 62332
```

```
Majority        TTGTCCTTTCCTAATATCTTGGGCAAAGATTCTGCTTGACATCTGCTTAATTTACAATCTGTATATTTTACATTCATATT
                        75850     75860     75870     75880     75890     75900     75910     75920
Human           TTATCCTTTGCTAATTTCTTGGGCAAAGATTCTCCTTGACATCTGCTTAATTTACAATCTGTATATTTTACATTCATATT 61436
Coquerel'sSifaka TCTTCCTCTGTTACAACAGAATGCTAAGATTTTTC---AGAGGTGAATAATTTA---T---CATAATGCACACT------ 49889
Ma'sNightMonkey TTATCCTTTCCTATTTTCTTGGGCAAAGATTTTCCTTGAC-TGTGTTTAATTTACAGTGTATATATTTTACATTCATATT 56903
VervetAGM       TTGTCCTTTCCTAATATCTTGGGCAAAGATTCTGCTTGACATCTGCTTAATTTACAATCTGTATATTTTACATTCATATT 63198
Gorrilla        TTATCCTTTCCTAATTTCTTGGGCAAAGATTCTCCTTGACATCTGCTTAATTTACAGTCTGTATATTTTACATTCATATT 61976
GrabEatingMacaqueTTGTCCTTTCCTAATATCTTGGGCAAAGATTCTGCTTGACATCTGCTTAATTTACAATCTGTATATTTTACATTCATATT 62390
Macaque         TTGTCCTTTCCTAATATCTTGGGCAAAGATTCTGCTTGACATCTGCTTAATTTACAATCTGTATATTTTACATTCATATT 63231
PigTailedMacaque TTGTCCTTTCCTAATATCTTGGGCAAAGATTCTGCTTGACATCTGCTTAATTTACAATCTGTATATTTTACATTCATATT 62121
Bonobo          TTATCCTTTCCTAATTTCTTGGGCAAAGATTCTCCTTGACATCTGCTTAATTTACAATCTGTATATTTTACATTCATATT 60534
OliveBaboon     TTGTCCTTTCCTAATATCTTGGGCAAAGATTCTGCTTGACATCTGCTTAATTTACAATCTGTATATTTTACATTCATGTT 61976
Orangutan       TTATCCTTTCCTAATTTCTTGGGCAAAGATTCTCCTTGACATCTGCTTAATTTACAATCTGTATATTTTACATTCATATT 62412

Majority        AATGGAGAATTTATGATGAAATCAGTGGTTTTAGGATGCTTTAGGAAAATCCTGCATAATCTACCAGGGTGGGGTT----
                        75930     75940     75950     75960     75970     75980     75990     76000
Human           AATGGAGAATTTATGATGAAATCAGTGGTTTTAGGATGCTTTAGGAAAATCCTGCATAATCTACCAGGGTGGGGTTATTT 61516
Coquerel'sSifaka --------ATCTGTAAGGTAGAGATTTTTTTCAGTCTACCTTTCAGATAGCCTTTCT----TTCCA--------TT---- 49945
Ma'sNightMonkey AATGGAGGATTTATGATGAAATCAATTATTTTAAGATGCTTTAGGAAAATCCTGCATAATCTACCAGG-TCAGGTTATTT 56982
VervetAGM       AATGGTGAATTTATGATGAAATCAGTGGTTTTAGGATGCTTTAGGAAAATCCTGCATAATCTACCAGGGTGGAGTT---- 63274
Gorrilla        AATGGAGAATTTATGATTAAATCAGTGGTTTTAGGATGCTTTAGGAAAATCCTGCATAATCTACCAGGGTGGGGTTATTT 62056
GrabEatingMacaqueAATGGAGAATTTATGATGAAATCAGTGGTTTTAGGATGCTTTAGGAAAATCCTGCATAATCTACCAGGGTGGAGTT---- 62466
Macaque         AATGGAGAATTTATGATGAAATCAGTGGTTTTAGGATGCTTTAGGAAAATCCTGCATAATCTACCAGGGTGGAGTT---- 63307
PigTailedMacaque AATGGAGAATTTATGATGAAATCAGTGGTTTTAGGATGCTTTAGGAAAATCCTGCATAATCTACCAGGGTAGAGTT---- 62197
Bonobo          AATGGAGAATTTATGATGAAATCAGTGGTTTTAGGATGCTTTAGGAAAATCCTGCATAATCTACCAGGGTGGGGTTATTT 60614
OliveBaboon     AATGGAGAATTTATGATGAAATCAGTGGTTTTAGGATGCTTTAGGAAAATCCTGCATAATCTACCAGGGTGGAGTT---- 62052
Orangutan       AATGGAGAATTTATGATGAAATCAGTGGTTTTAGGATGCTTTAGGAAAATCCTGCATAATATACCAGG--GGGGTTATTT 62490

Majority        GATATTGGTAAATAATTAGTCTGAAGTATTACATTAGTATTATAAGCTTAATTTTGTGTTGTTGATTTTGATAATTTTTC
                        76010     76020     76030     76040     76050     76060     76070     76080
Human           GATATTGGTAAATAATTAGTCTGAAGTATTACATTAGTATTATAAGCTTAATTTTGTGTTGTTGATTTTGATAATTTTTC 61596
Coquerel'sSifaka -GTGTTGCTACTCACTGGTTCTCAGGT--TA------T---ATAATCT-AATC-------AT--ACTATGATTGTCTTTT 50003
Ma'sNightMonkey GATATTCGTAAATAATTAGTCTAAAGTATTA-----GTAGTATAAGCTTAATTTTGTGTTGTTGATTCTGATAATTTTTC 57057
VervetAGM       GATAGTGGTAAATAATTAGTCTGAAATATTACATTAGTATTATAAGCTTAATTTTGTGTTGTTGATTTTGATAATTTTTC 63354
Gorrilla        GATATTGGTAAATAATTAGTCTGAAGTATTACATTAGTATTATAAGCTTAATTTTGTGTTGTTGATTTTGATAATTTTTC 62136
GrabEatingMacaqueGATAGTGGTAAATAATTAGTCTGAAATATTACATTAGTATTATAAGCTTAATTTTGTGTTGTTGATTTTGATAATTTTTC 62546
Macaque         GATAGTGGTAAATAATTAGTCTGAAATATTACATTAGTATTATAAGCTTAATTTTGTGTTGTTGATTTTGATAATTTTTC 63387
PigTailedMacaque GATAGTGGTAAATAATTAGTCTGAAATATTACATTAGTATTACAAGCTTAATTTTGTGTTGTTGATTTTGATAATTTTTC 62277
Bonobo          GATATTGGTAAATAATTAGTCTGAAGTATTACATTAGTATTATAAGCTTAATTTTGTGTTGTTGATTTTGATAATTTTTC 60694
OliveBaboon     GATAGTGGTAAATAATTAGTCTGAAATATTACATTAGTAGTATAAGCTTAATTTTGTGTTGTTGATTTTGATAATTTTTC 62132
Orangutan       GATATTGGTAAATAATTAGTCTGAAGTATTACATTAGTATTATAAGCTTAATTTTGTGTTGTTGATTTTGATAATTTTTC 62570
```

```
Majority          ATGACTTGCCAAAAATTAAAAAGTAGATATTTAAAGAATTTGGAAGTTATGGTTTCTCTCATCGTGTGTTAAATCTGATC
                          76090     76100     76110     76120     76130     76140     76150     76160
Human             ATGACTTGCCAAATATTAAAAAGTAGATATTTAAAGAATTTGGAAGTTAAGGTTTCTCTCAGTGTGTGTTAAATCTGATC 61676
Coquerel'sSifaka  CA------------------------------GGCAT-----AG----G-TTTCTCTGAACATCTGTTAAATCTGATC 50041
Ma'sNightMonkey   ATGACTTGCCAAAAATTAAAAAGTAGATATTTAAAGAATTTCGATGTTACGGTTTCTCTCAGCATGTGTTAAATCTGATC 57137
VervetAGM         ATGACTTGCCAAAAATTAAAAAGTAGATATTTAAAGAATTTGGAAGTTATGGTTTCTGTCATCGTGTGCTAAATCTGATC 63434
Gorrilla          ATGACTTGCCAAATATTAAAAAGTAGATATTTAAAGAATTTGGAAGTTAAGGTTTCTCTCAGTGTGTGTTAAATCTGATC 62216
GrabEatingMacaqueATGACTTGCCAAAAATTAAAAAGTAGATATTTAAAGAATTTGGAAGTTATGGTTTCTCTCATCGTGTGCTAAATCTGATC 62626
Macaque           ATGACTTGCCAAAAATTAAAAAGTAGATATTTAAAGAATTTGGAAGTTATGGTTTCTCTCATCGTGTGCTAAATCTGATC 63467
PigTailedMacaque  ATGACTTGCCAAAAATTAAAAAGTAGATATTTAAAGAATTTGGAAGTTATGGTTTCTCTCATCGTGTGCTAAATCTGATC 62357
Bonobo            ATGACTTGCCAAATATTAAAAAGTAGATATTTAAAGAATTTGGAAGTTAAGGTTTCTCTCAGTGTGTGTTAAATCTGATC 60774
OliveBaboon       ATGACTTGCCAAAAATTAAAAAGTAGATATTTAAAGAATTTGGAAGTTATGGTTTCTCTCATCGTGTGCTAAATCTGATC 62212
Orangutan         ATGACTTGCCAAAAATTGAAAAGTAGATATTTAAAGAATTTGGAAGTTAAGATTTCTCTCAGCGTGTGTTAAATCTGATC 62650

Majority          CCCAACAATCTCTTCAAAAAAGTAAGTTAT--AAATAGTAA--ACATTGTGA-AAAATGGAAATAGTTTTATTGTGGCTT
                          76170     76180     76190     76200     76210     76220     76230     76240
Human             CCCAACAATCTCTTCAAAAAAGTAAGTTAT--CAATAGTAA--ACGATGAGA-AAAATGGAAATAGTTTTATTTTGGCTT 61751
Coquerel'sSifaka  CCCAACAATCTTTTCAAAAAATTAAGTTATCTAAATAAAAAAAAAATTGCGATAAAATGGAAATAGTTTTATTGTGGCTT 50121
Ma'sNightMonkey   CCCAACAATCTCTTCAAAAAAGCAA------------------ACATTGAGA-AAAATGGAAATCGTTTTGTTGTGGCTT 57198
VervetAGM         CCCAACAATCTCTTCAAAAAAGTAAGTTAT--AAATAGTAA--ACATTCTGA-AAAATGGAAATGGTTTTATTGTGGCTT 63509
Gorrilla          CCCAACAATCTCTTCAAAAAAGTAAGTTAT--AAATAGTAA--ACAATGAGA-AAAATGGAAATAGTTTTATTTTGGCTT 62291
GrabEatingMacaqueCCCAACAATCTCTTCAAAAAAGTAAGTTAT--AAATAGTAA--ACATTCTGA-AAAATGGAAATAGTTTTATTGTGGCTT 62701
Macaque           CCCAACAATCTCTTCAAAAAAGTAAGTTAT--AAATAGTAA--ACATTCTGA-AAAATGGAAATAGTTTTATTGTGGCTT 63542
PigTailedMacaque  CCCAACAATCTCTTCAAAAAAGTAAGTTAT--AAATAGTAA--ACATTCTGA-AAAATGGAAATAGTTTTATTGTGGCTT 62432
Bonobo            CCCAACAATCTCTTCAAAAAAGTAAGTTAT--AAATAGTAA--ACAATGAGA-AAAATGGAAATAGTTTTATTTTGGCTT 60849
OliveBaboon       CCCAACAATCTCTTCAAAAAAGTAAGTTAT--AAATAGTAA--ACATTCTGA-AAAATGGAAATAGTTTTATTGTGGCTT 62287
Orangutan         CCCAACAATCTCTTCAAAAAAGTAAGTTAT--AAATAGTAA--ACAATGAGA-AAAATGGAAATAGTTTTATTTTGGCTT 62725

Majority          TTCATGTTTTTA---GTCCATATGCAATGTTTCATTGTGCTGCTTTAATTATGCTTTCTTTGCCTAATATCTTTCCTGTG
                          76250     76260     76270     76280     76290     76300     76310     76320
Human             TTCATGTTTTCA---GTCCATATGCAGTGTTTCATTGTGCTGCTTTAATTATGCTTTCTGTGCCTAATATCTTTCCTGTG 61828
Coquerel'sSifaka  TTTGTATTTTTC--AGTCCATATGCAGTGTTTCATTGTGCTGTTTTAACTATCCACTTTGTGTCTAATATCTTTCTTGTG 50199
Ma'sNightMonkey   TTCATGTTTTCAAAAGTCCATATGCGGTGTTTCATTGTGTTGGTTTAATTATGATTTCTATGCCTAATATCTTTCCTGTG 57278
VervetAGM         TTCATGTTTTTA---GTCCATATGCAATGTTTCATTGTGCTGCTATAATTATGCTTTCTTTGCCTAATATCTTTCCTGTG 63586
Gorrilla          TTCATGTTTTCA---GTCCATATGCAGTGTTTCATTGTGCTGCTTTAATTATGCTTTCTGTACCTAATATCTTTCCTGTG 62368
GrabEatingMacaqueTTCATGTTTTTA---GTCCATATGCAATGTTTCATTGTGCTGCTATAATTATGCTTTCTTTGCCTAATATCTTTCCTGTG 62778
Macaque           TTCATGTTTTTA---GTCCATATGCAATGTTTCATTGTGCTGCTATAATTATGCTTTCTTTGCCTAATATCTTTCCTGTG 63619
PigTailedMacaque  TTCATGTTTTTA---GTCCATATGCAATGTTTCATTGTGCTGCTATAATTATGCTTTCTTTGCCTAATATCTTTCCTGTG 62509
Bonobo            TTCATGTTTTCA---GTCCATATGCAGTGTTTCATTGTGCTGCTTTAATTATGCTTTCTGTGCCTAATATCTTTCCTGTG 60926
OliveBaboon       TTCATGTTTTTA---GTCCATATGCAATGTTTCATTGTGCTGCTATAATTATGCTTTATTTGCCTAATATCTTTCCTGTG 62364
Orangutan         TTCATGTTTTCG---GTCCATATGCAATGTTTCATTGTGCTGCTTTAATTATGCTTTCTGCGCCTAATATCTTTCCTGTG 62802
```

```
Majority         TTTTGATTTCCATGTTTTAGCCATAAACTTTAAATTTACATGAAAA-TTTTTTTTCTGATTGTTTTTGCTTACTGTCTAA

                         76330     76340     76350     76360     76370     76380     76390     76400

Human            TTTTGATTTCCATGTTTTAGCCATAAACTTTAAATTTACATGAAAACTTTTTTTTCTGATTGTTTTTGCTTACTGTCTAA 61908
Coquerel'sSifaka TTTTGATTATCATGTTTTAGCCATAGATTTTAAATTTATATGAAAA-TATTTTTCCTGATTGTTTTTGCTTACTGTCTAA 50278
Ma'sNightMonkey  TTTTGATTTTCGTGTTTTAGCCATAAATTTTAAATTTACATGAAAT-TTTTTTTTCTGAAGGTTTTTGCTTACTGTTTAA 57357
VervetAGM        TTTTGATTTCCATGTTTTAGCCATAAACTTCAAATTTACATGAAAA-TTTTTTTTCTGATTGTTTTTGCTTACTGTCTAA 63665
Gorrilla         TTTTGATTTCCATGTTTTAGCCATAAACTTTAAATTTACATGAAAACTTTTTTTTCTGATTGTTTTTGCTTACTGTCTAA 62448
GrabEatingMacaqueTTTTGATTTCCATGTTTTAGCCATAAACTTCAAATTTACATGAAAA-TTTTTTTTCTGATTGTTTTTGCTTACTGTCTAA 62857
Macaque          TTTTGATTTCCATGTTTTAGCCATAAACTTCAAATTTACATGAAAA-TTTTTTTTCTGATTGTTTTTGCTTACTGTCTAA 63698
PigTailedMacaque TTTTGATTTCCATGTTTTAGCCATAAACTTCAAATTTACATGAAAA-TTTTTTTTCTGATTGTTTTTGCTTACTGTCTAA 62588
Bonobo           TTTTGATTTCCATGTTTTAGCCATAAACTTTAAATTTACATGAAAACTTTTTTTTCTGATTGTTTTTGCTTACTGTCTAA 61006
OliveBaboon      TTTTGATTTCCATGTTTTAGCCATAAACTTCAAATTTACATGAAAT-TTTTTTTTCTGATTGTTTTTGCTTACTGTCTAA 62443
Orangutan        TTTTGATTTCCATGTTTTAGCCATAAACTTTAAATTTACTTGAAAA-TTCTTTTTCTGATTGTTTTTGCTTACTGTCTAA 62881

Majority         ACTTTCACTGTGAGTAGGCACATTTGGAAGGTCTTTCTGCAAATTGTCTTGAGCTTTTACTGTCAGAAATCTTTGCTCCA

                         76410     76420     76430     76440     76450     76460     76470     76480

Human            ACCTTCACTGTGAGTAGGCACATTTGGAAGGTCTTTCTGCAAATTATCTTGAGCTTTTAATGTCAGAAATCTTTGCTCCA 61988
Coquerel'sSifaka ACTTTCACTGTGAGTAGGCACATTTGAAAGGTCTTTCTGCAAATTATCTTGAGCTTTTACTGTCAGAAATCTTTGCTCCA 50358
Ma'sNightMonkey  ACTTTAACTGTGAGTAGGCATATTTAGAAGGTCGTTCTGCAAATCGTTTTGAGTTTTTACTGTCAGAAATCTTTGCTCCA 57437
VervetAGM        ACTTTCACTGTGAGTAGGCACATTTGGAAGGTCTTTCTGCAAATTGTCTTGAGCTTTTACTGTCAGAAATCTTAACTCCA 63745
Gorrilla         ACCTTCACTGTGAGTAGGCACATTTGGAAGGTCTTTCTGCAAATTATCTTGAGCTTTTAATGTCAGAAATCTTTGCTCCA 62528
GrabEatingMacaqueACTTTCACTGTGAGTAGGCACATTTGGAAGGTCTTTCTGCAAATTGTCTTGAGCTTTTACTGTCAGAAATCTTAACTCCA 62937
Macaque          ACTTTCACTGTGAGTAGGCACATTTGGAAGGTCTTTCTGCAAATTGTCTTGAGCTTTTACTGTCAGAAATCTTAACTCCA 63778
PigTailedMacaque ACTTTCACTGTGAGTAGGCACATTTGGAAGGTCTTTCTGCAAATTGTCTTGAGCTTTTACTGTCAGAAATCTTAACTCCA 62668
Bonobo           ACCTTCACTGTGAGTAGGCACATTTGGAAGGTCTTTCTGCAAATTATCTTGAGCTTTTAATGTCAGAAATCTTTGCTCCA 61086
OliveBaboon      ACTTTCACTGTGAGTAGGCACATTTGGAAGGTCTTTCTGCAAATTGTCTTGAGCTTTTACTGTCAGAAATCTTAACTCCA 62523
Orangutan        ACCTTCACTGTGAGTAGGCACATTTGGAAGGTCTTTCTGCAAATTGTCTTGAGCTTTTACTGTCAGAAATCTTTGCTTCA 62961

Majority         ACAAGATCACCGCATTCCTGATTGCTGAACCACAGGACTGGTTTGTGTGCTACTGCTATTTCTTGGGATTTTGCAAATAC

                         76490     76500     76510     76520     76530     76540     76550     76560

Human            ACAAGATCACCACATTCCTGATTGCTGAACCACAGGACTGGTTTGTGTGCTACTGCTATTTCTTGGGATTTTGCAAATAC 62068
Coquerel'sSifaka GCAAGATTACTGTGTTCCTGATTGCTGAACCACAGGACTGGTTTGTGTGCTCTTGTTATTTTCTGGAATTTTACAAATAT 50438
Ma'sNightMonkey  ACAAGCTCACTGCATTCCTGATTGCTGAATCATAGGACTGGTTTGTGTGCTACTGCTGTTTCTTGG-ATTTTGCAAATAC 57516
VervetAGM        ACAAGATCACCGCATTCCTGATTGCTGAACCACAGGACTGGTTTGTGTGCTACTGCT-TTTCTTGGGATTTTGCAAATAC 63824
Gorrilla         ACAAGATCACCACATTCCTGATTGCTGAACCACAGGACTGGTTTGTGTGCTACTGGTATTTCTTGGGATTTTGCAAATAT 62608
GrabEatingMacaqueACAAGATCACCGCATTCCTGATTGCTGAACCACAGGACTGGTTTGTGTGCTACTGCT-TTTCTTGGGATTTTGCAAATAC 63016
Macaque          ACAAGATCACCGCATTCCTGATTGCTGAACCACAGGACTGGTTTGTGTGCTACTGCT-TTTCTTGGGATTTTGCAAATAC 63857
PigTailedMacaque ACAAGATCACCGCATTCCTGATTGCTGAACCACAGGACTGGTTTGTGTGCTACTGCT-TTTCTTGGGATTTTGCAAATAC 62747
Bonobo           ACAAGATCACCACATTCCTGATTGCTGAACCACAGGACTGGTTTGTGTGCTACTACTATTTCTTGGGATTTTGCAAATAC 61166
OliveBaboon      ACAAGATCACCGCATTCCTGATTGCTGAACCACAGGACTGGTTTGTGTGCTACTGCT-TTTCTTGGGATTTTGCAAATAC 62602
Orangutan        ACAAGATCACCGCATTCCTGATTGCTGAACCACAGGACTGGTTTGTGTGCTACTGCCATTTCTTGGGATTTTGCAAATAT 63041
```

```
Majority          ATGAGCTTGAAGTGAGCTTGCATATATTTTTTAAATTTGATTTTACTCCCTTTCTTATTTGTGGTCACCT-ACTTTAGCC

                          76570     76580     76590     76600     76610     76620     76630     76640

Human             ATGAGCTTGAAGTGAGCTTGCATATATTTTTTAAATTTGATTTTACTCCCTTTCTTATTTGTGGTCACCT-CCTTTAACC 62147
Coquerel'sSifaka  ATGAGCATGAATTGAGCTTGTATATATATTTT-AATTTGATTT----TTACTCCTTATT-GTGGTCACCTGACTTTAGCC 50512
Ma'sNightMonkey   ATGAGCTTGACGTGAACCTGCATATATTTTTTGAATTTGATTT-ACTCCCTTTCTTATTTATGGTCACCT-GCTTTAGCC 57594
VervetAGM         ATGAGCTTGAAGTGAGCTTGCATATGTTTTTTAAATTTGGTTTTACTCCCTTTCTTATTTGTGGTCACCT-ACTTTAGTC 63903
Gorrilla          ATGAGCTTGAAGTGAGCTTGCATATATTTTTTAAATTTGATTTTACTCCCTTTCTTATTTGTGGTCACCT-CCTTTAACC 62687
GrabEatingMacaqueATGAGCTTGAAGTGAGCTTGCATATGTTTTTTAAATTTGATTTTACTCCCTTTCTTGTTTGTGGTCACCT-ACTTTAGTC 63095
Macaque           ATGAGCTTGAAGTGAGCTTGCATATGTTTTTTAAATTTGATTTTACTCCCTTTCTTGTTTGTGGTCACCT-ACTTTAGTC 63936
PigTailedMacaque  ATGAGCTTGAAGTGAGCTTGCATATGTTTTTTAAATTTGATTTTACTCCCTTTCTTGTTTGTGGTCACCT-ACTTTAGTC 62826
Bonobo            ATGAGCTTGAAGTGAGCTTGCATATATTTTTTAAATTTGATTTTACTCCCTTTCTTATTTGTGGTCACCT-CCTTTAACC 61245
OliveBaboon       ATGAGCTTGAAGTGAGCTTGCATATGTTTTTTAAATTTGATTTTACTCCCTTTCTTGTTTGTGGTCACCT-ACTTTAGTC 62681
Orangutan         ATGAGCTTGAAGTGAGCTTGCATATATTTTTTAAATTTGATTTTACTCCCTTTCTTATTTGTGGTCACCT-ACTTTAACC 63120

Majority          AACTATTTAAAATACCTGGATTTTTGTTCTCATGCATCCTCAGCTCCTATTTCTTTCCTCTTTTGCTAACAGATGATT--

                          76650     76660     76670     76680     76690     76700     76710     76720

Human             AACTATTTAAAATACCTGGATTTTTGCTCTCATACACCCTCAGCTCCTATTTCTTTCCTCTTTTGCCAACAGATGATT-- 62225
Coquerel'sSifaka  GGCTATTGAAAATGCCTGGATTCCTGTTTTCATGCATCCTCAGCTTTGGTTTCTCTCCTCTTTTGCTAACAGGTGGTTTT 50592
Ma'sNightMonkey   AACTATT----ATACCTGGATTTCTGTTCTCATGCACTCTT--CAGCTATTTCTTTCCTCTTTTGCTAAACAATGATT-- 57666
VervetAGM         AACTATTTAAAATACCTGGATTTTTGTTCTCATGCATCCTCAACTCCTATTTCTTTCCTCTTTTGCTAACAGATGATT-- 63981
Gorrilla          AACTATTTAAAATACCTGGATTTTTGCTCTCATACACCCTCAGCTCCTATTTCTTTCCTCTTTTGCTAACAGATGATT-- 62765
GrabEatingMacaqueAACTATTTAAAATACCTGGATTTTTGTTCTCATGCATCCTCAACTCCTATTTCTTTCCTCTTTTGCTAACAGATGATT-- 63173
Macaque           AACTATTTAAAATACCTGGATTTTTGTTCTCATGCATCCTCAACTCCTATTTCTTTCCTCTTTTGCTAACAGATGATT-- 64014
PigTailedMacaque  AACTATTTAAAATACCTGGATTTTTGTTCTCATGCATCCTCAACTCCTATTTCTTTCCTCTTTTGCTAACAGATGATT-- 62904
Bonobo            AACTATTTAAAATACCTGGATTTTTGCTCTCATACACCCTCAGCTCCTATTTCTTTCCTCTTTTGCCAACAGATCATT-- 61323
OliveBaboon       AACTATTTAAAATACCTGGATTTTTGTTCTCATGCATCCTCAACTCCTATTTCTTTCCTCTTTTGCTAACAGATGATT-- 62759
Orangutan         AACTATTTAAAATACCTGAATTTTTGTTCTCATACACCCTCAGCTCCTATTTCTTTCCTCTTTTGCTAACAGATGATT-- 63198

Majority          AATTTACATCTTGTTTGCTTATTTCTCTAGCTCTTGTACCTTAGGTATACTTGTGTATCTTCCACCTACATAAA------

                          76730     76740     76750     76760     76770     76780     76790     76800

Human             AATTTACATCTTGTTTGCTTATTTCTCTAGCTCTTGTACCTTAGGTATACTTCTGCATCTTCCACCTACATAAA------ 62299
Coquerel'sSifaka  AATGTACATGTTACTTGCTTATTTCTCTAGCTCTTACCGCTTAGGCATACTTGTGTATCTCCCATCTTCATAAA------ 50666
Ma'sNightMonkey   AATTTACATCTTACTTGCTTATTTCTGTAGCTTTTATACCTTACGTGTACTTGTGTATCTTCCACCTACATAAA------ 57740
VervetAGM         AATTTACATCTTGTTTGCTTATTTCTCTAGCTCTTGTACCTTAGGTATACTTGTGTATTTTCCACCTACATAAAATTTAT 64061
Gorrilla          AATTTACATCTTGTTTGCTTATTTCTCTAGCTCTTGTACCTTAGGTATACTTGTGCATCTTCCACCTACATAAA------ 62839
GrabEatingMacaqueAATTTACATCTTGTTTGCTTATTTCTCTAGCTCTTGTACCTTAGGTATACTTGTGTATTTTCCACCTACATAAAGTTTAT 63253
Macaque           AATTTACATCTTGTTTGCTTATTTCTCTAGCTCTTGTACCTTAGGTATACTTGTGTATTTTCCACCTACATAAAGTTTAT 64094
PigTailedMacaque  AATTTACATCTTGTTTGCTTATTTCTCTAGCTCTTGTACCTTAGGTATACTTGTGTATTTTCCACCTACATAAAGTTTAT 62984
Bonobo            AATTTACATCTTGTTTGCTTATTTCTCTAGCTCTTGTACCTTAGGTATACTTCTGCATCTTCCACCTACATGAA------ 61397
OliveBaboon       AATTTACATCTTGTTTGCTTATTTCTCTAGCTCTTGTACCTTAGGTATACTTGTGTATTTTCCACCTACATAAAGTTTAT 62839
Orangutan         AATTTACATCTTGTTTGCTTATTTCTGTAGCTCTCGTACCTTAGGTATACTTGTGCATCTTCCACCTACATAAA------ 63272
```

```
Majority        GTTTTTGTTTTTGTTTTT-----TAATGTTCAAAGGGA--AAATTTCAAAGGTTCCTGGAGGGCTTTCTAACAATTGGCA
                        76810     76820     76830     76840     76850     76860     76870     76880
Human           GTTTTTGTTTTTGTTTTT------AATGTTCAGAGGGA--AAATTTCAAAGGGTCCTGGAGGGCTTTCTAACAATTGGCA 62371
Coquerel'sSifaka A---ATGTTT----TTTA-----AAATGTTAAAAGAGG--AAATGTCCA---TTTTTGGAGTGCTTTCTCACGATTGGCA 50729
Ma'sNightMonkey GTTTTTTTTT----TTTT------AATATTCAAAGGGA--AATTTTCAAAGCTTCTTGGAGGACTTTCTAACAATTGGCA 57808
VervetAGM       GTTTTTGTTTTTGTTTTTTGTTTTGATGTTCAAAGGGAGAAAATTTCAAAGGTTCCTGGAGGGCTTTCTAACAATGGGCA 64141
Gorrilla        GTTTTTGTTTTTGTTTTT------AATGTTCAGAGGGA--AAATTTCAAAGGGTCCTGGAGGGCTTTCTAATAATTGGCA 62911
GrabEatingMacaqueGTTTTTGTTTTTGTTTTT-GTTTTAATGTTCAAAGGGAGAAAATTTCAAAGGTTCCTGGAGGGGTTTCTAACAATGGGCA 63332
Macaque         GTTTTTGTTTTTGTTTTT-GTTTTAATGTTCAAAGGGAGAAAATTTCAAAGGTTCCTGGAGGGGTTTCTAACAATGGGCA 64173
PigTailedMacaque GTTTTTGTTTTTGTTTTT-GTTTTAATGTTCAAAGGGAGAAAATTTCAAAGGTTCCTGGAGGGCTTTCTAACAATGGGCA 63063
Bonobo          GTTTTTGTTTTTGTTTTT------AATGTTCAGAGGGA--AAATTTCAAAGGGTCCTGGAGGGCTTTCTAACAATTGGCA 61469
OliveBaboon     GTTTTTGTTTTTGTTTTT-GTTTTAATGTTCAAAGGGAGAAAATTTCAAAGGTTCCTGGAGGGCTTTCTAACAATGGGCA 62918
Orangutan       GTTTTTGTTTTTGTTTTT------AATGTTCAAAGGGA--AAATTTCAAAGGTTCCTGGAGGGCTTTCTAACAATTGGCA 63344

Majority        TATTCATTGTTAGATAATTTGGCAGTCG-ACCTCAGTATTGCTGGGAAAGAATTCAGCTCCTAAAAAA-----CTTAGTG
                        76890     76900     76910     76920     76930     76940     76950     76960
Human           TATTCATTGTTAGATAATTTGGCAGTCGGACCTCAGTATTGCTGGGAAAGAATTCAACTCCTAAAAAA-----CTTAGTG 62446
Coquerel'sSifaka TATTCATTGT-AGATGATTTGGTAGTCT-ATTTCAGTGTTGCTTGGGAAGAACTCAGCCCCTAAAAAAGTTTACTTAGTG 50807
Ma'sNightMonkey TATTCATT---AGATAATTTGGCAGTCG-ACCTCAGTATTGCTGGGAAATAATTCAGCTCCTAAGAAA-----CTTAGTG 57879
VervetAGM       TATTAATTGTTAGATAATTTGGCAGTCG-CCCTCAGTATTGCTGGGAAAGAATTCAGCTCCTAAAAAA-----TATAGTG 64215
Gorrilla        TATTCATTGTTAGATAATTTGGCAGTCGGACCTCAGTATTGCTGGGAAAGAATTCAACTCCTAAAAAA-----CTTAGTG 62986
GrabEatingMacaqueTATTAATTGTTAGATAATTTGGCAGTCG-CCATCAGTATTGCTGGGAAAGAATTCAGCTCCTAAAAAA-----CATAGTG 63406
Macaque         TATTAATTGTTAGATAATTTGGCAGTCG-CCATCAGTATTGCTGGGAAAGAATTCAGCTCCTAAAAAA-----CATAGTG 64247
PigTailedMacaque TATTAATTGTTAGATAATTTGGCAGTCG-CCATCAGTATTGCTGGGAAAGAATTCAGCTCCTAAAAAA-----CATAGTG 63137
Bonobo          TATTCATTGTTAGATAATTTGGCAGTCGGACCTCAGTATTGCTGGGAAAGAATTCAACTCCTAAAAAA-----CTTAGTG 61544
OliveBaboon     TATTAATTGTTAGATAATTTGGCAGTCG-CCCTCAGTATTGCTGGGAAAGAATTCAGCTCCTAAAAAA-----CATAGTG 62992
Orangutan       TATTCATTGTTAGATAATTTGGCAGTCG-ACCTCAGTATTGCTGGGAAAGAATTCAGCTCCTAAAAAA-----CTTAGTG 63418

Majority        TGGGTTTAGTAATTTTGTATCAGCACT-----TTTAGCATTAACCTAGATACTGTGACTCAAAGCCAGATCTTTTCTCAT
                        76970     76980     76990     77000     77010     77020     77030     77040
Human           TGTGTTTAGTAATTTTGTATCAGCACT-----TTTAGCATTAACCTAGATACTGTGACTCAAACCCAGCTCTTTTCTCAT 62521
Coquerel'sSifaka AAGATTTGGTAATTTTGTATCGGTACTGTACTTTTAGCATTAACGTAGATAGTATGATTCAAAGCCATATTTTTTCTCAT 50887
Ma'sNightMonkey TGGGTTTAGTAATTTTGTATTAATACT-----TTTAGCATTAACCTAGATACTGTGACTCTAAGCCAGGTCTTTTCTCAT 57954
VervetAGM       TGGGTTTAGTAATTTTGTATCAGCACT-----TTTAGCATTAACCTAGATACTGTGACTCAAAGCCAGATCTTTTCTCAT 64290
Gorrilla        TATGTTTAGTAATTTTGTATCAGCACT-----TTCAGCATTAACCTAGATACTGTGACTCAAACCCAGCTCTTTTCTCAT 63061
GrabEatingMacaqueTGGGTTTAGTAATTTTGTATCAGCACT-----TTTAGCATTAACCTAGATACTGTGACTCAAAGCCAGATCTTTTCTCAT 63481
Macaque         TGGGTTTAGTAATTTTGTATCAGCACT-----TTTAGCATTAACCTAGATACTGTGACTCAAAGCCAGATCTTTTCTCAT 64322
PigTailedMacaque TGGGTTTAGTAATTTTGTATCAGCACT-----TTTAGCATTAACCTAGATACTGTGACTCAAAGCCAGATCTTTTCTCAT 63212
Bonobo          TATGTTTAGTAATTTTGTATCAGCACT-----TTTAGCATTAACCTAGATACTGTGACTCAAACCCAGCTCTTT-CTCAT 61618
OliveBaboon     TGGGTTTAGTAATTTTGTATCAGCACT-----TTTAGCATTAACCTAGATACTGTGACTCAAAGCCAGATCTTTTCTCAT 63067
Orangutan       TATGTTTAGTAATTTTGTATCAGCGCT-----TTTAGCATTAACCTAGATGCTGTGACTCAAACCCAGCTCTTTTCTCAT 63493
```

```
Majority         AAGGTATCATTTTCTCTTTGATAACATTGAAGTAGTCACCTAAGAGTGAATTTTGCTACTCATCTTTTGATATTTATCTG
                         77050     77060     77070     77080     77090     77100     77110     77120
Human            AAGGTATCATTTTCTCTTTGATAACATTGAAGTAGTCACCTAAGAGTGAATTTTGCTACTTATCTTTTGATATTTATCTA 62601
Coquerel'sSifaka A-----TCGTTCTTTGTTTGACAATATTGAAGTAGTCACCTAAGAGTCAATCATGCTACTCATCTTTTGATAATCGTATG 50962
Ma'sNightMonkey  AAGGTATCATTTTCTCTTTGATAATAATGAAGTAGTCACCTAAGAGTGAATTTTGCTACTCATCTTTTGATATTCATCTA 58034
VervetAGM        AAGGTATCATTTTCTCTTTGATAACATTGAAGTAGTCACCTAAGAGTGAATTTTGCTATTC--CTTTTGATACTTACCTG 64368
Gorrilla         AAGGTATCATTTTCTCTTTGATAACATTGAAGTAGTCACCTAAGAGTGAATTTTGCTACTTATCTTTTGATATTTATCTA 63141
GrabEatingMacaqueAAGGTATCATTTTCTCTTTGATAACATTGAAGTAGTCACCTAAGAGTGAATTTTGCTACTC--CTTTTGATACTTACCTG 63559
Macaque          AAGGTATCATTTTCTCTTTGATAACATTGAAGTAGTCACCTAAGAGTGAATTTTGCTACTC--CTTTTGATACTTACCTG 64400
PigTailedMacaque AAGGTATCATTTTCTCTTTGATAACATTGAAGTAGTCACCTAAGAGTGAATTTTGCTACTC--CTTTTGATACTTACCTG 63290
Bonobo           AAGGTATCATTTTCTCTTTGATAACATTGAAGTAGTCACCTAAGAGTGAATTTTGCTACTTATCTTTTGATATTTATCTA 61698
OliveBaboon      AAGGTATCATTTTCTCTTTGATAACATTGAAGTAGTCACCTAAGAGTGAATTTTGCTACTC--CTTTTGATACTTACCTG 63145
Orangutan        AAGGTATCATTTTCTCTTTGATCACATTGAAGTAGTCACCTAAGAGTGAATTTTGCTTCTTATCTTTTGATATTTATCTA 63573

Majority         TTGTGTAGAAACATCAGTTCTATTACTTATCTCACATTTTCCCTATAGGAATGCTACATTAGTAAATGAATACCTATCTT
                         77130     77140     77150     77160     77170     77180     77190     77200
Human            TTGTGTAGAAACATCAGTTCTATTACTTATCTCACATTTTCCCTATAGGAATGCTACATTAGTAAATGAATACCTATCTT 62681
Coquerel'sSifaka CTGTATAGAAACATAGATTCTATTACTTTTCTCACATTTTCCCTATAGGAATGCTACATGAGTAAAGTAATACCTATTTT 51042
Ma'sNightMonkey  TTGTGTAAAAACATCAGTTCTACTACTTACCTCACATTTTCCCTATAGGAATGCTATATTAGTAAATGAATACCCATCTT 58114
VervetAGM        TTGTGTAGAAACGTCAATTCTAT----TCTGTTACATTTTCCCTATAGGAATGCTACATTAGTAAATTAATACCTATCTT 64444
Gorrilla         TTGTGTAGAAACATCAATTCTATTACTTATCTCACATTTTCCCTATAGGAATGCTACGTTAGTAAATGAATACCTATCTT 63221
GrabEatingMacaqueTTGTGTAGAAATGTCAGTTCTATTACTTATCTTACATTTTCCCTATAGGAATGCTACATTAGTAAATAAATACCTATCTT 63639
Macaque          TTGTGTAGAAATGTCAGTTCTATTACTTATCTTACATTTTCCCTATAGGAATGCTACATTAGTAAATAAATACCTATCTT 64480
PigTailedMacaque TTGTGTAGAAATGTCAGTTCTATTACTTATCTTACATTTTCCCTATAGGAATGCTACATTAGTAAATAAATACATATCTT 63370
Bonobo           TTGTGTAGAAACATCAGTTCTATTACTTATCTCACATTTTCCCTATAGGAATGCTACATTAGTAAATGAATACCTATCTT 61778
OliveBaboon      TTATGTAGAAATGTCAATTCTATTACTTATCTTACATTTTCCCTATAGGAATGCTACATTAGTAAATTAATACCTATCTT 63225
Orangutan        TTGTGTAGAAACATCAATTCTATTACTTATCTCACATTTACCTATAGGAATACTACATTAGTAAATGAATACCTATCTT 63653

Majority         CAGTGCTTTGTTTGCCTTTACAC--CAGGGTATTGAATTAATAGTTATGTTATTCTGGCTGATAAAGTTGAT----ACAT
                         77210     77220     77230     77240     77250     77260     77270     77280
Human            CAGTGCTTTCTTCACCTTTACAC--CAGGGTATTCAATTAATAGTTATGTTATTCTGGCTGATAAAGTTGATTGATACAT 62759
Coquerel'sSifaka CAATGCTTTGTTTGCCTTTACACAACAGAGTATTGAATTTATAGTTATTTTATTCTGGCTGATAAAATTGAT----ACTT 51118
Ma'sNightMonkey  CTGTGCTTTGTTTGCCTTTACAG--CAGCGTATTGAATTAATAGTTATGTTATTCTGGCTGATAAAGTTGAT----ACAT 58188
VervetAGM        CAGTGCTTTGTTTGCCTTTACAC--CAGGGTGTTGAATTAATAGTTATGTTATTCTGGCTGATAAAGTTGAT----ACAT 64518
Gorrilla         CAGTGCTTTGTTCACCTTTACAC--CAGGGTATTCAATTAATAGTTATGTTATTCTGGCTGATAAAGTTGATTGATACAT 63299
GrabEatingMacaqueCAGTGCTTTGTTTGCCTTTACAC--CAGGGTGTTGAATTAATAGTTATGTTATTCTGGCTGATAAAGTTGAT----ACAT 63713
Macaque          CAGTGCTTTGTTTGCCTTTACAC--CAGGGTGTTGAATTAATAGTTATGTTATTCTGGCTGATAAAGTTGAT----ACAT 64554
PigTailedMacaque CAGTGCTTTGTTTGCCTTTACAC--CAGGGTGTTGAATTAATAGTTATGTTATTCTGGCTGATAAAGTTGAT----ACAT 63444
Bonobo           CAGTGCTTTCTTCACCTTTACAC--CAGGGTATTCAATTAATAGTTATGTTATTCTGGCTGATAAAGTTGATTGATACAT 61856
OliveBaboon      CAGTGCTTTGTTTGCCTTTACAC--CAGGGTGTTGAATTAATAGTTATGTTATTCTGGCTGATAAAGTTGAT----ACAT 63299
Orangutan        CAGTGGTTTGTTCGCCTTTACAC--CAGGGTATTGAATTAATAGTTATGTTATTCTGGCTGATAAAGTTGATTAATACAT 63731
```

```
Majority          GGCTTCCACTTTTATGTGATGATAGGTACCTTGCCTAACCCAAATGTAATTCAAATCCTGATGTGTATGAAGTCCCTTTG
                          77290     77300     77310     77320     77330     77340     77350     77360
Human             GGCTTCCACTTTTATGTGATGATAGGTACCTTGCCTAACCCAAATGTAATTCAAATCCTGATGTGTATGAACTCTCTTTC 62839
Coquerel'sSifaka  GGCTTCCACTTTTATGTGATGATAAGTGCCTTGCCTAACCTAAATGTAATTCATACCCTGATGTGTATGAAAT------G 51192
Ma'sNightMonkey   GGCTTCCACTGTTATGTGATGATGGGTACTTTGCCTAACCCAAATATAATTCAAATCCTGATGTGTATGAGGTCCCTTTG 58268
VervetAGM         GGCTTCCACTTTTATGTGATGATAGGTACCTTGCCTAACCCAAATGTAATTCATATCCTGATATGTATGAAGTCCCTTTG 64598
Gorrilla          GGCTTCCACTTTTATGTGATGATAGGTACCTTGCCTAACCCAAATGTAATTCAAATCCTGATGTGTATGAACTCTCTTTG 63379
GrabEatingMacaqueGGCTTCCACTTTTATGTGATGATAGGTACCTTGCCTAACCCAAATGTAATTCAAATCCTGATATGTATGAAGTCCCTTTG 63793
Macaque           GGCTTCCACTTTTACGTGATGATAGGTACCTTGCCTAACCCAAATGTAATTCAAATCCTGATATGTATGAAGTCCCTTTG 64634
PigTailedMacaque  GGCTTCCACTTTTATGTGATGATAGGTACCTTGCCTAACCCAAATGTAATTCAAATCCTGATATGTATGAAGTCCCTTTG 63524
Bonobo            GGCTTCCACTTCTATGTGATGATAGGTACCTTGCCTAACCCAAATGTAATTCAAATCCTGATGTGTATGAACTCTCTTTC 61936
OliveBaboon       GGCTTCCACTTTTATGTGATGATAGGTACCTTGCCTAACCCAAATGTAATTCAAATCCTGATATGTATGAAGTCCCTTTG 63379
Orangutan         GGCTTCCACTTTTATGTGATGATAGGTACCTTGCCTAACCCAAATGTAATTCAGATCCTGATGTGTATGAACTCTCTTTG 63811

Majority          AGTTTTTACCAGTGCTGTATTGAA-GTGGATGAACCTGTGTTGAATTATTTTCTAGGTTAGTTTTTAGCTAGGTAAAT-T
                          77370     77380     77390     77400     77410     77420     77430     77440
Human             AGTTTTTACCAGTGCTGTATTGAA-GTGGATGAATCTGTGTTGAATTATTTTCTAGGTTAGTTTTTAGGTAGGTAGAT-T 62917
Coquerel'sSifaka  AGTTTTTACCAGTACTGTATTGAAAGTGGGTGAACTTCTGTTGAATTATTTTTTAGGTTAGTTTTTAGCTTGGTAAATAC 51272
Ma'sNightMonkey   AGTTTTTACCAGTGCTGTATTTAA-GTGGATGAACCTGTGCTGAATCATGTTCTAGGTTAGTTTTTAGGTAGGTTAAT-T 58346
VervetAGM         AGGTTTTACCAGTGCTGTATTGAA-GTGGATGAACCTGTGTTGAATTATTTTCTAGGTTAGTTTTTAGCTAGGTAAAT-T 64676
Gorrilla          AGTTTTTACCAGTGCTGTATTGAA-GTGGATGAATCTGTGTTGAATTATTTTCTGTGTTAGTTTTTAGCTAGGTAGAT-T 63457
GrabEatingMacaqueAGTTTTTACCAGTGCTGTATTGAA-GTGGATGAACCTGTGTTGAATTATTTTCTAGGTTAGTTTTTAGCTAGGTAAAT-T 63871
Macaque           AGTTTTTACCAGTGCTGTATTGAA-GTGGATGAACCTGTGTTGAATTATTTTCTAGGTTAGTTTTTAGCTAGGTAAAT-T 64712
PigTailedMacaque  AGTTTTTACCAGTGCTGTGATGAA-GTGGATGAACCTGTGTTGAATTATTTTCTAGGTTAGTTTTTAGCTAGGTAAAT-T 63602
Bonobo            AGTTTTTACCAGTGCTGTATTGAA-GTGGATGAATCTGTGTTGAATTATTTTCTAGGTTAGTTTTTAGCTAGGTAGAT-T 62014
OliveBaboon       AGTTTTTACCAGTGCTGTATTGAA-GTGGATGAACCTGTGTTGAATTATTTTCTAGGTTAGTTTTTAGCTAGGTAAAT-T 63457
Orangutan         AGTTTTTACCAGTGCTGTATTGAA-GTGGATGAACCTGTGTTGAATTATTTTCTAGGTTAGTTTTTAGCTAGGTAAAT-T 63889

Majority          AGTCACGTAAAA----AATTCGTGTATTAAGAGAAAAGGTTTTATATTCCCAGAG-GTGAGAATATTTGAAT---TAAAA
                          77450     77460     77470     77480     77490     77500     77510     77520
Human             AGTCCATAAAAA----AATTCGTATATTAAGAGAAAAGGTTTTATATTCCCAGAG-GTGAGAATATTTGAAT---TAAAA 62989
Coquerel'sSifaka  AGTCACATAAAACTTAATTTTGTATATTAAGGAAAAAGGTTTTGTATTTCCAGAGAGTAGGAATAGTTGAATATTTGAAA 51352
Ma'sNightMonkey   AGTCACATAAAA----ATTTTGTGTATTAAGAGAAAAGGTTTTATATTCCCAGAG-GTGAGAATATTTGAAT---TAAAA 58418
VervetAGM         AGTCACGTAAAA----AATTTGTGTATTAAGAGAAAAGGTTTTATATTCCCAGAG-GTGAGAATATTTGAAT---TAAAA 64748
Gorrilla          AGTCCATAAAAA----AATTCGTGTATTAAGAGAAAAGGTTTTATATTCCCAGAG-GTGAGAATATTTGAAT---TAAAA 63529
GrabEatingMacaqueAGTCACGTAAAA----AATTCATGTATTAAGAGAAAAGGTTTTATATTCCCAGAG-GTGAGAATATTTGAAT---TAAAA 63943
Macaque           AGTCACGTAAAA----AATTCATGTATTAAGAGAAAAGGTTTTATATTCCCAGAG-GTGAGAATATTTGAAT---TAAAA 64784
PigTailedMacaque  AGTCACGTAAAA----AATTCATGTATTAAGAGAAAAGGTTTTATATTCCCAGAG-GTGAGAATATTTGAAT---TAAAA 63674
Bonobo            AGTCCATAAAAA----A-TTCGTATATTAAGAGAAAAGGTTTTATATTCCCAGAG-GTGAGAATATTTGAAT---TAAAA 62085
OliveBaboon       AGTCACGTAAAA----AATTCGTGTATTAAGAGAAAAGGTTTTATATTCCCAGAG-GTGAGAATATTTGAAT---TAAAA 63529
Orangutan         AGTCCATAAAAA----AATTCATGTATTAAGAGAAAAGGTTTTATATTCCCAGAG-GTGAGAATATTTGAAT---TAAAA 63961
```

```
Majority          GCTTTATATACTTAAGC-AACTGTAGAGGTTTACTTTTTCCATAACACGACATGTTTATTCTTTTAATGAAAAGTTAACA
                           77530     77540     77550     77560     77570     77580     77590     77600
Human             GCTTTATATACTTAAGC-AACTGTAGAGGTTTACTTTTTCCATAACACGACATGTTTATTCTTCTAATGAAAAGTTAACA 63068
Coquerel'sSifaka  GCTTTATATACTTAAGT-AGCTATAGAGGTTTACTTTCTCCATAGCAGGACGTGTTTATTCTAATACTGAAAAATCAACA 51431
Ma'sNightMonkey   -CTTTATATACTTAAGC----TGTAGAGGTTTACTGTCTCCATAACAGGACATGTTCATTCTTCTAACGAAAACTTAACA 58493
VervetAGM         GCTTTATATACTTAAGCTAACTGTAGAGGTTTACTTTTTCCATAACACGACATGTTTATTCTTTTAATGAGAAGTTAACA 64828
Gorrilla          GCTTTATATACTTAAGC-AACTGTAGAGGTTTACTTTTTCCATAACACGACATGTTTATTCTTCTAATGAAAAGTTAACA 63608
GrabEatingMacaque GCTTTATATACTTAAGCTAACTGTAGAGGTTTACTTTTTCCATAACACGACATGTTTATTCTTTTAATGAGAAGTTAACA 64023
Macaque           GCTTTATATACTTAAGCTAACTGTAGAGGTTTACTTTTTCCATAACACGACATGTTTATTCTTTTAATGAGAAGTTAACA 64864
PigTailedMacaque  GCTTTATATACTTAAGCTAACTGTAGAGGTTTACTTTTTCCATAACACGACATGTTTATTCTTTTAATGAGAAGTTAACA 63754
Bonobo            GCTTTATATACTTAAGC-AACTGTAGAGTTTACTTTTTCCATAACACGACATGTTTATTCTTCTAATGAAAAGTTAACA 62164
OliveBaboon       GCTTTATATACTTAAGCTAACTGTAGAGGTTTACTTTTTCCATAACACGACATGTTTATTCTTTTAATGAGAAGTTAACA 63609
Orangutan         GCTTTATATACTTAAGC-AACTGTAGAGGTTTACTTTTTCCATAACACGACATGTTTATTCTTCTAATGAAAAGTTAACG 64040

Majority          GTATGTTCACCTGTTCTCTAGTTTGTTTCCAGCTTATATCCAGCTGAACTA--GAAATGGTGATTATATAGGAGCAGAAA
                           77610     77620     77630     77640     77650     77660     77670     77680
Human             GTATGTTCACCTGTTCTCTAGTTTGTTTCCAGCTTATATCCAGCTGAACTA--GAAATGGTGATTATATAGGAGCAGAAA 63146
Coquerel'sSifaka  CTATATTTCCCCATTCTCTAGTTTGTTCCCTAGTT--ACCTAGATGTACTA--GAAATGGTAATTATACAGGAGCAGGAA 51507
Ma'sNightMonkey   ATATGTTCCCCTGTTCTCTAGTTTGTTAACAGCTTATAGCCAGCTGAACTA--GAAATGATGATTATATAGGAGCAGAAA 58571
VervetAGM         GTATGTTCACCTGTTCTCTAGTTTGTTTCCAGCTTATATCCAGCTGAACTA--CAAATGGTGATTATATAGGAGCAGAAA 64906
Gorrilla          GTATGTTCACCTGTTCTCTAGTTTGTTTCCAGCTTATATCCAGCTGAACTA--GGAATGGTGATTATATAGGAGCAGAAA 63686
GrabEatingMacaque GTATGTTCACCTGTTCTCTAGTTTGTTTCCAGCTTCTATCCAGCTGAACTATACAAATGGTGATTATATAGGAGCAGAAA 64103
Macaque           GTATGTTCACCTGTTCTCTAGTTTGTTTCCAGCTTCTATCCAGCTGAACTA--CAAATGGTGATTATATAGGAGCAGAAA 64942
PigTailedMacaque  GTATGTTCACCTGTTCTCTAGTTTGTTTCCAGCTTCTATCCAGCTGAACTA--CAAATGGTGATTATATAGGAGCAGAAA 63832
Bonobo            GTATGTTCACCTGTTCTCTAGTTTGTTTCCAGCTTATATCCAGCTGAACTA--GAAATGGTGGTTATATAGGAGCAGAAA 62242
OliveBaboon       GTATGTTCACCTGTTTTCTAGTTTGTTTCCAGCTTCTATCCAGCTGAACTA--CAAATGGTGATTATATAGGAGCAGAAA 63687
Orangutan         GTATGTTCACCTGTTCTCTAGTTTGTTTCCAGCTTATATCCAGCTGAACTA--GAAATGGTGATTATGTAGGAGCAGAAA 64118

Majority          AAA-TACAAGATAATCATAGCAGTAACATAGTCCTTTATAATTCATGTTGTATTTTCACATATGTGATCTTTAATTTACT
                           77690     77700     77710     77720     77730     77740     77750     77760
Human             AAG-TACAAGATAATCATAGCGGTAACATAGTCCTTTATAATTCATGATGTATTTTCACATATGTTGTCTTTAATTTACT 63225
Coquerel'sSifaka  AAAAACCAAGATAATCATAACAGCAGCAAAGTCTCATACAATTCACAGCATACTTTCACATATACGATCTTTTATTTAGT 51587
Ma'sNightMonkey   AA--TACAAGATAATCATAGCAGTAACATAGTCCTTTAAAATTCATGATGTATTTTAACATATGTGATCTTTAATTTACT 58649
VervetAGM         AAA-TACAAGATAATCATAGCAGTAACATAGTCCTTTAT-------GTTGTATTTTCACATTTGTGATCTTTAGTTTACT 64978
Gorrilla          AAG-TACAAGATAATCATAGCGGTAACATAGTCCTTTATAATTCATGATGTATTTTCACATATGTTGTCTTTAATTTACG 63765
GrabEatingMacaque AAA-TACAAGATAATCATAGCAGTAACATAGTCCTTTAT-------GTTGTATTTTCACATATGTGATCTTTAGTTTACT 64175
Macaque           AAA-TACAAGATAATCATAGCAGTAACATAGTCCTTTAT-------GTTGTATTTTCACATATGTGATCTTTAGTTTACT 65014
PigTailedMacaque  AAA-TACAAGATAATCATAGCAGTAACATAGTCCTTTAT-------GTTGTATTTTCACATATGTGATCTTTAGTTTACT 63904
Bonobo            AAG-TACAAGATAATCATAGCGGTAACATAGTCCTTTATAATTCATGATGTATTTTCACATATGTTGTCTTTAATTTACT 62321
OliveBaboon       AAAATACAAGATAATCGTAGCAGTAACATAGTCCTTTAT-------GTTGTATTTTCACATATGTGGTCTTTAGTTTACT 63760
Orangutan         AAA-TACAAGATAATCATAGCGGTAACATAGTCCTTTATAATTCATGATGTATTTTCATATATGTTGTCTTTAATTTACT 64197
```

```
Majority         CCTCATAATAACCCTGTGAAGAAGCATGACAGCCATTGTCATCATTCCTGTTTTCCCAATTAAGAAATTGAGATTCAGAG

                         77770     77780     77790     77800     77810     77820     77830     77840

Human            CCTCTTAATAACCCT-TGAAGAAGCATGACAGCCATTGTCATCATTCCTGTTTTCCCGATTAAGAAATTGAGATTCAGAG 63304
Coquerel'sSifaka CTT------AACTCTATGAGGAAGCATAGCAGCCATT---ATCATCTCTGTTTTCCAGCTTAAGAAATTGAGATTGAGGG 51658
Ma'sNightMonkey  CTTCATAATAACCCTATGAAGAAGCATGACAGCTATTGTCATTATTCCTATTTTCCAGAGTAAGAAATTGAGATTCAGAG 58729
VervetAGM        CCTCATAATAACCCTGTGAAGAACCATGACAGCCATTGTCATCATTCCTGTTTTCCCAATTAAGAAATTGAGATTCAGAG 65058
Gorrilla         CCTCTTAATAACCCTGTGAAGAAGCATGACAGCCATTGTCATCATTCCTGTTTTCCCGATTAAGAAATTGAGATTCAGAG 63845
GrabEatingMacaqueCCTCATAATAACCCTGTGAAGAACCATGACAGCCATTGTCATCATTCCTGTTTTCCCAATTAAGAAATTGAGATTCAGAG 64255
Macaque          CCTCATAATAACCCTGTGAAGAACCATGACAGCCATTGTCATCATTCCTGTTTTCCCAATTAAGAAATTGAGATTCAGAG 65094
PigTailedMacaque CCTCATAATAACCCTGTGAAGAACCATGACAGCCATTGTCATCATTCCTGTTTTCCCAATTAAGAAATTGAGATTCAGAG 63984
Bonobo           CCTCTTAATAACCCTGTGAAGAAGCATGACAGCCATTGTCATCATTCCTGTTTTCCCGATTAAGAAATTGAGATTCAGAG 62401
OliveBaboon      CCTCATAATAACCCTGTGAAGAACCATGACAGCCATTGTCATCATTCCTGTTTTCCCAATTAAGAAACTGAGATTCAGAG 63840
Orangutan        CCTCTTAATAACCCTGTGAAGAAGCATGACAGCCATTGTCATCATTCCTGTTTTCCCAATTAAGAAATTGAGATTCAGAG 64277

Majority         AGGTTAAACAACTTGCTTAAGATGACCTAAAAGAGTCTAGGCTTTTAATTAATCCTAGACCTGGCACTCGTCAGTTTCAG

                         77850     77860     77870     77880     77890     77900     77910     77920

Human            AGGTCAAATAACTTGCTTAAGATGACCGAAAAGAGTCTAGGCTTTTAATTAATCCTAGACCTGGCACTCGTCAGTTTCAG 63384
Coquerel'sSifaka AGGTTAAG--T--TGCTCAAGATGACGTAATTGAGTCTAGGCTTTT----AAGCCTAGACCTGACACTCTTCTGTTATAG 51730
Ma'sNightMonkey  AGGTTAAAAAACTTGCTTAAGATAACCTAATAGAGTCTAGGCTTTTAATTAATTCTAGACCTGGCACTCATCAATTTCAG 58809
VervetAGM        AGGTTAAACAACTTGCTTAAGATGACCTAAAAGACTCTAGGCTTTTAATTAATCCTAGACCTGGCACTTGTCAGTTTCAG 65138
Gorrilla         AGGTCAGATAACTTGCTTAAGATGACCTAAAAGAGTCTAGGCTTTTAATTAATCCTAGATCTGGCACTCGTCAGTTTCAG 63925
GrabEatingMacaqueAGGTTAAACAACTTGCTTAAGGTGACCTAAAAGACTCTAGGCTTTTAATTAATCCTAGACCTGGCACTTGTCAGTTTCAG 64335
Macaque          AGGTTAAACAACTTGCTTAAGGTGACCTAAAAGACTCTAGGCTTTTAATTAATCCTAGACCTGGCACTTGTCAGTTTCAG 65174
PigTailedMacaque AGGTTAAACAACTTGCTTAAGGTGACCTAAAAGACTCTAGGCTTTTAATTAATCCTAGACCTGGCACTTGTCAGTTTCAG 64064
Bonobo           AGGTCAAATAACTTGCTTAAGATGACCTAAAAGAGTCTAGGCTTTTAATTAATCCTAGACCTGGCACTCGTCAGTTTCAG 62481
OliveBaboon      AGGTTCAACAACTTGCTTAAGGTGACCTAAAAGACTCTAGGCTTTTAATTAATCCTAGACCTGGCACTTGTCAGTTTCAG 63920
Orangutan        AGGTTAAATAA----CTTAAGATGACCTAAAAGAGTCTAGGCTTTTAATTAATCCTAGACCTGGCACTCGTCAGTTTCAG 64353

Majority         AACTTTTCTGGAATGACATTGGATTTGCTAATTTCATCAAGATAATCTGATTGGGGGTGAACATTTCAGGTTAAGGAAA-

                         77930     77940     77950     77960     77970     77980     77990     78000

Human            AACTTTTCGGGAATGACATTGGATTTGCTAATTTCATCAAGATAATCTGATTGGGGGTGAACATTTCAGGTTAAGGAAA- 63463
Coquerel'sSifaka AACTTTCCTGTAATGACATTGGATTTCCTAATTTTATCAAGATATCCTGACTCAGGTTGAACACTTCAAGTTAAGAGTG- 51809
Ma'sNightMonkey  AACTTTTCTGGAATAACATTAGATTTGCTAATTTCATCAAGATAATCTGATTGGGGTTGAACATTTCAGGTTAAGGAAA- 58888
VervetAGM        AACTTTTCTGGAATGACATTGGATTTACTAATTTCATCAAGACACTCTGATTGGGGGTGAACATTTCAGGTTAAGGAAA- 65217
Gorrilla         AACTTTTCGGGAATGACATTGGATTTGCTAATTTCATCAAGATAATCTGATTGGGGGTGAACATTTCAGGTTAAGGAAA- 64004
GrabEatingMacaqueAACTTTTCTGGAATGACATTGGATTTACTAATTTCATCAAGACAGTCTGATTGGGGGTGAACATTTCAGGTTAAGGAAA- 64414
Macaque          AACTTTTCTGGAATGACATTGGATTTACTAATTTCATCAAGACAGTCTGATTGGGGGTGAACATTTCAGGTTAAGGAAA- 65253
PigTailedMacaque AACTTTTCTGGAATGACATTGGATTTACTAATTTCATCAAGACAGTCTGATTGGGGGTGAACATTTCAGGTTAAGAAAA- 64143
Bonobo           AACTTTTCGGGAATGACATTGGATTTGCTAATTTCATCAAGATAATCTGATTGGGGGTGAACATTTCAGGTTAAGGAAAT 62561
OliveBaboon      AACTTTTCTGGAATGACATTGGATTTACTAATTTCATCAAGACAGTCTGATTGGGGGTGAACATTTCAGGTTAAGGAAA- 63999
Orangutan        AACTTTTCTGGAATGACATTGGATTTGCTAATTTCATCAAGATAATCTGATTGGGGGTGAACATTTC------AGGAAA- 64426
```

```
Majority         ----------------ATAC----TTTTTTCTTTTTTGCCTCATTTTATGGGAATTGCTATTATGTGTTAGGATAATTTG

                         78010     78020     78030     78040     78050     78060     78070     78080

Human            ----------------ATACTAGTTTTTTTCTTTTTTGCCTCATTTTATGGGAATTGCTATTATGTGTTAGGATAATTTG 63527
Coquerel'sSifaka ----------------ATAC-------TAGTTTTTTTGTCCTATTTCATGGGAATTGCTATTGTACGCTGTAGT------ 51860
Ma'sNightMonkey  ----------------ATACTAGCTTTTTTCTTTTTTACCTCATTTTATGGGAATTGCTATTATGTGTTAGGATAATTTG 58952
VervetAGM        ----------------ATAG----TTTTTTCTTTTTTGCCTCATTTTATGGGAATTGCTATTATGTGTTAGGATAATTTG 65277
Gorrilla         ----------------ATACTAGTTTTTTTCTTTTTTGCCTCATTTTATGGGAATTGCTATTATGTGTTAGGATAATTTG 64068
GrabEatingMacaque----------------ATAG----TTTTTTCTTTTTTGCCTCATTTTATGGGAATTGCTATTATGTGTTAGGATAATTTG 64474
Macaque          ----------------ATAG----TTTTTTCTTTTTTGCCTCATTTTATGGGAATTGCTATTATGTGTTAGGATAATTTG 65313
PigTailedMacaque ----------------ATAG----TTTTTTCTTTTTTGCCTCATTTTATGGGAATTGCTATTATGTGTTAGGATAATTTG 64203
Bonobo           GTTTTTTTAAAAGAAAAAACTAGTTTTTT-CTTTTTTGCCTCATTTTATGGGAATTGCTATTATGTGTTAGGATAATTTG 62640
OliveBaboon      ----------------ATAG----TTTTTTCTTTTTTGCCTCATTTTATGGGAATTGCTATTATGTGTTAGGATAATTTG 64059
Orangutan        ----------------ATAC--ATTTTTTTCTTTTTTGCCTCATTTTATGGGAATTGCTATTATGTGTTAGGATAATTTG 64488

Majority         GTTTGAAGCAACTTTGTACAATGTGGCATCAAATAGCAGTTACCTTATAAAACTGTACTGAATCATATAAGTACTGCCAT

                         78090     78100     78110     78120     78130     78140     78150     78160

Human            GTTTGAAGCAACTTTGTACAATGTGGCATCAAATAGCAGTTACCTTCTAAAACTGTACTGAATCATATAAGTACTGCCAT 63607
Coquerel'sSifaka ----GAAGCAACGTCATGTAATGTGGCATCAAACTGCAGTTACCTTTGAAAACTATACCAATTCATATAAGTACTGCCAT 51936
Ma'sNightMonkey  GTTTGAAGCAACTTTGTACAGTGTAGCATCAAATAGCAGTTACTTCATAAAACTGTACTGATTCATATAAGTACTGCCAT 59032
VervetAGM        GTTTGAAGCAACTTTGTACAATGTGGCATCAAATAGCAGTTACCTTATAAAACTGTACTGAATCATCTAAGTACTGCTAT 65357
Gorrilla         GTTTGAAGCAACTTTGTACAATGTGGCATCAAATAGCAGTTACCTTATAAAACTGTACTGAATCATATAAGTACTGCCAT 64148
GrabEatingMacaqueGTTTGAAGCAGCTTTGTACAATGTGGCATCAAATAGCAGTTACCTTATAAAACTGTACTGAATCATCAAAGTACTGCCAT 64554
Macaque          GTTTGAAGCAGCTTTGTACAATGTGGCATCAAATAGCAGTTACCTTATAAAACTGTACTGAATCATCAAAGTACTGCCAT 65393
PigTailedMacaque GTTTGAAGCAGCTTTGTACAATGTGGCATCAAATAGCAGTTACCTTATAAAACTGTACTGAATCATCAAAGTACTGCCAT 64283
Bonobo           GTTTGAAGCAACTTTGTACAATGTGGCATCAAATAGCAGTTACCTTCTAAAACTGTACTGAATCATATAAGTACTGCCAT 62720
OliveBaboon      GTTTGAAGCAGTTTTGTACAATGTGGCATCAAATAGCAGTTACCTTATAAAACTGTACTGAATCATCAAAGTACTGCCAT 64139
Orangutan        GTTTGAAGCAACTTTGTACAATGTGGCATCAAATAGCAGTTACCTTATAAAACTGTACTTAATCATATAAGTACTGCCAT 64568

Majority         CCAGCAATAAATATATGTATAATAAAATTAACCTTATTGAGCAGAGTAGTGTGGTACTACTTTGGTAGGACTTCCTGTTT

                         78170     78180     78190     78200     78210     78220     78230     78240

Human            CCAGCAATAAATATATGTATAATAAAATTAACCTTATTGAGCAGAGTAGTGTGGTACTACTTTGGTAGGACTTCCTGTTT 63687
Coquerel'sSifaka ATAGCGATAAATACATGTAGAGCAAAATTAATTTTATTATGCATACTAGTATGGTACTACTTTGGTAGGACTGACTATAT 52016
Ma'sNightMonkey  CCAGCAATAAATATATGTATAATAAAATTAACCTTACTGAGCAGGGTAATATGGTACTACTTCAGTAGGACTTCCTGTTT 59112
VervetAGM        CCAGCAATAAATATATGTATAATAAAATTAACCTTATTGAGCAGAGTAGTGTGGTACTACTTTG-CAGGACTTCCTGTTT 65436
Gorrilla         CCAGCAATAAATATATGTATAATAAAATTAACCTTACTGAGCAGAGTAGTGTGGTACTACTTTGGTAGGACTTCCTGTTT 64228
GrabEatingMacaqueCCAGCAATAAATATATGTATAATAAAATTAACCTTATTGAGCAGAGTAGTGTGGCACTACTTTG-CAGGACTTCCTGTTT 64633
Macaque          CCAGCAATAAATATATGTATAATAAAATTAACCTTATTGAGCAGAGTAGTGTGGCACTACTTTG-CAGGACTTCCTGTTT 65472
PigTailedMacaque CCAGCAATAAATATCTGTATAATAAAATTAACCTTATTGAGCAGAGTAGTGTGGCACTACTTTG-CAGGACTTCCTGTTT 64362
Bonobo           CCAGCAATAAATGTATGTATAATAAAATTAACCTTATTGAGCAGAGTAGTGTGGTACTACTTTGGTAGGACTTCCTGTTT 62800
OliveBaboon      CCAGCAATAAATATATGTATAATAAAATTAACCTTATTGAGCAGAGTAGTGTGGCACTACTTTG-CAGGACTTCCTGTTT 64218
Orangutan        TCAGCAATAAATATATGTATAATAAAATTAACCTTATTGAGCAGAGTAGTGTGGTACTACTTTGGTAGGACTTCCTGTTT 64648
```

```
Majority          GTGATCCTTATTAGTTTTTTTAT-TAAATTAACCTATTTGCAATTTTTTACAATTAAAAGTATTAGCATGGATGCTTAGC
                          78250     78260     78270     78280     78290     78300     78310     78320
Human             GTGATCCTTATTAGTTTTTTT---AAAACTAACCTATTTGCAATTTTTAAAAATTAAAAGTATTAGCATGGATGCTTAGC 63764
Coquerel'sSifaka  TTGACCCTTACTAATTTTTTT---TAAATTA------TT------TTTTATAATTAAAAGTATTAGCATGGTT-TTTAAT 52080
Ma'sNightMonkey   GTGACCCTTATTAGTTTTTAA----AAATTAACTTATTTGCAATTATAAAAAATTAAAAGTATTAGCATGGATGCTTAGC 59188
VervetAGM         GTGATCCTTATTAGTTTTTTAAT-TAAATTAACATATTTGCAATTTTTTACAATTAAAAGTATTAGCATGGATGCTTAGC 65515
Gorrilla          GTGACCCTTATTAGTTTTTTT---AAAACTAACCTATTTGCAATTTTTAAAAATTAAAAGTATTAGCATGGATGCTTAGC 64305
GrabEatingMacaqueGTGATCCTTATTAATTTTTTTAT-TAAATTAACCTATTTGCAATTTTTTACAATTAAAAGTATTAGCATGGATGCTTAGC 64712
Macaque           GTGATCCTTATTAATTTTTTTAT-TAAATTAACCTATTTGCAATTTTTTACAATTAAAAGTATTAGCATGGATGCTTAGC 65551
PigTailedMacaque  GTGATCCTTATTAATTTTTTTAT-TAAATTAACCTATTTGCAATTTTTTACAATTAAAAGTATTAGCATGGATGCTTAGC 64441
Bonobo            GTGATCCTTATTAGTTTTTTT---TAAACTAACCTATTTGCAATTTTTAAAAATTGAAAGTATTAGCATGGATGCTTAGC 62877
OliveBaboon       GTGATCCTTATTAGTTTTTTTAT-TAAATTAACCTATTTGCAATTTTTTACAATTAAAAGTATTAGCATGGATGCTTAGC 64297
Orangutan         GTGACCCTTATTAGTTTTTTTTTTAAAATTAACCTATTTGCAATTTTTAAAAATTAAAAGTATTAGCATGGATGCTTAGC 64728

Majority          TGGTTG----TATTTTAGGTTGAAGTAGCATTTTCTATTTAAAGAAGATAATATTTGGGGGAAACTAATACATGGACAGC
                          78330     78340     78350     78360     78370     78380     78390     78400
Human             TGGTTG----TATTTTAGGTTGAAGTAGCATTTTCTATTTAAGGAAGATAATATTTGGGGGAAAGTAATACATGGACAGC 63840
Coquerel'sSifaka  TGGTTG----TATTTTAGATTTAAGTAGCATTTTCTATTTAAAGAAGATAGTTTCAGGTGGAAACTAATACAAATACAGC 52156
Ma'sNightMonkey   TGGTTG----TATTTTAGATTGAAGTAGCATTTTCTATTTAAGGAAGATAATATTTGGTGGAAACTAATACATGCACAGC 59264
VervetAGM         TGATTG----TATTTTAGGTTGAAGTAGCATTTTCTATTTAAAGAAGATAATATTTGGAGGAAACTAATACATGGACAGC 65591
Gorrilla          TGGTTG----TATTTTAGGTTGAAGTAGCATTTTCTATTTAAGGAAGATAATATTTGGGGGAAAGTAATACATGGACAGC 64381
GrabEatingMacaqueTGATTG----TATTTTAGGTTGAAGTAGCATTTTCTATTTAAAGAAGATAATATTTGGGGGAAACTAATACATGGACAGC 64788
Macaque           TGATTG----TATTTTAGGTTGAAGTAGCATTTTCTATTTAAAGAAGATAATATTTGGGGGAAACTAATACATGGACAGC 65627
PigTailedMacaque  TGATTG----TATTTTAGGTTGAAGTAGCATTTTCTATTTAAAGAAGATAATATTTGGGGGAAACTAATACATGGACAGC 64517
Bonobo            TGGTTG----TATTTTAGGTTGAAGTAGCATTTTCTATTTAAGGAAGATAATATTTGGGGGAAACTAACACATGGACAGC 62953
OliveBaboon       TGATTG----TATTTTAGGTTGAAGTAGCATTTTCTATTTAAAGAAGATAATATTTGGGGGAAACTAATACATGGACAGC 64373
Orangutan         TGGTTGGTTGTATTTTAGGTTGAAGTAGCATTTTCTATTTAAGGAAGATAATATTTGGGGGAAACTAATACATGGACAGC 64808

Majority          TGGAACAGCAAAATTTGATGTATGAAATTTGAATCCTTTTCCTGGTAGCTTTT-AAGTTCGAGTTTTTGTTGTTGAGTTT
                          78410     78420     78430     78440     78450     78460     78470     78480
Human             TGGAACAGCAAAATTTGATATATGAAATTTGAATCCTTTTCCTGGTAGCTTTT-AAGTTCGAGTTTTTGTTGTTGAGTTT 63919
Coquerel'sSifaka  TGGAACAGCAAAGTATGATGTATGAAATTTGAATTCTTTCCCTGGTAGCTTTT-ATGTGCAGGTTTTCATTGTTGAGTTT 52235
Ma'sNightMonkey   TA-AACAACAAAATAAGATGTATGAAATTTTAATCCTTTTCCTGGTGGCTTTT-AAGTTCGAGTTTTCGTTCTTTATTTT 59342
VervetAGM         TGGAATAGCAAAATTTGATGTATGAAATTGGAATCCTTTTCCTGCTAGCTTTT-AAGTTCGAGTTTTTGTTGTTGAATTT 65670
Gorrilla          TGGAACAGCAAAATTTGATATATGAAATTTGAATCCTTTTCCTGGTAGCTTTT-AAGTTTGAGTTTTTGTTGTTGAGTTT 64460
GrabEatingMacaqueTGGAATAGCAAAATTTGATGTATGAAATTGGAATCCTTTTCCTGGTAGCTTTTTAAGTTCGAGTTTTTGTTGTTGAATTT 64868
Macaque           TGGAATAGCAAAATTTGATGTATGAAATTGGAATCCTTTTCCTGGTAGCTTTTTAAGTTCGAGTTTTTGTTGTTGAATTT 65707
PigTailedMacaque  TGGAATAGCAAAATTTGATGTATGAAATTGGAATCCTTTTCCTGGTAGCTTTT-AAGTTCGAGTTTTTGTTGTTGAATTT 64596
Bonobo            TGGAACAGCAAAATTTGATATATGAAATTTGAATCCTTTTCCTGGTAGCTTTT-AAGTTCGAGTTTTTGTTGTTGAGTTT 63032
OliveBaboon       TGGAATAGCAAAATTTGATGTATGAAATTGGAATCCTTTTCCTGGTAGCTTTT-AAGTTCGAGTTTTTGTTGTTGAATTT 64452
Orangutan         TGGAACAGCAAAATTTGATATATGAAATTTGAATCCTTTTCCTGGTAGCTTTT-AAGTTCGAGTTTTTGTTGTTGAGTTT 64887
```

```
Majority          TATTA-TA-AAATTATACTTTCACTAATTTTTATTTTTTGTTTAAGATTGCTGATATACTTTATTCACTTTTACAGGCCC

                          78490     78500     78510     78520     78530     78540     78550     78560

Human             TATTACTA-AAATTATACTTTCACTAATTTTTATTTTTTGTTTAAGATTGCTGATATGCTTTATTCACTTTTACAGGCCC 63998
Coquerel'sSifaka  TATTGTCAGAAATCATACTTT-GCTAATTTTTATTCCTTATTTTAGATTGCTAATATACTTCATTCACTTTTACAGGCCC 52314
Ma'sNightMonkey   TATTATTA-AAATTATACTTTCACTAATTTTTATTCT----TTAAGATTGCTGATATACTTTATTCACTTTTACAGGCCC 59417
VervetAGM         TATTA----AAATTATACTTTCACTAATTTTTATGTTTTGTTTAAGATTGCTGATGTACTTTATTCACTTTTACAGGCCC 65746
Gorrilla          TATTACTA-AAATTATACTTTCACTAATTTTTATTTTTTGTTTAAGATTGCTGATATGCTTTATTCACTTTTACAGGCCC 64539
GrabEatingMacaqueTATTA----AAATTATACTTTCACTAATTTTTATTTTTTGTTTAAGATAGCTGATGTACTTTATTCACTTTTACAGGCCC 64944
Macaque           TATTA----AAATTATACTTTCACTAATTTTTATTTTTTGTTTAAGATAGCTGATGTACTTTATTCACTTTTACAGGCCC 65783
PigTailedMacaque  TATTA----AAATTATACTTTCACTAATTTTTATTTTTTGTTTAAGATTGCTGATATACTTTATTCACTTTTACAGGCCC 64672
Bonobo            TATTACTA-AAATTATACTTTCACTAATTTTTATTTTTTGTTTAAGATTGCTGATATGCTTTATTCACTTTTACAGGCCC 63111
OliveBaboon       TATTA----AAATTATACTTTCACTAATTTTTATTTTTTGTTTAAGATTGCTGATGTACTTTATTCACTTTTACAGGCCC 64528
Orangutan         TATTACTA-AAATTATACTTTCACTAATTTTTATTTTTTGTTTAAGATTGCTGATATGCTTTATTCACTTTTACAGGCCC 64966

Majority          TAGATGGTCAGAATATTTATAATGCCTGCTGTACCCTAAGGATTGATTTTTCCAAACTTGTGAATTTGAATGTAAAATAC

                          78570     78580     78590     78600     78610     78620     78630     78640

Human             TAGATGGTCAGAATATTTATAATGCCTGCTGTACCCTAAGGATTGATTTTTCCAAACTTGTGAATTTGAATGTAAAATAC 64078
Coquerel'sSifaka  TAGATGGTCAGAATATTTATAATGCTTGCTGTACCCTAAGGATTGATTTTTCCAAACTTGTGAATTTGAATGTAAAATAC 52394
Ma'sNightMonkey   TAGATGGTCAGAATATTTATAATGCCTGCTGTACCCTAAGGATTGATTTTTCCAAACTTGTGAATTTGAATGTAAAATAC 59497
VervetAGM         TAGATGGTCAGAATATTTATAATGCCTGCTGTACCCTAAGGATTGATTTTTCCAAACTTGTGAATTTGAATGTAAAATAC 65826
Gorrilla          TAGATGGTCAGAATATTTATAATGCCTGCTGTACCCTAAGGATTGATTTTTCCAAACTTGTGAATTTGAATGTAAAATAC 64619
GrabEatingMacaqueTAGATGGTCAGAATATTTATAATGCCTGCTGTACCCTAAGGATTGATTTTTCCAAACTTGTGAATTTGAATGTAAAATAC 65024
Macaque           TAGATGGTCAGAATATTTATAATGCCTGCTGTACCCTAAGGATTGATTTTTCCAAACTTGTGAATTTGAATGTAAAATAC 65863
PigTailedMacaque  TAGATGGTCAGAATATTTATAATGCCTGCTGTACCCTAAGGATTGATTTTTCCAAACTTGTGAATTTGAATGTAAAATAC 64752
Bonobo            TAGATGGTCAGAATATTTATAATGCCTGCTGTACCCTAAGGATTGATTTTTCCAAACTTGTGAATTTGAATGTAAAATAC 63191
OliveBaboon       TAGATGGTCAGAATATTTATAATGCCTGCTGTACCTTAAGGATTGATTTTTCCAAACTTGTGAATTTGAATGTAAAATAC 64608
Orangutan         TAGATGGTCAGAATATTTATAATGCCTGCTGTACCCTAAGGATTGATTTTTCCAAACTTGTGAATTTGAATGTAAAATAC 65046

Majority          AACAATGATAAAAGTAGGGATTATACTCGACCTGATCTTCCATCTGGGGATGGACAACCTGCATTGGACCCAGCTATTGC

                          78650     78660     78670     78680     78690     78700     78710     78720

Human             AACAATGATAAAAGTAGGGATTATACTCGACCTGATCTTCCATCTGGGGATGGACAACCTGCATTGGACCCAGCTATTGC 64158
Coquerel'sSifaka  AACAATGATAAAAGTAGGGATTATACTCGACCTGATCTTCCATCTGGGGATGGACAACCTGCATTGGACCCAGCTATTGC 52474
Ma'sNightMonkey   AACAATGATAAAAGTAGGGATTATACTCGACCCGATCTTCCCTCTGGGGATGGACAACCTGCATTGGACCCAGCTATTGC 59577
VervetAGM         AACAATGATAAAAGTAGGGATTATACTCGACCTGATCTTCCGTCTGGGGATGGACAACCTGCATTGGACCCAGCTATTGC 65906
Gorrilla          AACAATGATAAAAGTAGGGATTATACTCGACCTGATCTTCCATCTGGGGATGGACAACCTGCATTGGACCCAGCTATTGC 64699
GrabEatingMacaqueAACAATGATAAAAGTAGGGATTATACTCGACCTGATCTTCCGTCTGGGGATGGACAGCCTGCATTGGACCCAGCTATTGC 65104
Macaque           AACAATGATAAAAGTAGGGATTATACTCGACCTGATCTTCCGTCTGGGGATGGACAGCCTGCATTGGACCCAGCTATTGC 65943
PigTailedMacaque  AACAATGATAAAAGTAGGGATTATACTCGACCTGATCTTCCATCTGGGGATGGACAGCCTGCATTGGACCCAGCTATTGC 64832
Bonobo            AACAATGATAAAAGTAGGGATTATACTCGACCTGATCTTCCATCTGGGGATGGACAACCTGCATTGGACCCAGCTATTGC 63271
OliveBaboon       AACAATGATAAAAGTAGGGATTATACTCGACCTGATCTTCCGTCTGGGGATGGACAACCTGCATTGGACCCAGCTATTGC 64688
Orangutan         AACAATGATAAAAGTAGGGATTATACTCGACCTGATCTTCCATCTGGGGATGGACAACCTGCATTGGACCCAGCTATTGC 65126
```

```
Majority          TGCAGCATTTGCCAAGGAGACATCCCTCTTAGGTATGATTTTTATTGTCTTAACCACTTTTCTCCCATTTTGCCAAATGG

                          78730     78740     78750     78760     78770     78780     78790     78800

Human             TGCAGCATTTGCCAAGGAGACATCCCTCTTAGGTATGATTTTTATTGTCTTAACCACTTTTCTCCCATTTTGCCAAATGG 64238
Coquerel'sSifaka  TGCAGCATTTGCCAAGGAGACATCCCTCTTAGGTATGATTTTTATTGCCTGTACCACTTTTTTCCCATTTTGCCAAATGG 52554
Ma'sNightMonkey   TGCAGCATTTGCCAAGGAAACATCCCTCTTAGGTATGATTCTTATTGTCTTAACCACTTTTCTCTCATTTTGCCAAATGG 59657
VervetAGM         TGCAGCATTTGCCAAGGAGACATCTCTCTTAGGTATGATTTTTATTGTCTTAACCACTTTTCTCCCATTTTGCCGAATGG 65986
Gorrilla          TGCAGCATTTGCCAAGGAGACATCCCTCTTAGGTATGATTTTTATTGTCTTAACCACTTTTCTCCCATTTTGCCAAATGG 64779
GrabEatingMacaqueTGCAGCATTTGCCAAGGAGACATCTCTCTTAGGTATGATTTTTATTGGCTTAACCACTTTTCTCCCATTTTGCCAAATGG 65184
Macaque           TGCAGCATTTGCCAAGGAGACATCTCTCTTAGGTATGATTTTTATTGGCTTAACCACTTTTCTCCCATTTTGCCAAATGG 66023
PigTailedMacaque  TGCAGCATTTGCCAAGGAGACATCTCTCTTAGGTATGATTTTTATTGGCTTAACCACTTTTCTCCCATTTTGCCAAATGG 64912
Bonobo            TGCAGCATTTGCCAAGGAGACATCCCTCTTAGGTATGATTTTTATTGTCTTAACCACTTTTCTCCCATTTTGCCAAATGG 63351
OliveBaboon       TGCAGCATTTGCCAAGGAGACATCTCTCTTAGGTATGATTTTTATTGTCTTAACCACTTTTCTCCCATTTTGCCAAATGG 64768
Orangutan         TGCAGCATTTGCCAAGGAGACATCCCTCTTAGGTATGATTTTTATTGTCTTAACCACTTTTCTCCCATTTTGCCAAATGG 65206

Majority          AAAAGTA-----------CCAGTAAGTATAATGAATCCTCCCATTTTGGACCTTACCAAATTGTGTTAGGTTTCATGAGT

                          78810     78820     78830     78840     78850     78860     78870     78880

Human             AAAAGTA-----------CCAGTAAGTATAATGAATCCCCCCATTTTGGACCTTACCAAATTGTGTTAGGTTTCGTGAGT 64307
Coquerel'sSifaka  GAAAATGACTGTTAAATTCCAGTAAGTATAATGAATC-TCCCATTTTGGACCTTAACAAATTG--TTAGGTTTTATGAGT 52631
Ma'sNightMonkey   AAAAATA-----------CCAGTAAGTATAATGAATCACCCCATTTTGGACCTTACCAAATTGTGTTTGGTTTCATGAGT 59726
VervetAGM         AAAAGTA-----------CCAGTAAGTATAATGAATCCTTCCATTTTGGACCTTACCAAATTGTGTTAGGTTTCATGAGT 66055
Gorrilla          AAAAGTA-----------CCAGTAAGTATAATGAGTCCTCCCATTTTGAACCTTACCAAATTGTGTTAGGTTTCGTGAGT 64848
GrabEatingMacaqueAAAAGTA-----------CCAGTAAGTATAATGAATCCTTCCATTTTGGACCTTACCAAATTGTGTTAGGTTTCATGACT 65253
Macaque           AAAAGTA-----------CCAGTAAGTATAATGAATCCTTCCATTTTGGACCTTACCAAATTGTGTTAGGTTTCATGAGT 66092
PigTailedMacaque  AAAAGTA-----------CCAGTAAGTATAATGAATCCTTCCATTTTGGACCTTACCAAATTGTGTTAGGTTTCATGAGT 64981
Bonobo            AAAAGTA-----------CCAGTAAGTATAATGAATCCCCCCATTTTGGACCTTACCAAATTGTGTTAGGTTTCGTGAGT 63420
OliveBaboon       AAAAGTA-----------CCAGTAAGTATAATGAATCCTTCCATTTTGGACCTTACCAAATTGTGTTAGATTTCATGAGT 64837
Orangutan         AAAAGTA-----------CCAGTAAGTATAATGAATCCCCCCATTTTGGACCTTACCAACTTGTGTTAGGTTTCATGAGT 65275

Majority          TTTCTTTTT-CTCAAGTGAGA-AGGCATATGAATACTGTTAAGAAAAACCCAAAGTGTTCTTCATAAGTCTAATTAAAAT

                          78890     78900     78910     78920     78930     78940     78950     78960

Human             TTTCTTTTT-CTCAAGTGAGA-AGGCATATGAATACTGTTAAGAAAAACCCAAAGTATTCTTTATAAGTCTAATTAAAAT 64385
Coquerel'sSifaka  TTTGTTTTT-CTCAGGTGAGA-AGGCATATGTGTGCTTTTAGAAAAAACCTAAAGTGTCCTTCATAAGCATAATTAAAAT 52709
Ma'sNightMonkey   TTTATTTTT-CTCAAGTGAGACAGGCATATGAATACTGTTAAGAAAAACCCAAAGTGTTCTTCCTAAGTGTGATTAAAAT 59805
VervetAGM         TTTCTTTTT-CTCAAGTGAGA-AGGCATATTAATACTATTAAGAACAACCCAAAGTGTTCTCCGTAACTCTAAGTAAAAT 66133
Gorrilla          TTTCTTTTT-CTCAAGTGAGA-AGGCATATGAATACTGTTAAGAAAAACCCAAAGTGTTCTTCATAAGTCTAATTAAAAT 64926
GrabEatingMacaqueTTTCTTTTT-CTCAAGTGAGA-AGGCATATTAATACTGTTAAGAACAACCCAAAGTATTCTCCATAACTCTACGTAAAAT 65331
Macaque           TTTCTTTTT-CTTAAGTGAGA-AGGCATATTAATACTGTTAAGAACAACCCAAAGTGTTCTCCATAACTCTACGTAAAAT 66170
PigTailedMacaque  TTTCTTTTT-CTCAAGTGAGA-AGGCATATTAATACTGTTAAGAACAACCCAAAGTGTTCTCCGTAACTCTACGTAAAAT 65059
Bonobo            TTTCTTTTTTCTCAAGTGAGA-AGGCATATGAATACTGTTAAGAAAAACCCAAAGTATTCTTTATAAGTCTAATTAAAAT 63499
OliveBaboon       TTTCTTTTT-CTCAAGTGAGA-AGGCGTATTAACACTGTTAAGAACAACCCAAAGTGTTCTCCGTAACTCTACGTAAAAT 64915
Orangutan         TTTTTTTTT-CTCAAGTGAGA-AGGCATATGAATACTGTTAAGAAAAACCCAAAGTGTTCTTCATAAGTCTAATTAAAAT 65353
```

```
Majority         TAT--GTTAGTTTTGTTCCTTTTAATACCTTCAGCTTCTCTTCCATG--CCACTCTCCCAGAACAAAAGAAG--------

                         78970     78980     78990     79000     79010     79020     79030     79040

Human            TATATGTTAGTTTTGTTCCTTTTAATACCTTCAGCTTCTCTTCCATG--CCACTCTCCCAGAACAAAAGAAG-------- 64455
Coquerel'sSifaka TAT--ATTAGTTTTATTCCTTTTACCACCCTTTCCTTGTCTTTCTTTCACACCACTCCCAAAACAAAAGAAGTTCATTAG 52787
Ma'sNightMonkey  TAT--GTTAGTTTTGTTCCTTTTAATACCTTCACCTTTTCTTCCATGTGCTGTTTTCCCAGAACAAAAGAAG-------- 59875
VervetAGM        TAT--GTTAGTTTTGTTCCTTTTAATACCTTCAGCTTCTCTTCCATG--CCACTCGCCCAGAACAAAAGAAG-------- 66201
Gorrilla         TATATGTTAGTTTTGTTCCTTTTAATACCTTCAGCTTCTCTTCCATG--CCACTCTCCCAGAACAAAAGAAG-------- 64996
GrabEatingMacaqueTAT--GTTAGTTTTGTTCCTTTTAATACCTTCAGCTTCTCTTCCATG--CCACTCGCCCAGAACAAAAGAAG-------- 65399
Macaque          TAT--GTTAGTTTTGTTCCTTTTAATACCTTCAGCTTCTCTTCCATG--CCACTCGCCCAGAACAAAAGAAG-------- 66238
PigTailedMacaque TAT--GTTAGTTTTGTTCCTTTTAATACCTTTAGCTTCTCTTCCATG--CCACTCGCCCAGAACAAAAGAAG-------- 65127
Bonobo           TATATGTTAGTTTTGTTCCTTTTAATACCTTCAGCTTCTCTTCCATG--CCACTCTCCCAGAACAAAAGAAG-------- 63569
OliveBaboon      TAT--GTTAGTTTTGTTCCTTTTAATACCTTCAGCTTCTCTTCCATG--CCACTCGCCCAGAACAAAAGAAG-------- 64983
Orangutan        TATTTGTTAGTTTTGTTCCTTTTAATACCTTCAGCTTCTCTTCCATG--CCACTCTCCCAGAACAAAAGAAG-------- 65423

Majority         ---AAAAGCTCTAATGAAGAAATACTGTTGAATTCTTAATGCCATA----TTTACACTTTAAACTTGATGTCACTATGCC

                         79050     79060     79070     79080     79090     79100     79110     79120

Human            ---AAAAGCTCTAATGAAGAAATACTGTTGAATTCTTAATGCCATA----TTTACACTTTAAACTTGATGTCACTATGCC 64528
Coquerel'sSifaka AACAGAAGTTCTAATGGAGAAATACTGTTGAATTCTTAATGAAATAAATATTTACACTTTAAACT---TGTCATACTCTC 52864
Ma'sNightMonkey  ---GAAAGCTTTAATGAAGAATTACTGTTGAATTCTTAATGACATG----TTTACACTTTAAACTTTATGTCACTATGTC 59948
VervetAGM        ---AAAAGCTCTAATGAAGAAATACTGTTGAATTATTAATGCCATA----GTTACACTTTAAACTTGATGTTACTATGCC 66274
Gorrilla         ---AAAAGCTCTAATGAAGAAATACTGTTGAATTCTTAATGCCATA----TTTACACTTTAAACTTGATGTCACTATGCC 65069
GrabEatingMacaque---AAAAGCTCTAATGAAGAAATACTGTTGAATTCTTAATGCCATA----GTTACACTTTAAACTTGATGTCACTATGCC 65472
Macaque          ---AAAAGCTCTAATGAAGAAATACTGTTGAATTCTTAATGCCATA----GTTACACTTTAAACTTGATGTCACTATGCC 66311
PigTailedMacaque ---AAAAGCTCTAATGAAGAAATACTGTTGAATTCTTAATGCCATA----GTTACACTTTAAACTTGATGTCACTATGCC 65200
Bonobo           ---AAAAGCTCTAATGAAGAAATACTGTTGAATTCTTAATGCCATA----TTTACACTTTAAACTTGATGTCACTATGCC 63642
OliveBaboon      ---AAAAGCTCTAATGAAGAAATACTGTTGAATTCTTAATGCCATA----GTTACACTTTAAACTTGATGTCACTATGCC 65056
Orangutan        ---AAAAGCTCTAATGAAGAAATACTGTTGAATTTTTAATGCCATA----TTAACACTTTAAACTTGATGTCACTATGCC 65496

Majority         TA----AGTCTAGGCCTGAAGTTCTTGTGTGTGTTTTTCAGTGCTGATCTGATTGTTTTAATTTAGCATTG----CATGG

                         79130     79140     79150     79160     79170     79180     79190     79200

Human            TG----AGTCTAGGCCTAAAGTTCTTGTGTGTGTTTTTCAGTGCTGATCTGATTGTTTTAATTTAGCATTG----CATGG 64600
Coquerel'sSifaka TA---------AGGCT--AAGGCCCTGTGCCTGTTCTTTAGGGCTGATCTGATTGTTTAAGTTCAGTGCTG----CGTGG 52929
Ma'sNightMonkey  TA----AGTCTAGACCTGAAGTTCTTGTGTGTGTTTTTCAGTGCTGATTTGATTGTTTTAATTCATTATTG----CATGG 60020
VervetAGM        TA----AGTCTAGGCCTGAAGTTCTTGTGTGTGTTTTTCAGTGCTGATCTGATTGTTTGGATTTAGCATTG----CATGG 66346
Gorrilla         TG----AGTCTAGGCCTAAAGTTCTTGTGCGTGTTTTTCAGTGCTGATCTGATTGTTTTAATTTAGCATTG----CATGG 65141
GrabEatingMacaqueAA----AGTCTAGGCCCGAAGTTCTTGTGTGTGTTTTTCAGTGCTGATCTGATTGTTTTAATTTAGCATTGATTGCATGG 65548
Macaque          AA----AGTCTAGGCCCGAAGTTCTTGTGTGTGTTTTTCAGTGCTGATCTGATTGTTTTAATTTAGCATTG----CATGG 66383
PigTailedMacaque TA----AGTCTAGGCCCGAAGTTCTTGTGTGTGTTTTTCAGTGCTGATCTGATTGTTTTAATTTAGCATTG----CATGG 65272
Bonobo           TG----AGTCTAGGCCTAAAGTTCTTGTGCGTGTTTTTCAGTGCTGATCTGATTGTTTTAATTTAGCATTG----CATGG 63714
OliveBaboon      TA----AGTCTAGGCCTGAAGTTCTTGTGTGTGTTTTTCAGTGGTGATCTGATTGTTTTAATTTAGCATTG----CATGG 65128
Orangutan        TGCCTGAGTCTAGGCCTGAAGTTCTTGTGCGTGTTTTTCAGTGCTGATCTGATTGTTTTAATTTAGCATTG----CATGG 65572
```

```
Majority         AAACAGTGAAATGTTAAGAGACAAAACATACATAATGGGTGGAGGCATATGGGATTTTTTTT--GAAGCATATTGTAGT

                         79210     79220     79230     79240     79250     79260     79270     79280

Human            AAACAGTGAAATGTTAAGAGACAAAACATACATAATGGGTGGAGGCATATGGGATTTTTTTT---GAAGCATATTGTAGT 64677
Coquerel'sSifaka AAACAGTGAAATGTTAATAGACAAAACATATGTAATGAGAGTAGGCATACAGG--TGTTTTC---AAAGCATATTTTAGC 53004
Ma'sNightMonkey  AAACAGTGAAATGTTAAAATATAAAACATACATAATGGGTGGAGGCATATGGAATTTTTTTT--GAAGCATATTATAGT 60098
VervetAGM        AAACAGTGAAATATTAAGAGACAAAACATACATAATGGGTGGAGGCATGTGGGATTTTTTTT--GAAGCTTATTGTAGT 66424
Gorrilla         AAACAGTGAAATGTTAAGAGACAAAACATACATAATGGATGGAGGCATATGGGATTTTTTTT---GAAGCATATCGTAGT 65218
GrabEatingMacaqueAAACAGTGAAATATTAAGAGACAAAACATACATAATGGGTGGAGGCATGTAGGATTTTTTTTT-GAAGCATATTGTAGT 65627
Macaque          AAACAGTGAAATATTAAGAGACAAAACATACATAATGGGTGGAGGCATGTAGGATTTTTTTTT-GAAGCATATTGTAGT 66462
PigTailedMacaque AAACAGTGAAATATTAAGAGACAAAACATACATAATGGGTGGAGGCATGTAGGATTTTTTTTTTGAAGCATATTGTAGT 65352
Bonobo           AAACAGTGAAATGTTAAGAGACAAAACATACATAATGGGTGGAGACATATGGGATTTTTTTT---GAAGCATATTGTAGT 63791
OliveBaboon      AAACAGTGAAATATTAAGAGACAAAACATACATAATGGGTGGAGGCATGTAGGATTTTTTTT--GAAGCATATTGTAGT 65206
Orangutan        AAACAGTGAAATGTTAAGAGACAAAACATACATAATGGGTGGAGGCATATGGGATTTTTTTT---GAAGCATATTGTAGT 65649

Majority         GGCCTTT----AAAAACAATTTAAACTAGAAGAAATTTTCTGTAAAATTAATTTTGCTATTTCTTTGCATTTTGTCTTAA

                         79290     79300     79310     79320     79330     79340     79350     79360

Human            GGCCTTTTA-AAAAAACAATTTAAACTAGAAGAAATTTTCTGTAAAATTAATTTTGCTATTTCTTTGCATTTTGTCTTAA 64756
Coquerel'sSifaka CCCTTTC----AAAAATAATTTAAACTAGAAGAAATTTTCTATAAAGTTTATTTTGCTGTTTATTTCTATTTTGTCTTAA 53080
Ma'sNightMonkey  GCCCTTTTTTAAAAAACATTTTAAACTAGAA-AAATTTTCTGTAAAATTAGTTTTGCTATTTCTTTGCATTTTATCTTAA 60177
VervetAGM        GGCCTTT------AAACAATTCAAACTGGAAGAAATTTTCTGTAAAATTAATTTTGCTATTTCTTTGCATTTTGTCTTAA 66498
Gorrilla         GGCCTTTTA-AAAAAACTATTTAAACTAGAATAAATTTTCTGTAAAATTAATTTTGCTATTTCTTTGCATTTTGTCTTAA 65297
GrabEatingMacaqueGGCCTTT------AAACAATTCAAACTAGAAGAAATTTTCTGTAAAATTAATTTTGCTATTTCTTTGCATTTTGTCTTAA 65701
Macaque          GGCCTTT------AAACAATTCAAACTAGAAGAAATTTTCTGTAAAATTAATTTTGCTATTTCTTTGCATTTTGTCTTAA 66536
PigTailedMacaque GGCCTTT------AAACAATTCAAACTAGAAGAAATTTTCTGTAAAATTAATTTTGCTATTTCTTTGCATTTTGTCTTAA 65426
Bonobo           GGCCTTTTA-AAAAAACAATTTAAACTAGAAGAAATTTTCTGTAAAATTAATTTTGCTATTTCTTTGCATTTTGTCTTAA 63870
OliveBaboon      GGCCTTT------AAACAATTCAAACTAGAAGAAATTTTCTGTAAAATTAATTTTGCTATTTCTTTGCATTTTGTCTTAA 65280
Orangutan        GGCCTTTTA-AAAAAACAATTTACACTAGAAGAAATTTTCTGTAAAATTAATTTTGCTATTTCTTTGTGTTTTGTCTTAA 65728

Majority         TCATTTATATTTTACTTTGTACCACAATGGACTTGAGCCACCTAATAAAAA-TACAGTGCAAACATATGCAACTTATAGT

                         79370     79380     79390     79400     79410     79420     79430     79440

Human            TCATTTATATTTTACTTTGTACCACAATGGACTTGAGCCACCTAATAAAAA-TACAGTGCAAACATATGCAACTTATAGT 64835
Coquerel'sSifaka TC------ATTTCACTTTGTCCCACAATGGGTTTGAGCCACTTAATAAAAA-TACATT-CAAAAATATGCAGCTAATAGT 53152
Ma'sNightMonkey  TCATTTATATTTTACTTTGTACCACAATGGATTTGAGCCACCTAATAAAAA-TGCAGTGCAAACATATGCAACTTATAGT 60256
VervetAGM        TCATTTATATTTTACTTTGTACCACAATGGACTTGAGCCACCTAAAAAAA--TAAAGTGCAAGCATATGCAACTTATAGT 66576
Gorrilla         TCATTTATATTTTACTTTGTACCACAATGGACTTGAGCCACCTAATAAAAA-TACAGTGCAAACATATGCAACTTATAGT 65376
GrabEatingMacaqueTCATTTATATTTTACTTTGTACCACAATGGACTTGAGCCACCTAAAAAAAAATACCGTGCAAGCATATGCAGCTTATAGT 65781
Macaque          TCATTTATATTTTACTTTGTACCACAATGGACTTGAGCCACCTAAAAAAAAATACAGTGCAAGCATATGCAGCTTATAGT 66616
PigTailedMacaque TCATTTATATTTTACTTTGTACCACAATGGACTTGAGCCACTTAAAAAAAAATACAGTGCAAGCATATGCAGCTTATAGT 65506
Bonobo           TCATTTATATTTTACTTTGTACCACAATGGACTTGAGCCACCTAATAAAAA-TACAGTGCAAACATATGCAACTTATAGT 63949
OliveBaboon      TCATTTATATTTTACTTTGTACCACAATGGACTTGAGCCACCTAAAAAAA--TACAGTGCAAGCATATGCAGCTTATAGT 65358
Orangutan        TCATTTATATTTTACTTTGTACCACAATGGACTTGAGCCACCTAATAAAAA-TGCATTGCAAACATATGCAACTTATAGT 65807
```

```
Majority         TGATAGAGTGAAATCAAGATGAAGAAAAAAATAAAGGAAAATCTTATTTTGTAAGTAAAATGTGCAAAGTGATTTATA--

                         79450     79460     79470     79480     79490     79500     79510     79520

Human            TGATAGCGTGGAATCAAGATGAAGAAAAAAATAA-GGAAAATCTTACTTTGTAAGTAAAATGTGCAAAGTGATTTATAAG 64914
Coquerel'sSifaka TGATGGAGTCGAA-------GGGAGAAAAAACAAGGGAAAATGTTAATTTGTAAGTAAAATGAACGAAGTGATTTATG-- 53223
Ma'sNightMonkey  TGATAGAGTGGAATCACGATGAAGAAAAAAATAAAGAAAAATCTT--TT-GTAAGTAAAATGAGCA--GTGATTTATAAG 60331
VervetAGM        TAATAGAGTGAAATCAAGATGAAGAAAAAAATAAAGGAAAATCTTATTTTGTAAGTAAAATATGCAAAGTGATTT-TA-- 66653
Gorrilla         TGATAGAGTGGAATCAAGATGAAGAAAAAAATAAAGGAAAATCTTACTTTGTAAGTAAAATGTGCAAAGTGATTTATAAG 65456
GrabEatingMacaqueTGATAGAGTGAAATCAAGATGAAGAAAAAAATAAAGGAAAATCTTATTTTGTAAGTAAAATATGCAAAGTGATTT-TA-- 65858
Macaque          TGATAGAGTGAAATCAAGATGAAGAAAAAAATAAAGGAAAATCTTATTTTGTAAGTAAAATATGCAAAGTGATTT-TA-- 66693
PigTailedMacaque TGATAGAGTGAAATCAAGATGAAGAAAAAAATAAA----AATCTTATTTTGTAAGTAAAATATGCAAAGTGATTT-TA-- 65579
Bonobo           TGATAGAGTGAAATCAAGATGAAGAAAAAAATAAAGGAAAATCTTACTTTGTAAGTAAAATGTGCAAAGTGATTTATAAG 64029
OliveBaboon      TGATAGAGTGAAATCAAGATGAAGAAAAAAATAAAGGAAAATATTATTTTGTAAGTAAAATATGCAAAGTGATTT-TA-- 65435
Orangutan        TGATAGAGTGGAATCAAGATGAAGAAAAAAATAAAGGAAAATCTTACTTTGTAAGTAAAATGTGCAAAGTGATTTATAAG 65887

Majority         TTAAGATGGAATTTTAGAGTTTCCTTTTATTTGATG-------TATTTCTTGATTTCTAGTATTTGTAAGATTATCTTTA

                         79530     79540     79550     79560     79570     79580     79590     79600

Human            TTAAGATGGAATTTTAGAGTTTCCTTTTATTTGATG-------TATTTCTTGATTTCTAGTATTGGTAAGATTATCTTTA 64987
Coquerel'sSifaka TTAAGGTGGAATTTTACAGTTTTATTTGATGTGTTTAGGAAATTATTTCTTGATTTCTGGTGTTTGTATGATTATCTTTA 53303
Ma'sNightMonkey  TTAAGATGGAATTTTATAGTTTCCTTTTATTTGATG-------TATT---------CTGGTATTTATAAGATTATCTTTA 60395
VervetAGM        -TAAGATGGAATTGTAGAGTCTCCTTTTATTTGATG-------TATTTCTTGATTTCTAGTATTTGTAAGATTATCTTTA 66725
Gorrilla         TTAAGATGGAATTTTAGAGTTTCCTTTTATTTGATG-------TATTTCTTGATTTCTAGTATTGGTAAGATTATCTTTA 65529
GrabEatingMacaque-TAAGATGGAATTTTAGAGTCTCCTTTTATTTGATG-------TATTTCTTGATTTCTAGTATTTGTAAGATTATCTTTA 65930
Macaque          -TAAGATGGAATTTTAGAGTCTCCTTTTATTTGATG-------TATTTCTTGATTTCTAGTATTTGTAAGATTATCTTTA 66765
PigTailedMacaque -TAAGATGGAATTTTAGAGTCTCCTTTTATTTGATG-------TATTTCTTGATTTCTAGTATTTGTAAGATTATCTTTA 65651
Bonobo           TTAAGATGGAATTTTAGAGTTTCCTTTTATTTGATG-------TATTTCTTGATTTCTAGTATTGGTAAGATTATCTTTA 64102
OliveBaboon      -TAAGATGGAATTTTAGAGTCTCCTTTTATTTGATG-------TATTTCTTGATTTCTAGTATTTGTAAGATTATCTTTA 65507
Orangutan        TTAAGATGGAATTTTAGAGTTTCCTTTTATTTGATG-------TATTTCTTGATTTCTAGTATTTGTAAGATTATCTTTA 65960

Majority         AGTCATGATTTTTATACATATGGAACTCAAAATTGATAGTTGACAAACTAGCTTTTACAGTTTAGTTTATGTCCATCATT

                         79610     79620     79630     79640     79650     79660     79670     79680

Human            AGTCATGATTTTTATATATATGGAACTCAAAATTGATAGTTGACAAACTAGCTTTTACAATTTAGTTTATGTCTGTCATT 65067
Coquerel'sSifaka AGTCAGGGTTTTAATCCCCGTGGAACCCATAATTGGTAGTTGATACACTAACTCTTACAGTTTAGTTTACATCCATCATT 53383
Ma'sNightMonkey  AGTCTTGATTTTTATACATATGCAACTCATAATTGATAGTTGACA----AGCTCTTAAAGTTTAGCTTATGTCCATCATT 60471
VervetAGM        AGTCATGATTTTTATACTTATGGAACTCAAAATTGATAGTTGACAAACTACCTTTTACAGTTTAGTTTATGTCCATCATT 66805
Gorrilla         AGTCATGATTTTTATATATATGGAACTCAAAATTGATAGTTGACAAACTAGCTTTTACAATTTAGTTTATGTCTGTCATT 65609
GrabEatingMacaqueAGTCATGATTTTTATACATATGGAACTCAAAATTGATAGTTGACAAACTAGCTTTTACAGTTTAGTTTATGTCCATCATT 66010
Macaque          AGTCATGATTTTTATACATATGGAACTCAAAATTGATAGTTGACAAACTAGCTTTTACAGTTTAGTTTATGTCCATCATT 66845
PigTailedMacaque AGTCATGATTTTTATACATATGGAACTCAAAATTGATAGTTGACAAACTAGCTTTTACAGTTTAGTTTATGTCCATCATT 65731
Bonobo           AGTCATGATTTTTATATATATGGAACTCAAAATTGATAGTTGACAAACTAGCTTTTACAATTTAGTTTATGTCTGTCATT 64182
OliveBaboon      AGTCATGATTTTTATACATATGGAACTCAAAATTGATAGTTGACAAACTAGCTTTTACAGTTTAGTTTATGTCCATCATT 65587
Orangutan        AGTCATGATTTTTATATATATGGAACTCAAAATTGATAGTTGACAAACTAGCTTTTACAATTTAGTTTATGTCCATCATT 66040
```

```
Majority          TCAAAAAGAAAAA-ATCGTACAATTAAGTAGTTATTGGAAATATTTACTT---CCCAATATATAACAATCATGCCATCAG

                          79690     79700     79710     79720     79730     79740     79750     79760

Human             TCAAAA-GAAAAA-ATAGTATAATTAAGTAGTTATTGGAAATATTTACTT---CCCAATATATAACAGTCATGCCATCAG 65142
Coquerel'sSifaka  TAAAAA-AGTAT------TATAATGAAGTAATTCTTAGAAATATTTATTTAATGTGGATATATAACAATCATCCTATTCA 53456
Ma'sNightMonkey   TCAAAAAGAAAAA----GTACTATAATGTAGTTCTTGGAAATATTTACTTCTTCCCAATATATAACAATCATGCCATCAG 60547
VervetAGM         TAAAAAAGAAAAACATCGTACAATGAAGTAGTTATTGGAAATACTT-------CCGAGTGTATAACAATCATGCCATCAG 66878
Gorrilla          TCAAAAAGAAAAA-ACAGTATAATTAAGTAGTTATTGGAAATATTTACTT---CCCAATATATAACAGTCATGCCATCAG 65685
GrabEatingMacaqueTCAAAAAGAAAAAAAATCATACAATTAAGTAGTTATTGGAAATACTTACTT---CCCAGTATATAACAATCATGCCATCAG 66087
Macaque           TCAAAAAGAAAAAAAATCGTACAATTAAGTAGTTATTGGAAATACTTACTT---CCCAGTATATAACAATCATGCCATCAG 66922
PigTailedMacaque  TCAAAAAGAAAAAAAATCGTACAATTAAGTAGTTATTGGAAATACTTACTT---CCCAGTATATAACAATCATGCCATCAG 65808
Bonobo            TCAAAAAGAAAAA-ATAGTATAATTAAGTAGTTATTGGAAATATTTACTT---CCCAATATATAACAGTCATGCCATCAG 64258
OliveBaboon       TCAAAAAGAAAAAAAATCGTACAATGAAGTAGTTATTGGAAATACTTACTT---CCCAGTATATAACAATCATGCCATCAG 65664
Orangutan         TCAAAAAGAAAAA-ATAGTATAATGAAGTAGTTATTGGAAATATTTACTT---CCCAATATATAACAGTCATGCCATCAG 66116

Majority          TTTGTGAATCTGAAATCTGTAAGTGGTATATATGTAGAGAGAGATACTCT-AAGGCAGTTGATATTTGATTCTAGTAACT

                          79770     79780     79790     79800     79810     79820     79830     79840

Human             TTTGTGAATATGAAATCTGTAAGTGGTATATATGTAGAGAGAGATACTCT-AAGGCAGTTGGTATTTGATTCTGGTAACT 65221
Coquerel'sSifaka  CTTCTAAATATGAAATC--TAAGTGACACAAATGTGGAGAGAGACAATCT-AATGCACAAGAGA---------AACAGCT 53524
Ma'sNightMonkey   TTTGTGAACCTGAGATTGGTAAGTGGTATATGTGTAGAGAGAGATACTCT-AAGGCAGTTGATACTTAATTCTGGTAACT 60626
VervetAGM         TTTGTGAATCTGAAATCTGTAAGTGTTATATATGTAGAGAAAGATACTCT-AAGGCAGTTGATATTTGATTCTAGTAACT 66957
Gorrilla          TTTGTGAATATGAAATCTGTAAGTGGTATATATGTAGAGAGAGATACTCT-AAGGCAGTTGGTATTTGATTCTGGTAACT 65764
GrabEatingMacaqueTTTGTGAATCTGAAATCTGTAAGTGGTATATATGTAGAGAAATATACTCTTAAGGCAGTTGATATTTGATTCTAGTAACT 66167
Macaque           TTTGTGAATCTGAAATCTGTAAGTGGTATATATGTAGAGAAATATACTCTTAAGGCAGTTGATATTTGATTCTAGTAACT 67002
PigTailedMacaque  TTTGTGAATCTGAAATCTGTAAGTGGTATATATGTAGAGAAATATACTCTTAAGGCAGTTGATATTTGATTCTAGTAACT 65888
Bonobo            TTTGTGAATATGAAATCTGTAAGTGGTATATATGTAGAGAGAGATACTCT-AAGGCAGTTGGTATTTGATTCTGGTAACT 64337
OliveBaboon       TTTGTGAATCTGAAATCTGTAAGTGGTATATATGTAGAGAAAGATACTCT-AAGGCAGTTGATATTTGATTCTAGTAACT 65743
Orangutan         TTTGTGAATATGAAATCTGTAAGTGGTATATATATAGAGAGAGATACTCT-AAGGCAGTTGGTATTTGATTCTGGTAACT 66195

Majority          AATCAGAATTAACTACCTGTTAATTCTGGCAACTT----CAAACAAATCTGACCCTTTATTCGAATTTTC-TGGTATATC

                          79850     79860     79870     79880     79890     79900     79910     79920

Human             AATAAGAATTAACTACCTGTTAATTCTGGCAGCTG----CAAACAAATCTGACCCTTTATTCGAATTTTC-TGGTGTATC 65296
Coquerel'sSifaka  GAT---ATTTGA-----AATTAATTCTGGTGACTT----CAAACAAAACTGACCCTTTATTCGAATTTTCCTGATACATC 53592
Ma'sNightMonkey   AATCAGAATTAACTGTGTGTTAATTCCGGTAACTT----CAAATAAATCTGACCCTTTATTCGAATTTTC-TGGTATGTC 60701
VervetAGM         AATCAGAATTAACTACCTGTTAATTCTGGCAACTT--A---AACAAATCTGACCCTTTATTTGAATTTTC-TGGTATATC 67031
Gorrilla          AATCAGAATTAACTACCTGTTAATTCTGGCAACTG----CAAACAAATCTGACTCTTTATTCGAATTTTC-TGGTATATC 65839
GrabEatingMacaqueAATCAGAATTAACTACCTGTTAATTCTGGCAACTTTAAACAAACAAATCTGACCCTTTATTTGAATTTTC-TGGTATATC 66246
Macaque           AATCAGAATTAACTACCTGTTAATTCTGGCAACTTTAAACAAACAAATCTGACCCTTTATTTGAATTTTC-TGGTATATC 67081
PigTailedMacaque  AATCAGAATTAACTACCTGTTAATTCTGGCAACTTTAAACAAACAAATCTGACCCTTTATTTGAATTTTC-TGGTATATC 65967
Bonobo            AATCAGAATTAACTACCTGTTAATTCTGGCAGCTG----CAAACAAATCTGACCCTTTATTCGAATTTTC-TGGTATATC 64412
OliveBaboon       AATCAGAATTAACTACCTGTTAATTCTGGCAACTTTAAACAAATAAATCTGACCCTTTATTTGAATTTTC-TGTTATATC 65822
Orangutan         AATCAGAATTTACTACCTGTTAATTCTGGCAACTG----CAAACAAATCTGACCCTTTATTCGAATTTTC-TGGTGTATC 66270
```

```
Majority         -TAGCACAAGTCATAG-TGTTTATTATG--------ACTGTAGTAGATGCTTTAGGTCACTTACACTCTTTCCTGACAGA
                         79930     79940     79950     79960     79970     79980     79990     80000
Human            CTAGCAGAAGTCATAG-TGTTTATTATG--------ACTGTAGTAGATGCTTTAGGTCACTTACACTCTTTCCTGACAGA 65367
Coquerel'sSifaka -TGGCACGAGCCTTAGCTGTTTATCATG--------ACTCTAGTAGATACTTTGGGTTGCTTTCCCTTTTTCCTGACAAA 53663
Ma'sNightMonkey  -TAGCACAAGTCATAG-TGTTTATTATGTTATTATGACTGTAGTAGATGCTTTCAGTCACTTACACTCTTTCCTGACAGA 60779
VervetAGM        -TAGCACAAGTCATAG-TGTTTTTTATG--------ACTGCAGTAGATGCTTTAGGTCTCTTACACTCTTTCCTGAC--A 67099
Gorrilla         -TAGCAGAAGTCATAG-TGTTTATTATG--------ACTGTAGTAGATGCTTTAGGTCACTTACACTCTTTCCTGACAGA 65909
GrabEatingMacaque-TAGCACAAGTCATAG-TGTTTTTTATG--------ACTGCAGTAGATGCTTTAGGTCACTTACACTCTTTCCGGACAGA 66316
Macaque          -TAGCACAAGTCATAG-TGTTTTTTATG--------ACTGCAGTAGATGCTTTAGGTCACTTACACTCTTTCCGGACAGA 67151
PigTailedMacaque -TAGCACAAGTCATAG-TGTTTTTTATG--------ACTGCAGTAGATGCTTTAGGTCACTTACACTCTTTCCTGACAGA 66037
Bonobo           CTAGCAGAAGTCATAG-TGTTTATTATG--------ACTGTAGTAGATGCTTTAGGTCACTTACACTCTTTCCTGACAGA 64483
OliveBaboon      -TAGCACAAGTCATAG-TGTTTTTTATG--------ACTGCAGTAGATGCTTTAGGTCACTTACACTCTTTCCTGACAGA 65892
Orangutan        -TAGCACAAGTCATAG-TGTTTATTATG--------ACTGTAGTAGATACTTTAGGTCACTTACACTCTTTC-TGACAGA 66339

Majority         ATCTCTAC-----------------C-CCTCTGTGTGTGTCTAGCTTAATTCGTAATTCAAGTTAATAAGATTGAATATT
                         80010     80020     80030     80040     80050     80060     80070     80080
Human            ATCTCTAC-----------------TACCTCTGTGTGTGTCTAGCTTAATTCGTAATTCAAGTTAATAAGATTGAATATT 65430
Coquerel'sSifaka ATCTCTATCAATCAATGTGTGGGCCTAGTTTAAGTCTTAATTCCCTCAATTCTCCTTGCAAATTAATAAGATTGACTGTT 53743
Ma'sNightMonkey  ATCTCTGTGTGTTG----------CTAGCTTAATTCTTAATTCCCTTAATTTTTCTTTCAAGTTAATATAATTGAATATT 60849
VervetAGM        ATCTCTAC-----------------C-CCTCTGTGTGTGTCTAGCTGAATTCGTAATTCAAGTTAATAAGATTGAATATT 67161
Gorrilla         ATCTCTA--------------------CCTCTGTGTGTGTCTAGCTTAATTCGTAATTCAAGTTAATAAGATTGAATATT 65969
GrabEatingMacaqueATCTCTAC-----------------C-CCTCTGTGTGTGTCTAGCTGAATTTGTAATTCAAGTTAATAAGATTGAATATT 66378
Macaque          ATCTCTAC-----------------C-CCTCTGTGTGTGTCTAGCTGAATTTGTAATTCAAGTTAATAAGATTGAATATT 67213
PigTailedMacaque ATCTCTAC-----------------C-CCTCTGTGTGTGTCTAGCTGAATTCGTAATTCAAGTTAATAAGATTGAATATT 66099
Bonobo           ATCTCTA--------------------CCTCTGTGTGTGTCTAGCTTAATTCGTAATTCAAGTTAATAAGATTGAGTATT 64543
OliveBaboon      ATCTCTAC-----------------C-CCTCTGTGTGTGTCTAGCTGAATTCGTAATTCAAGTTAATAAGATTGAATATT 65954
Orangutan        ATCTCTA------------------------CCTCTGTGTGTCTAGCTTAATTCGTAATTCAAGTTAATAGGATTGAATATT 66397

Majority         TCACAGTGTGCAGTTTTTAAAGGCAAAGTCAAGAGTGAAACA-------GTGAAACATGGTTTTTGCTTATAATTATTAC
                         80090     80100     80110     80120     80130     80140     80150     80160
Human            TCACAGTGTGCAGTTTTTAAAGGCAAAGTCAGGAATGAAAGA-------GTGAAACATGGTTTTTGCTTATAATTATTAC 65503
Coquerel'sSifaka TCATAGTGTGCAGTTTTTAAAGGCAAAGTCAAGAGCGAGACAATGATGAGTAAAACATAGCCCTTGCTTATAATTATTAC 53823
Ma'sNightMonkey  TCACAGTGTGCAGGTTTTAAAGGCAAAGTCAAGA---------------GTGAAACATGGTCCTTGCTTATAATTATTAC 60914
VervetAGM        TCACAGTGTACAGTTTTTAAAGGCAAAGTCAAGAGTGAAACA-------GTGAAACATGGTTTTTGCTTATAATTACTAC 67234
Gorrilla         TCACAGTGTGCAGTTTTTAAAGGCAAAGTCAAGAATGAAAGA-------GTGAAACATGGTTTTTGCTTATAATTATTAC 66042
GrabEatingMacaqueTCACAGTATACAGTTTTTAAAGGCAAAGTCAAGAGTGAAACA-------GTGAAACATGGTTTTTGCTTATAATTACTAC 66451
Macaque          TCACAGTATACAGTTTTTAAAGGCAAAGTCAAGAGTGAAACA-------GTGAAACATGGTTTTTGCTTATAATTACTAC 67286
PigTailedMacaque TCACAGTATACAGTTTTTAAAGGCAAAGTCAAGAGTGAAACA-------GTGAAACATGGTTTTTGCTTATAATTACTAC 66172
Bonobo           TCACAGTGTGCAGTTTTTAAAGGCAAAGTCAGGAATGAAAGA-------GTGAAACATGGTTTTTGCTTATAATTATTAC 64616
OliveBaboon      TCACAGTGTACAGTTTTTAAAGGCAAAGTCAAGAGTGAAACA-------GTGAAACATGGTTTTTGCTTATAATTACTAC 66027
Orangutan        TCACAATGTGCAGTTTTTAAAGGCAAAGTCAAGAGTGAAAGA-------GTGAAACATGGTTTTTGCTTATAATTATTAC 66470
```

```
Majority         TATAGATTATTAAACTCTTTACTGATAGACATTGT--TATAGAAA-GAATCAGAATTTATTGAGTAGCTTCTAGGTCATA
                         80170     80180     80190     80200     80210     80220     80230     80240
Human            TATAGATTATTAAACTCTTTACTGATAGACATTGT--TTTAGAAA-GAATCAAAATTTATTGAGTAGCTTCTAGGTCATA 65580
Coquerel'sSifaka TACAGATTATTAAATTCTT-ACTGATGGAGATTATATTATAGAAAAGAGTCAACATATATCAAGTAGCTTCTAGGTCCTA 53902
Ma'sNightMonkey  TATAGATTATTAAACTCTTTACTGATAGAAGTTGT--TATAGAAAAGAATCAGAATTTATTGAGTAGCTTCTAGGTCATA 60992
VervetAGM        TATAGATTATTAAACTCTTTACTGATAGACATTGT--TATAGAAA-GAATCAGAATTTATTGAGTGGCTTCTAGGTCATA 67311
Gorrilla         TATAGATTATTAAACTCTTTACTGATAGACATTGT--TTTAGAAA-GAATCAAAATTTATTGAGTAGCTTCTAGGTCATA 66119
GrabEatingMacaqueTATAGATTATTAAACTCTTTACTGATAGACATTGT--TATAGAAA-GAATCAGAATTTATTGAGTGGCTTCTAGGTCATA 66528
Macaque          TATAGATTATTAAATTCTTTACTGATAGACATTGT--TATAGAAA-GAATCAGAATTTATTGAGTGGCTTCTAGGTCATA 67363
PigTailedMacaque TATAGATTATTAAACTCTTTACTGATAGACATTGT--TATAGAAA-GAATCAGAATTTATTGAGTGGCTTCTAGGTCATA 66249
Bonobo           TATAGATTATTAAACTCTTTACTGATAGACATTGT--TTTAGAAA-GAATCAAAATTTATTGAGTAGCTTCTAGGTCATA 64693
OliveBaboon      TATAGATTATTAAACTCTTTACTGATAGACATTGT--TATAGAAA-GAATCAGAATTTATTGAGTGGCTTCTAGGTCATA 66104
Orangutan        TAAAGATTATTAAAGTCTTCACTGATAGACATTGT--TTTAGAAA-GAATCAGAATTTATTGAGTAGCTTCTAGGTCATA 66547

Majority         AGCATTCAGCTAGGCACTTGGCAGATA-CATTACTTTAT---------TAAAATAGATGGTGTCATCAAGATTTTACATA
                         80250     80260     80270     80280     80290     80300     80310     80320
Human            TTCATTCAGCTAGGCACTTGGCAGATA-CATTACTTTGT---------TAAAATAGATGGTCTCATCAAGATTTTACATA 65650
Coquerel'sSifaka GACATTCTACTAAGCACTTGTCAGATAACGTTACTCAGAATACTCAGATAAAGTAGATGATGGCATCAAGATTTTACATG 53982
Ma'sNightMonkey  GGCATTCAGCTAGGTACTTGGTAGATA-CATTACTTTGT---------TAAAATAGGTGATGTCATCAAGATTTTACACA 61062
VervetAGM        AGCATTCAGCTAGGCACTTGACAGATA-CATTACTTTAT---------TAAAACAGATGGTGTCATCAAGATTTTACATA 67381
Gorrilla         TTCATTCAGCTAGGCACTTGGCAGATA-CATTACTTTAT---------TAAAATAAATGGTCTCATCAAGATTTTACATA 66189
GrabEatingMacaqueAGCATTCAGCTAGGCACTTGGCAGATA-CATTACTTTAT---------TAAAACAGATGGTGTCATCAAGATTTTACATA 66598
Macaque          AGCATTCAGCTAGGCACTTGGCAGATA-CATTACTTTAT---------TAAAACAGATGGTGTCATCAAGATTTTACATA 67433
PigTailedMacaque AGCATTCAGCTAGGCACTTGGCAGATA-CATTACTTTAT---------TAAAACAGATGGTGTCATCAAGATTTTACATA 66319
Bonobo           TTCATTCAGCTAGGCACTTGGCAGATA-CATTACTTTGT---------TAAAATAGATGATCTCATCAAGATTTTACATA 64763
OliveBaboon      AGCATTCAGCTAGGCACTTGGCAGATA-CATTACTTTAT---------TAAAACAGATGGTGTCATCAAGATTTTACATA 66174
Orangutan        GGCATTCAGCTAGGTACTTGGCAGATA-CGTTACTTTAT---------TAAAATAGATGGTGTCATCAAGATTTTACATA 66617

Majority         TAGTTTCTAAGAAGTTGAAAAATTCATAT-------------GAAACAATTTAAAGATAAAATACCAAGAAACTCCAG--
                         80330     80340     80350     80360     80370     80380     80390     80400
Human            TAGTTTCTAAGAAGTTGAAAAATTCACATGCAACTCATCAAAGAAACAATTTAAAGATAAAATACCAAGAAACTCCAGCT 65730
Coquerel'sSifaka CAGTTTCTGAGAAGTTAAAAAAATTCATGT----------------GCAGGCTGGGCACGGTGG-CTC----ACGCCTG-- 54039
Ma'sNightMonkey  TAGTTTCTAAGAAGTTGAAAAATTCACATGCAACTCATTAAAGAAACAATTTAAAAGGAAAATACCAAGAAACTCCGG-- 61140
VervetAGM        TAGTTTCTAAGAAGTTGAGAAATTCTTA--------------GAAACAATTTAAAGATAAAATACCCAGAAACTCCAG-- 67445
Gorrilla         TAGTTTCTAAGAAGTTGAAAAATTCACATGCAACTCATCAAAGAAACAATTTAAAGATAAAATACCAAGAAACTCCAGCT 66269
GrabEatingMacaqueTAGTTTCTAAGAAGTTGAAAAATTCTTA--------------GAAACAATTTAAAGATAAGATACCGAGCAACTCCAG-- 66662
Macaque          TAGTTTCTAAGAAGTTGAAAAATTCTTA--------------GAAACAATTTAAAGATAAAATACCGAGCAACTCCAG-- 67497
PigTailedMacaque TAGTTTCTAAGAAGTTGAAAAATTCTTA--------------GAAACAATTTAAAGATAAAATACCGAGAAACTCCAG-- 66383
Bonobo           TAGTTTCTAAGAAGTTGAAAAATTCACATGCAACTCATCAAAGAAACAATTTAAAGATAAAATACCAAGAAACTCCAGCT 64843
OliveBaboon      TAGTTTCTAAGAAGTTGAAAAATTCTTA--------------GAAACAATTTAAAGATAAAATACCGAGAAACTCCAG-- 66238
Orangutan        TAGTTTCTAAGAAGTTGAAAAATTCACATGCAACTCATCAAAGAAACAATTTAAAGATAAAATACCAAGAAACTCCAG-- 66695
```

```
Majority          --------------------------CTAGCATTAACAATGGACTAC--TAATC-ACTTCTTTGGACCATACTGAGCTAT

                           80410     80420     80430     80440     80450     80460     80470     80480

Human             AAAGATAAAATACCAAGAAACTCCAGCTAGTATTAACAATGGACTACAATAATC-ACTTCTTTGGACCCTACTGAGGTGT 65809
Coquerel'sSifaka ---------------------------TAATCCTAGCA---------------------CTCTGGG--AGACAGAGGTGG 54069
Ma'sNightMonkey   --------------------------CTAGCATAAACAATGGACTACAACAGTCCACTTCTTTGGACCCTACTGAGCTAT 61194
VervetAGM         --------------------------CTAGCATTAACAATGGACTAC--TAATC-ACTTCTTTGGACCATACTGAGCTAT 67496
Gorrilla          AAAGATAAAATAGCAAGAAACTCCAGCTAGTATTAACAATGGACTACAATAATC-ACTTCTTTGAACCCTACTGAGCTGT 66348
GrabEatingMacaque--------------------------CTAGCATTAACAATGGACTAC--TAATC-ACTTCTTTGGACCATACTGAGCTAT 66713
Macaque           --------------------------CTAGCGTTAACAATGGACTAC--TAATC-ACTTCTTTGGACCATACTGAGCTAT 67548
PigTailedMacaque --------------------------CTAGCATTAACAATGGACTAC--TAATC-ACTTCTTTGGACCATACTGAGCTAT 66434
Bonobo            AAAGATAAAATACCAAGAAACTCCAGCTAGTATTAACAATGGACTACAATAATC-ACTTCTTTGGACCCTACTGAGGTGT 64922
OliveBaboon       --------------------------CTAGCATTAACAATGGACTAC--TAATC-ACTTCTTTGGACCATACTGAGCTAT 66289
Orangutan         --------------------------CTAGTATTAACAATGGACTACAATAATC-ACTTCTTTGGACCCTACTGAGCTAT 66748

Majority          GTGTATTTGCCCATCTGAATATACTTAGATGTGCTTGGATCCTGGGTGGGAGGCTTGTTTAGAAGTCACCTGGATTCTTC

                           80490     80500     80510     80520     80530     80540     80550     80560

Human             GTGTATTTGCCCATCTGGATATACTTAGATGTGCTTGGATCCTGGGTGGGAGGCTTGGTTAGAAGTCACGTGGATTCTTC 65889
Coquerel'sSifaka GTGGATCGCTCAAGGTCAGGAGTTCGAGACCAGCCTC-AGCAAGAGCGAGA-----------------CCCCGTCTCTAC 54131
Ma'sNightMonkey   GTATATTTGCCCATCTGAGTATATTTAGATGTGTTTGGATCGTGGGTGGGAGGCATGGTTAGAAATCACCTGGATTCCTC 61274
VervetAGM         GTGTATTTGTCCATCTGAATATACTTAGATGCGCTTGGATCCTGGGTGGGAGGCTTGTTTAGAAGTCACCTGGATTCTTC 67576
Gorrilla          GTGTATTTGCCCATCTGGATATACTTAGATGTGCTTGGATCCTGGGTGGGAGGCTTGATTAGAAGTCACCTGGATTCTTC 66428
GrabEatingMacaqueGTGTATTTGCCCATCTGAATATACTTAGATGCGCTTGGATCCTGGGTGGGAGGCTTGTTTAGAAGTCACCTGGATTCTTC 66793
Macaque           GTGTATTTGCCCATCTGAATATACTTAGATGCGCTTGGATCCTGGGTGGGAGGCTTGTTTAGAAGTCACCTGGATTCTTC 67628
PigTailedMacaque GTGTATTTGCCCATCTGAATATACTTAGATGCGCTTGGATCCTGGGTGGGAGGCTTGTTTAGAAGTCACCTGGATTCTTC 66514
Bonobo            GTGTATTTGCCTATCTGGATATACTTAGATGTGCTTGGATCCTGGGTGGGAGGCTTGGTTAGAAGTCACCTGGATTCTTC 65002
OliveBaboon       GTGTATTTGCCCATCTGAATATACTTAGATGCGCTTGGATCCTGGGTGGGAGGCTTGTTTAGAAGTCACCTGGATTCTTC 66369
Orangutan         GTGTATTTGCCCATCTGAATATACTTAGATGTGCTTGGATCCTGGGTGGGAGGCTTGGTTAGAAGTCACCTGGATTCTTC 66828

Majority          TAGGGAATTTTTCAGCATTTTCCTTGTGATCTTATGACCTTGGTGAAACTTTTAAAACAATAATAAAGTCATTTT-----

                           80570     80580     80590     80600     80610     80620     80630     80640

Human             TAGGGAATTTATCACCATTTTTCTTGTGATCTTATGACCTTGCTGAAACTTTTAAAACAATAATAAAGTCATTTT----- 65964
Coquerel'sSifaka TAA--AAATAGAAAGAAATTATTTGGACAACTAAAAATCTATATAGAA-----AAAAAATTAGCCGGG-CATGGT----- 54198
Ma'sNightMonkey   TAGGGAATTCATCACCATTT-CCTTGCGATCTTAGGACCTTGGTGAAACTTCTAAAACAATATTAAAGTCATTTTAAATA 61353
VervetAGM         TCGGGAATTTTTCAGCATTTTCCTTGTGATCTCATGACCTTGGTGAAACTTTTAAAACAACAATAAAGTCATTTT----- 67651
Gorrilla          TAGGGAATTTATCATCATTTTTCTTGTGATCTTATGACCTTGCTGAAACTTTTAAAACAATAATAAAGTCATTTT----- 66503
GrabEatingMacaqueTCAGGAATTTTTCAGCATTTTCCTTGTGATCTTATGACCTTGGTGAAACTTTTAAAACAATAATAAAGTCATTTT----- 66868
Macaque           TCAGGAATTTTTCAGCATTTTCCTTGTGATCTTATGACCTTGGTGAAACTTTTAAAACAATAATAAAGTCATTTT----- 67703
PigTailedMacaque TCAGGAATTTTTCAGCATTTTCCTTGTGATCTTATGACCTTGGTGAAACTTTTAAAACAATAATAAAGTCATTTT----- 66589
Bonobo            TAGGGAATTTATCACCATTTTTCTTGTGATCTTATGACCTTGCTGAAACTTTTAAAACAATAATAAAGTCATTTT----- 65077
OliveBaboon       TCGGGAATTTTTCAGCATTTTCCTTGTGATCTTATGACCTTGGTGAAACTTT-AAAACAATAATAAAGTCATTTT----- 66443
Orangutan         TAGGTAATTTATCACCATTTTTCTTGTGATCTTATGACTTTGGTGAAACTTTTAAAACAATAATGAAGTCATTTT----- 66903
```

```
Majority          ------------------------------------------TTTTTT-CTTTCTTTGAGACGGAGTCTCACTCTGT
                          80650    80660    80670    80680    80690    80700    80710    80720
Human             ------------------------------------------TTTTCT-----CTTTGAGATGGAATCTCACTCTGT 65994
Coquerel'sSifaka  -----------------------------------------------G----GCGCACGTCTGTAGTCCCA----GC 54220
Ma'sNightMonkey   AAGATTATGAAGCCCTAGCACAGCTAGCTTCTGTCTTACTCTATGCTTTTCTTATTTTTTGAGACGGAGTCTTGCTCTGT 61433
VervetAGM         ------------------------------------------TTTTTTTCTTTCTTTGAGACAGAGTCTCACTCTGT 67686
Gorrilla          ------------------------------------------TTTTCT-----CTTTGAGATGGAGTCTCACTCTGT 66533
GrabEatingMacaque------------------------------------------TTTTTT-CTTTCTTTGAGACGGAGTCTCACTCTGT 66902
Macaque           ------------------------------------------TTTTTTTCTTTCTTTGAGACGGAGTCTCACTCTGT 67738
PigTailedMacaque  ------------------------------------------TTTTTT-CTTTCTTTGAGACGGAGTCTCACTCTGT 66623
Bonobo            ------------------------------------------TTTTCT-----CTTTGAGATGGAATCTCACTCTGT 65107
OliveBaboon       ------------------------------------------TTTTTT-CTTTCTTTGAGACGGAGTCTCACTCTGT 66477
Orangutan         ------------------------------------------TTTCTT-----CTTTGAGACGGAATCTCACCCTGT 66933

Majority          GGCCCAGGCTGGAGTGCAGTGGCGCAGTCTCGGCTCACTGCAACTTCCGCCTCCCGGGTTCAAGCGATTCTCCCGCCTCA
                          80730    80740    80750    80760    80770    80780    80790    80800
Human             CGCCCGTGTCAGAGTGTGGTGGCGCAGTCTCGGCTCACTGCAACTTCCGCCTCCCGGGTTCAAGCGATTCTCCCGCCTCA 66074
Coquerel'sSifaka  TACTCGGG--AGGCTGAGGCAGTA-------GGATTGCTGGAGCCCAGGAGTTTGAGGTTGCTGTGAG----CTAGGCTG 54287
Ma'sNightMonkey   CACCCAGGCTGGAGTGCAATGGCATAATCTTGGCTCACTGCAACTCCCGCCTCCCGGGTTCAAGTGATTCTCCCACATCA 61513
VervetAGM         GGCCCAGGCTGGAGTGCAGTGGCACAATCTCGGCTCACTGCAACTTCCACCTCCCAGGTTCAAGCGATTCTCCTGCCTCA 67766
Gorrilla          TGCCCGTGTCGGAGTGTGGTGGCGCAGTCTCGGCTCACTGCAACTTCCGCCTCCCGGGTTCGAGCGATTCTCCTGCCTCA 66613
GrabEatingMacaqueGGCCCAGGCTGGAATGCAGTGGCGCAGTCTTGGCTCACTGCAACTTCCACCTCCCGGGTTCAAGCGATTCTCCCGCCTCA 66982
Macaque           GGCCCAGGCTGGAATGCAGTGGCGCAGTCTTGGCTCACTGCAACTTCCACCTCCCGGGTTCAAGCGATTCTCCCGCCTCA 67818
PigTailedMacaque  GGCCCAGGCTGGAGTGCAGTGGCGCAGTCTTGGCTCACTGCAACTTCTACCTCCCGGGTTCAAGCGATTCTCCCGCCTCA 66703
Bonobo            CGCCCGTGTCAGAGTGTGGTGGCACAGTCTCGGCTCACTGCAACTTCCGCCTCCCGGGTTCAAGCGATTCTCCCGCCTCA 65187
OliveBaboon       GGCCCAGGCTGGAGTGCAGTGGCGCAGTCTCGGCTCACTGCAACTTCCACCTCCCGGGTTCAAGCGATTCTCCCGCCTCA 66557
Orangutan         CGCCCATATCGGAGTGTGGTGGCGCAGTCTCGGCTCACTGCAACTTCCGCCTCCCGGGTTCAAGTGATTCTCCCGCCTCA 67013

Majority          GCCTCCCGAGCATCTGGGACTACAGGCATGTACCACCACCCCTGGCTAATTTTTGT-GTTTTTTCGTAGAAATGAGATTT
                          80810    80820    80830    80840    80850    80860    80870    80880
Human             GCCTCCTGAGCATCTGGGACTACAGGCGTGTACCACCACCCCTGGCTAACTTTTGT-CTTTTTTCGTAGAGATGAGATTT 66153
Coquerel'sSifaka  ACGCCACGGCACTCT---ACTCCAGGCA-----------ACAGAGTTAGACTCTGT---CTCAAAAAAAAAAAAAAAATT 54350
Ma'sNightMonkey   GCCTCCCAAGTAGCTGGGATTACAGGTGTATGCCACCACACCTGGCTAATTTTTGT-ATTTTTTAGTAGAGATGAAGTTT 61592
VervetAGM         GCCTCCCGAGCATCTGGGACTACAGGCATGTAACACCACCCCTGGCTAATTTTTGT-GTTTTTTCGTAGAAATGAGATTT 67845
Gorrilla          GCCTCCTGAGCATCTGGGACTACAGGCGTGTACCACCACCCCTGGCTAACTTTTGT-CTTTTTTCATAGAGATGACATTT 66692
GrabEatingMacaqueTCCTCCCGAGCATCTGGGACTACAGGCATGTACCACCACCCC-GGCTAATTTTTGT-GTTTTTTCGTAGAAATGAGATTT 67060
Macaque           TCCTCCCGAGCATCTGGGACTACAGGCATGTACCACCACCCC-GGCTAATTTTTGT-GTTTTTTCGTAGAAATGAGATTT 67896
PigTailedMacaque  TCCTCCCGAGCATCTGGGACTACAGGCATGTACCACCACCCC-GGCTAATTTTTGT-GTTTTTTCGTAGAAATGAGATTT 66781
Bonobo            GCCTCCTGAGCATCTGGGACTACAGGCGTGTACCACCACTCCTGGCTAACTTTTGT-CTTTTTTCGTAGAGATGAGATTT 65266
OliveBaboon       TCCTCCCGAGCATCTGGGACTACAGGCATGTACCACCACCCCTGGCTAATTTTTGT-GTTTTTTCGTAGAAATGAGATTT 66636
Orangutan         GCCTCCCGAGCATCTGGGACTACAGGCGTGTACCACCACCCCTGGCTAATTTTTGTGTTTTTTTCGTAGAGGTGAGATTT 67093
```

```
Majority         CACGATGTTGGCCAGGCTGGTCTGGAACTCCTGATCTCAGGTGATCCGCCCGCCTCAGCCTCCCAAAGTGCTGGGATTA-
                         80890     80900     80910     80920     80930     80940     80950     80960
Human            CACCATGTTGGCCAGGCTGGTCTGGAACTCCTGATCTCAGGTGATCTGCCCGCCTCAGCCTCCCAAAGTGCTGAGATTA- 66232
Coquerel'sSifaka CAC------GTGCAGCTCATTCAAGAAACCAT--TTAAAGATAAAATACC-AA--GAA-ACTC--GAG--CTAGCATCAA 54414
Ma'sNightMonkey  CACCATCTTGGCCAGGCTCGTCTTGAACTCCTGGCCTTAGGTGATCTGCCTGCCTCAGCCTCCCAGAGTGCTGGGATTA- 61671
VervetAGM        CACGATGTTGGCCAGGCTGGTCTGGAACTCCTGATCTCAGGGGATCCGCCCGCCTCAGCCTCCCAAAGACCTGGGATTA- 67924
Gorrilla         CACCATGTTGGCCAGGCTGGTCTGGAACTCCTGATCTCAGGTGATCTGCCCGCCTCAGCCTCCCAAAGTGCTGAGATTA- 66771
GrabEatingMacaqueCACGATGTTGGCCAGGCTGGTCTGGAACTCCTGATCTCAGGGGATCCGCCCGCCTCAGCCTTCCAAAGAGCTGGGATTA- 67139
Macaque          CACGATGTTGGCCAGGCTGGTCTGGAACTCCTGATCTCAGGGGATCCGCCCGCCTCAGCCTTCCAAAGAGCTGGGATTA- 67975
PigTailedMacaque CACGATGTTGGCCAGGCTGGTCTGGAACTCCTGATCTCAGGGGATCCGCCCGCCTCAGCCTTCCAAAGAGCTGGGATTA- 66860
Bonobo           CACCATGTTGGCCAGGATGGTCTGGAACTCCTGATCTCAGGTGATCTGCCCGCCTCAGCCTCCCAAAGTGCTGAGATTA- 65345
OliveBaboon      CACGATGTTGGCCAGGCTGGTCTGGAACTCCTGATCTCAGGGGATCCGCCCGCCTCAGCCTCCCAAAGAGCTGGGATTA- 66715
Orangutan        CACCATGTTGGCCAGGCTGGTCTGGAACTCCTGATCTCAGGTGATCCGCCCACCTCAGCCTCCCAAAGTGCTGAGATTA- 67172

Majority         CAGGTGTGAGCCACCATGCCTGACAGCTTTTGTTCATTTTTTAAGTGTTTTACATTTTTACAT-TAAAAGCATGTAAATT
                         80970     80980     80990     81000     81010     81020     81030     81040
Human            CAGGTGTGAGCCACCATGCCTGGCCGCTTTTGTTCATTTTTTGAGCGTTGTACATTTTTACAT-TAAAGGCGTGTAAATT 66311
Coquerel'sSifaka CAAAGGATAGCAGTCACTCCTTACACCCT------AAG----GAGCTATGTGCATTTGCCCATGTGGGTATATTTAGATG 54484
Ma'sNightMonkey  CAGGCATGAGCCACCATGCCTGGCTGCTTTTGTTCATTTTTTAAGTATTCTACATTTTTACAC-TAAGAGTATGTAAATT 61750
VervetAGM        CAGGTGTGAGCCACCATACTTGACAGCTTTTGTTCATTTTTTAAGTGTTTTACCTTTTTACAT-TAAAAGCATGTAAATT 68003
Gorrilla         CAGTTGTGAGCCACCATGCCTGGCCGCTTTTGGTTGTTTTTTGAGCCTTGTACATTTTTACAT-TAAAGGCGTGTAAATT 66850
GrabEatingMacaqueCAGGTGTGAGCCACCATGCTTGACAGCTTTTGTTCATTTTTTAAGTGTTTTATGTTTTTACAT-TAAAAGCATGTAAATT 67218
Macaque          CAGGTGTGAGCCACCATGCTTGACAGCTTTTGTTCATTTTTAAAGTGTTTTATGTTTTTACAT-TAAAAGCATGTAAATT 68054
PigTailedMacaque CAGGTGTGAGCCACCATGCTTGACAGCTTTTGTTCATTTTTTAAGTGTTTTATGTTTTTACAT-TAAAAGCATGTAAATT 66939
Bonobo           CAGGTGTGAGCCACCATGCCTGGCCGCTTTTTTTCATTTTTTGAGCGT---ACATTTTTACAT-TAAAGGCGTGTAAATT 65421
OliveBaboon      CAGGTGTGAGCCACCATGCTTGACAGCTTTTGTTCATTTTTTAAGTGTTTTACGTTTTTACAT-TAAAAGCATGTAAATT 66794
Orangutan        CAGGTGTGAGCCACCATGCCTGGCCGCTTTTGTTCATTTTTTAAGTGTTCTACATTTTTACAT-TAAAGGCATGTAAATT 67251

Majority         AAGTTGCTCCCTTCGCCAAAAAA---------------------------------------------------------
                         81050     81060     81070     81080     81090     81100     81110     81120
Human            AAGTTGCTCGCTTTGCCAAAAAA--------------------------------------------------------- 66334
Coquerel'sSifaka GAGAGGGATCCCGGGTGGAGACA--------------------------------------------------------- 54507
Ma'sNightMonkey  AAGTTGCTCACTTGGTAACAAAA--------------------------------------------------------- 61773
VervetAGM        AACTTCCTCCCTTCACCAAAAAA---------------------------------TTGTCTAGTGTCTTTTTCCCTTCA 68050
Gorrilla         AAGTTGCTCCCTTCGCCAACAAA--------------------------------------------------------- 66873
GrabEatingMacaqueAAGTTCCTCCCTTCACCAAAAAA---------------------------------TTGTCTAGTGTCTTTTTCCCTTCA 67265
Macaque          AAGTTCCTCCCTTCACCAAAAAAATTGTCTAGTGTCTTTTTCCCTTCACCAAAAAATTGTCTAGTGTCTTTTTCCCTTCA 68134
PigTailedMacaque AAGTTCCTCCCTTCACCAAAAAA---------------------------------TTGTC--GTGTCTTTTTCCCTTCA 66984
Bonobo           AAGTTGCTCCCTTCGCCAAAAAA--------------------------------------------------------- 65444
OliveBaboon      AAGTTCCTCCCTTCACCAAAAAA---------------------------------TTGTCTAGTGTCTTTTTCCCTTCA 66841
Orangutan        AAGTTGCTCCCTTCGCCAAAAAC--------------------------------------------------------- 67274
```

```
Majority          ---AAAAATTGTCTAGTGTCTTTTTCCACTTCTGTGTTGAATTTTTAGTATAATGCATTTTTGTTTCTTCATCTCTGCC
                          81130     81140     81150     81160     81170     81180     81190     81200
Human             --------TTGTCTAGTGTCTTTTTCCACTTCTGTGTTGAATTTTTAGTATAACGCATTTTTGTTTCTTCATCTCTGCC 66406
Coquerel'sSifaka  ---------TGGTTAGAAGATACCTGGATTCTTCTGATGGAGAATTCATCACCG------T---TTTCCTTATG---ATC 54566
Ma'sNightMonkey   ---AAAAATTATCTGGTGTCTTTTTTCACTTCTGTGTTGAATTTTTAGTATAATGCATTTTTGTTTCTGCATGTCTGCC 61850
VervetAGM         CCAAAAAGTTGTCTAGTGTCTTTTTCCACTTCTGTGTTGAATTTTTAGTATAATGCATTTTTGTTTCTTCATCTCTGCC 68130
Gorrilla          --------TTGTCTAGTGTCTTTTTCCACTTCTGTGTTGAATTTTTAGTATAACGCATTTTTGTTTCTTCATCTCTGCC 66945
GrabEatingMacaqueCCAAAAAATTGTCTAGTGTCTTTTTCCACTTCTGTGTTGAATTTTTAGTATAATGCATTTTTGTTTCTTCATCTCTGCC 67345
Macaque           CCAAAAAATTGTCTAGTGTCTTTTTCCACTTCTGTGTTGAATTTTTAGTATAATGCATTTTTGTTTCTTCATCTCTGCC 68214
PigTailedMacaque  CCAAAAAATTGTCTAGTGTCTTTTTCCACTTCTGTGTTGAATTTTTAGTATAATGCATTTTTGTTTCTTCATCTCTGCC 67064
Bonobo            --------TTGTCTAGTGTCTTTTTCCACTTCTGTGTTGAATTTTTAGTATAACGCATTTTTGTTTCTTCATCTCTGCC 65516
OliveBaboon       CCAAAAAATTGTCTAGTGTCTTTTTCCACTTCTGTGTTGAATTTTTAGTATAATGCATTTTTGTTTCTTCATCTCTGCC 66921
Orangutan         --------TTGTCTAGTGTCTTTTTCCACTTCTGTGTTGAATGTTTAGTATAACGCATTTTTGTTTCTTTATCTCTGCC 67346

Majority          TCATAAGAAAAAGGAAAAAA-TGTTTAGCCTTTCCTAAGTGACTTCATTTTTT---------------------------
                          81210     81220     81230     81240     81250     81260     81270     81280
Human             TCATAAGAAAAAGGAAAAAG-TGTTTAGCCTTTCCTAAGTGACTTCTTTTTTTT-------------------------- 66459
Coquerel'sSifaka  TCATGACCTGAGTGAAAT---TTTTTAAAAAGTAATAATAAATTTTGCTTTT---------------------------- 54615
Ma'sNightMonkey   TCATAGAATAGAGGAAAAAAAATATTTAGCCTTTCCTAAATGACTTCATGTTTT--------------------------- 61903
VervetAGM         TCATAAGAAAAAGGAAAAAA-TGTTTAGCCTTTCCTAAGTGACTTCATTTTTT--------------------------- 68182
Gorrilla          TCATAAGAAAAAGGAAAAAG-TGTTTAGCCTTTCCTAAGTGACTTCTTTTTTTT-------------------------- 66998
GrabEatingMacaqueTCATAAGAAAAAGGAAAAAA-TGTTTAGCCTTTCCTAAGTGACTTCATTTTTTTTTTTTTTTTTTTTTTTTTTTGAGAC 67424
Macaque           TCATAAGAAAAAGGAAAAAA-TGTTTAGCCTTTCCTAAGTGACTTCATTTTTTTTTTTTTTTTTTT-------TTGAGAC 68286
PigTailedMacaque  TCATAAGAAAAAGGAAAAA--TGTTTAGCCTTTCCTAAGTGACTTCATTTTTT--------------------------- 67115
Bonobo            TCATAAGAAAAAGGAAAAAG-TGTTTAGCCTTTCCTAAGTGACTTCTTTTTTT--------------------------- 65568
OliveBaboon       TCATAAGAAAAAGGAAAAAA-TGTTTAGCCTTTCCTAAGTGACTTCATTT------------------------------ 66970
Orangutan         TCATAAGAAAAAGGAAAAAA-TGTTTAGCCTTTCCTAAGTGACTCCATTTTTTT-------------------------- 67399

Majority          --------------------------------------------------------------------------------
                          81290     81300     81310     81320     81330     81340     81350     81360
Human             -------------------------------------------------------------------------------- 66459
Coquerel'sSifaka  -------------------------------------------------------------------------------- 54615
Ma'sNightMonkey   -------------------------------------------------------------------------------- 61903
VervetAGM         -------------------------------------------------------------------------------- 68182
Gorrilla          -------------------------------------------------------------------------------- 66998
GrabEatingMacaqueGGAGTCTCGCTCTGTCACCCAGGCTGGAGTGCAGTGGCCGGATCTCAGCTCACTGCAAGCTCTGCCTCCCGGGTTTACGC 67504
Macaque           GGAGTCTCGCTCTGTCACCCAGGCTGGAGTGCAGTGGCCGGATCTCAGCTCACTGCAAGCTCTGCCTCCCGGGTTTACGC 68366
PigTailedMacaque  -------------------------------------------------------------------------------- 67115
Bonobo            -------------------------------------------------------------------------------- 65568
OliveBaboon       -------------------------------------------------------------------------------- 66970
Orangutan         -------------------------------------------------------------------------------- 67399
```

```
Majority          --------------------------------------------------------------------------------

                          81370     81380     81390     81400     81410     81420     81430     81440

Human             -------------------------------------------------------------------------------- 66459
Coquerel'sSifaka  -------------------------------------------------------------------------------- 54615
Ma'sNightMonkey   -------------------------------------------------------------------------------- 61903
VervetAGM         -------------------------------------------------------------------------------- 68182
Gorrilla          -------------------------------------------------------------------------------- 66998
GrabEatingMacaqueCATTCTCCTGCCTCAGCCTCCCGAGTAGCTGGGACTACAGGCACCCGCCATCTCCCCCGGCTAATTTTTTGTATTTTTTA 67584
Macaque           CATTCTCCTGCCTCAGCCTCCCGAGTAGCTGGGACTACAGGCGCCTGCCACCTCGCCCGGCTAATTTTTTGTATTTTTTA 68446
PigTailedMacaque  -------------------------------------------------------------------------------- 67115
Bonobo            -------------------------------------------------------------------------------- 65568
OliveBaboon       -------------------------------------------------------------------------------- 66970
Orangutan         -------------------------------------------------------------------------------- 67399

Majority          --------------------------------------------------------------------------------

                          81450     81460     81470     81480     81490     81500     81510     81520

Human             -------------------------------------------------------------------------------- 66459
Coquerel'sSifaka  -------------------------------------------------------------------------------- 54615
Ma'sNightMonkey   -------------------------------------------------------------------------------- 61903
VervetAGM         -------------------------------------------------------------------------------- 68182
Gorrilla          -------------------------------------------------------------------------------- 66998
GrabEatingMacaqueGTAGAGACGGGGTTTCACCGTGTTAGCCAGGATGGTCTCGATCTCCTGACCTCGTGATCCGCCCGTCTCGGCCTCCCAAA 67664
Macaque           CTAGAGACGGGGTTTCACCGTGTTAGCCAGGATGGTCTCGATCTCCTGACCTCGTGATCCGCCCGTCTCGGCCTCCCAAA 68526
PigTailedMacaque  -------------------------------------------------------------------------------- 67115
Bonobo            -------------------------------------------------------------------------------- 65568
OliveBaboon       -------------------------------------------------------------------------------- 66970
Orangutan         -------------------------------------------------------------------------------- 67399

Majority          ----------------------------------------------------ATCTGTTTTAATGTTTTTGCATCTTGTT

                          81530     81540     81550     81560     81570     81580     81590     81600

Human             ----------------------------------------------------ATCTGTTTTAATGTTTTTGCATCTTGTT 66487
Coquerel'sSifaka  ------------------------------------------------------------------------------TT 54617
Ma'sNightMonkey   ----------------------------------------------------ACCTGTTCGAATGTTTTTACATCTTGTT 61931
VervetAGM         ----------------------------------------------------ATCTGTTTTAATGTTTTTGCATCTTGTT 68210
Gorrilla          ----------------------------------------------------ATCTGTTTTAATGTTTTTGCATCTTGTT 67026
GrabEatingMacaqueGTGCTGGGATTACAGGCTTGAGCCACCGCGCCTGGCTGTGACTTCATTTTTTATCTGTTTTAATGTTTTTGCATCTTGTT 67744
Macaque           GTGCTGGGATTACAGGCTTGAGCCACCGCGCCCGGCTGTGACTTCATTTTTTATCTGTTTTAATGTTTTTGCATCTTGTT 68606
PigTailedMacaque  ----------------------------------------------------ATCTGTTTTAATGTTTTTGCATCTTGTT 67143
Bonobo            ----------------------------------------------------ATCTGTTTTAATGTTTTTGCATCTTGTT 65596
OliveBaboon       ----------------------------------------------------ATCTGTTTTAATGTTTCTGCATCTTGTT 66998
Orangutan         ----------------------------------------------------ATCTGTTTTAATGTTTTTGCATCTTGTT 67427
```

```
Majority          ATAACTGGGCTTTTTAAGTATACTCAAAAATAGGTTGAAAGTAGGTGAGTTAAAGGTTTTAAAAATCAGATAATGGACCC

                          81610     81620     81630     81640     81650     81660     81670     81680

Human             ATAACTGGGCTTTTTAACTATACTCAAAAATAGGTTAAAAGTAGGTGAGTTAAAGGTTTTAAAAATCAGATAATGGACCC 66567
Coquerel'sSifaka  TGAGACAGGGTCTCACTCTGTCACCTGGGCTAGAG-CGCGGTGGCGAGATCATAGCTCACAGCAACCTCAAACTC---CT 54693
Ma'sNightMonkey   ACAACTGGACTTTTAAAGTATACTCAAAAATAGGTTAAAAGTAGGTGAGTTACGGTTTTTAAACATTGTTTACAG----T 62007
VervetAGM         ATAACTGGGCTTTTTAAGTATACTCAAAAATAGGTTGAAAGTAGGTGAGTGAAAGGTTTTAAAAATCAGATAATGGACCC 68290
Gorrilla          ATAACTGGGCTTTTTAACTATACTCAAAAATAGGTTAAAAGTAGGTGAGTTAAAGGTTTTAAAAATCAGATAATGGACCC 67106
GrabEatingMacaqueATAACTGAGCTTTTTAAGTATACTCAAAAATAGGTTGAAAGTAGGTGAGTTAAAGGTTTTAAAAATCAGATAATGGACCC 67824
Macaque           ATAACTGAGCTTTTTAAGTATACTCAAAAATAGGTTGAAAGTAGGTGAGTTAAAGGTTTTAAAAATCAGATAATGGACCC 68686
PigTailedMacaque  ATAACTGAGCTTTTTAAGTATACTCAAAAATAGGTTGAAAGTAGGTGAGTTAAAGGTTTTAAAAATCAGATAATGGACCC 67223
Bonobo            ATAACTGGGCTTTTTAACTATACTCAAAAATAGGTTAAAAGTAGGTGAGTTAAAGGTTTTAAAAATCAGATAATGGACCC 65676
OliveBaboon       ATAACTGAGCTTTTTAAGTATACTCAAAAACAGGTTGAAAATAGGTGAGTTAAAGGTTTTAAAAATCAGATAATGGACCC 67078
Orangutan         ATAACTGGGCTTTTTAACTATACTAAAAAATAGGT-AAAAGTAGGTTAGTTAAAGGTTTTAAAAATCAGATAATGGACCC 67506

Majority          AAGTTTTGATAATTCACAAGAGATAACCATTTATTTTCATATTTTTTATGTAAATACATGTTTCTGTACAAAATTAAAAT

                          81690     81700     81710     81720     81730     81740     81750     81760

Human             TGGTTTTGATAATTCACAAGAGATAACCATTTATTTTCATATTTTTTATGTAAATACATGTTTCTGTACAAAATTAAAAT 66647
Coquerel'sSifaka  GGGCTCAAGCAATCCTACTGCCTCA---------------GCCTCCCAAGTAGCTG-------------------GGAC 54738
Ma'sNightMonkey   GACATCCAGTATGGCATGAGTGAGG--------T---CATGCCTTTTAGATGAATA-------------A---CTAAATC 62060
VervetAGM         AAGTTTTGATAATTCACAAGAGATAACCATTTATTTTTATATTTTTAATGTAAATACATGTTTCTGTACAAAATTAAAAT 68370
Gorrilla          TGGTTTTGATAATTCACAAGAGATAACCATTTATTTTCATATTTTTTATGTAAATACATGTTTCTGTACAAAATTAAAAT 67186
GrabEatingMacaqueAAGTTTTGATAATTCACAAGAGATAACCATTTATTTTAATATTTTTAATGTAAATACATGTTTTTGTACAAAATTAAAAT 67904
Macaque           AAGTTTTGATAATTCACAAGAGATAACCATTTATTTTAATATTTTTAATGTAAATACATGTTTTTGTACAAAATTAAAAT 68766
PigTailedMacaque  AAGTTTTGATAATTCACAAGAGATAACCATTTATTTTAATATTTTTAATGTAAATACATGTTTTTGTACAAAATTAAAAT 67303
Bonobo            TGGTTTTGATAATTCACAAGAGATAACCATTTATTTTCATATTTTTTATGTAAATACATGTTTCTTTACAAAATTAAAAT 65756
OliveBaboon       AAGTTTTGATAATTCACAAGAGATAACCATTTATTTTTATATTTTTAATGTAAATACATGTTTCTGTACAAAATTAAAAT 67158
Orangutan         TGGGTTTGATAATTCAAAAGAGATAACCGTTTATTTTTATATTTTTTATGTAAATACATGTTTCTGTACAAAATTAAAAT 67586

Majority          TGCTCTGTATTTGCAGATTTGCATTATCTTTTATTGTAATTGACATTTTACTTAGAATTTGTTTTAAAATATAGTTTTAA

                          81770     81780     81790     81800     81810     81820     81830     81840

Human             TGCTCTGTATTTGCAGATTTGCATTATCTTTTATTGTAATTGACATTTTACTTAGAATTTGTTTTAAAAGATAGCTTTAA 66727
Coquerel'sSifaka  TA-TGGGCATGCGC---------------------------CAC--CATGCCCAGC-TAATTTTTTCTATGTGTGTTTAG 54787
Ma'sNightMonkey   TTTTCTGTGTTT---------------CTATTCCTCTAATTA----TTTAATCAGA-TGACTTCAACAAAATAGTAGTGA 62120
VervetAGM         TGCTCTGTATTTGCAGATTTGCATTATCTTTTATTGTAATTGACATTTTACTTAGAATTTGTTTTAAAAGATAGTTTTAA 68450
Gorrilla          TGCTCTGTATTTGCAGATTTGCATTATCTTTTATTGTAATTGACATTTTACTTAGAATTTGTTTTAAAAGATAGCTTTAA 67266
GrabEatingMacaqueTGCTCTGTATTTGCAGATTTGCATTATCTTTTATTGTAATTGATATTTTACTTAGAATTTGTTTTAAAATATAGTTTTAA 67984
Macaque           TGCTCTGTATTTGCAGATTTGCATTATCTTTTATAGTAATTGATATTTTACTTAGAATTTGTTTTAAAATATAGTTTTAA 68846
PigTailedMacaque  TGCTCTGTATTTGCAGATTTGCATTATCTTTTATTGTAATTGATATTTTACTTAGAATTTGTTTTAAAATACAGTTTTAA 67383
Bonobo            TGCTCTGTATTTGCATATTTGCATTATGTTTTATTGTAATTGACATTTTACTTAGAATTTGTTTTAAAAGATAGCTTTAA 65836
OliveBaboon       TGCTCTGTATTTGCAGATTTGCATTATCTTTTATTGTAATTGATATTTTACTTAGAATTTGTTTTAAAATATAGTTTTAA 67238
Orangutan         TGCTCTGTATTTGCAGATTTGCATTATCTTTTAGTGTAATTGACATTTTACTTAGAATTTGTTTTAAAAGATAGTTTTAA 67666
```

```
Majority          TACCTGCGTAGCATGTTAGTGGATATGTTGTACCTTTGAAAGTGGACATGTAGGCTGTTCCATACTTTTTATTAAAACAC

                          81850     81860     81870     81880     81890     81900     81910     81920

Human             TACCTGCGTAGCATGTTCATGGGTATGCCATACCTTTGAAAGTGGACATGTAGGCTATTCCATACTTTTTATTAAAACAC 66807
Coquerel'sSifaka  TT---GTCCAGCTC----------------------------------------ATTTCTTTCTATTTTTAGTAAAGACAG 54825
Ma'sNightMonkey   TA------------------GGAT---------------------------TAATTTCCTGCTGAGTCTTCCTCAAA---- 62152
VervetAGM         TACCTGCGTAGCATGTTAGTGGATATGTTGTACCTTTGAAAGTGAACATGTAGGCTGTTCCATACTTTTTATTAAAACAC 68530
Gorrilla          TACCTGCGTAGCATGTTCATGGGTATGCCGTACCTTTGAAAGTGGACAAGTAGGCTATTCCATACTTTTTATTAAAACAC 67346
GrabEatingMacaqueTACCTGCATAGCATGTTAGTGGATATGTTGTACCTTTGAAAGTGGACATGTAGGCTGTTCCATACTTTTTATTAAAACAC 68064
Macaque           TACCTGCATAGCATGTTAGTGGATATGTTGTACCTTTGAAAGTGGACATGTAGGCTGTTCCATACTTTTTATTAAAACAC 68926
PigTailedMacaque  TACCTGCATAGCATGTTAGTGGATATGTTGTACCTTTGAAAGTGGACATGTAGGCTGTTCCATACTTTTTATTAAAACAC 67463
Bonobo            TACCTGCGTAGCATGTTCATGGGTATGCCATACCTTTGAAAGTGGACATGTAGGCTATTCCATACTTTTTATTAAAACAC 65916
OliveBaboon       TACCTGCATAGCATGTTAGTGGATATGTTGTACCTTTGAAAGTGGACATGTAGGCTGTTCCATATTTTTTATTAAAATAC 67318
Orangutan         TACCTGTGTAGCATGTTCATGAGTATGTCGTACCTTTGACAGTGGACATGTAGGCTATTCCATACTTTTTATTAAAACAC 67746

Majority          CCTTGTACTTTTTTGCCTCTCAAATTATTTTCTTAGACTAGATTCCTAGAATTAGAATTATTGGGTCAAAGAAAATGTTA

                          81930     81940     81950     81960     81970     81980     81990     82000

Human             CCTTGTACTTTTTTGCCTCTCAAATTATTTTCTTAGACTAGATTCCTAGAATTAGAATTATTGGGTCAAAGAAAATGTTA 66887
Coquerel'sSifaka  TCTCACTCTTGCT-------CAGTCTGGTCT-------TGAACTCCTGAGCT--------------------------- 54863
Ma'sNightMonkey   --TGGTACTTGTT-------CAAAATGCAAT---------AATTGATAGAAT---ATTTATT---TCAAAGATTTTGTAA 62208
VervetAGM         CCTTGTACTTTTTTGCCTCTCAAATTATTTTCTTAGATTAGATTCCTAGAATTAGAATTATTGGCTCAAAGAAAATGTTA 68610
Gorrilla          CCTTGTACTTTTTTGCCTGTCAAATTATTTTCTTAGACTAGATTCCTAGAATTAGAATTATTGGGTCAAAGAAAATGTTA 67426
GrabEatingMacaqueCCTTGTACTTTTTTGCCTCTCAAATTATTTTCTTAGACTAGATTCCTAGAATTAGAATTATTGGGTCAAAGAAAATGTTA 68144
Macaque           CCTTGTACTTTTTTGCCTCTCAAATTATTTTCTTAGACTAGATTCCTAGAATTAGAATTATTGGGTCAAAGAAAATGTTA 69006
PigTailedMacaque  CCTTGTACTTTTTTGCCTCTCAAATTATTTTCTTAGACTAGATTCCTAGAATTAGAATTATTGGGTCAAAGAAAATGTTA 67543
Bonobo            CCTTGTACTTTTTTGCCTCTCAAATTATTTTCTTAGACTAGATTCCTAGAATTAGAATTATTGGGTCAAAGAAAATGTTA 65996
OliveBaboon       CCTTGTACTTTTTTGCCTCTCAAATTATTTTCTTAGACTAGATTCCTAGAATTAGAATTATTGGGTCAAAGAAAATGTTA 67398
Orangutan         CCTTGTACTTTTTTGCCTCTCAAATTATTTTCTTAGACTAGATTCCTAGGATTAGAATTATTGGGTCAAAGAAAATGTTA 67826

Majority          AAGCCTATTTACATTATTTGCAAATTTCTTCATCTAAAGGTGATATTAACTTAAACTAACATTAATTATATATGAGGCTG

                          82010     82020     82030     82040     82050     82060     82070     82080

Human             AAGCCTGTTTACTTTATTTGCAAATTTCTTCATCTAAAGGTGATATTAACTTAAACTAACATTAATTATATATGAGGCTG 66967
Coquerel'sSifaka  --C------------------AAA---C-----------GAT----------CCA-------------------------- 54874
Ma'sNightMonkey   GAA----------C-----ACAAAT------AT--AA-GGTGATACT--CTTCTGCTAA--------ATATTTGTAGTTA 62254
VervetAGM         AAG-------ACATTATTTGCAAATTTCTTCATCTAAAGGTGATATTAACTTAAACTAACATTAATTATATATGAGGCTG 68683
Gorrilla          AAGCCTTTTTACTTTATTTGCAAATTTCTTCATCTAAAGGTGATATTAACTTGAACTAACAGTAATTATATATGAGGCTG 67506
GrabEatingMacaqueAAGCCTATTTACATTATTTGCAAATTTCTTCATCTAAAGGTGATATTAACTTAAACTAACATTAATTATATATGAGGCTG 68224
Macaque           AAGCCTATTTACATTATTTGCAAATTTCTTCATCTAAAGGTGATATTAACTTAAACTAACATTAATTATATATGAGGCTG 69086
PigTailedMacaque  AAGCCTATTTACATTATTTGCAAATTTCTTCATCTAAAGGTGATATTAACTTAAACTAACATTAATTATATATGAGGCTG 67623
Bonobo            AAGCGTATTTACTTTATTTGCAAATTTCTTCATCTAAAGGTGATATTAACTTAAACTAACATTAATTATATATGAGGCTG 66076
OliveBaboon       AAGCCTATTTACATTATTTGCAAATTTCTTCATCTAAAGGTGATATTAACTTAAACTAACATTAATTATATATGAGGCTG 67478
Orangutan         AAGCCTATTTACATTATTTGCAAATTTCTTCATCTAAAGGTGATATTAACTTAAACTAACATTAATTATATATGAGGCTG 67906
```

```
Majority         GGTGTCCTACTTTTTCCCCTACCAGCATATTATTTTTTAATTTCACAAGATTAATTTGATAGGAAAATGTCTATTATTTT

                         82090     82100     82110     82120     82130     82140     82150     82160

Human            GGTGTCCTACTTTTTCCCCTGCCAGCATACTATTTTTTAATTTCACAAGATTAATTTGATAGGCAAATGCCT---ATTTT 67044
Coquerel'sSifaka -----CCTGCCTTGGCCTCC--CAGAGTGCTAGGATTATAGGCTAAGATATTAATTTGATAAGAAAATGTCTA-----TT 54942
Ma'sNightMonkey  GGG---CTTCCCTTTACCTT----G--TATGATTTTTAGAATTAGCT------ACTTGACTG--AA-----------TTT 62306
VervetAGM        GGTGTCCTACTTTTTCCCCTACCAGCATATTATTTTTTAATTTCACAAGATTAATTTGATAGGAAAATGTCTATTATTTT 68763
Gorrilla         GGTGTCCTACTTTTTCCCCTGCCAGCATACTACTTTTTAATTTCACAAGATTAATTTGATAGGCAAATGTCTATTATTTT 67586
GrabEatingMacaqueGGTGTCCTACTTTTTCCCCTACCAGCATATTATTTTTTAATTTCACAAGATTAATTTGATAGGAAAATGTCTATTATTTT 68304
Macaque          GGTGTCCTACTTTTTCCCCTACCAGCATATTATTTTTTAATTTCACAAGATTAATTTGATAGGAAAATGTCTATTATTTT 69166
PigTailedMacaque GGTGTCCTACTTTTTCCCCTACCAGCATATTATTTTTTAATTTCACAAGATTAATTTGATAGGAAAATTTCTATTATTTT 67703
Bonobo           GGTGTCCTACTTTTTCCCCTGCCAGCATACTATTTTTTAATTTCACAAGATTAATTTGATAGGCAAATGTCTGTTATTTT 66156
OliveBaboon      GGTGTCCTACTTTTTCCCCTACCAGCATATTATTTTTTAATTTCACAAGATTAATTTGATAGGAAAATGTCTATTATTTT 67558
Orangutan        GGTGTCCTACTTTTTCCCCTACCAGCATACTATTTTTTAATTTCACAAGATTAATTTGATAGGAAAATGTCTA-----TT 67981

Majority         AATTTCTGTTAAGATTTAGTTTGCAGTCTTTAAATG-TTTATTTGACAGTCACAATTCTCTAAATTGCTTACCTGCATTC

                         82170     82180     82190     82200     82210     82220     82230     82240

Human            AATTTCTGTTAAGATTTAGTTTACAATCTTTAAATG-TTTATTTGATAGTCACAATTTGCTAAATTGCTTGCCTGCATTC 67123
Coquerel'sSifaka ATTTTCTTTTATAATCTAGTTTGCAGTTGTTAAGTG-CTTATTCAGTATTTACAATTCTCTAAATTGGTTATCTGCATTC 55021
Ma'sNightMonkey  AATTACC-TTAGAATCAGTACTATAAATTTTAATTGACTTATTTTACA-CAGTAATCTTTTAAG--ACTAACATTTATCT 62382
VervetAGM        AATTTCTGTTAAGATTTAGTTTGCAGTCTTTAAATG-TTTATTTGACAGTCACAATTCTCTAAATTGCTTACCTGCATTC 68842
Gorrilla         AATTTCTGTTAAGATTTAGTTTACAATCTTTAAATG-TTTATTTGATAGTCACAATTCGCTAAATTGCTTGCCTGCATTC 67665
GrabEatingMacaqueAATTTCTGTTAAGATTTAGTTTGCAGTCTTTAAATG-TTTATTTGACAGTCACAATTCTCTAAATTGCTTACCTGCATTC 68383
Macaque          AATTTCTGTTAAGATTTAGTTTGCAGTCTTTAAATG-TTTATTTGACAGTCACAATTCTCTAAATTGCTTACCTGCATTC 69245
PigTailedMacaque AATTTCTGTTAAGATTTAGTTTGCAGTCTTTAAATG-TTTATTTGACAGTCACAATTCTCTAAATTGCTTACCTGCATTC 67782
Bonobo           AATTTCTGTTAAGATTTAGTTTACAGTCTTTAAATG-TTTATTTGATAGTCACAATTCGCTAAATTGCTTGCCTGCATTC 66235
OliveBaboon      AATTTCTGTTAAGATTTAGTTTGCAGTCTTTAAATG-TTTATTTGACAGTCACAATTCTCTAAATTGCTTACCTTCATTC 67637
Orangutan        AATTTCTGTTAAGATTTAGTTTACAATCTTTAAATG-TTTATTTGATAGTCACAATTCTCTAAATTTCTTGCCTGCATTC 68060

Majority         TTTCTGTTAGGATGTACAGTAATTCTTATAAAATGCGTAGTAAGGATATTAATATGCATATTTTGGATGAGCCAAGCCTT

                         82250     82260     82270     82280     82290     82300     82310     82320

Human            TTTCTGTTAGGATGTACGGTAATTCTTATAAAATGCATAGTAAGGATATTAATATGCATATTTTGGATGAGCCAAGCCTT 67203
Coquerel'sSifaka CTTCTATTGGGATGTACAGTAATTCTTGTAAAATGCTTATAAATGATATCA-TGTTCATATTTGGTATGAGTTGAGCCTT 55100
Ma'sNightMonkey  CT-------GGTTTTTCTGT--TTCTTTTAA------TGCTAGGTGTCACA----G--TGTCTCAGA--AA-------TT 62432
VervetAGM        TTTCTGTTAGGATGTACAATAATTTTTTTAAAATGGGTAGTAAGGATATTAATATGCATATTTTGGATGAGCCAAGCCTT 68922
Gorrilla         TTTCTGTTAGGATGTACGGTAATTCTTATAAAATGCATAGGAAGGATATTAATATGCATATTTTGGATGAGCCAAGCCTT 67745
GrabEatingMacaqueTTTCTGTTAGGATGTACAATAATTTTTATAAAATGGGTAGTAAGGATATTAATATGCGTATTTTGGATAGGCCAAGCCTT 68463
Macaque          TTTCTGTTAGGATGTACAATAATTTTTATAAAATGGGTAGTAAGGATATTAATATGCGTATTTTGGATAGGCCAAGCCTT 69325
PigTailedMacaque TTTCTGTTAGGATGTACAATAATTTTTATAAAATGCGTAGTAAGGATATTAATATGCGTATTTTGGATAGGCCAAGCCTT 67862
Bonobo           TTTCTGTTAGGATGTATGGTAATTCTTATAAAATGCATAGTAAGGATATTAATATGCATATTTTGGATGAGCCAAGCCTT 66315
OliveBaboon      TTTCTGTTAGGATGTACAATAATTTTTATAAAATGGGTAGTAAGGATATTAATATGCGTATTTTGGATGAGCCAAGCCTT 67717
Orangutan        TTTCTGTTAGGATGTACAGTAATTCTCATAAAATGCATAGTAAGGATATTAATATGCATATTTTGGATGAGCCAAGCCTT 68140
```

```
Majority          CAGCTTTATTCACATATGTTAGA---GTCAGTTTTTGGAAAAGTATGTTTAAATAATCTTACCTTTTTAAATAATTGATC

                          82330     82340     82350     82360     82370     82380     82390     82400

Human             CAGCTTTATTCACTTATGTTAG----GTCAGTTTTTGGAAAAGTATTTTTCAATAATCT-CTCTTTTTAAATAATTGATC 67278
Coquerel'sSifaka  CAGCTTTATTCACCTATTTTACACTGGTCAGTTTTTGGAAAACTATTTTAAAATAATTT-ATCTCTTTAATGAATTGATC 55179
Ma'sNightMonkey   GTAATTTGTTCAAAGAAAGGAGA---CTGAGTATTTTGTAA--TGAGTTTAAA--ATCTGACATCT--AAATACATAATC 62503
VervetAGM         CAGCTTTATTCACATATGTTAG----GTCAGTTTTTGGAAAAGTATGTTTAAATAATCTTACCTTTTTAAATAATTGATC 68998
Gorrilla          CAGCTTTATTCATGTATGTTAG----GTCAGTTTTTGGAAAAGTATTTTTCAATAATCT-ATCTTTTTAAATAATTGATC 67820
GrabEatingMacaqueCAGCTTTATTCACATATGTTAGACAGGTCAGTTTTTGGAAAAGTATGTTTAAATAATCTTACCTTTTTAAATAATTGATC 68543
Macaque           CAGCTTTATTCACATATGTTAGACAGGTCAGTTTTTGGAAAAGTATGTTTAAATAATCTTACCTTTTTAAATAATTGATC 69405
PigTailedMacaque  CAGCTTTATTCACATATGTTAGACAGGTCAGTTTTTGGAAAAGTATGTTTAAATAATCTTACCTTTTTAAATAATTGATC 67942
Bonobo            CAGCTTTATTCACTTATGTTAG----GTCAGTTTTTGGAAAAGTATTTTTCAATAATCT-ATCTTTTTAAATAATTGATC 66390
OliveBaboon       CAGCTTTATTCACATATGTTAGACAGGTCAATTTTTGGAAAAGTATGTTTAAATAATCTTACCTTTTTAAATAATTGATC 67797
Orangutan         CAGCTTTGTTCATGTATGTTAG----GTCAGTTTTTGGAAAAGTATTTTTTAATAATCT-ATCTTTTTAAATAATTGATT 68215

Majority          TCAATCCCAAAGTATTCATTTGTAGGACATGAAGACACTATTTTCTTCTTCATACATTTGGTGAATATTTT--GATCTC-

                          82410     82420     82430     82440     82450     82460     82470     82480

Human             TCAATCCCAAAGTATTCATTTGTAGGACATGAAGACACAATTTTCTTCTTCATACGTTTGGTGAATATTTT--GATCTC- 67355
Coquerel'sSifaka  TCAATCCAAAGGTATCCATTTGCAGGACATGAAGATA----ATTTTTTTTCACACGTTTGGTGGATATTATATGATCTTT 55255
Ma'sNightMonkey   ACAAACCTGGCAGACT-ATTAGT----CATTCACATA-----TTCTGTTTCAAA--------GAACACGGT--GATCAA- 62562
VervetAGM         TCAATCCCAAAGTATTCATTTGTACCACATGAAGACACTATTTTCTTCTTCACACATTTGGTGAATATTT---GATCTC- 69074
Gorrilla          TCAATCCCAAAGTATTCATTTGTAGGACATGAAGACACAATTTTCTTCTTCATACGTTTGGTGAATATTTT--GATCTC- 67897
GrabEatingMacaqueTCAATCCCAAAGTATTCATTTGTACGACATGAAGACACTATTTTCTTCTTCATACATTTGGTGAATATTT---GATCTC- 68619
Macaque           TCAATCCCAAAGTATTCATTTGTACGACATGAAGACACTATTTTCTTCTTCATACATTTGGTGAATATTT---GATCTC- 69481
PigTailedMacaque  TCAATCCCAAAGTATTCATTTGTACGACATGAAGACACTATTTTCTTCTTCATACATTTGGTGAATATTT---GATCTC- 68018
Bonobo            TCAATCCCAAAGTATTCATTTGTAGGACATGAAGACACAATTTTCTTCTTCATACATTTGGTGAATATTTT--GATCTC- 66467
OliveBaboon       TCAATCCCAAAGTATTCATTTGTACGACATGAAGACACTATTTTCTTCTTCATACATTTGGTGAATATTT---GATCTC- 67873
Orangutan         TCAATCCCAAAGTATTCATTTGTAGGACATGAAGACACAATTTTCTTCTTCAGACATTTGGTGAATATTTT--GATCTC- 68292

Majority          ------AGTTTCC-----ACCTTTATTTTT----GCTTTTTAAACATTGTTTACAGTGATAT--CCAATATCGCATAAGT

                          82490     82500     82510     82520     82530     82540     82550     82560

Human             ------AGTTTCC-----ACTTCTCTTTTT----GCTTTTTAAACATTGTTTATGGTGATAT--CCAATATTGCATGAGT 67418
Coquerel'sSifaka  AGTAGGAATTCCTTAACTGTTTTGCTCTTTATGTGGTTTTCACACACCCTTTACAGTGACAT--CCAATATTGCATTAGT 55333
Ma'sNightMonkey   ------AAAT---------CCTTGAGATTA----TATATTGAGACATCGTT-ATAATGAT--------CATTTTAT---- 62610
VervetAGM         ------AGTTTCC-----ACCTTTATTTTT----GCTTTTTAAACATTGTTTACAGTGATAC--CCAATATCCCATAAGT 69137
Gorrilla          ------AGTTTCC-----ACTTTTCTTTTT----GCTTTTTAAACATTGTTTACGGTGATAT--CCAATATTGCATGAGT 67960
GrabEatingMacaque------AGTTTCC-----ACCTTTATTTTT----GCTTTTTAAACATTGTTTACAGTGATAC--CCAATATCGCATAAGT 68682
Macaque           ------AGTTTCC-----ACCTTTATTTTT----GCTTTTTAAACATTGTTTACAGTGATAC--CCAATATCGCATAAGT 69544
PigTailedMacaque  ------AGTTTCC-----ACCTTTATTTTT----GCTTTTTAAACATTGTTTACAGTGATAC--CCAATATCGCATAAGT 68081
Bonobo            ------AGTTTCC-----ACTTTTCTTTTT----GCTTTTTAAACATTGTTTACGGTGATAT--CCAATATTGCATGAGT 66530
OliveBaboon       ------AGTTTCC-----ACCTTTATTTTT----GCTTTTTAAACATTGTTTACAGTGATAC--CCAATATCGCGTAAGT 67936
Orangutan         ------AGTTTCC-----ACTTTTCTTTTT----GCTTTTTAAACATTGTTTACAGTGATATATCCAATATCGCATGGGT 68357
```

```
Majority          GAGG-TCATGCCTTTTAGAATAGTAA--------CTAAATCTTTTCCTCTA----ATTATTTAATCAGATGACTTCAA--

                        82570     82580     82590     82600     82610     82620     82630     82640

Human             GAGG-TCATGCCTTTTAGAATAATAA--------CTGAATCTTTTCTTCTA----ATTATTTAATCAGATGACTTCAG-- 67483
Coquerel'sSifaka  AAAG-TCATGCCTTTGGGAATAACTAAATCTTTTCCATCTTTCTTTCCCTACAATAATACTTGATCAGATGACTTCGAAG 55412
Ma'sNightMonkey   ------CATGC---TAGGAAGTGTAG--------TTAATTT--------------ATTAAGGGGT-AGATCACTTTTA-- 62656
VervetAGM         GAGG-ACATGCCTTTTAGAATAGTAA--------CTAAATCTTTTCCTCTA----ATTATTTAATCAGATGACTTCAA-- 69202
Gorrilla          GAGG-TCATGCCTTTTAGAATAATAA--------CTGAATCTTTCCCTCTA----ATTATTTAATCAGATGACTTCAG-- 68025
GrabEatingMacaqueGAGG-ACATGCCTTTTAGAATAGTAA--------CTAAATCTTTTCCTCTA----ATTATTTAATCAGATGACTTCAA-- 68747
Macaque           GAGG-ACATGCCTTTTAGAATAGTAA--------CTAAATCTTTTCCTCTA----ATTATTTAATCAGATGACTTCAA-- 69609
PigTailedMacaque  GAGGGACATGCCTTTTAGAATAGTAA--------CTAAATCTTTTCCTCTA----ATTATTTAATCAGATGACTTCAA-- 68147
Bonobo            GAGG-TCATGCCTTTTAGAATAATAA--------CTGAATCTTT-CTTCTA----ATTATTTAATCAGATGACTTCAG-- 66594
OliveBaboon       GAGG-ACATGCCTTTTAGAATAGTAA--------CTAAATCTTTTCCTCTA----ATTATTTAATCAGATGACTTCAA-- 68001
Orangutan         GAGG-TCATGCCTTTTAGAATAATAA--------CTGAATCTTTTCCGCTA----ATTATTTAATCAGATGACTTCAA-- 68422

Majority          ------CAAACTAGTATTGATGGGATTAACTTCCTGCTCAGTCTTCCTGAAACAGTACTTGTTCAAAATGAAATAATTGA

                        82650     82660     82670     82680     82690     82700     82710     82720

Human             ------CAAACTAATATTGGTGGGATTAACTTCCTGCTCAGTCTTCCAGTAACAGTACTTGTTCAAAATGAAATAATTGA 67557
Coquerel'sSifaka  TAAAACCAAACTACTATTGATGGGACTCATTGCCTGCTCAGTAGTCTTCAAACAGTTCTTGTTCAAAATGAAATAACTGA 55492
Ma'sNightMonkey   ------GAAAA----ATTGCTAGAAATAATTT----------------------------TTCATGATCATGTTGTCTA 62697
VervetAGM         ------CAAACTAGTATTGATGGGATTAACTTGCTGCTCAGTCTTCCTGAAACAGTACTTGTTCAAAATGAAATAATTGA 69276
Gorrilla          ------CAAACTAATATTGGTGGGATTAACTTCCTGCTCAGTCTTCCAGTAACAGTACTTGTTCAAAATGAAATAATTGA 68099
GrabEatingMacaque------CAAACTAGTATTGATGGGATTAACTTCCTGCTCAGTCTTCCTGAAACAGTACTTGTTCAAAATGAAGTCATTGA 68821
Macaque           ------CAAACTAGTATTGATGGGATTAACTTCCTGCTCAGTCTTCCTGAAACAGTACTTGTTCAAAATGAAGTCATTGA 69683
PigTailedMacaque  ------CAAACTAGTATTGATGGGATTAACTTCCTGCTCAGTCTTCCTGAAACAGTACTTGTTCAAAATGAAGTCATTGA 68221
Bonobo            ------CAAACTAATATTGGTGGGATTAACTTCCTGCTCAGTCTTCCAGTAACAGTACTTGTTCAAAATGAAATAATTGA 66668
OliveBaboon       ------CAAACTAGTATTGCTGGAATTAACTTCCTGCTCAGTCTTCCTGAAACAGTACTTGTTCAAAATGAAGTAATTGA 68075
Orangutan         ------CAAACTAGTATTGGTGGGATTAACTTCCTGCCCAGTCTTCCTGTAACAGTACTTGTTCAAAATGAAATAATTAA 68496

Majority          -----TA-----TTTGTTTCAAAAATTTTGTAAGAACACAAATATAAGGTGATACTCTTTTGCTAAATATTTGTGTTGGG

                        82730     82740     82750     82760     82770     82780     82790     82800

Human             TAGAGTA-----TTTATTTCAAAAATTTT-TAAGAACACAAATATAAGGTGATACTCTTTTGCTAAA-ATTTGTGGGGGG 67630
Coquerel'sSifaka  TAGAGTACCCCCTTTATTTCAAAGATTTTGTAAGAACACAAATATAAGGTGATAGTCTTCCACTAAATACTTGAG--GGG 55570
Ma'sNightMonkey   -----CA-----TTC----TAAAAATTAG---AGATATTGTATACAAAG-AATATTTATTT--TAGA------------G 62745
VervetAGM         ------A-----TTTGTTTCAAAAATTTTGTAAGAACACAAATATAAGGTGATACTCTTTTGCTAAATATTTGTGTTGGG 69345
Gorrilla          TAGAGTA-----TTTATTTCAAAAATTTTGTAAGAACACAAATATAAGGTGATACTCTTTTGCTAAA-ATTTGTGGGGGG 68173
GrabEatingMacaque------A-----TTTGTTTCAAAAATTTTGTAAGAACACAAATATAAGGTGATACTCTTTTGCTAAATATTTGTGTTGGG 68890
Macaque           ------A-----TTTGTTTCAAAAATTTTGTAAGAACACAAATATAAGGTGATACTCTTTTGCTAAATATTTGTGTTGGG 69752
PigTailedMacaque  ------A-----TTTGTTTCAAAAATTTTGTAAGAACACAAATATAAGGTGATACTCTTTTGCTAAATATTTGTGTTGGG 68290
Bonobo            TAGAGTA-----TTT----CAAAAATTTTGTAAGAACACAAATATAAGGTGATACTCTTTTGCTAAA-ATTTGTGGGGGG 66738
OliveBaboon       ------A-----TTTGTTTCAAAAATTTTGTAAGAACACAAATATAAGGTGATACTCTTTTGCTAAATATTTGTGTTGGG 68144
Orangutan         TATAGTA-----TTTATTTCAAAAATTTTGTAAGAACACAAATATAAGGTGATACTCTTTTGCTAAA-ATCTGTGGGAGG 68570
```

```
Majority        CTTC-CTTTACCTTGTATGATTTTTATATTAGATCTAGCTACTTGACTGAATTTAATTACTTTAGAATCAGTACTATAAA
                      82810     82820     82830     82840     82850     82860     82870     82880
Human           CTTCACTTTACCTTGTATGATTTTTATATTAGATCTAGCTACTTGACTGAATTTAATTACTTTAGAATCAGTACTATAAA 67710
Coquerel'sSifaka TTTC------------ATTCACTTTACAATATATCTAGCCACTTGATTGAATTTAATTACTATAGAATCAATATTATAAA 55638
Ma'sNightMonkey CTTT------TCTTTTATTAC----AGATTGAAT--AACAGCCGTTCTCATCATGAAGTTTTTAAAAGAAAGACAA---- 62809
VervetAGM       CTTT-CTTTACCTTGTATGATTTTTATATTAGATCTAGCTATTTGACTGAATTTAATTACTTTAGAATCAGTACTATAAA 69424
Gorrilla        CTTCACTTTACCTTGTGTGATTTTTATATTAGATCTAGCTACTTGACTGAATTTAATTACTTTAGAATCAGTACTATAAA 68253
GrabEatingMacaqueCTTC-CTTTACCTTGTATGATTTTTATATTAGATCTAGCTACTTGACTGAATTTAATTACTTTAGAATCAGTACTATAAA 68969
Macaque         CTTC-CTTTACCTTGTATGATTTTTATATTAGATCTAGCTACTTGACTGAATTTAATTACTTTAGAATCAGTACTATAAA 69831
PigTailedMacaque CTTC-CTTTACCTTGTATGATTTTTATATTAGATCTAGCTACTTGACTGAATTTAATTACTTTAGAATCAGTACTATAAA 68369
Bonobo          CTTCACTTTACCTTGTATGATTTTTATATTAGATCTAGCTACTTGACTGAATTTAATTACTTTAGAATCAGTACTATAAA 66818
OliveBaboon     CTTC-CTTTACCTTGTATGATTTTTATATTAGATCTAGCTACTTGACTGAATTTAATTACTTTAGAATCAGTACTATAAA 68223
Orangutan       ATTCACTTTACCTTGTATGATTTTTATATTAGATCTAGCTACTTGACTGAATTTAATTACTTTAGAATCAGTACTATAAA 68650

Majority        TTTTAATTGATTTATTTTACACAGTAATT--TTTTAAGACTAACATTTATGTCTGGCTTTTCTGTTTCCTTTAATGCTAG
                      82890     82900     82910     82920     82930     82940     82950     82960
Human           TTTTAATTGATTTATTTTACACAGTAATT--TTTTAAGACTAACATTTATGTCTGGCTTTTCTGTTTCCTTTAATGCTAG 67788
Coquerel'sSifaka TT----TTAACTTATTTTGCATAGTAATT--TTTTAAGACTAGCATTTATGTTTGGCTTCTCTGTTTCTTTTAATGCTAG 55712
Ma'sNightMonkey ----AACAGACTTTTTGTAGAAAATAATTGATGTTAAAATAGTTATCATTGGCTAGGTGAGGTGGCTC----A-CACCTG 62880
VervetAGM       TTTAAATTGATTTATTTTACACAGTACTT--TTTTAAGACTAACATTTATATCTGGCTTTTCTGTTTCCTTCAATGCTAG 69502
Gorrilla        TTTTAATTGATTTATTTTACACAGTAATT--TTTTAAGACTAACATTTATGTCTGGCTTTTCTGTTTCCTTTAATGCTAG 68331
GrabEatingMacaqueTTTGAATTGATTTATTTTCCACAGTACTT--TTTTAAGACTAACATTTATATCTGGCTTTTCTGTTTCCTTCAATGCTAG 69047
Macaque         TTTGAATTGATTTATTTTCCACAGTACTT--TTTTAAGACTAACATTTATATCTGGCTTTTCTGTTTCCTTCAATGCTAG 69909
PigTailedMacaque TTTGAATTGATTTATTTTCCACAGTACTT--TTTTAAGACTAACATTTATATCTGGCTTTTCTGTTTCCTTCAATGCTAG 68447
Bonobo          TTTTAATTGATTTATTTTACACAGTAATT--TTTTAAGACTAACATTTATGTCTGGCTTTTCTGTTTCCTTTAATGCTAG 66896
OliveBaboon     TTTGAATTTATTTATTTTACACAGTACTT--TTTTAAGACTAACATTTATATCTGGCTTTTCTGTTTCCTTCAATGCTAG 68301
Orangutan       TTTTAATCGATTTATTTTACGCAGTAATT--TTTTAAGACTAACATTTATGTCTGGCTTTTCTGTTTCCTTTAATGCTAG 68728

Majority        GTATCACAGTGTCTCAGAAAATGTAATTTGTTCAAAGAAAGGAGACTGA--GTACTTTGTAATGAGTTTAAAA-TCTGAC
                      82970     82980     82990     83000     83010     83020     83030     83040
Human           GTATCACAGTGTCTCAGAAAATGTAATTTGTTCAAAGAAAGGAGACTGA--GTGCTTTGTAATGAGTTTAAAA-TCTGAC 67865
Coquerel'sSifaka GGATTACAGTACCTCAGAAAATGTAATTTATTCAGAGAAGGGAGACCTACAGTGCTTTGTAATTAGTTAAAAA-TCTGAC 55791
Ma'sNightMonkey TAATCCCAGTACTTTGGGAGGCGGAG---GC-----GGGCGGATTATGA--G------GTAG-AAGTTCAAGA-----AC 62938
VervetAGM       GTATCATAGTGTCTCAGAAAATGTAATTTGTTCAAAGAAAGGAGACTGA--GTACTTTGTAATGAGTTTAAAAATCTGAC 69580
Gorrilla        GTATCACAGTGTCTCAGAAAATGTAATTTGTTCAAAGAAAGGAGACTGA--GTGCTTTGTAATGAGTTTAAAA-TCTGAC 68408
GrabEatingMacaqueGTATCATAGTGTCTCAGAAAATGTAATTTGTTCAAAGAAAGGAGACTGA--GTACTTTGTAATGAGTTTAAAAATCTGAC 69125
Macaque         GTATCATAGTGTCTCAGAAAATGTAATTTGTTCAAAGAAAGGAGACTGA--GTACTTTGTAATGAGTTTAAAAATCTGAC 69987
PigTailedMacaque GTATCATAGTGTCTCAGAAAATGTAATTTGTTCAAAGAAAGGAGACTGA--GTACTTTGTAATGAGTTTAAAAATCTGAC 68525
Bonobo          GTATCACAGTGTCTCAGAAAATGTAATTTGTTCAAAGAAAGGAGACTGA--GTGCTTTGTAATGAGTTTAAAA-TCTGAC 66973
OliveBaboon     GTATCATAGTGTCTCAGAAAATGTAATTTGTTCAAAGAAAGGAGACTGA--GTACTTTGTAATGAGTTTAAAAATCTGAC 68379
Orangutan       GTATCACAGTGTCTCAGAAAATGTAATTTGTTCAAAGAAAGGAGACTGA--G-----TGTAATGAGTTTAAAA-TCTGAC 68800
```

```
Majority          ATCTAAA-TACATAATCACAAACGTGGCAGACTATTAGTCATTCACATATTCTGTTTCAAAGAACACGGTGATCAAAAAT

                          83050     83060     83070     83080     83090     83100     83110     83120

Human             ATCTAAAATACATAATCACAAATGTGGCCGACTATTAGTCATTCACATATTCTGTTTCAAAGAACACAGTGATCAAAAAT 67945
Coquerel'sSifaka  CTCTAAA-TACTTAATCACAAACCTGGCAGACTATTAGTCATTTACATATTCTGTTTTAAAGAACAAGGTGATCAAAAAT 55870
Ma'sNightMonkey   AGCCTGG-----------CTAACATGGTGAAAC----------CCCAT-TTCTATT--AAAGAAAATTA----CAAAAAT 62990
VervetAGM         ATCTAAA-TACGTAATCACAAACGTGGCAGACTATTAGTCATTCACATATTCTGTTTCAGAGAACACGGTGATCAAAAAT 69659
Gorrilla          ATCTAAAATACATAATCACAAATGTGGCCGACTATTAGTCATTCATATATTCTGTTTCAAAGAACACGGTGATCAAAAAT 68488
GrabEatingMacaqueATCTAAA-TACATAATCACAAACATGGCAGACTATTAGTCATTCACATATTCTGTTTCAAAGAAAACGGTGATCAAAAAT 69204
Macaque           ATCTAAA-TACATAATCACAAACATGGCAGACTATTAGTCATTCACATATTCTGTTTCAAAGAAAACGGTGATCAAAAAT 70066
PigTailedMacaque  ATCTAAA-TACATAATCACAAACATGGCAGACTATTAGTCATTCACATATTCTGTTTCAAAGAAAACGGTGATCAAAAAT 68604
Bonobo            ATCTAAAATACATAATCACAAATGTGGCCAACTATTAGTCATTCACATATTCTGTTTCAAAGAACACAGTGATCAAAAAT 67053
OliveBaboon       ATCTAAA-TACATAATCACAAACGTGGCAGACTATTAGTCATTCACATATTCTGTTTCAAAGAACACGGTGATCAAAAAT 68458
Orangutan         ATCTAAAATACATAATCACAAACATGGCTGACTATTAGTCATTCGCATATTCTGTTTCAAAGAACACGGTGATCAAAAAT 68880

Majority          CCTTCAGATTATAAATTGAGATATCATTATAAT----CGTTTTCTCATGTTAAGAGGTGTAGTTAATTTATTAAGGGGTA

                          83130     83140     83150     83160     83170     83180     83190     83200

Human             CCTTCAGATTATAAATTGAGACATCATTATAATGGATCATTTTATCATGTTAAGAGGTGTAGTTAATTTATTAAGGGGTA 68025
Coquerel'sSifaka  CCTCGAGATTATAAATGGAGACATCATTATAAT-GATTATTTTATCATGTTAAGAAATGTAGTTAATTTATTAAGGGATA 55949
Ma'sNightMonkey   TAGCCAGAT------------------------------------------GTGGTGGCGTGCATCTGT--A------ 63018
VervetAGM         CCTTCAGATTATAAATTGAGATAAC---ATAAT----TGCTTTCTCATGTTAAGAGGTGTAGTTAATTTATTAAGGGGTA 69732
Gorrilla          CCTTCAGATTATAAATTGAGACATCATTATAATGGATCATTTTATCATGTTAAGAGGTGTAGTTAATTTATTAAGGGGTA 68568
GrabEatingMacaqueCCTTCAGATTATAAATTGAGATATCATTATCAT----CGTTTTCTCATGTTAAGAGGTGTAGTTAATTTATTAAGGGGTA 69280
Macaque           CCTTCAGATTATAAATTGAGATATCATTATCAT----CGTTTTCTCATGTTAAGAGGTGTAGTTAATTTATTAAGGGGTA 70142
PigTailedMacaque  CCTTCAGATTATAAATTGAGATATCATTATAAT----CGTTTTCTCATGTTAAGAGGTGTAGTTAATTTATTAAGGGGTA 68680
Bonobo            CCTTCAGATTATAAATTGAGACATCATTATAATAGATCATTTTATCATGTTAAGAGGTGTAGTTAATTTATTAAGGGGTA 67133
OliveBaboon       CCTTCAGATTATAAATTGAGATATCATTATAAT----CGTTTTCTCATGTTAAGAGGTGTAGTTAATTTATTAAGGGGTA 68534
Orangutan         CCTTCAGATTATAAATTGAGAC---ATTATAATGGATCATTTTATCATGTTAAGAGGTGTAGTTAATTTATTAAGGGGTA 68957

Majority          GATCACTT--AGAAAAGTTGCTGGAAGTAATTTTTCATGATCATGTTGTCTGCATTCTAAAAATTAGGAGAGAGAGTGTG

                          83210     83220     83230     83240     83250     83260     83270     83280

Human             GATCACTTTTAGAAAAAATTGCTGGAAGTAATTTTTCATGATCATGTTATCTACATTCTAAAAATTAGGAGAGAGACTGTG 68105
Coquerel'sSifaka  GTTCACTTTTAGAAAAAATTGCTAGAAGTCATTATTCATGATCATGTTGTCTACGTTTTAAAAATTAGGGCAGATAATGCA 56029
Ma'sNightMonkey   -ATCCC---------AGCTACTTGGGATATTGAGGCAGGAGCA--TTGCCTGAAACCGGAGGCGGAGG----TTGCTGTG 63082
VervetAGM         GATCACTT--AGAAAAGTTGCTGGAAGTAATTTTTCATGATCATGTTGTCTGCATTCTAAAAATTAGGAGAGAGAGTGTG 69810
Gorrilla          GATCACTTTTAGAAAAAATTGCTGGAAGTAATTTTTCATGATCATGTTATCTACATTCTAAAAATTAG-------AGTGTG 68641
GrabEatingMacaqueGATCACTT--AGAAAAGTTGCTGGAAGTAATTTTTCATGATCCTGTTGTCTGCATTCTAAAAATTAGGAGAGAGAGTGTG 69358
Macaque           GATCACTT--AGAAAAGTTGCTGGAAGTAATTTTTCATGATCCTGTTGTCTGCATTCTAAAAATTAGGAGAGAGAGTGTG 70220
PigTailedMacaque  GATCACTT--AGAAAAGTTGCTGGAAGTAATTTTTCATGATCCTGTTGTCTGCATTCTAAAAATTAGGAGAGAGAGTGTG 68758
Bonobo            GATCACTTTTAGAAAAAATTGCTGGAAGTAATTTTTCATGATCATGTTATCTACATTCTAAAAATTAGGAGAGAGAGTGTG 67213
OliveBaboon       GATCACTT--AGAAAAGTTGCTGGAAGTAATTTTTCATGATCATGTTGTCTGCATTCTAAAAATTAGGAGAAAGAGTGTG 68612
Orangutan         GATCACTTTTAGAAAAAATTGCTGGAAGTAATTTTTCATGATGATGTTATCTACATTCTAAAAATTAG-----AGAGTGTG 69032
```

```
Majority          TACAAAGAATATTTATTTTAGAGCTTTCCTTGTATTTCAAATTGA-----GTAACAGGCATTCTCATCATAAAGTTTTTA
                           83290     83300     83310     83320     83330     83340     83350     83360
Human             TACAAAGAGTGTTTATTTTAGAGCTTTCCTTGTATTTCAAATTGA-----ATAACAGGCATTCTCATCATAAAGTTTTTA 68180
Coquerel'sSifaka  TACAAAGAATAGTTACTTTAGAGCTTTGCATGTATTTTGAGTTGAATGAAGTAATAGGCATTCTCATCATAAGGTCTT-- 56107
Ma'sNightMonkey   AACTGAGA--------TTG--TGC---CATTGCACTCCAGCCTGG-----GCGACA------------ACAAGACTC--- 63129
VervetAGM         TACAAAGAATATTTATTTTAGAGCTTTCCTTGTATTTCAAATTGA-----GGAACAGGCATTCTCATCATAAAGTTTTTA 69885
Gorrilla          TACAAAGAGTATTTATTTTAGAGCTTTCCTTGTATTTCAAATTGA-----ATAACAGGCATTCTCATCATAAAGTTTTTA 68716
GrabEatingMacaqueTACAAAGAATATTTATTTTAGAGCTTTCCTTGTATTTCAAATTGA-----GGAACAGGCATTCTCATCATAAAGTTTTTA 69433
Macaque           TACAAAGAATATTTATTTTAGAGCTTTCCTTGTATTTCAAATTGA-----GGAACAGGCATTCTCATCATAAAGTTTTTA 70295
PigTailedMacaque  TACAAAGAATATTTATTTTAGAGCTTTCCTTGTATTTCAAATTGA-----GGAACAGGCATTCTCATCATAAAGTTTTTA 68833
Bonobo            TACAAAGAGTATTTATTTTAGAGCTTTCCTTGTATTTCAAATTGA-----ATAACAGGCATTCTCATCATAAAGTTTTTA 67288
OliveBaboon       TACAAAGAATATTTATTTTAGAGCTTTCCTTGTATTTCAAATTGA-----GGAACAGGCATTCTCATCATAAAGTTTTTA 68687
Orangutan         TACAAAGAGT----ATTTTAGAACTTTCCTTGTATTTCAAATTGA-----ATAACAGGCATTCTCATCATAAAGTTTTTA 69103

Majority          AAA-GAAAGGCAAAGCAGATCTTTTGTAGGAAATC-ATTGACGTTAAAATAGTTATCATTGTGAACAGATAC---AACAT
                           83370     83380     83390     83400     83410     83420     83430     83440
Human             AAA-GAAAGGCAAAGCAGACTTTCTGTAGGAAATC-ATTGACGTTAAAATAGTTATAATTGTGAACAGATAC---AACAT 68255
Coquerel'sSifaka  AAAAGAAAAGCAAAACAAACTTTTAATAAGAAATCTGTTGACATTAAAATATTTAAAATTGTGAACAGATTAGCAAACAT 56187
Ma'sNightMonkey   ---------------C--ATCTC----AAAAA-------------AAAAAAGTTATCATTGTGAACAGATAC---AACAT 63172
VervetAGM         AAAAGAAAGGCAAAGCAGATCTTTTGTAGGAAATC-ATTGACGTTAAAATAGTTATCATTATGAACAGATAC---AACAT 69961
Gorrilla          AAA-GAAAGGCAAAGCAGACTTTCTGTAGGA-----ATTGACGTTAAAATAGTTATAACTGTGAACAGATAG---AATAT 68787
GrabEatingMacaqueAAA-GAAAGGCAAAGCAGATCTTTTGTAGGAAATC-ATTGATGTTAAAATAGTTATCATTGTGAACAGATAC---AACAT 69508
Macaque           AAA-GAAAGGCAAAGCAGATCTTTTGTAGGAAATC-ATTGATGTTAAAATAGTTATCATTGTGAACAGATAC---AACAT 70370
PigTailedMacaque  AAA-GAAAGGCAAAGCAGATCTTTTGTAGGAAATC-ATTGATGTTAAAATAGTTATCATTGTGAACAGATAC---AACAT 68908
Bonobo            AAA-GAAAGGCAAAGCAGACTTT----AGGAAATC-ACTGACGTTAAAATAGTTATAATTGTGAACAGATAC---AACAT 67359
OliveBaboon       AAA-GAAAGGCAAAGCAGATCTTTTGTAGGAAATC-ATTGACGTTAAAATAGTTATCATTGTGAACAGATAC---AACAT 68762
Orangutan         AAA-GAAAGGCAAAGCAGACTTTTTGTAGGAAATC-ATTGACATTAAAATAGTTATAATTGTGAACAGATAC---AACAT 69178

Majority          TTATTCATGAAGGTAAACATGTAGGTCTTATAGAACATTGTTTCTCAAAATTTTGCTGCACATTGAAGTCACCAGATTCT
                           83450     83460     83470     83480     83490     83500     83510     83520
Human             TTATTCATGAAGGTAAACATGTAGGTCTTATAGAATATTGTTTCTCAAAATTTTGCTGCACGTTGAAGTCACCAGATTCT 68335
Coquerel'sSifaka  TTATTTATGAAGGTAAATATTTAGCCTTTACAGAACAGTGTTTTTCAAACTTTTGCTGCACGTTGAAGTTACTGGGCCCT 56267
Ma'sNightMonkey   TTATTCATGAAAGTAAACATGTAGGTCTTATAGAACAGTGTTTCTCAAA--------------------------CTGT 63225
VervetAGM         TTATTCATAAAGATAAACATGTCGGTCTTATAGAACATTGTTTTTCAAAATTTTGCTGCACATTGAAGTCACCAAATTCT 70041
Gorrilla          TTATTCATGAAGGTAAACATGTGGGTCTTATAGAACATTGTTTCTCAAAATTTTGCTGCACGTTGAAGTCACCAGATTCT 68867
GrabEatingMacaqueTTATTCATAAAGATAAACATGTAGGTCTTATAGAACATTGTTTCTCAAAATTTTGCTGCACATTGAAGTCACCAAATTCT 69588
Macaque           TTATTCATAAAGATAAACATGTAGGTCTTATAGAACATTGTTTCTCAAAATTTTGCTGCACATTGAAGTCACCAAATTCT 70450
PigTailedMacaque  TTATTCATAAAGATAAACATGTAGGTCTTATAGAACATTGTTTCTCAAAATTTTGCTGCACATTGAAGTCACCAAATTCT 68988
Bonobo            TTATTCATGAAGGTAAACATGTAGGTCTTATAGAACATTGTTTCTCAAAATTTTGCTGCACGTTGAAGTCACCAGATTCT 67439
OliveBaboon       TTATTCATAAAGATAAACATGTAGGTCTTATAGAACATTGTTTCTCAAAATTTTGCTGCACATTGAAGTCACCAAATTCT 68842
Orangutan         TTATTCATGAAGGTAAACATGTAGGTCTTATAGAACCTTGTTTCTCAAAATTTTGCTGCACATTGAAGTCACCAGATTCT 69258
```

```
Majority          ACCCCAGACTGAGAATAGTT--GGAGTGGGTCACAGGCATTCCTATGTTTTT-AGGATCTCAAGATGGTTTT-ACTGTGT

                           83530     83540     83550     83560     83570     83580     83590     83600

Human             ACCCCAGGCTGAGAATAGTTAAAGAGTGGGTCACAGGCATTCCTATGTTTTT-AGGATCTCAAGATGGTTTT-ACTATGT 68413
Coquerel'sSifaka  AACCCAGACTGTGAATCAGT-GGGAGTGAGTCTCTGGCATTCATATTTTTTTAAGATCCCAAGATGGTTTT-ACTTTGT 56345
Ma'sNightMonkey   ACCCCAGACTGAGAATAGTT--GGAGTGGGTCACAGGCATTCCTATGTTTTT-AGGATCTCAAAATGGTTTTACCTAGGT 63302
VervetAGM         ACCCCAGACTGAGAATAGTT--GGAGTGGGTCACAGGCATTCCTATGTTTTT-AGGATCTCAAGATGGTTTT-ACTGTGT 70117
Gorrilla          ACCCCAGGCTGAGAATAGTTAAAGAGTGGGTCACAGGCATTCCTATGTTTTT-AGGATCTCAAGATGGTTTT-ACTATGT 68945
GrabEatingMacaqueACCCCAGACTGAGAATAGTT--GGAGTGGGTCACAGGCATTCCTGTGTTTTT-AGGATCTCAAGATGGTTTT-ACTGTGT 69664
Macaque           ACCCCAGACTGAGAATAGTT--GGAGTGGGTCACAGGCATTCCTGTGTTTTT-AGGATCTCAAGATGGTTTT-ACTGTGT 70526
PigTailedMacaque  ACCCCAGACTGAGAATAGTT--GGAGTGGGTCACAGGCATTCCTGTGTTTTT-AGGATCTCAAGATGGTTTT-ACTGTGT 69064
Bonobo            ACCCCAGGCTGAGAATAGTTAAAGAGTGGGTCACAGGCATTCCTGTGTTTTT-AGGATCTCAAGATGGTTTT-ACTATGT 67517
OliveBaboon       ACCCCAGACTGAGAATAGTT--GGAGTGGGTCACAGACATTCCTATGTTTTT-AGGATCTCAAGATGGTTTT-ACTGTGT 68918
Orangutan         ATCCCAGGCTGAGAATA----AAGAGTGGGTCACAGGCATTCCTATGCTTTT-AGGATCTCAAGATGATTTTTACCATGT 69333

Majority          AGCAAGAGTTGAGAATTACTGCTACAGAAAATAACTTGTAACTTAATTAATGTCATTTAAATCTATTTTGGTACAGTTCT

                           83610     83620     83630     83640     83650     83660     83670     83680

Human             AGCAAGAGTTGAGAATTACTGCTACAGAAAAGAGCTTGTAACTT----AATGTCATTTAAATCTATTTTGGTACAGTTCT 68489
Coquerel'sSifaka  AGCGAGAGTTGAGGATTACTGCTATAGAAAAAAGTTTGTAATTTAATTCATGTCTTTTAAATCTGTCCTGGTACATATCT 56425
Ma'sNightMonkey   AGCAAGAGTTGAGAATTGCTGCTATAGAAAATAATTCATAATTT----AATGTCATTTAAATCTATTTTGGTACAGTTCT 63378
VervetAGM         AGCAAGAGTTGAGAATTACTGACACAGAAAATAACTCGTGACTTAATTAATGTCATTTAAATCTATTTTGGTACAGTTCT 70197
Gorrilla          AGCAAGAGTTGAGAATTACTGCTACAGAAAA--GCTTGTAACTT----AATGTCATTTAAATCTATTTTGGTGCAGTTCT 69019
GrabEatingMacaqueAGCAAGAGTTGAGAATTACTGACACAGAAAATAACTCGTGACTTAATTAATGTCATTTAAATCTATTTTGGTACAGTTCT 69744
Macaque           AGCAAGAGTTGAGAATTACTGACACAGAAAATAACTCGTGACTTAATTAATGTCATTTAAATCTATTTTGGTACAGTTCT 70606
PigTailedMacaque  AGCAAAAGTTGAGAATTA----CACAGAAAATAACTCGTGACTTAATTAATGTCATTTAAATCTATTTTGGTACAGTTCT 69140
Bonobo            AGCAAGAGTTGAGAATTACTGCTACAGAAAAGAGCTTGTAACTT----AATGTCATTTAAATCTATTTTGGTACAGTTCT 67593
OliveBaboon       AGCAAGAGTTGAGAATTACTGACACAGAAAATAACTTGTGACTTAATTAATGTCATTTAAATCTATTTTGGTACAGTTCT 68998
Orangutan         AGCAAGAGTTGAGAATTACTGCTGCAGAAAAGAGCTTGTAACTT----AATGTCGTTTAAATCTATTTTGGTACAGTTCT 69409

Majority          GCTGGCACAATTTTAATTAGCTGTTAAACTGAATTTTTTATTTTCTATCTGTTTGTGATACATAAAATTAACTGGAAAAC

                           83690     83700     83710     83720     83730     83740     83750     83760

Human             CCTGGCACAATTTTAATTAGCTGTTAAACTGAATTTATAATTTTCTATATGTTTGTGATACATAAAATTAACTGGAAAAC 68569
Coquerel'sSifaka  GTTGGTGCAGTATTGTTTAACTGTTAAACTGAATTT---ACTTTTTCTTTGTTTGTGATACATAAAATTAACAAGTTAAC 56502
Ma'sNightMonkey   GCTGGCACAATATTAATTAGCTGTTAAGCTGAATTTTTTATTTTTTATATGTTTGTGATATGTAAAATTAACTGGTAAAC 63458
VervetAGM         GGTGGCATAATTTTAATTAGCTGTTAAACTGAATTTTTTATTTTCTATCTATTTGTGATACATAAAATTAACTGGAAAAC 70277
Gorrilla          GCTAGCACAATTTTAATTAGCTGTTAAACTGAATTTATAATTTTCTGTATGTTTGTGATACTTAAAATTAACTGGAAAAC 69099
GrabEatingMacaqueGCTGGCATAATTTTAATTAGCTGTTAAACTGAATTTTTTATTTTCTATCTATTTGTGATACATAAAATTAACTGGAAAAC 69824
Macaque           ---GGCATAATTTTAATTAGCTGTTAAACTGAATTTTTTATTTTCTATCTATTTGTGATACATAAAATTAACTGGAAAAC 70683
PigTailedMacaque  GCTGGCATAATTTTAATTAGCTGTTAAACTGAATTTTTTATTTTCTATCT----GTGATACATAAAATTAACTGGAAAAC 69216
Bonobo            GCTGGCACAATTTTAATTAGCTGTTAAACTGAATTTATAATTTTCTATATGTTTGTGATACATAAAACTAACTGCAAAAC 67673
OliveBaboon       GCTGGCATAATTTTAATTAGCTGTTAAACTGAATTTTTTATTTTCTATCTATTTGTGATACATAAAATTAACTGGAAAAC 69078
Orangutan         GCT-GCACAATTTTAATTAGCTGTTAAACTGAATTTTTAATTTTCTGTATGTTTGTGATACATAAAATTAACTGGAAAAC 69488
```

```
Majority          TTATTT-----TGATTTT------CTTTC---TTTGTTACATTTGTATGGGTTATTCATTTGGTAATATTTATTAAT---

                          83770     83780     83790     83800     83810     83820     83830     83840

Human             TT---------TGATTTT------CTTTC---TTTGTTACATTTGTATGGGTTATTCATTTGGTAATATTTATTAAT--- 68628
Coquerel'sSifaka  TTATTTTTAAATGATTTCTGACAGCTTTGATTTTCTTTACTTTTGAATGGGTCATTCACATAGGAATATTTATTAGTATT 56582
Ma'sNightMonkey   TTATTT-----TCACTTT------CTTT-------GTTACATTTGAATGGGTTATTCATATGGTAATATTTATTAAT--- 63517
VervetAGM         TTATTT-----TGATTTT------CTTTC---TTTGTTACATTTGTATGGGTTATTCATTTGGTAATATTTATTAAT--- 70340
Gorrilla          TTACTT-----TGATTTT------CTTTC---TTTGTTACATTTGTATGGGTTATTCATTTGGTAATATTTATTAAT--- 69162
GrabEatingMacaqueTTATTT-----TGATTTT------CTTTC---TTTGTTACATTTGTATGGGTTATTCATTTGGTAATATTTATTAAT--- 69887
Macaque           TTATTT-----TGATTTT------CTTTC---TTTGTTACATTTGTATGGGTTATTCATTTGGTAATATTTATTAAT--- 70746
PigTailedMacaque  TTATTT-----TGATTTT------CTTTC---TTTGTTACATTTGTATGGGTTATTCATTTGGTAATATTTATTAAT--- 69279
Bonobo            TTACTT-----TGATTTT------CTTTC---TTTGTTACATTTGTATGGGTTATTCATTTGGTAATATTTATTAAT--- 67736
OliveBaboon       TTATTT-----TGATTTT------CTTT-------GTTACATTTGTATGGGTTATTCATTTGGTAATATTTATTAAT--- 69137
Orangutan         TTATTT-----TGATTTT------CTTT-------GTTACATTTTTATGGGTTATTCATTTGGTAATATTTATTAAT--- 69547

Majority          ----------TTATTATTAGAGATTTAAGCTATATGCTAATATAATGAATCCCATACACCTGTCATCTTGGTTCAATAGC

                          83850     83860     83870     83880     83890     83900     83910     83920

Human             ----------TTATTATTAGAGATTTCAGCTATTTGCTAATATAATGAATCCCATACACCTGTCATCTTGGTTCAATAGC 68698
Coquerel'sSifaka  TATTAACTTTTTATTATCAAGGATTTCAGTTATATACTAATATAATGAATTCCACATACCCATTATCATAGTTCAGTATT 56662
Ma'sNightMonkey   ----------TTATTATCAGAGATTTAAGCTATACACTAATATAATGAATCCCATACACCTGTCATCTTGGTTCAGTAGC 63587
VervetAGM         ----------TTATTATTAGAGATTTAAGCTGTATGCTAATATAACGAATCCCATACACCTGTCATCTTGGTTCAATAGC 70410
Gorrilla          ----------TTATTATTAGAGATTTCAGCTATATGCTAATATAATGAATCCCATACACCTGTCATCTTGGTTCAATAGC 69232
GrabEatingMacaque----------TTATTATTAGAGATTTAAGCTGTATGCTAATATAACGAATCCCATACACCTGTCATCTTGGTTCAATAGC 69957
Macaque           ----------TTATTATTAGAGATTTAAGCTGTATGCTAATATAACGAATCCCATACACCTGTCATCTTGGTTCAATAGC 70816
PigTailedMacaque  ----------TTATTATTAGAGATTTAAGCTGTATGCTAATATAACGAATCCCATACACCTGTCATCTTGGTTCAATAGC 69349
Bonobo            ----------TTATTATTAGAGATTTCAGCTATATGCTAATATAATGAATCCCATACACCTGTCATCTTGGTTCAATAGC 67806
OliveBaboon       ----------TTATTATTAGAGATTTAAGCTGTATGCTAATATAACGAATCCCATACACCTGTCATCTTGGTTCAATAGC 69207
Orangutan         ----------TTATTATTAGAAATTTCAGCTATATGCTAATATAATGAATCCCATACACCTGTCATCTTGGTTCAATAGT 69617

Majority          GCTGACACTTAGTTTTATCTATTCAGTTTTT--CTTTTGTATTCTAAAGCAAATCCTTGACATTATGACATGTCTTCTCA

                          83930     83940     83950     83960     83970     83980     83990     84000

Human             GCTGACACTTAGTTTTATCTATTCAGTTTTT--CTTTTGTGTTCTAAAGCAAATCCTTGACATTGTGACATGTCTTCTCA 68776
Coquerel'sSifaka  GCCTACATTTGTTTTTATCTATTCATTTTTTTTCTGTTGTGTTTTAGAACAAATCCTTGACATCATGACATGTCTTCTCA 56742
Ma'sNightMonkey   GCTGACA----GTTTTATCTATTCAGTTTTT--CTATTGTATTCTAAAGCAAATCCTTGA--TCATGACATGTCTTCTCA 63659
VervetAGM         GCTGACACTTAGTTTTATCTATTCAGTTTTT--CTTTTGTATTCAAAAGCAAATCCTTGACATTATGACATGTCTTCTCA 70488
Gorrilla          ACTGACACTTAGTTTTATCTATTCAGTTTTT--CTTTTGTGTTCTAAAGCAAATCCTTGACATTGTGACATGTCTTCTCA 69310
GrabEatingMacaqueGGTGACACTTAGTTTTATCTATTCAGTTTTT--CTTTTGTATTCTAAAGCAAATCCTTGACATTATGACATGTCTTCTCA 70035
Macaque           GGTGACACTTAGTTTTATCTATTCAGTTTTT--CTTTTGTATTCTAAAGCAAATCCTTGACATTATGACATGTCTTCTCA 70894
PigTailedMacaque  GCTGACACTTAGTTTTATCTATTCAGTTTTT--CTTTTGTATTCTAAAGCAAATCCTTGACATTATGACATGTCTTCTCA 69427
Bonobo            GCTGACACTTAGTTTTATCTATTCAGTTTTT--CTTTTGTGTTCTAAAGCAAATCCTTGACATTGTGACATGTCTTCTCA 67884
OliveBaboon       GCTGACACTTAGTTTTATCTATTCAGTTGTT--CTTTTGTATTCTAAAGCAGATCCTTGACATTATGACATGTCTTCTCA 69285
Orangutan         GCTGACACTTAGTTTTATCTATTCAGTTTTT--CTTTTGTATTCTAAAGCAAATCCTTGACATTGTGACATGTCTTCTCA 69695
```

```
Majority          AATATTACC-ATAGTATCTCTAAAATAATTACATTTTCTTACCAAACCATAATATGATTATTACACTTA-TCAAAATTGA

                          84010     84020     84030     84040     84050     84060     84070     84080

Human             AATATTACC-ATAGTATCTCTAAAATAATTACATTTTCTTACCAAACCATAATATGATTATTACACTTA-CCAGAATTGA 68854
Coquerel'sSifaka  AATATTACCCATAGTATCTCTAAAGTAATTACCTTTTCTTACCAAAC---AATATGATTTTCACAC-----CAAAATTGA 56814
Ma'sNightMonkey   TATATTACC-ATAGTTTCTCTAAAATAACTGTGTTTTCTCACCAAACCATACTATGATTATTACACTTA-TCAAAATTGA 63737
VervetAGM         AATATTACC-ATAGTATCTCTAAAATAATTACATTTTCTTACCAAACCATAATATGATTATTACACTTAATCAAAATTGA 70567
Gorrilla          AATATTACC-ATAGTATCTCTAAAATAATTACATTTTCTTACCAAACCATAATATGATTATTACACTTA-CCAAAATTGA 69388
GrabEatingMacaqueAATATTACC-ATAGTATCTCTAAAATAATTACATTTTCTTACCAAACCATAATATGATTATTACACTTAATCAAAATTGA 70114
Macaque           AATATTACC-ATAGTATCTCTAAAATAATTACATTTTCTTACCAAACCATAATATGATTATTACACTTAATCAAAATTGA 70973
PigTailedMacaque  AATATTACC-ATAGTATCTCTAAAATAATTACATTTTCTTACCAAACCATAATATGATTATTACACTTAATCAAAATTGA 69506
Bonobo            AATATTACC-ATAGTATCTCTAAAATAATTACATTTTCTTACCAAACCGTAATATGATTATTACACTTA-CCAGAATTGA 67962
OliveBaboon       AATATTACC-ATAGTATCTCTAAAATAATTACATTTTCTTACCAAACCATAATATGATTATTACACTTAATCAAAATTGA 69364
Orangutan         AATATTACC-ATAGTATCTCTAAAATAATTACATTTTCGTACCAAACCATAATATGATTATTACACTTA-CCAAAATTGA 69773

Majority          CAATGAT--TTCTTAATGTCATCAAATACCCAACTTACATTAAAATTTCCCCAATTATCTTGAAAATGCTTTTAAAGTTT

                          84090     84100     84110     84120     84130     84140     84150     84160

Human             CAATGAT--TTCTTAATGTCCTCAAATACCCAGCTTACATTAAAATTTCCCCAATTATTTTGAAAATGCTTTTAAAGTTT 68932
Coquerel'sSifaka  CAGTGACAATTCTTAATATCATCAAATACCCAACTTA--TTAAAATTTCCCTCTTTACCTCAAAAATGTTTTTTAGATTT 56892
Ma'sNightMonkey   CAATGAT--TTCCTAATATCATCAAATATCCTACTCACATTAAAATTTCCCCAATTATCCCGAAAATGCTTTTAAGGTTT 63815
VervetAGM         CAATGAT--TTCTTAATGTCATCAAATACCCAACTCACATTAAAACTTCCCCAATTATCTTGAAAATGCTTTTAAAGTTT 70645
Gorrilla          CAATGAT--TTCTTAATGTCCTCAAATACCCAGCTTACATTAAAATTTCCCCAATTATTTTGAAAATGCTTTTAAAGTTT 69466
GrabEatingMacaqueCAATGAT--TTCTTAATGTCATCAAATACCCAACTTACATTAAAATTTCCCCAATTATCTTGAAAATGCTTTTAAAGTTT 70192
Macaque           CAATGAT--TTCTTAATGTCATCAAATACCCAACTTACATTAAAATTTCCCCAATTATCTTGAAAATGCTTTTAAAGTTT 71051
PigTailedMacaque  CAGTGAT--TTCTTAATGTCATCAAATACCCAACTTACATTAAAATTTCCCCAATTATCTTGAAAATGCTTTTAAAGTTT 69584
Bonobo            CAATGAT--TTCTTAATGTCCTCAAATACCCAGCTTACATTAAAATTTCCCCAATTATTTTGAAAATGCTTTTAAAGTTT 68040
OliveBaboon       CAATGAT--TTCTTAATGTCATCAAATACCCAGCTTACATTAAAATTTCCCCAATTATCTTGAAAATGCTTTTAAAGTTT 69442
Orangutan         CAATGAT--TTCTTAATGTCATCAAATACCCAGCTTACATTAAAATTTCACCAATTATTTTGAAAATGCTTTTAAAGTTT 69851

Majority          GATTTGTTCAGAACAAGGTCCTAT-GGAGGGCCGCACATAGCATTTGGTTGTTTTATCTCATTAGTCTGTTTCAGTCTGT

                          84170     84180     84190     84200     84210     84220     84230     84240

Human             GATTTGTTCAGAACAAGGTCCTAT-GGAGGGCCGCACATAGTATTTGGTTGTTACATCTCATTAGTCTGTTTCAGTCTGT 69011
Coquerel'sSifaka  GATTTGTTCAAAATAGGATCCTGT-CAGGGGCCCCACATGGCATTTGGTGGTTGTGTCTCCCAAGTCTCTTTCAGTCTGT 56971
Ma'sNightMonkey   GATTTGTTCAGAACAAAGTCCTGT-GGAGGGCCGCACATAGTATTTGGTTAT---GTCTCATTAGTCTGATTCAGTCTGT 63891
VervetAGM         GATTTGTGCAGAACAAGGTCCTAT-GGAGGGCCGCACATAGCATTTGGTTGTTTTATCTCATTAGTCTGTTTCAGTCTGT 70724
Gorrilla          GATTTGTTCAGAACAAGGTCCTAT-GGAGGGCCGCACATAGTATTTGGTTGTTATATCTCATTAGTCTGTTTCAGTCTGT 69545
GrabEatingMacaqueGATTTGTTCAGAACAAGGTCCTAT-GGAGGGCCGCACATAGCATTTGGTTGTTTTATCTCATTAGTCTGTTTCAGTCTGT 70271
Macaque           GATTTGTTCAGAACAAGGTCCTAT-GGAGGGCCGCACATAGCATTTGGTTGTTTTATCTCATTAGTCTGTTTCAGTCTGT 71130
PigTailedMacaque  GATTTGTTCAGAACAAGGTCCTAT-GGAGGGCCGCACATAGCATTTGGTTGTTTTATCTCATTAGTCTGTTTCAGTCTGT 69663
Bonobo            GATTTGTTCAGAACAAGGTCCTAT-GGAGGGCCGCACATAGTATTTGGTTGTTATATCTCATTAGTCTGTTTCAGTCTGT 68119
OliveBaboon       GATTTGTTCAGAACAAGGTCCTAT-GGAGGGCCGCACATAGCATTTGGTTGTTTTATCTCATTAGTCTATTTCAGTCTGT 69521
Orangutan         GATTTGTTCAGAACAAGGTCCTATTGGAGGGCCGCACATAGTATTTGGTTGTTATATCTCATTAGTCTGTTTCAGTCTGT 69931
```

```
Majority          AATAGTCCTCTCCCTCTCTCTACTCCCAATCTCACATCCATTTACTTA-TGAAGTGACGTGGTTATCCTTTTAGAAAGTT

                          84250     84260     84270     84280     84290     84300     84310     84320

Human             AATAGTCCTCTCCCTCTCTCTACTCCCAATCTCTCATTCATTTACTTA-TGAAGTGATGTT----TCCTTTTAGAAAGTT 69086
Coquerel'sSifaka  TGTTACGGTC-CTTTCCCTTTACCCCCAACCCCATTTCCATTTACTTAATGAAGAGACCTAGTAATTCTTTTACAAAGTT 57050
Ma'sNightMonkey   AATGGTCCTGTCCCTCTCTCTACTCCCAGTCTCACTTCCATTTACTTA-TGAAGTGATGTGGTTATCCTTTTAGAAAGTT 63970
VervetAGM         AATAGTCCTCTTCCTTTCTCTACTCCCAATCTCACATCCGTTTACTTA-TGAAGTGACATGGTTATCCTTTTAGAAAGTT 70803
Gorrilla          AATAGTCCTCTCCCTCTCTCTACTCCCAATCTCACGTTCATTTACTTA-TGAAGTGATGTT----TCCTTTTAGAAAGTT 69620
GrabEatingMacaqueAATAGTCATCTCCCTTTCTCTACTCCCAATCTCACATCCGTTTACTTA-TGAAGTGACATGGTTATCCTTTTAGAAAGTT 70350
Macaque           AATAGTCATCTCCCTTTCTCTACTCCCAGTCTCACATCCGTTTACTTA-TGAAGTGACATGGTTATCCTTTTAGAAAGTT 71209
PigTailedMacaque  AATAGTCATCTCCCTTTCTCTACTCCCAATCTCACATCCGTTTACTTA-TGAAGTGACATGGTTATCCTTTTAGAAAGTT 69742
Bonobo            AATAGTCCTCTCCCTCTCTCTACTCCCAGTCTC--GTTCATTTACTTA-TGAAGTGATGTT----TCCTTTTAGAAAGTT 68192
OliveBaboon       AATAGTCCTCTCCCTTTCTCTACTCCCAATCTCACATCCGTTTACTTA-TGAAGTGACATGGTTATCCTTTTAGAAAGTT 69600
Orangutan         AATAGTCCTCTCCCTCTCTCTACTCCCAATCTCACGTCCATTTACTTA-TGAAGTGATGTGGTTATCCTTTTAGAAAGTT 70010

Majority          TCACAGATTAGACTAGTCTGGCTGTTTC-CTTAGAGTGTCATGTTAACTTGTT-CCTCTATCTGCTGCGTTTCTTATATA

                          84330     84340     84350     84360     84370     84380     84390     84400

Human             TCACAGATTAGACTAGTCTGGCTGTTTC-TTTAGAGTGTCATGTTAACTTGTT-CCTCTATCTGCTGTGTTTCTTATATA 69164
Coquerel'sSifaka  TCACAGATTAGATTGGTCTGGCTGTTTC-CTTGTAGTGTCATCTTGATAAAAT---T-TACAGTCCGGGTTTTTTTGATT 57125
Ma'sNightMonkey   TCACAGGTTAGACTAGTCTGACTGTTTC-CTTATAGTGTCATGTTAACTTCTT-CCTCTATCTGCTGCATTTTTTATGTA 64048
VervetAGM         TCACAGATTAGACTAGCCTTGCTGTTTC-CTTAGAGTGTCATGTTAACTTGTT-CCTCTATCTGCTGCGTTTCTTATATA 70881
Gorrilla          TCACAGATTAGACTAGTCTGGCTGTTTC-TTTAGAGTGTCATGTTAACTTGTT-CCTCTATCTGCTGCGTTTCTTATATA 69698
GrabEatingMacaqueTCACAGATTAGACTAGCCTTGCTGTTTC-CTTAGAGCGTCATGTTAACTTGTT-CCTCTATCTGCTGCGTTTCTTATATA 70428
Macaque           TCACAGATTAGACTAGCCTTGCTGTTTC-CTTAGAGCGTCATGTTAACTTGTT-CCTCTATCTGCTGCGTTTCTTATATA 71287
PigTailedMacaque  TCACAGATTAGACTAGCCTTGCTGTTTC-CTTAGAGCGTCATGTTAATTTGTT-CCTCTATCTGCTGCGTTTCTTATATA 69820
Bonobo            TCACAGATTAGACTAGTCTGGCTGTTTC-TTTAGAGTGTCATGTTAACTTGTT-CCTCTATCTGCTGCGTTTCTTATATA 68270
OliveBaboon       TCACAGATTAGACTAGCCTTGCTGTTTC-CTTAGAGTGTCATGTTAACTTGTTTCCTCTATCTGCTGCGTTTCTTATATA 69679
Orangutan         TCACAGATTAGACTAGTCTGGCTGTTTCCTTTAGAGTGTCATGTTAACTTGTT-CCTCTATCTGCTGCGTTTCTTATATA 70089

Majority          AACTTAAAGTTAGTTCCAAAGGCTTGATAAGATTTAGAGCCTGTTTTTTTTTTTT---------TTTTTCTTTTT----

                          84410     84420     84430     84440     84450     84460     84470     84480

Human             AACTTAAAGTTAGTTCCAAAGGCTTGATAAGATTTAGAGCC--TTTTTTTTTTTT------------------------- 69217
Coquerel'sSifaka  TTTTTTGATTTTTTTTTTAAGCAAGAATACTATATAGA----------------T------------------------- 57164
Ma'sNightMonkey   AACTTA----CAGTTCCAAAGGCTTGATAAGACTTAGAGCC-GTTTTTTTTTTTGGTATTATTGTTTTTTAAGCTGAAC 64123
VervetAGM         AACTTAAAGTTAGTTCCAAAGGCTTGATAAGATTTAGAGCCTGTTTTTTTTTCTTCTTCTTCTTTTTTTCTTTTT---- 70957
Gorrilla          AACTTAAAGTTAGTTCCAAAGGCTTGATAAGATTTAGAGCCTTTTTTTTTTTTTT------------------TT---- 69756
GrabEatingMacaqueAACTTAAAGTTAGTTCCAAAGGCTTGATAAGATTTAGAGCCTGTTTTTCTTTCTTT---------TTTTTCTTTTT---- 70495
Macaque           AACTTAAAGTTAGTTCCAAAGGCTTGATAAGATTTAGAGCCTGTTTTTCTTTCTTT---------TTTTTCTTTTT---- 71354
PigTailedMacaque  AACTTAAAGTTAGTTCCAAAGGCTTGATAAGATTTAGAGCCTGTTTTTCTTTCTTT---------TTTTTCTTTTT---- 69887
Bonobo            AACTTAAAGTTAGTTCCAAAGGCTTGATAAGATTTAGAGTCCTTTTTTTTTTTTT------------------------- 68325
OliveBaboon       AACTTAAAGTTAGTTCCAAAGGCTTGATAAGATTTAGAGCCTGTTTTTTTTTCTTT---------TTTTTCTTTTT---- 69746
Orangutan         AACTTAAAGTTAGTTCCAAAGGCTTGATAAGATTTAGAGAC---T--TTTTTTTT------------------------- 70139
```

```
Majority         --TTAAGTGTTTCTTGTGCTTCCTATTGTGTCTCAGTAGGAGACATCTAATATCTGGTTGCCCCACTTTTAGTGATTTTA
                          84490     84500     84510     84520     84530     84540     84550     84560
Human            ---GAAGTGTTTCTTGTGCTTCCTATTGTGTCTCAGTAGGAGACATCTAATATCTGGTTGCCCCACTTTTAGTGATTTTA 69294
Coquerel'sSifaka --------GTTCTTTGTGCTTCATATTATGTCTCAGTAAGAGGCAAATTATATCTGGTTATCCCACTTTTGGTGATTTTA 57236
Ma'sNightMonkey  TACTAAGTGTTTCTTGTGCTTTCGTTTGTGTCTCAGTAGGAGGCAGCTAGTAATTGGTTGCCCCACTTTTAGTGATTTTA 64203
VervetAGM        --TTAAGTGCTTCTTGTGCTTCCTATTGTGTCTCAGTAGGAGACATCTAGTATCTGGTTGCCCCACTTTTAGTGATTTTA 71035
Gorrilla         --GGAAGTGTTTCTTGTGCTTCCTATTGTGTCTCAGTAGGAGACATCTAATATCTGGTTGCCCCACTTTTAGTGATTTTA 69834
GrabEatingMacaque--TTAAGTGCTTCTTGTGCTTCCTATTGTGTCTCAGTAGGAGACATCTAATATCTGGTTGCCCCACTTTTAGTGATTTTA 70573
Macaque          --TTAAGTGCTTCTTGTGCTTCCTATTGTGTCTCAGTAGGAGACATCTAATATCTGGTTGCCCCACTTTTAGTGATTTTA 71432
PigTailedMacaque --TCAAGTGCTTCTTGTGCTTCCTATTGTGTCTCAGTAGGAGACATCTAATATCTGGTTGCCCCACTTTTAGTGATTTTA 69965
Bonobo           ---GAAGTGTTTCTTGTGCTTCCTATTGTGTCTCAGTAGGAGACATCTAATATCTGGTTGCCCCACTTTTAGTGATTTTA 68402
OliveBaboon      --TTAAGTGCTTCTTGTGCTTCCTATTGTGTCTCAGTAGGAGACATCTAATATCTGGTTGCCCCACTTTTAGTGATTTTA 69824
Orangutan        ---AAAGTGTTTCTTGTGCTTCCTATTGTGTCTCAGTAGGAGACATCTAATATCTGGTTGCTCCACTTTTAGTGATTTTA 70216

Majority         ATGATGCTTTAAGACTGATTGTGGATTTGGGTGTTGACCTTCTGATCCTTTCGTTACATTTTTTTCTGCTAATGATTTTA
                          84570     84580     84590     84600     84610     84620     84630     84640
Human            ATGATGCTTTAAGACTGATTGTGGATTTGGGTGTTGACATTCTGATCCTTTCGTTACTTTTTTTTCTACTAATGATTTTA 69374
Coquerel'sSifaka GTGATGCTTTAAGACTGGTTGTGG--------------------------------TTTT--------AATGATTTTA 57274
Ma'sNightMonkey  ATGATGCTTTAAGACTGATTGTGTATTTGAGTGTTGACATTCTGATCCTTTCATTACAGTTTTTCTACTAATGATTTTA 64283
VervetAGM        ATGATGCTTTAAGACTT-TTGTGGATTTGGGTGTTGACCTTCTGATCCTTTCATTACATTTTTTTCTGCTAATGATTTTA 71114
Gorrilla         ATGATGCTTTAAGACTGATTGTGGATTTGGGTGTTGACATTCTGATCCTTTCATTACTTTTTTTTCTACTAATGATTTTA 69914
GrabEatingMacaqueATGATGCTTTAAGACTT-TTGTGGATTTGGGTGTTGACCTTCTGATCCTTTCGTTACATTTTTTTCTGCTAATGATTTTA 70652
Macaque          ATGATGCTTTAAGACTT-TTGTGGATTTGGGTGTTGACCTTCTGATCCTTTCATTACATTTTTT-CTGCTAATGATTTTA 71510
PigTailedMacaque ATGATGCTTTAAGACTT-TTGTGGATTTGGGTGTTGACCTTCTGATGCTTTCGTTACATTTTTTTCTGCTAATGATTTTA 70044
Bonobo           ATGATGCTTTAAGACTGATTGTGGATTTGGGTGTTGACATTCTGATCCTTTCGTTACTTTTTTTTC---TAATGATTTTA 68479
OliveBaboon      ATGATGCTTTAAGACTT-TTGTGGATTTGGGTGTTGACCTTCTGATCCTTCCGTTACATTTTTTTCTGCTAATGATTTTA 69903
Orangutan        ATGATGCTTTAAGACTGATTGTGGATTTGGGTGTTGACATTCTGATCCTTTCGTTACATTTTTTTCTACTAATGATTTTA 70296

Majority         GTATCCACTAATGGTTGCTGCCTTAATTAATTTCACTAGTGGGTTGCAAATA-TTGCTTTTTCTCCCCTAATTCACATAT
                          84650     84660     84670     84680     84690     84700     84710     84720
Human            GTATCCAATAATGGTTGCTGCCTTAATTAATTTCACTAGTGGGTTGCAAATA-TTGCTTTTTCTCCCCTAATTCACATAT 69453
Coquerel'sSifaka ATATCCACTAATGATTGCTGCCTTAATTTATTTCATGGGCAGGGTGGTTACAGATACTGCAGCCCCCCCAATTCACATAC 57354
Ma'sNightMonkey  GTATTCACTAATGGTTGCTGCCTTAATTAATTTCACTAGTAGGTTGCAAATA-ATGCTTTTTTCTCCCCAATTCACGTAT 64362
VervetAGM        GTATACACTAATGGTTGCTGCTTTAATTAATTTCACTAGTGGGTTGCAAATA-TTGCTTTTTCTCCCCTAATTCACATAT 71193
Gorrilla         GTATCCAGTAATGGTTGCTGCCTTAATTAATTTCACTAGTGGGTTGCAAATA-TTGCTTTTTCTCCCCTAATTCACATAT 69993
GrabEatingMacaqueGTATACACTAATGGTTGCTACCTTAATTAATTTCACTAGTGGGTTGCAAATA-TTGCTTTTTCTCCCCTAATTCACATAT 70731
Macaque          GTATACACTAATGGTTGCTACCTTAATTAATTTCACTAGTGGGTTGCAAATA-TTGCTTTTTCTCCCCTAATTCACATAT 71589
PigTailedMacaque GTATACACTAATGGTTGCTACCTTAATTAATTTCACTAGTGGGTTGCAAATA-TTGCTTTTTCTCCCCTAATTCACATAT 70123
Bonobo           GTATCCAATAATGGTTGCTGCCTTAATTAATTTCACTAGTGGGTTGCAAATA-TTGCTTTTTCTCCCCTAATTCACATAT 68558
OliveBaboon      GTATACACTAATGGTTGCTACCTTAATTAATTTCACTAGTGGGTTGCAAATA-TTGCTTTTTCTCCCCTAATTCACATAT 69982
Orangutan        GTATCCAATAATGGTTGCTGCCTTAATTAATTTCACTAGTGGGTTGCAAATA-TTGCTTTTTCTCCCCTAATTCACATAT 70375
```

```
Majority         TCCTTCTCTACATTAATTAGCTGAAAATCTTTATAAAGTAGAATTTTCCTTCATCAACTAGAATTATCAACCTTGTAATT

                         84730     84740     84750     84760     84770     84780     84790     84800

Human            TCCTTCTCTACATTAATTAGCTGAAAATCTTTATAAAGTAGAATTTTCCTTCATCAACTAGAATTATCAACCTTGTAATT 69533
Coquerel'sSifaka TCCTTCT--GCATTAATTAGATGAAAATCTTTATAAAGTAGAAATTTTTGTTACCAGTTAGAGCTGTCTACTTTG----- 57427
Ma'sNightMonkey  TCCTTCTATACATTAATTAGCTGAAAATCTTTATAAAGTAGAATTTTCCTTCATCAACTAAAATTATCTACCTTGTAATT 64442
VervetAGM        TCCTTCTCTACATTAATTAGCTGAAAATCTTTATAAAGTAGAAGTTTCCTTCATCAACTAGAATTATCAACCTTGTAATT 71273
Gorrilla         TCCTTCTCTACATTAATTAGCTGAAAATCTTTATAAAGTAGAATTTTCCTTCATCAACTAGAATTATCAACCTTGTAATT 70073
GrabEatingMacaqueTCCTTCTCTACATTAATTAGCTGAAAATCTTTATAAAGTAGAATTTTCCTTCATCAACTAGAATTATCAACCTTGTAATT 70811
Macaque          TCCTTCTCTACATTAATTAGCTGAAAATCTTTATAAAGTAGAATTTTCCTTCATCAACTAGAATTATCAACCTTGTAATT 71669
PigTailedMacaque TCCTTCTCTACATTAATTAGCTGAAAATCTTTATAAAGTAGAATTTTCCTTCATCAACTAGAATTATCAACCTTGTAATT 70203
Bonobo           TCCTTCTCTACATTAATTAGCTGAAAATCTTTATAAAGTAGAATTTTCCTTCATCAACTAGAATTATCAACCTTGTAATT 68638
OliveBaboon      TCCTTCTCTACATTAATTAGCTGAAAATCTTTATAAAGTAGAATTTTCCTTCATCAACTAGAATTATCAACCTTGTAATT 70062
Orangutan        TCCTTTTCTACATTAATTAGCTGAAAATCTTTATAAAGTAGAATTTTCCTTCATCAACTAGAATTATCAACCTTGTAATT 70455

Majority         CAATTTGTGCAGGACAATTAGTTAAATTCTTGCATTTATTTGCTAATTTTA---------AGTTGTTGCCCAGCTTTTGT

                         84810     84820     84830     84840     84850     84860     84870     84880

Human            CAATTTGTGCAGGACAATTAGTTAAATTCTTGCATTTGTTTGCTAATTTTA---------AGTTGGTGCCCAGCTTTTGT 69604
Coquerel'sSifaka --ATTTGTGCAGAACAGTTAGTGAGATTCTTGCATTTATTTGCTAATTTTACCTGGAAAAAGTGGATGCCCAGCTTTTGT 57505
Ma'sNightMonkey  AAATTGGTGCAGGACAATCAGTTAAATTCTTGCATTTATTTGCTAATTTTA---------TGTTGGTGCTCAGCTTTTGT 64513
VervetAGM        CAATTTGTGCAGGACAATTAGTTAAATTCTTGCATTTATTTGCTAATTTTA---------AGTTTTTGCCCACCTTTTGT 71344
Gorrilla         CAATTTGTGCAGGACAATTAGTTAAATTCTTGCATTTGTTTGCTAACTTTA---------AGTTGGTGCCCAGCTTTTGT 70144
GrabEatingMacaqueCAATTTGTGCAGGACAATTAGTTAAATTCTTGCATTTATTTGCTAATTTTA---------AGTTTTTGCCCAGCTTTTGT 70882
Macaque          CAATTTGTGCAGGACAATTAGTTAAATTCTTGCATTTATTTGCTAATTTTA---------AGTTTTTGCCCAGCTTTTGT 71740
PigTailedMacaque CAATTTGTGCAGGACAATTAGTTAAATTCTTGCATTTATTTGCTAATTTTA---------AGTTTTTGCCCAGCTTTTGT 70274
Bonobo           CAATTTGTGCAGGACAATTAGTTAAATTCTTGCATTTGTTTGCTAATTTTA---------AGTTGGTGCCCAGCTTTTGT 68709
OliveBaboon      CAATTTGTGCAGGACAATTAGTTAAATTCTTGCATTTATTTGCTAATTTTC---------AGTTTTTGCCCAGCTTTTGT 70133
Orangutan        CAATTTGTTCAGGATAATTA----AATTCTTGGATTTATTTGCTAATTTTA---------AGTTGGTACCCAGCTTTTGT 70522

Majority         TTTCAGGTAAGTACTTAAACCAGCTTATGAACAGGTACATTATAGATTCTTCCCACTGTCTGCTTTAAACTTTTGAAACT

                         84890     84900     84910     84920     84930     84940     84950     84960

Human            TTTCAGGTAAGTACTTAAACCAGCTTATGAACAGGTACAGTATAGATTCTTCCCACTGTCTGCTTTAAACTTTTGAAACT 69684
Coquerel'sSifaka TTGAAAGTAAGTACTTAGAGCAGTTTCTGAATGAGTACAACATGGACTCTTCCCAACACGTGCTTGAAACTTTAGAAACT 57585
Ma'sNightMonkey  TTTCAGGTAAGTACTTAAACCAGCTTATGAACAGGTTCATTATAGATTCTTCCCACTGTCTGCTTTAAACTTTTGAAACT 64593
VervetAGM        TTTCAGGTAAGTACTTAAACCAGCTTATGAACAGGTACATTACAGATTCTTCCCACTGTCTGCTTTAAACTTTTGAAACT 71424
Gorrilla         TTTCAGGTAAGTACTTAAACCAGCTTATGAACAGGTACATTATAGATTCTTCCCACTGTCTGCTTTAAACTTTTGAAACT 70224
GrabEatingMacaqueTTTCAGGTAAGTACTTAAACCAGCTTATGAACAGGTACATTATAGATTCTTCCCACTGTCTGCTTTAAACTTTTGAAACT 70962
Macaque          TTTCAGGTAAGTACTTAAACCAGCTTATGAACAGGTACATTATAGATTCTTCCCACTGTCTGCTTTAAACTTTTGAAACT 71820
PigTailedMacaque TTTCAGGTAAGTACTTAAACCAGCTTATGAACAGGTACATTATAGATTCTTCCCACTGTCTGCTTTAAACTTTTGAAACT 70354
Bonobo           TTTCAGGTAAGTACTTAAACCAGCTTATGAACAGGTACAGTATAGATTCTTCCCACTGTCTGCTTTAAACTTTTGAAACT 68789
OliveBaboon      TTTCAGGTAAGTACTTAAACCAGCTTATGAACAGGTACGTTATAGATTCTTCCCACTGTCTGCTTTAAACTTTTGAAACT 70213
Orangutan        TTTCAGGTAAGTACTTAAACCAGCTTATGAACAGGTACATTATAGATTCTTCCCACTGTCTGCTTTAAACTTT-AAAACT 70601
```

```
Majority          GTCAGTTGTTTGCAGGTTCTAGTAGCCC-AGAGTGGTTTGAAATGGTGACCTTAGCCCAGATGT--------------AT

                          84970     84980     84990     85000     85010     85020     85030     85040

Human             GTCAGTTGTTTGCAGGTTCTGGTAGCCC-AGAGTGGTTTGAAATGGTGACCTTAGCCCAGAAGT--------------AT 69749
Coquerel'sSifaka  ATTGGTTGCTTGCAGGTTCTAGTAGCCACAGAGTGGTTTGAAATGGTGACCTTAGCTTAGAGGTAAGCATTTTTATTTAT 57665
Ma'sNightMonkey   GTCAGTTGCTTGCAGGTTTTAGTAGCCCCAGAGTGGTTTGAAATGGTGACCTTAGCCCAGAAGTGTGCAATTTTATTCAT 64673
VervetAGM         GTCAGTTGTTTGCAGGTGCTAGTAGCCC-AGAGTGGTTTGAAACGGTGACCTTAGCCCAGATGT--------------AT 71489
Gorrilla          GTCAGTTGTTTGCAGGTTCTGGTAGCCC-AGAGTGGTTTGAAATGGTGACCTTAGCCCAGATGT--------------AT 70289
GrabEatingMacaqueGTCAGTTGTTTGCAGGTTCTAGTAGCCC-AGAGTGGTTTGAAATGGTGACCTTAGCCCCGATGT--------------AT 71027
Macaque           GTCAGTTGTTTGCAGGTTCTAGTAGCCC-AGAGTGGTTTGAAATGGTGACCTTAGCCCCGATGT--------------AT 71885
PigTailedMacaque  GTCAGTTGTTTGCAGGTTCTAGTAGCCC-AGAGTGGTTTGAAATGGTGACCTTAGCCCCGATGT--------------AT 70419
Bonobo            GTCAGTTGTTTGCAGGTTCTGGTAGCCC-AGAGTGGTTTGAAATGGTGACCTTAGCCCAGATGT--------------AT 68854
OliveBaboon       GTCAGTTGTTTGCAGGTTCTGGTAGCCC-AGAGTGGTTTGAAATGGTGACCTTAGCCCCGATGT--------------AT 70278
Orangutan         GTCAGTTGTTTGCAGGTTCTAGTAGCCC-CGAGTGGTTTGAAATGGTGACCTTAGCCCAGATGT--------------AT 70666

Majority          GTAATTATTTAATTTGGCCTTGACCAATGGCCCAAAAAAGGAAGGGGGT----GGGGTGGATAAAGGACTAAACAAAATT

                          85050     85060     85070     85080     85090     85100     85110     85120

Human             GTAATTATTTAATTTGGCCTTGACCAGTGGCCCAAAAAAGGAAGGGGGT----GGGGTGGATAAAGGACTAAACAAAATT 69825
Coquerel'sSifaka  GTAATTATTTAATTTGTCCTTGTCCAGTGACCCAAAAAAGGAAAGGG--------GGTACTTAA-GGACTAAACAAAGTT 57736
Ma'sNightMonkey   ATAATTATTTAATTTGGCCTTGACCAATGGCCCAAAAAAGGAAGGGG-T----GGGTTGGATAAAGGACTAAACAAAATT 64748
VervetAGM         GTAATTATTTAATTTGGCCTTGAACAATGGCCCAAAAAAGGAAGGGGGT----CGGGTGGATAAAGGACTAAGCAAAATT 71565
Gorrilla          GTAATTATTTAATTTGGCCTTGACCAATGGCCCAAAAAAGGAAGGGGGT----GGGGTGGATAAAGGACTAAACAAAATT 70365
GrabEatingMacaqueGTAATTATTTAATTTGGCCTTGAACAATGGCCCAAAAAAGGAAGGGGGT----GGTGTGGATAAAGGACTAAGCAAAATT 71103
Macaque           GTAATTATTTAATTTGGCCTTGAACAATGGCCCAAAAAAGGAAGGGGGT----GGTGTGGATAAAGGACTAAGCAAAATT 71961
PigTailedMacaque  GTAATTATTTAATTTGGCCTTGAACAATGGCCCAAAAAAGGAAGGGGGT----GGGGTGGATAAAGGACTAAGCAAAATT 70495
Bonobo            GTAATTATTTAATTTGGCCTTGACCAGTGGCCCAAAAAAGGAAGGGGGT----GGGGTGGATAAAGGACTAAACAAAATT 68930
OliveBaboon       GTAATTATTTAATTTGGCCTTGAACAATGGCCCAAAAAAGGAAGGGGGT----GGGGTGGATAAAGGACTAAGCAAAATT 70354
Orangutan         TTAATTATTTAATTTGGCCTTGACCAGTGGCCCAAAAAAGGAAGGGGGTTGGTGGGATGGATAAATGACTAAACAAAATT 70746

Majority          AATTAAAATTTAGAAATCTTTCTCTCAGAAGGAATTCATGCCCATAAAAGAAAGA--CCAGGATACATTAATATTTT-CT

                          85130     85140     85150     85160     85170     85180     85190     85200

Human             AATTAAAATTTAGAAATTTTTCTCTCAGAAGGAATTCATGCCCATAAAACAAAGA--CCAGGATACATTCGTATTTT-AT 69902
Coquerel'sSifaka  AGTTAAAATATAGGTATCTTTCTCTCAAAAGGAATTTGTCCCCATAAAAAAAAAGAGCCAGGAAGAATGAATATTTTTCT 57816
Ma'sNightMonkey   AATTAAAATTTAGAAATTTTTGTC--AGAAGGAATTTACCCCCATAAAATAAAGA--GCAAGAAGTCTTAATATTTT-CT 64823
VervetAGM         AATTAAAATTTAGAAATCTTTCTCTCAGAAGGAATTCATGCCCATAAATGGAAGA--CCAGGATACATTAATATTTT-CT 71642
Gorrilla          AATTAAAATTTAGAAATTTTTCTCTCAGAAGGAATTCATGCCCATAAAACAAAGA--CCAGGATACATTCATATTTT-CT 70442
GrabEatingMacaqueAATTAAAATTTAGAAATCTTTCTCTCAGAAGGAATTCATGCCCATAAATGGAAGA--CCAGGATACATTAATATTTT-CT 71180
Macaque           AATTAAAATTTAGAAATCTTTCTCTCAGAAGGAATTCATGCCCATAAATGGAAGA--CCAGGATACATTAATATTTT-CT 72038
PigTailedMacaque  AATTAAAATTTAGAAATCTTTCTCTCAAAAGGAATTCATGCCCATAAATGGAAGA--CCAGGATACATTAATATTTT-CT 70572
Bonobo            AATTAAAATTTAGAAATTTTTCTCTCAGAAGGAATTCATGCCCATAAAACAAAGA--CCAGGATACATTCATATTTT-CT 69007
OliveBaboon       AATTAAAATTTAGAAATCTTTCTCTCAGAAGGAATTCATGCCCATAAATGGAAGA--CCAGGATACATTAATATTTT-CT 70431
Orangutan         AATTAAAATTTAGAAATTTTTCTCTCAGAAGGAATTCATGCCCATAAAACAAAGA--CCAGGATACATTCATATTTT-CT 70823
```

```
Majority          CAGTAAATACCAATAGATTATAAATCACTTTTCTTGTATACATAATTGTG-------AAAAAACCCATCCAGGTGTGCAT

                          85210     85220     85230     85240     85250     85260     85270     85280

Human             CGGTAAATACCAATAGATTATAAATCACTTTTCTCGTATACATAATTGTG-------AAAAAAAAAAACCATCCAGGTAT 69975
Coquerel'sSifaka  CAGTAAATACCAATAGATT-TAAATCACTTCTCCCTTCTAGCTTATTGTGAGGT---AAAAATAATATCCAGCTGAG--T 57890
Ma'sNightMonkey   CAGTAAATAAAAATAGGTTAAAAGTCACTTTTCTCGTATACATAATTGTGGAGGGAAAAAAAACCCATCCAGGTGTGCAT 64903
VervetAGM         CAGTAAATACCAATAGATTAAAAATCACTTTTCTTGTATACATAATTGTG-------AAAAAACCCATCCAGGTGTGCAT 71715
Gorrilla          CGGTAAATACCAATAGATTATAAATCATTTTTCTCGTATACATAATTGTG-------AAAAAAAAA--CCATCCAGGTAT 70513
GrabEatingMacaqueCAGTAAATACCAATAGATTATAAATCACTTTTCTTGTATACATAATTGTG-------AAAAAACCCATCCAGGTGTGCAT 71253
Macaque           CAGTAAATACCAATAGATTATAAATCACTTTTCTTGTATACATAATTGTG-------AAAAAACCCATCCAGGTGTGCAT 72111
PigTailedMacaque  CAGTAAATACCAATAGATTATAAATCACTTTTCTTGTATACATAATTGTG-------AAAAAGCCCATCCAGGTGTGCAT 70645
Bonobo            CGGTAAATACCAATAGATTATAAGTCACTTTTCTCGTATACATAATTGTG-------AAAAAAAAA--CCATCCAGGTAT 69078
OliveBaboon       CAGTAAATACCAATAGATTATAAATCACTTTTCTTGTATACATAATTGTG-------AAAAAACCCATCCAGGTGTGCAT 70504
Orangutan         CAGTAAATACCAATAGATTATAAAT-ACTTTTCTTGTATATATAATTGTG-------AAAAAAAAAAAACATCCGGGTAT 70895

Majority          GTGAGCTATTTATTCATTGAGAGGATTTACATCTCTTAGGTGTTTTTTGTTGTTGTATGTGTACTTTTGTGTAGTC----

                          85290     85300     85310     85320     85330     85340     85350     85360

Human             GCATGTTATTTATTCATTGAGAGGATATACATCTCTCAGGTGTTTTTTGTCGT---ATGTGTACTTTTGTGTAGTC---- 70048
Coquerel'sSifaka  GTGAGTTACCTATTCAATTAAAGGATTAACATCCCTTACGTGTTTTTTGTTGTTGTGTATATCTGTATGTGTAGTCAGTG 57970
Ma'sNightMonkey   ATTATCTATTTATCCACTGAGAGAATTAACATCCCTCAGGTGTTTGTTGTTGT---ATGTGTACTTT------------- 64967
VervetAGM         GTGAGCTGTTTATTCATTGAGAGGATTTAGGTCTCTTAGGTGCTTGTTGTTGTTGTATGTGTACTTTTGTGTAGTC---- 71791
Gorrilla          GCGTGTTATTTATTCATTGAGAGGATATACATCTCTCAGGTGTTTTTTGTCGT---ATGTGTACTTTTGTGTAGTC---- 70586
GrabEatingMacaqueGTGAGCTGTTTATTCATTGAGAGGATTTAGATCTTTTAGGTGTTTTTTGTTGTTGTATGTGTACTTTTGTGTAGTC---- 71329
Macaque           GTGAGCTGTTTATTCATTGAGAGGATTTAGATCTTTTAGGTGTTTTTTTTTGTTGTATGTGTACTTTTGTGTAGTC---- 72187
PigTailedMacaque  GTGAGCTGTTTATTCATTGAGAGGATTTAGATCTTTTAGGTGTTTTTTGTTGTTGTATGTGTACTTTTGTGTAGTC---- 70721
Bonobo            GCGTGTTATTTATTCATTGAGAGGATATACATCTCTCAGGTGTTTTTTGTCGT---ATGTGTACTTTTGTGTAGTC---- 69151
OliveBaboon       GTGAGCTGTTTATTCATTGAGAGGATTTAGATCTCTTAGGTGTTTTTTGTTGTTGTATGTATACTTTCGTGTAGTC---- 70580
Orangutan         GCATGTTATTTATTCATTGAGAGGATATACATCTCTCAGGTGTTTTTTGTTGTTGTATGTATACTTTTGTGTAGTC---- 70971

Majority          ---TGCCTGTT-TATTCTTTTTGAATAGGAT-----GATATGTACAATTAAAAAAA--ATCTTTTGTGAAATGTGGTTTT

                          85370     85380     85390     85400     85410     85420     85430     85440

Human             ---TGCCTGTT-TATTCTTTTTGAATAGGAT-----GATATGTACAATTAAAAAAA--ATCTTTTGTGAAATGTGGTTTT 70117
Coquerel'sSifaka  CTGTGTCTGTT-TATTCTTTTTGAATAGGAGTCCAGAAGATGTGCAGTAAAAACAGACATTTTTTGTGGAACATTATGTT 58049
Ma'sNightMonkey   -----------------TTTCAAATAGGAT-----GATATGTACAATTAAAC-----ATCTTTTGTGAAATGTGGTTTT 65019
VervetAGM         ---TGCCTGTT-TGTTCTTTTTGAATAGGAT-----GATATGTACAATTTAAAAAA--GTATTTTATGAAATTTGGCTTT 71860
Gorrilla          ---TGCCTGTT-TACTCTTTTTGAATAGGAT-----GATACGTACAATTAAAAAAA--ATCTTTTGTGAAATGTGGTTTT 70655
GrabEatingMacaque---TGCCTGTTCTATTCTTTTTGAATAGGAT-----GATATGTACAATTTTAAAAA--GTCTTTTGTGAAATGTGGCTTT 71399
Macaque           ---TGCCTGTTCTATTCTTTTTGGATAGGAT-----GATATGTACAATTTTAAAAA--GTCTTTTGTGAAATGTGGCTTT 72257
PigTailedMacaque  ---TGCCTGTTCTATTCTTTTTGAATAGGAT-----GATATGTACAATTTAAAAAA--GTCTTTTGTGAAATGTGGCTTT 70791
Bonobo            ---TGCCTGTT-TATTCTTTTTGAATAGGAT-----GATATGTACAATTAAAAAAA--ATCTTTTGTGAAATGTGGTTTT 69220
OliveBaboon       ---TGCCTGTTCTATTCTTTTTGAATAGGAT-----GATATGTACAATTTAAAAAA--GTCTTTTGTGAAATGTGGCTTT 70650
Orangutan         ---TGCCTGTT-TATTCTTTTTGAATAGGAT-----GATATGTACAATTAAAC-----ATCTTTTGTGAAATGTGGTTTT 71037
```

```
Majority          CTTAAATACATTATTGCATTTCATCTACAGTGATGAAGGGAAAAACTACAAAACTAGACAGATTGCAAATT----AGTAT
                          85450     85460     85470     85480     85490     85500     85510     85520
Human             CTTAAATACATTATTGCATTTCATACACAGTGATGAAGGGAAAAACTACAAAGCTAGACAGATTGCAAATT----AGTAT 70193
Coquerel'sSifaka  ATATATGTTATTAATATATTTCATC-ACAGCAATGAGGGGAAAAACTACAAAACTAGATAAATTGGAAAT-----AGTGT 58123
Ma'sNightMonkey   GTTAAATACATTATTATATTTCACCTACAGTGATGAAGGGAAAAACTACAAAGCTAGACAGATTGTAAATTAATTAGTAT 65099
VervetAGM         CTTAAATACATTATTGCATTTCATCTACAGTGATGAAGGGAAAAACTACAAAACTAGACAGATTACATATT----AGTAT 71936
Gorrilla          CTTAAATACATTATTGCATTTCATACACAGTGATGAAGGGAAAAACTACAAAGCTAGACAGATTGCAAATT----AGTAT 70731
GrabEatingMacaqueCTTAAATACATTATTGCATTTCATCTACAGTGATGAAGGGAAAAACTACAAAACTAGACAGATTACATATTAATTAGTAT 71479
Macaque           CTTAAATACATTATTGCATTTCATCTACAGTGATGAAGGGAAAAACTACAAAACTAGACAGATTACATATTAATTAGTAT 72337
PigTailedMacaque  CTTAAATACATTATTGCATTTCATCTACAGTGATGAAGGGAAAAACTACAAAACTAGACAGATTACGTATTAATTAGTAT 70871
Bonobo            CTTAAATACATTATTGCATTTCATACACAGTGATGAAGGGAAAAACTACAAAGCTAGACAGATTGTAAATT----AGTAT 69296
OliveBaboon       CTTAAATACATTATTGCATTTCATCTACAGTGATGAAGGGAAAAACTACAAAACTAGACAGATTACATATTAATTAGTAT 70730
Orangutan         -TAAAATACATTATTGCATTTCATCTACAGTGATGAAGGGAAAAACTACAAAGCTAGACAGATTGCAAATT----AGT-T 71111

Majority          TGCAGTAGTGAGGTAATAGAGTAATACCCATATATTTTCTTACAATATATGTAGGT-GATGCTTGTGTTCTTTACTTTTG
                          85530     85540     85550     85560     85570     85580     85590     85600
Human             TGCAGTAGTGAGGTAATAGAGCAATACCCAAATGTTTTCTTAAAATATATGTAGGT-GATGCCTGTGTTCTTTACTTTTG 70272
Coquerel'sSifaka  TGTAGCAGTGAGGTAACAGCA-GTCACCTATATATTTTGTTACAGTATATATAGAT-AATGCTGTGTTTTTTTTTCCTTG 58201
Ma'sNightMonkey   TACAGTAGTGAGGTAATAGAGCAATTCCCATGTATTTTCTTACAATGTATGTAGGTAGATACCTGTATT-TTTACTTTTG 65178
VervetAGM         TGCAGTAGTGAGGTAATAGAGTAATACTCATATATTTTCTTACAATATATGTAGGT-GATGCTTGTATTCCTTACTTTTG 72015
Gorrilla          TGCAGTAGTGAGGTAATAGAGCAATACCCAAATGTTTTCTTAAAATATATGTAGGT-GATGCCTGTGTTCTTTACATTTG 70810
GrabEatingMacaqueTGCAGTAGTGAGGTAATAGAGTAATACTGATACATTTTCTTACAATATATGTAGGT-GATGCTTGTGTTCTTTACTTTTG 71558
Macaque           TGCAGTAGTGAGGTAATAGAGTAATACTGATATATTTTCTTACAATATATGTAGGT-GATGCTTGTGTTCTTTACTTTTG 72416
PigTailedMacaque  TGCAGTAGTGAGGTAATAGAGTAATACTGATATATTTTCTTACAATATATGTAGGT-GATGCTTGTGTTCTTTACTTTTG 70950
Bonobo            TGCAGTAGTGAGGTAATAGAGCAATACCCAAATGTTTTCTTAAAATATATGTAGGT-GATGCCTGTGTTCTTTACTTTTG 69375
OliveBaboon       TGCAGTAGTGAGGTAATAGAGTAATACTCATATATTTTCTTACAATATATGTAGGT-GATGCTTGTGTTCTTTACTTTTG 70809
Orangutan         TGCAGTAGTGAGGTAATAGAGCAATACCCATATATTTTCTTACAATATATGTAGAT-GATGTCTGTGTTCTTTACTTCTG 71190

Majority          TGTGTGTGTGTGTGT-T------TTTTAAACAAGATTCATTGTAGTCTTTTAGATATGATTTAATAAATTTAGGCTGTTC
                          85610     85620     85630     85640     85650     85660     85670     85680
Human             TGTGTGTGTATGTGT--------TTTTAAACAAGATTCATTGTAGTCTTTTAGATATAATTTAATAAATTTAGGCTGTTC 70344
Coquerel'sSifaka  TATGAGTGTATGTGTTA----TAAAGGAAACAAGATTCACTGT--CTTTT--GATATTATTTAATACGTTAAGGATGTTC 58273
Ma'sNightMonkey   TGTATGTGTACATGTCT--TTTTAAGAGAACAAGATTCATTGTAGTCATTTAGATATAATTTAATAAATTTAGGCTGTTC 65256
VervetAGM         TGTGTGTGTGTGTGTGTGT----TTTTAAACAAGATTCATTATAGTCTTTCAGATATGATTTAATAAATTTAGGCTGTTC 72091
Gorrilla          TGTGTGT----GTGT--------TTTTAAACAAGATTCATTGTAGTCTTTTAGATATAATTTAATAAATTTAGGCTGTTC 70878
GrabEatingMacaqueTGTGTGTGTGTGTGT--------TTTTAAACAAGATTCATTATAGTCTTTCAGATATGATTTAATAAATTTAGGCTGTTC 71630
Macaque           TGTGTGTGTGTGTGTGTGT----TTTTAAACAAGATTCATTATAGTCTTTCAGATATGATTTAATAAATTTAGGCTGTTC 72492
PigTailedMacaque  TGTGTGTGTGTGTGTGT------TTTTAAACAAGATTCATTATAGTCTTTCAGATATGATTTAATAAATTTAGGCTGTTC 71024
Bonobo            TGTGTGTGTATGTGT--------TTTTAAACAAGATTCATTGTAGTCTTTTAGATATAATTTAATAAATTTAGGCTGTTC 69447
OliveBaboon       TGTGTGTGTGTGTGTGTGTGTGTGTTTTTAAACAAGATTCATTATAGTCTTTCAGATATGATTTAATAAATTTAGGCTGTTC 70889
Orangutan         TGTGTGTGTATGTGT--------TTTTAAACAAGATTCATTGTAGTCTTTTAGATATAATTTAATAAATTTAGGCTGTTC 71262
```

```
Majority          AGGGTTTCAGTATCCTACAAAGATGAATGCTGCTTCTAAGTTTTTGGGTTTCTTGAA----CCAGAGCTTAGAAAGAACA
                          85690     85700     85710     85720     85730     85740     85750     85760
Human             AGGGTTTCAGTATCCTACAAAGAAGAACGCTGCTTCTAAGTTTTTGGGTTTCTTGAA----CCAGAGCTTAGAAAGAACA 70420
Coquerel'sSifaka  AGGGTTTCAGTGTCCTCCAGAGATGAATGCCGTTTCAGAGTTTTGGGGTTTTTCAGTAGCCCCAGAACTTAAAAAGCACA 58353
Ma'sNightMonkey   AGGGTTTTAGCATCCTGCAAAGATGAGTATTGCTTCTAAGTTTTTGGTTTTCCTGAA----CCAGAGCTTAGAATGAACA 65332
VervetAGM         AGGGTTTC------CTACAAAGATGAATGCTGCTTCTAAGGTTTTGGGTTTCTTGAA----CCAGAGCTTAGAAAGAACA 72161
Gorrilla          AGGGTTTCAGTATCCTACAAAGAAGAATGCTGCTTCTAAGTTTTTGGGTTTCTTGAA----CCAGAGCTTAGAAAGAACA 70954
GrabEatingMacaqueAGGGTTTC------CTACAGAGATGAATGCTGCTTCTAAGGTTTTGGGTTTCTTGAA----CCAGAGCTTAGAAAGAACA 71700
Macaque           AGGGTTTC------CTACAGAGATGAATGCTGCTTCTAAGGTTTTGGGTTTCTTGAA----CCAGAGCTTAGAAAGAACA 72562
PigTailedMacaque  AGGGTTTC------CTACAGAGATGAATGCTGCTTCTAAGGTTTTGGGTTTCTTGAA----CCAGAGCTTAGAAAGAACA 71094
Bonobo            AGGGTTTCAGTATCCTACAAAGAAGAATGCTGCTTCTAAGTTTTTGGGTTTCTTGAA----CCAGAACTTAGAAAGAACA 69523
OliveBaboon       AGGGTTTC------CTACAGAGATGAATGCTGCTTCTAAGGTTTTGGGTTTCTTGAA----CCAGAGCTTAGAAAGAATA 70959
Orangutan         AGGGTTTCAGTATCCTACAAAGATGAATGCTGCTTCTAAGTTTTTGGGTTTCTTGAA----CCAGAGCTTAGAA----CA 71334

Majority          AAAATATGGCATGGTATTTTCATGGTTTAGTAAGGCTACTTTTAGTTAATTATTGTGATGTAGGCCGTTAA-GTAGACTT
                          85770     85780     85790     85800     85810     85820     85830     85840
Human             AAAATATGGCATGGTATTTTCATGGTTTAGTAAGGCTACTTTTAGTTAATTATTGTGATGTAGGCCATTAA-GTAGACTT 70499
Coquerel'sSifaka  AAAGTATTGCTTGATATATTCATGAGTTAGTATGGCTACCTTTAGTTAATTATTGTGATGTAGGCCATTAAAGTAGACTT 58433
Ma'sNightMonkey   AAAATATGGCTTGGTATTTTCATGGTTTAGTAAGGATACTTTTAGTTAATTATTCTGATGTAGTCTGTTAA-GTAGACTT 65411
VervetAGM         AGAATATGGCATGGTATTTTCGTGGTTTAGTAAGGCTACTTTTAGTTAATTATTGTGATGTAGGCCGTTAA-GTAGACTT 72240
Gorrilla          AAAATATGGCATGGTATTTTCATGGTTTAGTAAGGCTACTTTTAGTTAATTATTGTGATGTAGGCCATTAA-GTAGACTT 71033
GrabEatingMacaqueAGAATATGGCATGGTATTTTCGTGGTTTAGTAAGGCTACTTTTAGTTAATTATTGTGATGTAGGCCGTTAA-GTAGACTT 71779
Macaque           AGAATATGGCATGGTATTTTCGTGGTTTAGTAAGGCTACTTTTAGTTAATTATTGTGATGTAGGCCGTTAA-GTAGGCTT 72641
PigTailedMacaque  AGAATATGGCATGGTATTTTCGTGGTTTAGTAAGGCTACTTTTAGTTAATTATTGTGATGTAGGCCGTTAA-GTAGACTT 71173
Bonobo            AAAATATGGCATGGTATTTTCATGGTTTAGTAAGGCTACTTTTAGTTAATTATTGTGATGTAGGCCATTAA-GTAGACTT 69602
OliveBaboon       AGAATATGGCATGGTATTTTCGTGGTTTAGTAAGGCTACTTTTAGTTAATTATTGTGATGTAGGCTGTTAA-GTAGACTT 71038
Orangutan         AAAATATGGCATGGTATTTTCATGGTTTAGTAAGGCTACTTTTAGTTAATTATTGTGATGTAGGCCATTAA-GTAGACTT 71413

Majority          TT-CTAAAACGAAACCCCAGTGAAAAGGGGTTTTGAAGATACATATATACTGGTTTCTAAGAACTAGTCTTTTTGGATCA
                          85850     85860     85870     85880     85890     85900     85910     85920
Human             TT-CTAAAACGAAACCCCAGAGGAGAGGGGTTTTGAAGATACATATATACTGGTTTCTAAGAACTAATCTTTTTGGATCA 70578
Coquerel'sSifaka  TTTTTAAAAAGAAACCCCAGTAGGGGGGCAAGGGCAACATAAGTA-ACATAAATATCTG-----TACCCCCATAATAT-G 58506
Ma'sNightMonkey   TT-CTTAAA-GAAACCCCAGTGAAAAGGGGTTTTAAAGATACATATATATTAGTTTCTAAGAACTAGTCTTTTTGAATCA 65489
VervetAGM         ---CTAAAACGAAACCCCAGTGAAAAGGGGTTTTGAAGATGCATATATACTGGTTTCTAAGAACTAGGCTTTTTGGATCA 72317
Gorrilla          TT-CTAAAACGAAACCCCAGAGAAGAGGGGTTTTGAAGATACGTATATACTGGTTTCTAAGAACTAATCTTTTTGGATCA 71112
GrabEatingMacaqueCT-CTAAAACGAAACCCCAGTGAAAAGGGGTTTTGAAGATGCATATATACTGGTTTCTAAGAACTAGGCTTTTTGGATCA 71858
Macaque           CT-CTAAAACGAAACCCCAGTGAAAAGGGGTTTTGAAGATGCATATATACTGGTTTCTAAGAACTAGGCTTTTTGGATCA 72720
PigTailedMacaque  CT-CTAAAACGAAACCCCAGTGAAAAGGGGTTTTGAAGATGCATATATACTGGTTTCTAAGAACTAGGCTTTTTGGATCA 71252
Bonobo            TT-CTAAAACGAAACCCCAGAGGAGAGGGGTTTTGAAGATACATATATACTGGTTTCTAAGAACTAATCTTTTTGGATCA 69681
OliveBaboon       TT-CTAAAACGAAACCCCAGTGAAAAGGGGTTTTGAAGATGCATATATACTGGTTTCTAAGAACTAGGCTTTTTGGATCA 71117
Orangutan         TT-CTAAAACGAAACCCCAGAGAAGAGGGGTTTTGAAGATACATGTATACTGGTTTCTAAGAACTAATCTTTTTGGATCA 71492
```

```
Majority         TTGGTATTGGTAGAAGGGATTTGCTGTGTTTTCTGCCACCATTGAATCTCTGTATAAA-GTTTGGCT-CCACTATCAAAA

                         85930     85940     85950     85960     85970     85980     85990     86000

Human            TTGGTATTGGTAGAAGGGATTCGCTATGTTTTCTGCCACCATTGGATCTCTGTATAAA-GTTTGGCT-CCACTATCAAAA 70656
Coquerel'sSifaka CTGAAATAAAAAAAAAAAAAA-A----G----AAACCCCAGTGAAAAGGGTTTATAAAGGTCCCTATGCCAGTTTCTAAG 58577
Ma'sNightMonkey  CTGGTATTGGTAGAACGGATT-----------CTGCCACCATTGGATCGCTGTATAAAAGTTTGTCT-CCACTATCAAAA 65557
VervetAGM        TTGGTATTGGTAGAAGGGATTTGCTGTGTTTTCTGCCACCATTGAATCTCTGTAAAAA-GTTTGGCT-CCACTATCAAAA 72395
Gorrilla         TTGGTATTGGTAGAAGGGATTCGCTGTGTTTTCTGCCACCATTGGATCCAC-TATAAA-GTTTGGCT-CCACTATCAAAA 71189
GrabEatingMacaqueTTGGTATTGGTAGAAGGGATTTGCTGTGTTTTCTGCCGCCATTGAATCTCTGTATAAA-GTTTGGCT-CCGCTATCAAAA 71936
Macaque          TTGGTATTGGTAGAAGGGATTTGCTGTGTTTTCTGCCGCCATTGAATCTCTGTATAAA-GTTTGGCT-CCGCTATCAAAA 72798
PigTailedMacaque TTGGTATTGGTAGAAGGGATTTGCTGTGTTTTCTGCCGCCATTGAATCTCTGTATAAA-GTTTGGCT-CCGCTATCAAAA 71330
Bonobo           TTGGTATTGGTAGAAGGGATTCGCTATGTTTTCTGCCACCATTGGATCTCTGTATAAA-GTTTGGCT-CCACTATCAAAA 69759
OliveBaboon      TTGGTATTGGTAGAAGGGATTTGCTGTGTTTTCTGCCACCATTGAATCTCTGTATAAA-GTTTGGCT-CCGCTATCAAAA 71195
Orangutan        TTGGTATTGGTAGAAGGGATTCACTGTGTTTTCTGCCACCATTGGATCTCT--GTATA-GTTTGGCT-CCACTATCAAAA 71568

Majority         TGCTTAGATAGATCCCTTTGCAATAGTTGCCACCCTAGGAATAGCTCGAAAATTATTAAGTTAATACTAGTTCCTTAGAT

                         86010     86020     86030     86040     86050     86060     86070     86080

Human            TGCTTAGATAGATGCCTTTGCAATAGTTGCCACCCTAGGAATAGCTCAAAAATTATTAACTTAATACTGGTTACTTAAAT 70736
Coquerel'sSifaka AAGTGAGG--GGTTTTTTTG-GATCATTAGTATTAATAGAAGGGATTAGCTGTTTTTCTACCATTATTGGATCTCCATAT 58654
Ma'sNightMonkey  TGCTTAGATAGATCCCTTTGCAACAGTTGCCACCCTAGGAATAACTTGAAAATTACTAACTTAGTACTAATTACTTAGAT 65637
VervetAGM        TGCTTAGATAGATCCCTTTGCAATAGTTGCCACCCTAGGAATAGCTCGAAAATTATTAAGTTAATACTAGTTCCTTAGAT 72475
Gorrilla         TGCTTAGATAGATCCCTTTGCAATAGTTACCACCCTAGGAATAGCTCAAAAATTATTAACTTAATACTAGTTACTTAGAT 71269
GrabEatingMacaqueTGCTTAGATAGATCCCTTTGCAGTAGTTGCCACCCTAGGAATAGCTCGAAAATTATTAAGTTAATACTAGTTCCTTAGAT 72016
Macaque          TGCTTAGATAGATCCCTTTGCAGTAGTTGCCACCCTAGGAATAGCTCGAAAATTATTAAGTTAATACTAGTTCCTTAGAT 72878
PigTailedMacaque TGCTTAGATAGATCCCTTTGCAGTAGTTGCCACCCTAGGAATAGCTCTAAAATTATTAAGTTAATACTAGTTCCTTAGAT 71410
Bonobo           TGCTTAGATAGATCCCTTTGCAATAGTTGCCACCCTAGGAATAGCTCAAAAATTATTAACTTAATACTGGTTACTTAGAT 69839
OliveBaboon      TGCTTAGATAGATCCCTTTGCAATAGTTGCCACCCTAGGAATAGCTCGAAAATTATTAAGTTAATACTAGTTCCTTAGAT 71275
Orangutan        TGCTTAGATAGATCCCTTTGCAATAGTTGCCACCCTAGGAATAGCTAGAAAATTATTAACTTAATACTAGTTACTTAGAT 71648

Majority         TCCACCTCTATTCCTTGGAGTT-T--GTTTTTTGTTTTTTGTTTTTT-TTT--TTTT-GAGACGG--------------

                         86090     86100     86110     86120     86130     86140     86150     86160

Human            TCCACCTCTGTTGCTTGGAGTTGT--GCTTTTTTTTTTTTTTTTTTTTTTTG-------AGAT----------------- 70790
Coquerel'sSifaka AAAAATTTGA---C------------------------------------------------------------------ 58665
Ma'sNightMonkey  TCCACCTCT----------------------------------------------------------------------- 65646
VervetAGM        TCCACCTCTATTCCTTGGAGTT-T--GTTTTTTGTTTTTTGTTTTTTGTGTT-TTTTTGAGACGG--------------- 72536
Gorrilla         TCCACCTCTGTTGCTTGGAGTTGT--GTTTTTTTTGTTTTTTGTTGTTGTTGTTGTTGTTGTTGTTTTGTTTTTTTTTTT 71347
GrabEatingMacaqueTCCACCTCTATTCCTTGGAGTT-T--GTTTTTTGTTATTTGTTTTTT------TTTTTGAGACGG--------------- 72072
Macaque          TCCACCTCTATTCCTTGGAGTT-T--GTTTTTTGTTATTTGTTTTTT------TTTTTGAGACAG--------------- 72934
PigTailedMacaque TCCACCTCTATTCCTTGGAGTT-T--GTTTTTTGTTTTTTGTTTTTT------TTTT-GAGACGG--------------- 71465
Bonobo           TCCACCTCTGTTGCTTGGAGTTGTTTGTTTTTTTTTTTTTTTTTTTTTTTTTTTTTTTGTAGAT---------------- 69902
OliveBaboon      TCCACCTCTATTCCTTGGAGTT-T--GTTTTTTGTTTTTTGTTTTTTGTTTTTTTTTTGAGACGG--------------- 71337
Orangutan        TCCACCTCTGTTGCTTGGAGTTGT--TTTTTTTTTTTTTTTTTTTTTTTTTG-------AGAC----------------- 71702
```

```
Majority          AGTAGTCTCGCTCTGTCGCCCAGGCTGGAGTGCAGTGG--CGCGATCTCGGCTCACTGCAAGCTCCGCCTCCTGAGTTCA

                          86170     86180     86190     86200     86210     86220     86230     86240

Human             -AGAGTCTGGCTCTGTCACCCAGGCTGGAGTGCAGTGGCATGCAATCTCGGCTCACTGCAAGCTCCGCCTCCTGGGTTCA 70869
Coquerel'sSifaka  ------CT--CACTAT--C-------AGAATGC----------------------CTACA-----TAGATCCC---TTCG 58698
Ma'sNightMonkey   -------------------------------------------------------------------------------- 65646
VervetAGM         AGTCGTCTCGCTCTGTCGGCCAGGCTGGAGTGCAGTGG--CGCGATCTCAGCTCACTGCAACCTCCGTCTCCTGAGTTCA 72614
Gorrilla          GAGAGTCTGTCTCTGTCACCCAGGCTGGAGTGCAGTGGGGTGCAATCTCGGCTCACTGCAAGCTCCGCCTCCTGGGTTCA 71427
GrabEatingMacaqueAGTTGTCTCGCTCTGTCGCCCAGGCTGGAGTGCAGTGG--CGCGATCTCGGCTCACTGCAATCTCCGCCTCCTGAGTTCA 72150
Macaque           AGTTGTCTCGCTCTGTCGCCCAGGCTGGAGTGCAGTGG--CGCGATCTCGGCTCACTGCAATCTCCGCCTCCTGAGTTCA 73012
PigTailedMacaque  AGTTGTCTCGCTCTGTCGCCCAGGCTGGAGTGCATTGG--CGCGATCTCGGCTCACTGCAACCTCCGCCTCCTGAGTTCA 71543
Bonobo            -AGAGTCTGGCTCTGTCACCCAGGCTGGAGTGCAGTGGCATGCAATCTCGGCTCACTGCAAGCTCTGCCTCCTGGGTTCA 69981
OliveBaboon       AGTCGTCTCGCTCTGTCGCCCAGGCTGGAGTGCAGTGG--CGCGATCTCGGCTCACTGCAACCTCTGCCTCCTGAGTTCA 71415
Orangutan         -AGAGTCTGGCTCTGTCGTCCAGGCTGGAGTGCAGTGGTGCGCAATCTTGGCTCACTGCAAGCTCCGCCTCCTGGGTTCA 71781

Majority          CGCCATTCTCCTGCCTCAGCCTCCCGAGTAGCTGGGACTACAGGTGCCTGCCACCATGCCCGGCTAATTTCTTTGTATTT

                          86250     86260     86270     86280     86290     86300     86310     86320

Human             CGCCATTCTCCTGCCTCAGCCTCCCAAGTAGCTGGGACTACAGGCGCACGCCACCACGCCCAGCTAATTTCTTCATATTT 70949
Coquerel'sSifaka  TAATAGT-TCCTACCCTAG------GAATAACTAGAA----AAATA-----------------TTAATTT---------- 58740
Ma'sNightMonkey   -------------------------------------------------------------------------------- 65646
VervetAGM         TGCCATTCTCCTGCCTCAGCCTCCCGAGTAGCTGGGACTGCAGGTGCCTGCCACCATGCCCGGCTAATTTCTTTGTATTT 72694
Gorrilla          CGCCATTCTCCTGCCTCAGCCTCCCAAGTAGCTGGGACTACAGGTGCCCGCCACCAGGCCCAGCTAATTTCTTCGTATTT 71507
GrabEatingMacaqueCGCCATTCTCCTGCCTCAGCCTCC-GAGTAGCTGGGACTACAGGTGCCTGCCACCATGCCCGGCTAATTTCTTTGTATTT 72229
Macaque           CGCCATTCTCCTGCCTCAGCCTCC-GAGTAGCTGGGACTACAGGTGCCTGCCACCATGCCCGGCTAATTTCTTTGTATTT 73091
PigTailedMacaque  CGCCATTCTCCTGCCTCAGCCTCC-GAGTAGCTGGGACTACAGGTGCCTGCCACCATGCCCGGCTAATTTCTTTGTATTT 71622
Bonobo            CGCCATTCTCCTGCCTCAGCCTCCCAAGTAGCTGGGACTATAGGCGCCCGCCACCACGCCCAGCTAATTTCTTCGTATTT 70061
OliveBaboon       CGCCATTCTCCTGCCTCAGCCTCCCGAGTAGCTGGGACTACAGGTGCCTGCCACCATGCCCGGCTCATTTCTTTGTATTT 71495
Orangutan         CGCCATTCTCCTGCCTCAGCCTCCCAAGTAGCTGGGACTACAGGCACCCGCCACCACACCCAGCTAATTTCTTTGTATTT 71861

Majority          TTTT-AGTAGAGACGGGGTTTCACCGTGTTAGCCAGGATGGTCTTGAT----CCCTGACCTCGTGATCCTCCTGCCTTGG

                          86330     86340     86350     86360     86370     86380     86390     86400

Human             TTTTTAGTAGAGACGGGGTTTTACTGTGTTAGCCAGGATGGTCTTGAT---CTCCTGACCTCCTGATCCTCCTGCCTCGG 71026
Coquerel'sSifaka  -A-------------------------------AGAACAG---T------------------------------------ 58749
Ma'sNightMonkey   ---------------------------------------G---T------------------------------------ 65648
VervetAGM         TTTT-AGTAGAGACGGGGTTTCACCGTGTTAGCCAGGATGGTCTTGAT----CCCTGACCTCGTGATCCTCCCACCTTGG 72769
Gorrilla          TTTT-AGTAGAGACGGGGTTTTACTGTGTTAGCCAGGATGGTCTTGAT---CTCCTGACCTCCTGATCCTCCTGCCTCGG 71583
GrabEatingMacaqueTTTTTAGTAGAGACGGGGTTTCACCATGTTAGCCAGGATAGTCTTGAT----CCCTGACCTCGTGATCCTCCTGCCTTGG 72305
Macaque           TTTTTAGTAGAGACGGGGTTTCACCATGTTAGCCAGGATAGTCTTGAT----CCCTGACCTCGTGATCCTCCTGCCTTGG 73167
PigTailedMacaque  TTTTTAGTAGAGACGGGGTTTCACCATGTTAGCCAGGATAGTCTTGAT----CCCTGACCTCGTGATCCTCCTGCCTTGG 71698
Bonobo            TTTT-AGTAGAGATGGGGTTTTACTGTGTTAGCCAGGATGGTCTTGAT---CTCCTGACCTCCTGATCCTCCTGCCTCGG 70137
OliveBaboon       TTTTTAGTAGAGACGGGGTTTCACCGTGTTAGCCAGGATGGTCTTGAT----CCCTGACCTCGTGATCCTCCCGCCTTGG 71571
Orangutan         TTTT-AGTAGAGACGGGGTTTTACCATGTTAGCCAGGATGGTCTTGATGATCTCCTGACCTCCTGATCCTCCTGCCTCGG 71940
```

```
Majority          CCTCCCAAAGTACTGGGATTACAGGCGTGAGCAACCGCGCCTGGCCTGCTTAGAGTTTTTTATGTCATTATTTTGTGACA

                          86410     86420     86430     86440     86450     86460     86470     86480

Human             CCTCCCAAAGTGCTGGGATTACGGGCGTGAGCCACCGCGCCTAGCCTGCTTAGAGTTTTTTATGTAATTATTTTGTGACA 71106
Coquerel'sSifaka  ----------TACTTAGATT--------------CCATCTCTG--TTGCTTAGAGTT---CATGTCATTATTTTGTGGCA 58800
Ma'sNightMonkey   -------------T----------G-----------------------CTTAGAGTTTTTCATGTCATTATTTTGTGGCA 65682
VervetAGM         CCTCCCAAAGTACTGGGATTACAGGCGTGAGCACCCGCGCCTGGCCTGCTTAGAGTTTTTTATGTCATCATTTTGTGACA 72849
Gorrilla          CCTCCCAAAGTGCTGGGATTACGGGCATGAGCCACCGCGCCTAGCCTGCTTAGAGTTTTTTATGTAATTATTTTGTGACA 71663
GrabEatingMacaqueCCTCCCAAAGTACTGGGATTACAGGCGTGAGCAACCGCGCCTGGCCTGCTTAGAGTTTTTTATGTCATCATTTTGTGACA 72385
Macaque           CCTCCCAAAGTACTGGGATTACAGGCGTGAGCAACCGCGCCTGGCCTGCTTAGAGTTTTTTATGTCATCATTTTGTGACA 73247
PigTailedMacaque  CCTCCCAAAGTACTGGGATTACAGGCGTGAGCAACCGCGCCTGGCCTGCTTAGAGTTTTTTATGTCATCATTTTGTGACA 71778
Bonobo            CCTCCCAAAGTGCTGGGATTATGGGCGTGAGCCACCGTGCCTAGCCTGCTTAGAGTTTTTTATGTAATTATTTTGTGATG 70217
OliveBaboon       CCTCCCAAAGTACTGGGATTACAGGCGTGAGCAACCGCACCTGGCCTGCTTAGAGTTTTTTATGTCATCATTTTGTGACA 71651
Orangutan         CCTCCCAAAGTGCTGGGATTACAGGCATGAGCCACCGCGCCTGGCCTGCTTAGAGTTTTTTATGTAATTATTTTGTGGCA 72020

Majority          CAAACATTTAAAATACTGTAGCTTAGAATG-AAATTGTCTCAATCCTAGGAGCCAAAAGTT----TGATGAATATGAATA

                          86490     86500     86510     86520     86530     86540     86550     86560

Human             CAAACATTTAAAATACTGTAGCTTAGAATG-AAATTGTCTCAATCCTAGGAGCCAAAAGTT----TGATGAATATGAATA 71181
Coquerel'sSifaka  AAAACATTCAAGATACAATACTATAGAATGGAAATTCATTTAATCCTAGGAACGAAAAGTTGGTTTGATGCATAGGAATC 58880
Ma'sNightMonkey   CAAACATTTAAAATACTATAGTTTAGAATG-AAATTGTCTCAATCCCAGGAGCCAAAAGTT----GGAGGTGTATGAATA 65757
VervetAGM         CAAACATTTAAAATACTGTAGCTTAGAATG-AATT-GTCTCAATCCTAGGAGCCAAAAGTT----TGCTGAATATGAATA 72923
Gorrilla          CAAACATTTAAAATACTGTAGCTTAGAATG-AAATTGTCTCAATCCTAGGAGCCAAAAGTT----TGATGAATATGAATA 71738
GrabEatingMacaqueCAAACATTTAAAATACTGTAGCTTAGAATG-AAAT-GTCTCAATCCTAGGAGCCAAAAGTT----TGCTGAATATGAATA 72459
Macaque           CAAACATTTAAAATACTGTAGCTTAGAATG-AAAT-GTCTCAATCCTAGGAGCCAAAAGTT----TGCTGAATATGAATA 73321
PigTailedMacaque  CAAACATTTAAAATACTGTAGCTTAGAATG-AAAT-GTCTCAATCCTAGGAGCCAAAAGTT----TGCTGAATATGAATA 71852
Bonobo            CAAACATTTAAAATACTGTAGCTTAGAATG-AAATTGTCTCAATCCTAGGAGCCAAAAGTT----TGATGAATATGAATA 70292
OliveBaboon       CAAACATTTAAAATACTGTAGCTTAGAATG-AAAT-GTCTCAATCCTAGGAGCCAAAAGTT----TGCTGAATATGAATA 71725
Orangutan         CAAACATTTAAAATACTGTAGCTTAGAATG-AAATTGTCTCAATCCTAGGAGCCAAAAGTT----TGATGAATGTGAATA 72095

Majority          CTAATATTAGCTAACCATTTTTGTGTT--CTGTGTCAGGCA-----CTGTTCTTAGCCCTTTACAAGTAAGTTAATAC-A

                          86570     86580     86590     86600     86610     86620     86630     86640

Human             CTAATATTA----ACCATTTTTGTGTT--CTGTGTCAGGCA-----CTGTTCTTAGCCCTTTACAAGTAAGTTAATAC-A 71249
Coquerel'sSifaka  TTAATAATA----GCTGTTACTATGTATTGTGTGCCAGGCA-----CTATTCTTAGCCCTTTACATTTGAGTTAATCCTG 58951
Ma'sNightMonkey   TTAATACTAGCTAGCCATTTTTGTGTTTTCTATGTCAGGCA-----CTGTTCTTAGCCCTTTACAGATAAGTTAATAC-A 65831
VervetAGM         CTAATATTAGCTAACCATTTTTGTGTC--CTGTTTCAGGCACTCAACTGTTCTCAGCCCTTCACAAGTAA-----TAC-A 72995
Gorrilla          CTAATATTA----ACCATTTTTGTGTT--CTGTGTCAGGCA-----CTGTTCTTAGCCCTTTACAAGTAAGTTAATAC-A 71806
GrabEatingMacaqueCTAATATTAGCTAACCATTTTTCTGTC--CTGTTTCAGGCACTCAACTGTTCTTAGCCCTTCACAAGTAA-----TAC-A 72531
Macaque           CTAATATTAGCTAACCATTTTTCTGTC--CTGTTTCAGGCACTCAACTGTTCTTAGCCCTTCACAAGTAA-----TAC-A 73393
PigTailedMacaque  CTAATATTAGCTAACCATTTTTCTGTC--CTGTTTCAGGCACTCAACTGTTCTTAGCCCTTCACAAGTAA-----TAC-A 71924
Bonobo            CTAATATTA----ACCATTTTTGTGTT--CTGTGTCAGGCA-----CTGTTCTTAGCCCTTTACAAGTAAGTTAATAC-A 70360
OliveBaboon       CTAATATTAGCTAACCATTTTTCTGTC--CTGTTTCAGGCACTCAACTGTTCTTAGCCCTTCACAAGTAA-----TAC-A 71797
Orangutan         CTAATATTAGCTAACCATTTTTGTGTT--CTGTGTCAGGCA-----CTGTTCTTAGCCCTTTACAAGTAAGTTAATAC-A 72167
```

```
Majority        TCATCTCTAAATGTAGTTACCATTATCGATCCCATTTTACAGATTTGGAAAGTAGGGCACAAAGAACAGTTTGCTCAGAT
                        86650     86660     86670     86680     86690     86700     86710     86720
Human           TCATCTCTAAATGTAGTTACCATTATCGATCCCATTTTATAGATTTGGAAAGTAGGGTACAAAGAACAGTTTGCCCAGAT 71329
Coquerel'sSifaka GCATCTCTAAACACAGTTACCATTGTTGCCTCTATTTTGCAGATCTGAAAACTAGGGCACAAAGAGCAGTTTG-TCAGAT 59030
Ma'sNightMonkey TCATCTCTAAATGTAGTTAACATTATCAACCCTGTTGTACAGATTTGGAAAGTAGGGCACAAAGAGCAGTTTGCTCAGAT 65911
VervetAGM       TCATCTCTAAATGTAGTTACCATTATGGATCCCATTTTACAGATTTGGAAAGTAGGGCAGAAAGAACAGTTTGCTCAGAT 73075
Gorrilla        TCATATCTAAATGTAGTTACCATTATCGATCCCATTTTATAGATTTGGAAAGTAGGGTACAAAGAACAGTTTGCTCAGAT 71886
GrabEatingMacaqueTCATCTCTAAATGTAGTTACCATTATAGATCCCATTTTACAGATTTGGAAAGTAGGGCAGAAAGAACAGTTTGCTCAGAT 72611
Macaque         TCATCTCTAAATGTAGTTACCATTATAGATCCCATTTTACAGATTTGGAAAGTAGGGCAGAAAGAACAGTTTGCTCAGAT 73473
PigTailedMacaque TCATCTCTAAATGTAGTTACCATTATAGATCCCATTTTACAGATTTGGAAAGTAGGGCAGAAAGAACAGTTTGCTCAGAT 72004
Bonobo          TCATCTCTAAATGTAGTTACCATTATCGATCCCATTTTATAGATTTGGAAAGTAGGGTACAAAGAACAGTTTGCTCAGAT 70440
OliveBaboon     TCATCTCTAAATGTAGTTACCATTATGGATCCCATTTTACAGATTTGGAAAGTAGGACAGAAAGAACAGTTTGCTCAGAT 71877
Orangutan       TCATCTCTAAATGTAGTTACCATTATCGATCCCATTTTATAGATTTGGAAGGTAGGGCACAAAGAACAGTTTGCTCAGAT 72247

Majority        TCCCTTAACTTTTGAGTGGCCGAGCCCTAGCACTCAATGCCAGGTAGTCTAGTTCCAAAGTCCGTACTCCTAACTAATAA
                        86730     86740     86750     86760     86770     86780     86790     86800
Human           TCCTTTAACTTTTGAGTGGCCGAGCCCTAGCACTGAAAGCCAGGTAGTCTGGTTCCAAAGTCCGTACTCCTGACTAATAA 71409
Coquerel'sSifaka TCTCATAACTGGTGAGTGGCTGAGT--TAGTACTCAAAGCCAGATAGTCTGGCTGCAAAGTCCATGCTCCTTAGTAATCA 59108
Ma'sNightMonkey TCCCTCAACTTTTGAATGGCCCAGCCCAAGCACTCA--GTCAGGTAGTCTAGTTCCAAAGCCCATACTCCTAACTA---- 65985
VervetAGM       TCCCTTAACTTTTGAGTGACCAAGCCCTGGCACTCAATGCCAGGAAATCTACTTCCAAGGTCCGTACTCCTAACTAATAA 73155
Gorrilla        TCCTTTAACTTTTGAGTGGCCGAGCCCTAGCACTCAAAGCCAGGTAGTCTGGTTCCAAAGTCTGTACTCCTAACTAATAT 71966
GrabEatingMacaqueTCCCTTAACTTTTGAGTGACCAAGCCCTGGCACTCAATGCCAGGAAATCTAGTTCCAAGGTCCGTACTCCTAACTAATAA 72691
Macaque         TCCCTTAACTTTTGAGTGACCAAGCCCTGGCACTCAATGCCAGGAAATCTAGTTCCAAGGTCCGTACTCCTAACTAATAA 73553
PigTailedMacaque TCCCTTAACTTTTGAGTGACCAAGCCCTGGCACTCAATGCCAGGAAATCTAGTTCCAAGGTCCGTACTCCTAACTAATAA 72084
Bonobo          TCCTTTAACTTTTGAGTGGCCGAGCCCTAGCACTGAAAGCCAGGTAGTCTGGTTCCAAAGTCCATACTCCTAACTAATAA 70520
OliveBaboon     TCCCTTAACTTTTGAGTGACCAAGCCCTGGCACTCAATGCCAGGAAATCTAGTTCCAAGGTCCGTACTCCTAACTAATAA 71957
Orangutan       TCCTTTAACTTTTGAGTGGCCGAGCCCTAGCACTCAAAGCCAGGTAGTCTAGTTTCAAAGTCCGTACTCTTAACTAATAA 72327

Majority        ATAGTGGAATAAAAT------TAGATTAGCTTGACATTTTAGCCTGTCGTCTTCTCCCCTGTAGAATAGTTATCAAGAAT
                        86810     86820     86830     86840     86850     86860     86870     86880
Human           ATAGTGGAATAAAAT------TAGATTAGCTTGACATTTTAGCCTGTCGTCTTCTCACCTGTAGAATAGTTATCAAGAAT 71483
Coquerel'sSifaka ACAATAGAATAAAAGGTACTATAGGTTACCTTGGCCTTTTAGCTTGTCTTCCTTCCCCCTGTGAAATAGTTATCTAGAAT 59188
Ma'sNightMonkey ---GTGGAGTAAAAT------TAGATTAGCTTGACATTTTAGCCTGTCGTCTTCTTCCCCGTAGAATAGTTATCAATA-- 66054
VervetAGM       ATAGTGGAATAAAAT------TAGATTAGCTTGACATTTTAGCCTGTCATCTTCTCCCCTGTAGAATAGTTATCAAGAAT 73229
Gorrilla        ATAGTGGAATAAAAT------TAGATTAGCTTGACATTTTAGCCTGTCGTCTTCTCCCCTGTAGAATAGTTATCAAGAAT 72040
GrabEatingMacaqueATAGTGGAATAAAAT------TAGATTAGCTTGACATTTTAGCCTGTCATCTTCTCCCCTGTAAAATAGTTATCAAGAAT 72765
Macaque         ATAGTGGAATAAAAT------TAGATTAGCTTGACATTTTAGCCTGTCATCTTCTCCCCTGTAGAATAGTTATCAAGAAT 73627
PigTailedMacaque ATAGTGGAATAAAAT------TAGATTAGCTTGACATTTTAGCCTGTCATCTTCTCCCCTGTAGAATAGTTATCAAGAAT 72158
Bonobo          ATAGTGGAATAAAAT------TAGATTAGCTTGACATTTTAGCCTGTCGTCTTCTCACCTGTAGAATAGTTATCAAGAAT 70594
OliveBaboon     ATAGTGGAATAAAAT------TAGATTAGCTTGACATTTTAGCCTGTCATCTTCTCCCCTGTAGAATAGTTATCAAGAAT 72031
Orangutan       ATAGTGGAATAAAAT------TAGATTAGCTTGACATTTTAGCCGGTCGTCTTCTCCCCTGTAGAATAGTTATCAAGAAT 72401
```

```
Majority         AATAATATACATGTAGTAAAAATGTAAGTCCCATCATTTTAGCTTTCTTTTTTTTTTTTTTTTT----ACATACTAAGAT
                          86890     86900     86910     86920     86930     86940     86950     86960
Human            AGTAATATACATATAGTAAAAATTTAAGTCCCATCATTTTAGCTTGCTTTTTTTTT------------ACATACTAAGAT 71551
Coquerel'sSifaka AATGTCATACATGTAGCAAATATGTAAGTCCCATAGTTTTAATTTTCTTTTTTC--------------ACATACTAAGAT 59254
Ma'sNightMonkey  --CATTATACATGTAGTAAAAATGTAAGTCCCATCATTTTTGCTTTCTTCTTATTTTTTTTTTG----GCATACTAAGAT 66128
VervetAGM        AATAATATACATGTAGTAAAAATGTAAGTCCCATCATTTTAGCTTTCTTTTTTTTTTCTTTTTT----ACATAGTAAGAT 73305
Gorrilla         AGTAATATACATATAGTCAAAATTTAAGTCCCATCATTTTAGCTTACTTTTTTTTTTTTT--------ACATACTAAGAT 72112
GrabEatingMacaqueAATAATATACATGTAGTAAAAATGTAAGTCCCATCATTTTAGCTTTCTTTTTTTTTTTTTTTTTTTTTACATAGTAAGAT 72845
Macaque          AATAATATACATGTAGTAAAAATGTAAGTCCCATCATTTTAGCTTTCTTTCTTTTTTTTTTTTTTTT-ACATAGTAAGAT 73706
PigTailedMacaque AATAATATACATATAGTAAAAATGTAAGTCCCATCATTTTAGCTTTCTTTTTTTTTTTTTTT------ACATAGTAAGAT 72232
Bonobo           AGTAATATACATATAGTAAAAATTTAAGTCCCATCATTTTAGCTTGCTTTTTTTTTTT----------ACATACTAAGAT 70664
OliveBaboon      AATAATATACATGTAGTAAAAATGTAAGTCCCATCATTTTAGCTTTCTTTTTTTTTTTTTTTTTTTT-ACATAGTAAGAT 72110
Orangutan        AGTAATATACATATAGTAAAAATGTAAGTCCCATCATTTTAGCTTTCTTTTCTTTTTTTTTTTTTCC-ACATACTAAGAT 72480

Majority         ATTTTTTGTTACCAAGTCCATTCAGTATTTAGCCTGATTATCTTATTGATTAAAAAACCAAAAGTGAGTATTGTTCATAC
                          86970     86980     86990     87000     87010     87020     87030     87040
Human            ATTTTTTGTTACCAAGTCCATTAAGTATTTAGCCTGATTATCTTATTGATTAAAATACCAAAAATGTGTATTGTTCATGC 71631
Coquerel'sSifaka ATTTTTTGTTGCCAAGTCCATTCCGTGTTTAACCTGGTTATCTTAT-GATTACAAAACCAAA-GTGAGTATTGTTCTTAT 59332
Ma'sNightMonkey  ATTTTTTGTTACCAAGTCCATTCAGTATTTAGTCTGATCATCTTATTGGTTATAAAACCAAAAGTGAGTATTGTTGATAT 66208
VervetAGM        ATTTTTTGTTACCAAGTCCATTCAGTATTTAGCCTGATTATCTTATTGATTAAAAAATGAAAAGTGAGTATTGTTCATAC 73385
Gorrilla         ATTTTTTGTTACCAAGTCCATTAAGTATTTAGCCTGATTATCTTATTGATTAAAATACCAAAAATGAGTATTGTTCATGC 72192
GrabEatingMacaqueATTTTTTGTTACCAAGTCCATTCAGTATTTAGCCTGATTATCTTATTGATTAAAAAATGAAAAGTGAGTATTGTTCATAC 72925
Macaque          ATTTTTTGTTACCAAGTCCATTCAGTATTTAGCCTGATTATCTTATTGATTAAAAAATGAAAAGTGAGTATTGTTCATAC 73786
PigTailedMacaque ATTTTTTGTTACCAAGTCCATTCAGTATTTAGCCTGATTATCTTATTGATTAAAAAATGAAAAGTGAGTATTGTTCATAC 72312
Bonobo           -TTTTTTGTTACCAAGTCCATTAAGTATTTAGCCTGATTATCTTATTGATTAAAATACCAAAAATGTGTATTGTTCATGC 70743
OliveBaboon      ATTTTTTGTTACCAAGTCCATTCAGTATTTAGCCTGATTATCTTATTGATTAAAAAATGAAAAGTGAGTATTGTTCATAC 72190
Orangutan        ATTTTTTGTTACCAAGTCCATTCAGTATTTAGCCTGATTATCTTATTGATTAAAAAACCAAAAATGAGTATTGTTCATGC 72560

Majority         AT--TCATTCAAAATTGGGTACAAAACAGGGGTAGATATGTAACATGCAAATAAT--------AAACTGAACTTATATTT
                          87050     87060     87070     87080     87090     87100     87110     87120
Human            AT--TGATTCAAAATTGGGTACAAAACGGG--TAGATATGTAACATGCAAATAAT--------AAATTGAACTTATATTT 71699
Coquerel'sSifaka ATAGCCATTCAAAATTGTGTACAAAATAGGGATAGATACATAACATGCAAATCAAAATACAGTAAACTGAATTTATATTT 59412
Ma'sNightMonkey  AT--TCATTCAAATTTGGGTATAAAATAGGGGTGGATATGTAATATGCAAATCA---------AAACATAATAAACAATT 66277
VervetAGM        AT--TCATTCAAAATTGGGTACAAAACAAGGGTAGATATGTAACATGCAAATAAT--------AAACTGAACTTATATTT 73455
Gorrilla         AT--TGATTTAAAATTGGGTACAAAACAGGGGTAGATATGTAACATGCAAATAAT--------AAATTGAACTTATATTT 72262
GrabEatingMacaqueAT--TCATTCAAAATTGGGTACAAAACAAGGGTAGATATGTAACATGCAAATAAT--------AAACTGAACTTATATTT 72995
Macaque          AT--TCATTCAAAATTGGGTACAAAACAAGGGTAGATATGTAACATGCAAATAAT--------AAAC--AACTTATATTT 73854
PigTailedMacaque AT--TCATTCAAAATTGGGTACAAAACAAGGGTAGATATGTAACATGCAAATAAT--------AAACTGAACTTATATTT 72382
Bonobo           AT--TGATTCAAAATTGGGTACAAAACAGGAGTAGATATGTAACATGCAAATAAT--------AAATTGAACTTATATTT 70813
OliveBaboon      AT--TCATTCAAAATTGGGTACAAAACAAGGGTAGATATGTAACATGCAAATAAT--------AAACTGAACTTACATTT 72260
Orangutan        AT--TGATTCAAAATTGGGTACAAAACAGGGGTAAATATGTAACATGCAAATAAT--------AAACT------------ 72618
```

```
Majority         TTATCTTTTTGGCA----ACACATTAAAATTTATGTC----AATCTT-ATTTTTTCAAAATCCTTTTCTATAATATTTTA
                         87130     87140     87150     87160     87170     87180     87190     87200
Human            TTATCTTTTTGACAAACAACACGTTAAAATTTATGTC----AATCTT-ATTTTTTCAAAATCCTTTTCTA---TATTTTA 71771
Coquerel'sSifaka TTATTTTTTTATAAA-TTATACATTAACATTTATGTGCCAAAATCTCGATTTTCATAAAATTCTGTAATATTCCATT--A 59489
Ma'sNightMonkey  T--ATTTTTTATCA---AACACATTAAAATTTATATGTCAAAATCTT-ATTTTTTCAAAATCCTCTTCTATAATATT--- 66348
VervetAGM        TTATCTTTTTGGCA----AC--ATTAAAATTTATGTC----AATCTA-ATTTTTTCAAAATCTTTTTCTATAATATTTTA 73524
Gorrilla         TTATCTTTTTGACAAACAACACGTTAAAATTTATGTC----AATCTT-ATTTTTTCAAAATCCTTTTCTA---TATTTTA 72334
GrabEatingMacaqueTTATCTTTTTGGCA----ACACATTAAAATTTATGTC----AATCTA-ATTTTTTCAAAATCTTTTTCTATAATATTTTA 73066
Macaque          TTATCTTTTTGGCA----ACACATTAAAATTTATGTC----AATCTA-ATTTTTTCAAAATCTTTTTCTATAATATTTTA 73925
PigTailedMacaque TTATCTTTTTGGCA----ACACATTAAAATTTATGTC----AATCTA-ATTTTTTCAAAATCTTTTTCTATAATATTTTA 72453
Bonobo           TTATCTTTTTGACA----ACACGTTAAAATTTATGTC----AATCTT-ATTTTTTCAAAATCCTTTTCTA---TATT--- 70878
OliveBaboon      TTATCTTTTTGGCA----ACACATTAAAATTTATGTC----AATCTA-ATTTTTTCAAAATCTTTTTCTATAATATTTTA 72331
Orangutan        ---CCTTTTTGACAAACAACACATTAAAATTTATGTC----AATCTT-ATTTTTTCAAAATCCTTTTCTA---TATTTTA 72687
```

```
Majority         TTAAGACTAATTATAGCAAA-----TAGTAGCCATAAAATTAAACCAAAAAACTCATCTACTGCAG-ACATATTTGAAAG
                         87210     87220     87230     87240     87250     87260     87270     87280
Human            TTAAGACTAATTATAGCAAA-----TAATAGCCATAAAATTAAACCAAAAAACTCATCTACTGCAG-ACATATTTGAAAG 71845
Coquerel'sSifaka GTGAGACTAATTATAGCAAAGAAAATAATGGCCATAAAATCAAACCAAATAACTCATCTACTGCAA-AGATATTTGAAAG 59568
Ma'sNightMonkey  --AAGACTAATTATAGCAAA-----TA---GCCATAAAATTAAACCAAAAAACTCATCTATTGCAG-TCATATTTGAAAG 66417
VervetAGM        TTAAGACTAATTATAGCAAA-----TAGTAGCTGTAAAATTAAACCAAAAAACTTATCTACTGCAGGACATATTTGAAAG 73599
Gorrilla         TTAAGACTAATTATAGCAAA-----TAATAGCCATAAAATTAAACCAAAAAACTCATCTACTGCAG-ACATATTTGAAAG 72408
GrabEatingMacaqueTTAAGACTAATTATAGCAAA-----TAGTAGCTGTAAAATTAAACCAAAAAACTTATCTACTGCAGGACATATTTGAAAG 73141
Macaque          TTAAGACTAATTATAGCAAA-----TAGTAGCTGTAAAATTAAACCAAAAAACTTATCTACTGCAGGACATATTTGAAAG 74000
PigTailedMacaque TTAAGACTAATTATAGCAAA-----TAGTAGCTGTAAAATTAAACCAAAAAACTTGTCTACTGCAGGACATATTTGAAAG 72528
Bonobo           --AAGACTAATTATAGCAAA-----TAATAGCCATAAAATTAAACCAAAAAACTCATCTACTGCAG-ACATATTTGAAAG 70950
OliveBaboon      TTAAGACTAATTATAGCAAA-----TAGTAGCTGTAAAATTAAACCAAAAAACTTATCTACTGCAGGACATATTTGAAAG 72406
Orangutan        TTAAGACTAATTATAGCAAA-----TAATAGCCATAAAATTAAACAAAAA-CTCATCTGCTGCAG-ACATGTTTGAAAG 72760
```

```
Majority         CTATCCCCCAAGAAA-GACCAAAGT-AATCACATGTACATAAACATGCTA---GTCTTTCTCAGAAATATTAAAAATACA
                         87290     87300     87310     87320     87330     87340     87350     87360
Human            CTGTCCCCCAAGAAA-GACCAAAGT-AATCACACATACCTAAACATGCTA---GTATTTCCCAGAAATATTAAAAATACA 71920
Coquerel'sSifaka ATATCTTCCAAGAAA-GACTAAAAT-AATCACATTTGCACAAATATCCTAGAAGTATGTCCTAGAAATGTTAAAC-TACA 59645
Ma'sNightMonkey  CTATCCCCCCAGAAAAGACTAAAGT-AATCACACATACATAAACATGCTACAAGTATTTCCCAGAAATATTAAAAATACA 66496
VervetAGM        CTATCCCCCAAGAAA-GACCAAAGT-AATCACATGTACATAAACATGCTA---GTCTTTCTCAGAAATGTTAAAAATACA 73674
Gorrilla         CTATCCCCCAAGAAA-GACCA----------------------------------------------------------- 72428
GrabEatingMacaqueCTATCCCCCAAGAAA-GACCAAAGT-AATCACATGTACATAAACATGCTA---GTCTTTCTCAGAAATATTAAAAATACA 73216
Macaque          CTATCCCCCAAGAAA-GACCAAAGT-AATCACATGTACATAAACATGCTA---GTCTTTCTCAGAAATATTAAAAATACA 74075
PigTailedMacaque CTATCCCCCAAGAAA-GACCAAAGT-AATCACATGTACATAAACATGCTG---GTCTTTCTCAGAAATATTAAAAATACA 72603
Bonobo           CTATCCCCCAAGAAA-GACCAAAGT-AATCACACATACCTAAACATGCTA---GTATTTCCCAGAAATATTAAAAATACA 71025
OliveBaboon      CTATCCCTCAAGAAA-GACCAAAGT-AATCACATGTACATAAACATGCTA---GTCTTTCTCAGAAATATTAAAAATACA 72481
Orangutan        CTATCCCCCAAGAAA-GACCAAAGTTAATCACACATACCTAAACATGCTA---GTATTTCCCAGAAATATTAAAAATACA 72836
```

```
Majority          TAGATTTTAAAGCACATTGCTGAATGC----CATA---ACACCAAAATAAAAAATACATATTAATTAAAAATTAATATTT

                          87370     87380     87390     87400     87410     87420     87430     87440

Human             TAGATTTTGAAGCACATTGCTGAATGC----CATAGTAACACCAAACTAAAAAATACATGTAAATTAAAAATTAATATTT 71996
Coquerel'sSifaka  TAGATTTTAAAACACATCAATCAATGCATGCCATAGTAACACCAAAATAAA-----TATATAAATTAAAAACTAGTATTT 59720
Ma'sNightMonkey   TAGATTTTAAAGCACATTGCTGACTGC----CACACTAACACCAAAATAAAAAATACATGTAAATTAAAAATCAGTATTT 66572
VervetAGM         TAGATTTTAAAGCACATTGCTGAATGC----CATA---ACACCAAAATAAAAAATATGTATTAATTAAA----AATATTT 73743
Gorrilla          -------------------------------------------------------------------------------- 72428
GrabEatingMacaqueTAGATTTTAAAGCACATTGCTGAATGC----CATA---ACACCAAAATAAAAAATACATATTAATTAAAAATTAATATTT 73289
Macaque           TAGATTTTAAAGCACATTGCTGAATGC----CATA---ACACCAAAATAAAAAATACATATTAATTAAAAATTAATATTT 74148
PigTailedMacaque  TAGATTTTAAAGCACATTGCTGAATGC----CATA---ACACCAAAATAAAAAATATATATTAATTAAAAATTAATATTT 72676
Bonobo            TAGATTTTGAAGCACATTGCTGAATGC----CATAGTAACACCAAACTAAAAAATACATGTAAATTAAAAATTAATATTT 71101
OliveBaboon       TAGATTTTAAAGCACATTGCTGAATGC----CATA---ACACCAAAATAAAAAATATATATTAATTAAAAATTAATATTT 72554
Orangutan         TAGATTTTGAAGCACATTGCTGAATGC----CATAGTAACACCAAACTAAAAAATACATGTAAATTAAAAATTAATATTT 72912
```

```
Majority          TATT---TTCAACATAAA----TCAGAAATACTTGGTCAGAGGTCAC-------TACCTGTCCTAACT----GTCCCATA

                          87450     87460     87470     87480     87490     87500     87510     87520

Human             TATT---TTCAACATAAA----TCAGAAATACTTGGTTAGAGGTCACATGATAATACCTGTCCTAACT----GTCCCATA 72065
Coquerel'sSifaka  TCTCATGTCCAGCATAAA----TCAGAAATATTTGCTCAGAAATCATATGAAAATAGCTATCCTAACTCACTGTCCTATA 59796
Ma'sNightMonkey   TATTAGTTTCAACATAAACAAATCAGAAATATTTGGGCAGAGTTCACACGAAAATACCTATCCTAACT----GTCCTGTA 66648
VervetAGM         TATT---TTCAACATAAA----TCAGAAATACTTGATCAGAGGTCAC-------TACCTGTCCTAACT----GTCCCATA 73805
Gorrilla          -------------------------------------------------------------------------------- 72428
GrabEatingMacaqueTATT---TTCAACGTAAA----TCAGAAATACTTGGTCAGAGGTCAC-------TACCTGTCCTAACT----GTCCCATA 73351
Macaque           TATT---TTCAACGTAAA----TCAGAAATACTTGGTCAGAGGTCAC-------TACCTGTCCTAACT----GTCCCATA 74210
PigTailedMacaque  TATT---TTCAACGTAAA----TCAGAAATACTTGGTCAGAGGTCAC-------TACCTGTCCTAACT----GTCCCATA 72738
Bonobo            TATT---TTCAACATAAA----TCAGAAATACTTGGTTAGAGGTCACATGATAATACCTGTCCTAACT----GTCCCATA 71170
OliveBaboon       TATT---TTCAACATAAA----TCAGAAATACTTGGTCAGAGGTCAC-------TACCTGTCCTAACT----GTCCCATA 72616
Orangutan         TGTT---TTCAACATAAA----TCAGAAATACTTGGTCAGAGGTCACACAATAATGCCTGTCCTAACT----GTCTCATA 72981
```

```
Majority          ACTTTGACT-----------------------------------------TTCTCATTTCATGT--GTTTTAATATCTA

                          87530     87540     87550     87560     87570     87580     87590     87600

Human             ACTTCGGCT-----------------------------------------TTCTCATTTCATGT--GTTTTAATATCTA 72101
Coquerel'sSifaka  ACTTTGATTCTCTGAAAATGCCTATCCTAACTCACTGTCCTATAACTTTGATTCTTATTTGTGTCTGTCTTAATACCTA 59876
Ma'sNightMonkey   ACTTTGACT-----------------------------------------TTCTCGTTTCATGT--GTTTTAATATCTA 66684
VervetAGM         ACTTTGACT-----------------------------------------TTCTGATTTCATGT--GTTTTAATATCTA 73841
Gorrilla          -------------------------------------------------------------------------------- 72428
GrabEatingMacaqueACTTTTACT-----------------------------------------TTCTCATTTCATGT--GTTTTAATATCTA 73387
Macaque           ACTTTTACT-----------------------------------------TTCTCATTTCATGT--GTTTTAATATCTA 74246
PigTailedMacaque  ACTTTTACT-----------------------------------------TTCTCATTTCATGT--GTTTTAATATCTA 72774
Bonobo            ACTTCGGCT-----------------------------------------TTCTCATTTCATGT--GTTTTAATATCTA 71206
OliveBaboon       ACTTTGACT-----------------------------------------TGCTCATTTCATGT--GTTTTAGTATCTA 72652
Orangutan         ACTTTGGCT-----------------------------------------TTCTCATTTCATGT--GTTTTAATATCTA 73017
```

```
Majority        AGAAGTAAAAAATCGTTGGAGTGTGCTTCTT-TGTATGTACAATTTTA---AATGATGTTGACTTCTTTGGATTATTGAA

                        87610     87620     87630     87640     87650     87660     87670     87680

Human           AGAAGTAAAAAATAGTTGGAGCATGCCTCTT-TATATGTGCAATTTTA---AATTATGTTGACTTCTTTGGATTATTGAA 72177
Coquerel'sSifaka AGAAGTAAACTATAGTTGGAACATACCTTCTGTATGTGTACAGTCTTAGAAAATGATGTTGAGTT-TTTAGATTATTGAA 59955
Ma'sNightMonkey AGAAGTAAAAACTAGTTGGAGCATGCCTCTTCTCTATGTGTA----CA---A------CTGAGTTATTTGGATTTTTGAA 66751
VervetAGM       AGAAGTAAAAAATCGTTGGAGTGTGCTTCTT-TGTATGTACAATTTTA---AATGATGTTGACTTCTTTGGATTATCGAA 73917
Gorrilla        -------------------------------------------------------------------------------- 72428
GrabEatingMacaqueAGAAGTAAAAAATCGTTGGAATGTGCTTCTT-TGTATGTACAATTTTA---AATGATGTTGACTTCTTTGGATTATTGAA 73463
Macaque         AGAAGTAAAAAATCATTGGAATGTGCTTCTT-TGTATGTACAATTTTA---AATGATGTTGACTTCTTTGGATTATTGAA 74322
PigTailedMacaque AGAAGTAAAAAATCGTTGGAATGTGCTTCTT-TGTATGTACAATTTTA---AATGATGTTGACTTCTTTGGATTATTGAA 72850
Bonobo          AGAAGTAAAAAATAGTTGGAGCATGCCTCTT-TATATGTGCAATTTTA---AATTATGTTGACTTCTTTGGATTATTGAA 71282
OliveBaboon     AGAAGTAAAAAATCGTTGGAGTGTGCTTCTT-TGTATGTACAATTTTA---AATGATGTTGACTTCTTTGGATTATTGAA 72728
Orangutan       AGAAGTAAAAAATAGTTGGAGCATGCCTCTT-TATATGTGCAATTTTA---AATGATGTTGACTTCTTTGGATTATTGAA 73093

Majority        CTGTCCTCTACTTTGCTGTAGCTGAATTTTTAAAATGTTAATTTTTAAGTCTTAAACTGT--CTTTTGGAGGAAACATTG

                        87690     87700     87710     87720     87730     87740     87750     87760

Human           CTGTCCTCTGCTTTGCTATAGCTGAATTTTTAAAATGTTAATTTTGAAGTCTTAAAATGTGTCTTTTGGAAAAAACATTG 72257
Coquerel'sSifaka CTGTCCTCTACTTTATTGTAGCTGAATTTTTAAAATTTTAATTTTTAAATCTTAAGATGTCTCTTTTAGAAAAGAAGTTG 60035
Ma'sNightMonkey TTGTCCTCTACTTTCCTATAGCTTAATGCTTAAAATGTTAATTCTTAA-TTTTAAAATGTGTCTTTTGGAACAAACATTG 66830
VervetAGM       CTGTCCTCTACTTTGCTGTAGCTGAATTTTTAAAATGTTAATTTTTAAGTCTTAAACTGT--CGTTTGGAGGAAACATTG 73995
Gorrilla        -------------------------------------------------------------------------------- 72428
GrabEatingMacaqueCTGTCCTCTACTTTGCTGTAGCTGAATTTTTAAAATGTTAATTTTTAAGTCTTAAACTGT--CTTTTGGAGGAAACATTG 73541
Macaque         CTGTCCTCTACTTTGCTGTAGCTGAATTTTTAAAATGTTAATTTTTAAGTCTTAAACTGT--CTTTTGGAGGAAACATTG 74400
PigTailedMacaque CTGTCCTCTATTTTGCTGTAGCTGAATTTTTAAAATGTTAATTTTTAAGTCTTAAACTGT--CTTTTGGAGGAAACATTG 72928
Bonobo          CTGTCCTCTGCTTTGCTGTAGCTGAATTTTTAAAATGTTAATTTTGAAGTCTTAAAATGTGTCTTTTGGAAAAAACATTG 71362
OliveBaboon     CTGTCCTCTACTTTGCTGTAGCTGAATTTTTAAAATGTTAATTTTTAAGTCTTAAACTGT--CTTTTGGAGGAAACATTG 72806
Orangutan       CTGTCCTCTG-----CTATAGCTGAATTTTTAAAATGTTAATTTGAAGTCTTAAAATGTGTCTTTTGGAAAAAACATTG 73168

Majority        GAAGAGCTAAAGTAGATAGAGTT---------------------------------------------------------

                        87770     87780     87790     87800     87810     87820     87830     87840

Human           GAAGAGCTAAAGTAGATAGAGTTGTTCATTTAAATTGTTTATTTTTTATTTTATTTTATTTTTTTTAGTTGGAGTACTGG 72337
Coquerel'sSifaka GAAGAGCTGAGGTAGATAGAATTG-------------------------------------------------------- 60059
Ma'sNightMonkey GAAGAGCTGAAGTAGACAGAATTGTTCATTTAAAAATTTTACCACTGGATAAAATTTTTTTTT--------ACT-TTTT 66901
VervetAGM       GAAGAGCCAAAGTAGATAGAGTT--------------------------------------------------------- 74018
Gorrilla        -------------------------------------------------------------------------------- 72428
GrabEatingMacaqueGAAGAGCTAAAGTAGATAGAGTT--------------------------------------------------------- 73564
Macaque         GAAGAGCTAAAGTAGATAGAGTT--------------------------------------------------------- 74423
PigTailedMacaque GAAGAGCTAAAGTAGATAGAGTT--------------------------------------------------------- 72951
Bonobo          GAAGAGCTAAAGTAGATAGAGTTGTTCATTTAAATTGTTTATTTTTTATTTTATTTTATTTTTTT-AGTTGGAGTACTGG 71441
OliveBaboon     GAAGAGCTAAAGTAGATAGAGTT--------------------------------------------------------- 72829
Orangutan       GAAGAGCTAAAGTAGATAGAGTTGTTAATTTAAATTTTTTATTTTTTATTTTTCATTTTTTTTTTGAGATGGAGTCCTG- 73247
```

```
Majority          --------------------------------------------------------------------------------

                          87850     87860     87870     87880     87890     87900     87910     87920

Human             CTCTGTTGCCTGGGCTGGAGTGCAGTGGCGGGATCTCTGCTCACTGCAAGCTCCGCCTCCTGGGTTCACGCCATTCTGCC 72417
Coquerel'sSifaka  -------------------------------------------------------------------------------- 60059
Ma'sNightMonkey   TTTTTTTTTTTTTTTGACAGTCTCGCTCTGTAGCCTAGGCTGGAGTGCAGTGGCACGATCTTGCTTCACTGCAACCTCCG 66981
VervetAGM         -------------------------------------------------------------------------------- 74018
Gorrilla          -------------------------------------------------------------------------------- 72428
GrabEatingMacaque-------------------------------------------------------------------------------- 73564
Macaque           -------------------------------------------------------------------------------- 74423
PigTailedMacaque  -------------------------------------------------------------------------------- 72951
Bonobo            CTCTGTTGCCTGGGCTGGAGTGCAGTGGCGCGATCTCTGCTCACTGCAAGCTCTGCCTCCCGGGTTCACGCCATTCTGCC 71521
OliveBaboon       -------------------------------------------------------------------------------- 72829
Orangutan         CTCTGTTGCCCGGGCTGGAGTGCAGTGGCGCAGTCTCCGCTCACTACAAGCTCCGCCTCCCGGGTTCACGCCATTCTGCC 73327

Majority          --------------------------------------------------------------------------------

                          87930     87940     87950     87960     87970     87980     87990     88000

Human             GCCTCAGCCTCCTGAGTAGCTGGGGCTACAGGTGCCCTCCACCACACCTGGCTAATTTTTTTTGTAGTTTTAGTAGAGAC 72497
Coquerel'sSifaka  -------------------------------------------------------------------------------- 60059
Ma'sNightMonkey   CCTCCCGGGTCCAGGTTCAAGCAATTCTCCTGCCTCAGCCTCCCAGGGAGCTGGAATTACAGGCACACATCACCATGCCC 67061
VervetAGM         -------------------------------------------------------------------------------- 74018
Gorrilla          -------------------------------------------------------------------------------- 72428
GrabEatingMacaque-------------------------------------------------------------------------------- 73564
Macaque           -------------------------------------------------------------------------------- 74423
PigTailedMacaque  -------------------------------------------------------------------------------- 72951
Bonobo            GCCTCAGCCTCCTGAGTAGCTGGGGCTACAGGCGCCCTCCACCACACCCGGCTAATTTTTTTTGTAGTTTTAGTAGAGAC 71601
OliveBaboon       -------------------------------------------------------------------------------- 72829
Orangutan         GCCTCAGGCTCCTGAGTAGCTGGGGCTGCAGGCGCCCTCCACCACGCCCGGCTAATTTTTTTTGTAGTTTTAGTAGAGAC 73407

Majority          --------------------------------------------------------------------------------

                          88010     88020     88030     88040     88050     88060     88070     88080

Human             AGGCTTTCACCGTGTTAGCCAGGATGGTCTCAATCTCCTGACCTCGTGATCCGCCCACCTTGGTCTCCCAAAGTGCTGGG 72577
Coquerel'sSifaka  -------------------------------------------------------------------------------- 60059
Ma'sNightMonkey   AG---CTAATTTTTGTACTTTTAGTAGAGATGGGGTTTCACCCTTTTGGCCAAGCTAGTCTTGAACTCCTGACCTTGTGA 67138
VervetAGM         -------------------------------------------------------------------------------- 74018
Gorrilla          -------------------------------------------------------------------------------- 72428
GrabEatingMacaque-------------------------------------------------------------------------------- 73564
Macaque           -------------------------------------------------------------------------------- 74423
PigTailedMacaque  -------------------------------------------------------------------------------- 72951
Bonobo            GGGCTTTCACCGTGTTAGCCAGGATGGTCTCAATCTCCTGACCTCGTGATCTGCCCACCTTGGTCTCCCAAAGTGCTGGG 71681
OliveBaboon       -------------------------------------------------------------------------------- 72829
Orangutan         GGACTTTCACCGTGTTAGCCAGGATGGTCTCGATCTCCTGACCTCATGATCTGCCCACCTTGGTCTCCCAAAGTGCTGG- 73486
```

```
Majority          ------------------------------TATTTAAAATTT----TTTAACACTGGATAAAATTTTTACTTTTGAGTCA

                         88090      88100      88110      88120      88130      88140      88150      88160

Human             ATTACAGTCATGAGCCACCGTGCCTGGCCTCATTTAAAATTT----TTTAACACTGGGTAAAATTTTTATTTTTGAGTCA 72653
Coquerel'sSifaka  -----------------------------------------------------------------TTCA-TTT------A 60067
Ma'sNightMonkey   TCCATCCTCCACGGCCTCCCAAAGTGCTGGCATTATAGGTGTGAGCCACCATGCCTGGCA-AATTTTTACTTTTGAGTCA 67217
VervetAGM         ------------------------------TATTTAAAATTT----TTTAACACTGGATAAAATTTTTACTTTTGAGTCA 74064
Gorrilla          -------------------------------------------------------------------------------- 72428
GrabEatingMacaque------------------------------TATTTAAAATTT----TTTAACACTGGATAAAATTTTTACTTTTGAGTCA 73610
Macaque           ------------------------------TATTTAAAATTT----TTTAACACTGGATAAAATTTTTACTTTCGAGTCA 74469
PigTailedMacaque  ------------------------------TATTTAAAATTT----TTTAACACTGGATAAAATTTTTACTTTCGAGTCA 72997
Bonobo            ATTACAGTCATGAGCCACCGTGCCTGGCCTCATTTAAAATTT----TTTAACACTGGGTAAAATTTTTATTTTTGAGTCA 71757
OliveBaboon       ------------------------------TATTTAAAATTT----TTTAACACTGGATAAAATTTTTACTTTTGGGTCA 72875
Orangutan         --------CGTGAGCCACCGTGCCTGGCCTCATTTAAAATTT----TTTAACACTGGATACAATTTTTATTTTTGAGTCA 73554

Majority          CAAATTAATTTGAAGCTGTGTCTTTTAGAGAGCTTATTCAGCTCTTAATTCCATTATAAGTAATATTTGAATGAATGTGT

                         88170      88180      88190      88200      88210      88220      88230      88240

Human             CAAATTAATTTGAAGCTGTGTCTTTTAGAGAGCTTATTCAACTCTTAATTCCATTATAAATAATATTTGAATGAATGTGT 72733
Coquerel'sSifaka  CAAATTAATTTGAAAGTATATCCTTTAGAGAGCTTATTCAACT-TTGATTCCATTTTATATAATATTTGAATGAATGTGT 60146
Ma'sNightMonkey   CAAATTAATTTGAAGCTGTGTCTTTTGGAGAGCT-ATTCAACTCTTAATTCCATTATAAGTATTATTTAAATGAATGTGT 67296
VervetAGM         CAAATTAATTTGAAGCTGTGTCTTTTAGAGAGCTTATTCAGCTCTTAATTCCATTATAAGTAATATTTGAATGAATGTGT 74144
Gorrilla          -------------------------------------------------------------------------------- 72428
GrabEatingMacaqueCAAATTAATTTGAAGCTGTGTCTTTTAGAGAGCTTATTCAGCTCTTAATTTCATTATAAGTAATATTTGAATGAATGTGT 73690
Macaque           CAAATTAATTTGAAGCTGTGTCTTTTAGAGAGCTTATTCAGCTCTTAATTTCATTATAAGTAATATTTGAATGAATGTGT 74549
PigTailedMacaque  CAAATTAATTTGAAGCTGTGTCTTTTAGAGAGCTTATTCAGCTCTTAATTCCATTATAAGTAATATTTGAATGAATGTGT 73077
Bonobo            CAAATTAATTTGAAGCTGTGTCTTTTAGAGAGCTTATTCAACTCGTAATTCCATTATAAATAATATTTGAATGAATGTGT 71837
OliveBaboon       CAAATTAATTTGAAGCTGTGTCTTTTAGAGAGCTTATTCAGCTCTTAATTCCATTATAAGTAATATTTGAATGAATGTGT 72955
Orangutan         CAAATTAATTTGAAGCTGTGTCTTTTAGAGAGCTTATTCAACTCTTAATTCCATTATAAGTAATATTTGAATGAATGTGT 73634

Majority          TTTATATCTGGAATGATTTTTATCATCTGAGTAA-AGGAAATATGTTTGATGGTAGTTGG-GTACTTTGAAGGTATTACG

                         88250      88260      88270      88280      88290      88300      88310      88320

Human             TTTACATCTGGAATGATTTTTATCATCTGAGTAA-AGGAAATATGTTTGATGGTAGTTGG-GAACTTTGAAGGTATTACG 72811
Coquerel'sSifaka  TTTAAATCTGGAATGATTTTTATCTTCTGAGCAATGGGAAATATTTTTGGTGGTAATTGA-GTACTTTCAAG-TATTATG 60224
Ma'sNightMonkey   TTTACATCTGGAATAATTTTTATCATCTCAGTAA-AGGAAATACGTTTGATGGTAGTTGA-GTACTTTGAAGGTATTATG 67374
VervetAGM         TTTATATCTGGAATGATTTTTATCATCTGAGTAA-AGGAAATATGTTTGATGATAGTTGG-GTACTTGGAAGGTATTACG 74222
Gorrilla          -------------------------------------------------------------------------------- 72428
GrabEatingMacaqueTTTATATCTGGAGTGATTTTTATCATCTGAGTAA-GGGAAATATGTTTGATGGTAGTTGG-GTACTTGGAAGGTATTACG 73768
Macaque           TTTATATCTGGAGTGATTTTTATCATCTGAGTAA-AGGAAATATGTTTGATGGTAGTTGG-GTACTTGGAAGGTATTACG 74627
PigTailedMacaque  TTTATATCTGGAGTGATTTTTATCATCTGAGTAA-AGGAAATATGTTTGATGGTAGTTGG-GTACTTGGAAGGTATTACG 73155
Bonobo            TTTACATCTGGAATGATTTTTATCATCTGAGTAA-AGGAAATATGTTTGATGGTAGTTGG-GAACTTTGAAGGTATTACG 71915
OliveBaboon       TTTATATCTGGAATGATTTTTATCATCTGAGTAA-AGGAAATATGTTTGATGGTAGTTTGCGTACTTGGAAGGTATTACG 73034
Orangutan         TTTACATCTGGAATGATTTTTATCATCTGAGTAA-AGGAAATATGTTTGATGGTAGTTGG-GTACTTTGAAGGTATTACG 73712
```

```
Majority         GGTTACAGATG--------------AGAAAAAAATGTTACTACATCATTTCATGTTTACATACTGACCAGCATTTTCAGT

                         88330     88340     88350     88360     88370     88380     88390     88400

Human            GGCTACAGATG--------------AGAAAAAAATGTTACTACGTCATTTCATGTTTACATACTGACCAGCATTTTCAAT 72877
Coquerel'sSifaka GGTTACTGATGTGATGTAAACATTAGGAAAAACATGTTATTACATCAGTTCATAGTTACTTATTGATTGACATTCTCAGT 60304
Ma'sNightMonkey  GGCTACAGATG--------------AGAAAGAAATGTTACTACATCAGTTCCTATTTACATACTGACCAGCATTTTCGAT 67440
VervetAGM        GGTTACAGATG--------------AGAAAAAAATGTTACTACATCATTTCATGTTTACATACTGACCAGCATTTTCAGT 74288
Gorrilla         -------------------------------------------------------------------------------- 72428
GrabEatingMacaqueGGTTACAGATG--------------AGAAAAAAATGTTACTACATCATTTCATGTTTACATACTGACCAGCATTTTCAGT 73834
Macaque          GGTTACAGATG--------------AGAAAAAAATGTTACTACATCATTTCATGTTTACATACTGACCAGCATTTTCAGT 74693
PigTailedMacaque GGTTACAGATG--------------AGAAAAAAATGTTACTACATCATTTCATGTTTACATACTGACCAGCATTTTCAGT 73221
Bonobo           GGCTACAGATG--------------AGAAAAAAATGTTACTACGTCATTTCGTGTTTACATACTGACCAGCATTTTCAAT 71981
OliveBaboon      GGTTACAGATG--------------AGAAAAAAATGTTACTACATCATTTCATGTTTACATACTGACTAGCATTTTCAGT 73100
Orangutan        GGCTACAGATG--------------AGAAAAAAATGTTACTACGTCATTTCATGTTTACATACTGACCAGCATTTTCAAT 73778

Majority         GAAATACGCTTATTACTATAGGACTATAAAATGTTTTTCAAAGG------TTTAATTATCCAGGGCAGATAGTTAAGACT

                         88410     88420     88430     88440     88450     88460     88470     88480

Human            GAAATAAGCTTATTACTATAGGACTATAAAATGTTTTTCAAAGG------TTTAACTGTCCAGGGCAGATACTTAAGACT 72951
Coquerel'sSifaka GGAATA-GCTTATT-TTATAGGACTA--AAGTGTTACGCACAAGCCTTCTTTTAACTCTCCAAGGCAGAATTTTTATTTA 60380
Ma'sNightMonkey  GAAATACGCTTATTACTATAGGACTATAAAATGTTTTTCATAGG------TTTAACTATCCAGGGCAGATAGTTAAGACT 67514
VervetAGM        GAAATACGCTTATTACAATAGGACTATAAAATGTTTTTCAAAGG------TTTAATTATCCAGGGCAGATAGTTAAGACT 74362
Gorrilla         -------------------------------------------------------------------------------- 72428
GrabEatingMacaqueGAAATACGCTTATTACAATAGGACTATAAAATGTTTTTCAAAGG------TTTAATTATCCAGGGCAGATAGTTAAGACT 73908
Macaque          GAAATACGCTTATTACGATAGGACTATAAAATGTTTTTCAAAGG------TTTAATTATCCAGGGCAGATAGTTAAGACT 74767
PigTailedMacaque GAAATACGCTTATTACGATAGGACTATAAAATGTTTTTCAAAGG------TTTAATTATCCAGGGCAGATAGTTAAGACT 73295
Bonobo           GAAATAAGCTTATTACTATAGGACTATAAAATGTTTTTCAAAGG------TTTAACTGTCCAGGGCAGATACTTAAGACT 72055
OliveBaboon      GAAATACGCTTATTACAATAGGACTATAAAATGTTTTTTAAAGG------TTTAATTATCCAGGGCAGATAGTTAAGACT 73174
Orangutan        GAAATAACCTTATTACTATAGGACTATAAAATGTTTTCCAAAGG------TTTAACTGTCCTGGGCAGATAGTTAAGACT 73852

Majority         ATGTGATCATCCATTAAAAACTTTTCACATAGTCTTGCTTAAATGGATCCATTATGTTTACCCTTTATATTGTTCTCATC

                         88490     88500     88510     88520     88530     88540     88550     88560

Human            ATCTGATCATCCATTAAAAACTTTTCACATAGTCTTGCTTAAATGGATCCATTATGTTTACCCATTATATTGTTCTCAGC 73031
Coquerel'sSifaka ATATATAAAGTTCTGAATGGATGGTCACATAGTCTTGCTTAAACTGATCTATTATGATTATCCATCATACTGTTCTCAGC 60460
Ma'sNightMonkey  ATGTAATTATCTATTAAGAACTTTTCATATACTCTTGCTTAAATGGATCCATTATATTTACCTGTTGTATTGTTCTCAGC 67594
VervetAGM        ATGTGATCATCCATTAAAAACTTTTCACATAGTCTTGCTTAAATGGATCCATTATGTTTACTCTTTATATTGTCCTCATC 74442
Gorrilla         -------------------------------------------------------------------------------- 72428
GrabEatingMacaqueATGTGATCATCCATTAAAAACTTTTCACATAGTCTTGCTTAAATGGATCCATTATGTTTACCCTTTATATTGTCCTCATC 73988
Macaque          ATGTGATCATCCATTAAAAACTTTTCACATAGTCTTGCTTAAATGGATCCATTATGTTTACCCTTTATATTGTCCTCATC 74847
PigTailedMacaque ATGTGATCATCCATTAAAAACTTTTCACATAGTCTTGCTTAAATGGATCCATTATGTTTACTCTTTATATTGTCCTCATC 73375
Bonobo           ATCTGATCATCCATTAAAAACTTTTCACATAGTCTTGCTTAAATGGATCCATTATGTTTACCCATTATATTGTTCTCAGC 72135
OliveBaboon      ATGTGATCATCCATTAAAAACTTTTCACATAGTCTTGCTTAAATGGATCCATTATGTTTACCCTTTATATTGTCCTCATC 73254
Orangutan        ATGTGATCATCCATTAAAAACTTTTCACATAGTCTTGCTTAAATGGATCCATTATGTTTACCCATTATATTGTTCTCAGC 73932
```

```
Majority          TGTAGTTTTAAGAAATTTATTTCATTTGTTAATACTTACTTTCCATTACCTTCCCCTTTTCTATGACAATCTGTT-GATA
                          88570     88580     88590     88600     88610     88620     88630     88640
Human             TGTAGTTTTAAGAAATTTATTTCATTTGTTAATACTTACTTTCCATTACCTTCCCCTTTTCTGTGACAATCCGTT-GATA 73110
Coquerel'sSifaka  TGTAGTTTTAAGAAATTTCTTTCGTT-ATTAATCCTTTATTTACCTTCCGTTACCTTCCTCT-----------T------ 60522
Ma'sNightMonkey   TGTAGTTTTAAGAAATGTATTTAATTTATTAATACTTACCTTCCATTGCCTTCTCCATTTCTATGACAACCTGTCAGATA 67674
VervetAGM         TGTAGTTTTAAGAAATTTACTTCATTTGTTAATACTTACTTTCCATTACCTTCCCCTTTTCTATGACAATCTGTT-GATA 74521
Gorrilla          -------------------------------------------------------------------------------- 72428
GrabEatingMacaqueTGTAGTTTTAAGAAATTTACTTCGTTTGTTAATACTTACTTTCCGTTACCTTCCCCTTTTCTATGACAATCTGTT-GATA 74067
Macaque           TGTAGTTTTAAGAAATTTACTTCGTTTGTTAATACTTACTTTCCGTTACCTTCCCCTTTTCTATGACAATCTGTT-GATA 74926
PigTailedMacaque  TGTAGTTTTAAGAAATTTACTTCATTTGTTAATACTTACTTTCCGTTACCTTCCCCTTTTCTATGACAATCTGTT-GATA 73454
Bonobo            TGTAGTTTTAAGAAATTTATTTCATTTGTTAATACTTACTTTCCATTACCTTCCCCTTTTCTGTGACAATCTGTT-GATA 72214
OliveBaboon       TGTAGTTTTAAGAAATTTACTTCATTTGTTAATACTTACTTTCCGTTACCTTCCCCTTTTCTATGACAATCTGTT-GATA 73333
Orangutan         TGTAGTTTTAAGGAATTTATTTCATTTATTAATACTTACTTTCCATTACCTTCCCCTTTTCTATGACAGTCTGTT-GATG 74011

Majority          CTTGAAGACCTCTCTTGTATTCATCTTAGAGTTAATATTTTTAAGGCCAAACAGCCTAAGTTCTATGGTAATCAACTCCA
                          88650     88660     88670     88680     88690     88700     88710     88720
Human             CTTGAAGACCTCTCTTGTATTCATCTTAGGGTTAATATTTTTAAGGCCAAACAGCCTAAATTCTATGGTAATCAACTCCA 73190
Coquerel'sSifaka  -TTAAAGGC--C------ATACAGCTGGGA-----T---TCTA-----------------TCTGTGGTGATCAACTCCA 60567
Ma'sNightMonkey   CTTAAAGACCTCTCTCATATTCATCTTAGAGTTAGTATTTTTAAGGCCAAACAGCCTGAATTCTATGGTAATCAACCCCA 67754
VervetAGM         CTTGAAGACCTCTCTTGTATTCATCTTAGAGTTAATATTTTTAAGGCCAAACAGCCTAAGTTCTATGGTAATCAACTCCA 74601
Gorrilla          -------------------------------------------------------------------------------- 72428
GrabEatingMacaqueCTTGAAGACCTCTCTTGTATTCATCTTAGAGTTAATATTTTTAAGGCCAAACAGCCTAAGTTCTATGGTAATCAACTCCA 74147
Macaque           CTTGAAGACCTCTCTTGTATTCATCTTAGAGTTAATATTTTTAAGGCCAAACAGCCTAAGTTCTATGGTAATCAACTCCA 75006
PigTailedMacaque  CTTGAAGACCTCTCTTGTATTCATCTTAGAGTTAATATTTTTAAGGCCAAACAGCCTAAGTTCTATGGTAATCAACTCCA 73534
Bonobo            CTTGAAGACCTCTCTTGTATTCATCTTAGGGTTAATATTTTTAAGGCCAAACAGCCTAAATTCTATGGTAATCAACTCCA 72294
OliveBaboon       CTTGAAGACTTCTCTTGTATTCATCTTAGAGTTAATATTTTTAAGGCCAAACAGCCTAAGTTCTGTGGTAATCAACTCCA 73413
Orangutan         CTTGAAGACCTCTCTTGTATTCATCTTAGAGTTAATATTTTTAAGGCCAAACAGCCTAAATTCTATGGTAATCAACTCCA 74091

Majority          GCCTT-GCATAATTAAAATCTTCTGCATAAAGATAGGTTTAAATCAAATCAGATTGCAGATTTTATT--GAAGAAATT--
                          88730     88740     88750     88760     88770     88780     88790     88800
Human             GCCTT-GTGTAATGAAA-TCTTCTGCATAAAGATAGGTTTAAATCAAATCAGATTGCAGATTTTATT--GAAGAAATT-- 73264
Coquerel'sSifaka  GCCTTTGGATAATGAAGATTTTCTGTATAAAGATAGATTTAAATCAAATCAAGCCATAGGATTTATTTTGAAAAAATTTT 60647
Ma'sNightMonkey   GCCTTTGCATAATGAAAATCTTCGACATAAAGATAGGTTTAAATCAAATCACACTGTAGATTTTATT--GAAGAAATT-- 67830
VervetAGM         GCCTT-GCATAATTAAAATTTTCTGCATAAAGATAAGTTTAAATCAAATCAGATTGCAGATTTTATT--AAAGAAAAT-- 74676
Gorrilla          -------------------------------------------------------------------------------- 72428
GrabEatingMacaqueACTTT-GCATAATTAAAATCTTCTGCATAAAGATAAGTTTAAGTCAAATCAGATTGCAGATTTTATT--AAAGAAATT-- 74222
Macaque           ACTTT-GCATAATTAAAATCTTCTGCATAAAGATAAGTTTAAGTCAAATCAGATTGCAGATTTTATT--AAAGAAATT-- 75081
PigTailedMacaque  ACTTT-GCATAATTAAAATCTTCTGCATAAAGATAAGTTTAAGTCAAATCAGATTGCAGATTTTATT--AAAGAAATT-- 73609
Bonobo            GCCTT-GCGTAATGAAA-TCTTCTGCATAAAGATAGGTTTAAATCAAATCAGATTGCAGATTTTATT--GAAGAAATT-- 72368
OliveBaboon       ACTTT-GCATAATTAAAATCTTCTGCATAAAGATAAGTTTAAATCAAATCAGATTGCAGATTTTATT--AAAGAAATT-- 73488
Orangutan         GCCTT-GCATAATGAAAATCTTCTGCATAAAGATAGGTTTCAGTCAAATCAGATTGCAGATTTTATT--GAAGAAATT-- 74166
```

```
Majority          GTGTTTTTAAGAGTTGACAA--ATATATATTGTATGGCTAAAACAAAGAAAA----------------TACTTCTTTTGC

                          88810     88820     88830     88840     88850     88860     88870     88880

Human             GTGTTTTTAAGAGTTGACAA--ATATATGTTGTATGGCTAAAACAAAGAAAA----------------TACTTCTGTTGC 73326
Coquerel'sSifaka  GTGTTTTTAAGAGTTGATGGTTATATATATGGTATGGCCAAAACAAAGAAAAGCCTTCTAAAGAAGTGTATTACTGCTGC 60727
Ma'sNightMonkey   GTGTTTTTAAGAGTCGACAA--T------------------AGCAAAGAAAA----------------TACTTCTGCTGC 67874
VervetAGM         GTGTTTTTAAGAGTTGACAA--ATATATATCGTATGGCTAAAACAAAGAAAA----------------TACTTCTTTTGC 74738
Gorrilla          -------------------------------------------------------------------------------- 72428
GrabEatingMacaque GTGTTTTTAAGAGTTGACAA--ATATATATTGTATGGCTAAAACAAAGAAAA----------------TACTTCTTTTGC 74284
Macaque           GTGTTTTTAAGAGTTGACAA--ATATATATTGTATGGCTAAAACAAAGAAAA----------------TACTTCTTTTGC 75143
PigTailedMacaque  GTGTTTTTAAGAGTTGACAA--ATATATATTGTATGGCTAAAACAAAGAAAA----------------TACTTCTTTTGC 73671
Bonobo            GTGTTTTTAAGAGTTGACAA--ATATATATTGTATGGCTAAAACAAAGAAAA----------------TACTTCTGTTGC 72430
OliveBaboon       GTGTTTTTAAGAGTTGACAA--ATATATATTGTATGGCTAAAACA----AAA----------------TACTTCTTTTGC 73546
Orangutan         GTGTTTTTAAGAGTTGACAA--ATATGTATTGTATGGCTAAAACAAAGAAAA----------------TACTTCTGTTGC 74228

Majority          TTCTGCATTT-AGTAGAAGAAAAACTATATATGTTTTTGACCAAAGTATAAAATATGATTCTTTCCAGGGAGGTAAA--T

                          88890     88900     88910     88920     88930     88940     88950     88960

Human             TTCTGCATTT-AGTAGAAGAAAAACTATATATGTTTGTGACCAAAGTATAAAATATGATTCTTTCCAGGGAGGTAAAGGT 73405
Coquerel'sSifaka  TCCTGCAATT-AGTGGAAGAA-AACCGTATATGTTTGTGACCATAGTATAAAATGTGCCTCTTTCCAAGGAGGTAAAAAT 60805
Ma'sNightMonkey   TTCTGCATTT-AGTAGAGAAACAACTATATATGTTTGCGACCACAGTATAAAATGTGCTTCTTTCCAGGGAGGTAAAGGT 67953
VervetAGM         TTCTGCATTTTAGTAGAAGAAAAACTATATATGTTTTTGACCAAAGTATAAAAGATGATTCTTTCCAGGGAGGTAAA--T 74816
Gorrilla          -------------------------------------------------------------------------------- 72428
GrabEatingMacaque TTCTGCATTT-AGTAGAAGAAAAACTATATATGTTTTTGACCAAAGTATAAAAGATGATTCTTTCCAGGGAGGTAAA--T 74361
Macaque           TTCTGCATTT-AGTAGAAGAAAAACTATATATGTTTTTGACCAAAGTATAAAAGATGATTCTTTCCAGGGAGGTAAA--T 75220
PigTailedMacaque  TTCTGCATTT-AGTAGAAGAAAAACTATATATGTTTTTGACCAAAGTATAAAAGATGATTCTTTCCAGGGAGGTAAA--T 73748
Bonobo            TTCTGCATTT-AGTAGAAGAAAAACTATATATGTTTGTGACCAAAGTATAAAATATGATTCTTTCCAGGGAGGTAAAGGT 72509
OliveBaboon       TTCTGCATTT-AGTAGAAGAAAAACTATATATGTTTTTGACCAAAGTATAAAAGATGATTCTTTCCAGGGAGGTAAA--T 73623
Orangutan         TTCTGCATTT-AGTAGAAGAAAAACTATATATGTTTGTAACCAAAGTATAAAATATGATTCTTTCCAGGGGGGTAAAGGT 74307

Majority          TATGCAGAAGATTTTCACTAGCAGCTCTAAACGGC-------------------------------TATC----------

                          88970     88980     88990     89000     89010     89020     89030     89040

Human             TATGCAGAAGATTTTCACTAGCAGCTCTAAAAGGC-------------------------------TACC---------- 73444
Coquerel'sSifaka  CATGCAAA----TTTTGTCAGCAACTCTAAAAAGCATTAGATACATATAGAGCATTTCATTTAGATTCTTTCTTGATCTG 60881
Ma'sNightMonkey   TATGCACAAGATTTTCACTAGCAACTCTAAAAGACATTAGTTACATATATGAAATTTCATTTAGATTATCTCTTGACCTT 68033
VervetAGM         TATGCAGAAGATTTTCACTAGCAGCTCTAAACGGC-------------------------------TATC---------- 74855
Gorrilla          -------------------------------------------------------------------------------- 72428
GrabEatingMacaque TATGCAGAAGATTTTCACTAGCAGCTCTAAACGGC-------------------------------TATC---------- 74400
Macaque           TATGCAGAAGATTTTCACTAGCAGCTCTAAACGGC-------------------------------TATC---------- 75259
PigTailedMacaque  TATGTAGAAGATTTTCACTAGCAGCTCTAAACGGC-------------------------------TATC---------- 73787
Bonobo            TATGCAGAAGATTTTCACTAGCAGCTCTAAAAGGC-------------------------------TACC---------- 72548
OliveBaboon       TATGCAGAAGATTTTCACTAGCAGCTCTAAACGGC-------------------------------TATC---------- 73662
Orangutan         TATGCAGAAGATTTTCACTAGCAGCTCTAAAAGGC-------------------------------TACC---------- 74346
```

```
Majority          --------------------------------------------------------------------------------
                          89050     89060     89070     89080     89090     89100     89110     89120
Human             -------------------------------------------------------------------------------- 73444
Coquerel'sSifaka  TATTTTTCCTGAGTTATTTCTAACTTTATATTATTTTCCTGTTGATTTAACAGTGAAATCCTGTCTCTGAATCTA----- 60956
Ma'sNightMonkey   TTTTCCCCGAGTTTTTTTCTCATCATAATTCTGTCCCTTTCAGGTTTTTTTCTGTTGATCTTGATTTTAATTTAAAATTG 68113
VervetAGM         -------------------------------------------------------------------------------- 74855
Gorrilla          -------------------------------------------------------------------------------- 72428
GrabEatingMacaque -------------------------------------------------------------------------------- 74400
Macaque           -------------------------------------------------------------------------------- 75259
PigTailedMacaque  -------------------------------------------------------------------------------- 73787
Bonobo            -------------------------------------------------------------------------------- 72548
OliveBaboon       -------------------------------------------------------------------------------- 73662
Orangutan         -------------------------------------------------------------------------------- 74346

Majority          -----------------------------------------------------------------CTCAATTAATTGCCA
                          89130     89140     89150     89160     89170     89180     89190     89200
Human             -----------------------------------------------------------------CTCAATTAATTGCCA 73459
Coquerel'sSifaka  -----------------------CC----CTTATTTGCCTCCGTTTTCTAGATTTTTCTGCTATCCTTAAGTAATTGCCA 61009
Ma'sNightMonkey   GTACCCTTTTTCTCTGTCAGAATCCACCTTTCTATTGGCTCCAATTTCTAGGTTTTCTTCTTAGTCTCAATTAATTACCA 68193
VervetAGM         -----------------------------------------------------------------CTCAATTAATTGCCA 74870
Gorrilla          -------------------------------------------------------------------------------- 72428
GrabEatingMacaque -----------------------------------------------------------------CTCAATTAATTGCCA 74415
Macaque           -----------------------------------------------------------------CTCAATTAATTGCCA 75274
PigTailedMacaque  -----------------------------------------------------------------CTCAATTAATTGCCA 73802
Bonobo            -----------------------------------------------------------------GTCAAATAATTGCCA 72563
OliveBaboon       -----------------------------------------------------------------CTCAATTAATTGCCA 73677
Orangutan         -----------------------------------------------------------------CTCAATTAATTGCCG 74361

Majority          TGAACATTTCATAGCCCTAGAAGGATGTTGGCTCACTTCAGTGTCATCCTGGTTTATTCATTTATTGTATTATTCAGCAG
                          89210     89220     89230     89240     89250     89260     89270     89280
Human             TGAACATTTCATAGCCCTAGAAGGATGTTGGCTCATTTCAGTGTCATCCTGGTTTATTC-TTTATTGTATTATTCAGCAG 73538
Coquerel'sSifaka  TGGGCATTTCATATTCCTAGAAGGATACTGCCTCACTTCAGCGTTATCCTGGTTTATTAATTTATTGCATTATTCAGCAG 61089
Ma'sNightMonkey   TGAACATTTCATATCCCTAGAAGGATGTTGGCTCACTTCAGTGTCATC-TGGTTTATTCATTTGTTGTATTATTCAGGAG 68272
VervetAGM         TGAACATTTCATAGCCCTAGAAGGATGTTGGCTCACTTCAGTGTCATCCTGGTTTATTCGTTCATTGTATTATTCAGCAG 74950
Gorrilla          -------------------------------------------------------------------------------- 72428
GrabEatingMacaque TGAACATTTCATAGCCCTAGAAGGATGTTGGCTCACTTCAGTGTCATCCTGGTTTATTCATTCATTGTATTATTCAGCAG 74495
Macaque           TGAACATTTCATAGCCCTAGAAGGATGTTGGCTCACTTCAGTGTCATCCTGGTTTATTCATTCATTGTATTATTCAGCAG 75354
PigTailedMacaque  TGAACATTTCATAGCCCTAGAAGGATGTTAGCTCACTTCAGTGTCATCCTGGTTTATTCATTCATTGTATTATTCAGCAG 73882
Bonobo            TGAACATTTCATAGCCCTAGAAGGATGTTGGCTCATTTCAGTGTCATCCTGGTTTATTC-TTTATTGTATTATTCAGCAG 72642
OliveBaboon       TGAACATTTCATAGCCCTAGAAGGATGTTGGCTCACTTCAGTGTCATCCTGGTTTATTCATTCATTGTATTATTCAGCAG 73757
Orangutan         TGAACATTTCATAGCCCTAGAAGGATGTTGGCTCATTTCAGTGTCATCCTGGTTTATTCATTTATTGTATTATTCAGCAG 74441
```

```
Majority          TCATTTTAACACTGTGCTACACACTTCAGGGACTCAGAAGAATAACAGAGTTTCTGTTTTCATGAAGCTTCTCATC----

                         89290     89300     89310     89320     89330     89340     89350     89360

Human             TCATTTTAACACTATGCTAGACACTTTAGAGATTCAGAAGAGTAACAGGGTTTCTGTTCTCATGAAGCTT---ATC---- 73611
Coquerel'sSifaka  T--------------------CACTGCAGGGACCCGGAAGAATAACAAAGTTTCTGTCTTCATAAAGCTTACAATCTTGT 61149
Ma'sNightMonkey   TTGTTTTAACATTATGCTAGACACTGCAGGGACTCAGAAGAATAACGAGGTTTCTTTTCTCATAAAGCTTATAATC---- 68348
VervetAGM         TCATTTTAACACTGTGCTACACACTTCAGGGACTCAGAAGAATAACAGAGTTTCTGTTTTCATGAAGCTTCTCATC---- 75026
Gorrilla          -------------------------------------------------------------------------------- 72428
GrabEatingMacaqueTCATTTTAACACTGTGCTACACACTTCAGGGACTCAGGAGAATAACAGAGTTTCTGTTTTCGTGAAGCTTCTCATC---- 74571
Macaque           TCATTTTAACACTGTGCTACACACTTCAGGGACTCAGGAGAATAACAGAGTTTCTGTTTTCGTGAAGCTTCTCATC---- 75430
PigTailedMacaque TCATTTTAACACTGTGCTACACACTTCAGGGACTCAGGAGAATAACAGAGTTTCTGTTTTCGTGAAGCTTCTCATC---- 73958
Bonobo            TCATTTTAACACTATGCTAGACACTTTAGGGATTCAGAAGAGTAACAGGGTTTCTGTTCTCATGAAGCTT---ATC---- 72715
OliveBaboon       TCATTTTAACACTGTGCTACACACTTCAGGGACTCAGAAGAATAACAGAGTTTCTGTTTTCGTGAAGCTTCTCATC---- 73833
Orangutan         TCATTTTAACACTATGCTAGACACTGCAGGGATTCAGAAGAGTAACAGGGTTTCTGTTCTCATGAAGCTT---ATC---- 74514
```

```
Majority          AGAGACAGCAAACATATGAGTTAGATCTAATTGGAGGCAAACTGAAATATATAGTGGAGTTAGTGTGGTTATCAGCACAT

                         89370     89380     89390     89400     89410     89420     89430     89440

Human             AGAGACAGAAAACATATGAGTTAGATCTAATTGGAGGCAAACTGAAATATATAGTGGAGTTAGTGTGGTTATCAGCACAT 73691
Coquerel'sSifaka  GGAGGCAGCAAACACAAG---TAGCTCTGATTGGATGCAAATTGAAATATTTAATAGAGTCAGTGTGGT-ATTAGTACAT 61225
Ma'sNightMonkey   GGAGACAGCAAACAT--GAGTTAGATATAATTGGAGACAAAGTGAAATATATAGTGGAGTTAGTGTGGTTATTAGCACAT 68426
VervetAGM         AGAGACAGTAAACATATGAGTTAGATCTAATTGGAGGCAAACTGAAATATATAGTGGAGTTAGTGTGGTTATCAGCACAT 75106
Gorrilla          -------------------------------------------------------------------------------- 72428
GrabEatingMacaqueAGAGACAGCAAACATATGAGTTAGATCTAATTGGAGGCAAACTGAAATATATAGTGGAGTTAGTGTGGTTATCAGCACAT 74651
Macaque           AGAGACAGCAAACATATGAGTTAGATCTAATTGGAGGCAAACTGAAATATATAGTGGAGTTAGTGTGGTTATCAGCACAT 75510
PigTailedMacaque AGAGACAGCAAACATATGAGTTAGATCTAATTGGAGGCAAACTGAAATATATAGTGGAGTTAGTGTGGTTATCAGCACAT 74038
Bonobo            AGAGACAGAAAACATACGAGTTAGATCTAATTGGAGGCAAACTGAAATATATAGTGGAGTTAGTGTGGTTATCAGCACAC 72795
OliveBaboon       AGAGACAGCAAACATATGAGTTAGATCTAATTGGAGGCAAACTGAAATACATAGTGGAGTTAGTGTGGTTATCAGCACAT 73913
Orangutan         AGAGACAGCAAACATATGAGTTAGATCTAATTGGAGGCAAACTGAAATATATAGTGGAGTTAGTGTGGTTATCAGCACAT 74594
```

```
Majority          AA----ATGAGTGATCCATCAACAAAAGGAGAAATTGGGAGGGTTTTATGGGCCAAAAAGATAGCATGATTAAATGTGAT

                         89450     89460     89470     89480     89490     89500     89510     89520

Human             AA----ATGAGTGATCCATCAACAAAAGGAGAAATTGGGAGGGTTTTATGGGCCAAAA--ACAGCATGATTAAATGTGAT 73765
Coquerel'sSifaka  AATAGAATGAGTAATTCATCAAGAAAAGGAGAAATTGGGAGGGTTTTATGGACCAAAAATATAGCATGAGCAAATGTAAT 61305
Ma'sNightMonkey   AATGAAATGAGTGATCCATTAACAAAAGGAGAAATTGGGAGTGCTTTATGGGCCAAAAACATAGCATGATTAAATGTGAT 68506
VervetAGM         AA----ATGAGTGATCCATCAACAAAAGGAGAAATTGGGAGGGTTTTATGGGCCAAAAAGATAGCATGATTAAATGTGAT 75182
Gorrilla          -------------------------------------------------------------------------------- 72428
GrabEatingMacaqueAA----ATGAGTGATCCATCAACAAAAGGAGAAATTGGGAGGGTTTTATGGGCCAAAAAGATAGCGTGATTAAATGTGAT 74727
Macaque           AA----ATGAGTGATCCATCAACAAAAGGAGAAATTGGGAGGGTTTTATGGGCCAAAAAGATAGCATGATTAAATGTGAT 75586
PigTailedMacaque AA----ATGAGTGATCCATCAACAAAAGGAGAAATTGGGAGGGTTTTATGGGCCAAAAAGATAGCATGATTAAATGTGAT 74114
Bonobo            AA----ATGAGTGATCCATCAACAAAAGGAGAAATTGGGAGGGTTTTATGGGCCAAAA--ACAGCATGATTAAATGTGAT 72869
OliveBaboon       AA----ATGAGTGATCCATCAACAAAAGGAGAAATTGGGAGGGTTTTATGGGCCAAAAAGATAGCATGATTAAATGTGAT 73989
Orangutan         AA----ATGAGTGATCCATCAACAAAAGGAGAAATTGGGAGGGTTTTATGGGCCAAAA--ACAGC--------------- 74653
```

```
Majority          AGAGTATATGTCATGTTTTAGGAGTGATAAACATTCAGTTATGTGTGACGAATAGGATAATTGAAAAAATATGAAAGGTA
                          89530     89540     89550     89560     89570     89580     89590     89600
Human             AGAGTATATGTCATGTTTTAGGTGTGATGAACATTCAGTTATGTGTGACGAATAGGATAATTGAAAAAATATGAAAGGCT 73845
Coquerel'sSifaka  AAAGTATATACCATGTTTTAGAAGTGACAGATTTTCACTTACAAGTGACATACAGGGTAATTGGGAAAATACGAAATGTA 61385
Ma'sNightMonkey   AGAGTATATGTCATATTTTAGGAGTGACAAACATTCAGTTATGTGTGACGAACAGGATAATGGAAAAAA-ATGAAAGGTA 68585
VervetAGM         AGAGTATATGTCATGTTTTAGGAGTGATAAACATTCAGTTATGTGTGACGAATAGGATAATTGTAAAAATATGAAAGGTA 75262
Gorilla           -------------------------------------------------------------------------------- 72428
GrabEatingMacaque AGAGTATATGTCATGTTTTAGGAGTGATAAACATTCAGTTATGTGTGACGAATAGGATAATTGAAAAAATATGAAAGGTA 74807
Macaque           AGAGTATATGTCATGTTTTAGGAGTGATAAACATTCAGTTATGTGTGACGAATAGGATAATTGAAAAAATATGAAAGGTA 75666
PigTailedMacaque  AGAGTATATGTCATGTTTTAGGAGTGATAAACATTCAGTTATGTGTGACGAATAGGATAATTGAAAAAATATGAAAGGTA 74194
Bonobo            AGAGTATATGTCATGTTTTAGGAGTGATGAACATTCAGTTATGTGTGACGAATAGGATAATTGAAAAAATATGAAAGGCT 72949
OliveBaboon       AGAGTATATGTCATGTTTTAGGAGTGATAAACATTCAGTTATGTGTGACGAATAGGATAATTGAAAAAATATGAAAGGTA 74069
Orangutan         ---GTATACGTC-TGTTTTAGGAGTGATGAACATTCAGTTATGTGTGACGAATAGGATAATTGAAAAAATATGAAAGGTA 74729

Majority          ATGATGCCAGAAAG---TATTATGGGACAAGATCTTAAAACCGATGTT-ATCCAGGGAGTATGAATTTTATATGGGAATT
                          89610     89620     89630     89640     89650     89660     89670     89680
Human             ATGATGCCAGAAAG---TATTATGGGACAAGATCTTAAAACCAGTGTT-ACCCAGGGAGTATGAATTTAATATGGGAATT 73921
Coquerel'sSifaka  ATGATGCTAGAAAGAGCTATTAAAAGACAAGATCTTAGAACTGCTATT-CTCCAAGGAGTGTGAATTTTATGCAGGCATT 61464
Ma'sNightMonkey   ATGATGCCAGAAAG---TATTATGGGACAAGATCTTAAAACCGATGTTTATCCAAGGAGTATGAATTTTACATGGGAATT 68662
VervetAGM         ATGATGCCAGAAAG---TATTATGGGACAAGATCTTAAAACTGATGTT-ATCCAGGGAGTATGAATTTTATATGGGAATT 75338
Gorilla           -------------------------------------------GTGTT-ACCCAGGGAATATGAATTTTATATGGGAATT 72464
GrabEatingMacaque ATGATGCCAGAAAG---TATTATGGGACAAGATCTTAAAACCGATGTT-ATCCAGGGAGTATGAATTTTATATGGGAATT 74883
Macaque           ATGATGCCAGAAAG---TATTATGGGACAAGATCTTAAAACCGATGTT-ATCCAGGGAGTATGAATTTTATATGGGAATT 75742
PigTailedMacaque  ATGATGTCAGAAAG---TATTATGGGACAAGATCTTAAAACCGATGTT-ATCCAGGGAGTATGAATTTTATATGGGAATT 74270
Bonobo            ATGATGCCAGAAAG---TATTATGGGACAAGATCTTAAAACCAGTGTT-ACCCAGGGAGTATGAATTTTATATGGGAATT 73025
OliveBaboon       ATGATGCCAGAAAG---TATTATGGGACAAGATCTTAAAACCGATGTT-ATCCAGGGAGTATGAATTTTATATGGGAATT 74145
Orangutan         ATGATGCCAGAAAG---TATTATGGGACAAGATCTTAAAACCAGTGTT-ATCCAGGGAGTATGAATTTTATATGGGAATT 74805

Majority          CTTAAACTCCTTTATGACTGGAAGATGAGCGTCAG-AGTATCTGCGACCATTTGATGATATTATGTACCAAGTTTTAGAT
                          89690     89700     89710     89720     89730     89740     89750     89760
Human             CTTAAACTCCTTTATGACTGGAAGATGAGCATCAG-AGTGTCTGCGACCATTTGATGATATTATGTACCAAGTTTTAGAT 74000
Coquerel'sSifaka  CTTAGACTCCTTTAGGATCA-AACATGAACTTAAA-AGTATCGGTAACCATCTCAT---ATTGTGGACCACATTTTAGTT 61539
Ma'sNightMonkey   ATTGGACTCCTTTATGGCTAGAAGATGAGTGTCAGGAGTATCTGTGACCATTTGATGATGTTATGAACCAAGTTTTAGAT 68742
VervetAGM         CTTAAACTCCTTTTTGACTGGAAGATGAGCGTCTG-AGTATCTGCGACTATTTGATGATATTATGTACCAAGTTTTAGAT 75417
Gorilla           CTTAAACTCCTTTATGACTGGAAGATGAGCATCAG-AGTGTCTGCGACCATTTGATGATATTTTGTACCAAGTTTTAGAT 72543
GrabEatingMacaque CTTAAACTCCTTTTTGACTGGAAGATGAGCGTCTG-AGTATCTGTGACTATTTGATGATATTATGTACCAAGTTTTAGAT 74962
Macaque           CTTAAACTCCTTTTTGACTGGAAGATGAGCGTCTG-AGTATCTGTGACTATTTGATGATATTATGTACCAAGTTTTAGAT 75821
PigTailedMacaque  CTTAAACTCCTTTTTCACTGGAAGATGAGCGTCTG-AGTATCTGCGACTATTTGATGATATTATGTACCAAGTTTTAGAT 74349
Bonobo            CTTAAACTCCTTTATGACTGGAAGATGAGCATCAG-AGTGTCTGTGACCATTTGATGATATTATATACCAAGTTTTAGAT 73104
OliveBaboon       CTTAAACTCCTTTTTGACTGGAAGATGAGTGTCTG-AGTATCTGCGACTATTTGATGATATTATGTACCAAGTTTTAGAT 74224
Orangutan         CTTAAACTCCATTACAACTGGAAGATGAGTGTCAG-AGTATCTGCGACCATTTGATGATACTATGTACCAAGTTTTAGAT 74884
```

```
Majority          GTTTGGCTTTTTTCAGGTTATCAAAGTGGGGG--ATGAGTTAAGAACCATTGCTGTGAAGGATTCATCAACTATTTTTAG

                          89770     89780     89790     89800     89810     89820     89830     89840

Human             GTTTGGCTTTTTTCAGGTTATGAAAGCGGGGG--ATGAGTTAAGAACCACTGCTGTGAAGGATTCATCAACTATTTTTAG 74078
Coquerel'sSifaka  GTTTGGCTTTCATCAGATCATCCAAGGAG------TGAGTTAAGAACCCTTGCTGTGAAGGATTCATGAGCTGTTTTTAA 61613
Ma'sNightMonkey   GTTTGACTTCTTTCAGATTATCAAAGAGGG----ATGAGTTAAGAACCATTGCTGTGAAGGATTCATCA-CCATTTTTAA 68817
VervetAGM         GTTTGGCTTTTTTCAGGTTATCAAAGTGGGGG--ATGAGTTAAGAACCATTGTTGTGAAGGATTCATCAACTATTTTTAG 75495
Gorrilla          GTTTGGCTTTTTTCAGGTTATCAAAGTGGGGG--ATGAGTTAAGAACCAGTGCTGTGAAGGATTCATCAACTATTTTTAG 72621
GrabEatingMacaqueGTTTGGCTTTTTTCAGGTTATCAAAGTGGGGG--ATGAGTTAAGAACCATTGCTGTGAAGGATTCATCAACTATTTTTAG 75040
Macaque           GTTTGGCTTTTTTCAGGTTATCAAAGTGGGGG--ATGAGTTAAGAACCATTGCTGTGAAGGATTCATCAACTATTTTTAG 75899
PigTailedMacaque  GTTTGGCTTTTTTCAGGTTATCAAAGTGGGGG--ATGAGTTAAGAACCATTGCTGTGAAGGATTCATCAACTATTTTTAG 74427
Bonobo            GTTTGGCTTTTTTCAGGTTATCAAAGCGGGGG--ATGAGTTAAGAACCATTGCTGTGAAGGATTCATCAACTATTTTTAG 73182
OliveBaboon       GTTTGGCTTTTTTCAGGTTATCAAAGTGGGGG--ATGAGTTAAGAACCATTGCTATGAAGGATTCATCAACTATTTTTAG 74302
Orangutan         GTTTGGCTTTTTTCAGGTTATCAAAGTGGGGGGGATGAGTTAAGAACCATTGCTGTGAAGGATTCATCAACTATTTTTAG 74964

Majority          GCAGTTGGGTAAAAATGACCAATTTAGTTTTAAGAAA--CCAACTGTGGCTCCAGAGTATGTTGGAGAAGTGAAAATGGA

                          89850     89860     89870     89880     89890     89900     89910     89920

Human             GCAGTTGGGTAAAAATGACCAATTTAGTTTTAAGAAA--CTGACTGTGGCTCCAGAGTATGTTGGAGAAGTGAAAATGGA 74156
Coquerel'sSifaka  GCAGTTACATAAAAACGACCAGATTAGTTTTAAGAAAAATTAACTGTGGCTCCAGAGTATGCTGGAGAGGTGAAAATGGA 61693
Ma'sNightMonkey   GCAGTTGGGTAAAAATGACCAGTTTAGTTTTAAGAAA--CCAACTGTG---------TATGTTGGAGAAGTGAAAATGGA 68886
VervetAGM         GCAGTTGGGTAAAAATGAGCAATTTAGTTTTAAGAAA--CCAACTGCGGCTCCAGAGTATGTTGGAAAAGTGAAAATAGA 75573
Gorrilla          GCAGTTGGGTAAAAATGACCAATTTAGTTTTAAGAAA--CCGACTGTGGCTCCAGAGTATGTTGGAGAAGTGAAAATGGA 72699
GrabEatingMacaqueGCAGTTGGGTAAAAATGAGCAATTTAGTTTTAAGAAA--CCAACTGCGGCTCCAGAGTATGTTGGAAAAGTGAAAATAGA 75118
Macaque           GCAGTTGGGTAAAAATGAGCAATTTAGTTTTAAGAAA--CCAACTGCGGCTCCAGAGTATGTTGGAAAAGTGAAAATAGA 75977
PigTailedMacaque  GCAGTTGGGTAAAAATGAGCAATTTAGTTTTAAGAAA--CCAACTGCGGCTCCAGAGTATGTTGGAAAAGTGAAAATAGA 74505
Bonobo            GCAGTTGGGTAAAAATGACCAATTTAGTTTTAAGAAA--CTGACTGTGGCTCCAGAGTATGTTGGAGAAGTGAAAATGGA 73260
OliveBaboon       GCAGTTGGGTAAAAATGAGCAATTTAGTTTTAAGAAA--CCAACTGCGGCTCCAGAGTATGTTGGAAAAGTGAAAATAGA 74380
Orangutan         GCAGTTGGGTAAAAATGACCAATTTAGTTTTAAGAAA--CCGACTGTGGTTCCAGAGTATGTTGGAGAAGTGAAAATGGA 75042

Majority          GACTAGGAATAACAGGTAGGAGACTCTTAGTCTAATTAAGATGTAATTATAAATCTAAGCTAGGAACGTAAAATGAGAAT

                          89930     89940     89950     89960     89970     89980     89990     90000

Human             GACTAGGAATAACAGGTGGGAGACTATTAGTCTAATTAAGATGTAATTATAAATCTAAGCTAGGAACGTAAAATGAGAAT 74236
Coquerel'sSifaka  GACTAGGAATACCAGATAGGAGACT----GTCTAAGATAGATATAATTGTAAATCTAAACTAGGAACATGAAATGAGAAT 61769
Ma'sNightMonkey   GACTAAGCATTCCAGGTAGGATACTGTTAGTCTAAGTAAGATGTAAGTATAAATCTAGGTTAGGAGCATGAAATGAGAAT 68966
VervetAGM         GATTAGGAAAAACAGGTAGGAGACTCTTAGTTTAATTAAGATGTGATTATACATCTAAGCTAGGAACGTAAAATGAGAAT 75653
Gorrilla          GACTAGGAATAACAGGTGGGAGACTATTAGTCTAATTAAGATGTAATTATAAATCTAAGCTAGGAACGTAAAATGAGAAT 72779
GrabEatingMacaqueGATTAGGAAAAACAGGTAGGAGACTCTTAGTTTAATTAAGCTGTAATTATACATCTAAGCTAGAAATGTAAAATGAGAAT 75198
Macaque           GATTAGGAAAAACAGGTAGGAGACTCTTAGTTTAATTAAGCTGTAATTATACATCTAAGCTAGAAACGTAAAATGAGAAT 76057
PigTailedMacaque  GATTAGGAAAAACAGGTAGGAGACTCTTAGTTTAATTAAGATGTAATTATACATCTAAGCTAGAAACGTAAAATGAGAAT 74585
Bonobo            GACTAGGAATAACAGGTGGGAGACTATTAGTCTAATTAAGATGTAATTATAAATCTAAGCTAGGAACGTAAAATGAGAAT 73340
OliveBaboon       GATTAGGAAAAACAGGTAGGAGACTCTTAGTTTAATTAAGATGTAATTATACATCTAAGCTAGAAACGTAAAATGAGAAT 74460
Orangutan         GACTAGGAATAACAGGTAGGAGATTATTAGTCTAATTAAGATGTAATTATAAATCTAAGCTAGGAACGTAAAATGAGAAT 75122
```

```
Majority         GCAAAGTAAGAA----ATCTGGGGAAAATTATATGTAAAAGTAATAGGACTTGGCATCTTATTGATATGATTGCTTATGA

                         90010     90020     90030     90040     90050     90060     90070     90080

Human            GCAAAGTAAGAAACAAATATGGGGAAAATTATATGTAAAAGTAATAGGACTTGGCATCTTACTGATGTGATTGATTATGA 74316
Coquerel'sSifaka GCAAAGTAAGAA----ATATGGGGAAAATTATACATAAA-GTAATAGGACTTGGCATCTTAGTGATAGGATTGCTTATGA 61844
Ma'sNightMonkey  ACAAAGTAAGAA----ATGTGGGGAAAATTTCATGTAAAAGTAATAGGACTTGGCATCTTACTGATATGATTGCTTATGA 69042
VervetAGM        GCAAAGTAAGAA----ATCTGGGGAAAATTATATGTAAAAGTAATAGGACTTGGC-TCTTATTGATATGATAGCTTATGA 75728
Gorrilla         GCAAAGTAAGAAACAAATATGGGGAAAATTATATGTAAAAGTAATAGGACTTGGCATCTTACTGATGTGATTGATTATGA 72859
GrabEatingMacaqueGCAAAGTAAGAA----ATCTGGGGAAAATTATATGTAAAAGTAATAGGACTTGGC-TCTTATTGATATGATAGCTTATGA 75273
Macaque          GCAAAGTAAGAA----ATCTGGGGAAAATTATATGTAAAAGTAATAGGACTTGGC-TCTTATTGATATGATAGCTTATGA 76132
PigTailedMacaque GCAAAGTAAGAA----ATCTGGGGAAAATTATATGTAAAAGTAATAGGACTTGGC-TCTTATTGATATGATAGCTTATGA 74660
Bonobo           GCAAACTAAGAA----ATATGGGGAAAATTATATGGAAAAGTAATAGGACTTGGCATCTTATTGATGTGATTGATTATGA 73416
OliveBaboon      GCAAAGTAAGAA----ATCTGGGGAAAATTATATGTAAAAGTAATAGGACTTGGC-TCTTATTGATATGATAGCTTATGA 74535
Orangutan        GCAAAGTAAGAAACAAATATGGGGAAAATTATATGCAAAAGTAATAGGACTTGGCATCTTACTGATGTGATTGATGATGA 75202

Majority         GAAAAATGA-GCATGTGGAGGAGTCCACCGGACAGTAGGAAATTCAGCCTAAGACTTGGGCAAGAGTTCTGTGGAGTTGT

                         90090     90100     90110     90120     90130     90140     90150     90160

Human            GAAAAATGAAGCATGTGGAGGAGTCCACTGGACAGTAGGAAATTCAGCCTAAGACTTGGGTAAGAGTTCTGTGGAGTTGT 74396
Coquerel'sSifaka AGAGGGAA--GCATGTGGAGGAGTCCACTAGAGAGTAGGAAATTCAGCCTACT-CTTGGGCAGGAGTTCTGTGGGGTTGT 61921
Ma'sNightMonkey  GAAAAATGAAGCATGTGGAGGAGTCCACTGAGCAGTAGGAAATTCAGCTGAAGACTTGGGCAAGAGTTCTGTGGAATTGT 69122
VervetAGM        GAAAAATGT-------GGAGGAGTCCACCGGACAGTAGGAAATTCAGCCTAAGACTTGGGCAAGAGTTCTGTGGAATTGT 75801
Gorrilla         GAAAAATGAAGCATGTGGAGGAGTCCACCGGACAGTAGGAAATTCAGCCTAAGACTTGGGTAAGAGTTCTGTGGAGTTGT 72939
GrabEatingMacaqueGAAAAATG----------AGGAGTCCACCAGACAGTAGGAAATTCAGCCTAAGACTTGGGCAAGAGTTCTGTGGAGTTGT 75343
Macaque          GAAAAATG----------AGGAGTCCACCAGACAGTAGGAAATTCAGCCTAAGACTTGGGCAAGAGTTCTGTGGAGTTGT 76202
PigTailedMacaque GAAAAATGT-------GGAGGAGTCTACCAGACAGTAGGAAATTCAGCCTAAGACTTGGGCAAGAGTTCTGTGGAGTTGT 74733
Bonobo           GAAAAATGAAGCATGTGGAGGAGTCCACTGGACAGTAGGAAATTCAGCCTAAGACTTGGGTAAGAGTTCTGTGGAGTTGT 73496
OliveBaboon      GAAAAATGT-------GGAGGAGTCCACCAGACAGTAGGAAATTCAGCCTAAGACTTGGGCAAGAGTTCTGTGGAGTTGT 74608
Orangutan        GAAAAATGAAGCATGTGGAGGAGTCCACTGGACAGTAGGAAATTTGGCCTAAGACTTGGGCAAGAGTTCTGTGGAGTTGT 75282

Majority         GAATTCAGAGGCCAGAGATGTGATATTTAAAATTTTGGTTTAAGATTTCCCAGGTATAAGAAAGCAAGAGGATTAAAGCA

                         90170     90180     90190     90200     90210     90220     90230     90240

Human            GAATTCAGAGGCCAGAGATGTGATATTTAAAATTTTGGTTCAAGATTTCCCAGGTATAAGAAAGCAAGAGGATTAAAGCA 74476
Coquerel'sSifaka GAATTCACAGACCAGAGGTGTGATATTTGAAACACTGGCTTAAGCTTTTCCAGTTATAAGAGAGCAAGAGGATTAAAGCA 62001
Ma'sNightMonkey  GAATTCAGAGGCCAGAGATGTGGTACTTAAAATTTTGGTATAAGATTTCCCAGGTATAAAAAAGCAAGAAGATTAAAGCA 69202
VervetAGM        GTATTCAGAGGCCAGAGATGTGATGTTTAAAATTTTGATTTAAGATTTCCCAGGTATAAGAAAGCAAGAGGATTAAAGCA 75881
Gorrilla         GAATTCAGAGGCCAGACATGTGATATTTAAAATTTTGGTTTAAGATTTCCCAGGTAAAAGAAAGCAAGAGGATTAAAGCA 73019
GrabEatingMacaqueGTATTCAGAGGCCAGAGATGTGATGTTTAAAATTTTGATTTAAGATTTCCCAGGTATAAGAAAGCAAGAGGATTAAAGCA 75423
Macaque          GTATTCAGAGGCCAGAGATGTGATGTTTAAAATTTTGATTTAAGATTTCCCAGGTATAAGAAAGCAAGAGGATTAAAGCA 76282
PigTailedMacaque GTATTCAGAGGCCAGAGATGTGATGTTTAAAATTTTGATTTAAGATTTCCCAGGTATAAGAAAGCAAGAGGATTAAAGCA 74813
Bonobo           GAATTCAGAGGCCAGAGATGTGATATTTAAAATTTTGGTTTAAGATTTCCCAGGTATAAGAAAGCAAGAGGATTAAAGCA 73576
OliveBaboon      GTATTCAGAGGCCAGAGATGTGATGTTTAAAATTTTGATTTAAGATTTCCCAGGTATAAGAAAGCAAGAGGATTAAAGCA 74688
Orangutan        GAATTCAGAGGCCAGAGATGTGATATTTAAAATTTTTGTTTAAGATTTCCCAGGAATAAGAAAGCAAGAGGATTAAAGCA 75362
```

```
Majority          TT-GTAATTAAACTTTAAGCAGTGCATATTTATGTTATAGATAAGATAAACAAGAAATCTAGGGATCAAATAAATAGGAT

                          90250     90260     90270     90280     90290     90300     90310     90320

Human             TT-GTAATTAAACTTTAAGCAGTGCATATTTATGTTATAGATAAGATAAACAAGAAATCTAGGGATCAAATA----GGAT 74551
Coquerel'sSifaka  TT-GACATAAAAATATAAGCAGTGCACATTCATATTGTGGATAAAATAAA-TAGAAAT-TGAGGATTGAATAAA-----T 62073
Ma'sNightMonkey   TTTGTAATTAAACTTTAAGCAGTGCATATTTATGTTATAGATAAAATAAA-AAGGAATCTAGGGATCAGATAAATAGGAT 69281
VervetAGM         TT-GTAATTAAACTTTAAGCAGTGCATATTTATGTTATAGATAAGATAAACAAGAAATCTAGCAATCAAATACATAGGAT 75960
Gorrilla          TT-GTAATTAAACTTTAAGCAGTGCCTATTTATGTTATAGATAAGATAAACAAGAAATCTAGGGATCAGATAAATAGGAT 73098
GrabEatingMacaqueTT-GTAATTAAACTTTAAGCAGTGCATATTTATGTTATAGATAAGATAAACAAGAAGTCTAGCAATCAAATAAATAGGAT 75502
Macaque           TT-GTAATTAAACTTTAAGCAGTGCATATTTATGTTATAGATAAGATAAACAAGAAGTCTAGCAATCAAATAAATAGGAT 76361
PigTailedMacaque  TT-GTAATTAAACTTTAAGCAGTGCATATTTATGTTATAGATAAGATAAACAAGAAGTCTAGCAATCAAATAAATAGGAT 74892
Bonobo            TT-GTAATTAAACTTTAAGCAGTGCATATTTATGTTATAGATAAGATAAACAAGAAATTTAGGGATCAAATAAATAGGAT 73655
OliveBaboon       TT-GTAATTAAACTTTAAGCAGTGCATATTTATGTTATAGATAAGATAAACAAGAAGTCTAGCAATCAAATAAATAGGAT 74767
Orangutan         TT-GTAATTAAACTTTAAGCAGTGCATATTTATGTTATAGATAAGATAAACAAGAAATCTAGGGATCAAATAAATAGGAT 75441

Majority          TAAAATTAGT-AGTGATTATTCAGTACAGT--AGTTA-CATAAGATTATTCACAAGAGT--GTATAAATCAAATTACAAG

                          90330     90340     90350     90360     90370     90380     90390     90400

Human             TAAAATTAGT-AGTGATCATTCAGTACAGT--AGTTA-CGTACTGTTATTCACAAGAGT--ATATAAATCAAATTACAAG 74625
Coquerel'sSifaka  -AAAATTAGA-AATGGCTGTTCAGTCCAGT--AGTTAACATAATATTATTCACGAGAATAGGTATAAATCAAATTACAAG 62149
Ma'sNightMonkey   TAAAATTAGT-AGTAATTGTTGAGTACAGT--AGTTA-CATAAGATTATTCACAAGCAT--GTATAAATCAGATTACAAG 69355
VervetAGM         TAAAATTAGT-AGTGATTGTTCAGTACAGTGTAGTTA-CATAAGATTATTCACAAGAGT--GTATAAATCAAATTACAAT 76036
Gorrilla          TAAAATTAGT-AGTGATCATTCAGTACAGT--AGTTA-CGTACTGTTATTCACAAGAGT--ATATCAATCAAATTACAAG 73172
GrabEatingMacaqueTAAAATTAGT-AGTGATTATTCAGTACAGTGTAGTTA-CATAAGATTATTCACAAGAGT--GTATAAATCAAATTACAAT 75578
Macaque           TAAAATTAGT-AGCGATTATTCAGTACAGTGTAGTTA-CATAAGATTATTCACAAGAGT--GTATAAATCAAATTACAAT 76437
PigTailedMacaque  TAAAATTAGT-AGTGATTATTCAGTACAGTGTAGTTA-CATAAGATTATTCACAAGAGT--GTATAAATCAAATTACAAT 74968
Bonobo            TAAAATTAGT-AGTGATCATTCAGTACAGT--AGTTA-CGTACTGTTATTCACAAGAGT--ATATAAATCAAATTACAAG 73729
OliveBaboon       TAAAATTAGT-AGTGATT-TTCAGTACAGTGTAGTTA-CATAAGATTATTCACAAGAGT--GTATAAATCAAATTACAAT 74842
Orangutan         TAAAATTAGTTAGTGATTATTCAGTACAGT--AGTTA-CGTATGATTATTCACAAGAGT--GTATAAATCAAATTACAAG 75516

Majority          GGATTAAGGATATAAATGTGATGAGAAAGTA---ACTGTACTCTTTGAAGAAGTGTGCCATAGAAAGGAAGAAGAAATGG

                          90410     90420     90430     90440     90450     90460     90470     90480

Human             GAATTAAGGATATAAACGTGATAAGAAAGTATGCACTGTACTCTTTGAGGAAGTTTGCCATAGAAAGGAAGAAGAAATAG 74705
Coquerel'sSifaka  GAATCGGGGGTATAAATGTGATAAGAGCACA---CTTCTGCTGTTTAAAGAAGTGTGCCATGGAAAGGAAGAAGAAATGG 62226
Ma'sNightMonkey   GCATTAAGGATATAAATGTGATGAACAAGTAT--CTAATACTCTTTGAAGAAGTGTGCCATAGAAAGGAAGAAGAAATAG 69433
VervetAGM         GGATTAAGGATATAAATGTGATGAGAAAGTA---ACTGTACTCTTTGAAGAAGTGTGCCATAGAAAAGAAGAAGAAATGG 76113
Gorrilla          GAATTAAGGATATAAACGTGATAAGAAAGTATACACTGTACTCTTTGAGGAAGTGTGCCATAGAAAGGAAGAAGAAATAG 73252
GrabEatingMacaqueGGATTAAGGATATAAATATGATGAGAAAGTA---ACTGTACTCTTTGAAGAAGTGTGCCATAGAAAAGAAGAAGAAATGG 75655
Macaque           GGATTAAGGATATAAATATGATGAGAAAGTA---ACTGTACTCTTTGAAGAAGTGTGCCATAGAAAAGAAGAAGAAATGG 76514
PigTailedMacaque  GGATTAAGGATATAAATATGATGAGAAAGTA---ACTGTACTCTTTGAAGAAGTGTGCCATAGAAAAGAAGAAGAAATGG 75045
Bonobo            GAATTAAGGATATAAACGTGATAAGAAAGTATGCACTGTACTCTTTGCGGAAGTTTGCCATAGAAAGGAAGAAGAAATAG 73809
OliveBaboon       GGATTAAGGATATAAATGTGATGAGAAAGTA---ACTGTACTCTTTGAAGAAGTGTGCCATAGAAAAGAAGAAGAAATGG 74919
Orangutan         GAATTAAGGATATAAATGTGATAAGAAAGTATGCACTGTACTCTTTGAGGAAGTGTGCCATAGAAAGGAAGAAGAAATAG 75596
```

```
Majority          GATGGTAGTTCAGGAGTAAAGCAGGACCCAATGGGGGGAATGTTTGCAGTGAGGCAGTATGTATAATCATTTAAAACATG
                          90490     90500     90510     90520     90530     90540     90550     90560
Human             GATGGTAGATCAGAAGTAAAGCAGGACCCAGTGGGGGGAGTGTTTGCAGTGAGGCAGTATGTATAATCATTTAAAACATG 74785
Coquerel'sSifaka  GATGGTAGTTTAGGAGTAAAGCAGGACCGAA--GACGGAATATTTTCAGTGAAGCAGTATGCATAATCATTAAGAGCACG 62304
Ma'sNightMonkey   GATGGTAGTTCAGGAGTAAAGCAGGACCTGCTGGGGGGAATGTTTGCAGTGAGGTAGTATGTATAATCATTTAAAACATG 69513
VervetAGM         GATGGTAGTTCAGGAGTAAAGCAGGAACCAATGGAGGGAATGTTTGCAGTGAGGCAGTATGTATAATCATTTAAAACATG 76193
Gorrilla          GATGGTAGATCAGAAGTAAAGCAGGACCCAGTGGGGGGAATGTTTGCAGTGAGGCAGTATGTATAATGATTTAAAACATG 73332
GrabEatingMacaqueGGTGGTAGTTCAGGAGTAAAGTAGGAACCAATGGGGGGAATGTTTGCAGTGAGGCAGTATGTATAATCATTTAAAACATG 75735
Macaque           GGTGGTAGTTCAGGAGTAAAGTAGGAACCAATGGGGGGAATGTTTGCAGTGAGGCAGTATGTATAATCATTTAAAACATG 76594
PigTailedMacaque  GGTGGTAGTTCAGGAGTAAAGTAGGAACCAATGGGGGGAATGTTTGCAGTGAGGCAGTATGTATAATCATTTAAAACATG 75125
Bonobo            GATGGTAGATCAGAAGTAAAGCAGGACCCAGTGGGGGGAGTGTTTGCTGTGAGGCAGTATGTATAATCATTTAAAACATG 73889
OliveBaboon       GGTGGTAGTTCAGGAGTAAAGCAGGAACCAATGGGGGGAATGTTTGCAGTGAGGCAGTATGTATAATCATTTAAAACATG 74999
Orangutan         GATGGTAGATCAGAAGTAAAGCAGGACCCAATGGGGGGAATGTTTGCAGTGAGGCAGTATGTATAATCATTTAAAACATG 75676

Majority          GGCTTGGAGTCCTCTCAT-TTCCACGTTTGTAATGGACATAATGATAATAATACCTTTCATTTAAGGCTGTTGTGAGGAT
                          90570     90580     90590     90600     90610     90620     90630     90640
Human             GGTTTGGAGTCCTCTCAGGTTCCATGTTTGTAATGGACATAATGATAATAATCCCTTTCATTTAAGGCTGTTGTGAGGAT 74865
Coquerel'sSifaka  GGCTTCAAATCCTCTCAGCTTCCACATTTGTAATGGACATAATGATAATAATACCT---ATCTAAGGCTTTTGTGAAGAT 62381
Ma'sNightMonkey   GCCTTGAAATCCTCTCAATTTCCATGCTTGTAATGGGCATATTGATAATAATCCCTATCATTTAAGGCTGTTGTGAGGAT 69593
VervetAGM         GGCTTGGAGTCCTCTCAT-TTCCACGTTTG-----GACATAATGATAATAATACCTTCCATTTAAGGCTGTTGTGAGGAT 76267
Gorrilla          GGTTTGGAGTCCTCTCAGGTTCCATGTTTGTAATGGACATAATGATAATAATCCCTTTCTTTTAAGGCTGTTGTGAGGAT 73412
GrabEatingMacaqueGGCTTGGAGTCCTCTCAT-TTCCACATTTG-----GACATAATGATAATAATACCTTCCATTTAAGGCTGTTGTGAGGAT 75809
Macaque           GGCTTGGAGTCCTCTCAT-TTCCACATTTG-----GACATAATGATAATAATACCTTCCATTTAAGGCTGTTGTGAGGAT 76668
PigTailedMacaque  GGCTTGGAGTCCTCTCAT-TTCCACGTTTG-----GACATAATGATAATAATACCTTCCATTTAAGGCTGTTGTGAGGAT 75199
Bonobo            GGTTTGGAGTCCTCTCAGGTTCCATGTTTGTAATGGACATAATGATAATAATCCCTTTCATTTAAGGCTGTTGTGAGGAT 73969
OliveBaboon       GGCTTGGAGTCCTCTCAT-TTCCACGTTTG-----AACATAATGATAATAATACCTTCCATTTAAGGCTGTTGTGAGGAT 75073
Orangutan         GGCTTGGAGTCCTCTCAGGTTCCATGTTTGTAATGGACATAATGATAATAATCCCTTTCATTTAAGGCTGTTGTGAGGAT 75756

Majority          TAAATGTGTTAAT--GTGCAAAGAACTTTGTACAGTGCCTGGCATATAATAAATGCTTGCTACCTATTAAC-TAGTATTT
                          90650     90660     90670     90680     90690     90700     90710     90720
Human             TAAATGTGTTAAT--GTGCAAATAACTTTACACAGTGCCTGGTATATAATAAATGCTTGCTACCTATTAAC-TAGTATTT 74942
Coquerel'sSifaka  TAAATGTGTTCAT--ACATGGAGAACTTTGTACAGTGCCTGGCATATAATGAATGCTTGCTACATGTTAAC-TTGTATTT 62458
Ma'sNightMonkey   TAAATGTGTTAATATGTGCAAAGAACTTTGCACAGTGCCTGGCATATAGTAAATGCTTGCTACCTGTTAACCTAGTATTT 69673
VervetAGM         TAAATGTGTTAAT--GTGCAAAGAACTTTGTACAGTGCCTGTCATATAATAAATGCTCCCTACCTATTAAC-TAGTATTT 76344
Gorrilla          TAAATGTGTTAAT--GTGCAAATAACTTTACACAGTGCCTGGTATGTAATAAATGCTTGCTACCTATTAAC-TAGTACTT 73489
GrabEatingMacaqueTAAATGTGTTAAT--GTGCAAAGAACTTTGTACAGTGCCTGGCATATAATAAATGCTTGCTACCTATTAAC-TAGTATTT 75886
Macaque           TAAATGTGTTAAT--GTGCAAAGAACTTTGTACAGTGCCTGGCATATAATAAATGCTTGCTACCTATTAAC-TAGTATTT 76745
PigTailedMacaque  TAAATGTGTTAAT--GTGCAAAGAACTTTGTACAGTGCCTGGCATATAATAAATGCTTACTACCTGTTAAC-TAGTA--- 75273
Bonobo            TAAATGTGTTAAT--GTGCAAATAACTTTACACAGTGCCTGGTATATAATAAATGCTTGCTACCTATTAAC-TAGTATTT 74046
OliveBaboon       TAAATGTGTTAAT--GTGCAAAGAACTTTTTACAGTGCCTGGCATATAATAAATGCTCGCTACCTATTAAC-TAGTATTT 75150
Orangutan         TAAATGTGTTAAT--GTGCAAAGAACTTTGCACAGAGCCTGGCATATAATAAATGCTTGCTACCTATTAAC-TAGTATTT 75833
```

```
Majority          GTTTCTAAGGCTAGTTTAAGTCCTAGAATTGATTGTAA-GGATTAGATCAGGAGTATAGTGGACATGTTGGGATTTAAAT
                           90730     90740     90750     90760     90770     90780     90790     90800
Human             GTTTCTAAGGCTAATTTAAGTCCTAGAATTGATTGCAA-GGATTAGATCAGGAGTATAGTGGACATGTTGGGATTTAAAT 75021
Coquerel'sSifaka  ATTTCTGAG-----------TCCTAGAATTGATTATGATGGATTAGATTAAGAGTATGGTGGAAATGTTGGAGCGTGGAA 62527
Ma'sNightMonkey   GTTTCTAAGGCTAATTTAAGTCCTAGAATTGATTGTAA-GGATTAGATCAGTAGTATAATGGACATGTTAGGATTTAAAT 69752
VervetAGM         GTTTCTAAGGCTAGTTTAAGTCCTAGAATTGATTGTAA-GGATTAGATCAGGAGTATAGTGTACATGTTGGGATTTAAAT 76423
Gorrilla          GTTTCTAAGGCTAGTTTAAGTCCCAGAATTGATTGCAA-GGATTAGATCAGGAGTATGGTGGACATGTTGGGATTTAAAT 73568
GrabEatingMacaqueGTTTCTAAGGCTAGTTTAAGTCCTAGAATTGATTGTAA-GGATTAGATCAGGAGTATAGTGTACATGTTGGGATTTAAAT 75965
Macaque           GTTTCTAAGGCTAGTTTAAGTCCTAGAATTGATTGTAA-GGATTAGATCAGGAGTATAGTGTACATGTTGGGATTTAAAT 76824
PigTailedMacaque  --T------------------CCTAGAATTGATTGTAA-GGATTAGATCAGGAGTATAGTGTACATGTTGGGATTTAAAT 75332
Bonobo            GTTTCTAAGGCTAGTTTAAGTCCTAGAATTGATTGCAA-GGATTAGATCAGGAGTATAGTGGACATGTTGGGATTTAAAT 74125
OliveBaboon       GTTTCTAAGGCTAGTTTAAGTCCTAGAATTGATTGTAA-GGATTAGATCAGGAGTATAGTGTACATGTTGGGATTTAAAT 75229
Orangutan         GTTTCTAAGGCTAGTTTAAGTCCTAGAATTGATTGTAA-GGATTAGATCAGGAGTATAGTGGACATGTTGGGATTTAAAT 75912

Majority          AT------AGAGAGATATTTTTTAGGACCATTGTTAGAACCAGAAGAGATTTTTTATCAAGTTCACACAGAAATGTAGGT
                           90810     90820     90830     90840     90850     90860     90870     90880
Human             ATTTAAATATAGAGATGCTTTTTAGGACCATTGTTAGAACCAGAAGAGATTTTTTACCAAGTTCACACAGAAATGTAGGT 75101
Coquerel'sSifaka  AAT-----ATAGAGATAGTTTT-AGGTCCATTGTGAGGACTAGAAGGGATTTTT-ATCAAGTTCACATGGAAAT------ 62594
Ma'sNightMonkey   AT--------AGAGATACTTTTTAGGACCATTGTTAGAACCAGAAGGAATTTTTTATCAAGTTCACTTAGAAATGTAGGT 69824
VervetAGM         AT------AGAGATATATTTTTTAAAGACCATTGTTAGAACCAGAAGAGATTTTTTATCAAGTTCACACAGAAATGTAGGT 76497
Gorrilla          ATTTAAATATAGAGATGCTTTTTAGGACCATTGTTAGAACCAGAAGAGATTTTTTATCAAGTTCACACAGAAATGTAGGT 73648
GrabEatingMacaqueAT------AGAGATATATTTTTTAAGACCATTGTTAGAACCAGAAGAGATTTTTTATCAAGTTCACACAGAAATGTAGGT 76039
Macaque           AT------AGAGATATATTTTTTAAGACCATTGTTAGAACCAGAAGAGATTTTTTATCAAGTTCACACAGAAATGTAGGT 76898
PigTailedMacaque  AT------AGAGATATATTTTTTAAGACCATTGTTAGAACCAGAAGAGATTTTTTATCAAGTTCACACAGAAATGTAGGT 75406
Bonobo            ATTTAAATATAGAGATGCTTTTTAGGACCATTGTTAGAACCAGAAGAGATTTTTTATCAAGTTCACACAGAAATGTAGGT 74205
OliveBaboon       AT------AGAGATATATTTTTAAAGACCATTGTTAGAACCAGAAGAGATTTTTTATCAAGTTCACACAGAAATGTAGGT 75303
Orangutan         AC--------AGAGATGCTTTTTAGGACCATTGTTAGAACCAGAATAGATTTTTTATCAAGTTCACACAGAAATGTGGGT 75984

Majority          GCATTGGCTGGGCGTGGTGGCTCACACCTGCAATCCCAGCACTTTGGAAGGCTGAGGCAGGAGAATTGCTTGAGGCCAAG
                           90890     90900     90910     90920     90930     90940     90950     90960
Human             GCATTGGCTGGGCATGGTGGCTCACACCTGCAATCCCAGCACTTGGGAAGGCTGAGGCAGAAGAACTGCTTGAGGCCAAC 75181
Coquerel'sSifaka  ----------------------------------------------------------------------TGAGG----- 62599
Ma'sNightMonkey   GTATTGGCTGGGCATGGTGGCTCACACCTCCAGTCCCAGCTCTTTGGAAGGCTGAGGCAGCAGAATTGCTTGAGGCCAAG 69904
VervetAGM         GCATTGGCTGGGCGTAGTAACTCACACCTGCAATCCCAGCACTTTGGAAGGCTGAGGCAGGAGAATTGCTTGAGGCCAAG 76577
Gorrilla          GCATTGGCTGGGCATGGTGGCTCACACCTGCAATCCCAGCACTTGGGAAGGCTGAGGCAGAAGAACTGCTTGAGGCCAAG 73728
GrabEatingMacaqueGCATTGGCTGGGCGTAGTAACTCGCACCTGCAATCCCAGCACTTTGGAAGGCTGAGGCAGGAGAATTGCTTGAGGCCAAG 76119
Macaque           GCATTGGCTGGGCGTAGTAACTCGCACCTGCAATCCCAGCACTTTGGAAGGCTGAGGCAGGAGAATTGCTTGAGGCCAAG 76978
PigTailedMacaque  GCATTGGCTGGGCGTAGTAACTCGCACCTGCAATCCCAGCACTTTGGAAGGCTGAGGCAGGATAATTGCTTGAGGCCAAG 75486
Bonobo            GCATTGGCTGGGCATGGTGGCTCACACCTGCAATCCCAGCACTTGGGAAGGCTGAGGCAGAAGAACTGCTTGAGGCCAAC 74285
OliveBaboon       GCATTGGCTGGGCGTAGTAACTCGCACCTGCAATCCCAGCACTTTGGAAGGCTGAGGCAGGAGAATTGCTTGAGGCCAAG 75383
Orangutan         GCATTGGCTGGGCATGGTGGCTCACACCTGCAATCCCAGCACTTGGGAAGGCTGGGGCAGGAGAACTGCTTGAGGCCAAG 76064
```

```
Majority          ATTTTGAGACCATTCTGGGCAACCTCGCAAGACCCTATCTCCACCAAAAAAAAAAAAAAAA--------G----------

                        90970     90980     90990     91000     91010     91020     91030     91040

Human             ATTTTGAGACCAGCCTGGGCAACATATTAAGACCCCGTCTCCACCAAAAAAAAAAAAAAAA-----GAAG---------- 75246
Coquerel'sSifaka  --------------------------------------T----------------------------------------- 62600
Ma'sNightMonkey   ATTTTTAGACCAGCCTGGACAACATGGCAAGGCCCCATCTCCACCAAAAAAAAAAAAAAAAAAAAAAAAAAAAAAAAAAA 69984
VervetAGM         ATTTTGAGACCATTCTGGGCAACCTCGCAAGACCCTATCTCCACCAAAAAAAAAAAAAAG-------------------- 76636
Gorrilla          ATTTTGAGACCAGCCTGGGCAACATATTAAGACCCCGTCTCCACCAAAAAAAAAAAAAAAAGAA---GAAG---------- 73795
GrabEatingMacaque ATTTTGAGACCATTCTGGGCAACCTCGCAAGACCCTATCTCCACCAAAAAAAAAAAAAA----------G---------- 76179
Macaque           ATTTTGAGACCATTCTGGGCAACCTCGCAAGACCCTATCTCCACCAAAAAAAAAAAAAAAAAAAAAAAAAG---------- 77048
PigTailedMacaque  ATTTTGAGACCATTCTGGGCAACCTCGCAAGACCCTATCTCCACCAAAAAAAAAAAAAAAAA-------G---------- 75549
Bonobo            ATTTTGAGACCAGCCTGGGCAACATATTAAGACCCCGTCTCCACCAAAAAAAAAAAAGAGA---------G---------- 74346
OliveBaboon       ATTTTGAGACCATTCTGGGCAACCTCGCAAGACCCTATCTCCACCAAAAAAAAAAAAAAAA--------G---------- 75445
Orangutan         ATTTTGAGACTAGCCTGGGCAACATAGTAAGACCCCATCTCCACCAAAAAAAAAAAAAAAAAAAA-AAAG---------- 76133

Majority          --TAGGTGCAGAGCTGGAAGCAGAACCGAAATCATCAGTGTTACAGTCATTATTCTTTCCTGTCACCATTATATGTCTTT

                        91050     91060     91070     91080     91090     91100     91110     91120

Human             --TAGGTGCAGAGCTGGAAGCAGAACCGAAATCATCAGTGTTACAGTCATTATTCTTTCCTGTCACCATTATATGTCTTT 75324
Coquerel'sSifaka  -------GCAGAGTTGGAGCCAAAACTGAAATCATCAGCTTTACAGTCTTTATTCTTTCTTTTCACCATTCTGTGTTGTT 62673
Ma'sNightMonkey   ATTAGGTGCAGAGCTGGAAGCAGAACTGAAATAATCAGTTCTATAGTCTTT-TTCTTTCCTCTCACCATTATGTGTCTTT 70063
VervetAGM         --TAGATGCAGAGCTGGAAGCAGAACCGAAATCATCAGTGTTACAGTCATTATTCTTTCCTGTCACCATTATATGTCTTT 76714
Gorrilla          --TAGGTGCAGAGCTGGAAGCAGAACCGAAATCATCAGTGTTACAGTCATTATTCTTTCCTGTCACCATTATATGTCTTT 73873
GrabEatingMacaque --TAGATGCAGAGCTGGAAGCAGAACCGAAATCATCAGTGTTACAGTCATTATTCTTTCCTGTCACCATTATATGTCTTT 76257
Macaque           --TAGATGCAGAGCTGGAAGCAGAACCGAAATCATCAGTGTTACAGTCATTATTCTTTCCTGTCACCATTATATGTCTTT 77126
PigTailedMacaque  --TAGATGCAGAGCTGGAAGCAGAACCGAAATCATCAGTGTTACAGTCATTATTCTTTCCTGTCACCATTATATGTCTTT 75627
Bonobo            --TAGGTGCAGAGCTGGAAGCAGAACCGAAATCATCAGTGTTACAGTCATTATTCTTTCCTGTCACCATTATATGTCTTT 74424
OliveBaboon       --TAGATGCAGAGCTGGAAGCAGAACTGAAATCATCAGTTTTACAGTCATTATTCTTTCCTGTCACCATTATATGTCTTT 75523
Orangutan         --TAGGTGCAGAGCTGGAAGCAGAACCAAAATCATCGGTGTTACAGTGATTATTCTTTCCTGTCACTATTACATGTCTTT 76211

Majority          ATGAAGCAAGGGAGAAAGAAGAACAGATGAAAGAAGTGAGGATTTTGAAGTTGGTTGAAAAA--TTTGATTGAATTCTGA

                        91130     91140     91150     91160     91170     91180     91190     91200

Human             ATGAAGCAAGGGAGAAAGAAGAACAGATGAAAGAAGTGAGGATTTTGAAGTTGGTTGAAAGA--TTTGATTGAATTCTGA 75402
Coquerel'sSifaka  ACGAAGAAAGGGAAAAAG---AACA-----------TGAGGACTTTGAAGTTGGTTGAAAAAAATCTGAGAGAGTTCCAG 62739
Ma'sNightMonkey   ATAAAGAAAGGGAGAAGGAAGAACAGCTGAAAGCAGTGAGGATTTTGAAGGTAGCTGAAAAA--TTTGAGAGAATTCTGT 70141
VervetAGM         ATGAAGCAAGGGAGAAAGAAGAACAGATGAAAGAAGTGAGGATTTTGAAGTTGGTTGAAAAA--TTTGATTGAATTCTGA 76792
Gorrilla          ATGAAGCAAGGGAGAAAGAACAACAGATGAAAGAAGTGAGGATTTTGAGGTTGGTTGAAAGA--TTTGATTGAATTCTGA 73951
GrabEatingMacaque ATGAAGCAAGGGAGAAAGAAGAACAGATGAAAGAAGTGAGGATTTTGAAGTTGGTTGAAAAA--TTTGATTGAATTCTGA 76335
Macaque           ATGAAGCAAGGGAGAAAGAAGAACAGATGAAAGAAGTGAGGATTTTGAAGTTGGTTGAAAAA--TTTGATTGAATTCTGA 77204
PigTailedMacaque  ATGAAGCAAGGGAGAAAGAAGAACAGATGAAAGAAGTGAGGATTTTGAAGTTGGTTGAAAAA--TTTGATTGAATTCTGA 75705
Bonobo            ATGAAGCAAGGGAGAAAGAAGAACAGATGAAAGAAGTGAGGATTTTGAAGTTGGTTGAAAGA--TTTGATTGAATTCTGA 74502
OliveBaboon       ATGAAGCAAGGGAGAAAGAAGAACAGATGAAAGAAGTGAGGATTTTGAAGTTGGTTGAAAAA--TTTGATTGAATTCTGA 75601
Orangutan         ATGAAGCAAGGGAGAAAGAAGAACAGATGAAAGAAGTGAGGATTTTGAAGTTGGTTGAAAAA--TTTGATTGAATTCTGA 76289
```

```
Majority          TCTGAATATTATAAGACACTTGCTTAACA------AGTTGAAAGTAGGAAAGTAGACTT-AAGACTCTACTGGATTTGG-
                          91210     91220     91230     91240     91250     91260     91270     91280
Human             TCTAAAAATTATAAGGCACTTGTTTAACA------AGTTGAAAGTAGGAAAGTAGACAT-AAGACTCTACTAGATTTGG- 75474
Coquerel'sSifaka  TCTGAATATTATAAG--ATTTGCTGAACATATGGAGGTTGAAAGTAGGATAAAGGACTT---AAGTCTACTGAATTTGTT 62814
Ma'sNightMonkey   TCTGAATATTGTAAGACACTTGCTGAACA------AGTTGAAAGTAGGAAAGTAGACTT---AATACTACTGGATTTGG- 70211
VervetAGM         TCTGAATATTATAAGACACTTGCTTAACA------AGTTGAAAGTAGGAAAGTAGACTT-AAGACTCTATTGGATTTGG- 76864
Gorrilla          TGTGAAAATTATAAGGCACTTGTTTAACA------AGTTGAAAGTAGGAAAGTAGACTT-AAGACTCTACTAGATTTGG- 74023
GrabEatingMacaqueTCTGAATATTATAAGACACTTGCTTAACA------AGTTGAAAGTAGGAAAGTAGACTT-AAGACTCTGTTGGATTTGG- 76407
Macaque           TCTGAATATTATAAGACACTTGCTAAACA------AGTTGAAAGTAGGAAAGTAGACTTTAAGACTCTGTTGGATTTGG- 77277
PigTailedMacaque  TCTGAATATTATAAGACACTTGCTTAACA------AGTTGAAAGTAGGAAAGTAGACTT-AAGACTCTATTGGATTTGG- 75777
Bonobo            TCTAAAAATTATAAGGCACTTGTTTAACA------AGTTGAAAGTAGGAAAGTAGACTT-AAGACTCTACTAGATTTGG- 74574
OliveBaboon       TCTGAATATTATAAGACACTTGCTTAACA------AGTCGAAAGTAGGAAAGTAGACTT-AAGACTCTGTTGGATTTGG- 75673
Orangutan         TCTGAAAATTATAAGGCACTTGTTTAACA------AGTTGAAAGTAGGAAAGTAGACTT-AAGACTCTACTAGATTTGG- 76361

Majority          GGAAACTCTC--AAAAATGGACTGGAAATTCAGCTAAAAGTGGATAACAAAATATTTCTAGAATTAGCATTTGTGGGGTT
                          91290     91300     91310     91320     91330     91340     91350     91360
Human             GGAAACTCTC--AAAAATGGACTGGAAATTCAGCTAAAAGTGGATAACAAAATATTTCTAGAATTAGCATTTGTGGGGTG 75552
Coquerel'sSifaka  GGGAACTCTTTGGAAAATGGAGTAGGAAATCAGGTAAAAGTAGATAACAAAATATCTGTAGAATT--------------- 62879
Ma'sNightMonkey   GGGAACTCTC--AAAAATGGACTGAAAATTCAGCTAAAAGTGGATAACAAAATATTTCTAGAATTAGCCTTTGTGGGGCA 70289
VervetAGM         GGAAACTCTC--AAGAATGGACTGGAAATTCAGCTAAAAGTGGATTACAAAATATTTCTAGAATTAGCATTTGTGGGGTT 76942
Gorrilla          GGAAACTCTC--AAAAATGGACTGGTAATTCAGCTAAAAGTGGATAACAAAATATTTCTAGAATTAGCATTTGTGGGGTG 74101
GrabEatingMacaqueGGAAACTCTC--AAGAATGGACTGGAAATTCAGCTAAAAGTGGATTACAAAATATTTCTAGAATTAGCATTTGTGGGGTT 76485
Macaque           GGAAACTCTC--AAGAATGGACTGGAAATTCAGCTAAAAGTGGATTACAAAATATTTCTAGAATTAGCATTTGTGGGGTT 77355
PigTailedMacaque  GGAAACTCTC--AAGAATGGACTGGAAATTCAGCTAAAAGTGGATTACAAAATATTTCTAGAATTAGCATTTGTGGGGTT 75855
Bonobo            GGAAACTCTC--AAAAATGGACTGGAAATTCAGCTAAAAGTGGATAACAAAATATTTCTAGAGTTAGCATTTGTGGGGTG 74652
OliveBaboon       GGAAACTCTC--AAGAATGGACTAGAAATTCAGCTAAAAGTGGATTACAAAATATTTCTAGAATTAGCATTTGTGGGGTT 75751
Orangutan         GGAAACTCTC--AAAAATGGACTGGAAATTCAGCTAAAAGTGGATAACAAAATATTTCTAGAATTAGCATTTGTGGGGTG 76439

Majority          TGTGTGTTTTCACTCTAGTA-TTTGTCAAGCCCAGATGAAAGCATAGACAGAATATAAGACTGGATTTATCTAAGTCTGG
                          91370     91380     91390     91400     91410     91420     91430     91440
Human             TGTGTGTTTTCACTCTAGTA-TTTGTCAAGCCCAGATGAAAGCATAGACAGAATGTAAGACTGGATTTATCTAAGTCTGG 75631
Coquerel'sSifaka  ------TT---ACTCTAATA-CTTATCCAACCCATATGAAAGCATAGACACA-CATAAGGTTGAATTTGTCTAAGTCTGG 62948
Ma'sNightMonkey   TATGTGTTTTCACTCTCATAATTCATTAAGCCCACATGAAAGCATGGACAGAATGTAAGACTGGATTTATCTAAGTCTGA 70369
VervetAGM         TGTGTGTTTTCACTCTAATA-TTTGTCAAGCCGAGATGAAAGCGTAGACAGAATATAAGACTGGATTTATCTAAGTCTGG 77021
Gorrilla          TGTGTGTTTTCACTCTAATA-TTTGTCAAGCCCAGATGAAAGCATGGACAGAATGTAAGACTGGATTTATTTAAGTCTGG 74180
GrabEatingMacaqueTGTGTGTTTTCACTCAAGTA-TTTGTCAAGCCCAGATGAAAGTGTAGACAGAATATAAGACTGGATTTATCTAAGTCTGG 76564
Macaque           TGTGTGTTTTCACTCAAGTA-TTTGTCAAGCCCAGATGAAAGTGTAGACAGAATATAAGACTGGATTTATCTAAGTCTGG 77434
PigTailedMacaque  TGTGTGTTTTCACTCAAGTA-TTTGTCAAGCCCAGATGAAAGTGTAGACAGAATATAAGACTGGATTTATCTAAGTCTGG 75934
Bonobo            TGTGTGTTTTCACTCTAGTA-TTTGTCAAGCTCAGATGAAAGCATAGACAGAATGTAAGACTGGATTTATCTAAGTCTGG 74731
OliveBaboon       TGTGTGTTTTCACTCTAGTA-TTTGTCAAGCCCAGATGAAAGCATAGACAGACTATAAGACTGGATTTATCTAAGTCTGG 75830
Orangutan         TGTGTGTTTTCACTCTAATA-TTTGTCAAGCCCAGATGAAAGCATAGACAGAATGTAAGACTGGATTTATCTAAGTCTGG 76518
```

```
Majority          AATTGT-GTAACATTAAAGAAATAGTAGCAAATGAGCAGAGTGTTGGTCCAA---GCTTA-----------------GGC
                          91450    91460    91470    91480    91490    91500    91510    91520
Human             AATTGT-GTAACATTAAAGGAATAGTAGCAAATGAGCAGAGTGTTGGCTCAA---GCCTAAGCTTGAGCCTAAGCTTGAC 75707
Coquerel'sSifaka  AATTACAGTGTTAAAGGAATAGTAATAGCACATGAACAGAGTATTGGGCCAAAAGGCTTA-----------------GGC 63011
Ma'sNightMonkey   AATTGC-ATGACATCAAAGTAATAGTAGCAGATGAGCAGCATGTTGGCTCAA---GCTTA-----------------GGC 70428
VervetAGM         AATTGT-GTAACATTAAAGAAACAGTAGCAGATGAGCAGAGTGTTGGTCCAA---GCTTA-----------------GGC 77080
Gorrilla          AATTGT-GTAACATTAAAGGAATAGTAGCAAATGAGCAGAGTGTTGGCTCAA---GCCTAAGCTTGAGCCTAAGCTTGAC 74256
GrabEatingMacaqueAATTGT-GTAACATTAAAGAAATAGTAGCAAATGAGCAGAGTGTTGGTCCAA---GCTTA-----------------GGC 76623
Macaque           AATTGT-GTAACATTAAAGAAATAGTAGCAAATGAGCAGAGTGTTGGTCCAA---GCTTA-----------------GGC 77493
PigTailedMacaque  AATTGT-GTAACATTAAAGAAATAGTAGCAAATGAGCAGAGTGTTGGTCCAA---GCTTA-----------------GGC 75993
Bonobo            AATTGT-GTAACATTAAAGGAATAGTAGCAAATGAGCAGAGTGTTGGCTCAA---GCCTAAGCTTGAGCCTAAGCTTGAC 74807
OliveBaboon       AATTGT-GTAACATTAAAGAAATAGTAGCAAATGAGCAGAGTGTTGGTCCAA---GCTTA-----------------GGC 75889
Orangutan         AATTGT-GTAACATTAAAGGAATAGTAGCAAATGAGCAGAGTTTTGGCTGAA---GCTTA-----------------GAC 76577

Majority          TCTATGGTAAAGTCAAGTCAAGGGAGAATAGAAAGGG----GGTCACCATAAAGGTCAAAAGTGGGTTTAGTGGTTGTGT
                          91530    91540    91550    91560    91570    91580    91590    91600
Human             TCTATGGTAAAGTCAAGTCAAGGGAGAATAGAAAGGG----GGTCACCATAAAGGTCAAAAGTGGGTTTAGTGGTTGTGT 75783
Coquerel'sSifaka  TATATGGTAAAGTCAAGCCAAAGGAGAATAGAAAGGGAACTGGTCACTATAAAGGCCAAAAGTGGGTTCAGTG-TTGCGT 63090
Ma'sNightMonkey   TATATGGTAAAGTCAAGCCCAGGGAGCATATAAAGGAA-CTGGTCACCATAAAGGCCAAAAGTGTGTTTAGTGGTTATGT 70507
VervetAGM         TCTATGGTAAAGTCAAGTCAAGGGAGAATAGAAAGGG----GGTCACCATAAAGGTCAAAAGTGGGTTTAGTGGTTGTAT 77156
Gorrilla          CCTATGGTAAAGTCAAGTCAAGGGAGAATAGAAAGGG----GGTCACCATAAAGGTCAAAAGTGGGTTTAGTGGTTGTGT 74332
GrabEatingMacaqueTCTATGGTAAAGTCAAGTCAAGGGAGAATAGAAAGGG----GGTCACCATAAAAGTCAAAAGTGGGTTTAGTGGTTGTGT 76699
Macaque           TCTATGGTAAAGTCAAGTCAAGGGAGAATAGAAAGGG----GGTCACCATAAAAGTCAAAAGTGGGTTTAGTGGTTGTGT 77569
PigTailedMacaque  TCTATGGTAAAGTCAAGTCAAGGGAGAATAGAAAGGG----GGTCACCATAAAGGTCAAAAGTGGGTTTAGTGGTTGTGT 76069
Bonobo            TCTATGGTAAAGTCAAGTCAAGGGAGAATAGAAAGGG----GGTCACCATAAAGGTCAAAAGTGGGTTTAGTGGTTGTGT 74883
OliveBaboon       TCTATGGTAAAGTCAAGTCAAGGGAGAATAGAAAGGG----GGTCACCATAAAGGTCAAAAGTGGGTTTAGTGGTTGTGT 75965
Orangutan         TGTATGGTAAAGTCAAGTCAAGGGAGAATAGAAAGGG----GGTCACCATAAAGGTCAAAAGTGGGTTTAGTGGTTGTGT 76653

Majority          GGGAATAGGAGGATCAAGAAAAGAATGAAGTTAGGAAAGGAGATATAAGTGTT----GAATGACCATTACAAAAAGAGAC
                          91610    91620    91630    91640    91650    91660    91670    91680
Human             GGGAATAGGCAGATCAAGAAAAGAATGAAGTTAGGAAAGGAGATATAAGTGTT----GAATGACCATTACAAAAAGAGAC 75859
Coquerel'sSifaka  GGTAATAG---GATCAAGAA--GAGTGAAGTCAGGAAAGGGGATTCAAAGGTTAAATGAATGGCCATT-TAAAAAAAG-- 63162
Ma'sNightMonkey   GGGAATAGGAGGATCAAGAA--GAATGAAGTTAGGAAAGGAGATGTAAGTGTTACATGAATGACCATTACAAAAAGTGAC 70585
VervetAGM         GGGAATAGGAGGATGAAGAAAAGAAAGAAGTTAGGAAAGGAGATATAAGTGTT----GAATGACCATTACAAAAAGAGAC 77232
Gorrilla          GGGAATAGGCGGATCAAGAAAAGAATGAAGTTAGGAAAGGAGATATAAGTGTT----GAATGACCATTACAAAAGGAGAC 74408
GrabEatingMacaqueAGGAATAGGAGGATCAAGAAAAGAAAGAAGTTAGGAAAGGAGATATAAGTGTT----GAATGACCATTACAAAAAGAGAC 76775
Macaque           AGGAATAGGAGGATCAAGAAAAGAAAGAAGTTAGGAAAGGAGATATAAGTGTT----GAATGACCATTACAAAAAGAGAC 77645
PigTailedMacaque  GGGAATAGGAGGATCAAGAAAAGAAAGAAGTTAGGAAAGGAGATATAAGTGTT----GAATGACCATTACAAAAAGAGAC 76145
Bonobo            GGGAATAGGCGGATCAAGAAAAGAATGAAGTTAGGAAAGGAGATATAAGTGTT----GAATGACCATTACAAAAAGAGAC 74959
OliveBaboon       GGGAATAGGAGGATCAAGAAAAGAAAGAAGTTAGGAAAGGAGATATAAGTGTT----GAATGACCATTACAAAAAGAGAC 76041
Orangutan         GGGAATAGGGGGATCAAGAAAAGAATGAAGTTAGGAAAGGAGACATAAGTGTT----GAATGACCATTACAAAAAGAG-- 76727
```

```
Majority          AGAGGAAAGAAAAATCAAGATGTAGCAAAAGAAGTTGCTAATATGGATGGATGTAGTAAAAACCATGAAAGTAGATGTTT

                          91690     91700     91710     91720     91730     91740     91750     91760

Human             AGAGGAAAGAAAAATGAAGATGTATCAAAAGAAGTTGCTAATATGGATGG-------------C---AAAGTAGATGTTT 75923
Coquerel'sSifaka  AGAG--AAGAAAA----AGATATAGCAAAAGAAGTTGCTAAAATGGATTGGTATAGTAAAATCCATCAAAGTAAAAGTTT 63236
Ma'sNightMonkey   ACAGGAAAGAAAA----AGATGTAGCAAAAGAAGTTGCTAATATGGATTGATGTTGTAAAAACCATCAAAGTAGACGTTA 70661
VervetAGM         AGAGGAAAGAAAAATCAAGATGTAGCAAAAGAAGTTGCTAATACGGATGGATGTAGTAAAAACCATCAAAGTAGATGTTT 77312
Gorrilla          AGAGGAAAGAAAAATGAAGATGTATCAAAAGAAGTTGCTAATATGGATGG-------------C---AAAGTAGATGTTT 74472
GrabEatingMacaqueAGAGGAAAGAAAAATCAAGATGTAGCAAAAGAAGTTGCTAATATGGATGGATGTAGTAAAAACCATGAAAGTAGATGTTT 76855
Macaque           AGAGGAAAGAAAAATCAAGATGTAGCAAAAGAAGTTGCTAATATGGATGGATGTAGTAAAAACCATGAAAGTAGATGTTT 77725
PigTailedMacaque  AGAGGAAAGAAAAATCAAGATGTAGCAAAAGAAGTTGCTAATATGGATGGATGTAGTAAAAACCATGAAAGTAGATGTTT 76225
Bonobo            AGAGGAAAGAAAAATGAAGATGTATCAAAAGAAGTTGCTAATATGGATGG-------------C---AAAGTAGATGTTT 75023
OliveBaboon       AGAGGAAAGAAAAATCAAGATGTAGCAAAAGAAGTTGCTAATACGGATGGATGTAGTAAAAACCATGAAAGTAGATGTTT 76121
Orangutan         AGAGGAAAGAAAAATGAAGATGTATCAAAAGAAGTTGCTAATATGGATGG-------------C---AAAGTAGATGTTT 76791

Majority          TTAAGAAATCATGAGACGAGAGTCTTGGAAAAGTCATA--GGATGATGCAGG----GAAT---GGAGAAGAAGGAAATAA

                          91770     91780     91790     91800     91810     91820     91830     91840

Human             TTAAGAAATCATGAGACCAGAGTCTTGGAAAAGTCATA--GGATGATGCAGG----GAAT---GGAGAAGAGGGAAATAA 75994
Coquerel'sSifaka  TTAAGAAATCATGAGACTGTAGTATTGGAAAGATCACACAGGATTATGTAGGTAGGGACTAAGGGAGTAGAAGGGAATAA 63316
Ma'sNightMonkey   TTAAGAAATTGTGAGGCCAGCGTGTTGGAAAGGTCATA--GGATGATGCAGG----GACT---GGAGAAGAAGGAAATAA 70732
VervetAGM         TTAAGAAATCATGAGACGAGAGTCTTGGAAAAGTCATA--GGTTGATGCAGG----GAAT---GGAGAAGAAGGAAATAA 77383
Gorrilla          TTAAGAAATCATGAGACCAGAGTCTTGGAAAAGTCATA--GGATGATGCAGG----GAAT---GGAGAAGAAGGAAATAA 74543
GrabEatingMacaqueTTAAGAAATCATGAGACGAGAGTCTTGGAAAAGTCATA--GGTTGATGCAGG----GAAT---GGAGAAGAAGGAAATAA 76926
Macaque           TTAAGAAATCATGAGACGAGAGTCTTGGAAAAGTCATA--GGTTGATGCAGG----GAAT---GGAGAAGAAGGAAATAA 77796
PigTailedMacaque  TTAAGAAATCATGAGACGAGAGTCTTGGAAAAGTCATA--GGTTGATGCAGG----GAAT---GGAGAAGAAGGAAGTAA 76296
Bonobo            TTAAGAAATCATGAGACCAGAGTCTTGGAAAAGTCATA--GGATGATGCAGG----GAAT---GGAGAAGAGGGAAATAA 75094
OliveBaboon       TTAAGAAATCATGAGATGAGAGTCTTGGAAAAGTCATA--GGTTGATGCAGG----GAAT---GGAGAAGAAGGAAATAA 76192
Orangutan         TTAAGAAATCATGAGACCAGAGTCTTGGAAAAGTCATA--GGATGATGCAGG----GAAT---GGAGAAGAAGGAAATAA 76862

Majority          AGCCAGGTGCTGAAGTCTTTATGTAATGGGAGGAGATGTTCCAGTAATCCAATGGCTATTTTGATGGGAAAGAGTGTGGT

                          91850     91860     91870     91880     91890     91900     91910     91920

Human             AGCCAGGTGCTGAAGTCTTTATGTAATGGGAGGAGATGTTCCAGTAATCCAATGGCTATTTTGATGGGAAAGAGTGTGGT 76074
Coquerel'sSifaka  CACCAGATGCCAAAGGCTTTATGTAA----GGAAAGTGTCCCAGTGACCCAGTGGCTATTTTGATGAGAAAGATTGTGGT 63392
Ma'sNightMonkey   AACCAGGTGCTAAAGTCTTTATGTAATGAGTGAAGATGTCCCAGTGACCCAGTGGCTATTTTGATGGGAGAAAGTGTGTT 70812
VervetAGM         AGCCAGATGCTGAAGTCTTTATGTAATGGGAGGAGATGTTCCAGTAATCCAGTGGCTATTTTGATGGGAAAGAGTGTGGT 77463
Gorrilla          AGCCAGGTGCTGAAGTCTTTATGTAATGGGAGGAGATGTTCCAGTAACCCAATGGCTATTTTGATGGGAAAGAGTGTGGT 74623
GrabEatingMacaqueAGCCAGGTGCTGAAGTCTTTATGTAATGGGAGGAGATGTTCCAGTAATCCAATGGCTATTTTGATGGGAAAGAGTGTGGT 77006
Macaque           AGCCAGGTGCTGAAGTCTTTATGTAATGGGAGGAGATGTTCCAGTAATCCAATGGCTATTTTGATGGGAAAGAGTGTGGT 77876
PigTailedMacaque  AGCCAGGTGCTGAAGTCTTTATGTAATGGGAGGAGATGTTCCAGTAATCCAATGGCTATTTTGATGGGAAAGAGTGTGGT 76376
Bonobo            AGCCAGGTGCTGAAGTCTTTATCTAATGGGAGGAGATGTTCCAGTAATCCAATGGCTATTTTGATGGGAAAGAGTGTGGT 75174
OliveBaboon       AGCCAGGTGCTGAAGTCTTTATGTAATGGGAGGAGATGTTCCAGTAATCCAATGGCTATTTTGATGGGAAAGAGTGTGGT 76272
Orangutan         AGCCAGGTGCTGAAGTCTT----TAATGGGAGGAGATGTTCCAGTAACCCAGTGGCTATTTTGATGGGAAAGAGTGTGGT 76938
```

```
Majority          ATGATTAGGTGGCATTGACATCGGAAGCCATCCTCATTGATGGTGGTAGAACA---GCAGTTTGAAAGTAACATTGGGCG

                          91930     91940     91950     91960     91970     91980     91990     92000

Human             ATGATTGGGTGGCATTGACATCGGAAGCCATCCTCATTGATGGTGGTGGAACA---GCAGTTTGAAAGTAACATTGTGCG 76151
Coquerel'sSifaka  ATGATTAGATGGCATTGACTTCAAAAGCTGCCCCCATCAATGGTGGTAGAGCA---ATAGTGTGAAAGTAACATTGGGTG 63469
Ma'sNightMonkey   ATGATTAGGTGGTATTGACATCAGAAGTCATCCTCATTGATGGTGGTAGAACAACAGCAGTTTGAAAGTAACATTGGGTG 70892
VervetAGM         ATGATTAGGTGGCATTGGCATTGGAAACCAAACTTATTGATGGTGGTAGAACA---GCAGTTTGAAAGTAACATTGGGCG 77540
Gorrilla          ATGATTGGGTGGCATTGACATCGGAAGCCATCCTCATTGATGGTGGTGGAACC---GCAGTTTGAAAGTAACATTGTGCG 74700
GrabEatingMacaqueATGATTAGGTGGCATTGACATTGGAAACCAAACTTACTGATGGTGGTAGAACA---GCAGTTTGAAAGTAACATTGGGCG 77083
Macaque           ATGATTAGGTGGCATTGACATTGGAAACCAAACTTACTGATGGTGGTAGAACA---GCAGTTTGAAAGTAACATTGGGCG 77953
PigTailedMacaque  ATGATTAGGTGGCATTGACATTGGAAACCAAACTTATTGATGGTGGTAGAACA---GCAGTTTGAAAGTAACATTGGGCG 76453
Bonobo            ATGATTGGGTGGCGTTGACATCGGAAGCCATCCTCGTTGATGGTGGTGGAACA---GCAGTTTGAAAGTAACATTGTGCG 75251
OliveBaboon       ATGATTAGGTGGCATTGACATTGGAAACCAAACTTATTGATGGTGGTAGAACA---GCAGTTTGAAAGTAACATTGGGCG 76349
Orangutan         ATGATTGGGTGGCATTGACATCGGAAGCCATCCTCATCGATGGT---GGAACA---GCAGTTTGAAAGTAACATTGTGCG 77012

Majority          GTGAGGTAGAGTGATGCATGATGCATCCTTATTCTTTCCTTTGGAGAAAAGTTGAGGGAGACCAAAAATGACTTTTT-GA

                          92010     92020     92030     92040     92050     92060     92070     92080

Human             GTGAGGTAGAGTGGCACATGATGCATCCTTATTCTTACCTTTGGAGAAAAGTTGAGGGAGACCAAAAATGACTTTTT-GA 76230
Coquerel'sSifaka  GTGAGGTAGAATGATGAATAATGCATCTTTTTTCTTCCCTTTGG-GAAAGATTTAGGGAGATAAAAA-TGACTTTTT-GA 63546
Ma'sNightMonkey   GTGAGGTAAAGTGATGCGTGATGCATCCTTATTCTTTTCTTTGGAGAAAAGTTAAGGGAGACTAA---TGAATTTTT-GA 70968
VervetAGM         GTGAGGTGGAGTGATGCATGATGCATCCTTACTCTTTCCTTTGGAGAAAAGTTGAGGGAGACCAAAAATGACTTTTT-GA 77619
Gorrilla          GTGAGGTAGAGTGGCGCATGATGCATCCTTATTCTTTCCTTTGGAGAAAAGTTGAGGGAGACCAAAAATGACTTTTTTGA 74780
GrabEatingMacaqueGTGAGGTGGAGTGATGCATGATGCATCCTTATTCTTTCCTTTGGAGAAAAGTTGAGGGAGACCAAAAATGACTTTTT-GA 77162
Macaque           GTGAGGTGGAGTGATGCATGATGCATCCTTATTCTTTCCTTTGGAGAAAAGTTGAGGGAGACCAAAAATGACTTTTT-GA 78032
PigTailedMacaque  GTGAGGTGGAGTGATGCATGATGCATCCTTGTTCTTTCCTTTGGAGAAAAGTTGAGGGAGACCAAAAATGACTTTTT-GA 76532
Bonobo            GTGAGGTAGAGTGGCGCATGATGCATCCTTATTCTT-CCTTTGGAGAAAAGTTGAGGGAGACCAAAAATGACTTTTT-GA 75329
OliveBaboon       GTGAGCTGGAGTGATGCATGATGCATCCTTATTCTTTCCTTTGGAGAAAAGTTGAGGGAGACCAAAAATGACTTTTT-GA 76428
Orangutan         GTGAGGTAGAGTGGTGCATGATGCATCCTTTTTCTTTCCTTCGGAGAAAAGTTGATGGAGACCAAAAATGACTTTTT-GA 77091

Majority          GGGGATTGTAGAAG---TTTCATTAGAAGAA--AAGTAAGTTTTTAATTAAAAAGTTAATCTGAGGAACAGGTAGAATAA

                          92090     92100     92110     92120     92130     92140     92150     92160

Human             GGGAATTGTAGAAG---TTTCATTAGAAGAA--AAGTAAGTTTTTAATTAAAAAGTTAATCTGAGGAACAGGTAGAATAA 76305
Coquerel'sSifaka  GAGGGATGTAGAAGAAGTTTCATTAGAACAG--AGGTAAGCTTTTAATTAAAAAAATTTCTCTAAGGAACCAGCTGAACAA 63624
Ma'sNightMonkey   GGGGTTTGTAGAAG---TTTCATTGGTGGAAGAAAGTAAGTTTTTAATTAAAAAGTTTTTCTGAGGAACAGGTAGAGTAA 71045
VervetAGM         GGGGATTGTGGAAG---TTTCATTAGAAGAA--AAGTAAGTTTTTAATTAAAAAGTTAATCTGAGGAACAGGTAGAATAA 77694
Gorrilla          GGGGATTGTAGAAG---TTTCATTAGAAGAA--AAGTAAGTTTTTAATTAAAAAGTTAATCTGAGGAACAGGTAGAATAA 74855
GrabEatingMacaqueGGGGATTGTGGAAG---TTTCATTAGAAGAA--AAGTAAGTTTTTAATTAAAAAGTTAATCTGAGGAACAGGTAGAATAA 77237
Macaque           GGGGATTGTGGAAG---TTTCATTAGAAGAA--AAGTAAGTTTTTAATTAAAAAGTTAATCTGAGGAACAGGTAGAATAA 78107
PigTailedMacaque  GGGGATTGTGGAAG---TTTCATTAGAAGAA--AAGTAAGTTTTTAATTAAAAAGTTAATCTGAGGAACAGGTAGAATAA 76607
Bonobo            GGGGATTGTAGAGG---TTTCATTAGAAGAA--AAGTAAGTTTTTAATTAAAAAGTTAATCTGAGGAACAGGTAGAATAA 75404
OliveBaboon       GGGGATTGTGGAAG---TTTCATTAGAAGAA--AAATAAGTTTTTAATTAAAAAGTTAATCTGAGGAACACGTAGAATAA 76503
Orangutan         GGGGATTGTAGAAG---TTTCATTAGAAGAA--AAGTAAGTTTTTAATTAAAAAGTTAATCTGAGGAACAGGTAGAATAA 77166
```

```
Majority          AAGTGTAGTTGTTAATGGTAGAA--GAGAATGGATTCCATAGGGCAAAATAAGAACT-AAGGGAAGGTTGGTGGAAGAGG

                           92170     92180     92190     92200     92210     92220     92230     92240

Human             AAGTGTAGTTGTTAGTGGTAGAA--GAGAATGGATTCCATAGGGCAAAATAAGAACTCAAGGGAAGGTTGGTGGAAGAGG 76383
Coquerel'sSifaka  AAATGCAGTTGTTAATGGTAGAAAAGAGAATGGATTCTATACAGAAAAATTGGAATTAAAGGAAAGGTTGGCGGAAGAGG 63704
Ma'sNightMonkey   AAGT-T--------ATGGTAGAA--GAGAATGGATTCCATAGGGCAAAATAAGAACTGAAGGGAAGGTTGGTGGAAGAGG 71114
VervetAGM         AAGTGTAGCTGTTAATGGTAGAA--GAGAATGGATTCCATAAGGCAAAATAAGAACG-AAAGGAAGGTTGGTGGAAGAGA 77771
Gorrilla          AAATGTAGTTGTTAGTGGTAGAA--GAGAATGGATTCCATAGGGCAAAATAAGAACTCAAGGGAAGGTTGGTGGAAGAGG 74933
GrabEatingMacaqueAAGTGTAGCTGTTAATGATAGAA--GAGAATGGATTCCATAAGGCAAAATAAGAACG-AAAGGAAGATTGGTGGAAGAGA 77314
Macaque           AAGTGTAGCTGTTAATGATAGAA--GAGAATGGATTCCATAAGGCAAAATAAGAACG-AAAGGAAGATTGGTGGAAGAGA 78184
PigTailedMacaque  AAGTGTAGCTGTTAATGGTAGAA--GAGAATGGATTCCATAAGGCAAAATAAGAACG-AAAGGAAGGTTGGTGGAAGAGA 76684
Bonobo            AAGTGTAGTTGTTAGTGGTAGAA--GAGAATGGATTCCATAGGGCAAAATAAGAACTCAAGGGAAGGTTGGTGGAAGAGG 75482
OliveBaboon       AAGTGTAGCTGTTAATGGTAGAA--GAGAATGGATCCCATAAGGCAAAATAAGAACG-AAAGGAAGGTTGGTGGAAGAGA 76580
Orangutan         AAGTGTAGTTGTTAGTGGTAGAA--GAGAATGGATTCCATAGGGCAAAATAAGAACTCAAGGGAAGGTTGGTGGAAGAGG 77244

Majority          AAGAGGATTGAATTGTTTCAAGAAAGAATAGCAGTTGTCATGCTTATGAAAAGTAAAATTTTTATTTTCAAATCAGGAAA

                           92250     92260     92270     92280     92290     92300     92310     92320

Human             AAGAGGATTGAATTGTTTCAAGAAAGAATAGCAGTTGTCATCCTTATGAAAAGTAAAATTTTTATTTTCAAATCAGGAAA 76463
Coquerel'sSifaka  A------TTGGATTGTTG-AGGAAAGAATAGCAGTTGTCATTCTTATCTAAAGTAAAATTTTTATTTTCAGATCAGGAAA 63777
Ma'sNightMonkey   AAGAGAATTGAATTGTTTCAGGAAAGAATAGCATTTATCATGCTTATGAAAAGTAAAATTTTTATTTTCAAATCAGAAAA 71194
VervetAGM         AAGAGGATTGAATTGTTTCAAGAAAGAATAGCAGGTGTCATGCTTATGAAAAGTAAAATTTTTATTTTCAAATCAGGAAA 77851
Gorrilla          AAGAGGATTGAATTGTTTCAAGAAAGAATAGCAGTTGTCATCCTTATGAAAAGTAAAATTTTTATTTTCAAATCAGGAAA 75013
GrabEatingMacaqueAAGAGGATTGAATTGTTTCAAGAAAGAATAGCAGGTGTCATGCTTATGAAAAGTAAAATTTTTATTTTCAAATCAGGAAA 77394
Macaque           AAGAGGATTGAATTGTTTCAAGAAAGAATAGCAGGTGTCATGCTTATGAAAAGTAAAATTTTTATTTTCAAATCAGGAAA 78264
PigTailedMacaque  AAGAGGATTGAATTGTTTCAAGAAAGAATAGCAGGTGTCATGCTTATGAAAAGTAAAATTTTTATTTTCAAATCAGGAAA 76764
Bonobo            AAGAGGATTGAATTGTTTCAAGAAAGAATAGCAGTTGTCATCCTTATGAAAAGTAAAATTTTTATTTTCAAATCAGGAAA 75562
OliveBaboon       AAGAGGATTGAATTGTTTCAAGAAAGAATAGCAGGTGTCATGCTTATGAAAAGTAAAATTTTTATTTTCAAATCAGGAAA 76660
Orangutan         A-GAGGATTGAATTGTTTCAAGAAAGAATATCAGTTGTCATCCTTATGAAAAGTAAAATTTTTATTTTCAAATCAGGAAA 77323

Majority          TAAAATGTAAAATGTGCCTTCCAGACCCCTTGGTA---TACATGGAAGATTGGTTCTAGGACACACACAGTCCCACTCCC

                           92330     92340     92350     92360     92370     92380     92390     92400

Human             T-----GTAAAATGTGCCTTCCAGACCCCTTGGTGGTATACATGGGAGATTGGTTCTAGGACACACACAGTCCCATCCCC 76538
Coquerel'sSifaka  TAAAATATAAAAAATGCTGTCCTCAGCC---AGGA--CCCCATACAAGTATCCCCTCAATATCCATGGGGAATTGGTTCT 63852
Ma'sNightMonkey   TAAAATATAAAAAGTGCCTTCAAGACCCCTTGGTA---TGCATGGGAGATTGGTTCTAGGACACACACAGCCCCACGCCA 71271
VervetAGM         TAAAATGTAAAATGTGCCTTCCAGACCCCTTGGTA---TACATGGAAGATTGGTTCTAGGACACACACAGTCCCACTCCC 77928
Gorrilla          TAAAACGTAAAATGTGCCTTCCAGACCCCTTGGTGGTATACATGGGAGATTGGTTCTAGGACACACACAGTCCCATCCCC 75093
GrabEatingMacaqueTAAAATGTAAAATGTGCCTTCCAAACCCCTTGGTA---TACATGGAAGATTGGTTCTAGGACACACACAGTCCCACTCCC 77471
Macaque           TAAAATGTAAAATGTGCCTTCCAAACCCCTTGGTA---TACATGGAAGATTGGTTCTAGGACACACACAGTCCCACTCCC 78341
PigTailedMacaque  TAAAATGTAAAATGTGCCTTCCAAACCCCTTGGTA---TACATGGAAGATTGGTTCTAGGACACACACAGTCCTACTCCC 76841
Bonobo            TAAAACGTAAAATGTGCCTTCCAGACCCCTTGGTGGTATACATGGGAGATTGGTTCTAGGACACACACAGTCCCATCCCC 75642
OliveBaboon       TAAAATGTAAAATGTGCCTTCCAGACCCCTTGGTA---TACATGGAAGATTGGTTCTAGGACACACACAGTCCCACTCCC 76737
Orangutan         TAAAATGTAAAATGTGCCTTCCAGACCCCTTGGTG---TACATGGGAGATTGGTTCTAGGACACACACAGTCCCACCCCC 77400
```

```
Majority         CACC---TACCCCCGCCCCCACACACCCCCTGGATACTAAAATCCACTGATGCTCAAGTTCCTTGTATAAAATGGTATAG
                         92410    92420    92430    92440    92450    92460    92470    92480
Human            CACC-----CTCT-GACCCCATACACCCCCTGGATACTCAAATCCACTGATGCTCAAGTTCCTTGCATAAAATGGTATAG 76612
Coquerel'sSifaka AGGA----------ACTCCCC--TGCCTCCTGTATACCAGAATCCATGGATGCTCAAGTTCCTTATATAAAATGGTATAG 63920
Ma'sNightMonkey  CCCC---TACTCCCACTCCCAAACACACCCTGGATACTAAAATCCACTGATGCTCAAGTACCTTGTATAAAATGGTATAG 71348
VervetAGM        CACCGCCTACCCCCGCCCCCACACACCCCCTGGATACTAAAATCCACTGGTGCTCAAGTTCCTTGTATAAAACGGTATAG 78008
Gorrilla         CACC-----CTCT-GCCCCCATACAGCCCCTGGATACTCAAATCCACTGATGCTCAAGTTCCTTGTATAAAATGGTATAG 75167
GrabEatingMacaqueTACCGCCTACCCCCGCCCCCACATACCCCC-GGATACTAAAATCCACTGATGCTCAAGTTCCTTGTATAAAATGGTATAG 77550
Macaque          TACCGCCTACCCCCGCCCCCACATACCCCC-GGATACTAAAATCCACTGATGCTCAAGTTCCTTGTATAAAATGGTATAG 78420
PigTailedMacaque TACCGCCTACCCCCTCCCCCACACACCCCC-GGATACTAAAATCCACTGATGCTCAAGTTCCTTGTATAAAATGGTATAG 76920
Bonobo           CACC-----CTCT-GACCCCATACACCCCCTGGATACTCAAATCCACTGGTGCTCAAGTTCCTTGTATAAAATGGTATAG 75716
OliveBaboon      CACCGCCTACCCCCGCCCCCACACACCCCC-AGATACTAAAATCCACTGATGCTCAAGTTCCTTGTATAAAATGGTATAG 76816
Orangutan        CACC-----CCCT-GCCCCCATACACCCCCTGGATACTCAAATCCGCTGATGCTCAAGTTCCTTGTATAAAATGGTATAG 77474

Majority         TGTTTGCATATGACCTATACACAACCTCTTATATGTACTTTAAATCATCTCTAGATTACTTGTATTACCCAGTACAATAT
                         92490    92500    92510    92520    92530    92540    92550    92560
Human            TGTTTGCATGTGACCTATACACAACCTCTTATGTGTACTTTAAATCATCTCTAGATTACTTATATTACCCAGTACAATAT 76692
Coquerel'sSifaka TATTTGCATATCACTTATGCACCACCTCCTGTATATACTTTAAATCATCT-----CTACATGTAATACCTAGTACAACAT 63995
Ma'sNightMonkey  TGTTTGTATATGACCTATGCACAACCTCTTATATGTACTTTAAATCATCTCTAGATTACTTATATTACCCAAAACAATAT 71428
VervetAGM        TGTTTGCATATGACCTATACACAACCTCTTATATGTACTTTAAATCATCTTTAGATTACTTGTATTACCCAGTACAATAT 78088
Gorrilla         TGTTTGCATATGACCTATACACAACCTCTCATGTGTACTTTAAATCATCTCTAGATTACTTATATTACCCAGTACAATAT 75247
GrabEatingMacaqueTGTTTGCATATGACCTATACACAACCTCTTATATGTACTTTAAATCATCT--AGATTACTTGTATTACCCAGTACCATAT 77628
Macaque          TGTTTGCATATGACCTATACACAACCTCTTATATGTACTTTAAATCATCTTTAGATTACTTGTATTACCCAGTACCATAT 78500
PigTailedMacaque TGTTTGCATATGACCTATACACAACCTCTTATATGTACTTTAAATCATCTTTAGATTACTTGTATTACCCAGTACCATAT 77000
Bonobo           TGTTTGCATATGACCTATACACAACCTCTTATGTATACTTTAAATCATCTCTAGATTACTTATATTACCCAGTACAATAT 75796
OliveBaboon      TGTTTGCATATGACCTATACACAACCTCTTATATGTACTTTAAATCATCTTTAGATTACTTGTATTACCCAGTACAATAT 76896
Orangutan        TGTTTGCTTCTGACCTATACACAACCTCTTATATGTACTTTAAATCATCTCTAGATTACTTATATTACCCAGTACAATAT 77554

Majority         AAATGCTATGTAAATGGTTGTTATACTGTATTGTTTAGGGAATAA-TGACAAGAACAACTTTCTATACATTTGCAGTACA
                         92570    92580    92590    92600    92610    92620    92630    92640
Human            AAATGTTATGTAAATGGTTGTTATAGTGTATTGTTTAGGGAATAA-TGACAAGAACAACTTTCTATACATTTGCAGTACA 76771
Coquerel'sSifaka AAATGCTTTGTAAATAGTTGTTACACTGTATTGTTTAGGGAATAA-TGACAAGAAAAA----GTGTATATGTTCAGTACA 64070
Ma'sNightMonkey  AAATGCTATGTAAATGGTTGTTACACTGTATTGTTTAGATAATAAATGACAAGAGCAACTTTCTATATGTGTGCAGTACA 71508
VervetAGM        AAATGCTATGTAAATGGTTGTTATACTATATTGTTTAGGGAATAA-GGACAAGAACAACTTTCTATACATGTGCAGTACA 78167
Gorrilla         AAATGTTATGTAAATGGTTGTTATACTGTATTGTTTAGGGAATAA-TGACAAGAACAACTTTCTATACATTTGCAGTACA 75326
GrabEatingMacaqueAAATGCTATGTAAATGGTTGTTATACTATATTGTTTAGGGAGTAA-GGACAAGAACAACTTTCTATACATATGCAGTACA 77707
Macaque          AAATGCTATGTAAATGGTTGTTATACTATATTGTTTAGGGAATAA-GGACAAGAACAACTTTCTATACATATGCAGTACA 78579
PigTailedMacaque AAATGCTATGTAAATGGTTGTTATACTATATTGTTTAGGGAATAA-GGACAAGAACAACTTTCTATACATATGCAGTACA 77079
Bonobo           AAATGTTATGTAAATGGTTGTTATACTGTATTGTTTAGGGAATAA-TGACAAGAACAACTTTCTATACATTTGCAGTACA 75875
OliveBaboon      AAATGCTATGTAAATGGTTGTTATACTATATTGTTTGGGGAATAA-GGACAAGAACAACTTTCTATACATGTGCAGTACA 76975
Orangutan        AAACGTTATATAAATGGTTGTTATACTGTATTGTTTAGGGAATAA-TCACAAGAACAACTTTGTATACATTTGCAGTACA 77633
```

```
Majority          -------CCATCGTTTTC---CCCCCAAATATTTTTGATCCAAGGTTGGTTGAATCGGAACCCAGAGATACAGAGGGCTG

                          92650     92660     92670     92680     92690     92700     92710     92720

Human             -------CCATTGTTTTA---CCCCCAAATATTTTTGATCCAAGGTTGGTTGAATCGGAACCCAGAGATACAGAGGGCTG 76841
Coquerel'sSifaka  GATACAAGCATCATTTTTTTACCCCTGAATGTTTTTGATCTGAGGT-GGTTGAATT------CACAGATACAG------- 64136
Ma'sNightMonkey   GATGCAGCCATCGTTTTTT--CCCCCAAATATTTTTGATCCAAGGTTGGTTGAATCGGAACCCAGACCTATAGAGGGCTG 71586
VervetAGM         -------CCATCGTTTTC---CCCCCAAATATTTTT-ATCCAAGGTTGGTTGAATCGGAACCCAGAGATACAGAGGGCTG 78236
Gorrilla          -------CCATTGTTTTC---CCCCCAAATATTTTTGATCCAAGGTTGGTTGAATTGGAACCCAGAGATACAGAGGGCTG 75396
GrabEatingMacaque-------CCATCGTTTTC---CCCCCAAATATTTTT-ATCCAAGGTTGGTTGAATCGGAACCCAGAGATACAGAGGGCTG 77776
Macaque           -------CCATCGTTTTC---CCCCCAAATATTTTT-ATCCAAGGTTGGTTGAATCGGAACCCAGAGATACAGAGGGCTG 78648
PigTailedMacaque  -------CCATCGTTTTC---CCCCCAAATATTTTT-ATCCAAGGTTGGTTGAATCGGAACCCAGAGATACAGAGGGCTG 77148
Bonobo            -------CCATTGTTTTC---CCCCCAAATATTTTTGATCCAAGGTTGGTTGAATCGGAACCCAGAGATACAGAGGGCTG 75945
OliveBaboon       -------CCATCGTTTTC---CCCCCAAATATTTTT-ATCCAAGGTTGGTTGAATCGGAACCCAGAGATACAGAGGGCTG 77044
Orangutan         -------CCATTGTTTTC---CCCCCAAATATTTTTGATCCAAGGTTGGTTGAATCGGAACCCAGAGATACAGAGGGCTG 77703

Majority          ACTATACTTT-AAGAATTAGA---ATTAGCCAGTCGTGGTTGCAGGTGCCTGTAGTCCCAGCTACTCGGGAGGCTGAGTC

                          92730     92740     92750     92760     92770     92780     92790     92800

Human             ACTATACTTT-AAGAATTAGA---ATTAGCTGGGTGTGGTGGTGGGTGCCTGTAGTCCCAGCTACTCGGGAGGCTGACGC 76917
Coquerel'sSifaka  ------------------------------------------------G--------------------------- 64137
Ma'sNightMonkey   ACTATACTTT-AAGAATTAGATGGGGTGGCCAGGAGCGGTGGCTTACACCTGTAACCCCAGCACTTTGGGAGGCCAAGTA 71665
VervetAGM         ACTATACTTT-AAGAATTAGA---ATTAGCCAGTCATGGTTGCAGGTGCCTGTAGTCCCAGCTACTTGGGAGGCTGAGTC 78312
Gorrilla          ACTATACTTT-AAGAATTAGA---ATTAGCTGGGTGTGGTGGTCGGTGCCTGTAGTCCCAGCTACTCGGGAGGCTGACGC 75472
GrabEatingMacaqueACTATACTTT-AAGAATTAGA---ATTAGCCAGTCATGGTTGCAGGCGCCTGTAGTCCCAGCTGCTCCGGAGGCTGAGTC 77852
Macaque           ACTATACTTT-AAGAATTAGA---ATTAGCCAGTCATGGTTGCAGGCGCCTGTAGTCCCAGCTACTCCGGAGGCTGAGTC 78724
PigTailedMacaque  ACTATACTTT-AAGAATTAGA---ATTAGCCAGTCATGGTTGCAGGCGCCTGTAGTCCCAGCTACTCCGGAGGCTGAGTC 77224
Bonobo            ACTATACTTT-AAGAATTAGA---ATTAGCTGGGTGTGGTGGTGGGTGCCTGTAGTCCCAGCTACTCGGGAGGCTGACGC 76021
OliveBaboon       ACTATACTTT-AAGAATTAGA---ATTAGCCAGTCATGGTTGCAGGTGCCTGTAGTCCCAGCTACTCGGGAGGCTGAGTC 77120
Orangutan         ACCATACTTTTAAGAATTAGA---ATTAGCTGGGTGTGGTGGTGGGTGCGTGTAG-------------GGAGGCTGACGC 77767

Majority          AGGAGAATGGCATGAACCTGGGAGGTGGAGCTTGTAGT---GAGCCGAGATCGTGCCACTGCGCTCCAGCCTGGGCGACA

                          92810     92820     92830     92840     92850     92860     92870     92880

Human             AGGAGAAAGGCGTGAACCCGGGAGGTGGAGCTTGCAGT---GAGCCGAGATCGTGCCACTGCACTCCAGCCTGGGCGACA 76994
Coquerel'sSifaka  -------ATCCA-------GGGATATGGAG----------G------------GCCGCTGTACTTTA----------A 64169
Ma'sNightMonkey   GTTCAAAAACCAGCATGATGAGACTTGAGGTCAGTAGTTAAAAACCAGCATGATGAAACCCTGTCTCTACTAAGAATACA 71745
VervetAGM         AGGAGAATGGCATGAACCTGGGAGGTGGAGCTTATAGT---GAGCCGAGATCGTGCCACAGCGCTCCAGCCTGGGCGACA 78389
Gorrilla          AGGAGAAAGGCATGAACCCGGGAGGTGGAGCTTGCAGT---GAGCCGAGATCGTGCCACTGCACTCCAGCCTGGGCGACA 75549
GrabEatingMacaqueAGGAGAATGGCATGAACCTGGGAGGTGAAGCTTATAGT---GAGCCGAGATCGTGCCACAGCGCTCCAGCCTGGGCGACA 77929
Macaque           AGGAGAATGGCATGAACCTGGGAGGTGAAGCTTATAGT---GAGCCGAGATCGTGCCACAGCGCTCCAGCCTGGGCGACA 78801
PigTailedMacaque  AGGAGAATGGCATGAACCTGGGAGGTGAAGCTTATAGT---GAGCCGAGATCGTGCCACAGCGCTCCAGCCTGGGCGACA 77301
Bonobo            AGGAGAAAGGCGTGAACCCGGGAGGTGGAGCTTGCAGT---GAGCCGAGATCGTGCCACTGCACTCCAGCCTGGGCGACA 76098
OliveBaboon       CAGAGAATGGCATGAACCTGGGAGGTGAAGCTTATAGT---GAGCCGAGATTGTGCCACAGCGCTCCAGCCTGGGCGATA 77197
Orangutan         AGGAGAATGGCGTGAACCCGGGAGGTGGAGCTTGCAGT---GAGCCGAGATCGTGCCACTGCACTCCAGCCTGGGCGACA 77844
```

```
Majority         GAGCGAGACTCTGTCTCAAAAAAAAAAAAAAAAAAAAAAAAATTAGA-AGGTGCT---------------------------

                         92890     92900     92910     92920     92930     92940     92950     92960

Human            GAGCGAGACTCTGTCT----CTAAAAAAAAAAAAAAAAAAAATTAGA-AGGGGCT--------------------------- 77042
Coquerel'sSifaka GAATTAG---------------------A---AAAA---------------------------------------------- 64181
Ma'sNightMonkey  AAATTAG-CCCGATGTGATAGCAGGCGCCTGTAATCTCAGCTACTCAGGAGACTGAGGCAGGAGAATCACTTGAACCTGG 71824
VervetAGM        GAGCGAGACTCTGTCTCAAAAAAAAAAAAAAAAAAGGTA-------------GCT--------------------------- 78429
Gorrilla         AAGTGAGACTCTGTCTCCAAAAAAAAAAAAAAAAAAAAGAATTAGA-AGGGGCT--------------------------- 75601
GrabEatingMacaqueGAGCGAGACTCCGACTCAAAAAAAAAAAAAAAAAAA----TTAGA-AGGTGCT--------------------------- 77977
Macaque          GAGCGAGACTCCGACTCAAAAAAAAAAAAAAAAAAAAAAAAATTAGA-AGGTGCT--------------------------- 78853
PigTailedMacaque GACCGGGACTCCGACTCAAAAAAAAAAAAAAAAAAAAAAAAATTAGA-AGGTGCT--------------------------- 77353
Bonobo           GAGCGAGACTCTGTCTA-AACTAAAAAAAAANNNNNNNNNNNNN-NNNNNNTACGTCGTACTAAAAAACTAAAAAAAAN 76176
OliveBaboon      GAGCGAGACTCCGTCTCAAAAAAAAAAAAAAAAAGAAAAATTAGA-AGGTGCT--------------------------- 77249
Orangutan        GAGCGAGACTCTGTCTC-AAAAAAAAAAAAATACAAACAATTAGA-AGGGGCT--------------------------- 77895

Majority         -------------------------GTCAGGCGCGATGGTTCTTGCCT--------------------GTAATCCCAGCA

                         92970     92980     92990     93000     93010     93020     93030     93040

Human            -------------------------GTCAGGCGTGGTGTCTCATGCCTA--TAATCCCAGTCATGCCTGTAATCCCAGCA 77095
Coquerel'sSifaka ------------------------------------------GCCT-------------------------------- 64185
Ma'sNightMonkey  GAGGTGGAGGTTGCAGTGAGCTGAGATCACGCCATTGCACTCCAGCCTGGGCGACAGTGAAACTCCATCTCAAAAAAAAA 71904
VervetAGM        -------------------------GCCAGGCGCGATGGTTCTTGCCT--------------------GTAATGCCAGCA 78464
Gorrilla         -------------------------GTCAGGCGTGGTGGCTCATGCCTA--TAATCCCAGTCATGCCTATAATCCCAGCA 75654
GrabEatingMacaque-------------------------GCCAGGCGCGATGGTTCTTGCCT--------------------GTAATCCCAGCA 78012
Macaque          -------------------------GCCAGGCGCGATGGTTCTTGCCT--------------------GTAATCCCAGCA 78888
PigTailedMacaque -------------------------GCCAGGCGCGATGGTTCTTGCCT--------------------GTAATCCCAGCA 77388
Bonobo           AAAAAAAAAAGAATTAGAAGGGGCTGTCAGGCGTGGTGGCTCATGCCTA--TAATCCCAGTCATGCCTGTAATCCCAGCA 76254
OliveBaboon      -------------------------GCCAGGCGCGATGGTTCTTGCCT--------------------GTAATCCCAGCA 77284
Orangutan        -------------------------GTCAGGCATGGTGGCTCATGCCT--------------------GTAATCCCAGCA 77930

Majority         CTTTGGGAGGCTGAGGCGGGCATATCA-----CTTGAGGTGAGGAGTTTAAAACCAGCCTGGCCAACATGGTGAAACAAC

                         93050     93060     93070     93080     93090     93100     93110     93120

Human            CTTTGGGAGGCTAAGGTGGGCATATCA-----CTTGAGATCATGAGTTCAAAACCAGCCTGGCCAACATGGTGAAACA-C 77169
Coquerel'sSifaka -------------------------------------------------------------------------------- 64185
Ma'sNightMonkey  AAAAGGAAAAAAAAAAAAGAATTAGAAGGGACTTTGGAGTCCCACCACTAATCCTCTTCTGTTCAGTAGTACACAAGACT 71984
VervetAGM        CTTTGGGAGGCTGAGGCGGGCATGTCA-----CTTGAGGTGAGGAGTTTAAAACCAGCCTGGCCAACATGGTGAAACAAC 78539
Gorrilla         TTTTGGGAGGCTAAGGTGGGCATATCA-----CTTGAGGTCTTGAGTTCAAAACCAGCCTGGCCAACATGGTGAAACA-C 75728
GrabEatingMacaqueCTTTGGGAGGCTGAGGCGGGCATATCA-----CTTGAGGTGAGGAGTTTAAAACCAGCCTGGCCAACATGGTGAAACAAC 78087
Macaque          CTTTGGGAGGCTGAGGCGGGCATATCA-----CTTGAGGTGAGGAGTTTAAAACCAGCCTGGCCAACATGGTGAAACAAC 78963
PigTailedMacaque CTTTGGGAGGCTGAGGCGGGCATATCA-----CTTGAGGTGAGGAGTTTAAAACCAGCCTGGCCAACATGGTGAAACAAC 77463
Bonobo           CTTTGGGAGGCTAAGGTGGGCATATCA-----CTTGAGGTCATGAGTTCAAAACCAGCCTGGCCAACATGGTGAAACA-C 76328
OliveBaboon      CTTTGGGAGGCTGAGGCGGGCATATCA-----CTTGAGGTGAGGAGTTTAAAACCAGCCTGGCCAACATGGTGAAACAAC 77359
Orangutan        CTTTGGGAGGCTGAGGTGGGCATATCA-----CTTGAGGTCAGGAGTTCAAAACCAGCCTGGCCAACATGGTGAAACA-C 78004
```

```
Majority        CGTCTCTACTAAAAAT-ACAAAAATTAGCCAGGTGTGTTGGTGGGCGCCTGTAATCTTAGCTCCTCAGGAAGCTGAGTCA
                         93130     93140     93150     93160     93170     93180     93190     93200
Human           TGTCTCTACTAAAAAT-ACAAATATTAGCCAGGTGTGGTGGTGGGCGCCTGTAATCTTAGCTACTCAGGAGGCTGAGTCA 77248
Coquerel'sSifaka -------------T------------------------------------------------------------------ 64186
Ma'sNightMonkey ACTAACTACCAGATGTCATGAACAGGTAAAAGAAGTAAGGCCAAAAGAA-ATCATGTAAATTACTTTGGATGGAAGTTTT 72063
VervetAGM       CATCTCTACTAAAAAT-ACAAAAATTAGCCAGGTGTGTTGGTGGGCGCCTGTAATCTTAGCTCCTCAGGAAGCTGAGTCA 78618
Gorrilla        TGTCTGTACTAAAAAT-ACAAATACTAGCCAGGTGTGGTGGTGGGCGCCTGTAATCTTAGCTACTCAGGAGGCTGAGTCA 75807
GrabEatingMacaqueCGTCTCTACTAAAAAT-ACAAAAATTAGCCAGGTGTGTTGGT----------AATCTTAGCTCCTCAGGAAGCTGAGTCA 78156
Macaque         CGTCTCTACTAAAAAT-ACAAAAATTAGCCAGGTGTGTTGGT----------AATCTTAGCTCCTCAGGAAGCTGAGTCA 79032
PigTailedMacaque CGTCTCTACTAAAAAT-ACAAAAATTAGCCAGGTGTGTTGGTGGGTGCCTGTAATCTTAGCTCCTCAGGAAGCTGAGTCA 77542
Bonobo          TGTCTCTACTAAAAAT-ACAAATATTAGCCAGGTGTGGTGGTGGGCGCCTGTAATCTTAGCTACTCAGGAGGCTGAGTCA 76407
OliveBaboon     CGTCTCTACTAAAAAT-ACAAAAATTAGCCAGGTGTGTTGGTGGGCGCCTGTAATCTTAGCTCCTCGGGAAGCTGAGTCA 77438
Orangutan       TGTCTCTACTAAAAAT-ACAAATATTAGCCACGTGTGGTGGTGGGCGCCTGTAATCTTAGCTACTCAGGAGGCTGAGTCA 78083
```

```
Majority        GGAGAATCGCTTGAACCCAGGAGGTGGAGGTTGCAGTGAGCTG-AGATCATGCCATTGCACTCCAG------CCTGGGCG
                         93210     93220     93230     93240     93250     93260     93270     93280
Human           GGAGAATTGCTTGAACCCAGGAGGTGGAGGTTGCAGTGAGCTG-AGATCATGCCATTGCACTCCAG------CCTGTGTG 77321
Coquerel'sSifaka ------A---------------------------------------------------------G------------- 64188
Ma'sNightMonkey AGAGTGGGAAGTGGAAGGATAAATTTGACGTTCTTCAAACTCGTAGATTAAACTCTTACAAGTAAGGGGAACCTAAGGAG 72143
VervetAGM       GGAGAATCGCTCGAACCCAGGAGGTGGAGGTTGCAGTGAGCTG-AGATCATGCCATTGCACTCCAG------CCTGGGCG 78691
Gorrilla        GGAGAATTGCTTGAACCCAGGAGGTGGAGGTTGCAGTGAGCTG-AGATCATGCCATTGCACTCCAG------CCTGTGTG 75880
GrabEatingMacaqueGGAGAATCGCTTGAACCCAGGAGGTGGAGGTTGCAGTGAGCTG-AGATCATGCCATTGCACTCCAG------CCTGGGCA 78229
Macaque         GGAGAATCGCTTGAACCCAGGAGGTGGAGGTTGCAGTGAGCTG-AGATCATGCCATTGCACTCCAG------CCTGGGCG 79105
PigTailedMacaque GGAGAATCGCTTGAACCCAGGAGGTGGAGGTTGCAGTGAGCTG-AGATCATGCCATTGCACTCCAG------CCTGGGCG 77615
Bonobo          GGAGAATTGCTTGAACCGAGGAGGTGGAGGTTGCAGTGAGCTG-AGATCATGCCAT-GCACTCCAG------CCTGTGTG 76479
OliveBaboon     GGAGAATCGCTTGAACCCAGGAGGTGGAGGTTGCAGTAAGCTG-AGATCATGCCATTGCACTCCAG------CCTGGGCG 77511
Orangutan       GAAGAATTGCTTGAACCCAGGAGGTGGAGGTTGCAGTGAGCTG-AGATCATGCCATTGCACTCCAG------CCTGCGTG 78156
```

```
Majority        ACAGAGTGA---AACTCCAT--------CTCAAAAAAAAAAAAAAAAATAGAA---GGGGCTTTAG--AGTCCTACCACG
                         93290     93300     93310     93320     93330     93340     93350     93360
Human           ACAGAGTGA---AACTCCAT--------CTCAAAAATACAAAAAGAATTAGAA---AGGGCTTTAG--AGTCCCACCACT 77385
Coquerel'sSifaka --A-------------------------------------------------------------GTCCCACCACC 64200
Ma'sNightMonkey ACATGATGATTAAATGTAATAGGAGATACTGGAACAACAAAAAAGGGACATTAGATGCAAATTGAGGAAATCTGATAAAG 72223
VervetAGM       ACAGAGTGA---AACTCCAT--------CTCAAAAAAAAAAAAAAGAAGAAG-----GGGCTTTAG--AGTCCTACCACC 78753
Gorrilla        ACAGAGTGA---AACTCCAT--------CTCAAAAATACAAAAAGAATTAGAA---GGGGCTTTAG--AGTCCCACCACT 75944
GrabEatingMacaqueACAGAGTGA---AACTCCAT--------CTCAAAAAAAAAAAAAAAAAAAAA------GGCTTTAG--AGTCCTACCACG 78290
Macaque         ACAGAGTGA---AACTCCAT--------CTCAAAAAAAAAAAAAAAAAAAAAAAAAGGGGGCTTTAG--AGTCCTACCACG 79172
PigTailedMacaque ACAGAGCGA---AACTCTGT--------CTCAAAAAAAAAAAAAAAAAAGAA----GGGGCTTTAG--AGTCCTACCACG 77678
Bonobo          ACAGAGTGA---AACTCCAT--------CTCAAAAATACAAAAATAATTAGAA---AGGGCTTTAG--AGTCCCACCACT 76543
OliveBaboon     ACAGAGTGA---AACTCCAT--------CTCAAAAAAAAAAAAAAAAACAGAA---GGGGCTTTAG--AGTCCTACCACG 77575
Orangutan       ACAGAGTGA---AACTCCAT--------CTCAAA------AAAAGAATT--------------TGG--AGTCCCACCACT 78203
```

```
Majority          AATGCTCTTCTGTTCAGTAGTACACAAGACTAAT-TACTACCAGATGTTATGAACAGGGAAAAGAAGTAAGACCAAAAAA
                           93370     93380     93390     93400     93410     93420     93430     93440
Human             AATGCTCTTCTGTTCAGTAGTACACAAGACTAAT-AACTACGAGATGTTATGAACAGGGAAAAGAAGTAAGACCAAAAGA 77464
Coquerel'sSifaka  AGTCCTCTTCTGTTCATTAGTACACAAGATTGAT-AACTACCAGATGTCTTGAAGATGGAAAAGAAGACCAAAAGAGAAA 64279
Ma'sNightMonkey   AATTTTTTTTTTTTTTTTGAGACAGAGTCTTGCTCTGTTGCCAGCTGCCA-GGCTGGAGTGCAGTGGCACTACCTTGGCT 72302
VervetAGM         AATGCTCTTCTGTTCAGTAGTACACAAGACTAAT-TACTACCAGATGTTGTGAACAGGGAAAAGAAGTAAGACCAAAAAA 78832
Gorrilla          AATGCTCTTCTGTTCAGTAGTACACAAGACTAAT-AACTACCAGATGTTATGAACAGGGAAAAGAAGTAAGACCAAAAGA 76023
GrabEatingMacaqueAATGCTCTTCTGTTCAGTAGTACACAAGACTAAT-TACTACCAGATGT---GAACAGGGAAAAGAAGTAAGACCAAAAAA 78366
Macaque           AATGCTCTTCTGTTCAGTAGTACACAAGACTAAT-TACTACCAGATGT---GAACAGGGAAAAGAAGTAAGACCAAAAAA 79248
PigTailedMacaque  AATGCTCTTCTGTTCAGTAGTACACAAGACTAAT-TACTACCAGATGT---GAACAGGGAAAAGAAGTAAGACCAAAAAA 77754
Bonobo            AATGCTCTTCTGTTTAGTAGTACACAAGACGAAT-AACTACGAGATGTTATGAACAGGGAAAAGAAGTAAGACCAAAAGA 76622
OliveBaboon       AATGCTCTTCTGTTCAGTAGTACACAAGACTAAT-TACTACCAGATGTTGTGAACAAGGAAAAGAAGTAAGACCAAAAAA 77654
Orangutan         AATGCTCTTCTGTTCTGTAGTACACAAGGCTAAT-AACTACCAGGTGTTGTGAACAGGGAAAAGAAGTAAGACCAAAAGA 78282

Majority          AATCATAAATTACTT-----TGGATGGA--ACTT--------TTAGAAT----GGAAGATGGAGGGATAAATTTGA----
                           93450     93460     93470     93480     93490     93500     93510     93520
Human             AATCATAAATTACTT-----TGGATGGA--ACTT--------TTAGAAT----GGCAGATGGAGGGATAAATTTGA---- 77521
Coquerel'sSifaka  TAATATAAATGTCTT-----TGGCTGGA--AGTT--------TTAGAAT----GGGAAATGGAAGGATAAAACAAGTCCT 64340
Ma'sNightMonkey   CACTGCAACCTCTTCCTCCCTGGTTCAAGTACTTCTCCTGCCTTAACCTTTGAGTAGCTGGAATTACAGGTGTGCGC-- 72380
VervetAGM         AATCATAAATTACTT-----TGGATGGA--ACTT--------TTAGAAT----GGAAGATGGAGGGATAAATTTGA---- 78889
Gorrilla          AATCATAAATTACTT-----TGGATGGA--ACTT--------TTAGAAT----GGCAGATGGAGGGATAAATTTGA---- 76080
GrabEatingMacaqueAATCATAAATTACTT-----TGGATGGA--ACTT--------TTAGAAT----GGAAGATGGAGGGATAAATTTGA---- 78423
Macaque           AATCATAAATTACTT-----TGGATGGA--ACTT--------TTAGAAT----GGAAGATGGAGGGATAAATTTGA---- 79305
PigTailedMacaque  AATCATAAATTACTT-----TGGATGGA--ACTT--------TTAGAAT----GGAAGATGGAGGGATAAATTTGA---- 77811
Bonobo            AATCATAAATTACTT-----TGGATGGA--ACTT--------TTAGAAT----GGCAGATGGAGGGATAAATTTGA---- 76679
OliveBaboon       AATCATAAATTACTT-----TGGATGGA--ACTT--------TTAGAAT----GGAAGATGGAGGGATAAATTTGA---- 77711
Orangutan         AATCATAAATTACTT-----TGGATGGA--ACTT--------TTAGAAT----GGAAGATGGAGGGATAAATTTGA---- 78339

Majority          -CATTTTTCAGAATCATA-----AACTCTCACAAGTAAGGGGA----ACCTAAGGAGACAAGATGAT-TAGAT-------
                           93530     93540     93550     93560     93570     93580     93590     93600
Human             -CATTGTTCAAAATCATAGAGTAAACTCTCAAAAGTAAGGGGA----ACCTAAGGAGACAAGATGAT-TAAAT------- 77588
Coquerel'sSifaka  ACGTTCTTAAAAATCATAGATTAAACTGTCACAGTTTTAAGGT---CACCTAAGGATAAATGATGAC-TAAAT------- 64409
Ma'sNightMonkey   -CACCATGCCCAGCTAATTTTTGTATTTTTAATAGAGACGGGGTTTCACCATGTTGGCCACGATGGTCTCGATCTCTTGA 72459
VervetAGM         -CATTTTTCAGAATCATA-----AACTCTCACAAGTAAGGGGA----ACCTAAGGAAACAAGATGAT-TAGAT------- 78951
Gorrilla          -CATTGTTCAAAATCATAGAGTAAAC----------------------CTAAGGAGACAAGATGAT-TAAAT------- 76128
GrabEatingMacaque-TATTTTTCAGAATCATA-----AACTCTCACAAGTAAGGGGA----ACCTAAGGAAACAAGATGAT-TAGAT------- 78485
Macaque           -TATTTTTCAGAATCATA-----AACTCTCACAAGTAAGGGGA----ACCTAAGGAAACAAGATGAT-TAGAT------- 79367
PigTailedMacaque  -TATTTTTCAGAATCATA-----AACTCTCACAAGTAAGGGAA----ACCTAAGGAAACAAGATGAT-TAGAT------- 77873
Bonobo            -CATTGTTCAAAATCATAGAGTAAACTCTCTAAAGTAAGGGGA----ACCTAAGGAGACAAGATGAT-TAAAT------- 76746
OliveBaboon       -CATTTTTCAGAATCATA-----AACTCTCACAAGTAAGGGGA----ACCTAAGGAAACAAGATGAT-TAGAT------- 77773
Orangutan         -CATTCTTCAAAATCATA-----AACTCTCACAAGTAAGGGGA----ACCTAAGGAGACAAGATGAT-TAAAT------- 78401
```

```
Majority          ----GTAATAGGAGATACTGGAACAAAAAAAA-GGGGACATTAGATGCA-AATTGAGGAAATCTGA-CAAAGAATGGACT

                          93610     93620     93630     93640     93650     93660     93670     93680

Human             ----GTAATAGGAGATACTGGAACAACAAAAA-AGGGACATTAGATGCA-ATTTGAGGAAATCTGA-TAATG----GACT 77657
Coquerel'sSifaka  ----GTACTATGAGATCCTGGAATAAACAAAG----GATATTAGGTACA-AACTAAGGAAAGCTGAATAAAGAATGAACT 64480
Ma'sNightMonkey   CCTCGTAATCTGCCCACCTTGGCCTCCCAAAGTGCTGGGATTACAGGCGTGAGCCACCTTGCCCAG-CCAAGAATGGACT 72538
VervetAGM         ----GTAATAGGAGATACTGGAACAAAAATAA-GGGTACATTAGATGCA-AATTGAAGAAATCTGA-CAAAGAATGGACT 79024
Gorrilla          ----GTAATAGGAGATACTGGAACAACAAAAA-AGGGACATTAGATGCA-ATTTGAGGAAATCTGA-TAAAGAATGGACT 76201
GrabEatingMacaque ----GTAATAGGAGATACTGGACCAAAAAAAAGGGGGACATTAGATGCA-AATTGAAGAAATCTGA-CAAAGAATGGACT 78559
Macaque           ----GTAATAGGAGATACTGGACCAAAAAAAAGGGGGACATTAGATGCA-AATTGAAGAAATCTGA-CAAAGAATGGACT 79441
PigTailedMacaque  ----GTAATAGGAGATACTGGACCAAAAAAAAAGGGGACATTAGATGCA-AATTGAAGAAATCTGA-CAAAGAATGGACT 77947
Bonobo            ----GTAATAGGAGATACTGGAACAACAAAAA-AGGGACATTAGATGCA-ATTTGAGGAAATCTGA-TAAAGAATCGACT 76819
OliveBaboon       ----GTAATAGGAGATACTGGAACAAAAAAAA-GGGGACATTAGATGCA-AATTGAAGAAATCTGA-CAAAGAATGGACT 77846
Orangutan         ----GTAATAGGAGATACTGGAACAACAAAAA-AGGGACATTAGATGCA-ATTTGAGGAAATCTGA-TAAAGAATGGACT 78474

Majority          TTTGTTAATTTATCAATATTGGTTGATTAATTGCAACAACTAATAAATTGCAATACCATACTAATGTATGGTGCTAATAA

                          93690     93700     93710     93720     93730     93740     93750     93760

Human             TTTGTTGATTTATCGATATTGGTTGATTAATTGCAAGAACTAATAAATTGTAATACCATACTAATGTATGATGCTAATAA 77737
Coquerel'sSifaka  TGAG--------TTAATATTAT---ATCAAT---ACTGGTTAATTAATTGCAACACCATACTAATGTAT--TAATAATGG 64544
Ma'sNightMonkey   TTTG---ATTTATCAATATTGGTTGATTAATTGTAACAACTAATAAATTTCAACACCTTAC-AGTGTATGGTGCTAATAA 72614
VervetAGM         TTTGTTAATTTAGCAATATTGGTTGATTAATTGCAACAACTAATAAATTGCAATACCATACTAATGTATGGTGCTAATAA 79104
Gorrilla          TTTGTTAATTTATCAATATTGGTTGATTAATTGCAAGAACTAATAAATTGTAATACCATACTAATGTATGATGCTAATAA 76281
GrabEatingMacaque TTTGTTAATTTATCAATATTGGTTGATTAATTGCAACAACTAATAAATTGCAATACCATACTAATGTATGGTGCTAATAA 78639
Macaque           TTTGTTAATTTATCAATATTGGTTGATTAATTGCAACAACTAATAAATTGCAATACCATACTAATGTATGGTGCTAATAA 79521
PigTailedMacaque  TTTGTTAATTTATCAATATTGGTTGATTAATTGCAACAACTAATAAATTGCAATACCATACTAATGTATGGTGCTAATAA 78027
Bonobo            TTTGTTAATTTATCGATATTGGTTGATTAATTGCAAGAACTAATAAATTGTAATACCATACTAATGTATGATGCTAATAA 76899
OliveBaboon       TTTGTTAATTTATCAATATTGGTTGATTAATTGCAACAACTAATAAATTGCAATACCATACTAATGTATGGTGCTAATAA 77926
Orangutan         TTTGTTAATTTATCGATATTGGCTGATTAATTGCAACAACTAATAAATTGCAATACCATACTAATGTATGATGCTAATAA 78554

Majority          TGGGGGAACTGGGGTTTCCAGGTTATATAAGAACTTTGTAATGTCTTAAATTTGTTTGTAAATCTAAACCT--TTTAAAA

                          93770     93780     93790     93800     93810     93820     93830     93840

Human             TGGGGGAACTGCGGTTTCCAGGTTATATAAGAACTCTGTAATGTCTTAAATTTGTTTGTAAATCTAAACCT--TTTAAAA 77815
Coquerel'sSifaka  GGGAGGAAGTGGGGTGCTCAGGTCATAAAAGAAGCCTGTAAAGTTTTCAGGTTTCTGTAAATCTAAAACTGTTCTAAAA 64624
Ma'sNightMonkey   TGGGGGAATTGGGGTTTCCAGGTTATGTAAGAACTGTGTAATGTCTTAAATT--TTTGTAAATCTAAACCT--TTTAAAA 72690
VervetAGM         TGGGGGATCTGGGGTTTCCAGGTTATCTAAGAATGTTGTAATGTCTTAAATTTGTTTGTAAATCTAAACCT--TTTAAAA 79182
Gorrilla          TGGGGGAACTGCGGTTTCCAGGTTATATAAGAACTCTGTAATGTCTTAAATTTGTTTGTAAATCTAAACCT--TTTAAAA 76359
GrabEatingMacaque TGGGGGATCTGGGGTTTCCAGGTTGTATAAGAACATTGTAATGTCTTAAGTTTGTTTGTAAATCTAAACCT--TTTAAAA 78717
Macaque           TGGGGGATCTGGGGTTTCCAGGTTGTATAAGAACATTGTAATGTCTTAAGTTTGTTTGTAAATCTAAACCT--TTTAAAA 79599
PigTailedMacaque  TGGGGGATCTGGGGTTTCCAGGTTATATAAGAACGTTGTAATGTCTTAAGTTTGTTTGTAAATCTAAACCT--TTTAAAA 78105
Bonobo            TGGGGGAACTGCGGTTTCCAGGTTATATAAGAACTCTGTAATGTCTTAAATTTGTTTGTAAATCTAAACCT--TTTAAAA 76977
OliveBaboon       TGGGGGATCTGGGGTTTCCAGGTTATATAAGAACGTTGTAATGTCTTAAATTTGTTTGTAAATCTAAACCT--TTTAAAA 78004
Orangutan         TGGGGGAACTGGGGTTTCCAGGTTATATAAGAACTTTGTAATGTCTTAAATTTGTTTGTAAATCTAAACCT--TTTAAAA 78632
```

```
Majority          TAGCTAAA-AATTATAGAT---ACAATTTCTAAATGATTTAAT-TAACAAAACCAGTTTAATTCTTCAGTTGAAATGAGC

                         93850     93860     93870     93880     93890     93900     93910     93920

Human             TAGCTAAA-AATTATAGAT---ACAATTTCTAAATGATTTAAT-TAACAAAACCAGTTTAATTCTTCAGTTGAAATGAGC 77890
Coquerel'sSifaka  TAGAAAGTTAATTAAAAATTATAGAATTTGTAAATGATTTAAAATAAATTAATAAGTATAATTCTGTAGTTGAATCAGGC 64704
Ma'sNightMonkey   TACATAAA-AATTATATAT---ACAATTTCTAAATGATTTAAT-TAACAAAAACAG--TAATTCTTCAGTTGAAATGAAC 72763
VervetAGM         TAGCTAAA-AATTACAGAT---ACAATTTCTAAATGATTTAAT-TAACAAAACCAGTTTAATTCTTCAGTTGAAATGAGC 79257
Gorrilla          TAGCTAAA-AATTATAGAT---AGAATTTCTAAATGATTTAAT-TAACAAAACCAGTTTAATTCTTAAGTTGAAATGAGC 76434
GrabEatingMacaqueTAGCTAAA-AATTATAGAT---ACAATCTCTAAATGATTTAAT-TAACAAAACCAGTTTAATTCTTCAGTTGAAATGAGC 78792
Macaque           TAGCTAAA-AATTATAGAT---ACAATCTCTAAATGATTTAAT-TAACAAAACCAGTTTAATTCTTCAGTTGAAATGAGC 79674
PigTailedMacaque  TAGCTAAA-AATTATAGAT---ACAATTTCTAAATGATTTAAT-TAACAAAACCAGTTTAATTCTTCAGTTGAAATGAGC 78180
Bonobo            TAGCTAAA-AATTATAGAT---ACAATTTCTAAATGATTTAAT-TAACAAAACCAGTTTAATTCTTCAGTTGAAATGAGC 77052
OliveBaboon       TAGCTAAA-AATTATAGAT---ACAATTTCTAAATGATTTAAT-TAACAAAACCAGTTTAATTCTTCAGTTGAAATGAGC 78079
Orangutan         TAGCTAAA-AATTATAGAT---ACAATTTCTAAATGATTTAAT-TAACAAAACCAGTTTAATTCTTCAGTTGAAATGAGC 78707

Majority          AAGGTTAGGG-ATACCTGTGTGTTTCAGTGTAATAT---CTGAAAGGAAGAGAAT-AGATACATAATTTATATCACTTCT

                         93930     93940     93950     93960     93970     93980     93990     94000

Human             AAGGTTAGGG-ATACCTGTGTGTTTCAGTGTAATAT---CTGAAAGGAAAAGAAT-AGATACATAATTTATATCACTTCT 77965
Coquerel'sSifaka  AAAGTTAGGG-ATGCCTATATGTTTCAGTGAAATATGAAATGAAGGGAAGAGGGTTGGGTACATAATTTATATCACTTCT 64783
Ma'sNightMonkey   AAGGTTAGGGGATACCTATGTGTTTCAGTGTAATAT---ATGAAAG----AGAAT-AGATACATAATTTATATCACTTCT 72835
VervetAGM         AAGGTTAGGG-ATACCTGTGTGTTTCAGTGTAATAT---CGGAAAGGAAGAGAAC-AGATACATAATTTGTATCACTTCT 79332
Gorrilla          AAGGTTAGGG-ATACCTGTGTGTTTCAGTGTAATAT---CTGAAAGGAAAAGAAT-AGATACATAATTTATATCACTTCT 76509
GrabEatingMacaqueAAGGTTAGGG-ATACCTGTGTGTTTCAGTGTAATAT---CTGAAAGGAAGAGAAC-AGATACATAATTTGTATCACTTCT 78867
Macaque           AAGGTTAGGG-ATACCTGTGTGTTTTAGTGTAATAT---CTGAAAGGAAGAGAAC-AGATACATAATTTGTATCACTTCT 79749
PigTailedMacaque  AAGGTTAGGG-ATACCTGTGTGTTTCAGTGTAATAT---CTGAAAGGAAGAGAAC-AGATACATAATTTGTATCACTTCT 78255
Bonobo            AAGGTTAGGG-ATACCTGTGTGTTTCAGTGTAATAT---CTGAAAGGAAAAGAAT-AGATACATAATTTATATCACTTCT 77127
OliveBaboon       AAGGTTAGGG-ATACCTGTGTGTTTCAGTGTAATAT---CTGAAAGGAAGAGAAC-AGATACATAATTTGTATCACTTCT 78154
Orangutan         AAGGTTAGGG-ATACCTGTGTGTTTCAGTGTAATAT---CTGAAAGGAAAAGAAT-AGATACATAATTTATATCACTTCT 78782

Majority          ---CTCTGGACTTAGGGTCTCTTGTCATATACATGGCTGGGCATCCATCAGCTTCTGTGGAGGAAAGGTTAGTAATAATA

                         94010     94020     94030     94040     94050     94060     94070     94080

Human             ---CTCTGGACTTAGGGTCTCTTGTCATATACATGACTGGGCATCCATCAGCTTCAGTGGAGGAAAGGTTAGGAATAATA 78042
Coquerel'sSifaka  TCCCTCTGGACTTTGGGTCTCT-ATCATATACATGGCTGGGTGTCCGTCAGTTTCAGTGGATGAAGGGTTAGTAATAATA 64862
Ma'sNightMonkey   ---CTGTGGACTTAGGTTCTCTTGTCATATACATGGCTGGGCATCCATCAGTTTCCGTGGAGGAAAGGTTAGTAATAATA 72912
VervetAGM         ---GTCTGGACTTAGGGTCTCTTGTCATATACATGGCTGGGCATCCATCAGCTTCTGTGGAGGAAAGGTTAGTAGTAATA 79409
Gorrilla          ---CTCTGGACTTAGGGTCTCTTGTCATATACATGACTGGGCATCCATCAGCTTCAGTGGAGGAAAGGTTAGGAATAATA 76586
GrabEatingMacaque---GTCTGGACTTAGGGTCTCTTGTCATATACATGGCTGGGCATCCATCAGCTTCTGTGGAGGAAAGGTTAGTAATTATA 78944
Macaque           ---GTCTGGACTTAGGGTCTCTTGTCATATACATGGCTGGGCATCCATCAGCTTCTGTGGAGGAAAGGTTAGTAATTATA 79826
PigTailedMacaque  ---GTCTGGACTTAGGGTCTCTTGTCATATACATGGCTGGGCATCCATCAGCTTCTGTGGAGGAAAGGTTAGTAATTATA 78332
Bonobo            ---CTCTGGACTTAGGGTCTCTTGTCATATACATGACTGGGCATCCATCAGCTTCAGTGGAGGAAAGGTTAGGAATAATA 77204
OliveBaboon       ---GTCTGGACTTAGGGTCTCTTGTCATATACATGGCTGGGCATCCATCAGCTTCTGTGGAGGAAAGGTTAGTAATTATA 78231
Orangutan         ---CTCTGGACTTAGGGTCTCTTGTCATATACATGACTGGGCATCCATCAGCTTCAGTGGAGGAAACGTTAGGAATAATA 78859
```

```
Majority         --GTGTCAGAACTAACATGAGAATTTTAGGAGATTGCTGAGTCACTCAAAATGTCTGGGTGACCCATCTGATGAGGGCCA

                         94090     94100     94110     94120     94130     94140     94150     94160

Human            --GTGTCAGAACTAACATGAGAATTTTAGGAGATTACTGAGTCACTCAAAATGTCTGGGTGACCCAGCTGATGAGGGCCA 78120
Coquerel'sSifaka CAGTGTCAGAACTAACACAAGAATTTTGTAAAATTACTGA--TACTCAAGATGTCTGGTTGGCCCACCTGATGGGGATCG 64940
Ma'sNightMonkey  --GTGTCAGAACTAACATGAGAAATTTAGGAGATTGCTGAGTTAATCAAAATGTCTGGGTGACCCAGCTGATGTGGGCCA 72990
VervetAGM        --GTGTCAGAACTAACTTGAGAATTTTAGGAGATTGCTGAGTCAGTCACAGTGTCTGGGTGACCCATCTGATGAGGGCCA 79487
Gorrilla         --GTGTCAGAACTAACGTGAGAATTTTAGGAGATTACTGAGTCACTCAAAATGTCTGGGTGACCCAGCTGATGAGGGCCA 76664
GrabEatingMacaque--GTGTCAGAACTAACATGAGAATTTTAGGAGATTGCTGAGTCACTCACAGTGTCTGGGTGACCCATCTGATGAGGGCCA 79022
Macaque          --GTGTCAGAACTAACATGAGAATTTTAGGAGATTGCTGAGTCACTCACAGTGTCTGGGTGACCCATCTGATGAGGGCCA 79904
PigTailedMacaque --GTGTCAGAACTAACATGAGAATTTTAGGAGATTGCTGAGTCACTCACAGTGTCTGGGTGACCCATCTGATGAGGGCCA 78410
Bonobo           --GTGTCAGAACTAACATGAGAATTTTAGGAGATTACTGAGTCACTCAAAATGTCTGGGTGACCCAGCTGATGAGGGCCA 77282
OliveBaboon      --GTGTCAGAACTAACATGAGAATTTTAAGAGATTGCTGAGTCACTCACAGTGTCTGGGTGACCCATCTGATGAGGGCCA 78309
Orangutan        --GTGTCAGAACTAACATGAGAATTTTAGGAGATTACTGAGTCACGCAAAATGTCTGGGTGACCCAGCTGATGAGGGCCA 78937

Majority         TTTCACATGAAGAAGATTCCTGCAACACCTTCTTTTCTTCAGCACATGATCGTTTATGAAGCTCTTTTACATTATTTTAT

                         94170     94180     94190     94200     94210     94220     94230     94240

Human            TTTCACATGAAAAAGATTCCTGCAACACCTTCTTTTCTTTAGCACATGATCGTTTATGAAGCTCTTTTACATTGTTTTAT 78200
Coquerel'sSifaka TTTCATATGAATAGGATCCCTGCAACACCTT-----CTTCAACATAGGACAGTTTACAAAGCTCTTTTACATTATTTTAT 65015
Ma'sNightMonkey  TTTCACATGAAAAAGATTCCTGCAACACCTTATTTTCTTCAGCACATTGTAGTTTATGAAGCTCTTTTACATTATTTTAT 73070
VervetAGM        TTTCACATGAAGAAGATTCCTGCAGCACCTTCTTTTCTTCAGCACATGATCGTTTAGGAAGCTCTTTTACATTATTTTAT 79567
Gorrilla         TTTCACATGAAAAAGATTCCTGCAACACCTTCTTTTCTTTAGCACATGATCATGTATGAAGCTCTTTTACATTGTTTTAT 76744
GrabEatingMacaqueTTTCACATGAAGAAGATTCCTGCAGCACCTTCTTTTCTTCAGCACATGATCGTTTAGGAAGCTCTTTTACATTATTTTAT 79102
Macaque          TTTCACATGAAGAAGATTCCTGCAGCACCTTCTTTTCTTCAGCACATGATCGTTTAGGAAGCTCTTTTACATTATTTTAT 79984
PigTailedMacaque TTTCACATGAAGAAGATTCCTGCAGCACCTTCTTTTCTTCAGCACATGATCGTTTAGGAAGCTCTTTTACATTATTTTAT 78490
Bonobo           TTTCACATGAAAAAGATTCCTGCAACACCTTCTTTTCTTTAGCACATGATCGTTTATGAAGCTCTTTTACATTGTTTTAT 77362
OliveBaboon      TTTCACATGAAGAAGATTCCTGCAGCACTTTCTTTTCTTCAGCACATGATTGTTTAGGAAGCTCTTTTACATTATTTTAT 78389
Orangutan        TTTCACATGAAAAAGATTCCTGCAACACCTTCTTTTCTTCAGCACGTGATCGTTTATGAAGCTCTTTTACATTGTTTTAT 79017

Majority         CTTTATAACAATCCTCAGAGTAGATCAAGC--------------------------------------------------

                         94250     94260     94270     94280     94290     94300     94310     94320

Human            CTTTATAGCAATCCTCAGAATAGATCAAGCTTTTTTTT----TTTTTTTTTTGAGATGGAGTCTTGCTCTGTCGCCCAG 78276
Coquerel'sSifaka TCTTATAACAATCTTGGGAGTAAAACAGGC-------------------------------------------------- 65045
Ma'sNightMonkey  CCTTCTAACAATCCCTAGAATAGATCAAGC-------------------------------------------------- 73100
VervetAGM        CTTTATAACAATCCTCAGAGTAGATCGAG--------------------------------------------------- 79596
Gorrilla         CTTTATAGCAATCCTCAGAATAGATCAAGCTTTTTTTT-----TTTTT-TTTTGGAATGGAGTCTCGCTCTGTCGCCCAG 76818
GrabEatingMacaqueCTTTATAACAATCCTCAGAGTAGATCGAG--------------------------------------------------- 79131
Macaque          CTTTATAACAATCCTCAGAGTAGATCGAG--------------------------------------------------- 80013
PigTailedMacaque CTTTATAACAATCCTCAGAGTAGATCGAG--------------------------------------------------- 78519
Bonobo           CTTTATAGCAATCCTCAGAATAGATCAAGCTTTTTTTTGTTTTTTTTTTTTTTGAGATGGAGTCTCACTCTGTCGCCCAG 77442
OliveBaboon      CTTTATAACAATCCTCAGAGTAGATCGAG--------------------------------------------------- 78418
Orangutan        CTTTATAGCAACCCTCAGAGTAGATCAAGCTTTTTTTT-----TTTTTTTTTGAGACGGAGTCTCACTCTGTCGCCCAG 79092
```

```
Majority        --------------------------------------------------------------------------------
                        94330     94340     94350     94360     94370     94380     94390     94400
Human           GCTGGAGTGCAATGG----TGCGATCTCGGCTCACTGCAAACTCGGCCTCCCGGGTTCGTGCCATTCTTCTGCCTCAGCC 78352
Coquerel'sSifaka-------------------------------------------------------------------------------- 65045
Ma'sNightMonkey -------------------------------------------------------------------------------- 73100
VervetAGM       -------------------------------------------------------------------------------- 79596
Gorrilla        GCTGGAGTGCGATGGATGGCGTGATCTCGGCTCACTGCAAACTTGGCCTCCCGGGTTCATGCCATTCTTCTGCCTCAGCC 76898
GrabEatingMacaque-------------------------------------------------------------------------------- 79131
Macaque         -------------------------------------------------------------------------------- 80013
PigTailedMacaque-------------------------------------------------------------------------------- 78519
Bonobo          GCTGGAGTGCAATGG----CGCGATCTCGGCTCACTGCAAACTCCGCCTCCCGGGTTCGTGCCATTCTTCTGCCTCAGCC 77518
OliveBaboon     -------------------------------------------------------------------------------- 78418
Orangutan       GCTGGAGTGCAGTGG----TGCGATCTCGGC-------AAACTCAGCCTCCTGGGTTCATGCCATTCTCCTGCCTCAGCC 79161

Majority        -----------------------------------------------------T--------------------------
                        94410     94420     94430     94440     94450     94460     94470     94480
Human           TCCCGAGTAGCTGCTACTACAGGTGCCTGCCACCACGCCCAGCTAATTTTTTTGTATGTTTAGTAGAGATGGGGTTTCAT 78432
Coquerel'sSifaka-------------------------------------------------------------------------------- 65045
Ma'sNightMonkey -------------------------------------------------------------------------------- 73100
VervetAGM       -----------------------------------------------------T-------------------------- 79597
Gorrilla        TCCGGAGTAGCTGGGACTACAGGCGCCCGCCACCACGCC-AGCTAATTTTTTTGTATGTTTAGTAGAGACGGGGTTTCAT 76977
GrabEatingMacaque-----------------------------------------------------T-------------------------- 79132
Macaque         -----------------------------------------------------T-------------------------- 80014
PigTailedMacaque-----------------------------------------------------T-------------------------- 78520
Bonobo          TCCTGAGTAGCTGGGACTACAGGCGCCTGCCACCACGCCCAGCTAATTTTTT-GTATGTTTAGTAGAGACGGGGTTTCAT 77597
OliveBaboon     -----------------------------------------------------T-------------------------- 78419
Orangutan       TCCCGAGTAGCTGGGACTACTGGCGCCTGCCACCACACCCAGCTAATTTTTTTGTATATTTAGTAGAGATGGGGTTTCAT 79241

Majority        -------------------------------------------------ATTTTTATCCCTCCTTATCAATATGTGTAAA
                        94490     94500     94510     94520     94530     94540     94550     94560
Human           CGTGTTAGCCAGGATGGTCTCGATCTCCTGACCTTGTGATAGATCAAGTATTTTTATCCCTC-TCATCAACATGTGTAAA 78511
Coquerel'sSifaka-------------------------------------------------ATTTTTATCCCTCCTTATCAATGTGCTTAAA 65076
Ma'sNightMonkey -------------------------------------------------ATTTTTATTCCTCCTCATCAATATGTGTAAA 73131
VervetAGM       -------------------------------------------------ATTTTTATCCCTCCTTATCAGTATGTGAAAA 79628
Gorrilla        CGTGTTAGCCAGGATGGTCTCGATCTCCTGACCTTGTGATAGATCAAGTATTTTTATCCCTCCTCATCAACATGTGTAAA 77057
GrabEatingMacaque-------------------------------------------------ATTTTTATCCCTCCTTATCAATATGTGAAAA 79163
Macaque         -------------------------------------------------ATTTTTATCCCTCCTTATCAATATGTGAAAA 80045
PigTailedMacaque-------------------------------------------------ATTTTTATCCCTCCTTATCAATATGTGAAAA 78551
Bonobo          AGTGTTAGCCAGGATGGTCTCGATCTCCTGACCTTGTGATAGATCAAGTATTTTTATCCCTCCTCATCAACATGTGTAAA 77677
OliveBaboon     -------------------------------------------------ATTTTTATCCCTCCTTATCAATATGTGAAAA 78450
Orangutan       TATGTTAGCCAGGATGGTCTCGATCTCCTGACTTCGTGATAGATGAAGTATTTTTATCTCTCCTCATCAACATGTGTAAA 79321
```

```
Majority        CAGTTTTTTCCT-GCATATCTGCTGCCTTAGCTAAAGATGCAAATCATTAAAGTTAAAATTATAA--TTTCTGTAGCTAA
                        94570     94580     94590     94600     94610     94620     94630     94640
Human           CAGTTTTTTCCTCACATATCTGCTGCCTTAGCTAAAGATGCAAATCATTAAAGTTAAAATTATAA--TTTATGTAGCTAA 78589
Coquerel'sSifaka AGTGTTTTTTCCAGTATACTTGCTGCCTTAGCTAAAGATTTTAATAATAAAAATTAAAATTACAACTTTTTTATAGCTAA 65156
Ma'sNightMonkey CAGTTTTTTCCCAGCATATCTACTGCCTTA----AAGACGAAAATCATTGAAGTTAAAATTAAAA--TTTCTGTAACTAA 73205
VervetAGM       CAGTTTTTTCCT-GCATATCTGCTGCCTTAGCTAAAGATGCAAATTATTAAAGTTAAAATTTTAA--TTTCTGTAGCTAA 79705
Gorrilla        CAGTTTTTTCCCCACATATCTGCTGCCTTAGCTAAAGATGCAAATCATTAAAGTTAAAATTATAA--TTTATGTAGCTAA 77135
GrabEatingMacaqueCAGTTTTTTCCT-GCATATCTGCTGCCTTAGCTAAAGATGCAAATCATTAAAGTTAAAATTATAA--TTTCTGTAGCTAA 79240
Macaque         CAGTTTTTTCCT-GCATATCTGCTGCCTTAGCTAAAGATGCAAATCATTAAAGTTAAAATTATAA--TTTCTGTAGCTAA 80122
PigTailedMacaque CAGTTTTTTCCT-GCATATCTGCTGCCTTAGCTAAAGATGCAAATCATTAAAGTTAAAATTATAA--TTTCTGTAGCTAA 78628
Bonobo          CAGTTTTTCCCCCACATATCTGCTGCCTTAGCTAAAGATGCAAATCATTAAAGTTGAAATTATAA--TTTATGTAGCTAA 77755
OliveBaboon     CAGTTTTTTCCT-GCATATCTGCTGCCTTAGCTAAAGATGCAAATCATTAAAGTTAAAATTATAA--TTTCTGTAGCTAA 78527
Orangutan       CAGTTTTTTCCTCACGTATCTGCTGCCTTAGCTAAAGATGCAAATCATTAAAGTTAAAATTATAA--TTTCTGTAGCTAA 79399

Majority        AAGTAATCTG----TTAGAGCCAGTGTATACAATATGTGTAATATGCTTTCTATAACTTTCTCAAAGGGCCCATGGTGAG
                        94650     94660     94670     94680     94690     94700     94710     94720
Human           AAGTAATCTG----TTAGAGCCAGTGTATACAATATGTGTAATATGCTTTCTATAACTTTCTCAAAGGGCCCTTGGTAAG 78665
Coquerel'sSifaka AAGTAATCTAAATATTAGAGTCAGTGTATGTAATGTATGTAACACATTTTATATAACTCTTACAAAGGGACCATGGTAAA 65236
Ma'sNightMonkey AAAAAATCTG----TTAGAGCCAGGGTATATAATATGTGGAATATACTTCATATAACTTTTACAAAGGGCT-ATGGTGAG 73280
VervetAGM       AAGTAATCTG----TTAGAGCTGGTGTATACAATATGTTTAATATGCTTTCTATAACTTTCTCAAAGGGCCCACGGTGAG 79781
Gorrilla        AAGTAATCTG----TTAGAGCCAGTGTATACAATATGTGTAATATGCTTTCTATAACTTTCTCAAAGGGCCCGTGGTAAG 77211
GrabEatingMacaqueAAGTAATCTG----TTAGAGCCAGTGTATACAATATGTTTAATATGCTTTCTATAACTTTCTCAAAGGGCCCATGGTGAG 79316
Macaque         AAGTAATCTG----TTAGAGCCAGTGTATACAATATGTTTAATATGCTTTCTATAACTTTCTCAAAGGGCCCATGGTGAG 80198
PigTailedMacaque AAGTAATCTG----TTAGAGCCAGTGTATACAATATGTTTAATATGCTTTCTATAACTTTCTCAAAGGGCCCATGGTGAG 78704
Bonobo          AAGTAATCTG----TTAGAGCCAGTGTATACAATATGTGTAATATGCTTTCTATAACTTTCTCAAAGGGCCCGTGGTAAG 77831
OliveBaboon     AAGTAATCTG----TTAGAGCCAGTGTATACAATATGTTTAATATGCTTTCTATAACTTTCTCAAAGGGCCCATGGTGAG 78603
Orangutan       AAGTAATCTG----TTAGAGCCAGTGTATACAATACGTGTAATATGCTTTCTGCAACTTTCTCAAAGGGCCCATGGTGAG 79475

Majority        TTTATTGTCCTCT---------------CATTTAGCTGGGAGTTAAGAACAGAATTATTTTCTAGAATGTATTA-TTAT-
                        94730     94740     94750     94760     94770     94780     94790     94800
Human           TTTATTGTTTTCT---------------CATTTAGCTGGGAGTTAAGAACAGAACTATTTTCTAGAATGTATTAATTAT- 78729
Coquerel'sSifaka TTTATTGTCTTTAATAGCTGAGAGTTAATATTTATATGAAAAATACAGCGCAGATTTTTTTCCAGAATTTATCA-TTATT 65315
Ma'sNightMonkey TTTATTGTCCTCT---------------TATTTAGCTTGGAGTTAAGAACAGAATTATTTTATAGAATGTATTA-TTATG 73344
VervetAGM       TTTATTGTCCTCT---------------CATTTAGCTGGGAGTTCAGAACAGAATTATTTTCTAGAATGTATTA-AGAT- 79844
Gorrilla        TTTATTGTTCTCT---------------CATTTAGCTGGGAGTTAAGAACAGAACTATTTTCTAGAATGTATTAATTAT- 77275
GrabEatingMacaqueTTTATTGTCCTCT---------------CATTTAGCTGGGAGTTCAGAACAGAATTATTTTCTAGAATGTATTA-TGAT- 79379
Macaque         TTTATTGTCCTCT---------------CATTTAGCTGGGAGTTCAGAACAGAATTATTTTCTAGAATGTATTA-TGAT- 80261
PigTailedMacaque TTTATTGTCCTCT---------------CATTTAGCTGGGAGTTCAGAACAGAATTATTTTCTAGAATGTATTA-TGAT- 78767
Bonobo          TTTATTGTTTTCT---------------CATTTAGCTGGGAGTTAAGAACAGAACTGTTTTCTAGAATGTATTAATTAT- 77895
OliveBaboon     TTTATTGTCCTCT---------------CATTTAGCTGGGAGTTGAGAACAGAATTATTTTCTAGAATGTATTA-TGAT- 78666
Orangutan       TTTATTGTTCTCT---------------CATTTAGCTGGGAGTTAAGAACAGAATTATTTTCTAGAATGTATTAATTATG 79540
```

```
Majority          --ATAAATAGTGCATCATGAAATATTTTGTAGGTTTTTATATTTTTATTGCAGCAGACAGCATTTAGATTAAACCTAAAA
                          94810     94820     94830     94840     94850     94860     94870     94880
Human             --ATAAATAGTGCATCATGAAATATTTTGTAGGTTTTTATATTTTTATTGCAGCAGACAGCATTTAGATTAAACCTAAAA 78807
Coquerel'sSifaka GTATAAATAGTGCATCATGAAATATTTTGTAGGTTTTTATATTTTTATTGCAGCCGACAACATTTAGATTAAACCTAAGA 65395
Ma'sNightMonkey  ATACAAATAGGGCATCATGAAATATTTTGTAGATTTTTATGTTTTTATTGCAGCAGACAGCATTTAGATTAAACGTTAAA 73424
VervetAGM         --ATAAATATTGCATCATGAAATATTTTGTAGGTTTTTATATTTTTATTGCAGCAGACAGCATTGAGATTAAACCTAAAA 79922
Gorrilla          --ATAAATAGTGCATCATGAAATATTTTGTAGGTTTTTATATTTTTATTGCAGCAGACAGCATTTAGATTAAACCTAAAA 77353
GrabEatingMacaque--ATAAATAGTGCATCATGAAATATTTTGTAGGTTTGTATATTTTTATTGCAGCAGACAGTATTGAGATTAAACCTAAAA 79457
Macaque           --ATAAATAGTGCATCATGAAATATTTTGTAGGTTTGTATATTTTTATTGCAGCAGACAGTATTGAGATTAAACCTAAAA 80339
PigTailedMacaque --ATAAATAGTGCATCATGATATATTTTGTAGGTTTGTATATTTTTATTGCAGCAGACAGTATTGAGATTAAACCTAAAA 78845
Bonobo            --ATAAATAGTGCATCATGAAATATTTTGTAAGTGTTTATATTTTTATTGCAGCAGACAGCATTTAGATTAAACCTAAAA 77973
OliveBaboon       --ATAAATAGTGCATCATGAAATATTTTGTAGGTTTGTATATTTTTATTACAGCAGACAGCATTGAGATTAAACCTAAAA 78744
Orangutan         ATATAAATAATGCATCATGAAATATTTTGTAGGTTTTTATATTTTTATTGCAGCAGACAGCATTTAGATTAAACCTAAAA 79620

Majority          A-------TACCTGATTGACAAAGTAATTTTTCAGACAAAAGTATATTGAGGGAAATTGGAGAATCT----TCAGAGGCT
                          94890     94900     94910     94920     94930     94940     94950     94960
Human             A-------TACCTGATTGACAAAGTAGTTTTTCAGACAAAAGTATATTGAGGGAAATTGGAGAATCT----TCAGAGGCT 78876
Coquerel'sSifaka AAACATTATACCTGATTGACCAAGTAATTTTTCAGAGAAGAGTATGCTGAGGAAAATTGTGGAATTTATGCTCAGAGGCA 65475
Ma'sNightMonkey  A-------TACCTGATTGACAAAGTAATTTTTCAGAGAAAAGAATATTGAGGGAAATTGGAGA--CT----TCAGAGGCT 73491
VervetAGM         A-------TACCTGATTGACAAAGTAATTTTTCAGACAAAAGTATATTGAGGGAAATTGGAGAATCT----TCAGAGGCT 79991
Gorrilla          A-------TACCTGATTGACAAAGTAGTTTTTCAGACAAAAGTATATTGAGGGAAATTGGAGAATCT----TCAGAGGCT 77422
GrabEatingMacaqueA-------TACCTGATTGACAAAGTAATTTTTCAGACAAAAGTATATCGAGGGAAATTGGAGAATCT----TCAGAGGCT 79526
Macaque           A-------TACCTGATTGACAAAGTAATTTTTCAGACAAAAGTATATCGAGGGAAATTGGAGAATCT----TCAGAGGCT 80408
PigTailedMacaque A-------TACCTGATTGACAAAGTAATTTTTCAGACAAAAGTATATCGAGGGAAATTGGAGAATCT----TCAGAGGCT 78914
Bonobo            A-------TACCTGATTGACAAAGTAGTTTTTCAGACAAAAGTATATTGAGGGAAATTGGAGAATCT----TCAGAGGCT 78042
OliveBaboon       --------TACCTGATTGACAAAGTAATTTTTCAGACAAAAGTATATTGAGGGAAATTGGAGAATCT----TCAGAGGCT 78812
Orangutan         A-------TACCTGATTGACAAAGTAATTTTTCAGACAAAAGTATATTGAGGGAAATTGGAGAATCT----TCAGAGGCT 79689

Majority          TTAAAA--GTCCAGGAAAATCTTA-GACTACTCCAGTGATTTAGAACTGTGTAAAGGTAGCCTAAGTCGTTTTTCTAAGT
                          94970     94980     94990     95000     95010     95020     95030     95040
Human             TTAAAA--GTCCAGGAAAATCTTA-GACTACTCCAGTGATTTAGAACGGTGTAAAGATAGCCTAAGTAGTTTTTCTAAGT 78953
Coquerel'sSifaka TTAAAA--GTCCAATAAAAACTTAAGACTACTCCAGTGATTCAGAACTGTGTAAAGGTAGCTTAAGTAGTTTTTTTAGGA 65553
Ma'sNightMonkey  TTAAAA--GTCCAACAAAATCTTA-GACTACTCCAGTGATTTAGAACT--GTAAAGATAGCCTAAGTCGTTTTTCTAAGT 73566
VervetAGM         TTAAAA--GTCCAGGAAAATCTTA-GACTACTCCAGTGATTTAGAACTGAGTAAAGGTAGCCTAAGTCGT------AAGT 80062
Gorrilla          TTAAAA--GTCCAGGAAAATCTTA-GACTACTCCAGTGATTTAGAACTGTGTAAAGATAGCCTAAGTAGTTTTTCTAAGT 77499
GrabEatingMacaqueTTAAAA--GTCCAGGAAAATCTTA-GACTACTCCAGTGATTTAGAACTGTGTAAAGGTAGCCTAAGTCGTTTTTCTAAGT 79603
Macaque           TTAAAA--GTCCAGGAAAATCTTA-GACTACTCCAGTGATTTAGAACTGTGTAAAGGTAGCCTAAGTCGTTTTTCTAAGT 80485
PigTailedMacaque TTAAAAAAGTCCAGGAAAATCTTAAGACTACTCCAGTGATTTAGAACTGTGTAAAGGTAGCCTAAGTCGTTTTTCTAAGT 78994
Bonobo            TTAAAA--GTCCAGGAAAATCTTA-GACTACTCCAGTGATTTAGAACTGTGTAAAGATAGCCTAAGTAGTTTTTCTAAGT 78119
OliveBaboon       TTAAAA--GTCCAGGAAAATCTTA-GACTACTCCAGTGATTTAGAACTGTGTAAAGGTAGCCTAAGTCGTTTTTCTAAGT 78889
Orangutan         TTAAAA--GTCCAGGAAAATCTTA-GACTACTCCAGTGATTTAGAACTGTGTAAAGATAGCCTAAGTAGTTTTTCTAAGT 79766
```

```
Majority          TATCAGCCGAGGGGAGGAAATAATGAAAGAGAAATACATACTAGTGGCATTAAGGACAGGTGGTGGCT-CCTCTTACCTA

                          95050     95060     95070     95080     95090     95100     95110     95120

Human             TATCAGCCGCGGGGAGGAAATAATGAAAGAGAAATACATACGAGTGGCATTAAGGACAGGTGGTGGCT-TCTTTTACCTA 79032
Coquerel'sSifaka  TATCAGCCCAGGGGAGGAAATA-TGGTAGAGAAATAGATACTAGTGGCATTAAGGATAGATGGCGACTGCCCCTTATCTC 65632
Ma'sNightMonkey   TATCAGCTGAGGGGAGGAAATAATGAAAGAGAACTACATACTAG--GCATTAAGGACAGGTGGTGGCT-CCTCTTACCTG 73643
VervetAGM         TATCAGCCTAGGGGAGGAAATAATGAAAGAGAAATACATACTAGTGGGATTAAGGACAGGTGGTGGCT-CCTCTTCCCTA 80141
Gorrilla          TATCAGCCGCGGGGAGGAAATAAT-AAAGAGAAATACATACGAGTGGCATTAAGGACAGGTGGTGGCT-TCTTTTACCTA 77577
GrabEatingMacaqueTATCAGCTGAGGGGAGGAAATAATGAAAGAGAAATACATACTAGTGGGATTAAGGACAGGTGGTGGCT-CCTCTTACCTA 79682
Macaque           TATCAGCTGAGGGGAGGAAATAATGAAAGAGAAATACATACTAGTGGGATTAAGGACAGGTGGTGGCT-CCTCTTACCTA 80564
PigTailedMacaque  TATCAGCTGAGGGGAGGAAATAATGAAAGAGAAATACATACTAGTGGGATTAAGGACAGGTGGTGGCT-CCTCTTACCTA 79073
Bonobo            TATCAGCCGCGGGAAGGAAATAATGAAAGAGAAATACATACGAGTGGCATTAAGGACACGTGGTGGCT-TCTTTTACCTA 78198
OliveBaboon       TATCAGCCGAGGGGAGGAAATAATGAAAGAGAAATACATACTAGTGGGATTAAGGACAGGTGGTGGCT-CCTCTTACCTA 78968
Orangutan         TATGAGCCGAGGGGAGGAAATAATGAAAGAGAAATACATACGAGTGGCATTAAGGACAGGTGGTGGCT-CTTCTTACCTA 79845

Majority          TTAAAGCATTCTTACACCATACTATGAACTGTGTAAAAGGAAGGATCATTGGACATAATAGATCACCTGACAAGGTGGCC

                          95130     95140     95150     95160     95170     95180     95190     95200

Human             TTAAAGCATTCTTACACCATACTGTGAACTGTGTAAAAGGAAGGATCATTGGACATAATAGATTATCTGACAGGGTGGTC 79112
Coquerel'sSifaka  TTAAAGCACTCTTGCATCATACTATAGGCT---------GACCTACTACCTGACTTAGTAG---------TACAGTGGCC 65694
Ma'sNightMonkey   TTAAAACATTCTTACACCATACTATGAACTTTGTAAAAGGAAGGATCATTTGGCATAATAGATCACCTGACAGGGTGGCA 73723
VervetAGM         TTAAAGCATTCCTATACCATACTATGAACTGTGTAAAAGGAAGGATCATTGGACATAATAGATCACCTGACAAGGTGGCC 80221
Gorrilla          TTAAAGCATTCTTACACCATACTGTGAACTGTGTAAAAGGAAGGATCATTGGACATAATAGATTATCTGACAAGGTGGCC 77657
GrabEatingMacaqueTTAAAGCATTCTTACACCATACTATGAACTGTGTAAAAGGAAGGATCATTGGACATAATAGATCACCTGACAAGGTGGCC 79762
Macaque           TTAAAGCATTCTTACACCATACTATGAACTGTGTAAAAGGAAGGATCATTGGACATAATAGATCACCTGACAAGGTGGCC 80644
PigTailedMacaque  TTAAAGCATTCTTACACCATACTATGAACTGTGTAAAAGGAAGGATCATTGGACATAATAGATCACCTGACAAGGTGGCC 79153
Bonobo            TTAAAGCATTCTTACACCATACTGTGAACTGTGTAAAAGGAAGGATCATTGGACATAATAGATTATCTGACAAGGTGGCC 78278
OliveBaboon       TTAAAGCATTCTTACACCATACTATGAACTGTGTAAAAGGAAGGATCATTGGACATAATAGATCACCTGACAAGGTGCCC 79048
Orangutan         TTAAAGCATTCTTACACCATACTGTGAACTGTGTAAAAGGAAGGATCATTGGACATAATAGATTATCTGACAAGGTGGCC 79925

Majority          CAGAATGGTGGAAGTAAATCGACAAGTGAGGAGACTG--AATTAGCATTCAGACAG-----TGGGGACCTCACACCTTGC

                          95210     95220     95230     95240     95250     95260     95270     95280

Human             CGGAATGGTGGAAGTAAATCGACAGGTGAGGACACTG--AATTAGCATTCAGACAG-----TGGGGACCTCACACCTTGT 79185
Coquerel'sSifaka  CAGAATGGTGGAAGTAAGTGGACAAGTTAGGAGACTATCAATTAGCGTTAGTAAGGACATTTGGGGTCCTAACAGCTTAC 65774
Ma'sNightMonkey   AAGAATGGTGGAAGTAAATGGACAAGTGAGGAGACTA--AATTAGCATTCAGACAG-----TGGGGACCTCACAGCTTGC 73796
VervetAGM         CAGAATGGTGGAAGTAAATCGACAAGTGAGGAGACTG--AATTAGCATTCAGACAG-----TAGGGACCTCACACCTTGC 80294
Gorrilla          CTGAATGGTGGAAGTAAATCAACAAGTGAGGAGACTG--AATTAGCATTCAGACAG-----TGGGAACCTAACACCTTGC 77730
GrabEatingMacaqueCAGAATGGTGGAAGTAAATCGACAAGTGAGGAGACTG--AATTAGCATTCAGACAG-----TGGGGACCTCACACCTTGC 79835
Macaque           CAAAATGGTGGAAGTAAATCGACAAGTGAGGAGACTG--AATTAGCATTCAGACAG-----TGGGGACCTCACACCTTGC 80717
PigTailedMacaque  CAGAATGGTGGAAGTAAATCGACAAGTGAGGAGACTG--AATTAGCATTCAGACAG-----TGGGGACCTCACACCTTGC 79226
Bonobo            CGGAATGGTGGAAGTAAATCGACAAGTGAGGACACTG--AATTAGCATTCAGACAG-----TGGGGACCTCACACCTTGT 78351
OliveBaboon       CAGAATGGTGGAAGTAAATCGACAAGTGAGGAGACTG--AATTAGCATTCAGACAG-----TGGGGACCTCACACCTTGC 79121
Orangutan         CAGAATGGTGGAAGTAAATCGACAAGTGAGGAGACTG--AATTAGCATTCAGACAG-----TGGGGACCTCACACCTTGC 79998
```

```
Majority          ATTCCCCTTTTTGGTAAGATTTTTATTACCTCCTTAGTTGGAAAGAGGTAAATT-CATCTATTTTGAGCCCAAATTTTTC
                          95290     95300     95310     95320     95330     95340     95350     95360
Human             ATTCCCTTTTTTGGTAAGATTTTTATTACCTCCTTAG----AAAGAGGTAAATT-CATCTATTTCGAGCCCAAATTTTTC 79260
Coquerel'sSifaka  ATTCTACTTTTGGGTAAGATTTCCATTACCTCCTTATT-AGAAAGAGGTAAATTGTATCTATTTTGAACCCAAATTTTTC 65853
Ma'sNightMonkey   ATGAC--TTTTTGGTAAGATTTTTGTTACCTCCTTAGTTAGAAAGAGGTAAATT-CTTCTATTTTGAACCCAAATTTTTC 73873
VervetAGM         ATTCCCCTTTTTGGTAAGATTTTTATTACCTCCTTAGTTGGAAAGAGGTAAATT-CATCTATTTTGAGCCCAAATTTTTC 80373
Gorrilla          ATTCCCTTTTTTGGTAAGATTTTTATTAGCTCCTTAG----AAAGAGGTAAATT-CATCTATTTCGAACCCAAATTTTTC 77805
GrabEatingMacaqueATTCCCCTTTTTGGTAAGATTTTTATTACCTCCTTAGTTGGAAAGAGGTAAATT-CATCTATTTTGAGCCCAAATTTTTC 79914
Macaque           ATTCCCCTTTTTGGTAAGATTTTTATTACCTCCTTAGTTGGAAAGAGGTAAATT-CATCTATTTTGAGCCCAAATTTTTC 80796
PigTailedMacaque  ATTCCCCTTTTTGGTAAGATTTTTATTACCTCCTTAGTTGGAAAGAGGTAAATT-CATCTATTTTGAGCCCAAATTTTTC 79305
Bonobo            ATTCTCTTTTTTGGTAAGATTTTTATTACCTCCTTAG----AAAGAGGTAAATT-CATCTATTTTGAGCCCAAATTTTTC 78426
OliveBaboon       ATTCCCCTTTTTGGTAAGATTTTTATTACCTCCTTAGTTAGAAAGAGGTAAATT-CATCTATTTTGAGCCCAAATTTTTC 79200
Orangutan         ATTCCCTTTTTTGGTAAGATTTTTATTACCTCCTTAG----AAAGAGGTAAATT-CATCTATTTTGAGCCCAAATTTTTC 80073

Majority          TGACTAGAAGATTGGTAGTCTTATTTTTACTAAAGTACAAGATTATTTAGTAAAATATAGCATTCTCTGGCTT-------
                          95370     95380     95390     95400     95410     95420     95430     95440
Human             TGTCTAGAAGATTGGTAGTCTTATTTTTACTAAAGTACAAGATTATTTAGTAAAAGGTAGCATTCTCTGGCTT------- 79333
Coquerel'sSifaka  TCACTGGAAAATTAGTAGTCTTAATTTCACTTAAGCACCATATTGTTTATTAAAATATAGCATTCTCTGGCCT------- 65926
Ma'sNightMonkey   TCACTAGAAGATTGGTAGTCTTACTAA----AAAATACAAGATTGTTTAGTAAAATGTGGCATTCTCTGGCTTGCTTATT 73949
VervetAGM         TGACTAGAAGATTGGTAGTCTTATTTTTACTAAAGTGCAAGATTATTTAGTAAAATATAGCATTCTCAGCCCT------- 80446
Gorrilla          TGTCTAGAAGATTGGTAGTCTTATTTTTACTAAAGTACAAGATTATTTAGTAAAAGGTAGCGTTCTCTGGCTT------- 77878
GrabEatingMacaqueTGACTAGAAGATTGGTAGTCTTATTTTTACTAAAGTGCAAGATTATTTAGTAAAATATAGCATTCTCTGGCTT------- 79987
Macaque           TGACTAGAAGATTGGTAGTCTTATTTTTACTAAAGTGCAAGATTATTTAGTAAAATATAGCATTCTCTGGCTT------- 80869
PigTailedMacaque  TGACTAGAAGATTGGTAGTCTTATTTTTACTAAAGTGCAAGATTATTTAGTAAAATATAGCATTCTCTGGCTT------- 79378
Bonobo            TGTCTAGAAGATTGGTAGTCTTATTTTTACTAAAGTACAAGATTATTTAGTAAAAGGTAGCATTCTCTGGCTT------- 78499
OliveBaboon       TGACTAGAAGATTGGTAGTCTTATTTTTACTAAAGTGCAAGATTAGTTAGTAAAATATAGCATTCTCTGGCTT------- 79273
Orangutan         TCTCTAGAAGATTGGTAGTCTTATTTTTACTAAAGTACAAGATTGTTTAGTAAAAGGTAGCATTCTCTGGCTT------- 80146

Majority          --------------------------------------------------------------------------------
                          95450     95460     95470     95480     95490     95500     95510     95520
Human             -------------------------------------------------------------------------------- 79333
Coquerel'sSifaka  -------------------------------------------------------------------------------- 65926
Ma'sNightMonkey   TACTTACTTACTTACTTACTTACTTACTTACTTACTTACTTATTTATTTATTTTCAGACGGAGTCTTGCTTTGTCACCAG 74029
VervetAGM         -------------------------------------------------------------------------------- 80446
Gorrilla          -------------------------------------------------------------------------------- 77878
GrabEatingMacaque-------------------------------------------------------------------------------- 79987
Macaque           -------------------------------------------------------------------------------- 80869
PigTailedMacaque  -------------------------------------------------------------------------------- 79378
Bonobo            -------------------------------------------------------------------------------- 78499
OliveBaboon       -------------------------------------------------------------------------------- 79273
Orangutan         -------------------------------------------------------------------------------- 80146
```

```
Majority         --------------------------------------------------------------------------------

                         95530     95540     95550     95560     95570     95580     95590     95600

Human            -------------------------------------------------------------------------------- 79333
Coquerel'sSifaka -------------------------------------------------------------------------------- 65926
Ma'sNightMonkey  GCTGGAGTGCAGTGGCTGGATCTCAGCTCAGTGCAACCTCTGCCTTCTGGGTTCAAGTGATTCTCCTGCCTCAGTTTCCT 74109
VervetAGM        -------------------------------------------------------------------------------- 80446
Gorrilla         -------------------------------------------------------------------------------- 77878
GrabEatingMacaque-------------------------------------------------------------------------------- 79987
Macaque          -------------------------------------------------------------------------------- 80869
PigTailedMacaque -------------------------------------------------------------------------------- 79378
Bonobo           -------------------------------------------------------------------------------- 78499
OliveBaboon      -------------------------------------------------------------------------------- 79273
Orangutan        -------------------------------------------------------------------------------- 80146

Majority         ------------------------------------------TTTTTTTTTTTTT-------------------------

                         95610     95620     95630     95640     95650     95660     95670     95680

Human            ------------------------------------------TTTTTTTTTTTTT------------------------- 79346
Coquerel'sSifaka ------------------------------------------TTTTTTTTTTAAT------------------------- 65939
Ma'sNightMonkey  GAGCTGCTGGGACTACAAGTCTGTGGCACCACGCCCAGCTAATTTTTGTATTTTTAGTAGAGATGGGGTTTCACCATGTT 74189
VervetAGM        ------------------------------------------TTTTTTTTTTTTT------------------------- 80459
Gorrilla         ------------------------------------------TTTTTTTTTTTTT------------------------- 77891
GrabEatingMacaque------------------------------------------TTTTTTTTTTTTT------------------------- 80000
Macaque          ------------------------------------------TTTTTTTTTTTT-------------------------- 80881
PigTailedMacaque ------------------------------------------TTTTTTTTTTTTT------------------------- 79391
Bonobo           ------------------------------------------TTTTTTTTTTTTT------------------------- 78512
OliveBaboon      ------------------------------------------TTTTTTTTTTTTT------------------------- 79286
Orangutan        ------------------------------------------TTTTTTGTGTGTG------------------------- 80159

Majority         --------------------------------------------------------------------------------

                         95690     95700     95710     95720     95730     95740     95750     95760

Human            -------------------------------------------------------------------------------- 79346
Coquerel'sSifaka -------------------------------------------------------------------------------- 65939
Ma'sNightMonkey  GGCCAGGATGATCTCCATCTCTTGACCTCGTGATCCACCTGCCTCGGCCTCCCAAAGTGTTGGGATTATAGGCATGAGCC 74269
VervetAGM        -------------------------------------------------------------------------------- 80459
Gorrilla         -------------------------------------------------------------------------------- 77891
GrabEatingMacaque-------------------------------------------------------------------------------- 80000
Macaque          -------------------------------------------------------------------------------- 80881
PigTailedMacaque -------------------------------------------------------------------------------- 79391
Bonobo           -------------------------------------------------------------------------------- 78512
OliveBaboon      -------------------------------------------------------------------------------- 79286
Orangutan        -------------------------------------------------------------------------------- 80159
```

```
Majority          -------------TTTTT----------------AATGTTTCAGATCTGTCACACTAAAAA-GGGCTTTAGGCCAGGTGT
                           95770     95780     95790     95800     95810     95820     95830     95840
Human             -------------T-TTT----------------AATGTTTCAGATCTGTCACACTAAAAA-GGGCTTTAGACCAGGTGT 79395
Coquerel'sSifaka  -----------------------------------GTCTCTTGGGGCTACCACACTAAAAAAGGGCTTTACCCTAGATAT 65984
Ma'sNightMonkey   ACCACACCCAGCCTTTTTATTACTATTATTTTTTAATGTCTCAGGTCTGTCACACTAAAA--GGGCTTTAGGCCAGGCAT 74347
VervetAGM         -------------TTTTT--------------TAAATGTTTCAGATCTGTCACACTAAAAA-GGGCTTTAGGCCGGGTGT 80511
Gorrilla          -------------TCTTT----------------TATGTTTCAGATCTGTCACACTAAAAA-GGGCTTTAGGCCAGGTGT 77941
GrabEatingMacaque-------------TTA------------------AATGTTTCAGATCTGTCACACTAAAAA-GGGCTTTAGGCTGGGTGT 80048
Macaque           ----------------------------------AATGTTTCAGATCTGTCACACTAAAAA-GGGCTTTAGGCTGGGTGT 80926
PigTailedMacaque  -------------TTTTTTTT---------TTTTAATGTTTCAGATCTGTCACACTAAAAA-GGGCTTTAGGCTGGGTGT 79448
Bonobo            -------------T-TTT----------------AATGTTTCAGATCTGTCACACTAAAAA-GGGCTTTAGGCCAGGTGT 78561
OliveBaboon       -------------TTTT---------------GTAATGTTTCAGATCTGTCACACTAAAAA-GGGCTTTAGGCCGGGTGT 79337
Orangutan         -------------T---------------------GTGTTTCAGATCTATCACACTAAAAA-GGGCTTTAGGCCAGGTGT 80204
```

```
Majority          TGTGGCTCACACCTGTAATCCCAGCACTTTGAGAGGTCAAGGTGGAGGATTGCTTGAGGCCAGCAGTTTGAGACCAGCC
                           95850     95860     95870     95880     95890     95900     95910     95920
Human             TGTGGCTCACACCTGTAATCCCAGCACTTTGACAGGTCAAGGTGGAGGATTGCTGGAGGCCAGGAGTTTGAGACCAGCC 79475
Coquerel'sSifaka  TTTAATTCTAAATTATCATTTTA----------AATTCATTTTA-----ATTATTT----TTAAAATTCTC--ACCATTT 66043
Ma'sNightMonkey   T-TTGCTCACATCTGTAATCCCAGCACTTTGAGAGGTCAAGGTGGAGGATTGCTTGAGGCCAGCAGTTTGAGACCAGCC 74426
VervetAGM         TGTGGCTCACACCTGTAATCCCAGCACTTTGAGAGATCAAGGTGGAGGATTGCTTGAGGCCAGCAGTTTGAGACCAGCC 80591
Gorrilla          TGTGGCTCACACCTGTAATCCCAGCACTTTGAGAGGTCAAGGTGGAGGATTGCTTGAGGCCAGGAGTTTGAGACCAGCC 78021
GrabEatingMacaqueTGTGGCTCACCCCTGTAATCCCAGCACTTTGAGAGGTCAAGGTGGAGGATTGCTTGAGGCCAGCAGTTTGAGACCAGCC 80128
Macaque           TGTGGCTCACCCCTGTAATCCCAGCACTTTGAGAGGTCAAGGTGGAGGATTGCTTGAGGCCAGCAGTTTGAGACCAGCC 81006
PigTailedMacaque  TGTGGCTCACCCCTGTAATCCCAGCACTTTGAGAGGTCAAGGTGGAGGATTGCTTGAGGCCAGCAGTTTGAGACCAGCC 79528
Bonobo            TGTGGCTCACACCTGTAATCCCAGCACTTTGAGAGGTCAAGGTGGAGGATTGCTTGAGGCCAGGAGTTTGAGACCAGCC 78641
OliveBaboon       TGTGGCTCACCCCTGTAATCCCAGCACTTTGAGAGGTCAAGGTGGAGGATTGCTTGAGGCCAGCAGTTTGAGACCAGCC 79417
Orangutan         TGTGGCTCACACCTGTAATCCCAGCACTTTGAGAAGTCAAGGTGGAGGATTGCTTGAGGCCAGCAGTTTGAGACCAGCC 80284
```

```
Majority          TGAGCAACATAGTGAGATCTCAGTCTCTACTAAAAATAAAAA-----AAATTAGCCAGGCCTGGTGGCATGTGCCTGTAG
                           95930     95940     95950     95960     95970     95980     95990     96000
Human             TGAGCAACATAGTGAGATCTCAGTCTCTACTAAAAATAAAAA-----AAATTAGCCATGCCTGGTGGCATGTGCCTGTAG 79550
Coquerel'sSifaka  T----TCCATGATAAGGCTTGATGTTATGTTCTAAACAAT--------A-TTA-TGACACCAA--GTCAT----CTCCAG 66103
Ma'sNightMonkey   TGAGCAATGTAGTGAGATCTTGGTCTCTACTAAAACTAAAAAAAAAAAATTAGCCAGACCTG-TGGCATGTGCCTGTAG 74505
VervetAGM         TGAGCAACATAGTGAGATCTCAGTCTCTACTAAAAAGAAAAA-----AAATTAGCCAGGCCTGGTGGCATGTGCCTGTAG 80666
Gorrilla          TGAGCAACATAGTGAGATCTCAGTCTCTACTAAAAATAAAAA-----AAATTAGCCATGCCTGGTGGCATGTGCCTGTAG 78096
GrabEatingMacaqueTGAGCAACATAGTGAGATCTCAGTCTCTACTAAAAATAAAAA-----AAATTAGCCAGGCCTGGTGGCATGTGCCTGTAG 80203
Macaque           TGAGCAACATAGTGAGATCTCAGTCTCTACTAAAAATAAAAA-----AAATTAGCCAGGCCTGGTGGCATGTGCCTGTAG 81081
PigTailedMacaque  TGAGCAACATAGTGAGATCTCAGTCTCTACTAAAATTAAAAA-----AAATTAGCCAGGCCTGGTGGCATGTGCCTGTAG 79603
Bonobo            TGAGCAACATAGTGAGATCTCAGTCTCTACTAAAAATAAAAT-----AAATTAGCCATGCCTGGTGGCATGTGCCTGTAG 78716
OliveBaboon       TGAGCAACATAGTGAGATCTCAGTCTCTACTAAAAATAAAAA-----AAATTAGCCAGGCCTGGTGGCATGTGCCTGTAG 79492
Orangutan         TGAGCAACATAGTGAGATCTCAGTCTCTACTAAAAATAAA-------AATTTAGCCATGCGTGGTGGCATATGCCTGTAG 80357
```

```
Majority          TCCCAGCTACTTAGGAGGCTGAGGTGGGAGGATTGCCTGAGCCCAGAAGATAGAGACTGCAGTGAGCCATGATCATGCCA
                          96010     96020     96030     96040     96050     96060     96070     96080
Human             TCCCAGCTACTTAGTAGGCTGAGGTGGGAGGATTGCCTGAGCCCAGAAGATAGAAACAGCAGTGAGCCATGATCATGCCA 79630
Coquerel'sSifaka -------------------TGTAAAACCCAGATTTCAAGA----AAAAAATGTATTTTAAAAACAATTATT-TCTTAATA 66159
Ma'sNightMonkey   -----------------GCTGAGGTGGGAGGATTGCTTGATCCCAGAAAATCAAGACTGTAGTGAGCCAAGATCACGCCA 74568
VervetAGM         TCCCAGCTACTTAGGAGGCTGAGGTGGGAGGATTGCCTGAGCCCAGAAGATAGAGACTACAGTGAGCCATGATCATGCCA 80746
Gorrilla          TCCCAGCTACTTAGTAGGCTGAGGTGGGAGGATTGCCTGAGCCCAGAAGATAGAAACAGCAGTGAGCCATGATCATGCTA 78176
GrabEatingMacaqueTCCCAGCTACTTAGGAGGCTGAGGTGGGAGGATTGCCTGAGCCCAGAAGATAGAGACTGCAGTGAGCCATGATCATGCCA 80283
Macaque           TCCCAGCTACTTAGGAGGCTGAGGTGGGAGGATTGCCTGAGCCCAGAAGATAGAGACTGCAGTGAGCCATGATCATGCCA 81161
PigTailedMacaque TCCCAGCTACTTAGGAGGCTGAGGTGGGAGGATTGCCTGAGCCCAGAAGATAGAGACTGCAGTGAGCCATGATCATGCCA 79683
Bonobo            TCCCAGCTACTTAGTAGGCTGAGGTGGGAGGATTGCCTGAGCCCAGAAGATAGAAACAGCAGTGAGCCATGATCATGCCA 78796
OliveBaboon       TCCCAGCTACTTAGGAGGCTGAGGTGGGAGGATTGCCTGAGCCCAGAAGATAGAGACTGCAGTGAGCCATGATCATGCCA 79572
Orangutan         TCCCAGCTACTTAGTAGGCTGAGGTGGGAGGATTGCCTGAGCCCAGAAGATAGAAACTGCAGTGAGCCATGATCATGCCA 80437

Majority          CTGTACTCTAGCTTGGGTGACAGAGTGAACCTTGTCTCAAAAAATAAAGGGTGGGAAGCTTTAGCCTAGACTTTTAATT
                          96090     96100     96110     96120     96130     96140     96150     96160
Human             CTGCACTCTAGCCTGGGTGACAGAGCAAACCTTGTCTCAAAAAATAAAGGGTGGGAAGCTTTAGCCTAGGCTTTTAATT 79710
Coquerel'sSifaka TTTAAGGCTTTTTTAGGT----AAGTTTTTCTCACCTTTAAACTTTTTG-GTGAGTAATTTTTA---------TTGGATT 66225
Ma'sNightMonkey   CCACATTCTTGCCTGGGTGACAGAGCAAACCTTTTCTCAAAAAATGAAGAGTTGGGGAGCTTTAGCCTAGACTTATAATT 74648
VervetAGM         CTGTACTCTAGCTTGGGTGACAGAGTGAACCTTATCTCAAAAAATAAAGGGTGGGAAGCTTTAGCCTAGACTTTTAATT 80826
Gorrilla          CTGCACTCTAGCCTGGGTGACAGAGCAAACCTTGTCTCAAAAAATAAAGGGTGGGAAGCTTTAGCCTAGACTTTTAATT 78256
GrabEatingMacaqueCTGTACTCTAGCTTGGGTGACAGACTGAACCTTGTCTCAAAAAATAAAGGGTGCGGAAGCTTTAGCCTAGACTTTTAATT 80363
Macaque           CTGTACTCTAGCTTGGGTGACAGACTGAACCTTGTCTCAAAAAATAAAGGGTGGGAAGCTTTAGCCTAGACTTTTAATT 81241
PigTailedMacaque CTGTACTCTAGCTTGGGTGACAGACTGAACCTTGTCTCAAAAAATAAAGGGTGGGAAGCTTTAGCCTAGACTTTTAATT 79763
Bonobo            CTGCACTCTAGCCTGGGTGACAGAGCAAACCTTGTCTCAAAAAATAAAGGGTGGGAAGCTTTAGCCTAGACTTTTAATT 78876
OliveBaboon       CTGTACTCTAGCTTGGGTGACAGAGTGAACCTTGTCTCAAAAAATAAAGGGTGGGAAGCTTTAGCCTAGACTTTTAATT 79652
Orangutan         CTGCACTCTAGCCTGGGTGACAGAGCGAACCTTGTCTCAAAAAATAAAGGGTGGGAAGCTTTAGCCTAGACTTTTAATT 80517

Majority          CTCAA-TTACCATTATAAATTTATTTTAATTATTAACTAATTATTTTAATTCTCAATTAGTGTTTTTCCATGATAAGGCT
                          96170     96180     96190     96200     96210     96220     96230     96240
Human             CTCAA-TTACCATTATAAATTTATTTTAATTATTAACTAATTACTTTAATTCTCAATTACTATTTTTCCATGAAAAGGCT 79789
Coquerel'sSifaka GTTTA------ATTGTATGTTGGT----GGCACGGTAAAAGTATCTCAG-----AAGAAATAATTCTTCATAAAAA---T 66287
Ma'sNightMonkey   CTCAA-TTACCATTATAAGTTTA-----ATTATTAACTAATTATTTTAATTCTCAATTACTGTTTTTCCATGATAAGGCT 74722
VervetAGM         CTCAA-TTACCATTATAAATTTATTTTAATTATTAACTAATTATTTTAATTCTCAATTAGTGTTTTTCCATGATAAGGCT 80905
Gorrilla          CTCAA-TTACCATTATAAATTTATTTTAATTATTAACTAATTACTTTAATTCTCAATTACTATTTTTCCATGAGAAGGCT 78335
GrabEatingMacaqueCTCAAATTACCATTATAAATTTATTTTAATTATTAACTAATTATTTTAATTCTCAATTAGTGCTTTTCCATGATAAGGCT 80443
Macaque           CTCAAATTACCATTATAAATTTATTTTAATTATTAACTAATTATTTTAATTCTCAATTAGTGTTTTTCCATGATAAGGCT 81321
PigTailedMacaque CTCAAATTACCATTATAAATTTATTTTAATTATTAACTAATTATTTTAATTCTCAATTAGTGTGTTTCCATGATAAGGCT 79843
Bonobo            CTCAA-TTACCATTATAAATTTATTTTAATTATTAACTAATTACTTTAATTCTCAATTACTATTTTTCCATGAAAAGGCT 78955
OliveBaboon       CTCAA-TTACCATTATAAATTTATTTTAATTATTAACTAATTATTTTAATTCTCAATTAGTGTTTTTCCATGATAAGGCT 79731
Orangutan         CTCAA-TTACCATTATAAATTTATTTTAATTATTAACTAATTACTTTAATTCTCAATTACTGTTTTTCCATGATAAGGCT 80596
```

```
Majority          TGATGTTCTAAACAATATTATACCACCAAGTTATCTCTACTGGAAAACCCAGATTTCAAGAAA--AAAAAA-TGTATTTT

                          96250     96260     96270     96280     96290     96300     96310     96320

Human             TGATGTTCTAAACAGTATTATACCACCAAGTTATCTCTACTGGAAAACCCAGATTTCAAGAAA--AAAAAAATGTATTTT 79867
Coquerel'sSifaka  -GAAGTATTAACC----TTAGGCTTGTATGTT-------TAGATAGATATTGAATTTAAGTAG---ATTGA-TAAATTT- 66350
Ma'sNightMonkey   TGATGTTCTAAACGATATTATACCAGCA---T---------GGAAAACCCTGATTTCAAGAAATGAAATAAGTGTATTTT 74790
VervetAGM         TGATGTTCTAAACAATATCATACCACCAAGTTATCTCTACTGGAAAACCCAGATTTCAAGAAA--AAAAAA-TGTATTTT 80982
Gorrilla          TGATGTTCTAAACAGTATTATACCACCAAGTTATCCCTACTGGAAAACCCAGATTTCAAGAAA--AAAAAAATGTATTTT 78413
GrabEatingMacaqueTGATGTTCTAAACAATATCATACCACCAAGTTACCTCTGCTGGAAAACCCAGATTTCAAGAAA--AAAAAA-TGTATTTT 80520
Macaque           TGATGTTCTAAACAATATCATACCACCAAGTTACCTCTGCTGGAAAACCCAGATTTCAAGAAA--AAAAAA-TGTATTTT 81398
PigTailedMacaque  TGATGTTCTAAACAATATCATACCACCAAGTTACCTCTGCTGGAAAACCCAGATTTCAAGAAA--AAAAAAATGTATTTT 79921
Bonobo            TGATGTTCTAAACAGTATTATACCACCAAGTTATCTCTACTGGAAAACCCAGATTTCAAGAAA--AAAAAA-TGTATTTT 79032
OliveBaboon       TGATGTTCTAAACAATATCATACCACCAAGTTACCTCTGCTGGAAAACCCAGATTTCAAGGAA--AAAAA--TGTATTTT 79807
Orangutan         TGATGTTCTAAACAGTATTATACCACCAAGTTATCTCTACTGGAAAACCCAGATTTCAAGAAA--AAAAAA-TGTATTTT 80673

Majority          GAAAACAATTATGTCTTAAATATTTAAAATTCTTTTGACTTGTTTTT--ACCATCTTTAAATGTTTTTGGTAAGTAATT

                          96330     96340     96350     96360     96370     96380     96390     96400

Human             GAAAACAATTATGTCTTAAATATTTAAAATTCTTTTAAACATGTTTTT-AACCATCTTTAAATTTTTTTGGTAAGTAATT 79946
Coquerel'sSifaka  -G-TA--TGTATTTTATGAA--TTAGTAATTTTTCTTGTGATGT-------CAACAGGGAAAAATGAATAATAGGTGACT 66417
Ma'sNightMonkey   GAAAACAGTTGTCTTTT------TTTTGGTGGGGTGGGGGGAGTTACT-GATCACCTT--ATTATTTTTGCTGGGGCAGG 74861
VervetAGM         GGAAACAATTATGTCTTAAATATTTAAAATTCTTTTGACTGGTTTTC--ACCATCTTTAAATGTTTTTGGTAAGTAATT 81060
Gorrilla          GAAAACAATTATGTCTTAAATATTTAAAATTCTTTTAACATGTTTTTTAACCATCTTTAAATTTTTTTGGTAAGTAATT 78493
GrabEatingMacaqueGAAAACAATTATGTCTTAAATATTTAAAATTCTTTTGACTGGTTTTC--ACCATCTTTAAATGTTTTTGGTAAGTAATT 80598
Macaque           GAAAACAATTATGTCTTAAATATTTAAAATTCTTTTGACTGGTTTTC--ACCATCTTTAAATGTTTTTGGTAAGTAATT 81476
PigTailedMacaque  GAAAACAATTATGTCTTAAATATTTAAAATTCTTTTGACTGGTTTTC--ACCATCTTTAAATGTTTTTGGTAAGTAATT 79999
Bonobo            GAAAACAATTATGTCTTAAATATTTAAAATTCTTTTAAACATGTTTTT-AACCATCTTTAAATTTTTTTGGTAAGTGATT 79111
OliveBaboon       GAAAACAATTATGTCTTAAATATTTAAAATTCTTTTGACTGGTTTTC--ACCATCTTTAAATGTTTTTGGTAAGTAATT 79885
Orangutan         GAAAACAATTATGTCTTAAATATTTAAAATTCTCTTTAACATGTTTTT-AACCATCTTTAAATTTTTTTGGTAAGTAATT 80752

Majority          TTCA-TTAGATTATTTAAATGTATAGTGGCACGGTAGGGTTGTATCTCA-GATATGAAGCGATTTTTCATAAAAATA-AA

                          96410     96420     96430     96440     96450     96460     96470     96480

Human             TTCA-TTAGATTGTTAAAATGTATAGTGGCACGGTAGGGTTGTATCTCAAGATATGAAGCAGTTTTTCATAAAAATA-AA 80024
Coquerel'sSifaka  TGGAACTATAAATTTTGAAAGC-----------GTCAGTGTATAATTTA----ACTTAGCATTCTTTTTAAAAAATACAA 66482
Ma'sNightMonkey   CAGA-TCACAAGATCAAGAGGT-------CGAGACCATCCTGGCCAACATGGTGAAACCCCATCTCTACTAAAAATACAA 74933
VervetAGM         TTCA-TTAGATTATTTAAATGTATAGTGGCACGGTAGGGTTGTATCTCA-GATATGAAGTGATTTTTCATGAAAATA-AA 81137
Gorrilla          TTCA-TTAGATTGTTTAAATGTATAGTGGCATGGTAGGGTTGTATCTCAAGATATGAAGCAGTTTTTCATAAAAATA-AA 78571
GrabEatingMacaqueTTCA-TTAGATTATTTAAATGTATAGTGGCACAGTAGGGTTGTATCTCA-GATATGAAGTGATTTTTCATAAAAATA-AA 80675
Macaque           TTCA-TTAGATTATTTAAATGTATAGTGGCACAGTAGGGTTGTATCTCA-GATATGAAGTGATTTTTCATAAAAATA-AA 81553
PigTailedMacaque  TTCA-TTAGATTATTTAAATGTATAGTGGCACAGTAGGGTTGTATCTCA-GATATGAAGTGATTTTTCATAAAAATA-AA 80076
Bonobo            TTCA-TTAGATTGTTAAAATGTATAGTGGCACGGTAGGGTTGTATCTCAAGATATGAAGCAGTTTTTCATAAAAATA-AA 79189
OliveBaboon       TTCA-TTAGATTATTTAAATGTATAGTGGCACGGTAGGGTTGTATCTCA-GATATGAAGCGATTTTTCATAAAAATA-AA 79962
Orangutan         TTCA-TTAGATTGTTTAAATGTATAGTGGCACGGTAGGGTTGTATCTCAAGATATGAAGCAGTTTTTCATAAAAATA-AA 80830
```

```
Majority          GTATTAACCT--TAGGCTGGTATGTTTAGATCAATACTGAATTTAAATAGATTGAT-------AATTTTTTAAATATATT

                          96490     96500     96510     96520     96530     96540     96550     96560

Human             GTATTAACCT--TAGGCTGGTATGTTTAGATCAATACTGTATTTAAATAGATTGAT-------AATTTTTTA--TATATT 80093
Coquerel'sSifaka  ATACATATTA--TGTATT-GTATATTATGTTATATAACATAAATATGTATTATAAC--------ATAAATATAAAATGTA 66551
Ma'sNightMonkey   AAATTAGCTGGGTGTGATGGTGTGCACCTATAGTCCCAGCTACTGAGGAGGCTGAGGCAGGAGAATTGCTTGAACCTGGG 75013
VervetAGM         GTGTTAACCT--TAGGCCAGTATGTTTAGATCAATACTGAATTTAAATAGATTGAT-------AATTTTTTAAATATACT 81208
Gorrilla          GTATTAACCT--TAGGCTGGTATGTTTAGATCAATACTGTATTTAAATAGATTGAT-------AATTTTTTA--TATTTT 78640
GrabEatingMacaqueGTGTTAACCT--TAGGCCAGTATGTTTAGGTCAATACTGAATTTAAATAGATTGAT-------AATTTTTTAAATATACT 80746
Macaque           GTGTTAACCT--TAGGCCAGTATGTTTAGGTCAATACTGAATTTAAATAGATTGAT-------AATTTTTTAAATATACT 81624
PigTailedMacaque  GTGTTAACCT--TAGGCCAGTATGTTTAGGTCAATACTGAATTTAAATAGATTGAT-------AATTTTTTAAATATACT 80147
Bonobo            GTATTAACCT--TAGGCTGGTATGTTTAGATCAATACTGTATTTAAATAGATTGAT-------AATTTTTTA--TATATT 79258
OliveBaboon       GTGTTAACCT--TAGGCCAGTATGTTTAGATCAATACTGAATTTAAATAGATTGAT-------AATTTTTTAAATATACT 80033
Orangutan         GTATTAACCT--TAGGCTGGTATGTTTAGATCAATACTGAATTTAAATAGATTGAT-------AATTTTTTAA-TATATT 80900

Majority          TTATGAATTTGTAATTTTTCCTGAGATTTCAAGAGGGAAAAATGAGTAGGAGATGACTGAGAGCTGTAAAGTTTGGGAGT

                          96570     96580     96590     96600     96610     96620     96630     96640

Human             TTATGAATTTATACTTTTTCCTGAGATTTCAGGAGGGAAAAATGAGTAGGAGATGACTGAGAGCT-TAAAGTTTGGGAGT 80172
Coquerel'sSifaka  ATATAAATATATAAATTTTG--GAAGTGTCAGTAGTGTGTACTT---------TAACTCAGCATT--------------- 66605
Ma'sNightMonkey   AGGCGGAGGT-TGCAGTGAGCCAAAATCACGCCACTGCACTCCACCCTAGTGACAGAGCAAGACTGCA---TTTCAAAAA 75089
VervetAGM         TTATGAATTTGTAATTTTTCCTGAGATTTCAAGAGGGAAAAATGAGTAGGAGATGACTGAGAGCTATAAAGTTTGGGAGT 81288
Gorrilla          TTATGAATTTATAATTTTTCCTGAGATTTCAAGAGGGAAGAATGAGTAGGAGATGACTGAGAGCTGCAAAGTTTGGGAGT 78720
GrabEatingMacaqueTTATGAATTTGTAATTTTTCCTGAGATTTCAAGAGGGAAAAATGAGTAGGAGATGACTGAGAGCTGTAAAGTTTGGGAGT 80826
Macaque           TTATGAATTTGTAATTTTTCCTGAGATTTCAAGAGGGAAAAATGAGTAGGAGATGACTGAGAGCTGTAAAGTTTGGGAGT 81704
PigTailedMacaque  TTATGAATTTGTAATTTTTCCTGAGATTTCAAGAGGGAAAAATGAGTAGGAGATGACTGAGAGCTGTAAAGTTTGGGAGT 80227
Bonobo            TTATGAATTTATACTTTTTCCTGAGATTTCAAGAGGGAAAAATGAGTAGGAGATGACTGAGAGCT-TAAAGTTTGGGAGT 79337
OliveBaboon       TTATGAATTTGTAATTTTTCCTGAGATTTCAAGAGGGAAAAATGAGTAGGAGATGACTGAGAGCTGTAAAGTTTGGGAGT 80113
Orangutan         TTATGAATTTATAATTTTTCCTGAGATTTCAAGAGGGAAAAATGAGTAGGAGATGACGGAGAGCTGTAAAATTTGGGAGT 80980

Majority          GTCAATTAACTCAGCATTCTTTTAAAAAATATATGTCATATATTACAGCATTTTCTTTTATTTGAAGTGAGTAAATGTAT

                          96650     96660     96670     96680     96690     96700     96710     96720

Human             GTCAATTAACTCAGCATTCTTTTAAAAAACATGTGTCATATATTACAGCATTTTCTTTTATTTGAAGTGAGTAAATGTAT 80252
Coquerel'sSifaka  ----------TTA--TTTTTTAC----TAAATCTGTCATATATTACAGTATTTTCTTTTCTTTGAAGTGAGTAAATGCAT 66669
Ma'sNightMonkey   AAAAAAAAAAAAAATATATATAT---ATATATATGTCATATATTACAGCATTTTCTTTTATTTGAAGTGAGTAAATGTAT 75166
VervetAGM         GTCAATTAACTCAGCATTCTTTTAAAAAATATATGTCATATATTACAGCATTTTCTTTTATTTGAAGTGAGTAAATGTTA 81368
Gorrilla          GTCAATTAACTCAGCATTCTTTTAAAAAATATGTGTCATATATTACAGCATTTTCTATTATTTGAAGTGAGTAAATGTAT 78800
GrabEatingMacaqueGTCAATTAACTCATCATTCTTTTGAAAAATATATGTCATATATTACAGCATTTTCTTTTATTTGAAGTGAGTAAATGTTA 80906
Macaque           GTCAATTAACTCATCATTCTTTTGAAAAATATATGTCATATATTACAGCATTTTCTTTTATTTGAAGTGAGTAAATGTTA 81784
PigTailedMacaque  GTCAATTAACTCATCATTCATTTAAAATATATATGTCATATATTACAGCATTTTCTTTTATTTGAAGTGAGTAAATGTTA 80307
Bonobo            GTCAATTAACTCAGCATTCTTTTAAAAAATATGTGTCATATATTACAGCATTTTCTTTTATTTGAAGTGAGTAAATGTAT 79417
OliveBaboon       GTCAATTAACTCAGCATTCTTTTAAAAAATATATGTCATATATTACAGCATTTTCTTTTATTTGAAGTGAGTAAATGTTA 80193
Orangutan         GTCAATTAACTCAGCATTCTTTTAAAAAATATATGTCATATATTACAGCATTTTTT----TTTGAAGTGAGGAAATGTAT 81056
```

```
Majority         CTTTTTAAATTCCTTAGTAATTTTT-GAGCACTCCATATATACAAAGCATGTGAATATTTGGTAGCGTTTTACA-AATGT

                         96730     96740     96750     96760     96770     96780     96790     96800

Human            CTTTTTAAATTCCTTAGTAATTTTT-GAGCACTCCATATGTATAAAGCATGTGAATATTTGGTAGCATTTTACA-AATGT 80330
Coquerel'sSifaka CTTTT-AAATTCCTATGTACTTTTTTGAGCACTCCATATGTACAAAGCATAT--GTATATGATAGAGGTTTACATAATGT 66746
Ma'sNightMonkey  CTTTT-AAATTCCTTAATAATTTCT-GAGCACTCCATATATATAAAGCAT----ATATTTGGTAGCATTTTGCA-AATGT 75239
VervetAGM        TCTTTTAAGTTCCTTAGTAATTTTT-GAGCACTCCATATATACAAAGCATGTGAATATTTGGTAACGTTTTACA-AATGT 81446
Gorrilla         CTTTTTAAATTCCTTAGTAATTTTT-GAGCACTCCTTATGTATAAAGCATGTGAATATTTGGTAGCATTTTACA-AATGT 78878
GrabEatingMacaqueTCTTTTAAGTTCCTTAGTAGTTTTT-GAGGACTCCATATATACAAAGCATGTGAATATTTGGTAGCGTTTTACA-AATGT 80984
Macaque          TCTTTTAAGTTCCTTAGTAGTTTTT-GAGGACTCCATATATACAAAGCATGTGAATATTTGGTAGCGTTTTACA-AATGT 81862
PigTailedMacaque TCTTTTAAGTTCCTTAGTAGTTTTT-GAGGACTCCATATATACAAAGCATGTGAATATTTGGTAGCGTTTTACA-AATGT 80385
Bonobo           CTTTTTAAATTCCTTAGTAATTTTT-GAGCACTCCATATGTATAAAGCATGTGAATATTTGGTAGCATTTTACA-AATGT 79495
OliveBaboon      TCTTTTAAGTTCCTTAGTAATTTTT-GAGCACTCCATATATACAAAGCATGTGAATATTTGGTAGCGTTTTACA-AATGT 80271
Orangutan        CTTTT-AAATTCCTTAGTAATTTTT-GAGCACTCCATATGTACAAAGCATGTGAATATTTGGTAGCATTTTACA-AATGT 81133

Majority         -------CCAGAGATTTGTGAGAGTTCCTGAGATCCTCATAGGGGGCCTACAAAGTTTAGTATTACTTTTCATGGTAATA

                         96810     96820     96830     96840     96850     96860     96870     96880

Human            -------CCAGAGATTTGTGAGAGTTCCTGAGATCTTCATAGGGGGCCCACAAAGTTTAGTATTACTTTTCACGGTAATA 80403
Coquerel'sSifaka T----TTCCAGAGATCTTTGGGCGTTCCTGAGA-CCTCA-AGGAGGTCTACAAAGTG----AGAACTTTT-AGGATAATA 66815
Ma'sNightMonkey  TGGTTGTCCAGAGATCTGTGAGAGTTCCTGAGATCCTCATAGAGGGCCCACACAGTTTAGTATTACTTTTCACGGTAGTA 75319
VervetAGM        -------CCAGAGATTTGTGAGAGTTCCTGAGATCCTTATAGGGGGCCTACAAAGTTTAGTATTACTTTTCATGGTAATA 81519
Gorrilla         -------CCAGAGATTTGTGAGAGTTCCTGAGATCTTCATAGGGGGCCCACAAAGTTTAGTATTACTTTTCACGGTAATA 78951
GrabEatingMacaque-------CCAGAGATTTGTGAGAGTTCCCAAGATCCTTATAGGGGGCCTACAAAGTTTAGTATTACTTTTCATGGTAATA 81057
Macaque          -------CCAGAGATTTGTGAGAGTTCCCAAGATCCTTATAGGGGGCCTACAAAGTTTAGTATTACTTTTCATGGTAATA 81935
PigTailedMacaque -------CCAGAGATTTGTAAGAGTTCCCGAGATCCTTATAGGGGGCCTACAAAGTTTAGTATTACTTTTCATGCTAATA 80458
Bonobo           -------CCAGAGATTTGCGAGAGTTCCTGAGATCTTCATAGGGGGCCCACAAAGTTTAGTATTACTTTTCACGGTAATA 79568
OliveBaboon      -------CCAGAGATTTGTGAGAGTTCCTGAGATCCTTATAGGGGGCCTACAAAGTTTAGTATTACTTTTCATGGTAATA 80344
Orangutan        -------CCAGAGATTTGTGAGAGTTCCTGAGATCCTCATAGGGGGCCCACAAAGTT-AATATTACTTTTCATGGTAATA 81205

Majority         CTAAAATGTATTTTGCCTTTTTTTACTTT--TTCTCTTAATAGCGTACAGTG----GCATCACTCTGAAGGCTAATAGTA

                         96890     96900     96910     96920     96930     96940     96950     96960

Human            CTAAAGTGTATTTTGCCTCTTTTTACTTT--TTCTCTTAATAGCATACAGTG----GTAA----CTGAAGGCTAATAGTA 80473
Coquerel'sSifaka CTAAAATGTTTTTTGCCT-TTTTGATTCTCATTCTCTTAGTACAGTGGTGTTCCCTACATCATTCTGAAGACTAATAGAA 66894
Ma'sNightMonkey  CTAAAATGTATTTTGCCT-TTTTGACTTT--TTGTCTTAATAGCATACAGTG----GTATCACTCTGAAGGCTAATAGTA 75392
VervetAGM        CTAAAATATAGTTTGCCTTTTTTTACTTT--TTCTCTTAATAGCGTACAGTG----GCATCTCTCTGAAGGCTAATAGTA 81593
Gorrilla         CTAAAGTGTATTTTGCCTCTTTTTACTTT--TTCTCTTAATAGCATACAGTG----GTATGACTCTGAAGGCTAATAGTA 79025
GrabEatingMacaqueCTAAAATATAGTTTGCCTATTTTTACTTT--TTCTCTTAATAGCGTACAGTG----GCATCACTCTGAAGGCTAATAGTA 81131
Macaque          CTAAAATATAGTTTGCCTATTTTTACTTT--TTCTCTTAATAGCGTACAGTG----GCATCACTCTGAAGGCTAATAGTA 82009
PigTailedMacaque CTAAAATATAGTTTGCCTTTTTTTACTTT--TTCTCTTAATAGCGTACAGTG----GCATCACTCTGAAGGCTAATAGTA 80532
Bonobo           CTAAAGTGTATTTTGCCTCTTTTTACTTT--TTCTCTTAATAGCATACAGCG----GTAA----CTGAAGGCTAATAGTA 79638
OliveBaboon      CTAAAATATAGTTTGCCTTTTTTTACTTT--TTCTCTTAATAGCGTACAGTG----GCATCACTCTGAAGGCTAATAGTA 80418
Orangutan        CTAAAGTGTATTTTGCCTTTTTTTACTTT--TTGTCTTAATAGCATACAGTG----GTATTACTCTGAAGGCTAATAGTA 81279
```

```
Majority         TGTGTGTTTGTGTGCTTTA-----AA--AAGTTCGTGGTTTTGTCCGGGCACAGTGGCTCAGTCCTGTAATCCCAGCACT
                         96970     96980     96990     97000     97010     97020     97030     97040
Human            TGTGTGTTTATGTGCTTTA-----AA--AAGTTCGTGGTTTTGGCCAGGCGCAGTGGCTCAGACCTGTAATCCCAGCACT 80546
Coquerel'sSifaka TGTGTGAAAGTGTGACCTGTGCCTTAAGAAGTTCTTGGTTTTAATTTC-TGAAGTGATAAATATCAATAGATACAGC-CC 66972
Ma'sNightMonkey  TGTGTGAAAGTGTG----------A-----------------GGCCGGGCATGGTGGCTCACGCCTGTAATCCCAGCACT 75445
VervetAGM        TGTGTGTTTGTGTGCTTTA-----AA--AAGTTAGTGGTTTTGTCCGGGCACATTGGCTCAGTCCTGTAATCCCAGTACT 81666
Gorrilla         TGTGTGTTTATGTGCTTTA-----AA--AAGTTCGTGATTTTGGCCAGGCGCAGTGGCTCAGACCTGTAATCCCAGCACT 79098
GrabEatingMacaqueTGTGTGTTTGTGTGCTTTA-----AA--AAGTTAGTGGTTTTGTCCGGGCACATTGGCTCAGTCCTGTAATCCCAGTACT 81204
Macaque          TGTGTGTTTGTGTGCTTTA-----AATAAAGTTAGTGGTTTTGTCCGGGCACATTGGCTCAGTCCTGTAATCCCAGTACT 82084
PigTailedMacaque TGTGTGTTTGTGTGCTTTA-----AA--AAGTTAGTGGTTTTGTCCGGGCACATTGGCTCAGTCCTGTAATCCCAGTACT 80605
Bonobo           TGTGTGTTTATGTGCTTTA-----AA--AAGTTCGTGGTTTTGGCCAAGCGCAGTGGCTCAGACCTGTAATCCCAGCACT 79711
OliveBaboon      TGTGTATTTGTGTGCTTTA-----AA--AAGTTAGTGGTTTTGTCCGGGCACATTGGCTCAGTTCTGTAATCCCAGTACT 80491
Orangutan        TGTGTGTTTGTGTGCTTTA-----AA--AAGTTCATGGTTTTGGCCAGGTGCAGTGGCTCAGACCTGTAATCCCAGCACT 81352
```

```
Majority         TTGGGAGGCTGAGGCGGGCGGATCACC-TGAGGTCAGGAGTTCGAGACCA------GCCTG-GCCAACATGGTGAAACCC
                         97050     97060     97070     97080     97090     97100     97110     97120
Human            TTGGGAGGCTGAGGCAGGTGGATCACC-TGAGGTCAGGAGTTCAAGACCAAAACCAGCCTG-GCCAACATGGTGAAACCC 80624
Coquerel'sSifaka ACGTAAGCGAGAACTTTGTGGTCCTCAGTAATTTTAAGAGTGTAAAAGGG------TCCTGAGACCAAAAAGTCTGACAG 67046
Ma'sNightMonkey  TTGGGAGGCCGAGGCGAGTGGATCAC---GAGGTCAAGAGATTGAGACCA------TCCTG-GCCAACATGGTGAAACCC 75515
VervetAGM        TTGGGAGGCTGAGGCGGGCAGATCACC-TGAGGTCAGGAGTTCGAGACCA------GCCTG-GCCAACATGGTGAAACCC 81738
Gorrilla         TTGGGAGGCTGAGGCAGGTGGATCACC-TGAGGTCAGGAGTTCAAGACCAAAACCAGCCTG-GCCAACATGGTGAAGCCC 79176
GrabEatingMacaqueTTGGGAGGCTGAGGCGGGCAGATCACC-TGAGGTCAGGAGTTCGAGACCA------GCCTG-GCCAACATGGTGAAACCT 81276
Macaque          TTGGGAGGCTGAGGCGGGCAGATCACC-TGAGGTCAGGAGTTCGAGACCA------GCCTG-GCCAACATGGTGAAACCC 82156
PigTailedMacaque TTGGGAGGCTGAGGCGGGCAGATCACC-TGAGGTCAGGAGTTCGAGACCA------GCCTG-GCCAACATGGTGAAACCC 80677
Bonobo           TTGGGAGGCTGAGGCAGGTGGATCACC-TGAGGTCAGGAGTTCAAGACCAAAACCAGCCTG-GCCAACGTGGTGAAACCC 79789
OliveBaboon      TTGGGAGGCTGAGGCGGGCAGATCACC-TGAGGTCAGGAGTTCGAGACCA------GCCTG-GCCAAAATGGTGAAACCC 80563
Orangutan        TTGGGAGGCTGAGGCGGGCGGATCACC-TGAGGTCAGGAGTTCAAGACCA------GCCTG-GCCAACATGGTGAAACCC 81424
```

```
Majority         CATCTCTTCTAAAA----ATACAAAAATT-AGCCGGGCATGGTGGCGGGTGCCTGTAGTCTCAGCTACTCGAGAGGCTGA
                         97130     97140     97150     97160     97170     97180     97190     97200
Human            CATCTCTACTAAAA----ATACAAAAATT-AGCCAGGCATGGTAGTGGGTGCCTGTAGTCTCAGCTACTCGAGATGCTGA 80699
Coquerel'sSifaka CTTTTTCACTGTAG---GATACAAAGTTCCAGACAATTCTAGTAG-ATAAATGGGAGATACAGAGTATTTTAAT----AA 67118
Ma'sNightMonkey  CGTCTCTGCTAAAATATAAAACAAAAATT-AGTTGCGCCTGGTGGCTTACACCTGTAGTCTCAGCTACCCAGGAGACTGA 75594
VervetAGM        CATCTCTTCTAAAA----ATACAAAAATT-AGCCGGACATGGTGGCGGGTGCCTGTACTCTCAGCTACTCGAGAGGCTGA 81813
Gorrilla         CATCTCTACTAAAA----ATACAAAAATT-AGCCAGGCATGGTAGCGGGTGCCTGTAGTCTCAGCTACTCGAGATGCTGA 79251
GrabEatingMacaqueCATCTCTTCTAAAA----ATACAAAAATT-AGCCGGGCGTGGTGGCGGGTGCCTGTAGTCTCAGCTCCTCGAGAGGCTGA 81351
Macaque          CATCTCTTCTAAAA----ATACAAAAATT-AGCCGGGCGTGGTGGCGGGTGCCTGTAGTCTCAGCTCCTCGAGAGGCTGA 82231
PigTailedMacaque CATCTCTTCTAAAA----ATACAAAAATT-AGCCGGGCGTGGTGGCGGGTGCCTGTAGTCTCAGCTCCTCGAGAGGCTGA 80752
Bonobo           CATCTCTACTAAAA----ATACAAAAATT-AGCCAGGCATGGTAGTGGGTGCCTGTAGTCTCAGCTACTCGAGATGCTGA 79864
OliveBaboon      CATCTCTTCTAAAA----ATACAAAAATT-AGCCGGGCGTGGTGGTGGGTGCCTGTAGTCTCAGCTCCTCGAGAGGCTGA 80638
Orangutan        CATCTCTACTAAAA----ATACAAAAATC-AGCCAGGCATGGTGGCGGGTGCCTGTAGTCTCAGCTACTCGAGATGCTGA 81499
```

```
Majority          GGCAGGAGAAT-CGCTTGAAGCCAGGAGGTGGAGATTGCAGTGAGCCAAAATCTTGCCATTACACTCCA----GCCTGG-

                          97210     97220     97230     97240     97250     97260     97270     97280

Human             GGCAGGAGAAT-CACTTGAAGCTGGGAGGTGGAGGTTGCAGTGAGCCAAAATCTCGCCATTACACTCCA----GCCTGG- 80773
Coquerel'sSifaka  A-CAGTAGACTGTGTTTGGAGTCAAGAGATGTACAGAGAAAATGCTTGTGATCTGATTTCTGAGATTATTTTGATTTTG- 67196
Ma'sNightMonkey   GGCAGGAGAAT-TGCTTGAACCTGGGAGGCGGAGGTTGCAGTGAGCTGAGATCGTGCCACTGTACTCCATCCAGCCTGGC 75673
VervetAGM         GGCAGGAGAAT-CGCTTGAAGCCAGAAGGTGGAGATTGCAGTAAGCCAAAATCTTGCCATTACACTCCA----GCCTGG- 81887
Gorrilla          GGCAGGAGAAT-CACTTGAAGCTGGGAGGTGGAGGTTGCAGTGAGCCAAAATCTCGCCATTACACTCCA----GCCTGG- 79325
GrabEatingMacaqueGGCAGGAGAAT-CGCTTGAAGCCAGAAGGTGGAGATTGCAGTGAGCCAAAATCTTGCCATTACACTCCA----GCCTGG- 81425
Macaque           GGCAGGAGAAT-CGCTTGAAGCCAGAAGGTGGAGATTGCAGTGAGCCAAAATCTTGCCATTACACTCCA----GCCTGG- 82305
PigTailedMacaque  GGCAGGAGAAT-CGCTTGAAGCCAGAAGGTGGAGATTGCAGTGAGCCAAAATCTTGCCATTACACTCCA----GCCTGG- 80826
Bonobo            GGCAGGAGAAT-CACTTGAAGCTGGGAGGTGGAGGTTGCAGTGAGCCAAAATCTCGCCATTACACTCCA----GCCTGG- 79938
OliveBaboon       GGCAGGAGAAT-TGCTTGAAGCCAGAAGGTGGAGATTGCAGTGAGCCAAAATCTTGCCATTACACTCCA----GCCTGG- 80712
Orangutan         GGCAGGAGAAT-CACTTGAAGCCAGGAGGTGGAGGTTGCAGTGAGCCAAAATCTTGCCATTACACTCCA----GCCTGG- 81573

Majority          ----GGGACAAGAGCGAGACTCCGTCTCAAA------AAAAAAAAAAAAAAAAA----------------------GTTC

                          97290     97300     97310     97320     97330     97340     97350     97360

Human             ----GGGACAAGAGCAAGACTCCATCTCA--------AAAAAAAAAAAAAAAAA----------------------GTTA 80819
Coquerel'sSifaka  GCAGCTAATTACAATTAGTTTCTCAATTAAGTCTTTCTGGCTTAGTGAGCACTGAG---------------AATCACTTC 67261
Ma'sNightMonkey   GCCTGGTGACAGAGCAAGTCT-TGTCTCAAAAAAAGAAAAGAAAAGAAAGAAAGTGTGTTTGTGTGCTTTTAAAAAGTTC 75752
VervetAGM         ----GGGACAAGAGCGAGACTCCGTCTCAAAA-----AAAAAAAAAAAAAAAAA----------------------GTTC 81936
Gorrilla          ----GGGACAAGAGCAAGACTCCATCTCAAAAAAAAAAAAAAAAAAAAAAAAAA----------------------GTTA 79379
GrabEatingMacaque----GGGACAAGAGCGAGACTCTGTCTCA--------AAAAAAAAAAAA---------------------------GTTC 81466
Macaque           ----GGGACAAGAGCGAGACTCTGTCTCA--------AAAAAAAAAAAAAAAAAA----------------AAAAAGTTC 82357
PigTailedMacaque  ----GGGACAAGAGCGAGACTCCGTCTCA--------AAAAAAAAAAAAAAA------------------------GTTC 80870
Bonobo            ----GGGACAAGAGCAAGACTCCATCT----------ACAAAAAAAAAAAAAAA----------------------GTTA 79982
OliveBaboon       ----GGGACAAGAGCAAGACTCCGTCTCACAA-----AAAGAAAAAAAAAAAAAA----------------AAAAAATTC 80767
Orangutan         ----GGGACAATAGCGAGACTCCATGTCAAA------AAAAAAAAAAAAAAAAA----------------------GTTC 81621

Majority          TTGGTTTTAATTTCTGAAATGATAACCATTAATAGAACCCAAATT-------------------A---------------

                          97370     97380     97390     97400     97410     97420     97430     97440

Human             TTGGTTTTAATTTTGGAAATGACAACCATTAATAGAACCCACATTAGGCCGGGCGCGGTGGCTCATGCCTGTAATCCTAG 80899
Coquerel'sSifaka  TTGACTGTTAGAAGTGAAAAGA-AACCAGAAATTAAACT--GGTTT------------------AATTTTTTAT------ 67314
Ma'sNightMonkey   TTGGTTTTAATTTCTGAAATGATAACCATTAATAGAACCCAAATT-------------------A--------------- 75798
VervetAGM         TTGGTTTTAATTTCTGAAATGATAACCATTAATAGAACCCAAATT-------------------A--------------- 81982
Gorrilla          TTGGTTTTAATTTCGGAAATGACAACCATTAATAGAACCCACATT-------------------A--------------- 79425
GrabEatingMacaqueTTGGTTTTAATTTCTGAAATGATAACCATTAATAGAACCCAAATT-------------------A--------------- 81512
Macaque           TTGGTTTTAATTTCTGAAATGATAACCATTAATAGAACCCAAATT-------------------A--------------- 82403
PigTailedMacaque  TTGGTTTTAATTTCTGAAATGATAACCATTAATAGAACCCAAATT-------------------A--------------- 80916
Bonobo            TTGGTTTTAATTTCGGAAATGACAACCATTAATAGAACCCACATT-------------------A--------------- 80028
OliveBaboon       TTGGTTTTAATTTCTGAAATGATAACCATTAATAGAACCTAAATT-------------------A--------------- 80813
Orangutan         TTGGTTTTAATTTCGGAAATGACAACCATTAATAGAACCCACATT-------------------A--------------- 81667
```

```
Majority        --------------------------------------------------------------------------------

                         97450     97460     97470     97480     97490     97500     97510     97520

Human           CACTTTGGGAGGCCGAGACGGGTGGATCACGAGGTCAGGAGATCAAGACCATCTTGGCTAACACGGTGAAACCCCGTTTC 80979
Coquerel'sSifaka -------------------------------------------------------------------------------- 67314
Ma'sNightMonkey --------------------------------------------------------------------------------- 75798
VervetAGM       -------------------------------------------------------------------------------- 81982
Gorrilla        -------------------------------------------------------------------------------- 79425
GrabEatingMacaque-------------------------------------------------------------------------------- 81512
Macaque         -------------------------------------------------------------------------------- 82403
PigTailedMacaque -------------------------------------------------------------------------------- 80916
Bonobo          -------------------------------------------------------------------------------- 80028
OliveBaboon     -------------------------------------------------------------------------------- 80813
Orangutan       -------------------------------------------------------------------------------- 81667

Majority        --------------------------------------------------------------------------------

                         97530     97540     97550     97560     97570     97580     97590     97600

Human           TACTAAAAATACAAAAAATTAGCCGGGCGTGTTGGCGGGCGCCTGTAGTCCCAGCTACTTGGGAGGCTGAGGCAGGAGAA 81059
Coquerel'sSifaka -------------------------------------------------------------------------------- 67314
Ma'sNightMonkey -------------------------------------------------------------------------------- 75798
VervetAGM       -------------------------------------------------------------------------------- 81982
Gorrilla        -------------------------------------------------------------------------------- 79425
GrabEatingMacaque-------------------------------------------------------------------------------- 81512
Macaque         -------------------------------------------------------------------------------- 82403
PigTailedMacaque -------------------------------------------------------------------------------- 80916
Bonobo          -------------------------------------------------------------------------------- 80028
OliveBaboon     -------------------------------------------------------------------------------- 80813
Orangutan       -------------------------------------------------------------------------------- 81667

Majority        --------------------------------------------------------------------------------

                         97610     97620     97630     97640     97650     97660     97670     97680

Human           TGGCATGAACCTGGGAGGCGGAGCTTGCAGTGAGCCGAGATCACGCCACTGCACTCCAACCTGAGAGACACAGCGAGACT 81139
Coquerel'sSifaka --------------------------------------------------------------T---------G------- 67316
Ma'sNightMonkey -------------------------------------------------------------------------------- 75798
VervetAGM       -------------------------------------------------------------------------------- 81982
Gorrilla        -------------------------------------------------------------------------------- 79425
GrabEatingMacaque-------------------------------------------------------------------------------- 81512
Macaque         -------------------------------------------------------------------------------- 82403
PigTailedMacaque -------------------------------------------------------------------------------- 80916
Bonobo          -------------------------------------------------------------------------------- 80028
OliveBaboon     -------------------------------------------------------------------------------- 80813
Orangutan       -------------------------------------------------------------------------------- 81667
```

```
Majority         -----------------------------------------ACAAAAACTCCTTGTGATCCTCTATAGTTTTTAAGACT
                          97690     97700     97710     97720     97730     97740     97750     97760
Human            CCGTCTCGAAAAAAAAAAAAAAAAAAAAAAAGAACCCACATTAACAAAAACTCCTTGTGATTCTCCATAGTTTT-AAGACT 81218
Coquerel'sSifaka --------------------------------------TGGGAAAGAGTGGGTCCTATATTTTTGGATTTTTTTATTTT 67357
Ma'sNightMonkey  -----------------------------------------ACAAAAACTTCTTGTGATCCTCCATAGTTTTTAAGACT 75836
VervetAGM        -----------------------------------------ACAAAAACTCCTTGTGATCCTCTATAATTTTTAGGACT 82020
Gorrilla         -----------------------------------------ACAAAAACTCCTTGTGATTCTCCATAGTTTT-AAGACT 79462
GrabEatingMacaque-----------------------------------------ACAAAAACTCCTTGTGATCCTCTATAGTTTTTAAGACT 81550
Macaque          -----------------------------------------ACAAAAACTCCTTGTGATCCTCTATAGTTTTTAAGACT 82441
PigTailedMacaque -----------------------------------------ACAAAAACTCCTTGTGATCCTCTATAGTTTTTAAGACT 80954
Bonobo           -----------------------------------------ACAAAAACTCCTTGTGATTCTCCATAGTTTT-AAGACT 80065
OliveBaboon      -----------------------------------------ACAAAAACTCCTTGTGATCCTCTATAATTTTTAAGACT 80851
Orangutan        -----------------------------------------ACAAAAATTCCTTGTGATTCTCCATAGTTTT-AAGACT 81704

Majority         ATAAAGGGGGCCTGA--GACCAAAAAGTTTGAGAACGTTTTCACTGTGGGATACAAAGTTTAAGACAATTGTAGTTAAAT
                          97770     97780     97790     97800     97810     97820     97830     97840
Human            ATAAAGGGGAGCTGA--GACCAAAAAGTTTGAGAACTTTTTCACTGTGGGATACAAAGTTGAAGACAATTATAGTTAAAT 81296
Coquerel'sSifaka AAGAAAGTCTTTTTATTGTTAAAAGGGAACATGAGGCTGGGCGCGGTGGCTCAC---GCCTGTAATCCTAGCACTCTGGG 67434
Ma'sNightMonkey  TTAAAGGGAGCCCGA--GACCAAAAAGCTTGAGAAC-TTTTCACTGTGGGATACAAAGTTTAAGACAATTGTAGT-AAAT 75912
VervetAGM        ATAAAGGGAGCCTGA--GACCAAAAA-TTTGAGAACGTTTTCACTTTGGGATACAAAGTTTAAGACAATTGTAGTTAAAT 82097
Gorrilla         ATAAAGGGGACCTGA--GACCAAAAAGTTTGAGAACTTTTTCACTGTGGGATACAAAGTTTAAGACAATTATAGTTAAAT 79540
GrabEatingMacaqueATAAAGGGGGCCTGA--GACCAAAAA-TTTGAGAACGTTTTCACTTTGAGATACAAAGTTTAAGACAATTGTAGTTAAAT 81627
Macaque          ATAAAGGGGGCCTGA--GACCAAAAA-TTTGAGAACGTTTTCACTTTGGGATACAAAGTTTAAGACAATTGTAGTTAAAT 82518
PigTailedMacaque ATAAAGGGGGCCTGA--GACCAAAAA-TTTGAGAACGTTTTCACTTTGGGATACAAAGTTTAAGACAATTGTAGTTAAAT 81031
Bonobo           ATAAAGGGGAGCTGA--GACCAAAAAGTTTGAGAACTTTTTCACTGTGGGATACAAAGTTTAAGACAATTATAGTTAAAT 80143
OliveBaboon      ATAAAGGGGGCCTGA--GACCAAAAA-TTTGAGAACGTTTTCACTTTGGGATATAAAGTTTAAGACAATTGTAGTTAAAT 80928
Orangutan        ATAAAGGGGGCCTGA--GACCAAAAAGTTTGAGAACTTTTTCACTGT------------TTAAGACAATTATAGTTAAAT 81770

Majority         AAGTAGGAG-ATACAGAGTATTTTAA-TAAACAGTAGATGGTTATAAGAGTCAAGAGATGTACAGAGAAAATGCTAGTGA
                          97850     97860     97870     97880     97890     97900     97910     97920
Human            AAGCAGGAG-ATACAGAGTATTTTAA-TGAACAGTAGATGGTTATAAGAGTCAAGAGATGTACAGAGAAAATGCTAGTGA 81374
Coquerel'sSifaka AGGCTGAGGCGGGCAGATTGTTTGAGCTCAGGAGTTCAAGACCAGCCTGAGCAAGAGCGAGACCCCGTCTCTACTAAAAA 67514
Ma'sNightMonkey  AAGTAGGAG-ATACAGAGTATTTTAA-TAAACAGTGGATGGTTATAAGAGTCAAGAGGTGTACAGAGAAAATGCTAGTGA 75990
VervetAGM        AAGTAGGAG-ATA--GAGTATTCTAA-TAAACAGTAGATGGTTATAAGAGTCAAGAGATGTACAGAGAAAATGCTAGTGA 82173
Gorrilla         AAGCAGGAG-ATACAGAGTATTTTAA-TTAACAGTAGATGGTTATAAGAGTCAAGAGATGTACAGAGAAAATGCTAGTGA 79618
GrabEatingMacaqueAAGTAGGAG-ATACAGAGTATTTTAA-TAAACAGTAGATGGTTATAAGAGTCAAGAGATGTACAGAGAAAATGCTAGTGA 81705
Macaque          AAGTAGGAG-ATACAGAGTATTTTAA-TAAACAGTAGATGGTTATAAGAGTCAAGAGATGTACAGAGAAAATGCTAGTGA 82596
PigTailedMacaque AAGTAGGAG-ATACAGAGTATTTTAA-TAAACAGTAGATGGTTATAAGAGTCAAGAGATGTACAGAGAAAATGCTAGTGA 81109
Bonobo           AAGCAGGAG-AAACAGAGTATTTTAA-TGAACAGTAGATGGTTATAAGAGTCAAGAGATGTACAGAGAAAATGCTAGTGA 80221
OliveBaboon      AAGTAGGAG-ATACAGAGTATTTTAA-TAAACAGTAGATGGTTACAAAAGTCAAGAGATGTACAGAGAAAATGCTAGTGA 81006
Orangutan        AAGCAGGAG-ATACAGAGTATTTTAA-TTAACAGTAAATGGTTATAAGAGTCAAGAGATGTACAGAGAAAATGCTAGTGA 81848
```

```
Majority          TTAG-------TTTAGATTATTTTAA---TGGTAACTAATTATAATTCCACAGTTAAGATTTTTTGG-CTTAGTGG---G

                          97930     97940     97950     97960     97970     97980     97990     98000

Human             TTAGAG---TTTTTAGATTATTTTAATTTTGGTAACTAATTATAATTCCTCAGTTAAGATTTTTTGG-CTTAGTGG---G 81447
Coquerel'sSifaka  AAAATAGA---AAGAAATTATCTGGA--CAACTAAAAATATATATATATATAGAAAAAATTAGCTGGGCATGGTGGCGCG 67589
Ma'sNightMonkey   TTAGGAGGTTTTTAGATTATTTTAATTCTGGTAACTAATTATAATTACTCAGTTAAGATTTTTTGT-CTCAGTGG---G 76066
VervetAGM         TGAG---------CAGATTTTTTT------GGTAACTAATTTTAATTCCACAGTGAAGATTTTTTGG-CTTAGTGG---G 82234
Gorrilla          TTAGAG---TTTTTAGATTATTTTAATTTTGGTAACTAATTATAATTCCTCAGTTAAGATTTTTTGG-CTTAGTGG---G 79691
GrabEatingMacaqueTGAG---------CAGATTTTTTT------GGTAACTAATTTTAATTCCACAGTGAAGATTTTTTGG-TTTAGTGG---G 81766
Macaque           TGAG---------CAGATTTTTTT------GGTAACTAATTTTAATTCCACAGTGAAGATTTTTTGG-TTTAGTGG---G 82657
PigTailedMacaque  TGAG---------CAGATTTTTTT------GGTAACTAATTTTAATTCCACAGTGAAGATTTTTTGG-TTTAGTGG---G 81170
Bonobo            TTAGAG---TTTTTAGATTATTTTAATTTTGGTAACTAATTATAATTCCTCAGTTAAGATTTTTTGG-CTTAGTGG---G 80294
OliveBaboon       TGAG---------CAGATTTTTTT------GGTAACTAATTTTAATTCCACAGTGAAGATTTTTTGG-TTTAGTGG---G 81067
Orangutan         TTAGAT---TTTTTAGATTATTTTAATTTTGGTAACTAATTATAATTCCTCAGTTAAGATTTTTTGG-CTTAGTGG---A 81921

Majority          CCCTTAGAATTGCTTCTTCCTGTTAGGATTAAA-AGTGAAACCAGA-AATTAAAGTGATTTAATTTTTTTATTGTGGTAA

                          98010     98020     98030     98040     98050     98060     98070     98080

Human             CCCTTAGAATTACTTCTTCCTGTTAGAATTAAA-AGTGAAACCAGA-AATTAAAGTGATTTAATTTTTTTATTGTGGCAA 81525
Coquerel'sSifaka  TACCTGTAGTCCCAGCTACTTGGGAGGCTGAGGCAGTAGGATCGCTTGAGCCCAGGAGTTTGAGGTTGC---TGTGAGCT 67666
Ma'sNightMonkey   CCCTTATAATTGCTTCTTCCTGTTAGAATTAAA-AATGAAACTAGA-AATTAAAGTGATTTAATTTTTT-ATTGTGGGAA 76143
VervetAGM         CCCTTAGAATTGCTTCTTCCTGTTAGGATTAAA-AGGGAAACCAGA-AATTAAAATGATTTAATTTTTTTATTGTGGTAA 82312
Gorrilla          CCCTTAGAATTACTTCTTCCTGTTAGAATTAAA-AGTGAAACCAGA-AATTAAAGTGATTTAATTTTTTTATTGTGGCAA 79769
GrabEatingMacaqueCCCTTAGAATTGCTTCTTCCTGTTAGGATTAAA-AGTGAAACCAGA-AATTAAAATGATTTAATTTTTTTATTGTGGTAA 81844
Macaque           CCCTTAGAATTGCTTCTTCCTGTTAGGATTAAA-AGTGAAACCAGA-AATTAAAATGATTTAATTTTTTTATTGTGGTAA 82735
PigTailedMacaque  CCCTTAGAATTGCTTCTTCCTGTTAGGATTAAA-AGTGAAACCAGA-AATTAAAATGATTTAATTTTTTTATTTTGGTAA 81248
Bonobo            CCCTTAGAATTACTTCTTCCTGTTAGAATTAAA-AGTGAAACCAGA-AATTAAAGTGATTTAATTTTTT-ATTGTGGCAA 80371
OliveBaboon       CCCTTAGAATTGCTTCTTCCTGTTAGGATTAAA-AGTGAAACCAGA-AATTAAAATGATTTAATTTTTTTATTGTGGTAA 81145
Orangutan         CCCTTAGAATTACTTCTTCCTGTTAGAATTAAA-AGTGAAACCAGA-AATTAAAGTGATTTAATTTTTTTATTATGGCAA 81999

Majority          AGATCTAGTCCA-AATATCCTTGATGGACTTTTTAAAAAAA--CAGGCTTTGT-----ATTGTTAAAAAGGGAATTTGAG

                          98090     98100     98110     98120     98130     98140     98150     98160

Human             AGATCTAGTCCA-AATATCTTTGATGGACTTTTTGAAAA----CAGACTTT-------ATTGTTAAAAAGGGAATTTGAG 81593
Coquerel'sSifaka  AGGCTGACGCCACGGCACTCACTCTAGCCCGGGCAAAACT--CTGTCTCAAAAAAAAAAAAAAAAAAAAAGGAACATGAG 67744
Ma'sNightMonkey   AGATCTAGTCCA-AATATCTTTGTCAGACTTTTTAAAAA----CAGGCTTTTT-----ATTGTTAAAAAGGGAATTTGAG 76213
VervetAGM         AGATCTAGTCCA-AATATCCTTGATGGACTTTAAAAAAAAAAACAGGCTTTGT-----ATTGTTAAAAAGGGAATTTGAG 82386
Gorrilla          AGATCTAGTCCA-AATATCTTTGATGGACTTTTTGAAAA----CAGACTTTTT-----ATTGTTAAAAAGGGAATTTGAG 79839
GrabEatingMacaqueAGATCTAGTCCA-AATATCCTTGATGGACTTTTAAGAAAAA--CAGGCTTTGT-----ATTGTTAAAAAGAGAATTTGAG 81916
Macaque           AGATCTAGTCCA-AATATCCTTGATGGACTTTTAAAAAAAA--CAGGCTTTGT-----ATTGTTAAAAAGAGAATTTGAG 82807
PigTailedMacaque  AGATCTAGTCCA-AATATCCTTGATGGACTTTTAAAAAAAA--CAGGCTTTGT-----ATTGTTAAAAAGAGAATTTGAG 81320
Bonobo            AGATCTAGTCCA-AGTATCTTTGATGGACTTTTTGAAAA----CAGACTTTTT-----ATTGTTAAAAAGGGAATTTGAG 80441
OliveBaboon       AGATCTAGTCCA-AATATCCTTGATGGACTTTTAAAAAAAA--CAGGCTTTGT-----ATTGTTAAAAAGGGAATTTGAG 81217
Orangutan         AGATCTAGTCCA-AATATCTTTGATGGACTTTTTAAAAA----CAGACTTTTT-----ATTGTTAAAAAGGGAATTTGAG 82069
```

```
Majority          GCTTGGTACTGAAC-----TACTTCTGCATGCCAT--ATTGAAGTTGGTGGTTTTGTGTTTGTTT----------GCCAT
                          98170     98180     98190     98200     98210     98220     98230     98240
Human             GCTTGGTACTGAAC-----TTCTTCTGCATGCCAT--ATTGAAGTTGGTGGTTTTTTGTTTGTTT----------GCCAT 81656
Coquerel'sSifaka  GCTCAGTACTGAACATTTTTTCTTCTGTATGCCACGTATTGCAGTTGGTAGTTTTGCCTTTTTTTTGGGGGGGGGGGCAT 67824
Ma'sNightMonkey   GCTTGGTACTGAAC-----TACTTCTGCATGCCATGTATCGAAGTTGATGGTTTTGTATTTGTTT----------GCCAT 76278
VervetAGM         GCTTGGTACTGAAC-----AACTTCTGCATGCCAT--ATTGAAGTTGGTGGTTTTGTGTTTGTTT----------GCCAT 82449
Gorrilla          GCTTGGTACTGAAC-----TTCTTCTGCATGCCAT--ATTGAAGTTGGTGGTTTTTTGTTTGTTT----------GCCAT 79902
GrabEatingMacaqueGCTTGGTACTGAAC-----AACTTCTGCATGCCAT--ATTGAAGTTGGTGGTTTTGTGTTTGTTT----------GCCAT 81979
Macaque           GCTTGGTACTGAAC-----AACTTCTGCATGCCAT--ATTGAAGTTGGTGGTTTTGTGTTTGTTT----------GCCAT 82870
PigTailedMacaque  GCTTGGTACTGAAC-----AACTTCTGCATGCCAT--ATTGAAGTTGGTGGTTTTGTGTTTGTTT----------GCCAT 81383
Bonobo            GCTTGGTACTGAAC-----TTCTTCTGCATGCCAT--ATTGAAGTTGGTGGTTTTTTGTTTGTTT----------GCCAT 80504
OliveBaboon       GCTTGGTACTGAAC-----AACTTCTGCATGCCAC--ATTGAAGTTGGTGGTTTTGTGTTTGTTT----------GCTAT 81280
Orangutan         GCTTGGTACTGAAC-----TTCCTCTGCATGCCAT--ATTGAAGTTGGTCGTTTTTTGTTTGTTT----------GCCAT 82132

Majority          CAGTTGGATCTCATAAAGACTATGGAATTTAATAGAATTTTTAAGTTACCGTTTTT-CCTAAAAGGTAGCTAACTAAAGG
                          98250     98260     98270     98280     98290     98300     98310     98320
Human             CAGTGGGATCTCATACAGACTATGGAATTTAATAGAATTTTTAAGATACTGTTTTT-CCTAAAAGGTAGCTAACTAAAGG 81735
Coquerel'sSifaka  CAGCTGGATCTCATAAAGACTATGGAATTTAACAGAATTTTTAAGTTACTTTTTCT-ACTTAAAGGTAGCTAATTAAAGG 67903
Ma'sNightMonkey   CAGTTGGATCTCATAAAGACTATGGAATTTAATAGAATTTT-AGGTTACTGTTTTT-CCTAAAAGGTAGCTAATTAAAGG 76356
VervetAGM         CAGTTGGATCTCATAAAGACTATGGAATTTAATAGAATTTTTAAGTTACCATTTTTTCCTAAAAGGTAGCTAACTAAAGG 82529
Gorrilla          CAGTGGGATCTCATACAGACTATGGAATTTAATAGAATTTTTAAGATAGTGTTTTT-CCTAAAAGGTAGCTAGCTAAAGG 79981
GrabEatingMacaqueCAGTCGGATCTCATAAAGACTATGGAATTTAATAGAATTTTAAAGTTACCATTTTT-CCTAAAAGGTAGCTAACTGAAGG 82058
Macaque           CAGTCGGATCTCATAAAGACTATGGAATTTAATAGAATTTTAAAGTTACCATTTTT-CCTAAAAGGTAGCTAACTAAAGG 82949
PigTailedMacaque  CAGTCGGATCTCATAAAGACTACGGAATTTAATAGAATTTTAAAGTTACCATTTTT-CCTAAAAGGTAGCTAACTAAAGG 81462
Bonobo            CAGTGGGATCTCATACAGACTATGGAATTTAATAGAATTTTTAAGATACTGTTTTT-CCTAAAAGGTAGCTAACTAAAGG 80583
OliveBaboon       CGGTCGGATCTCATAAAGACTATGGAATTTAATAGAATTTTAAAGTTACCATTTTT-CCTAAAAGGTAGCTAACTAAAGG 81359
Orangutan         CAGTTGGATCTCATACAGACTATGGAATTTAATAGAATTTGTAAGATACCGTTTTT-TCTAAAAGGTAGCTAACTAAAGG 82211

Majority          CAGGAAAATAGGAGAATGATGCAACAATATAATGACTACATCTGGAGTTTCTGCTACTAATAAATAATTGTATTGTCAAC
                          98330     98340     98350     98360     98370     98380     98390     98400
Human             CAGGAAAATAG---AATGATGTAACAATATAATGACTACATCTGGAGTTTCTGCTACTAATAAATAATTGTATTGTCAAC 81812
Coquerel'sSifaka  CAGGAAAACAAGGGGATGATTTGACAATATAATGACTACATCTGGCGTTTCCACTATTATTAAATAATTGTGTTGTTGTT 67983
Ma'sNightMonkey   CAGGAAAATAAGGGAATGATTTGACAATATAATGACTACATCTGGAGTTGCTGCTACTAA-------TTGTATTGTCAAC 76429
VervetAGM         CAGGAAAATAGGAGAATGATGCAACAATATAATGACTACATCTGGAGTTTCTGCTACTAATAAATAATTGTATTGTCAAC 82609
Gorrilla          CAGGAAAATAGGAGAATGATGTAACAATATAATGACTACATCTGGAGTTTCTGTTACTAATAAATAATTGTATTGTCAAC 80061
GrabEatingMacaqueCAGGAAAATAGGAGAATGATGCAACAATATAATAACTACATCTGGAGTTTCTGCTACTAATAAATAATTGTATTGTCAAC 82138
Macaque           CAGGAAAATAGGAGAATGATGCAACAATATAATAACTACATCTGGAGTTTCTGCTACTAATAAATAATTGTATTGTCAAC 83029
PigTailedMacaque  CAGGAAAATAGGAGAATGATGCAACAATATAATAACTACATCTGGAGTTTCTGCTACTAATAAATAATTGTATTGTCAAC 81542
Bonobo            CAGGAAAATAG---AATGATGTAACAATATAATGACTACATCTGGAGTTTCTGCTACTAATAAATAATTGTATTGTCAAC 80660
OliveBaboon       CAGGAAAATAGGAGAATGATGCAACAATATAATGACTACATCTGGAGTTTCTGCTACTAATAAATAATTGTGTTGTCAAC 81439
Orangutan         CAGGAAAATAGGAGAATGATGCAACAATATAAGGACTATATCTGGAGTTTCTGCTACTAATAAATAATTGTATTGTCAAC 82291
```

```
Majority          --TTT-AGTCAAATATTCTGTGCATTCAGTCAGAGCTATCTGAAAATACTGATGAATAGTATTACA------GTGTGGGG

                        98410     98420     98430     98440     98450     98460     98470     98480

Human             --TTT-AGTCAAATATTCTGTGCATTCAGTCAGAGCTATCTGAAAATACTGACGAATAGTATTACA------ATATGGGG 81883
Coquerel'sSifaka  GACTTAGGTTAAATGTTCTGTGCATTCAAGCAAAGCTATCTGAAAATACTGAAGAACAATGTTAAAAATTAAGTGTGGAG 68063
Ma'sNightMonkey   --TTTTAGTTGAATATTTTATGCATTCAGTCAGAGTTATCTGAAAATACTGAAGAATAGTGTCACA------GTGTGGGG 76501
VervetAGM         --TTT-AGTCTAATATTCTGTACATTCAGTCAGAGCTATCTGAAAATACTGATGATTAGTATTACA------GTGTGGGG 82680
Gorrilla          --TTT-AGTCGAATATTCTGTGCATTCAGTCAGAGCTATCTGAAAATACTGACAAATAGTATTACA------ATGTGGGG 80132
GrabEatingMacaque--TTT-AGTCAAATATTCTGTGCATTCAGTCAGAGCTATCTGAAAATACTGATGAATAGTATTACA------GTGTGGGG 82209
Macaque           --TTT-AGTCAAATATTCTGTGCATTCAGTCAGAGCTATCTGAAAATACTGATGAATAGTATTACA------GTGTGGGG 83100
PigTailedMacaque  --TTT-AGTCAAATATTCTGTGCATTCAGTCAGAGCTATCTGAAAATACTGATGAATAGTATTACA------GTGTGGGG 81613
Bonobo            --TTT-AGTCGAATATTCTGTGCATTCAGTCAGAGCTATCTGAAAATACTGACAAATACTATTACA------ATATGGGG 80731
OliveBaboon       --TTT-AGTCAAATATTCTGTGCATTCAGTCAGAGCTATCTGAAAATACTGATGAATAGTATTACA------GTGTGGGG 81510
Orangutan         --TTT-AGTTGAATATTCTGTGCATTCAATCAGAGCTATCTGAAAATACTGACGAATAGTATTACA------GTGTAGGG 82362

Majority          AGGTCACTTCTCAAGACTGCAAGAAAAAAAA-TTGTTGTGTGTAGGAATGCATGCCTAGGAATTAGGAATGTAT------

                        98490     98500     98510     98520     98530     98540     98550     98560

Human             AGGTCACTTCTCAAGACTGCAAGAAAAAAAA-TTGTTGTGTGTAGGAATGTTTGCCTAGGAATTAGGAATGTAT------ 81956
Coquerel'sSifaka  AAGTCACTTTCCTAGAGTATGCAAGTTACA-------GTGTATGTGTGTGCGTGCGTAGGAATTAGGAGTGTATCTTTAT 68136
Ma'sNightMonkey   AGGTCACTTTTCAAGACTGTGCAAGAAAAAAGT-GTTGTGTGTAGGAATGCATGCAAAGGAATTAGGAATGTAT------ 76574
VervetAGM         AGGTCACTTCTCAAGACTGCAAGAAAAAAAAAATTGTTGTGTGTAGGAATGCATGCCTGGGAATTAGGAATGTAT------ 82754
Gorrilla          AGGTCACTTCTCAAGACTGCAAGAAAAAAAA-TTGTTGTGTGTAGGAATGCTTGCCTAGGAATTAGGAATGTAT------ 80205
GrabEatingMacaqueAGGTCACTTCTCAAGACTGCAAGAAAGAAAAGTTGTTGTGTGTAGGAATGCACGCCTGGGAATTAGGAATGTAT------ 82283
Macaque           AGGTCACTTCTCAAGACTGCAAGAAAAAAAAAATT---GTGTGTAGGAATGCATGCCTGGGAATTAGGAATGTAT------ 83171
PigTailedMacaque  AGGTCACTTCTCAAGACTGCAAGAAAAAAAAAATTGTTGTGTGTAGGAATGCATGCCTGGGAATTAGGAATGTAT------ 81687
Bonobo            AGGTCACTTCTCAAGACTGCAAGAAAAAAAA-TTGTTGTGTGTAGGAATGCTTGCCTAGGAATTAGGAATGTAT------ 80804
OliveBaboon       AGGTCACTTCTCAAGACTGCAAGAAAAAAAAAAAAATTGTGTGTAGGAATGCATGCCTGGGAATTAGGAATGTAT------ 81584
Orangutan         AGGTCACTTCTCAAGACTGCAAGAAAAAAA-----TTGTGTGTAGGAATGCATGCCTAGGAATTAGGAATGTAT------ 82431

Majority          ------ACTTGACAACAAATGAACATATCCATTGAATGTTAAATGAGAACAACTTATGGGGTGGATAGTGAGAGGTATAG

                        98570     98580     98590     98600     98610     98620     98630     98640

Human             ------ACTTGACAACAAGTGAACGTATCCATTGAATGTTAAATGAGAACAACTTATGGGGAGGATAGTGAGAGGTATAG 82030
Coquerel'sSifaka  ACTAGCACTTGGTAACGAATGAACTTATCCATTAAATGTTAAATGGAAATGACCTGTGAAGTGTAAAGCTG------TAG 68210
Ma'sNightMonkey   ------ACT-GATAACAAATGAACATATCCATTGAATGTTAGATGAGAACAGCTTACGGGGAGGATAGTGC---G----- 76639
VervetAGM         ------GCTTGACAACAAATGAACATATCTATTGAATGTTAAATGAGAACAACTTACGGGGTGGATAGTGAGAGGTATAG 82828
Gorrilla          ------ACTTGACAACAAGTGAACGTATCCATTGAATGTTAAATGAGAACAACTTATGGGGAGGATAGTGAGAGGTATAG 80279
GrabEatingMacaque------GCTTGACAACAAATGAACATATCTGTTGAATGTTAAATGAGAACAACTTACGGGGTGGATAGTGAGAGGTATAG 82357
Macaque           ------GCTTGACAACAAATGAACATATCTGTTGAATGTTAAATGAGAACAACTTACGGGGTGGATAGTGAGAGGTATAG 83245
PigTailedMacaque  ------GCTTGACAGCAAATGAACATATCTGTTGAATGTTAAATGAGAACAACTTATGGGGTGGATAGTGAGAGGTATAG 81761
Bonobo            ------ACTTGACAACAAGTGAACGTATCCATTGAATGTTAAATGAGAACAACTTATGGGGAGGATAGTGAGGGGTATAG 80878
OliveBaboon       ------GCTTGACAACAAATGAACATATCTGTTGAATGTTAAATGAGAACAACTTACGGGGTGGATAGTGAGAGGTATAG 81658
Orangutan         ------ACTTGACAACAAGTGAACATATCCATTTAATGTTAAATGAGAACAACTTATGGGGAGGATAGTGAGAGGTATAG 82505
```

```
Majority         TATTTAACATGGAGAAGTTAGTAATATAGTAGAATATTTGATTGAAAAGATCCACATTGGTGTATTCAAAGACACACTGA
                         98650     98660     98670     98680     98690     98700     98710     98720
Human            TATTTAACATGGAAAAGTTAGTAATATAGTAGAATATTTGGTTGAAAAGATCCACATTGGTGTATTCAAAGACACACTGA 82110
Coquerel'sSifaka TATTTAACATGGAGAAGTTAGTAATGCAGTAGAATAGTTGATTGAAAAGATCCACACTGGTGTATCCAAGGAGTCATCAA 68290
Ma'sNightMonkey  ----------------------------GTAGATTATTTGGTTGAAAAGATCCATACTGGTGTATTCAAAGACACACTGA 76691
VervetAGM        TATTTAATATGGAGAAGTTAGTAATATAGTAGAATATTTGATTGAAAAGATCCACATTGGTGTATTCAAAGACACACTGA 82908
Gorrilla         TATTTAACATGGAAAAGTTAGTAATATAGTAGAATATTTGGTTGAAAAGATCCACACTGGTATATTCAAAGACACACTGA 80359
GrabEatingMacaqueTATTTAACATGGAGAAGTTAGTAATATAGTAGAATATTTGATTGAAAAGATCCACATTGGTGTATTCAAAGACACACTGA 82437
Macaque          TATTTAACATGGAGAAGTTAGTAATATAGTAGAATATTTGATTGAAAAGATCCACATTGGTGTATTCAAAGACACACTGA 83325
PigTailedMacaque TATTTAACATGGAGAAGTTAGTAATATAGTAGAATATTTGATTGAAAAGATCCACATTGGTGTATTCAAAGACACACTGA 81841
Bonobo           TATTTAACATGGAAAAGTTAGTAATATAGTAGAATATTTGGTTGAAAAGATCCACATTGGTGTATTCAAAGACACACTGA 80958
OliveBaboon      TATTTAACATGGAGAAGTTAGTAATATAGTAGAATATTTGATTGAAAAGATCCACATTGGTGTATTCAAAGACACACTGA 81738
Orangutan        TATTTAACATGGAAAAGTTAGTAATATAGTAGAATATTTGGTTGAAAAGATTCACATTGGTGTATTCAAAGACACACTGA 82585

Majority         AGACTATTGTGCATTAGTAAGTATTAAACAT-GAGAATTTTTATTTTAAAATTAGATCCTAATTTCTGAGATTTACTTT
                         98730     98740     98750     98760     98770     98780     98790     98800
Human            AGACTATTGTGCATTAGTAAGTATTAAACAT-GAGAATTCTTAATTTTAAAATTGGACCCTAATTTCTGAGATTTACTTT 82189
Coquerel'sSifaka AGACTACTGTACATTAGTAAATATTAGACTTCGAGACTTTTCTATTTTAAAGTTAGATCCTTATTTCTGAGATTTACTTT 68370
Ma'sNightMonkey  AGATTGTTGTGCATTAGTAAGTATTAAACAC-AAGAATTTTCTACTTTAAAATTAGATCCTAATTTCTGGAATGTACTTT 76770
VervetAGM        AGACTATTATGCATTAGTAAGTATTAAACAT-GAGAATTTTTATTTTAAAGTTAGATTCTAATTTCTGAGATTTACTTT 82987
Gorrilla         AGACTATTGTGCATTAGTAAGTATTAAACAT-GAGAATTCTTAATTTTAAAATTGGACCCTAATTTCTGAGACTTACTTT 80438
GrabEatingMacaqueAGACTATTATGCATTAGTAAGTATTAAACAT-GAGAATTTTTTA------------------------------------ 82480
Macaque          AGACTATTATGCATTAGTAAGTATTAAACAT-GAGAATTTTTTA------------------------------------ 83368
PigTailedMacaque AGACTATTATGCATTAGTAAGTATTAAACAT-GAGAATTTTTATTTTAAAATTAGATTCTAATTTCTGAGATTTACTTT 81920
Bonobo           AGACTATTGTGCATTAGTAAGTATTAAACAT-GAGAATTCTTAATTTTAAAATTGGACCCTAATTTCTGAGATTTACTTT 81037
OliveBaboon      AGACTATTATGCATTAGTAAGTATTAAACAT-GAGAATTTTTATTTTAAAATTAGATTCTAATTTCTGAGATTTACTTT 81817
Orangutan        AGACTACTGTGCATTAGTAAGTATTAAACAT-GAGAATTCTTAATTTTAAAATTGGATCCTAATTTCTGAGATTTACTTT 82664

Majority         AAGAATAAAA-------GGGGATGAAACAAGATTGGCCATGAGTTGATCATTGTTAAAGCTGGGTGATGGCTACATAGAG
                         98810     98820     98830     98840     98850     98860     98870     98880
Human            AAGAATAAAA-------GGGGATGAAACGAGATTGGCCATGAGTTGATCATTGTTAAAGTTGGGTGATGGCTACTTAGAG 82262
Coquerel'sSifaka AAAAATAAAATGAGTGAGTGGATGAAACAAGATTGCCCATGAGTTG----TTGTTAAACCTGTGTAATGGGTATATGGGG 68446
Ma'sNightMonkey  AAGAACAAAATGGATAAGGAGATGAAACAAGACTGGCCATGAGTTGCTTATTGTTAAACCTGGGTGATGGCTACGTAGAG 76850
VervetAGM        AAGAATAAAA-------GGGGATGAAACAAGATTGGCCATGAGTTGATCATTGTTAAAGCTGGGTGATGGCTACATAGAG 83060
Gorrilla         AAGAATAAAA-------GGGGATGAAACGAGATTGGCCATGAGTTGATCATTGTTAAAGTTGGGTGATGGCTACATAGAG 80511
GrabEatingMacaque-AGAATAAAA-------GGGGATGAAACAAGATTGGCCATGAGTTCATCATTGTTAAAGCTGGGTGATGGCTACATAGAG 82552
Macaque          -AGAATAAAA-------GGGGATGAAACAAGATTGGCCATGAGTTCATCATTGTTAAAGCTGGGTGATGGCTACATAGAG 83440
PigTailedMacaque AAGAATAAAA-------GGGGATGAAACAAGATTGGCCATGAGTTCATCATTGTTAAAGCTGGGTGATGGCTACATAGAG 81993
Bonobo           AAGAATAAAA-------GGGGATGAAACGAGGTTGGCCATGAGTTGATCATTGTTAAAGTTGGGTGATGGCTACATAGAG 81110
OliveBaboon      AAGAATAAAA-------GCGTATGAAACAAGATTGGCCATGAGTTGATCATTGTTAAAGCTGAGTGATGGCTACATAGAA 81890
Orangutan        AAGAATAAAA-------GGGGATGAAATGAGATTGGCCATGAGTTGATCATTGTTAAAGTTGGGTGATGGCTACGTAGAG 82737
```

```
Majority        GTGCATTATTATAGTCTCTTT-ACTTTTGTATATGTTGGAAAT-CTTCCAAAACAAAATGTAAAGCCATAAAAAAG-GGT
                         98890     98900     98910     98920     98930     98940     98950     98960
Human           TTGCATTATTTTAGTCACTTTTACTTTTGTATATGTTGGAAAT-CTTCCAAAACAAAATGTATAGCCATAAAAAAAAAGGG 82341
Coquerel'sSifaka GTTCATTAGTCTAATCTTTTT-ACTTTTATATATGTTGGAAAC-TTTCCAAAGCAAAAAGTTGTATCAAAAAAAAAG-AAA 68523
Ma'sNightMonkey ATAGGTTATTCTAGTCTCTTT-ACTTTTGTATATGTTGGAAAT--TTTC----CAAAGTGTAAAGTCGTGAAAAAA-GAT 76922
VervetAGM       GTGCATTATTATAGTCTCTTT-ACTTTTGTATATGTTGGAAATTCTTCCAAAACAAAATGTAAAGCCATAAAAAAG--GT 83137
Gorrilla        GTGCATTATTTTAGTCACTTTTACTTTTGTGTATGTTGGAAAT-CTTCCAAAACAAAATGTATAGCCATAAAAAAAAAGGG 80590
GrabEatingMacaqueGTGCATTATTATAGTCTCTTT-ACTTTTGTATATGTTGGAAATTCTTCCAAAACAAAATGTAAAGCCATAAAAAAG--GT 82629
Macaque         GTGCATTATTATAGTCTCTTT-ACTTTTGTATATGTTGGAAATTCTTCCAAAACAAAATGTAAAGCCATAAAAAAG--GT 83517
PigTailedMacaque GTGCATTATTATAGTCTCTTT-ACTTTTGTATATGTTGGAAATTCTTCCAAAACAAAATGTAAAGCCATAAAAAAG--GT 82070
Bonobo          ATGCATTATTTTAGTCACTTTTACTTTTGTATATGTTGGAAAT-CTTCCAAAACAAAATGTATAGCCATAAAAAAA-GGG 81188
OliveBaboon     GTGCATTATTATAGTCTCTTT-ACTTTTGTATATGTTGGAAATTCTTCCAAAACAAAATGTAAAGCCATAAAAAAG--GT 81967
Orangutan       GTGCATTATTTTAGTCTCTTT-ACTTTTGTATATGTTGGAAAT-CTTCCAAAACAAAATGTATAGCCATAAAAAAA-GGG 82814

Majority        AGCAAAGTAAGAAAAAAAGA--GAGCCAAAAAAAAAGGGTATAGGCAAAAAGCTGTGAAAAGAAACTCATCAAGAGCAAG
                         98970     98980     98990     99000     99010     99020     99030     99040
Human           AGCAAAGTAAGAAAAAAAGG-AGAGCCTAAAAAAAAAGGTACAGGCAAAAAGGTGTGAAAAGAAACTCATCAAGAGCAAG 82420
Coquerel'sSifaka AGGGGAGAAATAGGAAAAGAAGAAGCTCCTAAAAAATGGCATGGGAAAAAAAGCTGTAATATAAACTCATCCAAAGCAAG 68603
Ma'sNightMonkey GGCAAAGAAAGAAAAAGAGATCCTAAAAAAAAAAAAAGGTATAGGCAAAAAGCTGTGAAAAAAGACTCATCAAGAGCAGA 77002
VervetAGM       AGCAAAGTAAGAAAAAAGGA--GAGCCAA-AAAAAAGGGTATAGACAAAAAGCTGTGAAAAGAAACTCATCAAGAGCAAG 83214
Gorrilla        AGCAAAGTAAGAAAAAAAGG-AGAGCCTAAAAAAAAAGGTACAGGCAAAAAGCTGTGAAAAGAAACTCATCAAGAGCAAG 80669
GrabEatingMacaqueAGCAAAGTAAGAAAAAAGGA--GAGCCAACAAAAAAGGGTATAGACAAAAAGCTGTGAAAAGAAACTCATCAAGAGCAAG 82707
Macaque         AGCAAAGTAAGAAAAAAGGA--GAGCCAACAAAAAAGGGTATAGACAAAAAGCTGTGAAAAGAAACTCATCAAGAGCAAG 83595
PigTailedMacaque AGCAAAGTAAGAAAAAAGGA--GAGCCAACAAAAACGGGTATAGACAAAGAGCTGTGAAAAGAAACTCATCAAGAGCAAG 82148
Bonobo          AGCAAAGTAAGAAAAAAAGG-AGAGCCTAAAAAAAA-GGTACAGGCAAAAAGCTGTGAAAAGAAACTCATCAAGAGCAAG 81266
OliveBaboon     AGCAAAGTAAGAAAAAAGGA--GAGCCAGCAAAAAAGGGTATAGACAAAAAGCTGTGAAAAGAAACTCATCAAGAGCAAG 82045
Orangutan       TGCAAAGTAAGAAAAAAAGGGAGAGCCTTAAAAAAA-GGTACAGGCAAAAAGCTGTGAAAAGAAACTGATCAAGAGCAAG 82893

Majority        AGTATTAAATGAGAAACATATG---CCACTGACAGAGTTCTC----AGAAGAGATAAAGGCTGAATTAAAAAGAGAT--G
                         99050     99060     99070     99080     99090     99100     99110     99120
Human           AGTATTAAATGAGAAACATATG---CCACTGACAGAGTTCTC----AGAAGAGATAAAGGCTGAATTAAAAAGAGAT--G 82491
Coquerel'sSifaka AATATTAAATGAGAACCATATGATTCCACTGATACAGTGCTTGGAGAGAAGGCATAAAGGCTGAATTATAGAACAAT--- 68680
Ma'sNightMonkey AGTGTTAAATGAGAATCATATG---CAACTGACAGAGTTCTC----AGAAGAGAGAAAGGCTGAATTAAAAGGTGATTGG 77075
VervetAGM       AGTATTAAATGAGAAACATATG---CCACTGACAGAGTTCTC----AGAAGAGATAAAGGCTGAATTAAAAAGAGAT--G 83285
Gorrilla        AGTATTAAATGAGAAACATATG---CCACTGACAGAGTTCTC----AGAAGAGATAAAGGCTGAATTAAAAAGAGAT--G 80740
GrabEatingMacaqueAGTATTAAATGAGAAACATATG---CCACTGACAGAGTTCTC----AGAAGAGATAAAGGCTGAATTAAAAAGAGAT--G 82778
Macaque         AGTATTAAATGAGAAACATATG---CCACTGACAGAGTTCTC----AGAAGAGATAAAGGCTGAATTAAAAAGAGAT--G 83666
PigTailedMacaque AGTATTAAATGAGAAACATATG---CCACTGACAGAGTTCTC----AGAAGAGATAAAGGCTGAATTAAAAAGAGAT--G 82219
Bonobo          AGTATTAAATGAGAAACATATG---CCACTGACAGAGTTCTC----AGAAGAGATAAAGGCTGAATTAAAAAGAGAT--G 81337
OliveBaboon     AGTATTAAATGAGAAACATATG---CCACTGACAGAGTTCTC----AGAAGAGATAAAGGCTGAATTAAAAAGAGAT--G 82116
Orangutan       AGTATTAAATGAGAAACAT--G---CCACTGACAGAGTTCTC----AGAAGAGATAAAGGCTGAATTAAAAAGAGAT--G 82962
```

```
Majority          GAGGGGAAAGTTTTGTAAGTCAAAAACTTAGGTTAAGGATTGTGAATATTAAAGAGCAC-----TTCAGACTGCCAATGA

                          99130     99140     99150     99160     99170     99180     99190     99200

Human             GAGGGGAAAGTTTTATAAGTCAAAAACTTAGGTTAAGGTTTGTGAATATTAAAGAGCGC-----TTCAGATTGCCAATGA 82566
Coquerel'sSifaka  --TTG-GAGG-----------AAAAACTTAGGTTAAGGGTTGTGAATATTAAAGACTGCCAGTATTCAGACTGCCAGTGA 68746
Ma'sNightMonkey   AGGAAAAAAGTTTTATAAGTCAAAAACTTAGGTTAAGGATTGTGAATATTAAAGAGCAC-----TTCTGACTGCCAGTGA 77150
VervetAGM         GAGGGGAAAGTTTCGTAAATCAAAAATTTAGGTTAAGGATTGTGAATATTAAAGAGCAC-----TTCAGACTGCCAATGA 83360
Gorrilla          GAGGGGAAAGTTTTATAAGTCAAAAACTTAGGTTAAGGATTGTGAATATTAAAGAGCAC-----TTCAGATTGCCGATGA 80815
GrabEatingMacaqueGAGGGGAAAGTTTCGTAAATCAAAAATTTAGGTTAAGGATTGTGAATATTAAAGAGCAC-----TTCAGACTGCCAATGA 82853
Macaque           GAGGGGAAAGTTTCGTAAATCAAAAATTTAGGTTAAGGATTGTGAATATTAAAGAGCAC-----TTCAGACTGCCAATGA 83741
PigTailedMacaque  AAGGGGAAAGTTTCGTAAATCAAAAATTTAGGTTAAGGATTGTGAATATTAAAGAGCAC-----TTCAGACTGCCAATGA 82294
Bonobo            GAGGGGAAAGTTTTATAAGTCAAAAACTTAGGTTAAGGATTGTGAATATTAAAGAGCAC-----TTCAGACTGCCAATGA 81412
OliveBaboon       GAGGGGAAAGTTTCGTAAATCAAAAATTTAGGTTAAGGATTGTGAATATTAAAGAGCAC-----TTCAGACTGCCAATGA 82191
Orangutan         GAGGGGAAAGTTTTATAAGTCAAAAACTTAGGTTAAGGATTGTGAATATTAAAGAGCAC-----TTCAGACTGCCAATGA 83037
```

```
Majority          AAGGTCATA----GTAGTTTCTATAAAGTAAAAAGATCAAAAACTTGACTT--AAAAGAGTAAGATCTCTAAAACCAAAG

                          99210     99220     99230     99240     99250     99260     99270     99280

Human             AAGGTCATA----GTAGTTTCTATAAAGTCAAAAGATCAAAAACTTGACTT--AAAAGAGTAAGATCTCTAAAACCAAAG 82640
Coquerel'sSifaka  AAGGTCATA----GTAATTTCTGTAAAGTAAAAAGGTCAAAAACTTAACT---AAAAGATTAAGATCTCTGAAACCAAAG 68819
Ma'sNightMonkey   AAGGTCATA----GTAGTTTCTATAAAGTAAAAAGATCAAAAACTTGACTT--AAAAGAATAAGATTTCTAAAACCGAAG 77224
VervetAGM         AAGGTCACA----GTAGTGTCTATAAAGTAAAAAGATCAAAAACTTGACTTTTAAAAGAGTAAGATCTCTAAAACCAAAG 83436
Gorrilla          AAGGTCATA----GTAGTTTCTATAAAGTAAAAAGATCAAAAACTTGACTT--AAAAGAGTAAGATCTCTAAAACCAAAG 80889
GrabEatingMacaqueAAGGTCACA----GTAGTGTCTATAAAGTAAAAAGATCAAAA-CTTGACTTTCAAAAGAGTAAGATCTCTAAAACCAAAG 82928
Macaque           AAGGTCACA----GTAGTGTCTATAAAGTAAAAAGATCAAAAACTTGACTTTCAAAAGAGTAAGATCTCTAAAACCAAAG 83817
PigTailedMacaque  AAGGTCACACAGTGTAGTGTCTATAAAGTAAAAAGATCAAAAACCTGACTTTCAAAAGAGTAAGATCTCTAAAACCAAAG 82374
Bonobo            AAGGTCATA----GTAGTTTCTATAAAGTAAAAAGATCAAAAACTTGACTT--AAAAGAGTAAGATCTCTAAAACCAAAG 81486
OliveBaboon       AAGGTCACA----GTAGTGTCTATAAAGTAAAAAGATCAAAAACTTGACTTTTAAAAGAGTAAGATCTCTAAAACCAAAG 82267
Orangutan         AAGGTCATA----GTAGTTTCTATAAAGTAAAAAGATCAAAAACTTGACTT--AAAAGAGTAAGAGCTCTAAAACCAAAG 83111
```

```
Majority          GTAACAGCAGATTCTTATGAAATGCACCAGAAAGCTGGAAAGAAAGAAGAAATAAAATGTGAACTTATGGAACAAGAGTG

                          99290     99300     99310     99320     99330     99340     99350     99360

Human             GTAACAGCAGATTCTTATGAAATGCACCAGAAAGCTGGAAAGAAAGAAGAAATAAAATGTGAACTTATGGAACAAGAGTG 82720
Coquerel'sSifaka  GTAACAACAGATGCTTATGAAATGCTCAAGAAGGCTGGAAAGAGAGAAGAAATAAAATGTGAACTTACCGAACGACAGTG 68899
Ma'sNightMonkey   ATAACAGCAGATTCTTATGAAATGCAACCGAAAGCTGGAAAGAAAGAAGAAATAAAATGTGAACTTATGGAACAAGAGTG 77304
VervetAGM         GTAACAGCAGATTCTTATGAAATGCACCAGAAAGCTGGAAAGAAAGAAGAAATAAAATGTGAACTTATGGAACAAGAGTG 83516
Gorrilla          GTAACAGCAGATTCTTATGAAATGCACCAGAAAGCTGGAAAGAAAGAAGAAATAAAATGTGAACTTATGGAACAAGAGTG 80969
GrabEatingMacaqueGTAACAGCAGATTCTTATGAAATGCACCAGAAAGCTGGAAAGAAAGAAGAAATAAAATGTGAACTTATGGAACAAGAGTG 83008
Macaque           GTAACAGCAGATTCTTATGAAATGCACCAGAAAGCTGGAAAGAAAGAAGAAATAAAATGTGAACTTATGGAACAAGAGTG 83897
PigTailedMacaque  GTAACAGCAGATTCTTATGAAATGCACCAGAAAGCTGGAAAGAAAGAAGAAATAAAATGTGAACTTATGGAACAAGAGTG 82454
Bonobo            GTAACAGCAGATTCTTACGAAATGCACCAGAAAGCTGGAAAGAAAGAAGAAATAAAATGTGAACTTATAGAACAAGAGTG 81566
OliveBaboon       GTAACAGCAGATTCTTATGAAATGCACCAGAAAGCTGGAAAGAAAGAAGAAATAAAATGTGAACTTATGGAACAAGAGTG 82347
Orangutan         GTAACAGCAGATTCTTATGAAATGCACCAGAAAGCTGGAAAGAAAGAAGAAATAAAATGTGAACTTATGGAACAAGAGTG 83191
```

```
Majority         GTAAGGGCCCGTTCTACCCAGGTATGTTATGAAATCAAATTCATTTAGTTTGGGTGTTTGTTCCTTTATGTTGGGAAGGT
                         99370     99380     99390     99400     99410     99420     99430     99440
Human            CTAAGGGCCTGTTCTACCCAGGTATGTTATGAAATCAAATTCATTTAGTTTGGGTGTTTGTTCCTTTATGTTGGGAAGAT 82800
Coquerel'sSifaka GTAAGGGCC----------AGGTATGTCATGAAATCAGCTTCATTTAGTTTGGGTGT-TGTTCCTTTATCATGGGAAGGT 68968
Ma'sNightMonkey  GTAAGGGCCCGTTCTATCCAGGTATGTCATGAAATCAACTTCATTTAGTTTGAGTGTTTGTTCCTTTATGCTGGGAAGGT 77384
VervetAGM        GTAAAGGCCCGTTCTACCCAGGTATGTTATGAAATCAAATTCATTTAGTTTGGGTGTGTGTTCCTCTATGTTGGGAAGGT 83596
Gorrilla         GTAAGGGCCTGTTCTACCCAGGTATGTTATGAAATCAAATTCATTTAGTTTGAGTGTTTGTTCCTTTATGTTGGGAAGAT 81049
GrabEatingMacaqueGTAAAGGCCCATTCTACCCAGGTATGTTATGAAATCAAATTCATTTAGTTTGGGTGTTTGTTCCTCTATGTTGGGAAGGT 83088
Macaque          GTAAAGGCCCGTTCTACCCAGGTATGTTATGAAATCAAATTCATTTAGTTTGGGTGTTTGTTCCTCTATGTTGGGAAGGT 83977
PigTailedMacaque GTAAAGGCCCGTTCTACCCAGGTATGTTATGAAATCAAATTCATTTAGTTTGGGTGTTTGTTCCTCTATGTTGGGAAGGT 82534
Bonobo           GTAAGGGCCTGTTCTACCCAGGTATGTTATGAAATCAAATTCATTTACTTTGGGTGTTTGTTCCTTTATGTTGGGAAGAT 81646
OliveBaboon      GTAAAGGCCCGTTCTACCCAGGTATGTTATGAAATCAGATTCATTTAGTTTGGGTGTTTGTTCCTCTATGTTGGGAAGGT 82427
Orangutan        GTAAGGGCCTGTTCTACCCAGGTATGTTATGAAATCAAATTCATTTAGTTTGGGTGTTTGTTCCTTTATGTTGGGAAGGT 83271

Majority         AAAAAAGAA------CATATAATTCGTTTCTTTAGTGAACCAATATTTAT-GAGAAATAAGCTAG-TGTGTAAGAATTTA
                         99450     99460     99470     99480     99490     99500     99510     99520
Human            AAAAAAGAA------CATATAATTCGTTTCTTGAGTGAACCAGTATTTAT-GAGAAATAAGCTGGGTGTGTAAGAATTTA 82873
Coquerel'sSifaka AAAAAAGAAAAAAAAACATGTAATTAATTTCTTTAGTGAACTAACATTTATTGAAAAATAAACTGG-TATGTAAGAATTTT 69047
Ma'sNightMonkey  AAAAAAGAA------CATATAACTTGTTTCTTTAATAAACCAATATTTAT-GAGTAATAAGCTAG-TGTGTAAGAATTTA 77456
VervetAGM        AAAAAAGAA------CAAACAATTGGTTTGTTTAGTGAACCAATATTTAT-GAGAAATAAGCTAG-TGTGTAAGAATTTA 83668
Gorrilla         AAAAAAGAA------CATATAATTCGTTTCTTGAGTGAACCAGTATTTAT-GAGAAATAAGCTGGGTGTGTAAGAATTTA 81122
GrabEatingMacaqueAAAAGAGAA------CATACAATTCATTTGTTTAGTGAACCAATATTTAT-GAGAAATAAGCTAG-TGTGTAAGAATTTA 83160
Macaque          AAAAAAGAA------CATACAATTCATTTGTTTAGTGAACCAATATTTAT-GAGAAATAAGCTAG-TGTGTAAGAATTTA 84049
PigTailedMacaque AAAAAAGAA------CATACAATTCATTTGTTTAGTGAACCAATATTTAT-GAGAAATAAGCTAG-TGTGTAAGAATTTA 82606
Bonobo           AAAAAAGAA------CATATAATTCGTTTCTTGAGTGAACCAGTATTTAT-GAGAAATAAGCTGGGTGTGTAAGAATTTA 81719
OliveBaboon      AAAAAAGAA------CATACAATTCGTTTGTTTAGTGAACCAATATTTAT-GAGAAATAAGCTAG-TGTGTAAGAATTTA 82499
Orangutan        AAAAAAGAA------CATATAATTCATTTCTTGAGGGAACCAGTATTTAT-GATAAATAAGCTGGGTGTGTAAGAATTTA 83344

Majority         AGGGATGAAAGTATTCCAAGTTGATTGGTAATTGAAAAGTAGATGAAGGGTGCTAACACAAAAGGGAATTCTGTCTAGAA
                         99530     99540     99550     99560     99570     99580     99590     99600
Human            AGGGATGAAAATATTCCAAGTTGATTGGTAATTGAAAAGTAGATGAACGGTGCTAACACAAAAGGGAATTCTGTCTAGAA 82953
Coquerel'sSifaka AGGGATGAAAGTATTCTGAGTTCATTTGTAATTGAAAAGTGGATAAAGGGTGCTAAGACAGAAGAGAATTCTATCTAGAA 69127
Ma'sNightMonkey  AGAGATTAAAGTATTCCAAGTTGATTGGTAATGGAAAAGTAGATGAAGGGTACTAACACAAAAGGGTTTTCTCTCTAGAA 77536
VervetAGM        AGGGATGAACGTATTCCAAGCTGATTGGTAATTGAAAAGTAGATGAAGGGTGCTAACACAAAAGGGAATTCTGTCTAGAA 83748
Gorrilla         AGGGATGAAAGTATTCCAAGTTGATTGGTAATTGAAAAGTAGATGAACGGTGCTAACACAAAAGGGAATTCTGTCTAGAA 81202
GrabEatingMacaqueAGGGATGAACGTATTCCAAGCTGATTGGTAATTGAAAAGTAGATGAAGGGTGCTAACACAAAAGGGAATTCTGTCTAGAA 83240
Macaque          AGGGATGAACGTATTCCAAGCTGATTGGTAATTGAAAAGTAGATGAAGGGTGCTAACACAAAAGGGAATTCTGTCTAGAA 84129
PigTailedMacaque AGGGATGAACGTATTCCAAGCTGATTGGTAATTGAAAAGTAGATGAAGGGTGCTAACACAAAAGGGAATTCTGTCTAGAA 82686
Bonobo           AGGGATGAAAGTATTCCAAGTTGATTGGTAATTGAAAAGTAGATGAACGGTGCTAACACAAAAGGGAATTCTGTCTAGAA 81799
OliveBaboon      AGGGATGAACGTATTCCAAGCTGATTGGTAATTGAAAAGTAGATGAAGGGTGCTAACACAAAAGGGAATTCTGTCTAGAA 82579
Orangutan        AAGGACGAAAGTATTCCAAGTTGATTGGTAATTGAAAAGTAGATGAAGGGTGCTAACA--AAAGGAAATTCTGTCTAGAA 83422
```

```
Majority          TAAATGACTGAAGATATGGT-GACTTAAAGATAGTAGGACAGCAATAAGACTGGAAACTAGAACAATA-------A---A

                          99610     99620     99630     99640     99650     99660     99670     99680

Human             TAAATGA----AAATATGGT-GACTTAAAGATAGTAGGACAGCAATAAGACTGGAAATTAGAACAATA-------AAATA 83021
Coquerel'sSifaka  TAAATGACCGAAGGTATGGAAGACCTAAAGATAATAGGACAACAATAAGACTGGAAACTAGAAAAATATTAATAGAAATA 69207
Ma'sNightMonkey   TAAGTGACTGAAGATATGGT-GACTTAAAGATAG---GACAATAATAAGACTGGAAACTACAACAATA-------A---A 77602
VervetAGM         TAGATGACTGAAGAGATGGT-GACTTAAAGGTAGTAGGACAGCAATAAGACTGGAAACTAGAACAATA-------A---A 83817
Gorrilla          TAAATGA----AAATATGGT-GACTTAAAGATAGTAGGACAGCAATAAGACTGGAAATTAGAACAATA-------AAATA 81270
GrabEatingMacaqueTAAATGACTGAAGAGATGGT-GACTTAAAGGTAGTAGGACAGCAGTAAGACTGGAAACTAGAACAATA-------A---A 83309
Macaque           TAAATGACTGAAGAGATGGT-GACTTAAAGGTAGTAGGACAGCAGTAAGACTGGAAACTAGAACAATA-------A---A 84198
PigTailedMacaque  TAAATGACTGAAGAGATGGT-GACTTAAAGGTAGTAGGACAGCAGTAAGACTGGAAACTAGAACAATA-------A---A 82755
Bonobo            TAAATGA----AAATATGGT-GACTTAAAGATAGTAGGACAGCAATAAGACTGGAAATTAGAACAATA-------AAATA 81867
OliveBaboon       TAAATGACTGAAGAGATGGT-GACTTAAAGGTAGTAGGACAGCAGTAAGACTGGAAACTAGAACAATA-------A---A 82648
Orangutan         TAAATGACTGAAAATATGGT-GACTTAAAGATAGTAGGACAGCAATAAGACTGGAAACTAGAACAA-----------TA 83489

Majority          ATAAGGAAATGATAACATAGATTGGGAAAGGATAGGAAAATACTGAAACAAAATCTGTGTTGTATAGTGATGGTTTCTTG

                          99690     99700     99710     99720     99730     99740     99750     99760

Human             AGAAGGAAATGATAACATAGATTGGGAAAGGATAGGAAAATACTGAAACAAAATCTGTGTTGTATAGTGATGGTTTCTTG 83101
Coquerel'sSifaka  AGAAGGAAATGGTAAAACACATTGGCAAAGGATAGGAACATACTGAAATAGAAAGTCTGTTACATAGTGACGGTTTCTCT 69287
Ma'sNightMonkey   ATAAGGAAATGGTAACATAGATTGGGAAAGGATAGGAAAATACTGAAACAAAATCTGTGTTGTATAGTGATGGTTTCTTG 77682
VervetAGM         ATAAGGAAATGATAACATAGATTGGGAAAGGATAGGAAAATACTGAAACAAAATCTGTGTT--ATAGTGATGGTTTCTTG 83895
Gorrilla          AGAAGGAAATGATAACATAGATTGGGAAAGGATAGGAAAATACTGAAACAAAATCTGTGTTGTATAGTGATGGTTTCTTG 81350
GrabEatingMacaqueATAAGGAAATGATAACATAGATTGGGAAAGGATAGGAAAATACTGAAACAAAATCTGTGTT--ATAGTGATGGTTTCTTG 83387
Macaque           ATAAGGAAATGATAACATAGATTGGGAAAGGATAGGAAAATACTGAAACAAAATCTGTGTT--ATAGTGATGGTTTCTTG 84276
PigTailedMacaque  ATAAGGAAATGATAACATAGATTGGGAAAGGATAGGAAAATACTGAAACAAAATCTGTGTT--ATAGTGATGGTTTCTTG 82833
Bonobo            AGAAGGAAATGATAACATAGATTGGGAAAGGATAGGAAAATACTGAAACAAAATCTGTGTTGTATAGTGATGGTTTCTTG 81947
OliveBaboon       ATAAGGAAATGATAACATAGATTGGGAAAGGATAGGAAAATACTGAAACAAAATCTGTGTT--ATAGTGATGGTTTCTTG 82726
Orangutan         AGAAGGAAATGATAACATAGATTGGGAAAGGATAGGAAAATACTGAAACAAAATCTGTGTTGTATTTTGATGGTTTCTTG 83569

Majority          TTCTTTCAACTGGGAGAGTGTTGTTTTGAAGAAACTTGTATT----ATAATTACTTGAGTGTATAATTAAGAAGGCAAGC

                          99770     99780     99790     99800     99810     99820     99830     99840

Human             TTCTTTCAACCGGGAGAGTGTTGTGTTGAAGGAACTTGTATTCATTTTAATTACTTGAGTGTATAATTAAGAAGGCAAGC 83181
Coquerel'sSifaka  TGCTTTCTACTGGGAGGGTATTGTACTGGAGAAACTT--ATT----ATAACTACTCAAGTGTAGAATTAAG---GCAAAC 69358
Ma'sNightMonkey   TTCATTCAACTGGTAGAGTATTGTGTTGAAGAAACTTGTATTCATTATAATTACTTGAGTGTGTAATTAAGAAGGCAAGC 77762
VervetAGM         TTCTTTGAACTGGGAGAGTGTTGTTTTGAAGAAACCTGTATT----ATAATTACTTGAGTGTATAATTAAGAAGGCAAGC 83971
Gorrilla          TTCTTTCAACCGGGAGAGTGTTGTGTTGAAGGAACTTGTATTCATTTTAATTACTTGAGTGTATAATTAAGAAGGCAAGC 81430
GrabEatingMacaqueTTCTTTGAACTGGAAGAGTGTTGTTTTGAAGAAACCTGTATT----ATAATTACTTGAGCGTATAATTAAGAAGGCAAGC 83463
Macaque           TTCTTTGAACTGGAAGAGTGTTGTTTTGAAGAAACCTGTATT----ATAATTACTTGAGCGTATAATTAAGAAGGCAAGC 84352
PigTailedMacaque  TTCTTTGAACTGGAAGAGTGTTGTTTTGAAGAAACCTGTATT----ATAATTACTTGAGTGTATAATTAAGAAGGCAAGC 82909
Bonobo            TTCTTTCAACCAGGAGAGTGTTGTGTTGAAGGAACTTGTATTCATTTTAATTACTTGAGTGTATAATTAAGAAGGCAAGC 82027
OliveBaboon       TTCTTTGAACTGGAAGAGTGTTGTTTTGAAGAAACCTGTATT----ATAATTACTTGAGTGTATAATTAAGAAGGCAAGC 82802
Orangutan         TTCTTTCAACTGGGAGAGTGTTGTGTTGAAGGAACTTGTATTCATTATAATTACTTGAGTGTATAATTAAG---GCAAGC 83646
```

```
Majority         ACATTTTGCTAAGATTTTCTTTA-----TTTGCATGAATTAAATAATGATGTTCAGAGTGATTTGTCTCTAGGAGCTGAA

                         99850     99860     99870     99880     99890     99900     99910     99920

Human            ACATTTTGCTAAGATTTTCTTTA-----TTTGCATGAATTAAATAATGATGTTCAGAGTGATTTGTCTCTAGGAGCTGAA 83256
Coquerel'sSifaka ACATTTTGCTAAGCTTTTCTTTAGGTTATTTGCATGAATTAAATAATGTT---CAGAGTGACTTGTCTCGAGGAACTGAA 69435
Ma'sNightMonkey  ACATTTTGCTAAGATTTTCTTTA-----TTTGCATGAATTAAATGATGAT---CAGAGTGATATGTC--TAGGAGTTGAA 77832
VervetAGM        ACATTTTGCTAAGATTTTCTTTA-----TTTGCATGAATTAAATAATGATGTTCAGAGTGATTTGTCTCTAGGAGCTGAA 84046
Gorrilla         ACATTTTGCTAAGATTTTCTTTA-----TTTGCATGAATTAAATAATGATGTTCAGAGTGATTTGTCTCTAGGAGCTGAA 81505
GrabEatingMacaqueACATTTTGCTAAGATTTTCTTTA-----TTTGCATGAATTAAATAATGATGTTCAGAGTGATTTGTCTCTAGGAGCTGAA 83538
Macaque          ACATTTTGCTAAGATTTTCTTTA-----TTTGCATGAATTAAATAATGATGTTCAGAGTGATTTGTCTCTAGGAGCTGAA 84427
PigTailedMacaque ACATTTTGCTAAGATTTTCTTTA-----TTTGCATGAATTAAATAATGATGTTCAGAGTGATTTGTCTCTAGGAGCTGAA 82984
Bonobo           ACATTTTGCTAAGATTTTCTTTA-----TTTGCATGAATTAAATAATGATGTTCAGAGTGATTTGTCTCTAGGAGCTGAA 82102
OliveBaboon      ACATTTTGCTAAGATTTTCTTTA-----TTTGCATGAATTAAATAATGATGTTCAGAGTGATTTGTCTCTAGGAGCTGAA 82877
Orangutan        ACATTTTGCTAAGATTTTCTTTA-----TTTCCATGAATTAAATAATGCTGTTCAGAGTGACTTGTCTCTAGGAGCTGAA 83721

Majority         AGACT--ACTATCTTTGTTTCATAAGCTGTAATTATTTGTCTGATTTCAAGTGTGAGTTTATGAAGAAGATAGCACTTGC

                         99930     99940     99950     99960     99970     99980     99990    100000

Human            AGACT--ACTACCTTTGTTTCATAAGCTGTAATTATTTG----ATTTCAAGTGTGAGTTTATGAAGAAGATAGCACTTGC 83330
Coquerel'sSifaka GGATTTTACTGTATTTGTTTCATAGG-TGTGATTATTTG----ATTTCAAGTGTGAGTTTATAAAGAAGATAGCACTTGT 69510
Ma'sNightMonkey  AGACT--ACTATCGTTGTTTCATAAGCTGTAATTATTTGTCTGATTTCAAGTGTGAGTTTATGAAGAAGATAGCACTTGC 77910
VervetAGM        ACACT--ACTATCTTTGTTTCTTAAGCTGTAATTATTTGTCTGATTTCAAGTGTGAGTTTATGAAGAAGATAGCACTTGC 84124
Gorrilla         AGACT--ACTACCTTTGTTTCATAAGCTGTAATTATTTG----ATTTCAAGTGTGAGTTTATGAAGAAGATAGCACTTGT 81579
GrabEatingMacaqueAGACT--ACTATCTTTGTTTCTTAAGCTGTAATTATTTGTCTGATTTCAAGTGTGGGTTTATGAAGAAGATAGCACTTGC 83616
Macaque          AGACT--ACTATCTTTGTTTCTTAAGCTGTAATTATTTGTCTGATTTCAAGTGTGGGTTTATGAAGAAGATAGCACTTGC 84505
PigTailedMacaque AGACT--ACTATCTTTGTTTCTTAAGCTGTAATTATTTGTCTGATTTCAAGTGTGAGTTTATGAAGAAGATAGCACTTGC 83062
Bonobo           AGACT--ACTACCTTTGTTTCATAAGCTGTAATTATTTG----ATTTCAAGTGTGAGTTTATGAAGAAGATAGCACTTGC 82176
OliveBaboon      AGACT--ACTATCTTTGTTTCTTAAGCTGTAATTATTTGTCTGATTTCAAGTGTGAGTTTATGAAGAAGATAGCACTTGC 82955
Orangutan        AGACT--ACTACCTTTGTTTCATAAGCTGTAATTATTTG----ATTTCAAGTGTGAGTTTATGAAGAAGATAGCACTTGC 83795

Majority         AGAAAAAAGTG-AATTACCATTAT----GTCGAAAGTGATACTTTTTTACATTTGGCTTATTACTGTAAATCATGGAAGT

                        100010    100020    100030    100040    100050    100060    100070    100080

Human            GGAAAAAAGTG-AATTACCATTAT----GTCGAAAGTGATACTTTTTTACATTTGGCCTTTTACTGTAAATCGTGGAAGT 83405
Coquerel'sSifaka AGAAAAAAGTATGAATGCTATTAT----GTCGAAGGTGAT--TTTTTTACATTTGGCTTCTCAGTATAAACCATGGGAGT 69584
Ma'sNightMonkey  AGAAAAAAGTG-AATTACCATTAT----GTC-AAAGTGATACTTTTCTACATTTGGCTTATTACTGTAAATCATGGAAGT 77984
VervetAGM        AGAAAATAGTG-AATTACCATTATGTATGTCGAAAGTGATACTTTTTTACATTTGGCTTATTACTGTAAATCATGGAAGT 84203
Gorrilla         GGAAAAAAGTG-AATTACCATTAT----GTCGAAAGTGATACTTTTTTACATTTGGCCTTTTACTGTAAATCGTGGAAGT 81654
GrabEatingMacaqueAGAAAATAGTG-AATTACCATTATGTATGTCGAAAGTGATACTTTTTTACATTTGGCTTATTACTGTAAATCATGGAAGT 83695
Macaque          AGAAAATAGTG-AATTACCATTATGTATGTCGAAAGTGATACTTTTTTACATTTGGCTTATTACTGTAAATCATGGAAGT 84584
PigTailedMacaque AGAAAATAGTG-AATTACCATTATGTATGTCGAAAGTGATACTTTTTTACATTTGGCTTATTACTGTAAATCATGGAAGT 83141
Bonobo           GGAAAAAAATG-AATTACCATTAT----GTCGAAAGTGATACTTTTTTACATTTGGCCTTTTACTGTAAATCGTGGAAGT 82251
OliveBaboon      AGAAAATAGTG-AATTACCATTATGTATGTCGAAAGTGATACTTTTAACATTTGGCTTATTACTGTAAATCATGGAAGT 83034
Orangutan        GGAAAAAAGTG-AATTACCATTAT----GTCGAAACTGATACTTCTTTACATTTGGCCTTTTACTGTAAATCATGGAAGT 83870
```

```
Majority        GAATTTTCCAAGCCTGTGACTTGGAATTATATTCTTCATGCTTTGTTTGCTTTTCCAGAGGAAATAGAGTATTATATATT

                      100090    100100    100110    100120    100130    100140    100150    100160

Human           GAATTTTCCAAGCCTGTGACTTGGAATTATATTTTTCATGCTTTGTTTGCTTTTCCAGAGGAAATAGAGTATTATATATT 83485
Coquerel'sSifaka GAATTTTCTGAGTCTCTGACTTGGAAATATATTCTTTATGCTTTGTTTGCTTTTCCTTAGGAAATGGAGAAATGCATATT 69664
Ma'sNightMonkey GAATTTTCCAAGCCTGTGACTTGGAATTAAATTCTTCACGCTTTGTTTGCTTTTCCAGAGAAAATAGAGTATTATGTATT 78064
VervetAGM       GAATTTTCCAAGCCTGTGACTTGGAATTATATTCTTCATGCTTTGTTTGCTTTTCCAGAGGAAATAGAGTATTATATATT 84283
Gorrilla        GAATTTTCCAAGCCTGTGACTTGGAATTATATTCTTCATGCTTTGTTTGCTTTTCCAGAGGAAATAGAGTATTATATATT 81734
GrabEatingMacaqueGAATTTTCCAAGCCTGTGACTTGGAATTATATTCTTCATGCTTTGTTTGCTTTTCCAGAGGAAATAGAGTATTATATATT 83775
Macaque         GAATTTTCCAAGCCTGTGACTTGGAATTATATTCTTCATGCTTTGTTTGCTTTTCCAGAGGAAATAGAGTATTATATATT 84664
PigTailedMacaque GAATTTTCCAAGCCTGTGACTTGGAATTATATTCTTCATGCTTTGTTTGCTTTTCCAGAGGAAATAGAGTATTATATATT 83221
Bonobo          GAATTTTCCAAGCCTGTGACTTGGAATTATATTCTTCATGCTTTGTTTGCTTTTCCAGAGGAAATAGAGTATTATATATT 82331
OliveBaboon     GAATTTTCCAAGCCTGTGACTTGGAATTATATTCTTCATGCTTTGTTTGCTTTTCCAGAGGAAATAGAGTATTATATATT 83114
Orangutan       GAATTTTCCAAGCCTGTGACTTGGAATTATATTCTTCATGCTTTGTTTGCTTTTCCAGAGGAAATAGAGTATTATATATT 83950

Majority        TGATATTTTAACATAATACAGGTATGTACTT-------------------------------------TAGTTCAAATAA

                      100170    100180    100190    100200    100210    100220    100230    100240

Human           TGATATTTTAACATAATACAGGTATGTACTT-------------------------------------TAGTTCAAATAA 83528
Coquerel'sSifaka TAAT-TTTTAACAT---ACAGGTATGTATCT-------------------------------------TAGCTGAAATAA 69703
Ma'sNightMonkey TGATATTTTAACATAATACAGGTATGTACTT-------------------------------------TAGTTCAAATAA 78107
VervetAGM       TGATATTTTAACATAATACAGGTATGTACTTCAATTAATGTAGTTCAATTAATGTAGTTCAATTAATGTAGTTCAATTAA 84363
Gorrilla        TGATATTTTAACATAATACAGGTATGTACTT-------------------------------------TAGTTCAAATAA 81777
GrabEatingMacaqueTGATATTTTAACATAATACAGGTATGTACTT-------------------------------------TAGTTCAATTAA 83818
Macaque         TGATATTTTAACATAATACAGGTATGTACTT-------------------------------------TAGTTCAATTAA 84707
PigTailedMacaque TGATATTTTAACATAATACAGGTATGTACTT-------------------------------------TAGTTCAATTAA 83264
Bonobo          TGATATTTTAACATAATACAGGTATGTACTT-------------------------------------TAGTTCAAATAA 82374
OliveBaboon     TCATATTTTAACATAATACAGGTATGTACTT-------------------------------------TAGTTCAATTAA 83157
Orangutan       TGATATTTTAACATAATACAGGTATGTACTT-------------------------------------TAGTTCAAATAA 83993

Majority        TT----GTTTTGTTGAAATCAATTATTTTTCTCTACTACACATCAGCAAACAATTTTTCTAAACACTATAAAAATTAACT

                      100250    100260    100270    100280    100290    100300    100310    100320

Human           TTAATGGTTTTGTTGAAATCAATTATTTTTCTTTACTACACATCAGCAAACAATTTTTCTAAACACTATAAAAATTAACT 83608
Coquerel'sSifaka TTAATGGTTTTGTTGAAATAAATTATCTTTCTCTACTACACATCAGCAAATAATTTTTATAAACA---TAAAAATTAACT 69780
Ma'sNightMonkey TTAATGGTTTTGTTGAAATCAGTTATTTTTCTCTACTACACATCAGCAAACAATTTTTCTAAACACTATAAAAATTAACT 78187
VervetAGM       TG----GTTTTGTTGAAATCGATTATTTTTCTCTACTACACATCAGCAAACAATTTTTCTAAACACTATAAAAATTAACT 84439
Gorrilla        TTAATGGTTTTGTTGAAATCAATTATTTTTCTCTACTACACATCAGCAAACAATTTTTCTAAACACTATAAAAATTAACT 81857
GrabEatingMacaqueTG----GTTTTGTTGAAATCGATTATTTTTCTCTACTACACATCAGCAAACAATTTTTCTAAACACTATAAAAATTAACT 83894
Macaque         TG----GTTTTGTTGAAATCGATTATTTTTCTCTACTACACATCAGCAAACAATTTTTCTAAACACTATAAAAATTAACT 84783
PigTailedMacaque TG----GTTTTGTTGAAATCGATTATTTTTCTCTACTACACATCAGCAAACAATTTTTCTAAACACTATAAAAATTAACT 83340
Bonobo          TT----GTTTTGTTGGAATCAATTATTTTTCTTTAC---ACATCAGCAAACAATTTTTCTAAACACTATAAAAATTAACT 82447
OliveBaboon     TG----GTTTTGTTGAAATCGATTATTTTTCTCTACTACACATCAGCAAACAATTTTTCTAAACACTATAAAAATTAACT 83233
Orangutan       TTAATGGTTTTGTTGAAATCAATTATTTTTCTCTACTACACATCAGCAAACAATTTTTCTAAACACTATAAAAATTAACT 84073
```

```
Majority          TTCAGGAACCATTTGGAATGGTCAGCCGTAAGCCATGCAGCCATCGTGCTTAAATTGGTTTTTGTAAACGACTTGTGTGT

                          100330    100340    100350    100360    100370    100380    100390    100400

Human             TTCAGGAACCATTTGGAATGGTCAGCCGTAAGCCATGCAGCCATCGTGCTTAAATTGGTTTTTGTAAACGACTTGTGTGT 83688
Coquerel'sSifaka  TTCAGGAGCCATTTGGAATGGTCAGCCGTAAGCCATGCAGCCATCATGCTTAAATTGGTTTTTATAAATGACTTGTGTGT 69860
Ma'sNightMonkey   TTCAGGAACCATTTGGAATGGTCAGCCGTAAGCCATGTAGCCCTCGTGCTTAAATTGGTTTTTGTAAACGACTTGTGTGT 78267
VervetAGM         TTCAGGAACCATTTGGAATGGTCAGCCGTAAGCCATGCAGCCATCGTGCTTAAATTGGTTTTTGTAAACGACTTGTGTGT 84519
Gorrilla          TTCAGGAACCATTTGGAATGGTCAGCCGTAAGCCATGCAGCCATCATGCTTAAATTGGTTTTTGTAAACGACTTGTGTGT 81937
GrabEatingMacaque TTCAGGAACCATTTGGAATGGTCAGCCGTAAGCCATGCAGCCATCGTGCTTAAATTGGTTTTTGTAAACGACTTGTGTGT 83974
Macaque           TTCAGGAACCATTTGGAATGGTCAGCCGTAAGCCATGCAGCCATCGTGCTTAAATTGGTTTTTGTAAACGACTTGTGTGT 84863
PigTailedMacaque  TTCAGGAACCATTTGGAATGGTCAGCCGTAAGCCATGCAGCCATCGTGCTTAAATTGGTTTTTGTAAACGACTTGTGTGT 83420
Bonobo            TTCAGGAACCATTTGGAATGGTCAGCCGTAAGCCATGCAGCCATCGTGCTTAAATTGGTTTTTGTAAACGACTTGTGTGT 82527
OliveBaboon       TTCAGGAACCATTTGGAATGGTCAGCCGTAAGCCATGCAGCCATCGTGCTTAAATTGGTTTTTGTAAACGACTTGTGTGT 83313
Orangutan         TTCAGGAACCATTTGGAATGGTCAGCCGTAAGCCATGCAGCCATCGTGCTTAAATTGGTTTTTGTAAACGACTTGTGTGT 84153

Majority          GTTTCGTAAAATATGCTTTATTTTAAAATTAGGGCTTCCTGTTGCAGCTGTTCCAGGAGCTCTGAGTCCTTTGGCTATTC

                          100410    100420    100430    100440    100450    100460    100470    100480

Human             GTTTCGTAAAATATGCTTTATTTTAAAATTAGGGCTTCCTGTTGCAGCTGTTCCAGGAGCTCTGAGTCCTTTGGCCATTC 83768
Coquerel'sSifaka  GTTTCGTAAAATATACTTTATTTTAAAATTAGGGCTTCCTGTTGCAGCTGTTCCAGGAGCTCTGAGTCCTTTGGCTATTC 69940
Ma'sNightMonkey   GTTTCGTAAAATATGCTTTATTTTAAAATTAGGGCTTCCTGTTGCAGCTGTTCCAGGAGCGCTGAGTCCTTTGGCTATTC 78347
VervetAGM         GTTTCGTAAAATATGCTTTATTTTAAAATTAGGGCTTCCTGTTGCAGCTGTTCCAGGAGCTCTGAGTCCTTTGGCTATTC 84599
Gorrilla          GTTTCGTAAAATATGCTTTATTTTAAAATTAGGGCTTCCTGTTGCAGCTGTTCCAGGAGCTCTGAGTCCTTTGGCCATTC 82017
GrabEatingMacaque GTTTCGTAAAATATGCTTTATTTTAAAATTAGGGCTTCCTGTTGCAGCTGTTCCAGGAGCTCTGAGTCCTTTGGCTATTC 84054
Macaque           GTTTCGTAAAATATGCTTTATTTTAAAATTAGGGCTTCCTGTTGCAGCTGTTCCAGGAGCTCTGAGTCCTTTGGCTATTC 84943
PigTailedMacaque  GTTTCGTAAAATATGCTTTATTTTAAAATTAGGGCTTCCTGTTGCAGCTGTTCCAGGAGCTCTGAGTCCTTTGGCTATTC 83500
Bonobo            GTTTCGTAAAATATGCTTTATTTTAAAATTAGGGCTTCCTGTTGCAGCTGTTCCAGGAGCTCTGAGTCCTTTGGCCATTC 82607
OliveBaboon       GTTTCGTAAAATATGCTTTATTTTAAAATTAGGGCTTCCTGTTGCAGCTGTTCCAGGAGCTCTGAGTCCTTTGGCTATTC 83393
Orangutan         GTTTCGTAAAATATGCTTTATTTTAAAATTAGGGCTTCCTGTTGCAGCTGTTCCAGGAGCTCTGAGTCCTTTGGCTATTC 84233

Majority          CAAATGCTGCTGCAGCAGCTGCTGCAGCTGCTGCTGGCCGAGTGGGTATGCCTGGAGTCTCAGCTGGTGGCAATACAGTC

                          100490    100500    100510    100520    100530    100540    100550    100560

Human             CAAATGCTGCTGCAGCAGCTGCTGCAGCTGCTGCTGGCCGAGTGGGTATGCCTGGAGTCTCAGCTGGTGGCAATACAGTC 83848
Coquerel'sSifaka  CAAATGCTGCTGCAGCAGCTGCTGCAGCTGCTGCTGGCCGAGTGGGTATGCCTGGAGTCTCAGCTGGTGGCAATACAGTC 70020
Ma'sNightMonkey   CAAATGCTGCTGCAGCAGCTGCTGCAGCTGCTGCTGGCCGAGTGGGTATGCCTGGAGTCTCAGCTGGTGGCAATACAGTC 78427
VervetAGM         CAAATGCTGCTGCAGCAGCTGCTGCAGCTGCTGCTGGCCGAGTGGGTATGCCTGGAGTCTCAGCTGGTGGCAATACAGTC 84679
Gorrilla          CAAATGCTGCTGCAGCAGCTGCTGCAGCTGCTGCTGGCCGAGTGGGTATGCCTGGAGTCTCAGCTGGTGGCAATACAGTC 82097
GrabEatingMacaque CAAATGCTGCTGCAGCAGCTGCTGCAGCTGCTGCTGGCCGAGTGGGTATGCCTGGAGTCTCAGCTGGTGGCAATACAGTC 84134
Macaque           CAAATGCTGCTGCAGCAGCTGCTGCAGCTGCTGCTGGCCGAGTGGGTATGCCTGGAGTCTCAGCTGGTGGCAATACAGTC 85023
PigTailedMacaque  CAAATGCTGCTGCAGCAGCTGCTGCAGCTGCTGCTGGCCGAGTGGGTATGCCTGGAGTCTCAGCTGGTGGCAATACAGTC 83580
Bonobo            CAAATGCTGCTGCAGCAGCTGCTGCAGCTGCTGCTGGCCGAGTGGGTATGCCTGGAGTCTCAGCTGGTGGCAATACAGTC 82687
OliveBaboon       CAAATGCTGCTGCAGCAGCTGCTGCAGCTGCTGCTGGCCGAGTGGGTATGCCTGGAGTCTCAGCTGGTGGCAATACAGTC 83473
Orangutan         CAAATGCTGCTGCAGCAGCTGCTGCAGCTGCTGCTGGCCGAGTGGGTATGCCTGGAGTCTCAGCTGGTGGCAATACAGTC 84313
```

```
Majority         CTGTTGGTTAGCAATTTAAATGAAGAGGTTAGTAAAATAATCTCTAATGTTTATTCTTTAACTCCATTTCATTCGTGAAA

                         100570    100580    100590    100600    100610    100620    100630    100640

Human            CTGTTGGTTAGCAATTTAAATGAAGAGGTTAGTAAAATAATCTCTAATGTTTATTCTTTAACTCCATTTCATTTGTGAAA 83928
Coquerel'sSifaka CTGTTGGTTAGCAATTTAAATGAAGAGGTTAGTAAAATAATTTCTAATGTTTATTCTTTAACTCCATTTCATTTGTGAAA 70100
Ma'sNightMonkey  CTGTTGGTTAGCAATTTAAATGAAGAGGTTAGTAAAATAATCTCTAATGTTTATTCTTTAACTCCATTTCATTTGTGAAA 78507
VervetAGM        CTGTTGGTTAGCAATTTAAATGAAGAGGTTAGTAAAATAATCTCTAATGTTTATTCTTTAACTCCATTTCATTCGTGAAA 84759
Gorrilla         CTGTTGGTTAGCAATTTAAATGAAGAGGTTAGTAAAATAATCTCTAATGTTTATTCTTTAACTCCATTTCATTTGTGAAA 82177
GrabEatingMacaqueCTGTTGGTTAGCAATTTAAATGAAGAGGTTAGTAAAATAATCTCTAATGTTTATTCTTTAATTCCATTTCATTCGTGAAA 84214
Macaque          CTGTTGGTTAGCAATTTAAATGAAGAGGTTAGTAAAATAATCTCTAATGTTTATTCTTTAATTCCATTTCATTCGTGAAA 85103
PigTailedMacaque CTGTTGGTTAGCAATTTAAATGAAGAGGTTAGTAAAATAATCTCTAATGTTTATTCTTTAACTCCATTTCATTCGTGAAA 83660
Bonobo           CTGTTGGTTAGCAATTTAAATGAAGAGGTTAGTAAAATAATCTCTAATGTTTATTCTTTAACTCCATTTCATTTGTGAAA 82767
OliveBaboon      CTGTTGGTTAGCAATTTAAATGAAGAGGTTAGTAAAATAATCTCTAATGTTTATTCTTTAACTCCATTTCATTCGTGAAA 83553
Orangutan        CTGTTGGTTAGCAATTTAAATGAAGAGGTTAGTAAAATAATCTCTAATGTTTATTCTTTAACTTCATTTCATTCGTGAAA 84393

Majority         GTTTTTCATGTTTATTTCATTTTGCACTTGCCTTTCTTTTTATGTACATTCATTAGTCAAAGTATTTTCTGTATGTTTCT

                         100650    100660    100670    100680    100690    100700    100710    100720

Human            GTTTTTCATGTTTATTTCATTTTGCACTTGCCTTTCTTTTTATGTACATTCATTAGTCAAAGTATTTTCTGTATGTTTCT 84008
Coquerel'sSifaka GTTTTTCATGTTTATTTCATTTTGCACTTGCCTTTCTTTTTATGTACATTCATTAGTCAAAGTATTTTCTGTATGTTTCT 70180
Ma'sNightMonkey  GTTTTTCATGTTTATTTCATTTTGCCCTTGCCTTTCTTTTTATGTACATTCATTAGTCAGAGTATTTTCTGTATGTTTCT 78587
VervetAGM        GTTTTTCATGTTTATTTCATTTTGCACTTGCCTTTCTTTTTATGTACATTCATTAGTCAAAGTATTTTCTGTATGTTTCT 84839
Gorrilla         GTTTTTCATGTTTATTTCATTTTGCACTTGCCTTTCTTTTTATGTACATTCATTAGTCAAAGTATTTTCTGTATGTTTCT 82257
GrabEatingMacaqueGTTTTTCATGTTTATTTCATTTTGCACTTGCCTTTCTTTTTATGTACATTCATTAGTCGAAGTATTTTCTGTATGTTTCT 84294
Macaque          GTTTTTCATGTTTATTTCATTTTGCACTTGCCTTTCTTTTTATGTACATTCATTAGTCGAAGTATTTTCTGTATGTTTCT 85183
PigTailedMacaque GTTTTTCATGTTTATTTCATTTTGCACTTGCCTTTCTTTTTATGTACATTCATTAGTCGAAGTATTTTCTGTATGTTTCT 83740
Bonobo           GTTTTTCATGTTTATTTCATTTTGCACTTGCCTTTCTTTTTATGTACATTCATTAGTCAAAGTATTTTCTGTATGTTTCT 82847
OliveBaboon      GTTTTTCATGTTTATTTCATTTTGCACTTGCCTTTCTTTTTATGTACATTCATTAGTCAAAGTATTTTCTGTATGTTTCT 83633
Orangutan        GTTTTTCATGTTTATTTCATTTTGCACTTGCCTTTCTTTTTATGTACATTCATTAGTCAAAGTATTTTCTGTATGTTTCT 84473

Majority         ACTTCTGAATAAAATCTATGTA-TTGTTTAGGTAATACTTTATTTCCTTAAACAGTCATGTCTCTTTATATCTATATCTA

                         100730    100740    100750    100760    100770    100780    100790    100800

Human            ACTTCTGAATAAAATCTACATA-TTGTTTAGGTAATACTTTATTTCCTTAAACAGTCATGTCTCTTTATATCTATATCTA 84087
Coquerel'sSifaka ACTTCTGAATAAAATCTATATACTTGTTTAGGTAATACTTTATTTCCTTAGACAGTCATATCTCTTTATATCTATAT--- 70257
Ma'sNightMonkey  ACTTCTGAATAAATTCTACGTA-TCGTTTAGGTAATACTTTATTTCCTTAGACAGTCATGTCTCTTTATATCTATATCTA 78666
VervetAGM        ACTTCTGAATAAAATCTATGTA-TTGTTTAGGTAATACTTTATTTCCTTAAACAGTCATGTCTCTTTATATCTATATCTA 84918
Gorrilla         ACTTCTGAATAAAATCTACGTA-TTGTTTAGGTAATACTTTATTTCCTTAAACAGTCATGTCTCTTTATATCTATATCTA 82336
GrabEatingMacaqueACTTCTGAATAAAATCTATGTA-TTGTTTAGGTAATACTTT---TCCTTAAACAGTCATGTCTCTTTATATCTATAT--- 84367
Macaque          ACTTCTGAATAAAATCTATGTA-TTGTTTAGGTAATACTTT---TCCTTAAACAGTCATGTCTCTTTATATCTATAT--- 85256
PigTailedMacaque ACTTCTGAATAAAATCTATGTA-TTGTTTAGGTAATACTTT---TCCTTAAACAGTCATGTCTCTTTATATCTATAT--- 83813
Bonobo           ACTTCTGAATAAAATCTACGTA-TGGTTTAGGTAATACTTTATTTCCTTAAACAGTCATGTCTCTTTATATCTATATCTA 82926
OliveBaboon      ACTTCTGAATAAAATCTATGTA-TTGTTTAGGTAATACTTTATTTCCTTAAACAGTCATGTCTCTTTATATCTATAT--- 83709
Orangutan        GCTTCTGAATAAAATCTACATA-TTGTTTAGGTAATACTTTATTTCCTTAAACAGTCATGTCTCTTTATATCTATATCTA 84552
```

```
Majority         TATATGTATATTTTTT-AACCTAAC---CTATTTTTCCTAAGAAAAATATGGTGAAGTACTGTAGTTTGGCTT---ATCT
                         100810    100820    100830    100840    100850    100860    100870    100880
Human            TATATGTATATTTTTT-AACCTAAC---CTATTTTTCCTAAGAAAAATATGGTGAAGTACTGTAGTTTGGCTT---ATCT 84160
Coquerel'sSifaka ---ATGTATATTTTTTTAACCTAACTACCTATTTTTCCTAAGAAAAATATGGTGAAGTACTGTAGTTTGGCTTTTTATCT 70334
Ma'sNightMonkey  TATATGTATATTTTTT-AACCTAAC---CTATTTTTCCTAAGAAAAATATGGTGAAGTACTGTAGTTTGGCTT---ATCT 78739
VervetAGM        TATTTGTATATTTTTT-AACCTAAC---CTATTTTTCCTAAGAAAAATATGGTGAAGTACTGTAGTTTGGCTT---ATCA 84991
Gorrilla         TATATGTATATTTTTT-AACCTAAC---CTATTTTTCCTAAGAAAAATATGGTGAAGTACTGTAGTTTGGCTT---ATCT 82409
GrabEatingMacaque---TTGTATATTTTTT-AACCTAAC---CTATTTTTCCTAAGAAAAATATGGTGAAGTACTGTAGTTTGGCTT---ATCA 84437
Macaque          ---TTGTATATTTTTT-AACCTAAC---CTATTTTTCCTAAGAAAAATATGGTGAAGTACTGTAGTTTGGCTT---ATCA 85326
PigTailedMacaque ---TTGTATATTTTTT-AACCTAAC---CTATTTTTCCTAAGAAAAATATGGTGAAGTACTGTAGTTTGGCTT---ATCA 83883
Bonobo           TATATGTATATTTTTT-AACCTAAC---CTATTTTTCCTAAGAAAAATATGGTGAAGTACTGTAGTTTGGCTT---ATCT 82999
OliveBaboon      ---TTGTATATTTTTT-AACCTAAC---CTATTTTTCCTAAGAAAAATATGGTGAAGTACTGTAGTTTGGCTT---ATCA 83779
Orangutan        TATATGTATATTTTTT-AACCTAAC---CTATTTTTCCTAAGAAAAATATGGTGAAGTACTGTAGTTTGGCTT---ATCT 84625

Majority         TTTTGTTGTTCTCAAAGGCAAATGTGATCTAATTTGCAAATTTAACTGTAAAACAATTTTGCTCTTTGCCTTTTCTAATT
                         100890    100900    100910    100920    100930    100940    100950    100960
Human            TTTTGTTGTTCTCAAAGGCAAATGTGATCTAATTTGCAAATTTAACTGTAAAACAATTTTGCTCTTTGCCTTTTCTAATT 84240
Coquerel'sSifaka TTTTGTTGATTTCAAAGGCAAATGTGATCTAATTTGCAAATTTAACTGTAAAACAATTTTGCTCTTTGCCTTTTCTAATT 70414
Ma'sNightMonkey  TTTTGTTGTTCTCAAAGGCAAATGTGATCTAATTTGCAAATTTAATTGTAAAACAATTTTGCTCTTTGCCTTTTCTAATT 78819
VervetAGM        TTTTGTTGTTCTCAAAGGCAAATGTGATCTAATTTGCAAATTTAACTGTAAAACAATTTTGCTCTTTGCCTTTTCTAATT 85071
Gorrilla         TTTTGTTGTTCTTAAAGGCAAATGTGATCTAATTTGCAAATTTAACTGTAAAACAATTTTGCTCTTTGCCTTTTCTAATT 82489
GrabEatingMacaqueTTTTGTTGTTCTCAAAGGCAAATGTGATCTAATTTGCAAATTTAACTGTAAAACAATTTTGCTCTTTGCCTTTTCTAATT 84517
Macaque          TTTTGTTGTTCTCAAAGGCAAATGTGATCTAATTTGCAAATTTAACTGTAAAACAATTTTGCTCTTTGCCTTTTCTAATT 85406
PigTailedMacaque TTTTGTTGTTCTCAAAGGCAAATGTGATCTAATTTGCAAATTTAACTGTAAAACAATTTTGCTCTTTGCCTTTTCTAATT 83963
Bonobo           TTTTGTTGTTCTCAAAGGCAAATGTGATCTAATTTGCAAATTTAACTGTAAAACAATTTTGCTCTTTGCCTTTTCTAATT 83079
OliveBaboon      TTTTGTTGTTCTCAAAGGCAAATGTGATCTAATTTGCAAATTTAACTGTAAAACAATTTTGCTCTTTGCCTTTTCTAATT 83859
Orangutan        TTTTGTTGTTCTCAAAGTCAAATGTGATCTAATTTGCAAATTTAACTGTAAAACAATTTTGCTCTTTGCCTTTTCTAATT 84705

Majority         ATTTGCTTGTTCATTTCATGCTTATATGTCATTGCATTTTTTTTAAATCTTATTTT-ATGTGAACTACTCTAATACATTT
                         100970    100980    100990    101000    101010    101020    101030    101040
Human            ATTTGCTTGTTCATTTCATGCTTATATGTCATTGCATTTTTTTTAAATCTTATTTT-ATGTGAACTACTCTAATACATTT 84319
Coquerel'sSifaka ATTTGCTTGTTCATTTCATGCTTATGTGTCATTGT-TTTTTTTTAAATCTTATTTT-ATGTGAACTACTCTAATACATTT 70492
Ma'sNightMonkey  ATTTGCTTGTTCATTTCATGCTTATATGTCATTGCATTTTTTTTAAATCTTATTTTTATGTGAACTACTCTAATACATTT 78899
VervetAGM        ATTTGCTTGTTCATTTCATGCTTATATGTCATTGCATTTTTTTTAAATCTTATTTT-ATGTGAACTACTCTAATACATTT 85150
Gorrilla         ATTTGCTTGTTCATTTCATGCTTATATGTCATTGCATTTTTTTTAAATCTTATTTT-ATGTGAACTACTCTAATACATTT 82568
GrabEatingMacaqueATTTGCTTGTTCATTTCATGCTTATATGTCATTGCATTTTTTTTAAATCTTATTTT-ATGTGAACTACTCTAATACATTT 84596
Macaque          ATTTGCTTGTTCATTTCATGCTTATATGTCATTGCATTTTTTTTAAATCTTATTTT-ATGTGAACTACTCTAATACATTT 85485
PigTailedMacaque ATTTGCTTGTTCATTTCATGCTTATATGTCATTGCATTTTTTTTAAATCTTATTTT-ATGTGAACTACTCTAATACATTT 84042
Bonobo           ATTTGCTTGTTCATTTCATGCTTATATGTCATTGCATTTTTTTTAAATCTTATTTT-ATGTGAACTACTCTAATACATTT 83158
OliveBaboon      ATTTGCTTGTTCATTTCATGCTTATATGTCATTGCATTTTTTTTAAATCTTATTTT-ATGTGAACTACTCTAATACATTT 83938
Orangutan        ATTTGCTTGTTCATTTCATGCTTATATGTCATTGCATTTTTTTTAAATCTTATTTT-ATGTGAACTACTCTAATACATTT 84784
```

```
Majority          TTCTTTGAAGGTTCTTCCAAAGATCTTGACGAGGCACTCTTCCAGTCTTTCTTAGTAATTTTTT-CTTTGCAGTTATTAG

                          101050    101060    101070    101080    101090    101100    101110    101120

Human             TTCTTTGAAGGTTCTTCCAAAGATCTTGACGAGGCACTCTTCCCGTCTTTCTTAGTAATTTTTT-CTTTGCAGTTATTAG 84398
Coquerel'sSifaka  TTCTTTGAAGGTTCTTCCAAAGATCTTGACGAGGCACTCTTCCAGTCTTTCTTAGTAATTTTTT-CTTTGCAGTTATTAG 70571
Ma'sNightMonkey   TTCTTTGAAGGTTCTTCCAAAGATCTTGACGAGGCACTCTTCCAGTCTTTCTTAGTAATTTTTT-CTTTGCAGTTATTAG 78978
VervetAGM         TTCTTTAAAGGTTCTTCCAAAGATCTTGACGAGGCACTCTTCCAGTCTTTCTTAGTAATTTTTT-CTTTGCAGTTATTAG 85229
Gorrilla          TTCTTTGAAGGTTCTTCCAAAGATCTTGACGAGGCACTCTTCCAGTCTTTCTTAGTAATTTTTTTCTTTGCAGTTATTAG 82648
GrabEatingMacaqueTTCTTTGAAGGTTCTTCCAAAGATCTTGACGAGGCACTCTTCCAGTCTTTCTTAGTAATTTTTT-CTTTGCAGTTATTAG 84675
Macaque           TTCTTTGAAGGTTCTTCCAAAGATCTTGACGAGGCACTCTTCCAGTCTTTCTTAGTAATTTTTT-CTTTGCAGTTATTAG 85564
PigTailedMacaque  TTCTTTGAAGGTTCTTCCAAAGATCTTGACGAGGCACTCTTCCAGTCTTTCTTAGTAATTTTTT-CTTTGCAGTTATTAG 84121
Bonobo            TTCTTTGAAGGTTCTTCCAAAGATCTTGACGAGGCACTCTTCCCGTCTTTCTTAGTAATTTTTT-CTTTGCAGTTATTAG 83237
OliveBaboon       TTCTTTGAAGGTTCTTCCAAAGATCTTGACGAGGCACTCTTCCAGTCTTTCTTAGTAATTTTTT-CTTTGCAGTTATTAG 84017
Orangutan         TTCTTTGAAGGTTCTTCCAAAGATCTTGACGAGGCACTCTTCCAGTCTTTCTTAGTAATTTTTT-CTTTGCAGTTATTAG 84863

Majority          TCATTGTCTTCTAAAAATATTTTTTAACTTTATTCCCCCACCCCTTCCTTCCTGACCCCCGACCCACCCCACCCACCTCA

                          101130    101140    101150    101160    101170    101180    101190    101200

Human             TCATTGTCTTCTAAAAATATTTTTTAACTTTATTCCCCCACCCCTTCCTTCCTTACC--------ACCCCCT-----CCA 84465
Coquerel'sSifaka  TTATTGTCTTCTAAAAATATTTTTCAACTTTATTCCCCTTTCCCTCCCCCCC---CC---------------------- 70625
Ma'sNightMonkey   TCATTGTCTTCTAAAAATATTTTTTAACTTTAA-CCCCCACCCCTTCCTCCCTGACCCCCTCCCCACCCCGCC-ACCCTG 79056
VervetAGM         TCATTGTCTTCTAAAAATATTTTTTAACTTTATTCCCCCACCCCTTCCTTCCTGACCCCCGACCCACCCCACCCACCTCA 85309
Gorrilla          TCATTGTCTTCTAAAAATATTTTTTAACTTTATTCCCCCACCCCTTCCTTCCTTACC--------ACCCTCC-----CCA 82715
GrabEatingMacaqueTCATTGTCTTCTAAAAATATTTTTTAACTTTATTCCCCTACCCCTTCCTTCCTGACCCCCGACCCACCCCACCCACCTCG 84755
Macaque           TCATTGTCTTCTAAAAATATTTTTTAACTTTATTCCCCTACCCCTTCCTTCCTGACCCCCGACCCACCCCACCCACCTCG 85644
PigTailedMacaque  TCATTGTCTTCTAAAAATATTTTTAAACTTTATTCCCCCACCCCTTCCTTCCTGACCCCCGACCCACCCCACCCACCTCA 84201
Bonobo            TCATTGTCTTCTAAAAATATTTTTTGACTTTATTCCCCCACCCCTTCCTTCCTTACC--------ACCCCC------CCA 83303
OliveBaboon       TCATTGTCTTCTAAAAATATTTTTTAACTTTATTCCCCCACCCCTTCCTTCCTGACCCCCGACCCACCCCACCCACCTCG 84097
Orangutan         TCATTGTCTTCTAAAAATATTTTTTAACTTTATTCCCCCAACCCTTCCTTCCTTACC--------ACCTCCCCCCACCCA 84935

Majority          CCCAGTTAAATAAAATTTATTGCTAAGTTATTTTCTTT-AGTTGTACATTTTATGTCTGGTATATTTTATTTTGAATGCT

                          101210    101220    101230    101240    101250    101260    101270    101280

Human             CCCAGTTAAATAAAATTTATTGCTAAGTTATTTTCTTT-AGTTGTACATTTTATGTCTGGTATATTTTATTTTGAATGCT 84544
Coquerel'sSifaka  ----GTTGAATAAAATTTATTGCTAAGTTATTTTATTT-AGTTGTACATTTTATGTCTGGTATATTTTATTTTGATTGCT 70700
Ma'sNightMonkey   CCCGGTTAAATAAAATTTATTGCTAAGTTGTTTTTTTTTAGTTGTACATTTTATGTCTGGTATATTTTATTTTGAATGCT 79136
VervetAGM         CCCAGTTAAATAAAATTTATTGCTAAGTTATTTTCTTT-AGTTGTACATTTTATGTCTGGTATATTTTATTTTGAATGCT 85388
Gorrilla          CCCAGTTAAATAAAATTTATTGCCAAGTTATTTTCTTT-AGTTGTACATTTTATGTCTGGTATATTTTATTTTGAATGCT 82794
GrabEatingMacaqueCCCAGTTAAATAAAATTTATTGCTAAGTTATTTTCTTT-AGTTGTACATTTTATGTCTGGTATATTTTATTTTGAATGCT 84834
Macaque           CCCAGTTAAATAAAATTTATTGCTAAGTTATTTTCTTT-AGTTGTACATTTTATGTCTGGTATATTTTATTTTGAATGCT 85723
PigTailedMacaque  CCCAGTTAAATAAAATTTATTGCTAAGTTATTTTCTTT-AGTTGTACATTTTATGTCTGGTATATTTTATTTTGAATGCT 84280
Bonobo            CCCAGTTAAATAAAATTTATTGCTAAGTTATTTTCTTT-AGTTGTACATTTTATGTCTGGTATATTTTATTTTGAATGCT 83382
OliveBaboon       CCCAGTTAAATAAAATTTATTGCTAAGTTATTTTCTTT-AGTTGTACATTTTATGTCTGGTATATTTTATTTTGAATGCT 84176
Orangutan         TCCAGTTAAATAAAATTTATTGCTAAGTTATTTTCTTT-AGTTGTACATTTTATGTCTGGTATATTTTATTTTGAATGCT 85014
```

```
Majority          ATGAAAGCTGGTATGAAATGTGGGAAGCTCAGTGTATCAGTTTATGATGTCTAATTTGAATATTTGTTTCATTTTTAATT
                          101290    101300    101310    101320    101330    101340    101350    101360
Human             ATGAAAGCTGGTATGAAATGTGGGAAGCTCAGTGTATCAGTTTATGATGTCTAATTTGAATATTTGTTTCATTTTTAATT 84624
Coquerel'sSifaka  ATGAAAGCTGGTATGAAATGTGGGAAGCTCAATGTATCAGTTTATGATGTCTAATTTGAATATTTGTTTCATTTTTAATT 70780
Ma'sNightMonkey   ATGAACGCTGGTATGAAATGTGGGAAGCTCAGTGTATCAGTTTATGATGTCTAATTTGAATATTTGTTTCATTTTTAATT 79216
VervetAGM         ATGAAAGCTGGTATGAAATGTGGGAAGCTCAGTGTATCAGTTTATGATGTCTAATTTGAAT--TTGTTTCATTTTTAATT 85466
Gorrilla          ATGAAAGCTGGTATGAAATGTGGGAAGCTCAGTGTATCAGTTTATGATGTCTAATTTGAATATTTGTTTCATTTTTAATT 82874
GrabEatingMacaqueATGAAAGCTGGTATGAAATGTGGGAAGCTCAGTGTATCAGTTTATGATGTCTAATTTGAAT--TTGTTTCATTTTTAATT 84912
Macaque           ATGAAAGCTGGTATGAAATGTGGGAAGCTCAGTGTATCAGTTTATGATGTCTAATTTGAAT--TTGTTTCATTTTTAATT 85801
PigTailedMacaque  ATGAAAGCTGGTATGAAATGTGGGAAGCTCAGTGTATCAGTTTATGATGTCTAATTTGAAT--TTGTTTCATTTTTAATT 84358
Bonobo            ATGAAAGCTGGTATGAAATGTGGGAAGCTCAGTGTATCAGTTTATGATGTCTAATTTGAATATTTGTTTCATTTTTAATT 83462
OliveBaboon       ATGAAAGCTGGTATGAAATGTGGGAAGCTCAGTGTATCAGTTTATGATGTCTAATTTGAAT--TTGTTTCATTTTTAATT 84254
Orangutan         ATGAAAGCTGGTATGAAATGTGGGAAGCTCAGTGTATCAGTTTATGATGTCTAATTTGAATATTTGTTTCATTTTTAATT 85094

Majority          GGCCTCTGTTAATATGAACATTA-GCTTATTTTATCCATTTAATACTATTATCATTCACAGTTCTGCATGCTAAAATGTT
                          101370    101380    101390    101400    101410    101420    101430    101440
Human             GGCCTCTGTTAATATGAACATTA-GCTTATTTTATCCATTTAATACTATTATCATTCACAGTTCTGCATGCTAAAATGTT 84703
Coquerel'sSifaka  GGCCT--GTTAATATGAACATTAAGCTTATTTTATC-ATTTAATACTATTATCATTCACAGTTCTGCATGCTAAAATGTT 70857
Ma'sNightMonkey   GGCCTCTGTTAATATGAACATTA-GCTTATTTTATC-ATTTAATACTATTATCATTCACAGTTCTGCATGCTAAAATGTT 79294
VervetAGM         GGCCTCTGTTAATATGAACATTA-GCTTATTTTATCCATTTAATACTATTATCATTCACAGTTCTGCATGCTAAAATGTT 85545
Gorrilla          GGCCTCTGTTAATATGAACATTA-GCTTATTTTATCCATTTAATACTATTATCATTCACAGTTCTGCATGCTAAAATGTT 82953
GrabEatingMacaqueGGCCTCTGTTAATATGAACATTA-GCTTATTTTATCCATTTAATACTATTATCATTCACAGTTCTGCATGCTAAAATGTT 84991
Macaque           GGCCTCTGTTAATATGAACATTA-GCTTATTTTATCCATTTAATACTATTATCATTCACAGTTCTGCATGCTAAAATGTT 85880
PigTailedMacaque  GGCCTCTGTTAATATGAACATTA-GCTTATTTTATCCATTTAATACTATTATCATTCACAGTTCTGCATGCTAAAATGTT 84437
Bonobo            GGCCTCTGTTAATATGAACATTA-GCTTATTTTATCCATTTAATACTATTATCATTCACAGTTCTGCATGCTAAAATGTT 83541
OliveBaboon       GGCCTCTGTTAATATGAACATTA-GCTTATTTTATCCATTTAATACTATTATCATTCACAGTTCTGCATGCTAAAATGTT 84333
Orangutan         GGCCTCTGTTAATATGAACATTA-GCTTATTTTATCCATTTAATACTATTATCATTCACAGTTCTGCATGCTAAAATGTT 85173

Majority          TGAATCAGAGTGCCTTTGTTTTTATTTTAAGACTTTTGCCTGCATTTCATAACCAGCCATGCTTATGCAGTT-AAAGTTC
                          101450    101460    101470    101480    101490    101500    101510    101520
Human             TGAATCAGAGTGCCTTTGTTTTTATTTTAAGACTTTTGCCTGCATTTCATAACCAGCCATGCTTATGCAGTT-AAAGTTC 84782
Coquerel'sSifaka  TGAATCAGAGTGCCTTTGTTTTTATTTTAAGACTTTTGCCTGCATTTCATAACCAGCCATGCTTATGCAGTT-AAAGTTC 70936
Ma'sNightMonkey   TGAATCAGAGTGCCTTTGTTTTTATTTTAAGACTTTTGCCTGCATTTCATAACCAGCCATGCTTATGCAGTTGAAAGTTC 79374
VervetAGM         TGAATCAGAGTGCCTTTGTTTTTGTTTTAAGACTTTTGCCTGCATTTCATAACCAGCCATGCTTATGCAGTT-AAAGTTC 85624
Gorrilla          TGAATCAGAGTGCCTTTGTTTTTATTTTAAGACTTTTGCCTGCATTTCATAACCAGCCATGCTTATGCAGTT-AAAGTTC 83032
GrabEatingMacaqueTGAATCAGAGTGCCTTTGTTTTTGTTTTAAGACTTTTGCCTGCATTTCATAACCAGCCATGCTTATGCAGTT-AAAGTTC 85070
Macaque           TGAATCAGAGTGCCTTTGTTTTTGTTTTAAGACTTTTGCCTGCATTTCATAACCAGCCATGCTTATGCAGTT-AAAGTTC 85959
PigTailedMacaque  TGAATCAGAGTGCCTTTGTTTTTGTTTTAAGACTTTTGCCTGCATTTCATAACCAGCCATGCTTATGCAGTT-AAAGTTC 84516
Bonobo            TGAATCAGAGTGCCTTTGTTTTTATTTTAAGACTTTTGCCTGCATTTCATAACCAGCCATGCTTATGCAGTT-AAAGTTC 83620
OliveBaboon       TGAATCAGAGTGCCTTTGTTTTTGTTTTAAGACTTTTGCCTGCATTTCATAACCAGCCATGCTTATGCAGTT-AAAGTTC 84412
Orangutan         TGAATCAGAGTGCCTTTGTTTTTATTTTAAGACTTTTGCCTGCATTTCATAACCAGCCATGCTTATGCAGTT-AAAGTTC 85252
```

```
Majority         AAAGTTTAAAATTCCTATGCATGCTTTCCTTCCCTATGTTGAGATGAAATGCTGTAATTTACTCTTTGATATAGATGTAC

                         101530    101540    101550    101560    101570    101580    101590    101600

Human            AAAGTTTAAAATTCCTATGCATGCTTTCCTTCCCTATGTTGAGATGAAATGCTGTAATTTACTCTTTGATATAGATGTAC 84862
Coquerel'sSifaka AAAGTTTAAAATTCCTATGCATGCTTTCCTTCCCTATGTTGAGATGAAATGCTGTAATTTACTCTTTGATATAGATGTAC 71016
Ma'sNightMonkey  AAAGTTTAAAATTCCTATGCATGCTTTCCTTCCCTATGTTGAGATGAAATGCTGTA-TTTACTCTTTGATATAGATGTAC 79453
VervetAGM        AAAGTTAAAAATTCCTATGCATGCTTTCCTTCCCTATGTTGAGATGAAATGCTGTAATTTACTCTTTGATATAGATGTAC 85704
Gorrilla         AAAGTTTAAAATTCCTATGCATGCTTTCCTTCCCTATGTTGAGATGAAATGCTGTAATTTACTCTTTGATATAGATGTAC 83112
GrabEatingMacaqueAAAGTTAAAAATTCCTATGCATGCTTTCCTTCCCTATGTTGAGATGAAATGCTGTAATTTACTCTTTGATATAGATGTAC 85150
Macaque          AAAGTTAAAAATTCCTATGCATGCTTTCCTTCCCTATGTTGAGATGAAATGCTGTAATTTACTCTTTGATATAGATGTAC 86039
PigTailedMacaque AAAGTTAAAAATTCCTATGCATGCTTTCCTTCCCTATGTTGAGATGAAATGCTGTAATTTACTCTTTGATATAGATGTAC 84596
Bonobo           AAAGTTTAAAATTCCTATGCATGCTTTCCTTCCCTATGTTGAGATGAAATGCTGTAATTTACTCTTTGATATAGATGTAC 83700
OliveBaboon      AAAGTTAAAAATTCCTATGCATGCTTTCCTTCCCTATGTTGAGATGAAATGCTGTAATTTACTCTTTGATATAGATGTAC 84492
Orangutan        AAAGTTTAAAATTCCTATGCATGCTTTCCTTCCCTATGTTGAGATGAAATGCTGTAATTTACTCTTTGATATAGATGTAC 85332

Majority         TTTACCCATATTTGTCTTGGATGACTACATTTTACTCAGTTTTTCTTGTACAGTACATAAACCAACCATTTTCTGACCAA

                         101610    101620    101630    101640    101650    101660    101670    101680

Human            TTTACCCATATTTGTCTTGGATGACTACATTTTACTCAGTTTTTCTTGTACAGTACATAAACCAACCATTTTCTGACCAA 84942
Coquerel'sSifaka TTTACCCATATTTGTCTTGGATGACTACATTTTACTCAGTTTTTCTTGTACAGTACATAAACCAACCATTTTCTGACCAA 71096
Ma'sNightMonkey  TTTACCCATATTTGTCTTGGATGACTACATTTTACTCAGTTTTTCTTGTACAGTACATAAACCAACCATTTTCTGACCAA 79533
VervetAGM        TTTACCCATATTTGTCTTGGATGACTACATTTTACTCAGTTTTTCTTGTACAGTACATAAACCAACCATTTTCTGACCAA 85784
Gorrilla         TTTACCCATATTTGTCTTGGATGACTACATTTTACTCAGTTTTTCTTGTACAGTACATAAACCAACCATTTTCTGACCAA 83192
GrabEatingMacaqueTTTACCCATATTTGTCTTGGATGACTACATTTTACTCAGTTTTTCTTGTACAGTACATAAACCAACCATTTTCTGACCAA 85230
Macaque          TTTACCCATATTTGTCTTGGATGACTACATTTTACTCAGTTTTTCTTGTACAGTACATAAACCAACCATTTTCTGACCAA 86119
PigTailedMacaque TTTACCCATATTTGTCTTGGATGACTACATTTTACTCAGTTTTTCTTGTACAGTACATAAACCAACCATTTTCTGACCAA 84676
Bonobo           TTTACCCATATTTGTCTTGGATGACTACATTTTACTCAGTTTTTCTTGTACAGTACATAAACCAACCATTTTCTGACCAA 83780
OliveBaboon      TTTACCCATATTTGTCTTGGATGACTACATTTTACTCAGTTTTTCTTGTACAGTACATAAACCAACCATTTTCTGACCAA 84572
Orangutan        TTTACCCATATTTGTCTTGGATGACTACATTTTACTCAGTTTTTCTTATACAGTACATAAACCAACCATTTTCTGACCAA 85412

Majority         ATTCCTGCATTTCCTATGTACTGACCTATATTTTATTTTGTTTTT---GTTCCCCAATTCCTTATTTTTTTCTTCTGCAT

                         101690    101700    101710    101720    101730    101740    101750    101760

Human            ATTCCTGCATTTCCTATGTACTGACCTATATTTTATTTTGTTTTT---GTTCCCCAATTCCTTATTTTTTTCTTCTGCAT 85019
Coquerel'sSifaka ATTCCTGCATTTCCTATGTACTGACCTATATTTTATTTTTTTTTTTTTGTTCCCCAATTCCTTATTTTTTTCTTCTGCAT 71176
Ma'sNightMonkey  ATTCCTGCATTTCCTATGTACTGACCTATATTTTATTTTGTTTTT---GTTCCCCAATTCCTTATTTTTTTCTTCTGCAT 79610
VervetAGM        ATTCCTGCATTTCCTATGTACTGACCTATATTTTATTTTGTTTTT---GTTCCCCAATTCCTTATTTTTTTCTTCTGCAT 85861
Gorrilla         ATTCCTGCATTTCCTATGTACTGACCTATATTTTATTTTGTTTTT---GTTCCCCAATTCCTTATTTTTTTCTTCTGCAT 83269
GrabEatingMacaqueATTCCTGCATTTCCTATGTACTGACCTATATTTTATTTTGTTTTT---GTTCCCCAATTCCTTATTTTTTTCTTCTGCAT 85307
Macaque          ATTCCTGCATTTCCTATGTACTGACCTATATTTTATTTTGTTTTT---GTTCCCCAATTCCTTATTTTTTTCTTCTGCAT 86196
PigTailedMacaque ATTCCTGCATTTCCTATGTACTGACCTATATTTTATTTTGTTTTT---GTTCCCCAATTCCTTATTTTTTTCTTCTGCAT 84753
Bonobo           ATTCCTGCATTTCCTATGTACTGACCTATATTTTATTTTGTTTTT---GTTCCCCAATTCCTTATTTTTTTCTTCTGCAT 83857
OliveBaboon      ATTCCTGCATTTCCTATGTACTGACCTATATTTTATTTTGTTTTT---GTTCCCCAATTCCTTATTTTTTTCTTCTGCAT 84649
Orangutan        ATTCCTGCATTTCCTATGTACTGACCTATATTTTATTTTGTTTTT---GTTCCCCAATTCCTTATTTTTTTCTTCTGCAT 85489
```

```
Majority        TGCTGTTTCCCTTCCCCATTTCATCCTTTTCCCTGTGTGTTCACCTTCCCTTTCCTTGTCTTTTCCCAAATGCCCATTCC
                        101770    101780    101790    101800    101810    101820    101830    101840
Human           TGCTGTTTCCCTTCCCCATTTCATCCTTTTCCCTGTGTGTTCACCTTCCCTTTCCTTGTCTTTTCCCAAATGCCCATTCC 85099
Coquerel'sSifaka TGCTGTTTCCCTTCCCCATTTCATCCTTTTCCCTGTGTGTTCACCTTCCCTTTCCTTGTCTTTTCCCAAATACCCATTCC 71256
Ma'sNightMonkey TGCTGTTTCCCTTCCCCATTTCATCCTTTTCCCTGTGTGTTCACCTTCCCTTTCCTTGTCTTTTCCCAAATGCCCATTCC 79690
VervetAGM       TGCTGTTTCCCTTCCCCATTTCATCCTTTTCCCTGTGTGTTCACCTTCCCTTTCCTTGTCTTTTCCCAAATGCCCATTCC 85941
Gorrilla        TGCTGTTTCCCTTCCCCATTTCATCCTTTTCCCTGTGTGTTCACCTTCCCTTTCCTTGTCTTTTCCCAAATGTCCATTCC 83349
GrabEatingMacaqueTGCTGTTTCCCTTCCCCATTTCATCCTTTTCCCTGTGTGTTCACCTTCCCTTTCCTTGTCTTTTCCCAAATGCCCATTCC 85387
Macaque         TGCTGTTTCCCTTCCCCATTTCATCCTTTTCCCTGTGTGTTCACCTTCCCTTTCCTTGTCTTTTCCCAAATGCCCATTCC 86276
PigTailedMacaque TGCTGTTTCCCTTCCCCATTTCATCCTTTTCCCTGTGTGTTCACCTTCCCTTTCCTTGTCTTTTCCCAAATGCCCATTCC 84833
Bonobo          TGCTGTTTCCCTTCCCCATTTCATCCTTTTCCCTGTGTGTTCACCTTCCCTTTCCTTGTCTTTTCCCAAATGCCCATTCC 83937
OliveBaboon     TGCTGTTTCCCTTCCCCATTTCATCCTTTTCCCTGTGTGTTCACCTTCCCTTTCCTTGTCTTTTCCCAAATGCCCATTCC 84729
Orangutan       TGCTGTTTCCCTTCCCCATTTCATCCTTTTCCCTGTGTGTTCACCTTCCCTTTCCTTGTCTTTTCCCAAATGCCCATTCC 85569
```

```
Majority        CTTCCCTGTCTTATCCTTTATTTTCCTTGTCCTTGTCTTCATTCCCTGTCTCCATTCCCTATGTTCATGCTTCTGTGCTT
                        101850    101860    101870    101880    101890    101900    101910    101920
Human           CTTCCCTGTCTTATCCTTTATTTTCCTTGTCCTTGTCTTCATTCCCTGTCTCCATTCCCTATGTTCATGCTTCTGTGCTT 85179
Coquerel'sSifaka CTTCCCTGTCTTATCCTTTATTTTCCTTGTCCTTGTCTTCATTCCCTGTCTCCATTCCCTATGTTCATGCTTCTGTGCTT 71336
Ma'sNightMonkey CTTCCCTGTCTTATCCTTTATTTTCCTTGTCCTTGTCTTCATTCCCTGTCTCCATTCCCTATGTTCATGCTTCTGTGCTT 79770
VervetAGM       CTTCCCTGTCTTATCCTTTATTTTCCTTGTCCTTGTCTTCATTCCCTGTCTCCATTCCCTATGTTCATGCTTCTGTGCTT 86021
Gorrilla        CTTCCCTGTCTTATCCTTTATTTTCCTTGTCCTTGTCTTCATTCCCTGTCTCCATTCCCTATGTTCATGCTTCTGTGCTT 83429
GrabEatingMacaqueCTTCCCTGTCTTATCCTTTATTTTCCTTGTCCTTGTCTTCATTCCCTGTCTCCATTCCCTATGTTCATGCTTCTGTGCTT 85467
Macaque         CTTCCCTGTCTTATCCTTTATTTTCCTTGTCCTTGTCTTCATTCCCTGTCTCCATTCCCTATGTTCATGCTTCTGTGCTT 86356
PigTailedMacaque CTTCCCTGTCTTATCCTTTATTTTCCTTGTCCTTGTCTTCATTCCCTGTCTCCATTCCCTATGTTCATGCTTCTGTGCTT 84913
Bonobo          CTTCCCTGTCTTATCCTTTATTTTCCTTGTCCTTGTCTTCATTCCCTGTCTCCATTCCCTATGTTCATGCTTCTGTGCTT 84017
OliveBaboon     CTTCCCTGTCTTATCCTTTATTTTCCTTGTCCTTGTCTTCATTCCCTGTCTCCATTCCCTATGTTCATGCTTCTGTGCTT 84809
Orangutan       CTTCCCTGTCTTATCCTTTATTTTCCTTGTCCTTGTCTTCATTCCCTGTCTCCATTCCCTATGTTCATGCTTCTGTGCTT 85649
```

```
Majority        GAACAAATGTTCCTCGGACCAACTTGCCCCAATTAACCGCCTTGAACCATGATCCATGACCACCTCACCATTCTGCGGGA
                        101930    101940    101950    101960    101970    101980    101990    102000
Human           GAACAAATGTTCCTCGGACCAACTTGCCCCAATTAACCGCCTTGAACCATGATCCATGACCACCTCACCATTCTGCGGGA 85259
Coquerel'sSifaka GAACAAATGTTCCTCGGACCAACTTGCCCCAATTAACCGCCTTGAACCATGATCCATGACCACCTCACCATTCTGCGGGA 71416
Ma'sNightMonkey GAACAAATGTTCCTCGGACCAACTTGCCCCAATTAACCGCCTTGAACCATGATCCATGACCACCTCACCATTCTGCGGGA 79850
VervetAGM       GAACAAATGTTCCTCGGACCAACTTGCCCCAATTAACCGCCTTGAACCATGATCCATGACCACCTCACCATTCTGCGGGA 86101
Gorrilla        GAACAAATGTTCCTCGGACCAACTTGCCCCAATTAACCGCCTTGAACCATGATCCATGACCACCTCACCATTCTGCGGGA 83509
GrabEatingMacaqueGAACAAATGTTCCTCGGACCAACTTGCCCCAATTAACCGCCTTGAACCATGATCCATGACCACCTCACCATTCTGCGGGA 85547
Macaque         GAACAAATGTTCCTCGGACCAACTTGCCCCAATTAACCGCCTTGAACCATGATCCATGACCACCTCACCATTCTGCGGGA 86436
PigTailedMacaque GAACAAATGTTCCTCGGACCAACTTGCCCCAATTAACCGCCTTGAACCATGATCCATGACCACCTCACCATTCTGCGGGA 84993
Bonobo          GAACAAATGTTCCTCGGACCAACTTGCCCCAATTAACCGCCTTGAACCATGATCCATGACCACCTCACCATTCTGCGGGA 84097
OliveBaboon     GAACAAATGTTCCTCGGACCAACTTGCCCCAATTAACCGCCTTGAACCATGATCCATGACCACCTCACCATTCTGCGGGA 84889
Orangutan       GAACAAATGTTCCTCGGACCAACTTGCCCCAATTAACCGCCTTGAACCATGATCCATGACCACCTCACCATTCTGCGGGA 85729
```

```
Majority        ACCACCCTTCGTTATGGATGATCTGTTCATCTCCGCTCTTCCTCGACTCTTCTCTCTTCTTGTCTTACGCTGCTTGCTCT

                      102010    102020    102030    102040    102050    102060    102070    102080

Human           ACCACCCTTCGTTATGGATGATCTGTTCATCTCCGCTCTTCCTCGACTCTTCTCTCTTCTTGTCTTACGCTGCTTGCTCT 85339
Coquerel'sSifaka ACCACCCTTCGTTATGGATGATCTGTTCATCTCCGCTCTTCCTCGACTCTTCTCTCTTCTTGTCTTACGCTGCTTGCTCT 71496
Ma'sNightMonkey ACCACCCTTCGTTATGGATGATCTGTTCATCTCCGCTCTTCCTCGACTCTTCTCTCTTCTTGTCTTACGCTGCTTGCTCT 79930
VervetAGM       ACCACCCTTCGTTATGGATGATCTGTTCATCTCCGCTCTTCCTCGACTCTTCTCTCTTCTTGTCTTACGCTGCTTGCTCT 86181
Gorrilla        ACCACCCTTCGTTATGGATGATCTGTTCATCTCCGCTCTTCCTCGACTCTTCTCTCTTCTTGTCTTACGCTGCTTGCTCT 83589
GrabEatingMacaqueACCACCCTTCGTTATGGATGATCTGTTCATCTCCGCTCTTCCTCGACTCTTCTCTCTTCTTGTCTTACGCTGCTTGCTCT 85627
Macaque         ACCACCCTTCGTTATGGATGATCTGTTCATCTCCGCTCTTCCTCGACTCTTCTCTCTTCTTGTCTTACGCTGCTTGCTCT 86516
PigTailedMacaque ACCACCCTTCGTTATGGATGATCTGTTCATCTCCGCTCTTCCTCGACTCTTCTCTCTTCTTGTCTTACGCTGCTTGCTCT 85073
Bonobo          ACCACCCTTCGTTATGGATGATCTGTTCATCTCCGCTCTTCCTCGACTCTTCTCTCTTCTTGTCTTACGCTGCTTGCTCT 84177
OliveBaboon     ACCACCCTTCGTTATGGATGATCTGTTCATCTCCGCTCTTCCTCGACTCTTCTCTCTTCTTGTCTTACGCTGCTTGCTCT 84969
Orangutan       ACCACCCTTCGTTATGGATGATCTGTTCATCTCCGCTCTTCCTCGACTCTTCTCTCTTCTTGTCTTACGCTGCTTGCTCT 85809

Majority        TCTCTCCTTCTAAAGATGGTTACGCCCCAAAGTCTGTTTACCCTCTTCGGTATGTTATTGTTAGCACTATACTTTTATTA

                      102090    102100    102110    102120    102130    102140    102150    102160

Human           TCTCTCCTTCTAAAGATGGTTACGCCCCAAAGTCTGTTTACCCTCTTCGGTATGTTATTGTTAGCACTATACTTTTATTA 85419
Coquerel'sSifaka TCTCTCCTTCTAAAGATGGTTACGCCCCAAAGTCTGTTTACCCTCTTCGGTATGTTATTGTTAGCACTATACTTTTATTA 71576
Ma'sNightMonkey TCTCTCCTTCTAAAGATGGTTACGCCCCAAAGTCTGTTTACCCTCTTCGGTATGTTATTGTTAGCACTATACTTTTATTA 80010
VervetAGM       TCTCTCCTTCTAAAGATGGTTACGCCCCAAAGTCTGTTTACCCTCTTCGGTATGTTATTGTTAGCACTATACTTTTATTA 86261
Gorrilla        TCTCTCCTTCTAAAGATGGTTACGCCCCAAAGTCTGTTTACCCTCTTCGGTATGTTATTGTTAGCACTATACTTTTATTA 83669
GrabEatingMacaqueTCTCTCCTTCTAAAGATGGTTACGCCCCAAAGTCTGTTTACCCTCTTCGGTATGTTATTGTTAGCACTATACTTTTATTA 85707
Macaque         TCTCTCCTTCTAAAGATGGTTACGCCCCAAAGTCTGTTTACCCTCTTCGGTATGTTATTGTTAGCACTATACTTTTATTA 86596
PigTailedMacaque TCTCTCCTTCTAAAGATGGTTACGCCCCAAAGTCTGTTTACCCTCTTCGGTATGTTATTGTTAGCACTATACTTTTATTA 85153
Bonobo          TCTCTCCTTCTAAAGATGGTTACGCCCCAAAGTCTGTTTACCCTCTTCGGTATGTTATTGTTAGCACTATACTTTTATTA 84257
OliveBaboon     TCTCTCCTTCTAAAGATGGTTACGCCCCAAAGTCTGTTTACCCTCTTCGGTATGTTATTGTTAGCACTATACTTTTATTA 85049
Orangutan       TCTCTCCTTCTAAAGATGGTTACGCCCCAAAGTCTGTTTACCCTCTTCGGTATGTTATTGTTAGCACTATACTTTTATTA 85889

Majority        TTGATTTGATTTTTGTTTCACCTTAATTCTTATTTGTAGCTAGCACTTTGGCTTAAAGTTGAATAGTAAATCTTTTGCTA

                      102170    102180    102190    102200    102210    102220    102230    102240

Human           TTGATTTGATTTTTGTTTCACCTTAATTCTTATTTGTAGCTAGCACTTTGGCTTAAAGTTGAATAGTAAATCTTTTGCTA 85499
Coquerel'sSifaka TTGATTTGATTTTTGTTTCACCTTAATTCTTATTTGTAGCTAGCACTTTGGCTTAAAGTTGAATAGTAAATCTTTTGCTA 71656
Ma'sNightMonkey TTGATTTGATTTTTGTTTCACCTTAATTCTTATTTGTAGCTAGCACTTTGGCTTAAAGTTGAATAGTAAATCTTTTGCTA 80090
VervetAGM       TTGATTTGATTTTTGTTTCACCTTAATTCTTATTTGTAGCTAGCACTTTGGCTTAAAGTTGAATAGTAAATCTTTTGCTA 86341
Gorrilla        TTGATTTGATTTTTGTTTCACCTTAATTCTTATTTGTAGCTAGCACTTTGGCTTAAAGTTGAATAGTAAATCTTTTGCTA 83749
GrabEatingMacaqueTTGATTTGATTTTTGTTTCACCTTAATTCTTATTTGTAGCTAGCACTTTGGCTTAAAGTTGAATAGTAAATCTTTTGCTA 85787
Macaque         TTGATTTGATTTTTGTTTCACCTTAATTCTTATTTGTAGCTAGCACTTTGGCTTAAAGTTGAATAGTAAATCTTTTGCTA 86676
PigTailedMacaque TTGATTTGATTTTTGTTTCACCTTAATTCTTATTTGTAGCTAGCACTTTGGCTTAAAGTTGAATAGTAAATCTTTTGCTA 85233
Bonobo          TTGATTTGATTTTTGTTTCACCTTAATTCTTATTTGTAGCTAGCACTTTGGCTTAAAGTTGAATAGTAAATCTTTTGCTA 84337
OliveBaboon     TTGATTTGATTTTTGTTTCACCTTAATTCTTATTTGTAGCTAGCACTTTGGCTTAAAGTTGAATAGTAAATCTTTTGCTA 85129
Orangutan       TTGATTTGATTTTTGTTTCACCTTAATTCTTATTTGTAGCTAGCACTTTGGCTTAAAGTTGAATAGTAAATCTTTTGCTA 85969
```

```
Majority         TTTTTCTTTGCTATTTAAAACTCTCCATAGACACAAAATTTGTTTTAATGCATGCTAATTTATTTTGCATGGTCTTTAAT

                         102250    102260    102270    102280    102290    102300    102310    102320

Human            TTTTTCTTTGCTATTTAAAACTCTCCATAGACACAAAATTTGTTTTAATGCATGCTAATTTATTTTGCATGGTCTTTAAT 85579
Coquerel'sSifaka TTTTTCTTTGCTATTTAAAACTCTCCATAGACACAAAATTTGTTTTAATGCATGCTGATTTATTTTGCATGGTCTTTAAT 71736
Ma'sNightMonkey  TTTTTCTTTGCTATTTAAAACTCTCCATAGACACAAAATTTGTTTTAATGCATGCTAATTTATTTTGCATGGTCTTTAAT 80170
VervetAGM        TTTTTCTTTGCTATTTAAAACTCTCCATAGACACAAAATTTGTTTTAATGCATGCTAATTTATTTTGCATGGTCTTTAAT 86421
Gorrilla         TTTTTCTTTGCTATTTAAAACTCTCCATAGACACAAAATTTGTTTTAATGCATGCTAATTTATTTTGCATGGTCTTTAAT 83829
GrabEatingMacaqueTTTTTCTTTGCTATTTAAAACTCTCCATAGACACAAAATTTGTTTTAATGCATGCTAATTTATTTTGCATGGTCTTTAAT 85867
Macaque          TTTTTCTTTGCTATTTAAAACTCTCCATAGACACAAAATTTGTTTTAATGCATGCTAATTTATTTTGCATGGTCTTTAAT 86756
PigTailedMacaque TTTTTCTTTGCTATTTAAAACTCTCCATAGACACAAAATTTGTTTTAATGCATGCTAATTTATTTTGCATGGTCTTTAAT 85313
Bonobo           TTTTTCTTTGCTATTTAAAACTCTCCATAGACACAAAATTTGTTTTAATGCATGCTAATTTATTTTGCATGGTCTTTAAT 84417
OliveBaboon      TTTTTCTTTGCTATTTAAAACTCTCCATAGACACAAAATTTGTTTTAATGCATGCTAATTTATTTTGCATGGTCTTTAAT 85209
Orangutan        TTTTTCTTTGCTATTTAAAACTCTCCATAGACACAAAATTTGTTTTAATGCATGCTAATTTATTTTGCATGGTCTTTAAT 86049

Majority         TTAATATCATTCACATAGCTTTGAGGGTTTATCAAAAATTATTCTTTTCAAAATTCACTGTTCAAAATCTTGATCTTCTT

                         102330    102340    102350    102360    102370    102380    102390    102400

Human            TTAATATCATTCACATAGCTTTGAGGGTTTATCAAAAATTATTCTTTTCAAAATTCACTGTTCAAAATCTTGATCTTCTT 85659
Coquerel'sSifaka TTAATATCATTCACATAGCTTTGAGGGTTTATCAAAAATTATTCTTTTCAAAATTCACTGTTCAAAATCTTGATCTTCTG 71816
Ma'sNightMonkey  TTAATATCATTCACATAGCTTTGAGGGTTTATCAAAAATTATTCTTTTCAAAATTCACTGTTCAAAATCTTGATCTTCTT 80250
VervetAGM        TTAATATCATTCACATAGCTTTGAGGGTTTATCAAAAATTATTCTTTTCAAAATTCACTGTTCAAAATCTTGATCTTCTT 86501
Gorrilla         TTAATATCATTCACATAGCTTTGAGGGTTTATCAAAAATTATTCTTTTCAAAATTCACTGTTCAAAATCTTGATCTTCTT 83909
GrabEatingMacaqueTTAATATCATTCACATAGCTTTGAGGGTTTATCAAAAATTATTCTTTTCAAAATTCACTGTTCAAAATCTTGATCTTCTT 85947
Macaque          TTAATATCATTCACATAGCTTTGAGGGTTTATCAAAAATTATTCTTTTCAAAATTCACTGTTCAAAATCTTGATCTTCTT 86836
PigTailedMacaque TTAATATCATTCACATAGCTTTGAGGGTTTATCAAAAATTATTCTTTTCAAAATTCACTGTTCAAAATCTTGATCTTCTT 85393
Bonobo           TTAATATCATTCACATAGCTTTGAGGGTTTATCAAAAATTATTCTTTTCAAAATTCACTGTTCAAAATCTTGATCTTCTT 84497
OliveBaboon      TTAATATCATTCACATAGCTTTGAGGGTTTATCAAAAATTATTCTTTTCAAAATTCACTGTTCAAAATCTTGATCTTCTT 85289
Orangutan        TTAATATCATTCACATAGCTTTGAGGGTTTATCAAAAATTATTCTTTTCAAAATTCACTGTTCAAAATCTTGATCTTCTT 86129

Majority         TATTCATTTGTGAGAATGATGAGATTGAATGAGTGATCATGTTGATGTCTGAATGTTTCATTGATATGTCAGAAAGATAA

                         102410    102420    102430    102440    102450    102460    102470    102480

Human            TATTCATTTGTGAGAATGATGAGATTGA----GTGATCATGTTGATGTCTGAATGTTTCATTGATATGTCAGAAAGATAA 85735
Coquerel'sSifaka TATTCATTTGTGAGAATGATGAGATTGAATGAGTGATCATGTTGATGTCTGAATGTTTCATTGATATGTCAGAAAGATAA 71896
Ma'sNightMonkey  TATTCATTTGTGAGAATGATGAGATTGAATGAGTGATCATGTTGATGTCTGAATGTTTCATTGATATGTCAGAAAAATAA 80330
VervetAGM        TATTCATTTGTGAGAATGATGAGATTGAATGAGTGATCATGTTGATGTCTGAATGTTTCATTGATATGTCAGAAAGATAA 86581
Gorrilla         TATTCATTTGTGAGAATGATGAGATTGA----GTGATCATGTTGATGTCTGAATGTTTCATTGATATGTCAGAAAGATAA 83985
GrabEatingMacaqueTATTCATTTGTGAGAATGATGAGATTGAATGAGTGATCATGTTGATGTCTGAATGTTTCATTGATATGTCAGAAAGATAA 86027
Macaque          TATTCATTTGTGAGAATGATGAGATTGAATGAGTGATCATGTTGATGTCTGAATGTTTCATTGATATGTCAGAAAGATAA 86916
PigTailedMacaque TATTCATTTGTGAGAATGATGAGATTGAATGAGTGATCATGTTGATGTCTGAATGTTTCATTGATATGTCAGAAAGATAA 85473
Bonobo           TATTCATTTGTGAGAATGATGAGATTGA----GTGATCATGTTGATGTCTGAATGTTTCATTGATATGTCAGAAAGATAA 84573
OliveBaboon      TATTCATTTGTGAGAATGATGAGATTGAATGAGTGATCATGTTGATGTCTGAATGTTTCATTGATATGTCAGAAAGATAA 85369
Orangutan        TATTCATTTGTGAGAATGATGAGATTGA----GTGATCATGTTGATGTCTGAATGTTTCATTGATATGTCAGAAAGATAA 86205
```

```
Majority        TCTTTAGGTGACTTTCACATAAAATTAATTCCATTTTTGGATTACCTCTCTGTGGCTCCAAAAAAGGTGTTTATGGAGAT

                        102490    102500    102510    102520    102530    102540    102550    102560

Human           TCTTTAGGTGACTTCCACATAAAATTAATTCCATTTTTGGATTACCTCTCTGTGGCTCCAAAAAAGGTGTTTATGGAGAT 85815
Coquerel'sSifaka TCTTTAGCTGACTTGCGCATAAAATTAATTCCATTTTTGGATTACCTCTCTGTGACTCCAAAAAAGGTGTTTATGGAGAT 71976
Ma'sNightMonkey TCTTTAGGTGACTTTCACATAAAATTAATTCCGTTTTTGGATTACCTCTCTGTGACTCCAAAAAAGGTGTTTATGGAGAT 80410
VervetAGM       TCTTTAGGTGACTTTCACATAAAATTAATTCCATTTTTGGATTACCTCTCTGTGGCTCCAAAAAAGGTGTTTATGGAGAT 86661
Gorrilla        TCTTTAGGTGACTTCCACATAAAATTAATTCCATTTTTGGATTACCTCTCTGTGGCTCCAAAAAAGGTGTTTATGGAGAT 84065
GrabEatingMacaqueTCTTTAGGTGACTTTCACATAAAATTAATTCCATTTTTGGATTACCTCTCTGTGGCTCCAAAAAAGGTGTTTATGGAGAT 86107
Macaque         TCTTTAGGTGACTTTCACATAAAATTAATTCCATTTTTGGATTACCTCTCTGTGGCTCCAAAAAAGGTGTTTATGGAGAT 86996
PigTailedMacaque TCTTTAGGTGACTTTCACATAAAATTAATTCCATTTTTGGATTACCTCTCTGTGGCTCCAAAAAAGGTGTTTATGGAGAT 85553
Bonobo          TCTTTAGGTGACTTCCACATAAAATTAATTCCATTTTTGGATTACCTCTCTGTGGCTCCAAAAAAGGTGTTTATGGAGAT 84653
OliveBaboon     TCTTTAGGTGACTTTCACATAAAATTAATTCCATTTTTGGATTACCTCTCTGTGGCTCCAAAAAAGGTGTTTATGGAGAT 85449
Orangutan       TCTTTAGGTGACTTTCACGTAAAATTAATTCCATTTTTGGATTACCTCTCTGTGGCTCCAAAAAAGGTGTTTATGGAGAT 86285

Majority        GTGCAGCGTGTGAAGATTTTGTACAATAAGAAAGACAGCGCTCTAATACAGATGGCTGATGGAAACCAGTCACAACTTGG

                        102570    102580    102590    102600    102610    102620    102630    102640

Human           GTGCAGCGTGTGAAGATTTTATACAATAAGAAAGACAGCGCTCTAATACAGATGGCTGATGGAAACCAATCACAACTTGG 85895
Coquerel'sSifaka GTGCAGCGTGTGAAGATTTTGTACAATAAGAAAGACAGTGCTCTAATACAGATGGCTGATGGAAACCAATCACAACTTGG 72056
Ma'sNightMonkey GTGCAGCGTGTGAAGATTTTGTACAATAAGAAAGACAGTGCTCTAATACAGATGGCTGATGGAAACCAGTCGCAACTTGG 80490
VervetAGM       GTGCAGCGTGTGAAGATTTTGTACAATAAGAAAGACAGCGCTCTAATACAGATGGCTGATGGAAACCAGTCACAACTTGG 86741
Gorrilla        GTGCAGCGTGTGAAGATTTTGTACAATAAGAAAGACAGCGCTCTAATACAGATGGCTGATGGAAACCAATCACAACTTGG 84145
GrabEatingMacaqueGTGCAGCGTGTGAAGATTTTGTACAATAAGAAAGACAGCGCTCTAATACAGATGGCTGATGGAAACCAGTCACAACTTGG 86187
Macaque         GTGCAGCGTGTGAAGATTTTGTACAATAAGAAAGACAGCGCTCTAATACAGATGGCTGATGGAAACCAGTCACAACTTGG 87076
PigTailedMacaque GTGCAGCGTGTGAAGATTTTGTACAATAAGAAAGACAGCGCTCTAATACAGATGGCTGATGGAAACCAGTCACAACTTGG 85633
Bonobo          GTGCAGCGTGTGAAGATTTTGTACAATAAGAAAGACAGCGCTCTAATACAGATGGCTGATGGAAACCAATCACAACTTGG 84733
OliveBaboon     GTGCAGCGTGTGAAGATTTTGTACAATAAGAAAGACAGCGCTCTAATACAGATGGCTGATGGAAACCAGTCACAACTTGG 85529
Orangutan       GTGCAGCGTGTGAAGATTTTGTACAATAAGAAAGACAGCGCTCTAATACAGATGGCTGATGGAAACCAATCACAACTTGG 86365

Majority        TAAGATTAAACTATGTTTTATCTATACATCTTCACTTCTGCTTTCAAATGCATA--ATGTGAATCTGTGAATAAAAATAA

                        102650    102660    102670    102680    102690    102700    102710    102720

Human           TAAGATTAAACTATGTTTTATCTATACATCTTCACTTCTGCTTTCAAATGCATA--ATGTGAATGTGCGAATAAAAATAA 85973
Coquerel'sSifaka TAAGACTAAACTATGTGTTATCTATACATCTTCAGTTCTGATTTCAAATACA-----T--AGTCC-----------ATAG 72118
Ma'sNightMonkey TAAGATTAAAT--TGTTTTATCTATACATCTTTAGTTCTGCTTTCAAATGCATAAAATGTGAATCTGCAAATAAAAATAA 80568
VervetAGM       TAAGATTAAACTATGTTTCATCTGTACATCTTCACTTCTGCTTTCAAATGCATA--ATGTGAATCTGTGAATAAAAATAA 86819
Gorrilla        TAAGATTAAACTATGTTTTATCTATACATCTTCACTTCTGCTTTCAAATGCATA--ATGTGAATCTGCGAATAAAAATAA 84223
GrabEatingMacaqueTAAGATTAAACTATGTTTCATCTGTACATCTTCACTTCTGCTTTCAAATGCGT---ATGTGAATCTGTGAATAAAAATAA 86264
Macaque         TAAGATTAAACTATGTTTCATCTGTACATCTTCACTTCTGCTTTCAAATGCGT---ATGTGAATCTGTGAATAAAAATAA 87153
PigTailedMacaque TAAGATTAAACTATGTTTCATCTGTACATCTTCACTTCTGCTTTCAAATGCGT---ATGTGAATCTGTGAATAAAAATAA 85710
Bonobo          TAAGATTAAACTATGTTTTATCTATACATCTTCACTTCTGCTTTCAAATGCATA--ATGTGAATCTGCGAATAAAAATAA 84811
OliveBaboon     TAAGATTAAACTATGTTTCATCTGTACGTCTTCACTTCTGCTTTCAAATGCA----ATGTGAATCTGTGAATAAAAATAA 85605
Orangutan       TAAGATTAAACTATATTTTATCTATACATCTTCACTTCTGCTTTCAAATGCATA--ATGTGAATCTGCGAATAAAAATAA 86443
```

```
Majority          ACTCCTTTACATCAGTAAGAAATTATTTATTTTAATGGCCAGGGCTCAAAATGTCATTTTAATGTGAATAGTTGTATGTG
                          102730    102740    102750    102760    102770    102780    102790    102800
Human             ACTCCTTTACATCAGTAAGAAATTATTT----TAATGGCCAGGGCTCAAAATGTCATTTTAATGTGAATAGTTGTATGTG 86049
Coquerel'sSifaka  AATCCTG---ATTAATAC-ACATTATTT----TAAT-------TCTCCTAAAGTCATTT-----------ATTGTA---- 72168
Ma'sNightMonkey   ACCCCTTTACATCAGTAAGAAATTATTTATTTTAATGGCCAGGGCTCAAAATGTCATTTTAATGTGAATAGTTGTATGTG 80648
VervetAGM         ACTCCTTTACATCAGTAAGAAATTATTTATTTTAATGGCCAGGGCTCAAAATGTCATTTTAATGTGAATAGTTGTATGTG 86899
Gorrilla          ACTCCTTTACATCAGTAAGAAATTATTT----TAATGGCCAGGGCTCAAAATGTCATTTTAATGTGAATAGTTGTATGTG 84299
GrabEatingMacaqueACTCCTTTACATCAGTAAGAAATTATTTATTTTAATGGCCAGGGCTCAAAATGTCATTTTAATGTGAATAGTTGTATGTG 86344
Macaque           ACTCCTTTACATCAGTAAGAAATTATTTATTTTAATGGCCAGGGCTCAAAATGTCATTTTAATGTGAATAGTTGTATGTG 87233
PigTailedMacaque  ACTCCTTTACATCAGTAAGAAATTATTTATTTTAATGGCCAGGGCTCAAAATGTCATTTTAATGTGAATAGTTGTATGTG 85790
Bonobo            ACTCCTTTACATCAGTAAGAAATTATTT----TAATGGCCAGGGCTCAAAATGTCATTTTAATGTGAATAGTTGTATGTG 84887
OliveBaboon       ACTCCTTTACATCAGTAAGAAATTATTTATTTTAATGGCCAGGGCTCAAAATGTCATTTTAATGTGAATAGTTGTATGTG 85685
Orangutan         ACTCCTTTACATCAGTAAAAAATTATTTATTTTAATGGCCAGGGCTCAAAATGTCATTTTAATGTGAATAGTTGTATGTG 86523

Majority          GTGAACATATTTGTATTTTTCTGCTATCCTCCTACTGTTTTCAAATGCACTCA-------TAATAGGAAAAGATTTTAG
                          102810    102820    102830    102840    102850    102860    102870    102880
Human             ACGAACATATTTGTATTTTTCTGCTATCCTCCTACTGTTTTCAAATGCACTCA-------TAATAGGAAGAGATTTTAG 86122
Coquerel'sSifaka  -----CGTATTTG---------GCT-----------------AA----ACTTAG------TAATGGAAAGAG------- 72199
Ma'sNightMonkey   ATGAACGTATTTGTGTTTTTCTGCTATCCTCCTCCTATTTCTCAAATGCACTCTAATGCTCTAATAGGAAAAGATTTTAG 80728
VervetAGM         GTGAACATATTTGTATTTTTCTGCTTTCCTCCTCCTGTTTTCAAATGCACTCA-------TAATAGGAAAAGATTTTAG 86972
Gorrilla          ACGAACATATTTGTATTTTTCTGCTATCCTCCTATTGTTTTCAAATGCACTCA-------TAATAGGAAGAGATTTTAG 84372
GrabEatingMacaqueGTGAACATATTTGTATTTTTCTGCTATCCTCCTACTGTTTTCAAATGCACTCA-------TAATAGGAAAAGATTTTAG 86417
Macaque           GTGAACATATTTGTATTTTTCTGCTATCCTCCTACTGTTTTCAAATGCACTCA-------TAATAGGAAAAGATTTTAG 87306
PigTailedMacaque  GTGAACATATTTGTATTTTTCTGCTATCCTCCTACTGTTTTCAAATGCACTCA-------TAATAGGAAAAGATTTTAG 85863
Bonobo            ACGAACATATTTGTATTTTTCTGCTATCCTCCTACTGTTTTCAAATGCACTCA-------TAATAGGAAGAGATTTTAG 84960
OliveBaboon       GTGAACATATTTGTATTTTTCTGCTATTCTCCTACTGTTTTCAGATGCACTCA-------TAATAGGAAAAGATTTTAG 85758
Orangutan         ATGAACATATTTGTATTTTTCTGCTATCCTCCTACTGTTTTCAAATCCACTCG-------TAATAGGAAAAGATTTTAG 86596

Majority          CTTCAAAATTGTAATAATAGTTTCACATGAGAAT-----TATTTATATTGAAGATTCATAGTGATTTTTATTCTAGCTAC
                          102890    102900    102910    102920    102930    102940    102950    102960
Human             CTTCAAAATTGTAATAATAGTTTCACATGAGAATTATATCATTTATATTGAAGATTCATAGTGATTTTTATTCTAGGTAC 86202
Coquerel'sSifaka  ---------------------T-CACATG-----------G--------------A---AG------------------- 72210
Ma'sNightMonkey   CTTTCAAATTGTAATG---GTTTCACATGAGAATTGTATCATTTATATTGAAAATCAGTAGTGATTTTTATTCCAGCTAC 80805
VervetAGM         CTTCAAAATTGTAATAATAGTTTCACATGAGAAT-----TATTTATTTTGAAGATTCATAGTGATTTTTATTCTAGCTAC 87047
Gorrilla          CTTCAAAATTGTAATAATAGTTTCACATGAGAATTATATCATTTATATTGAAGATTCATAGTGATTTTTATTCTAGCTAC 84452
GrabEatingMacaqueCTTCAAAATTGTAATAATAGTTTCACATGAGAAT-----TATTTATTTTGAAGATTCATAGTGATTTTTGTTCTAGCTAC 86492
Macaque           CTTCAAAATTGTAATAATAGTTTCACATGAGAAT-----TATTTATTTTGAAGATTCATAGTGATTTTTGTTCTAGCTAC 87381
PigTailedMacaque  CTTCAAAATTGTAATAATAGTTTCACATGAGAAT-----TATTTATTTTGAAGATTCATAGTGATTTTTGTTCTAGCTAC 85938
Bonobo            CTTCAAAATTGTAATAATAGTTTCACATGAGAATTATATCATTTATATTGAAGATTCATAGTGATTTTTATTCTAGCTAC 85040
OliveBaboon       CTTCAAAATTGTAATAATAGTTTCACATGAGAAT-----TATTT----TGAAGATTCATAGTGATTTTTGTTCTAGCTAC 85829
Orangutan         CTTCAAAATTGTAATAATAGTTTTACATGAGAAT-ATATCATTTATATTGAAGATTCATAGTGATTTTTATTCTAGCTAC 86675
```

```
Majority          TCCTTGTTAATAGAAGCTAACT----GTTCATATA-TCCATAGAATCCTTCTTAATGCCGTGTTATTTTAATTCTTGTAA

                        102970    102980    102990    103000    103010    103020    103030    103040

Human             TCCTTGTTAATAGAGGCTAACT----GTTCATACAGTCCATAGAATCCTTCTTAATGCCATGTTATTTTAATTCTTCTAA 86278
Coquerel'sSifaka  ------------------------------------------------------ATGCC------TTTTA---------- 72220
Ma'sNightMonkey   TCCTTGT-AATAGAAGCTAATTAATTGTTCATATAGTCCATAGAATCCTACCTAATGCCATGTTATTTTAATTCTTCTTA 80884
VervetAGM         TCCTTGTTAATAGAAGCTAACT----GTTCATATAGCCCATAGAATCCTCCTTAATGCCGTGTTATTTTAATTCTTGTAA 87123
Gorrilla          TCCTTGTTAATAGAGGCTAACT----GTTCATACAATCCATAGAATGCTTCTTAATGCCATGTTATTTTAATTCTTCTAA 84528
GrabEatingMacaque TCCTTGTTAATAGAAGCTAACT----GTTCATATA-CCCATAGAATCCTTCTTAATGCCGTGTTATTTTAATTCTTGTAA 86567
Macaque           TCCTTGTTAATAGAAGCTAACT----GTTCATATA-CCCATAGAATCTTTCTTAATGCCGTGTTATTTTAATTCTTGTAA 87456
PigTailedMacaque  TCCTTGTTAATAGAAGCTAACT----GTTCATATA-CCCATAGAATCCTTCTTAATGCCGTGTTATTTTAATTCTTGTAA 86013
Bonobo            TCCTTGTTAATAGAGGCTAACT----GTTCATACAGTCCATAGAATCCTTCTTAATGCCATGTTATTTTAATTCTTCTAA 85116
OliveBaboon       TCCTTGTTAATAGAAGCTAACT----GTTCATATA-CCCATAGAATCCTTCTTAATGCCGTGTTATTTTAATTCTTGTAA 85904
Orangutan         TCCTTGTTAATAGAGGCTAACT----GTTCATATATTCCATAGAATCCTTCTTAATGCCATGTTATTTTAATTCTTCTAA 86751

Majority          AGTCTTGCGATACCAGGTTCTTACAAAATGAAAAGCTTGTGCTAAAAA-CACTGTTGTTAATCCCTTTGGGAATCTTAAA

                        103050    103060    103070    103080    103090    103100    103110    103120

Human             AGTCTTGCGATACCAGGTTCTTAGAAAATGAAAAGCTTGTGCTAAAAA-CACTGTTGTTAATCCCTTTGGGAATCTTAAA 86357
Coquerel'sSifaka  ------GAGATACCAAGTTCTTACAAAATGAAAAACCTGTGCCAAAAG-CACTGTTGTTAATCCCTTTGGGAATCTTAAG 72293
Ma'sNightMonkey   AGTCTTGAGATACCAGGTTTTTACAAAATGAAAATCTGTGCTAAAAAACACTGTTGTTAATCCCTTTGGGAATCTTAAA 80964
VervetAGM         AGTCTTGCGATACCAGGTT--TACAAAATGAAAAGCT-GTGCTAAAAA-CACTGTTGTTAATCACTTTGGGAATCTTAAA 87199
Gorrilla          AGTCTTGCGATACCAGGTTCTTAGAAAATGAAAAGTTTGTGCTAAAAG-CACTGTTGTTAATCCCTTTGGGAATCTTAAA 84607
GrabEatingMacaque AGTCTTGCGATACCAGGTT--TACAAAATGAAAAGCTTGTGCTAAAAA-CACTGTTGTTCATCACTTTGGGAATCTTAAA 86644
Macaque           AGTCTTGCGATACCAGGTT--TACAAAATGAAAAGCTTGTGCTAAAAA-CACTGTTGTTAATCACTTTGGGAATCTTAAA 87533
PigTailedMacaque  AGTCTTGCGATACCAGGTT--TACAAAATGAAAAGCTTGTGCTAAAAA-CACTGTTGTTAATCACTTTGGGAATCTTAAA 86090
Bonobo            AGGCTTGCGATACCAGGTTCTTAGAAGATGAAAAGCTTGTGCTAAAAA-CACTGTTGTTAATCCCTTTGGGAATCTTAAA 85195
OliveBaboon       AGTCTTGCGATACCAGGTT--TACAAAATGAAAAGCTTGTGCTAAAAA-CACTGTTGTTAATCACTTTGGGAATCTTAAA 85981
Orangutan         AGTCTTGCGATACCAGGTTCTTACAAAATGAAAAGCTTGTGCTAAAAA-CACTGTTGTTAATCCCTTTGGGAATCTTAAA 86830

Majority          AGGTACTTTAGTCAAGTTACACTTTATATCATTACTTAAATAAGTAATTGATTATTTTAATTAAACTTA--TGTCATTTG

                        103130    103140    103150    103160    103170    103180    103190    103200

Human             AGGCACTTTAGTCAGGTTACACTTTATATCATTACTTAAATAAGTAATTGATTATTTTAATTAAACTTA--TGTCGTTTG 86435
Coquerel'sSifaka  ATGTACTTGAGTCAAGTTACACTTAA-ATCATTAGTTAGATAAATAATTGGTGATTTTAATTAAACTTACATCTCATTTG 72372
Ma'sNightMonkey   AGGTACTGTATTTAAGTTACGCTTTATTTCATTACTTAGATAAATAATTGGTTATTTTAATTAAACTTATATGTCATTTG 81044
VervetAGM         AGGTAGTTTAGTCAAGTTACCCTTTATATCATTACTTAAATAAGTAATTGATTATTTTAATTAAACTTA--TGTCATTTG 87277
Gorrilla          AGGTACTTTAGTCAGGTTACACTTTATATCATTACTTAAATAAGTAATTGATTATTTTAATTAAACTTA--TGTCATTTG 84685
GrabEatingMacaque AGGTAGTTTAGTCAAGTTACACTTTATATCATTACTTAAATAAGTAATTGATTATTTTAATTAAACTTA--TGTCATTTG 86722
Macaque           AGGTAGTTTAGTCAAGTTACACTTTATATCATTACTTAAATAAGTAATTGATTATTTTAATTAAACTTA--TGTCATTTG 87611
PigTailedMacaque  AGGTAGTTTAGTCAAGTTACACTTTATATCATTACTTAAATAAGTAATTGATTATTTTAATTAAACTTA--TGTCATTTG 86168
Bonobo            AGGCACTTTAGTCAGGTTACACTTTATATCATTACTTAAATAAGTAATTGATTATTTTAATTAAACTTA--TGTCGTTTG 85273
OliveBaboon       AGGTAGTTTAGTCAAGTTACACTTTATATCATTACTTAAATAAGTAATTGATTATTTTAATTAAACTTA--TGTCATTTG 86059
Orangutan         AGGTACTTTAGTCAAGTTACACTTTGTATCATTACTTAAATAAGTAATTGATTATTTTAATTAAACTTAA-TGTCATTTG 86909
```

```
Majority        ACTTCATACATAGTCGAAT-ACATAAATCTAGAACACACCTGCACATTCACACCAGTGATTGACTGTAAATGCCAGGATG
                        103210    103220    103230    103240    103250    103260    103270    103280
Human           ACTTCATACATCCTCGAAT-ACATAAATCTAGAACACACCTGCACATTCACACCAGTGATTGACTGTAAATGCCAGGATG 86514
Coquerel'sSifaka ACTTCATACACAGTCATGTCACATAAATCTGGAATATAACTGCATA----CAGTAGTGATTGACTATAAATATTAGTATG 72448
Ma'sNightMonkey GCTTCGTACATAGTCAAAT-ACGTAAATCTAAAACACACCTGCACATTCACACTAGTGATTGACCGTAAGTGCCAGGATG 81123
VervetAGM       ACTTCATACATAGTCGAAT-ACATAAATCTAGAACACACCTGCACATTCACACCAGTGATTGACTGTAAATGCCAGGATG 87356
Gorrilla        ACTTCATACATACTCGAAT-ACATAAATCTAGAACACACCTGCACATTCACACCAGTGATTGACTGTAAATGCCAGGATG 84764
GrabEatingMacaqueACTTCATACATAGTTGAAT-ACATAAATCTAGAACACACCTGCACATTCACACCAGTGATTGACTGTAAATGCCAGGATG 86801
Macaque         ACTTCATACATAGTTGAAT-ACATAAATCTAGAACACACCTGCACATTCACACCAGTGATTGACTGTAAATGCCAGGATG 87690
PigTailedMacaque ACTTCATACATAGTTGAAT-ACATAAATCTAGAACACACCTGCACATTCACACCAGTGATTGACTGTAAATGCCAGGATG 86247
Bonobo          ACTTCATACATACTTGAAT-ACATAAATCTAGAACACACCTGCACATTCACACCAGTGATTGACTGTAAATGCCAGGATG 85352
OliveBaboon     ACTTCATACATAGTCGAAT-ACATAAATCTAGAACACACCTGCACATTCACACCAGTGATTGACTGTAAATGCCAGGATG 86138
Orangutan       ACTTCATACATAGTCGAAT-ACATAAATCTAGAACACACCTGCACATTCACACCAGTGATTGACTGTAAATGCCAGGATG 86988

Majority        TTC-----TTACAGTTCTGTTCATTGAGATTCAGGGATAATGGTGCCTCAGACTGATTGTATTCACATTGTTAATTTGTA
                        103290    103300    103310    103320    103330    103340    103350    103360
Human           TTC-----TTACATTTCTGTTCATTGAGATTCAGGGATAATGGTGCCTCAGACTGATTGTATTCACATTGTTAATTTGTA 86589
Coquerel'sSifaka TTCATCACTTACAATTCTGTTCATTGAGATT-AGGGATGACGGTGCCTTAGAC----TGTATTTACATTGTTAATTTTGA 72523
Ma'sNightMonkey TTC-----TTA-GGTTTTGTTCATTGAGATTCAAGGATAGTGGTACCTCAGACTGATTGTATTCACATTGTTAATTTGTA 81197
VervetAGM       TTC-----TTACAGCTCTGTTCATTGAGATTCAGGGATAATGGTGCCTCAGACTAATTGTATTCACATTGTTAATTTGTA 87431
Gorrilla        TTC-----TTACATTTCTGTTCATTGAGATTCAGGGATAATGGTGCCTCAGACTGATTGTATTCACATTGTTAATTTGTA 84839
GrabEatingMacaqueTTC-----TTACAGCTCTGTTCATTGAGATTCAGGGATAATGGTGCCTCAGACTGATTGTATTCACATTGTTAATTTGTA 86876
Macaque         TTC-----TTACAGCTCTGTTCATTGAGATTCAGGGATAATGGTGCCTCAGACTGATTGTATTCACATTGTTAATTTGTA 87765
PigTailedMacaque TTC-----TTACAGCTCTGTTCATTGAGATTCAGGGATAATGGTGCCTCAGACTGATTGTATTCACATTGTTAATTTATA 86322
Bonobo          TTC-----TTACATTTCTGTTCATTGAGATTCAGGGATAATGGTGCCTCAGACTGATTGTATTCACATTGTTAATTTGTA 85427
OliveBaboon     TTC-----TTACAGCTCTGTTCGTTGAGATTCAGGGATAATGGTGCCTCAGACTGATTGTATTCACATTGTTAATTTGTA 86213
Orangutan       TTC-----TTACAGTTCTGTTCATTGAGATTCAGGGATAATGGTGCCTCAGACTGATTGTATTCACATTGTTAATTTGTA 87063

Majority        AATTTGCCATTTTTGATGTAAGGACGAGTGTATTTTTGCTTTTATATCCC--T---GAGACAGAGATGAACAACTTGTTA
                        103370    103380    103390    103400    103410    103420    103430    103440
Human           AATTTGCCATTTTTGATGTAAGGACGAGTGTATTTTTGC--T------------------CAGAGATGAACAACTTGCTA 86649
Coquerel'sSifaka AATTTGCCATTTTTGATGTAAGAACTAGTATATTTTTGCTGTTATAT-------GAAACTCAGAGATGAACAACGTGATA 72596
Ma'sNightMonkey AATTTGCCATTTTTGATGTAAGGACGAGTGTATTTCTGCTTTTATATCCCTATTGAGACTCAGAGATGAACAACTTATTA 81277
VervetAGM       AATTTGCCATTTTTGATGTTAGGACGAGTGTATTTTTGCTTTTATATCCCTCT---GAGACTCAGATGAACAACTTGTTA 87508
Gorrilla        AATTTGCCATTTTTGATGTAAGGACGAGCGTATTTTTGC--T------------------CAGAGATGAACAACTTGCTA 84899
GrabEatingMacaqueAATTTGCCATTTTTGATGTTAGGACGAGTGTATCTTTGCTTTTATATCCCCGT---GAGACTCAGATGAACAACTTGTTA 86953
Macaque         AATTTGCCATTTTTGATGTTAGGACGAGTGTATCTTTGCTTTTATATCCCCGT---GAGACTCAGATGAACAACTTGTTA 87842
PigTailedMacaque AATTTGCCATTTTTGATGTTAGGACGAGTGTATTTTTGCTTTTATATCCCTGT---GAGACTCAGATGAACAACTTGTTA 86399
Bonobo          AATTTGCCATTTTTGATGTAAGGACGAGTGTATTTTTGC--T------------------CAGAGATGAACAACTTGCTA 85487
OliveBaboon     AATTTGCCATTTTTGATGTTAGGACGAGTGTATTTTTGCTTTTATATCCCTGT---GAGACTCAGATGAACAACTTGTTA 86290
Orangutan       AATTTGCCATTTTTGATGTAAGGACGAGTGTATTTTTGC--T------------------CAGAGATGAACAACTTGCTA 87123
```

```
Majority          TACTAGACCAATTTTGGCCTTTGGCATCACATAGAAAATTTTGAATTTCAAACACATAATAGGTTTGAATTTTAGTGTTT

                          103450    103460    103470    103480    103490    103500    103510    103520

Human             TATTAGACCAATTTTGGCCTTTGACATCACATAGAAAATTTTGAATTTCAAACACATAATAGGTTTGAATTTTAGTGTTT 86729
Coquerel'sSifaka  TACTAGACCACCTTTGGCCTTTGGAATCAT----AAAAGTTGGAATTTCAAAC----AGTAAGTTTAAACTTTAGTGTT- 72667
Ma'sNightMonkey   TACTAGACCAATTTTGGTCTTTGGCATCACATAGAAAATTTTGAATTTCAAACAGAAAGTAAGTTTGAAGTTTAGTGTTC 81357
VervetAGM         -GCTAGACCAATTTTGGCCTTTGGCATCACATAGAAAATTTTGAATTTCAAACACATAATAGGTTTGAATTTTAGTGTTT 87587
Gorrilla          TACTAGACCAATTTTGGCCTTTGACATCACATAGAAAATTTTGAATTTCAAACACATAATAGGTTTGAATTTTAGTGTTT 84979
GrabEatingMacaqueCACTAGACTAATTTTGGCCTTTGGCATCACGTAGAAAATTTTGAATTTCAAACACATAATAGGTTTGAATTTTAGTGTTT 87033
Macaque           CACTAGACCAATTTTGGCCTTTGGCATCACGTAGAAAATTTTGAATTTCAAACACATAATAGGTTTGAATTTTAGTGTTT 87922
PigTailedMacaque  CACTAGACCAATTTTGGCCTTTGGCATCACGTAGAAAATTTTGAATTTCAAACACATAATAGGTTTGAATTTTAGTGTTT 86479
Bonobo            TACTAGACCAATTTTGGCCTTTGACATCACATAGAAAATTTTGAATTTCAAACACATAATAGGTTTGAATTTTAGTGTTT 85567
OliveBaboon       CGCTAGACCAATTTTGGCCTTTGGCATCACGTAGAAAATTTTGAATTTCAAACACATAATAGGTTTGAATTTTAGTGTTT 86370
Orangutan         TACTAGACCAATTTTGGCCTTTGACATCACATAGAAAATTTTGAATTTCAAACACATAATAGGTTTGAATTTTAGTGTTT 87203
```

```
Majority          CTTGGCAATAGTCTTAGCAACTCAGATGAGGTATAAAACTATCCATCTGGCTCTCCATTTTTAAAATGGTGGTATGTGTC

                          103530    103540    103550    103560    103570    103580    103590    103600

Human             CTTGGCAATAGTCTTAGCAACTCAGATGAGGTATAAAACTATCCATCTGGCTGTCCATTTTTAAAATGGTGGTATGTGTT 86809
Coquerel'sSifaka  CTTGGCAATGATCTTAGCAACTCAGACAAGGCATGAAACTATTCATCTGGTTCTCCATTTTTATAATGGTGATACCTATT 72747
Ma'sNightMonkey   CTTTCCAATAGTCTTAGAAGCTCAGATGAGGTATAAAGCTATCCATCTGGCTCTTCATTTTTAAAATGGTGGTACGTGTC 81437
VervetAGM         CTTGGCAATACTCTTAGCAACTCAGATGAGGCATAAAACTATCCATCTGGCTCTCCATTTTTAAAATGGTGGTATGTGTC 87667
Gorrilla          CTTGGCAATAGTCTTAGCAACTCAGATGAGGT--A----------TCTGGCTGTCCATTTTTAAAATGGTGGTATGTGTT 85047
GrabEatingMacaqueCTTGGCAATACTCTTAGCAACTCAGATGAGGTATAAAACTATCCATCTGGCTCTCCATTTTTAAAATGGTGGTATGTGTC 87113
Macaque           CTTGGCAATACTCTTAGCAACTCAGATGAGGTATAAAACTATCCATCTGGCTCTCCATTTTTAAAATGGTGGTATGTGTC 88002
PigTailedMacaque  CTTGGCAATACTCTTAGCAACTCAGATGAGGTATAAAACTATCCATCTGGCTCTCCATTTTTAAAATGGTGGTATGTGTC 86559
Bonobo            CTTGGCAATAGTCTTAGCAACTCAGATCAGGTATAAAACTATCCATCTGGCTGTCCATTTTTAAAATGGTGGTATGTGTT 85647
OliveBaboon       CTTGGCAATACTCTTAGCAACTCAGATGAGGTATAAAACTATCCATCTGGCTCTCCATTTTTAAAATGGTGGTATGTGTC 86450
Orangutan         CTTGGCAATAGTCTTAGCAACTCAGATGAGGTATAAAACTATCCATCTGGCTGTCCATTTTTAAAATGGTGGTATGTGTC 87283
```

```
Majority          TTAAAGAGGTATGAAAGTGCTGATTATGTCATGATCCTTAGTATATATTTTCCTCT------GTGTGCACTTCTGTACTC

                          103610    103620    103630    103640    103650    103660    103670    103680

Human             TTAAAGAGATACGAAAGTGCTGATTATGTCAAGATACTTAGTACATATTTTCCTCTTTCTCTGTGTGCACTTCTGTACTC 86889
Coquerel'sSifaka  TTAAA------TGAAAG----------------------------ACAT--GTA-------------A----------- 72766
Ma'sNightMonkey   TTAAAGAGATCTGAAAGTTCTGATCATTTCAAGATACTTAGTATATATTTTCCTCTTTCTCTGTGCACACTTCTGTACTC 81517
VervetAGM         TTACAGAGGTATGAAAGTGCTGATTATGTCATGATCCTTAGTATATATTTTCCTCT------GTGTGCACTTCTGTACTC 87741
Gorrilla          TTAAAGAGATATGAAAGTGCTGATTATGTCAAGATACTTAGTACATATTTTCCTCTTTCTCTGTGTGCACTTCTGTACTC 85127
GrabEatingMacaqueTTACAGAGGTATGAAAGTGCTGATTATGTCATGATCCTTAGTATATATTTTCCTCT------GTGTGCACTTCTGTACTC 87187
Macaque           TTACAGAGGTATGAAAGTGCTGATTATGTCATGATCCTTAGTATATATTTTCCTCT------GTGTGCACTTCTGTACTC 88076
PigTailedMacaque  TTACAGAGGTATGAAAGTGCTGATTATGTCATGATCCTTAGTATATATTTTCCTCT------GTGTGCACTTCTGTACTC 86633
Bonobo            TTAAAGAGATATGAAAGTGCTGATTATGTCAAGATACTTAGTACATATTTTCCTCTTTCTCTGTGTGCACTTCTGTACTC 85727
OliveBaboon       TTATAGAGGTATGAAAGTGCTGATTATGTCATGATCCTTAGTATATATTTTCCTCT------GTGTGCACTTCTGTACTC 86524
Orangutan         TTAAAGAGATATGAAAGTGCTGATTATGTCAAGATACTTAGTACATATTTTCCTCTTTCTCTGTGTGCACTTCTGTACTC 87363
```

```
Majority          TGGTTGGAATAGTCATTGCTGTTGCTTTCATATATGTTGTAAGTAGC-ACTTGACAGTACTTCATAATCA---GAGTGTT

                        103690    103700    103710    103720    103730    103740    103750    103760

Human             TGGTTGGAATAGTCATTGCTGTTGTTTTCATATATGTTGTAAGTAGC-ACTTAACAGTACTTCATAATAG---GAGTATT 86965
Coquerel'sSifaka  ------GACTGATGATA--------TGTCATATACATTGTAAGTAACCATTTGACAGTGCTTCATAGTCAACAGAACATT 72832
Ma'sNightMonkey   TGGTGGGAATAGTCATTACTCTTGTTTTTATATATATTGTAAGTAGC-ATTTGACAATACTTCATAGTGA---GAGTATT 81593
VervetAGM         TGGTTGGAATAGTAATTGCTGTTGCTTTCATACATGTTGTAAGTAGC-ACTTGGCAGTACTTCATAATCA---GAGTGTT 87817
Gorrilla          TGGTTGGAATAGTCATTGCTGTTGTTTTCATATATGTTGTAAGTAGC-ACTTAACAGTACTTCATAATAG---GAGTATT 85203
GrabEatingMacaqueTGGTTGGAATAGTAATTGCTGTTGCTTTCATACATGTTGTAAGTAGC-ACTTGGCAGTACTTCATAATCA---GAGTGTT 87263
Macaque           TGGTTGGAATAGTAATTGCTGTTGCTTTCATACATGTTGTAAGTAGC-ACTTGGCAGTACTTCATAATCA---GAGTGTT 88152
PigTailedMacaque  TGGTTGGAATAGTAATTGCTGTTGCTTTCATACATGTTGTAAGTAGC-ACTTGGCAGTACTTCATAATCA---GAGTGTT 86709
Bonobo            TGGTTGGAATAGTCATTGCTGTTGTTTTCATATATGTTGTAAGTAGC-ACTTAACAGTACTTCATAATAG---GAGTATT 85803
OliveBaboon       TGGTTGGAATAGTAATTGCTGTTGCTTTCATACATGTTGTAAGTAGC-ACTTGGCAGTACTTCATAATCA---GAGTGTT 86600
Orangutan         TGGTTGGAATAGTCATTGCTGTTGCTTTCATATATGTTGTAAGTAGC-ACTTAACAGTACTTCATAATAG---GAGTGTT 87439

Majority          CTCAAATATGTCATTTTTTTCCCTA--ATTGTTTGAGGTATGGGTGGCATTATGTGTCATTTAAATAAGGGATGCAGAGT

                        103770    103780    103790    103800    103810    103820    103830    103840

Human             CTCAAATATGTCATTTTTTTCCCTA--ATTGTTTGAGGTATGGGTGGCATT--GTGTCATTTAAATAAGGGATGCATAGT 87041
Coquerel'sSifaka  CTTAAATATGTCATTTTTTTTCTTGTTACCATTTGAGATCTGGGTGTCATTA-CCATCATATAAATAATGGATACAAAAT 72911
Ma'sNightMonkey   CTCAAATGTGTCATTTTTT-CCCTA--ATTGTTTGAGTTATGGGTGGCATTATGTGTCATTTAAATAAGGGATACAGAGT 81670
VervetAGM         CTCAAATATGTCATTTTTTCCCCTA--ATTGTTTGAGGTATGGGTGGCATTGTGTGTCATTTAAATAAAGGATGCAGAGT 87895
Gorrilla          CTCAAATATGTCATTTTTTTCCCTA--ATTGTTTGAGGTATGGGTGGCATT--GTGTCATTTAAATAAGGGATGCATAGT 85279
GrabEatingMacaqueCTCAAATATGTCATTTTTTCCCCTA--ATTGTTTGAGGTATGGGTGGCATTATGTGTCATTTAAATTAAGGATGCAGAGT 87341
Macaque           CTCAAATATGTCATTTTTTCCCCTA--ATTGTTTGAGGTATGGGTGGCATTATGTGTCATTTAAATTAAGGATGCAGAGT 88230
PigTailedMacaque  CTCAAATATGTCATTTTTTCCCCTA--ATTGTTTGAGGTATGGGTGGCATTATGTGTCATTTAAATAAAGGATGCAGAGT 86787
Bonobo            CTCAAATATGTCATTTTTTTCCCTA--ATTGTTTGAGGTATGGGTGGCATT--GTGTCATTTAAATAAGGGATGCATAGT 85879
OliveBaboon       CTCAAATATGTCATTTTTTCCCCTA--ATTGTTTGAGGTATGGGTGGCATTGTGTGTCATTTAAATAAAGGATGCAGAGT 86678
Orangutan         CTCAAATATGTCATTTTTTTCCCTA--ATTGTTTGAGGTATGGGTGGCATT--GTGTCATTTAAATAAGGAGTGCATAGT 87515

Majority          CTTTATGGTTGATAATTACCTGTGGTAGAATTACCTCAGAT-CTTCTAGATAGTTTTT---GTTTTATGTAAAGACTGTT

                        103850    103860    103870    103880    103890    103900    103910    103920

Human             CTTTATGGTTGGTAATTACCTGTGGTAGAATTACCTCAGAT-CTTCCAGATAGTTTTT---GTTTTATGTAAAGACTGTT 87117
Coquerel'sSifaka  CATTACGGTTGAGAATTTCCTAAGATAAAAAGAAACCAAATTCCTCTGGGTAGTTTTTTTGGTTTTATGTAAAGACTATT 72991
Ma'sNightMonkey   CTTTATGGTTGATAATTACTTGTGGTAGAATTACCTCAGAT-CTTCTGGATAGTTTTT---GTTTTATGTAAAGACTGTT 81746
VervetAGM         CTTTATGGTTGATAATTACCTGTGGTAGAGTTACCTCAGAT-CTTCTAGATAGTTTTT---GTTTTATGTAAAGAGTGTT 87971
Gorrilla          CTTTATGGTTGGTAATTACCTGCAGTAGAATTACCTCAGAT-CTTCCAGATAGTTTTT---GTTTTATGTAAAGACTGTT 85355
GrabEatingMacaqueCTTTATGGTTGATAATTACCTGTGGTAGAGTTACCTCAGAT-CTTCTAGATAGTTTTT---GTTTTATGTAAAGACTGTT 87417
Macaque           CTTTATGGTTGATAATTACCTGTGGTAGAGTTACCTCAGAT-CTTCTAGATAGTTTTT---GTTTTATGTAAAGACTGTT 88306
PigTailedMacaque  CTTTATGGTTGATAATTACCTGTGGTAGAGTTACCTCAGAT-CTTCTAGATAGTTTTT---GTTTTATGTAAAGACTGTT 86863
Bonobo            CTTTATGGTTGGTAATTACCTGTGGTAGAATTACCTCAGAT-CTTCCAGATAGTTTTT---GTTTTATGTAAAGACTGTT 85955
OliveBaboon       CTTTATGGTTGATAATTACCTGTGGTAGAGTTACCTCAGAT-CTTCTAGATAGTTTTT---GTTTTATGTAAAGACTGTT 86754
Orangutan         CTTTATGGTTGATAATTACCTGTGGTAGAATTACCTCAGAT-CTTCCAGATAGATTTT---GTTTTATGTA--GACTGTT 87589
```

```
Majority          TTAAGTAA----TATTTTAATTTTT-------------TGCCATACTTGTAGTCTGCTTGGGCTGCCATAACCAAAATAC
                          103930    103940    103950    103960    103970    103980    103990    104000
Human             TTATGTAA----TATTTTAATTTTT-------------TGCCATGCTCGTAGTCTGCTTGGGCTGCTGTAACCAAAATAC 87180
Coquerel'sSifaka  TTAAATAAGTGGTATTTTAATTTTTACCATACTTGTATTAGTGTACTTGGAGTCTGCTTGGGCTGCCATAA---AAATAC 73068
Ma'sNightMonkey   TTAAGTAA----TATTTTAATTTTT-------------TGCCATACTCGTAGTCTGCTTGGGCTACCGTAACCAAAATAC 81809
VervetAGM         TTAAGTAA----TACTTTAATTTTT-------------TGCCATACTTGTAGTCTACTTGGGCTGCCATGACCAAAATAC 88034
Gorrilla          TTATGTAA----TATTTTAATTTTT-------------TGCCATACTCGTAGTCTGCTTGGGCTGCTGTAACCAAAATAC 85418
GrabEatingMacaqueCTAAGTAA----TACTTTAATTTTT-------------TGCCATACTTGTAGTCTGCTTGGGCTGCCATAACCAAAATAC 87480
Macaque           CTAAGTAA----TACTTTAATTTTT-------------TGCCATACTTGTAGTCTGCTTGGGCTGCCATAACCAAAATAC 88369
PigTailedMacaque  CTAAGTAA----TACTTTAATTTTT-------------TGCCATACTTGTAGTCTGCTTGGGCTGCCAAAATCAAAATAC 86926
Bonobo            TTATGTAA----TATTTTAATTTTT-------------TGCCATACTCGTAGTCTGCTTGGGCTGCTGTAACCAAAATAC 86018
OliveBaboon       CTAAGTAA----TACTTTAATTTTT-------------TGCCATACTTGTAGTCTGCTTGGGCTGCCATAACCAAAATAC 86817
Orangutan         TTAAGTAA----TATTTTAATTTTT-------------TGCCATACTTGTAGTCTGCTTGGGCTGCTGTAACCAAAATAC 87652

Majority          CATAGACT-GAGTAGATGAAACAACAAAAATTTATTTCCTCAAAGTTTTGAGAAATAAAACTTTGGGGTCTAGAAGTCCC
                          104010    104020    104030    104040    104050    104060    104070    104080
Human             CATAGACT-GAGTAGATGAAACAACAAAAATGTATTTCTTCAAAGTTTTGAGAAATAAAACTTTGGGGTCTAGAAGTCCC 87259
Coquerel'sSifaka  CACAGACT-GGGTGACTTAAACACCAGAAATTTATTTTATCA--GTTT------------T-----------GGAGTCTC 73122
Ma'sNightMonkey   CAAAGACTTGAGCAGATGAAACAACAAAAATTTATTTTCACAAAATTTCGAGAAATAAAACT--GGGATCTAGAAGTCCC 81887
VervetAGM         CATGGACT-GAGTAGATGAAACAACGAAAATTTATTTCCTCAAAGTTTTCAGAAATAAAACTTTGGGGTCTAGAAGTCCC 88113
Gorrilla          CATAGACT-GAGTAGATGAAACAACAAAAATGTATTTCTTCAAAGTTTTGAGAAATAAAACTTTGGGGTCTTGAAGTCCC 85497
GrabEatingMacaqueCACGGACT-GAGTAGATGAAACAACGAAAATTTATTTCCTCAAAGTTTTCAGAAATAAAACTTTGGGGTCTAGAAGTCCC 87559
Macaque           CACGGACT-GAGTAGATGAAACAACGAAAATTTATTTCCTCAAAGTTTTCAGAAATAAAACTTTGGGGTCTAGAAGTCCC 88448
PigTailedMacaque  CACGGACT-GAGTAGATGAAACAACGAAAATTTATTTCCTCAAAGTTTTCAGAAATAAAACTTTGGGGTCTAGAAGTCCC 87005
Bonobo            CATAGACT-GAGTAGATGAAACAACAAAAATGTATTTCTTCAAAGTTTTGAGAAATAAAACTTTGGGGTCAAGAAGTCCC 86097
OliveBaboon       CACGGACT-GAGTAGATGAAACAATGAAAATTTATTTCCTCAAAGTTTTCAGAAATAAAACTTTGGGGTCTAGAAGTCCC 86896
Orangutan         CATAGACT-GAGTAGATGAAACAACAAAAATGTATTTCTTCAAAGTTTTGAGAAATAAAACTTTGGGGTCTAGAAGTCCC 87731

Majority          AGATGAAACTGCTAGCAGATT--GTTTCTGGTGAGGTCTCTCTT---GGCTTGCA-GCCAACCATCTTCCCC-ATGTGTC
                          104090    104100    104110    104120    104130    104140    104150    104160
Human             AGATGAAACTGCTAGCAGATT--GTTTCTGGTGAGGTCTCTCTT---GGCTTGCA-GCCAACCATCTTCCCC-CTGTGTC 87332
Coquerel'sSifaka  AGATCAAGGTGCCAACAGATTTGGTTTCTGATGAGGGCTCTCTTCTTGGCTTGCCTACAAGCCATCTTCTCTGTTGTGTC 73202
Ma'sNightMonkey   AGATGAAACTGCCAGCAGATT--GTTTCTGGTGAGGTCTCTCTT---GGTTTGCA-ACCAGCCATCTTCCCT-TTGTCTC 81960
VervetAGM         AGATGAAACTGCTAGCAGATT--GTTTCTGGTGAGGTGTCTCTT---GACTTGCA-GCCAACCATCTTCCCC-ATGTGTC 88186
Gorrilla          AGATGAAACTGCTAGCAGATT--GTTTCTGGTGAGGTCTCTCTT---GGCTTGCA-GCCGGCCATCTTCCCC-ATGTGTC 85570
GrabEatingMacaqueAGATGAGACTGCTAGCAGATT--GTTTCTGGTGAGGTCTCTCTT---GACTTGCA-GCCAACCATCTTCCCC-AAGTGTC 87632
Macaque           AGATGAGACTGCTAGCAGATT--GTTTCTGGTGAGGTCTCTCTT---GACTTGCA-GCCAACCATCTTCCCC-AAGTGTC 88521
PigTailedMacaque  AGATGAAACTGCTAGCAGATT--GTTTCTGGTGAGGTCTCTCTT---GACTTGCA-GCCAACCATCTTCCCC-AAGTGTC 87078
Bonobo            AGATGAAACTGCTAGCAGATT--GTTTCTGGTGAGGTCTCTCTT---GGCTTGCA-GCCAACCATCTTCCCC-ATGTGTC 86170
OliveBaboon       AGATGAAACTGCTAGCGGATT--GTTTCTGGTGAGGTCTCTCTT---GACTTGCA-GCCAACCATCTTCCCC-ATGTGTC 86969
Orangutan         AGATGAAACTGCTAGCAGATT--GTTTCTGGTGAGGTCTTTCTT---GGCTTGCA-GCCAACTGTCTTCCCC-ATGTGTC 87804
```

```
Majority          CTCATGTGGCCTTTCCACTGTGCAGGCACGTTAAGAGTCTCTTCCCCTTATAAGGATACTAGTCCTGTTGAATCAGGGCT
                          104170    104180    104190    104200    104210    104220    104230    104240
Human             CTCATGTGGCCTTTCCACTGTGCAAGCACGTTAAGAGTCTCTTCCCCTTGTAAGGATCCTAGTCCTGTTGAATCAGGGCT 87412
Coquerel'sSifaka  CTCACGTGGCCTTTTTGGTG-ACAGAGATCT------CTGCCTCTTCTTACAAGGACACCAGTTCTGTTGAATTAGGACT 73275
Ma'sNightMonkey   TTCATGTGGCCTTTCCACTGTGCAAGCACATTAAGAGTCTCTTCTTCTTATAAGGACACTAGTCCTGTTGAGTCAGGGCT 82040
VervetAGM         CTCATGTGGCCTTTCCACTGTGCAGGCGTGTTAAGAGTCTCTTCCCCTTATAAGGATACTAGTCCTGTTGAATCAGGGCT 88266
Gorrilla          CTCATGTGGCCTTTCCACTGTGCAAGCACGTTAAGAGTCTCTTCCCCTTGTAAGGATCCTAGTCCTGTTGAATCAGGGCT 85650
GrabEatingMacaqueCTCATGTGGCCTTTCCACTGTGCAGGCACGTTAAGAGTCTCTTCCCCTTATAAGGATACTAGTCCTGTTGAATCAGGGCT 87712
Macaque           CTCATGTGGCCTTTCCACTGTGCAGGCACGTTAAGAGTCTCTTCCCCTTATAAGGATACTAGTCCTGTTGAATCAGGGCT 88601
PigTailedMacaque  CTCATGTGGCCTTTCCACTGTGCAGGCTCGTTAAGAGTCTCTTCCCCTTATAAGGATACTAGTCCTGTTGAATCAGGGCT 87158
Bonobo            CTCATGTGGCCTTTCCACTGTGCAAGCACGTTAAGAGTCTCTTCCCCTTGTAAGGATCCTAGTCCTGTTGAATCAGGGCT 86250
OliveBaboon       CTCATGTGGCCTTTCCACTGTGCAGGCGCGTTAAGAGTCTCTTCCCCTTATAAGGATACTAGTCCTGTTGAATCAGGGCT 87049
Orangutan         CTCATGTGGCCTTTCCACTGTGCAAGTGCATGAAGAGTCTCTTCCCCTTATAAGGATACTAGTCCTGTTGAATCAGGGCT 87884

Majority          CTACCCTTAATGACCACATTTAACCTT--AATTACCTCCTTAAAGGTCCTGTTTTTAAGTACAGTTACATT-GGGGGTTA
                          104250    104260    104270    104280    104290    104300    104310    104320
Human             TTACCCTTAATGACCACATTTAACCTT--AATTACCTCCTTAAAGGTCCTGTTTTTAAGTACAGTTACATT-GGGGGTTA 87489
Coquerel'sSifaka  CTCCCCTAA--GACTTCATTTAACCTTGAAATTACCTTCTTAAAGGTCCCATCTTTAAATACAGTTACATTTGGGAGTTA 73353
Ma'sNightMonkey   CCACCCTTAATGAGCACATTTAACCTT--AATTACCTCCTTAAAGATCTTATTTTTAAACAT---TG-----GGGGGTTA 82110
VervetAGM         CTACTCTTAATGACCACATTTA-CCTG--AATTACCTCCTTAAAGGCCCTGTTTTTAAGTACCATTACATT-GGGGGTTA 88342
Gorrilla          TTACCCTTAATGACCACATTTAACCTT--AATTACCTCCTTAAAGGTCCTGTTTTTAAGTACAGTTACATT-GGGGGTTA 85727
GrabEatingMacaqueCTACTCTTAATGACCACATTTA-CCTG--AATTACCTCCTTAAAGGCCCTGTTTTTAAGTACAGTTACATT-GGGGGTTA 87788
Macaque           CTACTCTTAATGACCACATTTA-CCTG--AATTACCTCCTTAAAGGCCCTGTTTTTAAGTACAGTTACATT-GGGGGTTA 88677
PigTailedMacaque  CTACTCTTAATGACCACATTTA-CCTG--AATTACCTCCTTAAAGGCCCTGTTTTTAAGTACAGTTACATT-GGGGGTTA 87234
Bonobo            TTACCCTTAATGACCACATTTAACCTT--AATTACCTCCTTAAAGGTCCTGTTTTTAAGTACAGTTACATT-GGGGGTTA 86327
OliveBaboon       CTACTCTTAATGACCACATTTA-CCTG--AATTACTTCCTTAAAGGCCCTGTTTTTAAGTACAGTTACATT-GGGGGTTA 87125
Orangutan         CTACCCTTAATGACCACATTTAACCTT--AATTACCTCCTTAAAGGTCCTGTTTTTAAGTACAGTTACATT-GGGGGTTA 87961

Majority          GGACTTCAACATACAAATTTTGGGAGGACACAATTCAGT---TAATAGTACTTAAATTTGTAAATCATTTATCCC-AATT
                          104330    104340    104350    104360    104370    104380    104390    104400
Human             GGACTTCAACATATAAATTTGGGGAGGACACAATTCAGT---TAATAGTACTTAAATTTGTAAATCATTTATCCCAAATT 87566
Coquerel'sSifaka  GGGCTTCAACATACGAATCTGGGGA-AACACAATTCAGTCTGTTACATTACTTATATTTGGAAATCATTTATCCCCAATT 73432
Ma'sNightMonkey   GGAGTT---CATATGAATTTTGAGAGAACAAAATTCAGT---TTATAGTGCTTATATTTGTAAATCATTTATGCCCAATT 82184
VervetAGM         GGACTTCAACATACAAATTTTGGGAGGACACAATTGAGT---TAATAGAACTTAAATTTGTAAATCATTTAT-------- 88411
Gorrilla          GGACTTCAACATATAAATTTGGGGAGGACACAATTCAGT---TAATAGTACTTAAATTTGTAAATCATTTATCCCCAATT 85804
GrabEatingMacaqueGGACTTCAACATACAAATTTTGGGAGGACACAGTTGAGT---TAATAGTACTTAAATTTGTAAATCATTTAT-------- 87857
Macaque           GGACTTCAACATACAAATTTTGGGAGGACACAGTTGAGT---TAATAGTACTTAAATTTGTAAATCATTTAT-------- 88746
PigTailedMacaque  GGACTTCAACATACAAATTTTGGGAGGACACAGTTGAGT---TAATAGTACTTAAATTTGTAAATCATTTAT-------- 87303
Bonobo            GGACTTCAACATATAAATTTGGGGAGGACACAATTCAGT---TAATAGTACTTAAATTTGTAAATCATTTATCCCAAATT 86404
OliveBaboon       GGACTTCAACATACAAATTTTGGGAGGACACAGTTGAGT---TAATAGTACTTAAATTTGTAAATCATTTAT-------- 87194
Orangutan         GTACTTAAACATATAAATTTGGGGAGGACACAATTCAGT---TAATAGTACTTAAATTTGTAAATCATTTATCCCCAATT 88038
```

```
Majority          TTATAAGATATTGTCACTAAAGTTTTGACTTGCTAGTAA---------GGCTTATAATTTTTGGACTATATGTAGTGGTA

                        104410    104420    104430    104440    104450    104460    104470    104480

Human             TTATAAGATATTGTCACTAAAGTTTTGACTTGCTAGTAG---------GGCTTATAATTTTTGGACTATCTGTAGTGGTA 87637
Coquerel'sSifaka  TCTTAAGATACTATCACTAAAGTTTTGACTTGCTATTAATAGTAGCCTGGTTTATAATTTTTGGACTTTATGTAGTGGTT 73512
Ma'sNightMonkey   TTATAAGATATTGTCACTAAAGTTTTGACTTGCTAGTAG---------ACCTTATAATTTTTTGACTATATCTAGTGGTA 82255
VervetAGM         -----------TGTCATTAAAGTTTTGACTTGCTAGGAA---------GGCTTATAATTTTTGGACTATATATAGTGGTA 88471
Gorrilla          TTATAAGATATTGTCACTAAAGTTTTGACTTGCTAGTAG---------GGCTTATAATTTTTGGACTATCTGTAGTGGTA 85875
GrabEatingMacaque-----------TGTCATTAAAGTTTTGACTTGCTAGGAA---------GGCTTATAAGTTTTGGACTATATGTAGTGGTA 87917
Macaque           -----------TGTCATTAAAGTTTTGACTTGCTAGGAA---------GGCTTATAAGTTTTGGACTATATGTAGTGGTA 88806
PigTailedMacaque  -----------TGTCATTAAAGTTTTGACTTGCTAGGAA---------GGCTTATAAGTTTTGGACTATATGTAGTGGTA 87363
Bonobo            TTATAAGATATTGTCACTAAAGTTTTGACTTGCTAGTAG---------GGCTTATAATTTTTGGACTATCTGTAGTGGTA 86475
OliveBaboon       -----------TGTCATTAAAGTTTTGACTTGCTAGGAA---------GGCTTATAATTTTTGGACTATATGTAGTGATA 87254
Orangutan         TTATAAGATATTGTCACTAAAGTTTTGACTTGCTAGTAG---------GGCTTATAATTTTTGGACTATCTGTAGTGGTA 88109

Majority          AATAATTCATCATTTCTCATTGCTGCTAGGTAGATTTT-GCTTTCTTGAACTGTGTGACCATAAAAATATGTACTGACAC

                        104490    104500    104510    104520    104530    104540    104550    104560

Human             AATAATTCATCATTTCTCATTGCTGCTAGGTAGATTTT-GCTTTCTTGAACTGTGTGACCATAAAAGTATGTACTGACAC 87716
Coquerel'sSifaka  AATAATTTATTATTTCTCATTG---CTAGGTAGATACT-GCTTTCTTGAAGTGTATGACCATAAAAATA-GTATTCACAC 73587
Ma'sNightMonkey   AATAATTCATCATTTCTCATTGCTGCTAGGTAAATTTT-GCTTTCTTAAACTGTGTGACCATAAAAATATGTACTGACAC 82334
VervetAGM         AATAATTCAT---TTCTCATTGCTGCTAGGTAGATTTTTGCTTTCGTGAACTATGTGACCATAAAAATATGTACTGACAC 88548
Gorrilla          AATAATTCATCATTTCTCATTGCTGCTAGGTAGATTTT-GCTTTCTTGAACTGTGTGACCATAAAAGTATGTACTGACAC 85954
GrabEatingMacaqueAATAATTCAT---TTCTCATTGCTGCTAGGTAGATTTTTGCTTTCGTGAACTATGTGACCATAAAAATATGTACTGACAC 87994
Macaque           AATAATTCAT---TTCTCATTGCTGCTAGGTAGATTTTTGCTTTCGTGAACTATGTGACCATAAAAATATGTACTGACAC 88883
PigTailedMacaque  AATAATTCAT---TTCTCATTGCTGCTAGGTAGATTTTTGCTTTCGTGAGCTATGTGACCATAAAAATATGTACTGACAC 87440
Bonobo            AATAATTCATCATTTCTCATTGCTGCTAGGTAGATTTT-GCTTTCTTGAACTGTGTGACCATAAAAGTATGTACTGACAC 86554
OliveBaboon       AATAATTCAT---TTCTCATTGCTGCTAGGTAGATTTTTGCTTTCGTGAACTATGTGACCATAAAAATACGTACTGACAC 87331
Orangutan         AATAATTCATCGTTTCTCATTGCTGCTAGGTAGATTCT-GCTTTCTTGAACTGTGTGACCATAAAAATACGTACTGACAC 88188

Majority          CATC--ACTTCCTTTAAAACACACACACACACACACACCCCCACA--CCCACACA--AAACTCTGTGGTGTACTATTGAG

                        104570    104580    104590    104600    104610    104620    104630    104640

Human             CATC--ATTTCCTTTAAAACACACACACACAAACACACACACACA--CACGCA--------------------------- 87765
Coquerel'sSifaka  CATCTCAGTGCCTGTAAAACACGCACATACACTCACAGTCAAAT---CCTACAGG--AAACTCTAGGATGTGGTATTGAG 73662
Ma'sNightMonkey   TATT--ACTTCCTTTAAAACACACACTCGCTCTCGCTTTCTCTCTGCCCCACACACCAAACTCTATGGTGTACCATTGAG 82412
VervetAGM         CATC--ACTTCCTTTAAAACACACCCACATACACACACCCCCACA--CCCACACA--AAACTCTGTGGTGTACTATTGAG 88622
Gorrilla          CATC--ATTTCCTTTAAAACACACACACACACACACATACATACA--CTCACACA------------------------- 86005
GrabEatingMacaqueCATC--ACTTCCTTTAAAGCACACCCACACACACACACCCCCACA--CCCACACA--AAACTCTGTGGTGTACTATTGAG 88068
Macaque           CATC--ACTTCCTTTAAAGCACACCCACACACACACACCCCCACA--CCCACACA--AAACTCTGTGGTGTACTATTGAG 88957
PigTailedMacaque  CATC--ACTTCCTTTAAAGCACACCCACACACACACACCCCCACA--CCCACACA--AACATCTGTGGTGTACTATTGAG 87514
Bonobo            CATC--ATTTCCTTTAAAACACACACACACAAACACACACACACA--C-------------------------------- 86598
OliveBaboon       CATC--ACTTCCTTTAAAACACACCCACACACACACACCCCCACA--CCCACACA--AAACTCTGTGGTGTACTATTGAG 87405
Orangutan         CATC--ACTTCCTTTAAAACACACACACACACACACACACACACT--CCCACACA--AAACTCTATGGTGTACTATTGAG 88262
```

```
Majority          AACAAATAAA-TGAGGCCAAAACATTTTT----TT-GTTTCTGAACTCTATTCTT-ATTCTTTTTGGTTTTCAAGGGGAC
                          104650    104660    104670    104680    104690    104700    104710    104720
Human             -------------------------------------------------------------------------------- 87765
Coquerel'sSifaka  AAAAATGAGACTTAAAAAAAAAAATTTTTAATTTTGGTTTTTGGACTCTGTCCTC-ATTCTTTCTGGTTTTCAT-GGGAC 73740
Ma'sNightMonkey   AACAAATAAA-GGAGGCCAAAACATTTTT----TTTGTTTCTGGACTCTGTTCTT-ATTATTTTTGGTTTTCAAAGGGAC 82486
VervetAGM         AACAAATAAA-TGAGGCCAAAACATTTTT----TT-GTTTCTGAACTCTATTCTT-ATTCTTTTTGGTTTTCAAGGGGAC 88695
Gorrilla          -------------------------------------------------------------------------------- 86005
GrabEatingMacaqueAACAAATAAA-TGAGGCCAAAACATTTTT----TT-GTTTCTGAACTCTATTCTT-ATTCTTTTTGGTTTTCAAGGGGAC 88141
Macaque           AACAAATAAA-TGAGGCCAAAACATTTTT----TT-GTTTCTGAACTCTATTCTT-ATTCTTTTTGGTTTTCAAGGGGAC 89030
PigTailedMacaque  AACAAATAAA-TGAGGCCAAAACATTTTT----TT-GTTTCTGAACTCTATTCTTTATTCTTTTTGTTTTACAAGGGGAC 87588
Bonobo            -------------------------------------------------------------------------------- 86598
OliveBaboon       AACAAATCAG-TGAGGCCAAAACATTTTT----TT-GTTTCTGAACTCTATTCTT-ATTCTTTTTGGTTTTCAAGGGGAC 87478
Orangutan         AACAAATAAA-TGAGGCCAAAACATTTTT----TTTGTTTCTGTACTCTATTCTT-ATTCTTTTTGGTTTTCAAGGGGAC 88336

Majority          TCTATTTGTGAGTATTCATTGCTTTGTATTATAGGCAGATGTCTTTATGCTTAAGAGGTAACATGGGAAAAATAAGTGGC
                          104730    104740    104750    104760    104770    104780    104790    104800
Human             -------------------------------------------------------------------------------- 87765
Coquerel'sSifaka  TCTAGTTGGAAATATTCATTGCTTTATGTTACAGGCAGATGTCTTTATGTTTAAGAGGTAGCATGGGAGAAATAAATGAC 73820
Ma'sNightMonkey   TCT--TTGTGAGTATTCATTGCTTTATATTATAGGCAGA--T-------------------------------------- 82524
VervetAGM         TCTATTTGTGAGTATTCATTGCTTTGTATTATAGGCAGATGTCTTTATGCTTAAGAGGTAACATGGGAAAAATAAGTGGC 88775
Gorrilla          -------------------------------------------------------------------------------- 86005
GrabEatingMacaqueTCTATTTGTGAGTATTCATTGCTTTGTATTATAGGCAGATGTCTTTATGCTTAAGAGGTAACATGGGAAAAATAAGTGGC 88221
Macaque           TCTATTTGTGAGTATTCATTGCTTTGTATTATAGGCAGATGTCTTTATGCTTAAGAGGTAACATGGGAAAAATAAGTGGC 89110
PigTailedMacaque  TCTATTTGTGAGTATTCATTGCTTTGTATTATAGGCAGATGTCTTTATGCTTAAGAGGTAACATGGGAAAAATAAGTGGC 87668
Bonobo            -------------------------------------------------------------------------------- 86598
OliveBaboon       TCTATTTGTGAGTATTCATTGCTTTGTATTATAGGCAGATGTCTTTATGCTTAAGAGGTAACATGGGAAAACTAAGTGGC 87558
Orangutan         TC----TGTGAGTATTCATTGCTTTGTGTTATAGGCAGTTGTCTTTATGCTTAAGAGGTAACATGGGAAAAATAAATGAC 88412

Majority          AAGGAAAATTAAGAGAG---ATACTCAGAAATGTTTAAATGTGAATGGAACTGT---AAATCTTACAGGCCATGTTAAAT
                          104810    104820    104830    104840    104850    104860    104870    104880
Human             -------------------------------------------------------------------------------- 87765
Coquerel'sSifaka  AAGGGAAACTAAGAGAGGATATAGTCAGCAGTGTTTAAATGTGAATGAGATTGTTGCAGATCTTACAGGCGATGTTAAAT 73900
Ma'sNightMonkey   ----AA--TTAAGAGA-----TACTCAGTAATGTTTAAATGTGAATGGAATTGTTACAAATCTTACAGGCCATGTTAAAT 82593
VervetAGM         AAGGAAAATTGAGAGAG---ATACTCAGAAATGTTTAAATGTGAATGGAACTGT---AAATCTTACAGGCCATGTTAAAT 88849
Gorrilla          -------------------------------------------------------------------------------- 86005
GrabEatingMacaqueAAGGAAAATTAAGAGAG---ATACTCAGAAATGTTTAAATGTGAATGGAACTGT---AAATCTTACAGGCCATGTTAAAT 88295
Macaque           AAGGAAAATTAAGAGAG---ATACTCAGAAATGTTTAAATGTGAATGGAACTGT---AAATCTTACAGGCCATGTTAAAT 89184
PigTailedMacaque  AAGGAAAATTAAGAGAG---ATACTCAGAAATGTTTAAATGTGAATGGAACTGT---AAATCTTACAGGCCATGTTAAAT 87742
Bonobo            -------------------------------------------------------------------------------- 86598
OliveBaboon       AAGGAAAATTAAGAGAG---ATACTCAGAAATGTTTAAATGTGAATGGAACTGT---AAATCTTACAGGCCATGTTAAAT 87632
Orangutan         AAGGAAAATCAAGAGA-----TACTCAGTAATGTTTAAATGTGAATGGAATTGT---AAATCTTACAGGCCATGTTAAAT 88484
```

```
Majority          TGTTTCAGACCAGAGTTTTAAAATTTGTAAACATTAAGGTGTTTTCA----AATATTAATACTCTTAGAATTTTTCTTAG

                        104890    104900    104910    104920    104930    104940    104950    104960

Human             -------------------------------------------------------------------------------- 87765
Coquerel'sSifaka  CATTTCACACCAGAATTTTATAATTTTCAAACTTTAAGATGTTTCTTTGATAAACTTTGTCCCATTAGCCTATTTAATTG 73980
Ma'sNightMonkey   TGTTTCAGACCAGAGTTCTAAAAGTTGTAAACATTAAGATGTTTTTA----AAGATTAATATTATGTAAATCATTCTTAG 82669
VervetAGM         TGTTTCAGACCAGAGTTTTAAAATTTGTAAACATTAAGGTGTTTTCA----AATATTAATACTCTTAGAATTTTTCTTAG 88925
Gorrilla          -------------------------------------------------------------------------------- 86005
GrabEatingMacaqueTGTTTCAGACCAGAGTTTTAAAATTTGTAAACATTAAGGTGTTTTCA----AATATTAATACTCTTAGAATTTTTCTTAG 88371
Macaque           TGTTTCAGACCAGAGTTTTAAAATTTGTAAACATTAAGGTGTTTTCA----AATATTAATACTCTTAGAATTTTTCTTAG 89260
PigTailedMacaque  TGTTTCAGACCAGAGTTTTAAAATTTGTAAACATTAAGGTGTTTTCA----AATATTAATACTCTTAGAATTTTTCTTAG 87818
Bonobo            -------------------------------------------------------------------------------- 86598
OliveBaboon       TGTTTCAGACCAGAGTTTTAAAGTTTGTAAACATTAAGGTGTTTTCA----AATATTAATACTCTTAGAATTTTTCTTAG 87708
Orangutan         TGTTTCAGACCAGAGTTTTAAAATTTGCAAACATGAAGATGTTTTCA----AAGATTAATACTCTTAGAATTATTCTTAG 88560

Majority          A-GTATTATTGGGTAATATTTCTTAGAGTATTACTGTATAATTTGTAAATTCTCATCTTTAAAAGTAGTATATTGATCTC

                        104970    104980    104990    105000    105010    105020    105030    105040

Human             --------------------------------------------TACACTCCCAC------------------------- 87776
Coquerel'sSifaka  ATACAGTATTTTTTCAATTGAATATTTTAAGAATAATAAAATTTGTAAATTTTCATCTT-AAAAGCAGTATACTTATCTG 74059
Ma'sNightMonkey   A-GTATTATTGGGTAATAAT------------ACTGTATAATTTGTAAATTCACATCTTTAAAGATAGTATATTGATCTC 82736
VervetAGM         A-GTATTATTGGGTAATATTTCTTAGAGTATTACTGAATAATTTGTAAATTCTCATCTTTAAAAGTAGTATATTGATCTC 89004
Gorrilla          --------------------------------------------TACACTCCCAC------------------------- 86016
GrabEatingMacaqueA-GTATTATTGGGTAATATTTCTTAGAGTATTACTGTATAATTTGTAAATTCTCATCTTTAAAAGTAGTATATTGATCTC 88450
Macaque           A-GTATTATTGGGTAATATTTCTTAGAGTATTACTGTATAATTTGTAAATTCTCATCTTTAAAAGTAGTATATTGATCTC 89339
PigTailedMacaque  A-GTATTATTGGGTAATATTTCTTAGAGTATTACTGTATAATTTGTAAATTCTCATCTTTAAAAGTAGTATATTGATCTC 87897
Bonobo            ---------------------------------------------ACACTCCCAC------------------------- 86608
OliveBaboon       A-GTATTATTGGGTAATATTTCTTAGAGTATTACTGTATAATTTGTAAATTCTCATCTTTGAAAGTAGTATATTGATCTC 87787
Orangutan         A-GTATTATTGGGTAATAAT------------ACTGTATAATTTGTAAATTCTCATCTTTAAAAGTAGTATATTGATCTC 88627

Majority          CAA-GATTAAAATAAATTCTAACAAAATGCCAA--AGTAGTTTATTCATAGTAA----AATAATTTAGTTTCTCTAGTTC

                        105050    105060    105070    105080    105090    105100    105110    105120

Human             ---------------------ACAAAAC----------------TCTATGGT---------------------------- 87791
Coquerel'sSifaka  CACAGATT--------TTCTGAAAAAAA---------TAGTTCCTTTAGGGTAAGAATAATTGTCTAGATTCTTCAATTT 74122
Ma'sNightMonkey   CAAAAATTAAAATAAATTTTAACAAAATGCTGG--AGTAGTTTGTTCATAGTAA----TACA-----GTTTCTCTAGTTC 82805
VervetAGM         CAA-GATTAAAATAAATTCTAACAAAATGCCAACTAGTAGTTTATTCATAGTAA----AATAATTTAGTTTCTCTAGTTC 89079
Gorrilla          ---------------------ACAAAAC----------------TCTATGGT---------------------------- 86031
GrabEatingMacaqueCAA-GATTAAAATAAATTCTAACGAAATGCCAACTAGTAGTTTATTCATAGTAA----AATAATTTAGTTTCTCTGGTTC 88525
Macaque           CAA-GATTAAAATAAATTCTAACGAAATGCCAACTAGTAGTTTATTCATAGTAA----AATAATTGAGTTTCTCTGGTTC 89414
PigTailedMacaque  CAA-GATTAAAATAAATTCTAACGAAATGCCAACTAGTAGTTTATTCATAGTAA----AATAATTTAGTTTCTCTAGTTC 87972
Bonobo            ---------------------ACAAAAC----------------TCTATGGT---------------------------- 86623
OliveBaboon       CAA-GATTAAAATAAATTCTAACAAAATGCCAACTAGTAGTTTATTCATAGTAA----AATAATTTAGTTTCTCTAGTTC 87862
Orangutan         CAAAGATTAAAATAAATTCTAACAAAATGCCA---AATAGTTTATTCATAGTAA----AATAATTTAGTTTCTCCAGTTC 88700
```

```
Majority          ATGTTTTCAAGAACTATTT--T---TTTAAATGCCTGCTTTTAA-TATATAGCTACCATCTCAAAAGTAAA-----CTTA
                         105130    105140    105150    105160    105170    105180    105190    105200
Human             ----------GTACTATTT---------------------------------------------AGTA----------- 87804
Coquerel'sSifaka  ATGTTTTTACCAACTGCTTATAAAATGCCTCTGTTTTTAATTAAATATATAACTGTCATCTCAGAAGTTAAACTGATTTA 74202
Ma'sNightMonkey   ATGTTTTCAAGAATTATTTTTTAAATGCCCTAGTTTTTATTTAA-TATATAGCTGACATCTCAAAAATAAA-----CTTA 82879
VervetAGM         ATGTTTTCAAGAACTATTGAAC---TATAAATGCCTGCTTTTAA-TATATAGCTACCATCTCAAAAGTAAA-----CTTA 89150
Gorrilla          ----------GTACTATTT---------------------------------------------AGTA----------- 86044
GrabEatingMacaqueATGTTTTCAAGAACTATTT--T---TTTAAATGCCTGCTTTTAA-TATATAGCTACCATCTCAAAAGTAAA-----CTTA 88594
Macaque           ATGTTTTCAAGAACTATTT--T---TTTAAATGCCTACTTTTAA-TATATAGCTACCATCTCAAAAGTAAA-----CTTA 89483
PigTailedMacaque  ATGTTTTCAAGAACTATTT--T---TTTAAATGCCTGCTTTTAA-TATATAGCTACCATCTCAAAAGTAAA-----CTTA 88041
Bonobo            ----------GTACTATTT---------------------------------------------AGTA----------- 86636
OliveBaboon       ATGTTTTCAAGAACTATTT--T---TTTAAATGCCTGCTTTTAA-TATATAGCTACCATCTCAAAAGTAAA-----CTTA 87931
Orangutan         ATGTTT-CAAGAACTATTTT-----TTTAAATGCCTGCTTTTAA-TATATAGCCGCCGTCTCAAAAGTAAA-----CTTA 88768

Majority          GTTCTTATTTATAATAAGCCTTTAC-----AAAATTATATCAAAATGTTCTGTAAAGTGAGTGGAAATCAT-----TCTG
                         105210    105220    105230    105240    105250    105260    105270    105280
Human             ------------------------------------------------------------------------------TG 87806
Coquerel'sSifaka  GTTCTTGTTCGTAACCTTTACCTACTAAATTATATC-TATAAAAGTGTTCTA-AGAATGAGTGGAAATCATATTACCCTA 74280
Ma'sNightMonkey   GTTCTTGTTCATAATAAGCCTTTACTT-ACTAAATTATATCAAAATGTTCTGTAAAGTGAGTAGAAATCATATTATTCTG 82958
VervetAGM         GTTCTTATTTATAATAAGCCTTTACTTTACAAAATTATATCAGAATGTTCTGTAAAGTGAGTGGAAATCAT-----TCTG 89225
Gorrilla          ------------------------------------------------------------------------------TG 86046
GrabEatingMacaqueGTTCTTATTTATAATAAGCCTTTAC-----AAAATTATATCAAGATGTTCTGTAAAGTGAGTGGAAATCAT-----TCTG 88664
Macaque           GTTCTTATTTATAATAAGCCTTTAC-----AAAATTATATCAAGATGTTCTGTAAAGTGAGTGGAAATCAT-----TCTG 89553
PigTailedMacaque  GTTCTTATTTATAATAAGCCTTTAC-----AAAATTATATCAAAATGTTCTGTAAAGTGAGTGGAAATCAT-----TCTG 88111
Bonobo            ------------------------------------------------------------------------------TG 86638
OliveBaboon       GTTCTTATTTATCATAAGCCTTTACTTTACAAAATTATATCAAAATATTCTATAAAGTGAGTGGAAATCAT-----TCTG 88006
Orangutan         GTTCTTGTTTATAATAAGCCTGTGCTTTACAAAATTACATCAAAATGTTCTGTAAAGTGAGTGGAAATCAT-----TCTG 88843

Majority          GTGTTATGATATTTGAACAGTTTTT--GTGTATATTGGTTTTCACTTCCACTGTTATG-AGTAATGAGTAATTGCCACAC
                         105290    105300    105310    105320    105330    105340    105350    105360
Human             GTGT---------------------------------------------------------------------------- 87810
Coquerel'sSifaka  GTGTTATGGCACTTGAGAAGTTTTTAAGCGTGTGTATGTATTCACTTTTAAGTATGTG----TATACATTATTGTCATAA 74356
Ma'sNightMonkey   TGGTTATGATATCTGAACAATTTTT--GTATATACTGGTTTTCACTTACACTATCATG-AGTAATGAGTAATTGCTACAC 83035
VervetAGM         CTGTTACGATATTTGAACAATTTTT--GTGTATATTGGTTTTCACTTCCACTGTTATG-AGTAATGAGTAATTGCCACAC 89302
Gorrilla          GTGT---------------------------------------------------------------------------- 86050
GrabEatingMacaqueCTGTTATGATATTTGAACAGTTTTT--GTGTATATTGGTTTTCACTTCCACTGTTATG-AGTAATGAGTAATTGCCACAC 88741
Macaque           CTGTTATGATATTTGAACAGTTTTT--GTGTATATTGGTTTTCACTTCCACTGTTATG-AGTAATGAGTAATTGCCACAC 89630
PigTailedMacaque  CTGTTATGATATTTGAACAGTTTTT--GTGTATATTGGTTTTCACTTCCACTGTTACG-AGTAATGAGTAATTGCCACAC 88188
Bonobo            GTGT---------------------------------------------------------------------------- 86642
OliveBaboon       CTGTTATGATATTTGAACAGTTTTT--GTGTGTATTGGTTTTCACTTCCACTGTTATG-AGTAATGAGTAATTGCCACAC 88083
Orangutan         GTGTTATGATATTTGAACAATTTTT--GTATATATTGGTTTTCACTTCCACTGTTATGGAGTAATGAGTAATTGCCACAC 88921
```

```
Majority          TTTGGGG-CATTGCTGTTTTCATAAAAGAGAGATCTTACCCTCCAAAACAAAACAAGGGAAGGAGAGGGAGACAGCAAAT

                        105370    105380    105390    105400    105410    105420    105430    105440

Human             --------------------------------------------------------------------------------- 87810
Coquerel'sSifaka GT--AGTTAGTAATAAGTCACACTTTGAAGCATTGTTGGCCTC-ACAAAAGAGAGATCTTAAGAGATCTTCCCTCCAAAA 74433
Ma'sNightMonkey  TTTGAAGCATTCCTGGTTTTCATAAAACAGAGATCTTCTCCTCCAAAACAAA-CAAAGGCAAGAGAGGGAAACAGCAAAT 83114
VervetAGM        TTTGGGGGCATTGCTGTTTTCATAAAAGAGAGATCTTACCGTCCAAAACAAAACAAGGGAAGGAGAGGGAGACAGCAAAT 89382
Gorrilla         --------------------------------------------------------------------------------- 86050
GrabEatingMacaqueTTTGGGGTCATTGCTGTTTTCATAAAAGAGAGATCTTACCCTCCAAAACAAAACAAGGGAAGGAGAGGGAGACAGCAAAT 88821
Macaque          TTTGGGGTCATTGCTGTTTTCATAAAAGAGAGATCTTACCCTCCAAAACAAAACAAGGGAAGGAGAGGGAGACAGCAAAT 89710
PigTailedMacaque TTTGGGGGCATTGCTGTTTTCATAAAAGAGAGATCTTACCCTCCAAAACAAAACAAGGGAAGGAGAGGGAGACAGCAAAT 88268
Bonobo           --------------------------------------------------------------------------------- 86642
OliveBaboon      TTTGGGGGCATTGCTGTTTTCATAAAAGAGAGATCTTACCCTCCAAAACAAAACAAGGGAAGGAGAGGGAGACAGCAAAT 88163
Orangutan        TTTGGGG-TATTGCTGTTTTCATAAAAGAGACATCTAATCCTCCAAAACAAAACAAGGGAAAGAGAGGGAGACAGCAAAT 89000

Majority          TTATATGGAGAAAAATAGGGAAAA----ATTAAAAATTATGGTAGAATCAAATTCTGATGCATCAGTAATAGTGAAAAAT

                        105450    105460    105470    105480    105490    105500    105510    105520

Human            --------------------------------------------------------------------------------- 87810
Coquerel'sSifaka ACAAAGAAGGGAAGGAGGGGAAAACAGCAAATGAAATTATGATAGAGTCAAGACCTGATGCATCAATAATAATGACAAAT 74513
Ma'sNightMonkey  T-ATACAGAGAAAAATAGGGAAAA----ATAAGAAATTATAATAGAATCAAATTCTGATGCATCAGTAATGTTGAACAAT 83189
VervetAGM        TTATATGG----------GGAAAA----ATTAAAAATTATGGTAGAATCAAATTCTGATGCATCAGTAATAGTGAAAAAT 89448
Gorrilla         --------------------------------------------------------------------------------- 86050
GrabEatingMacaqueTTATATGGAGAAAAATAGGGAAAA----ATTAAAAATTATGGTAGAATCAAATTCTGATGCATCAGTAATAGTGAAAAAT 88897
Macaque          TTATATGGAGAAAAATAGGGAAAA----ATTAAAAATTATGGTAGAATCAAATTCTGATGCATCAGTAATAGTGAAAAAT 89786
PigTailedMacaque TTATATGGAGAAAAATAGGGAAAA----ATTAAAAATTATGGTAGAATCAAATTCTGATGCATCAGTAATAGTGAAAAAT 88344
Bonobo           --------------------------------------------------------------------------------- 86642
OliveBaboon      TTATATGGAGAAAAATAGGGAAAA----ATTAAAAATTATGGTAGAATCAAATTCTGATGCATCAGTAATAGTGAAAAAT 88239
Orangutan        T-ATATGGAGAAAAATAGGGAAAA----ATTAAAAATCATGGTAGAATCAAATTCTGATGCATCAGTAATAGTGAAAAAT 89075

Majority          GTAAAGGAATAAATTTGTCAACTAAAAAGATTGTCAAACTTGGTATTTAAAAAGCTACCTGTATAC-TTTTTACAAGAGA

                        105530    105540    105550    105560    105570    105580    105590    105600

Human            --------------------ACTAAAAAGATTATCAAACTTGGTATTTAAAAAGTTAGCTATATAC-TTTTTACAAGAGA 87869
Coquerel'sSifaka GTGAAGGAATAAATTCATCAATTAAAA-TATTGTCAGATTTGATTTTTGAAAAGTTCCCTGTATGC-TCTTTGCAACAGA 74591
Ma'sNightMonkey  GTGAAGGGATAAATTTGTCAGCTAAAAAGATTGGCAGACTTGATATTTAAAAAGCCACCTAAATGCATTTTTACAAGAGA 83269
VervetAGM        GTAAAGGAATAAATTTGTCAACTAAAAAGATTGTCAAACTTAGTATTTAAAAAGCTACCTGT---------TACAAGAGA 89519
Gorrilla         --------------------ACTAAAA-GATTGTCAAACTTGGTATTTAAAAAGTTAGCTATATAC-TTTTTACAAGAGA 86108
GrabEatingMacaqueGTAAAGGAATAAATTTGTTAACTAAAAAGATTGTCAAACTTAGTATTTAAAAAGCTACCTGTATAC-TTTTTACAAGAGA 88976
Macaque          GTAAAGGAATAAATTTGTTAACTAAAAAGATTGTCAAACTTAGTATTTAAAAAGCTACCTGTATAC-TTTTTACAAGAGA 89865
PigTailedMacaque GTAAAGGAATAAATTTGTTAACTAAAAAGATTGTCAAACTTAGTATTTAAAAAGCTACGTGTATAC-TTTTTACAAGAGA 88423
Bonobo           --------------------ACTAAAAAGATTATCAAACTTGGTATTTAAAAAGTTAGCTATATAC-TTTTTACAAGAGA 86701
OliveBaboon      GTAAAGGAATAAATTTGTCAACTAAAAAGATTGTCAAACTTAGTATTTAAAAAGCTACCTGTATAC-TTTTTACAAGAGA 88318
Orangutan        GTAAAGGAATAAATTTGTCAACTAAAAAGATTGTCAAACTTGGTATTTAAAAAGTTACCTATATAC-TTTTTACGAGGGA 89154
```

```
Majority         CATGTAAAGCAGAAGGATGT-GAAAGATTGAAAGGAAAAGGATGTAAAGAGATACTAATATACTGGGCAGATAGTAAGCA

                      105610    105620    105630    105640    105650    105660    105670    105680

Human            CATGTAAAGCAGAAGAATAT-GAAAGATTGAAAGGAAAAGGATGTAAAGAGATACTAATATACTGGACAGATAGTAAGCA 87948
Coquerel'sSifaka CGTATGAAGCACAAG-ATGTGGAAGGATTGAAAGCAAAGGGATGTAAAGAGAT------GTATTGGGCAGATAGTAAGCC 74664
Ma'sNightMonkey  CATGTAAAGCAGAAGGATATGGAAAGATTGAAAGGAAAGGGATATAAAGAGATACTGATATTTTGGGCATATAGTAAGCA 83349
VervetAGM        CATGTAAAGCAGAAGGATG--GAAAGATTGAAAAGAAAAGGATGTGAAGAGATACTAATATACTGGGCAGATAGTAAGCA 89597
Gorrilla         CATGTAAAGCAGAAGAATAT-GAAAGATTGAAAGGAAAAGGATGTAAAGAGATACTAATATACTGGACAGATAGTAAGCA 86187
GrabEatingMacaqueCATGTAAAGCAGAAGGATG--GAAAGATTGAAAGGAAAAGGATGTGAAGAGATACTAATATACTGGGCAGATAGTAAGCA 89054
Macaque          CATGTAAAGCAGAAGGATG--GAAAGATTGAAAGGAAAAGGATGTGAAGAGATACTAATATACTGGGCAGATAGTAAGCA 89943
PigTailedMacaque CATGTAAAGCAGAAGGATG--GAAAGATTGAAAGGAAAAGGATGTGAAGAGATACTAATATACTGGGCAGATAGTAAGCA 88501
Bonobo           CATGTAAAGCAGAAGAATAT-GAAAGATTGAAAGGAAAAGGATGTAAAGAGATACTAATATACTGTACAGATAGTAAGCA 86780
OliveBaboon      CATGTAAAGCAGAAGGATG--GAAAGATTGAAAGGAAAAGGATGTGAAGAGATACTAATATACTGGGCAGATAGTAAGCA 88396
Orangutan        CATGTAAAGCAGAAGGATAT-GAAAGATTGAAAGGAAAAGGATGTAAAGAGATACTAATATACTGGGCAGATAGTAAGCA 89233

Majority         AAACAAAACTTAAATACCTTTATTAACTTAAGACAGAGA-ATTGATAAA-GGATTTAGTTCACTAGGAAGATATAACAGT

                      105690    105700    105710    105720    105730    105740    105750    105760

Human            AGACAAAACTTAAATACCTTTATTAACTTAAGACAGA---ATTGATAAA-GGATTTAGTTCACTAGAAAGATATAACAGT 88024
Coquerel'sSifaka AAAGAAAACTCACGTGACTTTGCTAATTTAAGCCAAATGTATTGATAAA-AGATTTAGTTCACCAGGAAAATACAACAGT 74743
Ma'sNightMonkey  AAAGGAAACTTAAATACCTTTGTTAA-----GACATAGA-ATTGAGAAA-GGATTTGGTTCACTAGGAAGATGCAACAAT 83422
VervetAGM        AAACAAAACTTAAATACCTTTATTAACTTAAGACCGAGA-ATTGATAAA-GGATTTAGTTCACTGGGAAGATATAACAGT 89675
Gorrilla         AAACAAAACTTAAATACCTTTATTAACTTAAGACAGAGA-ATTGATAAA-GGATTTAGTTCACTAGAAAGATATAACAGT 86265
GrabEatingMacaqueAAACAAAACTTAAATACCTTTATTAACTTAAGACCGAGA-ATTGATAAA-GGATTTAGTTCACTGGGAAGATATAACAGT 89132
Macaque          AAACAAAACTTAAATACCTTTATTAACTTAAGACCGAGA-ATTGATAAA-GGATTTAGTTCACTGGGAAGATATAACAGT 90021
PigTailedMacaque AAACAAAACTTAAATACCTTTATTAACTTAAGACCGAGA-ATTGATAAA-GGATTTAGTTCACTGGGAAGATATAACAGT 88579
Bonobo           AGACAAAACTTAAATACCTTTATTAA-----GACAGA---ATTGATAAA-GGATTTAGTTCACTAGAAAGATATAACAGT 86851
OliveBaboon      AAACAAAACTTAAATACCTTTATT-----AAGACCGAGA-ATTGATAAA-GGATTTAGTTCACTGGGAAGATATAACAGT 88469
Orangutan        AAACAAAATTTAAATACCTTTATTAACTTAAGACAGAGA-ATTGATAAAAGGATTTAGTTCACTAGAAAGATATAACAGT 89312

Majority         TTGTGTATATGTGTGCCTAGTGA---AATAGCTTCAAAATATATAAATAATAAGTGGATAGAACTACAGGTAGACTTGAG

                      105770    105780    105790    105800    105810    105820    105830    105840

Human            TTATGTATATGTGTGCCTAGTGA---AATAGCCTCAAAATATATAAATAATAAGTAGATAGAACTACAGGTAGACTTGAG 88101
Coquerel'sSifaka TT-TATGTATGTATGTCTAGTGA---AATAACTTCAAAATGTATAAATCATAAATGGATAACGCTGCAGGTAGAATTGAG 74819
Ma'sNightMonkey  TTATATATATGTGGGCCTAGTGA---AATAGCCTCAAAATATATAAATAATAAATGGCTGGAACTACAGGTAAACTTGAG 83499
VervetAGM        TTGTGTATATGTGTGCCTAGTGA---AATAGCTTCAAAATACATAG----TAAGTGGATAGAACTACAGGTCGACTTGAG 89748
Gorrilla         TTATGTATATGTGTGCCTAGTGA---AATAGCCTCAAAATATATAAATAATAAGTAGATAGAACTACAGGTAGACTTGAG 86342
GrabEatingMacaqueTTGTGTATATGTGTGCCTAGTGA---AATAGCTTCAAAATACATAG----TAAGTGGATAGAACTACAGGTCGACTTGAG 89205
Macaque          TTGTGTATATGTGTGCCTAGTGA---AATAGCTTCAAAATACATAG----TAAGTGGATAGAACTACAGGTCGACTTGAG 90094
PigTailedMacaque TTGTGTATATGTGTGCCTAGTGA---AATAGCTTCAAAATACATAG----TAAGTGGATAGAACTACAGGTCGACTTGAG 88652
Bonobo           TTATGTATATGTGTGCCTAGTGA---AATAGCCTCAAAATATATAAATAATAAGTAGATAAAACTACAGGTAGACTTGAG 86928
OliveBaboon      TTGTGTATATGTGTGCCTAGTGATGAAATAGCTTCAAAATACATAG----TAAGTGGATAGAACTACAGGTCGACTTGAG 88545
Orangutan        TTATGTATATGTGTGCCTAGTGA---AATAGCCTCAAAATATATAAATAATAAGTGGATAGAACTACAGGTAGACTTGAG 89389
```

```
Majority         CAAATCTATTATAGTGGTGGATTTCAATTCACCTTTCTGAATTACATGTCAGACTGACCAAAAAAAGACAAAATCAACAA

                         105850    105860    105870    105880    105890    105900    105910    105920

Human            CAAATCTATTATAGTGGTGGATTTCAATTCACTTTTCTGAATTACATGTCAAACTGACCAAAAAAAGACAAAATCAACAA 88181
Coquerel'sSifaka TAAATCTATCATAGTGGTGGATTTCAACCCACTTTTCTGAATTATATGTCAAGCAGACCAAAAAAAAACAAA---AACAA 74896
Ma'sNightMonkey  CAAATCTGTTATAGTGGTGGATTTCAATTCACCTTTCTGAATTACATGTCACACTGACCAGAAAAAAAGAAAATCAACAA 83579
VervetAGM        CAAATCTATTATAGTGGTAGATTTCAGTTCACCTTTCTGAATTACATGTCAGACTGACCCAAAAAAGACAAAATCAACAA 89828
Gorrilla         CAAATCTATTATAGTGGTGGATTTCAATTCACCTTTCTGAATTACATGTCAAACTGACCAAAAAAAGACAAAATCAACAA 86422
GrabEatingMacaqueCAAATCTATTATAGTGGTGGATTTCAGTTCACCTTTCTGAATTACATGTCAGACTGACCCGAAAAAGACAAAATCAACAA 89285
Macaque          CAAATCTATTATAGTGGTGGATTTCAGTTCACCTTTCTGAATTACATGTCAGACTGACCCGAAAAAGACAAAATCAACAA 90174
PigTailedMacaque CAAATCTATTATAGTGGTGGATTTCAGTTCACCTTTCTGAATTACATGTCAGACTGACCCGAAAAAGACAAAATCAACAA 88732
Bonobo           CAAATCTATTATAGTGGTGGATTTCAATTCACTTTTCTGAATTACATGTCAAACTGACCAAAAAAAGACAAAATCAACAA 87008
OliveBaboon      CAAATCTATTATAGTGGTGGATTTCAGTTCACCTTTCTGAATTACATGTCAGACTGACCCAAAAAAGACAAAATCAACAA 88625
Orangutan        CAAATCTATTATAGTGGTGGATTTCAATTCACCTTTCTGAATTACATGTCAAACTGACCAAAAAAAGACAAAATCAACAA 89469

Majority         AGATACAAA-TGATACAAT-----TACCTTAACAGAATTATGTGTCTAA--GTTACGCATCCAACACAGTTTTT--AAAC

                         105930    105940    105950    105960    105970    105980    105990    106000

Human            AGATACAAA-TGATACAATACAATTACCTTAACAGAATTATGTGTCTAA--GTTATGCATCCAACACAGTTTTT--AAAC 88256
Coquerel'sSifaka AGATACAAAATGATACAAC--------------ACAATTCTCTGT-TAA------TGCATCCAGCACATTTTTTTAAAGC 74955
Ma'sNightMonkey  AGATACAAA-TTATACAACACAGTTACCTGCACAGAATTGTGTGTCTAAC-GGTATGCATCCAACACAGTTTTT--AAGC 83655
VervetAGM        AGATACAAA-TGATACAAT-----TACCTTAACAGAATTATGTGTCTAACAGTTACACATCCAATACAGTTTTT--AAAC 89900
Gorrilla         AGATACAAA-TGATACAATGCGATTACCTTAACAGAATTATGTGTCTAA--GTTATGCATCCAACACAGTTTTT--AAAC 86497
GrabEatingMacaqueAGATACAAA-TGATACAAT-----TACCTTAACAGAATTATGTGTCTAAAAGTTACACATCCAGTACAGTTTTT--AAAC 89357
Macaque          AGATACAAA-TGATACAAT-----TACCTTAACAGAATTATGTGTCTAAAAGTTACACATCCAGTACAGTTTTT--AAAC 90246
PigTailedMacaque AGATACAAA-TGATACAAT-----TACCTTAACAGAATTATGTGTCTAAAAGTTACACATCCAGTACAGTTTTT--AAAC 88804
Bonobo           AGATACAAA-TGATACAATACAATTACCTTAACAGAATTATGTGTCTAA--GTTATGCATCCAACACAGTTTTT--AAAC 87083
OliveBaboon      AGATACAAA-TGATACAAT-----TACCTTAACAGAATTATGTGTCTAAAAGTTACACATCCAGTACAGTTTTT--AAAC 88697
Orangutan        AGATACAAA-TGATACAATACAATTACCCTAACAGAATTATGTGTCTAA--GTTACGCATCCAACGCAATTTTT--AAAC 89544

Majority         ACATATGGAACATTTTTGAAAAATCAGTCACATACTAAACCATAAAGCAAGCTTCATCTAATTTCAAAGAGTATCA----

                         106010    106020    106030    106040    106050    106060    106070    106080

Human            GCATATGGAACATTTTTGAAAAATCAGTCACATACTAAACCATAAAGCAAGCTTCAGCTAATTTCAAAGAGTATCA---- 88332
Coquerel'sSifaka ACATATGGAACATTTA-AAAAAATTGATCATATACTAAACCATAAAACAAGCTTCAATTAATTTCAAAGAGTAAGTATCA 75034
Ma'sNightMonkey  ACATATGGAACATTTTTGAAAAATCAGTGACATACTAAACCGTAAAGCAAGCTTCAACTAATTTCAAAGAGTATCA---- 83731
VervetAGM        ACATATGGAACATTTTTGAAAAATCAGCCACATACTAAACCATAAAGCAAGCTTCATCTAATTTCACAGAGTATCA---- 89976
Gorrilla         GCATATGGAACATTTTTGAAAAATCAGTCACATACTAAACCATAAAGCAAGCTTCAGCTAATTTCAAAGAGTATCA---- 86573
GrabEatingMacaqueACATACGGAACATTTTTGAAAAATCAGCCACATACTAAACCATAAAGCAAGCTTCATCTAATTTCAAAGAGTATCA---- 89433
Macaque          ACATACGGAACATTTTTGAAAAATCAGCCACATACTAAACCATAAAGCAAGCTTCATCTAATTTCAAAGAGTATCAATCA 90326
PigTailedMacaque ACATATGGAACATTTTTGAAAAATCAGCCACATACTAAACCATAAAGCAAGCTTCATCTAATTTCAAAGGGTATCA---- 88880
Bonobo           GCATATGGAACATTTTTGAAAAATCAGTCACATACTAAACCATAAAGCAAGCTTCAGCTAATTTC--AGAGTATCA---- 87157
OliveBaboon      ACATATGGAACATTTTTGAAAAATCAGCCACATACTAAACCATAAAGCAAGTTTCATCTAATTTCAAAGAGTATCA---- 88773
Orangutan        GCATATGGAACATTTTTGAAATATCAGTCACATACTAAACCATAAAGCAAGCTTCAGCTAATTTCAAAGAGTAGCA---- 89620
```

```
Majority          TACAGACCTCATTCTCTGACTATTGCACAATTAAGTTAGAATTAATAA----CAAAAAGA-TGCATTTAAAATCT----G
                          106090    106100    106110    106120    106130    106140    106150    106160
Human             TACAGACCTCATTCT----CTATTGCACAAGTAAGTTAGAATTAATAA----CAAAAAGA-TGCATATAAAATCT----G 88399
Coquerel'sSifaka  TACAGACCTCATTCT----CTGTTGCA--A---AGTTAGAAATAATAA----CAGAAAAA-TACATTTAAAGTCTCCATG 75100
Ma'sNightMonkey   TACAGACCTTATTCTCTGACTATTGCACAATAGAGTTAGAATTAATAAATAACAAAAGGA-TACATTTAAAATCT----G 83806
VervetAGM         TACAGACCTCATTCTCTGACTATTGCACAATTAAGTTAGAATTAATAA----CAAAAAGA-TGCATTTAAAATCT----G 90047
Gorrilla          TACAGACCTCATTCT----CTATTGCACAAGTAAGTTAGAATTAATAA----CAAAAAGA-TGCATATAAAATCT----G 86640
GrabEatingMacaqueTACAGACCTCATTCTCTGACTATTGCACAATTAAGTTAGAATTAATAA----CAAAAAAAATGCATTTAAAATCT----G 89505
Macaque           TACAGACCTCATTCTCTGACTATTGCACAATTAAGTTAGAATTAATAA----CAAAAAAA-TGCATTTAAAATCT----G 90397
PigTailedMacaque  TACAGACCTCATTCTCTGACTATTGCACAATTAAGTTAGAATTAATAA----CAAAAAAA-TGCATTTAAAATCT----G 88951
Bonobo            TACAGACCTCATTCT----CCATTGCACAAGTAAGTTAGAATTAATAA----CAAAAAGA-TGCATATAAAATCT----G 87224
OliveBaboon       TACAGACCTCATTCTCTGACTATTGCACAATTAAGTTAGAATTAATAA----CAAAAAAA-TGCATTTAAAATCT----G 88844
Orangutan         TACAGACCTCATTCT----CTATTGCACAAGTAAGTTAGAATTAATAA----CAAAAAGA-TGCATATAAAATCT----G 89687

Majority          TATCCAGAGATTTAAAAATAAGTCTCAGTATTGCATGAGTCAAAAGTTTGTTTT--TTTGTTTT--------T-GTTTTT
                          106170    106180    106190    106200    106210    106220    106230    106240
Human             TATCCTGAGATTTAAAAATAAGTCTCAGTATTGCATGAGTCAGAAGTTTGTTTG--TTTGTTTG------------TTTT 88465
Coquerel'sSifaka  TACCTGGAAATTTAAAAGCAACTCCCAGTGTTTCATGGGTCGAAGAGGTG---G-----------------------TT 75153
Ma'sNightMonkey   TATCCAGAGACTTAAAAATAAGTCTCAGTATTGTATGAGTCAAAAGGTTTTTTTGGTTTGTTTT--------T-GTTTTT 83877
VervetAGM         TGTCCAGAGATGTAAAAATAAGTCTCAGTATTGCATGAGTCAAAAGTTTGTTTT--TTTGTTTTGTATTGTTTTGTTTTT 90125
Gorrilla          TATCCGGAGATTTAAAAATAAGTCTCAGTATTGCATGAGTCAAAAGTTTGTTTG--TTTGTTTG------------TTTT 86706
GrabEatingMacaqueTGTCCAGAGATGTAAAAATAAGTCTCAGTATTGCATGAGTCAAAAGTTTGTTTT--TTTGTTTTGTATTGTTTTGTTTTT 89583
Macaque           TGTCCAGAGATGTAAAAATAAGTCTCAGTATTGCATGAGTCAAAAGTTTGTTTT--TTTGTTTTGTATTGTTTTGTTTTT 90475
PigTailedMacaque  TGTCCAGAGATATAAAAATAAGTCTCAGTATTGCATGAGTCAAAAGTTTGTTTT--TTTGTTTTGTATTGTTTTGTTTTT 89029
Bonobo            TATCCTGAGATTTAAAAATAAGTCTCAGTATTGCATGAGTCAGAAGTTTGTTTG--TTTGTTTG------------TTTT 87290
OliveBaboon       TGTCCAGAGATGTAAAAATAAGTCTCAGTATTGCATGAGTCAAAAGTTTGTTTT--TTTGTTTTGTATTGTTTTGTTTTT 88922
Orangutan         TATCCTGAGATTTAAAAATAAGTCTCAGTATTGCATGAGTCAAAAGTTTG--------------------------TTTT 89741

Majority          TGAGACAG-GTCTTGCTCTGTCCCCCAGGCTGGAGTGCAGTGGT---ATTGTGGCTCACTGCAGCCTCGACCTCCCAGGC
                          106250    106260    106270    106280    106290    106300    106310    106320
Human             TGAGCCAGTGTCTTGCTCTGTCCCCCAGGGTGGAGTGCAGTGGTGCTATTGTGGCTCACTGCAGCCTCGACCTCCCAGGC 88545
Coquerel'sSifaka  TTAATGGGAGTTTAAA---ATACTTAAAACTAGATATTGGAAGT---------ACTATATGTAAATTAGATATCTGTGGT 75221
Ma'sNightMonkey   TGAGACAGGGTCTTGCCCTGTTGCCCAGGCTGGAGTGCAGTGGTGCTCTCATGGCTCGCTGCAGCCTTGACCTTCCAGGC 83957
VervetAGM         TGAGACAG-GTCTTGCTCTGTCCCCCAGGCTGGAGTGCAGTGGT---ATTGTGGCTCACTGCAGCCTCGACCTCCCAGGC 90201
Gorrilla          TGAGACAGTGTCTTGCTCTGTCCCCCAGGGTGGAGTGCAGTGGTGCTATTGTGGCTCACTGCAGCCTCGACCTCCCAGGC 86786
GrabEatingMacaqueTGAGACAG-GTCTTGCTCTGTCCCCCAGGCTGGAGTGCAGTGGT---ATTGTGGCTCACTGCAGCCTCGACCTCCCAGGC 89659
Macaque           TGAGACAG-GTCTTGCTCTGTCCCCCAGGCTGGAGTGCAGTGGT---ATTGTGGCTCACTGCAGCCTCGACCTCCCAGGC 90551
PigTailedMacaque  TGAGACAG-GTCTTGCTCTGTCCCCCAGGCTGGAGTGCAGTGGT---ATTGTGGCTCACTGCAGCCTCGACCTCCCAGGC 89105
Bonobo            TGAGCCAGTGTCTTGCTCTGTCCCCCAGGGTGGAGTGCAGTGGTGCTATTGTGGCTCACTGCAGCCTCGACCTCCCAGGC 87370
OliveBaboon       TGAGACAG-GTCTTGCTCTGTCCCCCAGGCTGGAGTGCAGTGGT---ATTGTGGCTCACTGCAGCCTCGACCTCCCAGGC 88998
Orangutan         TGAGACAGTGTCTTGCTCTGTCCCCCAGGGTGGAGTGCAGTGGTGCTATTGTGGCTCACTGCAGCCTCGACCTCCCAGGC 89821
```

```
Majority        TCAAGCAATCCTTGCACTTAAGCCTCCCAAGTAGCTAGGACTACAAGCATGCATCACTTGCCCGGCTAATTTATTATTAT

                        106330    106340    106350    106360    106370    106380    106390    106400

Human           TTAAGCAGTCCTTGCACTTAAGCCTCCAAAGTAGCTAGGACTAAAAGCATGCACCACCTGCCCGGCTAATTTATTATTAT 88625
Coquerel'sSifaka ACAGTAAAAGAGGTCTTTTGAA------AGAAAGTTTTAACTTTAAGGAAACAGCA--------------TAA------- 75274
Ma'sNightMonkey TCAAGCAGTCCTCCCACTTCAGCCTCCCAAGTAGCTAGGACTACAAGCAC-CACCA--TGCC------------------ 84016
VervetAGM       TCAAGCAACCCTTGAACTTAAGCCTCCCAAGTAGCTAGGACTACAAGCATGCATCACTTGCCCGGCTAATTTTTTTTTAT 90281
Gorrilla        TTAAGCAGTCCTTGCACTTAAGCCTCCGAAGTAGCTAGGACTAAAAGCATGCACCACCTGCCCGGCTAATTTATTATTAT 86866
GrabEatingMacaqueTCAAGCAATCCTTGTACTTAAGCCTCCCAAGTAGCTGGGACTACAAGCATGCATCACTTGCCCGGCTAATTTATTATTAT 89739
Macaque         TCAAGCAATCCTTGTACTTAAGCCTCCCAAGTAGCTGGGACTACAAGCATGCATCACTTGCCCGGCTAATTTATTATTAT 90631
PigTailedMacaque TCAAGCAATCCTTGTACTTAAGCCTCCCAAGTAGCTGGGACTACAAGCATGCATCACTTGCCCGGCTAATTTATTTTTAT 89185
Bonobo          TTAAGCAGTCCTTGCACTTAAGCCTCCGAAGTAGCTAGGACTAAAAGCATGCACCACCTGCCCGGCTAATTTATTATTAT 87450
OliveBaboon     TCAAGCAATCCTTGCACTTAAGCCTCCCAAGTAGCTAGGACTACAAGCATGCATCACTTGCCCGGCTAATTTTTTTATTAT 89078
Orangutan       TTAAGCAATCCTTGCACTTAAGCCTCCCAAGTAGCTAGGACTAAAAGCATGCACCACCTGCCCGGCTAATTTATTATTAT 89901

Majority        TATTATTTTT-GTAGAGATGAATTCTCACTGTGTTGCCCAGACGGAAGTTATTTTAATGGGAATATAAAAA-ACTTAAA

                        106410    106420    106430    106440    106450    106460    106470    106480

Human           TATTATTTTT-GTAGAGATGAGTTCTCACTGTGTTGCCCAGACAGAAGTTATTTTAATGGGAATATAAAAT-ACTTAAA 88703
Coquerel'sSifaka -----------------------------------ACCCAAGG---------------------G------------- 75283
Ma'sNightMonkey ----------T-----------------------GTCTAGAC------------------------------------ 84025
VervetAGM       TATTATTTTT-GTAGAGATGAATTCTCACTATGTTGCCCAGACGGAAGTTATTTTAATGGGAATATAAAAATACTTAAA 90360
Gorrilla        TATTTTTTTT-GTAGAGATGAGTTCTCACTGTGTTGCCCAGACAGAAGTTATTTTAATGGGAATATAAAAT-ACTTAAA 86944
GrabEatingMacaqueTATTATTTTT-GTAGAGATGAATTCTCACTCTGTTGCCCAGACGGAAGTTATTTTAATGGGAATATAAAAATACTTAAA 89818
Macaque         TATTATTTTT-GTAGAGATGAATTCTCACTCTGTTGCCCAGACGGAAGTTATTTTAATGGGAATATAAAAATACTTAAA 90710
PigTailedMacaque TATTATTTTT-GTAGAGATGAATTCTCACTATGTTGCCCAGACGGAAGTTATTTTAATGGGAATATAAAAATACTTAAA 89264
Bonobo          TATTATTTTT-GTAGAGATGAGTTCTCACTGTGTTGCCCAGACAGAAGTTATTTTAATGGGAATATAAAAT-ACTTAAA 87528
OliveBaboon     TATTATTTTT-GTAGAGATGAATTCTCACTATGTTGCCCAGATGGAAGTTATTTTAATGGGAATATAAAAATACTTAAA 89157
Orangutan       TATTTTTTTTTGTAGAGATGAGTTCT----GTGTTGCCCAGACAGAAGTTATTTTAATGGGAATATTAAA------AAA 89971

Majority        ATTAAGCGATAAAGTACTATATACATAT---TTAGATATATGTGGTACAGTGAAAGAGATGTCTTGACGGGAAGTTTTAA

                        106490    106500    106510    106520    106530    106540    106550    106560

Human           ATTAAGCGATAAAGTACTATATACAAAT---T-AGATATATGTGGTACAGTGAAAGAGAAGTCTTGACGGGAAGTTTTAA 88779
Coquerel'sSifaka ----------AAGG------------A-------GATA------------TTAAGGA----------------------- 75299
Ma'sNightMonkey --------------------------------------------------TTAAG------------------------ 84030
VervetAGM       ATTAAGCGATAAAGTACTATATACATATAAATTAGATATATGTGGTACAGTGAAAGAGATGTCTTGACGGGAAGTTTTAA 90440
Gorrilla        ATTAAGCGATAAAGTACTATATACAAAT---T-AGATATATGTGGTACAGTGAAAGAGAAGTCTTGACGGGAAGTTTTAA 87020
GrabEatingMacaqueATTAAGCGATAAAGTACTATATACATATAAATTAGATATATGTGGTACAGTGAAAGAGATG-----ACGGGAAGTTTTAA 89893
Macaque         ATTAAGCGATAAAGTACTATATACATATAAATTAGATATATGTGGTACAGTGAAAGAGATG-----ACGGGAAGTTTTAA 90785
PigTailedMacaque ATTAAGCGATAAAGTACTATATACATATAAATTAGATATATGTGGTACAGTGAAAGAGATGTCTTGACGGGAAGTTTTAA 89344
Bonobo          ATTAAGCGATAAAGTACTACATACAAAT---T-AGATATATGTGGTACAGTGAAAGAGAAGTCTTGACGGGAAGTTTTAA 87604
OliveBaboon     ATTAAGCGATAAAGTACTATATACATATAAATTAGATATATGTGGTACAGTGAAAGAGATGTCTTGAAGGGAAGTTTTAA 89237
Orangutan       ATTAAGCGATAAAGTACTATATACAAAT---TTAGATATATGTGGTACAGTGAAAGAGATGTCTTGATGGGAAGTTTTAA 90048
```

```
Majority          CTTT--AAGAATAACAGTGTTACCCCCAGGAAAGGAGATATTAAAGATAAGACTAGAAATCAGTGGTAT---AAAAGACA

                        106570    106580    106590    106600    106610    106620    106630    106640

Human             CTTT--AAGAATAACAGTGTTACCCCCAGGGAAGGAGATATTAAAGACAAGACTGGAAATCAATGGTAT---AAAAGACA 88854
Coquerel'sSifaka  CT-----AGAAT--CAGTGGTAT---------AGAAGA--------------C----------------------AAACA 75327
Ma'sNightMonkey   ------------------GTTGTC--------AGGAGA-----------------------A------------AAAATT 84049
VervetAGM         CTTTTTAAGAATAACAGTGTTACCCCCAGGAAAGGAGATATTAAAGATAAGACTAGAAATCAGTGGTAT---AAAAGACA 90517
Gorrilla          CTTT--AAGAATAACAGTGTTACCCCCAGGGAAGGAGATATTAAAGACAAGACTGGAAATCAATGGTATTTTAAAAGACA 87098
GrabEatingMacaqueCTTT--AAGAATAACAGTGTTACCCCCAGGAAAGGAGATATTAAAGATAAGACTAGAAATCAGTGGTAT---AAAAGACA 89968
Macaque           CTTT--AAGAATAACAGTGTTACCCCCAGGAAAGGAGATATTAAAGATAAGACTAGAAATCAGTGGTAT---AAAAGACA 90860
PigTailedMacaque  CTTT--AAGAATAACAGTGTTACCCCCAGGAAAGGAGATATTAAAGATAAGACTAGAAATCAGTGGTAT---AAAAGACA 89419
Bonobo            CTTT--AAGAATAACAGTGTTACCCCCAGGGAAGGAGATATTAAAGACAAGACTGGAAATCAATGGTAT---AAAAGACA 87679
OliveBaboon       CTTT--AAGAATAACAGTGTTACCCCCAGGAAAGGAGATATTAAAGATAAGACTAGAAATCAGTGGTAT---AAAAGACA 89312
Orangutan         CTTT--AAGAATAACAGTGTTACCCCCAGGGAAGGAGATATTAAAGACAAGACTAGAAATCAGTGGTAT---AAAAGACA 90123

Majority          GTTGAGAGAAAAAAAC-ATGCCACAAGTTGGGTTCTTTGAAAAG----ACTAAAGAAATGGACAAACATCTAAAGATTGA

                        106650    106660    106670    106680    106690    106700    106710    106720

Human             GTTGAGAGAAAAAAAC-ATGCCACAAGTTGGATTCTTTGAAAAG----ACTAAAAAAATAGACAAACATCTAAAGATTGA 88929
Coquerel'sSifaka  GTTGAGAGAAAAAAACGAAGCCAAAAGTTGAGTTCTTTGAAAAAGGCTAGTAATAAAATGGACAGGTGTCTGTTAA--GA 75405
Ma'sNightMonkey   GTCTAAATAAAAAGAC-ACACAAACAGCTGAG--CTTTGAGATA----ACGGAAGGA----------------------- 84099
VervetAGM         GTTGAGAGAAAAAAAC-ATGCCACAAGTTGGGTTCTTTGAAAAG----ACTAAAGAAATGGACAAACATCTAAAGATTGA 90592
Gorrilla          GTTGAGAGAAAAAAAC-ATGCCACAAGTTGGATTCTTTGAAAAG----ACTAAAAAAATAGACAAACACCTAAAGATTGA 87173
GrabEatingMacaqueGTTGAGAGAAAAAAAC-ATGCCACAAGTTGGGTTCTTTGAAAAG----ACTAAAGAAATGGACAAACATCTAAAGATTGA 90043
Macaque           GTTGAGAGAAAAAAAC-ATGCCACAAGTTGGGTTCTTTGAAAAG----ACTAAAGAAATGGACAAACATCTAAAGATTGT 90935
PigTailedMacaque  GTTGAGTGAAAAAAAC-ATGCCACAAGTTGGGTTCTTTGAAAAG----ACTAAAGAAATGGACAAACATCTAAAGATTGA 89494
Bonobo            GTTGAGAGAAAAAAAC-ATGCCACAAGTTGGATTCTTTGAAAAG----ACTAAAAAAATAGACAAACATCTAAAGATTGA 87754
OliveBaboon       GTTGAGAGAAAAAAAC-ATGCCACAAGTTGGGTTCTTTGAAAAG----ACTAAAGAAATGGACAAACATCTAAAGATTGA 89387
Orangutan         GTTGAGAGAAAAAAAC-ATGCCACAAGTTGGGTTCTTTGAAAAG----ACTAAAAAA-TAGACAAACATCTAAAGGTTGA 90197

Majority          TTAAAAAATGAGAGTGGTAACACAAAAAAA----TTTTTTGAATTATAAAGGGGAGAACCATTGATAAAGCTCCAGTTTC

                        106730    106740    106750    106760    106770    106780    106790    106800

Human             TTAAAAAATGAGAGTGGTAACACAAAAAAAT---TTTTTTGAATTATAAAGGGGAGAAGCATTGATAAAGCTCCAGTTTC 89006
Coquerel'sSifaka  TTAAGAAATGAGAGTG-TGGCACAAGTAA-----TATTTTGAATTATAAAGGGGAGAACTATTGATATAGCTCCAG-TTC 75478
Ma'sNightMonkey   ---AGGAATAAGA-------------A---------------A--ATAAA-----------------AAGTCCCAG---- 84125
VervetAGM         TTAAAACATGAGAGTGGTAACACA-AA------TTTTTTT-AATTATAAAGGGGAGAACCATTGCAAAAGCTCCAGTTTC 90664
Gorrilla          TTAAAAAACGAGAGTGGTAACACAAAAAAAA---TTTTTTGAATTATAAAGGGGAGAAGCATTGATAAAGCTCCAGTTTC 87250
GrabEatingMacaqueTTAAAACATGAGAGTGGTAACACAAAAAAA-TATTTTTTT-AATTATAAAGGGGAGAACCATTGAAAAAGCTCCAGTTTC 90121
Macaque           TTAAAACATGAGAGTGGTAACACAAAAAAA-TATTTTTTT-AATTATAAAGGGGAGAACCATTGAAAAAGCTCCAGTTTC 91013
PigTailedMacaque  CTAAAACATGAGAGTGGTAACACACAAAAA-TATTTTTTT-AATTATAAAGGGGAGAACCATTGAAAAAGCTCCAGTTTC 89572
Bonobo            TTAAAAAATGAGAGTGGTAACACAAAAAAAT---TTTTTTGAATTATAAAGGGAAGAAGCATTGATAAAGCTCCAGTTTC 87831
OliveBaboon       TTAAAACATGAGAGTGGTAACACAAAAAAAATATTTTTTTTAATTATAAAGGGGAGAACCATTGAAAAAGCTCCAGCTTC 89467
Orangutan         TTAAAAAATGAGAGTGGTAACACAAAAAAGTA--TTTTTTGAATTATAAAGGGGAGAACCATTGATAAAGCTCCAGTTTC 90275
```

```
Majority          CACATCATATAAATGCCACCTATATCTGTAAATTTCTTGAGGTCTTTTAAATTTTTTATTATTCATTAATA-TCTTCTTC

                        106810    106820    106830    106840    106850    106860    106870    106880

Human             CACATCATATAAACACCAC-TATGTCTGTAAATTTCTTGAGGTCTTTTAAAATTTTTATTATTCGTTAATAATCTCTTTC 89085
Coquerel'sSifaka  CACATTATATATA---CTTCTGTATCTGTAAACTTCTTGCTGTCTTTTGAA-TTTTCATAATTCATTAATA-TCTTCTTC 75553
Ma'sNightMonkey   -GCATAGTA--------AC---TGTATGCAGA------------------------------------------------ 84145
VervetAGM         CACATCATATAAATGCCACCTATATCCGTAAATTTCTTGAGGTCTTTTAAGTTTTTAATTATTCGTTAATA-TCTTCTTC 90743
Gorrilla          CACATCATATAAACACCAC-TATGTCTGTAAATTTCTTGAGGTCTTTTAAAATTTTTATTATTCGTTAATAATCTCTTTC 87329
GrabEatingMacaqueCACATCATATAAATGCCACCTATATCTGTAAATTTCTTGAGGTCTTTTAAATTTTTTATTATTCATTAATA-TCTTCTTC 90200
Macaque           CACATCATATAAATGCCACCTATATCTGTAAATTTCTTGAGGTCTTTTAAATTTTTTATTATTCATTAATA-TCTTCTTC 91092
PigTailedMacaque  CACATCATATAAATGCCACCTATATCTGTAAATTTCTTGAGGTCTTTTAAATTTTTTATTATTCATTAATA-TCTTCTTC 89651
Bonobo            CACATCATATAAACACCAC-TATGTCTGTAAATTTCTTGAGGTCTTTTAAAATTTT-ATTATTCGTTAATAATCTCTTTC 87909
OliveBaboon       CACATCATATAAATGCCACCTATATCTGTAAATTTCTTGAGGTCTTTTAAATTTTTTATTATTCATTAATA-TCTTCTTC 89546
Orangutan         CACATCATATAAACACCACCTATGTCTGTAAATTTCTTGAGGTATTTTAAAATTTCTATTATTCATTAATA-TCTCCTTC 90354

Majority          ATAGATGGATTAAGGGAAAGGTAAAAAA-TTACATTCAACCAAAAATGCCATTTCAATTGTGAAACATTGACTTTTTCCT

                        106890    106900    106910    106920    106930    106940    106950    106960

Human             ATAGATGGATTAAGGGAAATGTAAAAAA-TTACATTAAATCAAAAATGTCATTTCAATTGTGAAACATTGACTTTTTCCT 89164
Coquerel'sSifaka  AGAGATGGAATATGGTAAAGCTAAAAAA-TTATGTTCAGCCAAAAATGCTATTTCTACAGTGCAACATTGACTTTTTCCT 75632
Ma'sNightMonkey   ACAGATGAATGAC--------TGAAGAA---------------------------------------------------- 84165
VervetAGM         ATAGATCGATTAAGGGAAAGGTAAAAAAATTACATTCAACCAAAAATGCCATTTCAATTGTGAAACACTGACTTTTTCCT 90823
Gorrilla          ATAGATGGATTAAGGGAAAGGTAAAAAA-TTATATTAAATCAAAAATGCCATTTCAATTGTGAAACATTGACTTTTTCCT 87408
GrabEatingMacaqueATAGATGGATTAAGGGAAAGGTAAAAAAATTACATTCAACCAAAAATGCCATTTCAATTGTGAAACACTGACTTTTTCCT 90280
Macaque           ATAGATGGATTAAGGGAAAGGTAAAAAAATTACATTCAACCAAAAATGCCATTTCAATTGTGAAACACTGACTTTTTCCT 91172
PigTailedMacaque  ATAGATGGATTAAGGGAAAGGTAAAAAAATTACATTCAACCAAAAATGCCATTTCAGTTGTGAAACACTGACTTTTTCCT 89731
Bonobo            ATAGATGGATTAAGGGAAAGGTAAAAAA-TTATATTAAATCAAAAATGTCATTTCAATTGTGAAACATTGACTTTTTCCT 87988
OliveBaboon       ATAGATGGATTAAAGGAAAGGTAAAAAAATTACATTCAACCAAAAATGCCATTTCAATTGTGAAACACTGACTTTTTCCT 89626
Orangutan         ATAGATGGATTAAGGGAAAGGTAAAAAA-TTATATTCAATCAGAAATGCCATTTCAATTGTGAAACATTGACTTTTTCCT 90433

Majority          TCTTTATTATTGCCTTGTCCATTTATGCCATATTTCTTGGAATAGGAGACTTCAAAGTGTAAGATTTACATTGTTT-AAA

                        106970    106980    106990    107000    107010    107020    107030    107040

Human             T---TATTATTGCCTTGTCCATTTATGCCATATTTCTTGGAATAGGAGAGTTCAA-GTGTAAGATTCACATTGTTT-AAA 89239
Coquerel'sSifaka  CCTTTATTATTGCTTTGTCCGTTTATGCCATATTTATTGGAATAGTAGATTCCTA-GTATAAGATTCTCATTGTTTTAAA 75711
Ma'sNightMonkey   -----------------------------------TGGAA------------------------------------ 84170
VervetAGM         TCTTTATTATTGCCTTGTCCATTTATGTCATATTTCTTGGAATAGGAGACTTCAAAGTGTAAGATTTACATTGTTT-AAA 90902
Gorrilla          TCTTTATTATTGCCTTGTCCATTTATGCCATATTTCTTGGAATAGGAGACTTCAA-GTGTAAGATTCACATTGTTT-AAA 87486
GrabEatingMacaqueTCTTTATTATTGCCTTGTCCATTTATGCCATATTTCTTAGAATAGGAGACTTGAAAGTGTAAGATTTACATTGTTT-AAA 90359
Macaque           TCTTTATTATTGCCTTGTCCATTTATGCCATATTTCTTAGAATAGGAGACTTCAAAGTGTAAGATTTACATTGTTT-AAA 91251
PigTailedMacaque  TCTTTATTATTGCCTTGTCCATTTATGCCATATTTCTTGGAATAGGAGACTTCAAAGTGTAAGATTTACATTGTTT-AAA 89810
Bonobo            TCTTTATTATTGCCTTGTCCATTTATGCCACATTTCTTGGAATAGGAGACTTCAA-GTGTAAGATTCACATTGTTT-AAA 88066
OliveBaboon       TCTTTATTATTGCCTTGTCCATTTATGCCATATTTCTTGGAACAGGAGACTTCAAAGTGTAAGATTTACATTGTTT-AAA 89705
Orangutan         TCTTTATTATTGCCTTGTCCATTTATGCCATATTTCTTGGAATAGGAGACTTCAAAGTGTAAGATTCACATTGTTT-AAA 90512
```

```
Majority          ATTTTATTTATTTAAAATTAGTAATTCAGATAGTTTCCTTCTGCTCT-----GTTTACTCATTGTGTACTTTCAGTTTTT

                          107050    107060    107070    107080    107090    107100    107110    107120

Human             ATTTTATTCATTTAAAATTAGTAATTCAGGTAATTTCCTTCTGCTCT-----GTTTACTCCTTGTGTACTTTCAGTTTTT 89314
Coquerel'sSifaka  AAGTGATTCATTTAAAATTAGTGATTAAGATACTTTCCTTCTGCTTTATTTGTTTAATCCTTATATACCT-CAATTTTT 75790
Ma'sNightMonkey   ----------TTAAAAT---------AA--A--------------------------------------------- 84180
VervetAGM         ATTTTATTTATTTAAAATTAGTAATTCAGATAGTTTCCTTCTGCTCT-----GTTTACTCATTGTGTACTTTCAGTTTTT 90977
Gorrilla          ATTTTATTCATTTAAAATTAGTAATTCAGGTAATTTCCTTCTGCTCT-----GTTTACTCATTGTGTACTTTCAGTTTTT 87561
GrabEatingMacaqueATTTTATTTATTTAAAATTAGTAATTCAGATAGTTTTCTTCTGCTCT-----GTTTACTCATTGTGTACTTTCAGTTTTT 90434
Macaque           ATTTTATTTATTTAAAATTAGTAATTCAGATAGTTTTCTTCTGCTCT-----GTTTACTCATTGTGTACTTTCAGTTTTT 91326
PigTailedMacaque  ATTTTATTTATTTAAAATTAGTAATTCAGATAGTTTTATTCTGCTCT-----GTTTACTCATTGTGTACTTTCAGTTTTT 89885
Bonobo            ATTTTATTCATTTAAAATTAGTAATTCAGGTAATTTCCTTCTGCTCT-----GTTTACTCCTTGTGTACTTTCAGTTTTT 88141
OliveBaboon       ATTTTATTTATTTAAAATTAGTAATTCAGATAGTTTCCTTCTGCTCT-----GTTTACTCATTGTGTACTTTCAGTTTTT 89780
Orangutan         ATTTTATTCATTTAAAGTTAGTAATTCAGGTAGTTTCCTTCTGCTCT-----GTTTACTCATTGTGTACTTTCAGTTTTT 90587

Majority          GACAAAGC-TTTTGTTTTCTT---TTTT------ACTTAAAAACTTTGGTATT--------CTAATTATCCTCCCCCTTC

                          107130    107140    107150    107160    107170    107180    107190    107200

Human             GACAAAGC-TTTTGTTTTCTT---TTTT------ACTTAAAAATTTTGGTATT--------CTAATTATCATCCCCCTTC 89376
Coquerel'sSifaka  GGCAAAGTGTTTTGTTTTTTTGGGTTTTTTTTAAACTTAAAAACTTTGATATTTTAACTGCCACTCTATCCTCCCCTT-- 75868
Ma'sNightMonkey   -ACAAA-------------------------------------------AT------------------------- 84187
VervetAGM         GACAAAGC-TTTTGTTTTCTT---TTTT------ACTTAAAAACTTTGGTTCTATAATTATCTAATTATCCTCCCCCTTC 91047
Gorrilla          GACAAAGC-TTTTGTTTTCTT---TTTT------ACTTAAAAATTCTGGTATT--------CTAATTATCCTCCCCCTTC 87623
GrabEatingMacaqueGACAAA-C-CTTTGTTTTCTT---TTTT------ACTTAAAAACTTTGGTACT--------CTAATTATCCTCCCCCTTC 90495
Macaque           GACAAAGC-TTTTGTTTTCTT---TTTT------ACTTAAAAACTTTGGTACT--------CTAATTATCCTCCCCCTTC 91388
PigTailedMacaque  GACAAAGC-TTTTGTTTTCTT---TTTT------ACTTAAAAACTTTGGTACT--------CTAATTATCCTCCCCCTTC 89947
Bonobo            GACAAAGC-TTTTGTTTTCTT---TTTT------ACTTAAAAATTTTGGTATT--------CTAATTATCATCCCCCTTC 88203
OliveBaboon       GACAAAGC-TTTTGTTTTCTT---TTTT------ACTTAAAAACTTTGGTACT--------CTAATTATCCTCCCCCTTC 89842
Orangutan         GACAAAGC-TTCTGTTTTCTT---TTTT------AGTTAAATATTTTGGTATT--------CTAATTATCCTCCCCCTTC 90649

Majority          CCCTTTAGATCACTTCAGATCCTATTGT--CAGCAATCAGCTGGCTAGAA-CTCTC--ATTACCTGGCTACCTTCTGAGG

                          107210    107220    107230    107240    107250    107260    107270    107280

Human             CCCTTTAGATCACTTCAGATCCTATTGT--CAGCAATCAGCTGGCTAGAA-CTCTCTTGTTACCTGGCTACTTCCTGAGG 89453
Coquerel'sSifaka  --CTTTAAGTCACTTCAGATCTTTTTGTGTTAACAATCAGCTGACTTGAATCTCTCTTATTACCTGGCTACTGCCTGAGG 75946
Ma'sNightMonkey   ---------A--------------------------------GCCAAA----C-----------AGCTACCTCAGGAAG 84210
VervetAGM         CCCTTTAGATCACTTCAGATCCTATTGT--CAGCAATCAGCTGGCTAGAA-CTCTC--ATTACCTGGCTACCTTCTGAGG 91122
Gorrilla          CCCTTTAGATCACTTCAGATCCTATTGT--CAGCAATCAGCTGGCTAGAA-CTCTCTTGTTACCTGGCTACTTCCTGAGG 87700
GrabEatingMacaqueCCCTTTAGATCACTTCAGATCCTATTGT--CAGCAATCAGCTGGCTAGAA-CTCTC--ATTACCTGGCTACCTTCTGAGG 90570
Macaque           CCCTTTAGATCACTTCAGATCCTATTGT--CAGCAATCAGCTGGCTAGAA-CTCTC--ATTACCTGGCTACCTTCTGAGG 91463
PigTailedMacaque  CCCTTTAGATCACTTCAGATCCTATTGT--CAGCAATCAGCTGGCTAGAA-CTCTC--ATTACCTGGCTACCTTCTGAGG 90022
Bonobo            CCCTTTAGATCACTTCAGATCCTATTGT--CGGCAATCAGCTGGCTAGAA-CTCTCTTGTTACCTGGCTACTTCCTGAGG 88280
OliveBaboon       CCCTTTAGATCACTTCAGATCCTATTGT--CAGCAATCAGCTGGCTAGAA-CTCTC--ATTACCTGGCTACCTTCTGAGG 89917
Orangutan         CCCTTTAGATCACTTCAGATCCTATTGT--CAGCAATCAGCTGGCTAGAA-TTCTCTCGTTACCTGGCTACTTGCTGAGG 90726
```

```
Majority          TAGCTGTTTGGTTATTTTGCTTTATTTTAATCCATTCCTCTAT--CACTCATCTGTTCTGTATACAGTTACTATGCCTG
                        107290   107300   107310   107320   107330   107340   107350   107360
Human             TAGCTGTTTGGTTATTTAGCTTTATTTTAATCCATTCCTCTAT--CACTCATCTGTTCTGTATGCAGTTACTATGCCTG 89531
Coquerel'sSifaka  TAGCTGTTTGGTTATTTTGCTTTATTTTAATTCCATTCCTTTAT--CATCAGTCTCTTCTTTGTACAGTTACTGTGCATA 76024
Ma'sNightMonkey   TAG---------------------------------------------------------------------------- 84213
VervetAGM         TAGCTGTTTGGTTATTTTGCTTTATTCTAATCTCATTCCTCTAT--CACTCATCTGTTCTGTATACAGTTACTATGCCTG 91200
Gorrilla          TAGCTGTTTGGTTATTTTGCTTTATTTTAATCCATTCCTCTAT--CACTCATCTGTTCTGTATGCAATTACTATGCCTG 87778
GrabEatingMacaqueTAGCTGTTTGGTTATTTTGCTTTATTCTAATCCATTCCTCTAT--CACTCATCTGTTCTGTATACAGTTACTATGCCTG 90648
Macaque           TAGCTGTTTGGTTATTTTGCTTTATTCTAATCCATTCCTCTAT--CACTCATCTGTTCTGTATACAGTTACTATGCCTG 91541
PigTailedMacaque  TAGCTGTTTGGTTATATTGCTTTATTCTAATCCATTCCTCTAT--CACTCATCTGTTCTGTATACAGTTACTATGCCTG 90100
Bonobo            TAGCTGTTTGGTTATTTAGCTTTATTTTAATCCATTCCTCTAT--CACTCATCTGTTCTGTATGCAATTACTATGCCTG 88358
OliveBaboon       TAGCTGTTTGGTTATTTTGCTTTATTCTAATCCATTCCTCTAT--CACTCATCTGTTCTGTATACAGTTACTATGCCTG 89995
Orangutan         TAGCTGTTTGGTTATTTTGCTTTATTTTAATCCATTCCTCTATATCACTCATCTGTTCTGTATGCAATTACTATGCCTG 90806

Majority          GGACTTTTTATTTTCTTATTCCTTCCCTCCATTATCTCAAAGCTCAGCTGTTTGTGCTGTCTTTTTATTTAGACAGCTTT
                        107370   107380   107390   107400   107410   107420   107430   107440
Human             GGACTT---ATTTTCTTATTCTTTCCCTCCATTATCTCAAAGCTCTGCTGTTTGTGCTGTCTTTTTATTTAGACAGCTTT 89608
Coquerel'sSifaka  AGACTTTCTATTTTCTTATTCCTACCCTTCATTATCTCAAAGCTCAGCTGTTTGTGCAGTCTTTTCATTGAGATGGCTTT 76104
Ma'sNightMonkey   ------------------------------------------TCAGG-----------------TAATGAGAGAGATTC 84233
VervetAGM         GGACTTTTTATTTTCTTATTCGTTCCCTCCATTATCTCAAAGCTCAGCTGTTTGTGCTGTCTTTTTATTTAGACAGCTTT 91280
Gorrilla          GGACTT---ATTTTCTTATTCTTTCCCTCCATTATCTCAAAGCTCTGCTGTTTGTGCTGTCTTTTTATTTAGACAGCTTT 87855
GrabEatingMacaqueGGACTTTTTATTTTCTTATTCCTTCCCTCCATTATCTCAAAGCTCAGCTGTTTGTGCT----TTTTATTTAGACAGCTTT 90724
Macaque           GGACTTTTTATTTTCTTATTCCTTCCCTCCATTATCTCAAAGCTCAGCTGTTTGTGCT----TTTTATTTAGACAGCTTT 91617
PigTailedMacaque  GGACTTTTTATTTTCTTATTCCTTCCCTCCATTATCTCAAAGCTCAGCTGTTTGTGCTGTCTTTTTATTTAGACAGCTTT 90180
Bonobo            GGACTT---ATTTTCTTATTCTTTCCCTCCATTATCTCAAAGCTCTGCTGTTTGTGCTGTCTTTTTATTTAGACAGCTTT 88435
OliveBaboon       GGACTTTTTATTTTCTTATTCCGTCCCTCCATTATCTCAAAGCTCAGCTGTTTGTGCTGTCTTTTTATTTAGACAGCTTT 90075
Orangutan         GGACTTTTTATTTTCTTATTCCTTCCCTCCATTATCTCAAAGCTCTGCTGTTTGTGCTGTCTTTTTATTTAGACAGCTTT 90886

Majority          TTTTCTGACAACCTTAAGTCTACTTATTTATATCACTTTATTTCTCAAACTCATACTTAATAACACATTTTGTCTGAGGT
                        107450   107460   107470   107480   107490   107500   107510   107520
Human             TTTTCTGACAACGTTAAGTCTACTTATTTATATCACTTTATTTCTCAAACTCACACTTAATAACACATTTTATCTGAGGT 89688
Coquerel'sSifaka  CTTATGG-CAACCTTA-GTCTCCT-------------TTATTCCCCAAACTCATATATTATAATATGTTATGTCTGAAGT 76169
Ma'sNightMonkey   TCGCCAG--A--------------------------TGATTGCTGA----------TAATAGGA----T---CGGAAGT 84267
VervetAGM         TTTTCTGACAACCTTAAGTCTACTTATTTATATCACTTTATTTCTCAAACCCATACTTAATAACACATTTTGTCTGAGGT 91360
Gorrilla          TTTTCTGACAACCTTAAGTCTACTTATTTATATCACTTTATTTCTCAAACTCACACTTAATAACACATTTTGTCTGAGGT 87935
GrabEatingMacaqueTTTTCTGACAACCTTAAGTCTACTTATTTATATCACTTTATTTCTCAAACCCATACTTAATAACACATTTTGTCTGAGGT 90804
Macaque           TTTTCTGACAACCTTAAGTCTACTTATTTATATCACTTTATTTCTCAAACCCATACTTAATAACACATTTTGTCTGAGGT 91697
PigTailedMacaque  TTTTCTGACAACCTTAAGTCTACTTATTTATATCACTTTATTTCTCAAACCCATACTTAATAACACATTTTGTCTGAGGT 90260
Bonobo            TTTTCTGACAACCTGAAGTCTACTTATTTATATCACTTAATTTCTCAAACTCACACTTAATAACACATTTTGTCTGAGGT 88515
OliveBaboon       TTTTCTGACAACCTTAAGTCTACTTATTTATATCACTTTATTTCTCAAACCCATACTTAATAACACATTTTGTCTGAGGT 90155
Orangutan         TTTTCTGACAACCTTAAGTCTACTTATTTATATCACTTTATTTCTCAAACTCACACTTAATAACACATTTTGTCTGAGGT 90966
```

```
Majority         AGTAGCACTTTATCTAAACAAAAGTTTGTTCACTTAAAACAGTTATAGA-------------------------------
                       107530    107540    107550    107560    107570    107580    107590    107600
Human            AGT------TTATCTAAACAAAAGTTTGTTCACTTAAAATAGTTATAGA------------------------------- 89731
Coquerel'sSifaka A-TAGCACTTTATC---ATCGAAGCTTGTTCACTTAATAGAATTATAGG------------------------------- 76214
Ma'sNightMonkey  GAT----------CTAAAGGGGA-----------------------AGG------------------------------- 84283
VervetAGM        AGTAGCACTTTATCTAAACAAAAGTTTATTCACTTAAAACAGTTACAGA------------------------------- 91409
Gorrilla         AGT------TTATCTAAACAAAAGTTTGTTCACTTAAAATAGTTATAGA------------------------------- 87978
GrabEatingMacaqueAGTAGCACTTTATCTAAACAAAAGTTTATTCACTTAAAACAGTTACAGA------------------------------- 90853
Macaque          AGTAGCACTTTATCTAAACAAAAGTTTATTCACTTAAAACAGTTACAGA------------------------------- 91746
PigTailedMacaque AGTAGCACTTTATCTAAACAAAAGTTTATTCACTTAAAACAGTTACAGAGGCCGGGCGCGGTGGCTCAAGCCTGTAATCC 90340
Bonobo           AGT------TTATCTAAACGAAAGTTTGTTCACTTAAAATAGTTATAGA------------------------------- 88558
OliveBaboon      AGTAGCACTTTATCTAAACAAAAGTTTATTCACTTAAAACAGTTACAGA------------------------------- 90204
Orangutan        AGT------TTATCTAAACAAAAGTTTGTTCACTTAAAACAGTTATAGA------------------------------- 91009

Majority         --------------------------------------------------------------------------------
                       107610    107620    107630    107640    107650    107660    107670    107680
Human            -------------------------------------------------------------------------------- 89731
Coquerel'sSifaka -------------------------------------------------------------------------------- 76214
Ma'sNightMonkey  -------------------------------------------------------------------------------- 84283
VervetAGM        -------------------------------------------------------------------------------- 91409
Gorrilla         -------------------------------------------------------------------------------- 87978
GrabEatingMacaque-------------------------------------------------------------------------------- 90853
Macaque          -------------------------------------------------------------------------------- 91746
PigTailedMacaque CATCACTTTGGGAGGCCGAGATGGGCGGATCACGAGGTCAGGAGATCGAGACCATCCTGGCTAACACGGTGAAACCCCGT 90420
Bonobo           -------------------------------------------------------------------------------- 88558
OliveBaboon      -------------------------------------------------------------------------------- 90204
Orangutan        -------------------------------------------------------------------------------- 91009

Majority         --------------------------------------------------------------------------------
                       107690    107700    107710    107720    107730    107740    107750    107760
Human            -------------------------------------------------------------------------------- 89731
Coquerel'sSifaka -------------------------------------------------------------------------------- 76214
Ma'sNightMonkey  -------------------------------------------------------------------------------- 84283
VervetAGM        -------------------------------------------------------------------------------- 91409
Gorrilla         -------------------------------------------------------------------------------- 87978
GrabEatingMacaque-------------------------------------------------------------------------------- 90853
Macaque          -------------------------------------------------------------------------------- 91746
PigTailedMacaque CTCTACTAAGAAATACAAAAAATAGCCGGGCGAGGTGGCAGCGCCTGTAGTCCCAGCTACTCGGGAGGCTGAGGCCGGAG 90500
Bonobo           -------------------------------------------------------------------------------- 88558
OliveBaboon      -------------------------------------------------------------------------------- 90204
Orangutan        -------------------------------------------------------------------------------- 91009
```

```
Majority        --------------------------------------------------------------------------------

                       107770    107780    107790    107800    107810    107820    107830    107840

Human           -------------------------------------------------------------------------------- 89731
Coquerel'sSifaka -------------------------------------------------------------------------------- 76214
Ma'sNightMonkey -------------------------------------------------------------------------------- 84283
VervetAGM       -------------------------------------------------------------------------------- 91409
Gorrilla        -------------------------------------------------------------------------------- 87978
GrabEatingMacaque-------------------------------------------------------------------------------- 90853
Macaque         -------------------------------------------------------------------------------- 91746
PigTailedMacaque AATGGCGTGAACCCGGAGGTGGAGGTTGAGCTGAGATCCGGCCACTGCACTCCAGCCTGGGCTACAGAGCGAGACTCCGT 90580
Bonobo          -------------------------------------------------------------------------------- 88558
OliveBaboon     -------------------------------------------------------------------------------- 90204
Orangutan       -------------------------------------------------------------------------------- 91009

Majority        -----------------------------------------ACTTTAAAAT--TTTATAATTCAGTGGGTTTTATTGTAA

                       107850    107860    107870    107880    107890    107900    107910    107920

Human           -----------------------------------------ACTTTAAAAT--TTTATAATTCAGTGGGTTTTATTGTAA 89768
Coquerel'sSifaka ------------------------------------AC----TTTTAAAATGTTTTATAATTCAGTGGG----ATTATAA 76250
Ma'sNightMonkey -------------------------------------------------------------------------------- 84283
VervetAGM       -----------------------------------------ACTTTAAAAT--TTTATAATTCAGTGGGTTTTATTGTAA 91446
Gorrilla        -----------------------------------------ACTTTAAAAT--TTTATAATTCAGTGGGTTTTATTGTAA 88015
GrabEatingMacaque-----------------------------------------ACTTTAAAAT--TTTATAATTCAGTGGGTTTTATAGTAA 90890
Macaque         -----------------------------------------ACTTTAAAAT--TTTATAATTCAATGGGTTTTATTGTAA 91783
PigTailedMacaque CTCAAAACAAACAAACAAACAAACAAACAAACAGTTACAGAACTTTAAAAT--TTTATAATTCAGTGGGTTTTATTGTAA 90658
Bonobo          -----------------------------------------ACTTTAAAAT--TTTATAATTCAGTGGGTTTTATTGTAA 88595
OliveBaboon     -----------------------------------------ACTTTAAAAT--TTTATAAATCAGTGGGTTTTATTGTAA 90241
Orangutan       -----------------------------------------ACTTTAAAAT--TTTATAATTCATTGGGTTTTATTGTAA 91046

Majority        AAGGATTTTATGTACTGTCACTGTAGATAATT-------GGTTTTACAGGTGTAAGTGTAAATTTGCAAGTATATGAAAT

                       107930    107940    107950    107960    107970    107980    107990    108000

Human           AAGGATTTTATGTACTGTCACTGTAGATAATT-------GGTTTTACAGGTGTAAGCGTAAATTTGCAAGTATATGAAAT 89841
Coquerel'sSifaka AAGGATTTTATGTACCATCACTATAGATAATTAAAAATTGGTTTTATAAATGTTAGTGTAAATTTATAAGT--------- 76321
Ma'sNightMonkey -------------------------------------------------------------------------------- 84283
VervetAGM       AAGGATTTTATGTACTGTCACTGTAGATAATT-------GGTTTTACAGGTATAAGTGTAAATTTGTAAGTATATGAAAT 91519
Gorrilla        AAGGATTTTATGTACTGTCACTGTAGATAATT-------GGTTTTACAGGTGTAAGCGTAAATTTGCAAGTATATGAAAT 88088
GrabEatingMacaqueAAGGATTTTATGTACTGTCACTGTAGATAATT-------GGTTTTACAGGTATAAGTGTAAATTTGCAAGTATATGAAAT 90963
Macaque         AAGGATTTTATGTACTGTCACTGTAGATAATT-------GGTTTTACAGGTATAAGTGTAAATTTGCAAGTATATGAAAT 91856
PigTailedMacaque AAGGATTTTATGTACTGTCACTGTAGATAATT-------GGTTTTACAGGTATAAGTGTAAATTTGCAAGTATATGAAAT 90731
Bonobo          AAGGATTTTATGTACTGTCACTGTAGATAATT-------GGTTTTACAGGTGTAAGCGTAAATTTGCAAGTATATGAAAT 88668
OliveBaboon     AAGGATTTTATGTACTGTCACTGTAGATAATT-------GGTTTTACAGGTATAAGTGTAAATTTGCAAGTATATGAAAT 90314
Orangutan       AAGGATTTTATGTACTGTCACTGTAGATAATT-------GGTTTTACAGGTGTAAGCGTAAATTTGCAAGTATATGAAAT 91119
```

```
Majority          TAAGTATGTGAAATGCAGTGTGAGAAACAAATGAGA--ACATTCAGGCTTGTGAGAAGATTGTTTTTTTTT--TTTTCTT

                        108010    108020    108030    108040    108050    108060    108070    108080

Human             TAAGTATGTGAAATGCAGTGTGAGAAACAAATGAGA--AAATTCAGACTTGTGAGAAGATTGTTTTGTTTT--TTTTCTG 89917
Coquerel'sSifaka  ---GTATGTGAAATGTACTGTAAGAAACTAGTGAGAGAAGATTCAGGCCTGAGAGATGAT-ATTTTTTTTT---TTTTCC 76394
Ma'sNightMonkey   ---------------------GAAATGATAATTAGA--ATACCAGAGTTTTTAAGTAAA--------------------- 84319
VervetAGM         TAAGTATGTGAAATGCAGTGCGAGAAACAAATGAGA--ACATTCAGGCTTGTGAGAAGATTGTTTTTTTTTTTTTTTTTT 91597
Gorrilla          TAAGTATGTGAAATGCAGTGTGAGAAACAAATGAGA--AAATTCAGACTTGTGAGAAGATTGTTTTGTTTT--TTTTCTG 88164
GrabEatingMacaqueTAAGTATGTGAAATGCAGTGCGAGAAACAAATAAGA--ACATTCAGGCTTGTGAGAAGATTGTTGTTTTTT-TTTTTCTT 91040
Macaque           TAAGTATGTGAAATGCAGTGCGAGAAACAAATAAGA--ACATTCAGGCTTGTGAGAAGATTGTTGTTTTTT-TTTTT-TT 91932
PigTailedMacaque  TAAGTATGTGAAATGCAGTGCGAGAAACAAATAAGA--ACATTCAGGCTTGTGAGAAGATTGTTTTTTTTT-TTTTTTTC 90808
Bonobo            TAAGTATGTGAAATGCAGTGTGAGAAACAAATGAGA--AAATTCAGACTTGTGAGAAGATTGTTTTGTTTT--TTTTCTG 88744
OliveBaboon       TAAGTATGTGAAATGCAGTGCGAGAAACAAGTGAGA--ACATTCAGGCTTGTGAGAAGATTGTTTTGTTTTGTTTTTTTT 90392
Orangutan         TAAGTATGTGAAATGCAGTGTGAGAAACAAATGAGA--AAATTCAGGCTTGTGAGAAGATTGTTTTGGTTT--TTTTCTG 91195
```

```
Majority          CTGTTAGCCTTGGCGGAAAATCTGAGAAAACTTAAAATATATAACACTGGAGTGACAATCAGGAAGAGTGG--CCCCCCC

                        108090    108100    108110    108120    108130    108140    108150    108160

Human             TGCTTAGCCTTGGCGAAAAATCTGAGAAAACTTAAAATATATAACACTGGAGTGACAATCAGGAAGAGTGGACTCTTTTT 89997
Coquerel'sSifaka  CTGTTAGCCTCAGTTGAAAATAGAAAACTGTTACAAATATATA--A---------CAGT-----GTAGTG---------- 76448
Ma'sNightMonkey   ------A----------------AAGAAAACAAAAGCTTTGT-------------CA------AAAACTG--------A- 84349
VervetAGM         CTGTTAGCCTTGGCGGAAAATCTGAGAAAACTTAAAATATATAACACTGGAGTGACAATCAGGAATAGTG---CCCCCCC 91674
Gorrilla          TGCTTAGCCTTGGCGAAAAATCTGAGAAAACTTAAAATATATAACACTGGAGTGACAATCAGGAAGAGTGGACTCTTTTT 88244
GrabEatingMacaqueCTGTTAGCCTTGGCAGAAAATCTGAGAAAACTTAAAATATATAACACTGGAGTGACAATCAGGAAGAGTG---CCCCCCC 91117
Macaque           CTGTTAGCCTTGGCGGAAAATCTGAGAAAACTTAAAATATATAACACTGGAGTGACAATCAGGAAGAGTGGC-CCCCCCC 92011
PigTailedMacaque  CTGTTAGCCTTGGCGGAAAATCTGAGAAAACTTAAAATATATAACACTGGAGTGACAATCAGGAAGAGTG---CCCCCCC 90885
Bonobo            TGCTTAGCCTTCGCGAAAAATCTGAGAAAACTTAAAATATATAACACTGGAGTGACAATCAGGAAGAGTGGACTCTTTTT 88824
OliveBaboon       CTGTTAGCCTTGGCGGAAAATCTGAGAAAACTTAAAATATATAACACTGGAGTGACAATCAGGAAGAGTGGCCCCCCCCC 90472
Orangutan         TGCTTAGCCTTGGCGAAAAATCTGCGAAAACTTAAAGTGTATAACACTGGAGTGACAATCAGGAAGACTAGACTCTTTTT 91275
```

```
Majority          CCCTT---TTTTTTTAAACAAATTAGAGAAGTAAGTTCAGTGGCTAGTGGTTATCACAATAAGAAAAATTTAAGGAAGTT

                        108170    108180    108190    108200    108210    108220    108230    108240

Human             TTTTTTAATTTTTTTAAACAAATTAGGGAAGTAAGTTCAATGGCTAGTGGTTATCATAATAAGAAAAATTTAAGGAAGTT 90077
Coquerel'sSifaka  -----------------ACATTCAGGAAGAGTAAACT-----T------GTTTTTACA---------AATCAGGGAAGTT 76491
Ma'sNightMonkey   -------------------AATTAGGAAAGTAAGTTCAATGTCTTGTAGTTATAATAAGAAAAATAAATTAAGGAAGTT 84409
VervetAGM         CCCCC---CTTTTTTAAACAAATTAGAGAAGTAAGTTCAGTGGCTGGTGGTTATCACAATAAGAAAAATTTAAGCAAGTT 91751
Gorrilla          TTTA--AATTTTTTAAAACAAATTAGGGAAGTAAATTCAATGGCTAGTGGTTATCATAATAATAAAAATTTAAGGAAGTT 88322
GrabEatingMacaqueCCCTT---TGTTTTTAAACAAATTAGAGAAGTAAGTTCAGTGGCTAGTGGTTATCACAATAAGAAAAATTTAAGCAAGTT 91194
Macaque           CCCTT---TGTTGTTAAACAAATTAGAGAAGTAAGTTCAGTGGCTAGTGGTTATCACAATAAGAAAAATTTAAGCAAGTT 92088
PigTailedMacaque  CCCCT---TTTTTTTAAACAAATTAGAGAAGTAAGTTCAGTGGCTAGTGGTTATCACAATAAGAAAAATTTAAGCAAGTT 90962
Bonobo            TTTA--AATTTTTTAAAACAAATTAGGGAAGTAAGTTCAATGGCTAGTGGTTATCATAATAAGAAAAATTTAAGGAAGTT 88902
OliveBaboon       CCCTT---TTTTTTTAAACAAATTAGAGAAGTAAGTTCAGTGGCTGGTGGTTATCACAATAAGAAAAATTTAAGCAAGTT 90549
Orangutan         TTTT------TTA-AAACAAATTAGGGAAGTAAGTTCAATGGCTAGTGGTTATCATA---AGAAAAATTTAAGGAAGTT 91345
```

```
Majority          ATAGGTAAAATAA----TTTACTT----------TTCTGAATGGTACCTCATATTTTGACAGCATTATCCTTTTCAGATC

                        108250    108260    108270    108280    108290    108300    108310    108320

Human             ATAGGTAAAATAAGAATTTTACTTTTGTTTC-TTTTCTGAATGGTACCTCATATTTTGACAGCATTATCCTTTTCAGATC 90156
Coquerel'sSifaka  GTAAGTAAACAAAGAATTTAATTT----TTTTTTCCTGAATGGTATCTCATACTTTCACAGCATTATCCTTTTCAGATC 76567
Ma'sNightMonkey   ATAGGTAAAAGAA----TTTATAAGAATTTTGTTTTCTGAATGGTACCTCATATTTTGACAGCATTATCCTTTTCAGATC 84485
VervetAGM         ATAGGTAAAATAA----TTTACTT----------TTCTGAATGGTACCTCATTTTTTGACAGCATTATCCTTTTCAGGTC 91817
Gorrilla          ATAGGTAAAATAAGAATTTTACTTTTGTTTC-TTTTCTGAATGGTACCTCATATTTTGACAGCATTATCCTTTTCAGATC 88401
GrabEatingMacaqueATAGGTAAAATAA----TTTACTT----------TTCTGAATGGTACCTCATATTTTGACAGCATTATCCTTTTCAGGTC 91260
Macaque           ATAGGTAAAATAA----TTTACTT----------TTCTGAATGGTACCTCATATTTTGACAGCATTATCCTTTTCAGGTC 92154
PigTailedMacaque  ATAGGTAAAATAA----TTTACTT----------TTCTGAATGGTACCTCATATTTTGACAGCATTATCCTTTTCAGGTC 91028
Bonobo            ATAGGTAAAATAAGAATTTTACTTTTGTTTC-TTTTCTGAATGGTACCTCATATTTTGACAGCATTATCCTTTTCAGATC 88981
OliveBaboon       ATAGGTAAAATAA----TTTACTT----------TTCTGAATGGTACCTCATATTTTGACAGCATTATCCTTTTCAGGTC 90615
Orangutan         ACAGGTAAAATAAGAATTTAA-------------TTCTGAATGGTACCTCATATTTTGACAGCATTATCCTTTTCAGATC 91412

Majority          TGTTTTATATTTAATTGGTTTATTT-ATACATGTAAGTATGAT-AGATTCGTGGATTTAACCAGTTCTTTGTATCTTCTG

                        108330    108340    108350    108360    108370    108380    108390    108400

Human             TGTTTTATATTTAATTGGTTTATT--ATACATGTAAGTATGATTAGATTCATGGATTTAACCAGTTCTTTGTATCTTCTG 90234
Coquerel'sSifaka  TAT--TGTATTTAATTGGTTTATTTTATACATGTAAGTATGACAAGATTCATGGCTTTAACAAATTCTTGGTATCTTCTG 76645
Ma'sNightMonkey   TGTTTTGTATTTAATTGGCTTATTT-ATACATATAAGTATGA---GATTCATGGATTTAACAAATTCTTTGTATCTTCTG 84561
VervetAGM         TGTTTTATGTTTAATTGGTTTATTT-ATACATGTGAGTATGATGATATTCGTGGATTTAACCAGTTCTTTGTGTCTTCTG 91896
Gorrilla          TGTTTTATATTTAATTGGTTTATT--ATACATGTAAGTATGATTAGATTCGTGGATTTAACCAGTTCTTTGTATCTTCTG 88479
GrabEatingMacaqueTGTTTTATGTTTAATTGGTTTATTT-ATACATGTGAGTATGA---GATTCGTGGATTTAACCAGTTCTTTGTATCTTCTG 91336
Macaque           TGTTTTATGTTTAATTGGTTTATTT-ATACATGTGAGTATGA---GATTCGTGGATTTAACCAGTTCTTTGTATCTTCTG 92230
PigTailedMacaque  TGTTTTATGTTTAATTGGTTTATTT-ATACATGTGAGTATGA---GATTCGTGGATTTAACCAGTTCTTTGTATCTTCTG 91104
Bonobo            TGTTTTATATTTAATTGGTTTATT--ATACATGTAAGTATGATTAGATTCATGGATTTAACCAGTTCTTTGTATCTTCTG 89059
OliveBaboon       TGTTTTATGTTTAATTGGTTTATTT-ATACATGTGAGTATGATGAGATTCGTGGATTTAACCAGTTCTTTGTATCTTCTG 90694
Orangutan         TGTTTTATATTTAATTGGTTTATT--ATACATGTAAGTATGATGAGATTCGTGGATTTAACCAGTTCTTTGT-----CTG 91485

Majority          TGTCAAAATGTATACTGTAGTATACTGTTTATATTAGAAACTCTTTGAAAGTGGATGTTCTCTTCT--------CAGTTT

                        108410    108420    108430    108440    108450    108460    108470    108480

Human             TGTCAAAATGTATACTGTAGTATACTGTTTATATTAGAAACTCTTTAAAAGTGGATGTTCTATTCT--------TAATTT 90306
Coquerel'sSifaka  TGTCAGAATGTGTACCATAGTGTACTAATTGTATTAGAAACTCTTGGAGAGTAGATTCTATTGTCAGTTTGAAGCATTGT 76725
Ma'sNightMonkey   TGTCAGAAT-TATACTGTAGTATACCATTTAAACTAGAAACTCTTTGAAAGTGGATGTTCTATTTT--------CAATTT 84632
VervetAGM         TGTCAAAATGTATACTGTAGCATACTGTTTATATTAGAAACTCTTTGAAAGTGGATGTTCTCTTCT--------CAGTTT 91968
Gorrilla          TGTCAAAATGTATACTGTAGTATACTGTTTATATTAGAAACTCTTTGAAAGTGGATGTTCTATTCT--------TAATTT 88551
GrabEatingMacaqueTGTCAAAATGTATACTGTAGTATGCTGTTTATATTAGAAACTCTTTGAAAGTGGATGTTCTC-------------AGTTT 91403
Macaque           TGTCAAAATGTATACTGTAGTATGCTGTTTATATTAGAAACTCTTTGAAAGTGGATGTTCTC-------------AGTTT 92297
PigTailedMacaque  TGTCAAAATGTATACTGTAGTATACTGTTTATATTAGAAACTCTTTGAAAGTGGATGTTCTC-------------AGTTT 91171
Bonobo            TGTCAAAATGTATACTGTAGTATACTGTTTATATTAGAAACTCTTTGAAAGTGGATGTTCTATTCT--------TAATTT 89131
OliveBaboon       TGTCAAAATGTATACTGTAGTATACTGTTTATATTAGAAACTCTTTGAAAGTGGATGTTCTC-------------AGTTT 90761
Orangutan         TGTCAAAATGTATACCGTAGTATACTTTTTATATTAGAAACTCTTTGAAAGTGGATGTTCTATTCT--------CAATTT 91557
```

```
Majority        CCTGATCTTCC--------ACATTGT-----CTTTATCT-----CTCTGCTGTTTTTTA----TTTGTTTTT-------G

                      108490    108500    108510    108520    108530    108540    108550    108560

Human           CCTGATCTTCC--------ACATTGT-----CTTTATCTG-TAACTCTACTGTTTTTTG----TTGGTTTTT-------G 90361
Coquerel'sSifaka CTTTACCTCTCTGCTGCTAATACTTTAATGCCTTTATCCAGTTTCATTACAATCATTTAGACCTTCAATTTAGTCAAAAG 76805
Ma'sNightMonkey CCTGATCGTCC--------ACATT---------T-ATCT----------------------------------------- 84653
VervetAGM       CCTGATCTTCC--------ACATTGT-----CTTTATCT-----CTCTGCTGTTTTTTA----TTTGTTTTTTGTTTTTG 92026
Gorrilla        CCTGATCTTCC--------ACATTGT-----CTTTATCTG-TAACTCTACTGTTTTTTG----TTTGTTTTT-------G 88606
GrabEatingMacaqueCCTGATCTTCC--------ACATTGT-----CTTTATCT-----CTCTGCTGTTTTTTA----TTTGTTTTTG--TTTGG 91459
Macaque         CCTGATCTTCC--------ACATTGT-----CTTTATCT-----CTCTGCTGTTTTTTA----TTTGTTTTTG--TTTGG 92353
PigTailedMacaque CCTGATCTTCC--------ACATTGT-----CTTTATCT-----CTCTGCTGTTTTTTA----TTTGTTTTTG--TTTTG 91227
Bonobo          CCTGATCTTCC--------ACATTGT-----CTTTATCTG-TAACTCTACTGTTTTTTG----TTTGTTTTT-------G 89186
OliveBaboon     CCTGATCTTCC--------ACATTGT-----CTTTATCT-----CTCTGCTGTTTTTTA----TTTGTTTT--------- 90810
Orangutan       CCTGATCTTCT--------GCATTGT-----CTTTATCTG-TAACTCTACTGTTTTTTG----TTTGTTT---------- 91609

Majority        TTTTTGTTTTG--AGACGGAGTCTTGCTCTGTCACCCAGGCTGG--AGTGCA--GTGGCACCATCTC------------A

                      108570    108580    108590    108600    108610    108620    108630    108640

Human           TTTTTGTTTTG--AGACAGAGTCTCGCTCTGTCACCCAGGCTGG--AGTGCACTGTGGCACCATCTC------------A 90425
Coquerel'sSifaka TGTATGACTTATTAAAAATGGTCTTTTTTAGTTGTTGGGACTGGTAAATAAAACATAAAACTGTTTCTAATATCAGATCA 76885
Ma'sNightMonkey -------------------------------------------------------------------------------- 84653
VervetAGM       TTTTTGTTTTG--AGACGGAGTCTTGCTCTGTCACCCAGGCTGG--AGTGCA--GTGGCACCATCTC------------A 92088
Gorrilla        TTTTTGTTTTG--AGACAGAGTCTCGCTCTGTCACCCAGGCTGG--AGTGCACTGTGGCACCATCTC------------A 88670
GrabEatingMacaqueTTTTTGTTTTG--AGACGGAGTCTTGCTCTGTCACCCAGGCTGG--AGTGCA--GTGGCACCATCTC------------A 91521
Macaque         TTTTTGTTTTG--AGACGGAGTCTTGCTCTGTCACCCAGGCTGG--AGTGCA--GTGGCACCATCTC------------A 92415
PigTailedMacaque TTTTTGTTTTG--AGACGGAGTCTTGCTCTGTCACCCAGGCTGG--AGTGCA--GTGGCACCATCTC------------A 91289
Bonobo          TTTTTGTTTTG--AGACAGAGTCTCGCTCTGTCACCCAGGCTGG--AGTGCACTGTGGCACCATCTC------------A 89250
OliveBaboon     ----TGTTTTG--AGACGGAGTCTTGCTCTGTCACCCAGGCTGG--AGTGCA--GTGGCACCATCTC------------A 90868
Orangutan       -----GTTTTG--AGACAGAGTCTCGCTCTGTCACCCAGGCTGG--AGTGCACTGTGGCACCATCTC------------A 91668

Majority        GCTCACT----------GTAACCT-----CCACCTCCTGAATTCAA-GTGATTCTTCTGCCTCAGCCTCCTGAGTAGCTG

                      108650    108660    108670    108680    108690    108700    108710    108720

Human           GCTCACT----------GTAACCT-----CCGCCTCCTGGGTACAA-GCAATTCT---GCCTCAGCCTCCTGAGTAACTG 90486
Coquerel'sSifaka GTTCATTTAGTTTAAAAATAACCCAACAGCTACCTTAAGAAATAACTGTAATGTTCTGTGTTCACGCACTGCACTATGCG 76965
Ma'sNightMonkey -------------------------------------------------------------------------------- 84653
VervetAGM       GCTCACT----------GTAACCT-----CCACCTCCTGAATTCAA-GTGATTCTTCTGCCTCAGCCTCCTGAGTAGCTG 92152
Gorrilla        GCTCACT----------GTAACCT-----CCGCCTCCTGGGTACAA-GCAATTCT---GCCTCAGCCTCCTGAGTAACTG 88731
GrabEatingMacaqueGCTCACT----------GTAACCT-----CCACCTCCTGAATTCAA-GTGATTCTTCTGCCTCAGCCTCCTGAGTAGCTG 91585
Macaque         GCTCACT----------GTAACCT-----CCACCTCCTGAATTCAA-GTGATTCTTCTGCCTCAGCCTCCTGAGTAGCTG 92479
PigTailedMacaque GCTCACT----------GTAACCT-----CCACCTCCTGAATTCAA-GTGATTCTTCTGCCTCAGCCTCCTGAGTAGCTG 91353
Bonobo          GCTCACT----------GTAACCT-----CTGCCTCCTGGATACAA-GCAATTCT---GCCTCAGCCTCCTGAGTAACTG 89311
OliveBaboon     GCTCACT----------GTAACCT-----CCACCTCCTGAATTCAA-GTGATTCTTCTGCCTTAGCCTCCTGAGTAGCTG 90932
Orangutan       GCTCACT----------GTAACCT-----CCACCTCCTGGGTTCAA-GCAATTCTTCTGCCTCAGCCTCCTGAGTAGCTG 91732
```

```
Majority         --GGACTACAGGCACGAGCCACCATGCCTAGCTAATTT-----TTG--------TATTTT---TAGTAGAGACGGGGTGT

                       108730    108740    108750    108760    108770    108780    108790    108800

Human            --GGACCACAGGCACGAGCCACCACGCCCAGCTAATTT-----TTG--------TATTTT---TAGTAGAGATGGGGTGT 90548
Coquerel'sSifaka CCGGGGTAGAGAAAGTAATTCATGTCTCCATGTACTTTACAGTCTGAAGGAGAATATTGTAGATAATGACAATAAATTGT 77045
Ma'sNightMonkey  -------------------------------------------------------------------------------- 84653
VervetAGM        --GGACTACAGGCACGAGCCACCACGCCTGGCTAATTT-----TTG--------TATTTT---TAGTAGAGACGGGGTGT 92214
Gorrilla         --GGACCACAGGCACGAGTCACCACGCCCAGCTAATTT-----TTG--------TATTTT---TAGTAGAGATGGGGTGT 88793
GrabEatingMacaque--GGACTACAGGCACGAGCCACCATGCCTGGCTAATTT-----TTG--------TATTTT---TAGTAGAGACGGGGTGT 91647
Macaque          --GGACTACAGGCACGAGCCACCATGCCTGGCTAATTT-----TTG--------TATTTT---TAGTAGAGACGGGGTGT 92541
PigTailedMacaque --GGACTACAGGCACGAGCCACCATGCCTAGCTAATTT-----TTG--------TATTTT---TAGTAGAGACGGGGTGT 91415
Bonobo           --GGACCACAGGCACGAGCCACCACGCCCAGCTAATTT-----TTG--------TATTTT---TAGTAGAGATGGGGTGT 89373
OliveBaboon      --GGACTACAGGCACGAGCCACCATGCCTGGCTAATTT-----TTG--------TATTTT---TAGTAGAGACGGGGTGT 90994
Orangutan        --GGACCACAGGCACAAGCCACCACGCCCAGCTAATTT-----TTG--------TATTTT---TAGTAGAGACGGCGTGT 91794

Majority         CACCATAT----------TGGC-CAGGCTG-GTCT--TGAGCTCCTAGACCTTGT-----------GATCCGCCCACCTG

                       108810    108820    108830    108840    108850    108860    108870    108880

Human            TACCATAT----------TGGC-CAGGCTG-GTCT--CGAACTCCTAGACCTTGT-----------GATCCGCCCACCTC 90603
Coquerel'sSifaka GATGATAAACTTAAAAAATGGTACAGAGTACGTCTAGTGAGGTTCTGAACCTTTCCAGAGGATAAAGAAAGGCCTTCCTG 77125
Ma'sNightMonkey  -------------------------------------------------------------------------------- 84653
VervetAGM        CACCATAT----------TGGC-CAGGCTG-GTCT--TGAGCTCCTAGACCTCGT-----------GATCCGCCCACCTG 92269
Gorrilla         CACCATAT----------TGGC-CAGTCTG-GTCT--CGAACTCCTAGACCTTGT-----------GATCCGCCCACCTC 88848
GrabEatingMacaqueCACCATAT----------TGGC-CAGGCTG-GTCT--TGAGCTCCTAGACCTCGT-----------GATCCGCCCACCTG 91702
Macaque          CACCATAT----------TGGC-CAGGCTG-GTCT--TGAGCTCCTAGACCTCGT-----------GATCCGCCCACCTG 92596
PigTailedMacaque CACCATAT----------TGGC-CAGGCTG-GTCT--TGAGCTCCTAGACCTCGT-----------GATCCGCCCACCTG 91470
Bonobo           CACCATAT----------TGGC-CAGGCTG-GTCT--CAAACTCCTAGACCTTGT-----------GATCCGCCCACCTC 89428
OliveBaboon      CACCATAT----------TGGC-CAGGCTG-GTCT--TGAGCTCCTAGACCTCGT-----------GATCCGCCCACCTG 91049
Orangutan        CACCATAT----------TGGC-CAGGCTG-GTCT--CGAACTCCTAGACCTTGT-----------GATCCACCCACCTC 91849

Majority         GG-------CCTCCCAAAGTGCTGGGATTACAGGCGTGAGCCAACGCGCCTGGCATCTACTGTTAATACTTTAATGTTTT

                       108890    108900    108910    108920    108930    108940    108950    108960

Human            AG-------CCTCCCAAAGTGTTGGGATTACAGGCGTGAGCCAACGCGCCCGGCATCTACTGTTAATACTTTA------T 90670
Coquerel'sSifaka GAGAAGTGACTTTGAAGAATAAGTAGACTAAAAGGGAAAGGAAAGGACATAGGAAATTGTTATAGATAGAAGATCATGTA 77205
Ma'sNightMonkey  -------------------------------------------------------CTACTGTTAATACTTTCATGTTTT 84677
VervetAGM        GG-------CCTCCCAAAGTGCTGGGATTACAGGCGTGAGACAACACGCCTGGCATCTACTGTTA-TACTTTAATGTTTT 92341
Gorrilla         AG-------CCTCCCAAAGTGTTGGGATTACAGGCGTGAGCCAACGCGCCCGGCATCTACTGTTAATAGTTTAATGTTTT 88921
GrabEatingMacaqueGG-------CCTCCCAAAGTGCTGGGATTACAGGCGTGAGCCAACACGCCTGGCATCTACTGTTA-TACTTTAATGTTTT 91774
Macaque          GG-------CCTCCCAAAGTGCTGGGATTACAGGCGTGAGCCAACACGCCTGGCATCTACTGTTA-TACTTTAATGTTTT 92668
PigTailedMacaque GG-------CCTCCCAAAGTGCTGGGATTACAGGCGTGAGCCAACACGCCTGGCATCTACTGTTA-TACTTTAATGTTTT 91542
Bonobo           AG-------CCTCCCAAAGTGTTGGGATTACAGGCGTGAGCCAACGCGCCCGGCATCTACTGTTAATACTTTAATGTTTT 89501
OliveBaboon      AG-------CCTCCCAAAGTGCTGGGATTACAGGCGTGAGCCAACACGCCTGGCATCTACTGTTA-TATTTTAATGTTTT 91121
Orangutan        AG-------CCTCCCAAGGTGCTGGAAATCCAGGCGTGAGCCAACGCACCCGGCATCTACTATTAATACTTTAATGTTTT 91922
```

```
Majority         TATCTAGTTGATTATA---GTCATTTAGACCT------------TCAGTTTAGCCAAAGTGCATGACATATTAGAAATGG

                       108970    108980    108990    109000    109010    109020    109030    109040

Human            TATCTAGTTGATTATA---GTCATTTAGACCT------------TCAGTTTAGCCAAAGTGCATGACATATTAGAAATGG 90735
Coquerel'sSifaka CACAAGAATAAAAATAAGAGACACTTGCACTTAGTGTTAGGATATCTGTTTTGTATAGCTGGAGCATAAAGTGCAAGCAG 77285
Ma'sNightMonkey  TACCTAGTTGATTATA---GTCATTTAGACTT------------TCGGTTTAGCCAAAGTGCATGACATATTAGAAATGG 84742
VervetAGM        TTTCTAGTTGATTATA---GTCATTTAGACCT------------TCAGTTTAACCAAAGGGCATGACATACTAGAAATGG 92406
Gorrilla         TATCTAGTTGATTATA---GTCATTTAGACCT------------TCAGTTTAGCCAAAGTGCATGACATATTAGAAATGG 88986
GrabEatingMacaqueCATCTAGTTGATTATA---GTCATTTAGACCT------------TCAGTTTAACCAAAGGGCATGACATACTAGAAATGG 91839
Macaque          CATCTAGTTGATTATA---GTCATTTAGACCT------------TCAGTTTAACCAAAGGGCATGACATACTAGAAATGG 92733
PigTailedMacaque CATCTAGTTGATTATA---GTCATTTAGACCT------------TCAGTTTAACGAAAGGGCATGACATACTAGAAATGG 91607
Bonobo           TATCTAGTTGATTGTA---GTCATTTAGACCT------------TCAGTTTAGCCAAAGTGCATGACATATTAGAAATGG 89566
OliveBaboon      CATCTAGTTGATTATA---GTCATTTAGACCT------------TCAGTTTAACCAAAGGGCATGACATACTAGAAATGG 91186
Orangutan        TATCTAGTTGATTATA---GTCATTTAGACCT------------TCAGTTTAGCCAAAGTGCATGGCATATTAGAAATGG 91987

Majority         TCT--TTTTTAGTTATTTTGACT-----GGTGAATAAAA-----------CATGTAACTGT--TTCTAGCATCAGACCAG

                       109050    109060    109070    109080    109090    109100    109110    109120

Human            TCT--TTTTTAGTTATTTTGACT-----GGTGAATAAAA-----------CATATAACT----TTCTAGCATCAGACCAG 90793
Coquerel'sSifaka TATAGTACTAAGATACCATAAATTCCACGAGGGCTGGGATTGTGTCTTATCTTACCACTTTATTCCCAGCACTAGCACAG 77365
Ma'sNightMonkey  TCT--TTTT-AGTTATTGTGACT-----GGTGAATAAAA-----------CATATAATTGT--TTCTAGTATCAGACCAG 84801
VervetAGM        TCT--TTTTTAGTTATTTTGACT-----GGTGAATAAAA-----------CATGTAACTGT--TTCTAGCATCAGACCAG 92466
Gorrilla         TCT--TTTTTAGTTATTTTGACT-----GGTGAATAAAA-----------CATGTAACT----TTCTAGCATCAGACCAG 89044
GrabEatingMacaqueTCT--TTTTTAGTTATTTTGACT-----GGTGAATAAAA-----------CATGTAACTGT--TTCTAGCATCAGACCAG 91899
Macaque          TCT--TTTTTAGTTATTTTGACT-----GGTGAATAAAA-----------CATGTAACTGT--TTCTAGCATCAGACCAG 92793
PigTailedMacaque TCT--TTTTTAGTTATTTTGACT-----GGTGAATAAAA-----------CATGTAACTGT--TTCTAGCATCAGACCAG 91667
Bonobo           TCT--TTTTTAGTTATTTTGACT-----GGTGAATAAAA-----------CATATAACT----TTCTAGCATCAGACCAG 89624
OliveBaboon      TCT--TTTTTAGTTATTTTGACT-----GGTGAATAAAA-----------CATGTAACTGT--TTCTAGCATCAGACCAG 91246
Orangutan        TCT--TTTTTAGTTATTTTGACT-----GGTGAATAAAA-----------CATATAACTGT--TTGTAGCATCACACCAG 92047

Majority         TTCATTTAGTTTAAAAATGATCCAGCAG---CTATGTCAAGAAAT----AGTCATGTA-ATGTT---CTTTGTTCACACA

                       109130    109140    109150    109160    109170    109180    109190    109200

Human            TTCATTTAGTTTAAAAATGATCCAGCAG---CTATGTCAAGAAAT----AGTCATGTA-ATGTT---CTGTTTTCACACA 90862
Coquerel'sSifaka TG-ATTGATATTGCACATCATTGATAAAAATCTGCTCAAAGAAGCTGAAAGTTATATGCAGGTTGAGTATTGCTTATCCA 77444
Ma'sNightMonkey  TTCATTTAGTTTAAAAATGACCCAGCAG---CTATGTCAAGAAAT----AGTCATGTA-ATGTT---CTGTGTTCACACA 84870
VervetAGM        TTCATTTAGTTTAAAAATGATCCAGCAG---CTATGTCAAGAAAT----AGTAATGTA-ATGTT---CTTTGTTCACACA 92535
Gorrilla         TTCATTTAGTTTAAAAATGATCTAGCAG---CTATGTCAAGAAAT----AGTCATGTA-ATGTT---CTGTTTTCACACA 89113
GrabEatingMacaqueTTCATTTAGTTTAAAAATGATCCAGCAG---CTATGTCAAGAAAT----AGTCATGTA-ATGTT---CTTTGTTCACACA 91968
Macaque          TTCATTTAGTTTAAAAATGATCCAGCAG---CTATGTCAAGAAAT----AGTCATGTA-ATGTT---CTTTGTTCACACA 92862
PigTailedMacaque TTCATTTAGTTTAAAAATGATCCAGCAG---CTATGTCAAGAAAT----AGTCATGTA-ATGTT---CTTTGTTCACACA 91736
Bonobo           TTCATTTAGTTTAAAAATGATCCAGCAG---CTATGTCAAGAAAT----AGTCATGTA-ATGTT---CTGTTTTCACACA 89693
OliveBaboon      TTCATTTAGTTTAAAAATGATCCAGCAG---CTATGTCAAGAAAT----AGTCATGTA-ATGTT---CTTTGTTCACACA 91315
Orangutan        TTCGATTAGTTTAAAAATGATCCAGCAG---CTATGTCAAGAAAT----AGTCATGTA-ATGTT---CTGTTTTCACACA 92116
```

```
Majority           CTGCACTATGTTCTGGGG------TAGAG---ACAAAGGTTTGGATTTTGGAATATTTGCATTATACCAATTGAGCTTCC

                         109210    109220    109230    109240    109250    109260    109270    109280

Human              CTGCACTATGTTCTGGGG------TAGAG---ACAAAGGTTTGGATTTTGGAATATTTGCATTATACCAATTGAGCTTCC 90933
Coquerel'sSifaka   AAATGATAAGGGCCAGAAGTGTTTCAGAGTTGACAATTTTTCAGATTTTGGAATATTTGCATTATACCAGTTGAGCATCC 77524
Ma'sNightMonkey    CTGCACTATGTCCTGGGA------TAGAG---ACAAAGGTTAGGATTTTGTA-TATTTGCGTTATCCCAGTTGAGCTTCC 84940
VervetAGM          CTGCACTATGTTCTGGGG------TAGAG---GCAAAGGTTTGAATTTTGGAATATTTGCATTATACCAATTGAACTTCC 92606
Gorrilla           CTGCACTATGTTCTGGGG------TAGAG---ACAAAGGTTTGGATTTTGGAATATTTGCATTATACCAGTTGAGCTTCC 89184
GrabEatingMacaqueCTGCACTATGTTCTGGGG------TAGAG---GCAAAGGTTTGAATTTTGGAATATTTGCATTATACCAATTGAGCTTTC 92039
Macaque            CTGCACTATGTTCTGGGG------TAGAG---GCAAAGGTTTGAATTTTGGAATATTTGCATTATACCAATTGAGCTTTC 92933
PigTailedMacaque   CTGCACTATGTTCTGGGG------TAGAG---GCAAAGGTTTGAATTTTGGAATATTTGCATTATACCAGTTGAGCTTTC 91807
Bonobo             CTGCACTATGTTCTGGGG------TAGAG---ACAAAGGTTTGGATTTTGGAATATTTGCATTATACCAATTGAGCTTCC 89764
OliveBaboon        CTGCACTATGTTCTGGGG------TAGAG---GCAAAGGTTTGAATTTTGGAATATTTGCATTATACCAATTGAGCTTCC 91386
Orangutan          CTGCACTATGTTCTGGGG------TAGAG---ACAAAGTTTTGGATTTTGGAATATTTGCATTATACCAGTTGAGCTTCC 92187

Majority           CAAATCTGAAATGCTCCAATGAGCATTTCCC-TTGAGCATCTTGTTGGCATTCAGTTTTGGATTTTGGAACATTTCAGAT

                         109290    109300    109310    109320    109330    109340    109350    109360

Human              CAAATCTGAAATGTTCCAATGAGCATTTCCC-TTGAGCATCATGTTGGCCCTCAGTTTTGGATTTTGGAACATTTCAGAT 91012
Coquerel'sSifaka   CAAATCTGAAAATCCAAAATCAGAAATGCTC------TAGTGGGCACTCAAAAAGCTTTGAATCTTAGAGAATTTCAGAT 77598
Ma'sNightMonkey    CAAATCTGAAATGCTCCAATGAGCATTTCCC-TTGAACATAATGTTGGCACTCAGTTTTGGATTTTGGAACATTTGAGAT 85019
VervetAGM          CAAATCTGAAATGCTGCAGTGATCATTTCCCCTTGAGCAACTTGTTGGCATTCAGTTTTGGATTTTGGAACATTTCAGAT 92686
Gorrilla           CAAATCTGAAATGTTCCAATGAGCGTTTCCC-TTGAGCATCATGTTGGCCCTCAGTTTTGGATTTTGGAACATTTCAGAT 89263
GrabEatingMacaqueCAAATCTGAAATGCTCCAGTGATCATTTCCC-TTGAGCAACTTGTTGGCATTCAGTTTTGGATTTTGGAACATTTCAGAT 92118
Macaque            CAAATCTGAAATGCTCCAGTGATCATTTCCC-TTGAGCAACTTGTTGGCATTCAGTTTTGGATTTTGGAACATTTCAGAT 93012
PigTailedMacaque   CAAATCTGAAATGCTCCAGTGATCATTTCCC-TTGAGCAACTTGTTGGCATTCAGTTTTGGATTTTGGAACATTTCAGAT 91886
Bonobo             CAAATCTGAGATGTTCCAATGAGCATTTCCC-TTGAGCATCATGTTGGCCCTCAGTTTTGGATTTTGGAACATTTCAGAT 89843
OliveBaboon        CAAATCTGAAATGCTCCAGTGATCATTTCCC-TTGAGCAACTTGTTGGCATTCAGTTTTGGATTTTGGAACATTTCAGAT 91465
Orangutan          CAAATGTGAAATGCTCGAATGAGCATTTCCC-TTGAGCATCATGTTGGCACTCAGTTTTGGATTTTGGAACATTTCAGAT 92266

Majority           TTCAGATTTCTGCATTAAGGATACTCAACCTGTA-------CAGACTGGGAAAGGTATAAGCCATGTTAAAGAATGAGTT

                         109370    109380    109390    109400    109410    109420    109430    109440

Human              TTCAGATTTCTGCATTAAGGATACTCAACCTGTA-------CAGACTGGGAAAGGTATAAGCCATGTTAAAGAATGAGTT 91085
Coquerel'sSifaka   TTTGGATTTTTGAATTAAGGATACTCAACCTGTATGAGGGTCAGATTGGAAAAAGTCTAAGCCATTTTAAGGAATGA--T 77676
Ma'sNightMonkey    TTTGGATTTCTGCATTAAGGATACTCAACCTGTA-------CAGACTGGGAAAGGTACAAGTCATGTTAAATAATGAGTT 85092
VervetAGM          TTCAGATTTCTGCATTAAGGATACTCAACCTGTA-------CAGACTGGGAAAGGTATAAGCCATATTAAAGAATGAGTT 92759
Gorrilla           TTCAGATTTCTGCATTAAGGATACTCAACCTGTA-------CAGACTGGGAAAGGTATAAGCCATGTTAAAGAATGAGTT 89336
GrabEatingMacaqueTTCAGATTTCTGCATTAAGGATACTCAACCTGTA-------CAGACTGGGAAAGGTATAAGCCATATTAAAGAATGAGTT 92191
Macaque            TTCAGATTTCTGCATTAAGGATACTCAACCTGTA-------CAGACTGGGAAAGGTATAAGCCATATTAAAGAATGAGTT 93085
PigTailedMacaque   TTCAGATTTCTGCATGAAGGATACTCAACCTGTA-------CAGACTGGGAAAGGTATAAGCCATATTAAAGAATGAGTT 91959
Bonobo             TTCAGATTTCTGCATTAAGGATACTCAACCTGTA-------CAGACTGGGAAAGGTATAAGCCATGTTAAAGAATGAGTT 89916
OliveBaboon        TTCAGATTTCTGCATTAAGGATACTCAACCTGTA-------CAGACTGGGAAAGGTATAAGCCATATTAAAGAATGAGTT 91538
Orangutan          TTCAGATTTCTGCATTAAGGATACTCAACCTGTA-------CAGACTGGGAAAGGTATAAGCCATGTTAAAGAATGAGTT 92339
```

```
Majority        TCAACTTTGTCCTTAAGGGTTTTAAGCAGGTATTGACATGATCAGATTTGTAATTTACGAAGGTCATCAAGCTGCTGTAT

                      109450    109460    109470    109480    109490    109500    109510    109520

Human           TCAACTTTGTCCTTAAGGGTTTTAAGCAGGTATTGACGTGATCAGATTTGTAATTTATGAAGGTCATCAAGCTGCTGTAT 91165
Coquerel'sSifaka -AGACTTTGTCCCTA-AGGTTTTAAGCAGGTAATGGCATGATTAAATTTATAATTTGCAAAGGTCATCAAGCTGATATGT 77754
Ma'sNightMonkey TAGACTTTGTCCTTAAGGGTTTTAAGTAGGTATTGACATGATCAGATTTGTAATTTACAAACGTCATCATGCTGCTGTAT 85172
VervetAGM       TCAACTTTGTCCTGAAGAGTTTTAAGCATGTGTTGACATGATCAGATTTGTAATTTACGAAGGTCATCAAGCTGCTATAT 92839
Gorrilla        TCAACTTTGTCCTTAAGGGTTTTAAGCAGGTATTGACGTGATCAGATTTGTAATTTATGAAGGTCATCAAGCTGCTGTAT 89416
GrabEatingMacaqueTCAACTTTGTCCTT--GAGTTTTAAGCATGTGTTGACATGATCAGATTTGTAATTTACGAAGGTCATCAAGCTGCTGTAT 92269
Macaque         TCAACTTTGTCCTT--GAGTTTTAAGCATGTGTTGACATGATCAGATTTGTAATTTACGAAGGTCATCAAGCTGCTGTAT 93163
PigTailedMacaque TCAACTTTGTCCTT--GAGTTTTCAGCATGTGTTGACATGATCAGATTTGTAATTTACGAAGGTCATCAAGCTGCTGTAT 92037
Bonobo          TCAACTTTGTCCTTAAGGGTTTTAAGCAGGTATTGACGTGATCAGATTTGTAATTTATGAAGGTCATGAAGCTGCTGTAT 89996
OliveBaboon     TCAACTTTGTCCTTAAGAGTTTTAAGCATGTGTTGACATGATCAGATTTGTAATTTACGAAGGTCATCAAGCTGCTGTAT 91618
Orangutan       TCGACTTTGTCCTTAAGGGTTTTAAGCAGGTATTGACATGATCAGATTTGTAATTTATGAAGGTCATCAAGCTGCTGTAT 92419

Majority        AGAAAATGGATTGGAAAAAAA----AAAAA--TCCAACAGGAAGACAAATGATGA----GACTGAGTTATCCAGATAAAA

                      109530    109540    109550    109560    109570    109580    109590    109600

Human           AGAAAATGGATTGGAAAAAT-----TA-----TCCAACAGGAAGACAAAT-ATGA----GACTGAGTTATCCAGATAAAA 91230
Coquerel'sSifaka ATAGAATGGATTGAAAAAAATACAGAGAAATATCCAGTAGGAAGACAAATGGGGACACTGATTGAGTTATCCAGATAAAA 77834
Ma'sNightMonkey AGAAAATGGATTGGAAAAAAG----AATTA--TCCAACAGGAAGACAAATGAGGA----GACTGAGTTATCCAGATAAAA 85242
VervetAGM       AGAAAATGGATTGGAAAAAAA----AAAAA--TCCAACAGGAAGACAAACGATGA----GACTGAGGTATCCAGATAAAA 92909
Gorrilla        AGAAAATGGATTGGAAAAAA-----TA-----TCCAACAGGAAGACAAAT-AAGA----GACTGAGTTATCCAGATAAAA 89481
GrabEatingMacaqueAGAAAATGGATTGGAAAAAAA----AAAAAA-TCCAGCAGGAAGACAAACGATGA----GACTGAGGTATCCAGATAAAA 92340
Macaque         AGAAAATGGATTGGAAAAAAA----AAAAAAATCCAGCAGGAAGACAAACGATGA----GACTGAGGTATCCAGATAAAA 93235
PigTailedMacaque AGAAAATGGATTGGAAAAAAA----AAAA---TCCAGCAGGAAGACAAACGATGA----GACTGAGGTATCCAGATAAAA 92106
Bonobo          AGAAAATGGATTGGAAAAAAA----TA-----TCCAACAGGAAGACAAAT-ATGA----GACTGAGTTATCCAGATAAAA 90062
OliveBaboon     AGAAAATGGATTGGAAAAAAA----AAAAA--TCCAACAGGAAGACAAACGATGA----GACTGAGGTATCCAGATAAAA 91688
Orangutan       AGAAAATGGATTGGAAAAAA-----TA-----TCCAACAGGAAGACAAAT-ATGA----GACTGAGTTATCTAGATAAAA 92484

Majority        AATAATGGCAGTGAAAAGTAGAGAAAT----AGAACATGAATTTGAGAGATAATTAGGAGGAAGGCTATCACAGTATTTA

                      109610    109620    109630    109640    109650    109660    109670    109680

Human           AATAATGTCAGCGAGAAGTAGAGAAAC----AGATCATGAATTTGAGGGACAATTAGGAGGAAGGCTATCACAATATTTA 91306
Coquerel'sSifaka TATA------GTAAAAAGTGGAGAAATGGAGAGAACATGAATTTGAGAGACAGTTAGGAGAAGGAATATTACA--A--TA 77904
Ma'sNightMonkey AATAATGGCAGTGAAAAGTAGAGAAAT----AGAGCATGAATTTGAGAGATAATTAGGAGGAAGACTATCACAGGATTTA 85318
VervetAGM       AATAATGGCAGTGAAAAGTAGAGAAAT----AGAACATGAATTTGAGAGATAATTAGCAGGAAGGCTATCACAGTATTTA 92985
Gorrilla        AATAATGGCAGCGAGAAGTAGAGAAAC----AGATCATGAATTTGAGGGATAATTAGGAGGAAGGCTATCACAATATTTA 89557
GrabEatingMacaqueAATAATGGCAGTGAAAAGTAGAGAAAT----AGAACATGAATTTGAGAGATAATTAGGAGGAAGGCTATCACAGTATTTA 92416
Macaque         AATAATGGCAGTGAAAAGTAGAGAAAT----AGAACATGAATTTGAGAGATAATTAGGAGGAAGGCTATCACAGTATTTA 93311
PigTailedMacaque AATAATGGCAGTGAAAAGTAGAGAAAT----AGAACATGAATTTGAGAGATAATTAGGAGGAAGGCTATCACAGTATTTA 92182
Bonobo          AATAATGTCAGCGAGAAGTAGAGAAAC----AGATCATGAATTTGAGGGACAATTAGGAGGAAGGCTATCACAATATTTA 90138
OliveBaboon     AATAATGGCAGTGAAAAGTAGAGAAAT----AGAACATGAATTTGAGAGATAATTAGGAGGAAGGCTATCACAGTATTTA 91764
Orangutan       AATAATGGCAGTGAAAAGTAGAGAAAT----AGATCATGAATTTGAGGGACAATTAGGAGGAAGGCTATCGCAATATTTA 92560
```

```
Majority          GCAATTAGGATGTGTAACAGAAGGGAGAAAAAGAGCTAGGGATGATTATTAAGTTTCTTTCTTGTGTAGCTCACTATATG
                        109690    109700    109710    109720    109730    109740    109750    109760
Human             GCAATTAGGATGTGTAACAGAAGGGAGAAAAAGAGCTAGGGATGCTTATTAAGTTTCTTTCTTGTGTAGCTCACTATACG 91386
Coquerel'sSifaka  GCAATTAGGATGTGTAGCAGAAGGGAGAAAAAGAGAGATGACTA--TACTAAGTATCTTTCTTGGGTAGCTAAGTATATG 77982
Ma'sNightMonkey   GCAATTAGGATGTGGAGCAGGAGGGAGAAAAAGAGCTAGGGATTATTATTAAGTTTCTTCCTTGTGTAGCTAACTATACG 85398
VervetAGM         GCAATTAGGATATGTAACAGAAGGGAGAAAAAGAGCTAGGGATGATTATTAAGTTTCTT----GTGTAGCTCACTATATG 93061
Gorrilla          GCAATTAGGATGTGTAACAGAAGGGAGAAAAAGAGCTAGGGATGCTTATTAAGTTTCTTTCTTGTGTAGCTCACTATACG 89637
GrabEatingMacaqueGCAATTAGGATGTGTAACAGAAGGGAGAAAAAGAGCTAGGGATGATTATTAAGTTTCTT----GTGTAGCTCACTATATG 92492
Macaque           GCAATTAGGATGTGTAACAGAAGGGAGAAAAAGAGCTAGGGATGATTATTAAGTTTCTT----GTGTAGCTCACTATATG 93387
PigTailedMacaque  GCAATTAGGATGTGTAACAGAAGGGAGAAAAAGAGCTAGGGATGATTATTAAGTTTCTT----GTGTAGCTCACTATATG 92258
Bonobo            GCAATTAGGATGTGTAACAGAAGGGAGAAAAAGAGCTAGGGATGCTTATTAAGTTTCTTTCTTGTGTAGCTCACTATACG 90218
OliveBaboon       GCAATTAGGATGTGTAACAGAAGGGAGAAAAAGAGCTAGGGATGATTATTAAGTTTCTT----GTGTAGCTCACTATATG 91840
Orangutan         GCAATTAGGATGTGTAACAGAAGGGAGAAAAAGAGCTAGGGATGCTTATCAAGTTTCTTTCTAGTGTAGCTCACTATATA 92640

Majority          ATTTGTCAGCAGGATTGTTGAGGAAGATAGATATTCATAATCTGGATTTTCATTCTATGAAAGTAGCTGGCTTTTTTGCC
                        109770    109780    109790    109800    109810    109820    109830    109840
Human             ATTTGTTAGCAGGATTGTTGAGGAAGATAGATATTCATAATCTGGATTTTCATTCTATGAAAGTAGCTGGCTTTTTTGCC 91466
Coquerel'sSifaka  ATTTATCAGCAGGATTATTGAGGAAGGTGGATAGTCATAATCTGGATTTTTGTTCTGTGAAAGAAGCCAGCTTTTTTACC 78062
Ma'sNightMonkey   ATTTATCAGCAGGATTGTTGAGGAAGATAGATATTCATAATCTGAATTTTCATTCTTTGAAAGTAGCTGGCTTTT--GCT 85476
VervetAGM         ATTTGTCAGCAGGATTGTTGAGGAAGATAGATAGTCATAATCCGGATTTTCATTCTATGAAAGTAGCTGGCTTTTTTGCC 93141
Gorrilla          ATTTGTTAGCAGGATTGTTGAGGAAGATAGATATTCATAATCTGGATTTTCATTCTATGAAAGTAGCTGGCTTTTTTGCC 89717
GrabEatingMacaqueATTTGTCAGCAGGATTGTTGAGGAAGATAGATATTCATAATCTGGATTTTCATTCTATGAAAGTAGCTGGCTTTTTTGCC 92572
Macaque           ATTTGTCAGCAGGATTGTTGAGGAAGATAGATATTCATAATCTGGATTTTCATTCTATGAAAGTAGCTGGCTTTTTTGCC 93467
PigTailedMacaque  ATTTGTCAGCAGGATTGTTGAGGAAGATAGATATTCATAATCTGGATTTTCATTCTATGAAAGTAGCTGGCTTTTTTGCC 92338
Bonobo            ATTTGTTAGCAGGATTGTTGAGGAAGATAGATATTCATAATCTGGATTTTCATTCTATGAAAGTAGCTGGCTTTTTTGCC 90298
OliveBaboon       ATTTCTCAGCAGGATTGTTGAGGAAGATAGATATTCATAATCTGGATTTTCATTCTATGAAAGTAGCTGGCTTTTTTGCC 91920
Orangutan         ATTTGTTAGCAGGATTGTTGAGGAAGATAGATATTCATAATCTGGATTTTCATTCTATGAAAGTAGCTGGCTTTTTTGCC 92720

Majority          AGAATGCCTTGTCTAGTACAAATGAAAATGTACTT-AAGTCTTGTCACCAATGGCTATGGTAAATTGGTGATTTATTTTA
                        109850    109860    109870    109880    109890    109900    109910    109920
Human             AGAATGCCTTGTCTAGTTCAAATGAAAATGTACTT-AAGTCTTATCACCAGTGGCTATGGTAAATTGGTGATTTATTTTA 91545
Coquerel'sSifaka  AGAATATCTTATCTACTACAAGTGAAAATGGGATTGAAGTCTTGTCACCAATGGCTATGCTAAATTCATGGTTTATTTTA 78142
Ma'sNightMonkey   AGACTGCCTTATCTAGTACAAATGAAAATGCACTT-AGGCCTTGTCACCAATGGCTATGGTAAATTGGTGATTTATTTTT 85555
VervetAGM         AGAATGCCATGTCTAGTACAAATGAAAATGTACTT-AAGTCTTGTCACCAATGGCTATGGTAAATTGGTGATTTATTTTA 93220
Gorrilla          AGAATGCCTTGTCTAGTTCAAATGAAAATGTACTT-AAGTCTTATCACCAGTGGCTATGGTAAATTGGTGATTTATTTTA 89796
GrabEatingMacaqueAGAATGCCATGTCTAGTACAAATGAAAATGTACTT-AAGTCTTGTCACCAATGGCTATGGTAAATTGGTGATTTATTTTA 92651
Macaque           AGAATGCCATGTCTAGTACAAATGAAAATGTACTT-AAGTCTTGTCACCAATGGCTATGGTAAATTGGTGATTTATTTTA 93546
PigTailedMacaque  AGAATGCCATGTCTAGTACAAATGAAAATGTACTT-AAGTCTTGTCACCAATGGCTATGGTAAATTGGTGATTTATTTTA 92417
Bonobo            AGAATGCCTTGTCTAGTTCAAATGAAAATGTACTT-AAGTCTTATCACCAGTGGCCAGGGTAAATTGGTGATTTATTTTA 90377
OliveBaboon       AGAATGCCATGTCTAGTACAAATGAAAATGTACTT-AACTCTTGTCACCAATGGCTATGGTAAATTGGTGATTTATTTTA 91999
Orangutan         AGAATGCCTTGTCTAGTACAAATGAAAATGTACTT-AAGTCTTGTCACCAATGGCTATGGTAAATTGGTGATTTATTTTA 92799
```

```
Majority         AAATCATAAGAAAATTCAAATTTTTAAACTGTTACTTTAAAA----GAATTATTTTTGTTTTGAGCTTTGATATTGTTTG

                         109930    109940    109950    109960    109970    109980    109990    110000

Human            AAATCATAAGAAAATTCAAATTTTTAAACTGTTACGTTAAAA----GAATTATGTTTGTTTTGAGCTTTGATATTGTTTG 91621
Coquerel'sSifaka AAGTCATAAGAAAAT-CAGATTTT-AAACTATTATTTTAAAATAAGAATTATTTTTGTTTTGAACTTTGATATTGTTTA 78220
Ma'sNightMonkey  AAATCATTAGAAAATTCAAATTTTTAAACTGTTA-------------AAGTATTTTTGTTTTGAGCTTTGATATTGT--- 85619
VervetAGM        AAATCATAAGAAAATTCAAATTTTTAAGCTGTTACTTTAAAA----GAATTATTTTTGTTTTGAGCTTTGATATTGTTTG 93296
Gorrilla         AAATCATAAGAAAATTCAGATTTTTAAACTGTTACGTTAAAA----GAATTATGTTTGTTTTGAGCTTTGATATTGTTTG 89872
GrabEatingMacaqueAAATCATAAGAAAATTCAAATTTTTAAGCTGTTACTTTAAAA----GAATTATTTTTGTTTTGAGCTTTGATATTGTTTG 92727
Macaque          AAATCATAAGAAAATTCAAATTTTTAAGCTGTTACTTTAAAA----GAATTATTTTTGTTTTGAGCTTTGATATTGTTTG 93622
PigTailedMacaque AAATCATAAGAAAATTCAAATTTTTAAGCTGTTACTTTAAAA----GAATTATTTTTGTTTTGAGCTTTGATATTGTTTG 92493
Bonobo           AAATCATAAGAAAATTCAGATTTTTAAACTGTTACGTTAAAA----GAATTATGTTTGTTTTGAGCTTTGATATTGTTTG 90453
OliveBaboon      AAATCATAAAAAAATTCAAATTTTTAAGCTGTTACTTTAAAA----GAATTATTTTTGTTTTGAGCTTTGATATTGTTTG 92075
Orangutan        AAATCATAAGAAAATTCAAATTTTTAAACTGTTACTTTAAAA----GAATTATGTTTGTTTTGAGCTTTGATATTGTTTG 92875

Majority         TTAATAGACATGCTTTCTTTTT-ATAGCCATGAATCATCTTAATGGACAGAAAATGTATGGAAAAATTATTCGTGTTACT

                         110010    110020    110030    110040    110050    110060    110070    110080

Human            TTAATAGACATGCTTTCTTTTT-ATAGCCATGAATCATCTTAATGGACAGAAAATGTATGGAAAAATTATTCGTGTTACT 91700
Coquerel'sSifaka TTAATAAGCATTCTTTCTTTTT-ATAGCCATGAACCATCTTAATGGACAGAAAATGTATGGAAAAATTATTCGTGTTACT 78299
Ma'sNightMonkey  -TAATAGACATCTTTTTTTTTTTATAGCCATGAATCATCTTAATGGACAGAAAATGTATGGAAAAATTATTCGTGTTACT 85698
VervetAGM        TTAATAGACATACTTTCTTTTT-ATAGCCATGAATCATCTTAATGGACAGAAAATGTATGGAAAAATTATTCGTGTTACT 93375
Gorrilla         TTAATAGACATGCTTTCTTTTT-ATAGCCATGAATCATCTTAATGGACAGAAAATGTATGGAAAAATTATTCGTGTTACT 89951
GrabEatingMacaqueTTAATAGACATGCTTTCTTTTT-ATAGCCATGAATCATCTTAATGGACAGAAAATGTATGGAAAAATTATTCGTGTTACT 92806
Macaque          TTAATAGACATGCTTTCTTTTT-ATAGCCATGAATCATCTTAATGGACAGAAAATGTATGGAAAAATTATTCGTGTTACT 93701
PigTailedMacaque TTAATAGACATGCTTTCTTTTT-ATAGCCATGAATCATCTTAATGGACAGAAAATGTATGGAAAAATTATTCGTGTTACT 92572
Bonobo           TTAATAGACATGCTTTCTTTTT-ATAGCCATGAATCATCTTAATGGACAGAAAATGTATGGAAAAATTATTCGTGTTACT 90532
OliveBaboon      TTAATAGACATGCTTTCTTTTT-ATAGCCATGAATCATCTTAATGGACAGAAAATGTATGGAAAAATTATTCGTGTTACT 92154
Orangutan        TTAATAGACATGCTTTCTTTTT-ATAGCCATGAATCATCTTAATGGACAGAAAATGTATGGAAAAATTATTCGTGTTACT 92954

Majority         CTGTCTAAACATCAGACTGTACAACTACCTCGAGAGGGACTTGATGATCAAGGGCTAACAAAAGATTTTGGTAATTCCCC

                         110090    110100    110110    110120    110130    110140    110150    110160

Human            CTGTCTAAACATCAGACTGTACAGCTACCTCGAGAGGGACTTGATGATCAAGGGCTAACAAAAGATTTTGGTAATTCCCC 91780
Coquerel'sSifaka CTTTCTAAACATCAGACTGTACAACTACCTCGAGAGGGACTTGATGATCAAGGGCTAACAAAAGATTTTGGTAATTCCCC 78379
Ma'sNightMonkey  CTGTCCAAACATCAGACTGTACAACTACCTCGAGAGGGACTTGATGATCAAGGGCTAACAAAAGATTTTGGTAATTCCCC 85778
VervetAGM        CTGTCGAAACATCAGACTGTACAACTACCTCGAGAGGGACTTGATGATCAAGGGCTAACAAAAGATTTTGGAAATTCCCC 93455
Gorrilla         CTGTCTAAACATCAGACTGTACAGCTACCTCGAGAGGGACTTGATGATCAAGGGCTAACAAAAGATTTTGGTAATTCCCC 90031
GrabEatingMacaqueCTGTCGAAACATCAGACTGTGCAACTACCTCGAGAGGGACTTGATGATCAAGGGCTAACAAAAGATTTTGGGAATTCCCC 92886
Macaque          CTGTCGAAACATCAGACTGTACAACTACCTCGAGAGGGACTTGATGATCAAGGGCTAACAAAAGATTTTGGGAATTCCCC 93781
PigTailedMacaque CTGTCGAAACATCAGACTGTACAACTACCTCGAGAGGGACTTGATGATCAAGGGCTAACAAAAGATTTTGGGAATTCCCC 92652
Bonobo           CTGTCTAAACATCAGACTGTACAGCTACCTCGAGAGGGACTTGATGATCAAGGGCTAACAAAAGATTTTGGTAATTCCCC 90612
OliveBaboon      CTGTCGAAACATCAGACTGTACAACTACCTCGAGAGGGACTTGATGATCAAGGGCTAACAAAAGATTTTGGGAATTCCCC 92234
Orangutan        CTGTCTAAACATCAGACTGTACAGCTACCTCGAGAGGGACTTGATGATCAAGGGCTAACAAAAGATTTTGGTAATTCCCC 93034
```

```
Majority        ATTGCATCGTTTTAAGAAACCTGGATCCAAAAATTTTCAAAACATTTTTCCTCCTTCTGCCACTCTTCACCTATCTAATA
                        110170    110180    110190    110200    110210    110220    110230    110240

Human           ATTGCATCGTTTTAAGAAACCTGGATCCAAAAATTTTCAAAACATTTTTCCTCCTTCTGCCACCCTTCACCTATCTAATA 91860
Coquerel'sSifaka ATTGCATCGTTTTAAGAAACCTGGATCCAAAAATTTTCAGAACATTTTTCCTCCTTCTGCAACCCTTCACCTGTCTAATA 78459
Ma'sNightMonkey ATTGCATCGTTTTAAGAAACCTGGATCCAAAAATTTTCAAAACATTTTTCCTCCTTCTGCAACCCTTCACCTATCTAATA 85858
VervetAGM       ATTGCATCGTTTTAAGAAACCTGGATCCAAAAATTTTCAAAACATTTTTCCTCCTTCTGCCACTCTTCACCTATCTAATA 93535
Gorrilla        ATTGCATCGTTTTAAGAAACCCGGATCCAAAAATTTTCAAAACATTTTTCCTCCTTCTGCCACCCTTCACCTATCTAATA 90111
GrabEatingMacaqueATTGCATCGTTTTAAGAAACCTGGATCCAAAAATTTTCAAAACATTTTTCCTCCTTCTGCCACTCTTCACCTATCTAATA 92966
Macaque         ATTGCATCGTTTTAAGAAACCTGGATCCAAAAATTTTCAAAACATTTTTCCTCCTTCTGCCACTCTTCACCTATCTAATA 93861
PigTailedMacaque ATTGCATCGTTTTAAGAAACCTGGATCCAAAAATTTTCAAAACATTTTTCCTCCTTCTGCCACTCTTCACCTATCTAATA 92732
Bonobo          ATTGCATCGTTTTAAGAAACCTGGATCCAAAAATTTTCAAAACATTTTTCCTCCTTCTGCCACCCTTCACCTATCTAATA 90692
OliveBaboon     ATTGCATCGTTTTAAGAAACCTGGATCCAAAAATTTTCAAAACATTTTTCCTCCTTCTGCCACTCTTCACCTATCTAATA 92314
Orangutan       ATTGCATCGTTTTAAGAAACCTGGATCCAAAAATTTTCAAAACATTTTTCCTCCTTCTGCCACTCTTCACCTATCTAATA 93114

Majority        TCCCGTAAGTATATAAGCTAGAGTGTATTAAGGAGATACATTCTATTTTGATAAAATATGAAATTTATTATTAATCTTCA
                        110250    110260    110270    110280    110290    110300    110310    110320

Human           TCCCGTAAGTATATAAGCTAGAGTGTATT---GAGATACATTCTATTTTGATAAAATATGAAATTTATTCTTAATCTTCA 91937
Coquerel'sSifaka TCCCGTAAGTATTTAGGCTGGAGTTTATTAAAGGTATATATTTTATTTTAATAAAATATAAAATTTATTATTAATCTTTG 78539
Ma'sNightMonkey TCCCGTAAGTATATAAGCTAGAGTGTATTAAGGAGATGCATTCTGTTTTGATAAAATATGAAATTTATTATTAATCTTTA 85938
VervetAGM       TCCCGTAAGTTTATAAGCTAGAGTGTATTAAGGAGATACATTCTATTTTGATAAAATGTGAAATTTATTATTAATCTTCA 93615
Gorrilla        TCCCGTAAGTATATAAGCTAGAGTGTATT---GAGATACATTCTATTTTGATAAAATATGAAGTTTATTACTAATCTTCA 90188
GrabEatingMacaqueTCCCGTAAGTATATAAGCTAGAGTATATTAAGGAGATACATTCTATTTTGATAAAATGTGAAATTTATTATTAATCTTCA 93046
Macaque         TCCCGTAAGTATATAAGCTAGAGTATATTAAGGAGATACATTCTATTTTGATAAAATGTGAAATTTATTATTAATCTTCA 93941
PigTailedMacaque TCCCGTAAGTATATAAGCTAGAGTATATTAAGGAGATACATTCTATTTTGATAAAATGTGAAATTTATTATTAATCTTCA 92812
Bonobo          TCCCGTAAGTATATAAGCTAGAGTGTATT---GAGATACATTCTATTTTGATAAAATATGAAATTTATTATTAATCTTCA 90769
OliveBaboon     TCCCGTAAGTATATAAGCTAGAGTGTATTAAGGAGATACATTCTATTTTGATAAAATGTGAAATTTATTATTAATCTTCA 92394
Orangutan       TCCCGTAAGTATATAAGCTACAGTGTATTAAGGAGATACATTCTATTTTGATAAAATACGAAATTTATTATTAATCTTCA 93194

Majority        CTTTTTCTTCCCATTCAATTTTCCTAGTCCATCAGTAGCAGAAGAGGATCTACGAACACTGTTCGCTAACACTGGGGGCA
                        110330    110340    110350    110360    110370    110380    110390    110400

Human           CTTTTTCTTCCCATTCAATTTTCCTAGTCCATCAGTAGCAGAAGAGGATCTACGAACACTGTTCGCTAACACTGGGGGCA 92017
Coquerel'sSifaka CTTCTTTCCCCCATCCAATTTTCCTAGTCCTTCAGTAGCAGAAGAGGATCTACGAACACTGTTTGCTAACACTGGGGGCA 78619
Ma'sNightMonkey CTTTTTCTTCCCATTCAATTTTCCTAGTCCATCAGTAGCAGAAGAGGATCTACGAACACTGTTTGCTAACACTGGGGGCA 86018
VervetAGM       CTTTTTCTTCCCATTCAATTTTCCTAGTCCATCAGTAGCAGAAGAGGATCTACGAACACTGTTCGCTAACACTGGGGGCA 93695
Gorrilla        CTTTTTCTTCCCATTCAATTTTCCTAGTCCATCAGTAGCAGAAGAGGATCTACGAACACTGTTCGCTAACACTGGGGGCA 90268
GrabEatingMacaqueCTTTTTCTTCCCATTCAATTTTCCTAGTCCATCAGTAGCAGAAGAGGATCTACGAACACTGTTCGCTAACACTGGGGGCA 93126
Macaque         CTTTTTCTTCCCATTCAATTTTCCTAGTCCATCAGTAGCAGAAGAGGATCTACGAACACTGTTCGCTAACACTGGGGGCA 94021
PigTailedMacaque CTTTTTCTTCCCATTCAATTTTCCTAGTCCATCAGTAGCAGAAGAGGATCTACGAACACTGTTCGCTAACACTGGGGGCA 92892
Bonobo          CTTTTTCTTCCCATTCAATTTTCCTAGTCCATCAGTAGCAGAAGAGGATCTACGAACACTGTTCGCTAACACTGGGGGCA 90849
OliveBaboon     CTTTTTCTTCCCATTCAATTTTCCTAGTCCATCAGTAGCAGAAGAGGATCTACGAACACTGTTCGCTAACACTGGGGGCA 92474
Orangutan       CTTTTTCTTCCCATTCAATTTTCCTAGTCCATCAGTAGCAGAAGAGGATCTACGAACACTGTTCGCTAACACTGGGGGCA 93274
```

```
Majority          CTGTGAAAGCATTTAAGTTTTTTCAGTAAGCAAGCTTCCTTATCTTTAAATTAGTGACCTGATA------AAAGTTTTCT
                          110410    110420    110430    110440    110450    110460    110470    110480
Human             CTGTGAAAGCATTTAAGTTTTTTCAGTAAGCAAGCTTCCTTATCTTTAAATTAGTGACCTGATA------AAATTTTT-- 92089
Coquerel'sSifaka  CTGTGAAAGCATTTAAGTTTTTTCAGTAAGCAAGCTTCCTTGTCTTTAAATTAGTGACTTGATAGAAATAAAAGTTTTCT 78699
Ma'sNightMonkey   CGGTGAAAGCATTTAAGTTTTTTCAGTAAGCAAGCTTCCTTATCTTTAAATTAGTGACCCGATA------AATGTTTTCT 86092
VervetAGM         CTGTGAAAGCATTTAAGTTTTTTCAGTAAGCAAGCTTCCTTATCTTTAAATTAGTGACCTGATA------AAAGTTTTCT 93769
Gorrilla          CTGTGAAAGCATTTAAGTTTTTTCAGTAAGCAAGCTTCCTTATCTTTAAATTAGTGACCTGATA------AAATTTTTCT 90342
GrabEatingMacaqueCTGTGAAAGCATTTAAGTTTTTTCAGTAAGCAAGCTTCCTTATCTTTAAATTAGTGTCCTGATA------AAAGTTTTCT 93200
Macaque           CTGTGAAAGCATTTAAGTTTTTTCAGTAAGCAAGCTTCCTTATCTTTAAATTAGTGTCCTGATA------AAAGTTTTCT 94095
PigTailedMacaque  CTGTGAAAGCATTTAAGTTTTTTCAGTAAGCAAGCTTCCTTATCTTTAAATTAGTGTCCTGATA------AAAGTTTTCT 92966
Bonobo            CTGTGAAAGCATTTAAGTTTTTTCAGTAAGCAAGCTTCCTTATCTTTAAATTAGTGACCTGATA------AAATTTTTCT 90923
OliveBaboon       CTGTGAAAGCATTTAAGTTTTTTCAGTAAGCAAGCTTCCTTATCTTTAAATTAGTGACCTGAGA------AAAGTTTTCT 92548
Orangutan         CTGTGAAAGCATTTAAGTTTTTTCAGTAAGCAAGCTTCCTTATCTTTAAATTAGTGACCTGATA------AAATTTTTCT 93348
```

```
Majority          TTAAGTAGTTTTTATTCTTTTTGTTTATAGAAATTTTTGATTCTGGAATGGCCAAAATTCAAGTATTTAATAAGCCCTTT
                          110490    110500    110510    110520    110530    110540    110550    110560
Human             --AAGTAGTTTTTGTTCTTTTCGTTTATAGAAATTTTTGATTCCGGAATGGCCAAAATTCAAGTATTTAATAAGCCCTTT 92167
Coquerel'sSifaka  TCAAGTAGTTTTTATTCTTTTTGTTTATAGAAATTTTTGATTCTGGAATGGGCAAAATTCAAGTATTTAATGAGCCCTTC 78779
Ma'sNightMonkey   TTAAAGAGTTTTTATTCTTTTTGTTTATAAAAATGTTTTATTCTGGAATGGCCAAAATTCAAGTAGTTA------TCTTC 86166
VervetAGM         TTAAGTAGTTTTTATTCTTGTTGTTTGTAGAAATTTTTGATTCTGAAATGGCCAAAATTCAAGTATTTAATAAGCCATTT 93849
Gorrilla          TTAAGTAGTTTTTGTTCTTTTCGTTTATAGAAATTTTTGATTCCGGAATGGCCAAAATTCAAGTATTTAATAAGCCCTTT 90422
GrabEatingMacaqueTTAAGTAGTTTTTATTCTTGTTGTTTATAGAAATTTTTGATTCTGGAATGGCCAAAATTCAAGTATTTAATAAGCCATTT 93280
Macaque           TTAAGTAGTTTTTATTCTTGTTGTTTATAGAAATTTTTGATTCTGGAATGGCCAAAATTCAAGTATTTAATAAGCCATTT 94175
PigTailedMacaque  TTAAGTAGTTTTTATTCTTGTTGTTCATAGAAATTTTTGATTCTGGAATGGCCAAAATTCAAGTATTTAATAAGCCATTT 93046
Bonobo            TTAAGTAGTTTTTGTTCTTTTCGTTTATAGAAATTTTTGATTCCGGAATGGCCAAAATTCAAGTATTTAATAAGCCCTTT 91003
OliveBaboon       TTAAGTAGTTTTTATTCTTGTTGTTTATAGAAATTTTTGATTCTGGAATGGCCAAAATTCAAGTATTTAATAAGCCATTT 92628
Orangutan         TTAAGTAGTTTTTGTTCTTTTTGTTTATAGAAATTTTTGATTCTGGAATGGCCAAAATTCAAGTATTTAATAAGCCCTTT 93428
```

```
Majority          CTAATTTGTATGAAGTATCTAATTTTATAATTTTGTTTCAGAAGAGATCACAAAATGGCTCTTCTTCAGATGGCAACAGT
                          110570    110580    110590    110600    110610    110620    110630    110640
Human             CTAATTTGTATGAAGTGTCTAATTTTATAATTTTGTTTCAGAAGAGATCACAAAATGGCTCTTCTTCAGATGGCAACAGT 92247
Coquerel'sSifaka  CTAGTTTTTATGAAGTATCTAATTTCATAATTTTGTTTCAGAAGAGATCACAAAATGGCTCTTCTTCAGATGGCAACAGT 78859
Ma'sNightMonkey   CTAATTTTTAGAAAGTATCTAATTTTATAATTTTGTTTCAGAAGAGATCACAAAATGGCTCTTCTTCAGATGGCAACAGT 86246
VervetAGM         CTTATTTGTATGAAGTATCTGATTTTATAATTTTGTTTCAGAAGAGATCACAAAATGGCTCTTCTTCAGATGGCAACAGT 93929
Gorrilla          CTAATTTGTATGAAGTATCTAATTTTATAATTTTGTTTCAGAAGAGATCACAAAATGGCTCTTCTTCAGATGGCAACAGT 90502
GrabEatingMacaqueCTTATTTGTATGAAGTATCTGATTTTATAATTTTGTTTCAGAAGAGATCACAAAATGGCTCTTCTTCAGATGGCAACAGT 93360
Macaque           CTTATTTGTATGAAGTATCTGATTTTATAATTTTGTTTCAGAAGAGATCACAAAATGGCTCTTCTTCAGATGGCAACAGT 94255
PigTailedMacaque  CTTATTTGTATGAAGTATCTGATTTTATAATTTTGTTTCAGAAGAGATCACAAAATGGCTCTTCTTCAGATGGCAACAGT 93126
Bonobo            CTAATTTGTATGAAGTATCTAATTTTATAATTTTGTTTCAGAAGAGATCACAAAATGGCTCTTCTTCAGATGGCAACAGT 91083
OliveBaboon       CTTATTTGTATGAAGTATCTGATTTTATAATTTTGTTTCAGAAGAGATCACAAAATGGCTCTTCTTCAGATGGCAACAGT 92708
Orangutan         CTAATTTGTATAAAGTATCTAATTTTATAATTTTGTTTCAGAAGAGATCACAAAATGGCTCTTCTTCAGATGGCAACAGT 93508
```

```
Majority         GGAAGAAGCTATTCAGGCCTTGATTGATCTTCATAATTATAACCTTGGAGAAAACCATCATCTGAGAGTGTCTTTCTCCA
                          110650    110660    110670    110680    110690    110700    110710    110720
Human            GGAAGAAGCTATTCAGGCCTTGATTGATCTTCATAATTATAACCTTGGAGAAAACCATCATCTGAGAGTGTCTTTCTCCA 92327
Coquerel'sSifaka GGAAGAAGCTATCCAGGCCTTGATTGATCTTCATAATTATAACCTTGGTGAAAACCATCATCTGAGAGTGTCTTTCTCCA 78939
Ma'sNightMonkey  GGAAGAAGCTATTCAGGCCTTGATTGATCTTCATAATTATAACCTTGGAGAAAACCATCATCTGAGAGTGTCTTTCTCTA 86326
VervetAGM        GGAAGAAGCTATTCAGGCCTTGATTGATCTTCATAATTATAACCTTGGAGAAAATCATCATCTGAGAGTGTCTTTTTCCA 94009
Gorrilla         GGAAGAAGCTATTCAGGCCTTGATTGATCTTCATAATTATAACCTTGGAGAAAACCATCATCTGAGAGTGTCTTTCTCCA 90582
GrabEatingMacaqueGGAAGAAGCTATTCAGGCCTTGATTGATCTTCATAATTATAACCTTGGAGAAAATCATCATCTGAGAGTGTCTTTTTCCA 93440
Macaque          GGAAGAAGCTATTCAGGCCTTGATTGATCTTCATAATTATAACCTTGGAGAAAATCATCATCTGAGAGTGTCTTTTTCCA 94335
PigTailedMacaque GGAAGAAGCTATTCAGGCCTTGATTGATCTTCATAATTATAACCTTGGAGAAAATCATCATCTGAGAGTGTCTTTTTCCA 93206
Bonobo           GGAAGAAGCTATTCAGGCCTTGATTGATCTTCATAATTATAACCTTGGAGAAAACCATCATCTGAGAGTGTCTTTCTCCA 91163
OliveBaboon      GGAAGAAGCTATTCAGGCCTTGATTGATCTTCATAATTATAACCTTGGAGAAAATCATCATCTGAGAGTGTCTTTTTCCA 92788
Orangutan        AGAAGAAGCTATTCAGGCCTTGATTGATCTTCATAATTATAACCTTGGAGAAAACCATCATCTGAGAGTGTCTTTCTCCA 93588
```

```
Majority         AGTCAACAATTTAAAAAGGGGAAGATGAAGATTGGGGGTGAATCACATTGTTCAGTGTCATCACCTATT-GACTGTTCAG
                          110730    110740    110750    110760    110770    110780    110790    110800
Human            AGTCAACAATTTAAAAATGGGAAGATGAAGATTGGGGGTGAATCACATTGTTCAATGTCATCACCTATTTGACTGTTCAG 92407
Coquerel'sSifaka AGTCAACAATTTAAAAAGTGGGAGATGAAGATTGGGG-TGAATCACATTGTTCGGTGTCATCACCTATT-GACTGTTCAG 79017
Ma'sNightMonkey  AGTCAACAATTTAAAAAGGGGAAGATGAAGATTGGGGGTGAATCACATTGTTCAGTGTCATCACCTATT-GACTGTTCAG 86405
VervetAGM        AGTCAACAATTTAAAAAGGGGAAGATGAAGATTGGGGGTGAATCACATTGTTCAGTGTCATCACCTATT-GACTGTTCAG 94088
Gorrilla         AGTCAACAATTTAAAAATGGGAAGATGAAGATTGGGGGTGAATCACATTGTTCAGTGTCATCACCTATTTGACTGTTCAG 90662
GrabEatingMacaqueAGTCAACAATTTAAAAAGGGGAAGATGAAGATTGGGGGTGAATCACATTGTTCAGTGTCATCACCTATT-GACTGTTCAG 93519
Macaque          AGTCAACAATTTAAAAAGGGGAAGATGAAGATTGGGGGTGAATCACATTGTTCAGTGTCATCACCTATT-GACTGTTCAG 94414
PigTailedMacaque AGTCAACAATTTAAAAAGGGGAAGATGAAGATTGGGGGTGAATCACATTGTTCAGTGTCATCACCTATT-GACTGTTCAG 93285
Bonobo           AGTCAACAATTTAAAAATGGGAAGATGAAGATTGGGGGTGAATCACATTGTTCAATGTCATCACCTATTTGACTGTTCAG 91243
OliveBaboon      AGTCAACAATTTAAAAAGGGGAAGATGAAGATTGGGGGTGAATCACATTGTTCAGTGTCATCACCTATT-GACTGTTCAG 92867
Orangutan        AGTCAACAATTTAAAAATGGGAAGATGAAGATTGGGGGTGAATCACATTGTTCAATGTCATCACCTATTTGACTGTTCAG 93668
```

```
Majority         AAAAGTGGGGACCAGAGTTTGATTTTTTTTGTTTTTGTTTTTTTGGGGTTT-CTTTTTTTTTT-------CCATGCTGT
                          110810    110820    110830    110840    110850    110860    110870    110880
Human            AAAAGTGGGGACCAGAGTTTGATTTTTTTTGTTTTTGTTTTTTTGGGGTTT-CTTTTTTTTTT--------CCATGCTGT 92478
Coquerel'sSifaka AAAAGTGGGGACCAGAGTTTGATTTTTTTGTTAATT--TTTTTT--------CTTTTTTTC---------CCATGCTGT 79078
Ma'sNightMonkey  AAAAGTGGGGACCAGAGTTTGATTTTTTTTCTGT----TTTTTTGGGGTTTCCTTTTTTTTTTTTTTTCTCCCATGCTGT 86481
VervetAGM        AAAAGTGGGGACCAGAGTTTGATTTTTTTTGCTTTTGTTTTTTTGGGTTTTTTTTTTTTTTTTT-------CCATGCTGT 94161
Gorrilla         AAAAGTGGGGACCAGAGTTTGATTTTTTTTGTTTTTGTTTTTTTGGGGTTT-CTTTTTTTTT---------CCATGCTGT 90732
GrabEatingMacaqueAAAAGTGGGGACCAGAGTTTGATTTTTTTTGCTTTTGTTTTTTTGGG-----TTTTTTTTTTTT-------CCATGCTGT 93587
Macaque          AAAAGTGGGGACCAGAGTTTGATTTTTTTTGCTTTTGTTTTTTTGGG-----TTTTTTTTTTTT-------CCATGCTGT 94482
PigTailedMacaque AAAAGTGGGGACCAGAGTTTGATTTTTTTTGCTTTTGTTTTTTTGGGGT---TTTTTTTTTTTT-------CCATGCTGT 93355
Bonobo           AAAAGTGGGGACCAGAGTTTGATTTTTTTTGTTTTTGTTTTTTTGGGGTTT-CTTTTTTTTT---------CCATGCTGT 91313
OliveBaboon      AAAAGTGGGGACCAGAGTTTGATTTTTTTTGCTTTTGTTTTTTTGGGGT---TTTTTTTTTTTT-------CCATGCTGT 92937
Orangutan        AAAAGTGGGGACCAGAGTTTGATTTTTTTTGTTT----TCTTTTGGGGTTT-CTTTTTTTTTTT-------CCATGCTGT 93736
```

```
Majority         TATCATTCCTTGGTTATAAAATGAAATGGCATATGTAAAGGCAGAGTTGTTAACTGCTATATTTCATCTGTTCTATAGGG

                         110890    110900    110910    110920    110930    110940    110950    110960

Human            TATCATTCCTTGGTTATAAAATGAAATGGCATATGTAAAGGCAGAGTTGTTAACTGCTATATTTCATCTGTTCTATAGGG 92558
Coquerel'sSifaka TATCATTCCTTGGTTATGAAATGAAATGGCATATGTAAAGGCAGAGTTATTAACTGCTGTATTTCATCTGTTCTATAGGG 79158
Ma'sNightMonkey  TATCATTCCTTGGTTATAAAATGAAATGGCATATGTAAAGGCAGAGTTATCAACTGCTATATTTCATCTGTTCTATAGGG 86561
VervetAGM        TATCATTCCTTGGTTATAAAATGAAATGGCATATGTAAAGGCAGAGTTGTTAACTGCTATATTTCATCTGTTCTATAGGG 94241
Gorrilla         TATCATTCCTTGGTTATAAAATGAAATGGCATATGTAAAGGCAGAGTTGTTAACTGCTATATTTCATCTGTTCTATAGGG 90812
GrabEatingMacaqueTATCATTCCTTGGTTATAAAATGAAATGGCATATGTAAAGGCAGAGTTGTTAACTGCTATATTTCATCTGTTCTATAGGG 93667
Macaque          TATCATTCCTTGGTTATAAAATGAAATGGCATATGTAAAGGCAGAGTTGTTAACTGCTATATTTCATCTGTTCTATAGGG 94562
PigTailedMacaque TATCATTCCTTGGTTATAAAATGAAATGGCATATGTAAAGGCAGAGTTGTTAACTGCTATATTTCATCTGTTCTATAGGG 93435
Bonobo           TATCATTCCTTGGTTATAAAATGAAATGGCATATGTAAAGGCAGAGTTGTTAACTGCTATATTTCATCTGTTCTATAGGG 91393
OliveBaboon      TATCATTCCTTGGTTATAAAATGAAATGGCATATGTAAAGGCAGAATTGTTAACTGCTATATTTCATCTGTTCTATAGGG 93017
Orangutan        TATCATTCCTTGGTTATAAAATGAAATGGCATACGTAAAGGCAGAGTTGTTAACTGCTATATTTCATCTGTTCTATAGGG 93816

Majority         AAGCCATTTTTGTCTGTTTAAAATTTCAGTTTAATTTTGCTTTTTTTTTTTTTCTT------CCTTTCAACTTAGTTGACA

                         110970    110980    110990    111000    111010    111020    111030    111040

Human            AAGCCATTTT-GTCTGTTTAAAATTTCAGTTTAATTTTGCTTTTTTTTTTTTTTTTTTTTTTCCTTTCAACTTAGTTGACA 92637
Coquerel'sSifaka AAGCCATTTT-GTCTGTTTAAAATTTCAGTTTAATTTTGCTTTCTTTTTTC----------------AACTTAGTTGACA 79221
Ma'sNightMonkey  AAGCCATTTTTGTCTGTTTAAAATTTCGATTTAATTTTGCTTTTTTTTTTT--------------TCAACTTAGTTGACA 86627
VervetAGM        AAGCCATTTTTGTCTGTTTAAAATTTCAGTTTAATTTTGCTTTTTTTTTTTTTCTT------CCTTTCAACTTAGTTGACA 94315
Gorrilla         AAGCCATTTT-GTCTGTTTAAAATTTCAGTTTAATTTTGCTTTTTTTTTTTTTTT-------CCTTTCAACTTAGTTGACA 90884
GrabEatingMacaqueAAGCCATTTTTGTCTGTTTAAAATTTCAGTTTAATTTTGCTTTTTTTTTTT-CTT------CCTTTCAACTTAGTTGACA 93740
Macaque          AAGCCATTTTTGTCTGTTTAAAATTTCAGTTTAATTTTGCTTTTTTTTTTTTTCTT------CCTTTCAACTTAGTTGACA 94636
PigTailedMacaque AAGCCATTTTTGTCTGTTTAAAATTTCAGTTTAATTTTGCTTTTTTTTTTT-CTT------CCTTTCAACTTAGTTGACA 93508
Bonobo           AAGCCATTTT-GTCTGTTTAAAATTTCAGTTTAATTTTGCTTTTTTTTTTTTTTTTT---TTCCTTTCAACTTAGTTGACA 91469
OliveBaboon      AAGCCATTTTTGTCTGTTTAAAATTTCAGTTTAATTTTGCTTTTTTTTTTTTTCTT------CCTTTCAACTTAGTTGACA 93091
Orangutan        AAGCCATTTT-GTC--TTTAAAATTTCTGTTTAATTTTGCTTTTTTTTTTCTTT------------CAACTTAGTTGACA 93881

Majority         TACGTGCCTTAAAAAGGAAAACTAGTGTTGCTATTGTGCATTTACTAGAAAAAAGGAATTGGTTGTTTAGGGCACACTGT

                         111050    111060    111070    111080    111090    111100    111110    111120

Human            TACGTGCCTTAAAAAGGAAAACTAGTGTTGCTATTGTGCATTTACTAGAAAAAAGGAATTGGTTGTTTAGGGCACACTGT 92717
Coquerel'sSifaka TACGTGCCTTAAAAAGGAAAACTAGTGTTGCTATTGTGCATTTACTAGAAAAAAGGAATTGGTTGTCTAGGGCACATTGT 79301
Ma'sNightMonkey  TACGTGCCTTAAAAAGGAAAACTAGTGTTGCTATTGTGCATTTACTAGAAAAAAGGAATTGGTTGTTTAGGGCACACTGT 86707
VervetAGM        TACGTGCCTTAAAAAGGAAAACTAGTGTTGCTATTGTGCATTTACTAGAAAAAAGGAATTGGTTGTTTAGGGCACACTGT 94395
Gorrilla         TACGTGCCTTAAAAAGGAAAACTAGTGTTGCTATTGTGCATTTACTAGAAAAAAGGAATTGGTTGTTTAGGGCACACTGT 90964
GrabEatingMacaqueTACGTGCCTTAAAAAGGAAAACTAGTGTTGCTATTGTGCATTTACTAGAAAAAAGGAATTGGTTGTTTAGGGCACACTGT 93820
Macaque          TACGTGCCTTAAAAAGGAAAACTAGTGTTGCTATTGTGCATTTACTAGAAAAAAGGAATTGGTTGTTTAGGGCACACTGT 94716
PigTailedMacaque TACGTGCCTTAAAAAGGAAAACTAGTGTTGCTATTGTGCATTTACTAGAAAAAAGGAATTGGTTGTTTAGGGCACACTGT 93588
Bonobo           TATGTGCCTTAAAAAGGAAAACTAGTGTTGCTATTGTGCATTTACTAGAAAAAAGGAATTGGTTGTTTAGGGCACACTGT 91549
OliveBaboon      TACGTGCCTTAAAAAGGAAAACTAGTGTTGCTATTGTGCATTTACTAGAAAAAAGGAATTGGTTGTTTAGGGCACACTGT 93171
Orangutan        TACGTGCCTTAAAAAGGAAAACTAGTGTTGCTATTGTGCATTTACTAGAAAAAAGGAATTGGTTGTTTAGGGCACACTGT 93961
```

```
Majority          TATATGGGAATTAAAATATGTTTAGGCAGGGGTGTGTAAAAAGGTTAAGTTTTTGTTTCTCCTGCTTGGAACTTATTTTG
                          111130    111140    111150    111160    111170    111180    111190    111200
Human             TATATGGGAATTAAAATATGTTTAGGCAGGGGTGTGTAAAAAGGTTAAGTTTTTGTTTCTCCTGCTTGGAACTTATTTTG 92797
Coquerel'sSifaka  TATATGGGAATTAAAATATGTTTAGGCAGGGGTGTGTAAAAAGGTTAAGTTTTTGTTTCTCCTGCTTGGAATTTATTTTG 79381
Ma'sNightMonkey   TACATGGGAATTAAAATATGTTTAGGCAGGGGTGTGTAAAAAGGTTAAGTTTTTGTTTCTCCTGCTTGAAACTTATTTTG 86787
VervetAGM         TATATGGGAATTAAAATATGTTTAGGCAGGGGTGTGTAAAAAGGTTAAGTTTTTGTTTCTCCTGCTTGGAACTTATTTTG 94475
Gorrilla          TATATGGGAATTAAAATATGTTTAGGCAGGGGTGTGTAAAAAGGTTAAGTTTTTGTTTCTCCTGCTTGGAACTTATTTTG 91044
GrabEatingMacaqueTATATGGGAATTAAAATATGTTTAGGCAGGGGTGTGTAAAAAGGTTAAGTTTTTGTTTCTCCTGCTTGGAACTTATTTTG 93900
Macaque           TATATGGGAATTAAAATATGTTTAGGCAGGGGTGTGTAAAAAGGTTAAGTTTTTGTTTCTCCTGCTTGGAACTTATTTTG 94796
PigTailedMacaque  TATATGGGAATTAAAATATGTTTAGGCAGGGGTGTGTAAAAAGGTTAAGTTTTTGTTTCTCCTGCTTGGAACTTATTTTG 93668
Bonobo            TATATGGGAATTAAAATATGTTTAGGCAGGGGTGTGTAAAAAGGTTAAGTTTTTGTTTCTCCTGCTTGGAACTTATTTTG 91629
OliveBaboon       TATATGGGAATTAAAATATGTTTAGGCAGGGGTGTGTAAAAAGGTTAAGTTTTTGTTTCTCCTGCTTGGAACTTATTTTG 93251
Orangutan         TATATGGGAATTAAAATATGTTTAGGCAGGGGTGTGTAAAAAGGTTAAGTTTTTGTTTCTCCTGCTTGGAACTTATTTTG 94041

Majority          AATTACTGGCTTGTCACCTTTTTTTCTATTTAATCAAATAAGATACATGATATTGAAAGAATAAAGCAGCATTTTTAGTT
                          111210    111220    111230    111240    111250    111260    111270    111280
Human             AATTACTGGCTTGTCACCTTTTTTTCTATTTAATCAAATAAGATACATGATATTGAAAGAATAAAGCAGCATTTTTAGTT 92877
Coquerel'sSifaka  AATTATTGACTTGTCACATTATTTTCTATTTAATCAAATAAGATACATGCTACTGAAAGAATAAAGCAGCATTTTTAGGT 79461
Ma'sNightMonkey   AATTACTGGCTTGTCACCTTTTTTTCTATTTACTCAAATAAGATACATGATATTGAAAGAATAAAGCAGCATTTTTAGTT 86867
VervetAGM         AATTACTGGCTT-TCACCTTTTTTTGTATTTAATCAAATAAGATACATGATATTGAAAGAATAAAGCAGCATTTTTAGTT 94554
Gorrilla          AATTACTGGCTTGTCACCTTTTTTTCTATTTAATCAAATAAGATACATGATATTGAAAGAATAAAGCAGCATTTTTAGTT 91124
GrabEatingMacaqueAATTACTGGCTT-TCACCTTTTTTTGTATTTAATCAAATAAGATACATGATATTGAAAGAATAAAGCAGCATTTTTAGTT 93979
Macaque           AATTACTGGCTT-TCACCTTTTTTTGTATTTAATCAAATAAGATACATGATATTGAAAGAATAAAGCAGCATTTTTAGTT 94875
PigTailedMacaque  AATTACTGGCTT-TCACCTTTTTTTGTATTTAATCAAATAAGATACATGATATTGAAAGAATAAAGCAGCATTTTTAGTT 93747
Bonobo            AATTACTGGCTTGTCACCTTTTTTTCTATTTAATCAAATAAGATACATGATATTGAAAGAATAAAGCAGCATTTTTAGTT 91709
OliveBaboon       AATTACTGGCTT-TCACCTTTTTTTGTATTTAATCAAATAAGATACATGATATTGAAAGAATAAAGCAGCATTTTTAGTT 93330
Orangutan         AATTACTGGCTTGTCACCTTTTTTTCTATTTAATCAAATAAGATACATGATATTGAAAGAATAAAGCAGCATTTTTAGTT 94121

Majority          TTTACTACCTTAGGCTTTATTGCTTTAAAAACAACATTGGCCTTTTGTATCTCACAATTCTGGTCTAGATTCAGTTATGA
                          111290    111300    111310    111320    111330    111340    111350    111360
Human             TTTACTACCTTAGGCTTTATTGCTTTGAAAACAACATTGGCCTTTTGTATCTCACAATTCTGGTCTAGATTCAGTTATGA 92957
Coquerel'sSifaka  TTCACTACCTTAGGCTTTATTGCTTTAAAAA-AACATTGGCCTTTTGTATCTCACAATTCTGGTCTAGATTCAGTTATGA 79540
Ma'sNightMonkey   TTTACTACCTTAGGCTTTATTGCTTTAAAAA-AACATTGGCCTTTTGTATCTCACAATTCTGGTCTAGATTCAGTTATGA 86946
VervetAGM         TTTACTACCTTAGGCTTTATTGCTTTAAAAACAACATTGGCCTTTTGTATCTCACAATTCTGGTCTAGATTCAGTTATGA 94634
Gorrilla          TTTACTACCTTAGGCTTTATTGCTTTGAAAACAACATTGGCCTTTTGTATCTCACAATTCTGGTCTAGATTCAGTTATGA 91204
GrabEatingMacaqueTTTACTACCTTAGGCTTTATTGCTTTAAAAACAACATTGGCCTTTTGTATCTCACAATTCTGGTCTAGATTCAGTTATGA 94059
Macaque           TTTACTACCTTAGGCTTTATTGCTTTAAAAACAACATTGGCCTTTTGTATCTCACAATTCTGGTCTAGATTCAGTTATGA 94955
PigTailedMacaque  TTTACTACCTTAGGCTTTATTGCTTTAAAAACAACATTGGCCTTTTGTATCTCACAATTCTGGTCTAGATTCAGTTATGA 93827
Bonobo            TTTACTACCTTAGGCTTTATTGCTTTGAAAACAACATTGGCCTTTTGTATCTCACAATTCTGGTCTAGATTCAGTTATGA 91789
OliveBaboon       TTTACTACCTTAGGCTTTATTGCTTTAAAAACAACATTGGCCTTTTGTATCTCACAATTCTGGTCTAGATTCAGTTATGA 93410
Orangutan         TTTACTACCTTAGGCTTTATTGCTTTGAAAACAACATTGGCCTTTTGTATCTCACAATTCTGGTCTAGATTCAGTTATGA 94201
```

```
Majority          ATGTAGGCATTAGTTAAAATTAACAAGATGCAGAGTATTAATTTCTTAAGACAACAAAGTGATTTCTGTAAGTTTGAGCC

                          111370    111380    111390    111400    111410    111420    111430    111440

Human             ATGTAGGCATTAGTTAAAATTAACAAGATGCAGAGTATTAATTTCTTAAGACAACAAAGTGATTTCTGTAAGTTTGAGCC 93037
Coquerel'sSifaka  ATGTAGGCATTAGTTAAAATTAACAAGATGCAGAGTATTAATTTCTTAAGACAACAAAGTGATTTCTGTAAGTTTGAGCC 79620
Ma'sNightMonkey   ATGTAGGCATTAGTTAAAATTAACAAGATGCAGAGTATTAATTTCTTAAGACAACAAAGTGATTTCTGTAAGTTTGAGCC 87026
VervetAGM         ATGTAGGCATTAGTTAAAATTAACAAGATGCAGAGTATTAATTTCTTAAGACAACAAAGTGATTTCTGTAAGTTTGAGCC 94714
Gorrilla          ATGTAGGCATTAGTTAAAATTAACAAGATGCAGAGTATTAATTTCTTAAGACAACAAAGAGATTTCTGTAAGTTTGAGCC 91284
GrabEatingMacaqueATGTAGGCATTAGTTAAAATTAACAAGATGCAGAGTATTAATTTCTTAAGACAACAAAGTGATTTCTGTAAGTTTGAGCC 94139
Macaque           ATGTAGGCATTAGTTAAAATTAACAAGATGCAGAGTATTAATTTCTTAAGACAACAAAGTGAT                  95018
PigTailedMacaque  ATGTAGGCATTAGTTAAAATTAACAAGATGCAGAGTATTAATTTCTTAAGACAACAAAGTGATTTCTGTAAGTTTGAGCC 93907
Bonobo            ATGTAGGCATTAGTTAAAATTAACAAGATGCAGAGTATTAATTTCTTAAGACAACAAAGTGATTTCTGTAAGTTTGAGCC 91869
OliveBaboon       ATGTAGGCATTAGTTAAAATTAACAAGATGCAGAGTATTAATTTCTTAAGACAACAAAGTGATTTCTGTAAGTTTGAGCC 93490
Orangutan         ATGTAGGCATTAGTTAAAATTAACAAGATGCAGAGTATTAATTTCTTAAGACAACAAAGTGATTTCTGTAAGTTTGAGCC 94281
```

```
Majority          CTATGTGGAAAGCATTGTGGAATCTTAACCTTTTTGTACACACTCTTGTGGGACGTATCATATAAATGTCAGCACTAAGT

                          111450    111460    111470    111480    111490    111500    111510    111520

Human             CTATGTGGAAAGCATTGTGGAATCTTAACCTTTTCGTACACACTCTTGTGGGACGTATCATATAAATGTCAGCACTAAGT 93117
Coquerel'sSifaka  CTATGTGGAAAGCATTGTGGAATCTTAACCTTTTTGTACACACTCTTGTGGGACGTATCATATAAATGTCAGCACTAAGT 79700
Ma'sNightMonkey   CTATGTGGAAAGCATTGTGGAATCTTAACCTTTTTGTACACACTCTTGTGGGACGTATCATATAAATGTCAGCACTAAGT 87106
VervetAGM         CTATGTGGAAAGCATTGTGGAATCTTAACCTTTTTGTACACACTCTTGTGGGACGTATCATATAAATGTCAGCACTAAGT 94794
Gorrilla          CTATGTGGAAAGCATTGTGGAATCTTAACCTTTTTGTACACACTCTTGTGGGACGTATCATATAAATGTCAGCACTAAGT 91364
GrabEatingMacaqueCTATGTGGAAAGCATTGTGGAATCTTAACCTTTTTGTACACACTCTTGTGGGACGTATCATATAAATGTCAGCACTAAGT 94219
Macaque                                                                                            95018
PigTailedMacaque  CTATGTGGAAAGCATTGTGGAATCTTAACCTTTTTGTACACACTCTTGTGGGACGTATCATATAAATGTCAGCACTAAGT 93987
Bonobo            CTATGTGGAAAGCATTGTGGAATCTTAACCTTTTTGTACACACTCTTGTGGGACGTATCATATAAATGTCAGCACTAAGT 91949
OliveBaboon       CTATGTGGAAAGCATTGTGGAATCTTAACCTTTTTGTACACACTCTTGTGGGACGTATCATATAAATGTCAGCACTAAGT 93570
Orangutan         CTATGTGGAAAGCATTGTGGAATCTTAACCTTTTTGTACACACTCTTGTGGGACGTATCATATAAATGTCAGCACTAAGT 94361
```

```
Majority          AATGTCTTGTTTGTGGCTGAATATTTTTCGTAGATGTTTTTGAAGTTGACATGACTTACGTGCATTTAAATATATATTGC

                          111530    111540    111550    111560    111570    111580    111590    111600

Human             AATGTCTTGTTTGTGGCTGAATATTTTTCGTAGATGTTTTTGAAGTTGACATGACTTACGTGCATTTAAATATATATTGC 93197
Coquerel'sSifaka  AATGTCTTGTTTGTGGCTGAATATTTTTCGTAGATGTTTTTGAAGTTGACATGACTTATGTGCATTTAAATATATATTGC 79780
Ma'sNightMonkey   AATGTCTTGTTTGTGGCTGAATATTTTTCGTAGATGTTTTTGAAGTTGACATGACTTACGTGCATTTAAATATATATTGC 87186
VervetAGM         AATGTCTTGTTTGTGGCTGAATATTTTTCGTAGATGTTTTTGAAGTTGACATGACTTACGTGCATTTAAATATATATTGC 94874
Gorrilla          AATGTCTTGTTTGTGGCTGAATATTTTTCGTAGATGTTTTTGAAGTTGACATGACTTACGTGCATTTAAATATATATTGC 91444
GrabEatingMacaqueAATGTCTTGTTTGTGGCTGAATATTTTTCGTAGATGTTTTTGAAGTTGACATGACTTACGTGCATTTAAATATATATTGC 94299
Macaque                                                                                            95018
PigTailedMacaque  AATGTCTTGTTTGTGGCTGAATATTTTTCGTAGATGTTTTTGAAGTTGACATGACTTACGTGCATTTAAATATATATTGC 94067
Bonobo            AATGTCTTGTTTGTGGCTGAATATTTTTCGTAGATGTTTTTGAAGTTGACATGACTTACGTGCATTTAAATATATATTGC 92029
OliveBaboon       AATGTCTTGTTTGTGGCTGAATATTTTTCGTAGATGTTTTTGAAGTTGACATGACTTACGTGCATTTAAATATATATTGC 93650
Orangutan         AATGTCTTGTTTGTGGCTGAATATTTTTCGTAGATGTTTTTGAAGTTGACATGACTTACGTGCATTTAAATATATATTGC 94441
```

```
Majority          CATCCTTAGTTTGTAATTAAGATTTGGAATATGGTTGTGGATTTCTGAGCATGTGCAGACTGGTCTAGCTAGTTCAGGAA
                        111610    111620    111630    111640    111650    111660    111670    111680
Human             CATCCTTAGTTTGTAATTAAGATTTGGAATATGGTTGTGGATTTCTGAGCATGTGCAGACTGGTCTAGCTAGTTCAGGAA 93277
Coquerel'sSifaka  CATCCTTAGTTTGTAATTAAGATTTGGAATATGGTTGTGGATTTCTGAGCATGTGCAGACTGGTCTAGCTAGTTCAGAAA 79860
Ma'sNightMonkey   CATCCTTAGTTTGTAATTAAGATTTGGAATATGGTTGTGGATTTCTGAGCATGTGCAGACTGGTCTAGCTAGTTCAGGAA 87266
VervetAGM         CATCCTTAGTTTGTAATTAAGATTTGGAATATGGTTGTGGATTTCTGAGCATGTGCAGACTGGTCTAGCTAGTTCAGGAA 94954
Gorrilla          CATCCTTAGTTTGTAATTAAGATTTGGAATATGGTTGTGGATTTCTGAGCATGTGCAGACTGGTCTAGCTAGTTCAGGAA 91524
GrabEatingMacaqueCATCCTTAGTTTGTAATTAAGATTTGGAATATGGTTGTGGATTTCTGAGCATGTGCAGACTGGTCTAGCTAGTTCAGGAA 94379
Macaque                                                                                            95018
PigTailedMacaque  CATCCTTAGTTTGTAATTAAGATTTGGAATATGGTTGTGGATTTCTGAGCATGTGCAGACTGGTCTAGCTAGTTCAGGAA 94147
Bonobo            CATCCTTAGTTTGTAATTAAGATTTGGAATATGGTTGTGGATTTCTGAGCATGTGCAGACTGGTCTAGCTAGTTCAGGAA 92109
OliveBaboon       CATCCTTAGTTTGTAATTAAGATTTGGAATATGGTTGTGGATTTCTGAGCATGTGCAGACTGGTCTAGCTAGTTCAGGAA 93730
Orangutan         CATCCTTAGTTTGTAATTAAGATTTGGAATATGGTTGTGGATTTCTGAGCATGTGCAGACTGGTCTAGCTAGTTCAGGAA 94521

Majority          CTGGTGCATGTATTTTTCAAAGATAAAGAAAGTGTACTGCGAAAATATGCAGGAAGATTAATTTTGTGGCAGTTTTCTAA
                        111690    111700    111710    111720    111730    111740    111750    111760
Human             CTGGTGCATGTATTTTTCAAAGATAAAGAAAGTGTACTGCGAAAATATGCAGGAAGATTAATTTTGTGGCAGTTTTCTAA 93357
Coquerel'sSifaka  CTGGTGCATGTATTTTTCAAAGATAAAGAAAGTGTACTGCGGAAATTTGCAGGAAGATTAATTTTGTGGCAGTTTTCTAA 79940
Ma'sNightMonkey   CTGGTGCATGTATTTTTCAAAGATAAAGAAAGTGTACTGCGAAAATATGCAGGAAGATTAATTTTGTGGCAGTTTTCTAA 87346
VervetAGM         CTGGTGCATGTATTTTTCAAAGATAAAGAAAGTGTACTGCGAAAATATGCAGGAAGATTAATTTTGTGGCAGTTTTCTAA 95034
Gorrilla          CTGGTGCATGTATTTTTCAAAGATAAAGAAAGTGTACTGCGAAAATATGCAGGAAGATTAATTTTGTGGCAGTTTTCTAA 91604
GrabEatingMacaqueCTGGTGCATGTATTTTTCAAAGATAAAGAAAGTGTACTGCGAAAATATGCAGGAAGATTAATTTTGTGGCAGTTTTCTAA 94459
Macaque                                                                                            95018
PigTailedMacaque  CTGGTGCATGTATTTTTCAAAGATAAAGAAAGTGTACTGCGAAAATATGCAGGAAGATTAATTTTGTGGCAGTTTTCTAA 94227
Bonobo            CTGGTGCATGTATTTTTCAAAGATAAAGAAAGTGTACTGCGAAAATATGCAGGAAGATTAATTTTGTGGCAGTTTTCTAA 92189
OliveBaboon       CTGGTGCATGTATTTTTCAAAGATAAAGAAAGTGTACTGCGAAAATATGCAGGAAGATTAATTTTGTGGCAGTTTTCTAA 93810
Orangutan         CTGGTGCATGTATTTTTCAAAGATAAAGAAAGTGTACTGCGAAAATATGCAGGAAGATTAATTTTGTGGCAGTTTTCTAA 94601

Majority          AACTGACAACCAGGTGGGACCAAAGTTTATGTGCCTTTAGTCTTAATTTACCTTGCATTGTAATATTCAGTTTTAATAAA
                        111770    111780    111790    111800    111810    111820    111830    111840
Human             AACTGACAACCAGGTGGGACCAAAGTTTATGTGCCTTTAGTCTTAATTTACCTTGCATTGTAATATTCAGTTTTAATAAA 93437
Coquerel'sSifaka  AACTGACAACCAGGTGGGACCAAAGTTTATGTGCCTTTAGTCTTAATTTACCTTGCATTGTAATATTCAGTTTTAATAAA 80020
Ma'sNightMonkey   AACTGACAACCAGGTGGGACCAAAGTTTATGTGCCTTTAGTCTTAATTTACCTTGCATTGTAATATTCAGTTTTAATAAA 87426
VervetAGM         AACTGACAACCAGGTGGGACCAAAGTTTATGTGCCTTTAGTCTTAATTTACCTTGCATTGTAATATTCAGTTTTAATAAA 95114
Gorrilla          AGCTGACAACCAGGTGGGACCAAAGTTTATGTGCCTTTAGTCTTAATTTACCTTGCATTGTAATATTCAGTTTTAATAAA 91684
GrabEatingMacaqueAACTGACAACCAGGTGGGACCAAAGTTTATGTGCCTTTAGTCTTAATTTACCTTGCATTGTAATATTCAGTTTTAATAAA 94539
Macaque                                                                                            95018
PigTailedMacaque  AACTGACAACCAGGTGGGACCAAAGTTTATGTGCCTTTAGTCTTAATTTACCTTGCATTGTAATATTCAGTTTTAATAAA 94307
Bonobo            AACTGACAACCAGGTGGGACCAAAGTTTATGTGCCTTTAGTCTTAATTTACCTTGCATTGTAATATTCAGTTTTAATAAA 92269
OliveBaboon       AACTGACAACCAGGTGGGACCAAAGTTTATGTGCCTTTAGTCTTAATTTACCTTGCATTGTAATATTCAGTTTTAATAAA 93890
Orangutan         AACTGACAACCAGGTGGGACCAAAGTTTATGTGCCTTTAGTCTTAATTTACCTTGCATTGTAATATTCAGTTTTAATAAA 94681
```

```
Majority          TCTTCAAAATATTTTGTATTTAGGAATAGATCTGACTTTAATAAAAACATGGCTCAGAATCTACAGGTCAAATTAATTTG

                           111850    111860    111870    111880    111890    111900    111910    111920

Human             TCTTCAAAATATTTTGTATTTAGGAATAGATCTGACTTTAATAAAAACATGGCTCAGAATCTACAGGTCAAATTAATTTG 93517
Coquerel'sSifaka  TCTTCAAAATATTTTGTATTTAGGAATAGATCTGACTTTAATAAAAACATGGCTCAGAATCTACAGGTCAAATTAATTTG 80100
Ma'sNightMonkey   TCTTCAAAATATTTTGTATTTAGGAATAGATCTGACTTTAATAAAAACATGGCTCAGAATCTACAGGTCAAATTAATTTG 87506
VervetAGM         TCTTCAAAATATTTTGTATTTAGGAATAGATCTGACTTTAATAAAAACATGGCTCAGAATCTACAGGTCAAATTAATTTG 95194
Gorrilla          TCTTCAAAATATTTTGTATTTAGGAATAGATCTGACTTTAATAAAAACATGGCTCAGAATCTACAGGTCAAATTAATTTG 91764
GrabEatingMacaqueTCTTCAAAATATTTTGTATTTAGGAATAGATCTGACTTTAATAAAAACATGGCTCAGAATCTACAGGTCAAATTAATTTG 94619
Macaque                                                                                            95018
PigTailedMacaque  TCTTCAAAATATTTTGTATTTAGGAATAGATCTGACTTTAATAAAAACATGGCTCAGAATCTACAGGTCAAATTAATTTG 94387
Bonobo            TCTTCAAAATATTTTGTATTTAGGAATAGATCTGACTTTAATAAAAACATGGCTCAGAATCTACAGGTCAAATTAATTTG 92349
OliveBaboon       TCTTCAAAATATTTTGTATTTAGGAATAGATCTGACTTTAATAAAAACATGGCTCAGAATCTACAGGTCAAATTAATTTG 93970
Orangutan         TCTTCAAAATATTTTGTATTTAGGAATAGATCTGACTTTAATAAAAACATGGCTCAGAATCTACAGGTCAAATTAATTTG 94761

Majority          AACAGTTCTTGTCAATCTGAATTGTTGATTCTGTTTAAATGACCAATACTTTTTGAAATTGATGTACTTAGTTTCAAGAT

                           111930    111940    111950    111960    111970    111980    111990    112000

Human             AACAGTTCTTGTCAATCCGAATTGTTGATTCTGTTTAAATGACCAATACTTTTTGAAATTGATGTACTTAGTTTCAAGAT 93597
Coquerel'sSifaka  AACAGTTCTTGTCAATCTGAATTGTTGATTCTGTTTAAATGACCAATACTTTTTGAAATTGATGTACTTAGTTTCAAGAT 80180
Ma'sNightMonkey   AACAGTTCTTGTCAATCTGAATTGTTGATTCTGTTTAAATGACCAATACTTTTTGAAATTGATGTACTTAGTTTCAAGAT 87586
VervetAGM         AACAGTTCTTGTCAATCTGAATTGTTGATTCTGTTTAAATGACCAATACTTTTTGAAATTGATGTACTTAGTTTCAAGAT 95274
Gorrilla          AACAGTTCTTGTCAATCCGAATTGTTGATTCTGTTTAAATGACCAATACTTTTTGAAATTGATGTACTTAGTTTCAAGAT 91844
GrabEatingMacaqueAACAGTTCTTGTCAATCTGAATTGTTGATTCTGTTTAAATGACCAATACTTTTTGAAATTGATGTACTTAGTTTCAAGAT 94699
Macaque                                                                                            95018
PigTailedMacaque  AACAGTTCTTGTCAATCTGAATTGTTGATTCTGTTTAAATGACCAATACTTTTTGAAATTGATGTACTTAGTTTCAAGAT 94467
Bonobo            AACAGTTCTTGTCAATCCGAATTGTTGATTCTGTTTAAATGACCAATACTTTTTGAAATTGATGTACTTAGTTTCAAGAT 92429
OliveBaboon       AACAGTTCTTGTCAATCTGAATTGTTGATTCTGTTTAAATGACCAATACTTTTTGAAATTGATGTACTTAGTTTCAAGAT 94050
Orangutan         AACAGTTCTTGTCAATCTGAATTGTTGATTCTGTTTAAATGACCAATACTTTTTGAAATTGATGTACTTAGTTTCAAGAT 94841

Majority          TCATAGATTCTGTTATCTATGTAGACAGAATGGTCATGTATATTTTCTATTAGTTGAGTTTTTACATCTTTAGAAATGTA

                           112010    112020    112030    112040    112050    112060    112070    112080

Human             TCATAGATTCTGTTATCTATGTAGACAGAATGGTCATGTATATTTTCTATTAGTTGAGTTTTTACATCTTTAGAAATGTA 93677
Coquerel'sSifaka  TCATAGATTCTGTTATCTATGTAGACAGAATGGTCATGTATATTTTCTATTAGTTGAGTTTTTACATCTTTAGAAATGTA 80260
Ma'sNightMonkey   TCATAGATTCTGTTATCTATGTAGACAGAATGGTCATGTATATTTTCTATTAGTTGAGTTTTTACATCTTTAGAAATGTA 87666
VervetAGM         TCATAGATTCTGTTATCTATGTAGACAGAATGGTCATGTATATTTTCTATTAGTTGAGTTTTTACATCTTTAGAAATGTA 95354
Gorrilla          TCATAGATTCTGTTATCTATGTAGACAGAATGGTCATGTATATTTTCTATTAGTTGAGTTTTTACATCTTTAGAAATGTA 91924
GrabEatingMacaqueTCATAGATTCTGTTATCTATGTAGACAGAATGGTCATGTATATTTTCTATTAGTTGAGTTTTTACATCTTTAGAAATGTA 94779
Macaque                                                                                            95018
PigTailedMacaque  TCATAGATTCTGTTATCTATGTAGACAGAATGGTCATGTATATTTTCTATTAGTTGAGTTTTTACATCTTTAGAAATGTA 94547
Bonobo            TCATAGATTCTGTTATCTATGTAGACAGAATGGTCATGTATATTTTCTATTAGTTGAGTTTTTACATCTTTAGAAATGTA 92509
OliveBaboon       TCATAGATTCTGTTATCTATGTAGACAGAATGGTCATGTATATTTTCTATTAGTTGAGTTTTTACATCTTTAGAAATGTA 94130
Orangutan         TCATAGATTCTGTTATCTATGTAGACAGAATGGTCATGTATATTTTCTATTAGTTGAGTTTTTACATCTTTAGAAATGTA 94921
```

```
Majority          AAATTCAGTATAGTTTGAAAGCGGCACAATTAAAAATTAATTTTCTAACAAAGTTGGGAGGTTTGATGGTTGTTTAATTT

                          112090    112100    112110    112120    112130    112140    112150    112160

Human             AAATTCAGTATAGTTTGAAAGCGGCACAATTAAAAATTAATTTTCTAACAAAGTTGGGAGGTTTGATGGTTGTTTAATTT 93757
Coquerel'sSifaka  AAATTCAGTATAGTTTGAAAGCGGCACAATTAAAAATTAATTTTCTAACAAAGTTGGGAGGTTTGATGGTTGTTTAATTT 80340
Ma'sNightMonkey   AAATTCAGTATAGTTTGAAAGCGGCACAATTAAAAATTAATTTTCTAACAAAGTTGGGAGGTTTGATGGTTGTTTAATTT 87746
VervetAGM         AAATTCAGTATAGTTTGAAAGCGGCACAATTAAAAATTAATTTTCTAACAAAGTTGGGAGGTTTGATGGTTGTTTAATTT 95434
Gorrilla          AAATTCAGTATAGTTTGAAAGCGGCACAATTAAAAATTAATTTTCTAACAAAGTTGGGAGGTTTGATGGTTGTTTAATTT 92004
GrabEatingMacaqueAAATTCAGTATAGTTTGAAAGCGGCACAATTAAAAATTAATTTTCTAACAAAGTTGGGAGGTTTGATGGTTGTTTAATTT 94859
Macaque                                                                                            95018
PigTailedMacaque  AAATTCAGTATAGTTTGAAAGCGGCACAATTAAAAATTAATTTTCTAACAAAGTTGGGAGGTTTGATGGTTGTTTAATTT 94627
Bonobo            AAATTCAGTATAGTTTGAAAGCGGCACAATTAAAAATTAATTTTCTAACAAAGTTGGGAGGTTTGATGGTTGTTTAATTT 92589
OliveBaboon       AAATTCAGTATAGTTTGAAAGCGGCACAATTAAAAATTAATTTTCTAACAAAGTTGGGAGGTTTGATGGTGGTTTAATTT 94210
Orangutan         AAATTCAGTATAGTTTGAAAGCGGCACAATTAAAAATTAATTTTCTAACAAAGTTGGGAGGTTTGATGGTTGTTTAATTT 95001

Majority          CATTTTGTGTGTACTCTGCTTACCTCTGTAGCATGCTCAATAAACACTTCTGTAGCTCTATATTCACCTTTTCTGTCTTT

                          112170    112180    112190    112200    112210    112220    112230    112240

Human             CATTTTGTGTGTACTCTGCTTACCCCTGTAGCATGCTCAATAAACACTTCTGTAGCTCTATATTCACCTTTTCTGTCTTT 93837
Coquerel'sSifaka  CATTTTGTGTGTACTCTGCTTACCTCTGTAGCATGCTCAATAAACACTTCTGTAGCTCTGTATTCACCTTTTCTGTCTTT 80420
Ma'sNightMonkey   CATTTTGTGTGTACTCTGCTTACCTCTGTAGCATGCTCAATAAACACTTCTGTAGCTCTATATTCACCTTTTCTGTCTTT 87826
VervetAGM         CATTTTGTGTGTACTCTGCTTACCTCTGTAGCATGCTCAATAAACACTTCTGTAGCTCTATATTCACCTTTTCTGTCTTT 95514
Gorrilla          CATTTTGTGTGTACTCTGCTTACCTCTGTAGCATGCTCAATAAACACTTCTGTAGCTCTATATTCACCTTTTCTGTCTTT 92084
GrabEatingMacaqueCATTTTGTGTGTACTCTGCTTACCTCTGTAGCATGCTCAATAAACACTTCTGTAGCTCTATATTCACCTTTTCTGTCTTT 94939
Macaque                                                                                            95018
PigTailedMacaque  CATTTTGTGTGTACTCTGCTTACCTCTGTAGCATGCTCAATAAACACTTCTGTAGCTCTATATTCACCTTTTCTGTCTTT 94707
Bonobo            CATTTTGTGTGTACTCTGCTTACCCCTGTAGCATGCTCAATAAACACTTCTGTAGCTCTATATTCACCTTTTCTGTCTTT 92669
OliveBaboon       CATTTTGTGTGTACTCTGCTTACCTCTGTAGCATGCTCAATAAACACTTCTGTAGCTCTATATTCACCTTTTCTGTCTTT 94290
Orangutan         CATTTTGTGTGTACTCTGCTTACCTCTGTAGCATGCTCAATAAACACTTCTGTAGCTCTATATTCACCTTTTCTGTCTTT 95081

Majority          CTCTGCTGCCTTTT-CTCTCTCCTCTTCTTTGTTTTCACTCCACTGTGCTTCTGAATTCATGTTTATTCTCTGCCAGGGT

                          112250    112260    112270    112280    112290    112300    112310    112320

Human             CTCTGCTGCCTTTT-CTCTCTCCTCTTCTTTGTTTTCACTCCACTGTGCTTCTGAATTCATGTTTATTCTCTGCCAGGGT 93916
Coquerel'sSifaka  CTCTGCTGCCTTTTTCTCTCTCCTCTTCTTTGTTTTCACTCCACTGTGCTTCTGAATTCATGTTTATTCTCTGCCAGGGT 80500
Ma'sNightMonkey   CTCTGCTGCCTTTT-CTCTCTCCTCTTCTTTGTTTTCACTCCACTGTGCTTCTGAATTCATGTTTATTCTCTGCCAGGGT 87905
VervetAGM         CTCTGCTGCCTTTT-CTCTCTCCTCTTCTTTGTTTTCACTCCACTGTGCTTCTGAATTCATGTTTATTCTCTGCCAGGGT 95593
Gorrilla          CTCTGCTGCCTTTT-CTCTCTCCTCTTCTTTGTTTTCACTCCACTGTGCTTCTGAATTCATGTTTATTCTCTGCCAGGGT 92163
GrabEatingMacaqueCTCTGCTGCCTTTT-CTCTCTCCTCCTCTTTGTTTTCACTCCACTGTGCTTCTGAATTCATGTTTATTCTCTGCCAGGGT 95018
Macaque                                                                                            95018
PigTailedMacaque  CTCTGCTGCCTTTT-CTCTCTCCTCCTCTTTGTTTTCACTCCACTGTGCTTCTGAATTCATGTTTATTCTCTGCCAGGGT 94786
Bonobo            CTCTGCTGCCTTTT-CTCTCTCCTCTTCTTTGTTTTCACTCCACTGTGCTTCTGAATTCATGTTTATTCTCTGCCAGGGT 92748
OliveBaboon       CTCTGCTGCCTTTT-CTCTCTCCTCCTCTTTGTTTTCACTCCACTGTGCTTCTGAATTCATGTTTATTCTCTGCCAGGGT 94369
Orangutan         CTCTGCTGCCTTTT-CTCTCTCCTCTTCTTTGTTTTCACTCCACTGTGCTTCTGAATTCATGTTTATTCTCTGCCAGGGT 95160
```

```
Majority          GGGAAAGGAGTAATAATATTACAATTC-TATGGCTTTATACCATAAATAAATCTAGATGCTGTGAAAATATACCAGCTGG

                          112330    112340    112350    112360    112370    112380    112390    112400

Human             GGGAAAGGAGTAATAATATTACAATTC-TATGGCTTTATACCATAAATAAATCTAGATGCTGTGAAAATATACCAGCTGG 93995
Coquerel'sSifaka  GGGAAAGGAGTAATAATATTACAATTCCTATGGCTTTATACCATAAATAAATCTAGATGCTGTGAAAATATACCAGCTGG 80580
Ma'sNightMonkey   GGGAAAGGAGTAATAATATTACAATTC-TATGGCTTTATACCATAAATAAATCTAGATGCTGTGAAAATATACCAGCTGG 87984
VervetAGM         GGGAAAGGAGTAATAATATTACAATTC-TATGGCTTTATACCATAAATAAATCTAGATGCTGTGAAAATATACCAGCTGT 95672
Gorrilla          GGGAAAGGAGTAATAATATTACAATTC-TATGGCTTTATACCATAAATAAATCTAGATGCTGTGAAAATATACCAGCTGG 92242
GrabEatingMacaqueGGGAAAGGAGTAATAATATTACAATTC-TATGGCTTTATACCATAAATAAATCTAGATGCTGTGAAAATATACCAGCTGT 95097
Macaque                                                                                            95018
PigTailedMacaque  GGGAAAGGAGTAATAATATTACAATTC-TATGGCTTTATACCATAAATAAATCTAGATGCTGTGAAAATATACCAGCTGT 94865
Bonobo            GGGAAAGGAGTAATAATATTACAATTC-TATGGCTTTATACCATAAATAAATCTAGATGCTGTGAAAATATACCAGCTGG 92827
OliveBaboon       GGGAAAGGAGTAATAATAATACAATTC-TATGGCTTTATACCATAAATAAATCTAGATGCTGTGAAAATATACCAGCTGT 94448
Orangutan         GGGAAAGGAGTAATAATATTACAATTC-TATGGCTTTATACCATAAATAAATCTAGATGCTGTGAAAATATACCAGCTGG 95239

Majority          TTTTTTTT-AATTTAAAAGATGGTAACTGCTTTTCAGGAGGACACATATTAAACATTTCCCACCCTGTTATAATCTACTG

                          112410    112420    112430    112440    112450    112460    112470    112480

Human             TTTTTTTT-AATTTAAAAGATGGTAACTGCTTTTCAGGAGGACACATATTAAACATTTCCCACCCTGTTATAATCTACTG 94074
Coquerel'sSifaka  TTTTTTTT-AATTTAAAAGATGATAACTGCTTTTCAAGAGGACACATATTAAACATTTCCCACCCTGTTATAATCTACTG 80659
Ma'sNightMonkey   TTTTTTTT--AATTAAAAGATGGTGACTGCTTTTCAGGAGGACACATATTAAACATTTCCCACCCTGTTATAATCTACTG 88062
VervetAGM         TTTTTTTTTAACTTAAAAGATGGTAACTGCTTTTCAGGAGGACACATATTAAACATTTCCCACCCTGTTATAATCTACTG 95752
Gorrilla          TTTTTTTT-AATTTAAAAGATGGTAACTGCTTTTCAGGAGGACACATATTAAACATTTCCCACCCTGTTATAATCTACTG 92321
GrabEatingMacaqueTTTTTTTT-AATTTAAAAGATAGTAACTGCTTTTCAGGAGGACACATATTAAACATTTCCCACCCTGTTATAATCTACTG 95176
Macaque                                                                                            95018
PigTailedMacaque  TTTTTTTT-AATTTAAAAGATAGTAACTGCTTTTCAGGAGGACACATATTAAACATTTCCCACCCTGTTATAATCTACTG 94944
Bonobo            TTTTTTT--AATTTAAAAGATGGTAACTGCTTTTCAGGAGGACACATATTAAACATTTCCCACCCTGTTATAATCTACTG 92905
OliveBaboon       TTTTTTTT-AATTTAAAAGATAGTAACTGCTTTTCAGGAGGACACATATTAAACATTTCCCACCCTGTTATAATCTACTG 94527
Orangutan         TTTTTTTT-AATTTAAAAGATGGTAACTGCTTTTCAGGAGGACACATATTAAACATTTCCCACCCTGTTATAATCTACTG 95318

Majority          CTTTAAAGACATAACTTTTATTGTAGCTTGTTAATTCTATCTCTTTTGTTGTTGTTGTTTTTTTTTT---CCAGTAGAT

                          112490    112500    112510    112520    112530    112540    112550    112560

Human             CTTTAAAGACATAACTTTTATTGTAGCTTGTTAATTCTATCTCTTTTGTTCTTGTTGTTTTTTTTTTTTTTCCAGTAGAT 94154
Coquerel'sSifaka  CTTTAAAGACATAACTTTTATTGTAGCTTGTTAATTCTTTCTATCT--CTTTTGTTGTTTTTCTTTTTTTTCCAGTAGAT 80737
Ma'sNightMonkey   CTTTAAAGACATAACTTTTATTGTAGCTTGTTAATTCTATCTCTTTTGTTGTTGTTGTTTTTCTTTT----CCAGTAGAT 88138
VervetAGM         CTTTAAAGACATAACTTTTACTGTAGCTTGTTAATTCTATCTCTTTTGTTGTTGTTGTTGTTTTTTTT---CCAGTAGAT 95829
Gorrilla          CTTTAAAGACATAACTTTTATTGTAGCTTGTTAATTCTATCTCTTTTGTTCTTGTTGTTTTTTTTTT----CCAGTAGAT 92397
GrabEatingMacaqueCTTTAAAGACATAACTTTTACTGTAGCTTGTTAATTCTATCTCTTTTGTTGTTGTTGTTTTTTTTTTT---CCAGTAGAT 95253
Macaque                                                                                            95018
PigTailedMacaque  CTTTAAAGACATAACTTTTACTGTAGCTTGTTAATTCTATCTCTTTTGTTGTTGTTGTTGTTTTTTT----CCAGTAGAT 95020
Bonobo            CTTTAAAGACATAACTTTTATTGTAGCTTGTTAATTCTATCTCTTTTGTTCTTGTTGTTTTTTTTTT----CCAGTAGAT 92981
OliveBaboon       CTTTAAAGACATAACTTTTACTGTAGCTTGTTAATTCTATCTCTTTTGTTGTTGTTGTTGTTTTTTTT---CCAGTAGAT 94604
Orangutan         CTTTAAAGACATAACTTTTATTGTAGCTTGTTAATTCTATCTCTTTTGTTCTTGTTGTTTTTTTTTTTTT-CCAGTAGAT 95397
```

```
Majority         TTATGCACTAATAGATCTTTTGGATTTGCCATGCTCTCTTGCTGCAGTTTCATCTTTCATCTTTTGTGTCTGCTAAAGAT
                         112570    112580    112590    112600    112610    112620    112630    112640
Human            TTATGCACTAATAGATCTTTTGGATTTGCCATGCTCTCTTGCTGCAGTTTCATCTTTCATCTTTTGTGTCTGCTAAAGAT 94234
Coquerel'sSifaka TTATGCACTAATAGATCTTTTGGATTTGCCATGCTCTCTTGCTGCAGTTTCATCTTTCATCTTTTGTGTCTGCTAAAGAT 80817
Ma'sNightMonkey  TTCTGCACTAATAGATCTTT-GGATTTGCCATGCTCTCTTGCTGCAGTTTCATCTTTCATCTTTTTTGTCTGCTAAAGAT 88217
VervetAGM        TTATGCACTAATAGATCTTTTGGATTTGCCATGCTCTCTTGCTGCAGTTTCATCTTTCATCTTTTGTGTCTGCTAAAGAT 95909
Gorrilla         TTATGCACTAATAGATCTTTTGGATTTGCCATGCTCTCTTGCTGCAGTTTCATCTTTCATCTTTTGTGTCTGCTAAAGAT 92477
GrabEatingMacaqueTTATGCACTAATAGATCTTTTGGATTTGCCATGCTCTCTTGCTGCAGTTTCATCTTTCATCTTTTGTGTCTGCTAAAGAT 95333
Macaque                                                                                           95018
PigTailedMacaque TTATGCACTAATAGATCTTTTGGATTTGCCATGCTCTCTTGCTGCAGTTTCATCTTTCATCTTTTGTGTCTGCTAAAGAT 95100
Bonobo           TTATGCACTAATAGATCTTTTGGATTTGCCATGCTCTCTTGCTGCAGTTTCATCTTTCATCTTTTGTGTCTGCTAAAGAT 93061
OliveBaboon      TTATGCACTAATAGATCTTTTGGATTTGCCATGCTCTCTTGCTGCAGTTTCATCTTTCATCTTTTGTGTCTGCTAAAGAT 94684
Orangutan        TTATGCACTAATAGATCTTTTGGATTTGCCATGCTCTCTTGCTGCAGTTTCATCTTTCATCTTTTGTGTCTGCTAAAGAT 95477

Majority         TTCTTACTAA---TCTTA-GACTACCTT-GTGAGTTAACAAAAAGAACTTGATGTTACTGGGAAGAAAGAGCAGCATATC
                         112650    112660    112670    112680    112690    112700    112710    112720
Human            TTCTTACTAA---TCTTA-GACTACCTT-GTGAGTTAACAAAAAGAACTTGATATTACTGGGAAGAAAGAGCAGCATATC 94309
Coquerel'sSifaka TTCCTACTAA---TCTTA-GACTACTTAAGTTAGTTAACAAAGAGTACTTGATGTT---GGGAAGAAAGAGTATCATATC 80890
Ma'sNightMonkey  TTCTCACTAA---TCTTA-GACTACCTT-GTGAGTTAACAAAAAGAACTCGATGTTACTGGGAAGAAAGAGCAACATATC 88292
VervetAGM        TTCTTACTAATAATCTTA-GACTATCTT-GTGAGTTAACTAAAAGAACTTGATGTTACTGGGAAGAAAGAGCAGCATATC 95987
Gorrilla         TTCTTACTAA---TCTTAAGACTACCTT-GTGAGTTAACAAAAAGAACTTGATATTACTGGGAAGAAAGAGCAGCATATC 92553
GrabEatingMacaqueTTCTTACTAATAATCTTA-GACTATCTT-GTGAGTTAACTAAAAGAACTTGATGTTACTGGGAAGAAAGAGCAGCATATC 95411
Macaque                                                                                           95018
PigTailedMacaque TTCTTACTAATAATCTTA-GACTATCTT-GTGAGTTAACTAAAAGAACTTGATGTTACTGGGAAGAAAGAGCAGCATATC 95178
Bonobo           TTCTTACTAA---TCTTA-GACTACCTT-GTGAGTTAACAAAAAGAACTTGATATTACTGGGAAGAAAGAGCAGCATATC 93136
OliveBaboon      TTCTTACTAATAATCTTA-GACTATCTT-GTGAGTTAACTAAAAGAACTTGATGTTACTGGGAAGAAAGAGCAGCATATC 94762
Orangutan        TTCTTACTAA---TCTTA-GACTACCTT-GTGAGTTAACAAAAAGAACTTGATGTTACTGGGAAGAAAGAGCAGCATATC 95552

Majority         -------------GTCTGCTTTTGCTTTGGGTGGGAAGGATGATATACATTCAAGTATTTAAAAACTTA--GAGTAATTT
                         112730    112740    112750    112760    112770    112780    112790    112800
Human            TGCATATCAGCATATCTGCTTTTGCCTTGGGTGGGAAGAATGATATACATTCAAGTATTTAAAAACTTA--GAGTAATTT 94387
Coquerel'sSifaka -------------ATCTGCTTTTGCCTTGGGTGGGAAGAATGGTATATATTCAAGCATTTAAAAACAAG---GGTAATTG 80954
Ma'sNightMonkey  -------------ATCTGCTTTTGTTTTGGGTGGGAAGGATGATATACATTCAGGTGTTTAAAGACTTAAAGAGTAATTT 88359
VervetAGM        -------------GCCTGCTTTTGCTGTGGGTGGGAAGGATGATATACATTCAAGTATTTAAAAACTTA--GAGTAATTT 96052
Gorrilla         TGCATATCAGCATATCTGCTTTTGCCTTGGGTGGGAAGAATGATATACATTCAAGTATTTAAAAACTTA--GAGCAATTT 92631
GrabEatingMacaque-------------GCCTGCTTTTGCTGTGGGTGGGAAGGATGATACACATTCAAGTATTTAAAAACTTA--GAGTAATTT 95476
Macaque                                                                                           95018
PigTailedMacaque -------------GCCTGCTTTTGCTGTGGGTGGGAAGGATGATACACATTCAAGTATTTAAAAACTTA--GAGTAATTT 95243
Bonobo           TGCATATCAGCATATCTGCTTTTGCCTTGGGTGGGAAGAATGATATACATTCAAGTATTTAAAAACTTA--GAGTAATTT 93214
OliveBaboon      -------------GCCTGCTTTTGCTGTGGGTGGGAAGGATGATATACATTCAAGTATTTAAAAACTTA--GAGTAATTT 94827
Orangutan        -------------GTCTGCTTTTGCCTT                                                     95567
```

```
Majority          GCATTTAACAACTGAAGATGTTACTACTTTAACTTTTT-AAACAGTTACAATTAGGGTGCTATTTATATTCAAAATATTT

                           112810    112820    112830    112840    112850    112860    112870    112880

Human             GCATTTAACAACTGAGGATGTTACTACTGTAACTTGTT-AAGCAGTTACAATTAGGGTGCTATTTATATTCAAAATATTT 94466
Coquerel'sSifaka  GCATTTAACAACTGGAAATGTTACTACTTTCACTTTTTAAAACAGGTATGATTAGGGTGC------TATTCAAAATATTT 81028
Ma'sNightMonkey   GCATTTAACAACTGAAGA-GTTACTACTTTCACTTCTT-AAACAGTTACAATTAGGGTGCTATTTATATTCAAAATACTT 88437
VervetAGM         GCATTTAACAACTAAATATGTTACTACTTTAACTTTTT-AAACAGTTACAATTAGGGTGCTATTTATATTCAAAATATTT 96131
Gorrilla          GCATTTAACAACTGAGGATGTTACTACTGTAACTTGTT-AAACAGTTACAATTAGGGTGCTATTTATATTCAAAATATTT 92710
GrabEatingMacaqueGCATTTAACAACTAAACATGTTACTA-------------AACAGTTACAATTAGGGTGCTATTTATATTCAAAATATTT 95542
Macaque                                                                                            95018
PigTailedMacaque  GCATTTAACAACTAAACATGTTACTACTTTAACTT----AAACAGTTACAATTAGGGTGCTATTTATATTCAAAATATTT 95319
Bonobo            GCATTTAACAACTGAGGATGTTACTACTGTAACTTGT--AAGCAGTTACAATTAGGGTGCTATTTATATTCAAAATATTT 93292
OliveBaboon       GCATTTAACAACTAAACATGTTACTACTTTAACTTTTT-AAACAGTTACAATTAGGGTGCTATTTATATTCAAAATATTT 94906
Orangutan                                                                                          95567
```

```
Majority          TGG-TCAGTTTTTCTAAACAGATATATTGGTGTCAAATAACTTTGGAGTTATGCATG---TTTCTTAGGTTTGTTCTGTT

                           112890    112900    112910    112920    112930    112940    112950    112960

Human             CGG-TCAGTTTTTCTAAACAGATATATTGGTATCAAATAACTTTGGAGTTACACATG---TTTCTTAGGTTTGTTCTGTT 94542
Coquerel'sSifaka  TGGGCCACTTTTTCTAAATAGGTATACTGGTGTCAAATAACTTTGAAGTTATATATG---TTCCTTAAGTTTGTTCTGTT 81105
Ma'sNightMonkey   TGG-TCACTTTTTCTAAACAGATATATTGGTATCAAATAACTTTGAAGTTATGCATG---TTCCTTAGGTTTGTTCTGTT 88513
VervetAGM         TGG-TCAGTTTTTCTAAACAGGTATGTTGGTGTCAGATAACTTTGGAGTTATGCATGATGTTTCTTAGGTTTGCTCTGTT 96210
Gorrilla          TGG-TCAGTTTTTCTAAACAGATGTATTGATATCAAATAACTTTGGAGTTACACATG---TTTCTTAGGTTTGTTCTGTT 92786
GrabEatingMacaqueTGG-TCAGTTTTTCTAAACAGATATGTTGGTGTCAGATAACTTTGGAGTTATGCATGATGTTTCTTAGGTTTGCTCTGTT 95621
Macaque                                                                                            95018
PigTailedMacaque  TGG-TCAGTTTTTCTAAACAGATATGTTGGTGTCAGATAACTTTGGAGTTATGCATGATGTTTCTTAGGTTTGCTCTGTT 95398
Bonobo            TGG-TCAGTTTTTCTAAACAGATATATTGGTATCAAATAACTTTGGAGTTACACATG---TTTCTTAGGTTTGTTCTGTT 93368
OliveBaboon       TGG-TCAGTTTTTCTAAACAGATATGTTGGTGTCAGATAACTTTGGAGTTATGCATGATGTTTCTTAGGTTTGCTCTGTT 94985
Orangutan                                                                                          95567
```

```
Majority          ACAAAATATCCCACACTTATGCAAAACTTATACAT-AGGATAATAAAAAGTAAATGAAGGAXCATAXXXXXXXXXXXXXX

                           112970    112980    112990    113000    113010    113020    113030    113040

Human             ATAAAATATCCCACACTTATGCAAAACTTACACAT-AGGATAATAAAAAGTAAATGAAGGAACATAAAAT-ATGTTTTAC 94620
Coquerel'sSifaka  AC-----ATCTCACAATTACACAAAACTTACACATTAGGATAAT--AA--AAAATGAAGGAGCATAGG             81164
Ma'sNightMonkey   ATAAAATATCCCACAGTTATGCAAAAGTTATACAG-AAGATAA---AAAGTAAATGAAGGAACATAAAATTATGTTTAAA 88589
VervetAGM         ACAAAATATCCCACACTTATACAAAACTTATACGT-AGGATAATAAAAAGTAAATGAAGGAACATAAAAT-ACGTTTTAC 96288
Gorrilla          ATAAAATATCCCACACTTATGCAAAACTTATACAT-AGGAT                                        92826
GrabEatingMacaqueACAAAATATCCCACACTTATGCAAAACTTACACGT-AGGATAATAAAAAGTAAATGAAGGAACATA              95686
Macaque                                                                                            95018
PigTailedMacaque  ACAAAATATCCCACACTTATGCAAAACTTATACGT-AGGATAATAAAAAGTA                             95449
Bonobo            ATAAAATATCCCACACTTATGCAAAACTTATACAT-AGGATAAT                                     93411
OliveBaboon       ACAAAATATCCCACACTTATGCAAAACTTATAGGT-AGGATAATAAAAAGTAAATGAAGGAACATAAAAT-ATGTTTTAC 95063
Orangutan                                                                                          95567
```

```
Majority          XXXXXXXXXXXXXXXXXXXXXXXXXXXXXXXXXXXXXXXXXXXXXXXXXXXXXXXXXXXXXXXXXXXXXXXXXXXXXXXX

                         113050    113060    113070    113080    113090    113100    113110    113120

Human             TATACTTAATGTAGTAATTCAGTTACCTCCAAGAAAGAAGAATATAATAAGTAAAATAATTTTGTTTACACTAGTGTCAC 94700
Coquerel'sSifaka                                                                                   81164
Ma'sNightMonkey   TATACTATACTTAGTACAGTAATT                                                         88613
VervetAGM         TATACCTAATATAGTAATTCAGTTACCTCCAAGAAAGAAGAACATAGTAAGTAAAATAATTTTGTTTACACTAGTGTCAC 96368
Gorrilla                                                                                           92826
GrabEatingMacaque                                                                                  95686
Macaque                                                                                            95018
PigTailedMacaque                                                                                   95449
Bonobo                                                                                             93411
OliveBaboon       TATACCTAATGTAGTAATTCAGTTACCTCCAAGAAAGAAGAACATAGTAAGTAAAATAATTTTGTTTACACTAGTGTCAC 95143
Orangutan                                                                                          95567

Majority          XXXXXXXXXXXXXXXXXXXXXXXXXXXXXXXXXXXXXXXXXXXXXXXXXXXXXXXXXXXXXXXXXXXXXXXXXXXXXXXX

                         113130    113140    113150    113160    113170    113180    113190    113200

Human             TGTTGTCATCTGAAGAAGAACCATTAGAGTCAACAGTCTGGGATGATTTGGAAGCTAGGTGATCTGTGTTTTTTTCTTAA 94780
Coquerel'sSifaka                                                                                   81164
Ma'sNightMonkey                                                                                    88613
VervetAGM         TGTTGTCATCTGAAGAAGAACCATTAGAGTCAACAGTTTGGGATGATTTGGAAGCTAGGTGATCTGTGTTTTTTTCTTAA 96448
Gorrilla                                                                                           92826
GrabEatingMacaque                                                                                  95686
Macaque                                                                                            95018
PigTailedMacaque                                                                                   95449
Bonobo                                                                                             93411
OliveBaboon       TGTTGTCATCTGAAGAACCACCATTAGAGTCTACAGTTTGGGATGATTTGGAATTTAGGTGATATGTTTTTTTTTTTT-- 95221
Orangutan                                                                                          95567

Majority          XXXXXXXXXXXXXXXXXXXXXXXXXXXXXXXXXXXXXXXXXXXXXXXXXXXXXXXXXXXXXXXXXXXXXXXXXXXXXXXX

                         113210    113220    113230    113240    113250    113260    113270    113280

Human             AATAACATCGTAGCACAGAGGAACTCTACCAGCATCAGATAAGTCTAAAGTCTAAAAAACCAATACCAGGGTTAGTGAAT 94860
Coquerel'sSifaka                                                                                   81164
Ma'sNightMonkey                                                                                    88613
VervetAGM         AATAACATTGTAGCACAGAGGAACTCTAGCAACATCAGATATGTCTAAAGTCTAAGAAACCAGTCCCAGGGTTAGTGAAC 96528
Gorrilla                                                                                           92826
GrabEatingMacaque                                                                                  95686
Macaque                                                                                            95018
PigTailedMacaque                                                                                   95449
Bonobo                                                                                             93411
OliveBaboon       -------TTGTAGCACAGAGGAACTCTAGCAACATCAGATATGTCTAAAGTCTAA---ACGTAATCCCAGGTTAGTGTTG 95291
Orangutan                                                                                          95567
```

```
Majority          XXXXXXXXXXXXXXXXXXXXXXXXXXXXXXXXXXXXXXXXXXXXXXXXXXXXXXXXXXXXXXXXXXXXXXXXXXXXXXXX
                        113290    113300    113310    113320    113330    113340    113350    113360
Human             AGTAATTGAACAAAGCTTCTCAAGGTGAAGTTTTTCTCAGTAATTAGTGGTGGCTATAATATTTGAAAGTAGGTTCTGTC 94940
Coquerel'sSifaka                                                                                   81164
Ma'sNightMonkey                                                                                    88613
VervetAGM         AGTAATTGAACAAAGCTGCTCAAGGTGGCATTTTTCTCAGTAATGAGTGGTGGTGATAATATTTGAAAGTAGCTTCT-TC 96607
Gorrilla                                                                                           92826
GrabEatingMacaque                                                                                  95686
Macaque                                                                                            95018
PigTailedMacaque                                                                                   95449
Bonobo                                                                                             93411
OliveBaboon       TGAAC---AACAAAGCTGCTCAAGGTGGCGTTTTTCTCAGTAATGAGTGGTGGTGATAATATTTGAAAGTAGCTTCTGCC 95368
Orangutan                                                                                          95567

Majority          XXXXXXXXXXXXXXXXXXXXXXXXXXXXXXXXXXXXXXXXXXXXXXXXXXXXXXXXXXXXXXXXXXXXXXXXXXXXXXXX
                        113370    113380    113390    113400    113410    113420    113430    113440
Human             ACCTTTTCAGGTGCTCATTGTGGGTGGGATTGCCTTGGGAAGGAGAAACT-AAGTCCTGAGCTGTGTGTGTGGACTTAGT 95019
Coquerel'sSifaka                                                                                   81164
Ma'sNightMonkey                                                                                    88613
VervetAGM         ACCTTTTCAGGTGCTCATTGTGGGTGGCATTGCCTTGGGAAGGAGAAACTTAAGTCCCGAGCTGTGTGTGGGAACTTAGT 96687
Gorrilla                                                                                           92826
GrabEatingMacaque                                                                                  95686
Macaque                                                                                            95018
PigTailedMacaque                                                                                   95449
Bonobo                                                                                             93411
OliveBaboon       ACCTTTTCAGGTGCTCATTGTGGGTGGCATTGCCTTGGGAAGGAGAAACTTAAGTCCCGAGCTGTGTGTGTGGG---AAC 95445
Orangutan                                                                                          95567

Majority          XXXXXXXXXXXXXXXXXXXXXXXXXXXXXXXXXXXXXXXXXXXXXXXXXXXXXXXXXXXXXXXXXXXXXXXXXXXXXXXX
                        113450    113460    113470    113480    113490    113500    113510    113520
Human             TTGTGCCAGTTGCTAAGCTGGTGGGAGTTCCCAGTCTCCCTAGTGGGAGATAACCAGAAACCAGCTCTTCCTGC-CTAAG 95098
Coquerel'sSifaka                                                                                   81164
Ma'sNightMonkey                                                                                    88613
VervetAGM         TTGCGCCATTTGCTAAGCTGGTGAGAGTTCCCAATCTCCCTAGTGGGAGGTAACCAGAAACCAGCTCTTCTTGCCTAAGG 96767
Gorrilla                                                                                           92826
GrabEatingMacaque                                                                                  95686
Macaque                                                                                            95018
PigTailedMacaque                                                                                   95449
Bonobo                                                                                             93411
OliveBaboon       TTCTGTCATGCCTCATGTTGGTGAGA-TTCTTAATTCCCTAGGTGGGAGGTAACCAGAAACCAGCTCTGCCTGC-TGACT 95523
Orangutan                                                                                          95567
```

```
Majority          XXXXXXXXXXXXXXXXXXXXXXXXXXXXXXXXXXXXXXXXXXXXXXXXXXXXXXXXXXXXXXXXXXXXXXXXXXXXXXXX

                        113530    113540    113550    113560    113570    113580    113590    113600

Human             GACTACAGCAGATTCTATACTGTCTTACAGTATGCCCTAATACGGCCTTCCTTCCAAAGCACCAAGTCACTAAAGGGGGA 95178
Coquerel'sSifaka                                                                                   81164
Ma'sNightMonkey                                                                                    88613
VervetAGM         ACTTACAGCAGATTCTATACTCTCTTACACTATACCCCAATACAGCCTTCCTTCCAAAGCACCAAGTCAGTAAAGGGGGA 96847
Gorrilla                                                                                           92826
GrabEatingMacaque                                                                                  95686
Macaque                                                                                            95018
PigTailedMacaque                                                                                   95449
Bonobo                                                                                             93411
OliveBaboon       TCATGCAGCAGATTCTATACTCTCTTACACTATACCCCAATACAGCCTTCCTTCCAAAGCACCAAGTCAGTAAAGGGGGA 95603
Orangutan                                                                                          95567

Majority          XXXXXXXXXXXXXXXXXXXXXXXXXXXXXXXXXXXXXXXXXXXXXXXXXXXXXXXXXXXXXXXXXXXXXXXXXXXXXXXX

                        113610    113620    113630    113640    113650    113660    113670    113680

Human             TTTTGACATTCTAGAATATTTTTAATTCAGTACATCAAATTAACCCAGGCCTTGGCATATAGTGAATTGCTTATTAAAAG 95258
Coquerel'sSifaka                                                                                   81164
Ma'sNightMonkey                                                                                    88613
VervetAGM         TTTTGACATTCTGGAATATTTTTAATTCAGTACATGAAATTAACCCAGACCTTGGCATATAGCGAATTACTTATTAAAAG 96927
Gorrilla                                                                                           92826
GrabEatingMacaque                                                                                  95686
Macaque                                                                                            95018
PigTailedMacaque                                                                                   95449
Bonobo                                                                                             93411
OliveBaboon       ATTTGACATTCTGGAATACGGACCAGT------ATGAAATTAACTCAGACCCATATAGCTAATTGTTT--CTATTAAAAG 95675
Orangutan                                                                                          95567

Majority          XXXXXXXXXXXXXXXXXXXXXXXXXXXXXXXXXXXXXXXXXXXXXXXXXXXXXXXXXXXXXXXXXXXXXXXXXXXXXXXX

                        113690    113700    113710    113720    113730    113740    113750    113760

Human             TTAGGTAAAAATAAAAACAGCTTTGTCGTATATTAGTACTCTATGACTCTTAGAGTGAACAAGCTAACAAGTATTCTTAG 95338
Coquerel'sSifaka                                                                                   81164
Ma'sNightMonkey                                                                                    88613
VervetAGM         TTAGGTAAAAATAATAACAGCTTTGTTGTATATTAGTACTCTATGCCTCTTAGAGTGAACTAGCTAACAATGAAGTATTC 97007
Gorrilla                                                                                           92826
GrabEatingMacaque                                                                                  95686
Macaque                                                                                            95018
PigTailedMacaque                                                                                   95449
Bonobo                                                                                             93411
OliveBaboon       TTAGGTAAAAATAATAACAGCTTTGTTGTATATTAGTACTCTATGCCTCTTAGAGTGAACTAGCTAACAATGAAGTATTC 95755
Orangutan                                                                                          95567
```

```
Majority          XXXXXXXXXXXXXXXXXXXXXXXXXXXXXXXXXXXXXXXXXXXXXXXXXXXXXXXXXXXXXXXXXXXXXXXXXXXXXXXX

                           113770    113780    113790    113800    113810    113820    113830    113840

Human             GACTTAGCTGCTTTGTTAATAGAGGTCTTAACTTGAATATTTAGAAATAATCAGAAAAATTATTTTTTGGTATTGAAGAT 95418
Coquerel'sSifaka                                                                                   81164
Ma'sNightMonkey                                                                                    88613
VervetAGM         TTAGGACTCAGCTGCTTTGTTAATAGAGGTATTAACTTGAATATTTAAAAATAACTTTAATCAGAAAAATTATTTTTGAC 97087
Gorrilla                                                                                           92826
GrabEatingMacaque                                                                                  95686
Macaque                                                                                            95018
PigTailedMacaque                                                                                   95449
Bonobo                                                                                             93411
OliveBaboon       TTAGGACTCAGCTGCTTTGTTAATAGAGGTATTAACTTGAATATTTAAAAATAACTTTAATCAGAAAAATTATTTTTGAC 95835
Orangutan                                                                                          95567

Majority          XXXXXXXXXXXXXXXXXXXXXXXXXXXXXXXXXXXXXXXXXXXXXXXXXXXXXXXXXXXXXXXXXXXXXXXXXXXXXXXX

                           113850    113860    113870    113880    113890    113900    113910    113920

Human             GTATTTTGGAAATGGGTTTTAAAGTACCTACTTAAGGAAGAGATATCTACAGTTTATATTTAACGTTAGCATTTGAAAGC 95498
Coquerel'sSifaka                                                                                   81164
Ma'sNightMonkey                                                                                    88613
VervetAGM         ATTTAATAAGATGTATTTTGGAAATGGGTTTTAAAGCACCTACTTAAGGAAGAGATATCTACAGTTTAATGTTAGCATTT 97167
Gorrilla                                                                                           92826
GrabEatingMacaque                                                                                  95686
Macaque                                                                                            95018
PigTailedMacaque                                                                                   95449
Bonobo                                                                                             93411
OliveBaboon       ATTTAATAAGATGTATTTTGGAATTTTTTTTTTTTTTTGAGATTTGGAGTCTCGGCCTGT-CACTCAGGGCTGAGTGCAG 95914
Orangutan                                                                                          95567

Majority          XXXXXXXXXXXXXXXXXXXXXXXXXXXXXXXXXXXXXXXXXXXXXXXXXXXXXXXXXXXXXXXXXXXXXXXXXXXXXXXX

                           113930    113940    113950    113960    113970    113980    113990    114000

Human             AATGAAGAAAATTTTTCCTTAACCAGGAAGTGTAATGAATCATTATATTAGAGATTAAATTAACACTTTGGAAGCATTTT 95578
Coquerel'sSifaka                                                                                   81164
Ma'sNightMonkey                                                                                    88613
VervetAGM         GAAAGCAACGAAGAAAATTTTTGCTTAACTAGGAAGTGTAATGAATCATTAT                             97219
Gorrilla                                                                                           92826
GrabEatingMacaque                                                                                  95686
Macaque                                                                                            95018
PigTailedMacaque                                                                                   95449
Bonobo                                                                                             93411
OliveBaboon       GCCAGATTTCAGCTCCAGCCCGGCCTCCCAGGTTGCCTATCTTCTGCCTCAGCCTCCCCGAGAAGCTGGGACTACAGGCC 95994
Orangutan                                                                                          95567
```

```
Majority          XXXXXXXXXXXXXXXXXXXXXXXXXXXXXXXXXXXXXXXXXXXXXXXXXXXXXXXXXXXXXXXXXXXXXXXXXXXXXXXX

                          114010    114020    114030    114040    114050    114060    114070    114080

Human             AGCTCCTAGAGTTAAGGTTAGTATAGTGAAATTATGGTGATTAGCTTAATTCATTCATGTACATCTATTTGTATCACCAT 95658
Coquerel'sSifaka                                                                                   81164
Ma'sNightMonkey                                                                                    88613
VervetAGM                                                                                          97219
Gorrilla                                                                                           92826
GrabEatingMacaque                                                                                  95686
Macaque                                                                                            95018
PigTailedMacaque                                                                                   95449
Bonobo                                                                                             93411
OliveBaboon       CACCACCTCAGCCTCGCCAGTTTTTGTATTTTAGTAGAGATGGGGTTTCACCCCAGAGCTAGCCAGGATGGTCTCGACTT 96074
Orangutan                                                                                          95567

Majority          XXXXXXXXXXXXXXXXXXXXXXXXXXXXXXXXXXXXXXXXXXXXXXXXXXXXXXXXXXXXXXXXXXXXXXXXXXXXXXXX

                          114090    114100    114110    114120    114130    114140    114150    114160

Human             ATAATTACTAGGGAAAGGACAAATCACTTTTCAAAGCCTCAATGTTATTTGTAAAATGGAGGCAATAAGTTCTGCTCTGC 95738
Coquerel'sSifaka                                                                                   81164
Ma'sNightMonkey                                                                                    88613
VervetAGM                                                                                          97219
Gorrilla                                                                                           92826
GrabEatingMacaque                                                                                  95686
Macaque                                                                                            95018
PigTailedMacaque                                                                                   95449
Bonobo                                                                                             93411
OliveBaboon       CCCGACTCATGATCCCGCCTGGCCTCAGCCTCCCAAAGTGCTGGGATTACAGGCTTGAGCCACCGCGCCCAGCCGGAAAT 96154
Orangutan                                                                                          95567

Majority          XXXXXXXXXXXXXXXXXXXXXXXXXXXXXXXXXXXXXXXXXXXXXXXXXXXXXXXXXXXXXXXXXXXXXXXXXXXXXXXX

                          114170    114180    114190    114200    114210    114220    114230    114240

Human             TCCCCTCACAGGATTATTACAGAGGCTCAAATAGGATAAGTGAAGGAATGCTATGCAATTAGTTAACTAATGCCAGTTCC 95818
Coquerel'sSifaka                                                                                   81164
Ma'sNightMonkey                                                                                    88613
VervetAGM                                                                                          97219
Gorrilla                                                                                           92826
GrabEatingMacaque                                                                                  95686
Macaque                                                                                            95018
PigTailedMacaque                                                                                   95449
Bonobo                                                                                             93411
OliveBaboon       GGGTTCTAAAGCACCTACTTAAGGAAGAGATATCTACAGTTTAATGTTAGCATTTGAAAGCAACGAAGAAAATTTTTGCT 96234
Orangutan                                                                                          95567
```

```
Majority          XXXXXXXXXXXXXXXXXXXXXXXXXXXXXXXXXXXXXXXXXXXXXXXXXXXXXXXXXXXXXXXXXXXXXXXXXXXXXXXXXX

                       114250    114260    114270    114280    114290    114300    114310    114320

Human             CAGACTGCCAGATTTGCCCACAGTAAACTTCATTATCCACAATACATGTTATCCTAATATGTCTTTTTGCTACAAAGAAA 95898
Coquerel'sSifaka                                                                                   81164
Ma'sNightMonkey                                                                                    88613
VervetAGM                                                                                          97219
Gorrilla                                                                                           92826
GrabEatingMacaque                                                                                  95686
Macaque                                                                                            95018
PigTailedMacaque                                                                                   95449
Bonobo                                                                                             93411
OliveBaboon       TAACTAGGAAGTGTAATGAATCATTATATTAGAGATTAAATTAAGACTTTGGAAGCATTTTAGCCCGTAGAGTTAAGGTT 96314
Orangutan                                                                                          95567

Majority          XXXXXXXXXXXXXXXXXXXXXXXXXXXXXXXXXXXXXXXXXXXXXXXXXXXXXXXXXXXXXXXXXXXXXXXXXXXXXXXXXX

                       114330    114340    114350    114360    114370    114380    114390    114400

Human             TACTAATGTTTTAAAATTATTTACTAGTTTCATAAAGCAGAAAGATGTAAAATGCAGTTATAGAGAGCATCCCAAGCCAG 95978
Coquerel'sSifaka                                                                                   81164
Ma'sNightMonkey                                                                                    88613
VervetAGM                                                                                          97219
Gorrilla                                                                                           92826
GrabEatingMacaque                                                                                  95686
Macaque                                                                                            95018
PigTailedMacaque                                                                                   95449
Bonobo                                                                                             93411
OliveBaboon       AGTGTAGTGAAATTATGGTGATTAGCTTAATTCATTCATGTACATTTATTTGTATCTGTACCAGATAATTACTAGGTAAA 96394
Orangutan                                                                                          95567

Majority          XXXXXXXXXXXXXXXXXXXXXXXXXXXXXXXXXXXXXXXXXXXXXXXXXXXXXXXXXXXXXXXXXXXXXXXXXXXXXXXXXX

                       114410    114420    114430    114440    114450    114460    114470    114480

Human             ATGAAAAGAAACCATGGATTTAGCTTTATATTTTCTGTTTGAACTTGATAGCCCAATCATAAAATGCACATATTTAATTA 96058
Coquerel'sSifaka                                                                                   81164
Ma'sNightMonkey                                                                                    88613
VervetAGM                                                                                          97219
Gorrilla                                                                                           92826
GrabEatingMacaque                                                                                  95686
Macaque                                                                                            95018
PigTailedMacaque                                                                                   95449
Bonobo                                                                                             93411
OliveBaboon       GGACAAATCACTTCAAAGCCTCAGTATTATTTGTAAAATGGAGGCAATAAGTTCTGCTCTGCTCACCTCACAGGATTATT 96474
Orangutan                                                                                          95567
```

```
Majority          XXXXXXXXXXXXXXXXXXXXXXXXXXXXXXXXXXXXXXXXXXXXXXXXXXXXXXXXXXXXXXXXXXXXXXXXXXXXXXXX

                        114490    114500    114510    114520    114530    114540    114550    114560

Human             TAGACTGCACTTAAGATTTCATATGTATAAAAAAGTTGCTGTGAAACAGGTAATTTTAACACTCAGGAATTTCAAGGCTA 96138
Coquerel'sSifaka                                                                                   81164
Ma'sNightMonkey                                                                                    88613
VervetAGM                                                                                          97219
Gorrilla                                                                                           92826
GrabEatingMacaque                                                                                  95686
Macaque                                                                                            95018
PigTailedMacaque                                                                                   95449
Bonobo                                                                                             93411
OliveBaboon       ATAGAGGCTCAAATAGGATAAGTGAAGAAATGCTATGCAATTAGTAAACTGTTAGGCCAGAGAGTTCCCAGACTGCCAGA 96554
Orangutan                                                                                          95567

Majority          XXXXXXXXXXXXXXXXXXXXXXXXXXXXXXXXXXXXXXXXXXXXXXXXXXXXXXXXXXXXXXXXXXXXXXXXXXXXXXXX

                        114570    114580    114590    114600    114610    114620    114630    114640

Human             AGGAAGAAAGACATACTCTCATGAGCAGCTCTGAACTTCAAACAGGATTTTCTGGCTGCCTTAATATATTAAAATGGAGC 96218
Coquerel'sSifaka                                                                                   81164
Ma'sNightMonkey                                                                                    88613
VervetAGM                                                                                          97219
Gorrilla                                                                                           92826
GrabEatingMacaque                                                                                  95686
Macaque                                                                                            95018
PigTailedMacaque                                                                                   95449
Bonobo                                                                                             93411
OliveBaboon       TTTGCCCACAGTAAACTTCATTATCCGCAATACATGTTATCCTAATATGTCTTTTTGCTACGAAGAAATACTAATGTTTT 96634
Orangutan                                                                                          95567

Majority          XXXXXXXXXXXXXXXXXXXXXXXXXXXXXXXXXXXXXXXXXXXXXXXXXXXXXXXXXXXXXXXXXXXXXXXXXXXXXXXX

                        114650    114660    114670    114680    114690    114700    114710    114720

Human             CTAAATCTGAAAACGTTTTTTTCATTCAGAAGAAAGTACAGAATTCAGTGAAATTGAGTATACTAGGAAATCTGATGGCA 96298
Coquerel'sSifaka                                                                                   81164
Ma'sNightMonkey                                                                                    88613
VervetAGM                                                                                          97219
Gorrilla                                                                                           92826
GrabEatingMacaque                                                                                  95686
Macaque                                                                                            95018
PigTailedMacaque                                                                                   95449
Bonobo                                                                                             93411
OliveBaboon       AAAATTATTTACTAGTTTCATAAAGCAGAAAGATGTAAAATGCAGTTATAGAGAGCATCCCAAGCCAAACGAAAAGAAAC 96714
Orangutan                                                                                          95567
```

```
Majority          XXXXXXXXXXXXXXXXXXXXXXXXXXXXXXXXXXXXXXXXXXXXXXXXXXXXXXXXXXXXXXXXXXXXXXXXXXXXXXXX

                        114730    114740    114750    114760    114770    114780    114790    114800

Human             AGAATTCAGCAGAAAGTCTTAATTTTAAACCGTAATATTCATTGATTATGCAAAACCTTAGTTTTATAGTTATTCTTTGT 96378
Coquerel'sSifaka                                                                                  81164
Ma'sNightMonkey                                                                                   88613
VervetAGM                                                                                         97219
Gorrilla                                                                                          92826
GrabEatingMacaque                                                                                 95686
Macaque                                                                                           95018
PigTailedMacaque                                                                                  95449
Bonobo                                                                                            93411
OliveBaboon       CATGGATTTAGTTTTGTATTTTGTTTGAACTTGATAGCCCAATCATAAAATGCATATTTAATTATATTTAATTATAGACT 96794
Orangutan                                                                                         95567

Majority          XXXXXXXXXXXXXXXXXXXXXXXXXXXXXXXXXXXXXXXXXXXXXXXXXXXXXXXXXXXXXXXXXXXXXXXXXXXXXXXX

                        114810    114820    114830    114840    114850    114860    114870    114880

Human             TATTTTGAATAGCTTCCCTTTAAATTTTCTAGTTTCAGATTTTGCAAATAAAAGTGTACATGTTAAAGTTATAGAGTATT 96458
Coquerel'sSifaka                                                                                  81164
Ma'sNightMonkey                                                                                   88613
VervetAGM                                                                                         97219
Gorrilla                                                                                          92826
GrabEatingMacaque                                                                                 95686
Macaque                                                                                           95018
PigTailedMacaque                                                                                  95449
Bonobo                                                                                            93411
OliveBaboon       GCACTTAAGATTTCATATGTATAAAAAAGTTGCTGTGACACTTAGAGGTAATTCTAACACTCAGGAATTTCAAGGCTAAG 96874
Orangutan                                                                                         95567

Majority          XXXXXXXXXXXXXXXXXXXXXXXXXXXXXXXXXXXXXXXXXXXXXXXXXXXXXXXXXXXXXXXXXXXXXXXXXXXXXXXX

                        114890    114900    114910    114920    114930    114940    114950    114960

Human             AGATTATTCACATTAAGAACTGTTCAAATACGCGGAAAAATAAAACCGTAATGAACACCTATGCACTTCACTCAACTATA 96538
Coquerel'sSifaka                                                                                  81164
Ma'sNightMonkey                                                                                   88613
VervetAGM                                                                                         97219
Gorrilla                                                                                          92826
GrabEatingMacaque                                                                                 95686
Macaque                                                                                           95018
PigTailedMacaque                                                                                  95449
Bonobo                                                                                            93411
OliveBaboon       GAAGAAAGACATATTCTCACGAGCAGCTCTGAACTTCAAACAGGATTTTCTGGCTGCCTTAATATATTAAAATGGAGCCT 96954
Orangutan                                                                                         95567
```

```
Majority          XXXXXXXXXXXXXXXXXXXXXXXXXXXXXXXXXXXXXXXXXXXXXXXXXXXXXXXXXXXXXXXXXXXXXXXXXXXXXXXX

                          114970    114980    114990    115000    115010    115020    115030    115040

Human             TCACTACCTAACATATTCCATATTTTAAACGTAATTATATATTAGTTGAAAGAAAACGTTACAGAATGAACTGAAATCCC 96618
Coquerel'sSifaka                                                                                   81164
Ma'sNightMonkey                                                                                    88613
VervetAGM                                                                                          97219
Gorrilla                                                                                           92826
GrabEatingMacaque                                                                                  95686
Macaque                                                                                            95018
PigTailedMacaque                                                                                   95449
Bonobo                                                                                             93411
OliveBaboon       AAATCCAAAAACTTTTTTTCATCCAGAAGAAAGTACAGAATGCAGTGAAATTGAGTACACTAGAAATTCTGATGGCAAGA 97034
Orangutan                                                                                          95567

Majority          XXXXXXXXXXXXXXXXXXXXXXXXXXXXXXXXXXXXXXXXXXXXXXXXXXXXXXXXXXXXXXXXXXXXXXXXXXXXXXXX

                          115050    115060    115070    115080    115090    115100    115110    115120

Human             ATATGAACCTCTTTCTGATCTTAGTTCAAACCTTATTCTTTCCCAAGAGGTAATTGTTACCTTCAGTTTAGTATTTTATT 96698
Coquerel'sSifaka                                                                                   81164
Ma'sNightMonkey                                                                                    88613
VervetAGM                                                                                          97219
Gorrilla                                                                                           92826
GrabEatingMacaque                                                                                  95686
Macaque                                                                                            95018
PigTailedMacaque                                                                                   95449
Bonobo                                                                                             93411
OliveBaboon       ATTGAGCAGAAAGTCTTAAATTTAAACCATAATATTCATTGGTTATCTAAAACCTTCGTTTTATTCTTTGTTATTTTGAA 97114
Orangutan                                                                                          95567

Majority          XXXXXXXXXXXXXXXXXXXXXXXXXXXXXXXXXXXXXXXXXXXXXXXXXXXXXXXXXXXXXXXXXXXXXXXXXXXXXXXX

                          115130    115140    115150    115160    115170    115180    115190    115200

Human             ATTCAGTGGTTGCTTTTCAAGAGGAATTTGTCCTATATTCTGTAGGCTAAATTTCTTGTTTTGCCTAGTTATAAAAATGG 96778
Coquerel'sSifaka                                                                                   81164
Ma'sNightMonkey                                                                                    88613
VervetAGM                                                                                          97219
Gorrilla                                                                                           92826
GrabEatingMacaque                                                                                  95686
Macaque                                                                                            95018
PigTailedMacaque                                                                                   95449
Bonobo                                                                                             93411
OliveBaboon       TAGTTTCCCTTTAAATTTTTTAGTTTTGAATTTTGCAAATAAAAGTGTACATGTTAAAGTTACAGAGTATTAGATTATTC 97194
Orangutan                                                                                          95567
```

```
Majority           XXXXXXXXXXXXXXXXXXXXXXXXXXXXXXXXXXXXXXXXXXXXXXXXXXXXXXXXXXXXXXXXXXXXXXXXXXXXXXXX

                           115210    115220    115230    115240    115250    115260    115270    115280

Human              CAAATAATATGATATTGGGTGTTTAAATAGTACAATATTGTTCAAAACACACTTTATAATTTTTAACAGACTGTTTGGAC 96858
Coquerel'sSifaka                                                                                    81164
Ma'sNightMonkey                                                                                     88613
VervetAGM                                                                                           97219
Gorrilla                                                                                            92826
GrabEatingMacaque                                                                                   95686
Macaque                                                                                             95018
PigTailedMacaque                                                                                    95449
Bonobo                                                                                              93411
OliveBaboon        ACATTAAGAACTGTTCAAATACGCGGAAACATAAAACCATAATGAACACCTGTGCACTTCACTCAACTATATCACTACCT 97274
Orangutan                                                                                           95567

Majority           XXXXXXXXXXXXXXXXXXXXXXXXXXXXXXXXXXXXXXXXXXXXXXXXXXXXXXXXXXXXXXXXXXXXXXXXXXXXXXXX

                           115290    115300    115310    115320    115330    115340    115350    115360

Human              ATTGATAATTCCCCTATCAAATCTCATGACATTTAACCAATTTTTAACAAGGATTTTAAATTGTGTTTAAGAAATAAGTC 96938
Coquerel'sSifaka                                                                                    81164
Ma'sNightMonkey                                                                                     88613
VervetAGM                                                                                           97219
Gorrilla                                                                                            92826
GrabEatingMacaque                                                                                   95686
Macaque                                                                                             95018
PigTailedMacaque                                                                                    95449
Bonobo                                                                                              93411
OliveBaboon        TTTTCCATATTTTAAATGTAATTATATATTAGTTGAAAGGAAATGTTACAGAATGAACTGAAATCCCATATGAACCTCTT 97354
Orangutan                                                                                           95567

Majority           XXXXXXXXXXXXXXXXXXXXXXXXXXXXXXXXXXXXXXXXXXXXXXXXXXXXXXXXXXXXXXXXXXXXXXXXXXXXXXXX

                           115370    115380    115390    115400    115410    115420    115430    115440

Human              ATTTGGGCCTCAGGTTTCTCTTAAGGATGTAAACAAGGAGCTATATCATTGGTATTTTAGACGCTGAATTATGTGTAAGG 97018
Coquerel'sSifaka                                                                                    81164
Ma'sNightMonkey                                                                                     88613
VervetAGM                                                                                           97219
Gorrilla                                                                                            92826
GrabEatingMacaque                                                                                   95686
Macaque                                                                                             95018
PigTailedMacaque                                                                                    95449
Bonobo                                                                                              93411
OliveBaboon        TCTGATCTTAGTTCAAACCTTGTTCTTTCCCAAGAGGTAATTGTTCCCTTCAATTTAGTATTTTATTATTCAGTGGTTGC 97434
Orangutan                                                                                           95567
```

```
Majority          XXXXXXXXXXXXXXXXXXXXXXXXXXXXXXXXXXXXXXXXXXXXXXXXXXXXXXXXXXXXXXXXXXXXXXXXXXXXXXXX

                           115450    115460    115470    115480    115490    115500    115510    115520

Human             TCCTTAAATACAAACATACCCTTTGTAGCTTCACAGAGCCACCCGTTTTGCACTTCTCTCGCTTTTCTTCCTCCCTCTTT 97098
Coquerel'sSifaka                                                                                   81164
Ma'sNightMonkey                                                                                    88613
VervetAGM                                                                                          97219
Gorrilla                                                                                           92826
GrabEatingMacaque                                                                                  95686
Macaque                                                                                            95018
PigTailedMacaque                                                                                   95449
Bonobo                                                                                             93411
OliveBaboon       TTTTCAAGAGGAATTTGTCCTAAATTTCTTGTTCTGCCTAGTTATAAAAATGGCAAATAATATAATACTGGGTGTTTAAA 97514
Orangutan                                                                                          95567

Majority          XXXXXXXXXXXXXXXXXXXXXXXXXXXXXXXXXXXXXXXXXXXXXXXXXXXXXXXXXXXXXXXXXXXXXXXXXXXXXXXX

                           115530    115540    115550    115560    115570    115580    115590    115600

Human             GGCTCCCATTTCAATGGACAGAAAGACTATGTGGCATCTCTATTTTCATTCACTTGGTTTATTGTCTTGGTATGTTGTAT 97178
Coquerel'sSifaka                                                                                   81164
Ma'sNightMonkey                                                                                    88613
VervetAGM                                                                                          97219
Gorrilla                                                                                           92826
GrabEatingMacaque                                                                                  95686
Macaque                                                                                            95018
PigTailedMacaque                                                                                   95449
Bonobo                                                                                             93411
OliveBaboon       TAGTACAATATTTTATTGTTCAAAACACATTTTATAATTTTTAACAGATTGGACATTGATAATCCCCTTATCAAATCTCA 97594
Orangutan                                                                                          95567

Majority          XXXXXXXXXXXXXXXXXXXXXXXXXXXXXXXXXXXXXXXXXXXXXXXXXXXXXXXXXXXXXXXXXXXXXXXXXXXXXXXX

                           115610    115620    115630    115640    115650    115660    115670    115680

Human             AGGTTTTTGAATCAGAAAATTTTAACATTTTTAGATATTTCAAACATACAAAAAATAGAAGAATATTTAACAAACCCTTG 97258
Coquerel'sSifaka                                                                                   81164
Ma'sNightMonkey                                                                                    88613
VervetAGM                                                                                          97219
Gorrilla                                                                                           92826
GrabEatingMacaque                                                                                  95686
Macaque                                                                                            95018
PigTailedMacaque                                                                                   95449
Bonobo                                                                                             93411
OliveBaboon       TGACATGTAACCAATTTTTAATAAGGATTTTAAATCGTGTTTAAGAAATAAGTCATTTGGGCCCCAGGTTTCTCTTAAAT 97674
Orangutan                                                                                          95567
```

```
Majority          XXXXXXXXXXXXXXXXXXXXXXXXXXXXXXXXXXXXXXXXXXXXXXXXXXXXXXXXXXXXXXXXXXXXXXXXXXXXXXXX

                           115690    115700    115710    115720    115730    115740    115750    115760

Human             TCAGCTTCAGTTAGCAACTTAGATCTTCCTCGTTTCCTCCACCCAATTCTCATCTCCTTTATTAATGCAGATCCCAGATA 97338
Coquerel'sSifaka                                                                                   81164
Ma'sNightMonkey                                                                                    88613
VervetAGM                                                                                          97219
Gorrilla                                                                                           92826
GrabEatingMacaque                                                                                  95686
Macaque                                                                                            95018
PigTailedMacaque                                                                                   95449
Bonobo                                                                                             93411
OliveBaboon       ATATAAACAAGGAGCTATATCATTGGCATTTTAGAAGCTGAATTATGTGTAAGATCCTTAAATACAAACATACTCTTTGT 97754
Orangutan                                                                                          95567

Majority          XXXXXXXXXXXXXXXXXXXXXXXXXXXXXXXXXXXXXXXXXXXXXXXXXXXXXXXXXXXXXXXXXXXXXXXXXXXXXXXX

                           115770    115780    115790    115800    115810    115820    115830    115840

Human             TATTTTATCAAGAAATTTTGTAGCATATATTTGTGTTGTTTTATTCGTGGTCATAGTTGCTTTCTCTAATATCTAGACTA 97418
Coquerel'sSifaka                                                                                   81164
Ma'sNightMonkey                                                                                    88613
VervetAGM                                                                                          97219
Gorrilla                                                                                           92826
GrabEatingMacaque                                                                                  95686
Macaque                                                                                            95018
PigTailedMacaque                                                                                   95449
Bonobo                                                                                             93411
OliveBaboon       AGCTTCACAGAGCCACCCTTTTTGCACTTCTCCCCCTTTTCTTCCTCCCTCCTTTGCTCCCATTTCAATGGACAGAAAGA 97834
Orangutan                                                                                          95567

Majority          XXXXXXXXXXXXXXXXXXXXXXXXXXXXXXXXXXXXXXXXXXXXXXXXXXXXXXXXXXXXXXXXXXXXXXXXXXXXXXXX

                           115850    115860    115870    115880    115890    115900    115910    115920

Human             GCCTAGCTGGTCAATACTACTTTTCTTCAAATCCTAGAACCTCCCTATTAAAAATATAGTCACCAGACCAGTGGCAAATT 97498
Coquerel'sSifaka                                                                                   81164
Ma'sNightMonkey                                                                                    88613
VervetAGM                                                                                          97219
Gorrilla                                                                                           92826
GrabEatingMacaque                                                                                  95686
Macaque                                                                                            95018
PigTailedMacaque                                                                                   95449
Bonobo                                                                                             93411
OliveBaboon       CTATGCGGCATCTCTATTTTCATTCACTTGGTTTATTGTCTTGGTATGTTGTATAGGTTTTTGAATCAAAGGAATTTTTA 97914
Orangutan                                                                                          95567
```

```
Majority          XXXXXXXXXXXXXXXXXXXXXXXXXXXXXXXXXXXXXXXXXXXXXXXXXXXXXXXXXXXXXXXXXXXXXXXXXXXXXXXX

                        115930    115940    115950    115960    115970    115980    115990    116000

Human             AGTATCACCTAGGAGTTTATTAGAAACTCAGTCTATGAGCCTACTGAATCTACATTTTGACAAGATTGGCAGGTGATGCA 97578
Coquerel'sSifaka                                                                                   81164
Ma'sNightMonkey                                                                                    88613
VervetAGM                                                                                          97219
Gorrilla                                                                                           92826
GrabEatingMacaque                                                                                  95686
Macaque                                                                                            95018
PigTailedMacaque                                                                                   95449
Bonobo                                                                                             93411
OliveBaboon       ACATTTTAGATATTTCAAACATAGAAAAACTAGAAGAATGTAACAAACCCTTGTCAGCTTCAGTTAGCAACTTACATCTT 97994
Orangutan                                                                                          95567

Majority          XXXXXXXXXXXXXXXXXXXXXXXXXXXXXXXXXXXXXXXXXXXXXXXXXXXXXXXXXXXXXXXXXXXXXXXXXXXXXXXX

                        116010    116020    116030    116040    116050    116060    116070    116080

Human             TTTGGACATAAGTTGGAGAAGCACTCAATGCATTTTTTCAGTTCTATAAAAATCAGCTTCAAAGTTTTTTCCTGAGTGTG 97658
Coquerel'sSifaka                                                                                   81164
Ma'sNightMonkey                                                                                    88613
VervetAGM                                                                                          97219
Gorrilla                                                                                           92826
GrabEatingMacaque                                                                                  95686
Macaque                                                                                            95018
PigTailedMacaque                                                                                   95449
Bonobo                                                                                             93411
OliveBaboon       CCTCATTTCCTCCACCCAGTTCTCCTTTATTAATGTAAATCTTAGACATATTTTATCATGAAATTTTGTGGCATATATTT 98074
Orangutan                                                                                          95567

Majority          XXXXXXXXXXXXXXXXXXXXXXXXXXXXXXXXXXXXXXXXXXXXXXXXXXXXXXXXXXXXXXXXXXXXXXXXXXXXXXXX

                        116090    116100    116110    116120    116130    116140    116150    116160

Human             GAAATTATTTCATTAGTAATTTTAAGTTATGCAGATATAGTTAATACCAAGAAATAATTGAACTGAAAAGGAACCCGGAT 97738
Coquerel'sSifaka                                                                                   81164
Ma'sNightMonkey                                                                                    88613
VervetAGM                                                                                          97219
Gorrilla                                                                                           92826
GrabEatingMacaque                                                                                  95686
Macaque                                                                                            95018
PigTailedMacaque                                                                                   95449
Bonobo                                                                                             93411
OliveBaboon       GTGGTGTTTTATTCCTGGTCATAGTTGCTTTCTCTAATACCTGGATTAGCCTAGCTTGTCAATACTACTTCAAATCCTAG 98154
Orangutan                                                                                          95567
```

```
Majority          XXXXXXXXXXXXXXXXXXXXXXXXXXXXXXXXXXXXXXXXXXXXXXXXXXXXXXXXXXXXXXXXXXXXXXXXXXXXXXXX

                        116170    116180    116190    116200    116210    116220    116230    116240

Human             ATCTAGTTTTAACCAGCTGTTTTAAAGATAAACAGTTCTAGAGATCAAATGAATAGTTTAGCAACATATTTATTGTAACT 97818
Coquerel'sSifaka                                                                                   81164
Ma'sNightMonkey                                                                                    88613
VervetAGM                                                                                          97219
Gorrilla                                                                                           92826
GrabEatingMacaque                                                                                  95686
Macaque                                                                                            95018
PigTailedMacaque                                                                                   95449
Bonobo                                                                                             93411
OliveBaboon       AACCTCGCTATTAAAAATGCAGTCACCAAACCAGTGGCAAATTAATATCACCTAGGAGTTTATTAGAAACTCAATCTATG 98234
Orangutan                                                                                          95567

Majority          XXXXXXXXXXXXXXXXXXXXXXXXXXXXXXXXXXXXXXXXXXXXXXXXXXXXXXXXXXXXXXXXXXXXXXXXXXXXXXXX

                        116250    116260    116270    116280    116290    116300    116310    116320

Human             TACCTGTAGTTAGAATCCAAGTCCCTGGCTTTCCAATCCAGTCTTCAATGTATAATACTGATCAAAAGAAATACAGGCCT 97898
Coquerel'sSifaka                                                                                   81164
Ma'sNightMonkey                                                                                    88613
VervetAGM                                                                                          97219
Gorrilla                                                                                           92826
GrabEatingMacaque                                                                                  95686
Macaque                                                                                            95018
PigTailedMacaque                                                                                   95449
Bonobo                                                                                             93411
OliveBaboon       ATCCTACTGAATCTACACTTTGGCAAGATTTGGCAGGTGATGCATTTGGACATAAGTTGAAGAAGCACTCTTAATAGAAT 98314
Orangutan                                                                                          95567

Majority          XXXXXXXXXXXXXXXXXXXXXXXXXXXXXXXXXXXXXXXXXXXXXXXXXXXXXXXXXXXXXXXXXXXXXXXXXXXXXXXX

                        116330    116340    116350    116360    116370    116380    116390    116400

Human             GCTATTATTCATAAATTTAAACTATTATGTACAAGTTGATAGCTTATAATAGAAATCAGTATGATTAGTGTTAAAAGTCA 97978
Coquerel'sSifaka                                                                                   81164
Ma'sNightMonkey                                                                                    88613
VervetAGM                                                                                          97219
Gorrilla                                                                                           92826
GrabEatingMacaque                                                                                  95686
Macaque                                                                                            95018
PigTailedMacaque                                                                                   95449
Bonobo                                                                                             93411
OliveBaboon       GCGTTTTTTTCAGTTCTGTAAAAATCAGCTTCCAAGTTTTTTCCTGAGTGTGGAAATTATTTCTTAGTAATTTTAAGTTT 98394
Orangutan                                                                                          95567
```

```
Majority          XXXXXXXXXXXXXXXXXXXXXXXXXXXXXXXXXXXXXXXXXXXXXXXXXXXXXXXXXXXXXXXXXXXXXXXXXXXXXXXX

                        116410    116420    116430    116440    116450    116460    116470    116480

Human             AATTTAGTCTGACAGGGAAGGAATGTCCATGTGAACTGATTTTTTTTAATAGAATGCAAATTAATACTCAATTCCTGATT 98058
Coquerel'sSifaka                                                                                   81164
Ma'sNightMonkey                                                                                    88613
VervetAGM                                                                                          97219
Gorrilla                                                                                           92826
GrabEatingMacaque                                                                                  95686
Macaque                                                                                            95018
PigTailedMacaque                                                                                   95449
Bonobo                                                                                             93411
OliveBaboon       TGCAGATATGTAGTTAATAATACCAAGAAATAATTGAAAAGGAACCCGGATATCCAGTTTTAACCTCCTGTTTTAAAGAT 98474
Orangutan                                                                                          95567

Majority          XXXXXXXXXXXXXXXXXXXXXXXXXXXXXXXXXXXXXXXXXXXXXXXXXXXXXXXXXXXXXXXXXXXXXXXXXXXXXXXX

                        116490    116500    116510    116520    116530    116540    116550    116560

Human             TGTTTATTACATTTTGTGGTTTAATGAAATGATACTCAGGTCATTTTCATAAATAGCTTTTCTATTAATGCTTCCTCAGT 98138
Coquerel'sSifaka                                                                                   81164
Ma'sNightMonkey                                                                                    88613
VervetAGM                                                                                          97219
Gorrilla                                                                                           92826
GrabEatingMacaque                                                                                  95686
Macaque                                                                                            95018
PigTailedMacaque                                                                                   95449
Bonobo                                                                                             93411
OliveBaboon       AAACAGTTCTAGAGCTCAAATAAATAGTTTAGCAACATTTATTGTAACATACCTGTAGTTAGAATCCAAGTCCCTGGCTT 98554
Orangutan                                                                                          95567

Majority          XXXXXXXXXXXXXXXXXXXXXXXXXXXXXXXXXXXXXXXXXXXXXXXXXXXXXXXXXXXXXXXXXXXXXXXXXXXXXXXX

                        116570    116580    116590    116600    116610    116620    116630    116640

Human             CACATTCTCTATTCCTATTGCATAGCACAGTCTAACAGTTGAAATGCCTAAGCCTTACTGAATGATGACTGATATTCTCT 98218
Coquerel'sSifaka                                                                                   81164
Ma'sNightMonkey                                                                                    88613
VervetAGM                                                                                          97219
Gorrilla                                                                                           92826
GrabEatingMacaque                                                                                  95686
Macaque                                                                                            95018
PigTailedMacaque                                                                                   95449
Bonobo                                                                                             93411
OliveBaboon       TCCAATCCAGTCTTCAGTGTTTTACACTGATCAAAAGAAATACAGGCCTGCTATTATTCATAAATTTAAACTATTACGTA 98634
Orangutan                                                                                          95567
```

```
Majority          XXXXXXXXXXXXXXXXXXXXXXXXXXXXXXXXXXXXXXXXXXXXXXXXXXXXXXXXXXXXXXXXXXXXXXXXXXXXXXXX

                          116650    116660    116670    116680    116690    116700    116710    116720

Human             GTGAGAGTTAGAGCTGTACTATAAATATACATTACCTGTGCAAAGTATGCCTTCAGGGGCTATTCCCCATAACCTTAGCA 98298
Coquerel'sSifaka                                                                                   81164
Ma'sNightMonkey                                                                                    88613
VervetAGM                                                                                          97219
Gorrilla                                                                                           92826
GrabEatingMacaque                                                                                  95686
Macaque                                                                                            95018
PigTailedMacaque                                                                                   95449
Bonobo                                                                                             93411
OliveBaboon       CAAGTTGGTAGCTTATCATAGAAATCAGTATGATCAGTATTAAAAGTCAAATTTAACAGGGAAGGAATGTCCATGTGAAT 98714
Orangutan                                                                                          95567

Majority          XXXXXXXXXXXXXXXXXXXXXXXXXXXXXXXXXXXXXXXXXXXXXXXXXXXXXXXXXXXXXXXXXXXXXXXXXXXXXXXX

                          116730    116740    116750    116760    116770    116780    116790    116800

Human             AAATTTTAAAATATTCTTATGGCTAGCAATCTTATCTATTGTCATGTCTTACTATTTCCTGCTTACCACCCCCATTTCTC 98378
Coquerel'sSifaka                                                                                   81164
Ma'sNightMonkey                                                                                    88613
VervetAGM                                                                                          97219
Gorrilla                                                                                           92826
GrabEatingMacaque                                                                                  95686
Macaque                                                                                            95018
PigTailedMacaque                                                                                   95449
Bonobo                                                                                             93411
OliveBaboon       TGATTTTTTTAAATAGACTGCAAATTAGTACTCTTAATTCCTGATTTGTTTATTACATTTTGTGGTTTTATAAAATGATA 98794
Orangutan                                                                                          95567

Majority          XXXXXXXXXXXXXXXXXXXXXXXXXXXXXXXXXXXXXXXXXXXXXXXXXXXXXXXXXXXXXXXXXXXXXXXXXXXXXXXX

                          116810    116820    116830    116840    116850    116860    116870    116880

Human             TCTCAACCTTGTTGCTGCATTAGCTGTCCATCACTTAACACTGTTTATTTCTCTGTTTTTCTTCCATCATTCTCTTCTTT 98458
Coquerel'sSifaka                                                                                   81164
Ma'sNightMonkey                                                                                    88613
VervetAGM                                                                                          97219
Gorrilla                                                                                           92826
GrabEatingMacaque                                                                                  95686
Macaque                                                                                            95018
PigTailedMacaque                                                                                   95449
Bonobo                                                                                             93411
OliveBaboon       CTCGGGTCATTTTCGTAAATAGCTTTTCTACTCATTTAATGCTTCCTCAGTCACTTTTTCTACTATTGCATAGCACAGTC 98874
Orangutan                                                                                          95567
```

```
Majority          XXXXXXXXXXXXXXXXXXXXXXXXXXXXXXXXXXXXXXXXXXXXXXXXXXXXXXXXXXXXXXXXXXXXXXXXXXXXXXXX

                          116890    116900    116910    116920    116930    116940    116950    116960

Human             GCAACTAAATGATTTAGACTTTTTATAACCATAATCAACCAGTTAGATTCTTTTTATTTGTGGTTATTGAGTTGATAAAT 98538
Coquerel'sSifaka                                                                                   81164
Ma'sNightMonkey                                                                                    88613
VervetAGM                                                                                          97219
Gorrilla                                                                                           92826
GrabEatingMacaque                                                                                  95686
Macaque                                                                                            95018
PigTailedMacaque                                                                                   95449
Bonobo                                                                                             93411
OliveBaboon       TAACAGTTGAAATGCCTAAGCGTTACTGAATAATGACTCTGATATTCTCTCTGAGAGTTAAAGCTGTGCTATAAATATAC 98954
Orangutan                                                                                          95567

Majority          XXXXXXXXXXXXXXXXXXXXXXXXXXXXXXXXXXXXXXXXXXXXXXXXXXXXXXXXXXXXXXXXXXXXXXXXXXXXXXXX

                          116970    116980    116990    117000    117010    117020    117030    117040

Human             GATAGTGCATTTTATGTAATATTCAGGTGTTATGCTATTTTTATCTGGTTCACTTTAAAACCTCCCTCCCCCCACTCCCT 98618
Coquerel'sSifaka                                                                                   81164
Ma'sNightMonkey                                                                                    88613
VervetAGM                                                                                          97219
Gorrilla                                                                                           92826
GrabEatingMacaque                                                                                  95686
Macaque                                                                                            95018
PigTailedMacaque                                                                                   95449
Bonobo                                                                                             93411
OliveBaboon       ATTTACCTGTGCGAAGTATGCCTTCAGGGGCTATGCCCCATAACCTTAGCAAAATTTTAAAATATTCTTATGGCTAGCAA 99034
Orangutan                                                                                          95567

Majority          XXXXXXXXXXXXXXXXXXXXXXXXXXXXXXXXXXXXXXXXXXXXXXXXXXXXXXXXXXXXXXXXXXXXXXXXXXXXXXXX

                          117050    117060    117070    117080    117090    117100    117110    117120

Human             TTTTTTGTGGGTTTTTTTTTTATTATTATTATTTTCCAGTTTTGTGTTGTTTTGCTTTTTAACTTCTGAGATTGCAGTGG 98698
Coquerel'sSifaka                                                                                   81164
Ma'sNightMonkey                                                                                    88613
VervetAGM                                                                                          97219
Gorrilla                                                                                           92826
GrabEatingMacaque                                                                                  95686
Macaque                                                                                            95018
PigTailedMacaque                                                                                   95449
Bonobo                                                                                             93411
OliveBaboon       TTTTATCTATTGTCATGTCTTACAATTTCCTGCTTACCACCCCCATTTGTCTCTCAACCTTGTTGCTGCATTAGCTGTCC 99114
Orangutan                                                                                          95567
```

```
Majority          XXXXXXXXXXXXXXXXXXXXXXXXXXXXXXXXXXXXXXXXXXXXXXXXXXXXXXXXXXXXXXXXXXXXXXXXXXXXXXXX

                          117130    117140    117150    117160    117170    117180    117190    117200

Human             TTCCAGATGGTTGAAATCACTAGTAGTGATTTACATGAAGCATTCATGTATTTATTACTGGTATATTCAACTATGGTGGA 98778
Coquerel'sSifaka                                                                                   81164
Ma'sNightMonkey                                                                                    88613
VervetAGM                                                                                          97219
Gorrilla                                                                                           92826
GrabEatingMacaque                                                                                  95686
Macaque                                                                                            95018
PigTailedMacaque                                                                                   95449
Bonobo                                                                                             93411
OliveBaboon       ATCACTTAAAACTGTTTATTTCTGTTTTTCTTCCATCATTCTCTTTGCAACTACATGTCTTAGACTTTTTATAATCATAA 99194
Orangutan                                                                                          95567

Majority          XXXXXXXXXXXXXXXXXXXXXXXXXXXXXXXXXXXXXXXXXXXXXXXXXXXXXXXXXXXXXXXXXXXXXXXXXXXXXXXX

                          117210    117220    117230    117240    117250    117260    117270    117280

Human             GGGAAATTGGTCGAAGAATTACGGTTAAAGAGCTCCTTTAAAAAGGAGATTTGTAGAATTCTAAAATGTGATTCTGATTC 98858
Coquerel'sSifaka                                                                                   81164
Ma'sNightMonkey                                                                                    88613
VervetAGM                                                                                          97219
Gorrilla                                                                                           92826
GrabEatingMacaque                                                                                  95686
Macaque                                                                                            95018
PigTailedMacaque                                                                                   95449
Bonobo                                                                                             93411
OliveBaboon       TCAACCATAACCAGATTCTTTTTATTTGTGGTTAATGAGTTGACAAACAATAGTGCATTTTATGTAATAATCAGGTGTTT 99274
Orangutan                                                                                          95567

Majority          XXXXXXXXXXXXXXXXXXXXXXXXXXXXXXXXXXXXXXXXXXXXXXXXXXXXXXXXXXXXXXXXXXXXXXXXXXXXXXXX

                          117290    117300    117310    117320    117330    117340    117350    117360

Human             ATATAGTCATGATTCTTAGAAATTAGCTACAGGAAGAGATAGAAACATTGCTATAGTTGTCTTTGCCCTAAGTTTGGAGT 98938
Coquerel'sSifaka                                                                                   81164
Ma'sNightMonkey                                                                                    88613
VervetAGM                                                                                          97219
Gorrilla                                                                                           92826
GrabEatingMacaque                                                                                  95686
Macaque                                                                                            95018
PigTailedMacaque                                                                                   95449
Bonobo                                                                                             93411
OliveBaboon       TGCTATTTTTGTCTGGTTCAGCTTAAAATCTCCCTGCCCCCCACTCCCTTTTTTTGTGGGTTTTTAAAATATTATTACTA 99354
Orangutan                                                                                          95567
```

```
Majority          XXXXXXXXXXXXXXXXXXXXXXXXXXXXXXXXXXXXXXXXXXXXXXXXXXXXXXXXXXXXXXXXXXXXXXXXXXXXXXXX

                        117370    117380    117390    117400    117410    117420    117430    117440

Human             TTTATAGTGTAATATATATATAATAGCTCTAATTATAAATACACTTTTACTCTGGAAAAAGCATAGACATTGACATAAGA 99018
Coquerel'sSifaka                                                                                   81164
Ma'sNightMonkey                                                                                    88613
VervetAGM                                                                                          97219
Gorrilla                                                                                           92826
GrabEatingMacaque                                                                                  95686
Macaque                                                                                            95018
PigTailedMacaque                                                                                   95449
Bonobo                                                                                             93411
OliveBaboon       TTTTTTTCCGGTTTTGTTTCGCTTTTTAACTTCTGGGATTGCAGTGGTTCCAGATGGTTGAAATCACTAGTAGTGATTTA 99434
Orangutan                                                                                          95567

Majority          XXXXXXXXXXXXXXXXXXXXXXXXXXXXXXXXXXXXXXXXXXXXXXXXXXXXXXXXXXXXXXXXXXXXXXXXXXXXXXXX

                        117450    117460    117470    117480    117490    117500    117510    117520

Human             GACATTTAAAAACCATTATTTTCTCATCTGTAAAATGGAGAGAATTACTTCATATGTTCACTTATAAGGATTATGTGAAA 99098
Coquerel'sSifaka                                                                                   81164
Ma'sNightMonkey                                                                                    88613
VervetAGM                                                                                          97219
Gorrilla                                                                                           92826
GrabEatingMacaque                                                                                  95686
Macaque                                                                                            95018
PigTailedMacaque                                                                                   95449
Bonobo                                                                                             93411
OliveBaboon       CTTGACGCATTCATGTATTTATTACTGGCATATTCAACTATAATGGAGGGAAATTGGTCAAAGAATTAGGGTTAAAGAGC 99514
Orangutan                                                                                          95567

Majority          XXXXXXXXXXXXXXXXXXXXXXXXXXXXXXXXXXXXXXXXXXXXXXXXXXXXXXXXXXXXXXXXXXXXXXXXXXXXXXXX

                        117530    117540    117550    117560    117570    117580    117590    117600

Human             TACTGTTCCATTCCATTAGTAGTTTTTCTTTTCCTCTAATTGGGGGTGGGGGCAGTTTTTGGCATTCTATTTATGGAAGG 99178
Coquerel'sSifaka                                                                                   81164
Ma'sNightMonkey                                                                                    88613
VervetAGM                                                                                          97219
Gorrilla                                                                                           92826
GrabEatingMacaque                                                                                  95686
Macaque                                                                                            95018
PigTailedMacaque                                                                                   95449
Bonobo                                                                                             93411
OliveBaboon       TCCTATAAAAAGGAGATTTGTAGAATTCTAAAATGTGATTCCAATTCATATAGTCATGACTCTTAGAAATTAGCTACAGG 99594
Orangutan                                                                                          95567
```

```
Majority          XXXXXXXXXXXXXXXXXXXXXXXXXXXXXXXXXXXXXXXXXXXXXXXXXXXXXXXXXXXXXXXXXXXXXXXXXXXXXXXX
                          117610    117620    117630    117640    117650    117660    117670    117680
Human             TGTTTTTTTCCCTAAGATCATTTTGATGTCTCAGAAGACATTTGCTATGTACGTTGGACATTTGCCATGTACATTGTAGA 99258
Coquerel'sSifaka                                                                                   81164
Ma'sNightMonkey                                                                                    88613
VervetAGM                                                                                          97219
Gorrilla                                                                                           92826
GrabEatingMacaque                                                                                  95686
Macaque                                                                                            95018
PigTailedMacaque                                                                                   95449
Bonobo                                                                                             93411
OliveBaboon       GAGAGATAGTAACATTGCTATAGTTGTCTTTGCCCTAAATGTGGAGTTTTATAGTGTAAAATATATATAATAGCTCTAAT 99674
Orangutan                                                                                          95567

Majority          XXXXXXXXXXXXXXXXXXXXXXXXXXXXXXXXXXXXXXXXXXXXXXXXXXXXXXXXXXXXXXXXXXXXXXXXXXXXXXXX
                          117690    117700    117710    117720    117730    117740    117750    117760
Human             GAATACAAAGATAAACAGAACCACAGTACAGCTGGTTCTATTAGACATTGAAAATGCATATTCTAGCATATTTAATATAT 99338
Coquerel'sSifaka                                                                                   81164
Ma'sNightMonkey                                                                                    88613
VervetAGM                                                                                          97219
Gorrilla                                                                                           92826
GrabEatingMacaque                                                                                  95686
Macaque                                                                                            95018
PigTailedMacaque                                                                                   95449
Bonobo                                                                                             93411
OliveBaboon       TATAAATACACTTTTACTCTGGAAAAAACATAAGACATTGACATGAGACATTTAAAAACCATTATTTTCTCATCTGTAAA 99754
Orangutan                                                                                          95567

Majority          XXXXXXXXXXXXXXXXXXXXXXXXXXXXXXXXXXXXXXXXXXXXXXXXXXXXXXXXXXXXXXXXXXXXXXXXXXXXXXXX
                          117770    117780    117790    117800    117810    117820    117830    117840
Human             TAGGGAACAATTTAAGCATAATGTGAATTTCATGTTTACTGAAGATTTCATCCTGAGAAACAAATGAAAGCAGAAAATTA 99418
Coquerel'sSifaka                                                                                   81164
Ma'sNightMonkey                                                                                    88613
VervetAGM                                                                                          97219
Gorrilla                                                                                           92826
GrabEatingMacaque                                                                                  95686
Macaque                                                                                            95018
PigTailedMacaque                                                                                   95449
Bonobo                                                                                             93411
OliveBaboon       GTGGAGAGAATCACTTCATATGTTCACTTATAAGGATTATATACTGTTCCATTAGTAGTGTTTCTTTTCCTCTAATTAGG 99834
Orangutan                                                                                          95567
```

```
Majority           XXXXXXXXXXXXXXXXXXXXXXXXXXXXXXXXXXXXXXXXXXXXXXXXXXXXXXXXXXXXXXXXXXXXXXXXXXXXXXXX

                            117850    117860    117870    117880    117890    117900    117910    117920

Human              CAACCAACTGAACCAGCTGTGTAAGAAATATATATATGGTGTGCACACATGTACATACCTCAGACATCTATTAGCATTCT 99498
Coquerel'sSifaka                                                                                    81164
Ma'sNightMonkey                                                                                     88613
VervetAGM                                                                                           97219
Gorrilla                                                                                            92826
GrabEatingMacaque                                                                                   95686
Macaque                                                                                             95018
PigTailedMacaque                                                                                    95449
Bonobo                                                                                              93411
OliveBaboon        GGTGGGGGCAGTTTTTGGCATTTTATTTATGGAAGGTTTTTTTTTTCCCTAAAATCATTTTGACCTCTCAAAAGACATT 99914
Orangutan                                                                                           95567

Majority           XXXXXXXXXXXXXXXXXXXXXXXXXXXXXXXXXXXXXXXXXXXXXXXXXXXXXXXXXXXXXXXXXXXXXXXXXXXXXXXX

                            117930    117940    117950    117960    117970    117980    117990    118000

Human              CAGTTTTCCACAAGTTAAAAGCCACGCTCATCCATATCTGGTGCTAATAACTTCCTATCCAATTTCAGATAGCTGTTTTC 99578
Coquerel'sSifaka                                                                                    81164
Ma'sNightMonkey                                                                                     88613
VervetAGM                                                                                           97219
Gorrilla                                                                                            92826
GrabEatingMacaque                                                                                   95686
Macaque                                                                                             95018
PigTailedMacaque                                                                                    95449
Bonobo                                                                                              93411
OliveBaboon        TGCTATGTACGTTGGACATTTGCTATGTACATTGTAGAGAATACAAAGATAAACAGACACTGAAAATACAAATTTGTTCT 99994
Orangutan                                                                                           95567

Majority           XXXXXXXXXXXXXXXXXXXXXXXXXXXXXXXXXXXXXXXXXXXXXXXXXXXXXXXXXXXXXXXXXXXXXXXXXXXXXXXX

                            118010    118020    118030    118040    118050    118060    118070    118080

Human              AACACATCTGACACCTTCTTTTTTTCAAGCAAACTTCAGGTCTTTTTCAAGATAAAGTCATTTATAGTAGTGTCTATATA 99658
Coquerel'sSifaka                                                                                    81164
Ma'sNightMonkey                                                                                     88613
VervetAGM                                                                                           97219
Gorrilla                                                                                            92826
GrabEatingMacaque                                                                                   95686
Macaque                                                                                             95018
PigTailedMacaque                                                                                    95449
Bonobo                                                                                              93411
OliveBaboon        AGCATATTTAATATATTAGGGAACAATTTAAGCATAATGTGAATTTCATGTTTACTTAAGATTTCATCCTGAGAAACAAA 100074
Orangutan                                                                                           95567
```

```
Majority          XXXXXXXXXXXXXXXXXXXXXXXXXXXXXXXXXXXXXXXXXXXXXXXXXXXXXXXXXXXXXXXXXXXXXXXXXXXXXXXX

                        118090    118100    118110    118120    118130    118140    118150    118160

Human             TTTCTTAACCATTTAACATGTAAACTGTGCTGCCTTTTAAGAAATTAATTCTATCCTTTTTAGTGTCAGAGTTGAAGATT 99738
Coquerel'sSifaka                                                                                   81164
Ma'sNightMonkey                                                                                    88613
VervetAGM                                                                                          97219
Gorrilla                                                                                           92826
GrabEatingMacaque                                                                                  95686
Macaque                                                                                            95018
PigTailedMacaque                                                                                   95449
Bonobo                                                                                             93411
OliveBaboon       TGAAAGCAGGAAATTACAACCATTTTGAACCAGCTGTGGAAGAAAATATATATTTATCAGGTGTGCACACATGTACGTTC 100154
Orangutan                                                                                          95567

Majority          XXXXXXXXXXXXXXXXXXXXXXXXXXXXXXXXXXXXXXXXXXXXXXXXXXXXXXXXXXXXXXXXXXXXXXXXXXXXXXXX
```
[truncated: 567,053 more chars]
