# Supplementary material for: PTBP2 – a gene with relevance for both Anorexia nervosa and body weight regulation
Source: Transl Psychiatry. 2022 Jun 9;12:241. doi: 10.1038/s41398-022-02018-5 (PMC9184595; doi:10.1038/s41398-022-02018-5)
Supplement: Supplementary file 3 — gDNA alignment report (Laurasiatheria) [file 41398_2022_2018_MOESM3_ESM.pdf]

```
Majority     --------------------------------------------------------------------------------
                      10        20        30        40        50        60        70        80
Human        --------------------------------------------------------------------------------  0
PolarBear    --------------------------------------------------------------------------------  0
Cow          --------------------------------------------------------------------------------  0
Dog          --------------------------------------------------------------------------------  0
BelugaWhale  --------------------------------------------------------------------------------  0
Donkey       --------------------------------------------------------------------------------  0
Cat          AGAACATTTCCATCAGCCTAAAAAGAAACCTATGTTAATTTACAATTATTACCCATTTTGTCTGTAGGTATTTACCCTTT  80
Ferret       --------------------------------------------------------------------------------  0
Lion         --------------------------------------------------------------------------------  0
Vaquita      --------------------------------------------------------------------------------  0
Pig          --------------------------------------------------------------------------------  0

Majority     --------------------------------------------------------------------------------
                      90       100       110       120       130       140       150       160
Human        --------------------------------------------------------------------------------  0
PolarBear    --------------------------------------------------------------------------------  0
Cow          --------------------------------------------------------------------------------  0
Dog          --------------------------------------------------------------------------------  0
BelugaWhale  --------------------------------------------------------------------------------  0
Donkey       --------------------------------------------------------------------------------  0
Cat          CTGGGCATTTTGTATATGCTTTTATACCTTTGGAATTTACTTTATCTATTTTGAATTTATATCACCTATTCAAAGATACA  160
Ferret       --------------------------------------------------------------------------------  0
Lion         --------------------------------------------------------------------------------  0
Vaquita      --------------------------------------------------------------------------------  0
Pig          --------------------------------------------------------------------------------  0
```

```
Majority      --------------------------------------------------------------------------------

                            170       180       190       200       210       220       230       240

Human         --------------------------------------------------------------------------------  0
PolarBear     --------------------------------------------------------------------------------  0
Cow           --------------------------------------------------------------------------------  0
Dog           --------------------------------------------------------------------------------  0
BelugaWhale   --------------------------------------------------------------------------------  0
Donkey        --------------------------------------------------------------------------------  0
Cat           TATCAAAAAAATAAATGATCTAACTGATCACATTATTATTCTCTCTTCTCATAAGAAGAAACTGCTTTATGAAAACGTTA  240
Ferret        --------------------------------------------------------------------------------  0
Lion          --------------------------------------------------------------------------------  0
Vaquita       --------------------------------------------------------------------------------  0
Pig           --------------------------------------------------------------------------------  0

Majority      --------------------------------------------------------------------------------

                            250       260       270       280       290       300       310       320

Human         --------------------------------------------------------------------------------  0
PolarBear     --------------------------------------------------------------------------------  0
Cow           --------------------------------------------------------------------------------  0
Dog           --------------------------------------------------------------------------------  0
BelugaWhale   --------------------------------------------------------------------------------  0
Donkey        --------------------------------------------------------------------------------  0
Cat           AAGGAAGCCTTTGGTACTATATGCAGATGAGAGGAGACAGGAATAAAATCAGGACATTATGAACAAGATTCTATTTCCTA  320
Ferret        --------------------------------------------------------------------------------  0
Lion          --------------------------------------------------------------------------------  0
Vaquita       --------------------------------------------------------------------------------  0
Pig           --------------------------------------------------------------------------------  0
```

Majority	--	
	330 340 350 360 370 380 390 400	
Human	--	0
PolarBear	--	0
Cow	--	0
Dog	--	0
BelugaWhale	--	0
Donkey	--	0
Cat	CTTTTATTGCATCCATATTAGACCTCGTTTTGTCTTAGCATACACTGAAATTTAGATTTAATCATTGTTCACATAAGTGT	400
Ferret	--	0
Lion	--	0
Vaquita	--	0
Pig	--	0

Majority	--	
	410 420 430 440 450 460 470 480	
Human	--	0
PolarBear	--	0
Cow	--	0
Dog	--	0
BelugaWhale	--	0
Donkey	--	0
Cat	AAATCTCTGTAAAAGGTCAAAACATTTACCACTCAGAAGGGAAAGAACAAATTGTTACACTGAAAAAAGATATGGGTATG	480
Ferret	--	0
Lion	--	0
Vaquita	--	0
Pig	--	0

```
Majority     --------------------------------------------------------------------------------

                     490       500       510       520       530       540       550       560

Human        --------------------------------------------------------------------------------  0
PolarBear    --------------------------------------------------------------------------------  0
Cow          --------------------------------------------------------------------------------  0
Dog          --------------------------------------------------------------------------------  0
BelugaWhale  --------------------------------------------------------------------------------  0
Donkey       --------------------------------------------------------------------------------  0
Cat          AAAATAATACTGTACAGGCACTCACTTTTTATTGCCTTTTTTTTTTTAGGAATCCATAAACACTAATATAATCTTGTCAT  560
Ferret       --------------------------------------------------------------------------------  0
Lion         --------------------------------------------------------------------------------  0
Vaquita      --------------------------------------------------------------------------------  0
Pig          --------------------------------------------------------------------------------  0

Majority     --------------------------------------------------------------------------------

                     570       580       590       600       610       620       630       640

Human        --------------------------------------------------------------------------------  0
PolarBear    --------------------------------------------------------------------------------  0
Cow          --------------------------------------------------------------------------------  0
Dog          --------------------------------------------------------------------------------  0
BelugaWhale  --------------------------------------------------------------------------------  0
Donkey       --------------------------------------------------------------------------------  0
Cat          ATATTACAAAGGTTGGCCATGTGCTAATGTTGAGATTAATAATCTGTCTCATGGTTAACTCATAAAAATCATGTAAATTT  640
Ferret       --------------------------------------------------------------------------------  0
Lion         --------------------------------------------------------------------------------  0
Vaquita      --------------------------------------------------------------------------------  0
Pig          --------------------------------------------------------------------------------  0
```

Majority	--	
	650 660 670 680 690 700 710 720	
Human	--	0
PolarBear	--	0
Cow	--	0
Dog	--	0
BelugaWhale	--	0
Donkey	--	0
Cat	AGATTTGGATGAGGTTTCAAAACTTATCTATGATATTTTCCCAGATTTATTCCACTAGATACCAGCCAATCTAATTACTT	720
Ferret	--	0
Lion	--	0
Vaquita	--	0
Pig	--	0

Majority	--	
	730 740 750 760 770 780 790 800	
Human	--	0
PolarBear	--	0
Cow	--	0
Dog	---CCGCCGGGC	9
BelugaWhale	--	0
Donkey	--	0
Cat	CAATGACATCTTTAACCTTTGTGAAAATCTCTATATCGATTTATTTATATGAATACTGTTGATTGTTAACAATCCAAGAG	800
Ferret	--	0
Lion	--	0
Vaquita	--	0
Pig	--	0

```
Majority     --------------------------------------------------------------------------------
                               810       820       830       840       850       860       870       880
Human        --------------------------------------------------------------------------------  0
PolarBear    --------------------------------------------------------------------------------  0
Cow          --------------------------------------------------------------------------------  0
Dog          GCCGGCGCGCTCGGCTCTTTCCGCCGCCGCCGCTGCCGCGCGCTGCCCCGTGCGCTCCGGCACCTTCGGCAATTTCCGTC  89
BelugaWhale  --------------------------------------------------------------------------------  0
Donkey       --------------------------------------------------------------------------------  0
Cat          CTAAATTTGTTACCGGTTTCTTGTCGAGTGAATAACAGTTAATAAGTAAAAGAACTATGATGATAGCAGTCTTTATTGAA  880
Ferret       --------------------------------------------------------------------------------  0
Lion         --------------------------------------------------------------------------------  0
Vaquita      --------------------------------------------------------------------------------  0
Pig          --------------------------------------------------------------------------------  0

Majority     --------------------------------------------------------------------------------
                               890       900       910       920       930       940       950       960
Human        --------------------------------------------------------------------------------  0
PolarBear    --------------------------------------------------------------------------------  0
Cow          --------------------------------------------------------------------------------  0
Dog          GGGCCCCGGCCGCCATTTTCTCGCCGCTTGTGTGTCTCGCTGGCTGCGTGGCTCGGTTCCTGTGAGCGAAGCTTTGTC-C  168
BelugaWhale  ----------------------------------------------------------------GTTAACGAACATGTCT  16
Donkey       --------------------------------------------------------------------------------  0
Cat          TATTTACCATAACCCATCACTGAACTAGAGAGATACTTTACATACATTAAGGCATTTGGACCTCACAACCATCTAGGATG  960
Ferret       -------------------------------------------------------------ATGACTATTGTTTGTTCAT  19
Lion         --------------------------------------------------------------------------------  0
Vaquita      --------------------------------------------------------------------------------  0
Pig          ------------------------------------------------------TTTAACTGTCTTTAAGCAGCATGCTT  26
```

```
Majority     -----------------------------------------------TTTX-------XG---XTX--XC-TX--G-X-
                     970       980       990       1000      1010      1020      1030      1040
Human        --------------------------------------------------------------------------------  0
PolarBear    --------------------------------------------------------------------------------  0
Cow          -----------------------------------------------GACTTAGAGTTCTGCTTTACATACGTTAGGACT  33
Dog          GGTTCGGCAATGGA----CGGGTACGTAGTGCGGCCGGCGAGGAGGTGTGTGAGCGGGGCGCCGGGCCGTCCTGCCGCCC  244
BelugaWhale  AGTGTCACACAGCTACTAAATGAAAAGAGCTCAAATTTGAACCCAGTCTTTAATCAGCATGCTTTTGTGATATACATTGT  96
Donkey       --------------------------------------------------------------------------------  0
Cat          AAGTCCCTATTTTAAATCCCTTTTTCATTTTCAGTAATAAATCCCATATTTTCAGTAACAGAAACTTTTTCCTGGAGGAG  1040
Ferret       AGTCTTGCACTTTA----TTAGGAGAAAAAGCAAA-GAAAAATGTGTTTTTGTGAATATTGTAACTTC-TTTTGGCGCCT  93
Lion         ------------------------------------------------TTTTCAGTAACAGAAACTTTTTCCTGGAGGAG  32
Vaquita      --------------------------------------------------------------------------------  0
Pig          TTGTGATGTATGTTGTTGCATCAGCCTCACTCCTGACTCTATGCTGTATTTTGTCCTTTTGTCTCTTCCCCTTCATTTCA  106

Majority     T----XA-X-X-XCT---G-X-CX-XG-TX-X-X-T--XX-----TXXXXTTTXAAXTAGXA-TTXTTXAAXTXCXXTTT
                     1050      1060      1070      1080      1090      1100      1110      1120
Human        --------------------------------------------------------------------------------  0
PolarBear    --------------------------------------------------------------------------------  0
Cow          TTTAAGGTCAGTCATCTGGGATGAAGGTCTTATTTTTAA------TCCCATTTTTACTTACAGTAGAGGAAATCCCATTA  107
Dog          GGGCCGGGGTCGGCGGGGACCCCGCGGGCGGCCCCTCCCAG--GGCCGGGCTGCCGTTACCCAACCCCCGCCCCATCGCA  322
BelugaWhale  TGCATTAGCCTCACTCCTGACTCCATGTTGTATTTTATCCTTTTGTTTTATTTCTTATTTCACTTTTTATATT-CACTTT  175
Donkey       --------------------------------------------------------------------------------  0
Cat          TTAGTGAACATACCTAATGTCACACAGCTGCTAAATGAAAGAGCTCAAATCTGAAACTAGTTTTTGATCAACATGTTTTT  1120
Ferret       ACGGACAGGCCTCCCCTCATAAGATAGTTTGTCTTTGCCAATAGATCGATATTGATTGAGTCAATGTGGAGAATACAGAA  173
Lion         TTAGTGAACATACCTAATGTCACACAGCTGCTAAATGAAAGAGCTCAAATCTGAAACTAGTTTTTAATCAACATGTTTTT  112
Vaquita      -----------------------------------------TTTGTTTTATTTCTTATTTCACTTTTTATATTTCACTTT  39
Pig          TCTAGTACTTTATGTAAAGAACCTTAGCTTTTTTCTAGAAAGAGATGAGGTTTAAATAAGTAGGCATGGAGTTCCCGTCG  186
```

```
Majority     ----ATATTGATTXXTAAA--XX-XCCXTTGAXTXXTT--XTXGXAAGTGTTGG-GCATAXXTCTTC-AAG---XT--TG
                              1130      1140      1150      1160      1170      1180      1190      1200
Human        --------------------------------------------------------------------------------  0
PolarBear    --------------------------------------------------------------------------------  0
Cow          ----ACATCAGTTAATGAAACTTTTTCCTGGAGAGGTTAATGAACATGTCTAGTGTCACACATCTACTAAATGAAA-AGA  182
Dog          C---ACACCCCTCCCTCCG--CTTCCCCCGGCGGCTCCGCTCGGGAACCGGGGAAGAAAGCGGGCTCGGAGGCCGGGGCG  397
BelugaWhale  TTGTGTCTTGACTTGTAAAG----AACCTTAAATCTTTT-CTAGAAAGTGGTGGGGTTTAAATAAGC-AAGCATAT--TG  247
Donkey       --------------------------------------------------------------------------------  0
Cat          GTG-ATATACATTGCTACA--TTAGCCTCTTTTTGACTCCATGTTATATTTTTGTCCATTTGTCTTACTCCTTCTTATTT  1197
Ferret       A---ATTATGCTAGAGTTC--TTGCCCTTTGGGAGCTCACATATTAATTGTTGAAGGAAAATACATGTACAAAATCACAA  248
Lion         GTG-ATATACATTGTTACA--TTAGCCTCTTTTTGACTCCATGTTATATTTTTGTCCATTTGTCTTACTCC---TTATTT  186
Vaquita      TTGTGTCTTGACTTGTAAAG----AACCTTAAATCTTT--CTAGAAAGTGGTGGGGTTTAAATAAGC-AAGCATAT--TG  110
Pig          TGGCGCAGTGGTTAACGAA--TCCGACTAGGAACCATGAGGTGGCGGGTTCGGT-CCCTGCCCTTGCTCAGTGGGTTAAG  263

Majority     GCT-TTGXTTTTTTGTXXTCTATXGTATAXGTXGXGGCXXTXGAAGXGXTTTTGXXTTTTTGTGAXXXXX--ATGGTCXT
                              1210      1220      1230      1240      1250      1260      1270      1280
Human        --------------------------------------------------------------------------------  0
PolarBear    --------------------------------------------------------------------------------  0
Cow          GCTCTGATTTGAATCCAATCTTTAAT-CACCATGCTTTTGTGATATAGTTGTTGCATTAGTCTCACTTC----TGGTTCT  257
Dog          GCGGCGGGGCTGCGGGGGGCGACGGCGCGAGCGGCGGCCCCGGCGGGGGTGGGGTGGGGGCCCCGC--GGCGGCGGCCCG  475
BelugaWhale  ACTATTGTTTGTTTGTAGTCTTGTGCTTTATTAGGAGAAAAAGAAAGGAAAATGTGTTTTTGTGAGTATTATATTTTCTT  327
Donkey       -------------------TTGTGATATACGTTGTTGCACTAGCCTTTTTCCTGATTCCATGTTATATT----TTGTCCT  57
Cat          GACTTTGATATTTCGTCTAGTATTTTATATGTCTTGACTTGTAAAGAACTTTAAATCTTTTCTAGAAAGTGGAGGGTTTA  1277
Ferret       ATAGGTGAAAAGTGATTATTAATGGCATATAAAGGGCCCCTTATGTATTGTTAAGTCAATGGTGAA--GGCAAAGGGAAG  326
Lion         GACTTTGACATTTTGTCTAGTATTTTATATGTCTTGACTTGTAAAGAACTTTAAATCTTTTCTAGAAAGTGGAGGGTTTA  266
Vaquita      ACTATTGTTTGTTTGTAGTCTTTTGCTTTATTAGGAGAAAAAGAAAGAAAAATGTGTTTTTGTGAGTATTATATTTTCTT  190
Pig          GATCCGGCGTTGCCGTGAGCTGTGGTGTAGGTTGCAGACACGGCTGGGATCCTGCGTTGCTGTGGCTCTGGCGTAGGCCG  343
```

```
Majority     XTG-----X-X--CXTGATAATTCTTTGTTTGT----CTTXATXTAT-TXACGAGCT---------GCTTXAGXCATGXT
                      1290      1300      1310      1320      1330      1340      1350      1360
Human        --------------------------------------------------------------------------------  0
PolarBear    --------------------------------------------------------------------------------  0
Cow          ATG-TTGTATCACCCTTTTATTTCTTTATTTCA----CTTTATATATTTTACCTACT---------ACTTTTTGTGTCTT  323
Dog          GCCCTGCCCCCTCCGGGAGCCATTTCCATCCGA-GAGCCGGACCCGGGCCCCGGGCGGCCGCGCCGGCGGGAGCGAGGCC  554
BelugaWhale  TTG----------CCTCCTAAGGCCAGGTTTGT----CCCCATGCA----ACAGTTT---------GTTTTTGCCAGGAT  380
Donkey       TTT---G------TTTTATTCTTTCTTATTTCA----CTTTATTTCTTTCACCTAGT---------ACTTTATGTGTCTT  115
Cat          AATAAACAATCATATTGACTATTATTTGTTCAT-ACTCTTGTACTTTATTAGGAGAA---AAAATAGAGAAAAACGTGGT  1353
Ferret       GCAAAGGGAAGGCAAAGGGAAATATATATATAT-ATTTTTTTTATATCTCAGTAACA-------CAATTTATTTTATTTG  398
Lion         AATAAACAATCATATTGACTATTATTTGTTCAT-ACTCTTGTACTTTATTAGGAGAA---AAAATAGAGAAAAACGTGGT  342
Vaquita      TTG----------CCTCCTAAGGCCAGGTTTGC----CCCCATGCA----ACAGTTT---------GTTTTTGCCAGGAT  243
Pig          GTGGCTACAGCTCCGATTCAACCCCTGGCCTGGGAACCTCCATATGCCTCCGGAGCG---------GCCCAAGAAATAGC  414

Majority     TATG--AAGATXAAAAXAXTTAAXXXTATTTAAGXXAATXACGXGAC--CAAAXAAXXAXGCXATATTXACTXCTGTTTG
                      1370      1380      1390      1400      1410      1420      1430      1440
Human        --------------------------------------------------------------------------------  0
PolarBear    --------------------------------------------------------------------------------  0
Cow          GACT----TATAAAGAAACTTAAATGTTTTCTAGAAAGTGATGGGTC--TAAATAAGCAAGC-ACACTGACTATTGTCTG  396
Dog          TGTGGGGGGCGGCCGGGGGAGACGAGAGGGGAGGCAACGGCGTGACCCCCGGCCGCCCCCCCCCCCCCCCCCCCCG-GTC  633
BelugaWhale  AAAG-CAAGAGCTAATCAATCAACATTGGTTGAGTCATTGTGGGGAGTACAAAGAATTATGCTAGAGTTCCTGCCCTTTG  459
Donkey       ---------GTAAACAATCTTAAATCTTTTCTAGAAAGTGATGGGTT--TAAATAAAACAAGCATACTGACTACTGTTTG  184
Cat          TTTGTGAATATTATAACATCTTTTGCCATTTAAGG-ACAAGCTTCCCCTCATGGAGACAGTTTGTCTTTGACCATGCATC  1432
Ferret       TTTTTACAGATTTCGGGTTTTTTTTTAATTCAATTAATTAACATAATGTATTATTGGTTTCAGAGGTAGAGGTCAGTGAG  478
Lion         TTTGTGAATATTATAATATCTTTTGCCATTTAAGG-ACAAGCTTCCC-TCATGGAGACAGTTTGTCTTTGACCATCAATC  420
Vaquita      AAAG-CAAGAGCTAATCAATCAACATTGGTTGAGTCAATTTGGGGAATACAAAGAACTATGCTAGAGTTCCTGCCCTTTG  322
Pig          AACAACAACAACAAAAGACCAAAAAAAAAAAAAAAAAAAAATAAATAAATAAATAAGTAGGC-ATATTGACTATTGTTTG  493
```

```
Majority     T----GGTCTTATACATTXTGTAGAAGAXAGAAAAATAXAAXCGCGTTTCTGTXAATAGGCTACTTTGXT---------T
                               1450      1460      1470      1480      1490      1500      1510      1520
Human        --------------------------------------------------------------------------------  0
PolarBear    --------------------------------------------------------------------------------  0
Cow          TCCGTAGTCTTGTA-TTTTATTAGGAGAAAAAAAAAGAAAAACACGTTTTTGTGAATATTATAGTTTGGTCTTGGT---T  472
Dog          CCTCCGGCCTCCCCGGGCCTCCGGCCTCCGCGGCCCCCCGGCCTCCCTCCCCCTCCCGGCCCCCCCGGCCCGCCCCGGTT  713
BelugaWhale  G----GAGCTTACATATTTAATTGTTGAGTGA-AAATACATGTACAAAACCACAAACGGGCTTCCCTGGT---GGC---A  528
Donkey       TTTCTAATCTTGTACTTTATTAGGAGAAAAAAATAAAGAAAACGTGTTTTTGTGAATACTATAATTTC------------  252
Cat          AATATTGATTGAGTCAATATGGAGAATATAGAAAATTATGCTAGAGTTCCTGTCCTTTGGGAGCTTACATATTTAA---T  1509
Ferret       TCATCGGTCTTATATGATATGCGGCGCTCATTACATCATGAGCCCTCCTTAATATCCA--TCACCCAGGTAGCCCA---T  553
Lion         AATATCGATTGAGTCAATATGGAGAATACAGAAAATTATGCTAGAGTTCCTGTCCTTTGGGAGCTTACATATTTAA---T  497
Vaquita      G----GAGCTTACATATTTCATTGTTGAGTGA-AAATACATGTACAAAACCACAAACGGGCTTCCCTGGT---GGC---A  391
Pig          T----AGTGTTGTACTTTTACTAGGAGAATAAGAAAGAAAAACGTGTTTTTGTGACTACTCTAATTTG------------  557

Majority     X--XGX--XA-X----TTXX-C-X--CCGCCACCTXAGGACAGGTTTCCTG-CX-TXAXXGGGCAGTTTXTXTATGCCAA
                               1530      1540      1550      1560      1570      1580      1590      1600
Human        --------------------------------------------------------------------------------  0
PolarBear    --------------------------------------------------------------------------------  0
Cow          AACAGGATAAATTTTGTTTGTTTGTTTTGACACCTAAGGACAGGTTTCCTT----TGGTGGAGCAGTTTGTCTTTGCCAA  548
Dog          CCCCGGCCTCCCGGCTCCCCCCGGCTCCCCCGGCTCCCCCCGGCTCCCCCCGGCCTCCGCGGCCTGCCTCCCCCCTCCG-  792
BelugaWhale  CAGTGGTTGAGA---GTCCGCCTG--CCGATGCAGGGGACACGGGTTCGTGCCCCGGTCCGGGAGGCTCCCACATGCCGC  603
Donkey       -------------------T-----TTTGCCACCTAAGGACAGTTTTCCCT-----CATGGAACAGTTTGTCTTTGCCAA  303
Cat          TGTTGAGGGAAAATACATGTACAACACCACAAATATATGAAAAGTGATAGGACTGATAATGGGTCCTTTATATATTGTAA  1589
Ferret       CCCCCACCTCCCTCCCCTCCAGCAACCCTCAGTTTGTCTCCTGGGATTAAGAGTATTTCTTGTTTTATTTTTTCCTCTC-  632
Lion         TGTTGAGGGAAA-TACATGTACAAAACCACAAATATATGAAAAGTGATAGGACTGATAATGGGTCCTTTATATATTGTAA  576
Vaquita      CAGTGGTTGAGA---GTCCGCCTG--CCGATGCAGGGCACACGGGTTCGTGCCCCGGTCCGGGAGGCTCCCACATGCCGC  466
Pig          ----------------TT---------TTGCCACCTAAGGACAGGCTTCCTTCCCTTCATGGAACAGTTTGTCTTTGCCAA  613
```

```
Majority     GXCAAAGCTGGAGCCAATGAXTCAXTXX-AAXTGAGTCTXTGXXG-GX---XATATGGAGXXTTACGGXAGAG---TXCC
                                 1610      1620      1630      1640      1650      1660      1670      1680
Human        --------------------------------------------------------------------------------  0
PolarBear    --------------------------------------------------------------------------------  0
Cow          GATAAAACAAGAGCTAATGAATCAACATTGATTGAGTCACTGTGGGGG---AATACAGAGAATTATGATAGAG---TTCC  622
Dog          GCCGCCCCGGCCCGCCCTGGCTGGGGGAGATGCCGGTGGCGGGAGCGGGGGGTGGGGGGGCGGGGAGGCCGAGCGGCGGC  872
BelugaWhale  GGAGCGGCTAG-GCGTGTGAGCCATGGC-TGCTGAGCCTGCGCGTCCGGAGCCTGTGCTCCGCAACGGGAGAG---GCCA  678
Donkey       GATAAAGCAGGAGCCAATCAATCAATATTGATTGAGTCCATGCTG-GG---AATACAGAGAATTATGTTAGAG---TTCC  376
Cat          GTCAATGGTGAAGGCAAAGACATTGTTGGAAATAGATTTTTTAA---AATATTTATTTATTTTTGAATGAGAGAGCGAGA  1666
Ferret       TTCCCCTATGATCCTCTGTTTTGTTTCTTAAATTCCACATGTCAGTGAGATCATATGATATTTGGCTTTCTCAGATTGAC  712
Lion         GTCAATGGTGAAGGCAAAGGCATTGTTGGAAATAGATTTTTTTTTTTAATATTTATTTATTTTTGAAGGAGAG------A  650
Vaquita      GGGGCGGCTAG-GCGCGTGAGCCATGGC-TGCTGAGCCT---------------GTGCTGCGCAACGGGAGAG---GCCA  526
Pig          AATAAAGCAAGAGCTAATCAATCAA----AATTGACTCAATATGGGG----AATACAAAGGATGACGCTAGAA---TTGC  682

Majority     XAXCGATXGGXAGCAXACXTATGTAATAATAGAATXAAAAXAC-----------GTXCAAAGCCACAAACACGTGAAAAG
                                 1690      1700      1710      1720      1730      1740      1750      1760
Human        --------------------------------------------------------------------------------  0
PolarBear    --------------------------------------------------------------------------------  0
Cow          TGGCCTTTGG-AGCTTACATATGTAATTGTTGAGTGAAAATACC--T-------GTTCAAAACCACAAACGTTTGAAGAG  692
Dog          GGGAGGAAGGGGGAAGGCAGATGTCATACTCCTTTGTTTTCACTGGAGTCTGGTGGTGGGGGCGGG----AGACCGAAAG  948
BelugaWhale  CAACAATGAGAGGCCCGCGTACCGCAAAAAAAAAAAAAA----------------------GCCACAAACACGTGAAAAG  736
Donkey       TGCCCTTTGGGAGCTTACATATTTAATTGTTGAGTGAAAATACA---------TGTACAAAACCACAAATACATGAAAAG  447
Cat          GAGAGAGAGACAGAACACGAGCGGGG-AAGAGCAGAGAGAGAGGGAGACACAGAATTCAAAGCAGGGTCCAGGCTCTGGG  1745
Ferret       ATATTCTGTTTTTCATAACACTCTTTTGTCCCTTTTGTTCCATCCTCAT-TGTTGCAAATGGCAAA----ATTTCATTTT  787
Lion         GAGAGAGAGACAGAACACGAGGGGGGGAAGAGCAGAGAGAGAG--AGACACAGAATTCAAAGCAGGATCCAGGCTCTGGG  728
Vaquita      CAACGATGAGAGGCCCGCGTACCGTAAAAAAAAAAAAAAAAAAAAAAAAAAAAAAAAAAAAGGCACAAACACGTGAAAAG  606
Pig          TAACCTTTGGGAGCTTCCATATTTAATTCTTGAGTGAAAATACA--G-------GGACAAAGCCACAAATGCGTGAACAG  753
```

```
Majority     TGATTATTAATGGCACTGATAATGGGXCCTTTATGTATCAXAAGTXAATGATTAXGXCAAAGG-XXGAXXTTGTAGGAAA
                      1770      1780      1790      1800      1810      1820      1830      1840
Human        --------------------------------------------------------------------------------  0
PolarBear    --------------------------------------------------------------------------------  0
Cow          TGATTATTAATGGCACTGATAATGGGTCCTCTATGTATCACAAGTTAATGTTAACGGCAAAGGGAAGACAATGTAGGAAG  772
Dog          AAAAGAAAAAAAGCCTTTTTGTGGGGGCCTGAGGACGTTCCAGGCTTGGCAGAAGGGCCCAGAAACGAAATCGTCTGAAA  1028
BelugaWhale  TGATTATTAATGGCACTGATAATGGGCCCTTTATGTATCATAAGTCAATGATTAAGGCAAAGA--AGGCATTGTAGGAAA  814
Donkey       TGATTATTAATGGCTCTGGTAATGGGTCCTTTATGTATCATAAGTCAATGGTTAAGCTGAAGGGAGGGTGTTGTAGGAAA  527
Cat          CTGTCAGCACAGACCCCAATGCGGGGCTCAAACCCACAAACCGTGAGATCATGACCCCAACGGAATCATGACCCCAACTT  1825
Ferret       TTTTATTTTCAGATTTTTTTTAAACTTTTTATTTTTTATAAACATATATTTTTATCCCCAGGTGGTACAGGTCTGTGAAT  867
Lion         CTGTCAGCACAGGCCCCAATGCGGGGCTCAAACCCACAAACCGTGAGATCATGACCCCAACGGAATCATGACCCCAACTT  808
Vaquita      TGATTATTAATGGCACTGATAATGGGCCCTTTATGTATCATAAGTCAATGATTAAGGCAAAGA--AGGCATTGTAGGAAA  684
Pig          TGATTATTAATGGCATTGATAATGGGTCCTTTAC-TATCCTGAGT------------CAAAGG-----------------  803

Majority     CAX---GTGTTT--C--------------TTTXAAGXAXTTCCAATCCCTGAAGCAAATCAAGAATTTT--TAACATTTG
                      1850      1860      1870      1880      1890      1900      1910      1920
Human        --------------------------------------------------------------------------------  0
PolarBear    --------------------------------------------------------------------------------  0
Cow          TAATGTGTGTTTTTTTGTTTTTGTTTGTTTTTTAAGAAATTCTAATCCCTGAAGCAAATCAAGAATTCT--TAACACTTG  850
Dog          ACCCGAGTGTTTG-CGGTCGACCTCAGTCACACGGGCAGAGGCCAGCAGCCATGGCAGGAACGAGAGCC--CCCGGTCCA  1105
BelugaWhale  TAG---GTGTTTTTGT-------------TTTTAAGGAATCCCAATCCCTGAACCAAATCAAGAATTTT--TAACATTTG  876
Donkey       C-----GGATTTCTT--TT----------TTTCAAGGATTGCCAATCTCTGAGGCAAATCAAGAATTTT--TAATGTTTG  588
Cat          CACCGACTGAGC--CACCCAGGCGCTCCCGAAATAGATTTTTTTTTAAGGATTGCCAACCGCTGAGGTAAATAACATTTG  1903
Ferret       CACCAGGTTTACA-CACTTCACAGCACTCACCAAAGCACATCCCATCCCTGAGGTAAGTCAAGAATTTT--TAACATTTG  944
Lion         CACCGACTGAGC--CACCCAGGCGCCCCCGAAATAGATTTTTTTTTAAGGATTGCCAATCCCTGAGGTAAATAACATTTG  886
Vaquita      TAG---GTGTTTTTGT-------------TTTTAAGGAATCCCAATCCCTGAACCAAATCAAGAATTTT--TAACATTTG  746
Pig          ----------------------------------------T-CAATCCCTCAAGTAAATTAAGAATTTT--TAACATTTG  840
```

```
Majority     AGTGCTTTGAAGAGTTCT----------ATTTGGACAAGATGCTCTATAAGGTTT-GTCCAACTTAAAAA-TAATATAAT
                      1930      1940      1950      1960      1970      1980      1990      2000
Human        --------------------------------------------------------------------------------  0
PolarBear    --------------------------------------------------------------------------------  0
Cow          AGTGCTCTGAAGAGTTCT----------ATTTGGACCAGATGTTCTATAAGTTTT-GTTCAAGTTAAAAA-CAATGTAAT  918
Dog          CCCACCCAGGGCCGTTTTTTTTTAAGTCACTTTGACCAGTTTCTGGACAAACAAA-AAACAACTGCAAAA--GACAAAAA  1182
BelugaWhale  AGTGCTTTGAAGAGTTCT----------ATTTGGACAAGATGCTCTATAAGGTTT-GCCCAAGTTAAAAA-TAATATAAT  944
Donkey       AGTGCTTTGAAAAGTTCTATAACAAAATATTTGGACAAGATGCTCTTTCAGTTTTTGTCCAACTTCAAAAATTATATAAT  668
Cat          TATACTTTTAAGAGTTCTATAG-AAAATATTTGGACAAGATGCTCTTTAAGGTCT-GTCCACCTTAAAAA-TAGTATTAT  1980
Ferret       TGTGCTTTGAATAGTTCCATAGCAAAATATTTGGACAAGATGCTCTTTAAGGTTT-TTCCACCTTAAAAA-TAATATAAT  1022
Lion         TATACTTTTAAGAGTTCTATAGCAAAATATTTGGACAAGATGCTCTTTAAGGTCT-GTCCACCTTAAAAA-TAGTATTAT  964
Vaquita      AGTGCTTTGAAGAGTTCT----------ATTTGGACAAGATGCTCTATAAGGTTT-GCCCAAGTTAAAAA-TAATATAAT  814
Pig          AGGGCTTTGAAGAGTTCC----------ATTTGGCCAAGATGCTCTACAAGGTCT-GCCCAACTGAAAAAATAACATAAT  909

Majority     TTTATGAATXATACAGAAAGTACCATTTAGTACAGTGAAATTCCAAXTAXTAAAXGGGTAAGAGGTTTGCATGAAGGAGA
                      2010      2020      2030      2040      2050      2060      2070      2080
Human        --------------------------------------------------------------------------------  0
PolarBear    --------------------------------------------------------------------------------  0
Cow          TTTATGAATCAT----AAAGTAT-ATTTAGTACAGTGAAATTCCAAATAGTAAATGGATAAGAGATTTGTATGAAGGAGA  993
Dog          CAAAAAAACAAAAAACCAAAAACCACCCGAGCCGGAACCGTGCGGCGACTCTGAAAGGCCGAGGAGGCGCAGTGCCGCTG  1262
BelugaWhale  TTTATGAATCATACAGAAAGTACCATTTAGTACAGTGAAATTCCAAATAGTAAAACGATAAGAGGTTTGTATGCAGAAGA  1024
Donkey       TTTATTAATTATACCGAAGGTACCAGTTAGTACACTGAAATTCCAATTAGTACAGGGG-AAGAGGTTTCCCTGAAGGAGA  747
Cat          TTTATGAATTGTATAGAACGTACCGGTTAGTGCAGTGAAATACCAATCAATAAAGGAGTAACAGGGTTGCATGTGGGAGG  2060
Ferret       TTTGTGAATTATACAGAAAGTAGCATTTAGTACAGCGAAATTCCAATTAATAAAGGGGTAAGAGTGTTGCATGAAGGATG  1102
Lion         TTTATGAATTGTATAGAACGTAGCGGTTAGTGCAGTGAAATACCAATCAATAAAGGAGTAACAGGGTTGCATGTAGGAGG  1044
Vaquita      TTTATGAATCATACAGAAAGTACCATTTAGTACAGTGAAATTCCACATAGTAAAAGGATAAGAGGTTTGTATGCAGAAGA  894
Pig          TTTATGAATCATCCAGAAAATACCATTTAATACAGTGAAGTTCCAAATAATAAAAGGATAAGAGGTGTGTTTGAGGAAGA  989
```

Majority	TACAGGAAAACCC-XAGTGGGCATTGAGAGTAACTGGAGAGACAAAGGAATAT-TTTTCXTXTCTCAGXGT---TX-GT	
	2090 2100 2110 2120 2130 2140 2150 2160	
Human	--	0
PolarBear	--	0
Cow	TATCAGAAAACCCAGAGTGGTCATTGAGAGTAATTGGAGAGACAAAGAGAATAT-TTTTCATTTCC-AGGAT---TAGAG	1068
Dog	AGCGAGGCGAGGC-CGCGGCGCGGGGAGGCGGCCCGGCCCGGGGTCTCCGGGGT-CTC-CGGCGCTCGGCGTCTGAG-GG	1338
BelugaWhale	TACAGGAAAGCCCATAATAGGCATTGAGAGTAACTGGAGAGACAAGGGATATTTGTGTGTGTGTGCACGCGC--------	1096
Donkey	TATAGGAAAACCCAGAGCCGGCATTGAGGGTAACAGGAGAGACAGAGGAAATTT-TTCTCATTTCTCAGGAT---TAGAA	823
Cat	TATAGACCCAG----AGTGGGCACTGAGAGTAACTGGAGAAATACAGAGAATAC-TTGTCCTGTGTGTGTGTGTGTGTGT	2135
Ferret	CACAGGAAAACCA-GACTGGACACTGAGAGTAACTGGAGAAATATAAGGAATAT-TTTTCTTTTCTCAGTATTCTAA-GT	1179
Lion	TATAGACCCAG----AGTGGGCACTGAGAGTAACTGGAGAA-TACAGAGAATAC-TTGTCCTGTGTGTGTGTGTGTGTGT	1118
Vaquita	TACAGGAAAGCCCATAATAGGCATTGAGAGTAACTGGAGAGACAAGGGATATTTGTGTGTGTGTGCACGCGCACGCGCGT	974
Pig	TACAGGAAAACCCAGAGAAGCTATCCAGAGTAAGTGGATAGACAAAGGAATAT-TTTGCATTTCCCAAGAT---TAAGT	1065

Majority	GTGTGTGTGTGTGTGTG----TTGTGGTTGGGGGTG-AAGGGTGGAGGAGAGGGAAAATTGCTGGCAAA--AGTCACCC-	
	2170 2180 2190 2200 2210 2220 2230 2240	
Human	--	0
PolarBear	--	0
Cow	GTGTGCGTATGTGTGTG----TTGTGGCTGCGGGGG-AAGGACGGAGGATAGAGAAAA-------------GTCGCCC-	1128
Dog	GC----CCGCGGGGGCG------GTGCTGGGCGACC-GCGCCGCCGTCCGCAGCCCTGCCGGGGGCTTG--AAGCGGGT-	1404
BelugaWhale	----------GTGTGTGTGTGTTGTGGTTGGGGGGG-AAAGGTGGAGGAGAGAGAAAATTGCTGGCAAAGCAGTTACCC-	1164
Donkey	GCGCATTTGTGTGTGTG----TTGTGGTTGGGGGTG-TAGGGTGGAGCAGAGGAAAAGATGCTGGCAAAGCAGTCACTT-	897
Cat	GTGTGTGTGTGTGTGTGTGTGTTGTAGTTCGGGGTG-ATGGA----GAAGACGGTAAGTTGCTGGCCAG--AGTCACCCC	2208
Ferret	ACATTTCTGTTTGTGTT------GTGGTTGGGGGTG-ATGGGTGGAGAAGAGGGCAAATTGCTGGCAAAGTAGTCACCC-	1251
Lion	GTGTGTGTGTGTGTGTG----TTGTAGTTCGGGGTG-ATGGATGGAGAAGAGGGTAAGTTGCCGGCCAG--AGTCACCCC	1191
Vaquita	GTGTGTGTGTGTGTGTGTGTGTTGTGGTTGGGGGGGGAAAGGTGGAGGACAGAGAAAATTGCTGGCAAAGCAGTTACCC-	1053
Pig	GTGTGTGTGTGTGGGGGGGGGTTGTGGTTGGGAGTG-AAGGGTGGAGGAGAGAGAAAATTGCTGTTAAAGGAGTCACCT-	1143

```
Majority     ACTGAGCAAATTTTCCAAAAAATGGATGCCTTTTCAAACTTTGTTGAGCCTCTG--AAAAACATGTTAAAGG-AAATGTCC

                         2250      2260      2270      2280      2290      2300      2310      2320

Human        --------------------------------------------------------------------------------  0
PolarBear    --------------------------------------------------------------------------------  0
Cow          ACTGAGCAAATTTTTCAAAAACGGATGCCTTTTCAAAC---GTTGAGCCTCTGATAAAAACACTTTAAAGG-AATTGTCC  1204
Dog          GGTTCAGAAAGCCCCTGGAGTAGAATCCGTGGCAACATGCGGAGAGCCAAGTA--AAATACGAGTTTATTTTAAATGTTC  1482
BelugaWhale  ACTGAGCAAATTTTTCAAAAAATGGATGCCTTTTCAAACGTTGTAGAACCTCTGATAAAA-CATTTTAAAGG-AAATTTCC  1242
Donkey       CCTGAGCAAATTTTCCAAAAAATGGGTGCCTTCTCAGACTTTGTTGAGCCTCTCATAAAAACACTTTAAAGG-AAATGTCC  976
Cat          ACTAAGCACATTTCCCCAAAATGGATGCTTGTTCAAATTTTGTCGAGCCCCTG--AAAAACATGTTAAAGG-AAAGGTCC  2285
Ferret       ACTGAGCAAATTTCCCCCAAATGGATGCTTGTTCAAACTTTGTTGAACCTCTG--AAAAACATGTTAAAGA-AAAGGTCC  1328
Lion         ACTAAGCACATTTCCCCAAAATGGATGCTTGTTGAAATTTTGTCGAGCCCCTG--AAAAACATGTTAAAGG-AAAGGTCC  1268
Vaquita      ACTGAGCAAATTTTTCAAAAAATGGATGCCTTTTCAAACGTTGTAGAACCTCTGATAAAAACATTTTAAAGG-AAATTTCC  1132
Pig          ACTAAGCAAATTTTCCAAAAAATGGATGCCTTTTCAAACTTTGTTACGACTCTG-TAAAAACATGTTAAAGA-AAATGTCC  1221

Majority     AATATGCTATTAAAATTAAAATGGAGACT-GTGAGAGATA-----XAGAAAAGGGAGTCA-ATTTGXGTAGCCCATTTTA

                         2330      2340      2350      2360      2370      2380      2390      2400

Human        --------------------------------------------------------------------------------  0
PolarBear    --------------------------------------------------------------------------------  0
Cow          AATATGCTTTTAAAATTTAAAAGGAAACCAGTGAGAGATA-----TAGAAAAGGGAGTCG-ATTTGAGTAGCCCATCGTA  1278
Dog          GTGGAGTTTAGTGTTTTTTAAGACACATC-G--AGGGAAG-----GACATACCAAAGTTAGAGTAACAGACTTCACCCCA  1554
BelugaWhale  AATATGCTATTAAAAATAAAACAGAGACA-GTGAGAGATACTGTATAGAAAAGGGAGTCA-ATTTGAGTAGCCTTTCTTA  1320
Donkey       TATATGCTATTAAAATTATAATGGAGACC-ATGAGACATA-----CAGAAAAGGGAGTCA-ATTTGTGTAGCCCATTTTA  1049
Cat          AATATACTATTAGAATTAAAATGAAGACT-GTGAGATACA-----CAGAAAAGGGAGTCA-ATTTGTGTGGCCCATTTCA  2358
Ferret       AATATGGTATTAGAATTAAAATGGGGACT-G--AGATACA-----TGGAAAAGGGAGTTA-ATTTGTGTAGCCCATTTTA  1399
Lion         AATATACTATTAGAATTAAAATGAAGACT-GTGAGATACA-----CAGGAAAGGGAGTCA-ATTTGTGTGGCCCATTTCA  1341
Vaquita      AATATGCTATTAAAAATAAAACAGAGACA-GTGAGAGATACTGTATAGAAAAGGGAGTCA-ATTTGAGTAGCCTTTCTTA  1210
Pig          AATATGCTATTAAAAC------GGAGACT-GTGAGAGATA-----CAGAAAAGGCAATCA-ATTTGAGTCGCTCACTTTA  1288
```

```
Majority     ATGTTTCTTCCATCTCTAACC-A-TGGTGAAGACGGAGCTACCACTCCCATCCCCACTTGCCCTTTCAAGCCCCCAAATG
                      2410      2420      2430      2440      2450      2460      2470      2480
Human        --------------------------------------------------------------------TGCCTCCAAATG  12
PolarBear    --------------------------------------------------------------------------------  0
Cow          GTGTTTCCTCCATCTTTAACC-AACGGTAAAGATGGAGCTAGTACTTCCATCCCCACTTGCTCTTTCAAGCCTCCAAATG  1357
Dog          GAGGTTGTTTAGACATTGTTT-TCCAGTGAA-ACGTAGATCTAGAGAAGTTCACTATTCCTCCAGAGAAACCTGGAAACT  1632
BelugaWhale  ATGCTTCTTCCATCTCTAATC-AGTGGTGAAGGTGGAGCTACCGCTCCCATCCCCACTTGCTCTTTCAAGCCCCCAAATC  1399
Donkey       ACGTTTCTTCCATCGCTAGCC-ACTGGGGAAGACTGAGCCACCACTTCCATCTCCACCTCCCCTTTCAAGCTCCCAAACC  1128
Cat          ATGTTCCTTCCATCCCTAAC----CAATGAAGACTGAGGCACCACTCTCATCCCCACTTTCCCTTTCGAGCTCCCAAATG  2434
Ferret       ACGTTCCTTTCATCTCTAACC-AGTGGTGAAGACTGAGGTACCACTCCCATCCCTACTTTCCCTTTGAAGCCCCCAAATG  1478
Lion         ATGTTCCTTCCATCCCTAAC----CAATGAAGACTGAGGCACCACTCTCATCCCCACTTTCCCTTTCGAGCTCCCAAATG  1417
Vaquita      ATGCTTCTTCCATCTCTAATC-AGTGGTGAAGATGGAGCTACCGCTCCCATCCCCACTTGCTCTTTCAAGCCCCCAAATC  1289
Pig          ACGTTTCTTCCATCTCTAGCCCAATGCGGAAGATGGAGCTACCACTCCCATCCCCACCTGCTCTTTCAAGCCCCCGAAAG  1368

Majority     GTAGATCCTA-CTXTCGGTAGCAXTTTTTGTACCAACTCTTTAATACATCGCTCAACCTGTGTTCTTTTCCATCCAGGT-
                      2490      2500      2510      2520      2530      2540      2550      2560
Human        TTAAACTCC----------A--AC----TATTGTAGCATTTTAACACATGGCTCCATTTGTGTCTATTTCCACCTAGCGT  76
PolarBear    --------------------------------------------------------------------------------  0
Cow          GTAGATCTTA-CTGTTGGTAGCAGATTTCTTACCAACTTTTTAATACATCTCTCAACCTGTGTTCTTTTCCATCCAGGT-  1435
Dog          GGTGACTAGACTTGGCAGTGGAGTTCATGTTCCCTGAGTCTATCAAGATAGCTGAAGAGTTGGTTTAAGTCTTCCATTAC  1712
BelugaWhale  ATAGATCCTA-CCCTTGGTACCAGTTTTTGTACCAACTGTTTAATACATCGCTCAACCTGTGTTCTTTTCCATCCAGGT-  1477
Donkey       CTAGATCACAACTATCGGT----G-TTCTGTACCAACTCTTCAACACGT--CTCAACCTGGGTGCTTTCCCATCCAGCT-  1200
Cat          GTAGATCCTAACTATCGCT-GCATTTTTCATACCTACTCTTTATTACAAGGCTCCACCTGTGTTCTTTTCCATCCAAGGT  2513
Ferret       ATAGATCCTAACTTACGATAGCATTTTTCGTCCCTACTCTTTATCACACAGCTCAAGCGGTCTTCTTTTCCATCTAGGGC  1558
Lion         GTAGATCCTAACTATCGCT-GCATTTTTCATACCTATTCTTTATTACAAGGCTCCACCTGTGTTCTTTTCCATCCAAGGT  1496
Vaquita      ATAGATCCTA-CCCTTGGTACCAGTTTTTGTACCAACTCTTTAATACATCGCTCAACCTGTGTTCTTTTCCATCCAGGT-  1367
Pig          GTAGCTCCT------CGGTAGCACTTTTTGTACCAACTCTTTAATAAATCACTCAACCTCGGCTCTTTCCCAGCCAAGT-  1441
```

```
Majority     -ATTGTGACTATTTCCTACAGGTGCCCACTAATCATCAXCTGAGGGAAGTXTGACXCXTTXTCCXACTTGXCTTGGAACT
                              2570      2580      2590      2600      2610      2620      2630      2640
Human        -ATTGTGAATATTTTCTACAGGCGCACACTAATCACCAACTGAAGTGAGAATCACATTTTCCTCCACTTGGCTTGGTAC-  154
PolarBear    --------------------------------------------------------------------------------  0
Cow          -ATTGTGAGTATTTCCTACAGGTGCAGACTA---ATCAGCTGAGTGAGGTGAGAATCGTTATCTTCCTCCACTTGGAACT  1511
Dog          TAGTCTTAAGTGTAAAGCTTGGCAGAAATGGCACCACAGTGTTTGGAGTTTTTGTTCTTTTTGGTAGATAAGGGT-----  1787
BelugaWhale  -ATTGTGAATATTTCCTAGGGGTCCCCACTAATCATCAACTGAGTGAGGTGAGAATC-ACATCCTTCTCCACTTGGAACT  1555
Donkey       -ACGGTGACTATTTCCTACAGGCGCTCACTGATCCTCAACTGAGAGAATT---------TCCTCCACTTGGCATAGAACG  1270
Cat          -ACTGTGACTGTTTCCTGTAAGTGCTCATTA-TCATTAGCTGAGGTGAGAATTACATTCTCTCCCAGTTGGCAGGGAACT  2591
Ferret       -ATTGTGACTATTTCCTACAGGTGTCCACTAATCATCAGTTGAGGTGAGAATCACATTTTTCTCCAGCTGGCGTG-----  1632
Lion         -ACTGTGACTGTTTCCTGTAAGTGCTCATTA-TCATCAGCTGAGGTGAGAATTACATTCTC--CCAGTTGGCAGGGAACT  1572
Vaquita      -ATTGTGAATATTTCCTAGGGGTCCCCACTAATCATCAACTGAGTGAGGTGAGAATC-ACATCCTTCTCCACTTGGTACT  1445
Pig          -ACCCTGACCATCTCCTACAGGTGCCCACTCCTCATCAACCGATGCACCTGAGAGCC-CCATCTTCCTCCACTTGGAAGC  1519

Majority     XCAAGGGTCTGGAAGCAAAAGCC-TAG--GTTTCAGTCTGATG-----TTTACTTGCTTTGGAAA-TAT--TTTTTACTA
                              2650      2660      2670      2680      2690      2700      2710      2720
Human        -TACAGGTCTTGAAGCAAAAGCC-TGT--ATTTCAGTCTGAGGAG--TTCTACTTGCTTGAGAAAGTACTGTTGTTACCA  228
PolarBear    --------------------------------------------------------------------------------  0
Cow          TCAAGCGTCGGGAAGCAAAAGCCGTAT--GTTTCAATCTGATG-----TCTACTTGCTTCAGAAG-CACAGTTTTCACTA  1583
Dog          -CGTGGGAACAATACCAAAAGTT-CAGCGACTTTCATCTCAGG----TTTGA-AAGCAATTTTCC-ACA----AGACCGA  1855
BelugaWhale  TCAAGTGTCTGGAAGCAAAAGCG-TAT--GTTTCAATCTGATGATG--TGTACTTGCTTTGGAAA-TACTGTTTTTACTA  1629
Donkey       ACAAATATCTGGAAGCAAAAACC-----TGTTTCAGTCTGATGACA--TCTACTTGCTTTGAAAAGTATTGTTTTTACTA  1343
Cat          ACAAGGGTCTGGAAGCAAAAATC-TAG---GTTCAGTCTGATT----TGTACAAAGCAATGGTTT-CAT----TTAGTGG  2658
Ferret       -GAAGGGTCTGGAAGCAAAAGCC-TAG--GTTTGGGTCTGATT----CCTAATAAACAGTGGTCT-CAT----TCAATGG  1699
Lion         ACAAGGGTCTGGAAGCAAAAATC-TAG---GTTCAGTCTGATT----TGTACAAAGCAGTGGTTT-CAT----TTAGTGG  1639
Vaquita      TCAAGTGTCTGGAAGCAAAAGCG-TAT--GTTTCAATCTGATGATG--TGTACTTGCTTTGGAAA-TACTGTTTTTACTA  1519
Pig          TCAAGTGTCTGGAAGCAAAAGCC-TAG--GTTCCGGTCTGATGATGCCCTGACCTGCTTTGGAAA-TAT--TTTCTGCTA  1593
```

```
Majority     AGTAGTGGTCTTX--TAA-GGGGTAAAG---TGTTTGTTTGTTTXXXTTTTAGAGGATGG---XAGGGTCTGCAAATAAA
                      2730      2740      2750      2760      2770      2780      2790      2800
Human        AGTGGTCTCGTT---CAGTGGGGTAAAG------ATTTTTG------TTTTAGAGGCTGG--AGCCGGTCTGCAAATAAA  291
PolarBear    --------------------------------------------------------------------------------  0
Cow          AGCAGTGGTCTTGGTTAATGGGATAAAGTTTCTTTTGGTT-TTTGGTTTTGAGAGGATGG---AGGGGTCTGTTAATAAA  1659
Dog          AGTGTCGTTG------------ACCATC---TCTCTATTTGTTTTCCATATGCATGGTGACTGGAGGATTTATTAATGAT  1920
BelugaWhale  AGCAGTGGTCTTGGTTAACGGGGTAAAGTGTTGTTTGTTT-------TTTAAGAGGATGG---AGAGGTCTGCAAATAAA  1699
Donkey       AGTAGCGTTCTTCTTTAACGGGGTCAAG-----TTTTTTGATTTTTGTTTTAAAGGATGA---AAAGGGTCGCAAATAAC  1415
Cat          GGTAAAGGTTTTGTGTGTGTGTGTGTGTGTGTGTGTGTGTGTGTGTGTTTGAGAGGATGG--AGAGGGTCTGCAAATAAA  2736
Ferret       GGTAAAGGTG------------TTTTGT---TTTTTCTCTGTTTTTGTTTTAGAGGATGG--AGAGGGTCTGCAAATAAA  1762
Lion         GGTAAAGGTT------------GTGTGT----GTGTGTGTGTGTGTGTTTGAGAGGATGG--AGAGGGTCTGCAAATAAA  1701
Vaquita      AGCAGTGGTCTTGGTTAACGGGGTAAAATGTTGGTTGTTT-------TTTAAGAGGATGG---AGAGGTCTGCAAATAAA  1589
Pig          AGCAGCGGTCTTGGCTAATGGGATGAAGGTTTTTTTTTTGGTTTGCTTATTTAGAGATGG---AGCCGTCTGTAAATAGA  1670

Majority     AGATGGCAT--------AAGAGAAAAGCTATAAAATAG-TTTTTCCTTGGCCCXCGTAGGTCGAGGGT--AGCCAAGTTG
                      2810      2820      2830      2840      2850      2860      2870      2880
Human        AGATGGCTT--------AGGAGAAAAGCTATCCAATGT-TTTTTCCTTGGCCCGCATAGGTTGACGGT--AGCCAAGCTG  360
PolarBear    --------------------------------------------------------------------------------  0
Cow          AGATGGCATAGTAAAAAAAGAAAAAAGCTATGAAATAGTTTTTTTCTTGGCCCATCTAAGTCGACGGT--AGCCAAGTTG  1737
Dog          GAACTACTCT-----GAAAAAGTTTTAGAGCCCAACAG-TGATCCTTAGAACAACGAGGCTGAAACTT--TTTTTTTCTT  1992
BelugaWhale  AGATGGCATA-------GAAAAAAAAGCTATAAAATAG-TTTTTCCTTGGCCCGCCTAGGTCGAGGGGGGAGCCAACTTG  1771
Donkey       AGATGGCAC--------AGGAGGAAAGCCATAAAGTAG-TTTTTCCTTGGCCCCCATAGGTCGGGAGC--AGCCAAGCGG  1484
Cat          AGATGGCGT--------AGGAGGACAGCTATAAAATGG-TTTTTCCTTTGCCCAAGTAGGTCGAGGGT--GGCCAAGTTG  2805
Ferret       AGATGGCGT-------AAAAGGAAAAACTATCAAATAG-TTTTTCCTCGGCCCGGGTAGGTCGAGGGT--AGCCAAGCTG  1832
Lion         AGATGGCGT--------AGGAGGACAGCTATAAAATGG-TTTTCCCTTTGCCCAAGTAGGTCGAGGGT--GGCCAAGTTG  1770
Vaquita      AGATGGCATA-------GAAAAAAAAGCTATAAAATAG-TTTTTCCTTGGCCCGCCTAGGTCGAGGGGGGAGCCAACTTG  1661
Pig          AGACGGCAT--TG------GAAAAAAGCT-TCAAATTCGTTTTCCCTTGGCCC---------------------------  1714
```

```
Majority     -------GGACCTGGTTTGGAGGGAAAAGTGGAGGCT--GCCTGGGGGCAGTCCT-----GCGGTCCAGGGACCACCCTC
                              2890      2900      2910      2920      2930      2940      2950      2960
Human        -------GTACTTGGCTTGGAGTGAAAAGTGGAGGCT---GCCTGGGGGCGTCCT-----GCAGTC---AGGG-------  415
PolarBear    --------------------------------------------------------------------------------  0
Cow          -------GGACCTGCTTTGGAGGGAAAAGTGGAGGAG--GTCTTTGGGCAGTCCT-----GCGGTCCAGGGGACACCCTC  1803
Dog          TCTTTACAGAATTGTCACTGAGGTTGCAGTTGGCGTGAAGGTACGAAAAAATTGTTTGAAACTAATGAAAACCGAATTAC  2072
BelugaWhale  -------GAGCCTGATTTGGAGGGAAAAGTGGAGGCT--GCCTGGGGGGAGTCCT-----GCGGTCCAGTGACCAGGCTC  1837
Donkey       -------GGACTCGGTTTGGAGGGAAAAGTGGAAGCT--GCCTGGGGGCAGTCCT-----GCAGTCCCGGGGCGCCCCTC  1550
Cat          -------GGACCTGGTTTGGAGTTAAAAGTGGAGGCT--GCTTGGTGGCAGTCGT-----GCAGTTCTGGGACCACCCCC  2871
Ferret       -------GGACCTGCTTTGGAGAGAAAAGTGGAGGCT---GCCCGTGGTAGTCCTAAGGTGCGGTTCAAGGACCACCCTC  1902
Lion         -------GGACCTGGTTTGGAGTTAAAAGTGGAGGCT--GCTTGGTGGCAGTCGT-----GCAGTTCTGGGACCACCCCC  1836
Vaquita      -------GAGCCTGATTTGGAGGGAAAAGTGGAGGCT--GCCTGGGGGGAGTCCT-----GCGGTCCAGTGACCAGGCTC  1727
Pig          --------GACCCGATTTGGAAGCT--------------GTCTGAGGGCATTCCT-----GCGGCCCGGAGACCACCCTT  1767

Majority     CCGAGGTCCAG-----GGCAGTGGGTGTGGAGGGTTGGGAGGTGTXCCACCCACACCGCCCAAGGAXGGCA----TCAGG
                              2970      2980      2990      3000      3010      3020      3030      3040
Human        --------CAG----AGAGAGTGTG-GAGAGCTGTTGGGAGGTGTCCCACCCACGCCCCCACAAGGAGGGG---------  473
PolarBear    --------------------------------------------------------------------------------  0
Cow          CCGAGGTCCAG-----GGCAGTGGGTGTGGAGGGCTGGGTGCTAGTCCACCCACACCGCCCTGAGAGGGCA----ACAGG  1874
Dog          TCAATCTACTGTTATCATTAGTGGAAATTTTCAGCTAACTTTTGTAAGTTACAATTAACAAAAGTGAAGTG----TTTAA  2148
BelugaWhale  CTGAGGTCTAA-----GGCAGTGGGTGTGGAGGGTTGGGCGGTGTCGCACCCACAGCGCCCTAGGAGGGCA----ACAGG  1908
Donkey       CTGAGATCCAA-----GGCCCTGGCTGCGGAGGGTTGGGAGG-AGTCCGACCACACCACTCCCGGAGGACA-ACGGGGAC  1623
Cat          GTGAGATCCAG-----GGCAATGGGTGTGAAGGGTTGAGAGGTGTTCCATCCACGCTGCCCAAGAAAGACAGACATCAGG  2946
Ferret       CCAAGGTGCAG-----GGCAGTGGGTGTGGAGGGTTGAGAGGTGCACCACCCACGCCTCTCAGGAAAGACA----TCAGG  1973
Lion         GTGAGATCCAG-----GGCAATGGGTGTGAAGGGTTGAGAGGTGTTCCAACCACGCTGCCCAAGAAAGACA----TCAGG  1907
Vaquita      CCGAGGTCTAA-----GGCAGTGGGTGTGGAGGGTTGGGCGGTGTCGCACCCACAGCGCCCTAGGAGGGCA----GCAGG  1798
Pig          CCCACGTGCTG-----GGCAGCGGGCGTGGGGGGTCGGAGGC-GTCCCACCCGCAGCGCCAAG-GAGGGCC----ACAGG  1836
```

```
Majority     GACTTCGXAGAGGAACGTXCAAXAXXAAXCGAGXACGCCACGCTCCACAGCAGCAGGCCCGGGCCCAG------AGXGCG
                      3050      3060      3070      3080      3090      3100      3110      3120
Human        -------AAACC--AGGT-----------------CGCCGCGGTCTGCAGCAGAACGCCAGGTTCCAG------AGGACG  521
PolarBear    --------------------------------------------------------------------------------  0
Cow          GACTTCGAGGAGGAACGTACAATAGAAACCAAGCAAGCCACTCTGCTCAACAACAGGCCGAGGCCCAG------AGCGCA  1948
Dog          TTTCAAGAGTTGGATTATGTAG--------TTGATAATCTAAATGGACAAAAACTAAGTTTTTTTTAG------AAAG-G  2213
BelugaWhale  GACTTCGGAGAGGAACGTACAAAAGAAACGGAGCACGCCACGCTGCACAGCAGCAGGCCCCGGCCCAC------AGCACT  1982
Donkey       CACTTCTTCGAGGAGCATCCACGAGGAAGCCAGGGCGCCATGCTTCCCAGCGGCAGGCCCGGGCCCGGGCGCGGAGGGCG  1703
Cat          GACTTCTCAGAGGCGCGTGCAAAAAGAAGCTAGGCCGCCCTGCTCCACCGCAGCAGGCCTGGGCCCAC------AAGGCG  3020
Ferret       GACTTCTCAGTGCAAAACGCAG--------GAAGCCGCCGCGCTCTACATCAGCAGGCCCGGGCCCAG------AGGGCG  2039
Lion         GACTTCTCACAGGCGCGTGCAAAAAGAAGCTAGGCCGCCCTGCTCCACCGCAGCAGGCCTGCGCCCAC------A-----  1976
Vaquita      GACTTCGGAGAGGAACGTACAAAAGAAACGGAGCACGCCACGCTGCACAGCAGCAGGCCCCGGCCCAC------AGCGCT  1872
Pig          GATCTCGGCGAAGAAGCTACTAGAAAGAGCGCGCACGCCGCGCTCTCCAGCAGCAGGCCCGGGCGCAG------AGCGCG  1910

Majority     GAGGGCGGAGGGCGGAGGGCCCCAGGCGCGCGGCGGCGCGG----AG--------------GAGGCGACGGCGGCGGCGC
                      3130      3140      3150      3160      3170      3180      3190      3200
Human        GCAGGCGGAGGGCGGAGGGAGAAAGGCGCGCGAAGGCGCGG----AG--------------GAGGCGTCGGCTGCGGCGC  583
PolarBear    --------------------------------------------------------------------------------  0
Cow          AAGGGCGGAGGG-------CCCCAGGCGCGCGGCGCCGCGT----AG--------------GAGGCCACTGCGGCGGCGC  2003
Dog          CAGTCTGTGAGAATAACATCTTAAAGTAGGTATGAGTGCTGTTCAGG--------------GAATGAAAAATGCTTTCGG  2279
BelugaWhale  GAGAGCGGAGGGCGGAGGGCCTCACGCGCGCGTCGGCGCGT----AG--------------GAGGCGACGGCGGCTGCGC  2044
Donkey       GAGGGCGGAGGGCGGAGGGCCCCGGGCGCGCGGCGGCGCGGAGGCGGAGGCGGCAGCGGCAGCGGCGGCGGCGGCGGCGC  1783
Cat          GAGGGCGGAGGGCGGAGGGCCCCAGGCGCGCGGCCGCGCGGAG-GAA--------------GAGGCGGCGGCGACGGCGC  3085
Ferret       GAGGGCGAAGGGCGGAGGGCCCCCAGCGCGCGGCGGCGCGG----AG--------------GAAGCGACGGCGGCGGCGC  2101
Lion         --GGGCGGAGGGCGGAGGGCCCCAGGCGCGCGGCCGCGCGGAG-GAA--------------GAGGCGGCGGCAACGGCGC  2039
Vaquita      GAGAGCGGAGGGCGGAGGGCCTCACGCGCGCGGCGGCGCGT----AG--------------GAGGCGACGGCGGCTGCGC  1934
Pig          GAGGGCGGGGGGA------CCCCAGGCGCGCGGCGGCGCGT----AG--------------GCGGCGACGGCGGCGGCGC  1966
```

```
Majority     CCACTCCCCCTCGTCCCCGCGCCCCGCCGCTCGCCXCCCGCAGCCGGGCGCCGGCGCGCTCGGCTCTTTCCGCCGCCGCC
                      3210      3220      3230      3240      3250      3260      3270      3280
Human        CCACTCCCCCTAGTCCCAGCGCCCTGCCGCTCGCCTCCCGCTGCCGGGCGCCGGCGCGCTCGGCTCTTTCCGCCGCCGCC  663
PolarBear    --------------------------------------------------------------------------------  0
Cow          CCCCTCCCCGCCGTCCCCGCGCCCCGCCGCTCGCCGCCCGCAGCCGGGCGCCGGCGCGCTCGGCTCTTTCCGCCGCCGCC  2083
Dog          ATATAGAGCTTTGAGAATATATAAATAATAGCTCAGTTTTTTGAGGAGAGATTACGATTTTAAATAGATAAGTGATTTGA  2359
BelugaWhale  CCACTCCCCCTCGTCCCCGCGCCCCGCCGCTCGCCGCCCGCAGCCGGGCGCCGGCGCGCTCGGCTCTTTCCGCCGCCGCC  2124
Donkey       CCACTCCCCCTCGTCCCCGCGCCCGGCCGCTCGCCTCCCGCCGCCCGGCGCCGGCGCGCTCGGCTCTTTCCGCCGCCGCC  1863
Cat          CCACTCCCCCTCGTCCCCGCGCCCCGCCGCTCGCCTCCCGCAGCCGGGCGCCGGCGCGCTCGGCTCTTTCCGCCGCCGCC  3165
Ferret       CCACTCCCCCTCGTCCCCGCGCCCCGCCGCTCTCCTCCCGCAGCCGGGCGCCGGCGCGCTCGGGTCTTTCCGCCAGCGCC  2181
Lion         CCACTCCCCCTCGTCCCCGCGCCCCGCCGCTCGCCTCCCGCAGCCGGGCGCCGGCGCGCTCGGCTCTTTCCGCCGCCGCC  2119
Vaquita      CCACTCCCCCTCGTCCCCGCGCCCCGCCGCTCGCCGCCCGCAGCCGGGCGCCGGCGCGCTCGGCTCTTTCCGCCGCCGCC  2014
Pig          CCACTCCCCCTCGTCCCCGCGCCCCGCCGCTCGCCGCGCGCCGCCGGGCGCCGGCGCGCTCGGCTCTTTCCGCCGCCGCC  2046

Majority     GCTGCCGCGCGCTGCCCCGTGCGCTCCGGCACCTTCGGCAATTTCCGTCGGGCCCTGGCCGCCATTT-TCTCGCCGCTTG
                      3290      3300      3310      3320      3330      3340      3350      3360
Human        GCTGCCGCGCGGTGGCCCGTGCGCCTCGGCACCTTCGGCAATTTCCGTCGGGCCCCAGCCGCCATTT-TCTCGCCGCTTG  742
PolarBear    --------------------------------------------------------------------------------  0
Cow          GCTGCCACGCGCTGCCCCGTGCGCTCCGGCACCTTCGGCAATTTCCGTCGGGCTCTAGCCGCCATTT-TCTCTCCGCTTG  2162
Dog          CCAAAATCGTGTGATTTTATTAATCTAATATTTTTTTGTACTTTTAATTGTGAAGACTAAACTATGTATTTCAATAATGA  2439
BelugaWhale  GCTGCCGCGCGCTGCCCCGTGCGCTCCAGCACCTTCGGCAATTTCCGTCGGGACCTGGCCGCCATTT-TCTCTCCGCTTG  2203
Donkey       GCGGCCGCGCGCTGCCCCGTGCGCTCCGGCACCTTCGGCAATTTCCGTCGGGCCCCGGCCGCCATTT-TCTCGCCGCTTG  1942
Cat          GCTGCCGCGCGCTGCCCCGTGCGCTCCGACACCTTCGGCAATTTCCGTCGGGCCCTGGCCGCCATTT-TCTAGCCGCTTG  3244
Ferret       GCTGCCGCGCGCTGCCCTGTGCGCTCCGGCACCTTCGGCAATTTCCGTCAGGCCCTGGCCGCCATTT-TCTCGCCGCTTG  2260
Lion         GCTGCCGCGCGCTGCCCCGTGCGCTCCGACACCTTCGGCAATTTCCGTCGGGCCCTGGCCGCCATTT-TCTAGCCGCTTG  2198
Vaquita      GCTGCCGCGCGCTGCCCCGTGCGCTCCGGCACCTTCGGCAATTTCCGTCGGGATCTGGCCGCCATTT-TCTCTCCGCTTG  2093
Pig          GCCGCCGCGCGCTGCCCCGTGCGCTCCGCCAGCTTCGGCAATTTCCGTCGGGCCCTGGCCGCCATTT-TCTCTCCGCTTG  2125
```

```
Majority     TGTGTCTCGCXGGCTGCGTGGCTCGGTTCTTGTGAGCGAAGCTTTGTCCGGTTCGGCAATGGACGGGTAXGTAATGGGGC
                      3370      3380      3390      3400      3410      3420      3430      3440
Human        TGTGGCTCGCTGGCTGCGTGGCTCGGTTCTTGTGAGCGAAGCTTTGTCCGGTTCGGCAATGGACGGGTATGTAATCGGGC  822
PolarBear    --------------------------------------------------------------------------------  0
Cow          TGTGTCTCGCCGGCTGCGTGGCTCGGTTCTTGTGAGCGAAGCTTTGTTCGGTTCGGCAATGGACGGGTAGGTAACGGGTT  2242
Dog          GCCACATTAACAATCCGTTATCTTGATCCTTAAAAGAAATTTGTTTAGGCAATTAGCTTCAAATTAGAGTCAGAAGTTAA  2519
BelugaWhale  TGTGTCTCGCCGGCTGCGTGGCTCGGTTCTTGTGAGCGAAGCTTTGTCCGGTTCGGCAATGGACGGGTAGGTAACGAGGC  2283
Donkey       TGTGTCTCGCTGGCTGCGTGGCTCGGTTCTTGTGAGCGAAGCTTTGTCCGGTTCGGCAATGGACGGGTAGGTCCTGGGCC  2022
Cat          TGTGTCTCGCTGGCTGCGTGGCTCGGTTCTTGTGAGCGAAGCTTTGTCCGGTTCGGCAATGGACGGGTATGTAATGGGGC  3324
Ferret       TGTGTCTCGCTGGCTGCGTGGCTCGGTTCTTGTGAGCGAAGCTTTGTCCGGTTCGGCAATGGACGGGTATGTAATGGGGC  2340
Lion         TGTGTCTCGCTGGCTGCGTGGCTCGGTTCTTGTGAGCGAAGCTTTGTCCGGTTCGGCAATGGACGGGTATGTAATGGGGC  2278
Vaquita      TGTGTCTCGCCGGCTGCGTGGCTCGGTTCTTGTGAGCGAAGCTTTGTCCGGTTCGGCAATGGACGGGTAGGTAACGAGGC  2173
Pig          TGTGTCTCGCCGGCTGCGTGGCTCGGTTCTTGTGAGCGAAGCTTTGTCCGGTTCGGCAATGGACGGGTAGGTAACGGGGC  2205

Majority     CGGCGAGGAGGTGTGTG--AGAGGGGAGTXGGACCCXCCTTCGGCCCGGTCCGGGGTCGGGCAGAAACCCTCCGGCGGCC
                      3450      3460      3470      3480      3490      3500      3510      3520
Human        CGGCGAGAAGGTGTGTGTGAGAGAGGAGTTGGACCGTCCTTCGGCCCGGTCCCGGGCCGGGGAGAAACCCTCCCGCGGCC  902
PolarBear    --------------------------------------------------------------------------------  0
Cow          TGGCGAGGTGGTGTGTG--AGAGGGGAGTGGGGCCCGCCTGTGGCCCCGTCCGGGGTCGGGCAGAAACCCTCTGGCGGCC  2320
Dog          GAGCTGGTTTGTCTTTT--CCATTAAAGGAAAATAGGCCTT-----GTGGTACAGATCATTTGGAATCTTTCC--T---C  2587
BelugaWhale  CGGCGAGGAGGTGTGCG--AGAGGGGAGTGGGGCCCGCCTTCGGCCTGGTCCAGGGTCGGGCCGAAACCCCCCGGCGGTC  2361
Donkey       CGGCGAGGAGGCGTGCG--AGAGGGGAGCTGGAGCCTCCTTCGGCCCGGGCCGGGGTCGGGCAGGACCCCTCGG-CGGCC  2099
Cat          CGGGAAGGAAGTGTGTG--AGAGGGGATTTGGACCCTCCTTCGGCCCGGTTCGGGGTCGGCCAGAAACCCTCCGGCGGCC  3402
Ferret       CAGCGAGGAGGTGTGTA--CGAGGGGAGTCGTACGGTTCTTCGGCCGGGTCCGGGGTCGTCCAGAAACCCTCCGGCGGCC  2418
Lion         CGGGAAGGAAGTGTGTG--AGAGGGGATTTGGACCCTCCTTCGGCCCGGTTCGGGGTCGGCCAGAAACCCTCCGGCGGCC  2356
Vaquita      CGGCGAGGAGGTGTGCG--AGAGGGGAGTGGGGCCCGCCTTCGGCCTGGTCCAGGGTCGGGCCGAAACCCCCCGGCCGTC  2251
Pig          CGGCGAGGAGGTGTGTG--AGAGGGGAGCGGGGCCCGCCCTCGGCCCGGCCCGGGGTCCGACCGAAACCCTCCGGCGGCC  2283
```

```
Majority     CCTCCC-AGGGCTGGGCTGCCGTTACCCAACCCCCGCCCCATCGCACACACCCCTCCCTXCGCTTCCCCC-GGCGGGCTC
                               3530      3540      3550      3560      3570      3580      3590      3600
Human        CCTCCC-AGGGCTGGGCTGCCGTTACCCAACCCCCGCCCCATCGCACACACCCCTCCCTTTGCTTCCCCC-GGCGGGCTT  980
PolarBear    --------------------------------------------------------------------------------  0
Cow          CCTCCC-AGGGCTGGGCTGCGGTTACCCAACCCCCGCCCCATCGCACACACCCCTCCCTGGGCTTCCCCC-GGCGGGCTC  2398
Dog          ACTTTT-TGGCCAGGAAAACTACTTATTTAGATCTTCCTTCTAATGCTTTGGCCTTT-TCCTTGAATCCT-TGCACATTT  2664
BelugaWhale  CCTCCCCAGGGCTGGGCTGCAGTTACCCAACCCCCGCCCCATCGCACACACCCCTCCTTGGGCTTCCCCCTGGCGGGCTC  2441
Donkey       CCTCCC-GGGGCTGGGCTGCCGTTACCCAACCCCCGCCCCATCGCACACACCCCTCCCTCCGCTTCCCCC-GGCGGGCGC  2177
Cat          CCTCCC-AGGGCTGGGCTGCCGTTACCCAACCCCCGCCCCATCGCACACACCCCTCCCTTCGCTTCCCCC-GGCGGGCTC  3480
Ferret       CCTCCC-TGGGCTTGGCTGCCGTTACCCAACCCCCGCCCCATCGCACACACCCCTCCCTTCGTTTCCCCC-GGCGGGCTC  2496
Lion         CCTCCC-AGGGCTGGGCTGCCGTTACCCAACCCCCGCCCCATCGCACACACCCCTCCCTTCGCTTCCCCC-GGCGGGCTC  2434
Vaquita      CCTCCCCAGGGCTGGGCTGCCGTTACCCAACCCCCGCCCCATCGCACACACCCCTCCTTGGGCTTCCCCCTGGCGGGCTC  2331
Pig          CCTCCC-AGGGCTGGGCTGCCGTTACCCAACCCCCGCCCCATCGCACACACCCCTCCCCGGGCTTCCCCC-GGCGG-CTC  2360
```

```
Majority     TGGTCGAGAAAATGXGAAGAAAGCGGGCTXGTAGACTGGAGAGGCXTTGXGGCGGTGGCGGGGGCGACGACCCCGGAGAG
                               3610      3620      3630      3640      3650      3660      3670      3680
Human        TGGTCGAGAAAATGAGAAGAAAGCGGGCTTGGAGGCTGGGGAGGCATAGGGGCGATGGCGGGGGTGAGGATCCCGGAGTG  1060
PolarBear    --------------------------------------------------------------------------------  0
Cow          TGGTCGAGAAAACGGGAAGAAAGCGGACTCGTAGGCTT------------GGCCGGCATTGGGGCGACGACCCCGGAGAA  2466
Dog          TAACAGTGTACTATGTCAAAATTTTTGTTTGCAAACCTGTCTG---CTGTGTTGGGGCATGGTTCTTTGGTTTTTTAAAA  2741
BelugaWhale  TGGTCGAGAAAACGGGAAGAAAGCGGGCTTGTGGACTCGACAGGCGTTG-GGGAGACGGCGGGACGACGACCCCGGGGAG  2520
Donkey       TGGTCGGGAAGATGAGGAGAAAGCGGGCTCGGAGGC--------------------------------------------  2213
Cat          TGGTCGAGAAAATGAGAAGAAAGCGGGCTCGTAGACTGGAGAGGCATTGGGGCGATGGCGGGGGCGACGACCCCGGAGAG  3560
Ferret       TGGTCGAGAAAATGAGAAGAAAGCGGGCTTGTAGACTGGAGAGGCATTGGGGCGATGGCGGGGGCGACGACCTCGGAGGG  2576
Lion         TGGTCGAGAAAATGAGAAGAAAGCGGGCTCGTAGACTGGAGAGGCATTGGGGCGATGGCGGGGGCGACGACCCCGGAGAG  2514
Vaquita      TGGTCGAGAAAACGGGAAGAAAGCGGGCTTGTGGACTCGGCAGGCGTTG-GGGAGACGGCGGGACGACGACCCCGGGGAG  2410
Pig          GGGCCGGGAAAACGGGCAGAAAGCGGGCTCGGGGGCCCCGGGGGCGTGGGGGCCGCGGCGGGGGCGACGGCCCGGCGGAG  2440
```

```
Majority     GGAGCGGGCCCCGGCGXGGCGGCGGGGGATGGGGT---GGGXACCCCAGAACGTC----TGGCCCGGCCCTGCCCCC-TC
                              3690      3700      3710      3720      3730      3740      3750      3760
Human        GGAGCGGGCACCGGCTTGGCGGCGGGGGATGGGGT---GGGAACCCCATAACGTCTCCCCGGTCTGTCCCTGCCCCC-TC  1136
PolarBear    --------------------------------------------------------------------------------  0
Cow          GCAGCGGGTCTTGGAGGGGCGGCGAGGGATGCGGT---GGGGGCCCCAGAATGTC----TCGCCCGGCCCTTCCCCCCTC  2539
Dog          ATTTTCAGACCTGGCACATCTTTTAGGCCTAATAC---ACAAATAACTTGTTGA-----TGCAATGACGTTTCCCCCTTT  2813
BelugaWhale  ACAGCGGGTCCCGGCGGGGCAGCGGGGGATGGGCTCTGGGGGGTCCCAGGACGTC----TCGCCCGGCCCTGCCCCC-TC  2595
Donkey       AGGGGAGGCCCGGGGGCGACGGCGGGGGCCGGGGA----GGGACCCCCGAACGTC----TGGCCCGGCCCTGCCTCC-TC  2284
Cat          GGAGCGGGCACGGACTTGGCGGCGGGGGATGGGGT---GGGAACCCCAGAACGTC----TGGCCCGTCCCTGCCCCCTCT  3633
Ferret       GGAGCAGGCACCGGCTTGACGGCGGGGGATGGGGC---GGGAACCCCAGAACGCC----TGGCCCGTCCCTGCCCCCTCT  2649
Lion         GGAGCGGGCACGGACTTGGCGGCGGGGGATGGGGT---GGGAACCCCAGAACGTC----TGGCCCGTCCCTGCCCCCTCT  2587
Vaquita      ACAGCGGGTCCCGGCGGGGCAGCGGGGGATGGGCTCTGGGGGGTCCCAGGACGTC----TCGCCCGGCCCTGCCCCC-TC  2485
Pig          GGAGCGGGCCGCGGCGGGGCGGCGGGCGCTGGGGT---GGGGGCCCCGGG---------CCGCCCGGCCCTGCCCCC-TC  2507

Majority     TAGGAGCCATTTCGATCCGTAACCTGGGACCCGAACCCCGGGTXAGCGGCGCCTGTGGGAGCGAGTGTAGTGGCC-GAGA
                              3770      3780      3790      3800      3810      3820      3830      3840
Human        TAGGAGCCATTTCGATCCGTACCTTGGGACCCGACCCTTGGGTTAGCGGTGCCTGTGAGAGCGAGTGGGATGGGCAGAGA  1216
PolarBear    --------------------------------------------------------------------------------  0
Cow          TAGGAGCCATTTCGATCCGTAACCGGGGACCCGGACCCCGGACGAGCGGTTCCGGTGGGAGCGAGTGTAGCGGCCGGAGA  2619
Dog          CAAGTATGAATAGTCTTGGGAGGCTATAGTGGTAGTGACTGTT-GACAATATTTTTTGGAATTCTTAAAATTTC-----A  2887
BelugaWhale  TAGGAGCCATTTCGATCCGTAAACTGGGACCCGGACCGCGGGCGAGCGGCGCGGCTGGGAGCGGGTGTAGCGGCCGGAGA  2675
Donkey       AAGGAGCCATTTCGCTCTGTAACCTGGGACCCGAACCCGGGGGGAGCGGCGCCTG-GGGAGCGGGGGTAGGGAC--GAGA  2361
Cat          -AGGAGCCATTTCGATCCGTAACCTGGGACCCGAACCCTGGGT-AGCCGCGCCTGTGGGAGCGAATGTTGTGGCCAAAGA  3711
Ferret       TAGGAGCCATTTCGATCCGTAGCCTGGGACCCGAACCCTGGGT-AGTCGCGCCTGTGGAAGCGAGTGTAGTGCC-----A  2723
Lion         -AGGAGCCATTTCGATCCGTAACCTGGGACCCGAACCCTGGGT-AGCCGCGCCTGTGGGAGCGAATGTTGTGGCCAAAGA  2665
Vaquita      TAGGAGCCATTTCGATCCGTAACCTGGGACCCGGACCGCGGGCGAGTGGCGCGGCTGGGAGCGGGTGTAGCGGCCGGAGA  2565
Pig          ACGGAGCCATTTCGGCCCGTAACCCGGGACCCGAACCCCGGGCGAGCGGCGCCGGGGGGAGCGCGTGTGGCGGCCCGGGG  2587
```

```
Majority     GXGAGGCCTGTGT---GGGAGTC--------CCCAGGGGAGAGXAGAGAGGX-XCCGX-------------TGCACCCCA
                       3850      3860      3870      3880      3890      3900      3910      3920
Human        CAGGCCTTTGGATTGGGGGAGCC-------TCTAGGGGAAGAGGAGAGAGGCACCC-C----------ATGTGGACCCCA  1278
PolarBear    --------------------------------------------------------------------------------  0
Cow          GCGAGGCCTACGTTGGGGGAGGGGGGCGGCTCCCCGCTGAGAGAACAGAGGCCCCCCCCCTCCCCGGACCCCCTCCCTA  2699
Dog          TCTACAACAATGT---GATATTC--------CTTAGAATATCCTAAGGAGATATTT----------------TGTATTTC  2941
BelugaWhale  GCGAGGCCTGCGT---GGGAGTC-------TCCCTGCGGAGAGAGAGG------CCGCCCCCACCTAGGCCCCC--CCAA  2737
Donkey       GGGAGGCCTGGGT---GGGCCTC---------CCGGGGGAGAGCCGCG--G--CCCG---------------GGGCCCCC  2410
Cat          GAGAGGCCTGTGT---GGGGGTT--------GTTAGGGGATGGGAGAAAGGCGTCTGTG------------TGCACCCCA  3768
Ferret       CAGAGGCCTGTGT---GGGGGTG--------GCTAGGGGAGAGGAGAGAGGCATCTGTA------------TGCACCCCA  2780
Lion         GAGAGGCCTGTGT---GGGGGTT--------GTTAGGGGATGGGAGAAAGGCGTCTGTG------------TGCACCCCA  2722
Vaquita      GCGAGGCCTGCGT---GGGAGGC-------TCCCTGCGGAGAGAGAGG------CCGCCCCCACCTAGGCCCCT--CCAA  2627
Pig          GGGGGGGCAGGCCTGGGGGAGGG-------CCCCGGGGGGGAGAGCGG--------------------------------  2628

Majority     GCCGTCCTXAGCCTCCCCGGCCTCGCCCGGCCCGC-----CCTGGCTGGGGGAGATGCCGGTGGCGGGAGCTCCGGGGAA
                       3930      3940      3950      3960      3970      3980      3990      4000
Human        GCCATGAGCAACCTCTCCGGCCTCGCCCGGCCCTC-----CGTGGTCGGGAGAGATGCCGGTGGCGGGAGCTCCGGGGAA  1353
PolarBear    --------------------------------------------------------------------------------  0
Cow          GAAGTCCCAGGCCTTCTTGGCCCCGCCTGGCCCGC-----CCTGGCTGGGGGAGATGCCGGTGGCGGGAGCTCCGGGGAA  2774
Dog          TTTTTTCTGCCTTACTAAATATTTGTTCATACTTG-----ATATTTAGTCTTATTTGTTGAGTTCATATTACATAGATCA  3016
BelugaWhale  GCCGTCCCAAGCCTCCCCGGTTTCGCCCGGCCCGCCCCGCCCCGGCTGGGGGAGATGCCGGTGGCGGGAGCTCCCGGGAA  2817
Donkey       GGCCGGGGAGTCGTCCTCGGCGTCGCGCGGCCCGC------CGGGCGGGGGA--TGCCGGTGGCGGGAGCCCCCGGGAG  2482
Cat          GCCGTCCTCCACCTGCCCGGCCTCGCCCGGCGGGC-----CCAGGCTGGGGGAGATGCCGGTGGCGGGAGCTCGGGGGAA  3843
Ferret       GCCGTCCTCAGCCTCCCCGGCCTCGCCCGGCCCGC-----CCTGGCAGGGGGAGATGCCGGTGGCGGGAGCTCGGGGGAA  2855
Lion         GCCGTCCTCCATCTGCCGGGCCTCGCCCGGCGGGC-----CCAGGCTGGGGGAGATGCCGGTGGCGGGAGCTCGGGGGAA  2797
Vaquita      GCCGTCCCAAGCCTCCCCGGTTTCGCCCGGCCCGC-----CCCGGCTGCGGGAGATGCCGGTGGCGAGAGCTCCCGGGAA  2702
Pig          ---GCCCGTGGCCCCCGCGGCCGCCCGGGCCGCGC-----CCTGGCGGAGGGGGATGCGGGTGGCGGGAGCGC-GGGGAA  2699
```

```
Majority     AGCTAAGTGGGAGCCGCTGGGGAAGGGGGGGG-----GGGGXGGCGTGCGGCGGCGGGAGGAAG-GGGGAGGGCAGATGT
                      4010      4020      4030      4040      4050      4060      4070      4080
Human        AGCCTAGTGGGAGCCGCTGGGGAAGGGGGG----------AGGGCGTGCGGCGGCGGGAGGAAG-GGGGAGGGCAGATGT  1422
PolarBear    --------------------------------------------------------------------------------  0
Cow          AGCTAAGTGGGAGCCGCGGGGGAAGGGGGG------CGGGGAGGCGTGCGGCGGCGGGAGGAAG-GGGGAGGGCAGATGT  2847
Dog          GGGGAGAGAGAGTGAGCTTTTTCTGGGGAAGGCA---ATCTTACTTGGCAGTGATATTTTCTAA-GTTGAAG-------T  3085
BelugaWhale  GGCTGAGTGGGAGCCGCGGGGGAAGGGGGGGG----CGGGGAGGCGTGCGGTGGCGGGAGGAAG-GGGGAGGGCAGATGT  2892
Donkey       AGCTGAGTGGGAGCCGC-------GGGGAG-------------GCGTGCGGCGGCGGCGGCGGG----GAGGGCAGATGT  2538
Cat          AGCTAAGTGGGAGCCGCTGGGGAAGGGGGGGGGG-CGGGGAGGGCGTGCGGCGGCGGGAGGAAG-GGGGAAGGCAGATGT  3921
Ferret       AGCTAAGTGGGAGCCGCTGGGGAAGGGGGGGGCG---GGGAGGGCGTGCGGCGGCGCGAGGAAG-GGGGATG-------T  2924
Lion         AGCTAAGTGGGAGCCGCTGGGGAAGGGGGGGGGGGCGGGGGGGGGGGGGGGGGGGGGGAGGAAG-GGGGAAGGCAGATGT  2876
Vaquita      GGCTGAGTGGGAGCCGCGGGGGAAGGGGGGGGGG--CGGGGAGGCGTGCGGTGGCGGGAGGAAG-GGGGAGGGCAGATGT  2779
Pig          GGCGAAGTGGGAGCCGCGGGCGAAGGGGGG------CGGGGAGGCGCGCGGCGGCGGCGGGAGGAGGGGAGGGCAGATGT  2773

Majority     CATACTCCTTTGTTTTCACTXGAGTCTGGTAGTGGGGGCGGGAGGCTG-AAAAAATGCCTTTTT-GTGGGGCCTAAGAAC
                      4090      4100      4110      4120      4130      4140      4150      4160
Human        CATACTCCTTTGTTTTCATTTGAGTCTGGTAGTGGGGGCGGGAGGGAGGAAAAAATGCCTTTTT-GTGGGGACTGAAAAC  1501
PolarBear    --------------------------------------------------------------------------------  0
Cow          CACACTCTTTTGTTTTCACTGGAGTCTGCTAGTGGGGGCGGGAGGCTG-AAAAA-TGCCTTTCT-GTGCGGCCTAAGAAC  2924
Dog          AGT--TGCAGGGTTTGAAATTGGGAATAGTT-CATGAGTGCAGGATAT-AGAATGTAAAATTACCATTGAAATAGATATC  3161
BelugaWhale  CACACTCCTTTGTTTTCACTGGAGTCCGCTAGTGGGGGCGGGAGGCTGAAAAAAACGCCTTTTT-GTGTGGCCTAAGATC  2971
Donkey       CATACTCCTTTGTTTTCGCTGGAGTCTGGGAGTGGGGGCGGGAGGCGG-AAAAAATGCCTTTTT-GTGGGGCCTAAGAAC  2616
Cat          CATACTCCTTTGTTTTCACTTGAGTCTGGTAGTGGGGGCGGGAGACCG-AAAAAAA-TGCCTTTTTGTGGGGGCCTAAAC  3999
Ferret       CATACTCCTTTGTTTTCACTTGAGTCTGGTAGTGGGGGCGGGAGACAG-AAAAAATGCCTTTTTGTGGGGGCCTAAGAAC  3003
Lion         CATACTCCTTTGTTTTCACTTGAGTCTGGTAGTGGGGGCGGGAGACCG-AAAAAAAATGCCTTTTTGTGGGGGCCTAAAC  2955
Vaquita      CACACTCCTTTGTTTTCACTGGAGTCCGCTAGTGGGGGCGGGAGGCTGAAAAAA-CGCCTTTTT-GTGTGGCCTAAGATC  2857
Pig          CACACTCCTTTGTTTTCACTGGCGGCGGCTGGTGGGGGCGGGAGGCTGCAAAAA-GGCCTTTCTTGTGGGGCCTGCGAGC  2852
```

```
Majority     GTTCAAGGCTTGGCAAAAGGGCCCAGAAATTAAATCATCT--AAAAAACGAAGTGTTTGTGGTCGACATCAGTCACATGG
                               4170      4180      4190      4200      4210      4220      4230      4240
Human        ATTCAAGGCTTGGCAAAAGGGCCCAGAAATTAAATCATCT--AAAAAACGAAGTGTTTGAAGCCGACATCAGTCACATGG 1579
PolarBear    -------------------------------------------------------------------------------- 0
Cow          GTTGAAGGCTTGGCAGAAGGGCCCAGAAATTAAATCATCT-CAAAAAACGAAGTGTTTGTGGTGGACATCAGTCACATGG 3003
Dog          CTCTGTAATTTTGTATAAATGAATGAATGATGTAAGTATGG-AGAGAACGAACTGATAAGAGCCACCAGGAGTAGGCTGA 3240
BelugaWhale  GTTCCAAGCTTGGCAGAAGGGCCCAGCAATTAAATCATCT--CAAAAACGAAGTGTTTGTGGTCGACATCAGTCACATGG 3049
Donkey       ATTGGAGGCTTGGCAAGAGGGCCCAGAAATTAAATCATCT--CAGAAACGGAGTGTTTGTGGTCGGCATCAGTCACATGG 2694
Cat          GTTCAAGGCTTGGCAAAAGGGCCCAGAAATTAAATCATCTG-AAAAACCCAAGTGTTTGTGGTCGGCATCAGTCACATGG 4078
Ferret       ATTCAAGGCTTGGCAAAAGGGCCCAGAAATTAAATTATCTT-AAAAAATCAAGTGTTTGTGGTCGACATCAGTCACATGG 3082
Lion         GTTCAAGGCTTGGCAAAAGGGCCCAGAAATTAAATCATCTG-AAAAACCCAAGTGTTTGTGGTCGGCATCAGTCACATGG 3034
Vaquita      GTTCCAAGCTTGGCAGAAGGGCCCAGCAATTAAATCATCT--CAAAAACGAAGTGTTTGTGGTCGACATCAGTCACATGG 2935
Pig          CTGGGCGGCTTGGCAGAGGGGCCGCGGAATTCAGTCATCTGCACAAAACGAAGCGTTTGTGGTCGACATCAGTCACATGG 2932

Majority     GCAAAGGCCAACAGCCATGGCAGAAACAAGAGCAGATA-GTCAACAC-AGGGCCATCTTTTXAGCTACTTTGACCAGTTT
                               4250      4260      4270      4280      4290      4300      4310      4320
Human        GCAAAGGCCAACAGCAATGGCAGAAACAAGAGCAGATA-GTCAACAA-AGGGCTATCTCTTTAGCTACTTTGGTCAGTTT 1657
PolarBear    -------------------------------------------------------------------------------- 0
Cow          GCAAAGGCTAAGAGCTGTGGCAGAAAGAAGAGCAGATA-GTCAACAC-AGGGCCATCTTTTCAGCTACTTTGACCAGTTT 3081
Dog          CAGAAATGAGACAGAGAATCAGATGTCCAGTTATGGTTTATTGGTTT-AACAGTTTTCTATATAT-TAGTAGAGGAGA-- 3316
BelugaWhale  GCAAAGGCCAAGAGCCATGGCAGAAACAAGAGTAGATA-GTCAACAC-AGGGCCATCTTTTCAGCTACTTTGACCAGTTT 3127
Donkey       GCAGAGGCCAAAAGCCATGGCAGAGCCCAGAGCAGACA-GTCAACAC-AAGGCCATCTTTTTAGCTCCTTTGACCAGTTT 2772
Cat          GCAAAGGCCAACAGCCATGGCAGAAACAAGAGCAGATA-GTCAACAC-AGGGCCATTTTTTTTT--AGCCACTTTGAC-- 4152
Ferret       GCAAAGGCCAACAGCCACGGCAGAAACAAGAGCAGAGAAGTCAACAC-AGGGCCATTTTTTGGTC-ACTTTGACCAGTTT 3160
Lion         GCAAAGGCCAACAGCCATGGCAGAAACAAGAGCAGATA-GTCAACAC-AGGGCCATTTTTTTTTT-AGCCACTTTGAC-- 3109
Vaquita      GCAAAGGCCAAGAGCCATGGCAGAAACAAGAGTAGATA-GTCAACAC-AGGGCCATCTTTTCAGCTACTTTGACCAGTTT 3013
Pig          GCCGAGGCCACCAGCCATGGCAGAAACCAGAGCAGATA-GGCAGCACGAGGGCCGTCTCCTCGGCTACTTTGACCAGTTT 3011
```

```
Majority     CTGGAAAAACXAAAACAACT-CAAA-AAGCCAGAACCGTGCTGCATCTTTGAG--CX--AGTGGAGATAGAATGCTGAGT
                      4330      4340      4350      4360      4370      4380      4390      4400
Human        CTGGAAAAACGACTATAA-----A--AACCCAGAACCATGCTGCATCTTTGCGAACCTGAGTCAAGACGGAATGCTGAGT  1730
PolarBear    --------------------------------------------------------------------------------  0
Cow          CGGGAAAAACAAAAACAACTCTTAAAAAGCCAGAACCAT-CTGCACCTTTGAG------GCTGGCGATAGAATGCTAAGT  3154
Dog          --AAAAAATTGGAAAACAATGTAAA-AATAATTGCCAGCCGTTTTTTTTTAAT-CCATTTTTTAATATAAAAGTTTGAAT  3392
BelugaWhale  CTGGAAAAACAAAAACAACTCCAAA-AAGCCAGAGCCGTGCTGCATCTTTGAG------GCTGGCGGTAG--TGCTAAGT  3198
Donkey       CTGGAAAAACGAAAACAGCTACCCA-AAACCAGAACCGTGTTGCATCCCTGAGAGCCCGAGTGGAAATAGAATGCTGAGT  2851
Cat          ---CGGTTTCGGGAAAAA---CAAA-AAGCCAGAACCGTGCTGCATCTTTGAGAGCCCTAGTGCAGATAGAATGCTGAGT  4225
Ferret       CCGGAAAAACAAAAACAACTACAAA-AAGCAAGACCCGTGCTGCATCTTTGATATCCCGAGTGGAGACAGAATGCTGAGG  3239
Lion         ---CGGTTTCGGGAAAAA---CAAA-AAGCCAGAACCGTGCTGCATCTTTGAGAGCCCTAGTGCAGGTAGAATGCTGAGT  3182
Vaquita      CTGGAAAAACAAAAACAACTCCCAA-AAGCCAGAGCCGTGCTGCATCTTTGAG------GCTGGCGATAG--TGCTAAGT  3084
Pig          GTGGACCCACAAAAAGCAACTCCACCAAGCCCCAGCCGTGCTGCGTCTTTGAC------AGCGGCGGTAG--TGCCCAGC  3083

Majority     GAGGTGAGG-CAAGGGCTTTGGGAAATGATACGG-ACCXGGGTCTC--CCTGGTACTTAACGCTCCACATCTGAAAAGAC
                      4410      4420      4430      4440      4450      4460      4470      4480
Human        GAGGTGAGAGGCAAGACTGTG--AAATGGTAGGG-ACCGATGTCTC------GC-TCATATGCTTCACACCTGAAAAGAC  1800
PolarBear    --------------------------------------------------------------------------------  0
Cow          GAGGTGAGG-CAAAGGCTTCGGGAAATGATACGGGACCAGGGTTTC--CCTG-CACTTAACGCTCCACACCTGAAAAGAC  3230
Dog          AACTGAAGT-TTTGTTTTGCGGACTTTTCTAAGA-TGGTGGTTAAT--AACTTTTGCTTTGGAGTCATGCCTGTGTATAA  3468
BelugaWhale  GAGGTGAGG-CAAGGGCTTCGGAAAATGTTACGG-ACCAGGGTCTCTCCCTGGTACTTAACGCTCCCCATCTGAAAAGAC  3276
Donkey       GAGGTGAGG-CGAGGGCTTAGGAAAATGGTATGG-TCCGGGGTCTC--CCTGGTGCTCAACGCTCCACATCTGAAAGGAC  2927
Cat          GAGGTGAGGGCAAGGGCTTTGGGAAACGACCGGG-ACCGGGGTCTC--CCTGGTACTTAATGCTCTACATCTGAAAAGAC  4302
Ferret       GACGTGAGG-CAAGAGCTTTGGGAAATGATACTG-ACCCGGGTCTC--CCTGGTACTTAATGCTCTACATCTGAAAAGAC  3315
Lion         GAGGTGAGGGCAAGGGCTTTGGGAAGCGACCAGG-ACCGGGGTCTC--CCTGGTACTTAATGCTCTACATCTGAAAAGAC  3259
Vaquita      GAGGTGAGG-CAAGGGCTTCGGAAAATGATACGG-ACCAGGGTCTCTCCCTGGTACTTAACGCTCCCCATCTGAAAAGAC  3162
Pig          GAGGTGAGG-CCAGGGCTTTGGGCAGTGATGCGG-ACCAGGCTCTC--CGTGGTGCTTAACGCTCCACACCTGAAAAGTC  3159
```

```
Majority     TCTT-AATAATTTAACTCATTGTTAGAAAGGAGGCACCCTT-TAGGGCGAAAATAXAGCTGTATTCAGCCCGTTTGG---
                     4490      4500      4510      4520      4530      4540      4550      4560
Human        TCA---GTAATTTAACTCAATGTTAGAAAGGGGGCACGCTT-AAGGTCAAAAATACAGCAGTATGCAGCTCTTTTGG---  1873
PolarBear    --------------------------------------------------------------------------------  0
Cow          TCT--AATAATTTAACTCATTGTT--AAAGGAGGCACCCTT-TAGGGCGAAA-TGTAGCCGTATTCAGCCCGTTTGG---  3301
Dog          GTAA-TTAAGTTTTCATATGTATATATATTCTTTTAATGTG-CCTGGTTAAAATTCATAGTGGTAAGTATTTCTCAC---  3543
BelugaWhale  TCTTTAATAATTTAACTCATTGTTAGAAAGGAGGCACCCTT-TAGGGTGAAAAAATAGCCGTATTCATCCCGTTTGG---  3352
Donkey       TAA---ATAATTTAACTCATTGTTAGGAAGGAGGCACCCTT-TAGGCCGAAAATGCAGCTGTATTAAGCCCATTTGGCAG  3003
Cat          TCTT-AATGATTT----AATTGTTGGAAAGGAGGCACCCTT-GAGGGCGAAAAGAGAGCTGTATTGGGCCCATTTGG---  4373
Ferret       TCAA-AGTGATTTAGCTAATTGTTAGAAAAGAGGCACCCTT-GAGGGCGAAAATATAGCTCTATTCAGCCCTTCTGG---  3390
Lion         TCTT-AATGATTT----AATTGTTGGAAAGGAGGCACCCTT-GAGGGCGAAAAGAGAGCTGTATTGGGCCCATTTGG---  3330
Vaquita      TCTTTAATAATTTAACTCATTGTTAGAAAGGAGGCACCCTT-TAGGGTGAAAATATAGCCGTATTCATCCCGTTTGG---  3238
Pig          CCTT-AATAATTAAACTCGTTAG---GAAGGAGGCACCCCTGTAGGGCGAAAGTACAGCTGTAGCCAACTCGTT------  3229

Majority     GGGTATA--AAAACAGTATCATTTAAAAAGTACATTTATTAGAATCAGTGACA-GTATTTGAAGAGTCA--AGTAAAATA
                     4570      4580      4590      4600      4610      4620      4630      4640
Human        GAGTGTA--AACATAGTATCATTTAACAAGTACATTTATTAAAATCAGTGA---GTATTTGAAGAATCA-----ACAATT  1943
PolarBear    --------------------------------------------------------------------------------  0
Cow          GAGCATAGGAAAACAGTATCT-------------TTTATTGGAATCAGTGACT-GTATTTGAAGGGTCAGAAGTAAAATA  3367
Dog          TGAGTCCC-AGTCCTGTTTCTCAGGTGAGGTAATTACGTTAAATTTTTTCTGGTGCATTTTTTTCCTGA--AGTGTTTTT  3620
BelugaWhale  GGGCATAGGAAAAGAGTATCACTTAAAAAATACGTTTATTGGAATCATTGACA-GTATTTGAAGGGTCAGAAGTAAAATG  3431
Donkey       GGGGCTATAAACACAGTATCGTTTAAAAAGTACATGTATTAGAATCGGTGACA-GTGTTTGAAGAGTCA--AGTAAAATG  3080
Cat          GGGTAT---AAAAACGAATCATTTAAAAAGTACATCTATTAGAATCAGTGGCA-GTATATGAAGAGTCC--TGTAAGATA  4447
Ferret       GGGTTGTA-AAAACCGTATCATTTAAAGAGCGTATTTATTAGAATCAGTGACA-ACATACGAAGAGCCA--AATAAAATT  3466
Lion         GGGTAT---AAAAACGAATCATTTAAAAAGTACATCTAGTAGAATCAGTGGTA-GTATATGAAGAGTCC--TGTAAAATA  3404
Vaquita      GGGCATAGGAAAACAGTATCACTTAAAAAGTACGTTTATTGGAATCCTTGACA-GTATTTGAAGGGTCAGAAGTAAAATG  3317
Pig          ------GC---AACAAGTCCA-------------TTTATTGGGCTCGTTGGCA-GTGCTTGAAGGGTCAGGAGTAAAATA  3286
```

```
Majority     TGAGTTTATTTTAA----GCGTCTGGAGTTTGGTGT-TTTTAAAAGATATTAAGTGXA-GATCATATAAAAGTTA--ACA
                       4650      4660      4670      4680      4690      4700      4710      4720
Human        TGGTTAAGC-----------GCCTAGAGTTTGGTGTGTTTTAAAAGGTACTAAAAACAAGATTTTATAAAAGTTAACACA  2012
PolarBear    --------------------------------------------------------------------------------  0
Cow          CGAGGTTTTTTTAA----GCATCTGGATTTCGATGT-TTTTTAAGGATATCAAGTGCA-GATCTAGTAAAAGTTA--ACA  3439
Dog          TAGGTTTGTAT-------ATTATCAAATATTAGCAT-CCTTGCTTATTTACACAAATG-GGTTTTCCACAAAT----ACC  3687
BelugaWhale  TGAGTTTATTTTAA----TCATCTGGAGTTCGGTAT-TTTGTGAAGAAACTAAGTGCA-GGTCACATAAAAGTTA--ACA  3503
Donkey       AGAGTTTATTTTCACTAAGCGTCTGAAGTTTGGTGT-TTTTAAAAGACATTGAGTGAA-GACCATGTAAAAGTTA--CCA  3156
Cat          TGAGTTTATTTTAATTAAGTGTTTGGAGTTTGGTGT-TTTTAAAAGATATTAAGGGAA-GACCATATCAAAGTGA--ACA  4523
Ferret       TATTTTGAT----------TGTTTGGAGTTTAGTGT-TTTTAAAAGATATTATGGATA-GACCATATCAAAGT----TAA  3530
Lion         TGAGTTTATTTTAATTAAGTGTTTGGAGTTTGGTAT-TTTTAAAAGATATTAAGGGAA-GACCATATCAAAGTGA--ACA  3480
Vaquita      TGAGTTTATTTTAA----TCATCTGGAGTTCGGTAT-TTTGTGAAGAAACTAAGTGCA-GGTCACATAAAAGTTA--ACA  3389
Pig          CGAGTTTATTTTAA----GCGCCTGGGGTTGGGTGT-TTTTTAAAGAGAGTAACTGAA-GATCCTGTAAAAGTTA--GCA  3358

Majority     GAGTAACAAAAXTTAAATCATCCTAGAGGTTATCTAGAGATTATTTTCCAGATGAAAC-T-------AAATCCAGAGAAG
                       4730      4740      4750      4760      4770      4780      4790      4800
Human        GTCTCACAGAATTCAAATCATCCTAGATGTTACCTAAAGATTATTTTCCAGATGAAAC---------AAATCCAGAGAAG  2083
PolarBear    --------------------------------------------------------------------------------  0
Cow          GACTAACGAAATTTGACTCATCCTAGGTGTTCTCTAAAGATTATTTTCTAGATGAAAC---------AAATCCAGAT---  3507
Dog          ATGTGTACTGGCTCACTACTTGCCTTTTTTTAAAAAGATAGCTTTATTGAGAGAAAATTT-------GACATAAAATAAA  3760
BelugaWhale  GATTAACAAAATTTGAATCTTCCTAGAGGTTCTCTGGAGATTATTTTCCAGATGAAAC---------AAATCCAGAGAAG  3574
Donkey       G--TAACAGAACTTAAATCATCCTAGTGCTTATCTAGAGTTTATTTTCTAGAAAACAGATCATAGATAGATCTAGAGAAG  3234
Cat          AAGTCACCAAACTTAAGTTGTCCGAGAGGTTATCTAGAGGTAATTTTCCACATGAAATCT-------AGATCTAAAGAAG  4596
Ferret       CAGTAACAAAACTTACATTATCCTAGAGGTTATCTAGAGATTGTTTTCCACATGAAATGT-------AAATCTAGAGAAG  3603
Lion         AAGTAACCAAACTTAAGTTGTCCGAGAGGTTATCTAGAGATAATTTTCCACATGAAATCT-------AGATCTAAAGAAG  3553
Vaquita      GATTAACAAAATTTGAATCTTCCTAGAGGTTCTCTGGAGATTATTTTCCAGATGAAAC---------AAATCCAGAGAAG  3460
Pig          GACTAACAAAATTTAAATCACCCTCGAGATGCTCTAGAGAGCATTCTCCAGATGAAACCT-------AAGTCCAGAGAAA  3431
```

```
Majority     TTCACTATTT-CCCCAGATCAACATGGAAATTGATGGCAAAACT--TGCACTGGAATT---CATGTTTCCTGATTC----
                     4810      4820      4830      4840      4850      4860      4870      4880
Human        TTCAGTATTCCCCCCAGATCAACACGGAAATTGATGTCAAAACT--TGCACTAGAATT---CATGTTTCCTGATTCCTGG  2158
PolarBear    --------------------------------------------------------------------------------  0
Cow          TTCACTGTTTTCCCCAGATCAGCATGGAAATAGATGGCAAAATT--TGTATTGAATTT---CATGTTTCCTCATCC----  3578
Dog          CTGCACTTAT-TATTGGACATATGAATACACCTATGAGATCATTACCATAATGAAGAT---CATGT---CTATCAC----  3829
BelugaWhale  TTCACTCTTTCCCCCAGATCAATATGGAAATAGATGGCAAAACT--TGCCCTGGAATT---CATGTTTCCTGTTTC----  3645
Donkey       TTCAGTATTC--CCCAGATCAACATGAAAATTGATGGCAAAACT--TGCACTGGAATT---CACGTTCCGAGATTC----  3303
Cat          TTTGCTATTT-CCCCAGGTTAACATGAAAATTGACGGCAAAAGT-TGGCACTGGAATT---CATGTTTTCTGATTC----  4667
Ferret       TTCACTATTT-CACCAGATAAACATGGAAATTGATGACAGAACT-TAGCAGTGGAATT---GTTTC---CTGATTC----  3671
Lion         TTTGCTATTT-CCCCAGGTTAAAATGAAAATTGACGGCAAAAGT-TGGCACTGGAATT---CATGTTTTCTGATTC----  3624
Vaquita      TTCACTCTTTCCCCCAGATCAATATGGAAATAGATGGCAAAACT--TGCCCTGGAATT---CATGATTCCTGTTTC----  3531
Pig          TTCGCTATTG-CCCCAGCTCAACATGGAAACGGATGGTAAAACT--TGCTCTGGAATTACTCATGTTTCCTGATTC----  3504

Majority     ----TATCAAGATTACTAAAGAGTTGXTTTAAGTTTTAAATTATTAATCTAAGTGTACTGGCTTTG-T--AAATGGCATT
                     4890      4900      4910      4920      4930      4940      4950      4960
Human        TTCCTGTGGAGACTACTAAAGAGTT----TAAGCGTTAAATTATTAATCTCAGTATACTGGCATTG-TAGAAATGGCAT-  2232
PolarBear    --------------------------------------------------------------------------------  0
Cow          ----TATTAAGATTACTAAAGAGTTGAATTAAGTCTTAAATTATTAACCTAAGTGTAGCGGG--------ACATTATGT-  3645
Dog          ----CCCCAGAAGTA---------TCATTTTGCCCCTTGGTAATCCCTTCCTCCTGTCTGTCTTTC-CCCAAACAACAAT  3895
BelugaWhale  ----TATCAAGATTACTAAAGAGTT-----AAGTTTTAAATCA-------AAGGGTAAAGGG-------------GTATT  3696
Donkey       ----TATCAAGGTGACTAAAGAGTTGATTCAAATTTTAAATTACTAATCTAAGTGTACCAGTTTTG-TAAAAGTGACATT  3378
Cat          ----TGTCAGGATAACTAAAGAATTGTTTTAAGTTTTAAACTATTAATCTAAGTATACTAGCTTTG-TGGAAATGGCATT  4742
Ferret       ----TATCAAGATAAAGA----GTTGGTTTAAGTTTTAAATTAATAATTTAGGTGTACAAGCCTTG-AGTAAATGACATT  3742
Lion         ----TGTCAGGATAACTA---AATTGTTTTAAGTTTTAAACTATTAATCTAAGTATACTAGCTTTG-TGGAAATGGCATT  3696
Vaquita      ----TATCAAGATTACTAAAGAGTT-----AAGTTTTAAATCA-------AAGGGTAAAGGG-------------GTATT  3582
Pig          ----TATCAGAATTACTGAAGAGTTGAATTAAGTTTTAAAGTACTAATCTAAGTGTAACTAGTTTGGTAAAAAGGGGGTT  3580
```

```
Majority     ATAGTC-TTTGGGGTTTTGTTCTTTTTG-CTATTTAAAGTGTT-TTAAAAA--ATACCAXAAGTTTAGT-GAXTTTCATC
                               4970      4980      4990      5000      5010      5020      5030      5040
Human        --AGTGTTTTGGG-TTTTGTTCTTTTTGGCTATTGAATGTGTTGTTAAAAA--ATACCACAA-----G-------TTGTC  2295
PolarBear    --------------------------------------------------------------------------------  0
Cow          --AGGC-TTTGGGGTTTTGTTCTTTTTG-CTATTTGAAAT----TAAAAAA--AAAACACCGAAACTGT-GAGTTTCCTC  3714
Dog          TGATCC-TCT----CTCTGTCACTGTAG-GGTCAGTTTGCATTTTTTAGAA--TTTTAAGGGAGTCAGG--ATGTACCTC  3965
BelugaWhale  ATAGGC-TTTGGGGGCTTGTTCAGTTTG-CTATTTGAAGTG---TTAAAAA--ATACCAAAAGTGTGGT-GAGTTTCATC  3768
Donkey       GTAGTG-TTTGGGGTTTTGTTCTTTTTG-CTATTTAAAGTGTTAAAAAATACCATACCATAAGTTTAGT-GAATTTCATC  3455
Cat          ATAGTTTCTTGGGGTTTTGTTCTTTTTG-GTATTTAATAGGTTATTAAAAA--ATACCAGGAGTTTAGT--GATTTCATC  4817
Ferret       CTAGTG-TTTGGGGTTTTGTTCTTTTTG-ATATTTAAAGGGTTGTTTAAAA--AATACAGGAAGTTTAGTGAATTTCATC  3818
Lion         ATAGTT-CTTGGGGTTTTGTTCTTTTTG-ATATTTAATAGGTTATTAAAAA--ATACCATGAGTTTAGT--GATTTCATC  3770
Vaquita      ATAGGT-TTTGGGGGCTTGTTCAGTTTG-CTATTTGAAGTG---TTAAAAA--ATACAAAAAGTGTGGT-GAGTTTCATC  3654
Pig          AGAGGC-TTCGGGAGTTTGCTCTTTTTG-CTGTTTAAAGTG---TTGACAA--ATAGCAAAAGTGTAGT-GAGTTTCCTC  3652

Majority     TCACGTTTGAAAGTXATTTCTAGAAAACCAAGTTGATAATAGTACACXG-AACXATCAXTGA-----------T-TGTTT
                               5050      5060      5070      5080      5090      5100      5110      5120
Human        TCACATTTGAAAGTTACTTTTAGAAAACCAGGTTGGTAACAGTACAGGTAAGTCACGGATCATCTGTGGTATTTTTTATC  2375
PolarBear    --------------------------------------------------------------------------------  0
Cow          ACATGTTTGAAAGTCATTTCTAGAAAGCCAAGTTGATAATGTTCCACCG--ACCATCTTTGT----------GTGTGTTT  3782
Dog          TTTGGTTTTATTTCACTTACTCTAATTATT--TTGAGATTTACTCATGTTATCTATCAATAG-----------TCTATTT  4032
BelugaWhale  ACACCTTTGAAAGTCATTTCTAGAAAGCCAAGTTGATTATGTGCCACTG--ACCATCTTT----------------GTTT  3830
Donkey       TCACGTTTGAAA---ATTTCAAGAAAACTAAGTTGATAATAGTGCAAAG-AAGTGTCACTGACCATCTGTGTATTTGTTT  3531
Cat          TTAAGTTTGAAAGCAATTTTTAGAAAACTGAGTTTAAAATAGTACAAAG-AATTGTCACTGACCATCTGTGTATCTGTTT  4896
Ferret       TCAAGTTTGAAAGTAATTTCTAGAAAACAG--TTGAAAATAGTACAGGG-AGGTGTCACTGA-----------CTTGTTT  3884
Lion         TTAAGTTTGAAAGCAGTTTTTAGAAAACTGAGTTTAAAATAGTACAAAG-AATTGTCACTGACCATCTGTGTATCTGTTT  3849
Vaquita      ACACCTTTGAAAGTCATTTCTAGAAAGCCAAGTTGATTATGTGCCACTG--ACCATCTTT----------------GTTT  3716
Pig          TCACGTTTGAAAGTCATTTCTGGAAAGCCAGGTTGGTAACACATCACTG--ACCATCTTTTT----------GT-TGTTT  3719
```

```
Majority     TCCATCTGCATX---GTGAGTGGAAGATTTATGAATGATGAACTACCCT-AAAAAGTT-----TCAAACACTGAC-CATT
                                 5130      5140      5150      5160      5170      5180      5190      5200
Human        CCCATCTGTGTGTGAGTGGCTGGAAGATTTATGAATGATGGACTATCCTAAAAAGTTTTAGAGCCAAAGAGTGAGTGATT  2455
PolarBear    --------------------------------------------------------------------------------  0
Cow          TCCATCTGCATT---TTGAGTGGAAGATTTATGATTGATGAACTACCCT-AAAAAGTT-----TCTAACAGTGAC-CATT  3852
Dog          TTT-CTTGCTTA---GTAGTATTACATTTTATGGATATACAGCAATTTG-TTTATCCA-----TTCAACTCTTGG-GTTG  4101
BelugaWhale  TCTGTCTGCGTT---GTGAGTGGAAAAATTCATGAATGATGAACTACCCT-AAAAAGTT-----TCAAACAATGAC-CATT  3900
Donkey       TGCATCTGCATT---GTGAGTGGAAGGTTTATGAATGATGAACCACTCTTAAAAAGTTGAG--TCAAACAGTGAC-CATT  3605
Cat          TCCATTTGCATG---GTTAGTGGAAGATTTATGAATGGTTAACTACCC---TAAAGTT-----TTAGAGACCAAC-AGTG  4964
Ferret       TCCATTTGCATG---ATGAGTGGATGATTTATGAATGATGAACCACCCT-AAAAAGTT-----TTAGAGTCCAAC-AGTG  3954
Lion         TCCATTTGCATG---GTTAGTGGAAGATTTATGAATGGTGAACTACCC---TAAAGTT-----TTAGAGACCAAC-AGTG  3917
Vaquita      TCTGTCTGTGTT---GTGAGTAGAAAATTCATGAATGATGAACTACCCT-AAAAAGTT-----TCAAACAATGAC-CATT  3786
Pig          TCCATCTGCATC---GTGAGTGCAAGGTTTATGAATGATGACCCACCCT-AAAAAGTT-----TCTTTTGAGGA------  3784

Majority     AG---AATAACAAGAGGTTGAAACTTATTCTTTTTTTTTTT-----ACAGAATTGTCACTGAGGTTGCAGTTGGCGTGAA
                                 5210      5220      5230      5240      5250      5260      5270      5280
Human        AGAAGAATAACAGGAGGTTGAAACTACTTATTTTTTCTTT------GCAGAATCGTCACTGAGGTTGCAGTTGGCGTGAA  2529
PolarBear    --------------------------------------------------------------------------------  0
Cow          TG---AATAACTAGAGGTTGAAGCTTTTTGTTTGTTTGTTTT----ACAGAATTGTCACTGATGTTGCAGTTGGCGTAAA  3925
Dog          TTTCCAGTTTTTGACTATTGTAAATAAAGCTACTGTGAACA--TCCTTGTAAAAGTCTTTGTGTGAACTTTTCATTTCTC  4179
BelugaWhale  TG---AAGAACAAGAGGTTGAAACTTGTTTTTTTTCATT-------ACAGAATTGTCACTGAAGTTGCAGTTGGCGTGAA  3970
Donkey       AGAAGAATAACAAGGAGTTGAAACTAACTGTTTTTTTCTTT-----ACAGAATTGTCACTGAGGTTGCAGTTGGCGTGAA  3680
Cat          AT--CATTAAAAGAATAA-GAGGTTGAAACTATTTTTTTTT-CTTTACAGAATTGTCACTGAGGTTGCAGTTGGCGTCAA  5040
Ferret       AT--CATTAGAAAAATAATGAGGTTGAAACTATTTTTTTTC--TTCACAGAATTGTCACTGAGGTTGCAGTTGGCGTGAA  4030
Lion         AT--CATTAAAAGAATAA-GAGGTTGAAACTATTTTTTTTTTCTTTACAGAATTGTCACTGAGGTTGCAGTTGGCGTCAA  3994
Vaquita      TG---AAGAACAAGAGGTTGAAATTTGTTTTTTTTTATT-------ACAGAATTGTCACTGAAGTTGCAGTTGGCGTGAA  3856
Pig          ------ATAACAAGAGGTTGAGACTTGTTTCTTTTTTTTTTTCATTGCAGAATTGTTACTGAGGTTGCAGTTGGCGTGAA  3858
```

```
Majority     GGTAGGAAAAAATTGTTT-XAACTAATGAAAACTGGATTGCTGAATCTACTXT---TATTATCAA---X--TGGAAAXTT
                                 5290      5300      5310      5320      5330      5340      5350      5360
Human        GGTAGGAAAATACTAT-------GTTTGAAACTGGGATTGTTGGATCTATTAT---CATAATTAC------TGGAAAATT  2593
PolarBear    --------------------------------------------------------------------------------  0
Cow          GGTAGGAAAACAGTGTTTAAAACTAAGGAAAGCTGGGTTCCTCGATCTACTGAGTGTATTATCATTA---AAGGAAACTT  4002
Dog          TTGGGGAAATACCTTT----GATTGG-AATGACTGGATCGTTTGGTATGCATA---TGCTATTAACTTT--TTTAAAGTT  4249
BelugaWhale  GGTAGGAAAAAA--GTTTAAAACTAATGAAAACTGGATTCCTCAGTCAACTGAGTATATTATCATCATTAGTGGAAACTT  4048
Donkey       GGTAGGACAAGATTGA----AACTAATGAAAACTGGATTACTGCATCTGCTAT---TATCATTAA------TGGAAATTT  3747
Cat          GGTAGGAAAAAATTGTTTGCAACTAATGAAAACAGAATGGCTGAATCTACTAT---TACCATCAA---T--GGAAATTTT  5112
Ferret       GGTAGGAAAAAATTGA----AACTAATGAAAACTGAATTGCTGAATCTGTTGT---TACCATTGA------TGGAAATTT  4097
Lion         GGTAGGAAAAAATTGTTTGAAACTAATGAAAACAGAATGGCTGAATCTACTAT---TACCATCAA---C--GGAAATTTT  4066
Vaquita      GGTAGGAAAAAA--GTTTAAAACTAATGAAAACTGGATTCCTCAGTCAACTGAGCATATTATCATCATTAGTGGAAACTT  3934
Pig          GGTAGGAAAAGATTGCTTAAACCTAATGAAAACTGGATTCCTCAATCTGCTTTATGTATTATCATCATTGATAGAAGCTT  3938

Majority     TCAG--TTAGCTTTTGAAAACTATAATXXA------CAAAACXCAAGTGTAAAGTGTTTXXTTTAATTTTXAAAG--TTG
                                 5370      5380      5390      5400      5410      5420      5430      5440
Human        TTAGCTTTTGCTTTTGAAAACTATAACGTAGCT-AACAAAACCCAAGTGTAAAATGTTTCCTTTCATTTGTAAAG--TTG  2670
PolarBear    --------------------------------------------------------------------------------  0
Cow          TCTG--TTGACTTCTGAAAACTATAATATAGCTTACAAAAATTCCAGTGGAAAGTACTT-GTTGAATTTTTAACA--TTG  4077
Dog          TTGA--GAAACTCCAGAC-CATATT-CCAA------AATGG-----TTGTATTATTTTACATTGCTACCAAAAGGGTATG  4314
BelugaWhale  TCTG--TTGGCTTTTGAAAACTGTAATACAGCTT-ACAAAATTCAAGTGTAAAGTGTTTTGTTCAATTTTTAAAG--TTG  4123
Donkey       TCAC--TTAGCGTTTGAAAACTATAATATAGCT-AACAAAACCTAAATGTAAAGTGTTTTATTTAATTTTAAAAA--TTG  3822
Cat          TAAG--TTAGCTTTTGAAAGTTAGAGTTAA------CAAAACCCAAGTGTAAAGTGTTTCATTTAATTTTAAAAG--TTG  5182
Ferret       TCAG--TTAGCTTTTGAA-GTTACAGTTAA------CAAAA-----GTGTAAAGTGCTTTGTTGAATTTTAAAAAAGTTG  4163
Lion         TAAG--TTAGCTTTTGAAAGTTAGAGTTAA------CAAAACCCAAGTGTAAAGTGTTTCATTTAATTTTAAAAG--TTG  4136
Vaquita      TCTG--TTGGCTTTTGAAAACTATAATACAGCTT-ACAAAATTCAAGTGTAAAGTGTTTTGTTCAGTTTTTAAAG--TTG  4009
Pig          TCTG--TTGACTTTTAAAAACTGTAATTT------TCAAAACTCAAGTGCAGAGTATT---T----TTTCTAAAG--TTG  4001
```

```
Majority     GATTATATAAAT---TTTAATCTAAATAGACAACXX-XATAAGTTTTTCTAGAAAGG-CAGTCTGTGAGAATAATATTAX
                      5450      5460      5470      5480      5490      5500      5510      5520
Human        GACCATATAAAC------------AGTTTGTAATCACCATAAGTTTTTCTAGAACTGTCAGTCTGTAAGAATAACCTTTT  2738
PolarBear    --------------------------------------------------------------------------------  0
Cow          GTTTATATAAATAGTTTTAACCTAAATAGACAAC---CATAAATTTTTCTAGAAAAG-CAGTCTCTGAGAATAATATTTT  4153
Dog          AGATGTCCAGTC---CTTCCACATCCTCTCCAATCC--TTGAGATTGCCTTTGGCTG-TAGGCATTCTCGTAGGTGTGAA  4388
BelugaWhale  GTTTATATAAATAGTTGTAATCTAAATAGACAACAACCATAAATTTTTTTAGAAAGG-CAGTCTGTGAGAATAACATTTT  4202
Donkey       GATTATAAAAAT------AGTTTCACTAGACAACCACCATAAGTTTTTCTGGAAAGG-CAGTCTGTGAGAACATTTTAAA  3895
Cat          GATTATATAATT---TACAATCTAAATAGACAAAA---CTAAGTTTTTCTAGAA-----AGTCTGTGAGAACATTCTAAA  5251
Ferret       GGTTATATAGTT---TATAATCTAAATAGACAGAAA--CTTAGTTTTTCTAGAAAAG-CAGTCTGTGAGAACATTTTAAA  4237
Lion         GATTATATAATT---TATAATCTAAATAGACAAAA---CTAAGTTTTTCTAGAA-----AGTTTGTGAGAACATTCTAAA  4205
Vaquita      GTTTATATAAATAGTTTAATCTAAATAGACAACCACCATAAATTTTTTTAGAAAGG-CAGTCTGTGAGAATAACATTTT  4088
Pig          GCTCGTGTGAATAGTTTTAGCCTAAACAGACGAC---ATTCATACTTTTTCGAGAGG-CAGTTGGAGAGAGTAACATTCT  4077

Majority     AAA--XGGTATGAATGGTGTTCAAGGAATTGAAAATGC--TTTCAGACATGGCTTCTTGTGAATATA-AAATGATA---T
                      5530      5540      5550      5560      5570      5580      5590      5600
Human        AAAGTAAGTATGA---ATGATCAAGAAATTGAAAATGC--TTTCACACATGGCTTCTTGT-AATATA-AAACGATA---C  2808
PolarBear    --------------------------------------------------------------------------------  0
Cow          AAA----GTATGAATGGTGTTCAAGGAATTGAAAAAGC--TTATA--------TTCTTGTAAATATA-AAACGATAA---  4215
Dog          GTGG-TAATCTCC-TGTGGTTTTAAT--TTGCATTTCT--TAT---GTCTGTGATGTTAATTATCTTTTCATGTTC---T  4456
BelugaWhale  AAAATAGTTATGAATGGTGTTCAAGGAATTTAAAAAGC--TTTCAGATATGGCTTCTTGTGAATAGA-AAATAA------  4273
Donkey       CTA---GGTATGCATGGTACTCAGGGAATTGGAAATGC--TTTCAGACATGACTTCTTGTGAATATA-AAATGATA---T  3966
Cat          CTA---GGTATGAATGGTGTTCAGGGAATGAAAAATGC--TTTCAGACATGACTT---GTGAATATAAAAATGATA---T  5320
Ferret       AAGT-AGTTATGAATGCTGTTTAGGG--TTGAAAACCC--TATCAGACATGGCTTTTTATGAATATATAAATGATA---T  4309
Lion         GTA---GGTATGAATGGTGTTCAGGGAATTAAAAATGC--TTTCAGACATGACTTTTTGTGAATATAAAAATGATA---T  4277
Vaquita      AAAATAGTTATGAATGGTGTTCAAGGAATTTAAAAAGC--TTTCAGACATGGCTTCTTGTGAATAGA-AAATAA------  4159
Pig          AAACCAGGTATGAATGGTGGTCAAGGAATTAAAAAAACACTTTCAGACATGGCTTCTTATGAATATA-AAACGATACAGT  4156
```

```
Majority     CCCAATTTTCTGAGG-GGAAATTATGGTTTTAAATGGACAGATAA-GTGATTTGACCTAAATCATATAATTTTATTAATC
                              5610      5620      5630      5640      5650      5660      5670      5680
Human        CCCAATTTTCTGAGG-GGAAATAATGATTTTAAATGGATGG-TAA-GTGGTTTGACCTATATCTTATAATTTTGTTAATC  2885
PolarBear    --------------------------------------------------------------------------------  0
Cow          CCCAATTTTCTGAGG-GGAACTTATGGTTCTAAATGGACAGATAAAGTGATTTGACCTAAATGATATAATTATAGTAATA  4294
Dog          TTTTGCCAGCTGTAT-ATCATCTTTGATGAAGTGTGTCTATATCT-CTTGCCCACTTTTTAAATTGGATTACTGTTGAGT  4534
BelugaWhale  CCCAATTTTCTGAGA-GGATATGATGGTTTTAAATGGACAGATAA-GTGATTTGACCTAAATCATATAATTTTAGTAATC  4351
Donkey       CCCAGTTTTCTGAAGCGGAAATTATGGCTTTAAATGGCCAGATAAAGTGATTTGACCTAAATTATGTAATTTTGTTAACC  4046
Cat          CCCAATTTTCTGAGG-GGAAGTTATGGTTTTAAGTGGGCAGATAA-GTGATTTGACATAAGTCATATAATTTTATTAATC  5398
Ferret       CCCAGTTTTCTGAGG-GAAAATTATGGTTTTAAATGGGTAGATAT-ATGATTTGACCTAAATCATGTAATTTTGTTAATC  4387
Lion         CCCAATTTTCTGAGG-GGAAGTTATGGTTTTAAGTGGGCAGATAA-GTGATTTGACATAAATCATATAATTTTATTAATC  4355
Vaquita      CCCAATTTTCTGAGA-GGATATGATGGTTTTAAATGGACAGATAA-GTGATTTGACCTAAATCATATAATTTTAGTAATC  4237
Pig          TTTTTTTTTTTGAGG-GGAAATTACGGTTTTAAGTGGACAGATAA-GTGATTTGAGCTAAATCACA--------------  4220

Majority     TAAGATTTTTTT-ACT--X--XTTTTTGTTATGAAGACTAAAATATT--TTTGTCAATAATAAGCCACATTAGAAATCAG
                              5690      5700      5710      5720      5730      5740      5750      5760
Human        TAAGTTTTTTGTTTTTTATTACTTTTTGTCATGAAAATGAATATATT--TTGGTTAATAATAAAGCACATTAGAAATGAA  2963
PolarBear    --------------------------------------------------------------------------------  0
Cow          TAAGATTTTTTTCACTC-TGCTTTTCTGTTATGAAGTCTGAGATATT--TTCACTAGTAATAAGCTTCATTAGAAATCAG  4371
Dog          CTTAAGTCTTCA----------TTCTGGTTATAAGTCTTTTATCAGT--TATATGGTTAATATTTTCTACCATTTGT-AT  4601
BelugaWhale  TAAGATTTTTTTAGCTC-TGCTTTTGTCTTACGAAGACTAAAATATT--TTCACCAATAATAAGCCTCATTAGAAATCAG  4428
Donkey       TAAGAATATTTT--TTT-CTCTTCTCTCTTATGAGGACTAAAATATTTTTTTGTTAGTAATAAGCCACATTGGAAAT-AG  4122
Cat          TAAGTGTTTTTT-AAC------TTTTGATTATGAAGACTAAAA------TGTGTCAGTAGTGAGCCACATTAGAAATCAG  5465
Ferret       TAAGATTTTTTTAAAC------TTTTCATTATGAATACTAAAATATG--TATGTCAACAATGAACTACATTAGAAATCAG  4459
Lion         TAAGTGTTTCT--AAC------TTTTGATTATGAAGACTAAAA------TGTGTCCGTAGTGAGCCACATTAGAAATCAG  4421
Vaquita      TAAGGTTTTTTTAGCTC-TGCTTTTGTCTTACGAAGACTAAAATATT--TTCACCAATAATAAGCCTCATTAGAAATCAG  4314
Pig          ----ATTTTTTCCACTC-TCTTTTTTTGTCTTACAAT--GAGAAATT--TTTGCCAATAATAAGCCTCATTAGAA-TTAA  4290
```

```
Majority     -TTACCCTGATCTTTAAAAAGAA----GAAACTTGTTTAGGCAXTTAGCTTCAAATTXGAGTCAGAAGTTA-AGAGCTGG
                               5770      5780      5790      5800      5810      5820      5830      5840
Human        -TTACACTGATCCTTAAAAAGGA-------AGTTGTTGAGGCAGTTAGCTTCAAATTTGAATTAGAAGTTA-AGAGCTGG  3034
PolarBear    --------------------------------------------------------------------------------  0
Cow          TTTACCCTGATTTTTAAAGAGAATAAAGAAACTTGTTCAGGCAGTTAGCTTCAAATTTGAGTCAGAAGTTATAGAGTTGG  4451
Dog          -TTTCATTGTCTCGATAG-------------TGTCTTTTGAAGAACAGCAGTTTTTAATTCTGAAGAACTG-AAGTTTA-  4665
BelugaWhale  -TTGCTCTGATCTTTAAAGAGAA--AAGAAACTTGTTTAGGCAGTTAGCTTCAAATTTGAGTCAGAAGTTATAAAGCTGG  4505
Donkey       --TTACCTGACTCTTAAAAAGAA----AAGATGTGTTTAGGCAATCAGCTTCAAA--AGAGTCACAAGTTA-AGAGCTGG  4193
Cat          -TTATCTTGATCTTTAAAACAAGC---GAAACTTGTTTCAGTAATTAGCTTCAAATTAGAGTCAGAAGTTA-AGAGCTGG  5540
Ferret       -TTATCTTGATACTTAAA-------------CTTGTTTAGGCAATTAGCTTCAAATTAGAGTCAGAAGTTA-AAAGCTGG  4524
Lion         -TTATCTTGATCTTTAAAACAAGC---GAAACTTGTTTCAGTAATTAGCTTCAAATTAGAGTCAGAAGTTA-AGAGCTGG  4496
Vaquita      -TTGCTCTGATCTTTAAAGAGAA--AAGAAACTTGTTTAGGCAGTTAGCTTCAAATTTGAGTCAGAAGTTATAAAGCTGG  4391
Pig          GTTACCCTGATCTGTAAAAAGAA--AGGAAACCTGTTTAGGCAGTTGGCTTCAAATTTGAGTCAGAAATTAAAGAGCTGG  4368

Majority     TTTGTCTTTCTATTAAAGG------AAXATAGGCCTTAT-----------------------------------------
                               5850      5860      5870      5880      5890      5900      5910      5920
Human        TTTGTCTATTAAAGAAAGTCTTTATGGTAGAGATCCTTTGGAATCCTTCCTTTTTGGCTAAGAAAACTACTCATCGTTTA  3114
PolarBear    --------------------------------------------------------------------------------  0
Cow          TTTGTGCGTCTACTAAAGA------AAGTTAGTCCTTACATAGAGATTGTTTAGAATCTTTCGTCACTCACTTTGGCCAG  4525
Dog          -CCAATTTTATGTTGTATG------GCTCATTTCTAAGC-----------------------------------------  4697
BelugaWhale  TTTGTGTGTCTGTTAAAGA------AAGATAGGCCTTA------------------------------------------  4537
Donkey       CTTTTCTTGCTGTTAAAGG------GAAACAGGCCTTAT-----------------------------------------  4226
Cat          TTTGTCTTTCTATTAAAGG------AAAATAGCTGTTAT-----------------------------------------  5573
Ferret       TTTGGCTTTCCATTAAAGG------AAAATAGGCCTTAT-----------------------------------------  4557
Lion         TTTGTCTTTCTATTAAAGG------AAAATAGCTGTTAT-----------------------------------------  4529
Vaquita      TTTGTGTGTCTGTTAAAGA------AAGATAGGCCTTA------------------------------------------  4423
Pig          TCTGTGTGTCTATTAAGGG------AAGGTAGGCTTT-------------------------------------------  4399
```

```
Majority     -------------------------------GGTA--GAGATCGTTTGGAATCTT--XCTCXCT--CTTTTTGGCCAGGA

                               5930      5940      5950      5960      5970      5980      5990      6000

Human        AACTTTAGTTCTAATGCTTAACATTTTCTTGAGTACTTGCAACTTTTAATGTCATGATGTATTGTAATTTTTTTTTGTTT   3194
PolarBear    --------------------------------------------------------------------------------   0
Cow          AAAAACTATTCATACTTTAAACCTTAGATCTAGTGTAGAGATTGTTTAGAATCTTT-CCTCACT--CTCTTTGGCCAGAA   4602
Dog          -------------------------------CAAA--TTTGCCTAACAAAGATTT--TTT-TCT--CTTTTGGTTTTTCT   4739
BelugaWhale  -----------------------------T-GGTA--GAGATTGTTTGGAATCTTT-CCTCACT--CACTATGACCAGGA   4582
Donkey       -------------------------------GGTA--GAGGTTCTTTGGGATCTTT-CCTCACAGACTTTTTGGTCAGGA   4272
Cat          -------------------------------AGTA--CAGATCATTTGGAATCTT--TCCCTCA--CTTTTTGGCCAGGA   5616
Ferret       -------------------------------GGTA--CAGATAATTTGGAATCTT--TCC-TCA--CTTTCTGGCCAGGG   4599
Lion         -------------------------------GGTA--CAGATCATTTGGAATCTT--TCCCTCA--CTTTTTGGCCAGGA   4572
Vaquita      -----------------------------T-GGTA--GAGATCGTTTGGAATCTTT-CCTCACT--CACTATGGCCAGGA   4468
Pig          -----------------------------CTGGTG--GAGATTGTGTGGCACCT---CCTCACT--CTCTGTGGCCGGGA   4443

Majority     AAACTACTCXT-CTTTAAACCTTAGTTCTAATGCTTAGGCCTTTXAT-TTCCTTGAATCCTTGCAXATTTTAA---CATC

                               6010      6020      6030      6040      6050      6060      6070      6080

Human        TTTGTTTTTTTGAGACGGAGCCTCACTCTGTTGCCCAGGCTGGATGCAGTGGCACGATCTTGGCCCACTGCAACCTCTGC   3274
PolarBear    --------------------------------------------------------------------------------   0
Cow          AAACTATTCATACTTTAAACCTTAGATCTAGTGCTTAGGCCTTTCAT-TTCCTTGAATCCTTGTACATTTTAA---CATT   4678
Dog          AATAGAAGT---TTTACAGTTTTTGGGTTTGCATTTAAGTTTTTTTT-TTAGTTCATTTTTTTTTTAATACAGTG-CAAA   4814
BelugaWhale  AAACTACTCATACTTTAAACCTTAGTTCTAGTGCTTAGGCGTTTCAT-TTTCTTGAATCCTTGCATATTTTAA---CATC   4658
Donkey       AAACTACTCATTCTTTAAACCTTAGTTCTAATGCTTAGGCCTCTTGT-GTCCTTTAATCCTTGCACATTTTAA---CATC   4348
Cat          AAACTACTC---CTTTAGACCTTAGTTCTAATACTTAGGCCTTTGAT-TTCCTTGAATCGTTGCACATTTTAA---CAGC   5689
Ferret       AAACTACTC---CTTTAGACCTTAGTTCTAATGTTTAGGTCTTTTAT-TTCCTTGAATCCTTGCATATTTTAA---CAGA   4672
Lion         AAACTACTC---CTTTAGACCTTAGTTCTAATACTTAGGCCTTTGAT-TTCCTTGAATCGTTGCACATTTTAA---CAGC   4645
Vaquita      AAACTACTCATACTTTAAACCTTAGTTCTAGTGCTTAGGCGTTTCAT-TTTCTTGAATCCTTGCATATTTTAA---CATC   4544
Pig          AGACTACCCATTCTTTAAACCTCAATTCTAATGCATAGGCCTTTCAT-TTTCTTGCATCCTTGCATGTTTTGA---CATT   4519
```

```
Majority     GTXATXTXTTAXAA---TTTTTGTTTGXAAXCCTG--------TCTGCCCTGTTAGGGCATGGTCCTTGTCTTAAATCTT
                               6090      6100      6110      6120      6130      6140      6150      6160
Human        CTCCTGGGTTCAAGCAGTTCTCCTGCCTCAGCCTCCTGAGTAGCTGGGATTACAGGTGCGCACCACCACGCCCAGCTAAT  3354
PolarBear    --------------------------------------------------------------------------------  0
Cow          GGGATGTATTACAAA--TTTCTGTTTGTAAACCTT--------TCTGCCCTGTTAGGGCACGAT-----TCTTAAATCTT  4743
Dog          CTATAAGTCAACAT---TTTTTGTTTTCGTATATGG-----ATTTAAACTTCCAGGACCATTTGTTGAAAAGGCTATCCT  4886
BelugaWhale  GTGATTTATTATAA---ATTTTGTTTGTAAGCCTT--------TCTGCCCTCTTAGGGCATGGTTCTTGTCTTAAATCTT  4727
Donkey       ATCATGTCTTATAA---TTTTTGTTTGCAAGCCTG--------TCTGCCCTGTTAGGGCATGGTCCTTGTCTTACATCTT  4417
Cat          ATAATATGTTAAAA---TTTTTGTTTGCAAACCTG--------TCTGCCGTGTTTGGGCATGGTCCTTGTCTTCTGTCTT  5758
Ferret       TTAATATGTTAAAA---TTTTTGTTGGCAAACCTG--------TCTACCTTGTTGGGGCATGGTCCTTGTGTTCCCTTTT  4741
Lion         ATAATATGTTAAAA---TTTTTGTTTGCAAACCTG--------TCTGCCGTGTTTGGGCATGGTCCTTGTCTTCTGTCTT  4714
Vaquita      GTGATTTATTATAA---ATTTTGTTTGTAAGCCTT--------TCTGCCCTCTTAGGGCATGGTTCTTGTCTTAAATCTT  4613
Pig          GTGGTGTATTTTAA---TTTTTGTTTGTAAGCCTT--------TCTGCCCTGTGATGGCATGGTCCTTGTCTTAAATCTT  4588

Majority     TT-XAATTTTCCAAGC-----C-TGGCACAACATCTTCCAA-GCCTAGTAXTCAAATACTTG-TTG--GATGCAATGACA
                               6170      6180      6190      6200      6210      6220      6230      6240
Human        TTGTATTTTTAGTAGAAATGGCATTTCACCCTGTTGGTCAG--GCTGGTCTCAAACTCCTGACCTCATGATCTGCCTGCC  3432
PolarBear    --------------------------------------------------------------------------------  0
Cow          T---AGTTTTCCAAGC-----C-TGGCAAAATGTCTTTCAA-GCCTAGATCTCAAATACTTG-TTG--GAGGCAA-GACA  4809
Dog          TTGTCATTGAATTGGC-----T-TTGCACCTT-TGTCAAAA-ATCAACTATGTATATA-TGA-TTC--TAT-TTCTGTAC  4953
BelugaWhale  C---AATTTTCCAAGC-----C-TGACAC--CATCTTCCAA-GCCTAGTACTAAGATACTTG-TTG--GATGCAATGACA  4792
Donkey       TT-GAATTTTCCAAGT-------CTGCACAACATTTTCCAAAGCCTAGTACTCAGATACTTG-GTG--GATGCAATGACG  4486
Cat          TT-TAATTTTTCAAGC-----C-TGGCACAACATACTCCAA-ACCTGATATACAAATACTTG-TTG--GATGCAGTAACA  5827
Ferret       TTAAAATTTTTTCAAA-----C-CTGATACGT-TCTTACAG-GCCTTATACACAAATACTTG-TTG--AAG-CAATGACA  4809
Lion         TT-TAATTTTTCAAGC-----C-TGGCACAACATACTCCAA-ACCTGATATACAAATACTTG-TTG--GATGCAGTAACA  4783
Vaquita      C---AATTTTCCAAGC-----C-TGACAC--CATCTTCCAA-GCCTAGTACTAAGATACTTG-TTG--GATGCAATGACA  4678
Pig          TT-AAATTTTCTAAGCTTGGCC-TGGCACAACATCTTTCAA-GCTTGGTATTCAAATACTTGGCTG--GAAGCAATGATG  4663
```

```
Majority     TT---------------TCCCCCCTTCAAGTGTGAGTAGTCTTGAGAGGCTGTGTGC----TAT----AGTCCAGTTATT
                              6250      6260      6270      6280      6290      6300      6310      6320
Human        TCTTATGATCTGCCTGCCTTGACCTCCCAAAGTGCTGGGATTACAAGTGTGAGCCACCGTGCCCGGCCTGTATTGTCATT  3512
PolarBear    --------------------------------------------------------------------------------  0
Cow          TT---T------AC-----CTCC-TTCAAGTATGAGTGGTCACGAGAGGCAGTTTGC----TCTTGAGAGTCCAGTTATT  4870
Dog          TG---------------TATCCTTTTCCACTGACCCATTTTTATCCATCTAGATGCC----AAAAACAAACTGTGTTAAT  5014
BelugaWhale  TTGGATGCAGTGACATTTCCCCCCTTCAAGTGTGAGTAGTCTTGAGAGGCTGTGTGC----TTA-----GTCCCGTTATG  4863
Donkey       TT---------------TCCCCCCTTGAAGTGTGAGTAGTCATGAGAGGCTGTGTGC-----TT----AGCTCAGTGATG  4542
Cat          TT---------------TCCCCCTTTCAAATATGAGTAGTCTTGAGAGGCTGTGTCCTTAGTATAGTGATGGTGACTGTT  5892
Ferret       TT---------------TCCCCCTTTCAAGTGTGAGTAGTCTTGAGAGGATGGTGAC----TATAATATTTTTTGGAATT  4870
Lion         TT---------------TCCCCCTTTCAAGCGTGAGTAGTCTTGAGAGGCTGTGTCCGTAGTATAGTGATGGTGACTGTT  4848
Vaquita      TTGGATGCAGTGACATTTCCCCCCTTCAAGTGTGAGTAGTCTTGAGAGGCTGTGTGC----TTA-----GTCCAGTTATG  4749
Pig          TT---T------CC-----CCCCCTTCAAGTGTGCGTAGTCCTGAGAGGCTATGGTG----CCT----AGTCCAGTGATG  4721

Majority     C-TGATTTTTCTA--AATAATTTTTGGAATTCTTXAAATTTCATCCATAACXGTGTGATATTT---TTT-----------
                              6330      6340      6350      6360      6370      6380      6390      6400
Human        TTTCATTTGCAAGCCTCTCTGTCCTGAGAGGCTTTCTGCTTAGTCCAGTGATGCTGACTGTTCAAACATTTTTTCGAATT  3592
PolarBear    --------------------------------------------------------------------------------  0
Cow          CCTGACCATTCTA--AATAATTTTTGGAATTCTTAAAATTTCATCAATAACAATGTGATATTC---TTT-----------  4934
Dog          TATGATGACTTTAGTCTTGAAATCAAGCAGTCAT-GGTCCTCTTTGTTCTTTTTCAAAGTTGT---TTT-----------  5079
BelugaWhale  C-TGATTGTTCCA--AATAATTTTTGGAATTCTTAAAATTTCATCCATAACAGTGTGATATTA---TTC-----------  4926
Donkey       C-TGACTGGTCAC--AATACTTTTTGGAATTCTTGAAATTTTATCCTAAACAGTGTGAGATTC---TTT-----------  4605
Cat          CACAGTATTTTTTGTAATAGTCTTTAGAATACTTTAGGGAATATTTTGTATTTCCTTGCTTAT---TTTCTGTCCTCTGC  5969
Ferret       CTTAAAATTTCATCCACAACAATGTGGTATTCTTTAGAATACTTTAAGAGATATTTTGTTTTT---TCT-----------  4936
Lion         CACAGTATTTTTTGTGATAGTCTTTAGAATACTTTAGGGAATATTTTGTATTTCCTTGCTTAT---TTTCTGTCCTCTGC  4925
Vaquita      C-TGATTGTTCCA--AATAATTTTTGGAATTCTTAAAATTTCATCCGTAACAGTGTGATATTA---TTC-----------  4812
Pig          C-TGACTATTGCA--GCTAATTTTTGGAATTCCTAAAGTTTCATCCATAACAGTGTGATATT------------------  4780
```

```
Majority     ---XXAX--CX-TTGGAGXTATTTTGTGTTTTA--XGTTTXTTTTCTGTGCTTTXTT-TXTAGTXAAGTXAG-ACTTGTC

                      6410      6420      6430      6440      6450      6460      6470      6480

Human        CTTAAAATTGTATCCATAATGGGGGGAGGGGGAAGGGATAGCATTGGGAGATATAGGGGGGAGGGGGGAGGG-ATAGCTT  3671
PolarBear    --------------------------------------------------------------------------------  0
Cow          ---AGAACACT--TGGAGATATTTTGTGTTTTCT-TGCTTATTTTCTGTCCTCTCTT-TCTAGTAAATTAAGCACTTGTC  5007
Dog          ------------GAAAAACAACTTAGTACTGTG----TTTTTTTTTTTTGTTTTTTT-TTTTAATAAGAG----------  5132
BelugaWhale  ---AGAACGCTTTTGGAGATATTTTGCGTTTT----GTTTATTTTCTGTCCTCTGTA-TCTAGTAAACTAAGTACTTGTC  4998
Donkey       --------------AGAGTACTTTTGCGTTTTCT-TGCTTATTTTCCGTTCTCTACCCTATAGTGAATTAGATACTTGTC  4670
Cat          CCTGTAGTACACTGGATACTTTTCCATGCTTTAAATTTTTTTTTTTAATGTTTTATT-TATTTTTGAGAGAGAGAGGGAG  6048
Ferret       ------------TATTTTCTGTTCTCTGCCCTA----TAATGCACTAGATATTTGTC-TATACTTCAGTA----------  4989
Lion         CCTGTAGTGCACTGGATACTTTTCCATGCTTTAAATTTTTTTTTTTAATGTTTTATT-TATTTTTGAGAGAGAGAGGGAG  5004
Vaquita      ---AGAACGCTTTTGGAGATATTTTGCGTTTT----GCTTATTTCCTGTCCTTTGTA-TCTAGTAAACTAAGTACTTGTC  4884
Pig          ---GGAGAACC--TGG-GTTATTTTGTATTTTCT-TGCTTGTTTTCCATTCTCTGTA-TCTAGTAAATTGGAAACTTGTC  4852

Majority     CATGXT----XAXXATTX-X---T------------------GCTTGTCAAGTTCATAT-TATGTA-------GATTGGG

                      6490      6500      6510      6520      6530      6540      6550      6560

Human        TAGGAGATATACCTAATGCTAAATGACAAGTTAATGGGTGCAGCACACCAACATGGCACATGTATACATATGTAACAAAC  3751
PolarBear    --------------------------------------------------------------------------------  0
Cow          CACACTG---TACTATTTTGCCTT------------------ACCTGTTAAGTTCATAT-TATGTA-------GATTGAG  5058
Dog          --------------------------------------------------------------------------------  5132
BelugaWhale  CATGCTT---TAATATTTAG----------------------GCTTGTCAAGTTCATAT-TATGTA-------GATTGGG  5045
Donkey       CATGATT---TAACATGTAGGCTT------------------ATTTGTTGAGTTCATGT-TATGTA-------GATTGGG  4721
Cat          GGAGACAGAGCACCATTGGAGTGTGGGGCAGAGAGAGAGAGAGAGAGACAGACAGACAG-AATCTAAAGCAGGCTCCAGG  6127
Ferret       --------------------------------------------------------------------------------  4989
Lion         GGAGACAGAGCACCATTGGAGTGTGGGGCAGAGAGAGAGACAGAGAGAGAGACAGACAG-AATCTAAAGCAGGCTCCAGG  5083
Vaquita      CATGCTT---TAATATTTAG----------------------GCTTGTCAAGTTCATAT-TATGTAC---GTAGATTGGG  4935
Pig          CATGCT-----------------T------------------ATTTGCCAAGTTCATGT-TACATA-------GATTGGG  4889
```

```
Majority     GGAGAAXTXATGAGCX-CTTTGTGGGAAGXTGTATTTATTTTTATTTGGCAATGXTTTTTTC----A-GXTGAGAXTGT-
                      6570      6580      6590      6600      6610      6620      6630      6640
Human        CTGCACGTTGTACTCATGTACCCTAAAACTTAAAGTATAATAATGATAATAATAATTGTATCCATAATGATGTGGTGTTC  3831
PolarBear    --------------------------------------------------------------------------------  0
Cow          GGAGAAGTAATGAGCT-TTTTGTGGGAAGGTGTATTTATTTTTATTCAGCAATGGTATTTTC--------TAAGAGGGTC  5129
Dog          ----------------------------------TTTCTACAAAAAAG--------------------------------  5146
BelugaWhale  GGAGAAATGATGAGCT-CTTTGTGGGAAGGTGTATTTATTTTTATTTGGCAATGCTGTTTTCT---AAGTTGAGAGTGCA  5121
Donkey       GGAGAAATAATGAGCTTTTTTGGGGGAAGATATATTTCTTTCTATTTGGCACTGATAGTTTCT---AAGTTGAGATAGT-  4797
Cat          CTCTGAGCTGTCAGCA-CAGAGCCTAACACGGGACTTGAACCTATGAACTATGAGATCATGACCTGAGCCTGAGGTTATG  6206
Ferret       ----------------------------------TTTAGGCTTATTT---------------------------------  5002
Lion         CTCTGAGCTGTCAGCA-CAGAGCCCAACACGGGACTTGAACCTATGAACTATGAGATCATGACCTGAGCCTGAGGTTATG  5162
Vaquita      GGAGAAATGATGAGCT-CTTTGTGGGAAGGTGTATTTATTTTTATTTGGCAATGCTGTTTTCT---AAGTTGAGAGTGCA  5011
Pig          GGAGAAATAATGAG---TTTTGTGGGAAGATGTATTTATTTTTATTTGGCAGTGATTTTCTTA---A-GTTGAGAGTGCA  4962

Majority     A-XTGTAGAXA---X-G-X-----AX---CATCCAAXCX----------CC-CTTGAAATGAT-X--TX--X----X--
                      6650      6660      6670      6680      6690      6700      6710      6720
Human        TTTCAAATACTTTTGGGGATGGTACTGTGTTTTCTTGCTTATTTTCTGTCCTCTTTCCTATAGTGAAATAGATATTTGCC  3911
PolarBear    --------------------------------------------------------------------------------  0
Cow          AGATGTAGTTA-------------AC---CATCCAAGCA--------TATCTTCTGGAAGTGAA----------------  5169
Dog          -----------------------------------------------------CTTGCTAGGATTT--TATTGGAATTAA  5171
BelugaWhale  AGATGTAGATATTATAGTT-----AC---CATCCAAACAG----------CCTCTGGAAGTGAA----------------  5167
Donkey       ---TGTAGG--------------------A-----------------------TTAGAAATCAG----------------  4815
Cat          ACCTGAGTAAAAGTTGGATGCTTAAC---CAACTAAGCCATCCAGGCTCCCCACTTACCATGCTTTAATATTTAGGCTTA  6283
Ferret       -----------------------------------------------------TTTGAGTTCATAT--TACGTAGATTGG  5027
Lion         ACCTGAGTAAAAGTTGGATGCTTAAC---TAACTAAGCCATCCAGGCTCCCCACTTACCATGCTTTAATATTTAGGCTTA  5239
Vaquita      AGATGTAGATATTATAGTT-----AT---CATCCAAACAG----------CCTCTGGAAGTGAA----------------  5057
Pig          AAATGTAGATGTTGTAGTT-----AT---CATCCAAACAGA------TATCCCTTGAAAGTGAAGTT-TTTGTTTTTTTA  5027
```

```
Majority     --X------------------------------AX-XXXCX-XXX-XGG--XA-G-A-----XX-TGXX--TGXTATTTT
                      6730      6740      6750      6760      6770      6780      6790      6800
Human        CATTCTTTAGGCTTGTTTCTTGAGTTCATTTTGTACAGACTAAAGGAGGAATAATACGCTCTTTTTGGTGATAGTATTTT 3991
PolarBear    -------------------------------------------------------------------------------- 0
Cow          -------------------------------------------------------------------------------- 5169
Dog          CTTG------------------------AATATATAAAACAATTTGAGGCAAATTGACGTCCTAACAATCTTGAGTTTTT 5227
BelugaWhale  -------------------------------------------------------------------------------- 5167
Donkey       ----G----------------------------AATAGGTCAAGAGTGCAAGATGTA-----GA-TATTGTAGTTACCAT 4857
Cat          TTTGTTGAGTTTCTACTATGTACATTGGGGGAGAAAGAGCTTTTTCTGGAGAAGGCATTTTTATTTGGCAGTGTTATTTT 6363
Ferret       GGGA------------------------GAAATAGTGAGCTTTTTCGGGGGAAGGTATTCTTATTTGGCAATGATACTTT 5083
Lion         TTTGTTGAGTTTCTACTATGTACATTGGGGGAGAAAGAGCTTTTTCTGGGGAAGGCATTTTTATTTGGCAGTGTTATTTT 5319
Vaquita      -------------------------------------------------------------------------------- 5057
Pig          TTTTTTAAAGTTTATTTTCTTACAGCTGATTTCAGTGTTCCGCCAATTTCCGCTGTACAGCAAAGTGACCCAGTTATCCT 5107

Majority     XXAAXTXXA---X-TX-----XXXT-XXXTCXGGAATXX--XX--A----AX--X-----XXXXX---------------
                      6810      6820      6830      6840      6850      6860      6870      6880
Human        CCAAGTTGAGTTAGTCATGGGATTTAAAATCAGGAATAGCTAAAGATTGCAAAATGGTAGATGTTGTAGGTTCCATCCAA 4071
PolarBear    -------------------------------------------------------------------------------- 0
Cow          -------------------------------------------------------------------------------- 5169
Dog          CCACTCACAAACAATTA---TATCTCTCCGTTTACCTAGATCTTAAA---AGATTTCTTTTAGCAGTGTTTAATTTTCAT 5301
BelugaWhale  -------------------------------------------------------------------------------- 5167
Donkey       CCAAACAGA---------------TATCCTCTGGAA-------------------------------------------- 4878
Cat          GTAAGTTGAGGTAGTTGCAGGATTTGAAATCGGGAATAGCTCAAGAGTGCAAGATGTAAAATGTATAGTTA--------- 6434
Ferret       CTAAGTTAAGGTAGTTGAAGGATTTGAAATCTGGAATAGGCCAAAAGTGCAAAATACAGAATGTAGACTTAACATCCAAA 5163
Lion         GTAAGTTGAGGTAGTTGCAGGATTTGAAATCGGGAATAGCTCAAGAGTGCAAGATGTAAAATGTATAGTTACCATTCAGT 5399
Vaquita      -------------------------------------------------------------------------------- 5057
Pig          ACATATACATTCTTTTCCCCTTATTATCTTATATCATGTTCCATCAA---AAGTGATTGGATATAGCTCCCTGTGCTATA 5184
```

```
Majority     ---------XTX-----GAATTTTXTAAAAGAATAAATGATACAAGTCTGAGTTGTCXXCTGTTA--GTGGATGAGGAGA
                      6890      6900      6910      6920      6930      6940      6950      6960
Human        ----ACAG-ATTTCTGTAAATTTTGTAAAAGAATAAATGATACAAGTGTGAGTTGTCGACTGTTA--GTGGAAAAGGAGA  4144
PolarBear    --------------------------------------------------------------------------------  0
Cow          ---------------------TTTTTAAAAATATAAATGATGCAAGTCTGAGTTGTCA---ATTA--CTGGATAAGGAGA  5223
Dog          TG----TACAGTTTTTGTACGTTTTTGTTAGATT---TATCCCTTTTTTGTGTTTTTTGGTGCTATTGTAAATGATGTTG  5374
BelugaWhale  --------------------TTTTTTAAAAGAATAAATGATTCAAGTCTGAGTTGTCAGCTGTTA--CTGGATGAGGAGA  5225
Donkey       ----------GC-----GAATTTTGTAAAAGAATAGGTGATAGAAGTCTGAGTTATGC--TATTA--GTGGATGGGGAGA  4939
Cat          ------GGTATCCTGTGGAATTTTGTAAAAGAATGAATGACCCAAGTCTGAGTTGTTCACTGTTA--GTGGGTGAGGAGA  6506
Ferret       TT----GGTATCCTCTGGAATTTTGTAAAAGAATGAATGATACAAGTCTGAGTTGCCCACTATTAGTGTGGATGAGGAGA  5239
Lion         TAGGTAGGTATCCTGTGGAATTTTGTAAAAGAATGAATGACCCAAGTCTGAGTTGTTCACTGTTA--GTGGGTGAGGAGA  5477
Vaquita      --------------------TTTTTTAAAAGAATAAATGATTCATGTCTGAGTTGTCAGCTGTTA--CTGGATGAGGAGA  5115
Pig          CAGCTTATCCTGGATGTGAACTTTTTAAAAGAATAAATGATACAAGTCTGAATTGTCAGCTATTA--TTGGATGAGGAGA  5262

Majority     GAAGGAACTCAG-CAXGACCCAGAAXGAGXAG-----AGAGAAATGAGGAGACTCGGAATAGGATGTCCAGXTXTGGTTT
                      6970      6980      6990      7000      7010      7020      7030      7040
Human        GGA-GTTTGTAGACAAGGCTCAGAATGAGTAGACAGGAAAAAACTGAGGAGACTTGGAATAGGATGTCCAGGTGTAATGT  4223
PolarBear    --------------------------------------------------------------------------------  0
Cow          AAAGGAATTCA--CAAAACCTAGAACAAGCAG-----AAAGAAATGAAGATGCTTGGAAGAGGGTGTTCAGATGTGGTTT  5296
Dog          TTTTAAAAATCTCAAGTTTCCA---------------GGGGGCACCAGGAACTCC---ACTGA--GTTCAGGGTGACTCA  5434
BelugaWhale  AAAGGAACTCA--TAGGACCCTGAACAAGCAG-----AGAGAAATGAGGATGCCTGGAAGAGGATGTACATATTTGGTTT  5298
Donkey       GAAGAAACTCAGGTAAGACTGAGAATGAGTAGGCAT-AGAGAAATGAGGAGACTCGGAATCAGATGTCTAGATATGGTTT  5018
Cat          GAAGGAATTCAGACAGGAGCCACCATGAGTAGGCCC-AGAGATAGGAGGAGACTCAGGATTGGATGTCCAGGTATGGTTT  6585
Ferret       GAAGGAACTCAGACAAGAGGTA---------------AGAAAAATGAGAAAAAGA---ATTGGATGTCCAGGTATGGTTT  5301
Lion         GAAGGAATTCAGACAGGAGCCACCATGAGTTGGCCC-AGAGATAGGAGGAGACTCGGGATTGGATGTCCAGGTATGGTTT  5556
Vaquita      AAAGGAACTCA--TAGGACCCCAAACAAGCAG-----AGAGAAATGAGGATGCCTGGAGGAGGATGTACATATTTGGTTT  5188
Pig          GAAGGAACTCAA-CAAGACCCAGAACGACCAG-----AGAGAAATGAGAAGACTCAGAAGAGGATGTTCAGATTTGGTTT  5336
```

```
Majority     ATTGGXTGT------TTTCTGTGTTAGTAGTTTGAXGAGAAAAAAX--XTAGAAAATGATXTAAAAXTAATTGCCAACCA
                               7050      7060      7070      7080      7090      7100      7110      7120
Human        ATTGGTTACACCAG-TTTTTTTTTTTTTTTTTTTGA-GACAGAGTCTCACTCTTCGCCCAGGTGGAGTGCAGTGGCACGAT  4301
PolarBear    --------------------------------------------------------------------------------  0
Cow          ATTGGCAGT------TTTCTACATTAGTAGTTTGAAGAGGGAATGTTGTTAGAAAATTATGTTAAGTGGTT-GCTAGCCT  5369
Dog          GTGG-----------TTGAATGTTTGCCCTTGGCTCAGGTCGTGAT--CCTGGGGTCCTGGGATCGAGTTCTGCAGCAG  5500
BelugaWhale  ATTGGCAGT------TTTCTGTATTAGTAATTTGAAGAGAAAACA---TTAGAAAATGATATAAAAGTAATTGCTAGCCA  5369
Donkey       ATTGATTACACTAGTTTTCTATGTTAGTACTAGTTTGAGGAGAAAGCATTAGGAAATGATATAAAAATAATTGCTAACTA  5098
Cat          ATTGGTTCTACCAG-TTTCTGTATTAGTGGTTTGAGGAGAAAAAAAA--TGGAAAATGATGTAAAAATAATTGCCAACCA  6662
Ferret       TACC-----------AGTTTTGTTAGTGGTTCAAGGAGAAGAAAAT--TGGAAAATGATATGAAAATAATTGCCAACCT  5367
Lion         ATTGGTTCTACCAG-TTTCTGTGTTAGTGGTTTGAGGAGAAAAAAA---TGGAAAATGATGTAAAAATAATTGCCAACCA  5632
Vaquita      ACTGGCAGT------TTTCTGTATTAGTAGTTTG-----AAAACA---TTAGAAAATGATATAAAAGTAATTGCTAGCTA  5254
Pig          ATTGGCTGTGCCGATTTTCTGTGTTAGTAGTATGAAGAGAAAACA---TTAGGAAATGATATAAAAGCAAT-GTCTAATC  5412

Majority     TTTTTTTT-GTCCATTGTTTAATATAAAAGTTTGAATAACTGAAGTTXTGTTTT----XT-XXCTTTTCX-----AGTXG
                               7130      7140      7150      7160      7170      7180      7190      7200
Human        CCTGGCTCACTACAAGCTCCGCCTT-----CCGGGTTCACTCCATTCTTCTGCC-----TCAGCCTCCC-----GAGTAG  4366
PolarBear    --------------------------------------------------------------------------------  0
Cow          TTTTTTTT-GTCTGTTGTTTAATATAAAAGTTTGATAACTGAAGTTGTATTTTTATAATCTGCTGCTGCTGCTAAGTCG  5448
Dog          GCTCCCTA-TGGGGGGAGCCTGCTTCTCCCCCTGCCTGTGTCTCTGCCTCTCTT----TGTGTCTCTCATGAATAAATAA  5575
BelugaWhale  ATTTTTTT-GTCCAGTGTTTAATATAAAAGTTTGATAACTAAAGTTATGTTTT---------------------------  5422
Donkey       TTTTTTAT--CCC-TTGTTTAATATAAAAGTTTGAGTAACTCAAGTTATGCTCT--------------------------  5149
Cat          TTTTTTTTTATCCTTTGTTTAATATAAAT-TTTGAATAACCGAAGTTTTGTTTT----ATGAACTTTTCTGAGACAGTGG  6737
Ferret       TTTTTTTA-ATCCATTGTTGAACATAAAACTTTGAATAACTGGAGTTTTGTTTT----ACGAACTTTTCTGAGGTAGTGA  5442
Lion         TTTTTTTT-ATCCTTTGTTCAATATAAAAGTTTGAATAACCGAAGTTTTGTTTT----ATGAACTTTTCTGAGACAGTGG  5707
Vaquita      ATTTTTTT-GTCCAGTGTTTAATATAAAAGTTTGATAACTAAAGTTATGTTTT---------------------------  5307
Pig          TTTATCTT-GTCTGTTGTTTAAAGT-----TTTCAATAACTAAAATTATGTTTT----ATCTAATTTTCTAGGATAATGG  5482
```

```
Majority     XTAAXAATXTTTGCX--XXXXT-CA-GCXTXXXTA----AXGTX-TXXAGTT-X--X---XATXTAATTTTCT-------
                      7210      7220      7230      7240      7250      7260      7270      7280
Human        CTGGGACTACAGGCGCCCACCACCACGCCCGGCTA----ATTTTTTGTATTTTTAGTAGAGACGGGGTTTCAC-------  4435
PolarBear    --------------------------------------------------------------------------------  0
Cow          CTTCAGTCGTGTCCGACTCTGTGCAACCCTGTAAACGTCAGTCCACCAGGCTCCCCCATCCCTGGGATTCTCCAGGCAAG  5528
Dog          ATAA-AACCTTTAAAAAAAAAT-GAAGTAAAAAAA---AATGTTTCCGGTTATTAGT--GTAAGGA-ACTACT----ACA  5643
BelugaWhale  -----------------------A---------AA----------------T-------------AATTTTCT-------  5434
Donkey       -------------------------------------------------------------ATATGATTTTCT-------  5161
Cat          TTAAGAATTTTTGCTTTGGAGT-CATGCATACGTA---CAAGTAGTTGAGTTCTCATT-GTATATATATTTCT----ATG  6808
Ferret       TTAAGAATTTTTGCTTGGGAGT-GAAGCCTGTATA---TTAGTAATTAAGTTTTCAT--ATATATA-TTTTTT----TAA  5511
Lion         TTAAGAATTTTTGCTTTGGAGT-CATGCATATGTA---CAAGTAGTTAAGTTCTCATT-GTATATATATTTCT----ATG  5778
Vaquita      -----------------------A---------AA----------------T-------------AATTTTCT-------  5319
Pig          TTAAGAATTTTTGCTATGGCAT-CAGACCTATATA---CAAGTAATTAAGCT------TTCATGAAATTTTTT----TAA  5548

Majority     -X--XTXX---A-X--TTCXTXGTG---------T-T-X-XAAXTXC-A-TX-XXXX--T-AGGT-XAGX--XXXXTXTT
                      7290      7300      7310      7320      7330      7340      7350      7360
Human        CGTGTTAGCCAGGATGGTCTTGATCTCCTGACCTTGTGGTTACACCAGTTTTTTGTTAGTT-GTTTGAGGAGAACACTTT  4514
PolarBear    --------------------------------------------------------------------------------  0
Cow          AACACTGGAGTGGGCTGCCATTTCCTTCTCCA-ATGCATAAAAGTAAAAAGTGAAA--GTGAAGTCACTCAGTCG-TGTC  5604
Dog          GTTTTTTG--CATGT-TTTCTTGTG--------TTCTTCACTCTTGCCAAACTCACTTGTTAGATCTAGTAGCTTTTTGT  5712
BelugaWhale  --------------------------------------------------------------------------------  5434
Donkey       --------------------------------------------------------------------------------  5161
Cat          TGCCCTTAAAAAAAA-TTCAAAGTGGAAGGTATGTCTCCCCAAATCCCAGTCCTGTTTCTCAGGTGTAGGTAATTATGTT  6887
Ferret       TGTACATGGTTAAAA-TTCATAGTGGTAAATATTTCTCCCTGAGTTCTAGTCCTATTTCTCAGGTGTAGTTAATTATGTT  5590
Lion         TGCCCTTAAAAAAA--TTTAAAGTGGAAGGTATGTTTCCCCAAATCCCAGTCCTGTTTCTCAGGTGTAGGTAATTATGTT  5856
Vaquita      --------------------------------------------------------------------------------  5319
Pig          AAGGTAAAAGTAGGAGTTCCTAGCGTGGAGCG-GTGGAAACAAATCCGACTAGGACCCATGAGGTTGCAGGTTCAATCCC  5627
```

```
Majority      AXAXTXTXXXXGXT---------XTXX--C-CTGA-XXXTTX-X-XXT---TGTXXXXTXXXXXACAXTXX-AXX-X---
                           7370      7380      7390      7400      7410      7420      7430      7440
Human         AGAAGATGATAAAAAAAAATTGCAAATCGTTTTTAAAAATCCATTGTTTAATATAAAAGTCTGAATAATTAAAGT-----  4589
PolarBear     --------------------------------------------------------------------------------  0
Cow           CAACTCTTCTTGACCCCCATGGACTACAGC-CTACCAGGCTCCTCTGTCCATGGGATTTTCCAGGCAAGAGTACTGGAGT  5683
Dog           AGATACCATCAGAT---------TTTCTACATAGACTGCTTATTTTAT---TTCTTCATTTTTTATCTTAATACCAT---  5777
BelugaWhale   --------------------------------------------------------------------------------  5434
Donkey        --------------------------------------------------------------------------------  5161
Cat           AAATTTTTCTTGGTGCTTT----TTTTCCCTCTGAGGTGTTACCATTT---TGTATATTATCAAACATTAGCATCCT---  6957
Ferret        AAATTTTTTCTGGTGCA------TTTTTTTCCTGAAGTATTAATAGTT---TGTATATTATCAAACAGTGGTATCCT---  5658
Lion          AAATTTTTCCTGGTGCTTT----TTTCCCCTCTGAGGTGTTACCATTT---TGTATATTATCAAACATTAGCATCCT---  5926
Vaquita       --------------------------------------------------------------------------------  5319
Pig           TGGCTTTGCTCAGTGGGTTAAGGATCCAGCACTGCTGTGAGCTGTGGTGTAGGTTGCAGACACGGCTTGGACCTGGTGTT  5707

Majority      ----TGXTT-XXTXXX--G--XGGA-TTAXGXTTAAGAXTXTTTGCTTTGGTGTXGGA---TTTAAGTAC-AAGTAATTA
                           7450      7460      7470      7480      7490      7500      7510      7520
Human         ----TAATTTAATTTTCTG----GAATAATGGTTAAGAATTTTTGCTTTGGTGTCAGG---CCTGTGTAC-AAGTAATTA  4657
PolarBear     --------------------------------------------------------------------------------  0
Cow           GGGGTGCCTATAATTT--GCTAGGA-TAATGGTTAAGAATTTTTCCTTTGGCGTTGGA---TTTATGTGCCAAGTAATTA  5757
Dog           ----TTTTTTTTTTTTTGG--GCGACCCGACACCCTGCCTAGGTCCTGTGCGGCGGAAATCTAGTATTAGTGCTGAGTAG  5851
BelugaWhale   ------------------A---GGA-TAATGGTTAAGAATTTTTGCTTTGGCGTCAGA---CTTAGGTAC-AAGTAATTA  5488
Donkey        ---------------------AGGA--TACGATTAAGAGTTTTTATTTTGGTGTCAGA---CCTATGTAC-AAGTAATTA  5214
Cat           ----TGCTTACTTACACAG--ATGGATTCATAACACATGTACTGGCCCACATCTTGCCTTTTAAGAACATAACTTTATTG  7031
Ferret        ----TGCTTGTTTATACAG--ATGAGTTCATACCATGTGTACTAGCTCACATCTTGCCTTTTTAAAAAAATAACTTTACT  5732
Lion          ----TGCTTACTTACACAG--GTGGATTCATAACACATGTACTGGCCCACATCTTGCCTTTTAAAAACATAACTTTATTG  6000
Vaquita       ------------------A---GGA-TAATGGTTAAGAATTTTTGCTTTGGCGTCAGA---CTTAGGTAC-AAGTAATTA  5373
Pig           TCTGTGGCTGTGGTGTAGGCTGGCAGCTACAGCTCAGATTAGACCCCCTAGCCTGGGAACCTCCAGGTGCGACCCTAAAA  5787
```

```
Majority     AGATTTCATGAAATXTAAAAT---AAAATAATXXXTGTTTATGGTT-AAAAAAAAATTCAAAGTTAAAAGTATTTCTCCC
                                 7530      7540      7550      7560      7570      7580      7590      7600
Human        AGTGTTCTTTAAAACTTAAAACATTTGTGAATTTGCTTTAAAGACACTGATGCATGTGCATATTTAAAAAAATTCAGGCC  4737
PolarBear    --------------------------------------------------------------------------------  0
Cow          AGCTTTCATGAAATTTAAAA-----AAGTAATACATGTGTGTAGTT--AAAAAAAATTCAA-GTGAAAAATATTTCTCCC  5829
Dog          AAAAAAGGGATGAGAGCCAGT---CAAAAAAATTCTTTGTTCCCTTATATGAGGGGTCCTAACTCT-CCGGGGGAAAAAC  5927
BelugaWhale  AGCTTTTATGAAATTAAAAA----AAAAT-------GTGCATGGTT-AAAAAAAAATTTTAAGTGAAAAGTATTTCTCCC  5556
Donkey       AGCTTTCATAAAATTTTTTCTTTTTGTAAAAGTAATACATGCACGTGGTTAAAAAATTCAGAGTTGGAAGTATTTCTCCC  5294
Cat          AGATTCAATTTGATATAAAAT---AAACTGGACATATTTTAAGTATACAGCATCTATGAGACCATTATCACAATCAAGAT  7108
Ferret       GGAGCACCTGGGTGGCTCAGT---GGGTTAGCCTCTGCCTTCAGCTTGGGTCATGGTCTGAGGGTC-CTGGGATCGAGGC  5808
Lion         AGATTCAATTTGATATAAAAT---AAACTGGACATATTTTAAGTATACAGCATCTATGAGACCATTATCACAATCAAGAT  6077
Vaquita      AGCTTTTATGAAATTAAAAA----AAAATAAT----GTTCATGGTT-AAAAAAAAATTTAAAGTGAAAAGTATTTCTCCC  5444
Pig          AGATTAAAAAAAAAAAAAAAAGGTAAAAGTAATACATGTACATGTTTTAAAAAAAAATTCAAAGTGAAAAGTATTTCTCCC  5867
```

```
Majority     TGATATCCAGTCCTATTTCTTAGGTGTAGGTATTTATGTTAACATTXTTTTTX--GCATTTTTTX-CCTGAAATTAXTAG
                                 7610      7620      7630      7640      7650      7660      7670      7680
Human        GGGCGCGGTGGGTCACACCTGTAATCCCAGCACTTTGGGAGGCCGAGGCGGGC--GGATCACGAGTTCAGGAGAGACCAT  4815
PolarBear    --------------------------------------------------------------------------------  0
Cow          TGATTTCCAGTCCTTTTTCTTAGGTGTAGGTGATTATGTTAAAATTTTTTTTG-TGCATTTTTTTTCCTGAAATTACTAG  5908
Dog          CCACACAAACCCAAATGGATAATTTCTTGATATTATTTCTTTATTGTCTTAGG---GAATCAATGATTGGGGCTTAATGT  6004
BelugaWhale  TGATATCCAGTCCTGTTTCTTAGGTGTAGATAATTATGTTAACATTATTTTTGGTGCATTTTTT--CCTGAAATTATTAG  5634
Donkey       TGATGCCCAGTCCTATTCCTTAGGTATATGTAGTTATGTTAAAATTTTTCTGGTGGGATTTTTTC-CCTGAAATTATTAG  5373
Cat          AATGAACATATCATTTTGCCCTTTTGTAACCCTTCCTTCTGCCTTTCTTTCTC---CAACCACTGATCTGCTTTCTGTCA  7185
Ferret       CCGCATCAGGCTCTGCTCAGCAGGGAGCCTGCTTCCTCCTCTCTCTGTCTGCC---TCTCTGCCTACTTGTGATCTCCCT  5885
Lion         AATGAACATATCATTTTGCCCTTTTGTAACCCTTCCTTCTGCCTTTCTTTCTC---CAACCACTGATCTGCTTTCTGTCA  6154
Vaquita      TGATATCCAGTCCTGTTTCTTAGGTGTAGTTAATTATGTTAACATTATTTTTGGTGCATTTTTT--CCTGAAATTATTAG  5522
Pig          TGATTACCAGTTCTATATCTTAGGTATAGGTAGTTATGTTAAAAAGATCTTAC-TGCATTTTT---CCTGAAATTAATAG  5943
```

```
Majority     TTTATATATTACCACACATA---XACATCCTTGTTTATXAACAC----GXATTCATGCCATGTGTACTGGCXCATATCTT
                      7690      7700      7710      7720      7730      7740      7750      7760
Human        CCTGGCTAACACGGTGAAACCCTGTCTTTACTAAAAATACAAAAAATTAGCCGGGTGTGGTGACGGGTGCCTGTAGTCCC  4895
PolarBear    --------------------------------------------------------------------------------  0
Cow          TTTACATATTACCACACATATA-AACATCCTTGTTTAGTTACACAAATGTATTCATGCCATGTGTACTGGCCCATATTGT  5987
Dog          TTGTGGGCATTCCAGTGAAA----GTTGCACTGTGGGCCTGAT-----GTTTATTTTCTTTGTGAAGTTTTAAATGACAC  6075
BelugaWhale  TTTATATATTAGCACACATGTA-AACATCCTTGTTTAGCAACACAT--GGATTCATGCCGTGTGTACTGGTCCATATCTT  5711
Donkey       TTTATATATTACCACACATATA-AACATCCTTATTTATTTACACAAATGGATTCATACCATGTGTCCTGGCCCATATCTT  5452
Cat          CTATAGGGTGTTGGCATTTT----ATAGAATTTTAAATGAATCC----GTATGTACTCCCCTTTTATTTGGTCTTATTTC  7257
Ferret       CTCTGTCAAATAAATAAATA----AAATCTTTAAGAAAAAAAG-----ATTTAAAAACAACAGCAACAAAAAACGGAGCT  5956
Lion         CTATAGGTTGTTGGCATTTT----ATAGAATTTTAAATGAATCC----GCATGTACGCCCCTTTTATTTGGTCTTATTTC  6226
Vaquita      TTTATATATTAGCACACATGTA-AACATCCTTGTTTAGCAATACATATGGATTCATGCCGTGTGTACTGCTCCATATCTT  5601
Pig          TTTATTAATTACCACACATA---AATATTCTTGTTTACTTACA--AAAGCATTCATGCCATGTGTATTGGCTTACATCTT  6018

Majority     GCTXTAA-----AAATAA-XCTTTATTGAGATAXAATT-----TGACATAAACTGCAXATATTTAAGGTATACTAGTATT
                      7770      7780      7790      7800      7810      7820      7830      7840
Human        AGCTACTCGGGAGGCTGAGGCAGGAGAATGGCATGAACCTGCGAGGCAGAGGTTGCA-GTAAGCGGAGATTGC-ACCACT  4973
PolarBear    --------------------------------------------------------------------------------  0
Cow          GCCTTAA-----AAAGAA-GCTTTATTGAGATATAATT-----TGAGATCAACCACCTATATTTAAGGTATAC-AGTGTT  6055
Dog          GTTTAAT-----TTAT-----TTAATAGATACAGGACT----ATTCAGGTTATTTTTTCTGTTTATTTTTTATTATTTTT  6141
BelugaWhale  GTTGTTA-----AAAAAA-GCTTTATTGAGATACAATT-----TGACATAAACTGCATATATTTAAGGTATACTAGTGTT  5780
Donkey       GCCCTTTCTGGAAAGAAAAACTTTATTGAGATATAATT-----TGACATAAACTGCACGTATTTAAAGTATACAATGATT  5527
Cat          ACTGTG-------ATTAT--TTTGAAATTTACCCGTGTTGTCTGGCAATAGTCCATTCCTTTTTATTGCTTAGTAGTATT  7328
Ferret       TTTAAAA-----AAATAG--CTTTATTGAGATAAAATTTGATATAAAATAAACTTCACATATCTAAAGTGTACAATTTTT  6029
Lion         ACTGTGT-----GATTAT--TTTGAAGTTTACCCGTGTTGTCTGTCAATAGTCCATTCCTTTTTATTGCTTAGTAGTATT  6299
Vaquita      GTTGTTA-----AAAAAA-GCTTTATTGAGATACAATT-----TGACATAAACTGCATATATTTAAGGTATACTAGTGTT  5670
Pig          GCCTTAA-----AAAGAAAGCTTTATTGAGATATAATT-----TGACATAAACTGCAAATATTCAAGTTACAC-AGTATT  6087
```

```
Majority      TXAA-XATGTATACACCTATGAAACXATCACTA---XXAAGATAATGAAC------X----XXX-XAGXATCAXTTTGCT
                                7850      7860      7870      7880      7890      7900      7910      7920
Human         GCACTCCAGCCTGGGCGACAGAGACAGACTCTATCTCAAAAAAAAAAAAAAAAAAAAAAAAAAAAAAAAAAAATTCAAA  5053
PolarBear     --------------------------------------------------------------------------------  0
Cow           TTTA-CATATATACACCTATGAA-CCACTACTA---TCAGGGTAATGAAC------C----CCAGGAGTATCATTTTGCT  6120
Dog           TTAAATTTATTCATGAGAGACACACACACACAC------AGAGAGCAG-A---------------GACATAGGCAGAGGG  6199
BelugaWhale   TTGA-CATGTATACCCCTATGAAACTATCACCACAATCAAGATAATGAAC------C----CCAGAAGTATCATTTTGCC  5849
Donkey        TGACATATGTATACACCTATGAAATCATCATCACAATCAAGGTAATGAACCTACATCACCTCCAAAAGTATTAGTTTGCT  5607
Cat           ACAT-TTTATGGATATACAGCAATTTATCATTC------AACTCTTGATG---------------GACATTTGGTTTTTT  7386
Ferret        TGACATGTGAATACACTTAGGAGACCACTTAGG------AGACAACAA-A---------------GATAATGAACCCGTG  6087
Lion          ACAT-TTTATGGATATACAGCAATTTATCATTC------AACTCTTGATG---------------GACATTTGGTTTTTT  6357
Vaquita       TTGA-CATGTATACCCCTATGAAACTATCACCACAATCAAGATAATGAAC------C----CCAGAAGTATCATTTTGCC  5739
Pig           TCGA-CATGTGTACAACGATGAAACCATTAATAAAATCAAGACAATATCCATTGCTC----CCCAAAGTATCGCTTTGCC  6162

Majority      CTTTTATAAT----XXX-TCTXCCTTTCTTTCCCCAAGCAACCACTGAT-CTGCTTTCTGTCACTATA-GATTAGTTTGC
                                7930      7940      7950      7960      7970      7980      7990      8000
Human         CTGCTTCACAGCTTTTTTTGCTATTTCTTAGGTGTAGGTAATTG-TGTTAAAATTTTGTGCCTTTTTT-CTTGAAATTAT  5131
PolarBear     --------------------------------------------------------------------------------  0
Cow           CCTTTGTAATTACTCCCTTCTGCCTTTCTTTCCCCAGCCAACCACTGAT-CTGTTTTGTGTCACTGTA-GATTAGTTTGC  6198
Dog           AGAGGAGAA-------------GCAGGCTCTGTGCAGGCGGCC--CGAT-GGGGGA-CTGGATCCAGG-GATTCCAGGGT  6261
BelugaWhale   CTTTTGTAATCTCTCCCATCTGCCTTTCTTTCCCCAAGCAACCACTGAT-CTGCTTTCTGTCACTATA-GATGAGTTTGC  5927
Donkey        CCTTTGTAGTCCTTCCCATCTGCCTTTCTTTGCCCGAGCAACTGCTATTTCTGCTTTCTGTCACTATA-GGTTAATTTGC  5686
Cat           T--CCAGTTT--------TTG-ACTATTGTAAATAAAGCTGTGAACATT-CCTGTAAAAGTCTTTGTGTGAACTTTTCGT  7454
Ferret        TTAGTATTGT--------TTGCCCTGCCTTTCCTCAAGCAACCATTGAT-GTACTTTCTGTCACTACA-GGTTAGTTTGC  6157
Lion          TTTCCAGTTT--------TTG-ACTATTGTAAATAAAGCTGTGAACATT-CCTGTAAAAGTCTTTGTGTGAACTTTTCGT  6427
Vaquita       CTTTTGTAATCTCTCCCATCTGCCTTTCTTTCCCCAAGCAACCACTGAT-CTGCTTTCTGTCACTATA-GATGAGTTTGC  5817
Pig           CTTTTATAAT----CCCTTCCACATGCCTTTCACCCGGTAACCACTGAT-CTGCTTTCTGTCACTATA-GATTAATGCGC  6236
```

```
Majority     AT-TTTTTAGAATTTTAAACAAATXGTCATACAXTATXTCCCCTXTTTTTXXCTGGATTATTCCACTTAGCATAATTATT
                               8010      8020      8030      8040      8050      8060      8070      8080
Human        TAATTTATATATTAGCACACATAT--AAACATCTTTATTTACGTGATTGAATTCAAACTATTGTTTTGGCCCACATCTT-  5208
PolarBear    --------------------------------------------------------------------------------  0
Cow          AG-TTTTTAGAATTTTAAGCAAAGAGTCATACAGTATGTATCCTCTTTTTCCCTGGATCATTCCACTCAGCATTATT---  6274
Dog          CT-TGCCCTGAGCCAAAGGCAGATGCCCAACCACTGAACCACCTGGGCATCCCAGGTTATCTAGTTCTTAAGTGAACTTT  6340
BelugaWhale  AA-TTTTTAGAATTTTAAACAAATAGTCATACAGTATGTACCTTCTTTTTGCCTGGATTATTCCACTCAGCATATTTATT  6006
Donkey       ATATTTTTTTAATTTTGAATAAATGGAGTTAACACGTACCCTTTTCTTTGGTCTGGCTTATTGCACTTGGCATAATTATT  5766
Cat          TT-GTCTTGGGGAAATACCTTTGATGGGAATGGTTAGATCATATGATACACATATGTTCTTAACTTTTTAAGAAAATGCA  7533
Ferret       AT-TTTTAAAAATTTTAAATGAATCAAAATGTACCTCCCCCCCCTTCTTTTTTGGTCTTTTTTCCCTCAGTGTAATTATT  6236
Lion         TT-GTCTTGGGGAAATACCTTTGATGGGAATGGTTAGATCATATGATACACATATGTTCTTAACTTTTTAAGAAACTGCA  6506
Vaquita      AA-TTTTTAGAATTTTAAACAAATAGTCATACAGTATGTACCTTCTTTTTGCCTGGATTATTCCACTCAGCATATTTATT  5896
Pig          AA-TTTTTAGAATTTTAAACTTAG--TCGTACAATATGTACCCTCTTTTTGTCTGGATTATTCTACTTAGCATAATTATT  6313

Majority     T-GGAATTTCACCCATGTTXTGTATCAGTAGTXCATTCCTTTTTATTGCTTAGTAGTATTA-CATTGTATGGAXATACAG
                               8090      8100      8110      8120      8130      8140      8150      8160
Human        --GCCTTTTTTTTTTTTTTTTGAGTCGGAGTCTCCCTCTGTCATCAGGCTGGAGTGCAGTGGCATGATCTCGGCTTACTG  5286
PolarBear    --------------------------------------------------------------------------------  0
Cow          --GGAATTTCAGCCATGTTGTATATCAGTAGTTCATTCCTTTTTATTGCTTAGTAGTACTA-CATTGTACAGACATACAG  6351
Dog          G-ATAATTTGTACCTCCCATAGAGTTAGTC---CATTTCTTCCATTTGCATAG-AGTTGTT-TGTGAGACTACCTTTTTA  6414
BelugaWhale  TTGGGATTTCACCCATATTGTGTATCAGTAGTTCATTTCTTTTTATTGCTTTGTAGTATTG-CATTGTATGGATATACAG  6085
Donkey       T-TGAGATTCACCCATGTTGTGTATCAGTAGTCTCTTCCTTTTTATTGCTTATTAATATTA-TATTGTGCAGATATATAG  5844
Cat          G-ACTATTTTCCAAAGTGGTTGTATAATTTTA-CATTGCCACCAATAGGGTATGAGATTTC-CAGTCCTTCTGCCTCCTT  7610
Ferret       T-TGAGATTTACTTATGTTTGTCAATAGTC---CATTACTTTTTCTTGCTTAGTAATACTA-CATTTTATGGATATACAG  6311
Lion         G-ACTATTTTCCAAAGTGGTTGTATAATTTTA-CATTGCCACCAATAGGGTATGAGATTTC-CATTCCTTCTGCATCCTT  6583
Vaquita      TTGGGATTTCACCCATATTGTGTATCAGTAGTTCATTTCTTTTTATTGCTTTGTAATATTG-CATTGTATGGATATACAG  5975
Pig          TTGGGATTTCACTCATGTTGTGCATCAATAGTTCCTTCTTTTTTGTTGCTTAGTAATATTA-CCTTTTATGGTTATAAGG  6392
```

```
Majority     CAGTTTGTTTXTCCAXTCAACTCT---TXGACATTTGGGTTGTTTCCA-GTTTTTGGCTATTXTAAATA-AAGCTTGXGT
                              8170      8180      8190      8200      8210      8220      8230      8240
Human        CAACCT--CTGCCTCCTGGGTTC-----AGGCAATTCTCCTGCCTCAGCCTCCCTAGTAGCTGGGACTACAGGCACACGC  5359
PolarBear    --------------------------------------------------------------------------------  0
Cow          CAGTTTGTTTATCCACACAACTCTTGATGGACATTTGGATTGTTTCCA-ATTTGGGTCTATTATAAATA-AAGTTTCTGT  6429
Dog          TACTATTGATGTCTGTAGAATCTG---TAGTGATGTCACTCCTCTC---ATTGCTGATACCTACGATTT-GTCTTTTCTC  6487
BelugaWhale  CAATTTGTTGATCCACTCAACCCTTGATGGACATTTGGATTGTTTCCA-GTTTTGGGCTATAATAAGTA-AAGCTTCTGT  6163
Donkey       CAGTTTGTTTATCCATTCAACTCTTGATGGATATTTGGGTTATTTCCA-GTTTTTGGCTATTATAAAT-----CTGTTGT  5918
Cat          TCCAATGCTTGTCTTTCTGATTC----TAGACATTCTAGAGGGGGTGA-AGTGGTAAT-CTTGTGGTTT-TAATTTGCAT  7683
Ferret       CAGTTTGTTTATCCATTCAACTCT---TGAACATTTGAGTTGTTTCCA-GTTTTTGAATATTGTAAATA-ATGCTGGCGT  6386
Lion         TCCAATGCTTGTCTTTCTGATTC----TAGACATTCTAGAGGGGGTGA-AGTGGTAAT-CTTGTGGTTT-TAATTTGCAT  6656
Vaquita      CAGTTTGTTTATCCACTCAACTCTTGATGGACATTTGGATTGTTTCCA-GTTTTGGGCTATAATAAGTA-AAGCTTCTGT  6053
Pig          CAATTTATTTG-CCACTCAACTCTTGATATATATTTGGGTTGTTTCCA-GTTTTGGGCTATTGTAAATA-AAGCTTGTAT  6469

Majority     GAACATTCXTGTG----XATGTCTTTTTTTTXATA-TATXCTTTC-----------------------------------
                              8250      8260      8270      8280      8290      8300      8310      8320
Human        CGCCATGCCAGACTAATTTTTTTTTTTTTTTCGAGACGGAGTCTCGC---------------------------------  5406
PolarBear    --------------------------------------------------------------------------------  0
Cow          GCAGGTCTTTTTGTAAACAAATGCTTTCATTTCCAGTTTACTGCCC--------------------GTG-----------  6478
Dog          TTTTTTCCTTGTCACTTTAGCTAGAGATTATTTTATTAA-----------------------------------------  6526
BelugaWhale  GAACATTCCTGTG----CAGGTCTTTTTGTGAATG-TAAGCTTTC-----------------------------------  6203
Donkey       GAACATTCCTGTA----CAAGTCTTTGTATGAACA-TATGCTTTC-----------------------------------  5958
Cat          TTCACTAATGTCTGTTGTGTTAAATATCTTTTC-----------------------------------------------  7716
Ferret       GGATATACTTACAAAAGTCTGTGGGAACTTTCATATC-------------------------------------------  6423
Lion         TTCACCAATGTCTGTTATGTTAAATATCTTTTCTTAGTTAGTTAGTTAGTTAGTTAATCTTAGTTAGTTAGTTAGGTAAT  6736
Vaquita      GAACATTCCTGTG----CAGGTCTTTTTGTGAATG-TAAGCTTTC-----------------------------------  6093
Pig          GAACATTCCTGTG----CAAGTCTTTTTATGAACA-TATGCTTTC-----------------------------------  6509
```

```
Majority     -------------------------------ATXTCXCTTGXGXX---------AATACCTATX-ATTGGAATGTCTAGA
                      8330      8340      8350      8360      8370      8380      8390      8400
Human        ------------------------CCGGTCTCCCAGGCTGGAGTGCAGTGTCGCGATCTCTGCTCACTGCAACCTCTGCC  5462
PolarBear    --------------------------------------------------------------------------------  0
Cow          -----------------------------GCATCCCCCATCTACATCCTCTC-TAGTGCTTGTT-ACTGTTTTTTCTTAA  6527
Dog          ---------------------------------------------TGTTCTCTAAATATCACCT-TTTGGTTTCTCTGAA  6560
BelugaWhale  -------------------------------ATTTCTCTTGGGGA---------AACACCTATG-ATTGGAATGGCTAGA  6242
Donkey       -------------------------------ATTTCTCTTGGGGG---------AATACCTATG-ATTGAAATGGCTGGC  5997
Cat          -----------------------------------------------------ATGTGTTTTCT-TTTGCCAGCTGCATA  7742
Ferret       ---------------------------------------------TCTTGGGGAAATACCTTTG-ATTGGAATGCCTGGA  6457
Lion         CTTGTGGTTTTAATTTGCATTTCACCAATGTCTGTTATGTTAAATATCTTTTCATGTGTTTTCT-TTTGCCAGCTGCATA  6815
Vaquita      -------------------------------ATTTCTCTTGGGGA---------AATACCTATG-ATTGGAATGGCTAGA  6132
Pig          -------------------------------ATTTCTCTTGGGGA---------AATACCTATG-ATTGGAATGGCTAGA  6548

Majority     TCAT--ATGGTTAGGCATATGTX--------CT--TTAACTTTTTAAGAAACTGCAAACTATTTTCXAAA--G--XTTGG
                      8410      8420      8430      8440      8450      8460      8470      8480
Human        TCCTGGGTTCACGCCATTCTCCTGTCTCAGCCTCCCAAGTAGCTGGGACTACAGGCGCCTGCCACCACGCCTGGCTTCTT  5542
PolarBear    --------------------------------------------------------------------------------  0
Cow          TTATAGACAGTCTGATGGATGTG------AACTGGTAATCTCTTTGTGGTTTTATTTTTCATTTTTCTAGT-GTCTTTGG  6600
Dog          TGTTCT--GTTTCTATCTGTTTT---ATTTTCTGCTTGCATTTAGTTCTTTTTTCAATTTATGTTGAAGCTTGAGGTCAT  6635
BelugaWhale  CCAT--ATGGTTAGGCATATGT-------------TTAACTTTTTAAGAAACTGCAAACTATTTTCTAAA------GTGG  6301
Donkey       TCAT--ATGGTAG-GCATATGT-------------TTAACTTTTTAAGAAACGGCCAACTATTTTCCGTA------GTGG  6055
Cat          TCATCTTTGGTGAAGTATCTCTTGAGATTGCCTGCCCACTTTTTAAATTGGGTTACTATTGAGTTTTAAGAAGCCTTCAT  7822
Ferret       TCATAT--GGTAG-GCATATGCT---TTTAACTTTTTAAAGTTTTAAGAAACGGCAGACCATTTTCCAAAGTGGTTGTAC  6531
Lion         TCATCTTTGGTGAAGTATCTCTTGAGATCGCCTGCCCACTTTTTAAATTGGGTTACTATTGAGTTTTAAGAAGCCTTCAT  6895
Vaquita      CCAT--ATGGTTAGGCATATGT-------------TTAACTTTTTAAGAAACTGCAAACTATTTTCTAAA------GTGG  6191
Pig          TCAT--ATGGTTTGGCATATGT-------------TTAACTTTTTAAGAAACTGCAAACTATTTTCCAAA------CTGG  6607
```

```
Majority     T-TTATAC-TATTTTAACAT------TGCXATCAGCAGGXTATGAGATTTTCXGTC----GTCXACATC-XXTTCCAGTG
                              8490      8500      8510      8520      8530      8540      8550      8560
Human        TTTTGTATATTCAGTAGAGACAGAGTTTCACCATGTTAGCCAGGATAGTCTCTATCTCCTGACCTCGTGATCTGCCTGCC 5622
PolarBear    -------------------------------------------------------------------------------- 0
Cow          TGTTGAACATCTTTTCATGG------ACTTTTTTGCAAGCTATATGTCATT----------GGTTAAGTC-TATTTGAATG 6664
Dog          TGACATACCTTTTCTAACATAG-AGGCTTAATGC-TAGGTGTTTCCCTTTTAAGTA------CAGCTTT-CATAGCATTT 6706
BelugaWhale  --TTATAC-TATTTTA-CAT------TGCCACCAGCAGGGTATGAGATTTCCAGTC---CATCTACATC-CTCTCCAGTG 6367
Donkey       --TTATAC-TATTTT-ACGT------TGCCACCAGCAGTGTATGAGATTTCCAGTC---CTTGCACATC-CTCTCCAATG 6121
Cat          TCTGGTTATAAGTCCTTTATCAGATATATGGTTCATAGATTTTTTTTTTTTTTTTTTTTTGCCAATTGT-AATTTCATTC 7901
Ferret       TATTTTACATTGCCACCAGTAGGATGTGAGATAT-CCAGTCCTTCCATATTCTCTC------CAATACC-TGTGATTGTT 6603
Lion         TCTGGTTATAAGTCCTTTATCAGATATATGGTTCATAGATTTTTTTTTTTTTTTTT---TGCCATTTGT-AATTTCATTC 6971
Vaquita      --TTATAC-TATTTT------------GCCACCAGCAGGGTATGAGATTTCCAGTC---CATCTACATC-CTCTCCAGTG 6252
Pig          --TTATAC-TCTTTTAACAT------TCCTATCAGCAGGGTATGAGATTTC----------ATCTGTATC-TTTTCTAGTG 6668

Majority     XCTGXTTTCTGACTTTT----XTTTXXAXTATTTAAATCTGAGTGAATTTGAAATX----X-TXXTTATGG--TTTTCAT
                              8570      8580      8590      8600      8610      8620      8630      8640
Human        TCGGCCTCCCAAAGTGCTGGGATTACAAGCGTGAGCCACCGCGCCTAGCCAAAATGCCAGACTAATTTTTGTATTTTTAG 5702
PolarBear    -------------------------------------------------------------------------------- 0
Cow          TCTGCTTTTCAAATTGA-----TTT-ATTTCTTTAAGTTTTGAGAAGTCTACATTC------TGGTTACAGGTTCTTTAT 6732
Dog          CTTAAATTTTGACACGCACAAGTATGTTTTTTGTGTATAGCATCAAGTTGGAATTTGATTTTTACATCCAACTTACACCC 6786
BelugaWhale  CCTGTTGTCTGGTTTTT-----TTT-GATTATAGAGATTCTAGTGGATGTGAAGTCGTAATGTCTTTGTAG--TTTTCAT 6439
Donkey       CCTGTCGTCTGTCTTTG----ATTCTCGACATTCTCGTGGGTGTGAAGTGGTAATC----------TTCTGG--TTTTAAT 6186
Cat          TCTCAGTAGTGTCTTTTAAAGAACAGCAATTTTTAATTCTGATGACCTTTACCATTTTTGTCTTCTTATGGCTTGTGCTT 7981
Ferret       TTTGATTGGAGACATTCTGGTAGGTGTGGAGTGGTAATCTCCTTGTGGTTTTAATTTGCATTTCTCTAATATCTATGATA 6683
Lion         TCTCAGTAGTGTCTTTTAAAGAACAGCAATTTTTAATTCTGATGACCTTTACCATTTTTGTCTTCTTATGGCTTGTACTT 7051
Vaquita      CCTGTTGTCTGGTTTTT-----TTTTGATGATAGAGATTCTAGTGGATGTGAAGT------------GTGG--TTTTCAT 6313
Pig          CCTGTTCTT--GATTA-------T--AGACATTAAAGTGGATGTGAAATAGTAATC------TCTTTGTGG--TTTTAAT 6729
```

```
Majority     T--XTGCATT-TTTATAAXATCTT----------TGGT-------GXTXXACTTC--TTTTCATGGGCTTTTTXC-----
                      8650      8660      8670      8680      8690      8700      8710      8720
Human        TAGAGACAGGGTTT-CACCATTTTGGCCAGGA--TGGTCTTGATCTCTTGACTCCATCATCCGCCCGCCTTGACTTCCCA  5779
PolarBear    --------------------------------------------------------------------------------  0
Cow          CAGATATATGGTTTGCAGTATTTT----------CTGCCTTTTTTGTTTTGATTC--TCTCAATGGTGTTTTGAA-----  6795
Dog          CCTTCTTTTGACTAATAGCATTTT----------AAAACGATTTTGCTGTTTTTC--TATTTATGCCTTGTGTTCTTCCC  6854
BelugaWhale  T---TGCATT-TCTCTAATGTCTT----------TGGT-------GCTGAACAT----TTTCATGGGCTGTTTTC-----  6489
Donkey       T---AGCGTT-TCTGTAATGTGTA----------TGAT-------GTTGAACATC--CTTTCATGTGCTTTCTGC-----  6238
Cat          TTCATGTCTTGTCTAAGCCATCTTTGCCTAACCCAAGGTCAGAAAGATTTTTTTC--CTTTGATTTGTAATAGAAGTTTC  8059
Ferret       TTGATTATCTTTTCAT-GTACTTT----------AAAAAAAAATTATTTATTTAT--TTGACAGAGATCACAAGTAGATG  6750
Lion         TTCAGGTCTTGTTTAAGCCATCTTTGCCTAACCCAAGGTCACAAAGATTTTTTTT--CTTTGATTTGTAATAGAAGTTTC  7129
Vaquita      T---TGCATT-TCTCTAATGTCTT----------TGGT-------GCTGAACATC--TTTTCATGGGCTGTTTTC-----  6365
Pig          T---TACATT-TTTGTAACATCTG----------TGGC-------GTTGGACATC--TTTTCATGGGCTTTT--------  6778

Majority     AAXTTGX-TGTTTCAXCTTTGGTGCA------------------GTGTTTTTTAAATX----XT-TTTTTGTTCACTTTT
                      8730      8740      8750      8760      8770      8780      8790      8800
Human        AAGTGTTGGGATTACAGGCTTGAGCCACCGCGTCCGGCCCATATCTTGCCTTTAAAAAA--AATAGCTTTATTGAGATAT  5857
PolarBear    --------------------------------------------------------------------------------  0
Cow          AAATAGCATTTTTTAATTCTGATGAATTTAAGTTTACCAG---GTGTTTGCTAGTGTGGCTCATGCTTTTGGTCATATCT  6872
Dog          GTATTCC-TGCTTCTGCTTTTGTGCATACGGTACTACTGTTT---AATTCCCTATATGTCTTGTTAGTTGATTAGCTTGT  6930
BelugaWhale  AAGCTG--TGTATCATCTTTGGTACA------------------GTGTCTTTTAAAT--------TTCTTGTCCACTTTT  6542
Donkey       CAGCTG--AATATCGTCTTTGGTGAA------------------GTGTCTGTTTCAAT--------CTCTTGCCTACTTTT  6291
Cat          ATAGTTTTGGGTTTTGCATTTGAGTTTTTTTTTTTCTTTCTTTTCTTTTTTTAATATGGTGCATTTCTTTTTTGGGGGGT  8139
Ferret       GAGAGGC-AGGCAAAGAGAGAGAGGAAGAAGCAG-GCTCCCT---GCTGAGCAGAGAGGCTGATGTGGGGCTCCATCCCA  6825
Lion         ATAGTTT-GGGTTTTGCATTTGAGGTTTTTTTTTTTTTT------TTTTTTTAATATGGTGCATTTCTTTTTTGGGGGGT  7202
Vaquita      AAGCTG--TGTATCATCTTTGGTACA------------------GTGTCTTTTAAAT--------TTCTTGTACACTTTT  6418
Pig          AAATTTTCTGTGTCATCTTTGGTGCA------------------GTGTCTGTTTAAAT--------CTCTTGCCCACATTT  6833
```

```
Majority     XAAA--TTGATTTATXXC---CTTTTTTTXAGTTTTGAGXXGTCTACCTTTTAX-X--CGTTACAXCTXCTTT-------
                      8810      8820      8830      8840      8850      8860      8870      8880
Human        AATTTGACATAAAATAAACTACCCATATTAAATATACAATGTTTTATCTCATATATACCCTTGTGAAACCACTGCAATCA  5937
PolarBear    --------------------------------------------------------------------------------  0
Cow          AAGT--CATCTTTAGGAT---CTTCCCTGGTGGCTCAGACGGTAAAGCATCTGCCT-ACAGTACCTACCATGTGGGAG--  6944
Dog          GATTCTTTGTTATATTAATGGCTGTTTTTAGGAGTTGTAGCATAGATCTTTAACTTAACCGTAGTTCTTCCCTCAAGG--  7008
BelugaWhale  TAAA--TTGATTTA--------TTTCTTTGAGTTTTGAGACGTCTACATTTCTG-----GTTACAAGTTCTTT-------  6600
Donkey       TTAA--TTGGGTTATTTCT--TTATTATTGACTTTTGAGAAGTCTTCATTCTG------GTTACGAGTCTTTT-------  6354
Cat          ATGGAGTTAAACTTTTTCCAGCACTGTTGAAAAGGCTGTCTTTTGTTCATTGAATT-GCTTTGCACCTTTGTCAAAAATC  8218
Ferret       GAACCCTGGGAATATGAC---CTGAGCTGAAGGCAGAAGCTTTAACCCACTGAGCCAGCCAGGCCCCCCTTTTCATGT--  6900
Lion         ATGGAGTTAAACTTTTTCCAGCACTGTTGAAAAGGCTGTCTTTTGTTCATTGAATTCGCTTTGCACCTTTGTCAAAAATC  7282
Vaquita      TAAA--TTGATTTA--------TTTCTTTGAGTTTTGAGAAGTCTACATTTCTG-----GTTACAAGTTCTTT-------  6476
Pig          TAAA--TTGATTT----T---TTTCTTTTGAGTTTTGAGAAGTCTACATTCTG------GGTACCCATCCTCT-------  6891

Majority     ----ATCXGA--T-XTXT---X-GXTTTGTAATATTTTCTG-------------CCACTTTTGXTTTXATTCXCXTCX--
                      8890      8900      8910      8920      8930      8940      8950      8960
Human        AGATAATGAACATCTATCACTCCCTACAGTATCATCTTGCGCCTTTATAGTCCCTCCTTTCTGCTTTTCTTTCCCCAAGG  6017
PolarBear    --------------------------------------------------------------------------------  0
Cow          ----ACCCGGGTT-CAATCCCTCGGTCGGGAAGATCTCCTGGAGAAGGAAATGGCAACCCACTCCAGTATTCTTGCCTGG  7019
Dog          -AGTATTTATGCCACTTCACATAAATTATGTGAGTATTAGAATACTTTATTTCTCCTCTCTTGGCATCTGTGCTATTGTT  7087
BelugaWhale  ----ATCAGA--T-ATAT-----GGTTTAAAATATTTTCTG-------------CCACTTGTGTTTTCATTCTCTTC---  6652
Donkey       ----ATCAGATACATGGTT----TACAGATATTATTTTCTG-------------CCACTTAATATTTTCATTCTCTCA--  6411
Cat          AGCTATCTGTATGAGTGGTTCTATTTCTGTACTGTATCCTTTTCCATTGACCCATTTTTGTCCATGTAGATGCCAAAAAC  8298
Ferret       -ACTTTTTGCCAC-CTGTATATCATCTTGGATGATGTGTCTCTGTATCACTTGCCCACTTTTTGAATTGGGTTACTGTTG  6978
Lion         AGCTATCTGTATGAGTGGTTCTATTTCTGTACTGTATCCTTTTCCATTGACCCATTTTTGTCCATGTAGATGCCAAAAAC  7362
Vaquita      ----ATCAGA--T-ATAT-----GGTTTAAAATATTTTCTG-------------CCACTTGTGTTTTCATTCTCTTC---  6528
Pig          ----ATCAGA--T-AAAT-----GGTTTGAAATATTTTCTG-------------CCACTTATGTTTGCACTCTCTC----  6942
```

```
Majority     A--CAXTGTCTTGATTAXTGXTA--GTXTAGTAGTTTTTAATTC-----X-------X-------X--------------
                              8970      8980      8990      9000      9010      9020      9030      9040
Human        AACCACTGATCTGCTTTCTGTCACTATAGATTAGTTCACACTTTTTAGAATTTTAGATAA-----ATGTAATCATATGGT  6092
PolarBear    --------------------------------------------------------------------------------  0
Cow          A--AAATCCCATGGATGGTGGCA--CC-TGGTAGGCTACAGTCCATGAGGTCGCAAAG-------AGTCGGACAC---GA  7084
Dog          A--TACATCTTCCGTAGATGTTA-TAAATTCTATTGTTTGTTTATTGAAACAGCCAAT------TACCTTTTTTAAAAAA  7158
BelugaWhale  A--CAGTGTTTTGAAGAGCAGTA--GTGCAGCAGTTTTTAATTC------------------------------------  6692
Donkey       A--CAGTGTCTTGA-----AGAA-----CAGCAGTTTTCAATTC------------------------------------  6443
Cat          A--TACTGTCTTAATTACTGTGACTTTATGTTAAGGCTTGACATCAAGCAATCATGGTTTTCTTTATTTTTTTTT-AAAG  8375
Ferret       A--CTTTTAAGAGGTCTTCATTC-TGGTTATAAGTCCTTAATCAGATACATGGTTAATA-----TTTCCTACTATTTGTG  7050
Lion         A--TACTGTCTTAATTACTGTGACTTTATGTTAAGGCTTGACATCAAGCAATCATGGTTTTCTTTATTTTTTTTTTAAAG  7440
Vaquita      A--CAGTGTTTTGAAGAGCAGTA--GTGCAGCAGTTTTTAATTC------------------------------------  6568
Pig          A--AAGTATCTTGG-----GGAA--C---AGTAGTTTTTAATTC------------------------------------  6974

Majority     -TGACXAAXTTCAGTTAX-CXXXTGXTAT-XTTAGAGGATTXXTTTTT-X---AGTGAGGCTTGC--GTXTTTTATTAXA
                              9050      9060      9070      9080      9090      9100      9110      9120
Human        ATGTACATTTTTGGTC-----TGGGTTAGTCCACTCAGAATAATTATT------TTGAAATTCACCCATGTTGTCTTTCA  6161
PolarBear    --------------------------------------------------------------------------------  0
Cow          CTGAGCGACTTCACTCACTCACTCATTGT-CTTTACGGCTTCCCTGGTGGCTCAGTGAGGGCTTC--TCAGTGGATCAGG  7161
Dog          ATATCTTA-TTTATTCATTCATGAGAGACACAGAGAGGCAGAGACAATAGGCGAGAGGGATATGC-GGTCTCCCTTCACT  7236
BelugaWhale  -CGATGAATTTCAGTTTACCAGGTGCTCTGTGGTGTGTATTTTTTTTTGGTTTGGTGTGGCTCAT--GCTTTTGGTCATA  6769
Donkey       -TGACGAACTTCAGT----------------TTACCAATTTTTATCTC------GTATGGATCGT------GCTTTTA--  6492
Cat          TTGTTTTGGCTCTTCTATGTCCTTCATGTTTTTATAGGAGTCAGTTTCTAC--AGAAAAGCTTGCTGGGATTTTATTGGA  8453
Ferret       TTAGCTTT-CTCAGTAATGCCTTTTAAAGAACAGCAGTTTTCAATTCTAATGAACTGAAGTTTGCCAATTTTATCTTGTA  7129
Lion         TTGTATTGGCTCTTCTATGTCCTTCATATTTTTATAGGAGTCAGTTTCTAC--AGAAAAGCTTGCTGGGATTTTATTGGA  7518
Vaquita      -CGATGAATTTCAGTTTACCAGGTGTTCTGTGGTGTGTATTTTTTTTTGGTTTGGTGTGGCTCAT--GCTTTTGGTCATA  6645
Pig          -TGACGAACTTCAGT----------TTAC-CAAGGTGGTTTTTTT------TTGGCAGGGGGTAG--GTGTTGGATTA--  7032
```

```
Majority      XGTAACCXAA---TA-TTXXAXATCTAAGXCATA-TTGAXXTXATCXCAAGGTTACAAATXGTXXTTCTTT---X-AXTT
                       9130      9140      9150      9160      9170      9180      9190      9200
Human         A-TAGTCCATT--CTGTTTTATTGCTTAGTAATAGTACACTTTATGGCTATACAGCAATTTGTTAATCCATTCAAGTCTT  6238
PolarBear     --------------------------------------------------------------------------------  0
Cow           GGTAAAGAAAC--CACCTGCAATGCAGAA-GATA-CAGGTTTGATCCCTGGGTTAGGAAGATTCCCTGGAGA--AGGAAA  7235
Dog           GGTAGCCTGA----AATGGCATTACTAAGA------TTCCCGGAC--TAGGACCACCAGCTGCCTGTATGACCCAGTATA  7304
BelugaWhale   TATAAGCCATC--TTTGTGTAACCCAAGGTCACA-ATGATTTTCTTCTCTGTTTTCTAATAGAAGTTTTAC-----AGTT  6841
Donkey        --G-------------TGTCGTATCTAAGCCATC-TTTGTCTAACC-CAAGGTCACAAAGATTTCTCCTTT---------  6546
Cat           AGTAACTTGAATGTAAAAAGCAATTTGAGGTAAA-TTGACATCATGACAATGTTGAAGTTTCCCACTCATGAACACAATT  8532
Ferret        GGTCTCCTGCTTTTGTTGTCATATCTAAGCCATC-TTTGCCTAACC-CAAGGTCACAAGATGTTCTCCTTTGATTTGTGA  7207
Lion          AGTAACTTGAATGTAGAAAGCAATTTGAGGTAAA-TTGACATCCTGACAATGTTGAAGTTTCCCACTCATGAACACAGTT  7597
Vaquita       TCTAAGCCATC--TTCGTGTAACCCAAGGTCACG-ATGATTTTCTTCTCTGTTTTCTAATAGAAGTTTTAC-----AGTT  6717
Pig           -------CA-------CTTTGTCACAAAC-CATC-TTTGTCTGA-CCCAAGGTCACAAAGATTTTTCCTTT-----GTTT  7090

Majority      TXTXXATTT-------GCTTTT-TXTTTTCTTTTAGTAATTTTTXTGTTXTGTTAAATTTTTTXTGT---------AGAG
                       9210      9220      9230      9240      9250      9260      9270      9280
Human         GAGGGACATTTGTATTGTTTTC-CGTTTTGAGCTAATAAAAATATAGTT--GTGAACATTACTGTACA--------AGTC  6307
PolarBear     --------------------------------------------------------------------------------  0
Cow           TGGCAACCC-------ACTCTAGTATTTTTCCTTGGTAAATACCATGGACAGAGGAGCCTGGTGGGTT--------GCAG  7300
Dog           TATTTATCT---GTAAACATTGTAATTATCAGGGTGGATTCTTTTTTTTTTTTTTTTTTGTTTTTAC---------AGGA  7372
BelugaWhale   TTTAGTTTT-------GCATTTAAGTCTTTTTGAATTAATTTTTTTAATATGGTGCAATGTATGAATC--------AAGG  6906
Donkey        -----GTTT---------TCT-----------------------------------------------------------  6553
Cat           TATCCATTTACTTAGATCTTCTTTAATTTCTTTTAGCAGTGTTTAGGTTTTTTTAAATTTATTTTGAGAGAGAGTGAGAG  8612
Ferret        TTGAAGTTT---TACAGTTTTTGGTTTTGCATTTAAGTTTCTTTGAGTTAATTTTTTTTTTTTTTGT---------AGAT  7275
Lion          TATCCATTTATTTAGATCTTCTTTAATTTCTTTTAGCAGTGTTTAGGTTTTTTTAAATTTATTTTGAGAGAGAGTGAGAG  7677
Vaquita       TTTAGTTTT-------GCATTTAAGTCTTTTTGAATTAATTTTTTTAATATGGTGCAATGTATGAATC--------AAGG  6782
Pig           TTTTAATTT-------TTTTT--TGTTTTGTTTTGGCTGCTCCCAGATCCTGTGAAAGTTCATAGGCC--------AGAT  7153
```

```
Majority     TTTATXTGGTTXCAAGA-XGAGATTX-XXT-AXAGAG-AGACX-TGAGTGAGAATXCTAGGTCXTT-GTGGAACTX--XT
                      9290      9300      9310      9320      9330      9340      9350      9360
Human        TTTGTATGGACATATGCTTTCATTTCTCTTGGTGGGGCAGGCTATGATTGGAAAGTCTGGATCATGTGATAGGCATATGT  6387
PolarBear    --------------------------------------------------------------------------------  0
Cow          TCCATGGAGTCACAAAA--GAGTCTGAAGTGACTTAG-TGAC--TAAGTAATAAAAACAGGTCATT-GTGAAACC-----  7369
Dog          TTTATTTACTTTGATGAATGAGAG-------ACAGGT-AGAGATCGAAAGAGAGTCAGACGACACCGGCAGAGTTCCGTG  7444
BelugaWhale  TTTTTTGGGTTTCCATATGGACATTCACTTTTTCTAG-CAACACTCATTGAAAAGACTATTTTTTTCATTGAACTGGCTT  6985
Donkey       -------------------------------------------------A------------------------------  6554
Cat          TGCACATGTGTGCAGGAATGAGGGAGGGGC-AGAGGG-AAGGGGAGAGAGAGAATCCCAAGCAGGCTCTGTACTGACAGT  8690
Ferret       TTTATTTCTTTATTTGACAGAGATC------ACAAGT-AGACAAAGTGATAGGCAGCGGGGTTGGGGGGGGGAGCTGAGC  7348
Lion         TGCACATGTGTGCAGGCATGAGGGAGGGGC-AGAGAG-AAGGGGAGAGAGAGAATCCCAAGCGGGCTCTGTACTGACAGT  7755
Vaquita      TTTTTTGGGTTTCCATATGGACATTCACTTTTTCTAG-CAACACTCATTGAAAAGACTATTTTTTTCATTGAACTGGCTT  6861
Pig          ATCGAACCCATGCCACA--GCAGTTGCCTTGAGTCAC-TGA-----AGTGACAATGCTGGATCCTT-AACCCACTG----  7220

Majority     --CACGXCCCATGXAXXTCT-TAACCTATGTA--CXTTTGXATCA--TTATCTAAGXTXXAGTCATGC-------ATTTA
                      9370      9380      9390      9400      9410      9420      9430      9440
Human        TTAACTTTTTAAGGAACTGCAAACTTTTTAAAAGTAGTTGTACCATCTTACAT-TGCCATCAGCATGGTATGAAATTTCC  6466
PolarBear    --------------------------------------------------------------------------------  0
Cow          --CAGG-GTCACAAAGATT--TCTCCTTTGT---TTTCTGATAGA--TTTTATAGTTTTTAGTTTTAC-------ATTTA  7432
Dog          GAGCCGCCCCATG-GGACTTTGATCCCCGGGA--CTCCAGGATCAGATTCCCCAGGCCAAATGCATGC-------GCTAA  7514
BelugaWhale  TGCACCTGTATCAAAAATCAATTATGTATGTA--CGTATGATTCTGTTTCTGTACTGTATTCTCGTCC-------ATT-G  7055
Donkey       ----------ATAGAAGTTT-TAT------------------------------AGTTTAGGTTTTGC-------ATTAA  6586
Cat          GAGGAGCCCGATGCAGGGCTCGAACCCACAAA--CCGTGAGATCA--TGATCTGAGCCGAAATCAGGCACTGGATGCTTA  8766
Ferret       AGAGAGCCCCATG-TGGGTCTTGATCCCAGGA--CCCTGAGATCA--TGACCTAAGTGTAAGGCAGAG-------GCTTA  7416
Lion         GAGGAGCCCGATGCAGGGCTCGAACCCACAAA--CCGTGAGATCA--TGATCTGAGCCGAAATCAGGCACTGGATGCTTA  7831
Vaquita      TGCACCTGTATCAAAAATCAATTATGTATGTA--CGTATGATTCTGTTTCTGTACTGTATTCTCGTCC-------ATT-G  6931
Pig          --CACC-ACCGAGGAACTCC-TCTCCTTTGT---CTTCTAAAAGAAGTTTCATAGTTTTTAGTTTTGC-------ATTTA  7286
```

```
Majority     ACCCATT------TXTAGGTACCTCTT----------TTAATATGGTXXAXTGTCTGAATAAATT---------------
                      9450      9460      9470      9480      9490      9500      9510      9520
Human        AGTCCTTCCACATCCTCTTTAATGCCTGTTATCTTCTGTTTTTGATTATAGATATTGTAGTGTATATGAAGTGATATTTC  6546
PolarBear    --------------------------------------------------------------------------------  0
Cow          AGTTCTT------TTTAACT---TTTT----------TTAATATGATGCAATGTATGAATCAAGA---------------  7478
Dog          AACCACTGAGCCAACGCGGTATCCCTC----------GGCGGGGGGTATGATCCCTTACCAGATT---------------  7569
BelugaWhale  ACCCATT------TTTGTCTACCTAGT----------AGCCAAAAGCGTACTGTCTTAATTACTG---------------  7104
Donkey       AGTCTTT------TTGAGTTAATTTTT----------TGAATATGGTGCAAAGTCTGAATCAACG---------------  6635
Cat          ACCCATTGAGCCACCCAGGCGCCCCAGCAGTGTTTAGTTTTCATTGTACAGTTTTTGTACATTTT-GTCATATTTATTCC  8845
Ferret       ACCCACTGAGCCACCCAGGTGCCCCAT----------TTAATTTTTTTTAATATGTGCAAAAATA---------------  7471
Lion         ACCCACTGAGCCACCTAGGCGCCCCAGCAGTGCTTAGTTTTCATTGTACAGTTTTTGGACATTTTTGTCAGATTTATTCC  7911
Vaquita      ACCCATT------TTTGTCTACCTAGT----------AGCCAAAAGCGTACTGTCTTAATTACTG---------------  6980
Pig          AGTCTTT------TTGAGTTAATTTTT----------TTAATACGGTCCAGTGTATGAATCAACT---------------  7335

Majority     ----------TTTTTTGGTGTTG--------GATATGGACATXAACTTTTTXTXGCATCATTTGTTGAAAAGACTATXCT
                      9530      9540      9550      9560      9570      9580      9590      9600
Human        ACTGTGGATTTAATTTG-CATTCCTCTAATATCTATGATGTTGGACATCTTTTCGTGTGCTTTTTGTCAGCTGTTATCTT  6625
PolarBear    --------------------------------------------------------------------------------  0
Cow          ----------TTTTTTGGATTTG--------AATATGGAAATTCACTTTTTTTAGCATGATTTGTTGAAAAGACTTTTTT  7540
Dog          --CCCAGAGGCGTCTCG--------------GCTGTG--C--AAACTCTCTTTGGTATTGTTTGCTGTAGAAAAACGACT  7629
BelugaWhale  ----------TAGTTTATATTA---------AATCTTGAAATCAAGTAGAACTTTGTTCTTTTTCAAAGTTGTTTTGGCT  7166
Donkey       ----------TTTTTTGGTGTTG--------AATATGGACATCCACTTTTTCCGACACCATTCGTTGAACAGGCTGTCCT  6697
Cat          TATTTCAGTGTTTTTTGGTGCTGTTATAAATGGTATTTTTAAAATCTCAGTTTCTAGTTAGTGGTGGAGAAAAATACAAA  8925
Ferret       --AGTCAGCATTTTTTGTTTCGG--------GGTATGGACTTAAACTTTTTCCAGCACCATTTGTTGAAAAGGCTGTCCT  7541
Lion         TATTTCAGTATTTTTTGGTGCTGTTATAAATGGTATTTTTAAAATCTCAGTTTCTAGTTAGTAGTGGAGAAAAACACAAA  7991
Vaquita      ----------TAGTTTATATTA---------AATCTTGAAATCAAGTAGAACTTTGTTCTTTTTCAAAGTTGTTTTGGCT  7042
Pig          ----------TTTTTGGGAGTTG--------GATGTGGACATCCACTTGTTCTAGCACCATTTGTTGAAAAGACTATTCT  7397
```

```
Majority     TTTTTTGTTGAXTXXGCTTTXCATCTTTGTCAAAAACXA---CAXTTATTTATGTATGTT----XTTTTTTTGAAXTGTA
                      9610      9620      9630      9640      9650      9660      9670      9680
Human        T--GGTGAAGTGTCTGTTTATATCTTTAGCCCAATTTTT---TATTTGGTTATTTATTTTAAATTGAGTTTTGAGAGGTC 6700
PolarBear    -------------------------------------------------------------------------------- 0
Cow          TTTCTAATTGAATTGGCTGTGCATCTTGGTCAGAAACAA---TTATTAT--CTACATATG-----ATTCTTTGTACTGTA 7610
Dog          T--ATTGTGGTCTCTGTGTGGACTCCCTGTC-----------TACTTACTTAGGAGTTTT---TTTGGTTTTGAAGTTTG 7693
BelugaWhale  CTTCTGGGTCCTTCACATTTTCCTGTGAGTCAGTTTCTA---CAAAAAGGCCTGCTGGGA-----TTTAATTGAAATTAA 7238
Donkey       TTCTCCCTTGAATTGGCTTTGCACCTTTGTCAAAAAT-----CACTTATCTATGTATGTATG-ATTCTATCTGTACTGGA 6771
Cat          TATTTTGTGTATTGTTCTTGTATTATTCACTCTTGCCAAACTCACTTGTTAAGATATAATAG-CTTTTTTTGTAGGCGCT 9004
Ferret       TTGTTCATTGAATTGGCTTTGCACCTTTGTCAAAAATCAAC-TATCTTTTTATGCATCAT---TCTATTTCTGTACTGTA 7617
Lion         TATTTTGTGTATTGTTCTTGTATTATTCACTCTTGCCAAACTCACTTGTTAAGATATAATAG-CTTTTTTTGTAGACGCT 8070
Vaquita      CTTCTGGGTCCTTCACATTTTCCTGTGAGTCAGTTTCTA---CAAAAAGGCCTGCTGGGA-----TTTAATTGAAATTAA 7114
Pig          TTCTCCATTGAATTGGCTTTGCACCTTTGTCAAAAATCA---ATAATA-----GTATGT------ACCTGTGATACTGTA 7463

Majority     TTCGTTTXCTTTGACCAATTTXTGTXAATTTAXXX-CCXAAAAXTGXATTCTCTTA-TTXTTGT-------XTTATATTA
                      9690      9700      9710      9720      9730      9740      9750      9760
Human        TTCATTGTGGTTACAAGTCCTTTATCAGCCATAAGGCCTGCAAATATTTTCTGCCACTCATCTTCTTAT-TCTCGCATTG 6779
PolarBear    -------------------------------------------------------------------------------- 0
Cow          TTCTTTTTCATTGACCCGTTTTTGTCTATTTAGTA-GCCAAAAATGTACTATCCTA-CT-TAGT-------TTTATATCA 7680
Dog          GCCGTTTTCTCCACCCAAGCTGTGCCATGGCCAGAGCCTGTGCCATGGCCCGGTC--TTTC--T-------CTTAAGAGA 7762
BelugaWhale  CTTGAATCAGTAGAGCAATTTGGGGAAAATTAACATCTTAATGTTGAGTTTTCTCA-TCCATGA-------ACACAATTT 7310
Donkey       GTCGT-TCCATTGGCT-G--TCTATTAGAT------GCCAAAAACATACTGTCTTAACTGCTGTG----ACTTTATGTGA 6837
Cat          ATCAGGTTTTCTACATAGACCGTCTTACTTTTTCCTTTCCTATCTGGATGCCGTTT-TTTTCCTGTGATATCTTAGTACA 9083
Ferret       TCCTTTTCTTTTGACCCATTTTTGTCCTTCCAGATGCCTAAAACCTGATGTCTTAA-TTACTATG----ATTTTATATTA 7692
Lion         ATCAGGTTTTCTACATAGACCATCTTACTTTTTCCTTTCCTATCTGGATGCCGTTT-TTTTCCTGTGATATCTTAGTACA 8149
Vaquita      CTTGAATCAGTAGAGCAATTTGGGGAAAAT------CTTAATGTTGAGTTTTCTCA-TCCATGA-------ACACAATTT 7180
Pig          TTCTTTTCCATTGACCCATTTTTGTTTATTTAGTT-GCCAAAAACATACCGTCTTA-CTATAGT-------TTTATATGA 7534
```

```
Majority     A----GTCTTGAAXT---XCAXTTAGGXX------C---TTTXTTTTTTTTCAGAATTGTTTTGGCTTTTCXTGTGT---

                      9770      9780      9790      9800      9810      9820      9830      9840

Human        T----CTTTTGAAGAGCAACAGTTATTAAA----TCTGATGGACTCCAGTTTACCAATTTTTTCTTTTATGGATTGTA--  6849
PolarBear    --------------------------------------------------------------------------------  0
Cow          A----ATCTTGAAAT----AAAGTGGAAC----------TGTGTTTGTTTTCAAAGTTGTTTTGGCTCTTC-TGGGT---  7738
Dog          G-ACTAAGCTGGAG----GCAGTCATGGA------CTCATTCATTTGTTTTTTGAAT---TTCAGGGATCAATGTCC---  7825
BelugaWhale  A----TCTTTCCATT----TACTTAGGTC----------TTTCATTTCTTTCAGCATTGTTTTAGTTTTCACTGTAT---  7369
Donkey       A----GTCTTAAAATCAAGTAGTCATGATC--CTCCAACTTTGTGCTTTTTCAAAGTTGTTTTGACTCCGGGTCCTT---  6908
Cat          GTGCTGAATAGAAGTGATGATATTAGGGTGAACACCTAAATGCTATTCTTTCTTAAATGTTTGGGCAGTTCACCAGTGAA  9163
Ferret       A-ATTGTCTTGAAATCTAGCAGTCATGGT------CCTCTTTGTTCTTTCTCAGAGTTGTTTTGGCTCTTCCCATGT---  7762
Lion         GTGCTGAATAGAAGTGATGATATTAGGGTGAACCCCTAAATGCTATTCTTTCTTAAATGTTTGGGCAATTCACCAG----  8225
Vaquita      A----TCTTTCCATT----TACTTAGGTC----------TTTCATTTCTTTCAGCATTGTTTTAGTTTTCACTGTAT---  7239
Pig          A----GTCTTGAAAT----CAAGTAGAAC----------TTTGCTGTTTTTCAAATTTGTTTTGACTTTT--TGGGT---  7591

Majority     --------------------TTXTGTTTTTXTATGAXTCAATTXXTCCATA-AAXXXGCTTGCTXGGTT-----TTAATT

                      9850      9860      9870      9880      9890      9900      9910      9920

Human        -----------------GTTTTGGGGTCTTGTCTGAGCCATTTTTGCCTGACTGTAAGCCATGAAGATTT---TTTTCCT  6909
PolarBear    --------------------------------------------------------------------------------  0
Cow          -----------------CCTTTACATTTTCCTATGAGTCAGTT--TCTACA-GAAAGGCCTGCTGGAAT-----TTAATT  7793
Dog          ----------------------ATCATTGTCTGATGTTCACTGGTTTTG---AAAACCATTGTTATG-------TACATC  7873
BelugaWhale  -----------------AGATTTTGTATTTTTTTGGTCAGATTTATCCTTA-TTTTGTGTTTTTTGGTG-----ATATTT  7426
Donkey       ---------------------TATGTTTTCATATGAGCCAGTT--TCTACAAAAAATGCCTGCTGAGAT-----TTTATT  6960
Cat          GTTGTGTGGGCCTGATGTTGTTTTCTTTGTGGAGGTTTTAAATGACACATTTAGTTTATTTAATAGATCCAGGACTACTC  9243
Ferret       ----------------------CCTTTTGTATATGAGTCAATTTCTACA---AAAACGCTTGCTGGGAT-----TTTATT  7812
Lion         ------T-----------T---TTCTTTGTGGAGGTTTTAAATGACACATTTAGTTTATTTAATAGATCCAGGACTATTC  8285
Vaquita      -----------------AGATTTTGTACTTTTTTGGTCAGATTTATCCTTA-TTTTGTGTTTTTTGGTG-----CTATTT  7296
Pig          -----------------TCTTTACGTTTTCTTATGAGTCAGTT--TCTACA-CAATAGCCTGCTGGGAT-----TTAATT  7646
```

```
Majority     TAAATTAATTTGAATTXTTAAXGCAATTTTGGTXAXAT-AGCATCTGA---GXGTTXGXCTXTCXXACCCAXGXACATAA
                      9930      9940      9950      9960      9970      9980      9990     10000
Human        TTTGTTTGTTAATAATAATAATAATTTTTTTTTTAGAGACAGGACCTGA-----CTCTGTCACCCCAGCTGGAGTGCAGTG  6984
PolarBear    --------------------------------------------------------------------------------  0
Cow          GAAATTAACTTGAATCAATAGAGCAATTTGGGGAAAATTAACATCTTA---ATGTTGAGTTTTTCCACCCATGAACACAA  7870
Dog          AGGTGTGATTTTTTTTTTTTTTTAAGTTCTTTTAATAGGGTAAACTGGTGTGTGTTACATCACCTTGCCTGGAGGTAGAA  7953
BelugaWhale  TAAAGTAATTTAAAAAATCAGGGCTTCCCTGGTGGCGC-AGTGGTTGA---GAGTCTGCCTGCCGATGCAGGGGACATGG  7502
Donkey       TTAATTAACTTGAGTCTATAAAACAATTTGGAAAAGATTGACATCTTAATAGTGTTGAGTTTTTCCATCCATGAACACAA  7040
Cat          AGGTTATCTATTTCTTCTTAAGTGAACTTTGGTGATTTGTGCCTTCCGTGTGAATTAGTCCATTTCATCCAAGTTATTGA  9323
Ferret       GGAGTTAACTTGAATGTGTAAAGCAGTTTGAGGTAAACTGACATCCTAACAGTCTTGAGTTTTCAAACTCATGAACACAA  7892
Lion         AAGTTATCTATTTCTTCTTAAGTGAACTTTGGTGATTTGTGCCTTCCGTGTGAATTAGTCCATTTCATCCAAGTTACTGA  8365
Vaquita      TAAAGTAATTTAAAAAATCAGGGCTTCCCTGGTGGCGC-AGTGGTTGA---GAGTCCGCCTGCCGATGCAGGGGACGTGG  7372
Pig          GAAATTAACTTGAATCTGTAGAGCAGTTT-------------------------------------------------AA  7677

Majority     GTXCATTTXCXX----TTTATTATGATCTTTTATTXXTTGAAGCXGTTGXTXTTTTTAG-XT-T-X-XX---GCCATTTT
                     10010     10020     10030     10040     10050     10060     10070     10080
Human        GTGCAATCGTAG----CTCATGATAACCTTGAACTCCTGGACACAAGGGCTCCTGCTTCAGCCTCCCAAGTAGCTAGGAC  7060
PolarBear    --------------------------------------------------------------------------------  0
Cow          ATT-ATCTTAACA---TTTAATTCAATCTTTTACTTTTTGGAGCAGTTATATTTTCACT---------------------  7925
Dog          GTCCATCTATTT----TTTTTTTTCACTTTTTTTTAGAAAATTTAATAATGTTTGTCTGCATTTTTTTATCTGCTATCTG  8029
BelugaWhale  GTTCGTGTCCTGG---TCTGGGAAGATCCCACATGCCGTGGAGCGGCTGGGTCTGTGAGCCATGGTCTGTGAGCCATGGC  7579
Donkey       TTTATTTCTCCCGTAGATCATCTTTAATCTCTTTCA---GCAGTGTTTTTAGTTTTCACTATACAGGTTTTGCACATTTT  7117
Cat          GTGTATTTGCATAGAGTTGTTTATGATAATTACCTTTTT-ATACTGTTGATGTCTGTAGAATCTGTAGTGCTGTCACTTT  9402
Ferret       CTCCTCTTTCTC----TTTACCTAGATCTTCTTTTAATTTCCTCCAGAAGTGTTTTTAGTGTATAGTTTCTGTACATTTT  7968
Lion         GTGTATTTGCATAGAGTTGTTTATGATAATTACCTTTTTTATACTGTTGATGTCTGTAGAATCTGTAGTGCTGTCACTTT  8445
Vaquita      GTTCGTGTCCTGG---TCCAGGAAGATGCCACATGCCACGGAGCGGCTGGGTCTGTGAG--------------CCATGGC  7435
Pig          ATG-AT---------------------------------G-AG-------------------------------------  7685
```

Majority	X-TXX--C-------TTGGXCATXTGTGXCTTTT-------ATTTGTTXTAATTCTGGGTGGTG-X-----T------TT	
	10090 10100 10110 10120 10130 10140 10150 10160	
Human	TATAGGCACACAGCACCATGCCCAGCTAACTTTT-----AAATTTTTTATAGACCCGGGGTCTCTCT---GTTTTGCCCA	7132
PolarBear	--	0
Cow	--------------ATACAGATTTTTTGTCTTTTTACTCAGATTTATCCTTATTTTGGGTTTTTTTTTTGGTGCTATTCT	7991
Dog	CTTCATTC-------TTTGTCATATGG-ACACAC--------TGCAATATAGTTCCTACTTTGG--------------TG	8079
BelugaWhale	CGCTGAGCCTGCGCGTCTGGAACCTGTG-CTCCGCAGCGGGAGAGGCCACAGCAGTGAGAGGCCCGCGTACTGCAAAAAA	7658
Donkey	---------------TTGGTCAGCTTTATCTC-T-------ATTTCATGTTTTTTTGGATGCTATT--------------	7160
Cat	TTTCATTCCTG-GTATTGATCATTTGTGTCTTTTCTCTTTTTTTCATTGTCAGTCTAGCTAGAGAT-----TATAGATTT	9476
Ferret	TGTCAGAT-------TTATCCCTATTTCATGTTT--------TTTGGTGCTATTGTAAATGGTG--------------AT	8019
Lion	TTTCATTCCTG-ATATTGATCATTTGTGTCTTTT-------TTTCGTTGTCAGTCTAGCTAGAGAT-----TATTGATTT	8512
Vaquita	CGCTGAGCCTGCGCGTCCGGAACCTGTG-CTCCGCAGCGGGAGAGGCCACAACAGTGAGAGGCCCGCGTACTGCAAAATA	7514
Pig	--	7685

Majority	XAXTAATAATXTAX---AATXTCCAGTTGTTATTAXTATATAGAXXTX-AAGATTATTTTTGTXCATTGCTCTTGTATTX	
	10170 10180 10190 10200 10210 10220 10230 10240	
Human	GGCTGGTGTTGAACTCCTGGCCTCAAGCGATCTTTCTGCCTCAGCCTTCCAAAGTGTTGGGATTAAAGG--CATGAGCCA	7210
PolarBear	--	0
Cow	AAGTAATATTTAAAACAAATGTCCATTTGTTATTAATATATAGAAATA-AAGATTATTTTTGCCCATTGCTTTTGTATTT	8070
Dog	GCGTTATGGTTT------GATTCCAGTTTTTATCAG-GTAGCAG-----ACTGGCGATATTTTCCATTG--CAGGTACAA	8145
BelugaWhale	AAGAAAAAA--AAATCAAATGTGTATTTGTTATTAATATATAGAAATA-CAGATTATTTATGCCCATTGCTCTTGTATTC	7735
Donkey	--TTAAAAAATTC----AATTTCCAGTTGTTA---GTGTATAGAAATACAA-ATGATTTTTGCATATTGCTCTTTTATTC	7230
Cat	TATTAATAGTCTCC---AAAAATCAGCTTTTGGTTTCATTGAGTTTTCTATTTCCTATTTTATTGAAAACTACTGTTTTA	9553
Ferret	GATTTAAAATTTTC---AGTTTCCAGTTGTTACTAGTGTAAAGA-----AATACAAATATTTTTGCATGTTCTTGTATTC	8091
Lion	TGTTAATAGTCTCA---AAAAATCAGCTTTTGGTTTCATTGAGTTTTCTGTGTCCTATTTTATTGATAACTACTGTTTTA	8589
Vaquita	AAGAAAAAA--AAATCAAATGTGTATTTGTTATTAATATATAGAAATA-CAGATTATTTATGCCCATTGCTCTTGTATTC	7591
Pig	--	7685

```
Majority     ATCACCTTTGCCAAACTCXTTTATTAXTXTTXA---CAGCTXTTTTGTAGATGCCATCXGATTTTXTATATTTACCTTTT
                       10250     10260     10270     10280     10290     10300     10310     10320
Human        CTACACTTGGCCACCTTTGTTTTCTAATAGAAGTTTTATACTTTGGTTTAACATTTGGGTCTCTTTCATTTTGACTTAAT  7290
PolarBear    --------------------------------------------------------------------------------  0
Cow          AGCACATTTGCCAGACATTTTTATTACAGTATA---CAGCTTTTTGGTAGATGCCATCAGATTTTCTGTGTACACTGTTT  8147
Dog          ATCAGTCTGCGTGTCACAGTGAAATCACAATTA---CAAAGTTTATGAAAACCACATTGGCAAAAGGATTCTTCACTAAT  8222
BelugaWhale  AGCACCTTTGCCAAACTCATTTATTAGATCGAA---CAGCTATTTGGTAGATGCCGTCAGATTTTATATATACACTGTTC  7812
Donkey       TGCACTCTTGCCAAACTCACTTAATAG-ATCTAA--TAGCTGTTTTGTAGACGCCGTCAGATTTTCTACATAGACCATTT  7307
Cat          ATTTTCTTCTGCTTTGCATTTAGTTCTTTTTCAACTTAGTTATGTTGAAGCTGGTATTGTTGACGTGATACTTTTCTAAC  9633
Ferret       TTTACTCTTGCTAAACTCACTTGTAGATACCGA---CATTTTTTTTGTAGATATCAGATTTTCACACATACTTACCTTTT  8168
Lion         ATTTTCTTCTGCTTTGCATTTAGTTCTTTTTCAAGTTAGTTATGTTGAAGCTGGTATTGTTGACGTGATACTTTTCTAAC  8669
Vaquita      AGCACCTTTGCCAAACTCATTTATTAGATCGAA---CAACTATTTGGTAGATGCCATCAGATTTTATATATACACTGTTC  7668
Pig          -AG--------CAGGCATTCTT----------------------------------------------------------  7698

Majority     A----TCXTATTTTATAXCTGGATGTCTTTTTTTTTTTTTTTC-T-X--XCATCAA-TTGTTCAATGTTTAAAT--GTATT
                       10330     10340     10350     10360     10370     10380     10390     10400
Human        AT--TTTGAATATGGTA-CAAAGTGTGGGTCAGAAGTATTTGGTT-TGCATGGGGATATCCAGTGTTTACAGCATCATT  7366
PolarBear    --------------------------------------------------------------------------------  0
Cow          A----TTATATTTTCTATCTGAATGTCTTTTCTTTTTCTTACCTAT-GGCATTTAGTTGTTCAGTCACTAAGTATGAAAA  8222
Dog          -----TTGTTTTCCACAACTGGATATGGGAGTGCCTGTTTCT-------CTACATCTTTACCCATTTCTGG----GTTTT  8286
BelugaWhale  A----TCATATTTATTATCTGGATGCCTTTTGTTTTTCTTGCCTGT-GGCATCTA---GTACAATATTTAAATG-ATGAG  7883
Donkey       ATGTATCTTCTCTTCTATCTGGATACCGTTTTTTTCTTGTTCTTGCCTATGGCATCTAATACAATGATGAATA--GAAGT  7385
Cat          A----TAGAGGTTTAGTGCTATATATTTTCCTTTTTTTATTT-----AATTAAAATTTTTTTAATGTTTTTAT--TTATT  9702
Ferret       -----TCATTTCCTGT--CTGGATGCCGTTTTTTCCTGCCTG-------TGGCATCTTGTGCAGTGCTGAGTA--GAAGT  8232
Lion         A----TAGAGGTTTAGTGCTATGTATTTTCCTTTTTTTATTTTTTT-AATTAAAAATTTTTTAATGTTTTTAT--TTATT  8742
Vaquita      A----TCATATTTATTATCTGGATGCCTTTTGTTTTTCTTGCCTGT-GGCATCTA---GTACAATATTTAAATA-ATGAG  7739
Pig          -----------------------------------------T-------------GTTCAAT---T-----------  7707
```

```
Majority     AGXXAXAXXTAGXATGTTT-C--------------X-TTCATGGTXTTAGAGT-AXATAC---CCTAAATGA---XATTT
                      10410     10420     10430     10440     10450     10460     10470     10480
Human        TGTTGAAAAGACTATCCTTTCTCCATTCACTTGGCTTTGCAGCTTCATGAAAAATCACTTATGTCTGTATGTGTGATTTT 7446
PolarBear    -------------------------------------------------------------------------------- 0
Cow          AGCATCTAGTGCAGTGTTTAAATAGGGAGAGCAAGCATTCTTTGTTCCTGAATTAAATAC---CTTAAATGACGTTATTT 8299
Dog          ATAAAAATTTAGAATGTTAGC--------------TAATTATAGGCTAAAAAGGTG--------CTTTATAG----TTTA 8340
BelugaWhale  AGCAGACCTTCTTACTTTT------------------TTCCTGATATTAGGGT-AAATAC---CTTAAATGA---TATTT 7938
Donkey       GATGAGAACAGGTGACTTGAC-TT-----------TGTTCCTGGTAGTAGGGT-AAACAA---CCTAAATGATATTCTTT 7449
Cat          TTGGAGAGACAGAGCGTGAGC--------------AGGGGAGGGGCAGAGAGAGGGAGAC---ACAGAATCT----GAAG 9761
Ferret       GATGAGAGCAAGCATCTTTAC--------------TATGTTCCCTATGTCAGGGCGAACC---CCTATATGA----TATT 8291
Lion         TTGGAGAGACAGAGTGTGAGC--------------AGGGGAGGGGCAGAGAGAGGGAGAC---ACAGAATCT----GAAG 8801
Vaquita      AGCAGACCTTCTTACTTTT------------------TTCCTGATATTAGGGT-AAATAC---CTTAAATGA---TATTT 7794
Pig          ----------------------------------------------CTAGAAT-ACATAT---CCTAAGTGA---TATTT 7734

Majority     TTTTCTTAAATGTTTGGTATA----------GTTCAXXAGTGAAGCTGTGTGGACTTGATGTTT-T--XCTTTGTGGGAA
                      10490     10500     10510     10520     10530     10540     10550     10560
Human        ATGTCTGTACTCTTTTTTGTATTGACCCATTTTCCTTTATCTAGATGCTGGAAACATACTGTCT-TAATCATTGTAGCTT 7525
PolarBear    -------------------------------------------------------------------------------- 0
Cow          TTTTCCTAAATGTTTGGTACA----------GTTCACCAGTGAAGCTGTATGGGTTTGCTGTTT-------TTGTGGGAA 8362
Dog          TATTTTGAAATGGTTGAGAT--------------TTTAAG---AATTTGAGAGTTTTAAATTTCTTGCCCTATGAACTAC 8403
BelugaWhale  TTTTCTTAAAAGTTTGGTACA----------GTTCACTAGTGAAGCTGTGTGGGCCTGGTGTTT----TCTTTGTGGGAA 8004
Donkey       CTTTCTTAAATGTTGGGTACA----------ATTCACCAGTGAAGCTGTGTAGACTGGTGTGTT------TTTGC----- 7508
Cat          CAGGCTCCAGGTTTTGAGCTGTTAGC-----ACATCTGGGCTTGAACTTGTGAACTGAAAGATCATGACCTGCGCCAAAG 9836
Ferret       ATTTCTTAAATGTTTGGGGGGG---------GCACCTGGG---TGGCTCAGTGGGTTAAAGCCTCTGCCTTCGGCTCAGG 8359
Lion         CAGGCTCCAGGTTTTGAGCTGTTAGC-----ACAGCTGGGCTTGAACTTGTGAACTGCAAGATCATGACCTGAGCCAAAG 8876
Vaquita      TTTTCTTAAAAGTTTGGTACA----------GTTCACTAGTGAAGCTGTGTGGGCCTGGTGTTT----TCTTTGTGGGAA 7860
Pig          TTTTCTTAAATGTTTGGTATA----------GTTCACCAGTGAAGTTGTGTGGACCTGGTGTTTATTTTCTTTGTGGGGA 7804
```

```
Majority     XXTTTTAAATX-CAGATTCAATTTATTGAATAGXTXCAGGAXTATTC----ACATTATXTX---XXTTTTGAGTGAGCTT
                       10570     10580     10590     10600     10610     10620     10630     10640
Human        TATATTGAGTC-TTGAAACCACTTACTTAGTCTTGGCCCTCCAACTCTG-TTCTTTTTCAAAGTTGTTTTTAACTCTTCT  7603
PolarBear    --------------------------------------------------------------------------------  0
Cow          GGTTTTAAACTACACATTCAATTTATTGAATAGATACAGGATTATTC----AGATTATCC------TTTTGAGTGAGCTT  8432
Dog          CCCTCTCTATACCTGACCTATTTTAATGATCATCTCTGTTAGATTTC---TGTGTAAAAAGTGGTAGATTCATTGTAGGT  8480
BelugaWhale  GGTTTTAAAC--CACATTCAATTTATTGAATAGATATAGGACTATTC----ACATTATTC--------TGGAGTGAGCTT  8070
Donkey       --TTT---------GTTTCATTTTATTTAATGGGT--AGAACTATTT----AGGTTATGTTT--CTTCCTGAGTGAGCTT  7569
Cat          TCGGACGCTTCACTGA-CTAAGCCATCGAGGTGCCCCTATATATTAC---CCTCCTAAGTACAGCTTTCGTGGCCTCTCT  9912
Ferret       TCATG---ATCCCAGGATTCTGGGATCGAGCCCCTCATCGGGCTCTC---TGCTCAGCAGGGAGCCTGCTCTCTCTCTGC  8433
Lion         TCAGATGTTTCACTGA-CTAAGCCACCGAGGTGCCCCTATATATTAC---CCTCCTAAGTACAGCTTTCGTGGCCTCTCT  8952
Vaquita      GGTTTTAAAC--CACATTCAATTTATTGAATAGATATAGGACTATTC----ACATTATTC--------TGGAGTGAGCTT  7926
Pig          GGTTTTAAACTACACATTAAATTTACTGCATGGATACAGGATTGTTCCGATAGATTACCTATTTTTTCTTGAGTGAGCTC  7884

Majority     TGGTAATTT-TGTTTTC-ATAGA-XTTAGTCTXTTTCATCAAAXTTGCTGAA-----ATXCAXAG--GGTTX--TGTTTT
                       10650     10660     10670     10680     10690     10700     10710     10720
Human        GAGTCCTTTGTATTTTC-ATATGAGTCAGT---TCTTAGAAAAACTGCTTGGATTTTACTGGATTTGACTTGAATCTGTA  7679
PolarBear    --------------------------------------------------------------------------------  0
Cow          TGGCAATTT-TGTTTTC-ATAGA-ATTAGTCTGTTTCATCAAAGTTGCTAAACTCTTCATCAAAGTTGCTAAGCTTTTTC  8509
Dog          ACATTTTCT-TACATTCTTTGAA--TTTGAGGTTTTTCATGTAGTTTTAGGT-----ATATAGATTTATTTA--TATTAA  8550
BelugaWhale  TGGTAATTT-TGTTTTC-ATAGA-ATTAGTCTGTTTCATCAAAGTTGCTGAACTTATTTGCATAG--GGTTGTTTGTTGT  8145
Donkey       TGGTAATTTGTGTCGTCCGTAGA-CTTAGTCCATTTT-TCCAAATTATTGAA----------TG----------TATTTA  7627
Cat          CAGATTTTT-TTTTTTTTTTTAA-GTTTATTCATTTCGAGAGAAAGAGAGAGAGAGAACCCCAAGCAGGCTC--TGCACT  9988
Ferret       CTGCTTCTC-TGCCTACTTGTGA--TCTCTGTCTGTCAAATAAATAAATAAA-----ATCTTTAAAAAAAAA--TGTTTG  8503
Lion         CAGATTTTT-TTTTTTTTTTTAAAGTTTATTCATTTCGAGAGAAAGAGAGAG----AACCCCAAGCAGGCTC--TGCACT  9025
Vaquita      TGGTAATTT-TGTTTTC-ATAGA-ATTAGTCTGTTTCATCAAAGTTGCTGAACTTATTTGCATAG--GGTTGTTTGTTGT  8001
Pig          TGGTAATTT-TGTCTTCCATAGG-TTTAGTCTCTTTCATCTAAGTTGCTGA-----------------------------  7933
```

```
Majority     GTXACAGTTGTTX-------------X---X----G----------XX--X----X----T------------X----XX

                      10730     10740     10750     10760     10770     10780     10790     10800

Human        GAGCAAATTTGGG-----GAAAAT--TGGCATCATGACTACAATTGATGTCTCTATTAGGTCTTTAATTTCTTTAAGCAT  7752
PolarBear    --------------------------------------------------------------------------------  0
Cow          ATCACAGTTGCTA----------------------------------------------------------------AAC  8525
Dog          GTATAATTTATTT-------------TTTTAGTTTGATCTGAAACATTTTTAGCTTCAGTTTTATCTTTTAAATGGGATT  8617
BelugaWhale  ATTACATTTTT---------------------------------------------------------------------  8156
Donkey       CATGGAGTTGTAA-------------------------------------------------------------------  7640
Cat          GTCAGTGCGGAGC-------------TGGAACTTGGGAACCGTGAAATCATGACCTGAGCTGAAATCAAGAGATGCATAC  10055
Ferret       GGGGCAATTCACC-------------AGTGAAATTGTGTGGGCCTGATGTTTGTTTTCTTTGTGGAAGTT---TTAAATG  8567
Lion         GTCAGTGCAGAGCCGGATGTGGGGC-TGGAACTCGGGAACCATGAAATCATGACCTGAGCTGAAATCAAGAGATCCATAC  9104
Vaquita      ATTACATTTTTTTTTTTTTTTTTTTTTTGCTGTACGCGGGCCTCTCACTGTTGTGGCCTCTCCTGTTGCGGAGCACAGGC  8081
Pig          ----CACTTATT--------------------------------------------------------------------  7941

Majority     TX-------X------------X-----X-------------------T---TAAXTAGGAX-T---X---T-X------

                      10810     10820     10830     10840     10850     10860     10870     10880

Human        TTTTTTTTTTTTTTTCAGTGCACACGTTTTGTGCATTGTTTGATTAGATTTACCCATAAGTATTTTATGTATTTTGAAGT  7832
PolarBear    --------------------------------------------------------------------------------  0
Cow          T--------------T-----------------------------------ATTTGTAGGGT------------------  8538
Dog          ATAATAGTTTTGAAGATT-AAACGTGATTATACAGAAAAAG-TATAGTTTCACAACTAGGTCTTAAGTTAATGTTGATTT  8695
BelugaWhale  --------------------------------------------------------------------------------  8156
Donkey       -------------------------------------------A----T----GA-TAATAC------------------  7650
Cat          TTAACTGACTGAGCTACCCAAGCTGAGTTACCTAAGCACCCTTGCATCTCTCTAATTTTGACATGCATACTTTTTATTCA  10135
Ferret       ATACCTTTATTTAATTAG-ATATAGGACTATTTGGGTTATC-TTTTTCTTCTTAAGTGAGCTTTGA-TAAATTTGTACCT  8644
Lion         TTAACCGGCTGAGCTACCCAAGCTGAGTTACCTAAGCACCCCTGCATCTCTCTAATTTTGACATGCATACTTTTTATTCA  9184
Vaquita      TCCGGACGTGCAGGCTCAGTGACCATGGCTCACGGGCCCAGCCACTCCACGGCATGTGGGATCTTCTGGACTGGGGCACG  8161
Pig          ----------------------------------------------------TGCATAGGGT------------------  7951
```

```
Majority     ------TXTTCATTXTATTG-X-------X------------T----X----------X-X-----T--------TTTTA
                      10890     10900     10910     10920     10930     10940     10950     10960
Human        AC----TGTAAATGATATTTTAAAAACTTAATTTCCAATTGTTTGCTAG-----TG--TATAGAAATGAT---TATGGCA  7898
PolarBear    --------------------------------------------------------------------------------  0
Cow          ------TGTTTGTTGTATTGG---------------------------------------------------AT-TTTTA  8560
Dog          ATACTACATACAGAATATCCAAA-TTTTAGAGCTTTAGGATATGTTGA--AATTTCATTTTTTTGTTATCT----TTATA  8768
BelugaWhale  -------------------------------------------------------------------------------A  8157
Donkey       --------CTTTTTATACTG---------------------------------------------------------TTA  7665
Cat          GTTCAGAATGCATTGTAATACATTTTTCTGCCCCATGTGTTATGAGAAGTATGTTATTTATTTAATTGCCAAATATTATG  10215
Ferret       TCTGTAGAATTAGTCCATTTCA--TCCAAGTTACTGAATTTATTTGCA--TAGTCATTTATGATACTACCC----TTTTA  8716
Lion         GTTCAAAATGCATTGTAATACATTTTTCTGCCCCATGTGTTATGAGAAGTATGTTATTTATTTAATTGCCAAATATTATG  9264
Vaquita      AACCCGTGTCCCCTGCATCGGC--AGGCAGACTCTCAACCACTGCGCCACCAGGGAAGCCCTGTTTTATTACAT-TTTTA  8238
Pig          ------TGTTCATGATATTG---------------------------------------------------------TTA  7968

Majority     TATTTTTAATATATGTAGAATCTGTAGTGATGTCACTTCTCTCATTC------CTXATATTGATAGTTXGTGTCTTTTCC
                      10970     10980     10990     11000     11010     11020     11030     11040
Human        TATTGCTCATATGTTCTGAACCTTTGCTAAACTCATTTAGTAGATCTAATAGTTTTTTTTATATATTTCATCAGATTTGC  7978
PolarBear    --------------------------------------------------------------------------------  0
Cow          TATTTTTA--ATAACTAGAATCTATAGTGATGTCATCTCTCTCATTT------CTGATATTGATAATTAGTGTCTTTTCC  8632
Dog          AATTTGCTGTAGTTACATCCCTTGTTTTTAGACCCCTGTCCTTTTTG------GTTCTGCTTAAGGGAGATCCTAACAGG  8842
BelugaWhale  TATTTTTATTATCTGTAGAATCTATAGTGATGTTGCTTCTCTCGTTC------CTGGTATTGATA----GTGTCTTTTCC  8227
Donkey       TACTTTTGGTATTTGTAGAGTCTGCAGTGATTTCA--TCTCTCATTC------CTGATGCTGGTAATTTGTTTCTTCTCT  7737
Cat          AGTGTTCCATAGATGTTTGTTACTTTTCTCGTTTAATTTTTTTTTTA------GTTTTTGAGAGAGCTAGAGCCCTCGCG  10289
Ferret       TACTCTTGATGTCTGTAGAATCTGTAGTGATGTCACTTCTCTCATTC------CTTATACTGATAATTTGTCTTTTTTTT  8790
Lion         AGTGTTCCATAGATGTTTGTTACTTTTTTCATTTAATTTTTTTTTTTTTAGTTTGTTTTTGAGAGAGCTAGAGCCCACGTG  9344
Vaquita      TATTTTTATTATCTGTAGAATCTATAGTGATGTTGCTTCTCTCGTTC------CTGGTATTGATA----GTGTCTTTTCC  8308
Pig          TATTTTTA--ATATGTATAGTCTGTAGTGATGGCGCCTTTCTCGTTC------CTGATATTGATAGTTTGTGTCTTTTCT  8040
```

```
Majority     CTTT------------XTCAGTCTGGCTAGAGA----TTXATTGATTTTATTAATATAAATCTXAAAT-XAGCCTTTGGX
                     11050     11060     11070     11080     11090     11100     11110     11120
Human        TTGAGAGGGAGTCTCAGTCACCCAGGCTGGAG-----TGCAGTGACGTGATCTTGGCTCACTGCAACCTGCACCTCCTGG  8053
PolarBear    --------------------------------------------------------------------------------  0
Cow          TTT-------------ATCAGTCTGTCTAGAA----ATACATTGATTTTATTAATTTAAATA----AT-CAGCTTTTGGC  8690
Dog          AAAT------T------CCGGGAATTTTTAATA---------AGAGGTTTGTTGCATCTCTTTAAAAT--GTTTTTTTAC  8899
BelugaWhale  CT--------------ATCAGTCTGGCTAAAGGTTTATTTATTGATTTCATTAATATAAAACAGAAAT-CAGCCTTTGGT  8292
Donkey       TTTTT-----TTCCTTGTCAGTCTGGCTGGAGG----TTTATTGATTTTATTAATATTCTCC-AAAACTCAGCTTTTAGT  7807
Cat          CACTCGTGCACATGTGCACATGCACGTGGGAGAG--GGGCAGAGAGAGAGGGAGACACACTCTGAAAC--AGGCTCCAAG  10365
Ferret       CCTT------G------TCATTTTAGCTAGAGA---------TTAATTTGTTAATATTCTCCAAAAAT--CATCTTTTGG  8847
Lion         CACT--------TGCGCACACGCATGTGGGAGAG--GGGCAGAGAGAGAGGGAGACACACTCTGAAAC--AGGCTCCAAG  9412
Vaquita      CT--------------ATCAGTCTGGCTAAAGATTGATTTATTGATTTTATTAATATAAAACAGAAAT-CAGCTTTTGGT  8373
Pig          CTTTTT----TTCATTGTCAGTCTGGCTAGAG----TTTTATCGATTTTATTAATAAAAATATAAAATGCAGCCTTTGCT  8112

Majority     TTCATXGAXTTXTCTC-----TTTTCTGTTTCCTATTTTATTGATTTCTGCTGTTTTAATXTTXTTCTGCTTTGTATTTA
                     11130     11140     11150     11160     11170     11180     11190     11200
Human        GTCAAGCAATTCTCCT-----GCCTCAGCCTCATAAGTAGCTG-GGACTGCAGGTGCACACCACCACGCCCAGCTAATTT  8127
PolarBear    --------------------------------------------------------------------------------  0
Cow          TTCATTGAATTTTCTATG---TTTTCCATTTCCCATCTTATTGATTTCTGCTGTTTGAATGTT---CTGCTTTGTATTTA  8764
Dog          AT--------TCTTTGTG---ATTTGTAATT--AATTTCAG---GGTTTCTTTCTATACCCTTAACCATTTTCCACTGCG  8963
BelugaWhale  TTCATGGAGTTTTCTC------------TTTCCCATTTTATTGATTTCTTCTGTTTTAATGTTTTTCTGCTTTGTATTTA  8360
Donkey       TTCATTGGATTTTCTCTGTTGTTTTCTGTTTCCTATTTTATTGATTTCTGCTGTTTTAATTTTCTTGTGCTTTGTATTTT  7887
Cat          CTCCAAGCTGTCAGCACA---GAACCTGATACGGGGCTCGAATCCATGAACTGTGAGATAATGACCTGGGCCGAAGTTGG  10442
Ferret       TT--------TCACTGCG---TTTTCTGTTTCCTATTTTATT--GATCTGCTGTTTTAGTTTTCTTCTGCTTTCCATTTA  8914
Lion         CTCCAAGCTGTCAGCACA---GAACCTGATACGGGGCTCGAATCCATGAACTGTGAGATAATGACCTGGGCTGAAGTTGG  9489
Vaquita      TTCATGGAGTTTTCTC------------TTTCCCATTTTATTGATTGCTTCTGTTTTAATGTTTTTCTGCTTTGTATTTA  8441
Pig          TTCATTGAGTTTTCTC-----TTTTCTGTTAGCTATTTTATTGATTTCTGCTGTTTTAGTTTT---CTGCTTTGTATTTA  8184
```

```
Majority     GTTTGXTTTCXXCTTAXTTAX-----CATTGAXGTTGATGTCATTGACATGATATCTTT----TCTAAT-ATAGA--GGT
                      11210     11220     11230     11240     11250     11260     11270     11280
Human        TTGCATTTTCAGTTGAGGCAGGGTTTCACC-ATGTTGGCCAGGCTGGTCTGGAACTCCTGACCTCAAGTGATACACTTAC  8206
PolarBear    --------------------------------------------------------------------------------  0
Cow          GTTTGCTTTTTTCCTATTTAA-----TATTAAAGTTGATGTAGTTGACACGGTATCTTT----TCTAAT-ACAGA--GGA  8832
Dog          AAATCTTTGCAAAATAATTG-------GCCTTTAGTGAAAACAACTGCCTGAAGTGTTT----CCATAT-CAGGA--ATT  9029
BelugaWhale  GTTTGCTCTCTTCTTAGTTAA-----CATTGAAGCTGATGTAGTTGACA---TATCTTT----TCTAAT-ATAGAGAGGT  8427
Donkey       GTTTACTTTTTCTCCTTTGTTA----CATTGAAGCTGATGTCATTGACACGATACCTTT----TCTAAT-ATACA--AGT  7956
Cat          GTGCTTAACCAACAGATGCC--------CCAATGTGTCTATTATTTTAATTCAATTCCA----TTGTGG-CTAGA--GAA  10507
Ferret       GTTCTTTTCCAACTTAGTTAT------GTTGATGCTGGTATCATTAATATGATACCTCT----TCTAAT-ATAGA--GGC  8981
Lion         GTGCTTAACCAACAGATGCCTAGACGCCCCAATGTGTCTATTATTTTAATTCAATTCCA----TTGTGG-CTAGA--GAA  9562
Vaquita      GTTTGCTCTCTTCTTAGTTAA-----CATTGAAGTTGATGTAGTTGACA---TATCTTT----TCTAAT-ATAGAGAGGT  8508
Pig          GTTTGCTCTCTTCCTGCTTAGGTA-GCATTTAAGTTGATGTCATTGACATGATACCTTT----TCTAAT-TCAGA--GGT  8256

Majority     TTAATGCT----ATGTATTTX-CATXTAAXTATTGCTT-----TXATGGC--ATCCCTCAAATTTTGACATGTATA----
                      11290     11300     11310     11320     11330     11340     11350     11360
Human        CTAGGCCTCCCAAAGTGTTAAGATTACAGGCGTGAGCCACCACCCCCAGCCTATGCCATTAAATTTTCTACATAGA---T  8283
PolarBear    --------------------------------------------------------------------------------  0
Cow          TTAATGTT----ATGTATTTT-CATACAAATATTGCTT-----TTATGGC--ATCCCTCAAGTTTTGACATGTAAG----  8896
Dog          CCTAGATT----ATAACTCGTTTGTTGAAATATTAACT-----CTTTGG------ATAGATGTTTTAAATAGTGAAAATT  9094
BelugaWhale  TTAATGCC----ATGTATTT--CATACAAGTGTTGCTT-----TCATGGC--ATCCCTCAAATTTTGACATTTATA----  8490
Donkey       TTAGTGCT----ATATAGTTC-CCTTTAAATACTGCTT-----TCATGGC--ATCTCTCAAATTTTGACATGTG------  8018
Cat          CATATTTT----AAGACTTGAATCTTTGTAAATTTGTTGAGACTTGTTTT--ATGATCCAAATATCATATATCTTAAGTT  10581
Ferret       TTACTGCT----TTGTATTTCCCTTTTAAGTACAGCTT-----TCATGGC--ATCTCTCAAATTTTGACATGCATACTTT  9050
Lion         CATACTGT----AAGACTTGAATCTTTGTAAATTTGTTGAGACTTGTTTT--ATGATCCAAATATCACATATCTTAAGTT  9636
Vaquita      TTAATGCC----ATGTATTT--CATACATGTGTTGCTT-----TCATGGC--ATCCCTCAAATTTTGACATTTATA----  8571
Pig          TTAATGCT----ATGTGTTTT-CATATAAGTGTTGCTC-----TTATGGC--ATCCCTCAAAATTTGACTTGTG------  8318
```

```
Majority      ----XTTCTTTTCATTCAGTTCAAAATA-T--XCATT--X----X-XT---X-------TTATTATTTTTTT--------

                        11370     11380     11390     11400     11410     11420     11430     11440

Human         --AATTTATACCCAATTGTTTTTAAATAGTTTTCTTTCTTTTCTATCTGGATGCCATTTTTTGTCTCCTTTTTT-TGTCT   8360
PolarBear     --------------------------------------------------------------------------------   0
Cow           ----CTTCTTTTCATTCAGTTCAGAGTA-----CAT-----------------------CTACCATGTTT----------   8934
Dog           GTAGTAGTTTTCCCTTTTTCCGGGTACACTACCCAAATGACGCCTTTTAAAAAAAAACGGTGCTACTTTATA------CG   9168
BelugaWhale   ----CTTCTTTTCATTCAGTTCAAAATA-----CATT----------------------CCAATATGCTTTT--------   8531
Donkey        -----TACTTTTCATTCTGTTCAAAATA-----CATT----------------------CTAATAGTTTT----------   8056
Cat           TTATGTGCACTTGATAGAGATGTGTATTCTGCTCCTGGGTAGTGTTTTATAAATGTCATTTGTTTATTGATAATGTTGTT   10661
Ferret        TTATTCAGCTCAAATACATTCTGATATACTTTTGACCCATGTATTATTCAGAAGTATGTTATTTATTTAATT------GC   9124
Lion          TTATGTGCACTTGATAGAGATGTGTATTCTGCTCTTGGGTAGTGTTTTATAAATGTCATTTGTTTATTGATAATGTTGTT   9716
Vaquita       ----CTTCTTTTCATTCAGTTCAAAATA-----CATT----------------------CCAATATGCTTTT--------   8612
Pig           -----TACTTTTCATTCAGCTCAAAATAATCCTCATGTTTTTATTATTAATATAATTTGTTCTTATATTTTT--------   8385

Majority      GAATTTCTTTGACCCATGGATTATTGAGAA-TATXTTATTTAATTTCCATGTATTTXGAATTTTCXATGTAXCTTTCT--

                        11450     11460     11470     11480     11490     11500     11510     11520

Human         GTGGTATCCAGTACAATGTTTAATAGAAGTGTTGAGAGAATATCTTTTCCTTGCTCCTGATATTAGGGTGAGAACTCTAA   8440
PolarBear     --------------------------------------------------------------------------------   0
Cow           --ATTTCTTTGACCCATGGATTATTGAGAGGTATGTTATTTAATTTCCAGGTATTTGGAATTTTCCATATACTTTT----   9008
Dog           GAAATAGTTTGTTACAAGCAGAAGTGGTTA-GCAACT-TTCCAACTGATTTCATTTATGTTTTTGAAAGCACCTACT---   9243
BelugaWhale   TTATTTCTTTGACCCATGGATTATTGAGAAGTATGTTATTTGATTTCCAAGTATTTGGAATTTTCCATGTATATTTCTG-   8610
Donkey        --ATCTCTTTGACCCATGGGCTGTGGAGAA-----GCATTTAATTACCAAATAATTTAAGTTTTCTATATATCTTTC---   8126
Cat           GAAGTGATCTCTGTCCTTCTTGATTTTCTG-TGTATTGTGCTATCACATTGAGAGAAGGATATTGAAATTTCCTCTTTTA   10740
Ferret        CAGATATTTGGAGTTTTCCATAGATGTTTC-TGTTAC-TCTAATTTCATTGTAGCTAGAGGACATATTGTAAGACTT---   9199
Lion          GAAGTGATCTCTGTCCTTCTTGATTTTCTG-TGTATTGTGCTATCACATTGAGAGAAGGATATTGAAATTTCCTCTTTTA   9795
Vaquita       TTATTTCTTTGACCCATGGATTATTGAGAAGTATGTTATTTGATTTCCAAGTATTTGGAATTTTCCATGTATTTTTCTG-   8691
Pig           AAATTTCTTTGACCTGTGGGTTATTGAGAA--ATGTTATGTAATTTCCAGCTATTTGGAATTTTCCATATATTTTTCTA-   8462
```

```
Majority      --TATTTATTTCTAATTTAATTCXATTGTXXCTAGAGAAX----ATXTTG-AAGACTTGAATXTTTGTAA---TTTATTT
                           11530     11540     11550     11560     11570     11580     11590     11600
Human         --AATTTCTTCCTTAACTGACACAGTTCA--CTAGTGAAGCCATGTGGGGCCAGATGTTTGT-TTTGTCGGGAGGTTTTA  8515
PolarBear     --------------------------------------------------------------------------------  0
Cow           --TGTTTGTTTCTAATTTAATTC-ATTGTGCCTGGAGAACAT--ATTTTGGAAGACTTGAATCTTTGTAAT--TTTTTCT  9081
Dog           --GGTTTGTACATTGCTTCCC----------CTA-------------------------------------CTTTCTT  9272
BelugaWhale   -TTATTTATTTCTAATTTAATTCCATTGTGCTTAGAGAAC----ATTTTGTAAGACTTGAATCTTTGTAA----TTATTT  8681
Donkey        ----TGTTGTTCTAATTTAATTCCATTGAAGCTAGAGAAAAT--AGTTTGTAAGACTTGAATCTTTGAAA--ATTTGTTG  8198
Cat           CCGGTGCATGTCTAAGTTTCTCCTTGCAGTTCCAGTATGTATGTATGTATGTATGTATGTATGTATGTATTTATTTATTT  10820
Ferret        --GAATCGTTTGTAAGTTTGT----------TGAG----------------------------A--------CTTGTTT  9230
Lion          CCTGTGCATGTCTAAGTTTCTGCTTGCAGTTCCA------------------------------GTTTTTATTTATTT  9843
Vaquita       -TTATTTATTTCTAATTTAATTCCATTGTGCTTAGAGAAC----ATTTTGTAAGACTTGAATCTTTGTAA----TTATTT  8762
Pig           -TTATTTATTCCAGATTTAATTCCATTGTGCCTAGAGGACGT--ATATTCAAACACTTGAGTCTTTGTGAC--TTTGTTT  8537

Majority      XATTGTTT-----TATGGTTTAAATATXATATATCCTGT-------TTTATGTGXTTTTGATAGGGA-TGTGTATTCTGT
                           11610     11620     11630     11640     11650     11660     11670     11680
Human         AACTACATATTCAACTTATTTAATAGATATAGGGCTATTCAGTTTGTTTAGTTCTTCTTGAGTGAACTTTGGTAATTTGT  8595
PolarBear     --------------------------------------------------------------------------------  0
Cow           TA---TTT-----TATGGTTTAGATATTATATATCCTGTT--AGT-TTTAAGTGCTTT--ATAGAGA-GGTGTATTCTGT  9147
Dog           AACTGTTT-------AAATTTGGCTAGAGTATATGAA--------------AAGTTACCAACATTGGCTTTTTAGATAGT  9331
BelugaWhale   TATTGTTT-----TATGGTTCAAATAT---ATATCCTGT-------TTTAAGTGCATTTGATAGGGA-TGTGCATTCTGT  8745
Donkey        AGACTTATTT---TATGGTCCAAATGTCATATATCCTCTT---AGTTTTATGTGCGCTTGATAGGGG-TGTGTATTCTGC  8271
Cat           ATTTATTTATGTTTTAAGTTTATGATTAGTACGTGAATGTTTAGGATTACTATGTCTTCTTTGTGCATTGACTCCTTTTT  10900
Ferret        TATCACCC-------AAATATGTAACTTACATTTTAC--------------ATGTATTTAATAGGGG-TGTGTATTCTGC  9288
Lion          ATTTTTT--T---T-AAGTTTATGATTAGTACATAAATGTTTAGGATTACTATGTCTTCTTTGTGCATTGACTCCTTTTT  9917
Vaquita       TATTGTTT-----TATGGTTCAAATAT---ATATCCTGT-------TTTAAGTGCATTTGATAGGGA-TGTGCATTCTGT  8826
Pig           TACTGTTT-----TATGGTTCAGACATCATATATCCTGTC--AGT-TTTATGTGCTTTTGATAGGGA-TGTGTATTCTGT  8608
```

```
Majority     TATTGTG---TAXTGTGTTCTTTACATXT---TTAGGTCATGTTTATTGATATTGTTGTTCATGATATCTGTCT-TTCCT
                         11690     11700     11710     11720     11730     11740     11750     11760
Human        ATCTTCTGTAGAACTAGTCCATTTCCTTTAAGTTGCTGAATGTATTTGTGTAGAGTTGTTCATGATACTACTATTTTTTT  8675
PolarBear    --------------------------------------------------------------------------------  0
Cow          TGTTGGG---CGATGTGTTCTGTATATGTTAATTAGGTCGTGCTTATTGGTAATGTTGTTCAAGTGAT--GTCT-TTCT-  9220
Dog          TGTAATG---AAATAATGCTCTTACTTTTT--TTGTAACGATTGAACATAACTGGTTGTTTATAATAGGTGTGTTATTAA  9406
BelugaWhale  TATTGT-------TGTGTTCTATGCATGTCAATTAGGTCATGTTTATTGGTAGTGTTGCTCAAGTGATCTGCCT-TTCCT  8817
Donkey       TGCTGTTCGGTGGTATGTTCTGTAAATG----TCAGGTCAAGTTTATTGATAATGTTCAA-GTCATATCTGTCC-TTCCT  8345
Cat          TATTATG---TAGTGACCATTTTATCCTTG--TAATACTCTTTGCTTTAAAATCTACTTTTATGATATAGCCAT-CTGGC  10974
Ferret       TGTTGGG---TAGTGTTCTATAAATATCAA--TTAGATTAAGTTTATTGATAATGTTGAC-GTGATTTCTGTATGGGAGT  9362
Lion         TATTATG---TAGTGACCATTTTATCCTTGG-TAATACTCTTTGCTTTAAAATCTACTTTTATGATATAGCCATTCTGGC  9993
Vaquita      TATTGT-------TGTGTTCTGTGCATGTCAATTAGGTCATGTTTATTGGTAGTGTTGCTCAAGTGAGCTGCCT-TTCCT  8898
Pig          TACTGGG---TGATGTGCTCTATACGT-----TCCGGTTATGTTTGTTGATATTGTTGCTCACAAGATCTGTCT-TTCCT  8679

Majority     GATTTTTTXXXT---GTT---GTTCTGTCACTTTTTGAXAXAAXAAXATTGAAATCTCX-TATATT-TT---XTGCATTX
                         11770     11780     11790     11800     11810     11820     11830     11840
Human        TTTTTTTTTTTTTTTTGAGACGGAGCCTTGCTCTGTCGCCCGGGCTGGAGTGCAGTGGCACCATCTCGGCTCAATGCAACC  8755
PolarBear    --------------------------------------------------------------------------------  0
Cow          ---------------ATT---G--CTGTCACTTACTGAAAAAATGACATTGAAATCT----ATATT------GTGCGTTC  9270
Dog          AAATGCTTTGCTCAGGTT----TTTTAGAATAGTATCTATTTCTTAAAATGCTATATATATATATATAT---ATATATTA  9479
BelugaWhale  GATTTTTT-------ATT---GTTCTGTCACTTATTGAGAAAACAATATTGAAATCTCC-TATATT------GTGCATTC  8880
Donkey       GATTTCTGTCT---ATT---GTTTATCCCTTTTTGAGAGAAGAATATTGAAATCTCCTCCTATACTT---GTGCATTT  8416
Cat          TTTCTTTTGAATAGGGTTCACATGGTATATCTTTTTCCATTCTTTTACTTTCAACCTATTTGTGTCCTT---AGATTTAA  11051
Ferret       ATCTGTTTCTCCACACCC----TTTTGCAGTTCTGGGTTTTAGAAAAATTTAGACTGTTAGTTAATTTT---AGGCTAAA  9435
Lion         TTTCTTTTGAATAGGGTTCACATGGCATATCTTTTTCCATTCTTTTACTTTCAACCTATTTGTGTCCTT---AGATTTAA  10070
Vaquita      GATTTTTT-------ATT---GTTCTGTCACTTACTGAGAAAACAATATTGAAATCTCC-TATATT------GTGCATTC  8961
Pig          GATTTTATGTCTATGGTT---GTTCTGTCACTTGTTGAGAAAACAATATTGAAATCTCC-TATATTTTT---GTGCATTT  8752
```

```
Majority     GTTTGTTTTT----XCTTGCAXTTCTACCAGTTT-----------TACXTTATATTTTTGGXAT-TATGATTAGTXCATA

                       11850     11860     11870     11880     11890     11900     11910     11920

Human        TCCACGTCCCGGGTTCACGCGGTTCTCCTGCCTCAGCATCCCAAGTAGCTGGGATCACAGGCACACACCACCACACCTGG  8835
PolarBear    --------------------------------------------------------------------------------  0
Cow          GTTTGTTTTG----CCTTGTGTTTCTATCATTTTC-----------ACTTTATATTTTTGAAAT-TACAATTAGTACATA  9334
Dog          TATACACACAC---ACACACATGTGTATAGCATG-----------CACATGCATCCTTAGGTAT-TTGTATCAGAATTTC  9544
BelugaWhale  GTTTGTTTTT----CCTTGCAGTTCTACCAGTTTT----------TGCTTTATGTTTTTGAAGT-TATAATTAGTACCTA  8945
Donkey       GTCTGTTTCT----GCTTAAAGTTCTACCAGTTTT----------TGCTTTATGTTTTTTGAAG-TATGATGAGCACATA  8481
Cat          AATGTGTTTTT---TGTAGATAGCATAAAGTTGGAATTCCATTTTTACATCCAACTTACACTCT-CTTGTTTTGACTAAT  11127
Ferret       AGAGTGCTTTT---TATAATTTACGTTTTTGAAA-----------TGGATGAGATTTGACCTTA-TATGTCTTGTCCTGT  9500
Lion         AATGTGTTTTT---TGTAGATAGCATAAAGTTGGAATTCCATTTTTACATCCAACTTACACTCT-CTTGTTTTGACTAAT  10146
Vaquita      GTTTGTTTTTT---CCTTGCAGTTCTAACAGTTTT----------TGCTTTATGTTTTTGAAGT-TATAATTAGTACATA  9027
Pig          GTCTGTTTTT----CCTTGCATATCTACCAGTTT---------------TTATGTTTTTTG----TAACATTAGTCCATA  8809

Majority     AAXGT-----TTAXAATTACT------AXGTCCTCTTTATTTATTGACCXCTTTATTATTTTGCA-GTGACCTTTTTTAT

                       11930     11940     11950     11960     11970     11980     11990     12000

Human        CTAATT--TTTTGTATTTTTAGTAGAGACGGGGTTTCACTATGTTGGCCAGGTTGGTCTCGAACTTGTGATCTGCCCGCC  8913
PolarBear    --------------------------------------------------------------------------------  0
Cow          AATGT-----TTAAGATTACT------ATGTCCTCTTGATATATTCACCTCTTTATTATTATACA-GTGACCATTTTTAT  9402
Dog          AAG-------TTGAAGGCATT-----TTTAGTGTGTCTGTTAAGTGACAACATTGTTAACTTGTA-ATTCAAAGACTGA-  9610
BelugaWhale  AACGT-----TCAGGATTACT------ATGTCCTCTTGATTCATTGACCCCTTTATTATTATGCA-GTGACCTTTTTTAT  9013
Donkey       AGTGT-----TTAGGATTACTGTGTCCTCTTGATTATTATGCAGTTACCATTTTTATCCCTGGTA-ATATTCTTTGCTTT  8555
Cat          AAGATTAGTTTTAAAACTACT----TTGCTGTTTTTCTGTTTGTGCTTTGTGTTCTTTGTTCCCT-TTTTCTGCTATCTT  11202
Ferret       GAA-------CTGCCTCTATA-----TACCTGACCTATCTTAATGTTCATTTTTATTAGATTTCT-GTGTAAAAATTGAC  9567
Lion         AAGATTAGTTTTAAAACTACTAC-TTTGCTGTTTTTCTGTTTGTGCTTTGTGTTCTTTGTTCCCT-TTTTCTGCTGTCTT  10224
Vaquita      AACGT-----TCAGGATTACT------ATGTCCTCTTGATTCATTGACCCCTTTATTATTATGCA-GTGACCTTTTTTAT  9095
Pig          AATGT-----TTAGAATTATT------ATGTCCTCTTTATTTGTTGATCTCTTTATTATTATGCA-GTGACCGTTTTTAT  8877
```

```
Majority     --G--X-XATX-T--TTX-XXTXA--XXTA--------------TXTT-TXAXXXGA-----TTXX---T-TXGTTXX-
                       12010      12020      12030      12040      12050      12060      12070      12080
Human        CTGCCTCACAAAGTGCTGGGATTACAGGCGTGAGCCACTGCGCCCAGCCATGATACTACATTTTTACACTTTTGGTATCT 8993
PolarBear    -------------------------------------------------------------------------------- 0
Cow          TCATGGTAATCTTCTTTGCTTTAAGGTATAC-------------TTTTTCTAATTATACTCATCTTGGCTT-TGTTTTGA 9468
Dog          --GAATAAATGCTCCTTGAGTGTAGAGGTAG-------------GGACC-TTATTTGA---ACTATGGTTT-TAGTGCAC 9670
BelugaWhale  -------------------------------------------------------------------------------- 9013
Donkey       AACATCTAGTTTTTCTAATAGTTATTAATACAGTCATCTCAGCTTTCTTTTGAATAGAGTTCACATTGTATATCTTTTTC 8635
Cat          --GGATGGATACTGTTTTAATTCCATTATAT-------------CTCTTGTTAACTGATTAGCTTATAACTCTGGTTATA 11267
Ferret       T-GATACCATATTCATTGTAGGTACATTTTC-------------TTCTT-TTATTTGA---ATTTTTTTCCCTAGCTTTG 9629
Lion         TTGGATGGATAATGTTTTAATTCCATTATAT-------------CTCTTGTTAACTGATTAGCTTATAACTCTGGTTATA 10291
Vaquita      -------------------------------------------------------------------------------- 9095
Pig          -------------------------------------------------------------------------------- 8877

Majority     -T-X----C---XT-A----GTCXTTTTACXXXT-TTTAATTTAAATTTATT-TT--XATCATTXAXTTTTXAXTTTATT
                       12090      12100      12110      12120      12130      12140      12150      12160
Human        GTTGAATCC---TTAGTGATGTCAGCTTTCTCATTCCCAGTAGG-GATAATTTGTGTCATCTTTTTTCCTTGTCTGTCTG 9069
PolarBear    -------------------------------------------------------------------------------- 0
Cow          GTAGAGTTCA-CATGG-TTTATCTTTTTCCATTGTGTTACTTTCAGACTATT-TG--TATCCTTCAGTTTAAAGTGTAGT 9543
Dog          CTCCTCCACG-CCCCA-T--GTGATTATATACTTACTCAGGGTAAGGTTATT--T--AATGACGGGACTCCCCATTCAAC 9742
BelugaWhale  ------------------C----------C---------CTGGTAATCTATT-T---TGCTTTAAAATTTTTAAAA-ATT 9051
Donkey       CATTCTTTCAGTTTCAACCTGTTTGTGTCCTTAGATTTAAAATATGTTTCTTGTAGACAGCATGAAGTTGGAACTTGATT 8715
Cat          TTGGTGGCTGGTTTTA-GAAGTTGTATCATACATCTCTAACTTA-AGTCTTCCTT--CAAGACTTATTTCTTCTCTCTTG 11343
Ferret       TGCAGTGACGGTATCA-T--ATCCAATGAGGTTTATCCAAGGCACTATTATTGCT--AATTGGATATTTTTTCCTTTGGG 9704
Lion         TTGGTGGCTGGTTTTA-GGAGTTGTATCATACATCTTTAACTTACAGTCTTCCTT--CAAGACTTATTTCTTCTCTCTTG 10368
Vaquita      ------------------C----------C---------CTAGTAATCTATT-T---TGCTTTAAAATTTTTAAAAA-ATT 9133
Pig          --------------------ATCTTTTTCA-----GTTATTTTAAATCTATT-TG--TATCCTTAAATTTAAAGTGTATT 8929
```

```
Majority     TCTTGTXT-CT---A-TTGXTXXXXXXXAXXTX----XTGTXTA---CTTTAGCAGTAATATTTCTATTXXGTXTTCAXG
                               12170      12180      12190      12200      12210      12220      12230      12240
Human        GATTGAAGTTT----ATTGATTTTATTAATCTCCAAAATACCAG---CTTTTGGTTTGAGTTTTCTCTGCTGTTTTTCTG  9142
PolarBear    --------------------------------------------------------------------------------  0
Cow          TCTTGTAGACT---A-CAGGTGGTCAGAGTATTG-GAGCTTCAG---CTTCAGCATCAGTCCTTCCAATGAATATTCAGG  9615
Dog          GCTCGGCTCTT---TCATTATGCTACATAAATCTCGGATGCGCA---TTTATACAGTAAAAAAGATATGGAGTAAACACG  9816
BelugaWhale  TCTTT--------------------------------G---------CTTT----------TTTTTAA-----------  9068
Donkey       TCTTTCTTTCTTTGAGGAAGAGTTGCCCTAAGCTAACATCTGCACCAGTCTTCCTCTACTTTGTGTGTGGGGTGCTCACA  8795
Cat          GCCTCTGTACT---A-TTGGTATCATATATTTTACTTTTGTATA---TGTTACAAGTCCTATAT-TATTTGTTGAAACAG  11415
Ferret       TTTCCTCAGGT---AGTTTTGGGCATATAGATTT-ATTTGTATT---ATTAAGTAGTATAATTTCTTCTTTTTTTTGTAG  9777
Lion         GCCTCTGTACT---A-TTGGTATCGTATATTTTACTTTTGTATA---TGTTACATGTCATATAT-TATTTGTTGAAACAG  10440
Vaquita      TCTTT--------------------------------G---------CTTTC------ATTTTTTTAA-----------  9154
Pig          TCTTGGAG-TT---C-CCA--------------------TTGTG---GTTCAGCGGGAATGAATCTGACCAGTATCCATG  8981

Majority     -X-X-T-T-TXTT-AAXATTXXC------XGTTTXATXTXTTTTCTXGTAXXXXGXAXAATAGA--TXT-T-XXXAACTT
                               12250      12260      12270      12280      12290      12300      12310      12320
Human        TTTTCTATTTTATTGATTTCTGCT------ATTTTTATTTTGTTTTGCTTTGTGGTTAGTTTGATCTTCATTTTCTACTT  9216
PolarBear    --------------------------------------------------------------------------------  0
Cow          GTTCATTT-TCTTTAGGATTGAC-----TGGTTTGATCTCCTTGCT-GTTCAGGGAACTCTGAACAGTTTTCTCCAGCAC  9688
Dog          CGCAATTT-TATTTCACACTAGC------ATCTCTCATTTTTATCTGGAGATACACAAAAGAGCTGTATCTTTTAGAACT  9889
BelugaWhale  ---------------------A--------------TTTCTTTGCT----------------------T-----------  9080
Donkey       GCATGGCTGTTGAGTGGGGTATGTCCGCACGTGGGATCTGAACCCGTGAACCCGGGCCACTGAAGCAGTGTCCAGAACTT  8875
Cat          CCAATTATCTTTTAAATACTACTTAAACAATAAGAAAAATATTTTCTGTATTACTCACAACACAGTTGTGTGCATGCATT  11495
Ferret       TTTATTTAATCTTGAACATTTTT------AGCTTCTTCCATCTTTTAAAAAGGATTATAATAGCTTTGAAGATTAAATCT  9851
Lion         CTAATTATCTTTTAAATACTACTTAAACAATAAGAAAAATATTTTCTATATTAATCATAACACAGTTGTGTGCATGCATT  10520
Vaquita      ---------------------A--------------TTTCTTTGCT----------------------T-----------  9166
Pig          --------------AGGAT--GC-----AGGTTTGATCTCTGGCCTTGCTCAGTGGGTTAAGGA----T----CCAGCGT  9032
```

```
Majority      TAXTATT-XGX-X-AXTATX-------TXTAAGTCXT-XXT-XXT-XXAC----TTXGTACTTTGTXT-XGAC------

                       12330     12340     12350     12360     12370     12380     12390     12400

Human         AGTTAACATTGAAGTTAATGTCATTAACTTAGTACTTTTACTTATATAGGG--ATTTATTGCTGTATGTTTCCCTTTTAA  9294
PolarBear     --------------------------------------------------------------------------------  0
Cow           CACAGTTCAAAAATACCATTT------TTTGAGTGCTCAGCCTTCTTCAC---AGTCCAACTCTCACATCTGTC------  9753
Dog           TCATATGAAGAGAATCGATT-------AAGAAAGCACAACTAGATCTTAA-----TAAGCACTATGTGCAACAT------  9951
BelugaWhale   TACTTTT---------------------------------------------------------------T--C------  9089
Donkey        TAACCACTAGGCCATGGGGCCAGCCCCTGAACTTAATTTTTGAATCCAAGCGTACACCCTCTTTTGATTAACACCATGTA  8955
Cat           GAATATTCGGGGGGATTTTCCACAGATTCCCAGAGCTGCCTCTGTGCAACTCTCTTTGGTACTTTGCTGCAGAAACCCTA  11575
Ferret        GATTATGTAGAAAAAGTATTTTTCACAACTAGGTCTTCAGTTAATGTTAAC-TGATTTACACTACATACAGAATATTCAA  9930
Lion          GAATTTTCGGGGGGATTTTCCACAGATTCCCAGAGCTGCCTCTGTGCAACTCTCTTTGGTACTTTGCTTCAGAAACCCTA  10600
Vaquita       TACTTTT---------------------------------------------------------------T--C------  9175
Pig           TGCTGT---GA---GCTGTGG------TGTAGGTCATAGGC-------AC---GATGCGGATCTGGTGT--GAC------  9082

Majority      ----TTTTGXTATXACXGGAGTC-TXA-CTCTT------TXT-TAAAXTTTXGCTATCTTCTXAATATTGTTTC-X-T--

                       12410     12420     12430     12440     12450     12460     12470     12480

Human         ---GTACCGCTTTCACGGCATTCCTCAAATTTT----GACACGTATATTTTTATTCACTTCAAAATACTTTTTTAAATAT  9367
PolarBear     --------------------------------------------------------------------------------  0
Cow           ----CATGATTACTGGAAAAACC-ATAGCTTTG---ACTATATGAACCTTTGTTGACCAAGTGATGTCTCTGCTTTTT-  9823
Dog           ----TTTGGCTCTCAATAGGGTGAGGATTAAGT------TTGGTAACGGCTCCGAGTAGACTTCACAATAAGTAGGGT--  10019
BelugaWhale   ----TGATATTAATACAGCCATC-----------------T--------TGGCTTTCTTTTGAATACAGTTTA------  9133
Donkey        CAGTTAATGTTATTACTAATGTGGTTAGTTTTACAACTACCACGTTGCTGTTTGTATATTCCTTGTGTTCTTCCCTCTTT  9035
Cat           CTTGCTTTGGTCTCCTTGGACTCATGTCCTCTTAGGGAGTTTTTGAAGTTTGGCCATTTTCTCCACCTGGGCTGTGCC--  11653
Ferret        ATTTTTTAGCTTTAGGATATGTTGAAATTTCAT------TTTTCATTATCTTTATAAATTTGCTGTAGTTACTCAGTT--  10002
Lion          CTTGCTTTGGTCTCCTTGGACTCATGTCCTCTTAGGGAGTTTTTGAAGTTTGGCCATTTTCTCCACCTGGGCTGTGCC--  10678
Vaquita       ----TGATATTAATACAGCCATC-----------------T--------TGGCTTTCTTTTGAATACAGTTTA------  9219
Pig           ----TGGGGCTGTGGCCAGCGTCTGCAACTCTG----ATT---CAACCCTTACCTAGCCTGGGAATCTCCCTTC------  9145
```

```
Majority     ----XTXTGCTX-TTTCTTTTTGCATAATCTGTXTTTAAXATAAATTGTXTXTTXTAATTTXXTGXXTTTAAAGTTTXTT
                      12490      12500      12510      12520      12530      12540      12550      12560
Human        TTCTTTGACCCA-TGGGTTATTGGAAAGTATGTTATTTATGTTATTTAATTTCCAAATATTTGGAGTTTTCCATATATCT  9446
PolarBear    --------------------------------------------------------------------------------  0
Cow          ---AATGTGCTGTCTAGTTTTGTCATAGCTTTTCTTCCAAGGAGCTAGTGTCTTTTAATTTCATGGCTGCAGTCTTTGTC  9900
Dog          ---GATATGTGACATAGTATCAGGGTAACCAGTTTTGGAGAGAAATTGTGATATAACAAGTCGTAGATGTTGAAGGGTCT  10096
BelugaWhale  -----CATGGTA-TATCTTTTTCCATTATCTTACTTTCAGACTATTTGTATCCTTAGAT--------TTTAAAGTGTATT  9199
Donkey       TCTCTTCTTCTGCTTTCTTTTGGATTGGTTTTTTTTTTTTCCATTTTATCTCTTGTTAGCTGATTAGCTTATAACTCGTT  9115
Cat          ---ATGGCCTGGTCTTTCAAGATAGTAAGCTGGGGCAATCATGGACTGACCTTACTTGTTTTCTGAATTTCAGGGATCAT  11730
Ferret       ---GATTTGAGACTCCTTTTTTGGTTAGCCTAGTGAGAGTATAAAAGGAAGTTTATAAATTTTTAATAAGAGATCTGCAT  10079
Lion         ---ATGGCCTGGTCTTTCAAGATAGTAAGCTGGGGCAATCATGGACTGACCT----TGTTTTCTGAATTTCAGGGATCAT  10751
Vaquita      -----CATGGTA-TATCTTTTTCCATTATCTTACTTTCAGACTATTTGTATCCTTAGAT--------TTTAAAGTGTATT  9285
Pig          ----ATATGCTG-CGGCTGTGGCCCTAAAAAGACAAAAAAAAAAAAAGTGTCTTGTAG----ATGGC-ATAAAATAGACT  9215

Majority     TGC------TTGTAGGGTXTXTAAAATAXATTCT-XCXTXTGATTTXAXTXTTTCAXXTTTATTTXTTAXXXX--AATTG
                      12570      12580      12590      12600      12610      12620      12630      12640
Human        TTCTGTTATGTATTCATAATTTTAGTTTTATTCTGGCCAGAGAACATATTTTCTAAGACTTGGATCTTTGTAAA-CATGG  9525
PolarBear    --------------------------------------------------------------------------------  0
Cow          TGCAGTTATTTTGGAGCCCAGGAAAATAAAATCTGTCACTTGTTTGCACTTTTTCGCCTCTATTTGCCATTAAG-TGATG  9979
Dog          CCTCT----TTATTGGGTATGCATGATTTATGGCAAATTTGTCGTTGACTGGGTGCGCACAAAATACGAACAA--AACCC  10170
BelugaWhale  TCC-------TGTAGACAGCATAAAATAGAGACT------TGATT-------------TTTA------------------  9235
Donkey       GTTTAGTTATGCTAGTGGCTGTCTTACGATTTATAGCATACATCTCTAATGTATCACAGTCTTCCCTCAAGGAGTATTTG  9195
Cat          TGTCCATCATTGTCTGGTTCTTGAAAACCATTGTTACACATGTCCTGTGTAATTTTTTTTTTTTTTTTTTTTTTTAAGTT  11810
Ferret       TTCTT----TAAAATGGTTTTTTACATTTCTTGTAATTTGTAATTTCAGGGTTTCATTTCTATACCCTTAACC--ATTTG  10153
Lion         TGTCCATCATTGTCTGGTTCTTGAAAACCATTGTTACACGTGTCATGTGTAAATTTTTTTTTTTTTTTA------AAGTT  10825
Vaquita      TAC-------TGTAGACAGCATAAAATAGAGACT------TGATT-------------TTTA------------------  9321
Pig          TG-----ATTTTTAAATCCTGTTTTATACGCTCT----TTTGATTAAGACCCTGCACATTCAGT-GTTAATATA-TATTA  9284
```

Majority	XCAXTGGXXA-ATTXTATCXTAXTACXTXXX-TTTTGACTTTTA-XXCAATTCTTTCACTXTTXTCTTTGTTTXAAXAAX	
	12650 12660 12670 12680 12690 12700 12710 12720	
Human	AGACTTGTT--TTGTGGTCCAAATATATATGTTGTTAAGTATTCACTGAATACTCTGATGATTCTCTGCAGATTTGGAGA	9603
PolarBear	--	0
Cow	GGACTGGATG-ATATGATCTTAGTTTTTTTTATTTTGAGTTTCAAGCCAACTTTTTCACTCTCCATTTTTTACCCTCATC	10058
Dog	AAAGTGAAGA-GCTTTTGTATGAGAGATGG--TAGGTAGGTATATACTGGTTGTATCAATGTAGTCTGAGGCGCAAGAGA	10247
BelugaWhale	----A------ATCCTATCTTACACCCTCTT---TTGGTTGTTG----AGACCCTTCACATTCAATATTGTTATATTAAT	9298
Donkey	CCACTTCACATATACTATGCGAATATTAGAAGATTTCTCTTCCTTGGCGTTTGTACTATTGTTATCATACATTTTACTTC	9275
Cat	TCAGTGGGAA-ACTCGTGCATATTACACCG--TCTTGCCTGGAAGTACAAGTCCACCTACTTTTTCCTTTTTTCAAAAAA	11887
Ferret	CCACTGTGAA-ATCTTTGCAAAGTAATTGGA-CTGTTACTTGGAAAACAAAAATTCCTGAAGTGATTGCATAGTAATTCC	10231
Lion	TCAGTGGGAA-ACTCGTGCATATTACACTG--TCTTGCCTGGAAGTACAAGTCCACCTATTTTTTCCTTTTTTCAAAAAA	10902
Vaquita	----A------ATCCTATCTTACACCCTCTTCTTTTGGTTGTTG----AGACCCTTCACAATCAATATTGTTATATTAAT	9387
Pig	ACA-TGGTTA-GTTTTATTTTATTTATTTATTTTTTGTCTTTTT-GCCATTTCTTGGGCTGCTCCGTCGGAAACTCCGAC	9361

Majority	AAGTTAAGTTXTXTAX-CTXTACTTTGTTATTTTXTXCCTT-TATGTTCTXTAGT--X--X--A-AAT--CXTTTTTATX	
	12730 12740 12750 12760 12770 12780 12790 12800	
Human	AATCTCTGTGCAGTT--CTTTTCTCTGGTACTTTGACCTGTATGTAAACTCTAGT------T-ACTTTGGTCTTCTCAGG	9674
PolarBear	--	0
Cow	AGGAGAAGGCAATGGCACTCCACTCCACTACTCTTGCCTGG-AAAATCCCATGGA--CGGAGGAGCCTGGTAGGCTGCAG	10135
Dog	AGATGGCACATACCAG-CAGTAGGTTGTAAAGAAGGCCCCCCTTAAGTGCCAGGG--TATACAAAAAT----TTGTTATT	10320
BelugaWhale	ATGGTTAGTTTTAAAAGTACCACTTTGTTGTTTTGTCTCT--TATGTTCTTCATT--C------------CCTTTTTTTT	9362
Donkey	TACATATGTTATAAATTCTATTCTGCATTATTTTTGTTTGTTTAAGCAGCTAATTATCTTTTAAAAAGATTTAAATAATA	9355
Cat	AAATTACATAATGTGGTCTGTAGTTCTAGATCTGCCTCATTCTTTGTTATATGGC--TACACCACAATGTCGTTTCTATG	11965
Ferret	AGATGATACTCAGTA--TGGTAAAATGTTAACTTTTTGGGTGGAAATTTTAA------ATAGTGAAAT----TTGTTACA	10299
Lion	AA-TTACATAATGTGGTCTGTAGTTCTAGATCTGCCTCATTCTTTGTTATATGGC--TACACCACGATGTAGTTTCTATG	10979
Vaquita	ATGGTTAGTTTTAAAAGTACCACTTTGTTGTTTTGTCTCT--TATGTTCTTCATT--C------------CCTTTTTTTT	9451
Pig	ATGGTTAGTTTTAGAAGTACCACTTTGTTGTTTTTTTTCTG-TTTGTCCCTTATT--T-------TCTTTCTTCCTTTTT	9431

```
Majority     TTCCTGCTXTCTTTTGAATTXAGTXAG-XTTTAAXXAXTXCATTTTAATCTTTXTAGCT---GAGTXGCTTACATATGTX
                      12810     12820     12830     12840     12850     12860     12870     12880
Human        CTCTTGGCTCTTTCACAATTAAAGTAGTCTTTGAGGCTCAGCCTGCTTTCCTCATAGCTATGCTATGGCCTGGACACTCA  9754
PolarBear    --------------------------------------------------------------------------------  0
Cow          CCCATGGGGTCTCTAGAGTCGGACACA-ACTGAGCGACTTCACTTTCA-CTTTTCACTT---TCATGCATTGGAGAAGGA  10210
Dog          TTACCATCGCTGTAGGCACTATTCTA----TTAGTAACTGCGTATAACCATACATAAC----AAGTATCTAGCATACTGT  10392
BelugaWhale  TTCCTGCTTTCTTTTGGATTGAGTAAA-TTTTT-TAATTCCATTTTAATCTCTTTAGCT---GATTAGCTTATAGCTGTT  9437
Donkey       ACAAAACTAACTTCTGCATTACCTGTGACATTGTTAGGTGCATGCATTGAATACTAGGGT--GGCGGAATTCACTGCAGA  9433
Cat          TTGATGACATAGTTTGATTCTAGTTTGCAGTTAAAGAGTATGTGGAAATATTTACAATGG--AAGTAAAAATCCTATGTC  12043
Ferret       GTTCTCTTACTAAATGAACTAAAGGAAGCCTTTAAGACTGGACAATTAATAAAGAATT----TAGTGGTTAAAATAGGTT  10375
Lion         TTGATGACATAGTTTGATTCTAGTTTGTAGTTAAAGAGTATGTGGAAATATTTACAATGG--AAGTAAAAATCCTGTGTC  11057
Vaquita      TTCCTGCTTTCTTTTGGATTGAGTAAA-TTTTT-TAATTCCATTTTAATCTCTTTAGCT---GATTAGCTTATAGCTGTT  9526
Pig          TTTCTGCTTTCTTTTGGATTGAGTATG-TTTTAAAAA-TCCATTTTAT-CTGGTTAGCT---GATTGGCCTACATATATA  9505

Majority     TXTTX-AATTT--TXTTGTGXXXGTXG--CTGTTATAXG-AATGCAXCXTGGAATTTTXATTTACCTCAGTCT-----GX
                      12890     12900     12910     12920     12930     12940     12950     12960
Human        AGGGAGTATAAGCTGAGGCAAACATGGACTCATTTGTTTTCTAACTTTCAGGGATTATTGTCCATCATTGCCTGATGTCC  9834
PolarBear    --------------------------------------------------------------------------------  0
Cow          AATGGCAACCCACTCCAGTGTTTTTGC--CTGGAGAATC-CCAGGGTCGGCGGAGTCTGGTGGGCTGCCGTCTA---TGG  10284
Dog          TATTCTGCTTT-TTTTTGGAATTGTGA--GAAATAAACGAGAAGAAGTGGGGAAGAATGAATAAGTT--CCAAA---AGG  10464
BelugaWhale  T------------TGTTATGTTGATAG--CTGTTTTAGGATTTATAGCATACATCTTTAATTTATCACAGTCT-------  9496
Donkey       TTTCCGAAGTTCTCTCTGTGCAACTCT--CTTCTCTGGT-ACTCTGCCGTAGAAACTCTAGTTGCTTTGGTCTCCTTGGA  9510
Cat          AAATGAAATTT-ACAGTTTAAAAGTTT--ATGAAAACCAGAATGCCAAAAGGATTCTTTCCACAACTATGTATG---GGA  12117
Ferret       GTTAGCATTTT-CCAAGTTGGATTTCA--CTAGTATTTTGAAAGCACTGTTGTAAAGTGCTGAACTTAACTGTA---TAA  10449
Lion         AAATGAAATTT-ACAGTTAAAAAGTTT--ATGAAAACCAGAATGCCAAAAGGATTCTTTCCACAACTATGTATG---GGA  11131
Vaquita      T------------TGTTATGTTGATAG--CTGTTTTAGGATTTATAGCATACATCTTTAATTTATCACAGTCT-------  9585
Pig          T-TT---------TCTCACGTTGGTAG--CTGTTATAAG-CCTACATC-------TCTGATTTACCGCAGTCT-------  9558
```

```
Majority     X-TCXC----------------------------TX--GTAGXATTTA-XXXACTX--CA------------A----X-C
                     12970     12980     12990     13000     13010     13020     13030     13040
Human        AGTGTC-TTGAAAAGCAATTATTCTGTATAGTTGCTTGATTGTTTGGTGTTGTTTCGGCGAGGCAAATCTGGGTCATGTT  9913
PolarBear    --------------------------------------------------------------------------------  0
Cow          GGTCGCACAG----------------------AGTCCGACAGGACTGAAGTGACTTAGCAGTAGCAG-TAGCAGTGGCTC  10341
Dog          AATTGGCTTT----------------------------------------------------------------------  10474
BelugaWhale  --TAAC----------------------------TCAAGTAGTATTTATGCTACTTTACA-----------TA----TAC  9531
Donkey       CCCTTGGCTTTCTCATCACAGAATGTTTTTGAAGTTCGGTGTGGTTTCCCCTTCTGAGCTGTGTCATGGCCTGAAATTTC  9590
Cat          ATGCCTGTTTCTCCACACCCTTATTAATTCTGGGTTTTGAACAATTTAGAGTATCACCTAATTTTAGGCTAAAAAATTGT  12197
Ferret       GTTTTGGTAT----------------------------------------------------------------------  10459
Lion         ATGCCTG-------------------------------G-----------------------------------------  11139
Vaquita      --TAAC----------------------------TCAAGTAGTATTTATGCTACTTTACA-----------TA----TAC  9620
Pig          --TCCC----------------------------TCCAGTAGTATTTATGCCACTTTACA---------T--A----CGC  9593

Majority     TXTXX-----GAATATTAGAATAXTTCT----XCXXXTTTG--XT-XGTAXTATXXTTAT--------------------
                     13050     13060     13070     13080     13090     13100     13110     13120
Human        ATTCTGTCTTGACTGGAAGTAGAAGTCCACTGTTTTTTTTTAATTACATAATATGGTCTCAGCAGTTGTAGATCTACCTC  9993
PolarBear    --------------------------------------------------------------------------------  0
Cow          TTTGCCATTAAAGTATTAGAATACTTCT----CCCCTTTTGGCCTGTGTACTATTGTTATT-------------------  10398
Dog          ----------ACTCTGTGGA------------------------------------------------------------  10484
BelugaWhale  TATGT-----GAATATTAGAATACTTCT----CCCCCTTTGGCCTCTGTACTATTATTAT--------------------  9582
Donkey       TCTCAAAGGAGTAAGCTGGGGCAATCATGGACTCTCATTTGCTTTCTGAGTCTTAGTGATCATTGTCCATTGTTGTCTGA  9670
Cat          TTTACAATTTATATTTTGAAATGGGTGA----GATTTTGAGAATTTGACTTTATATATCTTGTCCTGTGAACTGCCTTTT  12273
Ferret       ----------AGCTCTTGAA------------------------------------------------------------  10469
Lion         ------------------GA------------------------------------------------------------  11141
Vaquita      TATGT-----GAATATTAGAATACTTCT----CCCCCTTTGTCCTCTGTACTATTATTAT--------------------  9671
Pig          TTTGT-----GAATATTAGAGTACTTCT----CCCTTCCTAGTCCAAGTACTGTTGTTAT--------------------  9644
```

```
Majority     ---------------------------T-XXXATTTTACTTCTAXGTXTTATAAATTCTATA-TGCATTCT--------T

                      13130     13140     13150     13160     13170     13180     13190     13200

Human        ATTCTTTGTTAATGACAATGGAGG--ATACTCGTGTGGCTGCACCACAGTGTAGTTACTGGA-CATACCACAAT-----T  10065
PolarBear    --------------------------------------------------------------------------------  0
Cow          --------------------------AT-AACATTGTACTTCTACATGTTATAAATTCTATAGCGCATTCT--------T  10443
Dog          ---------------------------T--TTATTGTAGTGCTTTGAATTCAGAATACCAAA-TGAA------------G  10522
BelugaWhale  -----------------------------AACATTTTACTTCTACGTGTTATAAATTCTATAAGGCATTCT--------T  9625
Donkey       TGTCCAGTGGTCTTGAAAGCAATATTTTATATATTTTGTGTGGTTTGTTAGTTGTTTCAAGAGGGAATACTGGTCCATCT  9750
Cat          TGTATACCTGACCTATTTTAATGATCATCTTTGTTACACGTCTGTGTAAAATTGATTTCCTG-TACACATT--------G  12344
Ferret       ---------------------------T--TTATCAACATTGGCTGTTTAGATAGTTGCAA--TGAA------------G  10506
Lion         --------------A------------TCTTTGTTACACTTCTGTGTAAAATTGATTTCCTG-TACACATT--------G  11186
Vaquita      -----------------------------AACATTTTACTTCTACGTGTTATAAATTCTATACGGCATTCT--------T  9714
Pig          -----------------------------GACATTTTACTCCCACATGTTATAAATTCTACACTGCTTTCT--------T  9687

Majority     TAXGTTXXTTTTXTT------AGXCATTTATCTTTTTAAXTGATTTAXAXAAXAT--GATAAATAXXXT----TC-ACXT

                      13210     13220     13230     13240     13250     13260     13270     13280

Human        TAGTTTCCTACATCGATGGACATTTAGGTTTGATTCCAGTTTGTTGTTATAACAT---GTGGACATATT-----TAGAAT  10137
PolarBear    --------------------------------------------------------------------------------  0
Cow          TAAGT----------------AGCCATTTATCTTTTTAAAAGACTTAAACAACAA--GAGAAATA-----------ACCT  10494
Dog          TATCTTTAAACTGTT------ATATCTTAATCTCCGTATTTGTGTTCTGTGTGCT--GCTGTGAAGTAT----TC-ATAG  10589
BelugaWhale  TTTATTTGTTTAAAC------AGCCATTTATCTTTTTAAAAGATTTAAACAA---------GACAACCT----TCTACTT  9686
Donkey       TAGATTATCTTGGCTGGAAGTAGAAATCCACCTTTTTTATTTTTTTAAAAATTGCATAGTAAGATCTCTGCAGTTACCGA  9830
Cat          TAGGTACATTTTCTT------ACAGATTATTACTTTGAATTTCTTCATGTAGTGTTGGACATATAGATT----TA-TTTG  12413
Ferret       TAA-TGCCAACTGTT------ACTTGTTAACAATTGAACATAAGTGATTGTTTAT--AATAGGTATGTT----AC-TGAA  10572
Lion         TAGGTACATTTTCTT------ACAGATTATTACTTTGAATTTCTTCATGTAGTGTTGGACATATAGATT----TA-TTTG  11255
Vaquita      TTTATTTGTTTAAAC------AGCCATTTATCTTTTTAAAAGATTTAAACAACAA--GACAAATAATCT----TCTACTT  9782
Pig          TGTGTTT-----AAA------AGCCAGTTATCTTTTTAAAAGATTTAAACAACAA--GAAAGTAT-CT----TCTACAT  9749
```

```
Majority     TATXCTTAACATATTTAXGXATATXCAXTGTATACTTAXGXAGGTTTCTCTGCAGAT-TTXCA---GAXTTCTCTTTGGG
                     13290     13300     13310     13320     13330     13340     13350     13360

Human        AGAAGTAAAAATGCTGTGTCAAATGAAATTTATACTTAAAGAGTTACTGAAAACCACAATGCCA--GAACACTCATTTGT  10215
PolarBear    --------------------------------------------------------------------------------  0
Cow          TCTACTTTACATAGTTATGCACGTGCATTGAATACTCAGGGAGGATTCTCTGCTGACCTTCCA---GAGTTCTCTTTGTG  10571
Dog          CTTTCTTAA----TTTAGTTTTAGA-----CATATTGCATTAAGGGCTAATGTGGGTACGTAA---TATTTTTACTTTAG  10657
BelugaWhale  TACCCTTAACATAGTTATGCACATGCATTGGATACTTAGGGAGGATTCTCTGCAGAT-TTCCA---GAGTTCTCTCCGTG  9762
Donkey       TCTCTCTCATTCTTTGTTGTATGAACGAACCACAATGTAGTTCCTACATTGGTGGACATTTTGATTTGATTCCAGTTTGC  9910
Cat          TATTATTAAGGAGTATAGTTTTTT--TATTTGATCTTAAATGTTTTTAGCTTCAGGTCCTTTA---CTTTTTAGATGAGA  12488
Ferret       AACTTTTAT----TCAGGATTT--------TAGATTCTGATGTTTCTTAAAATGCTTTATAGA---TATTCATGCTTAGG  10637
Lion         TATTATTAAGGAGTATAGTTTTTTATTATTTGATCTTAAATGTTTTTAGCTTCAGGTCCTTTA---CTTTTTAGATGAGA  11332
Vaquita      TACCCTTAACATAGTTATGCACATGCATTGGATACTTAGGGAGGATTCTCTGCAGAT-TTCCA---GAGTTCTCTCCGTG  9858
Pig          TCCCCTTAACATAGTTATGCACATACATTGAATACTCAGGGAGGAGTCTCCGAAGAT-TTCCA---GAGTTCTCTCTGTA  9825

Majority     XAGTAATATTTX-GAACTTTXCXATAXAATATGTAGTTACTTTGTT--TXTTGGATT-CATGACT--TXAXTAAATGTXA
                     13370     13380     13390     13400     13410     13420     13430     13440

Human        TTTTCACAAGTGTGTATGGGAGTGCCTATTTTTCCATACCCTTGGCAATTCTAGGTTTTATCAAAATTTAAGAATACTAG  10295
PolarBear    --------------------------------------------------------------------------------  0
Cow          CAGCTCTCTGTT-GAACTCTGCCAAACAAACTTGAGCTGCTTTGAT---CTTGGATT-TTTGACT----TTTAACTGTCA  10642
Dog          AAGTAATA-----AAGTGTCACAAAAGCTTACGAAAATAAATTGTT--TTTTGAG-GACAAAAC------ACAATTTTAA  10723
BelugaWhale  CAGCTGTCTTTG-GAACTCTGCCATACAAACTCTAGTTGCTTTGGTCCCCTTGGATT-CTTGCCTCTTTCTTAACTGTCA  9840
Donkey       AAGTAAAAAGTGTGGAAGTATTTAAAATATAAGTAAAAATGCTGTGTCAAATGAATTTTGCAGTTAAAAAGTTTATGAAA  9990
Cat          TTATAATAGTTTTGAAGATTAAATGTGATTATGCATAAAAAGTATT--TTTCACAAACCAGGTCC--TCAGTTAATATTA  12564
Ferret       TATTTACATT---AGAATTTCCAGTTGCAGACTTTTTTAGTGTGTC--TACTAAATGACAACATT-----GTTAATTTAA  10707
Lion         TTATAATAGTTTTGAAGATTAAATGTGATTATGCATAAAAAGTATT--TTTCACAAACCAGGTCC--TCAGTTAATATTA  11408
Vaquita      CAGCTGTCTTTG-GAACTCTGCCATACAAACTCTAGTTGCTTTGGTCCCCTTGGATT-CTTGCCTCTTTCTTAACTGTCA  9936
Pig          CAGCACTCTG---GAACCCTGCCGTACCAGGTCTAGTTGCTTTGGTATCCTTGGATTCTTTGGCTCTTTTTTAGTTCTCA  9902
```

Majority	GXXA--GTTT-TCAAAXGTTAACCTATXTTTCCXTATTTGAGCTGTGCCTTGTCC-------TGGAATTTXTC--ATTTC	
	13450 13460 13470 13480 13490 13500 13510 13520	
Human	CTTATTTTACACTAAAAATTATTTTGTAATTTATATTTTGAAATGGGTGAGGTTTTTCAGATTTGGTTATTTAT-ATTTC	10374
PolarBear	--	0
Cow	GGGA--ATTT-CTGAAGTTCAACCCAG-TTTCCTTATTGGAGCTGGGCCTTGACC-------TGGACTTTCTC--AAGCT	10709
Dog	CCCA--GTTT-GTAAAAGAAAACATATAAAATGATAGTAAAAAAAAACCAAATAA-------AAGAATATACA--ATATC	10791
BelugaWhale	GGGA--GTTT-TCCAGGTTCGACCTGG-TTTCCTTATCTGAGCTGTGCCTTGACC-------TGGACTTTTTCTCAAGTG	9909
Donkey	ACCACAGTGC-CAAAAGGATTCCTCACAAGTGTATATGAGAGTGCCTGTTTTTCCACACCTTTAGAAATTCTGGGTTTTA	10069
Cat	ACTG--ATTT-GCACTACATATAGAATATCTGAATTTTATAACTTTAGGGTTTGT-------TGAAATTTCAT--TTTTC	12632
Ferret	TTTG--TAAC-TCAAAGGCTGAGGGAATATGTGCTAATTGAGGGTAGAGGTATTC-------AACAATAAGTT--GTTTT	10775
Lion	ACTG--ATTT-GCACTACATATAGAATATCTGAATTTTATAACTTTAGGGTTTGT-------TGAAATTTCAT--TTTTC	11476
Vaquita	GGGA--GTTT-TCCAAGTTCGACCTGG-TTTCCTTATCTGAGCTGTGCCTTGACC-------TGGACTTTTTCTCAAGTG	10005
Pig	GGGA--GTTT-TCAAAAGTTGACCCA--TTTCCCTGTCTGAGTTGTGCCATGACC-------TGGACTTTTTCTTAAGTG	9970

Majority	AXTAAXCTXGGACXATCAAXG-CCXX-----XXC-AGXTXGTTTTAT-AXT-XT------XTCATTGXTCATCATTGTCT	
	13530 13540 13550 13560 13570 13580 13590 13600	
Human	ATTTTCTGTGAACTGCCTGTTTGTGTACTGGACCTAGTTAATTCAAATATATATATTTGAATTAAGTGATTTTATTGTTT	10454
PolarBear	--	0
Cow	AGTAAGCTGGGACAGTCA--------------------TGGCTTATC--------------CCATGGTGCATCGTTGTCA	10755
Dog	AAAAACTTGAAAACAACAAGA-ACAC---------ACCAAATCTG--------T--------CCACAAGCTCGAACATGC	10845
BelugaWhale	AGTAAGCTGGGACGATGATGG-CCTTAC---CCCATGGTTGTTTTCTGACTCTGGG--GGATCATTGTTCATCATTGTCT	9983
Donkey	TAAAAATTTAGACTATTAGGT-AATTTTAGGTTAAAAAAAGTTTTATAATTTTGAA--ATGGATGAGATTTTCAAAATTT	10146
Cat	ATTACCCTTATAAATTTGATG-TCGCTA---CTC-AGTTGATTTTAGACTT---------CTTTTTGGTTAGCTTAGTGA	12698
Ferret	ATTGCTCCTCTATCATCAATA-GTACCT---GTT-AAGTAGTACA--------T--------ACTCAGTAAATAATTAAT	10834
Lion	ATTACCCTTATAAATTTGATG-TAGCTA---CTC-AGTTGATTTTAGACTTGTTT-----CTTTTTGGTTAGCTTAGTGA	11546
Vaquita	AGTAAGCTGGGATGATGATGG-CCTTAC---CCCATGGTGGTTTTCTGACTCTGGG--GGATCATTGTTCATCATTGTCT	10079
Pig	AGTAAGCTGGGACCATCAC---C---------------TCGCTTGTTGACTCTCAG--GAATCATTGTCCACCATTGTCT	10030

```
Majority     GATGTCCAATGGTCTTGAAAAACATTTTTCTATAXATTTTXTAAXXTTTTTATTTA----TT-X------TTTCTXAGXT

                       13610     13620     13630     13640     13650     13660     13670     13680

Human        AAAGTCCATTTGATGTGTAAACATTTTTAGCCTCAGCTTCTTCACCTTTTAAATTAGG-ATTTTAATAGTTCCACAAACT  10533
PolarBear    --------------------------------------------------------------------------------  0
Cow          AGTGTCCAGTGGTCTTGAAAAACAGTGTTGTATAAATTATGTATGACTTTTTTGTT--------------TTCCTAAGTA  10821
Dog          CCCCCCAAAAAGAAAGGGGAACGTAGATACAAAAAGGTTTTTTAACATATAAAAGA---------------AGCAGACCA  10910
BelugaWhale  GATGTCCAGTGGTCTTGAAGAACATCGTTCTATATATTATGTGTGATTTTCTTTTTC---TTTTATTTTTTTTCTCAGTT  10060
Donkey       GACTTTATATTTCTTTTCCTGTGGACTGCCTCTTTGTATCCGTGACCTATTTTAAGGATCTTTATTCTTGTTTATTACGA  10226
Cat          GAGTATAAAAAGAAATTTGTGAGTTTTCTT-AAAGATTTTTAAAAAAATTTATTTATTTATTCTGGGTGGGGGCAGAAAG  12777
Ferret       GACTCCTAAAGGTCTCTGGCTTCAGTACTCTTATAATCTTGTAAGCCACTTATCAA---------------TAAAAAGAT  10899
Lion         GAGTATAAAAATAAATTTGTGAGTTTTTTTTAAAGATTTTTTAAAAAATTTATTT----ATTCTGCGTGGGAGCAGAAAG  11622
Vaquita      GATGTCCAGTGGTCTTGAAGAACATCATTCTATATATTATGTGTGATTTTCTTTTTC---TTTTATTATTTTTCTCAGTT  10156
Pig          GATGTCCAGTGGTCTTGAAAATCATTGTTCTCTGTGTTTTCTTTCCTTCCTCCCTC--------------TCTCTTCTCC  10096

Majority     XGXCAXAXTGGTTXATTTTATACXCCTTCTTAGT--TAGXATTAGTAGTTAAXTTTX----AAAAAXATAATTXAGTXAT

                       13690     13700     13710     13720     13730     13740     13750     13760

Human        AGGTCTAATGTTAATGTATTTACATTTAGAATATCCAAGTTTTTATGGCTTTAGGATACAAATGAAATCTCATTTTCCAT  10613
PolarBear    --------------------------------------------------------------------------------  0
Cow          GGGCAGATTGGTCCAT-TGTTACACCTTCTTGGC--TAGAAGTAGAAGTTCACTTA------AAAATTTTACTTAATATG  10892
Dog          GGACCAAACTGGTGAATGTGAACGTTAGGAAAGT--TAGGAAAAGTGGACACGAAGG----AAGAACACAATTAGG----  10980
BelugaWhale  GGGCAAAGTGGCTCATGTTATA--CCTTCTTGGC--CAAAATTAGAAGTTCACTTT------AAAAAAAAATTATGTAAT  10130
Donkey       CTCTATAAAAATTGATTTCATATGCATCGTAAGTACTTTTTCTTACAGTTTATTATTCCTGATTTTCTTCGTATAGTTTT  10306
Cat          AGAGGGAGAGAGAATTCTTAGCAGGCTCCACACT--CAGCACAGAGTCTGACAAGGGGCTTAATCTCATGATCAGGAGAT  12855
Ferret       AGACACCAATTTTATTTTACAACTTTCTTCCTTT--TAGCT---GTAAATATGAAAG----AGCTATGTCTTTTAG----  10966
Lion         AGAGGGAGAGAGAATTCTTAGCAGGCTCCACATT--CAGCACAGAGTCTGACAAGGGGCTTAAACTCATGATCAGGAGAT  11700
Vaquita      GGGCAAAGTGGCTGATGTTATATACCTTCTTGGC--CAAAATTAGAAGTTCACTTTT---TAAAAAAAAAATTATGTAAT  10231
Pig          TCCCCCACTTCTTGCC-CCAAAGAAGTTCTTTTT--TTTTTTTTTTTTTTTTTTTT------TTAAATT-ACGTATTCTG  10166
```

```
Majority     GACTXTGTAATTATAGATTAATXGT--AGTCTTTXXXCX--ATTGATGCACCXCA-XGTAGTTGCTTAACATATGAAGAT
                       13770     13780     13790     13800     13810     13820     13830     13840
Human        TATTTTATAAATTTGCTGTATTCAGCTTTTCTTGATTTTAGACTTTTTTTTGTTAGCTCAGTGAGACTATTAAGAAAGAA  10693
PolarBear    --------------------------------------------------------------------------------  0
Cow          GTCTCTGCAATTATAGTTTT--------GTCTTGTTAC---ATTGGTGCCTCACACTGTAGTTGCTTTACACATCACAGT  10961
Dog          TACCAT-TGA----AACTAATGAAA-GATTACTAGA--A--ATACAGACAAGTTA--ATCATTGATGAACGGATGCACCT  11048
BelugaWhale  ATTTCTGCAGTAATAGATTTATCTT--ATTCTTTGTTCT--GTGGATGCACCACAGTGTAGTTGCTTGACATACCAAAAT  10206
Donkey       GGGCATAAAGATATATTTGAATTGTTAAGTAGTATACTCTTATTTATTTTTGTCTATTTGATACTTAAACGTTTTTAGTT  10386
Cat          CACGATCTGAGCTGAAATCACAAGTTGGGTGCTTAACCA--ACTGAGCCACCCAG--GTGCCTCAGAAATTTGTGAATTT  12931
Ferret       GACCTTGTGT----AGTTGAAGAGATGAGTACCTAA--C--ATTTAAGCATGT----GCCATATGGCCATGATAGGAGAG  11034
Lion         CACGACCTGAGCTGAAATCACAAGTTGGGTGCTTAACCA--ACTGAGCCACCCAG--GCGCCTCGGAAATTTATGAATTT  11776
Vaquita      ATTTCTGCAGTAATAGATTTATCTT--ATTCTTTGTTCT--ATGGATGCACCACAGTGTAGTTGCTTGAAATACCACGAT  10307
Pig          GTCTCTGCAGTTAGAGATATACCTC--AGTCTTTGTAT---GTGGATGCACCACAGTGTAGTTGCTTGACACACAACACT  10241

Majority     TTAGTTTCACATTG-ATTAACATTTTXGTTTAATTCCXTTTG-----CAGTTAAAAXT-----TGTAATATATTX---AA
                       13850     13860     13870     13880     13890     13900     13910     13920
Human        TATTTGTGAGTTTGTAATAGGAGATTTGTTGCATTTCTTTAAAATGGCTTTTTATATTCT--TCGTAATTTGTA----AT  10767
PolarBear    --------------------------------------------------------------------------------  0
Cow          TGAGTTCCACATCG-ACTAATATTTTGATTTAATTATTTTTG-----CAGTTAACAA------GTGAGAATATTA---AA  11026
Dog          TATATGACGAT-TA-ACTATCATCATTCAAGGGATACCAAATTGC--TAAGAACGATT-----TGTGAACTATCT---CA  11116
BelugaWhale  TTAGATCCACATTG-ATGAACATTTTGGTTTGATTCCCTTTG-----CAGTTAAAAAT-----AGAAGCATACTA---AA  10272
Donkey       TCAGTTTCTTCATC-TTTAAAATTGGGATTATAATAGTTTTGAATAAAAAATGTGATTATGCATAAAAAGTATTTTCCAC  10465
Cat          TTAATGAAGAGATT-TGTTGCATTTCTGTAAAATGGTTTTTTTGT--TCTTTGTAATT-----TGTAATTAACTT---CA  13000
Ferret       ATTGGTTTGGT-TT-GGTAGGATCTGAGTAGAGTTCACAAA-------AGTAGTGAAT-----TGTGATATATG----CA  11096
Lion         TTAATAAAGAGATT-TGTTGCATTTCTGTAAAATGGTTTTTTTGT--TCTTTGTAATT-----TGTAATTAACTT---CA  11845
Vaquita      TTAGATCCACATTG-ATGAACATTTTGGTTTGATTCCCTTTG-----CAGTTAAAAAT-----AGAAGCATACTA---AA  10373
Pig          TTGGTTTCACATTG-ATGAACATTTTAGTCTAATTCCCTTTG-----CAGTTAAAAA------GTGGACATATTA---AA  10306
```

```
Majority     AAXAGAAGTATXAATGCTGTGT-CAAATGAXGTT-ACXGTTAAAAAATTTTTAAAAXCCAXTTTGXCAXGAAXTTXTTXA
                       13930     13940     13950     13960     13970     13980     13990     14000
Human        ATCAGGGGTGTCATTTCTGTACTCTTAACCATTTTGCCAATGTGAAATCCTTGCAAAGTAATCGGCCTATTACTTGGGAA  10847
PolarBear    --------------------------------------------------------------------------------  0
Cow          AATAAAAGTAAAAATACTGTGT-CAAATTAAATT-ACAGCTAAAGAGTTTATTAAAGCCACAATGCCAAGAGGATTCTTA  11104
Dog          CCCTCGACTTTTCGTCCCCTCC-CGGGATGTTGT-GCCGCGGGGAAAATTTCAACATACTGTTCGCCTAGAACTTTTTTT  11194
BelugaWhale  AATAGAAGTAAAAATGCAGTGT-CAAATGAAATTTACAGTTAAAAAGTTTGTTAAAACCACAATACCACAAGTTTCCTTA  10351
Donkey       AAGCTAGGTCCTCAGTTAATGT-TAACTGATTTATGCTACATATAGAATATCCAGATTTTATAGCTTTGGGATATGTTGA  10544
Cat          TGGTTTCATTTCTATACTCTTA-ACCATTTTGCC-ACTGAAATCGCAGTATAATCGGCCTTTTACTGAGCAAAACAGAAA  13078
Ferret       GTGTAAATAATTTGGGATAGGA-GGTAAGTTGTA-TATGTTGAGGGAATGACATGGCCAAAGTTGTGTTGATGGACTGGC  11174
Lion         TGGTTTCATTTTTATACTCTTA-ACCATTTTGCC-ACTGAAATCGCAGTGTAATCGGCCTTTTACTGAGGAAAACAGAAA  11923
Vaquita      AATAGAAGTAAAAATGCAGTGT-CAAATGAAATTTACAGTTAAAAAGTTTGTTAAAACCACAATGCCACAAGTTTCCTTA  10452
Pig          AATAGAAATAAAAATGCTGTAC-CAAATGAAGTTTACAGTTAAAAAATTTG--GAGTTCCTGTTGTGGCGCAGTGGTTAA  10383

Majority     CTXATTTGTTTX--------XTACAGATTAXTXTGGGATTGCAXXTTTTTCGGTACXA---TTXTCAATTTTTGGATXTA
                       14010     14020     14030     14040     14050     14060     14070     14080
Human        GACAGAATTGTATGAAATGACTGTAGAATAATTCAGGATGGTCCCCAATACGGTAAAA---TGTTAACTTTTTAGATGTA  10924
PolarBear    --------------------------------------------------------------------------------  0
Cow          CTCATTTGTTTT--------CTACAATTGTGTATGGGAGTGCTTGTTTTTCTGTACCC---TTACCAATTCTGAGTTTTA  11173
Dog          TTTTTTTCTTCG--------TTTTTTTTTTATGTAA-ATTCTTAAGTTTTCAACTCTA---CTTAGAATTTATATATATA  11262
BelugaWhale  CTCATTTGTTTT--------CTACAGTTGTGTATGGGAGTGCCTGTTCTTCGACACCC---TTACCAATCGTGGGTTTTA  10420
Donkey       CATTTCATTTTTCATGACCCTTATGAATTGCTGTGGTTATTCAGTTCTCATTGTTTTAGACTACTTTATTTTTGGTTAGC  10624
Cat          TGAATGAAGTGA--------TTGCACAGTAATTCAAGATTATACTCCTTGTGGTAAAA---TGTTAACTTTTTGGATGGA  13147
Ferret       GCAAATTCACAA--------ACACAGAATAGCTTCT-GTAGCAATAGGTTCGTTCGTA---TAAGGGTTTGTTGTATGGA  11242
Lion         TGAATGAAGTGA--------TTGCACAGTAATTCAAGATTATACTCCTTGTGGTAAAA---TGTTAACTTTTTGAATGGA  11992
Vaquita      CTCATTTGTTTT--------CTACAGTTGTGTATGGGAGCGCCTGTTCTTCTACACCC---TTACCAATCCTGGGTTTTA  10521
Pig          CGAATCCG--------------ACTAGGAACCATGAGGTTGCGGGTT------------------CGATCCCTGGCCTTG  10431
```

```
Majority     -ATGX--A--AXXGTXAAATATGAGCTAGTTTTXATGTXAAATTAA-TXTTAAATGTATXXTT----ATAXGTG-GATTA
                       14090     14100     14110     14120     14130     14140     14150     14160
Human        GATTTTAAGTATGGTGAAATTTGTAGCAGTGTTCTTTAGTAAGTTAACTATTAATATATCAAGACCTATAACTG-GACAA  11003
PolarBear    --------------------------------------------------------------------------------  0
Cow          -ATGA--A--AATTTAGAATATTAGCTAATTTTAGGGTAAAAAAAGCTTTGTAATTTATATTTTGAAATAGGTGAGATTT  11248
Dog          GATG--------AATCTAATATATATTTTTTTT-ATATTTTTTTGTTTTTTTTTTTTTTTTTT-------------GTTA  11320
BelugaWhale  -ATAA--A--AATTTAGAATACTAGCTAATTTCACGGTAAAAAAAA----AAGTTTTGTAATTTGAAATGGGTGAGATTT  10491
Donkey       TTAGTGAG--ATGATAAAACAAGAAAGGAAATTGGTGTATTTTTAA-TAAGAGGTGTGTTGTGTTTCTTTAGAATGGTTT  10701
Cat          TATTTTAA--ACAGTGAAATTGGTGCTAGCTCTCATACTAAATTAACTACTAAATGAAGCCTT----TAAAACTGGACAA  13221
Ferret       TATG--------AGAGAAAAAGAAGATGGCAGT-ATGTTGTAGAAAGCCTCAAGTGCAG-----------------GTTA  11296
Lion         TATTTTAA--ACCGTGAAATTGGTGCTAGCTCTCATACTAAATTAACTACTAAATGAAGCCTT----TAAAACTGGACAA  12066
Vaquita      -ATAA--A--AATTTAGAATACTAGCTAATTTCACGGTAAAGAAAA----AAGTTTTGTAATTTGAAATGGGTGAGATTT  10592
Pig          -CTCA--G--TGGGTTAAGGAT---CCGGCGTTGCCGTGAG-------CTGTGGTGTAGGTT-----GCAGACGCGGCTC  10491

Majority     TXAAATTTTT----ATTTXTTATTTXXATGAATTXXCTTTTTGXATATXGGA-----TCTAXTTCXAAXATTAT---CXX
                       14170     14180     14190     14200     14210     14220     14230     14240
Human        CTAATAAATAACCTGTTTCTTAAAATGTTAGCTTTTTCTAAATATTCTGAAA---GTACTGGTTGTAAATTGCTGAACTC  11080
PolarBear    --------------------------------------------------------------------------------  0
Cow          TAAAACTTTG----GCTTTCTGTTTCTGTGAACTGCCTCTTTGAATATGGGA-----TCTATTTTAATGATTAT---CAT  11316
Dog          TTATGTTTTT----TTTTTTTTTTTTTTTTTTTTTTTTTTTTTTTTTTTTGT-----TTTTTTTTTTTTTTTTTTTTTTT  11391
BelugaWhale  TCAAAATTTG----ACTTTCTATTTCTGTGAACTGCCTCTTTGTATATGTGA-----TCTATTTCAATGATTAT---CTT  10559
Donkey       CTATGTTCTTTGTAATTTGTAATTTCAAGGGCTTTATTTCTCTACTCTTAACCATTTCCCACTGCGAAATCTTTGCGGCA  10781
Cat          TAGAGGACAT----ATTTGTTAAAATAAGTTATTAGCATTTTCCATGTTGGA-----TCTCACTGGTATTTAGAAAGCAC  13292
Ferret       TGAAATTTGT----ACTTCATCTGGTAAGCACTACTCTGTATACCTTTAAGT-----TCCCATTCATAAAAGTAGCTCAC  11367
Lion         TAGAGGACAT----ATTTGTTAAAATAAGTTATTAGCATTTTCCATGTTGGA-----TCTCACTGGTATATAGAAAGCAC  12137
Vaquita      TCAAAATTTG----ACTTTCTATTTCTGTGAACTGCCTCTTTGTATATGTGA-----TCTATTTCAATGATTAT---CTT  10660
Pig          --GGATCCCG----CTTTGCTGTGGCCCTGGCGTAGGCCAGTGGCTACAGC------TCCGATTTGACCCCTA-------  10552
```

```
Majority     TTTTGTTAXATTTCTATATAAAAATAAA---------TTAGATTCAAATXCATXAXAGATAGTTTTTCATAXAATTAA--
                      14250     14260     14270     14280     14290     14300     14310     14320
Human        ACAATTTAAATTATGATATGACTATTGA--------ATTCATCCTCATTGGCTTGCATAGAAAATTACAATCAAATAATG  11152
PolarBear    --------------------------------------------------------------------------------  0
Cow          GTTTGTCC-AACTCTATATATAAAGATA---------ATTGATTCATATGCATTATAGGTACTTTTTCCTACAGTTTT--  11384
Dog          TTTTTTTTTTTTTTTTTGTGAGAATTAA---------TAACCATCATGT--AGTGGGGAGAGTGAGACAGG-AAGAAA--  11457
BelugaWhale  GTTTGTTACAACTCTATATAAAAAGAAA---------ATTGATTCATATACATCATAGGTACTTTTTCTTACAGTTT---  10627
Donkey       TAATGCAGCAATTCCTTGGAAAGACAGAAAGTATGCAGTGGTCGCAGAATAATACAAGATGGTACTCGATATGGTAAAAC  10861
Cat          TGTTTGTAAATTGCTGAACTTAACTGTT---------TGAGATGTGAATTCACCAACATGGGCTTTTTAGATAATTGC--  13361
Ferret       TATTATGCTGTTTTTGGAATTTGGAGAA---------TGAACTCTGAAG--ATTGGTTTTGGTATTACATG-GAATGC--  11433
Lion         TGTTTGTAAATTGCTGAACTTAAC-------------TGAGATGTGAATTCACCAACGTGGGCTTTTTAGATAATTGC--  12202
Vaquita      GTTTGTTACAACTCTATATAAAAAGAAA---------ATTGTTTCATATACATCATAGGTACTTTTTCTTACAGTTT---  10728
Pig          GCCTGGGA-ACCTCCATATGCCGCTGGA---------GT-GGCCCAAAGAAATAGCAAAAAGACAAATAAATAAATAA--  10619

Majority     ATTAATTTAATGX-------------------TGATXGAATTTAAXTG-TXXTTXXTAGTAGGTTTGTTA----XAAAXT
                      14330     14340     14350     14360     14370     14380     14390     14400
Human        CTAAATGTAACTTTTTTG----------GTAATAGTTGTGTGTAACTGGTTGTTCATTATAGATGTGTTA----TTCAAA  11218
PolarBear    --------------------------------------------------------------------------------  0
Cow          ATTACTTTTTTGATACA-------------GTTTTGGGCATTTAGATGTTGTTGCCCACCAGGCTCGCTGG-TCCATGGG  11450
Dog          TCTAAGTTAGAACG------------------TACCCCAAACAAAAGAATGAGTCGGTGGAGGCACATCT---CACAAAT  11516
BelugaWhale  ATTACTTT--------------------------------TTTACCT----------------------C-----ATACA  10648
Donkey       GTTAACTTTTTGGATGCCTATTTTAAGTATGGTGAAATTTGTGGCAGTACTGTTTTTAGTAAGTTAACTAA-ATGAAGCC  10940
Cat          AATGAAATAATGCTAGATGTTACTTTTTGTAACGATTGAATATAACTGGTTGTTTATAGTAAGTATGTTATTAAAAACTT  13441
Ferret       TATGAATTAGAATAA-----------------TGAGTCTTTGTAAATGTTAATATTTTGATCTCTTATTT---GGTCGAT  11493
Lion         AATGAAATAATGC--------------------GATCGAATATAACTGGTTGTTTATAATAAGTGTGTTA---AAAACTT  12259
Vaquita      ATTACTTT--------------------------------TTTACCT----------------------C-----ATACA  10749
Pig          ATAAAGTTAAAAAAAAA-------------ATTTGTGAAACCTACAA-----TGCCGAAAGGATTCTT----ACTAATTT  10677
```

```
Majority     GTTTTTXGXXTXTXAGTATTGGAGTTXTT--TTTT---------------------------------------------
                      14410     14420     14430     14440     14450     14460     14470     14480
Human        ACTTTGGGTTTTTAGGTTTTGACTTTTCTTAAAATACTTTTCTTAAATGCTACTTAATGCTTTCTCTAGGGTATTTTTAT  11298
PolarBear    --------------------------------------------------------------------------------  0
Cow          GTTCTCCAGGCAAGAATACTGGAGTGGGTAGTCATTCTCTTCTCTGGGGGATTCTCCCAAC-------------------  11511
Dog          GTGTTTAACGTCATAACCCTTAGGTTGTTTGGCTG---------------------------------------------  11551
BelugaWhale  GTTTT------GGGCATCTAGGTGTG------------------------------------------------------  10668
Donkey       CTTATAACTGGACAGTTAATAAAGGACCTGTTTGTTAACATAAGTTGTTGGCTTTTCCAAGTTGGATTTTACTGGTATTT  11020
Cat          CTGTTTGGGCTTTTAGAGTGTGATTTTTCTTTTTTTTTTTTTTTAATTTTTTTTCTTCAACGTTTTTATTTTTATTTTTG  13521
Ferret       TTGTATTGCTAAATTGTAATAGCTTTCTTAGTTTT---------------------------------------------  11528
Lion         CTGTTTGGACTTTTAGAGTGTGATTTTTC---TT---------------------------------A------------  12291
Vaquita      GTTTT------GGGCATCTAGGTGTG------------------------------------------------------  10769
Pig          GTTTTCTACAACTGTGTATGGGAGTGCTTATTTTTCCTTCCCT-------------T--A--------------------  10722

Majority     --------------------------------------------------------------------------------
                      14490     14500     14510     14520     14530     14540     14550     14560
Human        TAGAATTTCAATTAACAGATATTTTGATGTCTGCGAAATGACAGTACTATGTTAGAAATTGATGTATTGGCTCC--TT--  11374
PolarBear    --------------------------------------------------------------------------------  0
Cow          --------------------------------------------------------------------------------  11511
Dog          --------------------------------------------------------------------------------  11551
BelugaWhale  --------------------------------------------------------------------------------  10668
Donkey       TGAAAGCACTGTTTGTAAGTTGCTGAATGCACAGTTTGAGTTGTGTTGTGGTTATTGAATTCACCATCATTGGCTTTCTT  11100
Cat          GGACAGAGAGAGACAGAGCATGAACGGGCGAGGGGCAGAGAGAGAGGGAGACACAGAATCGGAAACAGGCTCCAGGCTCT  13601
Ferret       --------------------------------------------------------------------------------  11528
Lion         --------------------------------------------------------------------------------  12291
Vaquita      --------------------------------------------------------------------------------  10769
Pig          --------------------------------------------------------------------------------  10722
```

Sequence	14570 – 14640	
Majority	--	
Human	--------------GAGGGCAGTGACCCTATTTGTTATGTTTGTTCCTCAATCATTAATAGTACACATATTAAATAGTAC	11440
PolarBear	--	0
Cow	--	11511
Dog	--	11551
BelugaWhale	--	10668
Donkey	AGATAATTGCAGTGAAATAATGCTGAACATTACTTTTTTTGTAATAGCTGAACATAACCGGTTGTTTATAATAGGTATGC	11180
Cat	GAGCCATCAGCCCAGAGCCTGACGCAGGGCTCGAACTCACGGACCGCGAGATCGTGACCTGGCTGAAGTCGGACGCTTAA	13681
Ferret	--	11528
Lion	--	12291
Vaquita	--	10769
Pig	--	10722

Sequence	14650 – 14720	
Majority	---------------------------------------AAATGCAAXATATTCATGCTTAXXTGAGX--AXX-XAAXT-	
Human	ATATACACTTAATATTTAATAACTCTTAGAAGTGTCATAGGCTTTAATATGCTCATACTCTAATGAGCCATTTATCAATA	11520
PolarBear	--	0
Cow	-------------CCAGGAATCGAACCTGTGTCTCCTGCATTGCAGGAAGATTCTTTACCATCTGAGCCACCAGGGAAGC	11578
Dog	---------------------------------------AAATTCAAGAGCCTAATGCTTTAACTGACTGAGACCACCAG	11592
BelugaWhale	---T--TTGAA-------------	10674
Donkey	TATTAAAAGCTTTGGGTTTTTAGGTTTTGATTTTTCTTAAAATGCTACATATCCTTGTTAGGATATTTATATAAGAATTT	11260
Cat	CCGACTGAGCCACCCAGGCGCCCCGTGTGATTTTTCTTAAAATGCTACATATTCATGCTTAGGTGTTTATATTTGAATTG	13761
Ferret	---------------------------------------AGATGTACTGTAGTAGGGGTTAACTTGGCTAAGTAATATTT	11569
Lion	---------------------------------------AAATGCTACATATGCATGCTTAGGTGTTTATATTTGAATTG	12332
Vaquita	---T--TTGAA-------------	10775
Pig	-------------------ACCAATTCTGGGTTTTATAAATACTTAGAATATTTGCTA--ATTTTAG--------G--G-	10770

```
Majority     XXA-TT--A--TAATXATATAGTGTAATTTATAATTTTC-AXXXT---XTXXTCTCTGATAXTTGAAAAACTTTXTTAAT
                       14730     14740     14750     14760     14770     14780     14790     14800
Human        AAAAGATAA-ATAACAAGACTATGTCTTTTAGGACTTTAGTTAGAGAAATAAGGACTCAAGGTTT--TAATTAAGTGCTA  11597
PolarBear    --------------------------------------------------------------------------------  0
Cow          CCATTTGAA--TTATTAAGTAGTATAATTCAGCTTTTTTTCCTGT---TTGATACTTAAACATTTTAAAACATTTTAAAC  11653
Dog          GCACCACTG--TATTATTATTTTATAATTTGTAACTTGATGGTGT---TGTGTCTCTAACAGAAGGGGGGTCTGTCTCCT  11667
BelugaWhale  -----------TTATGAAGTAGTATAATTTATTTTTTTT------------GTCTCTGATACTT---AAACATTTTTAAT  10728
Donkey       CAAATTACAGATAGATTTATAGTGTATCTACTAAATGACAGTGCTCTGTTAGAAACTGGTGATTCAAAGGCACTGAGACA  11340
Cat          CAAGTTACA--GACATTTATAGTGTGTCTACAAAATGCCAACACT---GTTAACTGGCAATTCAGAAACACTGAGAATAT  13836
Ferret       TTACTTTAG--AAGTAATAAAGTGTCACA-GAAACTTACGACAGA---TGAGTGTTT--TTGAAGAATGATTCATTTCAT  11641
Lion         CAAGTTGCA--GACATTTATAGTGTGTCTACAAAATGCCAACACT---GTTAACTGGCAGTTCAGAAACACTGAGAATAT  12407
Vaquita      -----------TTATGAAGTAGTATAATTTATTTTTTTTT----------TGTCTCTGATACTT---AAACATTTTTAAT  10830
Pig          -----------TAAAAAAGTTTTGTAATTCATATTTTG--A---------AATGAGTGAGAATTTAAAAATTTTGACTAT  10828

Majority     TTXTGTTTCTTTAGAAXXGAXAXGACXAATTXTGXXTATTATGXXXGTTTAT--ATXAATAGTATTTTTTATGAAGTACA
                       14810     14820     14830     14840     14850     14860     14870     14880
Human        TATGGCTACAGTAGGAAAGATTGGTTTGGGTTGGTAGGCTCAGAATAGTGGTCTGTGACCTAGCTAGATTGGGAGATAGG  11677
PolarBear    --------------------------------------------------------------------------------  0
Cow          TTAAGTTTCTTTAGAATTGAGATGACAAACTTTGAATATTAAGTGTGATTGTACATAAAAAGTATTTTCCATGAACTAAG  11733
Dog          TTTTTTTTTCCTAGAAAAACATAAACCAACGAATATTAGGATATCCAAAAAAA-ATGAACAATTCCCGTAATGCTTACCA  11746
BelugaWhale  TTCAGTTTCTTTAAAATTGAGATTATAAATTCTGAAGATTAAAAGTGTTTATGCATAAAAAGTATTTTCCGTGAATTAGG  10808
Donkey       ATATATGCTCCT-TGAGGGCAGAGACC-TTACTTGTTTTATTGCTCCTCCG----TCAGTAGTACATGTTAAACAGTACA  11414
Cat          GTGCTCTTTGAGGGTAGAGGTAGGGACCTTATTTGTTTTATTGCTCTTCCATC-ATCAGTAGTATGTGTTAAGTAGTACA  13915
Ferret       ATATTGTGAATTTTGAACATAGAAAAAGGCAATAGTTATCATGAACCAATAT---TTAACCTGTCACTTAGTCCCAATAA  11718
Lion         GTGCTCTTTGAGGGTAGAGGTAGGGACCTTATTTGTTTTATTGTTCTTCCATC-ATCAGTAGTATGTGTTAAGTAGTACA  12486
Vaquita      TTCAGTTTCTTTAAAATTGAGATTATAAATTCTGAAGATTAAAAGTGTTTATGCATAAAAAGTATTTTCCATGAATTAGG  10910
Pig          TTCTGTTTCTGT-GAACTG---C--C----TCTGTATATGAGGTCTGTTTT------AAT-GATTTCTTTATCTTGTTAC  10891
```

```
Majority     TACTCXGTXAATXTTAAACAATTTXTAXTAGATATA--GCATXAATATTCTTTTTATC-X-XX-XXTA--TTTXTAXXT-
                       14890     14900     14910     14920     14930     14940     14950     14960
Human        AGAAAGAGTGGAGAGCAACAGGCGTTGAGAGAAGGACGTAACCAAAGTTGTGTCTATAGGTTGGCATAAATTTACAGCCA 11757
PolarBear    -------------------------------------------------------------------------------- 0
Cow          TCCTTTATTAATGTTAACCAATTTATACTACATATA--GAATATCCAGTTTTTGTAGCTC-TGGGCTA--TGTTGAAATC 11808
Dog          TAAGAGGATAAAAGGAACCAATTGTGGGTAAAAAT----TGTAATTGTTCATAACATC-C-TAGAATT--TTTTTTTGC- 11817
BelugaWhale  TCCTTTGTTAATGTTAACCAATTTATACTACATAAA--GAACAAACAATTTTTATA------------------------ 10862
Donkey       TACTCAGTAAATATTTAATGGTTCTTTAAGAGTCTCTGGCTTCAATACACTCATAATCTCATGAGCCA--TTTACCAGTA 11492
Cat          TACACACTAAATATTTAATAACTCCTAATGGGTCTG--GCTTCAGTATGCTTTTCATC-T-CAAGCCA--CTTATCAGTG 13989
Ferret       TCATCAATAAACCATTGGTAAAATTTGT--GATAT----CCACTCTACTCTTCTCGTC-C-CGTAGTA--TTTTGAGAC- 11787
Lion         TACACACTAAATATTTAATAACTCCTAATGGGTCTG--GCTTCAGTATGCTTATCATC-T-CAAGCCA--CTTATCAGTG 12560
Vaquita      TCCTTTGTTAATGTTAACCAATTTATACTACATAAA--GAACAAACAATTTTTATA------------------------ 10964
Pig          AACTGTGTAAAA---AGAAAATTGATGC-ATATGCA--TCATAGGTACTTTTCTTA-----------TA--T-TTTA--TT 10950

Majority     --AAXTTXTA-----CATXXXTTTTTTTTCATXACATTCATCTTXTTTA-TGAAGAXCCAAAAXAGXT-TGAXTTCTAGG
                       14970     14980     14990     15000     15010     15020     15030     15040
Human        TTGATCAGTGAGTTGAATAGTTTGGCCTTTATGGCTAACAGCTTGTATATGGATATGAGAGAAAAGATAGAATGGCAGAT 11837
PolarBear    -------------------------------------------------------------------------------- 0
Cow          TCATTTTTCATTATCCATATGAATGTTTTCATTAATTTCAGTTCAGTTAGTTCAGTACCTCAGTTGTGTTCAACTCTTTG 11888
Dog          --AGGTTTT-----TTTTTTTTTTTTTGTGGAAGGGGGAGATCAGGATCAAATAAGCAGTGACACATTTGCAATATGAATG 11890
BelugaWhale  -------------------------------------------------------------------------------- 10862
Donkey       AAAAGATAAA-----CACCAGTTTTATTGTACCACATTCTCCCTTTTTGCTGGAGATCCAAAAGAGTTATGTCTTCTAGG 11567
Cat          --AAAAGATAA---ACATCAATTTTATTTCATAACATTCTTCCTTTTTGCTGGAGATCCAAAAGAGCTGTGTCTTCTAGG 14064
Ferret       --AAGTTGCAA---TTACTGTATTTTCATTATTACTGTTATTTTTTTTTTTAAAGAGAGAGAGTACTTCAGCAGGTGGGG 11862
Lion         --AAAAGATAA---ACATCAATTTTATTTCATAACATTCTTCCTATTTGCTGGAGATCCAAAAGAGCTGTGTCTTCTAGG 12635
Vaquita      -------------------------------------------------------------------------------- 10964
Pig          --ACTTTTTAC--TCCATAGAGTTTTGGGCATTAGATGTATTTGAACTA-TTAAGTAGTATAATT----TCATTTTTTTG 11021
```

Majority	AG-T--XXX-G-TXA-XAX---A-X-XXX--TXAAGXXXGTXCXTXAXXCTX-------GGA---TA-TXTGXTTTGGTX	
	15050 15060 15070 15080 15090 15100 15110 15120	
Human	AGGTTATATAGAAGACCTCAATTACAGGTAGTGAAATTTGTACTTCAT-CTGGTAGGCATTATTCTATATACCTGTAATT	11916
PolarBear	--	0
Cow	TGACCCCCACGGACTGCAGCACATCAGGCCTCCCTGTCCATCACCAACTCCT-------GGAGTTTACTCAAACTCATGT	11961
Dog	AGATGCCTGTGGTAGGTCTTTTAAATATG--TTAAGTCCGTTCACTTGGCTG-------GGGCTTGCTTCTCATCTGTTC	11961
BelugaWhale	--	10862
Donkey	ACCTTGTCTAGTTGAAGAG---ATAGAAGGTTTAAGCAAGTTCCACATGGCTACAGTAGGAGA---AATTTGGGTTGGTA	11641
Cat	ACCTCATCTGGTTAAAGAGATAACACAAGGTTTAAGCAAGTGCCTATGGCTGTTATAAAAGAGATTAGTCTGGTTTGGTA	14144
Ferret	AGGGGCATAGGATAAGGAAGGGGAGAGAA--AGAATCTTAAGCATGGAGCCT-------GACATGGGACTTGATTTCATG	11933
Lion	ACCTCATCTGGTTAAAGAGATAACACAAGGTTTAAGCAAGTGCCTATGACTGTTATAAAAGA--TTAGTCTGGTTTGGTA	12713
Vaquita	--	10964
Pig	GG--------------------------------GTCTATT--TGATACTT-------GAA---TGCTTATCTTTAGTT	11056

Majority	G---CAXAGTAGAX-TAACG-AA-X--TX--TTTTGAXXT-T-X-TT--XXXXTX--TG---AXAX-AX-TGAXTCCXAX	
	15130 15140 15150 15160 15170 15180 15190 15200	
Human	T--TCCACATAAAAGTAGCTCATTACTCTGCTTTCTTTCTTTCTTTTTTTTTTTTTTTTGCTATAGCAAATTTATTGTAAC	11994
PolarBear	--	0
Cow	C---CATTGAGTCGGTGATGCCATCCAACCATCTCATCTTCTGCCGA-GCCCTTCTCCTCCCACCTTCAATCTTTCCCAG	12037
Dog	G---CGTAGTAGCATCAGCAGACAT--TTGTTTTTGAAGAAAAACGT-GTTGGTACCTGATACTAGAGACTGTCCCCCCC	12035
BelugaWhale	--	10862
Donkey	GCATCAGAGTAGATTTCATGAAATTAGTGGGTTGTGACCTGTGCTTTTGTAAATAGGTTGGGAGAGGAGATGAATACTAT	11721
Cat	GGATCAGAGTAGACCTCACGAAAGTAGTGAGTTGTGACCTGTACATT-AAAAATAGTTGGGGAGAGGAGGTGAGTAGTGT	14223
Ferret	A---CCCTGAGATCATAACCTGAAC--TGAAATCAAGAGTCAGATTC-TTAAATGACTGA-----------GCCACCCAG	11996
Lion	GGATCAGAGTAGACCTCACGAAAGTAGTGAGTTGTGACCTATACATT-AAAAATAGTTGGGGAGAGGAGGTGAGTAGTGT	12792
Vaquita	--	10964
Pig	T---CATTGAGAGTATAGTA-------A---TTTTAA-------------------------AGATTAAATGTGACTGTG	11098

```
Majority     AXX--GAGGGATTGCCXTXGCCXXAGXTGTGTT----X----------------XGCTX-CAXAATATTAGXAGCATTGX

                       15210     15220     15230     15240     15250     15260     15270     15280

Human        AACACAAAGTATTATCTTATAGTTTTGTAGGTGAGAAATCTGACACAGGTGGGCTAAAATCAAGGTGTTAGCAGGGCTGT  12074
PolarBear    --------------------------------------------------------------------------------  0
Cow          CATCAGGGTCTTTTCAAATGAGTCAGCTCTTCTCATCA----------------GGTGGCCAAAGTATTGGGAGTTTCAC  12101
Dog          CCCCCCCCGGACCGCCCCCCCCCCCACCACCCG----C----------------CGCCCCCTCGTTCTCGTGTCTATTGC  12095
BelugaWhale  -------------------------------------------------------------G------------CTTT--  10867
Donkey       ATGTTGAGGGAATGACGTGGCCAAAGTTGTGTTGATAGC-TGGCACCAGTTCACAGCCATTGAAACAGTAAGCTGAATAA  11800
Cat          ATGTTGAGGGAATGACATGGCCAGAGTTGTGTTGAT-G----------------GGCTGGCACAAATTGAGAACCACTGA  14286
Ferret       GCACCCCTGTATTACTTTATTTGTAAATGTTTT----G----------------GTGTGTCTCTAAAAGGAAAGGACTCT  12056
Lion         ATGGTGAGGGAATGACATGGCCAGAGTTGTGATAAT-G----------------GGCTGGCACAAATTGAGAACCACTGA  12855
Vaquita      -------------------------------------------------------------G------------CTTTT-  10970
Pig          CAT--GAAATATTTCCCATGAACTAAGTCTTCT----------------------ATT-----AGTATTAACTGATTTAT  11149

Majority     ATAGXGTXT-X-TC-X------XTGTATTXGTXTTTXA----AAXATTXXX-XXGGGTTXTTXGAAATCTCXXG---AXX

                       15290     15300     15310     15320     15330     15340     15350     15360

Human        GTTCCTTTTTGCAGGATCTGGGGGGAATTTGTTTCCT----TGACATAACTCGGCTGTTAGCTTCATCTTCAAAGAAGGA  12150
PolarBear    --------------------------------------------------------------------------------  0
Cow          CTTCAGCATCAGTCCTTCCAATGAGTATTCAGGCCTGA---TTTCCTTTAGGATGGACTGGTTGAATCTCCTTGCA-GTC  12177
Dog          AAG---TGTGTTTCTG------ATGTTGTCATATTTAA----AAAATTGTCCAGGTGTCATTGCTCATGTTGGG---AGT  12159
BelugaWhale  --AGGGTAT---------------------------------------------G------TTGAAATCTC------AT-  10887
Donkey       TTTGGCTTTTATGGTGTATGGTTTGTATATGTGTATATAGGAAAGGATGCAGGTAGATTGTAGGAGGGCTCAAGTACAGG  11880
Cat          ATAGCTTTTGTGTCAG----ATTTGTATATGAATTTGAGAGAAAGATGGCACATAGGTTGTAGAAGGTCTCAAGTGCAGG  14362
Ferret       C-----TCTTTCTCTC------CTGTTTCCTTTTTTAA----AAAAATATAATCACAATACTGTTAATTCCTTG---ATG  12118
Lion         ATAGCTTTTGTGTCAGTA-GATTTGTATATGAATTTGAGAGAAAGATGGCATATAGGTTGTAGAAGGTCTCAAGTGCAGG  12934
Vaquita      --AGGGTAT---------------------------------------------G------TTGAAATCTC------AT-  10990
Pig          ATACTGTAT----------ATAAAATATTCAATTTTTA---TAGCTTT------GGGGTAATTGAAATTTC------AT-  11203
```

```
Majority     XTAXXAAXT-TX-X-TTXXTTCTTTAGXCTCATTTCTGTAAAGAATT---AGTTCCTCTGTATXCA----TGCTTXTAGT
                      15370     15380     15390     15400     15410     15420     15430     15440
Human        ATGGCAAA--GAAGACTCTGTCTCACATCACATCACTCCGACACATTCT-TGTGCCTCCGTTTCC-----CACTTACAGG  12222
PolarBear    --------------------------------------------------------------------------------  0
Cow          CAAGGGACTCTCAAGAGTCTTCTCCAACACCACAGTTCAAAAGTATA---AATTCTTTGGCACTTAGCCTTCTTTATAGA  12254
Dog          CTAGCGACTATCCTATTCGTTCTTTGTGTGTTGTTTTGTAAATAGTT-TAAGTGGTAG-GTCT--------GTTTTGTTT  12229
BelugaWhale  ----------TT-------TTCATTATCCTTATAAATGTGCTGTAG----ACTTACTCAGTTCCCAA---TGATTTTAGA  10943
Donkey       TTATGAAATTTGTGCTTCATCTGGTGGGCGCTATTCTATAAACCTGT--AAATCTCCATGTATAAG---TAGCTCATGTT  11955
Cat          TTATCAAATCTGTACTTCATCTGGTAGGCACTATTCTGTAAATACTGGTAAGTTCCCATACATAAAAAGTAGCTCATTAT  14442
Ferret       TTATCAAGTGCCAATTCTATGTTAAAATTTTACTTGACTCATGAAAG-TCAGTGTTTTTGCAG--------GTTTTTTTT  12189
Lion         TTATGAAATCTGTACTTGATCTGGTAGGCACTATTCTGTAAATACTGGTAAGTTCCCATACATAAAAAGTAGCTCATTAT  13014
Vaquita      ----------TT-------TTCATTATCCTTATAAATGTGCTGTAG----ACTTACTCAGTTCTCAA---TGATTTTAGA  11046
Pig          ----------TT-------TTCATTAGCCTCATAAAT---ATGCTGT---AGTTACTCGGTTCTCA---TTGATTTTAGA  11257
```

```
Majority     TTCCTTTTTATTAGGATAGGTXGATXAGACTCTAAAAXGAXAAAGGT----AATXTGTGGATTTTTAXTATTXAGATTGT
                      15450     15460     15470     15480     15490     15500     15510     15520
Human        GACCTTGTGATTACAATGGGCCCAACTACATAATAGAGGATAAATGTT--TAAATTACAAATTAATGAAGTGGCAACTGA  12300
PolarBear    --------------------------------------------------------------------------------  0
Cow          CCCCTTTTTATTTGGTTAGGTCAGTGAGACTGTGAAACA-GAAAGGA----AATTGGTAGATTTT-AATACAAAGTTTGT  12328
Dog          TTGTTTTGTTCCACGCTAGAAAGACCTTGCTGTTTGATTGTGTGTGT-----GTATTTTGGTTAATTTTTGTGGAATTGT  12304
BelugaWhale  CTCCTTTGTATTTGGTCAGGTCAGTGAGACTGTAAAACAAGAAAGGA----AATTTGTGAATTTTTAATATGAAGTTTGT  11019
Donkey       TCTGCTGTCTTTAGAATATGGAGACTGAGTTCCAAAAAGATTGGGTTTGGGACTACGCAGAACACTATGAATTAGAAGCA  12035
Cat          TATCCTCTTGTTAGAATTTGGAGAATTAACTCCAAGAAGACTGGCTTTGGTATTACGTGGGGTGCTATAAATTAGAATCA  14522
Ferret       TTTTTTTTTAGTGGGAGGGGAAGATCAGGATCAAGTAAGATGAACACATTCAGTATATCGATATGCCTGTTTTATTTCTT  12269
Lion         TATCCTCTTGTTAGAATTTGGAGAATTAACTCCAAAAAGACTGGCTTTGGTATTACGTGGGGTGCTATAAATTAGAATCA  13094
Vaquita      CTCCTTTGTATTTGGTCAGGTCAGTGAGACT--AAAACAAGAAAGGA----AATTTGTGAATTTTTAATATGAAGTTTGT  11120
Pig          GTCCTTTCTATTTGGTTAGGTCACTAAGACTATCAAACAAGAAAGGA----CATTTGAGAATTTTTAATATGAAGATTGT  11333
```

```
Majority     TGCATTTCTTX-AAXTXATTTTTTTAXATXXTTTGTXATTTGTTTXXXTT-XCAGAGTTTTA-ATTTCTGTACTTTTAAC
                       15530     15540     15550     15560     15570     15580     15590     15600
Human        AACAAGTTTT--AACCTATTCTCAAGAAAGGGCTTTGCTGGAATGGTGGC-TCACACCTGTA-ATCCCAGAACTTAGGGA  12376
PolarBear    --------------------------------------------------------------------------------  0
Cow          TGCATTTCTTAAAAAAAATTTTTTTTAATTCTTTGTAATTTGTT-------TCAGAGGTTT--ATTTCTATACTTTTAAC  12399
Dog          GTGGTATGTT--ATGTCTCAATGAG----CGCGCTTGTCATCACTTCTTTTCCATTGTTTTGTGCTTCAGCATAGCCGG-  12377
BelugaWhale  TGCATTTGTTTAAAATG-TTTTTTTATGTTCTTTGTAATTTGTT-------TCAGAGGTTTC-ATTTCTGTACTCTTAAC  11090
Donkey       TGAGTTTCTAT-AATTAACTTTTTTA-A--TCTCTTATTTCGTCTCGGTTGCTAAACTGTAATAGCTTTCTTAATTTAGT  12111
Cat          TGAGTCTCTA--AATTTAAAATTTAAACTCTTATGTGGTCTCTTTGTGTTGCTAAAGTGTAATATTTAGTTTTGGACATA  14600
Ferret       TTAATCTGCA--AGTTCTCCTTTCA----CTCCTGTGGGTTTTTTGGTTTTCTTTTGTTTTACATTGCATTTTATTTT--  12341
Lion         TGAATCTCTATAAATTTAAAATATAAACTCTTATGTGGTCTATTTGTGTTGCTAAAGTGTAATATTTAGTTTTGGACATA  13174
Vaquita      TGCATTTGTTTAAAATG-TTTTTTTATGTTCTTTGTAATTTGTT-------TCAGAGGTTTC-ATTTCTGTACTCTTAAC  11191
Pig          TGCATTTCTTTAAAATGGTTTTTTTATACTCTTTGTAATTTGTTACTAATTTCAGAGGTTTC-ATGTATGTACTCTTAAC  11412
```

```
Majority     CXTTTTGACAGTXTGAAATTCTTGCAGAATTATT----GGTCXAAATAGTTAG---TTTTTTXGAAGTTAAATTTCACAA
                       15610     15620     15630     15640     15650     15660     15670     15680
Human        GGCTGAGGCAGGCAGATCACTTGAGCCCAGGAGTTTGAGACCAGCCTGGTCCT---CATAGCAAGACCCCATCTCTACAA  12453
PolarBear    --------------------------------------------------------------------------------  0
Cow          CATTTTGCC--TCTGAAATTCTTGCAGAATAATG----GGCCCAAATAGTTAG---CTTTTTTTGGGTTAAATTTCACTA  12470
Dog          --CTTGGTAAACACTGTCGTTCACCAGTGTTCTTAACAGATCTATAAAAGAAGGTGTATCTTAAAGGTTGCACTTAAAAG  12455
BelugaWhale  CATTTTTGC-CTTTGAAATTCTTGCAGAATAATG----GGCCCAAGTAGTTAG---CTTTTTTCAAGTTAAATTTCACTA  11162
Donkey       --TTTAGACATATTGCATTAGGAGCTAAGGTGGTT---AAGTAATATTTTTAC---TTTAGAAGTAATAAAGTATCACAG  12183
Cat          CTGCATTAGGGCCTTAAGGTGGTTAAGTAATATTTTTACTTTAGAAGTGGTAAAAGTATCACAGAAACTTATCATGAAAG  14680
Ferret       --CGAAGAAAACTTGTTGGTCCCACAGAGTTGCC--CCAGTCTGCAACTACTGATTGTGTTCTGATGTTGTAATTTAAAA  12417
Lion         CTGCATTAGGGGCTTAAGGTGGTTAAGTAATATTTTTACTTTAGAAGTGGTAAAGGTATCACAGAAACTTATCATGAAAG  13254
Vaquita      CATTTTTGC-CTTTGAAATTCTTGCAGAATAATG----GGTCCAAGTAGTTAG---CTTTTTTCAAGTTAAATTTCACTA  11263
Pig          CATTTTGCCATTGTGAAATTCTTGCAAAATAATA----GGCCCAAGTAGTTAGGTTTTTTTTTTAAGTTAAATTTCACTA  11488
```

```
Majority     XAATTTTTAAAACACAGGT-XATTAA-TGTTGAATGTA------GTTTXAAXTTXTGATATGGCTATTTTATTCATAXTT

                      15690     15700     15710     15720     15730     15740     15750     15760

Human        AAACTTTTAAAAATTAGATAGATCTG--GTGGCATGGGCCTGTAGTCCCAGCTACTTGGGAGGCTGAGACAAGAGGATCA  12531
PolarBear    --------------------------------------------------------------------------------  0
Cow          GAATTTTGAAAGCACCAGT-TGTAAACTGTTGAATGCAAAGACAGTTT-AAGTTGTAATATGGGTACTTTATTCAGCATC  12548
Dog          TTGCATAATGGAAACCCGT--ATTAAATGTTAAATGTA------ACTTGAAAATTTTTGATGGTGGGACAAGGAATGGTT  12527
BelugaWhale  GAATTTTGAAAGCACTGGT-TGTAAATTGCTGAATGCA----CAGTTTTAAGTTGTGATATGACTATTTTATTCATCATC  11237
Donkey       AAAGTTATGAAAGACATGTGTTTTGAGCAGTGATTGTT-------TTTATAACTATGGTG-GTGAATTTTGAACATAGAA  12255
Cat          ATGTGTTTTTGAGCAATG---ATTT--TTTTATACCTA------TTGTGAAATTTGAACATAGAAAAAGACCTGATAGTT  14749
Ferret       T-GCTCATGTATCACGGGT--ATTGGATCTAGAGACTA------TCATATTCTCTTTTTGCTTGTTTTTTGGTAATATTT  12488
Lion         ATGTGTTTTTGAACAATG---ATTT--TTT-ATACCTA------TTGTGAAATTTGAACATAGAAAAAGACCTGATAGTT  13322
Vaquita      GAATTTTGAAAGCACTGGT-TGTAAATTGCTGAATGCA----CAGTTTTAAGTTGTGATATGACTATTTTATTCATCATC  11338
Pig          GTATTTTGAAAATACTGGT-T---AATTGCAGAATGCA----CAGTTT-AAGTTGTGATGTGGCTA-TTTATTCATCATT  11558

Majority     ATTG-GCXTTCTTAGXXAATTACAGTXAAXTATTACTAAXGAATCATTTTTTG--TAGTAGTXGA-ACATAACXXATTXA

                      15770     15780     15790     15800     15810     15820     15830     15840

Human        CCTGAGC---C--CAG--GCGGCAGTGAGCTATGATTGTATCACTACACTCCA--GCCTGGGCGAGAGGAGATCCAGTCT  12602
PolarBear    --------------------------------------------------------------------------------  0
Cow          ATTG-GCTTTCCTAGGTAATTGCAATAAAATAGTACTAAACATAACCTTTTTA-ATAGTAGTTGA-ATATAACTGATTGA  12625
Dog          TTC--GC----TGAATGTATTGTTGTCAATTAGAACAGGTGATTCAGATTTGC--TAGTCCTGAA-GGCTAGGGTT-TGA  12597
BelugaWhale  ATTG-GCTTTCCTAGACATTTACAGTAAAATAATGCTAAAGGAAACTTTTTTG--TAATAGTTGA-ACATAACTGATTTA  11313
Donkey       CAAAGACATT---A--TTTTTATCATGAACCATTATTTGTCACTCAGTCCCAA--CAGTCATCAACACACAACCCATTGC  12328
Cat          ATGAACCACTATTTGTCACTTAGTCCCAATAATCATCAATAAACCATTGCTAG--AATTTGTGAT-ATTCACTGTACTTT  14826
Ferret       TATAAGC----TGTGGTATGTTTTTTTCATTAGAAGACATGTGTTGCATGATG--TGTGTGTGCG-TGTGTGTGTA-TGT  12560
Lion         ATGAACCACTATTTGTCACTTAGTCCCAATAATCATCAATAAACCATTGCTAG--AATTTGTGAT-ATTCACTGTACTTT  13399
Vaquita      ATTG-GCTTTCCTAGACAATTACAGTAAAATAATGCTAAAGAAAACTTTTTTT--TAATAGTTGA-ACATAACTGATTTA  11414
Pig          ATTG-GCATTCTTGGATAATTGCAGTGAAATAATGCTAAAGATTTTTTTTTTTTGTGGTAGTTGA-ACATAACTGATTCA  11636
```

```
Majority     TAACAGXTATGTTAAAGAAAAACTGT-TTGTTTGGGTTTTTAGXTTTTGAGXTTTCTTA-AAATATTACATAATXCTTGC
                       15850     15860     15870     15880     15890     15900     15910     15920
Human        CAAAAAAAAAGGAAAAGGGCTTTCCTGAGGGTCAAATGAAGTACAGTTGACCCTTGAACAACATGATTT-TGAATTACAC  12681
PolarBear    --------------------------------------------------------------------------------  0
Cow          TGACCAGTATTTTAACCAAAAACTGTGTTATTCGGGTTTTTAGGTTTTGAGTTTTCTTA-AAATGTTACATAATTCTTGC  12704
Dog          CCTCTATAACATTGAAGGTAATTTCT-GAGCTTTAGTTTTAACTAATTTTTATATTTTT-AAATATTTTATTTATTTATT  12675
BelugaWhale  TAACAGGTGGGTTA-AGAAAAACTGTGTTGTTCGGGTTTTTAGATTTTGAGTTTTCTTA-AAACATTACATAATGCTCGC  11391
Donkey       TAAAACTTACAACATCCATACTCTGCTTTTCTTCTCCTGCGTTATTTTGAGACAAATTCCAGATGTATCATTTCATCTGT  12408
Cat          TCTCC--TATGTTATTAAAAAAATTT-TTTTTATGTTTATTCATTTTTGAAAGACAGAG-AGAAAGAGCACAAGCAGGGG  14902
Ferret       ATAGTGTTATTCCCAGTAATATTTCT-TGTAATAACTTCTTTCTCCTTGTTTGTGGTCA-GCGTAGTCTGTGCGCGGTAA  12638
Lion         TCTATGTTATTTAAAAAAAAAATTTT-TTTTTATGTTTATTCATTTTTGAAAGACAGAG-AGAAAGAGCACAAGCAGGGG  13477
Vaquita      TAACAGGTAGGTTA-AGAAAAACTGTGTTGTTTGGGTTTTTAGATTTTGAATTTTCTTA-AAACATTACATAATGCTTGC  11492
Pig          TAACAGGTGTGTTA-CCAAAAACTGT--TGTTTGGGTTTTTAGATTTTGAGCTTTCTTT-AAATGTTACATAATACTTGT  11712

Majority     TXGGGTATTTATATTAGGATTTAAAAAXAGAGAAATXTGTGCG--XGTCTCXG-AGXXXXXTGGACAGXXCXX-XXGTGX
                       15930     15940     15950     15960     15970     15980     15990     16000
Human        AGGTCCACTTATGCATAGATCCACTTATACAGAATTTTCTTCTC-CCTCTTCCACCCAAGACAGAAAGACCAACTCGTCC  12760
PolarBear    --------------------------------------------------------------------------------  0
Cow          TAGGGTATTTATGTTAGAATTTCAAATTAAAGACATTTGTACTCTTGCCTGGAGAATCCCATGGACAGGGGAGCCTGGTA  12784
Dog          TGACAGAACAAGCAGGGGAAGCAGCAGAGGAAGAGGGAGAAGC--TGGCTCCC-TGCCGAGCAGGGAGCCCCATGCAGGG  12752
BelugaWhale  TAGGGTATTTGTATTAGAATTTCAAATTAGAGACATCAGTGTG----TCTGCT---------------------------  11440
Donkey       AAGTGTTTTGGTGTGTCTCTTGAAAAAATAAGGACTCTTGGGG--TGGCCTTGTGGTGTAGTGGTTAAGTTTGCATGCTC  12486
Cat          TGGGGCGGAGAGAAAGGGGGACACAGAAACTGAAACAGGCTCC--AGGCTCTG-AGCTGTCAGCACAGAGCCCGACATGG  14979
Ferret       ACATCTGTTCATCAGTTCTCTTAAAGAATATGTAAAAGAAGTG--TATCTTAA-AGTTCACTTATAATTTGTATAATAGA  12715
Lion         TGGGGCGGAGAGAAAGGGGGACACAGAAACTGAAACAGGCTCC--AGGCTCTG-AGCTGTCCGCACAGAGCCCGATGTGG  13554
Vaquita      TAGGGTATTTGTATTAGAATTTCAAATTAGAGACATCAGTGTG----TCTGCT---------------------------  11541
Pig          TTGGGTATTCATATTAAGATTTTAAATTAGAGACCTTTATAGT---GTCTCT--AATGCCATAGACAGGAAGG--AGTTA  11785
```

```
Majority     GGCT---XXAXX-CA-G--XT-XXXGAXXAT-X-CXTGAGXCGXAX-AAXATGXTTX-X-XX-T---XXX---X--GCAX
                      16010     16020     16030     16040     16050     16060     16070     16080
Human        TCTTCCTCCTTAGCCTACTCAGCGTGAAGACAGTCAGGATGAAGACCTTTATTATGACTGATCTCCAAAAAGATTGGCTT  12840
PolarBear    --------------------------------------------------------------------------------  0
Cow          GGCT---GCAGTCCATGGGGTCACTAAGAAT-TGGACACGACTGAGGGACTTCACTTTTCACTTTT-TACTTTTATGCAT  12859
Dog          GGCT---GGATCCCA-GGACCTTGGGATCAT-GCCCTGAGCTGAAGGCAGATGCTTAACCAACT---AGCCACTCAGCAC  12824
BelugaWhale  ----------------------------------G------------AATGTCA----------------------GCAC  11452
Donkey       TGCTTTGGCAGCCCAGAGTTTACATGTTCAGATCCCTGGTGCAGACCTACACACCACTCATTAAGC-CATGCTGTGGCAG  12565
Cat          GGCT---CGAACTCATGAACTGCGAGATCAT-GACCTGAGCCGAAGTCGGATGCTTAACTGACTG--AGCCTCCCAGGCG  15053
Ferret       AGCA---CAATATTT-AATGTAAATTGAAGT-TATTGATGGTGGACAAGAATGTTTTTTCAA-T---GGAGGTTTTGTCA  12786
Lion         GGCT---CGAACTCACGAACTGCGAGATCAT-GACCTGAGCCGAAGTCGGATGCTTAACTGACTG--AGCCTCCCAGGCG  13628
Vaquita      ----------------------------------G------------AATGTCA----------------------GCAC  11553
Pig          T-CT---GTAG--CATG-------------------CACAACTAACAAA---------------------------GCTT  11813

Majority     TGTX--X---A-X---ATTXTAAXXTGXGXT----X-X-XXXGTATXTXAAXA-XXAT-X--X----XG--XTAT--AG-
                      16090     16100     16110     16120     16130     16140     16150     16160
Human        CAGTATTTCATGGAGTGCTATGAATTAGAATCCTGAGTCTCTATAT-TGTTAATTATTTTAGTCTCTTGGTCTACGTGTG  12919
PolarBear    --------------------------------------------------------------------------------  0
Cow          TGGCGAAGGAAATGGCAACCCACTCCAGTGTTCTTGCTTGGAGAATCTGAGGGACGGGGGAGCCTGGTGGGCTGCCTTCT  12939
Dog          CCTTAATTGAACAA--ATTTTAAAATGGACTT--AATAGGATCTACTTCAAAAGTCATTTTAAC-ACGGAATTATCAAGC  12899
BelugaWhale  TGT-----------------------G---T-----------T-----AAAGA-------------------------C-  11464
Donkey       TGTCCCACATGCAAA-ATAGAAGATTGGGACAGACGTTAGATCTTCCTCACCAAAAAAAAAAAAAACCAAAACAGAGAGA  12644
Cat          CCCCTCTCCTATGTT-ATTTTGAGATGAGTAG--CAGATACAGTATTTTACTTGTAATGTTTTGGTGTGTCTCATAAAGA  15130
Ferret       AGTTAAGAACATGT--GATCTAGAATTTGCT---AGTAAGCTAGGTTTTGGCCTCTATAACGTTGAAGGTAATCTCTAAG  12861
Lion         CCCCTCTCCTATGTT-ATTTTGAGATGAGTAG--CAGATACAGTATTTTACTTGTAATGTTTTGGTGTGTCTCATAAAGA  13705
Vaquita      TGT-----------------------G---T-----------T-----AAAGA-------------------------C-  11565
Pig          TAG-------------AATCCAC-----AAT-------------ATATAAAAA------------------CTCC-----  11839
```

```
Majority     XTAGAGTTXATTTAXAXTTTTXT-XATXTXAXXXTA---ATAXXXX-----ACAXTAAAGTXX-----XTTTXATGAX--

                       16170     16180     16190     16200     16210     16220     16230     16240

Human        TTCCTAAACTGTAGTAGCTTTCTTAATTTAGTTCTAGGCATACTACATTAGGGGATAAGGTGGTTAAGTGATATTTTCAC 12999
PolarBear    -------------------------------------------------------------------------------- 0
Cow          ATTGGGGTCACACGGAGTCGGAC-ACGACTGAAGTG---ACTTAGCAG-CAGCAGCACAGTGTGT-CTACTAAATGACAG 13013
Dog          TTACCGTAAAGCTACGAATTAGTTTATTGGGTGGTA---GAATGACTA--TATAATGTGCTTCA----AAGTGATGATCA 12970
BelugaWhale  ----TGGTGATTCAGAGACA------------------------------------------------------------ 11480
Donkey       A-AAATAAGGCTTATCTTTCTCTCTTTTTTTTTTTTA---GCAAAAGAAACAAAACTATAACTGCAATATTATTATGACAC 12720
Cat          TAAGAATTTTTTTTTTTTTTTTT-GATATAACCAGA---ATACTGTTAAGGACAGAAAAAAAAAAAACATTCCTTAATGT 15206
Ferret       CTTTAGTTTAACAAATTTTTAAGTGGTTTTAATTTA---GTAGGACC---TACCTCAAAGTTAA----TTTTGAAGATGA 12931
Lion         TAAGATTTTTTTTTTTTTTTTTTTGATATAACCACA---ATACTGTTA-GGACAGAAAAAAAA---ACATTCCTTAATGT 13778
Vaquita      ----TGGTGATTCAGAGACA------------------------------------------------------------ 11581
Pig          -TAGAAATCAGTAAGAAATAAAA-ACTATGAAGTTG---ACTTC--------TATTAAAGTGCTC-CTTCTAAATGA--- 11902

Majority     XAX-AXGX-AXXAATT------XTAAAXXTATTAAGA--TATATGTGTCT---GGGTXGXGAXCTTAATTGTTTXATTGA

                       16250     16260     16270     16280     16290     16300     16310     16320

Human        TTTAAAAGTAATAAGGTGTCACAGAAAGTTATCACGAGATTTTTTGAGCAGTACTTCTTTTTTAAAACTATTATAATGA 13079
PolarBear    -------------------------------------------------------------------------------- 0
Cow          CACTATGTTAAAAACTGGTGATTCAAAGACACTAAGA-ATATATGTGTCTTGAGAGCAGAGACCTTACTTATTTTGTTGA 13092
Dog          TGCTGGGTGACTGACT-------ATCAGCTCTTTATA-CCACCTCCTCCC---AGATTTGGACAGGAACACCTCAGTTGA 13039
BelugaWhale  --------------T--------TAAGAA---TAGAA--TATATGTGTCTTGAGGGCAGGGACCTTTTTTGTTTTATTGT 11533
Donkey       CAAAAAGCTAACAGTT----GCTTAATGTTATCAAGTGCCAAATTTGCACTAAAATTTCTAATTGTCCTATAAAAGTCAT 12796
Cat          TATCAAGTGCCAAATT------TTAAAATTTTTAATT-TTTGGGGTGCCT---GGGTGGCTCAGTCAGTTGAGCATCCGA 15276
Ferret       GATAACACATAAAATT-------TAGCACAGAAGGGC-ACATAAGTGCTC---AAATGATGATCATGATTGTTACTATAG 13000
Lion         TATCAAGTGCCAAATTAATG--TTAAAATTTTTAATT-TG-GGGGCGTCT---GGGTGGCTCAGTCAGTTGAGCATCCGA 13851
Vaquita      --------------T--------TAAGAA---TAGAA--TATATGTGTCTTGAGGGCAGGGACCTTTTTTGTTTTATTGT 11634
Pig          ---------G--A-TTTACAGTTTAAAGA-ACTACG----ACAGGTATC------------ATCATCATCATCTGAGCAC 11953
```

```
Majority     TTXTXTATCATAXGTAGTTCTTATXGTATXGTAXATACGAGGXATATATXTAATXA--CTTTTTGACAG-TCTCTGGCTT
                      16330     16340     16350     16360     16370     16380     16390     16400
Human        ATTTTAGACACAATAGTATCATGAACCATTGTGTATCTATCATTCAGTCCCCAAAACCATTAGTAACCCATAGCTAAAAT  13159
PolarBear    --------------------------------------------------------------------------------  0
Cow          TTTTCCATCATTGGTAGTACATATTACATAGTACATAGT----ATA--CTTAATGA--CTCTTGGAAGG-TCTCTGGCTT  13163
Dog          TTCCAAAA-ATAGAGAGTTTTTATGGTCTGTATTAGACGAGGACCAGGTTTTTTAA--GTGTTTAAAA--AAAAAAGTTA  13114
BelugaWhale  TTCTGTGTCATCAGTAGTGCCTGTTAAATAGTACATACTCAGTATATATTTAATGA--CTATTGAAGGG-TCTCTGGCTT  11610
Donkey       AGTGTTATTACAGGGC-TTTTTTTAGTGGGGGATGGATCAGGATCAGATAGGATCAACACATTTGATTAATATGCCTTTT  12875
Cat          CTTTAGCTTATGTCATGATCTCATGGTCTGT-GAGTTCGAGTCATGTGTCCGGTTC--TGTGCTGACAGCTCAGAGCCTG  15353
Ferret       TTCTTTAT-ATACCTTATTTGGAAGGTGCACCACCTCTGGATGCCAAATAATATAA--ATTTTTTACA--TAGCTATTAG  13075
Lion         CTTTAGCTTAGGTCATGATCTCATGGTCTGTTGAGTTCGAGTCCTGTGTCCGGTTC--TGTGCTGACAGCTCAGAGCCTG  13929
Vaquita      TTCTGTGTCATCAGTAGTGCCTGTTAAATAGTACATACTCAGTATATATTTAATGA--CTATTGAAGGG-TCTCTGGCTT  11711
Pig          TTCTGTACCATTC------TGTGCTAAATTTTTTATACA----TTA--TCTAAT----CTTCAAGACAA-TTTTTGAGGT  12016

Majority     AAATATGCTTATA-ATTTCATGAACCXCTXATTTGTAAAACGGT-AATGXCAATTTTATTG---AXTCTTCTTCTCAATT
                      16410     16420     16430     16440     16450     16460     16470     16480
Human        GATGATATCCACACTCTGCTTTTCCACTTCTGTTATTTTGAGATAAATTTCAGATATTCATATTGTTTTATCTGTAAAGG  13239
PolarBear    --------------------------------------------------------------------------------  0
Cow          CAATATGCTCATA-ATCTCATGAGCTACTTATCAGTAAAAAGGTCAATGCCAATTTTATTGCTTAACCTTCTTCCTTTTC  13242
Dog          CAATGTTGTT-----TTTTATTGAAGT-GCATTTAACATACAGT-ATTATAGTAGTTTCAA---AGGGTACATATCAATT  13184
BelugaWhale  CAACATGCCCATA-ATCTCATGAGCCACTTATCAGTAAAAAGGTAAATACCAATTTTATTGCACAACATTCTTCCTTTTT  11689
Donkey       AAATTTCTTTTAA-TCTACCTACAAGTTCTCCTTCCATCCCTGTGCTTGCACAAAATAATCTCTGAGCTTTTTTTTAATG  12954
Cat          AGGTCTGCTTCTG-ATTCTGTGGATCCCCCTCTCTCTGCCCCTC-CACCTCTTGTGCTCTG---TGTCTCTCTCTCAAAA  15428
Ferret       ACATAATTTT-----TTGTCTAAAGTTTAAATTTTTTAAATTGA-AGTGTAGTTAACATAT---AGTATTAATTTCAAGT  13146
Lion         AGGTCTGCTTCTG-ATTTTGTGGATCCCCCTCTCTCTGCCCCTC-CACCTCTTGTGCTCTG---TGTCTCTCTCTCAAAA  14004
Vaquita      CAACATGCCCATA-ATCTCATGAGCCACTTATCAGTAAAAAGGTAAATACCAATTTTATTGCACAACATTCTTCCTTTTT  11790
Pig          AGATCCTGTTAAA-ACCGCTTAA-------AACTGTAAAACTGA-AACGCAGAGGTTAT-----------CTTGTCCAAC  12076
```

```
Majority     ACTAXAXATAXAA----AAXAACTGTATCTTCTATAACCTXATXTATTAAATGACATAXATATAXXAXXGTGAXXTTTAX
                       16490     16500     16510     16520     16530     16540     16550     16560
Human        CTTCAGTGTATATCTTTAAAGAATAGGGACTCTCCTCTCCTTTTTA--AAATATAATCAAAAGACTGTTAGGACACACAC  13317
PolarBear    --------------------------------------------------------------------------------  0
Cow          CCTGGAGATCCAA----AAGAGCTATATCTTCTAGGACCTCATCTAGTTAAAGACATAAATATACTAATTCAAGGTTTAC  13318
Dog          AATATTAAAAAAA----AGTTTTTGTTTTTTTTTTTAAGTCGCGTTATCAGTCCTCGTTTGTAAAT-GTAGTGGTGCTTAA  13259
BelugaWhale  GCTGGAGATTCAA----AAGAGCTGTATCTTCTAGAACCTCATCTAGTTGAAGAAACAAATATACA------AGGTTTAA  11759
Donkey       AATTTTTAAATGGGCTTAATAATTAAGACCTATGTCAAGTTATTTTCAAGATAAGAT-TATATGTAAAAGAGTTAGCACA  13033
Cat          ACTAAATAAAAAC----ATAAAACGAAAATTGAATAAAAAAACATTTTAATTGTCTCATAAAGGTCAAAGTATTTGTTAC  15504
Ferret       GTACATCATTTAA----AAATAGTTTATTTTAATTATTAATTTTTTTTTTAATAATATCTGTGCCT-AGTGTGGGATTGAA  13221
Lion         ACTAAATAAAAAC----ATAAAAAGAAAATTGAATAAAAAAACATTTTAATTGTCTCATAAAGGTCAAAGTATTTGTTAC  14080
Vaquita      GCTGGAGATCCAA----AAGAGCTGTATCTTCTAGAACCTCATCTAGTTGAAGAAACAAATATACA------AGGTTTAA  11860
Pig          ATTAGAGATC--------AGTCCTA-ATCTGTTAAGTCTGAATC--------------A-CATGCT--T-TTAAATTGAC  12129

Majority     AXAAATGCC--XXATCTXCXATTAAGGAXTATTTXTTTTTT---X-TXAGTGAG-X-GTXTXAT-AX---XGTGGATTXT
                       16570     16580     16590     16600     16610     16620     16630     16640
Human        ACAAAGTT-----ACCTCATATTATAAAATGTCAAATTTGTATTAAAATTTCTAATTGTCTCATAAAAGTCATAGTATTT  13392
PolarBear    --------------------------------------------------------------------------------  0
Cow          AAAAATGCCCTAAAGCTAC-AGTAGGGAACATTAATTGGTAGAATCAGAGTAG----ATTTCATGAAATTAGTGGATTGT  13393
Dog          GAGTACAAG--GAACCCTGAATCAAGAGTTGCATGTTTTTC-----TAGCTGAGCCAGC-----------AGAGCCCCCT  13321
BelugaWhale  GCAAATGCCCTATGGCTACGTTTAGGGAAGATTGGTTGGTAGAATCAGAGTAGATAGATTTCATGAAATCAGTGGATTGT  11839
Donkey       GAATGGCACATAAGTGCTCAAATGATGATGATTGTTATTGTAGTTTTAAATCTGTAAATCTGATTTGGAAGGAACACTTT  13113
Cat          AGGG----------TTTTTTGTTGTGGTTTTTTTTTTTTTT-----TTGGTGAGAAGGAGAGATCAG---GATGAAACAA  15566
Ferret       ACACAT-----GATTCCATAATGAAGAGTCACATGCCCCTT-----TCACTGAGCCAGCCA---------GGTGCCCTCT  13282
Lion         AGGGGTTTTTGTTTTTTTTTTTTTTGTTTTTTTTTTTTTTGTTTTTTGGTGAGAAGGAGAGATCAG---GATGAAACAA  14157
Vaquita      GCAAATGCCCTATGGCTACGTTTAGGGAAGATTGATTGGTAGAATCAGAGTAG----ATTTCATGAAATCAGTGGATTGT  11936
Pig          AAGACCGCC----A----C-TGAAAAGAACATT---------------------------------------TCTT  12157
```

Majority	GACCAGTATATT--XTAAATTTTTAAGAG----AGTGXGXAGTTTATGTTAA--XAAAXXCATGTCAXATATTA----GT	
	16650 16660 16670 16680 16690 16700 16710 16720	
Human	TTATAGGGTTTTT------TTTTTTAGTG----GGGGAAGGAGTCACGATAAAATAAGATCAGCAAATTTAACAGGTCGT	13462
PolarBear	--	0
Cow	GACCAGTACAT---GTAAATGTTTGGGTGCAGAACTGAGCAATATATGTTGAGGGAAAGACATGTCTAAAGTTG---TGT	13467
Dog	CAAGTGTATATT-----AGTTTTTAAGAG----AGCCTGGAGTCCATGTTAAA-AATA-GCTAGTCAGATAACA----CT	13386
BelugaWhale	GACCTGTGTATT--GTAAATGTTTGGGAGCAGAAGTGAGCAGTATATGCTGAGGGGAAGACATGGCCAAAGTTT---TGT	11914
Donkey	AATCGAGGTAAAATATTACAAATTAATTTTTATGAGTGTGGAGTCAGGTTAA--AAATAGCTAGTCAGATAGCA----CT	13187
Cat	GATGAGCAAATTCAATAGGTTGATAAGCC----TTTTTTTAGTTTTTTTTTTT-AATCTGCATGTTATCCTTTC----GT	15637
Ferret	CGAGTGTACATTT--TAAATTTTTAAGAG----AACCTGGAGTCTGTGTTAAA-GAGATAACACTCAGATAACA----CT	13351
Lion	GATGAGCAAATTCAATAGGTTGATAAGCC----TTTTTTTAGTTTTTTTTTT--AATCTGCATGTTATCCTTTCACTCGT	14231
Vaquita	GACCTCTGTATT--GTAAATGTTTGGGAGCAGAAGTGAGCAGTATATGCTGAGGGGAAGACGTGGCCAAAGTTT---TGT	12011
Pig	GTCCACCATCA---ATAA---TTC---------------CAATTTATGTT-----AAA-------------------TCT	12192

Majority	XAAXAG-GCTTTXATAAAXX-XXAX-TA-AX--XACT---XT---C-X-TTXXATTTT-XTACAXAGAGCATG--AG-X-	
	16730 16740 16750 16760 16770 16780 16790 16800	
Human	TATTACCTCTTAAATATCTTTTAATGTACAAGCT-CTCCGTTCCTGTTTTTTGTTTTTTTTTTAAGTTGCATTTCATTTG	13541
PolarBear	--	0
Cow	CAATAG-GCTGGCACAGATTCATAACCATTGAACAATCAGCTGAATCGTTTGAATTTTACTGCAGAGTTTGTATGTGGGA	13546
Dog	AAAGATAGAATTAGTGAACTGAAAGATAAAAATTACTCTGTTTAACAGATAGCAACAAGCGAGATGAAAGATCGTAGAAG	13466
BelugaWhale	CAATAG-GCTGGCACA-------------------------------A-----ATT------CATAGCCCTTG-------	11944
Donkey	AAAGGA-GAATTTGTGAATAGTAAAATAAAATTTACT--------C----AGAATTTA---ACACAGAGCAACAAAGGGC	13251
Cat	GAAGTTTGTTTTTTTTAACATTGCATTTTATTAAACATTTTTTTTCATGTTTTATTTT-TTAGAGAGAGGGAGACAGAGA	15716
Ferret	AAAGAGAGAATTGGTTAACTGAAAGATAAAAGTTACTTGATTTAACAAAGAGCAACAAAAAGATGAAAATAGTTGAGGGG	13431
Lion	GAAGTT--TTTTTTTTAACATTGCATTTTATTAAACATTTTTTTTCATGTTTTATTTTATTTTTGAGAGAGAGAGAGACA	14309
Vaquita	CAGTAG-GCTGGCACA-------------------------------A-----ATT------CATAGCCCTTG-------	12041
Pig	TAATA---------C----------------------------------TTTATTTCTGTTACATAAATCTTA-------	12222

```
Majority     --GAXAGA-A--X-GGXA-GGA--XX-AXT--XAA-X-XX-ATCAXACTTTX-X-T-TCAG-A--X------X-A-A--X
                       16810     16820     16830     16840     16850     16860     16870     16880
Human        TTGAAGAAAACTGGTTATTGGTCTTACACAGTTGCCTATAGACTACATTTACTGATTGCGTTATGATTTTGTAATTTAGT  13621
PolarBear    --------------------------------------------------------------------------------  0
Cow          GGGAGAGAAAATATAGGATGTCAGATAGGTAGGCAGA-AAGATTGTACTTCATGTGGTAGGCAGTATTCC--ATATACCT  13623
Dog          GGGGTAAATATTGAGGAAAGGAAAGACATTTATAAGATAGTACCGTAATTTAAGGTTTCAGAAACGAGAA--ACGAAATA  13544
BelugaWhale  ----------------------------------------ACCA-ATTT-------------------------------  11952
Donkey       TTAAGGGACATGGAGGAAAGGAGGTGAAATTCTAACA-AACATCTGA--TTAGAGTTTCAGGAAGAGAAATTAAAGA--T  13326
Cat          CAGAGAGGGAGCAGGGGAGGGACAAAGAATCTGAATCAGGCTTCAGGCTCTGAGCTATCAGCACAGAGCCCTGCACAGAG  15796
Ferret       TTAAAAGATACAGAGGAAAGAAAGAGGAATTCTAACA-AGTATCTAA--TTAGAGTACCAGAAAGAGAAA--GTAAAGAT  13506
Lion         GAGAGAGGGAGCAGGGGAGGGACAAAGAATCTGAATCAGGCTTCAGGCTCTGAACTATCAGCACAGAGCCCTGCACAGAG  14389
Vaquita      ----------------------------------------AACA-ATTT-------------------------------  12049
Pig          ----------------------------------------ATCATACTT-------------------------------  12231

Majority     --X-A-XXGAGGT-GXXXX-T-A-AXGTATTAAGATCTTTACAXXXGXAGAXXTTGTXTGATAXA---GXTXAAXTXTXX
                       16890     16900     16910     16920     16930     16940     16950     16960
Human        ATGTTTTCCATATTTCCAGTAAATTGGTAGTTAGATCTAGAGACTTGCTCAGGGTCTCTATTTTTTTGGTAATATTTTTA  13701
PolarBear    --------------------------------------------------------------------------------  0
Cow          GTTGAGTTCAGTTCGGTTGCTCAGTTGTGTCTGGCTCTTTGCAACCCCATGGACTGCAGCACACC-AGGCTTCCCTGTCC  13702
Dog          TTGCAGCGGAAGTAGGCTA-TGACAAATTTGAAGATTCATAT-ATGGTCCAACAATTGATTTGAACCTCCTGAATTATTA  13622
BelugaWhale  ----A-----GCT-------------GAATGATGGATTTTACAGC-GAAGGGTTTGTGTAATATA---------------  11994
Donkey       TGT--AGAGAAGCAAAATT-TGAAAAGCTAAAAAGTGATAAT-TTTTCAAAATTGGTGAGCTACA-----TGAATTCTTA  13397
Cat          CCTGATGCGGGGCTGGAAA-TCACAGGCAGTAAGATCATGACCTGAGATGAAGTCGGATGCTTAACCAGCTAAGCCACCC  15875
Ferret       TGCAAAGAGAAGCAGAATA-TGAAAGTTTAAAATAGAAGAGTCATAATTTTCCAGAATTGATGAGCTTTATGCATTTTTA  13585
Lion         CCTGATGCGGGGCTGGAAA-TCACAGGCAGTAAGCTCATGACCTGAGATGAAGTCGGATGCTTAACCAGCTAAGCCACCC  14468
Vaquita      ----A-----GCT-------------GAATGATGGATTTTACAGC-GAAGGGTTTGTGTAATATA---------------  12091
Pig          -------TCATTT--------------TTTTTTGCTCTTTATAGCTGCAC---CTGCAGCATATAGAAGTTTCCCTGG--  12285
```

```
Majority     AX----XX---XGAXAGX-A-XTTXXXX-------XGTXX-AATCXAAGA-GXAG--C--C-A-XCXX-AA----X--XT

                      16970     16980     16990     17000     17010     17020     17030     17040

Human        AGTGGTGGGTTTTTATTTCTGTTAGACATGGTTTCTGAC-TTTTCGTGTATGTCA--TGTTTTTCCCAGGAACATTTCTT  13778
PolarBear    --------------------------------------------------------------------------------  0
Cow          ATCGCCAACTCCTAGAGTTTACTCAAACTCATGTCCGTTG-AGTCGGTGATGCTGTCCAACCATCTCATCCT--CTG-TT  13778
Dog          AAGTTGGGATGCAAGGAAAACCTTAGGTACATGTACATCACAGTGAAAGGTGCAGATGGACCAGCCAAAAGAGAGAGAA-  13701
BelugaWhale  -----------TGAGAG-------------------G----GAGAGAAAAGATAG---------------A-------AT  12018
Donkey       GATTTAGGACACACAAGAAAACTTAGACA------TGTCAACATGAAAGA-GCAGAACACCAAAGACTCAA-----AGAA  13465
Cat          AGGCGTCTCATTGCATTTTATTTTTGAAGAAAACTTGTTGATTCCACAGA-ATTGCCCCCCGAGTCTGCATCTACTAATT  15954
Ferret       GGTTTAGGAAGCATAAGAAAACTTAAGACC-----CATCATAGTTAAAGA-GCAGACCATCAGACCCAGAG---------  13650
Lion         AGGCGTCTCATTGCATTTTATTTTTGAAGAATACTTGTTGATTCCACAGA-ATTGCCCCCCGAGTCTGCATCTACTAATT  14547
Vaquita      -----------TGGGAG-------------------G----GAGAGAAAAAATAG---------------A-------AT  12115
Pig          -----------CTAGGG-------------------GTTG-AATTGGAGCTGCAG-------------------CTG-CT  12314

Majority     GT-CT-TXA-GC-GXXATXGAAAA--X------ATXXCXTAATTAGAGXX---TXCXGAAAATATCAXXTXTTTT-ATXT

                      17050     17060     17070     17080     17090     17100     17110     17120

Human        ATACTTTACCTTGTTTGTGCTAGGCAT------AGTCTGTGCTCAATAATTGTCTCCTTTTTTTTTCTTTTTTTTTTTTT  13852
PolarBear    --------------------------------------------------------------------------------  0
Cow          GTCCCCTTCTCCTCCTACCTTCAATCT------TTCCCAGCATCAGGGT--CTTTTCAAATGAGTCAGCTCTTCTCATCA  13850
Dog          GTTCTATAAAGCAGCCATAGAAAAATC----GTATTTCCTAACAAGAATAACGAACTGGA--GACCATATGCATT-CTTT  13774
BelugaWhale  G------------G---CCGATAG-----------------GTTGGAG----------AAAATTTCAAGTATTTC-ATGT  12055
Donkey       GATCTTTAAAGCAGCCAGAGGAAAATC----AGATTCCCTAAGAAGAACTAAGAAATAGAAAGACACAGTGTGCTTCTTC  13541
Cat          GTGTTCTGATGTTATAATTTAAAATGTTCATGTATCTGTCAATTGGTGTTGGATCTAGAGACTATCATATTCTTT-TTGT  16033
Ferret       -CTCTTTAAAGCAGCCATAGAAAAATC----AGATTTCTTAAGAAAGAATAAAAACTGGAAAGATACAAAATGCT-GCTT  13724
Lion         GTGTTCTGATGTTATAATTTAAAATGTTCATGTATCTGTCAATTGGTGTTGGATCTAGAGACTGTCATATTCTTT-TTGT  14626
Vaquita      G------------G---CCGATAG-----------------GTTGGAG----------AAAATTTCAAGTATTTC-ATGT  12152
Pig          G---------CC---TACGCACAG------------CCACAATCAGA------TCCAAGCTGTGCCGGGTCTGCA-ACCT  12363
```

```
Majority     --GTTCGTCXGGATXXAXTAXATA-AG--AXAGTATCTTTAAATXXXAGAAGCXAAATAX--ACTAXTTTA-TTTAGGAT
                       17130     17140     17150     17160     17170     17180     17190     17200
Human        TTTTTTCCTGCTTTTTGAGAGACA-----AGAGTCTTGCCCTGTTGCCCAGGCTGGA-GTGCAGTGGCCCAATCTCAGCT  13926
PolarBear    --------------------------------------------------------------------------------  0
Cow          --GGTGGCCAAAGTATTGGAGTTACAGCTTCAGCATCAGTCCTTCCAATGAACATTCTGG--ACTGATTTCCTTTAGGAT  13926
Dog          --CAGCAAGGAGATCCAGAATATA-TGGAAAAGTATCTTCAAATGTGAATGAATAACTA---ACTATTTTATTATATAGT  13848
BelugaWhale  --GGTAGGCAGTATTCCATATATCTG---TAAGCTTCCATCCATGAAAGTAGCTCATTAT--TCTGCTATC-TTTAGAAT  12127
Donkey       AGCAACATGGGAATCCAGAAGATAGTGGGAGAGTATCTGCAAAGTGTTGAGTGAAAATAACTATCCATTTATTATTGCAT  13621
Cat          --TTGTTTTTGGTAATATTTTATA-AGTGGTGGTATGTTTTTACATTAGAAGACATAT----TTGATTTTTGTGTGTGTC  16106
Ferret       --CAACATAGGGGTGCAGAAGATAGTGAAATAGTATCTTCAAGGTGTTGAATAAAAATA---ACTACCT-ATTCTATAAT  13798
Lion         --TTGTTTTTGGTAATATTTTATA-AGTGGTGGTATGTTTTTACATTAGAAGACATATAT--TTGATTTTTGTGTGTGTC  14701
Vaquita      --GGTAGGCAGTATTCCATATATCTG---TAAGCTTCCATACATGAAAGTAGCTCATTAT--TCTGCTATC-TTTAGAAT  12224
Pig          --GTACGCCACAGC-TCATGGCTACA---CCAGGACC--TTAATCCACTGAGCA----AG--GCCAG---------GGAT  12420

Majority     GGG-XX-TT-XTX-C-XTXXXAATCCAAATXATACTAXATXGTXXGXTTCXT-AGTACXXTATTGTACCATCAXCTX--X
                       17210     17220     17230     17240     17250     17260     17270     17280
Human        CAGTGCAACCTCTGCCTCCCGGATTCAAGCGATTCTCCTGCCTCAGCTTCCCAAGTAGCTGGAACTACAGGCGCCTGC-C  14005
PolarBear    --------------------------------------------------------------------------------  0
Cow          GGACTGGTTGAATCTCCTTGCAGTCCAAGGGACTCTCAAGAGTCTTCTCCAACACCACAGTTCAAAAGCATCAATTCTTC  14006
Dog          CGCTAAATTGTTCACGTTAAAGAGCAAAATAAAAATAT---GACATTTTCATAACACCCTTCTTCTTCTTCCACTTCTTC  13925
BelugaWhale  GTG-----------G------AGACTGA--GCTGCAAAATAGTTGGCTTTGG---TACTGTATGG-AGCATCA--TG---  12179
Donkey       AATCAGCTAAATTATCACT-TAAGGAGGAGAACAAAATAATGGCATTTTCAGAAACACTCCCTTTTTCCCCCAAAAAGGC  13700
Cat          TGTAATGTTATTCCCAATAATATTTCTTATAATAACTTCTTTCACCTTTTTTGTGTTCATCATTGTCTGCTCAGCATAGT  16186
Ferret       CAGCTTAATTATCACTTAGAAAAACAAAATAGGAGTGCCTGGGTGGCTCAGTGAGTTAAAGCCTCTGCCTTCGGTTTAGG  13878
Lion         TGTAATGTTATTCCCAATAATATTTCTTATAATAACTTCTTTCACCTTTTTTGTGTTCATCATTGTCTGCTCAGCATAGT  14781
Vaquita      GTG-----------G------AGACTGA--GCTGCAAAATAGTTGGCTTTGG---TACTGTATGG-AGCATCA--TG---  12276
Pig          GGA--A----------CCCGCATCCTTACGGATACTAATTGGATTCTTAC----CCTCTGAGCCACAACAGGAACTC---  12481
```

```
Majority     XCX--A-CXC-TTTGTTTTGAGTATAAT--XAAXCAT--GTX-XTGACXTTT-TCX--AXGX--ACXTGXAAATTTCTTX
                              17290     17300     17310     17320     17330     17340     17350     17360
Human        ACTACACCTGGCTAATTTTTGTGTTTTTAGTAGGGATGGGGTTTCACCACGT-TGGCCAGGCTGACTTGGAACTCCTGAC  14084
PolarBear    --------------------------------------------------------------------------------  0
Cow          TGTGCT-CATCTTTGTTTGTAGTCCAACTCTTCACATCCATACATGACTACTGGAAAACCATATACCTGTAAGCTTCCAT  14085
Dog          CCTTTC-CGCTCCGTCCTCTGACCCCCA-CAAAAGACA-GTTGGGGAAATTTATCAACAGACCCATCGAAGAATTTCTAG  14002
BelugaWhale  ---------A-----ATTAGAATATAGT------CGT-------TAACTATT--------------C---TGATCTCTTA  12215
Donkey       AATTGAGGAAATTTATCAACAGACCCACTGAAGGAATTTCTAGAGGTTGTACTTCAAGAAGAAGGCCAGT-GACTCCAGA  13779
Cat          TCAGTA-GACATCTGTTATCAGTATTCT-TAAAGGATATGTAAAAGAAATGTATCTTAAAGTTCACTTAA--TTTATATG  16262
Ferret       TCATGA-TCCCAGGGTCCTGGGATCGAG-CCCCACATC-GGGCTCTCTGCTCAGCAGGAAGTCGGCTTCCTCCTCTCTCT  13955
Lion         TCAGTA-GACATCTGTTATCAGTATTCT-TAAAGGATATGTAAAAGAAATGTATCTTAAAGTTCACTTAAAATTTATATG  14859
Vaquita      ---------A-----ATTAGAATATAGT------CGT-------TAACTATT--------------C---TAATCTCTTA  12312
Pig          -------C---CTTAATTGGACTTTAAC----AACAT---------ACTTCT--------------TTGTAGATATCCTT  12524

Majority     ATG------TAG-XTCATA-TT----T--TAXATTGTGXTTACTGAACGTAAAAGXC-------TTAGTX-TXGTCTTAA
                              17370     17380     17390     17400     17410     17420     17430     17440
Human        CTCAAGTGATCTGCCCACC-TTGGCTTCCCAAAGTGCTGGGATTATAGGCATGAGCC-------ACTGTGCCTGGCCAAT  14156
PolarBear    --------------------------------------------------------------------------------  0
Cow          ATATGAAAGTAG-CTCATTATTCTATTTTTAGAATGTCTCAACTGAGCTGCAAAGTCCTTAGCTTTGGTATTATATGGAG  14164
Dog          AGG-----------TTGTACTTTAGAA--AGAATACTGGTAACTCCAGGAAGGAGGA------TTTAGTAGGAAAATCAA  14063
BelugaWhale  CT-------TGG-TCCATT----------T-----GTGTTC-CTGAACTGTAATAGCT-------------T--TCTTAA  12256
Donkey       AAGGAGGTCTGAGGTAGAAGTAACGTT--AAGCAAGAAATTGGTAAGCCTGTGGGTAAATTTAATTACTGACTGTATAAG  13857
Cat          ATGGAAAGACAGTATTAAAGTTCAACT--TAAATTGTAGTTATTGATGGTAGACAAGAATT-TTTTAGTGGAGGTCTTGT  16339
Ferret       CTG-----------CCTTCCTCTCTGC--CTGCTTGTGATCTCTCTCTGTCAAACAA------ATAAATAAAATCTTTAA  14016
Lion         ATGGAAAGACAGTATTAAAGTTCAACT--TAAATTGTAGTTATTGATGGTAGACAAGAATT-TTTTAGTGGAGGTCTTGT  14936
Vaquita      CT-------TGG-TCCATT----------T-----GTGTTC-CTGAACTGTAATAGCT-------------T--TCTTAA  12353
Pig          A----AGAATA--CTGAT-------------GAACGTGTTTATTGAGC----AAG--C-------------TGTACTGAA  12566
```

```
Majority     XAA-X--------T-X-----XX-XXXG-XX-X-A---XXTAG----XCTAXX--AXT-AAXG-XATX--XAAGTTTXAG
                      17450     17460     17470     17480     17490     17500     17510     17520
Human        AAG----------TGT-----CTCTTAATGAGTATTCTTAAAGGATATCTAAA--AAGAAATGTAACTTAAAATTCACAT  14219
PolarBear    --------------------------------------------------------------------------------  0
Cow          CACTATGATTTAGAATCATGAGCCTCTGTAGTTAACTGTTTAGATATCTTATTTGGTTCATTTGTGTTGCTAAATTGAAA  14244
Dog          AACAAG-----GTTTAACCCCCGTACCGTGATTCAGGTATTATGGTTCCCGGTTTCCTTCTTGCTGTTATTACCTAAGAA  14138
BelugaWhale  TTT--------------------------------------------------------------------A-GTTTCAG  12267
Donkey       AAAATAATAGCAGTGATGGTTTGATTTGAGGTTTAATAAATAGAA--CTAAAAATATTAAATCATAACAACATGTGAGGG  13935
Cat          CAAGAGCATGTGATTTAGAATTTGCTAGCAGGCTAGAGTATGGCCTCTCTATAACATTGAAGGTAATCTCTGAGCTTTAG  16419
Ferret       AGAAAA-----AATAGTGACATTGTCAGAAACACATCAAGCACCTCCTCTCTGTCAAGAAAAGCAATTGGGGAAATTGAT  14091
Lion         CAAGAGCATGTGATTTAGAATTTGCTAGCAGGCTAGAGTATGGCCTCTCTATAACATTGAAGGTAATCTCTGAGCTTTAG  15016
Vaquita      TTT--------------------------------------------------------------------A-GTTTCAG  12364
Pig          CAC--------------------------------------------------------------------AAGGTGAAG  12578

Majority     AXTAACAGAGTT-X-XA-X-T-----TAGG---TXX--AAGAAXTAAGGTTGTTXGXTAAXTATTTTTACTTGTAAAGT-
                      17530     17540     17550     17560     17570     17580     17590     17600
Human        AATAGTAATACTATACAGTATTAACATTTAATGTAAGTTGGAACTATTGATGGTGGACAAGAATGTTTTCTTGTCAAGA-  14298
PolarBear    --------------------------------------------------------------------------------  0
Cow          GTTAAATAAGTTTCCTAATTTAGTTTTAGCTATTCATGAAGGACTAAGGTGGTTAGGTAAATTTTTTTACTTGAAAAGT-  14323
Dog          AATTA-AGGGCAAGGATTGTTTTGATTTGGGGGTGA--AAAAATAGAAATCACATATTCAATCAGAGTCCCTTTCACGC-  14214
BelugaWhale  ACATACTTCATT--------------------------AAGGGCTAAGGTGGTTAGGTAA-TATTTTTACTTTAAAAGT-  12319
Donkey       AGTAGTAGAGTAAATGAAGTT-----TAAGTTCTGATGACCTAGTATTGTTTTGAGCTC--TCTTGTCCATTATGGAGCC  14008
Cat          TTTAACAGATTTTTAAATGGTCTTAATAGGACTTACCTCAAAAGTTATTTTGGAGATTAAATGAAATAACATGTAAGATT  16499
Ferret       CAGCA-GACCCACTGAAGAATTTCTAGAGGTTGTACTTTAGAAAGTAGGTCGATGACTCCAGAAAGGAGGTTTTGAGGT-  14169
Lion         TTTAACAGATTTTTAAATGGTCTTAATAGGACTTACCTCAAAAGTTGTTTTGGAGATTAAATGAAATAACATGTAAGATT  15096
Vaquita      ACATACTTCATT--------------------------AAGGGCTAAGGTGGTTAGGTAA-TATTTTTACTTTAAAAGT-  12416
Pig          ACAAAGTTAG----------------------------AACAAGTAAGG--------------TTCTTGACAGAAACAC-  12615
```

```
Majority     --------AATAAAATATAAGT--AXAGATGAGGAAGGTATGTGTTTTTGXAXATTGATTCGTTTTACAXCTGATXTX--
                      17610     17620     17630     17640     17650     17660     17670     17680
Human        -------ACATACGATTTAGAATGAAAA--GATGAGGGTTTGACTTTTATAGCATTGAACAGGTTAACCTCTGAGCT---  14366
PolarBear    --------------------------------------------------------------------------------  0
Cow          --------AATAAAATATAATCAAAAAGTTATGGAAGAAATGTGTTTTTGAGAAATGATTCTTGTTATAGCTATTGTG--  14393
Dog          ---------ATAGTAGTTTGGTACATTGATGTTGAAGGTCTTGATGATTCTAGCTCTTACTACGCTGCTGTT--------  14277
BelugaWhale  --------AATAAAATACA-----ACAGTTATGAAAGGTATGTGTTTTTGAGCAATGATTCGTCTTATAACTGTTGTGG-  12385
Donkey       ACT-----AGTTGTATGTGGTT--ATTGAGCACTTGACACGTGGCTAGTCTAAATTGATCTGTTCCAATTGTAAAATACA  14081
Cat          TAGCACAGAATGGCACATAAGTGCTCAAATGAGGATCATGGTTGTTACTGTAGCTCTTTACTCTGTACACCTGATTTGGG  16579
Ferret       ---------GGGAGAAGTAAGGTTAACCAAGAAATTGGTAGGACTGTGGGTAAATTTAATTGCAGTATAA----------  14230
Lion         TAGCACAGAATGGCACATAAGTGCTCAAATGAGGATCGTGGTTGTTACTGTAGCTCTTTACTCTGTACACCTGATTTGGG  15176
Vaquita      --------AATAAAATACA-----ACAGTTATGAAAGGTATGTGTTTTTGAGCAATGATTCGTCTTATAACTGTTGTGG-  12482
Pig          --------CATAAA-TATATGCAAAAA---AAGCAGATATCACGTCTTCCAATAGAAAAACTTATCACTGCTTGTAT---  12680

Majority     --XXAATTTTXAACAT---------TAAAAAAAGXTTXAGTTGTATXAXATAGTGTTATTTTTATXTTAXXAAGTTXAAA
                      17690     17700     17710     17720     17730     17740     17750     17760
Human        --TCAGTTTTATACATT--------TTTAAATGGTTTCAGTTGTA---CCTACTTCCAAAATTATTTTGAATG--TTAGA  14431
PolarBear    --------------------------------------------------------------------------------  0
Cow          ----AATTTTGAACTT---------AGAAAAAAGACATAGTAGTATTATAAACTGTTATT----TGTCACTCAGCCCAAA  14456
Dog          --GTACCTTATAGTAT-----------TGAGCACGTTGACCTGGATAGTCTAAACCTATCTCCAAATTGCAAAGTGCACA  14344
BelugaWhale  --TGAATTTTAAACAT---------TAAAAAAAGACACAGTAGTATCACAAAGCGTTATTTATATGTCACTCAGCCCCAA  12454
Donkey       CGTGGATTTCTAAGACTTCATGGAAAAAAAGAAGGTA-ATTTTTATTACATGTTGAAATGATAATATTTTCAGTTAAATA  14160
Cat          AGGAACACCACAGTGGACTCCCAGATAGGAACTTTTTTACCTGGCTGTTAGAGACAAAATTTTTTCTTTAAAAATTAAAA  16659
Ferret       --GAAAGTAATGGCAG-----------TGATTGATTTGGATTTTAAAAAATAGGACTAAAAATATTATATCATAATAACA  14297
Lion         AGGAGCACCACAGTGGACTCCCAAATACGAACTTTTTTAACTGGCTATTAGAGACAAAATTTTTTCTTTAAAAATTAAAA  15256
Vaquita      --TGAATTTTAAACAT---------TAAAAAAAGACACAGTAGTATCACGAAGCGTTATTTATATGTCACTCAGCCCCAA  12551
Pig          ------TTCTGAA------------AACAAAAAGA-------------A-TCTGTTATC----TCTAGATC--------  12715
```

```
Majority     CAA-----XATXAAXAA-ACAACTTATTGCTA-----XAAXTTAXGXGATCCXXAC-CTACTTTGXTCCGTGTAGATAAT
                     17770     17780     17790     17800     17810     17820     17830     17840
Human        TGAG-----ATGAGGAA-AAGACTTATGCTGAAAGGATGAGAATGGTTGTTGTTGTAGTTCTTTAATCTGTAAATCTACA  14505
PolarBear    --------------------------------------------------------------------------------  0
Cow          CAA---T-CATCAACAA-ACAACCCATTGCTA-----AAATTTAGGCTATCCACACTCTACTTTTCTCCTCCTCTGTTAT  14526
Dog          TA--------TAAGGGA-TCCCTGTGTGGCCG-----CACGGTTGGGGAGCCTGGC----CTGTGGCCCGTGCTGTGAAT  14406
BelugaWhale  CAA---T-CATCAACGA-ACAACCCATTGCTA-----AAACTTAGGAGATCCACACTCTACTTTTCTCCTCCTGTATTAT  12524
Donkey       AAATATATCATTTGTATCACCTTTTCTTTTTACTTTTTAAAATATGGCTACTAGAG-CTATTAGAAAATTTGTAGG--AT  14237
Cat          TTAAAATTTTTAAAAAG-TTTATTTATTTTTGAGAGAGAGTGTATGCACGCATGTGGCAGGTAGGGGAGGGGCAGAGAGA  16738
Ferret       CA--------AAAGGGA-GTATCGAGTAGCTGATGTTTAAGTTCTGACATTGTAGTATTGTTTTGAGCTGTGTTGTCCAT  14368
Lion         TT------TTTAAAAAG-TTTATTTATTTTTGAGAGAGAGTGTATGCACACATGTGCCAGGTAGGGGAGGGGCAGAGAGA  15329
Vaquita      CAG---T-CATCAACGA-ACAACCCATTGCTA-----AAACTTAGGAGATCCACACTCTACTTTTCTCCTCCTGTATTAT  12621
Pig          CAA---C-TATCAAT----TGACG------------------TATATGAACAACA------TT-----------------  12746

Majority     TXTGGXAAC----AATTXCAGGCTATTXGTXTTCAAXCATAGATTCTCTGXTGXGTGG--CXTTXAAAGXTAXCGXCAGT
                     17850     17860     17870     17880     17890     17900     17910     17920
Human        TTTGGAAGGAAC-ACTTTTATGGAAATCAAATACAGTAAATTAATTTCTAAGAGGTCCTGGAGTTCAGGTTAAAAATAGC  14584
PolarBear    --------------------------------------------------------------------------------  0
Cow          TTTGAGAAA----AATTCCGGG-TATTTGTATTCATTCGAAAGTACTTTATTGTGTCT--CTTTTAAAGATAGAGTCAGT  14599
Dog          CCTGGAGAC-----------ACTTTCGAATCCCCACGTCTGGGCTCCCGGGGGCATGG--AAGCCTGCTTCGCCTCTGGT  14473
BelugaWhale  TTTGAGAAA----AATTCCAGGTTATTTGT-TTCATCCATAAATGTTTTGGTGTGTCT--CTTTAAAAGATAGGGTCAGT  12597
Donkey       TATGTGTGTT---GCTCACTTTCCATTTCT-TTCGGACAGTGTCAGTCTTGAGGGTGGAAATTTAAAGTCCAAAGTGAAT  14313
Cat          GAGGGAGACGAGGATCTGAAGCCAGCTCCCCGCTGACAGCAGGGTCTCTGATGCAGGGCTCAAACTCATGAGCCGCAAGA  16818
Ferret       TATAGTAGC-----------AACTAGTCATATATGATTATTGAGCACTTGATGTGTGACTAGTCTAAACTAATCTCCAGT  14437
Lion         GAGGGAGACTAGGATCTGAAGCCAGCTCCCCGCTGACAGCAGAGTCTCTGATGCGGGGCTCAAACTCATGAGCCACAAGA  15409
Vaquita      TTTGAGAAA----AATTCCAGGTTATTTGT-TTCATCCATAAATGTTTTGGTCTGTCT--CTTTAAAAGATAGGGTCAGT  12694
Pig          -------------AATTATAA--TATCAGAATACAATCAGGAGTTGCAAACTGCGT------------G-----GGCAAC  12794
```

```
Majority     TCTCTXT-----C--A--TCAXT-TTCTXAXTTAAXGAAATAAAAXCAXAGTXCACTTA--TTAAATCTCTCAAAGAA--
                      17930     17940     17950     17960     17970     17980     17990     18000
Human        CTCAGACAGCACTAAATATCTTTATTAGTGAACAAAAAAATAAAAATAACTTAGAATTTAGCACAGAGAAGCAAAGAGAT  14664
PolarBear    --------------------------------------------------------------------------------  0
Cow          TCTCTCTGTCTACCCTCCTCCCT-CTCTGATTTAAAGAAATACAACCACAGTGCAC----------TCCCTCCAAAAG--  14666
Dog          CCTCT-------CTGATGTCCTC-TGCATCTTTCCCGCGTTCCTCACCCCCCCCCCCCC--CCACCTCTCTCTGTGAC-T  14542
BelugaWhale  CCTCCCT------------CTCT-TTCTGTTTTAAAGAAATACAATCACAGTACTGTTA--GTGCACCCCCCC------  12656
Donkey       GAGATCAGTTTACATTTTAAATT-GTAAGGGTGAGCACAGTAAAAATAGGGATAAAGTG--TATAATTTCTAAAATAA--  14388
Cat          TCATTATCTGAGCTGAAGTCAAA-TGCTTAACTGACTGAACCACACTGGTGCCCCCAAA--TTAAATTTTTTATTGAAGT  16895
Ferret       TGTAAA-----ATGCACATAAAT-TTCCAAAACAGTATGATAAAAAAAGAATGTAATTT--TTATACCTATTACATAT-T  14508
Lion         TCATTATCTGAGCTGAAGTCAAA-TGCTTAACTGACTGAACCACACTGGTGCCCCCAAA--TTAAATTTTTTATTGAAGT  15486
Vaquita      CCTCCCT------------CTCT-TTCTGTTTTAAAGAAATACAATCACAGTACTGTTA--GTGCACCCCCCCCCAAA--  12757
Pig          TCTCT------------------------A----A-GAACAATAACCA--GT--------------TTTCTTCAAAAA--  12827

Majority     -XAAATAGCAXAAAT-TGXATAATGTTCTXAAGTACXXAATTTXTTX---AAAGATTACTATTTXT-TX-TAAAAXTCAT
                      18010     18020     18030     18040     18050     18060     18070     18080
Human        GAAAATAGGAGAAGCGTTAAAAATGTGTTGGAAAGGAAGAGAAGTTCTAACAAACATCTAATTAGCGTTCCAGAAATTAA  14744
PolarBear    --------------------------------------------------------------------------------  0
Cow          -AAAAAAGCAGCAAT-TGCTTAATGTTCTCAAGTGCCAAATTTGTGT---TAAGATTTCTATTTGTCTTGTAAAAGTTGT  14741
Dog          ATCATAAATAAAAAA-TAAAATAAAATAAAGTGCACATAAATTTCCA---AGAC-TTACTAGT---------ATGATAAA  14608
BelugaWhale  -AAAATAGCAACAAT-TGCTTAATGTTCTTAAGTGCCAGATTTGT------AAGATTTCTATTTGTCTCATGAAAGTCAT  12728
Donkey       -TTGGGGGTGGGAAG--ATGTATAATCCAAAAGAAGTCAGGAAAAAG---AAAAGTAAAAGAGAATAGGAAACAAAATGA  14462
Cat          ATAGTTGACAAACAT-AGTATTAGTTTCAGGTGTACATATTTTTTTTTCAATAGCTTACTAATTTTTTTTTAGTGATCTC  16974
Ferret       TACAATATTTTGAAT-TAAATATTATTTGTATCCTTTTTACTTTCTA---AAATATGACTATT---------ACAACATT  14575
Lion         ATAGTTGACATACAT-AGTATTAGTTTCAGGTGTACATAAGTTTTTT-CAATAGCTTACTAATTTTTTTTTAGTAGTCTC  15564
Vaquita      -AAAATAGCAACAGT-TGCTTAATGTTCTTAAGTGCCAGATTTGT------AAGATTTCTATTTGTCTCATGAAAGTCAT  12829
Pig          -TAAAATGTAG---------------TTATCAAGAACC----TTATAG---TAA-----------------TAAAAGG---  12865
```

```
Majority     AGTAATTTTAAX----TAATTTTTAGTGATATGXAGGGXTCA--ATCAA-ATAXTAACATATTTTTTAAAT---------
                       18090     18100     18110     18120     18130     18140     18150     18160
Human        AGATTGCAGAAAGGCAAAATTTGAAATGCTAAAGGCTGATAATTTTTCA-GAATTGGTGAGCTATATGAGTTCTTAGATT  14823
PolarBear    --------------------------------------------------------------------------------  0
Cow          AGTACGTTTACTGTCTTTATTTTTAGTGGTATGGAAGGTTCAGGATCAA-AGATCAACACATTCAGTAAAT---------  14811
Dog          AA-AAGAATA------TAATTTTTATACCTATTA----------------------CATGT-T----------------  14641
BelugaWhale  AATATTTTTACAGAGTTCATTTTTCGTGGTGGGGAGGGATCAGGATCAA-ATAAGAACACATTCAGTAAATTGACATGCC  12807
Donkey       GCTCATTTTAATAGTATTAAGAGTAGCTCTATGGAGGATTCCTTTGAGCCTTTAAAAGTTAGAATTTTCGAGGGTAGGGA  14542
Cat          TGTAATCTCAAG----TAATCTTTAGTAATCTCAAGACCCCGAAATCAAAGGAGTCGCATGCTTTTCCAAGCTGAGCCAG  17050
Ferret       TGTAAGATCT------TAAGTGTTGCTCATCTTA----------------------CATTTCTTTTGGAC----------  14617
Lion         TGTAATCTCAAA----TAATCTTTAGTAATCTCAAGACCCCAAAATCAAAGGAGTCGCATGCTTTTCCAAGCTGAGCCAG  15640
Vaquita      AATATTTTTACAGAGTTCATTTTTCGTGGTGGGGAGGGATCAGGATCAA-ATAAGAACACATTCAGTAAATTGACATGCC  12908
Pig          AGAATTTGTAG--ACTGAA-----AGGAATTTGAAAGGC-----------ATATTAATCTATCAAATTGA----------  12917

Majority     ---GAXACTXCTTTAAATCTACAAAATTTCXTTTTA-X--XXTGXXTTTTTTAATAATTACATTTTTTTXTTCAAGA---
                       18170     18180     18190     18200     18210     18220     18230     18240
Human        TAGGAGGCACAAGAAAACCTGGACACATAACAGTAAAAGAGCAGAGCACCAAAGACATAGAGATCTTTAAAGCAGCCAGA  14903
PolarBear    --------------------------------------------------------------------------------  0
Cow          ----AAATTTCTTTTGATCTGCAAATTATCCTTTTATCCTTGTGAGGCTTTT-ATAATTACATTTTATTTTTGAAGA---  14883
Dog          ---GATAATATTTTGGATTAAATAAAATATTTTTGT-----ATCCTTTTCTTTTTAATTTCTAAAATATGGTTACT----  14709
BelugaWhale  TTTTAAATTTCTTTCACTTTACGAATTCTCCTTTTATCACTGTGAGGTTTTCATAATTACATTTTATTTTTGAAGA---  12884
Donkey       CTTGGGGCTCATTCTAACCCT---AGTTACCTCCCAAAG-TCTCTATCTCCAAATACCATCACGTTGTGGTTCACCGTAT  14618
Cat          CCAGGTATCCCCTCAAGTGTACATAAGTTTTTTTTT------TTTTTTTTTTAACATTTATTTATCTTTGAGAGAGAG--  17122
Ferret       ---ATTACCAGTCTGGAGGATGGAAATTTCTTTTTT-----TTTTTTTTTTTTAAGATTTTATTTATTTATCCAACAG--  14687
Lion         CCAGGTACCCCCTCAAGTGTACATAAGTTTTTTTTTGT--TTTTTTTTTTTTAACATTTATTTATTTTTGAGAGAGAGAG  15718
Vaquita      TTTTAAATTTCTTTCACTTTACGAATTCTCCTTTTATCACTGTGAGGTTTTCATAATTACATTTTATTTTTGAAGA---  12985
Pig          ----AAACCTAT----ACCTG--AGGTTTTCTCCACC--------GTATCT-GCCATTCCATCTTCTCTCTCA------  12972
```

```
Majority     -------AAAXTXATTGTAGG--TAGTACAAAACAGTCCGAA-TATGCTTAAACAGATTCXAT-TCTG---------AXT

                        18250      18260      18270      18280      18290      18300      18310      18320

Human        GAAAATCAGATTCCCTAAAGAATAAGAACTAGAAAGTCCTAA-TATGCTTCATCAGCATGGGCATCCAGAAGATAGTGGA  14982
PolarBear    --------------------------------------------------------------------------------  0
Cow          -------AAACTGATTGTTGG--TGGTACAGAGTTATCCGTAGTCTGCATCTACTGATTGTAT-TCTG---------A-T  14943
Dog          -------ACAACATTTATAAG-ATTGTGTTGTACATCTTACATTTCTTTTGGACAGTACCAAT-CCGG---------AGG  14771
BelugaWhale  -------AAACTGGTTGTTGG--TCTTACAGAGTTGTCCATAGTCTGCATATACTGATTGTAT-TCTG---------A-T  12944
Donkey       GAATTTGAGGGCTCATAAACATTTAGTCCATAACACCCTGCA-TATACTTCTCCCACTTCCAC-CCCAGGTAACCACTAT  14696
Cat          ------CATGAACAGGGGAGGGTCAGATGAAGAGGGAGACACAGAATCTGAAACAGGCTCCAG-GCTCTGAGCTGTCAGC  17195
Ferret       -------ACAGAGATCACAGGCAGAGAGGCAGGCAGAGAGAGAGAGGAGGAAGCAGGCTCCGT-GCTG---------AGC  14750
Lion         AGAGAGCATGAACAGGGGAGGGCCAGAGGAAGAGGGAGACACAGAATCTGAAACAGGCTCCAG-GCTCTGAGCTGTCAGC  15797
Vaquita      -------AAACTGGTTGTTGG--TCTTACAGAGTTGTCCATAGTCTGCATATACTGATTGTAT-TCTG---------A-T  13045
Pig          ------------C-------------TCCCAAACCCTTTGC------CATAAATCCAAATTAT-TCTG---------C-T  13010

Majority     ATAGAAATTGATXTGTXGTCCATATCXAT--CAGTTTGXGGATATXTCTTXAGCCXATXXCGTTCXCTTAXCXTTXTTAG

                        18330      18340      18350      18360      18370      18380      18390      18400

Human        ATAATATTTTCAATGTGTTGAGTGAAAATAGCTGCCCACCTGTTATTTTATAGCCAGCCAAATTATCATATAAGGAGAAG  15062
PolarBear    --------------------------------------------------------------------------------  0
Cow          GTTACAATTATTATGTTCTCCATATGTGT--CAGTTGGTGGATGGATCTA-AGGCCATCACGTTCTCTTG--ATTTTTAG  15018
Dog          ATAGAAATTT---CAGGTCCAAAATGAAT--CAGATTAAGTTTACGTCTTAAATTGTAACGGTAAACTTTACCATTGTTA  14846
BelugaWhale  GTTATAATTGTTATGTTGTCCATATA-GT--CAGTTGGTAGATGGATCTACAGACCATCAGGTTCTCTTTCTGTTTTTAG  13021
Donkey       CCTGAAGTTG--GTGTTTGCCATTCCTAT--GAATTTCTTTATACCTTTGCTGCATGTATTGTTCGTATA-TTTTCTTCC  14771
Cat          ACAGAGCCCGACGCGGGGCTCGAACCCACA-GACCGCGAGATCATGACCTGAGCCGAAGTTGGACGCTTAACTGACTGAG  17274
Ferret       AGAGAGCCTGATGCGGGCCTCGATCCCAG--GACCCTGAGATCATGACCTGAGCAGAAGGCAGTGGCTTAACCCACTAAG  14828
Lion         ACAGAGCCTGACGCGGGGCTCGAACCCACA-GACTGCGAGATCGTGACCTGAGCCGAAGTCGGATGCTTAACTGACTGAG  15876
Vaquita      GTTATAATTGTTATGTTGTCCATATA-GT--CGGTTGGTAGATGGATCTACAGACCATCAGGTTCTCTTTCTGTTTTTAG  13122
Pig          G--GCAGTTCAACGGCTGTGAATTTGTGC--CAACTTACTGATATAACTT-TGGCCATGTCATCCCCTCA--AC------  13077
```

```
Majority     --CXATATAXTATXAGTXGT-G----ATATGTTT-XXXXTTAAAAGATAT---TTGAXXXCTGGTT----XTTTGXTTTG
                      18410     18420     18430     18440     18450     18460     18470     18480
Human        AACAAAATAAAGACACTTTTTGAAACACGTCATTCTGCCTCCAAAAAAGCAATTTAGGAAATGTATCAACAGACCCACTG  15142
PolarBear    --------------------------------------------------------------------------------  0
Cow          --TAATATTCTGTAAGTGGT-G----GAATGTTTTTCTGTTAAAAGATAC---ATGATAGCTGATTT----TTTTTTTTG  15084
Dog          --CCAGATAAAAATAGGGGT-AGAGTGTATACCT----TCTAAAATAATT--GGAGGATGAGGGTGACTAAAATGAAAGG  14917
BelugaWhale  --TAATATTTTATAAGTGGT-G----ATATGTTTTTCCATTAGAAGATAT---ATGATATCTGA-------TTTTTTTTG  13084
Donkey       --CTAGACAATATATGTTTT-GTTTTACATGTTTAAACTATATA-TATGTGCTGTCATGCTGTGTGTATTCTCTAGCTTG  14847
Cat          --CCACCCAGGCGCCCCTGT-A---CATAAGTTT-----TTAAGAGAGCT---TTGAGTCCAGGTTA-AAAATAGCTGGG  17339
Ferret       --CCACCCAGGCGCCCAAGG-AGGATGGAAATTTCAAGTCCAAAGTGAATCAGTTAAGTTTACATTTTAAAATTGTAATG  14905
Lion         --CCACCCAGGCGCCCCTGT-A---CATAAGTTT-----TTAAGAGAGCT---TTGAGTCCAGGTTA-AAAATAGCTGGG  15941
Vaquita      --TAATATTTTATAAGTGGT-G----ATATGTTTTTCCATTAGAAGATAT---ATGATATCTGT-------TTTTTTTTG  13185
Pig          ----ATATACTGCTCA--------------GTTCCACTCCCAAA--------------CA-----------TTTACAACG  13114

Majority     CACATXTCTXTAXAXXX-XTATTXCAAGAATATXAXXTAAAAAATATTTGTAAXATXTGTTTATGCACAXXAAAGGCXAA
                      18490     18500     18510     18520     18530     18540     18550     18560
Human        AAGGAATTTCTGGAGTTTGTACTTCAGGAAGA--AAGCCAATGACACCAGAAAGGAAGTCTGATATAGAAGTGAGGCTAA  15220
PolarBear    --------------------------------------------------------------------------------  0
Cow          CATACGTCTGTGT------TTTTTCAAGAACATTCGTTTAAATAACTTTGTTAAACCTGTTTATGCTCAGCATAGTCTTC  15158
Dog          CACATAATCCAAAAGAAGCAGGAGAAGAGGAAAAGGTTGAGAAACGCCTCAGGGACGCAC---AGTGCATTTCATTTCAT  14994
BelugaWhale  CATATGTCTGTGTGATGTTTTTTCCAAGAACATTTTGTAATAACTTTTTCTTCAATTTGTTTATGCTCAGCATAGTTTGC  13164
Donkey       CTCTTTTTGTTCAA-----TATTTTGGTTTTTTTAACCTAAGTTTATATGTGGGTCTAATTCATTTATTTTCATTGCCAT  14922
Cat          CAGATAACCCTACAGAGAGAATTAGTAAATTAAAACATAAAAATTACTCAGAATTTAACACAGAGCAACAAAAAAGCAGA  17419
Ferret       GTCAGCATAGTAAAAATAGGGATAAGGGCATATAACTTCTAAGATAATTGGAGGATGGGGGTGACTAAAATTAAAGTCAT  14985
Lion         CAGATAACCCTACAGAGAGAATTAGTAAATTAAAACATAAAAATTACTCAGAATTTAACACAGAGCAACAAAAAAGCAGA  16021
Vaquita      CATATGTCTGTGTGATGTTTTTTCCAAGAACATTTTGTAATAACTTTTTCTTCAATTTGTTTATGCTCAGCATAGTTTGC  13265
Pig          CACAGGTC---A----------------------CGATGCACTAATTTCATGAAATCTACT----CTCTGATCCTGCCAA  13165
```

```
Majority     TCAAAAAATATCTXTGXAACAGTAXTXTTAX---ATTXT----------XGA-XXT--TAXXT-TA--TX-T--X-XXXT
                      18570     18580     18590     18600     18610     18620     18630     18640
Human        TCAAGAAATTGGTAGGTCT--GTGAGTAAATCTAATTACTGACCATATAAGAATGTAATAGCAGTGATTGATAAGGGGTT 15298
PolarBear    -------------------------------------------------------------------------------- 0
Cow          TCGATAAGTATCTGTTCATCAGTATTCTT--------------------------------------------------- 15187
Dog          CTTAATAGTGATTTAGGAA--AAGGGTCTGTGGGATCCT--------TTTGAGTCT-TTAAATGTAGAATTTTGGAGGGT 15063
BelugaWhale  TCAATAGACATCTGTTTGTCAGTATTCTT--------------------------------------------------- 13193
Donkey       ATAGTATATGATTATATGAATATACCGTTACCCTGTTGACAAATATTGAGGTTGTTTCTAATTTTTTCCTGTTACAAATA 15002
Cat          GGTTAAAAGAGGAAAGGAAGAGAAATTCTAATAAATTATCTAGACTACCAGAAAGTGAAAGTTAAAGATTGCAGGGAAGT 17499
Ferret       CCAAAAGAAGTTGGGGGAATAAAGGGAAAATAATAGTAT--------GCAAAGTGAATTCATTTTAATTGTTTTAAAGGT 15057
Lion         GGTTAAAAGAGGAAAGGAAGAGAAATTCTAACAAATTAT-----CTACCAGAAAGTGAAAGTTAAAGATTGCAGGGAAGT 16096
Vaquita      TCAATAGACATCTGTTTGTCAGTATTCTTTTTTTTTTTT--------TTTTTTTTT-TTAAATTTATTTATTCATTTATT 13336
Pig          CCAAAACGAATCTCCCCCACTGTAGTCAT--------------------------------------------------- 13194

Majority     X--XX-T-XXX---T----XXXXX-TA---X-XCAAAGTTTAT-A-ATTCAXXTAT-XTTAGAX-T-XGA----XXX--A
                      18650     18660     18670     18680     18690     18700     18710     18720
Human        TAAAAATAGAAT--TAAAAATATTAAATCATAACAATATGTGAGAGAGTGGTAGAGACACTGGAGTTTGAGTTCTGATGA 15376
PolarBear    -------------------------------------------------------------------------------- 0
Cow          ----------------------------------AAAGGATAT---ATGCAAAGAT--GGAAAG---------------- 15212
Dog          TGGACCTTAGGTACCCCTCCCCCTTTATCCTCCCGAAGTCTCC-ATTGTCCCACATACCATGACATTCGGGCGTTAACAA 15142
BelugaWhale  -------------------------------------------------------------------------------- 13193
Donkey       GCTGCTCTGAGCATTCTTGTGCATATA----CCAATAGATGTGAATATTCGAGTTTCTCTAGAAGTGGAATTGCTAGATT 15078
Cat          GGAATATGAAAGTTTAAAAACCTCCTAATTTACCAAAATTGATGAGCTTCATGAATTCTTAGATTTAGGAAGCACAAGAA 17579
Ferret       TCTGTGGAGGATTCCTTTGAAAATGTAGAATTTTGGAGGGCAT-AGAGTTGGGTCT-CATTGAACCCTGATTATCTCCCA 15135
Lion         GGAATATGAAAGTTTAAAAACCTCCTAATTTACCAAAATTGATGAGCTTCATGAATTCTTAGATTTAGGAAGCACAAGAA 16176
Vaquita      TATTTTTGGCTGTGTTGGGTCTTCGTTTCTGTGCGAGGGCTTT---CTCTAGTTGT--GGCAAGCGGGGGTCACTCTTCA 13411
Pig          ----------------------------------AGGGCATTT---GCTTAAACCT--TG-------------------- 13215
```

```
Majority     ---X---X--X-T-------A--X-X---G-CATT-------C-----X-XTXXTACAXA-X---XG-XXXXX-X---TX
                      18730     18740     18750     18760     18770     18780     18790     18800
Human        -TCTAGTATTGATTGGAGCTGTGTTGTCAGTTACAGTAGCTACTAGTCATTTGTTACTGAGC----ACTTGACATGTATG 15451
PolarBear    -------------------------------------------------------------------------------- 0
Cow          ------------------------------------------------------TCCAAA-------------------- 15218
Dog          CTTCAAGCATCGTGA-----ATTTGGGGGGTCATAATCACTTCACGTCCACTTGCACTCATCTGTATCATACCTTTCCTC 15217
BelugaWhale  -------------------------------------------------------------------------------- 13193
Donkey       ATATGGTGTGTCTGTTTTCAAGTTGATTGGATATTGAC----TT--GTTGCTTTCCTAAGTGGTTAGGCTAGTTTATACT 15152
Cat          AACCTAGCCACATCACAGTGAAAGAGCAAGCCATTAGACACACGGAAGAGCTTTAAAGCAGCCATAGAAAAACCAGATTT 17659
Ferret       AAGTCTCCATTTTCC-----AGATACCATGACATTGGGAGTTATAACTTCAGCATGTGAATTTTAGGCATCATTAACGTT 15210
Lion         AACCTAGCCACGTCACAGTGAAAGAGCAAGCCATTAGACACACGGAAGAGCTTTAAAGCAGCCATAGAAAAACCAGATTT 16256
Vaquita      TCGCGGTGCGCGGGCCTCTCACTGTCGCGGCCTCTCTTGCTGCGGAGCACAGGCTCCAGACGCGCAGAGCTCAGTATTTG 13491
Pig          --------------------------------------------------------TA---------------------- 13217

Majority     CXXXX-A-XG-X--X-X-AX--TX--X--X---XX--CTT-XX--A--A---X---X-X---AXX-TG-TX-XXX-XT-X
                      18810     18820     18830     18840     18850     18860     18870     18880
Human        TAGTTTAAGTTGATCTTCTACTTAATTGAAAAATACACATTTCTGAGCCTTAGTCTGAAAGAAGAATGTAAAATATCCCA 15531
PolarBear    -------------------------------------------------------------------------------- 0
Cow          -------------------------------------------------------------------------------- 15218
Dog          CCGTCCACTCCCCCAGTAACCACTCTTGAATTTGGTGTTTGATGCAGTACTGTGTAACTTTCAATGTGTTATTTACTTTT 15297
BelugaWhale  -------------------------------------------------------------------------------- 13193
Donkey       CCATTAGCAGTGAATGAGAATATCTGTTTCTGCACATCCTTTCTAATAATTGGCATTGTTGAACTTTTTAATTGTTGTCA 15232
Cat          CCCAAGAAGGAGT-AACAACTGTAAAGATACAATACGCTTCTTCAGCAACATGGGGGTCCAGAAGATGGTGAAATAGTAT 17738
Ferret       CAGTTCATTGCATCCTCCAT-ATACTTTCCCTCTATTTTTACCTTAGGTAACCATTATCTTGAATTTGGTGTTTACCTTT 15289
Lion         CCCAAGAAGGAGT-AACAACTGTAAAGATACAATATGCTTCTTCAGCAACATGGGGATCCAGAAGATGGTGAAATAGTAT 16335
Vaquita      TAGCTCACGGGCCCAGCTGCTCCGTGGCACGTGGGATCTTCCCAGACCAGGGCTTGAACCCATGTCCCCTGCATTGGCAG 13571
Pig          -------------------------------------------------------------------------------- 13217
```

```
Majority     -XX-X-X-T-XT--X--XX--TA--T-------X-T--TX-X-TATTTTXXTTAAT-ATAATGTAAAAAGAAGTATAAXT

                       18890     18900     18910     18920     18930     18940     18950     18960

Human        AGTTTTCATATTGGTTACATGTACAGCGGATAATATTTTGGTAT-TTTGGGTTAAATGAAATACATTATG-TGTGTTGCC  15609
PolarBear    --------------------------------------------------------------------------------  0
Cow          -------------------------------------------TAAAATTTTTATA-ATAAAGCAGAGTCGGACACGACT  15254
Dog          TTGTTGTATGTTCAACAAAGATATATGGTT---TGTTTTTACATGGTTGAACTAGT-ATAGTATAATACTACAAAGAGAA  15373
BelugaWhale  ----------------------------------------------------AAAG-GATTTGTACAAAGAAGTATGTCT  13220
Donkey       TTCTTATGTACATGTGAATGGTACAT--------TA--------GATTGTTTTAATTTGAATTCCGTAAGTATTAGTATT  15296
Cat          CTTCAAGGTGTTGAATGAAAATAACTACCTACATGTTATTTTGTAGTCCGCTAAATTATCACTTAAAGAGAAGAACAAAA  17818
Ferret       CCTGTGTATACTTTCCCTGTGTGTGTTGTT---TATCTTTTTGTATATTAAGCAAT-ATATGGTTTTGTTTTGCATGTTT  15365
Lion         CTTCAAGGTGTTGAATGAAAACAAGTACCTACCTGTTATTTTGTAGTCTGCTAAATTATCACTTAAAGAGAAGAACAAAA  16415
Vaquita      GCAGACTCTCAACCACTGCGCCACCAGGGAAGCCCTGTTTGTCAGTATTCTTAAAG-GATATGTACAAAGAAGTATATCT  13650
Pig          -------------------------------------------TTTATTTCTT------AAAACTAGATGAAGCATAAAA  13248

Majority     TAXTXATCTCATTAACX--X-TTCTXTAXXAXCAGTAXCTTXAAATGXAATATAGATT--GGATTTACTXATTATGTTAA

                       18970     18980     18990     19000     19010     19020     19030     19040

Human        TTTTCCTCTTTTTTCCTTTCCTTTTTTTAAGATAGGGTCTTGCTCTGTCCACCAGGCT--GGAGTGCCGTGGCATGATCA  15687
PolarBear    --------------------------------------------------------------------------------  0
Cow          GAAGCGACTTAGCAGAA-----GCAGCAGCAGCAGTAT--TAAAATGTAATATAGATT--GGAGTTATTGATGATGAAAA  15325
Dog          AATGTGCCCCTATGC----TTGTGTGTAGTCTCGGTAACTTGCTTTCCTGTTAAATTA-TGTATTTTGGTATTTTTTTTA  15448
BelugaWhale  TGAAAGTCCAATTAAATT---TTCTATAATAGAAATATATTAAAATTGAACATAAATT--GGCATTACTGATGGTGGACA  13295
Donkey       TTCTTAGTTTATTGGCCATTCTTGTTTCTTGTCTGTTAAATGCTAGTTCATATAGTCTGTCCATTTTCTTTCTTTCTT--  15374
Cat          TAGTTATTTCAGAAACCCCTTTTCCCTTCACCCTCCCCCAAAAAAGGCAATTGAGGAAATTTATCAACAGACTCAGTGAA  17898
Ferret       AGCTTATGTCTGGGCCACATACTGTGTGTATTCTGTAACTTGTCCTTTCCGTTTGTAT-TAGTTTTACTTTTTAACCTAA  15444
Lion         TAGTCATTTCAGAAACCCCTTTTCCCTTCACCCTCCCCCAAAAAAGGCAATTGAGGAAATTTATCAACAGACTCACTGAA  16495
Vaquita      TGAAAGTCCAATTAAATT---TTCTATAATAGAAATATATTAAAATTGAACATAAATT--GGCATTACTGATGGTGGACA  13725
Pig          CATACAGCTCTTCTGGA-----TCTCTAGCAAAAAGAA--TGAT-TGCAATA-AAACT--GATATTATCTCTT-------  13310
```

```
Majority     XGATATXTTTTGTTAATGAAGGTCCXGTCAAATTAATAACACCTGATXTXGAATTAACAGACTX-A-XXTAACGTTAACA
                       19050     19060     19070     19080     19090     19100     19110     19120
Human        TGGCAGCCTCAACCTCCTGGGCTCCAGTGACCCTCCCA-CCTCAGCCTCCCAAGTAGCTGGG-----ACTACAGTTGTGT  15761
PolarBear    --------------------------------------------------------------------------------  0
Cow          AGAAATGTTTTGTCAGTGTAGGTCTTGTCAAATTAAAAACACATGATTTAGAATTAACAGAC---------GA-GTTAACA  15396
Dog          CTTTATCTATGCCTATAACAACGTCCTTCACTTCAGTGCCATCTGTACTTTGACTATATAAGTGTACCATAACCCTATTG  15528
BelugaWhale  AGAAAT-TTTTTTCAATGAAGGGCTTGTCAAATTAAAAGCACGTGATGTAGAATTAACAGACTAGAGTTGGCCTCTAACA  13374
Donkey       -TCTTTTTTTTGCTGAGGAAGATTAGCCC--TGAGCTAACATCTGTTGC-CAATCTTCCGTCTTTTTTTTCTTGTGGAAG  15450
Cat          GAATTTCTAGAGTTTATGCTGTAGGAAGAAAGCTGATGACTCCAGAAAGGAGGTTTGGGGTAGGAAAAGTAAGGTTAACC  17978
Ferret       TCTTATATGTGGCTCTAATTCATTCGCT---TTCATTGCCATTTGGTATGTGATTATG--AGTGTACCATTACCCTATTG  15519
Lion         GAATTTCTAGAGTTTATGCTGTAGGAAGAAAGCTGATGACTCCAGAAAGGAGGTTTGGGGTAGGAAAAGTAAGGTTAACC  16575
Vaquita      AGAAAT-TTTTTTCAATGAAGGGCTTGTCAAATTAAAA-CACGTGATGTAGAATTAACAGACTAGAGTTGGCCTCTAACA  13803
Pig          -------TTTTATTGATAAGTGACCCATAAGATTATCAGCACATT-----GAAGCCAGACAC---------CC-TTCCGGA  13369

Majority     ATGAA-----TAAGGTAGTTTCTGAGTTTTXGTTTTTACACTTTGAGAATGX-TTTAACXGGXATTATXTC-AXAAGTTG
                       19130     19140     19150     19160     19170     19180     19190     19200
Human        GCCACCACACCTGGCTAATTTTTGAGTTTTTTTTTGTAGAGACAGCGTCTCGCTGTATCGCTTAG-GCTGGTCTCAAACT  15840
PolarBear    --------------------------------------------------------------------------------  0
Cow          TTGAA-----TAAGGTAGCCTCTGAGTTTCAGTTTTACAGCTTTTAAAATGGGTTTAACGGGACCTATTTC-AAAAGTTG  15470
Dog          ACAAA--TATTGAGGTGGTTTCCAGTTTTTTCCTGTTATAAATTGTGAGTAT-TATTTTGCATAT-ATCAATAAAAGTCA  15604
BelugaWhale  TTGAA-----CAAGGTAACCTCTGAGTTTCAGTTTTTCAACTTTTAAGATGGGTTTAACAGGACCTACCTC-AGAAATTA  13448
Donkey       ATTAG--CCCTGAGCCAGCATTTGTGCCATTCTTCCTCCACTTT----------TTATATGTGGGTTATCACCACAGCATG  15519
Cat          AAGAAGTTGGTAGGCCTGTGGGTAAATATAATTATTGACAGTATGAGAAAGT-GATAGCAGTGATTGTTTG-ATTTGGGG  18056
Ferret       ATAAA--TACTGAGGTTGTTTCTAAATTTTTCCTGTTACAAATTGTGAGTAT-TCTTGTGCATATTATCAATAATTGTTA  15596
Lion         AAGAAGTTGGTAGGCCGGTGGGTAAATATAATTATTGACAGTATGAGAAAGT-GATAGCAGTGATTGTTTG-ATTTGGGG  16653
Vaquita      CTGAA-----CAAGGTAACCTCTGAGTTTCAGTTTTACAACTTTTAAGATGGGTTTAACAGGACCTACCTC-AGAAATTA  13877
Pig          GTCAT-----TAAATTA-TTCCTGAGTATGTGCT-------------A-----TTTAACATGCACTATT-------GATG  13418
```

```
Majority     TXTT--AAATTGAACTATAXGTAAAATAACTAG---ATXATAGTGXGTXAXTG-CTCAATXXAGTTTGAXXTTTGATXTT
                      19210     19220     19230     19240     19250     19260     19270     19280
Human        CCT---GGGCTCAAGTGATCCTCCTGCT-TTAGCCTTGCAAAGTGCTGGGATTACAGGACTGAGTCTGGCCACCTTTTTC  15916
PolarBear    --------------------------------------------------------------------------------  0
Cow          TCTTG---ATTGAACAATATGTAAAAGAATTAGCACAACATGGTACATAACTA-CTCAAATGATGGTGATGCTTTTTATT  15546
Dog          CTTTTCAAATGTCTCTGGAATTGGAATCACTGG---ATTTTAGTGTCTGTTTT--TCATTTCAAGTTGA--TTGGATATT  15677
BelugaWhale  TGTTGAAGATTGAATAATATGTAAAAGATTTAGCACAGAATGGC-CGTAAGTG-CTCAAA------TGATGCTTGGTATT  13520
Donkey       GCT---GAACGCTGGTGTAGGTCCATACCCAAG---ATCTGAACCTGCGAACCCGGGCTGCCAAAGTGGAATGTGCCGAA  15593
Cat          TTTAAGAAGTTGAACTAAAAATATTATAACAAC---GTGTAAGAGAGTAGTTGAGTAACTGATGTTTAAGTTCTGATGCT  18133
Ferret       CTATTCAAGTTTCTCTGGAGGTGGAATCACTGG---ATTGTAGTGTGTGTCTA--T--TTTCAATTTAG--TTACATGTT  15667
Lion         TTTAAAAAGTTGAACTAAAAATATTATAACAAC---GTGTAAGAGAGTAGTTGAGTAACTGATGTTTAAGTTCTGATGCT  16730
Vaquita      TGTTGAAGATTGAATAATATGTAAAAGATTTAGCACAGAATGGC-CGTAAGTG-CTCAAA------TGATGCTTGGTATT  13949
Pig          TC--------GGAAGAACA-GTAAAACA--------AATAAGGTCTCCGCC---CTCAAG-AAGCATGTGTTCTATTCTT  13477

Majority     G----TAGTTXTTTAAXXTGTAGXXCC--CATTTAXTTGCTGCCCTTTATTAGTAGTCAATGXG-X-T-----------
                      19290     19300     19310     19320     19330     19340     19350     19360
Human        TTTCTTTCTTTTTTTTTTAGACGGAGTCTCCCTTTGTTGCCCAGGCTGGAGTACAGTGGCTCTATCTCTGCTTCCTGCAA  15996
PolarBear    --------------------------------------------------------------------------------  0
Cow          A----TAGTTCTTTAATCTGTAAGTGAG-CGAGTAAGTGAAGTCGCTCAGTCGTGTCCGACTCTTTGT----------G  15610
Dog          GATGTTATATTTCTAAGTGGTTGTGCAT-CACTTATATACTCCAA--TACGAGTAGTGTGTGAGAATTCCTGTTTCTGTT  15754
BelugaWhale  G----TAGTTCTTTAATTTGTAAATCC--TATTTAGAAGGAGCCCTTTATTAGAAGTCAACTTT----------------  13578
Donkey       C----TTAACCTCTATGCCACAGGGCTGGCCCCTGTCCATTTTCTTTTTAATGTAGTTTTTATCTTTTTC--------TT  15661
Cat          TCTAATATTGTTTTGAGCTATGGTGTCC-ATTATAGTAGCCACTAGTCATACGTAGTCATTGAGCACTTG---ACATATG  18209
Ferret       G------CATTTCTCAGTGGTTGTAC-------TAATTGATACTC--TACTAGCAGTGTATGAGAAAT------------  15720
Lion         TCTAATATTGTTTTGAGCTATGGTGTCC-ATTATAATAGCCACTCGTCATACGTAGTCATTGAGCACTTG---ACATATG  16806
Vaquita      G----TAGTTCTTTAATTTGTAAATCC--TATTTAGAAGGAGCCCTTTATTAGAAGTCAACTTT----------------  14007
Pig          A----GTGTCCTTGAATC-ACCAGTTTT-TAAAAGAGTGCTGTCATTTA---GTGGCA----------------------  13526
```

```
Majority     --X--TCTGAXTXTATTTT---ATGTTACXCATTC-XTGGAXCTTTT--A-X--GTATAX--A-XA-XGCXTGCTATTGT

                       19370     19380     19390     19400     19410     19420     19430     19440

Human        GCTCTGCCTCCTGGGTTC----ACGCCATTCTCCTGCCTCAGCCTCCCGAGTAGCTGGGACTACAGGCGCCTGCCACCAC 16072
PolarBear    -------------------------------------------------------------------------------- 0
Cow          ATCCCATGGACTGTAGCCTACCAGGCTCCTCCTTCCATGGAATTTTCCAGGCAAGAATACT-G-GAGTGGTTGCCATTTC 15688
Dog          CTGTGTCTGCCCATATTCCTTGATACTGGGCAATA-GTTGGACTTTGTTGTCAGTTGTAA--G-GACCCCGTTGAATGGT 15830
BelugaWhale  -----GAAGTTTTTATTTT---TTGTTACTGATTTCTAGGAGTTTTT-------ATATATT-G----TGGGTACTAAAGC 13638
Donkey       AGTGATTCCTAGGAGTTTTTGTATATTATGGACAC--TAAAGCTTTGTTATATGTATTACAGA-TAACTCCTTCCTGTCT 15738
Cat          GCTAGTCTAAATTGATCTCCAGTTGTAAAATGTAC-ATAAACTTCCAAGACTTAGTAT----A-AAAAAAATGTAATTTT 18283
Ferret       -TGTTTCTGCACATCTTTTCTGATAATTGACATT--GTTGGACTTTTTAATTGTCCGTCT--A-AGGAACATGTTAATGG 15794
Lion         GCTAGTCTAAATTGATCTCCAGTTGTAAAATGTAC-ATAAACGTCCAAGACTTAGTATGATAA-AAAAAAATGTAATTTT 16884
Vaquita      -----GAAGTTTTTATTTT---TTGTTACTGATTTCTAGGAGTTTTT-------ATATATT-G----TGGGTACTAAAGT 14067
Pig          -------AGACTGGATCC----AAGCTGCATCT-----GCAACCTT-------------------------TGCTACACC 13565

Majority     TTTXTTATTTAXTTTTX--AXT-----GGGTAXAAATAAAATXTT--TXCTXTXTTTGTCTTXTACTAATTTCGXTACTG

                       19450     19460     19470     19480     19490     19500     19510     19520

Human        ACCCGGCTAATTTTTTGTATTTTTAGTAGAGATGGGTTTTACCATGGTCTCGATCTCCTGACCTCCTGATCTGCCCACCC 16152
PolarBear    -------------------------------------------------------------------------------- 0
Cow          TTTCTCCAAAAGATCTT--CCCGACCCAGGGATCGAACCGAGGTC--TCCTGCATTGTAGGCAGACGCTTTAC-CGTCTG 15763
Dog          TTATCTGTTTTAGTTTG--AATTCCCGGAGTATGAATGATACATT--TTCTTAGTT-------CACTCATGTTTCTTCTC 15899
BelugaWhale  TTTGTTAGTTATATGT------------GGTACAGATAAATTCCT--TCCTGTCTGTGGCTTGATTTTTTTGCATTGATG 13704
Donkey       GTGGCTTCTTTTTTTTTACATTGAT--GAATAGAAATTGGAAAGT--TTTTTTATTTAAAGGTTGGCAACTGAGCAACAA 15814
Cat          ATATTTATTACATGTTAGTATTATTTTGTGTTCAATAAAATTGTG--TTCTTTTTTTGTCTTTTAAAAATATGGTTATTA 18361
Ferret       TGTCTTGTTTAAGCTTG--AATTCCCTGAGTATGAATGATACATT--TTCTTAGTTTATAGGCCACTAATGTTGTTACTC 15870
Lion         ATATTTATTACATGTTGATATTATTTTGTGTTCAATAAAATTGTG--TTCTTTTTTTGTCTTTTAAAAATATGGTTATTA 16962
Vaquita      TTTGTTAGTTATATAT------------GGTACAGATAAATTCCT--TCCTGTCTGTGGCTTGATTTTTTTGCATTGATG 14133
Pig          TTG---C----------------------------AGCAA-------TCCTGCATC-----------CTTAAC-CCACTG 13595
```

```
Majority     AXTCATGTAAXAXATCTTTTXATTXAATCTGXGCATCCT---ATXXA-CTCTTTTTXA--XGTTTCTTTTAGAXTXTXC-
                      19530     19540     19550     19560     19570     19580     19590     19600
Human        TGGCCTCCCAAAGTGCTGGG-ATTACAGGCGTGAGCCACTGCGACCGGCCCACTTTTTCTTTTTACTTTTAAAAATGTGG 16231
PolarBear    -------------------------------------------------------------------------------- 0
Cow          AGCCACCAGGGAAGTCCTG----TAAATCTGTAAATCCTAAGAAGGAGCACTTTATTAGAAGTCAGCTTCAAAGTTTTCA 15839
Dog          -----TGTTAAGTACCTGTTCATATAGTCTGTTCGTTAT---TTTCA-TCCTTTTTAAAGTGTAGCTTTTAAATATTACA 15970
BelugaWhale  AGTAGATTAGGAAATTTTAACATTAAATTTACGTTTT------------------------------------------- 13741
Donkey       C-TGTTGCCAGTCTTCTTTTTTTTTCCCCTTCGTCTCCCCAAAGCCATCCAGTACATAGTTGTATATTCTAGA-TGTGA- 15891
Cat          GAACATTTAAAAGATCGTCTTACATTATAAGATCATCCT---ATATG-CTCATCTTAC---ATTTCTTTTGGACTGTACC 18434
Ferret       -----TGTTAAATACCTGTTTATACAGTCAGTCCATT-----------TTCTTTTTAA--TGTAATTTTTAGTCTTTT-- 15930
Lion         GAACATTTAAAAGATCGTCTTACATTATAAGATCATCCT---ATATG-CTCATCTTAC---ATTTCTTTTGGACTGTACC 17035
Vaquita      AATAGAGTAGGAAATTTTAACATTAAATTTACGTTTTTTTTTTTTTTTTTTTTTTTTTTTTTTTTGCAGTACGCGGGCCT 14213
Pig          AGTGATGCCAGATGTCT--------AACCTGCA--TCCTCACATAGA-CACT---------------------------- 13636

Majority     --XXT-T---T-TXTGGGTTTTTATATATTGAAGXXA-TAAXXTXAATTTGTATTXXXAXATGGTAAXTCT------CAX
                      19610     19620     19630     19640     19650     19660     19670     19680
Human        CTAATAGAAATTTATGAGATTATATTTATGGTTCATACTACGTTTCTTTTGGACAGTGCCAGAGTGAATCAGATAAGCTT 16311
PolarBear    -------------------------------------------------------------------------------- 0
Cow          TTTATTT---TCTTGGAGTTTTTATATATTGATTTATGGAACTTTTCCTTTTATTGATTCTTAGGAGTTTT------TAT 15910
Dog          TACATGT---CCTATAGGTGTTGATATATTGCGGATACTACTAACAATTTTTACTAAAACTTGATAAGACT------ACT 16041
BelugaWhale  -------------------------------------------------------------------------------- 13741
Donkey       -GTGCCT---CT----GGTTGTGCTATGTGGGATGCC--G-CCTCAACGTGGCCTGATGAGTGGTGCTACT------TCC 15954
Cat          AGTCTGG---AGGGTGGAAATTTTAAGACTAAAGTGAATCAGATAAATGTGTATTTTAAAATTGTAAGGGT------CAA 18505
Ferret       ------T---TCTGGGAGTTTTTATATATTGTAGATACTAAAGCCAGGTTGCTTACATATACTGTAATTAA------CTC 15995
Lion         AGTCTGG---AGGGTGGAAATTTTAAGACTAAAGTGAATCAGATAAATTTATATTTTAAAATTGTAAGGGT------CAA 17106
Vaquita      CTCACCA---T-TGTGGCCTCTCCCGTTGCGGAGCACAGGCTCCGGACGCGCAGGCTCAGCGGCCATGGCT------CAC 14283
Pig          --------------GGGTCCTTAACCTGCTGAGCCAC--AA-------------------T-GGGAATTCT------TA- 13673
```

```
Majority     GXAXXTAAX---CTAG-GXTTGXATXXAT-XXTTXT----------TAATTGGXGAATXGGGGXTA-TGAAATTXXAX-C

                     19690     19700     19710     19720     19730     19740     19750     19760

Human        GCATTTTAAAATCCTAAGGGTAAATGCAATAGAGATAGAACGCAAATAATTGGGGAGGGGGGTTGACTGAAATTAAAGAT 16391
PolarBear    -------------------------------------------------------------------------------- 0
Cow          GAATTGTGGGTACTAAAGCTTTGTTGTACAAGGTACAGA------TAAACTTCCTGTCTGTGGCTATTTTTTTTTTAA-T 15983
Dog          ATAGTTAACT--CCTGTCTGTGGCTGTTTTGTTTTT----------GCATTGATGAACAGAAGTTG--GAAATTT-AA-C 16105
BelugaWhale  -------------------------------------------------------------------------------- 13741
Donkey       GCACCCAGGATCCAAACCAGTGAAATCCTGGGCTGC---------CGAAGTGGAGTGTGCGAACTTAACCACTCAGCCAC 16025
Cat          CACAGTAAAT--ATAGGGATAGAATATATAACTTCTAAA------ATAATTGGAGAATGGGGGTGACTAAAATTAAAG-C 18576
Ferret       ATGTCTATGG--CTTGTTTTTTGTT------TTTTT----------TAATTGATGAATAGAATTTG--GAAATTTTAA-C 16054
Lion         CACAGTAAAC--ATAGGGATAGAATATATAACTTCTAAA------ATAATTGGAGAATTGGGGCAACTAAAATTAAAGAC 17178
Vaquita      GGGCCCAGCCGCTCCGCGGCATGTGGGATCTTCCCGGAC------CGGGGCACGAACCCGCGTCCCCTGCATCGGCAG-G 14356
Pig          ---------------------------G--------------------A-----------T-------TTTAGTTTAA-- 13687

Majority     ATXAXATTCAAA----TTCXGGXXTAAA----------AAAXX--AAATATGTTTTAAT-TTGAAGAAAT-TTTTATTAA

                     19770     19780     19790     19800     19810     19820     19830     19840

Human        GTATAATCCAAAAGAAGGCAAAAAAAAAGGAAAGACACAAAGTGAGCTTATATGTTAATAGCGTGGAAAGGTTCTATGAA 16471
PolarBear    -------------------------------------------------------------------------------- 0
Cow          TG-ATGGTTAGA----TTTGGAAATACT----------AAC-ATTAAATTTGTTTTAGC-TTTAGGAAAT-TTGTATTAA 16045
Dog          ATCATTTTTATG----TTTTGGCTTAAG----------AAAATAGATTTGGTCTTTAAA-CCTCACTCAG-GT--GTTAA 16167
BelugaWhale  --------------------------------------------------------AGT-TTTAAGAAAT-CTTTCTTAA 13763
Donkey       GCGGCCAGCCCAGAAGTTGGAAATTTTA----------ACATCACATTTATGTTTTAGT-CTGAAGAAATCTTTTTTTTA 16094
Cat          ATCTAATCCAAAAGAATTCAGGGGGGAA----------AAAAGTAAAAAAGGTCACAAA-GTGAACTCAT-TTTAATGGT 18644
Ferret       ATCACATTTATG----TTTTGGTTTTAA----------GAAAT-----TTTTCTTTAAC-TCGGGTTC--------TTAC 16106
Lion         ATCTAATCCAAAAGAATTCAGGGGGGAA----------AAAAGTAAAAAAGGTCACAAA-GTGAACTCAT-TTTAATGGT 17246
Vaquita      CGGACTCTCAAC----CACTGCGCCACC----------AGGGAAGCCCTACGTTTTAGT-TTTAAGAAAT-CTTTCTTAA 14420
Pig          ---------------------------------------------GAACTTGTTCTT---------AAAC-TTG----A- 13707
```

```
Majority     A-----TTCAGGGTXTTTXAXT-TATAGTXTTTTXGAXTTTATTTTGAAXATTTAAAAATTTTGATTTT-CACATTTAGG
                       19850     19860     19870     19880     19890     19900     19910     19920
Human        GGGTTGTTTAGAGCCTTTAAGGGCAAAATTTTCTTGTCTGGTTGCAGGAAAGAGTGGCATTGTTTTTGTTCACTTTTAAA  16551
PolarBear    --------------------------------------------------------------------------------  0
Cow          A-----TT---GATCTTTTA---TATAGTCTTCCAGATTTTATTTTGAACATTTAAAAATTTTGATTTT-CACATTTAGG  16113
Dog          C-----TACAGTAATTTCTGTTTGCCAGTTTTGATACAGTAGATTTTAT-TTGGAAAGTTTTGAAAGTT-TGTTTTTCAC  16240
BelugaWhale  A-----CTCAAGGTTTTATACTATATAGCCTTCTGGATTTTATTTTGAAAATTTTTAAATTTTGATTTT-CACATTTAGG  13837
Donkey       A-----CTCAGGTTCTTACA---TACAACCTTTGGGATTTTATTTTGAAAGTTTAAAAATTTTGATTTTTCACGTTTAGG  16166
Cat          G-----TTAGGAAAGGTTCGTTGGAGGGTTTCTTT----CAGCCTTTAA-ATGTAGAATTTTGAAGGTA-TGGAGTTGGA  18713
Ferret       A-----TACGGTGACTTTATTTTGAAAGTTTTTT--------------------GAAATTTTTGGTTTT-CACATTTAAG  16160
Lion         G-----TTAGGAAAGGTTCATTGGAGGGTTCCTTT----CAGCCTTTAA-ATGTAGAATTTTGAAGGTA-TGGAGTTGGA  17315
Vaquita      A-----CTCAAGGTTTTATACTATATAGTCTTCTGGATTTTATTTTGAAAATTTTTAAATTTTGATTTT-CACATTTAGG  14494
Pig          -----------GGTTTTACA---TATAGTCCTTTGGACTTTATTTTGAAATTTTTTTAATTTTTATTTT-CATATTTAGG  13772

Majority     G-CCTCAGTCTACCTGXAATTTAATTTTTGTGTAXX-TXTTAX-AAAG-TTAAATGATTTTTT-XC--XTTATGGXTAAC
                       19930     19940     19950     19960     19970     19980     19990     20000
Human        GCTACAAAAACAAGTAATGATGAGGAAAATTGGGACATGTTGAGGAAG--TAAGTAATTTTTTGGAA-TGTAGGTTGAGT  16628
PolarBear    --------------------------------------------------------------------------------  0
Cow          G-CCTCAGTCTACTTGAAATTTAATTTTAGTGT-----GTTAT-AAAGGTTACATGGTTTTTTTCCC-CTTATGGCTAAC  16185
Dog          A-TTTAAGGCCTCCTGGAATTTAAGTTTTGTGTAGTATATGA--AAAGGTTTAATGATATTTTTCT--CTTACAGATAAC  16315
BelugaWhale  G-CCTCAGTTTACCTGTAATTTAATTTTTGTGTAA-------------------TGTGTTGTA-------AATGGCTAAC  13890
Donkey       G-CCTCAACCTACCTGGAATTTAATTTTTGTGTAGTGTATGGT-AAAGATTTAATGATCATTC--C--CTTATGGTTAAT  16240
Cat          C-TTAATTTAACCCTAATTATCTCCCAGAGTCTCCATTTTCAAATACCATCACATTGGGAATTACAA-TTTGAATGTATG  18791
Ferret       G-CCTCAGTCTACCTGGAATTTAATTTTTGTATAGTGTATTATCAAGGTTTAAATGATTTCTTCCCCCCTTATGGGTAAC  16239
Lion         C-TTAATTTAACCCTAATTATCTCCCAAAGTCTCCATTTTCAAATACCATCACATTGGGAATTACAA-TTTGAATGTATG  17393
Vaquita      G-CCTCAGTTTACCTATAATTTAATTTTTGTGTAA-------------------TGTGTTGTA-------AATGGCTAAC  14547
Pig          --CATCAGTCTACTTGGAATTTAATTTTTGTGTACTCTGTTGT-AAAGGTTTAATGATTTTT---CC-TTTATGGTTAAC  13845
```

```
Majority     AAATTTGGTACAAACTCA-ATTGAATAGTTCATGTTTT---------CCCACTGATCTG---------------CAGTGT
                      20010     20020     20030     20040     20050     20060     20070     20080
Human        TAGAGTAGTAGAAGACAAAATTTAAAAAGTAGTTTTGACAGTAATATGGAGAGCTTTGAGTGCAAAGGATTTGCAGTGA  16708
PolarBear    --------------------------------------------------------------------------------  0
Cow          AAATTTGCTACAAACTTG-ATTGAT---TTCATGTTTTT--------CCTACTGATCTG---------------CAGTAT  16238
Dog          AAACTTTGTACCAACTTA-ATTGAATAGTTCGTGTTTTA---T-TTTCCCACTACTATAATCTT-GCCGTTTGTCACTCG  16389
BelugaWhale  AAATCTGGTACAAACTCA-GTTGAATGGTTCATATTTT---------CCTACTGATCTG---------------CAGTGT  13945
Donkey       AAATTTAGTACCAACTCA-GTTGAATAGCTCATGTGTTT-------TTCCACTGATGTG---------------CAGTGT  16297
Cat          AATTTTGGTGGTCATTAACATTCAGTTCGTTGCATCCTA---TATTTCCCTCCACTTCCACCCCAGGTAACCGCTATCTT  18868
Ferret       AGATTTAGTATCCACTTA-ATTGAATAGTTCATGTTTT------TTCCCCAGTGATCTG---------------CAGTGT  16297
Lion         AATTTTGGTGGTCATTAACATTCAGTTCATTGCATCCTG---TATTTCCCTCCACTTCCACCCCAGGTAACCACTATCTT  17470
Vaquita      AAATCTGGTACAAACTCA-GTTGAATGGTTCATATTTT---------CCTACTGATCTG---------------CAGTGT  14602
Pig          AAATTTG--AATAGTTCA-GTAG-----TTCATGTTTTT--------CCCACAAATCTG---------------CAGTGT  13894

Majority     TAGTTGTC--AGGTTXCCAT-ATATGTGTGAAACTGTCCCTGGGCTATCTCTTCTGTTTCATTGTTTGTXTCTTTC----
                      20090     20100     20110     20120     20130     20140     20150     20160
Human        GGACCTTTGAAAATTGAACCTGCTTTCTCCTATCAGTATTTATGTTTA--AATAAACTTGATTCTTTGTGAGTATTGTTA  16786
PolarBear    --------------------------------------------------------------------------------  0
Cow          TAGTTGTC--GTGTTGCCAT-ATATGTGAGAAACTT-CCCTGGGCTATCTCTTCTGTTTCATTGTTTGTGACTATCG---  16311
Dog          CTGTTATC--AGGTTGCCAT-ATATGCATGGAACTATTTCTGGGTTA-----TCTGTTTTATTGTTTGTCTTTTCC----  16457
BelugaWhale  TAGTTGTC--AGGTTTCCAT-ATATGTGTGAAACTTCCCCTGGGCTATCTCTTTTGTTTCATTGTCTATTACTGTC----  14018
Donkey       CAGCTGTC--AGATTTCTGTTATATGCATGGAACTG-TTTTGAGCTATCTCTTCTCTTTCATTATTTATCTATTCCTGTA  16374
Cat          GAATTTGG--TGTTTGCCAT-TCCTGTGAATGTCTGTACTTTCCCTGTGTG-TGTTATTTATGTTTTGTGTCATAA----  18940
Ferret       CAGCTCTC--AGGTTTCCAT-ACATGCATGGAACTGTTTCTGGACTATCTATTCTGTTTCATTGTTTATTTGTACC----  16370
Lion         GAATTTGG--TGTTTGCCAT-TCCTGTGAATGTCTGTACTTTCCCTGTGTG-TGTTATTTATGTTTTGTGTCATAA----  17542
Vaquita      TAGTTGTC--AGGTTTCCAT-ATATGTGTGAAACTTTCCCTGGGCTATCTCTTTTGTTTCATTGTCTATTACTGTC----  14675
Pig          TTGCTGTC--AGGTTTCCAT-TTA-GTATGAAACTTTCCCTGAGCTATCTGTTATGTTTCATTGTCTTTTCCTGTC----  13966
```

```
Majority     --X-XX--ACAATGTCTTAATTTAATGTAGCATCXXAGTAAXTTTTGG---XXAATCTTGXAGXGTG-XATTTTXCAACT
                        20170     20180     20190     20200     20210     20220     20230     20240
Human        TATATGTTTTCATAACTACTGTTGTAGTAACGTTTCTTTTTTTTTTACTAGCTTGCTTTCATGCCACGCTGTTGATTAAAT  16866
PolarBear    --------------------------------------------------------------------------------  0
Cow          CTATCAATACA-TGTCTTAATTTAATGTAGCATCATGATAAATCCTGGC-TGGAATTTTGGTGAGTA-AATTTCACAACT  16388
Dog          --------ACAGTGTCTTAATTTACTGTAGCGTCTTGGAGTCTTGTGGA-ATGATTTTTACAACTA--TGTTCTTCAAAA  16526
BelugaWhale  TTACTACCCAAATGTCTGAATTTAATGTGGCATCACAGTAAGTTTTGG-----AATCTTGGAGAGTG-AATTTTGCAACT  14092
Donkey       CCAGTACCACAGTGTCTTAATTTAGTGTAGCATTACAGTAAATCTTGG-----AATCTCGTAGAGTG-AATTTCATAACT  16448
Cat          --------GCAATATATGGTTTTGTTCTA-CATGTTTAAACTATGTATATGTGCCACATGCTGCTTG-TATTCTCTAACT  19010
Ferret       --------ATTGTGTCTTAATTTACTGAAGCATCTTGGAATCTTGTGGG-GTGAATTTCACAACTT--TGTTCTTCAGAA  16439
Lion         --------GCAATATATGGTTTTGTTTTA-CATGTTTAAACTGTGTATATGTGCCACATGCTGCTTG-TATTCTCTAACT  17612
Vaquita      TTACTACCCAAATGTCTGAATTTAATGTGGCATCACAGTAAGTTTTGG-----AATCTTGGAGAGTG-AATTTTGCAACT  14749
Pig          CCACTACCACAATGTCTTAACTTAATGCAACATCACAGTAAGTCTTGA-----AATCTTGGAGAGTA-AATTTTCCAACT  14040

Majority     TTXT---------TTTTTTXXXCATTTTCTTTATT--ATATXXTTTTTTAAG-ATCAGTTGGCTAX--GTTCCATGAAGA
                        20250     20260     20270     20280     20290     20300     20310     20320
Human        ATTTATGGGCCATTTTAAGGCTTCTAATCTTCATTTGTGATGAACTTTTGAGGAAGAGTTAGGTTTTGTAAGTGTTGAAC  16946
PolarBear    --------------------------------------------------------------------------------  0
Cow          T------------TT-CTTAAAAACTGTCCTTG----GCATTTTTTTTCAT---TTATTTATATA-----TACA--AATA  16441
Dog          TTGT---------CTTGA----CATTTTCTTTATC--ATGTG-TATTTTAAG-ATCAATTGGCTG--AGTTCCATGAAGA  16587
BelugaWhale  TCAAA-------ATTGTCTTGGCATTTTCTTCACTGTATATATATTTTTAAG-ATCAGTTGGCTAA--GTTCCATGAAGA  14162
Donkey       TTGTTCTTCAAAATTGTCTTGGCATTTTCTTTAAC--ATAGG-TATTTTAAG-ATCAGTTGGCTAA--GTTCCATGAAAA  16522
Cat          TGTT---------CTTTTTGTTCAATGTTATTATT--TTTTAATTTTTTAAC-GTTTGTTTACTTTTGAGAGAGCACAAA  19078
Ferret       TTGT---------TTTGAAGAACATTTTCTTTATC--ATGTG-TATTTTAAG-ATCATTTGGCTG--AGTTCCGTGAAGA  16504
Lion         TGTT---------CTTTTTGTTCAATGTTATTATT--TTTTAATTTTTTAAC-GTTTGTTTACTTTTGAGAGAGCACAAA  17680
Vaquita      TCAAA-------ATTGTCTTGGCATTTTCTTCACTGTATATATATTTTTAAG-ATCAGTTGGCTAA--GTTCCATGAAGA  14819
Pig          T------------TC-CTTTAAAATTGTC-TCG----GCATTTTCTTTTAAG-ATCAATTAAATAA--GTTCTA--AAGA  14097
```

```
Majority     CXTTTXAGGTTTTGATTXAA--TGTACTAAATTTATAAGTTAATTGGG----TXXXGTATTTGCATTATCAA-AAAATAA
                               20330     20340     20350     20360     20370     20380     20390     20400
Human        TCCAATAAACACAGTTCCAC--TTTCCTTAACTTCTCAGAAAGATACTTTGCTATTTGATACAGATAGTTGTCTAGGCAA  17024
PolarBear    --------------------------------------------------------------------------------  0
Cow          TATATATGTGTTTGTGTGTA--TATACAAGAT----AAGGTGGCTGGG----TTCCATGAAGACTTTT-----GATTTTT  16506
Dog          CACTTTGGGTTTTGATTAAA--TTGACTAAGTTTATAAGATAATTGGG----GGGAGTATTTACATTATCAATAAAATAA  16661
BelugaWhale  CTTTTCAGGTTTTGATTATAGTTGTACTAAATTTATAAGTTAATTGGAGA-GTATTGTATTTGTATTATCAAAAAAATAA  14241
Donkey       CTGTTTGGGTTTAAATTGCA------CTAAATTTGTCTGTTAATTGAGGAAGTGTTGTATTTGCATTATCAA-TAAATAA  16595
Cat          CAGGGGAGGGGCAGAGAGAG--AGGGAGAAACAGAATCTGAAGCAGTC----TCCAGGCTCTGAGCTGTCAGCACAGAAC  19152
Ferret       C-TTTGGGTTTCTAGTGTAT--TGCACTAAGTTTATAAGATAAT-GGG----GGGAGTATTTACATTACCAATACAATAA  16576
Lion         CAGGGGAGGGGCAGAGAGAG--AGGGAGAAACAGAATCTGAAGCAGGC----TGCAGGCTCTGAGCTGTCAGCACAGAAC  17754
Vaquita      CTTCTCAGGTTTTGATTATAGTTGTACTAAATTTATAAGTTAATTGGAGA-GTATTGTATTTGTATTATCAAAAAAATAA  14898
Pig          GTTTTCAGGTTTTGATTATAATTGTATTAAATTTATAAGTTAATTGGGGA-GTACCATATTTGCATTATCAA-AAAATAA  14175

Majority     GATG---GCAGTGCTTGAGCTCAXAGTGAXAGATTXCTTATAACAT--------------TTCTTXCX----TXATTTXC
                               20410     20420     20430     20440     20450     20460     20470     20480
Human        GCTATTAGCAGGGTTTCAGGTAAGACATATGTGTTTGCTATTTGTTTTAA-------GCACTCTTCCC----TCATTTAT  17093
PolarBear    --------------------------------------------------------------------------------  0
Cow          GATT---GTAGTTGT----ACTACATTTATAAATTAATTGGAGAGC--------------ATATTTC---------TTCT  16556
Dog          GATG---GCAGTACTTGAACTCAGAGGGAGAGA---ACTATAACGT--------------TTTTTTTTTGAAAAATTTAC  16721
BelugaWhale  GATG---GCAGAGCTGGAGAGAACAGTGATACTTTTCTTTGAAGAA--------------TTTTTCCT----TTACTTCT  14300
Donkey       GATG---ACAATGCTTGAGCTCAAAGACAGAGA---AGTATAACAT--------------TTCTTT---GAAGAATTTC  16652
Cat          CTGAT--GCAGGGCTTGAACTCTGACTGTGAAATAGGTCATGACCTGAGCCAAAGTCAGATGCTTAACCAACTGAGCCAC  19230
Ferret       GATG---GCAATACTTGAACTCAAAGGGAGAGA---CCTATAACAT--------------CTCTTT---GAAAAATTTA  16633
Lion         CTGAT--GCAGGGCTCGAACTCTGACTGTGAGATAGGTCATGACCTGAGCCAAAGTCAGACGCTTAACCAACTGAGCCAC  17832
Vaquita      GATG---GCAGAGCTGGAGAGAACAGTGATAGTTTTCTTTGAAGAA--------------TTTTTCCT----TTACTTCT  14957
Pig          GATG---GTAGTGCTTGAGAGAACAATGATATATTTCTTTGAAGAA--------------TTTTCCCC----TTATTTCC  14234
```

```
Majority     CCATGTTXTTTTXTTTAXXTTXTT-T---X---X-XATXCTTAXTGXAGAXA-CTTAATGCTTTAGGCAAAAAGGAAAAT
                               20490      20500      20510      20520      20530      20540      20550      20560
Human        TCAAATTGTAGCAAACATCTACTACACCCATTTCCAGTTCTTTGTCTTAATAGTTCAACTATTATAAAAAGGAGTGGTAT  17173
PolarBear    --------------------------------------------------------------------------------  0
Cow          CCATGATATTTTCTTT--ATT-TG-T-AAAA--GACATGCTCAATGCAGAAA-CTTGATGCTTTAGGCAAAAAGGAAAAT  16628
Dog          CTCTGTTTTTCTGTGTAATATTTTCT-------CTAATTATA-GAGGTTATG-CTTAATGCTTTAGGCAAAAAGGAAAAT  16792
BelugaWhale  CCATACTATTTTGTTTA-------------------ATGCTTAATGCAGAAA-CTTAATGCTTTAGGCAAAAAGGAAAAT  14360
Donkey       CCCCTTCTTTCTCATGATGTTCTAATTATGAAAGACATGCTTAACATAGAAAACTTATTGCTTTAGGCAG--AGGAAAAT  16730
Cat          CCAGGTCCCCATTTTTGTTTTTTTTTTTAACCCACGTTTACATGTGGCTCTA-ATTCATTTTCATTGCCATATGGTATAT  19309
Ferret       CTCT-TTCTTCCATATAACATTTTCT-------CTAATTATA-GAGGTTATG-CTTAATGCTTTAGGCAAAAAGGAAAAT  16703
Lion         CCAGGTCCCCGTTTTTGTTTTTTT---TAACCCAGGTTTACATGTGGCTCTA-ATTCATTTTCATTGCCATATGGTATAT  17908
Vaquita      CCATACTATTTTGTTTA-------------------ATGCTTAATGCAGAAA-CGTAATGCTTTAGGCAAAAAGGAAAGT  15017
Pig          CTATGATTTTTTTTTTTAATTATA-T-AAAA--AGCATGCTTGATGCAGAAA-C--AGCGTTTTAGGCAAAAAGGAAAGT  14307

Majority     AAATTAA----CCGGTAATTTTACCAAAGCTAATCAGCATTGTATGTA-----CTTCCCATCATTTT-XCCTATATXTTA
                               20570      20580      20590      20600      20610      20620      20630      20640
Human        ATATATA----TATAAACTCTTGCTTGGCTTTGGTTTGATTGAATTAATA---AGGTGAGTTTTTTTTGTTTGTTTGTTT  17246
PolarBear    --------------------------------------------------------------------------------  0
Cow          AAGTCA-----CTGGCAGTTTTACCAAAGCTAAT--GCATTGTA---------CTTCCCATCATTTG-TCCTACATGTTG  16691
Dog          AGATGAC-----CGGAGACTTTACCCAAGTTAATCAGCACCATACGTA-----CTTCGCATCATTTC-TGCTACATATCA  16861
BelugaWhale  AAATTAATTCACTGATAATTTTACCAAAGCTAATCAGCACTGTATGTA-----CTTCCCATCATTTT-CCCTATATGTTA  14434
Donkey       AAAATAAACCACCAGTAATTTTACCAAAGTTAATCAACATTGTATGTA-----CTTCCTATCATTTT--CCACTATGTTA  16803
Cat          GATTATGTG---AATGTACCTTACCCTA-CTGACAAATATCGAGGTTG-----TTTCTAATTTTTTCCTGTTATAAATTA  19380
Ferret       AAATTAAAGCAACAGTGATTTTACCCAAGTTAATTAGCACTATACGTA-----CTTC-CATCATTTC-CCCTGTGTATTA  16776
Lion         GATTATGTG---AATGTACCTTACCCTA-CTGACAAATATTGAGGTTG-----TTTCTAATTTTTTCCTGTTATAAATTA  17979
Vaquita      AAATTAATTCACTGATAATTTTACCAAAGCTAATCAGGACTGTATGTA-----CTTCCCATCATTTT-CCCTACATGTTA  15091
Pig          AAAT-------CCGGTAATTTT-CCAAAGCTAATCAGCATTGTATGTACCTCCCATCCCATCATTTT-CCCTACATATTA  14378
```

```
Majority      ----CAAGXATTTTAAACAGCT--TATGTGTAAXGXATGAGGAAAGAGAGATGCTTXTTT------AATAGTTCXATTTC
                       20650     20660     20670     20680     20690     20700     20710     20720
Human         TGAGATGGAGTCTCGCTCTGTTGCCCAGGCTGGAGTGCAGTGGTGGGATCTCGGCTCACT-------GCAGGCCATTCTC  17319
PolarBear     --------------------------------------------------------------------------------  0
Cow           ----CAAGGATTTTAAACAACT--TGTGTGTAAGGCATGAGGAAAGAGAGAGCCTTTTTTTTGACAAATAGTTTGATTTC  16765
Dog           ----TAAGAATTTTAAGCAGCACATATATGTAAAGGGTGAAAAAAGAGGGATACTTTG--------AATAGTTCAATTTC  16929
BelugaWhale   ----GAAGGATTTTTAAAAGCT--TATGTGTA-GGCATGAGGAAAGAGAGATGCTTCTTT------GCCAGTTTGATTTT  14501
Donkey        ----CAAGAATTTTAAGCAGCT--TATGTGTAAGGAATGAGGAGAGGGGGATACTTCTTT--GACAAATACTTCATTTTC  16875
Cat           CTGCTGTGAGTATTCTTGTGCATGCATTAATAAATGCCACTATTCAGGTTTCTCTGGAAGT---GGAATTGCTGGATTAT  19457
Ferret        ----CAAGGATTTTAAGCAGCT--TATATGTAAAGGGTGAGGAAAGAGGGATACTTTGAC-----AAATAGTTGAATTTC  16845
Lion          CTGCTGTGAGTATTCTTGTGCATGCATTAATAAATGCCACTATTCAGGTTTCTCTGGAAGT---GGAATTGCTGGATTAT  18056
Vaquita       ----GAAGGATTTTTAAAAGCT--TATGTGTA-GGCATGAGGAAAGAGAGATGCTTCTTT------GCCAGTTTGATTTT  15158
Pig           ----CAAAGATTTTAAACAGCT--TAGGTGTAAGGCATGAGGAACAAGAGATGCTTTTTT--GACATATAGTTCAGTTTC  14450

Majority      TT-CCAAAGTTATT-----GXTTTATTX-XT-XT----------X--------X----ATX--AGTXTT--TTTXTATAG
                       20730     20740     20750     20760     20770     20780     20790     20800
Human         CTGCCTCAGCCTCC-----CGAGTAGCTGGGACTGCAGGTGCCTGCCACCACACCTGGCTA--ATTTTTTGTATTTTTAG  17392
PolarBear     --------------------------------------------------------------------------------  0
Cow           TT-CCAAAGTTATTTTTCAGATTTATTTCTTTTTAAT-TGGAGGATAATTGTCTCTCAATAT-AGTATTGGCTTCTGCAA  16842
Dog           CT-TCAAAGTTACT-----GGTTTATTTAGTGTTTTC---CTGAAAATCATGTTTTTATTG--GGCTTCAGATTTCTCAG  16998
BelugaWhale   TTTCCAAAGTTATT-----GATTTATTC------------------------------A-----GTACT--TTTCTATT-  14538
Donkey        TT-CCAAAGTTATT------GTTTATTC-----------------------------------ACTACT--TTCCTAGAA  16911
Cat           AT-TGTGTGTCTGT-----TTGCAAGTTGATATTGACACGTTGCATTCCTAAATGGTTGTGCTAATTTATACTTTACTAG  19531
Ferret        TT-TCAAAGTTAGT-----GGTTTATTCAGTGTTTTC---CTAGAAAATTAGGTTTTACTG--GGCTTCAGATTTCTCAG  16914
Lion          AT-TGTGTGTCTAT-----TTGCAAGTTGATATTGACATGTTGCATTCCTAAATGGTTGTGCTAATTTATACTTTACTAG  18130
Vaquita       TTTCCAAAGTTATT-----GATTTATTC------------------------------A-----GTACT--TTTCTATT-  15195
Pig           TT-CTAAGATCATT-----GATTTATTCAGT--T------------------------ATTC-AGTACT--TTCCTACAC  14495
```

```
Majority     XATTGTX------XX----T-X--X-------------XX-----G-----XX-------------------X-X-TX
                      20810     20820     20830     20840     20850     20860     20870     20880
Human        TAGAGATGGGGTTTCACCGTGTTAGCCAGGATGGTCTAGATCTCCTGACCTTGTGATCCGCCCGCCTCGGAAATACTTTG 17472
PolarBear    -------------------------------------------------------------------------------- 0
Cow          TACAACAACATGAATCAGCCATAGGTATACATGTCCCCTCCCTCTTGATCCATGCTCCCACCTCTCATCAAAGTTTACTG 16922
Dog          AATTGTATAACATTTTTTAATTCTCCTCTT--------CATATTCTGTTTATTTCTCTTGTCAGAGAGCTTATTTTATTA 17070
BelugaWhale  -------------------------------------------------------------------------------- 14538
Donkey       AATTAT------------CT------------------------------------------------------------ 16919
Cat          CAGTGTATGAGAATTCTTGTTTCTGTACGTCT-TCTCTGATAATTGGCGTTGTTGGAATTTTTAATTGTCAGACTGAGGA 19610
Ferret       AATTGTATAGCATTTTGTATTGCTGATATTAA-ATATTTATATTCTGTTTATTTCTCTTGTCAGAGA---CATTTCACTA 16990
Lion         CAGTGTATGAGAATTCTTGTTTCTGTACGTCT-TCTCTGATAATTGGCGTTGTTGGAATTTTTAATTGTCAGACTGAGGA 18209
Vaquita      -------------------------------------------------------------------------------- 15195
Pig          CATTAC-----------------------------------------------------------------------CTG 14504

Majority     XTTTGTTXAXXXX-TCAGXTTTTTX-------------AGAATTTTATAGCATTTTTTAATTCTCXCTTTTTATA-TTTT
                      20890     20900     20910     20920     20930     20940     20950     20960
Human        CATTGTTGGATTGATTGGGTTTGAGCCCCT---------GTTGTACTTAAGTGATTATGAACAAGTCTTTTATAATCTGA 17543
PolarBear    -------------------------------------------------------------------------------- 0
Cow          ATTTATTCGGTTATTCAGTACTTTGCTAT---TTTC--AGAATTTTATAGCATTTTTTAATTCTCCCCTTTTTTA-TTTT 16996
Dog          GTCTGTACAAAGA-CCAGCTTTTC----------------------CATAATTCTTTAATC-TCTGTTTTTATCATTTT 17125
BelugaWhale  --------------T-------CCC-------------AGAATTTTATAGCATTTTAAAATTCTGCCTTTTTAAA-TTTT 14583
Donkey       GTTTACTGGGTT--TCATATTTCTC-------------AGAATTGTATAGCAGTTTTGAATTCTCTCCTTTGTTTCCTCC 16984
Cat          ACATGTGAATAGATCTCTTGTTTTACTTTGAATTTCCTGAGAATGGATACATTTTCTTATTTATAGGCCATTCTTGTTAA 19690
Ferret       GTCTGTACAAAGAACCAACTTTTCTATTTTTTTTTTT------GTTTCCATAATTCATTAGTC-TCTGTTTTTATC-TTTA 17062
Lion         ACATGTGAATAGATCTCTTGTTTTAGTTTGAATTCCCTGAGAATGGATACATTTTCTTATTTATAGGCCGTTCTTGTTAA 18289
Vaquita      --------------T-------CCC-------------AGAATTTTATAGCATTTTAAAATTCTCCCTTTTAAAA-TTTT 15240
Pig          TTTTATTGAGTT--TGAGATTTCTG-------------AGAATTTTATAGCATTTTTTAATTCTCCCTTTTAAAAGTTTT 14569
```

```
Majority     TTTTCTCATTACAXATTTATATTTTA---TXTTTTT-XXTTTTATTTTT----CTTXTXAAAGACCTTATTTXACXGGTT

                     20970     20980     20990     21000     21010     21020     21030     21040

Human        ATTTCCCTTTCCTAGTTTGTAATATTGAGGATAA-TAATTTCTAACTTGCATGATTGTTGTGAAGATTAAATAAGACAAT  17622
PolarBear    --------------------------------------------------------------------------------  0
Cow          TGCTAAAAGCACATATTCATATTTTA---TCC-------TTTAATTTTT----CTTGTCAGAGGCCTTATTTTACTGGTT  17062
Dog          TTTTCTCACACTAGATTAATTTTGTC---TTTTAGTACACTTTAGATCCTGAACTCATTGACTTTCTTGTTTAAAAAATG  17202
BelugaWhale  TATTAACAGTACACATTCATATTTTA---TCCTTTTGCTTTTTTTTTTTCTTTCTTATCAGAGACCTTATTTTACTGGTC  14660
Donkey       TGTACTAATATTAAACAT---CTGTA-------------TTTTCTTTCT----CTTGTCAGAGACGTTATTTTATTGGTC  17044
Cat          ATACCTGTTCATGGATATAGTCTATCCATTTTCTTTTTAATGTAGTTTTTATCCTTTTTTAAATTGTTTCCTAGGAGTTT  19770
Ferret       TTTTTTTGCTTCATGCCAGACTTGTT---TTTTAGTCCACTTTAGATCCTGAACTCATTGACTTTCTTGTTTAAAAAATA  17139
Lion         ATACCTGTTCATGGATAGAGTCTATCCATTTTCTTTTTAATGTAGTTTTTATCCTTTTTTAAGTTGTTTCCTAGGAGTTT  18369
Vaquita      TATTAACAGTACACATTCATATTTTA---TCCTTTTGCTTTTTTTTTTT---TCTTATCAGAGACCTTATTTTACTGGTG  15314
Pig          CATTAATATTTCGTATGTGTATTTTA---CCCATTT--TCTTTGTTTTT----CTTGTCAAAGACCTTATTTTACTGGTC  14640

Majority     TTTTCAAAGTACCAGCTTTTGX-AATTTTTXTTTTTTGTTTCATXTTTXACTAATTTCTGTTTTTXTCTTTTTTTTTCCA

                     21050     21060     21070     21080     21090     21100     21110     21120

Human        GATAT-TGGTAGGAGCTCTTTATAATCTTTCTCTCTTATGTACAATTCAAATATATGTCGGTTTAAATACCAAAATACCA  17701
PolarBear    --------------------------------------------------------------------------------  0
Cow          TTTTCAAAGTACCACCTTCTGT-AATTTTTTT--CATATTTCA-------CTAATTTCTGTTTTTATCTCTTTCTACCCA  17132
Dog          TGTTCAAGATTAAGGAAGATTA-GGTGATACTTTTGAGAATAAGGTTTTTTTTTTTTTTTTTCTTTAATGATAGTCACAG  17281
BelugaWhale  TTTTCAAAGTACCAGCTTTTGTTAATTTTTTTTGTTTGTTTCATGATTCACTAATTTATGTTTTTGTCTTTTTTTGCCCA  14740
Donkey       TTTTCAGAGAACCAACTTTTGA-ATTTTTTTTGTTTTGTTTCGTAATTCACTAATTTCTATTTTTATCTTTCTGCTTC-A  17122
Cat          TATACATTATGGGCCGTAAAGC-CATGTTACTTACATACATTAACTCCTGTCTGTGGCTTGTGTTTTTTTTTTTTTTTTT  19849
Ferret       CATTCAAGATGAAGGAAGATTA-GGTGATACTTTTGGCAATAAGTTTTTGCTTTTAGAGGTCTCTTT-------------  17205
Lion         TATACATTATGGGTCCTAAAGC-CATGTTACTTATGTACATTAACTCCTGTCTGTGGCTTGTGGTTTTTTTTTTTTTC-  18447
Vaquita      TTTTCAAAGTACCAGCTTTTGTTAATTTTTTTTGTTTGTTTCATGATTCACTAATTTATGCTTTTGTCTTTTTTTGCCCA  15394
Pig          TTTTCAAAGTACCAGCTTTTGTTAATTTTTTT--TTTGTTTCATAATTCTCTAATTTCTGTTTTTATCTTCTGCTTTC--  14716
```

```
Majority     -XCTAGATAXATTTTGGTA-ATT-X-XA--TTTTATGTATACTTTA---------A-X------XATXCTGAACATGTTT
                               21130     21140     21150     21160     21170     21180     21190     21200
Human        CTTTAAAAAATCTGGGCTGAAATGGACACTTCTAATGTAGGAGTAGTGCTTTAGAAAATAATGCTTTGTTTCGTATTCTT  17781
PolarBear    --------------------------------------------------------------------------------  0
Cow          -GCTAGATTTGTTTTATTA-----------TTTTTTGTC-ACTTT----------A--------CATGCTGAACATGTTT  17181
Dog          AGAGAGAGAGAGAGGGGCAGAGACACAG--GCAGAGGGAGAAGCAGGCTCTATGCACCGGAAGCCTGACGTGGGATTTGA  17359
BelugaWhale  CACTAGATCTATTTTACTGTATT-ATTA--TTTTTTGTCTACTTTAA--------ATT-----CCATCCCGAACATGTTT  14804
Donkey       CGCTAGATTTGTTCTAGTG--TT----A--TTTTTTGTCCACTTTAA----------AGTAG---ATCCTGAACTTGTTT  17181
Cat          AATTGAATAGAATTTGGAAATTTCACTG--TCACATTTATGTTTTAGTTCTAAGAAATTTTTCTTTAACTCAAGATCTTA  19927
Ferret       -------------------------TAA--GTTGAGAAATAATC----------------------GACATATAATGCTG  17236
Lion         -GTTGAATAGAATTTGGAAATTTTAATG--TCACATTTATGTTTTAGTTCTAAGAAATTTTTCTTTAACTCAAGATCTTA  18524
Vaquita      CACTAGATCTATTTTACTGTATT-ATTA--TTTTTTGTCCACTTTAA--------ATT-----CCATCCCGAACATGTTT  15458
Pig          -ACTAGATTGATTTTGTTACAT--------TTTTCTGTTGACTTT----------A--------TATCCTGAACACATTT  14769

Majority     XTTTTCGTGTTTAAAAXTGCXTTCAAGATXAAGAAAXATXAGTTXATAXTTTGGXCA----GTAAGCTTTGAATTTTAGA
                               21210     21220     21230     21240     21250     21260     21270     21280
Human        TTCCTAGTATTTTATGTCTAGTAACTCATAAGGAAAAAATGATCAGATAAGTATATTTT---TTAGATGCTGATTACAGC  17858
PolarBear    --------------------------------------------------------------------------------  0
Cow          ATTTTCTTATTTAAAAATGCATTTAAGATTAAGAAAAATCAGTTGATACGTTTGGCA----GTAAGCTTTGATTTGTAGA  17257
Dog          TCCCGGGTCTCCAGGATCGCGCCCTGGGCCAAAGGCAGGCGCTAAACCGCTGCGCCACCCAGGAATCCCTGAGAATAAGT  17439
BelugaWhale  A------------AAAATGCATTCAAGGTGAAGAAAGATCAGTTGATAGTTTGGGCA----GTAAGCTTTGACTTTTAGA  14868
Donkey       ATTTTCCTGTTTAAAAATGCAGTCAAGATTAAGAAAGATCAGTTGATACTTTGGCGT-----TAAGTTTTGACTTTTAGA  17256
Cat          TATATAGTGGTTTTTGTCATGAAAGTTTTGAAATTTTGGTTTTCACATTTAGGGCCTC---AGTCTACCTGGAATTTAAC  20004
Ferret       TTTCACATGTACAGCATAATGATTTGATATATGTGTATGTTATAAAACAGT--------------TACCTGAGTAGGTTT  17302
Lion         TATATAGTGGTTTTTATCATGAAAGTTTTGAAGTTTTGGTTTTCACATTTAGGGCCTC---AGTCTACCTGGAATTTAAC  18601
Vaquita      ATTTTCTTGTTTAAAAATGCATTCAAGGTGAAGAAAGATCAGTTGATAGTTTGGGCA----GTAAGCTTTGACTTTTAGA  15534
Pig          ATTTTCATGTTTAAAAATGCATTCAAGATTAAGAAAGATTAGTTGATACCTTTGGCA----GTAAGCTTTGAATTTTAGA  14845
```

```
Majority      GTT----CATTTTTXTTTTTTXTTTT--TXX-AAGATTGAGGTATAATTGAT-XATAAAAAAXTGTATTATTT---G-GT
                              21290     21300     21310     21320     21330     21340     21350     21360
Human         ATTATTTAAATAGTAGAAATAACAACTGTTCAGTGATAAGACAATGAATAAATTCTGTTCATAAACATTACAAATGTTTT  17938
PolarBear     --------------------------------------------------------------------------------  0
Cow           GTT--CTCTTTTTCCTTTTTTTTTTT------GAGATTGTGATATAATTGAC--ATATAACATTATATTATTTTCAG-GT  17326
Dog           TTT----TAAAGTTCTT-CTTTTTCTGTTTTTTATGTTGAGCTATAATTTACCTATAACT--CTTGTTTGACT---T-GT  17508
BelugaWhale   GTT--CCCTTTTATCTTTTTTTTTTTTTTTTTAAGATTGAGGTGTAATTGAT-GTAGAAACATTGTGTTATTTTCAG-GT  14944
Donkey        GTT------------CTTTCTCT----------TTGTTGAGATATAATTGCT--ATATAAAATTGTAATGTTTCAGGTGT  17312
Cat           TTTAGTATATGATAAAGGTTTAATGATTTTTCCCCCCTTATGAATAACCCATTTAGTACCAACTTAATTGAAT---A-GT  20080
Ferret        AGT----TAA-----------------CTATATGTGGTGATAAATGTATCACC---------------------------  17334
Lion          TTT-----ATGATAAAG-TTTAATGATTTTTCCCCCCTTATGAATAACCCATTTAGTACCAACTTAATTGAAT---A-GT  18671
Vaquita       GTT--CCCTTTTATCTTTTTTATTTT-----TAAGATTGAGGTGTAATTGAT-GTAGAAACATTGTGTTATTTTCAG-GT  15605
Pig           -------CTTTTTAACTTTTTTTTTT------AAGATTAAGCTACAGTTGACCAAAAAAAAACTGTATTATTT-CGG-GT  14910

Majority      ACA-CATAATGATTTGATGTATGTATA-TA--XTGTGAAATAATTACCAGAA-TAAGTTTAGTTAAXATCCATCACCACA
                              21370     21380     21390     21400     21410     21420     21430     21440
Human         TTA-AATATTTTTCATATGTGTGAAAAGTAGGATTAAAAATAACTTTGACAAATGCATACAATTTATATGTGTCCCCAAA  18017
PolarBear     --------------------------------------------------------------------------------  0
Cow           ATA-CAGCATGATTTGATGTACGTATA-AAAATTGTGAAACAATTACCAGAA-TAAGTATAGTAAACATCCATTGCCACA  17403
Dog           ACA-CATAATGATTTGATATATGTGTA-TGTTA--TGAAACAATTACCAAGA-TAAGTTTATTTAATATCTATCACAATA  17583
BelugaWhale   GCA-CAAAATGAATTGATGTATGTATA-TA--TTGTGAAATGATTACCAGAA-TAAGTTTAGTTAACATCCATCACCACA  15019
Donkey        ACAACATAATAATTTGATATATGTGTG-TA--TTGTGAAACAATTACCAGAA-TAAGTTTAGTTAACATCCATCCCCGCA  17388
Cat           TCA-TGTTTTTTCCCCACTGATCTGCAGTGTCACCTGTCAGATTTCCATAGA-TGGATGGAACTGTTTCTGGGCTATCTG  20158
Ferret        ACA-CATAGTTAAAAAAATTTTTTCCT-TATAA--TGAGAATTTTTAAGATT-TTACTCTATTAGCTCTTCAACGCA-TA  17408
Lion          TCA-TGTTTTTTCCCCACTGATCTGCAGTGTCAGCTGTCAGATTTCCATAGA-TGGATGGAACTGTTTCTGGGCTATCTG  18749
Vaquita       GCA-CAACATGAATTGATGTATGTATA-TA--TTGTGAAATGATTACCAGAA-TAAGTTTAGTTAACATCCATCACCACA  15680
Pig           GCA-GAACATGATTTGATACATGTATA-TA--TTGTGAAATGAT-ACTAGA--TAAGTTTAGTTAACATCTGTCACCACA  14983
```

```
Majority     CATAGTTAAATTTTTTTC----CTTGTGATGAGAAGTTXTAAXGTTTATTXXXX-AX--TCTCGX---A-----T--T--

                     21450     21460     21470     21480     21490     21500     21510     21520

Human        AGCTGGAGGGCAGTGTACCAAAATAG--AGTAGTTTTCTCCAGGTTGATGGGATTAGTTTTTTGTAATTCTTTATATATT  18095
PolarBear    --------------------------------------------------------------------------------  0
Cow          CATAGTTAAATTTTTTTC----CTTGTGCTGAGAACTTTTAAGATTTACTTTAGGAAACTTTTGGAGAAGGCAATGGCAC  17479
Dog          TAACATTAAAATTTTTTC----GT----TATGAGAATTTTAAAGATTTTTATTAGCACTTTGAAATGTACAATATAGTAT  17655
BelugaWhale  CATAGTTAAATTATTTTC----CTTGTGATGGGAATTTCTAAGATTTAT----------TCTCTG---------------  15070
Donkey       CATAGCTACAATTTTTTTTGGACTTGTGATGAGAAGTTTTAAAATTTACTCTCTCAGCAACTTTCAGATATACAGTATTG  17468
Cat          TTCTGTTTCATTGTTCGT----CTGTCCTGTACCAGTACCACAGTGTCTTAATTTAGTGTAGCGTCACAGTAAGTCTTGG  20234
Ferret       CAATGCAAAAAC----------------------AGTAACAGCGCTGTTCATTGCGTCTCCAGGATTCAT----TTATCT  17462
Lion         TTCTGTTTCATTGTTTGT----CTATCCTGTACCAGTACCACAGTGTCTTAATTTAGTGTAGCGTCACAGTAAGTCTTGG  18825
Vaquita      CATAGTTAAATTATTTTC----CTTGTGATGGGAATTTCTAAGATTTAT----------TCTCTG---------------  15731
Pig          TATAGTTAATTTTTTTCT----CTTGTGATGAGAA-----------------------T-TC------------------  15017

Majority     X-X--T-TAXX---X-TXXXA-A--TT--ATC-TXAXGX-X-X--TXXXX--TX--TXX-----TXTGTXT-TT--XAXC

                     21530     21540     21550     21560     21570     21580     21590     21600

Human        TTCTCTATATTCCACATTTTAAGTATTTTATGATAAAGTTATTAATATTGATAAAGTTATTAATGTTGCTTAATTGATTA  18175
PolarBear    --------------------------------------------------------------------------------  0
Cow          CCCTGTCCAGTACTCTTCTTGCCTGGAAAATCCCATGGACGGAGGAGCCTGGTAGGCTA--CAGTCCATGGGGTCGCTGA  17557
Dog          TTTTATTTACAGTAACTGCTATACGTTGCATCCTCAGGATTCATTTATCTTATAACTGC--AAGTTTGTATGTTTTGACC  17733
BelugaWhale  --------------------------------------------------------------------------------  15070
Donkey       TTAACTCTAGTCACCATTCTGTACATTACATCCCCAGGACTCAGATATTTTATAACTGC--AAGTTTGTACCTTTTGATC  17546
Cat          AACCTTGTAGAGTGAATTTCACAACTTTGTTCTTCAAAATTGTCTTGGCACTTTCTTTA--CCATGTGTATTTTAAGATC  20312
Ferret       TGTAACTGCCAACAA--AACATAACTTGTATGTTTTGG--CCATCCATTTTGTCTATCC-----TTTACCTCTGGCAACC  17533
Lion         AACCTTGTAGAGTGAATTTCACAACTTTGTTCTTCAAAATTGTCTTGGCACTTTCTTTA--CCATGTGTATTTTAAGATC  18903
Vaquita      --------------------------------------------------------------------------------  15731
Pig          --------------------------------------------------------------------------------  15017
```

```
Majority     A-XX---X-X-TTXXXTX-AX-X-----------------XXX-CXXX-A-XTTTXG-A-XT-X---------CTTCTTT
                       21610     21620     21630     21640     21650     21660     21670     21680
Human        AACCCTGATATTTTCTTGTTTCTTTAAAAAACGTTGCATAACTTGATATAAATCTTTTTAATTGTACAAAAAAACACATA  18255
PolarBear    --------------------------------------------------------------------------------  0
Cow          GAGTCGGACACGACTGAGCGA-CTTCAC----TTTCACTTTTCACTTTCATGCATTGGAGAAGGAAATGGCAACCCACTC  17632
Dog          ACCTTCATCCTTTTTGTCCATCCTCTAC--TTCTGGCAGCTTTCCATCTGTTTTTTGTATCTATAAATTTAGGTTTTGTT  17811
BelugaWhale  -------------------------------------------------------------------------CAACTTT  15077
Donkey       ACCTTCACCCATTTTGCCCATCC-----------------TCCACTCGCACCTTTGGCAACCATCAGT-----CTGTTCT  17604
Cat          AGTTGGCTGAGTTCCATGAAGACTTTTTGGTTTTGATTAAATTGCACTAAGTTTGGGGAGCTTGACTGGTTCAGTTGGTT  20392
Ferret       ACCCATTTGTTTTTTGTATAT-------------GTGAGTTCAGTGTTTAGTTTTTGTTTTTGT------------TTTT  17588
Lion         AGTCGGCTGAGTTCCATGAAGACTTTTTGGTTTTGATTAAATTGCACTAAGTTTGGGGAGCTTGACTGGTTCAGTTGGTT  18983
Vaquita      -------------------------------------------------------------------------CAGCTTT  15738
Pig          -------------------------------------------------------------------------CCCCCCC  15024

Majority     CAG-ATX-XA---T--X------X-XG--XX--------X-GT--------X-----------GTACXCAGT-----X--
                       21690     21700     21710     21720     21730     21740     21750     21760
Human        AAATTTACTATCTTAACCATTTTTAAGTGTACACTTAAATTGTTTAATTATTCTTATA----CGTACTTAATAATATTAC  18331
PolarBear    --------------------------------------------------------------------------------  0
Cow          CAGTGTTCTTGCCTGGAGAATCCCAGGGACGGGGGAGCCTGGTAGGCTACCGTCTATGGGGTCACACACAGTCGGACACG  17712
Dog          TAAAATTAGATTTTACATATAAGTGAGATCAT-ATGTG-TAGTATTTGTCTTTCTGTA---TCGGATTCAGTATAATAA-  17885
BelugaWhale  CAG------------------------------------------------------------ATATACAGT--------  15089
Donkey       CTGCATCTAA-----------------------------------------------------GAGTTCAGT--------  17623
Cat          AAGCATCTGACTCTTGATTCGGCTCAGGTCATGATCTCATGGTTCGTGAGATGGAGCC---CTGTGCAAGGCTCTGCACT  20469
Ferret       TTAAATCAGATTTTACATGTAAGTGAGATCAT-ACATAACAGTATTTGTCTTTCTCT-------GACTTAGCATAATA--  17658
Lion         GAGCATCTGACTCTTGATTCGGCTCAGGTCATGATCTCATGGTTCGTGAGATGGAGCC---CTGTGCCAGGCTCTGCACT  19060
Vaquita      CAG------------------------------------------------------------ATATACAGT--------  15750
Pig          C-------------------------------------------------------------------------------  15025
```

```
Majority     ------X--------X---G-------X-----------------X-----TXXX--T-TTTGTTTTTTTTTXCAACATX
                      21770     21780     21790     21800     21810     21820     21830     21840
Human        ATTGCAAAACCCATCTCCAGAACTTTTTGGAGAACTGAAACTCTATACTGATTTTAAAACTCCCTCTTTTGTCCTTCTGC  18411
PolarBear    --------------------------------------------------------------------------------  0
Cow          ACTGAAGTGACTTAGCGTAGCGTAGTGTAGCATAGCGTAGCGTTAGGCAACTTTCAAATATTGTTAACTATATCAGCATG  17792
Dog          -CCCTCAAGGCCCATCCATGTCATAAATGATAGGGT-TTCCGTTTTTTTTTTTTTTTTTTTTTTTTTTTTTTCTAATGGC  17963
BelugaWhale  -----------------------------------------------------------ATTGTTAACTATATCAGCATG  15110
Donkey       --------------TT-----------------------------TTTTTGTTTGCTTGTCTGTCTTTTGTGAGGAAGAT  17660
Cat          GACTGCACAGTGTGCTTGGGATTCTCTTTCTGCCCC-TCCCCTGCTCCCGTGCATGCTCTCCCTCTGTCTCTCAAACATT  20548
Ferret       -TCCTCAAGGTCCATCCATGTTGTTGTAAATGG------------------TAGGATTTCCTTTTTTTTGTTTAAATGCC  17719
Lion         GACTGCACAGCGTGCTTGGGATTCTCTTTCTGCCCC-TCCCCTGCTCCCATGCATGCTCTCCCTCTGTCTCTCAAACATT  19139
Vaquita      -----------------------------------------------------------ATTGTTAACTATATCAGCATG  15771
Pig          --------------------------------------------------------------------------------  15025

Majority     TGXAXXATXTTX-AXX-XX-ATTGTCAXTATTCATTTAT-TTATTAATT-X-------X--X------X-G-----GGT
                      21850     21860     21870     21880     21890     21900     21910     21920
Human        TGG----TAACCAGCATTCTATTTTCTGTGTCTATGAATTTGACTACTTTAAA-------TACCTCACATA------AGT  18474
PolarBear    --------------------------------------------------------------------------------  0
Cow          TGTTACATGGTCAGGGCTA-ATTTATGGGCTTCCTTGGT-AGCTCAGCTGGTAAACAATCCGCCTGCAGTGTGGGA-GAT  17869
Dog          TGAATAATATTCCACACCACATTGTCTTTATCCATTCAT-TCATTGATGGACATTTAGGTTAGTTTCCATGTCTT--GGT  18040
BelugaWhale  CGGTACATTAT---------ATTGTCAGGAGTCATTTAT-GTCATAACT-------------------------------  15149
Donkey       TGGCCCTGAGCTAACATC-TGTTGCCAATCTTCATCTTTTTGCTTGAGG--------AG---------G-ATT----GTC  17717
Cat          TAAAGAAGTTTATAGGATA-ATTGTAATATTATATTTACATTATCAATAAAATAAGATGGCAGTGCTTGAGCTCAAAGGT  20627
Ferret       TGAATAATATTCCACACCACATTGTCTTTATCCATTCAT-CCATCGAAGGACATTTAGGTTAGTTTCCATGGCTT--GGT  17796
Lion         TAAAGAAGTTTATAGGATA-ATTGTAATATTATATTTACATTATCAATAAAATAAGATGGCAGTGCTTGAGCTCAAAGGT  19218
Vaquita      CGGTACATTAT---------ATTGTCAGGAGTCATTTAT-GTCATAACT-------------------------------  15810
Pig          --------------------------------------------------------------------------------  15025
```

Majority	XGGAAXTT-TAXCATTTGACA---TCAXCCTTCXCTTXTT-AT-X-ATXTTTXCCCXCTACAGAGTTXTTXCCTGXAGXG	
	21930 21940 21950 21960 21970 21980 21990 22000	
Human	GGAATTATACAGTATAGGTTAA--GCATCCCTAATCTGAAAATCCAAAGTCTGAATGCTACAAAATCTGAAACTGGTGTG	18552
PolarBear	--	0
Cow	CTGAGTTTGTTCCCTCGGTTG---GCAAGATCCCCCAGATGAGGGAACGGCTACCCACTGCAGTATTCTGGCCTGGGAAA	17946
Dog	TGTAAATT-CTGCATTGAACA---TGGGGATGCAGGTATCTTTGAGATAGTGATATCCTTCAGATAAATTCCCAGAAGTG	18116
BelugaWhale	CTGAGTTTGTACCTTTTGACC---ACCACCTTCACTAATT-------CCTATCCCCCCT---CCGTCCCTCCCTAAAAGG	15216
Donkey	ACTGAGCT--AACGTCTGTC----CCAGTCTTCTCTTGTTTTT-ACGTGGGATGCCACCACA---CTGTGGCTTGGTGAG	17787
Cat	AGGAAACTGTAACATTTGAAAAATTTACCTCTTTCTTCCCCATAATATTTTCTGCAATTATAGAGGTTATGCTTAATGCG	20707
Ferret	TGTAAATT-TTGCAGTGGCCAA--TAGGCATCTTTTTTGAGATCGTGATTTCCTCTCCTTCAGGTATATTCCCAGGAGTG	17873
Lion	AGGAAACT--AACATTTGAAAAATTTATCTCTTTCTTCCCCATAATATTTTCTGCAATTATAGAGGTTATGCTTAATGCA	19296
Vaquita	CTGAGTTTGTACCTTTTGACC---ACCACCTTCACTAATT-------CCTATCCCCCCTACGTCGTCCCTCCCTAAAAGG	15880
Pig	-------------------------------CCCCCA--------------TACCCGCTACA---T--------GGAAAA	15049

Majority	X----------X--TX-TAGXTTATATTAC-X------------X-TTXGACAAAATTGX---------X-----ACXTT	
	22010 22020 22030 22040 22050 22060 22070 22080	
Human	TGTTTTTTTTTTTTTTTTTTTTTTTTTTGAGACGGAGTCTTACTCTGTCGCCCAGGCTGGAGTGCAGTGGCGCG-ATCTC	18631
PolarBear	--	0
Cow	TGGACAGAGGGGTCTGGCAGGCTACAGTCCATGGGGTCGCAAAGAGTTGGACACGACTGAGCGACTTCCACTTTCACTTT	18026
Dog	----------GGATTGCTGGATTATATAATTAT-----------ACTTTTAAAAATTTGG---------GGGAGAACCTT	18166
BelugaWhale	C----------------CTGGCTGTGCTTC-----------------TGGATACCACTG-------------------TT	15244
Donkey	C-----------AGTGCTAGGT--C--------------------CGTGCCCAGGATTCA--------------GACCTA	17820
Cat	TTAGCCAAAAAGGAAAGTAAATTAAATCACCAGTG---------ATTTTACCAAAGTTAATCAGCACT-GTATGCACTTC	20777
Ferret	----------GGATTGCTAGATTGTATAATGAT-----------ACTTTAACAAAATTCGTGGGTG---GGGGGGACCTT	17929
Lion	TTAGCCAAAAAGGAAAGTAAATTAAATCACCAGTG---------ATTTTACCGAAGTTAATCAGCACT-GTATATACTTC	19366
Vaquita	C----------------CTGGCTGTGCTTC-----------------TGGATACCACTG-------------------TT	15908
Pig	--	15049

```
Majority     TTCTC-TTXCX----------A-A--GGTTTCTGCCAGCTTA-XX----AX----AG-X-A-XXGXGTGTTTCXXTTTTC
                               22090     22100     22110     22120     22130     22140     22150     22160
Human        TGCTCACTGCAA-GCTCCACCACCCGGGTTCACGCCATCTTCCTGAGTAGCTGGGACTACAGGCGCCCGCCACCACGCCC  18710
PolarBear    --------------------------------------------------------------------------------  0
Cow          TTCACTTTATGTCAGAATTCCAAGTTTGTACCTTTTGGCCACCATCTTTATTTACTGCCCCAGACTGTGCTTCGGAATTC  18106
Dog          TACTGTTTTCC----------ATAGGGGCTGCA-CCAGTTTACATTCCTACCAGTAGTGAATAAGGG-ATCTCTTTTCCC  18234
BelugaWhale  TTC-----------------------TGTATCTGTGAGTTCA------------------------GTGTTTTGGGTTTT  15277
Donkey       TGAACCCTGGGC-----TCCCAAAGCAGAGTCTGCAAACTTA-------ACCACTATGCCACCAGACTGGTCTGGTTTTT  17888
Cat          CCATCATTTCCTCTATATATTATAAAGATTTTAAGCAGCTTGTATATAAAGGGTGAGGAAAGAGGGACATGTCTTTGATA  20857
Ferret       TACTGTTTTCC----------ATAGTGGCTGC--CCAGTTTACATTCCTACCAGTAGTATACAAGGA-ATCTCTTTTTTC  17996
Lion         CCATCATTTCCTCTATATATTACAAAGATTTTATGCAGCTTATATATAAAGGGTGAGGAAAGAGGGATACGTCTTTGATA  19446
Vaquita      TTC-----------------------TGTATCTGTGAGTTCA------------------------GTGTTTTGGGTTTT  15941
Pig          ---------------------------GTTCCTCCCGGCCA------------------------------G--------  15064

Majority     TATX--T-----TTTTTAXAAAT-TACATX---TTTAXTTCATACXGTGTTT-XCTXTCTCT--TTXTAACTXGTTTCAX
                               22170     22180     22190     22200     22210     22220     22230     22240
Human        GGCTAATT---TTTTGTATATATATATATATTTTTTAGTAGAGACGGGGTTTCACTGTAGCCAGGATGGACTCTTATCTC  18787
PolarBear    --------------------------------------------------------------------------------  0
Cow          AGTGTTTTGCGTTTTTTAAGAGTTTACAGGTAAATGAGATCATACAGTGTTTGTCTCTCTCTCTCTCTGACTTATTTCAC  18186
Dog          TACA--------TTCTTATAAAA-TACTT----TTTATTTATTGTTTTTTTTTTGTAATAACCATTATAACAGGTGTGAG  18301
BelugaWhale  TGTTTGTT----TTTTTAAGAATCTACATATAAATGAGATCATATGGTGTTTGTCTTTCTCT------GACTTGTTTCAC  15347
Donkey       TAT---------TTTTTA--GTTCAACATATAAGTGACATCTTACAGTATTTGTTTTTCTCT-GTTTTATTTTGCATAGT  17956
Cat          AATAGTTCA-ATTTCTTCCAAAGTTACTAG---TTTATTTACTGCTTTCTTA--GAAAATTATATTTTACTGGGTTTCAG  20931
Ferret       CACA--------TTCTCACCAA--TACTT----TTTATTTCTTGTCTTTTTG--ATAATAGCCTTTCTAACAGGTGTGAG  18060
Lion         AATGGTTCA-ATTTCTTCCAAAGTTACTAG---TTTATTTACTGCTTTCTTA--GACAATTATATTTTACTGGGCTTCAG  19520
Vaquita      TGTTTGTT----TTTTTAAGAATCTACATATAAATGAGATCATATGGTGTTTGTCTTTCTCT------GACTTGTTTCAC  16011
Pig          --------------------------------------------------------------------------------  15064
```

```
Majority     XTAGCCC-ATGACCXCATT-----GTTGTTTCAAATTGCATXATTTTXCT-XTTT-TGXTGCTGAGTAXTXTTT-A----

                       22250     22260     22270     22280     22290     22300     22310     22320

Human        CCGACCTCGTGATCCGCCTGCCTCGGCCTCCCAAAGTGC-TGGGATTACAGGCCTGAGCCACCGCGC-CTGGCC------  18859
PolarBear    --------------------------------------------------------------------------------  0
Cow          TTAGCCCTATGACCTCATTTGCATGTTGTTGCAAATGGTAAATTCTTCTT----T-TATAGCTGT------TATGAATGT  18255
Dog          GTGATAG-ATA-CCCCATT-----GTGATTTTAATTTGCATTACTCTGATGATTCGTGATGTTGACCACTTTTTTA--C-  18371
BelugaWhale  TTAGCCTAATGACCTCATTTGCATGTTGTTGCAAATGGTAGGATTTTCTTCTGTT-TATAGCTGAATAGTATTTCATTGT  15426
Donkey       ATAATGCCCTCAAGACCCATCCATGTTGTCACAAATGACAAGATTTTCCT--TTTTTATGGCTGAATAATATTCCACTAT  18034
Cat          ATATCTC-AGAATTCTATA-----GCATTTTTAAATTCCTTTTTGTTGCTAATTT-TGATCATTTGTATTCTGTTT----  21000
Ferret       GTGGTA------CTTCATT-----GTGGTTTTGAATTGCATTACTCTGATGATCTGTGATGTTGAGTACTTT--------  18121
Lion         ATATCTC-CGAATTCTATA-----GCATTTTTAAATTCCTTTTTATTGCTAATTT-TGATAATTTGTATTCTGTTTATTT  19593
Vaquita      TTAGCCTAATGACCTCATTTGCATGTTGTTGCAAATGGTAGGATTTTCTTCTGTT-TATAGCTGGATAATATTTCATTGT  16090
Pig          --------------------------------------------------------------------------------  15064

Majority     -T--ATTXACCACXTTTTX--------TTTAC-XGTTGXTX-AXACCTATGTTXTTTCCATXTTTTTTCTTTT-----TX

                       22330     22340     22350     22360     22370     22380     22390     22400

Human        ----AATCTGAAACTTTTTGAGCA---CTTACTTGATGCTCAAAGGAAGTGCTTAGTGGAGCATTTTGGATTTTAGATTT  18932
PolarBear    --------------------------------------------------------------------------------  0
Cow          GTAGATTAACCACATTTTC--------TTCATCCATTGATGGAGACCTAAGTTGTTTCC-----------CT-------G  18309
Dog          -----ATGATGACCACTTT--------CTTAC----------ATACCAAGTGGCCATCAGCCTTTTTTCTTTG-------  18421
BelugaWhale  ATAGATTAACCACATTTTC--------TTCATGTGTTGATGGAGACCTAGGTTGTTCCCATGTCTTGACTGTTGTAAATA  15498
Donkey       ATGTATATACCACAATTTCTTGATCCATTCACCTACTGATGGACACTTATATTGTTTGCATGTCTTGGTTGTTGTAAATA  18114
Cat          CTCTTGTCAGAGACCTTAT--------TTTACTGGTCTGTA-AAAAGAATCAGCTTTTCTTATTTTTTTTTT---GTTTC  21068
Ferret       ---------------------------CTTAC----------ATACTTATTGGCCATATACCTGTCTTCTTTG-------  18157
Lion         CTCTTGTCAGAGACCTTAT--------TTTACTGGTCTGTA-AAAAGAATCAGCTTTTCTTATTTTTTTTTTTTTGTTTC  19664
Vaquita      ATAGATTAACCACATTTTC--------TTCATGTGTTGATGGAGACCTAGGTTGTTCCCATGTCTTGACTGTTGTAAATA  16162
Pig          -------------------------------------------------------------------------------G  15065
```

```
Majority     GTGXTATAATXAATCTGGGG-TGTAXXTATCTTTTATTGAXTGTGATTTTATTTCTTTCATATAAATTCXTXGXA-TGGA

                             22410     22420     22430     22440     22450     22460     22470     22480

Human        TCAGATTAGGAATGCTCAGGTTGTGTTTGTCCTTTTGTGACTG-GCTTATATTCACTTAATATAATGTCCTCAGGTCCA  19011
PolarBear    --------------------------------------------------------------------------------  0
Cow          GTGCTACAGTGAACATGGGGGTGCAAATGTCT-----AGATAGTGATTTTATTTCCTTTAGATAAATATCCAGAAATGGC  18384
Dog          GAAAGATGTCTATTCAGATCATCTGCCCATTTTTAATTAGATTTTTTCAACCCTGAGTCATATGAATTCATTGTAT----  18497
BelugaWhale  GTGCTACAATGAATACAGGG-TGCAGATATCTTTTATAGATAGTGATTTTATTTCTTTCGGGTAAATACTTAGG--TGGT  15575
Donkey       ATGCTGCACTGAACATGGAGGTGCAGATATCTTTTCAATACAGTGATTTTATTTCCTTCAGATAAATACCCAGAAGTGAG  18194
Cat          TGTAATTGATTAATCTGTGCTTTTATCCTTATTTTTCTGCCTCACATGAAATTTATTTTATTGTCTTTTAGTCTACTTGA  21148
Ferret       GAAAGATGTTTATTCAGAGCATCTGCCCATTTTTAATT-GTTTTACTTTTTTCTGAGTCATATAAATTCTTTATATTATA  18236
Lion         TGTAATTGATTAATCTGTGCTTTTATCCTTATTTTTCTGCCTCACATGAGATTTATTTTATTGTCTTTTAGTCCACTTGA  19744
Vaquita      GTGCTACAATGAATACAGGG-TGCAGATATCTTTTATAGATAGTGATTTTATTTCTTTCGGGTAAATACTTAGG--TGGT  16239
Pig          GA----------------------------------------------------------------------G------C  15069

Majority     ATTXTTGGATCATATAATTTTACTTTTTAAAATTTTTTGAGGA---TGXATTCCATTXTCTTTAGTGGATACATCAATXT

                             22490     22500     22510     22520     22530     22540     22550     22560

Human        TCCATTTTGTAGCATGTGACAAGATTTCCTCCCTTTTTAAGG---CTACATAGCATTCCATT--GTGG-TACACAAAAGT  19085
PolarBear    --------------------------------------------------------------------------------  0
Cow          ATTACTGGATCATGCAGTTTTACTTTGTAAAATTTTTTGAGAAACTTGCATAGTGTTTTCCTTAGTGGGTACATCAGTTT  18464
Dog          -ATTTTGGATATACCATAGTATCCTCTTAGTGTCCCCCATGGG---TACATTCCAACATCTGTAGTGGATGCTTAAAACT  18573
BelugaWhale  ATTGTTGGATCATACAGTTGTACTTTTTCAAATTTTTTGAGGAACCTGTTTTCC-TTTTCCTTAGTGGGTACACCAATTT  15654
Donkey       ATTGCTGGATCATGTAATAGTACTTT-TAAAAGTTTTTGAAGAACCTCTATACTATTTTCCATAGTGGCTACATCAGTTT  18273
Cat          GATCCTGAACTCACTGACTTTCTTGTTTAAAAATGGATTCAGG---GGGCGCTTAGTCTGTTGAGCGTACGATTTTGGCT  21225
Ferret       ATTCTTGAATATTATCAAGTACCATAGTATCCCCTTATTCCCA---GGGGGTACATTCTATCCAGTGGATGCCTAAAACT  18313
Lion         GATCCTGAACTCATTGACTTTCTTGTTTAAAAATGGATTCAGG---GGGCGCTCAGTCTGTTGAGCATACGATTTTGGCT  19821
Vaquita      ATTGTTGGATCATACAGTTGTACTTTTTAAAATTTTTTGAGGAACCTGTTTTCC-TTTTCCTTAGTGGGTACACCAATTT  16318
Pig          -------GAAC-----------------------TTGGG-----------------------------------------  15078
```

```
Majority     AXATTCXXATCATCAXTGCXCATG--TXTXCCCTXTTTTTCACXT-XXTGTCAATACTTGTTATAXCTTXTAATTTXCAT
                       22570     22580     22590     22600     22610     22620     22630     22640
Human        TCATTTGAAGCCCCCTGTGTCATA---ACTCAGACTTCCATAACAAAATACCATAGACTG-AGTGACTTAAAAAACAGAG  19161
PolarBear    --------------------------------------------------------------------------------  0
Cow          ACATTTCCATCGTCATTGCGCAAG--TGTTCCCATTTCTTCAAATCCCTGCCAATGCTTGTTATTTCTTTTTGATAGCAT  18542
Dog          GA----ATAATATCTAATTCTATG--TATATGCTGTTTTTTACT--TGTATGTATACCTGTGGTAAAGTTTAATTTACAA  18645
BelugaWhale  ACATTCCCATCATCAGTGCACAAG--TCTTCCTTTTTCTCCACATCCTCAACAGTGCTTGTTATTTCTTATTTTTTTTGT  15732
Donkey       GTATTCCCATCAACAGTGCCCAAGGGT---TCCT-TTTTCTATTTCCTTGCCAACATTTGTTACCTCTTGTCTTTTTGGT  18349
Cat          CAGGTCATGATCTCATGGTTCATGAGTTCAAGCCCTATGTCAGGC-TCTGTGTTGACAGCCCAGAACCTGGAACCTGCTT  21304
Ferret       AA----ATAGTACCTAATCCTATA--TATATTGAGTTTTTTGCTG-TACATGCATACTTGT---AAACTTTAACTTACAA  18383
Lion         CAGGTCATGATCTCACGGTTCATGATTTCAAGCCCTATGTCAGGC-TCTGTGTTGACAGCTCAGAGCCTGGAACCTGCTT  19900
Vaquita      ACATTCCCATCATCAGTGCACAAG--TCTTCCTTTTTCTCCACATCCTCGACAGTGCTTGTTATTTCTTATTTTTTTGGT  16396
Pig          --------------------------------------------------------------------------------  15078

Majority     AATATCCATTCTAACAGGTGTGAGGXGATACCTCAT-GTGATTTTGATTTGXATXTC-CAGXTAAXTGATXXTGAXCACX
                       22650     22660     22670     22680     22690     22700     22710     22720
Human        ATTCGTT-TTCTCACAGTTCTGGAAACTGGGAAGTCCAAGATCAAGGTACCAGCAAGGCAACTTTCCTTCTGAGGCCACT  19240
PolarBear    --------------------------------------------------------------------------------  0
Cow          C-CATCCAC-CTAACAGGTGTGAGGTGATACTTCATTGTGATTTTGATTGGCATTTCACTGATCCATGATT--GAGTACC  18618
Dog          ATTAGGCACAGTAAGAGATTAATAACAATAG------AATAGTACTTTTTATAT-----AGCTAGGTGAATGTGATCTCT  18714
BelugaWhale  AATATCCATTCTAACAGGTGTGAGGTGATATCTCATTGTGGTTTTGATTGGCATTTCCCTGATTAGTGATGTTGAGCACC  15812
Donkey       AATAGCCATTCTAACAGGTGTGAGGTGATACCTCATTGTGCTTTTGATTTGCATTACTCTGATGATTAGTGATGTTTGAG  18429
Cat          CAGATTCTGTGTCTCCTCTCTTTGCCCCTTCTCTGC-GTGCGTGCTCTCTCTGTCTCTAAACTAAATAAACATTAAAAAA  21383
Ferret       ATGAGGTACAGTAAGTGATTAGCAACAATAACTAAT-AAAATAGTACCATTTAT-----AGTTAGGTGAATTTGATCTCT  18457
Lion         CAGGTTCTGTGTCTCCTCTCTTTGCCCCTTCTCTGC-GTGCGTGCTCTCTCTGTCTCTAAACTAAATAAACATTAAAAAA  19979
Vaquita      AATATCCATTCTAACAGGTGTGAGGTGATATCTCATTGTGGTTTTGATTGGCATTTCCCTGATTAGTGATGTTGAGCACC  16476
Pig          ----TC-------ACAGGAGTGA----------------------------------------CCATGCTG--GA-----  15100
```

```
Majority     TTT--TCXC---G--TXCCTXTTXGTACTGTGTXTGTCTT--------XTGATGAAAT----TGGCTATGCGXXXAGATT
                      22730     22740     22750     22760     22770     22780     22790     22800
Human        TCTCTTGAAGTGAGGCCACCATTT-TACTTTGTGTGCCTC---------ACATGACCTT--TTCTTTGTGAGCACACTTT  19308
PolarBear    --------------------------------------------------------------------------------  0
Cow          TTT--TCGT---G--TACCTATTGGTCATTTGTATGTCAA--------TTTATGTCCTC---TGCCCATTCTTTAATCTT  18680
Dog          T----TCA----G----ACTAATTGCACTGTACTCATCTTTCTTG--TGATATGAGATAAAATGGCTATGCGATGACATA  18780
BelugaWhale  TTT--TCAT---G--TTCCTGTTGGTTGTTTGTGTGTCTT--------TGGAAGAA------TGTCTACTGT---GTCTT  15868
Donkey       CATCTTTTC---ACATACCTGTTGGCCTTGTGTGTGTCTT------------TGGAA------AGACATCTGTTCAGATC  18488
Cat          TTTTTTTTCTCAGACTGTCTTATAGTACTGTACTCACCCT-------TGTGATGAAATA--ATGGCTATGCGAGGAGATG  21454
Ferret       CTCTCTCTCTGAGAATGTTTTATTGTACTGTACTTACTCACCCTTCTTGTGATTGGATAAAATGGCTACGTGATGAGATG  18537
Lion         TTTTTTTTCTCAGACTGTCTTATTGTACTGTACTCACCCT-------TGTGATGAAATA--ATGGCTATGCAAGGAGATG  20050
Vaquita      TTT--TCAT---G--TTCCTGTTGGTTGTTTGTGTGTCTT--------TGGAAGAA------TGTCTGTTGT---GTCTT  16532
Pig          --------------------------------------------------------------------------------  15100

Majority     AAGTGAG---AATGATXTAGG--CATTGTXATXTXTTXTCAGAATAGTATXXATCTTXTGA---ATACTTTAGAX-AATG
                      22810     22820     22830     22840     22850     22860     22870     22880
Human        GGGGGAG-TGGGTGGCAGAAGCTCTCTGGTATTTCTTCTCATAAAGGCATTAATCCCATCATGAAGGCCTTACTCTCATG  19387
PolarBear    --------------------------------------------------------------------------------  0
Cow          TAATGTT---TATTTTTAATCTTTAATGTTTATTTTTT-CTGAGTTCTATGAGTTTCTTGT---ATATTTTTGAT-ATTG  18752
Dog          AAGTGAGGTGAATGATGTAGA--CATTGTGATGTAGGATTAGGATGCTTTCGACCTTCTGA-CAATACTTCAGAAGAAGG  18857
BelugaWhale  C--TGCT---CATTCTTTGATCATACTGTTCATTTTTTTCTGAGTAGTATGAGTTCTTT-------ATTTTTGAT-ACTA  15935
Donkey       CTCTGCC---CATTTTTTA-----ATTGAATTCTTTTTTCTGAGTTGTATGTGTTCTTTAT---ATGTTTTAGAT-ATTA  18556
Cat          AAGTGAGGTGAATGACATAGGTGCATTGTGATGTAGGATTAGGATACTCTTGACCTTCTGA-CAATACCTCAGAAGAAGG  21533
Ferret       AAGTGAG-TGAATGACACAGG--CATTGTGATGTAGGATTAGGATAGTATTGACCTTCTGAACAGTACTTCCGAAGAGGG  18614
Lion         AAGTGAGGTGAATGACATAGGTGCATTGTGATGTAGGATTAGGATGCTCTTGACCTTCTGA-CAATACCTCAGAAGAAGG  20129
Vaquita      C--TGCT---CATTCTTTGATCATACTGTTCATTTTTT-CTGAGTAGTATGAGTTCTTT-------ATTTTTGAT-ACTA  16598
Pig          --------------------------------------------------------------------------------  15100
```

```
Majority     ATCTTCTAXXTXXXXTCTXTAGTTAXCTAX-X-XAATTCAA--T----------------X--XX-TA-XAXXT----X

                       22890     22900     22910     22920     22930     22940     22950     22960

Human        ATCTCATG---AAATCCTCCAGTTAACTCCTAAAGGCCCTGTTTCAAAATTGCATCACATTTGGGGTTAGGACTTCAGCA  19464
PolarBear    --------------------------------------------------------------------------------  0
Cow          ACATTTCACGTATGATGTGCAGATTTTTCC-TTCCATCCATCTTGTTGAT-GGTTTTCTTCACCCTACAGAAGCTAAAGT  18830
Dog          ATCATATGGTT---GACCATGGGTAACTA----AAATTATAGCTA------AGAGGAGACAATTGTATATGACTTGC-AA  18923
BelugaWhale  ACTTTCTATATATGATTTGTAAATATTTTC-TCCCATTCAG---------------------------------------  15975
Donkey       ACCTCTTG--TCAGATATATGGTTTGC-------AATTAT-------------------------T------------  18588
Cat          ATCATCTACTTCCAGGCTATGGTTGGCCATGAGTAACTGAAATTATAGGTAAGAGGGGACAACTGTATATGACTTGA-AA  21612
Ferret       ATCATCTGCTTCCAGACTGCAGTTGACCATGTGAAATTACATGTAGAT---AAGAGGGGCAACTGTTTATGACTTGG-AA  18690
Lion         ATCATCTACTTCCAGGCTATGGTTGGCCATGAGTAACTGAAATTATAGGTAAGAGGGGACAACTGTATATGACTT---AA  20206
Vaquita      ACTTTCTATATATGATTTGTAAATATTTTC-TCCCATTCAG---------------------------------------  16638
Pig          --------------------------------------------------------------------------------  15100

Majority     -X---XT-X--X-TTCTX-XGGTCXTTAGGTTGCCTTT-C--TTGXTGTCAAATCCAAAAAA-TCATTGCCXAGACT--T

                       22970     22980     22990     23000     23010     23020     23030     23040

Human        TGTGATTTTTGGGGAAACATAAGCATTGAGTTGATAATACCCTGCATATAGTTGACCCATACTTCTCCACTCCAGGTAAT  19544
PolarBear    --------------------------------------------------------------------------------  0
Cow          TTGACATAGTCCTAATTACTGTTGCTTTTGTTGCCTTTGCTTTTGGTGTCAAATCCAAAAAG-TCACTGCTAAGATTGAT  18909
Dog          ATATTTTCTCCCATTCTGTAGGTCCTCAGGTTGCCTTTGCTTTTGGTGTCAAATCCAAAAAAATCATTGCCTAGACTAAT  19003
BelugaWhale  --------------------------TAGGTTGCCTTTTC----------------------------------------  15989
Donkey       -------------TTCTCCTGGTCTGTCATTTGCCTTC-CTTTTAGTGTCGAATACAAAAAA-TGGTTGCCAAGACCAAT  18653
Cat          ATATTTTCTCCCATTCTGTAGGTCCTCAGGTTGCCT------TTGGTGTCAAATCCAAAATA-TCATTGCCTAGACTCTT  21685
Ferret       ATATTTTCTCACATTCTGGAGGTCTTTAGGTTGCCTTTGCTTTTG-TGTCAAATCCAAAAAA-TCATTACCTAGGCTG-T  18767
Lion         ATATTTTCTCCCATTCTGTAGGTCCTCAGGTTGCCT------TTGGTGTCAAATCCAAAATA-TCATTGCCTAGACCCTT  20279
Vaquita      --------------------------TAGGTTGCCTTTTC----------------------------------------  16652
Pig          ---------------------------TGATTA-----------------------------------------------  15106
```

```
Majority     XTTAAGGAGCTTA-X-----CCTXXXTTTTCTT-T--GAGTTTTATGXTTTCAG-TXTTATXTTCAX-TXTXTXA---AT
                      23050     23060     23070     23080     23090     23100     23110     23120
Human        --TACTATACTAAATTTGATGTTTACCATTCCCATAAATTTCTCATTTACTACATGTTTATGTATTTTTATCTTAAGCAA  19622
PolarBear    --------------------------------------------------------------------------------  0
Cow          TTCAAGGAGCTTACTTACTACTTACATTTTTTC-TGGGAGTTTTATAGTTTCAGCCCTTATGTTCAAATTTTTAATATAT  18988
Dog          GTTGAGGAGTTTATC-----CCTGTATTTTCTTCTAGGAGTTTTATGGTTTTAGATTTTATATTCAATTATTTAACTTAT  19078
BelugaWhale  ------------------------------------------------------------------------------AT  15991
Donkey       GTCAGGGTGCTTTTTT----CCTATGTCTTCTT-GAAGAGTTTTATGGTTTCAGGTCTTATGTTCA------------AT  18716
Cat          GTTAAGGAGCTTAGCA----CCTAAGTTTTCTT-T--GAGTTTTATGCTTTCAGATCTTATATTCAAGTATTTAATTCAT  21758
Ferret       GTTAAGGAGCTTATCA----CCTGTGTTTTTTTCTAGGAGTTTTATGGTTTCAGGTCTTATGTTCAAGTATTTAACCTGT  18843
Lion         GTTAAGGAGCTTAGCA----CCTATGTTTTCTT-T--GAGTTTTATGCTTTCAGATTTTATATTCAAGTATTTAATTCAT  20352
Vaquita      ------------------------------------------------------------------------------AT  16654
Pig          ------------------------------------------------------------------------------AT  15108
```

```
Majority     TTTGAGTTX-TTTTTGTGTXTGXT-TXAGXTAGTXGTCTAGTTTCATTCTTTT-XXATGTGGCTGTXCAXTTTTC-----
                      23130     23140     23150     23160     23170     23180     23190     23200
Human        TATATGGT-TTTGTTTTGCATG-TTTAAACTATAGATGTACTATCATGCTGTATGTATTCTGTAATTTGCTTTTCTTG-T  19699
PolarBear    --------------------------------------------------------------------------------  0
Cow          TTTGAGTTAATTTTTATGTGTGATGAAAGATGGTGGTCCAGTTTCTTTCTTCT-GCATGAGACTGTCCAGGTTTC-----  19062
Dog          TTTGAGTTGATTTTTGTGTGTGGTGTGAAGTAGTAGTCTAGTTTCATTCTTTT-ACATGTGGCTATTTTCTTAGCATCAT  19157
BelugaWhale  CTTGT------------------------TGATGGGT----------------------------TCCT-----------  16008
Donkey       TTTGAGTTACTTTGTGTGTGTGGTGTAAGACAGTAGTCTAGTCTCATTCTTTT-GCATGTGGCTGTCCATTTTTC-----  18790
Cat          TTTGAGTTAGTTTTTGTGTATAGTTTGAGATAGTAGTCTAGTTTCATTCTTTTTATATGTGGCTGTTCAGTTTTC-----  21833
Ferret       TTTGAGTTAATTTTTGTGTATGGTATGAGGTAGTATTCTAGTTTCATTCTTTT-GCATGTAGCTGTTCAGTTTTC-----  18917
Lion         TTTGAGTTAGTTTTTGTGTATAGTTTGAGATAGTAGTCTAGTTTCATTCTTTTTATATGTGGCTGTTCAGTTTTC-----  20427
Vaquita      CTTGT------------------------TGATGGGT----------------------------TCCT-----------  16671
Pig          CT----------------------------GGTGGG------------------------------CCA-----------  15119
```

```
Majority     ------------XXAXXAXCATTTA-TGAAGAGAXT----TTTCXCCATTGT-ATATTTTXGGCTXCTTTGTCATAAATT

                      23210     23220     23230     23240     23250     23260     23270     23280

Human        TCAGT--G----TTATTTTTGTTAAGTTTATATATATATATATTTTCATTGCCATATAGTATATGATATATGAATATATC  19773
PolarBear    --------------------------------------------------------------------------------  0
Cow          ------------TCAGCATCATTT--TGAAGAGACTGTTTTTTCTCCACGGT-GTATGTT-GGCTCCTTCTGTGTAAACT  19126
Dog          TTACTGAAGAGATTGTTTTGTTTTACTGAAGAGATTGTTGTGTCTCCATTGT-ATGTTTTTAGCTGCTTTGTCATAAATT  19236
BelugaWhale  --------------------------------------------------------------------------------  16008
Donkey       ------------CTAACAGCATTTATTGAAGAGAATGTC-TTTCCCCATTGT-ATATTCTTGGCTCCTTTGTCATAAATT  18856
Cat          ------------CCAGCACCATTTATTGAAGAGACT---GTTTCCCCATTGT-ATATTTTTGGCTCCTTTGTCATAAATT  21897
Ferret       ------------CTAGTATCATTTATTGAATAGACT-TTGTTTCCCCATTTT-ATGTTTTTGGCTTCTTTGTCATAAACT  18983
Lion         ------------CCAGCACCATTTATTGAAGAGACT---GTTTCCCCATTGT-ATATTTTTGCCTCCTTTGTCATAAATT  20491
Vaquita      --------------------------------------------------------------------------------  16671
Pig          --------------------------------------------------------------------------------  15119

Majority     AAT----------------------------------------TGACTCTGTATGTGTGGTXA--TTTCTATTCXAT--T

                      23290     23300     23310     23320     23330     23340     23350     23360

Human        ATCTTGACAAATGAGTTAATATTTGACGAGATTGATTTGTCAAATATTTTAAATTTTTTTCCATTACAAACTGCTACTGT  19853
PolarBear    --------------------------------------------------------------------------------  0
Cow          GAT----------------------------------------TGACCATGGATTATTTCTGA--ACTCAGTACAAT--T  19162
Dog          AAT----------------------------------------TGACCCTGTATGTGTGGATATTTTTCTCTTCCAT--T  19274
BelugaWhale  -------------------------------------------TTGCTCTGCAGCAGCTAAAA--GTTCGATA------T  16037
Donkey       AAT--------------------------TGACCAGATATACATGAGTTTATTTCTGGGCTCTCCATGCTATTCTCT--T  18908
Cat          GACCGTGTGTGTGTGTGTGTGTGTGTGTGTGTGTGTGTGTGTATGTATATGTATGTATGGTTGCATTTCTTTTCTGT--T  21975
Ferret       AAT----------------------------------------TGACCCTAAATATGTGGGA---TTTTTGTTCCAC--T  19018
Lion         AAT--TGACCATATATGTGTGTGTGTGTGTGTGTGTGTGTGTATGTATATGTATGTATGGTTGCATTTCTTTTCTAT--T  20567
Vaquita      -------------------------------------------TTGCTCTGCAGCAGCTAAAA--GTTTGATA------T  16700
Pig          ----------------------------------------------CCGTGGACGACC----------------------  15131
```

```
Majority     AATCTATX-TAT-C--GTTTTTATGCCXGTACCATX-TG-TTTTGATTACT------------AGT--TTXAAATXAGGX

                       23370     23380     23390     23400     23410     23420     23430     23440

Human        AAGCATTAATATGCATATTCTAACGTGTTAATATTAGAGTTTCTCATAGATTGTGTCTTTTCAAATCGATTGAAT--ATT  19931
PolarBear    --------------------------------------------------------------------------------  0
Cow          AATCTATAATAG-TCTGCTTTTATGCCCATATTGTACTG-TTTTGAGTTACTCTTTATATTACAGT--TTAAGACCAAGA  19238
Dog          GATCTGTG-TAT-CAAGTTTTTATGCCCGTGCCATCCTG-TTTTGAGTACT------------GGT--TTGAAATCAGGA  19337
BelugaWhale  AGTCCA-AAT----------TTAT----------------TTTTG-----------------------------------  16055
Donkey       GACCT----TTG-TGGGTTTTTATGGCAGTACCATATTG-TTTTGATTACTGTAGTTTTGTGATACAGCTTGAATCAGGA  18982
Cat          GATCTATG-TAT-CTAGTTTTTAGGCCAGTACCATCGTG-TTTTGATTACT------------AGT--TTGAAATCAGGG  22038
Ferret       AATCTGTG-TAT-CTGGTTTTTATGCCAGTACCATCCTAATTTTGATTACT------------AGT--TTGAAATCAGGA  19082
Lion         GATCTATG-TAT-CTAGTTTTTATGCCAGTACCATCGTG-TTTTGATTACT------------AGT--TTGAAATTAGGG  20630
Vaquita      AGTCCA-AAT----------TTAT----------------TTTTG-----------------------------------  16718
Pig          ACTC-----T-------------------------------TTTG-----------------------------------  15140

Majority     AGTGTGATGCCTXCA-CTT--TTGTTCTTTXTCAAGATTGCTTTGGCTCTTXAGGGTCCTTTGTGGTTCCATACAAATAT

                       23450     23460     23470     23480     23490     23500     23510     23520

Human        AACATATTGCTTTCA--TAGGTAATTATAT-TAATTATACTGTTAGCGCTGCATGTAGATTCTTGTTTCTATACATTCTT  20008
PolarBear    --------------------------------------------------------------------------------  0
Cow          GGTGTGCTGCCTCCA-CTT--TTGTTCTTTCTCACGATTGCTTTGGTTCTTAAGGATTTTTTGTTGTTTCGTATAGACTT  19315
Dog          AGTGTGAGGCCTCTAGCTT---TGTTCTTTTTCAAGATTTCTTTGGCTATTTGGGATCCTTCACAGTTCCATACAAATA-  19413
BelugaWhale  ----------------CTT--TTGTTGTCT-------TTGCTTTTGGTGTCAAAGCCAAAAAGTCATTGC-TAAGACTGA  16109
Donkey       AGTGTGATGCCTCCAGCTTTGTTCTTCTTTCTCAAGATTGCTTTGGTTGTTCGGGGTCCTTTGGGGTTCCATACAAATCT  19062
Cat          AGTGTGATGCCTTCAGCTTTGTTCTT---TATCAAGATTGCTTTAACTCTTCAGGGTCCTTTGTGGTTCTGTACAAATAG  22115
Ferret       AGTGCGATGCCTCCGGCTTTCTTGTTATTTCTCAAAATCACTTCGGCTATATGGG-TCCTTTGCAGTTCCATACAAATAG  19161
Lion         AGTGTGATGCCTTCAGCTTTGTTCTTCTTTATCAAGATTGCTTTAACTCTTCAGGGTCCTTTGTGGTTCCGTACAAATAG  20710
Vaquita      ----------------CTT--TTGTTGTCT-------TTGCTTTTGGTGTCAAAGCCAAAAAGTCATTGC-TAAGACTGA  16772
Pig          ---------------------TTGTTCTTTCTCA--ATTGTTTTGGTTCTTAGGGGTCCTTGGTGGTTCCATATAAATTT  15197
```

```
Majority     TAGGATTGTGTTTTCTATT-TXTGTGAAAAATGCCXTTGGAATTTTGATAGG--GATTGCATTGAATCT-GTAGATTGCT

                       23530     23540     23550     23560     23570     23580     23590     23600

Human        TGAAAATCGGCATTGTTGGACTTTTTAATTTTGCTGATCAAATGTACATGGGAAGGATATCTTGTGGTT-TTAATTTGCA  20087
PolarBear    --------------------------------------------------------------------------------  0
Cow          TAGGATTTTTTTTTTTCTAT-TTTATGAAAAATGTGATTGGAATTTTGAT-----GATTGCATTGAATCT-GTAGATTGCT  19388
Dog          --GGATTGTGT--TCTAT--TTTGTGAAAAATGCCGTTGGAATTTTGATAGG--GATTGCATTGAATCT-GTAGATTGCT  19484
BelugaWhale  TGCCAAGGGGCTTA---GT-TATACTAAA----TCCTTAG---TTTGATAAG--GATTGCATTGAATCT-GTAGATTGTT  16175
Donkey       CAGCATTTTTTTTTCTATT-TCTGTGAAAAATGCCATTGGAATTTTGATAGG--GATTGCATGGAATCT-GCAGATTGCA  19138
Cat          TAGGATTGTGTGTTCTATT-TCTGTGAAAAATGCTGTTGGAATTTTGATAGG--GATTGCATTGAATTT-GTAGATTGCT  22191
Ferret       TAGGATTGTGTAGTCTATG-TCTGTGAAAAATGCCACTGGAATTTTGATAGG--GATTACATTGAAACT---AGATTGCT  19235
Lion         TAGGATTGTGTGTTCTATT-TCTGTGAAAAATGCTGTTGGAATTTTGATAGG--GATTGCATTGAATTTTGTAGATTGCT  20787
Vaquita      TGCCAAGGGGCTTA---GT-TATACTAAA----TCCTTAG---TTTGATAAG--GATTGCATTGAATCT-GTAGATTGTT  16838
Pig          TGGGATTGTCTTTT-CTGT-TTTGTGAAAAATGCCATTGGAATTTTGATAAG--GATTACATTAAATCT-TTAGATAGCT  15272

Majority     TTGGGTAGTA-TG-AACATTTTAACAXTA-TXATTCTT--CCAXTCCATGAGCAXAGA--------ATATCTTTCCA---

                       23610     23620     23630     23640     23650     23660     23670     23680

Human        TTCCCTATTAATGCAAAAAAAAAAAAAACATTGATTCCTG-GGAGTTTTATATACTGTGCAGACTAATTGTTTTGTTATT  20166
PolarBear    --------------------------------------------------------------------------------  0
Cow          CTGAGTTGTA-TG-GGCATTTTAACAGTA----TACTT--CCAATCCATGAGCATAAATGGAAAGAATATCTTTCCA---  19457
Dog          TTGTGTAGTA-TA-AACACTTTAACAGTATTGATTTTC--CCAATCCCTGAGCACAAA--------ATGTCTTTCTA---  19549
BelugaWhale  TTGGGTAGTG-TG-AACATTTTAACAGTA----TTCTT--CCAGTCCATGAGCATGGA--------ATATCTTTCCACTT  16239
Donkey       TTGGGAAGAA-TG-AACATTTTGACAATATTAATTCTT--CCAGCCCATAGGCATGGA--------ATATCTTTCTGTTT  19206
Cat          TTGGGTAGTA-TG-AACATTTTAATGATACTGATTTTTTTCCAAACCAGGAGCACAGA--------ATGTCTTTCCA---  22258
Ferret       TTGGGTAGTA-TG-AACA-TTTAACAGTATTGATTTTT--CTAATCCGTGGGCACAAA--------ATGTCTTTCCA---  19299
Lion         TTGGGTAGTA-TG-AACATTTTAATGATACTGATTTTTTTCCAAACCAGGAGCACAGA--------ATGTCTTTCCA---  20854
Vaquita      TTGGGTAGTG-TG-AACATTTTAACAGTA----TTCTT--CCAGTCCATGAGCATGGA--------ATATCTTTCCACTT  16902
Pig          TGGGGTAGTA-TG-G--------ATAATA----TTCTT--CCAGTCCAGGAGCATGCA--------ATATCTTTCCA---  15325
```

```
Majority     -TXTGTATTTXCTTCAA-TTTTTTTCATCAXTXTXTTAXATXTTTTTAACTTTXX-AX-XT--TTATGAAAGXTTCAAGA
                        23690     23700     23710     23720     23730     23740     23750     23760
Human        TTTTTTGGTTTGTTTGTTTTGTTTTGTTTCATTTTTGTTTTTGTTTTGAGAGACAGAGTCTCACTGTGTCATCCAGGCTG  20246
PolarBear    --------------------------------------------------------------------------------  0
Cow          -TTTGCATCTTCTTCAA-TTTCTTCCATCAGTGTCTTTTACCTTTTTCACTTTTTTAAGCT--TTGTGAAAGCTTCAAAA  19533
Dog          ---TATATTTGTGTCAA--TTTTTTTCA----TTTTTA----TTTTTTAAACTTCA--------TTATTAAACTTTCAAGA  19608
BelugaWhale  ATTTGTGTCTTCTTCAA-TGTGTTTCATCAACGTCTTACAGCTTTTTCACTTTTT-AAGCT--TTGTGAAAGCTTCAAGG  16315
Donkey       ATGTGTGTTGTCTTCAA-TTTCTTTCATCAGTGTCTTGCATCTTTTTATTTTTTTAAACTTCCTTATAAAAGTTTCAAGA  19285
Cat          ---TTTATTTGTGTCAA--TTTTTTTTTCCCTTTTTAAATTTTTTTAAAACTTCA--------TTATTAAACTTTCAAGA  22325
Ferret       ---TATATTTGT--CAA--TTTTTTTCA----TTTTAA----TTTTTTAAGCTTCA--------TTTTTAAACATTCAAGA  19356
Lion         ---TTTATTTGTGTCAA--TTTTTTTT-CCCTTTTTAAATTTTTTTAAAACTTCA--------TTATTAAACTTTCAAGA  20920
Vaquita      ATTTGTGTCTTCTTCAA-TGTGTTTCATCAACGTCTTACAGCTTTTTCACTTTTTTAAGCT--TTGTGAAAGCTTCAGGG  16979
Pig          -TTTATTTGTGCTTTGAGTTTCTTTCATCACTGTCTTTTATCTTTTTCACTTTTTTAAGCT--TTACGAAGG-TTTAAGA  15401

Majority     T-ATACAAAAGTAGXGTGAATGGTA------TAATAATACACTA----XTXXCCTTCCTGTTGGAGAATATTAAAGTAAA
                        23770     23780     23790     23800     23810     23820     23830     23840
Human        GAGTGCAGTGGCTCGGTCACAGTCACTGCAGTCTTGACATCCCAGGCTCTAGCAATTCTCTCACCTTTGTTTCCTGAGTA  20326
PolarBear    --------------------------------------------------------------------------------  0
Cow          T-GTACAAAAGTGGGGTGAATGGTA------TAATAGTACACTT--C-TTTGCCTTCCTTTGGAAGAATATTAAAGCAAA  19603
Dog          T-ACACAAAAATAGTATGAATGGTA------CAGTGATACGCAA------ATCCTTTCTTTTGGGGAATATTAAAGTAAA  19675
BelugaWhale  T-ATACAAAAGTAGGGTGAATGGTA------TAATAATATACTT--CCTTTGCCTTCCTGTGGGAGAATATTAAAGTAAA  16386
Donkey       T-ACACAGAAGTAGTGTGAATGGTA---------TAATACTCTT--CTTTTTCCTTCCTGTTGGAGAATATTAAAGCACA  19353
Cat          TACATAAAAAGTAGTGTGAATGGTAATGGTACAGTAATACACAA------ATCCTTCCTGTTGGAGAATATTAAAGTAAA  22399
Ferret       TCACATAAAAGTGGTATGACTGGTA------CAGTAATACACAA------ATCCTTCCTTTTGGAGAACATTAAAGGAAA  19424
Lion         TACATAAAAAGGAGTGTGAATGGTAATGGTACAATAATACACAA------ATCCTTCCTGTTGGATAATATTAAAGTAAA  20994
Vaquita      T-ATACAAAAGTAGGGTGAATGGTA------TAATAATATACTT--CCTTTGCCTTCCTGTGGGAGAATATTAAAGTAAA  17050
Pig          T-GTAAAAAAGTAGGATGAATGGTA------TAATAATGTACT-----TTTGCCTTCCACTGGGAGAATATTAAAGCAAA  15469
```

```
Majority     TCTCAGAGATCAT-TTTATATCATT---CCTAAATACTTTATTGTGTGTCTCTAACAGATATAATCTCTTAAGGAT---G

                       23850     23860     23870     23880     23890     23900     23910     23920

Human        GCTGGGACTACAGGAATACACCACTATGCCCAGCTAATTTTTCAATTTTTTTGTAGAGATGGAGTCTCACTGTATTACCC  20406
PolarBear    --------------------------------------------------------------------------------  0
Cow          TCTTAGCGATGAT-TTTATATCATT---CCTAAATATTTCAATGTGTATCTCTAACAGATACAGTCTCTTGAA------G  19673
Dog          TCTCAGAGGTCAT-TTCATACCATC---CCTAAATATCTTATTGTGTGTCTC------ATATAATCTCCTAAGGAT---T  19742
BelugaWhale  TCTCAGTGATTAT------ATCATT---CCTAAATACTTCAATGTGTATTTCTAGTAGATATAATCTCTTAAAGAT---G  16454
Donkey       TCTCAAAGATCAT-TTCA---TATT---ACCAAATGCTT--GTGTGTGTTTCTAAC--ATATACTCTCTTAAAGAT---G  19419
Cat          TCTCAGAGATCAT-TTCATGTCCTC---ACTAAATACTTTATCGTGTGTCTCTAACA-TAATAATCTCCTAAGGAT---G  22471
Ferret       TCT---AGATAAT-TTTATACCATC---CCTAAGTATTTTATTGTGTGTCTCTAACAGATGTAATCTCCTCAGGAT---G  19494
Lion         TCTCAGAGATCAT-TTCATGTCCTC---ACTCAATACTTTATTGTGTGTCTCTAACA-TAATAATCTCCTAAGGAT---G  21066
Vaquita      TCTCAGTGATTAT-TTTATATCATT---CCTAAGTACTTCAATGTGTATTTCTAATAGATATAATCTCTTAAAGAT---G  17123
Pig          TCTCAGAGGTTGTGTTTATATCACT---CCTAAATACTTCAGTGTTTGTATCTAATAGATATATTCTGTAAAGA-T---G  15542

Majority     ATATTTATACC--AXT-TTATACCT-AATAAAATTT--AAAATAATTTXTTAATAT------T-XXXTAATXTCTAG---

                       23930     23940     23950     23960     23970     23980     23990     24000

Human        AGGTTTGTTGCGAACTCCCAGGCTCAAGCAGTTCTCCCGCATTGGCCTCCTAAAGTGCTGAGATTACAGGCGTGAGTCGC  20486
PolarBear    --------------------------------------------------------------------------------  0
Cow          ATATTT-----------TTATACAT-AATAAAATTT--TAAAGAATTTTTTGTTGT------T--GTTTAAGTGTAG---  19728
Dog          ATATTGGTACC--ATTATTAAACCTAAATAAAATTT--AAAATAATTTCTTAATAT------T-ATCTAATTCCCAG-AG  19810
BelugaWhale  ACATTTATATC--A---TCACACCT-AATAAAATTT--TAAATAAGTTTTTCCTTT------TTTATTGAAGTATAT---  16517
Donkey       CCATTGATACA--ATTATTATACCT-AATAAAATTT--AAAATAATTTATTAATAT------CCTCTAATCTCCTAGTGG  19488
Cat          ATATTGATACC--ATTATTATACCTTAATAAAATTT--AAAATAATTTCTTAATA----------TCTAATTTCCAG---  22534
Ferret       ATATTCATACC--ATTGTTATACTTAAATAAAATAT--AAAATAGTTTGTTAATAT------A-ATCTAATTTCCAATAG  19563
Lion         ATATTGATACC--ATTATTATACCTTAATAAAATTT--AAAATAATTTCTTAATA----------TCTAATTTCTAG---  21129
Vaquita      ACATTTATATC--A---TCACACCT-AATAAAATTT--TAAATAAGTTTTTCCTTT------TTTATTGAAGTATAT---  17186
Pig          ATATTTATATC--A---TTGTACCT-AATAAAATTT--AAAATAACTTTTTATTTT------TTAATTGATGTAAAG---  15605
```

```
Majority      TAGTCATAATTTAXTATXTXCXTTAAAGATGT--C------GTA---CAXCXTTATGAXTCAAXATTTAAAGAC--AXTA
                        24010     24020     24030     24040     24050     24060     24070     24080
Human         TGTGCCTGGCCTGTTTTGTTAGTTACATGTCCTACAGATAACTCTCTCCTCTCTCTGACCTGTTTTTTTTACACTGATGA  20566
PolarBear     --------------------------------------------------------------------------------  0
Cow           TTGACATACTGTATTATG---ATTTAGGA------------GTA---CAACATAATGATTCAACATTTATACAT--ATTA  19788
Dog           TAGTCAAAATTTCCCAGTTCCTTCAAAGATGT--C------TTTTTTCCCCTTTTCAAATCAGGAATTAAGGAC--AGTC  19880
BelugaWhale   TTGACATACAATATTATGTTAGTTTTAGGT-----------GTAA--TAACATAATGATTCAAAATTTACATAC--ATTA  16582
Donkey        TAGTCAAAATTTTCTAGTTGCCTCAAAAATAT--CTTTCACGTTTCTTTTCCTTTCAAATCAAGAACCAAGGAA--AGTC  19564
Cat           CAGTAAGAATTTTCCATTTGCCTCAGAAATGT--C------TTGG--CGCCTGGGTGGCTCAGT----TAAG----TGTC  22596
Ferret        TAGTCCAAATTTCCCAATTGCCTTGAAAATGT--C------TTTC--CCCCCTTTCAAATCAGGAATCAAGGGA--AGTT  19631
Lion          CAGTAAGAATTTTCCATTTGCCTCAGAAATGT--C------TTGG--CGCCTGGGTGGCTCAGTCAGTTAAG----TGTC  21195
Vaquita       TTGACATACAATATTATGTTAGTTTTAGGT-----------GTA---TAACATAATGATTCAAAATTTACATAC--ATTA  17250
Pig           TTGACATAAAATATTATGTTAGTTCCAGGTGT--T------GTA---CAACATCATGAGTCAACATTTACATAC--ATTA  15672

Majority      CAAATTXAXXA-TTTAAGT---------CATXTGTCAXXATATAAAATTTTTA--------C-TATTACATTCXCXATAG
                        24090     24100     24110     24120     24130     24140     24150     24160
Human         ATAGTTGTAAATTTTAATGGGACATTTATGTTTTTCTTTTAAAAAAACATTTA-------ACCTAAGGCCTTACGTATAG  20639
PolarBear     --------------------------------------------------------------------------------  0
Cow           CAAATTGGTCACTGTAAATCTAGTAG-TCATTTGTGACCATATAAAATTATTACAATATTATT-ATTACATTCCCCATGC  19866
Dog           AATATTTAACA-TCTAGTT---------AATGTTTAGTTAATTTAATGTTTAGTTA-ATGACTTTTAAGTCTCTTTATAA  19949
BelugaWhale   CAGATTGATCACCATAAC----------CATCTGTCACTGTAAAAAATTATTA-----------ATTATATTCCCCAAGC  16641
Donkey        AGAATGTTGAATTGGTTGATACCTTTTATATCTGTAATCAGTTTATCTTCCCAC----T--CACCTTTTTTTCTTTGTTA  19638
Cat           TGACTTTGG---CTCAGGT---------CATGATCACACAGTTTGTGGGTTTG--A-GCCCCGTGTCAGGCTCTGCGCAG  22661
Ferret        GATATGTTG-A-TTTAGTT---------AATGTCTTTTTAAAAAAATTTTT------------TTTAACTTTTTTTAAAG  19688
Lion          TGACTTTGG---CTCAGGT---------CATGATCGCACAGTTTGTGGGTTTG--A-GCTCCGTGTCAGGCTCTGCGCAG  21260
Vaquita       CAGATTGATCACCATAAA----------CATCTGTCACTGTAAAAAATTATTA-----------ATTATATTTCCCAAGC  17309
Pig           CAAATTGATAGTGGTAAGTCTATTAAACCATCTGTCACCATACAGAGGTATTACAATACTGTTGACTGCATTCCCTGTGG  15752
```

```
Majority     XGTACATTACATCCTAGTAACTTATTTATATTXTXX-TGTAAGTTTGTATCAC-TTAATCXCCXTC--ACTTATTTTGTC
                      24170     24180     24190     24200     24210     24220     24230     24240
Human        GCTTGTGTATTTTATTTTGAAAGTTTTAAAATTTTGATTTTCACATTTATGGCGTTAATCTACCTGGAATTTATTCTTTG  20719
PolarBear    --------------------------------------------------------------------------------  0
Cow          TGTGCATTACATCCCAGTAACTTACTTATAAC-----TGGAAGTTTGTATCAC-TTAATTACTTTC--ACTTACTTTGCC  19938
Dog          ATCAATTTACCCTCTTCTTCCCCTTTATCATTTTTTCTTTTACTGCTTATTAG-AGAAACTGTGTC--ATCTATTCTGTA  20026
BelugaWhale  TGTACATTACATCCCAGTAACTTATTTATTTTATAACTGGAAGTTGGTATCAC-TTAATCCCCTTC--ACTTATTTTGCC  16718
Donkey       GAGAAACTGGGTCAT-CTATTCTGTACAATTTTCCAGTTTCTTGATTTGCTGA-TTATACCTCCAT--GTTGTTGTTTTA  19714
Cat          AC-AGCTCAGAACCTAGAACCTGCTTCAGATTCTGTGTCTCCCTCTCTCTCTC-TGCCACTTTCCT--GCTTGTGCTGTC  22737
Ferret       AT------------------------------------------------------------------------------  19690
Lion         AC-AGCTCAGAACCTAGAACCTGCTTCAGATTCTGTGTCTCCCTCGCTCTCTC-TGCCCCTTTCCT--GCTTGTGCTGTC  21336
Vaquita      TGTACATTACATCCCAGTAACTTATTTATTTTATAACTGGAAGTTGGTATCAC-TTAATCCCCTTC--ACTTATTTTGCC  17386
Pig          TGTACATTACATCGCAGTAACTTATTTATTTCATAG-TGGAAGTTTGT--CAG-TTAATCCCCTTC--ACTTATTTTGTC  15826

Majority     TAX------ACCTCACC--T-------------------T-------------------------------C--X-X---
                      24250     24260     24270     24280     24290     24300     24310     24320
Human        TGTA-GCATATGTTACAGGT--T--TAATTA--TTT--TTTCTATATGGATAGCAAGCTTAGTATGTACTTGATTGAATA  20790
PolarBear    --------------------------------------------------------------------------------  0
Cow          CATC---CCACCCCACCACT-AAAATAATTTTTTCAAGTTACTTCTTTCTGGTTACTCCGGGTTTTCGTTGCTGTCTTAG  20014
Dog          TGATTTTCCACTTTGTTGATTTTGATGATTATACCTCTATGTCATTTTACACATTCCTCATATCCTCTTTCCTTTGATGA  20106
BelugaWhale  CA-------ATCCCACC---------------------------------------------------------------  16728
Donkey       TAT----GTACCTCATATCT-----TG----T-------TT---------------CCTGTAAACTTAACAGTTAGATGA  19759
Cat          TCTCTGAATAAATAAACATTTAAAAAAAAAATGTCTTTTTTTCCTCTTTCAAATCAGGATCCAAGGAAAGTCAGTATC-T  22816
Ferret       --------------------------------------------------------------------------------  19690
Lion         TCTCTGAATAAATAAACATT-AAAAAAAAAATGTGTTTTTTTC-TCTTTCAAATCAGATTCCAAGGAAAGTCAGTATCCT  21414
Vaquita      CA-------ATCCCACC---------------------------------------------------------------  17396
Pig          CA-------ACGTCACC------------------------------------------------------CCA--T---  15840
```

```
Majority     X--XXX---X----C-----XX---X-T-X--------XX----X--------------------TXX----X----C-C
                      24330     24340     24350     24360     24370     24380     24390     24400
Human        GCTCAGCTTTTCTCCAATGATCTGCTTTCTCAGCTCTTCAGGTTTCCGTATATTCACAGAACCATTTCTGTGCTATGTCT  20870
PolarBear    --------------------------------------------------------------------------------  0
Cow          GCTTTCTCTAGTTGCGGTGTGTGGGCTTCTCATTGTGGTGGCCTCTCTTGTTGTGGAGCAGGGACTCTATAATGGGCT-C  20093
Dog          GGCTTGACCAGATTCAG-GTTTAAATTTTA-GCAGGGATA--TTTTGTGGATATTCTCTATGTAATTCCTATTGTA-T-C  20180
BelugaWhale  -----------------------------------------------------------------------------C-C  16730
Donkey       AGCTTGATCAGATTCA--GGTTTAATTTTTAGCAAGAATA---TTTTTTTAGATATACTCTGTAATTCCTGTTGTATCAC  19834
Cat          GGTTTGGTTAACGTC---TTTTAAGTCTCT-ATAAAGATA--ACTATGAATCAGTTTACCTTCCATTCCCTTTATT-G-C  22888
Ferret       --------------------------------------------------------------------------------  19690
Lion         GATTTGGTTAACGTC---TTTTAAGTCTCT-ATAAAGATA--ACTATGAATCAGTTTACCTTCCATTCCCTTTATT-G-C  21486
Vaquita      -----------------------------------------------------------------------------C-C  17398
Pig          -----------------------------------------------------------------------------C-C  15842

Majority     ---------------TT-XXTTAXTTCTTX-T------X-----X--------------X------X----TX-----T-
                      24410     24420     24430     24440     24450     24460     24470     24480
Human        TGTAGCAGTACAACAGTGTCTTAATTACTTATGGCATCAGCATTAAGTTTTGGAATCTTGTACAGTGAGTGTCAAAACTT  20950
PolarBear    --------------------------------------------------------------------------------  0
Cow          AGTTGTTCTGGCGCATACACTCAGTTCCTCTTCTGCATGTGGAATCTTCCCGGACCAGGGATCAAAC-CCGTGTGCCCTA  20172
Dog          A--CAACAGGAGGAATATATTTATTTCTTTGTAGTGATAACATTTATCAGTGGATTTGGGTTTTGTCATTGTGATGTATC  20258
BelugaWhale  --------------------------------------------------------------------------------  16730
Donkey       ATCAGGAAGAACATGTTTAGTTACCTCTTT-TGATGTTAGCATTTATCAATAGGTTTAGTTGTTGTCAGCTTGATGCATC  19913
Cat          T--TT----------TTCTTTTATTGCTTATTAGAGAAACTGGGTCATCTATTCTGTACGGTTTTCCACTTTGTTGATTT  22956
Ferret       ---------------TTTATTTATTTTTTTG-------------------------------------------------  19706
Lion         T--TT----------TTCTTTTATTGCTTATTAGAGAAACCGGGTCATCTATTCTGTACGGTTTTCCACTTTGTTGATTT  21554
Vaquita      --------------------------------------------------------------------------------  17398
Pig          -----------------------------------------------------------------------------C--  15843
```

```
Majority     -ATTA-A-XXT-XT-XCXXXTX-XX-----------------T--CTAAXATAATTTCXTAATGTCATGTAAT----GTC
                      24490     24500     24510     24520     24530     24540     24550     24560
Human        TGTTTTTCAAAATTGTCTTGGTGTTTTCTTCACTGCATTTATT--TTAAGATCATTTGGCTAAGTTCTGTGAAGGCTCTT  21028
PolarBear    --------------------------------------------------------------------------------  0
Cow          CATTAGCAGGTGGGGTCTTATCCACTGTTAGAACTATGGAAGTCCCTAAAATAATTTCCCAATGCCATGTAAT----CTC  20248
Dog          CAGTATAGAGTGCTCCAAAATCCTTTTACCTTCGTTTTTTTTTTTTTTAAAGAATTTTATTTATTCAGAAAGA----GAG  20334
BelugaWhale  -ATCA---------------------------------------CTAAAATAATTTCTTAATATCATCTAAT----GTC  16765
Donkey       TGGTGTAGAGTTCTCCATCATCCTT----------------GTAGCTAA--TGATTGCATAATTTCATTAAGT----ATT  19971
Cat          TGGTG-ATTATACTTCCATGTCATTGTTTTACATGTTCCTCATGTCCTA-----TTTCCTGAGGTGAGGCTTG----ATC  23026
Ferret       ------ACAGAGATCACAAGT---------------------------------------------AGGCAGA----GAG  19731
Lion         TGGTG-ATTATACTTCCATGTCATTGTTTTACATGTTCCTCATGTCCTG-----TTTCCTGAGGTGAGGCTTG----ATC  21624
Vaquita      -ATCA---------------------------------------CTAAAATAATTTCTTAATATCATCTAAT----GTC  17433
Pig          -ATTA---------------------------------------CTAAAATAATTTCTTAATATCATCTAA-----CTC  15877

Majority     XXACAXXAGTTAA-ATTTTCCCAGXXAXTTTAAGGAAATCTTCTGTATXTTCTTTTTGXATCAXGAATCAAGGAAAGTCA
                      24570     24580     24590     24600     24610     24620     24630     24640
Human        TGGGTCTTATTTATACTTGCCTTAAATTTAGTAGTTAATTGGGAGAACATTATAGCTGCATTATCAAAATAAAATGGCAA  21108
PolarBear    --------------------------------------------------------------------------------  0
Cow          CAACAGTAGTAATCAATTTCCTGATCACTTTAAAGAAATCTTGTGTATTTTCTTTTTAAATCAGGGATCAAGGAAAGTCA  20328
Dog          AGAGAAACAGAGACATAGGCAGAGGGAGAAGCAGGCTCCATGCAGGAAGCCCGATATGGGGCTCAGTCCCAGGACTCTGG  20414
BelugaWhale  TAACAGTAGTCAA-AGTTTCCCAGTCACTTTAAAGAAGTCTTTTATATTTTCTCTTTGAATCAGGGATCAAGGAAAGTCA  16844
Donkey       GCAAAAATGATGATATTCTCTCCCCTCTTCCCCG---CTCCCCTCCATATGTTTGTTAGAATTCTTCTAAGAACTTTTTC  20048
Cat          AGATTCAGGTTTAATTTTTAGCAGGAATATTTTGTAGATGCACTGTGTGTTTTTCCTGTTGTATCACACCAGGAGGAACA  23106
Ferret       GCAGGCAGAGAGAGAGAAGGGGAAGCAGGCTCCC-CACCGAGCAGAGAGCCCCATGTGGGGCTTGATCCCAGGACTCTGA  19810
Lion         AGATTCAGGTTTAATTTTTAGCAGGAATATTTTGTAGATGCACTGTGTGTTTTTCCTGTTGTATCACACCAGGAGGAACA  21704
Vaquita      TAACAGTAGTCAA-AGTTTCCCAGTCACTTTAAAGAAGTCTTTTATATTTTCTCTTTGAATCAGGGATCAAGGAAAGTCA  17512
Pig          CAACAGTAGTCAG-AATTTCCCAGTAACTTCAAAAAAGTCTTTTATATTTTCCTTTTGCATCGGGAACCAGGGAAAGTCA  15956
```

```
Majority     GATXX-ATGATCTGXT---T-GTGAAGTATAXATTTX-XTXGX-G--CT--XX-CTTCACCCTCCTGTTTTCXTGATTT-
                        24650     24660     24670     24680     24690     24700     24710     24720
Human        TTTTTTTTGAGCTGAAAGGCAGAGAACTATAACATTTCTTTGAAGAATT-----TTCCACTTTCTTCTTCATGGTATTTT  21183
PolarBear    --------------------------------------------------------------------------------  0
Cow          GCTTA-CTGATTTGGTAC-T-TTAAGGTCTGTATCCCTGCTGGAGGGGCA-TGACAACCCACTCCAGTATTCTTGCTTGG  20404
Dog          GATC--ATGCCCTGAT---G-CTCAACTGCAGAGTCACCCAGGCATCCT-------CTACCTTCTGGTTTTTATAATGG-  20480
BelugaWhale  ACATG-TTGATTTGGTTC-T-TTTAAGTCTATTTAT--GTCAG-----TT-TTCCTTCTCCCTCCCCCTTTCTTGATTT-  16912
Donkey       CATCAAATAATTTGGTCA-TGCTGAAATACCCTTTTTATAAGAAAAGCAGGATAAGATTTTGACTTAAAAAAAAAATTTC  20127
Cat          TATCTCATTATCTTTT---T-GTGACGTTAACATTTATCAGGAGGTTCAGGTTTGGTTAGCATGATGTATCCAGTATAG-  23181
Ferret       GATC--ATGGCCTGAA---T-GTAAGGCAGAGGCTTAACCCACTGAGC-----------CACCCAGGTTCCCCTAGTG--  19871
Lion         TATCTCATTATCTTTT---T-GTGACGTTAACATTTATCAGGAGGTTCAGGTTTGGTTAGCATGATGTATCCAGTATAGA  21780
Vaquita      GCATG-TTGATTTGGTTC-T-TTTAAGTCTATTTAT--GTCAG-----TT-TTCCTTCTCCCTCCCCCTTTCTTGATTT-  17580
Pig          GCATG-TTGAAATGAT------TAAGGTATAAGTCA--GTTTT-----------CTTCCCCCTCCCCTTTTCTTGATTT-  16015

Majority     -----------ATTCTTTATTAA--XXTTAATCTGX--XGTCAATAATTX-X-T-TTGTTGAGTTTTXCGA-AAXGXTGA
                        24730     24740     24750     24760     24770     24780     24790     24800
Human        CCCTAAGAGACATGCTTAATGCAGAAAATATATTAAAAGGATTTTAAACAGCTTATATGTAAGGGATGAGGGAACAGGGA  21263
PolarBear    --------------------------------------------------------------------------------  0
Cow          ---------AGAATCCTCATGGACAGAGGAGCCTGGC-GGGCTATAGTTCATAGGGTGGAAAAGAGTCAGACACAACTGA  20474
Dog          -----TTTAAAACGATTTATTAATCATTTAAAATGATTAAT-AATGACTGCAGTTTCATTGAGCATTGCAAAAATGGTGA  20554
BelugaWhale  -------------TCTTTGTTAG----AGAAACTG---GGTCATCTATTC------TGTACAATTTTCTGA--------A  16958
Donkey       CCGTTGAGTTGGTACTCTAGCAAACTCT-GGTGTAACCAGTGTCCCTCTTTATTCTTGTTAGTGTCTTTGTGAAGGCAGG  20206
Cat          -TGCTCCCATTATCCTTTCATACCTTCTTGATTTTATTAGTTAATAATTGCAGTTTCGTTGGGTATTGCAAAAATGGTGA  23260
Ferret       ----------AATGCCTTTTAAGTCTCTTTATA-----AAG-AGAAACTGGGTCATCTATGTGATTTTCTACTTTGTTGA  19935
Lion         GTGCTCCCATTATCCTTTCATACCTTCTTGATTTTATTAGTTAATAATTGCAGTTTCGTTGGGTATTGCAAAAATGGTGA  21860
Vaquita      -------------TCTTTGTTAG----AGAAACTG---GGTCATCTATTC------TGTACAATTTTCTGA--------A  17626
Pig          -------------TCTTTGTTGA----G-AATCTG---GGTCTACTGTTC------TGTACAATTTTCAGA--------A  16060
```

```
Majority     TAT-----X-TATACTTX--XXX------GTTXATTA-TACTTTXATGTTGTTAXTTTATATGTTTCTCATACCGTATTT
                     24810     24820     24830     24840     24850     24860     24870     24880
Human        TACTTGGACTAATATTTCAATATTTCTAAAATTATTGGTTTATTGAGTACGTTCCTAGAAAACTGTTTTATTGAAAGCTT  21343
PolarBear    --------------------------------------------------------------------------------  0
Cow          AGCGACATAGCATGCATA--CAAGCA--AGTCTATTAATAGGACAGTTTTGTTTTTTT-TTTTTTTCTCTCCCCCTTTAT  20549
Dog          TATTCTTTTTTACCCCTG--CATATTTTAGTTAGATTTTTCT-----TTTTTTACATCAAATATTTGTCATACTGAAATA  20627
BelugaWhale  AAT-------TATAATT---TT-------GCTGATTA-TACCTTCATGTTGTCATTTTGTGTGTTTCCCATGTCTTGTAT  17020
Donkey       GAT----TTTTACGTTTA--TTT-------TCCAGTCTTATTGCAATGGTTCTTCTTTTCAAAATTCTTGAATTGTTTTA  20273
Cat          TCCACTCCTCCCCCCGCC--CCCGCCTATTTTAGATTTTTCTGTTAACTTTTTACGTCAGTATTTTGTCATACTGGAATT  23338
Ferret       TTTTGGTGACTATACCTC--CATGTC---------------------------ATTTGGTATGTTCCTCATACC------  19980
Lion         TCCACTCCTCCCCCTGCC--CCCGCCTATTTTAGATTTTTCTGTTAACTTTTTACGTCAGTATTTTGTCATACTGGAATT  21938
Vaquita      AAT-------TATAATT---TT-------GCTGATTA-TACCTTCATGTTGTCATTTTGTGTGTTTCCCATGTCTTATAT  17688
Pig          AGT-------CTTACTT---TT-------GCCGATTA-TACCTCCATGTTGTCATTTG-TGTGTTTCTCATATCCTGTAT  16121

Majority     ----TTTCTGT--X----ATACT------------T---TGTAGTT-X----A-XATGX-GCTTXXX--GATT-------
                     24890     24900     24910     24920     24930     24940     24950     24960
Human        CAAATTTCTCAGAATTGTATAGCATTTTTGAATTCTCTCTTTTATTACTAATATCAAGCACCTATGTTTTCTCCTATTTC  21423
PolarBear    --------------------------------------------------------------------------------  0
Cow          TGATTTTCTGTTAGAG--AAACTGGGTCATC-TATTCTGTACAATTTTTTG-AAAATTATGCTTTTGCTGATTTTACCTC  20625
Dog          CCCTTTTTTGTAAAAAACAGGGTAGATTTTGACTTTAAAAACGTTTCCTGATGAGTTGGTACTGTAGCAGCCTCCATTTT  20707
BelugaWhale  ----TTCCTGT-------GTACT----------------TGTAGTT------A-GATGAGGCTTGATTGGATT-------  17059
Donkey       ----TCTTGGGCCAGCAGGAGCT------CA-TGACACAAGTAGCTTT-GATATTTACTTGCTTTGAAGAATC--A----  20335
Cat          CCCTTTTTATAAGAAAACAGGCT------T----CTACATTTATTTCC-------------C------------------  23377
Ferret       --------------------------------------------------------------------------------  19980
Lion         CCCTTTTTATAAGAAAACAGGCT------T----CTACATTTATTTCC-------------C------------------  21977
Vaquita      ----TTCCTGT-------GTACT----------------TGTGGTT------A-GATGAGGCTTGATTAGATT-------  17727
Pig          ----TTCCTG-----G--GTACT------------T----GTAGT--T----A-GATGAGGCTTGATCAGATT---C---  16161
```

```
Majority     ----------------X---------XXAAX------------A--------------------------TT-XXXTG-T
                              24970     24980     24990     25000     25010     25020     25030     25040
Human        TTGATTTGTCTTGTCACAGAATTTATTTTACTGGTCTTTTCAAAGAACCAACCTTTTTACTTGTTTTCTTTTTTGTTGGG  21503
PolarBear    --------------------------------------------------------------------------------  0
Cow          T---------ATGTTGTCATTTTTT-TCTATTCCTCATATCCTTTAT-CTCTTGTGTA---CTTGTAG--TTAGGTAA-T  20688
Dog          AGCCAGTGTGAAGTTTTTGTTGTTA-AGAATCATTGTGATGGCAAGGACTTTTACATTAATTTTCCAGTCTTAGGATGAT  20786
BelugaWhale  ----------------C-----------AG--------------------------------------------------  17062
Donkey       ------------TTTCCTGCT--TC-AGACTTTTCATTAACCATTTCTTTAAAAAGTGCTGGTTTCTTTGGGTGGGAAGT  20400
Cat          --------------------------AGTCT--T---------A--------------------------TTGCAATGAT  23394
Ferret       --------------------------------------------------------------------------------  19980
Lion         --------------------------AGTCT--T---------A--------------------------TTGCAATGAT  21994
Vaquita      ----------------C-----------AG--------------------------------------------------  17730
Pig          ----------AGGTT-TGATTTTTA-GCAA------GAATGTTAAAT-GT--TAAAT-----TTTTAG--CAAGAATG-T  16212

Majority     -AX-CTTXAXXX--X-------XTTTAATTTTGAGCAAGTATGATCTC----ATXACACCTGTAXCTTTGAXAGTXACTT
                              25050     25060     25070     25080     25090     25100     25110     25120
Human        GGCAGTGGAGGGATTCACTTTAATTTGATCCTGAACTCCTTTATTTTCTTTTAAAATGCATTCAAGGTTAAAGGTAATCA  21583
PolarBear    --------------------------------------------------------------------------------  0
Cow          AAGACTTGATCAGAT--TCAA-GTTTAATTTTCAGCAAGAATACTTTGT-AAATGTACGGTGTAATTTCTAATGTATCAC  20764
Dog          CATTCTTTAAAAAGTCTTTG---TTTCATCTAGAGCCAGTGAGAGCTC----ATAACACAGGTAGCTTTGATACTTACTT  20859
BelugaWhale  ----------------------GTTTAATTTTTAGCAAGAATATTTTAT-AAATGTACTCTGTAATTTCTAATGTATCAC  17119
Donkey       GGTCCTTAGAGACCA---C----AATAATCTTGGGCCAGCAGGAGCTC----ATGACACAAGTAGCTTTGATATTTACTT  20469
Cat          CATTCTTTAAGAAGTCTTTAATTTTTCATTTTGAGCTATTGAGAGCTT----ATAACACCAGTAGCTATGATACTTACTT  23470
Ferret       -----------------------TTATTTCTTGAGCCAGTAAGAACTC----GTAACACGGATAGCTTTGATACTTACTT  20033
Lion         CATTCTTTAAGAAGTCTTTAATTTTTCATTTTGAGCTGTTGAGAGCTT----ATAACACCAGTAGCTATGATACTTACTT  22070
Vaquita      ----------------------GTTTAATTTTTAGCAAGAATATTTTAT-AAATGTACTCTGTAATTTCTAATGTATCAC  17787
Pig          TAAATTTTGTT-----------GTTAACTTTTTAGCAAGAATGTT-----AAATGTACTCTGTAATTTCTAATGTATCAC  16276
```

```
Majority     GCTT-GAAGXATCATTTCTTGTTXCAXATTTGTXA-TXGCCATTTCTTTAAGAAAAG---CTGGTTTCTTTGGGTGGGT-
                       25130     25140     25150     25160     25170     25180     25190     25200
Human        GCTG-ATAGCTTTGGCTTTTTTTTTTTTTTTTTACTTTTAGAGTTCTCTTTTTATTG---TAAACTTCATTTAAAGGTTT  21659
PolarBear    --------------------------------------------------------------------------------  0
Cow          CCTA-GAAGGAACATGCCTAGTTATCTTTTTGTGA--TGTT---------------------GACATTTATTAGTAGGTT  20820
Dog          GCTT-GAAGTAGGATTTCTTGTTCCAGACCTTTTGTTGGCCACTTCTTTAAGAAAAT--TCTGGTTCCTTTGGGTGGGT-  20935
BelugaWhale  ACCA-GGAGGAACACGTCTAGTTACCTCTTTGTGA--TGTTTTATCAGTAGGTTCAG-TACTGACATTTATCAGTAGGTT  17195
Donkey       GCTTTGAAGAATCATTTCCTGCTTCAGACTTTTCATTAGCCATTTCTTTAAAAAGTG---CTGGTTTCTTTGGGTGGGA-  20545
Cat          GCTT-GAAGTATCATTTCTTGTTCTAGATC--T---TAGCCATTTCTTTAAAAAAAAATCCTGGTTTCTTTGGGTGAGT-  23543
Ferret       GCTT-GAAGTACCATTTCTTGGTCCAGACCCCT-ATTAGCCATTTCTTTATGAAAAT--TCTGGTTCCTCTGGGTGGGT-  20108
Lion         GCTT-GAAGTATCATTTCTTGTTCTAGATC--T---TAGCCATTTCTTTAAAAAAAA-TCCTGGTTTCTTTGGGTGAGT-  22142
Vaquita      ACCA-GGAGGAACATGTCTAGTTACCTCTTTGTGA--TGTTTTATCAGTAGGTTCAG-TACTGACATTTATCAGTAGCTT  17863
Pig          TCTG-GAAGGAGTATGTCTAGTTACTTCTTTGTG----GT----------------------------------GTAGGTT  16318

Majority     -AGGGGTCCTXAXGAGACTACA-GTAATGT-GAGTGCAAA-XTGCTAATTGTTGTCAGGTGGTCTTTA-TTCAAAGCT-T
                       25210     25220     25230     25240     25250     25260     25270     25280
Human        TAAG---CTACACAAAAGTAGAGGGAATG--GTATATAAT-ACACTTTTTTCTGTTGGATAATATTAATGCAAATCTCAG  21733
PolarBear    --------------------------------------------------------------------------------  0
Cow          CAGGTGTTCTCTGCTTGATGCATCCAGTATAGAGTGCTCC-ATCATCTGTCTGTACTAGTGGGTTTTA----GTAGCT-A  20894
Dog          -AG---TCCTTA-GAGACCAC----AATGT-GGGTGCCAACGTGCTAATTGTTGTAAGGTTGTTACTACTTCAAAGCTCT  21005
BelugaWhale  CAAGTGTCCTCAGCTTGATGCATCCAGTGTAGAGTGCTCTTATCATCC-TTTCACCTAGTGGGCTTTA----GTAGCT-G  17269
Donkey       -AGTGGTCCTTA-GAGACCACA-ATAATGT-GGGTGCAAAGGTGCTAATTGTGTTCAGGTTGTCTTTGCTTCAAGGCCCT  20621
Cat          -AGTGGTCCTTAAGAGACTACC-GTAAGGT-GGGTGCAAAGGTGCTAATTGCTGTTAGGTGGTCATTACTTCAAAGGCCT  23620
Ferret       -AGTGGTCCTTA-GAGACCACA-GTAATGT-GAGAGCAAAGGTGTTAATTGCTGTTAGGTTGTTACTACTTCAAAGCT-T  20183
Lion         -AGTGGTCCTTAAGAGACCACA-GTAATGT-GGGTGCAAAGGTGCTAATTGCTGTTAGGTGGTCA----TTCAAAGCCCT  22215
Vaquita      CAAGTGTCCTCAGCTTGATGCATCCAGTGTAGAGTGCTCT-ATCATCC-TTTCACCTAGTGGGCTTTA----GTAGCT-G  17936
Pig          AAGGCCTCCTCAGCTGGATGTATGGAGTAGAGAGTGCCAT-ATCATCC-TTTCCCCTAGTGGGTTTTATTTAGTAGTT-G  16395
```

```
Majority     TTXAGTGAATAAAGCXAGXAGXTATTTXAAAA---AXX----X-TGTGAGXTCATTT-TTATTTCCAGXATA--TTTTAC
                              25290     25300     25310     25320     25330     25340     25350     25360
Human        ATCATTTCATATAATCTGCAGATATATCAGAATGTGTGTCTCTCTATTAAATGTGACATTGGTACCATTATAAAGTTTAA  21813
PolarBear    --------------------------------------------------------------------------------  0
Cow          ATAATTGCATAATTCCACTGGGTATTGCAAAA---ATG-------GTGATAC------TCTTCTCCTGCATA--TGTTAC  20956
Dog          TTTAGTAGATTAAGCTAGCAG-TGTTTTGAGG---GCA-GATACTTTGGGCTCATTTGTTATTTCCAGTTCGTATTTTAC  21080
BelugaWhale  ATAATTGAGTAATTTCATTGGGTATTGCAAAA---ATG-------GTGATATTATTT-TTTTCTCCTGCATA--TATTTT  17336
Donkey       TTCAGTGGACAGAGCTAACAAAT-TTATAGAA---ATA---CATTGTAAGCCTGT---GTATTTCCAAT-------TCAT  20684
Cat          TTTAGTGAACAGAGCTAGCAAATAATTTGGAG---ACATAATATTGTGAGCTCATTTGTTATTTCCAATTTA-ACTTTAC  23696
Ferret       TTTAGTGGACAGAGCTAGTAG-TGTTTTGAAG---ACA-AATACTGTGAGCTCATTTGTTATTTTCAGCTTATATTTAAC  20258
Lion         TTTAGTGAACAGAGCTAGCAAATAATTTGGAG---ACATAATATTGTGAGCTCATTTGTTATTTCCAATATA-ACTTTAC  22291
Vaquita      ATAATTGAGTAATTTCATTGGGTATTGCAAAA---ATG-------GTGATACTATTT-TTTTCTCCTGCATA--TATTTT  18003
Pig          GTAACTGCATAATCTCAGTGGGTCTTACAAAA---ACG-------GTGGTAG------TCTTATCACCC-------CTGC  16452

Majority     -------TTTTTAT-TTTTCTGA--TTTXXTATTX-GXACCTXTTGTATXAATTATXTAATTTTGCTT-XTAAXACTAX-
                              25370     25380     25390     25400     25410     25420     25430     25440
Human        AATAATTTCTTAATGTCGTCTAATCTCCAGTATTCAGAATACTTTCTTTTCATTATTTTATTTTTCTTGCCTTTTCTTT-  21892
PolarBear    --------------------------------------------------------------------------------  0
Cow          -------TTAGTAT-TTTTCTA---TTCAGAA----GAACTTTTCCTCTCAAATATTTGGTTTTACCA--AAACACCCT-  21018
Dog          ------ATTTTTAT-TCTTCTGA--TTTTATATTT-GTATCTCTTGTACG--TTGCATAATTTCGCTT-TTAATATTAA-  21146
BelugaWhale  -------TAAATAT-TTTTCTG----TTGGAA----GAACTTTTTTCGTCAAATATTTAGTCATACTG--AAACACCCT-  17397
Donkey       -------ATTTAAT-TATACAA----CTTATATTT-GTGTGTCTTGTACAT-TGAAAAAATCTTGCTTGCTAATGTTAA-  20749
Cat          -----AGTTTTTAC-TTTTCTGA--TTTTATATTT-CTACCTCTTGTATAAATTGAATAATTTTGCTTGCTAACATTAAA  23767
Ferret       TTTATAATTTTTAC-TTTTCTGA--TTTAATATTT-GTACCTCTTGTACA--TTGAATGTTTTTGCTTGCTAATAGTAA-  20331
Lion         -----AGTTTTTAC-TTTTCTGA--TTTTATATTT-CTACCTCTTGTATAAATTGAATAATTTTGCTTGCTAATATTAAA  22362
Vaquita      -------TAAATAT-TTTTCTG----TTGGAA----GAACTTTTTTCATCAAATATTTGGTCATACTG--AAACACCCT-  18064
Pig          -------ATGTTAT-TCTTCTAA--TTTAGCA----GAACATTTTTCATCAAATATTTGGTCATACCA--AAACACCTT-  16515
```

```
Majority     ---------TATTAAXAGATATXTAGACTAGXTTTTTA--TAAAAAAAXXTXACTATTAAACTGXT--ATTTTAATTATA
                      25450     25460     25470     25480     25490     25500     25510     25520
Human        -------TTTGTT-GTTGTTAGATAAAGTGAGTCATC---TGTACAATTTCCACTTTCTGGCTGGGC-GTGGTGGCTCAC 21960
PolarBear    -------------------------------------------------------------------------------- 0
Cow          ---------TATTAAAAGAAAAACAGGAGAGATTTTTG---CTAAAAATTTTCCTTATGAGCTGCT--AACTCTAGCCTA 21084
Dog          ----CAA--TAT---TACATATGTATACTAGTTTTTTG--AAAAGGAGCATCAATATTATACTGAT--ATTGTAATTACT 21213
BelugaWhale  ---------TACCAAAAGAAAAGCAGGGTAGATTTTCAGTTAAAAATTTTTTCCTCATGAGCTGGT--AACTCCAG--TA 17464
Donkey       ---------CATTATTACATGTATATACTAGGTTTTTAAAAAGTATTACACTAACATTGTAATTGTTGAATATAGTTTTA 20820
Cat          ATTGTAATATATGTACATATATATACACTAGTTA------AAAAGGAACATCAGTATTACACTGAC--ATTATAATTACT 23839
Ferret       ----CAAAATAC---TGCATATGTATACTAGTTTTTTT--TAAAGGAACATCAGTACTACACTGAC--ATTGTAATTACT 20400
Lion         ATTGTAACATATGTACATATATATACACTAGTTAGTTA--AAAAGGAACATCAGTATTACACTGAC--ATTATAATTACT 22438
Vaquita      ---------TATCAAAAGAAAAGCAGGGTAGATTTTCAGTTAAAAAATTTTTGCTCATGAGCTGGT--AACTCCAG--TA 18131
Pig          ---------TATTAAAAGAAAAGCAGGATAGATTTTTCACTTCAAAAATTTTTACTTTAAAAAGCC--A-CTCCACTGTA 16583

Majority     XACTACAGTTTAAXGT---T---TCTTTGAAGTTXATXTTGT------CTTATAGTGXAGXCACAGAATTTTAAAGTTTA
                      25530     25540     25550     25560     25570     25580     25590     25600
Human        GCCTGTAATCCCAGCA--C----TTCGGGAGGCTGAGGCGGG-----CAGATCACGAGGTCA-GGAGATCAAGACCATT 22027
PolarBear    -------------------------------------------------------------------------------- 0
Cow          AACTCCAGTACAACGTGAAT---TCTTTATTGTTGATGAAAGT----GTTATTTTGAAGGCAAAGATTTTTACATTTAG 21156
Dog          GACGATAGGTAAAGGT---T---TCTTTGTGGTTATTTTTGTT----CTTACAGTATAATTACAGAATTCCAAAGTTTA 21282
BelugaWhale  A-CTCCAGTGTAATGTGAGT---TCTTTAACATTGATGTTAG-----GTCGTTGTGAAGGCAAGGATTTTTACGTTTAT 17534
Donkey       AGATTTCTTTGCAGTTATTTTTATCCTTAAAATGTATTCCACGAAGAGTTTATAGTATAGTTACAGAATTTTAAAGTTTA 20900
Cat          GAATACAGTTTAAACT---T---CCTTTGCAGTTATTTTTGTC----CTTATAGTATAAAAACAGAATATCAAAGTTTA 23908
Ferret       GAATATAGTTTGAGGT---T---TCTTTGCAGTTATTTTTGTC----CTAATAGTGTAATTACAGAATTCTGAAGTTTA 20469
Lion         GAATACAGTTTAATGT---T---TCTTTGCAGTTATTTTTGTC----CTTATAGTATAAAAACAGAATATCAAAGTTTA 22507
Vaquita      A-CTCCAGTGTAATGTGAGT---TCTTTAACATTGATGTTAG-----GTCGTTGTGAAGGCAAGGATTTTTACGTTTAT 18201
Pig          A-CTACTGTGCGTTCT-------TTGTTGTTGTTCATGTCTTT----CTCGCCATGAAGGCAAGGATTTTTACATTTAT 16650
```

```
Majority     TTGACAGAXTTXTTXCXGTGGTTATTCXACTAAXAXXAT--TTAGXTTTXXTXGTTTCXTXTXGGTTXXGAXTTTTAATX
                               25610     25620     25630     25640     25650     25660     25670     25680
Human        TTGGCTAACACAGTGAAAACCTGTCTCTACTAAAAATACGAAAAAATTAGCCAGGC--GTGGTGGGGGGCACCTGTAGTC  22105
PolarBear    --------------------------------------------------------------------------------  0
Cow          T-------CTTATTGCAGTGATCATTTCTTTAAAAATGT--TTGAATTTTCTCATCTTGCGCCATCAGCAGCTCATAATA  21227
Dog          TTGAGAGATTTCTCT-TGTGGTTGTACTACCAAGTTGA----TAGGTTTAATGGTTTCATTTTGGTTTGGATTTTTAGGG  21357
BelugaWhale  T--CCAGTTTTATTGCAATGATCATTCCTTTAAAAATGT--TTAAATTTTCTC-----GGGCCAGCAGCAGCTCATAATA  17605
Donkey       TTGAGAGAATTCTATTTGTAGTTCTCCCACTAAGCTGATAGTTAGGTTTATTTGTTTAATTTTGGTTTTGATTTTCACTT  20980
Cat          TTGAAAGAATTCTGTGTGTGGTTCTACTACCAAGCTGA----TAGGTTTCATGTTTTCATTT-GGTTTTGATTTTTAGGG  23983
Ferret       TTGAGAGAATTCTATGTGTAGTTGTACTACCAAGTTGATAGTTAGGCTTAATGGTTTCATTTTGCTTTTGATTTTTAAGG  20549
Lion         TTGAAAGAATTCTGTGTGTGGTTCTACTACCAAGCTGA----TAGGTTTCATGTTTTCATTT-GGTTTTGATTTTTAGGG  22582
Vaquita      T--CCAGTCTTATTGCAATGATCATTCCTTTAAAAATGT--TTAAATTTTCTCATCTTGGGCCAGCAGCAGCTCATAATA  18277
Pig          TTTCCAGACTTATTGCAAT-ATCAGTCCTTAAAAAATGT--TTACATTTG-TCATCTTGGGCCAGCAGCAGCACATAATA  16726

Majority     XXTGTAATTTTG-X------AXCTXAAGXATAATTTTTTCXACTXAAAXTTAAATTA-XCTACATTTATAXAAATC---G
                               25690     25700     25710     25720     25730     25740     25750     25760
Human        CCAGCTACTCGGGAGGCTGAGGCAGGAGAATGGTGTGAACCCGGGAGGCGGAGCTTGCAGTGGCGCCACTGGACTCCAGC  22185
PolarBear    --------------------------------------------------------------------------------  0
Cow          TCACTAGTTTTGCT--CTTTACCTAAAGTATTATTTTTTTCTCCAAAATTTTAGTTA-GCTG---------GAAAT---C  21292
Dog          ATTCCACC------------------------------CAAGTGAAAAGTAAA----ACTCCATTTATAATGATC---G  21399
BelugaWhale  CCGGTAATTTTGAT--ACTTACCTTAAGTATCATTTCTTCCTCCAAAACTTCAATTA-GCCATTTCTTT--AAAAA---G  17677
Donkey       TTGGCTAAATT------T---TCCAAAGCCAAAACTGTAAAAAAAAAAAAAAAAAAAAAGCCACGTTGATAGAAGTA----  21047
Cat          ATTTTTTTTCCCCC--TAACATTTTTGGCTAAATTTTCCCAAATCAAAAGTAAAATAAACTACATTTATAGAAGTCT-GG  24060
Ferret       ATTCCCCC------------ATCTTTGGCTTAATTTTCCCAAGTGAAAGTAAAAC-AAACTACATTTATAATGATC---C  20613
Lion         ATTTTTTC-CCCCC--TAACATTTTTGGCTAAATTTTCCCAAATCAAAAGTAAAATAAACTACATTTATAGAAGTCT-GG  22658
Vaquita      CCGGTAATTTTGAT--ACTTACCTAAAGTATCATTTCTTCCTCCAAAACTTCAATTA-GCCATTTCTTT--AAAAA---G  18349
Pig          CCAGTAGTTTTGAT------ACCTA--GTATCATTTGTTCCTCAGAGACTTTAATTA-GCCATTTCTTTAAAAAGC---C  16794
```

```
Majority     XTAGGTCTTXTTGTCXXXXCTXGTXTTCTGTGXCTATACCAXTATAGGTGXX---GTTTXCATTAATTTTX--GGTTTGT
                      25770     25780     25790     25800     25810     25820     25830     25840
Human        CTGGGTGACAGAGCAAGACTCCGTCTTAAAAAAAAAAAAAAAATTCCACTTTCTGGATTCTGCTGATTGTACCGCTGTGT  22265
PolarBear    --------------------------------------------------------------------------------  0
Cow          ATGGGTCCTTTAGTGGGACCTGGTCTTTAGAGACAATAATAATGTGGGTGCAAAGGTGCCAATGTCTTTTA--GGTTTGT  21370
Dog          TTAAA--TTCTTATCCACTCTAATTTGCTTTTTCTATCCCATTATAGCT------ATTTTCATTAATATTT--GGTTTAT  21469
BelugaWhale  AATGGTCCTTTGGTGAGACCTGGTCCTTAGAGACCACAGTAATGTGGGTGCAAAGGTGCCAATTGCTTTTA--GGTT-GT  17754
Donkey       -------TAGTTGTCTACTCTCCTTTGCTTTTTCTCTCCCCTTA---GT------GTTTTCACTAATTTTT--GGTTTCT  21109
Cat          TTAATACTTCCTGTCCACTCTCCTTTGCTTTGTCTATCCCATTATAGGTGTC---ATTTTCATCAATTTTT--GGCTTAT  24135
Ferret       TTAAA--TTCATGTCTACTCTAATTTTCTGTTTCTATACCATTATATC-------ATTTTCATTAATTTTT--GGTTTTA  20682
Lion         TTAATACTTCCTGTCCACTCTCCTTTGCTTTGTCTATCCCATTATAGGTGTC---ATTTTCATCAATTTTT--GGCTTAT  22733
Vaquita      AATGGTCCTTTGGTGAGACCTGGTCCTTAGCGACCACAGTAATGTGGGTGCAAAGGTGCCAGTTGCTTTTA--GGTT-GT  18426
Pig          ATGGGTCCTTTGGTAGGACCTGGTCCTTGGAGACCACAATGATGTAGGTGCACAGGTCCCAAT-TGCTTTA--GGTT-GT  16870

Majority     TXTTXTTTCXAG--ATGTXTTXXAATXTXAAAXGCTTXCTACATTCTCXT---TCCXATXAAXXTAXAGAGAX---GTTX
                      25850     25860     25870     25880     25890     25900     25910     25920
Human        TGTCATTTCATA---TGTTCTTCATGTAAACAAGTTTACCTGAAATACAGGGGTTTGATCAGACTCTGGTGTAATTTTTA  22342
PolarBear    --------------------------------------------------------------------------------  0
Cow          TATTGTTTCCAA--ATTTTTT-CACTTGAGAAAGCTAATAAAATA------AGACATATATATCTGTGTTCAT----TTC  21437
Dog          CCTTCTTTATCG--A-ATATAAACATGTGAATTGCTTCCTGCATTCTTTC---ACCTGGGCAGTGAAAGAGAA--AGAGG  21541
BelugaWhale  TATTGTTTCAAG--GTTTTTTTCAATTCACAAAGCTAACAAAAATTTTTTGAGACAAATGTATTTGAACTCATTTATTTC  17832
Donkey       TCTTTTATTTAA---TGTAAGCAAATGTGAATTTCTTCCTACATCCTCCC---TCCTACTACCTTAGAGAAAAAGTGGCA  21183
Cat          CCTTCTTTTGTGTAATGTAAACAAATGTGAATTGCTTCCTACATTCTCCT---TCCCATCACCCTAGAGAGA----GTGA  24208
Ferret       TACTCTTACGCG--ATATAAACAAACGTGAACTGCTTCCTACATTCTCCC---TCCCATCACCCTAGAGAGAA--AGTGG  20755
Lion         CCTTCTTTTGTGTAATGTAAACAAATGTGAATTGCTTCCTACATTCTCCT---TCCCATCACCCTAGAGAGA----GTGA  22806
Vaquita      TATTGTTTCAAG--ATTTTTT-CAATTCACAAAGCTAACAAAATTTTTTTGAGACAAATGTATTTGAACTCATTTATTTC  18503
Pig          TGTAGTTTC-AG--GCCTTTT-CAATTGACAAAACTAACCAGATTTTTTTCAGGTAAGTGTATTTGAGCGCAT----TTC  16942
```

```
Majority     CAGTGTATACAXAATXTTACAGCTTTXCCT--TCCTGTTTATTGGTAAGTATATCCTGXAXAT---TGCATGGTXTTGCT
                      25930     25940     25950     25960     25970     25980     25990     26000
Human        CCAAGAATATTTTGCAGATGTACTCTATGTAATTCCTGTTGTATGTCAGCTTGCTCCATCTATTACAGAGCGCATCATCC  22422
PolarBear    --------------------------------------------------------------------------------  0
Cow          CAATTCGTACATAATTTTACTGTGTTTCCTTTTCCAATTTTTTTGTTGGTATCTCTTGTAGGT---TGAAAATTCTTGCT  21514
Dog          CAGTGTATTCAGAGTATTAATGCTAAG--------TGTATCCTGGAAATCACACCATAGCTA----TACATGGACAT---  21606
BelugaWhale  CAGTTCGTATGTAATTTTACAATTTTTCCTCTTCCAGTTTTTT-GTTTGTATCTCTTGTATGT---TGTAAACTCTTGCT  17908
Donkey       CAGTATACACAGGGTTTTATATCTTGCCTT--TTCTGCTTAATGATAAATATATCCTGGAGATCACGCCATTGCTATATA  21261
Cat          CAGTGTATACAGGTTATAACAGCTACAACT--TCCTTTTTAATGATAAGTTTATCCTGGAGATCACTCCATGGCTGTACA  24286
Ferret       CAGTGTATACAGACTATAACAGCTATAACT--TCCTTTTTAATGGTAAGTGTATCCTGGAAATCATTCCATGGTTAT---  20830
Lion         CAGTGTATACAGGTTATAACAGCTACAACT--TCCTTTTTAATGATAAGTTTATCCTGGAGATCACTCCATGGCTGTACA  22884
Vaquita      CAGTTCGTATGTAATTTTACAATTTTTCCTCTTCCAGTTTTTT-GTTTGTATCTCTTGTATGT---TGTAAACTCTTGCT  18579
Pig          CAATTCATATTTAATTTTATGATTTTTCCTATTCCAGTTTCTT-GTTTGTATCTTTTGTACAT---TGAAAACTCTTGCT  17018

Majority     ------------TXTTGTTATTTTTXTTTTXTTTXTACTXCTGTATAGAAAATACATGAGTGXXTCT-----ATTCXCAA
                      26010     26020     26030     26040     26050     26060     26070     26080
Human        TTTCACCTAATAGTTTTTAGTAGCTAATGATTGCATACTT-TTATTAGGTATTGCAAAAGTGGCGCTATTCTTTTTTTCT  22501
PolarBear    --------------------------------------------------------------------------------  0
Cow          AA-------------TGTTAACATAATTGCATATATATTT-CTTTAAAAAGCAACATCAGTGGTAACATTGTAGTATATT  21580
Dog          ------------TTTTTTTTTTTTTTTTTTTTGTGTATTGCTGTATAGAAAGTATATGAGTACCTCT-----GTTCCCAA  21669
BelugaWhale  TGC---------TAGTGTTAACATAATTGCATTTATACTAGTTTCTGTAAAAAGCAACATCAGTGTT-----AACATTAA  17974
Donkey       ------------TGGAAAATTTTCTCATTTCTTTGTCCTGCTGAATAGAAAGTACGAGTGTGCTTCT-----CTTCCCCA  21324
Cat          GGGAAATTTTGTCTTTTTTTTTTTTTTTTTTTTTGTACTGCTGTATAGAAAATATATGAGTGCCTCC-----ATTCCCGA  24361
Ferret       ------------AAATGGAATTTTTTTTTTCCTGGTACCACTATATAGAAAGTACATGAATGTCTCG-----GTTCCCAG  20893
Lion         GGGAAATTTTGTTTTTTTTTTTTTTTTTTTTTTTGTACTGCTGTATAGAAAATATATGAGTGCCTCC-----ATTCCCGA  22959
Vaquita      TGC---------TAGTGTTAACATAATTGCATATATACTAGTTTCTGTAAAAAGCAACATCAGTGTT-----AACATTAA  18645
Pig          ---------------TGCTAATGT--TTACATTTATTGCA-TGTATACTAGTTTTTTTTTTTTTTTTTAAGAGACATTAA  17080
```

```
Majority     TAGXGTXTTAAATTTXT--AGAATA-TATTGTTCAGTXATTXGGGTTTTGCAAGTCTAATTTATGAXXTXGCAXCXGTAT
                      26090     26100     26110     26120     26130     26140     26150     26160
Human        GTGTATATTAGAATTCTTAAGAATTATATCCATCAACTGTTTGGTCATACGTAAGTACCTTTTTATAAGAAAAGCAGGAG 22581
PolarBear    -------------------------------------------------------------------------------- 0
Cow          TTTTGTAATATAATTTT--AAGATT-TGTTTTGCAATTATTTTTGTCCTCAAAGTGTATTTCATGAAAAATGTATAGTAT 21657
Dog          TAACCTGTCCAAT------AGAATA-TATTGTTCAGTGATTGGAGTTTTGCTAGTCTGATATATGAGATGGCACCTTTAT 21742
BelugaWhale  CATTGTAATACAGTTTT--AAGAT---GTTT-GCAGTTATTTTTGTCCTTAAAGTGTATTTTGCAAAGAATGTATAGTAA 18048
Donkey       TAGCCTGCCCAAC------AGAGTA-TACTGTTAAATGATTGGGGCTTTGCCACTCTGATGGGTGAGATGGCATCTAAGT 21397
Cat          TAGCTTGTTGAAT------AGAGTA-TATTGTTCAGTGATTGGGGTTTTGCCAGTCTGATAGATGAGGTGGCACCTAAAT 24434
Ferret       TAGCCTGTCCAATTTTT--AGAGTA-TATTGTTCAGTGATTGGGGTTTTGCTAGTCTAACACATGAGATGGCAC-TTAAA 20969
Lion         TAGCTTGTTGAAT------AGAGTA-TATTGTTCAGTGATTGGGGTTTTGCCAGTCTGATAGATGAGGTGGCACCTAAAT 23032
Vaquita      CATTGTAATACAGTTTT--AAGAT---GTTT-GCAGTTATTTTTGTCCTTAAAGTGTATTTTGCAAAGAATGTATAGTGA 18719
Pig          CATTGTAATATAGTTTT--AGGATT-TGTTC-ACAGTTATTTTTGTCCTTAAAATGCATTTCGCAAAGAATGTATAGTAT 17156

Majority     --AGTATTAATTXGAAT--XTTTT-T--TXTXA-CAAGAT-AAAC-XC--TTTTC----AGGTTAATTT--------TGT
                      26170     26180     26190     26200     26210     26220     26230     26240
Human        AGAATTTGACTTCTTAAGTTTTCTGATGAGTTGTTACCCTAAAACCTCCAGTATAATCAATATGAGTTCCTTTTTGTTGC 22661
PolarBear    -------------------------------------------------------------------------------- 0
Cow          TGGGTTTGCCAACAGGTTCATTTGTTTTTTTCCATAAGATGACTCTAGTATTGCT----TAGTTATCTT--------TGT 21725
Dog          T--GTATTAATTTGAAT--GTTTT-TATTGTGAGCAAGATAAAAACAG--TTTTC----AGTTTAATCT--------TCT 21803
BelugaWhale  --AATC----A-CAG----AATTTTT----------AAAT----------CTATT----GAGATAATTC--------TGT 18085
Donkey       CTAGTATTAATTTGAATGTGTTTT-TAATGTGAGCAAGATTAAACAGCTTTTCACTTGAAGGATAATTTCCATTTCATGT 21476
Cat          GTAGTATTAATTTGAATTTTTTT----CTATGATCAGGGTTAAACAGC--TTTTC----ATATTAATCT--------TTT 24496
Ferret       T--GTATTAATTTGAATTGGTTTT-TATTGTAAGCAAGATGAAATGGC--TTTTC----AGTTTAATCC--------TTT 21032
Lion         GTAGTATTAATTTGAATTTGTTTTGTTCTATGATCAGGGTTAAACAGC--TTTTC----ATATTAATCT--------TTT 23098
Vaquita      --AATC----A-CAG----AATTTTT----------AAAT----------CTATT----GAGATAATTC--------TGT 18756
Pig          --AGTT----A-CAG----AATTT----T------AAAGT-------CTATTGCC----AG---AATTT--------TGT 17193
```

```
Majority     GCCXAG-----------------TTTTTTAA-XXXGXAACTAGTCTTTCTAXATTTTTATAA-CAXTCTGCATTT----X
                       26250     26260     26270     26280     26290     26300     26310     26320
Human        TGTTAGTATCGTCGCAACAGCAAAGAGTTTAATAACATTTATTTTCTAGTGTATTGCAGTAATCATTCTTCTTTTTTTTA  22741
PolarBear    --------------------------------------------------------------------------------  0
Cow          ATTAAC-----------------TTCAGTTGAAACAATTTTGGTAGTTGTTTTTTTTTA--A-CTTTGTTCACTTGAAAC  21785
Dog          GCCTATATAT-------------TTTTTTTTAATGGAGTAACTGGTCTTTCTAAATTTTCACAG-TAGTCTGTATTT----G  21867
BelugaWhale  GTGCGG-----------------TTCTACC---ATCAAGCTAATAGTTAGGTTTATTTGTTA-CATTTTGGTTTT----C  18140
Donkey       ACTGAGAACTAATCTTTTGCCTGTTTTTTTAAAGAGTAACTTGTCTTTCTAAATTTTCATAA-TGGTCTGTATTT----C  21551
Cat          GCCCAG----------------TTTTTTAAATGGGGTAACTAGTCTTTCTAAAATTTTATAA-TCGTCTGCATTT----G  24555
Ferret       GCCTAT-----------------TTTTTTAAGGGGGTAAT--GTCTTTCTAAATTTTCTCA---AGTCTGTATTT----T  21086
Lion         GCCCAG----------------TTTTTTAAATGGGGTAACTAGTCTTTCTAAAATTTTATAA-TAGTCTGCATTT----G  23157
Vaquita      GTGCGG-----------------TTCTACC---ACCAAGCTAATAGTTAGGTTTATTTGTTA-CATTTTGGTTTT----C  18811
Pig          GTGTGG-----------------TTCTACC---ACCAAATTGGTAGTTAAGTTTGTT----A-CATTGTGGTTTTAAGAG  17248

Majority     AAXTTTAX--ATTXTXTTTTTGATCXAAG-----TXATT-TAXGAACTATTTCAAAAGTTCATXT-ATAAAACTAXXAAA
                       26330     26340     26350     26360     26370     26380     26390     26400
Human        AATTTCTAAGCTGTTTTATTAAATGAAAAGAGAACAATGCTAAGCAGCTTGTATGGTGTGTGTGTTGTGTGGGTTTTTAT  22821
PolarBear    --------------------------------------------------------------------------------  0
Cow          AATTTTGTTAGATTGTATTGTGACAGCTG-----TCATA-TCAGCATGCATTTTAAAGAAAACTTATTAAAATTGGTGAA  21859
Dog          AAGTTCAT--AAGTACTTTTTGATCAAAG-----TTATT-TAGAAACTATTTGAAAATTTCATCTGATTAAACTA-----  21934
BelugaWhale  ATTTTTA--GGGAATTTTTTTCATG-------------T--TGAATAAATTTTCCAAAG--------TCAAAACTGCAAAA  18196
Donkey       AAGTTTA--GATTCTCTTTTTGACCCAAG-----TCATT-TAGGAACTATTTCAAAAGTTCTTCTAATGAAACTAAAATA  21623
Cat          AAGTTTAT--ATTCTCATTTTGATCCAAG-----TTATA-TAGGAACTATTTCAAAATTTCATCTGATTAAAACATTGAA  24627
Ferret       AAGTTTAT--ATGCTCATTTTGATCCAAG-----TTATT-TGGGAACCATTTCAAAATTTCATTTAATCAATCTA-----  21153
Lion         AAGTTTAT--ATTCTCATTTTGATCCAAG-----TTATA-TAGGAATTATTTCAAAATTTCATCTGATTAAAACATTGAA  23229
Vaquita      ATTTTTA--GGGAATTTTTTTCATG-------------T--TGAATAAATTTTCCAAAG--------TCAGAACTGCAAAA  18867
Pig          AGTTT-A------TGTTTTTTCAC-------------TT-TGACTAAATTTTCCAAAG--------TGAAAACTGAAAAA  17299
```

```
Majority     --------------CAA-TTACA-T----XATGGATGTXTXXTCXACXXTTXCTGTXCAXTCTACTTTAXTATTTCAXX-
                      26410     26420     26430     26440     26450     26460     26470     26480
Human        TTTGTATGAATGTTAAAAACACGGTGGCTAAAGCCTGTAATCCCAGCATTTTGGGAGGCCAAGGTGGTTGGATCACAAGG 22901
PolarBear    -------------------------------------------------------------------------------- 0
Cow          TTTTTGTGCAG--CCATTTTACAGTTGAGGATGGAAGGGAAAGCAACATTTTCGGCATATTATGCTTTATTATTTCAAGG 21937
Dog          -------------------------------------------------------------------------------- 21934
BelugaWhale  --------------CAAACTACATT----CATAGATGTCTAGTCTTTCACTCCTGTCCACTCTACTTTGCTTTTTCTGT- 18257
Donkey       GTTGAGTTTCGGTCTGTTTTTTTATTTTCTCTCTTCTTTTCATTAAAATGTCTTACTGCGTCTGACCAAATATTATGGTC 21703
Cat          TTTTCAGAGTG--CTGCCTGGGTAGCTCAGCTGGTTAAGTGTCTGACTCTTTGATTTCAGCTCAGGTCATGATCTCAGGG 24705
Ferret       -------------------------------------------------------------------------------- 21153
Lion         TTTTCAGAGCG--CTGCCTGGGTAGCTCAGCTGGTTAAGTGTTTGACTCTTTGATTTCAGCTCAGGTCATGATCTCAGGG 23307
Vaquita      --------------CAAGCTACATT----CATAGATGTCTAGTCTTTCACTCCTGTCCACTCTACTTTGCTTTTTCTAT- 18928
Pig          --------------CAAGCTACA-T---GCATAGAAGTCTGGTCTTCTACTCCTGTATATTCTACTTTGCTTTTTCTAT- 17360

Majority     ---X-A------------X-----------C--X-X--X---C----TCT-----------------TATAGAXAT-TTA
                      26490     26500     26510     26520     26530     26540     26550     26560
Human        TCAGGAGCTTGAGACCAGCCTGGCTCTACTAAAAATAAAATGTG-GTGAAACCCCGTCTCTACTAAAAATACAAAAATTA 22980
PolarBear    -------------------------------------------------------------------------------- 0
Cow          AAGGTAAAATGCAAC---TGAAAC-----ACAAAAAACGAC-T----TGTGTAGT-----------ATATGGAGAAGGTG 21993
Dog          -------------------------------------------------------------------------------- 21934
BelugaWhale  ------------------------------C-----------C----CCT-----------------TATAGGTAT-CTA 18274
Donkey       TATACAGTTTTTTCTTTATGGAATTTATTGAGGTATTCATTACATAGTATGTGATCTACTTTTGTGACTTGTAGATATTT 21783
Cat          TCGGGAGATCGAGCCTCTTTCTGCCCCTTACCCACTGTATCTC----TCTCTATTTAAAAAAAAAAAAAAAAAAAGGTAA 24781
Ferret       -------------------------------------------------------------------------------- 21153
Lion         TCGGGAGATCGAGCCTCTTTCTGCCCCTTACCCACTGTATCTC----TCTCTATT-----AAAAGAAAAAAAAAAGGTAA 23378
Vaquita      ------------------------------C-----------C----CCT-----------------TATAGGTAT-CTA 18945
Pig          -AGGTATA----------TAGAA---------AAATATATC-T----TCT-----------------TATAGATATGTTT 17398
```

```
Majority     -X-TA-AATAGTTGAATTTTXTTT-ATTCTT--TTAXCCAAXTGAXCAAAATGT--GAATTTGTTTGAGCAXXTATGGTT
                       26570     26580     26590     26600     26610     26620     26630     26640
Human        GCCCTTTGTTGTTGCACGCGCCTATAGTCCCAGCTACTCGGGAGGCTGAGGCAGAAAAATTGCTTGAACCTGGGGAGGCT  23060
PolarBear    --------------------------------------------------------------------------------  0
Cow          CTGTG-CCTGGTCAAATGTGTCAAAAGTGGT--TAATCGAAGTTTTCATGCTGG--GGATTTTTTGCTGGATGAGTAGGT  22068
Dog          ----A-AACAATTTAATTTTTTTCT-ATTTTT--TTCCCC---TCATCAAAATGTCTTACTGTGTTTGAGCAAATATGGTT  22003
BelugaWhale  TTTTC-ACTAGTT---TTTGGCTT-ATTCTT--TTATTTAATAGAAGTAAATGT--GAATTTCTTTTTACGTGCTAGCTC  18345
Donkey       AAATACAGTGGGTAAATTGTATATAACTGAA--ATATGCAAGTGCGGAATTTAAAAAACCATGGTACACCA-ATGTACTA  21860
Cat          AATAA-AACAATTGAATTTTGTTC-AATTTT--TTTCCCC--T---CAAAATATCTTATTCTGTTTGAGCAAATATGGTT  24852
Ferret       ----A-AACAATTGAATTTTTATT-TTTCTT--TTTCCCCATCAAACAAAATGTCTTACTGTGTTTGAGCAAATATGGTT  21225
Lion         ------AACAATTGAATTTTGTTC-AGTTTT--TTTCCCC--T---CAAAAT-----ATTCTGTTTGAGCAAATATGGTT  23439
Vaquita      TTTTC-ACTAGTT---TTTGGCTT-ATTCTT--TTATTTAATAGAAGTAAATGT--GAATTTCTTTTTACGTGCTAGCTC  19016
Pig          TTATT-AATGTTTGG-TTTATTTTGACTCTT--TTATTTAATAGATGAAAATGT--GGATGTCTTTCTGCATGCCCCCTC  17472

Majority     TGAACAATTTAGTCTXT------ATGGAGXATATTGAGXTATTCATTXCXTA--GXTTATATTXTGCCTTTGTGXXTTTA
                       26650     26660     26670     26680     26690     26700     26710     26720
Human        GAGGCAGAAAAATTACT-------TGAACCCGG--GAGACAGAGGTTGCAGCGAGCCGAGATTGCGCCACTGCACTCCAG  23131
PolarBear    --------------------------------------------------------------------------------  0
Cow          TGGGTAGACCAGTTGAAGTCCATAGTGATCAAATTGAGACATTCATTGAGAACAGTTGATATTACACCATGTGGGAGATA  22148
Dog          TGTACAATTC-TTCTTT------ATGGAGTTTATTGAGTTACTCATTACAGA--GATTATGATCTGCTTTTGTGACTTTC  22074
BelugaWhale  CCACCATCTTAGGGAAA------AGGTAGCACA-----GAATACACAGGGT----TTAATATCTTACC-TTTTTTACTTA  18409
Donkey       GAATGCTATGAAGCTGTTGATAAATGAAGTAGATTA--GTATGTACTGAATT---AAAAAATCTTTAAGCTTTGTTATTA  21935
Cat          TGCACAATTTCTTCTTT------ATGTAATTTATTGAGTTACTCATTACATA--GATTATGATCTGCTTTTGAGACTTTC  24924
Ferret       TGTACAATT---TCTTT------ATGGAATTTATTGAGTTACTGGTTATGTA--GCTTATGATCTGCTTTTGTGACTTTC  21294
Lion         TGCACAATTTCTTCTTT------ATGGAATTTATTGAGTTACTCATTACATA--GATTATGATCTGCTTTTGAGACTTTC  23511
Vaquita      CCACCATCTTAGGGAAA------AGGTAGCACA-----GAATACACAAGGT----TTAATATCTTACC-TTTTTTACTTA  19080
Pig          CGATCACTTTAGTGAAA------AGGGAGCACA-----GAATACACAGGGT----TTTATATCTTTTCCTTCTTTATTTA  17537
```

```
Majority      AXTGATAXATAAACACXXGGA--AXCATTXCATATAACTGXATCXTA--XAGTTTXAGTATTTAAAXAATAXTAGTXTAT
                             26730     26740     26750     26760     26770     26780     26790     26800
Human         CCTGGGCAACAGACTGAGATTCCATCTAAAAACAAAACCATGTATA----TATGTAAGTTTGAGGAAAAGAGTTGTATGA  23207
PolarBear     --------------------------------------------------------------------------------  0
Cow           GCTGACATACTCAAAATAGTCAGATCAAGCCTTGAAAATGGGTGGCAC-CAGCTTGGTTATGTTAATTGTTTTGATGTCT  22227
Dog           CTAGATATTTAAATACAAGGA--TGAGTTGCACATAACTGAAACATAT-CAGTTGAAGAATTTAAAAAATCATAGTATAC  22151
BelugaWhale   A-TGAGAAATACACTCTGGAA--ATCACTCCATGGGTATATATGGAA---AATCTCATT----TCTTTATACCACTGTAT  18479
Donkey        AATGAAAAGGGGACAATGCTA--AGCAGCACATAGAGCATGATCCTGTTTTGTATCAATATTAAAATCATGTAGATGTAT  22013
Cat           ACAGGTATTTAAATACAAGGA--TGAATTGCACATAACTGAAACATAT-CAGTTGGAGAATTTAAAAAACCGTAGTATAC  25001
Ferret        ATGAGTATTTAAATACAAGGA--TGAATTGCATATAACTGAAACATAA-CAGCTTGAGAACTTAAAAAACTATGATACAC  21371
Lion          ACAGGTATTTAAATACAAGGA--TGAATTGCACATAACTGAAACAT---CAGTTGGAGAATTTAAAAAACCGTAGTATAC  23586
Vaquita       A-TGAGAAATACACTCTGGAA--ATCACTCCATGGGTATATATGGAA---AATCTCATT----TCTTTATACCACTGTAT  19150
Pig           A-TGATAAATATACATTGGCT--ATCACTCCATGATTGTGTATGGAA---AATCTCATT----TCTTTGTACTGTGGTAT  17607

Majority      AAATATAATAGAATXXCATGAAXTXX-TGTTGATAAAATGAAGTATATTTATAXTGTTXAATTTTTTT-----GAATXTC
                             26810     26820     26830     26840     26850     26860     26870     26880
Human         AAATTCAGGATAATTAATAAAAGTAAACGAGAACTTCTTTACATATATATATATTTTTTAATTTTCTC--AGTTAATGTT  23285
PolarBear     --------------------------------------------------------------------------------  0
Cow           GGGTTCTACATAAATGAAAAAAACCT-TCTTGATGCTGTTCTCTACTTGGACATAGTGAAAACGTTCT-----GTTTTTA  22301
Dog           CAATATATTAGAATGCTATGTGAAGC-TGTTGGTAAAATGAAGTACATTAGTAGTGTTGAGTTTTTTTC---AGAATCTC  22227
BelugaWhale   AGAAAGTATGCAAGTGCCTCAG-TTC-CCCCTAGCCAT---ATTATA-GTATATTGTCAAATGCTTGG-----GGTTTTG  18548
Donkey        GTAAATTTGGGAAAGACAATTAAATT-TAAGGATAATAAATTTTGGACTGAGGGAGGTCTTTCTCTTTACATTTTCTGTC  22092
Cat           TAATATACTAGAATGCTACGAAGC---TGTTGATAAAATGAACTCGATTAGTAGTGTTGATCTTTTTTTAAAAAAATCTC  25078
Ferret        CAGTATACTAGAATGCGATGAAAA---TGTTGATAAAATGAAGTATATTTGTAGTGTTGAGTTTTTAA----AGAATCTC  21444
Lion          TAATATACTAGAATGCTACGAAGC---TGTTGATAAAATGAACTAGATTAGTAGTGTTGATCTTTTTTTTAAAAAATCTC  23663
Vaquita       AGAAAGTATGCAAGTGCCTCAG-TTC-CCCCTAGCCAA---AGTATA-GTATATTGTCAAATGCTTGG-----GGTTTTA  19219
Pig           AGAAAGCATATAAGTGCCTTAG-TTC-CCTGTATCCTGCCCAGTACA-ATATATTGTCAAATGGTTGG-----GGTTTTG  17679
```

Majority	TAA--TGTTTTGA--TAAATGAAATGAXATTGGTXTTAAGXAGTAAXTXTGXTTTGATTC---TATTGTG----ATGAAT	
	26890 26900 26910 26920 26930 26940 26950 26960	
Human	TGAATTTTTTTAA--CGTAGAAAATGTCAATGGGGTTATCTGGGGAGAAAGATTATAGTCCATGAGTCCCCCTGTTGTAT	23363
PolarBear	--	0
Cow	AAACAAACTGTGA--CAAGCAGTGAAAAGTGGATACTGTACAATCAGGTGGAATGGAAGA---GATTGTGGGACAAGAGA	22376
Dog	TTAGCTGTTTTAT--TAAATGAAAGGAAAGCAGTGTGAAGCAGCATGTATGGTATGATTA---TGTTTTG---TATGAAT	22299
BelugaWhale	TC----AGTCTGA--GAGGTGAAATGGCATCTAAATCTAGTATTAATTTGAATTTGTTTT---TACTGTG----AGCAAG	18615
Donkey	TTTTTGGATTTGAGTTTTATAAATTAATATTTGGAATTGATAATTATTGTTGAATGTTACCAATACTGTTGCATATGTCT	22172
Cat	TAAGCTGTTTTAT--TAAATGAAAGGAGAATGGTGTTAAGCAGCAGGTATGGTGTGATTC---TGTTTTG---TATGAAT	25150
Ferret	TAAACTGTTTTGT--TAAATGAAAGGAAGATGGTGTGAAGCAGCATTTGTGGTGTGATTC---TCTTCT-----ATGAAT	21514
Lion	TAAGATGTTTTAT--TAAATGAAAGGAGAATGGTGTTAAGCAGCAGGTATGGTGTGATT-----GTTTTG---TATGAAT	23733
Vaquita	TC----AGTCTGA--GAGGTGAAATGACATCTAAATCTAGTATTAATTTGAATTTGTTTT---TACTGTG----AGCAAG	19286
Pig	CT----GGTGTGA--TAGGTGAGATGGTGTCTAAATCCTGTATTAATTTGAACTTGTTTT---TACTATG----AGGAAG	17746

Majority	ATTAAAXAACT-------X---X--AXTTTTTGTTXAGGAATAATT----TTTATTTCATATATTTTGXAXT--GAAGAA	
	26970 26980 26990 27000 27010 27020 27030 27040	
Human	ATATACCTGTATTAAAATATGCAATAAATGCTATTTTAAAAAATGT----CTTATTACATAGAACAGAAG---ACCAGAA	23436
PolarBear	--	0
Cow	GTTGAAGTACCACCAGCCAAACACCAGTCTTCATCCA-AAGAAAGT----GATGTTGTATATATGGTGGGATTGGAAAGC	22451
Dog	ATTAGAAACA--------------TAATTTTTGGAAAAGAATTGTA---AATTTAATGATAAATTTTGGACTGAGGAGAA	22362
BelugaWhale	ATGAAACAACT----------------TTTCTGTTTACGGATAATT----TCCATTTCGTGTGTGCTGAA----AACTAA	18671
Donkey	ATACGTTCCTTTTTAACCCCCTCTTACTCTGCCATGAGTAGTGACTGCCATGAATTATTTATGTCTTATTCTTTCATATA	22252
Cat	ATTAAAAATGTA---GATATATATACATTTTTGGAAAGGAATTGTATTGAATTTAAGGACAAATTTTGGACTGTGGAGAA	25227
Ferret	ATTAGAAACAGA-----TAAGTATAAGTTTTTGAAAAGGAATTATA---ATAAATTTAATGATTTTTGAGCTGGGGAGAA	21586
Lion	ATTAAAAATGTA---GATATATATACATTTTTGGAAAGGAATTGTATTGAATTTAAGGACAAATTTTGGACTGTGGAGAA	23810
Vaquita	ATGAAACAACT----------------TTTCTGTTTACGGATAATT----TCCATTTCGTGTGTGCTGAA----AACTAA	19342
Pig	ATTGAACAACT----------------TTTCAGTTTA-AGATAATT----TCTGCTTCATGTACCGAG------AAGAGA	17799

Majority	TTTTATCTTTT-----CTXAXTTTTTGXACT----TGAATTXTCTA-CTAAATAT-TTGTAATTAXTTG--TATTTG---	
	27050 27060 27070 27080 27090 27100 27110 27120	
Human	TACTAGCTCTCCACGTCTTATGTCTTG-ATCAGTGAAGATTAGCTAGCAAAACAACCATAACTTAGGGATAGCTTTATTT	23515
PolarBear	--	0
Cow	AGTTATCTAATAAGAGCTCCCTCTGGGGAACCAAATGATTAATTTCAATAAATAT-TGCTACCAATTAGACCAACTG---	22527
Dog	TTTTACATTTT-----CT--CTTTTTGGACT----TGAATTTTCTA----AATAT-TTGGAATTAAT-------------	22413
BelugaWhale	TCTTATCTCTT----GCCTATTTTTTT-------ATGAGTAACTGGTCTAAATTT-TCATAGGAATCTG--TATATG---	18734
Donkey	TATGATTTTGTCCTGTCAAGCTGCTCC--ATGAGGTTATTCACTGGGCCAAGTTTCTTTTATCTTGTTGCTTCATTATTC	22330
Cat	CTTTACGTTTG-----C-ATCATTTTGGACT----TGAAGTTTCTAAATTAATAT-TTGGAATTAATTA--ATATGG---	25291
Ferret	CTTCACATTTT-----CTAATTTTTTGGACT----TGAATTTTCTA----AATAT-TTGGAATTAATTA--ATT--G---	21645
Lion	CTTTATGTTTT-----CTATCATTTTGGACT----TGAAGTTTCTA----AATAT-TTGGAATTAATTA--ATATGG---	23871
Vaquita	TCTTATCTCTT----GCCTATTTTTTT-------ATGAGTAACTGGTCTAAATTT-TCATAGGAATCTG--TATTTG---	19405
Pig	CCTTATCTCTT----GCCTATTTTTTGTAAC----TGATCTTTCTGTCTAAATTT-TTATAGTAATCTG--TATTTG---	17865

Majority	----AAXGTTXXXX-TTCTATTGTATXTGTTTATGXGTTCTTTXTAATCTCTCTXAAACTCTXCTXTGX-TAATGACTXC	
	27130 27140 27150 27160 27170 27180 27190 27200	
Human	GTGGGAAGTGTCATTTAGTATGTCATGTATTA-AAAATCATTTGTAGTCTTTCAGAGAGCATCTTATC----TTTCTTGT	23590
PolarBear	--	0
Cow	----AAAGGAGCACCCAATAAAAAGTGTGTGGATTTATTCAACAGAAAATGCAT-AATCTTCTATCAGGATAATGCAATA	22602
Dog	----AATGTTATCAATTCTGTTGAATATGCTTATGTATTCTTTTTAATCTCCCTTCTACTCTGCCATGAGTAGTGACTGC	22489
BelugaWhale	----AAGG-------TTTGATTCTATTTTTGGCTCAGGTTTTTAGGAACAGTTTCAAAATTTTATCTG---AATAACCTA	18800
Donkey	CCT-AAGCTTTTGTTCTTCTCTGCCTGGTCTGATGATGTATAATTGTACTCTCTCCCCCTCCCCCCCAAATTATTCTCTC	22409
Cat	----AATGTTTTCAGTGCTGTTGTATATGCTTATGTGTTCCTTTTAATATCCCT-CTACCCTGCTATGAGTAGTGACTGC	25366
Ferret	----AATATTACCAATGCTATTGCATATGGTT-TGTGTTCTTTTTAATTTCCCTTCTACCCTGCCATGAGTAGTGACTGC	21720
Lion	----AATGTTTTCAGTGCTGTTGTATATGCTTATGTGTT------------------ACCCTGCTATGAGTAGTGACTGC	23929
Vaquita	----AAGG-------TTTGATTCTATTTTTGACTCAGGGTTTTAGGAACAGTTTCAAAATTTTATCTG---AATAAACTA	19471
Pig	----AAGC---------ATAGATAATGTTTTCCCCAAGTTTTTAGGATTTATTTCAAATTTTTATCTG---AATGAATTA	17929

```
Majority     CAXXAATTATATTXTCTTTXTA-----XTAT----TTTTGTCAC-T--X-ATX-CAAAAAT-TAT---TGT-TATXTTTT
                             27210     27220     27230     27240     27250     27260     27270     27280
Human        AGGTGATAGTATTGGCTATCTATGTAGTGTCTTAAGTTCTTTAAAT---TATCCTTATTCTTACTTTTTGTGTACATTGT 23667
PolarBear    -------------------------------------------------------------------------------- 0
Cow          CCGCATGTTTATTTGATGACCA-----GCCC----CCCCCCCCCCC--AAAAAAAAAAAAAAAAC---TAT-TACAACTT 22667
Dog          CAAGAATTATTTTATCCTTTTC-----ATATGTGATTTTGTCACATGTATATGGCAAAAATGTAT---TGT-TTTGTGTT 22560
BelugaWhale  CAACGATTAGATTTTGTT-CTA-----TTTT----TCTTTTTTC------------------------TCT-TAT--TTT 18843
Donkey       CTGAGATTTGTCTGTTTTGTTAAAAAAAAATCAACTTTTGGTTTTCTTGGTCTTCTTCAGTTTATCATTGT-TATCTGTA 22488
Cat          CCAGAATTATTTTATCTTTTTC-----GTAT-----TTTGTCATGT--AAATGACAAAAATGTAT---TGT-TTTATATT 25430
Ferret       CAGGAATTATATTATCCTTTT------ATATATGAATTTGTCATAT--ATATGGCAAAAATATAT---TAT-TCTGTGTT 21788
Lion         CCAGAATTATTTTATCTTTTTC-----ATAT-----TTTGTCATGT--AAATGACAAAAATGTAT---TGT-TTTATATT 23993
Vaquita      CAACGATTAGATTTTGTT-CTA-----TTTT----TCTTTTTTC------------------------TCT-TAT--TTT 19514
Pig          CAGCAATTGGATTTGGGTTCTA-----TTTT----TCTTTTCTC------------------------TGT-TAT--TTT 17973

Majority     TTTTAAAATATTXTGAXA---ATGGTATXCC-XATTTT-TGTTTTTTA-TGACTTXTTTT----TTTTACAATTTXTTTA
                             27290     27300     27310     27320     27330     27340     27350     27360
Human        TTATAAAATAATGTGATCATCTTAATCTACCATTTTGATTATGGTTTGATCATTCTTTTACTATTTCCACTTGTTAGCAA 23747
PolarBear    -------------------------------------------------------------------------------- 0
Cow          ATCTGGAAAGTTCTGAT-----TGATCAGCTGTATTC---ACCAGATA-TTGCACCTTTG----GATTTCCATTTATTTT 22734
Dog          TTCTTGAGTCTTAC-AAA---ATGGTATGCC-CATTTTATGTTTTCCA-TGACTTGTGT-----TTTAACAATGGTTTTA 22629
BelugaWhale  CATTAAAATATTTTTATT---GTGTTTGACCAGATATTATGATCTGTA-TGATTTCTCCT----CTATGGAACTTACTGA 18915
Donkey       GTCTGTATTTTTATTGGTTGTATGAACTAGTGCTTTCACAGCTTCTTG-TAGGTAGGTAGCATACACTTCACCTGTTCAG 22567
Cat          TTT---AATTTTGTGAAA---ATGGTATGCC-TATTTTCTGTTTCTC--TCACTTGTGTTTTACTTTAACAGTGTTTTTA 25501
Ferret       TTCTTGAGTCTTATGAAA---ATGGTATGCC-CATTTTATGTTTTATG-TGACTTAATTTTTTATTTAACGATGTTTTTA 21863
Lion         TTT---AGTTTTGTGAAA---ATGGTATGCC-TGTTTTCTGTTTCTC--TCACTTGTGTTTTACCTTGACAGTGTTTTTA 24064
Vaquita      CATTAAAATATTTTTATT---GTGTTTGACCAGATAT---GATCTGTA-TGATTTCTCCT----CTATGGAACTTACTGA 19583
Pig          CATTAAAACATCTTG-------TGTTTGACCACATAC---CACCTATA-CAGTTTCTTCT----TTATGGAACTTAGTGG 18038
```

```
Majority     AXTATTAXCXATXXTTTTXTXXTGAGTTATATXATAX-----ATCTTXXTACTXTTGTATAAATCT----TTT---AAAA

                       27370     27380     27390     27400     27410     27420     27430     27440

Human        AGCCTTTATACTTACTAAATTATGATAAAAATGATTATTATAACATGCCCGGTTTTACTTAAATGTCTTATTT---A-AA  23823
PolarBear    --------------------------------------------------------------------------------  0
Cow          GGTCTTTACAAAATTCACCTAATGGAAAAGATTTCAA-----TTCCCTAGAAGACTGTAAAAAGCA--CCTGG---AACA  22804
Dog          ATTATTAGCCATGTTTTTGTGTATAGTTATATAATA------CTCATTGTAGTATAATATTAATCTATCTGTT---AAAA  22700
BelugaWhale  GGTATTCATTACA-TAGTAT-GTGATCCACATTTGTG-----ACTTACATACTCATGTATAAA-----CATAA---AATA  18980
Donkey       CTTCTTGGCAACACCTCTTTGTTCAAGGAGACCAGAGTGAGTACCTTCCTTCCTCTCCAAGAATCTAGTTTCCTCAGGA  22647
Cat          ATTATTAGCCATGCTTTTGTAAATAGTTATGTAATAA-----CTCTTTGCAATGTAGTATTCATCC----GTT---AAAA  25569
Ferret       ATTATTAGCTATGCTTTTGT----AGTTATACAATA------TTCATTGCAGTATAGTATTAATCT----GTG---AAAA  21926
Lion         ATTATTAGCCATGCTTTTGTATATAGTTATGTAATA------CTCTTTGCAATGTAGTATTCATCT----GTT---AAAA  24131
Vaquita      GGTATTCATTACA-TAGTAT-GTGATCCACTTTTGTG-----ACTTACATACTCATGTATAAA-----CATAA---AATA  19648
Pig          GGTATTCAC-----T-ACATAGTATGTAACTTTTTTG-----ATTTACATACTGATGTAAAGA---------------CA  18092

Majority     TXTXCTXATGTATATCX--TTTCTTGTTGXAXTTCTTTTGGGTCATGTXTCXTXXTTGAXTX-AAGAATAATGCTGCTC-

                       27450     27460     27470     27480     27490     27500     27510     27520

Human        CGTTCTGATTGTTATTC--TTTCTTATTCAGGTTTCTTATTGTCATGTTTTATGTCTGTTTTCAGGTGTAATTGTCCTC-  23900
PolarBear    --------------------------------------------------------------------------------  0
Cow          GTTCTTTACTCAAAACAAGTTTTTAGTTATAGAATTATGAAGTTATGCCTGA-AAAATGGCA-GAAAATAGTGGAACAAA  22882
Dog          TACCCTGATGTGTCTCT--TTTCCTGTTGGAATTCACCTTGGTCATTTACTATTTTTGACTT-AAGAACAATGCTGCTC-  22776
BelugaWhale  TTTTCTTCTGAAAAAT---TTTAAAGTCTTAT--CTGTTGCGTT-TGTCTCTTAATTCATCA-GAAAAAAAT--------  19045
Donkey       ACTTCATATTTGCATCCT-TCCCCCGTGACTTTTGTTATGGTTCCCTCTTCTCCATAGGAGAAAGTCTTTGTGCTGCTCT  22726
Cat          TACCCTGATCTATCTCT--TTTCTTGTTGGAATTCCTCTGGGTCATTTACTGTTTTTGACTTTAAGAATAATGCTGCTC-  25646
Ferret       TACCCTGATGTATTTCT--CTTCTTGTTGGAATTCAACTGGCTCATATACCATTTT-GACTT-AAGAATAGTGCTGCTC-  22001
Lion         TACCCTGATCTATCTCT--TTTCTTGTTGGAATTCCTCTGGGTCATTTACTGTTTTTGACTTTAAGAATAATGCTGCTC-  24208
Vaquita      TTTTCTTCTGAAAAAT---TTTAAAGTCTTAT--CTGTTGCGTT-TGTCTCTTAATTCATCA-GAAAAAAAT--------  19713
Pig          TTTTCTTATAAAAAAC---TGTAAAGTTTTAT--TTATTACGTT-TGTCTCT-AATTCATCA-GAAAAAAAT--------  18156
```

```
Majority     ----XXAACATATTTATT-XACTTCTTGGT------XCAXGTGGGCAACAGTTTTTGXTTGTA-------TAXTTCTAAG
                     27530     27540     27550     27560     27570     27580     27590     27600
Human        -T----CACAAATGTATCC-CATTTTGGGTGACAAAATACCTGATCACCTTATCCTTATAGTT-------TTCTCCCTAG  23967
PolarBear    --------------------------------------------------------------------------------  0
Cow          ACAGTGAATGTGTTCAGTAAAGTTCTTGGT------AAAAATGAAAAATGTGTCTTTTATTTT-------TACTTAAAAA  22949
Dog          ----TGAACTTTTTTTTTTTACTACTTGGT------ACATGTGTGCAAAAGTTATTGCGAGTA-------TA----TAAG  22835
BelugaWhale  --------TTAATTCATTTATTGTACAAG-----------GTGGGGATCCAATTTCATTTTTC--------TTCTGTCTTA  19099
Donkey       CAGTCCTGAAACCTAGCAGAGCCTGGGAGTGTTGTCACCCGCAGCCTTCAGTTTTTGCTTGCCGTCCTGGTAGTTCCAGC  22806
Cat          ----TGAACATATTTGT---ACTACTTGGT------TCATGTGTGCAAAAGTTTTTGAGGGTA-------CATATCTAAG  25706
Ferret       ----TGAACACTTTTGT---ACCACTTGGT------GCATGTCGGCAAGTTTTTTAGAGTATA-------TAT--CTAAG  22059
Lion         ----TGAACATATTTGT---ACTACTTGGT------TCATGTGTGCAAAAGTTTTTGAGGGTA-------CATATCTAAG  24268
Vaquita      --------TTAATTCATTTATTGTACAAG-----------GTGGGGATCCAATTTCATTTTTC--------TTCTGTCTTA  19767
Pig          ---------TT-TTAACTAATCATATTAG--------G---TGGGGATTCAGTTTTTTTTTTT-------TGTTCTTATA  18208

Majority     GAXATATATTGCTAXGATX-XTACA--GTXTGTXXATXTTTTTXAGGTAATA--AAGAATATTTATTT-XX----G-TA-
                     27610     27620     27630     27640     27650     27660     27670     27680
Human        AATCTTTTTTGTTATATTAATCAAAGTTTATTTGTGAGAATGTTATGAAATAT-AAGAAAATGAACATCA---T------  24037
PolarBear    --------------------------------------------------------------------------------  0
Cow          CAAATTTTTTGGTTAGACCAATACA--ATTACAGGGTTTTTTTTAAAGTTTAT-TGAAATAATTTTGT---------GT-  23016
Dog          AATAAAAATTGCTAGGATG--TAGA--GTATGTTGAACTTTGCAGGGTAGTA--TAGATTTTTTT---------------  22894
BelugaWhale  GAGATAGCCAGTTAAAAT--ATGCA--GTGTCA---TTTTTTTTTCACATTAC-AGATACATATATTTTAG----GGTA-  19166
Donkey       AACTCTTCCATCTGGGGATGATAGATGGTAGTTGGATGGGATTATGATGATAAACAGAATGTCCAATAACCAAGAGGAAA  22886
Cat          GACAAGTATTGCTAGGATG--TACA--GTGTGTTAAACTTCATAAGGTAGTA--CAGATTATTTGTTGGTGTTTTGCTC-  25779
Ferret       AATAAATAGTTCTACAATGTGTAGA--GTATGTTGAGCTTTTCAAGGTAATAC-TAGATTATTTTTCAAAGTATTGCTC-  22135
Lion         GACAAGTATTGCTAGGATG--TACA--GTGTGTTAAACTTCATAAGGTAGTA--CAGATTATTTGTCGGTGTTTTGCTC-  24341
Vaquita      GAGATAGCCAGTTAAAAT--ATGCA--GTGTCA---TTTTTTTT-CACATTAC-AGATATATATATTTTAG----GGTA-  19833
Pig          GAGATAATTAAAATATAT-GGTACC--ATTTTT---TTCCTATTAAGATATAT-AAGTATATATACTT---------TA-  18271
```

```
Majority     --------XAGTTTATG-CCAATAXAAGAAXATAAGAATXCTXACTXTT--ATATTCTXATCAXCA-------TTGXTCA
                           27690     27700     27710     27720     27730     27740     27750     27760
Human        --------GAACCTGTCACTCAAATAAAAAAACTTGAATGTTACCAATACTATTGTATATTCCATATGCAGCAATATTGC 24109
PolarBear    -------------------------------------------------------------------------------- 0
Cow          --------GTGGTTCTG-CCATCACCCTAATAGTTAGAT-TTGTTTGTT--ACATTTTGATTTTCAT------TTTTAGG 23078
Dog          --------CAATGTAT----AACAGTAGTATATAAGAATTCTTCGTGTTTTAGATTCTCATCAAGAA------TTGGTCA 22956
BelugaWhale  --------AAGGCTAAG-CCACTATAACAAAATGGTAATGCAGAACATA--GTGGTGTAAATAAC--------CT--AGA 19225
Donkey       AAAAAAAGAAATTTATG-TCACTGGAATGAATGCAAAATGTCGTTTAAACTGTACTTTTGTACTTATATTTGTCTGTGCT 22965
Cat          --------CAGTTAATG-TTAACAGTAGTATACAAGAGTTCTTACTGTTTCAGATTCTCATCAGCAC------TTGGTCA 25844
Ferret       --------CAATTTAT----GATAATAGTATATAAGAGTTCTTGCCGTTTCACATTCTCATCAGCCC------TTGGTCA 22197
Lion         --------CAGTTAATG-TTAACAGTAGTATACAAGAGTTCTTACTGTTTCAGATTCTCATCAGCAC------TTGGTCA 24406
Vaquita      --------AAGGCTAAG-CCACTATAACAAAATGGTAATGCAGAACATA--GTGGTGTAAATAAC--------CT--AGA 19892
Pig          --------AAGACTATG-CAACTGTAACAAAATGCAAA----ACATAA---ACAGCCTAAACAGC--------CT--ACA 18325

Majority     AGTTTTTTTTTCA------TXAGTXXATGXC-ATXTTGGTXTXTAAX-AAXTTCTXTXGTCTTXXTTT--ATATXXTCXT
                           27770     27780     27790     27800     27810     27820     27830     27840
Human        TTTTTTTTTTTTTAGAGTCTTGCTCTGATACCCACGCTGGAGTGCAGTGGTGTCATCTCGGCTCACAGCAAGCTTCGCCTC 24189
PolarBear    -------------------------------------------------------------------------------- 0
Cow          GAATAGTTTTCACTT---TGAATAAATTTT-CCAGAGTTGCAAAAC-AAGCCACATTCATAGATGTC--TTCTACTCCT 23151
Dog          AGTTTTAAATTCC------TTAATATTTGCT-ATTTTGGTATGTAAT-ATTTTCTTTGGTCTTTTTTT--ATATTTTCTT 23026
BelugaWhale  AGTTGATTTTTCA------TAAGTGAAAGTC-CTGCAGTTGTAAGA--AAATGGTATCAGT--ACGCT--CCATGAGGTT 19292
Donkey       GATTTCTTTCTCTAATTGGTATGTAGATTGT-ATCTTCCTATATTA--GTATTTTCCTAATTGTTTTT--TTGGGATGCT 23040
Cat          AGTGTTAAATTCC------TTGGTTTTTGCC-ATCTTGGTATGTAGT-AATTTCTTTGGTCTTTCTT---ATATTTCCCT 25913
Ferret       AGTATTAAATTCA------TTAATTTTTAAG-ATTTTAGTGTGTAAT-ATTTTCTTTGGTCTTGCTTT--ATATTTTCCT 22267
Lion         AGTGTTAAATTCC------TTGGTTTTTGCC-ATCTTGGTATGTAAT-AATTTCTTTGGTCTTTCTTT--ATATTTCCCT 24476
Vaquita      AGTTGATTTTTCA------TAAGTGAAAGTC-CTGCAGTTGTAAGA--AAATGGTATCAGT--ATGCT--CCATGAGGTT 19959
Pig          AGTTTATTTTTCA------TAAGCAAAAGTT-CTACAGCTATAAGA--AAATGATATCAA--ACTGTT--CCATGAGGTT 18392
```

```
Majority     AXTTAXTAATXX--GGXTAC---XTTTTTTTCCC-----CAAXATGTTTATGTT--X---TAAX-----GTTTTGAT---
                       27850     27860     27870     27880     27890     27900     27910     27920
Human        CCTGGTTCACCCCATTCTCCTGCCTCAGCCTCCCGAGTAGCTGGGACTTCAGGCACCCGCCACCA----CACCCGGCTAA  24265
PolarBear    --------------------------------------------------------------------------------  0
Cow          GTTCATTCTACTTTGCTTATT--GTATTCCCCTTA----CAAAGGGTTTCTACT-TTCATTAAT-----TTTTTGTTTAT  23219
Dog          AATTACTAATTTA-------------T-TTTCCCC----TAACATGTTTATGAC-CAT--TAGT-----GTTTCT-----  23075
BelugaWhale  ATTCACTG------GGCTAA---GTTTTTTTCAT---------CTGTTTTTTTT----CATGAC-----CTAATGAT---  19342
Donkey       AGTTTCTCATGAAGTGGTCATTGTTACTATACCAGAGGAAAAATGGTCTGTGGTCATATTTAACAAGGGGCATCGATGCT  23120
Cat          AATTATTAATGAGGCGTTACATCTTTATTTTCTCC----CGACATACTTATTTT-TTTTTTAAT-----TTTTTTTTCAA  25983
Ferret       TATTATTAGTGAAGTGTTACATTTTATTTTTCCCT----CAATATGTTTATGGC-CAT--TAGT-----GTTTCTAC---  22332
Lion         AATTATTAATGAGGCGTTACATCTTTATTTTCCCC----CGACATATTTA----------T-------------------  24523
Vaquita      ATTCACTG------GACTAA---GTTTTTTTCAT---------CTGTTTTTTTT----CATGAC-----CTAATGAT---  20009
Pig          ATTCACTG------GGCTAGG--GTTTTTTCCAT-----CAGGTTGCTTCAGTTATTCCGTGAG-----GTTTTGTT--T  18452

Majority     ---TXTGTATAATTA----XAAT---TATTTTTTTTT-----T--GXTXXTTA----CTTA---X-A-TXTAACCCX--T
                       27930     27940     27950     27960     27970     27980     27990     28000
Human        TTTTTTGAATTTTTAGTAGAGACGGGGTTTCACCCTGTTAGCCAGGATGGTTTTGATCTCATG-ACCTCGTGATCCGTCT  24344
PolarBear    --------------------------------------------------------------------------------  0
Cow          TCTTCTATTTAATAGAAGCAAATGTGAGTTTTTCTGCATGCT--CCCTCCTAGCATCTTAGAGAAAATATAGCACAGAA  23297
Dog          ---TTTGTGAAATTTTATGAAATACCTATTTGTTTCTT----T--GCCTAATAAAAATTTG---A-A-TTTAACCAG--T  23139
BelugaWhale  ------ATATAATTA-----------TGTTTTTTTT-----GT--TTTGCTTA----CCTA-----ATTCTCACCCAAAG  19389
Donkey       CGATGCTTATCTTAACACTCAATTTCTTTCTTTGATTGAATATAAGATCAATTGACGTCTATC-TTAGATGAATTAAATT  23199
Cat          CGTTTATTTATTTTTTGGGACAGAGAGAGACAGAGCAT----A--AATGGGGGAGGGGCAG---AGA-GAGAGGGAG--A  26051
Ferret       --TTTTGTGAAATTTTGTCAAATACCTACTTGTATCTT----T--GCCTAATAAAAATTTG---A-A-TCTAACCAG--T  22397
Lion         --------------------------------------------------------------------------------  24523
Vaquita      ------ATATAATTA-----------TGTTTTTTTTT----GT--TTTGCTTA----CCTA-----ATTCTCACCCAAAG  20057
Pig          TTGTCTGCATGCCTGAT---AATGTGTAATTATGCTG-----T--CTTTTTTC----CTTA-----A---TAGTTCT---  18507
```

```
Majority     TACX-AG-GTTTAT---TATGTTGTATTGTTTTXATXATXTTAA--AT-----TTAXGTTTTAXTXAT-ACT-CT-----
                    28010     28020     28030     28040     28050     28060     28070     28080
Human        GCCTCGGCCTCCCAAAGTGC-TGGGATTATAGGTGTGAGCTACTGCGCCCAGCCCA-ATATTGCTTTTTAATCCCCCTTC  24422
PolarBear    --------------------------------------------------------------------------------  0
Cow          TACACAGGGTTTAA---TATCTTGACTTTTTAACATAATGTTAA--ACATATCCTAGAAGGTAAATAT-ACC-CTGGAAA  23370
Dog          TACATAGTTTTT-----TATGTATCA--TTGTTGCTGATTCTCT--ATTGGTTAT------TGCTAAT-ATT-CTCTC--  23200
BelugaWhale  TTT-----GTCTAT---TCTGTTGTCTTGTTTTCAAAAAATCAA--AT-----TTTGGTTTTATTGAT-CCT-CT-----  19447
Donkey       TAAAAAACACTTATTCTTATAAGAGATTGTTATTCTGAGTTTAAGTACTACTAATGTGTTTCATCAGCCACTACTGTAGA  23279
Cat          CACAGAATCGGAAA---CAGGCTCCAGGCTCTGAGCCATCAGCC--CAGAGCCTGACGCGGGGCTCAA-ACT-CACGG--  26122
Ferret       TTTGTAGTTTTTTT---TATGTATTA--TTGTTACTGATTTTCT--ATTGGTTATATGTGTTGCTAAT-ATT-CTCTC--  22466
Lion         --------------------------------------------------------------------------------  24523
Vaquita      TTT-----GTCTAT---TCTGTTGTCTTGTTTTCAAAAAATCAA--AT-----TTTGGTTTTATTGAT-CCT-CT-----  20115
Pig          TACTCAGAGTTTGT---TTTGTTGTCTTGTTTTTAAAAAATCAG--AT-----TTTGGTTTTATTGAT-CCT-CT-----  18570

Majority     TTAGTTTGTAXTTX-XATATGGX------TTTTATTTTTTTAT------TGGXT-GAATG--------T---X---X-A-
                    28090     28100     28110     28120     28130     28140     28150     28160
Human        TTACCCTGCCATGGGTAGGTGGCTGCCACAACTTTTGCCTTATTTTTTCAATATGGATTTT--TCATGTAAATGACAAAA  24500
PolarBear    --------------------------------------------------------------------------------  0
Cow          TCACTTCATTGCTA-TATATGGAAAATCTTCTCATTTCTTTATACTGC-TGGTTAGAATGCAGATTCCTCAGTTCCCCAT  23448
Dog          ACAGTTTGTAGCTT-TACTTGGCA---CTTCTTAATGGTGTGTTTTGA-TGAACAGAACCTGCTAATTTTAAT-GTATAG  23274
BelugaWhale  TCGGTATGTCATTG---TATGTA------TTTTATATTTTTAT------TGGTT-GTATG--------------------  19491
Donkey       ATACCCTTCAATCACTATGCTGTTGAGTCATTCGATTTTATATCCCAAGTTAAACAACTGTGCCAGGGTATTTCAAATAA  23359
Cat          ACCGCGAG-ATCGT-GACCTGGTT---GAAGTCGGATGCTTAACCGAC-TGCGCCACCCAGGCGCCCCTCTCC-CGACAT  26195
Ferret       CTAGTTGGTAGTTT-TCCTTGGCA---CTACTTAATGCTGTGTTTTGA-TGAACAAAAGCTGCTAATTTTAAT-GAATAG  22540
Lion         --------------------------------------------------------------------------------  24523
Vaquita      TTGGTATGTCATTG---TATGTA------TTTTATATTTTTAT------TGGTT-GTATG--------------------  20159
Pig          TTGGTGTGTTGTTA--ATATGTG------TTTTGTATTTTTAT------TGGTT-GAATG--------------------  18615
```

```
Majority      AG---TTTX--XXXX--XXTXXA-TTGCTTGXXCAATTTTTAGTAXXAX-TXTTX-XXT-TTCGTATGTTXAGCATTXAG
                            28170     28180     28190     28200     28210     28220     28230     28240
Human         ATATATTT---AGTTTTTTTTTAGTTTATGGAAACGGTATGCCTATATAATGTTTTCCATGTCTTGTGTT-TTAATTCAA  24576
PolarBear     --------------------------------------------------------------------------------  0
Cow           AG---CCTGC-CCAGCATATTGAAATGCTTGGAATTTTGCTAGTCTGAGAAGTGAGATGGCATATATGTCTAGTATTAAT  23524
Dog           AG---TTT-A-ATATGGCCACAAATTCCCTGTGCCATCATAATTAAAAT-TATT--TGTGTTCTTTTTCTACCTGTTTGG  23346
BelugaWhale   AG---TTTG----------------TGCTTTCACAGCTTTTAGC-----------------AGGTAGGT--AGCACTCAG  19533
Donkey        AACGTTCTGTGAGATAACATGGAAATGTTAACGTTATCAGTACTGTAGAAGCACTTCTTTTGCCTATAATAGTGGTTAAG  23439
Cat           AC---TTA-T-GGTCATTAGTGTTTCTTTTGTGAAATTTTATGAAATATCTATTTATGTCTTTGCCTAATAAAAATTTGA  26270
Ferret        AG---TTTTA-ATATGGCTACAAGTTCTCTGTGCCATCATAATTAAAAT-TATTTATGTGTTCTTTTTCTACCCATTTAA  22615
Lion          -----------GGTCATTAGTGTTTCTTTTGTGAAATTTTATGAAATATCTATTTATGTCTTTGCCTAATAAAAATTTGA  24592
Vaquita       AG---TTTG----------------TGCTTTCACAGCTTTTAGC-----------------AGGTAGGT--AGCACTCAG  20201
Pig           AG---TCTG----------T------GCCTTCACAGTTTCTAGT-----------------AGATATGT--AGCACTCAG  18657

Majority      TTXTXTTCCTT--------TTTATCXTTTTT-A---TTTGXTAXTGATTGTXXGAAATXTXTAX-------ATGAGT-TG
                            28250     28260     28270     28280     28290     28300     28310     28320
Human         TAATGATTCTAATAAATAGCCGTATTTTTTGGATAGTTTTATAATACTCATTGCTGTGATATACTCTTTTGTGAAAATAC  24656
PolarBear     --------------------------------------------------------------------------------  0
Cow           TTTTTTTCTTT--------TCTTTTTTTTTTTAAACTGTGATCAAGATTAAGGGTAATTTTCATT----TCATGTGC-TG  23591
Dog           ATCTTTCCC----------TTGTTCTTATGGCAGTCTTAGCTGTTGATTGTTGGAAATCTTT----------TT--T---  23401
BelugaWhale   TCCACCTCCTT--------TGTAGCACCTTT------GTGTTTGTGGAGGCCAGAAATAC-----------ATGAG--TG  19586
Donkey        CTTTGTACTCACTGGAAAAGCAAACATAGTTGCCTTTTTATTAAAAAAAGTGATACATTTATAAATATCTCAAAATTGCA  23519
Cat           ATTTAACCAAC--------TTTATAGTTTTTTATGTATTGCTATTGATTCTCTGTTATTTATGTTACTCATATCATTTCT  26342
Ferret        ATATTTCC-----------TTGTTCTTTTGGGAATCTTAGCTGTTGATTGTTGGAAATCTTTAAAGCAGTCTCTAATAAT  22684
Lion          ATTTAACCAAC--------TTTATAGTTTTTTATGTATTGCTATTGATTC-CTGTTATTTATGTTACTCATATCATTTCC  24663
Vaquita       TCCACCTCCTT--------TGTAGCACCTTT------GTGTTCGTGGAGGCCAGAAATAC-----------ATGAG--TG  20254
Pig           TCCACCTCCTT--------GGTAGCACCTTT------GTGTTGAAGAAGGCTAGAAATACTCAT-------ATGAG--TA  18714
```

```
Majority     AXAATTXXXCTTTTCCTTTXTATTTGAGAATCTGTTT-TTXTTATXGAAATTGATATTCACXTXXX--G---AXXGX--X
                       28330     28340     28350     28360     28370     28380     28390     28400
Human        CCATTTTATCTTTTATTGGACATTTGGGTTGATGGACATTTGAATATTTAAAGATTTTGACTTAGGCCGGGCGTGGTGGC  24736
PolarBear    --------------------------------------------------------------------------------  0
Cow          AAAACTAATCTCTTGCTGCTTATTTTATAAGTAACTT-GTCTTTTAAAGTTTGTTATTACTCTGTGAGGTGTAGAGTCAT  23670
Dog          --T-TTTTAATTTTTTTATTTATTTATGATA-G------TCACACAGAGAGAGAGAGGCAGAGACACAGGCAGAGGGAGA  23471
BelugaWhale  AGAAC----CT--TCCTTTCTCTCTGAGAATCTAATT-TTCCTCAGGAACTTGATATTCACATC----------------  19643
Donkey       AATAATTTGATATTGACTCAGTTATGAAAGCTGTTAAATTTTCTTAGCAGGTGTAAAATTATTTGAGGGAGAAGAAAGAC  23599
Cat          TAGTTTGTAGATTTCCTTTTCACTTAATACTGTGT----TTTGATGAATAAAAACTGCTAATTTTAATGTATAGAGTTTT  26418
Ferret       GGTTTTTAAGTTTTCATCTCTTGTTGGGATACAGTTT-CTTTTATAGAGATTTTGTGGCCGCTCTGTAGGAGAATTCATT  22763
Lion         TAGTTTGTAGATTTCCTTTTCACTTAATACTGTGT----TTTGATGAATAAAAACTGTTAATTTTAGTGTATAGAGTTTT  24739
Vaquita      AGAAC----CT--TCCTTTCTCTCTGAGAATCTAATT-TTCCTCAGGAACTTGATATTCACATC----------------  20311
Pig          AGAAC----CT--TCCTTTCTCTTTGAGAATCTGATT-TTCCTCAGGAACTTGATATTCCCAT-----------------  18770

Majority     A-CATGTCCCATGTCTTTCTAT--CXTGXTTCXATA----CTTXATCAG--AXX-------AGATATTTXTGGXGXTTXT
                       28410     28420     28430     28440     28450     28460     28470     28480
Human        T-CAAGCTTGTAATCCCAGCACTTTGGGAGGCCAGGGCGGGCAGATCACAAGGT----CAGGAGTTTGAGACCAGCCTGG  24811
PolarBear    --------------------------------------------------------------------------------  0
Cow          TTTTTGGCTCAAATTTTTAAAA--ACTGTTCCAAAA----TCTTATCTG--AAT-------AAATAACAATTAGATTTTG  23735
Dog          AGCAGGCTCCATGCACTGGGAG--CCCGACGTGGGA----TTCGA------------TCCCGGGTCTCCAGGATCGCGCC  23533
BelugaWhale  --CTTGTCCCATGTCTTTCTGT--CATGGTTCTCT-----CTTCTTCAG--AGG-------AGAAAGTTGTTGGGCTAGT  19705
Donkey       CACAAGTACAAAATTTGAAAGAAACCAAAAACTAAAAATACTTTGTGATTCATGAAGATGCATTGCAGGAAGGAGAAAGA  23679
Cat          AATATGG-CCATGAGTTCCCA----TTGCCATCATA----ATTAAAA--------------TTATTTATGTGTTCTTTTT  26475
Ferret       AGTAGTTTTCATGGTTTATTTT--CTTGTGTTTTAA----CTTAAAAAGTCGCACATATTCAGATCATGAAGATATTTTC  22837
Lion         AATATGG-CCATGAGTTCCCA----TTGCCATCATA----ATTAAAA--------------TTATTTATGTGTTCTTTTT  24796
Vaquita      --CTTGTCCCATGTCTTTCTGT--CATGGTTCTCT-----CTTTTCCAG--AGG-------AGAAAGTTGTTGGGCTAGT  20373
Pig          C-CTTGTCCCATGTCTTTCTGT--CATGGTTCCC------TCTCTCCAG--AGG-------AGAAATTCATTGGGCACCT  18832
```

```
Majority      CTAAGTXTTGAAACCTTTCATAGCTCXTAAAGATACCATGXTTX-XXAAATGTTX--AX---------TCTTTXTTTT--
                        28490     28500     28510     28520     28530     28540     28550     28560
Human         CCAACATAGTGAAACCCCCATCTCTACTAAAAATACCATAATTAGCGGGGTATGGTGGTGGG------TGCCTGTAGTCC  24885
PolarBear     --------------------------------------------------------------------------------  0
Cow           TTCTGTTTTTCTCTTCTCTTACTTTCATTAAAATATCATGTTTGATCAAATATTACGATCTGTACAATTTCTTATTTTTG  23815
Dog           CTAGGCCAAAGGCAGGCGCTAAACTGCTGCACCACCCAGGGATC-CCGATTGTTGGAAA---------TCT------T--  23595
BelugaWhale   CTCAGTCCTGAAACCTGTCAGAGCCCA--AGGA--CTGTGATA----AGATGTTA-------------TCTCTGTTTTTC  19764
Donkey        GCATGTCCTGTCACCACACATCAAAATTACAGGCTTTCTTTTTTACTTAATGGACTTAGGTTCCACACCCTTCATCTCCA  23759
Cat           CCACCATTTGAATCTTTGCCTTGTTCTTATTGATATCTTTGCTG-TTGATTGTTGGAAA---------TCTT--------  26537
Ferret        ATACATTACAAAAATTTTTAAAGTTATGCCTTTTACAATGGTCT-CTAATTCATCAGAAATTTA----TTTTTTGTTTGT  22912
Lion          CCACCATTTGAATCTTTGCCTTGTTCTTATTGATATCTTTGCTG-TTGATTGTTGGAAA---------TCTT--------  24858
Vaquita       CTCAGTCCTGAAACCTGTCAGAGCCCA--AGGA--CTGTGATA----AGATGTTA-------------TCTCTGTTTTTT  20432
Pig           CTCAGTCTTGAAATCTTTCAGAGCTCACGAAGA--CTATGATATAT-AAATGTTA-------------TCTCTGTTTTGC  18896

Majority      XAA------TXAGGAAGCXATGXCTTAGAATAXXTTTTA---TTTXCCAXXTACATA-GTXGTTGAXAXXTGAXXT-XTA
                        28570     28580     28590     28600     28610     28620     28630     28640
Human         CAGCT--ACTCAGGAGGCTGAGACAGGAGAATCGCTTGA----ACCCAGGAGGCGGAGGTTGCAGTGAGCTGAGACCGCG  24959
PolarBear     --------------------------------------------------------------------------------  0
Cow           AAACT-TATTGAGGTATTCATTACATAGTATGTGATCCACT-TTTGTGACTTACACTCATAGATAAAAAGATATTTTCTG  23893
Dog           ---------TAAAG--C-AATCTCTAATAATGGTTTTTA---AGTT-----TTCATCTATTGTTGGGATA--GTTTTTTT  23653
BelugaWhale   AAA------TGAGTAAGTTAAGGTTTAGAACA-GGTAAGTAATTTACCAGTGACATA-GTAGGTGAAAGCTGAA---CTA  19833
Donkey        GAATT--ACTTATGAATCCACTGGGTTGGAAGGATTTTG---ACTCACAAGTAAGCAAACATCTAAAGAGTAAACTGGTA  23834
Cat           ---------TAAAG---CCATCTCTAGTAATAGTTTTTA---TGTT-----TTCATCTGTTGTTGGGATACAGTTTCTTT  26597
Ferret        GTATTGTGTTAAGGATCCAATTTCTTTTTATCTTTTTTT---CTTCCCCCCCTCCCCCTTTATAGAGATA--GCCAGTTA  22987
Lion          ---------TAAAG---CCATCTCTAGTAATAGTTTTTA---TGTT-----TTCATCTGTTGTTGGGATACAGTTTCTTT  24918
Vaquita       AAA------TGAGTAAGTTAAGGTTTAGAACA-GGTAAGTAATTTACCAGTGACATA-GTAGGTGAAAGCTGAA---CTA  20501
Pig           AAA------TGAGGAAGTTAAGGCTTAGAATA--GTTCA---TTTACCAGTTACACA-GTAGGTGGAAGCTGAA---CTG  18961
```

```
Majority     CAGAXACATTTX-X--T-XTTGCCATATTGTATGATTATCXTAXAXAXAAAXAXAA-TX-XTXAAT-CA--XXAX-A-TX
                      28650     28660     28670     28680     28690     28700     28710     28720
Human        CCATTACACTCCAGCCTGTGTGACAGACTGAGACTCCATCTCAAAAACAAACAAAAAAAAAACAACAACAAAAAAAGATTT  25039
PolarBear    --------------------------------------------------------------------------------  0
Cow          CTGAAAAATTTTAA----AGTCTTATCTATTCTGTTTCTAATTCATCAAAAAATATTTTAGTTACTACACTGTTTAGGTG  23969
Dog          AATAGAGATTT-----T-GTGTCCACTCTGTAGGAGAATTTA----------T--------------------------C  23691
BelugaWhale  CAGAATCCTTTTTCTTA-AATGCCATATGGCATAATTGTCATAGAAACAAAGAAAACTAGGTTAATTCATCACATCAATC  19912
Donkey       ATATGTATTTGCACAGA-GGTCAAATATACTCTTGAAAACTAGAAGAAGGCAATGTTTTTTGAGAAGAAGGGTAGTTGTT  23913
Cat          TAGAGAAATTT-----T-GTGGCCACTCTCTAGGAGTATTT---------------------------------------  26632
Ferret       AAATTACAGTTCCA--T-TTCTTCACATTTTAGGTAAATAAAAGGGTGAAGGCTAAGTTACTGTATTAAATGCAGAACAT  23064
Lion         TAGAGAAATTT-----T-GTGGCTACTCTGTAGGAGTATTT---------------------------------------  24953
Vaquita      CAGAATCCTTTTTCTTA-AATGCCATATGGCGTAATTGTCATAGAAACAAAGAAAACTAGGTTAATTCATCACATCAATC  20580
Pig          CAGAATCCTTTTTCTT--AATGCTATATTGCATACTTGTCATAGAAACAGAGAAAACTTGGTTAATCCATCACATCAATG  19039

Majority     GX--TTXAACAXGAATAATTTTCT----TATTXTATAACX---XX-XGG-TTAAXAATTCGTTAXATTTC---A-XATCA
                      28730     28740     28750     28760     28770     28780     28790     28800
Human        TGACTTTAAGAACAATACTGCTGTGAACTTCTTTGTACTTGTTTCCTGGTGCATTTATGCAAGCGTTTTTGAGAGGGTAT  25119
PolarBear    --------------------------------------------------------------------------------  0
Cow          GGG-ATCCAATTTTATTTCTTTTT----GCTTATATAGAGCT-AGTCAGTTGAAGTATGCAGTGTCTTTTTTTTCTATTA  24043
Dog          AGTATTTTTCATGATTCATTTTCT----TGTGTTCTAAA-------C---TTAAGAAGTCCTTGCATACTCAAATCATGA  23757
BelugaWhale  GA--CTAAAGAAGAAAAATCAT------GATCATATCACTAG-ATGAGGATAAAACATTTGACAGAATTC------AGCA  19977
Donkey       AATAATCAGCATTCATTTATTTATT---TATTCAACAATTATGTGTGGACCCTGTGCTTGGTTGACACTTGGAAATATTA  23990
Cat          ----TCTCACACGGTTCATTTTCT----TGTGTTATAAC----------TTAAAAAGTCCTTACATGACCTGAGGGTCA  26693
Ferret       AGTGTTATAAATAAGTTTATTTCT----CATAAGCTAAAGTCCTGCTGGTTTAAGAAAATGGTATCAGTCTCCTTGATGA  23140
Lion         ----TCTCACACGGTTCATTTTCT----TGTGTTATAAC----------TTAAAAAGTCCTTACATGACCTGAGGGTCA  25014
Vaquita      GA--CCAAAGAAGAAAAATCATAT----GATCATATCACTAG-ATGAGGATAAAACATTTGACAGAATTC------AGCA  20647
Pig          GA--ATAAAAAAGAAAAATTATGT----GATTGTATGGTG---AGGAAAAAGAAGTCT-CAGTAA-----------ATTA  19098
```

```
Majority     XXXAX---X-ATG---A---XT-XX---XX--TA--XXAXAXXTTCAXX--TAXCAAAAX-TTTT--XAC-----A-X-A
                      28810     28820     28830     28840     28850     28860     28870     28880
Human        ATTTAAAGGTATAGGTATATATTACTAGTGTGTAAAATAAACTTTATGAGACAGTACTAGATTATTTTCCAGTGTATTGT  25199
PolarBear    --------------------------------------------------------------------------------  0
Cow          CCCGTATTACAGGGGTATGTATCAGTTCAGTTCAGTTTAGTCGCTCAGTCGTGTCCGAAT-CTTTGCGACC--CCATGAA  24120
Dog          AG------------------------------------ATTATTTCATACATTACAAAAA-TTTT---------------  23785
BelugaWhale  CCCAT---TCATGATAAAAATTCTCACTAAACTAGAACAGAGGGGA------ACTTCAAC-TT----GAC-----AAAGA  20038
Donkey       TAGTGGTATAGTGACTACTGATGTGGGCCAGATACTTTACCTGGTAAGCACTATCCATGTATTACCACATTTAATCTCAA  24070
Cat          TAAAG---------------------------------ATATCTTCATATGTTACAGAAG-TTTT---------------  26724
Ferret       GGTTA---TTATTCACTGCTCCAGCTTTCTTTCACCTTGTTGCTTCATTATTTTCTAAGC-TTTTGTTCTTGTCTGCATG  23216
Lion         TAAAG---------------------------------GTATCTTCATACGTTACAAAA--TTTT---------------  25044
Vaquita      CCTAT---TCATGATAAAAATTCTCACTAAACTAGAACAGAGGGGA------ACTTCAAC-TT----GAC-----AAAGA  20708
Pig          --------GGATGGAAAGGAACTTGCTCAACTTGACAAAGAACATC-----TACCAGAA-------AGACC--CTAAAAA  19156

Majority     -XXTAAX-A--T----X-AAGTCATXCTTATGATATTTAXCTCT-----AACCCCTCTCACACTTAGX-T----------
                      28890     28900     28910     28920     28930     28940     28950     28960
Human        TCCTATTTATGTTCTCACCAGTAGTGCGTAGGAGTTCTTGCTGTTTCAGATCCCCACCAGCACTTGGCTAGGTATTTAAT  25279
PolarBear    --------------------------------------------------------------------------------  0
Cow          TTGCAGC-ACGC----C-AGGCCTCCCTGTCCATCACCAACTCCCG---GAGTTCACTCAGACTCAAGTC----------  24181
Dog          ---TAA-------------AGTTATACATTTTATATTTGTCTCT-----AATCCATCAGAAAATTA---T----------  23831
BelugaWhale  ACCCTAC-AGCT----A-ACATCATATTAATGGTGAGAAACTCC-------ACACTTTCTCATGAAGA------------  20093
Donkey       GGACTGTGATGT----GGACGTTATCCCTGTTGTATATATGAGGAAGTTAAGGCTTTGAATAGTTAATTTACCAGTTACA  24146
Cat          ---TAA-------------ATTAACGCCTCTTACATTTGTCTGT-----AACCCATCAGAAAATTACTAT----------  26773
Ferret       GTCTGATCATAT----A-TAATTGTGCTGTTTATTTTTTCCTTT-----GGCTATTCTCACACTGAGGTT----------  23276
Lion         ---TAA-------------ATTAATGCCTCTGACATTTGTCT-------AACCCATCAGAAAATTACTAT----------  25091
Vaquita      ACCCTAC-AGCT----A-ACATCATATAAATGGTGAGAAACTCC-------ACACTTTCTCATGAAGA------------  20763
Pig          ACAAAACTAAAT----G-ACGTCATATTGATGGTCAGAAACTC-------AGCACTTTCCTACTAAGA------------  19212
```

```
Majority     TTTCTAGTXCTTGAXA-----TAGATAXAGC-XCXTXTTCACTTTTTTTCAACCTTCTCXTGXT--XXXXATTACTTXCA
                              28970     28980     28990     29000     29010     29020     29030     29040
Human        TTCTTAATTTTTAACAATTT-GGTGTGTAGTATATTCTCTGGTCTTCCTTTGTCTTACCCTGAT-TATTAATGAGGTCTT  25357
PolarBear    --------------------------------------------------------------------------------  0
Cow          TTTCGAGTCCGTGAT------GCCATCCAGCCATCTCATCCTCTGTCGTCCCCTTCTCCTCCTGCCACCAATCCCTCCCA  24255
Dog          TTTTTGTTTATTGAAG-----TAGATAGGGATCCAATTTCCTTTTTTTTTTTTCTCCCTGTGTAGAGATAGCTAGTTAAA  23906
BelugaWhale  --TCAAGTACAAGGCA-----AGGATGTCCC-TCCTCACCACTTCTTTTCAACATCCTCCTGGA------ATTCCTTGCT  20159
Donkey       TACCTAGTAGGTGGAAGCTGGACTATAGAACCCTTTTGTTTCTTTTTAAAATTATTTTATTGAGGTCATATTGGCTTATA  24226
Cat          TTTTTGTGTATTGTAT-----TAGATAGGGATCCAATTTCAATTTTTGT-GACCTTATAGAGATAACCAGTTAAATAAAA  26847
Ferret       TGTCTGTTCTTTTATT-----TTGTTTAAAACTCAATTTTGTTTTTTATTGATCCTTTCAGTGTATCCTTGCCATCCGTA  23351
Lion         TTTTTGTGTATTGTAT-----TAGATAGGGATCCAATTTCAATTTTTGT-GACCTTATAGAGATAACCAGTTAAATAAAA  25165
Vaquita      --TCAAGTACAAGGCA-----AGGATGTCCC-TCCTCACCACTTCTTTTCAACATCCTCCTGGA------ATTCCTTGCT  20829
Pig          --TCAAGTACAAGACA-----GACATCTAGC-ATCTCAGCACTTCTTTTCAACATTCTCTTGGG------ATTCCTTGCT  19278

Majority     ATTGCAGTGAXATTTCXXCAXXT--TA--ATAGGTA----TATATATGTXGGGGA-AAGGXATAGAA-XXGTAX-TTTXT
                              29050     29060     29070     29080     29090     29100     29110     29120
Human        ACATCTTTTTTCCCCCCACATTTATGGCCATGGATG----TTTCTCTTTTGTGAA-ATTCTGTTAAATGCCTATTTGTGT  25432
PolarBear    --------------------------------------------------------------------------------  0
Cow          GCATCAGAGTCTTTTCAGTGAG-------TCAGCTC----TTCGCATGAGGTGGCCAAAGTACTGGAGTTCCAGCTTTAG  24324
Dog          ATTGCAGTGC-ATTTCTTCACAT--TA---TAGGTA----AATATGTGTTAGGGTGAAGGCTAAGTTACTGTAT-TAAA-  23974
BelugaWhale  GATGCAGTAAGGCAAGAAAAGGAAATT--AAAGGTA----TACAGATTGAGAAGG-AAGGTATAAAA--------CTTGT  20224
Donkey       ACATTGTGTAATTTTGGGTGTACATTATTATATATCAGTTTCTGTATGTACTCCATCGTGCTCACCACTAATAGTCTAGT  24306
Cat          ATGGCAGTGCCATTTCTTCACAT--TA---CAGGTA----AATATGTGTTAGGGTGAAGGCTAAGTTACTGTAA-TAAAA  26917
Ferret       GTCTGTATTT-ATTTATTTATTTATTTATTTACTTA----TTTATTTGACAGAGAGAGATCACAGAGGCAGGCA-GAGA-  23424
Lion         ATGGCAGTGCCATTTCTTCACAT--TA---CGGGTA----AATATGTGTTAGGGTGAAGGCTAAGTTACTGTAA-TAAAA  25235
Vaquita      AATGCAGTAAGGCAAGAAAAGGAAATT--AAAGGTA----TACAGATTGAGAAGG-AAGGTATAAAA--------CTTGT  20894
Pig          GATGCAGTAAAGCAAGAAAAGG-------AAAGGT-----TACAGATGGAGAAGA-AAAGTATAAAA---CCATTCTTGT  19342
```

```
Majority     TXTAXXXCXTTGTAGAAGACAT--------XGXTAGTCTATTT-ACAAXAT-CCAAXXCCXTXX-CTGATXA--TTTATG
                              29130     29140     29150     29160     29170     29180     29190     29200
Human        TTTT-TGCTTGATAAAATGTGAGCTTAT-TATTTAACTGATTTTGTAGTTTTAAAAAATTGCTGGGGGCGGTGGCTTGCG  25510
PolarBear    --------------------------------------------------------------------------------  0
Cow          CATCATTCCTTCCAAAGAACACCCA----GGGCTGATCTCCTTCAGAATGGACTGGTTGGATCTCCTTGCAG--TCCATG  24398
Dog          TGCAGAACATTGTGGTATAAAT-------AGACAAGTTTATTTTTCATAAGGCAAAGTCC------TCTTGG--TTTAAG  24039
BelugaWhale  ---------TTGCAGATGACAT---------GATAGTCTATGT-ACAACAT-CCAAAAGAATTGACAAAAAA-TTTCATG  20283
Donkey       TTTTATCCGTCACCATACATATGTGCCCCTTTACAACCCTTTCGCCCGCACTCCCACTCCCTTCTCTGGTAACCACTATC  24386
Cat          TGCAGAACATAGTGGCAGAAAT-------AGGCAAGTTTATTTCTCATAAGCCAAAGTCC------TGCTGG--CTTAA-  26981
Ferret       GAGAGAGAGGNNNNNNNNNNNN-------NNNNNNNNATGCGGGACTCAATCCCAGGACCCTGAGATCATGA--CCTGAG  23495
Lion         TGCAGAACATAGTGGCAGAAAT-------AGGCAAGTTTATTTCTCATAAGCCAAAGTCC------TGCTGG--CTTAA-  25299
Vaquita      ---------TTGCAGATGACAT---------GATAGTCTATGT-ACAACAT-CCAAAAGAATTGACAAAAAA-TTTCATG  20953
Pig          TATCATGTCTTGTAGATGACAT---------GATAACCTATGT-AGAACAT-TCAAAAGGATCGACAAAAAAATTTCATG  19411

Majority     TA----------AXTGGTATCAGTTTATTCCATGAXTGC-AXTTCACGAGGTTAATTTXTT--TCTG---GACTXAXTTT
                              29210     29220     29230     29240     29250     29260     29270     29280
Human        TC----------TGTAATCTCAGCTTTTTGGGAGGCTGAAGGAGGAGGA--TTGCTTGTGGGGCCCAGGAGTTTGAGGCT  25578
PolarBear    --------------------------------------------------------------------------------  0
Cow          GG----------ACTCTCAAGAGTCTTCTCCAACACCAC-AGTTCAAAAGCATCAGTTCTT---CGG---CACTCAGCTT  24461
Dog          AA----------AATGGTATCAGTCTGCTCCATCA-GGT-TATTCA-----------------CTG---GGCCAACTTT  24086
BelugaWhale  TA----------ACTGATAAGTAGTTATTGCAGGATTGC-AGGGTACAAGGTTAATATATTAATAGG---AAAGTAAATT  20349
Donkey       TGTTCTCTTTATCTATGTGTTTATCTTCCCCATATGAGTGAAATCATGTGGTGGTTGTCTTTCTCTGTCTGCCTTATTTT  24466
Cat          ------------AATGGTATCAGTTTGTTCCATAAAGGT-TATTCACTAGGCCAGCTTCATCTTGTG---GCTTCATTAT  27045
Ferret       CC----------GAAGGCAGCGGCTTAACCCACTG-AGC-CACCCAGGCGCCCCA-TGAGCCACCCA---GGCGCCCCGT  23559
Lion         ------------AATGGTATCAGTTTGTACCATAAAGGT-TATTCACTAGGCCAGCTTCATCTTGTG---GCTTCCTTAT  25363
Vaquita      TA----------ACTGATAAGTAGTTATTTCAGGATTGC-AGGGTACAAGGTTAATATATTAATAGG---AAAGTAAATT  21019
Pig          GA----------ACTGATAAGTGATTATTGCAAGATTGC-AGGGTATGAGGTTAAAATA-----CAG---AAGTTAA-TT  19471
```

```
Majority     GCTTTACXGTATAAXTGTTGT-GTGCATGXXTAATTTGAXGATAAAX-CAAXATTXTGTTTXXXTTAGCACCCCC---AA
                              29290     29300     29310     29320     29330     29340     29350     29360
Human        GCAGTGAGCTATGATCGTTCCATTGCACTC-TAGCTTGGTGACAGAG-CAAGACTTTGTCTGAAAAAAAAAAAAAAAAAA  25656
PolarBear    --------------------------------------------------------------------------------  0
Cow          TCTTCACAGTCCAACTCTCAC-ATCCATACACGACCACAGGAAAAAC-CATAGCCTTGACTAGACGGACCTTTGTTGGCA  24539
Dog          CTTTCA------TCTTGTTGC-TTCATTA-------------T-TTTTTAAGCTTTTGTTTTGT----------------  24129
BelugaWhale  GCTTTCCTGTATACCAGTGGT-GAGCAAGTGCAATTTGAAATTAAAAACAAAATACAGTTTACATTAGCACCCCC---AA  20425
Donkey       GCTTGACATAATAACCCTCACAGTCCATCCATGTTGCTGCAAATGGGATGAGTGTCTTTTTTTTTATGGCTTAGTCATTGT  24546
Cat          TTCCTAAGCTTTTGTTCTTGT-CTGCATGGTTTGGTGATGTATAATTGTGCTGTCTTGTTTCATTTTTTTCCTTT---GA  27121
Ferret       AGTCTG------TATTTTTAT-TGCGTTGGATGAGTTAGTGCTTCCCCAAAGCTTCTGGTAGGTAGCTAGCACCC---AG  23629
Lion         TTACTAAGCTTTTGTTCTTGT-CTGCATGGTTTGGTGATGTATAATTGTGCTGTCTTGTTTCATTTTTTTCCTTT---GA  25439
Vaquita      GCTTTCCTGTATACCAGTGGT-GAGCAAGTGCAATTTGAAATTAAAAACAAAATACAGTTTACATTAGCACCCCC---AA  21095
Pig          GCTTTCCTGTATATCAGTGAT-GAACAAGTGCAATTTGAAGTTAAAA-CAAAATACCATTTGCATTAGCACCCCC---AA  19546

Majority     AXXTTATCTXCTTXG--TGTAAAT---CTAXGAAXAT----AXAAATAXXCTCT-------XXATXAAGTT--X-XXXTT
                              29370     29380     29390     29400     29410     29420     29430     29440
Human        GTGTGTGTTTTACAGTTACTGATTGTCTTTTGGTTATTTGTGTTGCTGGTGTCTTTTCCCAGTTTGTAGCTTGCCTTGTC  25736
PolarBear    --------------------------------------------------------------------------------  0
Cow          AAGTAATGTCTCTGCT-TTTCAATATGCTATCTAGGTT---GGTCATAACTTTT-------CTTCCAAGGAGTAAGCGTT  24608
Dog          ----TGTCTGTGTGG--------T---CTGATAATAG----ATAATTGTACTC------------TCTGTTTTTATTTTT  24178
BelugaWhale  ATTTGAAAATCTTAGG-TATAAAT---CTAACAAAAT----ACATATAAGATCT-------ACATGAGGAC---------  20481
Donkey       CCGTTGTGTATATACACTGTATCTTCTTTATGCATTTATCAGTTGATGGGCTCTTGGGTTGCTTCCATGTCTTCGCTGTT  24626
Cat          TCATTCTCAACCTGA--GGTTTGT---CAAGGACACC----AGGAATAGTCATG-------TGAGTGAGTTCCTTCCTTT  27185
Ferret       AGCATGTCTCCTTGGC-AGCACCT---CTATGTTTAA----AGAAATACTCAC------------TGAGTACCTGCTTTT  23689
Lion         TCATTCTCAACCTGA--GGTTTGT---CAAGGACACC----AGGAATAGTCATG-------TGAGTGAGTTCCTTCCTTT  25503
Vaquita      ATTTGAAAATCTTAGG-TATAAAT---CTAACAAAAT----ACATATAAGATCT-------ACATGAGGAC---------  21151
Pig          AATTGAAGTGCTTAAG-TGTAAAT---CTAAAAAGGT----GTATATAAGATCT-------ACATGAGGAA---------  19602
```

```
Majority     --------TXACTATATAG-------------CTTTTATCATXAAXTXCAX-----TXGGA---AXCCTTAXACXTA---
                      29450     29460     29470     29480     29490     29500     29510     29520
Human        ACTTCTTTATGCTGTCTTGTCTGTCTGTCTGTCTGTCTCCCTCCCTCCCTCCCCCTCCCTCCGTCCGTCTGTCCCTCCC-  25815
PolarBear    --------------------------------------------------------------------------------  0
Cow          -----TTTTAATTTCAGGG-------------CTGCAGTCACCATCTGCAGTGATTTTGGA---GCCCCCAAAAATA---  24664
Dog          -----T--T-----------------------CTCTTTTGAT--------------T-------ATTCTCACACC-----  24202
BelugaWhale  ---------AACTACAAAA-------------CTTTGATAAATGAAACCAA-------AGA---ACCAA-ATAAATG---  20525
Donkey       GTGAATGATGAACATATGGGTGCATAAATTTCCTTGTATTATTGATTTCATGTTCTTTGGAAAAATACCCAGTGATAGGG  24706
Cat          --------TGAAAATCTAG-------------TTTTTCTCAGGAACTTG-------TTGGC---ATCCTTGCCCC-A---  27230
Ferret       -----C--TGAGAATCTAG-------------CTTTTCTCATGTACTTGATGTTGGTTGGC---ATCCTTGCCCCCA---  23743
Lion         -----C--TGAAAATCTAG-------------TTTTTCTCAGGAATTTGATG----TTGGC---ATCCTTGCCCC-A---  25552
Vaquita      ---------AACTACAAAA-------------CTTTGATAAATGAAACCAA-------AGA---ACCAA-ATAAATG---  21195
Pig          ---------AACTATAAAA-------------CTCTGATGAACAAAACCAG-------AGA---ACCAAAATAAATG---  19647

Majority     TGGCTTGAT---ATXCTGTTTXCATCXTT-------CCAGAAAAACTCXTTTTTGTGXAGATXTCAGTXCTXCAAXATTT
                      29530     29540     29550     29560     29570     29580     29590     29600
Human        TCCCTCCCTCCCTCCCTCCCTCCCTCCCT------CCCTCCCTTCCTTCCTTCCTTCCTTCCTTCCTTCCTACTTTACTT  25889
PolarBear    --------------------------------------------------------------------------------  0
Cow          AAGTCTGAC---ACT--GTTTCCACTGTT----TCCCCATCTATTTCCCATGAAGTGATGGGACCAGATCTTCATTTTTT  24735
Dog          ---CTGAGG---TTGTTCTTTTCTGTTTT----T--TTTTAAAAACTCGCTTTTTGGTGGG----GGTCCCGGGGTGCAG  24266
BelugaWhale  TGGATAGAT---ATTCCACGTTCATGGAT---------AGGAAGACTCAGTATTGTTAAGATGTCAGTTCTTCTGAAATT  20593
Donkey       TAGCTGGATCATATAGTGTTTTTATTTTTAATTTGTTGAGAAATCTCCATACTGTTTTCCATAGTGGCTGTACCAGTTTG  24786
Cat          TGACTTTCT---GTCGTGGTTCCTTCT---------CCAGAAAAGAAAGTCTTCGGGCAGTTCTTAGTCCTGAAACCTGG  27298
Ferret       TGACTTTGG---GTCATGGTTCCTTCCTC----T--CCAGAAAAGAAAGTCTTTGGGCAGTTCTCAGTCCTGAAACCTAA  23814
Lion         TGACTTTCT---GTCGTGGTTCCTTCTTC----T--CCAGAAAAGAAAGTCTTTGGGCAGTTCTTAGTCCTGAAACCTGG  25623
Vaquita      TGGGTAGAT---ATTCCACGTTCATGGAT---------AGGAAGACTCAGTATTGTTAAGATGTCAGTTCTTCTGAAATT  21263
Pig          TGGGTAGAT---ATTC-ATGTTCATGGAT---------AGGGAGACTCAATGTGGTCAAGATGTCAGCTCTTCCCAAATT  19714
```

```
Majority     CAGAGCC--GXXATTTTAGTCAAX----TTXAGTCCCTGTTTAATXTXCAGCAXG----TTGXTAGXTXXTGTXGATCTT
                      29610     29620     29630     29640     29650     29660     29670     29680
Human        TAGATTCAGGGTTACATGTGCAGGTTTGTTACATGGGTATATGGCATGAAGCTGAGG--TTTGCAATACAAGTGATCCTG  25967
PolarBear    --------------------------------------------------------------------------------  0
Cow          GAAAGTT--GAGTTTTCAGCCAACTTTTTCACTCTCCTCTTTAACCTTCATCAAGAGGCTTTTTAGTTCTTCTTCACTTT  24813
Dog          CAGCGT------ATTTCAGAGCGC----CTAGGCCGCGGCCCAG----------------GGGCCGATCCTGGAGACACG  24320
BelugaWhale  GA---------TATATAAATTCAG----TGCAGTCCCAGTCAAAATTCCAGCAAG----TAGTTA-----TATGGATGTC  20651
Donkey       CATTCCCACCAGCAGTGTACCAAGGA--TTCCCTCTTTGCATCTTCTCCAACATTTGTTGTTTTTTGTCTTGTTAATTGT  24864
Cat          CAGAGCCCAGG-ACTCTTGTCAGT----CTCAGCCTCCAGTTACT----GCTCGCCTTCCTGGTAGTTTCAGCAGCTCTT  27369
Ferret       CAGAGCCCAGGGACTCTAGTCAAC----CTACCTCAGTGTTCAGTTCTTGCTTGCCTTCTGGGTAGCTTCAGCAGGCCTT  23890
Lion         CAGAGCCCAGG-ACTCTTGTCAGC----CTCAGCCTCCAGTTATT----GCTTGCCTTCCTGGTAGTTTCAGCAGCTCTT  25694
Vaquita      GA---------TATATAAATTCAG----TGCAGTCCCAGTCAAAATTCCAGCAAG----TAGTTA-----TATGGATGTC  21321
Pig          GA--TCT--GATCTATAGATTAAA----TGAAATTCCAGTAAAAATTCCAGCAAG----TTTTTA-----TGTGCGTATG  19777
```

```
Majority     A-AC-CTGAGAGTGATGTAAGGTACTTATA-GGAXAXGTXAAAA----ACCCAGXAAXX---------------A--XTT
                      29690     29700     29710     29720     29730     29740     29750     29760
Human        TCAGCCAGAGAGTGAGCTCAGTACCCAACAGTTAGATTTACAATCCTTTCTCCCTCCCCCC-------ACCACTAGTTCC  26040
PolarBear    --------------------------------------------------------------------------------  0
Cow          ATGCCATAAGGGTGGTGTCATCTGCATATCTGAGGTTATTGATATTTCTCCCTGCAATCTTGATTCCAGCTTGTGCTTCT  24893
Dog          GGAT-CGGAATCCCACGTCAGGGCCTC-----------------------------------------------TCCCTT  24352
BelugaWhale  A----ACAAGCTGATTCTGAAATTTATAT--GGAAAAGCAAAAG----ACCCAGAAAGCC--------------------  20701
Donkey       AGCCATTCTGACAGGTGTAAGGTGATAGCTCATTGTAGTTTTTATTT-GCATTTCCCTAATAGTGATGTTGCACATCTTT  24943
Cat          CCAC-CTGGGAGTGATGGATGGTACTTGGATTGTACTATTATGATGA-ACATAATACCTAATAATAAAATTAAAAAAATT  27447
Ferret       CTAC-CTGGGAGTGGTAGATGGTACTTAGA--------------------------------------------AACCTT  23925
Lion         CCAC-CTGGGAGTGACGAATGGTACTTGGATTGTACTATTATGATGA-ACATAATATCTAATAATAAAATTTAAAAAATT  25772
Vaquita      A----ACAAGCTGATTCTGAAATTTATAT--GGAAAAGCAAAAG----ACCCAGAAAGCC--------------------  21371
Pig          A----ATGAATGGATTCTAAAGTATATATATGGAGAAGCAAAAG----ACCCCGGAAT---------AGC-------C--  19831
```

```
Majority     TCAAXCACAGTATTGA-GGTAAAATAXX-TTTXX-AAA-GTTTTXGTACTXATGCX-XT-T-TACXCCCTTTTCXTTTXT

                       29770     29780     29790     29800     29810     29820     29830     29840

Human        CAGTGCATATTGTTGC-GGATTAATACAGATTCTTAAT--GTATAGTTTTAATGTGGCCACAAATTCCCTTTGCATCTTA  26117
PolarBear    --------------------------------------------------------------------------------  0
Cow          TCTAGCCCAGTGTTTCTCATGATGTACTCTGCATAGAA-GTTAAAGAAGCAGGGTGACAGTATACAGCCTTGATGTACTC  24972
Dog          TCATGGGCCTATCTC----TTCTCCATTCCTTGC------TTTCTCTCCTTCTGCCTCTGCCTGTGTCCCTTACCTCTCT  24422
BelugaWhale  ---AACACAGTATTGAAGGAGAATAAC--------AAA-GTTGGAGGACTGATGC-------CACTCAACTTCCAAG---  20759
Donkey       TCATGTGCCTCTTGGCCATCTGTATATCTTTGGAAAAATGTTCATATCCTCAGTCCACTTTTTGACTGGGTTGTTTTGTT  25023
Cat          TCAAATACTGGAATGATTGTAAAATGTCATTTAAATGT-GCTTTTATACTTAATCCTATATGTTTATGCTTATTTTTTCT  27526
Ferret       TCAAGTACTGGAATA----TAAAACGTCATTTAA------TTTTTCTGCTCATACTATTTTCTACACCTATTTTTTTTCT  23995
Lion         TCAAATACTGGAATGATTGTAAAATGTCATTTAAATGT-GCTTTTATACTTA-TCCTATGTGTTTACGCTTATGTTTTCT  25850
Vaquita      ---AACACAGTATTGAAGGAGAATAAC--------AAA-GTTGGAGGACTGATGC-------CACTCAACTTCCAAG---  21429
Pig          ---AACACAGTACTGAAGGAAAAGAAC--------AAA-GTTGGAGGACTGATGT-------TACTAATTTTTCAAGCTC  19892

Majority     XTT--ATTTAATTTGTAT-------CTAX-----XXAGTAXTT----TCATGAXTG------X-X-TATGATTATTGXTX

                       29850     29860     29870     29880     29890     29900     29910     29920

Human        GATAAAAATAATTTATATGA---TCCCATTCTTTACACATTCCAAATTTTCCTTTGCTCTTCTGGACATCTTCCATGTTG  26194
PolarBear    --------------------------------------------------------------------------------  0
Cow          CTT--TTCCTATTTGGAA-------CCAG-----TCTGTTGTT----CCATGTCCAG--------TTCTAACTGTTGCTT  25026
Dog          CTC--------------------------------TC--TCTC----TCTCTCTGG------GTGTGTTG-TGACTACAT  24457
BelugaWhale  -----ATTTAGTATGAAG-------ATA-------CAGTGAT------CATGACAG----------TGTGGTTTTGGTGA  20804
Donkey       GTTGTTGTTAAGTTGTATGAGTTCTTTATATATTTTGGAAATTAACCCCGTGTCAGATACATGATTTGCAAATATTTTCT  25103
Cat          TTGACGTATAAGTTGTAT-------CTTTCCATGTTAGCATTT----TTCTGATTGTTTT-TGTGGGATACTAATTTCTC  27594
Ferret       TTTACATGCAGATTGTAT-------CTTTGCATATTAACATTT----TCCTGATTT------GTGGGATGTTCACTTCTC  24058
Lion         TTGACGTATAAGTTGTAT-------CTTTCCATGTTAGCATTT----TTCTGATTGTTTT-TGTGGGATACTAATTTCTC  25918
Vaquita      -----ATTTAGTATGAAG-------ATA-------CAGTGAT------CATGACAG----------TGTGGTTTTGGTGA  21474
Pig          AAG--ATTTAATATGAAG-------CTA-------CGGTAAT------CAAGACTG----------TCTGATTTTGGTGA  19940
```

```
Majority      AAGAATAGXXAATTTGATCT------AAGAAACAGATTAXATXGTCAXGXATTAAAAAGCXCXTCATAAA-------TAT

                            29930     29940     29950     29960     29970     29980     29990     30000

Human         ATTGCTGGAAGTCTTGAAGG------CAATCCCAAATAACAGTTTTTAGCTTTTCAACTGTTGCTGTGATACGGTTTCTT  26268
PolarBear     --------------------------------------------------------------------------------  0
Cow           CCTGACCTGCATATAGGTTTCTC---AAGAGGCAGGTCAGGTGGTCTGGTATTCCCATGCCTTTCAGAAC-------TTT  25096
Dog           AAAAAAAAAAAATTAAAAAAAAAAAAAAAAAAAAAAAAAAAAAAAAAAAAAACAAAAAAAGAAAAAAAAAA-------AAC  24530
BelugaWhale   AAGAATAGACAGGTTGATCA------GTGAAACAGAATGGAGAGCCCAGAATAGAGCCCCCCCCCATAAA-------TAT  20871
Donkey        CCTAGTAGGTGGGTTG-TCTTTTT--GTTTTGTTCATGGTTTCCTCTGCCATGCCGAAGCTTTTTAGTCTGATGTAGTCC  25180
Cat           ATGAAATGGTCATT------ATA--CAGGAAAAAGTTTCTGTGGTCAGATTTTAAAAAGCACGTCAATGCGTATCTTAAC  27666
Ferret        ACGAAATGGTTATTTTTATTACACAGGAGAAAAAGTTTCTGTGGTCAGATTTTAAAAGGGACATCAGTGTCTATCTTAAT  24138
Lion          ATGAAATGGTCATT------ATA--CAGGAAAAAGTTTCTGTGGTCAGATTTTAAAAAGCACGTCAATGCGTATCTTAAC  25990
Vaquita       AAGAATAGACAGGTTGATCA------GTGAAACAGAATGGAGAGCCCAGAATAGAGCCCCCCCCCATAAA-------TAT  21541
Pig           AAGAATGGACAATTTGATCT------ACGAAACAGATTAGAGAGCCCAGAAT---AGAGCCCTCCATAAA-------TAT  20004

Majority      ACTCAATTTATTTTTGX--XA--XA-TCTAAGXTTAATTGACATXCATCTTAGCXGAGXTAX---T-AXXAX---TXTCT

                            30010     30020     30030     30040     30050     30060     30070     30080

Human         ATAGAGATTTTTGTAGTAGAATTTATCAGTATTTTCTTTCATGTTCAGTTTTCTTGTGTCTTAATTTAAAAATCCTTACA  26348
PolarBear     --------------------------------------------------------------------------------  0
Cow           CCACAG-TTTCTTGTGATCCACACAGTCAAAGGTTTTGGCATAGTCAATAAAGCAGAAATAGATGTTTTTCTGGAACTCT  25175
Dog           ACTCATCTTGTTTTTT----------TTTATTAATAATTGTTAGTGGTAATCATTGGCAAC----CCAATAGTCTTATTT  24596
BelugaWhale   AGTCAACTTATTTTTGA---------CAAAGG-------------A-----GCAAAGGTAG---------------TCT  20908
Donkey        CATTTGTGTATTTGTTTCTTTTATTTCCTTTTCCTGAGTAGACATGGTATTCGAAAAGATG--TACTGCCTGTATTTTCT  25258
Cat           ACTTCATTTCTTTT-----AACGGAATCTAAAATTAATTGACATCCATCTTAGATGAATTAATTATAAAAATATGTATTT  27741
Ferret        ACTTAATTTCCTTTTT---AATTGAATCTACCATTACTTGACATCCATCTTAGGTGAATTT----TGAGAATACCTATTT  24211
Lion          ACTTCATTTCTTTTTTT--AACGGAATCTAAAATCAATTGACATCCATCTTAGATGAATTAATTATAAAAATATGCATTT  26068
Vaquita       AGTCAACTTATTTTTGA---------CAAAGG-------------A-----GCAAAGGTAG---------------TCT  21578
Pig           AGTCAT-TTATTTTTGA--CAGAGGCTCAAAGGCAATACAGCGG--AACAAAGCAAAGATAG---------------TCT  20064
```

```
Majority     TTCX-AX-GXTTGTXAT--X--AXTTXAXTTTCAATTTAGTT-T---GTTXX--TXX---------A-XX-TGTGGAACA
                      30090     30100     30110     30120     30130     30140     30150     30160
Human        TACTCAGAGTTCATAAA--GATACTATATTTTCTTCTGAAATTTTAAAAAATTTTGCTTTTTATATTTATCAGAAAATTA  26426
PolarBear    --------------------------------------------------------------------------------  0
Cow          CTTGCTTTTTCCATGAT-CCAGCGGATGTTGGCAATTTGGTC-TCTTGTTCCTCTGCCTTTTCTAAAACCAGCTTGAACA  25253
Dog          TTTTATTGGTTTGTTAT----GAGTTTAGTGTTAGTTAAGTT-CGT-GCTCAAATAGT--------AGAATTGCAAAATA  24662
BelugaWhale  CCC--------------------------CAACAAGTGGCT------------------------------AGTGGAACA  20932
Donkey       TCTAGGAGTTTTGTGGTTTCAGGTCTTGAAGTCAAGATTGTAATCCATTTTGAGTTAATTTTTGTGTTTGGTGTATGATA  25338
Cat          TTAGTAGAGATTGTTATTCCGAATTTAAGTTCTAATTTAGTT-C---GTTTCCATAGCC-------AGTATTGTGGAACA  27810
Ferret       TTAGTAGGGATTGTTAATCCAAATTTAAGTTTTAATTCAGTT-TTT-GTTTCAGCAGTC-------ACTACTGTGGAACA  24282
Lion         TTAGTAGAGATTGTTATTCCGAATTTAAGTTCTAATTTAGTT-T---GTTTCCATAGCC-------AGTATTGTAGAACA  26137
Vaquita      CCC--------------------------CAACAAGTGGCT------------------------------ACTGGAACA  21602
Pig          CTC--T---------------------G---ACAAGT-GGT------------------------------ACTGGGACA  20087

Majority     ACTGTCAATXC--XXTTT-XTGXATX-C----TTGAXTTTX-XAAXTTTTXCATGTTAAGCATTAGTCTCTTXATATX-A
                      30170     30180     30190     30200     30210     30220     30230     30240
Human        TTTTTGTGTATTGTATTTAGTAGGGATCTGGTTTCATTTTTTTTGCCCTTATTAAGATAGTCAATTAAATTGAAATCTCTG  26506
PolarBear    --------------------------------------------------------------------------------  0
Cow          TCTGGAAGTTCATGGTTC-ACGTATTGC----TGAAGCCTG--GCTTAGAGAATTTTGAGCATTACTTTACTAGCGTGTG  25326
Dog          ACTCTTGATATTCTGCTCTCTGGATCTC----TCGGACTTAGTTACTTTGGCAGGCTGAAGGCTTGTTATTGTTTTTAAA  24738
BelugaWhale  GCTGAACATAC--------ATGTA---C----AGAAGAATG--AATTGAGACACA------------CTCTTCACA---A  20980
Donkey       ATGGTCTACTTTCATTCTTTTGCATGTTGCTGTCCAGTTTTCCCAACACTATTTATTGAAGAGACTTCCCTTTCTCCATT  25418
Cat          ACCATCAATCACGATTCTGTTGAGTCAT----TTGATTTTA-TATCTTTTCCAAGTTAAGCATCAGTGTCAAGATATAGC  27885
Ferret       TCTATCAATCACTATTTTGTTGAGTCAT----TTGGTTGTTTTTTTTTTTTTTTTTTAAGATTTTATTTATTTATTTGAC  24358
Lion         ACCATCAATCACGATTCTGTTGAGTCAT----TTGATTTTA-TATCTTTTCCAAGTTAAGCATCAGTGTCAAGATATAGC  26212
Vaquita      GCTGAACATAC--------ATGTA---C----AGAAGAATG--AATTGAGACACAG---GTATTACACTCTTCAAA---A  21659
Pig          ACTGACTGTCC-----TC-ATGCA-----------------------AAAGAATG--AAACGACATACCCTTCACA---A  20133
```

```
Majority     AAATAAACTXAAC--XXXCAGXAXAGAT--GTAAAXATTAAATGTAAAGCTA----XAXACAATTAXXAGATXAGGXAAX
                     30250     30260     30270     30280     30290     30300     30310     30320
Human        TAATGACTTTTTCA----TATTACAGGTATATATATCGTAAGGCAAAGGCTG-----AAAAAACTATCATAACAA---AA  26574
PolarBear    --------------------------------------------------------------------------------  0
Cow          AGATGAGTGCAATT-GTGCGGTAGGGATATGTATATGTTAG-TGTAAAGTCT-----AAGCCACTGTAACAAAATGCAAA  25399
Dog          TGTTATACTCTTCT-GGCCAGCATTTAT--GTGT-----GATTTTAATTCATG---TGTGAAATTATTTGAGTGGATA--  24805
BelugaWhale  AAATTAACTCAAA--ATGGATCATAGACC--TAAATATAGAATGCAGAACTAT---AAAACTTTTAGAAGATAAGGTAAG  21053
Donkey       GTATGTTCTTGGCTCCATTGTCGAAGATTTGCTGTCCCTAGATATGTGATTTTATTTCTGGGCTTTCAGTACTGTTCCAT  25498
Cat          AAATAAAATTGTCC-TATCAGTTAATGT--GGAAACATTAACGTTATTAGTAC---TATAGAATCACTTATTTTGGGCAT  27959
Ferret       AGACAGAGATCACA-AGCAGGCAGAGAG--GCAGACA--GAGAGGAAGGGAAA---CAGGCGCCCTGTAGAGCAGAGAAC  24430
Lion         AAATAAAATTGTCC-TATAAGTTAATGT--GGAAACATTAATGTTATTGGTAC---TATAGAATCACTTATTTTGGGCAT  26286
Vaquita      AAATTAACTCAAA--ATGGATCATAGACC--TAAATATAGAATGCAGAACTAT---AAAACTTTCAGAAGATAAGGTAAG  21732
Pig          AAATTAACTCAA-------GGT--GGAT---CACAGACTAGATGTAAAGT-------------------GCGAAAT--AAG  20181

Majority     AGAAGAACTAGGTGG-TTAXATAXX-TXXAAGXTXTXTTTTTAGXX---XAX-X-TAXAACTTTAATTAAX--------T
                     30330     30340     30350     30360     30370     30380     30390     30400
Human        TGCAGAATATGGTGGCATAAATAAAAGAGTTTTTTTGTTTCTTTTTCTGTCATAGCAGAAGTCCTGTTGGTATAAGAAAG  26654
PolarBear    --------------------------------------------------------------------------------  0
Cow          TGAAGAACATAGTGGCATAAATAGCCTAGAAGTTTATTTTTCATAA---AAGTCCTGCAACTGTAAGAAAAT---GGTAT  25473
Dog          TGGATGATTAAGCCG-CCACACAAATATAAGCATTTTTTTAAAACC---AAGTAGACAAGACTTGGCCAATT---CCATT  24878
BelugaWhale  AGAAGATCTAGATGAGTTGGGTAT----AAAGATGTCTTTTTAGG----------TACCACACCAAAGA-----------  21108
Donkey       TGATATACGTGTCTGTTTTTGTGCCAGTACCATGCTGTTTTGATGACTGTAGCTTTGTAGTATATTTTGAAGTCAGCGAT  25578
Cat          AGTAGTGGTAAGCTT--TATATTCAGTGGAAAAGCCAACATAACTT---TAAAAAAAAATTTTTTTTTAATGTTTATTTT  28034
Ferret       CCAATGCAGAGCTTGATCCCAGGACCCTGGGGTCATGACTTGAGCT---GAAGGCAGAGGCTTTAACCCACTGAGCCACC  24507
Lion         AGTAGTGGTAAGCTT--TATATTCAGTGGAAAGGCCAACATAACTT---TAAAAAAAAATTTTTTTTTAATGTTTATTTT  26361
Vaquita      AGAAGATCTAGATGAGTTGGGTAT----AAAGATGTCTTTTTAGG----------TACCACACCAAAGA-----------  21787
Pig          AGAAAATCTAGATGACTTAGGTA---TAAAAGGTATCTTTTTAGA----------TGCAACACCAAAGA-----------  20237
```

```
Majority     CATXATCCCTCAAGC------GATTAATTTXTAAGCTGXGTTTXTGTAAAATXXXTAATTT-X--TXGX-TCCAGG--X-
                      30410     30420     30430     30440     30450     30460     30470     30480
Human        TGGTATCAGTCTGCTCCATAAGGTTATTCACTGGGCCAAGTTTCTTTCATATTGTTGCTTC---ACTGATCCCTAGGCTT  26731
PolarBear    --------------------------------------------------------------------------------  0
Cow          CATTATGCTCCATGA------GATTATTCACTGAGCTAAGTTTTTTTCATCTGGTTGCTTTCAGTTTTTTCCCTGAGCTT  25547
Dog          CACCACACCACCCCT------CATCAATATTCAT--------------GAACATATAATTGTAGAGAGG-ACAAGG----  24933
BelugaWhale  CATAATCCATGAAAA------AAATAATTTATAAGCTGGACTTTTGCAAAA-----------------------------  21153
Donkey       TGTTTTGCCTCCAGCTTTGTTGTTTTTTTCTTAAGTTTGCTTTGGCTATTAGGGGTCTTTTGTTGTTCCTTATAAATTTT  25658
Cat          TGACAGAGCGTGAGC------GAGTGGGGGAGGGGCAGAGAGAGAGGGAGACACAGAATCTGAAGCAGGCTCCAGGC-TC  28107
Ferret       CAGGAGCCCTCAGTC------ATTTGATTTTTATCTCTCTTCCAGCTTAAGTGTCTATGCCAAGATAGA-TCAAGT----  24576
Lion         TGACAGAGTGTGAGCAAGCATGAGCGGGGGAGGGGCAGAGAGAGAGGGAGACACAGAATCTGAAGCAGGCTCCAGGC-TC  26440
Vaquita      CATAATCCATGAAAA------AAATAATTTATAAGCTGGACTTTTGCAAAA-----------------------------  21832
Pig          CCTAATCCATGAAGG------AAATAATTGTTAAGCCGGACTTCATTAAAATCAGTAATTT----TTGCTCTGTGAAAGT  20307

Majority     T-GXTTGTXXGXATXGXGXCTGACGAG-ATACAAGTAXTXXCAGXXT----ATTAGA--ATXAC--TTGCTTT--XXT-G
                      30490     30500     30510     30520     30530     30540     30550     30560
Human        TTGTTCTTGGCTATGTGGTCTGTTGAT-GTGTGATTGTGCTCTTCTCCTTGTTTAAAAAAAAAGTTTTCCCTGTATTTGT  26810
PolarBear    --------------------------------------------------------------------------------  0
Cow          TTATTCTTATGTGCATGGATTAATGAG-GTATAAGTAGCTATCTTTT----TTTGTTTTGTCTCTTTTGTTGTCTTTTTG  25622
Dog          TAGAGGGTGGGAGTCGGTCCTGACACC-ACACTCTTCATGTCAGAGT----AG-AAA--GTAAC--AGGCTTC--TTTTA  25001
BelugaWhale  --G-------------------------ATACA-------------------------------------TGT------G  21163
Donkey       AGGATTCTTTGTTCTATTTCTGTGAAGAATGTCATTGCGATTCTGATTGGGATTCCATTGAATCTGTAGATTGCTTTAGG  25738
Cat          TGAGCTGTCAGCATGGAGCCCAACGCG-GAACCTGAACTCACAGACT----GTGAGATCATGACCTGGGCCCA--AGTCG  28180
Ferret       AAAACTGTGCCATCAGTTAATGTGGAA-ACATTAATATTTTCAGTAC----AATAGA--ATCAC--TTATTTT--GGGCA  24645
Lion         TGAGCTGTCAGCATGGAGCCCAACGCG-GAGCCTGAACTCACAGCCC----GTGAGATCATGACCTGGGCCCA--AGTCG  26513
Vaquita      --G-------------------------ATACA-------------------------------------TGT------G  21842
Pig          C-GGTATCAAGAAAATTAGAAGACAAG-CCACAGGCTGGGAGAAAAT----ATTTGCAGAAGATACATGTTAT-------  20374
```

```
Majority     -AAXATCAAGGGCTT-A-X---XTTAGTCAA------AXTATACAAATAATTTTXAAAAT-------TATTTAATAATAT
                     30570     30580     30590     30600     30610     30620     30630     30640
Human        GTATTTCATTGTCTTTTTTTTTTAAAGTCAGCTTTTGATTTTACTGATCCTCTTCATTGTACCATTGTTTTCTCTATTTT 26890
PolarBear    -------------------------------------------------------------------------------- 0
Cow          AAAAATCAAAAGCTTGA-TTTTATTGATCCT------CTTTTGTGTGTCATTGTTAAATT-------TATTTTGTATTTT 25688
Dog          TACTTCAGTAGATCTAA-GA--T-------------ATCTCACTCCTCATCTCCAGAAT-------TACATGAATCCAC 25057
BelugaWhale  ----ATAAAGGGCT--------GTTATCCAA------AATATACAAAAAACTTTTAAACT-------CAGC-AATAAGAA 21217
Donkey       TAATATGGATGTTTTAACTATGTTTAGTCTT------CTAATTCTTGAACATGGAATATCT---TTCCATTTGTTTATTT 25809
Cat          GACGCTCA-CCGACTGA-GC--CACCCAGGC------ACCCCATAACTTTTTTTAAAAATA---ATGTTTATAAACATAT 28247
Ferret       TAGAATGGTGAGCTTTA-TA--CTCAGTGGA------AAGACCAACATAATTTAAAAAAA-------TGTTTATAAACAT 24709
Lion         GACGCTCAACCGACTGA-GC--CACCCAGGC------ACCCCATAACTTTTTTAAAAAATA---ATGTTTATAAACATAT 26581
Vaquita      ----ATAAAGGGCT--------GTTATCCAA------AATATACAAAAAACTTTTAAACT-------CAGC-AATAAGAA 21896
Pig          -AAAACGGTGGGCT--------GTTAGCCAA------AATATATGTAGAATTTTTAAA----------AATTAGCAGTAA 20429

Majority     TATAAGTAGXXTTAGTAAAXATATCA-AXAATTTTAAATA--TAGATATTTCACX---------GACACGATTTTCAAXG
                     30650     30660     30670     30680     30690     30700     30710     30720
Human        TATTGGTTGCGTGAGTCAGGGCTTTTGCAAGTCTTTAATAGGTAGGTGGTATTCAGTCCACTTCTTCATCATCTTGGTGG 26970
PolarBear    -------------------------------------------------------------------------------- 0
Cow          TATTGGTTGTGTGAGTTTGTGCTCTC-ATGGTGTTCAGCAGGTAGATTCCTCTCT-T-------CGTGGAATCTTCAAAG 25759
Dog          TGGAGGAAGGAGAGGAAGGATTTTTG-AATTTTTATATTTTTTTTGTTTTTGACT-C-------AACACAAACATCCTAA 25128
BelugaWhale  AACAACCCG-ATTAGGAAATAGACCA-AGGAACTTAAC-----AGACATCTCACC-A-------AAGAAGATATACAGA- 21281
Donkey       CTTTGATTTCTTTCAATAACGTGTT--GTAGTTTTCATTGTATAGGTCTTTCACC-TCCTGGGTGAAATGTATTCCAAGA 25886
Cat          TTTCAGTAATTTTGGTAAAAATATCA-AGAATTGCAAATAATTTTATATTCTACT---------GACTCTGTTATGAAGG 28317
Ferret       TTTCAGTAGTTTTGGTAAAAATATCT-AGAATTTCAAATAACTTGATATTCTGCT---------GACTCAGATATGAAGG 24779
Lion         TTTCAGTAATTTTGGTAAAAATATCA-AGAATTGCAAATAATTTTATATTCT------------GACTCTGTTATGAAGG 26648
Vaquita      AACAACCCG-ATTAGGAAATAGACCA-AAAAACTTAAC-----AGACATCTCACC-A-------AAGAAGATATACAGA- 21960
Pig          GAAAACTCG-ATTAGGAAATGAACCA-AAGGCCTTAAG-----AGACACCTCACC-A-------GAGAAGATATGCAGA- 20493
```

```
Majority     XGGTTAATACTXATXTGATAAG--GTACT-X-T---TXAXAT-CATTTGTCX-ATTAAGGAAATGCAX---X-AACTXAA
                               30730     30740     30750     30760     30770     30780     30790     30800
Human        CACCTCTGTTTAAGGAGACCAGGAATACTCACCTGGGTACGTACCTTTCTTCCTCTCTGATAATCTATTTTTCCTCAGGA  27050
PolarBear    --------------------------------------------------------------------------------  0
Cow          TGAGAAATACTGGAGTGGGTTG--CCATTTCCTTCTCCAGGG-GATCTTCCCAATCCAGGGATTGAACCTGC-ATCTCTT  25835
Dog          CATTGTAAACTGATAAGAT-----GTATTTCAAA--AGAAGT-CACGTCTCACTTTTTTGAAAAGTGTTGGG-AAAGTGG  25199
BelugaWhale  TGGAAAATAAGCATATGAAAAG--ATGCT-------TCACAT-CAAATGTC--ATTAAGGAAATGCA------AACTGAA  21343
Donkey       TATTTTATTCTTTT-TGTTGCA--GTTCTCTTTTTCCTAGTT-CATTATTAGTGTGTAG-AAATGCAACGGA-TTTTTAT  25960
Cat          CTGTTAAACCTACTGTAGTTTA--GCAGCATGTG--TGAAAT-CATTTGAGGGAGAAAC-AAGACCACAAAT-AGAATG-  28389
Ferret       CTGTTAAACCTGTTCTTGTA----GCAGCATGTG--TGAAAT-CATTTGAAGGAGAAAAAAAGCCCACAAAT-AAAGGAT  24851
Lion         CTGTTAAACCTACTGTATTTTA--GCAGCATGTG--TGAAAT-CATTTGAGGGAGAAAC-AAGACCACAAAT-AGAATG-  26720
Vaquita      TGGAAAATAAGCATATGAAAAG--ATGCT-------TCACAT-CATATGTC--ATTAAGGAAATGCA------AACTGAA  22022
Pig          TGGCAAATAAGCATATGAAAAG--ATATT-------CCACAT-CATATACC--GTTAAGGAAATGCA------AACTAAA  20555
```

```
Majority     ATATCXTTTTTAT--XX-------XXTGCT-X---T-XTTACTTCCTGAAXAGCC-TX-T---X---XX-AGTGTCXTGA
                               30810     30820     30830     30840     30850     30860     30870     30880
Human        ACTTAATATTTGTGTCCTTGCCCGGTGTCTTTCTGTCATGGTTCCCTAGGCTGCCATCTCCACAGGAGAAAATGTTTGGT  27130
PolarBear    --------------------------------------------------------------------------------  0
Cow          ACATCTCCTGTATTGGCA-GGTGGATTCTTTACCACTTGCATCACCTGGGAAGTCCATATTC-ATGTATAGAAATACTCA  25913
Dog          TGGTTTTTTTTGAGGAAC------GATGGG-ACAATTGTTAATTACAAAACATTCATCTTCA-CTTTATTGTTGTCCCAA  25271
BelugaWhale  ATAACAATGAGAT-----------ACTGCT--------ACACTGTCAGAATAGCC---------------AGAGTCTGGA  21389
Donkey       GTTGATTTTGTACCCTGCAACTCTGTTGTAGTTGTTGATTATTTCTCATAGTTTTCTGGTAG-ATTCTTTAGGGTTTTCT  26039
Cat          ---------TTAAAAAAA-------------AAATTAATTCGTTAATGAACATATATTGTAG-GAAGGAGAGTGTTCTGC  28446
Ferret       TTTTTTTTTTTAAGAAATCAACAAGACGTT-GTCATTCCTACTTCCTACATACATTGTAGGA-AGGAGAGGGTGTCCTGC  24929
Lion         ---------TTAAAAAAA-------------AAATTAATTCATTAATGAACATATGTTGTAG-GAAGGAGAGTGTTCTGC  26777
Vaquita      ATAACAATGAGAT-----------ACTGCT--------ACACTGTCAGAATAGCC---------------AGAGTCTGGA  22068
Pig          ACAACACTAGAAT-----------ACTGCT--------ACACTATCAGAATAGCC---------------AAAATCTGGA  20601
```

```
Majority     ACACXGXAAATCATAAATXCTGGCXAXGATX-----TAGAXTCAGXGAAATCTXAX----X-AT-TCCTGATGCCTTAT-
                      30890     30900     30910     30920     30930     30940     30950     30960
Human        TAGTTCTCAGTCCCAAACCATAGCAGAGCCC----AGAGACTCTTGTCACCCTCAGCCTTCAGTCCTTGGTTGCCTTCTT  27206
PolarBear    --------------------------------------------------------------------------------  0
Cow          ATATTGTCAGGTGTCACTTCTTCCCAAAATGATCTATAGAGTCAGTGCAATCCCAGTCA-AAAT-TCCAGCAA-GTTAT-  25989
Dog          ACA-ATTGTTTGTGAACCCTTTAGTGTGGTT-----TCGTGACACTTTGGGGTACT------AT-CGTAGAGGTGACA--  25336
BelugaWhale  ACACTGGAAACCATAAATGCTGGCAAGTATG---TGGAGTGTCAG-GAATTCTCAT----ATAT-TGGGAATGCAAAAT-  21459
Donkey       ATATATAGAATCATAGCTCCTGACAACAGCAAGAGTTTTACTTTGTCCTTTCCAATTTGTATATCTTTAAATATTTCTTT  26119
Cat          ACACCACACATCAGAATTACAGGCT----TT-----TATACTTCAGAAGACCTAAG------AT-TCCTCACCCCTTATC  28510
Ferret       ATGCATCACATCAGGATTACAGGCTTTCTTT-----TATACTTAAGTAGATCTAAG------AT-TCCTCACTCCTCATC  24997
Lion         ACACCACACATCAGAATTACAGGCT----TT-----TATACTTCAGAAGACCTAAG------AT-TCCTCACCCCTTATC  26841
Vaquita      ACACTGGAAACCATAAATGCTGGCAAGGATG---TGCAGTGTCAG-GAATGCTCAT----ATAT-TGGGAATGCAAAAT-  22138
Pig          ACACAGGCAACCATAAATGCTGGTGAGGATG-----TGGAG-CATTGGAAACTCAT----ACAT-TCCTGGTG-GGAGT-  20668

Majority     TACATAGCTACTTAX----------GAACXC--XXTGGAGTTTAXCTATT----------TATTACAACAAACAACA---
                      30970     30980     30990     31000     31010     31020     31030     31040
Human        CGCAGACCTGGCAGCTCTTCCACCTGAAAGTGATACATGGTATTAATAGTTGGACTAGACTATTATGATAAACAGAGTAT  27286
PolarBear    --------------------------------------------------------------------------------  0
Cow          TATATGGGTATCAAC----------ATATTGATTGTGAAGTTTATATGGAGAAGCAAAAACTCCAGAATAGCCAACA---  26056
Dog          --CACTGCTGAATGT----------GAGCCC--ACGGCATTCATGCTGTC------------TTCTGACTAATCACA---  25387
BelugaWhale  TATATAGCCACTTTG----------GAAA-------ACAGTTTGGCTCT-----------TCTTACAAAACTGACCA---  21508
Donkey       TTCTTGCCTAATTGCTCTGCA----GAGCACTTTTTCTTAATGCCATATTGCATACTTATCATAAAAACAGAGAACAC--  26193
Cat          TCCAGAGTTACTTAT----------GAACCCCCACTGGTATGGAAGGATTAT--------GACTCCCGCAAACATCT---  28569
Ferret       TCCAGATTTACTTAT----------GAATCC--ACTGGGATGGAAGCATT------------TTGACGCATACATCT---  25050
Lion         TCCAGAGTTACTTAT----------GAACCCCCACTGGTATGGAAGGATTAT--------GACTCCTGCAAACATCT---  26900
Vaquita      TATATAGCCACTTTG----------GAAA-------ACAGTTTGGCTCT-----------TCTTACAAAACTGACCA---  22187
Pig          TACATAGCTACTTTC----------AAA---------GAGTTTGGCTG------------TTCTTAAATAGTGAACA---  20714
```

```
Majority     --AAAAAGTAAAGTAXTATAA---AXT------------T--AAACAACTCXAX-AXTTTGTTTTAAXCTATAGGAGTCT
                      31050     31060     31070     31080     31090     31100     31110     31120
Human        CTAATAACCATGAGAATAAAAAACAAGAAATTTGTAACATTGAAATGAGTGCTA-AATGGCCTTTAATCTGTACTTGCTT  27365
PolarBear    --------------------------------------------------------------------------------  0
Cow          --CAGTATTGAAGGAGAATAACAAAGTTGGAGGACTGATACTACTCAACTTCAAGATTTAATATGAAGCTACAGTAATCA  26134
Dog          --AAAAATCACTGTGGTTTAG---AAT------------TATCAACAACTTAATCAACTTAATCCCAGATTTGGATGATT  25450
BelugaWhale  --TACTCGTACTGTACGATCC---AG-------------------CAACTCG-----TTGGTATTTATCCAAAGGAGTTT  21559
Donkey       -TTGGGCCAGCCCCAGTATAGCCTAGTGCTTAAGTTCAGCTTGCTCCACTTCAGTGGCCTGGGTTTGGTTCCCAGGCGCT  26272
Cat          --AAAAAGTAAACTAATAATATA-TATA-------TACATGAAAACAGGTCAAGTATACTTTTGAAAAGTATGGGAGTCT  28639
Ferret       --AAAAAGCAAACTGATAAGACC-TAT------------TGAAAAGAATTCAGGTATACTTTTGAAAAGTATGGGAGTCT  25115
Lion         --AAAAAGTAAACTAATAATATA-TATA-------TACATGAAAACAGGTCAAGTATACTTTTGAAAAGTATGGGAGTCT  26970
Vaquita      --TACTCGTACTGTACGATCC---AG-------------------CAACTCG-----TTGGTATTTATCCAAAGGAGTTT  22238
Pig          --TACTCTTAAAGAAGCA-------G-------------------CAACTCC-----TTGGTATTTACCCAGAGGAATGG  20761

Majority     ATGX----T-AGTTTTTGTGCAXXTXX---AATAXAAAXCTGCAXXTAXAXCTTAATAAACA-XGATATATXCACXCTGG
                      31130     31140     31150     31160     31170     31180     31190     31200
Human        TTGCACATTTTTTTTTAAGTGGTTTCTTTCTCTGACGTGTAGAGTGTATCTTCCTATTTCTGAATTTTCTTGTTTCTGTG  27445
PolarBear    --------------------------------------------------------------------------------  0
Cow          AGGCAGCCT-GGTTTTCGTGAAACAGAA--TAGAGAGCCCAGCATAGAGCCCCCTATAAATATAGTCAAACCTATTTTTG  26211
Dog          TTGTAAAT--GTGTTCTCTCCATATATG--AATGAGGA-ATATTCTAAAGCTTAGAATAGTTAAGAAATTT--ACTAGGT  25523
BelugaWhale  AA--------AACTTACGTATA--------CACAGAAACCTGCACGTGGATCTTAATAG-CA--GCTTTATTCATAATAG  21620
Donkey       GACCCACACTGCTCTGTTAGCAGCCA----TGCTGTACTGGCAGCCCACACACTGAAAAATAGAGGAATATTGGCACAGA  26348
Cat          GTGTTTTCTGAGAACTTGGGCAACTGTT--AATAAT---CAGCATTCATTCATTAGTCAACAGTTATATATGGACCCTGT  28714
Ferret       TTGTTTTTTAAGAACTAGGGCAATTGTT--AATAAGCA-TTCATTCACTTATTTAGTTAGCAATTACATGTGCACCCTGT  25192
Lion         GTGTTTTCTGAGAACTTGGGCAACTGTT--AATAATAATCAGCATTCATTGATTAGTCAACAGTTATATATGGACCCTGT  27048
Vaquita      AA--------AACTTACGTATA--------CACAGAAACCTGCACGTGGATCTTAATAG-CA--GCTTTATTCATAATAG  22299
Pig          AA------T-ACTTTATGTACA--------CATGGAAACCTGCACATAGATCTTAATAGGCA--GCTTCATTCACAGTTG  20824
```

```
Majority     GCXTXGCTGGXAXXX-AXAAXAXX--X---AXGAT-GTCTTTCTGXTCGGTGCXAATGGXCTXA-TAAACTXXATGTGXX

                      31210     31220     31230     31240     31250     31260     31270     31280

Human        GGATGTTAGTTTCTCTTGAAATGGTCATTGATATTACTCAGGAAGAAAAGTTTCTGTGGTCAGATTTAATAAGAGATATC  27525
PolarBear    --------------------------------------------------------------------------------  0
Cow          ACAAAGAGGCAAAGGCAAAACAGTGGAGCAAAGATAGTCTTTCTGACAGGTGCTAGTGGAACAACTGAAATCCATGTGCA  26291
Dog          ACTTGGCTGGCAGGTGG-AAG----------------CTAAACTATCGGAGCCCCTTTTCTTT-TTTTTTTTTTTTTTTT  25584
BelugaWhale  GCAAAACATGGAAG--CAACCAA---------GAT-GTCTTTCAG-TAGGTG--AATGGATTAA-CAAACTG-TGGTACG  21683
Donkey       TGTTAGCTCAGGGTGAATCTTCCTCAGCAAAAAACAAAAAACAAAAACAAAGAAAACTAGGTTAATTCATCACATGAACA  26428
Cat          GCTTGGTTGACACTTCGGAATATTATAGTGAACACTGCTGCGGTGGGCTAGGCACACTGTCTGG-TAACCACTGTTTGTG  28793
Ferret       GCTTGGTTGTCACTTGGGAATATTGTAGTGAACACTGCCCATGTGGGCCAGGCATGCTGTCTGG-TAATAAGTGTTTGTG  25271
Lion         GCTTGGTTGACACTTCAGAATATTATAGTGAACACTGCTGCGGTGGGCTAGGCACAC----TGG-TAACCACTGTTTGTG  27123
Vaquita      GCAAAACATGGAAG--CAACCAA---------GAT-GTCTTTCAG-TAGGTG--AATGGATTAA-TAGACTG-TGGTGCA  22362
Pig          CCAAAACGTGGAAG--AAATCAG---------GAT-GTCCTTCTG-TGGGTG--AATGGATTAA-TAAAGT--ATGTACA  20886

Majority     TXTXA-AX-ATTTAAACAXA--T-GXTTTTTXTATAAATATTAAATCAATATXATTAAT-GAG-CXATTTAAAACTTATG

                      31290     31300     31310     31320     31330     31340     31350     31360

Human        TATGCTAGTCTTAACATTTA--ATTTCTTTTAGTTGAATCTAAAATTGATATCATTCTTAGAT-TAATTAAAAATTTTTT  27602
PolarBear    --------------------------------------------------------------------------------  0
Cow          AAAGAATGAATTGAGACAGACGTTAAACTTTTCACAAAAATTAACTCAAGGTGGATCATAGAC-CTAAATATAAAATGCT  26370
Dog          TTTTTTTTTTTTTATTTTTT--TTTTTTTTTTTTTTTTTTTGGAGCCCCTTTTCTTAAT-GCC-ATATTGCATACTTGTC  25660
BelugaWhale  ----------TCCCAACAA---T-GGGGTAATTATCAGCAGTAAAACAAAATGAGTTATAAAG-CCATGAAAAGG-CATG  21747
Donkey       GACTAGAAAAAAGGAAAAATCACATGATCATATCAGTAGATGCAGAGAAAGTATTTGACAGAATCCAACACCCATTCATG  26508
Cat          TGTTACAGCATTTAATCCCA--AGGATTTTAATGTAAATACTTTCTTCATATTACAAAT-GAG-GAAGTTAAAACTTAGG  28869
Ferret       TATCATAACATTTAATCCCA--AGGATTTTGATGTAAATGTTCTGTCCATGTTATGAAT-GAG-GAAGCTTAGAATAGTT  25347
Lion         TGTTACAGCATTTAATCCCA--AGGATTTTAATGTAAATACTTTCTCCATATTACAAAT-GAG-GAAGTTAAAACCTAAG  27199
Vaquita      ----------TCCCAACAA---T-GGGGTAATTATCAGCAGTAAAACAAAACGAGTTATCAAG-CCATGAAAAGG-CATG  22426
Pig          ----------TCCAGGTAG---T-GGAGTATTTATTAGCAGTAAAACAAAATGAGTTTT------CAAGTCC----TGCA  20942
```

```
Majority     AXAGTAAXTTACTACTTXAAAAGCAGGTTGGTAAATXATATAATTCAAX-----TTXTGATAATGCTATATXGCATAXXT
                              31370     31380     31390     31400     31410     31420     31430     31440
Human        AAAGAGCATGTATTCTTTAGTATTTTGATTCTAAGTACCACTAATTTGATTTGTTTCAACAGTCATTAATGTGGGGCAGT  27682
PolarBear    --------------------------------------------------------------------------------  0
Cow          AAACTATAAAATTTTTAGAAAATAATGTGGGAGAAAAATCTCGATAATTA---GGTACAAAGGTATCTTTTCAGGTACAA  26447
Dog          ATA-CAA---CACAATGAAAAATTAGGTTTGTTAATAATATAATCTAA-----TTGTAGGTCACATCAAACAACAACATA  25731
BelugaWhale  AGGGGTCCGTGGTACCATAAATGCAAATTACTAAATGAAAGAAGCCAAC--------TGAGAATGCTACATACTGTGTGA  21819
Donkey       ATA--AAAACTCTTTCAGTAACCTAGGAGTAGAGGGGAACTTCCTCAACTTGATAAAGGGCATCTACAAAAAAACTATAG  26586
Cat          ATAATTATTTACTAGTTACATAGCTGGTAGGTGGAAACTAGACTACAGAGCCCCTTTTCTTAATGCAATATTGCATACTT  28949
Ferret       AAG-TAATTTCCTAGTTACATAGCTGGTAGGTGGAAGCTAAACTATGAG-CACCTTTTCTTAATGCTATATTGCATTCTT  25425
Lion         ATAGTTATTTACTAGTTACATAGCTGGTAGGTGGAAACTAAACTACAGAGCCCCTTTTCTTAATGCAATATTGCATACTT  27279
Vaquita      AGGGGTACGTGGTACCATAAATGCAAATTACTAAATGAAAGAAGCCAAC--------TGAGAATGCTACATACTATGTGA  22498
Pig          GAGGCATGGGAGTACCTTAAATGCATATTACTAAATGAAGTCAATC-----------TGAGAATGTTATATCCTATGTGT  21011

Majority     TTCCAA-AAACATAA-TXAAAAA-AAGTXAATATTTA-XAGCAACAATAAX----AAXAXAAXXAATT----XX-X----
                              31450     31460     31470     31480     31490     31500     31510     31520
Human        CATCAATCACTACCATTGATCATTTATTTCATATACATTGTAAGTTAAATGACCAGGCTGAAATAATTCAGATGAAATTA  27762
PolarBear    --------------------------------------------------------------------------------  0
Cow          CACCAA-AGACATAA-TCCAAGA-AAGACATAATTGATTAGCTGGACTTGATT--AAAATCAGTAATTTTTTTGTC----  26518
Dog          CTAAAA-AAACAAGT-TCAAAGTCACGATGATAATAATCAGCAACAGTAGGTG--AATAAGAAAAAAA------------  25795
BelugaWhale  TTCCAG-CTATAT---TCTGAAA-AAGGCAAAACTGA--GGAGACAACAAA----AAGATAAGGGGTT------------  21876
Donkey       TACTGTCATACTTAA-TGGTGAAAAACTCAATGCTTTCTCCCTAAGATCAGGTACAAGACAAGTATGTCCCCTCTCACCA  26665
Cat          GTC-AT-AAAAACAA-TGAAAACTAGGTTAATCTATTACATCAGTAGACTGAA--AAAAGAAAAATCACATTTGACAAA-  29023
Ferret       TGCTGT-AAAAACAA-TGAAAATTAGGATAATCTCTCACATCAACAGTCCAGA--AAAAGAAAAAAAT------------  25489
Lion         GT-----AAAAACAA-TGAAAACTAGGTTAATCTATTACATCAGTAGACTGAA--AAAAGAAAAATCACATTTGACAAA-  27350
Vaquita      TTCCAG-CTATAT---TCTGAAA-AAGGCAAAACTGA--GGAGACAACAAA----AAGGTCAGGGGTT------------  22555
Pig          TTCCAA-CCACGT---TCTTGAG-AAGGCAAAATTA--TGGATACAACAAA----AAGATCAGTGGTTGCCAGGGC----  21076
```

```
Majority     ---------XACATGXGTTTAGAAGAAXAGCCAGAXX-AGAX---XA--------XXTTGAT--AX----------A-T-

                     31530     31540     31550     31560     31570     31580     31590     31600

Human        TTCTGTCAGTTAATGTGGTAATGTTAACATTTATCAGTAGTGCTGAATCACTTCTTTTGGGCATAATAGTGGTAAGCTTT  27842
PolarBear    --------------------------------------------------------------------------------  0
Cow          ------TGTGAAATATATCTAGAAGACAAGCCACAGACTTGGAGAAAATACTTGCAAATAATACACGGAGCAAAGGGCTA  26592
Dog          ---------AGCATTTGTTTTGGGCAGAATCCAACATCAGTT--------------TGTGAT------------------  25834
BelugaWhale  ---------GGGAGGAGTTTGGATGAATAGGCAGAG--------------------------------------------  21903
Donkey       CCACTTTTCAACATTCTACTGGAATTCTTGCTAATGTGGTAAGGCAAGAA----AAGAAAATAAAAAGATACACAGATTG  26741
Cat          ------TCTAATATCCACTTATGACAGAGGCTAGAAATAGATGAGAACTTCCTCAACTTGATGAAAAATATCTACAAAAA  29097
Ferret       ---------CACATGATAATAGCAATAGATGCAGAAAAAGCA--------------TTTGATGGAATCCAGCATCCACT-  25545
Lion         ------TCTAACATCCACTTATGACAAAGGCTAGAAATAGATGAGAACTTCCTCAACTTGATGAAAAATATCTACAAAAA  27424
Vaquita      ---------GGGAGGAGTTTGGATGAATAGGCAGAG--------------------------------------------  22582
Pig          ------C-TGGAACAGGGTTGGATGAATAGGCAGAG---CACAGAGATTATTTTTAAATT-TTT------------ATTA  21133

Majority     -TA-AAAAACTAXAXATAXCTTC--TXGCTTTATGX-XAXAAACTCAACXTTXTT-GAAAAACATCTACAA-----G-GA

                     31610     31620     31630     31640     31650     31660     31670     31680

Human        TTGCTCAATGCAAAGACAGGCATAATTGCTTTTTTCAAATAAAAATGTAATTTGTAAAAATACTCCAGTAATTTTTGTAA  27922
PolarBear    ------------------------------------------------------T-GAAAAACATCTACAG-----G-GG  19
Cow          TTATCTAAAATACCCAGAACTTT--CAAACTCAGCAGTAAGAAAGCAACCTGATTAGAAAGTGGACCAAAAAA-TTT-GA  26668
Dog          ----AAAAACTAGAAATAGTTTC--CAGTTTTAGGAGAACCTCCTCAACCTGGT--GAAGAACGTCTACAA-----A-GA  25900
BelugaWhale  --------------------------------------------------------------------------------  21903
Donkey       TGAAAGAAGACATGAAACTCTCG--TTGTTTGCAGATGACATGATAGTCTATGTTTGAAATTCAAA-AGAATCAACA-AA  26817
Cat          ATAAAAAACCTACAGGTAACATC--GAACTTAATGGTGAGAAACTCAACACTTTC-TAAGATTAGGTACAAGGAAAG-GA  29173
Ferret       -TACAAAAACTAGAAGTAGTTTC--TAGTTTTAGGAGAACTTTCTCAGCTTGATG-AAAAAACATCTAC-------G-GG  25613
Lion         ATAAAAAACCTACAGGTAACATC--GTACTTAATGGTGAGAAACTCAACACTTTC-TAAGATTAGGTACAAGGAAAG-GA  27500
Vaquita      --------------------------------------------------------------------------------  22582
Pig          TTATTATTATTTTTTTGGTCTTT--TTGCCTTTTC--TAGGGCTGCTCCCGCGGCATGTGGAGGTTCCCAGG--CTA-GG  21206
```

Majority	CACCXCATCA------ATXXATTXATTTCAGCATC-------AAXXXTT-X-TXAXXTCAT-------------GGTACA	
	31690 31700 31710 31720 31730 31740 31750 31760	
Human	AATCTCAAAATTGCAAATAATTTGACGTTATTCTCAGTAATGAAGGCT--GTTAAATCTCCTCTCTTAACGGCAGGTATG	28000
PolarBear	TGCCTGGGTG------GCTCATTCAGTTAAGCATC-------TACCTTTGGCTCAGGTCAT-------------GATCCC	73
Cow	CACCTCACCAAAGAAGATATACAGATGGCAGATACACATATGAAAAGATGCTCCACATCATATGTCATAAAGTAAATACA	26748
Dog	AACTAAAACAT-----ACTTAATGGTGACAAAATC-------AGCACTTTA-TAAGATTA--------------GGTACA	25953
BelugaWhale	--C------A--------------------	21905
Donkey	ATCTCCTTGAACTAATAAGCAATTATTGCAGGATTGCAGGATACAAGGTTAAT-ATACAAGTCAATTGCATTCCTGTATA	26896
Cat	CATTCCATCTC-----ATCACTTCCTTTCAACATCCTATTGGAAGCTTTTGCTAATGTGGT-------------AGGGCA	29235
Ferret	CACCTGGGTG------GCTCAGTCGATTAAGCGTC-------TGCTCTT---TCAGGTCAT-------------GGTCCC	25664
Lion	CATTCCATCTC-----ATCACTTCCTTTCAACATCCTATTGGAAGCTTTTGCTAATGTGGT-------------AGGGCA	27562
Vaquita	--C------A--------------------	22584
Pig	GGTCCAATCGGAGC-----TGTAGCTGCCAG---CGCTTGCCAGAG------CCACAGCA------ACACAG--GATCTG	21264

Majority	AGAXXXXAGGAATGGAACXXAG-ATCAGGXXCXCAGAXXXX--A------------X---X-CTCAACATXCTATXCCXA	
	31770 31780 31790 31800 31810 31820 31830 31840	
Human	AAATCATTTGAGGAAGAAAAAAGTTCATAAATACAAACTTAAAAAAAAATGGA--AAGACTTTTCCATGCAAAAAGATGC	28078
PolarBear	AGGGTCCTGGGATGGAGCCCCGCATTGGGCTCCTTGCTCGGCGGGGAGCCTGCTTTG---CCCTCTGCCTACCACTCCCC	150
Cow	AACGGAAACAACAGTGAAATACTGCTACACTATCAGAATAGCCAAAATACTGTTTCAGAATAGTTAAAACACTGGCACCC	26828
Dog	AGG---CAAGGATGTTGCCTCTTATCAGTTCCTTT-----------------------------CAACATTCTATTGCAA	26001
BelugaWhale	--	21905
Donkey	CAAATGAACAAGTGCAATTTGAAATTAAAAACACAATACCATTTACA-------TTAGCCCCCCAAAAATGAAATACTTA	26969
Cat	AGAAAAGAGAAAAGGTACACAG-ATCAAGAAAGAAGAGATAAAAT------TGTTCA---CAGATGACATGGTATATGTA	29305
Ferret	AGGGTCCTGGGAGGGAGCCCTGCATCAGGCTCTCTGCCTG----------------------CTCTGCCTGCCACTCCCT	25722
Lion	AGAAAAGAGAAAAGGTACACAG-ATCAAGAAAGAAGAGATAAAATCG---TTGTTCA---CAGATGACATGGTATATGTA	27635
Vaquita	--	22584
Pig	AGCCG---CGTCTGCAACCTACAGCACGGCTCACAGCAACGCCAGA--------------TCCTCAACCCACTGAGC---	21324

```
Majority     GTAAATGTGAAXGAX----TGAAATXTXTCTAGXACAAATAAAT----AATXAAAAT-CTX-----XAAAA---------

                       31850     31860     31870     31880     31890     31900     31910     31920

Human        ATTGCAG-GAAGGAGGAAGAGTGTCCTTCATAGCACACCACAAGTCAGTATTAGAGGCTTGCTTTTCT-ACTTGA---GT  28153
PolarBear    GTGCTTGTGTGTGCA----TGCTCTCTCTGTCTGACAAATAAAT----AAATAAAATGTT-------AAAA---------  206
Cow          GTAAAGGCCGGGGAGGATATTGAGTGTCAGGAAATCTCATAGATTCCTGGTGGGAATGCAGAATTATATAGCCACTTTGG  26908
Dog          GTTTTTGCTAATGTG-----GTGTTTTTCTTAGGGCAAAAAAAC----AAAAAAAA------------AAA---------  26051
BelugaWhale  -----------------------------------------C--------------------------------------  21906
Donkey       GTATAAATCTAACAAAATATAAATAAGATCTATGTGAGGAAAAT-----GACAAAACTCTGATGAACGAAACCAAAGGGC  27044
Cat          GAAAATCTGAAAGAAC---TGAAAAAAATCTAGAACTAAGAAGC----AATAATTGCACTG--TTGCAGGATTC------  29370
Ferret       GTGCTTGTGCATGA------GCCATTTCTCTCTGACAAATAAAT----AAATAAAATCTT-------TAAA---------  25776
Lion         GAAAATCTGAAAGAAC---TGAAAAAAATCTAGAACTAAGAAGC----AATAATTGCACTG--TTGCAGGATTC------  27700
Vaquita      -----------------------------------------C--------------------------------------  22585
Pig          ---AAGGCCAGGGA-----CCGAACCCTC--A-ACCTCAT-GGTTCCTAGTCGGATTTGTTAACCACTGAGCCGTGACGG  21392

Majority     AAAATCAAXAXAGAAAXTTXAXCTTAGTGX--AXATAXTGG---TGTATAATCTCAAXAC-TXATAAAATATATAT----

                       31930     31940     31950     31960     31970     31980     31990     32000

Human        AGACCCAAGATTCCCCACCTATCGTCTTCCAAAGTTACTTGTGATCCACTGCAATCACAAGTTTTACAGTGTGTATACTC  28233
PolarBear    AAAATCTACAAAGAAACTACAGCTAACAT---ACTTAATGG----TCAGAAACTCAACACTTTCTAAGATTAGGTA----  275
Cow          AAAACAAATTAGCTGTTTTTACAAAGCTGA--ACATACTTGTACTATATGATCCCATAACCTCTTGATACTTATTCAAAG  26986
Dog          AAAAAAAACAAAAAAACAAAAAAAAAAAA---AAAAAAAAA----AAAAAAAAAAAACACAAAAAAAAA-----------  26113
BelugaWhale  ---------AGAGGATTTTTAAGGTAGTGA--AGATACTG----TGTATGATGTTACAA-----TGACGGATATGT----  21962
Donkey       TAAATAAATAGAGTTGTTCCATGTTTGTGG--ATAGGAAGACTCAGTATTATCACGATGT-TAGTAATTCCCATCTCGAT  27121
Cat          AAGGTTCATATACAAACATCGGCTGCTTTCCTATATATCAGTGGTGAACAAGTGCAATTTGAGATTAAAAACACAA----  29446
Ferret       AAACTCTACAAAGAAACTACAGCTAACATCATACTTAATGT----GCAGAAACTCAGCACTTTGTAGGA-----------  25841
Lion         AAGGTTCATATACAAACATCGGCTGCTTTCCTATATATCAGTGGTGAACAAGTGCAATTTGAGATTAAAAACACAG----  27776
Vaquita      ---------AGAGGATTTTTAAGGTAGTGA--AGATACTG----TGTATGATGTTATAA-----TGACGGATATGT----  22641
Pig          GAACTCCCCAGAGAATTTTTAAGGTAGTGA--AAATATTT----TGTATGATGTTATTA--TAATGATGTGTATC-----  21459
```

```
Majority     -TATTACA--AATATATCCAAACACATAAATATACAAATGTATAATATTGAAAX-AATATACATAAGTXXTAAGACATGA
                      32010     32020     32030     32040     32050     32060     32070     32080
Human        TAGGTGTATGGAAGGATTTGAATTCACAAATAGTCAAACATCAGAAGAATAAGCTGATAATAATACATTTGAAAAGAGGT  28313
PolarBear    -CAAGGCA--AAGACATTCTCTCTCATCAGTTCCTTTCAACATTCTGTTGGAAC-TTTTTGCTCATGTGGTAGGGCAAGA  351
Cow          GTGTTGAA--AACTTACGTATACACAGAAACCTGCACATGTATCTTAACAGCAGCTTTATTCATAATTGCCAAAACATGA  27064
Dog          -----AAA--AAAAAAAAAAAAAAAAAAAAAAAAAAAAAAAAAAAAACAAAAAA-AAAAAAAAAAAAAAAAAAAACAAGG  26185
BelugaWhale  -CATTACA--CATTTGTCCAAACCCTTAGGCATTAAAATGTACAGTATTAA-------ACCCTTAG----------GTAA  22022
Donkey       TTATAGATTCAGTGCAATCCTAATCACAATTCCAGCAAGTTATTTTATGGATATCAGCAAACATTCTGAAGTTTATATGG  27201
Cat          -TATCACT--GACAGAACCGCAGAAAGTGAAATACCTAGGTATAAATCTGACAA-AATATATAGAAGATCTATGTGAGGA  29522
Ferret       ----GGCA--AGGACATTCCCTCTCATCAGTTCCTTTAAACATTCTATTGGAAG-TTTTTGCTGATGTGGTAGGGCAAGG  25914
Lion         -TATCACT--GACAGAACCGCAGAAAGTGAAATACCTAGGTATAAATCTGACAA-AATATATAGAAGATCTATGTGAGGA  27852
Vaquita      -CATTACA--CATTTGTCCAAACCCTTAGGTATTAAAATGTACAGTATTAA-------ACCCTTAG----------GTAA  22701
Pig          --ATTACA--TATTTATCCAAACACATTGA-------GTGTACAATA-------------TCATGA------A----TGA  21505

Majority     AAAGAACAAAAXT-------TTGAGTAC-----------X--AGAXCAAGAXGCAAAAXAA----AGAXTTATC----GT
                      32090     32100     32110     32120     32130     32140     32150     32160
Human        CAAGTATATTTTTGAAAACTAGGAGAGTTAAAGTGTTTTTGTGAACTAAGGAACAAAT-AA----GGCATTGACAATATT  28388
PolarBear    AAAGGA-AATAAA-------AGGTATAC--------------AGATCAAGAAGGAAAACAT----AAAATTGTT----GT  401
Cow          AATCAATCAAGATGTCCTACTTCAGTAGGTGAATGGATTAGTAAACTGTGGTACATAACAATGTGGTATTTATC----AG  27140
Dog          AAAAAATAAAGGG-------ATGGATACC-------------AGATCAAGATGGTAGAGAAC--AAAAATTATT----GT  26239
BelugaWhale  ACAGAACT------------TTGGGTG------------A-----TTATGATGTGTCAAAAT---AGGTTCATC----CT  22066
Donkey       AGA-GGCAAAAGACTCAGTATCCAACACAGTATTGA---AAGAGAACAAAATTGGAGCACTGACACTACCCGACTTCGAC  27277
Cat          AAACTGCAAAACT-------CTGATTAAT-------------GAACCAAAAACCAAAATAAATGGAGAGATATTCCATGT  29582
Ferret       AAAGGA-GACAAA-------AGGTATAC--------------AGATCAAGAAGCAAAACAT----AAAATTATT----GT  25964
Lion         AAACTGCAAAACT-------CTGATTAAT-------------GAACCAAAAACCAAAATAAATGGAGAGATATTCCATGT  27912
Vaquita      ACAGAACT------------TTGGGTG------------A-----TTATGATGTGTCAAAAT---AGGTTCATC----CT  22745
Pig          AC----TC------------TTGGGTA----A-------AGTGGACTTTGGGTGATAA-----TGATATTCATC----CT  21549
```

```
Majority     TTGTAG-ATA----GACTTAXTA--GCCTGXGTAAAGATTXTGAAATAATTGACAA----G--AX--AXTGTGAAXTTAX
                            32170     32180     32190     32200     32210     32220     32230     32240
Human        TTACTTGATTAGGAATTTTGTTC--AGGTGAGTAAAACTTTAAGCATCACTTAAGTTTTGTTTTTGTTTCTTACTTTTAG 28466
PolarBear    TTGCAG-AT-----GACGTGATG--GCCTGTGTAGAAAATCTGAAAGAAATGACAATCTGG--AACTAATAAGCAATTAC 471
Cow          CAGTAAAACAAAATGAATTTTCA--AGCGATGAAAAGGCATGGGGGTACCTGGTAGCATAA--AT--GCAGATTACTAAA 27214
Dog          TCACAG-AT-----AACTTGATA--GCCTCTGTAGAGAATCTGAAAGAAGTGACAA----A--AA--AATCTGGAATTGA 26303
BelugaWhale  TGGTG--ATA----AATGTACCA--GCCTGGTGGAGGATGTTG--ATAATGGGGGA------------------------ 22112
Donkey       TTACTATGAAGTTAGCGTTATCAA-GACAGTGTGGTTTTAGTGAAAGAATAGACAAATAGATCAATGGAATAGAATAGAA 27356
Cat          TTGTGG-ATAGTAAGACTCAGTATTGTCACGATACCGGTTCTTTCCAGCTTGACGTAC-AG--ATTTAGTGCAATCCTAG 29658
Ferret       TCGCAG-AT-----GACTTGATA--GCCTGTGTAGAAAATCTGAAAGAAATGACAA-------AA--AGTCTGGAACTAA 26027
Lion         TTGTGG-ATAGTAAGACTCAGTATTGTCACGATACTGGTTCTTTCCAGCTTGACGTAC-AG--ATTTAGTGCAATCCTAG 27988
Vaquita      TGGTG--ATA----AATGTACCA--GCCTGGTGGAGGATGTTG--ATAATGGGGGA------------------------ 22791
Pig          TGGTA--ATA----GGTGTACCA--GTCTGGTAGAAGATGTTG--AT--CAGGGAG----G--AG--GTTATGTATGTGG 21609

Majority     TG---XACTATXGCAAGTTAGXAXATXTATAT--ACX--XAGTTTCTAACTTTTCTGTGX----G-----X--AXCATTG
                            32250     32260     32270     32280     32290     32300     32310     32320
Human        AATATCTGATCTGGACTTCTGTGGATTTATGGAAGAGCTGGCTTTATTTCTCTATCCTGTGGGAGGG---GCAAACTTTG 28543
PolarBear    TG---CACTGTTGCAAGATACAAGGTTAATAT--ACA-GAAGTCAGTTGCTTTTCTGTAT-------------ATCAGTG 532
Cow          TG----GAAGAAGCCAATTGAGAATGCTGCAT--ACTGTAAGATTCCACCTGTATTCTGAAAAAG-G--CAAAACTATGG 27285
Dog          TAAGCCAGTATTGCACAGTTGCTTTTCTGTAT--ACCAGGAGTGAAAAAGGAAACTGCA--------------GTTTTTG 26367
BelugaWhale  -------------------------------------------------------------------------------- 22112
Donkey       AGCCCAGAAATAGGGAGCCCGGAGTAGAGCTCCCCATTAGTGTAGTCAACTTATCTCTGACAAAGGAGCAAAGGCAATGC 27436
Cat          CCCA-AATTACAGCAAGTTATTTTATGGATATCAACAAACAGATTCTAAAGTTTCTGTGGAGAGGCA--GAAGACCCCAG 29735
Ferret       TG---CACTATTGCAAGATACAAAGTTAATAT--AC---AAGTCACTTGCTTTTCTATGT-------------ATCAGTG 26086
Lion         TCCC-AATTACAGCAAGTTATTTTATGGATATCAACAAACGGATTCTAAAGTTTCTGTGGAGAGGCA--GAAGACCCCAG 28065
Vaquita      -------------------------------------------------------------------------------- 22791
Pig          TG----GCAGTGGGTATGTGGGAAATCTGTTT--CCT------TCTCAATTTTATTATAAACCGG-A--AAGTGTTCT-- 21672
```

```
Majority     A---XA-TAAATXCAGT---TTGAAATAAAATTGAA-----------X--------A--ATXTXXGXA-T----------
                      32330     32340     32350     32360     32370     32380     32390     32400
Human        ATTTTG-TATGTGGAGTAACTTGACCTTCAGTTTTTTTGGTCTGAGAACAAAGGCCAAAAAATATGTAACAGTTA--ATA  28620
PolarBear    GT--GAACAAACGCAGT---TTGAAATGAAAAA-----------------------------------------------  560
Cow          AGACAACAAAATTCAGGAGGTTGGAAGGAGTTTGGATGAAAATAGCCAGAGCACAGAGGATTTTGGGGGTAGTAAAAATA  27365
Dog          A----AATTAATTAAAT---TTGAAATTAAATTTAA--------------------A--ATCGCAATATT----------  26408
BelugaWhale  --------------------------------------------------------------------------------  22112
Donkey       AATGGAGGAAAGACAGCCTCTTTAACAAATGCTGCTGGAACAACTGGACATCTACTGGAACAACTGGAATTGAGACACAG  27516
Cat          AA--TAGTCAACACAATA--TTGAAGAAGAACAGAATCGGAGGACTGATCCTGCCCA--ATTTCAGCACTTACTGTGAAG  29809
Ferret       GT--GAACAAATGTAGT---TTGAAATAAAAAAAAA--------------------A--ATTCTTTTTTT----------  26129
Lion         AA--TAGTCAACACAATA--TTGAAGAAGAACAGAATCGGAGGACTGATCCTGCCCA--ATTTCAGCACTTACTGTGAAG  28139
Vaquita      --------------------------------------------------------------------------------  22791
Pig          ----AA---AATTTTTTTTTTTGGGGGGTCCTTTTAGGGCTATA-CCTGTGGGCATA-----T-GGAGGTTCCCAGGCTA  21738

Majority     -----XTA-----------T---X--TX--X-------AXX-X-XTACAAATAXXXC-CAGAAX-A--AXAXG-ATXCTA
                      32410     32420     32430     32440     32450     32460     32470     32480
Human        ACGTAATTCACTTACTTAGTCAATAAATATGTGGGGACATTGTACTTGGTTGACACTTGGGAATATTGTAGTGAGCACTA  28700
PolarBear    ---------------------------------------------TACAG------------------------------  565
Cow          ATGTTAAAAAAAAAAATGTTAAAAGATGGATAT-----ATGTTACTACACATTTGTC-CAGACCCACTGAATGTACAGTA  27439
Dog          -----GTT-------------------------------------TGCAAAAAAAACCCAGAGAGAACAGGCAAATGATG  26446
BelugaWhale  --------------------------------------------------------------------------------  22112
Donkey       ATGTTACACCCTTCACAAAAATTAACTCAAAATGTATCATAGACCTAAATGTAAAATGCAAAACTAGTAGACG-ATAACA  27595
Cat          CTACCGTAATTGAGA-CAGTGTGGTTTTGGTGA-----AAGAATAGACAAATAGGTCAGTGGAACAGAATAGAGATCCTA  29883
Ferret       -----TTTTTTTAAG-TGATAGAAGTATATTAA-----GTGAAGATACAGAAAAAGCTCTCAAGAGTGAGAGGGGTCCCA  26198
Lion         CTACCGTAATTGAGA-CAGTGTGGTTTTGGTGA-----AAGAATAGACAAATAGGTCAGTGGAGCAGAATAGAGATCCTA  28213
Vaquita      --------------------------------------------------------------------------------  22791
Pig          GGGGTTAA--A--------TCAGAGCTGTAGCT-----ACTGGCCTGCAC-CATGGC-CACAGC-----AATGTGGGGT-  21795
```

```
Majority     -XA-AGCXXXCCCTG-AGTX-TX-----------TGCTCATG-ACXATGCAAX--CAAGA-CAGTGAAATAAXX------
                      32490     32500     32510     32520     32530     32540     32550     32560
Human        CTGATGTGAGCCAGGCACTTTGCCTGGTAATCACTGTTCGTGTATAATTTAATCCTAAGGGCTGTGATGTAGATGTTATC  28780
PolarBear    ----------------------------------TACTCTTG-AC--AGCACC--CCAGA-AAATGAAATAAATA-----  600
Cow          TTA-AACCTTAATTAAAGTATTAAACTGGACTTTGGGTGATT-ATGATACATTAATAATC-TTTGGTAATAAATG-TACC  27515
Dog          --AAAAC-TAACCTA-GGTAATAA----------ATCTACTA-ACCAAACAAA--CCATA-GAGAAGAAGAG--------  26500
BelugaWhale  --------------------------------------------------------------------------------  22112
Donkey       TAGGAGAAAACCTAGATGTCCTTGGATGTGATGATGCCTTTT-TAGATACAACACCAAAC-ATACAATCCATGAAAGAAA  27673
Cat          GTATAGAGCCCCCTGTAAACGTAG-----CTTATCTTTGATG-ACAGAGCAAAGGCAAGA-CAGTGGAGCAAATAATGGC  29956
Ferret       --ACAGCGTTGCCTA-AAACACCA----------TACTCTTG-AC--TGCATC--CCAGA-AAATGAAATAG--------  26251
Lion         GTATAGAGCCCCCTGTAAACATAGTC-AGCTTATCTTTGATG-ACAGAGCAAAGGCAAGA-CAGTGGAGCAAATA-TGGC  28289
Vaquita      --------------------------------------------------------------------------------  22791
Pig          CTG-AGCCGTGTCTGCAGC------CTACACCACAGCTCATG-GCAATGCAG----GATC-CCT-------------A--  21847

Majority     --XCTA--A-AXAXAAG------XCTGAAATAAXTGXACATCTAXTGT--GAXAAAGTATAXGXGXAATCTG-----AAT
                      32570     32580     32590     32600     32610     32620     32630     32640
Human        CCTGTTTTACAAATAAAGAACTTCAGGCTTAGAATGATTAAGTACTGTTACCTAACTGATAAGTGGAAGCTG-----AGC  28855
PolarBear    --CCTA--G-GTATAAA------TCTAACACAA----A-ATATA------GAAGATCTATGTGAGGAA---------AAC  649
Cow          AGTCTGGTG-GAGGATGCTGATAATGGAGGAGGCTGTGCATGTGTGGTGGCAGGGAGTATATGAGAAATCTGTTC---CT  27591
Dog          -----A--A-AGAGAAG------CCGAGAAGGA----AGATCTG------GAGGAGGAAAAAAAGGAACTAA-----AAC  26551
BelugaWhale  --------------------------G-----GCGACGCATCTTTGGTAGCAGCGGGTATATGGGAAATCTGTTCCTTCT  22161
Donkey       TAATTGATA-A-ACTGGACGTCATTAAAATTAA--AATCTTCTACTCTGTGAAAGACTATCAAGAAAATCAGAAAGCAAG  27749
Cat          GTTTT---A-ACAAATGGCT---GCTGGAATAATTGGACATCTACTT---GCAAAAGAATGAATCCAGACGC-----AGT  30021
Ferret       ---CTA--G-GCATTAA------TCTAACACAA----A-ATATA------GAAGATCTTTGTGAGGAA---------AAC  26299
Lion         GTTTTT--A-ACAAATG-CT---GCTGGAATAATTGGACATCTACTT---GCAAAAGAATGAATCCAGACGC-----AGT  28354
Vaquita      --------------------------G-----GTGACGCATCTTTGGTGGCAGCGGGTATATGGGAAATCTGTTCCTTCT  22840
Pig          -ACCCACTG-AGAGAGGCC---------AGGGATTGAACTCAGGTCCTCATGGATACTACTCAGG-------------TT  21903
```

```
Majority     TCXCAAXCTTCTGATAAATXX----AACAAXXXT--XX-XXAAACXTTAAAAA-AAA--CAAAXTAGGAAACATTTTCGA
                      32650     32660     32670     32680     32690     32700     32710     32720
Human        TC-CAGAGCACCCTTTCTTAA----TACAGTATTTTCTACTTGTCATGAAAACATGAAGAAAGCTAGGAAACATTTTCTG  28930
PolarBear    TA-CAAAGCTCTGATGACTGG----AACAA-----------AAAC---------AAA---ATAAATGGAGAGATATTCCA  701
Cow          TCTCAATTTTATTTTAAATTT----AACAAGTGT--TCTAAAAAAATTAAAAAGAAAA-CAAACTAGGAAACATTTTTGT  27664
Dog          TA-CAAAATTCTGATGAATGA----AACCA-----------AAACAACCAACCCAAA---ACAAATGGAGAGATATTCCA  26612
BelugaWhale  TCTCAATTTTATTTTAAACCT----AA-AAGTGT--TCTAAAAAATTTTAAAA------AATACTAGGAAAT-TTTTTGT  22227
Donkey       CCACAGACTGGTAAAAAATATTTGCAAAAGATACATCTGGGGGTGGCCCAGTGGCACAACAGTTAAGTGCGCATGTTCTA  27829
Cat          TAACACTCTTCTGAAAAATTA----ACTCAAAGTATATCATAAACCTAAATGTAAAATGCAAAACTGTAAAATTCTTAGA  30097
Ferret       TA-CAAAATTCTGATGAATGA----AATAA-----------AACCC--------AAA---ATAAGTGGAGAGATAGTCCA  26352
Lion         TAACACTCTTCTGAAAAATTA----ACTCA-------T---AAACCTAAATGTAAAATGCAAAACTGTAAAATTCTTAGA  28420
Vaquita      TCTCAATTTTATTTTAAACCT----AA-AAGTGT--TCTAAAAAATTTTTAAA------AATACTAGGAAAT-TTTTTGT  22906
Pig          CCTTAACCTC---TGAGCCAT----GACAGGAAC--TCCTAAATTTTTTAAAA-AATC-CAAACTAGGAAGCATTTTTAT  21972

Majority     TGTTGATAGXGAA-------------XAAATCATXTATATXAXCAGG-------XTGTTAATCCTT----------AGCA
                      32730     32740     32750     32760     32770     32780     32790     32800
Human        TGTTGATAATGAG-------------CCAATAATGTGTATCAGTGGT--------TCTTAAACTTT----------AGCA  28979
PolarBear    TGTTTGTGAATA--------------GAAGACTCA-ATATTGTCAAG-------ATGTCAGTTCTTTCC-------AACT  752
Cow          TGTTGATAGTTAA-------------CCAGTAATGTTTATCAGCAGT--------TCTTAAACTTT----------AGCA  27713
Dog          TGTTTGTGGATAG-------------GAAGACTCA-ATATTGTCAAG-------ATGTCAGTTCT-----------AACT  26660
BelugaWhale  TGTTGATAGTGAA-------------CCAATAATGGATGTCAGCAGT--------TCTTAAACTTC----------AGCA  22276
Donkey       CTTTGGTGGCCTGGGGTTTGCCAGTTTGGATCCCCGGTGCGGACATGGCACTGCTTGTCAAGCCATGCTGTGGTA-GGCA  27908
Cat          AGATAATATAGGA-------------GAAAACCTATATGATCTTGGGT------ATGATGATGCCTTTTTAGATATAACA  30158
Ferret       TGTTTGTGGATAA-------------GAAGCCTCA-GTAGTGTCAAG-------ATGTCAGTTCTTTCC-------AAAT  26404
Lion         AGATAATATAGGA-------------GAAAACCTATATGATCTTGGGT------ATGATGATGCCTTTTTAGATATAACA  28481
Vaquita      TGTTGATAGTGAA-------------CCAATAATGGATGTCAGCAGT--------TCTTAAACTTC----------AGCA  22955
Pig          TGTTGCTAGTGAA-------------CGAGTAATGTATATCAGTAGT--------TCTTAACCTTT----------GGCA  22021
```

```
Majority     TGCAXCAGAAACAXCXGGAAAXCTTXTTXAAAAA-CTTAGXTCA-GTTGGATCCTATXTA-CCAXTGTTTTXTTCAGTAG
                      32810     32820     32830     32840     32850     32860     32870     32880
Human        TGCATCAGAAACACTTGGGGATTTTTTTTTTTAAACCTGGGGT-TTTTAGATCCCACCCTCAGAGTTTCTTATTCAGTAG  29058
PolarBear    TGATCTATAGATTTAATGCAATCCCAGTCAAAA---TTAGAGCAGGTTTTTTTGTAGATA-TCAGCAGGTGGATTCTAAA  828
Cow          TTCATTAGAAGCACCTGGAGGGCTTGTTAAGGAA-CTTAGGTT--GTTGGATCCTGTCT--CCATTGTTTTCTTCAGTAG  27788
Dog          TGATCTATAGATTTAATGCAATCCCAGTCAAAA---TTAGAGCAAGTTGATTT-TAGAGA-ATGGTTATCAACCAAACAG  26735
BelugaWhale  TGCATCAGAAGCATCTGAAAGACTTGTTAA--AA-CTCAGGTT--GTTGGATCCTGTCT--CCATTGTTCTGTTCAGTAT  22349
Donkey       TCCCACATATAAAATGGGGGAAGACGGGCACAGATATTAGCTCAGGGCCAGTCTTCCTCA-GCAAAAAGAGGAGGATGGG  27987
Cat          CCAACGACATAGTCCATGAAAATATAATTGATAATGCTGGACCACATTAAAAT-TAAAAA-CTTCTCTTTAAAAGACAGT  30236
Ferret       TGATCTATAGATTTAATGTAGTCCCATTCAGAC---TCAGAGCAAATTATTTTCTGGATA-TCAGCAAGCGGTTTCTGAA  26480
Lion         CCAACGACACAGTCCATGAAAATATAATTGATA-TGCTGGACCACATTAAAAT-TAAAAA-CTTCTCTTTAAAAGACAGT  28558
Vaquita      TGCATCAGAAGCATCTGAAAGGCTTGTTAA--AA-CTCAGGTT--GTTGGATCCTGTCT--CCATTGTTCTGTTCAGTAT  23028
Pig          CGCATCAGAAGCACCTGGAGAGCTTGTTAA--A--CTCAGGTT--GTTGGACCTTATCCG-TCATTGTTTTATTTAGTAG  22094

Majority     --GTCTAGGXTAG-ATCXGGGAACATATGTTGCTXAAGAAATXXCCAGGTCXA--------------XATACTGCTCTAG
                      32890     32900     32910     32920     32930     32940     32950     32960
Human        --GTCTGGGATTGGACCTGAGAACATATACTGTC-AGTAAGCTTCCAGGTCTAATCTTAGGATTAGGGACCACACTTTGA  29135
PolarBear    --GTTTCTGGGA-----AGG-----------GCAGAAGATCCAAAATAGCCAA--------------CACAGTACTGAAG  876
Cow          --GTCTAGGATGG-ATCTGAGAACATATTTCGCTAAGGAAATTTCCAGGTTTTT-------------TATATTGCTCTAG  27852
Dog          --ACGAAAAGGA-----GGG-----------GCAGAAGCCAGAAACAGATTCG--------------AGCGAAAGTTTCT  26783
BelugaWhale  --ATCTAGGATGG-ATCTGAGAACGTATGTTGCTAAGGAAATTTCCACGTCT---------------AATGCTGCTCTAG  22411
Donkey       CAGTGGATGTTAG-CTCAGGGCTAATCTTCCTCAAAAAAAAAAAAGAGATAAATCT-----------GATAAAGGACTGT  28055
Cat          --ATCAAGAGAAT-TAGAAGCTAAGCCCCAGACTGGAGAAGGCATTTGCAAAA--------------GATACATCTGATA  30299
Ferret       --GTTTCTAGGC-----AGGTTATGGGGGACACAGAAGATACAGAATAGCCAA--------------CACAATATTGAGG  26539
Lion         --ATCAAGAGAAT-TAGAAGCTAAGCCCCAGACTGGAGAAGGCATTTGCAAAA--------------GATACATCTGATA  28621
Vaquita      --ATCTAGGATGG-ATCTGAGAACATACGTTGCTAAGGAAATTTCCACGTCT---------------AATGCCGCTCTAG  23090
Pig          --GTGTTGGAAAG-ATGTGACATCATATGTTGCTAAGCAGGTGTCCAGGTCT---------------AATGCTGCTCTAG  22156
```

```
Majority     XAACCAAAA----ATTGA--GAGXCCTTAAAGTATCTAAC------TTCXGTAXATAATATCAAG------------TCX
                       32970     32980     32990     33000     33010     33020     33030     33040
Human        GAATGATTAGAGTATTCAATTAGATCTTAGGGACTACACTGTGAGAATCAGTAATGTATACAATTCTGCTCATCATGTTC  29215
PolarBear    AAAAACAGA----ATTGG---AGGACTGACACTACCCAAC------TTCAGCACTTACTGTGAAG------------CT-  930
Cow          GGACCACAA----TTTAA--GGATCTCTTAAGTATCTAAT-------TCTGTATG-----TCATG------------TCC  27902
Dog          GAAGGGAAA----A--GA---GGGGCAGAAGACCCAGAAC------CAGAGTAGCCAAGACTAAG------------TAG  26836
BelugaWhale  GGACCACAC----TTTAA--GGATCTCTAAAGTATCTAAT-------TCTGTATATCATATCATG------------TCT  22466
Donkey       TACCCAAAA----TATGCA-AAGAACTCTTAAAATTCAACA----ATAAGAAAAATCTGATTAAAA--------AAATGA  28118
Cat          AAGGACAAA----ACAGACCAAGAACTCTTAGAACTTAACA----GTGGGAAAACAATTAGAAAA------------TGG  30359
Ferret       AAAAACAAG----ACTGT---AGGCTTGACACTACCTAAC------TTCAGCACTTACTGTGAAG------------CT-  26593
Lion         AAGGACAAA----ACAGATCAAGAACTCTTAGAACTTAACA----GTGGGAAAACAATTAGAAAA------------TGG  28681
Vaquita      GGACCACAC----TTTAA--GAATCTCTAAAGTATCTAAT-------TCTGTATATCATATCATG------------TCT  23145
Pig          AGACCACA-----CTTGA--GGATCTCTAAAGCATCTAAT-------TCTGTGTA-----TCATG------------TCC  22205

Majority     ACATAAAACAGTACCATGCTGTTTCTTCCATACCXTGAACAAAXAATTAAXTAGGATATAAAAAXGXTXTCAACATXXTA
                       33050     33060     33070     33080     33090     33100     33110     33120
Human        TTATAAAACTATGCCATGCCAGATCTTCCATATCTTGATTAGAAATTGGTTAAGAAATTAGGCTTGGTGTAAATACAGTA  29295
PolarBear    ACATTAGTCAAGACAGTGTGGTTTTGGGGAAAGAATAGACAAATAGGTCAGTGGAACAGAATGGAGAGCCCAGTATAGAG  1010
Cow          TTATAAAACAGTGCCATGCTGTATCTTCCATATCTTGATTAAAAAATTAAAGATGATGTAAATGTGACTTGATACTGTGT  27982
Dog          ACAAAACTGAGTATTATTATTTTTTTTTAATAGCCAAGACAG---TATTAGTATTAAAGAAAAAAAAAAGCTACAGGGGA  26913
BelugaWhale  TTATAAAACAGTGCCATGCTGAATCTTCCATACTTTGATTAAAAAATTAAATATGATGTAAATATGGTATTAACATGACT  22546
Donkey       ACCAAAAACCTTAACAGACACCTAACCAAAGAAGATATACAGATGACAAATAAGCATATGAAAAGATGCTTCACATCATA  28198
Cat          ACCAAAGACCGTAATAGTCACTTCACCAAAGACGACGTACAGATGGCAAATAAGCATATGAAAAGATATTCCACAAAATA  30439
Ferret       ACAATAATCAAGAGAATTTTTTTCTGGTGAAAAAATAGACAAATAGGTCAGTGGAACAGAATAGAGAGCCCAGTGTAGAG  26673
Lion         ACCGAAGACCGTAATAGTCACTTCACCAAAGACGACGTACAGATGGCAAAGAAGCATATGAAAAGATATTCCACAAAATA  28761
Vaquita      TTATAAAACAGTGTCATGCTGAATCTTCCATACTTTGATTAAAAAATTAAATATGATGTAAACATGGTATTAACGTGACT  23225
Pig          TTATAAAACAATGCCATGCTGAATCGTCCATACCTTGATCAAAAAATTAAATGTGATATAGATAGAGTGTTAATGTGGCT  22285
```

```
Majority     TGXCAXTXAGGTAXTGTTXATT-------------TAATTCTTGTATATCXCTXAXTTTXATGCXAAXATGTAGAAAXCT
                           33130     33140     33150     33160     33170     33180     33190     33200
Human        TCAATGTGGCTTGAAACTGGTTG---TATTTTTCTGTTTTTTTTTTTCTTTGTGCATTTGATGCTAATAAGTAGAAAGCT   29372
PolarBear    CCCCC-GTAAATATAGTCAACT-------------CATCTTTGACAAAGGCACAAATGCAATACCA---TGGAGCAAAT-   1072
Cow          T----------T----TTATTT-------------TTCTTCTTGTATGTCTCTGAGCTTGATGTTAATAAATAGTTTGAT   28035
Dog          TGCCC---ATGGGTTGGCGAC--------------AAGCAACAGCGGTTGGGTTGGTTTTTTGCGG---TTGGTTTTTTT   26973
BelugaWhale  TGATACTATGTTTTTATTTTTC-------------TCATGCTTGTATTTCTGTGAACTTGATGCTAGCAAGTAGAAAGCT   22613
Donkey       TGTCATCAAGGAAATACAGATTAAAACAACAATGAGATAACTACTATACACCTATCAGAATAGCCAAAATCTGGAACACT   28278
Cat          TGTCATTGAGGAAATGTAAACT-------------AAAACAAGATACACACCTATCAGAATTGCCAAAATGTGGAGCACT   30506
Ferret       CCCCCTGTAAGTACAGTTGACT-------------AATCTTTGACAAAGGCTCAAGTGCAATACCA---TGGAGCAAAT-   26736
Lion         TGTCATTGAGGAAATGTGAACT-------------GAAACAAGATACACACCTATCAGAATTGCCAAAATGTGGAGCACT   28828
Vaquita      TGATACTATGTTTTTATTTTTC-------------TCATGCTTGTATTTCTGTGAACTTGATGCTAGCAAGTAGAAAGCT   23292
Pig          C----------A----TTTTTG-------------TCATGCTTGTAATTCTCTGAGTTTGATACTAATGAGTAGGAAGCT   22338

Majority     XXCAAAXTXAT--AXGGAGATGAXGTATGGAXATXTTTGGATCATTCTTATATTX--TGTAATAGAATTTATTAAGGTAT
                           33210     33220     33230     33240     33250     33260     33270     33280
Human        CTTAAAATATTTAAGGGAGACAACCTATGGATATCTGTTTACTCTTCTCATATTTGTTGTAATACAGTTTATTCATTATG   29452
PolarBear    -ACAGTCTCAT--TAGCAAATGCTGC-TGGAA-CAATTGGA-----CATCCACT---TGCAAAAGAATGAATCGAGACAC   1139
Cow          TTTAAAACAATG-AGGACAATGACATACGAATATTT----ATCATTTTTATATT----GTAATACAGCTTATTAAAGAAT   28106
Dog          GGCGGGTTGGT--TTGGCGACCCTGCCTTTGGGCTTGTGGC-----CGTATGTT---AGGGCGTGGATCCGTTAGGGC-C   27042
BelugaWhale  TTTTAAA------AAGGGGATGACGTATAAATATTTTTTCATCACTGTGATACTTATTATAATACAGCTTATTAAAGTAT   22687
Donkey       GACAACCATAA--GTGCTGATGAGGATGTGGAGTAACAGAAACCCTCATACGTTG--CTGGTGGGAATACAAAATGGTAT   28354
Cat          GACAATCTTTA--ATGCTGATGAGGATGTGAAGCAACAGGAATTTTCATATGTTG--TTAGTGGGAATGCAAAATGGTAT   30582
Ferret       -ACAGTCTCAT--TAATAAATGCTGC-TGGAAACAATTGGA-----CATCCACT---TGGAAAAGAATGAATTGAGACAC   26804
Lion         GACAGTCTTTA--ATGCTGACGAGGATGTGAAGCAACAGGAATTTTCGTATGTTG--TTGGTGGGAATGCAAAATGGTAT   28904
Vaquita      TTTTAAA------AAGGGGATGACGTATAAATATTTTTTCATCACTGTGATACTTATTATAATACAGCTTATTAAAGTAT   23366
Pig          TTTAAAACAATT-AGGGAGATGATGTATGGATATTT----ATCATTGTTATTTTT--TATAATATAGCTTGTT---GTAT   22408
```

```
Majority     AXTTAXXACACAAAXTA--ACATXAATTTTATGGXXAATTAAGATTGCTTTGTACTTTCT--AATGCTTTGATAGXTTAT
                              33290     33300     33310     33320     33330     33340     33350     33360
Human        AGTTTAAATACAAATTA--GCGTAAAGCTTATGGTTAATTTGGATTTT-TTGGACTTTCTGAAATGCTTTGATAATTCAT  29529
PolarBear    AGTTAACACTCTTAA----AAATTAATTCCATGG--ATTTAAGGTTCCTCCTTATCTTTT--CATGGTTTGATAGCCTGT  1211
Cow          GAGTTTAACACTGATTA--GCATGAAGTTTATGGTTAATTAGAATTGTTTTGGACTTTCTGAAATGCTC-GATAAATCAT  28183
Dog          TGATAATGCAGAGACCTG-GGATCGAATCCCCAC--ATC--AGGCTCC-CGGTGCATTTT--TTTTATGGAGTTGC-TGC  27113
BelugaWhale  GAGTTTAACACAAATTA--ACATAAATTTTATGGTTAATTTGGATTGTTTTGGACTTTGTAAAATGCTTTGGTAAATTAT  22765
Donkey       CGTCACTTTGGAAGACAATTTG-GCAGTTCTTACAAAACTAAACATACGCTTAGCATACG---ATCCAGCAATCGCATTC  28430
Cat          AATCACTTTGAAAAACAGTTTGGCAGTTTTATAA--AACTAAATGTAC-TCTTACCACAC--AATCCAGCAATTGTGCAT  30657
Ferret       AGTTAATGCTCTAAAGAA-AAACTAATTCCATGG--ATTTAAGGTTTCTCCTTATCTTTT--CATGGTTTGATAGCCTGT  26879
Lion         AATCACTTTGAAAAACAGTTTGGCAGTTTTATAA--AACTAAATGTAC-GCTTACCATAC--AATGCAGCAATTGTGCAT  28979
Vaquita      GAGTTTAACACAAATTA--ACATAAATTTTATGGTTAATTTGGATTGTTTTGGACTTTGTAAAATGCTTTGGTAAATTAT  23444
Pig          GTGTTTAACACAAATTA--ACA--AAGTTTATAGTTAATTTGGATTGTTTTGAACTTTCTGAAATGCTCTGGTAAATTAC  22484

Majority     CTTGTTTTTACCTCXTXT---TTX--ATTTTGGTGTTTGCACTXAA---CTTCGTACTGATTT-T-------T-TXTAAA
                              33370     33380     33390     33400     33410     33420     33430     33440
Human        TGCCTCCTTTGCACATTT---TTTTCATTTTGATGGTTGCATTTAACTTCTTTGTACTGATTT-----------TTTAAA  29595
PolarBear    TTTGTT-TTACTTCTGAAAAAT----ATCCCATTGTCTGGACATA----CCACAGTTTAGTTA-T-------TCGTTCAG  1274
Cow          CTCCTTTGTATATTTTTT---TTTCCATTTTGGTAGTTGTATTCAA---CTTCGTACTGTTTG-----------TCTAAA  28246
Dog          CTTGCT-TCTCCCC-------T----CTGCCTGTGTC-ACTCTAG----CCGCTCTCTCTCCT-C-------TCTGTGAC  27168
BelugaWhale  CTCCTTTGTACATTTTTT---TCC--ACTGTGGTGGTTGCATTCAA---CTTTGTCCTGATTG-----------TCTAAA  22826
Donkey       CTTGGTATTTACCGAAATGGGTTGA-AAACTTATGTTCACACAAAAAC-CTGCACACAGATGTTTATAGCAGCTTTTATT  28508
Cat          CTTGGTATTTACCCAGAGGAGTTGAAAACTTAATGTACACACAAAACC-CTGCACACAGATAT-T-------TATAGCAG  30728
Ferret       TTTGTT-TTACTTCAGAAAAAT----ATCCCATTGTCTGGACATA----CCACCGTTTAGTTA-T-------TCATTCAA  26942
Lion         CTTGGTATTTACCCAGAGGAGTTGAAAACTTAATGTACACACAAAACC-CTGCACACAGATAT-T-------TATAGCAG  29050
Vaquita      CTCCTTTGTACATTTTTT---TCC--ACTTTGGTGGTTGCATTCAA---CTTTGTCCTGATTG-----------TCTAAA  23505
Pig          CTTCTTTGTACATTTTTT---TCT--GTTTTGGTGGTTGCATTCAA---CTTTGTACTGATTGGT-------TGTCTAAA  22549
```

```
Majority      TCTGCTAGTXAXGAXTTAG---XTATT-TTAAATATTAAT-----XC-XTXTATGTAXGTATAXAXATAATAAAAAT-AX
                        33450     33460     33470     33480     33490     33500     33510     33520
Human         TCAGCCAGTATTAATTTAA--CCTATT-TTCAATACTAAT------------ATCCATGTATGTAAATATCCATAACTAA  29660
PolarBear     TCTACTGA--AAGACTTTTGGTTGCTC-CCAAGTTTTGAC------------AACTATGAATAAGGATGCTATAAAT-AT  1338
Cow           TCAGCTAGTATTGATTTAG---TTATT-TTAAATATTGCTT---ACCAATGTATGTAAGTATACACATACTTCAAAT-AG  28318
Dog           TATCATAA--ATAAAATATA--------AAAAAAAAAAAA------------AAAAATTAATTAAAAAAACAAAAAA-AA  27225
BelugaWhale   TCAGCTAGTGTTGATTTAG---TTATT-TTAAATATTAA------CCCATGTATGTAAGTATACACATAATTAAAAT-AT  22895
Donkey        CATAATTGCCAAAACTTGGAAACAACCAAGAGGTCACAAGGTGAAGCTTTTTCAGTAGGTGAATGGATGATTAAACTGTA  28588
Cat           CCTTATAGTCAAAACTTGGGAACATCC-AAAAGTGTTTGTG---GTATGTCTAGACACTGGGATAATTTTCAGAAGT-AA  30803
Ferret        TCTCCTGA--AAGACTTGA---------CCAAGTCTTGAC------------AACTATTAATAAGGTTGCTATAAGT-AT  26998
Lion          CCTTATAGTCAAAACTTGGGAGCATCC-AAAAGTCTTTGTG---GTATGTCTAGACACTGGGATAATTTTCAGAAGT-AA  29125
Vaquita       TCAGCTAGTGTTGATTTAG---TTATT-TTAAATATTAATA---ACCCATGTATGTAAGTATACACATAATTAAGAT-AT  23577
Pig           TCAGCTAGTGTCGATTT-------ATT-TTAAACATTAAT----ACCCATGTATGTAAGTATACACATA---------AT  22608

Majority      GTXAA-ACAGTTXATTTAGCTXTTTTAAA-TATTAGT-AGCTCACACATACTTTTGT----A--------AGGTACAXA-
                        33530     33540     33550     33560     33570     33580     33590     33600
Human         GGCAAAACAGTTGATTTTAGCTAGTTTAAATATT----AGTCCATTCATATTTTTGT-------------AAGTACATAA  29723
PolarBear     CTGTGTACAGTGTTTTTTGTGTATATCA----------AATTTTCAACTCCTTTGGG-------------AGTTGAAGA-  1394
Cow           GCAAA-ACAGTTGATTTAGCTGTTTTAAA-TATTAGTGTGCTCATATGTACTTTTGTAAGTACGCACATTACGTACGTA-  28395
Dog           AAAAA---AGCTTTTTTTTTTTTTTTTTAC--------AGTAATCAGATAGTGTAGGTT--T--------TGGTGTTGT-  27282
BelugaWhale   GCAAA-ACAGTTGATTTAGCTATTTTAAA-TATTAGTCTGCTCACACATACTTTTGT-------------ATGTACACA-  22959
Donkey        GTACACCCAGACAATGGAATATTTTTCAG----CAGTAACTCAAAATGAGCTATTGAGC-CATGAGAAAGGACCTTAAA-  28662
Cat           AAGAAAATGACCTATCAAGCCAAGAAAAGATAAGGGTGAGCCTTAAATCCATGTTACTA--AATGAA---AGAAGCCAT-  30877
Ferret        CTGTG--CAGGGTTTGTGTGTACATTG-----------AATTTTCAGTCCCTTTGGGTA--A--------ATACCAAGG-  27055
Lion          AAGAAAATGACCTATCAAGCCAAGAAAAGATAAGGGGGAACCTTAAATCCATGTTACTA--AATGAA---AGAAGCCAT-  29199
Vaquita       GCAAA-ACAGTTGATTTAGCTATTTTAAA-TATTAGTCTGCACACACATACTTTTGT-------------ATGTACACA-  23641
Pig           GTGAA-ACAGTTGATTTAGCTATTTTAAA-TATTAGTTTGCTCACACATACTTTC------A--------AGGTACGTA-  22671
```

```
Majority     TATTTAAAAAATAGATAAAXTAACATTGGX-AGTTTXAACA-TXTXTGXAGTTCCAAAAAT--ATAX-XX--XAXAACTT
                              33610     33620     33630     33640     33650     33660     33670     33680
Human        CATTTTCAAAATAGCTAAAATGTCACTGGCTGGTTTGAACA-ACTGTGCAGTTACCGAAGAG-ATG---------AATGT  29792
PolarBear    TGCATAATTGTTGGATTGTGT------AAG-AGTATATTTGTTTTATAAAACTTTCAAACT--ACTTTTCAAAGTAGCTC  1465
Cow          CACTTA-AAAACAGCTGAAACAACATTGCTTGGTTTGAACAAATTGTGTAGTTCTAAGAAC--ATATGAACATACGATTC  28472
Dog          TTTTTGAAGAACAAATAGGTCAG---TGGA-ACGATGGAAAGAATAGAGGGCCCTCGTAAT--AGATCCTGTGATAGCCC  27356
BelugaWhale  CATTTTTAAAATAGTTGAAACAACATTGGTTGGTTTGAACA---ACTGCAGTTCTGAGAAC--ATA--------CGATTT  23026
Donkey       TCCATATTAAGTGAAAGAAGTCACTCTGAGAAGGCTACATA--CTGTATGATTCCAACTATATAATGTTCTGGAAAAGGC  28740
Cat          TCTACGAAGTCTACATACTGTATGATTCTA-ACTTTATGACGTCCTGGAAAAGGCAGAACTCTAGAGACCGTAAAAACTT  30956
Ferret       TGCACAATTGTTGGATTGTGTGG---TAAG-AGTGTATTTGTTTTATAAAACTTCCAAACT--ATTTTCTAAAGTAACTC  27129
Lion         TCTGAGAAGTCTACATACTGTATGATTCTA-ACTATACAACGTCCTGGAAAAGGCAGAACTCTAGAGACCGTAAAAACTT  29278
Vaquita      CATTTTTAAAATAGTTGAAACAACATTGGTTGGTTTGAACA---ACTGCAGTTCTGAGAAC--ATA--------CGACTT  23708
Pig          CATTTA-AAAATAGCTAAAACAGTGTTG--------------GTTTTGCAGTTCCAAAAAC--AT----------GATTT  22724
```

```
Majority     AT-AACCTTATCAGTAAAGGATTTA-----AAGXAAXGATGAA-------TATAGATGAGCAGAAAGAAXTTTTTXAGTC
                              33690     33700     33710     33720     33730     33740     33750     33760
Human        AT-AACCTTTTCTGTAAAGAATTTT-----AAGACATGACCT--------TATTTTTTTGCAGAAAGAAATCTCTTCATT  29858
PolarBear    C---CCCTCACCAGAGATGGGGTTC-----GGGGAAGGATGAA-------TAAAGCAGAGCATAGAGAATTTTTT-AGGG  1529
Cow          ATGTGATTTTTCTTTAAAGGATTTG-----AAAACATGATTG--TCTCTTCTTATATTGGCAGAAAGAAATTTCTCATTC  28545
Dog          C---CCCT----GTAAATATATATC-----AACTTACCTTTGA-------CAAAG--GAGCAAATACAATACTGC--GGA  27413
BelugaWhale  AT-AACCTTTTCTTTAAAGGATTTA-----AAGACATGATTCA--ATCCCTTTATATTGGTAGAAGGAAATTTCTCATTC  23098
Donkey       AAAACTGGAGACAGTAAAAAGATCAGTGGTTGGGAGGAGTAGGTTGGAGGGAGGGATGAATAGGCAGAGCACAGAGGATG  28820
Cat          AA-GTGGTCACCAGGGATGGGGTT------GGGGAAGGATGAA-------TAAAGCAGAGCACAGAGAATTTCTT-AGGT  31021
Ferret       C---TCCTCACCAGGGATAGGGTTA-----GGGGAAGGATGAA-------TAGAGCAGAGCATAGATAATTTTTTTAGGG  27194
Lion         AA-GTGATCACCAGGGATGGGGTT------GGGGAAGGATGAA-------TAAAGCAGAGCACAGAGAATTTCTT-AGGT  29343
Vaquita      AT-AACCTTTTCTTTAAAGGATTTA-----AAGACATGATTCA--ATCCCTTTATATTGGTAGAAGGAAATTTCTCATTC  23780
Pig          AT-CATCTTATCACTAAAGGATTTA-----AAGATGTGATGCATTCTCTTTTTATATTAGCAGGAGGAAATGTTCCATTC  22798
```


Majority	CAXAGAATAT-G-XTXTGTTTACAATATTCTAXCGATXGAAATATAXC-TTACXTATTTGTTCAAAXXTAATGAATXTAA	
	33770 33780 33790 33800 33810 33820 33830 33840	
Human	CCAAAGATTTTGCCTTTGCTTTCGGTGTTCTAGCCACTGAAGTATA----TACCTGTTTA----ATTTTAATAATGATGA	29930
PolarBear	CAGTGAAAAT----ACTTTATACAACATA---ATGATAGAAGTATGTCCTTACACATTTGTTCAAACCCATCGAATGTAT	1602
Cow	CATAGATTTT-GCCTTTGCTTACAGTGTTCTAGCCATTGAAACATA----CACCTGTTTAA----TTTCAATGATTATGA	28616
Dog	TCAGGTACAT----TCTCATTAACAAATGCTGCTGG-AACAGTTGGACATCACTTACTTG--CAAAAGGAT-GAAT-CAA	27484
BelugaWhale	CATAGATTTTTGCCTTTGTTTACAGTGTTCTAGCCATTAGAATATA----TACCTAATTGTTTAATTTTAATGATTATGA	23174
Donkey	TATAGGGTAGTGAAAATACTCGATATATTATAACGATGGATATGTGTCATCGTACGTTTGTCTAAACCCACTGAATGTAC	28900
Cat	CAATGAAAAT----CCTCTATATGATATTATAATGATAGAAATACATCCTAACACATTTGTTCAAACCTGGTGAATGTAC	31097
Ferret	CAGTGAAAAT----ACTGTATACAACATAGTAATGATAGAAATATGTCGTTATATATTTGTTCAAACCCATTGAATGTAC	27270
Lion	CAGTGAAAAT----CCTCTATATGATATTATAATGATAGAAATACATCCTAACACATTTGTTCAAACCTGCTGAATGTAC	29419
Vaquita	CATAGATTTTTGCCTTTGTTTACAGTGTTCTAGCCATTAGAATATA----TACCTAATTGTTTAATTTTAATGATTATGA	23856
Pig	CAAGATTTTT-GTCTTTGTTTACAGTGTTCTAGCCATTTAAATACA----TACCTAATTG------TTTAATGATTATGA	22867

Majority	AAX-ATCAATAGTCCACCTCAT-GGTAAACTG--GATTTTCAGTGATTATGAXAXGTXCTTATXTGCTGA----XGACAA	
	33850 33860 33870 33880 33890 33900 33910 33920	
Human	AGATATTGATA-TCTAACTCA----------------------C--CAGAATGAAGTTCTAATTTTCTAA----AGACCT	29981
PolarBear	AAC-ATCAAGAGTGAACCCTCG-GGTAAACTG--GATTTTCAGTGATTATGATGTGTCATTGCAGGTTGATCCTTGG-AA	1677
Cow	AG--ATGAGTTGTCCAGATCA---T------------T---------AGTGTGAAGTTCTCATTTTCTGA----AGACCT	28666
Dog	GAC-ACTGCTAAC--ATCTTAC-AAAAAAATT--AACTCAAGATGTATTAGAAACCTAAATATAAGCTG-----CGA-AA	27552
BelugaWhale	AA--ATGAATTGTCCAGCTCAT-GTAAATGAGT-TGTCCAACTCACCATTGTGAAGTTCTCATTTTCTAA----AGACCT	23246
Donkey	AAC-ATCAAGAGTGAACTTCTTAGGTAAACTGTGGATTTTGGGTGCTTATGATGTGTCAGTATAGGTTCATCCTTTGTAA	28979
Cat	AAC-ATCAATA-----CCCTCC-GGTAAACTGCAGACTTTCAGTGATTATGATGTGTCCATGCGGGTTGATCCTTGGTAA	31170
Ferret	AG--ATCAAGAGTGAACCCTCA-GGTAAACTG--GATTTTCAGTGATTATGATGTGTCACTGCAAGTTGATCCTTGGTAA	27345
Lion	AAC-ATCAATA-----CCCTCT-GGTAAACTGCAGACTTTCAGTGATTATGATGTGTCCATGCAGGTTGATCCTTGGTAA	29492
Vaquita	AA--ATGAATTGTCCAGCTCAT-GTAAATGAGT-TGTCCAACTCACCATTGTGAAGTTCTCATTTTCTAA----AGACCT	23928
Pig	AA--ATGAGTTATTCAGCTCA---G------------C---------AGAGTGAAGTTCTCATTTTCTAA----AGACCT	22917

```
Majority     GAATTX-ACCTCTATGATGAGAGGT-TTTGAGGATGAXGGATTCAATGAATGTXTGTGGG----XAGXGTATAGATGTTT
                       33930     33940     33950     33960     33970     33980     33990     34000
Human        GAATT--ATCTGGATGAATAAAGGACCATTGGGATGGAGGATTTATAGAACGTTTCTGAG-----AAAGTCTAGCTGTTG  30054
PolarBear    TAGCT--ACCACTTTGTTGGGAGGT-TTTGGTAATGAGGGATGCGATGTATGTATGGGGGCAGGGAGTGTATAGAAATCT  1754
Cow          GAGTT--ATCTGGATGAGTAAAGGA-TATGAGGTTGAAGGATTCG--GAGTGTCTGTGAG-----AAAGTTCAACTGCTT  28736
Dog          CTGTAG-ACCTCTTAGAAGATAAGA-GGAGGGGAGAACAACCTAGATG-ACCTTTTTCGGTTATTAGCGTATATTTATAC  27629
BelugaWhale  GAATT--ATCTGGATGAGTAAAGGGCTATGAGGCTGAAGGATTCAC--AGTGTCTGTGAG-----AAAGTCCAGGTGCTG  23317
Donkey       TAAATGTACCACTCTTTTGGGCAGT--GTGGTAATGAGGAAGGCTAT--GCGTTTGTGGGGGTAGAGTGTATGAGAAATT  29055
Cat          AAACTGTACCACTGTGGTGGGAGTT-GTTGGTCATGAGGGAGGCTATGTATGTATGGGGGAAGGGAGTAAATAGAAGTCT  31249
Ferret       TAACTGTACCACTGTGCTGGGAGGT-TTTGATAATGAGGGATACAATGTATGTTTGGGGACAGGGAGTATATAGAAATCT  27424
Lion         AAACTGTACCACTGTGGTGGGAGTT-GTTGATCATGAGGGAGGCTATGTATGTATGGGGGAAGGGAGTAAATAGAAGTCT  29571
Vaquita      GAGTT--ATCTGGATGAGTAAAGGGCTATGAGGCTGAAGGATTCAC--AGTGTCTGTGAG-----AAAGTCCAGGTGCTG  23999
Pig          GAGTTT-ATCTGGATGAATAGAGGGCTTTGAGGTTGGAGGATTCACAGAATGTCTCT----------AAGTCCAGCTTCTT  22987

Majority     CXG-----XXXTTXCTTATTTTTATTAAAAAXCTAAAGCAGCTCTXXAAAACTTATA----ATTAXGAAAXCT-X---TC
                       34010     34020     34030     34040     34050     34060     34070     34080
Human        TGG-----CTTTGGTTTGTTTGT---AAACAATTGAGATAGCTTTCCAAAGCTTTTGC----TTATAGAATCTAA---TC  30119
PolarBear    CTGTTG-TCGCCTTCTCATTTTTATTGTAAACCTAAAACTGCTCTTAAAAAA-------------AGAGA--------AC  1812
Cow          TGG-----TATTTGCTTATT------AAAAAATTGATGTAGTTTTCCAAAGCTTTTA----ATTATGAAACCTGG---TC  28798
Dog          CT---------TTTTTTAGTTTTATAACCAAAAGACCTCAATCCATGAAAAA------------AAACAA--------TT  27680
BelugaWhale  TGG-----CATTAGTTTGTTTATTT-AAAAAAATGATGTAGTTCTCCAAGGCTTCTG----ACTATGAAACCTAA---TC  23384
Donkey       CCTT----TTCCTCCTCAATTTTATTGTAAACCTATAACTGCTCTAAAAAAATAA-A----ACTAGGAAA---------C  29117
Cat          CTGTTGGCTGCCTTCTCAATTTTCTTGCAAACCTAAATCTGCTCTTAAAAATTAAAAGAAAAAAAAGAAAGCTTGGAAAC  31329
Ferret       CTGTTG-CTGCCTTCTCATTTTTATTGTAAACCTAAAACTGCTCTTAAAAAATAAAAGCACAAAAAGAAA--------AC  27495
Lion         CTGTTGGCTGCCTTCTCAATTTTCTTGCAAACCTAAAGCTGCTCTTAAAAATTAAAAGAAAAAAAAGAAAGCTTGGAAAC  29651
Vaquita      TGG-----CATTAGTTTGTTTATTT-AAAAAAATGATGTAGTTCTCCAAGGCTTCTG----ATTATGAAACCTAA---TC  24066
Pig          TGG-----CATTAGTTTGTTGATTAAAAAAAGTTGATGCAGTTTTCCAGAGCTTTTG----ATTATGGAACCTTG---TC  23055
```

```
Majority     ATTTTTACTATTGATTGTCAATXAGX-----AT-----------TTAGGTGAAAGGAAGGA--AGGAAACACTGTTGGCT
                      34090     34100     34110     34120     34130     34140     34150     34160
Human        ACTTTTTCTATTTATTGTCTTTTGGG-----------------CTTAGAGTAAAGAAGGGA--AGGGAACACTGTTGCCT  30180
PolarBear    ATTTTTATTGTTGATAGTGAACCAAC-----AC-----------TTAGGTGAAAGGAAAGA--AGGAAAAACTGTTGGCT  1874
Cow          ATTTTTCCTATTTATTGCCTTTTGGA-----CT------------TGGGTTAATGGAAGGA--AGGACCTACTGTTGGCT  28859
Dog          GATTGTGCTCTGGATTACAAATTAA---------------------AAGTAAAAAGACTCT--TGCTCTTATA-AAGACA  27736
BelugaWhale  AGTTTTCCTATTTATTGTCTATTGGA-----CTA------------GGGTGAATGGAAGGA--AGGAAACACTGTTGGCT  23445
Donkey       ATTTTTATTGTTGATAGTAAACCAATGATGCATATCAGTGGTACTTAAACTTTAGCATGCATCAGAAGCACCTGGAGGGG  29197
Cat          ATTTTTATTATTGGTAGTGAGCCAGT-----AC-----------TTAGGTGAAAGGAAGAA--AGGAAGCCCTGTTGGCT  31391
Ferret       ATTTTTATTGTTGATAGTGAACCAAT-----AC-----------TTAGGTGAAAGGAAAGA--AGGAAAAACT-TAGGCT  27556
Lion         ATTTTTATTATTGGTAGTGAGCCAAT-----AC-----------TTAGGTGAAAGGAAGAA--AGGAAGCCCTGTTGGCT  29713
Vaquita      AGTTTTCCTATTTATTGTCTGTTGGA-----CTA------------GGGTGAATGGAAGGA--AGGAAACACTGTTGGCT  24127
Pig          ATTTTTCCTGTTTCTTGTCCCTAAGA-----TTATAAATTGTCTTTGGGTGAAAGGAAGGA--AGGAAACACTGTTGGTT  23128

Majority     TTGTGGAAAAGGAAGAGAG-TGGACAXGAGAGT--------GGAGTTAATTTTTT--ATTT-TAGGGATTAAAX----TT
                      34170     34180     34190     34200     34210     34220     34230     34240
Human        TTGTGAGAATGGGAGAGGGATAGATGGGAGAGTAGAGGGATGAAGTTAATTTTTT--ATTA-TAGGGATTAACA----TT  30253
PolarBear    TTGTGGAAAAGGGAGACAGATTGACAAGAAAGT--------GCAGTTAATTTTCT--ATTT-TAGGGGTTAAAA----TT  1939
Cow          TTGTGAGAAAGGAAGTTAC--GGATAGGAGAGT--------GGAGTTAACTTATT--GTTT-CAGGAATTAAA-----TT  28921
Dog          TTATCAAGAGAAATAGAAGATGAGCCACAGACT--------GGAGAAAAT-------ATTTGCAGAAGATGTG-----TC  27796
BelugaWhale  TTGTGGAAAAGGAAGAGAT--GGATAGGAGAGT--------GGAGTTAACTTT----ATTT-TAGGGATTAAA-----TT  23505
Donkey       TTGTTAAAACTCAGGTTGC-TGGACTCTACCCTCA------GGGTTTTATTCAGTAGATCTGAGTGGGTCTGAGAACATC  29270
Cat          TTGTGGAAAAGAGAGAGA--TTGACAAGAAAGT--------TAATTTTCTATTTT--AGTGGTTAAACTTTTAAAATATT  31459
Ferret       TTGTGGAAAAAGGAGAAAGATTGACAAGAAAGT--------AGAGTTAATTTTTC--TTTTGTAGGGGTTGAAA----TT  27622
Lion         TTGTGGAAAAGAGAGA----TTGACAAGAAAGT--------TAATTTTCTATTTT--AGTGGTTACAATTTTAAAATATT  29779
Vaquita      TTGTGGAAAAGGAAGAGAT--GGATAGGAGAGT--------GGAGTTAACTTTTT--ATTT-TAGGGATTAAA-----TT  24189
Pig          TTGTGGAAAAGGAAGAGATCTGGATGGGAGAGT--------GGAGTTAACTTTTT--ATTT-TAGGGATTGAA-----AT  23192
```

```
Majority     TTTAACTA-----TTCCCAATAGTGTA-------ACACCTGACATTT------------GTTTTTCAGTGAATCXATTTG
                       34250     34260     34270     34280     34290     34300     34310     34320
Human        TTTAACTA-----TTCCCAATAGTGTA-------ACATTTGACATTT----------------------------AAGTG  30293
PolarBear    TAAAACTA-----TTCCCAATAATGTA-------ACAGCTGACGTTT------------GCTTCTCAGTGAAT--ATTTG  1993
Cow          TTTTTTTA-----TTCCCAGTAGTGTA-------ACACCTGACATTT----------------TTCAGTGACACTATTTG  28973
Dog          TGATAAAG-----GACAAAACAGATCA-------ACTCTTAGAACTC------------AACAGTGAGAAAACCCAATTA  27852
BelugaWhale  TTT---TA-----TTCCCAGTAGTGTA-------ACACCTGACATTTGTTTTGACATTTGTTTTTCAGTGACACTGTTTG  23570
Donkey       TTTTGCTAACAAGTTTCCAATCTAATGCTGGTCTAGGCACCACACTT---TGAGAATCCCTAAAGTATCAAATCTGTTCG  29347
Cat          TTAAAATA-----CTGCCAGTAGTGTATGTA---ACACCTGACATTT------------GTTTTTCAGCGAAT--ACTTG  31517
Ferret       TTAAGCTA-----TTCCCAATAGTATA-------ACACCTGACATT-------------GTTTTTCAGTGAAT--ATTTG  27675
Lion         TTAAAATA-----CTGCCAATAGTGTATGTA---ACACCTGACATTT------------GTTTTTCAGCGAAT--ACTTG  29837
Vaquita      TTT---TA-----TTCCCAGTAGTGTA-------ACACCTGACATTTGTTTTGACATTTGTTTTTCAGTGACACTGTTTG  24254
Pig          GTT---TG-----TTTTCAATGGTATA-------ACACCTGACATTTGT--------------TTTCCAGTAACTATTTG  23243

Majority     AAAAGT-AC-AGATAAGTGA--------CAGA--TTAATATTTTTAAATTTAAAGAGGTCTTATGCTAATXTGTTTTT--
                       34330     34340     34350     34360     34370     34380     34390     34400
Human        AAAGGC-----------TTT--------TAGA--TTAATATTTTTAAATTTAAAGATGTCTTATGCTAATTTGTTTTT--  30350
PolarBear    AAAAAT-AT-AGATAAGTGA--------CAGA--TTCATATTTTTAAAGTTAAAAATGTCTTATGCTAACGTGTTTTT--  2059
Cow          AAAAGTTAC-AGAGAAGTGA----CTTTCAGA--TTAACATTTTTAAATTTAAAGGGGTCTTACACTAATTTGTTCTT--  29044
Dog          AAAAAT-GG-GTCTAAGACT--------TTAG--TAGACACTTC---ACCCAAGAAGATGTACAGGTGACAAATAAGC--  27915
BelugaWhale  AAAAGTTACCAGATAAGTGACAGACTTTCAGA--TTAATATTTTTAAATGTAAAGGGGTCTTATGCTAATTTGTTCTT--  23646
Donkey       TCATGTCCTTTTAAAAGTATGCCATACTGAATCTTCCATGCCTTGATAACAAATAAGGTATGATGCAAGTATGGTATTAA  29427
Cat          GAAAAT-AC-AGATAAGTGA--------TAGA--TTAATATTTTTAAATTTAAAGATAACTTATGTTAATGTGTTT----  31581
Ferret       AAAAAT-AT-AGATAAGTGA--------CAGG--TTCGTATTTTTAAAGTCAAAGATGTCTTATGCTAATGTGTTTTTTT  27743
Lion         GAAAA--AC-AGATAAGTGA--------TGGA--TTAATATTTTTAAGTTTAAAGATAACTTATGTTAATGTGTTTCT--  29902
Vaquita      AAAAGTTACCAGATAAGTGACAGACTTTCAGA--TTAATATTTTTAAATGTAAAGGGGTCTTATGCTAATTTGTTCTT--  24330
Pig          AAAAGTTAT-GAATAAGTGAAAGACTTACAGA--TTAAAATCTTTAAATTTAAAGGGATTT------ATTCTGTTTTT--  23312
```

```
Majority     ----------------GTTTTTATAGAGAGGATCTGACGAACTACTCTCAGGCAGT-GTTCTCAGT--AGTCCAAA--CT
                     34410     34420     34430     34440     34450     34460     34470     34480
Human        ----------------GTTTTCATAGAGAGGATCTGACGAACTACTCTCAGGCAGT-GTTCTCAGT--AGTCCGAA--CT  30409
PolarBear    ----------------GTTTTTATAGAGAGGATCTGACGAACTACTCTCAGGCAGT-GTTCTCAGT--AGTCCAAA--CT  2118
Cow          ----------------GTTTTTATAGAGAGGATCTGACGAACTACTCTCAGGCAGT-GTTCTCAGT--AGTCCAAA--CT  29103
Dog          ------ACATGGAAAGATGGCACCACAAATCATCAAGCATAGGGCATTCAAGGAAA-AACGCAAATTAAAACAAGA--TA  27986
BelugaWhale  ----------------GTTTTAACAGAGAGGATCTGACGAACTACTCTCAGGCAGT-GTTCTCAGT--AGTCCAAA--CT  23705
Donkey       TGTGGCTCAATGCTATGTTTTTATTTTTCTCATGCT-TGTATTTCTCTTGAGCATTTGATGCTAGT-GAGTAGAAAGCTT  29505
Cat          ---------------GTTTTTTCTAGAGAGGATCTGACGAACTACTCTCAGGCAGT-GTTCTCAGT--AGTCCAAA--CT  31641
Ferret       TGTTTTGTTTTGTTTTGTTTTTATAGAGAGGATCTGACGAACTACTCTCAGGCAGT-GTTCTCAGT--AGTCCAAA--CT  27818
Lion         ---------------GTTTTTTCTAGAGAGGATCTGACGAACTACTCTCAGGCAGT-GTTCTCAGT--AGTCCAAA--CT  29962
Vaquita      ----------------GTTTTAACAGAGAGGATCTGACGAACTACTCTCAGGCAGT-GTTCTCAGT--AGTCCAAA--CT  24389
Pig          ----------------GTTTTTCTAGAGAGGATCTGACGAACTACTCTCAGGCAGT-GTTCTCAGT--AGTCCAAA--CT  23371

Majority     CTAATATGAGCAGCATGGTG-GTTACAGGTAAGTGTTA-CTCTCTT---------AACAXTX-T---TCTCATTA-TTTG
                     34490     34500     34510     34520     34530     34540     34550     34560
Human        CTAATATGAGCAGCATGGTA-GTTACAGGTAAGTGTTA-CTCTCTT---------AGCAG---T--CTGTCAT----TTG  30469
PolarBear    CTAATATGAGCAGCATGGTG-GTTACAGGTAAGTGTTT-CTTTCTT---------AACAGTGTT--GCTTAATTA-TTTG  2184
Cow          CTAACATGACCAGCATGGTG-GTTACAGGTAAGTGTTA-CTCTCCT---------AACA----G---TCTCATTA-TTTG  29164
Dog          CTACCACACAT-GTATCAGA-ATAGCCAAAATGTATTT-AGAACTG---------AATC-TGAA--ACCCCATAA-ATGC  28050
BelugaWhale  CTAACATGAGCAGCATGGTG-GTTACAGGTAAGTGTTAACTCTCTT---------AACA----G---TCTCATTA-TTTG  23767
Donkey       TTAAAACAATTTGGGAGATGAGCTACAAATATTTGTTTACCTTTTTGATCTTTGTTATAATACAGCTTATTATAAATTTA  29585
Cat          CTAATATGAGCAGCATGGTG-GTTACAGGTAAGTGTTA-CTCTCTT---------AACAGTGTTTAATTTAATTA-TTTG  31709
Ferret       CTAATATGAGCAGCATGGTG-GTTACAGGTAAGTGTTT-CTTTCTT---------AACAGTGTT--GCTTAATTA-TTTG  27884
Lion         CTAATATGAGCAGCATGGTG-GTTACAGGTAAGTGTTA-CTCTCTT---------AACAGTGTTTAATTTAATTA-TTTG  30030
Vaquita      CTAACATGAGCAGCATGGTG-GTTACAGGTAAGTGTTAACTCTCTT---------AACA----G---TCTCATTA-TTTG  24451
Pig          CTAATATGAGCAGCATGGTG-GTTACAGGTGAGTGTTG-CTCTCTTT-------TAACA----G---TCTCAT----TTG  23431
```

```
Majority     CCA------TTTTATGGGGCAGAATGTTAAAA--------CTAAAATTTAGT---GTGTTTTACCAG--AATAGCCTTTX
                      34570     34580     34590     34600     34610     34620     34630     34640
Human        CCA------TTTTAGGGGGCAGAATGTTAAAA--------CTCAAATTTAAC--CCTGTTATACCAG--GAAAGCCTTTC  30531
PolarBear    CCA------TTTTATGGGGCATAATGTTAAAA--------CTAAAATTTA-----GTCTTTTATCAG--AATAGCCTTTT  2243
Cow          CCA------TTTTGCAGGGCAGAATGTTACA-------------AGTTTGGT---GTGCTTTACCAG--AGTAGCCTTTG  29220
Dog          TGA------CAAGGAGGAGTGTGAAGCAAGCA--------CAGGAATTAGTC---ATATGTGTTGAGTTGATGTGAAATG  28113
BelugaWhale  CCA------TTTTGCAGAGCAGAATGTTAAA-------------AGTTTAGT---GTGTTTTACCAG--AATAGCCATTA  23823
Donkey       ACAACAAATTAGCATAAGGTTTATGGTTAATTTGGATTGTCTTGAACTTCCTGAGATGCTTTGATAAATTGTCTCCTTTA  29665
Cat          CCA------TTTTATGGGTCAGAATATTAAAA--------CTAATATTTAGT---CTGCTTTACCAG--AATAGCCTTTT  31770
Ferret       CCA------TTTTATGGGGCAGAATATTAAAA--------CTAAAATTTATA---GTCTTTTGTGAG--AATAGCCTTTT  27945
Lion         CCA------TTTTATGGGTCAGAATATTAAAA--------CTAATATTTAGT---CTGCTTTACCAG--AATAGCCTTTT  30091
Vaquita      CCA------TTTTGCAGAGCAGAATGTTAAA-------------AGTTTAGT---GTGTTTTACCAG--AATAGCCTTTA  24507
Pig          CCA------TTTTACAGGGCAGAATGTTAAAC--------CTGAAGTTTAGT---GTGTTTTA--AT--AGCCCTTGTTA  23490

Majority     TTATTAXATGCT-----------ATX-TTXTGTTGAAACATTTTA----GA--GAAATTTTACTAAAGTAT---T-ATAT
                      34650     34660     34670     34680     34690     34700     34710     34720
Human        TTTTTAAATGCG-----------------TGTTGAAACATTTTA----GAG-TAAATTTTACTAAAGTAT-----ATAT  30583
PolarBear    ATAATTTATATTAA---------ATGTTTCTGTTGAAATGCGACA----GG---AAACTTTACTAGAGTATATAT-ATGT  2306
Cow          TTTTTAAATGCT---------------TTGTGTTGAAACATTTTA----GAG-GAAATTTTGCTGTAGTAG-----ATAT  29275
Dog          CGAATGCAGACTGGTT-T-----ATAGTCCCTTTGGAAAATAGTT----TGG--CAGTTTTATAGAACTAGCTAT-ACTT  28180
BelugaWhale  TTTTTAAATACT---------------CTGTGTTGAAACAGTTTA----GA--GAAATTTTACTATAGTAT-----GTAT  23877
Donkey       -TATTATACATTTTTTCATTTTGATGGTTGCATTCAACTTCTTTATTCTGACTGGTTTTAAATCAACTACTGTTAATTT  29744
Cat          ATAATTTATGTTAAATGTTTTAAATGTTCCTGTTGGAGAAAAAAA----AA---TGTTTTTGTTGAAACAC---T-ACAG  31839
Ferret       CTAATTTATGTTAAGTGTTTTAAATGTTTCTGTTGAAATGCTGCA----GAGTAAAATTTTAATAGAGTATATAT-GTGT  28020
Lion         ATAATTTATGGTAAATGTTTTAAATGTTCCTGTTGGA-AAAAAGA----AA---TGTTTCTGTTGAAACAC---T-ACAG  30159
Vaquita      TTTTTAAATACT---------------CTGTGTTGAAACAGTTTA----GA--GAAATTTTACTATAGTAT-----GTAT  24561
Pig          TTTTTAAATGCT---------------CTGTATTGAAACATTTTA----GAA-GAAATTTTACTATAGTAT-----ATAT  23545
```

```
Majority     X--------XA-XXGTGTCAAGTG-----ATAX---X---X---XXTAAAAAACCTAAAGTTGGTGACTTTTGTGGACAA
                      34730     34740     34750     34760     34770     34780     34790     34800
Human        --------------GAGCCGAGTGGAAAAAAAA--------------AAAAAACCTGAAGCTGGTAACTTTGGTGAACAA  30635
PolarBear    AAAAAAAAAAAAGTGTGTAAGGTGGGTAAAAATTTGTACTAAGAAGTAAAAAACCTAAACTTGGTGACTTCTGTGGGCAA  2386
Cow          --------------GTATCAAGTGG-----AA---------------AAAAAAACTGAAGTGGGTG--TCTTGTGGTCAA  29319
Dog          TTACC--AATAATTGTGCACCTTG-----GTATTTACCTAAAGAAGTTGAAAATTTACACACA--AAATCCTGGGCATAA  28251
BelugaWhale  --------------GTGTCAAGTG-------A---------------AAAAACCTTGAAGTGGGTGA--TTTGTGGGCAA  23919
Donkey       ACCTATTTTAAATATTAATACCCATGTATGTAAATATACACATACTTAAAATAGGCAAAACAGTTGA-TTTAGCTATTTT  29823
Cat          AG------GAAGTTTTATTAGAGT-----ATATATGTGTCA---AGTAAAATACCTAAAATTGGTGACTTTTGTGGACAA  31905
Ferret       AA------AAAATTGTGTAAAGTGTGTAAAGAAT-GTATTA--AAGTAAAAAACCTAAACTTGGTGACTTCTGTGGACAA  28091
Lion         AG------GAAGTTTTATTAGAGT-----ATATATGTGTCA---AGTAAAATACCTAAAATTGGTCACTTTTGTGGAGAA  30225
Vaquita      --------------GTGTCAAGTG-------A---------------AAAAACCTTGAAGTGGGTGA--TTTGTGGGAAA  24603
Pig          --------------GTGTCAAGTG-----------------------AAAAAACCTGAAGTGGGTGACTTTTGTGGGGAA  23588

Majority     GTA-CTAAGTC---------------------------------------------------------------------
                      34810     34820     34830     34840     34850     34860     34870     34880
Human        GT----TACTC---------------------------------------------------------------------  30642
PolarBear    GTACCTAAGT----------------------------------------------------------------------  2396
Cow          GTAATTAAATC---------------------------------------------------------------------  29330
Dog          TTACGGCAGCCATATTCATAG----------------TT-----------------------------------------  28274
BelugaWhale  GTAACTAAATC---------------------------------------------------------------------  23930
Donkey       CAATATTAGTCTATTCACACATTTTTGTAAACATACACTTTTTAAAAATAGGTAAAACAACACTGACTGGTTTGAACAAC  29903
Cat          GTA-GTAAGTG---------------------------------------------------------------------  31915
Ferret       GTACCTAACTAAGTCTTATTGCCTTTTTTTAAAAGATTTTAATTTATTTATTTGACAGACAGATCACAAGTAGGCAGAGA  28171
Lion         GT-----AGTG---------------------------------------------------------------------  30231
Vaquita      GTA-----------------------------------------------------------------------------  24606
Pig          GTAACTAAATC---------------------------------------------------------------------  23599
```

```
Majority     --------------------------------------------------------------------------------
                       34890     34900     34910     34920     34930     34940     34950     34960
Human        --------------------------------------------------------------------------------  30642
PolarBear    --------------------------------------------------------------------------------  2396
Cow          --------------------------------------------------------------------------------  29330
Dog          ---------------------------------------------------GT---------------------C------  28277
BelugaWhale  --------------------------------------------------------------------------------  23930
Donkey       TGTGCAGTTCCCAGAACGTATTAATTTATAACCCTTTCTCTGAGGGATTTAAAGACATGATTTAATCTCTTTTTATACTG  29983
Cat          --------------------------------------------------------------------------------  31915
Ferret       GGCAGGCAGAGAGAGAAGAGGAAGCAGGCTCCCTGCTGAGCAGAGAGCCCGATGTGGGGCTCAATCCCAGGGTCCTGGGA  28251
Lion         --------------------------------------------------------------------------------  30231
Vaquita      --------------------------------------------------------------------------------  24606
Pig          --------------------------------------------------------------------------------  23599

Majority     --------X-----------------------------------------------------------------------
                       34970     34980     34990     35000     35010     35020     35030     35040
Human        --------------------------------------------------------------------------------  30642
PolarBear    --------------------------------------------------------------------------------  2396
Cow          --------TT----------------------------------------------------------------------  29332
Dog          --AAAACTTGGG--------------------------------------------------------------------  28287
BelugaWhale  --------TG----------------------------------------------------------------------  23932
Donkey       GCAGGAAGAAATTTCTCATTCCATAGATTTTGCTTTTGCTTTCAGTGTTGTAGCCACTGAAGTATATACCTAATTGTTAA  30063
Cat          --------------------------------------------------------------------------------  31915
Ferret       TCATGACTTGAGCCGAAGGCAGAGGCTTTAACCCACTGAGCCACCCAGGCGCCCCTTATTGCCTTTTTTTTTTAATATTT  28331
Lion         --------------------------------------------------------------------------------  30231
Vaquita      --------------------------------------------------------------------------------  24606
Pig          --------TG----------------------------------------------------------------------  23601
```

```
Majority     --------------------------------------------------------------------------------
                             35050     35060     35070     35080     35090     35100     35110     35120
Human        --------------------------------------------------------------------------------  30642
PolarBear    --------------------------------------------------------------------------------  2396
Cow          --------------------------------------------------------------------------------  29332
Dog          ---------------------------------------------------------------------------G----  28288
BelugaWhale  --------------------------------------------------------------------------------  23932
Donkey       TTTTAATGATGATCATGAAGACTAGTTGTCCAATTCACCAGTATAAAGTTCTGATTTTCTGAAGTTTCTGATTTTCTCAG  30143
Cat          --------------------------------------------------------------------------------  31915
Ferret       TATTTATTTATCAGAGGGAGAGAGGGGGAGAGAGCGAGCACAGGCAGACAGAATGGCAGGCAGAGGGAGAAGCAGGCTTC  28411
Lion         --------------------------------------------------------------------------------  30231
Vaquita      --------------------------------------------------------------------------------  24606
Pig          --------------------------------------------------------------------------------  23601

Majority     --------------------------------------------------------------------------------
                             35130     35140     35150     35160     35170     35180     35190     35200
Human        ---------------------------------------------TT---------------------------------  30644
PolarBear    --------------------------------------------------------------------------------  2396
Cow          --------------------------------------------------------------------------------  29332
Dog          ------AGCAAG--------------------------------------------------------------------  28294
BelugaWhale  --------------------------------------------------------------------------------  23932
Donkey       TTATCTGGATGATTAAAGGCTGTAGGGTTGGAGGAGTCAGAGTATTTGTGAAAAAGTCCAGTGCCTGTGGCATTAGTTTG  30223
Cat          --------------------------------------------------------------------------------  31915
Ferret       CCGCTGAGCAAGGAGCCCGATGCAGGACTCGATCCCAGGACGCTGGGATCATGACCTGAGCCGAAGGCAGCTGCTTAACC  28491
Lion         --------------------------------------------------------------------------------  30231
Vaquita      --------------------------------------------------------------------------------  24606
Pig          --------------------------------------------------------------------------------  23601
```

```
Majority     -----------------------XTXXTTXCXTTTTTTTAATCACAAAAATATAACCTTTTTTTCTTTCA-TGTT--ACA

                      35210     35220     35230     35240     35250     35260     35270     35280

Human        -----------------------AAATTTGATTGCTTTTCATTTTTGAACCACAAATGTAATTCTGTCTCACATT---AT  30698
PolarBear    -----------------------CTGATTGCCTTTTTTAAATCAAAAAAATATAACCA-TTTTTCTTTCA-TGTT--CTA  2449
Cow          -----------------------ACAGCTTTTTTTTTTTAATGACAAATGTA-ACCTTTTTTTTCTTT---CATTT-ACA  29384
Dog          -----------------------CAAAACTCTCTCAGTAGATTGAATGAAT--AACTA-AACTGTGGTAA-GTCT--AGA  28345
BelugaWhale  -----------------------A-------TTGCTTTTAATCACAAAAATGTAACCTTTTTTTCTTTCA-TGTT--ACA  23979
Donkey       TTTATGAAAAAATTGAGGTGGTTCTCCAAAGCTTTTTTTGATTATGGAACCTAATCATTTTTCCTATTTATTGTCTTCTA  30303
Cat          -----------------------TTTTTGCCCTTTCTTTAATCACAAAAATATAACC--TTTTCCTTTCA-TGTT--AAA  31967
Ferret       AACTGAGCCACCCAGGCGTCCCCCTTATTGCCTTTTTTAAATCCCAAAAATATAAACC-TTTTTCTTTCA-TGTT--CTA  28567
Lion         -----------------------TTTTTGCCCTTTCTTTAATCACAAAAATATAAAC--TTTTCCTTTCA-TGTT--AAA  30283
Vaquita      -------------------------------TTGCTTTTAATCACAAAAATGTAACCTTTTTTTCTTTCA-TGTT--ACA  24652
Pig          -----------------------A--------TTGCTTTAAAAACAAACAAA-ACCCTTTTTTTCTTTGG-TGTTA-ACA  23647

Majority     AGTTCCCTCA-XTCTXACCCC--TTTTGTACTTTTGTTTTTAGXTTTTATGTT----TGXGTGTCATA---AAAT--ATT

                      35290     35300     35310     35320     35330     35340     35350     35360

Human        AGTCCCTTCATCTGCCCCTCTGGTATTTTTATTTTGTTTTTATTTTTTGTGTT----TGTGTGTCATA---TAAT--ATT  30769
PolarBear    AGTCCCCTCT-TTCTTGCCCC--TTTTGTACTTTTCTTTTTGGTTTTTGTGTT----TGCACGTCTTA---GAAT--ATT  2517
Cow          AGTTTCCTCA-CTCTCACT----TTTTGTACTTGCATGTTTAGCTTTCATGTT----TCTTTGTCATA---AAAT--ATT  29450
Dog          CAGTGGGATA-TTTTTCAAAA--GTTTTTACTTTTGAAATGGGCTTTCAAACCA---TGAAAAGATTG---AGGC--ACC  28414
BelugaWhale  AGTTCCCTCA-CTCTCACTCC--TTTTGTACTTTTGTGCTTAGCTTCTATTGT----G---TGTCATA---AAAT--ATT  24044
Donkey       GACTTGGGTGAAAGGAAGGAA--GGAAAAACTGTTGGTTTTGCGGAATAGGGAGAGATTGATAGGAGAGTGGAGTTAATT  30381
Cat          AGTCCCCTCA-TTCTTGCCCC--TTTTATCCTTTTGTTTTTAGTTTTTGTGTT----TGAGTGTCTTACAGAAAA--ATT  32038
Ferret       AATCTTCCCA-TTCTTGCCC---TTTTGTATTTTTGTTTTTAGTTTTTGTGTT----TGCGTGTCTTA---GAAT--ATT  28634
Lion         AGTCCCCTCA-TTCTTGCCCC--TTTTATCCTTTTGTTTTTAGTTTTTGTGTT----TGAGTGTCTTACAGAAAA--ATT  30354
Vaquita      AGTTCCCTCA-CTCTCACTCC--TTTTGTACTTTTGTGCTTAGCTTCTATTGT----G---TGTCATA---AAAT--ATT  24717
Pig          AATTTCCTCAACTATCACCCC--TTTTGTACTTTGTGTTTTCGCTTTTATGTT----T----GTCATA---AAAT--ATT  23712
```

```
Majority     TTTXATXTAAGTATTTCTT----------------CTCXATCCCGXAA-----ATCCTAAGXCXCAXAGTGTATGX-TGA
                      35370     35380     35390     35400     35410     35420     35430     35440
Human        TTTACTTTAAGTATTTAT-----------------------GCCACAA-----ACCCTAAAACCCAAATTTTAAGT---A  30818
PolarBear    TTTTACATA-GTCCCCGT------------------GCCTCCCTGAGAGATAAGTTCCAAGACTTGCAGGGAATGCCTGA  2578
Cow          TTTACTGTAAGTGTTTCTT----------------CTCAGTCCCACAA-----ATCCTACAGCCCCAAGTACAA------  29503
Dog          TTAAGTCTATGTTATTAA--------GGGA-AAGGAGCCATTCTGAGA----------AGGCTACATACTGTATGTATGG  28475
BelugaWhale  TTCATTTTAAGTATTTCTT----------------CTCTATCCCATAA-----ATCCTAAAGCCCAAACTGTAAAA---A  24100
Donkey       TTTTATTTTAGGGATTAAAT---TTTTTAACTATTTCCAGTAGTGTAATACCTGACGTTTGTTTTTCGGTGAATATTTGA  30458
Cat          TTTTATAATAGTTCCCCCC-----------------ACCTCCCTGGGC-ATGTGTTCCAAGATTTGCAGTGGATGCCTGA  32100
Ferret       TTTTACATA-GTCCTCATCAACCCCCCGCCCACCCCACTCTTCTGAGACATATGTTCCAAGACTCACAGTGGATGCCTGA  28713
Lion         TTTTATAATAGTTCCCCCCC----------------ACCTCCCCGGGC-TTGTTTTCCAAGTTTTGCAGTGGATGCCTGA  30417
Vaquita      TTTATTTTAAGTATTTCTT----------------CTCTATCCCATAA-----ATCCTAAAGCCCAAACTCTAAAA---A  24773
Pig          TTT----TAAGTATTTCTT----------------CTCTATCCCACAA-----ATCCTAAAGCCCAAACTATAAAT---A  23764

Majority     AA--CTXXAGXTAGT--ACAAGACTC-AGATXCATAGTTGT----------TTTTCXTATAXXTXXXXGC-------T-X
                      35450     35460     35470     35480     35490     35500     35510     35520
Human        AAAACCG------------AAGAATTCAGCTACATAGTTGTTTAAAACCATTGCAACCATAGTT----GC-----TATGG  30877
PolarBear    AA--CTGCAGGTAGT--ACAAAACCCTAAATATACTATGCT----------TTTTCCTATACATACATAC----CTATGA  2640
Cow          ---------------------GATTC-AGTTGCATAGTTGT----------CTTTTTTATG--T----GG-------T--  29536
Dog          AA--CTATATGCCAT--CCTGGA----AAAGGCACCAAACC----------TAGAGAGACA-GTCCAAAGTTACAAAGAT  28536
BelugaWhale  AA---AAA---TACC--ATAAGATTC-AGTTGCATAGCTGT----------TTTTTT-ATG--T----GC-------T--  24145
Donkey       AAAGTTACAGATAAGTGACAGGCCTTCAGATTAATGTTTTTAAATTTAAAGTTGTCTTATGCTAATGTGTTTGTTT-TTA  30537
Cat          AA--CTGTAGATGGT--ACAAAACCCTATATATACAGTGTT----------TTTTCCTGTACATACATAT-------CTG  32159
Ferret       AA--CTGCAGGTGGT--ATAAAACCCTAAATATACTATGCT----------TTTTCCTATACATACATACATACCTATGA  28779
Lion         AA--CTGTAGATGGT--ACAAAACCCTGTATATACAGTGTT----------TTTTCCTGTACATACATAT-------CTA  30476
Vaquita      AA---AAAAAATACC--ATAAGATTC-AGTTGCATAGCTGT----------TTTTTTTATG--T----GC-------T--  24822
Pig          AA---TAG-----C---AAAAGATTC-AGTTGCATAGTTGT----------TTTTTTAATG--T----GC-------T--  23807
```

```
Majority     TXAAGCTTATTTTA-XXXCTGTGCA-XTXACAGTATTXTCAACXCTTXAAAXTGGXATACTT--A--AXXATATXX--XA
                       35530     35540     35550     35560     35570     35580     35590     35600
Human        TTTTAACTAATT------TTTTGCT-GAAACAGTATTTT-AAT--TCTGAGTTGATCCTTTT--------ATGT------  30933
PolarBear    TAAAGCTTAGTTTATAAACTGGGCATATTAAGAAATTAACAAC---TAAAAATGGAACAATG--ATAACAATATAGTATA  2715
Cow          TTCAATATGTTT------CTTTGCT-GTAACAGTATTTT-AATTCTTTGAATTGACTTCCTT---------TAT------  29593
Dog          TTAAGGTTGGGGAGAAGGATATGAA-ATTATAATAATAAAAGC--CTGAGAATAGAGTTTTTC-AGGACAACATGG-AAA  28611
BelugaWhale  TGAAACCTTTTTTT----CTGTGTG-GTAACAGTATTTTTAACTCTTTGGATTGGTTTCCTT---------TAT------  24205
Donkey       TAGAGAGGATCTGACGAACTGCTCT-CTGGCAGTGTTCTCAGTAGTCCAAACTCGAATATGAGCAGCATGGTGGTTACAG  30616
Cat          TAAAGCTTAATTTATAAATTAGGCACATTAAGAGATTAACGAAAACTAAAAATAGGACAGTG--ATAACAATAAACTGTA  32237
Ferret       TAAAGCTTAGTTTATAAATTGGGCATATTAAGAGATTAACAACAACTAAAAATGGAAGAATG--ATAACAGTATAGTATT  28857
Lion         TAAAGCTTAATTTATAAATTAGGCACATTAAGAGATTAACGAAAACTAAAAATAGGACAGTG--ATAACAATAAACTGTA  30554
Vaquita      TTAAATCTTTTTTT----CTGTGTG-GTAACAGTATTTTTAACTCTTTGGATTGGTTTCCTT---------TAT------  24882
Pig          TTCAGTCTTTTTT-----CTTTGCT-GTAACAGTATTTT-AATTCTTGGAATTGATTTCCTT---------TATGG-GAA  23870

Majority     ATAAAAG---------------------------------------T-XA---XXX-TTTGTXTTXX--XG
                       35610     35620     35630     35640     35650     35660     35670     35680
Human        ---TGT--------------------------------------------------------------------------  30936
PolarBear    ATAAAAGGA---------------------------------------TGTACTCACCCTTTGTCTACTGATG  2749
Cow          --------------------------------------------------------------------------------  29593
Dog          ATATTTG-----------------------------------------TATAACAACCATTCTTCTGATATAG  28643
BelugaWhale  --------------------------------------------------------------------------------  24205
Donkey       GTAAGTGTTACTCTTATCAGGCTGTCACTCTTTGCCATTTTTTTGGGGCAGAGTATTAAACTGTAGTCTGTTTTACCAGA  30696
Cat          ATAAAAGCTATGTGAATGTGCATACTCTCTCCCTCTGTCTCTCAAAATATTGTATTGTACTCACCCTTTGTCTTCCTGTG  32317
Ferret       ATGAAGGG----------------------------------------TGTACTCACCCTTTGTCTTCCTAAG  28890
Lion         ATAAAAGCTATGTGAATGTGCATACTCTCTCCCTCTGTCTCTCAAAATATTGTATTATACTCACCCTTTGTTTTCCTGTG  30634
Vaquita      --------------------------------------------------------------------------------  24882
Pig          ATGTAAG--------------------------------------------------------------ATGATTTTGCTTC  23890
```

```
Majority     ATX---TXAXXT-TTAAAXTGGXT--XX---X-----TTAXXTGAAXXTA-ATTXAATGATGT-------A----X-XX-
                             35690     35700     35710     35720     35730     35740     35750     35760
Human        -------------------------------------CTACCTGCATATT--TAAGATGATTT-------GT--------  30962
PolarBear    ATGATGTGAGATGATAAAATGGCT--GTGTGA-----TAAGATGAAGCTA-GGTGAATAATGT-------A--GGCATTG  2812
Cow          -------------------------------------TTACTTGAAA----ATGTAAG-ATTT-------G---------  29615
Dog          ATAATGTCATTTATTAAATTTGTTCAACCCAG-----TGAATTGTACAAA-ACATCAAGATGT-------G--AAACCTA  28708
BelugaWhale  -------------TTA-------T-------------TTACTTGGAAAT--ATAAAATGATTT-------A---------  24234
Donkey       ATAACTTTTATTTTTAAATGCTTTCTGTTGAAAAATTTTAGAGGAAATTTTACTAGAGTATGTCAAATGAAAAAACCTGA  30776
Cat          ATGGTGTGAGATTATAAAATGGCT--GTGTGA-----TGAGATGAAGCAA-GGTGAATGATGT-------ATAGGCATTG  32382
Ferret       ATG---TGAGATGAAGAGATGGTT--GTGTGA-----TAAGATGAAGCTA-GGTGAGTGATGT-------A--GGCGTTG  28950
Lion         ATGGTGTGAGATTATAAAATGGCT--GTGTGA-----TGAGATGAAGCAA-GGTGAATGATGT-------ATAGGCATTG  30699
Vaquita      -------------TTA-------T-------------TTACTTGGAAAT--ATAAAATGATTT-------A---------  24911
Pig          ATC---TTCATAATTAGGGTGTTT-------------TTTCTTGAACTTT-ATTTATGTATTT-------A---------  23937

Majority     -XXTTTAATCTTXAG-TXC-----------------X-TXT-XX--XX-------TX-TGXXXCXGGAXTXTGGTTGACT
                             35770     35780     35790     35800     35810     35820     35830     35840
Human        ---TTTAATCTTCA---------------------------------------------------AAGTTAGAGTTAAAA  30988
PolarBear    TGATACAGTGTTAAGCTAC---------------AGTATTTTAGAAGGATGATCATC-TGCTTCTGGTCTGTGGTTGACA  2876
Cow          ---TTTAATCTT--------------------------------------------------------------------  29624
Dog          CAGTTTAACTTGTGATTTC---------------CCAGTTTCAGTGAACTATGATTGATGTGTCCAGACGCAAGTTGATC  28773
BelugaWhale  ---TTTAACCTTCAT-----------------------------------------------------------------  24246
Donkey       AGATGGTGACTTCTGTGGGCAAGTAACTAAGTCTGATTTTTTTTGTATCACTTAGTAGTATAACCCTTTTCTTTCATGTT  30856
Cat          TGATACAGTGTTAGGGTACTGTTGGCCTTTTGACAGTATTTTAGAAGGAGGATCATC-TGCTTCTGGATCATGGTTGACT  32461
Ferret       TGATACAATGTTAAGGTAC---------------AGTATCTCAGAAGG-------TC-TGCTCCTGGGCTGTGATTGACA  29007
Lion         TGATACAGTGTTAGGGTACTGTTGGCCTTTTGACAGTATTTTAGAAGGAGGATCATC-TGCTTCTGGATCATGGTTGACT  30778
Vaquita      ---TTTAATCTTCA------------------------------------------------------------------  24922
Pig          ---TTTTGTCTTTTTTGGT---------------------------CT-------TT-TAGGGCCACACCCGTGTCATAT  23979
```

```
Majority     AXTGGT------AXC----XXX-XXX-G-AX---AAACTGCA--GATXAXGXGXATTACXATGTTTTAAGTATTCTCTAT
                      35850     35860     35870     35880     35890     35900     35910     35920
Human        A---------------------------------AAATTGTA---T--ATGTCTGAAATAATGTTGTTTGTAGCATCTGT  31030
PolarBear    ACTGGT------AACTG--AAACCATGGAAAACAAAGCTGCA--GATAAAGGGAATTACTGTGTTTTAAGTATTTGATAT  2946
Cow          --------------------------------------------------------------------------------  29624
Dog          ATTGGTCAAG--AACTTGTAAGACAGGGCAAGGAAGGTTTT---GATAATGAGGGATACAATGTATGATGTGATGCGTGG  28848
BelugaWhale  --------------------------------------------------------------------------------  24246
Donkey       GTAAGTCCCCTCATTCTTGCCACTTTTGTACTTTTGTTTTTAGTGTTTGTGTGTCTTAGAATAATTTTAGTATTCTCTAT  30936
Cat          ACTGGT------ACCTG--AAACCATAGAAAGTGAAACAGCA--GATAAAGGGAATTACTATATTTGAAGTATTCTCTAT  32531
Ferret       ACTGGT------AACTG--AAACCATGGAAAGCAAAACTGCA--GATAAAGGGAATTACTGTGTTTTAAGTATTTTGCAT  29077
Lion         ACTGGT------AAC----AAATCATGGAAAGTGAAACAGCA--GATAAAGGGAATTACTATATTTGAAGTATTCTCTAT  30846
Vaquita      --------------------------------------------------------------------------------  24922
Pig          GGAGGT-------TC-----------------CCAGGCTAC-----------G-GATCCAGTCGGAACTGTAGTCACCAG  24023

Majority     CCCAXA--AGCXCXAAAGCCAAACTAAATAXAAXXAXXAA--GATTXAXCTA-ATAGTTXTCXTTT-T-TGTGXTTTCA-
                      35930     35940     35950     35960     35970     35980     35990     36000
Human        C-------------A--GTAAAACAATATA-GA-----------------------------------------------  31047
PolarBear    CCCATA--AGCCCTAAAGCCGAACTAAATAAAAATAAAAA--GATTCAGCTGTATAGTTGTCTTTTTTGTGTGCTTTCA-  3021
Cow          ---------------------CACGGGGTA--------------------------------------------------  29633
Dog          GGCGTAGTAGAGTAAGAATAGAGTCAAGAGTAGAGAGTAAG-AGTACATTTTTCTAAGAACAAAGTCTCTGTG-TGCCA-  28925
BelugaWhale  --------------------AGTTAGGGTG--------------------------------------------------  24256
Donkey       CCCACAA-GTCTTAAAGCCCAAACTGTAAATAAAAACCAAAAGATTCAACTACATAGTTGTCTTTTTTGTGTACTTTCAA  31015
Cat          CCCACA--AGCTCTAAAGCCCAACTAAATAAAAGTCAAAA--GATTGAGCTAGATAGTTTTCTTTTCTGTGTGCTTTCAA  32607
Ferret       CCCACA--AGCTCTAAAGCCCAACTAAATAAAAATAAAAAAAGATTCAGCTACATAGTTGTCTTTTTT-TGTGTTTTCA-  29153
Lion         CCCACA--AGCTCTAAAGCCCAACTAAATAAAAGTCAAAA--GATTGAGCTAGATAGTTTTCATTTCTGTGTGCTTTCAA  30922
Vaquita      -----------------------TAGGGTG--------------------------------------------------  24929
Pig          CCTATGCCAGAGCCACAGCAACACGGGATCCAAGCAGCAT--CTGCAACCTACACCACGGCTCATGGC-AACGCCGGATC  24100
```

```
Majority     CCT-TTTTTCTTTGCTXTAACAXTATTTTAATTCTTTXAATTG-GTTXCTTAATGT---------------------T-T

                       36010     36020     36030     36040     36050     36060     36070     36080

Human        ------------------------------------------------CCAAAT--------------------------  31053
PolarBear    CCCTTTTTTCTTTGCTGTAACAGTATTTTAATTCTTTGAATTGCATTCCTTAATGT---------------------TAT  3080
Cow          --------------------------------------------------------------------------------  29633
Dog          CCTCTCTTTTATTATTAACCTAAAATACTGCTTCTTAAAAATATTAACAAAAATGA---------------------GTT  28984
BelugaWhale  --------------------------------------------------------------------------------  24256
Donkey       TGT-TTTTTCTCTGCCTTAACAGTATTTTAATTCTTTTAATTGAGTTCCTTTATTTACATACTTGGAAATTACTTCATCT  31094
Cat          CCTTTTTTTCTTTGCTATAACAATATTTTAATTCTTTGAATTGGGTTTTTTAATGT---------------------TTT  32666
Ferret       CCCTTTTTTCTTTGCTCTAATAATATTTTAATTGTTTGAATTGAGTTCCTTAATGT---------------------TCT  29212
Lion         CCTTTTTTTCTTTGCTATAACAATATTTTAATTCTTTGAATTGGGTTTTTTAATGT---------------------TTT  30981
Vaquita      --------------------------------------------------------------------------------  24929
Pig          CTTAACCCACTGAGTGAGGCCAGGGATCGAACCCACATCGTCATGGATGCTAGTTG---------------------GGT  24159

Majority     XTXC-XATXATCTTXXTA---T--X-X-TTTAAATTTTAT----ATGTTTGAAAT----AATAXTGTTGGTTGTTTGTAA

                       36090     36100     36110     36120     36130     36140     36150     36160

Human        ----------------------------TTTAGACCTTAGTTTTATGGCTGATG-----GGCCAAATTTTTTTT---TGT  31097
PolarBear    CTCCCTATTATCTTCATACT-TAGGGTATTTTTTTTTTAT----ATGTCTGAAAT----AACATTGTTGGTTGT----AA  3147
Cow          ----------------------------TTTAAACTTTAT----ATGTTTGAAAT----AAT-----TGGTTGGTTGTAT  29672
Dog          GTATTGGTGTGCCTGGT-------GAAACCCAGATGGTTA----GCCTCACACCT----AACCTATTTCACGGA--AAAA  29047
BelugaWhale  ----------------------------TTTAAGCTTTAT----ATGTTTGAAAC----ATTACTGTTGGTTGGTTGTAA  24300
Donkey       ACTTGGATGATTTATTTAATCTTCATAGTTAAGGTTTTATTCTTACATTGTATGTCTGCAGTAACGTTGGTTGTTTGTAA  31174
Cat          CTCCATATTATCTTCATAGGGTATTTTTCTTAAATTGTAT----ATATGTCTGA-----AATAAAGTTGGTTGGTTGTAA  32737
Ferret       CTCCCTATGATCTTCATACT-TAGGATATTTTTCTTGTAT----ATGTGTGAAGT----AACATTGTTAGTTGTTTGTAA  29283
Lion         CTCCATATTATCTTCATAGGGTATTTTTCTTAAATTGTAT----ATATGTCTGA-----AATAAAGTTGGTTGTGTGTAA  31052
Vaquita      ----------------------------TTTAAGCTTTAT----ATGTTTGAAAC----ATTACTGTTGGTTGGTTGTAA  24973
Pig          TCGCTAACTGCTGAGCCACGATGGGAACTCTGAACTTTA-----ATGTTCGAAAC----AGTATTGTTGGTTGTTTGTAG  24230
```

```
Majority     TGTCTGTCT--AAATAAATATAAACTAATTTTTAGACCTTAG-TTTTATGGCCCAAAXXXXAAA-TTTGAATXTGATGAT

                     36170     36180     36190     36200     36210     36220     36230     36240

Human        ATTTTGTTT--AGTTTTGCTTGAATTGACGT----GTTCTCATCTGAATGGGCCAAATTTTGAA-TTTGATTATTATTAT  31170
PolarBear    TGTTTGTCT--AAACAAATGTAAACTAATTTTTGGACCCTAG-TTTTATGGACCAAAATTTGAA-TCTGATGATGATGAT  3223
Cow          TGTCTGTCTGTAAATA--TGTGGACCAATATTTAGATCTTAGATTTT-TGGCCACTGGACCAAA-TTTGAATCCGATGAT  29748
Dog          AGAAAGGAA--AAAAAAAAAAAAAATG----------TTTTGG-CTTTGTGGGAGAAAACAAAGA-GGGGA-GGGGAGATT  29112
BelugaWhale  TGTCTGTCTATAAATAAATACAGACTAATTTTTAGAGCTTAG-TTTTATGGCCAGTGGACCAAA-TATGAATCTGATGAT  24378
Donkey       TGTCTGTTTGTAAATAAATATAGACTAATTTTTAGACCTTAG-TTTTATGACTGATGGACCAAAATTTTAATCTGATGAT  31253
Cat          GGTCTGTCT--AAACAAATATAAACTAATTTTTAGACTCTTG-TTTTATGGATCAAAATTTGAT-ATGGG-GATAATGAT  32812
Ferret       TGTCTGTCT--AAACAAATATAAACT---C--TAG--TTTAG-TTTTATGGACCAAAATTTGAA-TCTGATGATAATGAT  29352
Lion         GGTCTGTCT--AAACAAATATAAACTAATTTTTAGACTCTTG-TTTTATGGATCAAAATTTGAT-ATGGG-GATAATGAT  31127
Vaquita      TGTCTGTCTCTAAATAAATACAGACTAATTTTTAGAGCTTAG-TTTTATGGCCAGTGGACCAAA-TATGAATCTGATGAT  25051
Pig          TATCTGTCTATAAATAA-TATGAACTGATTTTTAGACCTTAATTTTTATGGCCAGTGGACCAAA-TTTGAATCTGATGAT  24308

Majority     GATXA-----------------A-TGAATTAGTTTGCCTTATAACCCTACATTTTACATTGTTTACCTAAAACCAAAGTT

                     36250     36260     36270     36280     36290     36300     36310     36320

Human        TAT---------------------CAAATGAATTTACCTCACAACTCCACATTTTACATTATTTACCTGAAACCAAAGTT  31229
PolarBear    GATGATGACGACAACGACGACAACTGGATTAGTTTACCTTATAACCCTGCATTTTACATTGTTTACCTAAAACCAAAGTT  3303
Cow          GA--------------------A-TGAATTAGTTTGCTTTATAACTGTACATTTTATGTTATTTACCTTAA-CCAAGGTT  29806
Dog          GGACAGGAGAGGAGAGA-GAAACGAGAGATGGAGAGGGAGAGGGAATTGAACATGAGAGTG---GAGGAGAGGCGAGATT  29188
BelugaWhale  GA--------------------C-TGAATTAGTTTGCCTTATAACCCTACATTTTATATTATT-ACCTAAAGCCAAAGTT  24436
Donkey       GATGATG---------------ATTGAAGTAGTTTGCGT-AAGACCTTACATTTTACGTTGTTTACTTAAAATCAAAGTT  31317
Cat          GATGATAATGACGAT------GAATTAATTATTCTGCCT-ATACCCCTGCATTTTACATTGTTTACCTAAAACCGAAGTT  32885
Ferret       GATGACGACAACAAC------AACTGAATTAGTTTACCTTATAACCCTGCATTTTACATTGTTTACCTAAAATCAGAGTT  29426
Lion         GATGATAATGACGAT------GAATTAATTATTCTGCCT-ATACCCCTGCATTTTACACTGTTTACCTAAAACCGAAGTT  31200
Vaquita      ------------------------TGAATTAGTTTGCCTTATAACCCTACATTTTATATTATT-ACCTAAAGCCAAAGTT  25106
Pig          -----------------------------TAGTTTGTCTTACAGCCCTACATTTCATATTATTTACCTAAAACCAGAGTT  24359
```

```
Majority     CCTTTTTTCTAAACCCTTTCCTGAATTACTTGCTTGAGGAATTAATTGTATTGGGCAATAAAAGGATTGTAAAAGACTXG
                              36330     36340     36350     36360     36370     36380     36390     36400
Human        CCTTTTTCTGTATTTTAACTTTGAATTATTTGCTTGAAGAACAAATTATATTTGGCAATAAAAGGATCATGAAAGACTAG  31309
PolarBear    CCTTT--------------CCTGAATTACTTACTTGAGGAATTAATTGTATTGAATAGTAAAAGGGCTGTAAAAGACTGG  3369
Cow          TCTTTTTACTAAACCCTTTCCTAAATTACTTGTTTGAGGAATCAA----ATTGGGCAATGTAAAGATTGTAAAGGAATGA  29882
Dog          ----------------------GAGATAGAGAACAGGGAGGACAGGGGGAGAGGATTGAGACAAA----TAGGAGATT-G  29241
BelugaWhale  TCTTTTTTCTAAACCCTTTTCTGAATTACTTGTTTTAGGAATCGT----ATTGGGCAATATAAAGATTGTAAAAGAATGG  24512
Donkey       -CTTTTATCTAAACCGTCTGCTGAATTACCTACTTGAGGAATTAATTGTATTGGGCAATAAAGGGATTGTAAAAGACTGG  31396
Cat          CCTTTTTTCTGAACCCTTTCCTGAATTACTTGCTTGAGGAATTAATTGTTTTGGGTAGTAAAAGGATTGTAAAAAAACTAG  32965
Ferret       CCTTT--------------CGTGAATTACGTTCTTGAGGAATTAATTGTATTGGATAGTAAAAGGGTTGTAAAAGACTAG  29492
Lion         CCTTTTTTCTGAACCCTTTCCTGAATTACTTGCTTGAGGAATTAATTGTTTTGGGTAGTAAAAGGATTGTAAAAAAACTAG  31280
Vaquita      TCTTTTTTCTAAACCCTTTTCTGAATTACTTGTTTTAGGAATCGT----ATTGGGCAATATAAAGATTGTAAAAGAATGG  25182
Pig          TCTCTTTTCTGAACCCTTTCCAGAGTTACCTGTTTGAGGAATTAATTATATTGGGCAATACAAAGATTATAAAAGAATAG  24439

Majority     AAG-CATTTTGATTTXCTTCAGGTAGCTATTGCTTATGTATATGTACACCTXAGCACACACATACA--------CATXCC
                              36410     36420     36430     36440     36450     36460     36470     36480
Human        AAGACATTTTGATTTCCCTTAGGTAGCTGTGCTTAATGTATACATACATCTTGACACACACAAACACAAACACACATCTC  31389
PolarBear    AAG-CATTTTGATTTACTCCAGGTAGCTATTGCTTATGAATATGTACACCTCAGCACACACATACA--------CATGTC  3440
Cow          AAG-CATTTTGATTTTCTTTAGATAACCAC-ACCCATGTATATGTACACCTCAGTGCACACACACA--------GACC-C  29951
Dog          AGG-AGCATTGACCCAAACAAGGGAGTT------TACAT-TTTCTTTGTTTTAGGGTTTTTAT-----------TTTTTT  29302
BelugaWhale  AAG-CATTTTGATTTCCTTCAGGTAGCCAT-GTTTGTGTATATGTACACCTCAGTGCACATGTACA--------CATCCC  24582
Donkey       AAG-CATTTTAATTTCCTTCATATAGCCATTGCTTGTGTATATGTACACTGTGGCACGCAGATGCA--------CGTTTC  31467
Cat          AAG-CATTTTGATTTATTTCAGGTAGCCATTGCTTATGTATAGGTGCGCCTTGGCACACACATACA--------TATGCC  33036
Ferret       AAG-CATTTTGATTTACTTTAGGTAGCTATTGCTTATGT-TATGTGCACTTAAGCACGCACATGCA--------CATGCC  29562
Lion         AAG-CATTTTGATTTATTTCAGGTAGCTATTGCTTATGTATAGGTGTGCCTTGGCACACACATACA--------TATGCC  31351
Vaquita      AAG-CATTTTGATTTCCTTCAGGTAGCCAT-GCTTGTGTATATGTACACCTCAGTGCACATGTACA--------CATCCC  25252
Pig          AAG-TACTTTAATTTCCTTCAGGTAGCTGTTGCTTGTATGTATGTATACCTCAGTGCACATATACA--------TGTAAC  24510
```

```
Majority      TGACAGTTCAGTTAAGTAAAAATTATAATGAAGTACTTAXCAAATATAGTCATAATGATGTGAAAATGAATTTGTCTACA
                                36490     36500     36510     36520     36530     36540     36550     36560
Human         TGACAATTCAGTTAACTAAAAATTATAAGG----ACTTA--ACAGATAATAGTCATGATGTGAAGATAAATTTGTCAACA  31463
PolarBear     TGGCAGTTCAGTTAAATAAAAACTGTAATGAAGTATTTACCATATCTAGTCATAATAATGTGAAAATGAATTTGCCTACA  3520
Cow           TGCAAGTT-----AAATACAAATTACAATGAAGTATTCTCCAAATATAGTTATAATAATGATAGGATGAATTTTTCTTCA  30026
Dog           TAAAAATTT--TAAACTATTCCTAATAGTGTAACACCTG--ACATTTGTTTTTCATGGTGGAATAGTTTTCTTGAAAAAA  29378
BelugaWhale   TGACAATT-----AAGTAAAAATTATAATGAAGTATTTTTCAAATATAGTTATAATGATGTTAAGATGAATTTTTCTGCA  24657
Donkey        TGACAGTTCAGTTAAGTAAAAACTAAAATGAAGTACTTACCTAGTATAGTCATAATGAGGTGAAAATGAATTTGTCCACA  31547
Cat           TGGCAGTTCAGTTAAGTAAAAACTATAATGAAGTACTTA----ACATAGTCATAATGATGTGATAATGAATTTGCCCACA  33112
Ferret        TGGCAGTTCAGTTAAATAAAAACGGTAATGAAGTACTTACCAAATATGGTCATAATAGTGTGGAAATGTATTTGCCCACA  29642
Lion          TGGCAGTTCAGTTAAGTAAAAACTATAATGAAGTACTTA----ACATAGTCATAATGATGTGATAATGAATTTGCCCACA  31427
Vaquita       TGACAATT-----AATTAAAAATTATAATGAAGTATTTTTCAAA------TATAATGATGTTAAGATGAATTTTTCTGCA  25321
Pig           TGACAGTT-----AAGTAAAAATTACAGTGAAATACTTAGTAAATATAGTCATAACGATGTTAAGGTGAATTTTTCTGCA  24585

Majority      C-ATCATTATTATTGACTTTTCCATGTXA-XATTACAAAGCAAAA-TCACTAGGCCATGATTTT--GATAGTCAAAGTAC
                                36570     36580     36590     36600     36610     36620     36630     36640
Human         G-GTCATTATCATTGATTTTGCTATGTAATGATTACAAAGCAAAA-TCACTGGGCCTTGATTTT--GATAGTCAAAGTAC  31539
PolarBear     C-ATTATTATTATTGGCTTTTCCATG-----AGTACAAAGCAAAA-TCACTAGGCCATGATTTT--GATAGTCAAAGTAT  3591
Cow           C-ATTGTCATTACTGAGTTTTCCATGTCACAATTACAAAACAAAA-TTACTAGGCCATGATTTT--GAAAGTCAGGGTAC  30102
Dog           CCAGCAGATTAGTTGGACCATTTATA-----TTTTTAAATTTTAA-A-AAT--GTTCTGATGTTTATATA-TGTTTGTTT  29448
BelugaWhale   C-ATCATCATTACTGAGTTTTCCATGTCATGATTACAAAGCAAAG-TCACTAGGCCATGATTTT--GATAGTTAAGGTAC  24733
Donkey        C-ATCATTAGTACTGACTTTTGCATGTCATGATTACAAAG-AAAA-TCACTAGGCCATGGTTTT--GATAGTCAAAGTAC  31622
Cat           C-ATCATTATTATTGACTTTTCCGTG-----ATTACAAAACAAAA-TCACTAGGCCATGATTTT--GATAGTCAAAATAC  33183
Ferret        C-ATCATTATTATTGACTTTTCCATG-----ATTATAAAACAAAG-TCACTAGGCCATAGTTTTTTGATAGTCAAAGTAT  29715
Lion          C-ATCATTATTATTGACTTTTCCGTG-----ATTACAAAACAAAA-TCACTAGGCCATGATTTT--GATAGTCAAAATAC  31498
Vaquita       C-ATCATCATTACTGAGTTTTCCATGTCATGATTACAAAGCAAAG-TCACTAGGCCATGATTTT--GATAGTTAAGGTAC  25397
Pig           C-ATAATTATTACTGAGTTTTCCATGTCAGGATTACAAAGCAAAAATCACTAGGCCATGGTTTT--AATACTCAAGGTAT  24662
```

```
Majority     CATGTACAAATAATTGAACAAATAGG--CAAATGTCTGTAACTCTGCTTTGCCAGTTTXATTTTTTTAAAACT--AATAC
                       36650     36660     36670     36680     36690     36700     36710     36720
Human        CATGTACACATAATTGAACAAATAGG--CAGAAGTGTGTAACTCTGGTTTGTCGGTT-TCTCTTTTTGTTTTTACAATGC  31616
PolarBear    GGTATACAAATAATTAAACAGGTT----CAAATGTCTGTAATGCTGCTTTGCTA----TCATTTTTTAGAAC---AATAT  3660
Cow          CATGTGCAAATAATTGAACAAACAAGCTCAGATGTCTGTATCTCTGC-----CAGTTTCATTTTTTTGAAACT--AC---  30172
Dog          TTTACAGAGAGGATCTGACGAATA----CCACATCCTTCAGGCAGTCCAAGTTGTTTCTCATTAGTCCCAACTCGAATAT  29524
BelugaWhale  CATGTACAAATAATTGAACAAACAGGCTCAAATATCTGTATCTCTGCTTTGCCAGTTTCATGTTTTTTGAAAT--AGTAC  24811
Donkey       CATGTACAAATAATTGAACAAATAGGCTCAAATGTCTGTAACTCTGCTCTGCTGGTTTAATTTTCTTAAAACT--ATACA  31700
Cat          TGTGTACAAATAATTGAACAAATAGG--TTCAAATGTCTAACGCTGCTTTGCCACTTTGATTTTTTTAAAAC---AATTC  33258
Ferret       GGTATACAAATAATTAAACAGGTT----CAAATGTCTGTAATGGTGCTTTGCTAGTT-TCATTTTTTAAAAC---AATAT  29787
Lion         TGTGTACAAATAATTGAACAAATAGG--TTCAAATGTCTAACGCTGCTTTGCCACTTTGATTTTTTTAAAAC---AATAC  31573
Vaquita      CATGTACAAATAATTGAACAAACAGGCACAAATATCTGTATCTCTGCTTTGCCAGTTACATGTTTTTTGAAAT--AGTAC  25475
Pig          CATGTACAAATAATTGAACAAACAGGCTCTAACGTCTGTACCTCTGCTTTGCCAGTTTCATTTTTTTTGAAAC--A----  24736

Majority     ATATAGTGATGXATAATGGAAA--TGTTTT-AAA--AATT----AATATGTAGGGCTA-TGACAGAGTAACTGXTAGCAG
                       36730     36740     36750     36760     36770     36780     36790     36800
Human        ATACTGTGATCGATAATGGGAAATTAATTTGGAATTAATTTCCAAATATTTAGGGCTAGTGACAGAGTAACTAGTAGCAG  31696
PolarBear    ATATAGTGATGAATAGTTGAAA--CATTTT-AAA--AATT----AATATGTAGGGCTA-TGACAGAATAACTGATAGCAG  3730
Cow          GTTTAATGATGGATAATGGAAA--TGTTTT-AAA--AACT----GATTCGTAGGGCTG-TGACAGACTA-CTGGTAGCAG  30241
Dog          GAGCCGC-ATGCATGGTGGTTA--CAGGT--AAG--TGTT----TCTCTGTTAA-CAA-TGCTTAATTATTTGCCATTAA  29591
BelugaWhale  ATTTAATGATTGATAATGGAAA--TGTTTT-CTA--AATT----AATATGTAGGGCTA-TGACAGAGTA-CTGGTAGCAG  24880
Donkey       TAGTAGCAATTGATAATGGAAA--TGTTTT-AAA--AATT----AATATGTAGGGCTA-TGACAGAGTAACTAGTAGCAA  31770
Cat          ATATAGTGATGAATAGTTGAAA--TGTTTT-AAA--AATT----AGTATGTAGGGCTA-TGACAGAATAACTGATAGCAC  33328
Ferret       ATGTAGTGATGAATAGTTGAAA--CATTTTTAAA--AATT----AATATGTAGGGCTA-TGACAGAATAACTGATAATAG  29858
Lion         ATATAGTGATGAATAGTTGAAA--TGTTTT-AAA--AATT----AGTATGTAGGGCTA-TGACAGAATAACTGATAGCAC  31643
Vaquita      ATTTAATGATTGATAATGGAAA--TGTTTT-CTA--AATT----AATATGTAGGGCTG-TGACAGAGTA-CTGGTAGCAG  25544
Pig          GAACATT--TTAATAAAGGAGA--CATTTT-TAA--AATT----GATGTATAGGACTT-CGACAGAGTA-CTGATAGCAG  24803
```

```
Majority     CCTTTCCTTCTX--TTTCAATTT--CCCCCCAAAATCAAAAACTATAAAACTGAATAAAATATATGGGACAACTGTTGTC
                       36810     36820     36830     36840     36850     36860     36870     36880
Human        CCTTGGCCCCTT--TTTCAGTCT--CCCCCCAAAACCAAAAAGTATGTAACTGAATAAAATACGTGGAACAACTGTTGTT  31772
PolarBear    CTTTCCCTTCTCCCACTGATATG--CCCTCCAAAATCAACAACTATGAAACTGTGTAAAATATATTGGACAACTGTTGTC  3808
Cow          CCTTTCCTTC----TTTCCATCT--TCCCTCAAAATGGAAAACTATAAAATAGAGTAAAATATGTAGGACAGCTGTTGTC  30315
Dog          AATGTCAAA-----ACTAAAATT--TAGTCTTTTATAGAAATTTAGCACTTTTTATTTAATTTATGTTAAGGGTGCTTTT  29664
BelugaWhale  CCTTTCCTTC----TTTCCATCT--TTCCCCAAAATCAAAAACTACAAAACTGAATAAAATATATGGGACAACTGTTGTC  24954
Donkey       CCTTTCCTTCTCTCCTCATGTTCACCCCCCCTGTATCAAACACTGTAAAACTGAGTGAAATATATGGGACACTTGTTGTC  31850
Cat          CCTTTCCTTCTCC-TTCCATGTG--CCCTCCAAAAAAAAATAAT----AACTACA---AAACTGTATAAAATATATTGTC  33398
Ferret       ACTTCCCTTCTCCCCCTGAGATG--CCCTCCAAAATGAAAAACT--GAAATTGTATAAAGTATATTGGAAAACTGTTGTC  29934
Lion         CTTTTCCTTCTCC-TTCCATGTG--CCCTCCAAAAAAAAATAAT----AACTACA---AAACTGTATAAAATATATTGTC  31713
Vaquita      CCTTTCCTTC----TTTCCATCT--TTCCCCAAAATCAAAAACTACAAAACTGAATAAAATATATGGAACAACTGTTGTC  25618
Pig          TA------------------CT--TCTCCCAAAATTGAAAACTGTAAAACTGAATAAAATATCTGGGACAACTAATGTC  24862

Majority     TGGAATTGATGAGCAGACAGTACAAGATAA---TGATCCCTGAGAGAAGGGAA-----------ACATATGAGGTGAGTC
                       36890     36900     36910     36920     36930     36940     36950     36960
Human        TGGAAGTGATGAGCAGACGGTATAAGATCA------TCCTTGAGAGAAGGGGT-----------ACACGTGAGGTAAGTA  31835
PolarBear    TAGAATTGATGAGCAGACAGTACTAGATAA---TGATCCCTGAAAGAAGGGAATAGGGTCATTATTATATGAGGTGAATC  3885
Cow          TGGAATTGATGAGTAAATAGTATAGGATGG---TGATCCTTGAGATAAAGGAA-----------ACATGTGAAATGAACC  30381
Dog          TAAGATCGCGCTGCTGAGTGTTTCGTTTAA---ACACTAC--AGCAAATTGTACTAGATG--TAATATATGGTGTCAAAT  29737
BelugaWhale  TGGAATTGATGAGTAAATAGTACAAGATAA---TGAAACCTGAGATAAGGGAA-----------ACATATGAAGTGAGTC  25020
Donkey       TGGAATTGATGAGCAGACACTACAAGATAACAATGATCCTTGAGAGAAGGGAAT--A---------AAATGAGGTGAGTC  31919
Cat          TTGAATCGATGAGCAGACAGTACCAGATAA---AGATCCCTGAGAG---GGAA---AT-C-------CATGAGGGGAGTC  33461
Ferret       TGGAATTAATGAACAGACAGTAGTAGATAA---TGATCTCTGAAAGAAGGGAATAAGATCATTATCACATGAGGTGAATC  30011
Lion         TTGAATTGATGAGCAGACAGTACCAGATAA---AGATCCCTGAGAGAAGGGAA---AT-C-------CATGAGGGGAGTC  31779
Vaquita      TGGAATTGATGAGTAAATAGTACAAGATAA---TGAAACCTGAGATAAGGGAA-----------ACATATGAAGTGAGTC  25684
Pig          TGGAATTGATGAATAAATAGTACAGGGTAA---CGTTCCTTGAGATAAGGAAA-----------ATGAATGAGGTGAGTT  24928
```

```
Majority     TCATGAXCA--CTTTGGCTTTCTGCCTTGGGGTACCCTACTGTTGTAGCACAGGGAGGG-GGAACCTAAGCAGAGCATGG
                      36970     36980     36990     37000     37010     37020     37030     37040
Human        TCATGAACA--CTCTGGCTTTCTGTCTTGGACTATTTTACTGCTCTAAACCACAGAGAA-GTGGAGAGCCCAGAGTATAC  31912
PolarBear    CCATAATCA--CTTTGGCTTTCTTCCTTGGGGTACCCTGCTATTACAGCATAGGAAAGG-GGAATCTAAGCAGAGTATGG  3962
Cow          TCATAG-----TTTGGCTTTTCTCCTTTGAGGTACCCTACTGTTGTAGCACATGGAGGT-AGAACCCAAGCAGAGCATGA  30455
Dog          GTAAAAAAA--AA--AAAAACCTGAATTGGGTGAC--T------------TTCTTTCGG-GGCAACTAACTGAATCTTG-  29797
BelugaWhale  TCATGAATA-CTTCAGCTTTTCTGCTTTGGGGTACCCTACTGTTGTAGCACATGGAGGT-GGAGCCGAAGTAAAGCATGG  25098
Donkey       TTGTGGTCA--CTTTGGCTGTCTGCCTTGGGGTACCCTACTGTGGTAGCACAGGGAGGGTGGAGCCTAAGCAGAGCATGG  31997
Cat          GCATGATCG--TTTTGGCTTTCTGCCTTGGGCTACCCTATTCTTGTAGCACAGGAAAGG-AGAATCTAAGCAGAGCATGG  33538
Ferret       CCATAATCA--CTTTGGCTTCCTGCCTTGGGTTAC----------------AGGAAAGG-GGAATCTAAGCAGAGTATGG  30072
Lion         CCATGATCG--CTTTGGCTTTCTGCCTTGGGCTACCCTATTGTTGTAGCACAGGAAAGG-AGAATCTAAGCAGAGCATGG  31856
Vaquita      TCATGAATA-CTTTAGCTTTTCTGCTTTGGGGTACCCTACTGTTGTAGCACATGGAGGT-GGAGCCGAAGTAAAGCATGG  25762
Pig          CCATGAATAACCTTGGTTTTTCAGCATGGGAGTACCTTACTGTTGTAGCACATGGAGGT-GGAGCCCAATCAGAGCCTGG  25007

Majority     AGGTCTCACTGAGCTGAAGAGACTXAGATTGGAATTCAAAGCTGCTCAGACATCTGGGATTTATGGAG------------
                      37050     37060     37070     37080     37090     37100     37110     37120
Human        AGGTCTCACTGAGCTGAAGAGACCAAGAGTGGGTGTCAGGGCTGCCCAATCATTTGGAATTTATGGAG------------  31980
PolarBear    AAGTCTCACTGAGCCAAAAGACAGA---TTGGAATTCA--GCTGCTCAGACATCTAGGATTTGTGGAG------------  4025
Cow          AGGTTTCACTGTGCTGAAGAGAATAAGATTGGTATTCAGAGCTTCTGAGCTATTTGGAATTTATGGAG------------  30523
Dog          -AGTCT-GCTGATTGTCATTTTC-----TTATAACCAAAAAATGATAACTCTTTCTAGGTTTATATTA------------  29858
BelugaWhale  AGGTCTCACTGTGCTGAAGAGACTAAGATTGGAATTCAAAGCTGCTGAGACATGTGGGATTTATGGAG------------  25166
Donkey       AGGTCTGATTGAGTTGAAGAAACTGAGATTGGAACTTAGAGCTGCTTAGACATCTGAGATTTGTGGAGCTGCTTAGACAT  32077
Cat          AGGTCTCACTGAGCTGGAAAGACAGA--CTGGAATTCAAAGCTGCTCAGACATCTGGGATTTGCG---------------  33601
Ferret       AAGTCTTACTGAGCTTGAATTCCTGCT-TTGGAATTCAAAGCTGTTCAGACATCTGGGATTTGTGGAG------------  30139
Lion         AGGTCTCACTGAGCTGGAAAGATAGA--CTGGAATTCAAAGCTGCTCAGACATCTGGGATTTGTG---------------  31919
Vaquita      AGGTCTCACTGTGCTGAAGAGACTAAGATTGGAATTCAAAGCTGCTGAGACATGTGGGATTTATGGAG------------  25830
Pig          AGGCCTCACTGCAGTCCAGAGACTGAGATTGAGACAC--------T-------TCGAAGTTTATGAAG------------  25060
```

```
Majority     --AAGGXTCCTGA-AAAAGGAAGCTGTCCAGGGGTTT---GGGCATAGGGCAGGGGGCAGAATTCAGTGTGGGGATTC--
                              37130     37140     37150     37160     37170     37180     37190     37200
Human        ---AAGTCCTGGAGAAAAGAGAGCTGTCTAGGGGTTT---AGGAATAGAGCAGGGGGCAGAATTTAGTATGGTCATTTC-  32053
PolarBear    --AACGGTCCTAA-AAAAGGAAGCTCTCCAGGGATTG---GAGCATAGGGCAGGGGTGAAAATTCAGTGTGGGGATTC--  4097
Cow          --GAGGATCCTGA-AAAAGGAATTCGTCCAAGGGTTT---GGGCATAGGTCAGAGGGCAGAATTCAGTGTAGGGATTCAA  30597
Dog          --TAAGTCCC----ATAAGATCTTGTTCCGCGTTTTT---TGTTGTACTTTTGTTTCTTAGTTTTTGGGTGTTAGTCT--  29927
BelugaWhale  --AAGGATCCTGA-AAAAGGAACTTGTCCAGGGGTTT---GGGCATAGGTTGGAGGGCAGAATTTGGTGTGGGGATTCTT  25240
Donkey       CTGGGATTTGTGG-AGAAGGGTCCTGAAAAAAGTTGTCCAGGGCTTAGGGCAGGGGACAGAATTCGGTGTGGGGATTC--  32154
Cat          --AAAGGTCCTGA-AAA--GAAGCTCTCCAGGGGTTT---GGGCATTTTGCAGGGGTCACAATTCAGTATGGTGATTC--  33671
Ferret       --AAGGGCCCTGG-AAAAGGTAGCTGTCCAGAGGTTT---AAGCATAGAGCAGGGATCAGAATTCAATGTGGGAATTC--  30211
Lion         --AAGGGTCCTGA-AAAAGGAAGCTCTCCAGGGGTTT---GGGCATTTTGCAGGGGTCAGAATTCAGTATGGTGATTC--  31991
Vaquita      --AAGGATCCTGA-AAAAGGAACTTGTTCAGGGGTTT---GGGCATAGGTTGGAGGGCAGAATTTGGTGTGGGGATTCTT  25904
Pig          --AAGGATCCTGA-AAAAGAAACTTGTCTAGGGCTTT---GGGCATAGGTCATAGGGCAGGTTTCAATGTGGGGACTCCC  25134

Majority     ACTTGATTCA-GAATTTTGAATAATXGCTGGGCTGCCTGTGTGCAGGTTGAGA-CTCTGCATGGTCTGTCAGAAATGA-C
                              37210     37220     37230     37240     37250     37260     37270     37280
Human        ACTTCATTCA-CAGTTTTGGATAATGACTTGGTTACATGTGTGCAGGTTGAGG-CTTTGCCAGGTCTGTCAGAAATTG-T  32130
PolarBear    ATTTGATTCAGTGTTTTTGAATAATAGCTGGGCTGCCTGTGTGCAGTTGGGAAAAACTGCATGGTCTGTCAGAACTGA-C  4176
Cow          AAT-----CA-GAATTTTGAATAATGACTGGACTGCATATGTGCAGGTTAACA--CACTCATGGTCTGTCAGGAATGA-C  30668
Dog          TCTTAGTATA-TATTTTAACATAGTA------CTGCCTCCGAGT--CTTGTAACATATCCAAGAC---TCGGAGTGGATG  29995
BelugaWhale  ACTTAATTCA-GAGTTTTGAATAATGGCTGGGCTGCATATGTGTAGGTTGAGA--CTCTCCTGGTCTATCAGGAATGA-C  25316
Donkey       ACTTCATTTA-GAATTTTGAATAATGCCTGGGCTGTATGTGTACAGGTTGGGA-CTCTGCATGGTCTATCAGAAATGA-C  32231
Cat          ACTTGATTCA-GAATTTTGAATAATAGCTGGGCTGCCTGTATACAGGTTGAGAACTCTGCATGATCTGTCAGAATGTA-C  33749
Ferret       ATTTGATTGAGCATTTTTGAATAATAGCTGGGCTCCCTGTGTGCAGGTTGAGAACTCTCCATGGTCTGTCAGAACTGAGC  30291
Lion         ACTTGATTCA-GAATTTTGAATAATAGCTGGGCTGCCTGTATACAGGTTGAGAACTCTGCATGATCTGTCAGAACGGA-C  32069
Vaquita      ACTTAATTCA-GAGTTTTGAATAATGGCTGGGCTGCATATGTGTAGGTTGAGA--CTCTCCTGGTCTATCAGAAATGA-C  25980
Pig          ACTTCATTCA-GAATTTGGAATAACTACTGGGCTGCCGACGTGTAGATTGAAA--TTTTAATGGTTTATCAGAAATGA-C  25210
```

```
Majority     CTGCTCTGGGGCTGA-GAGCTAGAATGGTGA---TTCCAAAGGTTGTGCAGTGCTCTGAGACATTGG---AGTTCTGGGA
                               37290     37300     37310     37320     37330     37340     37350     37360
Human        CTGCTCTGGGGCTAA---------ATGGTGA---TTCCATAGGTTGCACGGTACTCTGAAACATTGG---AGTTCTGAGA  32195
PolarBear    CTGCTTTGGGGCTGA-GAGCTAGAATGGTGG--TTTCAAAAGATTGTTTAGTGCTCTGAGAGCTTAG---AGTTCTGGGA  4250
Cow          CTACTGTGGGGCTGA-GAGTTAGAATGGTGA---TTCCATAGATTGTACAGTGCTCTGAGACATTGG---AGTTCTGGAA  30741
Dog          CCTCTTGGGGACTCCTGAAAATGCACGGTTGGTTTTTACAAAACCCTAAAATCTACTACCACCTTTT---TTTCCTTAAT  30072
BelugaWhale  CAACTGTGGG--TCA-GAGCTAGAATGGTGA---TTCCATAGGTTGTGCAGTGCTCTGAGACATTGG---AGTTCTGGGT  25387
Donkey       CTGCACTGGGGCTAA-GAGCTAGAATGGTGA---TTCCAAAAGTTGTGCAGTGCTCTGAGACATTTG---AGTTCTGGGA  32304
Cat          CTGTTCTGGGGTTGA-GAGCTAAAGTGGTGA---TTCCCAAGGTTCTGTAGTGCTCTGAGATCTTGGG--AGTTCTGGGA  33823
Ferret       CTGCTTTGGGACTGA-GAGCTAGAATGGTGG--TTTCAAAAGGTTGTTTAGTGCTCTGAGACCTTAG---AGTTCTGGAA  30365
Lion         CTGTTCTGGGGTTGA-GAGCTAAAGTGGTGA---TTCCCAAGGTTCTGTAGTGCTCTGAGATCTTGGG--AGTTCTGGGA  32143
Vaquita      CAACTGTGGG--TCA-GAGCTAGAATGGTGA---TTCCATAGGTTGTGCAGTGCTCTGAGACATTGG---AGTTCTTCAT  26051
Pig          CTACTCTGGGGTTAA-GAGCCAGAATGATGA---TTCC-TGGGCTTTGCAGTGCTCTGAGATATTGGCATAGATATTGGA  25285

Majority     A---GTCAGAATGCAGACATCTTGXAGAGAACCTCAGGCATTCAXTTTAGACTACAGAAAGCCCAGGCCTTAAGA---GA
                               37370     37380     37390     37400     37410     37420     37430     37440
Human        A---GTCAGAATGCAGACATATTGCAGGGTACTTCAAACACTCCATTTAGACTACAGAAAGCCTGGACCTGAATA---GA  32269
PolarBear    A---GTCAGAATACAGACATCTTGGAGACAACCTCAAGCATTTAATTTAGACTACAGAAAGCCCAGTCCTTAAGA---GA  4324
Cow          A---GTTAGAATGTATACATCTTGTGGATTACCTCAAACATTCTGTTTAGGCTGCAGAATGCTCAGGCTTTCAGA---GA  30815
Dog          ATACGTACGCTTTCGGGTCTTACGGCTTGCTTTTCTGGTATGTATATTAGGCACATGAAGAGAGAGCTTTAACCA---AC  30149
BelugaWhale  A---GTTAGAATGCATATGTCTTGCAGATAGAC------ATTCTGTTTAGACTGCAGAAAGCTCAGGTCTTAAGA---GA  25455
Donkey       A---GTCAGAATGCAGACATCTTGCAGAGTTCCTCAGACATTCAGTTTAAACTACAAAAAGCCTAGGCTTTAAGA---GA  32378
Cat          A---GTCAGAATGCAGACATCTTGGAGAGAACCTCAGGCATTCAATTTAGACTACAGAAAGCCCAGTCCTTAAGA---GA  33897
Ferret       A---GTCAGAATGCAGACATCTTGGCGAGAAACTCAGGCATTTAATTTAGACTACAGAAAGCCCAGTCTTGTAGA---AA  30439
Lion         A---GTCAGAATGCAGACATCTTGGAGAGAACCTCAGGCATTCAATTTAGACTACTGAAAGCCCAGTCTTTAAGA---GA  32217
Vaquita      A---GTTAGAATGCATATGTCTTGCAGATAGAT------ATTCTGTTTAGACTGCAAAAAGCTCAGGTCTTAAGA---GA  26119
Pig          G---GTCAGAATGCACATGTCTTGCAGAGTACC----ACATTCAGTTTAG-CTACAGAAAGCTCAGGCCTTAAGAAGAGA  25357
```

```
Majority     AAGGATCACACACTATAGTAAGTGCCAACCT-----TAAGACTAAG--TTAAGAAACTG---AGATAGT-----------
                               37450     37460     37470     37480     37490     37500     37510     37520
Human        AAGGATCACACCCTATAGGAAGTGCCATCTATAAGACTGAGATAAAAACCAGAAACCTAGCCCGATAGGAAGTGAAGGAT  32349
PolarBear    GTGGATCACACACTATAGCAAGTGCCAACCT-----TAGGACTAAG--TTAAGAAACTG---ATATAGT-----------  4383
Cow          AAGGATCATACACTAATACTAATAGTAAGTGA----CAATCATAAGA-CTAAGGGACTG---TGATAGT-----------  30876
Dog          GCGTAACTCCTCCCCTCACAAA---CAAAAT-----GAACGGTAGG--TAATAATAC-----AAGTCAA-----------  30203
BelugaWhale  AAGGATCATACACTATAGTAAGTGACAGCCA-----TAAGACTAAG--TTAATAAACTC---TGATAGT-----------  25514
Donkey       AAGAATCACTCACTGTACTAAGTGCCAACAACCA--TAATACCAAG--TTAAGAAACTG---AGATAGT-----------  32440
Cat          ATGGATCACCCATTACACTAAGCACCAACCT-----TAAAACAAAGAGTGAAGAAGCT----AGATAGT-----------  33957
Ferret       ATGGATCACACACTATATTAAGTGCCAACCT-----TAAGACTAAG--TTAAGAAACTG---AGATAGT-----------  30498
Lion         ATGGATCACCCATTACACTAAGCACCAACCT-----TAAAAGGAAGAGTGAAGAAGCT----AGATAGT-----------  32277
Vaquita      AAGGATCATACACTATAGTAAGTGACAGCCA-----TAAGACTAAG--TTAATAAACTC---TGATAGT-----------  26178
Pig          AAGGATCACACACTATAGTAAGTGACGGCCGT----TAAGACTAAG--TTAAGAAATAG---TGATAGT-----------  25417

Majority     -------CTAGCCTGACAAG--AC--ATGAAGGATTCACCAGTAATTT-AATTGCCTGCCTAAAXAAAACAATACCCCCT
                               37530     37540     37550     37560     37570     37580     37590     37600
Human        TCACCAGTTAATCTAACCAGTAACTGATGAAGGATTAATCAGTAATTT-AGTTGCCTTCCAAAACAAAACAACACCCCC-  32427
PolarBear    -------TTAGCCTGACAAC--AC--ATTAAGGAT-CACCAGTAATTT-AGTTGCTTGCCTAAAGAAAACAATACCTCCT  4450
Cow          -------CCAGTCTGACAAG--AC--GTGAAGAATTCACCAGTAATTT-AATTGCCTGCCTAAATAAAACAGTATCCCCT  30944
Dog          -------CCAATATGACTGT--A---ATTAAAG------CGGTAATCC-----ATCCACTGTGGTGCACTCTTGTTGATG  30260
BelugaWhale  -------CCAGCCTGACAAG--AC--ATGAAGGATTCACCAGTAATTG-AATTGCCTGCCTAAATAAAACAATGTGCCCT  25582
Donkey       -------CTAGCCTGACAAG--AC--ATGAAGGCTTCATCAGTAGTTT-GATTGCCTGCCTAAACAAAACAACATCCCCT  32508
Cat          -------TTGGCCTGACAAG--AC--AATAAGGAT-CACCAGTAATTT-AATTGCTTGCCTAAACAAAGCCACACCCCCT  34024
Ferret       -------TTAGCCTGACAAC--AC--ATTAAGGAT-CACCAGTAATTTTAATTGCTTGCCTAAACCAAACAGTACCCACT  30566
Lion         -------TTGGCCTGACAAG--AC--AATAAGGAT-CACCAGTAATTT-AATTGCTTGCCTAAACAAAACCACACCCCCT  32344
Vaquita      -------CCAGCCTGACAAG--AC--ATGAAGGATTCACCAGTAGTTG-AATTGCCTGCCTAAATAAAACAATGTGCCCT  26246
Pig          -------CCACCCTGACAAG--AT--GTGAAGGATTCACCAGTGATTT-AATTGCCTGCCTAAATAAAACAACACATTGT  25485
```

```
Majority     AAGGAAGACA---TGATTCAGTCT----TCCGXCAACTATCATCCACAATATCCTGCAT---------------------

                               37610     37620     37630     37640     37650     37660     37670     37680

Human        AAGAAAGACA---TGATATAGTTT----TCCTGCAACTATCTTCCATAATATCTAGTAT---------------------  32479
PolarBear    AAGGAAGACA---TGATTCAGTCTTTATTTTGTGAACCGTCATTCACAGTATCCACCAT---------------------  4506
Cow          AAGGGAGACAACATGATTCAGTCT----TCCCACAATT---ATCCACAATATCCTACCT---------------------  30996
Dog          TATGTGGGTG---TGATTAAATGT--GTCTGGTCTTCTGTGATTAAGA-TGAAAATGAGGTGCATTGAC-----------  30323
BelugaWhale  AAGGAAGACA-CATGATTCAGTCT----TCCCACAACTATCATCCACAATATCCTGCAT---------------------  25636
Donkey       AGGGAAGACC--ATGATTCAGTCT----TCCTACAACTATCATCCATAATATCCTGCAT---------------------  32561
Cat          AAGGAAGACA---TGTCTGAGTCTT-TTTTTGTGAACTATCATTCATAATATCCCCCGT---------------------  34079
Ferret       AAGGAAGACA---TGATTCAGTCTTCATTTTGTGAACTGTCATTGACACTATCCACCATTGTCATTCACAATATCTACCA  30643
Lion         AAGGAAGACA---TGACTCAGTCTT-TTTTTGTGAACTATCATTCATAATATCCCCCAT---------------------  32399
Vaquita      AAGGAAGACAGCATGATTCAGTCT----TCCCACAACTATCATCCACAATATCCTGCAT---------------------  26301
Pig          AAGAAAGACAGCATAATTCAGTCT----TCCCACAACTGTCATCTACAGTATCCTGCAT---------------------  25540

Majority     ------ACCATCAAAAATTACAAATTATGTGAAG---CATGX-AAXATTCCATXAAXTAGAGA-AGCAT-T-CAATAGGA

                               37690     37700     37710     37720     37730     37740     37750     37760

Human        ------ACCATCAAAAATTACAAGTGATGTGCAGAAGCAGGAAAGTAACCCATGAAATATAAAGAACACATTTAATAGGA  32553
PolarBear    ------ACCATCAAAAATTGCAAATTATGGAAAG---CATGAGAACATTCCATAAAATTGAGA-AGCAT-T-AAATAGGA  4574
Cow          ------AC-ATCAGATGTTA-AAATCATGTAAAG---------------------A--------ACCAT-T-CAATAAGA  31037
Dog          -TGGCTGTTGAATGAGGTAGGACATTGTGAGTAG---CGCGCTAGTGTTGT-TAAGCTACAGT-ATTTT-TCGAATAACA  30396
BelugaWhale  ------ACCATCAAATTTTACGAATCCTTTGAAG---------------------A--------AGCAT-T-CAATAAGA  25679
Donkey       ------ACCATCAAAAATTATAAATCGTGTGAAGAAGCATGAAAAAATTCCATAAATTAGAGA-AGCAG-CATGCAATAG  32633
Cat          ------ACCATCAAAAATTACAAATTATGGAAAGAAGCATGAAAACATTCCATCTAATAGAGA-AGCAT-T-CAACAGGA  34150
Ferret       ATATCCACCATTAGAAATCACAAATTATGGAAAG---CATG--AACATTCCATAAAGTAGAGA-AGCAT-T-AAATAGGA  30715
Lion         ------ACCATCAAAAATTACAAATTATGGAAAGAAGCATGAAAACATTCCATCTAATAGAGA-AGCAT-T-CAACAGGA  32470
Vaquita      ------ACCATCAAATTTTACGAATCCTTTGAAG---------------------A--------AGCAT-T-CAATAAGA  26344
Pig          ------ACCATCAAATACTACAAATTTTGTGGAA---------------------A--------AACAA-T-CAGTAGGA  25583
```

```
Majority     GCAGACXCCAAAATGACC-TAGATATTGGAAT-TAGCAGACAAGGACTATAAATATTT-TTAAAGCTATAAAGGAAAATA
                     37770     37780     37790     37800     37810     37820     37830     37840
Human        ACAGATTCCAAAATGACC-TAGA-GTTAGAAT-TAGCAGACAAGGACGATAAGTAT---TTTAAGCTGTAAAGGAAAAGA  32627
PolarBear    CCAGACACAAGAATGATC-TAGATAATGGAAT-TGACAGACAAGGACTATAAATATTT-TTAAAGCTATAAAGGAAAATA  4651
Cow          ATAGACCTCAAAATGACC-TAAATACTGGAAT-TTGCAGACAGAGACTATAAGTATTT-TTAAAGCTGTAAAAGAAAATA  31114
Dog          GGAGGGATTCATCGGCTTCTGGAATATGGTTTGCAACTAGCAGGGGGTAAGGAC-----CTGAGGATA-ACTTGGAAAGG  30470
BelugaWhale  ATAGACCTCAAAATGACC-TTGATATTGGAAT-TAGCAGACAAGGACTATATATATTT-TTAAAGCTATAAAAGAAAATA  25756
Donkey       TAAGACCCCAAAATGACC-TAGATATTGGATT--AGCAGATAGAGATTATAAATATTTTTTAAAGCTATAAAGGAAAATC  32710
Cat          GCGGACACCAAAATGACC-TAGATAGTGGAAT-TAACACACAAGGATTATAGATATTT-TTAAAGCTATAAAGGAAAATA  34227
Ferret       GCAGACACCAAAATGAT---AGATCATGAAAT-TAACAGACAAGGACTATAAATATTT-TTAAAGCTATAAAGGAAAATA  30790
Lion         GCGGACACCAAAGTGACC-TAGATAGTGGAAT-TAACACACAAGGATTATAGATATTT-TTAAAGCTATAAAGGAAAATG  32547
Vaquita      GTAGACCTCAAAATGACC-TTGATATTGGAAT-TAGCAGACAAGGACTATATATATTT-TTAAAGCTATAAAAGAAAATA  26421
Pig          ATAGACCTCAGTATGACC-TAGATATTGGAAT-TAGCAGATAAGGACTATGAATACGT-TTAAAGCTATAAAAGGAAATA  25660

Majority     TT----TAATGAGTGA--ACATGGAGGAATGTGTTGAAAXAG--------CTATAAAGAAAGC-TATAGAAA-TGTTGTA
                     37850     37860     37870     37880     37890     37900     37910     37920
Human        TTGAAATAATGAGTG-----ATGGAGGAGTGTGTTG-AAGAGATAAAAAACTATAAAGAAAGCATGTAGAAA-TATTCTA  32700
PolarBear    TTAAA-TAGTGAGTGA--ACACGTAGAGGCATGTGTTAAGAG--------AGAAAAGAGAAGT-AGAGAGCA-TACAGAA  4718
Cow          TT----GAAAGAATAA--ACATGGAAGAATGTGTTGAGAAAG--------CTATAAAGAAAGTATATAGAAA-TGCTGTA  31179
Dog          TCAGG-TAGCGAATTG--GTCTATAGTAG------ACAGGGC--------ATATACTATGTTT---TTTGTA-TGTCTAT  30529
BelugaWhale  TT----AAAATAAT----ACATGGAAGAATGTGTTGAGAAAAGAA-----CTATAAAGAAAGCATATAGAAA-TGCTCTA  25822
Donkey       TTGAAATGATGAGTGAACGCTTGGAGGAATATGTTAAGAAAGAAAGAGAACTATAAAGAAAGCATATAGAAAATGTTGTA  32790
Cat          T-----TAGTGAGTGA--ACACACAGAGA---------------------------CTGTG-----------TGTTGAT  34261
Ferret       CTATG-TAGTGAGTGA--ATACATAGAGGTATGTGTTAAGAG--------AGAAAAGAGAGG-----GAGCA-TATAGAA  30853
Lion         C-----TAGTGAGTGA--ACACACAGAGA---------------------------CTGTG-----------TGTTGAT  32581
Vaquita      TT----GAAATAATGA--ACATGGAAGAATGTGTTGAGAAAAGAA-----CTATAAAGAAAGCATATAGAAA-TGCTCTA  26489
Pig          TTTTGCAAAATAATGA--ACATGGAAGAATGTGTTGAAACAA--------CTGTAAAGAAAGC-TATAGAAG-TGCTCTA  25728
```

```
Majority     ATTGAAAAA----------A--XAATATTTGGAATGAAAAATTCACTXGTTGAGACTAACAGCAGXTTGGAGACAACTGA
                               37930     37940     37950     37960     37970     37980     37990     38000
Human        ATTGAAAAATACAGTTTTTG--AAATAGTTGGGAC---TGATTCACTGGTTGGAACTAGTAGCAGATTGGAGACAATAGA  32775
PolarBear    ATCT--GAA----------A---TATATTTGAGATGAAAAATTC----ACTGGGGCTAACAGCAGATTGGAGACAACTAA  4779
Cow          ACTGAAAAATACAATATTCACACCATATTTGGAATGGAAAATTCACTGATTGAGACTAACAGAAGTTTGGAGACAACTGA  31259
Dog          ATCTCCCCC----------A---CCTTTCCACCAAGTCAAACA-----GGAAAGTCCAACT----GGCGGAAGCAATCAA  30587
BelugaWhale  ATTGAAAAA--------T---ACAAAATTTGGAATGAAAAATTCACTGGTTGAGAGTAACAGCAGTTTGAAGACAACTGA  25891
Donkey       ACTGAA-AA----------ATACAGTATGTGAAATGAAAATTTCACTGGTTGGGACTAACATCAGATTGCAGACAACTGA  32859
Cat          GTTT---------------A---TATGACAACAG----CACTAA----GTGAAGATAAATA-------GAATTTTACAGT  34308
Ferret       ATCTCTGAA----------A---TATATTTGAGATGAAAAACTC----ACTGGGGCTAACA---GACTGGGGACAACTGG  30913
Lion         GTTT---------------A---TATGACAACAGTTAGCACTAA----GTGAAGATAAATA-------GAATTTTACAGT  32632
Vaquita      ATTGAAAAA--------T---ACAAAATTTGGAATGAAAAATTCACTGGTTGAGAGTAACAGCAGTTTGACGACTACTGA  26558
Pig          ATTGGAAAA----------ACACAGTATTTGGAATGAAAAGTTAACTCTTTGAGACTAACAGCCGTTTGGAGACAACTGA  25798

Majority     AGAAAGGTTATTGAACTTGAA-TACAGATGGATAGAAAX-ATTCAGTTTCCCAGAGAGAG-AAAAXTATATCTTT-----
                               38010     38020     38030     38040     38050     38060     38070     38080
Human        AGAAATGTTAGTGAACTTGAA-TACTGTTGAATACAGTG-ATTAAATCTGTCAGAGAGGA-GAAAGTATATCTAAT----  32848
PolarBear    AGAAAGGTTTTTGAACTTGAA-TACACATGGATAGAAAA-ATTAAGTTTCCCAGAGAGAA-GAAAATCTATCAT----AG  4852
Cow          AGAAAGGTTAGTGAACTTGAG-TACAGATTGACAGAAAGTATTCAGTT-TCCAGAGAGAATAAAAGTATGTCTTT-----  31332
Dog          ACCAGA-TCATCGCACCTGAGCTGGATGTGAGAGGCCAA-AGTTGTATGCTTGTTGTGTG-AACTTTGAACCTG------  30658
BelugaWhale  AGAAAGGTTAATGAACTTGAA-TACAGATTGATAGAAATTACTCAGTTTTCCAGAGAGAGTAAAAGTATATCTTTG----  25966
Donkey       AGAAAGGTTAGTAAACTTGAG-TACAGACGAACAGAAATTATTCAGTTTACCAGAGAGAA-CAAAATATATGTTTG----  32933
Cat          TGTAAGGTTTTTA---TT----TACATG-AAGTGATAGA-ATATTAACTCTGACTTAAAG-TAGATTCTATTAA------  34372
Ferret       AGAAAAGTTATTGAACTTGAA-TACACATGGATAGAA---ATTAAGTTTCCCAGAGAGAA-GAAAATGAATCAGTCTAAT  30988
Lion         TGTAAGGTTTTTA---TTATT-TACATG-AAGTGATAGA-ATATTAACTCTAACTTAAAG-TAGATTCTATTAA------  32699
Vaquita      AGAAAGGTCAATGAACTTGAA-TACAGATTGATAGAAATTACTCAGTTTTCCAGAGAGAGTAAAAGTATATCTTTG----  26633
Pig          AGAAAGGTTAATGAACTTGAG-TACAGATTGATAGAAATTATTCAGTTATCCAGAAAGGGTAAAAATATATCTTTA----  25873
```

```
Majority     GGACGATTAAGCAGTCTAACATA-CATGTAA---TTGGAGTTTCA--GAGAGGGAATAAA--------------GAGAAT
                     38090     38100     38110     38120     38130     38140     38150     38160
Human        GGACAATGAAGCAGTCTGACATGTAAGTTGAATTTCAGAGTGAGAATGTGGGGGAATAAAACATCTATCTGTTTGAGATA 32928
PolarBear    GGACAATTAAACTGTCTAATACA-CGTATAA---TTGGAGTTCCA--GAGAGGAAATAAA--------------GAGAAT 4912
Cow          GGATGATCAGGCAGTCTAACGTA-TGTGTCC---TTGGAGTTTTC-AGAGAGGGAATGAA--------------GAGAAT 31393
Dog          GTTTGGTTGCGCGGT------AG-CGTGTGA---TCAAGATGTTA--TTGTGTGAATTAT--------------TGAATT 30712
BelugaWhale  GGGAGATCAAGTAGTCTAACATA-CATGTAA---TTGCAGTTTCA--GAGAGGGAGTAAA--------------GAGAAT 26026
Donkey       GGACAATCAAGCAGTCCAACATA-TGTGTAA---TTGGAGTTCTA--GAGTGGGAGTAAA--------------GAGAAT 32993
Cat          ----GGTTGCATACT---------GAAATAA---TTAAAA----------AAAACATACAA--------------GGAAGT 34413
Ferret       ATATGTATAAGCTGTCTA-TAAA-TGTATAA---TTGGAGTTCCA--GTGAGGAAGTAAA--------------GAGAAT 31047
Lion         ----GGTTGCATACT---------GAAATAA---TTAAAA----------AAA-CATACAA--------------GGAAGT 32739
Vaquita      GGGAGATCAAGTAGTCTAACATA-CATGTAA---TTGCAGTTTCA--GAGAGGGAGTAAA--------------GAGAAT 26693
Pig          GGACAGTTGAGCAGTCTAATATAACATGCAA---TTGGAGTTTCAGAGAGAGAGAATAAA--------------GAGAAT 25936

Majority     GGXG---CCAAATATGT---TAATTGAAGCAGTAA----TAGCTGAAAACTTCTAAAATTTGAAGAAAAATAT-CAAGTA
                     38170     38180     38190     38200     38210     38220     38230     38240
Human        GTAATGACTGAGAACTTCTAAAATTTGAATAAAATATATCAACTTACATTCCTAAGAAGCTCAGGTAAACTCCTCATGTA 33008
PolarBear    GGGG---CCAAGTATGTG--TAATTGAAGCAGTAA----TGGCTGAAAACTTCTAAAATTTTAAGAAAAATAT-CAAGTT 4982
Cow          GTTG---CCAAATATGTCTATAATTGAAGCATTAA----TAACTGAAAATGTCTAAAATCTGAAGAAAAATGC-TAGGTA 31465
Dog          GTGAGTTTCAATAATGTG--ATGCTGCCATAGTTC----TCTTTCATAGCTTGCGGGGTCTTTGGTGGAGTA---AATGT 30783
BelugaWhale  GGTG---CCAAATATAT--GTAATTGAAGCAGTAA----CAGTTGAAAACTTCTAAAATTTGAAGAAAACTGT-CAGGTA 26096
Donkey       GGGG---CCAAATATATT-GTAATTGAGACAGAGAC---TTGCTGAAAACTTAAAACATTTGAAGAAAAATAT-CAAGTA 33065
Cat          ACAG---TTAAAAGTGA---ATAGAGAAAAGGTTG----GGAGGACTGATTCCCTGTTTTCAAGTTACAATA---AAGCT 34480
Ferret       ACAG---CCAAGTATGT---TAGTTCAAGTAGTAA----TGGTGGAAAACTTCTAAAATTTTAAGAAAAATAT-CAAGTT 31116
Lion         ACAG---TTAAAAGTGA---ATAAAGAAAAGGTTG----GGAGGACTGATTCCCTGTTTTCAAGTTACAATA---AAGCT 32806
Vaquita      GGTG---CCAAATATAT--GTAATTGAAGCAGTAA----TAGTTGAAAACTTCTAAAATTTGAAGAAAACTGT-CAGTTA 26763
Pig          GGGG---CCAGATGTATACGTAATTGAAGCAGTAA----TAGCTGAAAACTTCTAAAATTTGAAGAAAAATAT-CAAGTA 26008
```

```
Majority     GGATAAATAAAA-AGAAACATACTXAGGCACATAATAGTCAAA-ATATTGAAAACTX-ATAATAAAGAGCAAATCT-TAC
                     38250     38260     38270     38280     38290     38300     38310     38320
Human        GGCTAAATAAAA-AGAACCATACTTAGACACATAGTAGTCAAA-ATATTGAAAACTA-GAAATGAAGAGCAAATCTAAAC  33085
PolarBear    GGGTAAATAAAA-AGAACCACGCTTAGGCACATAATAGTCAAA-ATATTGAAAATCTAATGATAAAGAGCAAATCT-TAC  5059
Cow          GGATAAGAAAAA-AGAAACATTTTGTTGTTCA-GTCACTAAGTCATGTCAGCCTCT---TTGTGACCCCGT-GGAC-TG-  31537
Dog          GTGTATGTGCTA-AGTGACGTAATAAGACAATAAATTGTCGGT-TGTTTGTAATGTCCAGTCTACTAAACAA---C-TAC  30857
BelugaWhale  GGATAAGTAAAA-AGAAACATACCAAGGCACACAATAATCAAA-ATATTGAAAACTA-ATAATAAAGAGAAAATCT-TAT  26172
Donkey       GGATAGATAAAA-AGAAACACATTTAGGTAGTTAATAGTCAAT-ATATTGAAAACTA-ATGATAAAGAACAAATCT-TTA  33141
Cat          GAGTAATCAAAACAGTATCATAGTGGCATAAAGAAAGATAAAT-AGAT---GAA-T--AGAACAGAATAGAA-----TCC  34548
Ferret       GGATAAATAAAA-AGAACCATACTTAGGCACATAATAGTCAAA-ATATTGAAAA-CTAATGATAAAGAGCAAATCT-TAC  31192
Lion         GAGTAATCAAAACAGTATCATAGTGGCATAAAGAAAGATAAAT-AGAT---GAA-T--AGAACAGAATAGAA-----TCC  32874
Vaquita      GGATAAATAAAA-AGAAACATACCAAGGCACACAATAATCAAA-ATATTGAAAACTA-ATAATAAAGAGAAAATCT-TAC  26839
Pig          GGATAAGTAAAA-AGAAATATACCAAGGCACATAACAGTCAAA-ATATTGAAAACT---TAATAAGCATCTAGGAA-AA-  26081

Majority     AGTCAXGCAAAAAAAATCTAAC----GACX--CXCCCXXATTXAAXTXAAAGACAXA--CGAACTTTAGXTTGAATAGAA
                     38330     38340     38350     38360     38370     38380     38390     38400
Human        AGCTGCGAAAAAAAACGTCTAACAGGAGACCAACACCTCGTATTAAAGGGGGAAAAAAAGCGAATTTC--TTTAGACTGAA  33163
PolarBear    AGTCAGGAAGAAGCCATCTCACAGGAGACCAACACCTTCACTAAAAGGAAAAAAAAG--CTAACTTTAGATTGAATAGAA  5137
Cow          CATTACTCCAGGCATCCCTGTCCTTCACCAG-CTCCCAGAGTTTGCTCAGACTCA----TGTCCATTGAGTCA---GTGA  31609
Dog          AG--AGTTAACAAATTTTTTAT----GG------CCCTGAGTTGTTTTGTGCAC-----CCAAGTGTATTTGAATCTGGA  30920
BelugaWhale  AAGCAAGCAAGAAAAAGATATCTCACAGGAGACTAACACCTTGCATTAAAAGAAAAAAAAGTTCTTTAGGCTGGATGGAA  26252
Donkey       AAACAAGCAGGAAAAAGACATC-------------TCACAGT--------AGACTGA--C--ACCTTGCACTAAA-AGAA  33195
Cat          AG--------AAATAAACCTAC-----A---------TAATT------ATGGAC-------AACTGGTTTTTGACTAAGG  34593
Ferret       AGTCAGGAAAAAGCCATTTCACAGGTGACCAACACCTTTACTAAAGGGGGGGAT--G--CTAACTTTGGATTGAATAGAA  31268
Lion         AG--------AAATAAACCTAC-----A---------TAATT------ATGGAC-------AACTGGTTTTTGACTAAGG  32919
Vaquita      AAGCAAGCAAGAAAAAGATATCTCACAGGAGACTAACACCTTGCATTAAAAGAAAAAAAAGTTCTTTAGGCTGAATGGAA  26919
Pig          AGTTATCTCACAGGAGACTGACGCTTTGCA--TTCAAAGAAAAAAACAAAACAGAAA--CGTTCTTTAGGTTGAACGGAA  26157
```

```
Majority     TGACACTAGGCAAAAACTTGGATCTGCAGGAAATAA-CAAGGAGTACCAGGAATGGTAAATATATGTGTXAA----TTAA
                       38410     38420     38430     38440     38450     38460     38470     38480
Human        TGGC--------ATAACTTGGCTCTACAGGGGATAA--GAGGAGTACTGGGAATGGTTAATATGTGTATAAA--TTTTAA  33231
PolarBear    TGATACTACGTAAAAACTTGGATTTGCAGGAGGTAAACCAGGAGTACCGGGAATGGTAAATGTGTGTGTAAA----TTAA  5213
Cow          TGCCATCCAACTGTCTCATCCTCTTGTCGCCCCCTT-C---TTCTGCCCTCAGTCCT-------TGCCAGCA----TCAG  31674
Dog          TGATGATGAGGAGGGAGGAGAGGAAGGAAGGAGGAGGAGGAAGACACTGAAATTAGTTGGCGTATAATCGTG----ACAG  30996
BelugaWhale  TAGCACTAGACAAAACCTTGGATCTGCAGGAAATAA-CAAGGAGTACCAGGAATGGTAAATAAATGTGCGAAAATTTTAA  26331
Donkey       AGAAAAAAGCTGATTTGTTTAGGCTGAATGGAATGA--------CACTAGAAAAAAG-----------------TTAA  33248
Cat          TATCCGTATGCAAAAACCAAAA-----ACAAAACAC-CAAAACACACTTGGATGTGT-ACCCCATAC-CATA----TT-G  34660
Ferret       TGACACTATGTAAAAACTTGGATTTGCAGGAAGTAA-CCAGGAGTACCAGGAATGGCAAATATGTGTGTAAA----TTAG  31343
Lion         TATC--TATGCAAAAACCAAAA-----ACAAAACAC-CAAAACACACTTGGATGTGT-ACCCCATAC-CATA----TT-G  32984
Vaquita      TAGCACTAGACAAAACCTTGGATCTGCAGGAAATAA-CAAGGAGTACCAGGAATGGTAAATAGATGTGTGAAAATTTTAA  26998
Pig          TGACACTAGACAGTAACTTGGATCTATAGGAAGTAA-C---GAGTACCAGGAATGGCAGAGGTGTGTATGAA----TTAA  26229

Majority     AAAGAT-TCTCTTTTAATCCTTTAAATTTCTTTGAAAGA--CTGT--CTXTXAAAAXAAAAAAACTTTGTATTATG-TGG
                       38490     38500     38510     38520     38530     38540     38550     38560
Human        AAGGGC-TCTCTCTTTTCCTATCAG-TTTCTTTGAAAGC--------CTTTATGTAAACAACAACTTTGTATTATG-GGG  33300
PolarBear    AAAAGC-TCTCTTTTAATCCTTTAAATCTCTTTGAAAGA--CTGTT-TACGTACATTTAAAAAAAAAAACAT--------  5281
Cow          AA-----TCTTTTCTAATCAGTAGGTTCTAGACATCAGG--TGAC--CAGCGTATTGAAG-----CTTCAACAAAGATGC  31740
Dog          TTTTAC-ATTTTTTAGCCCAGTCAAAAACCTTT--------TTGCT-TTTTGGGTGCACGTAAACCTTGTAC--------  31058
BelugaWhale  AAAGCTATCTCTTTTACTCCTTTAAACTTCTTTGAAAGA--CTGT--TTAGGGATACAGAAAACCTTTGAATTATG-TGG  26406
Donkey       AAAGACTCCTCTTTTAATCCATTAAATTTCTTTGAAAGACACTGTTTCTGTATAAAGAAAAAAACTTTGTATT----TGG  33324
Cat          AAGAAT-TCACTCTGAGTGGACCTAGAAGAAAC------------------ATAGTAAAAAAATTTTGTGACCT-----  34715
Ferret       AAAGAT-TTTCTTTTATTCCTTTAAATCTCTTTGAAAGA--CTGTA-CATTAAAAAAAAAAAAACTTTGTATTATGTGGT  31419
Lion         AAGAAT-TAACTCTGAGTGGACCTAGAAGAAAC------------------ATAGTAAAAAAATTTTGTGACCT-----  33039
Vaquita      AAAGCTATCTCTTTTACTCCTTTAAACTTCTTTGAAAGA--CTGT--TTAGGGATACAGAAAACCTTTGAATTATG-TGG  27073
Pig          AAGGC--TATCTTTTAATCCTTTGTCTTTGAAAGACTGT--TTAT--CAAAAAAACAAAAAAACCTTTAAATGATGGTGG  26303
```

```
Majority     TT-ATTATGTAC--GTAGATGTTTATATGAC-AACAXTTAGCACAAAGTGAAXATAAAXGG--AAATTTACAGG---AAX
                       38570     38580     38590     38600     38610     38620     38630     38640
Human        TTTATTATGTAA--ATGGATGTTTATATGAC-AACAGTTAGTACAAAGTGAAGATAAATGG--AATTCTTCAGTGGTAAG  33375
PolarBear    ------ATGTAT--GTAGATGTTTCTGTGAC-AACAG----CACTAAGTGAAAGTAGAATTTTACAGTTGTAGG------  5342
Cow          ATAGTTAAAAGCTAGTACAAATTTGGAGGACTGACTCCTGTTTTGAAGTTATTGTAAACCCT-AAAGTAATCAA---AAC  31816
Dog          -------TGAGC--GTGGACTTGCTTGAGGG-TATAGT--ATAGTAAACGATTGTAAAA----GGACCTATCAG------  31116
BelugaWhale  TTCATTCTGTAC--ATAGATGTTTATATGAC-AATGATTAGCACAAAGTGAAGATAAATGG--AATTTTACAGTT-TAAG  26480
Donkey       TTTATTATGTGT--ATAGATGTTTATATGAC-AGCAATTAGCACAAAGTAAAGATAAATGG--AATTTTACAGTTGTAAG  33399
Cat          ----TGGATTAG--GCAGACGTTTCTTTGAT-AGGAC----CCCAAAAGTACTATAAAA----G-AACAAGTGG------  34773
Ferret       TTATTTATATAT--GTAGATGTTCTTAGAAC-AACAGTTAGCACTAAATGAAAATAGAATTTTACAGTTACAGG------  31490
Lion         ----TGGATTAG--GCAGACGTTTCTTTGAT-AGGAC----CCCAAAAGTACTATAAAA----G-AACAAGTGG------  33097
Vaquita      TTCATTCTGTAC--ATAGATGTTTATATGAC-AGTGATTAGCACAAAGTGAAGATAAATGG--AATTTTACAGTT-TAAG  27147
Pig          TTTATTCTGTAC--GTAGAGTTTTATGTGAC-AACAATTAGCACAAAGTGAACTTAAGTGG--AATTTTATAGAT-TAAG  26377

Majority     GTTTTTATATTTTACATGAAATGATAGAGTATTAACTCTTACATAX--------------GAATATTX-GATAAXTTAAA
                       38650     38660     38670     38680     38690     38700     38710     38720
Human        GTTCTTATATTTTACCTGAAGTGATACAGTGTTAACTCTAACATAAAA---AGATTCTGTTAAGGTT---GCATATCAAA  33449
PolarBear    GCTTTTATTATTTATATGAAAAGATAGAATATTAACTTGAACATAGACTCTTTTAAGGTTGCATATTGAAATGATCTCCA  5422
Cow          AATATTATATTA-ACGTAAAGGTACATAG-ATGAG---TAGAACA---------------GAATA----GAGAGTCCAAG  31872
Dog          AGTACCACTTTTGATTTGCTTCAGTGGTGGCTTAGCTATTGCTTA--------------TGTATAT----ATACACCTTG  31178
BelugaWhale  GTTTTTATATTA--CATGAAATGATAGATTATTAACTCTTACATAA--------------GAATATTCTGGTAAATTAAA  26544
Donkey       GTTCTTATATTTTACATGAAATGAAAGAATATTAACTCTAACACAA--------------GTAGATTCTAGTAAGTTAAG  33465
Cat          GATTTCATCAGCT-TT--------TAGACTCTAAGTTCTTTTGCAG----------------ATACTG--ATAGTAGAAT  34826
Ferret       GTTTTTATTATTTATATGAAATGATAGAATATTAACTCTTATGTAGATTCT---AAGGCTATATATTGAGATAATTGCCA  31567
Lion         GATTTCATCAGCT-TT--------TAGACTCTAAGTTCTTTTGCAG----------------ATACTG--ATAGTAGAAT  33150
Vaquita      GTTTTTATATTA--CATGAAATGATAGATTATTAACTCTTACATAA--------------GAATATTCTGGTAAATTAAA  27211
Pig          ATTCTTAAATTTTACATGAAGTGATAGAGTATTAACTCTTACATG---------------GAATATCCTGATAAACTAAG  26442
```

```
Majority     G---AAAATAXAAACAATXTCTATAAAGXA-GTAXAATTA----------TAAATAGAGAGAAAGTTGGAGGACTGATTC
                      38730     38740     38750     38760     38770     38780     38790     38800
Human        ATTCTAAGTGTAATCTCATTTAAAAAGATAAAAAGAAGTATAACGAAAAGCCAATAGAGGAAAAATTGGAGCATTGACTA  33529
PolarBear    A---AAAAAAAAAAAAAAAAGATACAAGGAA-GTACAGTTAAGTG------TGAATAGAGAGAAAGTTGGAGGACTGATTC  5492
Cow          A--AAAAAGCCACACAATTAT-----------------------------------------------------------  31891
Dog          G---TGCACACGTAC---ACATGCCTAGCA-GTTCAGTT-----------TAAAAAGAAAAACTATACGAAGTTTATTTA  31240
BelugaWhale  GTTGCATATTGAAACAATGTCTTTAAAAAG-GTACAAAGACTTACATTCAAAAGCAAATAGAAAGTTGGAGGGCTGACTC  26623
Donkey       GTTGCGTATTGAAATAATCTCTAAAAAATACAAAGAAATACAGTTAAAAGCAAAGAGAGGGGATGTTGGAGGACTGACT-  33544
Cat          G---AAAACACAAAC------CATAGAGTA-GCAGAATA-----------TCTTTACAGAGCATAT-GGAACTTTGATCC  34884
Ferret       A---AAAAAAAAAAA---AGATACAAGGAA-GTATTGTTAAATG------TGAATAGAGAAAAAGTTGGAGGACTGATTT  31634
Lion         G---AAAACACAAAC------CATAGAGTA-GCAGAATA-----------TCTTTACAGAGCATAT-GGAACTTTGATCC  33208
Vaquita      GTTGCATATTGAAACAATGTCTTTAAAAAA-GTACAAAGACTTACATTCAAAAGCAAATAGAAAGTTGGAGGGCTGACTC  27290
Pig          GTTGCATATTGAAACAGTGTC-----------------------------------------------------------  26463

Majority     CCAATTTTCAAX--T-TAATATAAGACTGAGTAATCAAAACAATGTCATAT----XGGCATAAAGAAAGA-AAATAGATT
                      38810     38820     38830     38840     38850     38860     38870     38880
Human        CCAGTTTTCAAGAAT-TACTGCAAAGCTACGGTAATTAAAAATAGCATCAT-----AATA-ACATAAAGATAAATAGGTC  33602
PolarBear    CCTGTTTTCAAATGT-TAATATGAAGCTGAGAAATCAAAACAATGCCATAT----TGGCATAAAGAAAGATAAATAGATT  5567
Cow          --------------------------------------------------------------------------------  31891
Dog          CCAAAATATA---GT-CCATACGTGACTGTGTAAATGGAATTTTGCCTTGC----AGATACTTTTATTGA-TTTTTCATT  31311
BelugaWhale  CTGCTTTTAAAAGTTATTGTAAACCATACAGTAATCAAAACAAAGTCGTATTTATTAGCATAAAGAAAGATAAATAGATT  26703
Donkey       CTGTTTTCCAAAGTTATTGTA-AAGCTCCAGTAGTCACAATAATGTCACAT----TGGCATAAAGAAAGATAAATAGATC  33619
Cat          AGAATATATA------TAAATTCAGACTGTATAAATGCTAAAACTTAA-------GGCAACAAACAACC---------T-  34941
Ferret       CCTATTTTCAAACAT-TATTATAAAGCTGAGTAGTCAAAACAATGTCACAT----TAGCATAAAGAAAG--AAATAGATT  31707
Lion         AGAATATATA------TAAATTCAGACTGTATAAATGCTAAAACTTAATAA----GGCAACAAACAACC---------T-  33268
Vaquita      CTGCTTTTAAAAGTTATTGTAAACCATACAGTAATCAAAACAAAGTCGTATT----AGCATAAAGAAAGATAAATAGATT  27366
Pig          --------------------------------------------------------------------------------  26463
```

```
Majority     AATGGAACAGAATAGA---XCCAGAAAT----X-XCATAATTATGGACAACTGGTTTTTGACAAAGGTACAAAGXCAXTT

             38890  38900  38910  38920  38930  38940  38950  38960

Human        AGTGAGACAGAGCAGAGAGTTCAGAAGTTAACTCACACAATTATGGATAACTGATTTTTGACAAAGGTGCAAAGTCAATT  33682
PolarBear    AATAGAACAGAATAGAA--TCCAGAAAT----CTACATAATTATGGACAGCTGTTTTTTGACTAAGATACCCATATGTAA  5641
Cow          --------------------------------------------GGACAACCGGTTTTTGACAAAGGTACAAAGGCAGTT  31927
Dog          GATTTACAATACAAGCC--CCCCCAAAT-------TATCACTA--GATCATTGATTTTGATTAGCCATAGCC-CAAGTTC  31379
BelugaWhale  AATGGAACAGAATAGAGAGTCCGGAAATAAAGCCACATAGTTATGGACAACTGGTTTTTGACAGAGGCACAAAGGCAATT  26783
Donkey       AGTGGAACAGAATAGA----CTTAAAATAAACCCACGCAATAATGGACAACTGGTTTTTGACGGATGCACAAAGGCAATT  33695
Cat          AGTGTAATAAATTGG------CAAAAAT-----------ACTT--GATAGTT----------CAGCAAAAACATATGGCA  34992
Ferret       AATAGAACAGAATAGAA--TCCAGAAATAAACCTGTGTAATTATGGACAGCTAGTTTTTGACTAAGGTAGCCATATGTTT  31785
Lion         AGTGTAATAAATTGG------CAAAAAT-----------ACTT--GATAGTT----------CAGCAAAAACATATGGCA  33319
Vaquita      AATGGAACAGAATAGAGAGTCCGGAAATAAAGCCACATAGTTATGGACAACTGGTTTTTGACAGAGGCACAAAGGCAATT  27446
Pig          --------------------------------------------GGGAGTTCCCGTCGTGGCGCAGTGGTTAACGAATCC  26499

Majority     CAGTAGAXXCAAAAAAAAXAGAATTTTCAXC--AGXXXTCTGTAACA--TAGXATATCTAT---GTGCCAACXTGAACTGC

             38970  38980  38990  39000  39010  39020  39030  39040

Human        CAGTGGAGAAAAAGTAG----ACTTCCAACAAAGGGTGCTACAGCAGTTGGAGCTATCCGTATGCAAAAAAATGATCTTG  33758
PolarBear    AAAAAAAAACAAGAAAACAAAAAAAAACTT--GAACCTGTATCTCA--TACCATAGTTAA---GAACTAGCTCTGAGTGG  5714
Cow          CAGTAGAGGGGAAAAAATAGATTTTTCAG--CAGAGTTGTATAACAG-TAAGATATCCAT---ATGCTGCCAACAGCAAC  32001
Dog          TATGGTGT-CAAAATAATTAAAGT--GGTC--AGATGTCTGTAACG--CTGCTTTGCTA----GTTTCATTTTTAAAAAC  31448
BelugaWhale  CAGTGGAGGAAAAAAA-TAGATTTTTCAGCAAAGGATTCTGTAACAA-TAGGCTATCCATGTGCTGCCAACAACAGCAGC  26861
Donkey       CAATGGAGAGAGAAGA---GACTTTTCAGCAAAGGGTGCTGTAACAGTTGGGTTATCTAT---ATGCAAAAACAAAAGAC  33769
Cat          AATAAGTA-CATGAAAA--------AGAT----GCTCTATGTCATT--AATCAT---TA----GATGCAAATTGAAGTGA  35050
Ferret       AAACAAAA-CAAAACAA-AAAAACAAAGTT--GGATCTATATCTCA--AACCATAGTTAA---GAATTATCTCTAAGTGG  31856
Lion         AATAAGCA-CATGAAAA--------CGAT----GCTCTATGTCATT--AATCAT---TA----GATGCAAATTGAAGTGA  33377
Vaquita      CAGTGGAGGAAATAA--TAGATTTTTCAGCAAAGGATTCTGTAACAA-TAGGCTATCCATATGCTGCCAACAACAGCAGC  27523
Pig          GACTAGGAACCATGAGGTTGCGGGTTCGGT-CCCTGCCCTTGCTCAG-TGGGTTAACGATCCGGCGTTGCCGTGAGCTGT  26577
```

```
Majority     AAXAT----------------------------------ACATGCTGCAAATAAAA-TTGA-------------------
                       39050     39060     39070     39080     39090     39100     39110     39120
Human        GATCTG-T----------------------------ATCTCACACTGCATATGAAAATTAACTCCAA--------ATGGA 33801
PolarBear    ACCAT----------------------------------ACATGATGTAAATAAAA-TCTA------------------- 5740
Cow          AGCA---------------------------------------GCAGCAAACTTG--TTGG------------------- 32021
Dog          AATAT----------------------------------ATATAGTATGAATAG---T---------------------- 31469
BelugaWhale  AACAAGCTATCGATACTTGGATCTATACTTCATACAAAAATGAGCTCCAAATGGACTTTGATGTAAATGTAGAACCTAAA 26941
Donkey       AAAAAACTTGGGTCT-----AT--------------ACCTCATACGATATACAAAAATTAGCTCCAG----------GTG 33820
Cat          GATA-----------------------------------ACATTACGCACCTGTTA-T---------------------- 35072
Ferret       GCCAT----------------------------------ACATGATGTAAATAAAA-TCTA------------------- 31882
Lion         GATA-----------------------------------ACATTACGCACCTATTA-T---------------------- 33399
Vaquita      AACAAGCTATCGATACTTGGATCTATACTTCATACAAAAATGAGCTCCAAATGGACTTTGATGTAAATGTAGAACCTAAA 27603
Pig          GGTGT------------------------------------AGGTCGCAGACACGGCTCGG------------------- 26602

Majority     -------------------------A-ATTAXAAAATCTTTAGGAGATTGAGTAGAX---------XATXTTT-X-----
                       39130     39140     39150     39160     39170     39180     39190     39200
Human        TCATG---GCCTAATGTAAAACCAAAAATTATAAAATCTCTAGAAAAAAATACAGAAAATCTTCGTGACTTTGGATTAAG 33878
PolarBear    -------------------------AAATTATAAAACTTCTAGAAGACATAGTAGAA---------AATCTTTGTT---- 5782
Cow          ----------------------------------A-TCTATGTTTTGTTTAGTT---------------GTTA------- 32044
Dog          -----------------------------TG--AAACATTTAAAAAATTAATTACAT---------AGCCCC-------- 31501
BelugaWhale  AATTATAAAACTTCTAGAAGAAAGCATAGTAGAAAATCTTTGTGACCTTGGGTTATGCA-----GAGATTTCTTTGCATA 27016
Donkey       GACCATGACGTAAATGTAGAACCTAAAATTATAAAACTTGTAGGAGAAAACATAGTAGA------AAATCTTTGTGGCCT 33894
Cat          ---------------------------AATGGCTAATATTTAAGAGATTGACCATA------------------------ 35101
Ferret       -------------------------AAATTATAAAGCTTCTAGAAGACATAGTAGAA---------AATCTTTATG---- 31924
Lion         ---------------------------AATGGCTAATATTTAAGAGATTGACCATA------------------------ 33428
Vaquita      AATTATAAAACTTCTAGAAGAAAGCATAGTAGAAAATCTTTGTGACCTTGGGTTATGCA-----GAGATTTCTTTA---- 27674
Pig          ----------------------------------A-TCC-TGCGTTGCTGTGCT---------------TCTG------- 26624
```

```
Majority     -------CTATGTGATGXGACCC--CAAAAGCACAATCTATGAAAGAACAAATGGXACTTXAT-CAGAXTTTAAAAXTTA
                      39210     39220     39230     39240     39250     39260     39270     39280
Human        CAGGAATGTATTTGATATGACCC--TAAAAGCACAATCTATTAAA----AAATGGAATTTGACATCAGAATTAAAATCTT  33952
PolarBear    ------ACCTTGGGTTAGGACCC--CAAAAGCATAATCTATAAAAGAACAAATGGAACTTCAT-CAGGTTTTAAAACTTA  5853
Cow          --------------ATTTGACCC--CAAAAGCATGGTCTGTGAGAGAACAAATGGGATTTGAT-CAGTTTTAAAAACTTA  32107
Dog          -------CCCTATGACAGGA------AATAC--TGATTT---------CAAGC----CTTTAC-CTCCCCTTGAG--T-A  31549
BelugaWhale  ATCTATGAAAGATTATGCAATCTATGAAAAGCACAATCTATGAAAGAACAAATGGGACTTCAT-TAGTTTTAAAAACTTA  27095
Donkey       TGGGTTAAGCAGAAATTTGATATCTCAAAAGCACAGTCTGCGAAAGAATAAATA-----------GGACTTCATCA----  33959
Cat          -------CTAAGTGTTGGAA------A---G---GATGT----------GGAT------------GAACTGTAAC-----  35135
Ferret       ------ACTTTGGGTTAGGACCC--TAAAAGCATAATCTATAAAAGAACAAATGGGACTTTGT-GAGATTTTAAAATTTA  31995
Lion         -------CTAAGTGTTGGAA------A---G---GATGT----------GGAT------------GAACTGTAAC-----  33462
Vaquita      --------------ACGTGACCC--CAAAAGCACAATCTATGAAAGAACAAATGGGACTTCAT-TAGTTTTAAAAACTTA  27737
Pig          --------------GCGTAGGCC--GGTGGCTACAG-CTCCGATTCAACCCCTAGCCTGGGAA-CCTCCATATGCCGCGG  26686

Majority     -XGTTXTTCAAAAGACA--GXTA-GAGAATGAXAAGATAAGCCATAGAGTGG-TGAAXATCTXTAXAAA-----------
                      39290     39300     39310     39320     39330     39340     39350     39360
Human        CTGCCATTCAAAAGAAACTGCTA-GGGAATGAAAAGACAAGCCATAAACTGGAAGAACATCATAACAAA-----------  34020
PolarBear    AAGTTTTTCAGAAGACATTGATAGTAGAATGAAAAAACAAGCCATAGAATATCTGAACATCTTTAT-AA-----------  5921
Cow          ACATTATTCAAAATACA--GTTAAGAGAATAAAAAGACAAGCCATAGAGTGG-CAGACATCTTTACAAA-----------  32173
Dog          TGGTCCCCTCCAATA----GGAAAAAAAATATGAAGCTGTATCA-AATATAT-TGGATAGCTGTT---------------  31608
BelugaWhale  -CATTATTCAAAAGACATTGTTAAGAGAATGAGAAGATAAGCCACGGAGTGG-CAGATCTTTACAAAGATGTCTGATACA  27173
Donkey       -----GTTTAAAAAA-----------GAACAAAAAGACAAGCCATGGAGAGGCAGAATGTCTTTACAAA-----------  34012
Cat          -----TCTCATGTTG-------------GTGAGAATGTAAA-C------T---TGAAGAACTACT---------------  35172
Ferret       AAGTATTTCAAAAGACATTGATAGTGGAATGAAAAAATAAGCCGTAAAATATCTGAACATCTTTCTGAA-----------  32064
Lion         -----TCTCATGTTG-------------GTGAGAACGTAAA-C------T---TGAAGAACTACT---------------  33499
Vaquita      -CATTATTCAAAAGACATTGTTAAGAGAATGAGAAGATAAGCCATGGAGTGG-CAGATCTTTACAAAGATGTCTGATACA  27815
Pig          GAGCGGCCCAAGGAATA--GC---AACAACAACAACAAAAGACAAAAAGACA-AAAAAAAAAAAAAGAA-----------  26749
```

```
Majority     -------------GCATATAGCTGATAAAGGATTTTXATCCAGAATATAXATAAATTCAGACTATXTAXA--------CT
                      39370     39380     39390     39400     39410     39420     39430     39440
Human        -------------GCATATAGCTGATAAAGGACTTCTATCCAGAATATTAAAAGATTCAGTATATGTAAA--A------T  34079
PolarBear    -------------GCATATGGCTGGCAAAGAACTTTGATTCAGAATATATATAAATTTGGACTGTATAAACG------CT  5982
Cow          -------------GCATGCAGTTGATAAAGGATTTTTATCCAGAATAAAAAT----------------------------  32212
Dog          -------------GCCTGGGATTGATGAATAGGAATAATAC------TACGCCGTACTAGATTAC-TGATCC------CT  31662
BelugaWhale  TATACATATGTATATATATAGCTGATAAAGGATTTTTATCCAGAATATACATAAATTCAGACTACATATATAA----ACT  27249
Donkey       -------------GCATAAAGCTGATAAAGGA--CTTGTCTAGAATATATATAAATTCAGACTATATAGACTCCAAAACT  34077
Cat          ---------------------TGGACAAA-----ATAATCT--------------TGCAGATTCT--------------T  35198
Ferret       -------------GCATATAGCTGATAAAGAACTTTAATCCAAGGTATATATAAATTTGGACTATATAAGCT------CT  32125
Lion         ---------------------TGGACAAA-----ATAATCT--------------TGCAGATTCT--------------T  33525
Vaquita      TATACATATGTATATATATAGCTGATAAAGGATTTTTATCCAGAATGTACATAAATTCAGACTACATATATAA----ACT  27891
Pig          -------------ACA-GTGTCTTAAAAAAGATACAAA---GAGGTGTAGTT----------------------------  26784

Majority     AAA-AAXC----XAXTAAACA--ACCAAATGAAGTAAATTGACAAATTXTXXTTGAT---XCAGCAGGAA-------CXC
                      39450     39460     39470     39480     39490     39500     39510     39520
Human        CAGCAGTC-AAGCAACAAACT--ACTCAATG-AATTGGCCAAAATATTTGAACAGGTAGTACAGCACAAAAGATACACAG  34155
PolarBear    AAA-AATGAAGGCAACAAACA--ACCCAATGTAATAAATTGGCAAAAAGTACTTGAT----CAGCAAAAA-------CAT  6048
Cow          ---------------TAAAG-------------------TGAGACA---------------ACATTGC------------  32231
Dog          GAG-AAAAGGGGAAAAAAAAA--ATAACACGAAGTGAAATCCCTTTTCATTTTGGCC---TCTCCTGCAC-------CTT  31729
BelugaWhale  CTA-AAACTCAACAATAAGGC--AGCAAACAACCCAATTTGATAAATTGGCAAAAGTAATTGAATTGGTC-------AGC  27319
Donkey       GAACAGGTGAGGCAACAAAAACAACCCAACTTAGTAAATTGACAAAA-ATACTTGATAGTTCATCAAGAAAGATACACAA  34156
Cat          AAA-AAGT-------TAAACT--ACCAAATGACGTTTGTCAGCTGTTTATCTCTTAG------GCATTTA-------TGC  35255
Ferret       AAA-ACTC-------TAAACA--ACCCAATGTAATAAATTGGCAAAA-GTACTTGATAGTTCAGCAAAAA-------CAT  32187
Lion         AAA-AAGT-------TAAACT--ACCAAATGACGTTTGTCAGCTGTTTATCTCTTAG------GCATTTA-------TGC  33582
Vaquita      CTA-AAACTCAACAGTAAGGC--AGCAAACAACCCAATTTGATAAATTGGCAAAAGTAATTGAATTGGTC-------AGC  27961
Pig          ---------------TAAGGC------------------TAATAGA---------------AAATTGGAG----------  26806
```

```
Majority     ATGGAAAATAAACACATGAXAA---TGCTCAT----TTAAXAATCATXAGAGAAATGTATCTTAAAAXTAAAGTXAGGTA
                      39530     39540     39550     39560     39570     39580     39590     39600
Human        ATGGTAAGTAAGCACGTGAAAAAGCTGGTCAGC--ATCATTAGTTATTAGGGAAATGAAAATTAAAACTACAGTGAGATA  34233
PolarBear    GTGGCAAATAAAGACATGAAAATCGTGCTCAGT---ATAGTAATCATTAGAGAAATGTAGATTAAAACTAAAGTGAGAAA  6125
Cow          --------------------------------------------------------CTATCTCTACAACAGC--------  32247
Dog          GGAGCGGGTTTAGTGCCCTTAGT--TGCCCTAT---TTAA-AATCACCTGTGGCACGT--CTTAAAAAAAAAGTACAGGT  31801
BelugaWhale  AAAAAAGATATACAGATGACAAGTAAGCACATG---AAAAAGATGTTCAGCATCATTTGTCGTTAGAGAAATGCAAAGTG  27396
Donkey       ATGGTAAATAAGCATATGAAAAAGATGCTTACAGCATCATTAATCATTAGAGAAATGCAAATTAAAACTAAAGTGAGATA  34236
Cat          ATGAAAAATGAAGGCAT---A----TGTTCAT----TTAAAAAAAACCCCAGGGGCGC--CTGGGTGGCGCAGT-CGGTT  35321
Ferret       ATGGCAAATAAGCACATGCAAAAGATGCTCAA----GTATTAATCATTAGAGAAATGTAGGTTAAAACTTAAGTGAGGTA  32263
Lion         ATGAAAAATGAAGGCAT---A----TGTTCAT----TTAAAAAAA-----------------------------------  33616
Vaquita      AAAAAAGATGTACAGATGACAAGTAAGCACATG---AAAAAGATGTTCAGCATCATTTGTCGTCAGAGAAATGCAAAGTG  28038
Pig          -----------------G-------G------------------------------CTGTTTTCAAAGGATTG-------  26825

Majority     AXATTAC----XXC-TTTTTAXAATG-CTAAAATTTAAAAGACTG--ATCATACTA----AGTGTTGGAAAGGATGTG-G
                      39610     39620     39630     39640     39650     39660     39670     39680
Human        ATATTACAA---ACCTATTAATAGTGACTAAAATTAAAAAGAGTGCCATCTTGGAAAGCATGAGTTGGGAAGAATGTGGA  34310
PolarBear    ACATTAC----CCCCTTATTATAATGGCTAAAATTTAAAAGATTG--ACCATACT----GAGTGTTGGAAAAGATGTG-G  6194
Cow          ----------------------------TAATATTTTAAAGAATG--ACCATACTA----AGTGTTGAAAAAGATGTGAG  32293
Dog          GAATCCC----TTTTTTTTTTTTTTAAAAAATATTTTATTTATTC--AGAGAGCAGAG-TCTCTTTAGAGAGAGAGCG-G  31873
BelugaWhale  AGATAACATTACACATCTCTGCAACAGCTAAAATTTGAAAGACTG--ATCATACTA----AGTGTTGGAAAGGATGTGGG  27470
Donkey       ACATTACA----CACCTATTATAATGGCTAAAATTTAAAAGACTG--CCTGTACT------GTGTTGGAAAGGATATGGA  34304
Cat          AGGTGTC----CGA-CTTCAGCCAGG-TCACGATCTCGCGGTCCG--GGAGTTCGAGCCCCACGTCGGGCTCTGGGCT-G  35392
Ferret       ACATTAC----CCC-TTATTACAATG-CTAAAATTTAAAAGATTG--TAAAGATTTAA-AAGTATTGGAAAGGATGTG-G  32333
Lion         --------------------------------------------------------------------------------  33616
Vaquita      AGATAACATTACACATCTCTGCAACAGCTAAAATTTGAAAGACTG--ATCATACTA----AGTGTTGGAAAGGATGCGGG  28112
Pig          ----------------------------TAAAGCTAATAAAAGTG--TTCATATTGT---AAAGTAATCAAAACTGGCCT  26872
```

```
Majority     XGAACTGTAAAACTCA-TATACTGTTGATGGGAATGTAAAATTXTAGAACTACTTTGGAAAACAATTTGGCA---GTTTC
                               39690     39700     39710     39720     39730     39740     39750     39760
Human        AGAATGTTAATATTCACAATACTGCTGATGGGAATGTAAAATCGTAG---TACTTTGGAAAACAATCTGGTA---GTTTC  34384
PolarBear    A-AGAACCATAACTCT-TATACTGTTGGTGGGAATGTAAACTTGTAGAACTGCTTGGGCAAATAATCTGACA---GTTTC  6269
Cow          GGTACTGTAACACTCA-TATACTGTTGATGGGAATGTAAAATT-TAGAACTATTTTGGAAAACAGGTTGGCA---GTTTC  32368
Dog          GCAGGAGAGTAGATGA-GAGACGGCCAG------CAGAGACC---ATAAGTGCAGAGGGAGAAAAGAAGGAA---GAGTT  31940
BelugaWhale  GGAACTGTAACACTCA-TATACTGTTGATGGGAAAGTAAAATTGTAGAACTACTTTGGAAAACAATTTGGCA---GTTTC  27546
Donkey       GGAACTGTAACACTCA-TATATTGTTGATGGAAATGTAAAATTATAGAACTACGTTGGAAAACAATTTGGCA---GTTTC  34380
Cat          A-TGGCTCAGAGCCTG-GAGCCTGTTTC---------CGATTC-TGTGTCTCCCTCTCTCTCTGCCCCTCCC---CTGTT  35457
Ferret       A-TGAACCATAGCTCA-TATACTGTTGG---AAATGTAAACTTGTAGAACTACTTGGGCAAGTAATCAGGCC---ATTTC  32405
Lion         --------------------------------------------------------------------------------  33616
Vaquita      GGAACTGTAACACTCA-TATACTGTTGATGGGAAAGTAAAATTGTACAACTACTTTAGAAAACAATTTGGCA---GTTTC  28188
Pig          ATTAGCATAAAGATAA-ATAGTAGTAGAGCAAAATAGAGAATC-CAGAAATAAACCCACACAATTATGGACACCTGGTTT  26950

Majority     TTAAAA--GTTAAACATXTA---CXGTATGATCCAGCTATTTGTCTCTTAGGCATTTAXCCAAGXAAAATGAAGGCATXT
                               39770     39780     39790     39800     39810     39820     39830     39840
Human        TC------------CAT-----------GATCCAGGTTATTTGTCTCCTAAGCATTTACCCAAGAGAAAAGAAGGCATCT  34441
PolarBear    TTAAAAA-CTTGAACATCTA---TGAAATGATGCAGCTGTTTGTCTCTTAGGCATTGATGCAGGAAAAATGAAGACAT--  6343
Cow          TTAAAACTGTTAAACATTTA---CAGTATGATCCAACTTTGT-TTCCTAGGTACTTAACCCAAGAAAAATGGTAGCATAT  32444
Dog          CAGCAA------GGCTCCAA---TGCGGGACTCGAGTCCGATCACCCTTTGTGACTCTTCCATTGAACAT---GCCCTGG  32008
BelugaWhale  TTAAAAA-GTTAAACATTTA---CTGTATGATCCAGCTATTTGTTTCCTAGGTGCTTACCCAAGGAATATGAAGGCATAT  27622
Donkey       TGAA----GTTAAGTATGTA---CTGTATGATCCAGCTGTTTGTCTCCTGTGCAAGAGGAGAAAGAAAACCCAAGAAA--  34451
Cat          CATGCT--CT---GTCTCTC---T-CTGTCCCAAAAATAAATAAACGTTGAAAA---AAAAAAAGAAAATAAAAAAAA--  35523
Ferret       TTAAAA--TTCAAACATCTA---TGAAATGATGCAGCTGTTTGTCTCTTAGGCATTTATGCATGAGAAATGAAGGCATAT  32480
Lion         -----------------------------C--------------------------------------------------  33617
Vaquita      TTAAAAA-GTTAAACATTTA---CTGTATGATCCAGCTATTTGTTTCCTAGGTGCTTACCCAAGGAATATGAAGGCATAT  28264
Pig          TCAATAAAGGTACAAAGTCAATTCAGTGGAAGAAAAAATTAAATTTTTCAGTAAAGGGTTCTATAACAATAA-GTTATCC  27029
```

```
Majority     ACTCA-------------TTAAAAAACTC--ATATGAATGTTCATAGTAGCTTTXTTTGT-AATATCCAAAXCCCAAAAC

                      39850     39860     39870     39880     39890     39900     39910     39920

Human        ATTCA-------------TTAAAAAACTCTCATGTTAATGTTCATAGCCATTTTATTTGT-AATCACCAAAATGTGAAAC  34507
PolarBear    GTTCATT--AAAAAAAGCTTAAAAACTTA--AGATGAATGTTCATAGTAGCTCTTTTTGTTAATACCCAAAGCCCAAAAC  6419
Cow          ACTCA-------------TCA-----CAC--ATGTAAATGTTTATAGTAGTTTTATTTGT-AATATCTAAAGCCTAGAAC  32503
Dog          GCCCA----AA---GGGCTTCAGGACCC-----TCAACTGCTTGTAGGGTTCCACCCCAGGGAGGTCCCGAAC---AAGT  32073
BelugaWhale  ACTCA-------------TTAAAACACTC--ATATGAATGTTCATAGTAGCTTTATTCGT-AATATCTAAAATCCAGAAC  27686
Donkey       AATGAGGGCATATATTCCTTAAAAAACTTGTATATGAATGTTCATAGTAGCTTTTTTTGT-ACCATTGAAGACCCAAAAC  34530
Cat          ACCCA--------AAAAACTAAAAAACTC--AGTTGAATGTTCATAATAGCTTGTTT-GT-AATAAGCAAAGCCATAAAC  35591
Ferret       GTTCACTTAAAAAAAAAACTAAAAAACTA--AGATGAATGTTCATAGTAGCTTTTTTTGT-AATACTCAAAGCCCAAAAC  32557
Lion         --CCA--------AAAAACTAAAAAAATC--AGTTGAATGTTCATAATAGCTTGTTT-GT-AATAAGCAAAGCCATAAAC  33683
Vaquita      ACTCA-------------TTAAAACACTC--ATATGAATGTTCATAGTAGCTTTATTCGT-AATATCTAAAATCCAGAAC  28328
Pig          ATTTA-------------CTG-----CCA--AAAACAACAGCAGCAAACGACTTAGAGCT-ATGGACTTCATACTGAAAT  27088

Majority     AATCCAAATGTCCATCAACAGATGAATGGATAAATA-GATTGCTGTTTTGGCATTGTATTCCATTGCATACAXTGAAATA

                      39930     39940     39950     39960     39970     39980     39990     40000

Human        AA-----ACATTCATCAACAGATGAACTAATAAGTA-GATTCCTGTTTTGGCATCATATGCTTTTCCATACAGTGGAATA  34581
PolarBear    AATCCAGATGTCCATCAACAGCTGAATGGATAAATG-GATTGCAGTTTTGACATTGTATTCCATTGCATACTATGAACTA  6498
Cow          AACCCAAGGGTCCATCAGT-GATGAATGGATAAATG-AATGACTCTTTTGGCATTGAATTTCATGA-ATACAGTGAAATG  32580
Dog          ACTATTATTACCAACCTAACATCAAAACACTAAACT-AGTCACATTGCTTAAAT-GTACTCCATTG-GTTCAAAGAAAAA  32150
BelugaWhale  AACCCAAGTGTCCATCAGCAGATGAATATATAAAAA-GATAGCTCTTTTGGTATTGAATTCCATTGCATACAGTAAAATA  27765
Donkey       TACCCAAATGTCCATCAACAGATGAATGGATAAAAGTGATTACTGTTTGGGCATTGTATTCTATTGCATACATTGAAATG  34610
Cat          AATCCACATGTCCATCAACAGATGAATGGATAAATA-GATTGCAATTTTGGCCTTGTATTCCTTTGGATACAATGAAATA  35670
Ferret       AATCTAGATGTCTACTAGTAGCTGAATAGATAAATA-GATTGTAGTTTTGGCATTGTATTCCATTTAATATCATGAAATA  32636
Lion         AATCCACATGTCCATCAACAGATGAATGGATAAATA-GATTGCAATTTTGGCCTCGTATTCCTTTGCATACAATGAAATA  33762
Vaquita      AACCCAAGTGTCCATCAGCAGATGAATATATAAAAA-GATAGCTCTTTTGGTATTGAATTCCATTGCATACAGTAAAATA  28407
Pig          GAGCTCCAGATGGACTTTT-GAT------ACAAATG-TAGAACTTGAAAATTATAAAACTTCTAAG-AGAAAGCATAGTA  27159
```

```
Majority     CTA---CTCAGCCATATAGAXTXAGCT---------------------ATTXXTCTXTTA------------XTG-----
                      40010     40020     40030     40040     40050     40060     40070     40080
Human        CAA-----CAGCCATGTAGAATGAGCTATTGATAGACACAACAGCAAGAATGAATCTCCAAACAATTATGTTATGTAAAA   34656
PolarBear    CCA---CTCAGTCATAGAGAATAAGCT---------------------ATTTTTTTAAAG-------T----AAGC----   6539
Cow          CTG---CTCAGCCATATAGAGTGACCT---------------------ATTGTTCTATGA---------GCTTCG-----   32622
Dog          TCT---CTTTGTT------ACTAATC----------------------ACTA----------------------------   32171
BelugaWhale  CTG---CTTGGCCATATAGAGTGACCTATGTTACATGCAGCAACGGGGATAAATCTCTTAAATAATTATGCTCTG--AAA   27840
Donkey       CTA------AGCCATACAGAGTGAGC------------------------------------------------------   34630
Cat          CTA---CTCAGACATGTAGAATAAGCT---------------------ATTTTTCTTTTA-------T----GAAA----   35711
Ferret       CCA---CTCAGTCCTAGAGAATAATCT---------------------ATTT--T-------------------------   32665
Lion         CTA---CTCAGACATGTAGAATAAGCT---------------------ATTTTTCTTTTA-------T----GAAA----   33803
Vaquita      CTG---CTTGGCCATATAGAGTGACCTGTGTTACATGCAGCAGCAGGGATAAATCTCTTAAATAATTATGCTCTGTGAAA   28484
Pig          GGAAATCTTTGTGACCTTGGGTTAGGC---------------------AGAAATTTCTTT---------GCTGTGA----   27205

Majority     -------------------------------------------------CTG------------------CG--------
                      40090     40100     40110     40120     40130     40140     40150     40160
Human        GATGGGAGAAAAAA----------------AG---------ACTACATCCTG----------------TATGATTCCATT   34695
PolarBear    ------------------------------------------------TCTC------------------CG--------   6545
Cow          -------------------------------------------------CTAG---------------T-GG--------   32629
Dog          -----------------------------------------------------------------------G--------   32172
BelugaWhale  GACGGGGAAAAAAAGAGTATGTCCGGTATGAGTCTATTTATATAAAACTCTAGGAGATTGCATATTAAAGCAAATTGGTG   27920
Donkey       --------------------------------------------------------------------------------   34630
Cat          ------------------------------------------------GCTG----------------------------   35715
Ferret       -------------------------------------------------C--------------------CG--------   32668
Lion         ------------------------------------------------GCTGTC----------------TG--------   33811
Vaquita      GATGGGAAAAAAAAGAGTATGTCCTGTATCAGTCTATTTATATAAAACTCTAAGAGATTGCATATTAAAGCAGATTGGTG   28564
Pig          -----------------------------------------------CCCTGA---------------A-AG--------   27214
```

Majority	---------C-CC-----------------------A-XXGTGGAGATTGXATTCATGAC-CTXAGGA--TCAAGA----	
	40170 40180 40190 40200 40210 40220 40230 40240	
Human	TATATAAAACTCTGGGAAATGC------------AAACTAATCTAAAGCAAATTCATGGTTCTTAGGAGTTTAAGA---G	34760
PolarBear	---------C-CC-----------------------A-TCGTTGTGCTTGAACTGATGAC-CT-GGGA--TCAAGA----	6583
Cow	---------C-TC-----------------------AGTGGTAAAGAACCCGCCTGCCAA-TACAGGAGGTGTGGG----	32671
Dog	---------C-TC-----------------------A-GTGTTGGGGTCTCAGT--TGAG-CT-------TTGAAA----	32204
BelugaWhale	GTTGCTTGGAATTTAGAGGTGGGTAGAGCAAGGGAGAGGGTTGTAAAGGGCATAAGGAAA-TTTGGGGGTGATGGATATG	27999
Donkey	--	34630
Cat	---------C-CC-----------------------A-ATGTGGGGCTTGAATTCATGAC-CCCAACA--TCAAGA----	35754
Ferret	---------C-CC-----------------------A-GCGTGGGGCTTAAACTCATGAC-CTCAGGA--TCAAGAA--G	32709
Lion	---------C-CC-----------------------A-ATGTGGGGCTTGAATTCATGAC-CCCAACA--TCAAGA----	33850
Vaquita	GTTGCTTGGAATTTAGAGGTGGGTAGAGCAAGGGAGAGGGTTGTAAAGGGCATAAGGAAA-TTTGGGGGTGATGGATATG	28643
Pig	---------C-GC-----------------------AGTCTATAAAAGAACACATGGGAC-CTTATCAGTTTTAAA----	27256

Majority	-G-----XCTTATGCTGTA---TTGACXXA-------X-ACX---X-ACTC--C-XAGAGTAA----XATXXTGATXTAT	
	40250 40260 40270 40280 40290 40300 40310 40320	
Human	TGGGAAAGGACAGGCAGGAGGGGTGGCAAA---GGGACACAGGAAAACTC----GGGAGTGA------TGGACATATAA	34826
PolarBear	-G-----TCTCCTGCTGTA---CCGACTGA-----GCCAACCA--G-ACTC--C-TAGAATAA----GCCACTGATACAT	6639
Cow	TT-----CCAT-TCCTGGG---TTGAG--A----------A-----G-ATCCCCTGGAGAATGA----AGTGGCAACCTAC	32721
Dog	----------CTTGTTGCT---TGGGTT--------CCTAC----A-ACTG----TAGGGTTG----TATTGTCATGTGT	32250
BelugaWhale	TATATTCCCTTATGCTGAAGGTTTCACAGATGTAAATATATGTCAAAACTTGTCAGATTGTAACTTCATAGACATCTTAT	28079
Donkey	---T-------ATTGATCTGT	34641
Cat	-G-----TCTTATGCTGTA---CTGAGCC----------GCC-----ACTC--C-TGGAATAA----GATATTGATGCAT	35803
Ferret	AG-----TCTCATGCTCTA---CCAGCTGA-----GCCAGCCA--G-ACTCGCC-TAGAGTAA----GCTACTGATATAT	32768
Lion	-G-----TCTTATGCTGTA---CTGAGCCA-----GTCAGCC-----ACTC--C-TAGAATAA----GATATTGATGCAT	33904
Vaquita	TATATTCCCTTATGCTGAAGGTTTCACAGATGTAAATATATGTCAAAACTTGTCAGATTGTAACTTCATAGACATCTTAT	28723
Pig	AA-----CTTA-GGCTA-----TTCAA--A----------A-----G-ACTGTTAAGAGAGTGA----AAAGACAAGCCAC	27304

```
Majority      GTAATAGCAGGGATAAATCTCXAAATA-------------------ATAAXGCTGTGTAAAAGAAATAG-TXT--G-ATA
                                  40330     40340     40350     40360     40370     40380     40390     40400
Human         ATTATTGTGATGGTGGTTTTATGGATGTATGCATATGTCAAAACTTGTCAAATTTTATAATTTAAATACATATACACATC  34906
PolarBear     GTAGTAGCAGGGATAAATCTCCAAATA-------------------ATTATGCTGTG--AAAGAAGTGG------G-AAA  6691
Cow           TC--CAGTATTCTTGCTTGGGAAATCCCAT-----------GG---ACAGAGGAGTCTGATGGGCTACGGTCTGTG-GTG  32784
Dog           GTGGCACATGGTGTTGTTTTCAAAAT--------------------TCA---------AAAGGACCCG------T-ATC  32293
BelugaWhale   TTTATGTGAATTGTACCTCAAAAAGACTATTTAAAAAGAAAAGCCCATAGAGGAATTAAAATGGAATAC-TATCAA-ATA  28157
Donkey        GCAACAGCAGGGATAAATCTCCAAATA-------------------ATTATGCTGTGTGAAAGAAGCAA--------GGA  34694
Cat           GTAATAGCAGGGATAAATACCCAAATA-------------------ATGCTACTGTGTAAAAGAAATGGGCGGGGG-GGG  35863
Ferret        GTAGTAGCAGGGATAAATGTCCAAATA-------------------ATTATGCTGTGTGAAAGAAGTGG------G-AAA  32822
Lion          GTAATAGCAGGGATAAATACCCAAATA-------------------ATGCTACTCTGTAAAAGAAATGGACGGGGG-AGG  33964
Vaquita       TTTATGTGAATTGTACCTCAAAAAGACTATTTAAAAAGAAAAGCCCATAGAGGAATTAAAATGCAATAC-TATCAA-ATA  28801
Pig           GGAGTGGCAGACAT-CTTCACAAAGCATGT-----------AGCTTATAAAGATCTTTATCCAGAATATATATAAA-TTC  27371

Majority      AAGGAATAXATTCAGTATGAAT--CXATTTATATAAAAXACAAGGAXATACAXGCTAAAX-----CA-------------
                                  40410     40420     40430     40440     40450     40460     40470     40480
Human         CTAGAATGGCCGAACTTCAGAACATTGATAACACCAAATGCTGGCAAGGATGTGGAGCAA-----CAGGAACTCT--CAC  34979
PolarBear     AAGGAGTACATTCTGTGTGATT--CCATTTATATAAAACACAAGGAAATGCAAACTAAA------CA-------------  6750
Cow           TTGCAACAGAGTCAGACCCAA---CTTAGCAACTAAACAACAACAAAGCAAAGGG----------CA-------------  32838
Dog           AAAGAGTA-----------------AGTGAAGAAGAATTGCAAGATTCCCTTTTTAACT-----CA-------------  32337
BelugaWhale   TAGCAAGAGAAGAACAACAAAACACTGGACAGTTAGAAAATAAAGAGCTAGGTGTTAAACCCAGTCATAAAGGTAAATGT  28237
Donkey        AAAAAATACATTTTGTATGATT--CCATTTATATGAAGCACTAGGAAATGCAAGCCAAAAGTA--CAT---------TGG  34761
Cat           GGGGAGTACATTCTGTATGAAT--TCATTTATATAAAACCCAAGGAGATACAAACTGAAG-----CA-------------  35923
Ferret        AAGGAGTA---------TGATT--CCATTTATATAAAATGCAAGGAGACGCAAACTAAAG-----CA-------------  32873
Lion          GGA-AATACATTCTGTATGAAT--TCATTTATATAAAACCCAAGGAGATACAAACTGAAG-----CA-------------  34023
Vaquita       TAGCAAGAGAAGAACAACAAAACACTGGACAGTTAGAAAATAAAGAGCTAGGTGTTAAACCCAGTCATAAATGTAAATGT  28881
Pig           AGACTACATATGTAGTCTAAAA--CTCAACAGTAAGGCAACAAACAACCTAATGT----------AA-------------  27426
```

```
Majority     -----GTTTGGAT--------------TXGGGAGTGGGGAGGXGCGXGGGGGAGGGGGTTGTXAAGAGTATAAGGAAATT

                      40490     40500     40510     40520     40530     40540     40550     40560

Human        TTACTGCTCAGTGGAA-----------TGCAGAGTGGTACAGCCACTTTGGAAGACAATTTGGCAATTTCTTAGAAAACT  35048
PolarBear    -----GTTTGGAT--------------TTGGGGGTGGGGATGTCCGAAGGGGACGTGGTTGCAAAGAGTAAAAGGAGATT  6811
Cow          --------TGGG---------------GCGGGGGGAGCGGGGAAGGGGAGGAGGGGAT-TGTAAAGGACATAAGGAATTT  32894
Dog          -----GTTT------------------TTTAAACT----ATCACCCTTGAGTACAGAGTTGTGCAG--TAAAAGTATGAG  32388
BelugaWhale  AAATTGGTTAAACTAACATAGTTTAGTGCAGTAACCACTAGTTTTGTGAGACTGTGGAGCATGTGGAATGTGACTACTCC  28317
Donkey       TGGTTGCTTGGAAT-------------TCAGAGGTGGGGAAGAGTAAGGAGGAGG-TGTTGCCAAAGGCACAAAGAAATT  34827
Cat          -----GATTGGAT--------------TTGGGGGTGGGGAGGAGCCAGGGGGATG-GGTTGCAAAGGGTACAAGGAAATT  35983
Ferret       -----GTTTGGAT--------------TTGGGTGTGGGGAAGTGCAAGGGGGACATGGTTGTGAAGAGTAAAAGGAAATT  32934
Lion         -----GATTGGAT--------------TTGGGAGTGGGGAGGAGCAAGGGGGATG-GGTTGCAAAGGGTACAAGGAAATT  34083
Vaquita      AAATTGGTTAAACTAACATAGTTTAGTGCAGTAACCACTAGTTTTGTGAGACTGTGGAGCATGTGGAATGTGACTACTCC  28961
Pig          --------TAAATTA------------GCAAAAATAATTGAATTGGTCAGTAGAAAAGATATACAGAAGGTAAGTAAGCA  27486

Majority     TX---GGGXTG---ATGGATATGTATAXTTTTAAAAGCTG--------GAAGATTTCATAGATGTAT-GCATAT-ATCAA

                      40570     40580     40590     40600     40610     40620     40630     40640

Human        ATA--CGGCCGGGCACAGTGGCTTACGCCTGTAATCCCAGCGCTTTGGGAAGCCAAGGCAGGCGGATCACCTGAGGTCAG  35126
PolarBear    TG---GGGGTG---AT--ATATATATACTTACAGTATTTCTTAT---AGAAGATTTCATGGATGTAT-GAATAT-ATCAA  6878
Cow          G----GAGATG---ATGGATATGTATATTCTTTTATGCTG--------A-AGGTTTCATAGA--------------TGTA  32944
Dog          TG---TTGATG---AA-----TATATAAATATAAAAGCTC--------AGAAA--CC-TGAAAATAT-AAA----ATAAA  32441
BelugaWhale  TAACTGAGATGTAAATGGGTTTGTGTGTTGTTCAAAGACGTA---CCAAAAAAAACCACAAAATAAT-CTCATTTTTTAA  28393
Donkey       TG---GGGGTG---GTGGGTATGTATATCTCTTATGGT----------GAAGGTTTCATAGAGGAAT-ACATAG-GTCAA  34889
Cat          TA---GGGGTG---AATGATATATATACTCTCTTAGGGT---------GAAGATGTCATGGATGTTT-GCATAT-GTCAG  36046
Ferret       TG---GGGGTG---AGGGATATGTATACTTTTAGTATTTCTTAG---AGAAGATTTCATGGATGTGT-GCATAT-ATCAA  33003
Lion         TA---GGGGTG---AATGATATATATACTTTCTTAGGGT---------GAAGATTTCGTGGATGTTT-GCATAT-ATCAG  34146
Vaquita      TAACTGAGATGTAAATGGGTTTGTGTGTTGTTCAAAGACGTAGTACCAAAAAAAACCACAGAGTAAC-CTCATTTTTTAA  29040
Pig          T----GCGAAG---AAGATGCTCAGCATCTTTAGTCACTG--------G-AGAAAT-GCAAA--------------TTAA  27535
```

Majority	AAXTT--XTXXAATT---GTATXCXT-TAAATATATGAAGXTTATTGCATGTGAA-----TA---AAACTTCAAAA----	
	40650 40660 40670 40680 40690 40700 40710 40720	
Human	AAGTTCAAAACCAGCCTGGCCAACATGGAGAAACCCGATCTCTACTAAAAAATACAAAATTAGCTAGGCGTGGTGGTGCA	35206
PolarBear	ACCTT--GTCAAATT---GTATGCCT-TAAATATATGCAGCTTATTGTATGTGAA-----TA---AAACCTCAAAAGGGC	6944
Cow	AGCAT--------AT---GTCAA----AACTTGTCAGACGATTGTAGCTTTATA------TG---AAGCTTATTTTACG-	32999
Dog	A---------------------------AAATATTTAAATTTTAATTCTTATTA------TA---AACTTTTGATG----	32481
BelugaWhale	AGTATTGATTTATAT---GTTGATATGGTAATATTTCAGATATATTGGGTTAAAATATATTATTAAAATTAATTTATTTG	28470
Donkey	AATTT--GTCACATT---GTAGGCTT-TAAATATATGCAGCTTATTGTATGTGAAG----TA----TATATCAAAA----	34951
Cat	AACTT--GTCAAATT---GTATGCTT-TAAATGTATGCAGCTTATTGCATGTGAA-----TA---ATACCTCAGAA----	36108
Ferret	A----------AATT---GTATGCCT-TAAATATATGCAGCTTATTGTATGTGAA-----TA---AA-CCTCAAAA----	33056
Lion	AACTT--GTCAAATT---GTATACTT-TAAATGTATGCAGCTTATTGCATGTGAA-----TA---ATACCTCAGAA----	34208
Vaquita	AATATTGATTTATAT---GTTGATATGGTAATATTTCAGATATATTGGGTTAAAATATATTATTAAAATTAGTTTATTTG	29117
Pig	AGTGA--------AA---GTAAG----ATAACATTACACATCTAT-GCAATAGC------TA---AAATTTAAAAAACTG	27590

Majority	----X--XX-XXAX-X-X--XXAGACTGTTTAAAA--TGGA-AAACTCTTAX-X-------------------------A	
	40730 40740 40750 40760 40770 40780 40790 40800	
Human	TGCCTGTAATCCCA-GCCACTCAGGAGGCTGAAGCA--GGAGAATTACTTGAAC-----------------------CCA	35260
PolarBear	TGTTTAAAAAGAAAAAGCCCATAGATGGATTAAAA--TGGA-ATACTATTGA-A---------TATTCTA---------T	7002
Cow	----TGAAT-CATACCTCAGAGACACTGTTTAAAA--TGAA-AAACCCGTAG---------------------------A	33044
Dog	---------------------TAGA-AGTCATAAA--TAGA-ATATTACTAAAA---------CTTATAA---------G	32518
BelugaWhale	CAACTATGTGCCAGTAATATGGACAACCTGGAAGAAATGGACAAATTCTTAGAAAAGCACAACCTTCTGAGACTGAACCA	28550
Donkey	----------------------AGGCTGTTTAAAA---AGAAAAGCCCGTAG---------------------------A	34979
Cat	----------------------AGGCTGTTTAAAA--GGAA-AAA--GCCTA----------------------------	36133
Ferret	--------AAAAAAAAGCCCATAGACAGTTTAAAA--TGGA-ATACTGTCGGGA---------TGCCTGG---------G	33107
Lion	----------------------GGGCTGTTTAAAA--GGAA-AAA--GCCCA----------------------------	34233
Vaquita	CAACTATATGCCAGTAATGTGGACAACCTGGAAGAAATGGACAAATTCTTAGAAAAGCACAACCTTCTGAGACTGAACCA	29197
Pig	--ACCATAT-TAAGTGTTGGAAAAGATATGGAGGAGTTGTA-ACACTCGTA----------------------------A	27638

```
Majority     GGAAGTAAT-XGTAAAATAA--------XTCAATAATATCA-----------------------------------AAT
                      40810     40820     40830     40840     40850     40860     40870     40880
Human        GGAGGTGGAGGTTGCAGTGAGCCAGGATCACGCCCATTTCACTCCA----------------------ACC---TGGGC  35314
PolarBear    TAGTGCAGT--GCAAGACAA----GACCACCACCAAAAACA----C--------------------------------TGGGT  7043
Cow          GGAATTAAAATGGA--ATACT-------GTCA--AATATTG---------------------------------------  33074
Dog          TGG-ACATT--GCAGAACAT---TTCTTTTGGATATTACTA--ATC------------------------------TAAGA  32561
BelugaWhale  GGAAGAAATAGAAAATATAAACAAACTGGTCATAAGCACTGAAATTGAAACTGTGATTAAAAATCTTCCAACACACAAAA  28630
Donkey       GGAATTAAC-------ATGG------------AATACTATCA-----------------------------------GAT  35005
Cat          TGGAGGAAT---TAAAATGG------------AATATTATCA-----------------------------------AAT  36163
Ferret       TGGCTCAGTTGGTTAAGCAGC--TGCCTTCGGCTCAGGTCATGATC-----------------------------CCAGC  33156
Lion         TGGAGGAAT---TAAAATGG------------AATATTATCA-----------------------------------AAT  34263
Vaquita      GGAAGAAATAGAAAATATAAACAGACTAGTCATAAGCACTGAAATTGAAACTGTGATTAAAAATCTTCCAACACACAAAA  29277
Pig          GCAATTGATGGGAAC-ATAAA-------ATTGT-AGAACTG---------------------------------------  27670

Majority     ATCCAGGG--ATAGXGCXTXXCAA--------X--------TTTACAG-----XX--------X------C-GA-----X
                      40890     40900     40910     40920     40930     40940     40950     40960
Human        AACAAGAGCGAGACTCCATCTCAAATATACTTTCATTGTATGATCCAGCAGTTGTGCTCCCTGGTATTTGCCTAAAGGAG  35394
PolarBear    AGTTAGGA--ATAGCACATTTCAA-GTGCTCATAGC-----TTCACATAGCTACTGAT-----TA--CTACAGA-----T  7103
Cow          --CAAGAGCAGAACAACAAAACAC----------------TGGACAG-------------------------------T  33104
Dog          ATTAAGAG--GCAAAGGTTGTCAG-----ACTGGAT-----TAAAAAGCAAGGCCACC-----TAAACTGCCA------C  32618
BelugaWhale  GTCCAGGACCAGATGGCTTCACAGGTGAATTCTATCACACATTTACAGAAGAGCTAACATCCATCCTTCTCAAACTCTTC  28710
Donkey       ATTTTAT----TAATACTTTGCAA-------------------------------------------------------G  35026
Cat          ATTCTGT----TAGTGCAGTGCAA-------------------------------------------GA-----G  36186
Ferret       GTCCTGGG--ATCGAGTCCCACATCGGGCTCCTTGC-----TCGGCAGGGAGCCTGCT-----TCTCCCTCTGC-----C  33219
Lion         ATTCTGT----TAGTGCAGTGCAA-------------------------------------------GA-----G  34286
Vaquita      GTCCAGGACCAGATGGCTTCACAGGTGAATTCTATCACACATTTACAGAAGAGCTAACACCCATCCTTCTCAAAGTCTTC  29357
Pig          --CTTTGG-AAAGCAATTTGGCAG-----------------TTTTC----------------------------------T  27697
```

```
Majority     TAGAAAACTGX-----------------------------------TTTAXCCAGXTGCC--AACTAG-XAAAAA
                      40970     40980     40990     41000     41010     41020     41030     41040
Human        TTGAAAACTTAGTCTGCGCAGAAAGTAGCACATGGATGTTTATAGCACCTTTCTTTATAGTTGCCAGAACTTCATAGCAA  35474
PolarBear    TAAGGTTTTGG------------------------------GTTTGTTTTTTTTTTTTTGCC--AATTTT--ACACA  7145
Cow          TAGAAAATT-------------------------------------AATGGGTAGATGTTAAACCCAGTCATAAA  33142
Dog          TACACCTCTGG-----------------------------------CTATAGTCCATCGGA--GGCGAAGGACAGG  32657
BelugaWhale  CAAAAAATTGCAGAGGAAGGAACACTCCCAAACTCATTCTGTGAGGCCACCATCACCCTGATACCAAAACCAGACAAAGA  28790
Donkey       -AGAAGAATA-----------------------------------ACAACAAAACACC-----------AGA  35051
Cat          AAGAACACCAC-----------------------------------GAAAACACCAGGC--AATAAG---AAAA  36220
Ferret       CACCATTCTGTCTGC-----------------------CTGTGCTCGCTCTCTCCCCCTCTC--TCTTTCTGAGAAA  33271
Lion         AAGAACACCAT-----------------------------------GAAAACACCAGGC--AATAAG---AAAG  34320
Vaquita      CAAAAAATTGCAGAGGAAGGAACACTCCCAAACTCATTCTGTGAGGCCACCATCACCCTGATACCAAAACCAGACAAAGA  29437
Pig          TAAAAAAGA-------------------------------------TTCAGCTATTTGTC--TCCTAGGCATTTA  27733

Majority     TAAATAXAA-ATTAAATAGATA--------ACTGAT-AAXATAAATGTAGATXA----X--XXTAT-------A------
                      41050     41060     41070     41080     41090     41100     41110     41120
Human        CCAAGAAGTCCTTTAGGAGGTG---AATGGCCAAATAAACTGTGGTGTATATTCAGACAGTATAATGTTAATCAG-----  35546
PolarBear    TCAGTTTAGTGTTTAGAAGTTA-----------GTTTAACATCTATTTATTTTC-----TAACTAC-----CCAG--TAA  7202
Cow          T-CTCTAAATGTAAATTGGCTA-------AACTAAC--ATAGTCTCATAGTAACCATTGGTTATAT-------------  33198
Dog          AGGATAGAA-ATCGAGGAGAAA-----------GGGGGAAACGAGGGGAGGGAA---------TGG-----GGAG----G  32707
BelugaWhale  TACTACAAAAAAAGATTACAGACCAATGTAACTGATGAACATAGATGCAGAAATCCTCAACAAAATACTAGCAAATAGAA  28870
Donkey       CAATTAGAA-AATAAATAGCTA-------GA-TGTTAAACGTAAACCCATTGTA--------------------------  35096
Cat          TAAACAGAT-GTTAAACATAAA---------------TGTGTAAATGTAAATTA--------------------------  36258
Ferret       TAAATAAAATCTTAAAAAAAAATGGAA--TACTATCAAATATTAGTGCAGTGCAAAAAATAGATGT-----TAAAC-TAA  33343
Lion         TAAATAGAT-GTTAAACATAAA---------------TGTGTAAATGTAAATTA--------------------------  34358
Vaquita      TACTACAAAAAAAGATTACAGACCAATGTAACTGATGAACATAGATGCAAAAATCCTCAACAAAATACTAGCAAATAGAA  29517
Pig          C-CCAAGAAGAATGAAGGCGTA-------GACTCAT--ATATGAATGTTCATAGCTTTATTTGTA---------------  27788
```

```
Majority     ------XA-ACAAAAAAAAGATXXAAAAGCAXAXTGA--------------------------------CAAAAXAAAX
                      41130     41140     41150     41160     41170     41180     41190     41200
Human        ---CACTAAATGGAAATGAGCTATCAAGCCATGGAGAACCTTCAGT----------------GAACATTACTAAATAAAA  35607
PolarBear    CTTCTAAACACTAAACTAATGTGTAAAAGTAAATTGG---------------------------------CAAAAAAAAC  7249
Cow          -----------------AAGGCTGTAGAGCATG-------------------------------------CAAAATGT--  33222
Dog          ----GGAAGAGGAGGAGAAGATAGGGGAAAAGACGGA---------------------------------CAAGAGAAGA  32750
BelugaWhale  TCCAACAACACATTAAAAGGATCATACACCATGATCAAGTGGGATTTATGCCAGGGATGCAGGGATTCTTCAATATATGC  28950
Donkey       -----------------AGTCTGTAAATGTAAATTGG---------------------------------CAAAACTA--  35124
Cat          ---------GCAAAAAAAACAACAAAAAACAAACAAA---------------------------------CAAAAAAAAC  36296
Ferret       CTTCTCAACACAAAACTAATGTGTAAAAGTAAACTGG---------------------------------CAAAAAAAAA  33390
Lion         ---------GCAAAAAAAA----AAAAAACCAA--------------------------------------AAAAAAACA  34387
Vaquita      TCCAACAACACATTAAAAGGATCATACAGCATGATCAAGTGGGATTTATCCCAGGGATGCAAGGAGCCTTCAGTATATGC  29597
Pig          -----------------ATATCTGAAGGCATAA-------------------------------------CAACAGTT--  27813

Majority     XAAA-A-X-----AAAXACTTTAXTXTA-ATGXAGTAAXCAATA-------GCTGTATGAAGCAAT-GAACACTGGAA--
                      41210     41220     41230     41240     41250     41260     41270     41280
Human        GAAGCCACTCTGAAAATTCTGCATACCATATGATTCCAACTATATGACAT-TCTGGAAAAAGCAA---AATTATGGAG--  35681
PolarBear    GC----------CCAAAACCTTAATCT----GTAGTAATCAGT--------GCTATGTGAAGCTAT-GAGCACTTGAA--  7304
Cow          -GACTACTC----------------TTA-ATTGATAAGTGAATGGG-------TGTGTGCTTCAA--AGACGTAGTA---  33272
Dog          GGGAAGGATA--TAGACACCTGAAAACA-A-AAACGGACCAGA--------AGAAAGAATAGTAAT-AATCTATGCAG--  32815
BelugaWhale  AAATCAGTCAGTGTGATACACCATATTA-GCAAATTGAAGAATGAAAACCATATGATCATCTCAAT-AGATGCAGAAAAA  29028
Donkey       -----A-------------TTTAGTTTA-ATGTGGTAACAGCTA-------GCCATATGAGGCCATTGAGCACTTGGA--  35176
Cat          AA-A---------AAACCCTTTAATCTG-CTGTAGTAACCACTA-------GCTGTATGAGGCCGT-GAAC--TGT----  36351
Ferret       AAAAAAAAAA--CCAAAACCTTATTCTA-GTGTAGTAATCACTAGC---TAGCTGTATGAAGCCAT-GAACACTTGAA--  33461
Lion         GA-A---------AAACCCTTTAATCTG-CTGTAGTAACCCCTA-------GCTATATGAGGCCAT-GAAC--TGT----  34442
Vaquita      AAATCAATCCATGTGATAAACCATATTA-ACAAACTGAAGAATAAAACCCATATGATCATCTCAAT-AGATGCAGAAAAA  29675
Pig          -AAGTGTTTA---------------TCA-GGGAATGAATGGATGGA-------TGGCTGTTTT-----GTCATTGAA---  27861
```

```
Majority     -ATGT--AXTATTCCXAATXG----------------------------------------------------------

                      41290     41300     41310     41320     41330     41340     41350     41360

Human        -ACAGTAAAAATCAGTGGTTGCTAGGGTTTAGGGGTGGAGGGAGGGATGAACTGGTGGAGTACAGAGGATTTTAGGGTAC  35760
PolarBear    -ATGT--GCTATTCCTAATTG----------------------------------------------------------  7322
Cow          -CTGA--AAAAATAAGGATGT----------------------------------------------------------  33290
Dog          -AC----GACAAGCACAA-------------------------------------------------------------  32828
BelugaWhale  GCTTT-GACAAGATTCAACACCATTTATGGTAAAAACTCTCCAGAAAGTGGGCATAGAAGGAATCTA-----ATATAATA  29102
Donkey       -ATGT-GACTACCCCTAATTG----------------------------------------------------------  35195
Cat          -GC------TGTTCCTAATGG----------------------------------------------------------  36365
Ferret       -ATGT--GATATTCCCAGTTG----------------------------------------------------------  33479
Lion         -GC------TGTTCCTAATGG----------------------------------------------------------  34456
Vaquita      GCTTTTGACAAAATTCAACAC----------AAAAACTCTTCAGAAAGTGGGCATAGAGGGAACCTACCTCAACATAATG  29745
Pig          -TTCC--ATTGCATACAGTGA----------------------------------------------------------  27879

Majority     --------------------------AGATGTATXTCTG----------ATGCATGTGTGTTTCAAAGA-TATAGTAX--

                      41370     41380     41390     41400     41410     41420     41430     41440

Human        TGAAAATATTCTGCATGATACTATAATGATAGATACATG----TCATACATTTTTCCA-AACCCATAGAATGTAAGACAC  35835
PolarBear    --------------------------AGATGTATATGTG----------GTGCATGTGTGTTTCAAAGA-CGTAGTA---  7362
Cow          --------------------------AA---------A-------------------ACATCTCAAAAAATTTTAAAA--  33314
Dog          --------------------------AAAAGTTAATCTG----------ATTATTGTA-GCTTCAGA-----CAGAAT--  32864
BelugaWhale  AAGGCCATATAAAACAAACCCACAGCAAACATCATTCTCAATGGTGAAAAACTGAAAGCGTTTCCTCTAAGATCAGGA--  29180
Donkey       --------------------------AGATGTATTTGGG-----------AGGACGTGTGTTTCAAAGA-TGTAGTA---  35234
Cat          --------------------------AGATGTATGTGTGGAGAT---GTATGCCTTTGTGTTTCAAAAA-CATAGTA---  36412
Ferret       --------------------------AGATGTATGTCTG----------GTGCATGTGTGTTTCAAAGA-TGTAGTAA--  33520
Lion         --------------------------AGATGT-----------------ATGCCTTTGTGTTTCAAAAA-CATAGTA---  34489
Vaquita      AAGGCCATATATGGCAGACCCACAGCAAACATCATTCTCAATGGTGAAAAACTGAAAGCATTTCCTCTAAGATCAGGA--  29823
Pig          --------------------------AG---------T-------------------ACTGCTCAGCCA-TATGGAGT--  27902
```

```
Majority     TCAAAAXTAG----------------AATGTTAAATAATTTATTTTTGAAAATATTGAXT------XTATGTTAATATGG
                      41450     41460     41470     41480     41490     41500     41510     41520
Human        TTGGAGTGAACCTGGAGGTAACTACAGACTTTGAGTGATTATAATGTGTCAGTGTAGGTTCATCAGTTGTAACAATGTGC  35915
PolarBear    TCAAAAATAG----------------AATGTAAAATACTTGATTTTTAAGAATAATG----------TGTGTTGATACGG  7416
Cow          CTA----------------------CTGA--TTAT--ATGTTGATGTGGTGGCTCAGATT------GTAAAGAATCTGCC  33362
Dog          TCAAGACAAG----------------GATTGTTA----CTAATATTAAAACATTTCAT---------AGTGTTAAAGGGT  32915
BelugaWhale  ACAAGACAAGGATGTCCACTCTCGCCACTATTATTCAACATAGTTTTGGAAGTCCTAGCCACAACAGTCAAAGAAGAAAA  29260
Donkey       TCAAA---------------------AAAGAAGAA----TCTCATTAAAAAATCTTA------------CGTTGATACAG  35277
Cat          TCAAAAGTAAT---------------AATGTAAAATATTTCATTTTTTAAAATATTGACT------ATATGTTGATATGG  36471
Ferret       TCAGAAGTAGTA------------AGAATGCAAAATACTTAATTTTTAAAAATATTGATT------GTATGTTGATATGG  33582
Lion         TCAAAAGTAAT---------------AATGTAAAATATTTCATTTTTTAAAATATTGACT------ATATGTTGATATGG  34548
Vaquita      ACAAGACAAGGATGTCCTCTCTTGGCACTATTATTCAACATAGTTTTGGAAGTCCTAGCCATGGCAATCAGAGAAGAAAA  29903
Pig          GAA----------------------CTGT--TGCT--ACATGTATCAGCAGGGATAAATC------TCCAAATAATTATG  27950

Majority     TAATATTTCAGATATATTGATTXGAA---------------A---TCTATTX---G-A--AATTAATTTCATTCCT----
                      41530     41540     41550     41560     41570     41580     41590     41600
Human        CATCTGGTGGGAGATGTTGATAAAGG---------------GGAGGCTATTTGGGGCAGGAGGCAGGGAAATCTCTCTGC  35980
PolarBear    TAATATTTCAGATATATTGAACTGAA---------------A---TATATTA-----A--AATTAATTTCACTCCT----  7467
Cow          TGCAATGCAAGAGACCCAGGTTCGA------------------TCCCTGG-----G------TCGGCAAGATCC------  33407
Dog          TAATCTATTGGAAAACATTTATATAA---------------C---AGTAATA--------ACAGAGCCTCAGAGTT----  32965
BelugaWhale  AGAAATACAAGGAATCCAAATTAGAAAAGAAGAAGTTAAACTGTCACTGTTTGCAG-ATGACTTGATACTATACATGGAT  29339
Donkey       TAATATTTCAGATATATTGAGTTCAA--------------------ATATTGA-------AATTAATTTCACTCCT----  35326
Cat          TAATATTTCAGATATATTGATCTAAA---------------A---TCTATTCTT-A-A--AATTAATTTCACTCCT----  36525
Ferret       TAGTATTTCAGATATATTGAACTGAA---------------A---TATATTATT-A-A--AATTAATTTTATTCCT----  33636
Lion         TAATATTTCAGGTATATTGATCTAAA---------------A---TCTATTCTT-A-A--AATTAATTTCACTCCT----  34602
Vaquita      AGAAATAAAAGGAACCCAAATTAGAAAAGAAGAAGTTAAACTGTCACTGTTTGCAG-ATGACTTGATACTATACATGGAT  29982
Pig          TGAAAGATGGGGAAAAAAGAATACA------------------TCTGTAT-----G-ATTCATTTATATAAAAC------  28000
```

Majority	--TXTTAXATATTG----ATATGGTTAATAGAAXATTTAAAXTTATXTGAC----TTGCAGAAT----------------	
	41610 41620 41630 41640 41650 41660 41670 41680	
Human	TTTCCTCTCCATTTTGCTGTGTACCTAAACTTACTCTGAAAAATGTCTTTGAAAATTTACTCAT-----------G----	36045
PolarBear	--TTTTACTTTTTG----GTATGGTTAATAGAATATTTAAGATTATATGAC----TTGCTGAAC----------------	7521
Cow	--C-CTGGAGAAGG----AAA----TGGCAAATCAATCCAGTGTTCTTGCC-----TGGAGAAT----------------	33455
Dog	--CATTAAATAT------GTAAAACTAAAGAGAGAAACAAATCTATAATCA----TTATTGGAT----------------	33017
BelugaWhale	AATCCTAAAGATGCCACCAGAAAACTAGTAGAGCTAATCAATGAATTTGGTAAAGTTGCAGGATACAAAATTAAAGCACA	29419
Donkey	--TTTTACTTTTTG----ATATGGTTACTAGAATATTTAAAGTT--GTGTC----TTTCAGTCT----------------	35378
Cat	--CATT---TTTTG----ACATGGTTAATGGAATATTTAAAATTACATGAC----TTGCAGAAT----------------	36576
Ferret	--TTTTACTCTTTG----ATGTGGTTAATAGAATATTTAAGATTATGTGAT----TTGCAGAAC----------------	33690
Lion	--CATT---TTTTG----ACGTGGTTAATAGAATATTTAAAATTACATGAC----TTGCAGAAT----------------	34653
Vaquita	AATCCTAAAGATGCCACCAGAAAACTAGTAGAGCTAATCAATGAATTTGGTAAAGTTGCAGGATACAAAATTAAAGCACA	30062
Pig	--T-CTAG-GATAT----GCA----TGTTAAAGCAGACTGGTGG--TTGCT-----TGG---------------------	28040

Majority	------------------ATTTCXT-------TTAGAC--XTTAGTGGTCTAAGAAG-----------------TCCAAT	
	41690 41700 41710 41720 41730 41740 41750 41760	
Human	------CGCTTGCATTGCATTTCTT-------TTGGAT--ATTACTGGTCTAAGTAG-----------------TCCAGT	36093
PolarBear	------------------ATTTCTT-------TTGGAC--ATTACTGATCTAAGAAT-----------------TCCAAT	7557
Cow	------T---------CCATG----------GACAGAG--GAGAGAAGCCTGGTGGG---CTATAGT---CCATGGGGT	33501
Dog	------------------ATTT-----------TAACA--GTAATTGATTTAACAG-------------------TAAT	33046
BelugaWhale	GAAATCTCTTGCATTCCTATGCACTAACAAAGAAAGATCAGAAAGAGAAATGAAGGAAACGATCCCGTTCACCATTGTAA	29499
Donkey	-------------------TTT----------TTGGAC--ACTACTGGTGTAGGAAA-----------------TCCAAT	35410
Cat	------------------ATTTCCT-------GTGGAC--TTTAGTGGTCTAAGAAG-----------------TCCAAT	36612
Ferret	------------------ATTTCTT-------TCAGAC--ATTACTGATCTAAGAAG-----------------TCCAGT	33726
Lion	------------------ATTTCCT-------TTGGAC--ATTAGTGGTCTAAGAAG-----------------TCCAAT	34689
Vaquita	GAAATCTCTTGCATTCCTATGCACTAACAAAGAAAGATCAGAAAGAGAAATGAAGGAAACAATCCCGTTCACCATTGTAA	30142
Pig	----------------C-AT------------ATAGAG--GTGGGAAGAATGG----------------------GGGT	28066

```
Majority     TAAAAGGCAX-----------------------------GATTGTCAGACTGGA---TTAAAAAGCAAGXGCTXACTXXA

                      41770     41780     41790     41800     41810     41820     41830     41840

Human        TAAAAGACAA-------------------A---------GATTGTCAGACTTGA---TTAAAAAGCAAGGCCTAACTACA  36142
PolarBear    TAAAAGTCAG------A----------------------GATTGTCAGACTGGG---TTAAAAAGCAAGACCTAACTGCA  7606
Cow          TGCAAAGAGT--------CA----------------------AACATGACT-------GAGTGACTAACACTTCACTTTC  33544
Dog          TGATAG---------------------------------GACAGTTAGATCT-----TTAAATACATAGAGGGAGTTTT-  33087
BelugaWhale  TAAAAAGAATAAAATACCTAGGAATAAACCTATCTAAGGAGGTAAAAGACGTGTACTCAGAAAACTATAAGACGCTGATG  29579
Donkey       TAAAAGGCAA---------A-------------------GATTGTGAAACTGGA---TTAAAAAGCAAGGCTTAACTTCA  35459
Cat          TAAAAGGCAA------A----------------------GATTGTCAGACTGGA---TTAAAAAGCAAGGTCTGACTGCA  36661
Ferret       TAAAGG-CAG------A----------------------GATTGTCAGACTGGA---TTGTCAGACTGGTGCTAACTGTA  33774
Lion         TAAAAGGTAA------A----------------------GATTGTCAGACTGGA---TTAAAAAGCAAGGTCTGACTGCA  34738
Vaquita      TGAAAAGAATAAAATACCTAGGAATAAACCTATCTAAGGAGGTAAAAGACGTGTACTCAGAAAACTATAAGACGCTGATG  30222
Pig          GAGGAAGGGT--------CA----------------------TAAAAGACA-------AGGAAATTTGGGGGTGATATGT  28109

Majority     XTATCTATXA------CTAGAGTCAGATTGAGGTATA---------------------X---AGATAT------------

                      41850     41860     41870     41880     41890     41900     41910     41920

Human        CTGTCAAT-A------CGAGGGTCAGGTAGAAATATC----A--------------------AGATGC------------  36179
PolarBear    GTACCTATTA------CTAGAGTCAGACTGAGGTAAA---------------------A---GGACAC------------  7644
Cow          ACAGGAATA-------TTTCAGATATATTGAGTTAAA-----------------------ATATATATC-----------  33583
Dog          -TTTTTTTTT------TTTGAACAATGGTATACTTCT---------------------T---CAGCTG------------  33124
BelugaWhale  AAAGAAATCAAAGATGACACAAACAGATGGAGATATATACCATGTTCTTGGATTGGAAAAATCAATATTGTGAAAATGAC  29659
Donkey       CTGTCTGCTA------CTAGAGTCAGATTGAAGTAAA-------------A-----------AGACAC------------  35497
Cat          GTATCTT---------CTAGAGTCAGGTTGAAGTACA---------------------C---AGAT--------------  36694
Ferret       GTACCTATTA------CTAGAGTCAGACTGAGGTAGA---------------------A---AGACAG------------  33812
Lion         GTATTTT---------CTAGAGTCAGGTTGAAGTACA---------------------C---AGAT--------------  34771
Vaquita      AAAGAAATCAAAGATGACACAAACAGATGGAGATATATACCATGTTCTTGGATTGGAAAAATCAATATTGTGAAAATGAC  30302
Pig          AAAAAGAT--------TCTCTGACG-GTGAAGGTTTA-----------------------ATAGATGT------------  28145
```

Majority	----------------------AGATTGAAAGTAAAAAGGATGGAAAATAATATT---CTGTGCAAACAACAAGCACAAG	
	41930 41940 41950 41960 41970 41980 41990 42000	
Human	----------------------AGGTTGAAAGTAAAAAG-ATGGAAAATGATATT---CCGTGCAAACAGTAAGCACAAG	36233
PolarBear	----------------------AGACTGAAAGTAAAAAGGATGGAAAATAATATT---CTGTGTGGACAGCAAGCACAAG	7699
Cow	----------------------AAAGTTAATTTCACTTTCTTTAACTTCTCCATACGGTT---------------ACTAG	33626
Dog	----------------------GGCTTTAAACACTCTTTCAGGGAGGAAACTATA---CCTTGCCTGCAG-AATTACATA	33178
BelugaWhale	TATACTACCCAAAGCAATCTACAGATTCAGTACAACCCCTATCAAATTACCAATGGCATTTTTTATAGAACGAGAACAAA	29739
Donkey	----------------------AGATTGAAAGTAAAAGGGATGGAAAATAATATT---CCATGCAAATAGTAGACACAAG	35552
Cat	----------------------AGGCTGAAAGTAAAGAGGATTGAAAATAATACT---CTGTGCAAACAACAAGCACAAG	36749
Ferret	----------------------AGAATGAAAGTAAAAAGGGTGAAAAGTAATAT----CTGTGCAAACAACAAGCACAAG	33866
Lion	----------------------AGGCTGAAAGTAAAGAGGATTGAAAATAATATT---CTGTGCAAACAACAAGCACAAG	34826
Vaquita	TATACTACCCAAAGCAATCTACAGATTCAGTACAATCCCTATCAAATTACCAATGGCATTTTTTATAGAACTAGAACAAA	30382
Pig	----------------------AAACATACGTTAAAACCTGTCAAATTGTACACT---TT---------------ATATG	28185

Majority	AAAGTTGAX-TGATTATXTTAGCXTCAXACAGX---TAGAATTCAAGAC----X----GGAGTATTACCAGATAX-----	
	42010 42020 42030 42040 42050 42060 42070 42080	
Human	AAAGTTGATATGATTATATTA---CTAGACAGA----AAAATTTAAGAC----------AAATATTACCAGATAT-----	36291
PolarBear	AAAGTTGATCTGATTACATTAACTTCAGACAGAG--TAGAATTCAAGAC---------GGAGTATTACCAGATAT-----	7763
Cow	AAACTTAA--AA----GTATG---------------TGACTTATATATA---TTTTTTGGATATTAGTGG----------	33672
Dog	AACTTTGTCAAAGTGTACATAGCCGTTCACGGGG--TTTTATTATAGGAC---T----GACGTTATATCAGGACCA----	33245
BelugaWhale	AAATCTTA--AAATTTCTATGGCGACACAGAAGACCCTGAATAGCCAAAGGAGTCTTGAGGGAAAAAAAGGGAGCTGGAG	29817
Donkey	AAGGTTAATGTGATTGTATTAACATCAGATAG----TAGAATTCAAGACA-------AGAAATATTACCAGATA------	35615
Cat	AAAGTTCATCTGATTATATTAGCCT----CAGAG--TAAAATTCAAGAC----A----GGAGTATTACCAGATAT-----	36810
Ferret	AAAGTTGA---GATTATTTTAACTTCAGTCAG----TAGAATTCAAGACC---A----GGAGTATTACCAGATGC-----	33927
Lion	AAAGTTCATCTGATTATATTAGCCT----CAGAG--TAAAATTCAAGAC----A----GGAGTATTACCAGATAT-----	34887
Vaquita	AAATCTTA--AAATTTCTATGGCGACACAGAAGACCCTGAATAGCCAAAGGAGTCTTGAGGGAAAAAAAGGGAGCTGGAG	30460
Pig	CAGCTTT---------TTATG---------------TGAATTGTGC---------CTCATAAAAGAAAAG----------	28223

```
Majority     -----------------T-----------AAAAAAAGCATATTTTAT---------------------GTGXTXAAAGGG
                      42090     42100     42110     42120     42130     42140     42150     42160
Human        ------------------------------AAAAAAGCATATTTCAC---------------------AATGTTGAAGGG  36320
PolarBear    -----------------A-----------AAAAAAAGCATATTTCAT---------------------GTGTTTAAAGTG  7794
Cow          ---------T------CT------------AAGAAGTTCAATTAA----------A--------------AGACAAAGTC  33701
Dog          ---------T------TT-----------AAAATAAACTCCCTGGAC---------------------TTTTTCAAAATG  33278
BelugaWhale  GAATCAGACTCGCTGACTTCAGACTAGACTACAAAGCTACAGTAATCAAGACAATATGGTACTGGCACAAAAACAGAAGT  29897
Donkey       -----------------------------GGAAAAAGCATATTGCATA--------------------GTGCTGAAAGGG  35646
Cat          -----------------------------AAAAATACCATATTTTGTA--------------------GTGCTTAAAGGG  36841
Ferret       -----------------T-----------AAAAAA-GCATCTTTTGT---------------------GTGTTTAAAGGG  33957
Lion         -----------------------------AAAAATATCATATTTTGTA--------------------GTGCTTAAAGGG  34918
Vaquita      GAATCAGACTTGCTGACT-------AGACTACAAAGCTACAGTAATCAAGACAATATGGTACTGGCACAAAAACAGAAGT  30533
Pig          ---------C------CT------------ACAGTGGAATACTAT----------C--------------AAACATAGCA  28252

Majority     TTAAT----------ATATTAGAAAACAC------------TTTGTACXXC-GTAATAACAGA-CT--------------
                      42170     42180     42190     42200     42210     42220     42230     42240
Human        TCAGT----------TTATCAGAAAACAT-----TTCTTAATTTGTATGCCAATAATAACAAA-----------------  36368
PolarBear    TTAAT----------ATATTAGAAAACA-------------TTTGTATGTC-AGTAATACAGAGCT--------------  7836
Cow          ATAGT----------GGATTAAAAAGTAC----------------TACCT-------AACT-------------------  33729
Dog          TGCTG----------AAGTTGACAAATGG------------CCCATATATC--TAATTATAG------------------  33316
BelugaWhale  ATAAATCAATGGAACAGGATAGAAAGCCCAG-AGATAAACCCACGCACCTC-TGGTCAACTGATCTATGACAAAGGAGGC  29975
Donkey       TCAAT----------ATTTCAGAAAACATAATATTTCTTAATTTGTATGCTAGTAATAACAGAACT--------------  35702
Cat          TTAAT----------ATATTGAAAAACAC------------TTTATAC-CA-GTAATAACAGAGCT--------------  36883
Ferret       TTAAT----------ATATTGGAAAACG-------------TTTGTATACT-GTAATAACAGAACT--------------  33999
Lion         TTAAT----------ATATTGAAAAACAC------------TTTATAC-CA-GTAATAACAGACCT--------------  34960
Vaquita      ATAAATCAATGGAACAGGATAGAAAGCCCAG-AGATAAACCCACGCACCTC-TGGTCAACTGATCTATGACAAAGGAGGC  30611
Pig          AGAGA----------AGAACAACAAGT------------------CACTG-------GAC--------------------  28277
```

```
Majority     ------------------------------------------------TCAAAGTTTATTAAAAAAT--------XTAAA
                             42250     42260     42270     42280     42290     42300     42310     42320
Human        ------------------------------------------------TCAAAGTATATTAAGAAAAACAT---CACAGA  36397
PolarBear    ------------------------------------------------TTAGAGTTCATTAAATAAC-------------  7855
Cow          ------------------------------------------------ACACTCTCTACTGCTGAA---------GTCAG  33752
Dog          -------------------------------------------------TGGAATTTAAGTAGAAAT-------------  33334
BelugaWhale  AAGGATATACAGTGGAGAAAAGATAGTCTCTTCAATAAGTGGTCCTGGGAAAACTGGACAGCTACATGTAAATGAATGAA  30055
Donkey       ------------------------------------------------TCCGAGTACATTAAAAAAAT--G----ATAGA  35728
Cat          ------------------------------------------------TCAAAGTTTATGACAAAATACAT----GTATG  36911
Ferret       ------------------------------------------------TTAGCATTCATTAAATATA-------------  34018
Lion         ------------------------------------------------TCAAAGTTTATGAAAAAATACAC----GTATG  34988
Vaquita      AAGGATATACAGTGGAGAAAAGATAGTCTCTTCAATAAGTGGTCCTGGGAAAACTGGACAGCTACATGTAAATGAATGAA  30691
Pig          ------------------------------------------------AGAAAATAAATTGCTAGAT--------GTTAA  28301

Majority     AXTAAAX------------------ACAGAAATAAA----TCTATAATCATAGTTGGATATTTTAAXATTGAT-------
                             42330     42340     42350     42360     42370     42380     42390     42400
Human        ACTAAG------------------TAGAGAAACAAA--AATCCACAATCATAGCCTGGTTTTTAAAACATA---------  36449
PolarBear    --TAAGG------------------AGAGAAACAAG----TCTGTAATCATTGTTGGATATTTTAACATTAATT------  7905
Cow          GTTAAA-----------------GTACAGAGAT--A-------CAGATTGGAAA---ATATTTCATGC-AGATAGTA---  33799
Dog          --CAAAA------------------ACAGTAAGAAA----TCTAGGAA-ATGACCAAATATTTAGAAATTGAAC------  33383
BelugaWhale  ATTAGAACACTCCCTAACACCATACACAAAAATAAATTCAAAATGGATTAGAGACCTAAATGTAAGGCCAGGCACTATAA  30135
Donkey       ACTAAAG------------------AGAGAAACAGA--AATCTGCAATCATAGTTGGATATTTTAACATTCCT-------  35781
Cat          TGTGAAGT-------------T---AAAGAGAGAAACCAGTCTATAATCATTGTTGGATATTTTAACATT----------  36965
Ferret       --TAAAGC-------------C---AAAGAGAAAAA----TCTATAATCATTGTTGGATATTTTAAAATTAGTT------  34070
Lion         TGTAAAAC-------------T---AAAGAGAGAAACCAGTCTATAATCATTGTTGGATATTTTAACATT----------  35042
Vaquita      ATTAGAACACTCCCTAACACCATACACAAAAATAAATTCAAAATGGATTAGAGACGTAAATGTAAGGCCAGGCACTATAA  30771
Pig          ACTGAA-----------------ACACAAA-AT--G-------TAAATTAGCTA---A-ACTTAACGT-AGTCT------  28343
```

```
Majority     --------------AATATAXXAG--------------------------------------------------------
                      42410     42420     42430     42440     42450     42460     42470     42480
Human        -------------ATTTATGG----------------------------------------------------------- 36457
PolarBear    --------------GATTTAACAG-------------------------------------------------------- 7915
Cow          --------------AGCACAGGAAA----------G---------------------------------T---T------ 33813
Dog          --------------CTTGCATAAT-------------------------------------------------------- 33393
BelugaWhale  AACTCGTAGAGGAAAACGTAGGAACAACACTCTTTGACATAAATCAAAGCAAGATCTTCTTTGATCCACCTCCTAGAGTA 30215
Donkey       ----------------CACAA----------------------------------------------------------- 35786
Cat          ----------------TCTTACAG-------------------------------------------------------- 36973
Ferret       --------------GATTTAACAG-------------------------------------------------------- 34080
Lion         ----------------TCTTATAG-------------------------------------------------------- 35050
Vaquita      AACTCGTAGAGGAAAACGTAGGAACAACACTCTTTGACATAAATCAAAGCAAGATCTTCTTTGATCCACCTCCTAGAGTA 30851
Pig          --------------AATACAG----------------------------------------------------------- 28350

Majority     -------TAATTGATAGGACA---------------------------AGATCTT----------------------TAA
                      42490     42500     42510     42520     42530     42540     42550     42560
Human        -------CAATTGATAGGATAGTA-------------------C-----AGTCCT-----------------AGCCCCCA 36489
PolarBear    -------TAACCGATAGGACAGTT------------------------AGATCTT----------------------TAA 7942
Cow          -------TGTGTAATTG----------------------------------TATTG----------------ACA--TCA 33834
Dog          -------GTCCCGAACGAACA---------------------------AGTTGTT----------------------TGT 33417
BelugaWhale  ATGGAAATAAAAACCAGAATAAACAAATGGGACCTAATGAAACTTAAAAGCTTTTGCAAAGCAAAGGAAACTACAAACAA 30295
Donkey       -------TAATTGATAGGACAATT------------------------AGATCTTT---------------------AAA 35814
Cat          -------TAATTGATAAGACA----------------------------GATCTT----------------------TAA 36996
Ferret       -------TAATTGATAGGACAATT------------------------AGATCTT----------------------TAA 34107
Lion         -------TAATTGATAAGACA----------------------------GATCTT----------------------TAA 35073
Vaquita      ATGGAAATAAAAACCAGAATAAACAAATGGGACCTAATGAAACTTAAAAGCTTTTGCAAAGCAAAGGAAACTACAAACAA 30931
Pig          -------TGACCACTAG----------------------------------TTTT------------------------- 28364
```

```
Majority     AATAAATA-X-XXA--ATTXGAACAGXGXT-AACAT------A------------C---TGGGXTTAACTCATATTXAGA
                     42570     42580     42590     42600     42610     42620     42630     42640
Human        AATAAAAAGATACATAATTTGAAAAGTGCT-ATCAT--G--------------------TTGGCTTAACTCACATTTATA  36546
PolarBear    AATATTTT---------TTTAAACAATGT---CCTTC-----A-----------GC---TGGGCTTAATTCATATTCAGG  7991
Cow          GATGAATT-GGAA---TTCAAGACAAGG---AGTAT------C------------------AGTTGTATTGGTA-TAAAA  33882
Dog          TGTAAATT--------------ACAAGGA-------------------------------AGATATCACG-ATATA-AGG  33450
BelugaWhale  GATGAAAA-GACAACCCTCAGAATGGGAGAAAATATTTGCAAATGAATCAGTGGACAAAGGATTAATCTCCAAAATATGT  30374
Donkey       AAAAAGTACATAGAAGATTTGAACAGTCCTGTCAAT-------------------CAGCTTGGCTTAATTGACATTTATA  35875
Cat          AATAAATA----GAGGATTTGAACAGTGCTGAACATT----CA-----------GC---TGGGCTTAACTCACATTCACA  37054
Ferret       AATAGGTACATAGAGAGTTTGAACAGTGTTGTCCTTT-----A-----------GC---TGGACTTAACTCATATTCAGG  34168
Lion         AATAAATA----GAGGATTACAA-AGCTGTCATCATCAAGACA-----------GCA--TGGTATTGGCATAAAAACAGA  35135
Vaquita      GATGAAAA-GACAACCCTCAGAATGGGAGAAAATATTTGCAAATGAATCAGTGGACAAAGGATTAATCTCCAAAATATGT  31010
Pig          -ATGAG---GCCA---TTGAGCATGTGG---AATGT------A------------------ACTACTCCTAATAGTAAGT  28410

Majority     AAACTXTACCCT------AAXTTCA---GAATAACTX-TCAAGTGXACATAGAAXC--AA-------AATXAAATXXCT-
                     42650     42660     42670     42680     42690     42700     42710     42720
Human        AAACTATACCCAG----TAACTGTA---AAATAACT--TCAAGTGTACATAGAACATTCAC------AATGATAGACCT-  36610
PolarBear    AAACTACAACCT------GACTTCA---GAATAACTTTTCAAGTGTACATAGCACC--AT-------GATAGACTGACT-  8052
Cow          AGACATATTT-----CTTAGTGTCA------A-AA-----GGGTCAATATATTGG---AA-------AACAACGTTCCT-  33934
Dog          GAATATTTTTCT------GATT------GAATAA------AAGTGAAAATA-TATC--AA-------AATTTGATGGCT-  33501
BelugaWhale  AAACAGCTTAAGCAGCTCAATATTAGAAAAATAAACAACCTAATCAAAAAATGGGCAGAAGACCTAAATAGACATTTCTC  30454
Donkey       AAACTTTACCTAA----TAACTGCA---GAATAACTTTTCAAGTGTACATAGAATATTTAC------CATGAGACCA---  35939
Cat          AAACTATACCCT------AACTGCA---GAAAAACTTCTCAAGTGTATATAGCACC--AT-------GATCGACTA----  37112
Ferret       AAACTGTACCCT------GACTTCA---GAATAACTTTTCAAGTGTACATAGCACT--GT-------GATAGACTGTCT-  34229
Lion         CACATAGACCAATG---GAATAGAA---TAGAAACCCCAGAACTAGACCCACAAACGTAT-------GGCCAACTCA-T-  35200
Vaquita      AAACAGCTTAAGCAGCTCAATATTAGAAAAATAAACAACCTAATCAAAAAATGGGCAGAAGACCTAAATTGACATTTCTC  31090
Pig          AAATGGGTGT-----TTCAAAGACA------T-AG-----TAACAAAGAAATAGG---AA-------TGTAAGATACCT-  28462
```

```
Majority     ----------------------------------------TATTTTAGAXCATAAAATAA--------------------
                     42730     42740     42750     42760     42770     42780     42790     42800
Human        ----------------------------------------TATATTGGATTATAAAATAA-------------------- 36630
PolarBear    ------------------------------------A---TATATTAGGACATAAAATAG-------------------- 8073
Cow          --------------------------------------TAATCTGTATATTAGTAATAA--------------------- 33955
Dog          ------------------------------------G---TAATGCAAGGGA-AATACAT-------------------- 33521
BelugaWhale  CAAAGAAGACATACAGATGGCCAAGAAGCACGTGAAAACCTACTCAACATCACTAATTATTAGAGAAATGCAAATCAAAG 30534
Donkey       ----------------------------------------TATACTAGGACATAAAATAA-------------------- 35959
Cat          ----------------------------------------TATATTAGGGCATAAAATAA-------------------- 37132
Ferret       ------------------------------------A---TATTTTAGGACATAAAATAA-------------------- 34250
Lion         ------------------------------------C---TTTGACAAAGCAGGAAAGAA-------------------- 35221
Vaquita      CAAAGAAGACATACAGATGGCCAAGAAGCACATGAAAACCTACTCAACATCACTAATTATTAGAGAAATGCAAATCAAAG 31170
Pig          --------------------------------------CTTTTTTTAAAATATTGATTGT-------------------- 28484

Majority     -------CTXAATAGATTTCAAATGAXTGAAA-------------GCXAATATAGCA-----------------------
                     42810     42820     42830     42840     42850     42860     42870     42880
Human        -------CTCAATAAATTTCAAATGATTGACA-------------GCTAATGTATCT----------------------- 36667
PolarBear    -------CTCAATAGATTTCAAATGGCTGAAAA------------GCTCATATAGTA----------------------- 8111
Cow          ---CAGTTCAAGAACATTTTAAAAATGACAAA---------AAGAGAAATCTA--------------------------- 33996
Dog          -T-----TAGGGGGGAAGAAAAATGTTCGAAGA------------GCAAACGT--------------------------- 33556
BelugaWhale  CTACAGTGAGGTATCACCTCAGACTAGTTAGAAGGGGCATCATCAGAAAATCTACAAACAACAAATGCTGGAGAGGGTGT 30614
Donkey       -------CTCAATAAATCTCAAATGATTGAAC-------------TCTCATATAGCA----------------------- 35996
Cat          ------------TAGATTTCAAATGACTGAAA-------------GCTTATATAGTA----------------------- 37164
Ferret       -A-----CTCAATAGATTTCAAATGGCTGAGA-------------GCTCATGTAGTA----------------------- 34288
Lion         ------------CATCCAATGGAAAAAAGACA-------------GTCTCTTTAACA----------------------- 35253
Vaquita      CTACAGTGAGGTATCACCTCGGACTAGTTAGAAGGGGCATCATCAGAAAATCTACCAACAACA--TGCTGGAGAGGGTGT 31248
Pig          ---CGATATGGTAATATTTCAGATATATTAGA---------TTAAAATATAT---------------------------- 28524
```

```
Majority     -------------------TCTGATTXTAGT-------------------------------------------------
                       42890     42900     42910     42920     42930     42940     42950     42960
Human        ------------G-----ACTGACTATAGT-------------------------------------------------  36680
PolarBear    -------------------TCTGATTATAGT-------------------------------------------------  8123
Cow          ---------------------CAATTTT----------------------------------------------------  34003
Dog          -------------------TTTGGTCTTCCT-------------------------------------------------  33568
BelugaWhale  GGAGAAAAGGGAACCCTTTTGCACTGTTGGTGGGAATGTAGATTGATACAGCCACTATGGAGAACAGTATGGAGGTTCCT  30694
Donkey       -------------------CCTGATTATAGT-------------------------------------------------  36008
Cat          -------------------TCTGATCATAGT-------------------------------------------------  37176
Ferret       -------------------TCTGATTATAGT-------------------------------------------------  34300
Lion         -------------------AATGGTGCTGGG-------------------------------------------------  35265
Vaquita      GGAGAAAAGGGAACCCTTTTGCACTGTTGGTGGGAATGTAGATTGGTACAGCCACTATGGAGAACAGTATGGAGGTTCCT  31328
Pig          ---------------------TATTATT----------------------------------------------------  28531

Majority     ---AAAATTAAAXTAGGAA-------------------TCXAAAACTGX-------XC------AA------TCTAXGA
                       42970     42980     42990     43000     43010     43020     43030     43040
Human        ---GGAATTAAATTAGAAA-------------------TCAAAAATAGA--------CA-------------TCTAGGA  36716
PolarBear    ---GGAATTTAAGTAGAAC-------------------TCAAAAACAGAAA---------------------TCTAAGA  8159
Cow          ---AAATTTAAAACTGGAC-----------------ATTTTAATATTG--------TTCATAGTAA------T-CAAGA  34047
Dog          ---AATATCCATGTA-------------------------TAGAAGCTG------------------------CTTATA  33595
BelugaWhale  TAAAAAACTAAAAGCAGAATTAACATATGACCCAGCAGTCCCACTACTGGGCGTATATCCAGAGAAACCATAATTCAGAA  30774
Donkey       ---GAAATTTAATTAGAAA-------------------TCCAAAACAG--------T-----ATGAAA----TCTAGGA  36048
Cat          ---AGAATTTAAGTAAGAATCACCAAAT---------ATTTGAAAATTGAAC--------TACACAA------TTCTGAA  37230
Ferret       ---GAAATTTAAGTAGGAA-------------------TCAAAAACAGTAA------G-----GGA------TCTGAGA  34340
Lion         ---AGAACT-GGACAGCAACATGCAGAA---------GGTTGAAACTAGACCACTT-TCTCACACCA------TTCACAA  35325
Vaquita      TAAAAAACTAAAAGCAGAATTACCATATGACCCAGCAGTCCCACTACTGGGCGTATATCCAGAGAAACCATAATTCAGAA  31408
Pig          ---AAAATTAATTTCAGGG------------------AGTTCTCTGATG--------GCCTAGCGGG------T-TGAGG  28575
```

```
Majority     AAATACA-------AAATATTX-------AGAAATTTTACTA------------TGAAA--XCCTGAAXAXXA-T-----
                      43050     43060     43070     43080     43090     43100     43110     43120

Human        AAGTACT-------AAATATT--------AGAAATTACACAA-------------------TCGTGAACAATACA-----  36757
PolarBear    AATTACC-------AAATA-TTT------AGAAATTGAACTA------------CGTAA--TTCTGAACATGA-------  8204
Cow          AGACAAAT-----------------------CAATCTTAAAATATA-----CATAGAAG--ATTTGAACAG---T-----  34089
Dog          TCTTAGA-------AAAAA----------AAAAGTTTTCCC-------------T------CCCTCAAGAAAA-------  33633
BelugaWhale  AGACACATGTACTTCAGTGTTGACTGCAGCACTATTTTACAATAGCCAGGTCATGGAAGCAACCTAAATGCCCATCGACA  30854
Donkey       AATAACT-------AAATATTT-------AGAAATTGAGCTA------------CACAATTCCTTTTTTTTTTT------  36096
Cat          TAATACA----TGAAGATATAAA------GGGCATCTGACTGAATGAA---AAGTGAAA--ATATAATAAAATTTG----  37291
Ferret       AATTACC-------AAATAATTT------AGAAATTGAACTA------------TGTAA--TTCTGAACAATACA-----  34388
Lion         AAATAAAC---TCAAAATGGATA------AAGGACCTGAATG--TGAG---ACAGGAAA--CCATCAAAACCTTAG----  35385
Vaquita      AGACACATGTACTTCAGTGTTGACTGCAGCACTATTTTACAATAGCCAGGTCATGGAAGCAACCTAAATGCCCATCAACA  31488
Pig          CTCCTG-------------------------CGTTCTCAC-----------CGCTGTGG--CTCTGGTTGC---T-----  28609

Majority     ------XGGXTAAAGAAAGTGTXATX-----------------------X-XC------------------X---T----
                      43130     43140     43150     43160     43170     43180     43190     43200

Human        ------TGGATTATAGAAGTGTTACCA--------------------------CAA------------------------  36781
PolarBear    --------ATTAAGGAAAATACAAGGG------------------------------------------------T----  8224
Cow          --------------G---CTGTTG----------------------GCTCGGC-----------------TTAACT----  34109
Dog          -------GGAAAAGAAAACTACAATC------------------------------------------------------  33652
BelugaWhale  GACAAATGGGTAAAGAAGATGTGGTACATATATAGAATGAAATATTACTCAGCCATAAAAAGGAACAAAATTGGGTCGTT  30934
Donkey       -------GGT--AAGATTTTGTT---------------------------------------------------------  36110
Cat          -TTGGCTCATTTGAGAAAGTACATGGGGA-----------------AAATGTCTAT------GTATATACCCATAT----  37343
Ferret       ------TGACTTAAGAAAGTATAAGG------------------------------------------------------  34408
Lion         -AGGAGAAAGCAGGAAAAGACCTCTCTGA-----------------CCTCAGCCGT------AGCAATCTCTTACT----  35437
Vaquita      GACAAATGGGTAAAGAAGATGTGGTACATATATAGAATGAAATATTACTCAGCCATAAAAAGGAACAAAATTGGGTCGTT  31568
Pig          --------------G---CTGTG--------------------------CGGC-----------------GTAGGT----  28625
```

```
Majority     ------------X-TATATXTXTAAXTG--------AATXGAAGT--------A-----AAAAT----------------
                      43210     43220     43230     43240     43250     43260     43270     43280
Human        -----------GGACATATTTTTGACTG--------AATGAAAATATATCAAAATTTGTTGGATACTACCC------AAG  36836
PolarBear    -----------GGGTATCTTTATGACTG--------AATAAAAGT--------G-----AAAAT----------------  8256
Cow          --------------TATATTTATAAAAC---------AT-GCAGTAA--------CTGAAGAAT---AA-----------  34143
Dog          ---------------AATGCCATAGTTA--------TCAGGAAGG--------A-----ATGAT----------------  33680
BelugaWhale  TGTAGAGACATGGATGGATCTAGAGACT--CATACAGAGTGAAGTAAATCAGAAAGTGAAAAACAGATATCGTATAAACG  31012
Donkey       --------------TTTCCTCCTTCTCC----------CCAAAGC----T-----CCCCAGTAC----------------  36141
Cat          -----------TAGAAAATACCTACTTT--------AGAAAAAGC--------T-----TTAGAGG--------------  37377
Ferret       --------------TATCTTTCTGATTG--------AATAAAAGT--------G-----AAAAT----------------  34437
Lion         -----------CGGCACATCCCCAAAGG--------CAAGGGAAT--------T-----AAAAGCA--------------  35471
Vaquita      TGTAGAGACGTGGATGGATCTAGAGACTGTCATACAGAGTGAAGTAAATCAGAAAGTGAAAAACAGATATCGTATAAACG  31648
Pig          --------------TCAATCCCTGGCCC---------AG-GAATT----------CCCACATAC---TG-----------  28657

Majority     ----TG-ATGGCAXAAATAGT-------AT--A-A-AATAAXTGTAXXGXAAACAAGXAXAXAAAGG-------------
                      43290     43300     43310     43320     43330     43340     43350     43360
Human        GGTGTGTATGGTGAAAAGTAT--------GTCTTCAAATACCTATATTACAAAAAAAGGTTTAAAACTCC--------C-  36899
PolarBear    -------ATATCACAATTTGT-----------------TGGCTGTAATGCAAGAAAGTACCTGAGGG-------------  8299
Cow          --CTTTTTAAGCCAATATAGG-------ATATTCAACATGATTTTA-AAATAACTTACAGTAAATTT------------C  34201
Dog          -------ATGATGCAAGTAG----------------------GTAGAGGAAACCACTACAGATCGG-------------  33717
BelugaWhale  CATTTGCAGGGCAGAAATAGAGACACAGATGTAGAGAACAAACATATGGACAACAAGGGGAGAAACTGGTGGAGTGGTGC  31092
Donkey       -------ATAGTTGTATATAT--------T------T-TAGTTGTGAGTCCTTCTAGTTGTGGT----------------  36183
Cat          TGCCTGAGTGGCTCAGTCAGT--TAAGCATCAAACTCTTGATTTCGGCTCAGGTCAGGATCTCATGGTTCGGGAGTTCAA  37455
Ferret       -------ATATCAAAATTTGT-----------------TGGCTGTAATGCAAGAAAGTACATGGGGGG------------  34481
Lion         AAAGTGAATTACTGGGACCTTA-TGAAGATAAAAAGCTTCTGCACAGCAAAGGAAACAACCAACAAAACTAAAAGGCAAC  35550
Vaquita      CATTTGCAGGGCAGAAATAGAGACACAGATGTAGAGAACAAACGTATGGACAACAAGGGGAGAAAGTGGTGGAGTGGTGC  31728
Pig          --CCTGTGTGGCAAAAAAGAA-------AAAAAAAAAAAAAATACA-AAAAAACAAAAAAAAAA----------------  28711
```

```
Majority     ------------AAXATXAGATTACAC-----XX--X--X---X--A----------------X--------------
                      43370     43380     43390     43400     43410     43420     43430     43440
Human        ------------AATTTAAGACTGACC------------------AAG----------------------G--------  36918
PolarBear    ------------AAAATATGTCTACAC------------C---------------------------------------  8315
Cow          -----------AAATGATAGA--AAGCTGCTTCTCATTATCCTAGGAG---------------TTAAC-----------T  34242
Dog          ------------AGAAAGGGAATAGA------------------------------------------------------  33731
BelugaWhale  TGGTAGGAT-GAATTGGGAGATTGGGATTGACATGTATACACTAATATGTATAAAATGGATAACTAATAAGAACCTGCTG  31171
Donkey       -------------ACATGGGATGCTTCCCC--------------------------------------------------  36200
Cat          GCCTTGTGTCAGGTTCTACGTTGACAGTGTGGATGGAGCTTGCTTGAGATTCTCTCTCTCCCTGTCTGTCTGCCCCTCTG  37535
Ferret       ------------AAAATATGTCTACAC------------C---------------------------------------  34497
Lion         CAACGGAATGGGAAAAGATATTTACAA--ATGACACA--TCGGACAAAGGGCTAGTATCCAAAATCTATAAAGAGCTCAC  35626
Vaquita      TGGTAGGAT-GAATTGGGAGATTGGGATTGACATGTATACACTAATATGTATAAAATGGATAACTAATAAGAACCTGCTG  31807
Pig          -----------AAAAGAGAGA--GAACCTGAAATAAACGTTGCAACTC---------------TCATT-----------T  28752

Majority     --X---AXAAAAACTTAAATTCACXAX-XT-X-AXTX-ATGCXXATATTAXTACTAAAAAATTTA-----ATTATGCXXX
                      43450     43460     43470     43480     43490     43500     43510     43520
Human        ------AAAAAGACATAAATTACCAATACTGGAAATCAAGGTCGAGATAATCACTTTCAGAGAAAGTGGATATATGCAGA  36992
PolarBear    -------TATATACGTATATTAGAAA------------ATGCTTATATTAGAA--AAAAGGTTTA-----ATAATCCT--  8367
Cow          AGAA--ATAAAAAAC-AGTAAGAAATC-TAGGAAATTACCAAATATTTAGAAATTTCCATAATTCTGTACAGCACATGAT  34318
Dog          ----------AAACTTGTACT-------------------GCCAGTATCAATA--GTAGAGTTAC-----GTTA------  33769
BelugaWhale  AAATTCAAAAAAAATTAAGTTCACTCC-CTTTTACTTTTTG---ATGTGGTTACTAGAATACTTAA--AATTTATGTGGC  31245
Donkey       -----A--GCATGGCCCGGTCCA---------------CGACCAGGATTTGAACCGCTGAAACCC-------TGGGCTGC  36251
Cat          CTGCTTGTACATATGTATACTCTCCTT-GTCTCTCTC-AAATAAGCATTTTTT-TAAAAAGTTTC-----ATCAGTCAAG  37607
Ferret       -------TATATACTTATATTAGAAA------------AGGCTTATATTAGGC--AAAAAGTTTA-----ATAATCCTCT  34551
Lion         CAAACTCCACACCCGAAAAACAAATAA-CCCAGTGAAGAAATGGGCAGAAAACATGAATAGACAC-----TTC--TCTAA  35698
Vaquita      AAATTCAAAAAAAATTAAGTTCACTCC-CTTTTACTTTTTG---ATGTGGTTACTAGAATACTTAA--AATTTATGTGGC  31881
Pig          AAAA--AAAAAAATT-AATTTCACTCC-TTTTTACTTTCTG---GTATGGGTACTACAATATTTA---AAATTATATGAT  28822
```

```
Majority     TTATXATATXTATATTA---TGAATA-TCAGGAATXXAAGAAGXGXTATCAA--TAC--AAXATCXTACAGAATATTAAT

                      43530     43540     43550     43560     43570     43580     43590     43600

Human        GTATAATA-GTATAACA---TAAATAATATAAAGTGGAGATAGCACTTTCAATAAAAGCAGAGATATATAGAAT-ATAAT  37067
PolarBear    --ATCAGAACTATAATG---CCAATA-TCAGGAATGAAAGTAGAGGTACCAG--TAC---GGATCCTACAGAATGTTAAT  8436
Cow          TCATAGGATATTTCTGACAGTGAAAA-TGAAAACATAACAAAATTGTATTTCCTTG--GAATATAGAAGAAAATATAGAA  34395
Dog          --GTTAGTAGTAT-------CAGGTA-CCAGTAA--GCAGGAGGGTTACCAACCTAT---AGATCCTACAGAATGTTAAT  33834
BelugaWhale  TTTTAGTATGTTTCTTT---TGGACA-TTTGTAGTCTAAGAAGTCCAGTTAAAAGGCAAAGAATATCATACTGGATTAAA  31321
Donkey       TGAAGCAGAGCGTGCGAACTTAACCA-CTCGGCCACGGGGCCGGCCCCTGAAC-TACACAATTCTGAACAACACATGAAT  36329
Cat          AAAAGAAAGCTATATTA---CCAATA-TCAGGAATGAAAGTAGAGGTACCAC--TAC---AGATCCTACAGAA--CTAAC  37676
Ferret       --GTCAAAACTATAATA---CTGATA-TCAGGAATGAAAGTAGAGGTACCAG--TAC---AGATCCTACAGAATATTAAT  34620
Lion         AGAAGACATCCGGATGG---CCAA----CAGGCACATGAAAAGATGTTCAAC--GTC---GCTCCTTATCAGG--GAAAT  35764
Vaquita      TTTTAGTATGTTTCTTT---TGGACA-TTTGTAGTCTAAGAAGTCCAGTTAAAAGGCAAAGAATATCATACTGGATTAAA  31957
Pig          CTGCGGTATCTTTCTTT---TGAACA-TTCGTAGTCTAAGAAGTTCTATTAGGAGGCAGAAAATGTCAGACTGTATTAAA  28898

Majority     AGG-AAAATAAGACAATAXTACAAACTGCAG----TAATGCCAXTAAATT-CAGCAACTTAXGTGAAAAAGATAAAAXCC

                      43610     43620     43630     43640     43650     43660     43670     43680

Human        AGG-AAAATAAGTCAATATTAGTAATGTTGT----TAAGGCTAACAAATT-CAGCAACTTAGATGAAAAGTTTCTTGACA  37141
PolarBear    AGG-AAAATAAGGCAATATTACAAACGGCAG----TAATGCCAATAAATT-CAGCAACTTAGGTGAAATAGACAAAATCC  8510
Cow          ATATTCCCCACATCACCTCCAAGAAGTGCGTGGGGGGAAATAGTTTTGTACCTATATTAGATACTTATCTCTTATCTACC  34475
Dog          GGGGAAAATGAGACAATGTTACAAACAGCAAGCAGTAATGCCAATAGTTT-CACCAACTTAGGTGAAATAGACAAAATCC  33913
BelugaWhale  AAGTAAGACCTAATTACGCTGTCTACTGCTGTCGTCAGG----TTGAAGTACAGAGACACAAGTTGAAAG--TAAAAGGG  31395
Donkey       CAA-GATATAAGGGCATATTTCTGACTGAATG---AAAATATATCAAAATTTGTTAACTGCAACCCAAGAAATGGAGAGA  36405
Cat          AGG-AAAATAAGGCAGTACCACAAAGAGTAG----TAATGCCAATAAATT-CAGCAACCTAGGTGATATAGACAAGATCC  37750
Ferret       AGG-AAAATAAGGCAATATTACAAGCGGCAA----CAATGCCAATAAATT-CAGCAACTTAGGTGAAATAGACAAAATCC  34694
Lion         AC--AAATCAAAACCACACT-CAGATACCACC---TCACGCCAGTCAG----AGTGGCCAAAATGAAGAAATCAGGAGAC  35834
Vaquita      AAGTAAGACCTAATTACGGTGTCCACTGCTGTCGTCAGG----TTGAAGTACAGAGACACAAGTTGAAAG--TAAAAGGG  32031
Pig          AAGCAATACCGAACTACACTGTCCAGTGCTCTAGTCAGACTGCTTGAAGTACAGAGACACAAATTGAAAG--TAAAAAGG  28976
```

```
Majority     TTXAGAAACCTTATTTXAXG--A----X------AAGAAAAXTGATATXA-------ATACTATTACTXCCATAXXAAAT
                      43690     43700     43710     43720     43730     43740     43750     43760
Human        AATGCAAAACTGACTTAAGTATATAGCT--------TAACAGCTATATACT------ACTGTATTATACAAATTGGAATT  37207
PolarBear    TTCAGAAACCTAATT------------------TATCAAAAGTGACAT---------AAACTATTACT-CCATAATATAT  8562
Cow          TTTGTTGGCTCTATGAGCTTAAAAATATTA--AAAAAAATAATGGTTTGAA-----ATTCCTTTCGAGAA-AGA-AAAGT  34546
Dog          TTCATAAACCTAATTATAATTAACCCCCCC--CCAAGAGAACTGACAACCTTACAAAAAACTATTACT-CCATAA--AAT  33988
BelugaWhale  ATGGAGAATAATATTTCACGCAAACAGTAAGCACAAGAAAGTTGATGTGA--------TTGTATTAACATCAGATGAAGT  31467
Donkey       GAGAGAAA-AATATGT--------------------CTATACCTATATTA------GATACATTTATACCTATATTAGAA  36458
Cat          TTCACAAACCTAACTTAGCA----------------AAACACTGACAT---------AAACTGTTACT-CCATAATATAT  37804
Ferret       TTTAGAAACCTAATT------------------TATCAAAAGTGATAT---------AAA------CT-CCATAATAAAT  34740
Lion         TATAGATGCTGGAGAGGATGT-------------GGAGAAACAGGAAC---------CCTCTTGCACTGTTGGTGGGAAT  35892
Vaquita      ATGGAAAATAATATTTCACGCAAACAGTAAGCACAAGAAAGTTGATGTGA--------TTGTATTAACATCAGATGAAGT  32103
Pig          ATGGAAAATAATATTCCATGCGAAGAGTAA--ATAAGAAAGGTGGTATGA--------TCGTGTTAACAT-AGATAAAGT  29045

Majority     ACAAATTAXGTCTATGACTATTATXAAAAATAAAATGG--AAATTTCTTXCAGXCAGAAAGA-TC-AGATATXGGAAAX-
                      43770     43780     43790     43800     43810     43820     43830     43840
Human        TATAATTAAAA-AAATCTTTCCACAAAGATTACTCTAGTCATAGATGGTTTCACTATTGAATTTTATGAAATTTTAAAGG  37286
PolarBear    ACAGATTAGCTCTATGTCTATTATAAAAAATAAACTTA--AAATTTTTC-CACAAAGA----------------------  8617
Cow          GAGAGTGAAGTCACTCAGTCATGTCCGACCCTTAGCGACCCCATGGACTGCAGCCTACCAGGCTC-CTGTGTCCATGGGA  34625
Dog          ACAGATTATGTCTGTGTCCATTATAAAAATCAGATTTA--TAATTTTTTTCATCCACCAAAG------A----T------  34050
BelugaWhale  AGAATTCAAGACAAGGAGTATTACCGGGTATAAAAAGG-CATATTTCTTAGGGTC-GAAAGAGTCAATATATTGGAAAAT  31545
Donkey       AGAAGGAAGGTTTAAAACTCCTATCAAGAAAAGAAAGGAAACGTAAATTACCAATATTGGGA-----G------------  36521
Cat          ACAGATTAGCTCTATGTCTATTATAAAAATTAGATTAG--TAATCTTCT-CACAAAGATGACTCC-AGGCATAGGTGATT  37880
Ferret       ACAGATTAGCTCTGTGTCTTTTATAAAAATTGAACTTA--AAATTTCTC-CATGAAGGGACGCCT-GGGTGGCTCAGTTG  34816
Lion         GCAAATTGG---TGCAGCCGCTCTGGAAAGCAGTGTGG---AGGTTCCT-CAGAAAATTAAAAAT-AGACCTACCCTAT-  35963
Vaquita      AGAATTCAAGACAAGGAGTATTACCGGGTATAAAAAGG-CATATTTCTTAGGGTC-GAAAGAGTCAATATATTGGAAAAT  32181
Pig          AAGAGTTGAGACAAGGAGTATAACCAGGTATAAAAAAGGTTTATTTCTTAGTGCCTCAAAGAACCACTATATCGGAAAAC  29125
```

```
Majority     ---ACAXTXXTTAATTTAGATATTAG-ATXAACAATTTCAAXGTACAGTTTAAGAAA--ACAAAA---XATAGGAX---C
                               43850     43860     43870     43880     43890     43900     43910     43920
Human        AATACTAGCTGTCTTTGAAAATGGAGGAGGGAATACTACCCTGTTCACTTTATGAAACCACTATAACTGACAGCAAAACC 37366
PolarBear    ----------TTACTTCAGGCAT-AG-GT----GATTTCA-------------------CT------CATAGG------ 8649
Cow          T--TCTCCAGGCAAGAGTAATGGAGTGGGGTGCCATTTCCTTCTCCAGTGCATGAAA--GTGAAA---AGTGAAAG--TG 34696
Dog          ----------TTACTCTAGGTACTAG-GTGTG-AATTTCAA-----TG-----------C-------CACAGG------ 34088
BelugaWhale  ATAACATTCCTTTATTTGTATATTAGTAATAACAGTTTCAAAGTACATTTTAAAAAATGACAAAATTAAAAGAGAA--GC 31623
Donkey       -------TGAATAGTAGAGATGCCA---CTTACAGATCCTACAGAATGTAATAGGA-------A----AATAAGG----- 36575
Cat          TCACTCATAGGTGAGTTTACTCAGAT-ATGGGTGGTTTCACTGTTGTATTTTATGAAA-CTTCAA---CATAGGA----A 37951
Ferret       -GTTAAGCAGCTGCCTTCGGCTC-AG-GTCAT-GATCCCAGCGTCCTGGGATCGAGTC-CCA------CATCGGGC--TC 34883
Lion         -GACCCAGCAATAGCACTGCTAGGA---------ATTTATCCAAGGGATACAGGAGTA-CTGATG---CATAGGGG--CA 36027
Vaquita      ATAACATTCCTTTATTTGTATATTAGTAATAACAGTTTCAAAGTACATTTTAAAAAATGACAAAATTAAAAGAGAA--GC 32259
Pig          A--ACATTACTTAATTTGTATATTAGTAATAACAGTTTCAAGGTACATTTAAAAAAA--ATAACA---AGAGTGAA--TC 29196

Majority     AGAGAAXTCXA--ATXTTAAATTTXCAATT---TATTTTAACATTCXACXTA-TAAX---ATAGXACGAXTTXATTTTAA
                               43930     43940     43950     43960     43970     43980     43990     44000
Human        TGACAAGGGTAATATATCAAGATTTCAGACTAGTATTTC--TCATTAACCTAAATGCATATATTCTTAAGAAAATGTTGG 37444
PolarBear    -----------------------------------------------------------------TGATTTTACTC--- 8660
Cow          AAGTCGCTCAGTCGTGTCTGACCCTTAGT--GACACCATGGACTGCAGCCTACCAGCCTCCTACGTCCATGGGATTCTCC 34774
Dog          --------------------------------------------------------------TG--AGTTTGTACTCAG- 34103
BelugaWhale  AGAGAAATCTACAATTTAAAATTTACAATTGGCTATTTTAACATTCCTCATAGTAAT-CAGTAGGACGAATTGATCTTAA 31702
Donkey       ----------------C--AATCTTACAG-------------------------------ACAATGCTAATATTCTAA 36604
Cat          ATAATAGCC----ATCTTCAGTATACTGCT---TTTAGAAAAAGAGAAGCA--GAGA---ATACTCCAAATTCACTTTGT 38019
Ferret       CTTGCTCCGCAGGGAGCCTGCTTCTCCCTC---TGCCTCTGCCTTCCACTGTCTGCC---TGTGCTCGCTTTCACTCTCT 34957
Lion         CCTGTACCCCA--ATGTTTATAGCGGCACT---CTCAACAATAGCCAAATTATGGAA---AGAGCCTAAATGTCCATCA- 36098
Vaquita      AGAGAAATCTACAATTTTAAATTTACAATTGGCTATTTTAACATTCCTCACAGTAAT-CAATAGGACGAATTGATCTTAA 32338
Pig          AGGGAAATCTACAATATTAAATTGAAAATTGGATATTTTAACATTCCCCATAGTAAT-TGATAGAACAAATAGATCTTAA 29275
```

```
Majority     A--A------XXTATAGGATTTGTAAAAAXATG--A--X-AG------CTTAAATCACXXTTATAAAAXATC-CAATGAC
                      44010     44020     44030     44040     44050     44060     44070     44080
Human        TAAATCACCTCCGGTAGAATATGAGAGACAATTCTG----AGA-----CTTATCGCAGGATTGTAAGGTTTGTTTAACAT  37515
PolarBear    --------------------AAGTATG------------AAGC----TTCAAAGTAAGAAATAATAG-----CCAT---  8695
Cow          A--G-------GCAAGAGTACTGGAGTGGGGTGCCATTTAAGAAAAAACATAAATTACTGGTAGCAGTAGTGGAAGTGGC  34845
Dog          -----------------AATGCGTGTACAATGA--A----GGCT----CTCAGAGTATGAACATAAAT---AGCCAT-A-  34151
BelugaWhale  AAAAACAATACGTAGAGGATTTGAACAATGCTGTCAGCTTGG------CTTAACTCACATTTACAAGACATC-CAATAAC  31775
Donkey       A----------TTCAACAACTTAGATGCAGT---------AG------ACAAATTTACTTTCACAAACTTACAAAAT---  36656
Cat          GATG------CTGCTATGACTGATACCAAAACCTAACAAAGAGG----ATATAAGAAATCAAG-ATTTCAAACCAATATT  38088
Ferret       ---C------TCTCTCTGACAAATAAATAAATA--A----AATC----TTTAAAAAAAAAAAAAAAAAATTTCTCCATGAA  35018
Lion         ---A------CTGATG-AATGGATAAAGAAATT----GTGGTTT----ATATACACAATGGAATACTACATGGCAATGAG  36160
Vaquita      AAAAACAATACGTAGAGGATTTGAACAATGCTGTCAGCTTGG------CTTAAGTCACATTTACAAGACATC-CAATAAC  32411
Pig          A--G-------ATTTAGGATTTGAAAAGTGCTGTCAGTTTAG------CTTAACTCACATTTATGAAATGTT-AAATGAC  29339

Majority     XX-AAAAXAAXXTATTAXATAT--ATXXXATAT---TCAATGTGATATTAAAATAXCTXA----AAATTTCAX-----TG
                      44090     44100     44110     44120     44130     44140     44150     44160
Human        TTGAAAATGAACTCAGTGTAATTTGTCACAATGATAGAATGAAGAAAGAAGAATTTCTCTCAGAAAACTAGGAAGAGAAG  37595
PolarBear    -------------------------------------------------------------------CTTCGG-----TA  8703
Cow          GGTATTACTATAGATCCTATAC-CGTGATCTATAGATCCTTGTTATAGGAAAATAAGACA----GTATTACAGGCATCAG  34920
Dog          ------------------------------------------------TTCAATATAC------TT-TTTTAG-----TA  34171
BelugaWhale  TGCACAATAGCCTTTTAAATGTACATAGGATAT---TCAACGTGATGTTAAAATAACTTACAATAAATTTCAAATGATTG  31852
Donkey       ------------------------------------C--TATTATTCTATAATAGAT------ACATATTAG----CTG  36687
Cat          TTTCATTAACATAAATGCAGAT--ATTCTTAAC---AAAATGTTAGATTATCATATCTGG----CAGTTTGTA-----AA  38154
Ferret       GATTACTCAAGGCATAGGTGAT--TTCATTCAG---GTTATGAAGCTTCAAAGTATACTT----TTTCTTCAG-----TA  35084
Lion         ----AAAAAATGAAATATGGCC--TTTTGTAGC---AACATG---G---ATGGAACTGGA----GAGTGTG--------A  36213
Vaquita      TGCACAATAGCCTTTTAAATGTACATAGGATAT---TCAATATGATGTTAAAATAACTTACAATAAATTTCAAATGATTG  32488
Pig          AGCAGAACAACTTTTCAGGTGTACATAGAATAT---TCAGCATGACATGAAAATAATTCT----AAATTTCAAATGCTTG  29412
```

```
Majority     TAA-TXTTATATAXTAACTGATTG--AAXXAGGGAG-ATTTACTXXAAATTCTA-ACTTTA--AAGTGATATTG-TATAT
                       44170     44180     44190     44200     44210     44220     44230     44240
Human        GAGCTTTTACAATGTAAATAAGGGGTATCTACTAAGAACCTACAACAAATTTCATACTTAATGGAGAAATATTG-AATGT  37674
PolarBear    TAC-TTTTTTA-GAAAA--TAGAG--AAGCAGGGA--A--TATTGCAAATTCT--ACTTTA-----TGATGCTG-TGTAG  8765
Cow          TAATGCCAATAAATTCAACAAATG--AAATAGAGAG-TTTCCTTTATAAATCTA-GTTTAAAAAACCAATATTT-TAGAT  34995
Dog          GAA-GATTGAG-AGAATGTTAGAA--AATCATGGA--AAATACTCCAAATTC---ACGTTA-----TGATCCTGCTATAA  34237
BelugaWhale  AAG--CTCATATAGTACCTGATTG-----TAGTGGG-ATTTA--CAGAAGTCTA-GGA-AATTACCAAATATTTAGAAAT  31920
Donkey       CGTATTTACTATAAAAATTGAAC-----TCATGGGG-CTGGCCCAGTGGCATAG-TCGTTAAGTTTGGATGCTC-TGCTT  36759
Cat          TGA-TAATTCATGATTATTAATTG--AGGCGGGCCC-AAGGATTGTAAGGTTTA-ACATTTCAAAGTGAACTCAGTGTAA  38229
Ferret       TAC-TTTTTCA-GAAAA--TAGAG--AAGCAGGGA--A--TACTGCAAATTTT--ACTTTA-----TGATGCTGCTATAA  35147
Lion         TGC-TAAGTGA-AATAAGCCATAC--AGAGAAAGAC-AGATACCATATGGTTTC-ACTCTT--ATGTGGATCCTGAGAAA  36285
Vaquita      AAG--CTCATATAGTACCTGATTA-----TAGTGGG-ATTTA--CAGAAATCTA-GGA-AATTACCAAATATTTAGAAAT  32556
Pig          AAA-GCTCATATAGTATCTGATT----A-TAGTGAA-ATTTGACTAGAAATCAA-GAATCAGTAAGAAATCTAG-GAAAT  29483
```

```
Majority     TTXCTX-XCATAATCAAAXACAGAAGATX-AAGAAAXCXATAATGGXAATAXATATGAXTGAATGAA-------------
                       44250     44260     44270     44280     44290     44300     44310     44320
Human        TTTCTTCTTAAAAACAGAAATGAAGCA---AGGATGTCTGTTTTCTCAATTTTTCCCCTTAGCTTTCTGCTTACATTAAG  37751
PolarBear    C-------------------------------------------------------------------------------  8766
Cow          TACCTCTCTATATGTCAATAGATAGTATTGTAGGCATCAGTAATGCCAATAAATTCAACA-AGTGAA-------------  35061
Dog          TTGATA-CCAAAACCTAACAAAGA---TGTAAGAAATCAAGATTTCAAACTAATATTTTTCATTAAC-------------  34300
BelugaWhale  TTACAC--AATTCTGAAAAGCACATGAATCACAGAAGATATAAAGGGCATATTTTTGACTGAATGAAAATACATCATATT  31998
Donkey       CAGCGGCCCAGGGTTC---ACAGGTTCT----GATCCCAGGTGTAGACCTACATACCGCTCATCAA--------------  36818
Cat          TT--TA-TCACAATAATAGAATGAAGGGGAAAAAATGTCAATAGGAGAATAGGACAGATACAATAGG-------------  38293
Ferret       CTGATG-CCAAAATCTAACAAAGAAGATGTAAGGAGTCAAGGTTTCAAACTAATATATTTTATTAAC-------------  35213
Lion         CG--TA-ACAGAAACCCATGGGGGAGGGGAAGGAAAA--AAAAAAAAAAAAAAGAGGTTAGAGTGGG-------------  36347
Vaquita      TTACAC--AATTCTGAAAAGCACATGAATCACAGACGATATAAAGGGCATATTTTTGACTGAATGAAAATACATCATATT  32634
Pig          TACCA---AATAGAAACTTACACAATTCTGAACAAAGGTGTAAAGGGTATATTTCTGACAGAATGAA-------------  29547
```

```
Majority     ---------------ATAAATACACATCTAXATTTT-TTTGAAAXAXAGAAA-----XTAGXAA-XXXTXCT--------
                      44330     44340     44350     44360     44370     44380     44390     44400
Human        GCAG------GTGAAATAAAAG-ACATAAAGATTGTAAAGGAAGAAAGAAAACTGTCTTATTCACCATTCAACATGCTTT  37824
PolarBear    --------------------------------------------------------------------------------  8766
Cow          -----------CTAGACAAATTCCCTTACAAATCTAGTTTAAAAAACTGATA-----ATAGTAG-ATTACCT--------  35116
Dog          ---------------ATAAATGC---------------------------------------------------------  34308
BelugaWhale  TTAATCATACACCAAATGAAAATACATCTATATTTT-CTTGGAATATAGAAGAAAATATAGAAATATTTTCCCCACAAGA  32077
Donkey       ---------------GCCATGCTGTGGTGGCGTCCTATGTACAAAGTAG----------A--------------------  36853
Cat          ---------------ACAGATACAGCATTTCATTCATGATAAAACTCAGAAAACT-GGTAAGGG---AACTTC-------  38347
Ferret       ---------------ACAAATGCAGGCATTTTTTTTTTTTTAATTTTAAAAATTT-TTTAATGT-ATAATGTT-------  35269
Lion         ---------------AGAGAGCCAAAGCATAAGAGACTGTTAAAAACCGAGAACA-AACTGAGG---GTTGAT-------  36401
Vaquita      TTAGTCATACACCAAATGAAAATACATCTATATTTT-CTTGGAATATAGAAGAAAATATAGAAATATTTTCCCCACAAGA  32713
Pig          -----------C---ATGAAGATACATCAGAATTTG--TTGGAATGCAGCC-------TAGGAA-AT-GCCT--------  29594

Majority     ---------GXGAGATGTAGTTCTATACATATATTAX--------TX-XTATATTAXAAAATATCTATTTTAGCAAAAAA
                      44410     44420     44430     44440     44450     44460     44470     44480
Human        TTAAAGTAGAAGACCTGAAGGACTCTACAACAGATTTGGTAGAACTA-ATAAGTGAATTTAGAATAATTATAGGATACAA  37903
PolarBear    ------------AGCTG----------------------------------------------------ATGGCA-----  8777
Cow          ---------CTTATATGTTATTATAGATATAGGTTAT-----CTTTATATCTATTATCAAAATTGAATTTTTAATTAAAG  35182
Dog          ------------AGATAT-------------------------------------------------TCTTAACAA----  34323
BelugaWhale  AGTGCATGGGGGGAAAATAGTTCTATATCTATATTAGTACTTATCTACCTACATTGGAAAATATCTGTAGTAGAAAAAAA  32157
Donkey       ------------GGAAG-------A-------TTG-----------------GC--------ATGGATGTTAGCTCAGG-  36881
Cat          ---------CACAGATTGAGTGAGGGACATCCAGTAAGA---------GCCTAAAACAAATTCCATTCTTTAATGGTGAA  38409
Ferret       ---------ATTAGCTGTGGGGGTATAGGTCTGTGAATCTCCAGGTTTACACACTTCACAGCACTCACCATAGCACATAC  35340
Lion         ---------GGGGGGTGGGAGGGAGGGCAGGGTGGGTGAT--------GGGTATTGAGGAGGGCACCTTTTG--GGATGA  36462
Vaquita      AGTGCATGGGGGGAAAATAGTTCTATATCTATATTAGTACTTATCTACCTACATTGGAAAATATCTGTAGTAGAAAAAAA  32793
Pig          ---------GAGGAAAATTGTTCTATCCATATATTAGA--TACTTTACCTATATTAGAAAATATCTATGTTAGGGAAAAG  29663
```

```
Majority     G----GTTTXAAAXTTTTATXAX----GAATXXGXAX--ATAAATTXTCAGTATXXGGAATXXAAXTXX--AGATXTCAC
                         44490     44500     44510     44520     44530     44540     44550     44560
Human        GTTCTATGTACAGAAATAGTCTCCTTATACTTGGCA---GCAAATTCTTAGAAAATGAAATTTTAAAAAATCTGTTTATA  37980
PolarBear    --------------------------------------------------------------------------------  8777
Cow          T----GTT--AGTCTCTCAGTT-----GTCTCTGAGTCTTTGCTTTGTGACCCTGTGGACTATAGCCTGCCAGGCCCCAC  35251
Dog          --------------------------------------------------------------------------------  34323
BelugaWhale  GAAAGGTTTAAAATTTCTGTTAAGAAAGAAAAAGGAAACATAAATTACCAGAATCAGGAATGAAAGTGG--AGATATCAC  32235
Donkey       --------------------------------------------------------------------------------  36881
Cat          A----TATTGAATGTTTTCTCTTTATTTATTTT-TATTTATTTTTATTTTTTATTTTATTTATTTTTT--TTATATATGA  38482
Ferret       C----CTTCCCAATGTCCATAACCCCACCACCCTCAAGTGCAGACGTTCTTTTTTTTTTTTTTAATTTA-ATTTCTTTTC  35415
Lion         G----CACTGGGTGTTGTATGGAAACCAATTTGACAGTAAATTTCATATATTAAAAAATAAAAATAAAA-TTAAAAAAAA  36537
Vaquita      GAACGGTTTACAATTTCTGTTAAGAAAGAAAAAGAAAACATAAATTACCAGAATCAGGAATGAAAGTGG--AGATATCAC  32871
Pig          G----GTTTAAAACTCTTATCAA----GAAAAAGAAAGCATAAATTATCAGTATCAGGACTGAAAGTGG--AGATGGCAC  29733

Majority     AATAXATC---CTAXAXAGT--GTAATACXATAGXXAGAXXXTAT----AACATCXGTCATCCTGA-AAACCCA-X----
                         44570     44580     44590     44600     44610     44620     44630     44640
Human        AAAGTATCAGACAAAATATCAGGAAAAAAATTTGACAAAAGATATGTAAGACATCTACACCAATATGAAACACTGC----  38056
PolarBear    -------------------------------------------------AACTCGTGCC------A-AAACCTA------  8795
Cow          TGTCCATGGAATTGTACAGGCAGGAATACTGGAGTGGGTAGCTGT-------TCCCTTCTCCGTGGGATCTTC-------  35317
Dog          -------------------------------------------------AATGTTAGCA-----GA-TCATCTA------  34342
BelugaWhale  TACAGATC---CTACACAGT--GTAATAGGACAGAAAGACAGTATCACAAGCATCGGTAATGCTGATAAATTCAGCAACT  32310
Donkey       ----------------------------------------------------GCCAGTCTTCCTCACACACATA------  36903
Cat          AATTTATTGACAAAT-TGGTT-TCCATACAACACCCAGTGCTCATCCC-AAAAGGTGCCCTCCTCA-ATACCCATCA--C  38556
Ferret       AGTGTAACAGAATTCATTGTT-TATGCACCATACCCAGTGCTCTATGC-AATATGTGCCCTCCATA-ATACCCACCA--C  35490
Lion         AATAAATAAATAGA---GGAT-TTGA-ACAGTGCTGAACATTCAGCTG-GGCTTAACTCACATTCACAAAACTATAC--C  36609
Vaquita      TACAGATC---CTACACAGT--GTAATAGGACAGAAAGACAGTATCACAAGCATCGGTAATGCTGATAAATTCAGCAACT  32946
Pig          AGCAGATG---CTACACAGT--GTAATAGGATAGTGAGATAGTAT-------ATTACTAACATTGGTAAC----------  29791
```

```
Majority      ---------X------X--X-T-------XCTXATAAAXCAAAXXTG----------T-------A-AXTACAATATTAT
                       44650     44660     44670     44680     44690     44700     44710     44720
Human         ---------TA-AGAGAAGTTTTAAAAAGACTTACATGAAGACGTAGACCATGTTCATGAATTAGAAGGCTCAATATGTT  38126
PolarBear     --------------------------------------------------------------------------------  8795
Cow           CC-------------------------AGCCCAGGGATCAAACCTG-----------------GGTTTCCTGCATTGA  35353
Dog           --------------------------------------------------------------------------------  34342
BelugaWhale   TAGGTGAAATAGACAAACTCTTTTATAAATCTAGTGAACCAAAATTGATATAATAGATTATCTCTATATTATAATATTAT  32390
Donkey        -----------------------------------------------------------------------CAGAAAAAT  36912
Cat           CCACC----CTCCCCTC--CCTCTCACCCCCCATCAACCCTCAGTTTGTTCTCAATTTTTAACA--GTCTCTTATGCTTT  38628
Ferret        CAGGC----TCCCCCAA--CCTCCCACTCCCTGCCCCTTCAAAACCGTCAGATTGTTTTTCACAGTCCATAGTCTCTCAT  35564
Lion          CTAAC----TGCAGAAAAACTTGTCAAGTGTATATAGCACCATGATCGACTATATATTAGGACATAAAATAATAGATTTC  36685
Vaquita       TAGGTGAAATAGACAAACTCTTTTATAAATCTAGTGAACCAAAATTGATATAATAGATTATCTTTATATTATAATATTAT  33026
Pig           ------------------------------GCTGATAAATTCAAC--------------------A-ACGTCAGTAAAAT  29820

Majority      AGATAXA---TACCTTXAX-TCTXTTATAACAATXXATTTTTTAATAXT-XXX----TXXTTXXXXGAAGATTACTC-AX
                       44730     44740     44750     44760     44770     44780     44790     44800
Human         AACATGCCAGTTCTCCCTAAATTGGGATGGCATTGAATCTATAAAGACTTATAAA-----TTGAGAGTACATTCTAAAAT  38201
PolarBear     --------------------------------------------------------------------------------  8795
Cow           AGGCAGA---TTCTTTACTGTCTGAGCCACCAGGAAAGCCCTAAGTTA---------TTTTTCCACAAAGATTACTC-AA  35420
Dog           --------------------------------------------------------------------------------  34342
BelugaWhale   AGATATAGATTACCTTTAT------TATCAAAATTGAATTTTTAATAATAAAAAACATCTTTTCACGGAGATTACTC-AG  32463
Donkey        GAAT-----------------TC---ATGATAAAAAATGTTTC--CACT----------------GA---TTACTTCGG  36950
Cat           GGCTCTCTCCCACTCTAACCTCTTTTTTTTTTTTTTTTCCTTCCCCTCCCCCATGGGTTCCTG-TTAAGTTTCTCAGGAT  38707
Ferret        GGGTTCATCTCCCCCTCCAATTTCTCTCAACTCCCTTCTCCTCTCCATCTCCCCATGTCCTCCATGTTATTTCTTATGCT  35644
Lion          AAATGACTGAAAGCTCATATAGTATCTGATCATAGTAGAATTTGAATCACCAAATATTTGAAAATTGAACTACACAAT-T  36764
Vaquita       AGATATAGATTACCTTTATCTCTGTTATCAAAATTGAATTTTTAATAATAAAAAACATCTTTTCACGGAGATTACTC-AG  33105
Pig           AGGTAAA---TACCTTTACATCTGTTATAAAGATGGACTTTTTAGTAAAAAA-----TCTTTGCGTGAAGATTACTCCAG  29892
```

```
Majority     GCATAXGTGATTTCACTCXXA-TATGXTAATTACXT-TAGGTGGTTTTACTCAGGCATGGXTGGTTXCACTGTTGAATTT
                      44810     44820     44830     44840     44850     44860     44870     44880
Human        TTATGTGGAAATAAAAAGGAGCTAGGACAATTA-----AAACCATTTTGAAAAAGTAAATTTTGCCACAGTTCTCAAGAC  38276
PolarBear    --ACAAAGAAG---------A-TATGAGAAAT---------------------------CAAGAT---------------  8821
Cow          GCATAGGTGATTTCACTGGTC-AGTGGTTTTCTCTTGAAGGTGGTTTTACTCGGGTATAGGTGGTTTCACTGTTGAATTG  35499
Dog          --ACAA-TATAT--------G-AATGATAATT---------------------------CATGATTAT-T----------  34372
BelugaWhale  GCATAGGTGATTTCACTCGTA-GGTGGTTTTCACTTGTAGGTGGTTTTACTCAGGCATGGGTGGTTTCACTGTTGAATTT  32542
Donkey       GCATGGGTGGTTTCACTCAGG-CATAAGTAAT-------------TTCACTCATAGATGGGTGGTTTCACTGTTAAATTT  37016
Cat          CCACATAAGAGTGAAACC--A-TACGGTATCTGTCTTTCTCTGTATGGCTTATTTCACTTAGCATCACACTCTCCAGTTC  38784
Ferret       CCACAAATAAGTGAAACC--A-TATGATAATTGGCTTTCTCTGCTTGACTTATTTCACTCAGCATAATCTCTTCCAGTCC  35721
Lion         CTGAATAATACATGGATTGAA-CATATTAAGGGCATCTTTCTGACTGAATGAAAGTGAA-AATATTAAAATTTGTTGGCT  36842
Vaquita      GCATAGGTGATTTCACTCGTA-GGTGGTTTTCACTTGTAGGTGGTTTTACTCAGGCATGGGCGGTTTCACTGTTGAATTT  33184
Pig          GCATATGTGATTTCACT--T-----GGCTTTCACTCATAGGTGGTTTTACTCAGGCATGGGCGGTTTCACTACTGAATT-  29964

Majority     TATGAAA----AAAGXAAXAAATAATAXXCATCTTTTAACCTTTTTC---------AGAAAA------AGAGAAXAXXX-
                      44890     44900     44910     44920     44930     44940     44950     44960
Human        TCACTAC----AAACCTGTAGTTAATCCAAAACAGTTTACTACTAGC---------ATAGTTAGTGACACAGAATAGCGT  38343
PolarBear    -----------TTGAAAC-----------------------------------------------------TAATATTT-  8836
Cow          TATGAAA----TGTCAAAGAAATAATAGCCATCTTTTAACCTTTT-----------AGACAA------AGAGAAGAA---  35555
Dog          A---------ATTGAAGC------------------------TTACC---------CAAGGA------TTATAAGGTAT-  34403
BelugaWhale  TATGAAACTTCAAAGTAAGAAATAATAGCCATCTTTTAACCTTTTTT---------AGAAAA------GGAGAAGGG---  32604
Donkey       TATGAAACTTGAAAGTAAGAAATAATAATCTTCAGTATACATTTTT----------AGAAAA-------TAGAGAAACGG  37079
Cat          CATCCAC----GTTGCTACAAAAGGC---CATATTTCATTTTTTCTC---------ATTGCC------ACGTAATATTCC  38842
Ferret       CTTCCAT----GTTGATACAAAAGTT---GGGTATTCATCCTTTCTG---------ATGGAG------GCATAATACTCC  35779
Lion         CATTTGA----GAAAGTACATGGGGAAAATACGTCTATGTATATACC---------CATATT------AGAAAATACCTA  36903
Vaquita      TATGAAACTTCAAAGTAAGAAATAATAGCCATCTTTTAACCTTTTTT---------AGAAAA------AGAGAAGGG---  33246
Pig          TTTGAAC----AAAGTTAGAAATGATAGCCATCTTTTAACTTTTTTTTTTTTTTTTAGAAAATACAGAAGGGAAGAAAAT  30040
```

```
Majority     ---------TTXCTCACTAAXTCAXTTTATXAAGCTACCGAAAX---AAXCTAACXAAGTTAATATAAGAAAT----XTT
                      44970     44980     44990     45000     45010     45020     45030     45040
Human        ---------TCAGGGAATAACTCATGCATTAAAGTTGCCTTGAT----CACTCAGTGAAGAAAGAGAAGGCCTT---TCA  38407
PolarBear    -------------------------------------------------------TTCATTAA-----------------  8844
Cow          ----------TGCTCTTTAATTCACTTTATGAAGCTACTGAAAT---AATCTAACAAAGATAATATAAGAAATACAGATT  35622
Dog          -------------------------------------------------ATTTA-GAAATTGAACTG-------------  34420
BelugaWhale  -------GGATACTCCCTAATTCACTTTATGAAGCTACCAAAACCAAAATATAACAAAGATAATATAAGAAATCAGGATT  32677
Donkey       GGTACTCCCTGGTTCACTTTGTGAAGCTATACTATAACTGACACCAAAACCTAACAAAGATAATATAAGAAATGAAGTTT  37159
Cat          --------ATTGTGTATA-------TAAACCACAATTTCTTTAT---CCATTCA-TCAGTTGATGGACA--------TTT  38895
Ferret       --------ATAGTGTATAGTGTATATGGACCACATCTTCCTTAT---ACATTCA-TCCGTTGAAGGGCATCTTGGTTCCC  35847
Lion         --------CTTTAGAAAA-------AGCTTTAGAGGTGCCTGAG---TGGCTCAGTCAGTTAAGCATCAAAC-----TCT  36960
Vaquita      -------GGATACTCCCTAATTCACTTTATGAAGCTACCAAAACCAAAATCTAACAAAGATAATATAAGAAATCAGGATT  33319
Pig          GGGGGGGGGGCACTTGCTAATTCACTTTATAAAGCCACTGAAACCAAAATCTAACAAAGGTAATACAAGAAATCAAAATT  30120

Majority     TCACACT-AATATTTTTCATGAACATAAATGCAGACATTCTTAACAAAATXTTAGCAAATCACAT-TCAGCAGTATGTA-
                      45050     45060     45070     45080     45090     45100     45110     45120
Human        ACAGATGTGCTAGAACAGCTGAATATACATACAAAAAAGC--AAAAGAAACTTAA---ACCCTACATCA-TTCTGTATAA  38481
PolarBear    -----------------------CATAAATTCAGACATTCTTAACAAAGTGTTAG---ATCACAT-CTGGCAATATGTA-  8896
Cow          TCACACT-AATACTTTTCATTTACATAAATGTGGACATTCTTCACAAGATATTAGCAAATCACAT-TCAGCAGTATGTA-  35699
Dog          -----------------------CATAATTCTGAACAACACATAAAGAAGATAAAAGGGTAACTTTCTGACTGAATGAA-  34476
BelugaWhale  TCACACT-AACATTTTTCATTAACATAAATGCAGACATTCTTAACAAGATATTAGCAAATCACTT-TCAGCAGTATGTA-  32754
Donkey       TCAGACTTAATATTTTTCATTAATGTAAATGCAGACGTT--TAACAAAATGCTGGCAAATCACATCC------TATATA-  37230
Cat          AGGCTCT-TTCCATAATTTGGCT---ATTGTTGAGAGTGCTGCTATGAACATTGG---GGTACAAGT-GGCCCTATGCAT  38967
Ferret       TCCACAG-TTTGGCCACCGTGGCCATAAATGCAGACATTCTTAACAAAATGTTAGCAGATTACAT-CCAGCAATATGTA-  35924
Lion         TGATTTC-GGCTCAGGTCAGGATCTCATGGTTCAGGAGTTCAAGCCTTGTGTCAG---GTTCTACGTTGACAGTGTGGAT  37036
Vaquita      TCACACT-AACATTTTTCATTAACATAAATGCAGACATTCTTAACAAGATATTAGCAAATCACTT-TCAGCAGTATGTA-  33396
Pig          TCATAGT-AATATCTTTCATGAACATAAATACAGGTGTTCGTAACAAGATGTTAGCAAATCACAT-CCAGCAGTACATG-  30197
```

```
Majority     AAAGATAATTC-ATGATGACTAATTGAGGCTTACCTCXTGATTGTAAGGTT-TAACATTT-GAAAATGAAXTCA--XTAT

                      45130     45140     45150     45160     45170     45180     45190     45200

Human        AACAATGAGATTA--ATTATATAAAAACTAATATATAGAGTTTTTAGGAA---AAAGCATAGGATATACTTGCAA-CTTT  38555
PolarBear    AATGATAATTC-ATGATTATTAATTGAGGCTTACGTGAGGATTGTAAGGTT-TAACATTAAGCAAGTGAACTCA--GTAT  8972
Cow          AAAGATAATTC-ATGATGACTAATTGAGATTTACCCCTTGATTGTAGGGTT-TATCAGTG-GCAAATGAACTCT--ACAT  35774
Dog          AGTGAAAAGGGTA-ACTTTCTGACTGAA-TGAAAGTGAAAAGGGTAACTTTCTGACTGAATGAAAGTGAAAATA--TATC  34552
BelugaWhale  AAAGGTAATTC-ATGATGACTAATTGAGGCTTACCTCTTGATTGTAAAAT---AACAGTC-GAAAAGGAATTCA--GTGT  32827
Donkey       AAAGATAATTC-ATGATGACTGACTGAG-AGTACCCCAGGATTGTTAAGTTTTAATATTT-GAAAATGAATTCAAATTAT  37307
Cat          CAGTACTCCTGTA-----TCCCTTGGATAAATTCCTAGCAGTGCTATTGCTGGGTCATAGGGTAGGTCTATTTT--TAAT  39040
Ferret       AATAATAATTCGATAATTATTAATTGAGGCATACTGGAGGATTGTAAGTTT-TAACATTT-GCAAGTGAACTCA--GTAT  36000
Lion         GGAGCCTGCTTGAGATTCTCTCTCTCCCTGTCTGTCTGCCCCTCCGCTGCTTGTGCATATGTATGCTCTCCTTG--TCTC  37114
Vaquita      AAAGGTAATTC-ATGATGACTAATTGAGGCTTACCTCTTGATTGTAAGGTT-TAACAGTC-GAAAAGGAATTCA--GTGT  33471
Pig          AGTGATAATTT-GTGATGACTGATTGAGGCTTACCCCTTGATGGTGAGGTT-TAGCAATT-GAAGATGAACTCA--CTGT  30272

Majority     AATTTA--TCACAA--TAATAGAATAAAGG--------AGGAAAAAAAATCAT---GTTAATAGAAGCAGXAAAAX-GCA

                      45210     45220     45230     45240     45250     45260     45270     45280

Human        GGGATAAATGAAGATTTCTTAAAATAGAAAAATCTTAAAGGAATATATAAACTTGACTCCATCAAAATGGGAAATTTCTG  38635
PolarBear    AATTTA--TCACAA--TAATAGAATGAAGGTGCGGGGTGGGGGGGGGAATCAT---GTCACTAGAAGGAT-AAAAA-GCA  9043
Cow          AAT------CACAG--TAATAGAATAACAG--------AGGGGAAAAAATCATCCAGTTAATAGAAGCAGAAAAA--GCA  35836
Dog          AGTCTAATTTATAA--TAATAGAATGAAGG----------AGAAAAAAATCAT---TTTAATAGAAGGAA-AAAAA-GCA  34615
BelugaWhale  AATTTA--TCACAA--TA---GAATAACGG--------AGGAAAAAGAATCATCATGTTAATAGAAGCAGAAAAG--GCG  32890
Donkey       AATTTA--TCACAA--TGAGAGACTAAAGG--------AGAAAAGAACATTCT---ATCAATAGAAGCAGGAAAA--GCA  37370
Cat          TTTCTGAGGAAC----CTCCACACTGCTTTCCAGAGCGGCTGCACCAATTTGC---ATTCGCACCAACAGTGCAAG-AGG  39112
Ferret       AATTTA--TCACAA--TAATAGAATGAAGA--------AGAAAAAAATTTCAT---GTCAATAGAAAGAG-AAAAA-GCA  36063
Lion         TCTCAAATAAGCATTTTTTTAAAAAGTTTAATCTGTCAAGAAAAGAAAGCTAT---ATTACCAATATCAG-GAATG-AAA  37189
Vaquita      AATTTA--TCACAA--TA---GAATAATGG--------AGGAAAAAGAATCATCATGTTAATAGAAGCAGAAAAG--GCA  33534
Pig          AATTTA--TCACAG--TAGTGGAATAAAGG--------AGGAAGAAAAATCAT---GTTAATAGAAGCAGAAAAAA-GCA  30336
```

```
Majority     TTTGACAAAA--TACAGCATCCATTCATGATAAAAAXTTCTCAGAAAACTAGCAAGA-TAAGGGAACTTCCACAAGGTAX
                      45290     45300     45310     45320     45330     45340     45350     45360
Human        CTTCATCAAAA-GACATTGTTAAGACATTGAAAAGACAAGGCACAGACTTGGAGAATATAATATTTGTGTATCAATACAT  38714
PolarBear    TTTGATAAA---TACAGCATTCATTCATGATAAAAATCTCTCAGATAACTAGCAAGG-TAAGGGAACTTCCACAAGGTGT  9119
Cow          TTTGACAAAAAATACAGCATCTACTCATGGTAAAAAGTTCTCAGAAAGCTAACAAGA-AAAGGGACCTTCTGCAGGATAC  35915
Dog          TTTGATAAAA--TACAACATTCATACATGATAAAAGTCTTCCAGA-AGCTAGCAAGAATAAAGGAACTTTCACAACGTG-  34691
BelugaWhale  TTTGACAAAAA-TACAGCATCCATTCATGATAAAAAGTTCTCAGAAAACTAATAAGA-AAGGGGAACTTCTACAGGGTAG  32968
Donkey       TTTGACAAA---TACAGCATGCATTCATGATCAAAATCTCTCAGAAAACTAGCAAGG-GAAGGGAACTTCCACAAGG---  37443
Cat          GTTCCCGTTT--CTCCACATCCTCTCCAGCATCTA--TAGTCTCCTGATTTGTTC-ATTTTGGCCACTCTGACTGGTGTG  39187
Ferret       TTTGATAAA---TACAATTTTCATTCATGATAAAAATCTC--AGAAAACTAGCAAGA-TAAGGGAACTTCCACAAGGCG-  36136
Lion         GTAGAGGTAC--CACTACAGATCCTACAGAAACTAACTGGAAAATAAGGCAGTACTGCAAAGAGTAGTAATACCAATAAA  37267
Vaquita      TTTGACAAAAA-TACAGCATCCATTCATGATTAAAAGTTCTCAGAAAACTAATAAGA-AAGGGGAACTTCTACAGGGTAG  33612
Pig          TTTGACAAAAA-CATAGCATCCATTCATGAGAAAATGTAATCAGAAAACTTA------GCAAGGAGTTCCCGTCGTG-GC  30408

Majority     ACATGAGGCXTXTATT----AAGAGCCTAAAACAAA--TTCCATTCXT-XATXXTGA--AATATTGAATGTTTTX----T
                      45370     45380     45390     45400     45410     45420     45430     45440
Human        GCATATGAC------T--------GTATTAGTCCATTTTCATGCTGCT-GATAAAGA--CGTACCCGAGACTGGGCGGGC  38777
PolarBear    GAATAAGGGGCGTCT-----AAGAGCCTAAAACAAA--TTCCAATATTTAAT---GA--AGTATTGAATGTTTTC----T  9183
Cow          ACA-GAGGGATCTACT----AAGAGCCTACAACAAA--TTTCATTCGT-GATAATGG--AACACTGAATATTTTC----T  35981
Dog          -AATAAGGTGCACCTTATCAAAGAGACTAAAACAA---TTCCATTCTTTTATGGTAA--AATATTGAATATTTTC----T  34761
BelugaWhale  ACAAGGGGCATCTACT----AAGAGCCTACAATAAA--TTTCATTCAT-AATGGTGA--AATATTGAATGTTTT-----T  33034
Donkey       -----------------------------------------TTTCCC---AGC--T-----TTTTGCTTACCTT------  37466
Cat          AAGTGATACCTGAGTG------TGGTTTTGATTTGT-----ATTTCCCTGATAAGGAGCGACGCTGAACATCTTT----T  39252
Ferret       -AATAAAGGTCTAAT-----AAGAGCCTAAAACAAA--TTCCATTCTTTAATAGTGA--AATACTGAATGTTTTT----T  36202
Lion         TTCAGCAACCTAGGTGA---TATAGACAAGATCCTTCACAAACCTAACTTACTAAAACTGACATGAACTGTTACT----C  37340
Vaquita      ACAAGGGGCATCTACT----AAGAGCCTACAATAAA--TTTCATTCAT-AATGGTGA--AATATTGAATGTTTTCTTTTT  33683
Pig          GCA-GTGGT------------------TA--ACGAA--TCCGACTAG--GTTGCGGG--TTCAGTCCCTGTCCTT----G  30457
```

```
Majority     CTTTAA--AAATAGGAATGAAGTA-A-GGATGTCTATTTTCAXAATXTTXC-AGCTXT-TGCTTACATTAAGGXATGTAX
                       45450     45460     45470     45480     45490     45500     45510     45520
Human        AATTTATGAA--AGAAACCAGTTTAATGAATTTACAATTCTATGTGACTGGGGAAGCCTCATAATCATGGTGGAAGGCAA 38855
PolarBear    CTTTAA--AAACAGGAATGAGGTA-AAGGATGTCTGTTTTCACAATTTTCCCAGCTTTTTGCTTACATTAGGGCAAGTAG 9260
Cow          TCTTAA--AATTAGGAATGAAGT--AAGGATGTGTCTTCCCTGAATATTTCTAGCTTT-TACTTTCTTTAAGGCAAGTAA 36056
Dog          CTTGAA--AAACAGGAATGAAGTA-AGGATATTCTGTTTTCACAATTTTTCCAGCTTCTTGCTTGTATTAGGGCAAGTAG 34838
BelugaWhale  TTTTCC--ACTTTAAATTCAATTTTATTGAGGTATAATTTCCGTACA-----ATAAAT-TGCATCCATTTAA-ATTGTAC 33105
Donkey       --------------GAGGTAAGTAAA---ATATTACCTTAGGTAA------------------ATATTACTAAAGGTAA 37510
Cat          CATG-T--GCCTGTTGGCCATCCG-G---ATGTCTTCTTTAGAGAAGTGTCTATTCATGTTTTC-TGCCCATTTCTTCAC 39324
Ferret       CTTGAATTAAACAGGAATGAAGTA-A-GAATTTCTGTGTTCACAATTTTTCCAACTTTTTGCTTACATAAGTGCATGTAG 36280
Lion         CATAAT--ATATACAGATTAGCTC-T---ATGTCTATTATAAAAATTAGATTAGTAATCTTCTCACAAAGATTACTCCAG 37414
Vaquita      TTTTCC--ACTTTAAATTCAATTTTATTGAGGTATAATTTCCATACA-----ATAAAT-TGCATCCATTTAA-ATTGTAC 33754
Pig          CTC------------AGTGGGTT--AACGATCCGGCGTTGCCGTG-------AGCTGT-AGTGTAGGTT--G-CAGGTAC 30512

Majority     AGTA-----AXXA-CATAAAACTTAGAAAGGXAGAAGTAAAACTGTCXTATTCAGAGTCAACATATT--TTTTATXTTAG
                       45530     45540     45550     45560     45570     45580     45590     45600
Human        GGAGGAGCAAGTCACATCTTACGTAGATGGCAACAGGCAAAGAAAAGAGAGCTTGTGCGGGGAAACT--CTCCCCTTTTT 38933
PolarBear    AATA-----AAAAGCATAAAGTTTAAAAA---AGAAGTAAAACTATCTTATTCACAGTCAACATTTTTATTTTTTTTTTT 9332
Cow          AATAA-ATAAAAAGCATAAAGTTTAGAAAGGAAGAAGTAAAACTGTCTTATTCATAGTCAACATGAT--TTTTAAAATAG 36133
Dog          AATA-----AAA--TACAAAACTTAAAATGGAAGAAATAAAACTATCTTATTCACAA-CATGGTTTT---CATTTTTTTG 34907
BelugaWhale  AGTCAGATAAGTATTGAAAGATGTATACACTCATGAAATCA-CTGACAGTCAAAATATATATCCATC--ACACATACTAG 33182
Donkey       AG-----------CATAAATATTGGAAA----------AGACTGTCTTATTCATAGTCAACATGAT--TTTTAAAACAG 37566
Cat          ---T-----GGGT-TATTTGTTTTTCGGGTGTGGAGTTTGGTGAGCTCTTTATAGATTTTGGATACT-AGCGCTTTGTCT 39394
Ferret       AATA-----AAACCCATAAATATTAAAAAGGTAGAAGTCAAACTATCTTATTCACAGTCAGCATTTT--GTTTTTATTTT 36353
Lion         GCGT-----AGGT-GATTTCACTCATAGGTG---AGTTTACTCAGATATGGGTGGTTTC-----ACT-GTTGTATTTTAT 37479
Vaquita      AGTCAGATAAGTATTGAAAGACGTATACACCCATGAAATCA-CTGACAGTCAAAATATATATCCATC--ACACATACTAG 33831
Pig          AGTT---CGGATCCCGTGTTGCT--GTGGCTCTGGCGTAGG-CCGGCAGCTACAGCTCCGATTGGAC--CCCTAGCCTGG 30584
```

```
Majority     GAAACTCAAA-----------TGTCTTATATXACTGTXXXATXXXXTXXXT---AATTACAAAXA------------AXT
                      45610     45620     45630     45640     45650     45660     45670     45680
Human        AAAACCATGA------------GACTTACTTCACTGTCACGAGAACAGCATTGGAAAGACCTGCCCCCATGATTCAGTTA  39001
PolarBear    GAAATTGAGT-----------TTATTTGTAT-----------------------AATTACAGAGA------------AGT  9366
Cow          AAAACTCAAAGGAATCCAGGTGATTTTTTAGAACAGATACAGGATACAGTTCA-ACATACAAAGATAGTTTTTTCTGATA  36212
Dog          GAAATTGAAT-----------TATTTTATAG-----------------------AATTACAAGTA------------TAG  34941
BelugaWhale  ATTCCTCATAGCCCTTT--GTAGCCATCCCTTCCTGTTTTATCCCCTTTCC---AATCACTGATCT-GCTTTTTGTCACT  33256
Donkey       AAAACCCAAA--------------GGAATCT---------------------------ACAA---------------AGT  37590
Cat          GATATGTCAT-----------TTGCGAATATCTTTTCC-CATTCCGTTGGTTGCCTTTT-----A------------GTT  39445
Ferret       GAAATTGAGT-----------TTAATTTTATTATATTTATTACATTTGTTTATTAATTACAGAGA------------AGT  36410
Lion         GAAACTTCAA-----------AGTAGGAAATAATAGCCATCTTCAGTATACTGCTTTTAGAAAAA------------GAG  37536
Vaquita      ATTCCTCATAGCCCTTT--GTAGCCATCCCTTCCTGTTTTATCCCCTTCCC---AATCACTGATCT-GCTTTTTGTCACT  33905
Pig          GAACCTCCAT------------ATGCCGTGGGAGCGGCCCAAGAAATAGCA-A-AAAGACAAAAA------------ACA  30638

Majority     ATX----XAGTTTC-----TAXC-XACTTXXXAATACTACA-----XTACG-----------------------------
                      45690     45700     45710     45720     45730     45740     45750     45760
Human        ACTCCCACCAGGTCCCTCCCACAACACATGGGAATTCAAGAGG-AGATGTGGGTGGGGACACAGCCAAACCATATCAATG  39080
PolarBear    GTAG---ATGCTTC-----TGGC--AGTTCACGATACTACA------TAGG-----------------------------  9401
Cow          GTCTTAGCAAATTTTTGGACAATGAACTTTTTAAAAATCCATTTATGGAAACTTT-----AG-AAAGAAAAAATATCAAG  36286
Dog          AT-------GTTTC-----TGGC--AGTTCACAATATTTCA------TACG-----------------------------  34972
BelugaWhale  ATTA-ATTAGTTTGAATTCTATAGAATTTTGTATAAACAGAATCATGTACT-----------------------------  33306
Donkey       GT-------------------------TTGGTAGAACTA-------GTAAG-T---------------------------  37610
Cat          TTGT---TGGTTGT-----TTCC----TTTGCTGTGCAGAAG-----CTTT-----------------------------  39479
Ferret       GT-------GTTTC-----TGGC--AATTCACAGTACTGCA------TATG-----------------------------  36441
Lion         AAGC---AGAGTAT-----ACTCCAAATTCACTTTGTGATGC-----TGCT-----------------------------  37574
Vaquita      ATTA-ATTAGTTTGAATTCTATAGAATTTTGTATAAACAGAATCATGTACTGAATTCTATAGAATTTTGTATAAACAGAA  33984
Pig          AAC--AACAAC------AACAAAAAACTTAATAAGAA---A-----GGAAACTT---------GTATAAGGTAGACAAGG  30693
```

```
Majority     -------A----TATTTGG--TTATTATATTXAAATAACTGACTTTX---TATAAAAXTGATTTGC-------ATTTGTX
                       45770     45780     45790     45800     45810     45820     45830     45840
Human        ACAAAGGACTTGTATCCCAAAGGACTTGTAAAAAAAAAACTCCTCCAATCAATAAATATGAGCAGC-CC---AATTTTTT  39156
PolarBear    -------G----TATTTGG--TTCCTATTTTTAAATATCTGACTTTG---TATAAACTTGCTATTC-------GACTCT-  9457
Cow          AAATTTAACAAAAAGTTGCATGTGTTATACTAAGGAGACTGAACTTT---TTTAGAAAAGATTTACACTT-TAGAAAGGA  36362
Dog          -------G----TATTTGG--TTGCTATACTTAAATATCTGACTTTG---TACAAACCTGCTTTTC-------AGCTCTT  35029
BelugaWhale  ---------ATGTACTCTTCTGTGTTGTACTGCTTTCACTTCCTTTCCTCTTTACTGCTGAGTAGTGTTTGCATCTTGTT  33377
Donkey       ------------GAATT----TCAAATGGTTACAAGATACAAGTTCAATATACAAAAATAAATTGT-------TTCCTGA  37667
Cat          -------T----TATCT----TCATAAGGTCCCAGTAATTCACTTTT---GCTTTAATTCCCTTGC-------CTTTGGG  39534
Ferret       -------A----TATTTGG--TTACTATTTTTAAATATCTGACTTCG---TATTAACTTGCCATTC-------AACT---  36495
Lion         -------A----TAACTGATACCAGAGGATATAAGAAATCAAGATTT---CAAACCAATATTTTTC-------ATTAACA  37633
Vaquita      TCATGTACTATGTACTCTTCTGTGTTTTACTGCTTTCACTTCCTTTCCTCTTTACTGCTGAGTAGTGTTTGCATTTTGTT  34064
Pig          GACGTCTACAAGAAATTTCATTCATAATGGCAAA-ATACTGAATTCC---TTTAAATCAGGATGA-------AGTAAGGA  30762

Majority     TATTTGTTX-------XGTAATTTTTT----------------AXA--AAATGTAAATTAAACTAATXAXAGXX-----
                       45850     45860     45870     45880     45890     45900     45910     45920
Human        TTTAAGTGGCAAAAGAAGTGACATTATAAGGATGGGTAACAAGCACATGAAAAGATAATGTTAGTCATCAGGTGATCACA  39236
PolarBear    -ATTTGCTA-------TGTAATTTTCT----------------TT---AAATGCAACTTATGTAAGTT-----------  9498
Cow          TTTTTGTTCTGT--TTGGTGGTTTTTT--TTTTTTTTTTTTAGAAGGC-AGAGGCAAATTGGAGATATCAGGGTCCTTAA  36437
Dog          TATTTGCTA-------TGTAATTTTTT----------------A----AAATTTAAATTCAACTAATGTAAGTT-----  35076
BelugaWhale  TATTCATTCAGCCATTGATGGACACTTAAGTCATTTCCCTTTGGGGAC-TATTATGAGTAAAACTGTTAGGAGCAATCAT  33456
Donkey       TATTGGTA---------GCAAATTATT----------------------GGAAAATGAAATTTTTAAAC--------  37705
Cat          GATGTGTTG-------AGTAAGAGATT----------------GCTG-CAGCTGAGGTCAGAGAGGTCTTTTCC-----  39584
Ferret       -ATTTGCTA-------TGTAATTTTTT----------------AA---AAATTTAAATTCAACTAATTTA---------  36538
Lion         TAAATGCAG-------ATATTCTTAAC----------------AAAA-TGTTAGATTTTAGAATTATCATTTGT-----  37683
Vaquita      TATTCATTCAGCCATTGATGGACACTTAAGTCATTTCCCTTTGGGGAC-TATTATGAGTAAAACTGTTAGGAGCAATCAT  34143
Pig          TATCTGCT------TCCGCAATTTTTT--TTCTACCTTTTGCTTACAT-TAAGGCAAGCAAAATAAATAAAAAGCATAAA  30833
```

```
Majority     AGTTAACAX--TAAXGTGTAGXAT----------T--TGTTTCAX---------A-AATTTAATGATTGAXCAAT-----
                     45930     45940     45950     45960     45970     45980     45990     46000
Human        AGTCAAAACCATTAGAGATAGAATTTACACCAACTAGAATGGCTA---------A-AATTAAATAGATTGGCAAT--ACC  39304
PolarBear    AGTTAACA---TATAGTGTAGTAT----------T--GGTTTCAG-----------AATTGAGTGATTCATCACT-----  9547
Cow          TTTTAGAGTC-TGAGAAGTAGAATATGGAGAAAATGATGTATCAAGAAATTAGAAGAATTTCTGAGTTGGGAAGTAGACT  36516
Dog          AGTTAACT---TACGGCGTAGTAT----------T--AGTTTCAGG-------AGAATTTAGTGATGGATTCAT-----  35128
BelugaWhale  GTTCAAGTCT-TTACGTGAACATCC-------ACTGTTATTCCTCTTG---AGTAAATGCTGAGGAGTGGAATGCCTCTT  33525
Donkey       TCTTCACA----AAAACATAAGAC------------------AA-----------AAAACAAGGAATAAGT--------  37743
Cat          TGCTTTCTCC-TCTAAGGTTTTGA----------T--GGTTTCCTGTC-T---CACATTTAGGTCCTTTATCCAT-----  39642
Ferret       AGTTAACA---TAAAGTATAGTAT----------T--GGTTTCAG-----------AATTTAATGATTCATCACT-----  36587
Lion         AAATGATAAT-TCATGATTATTAA----------T--TGAGGCGGACC-C---AAGGATTGTAAGGTTTAAC-AT-----  37740
Vaquita      GTTCAAGTCT-TTACGTGAACATCC-------ACTGTTATTCCTCTTG---AGTAAATGCTGAGGAGTGGAATGCCTGTT  34212
Pig          GATCAGAAAG-GAAGACGTAAAAC----------TGTCTTGTCTACAG-TTAACGTGATTTTTAAAATAGAAAAC-----  30896

Majority     -----TTGXAGGAATATXTAGAACXTTATT------TCAATAAGTAACCX----TTCAATA--XCCATTACC------CA
                     46010     46020     46030     46040     46050     46060     46070     46080
Human        GAATGTTGATG-AAGATGTGGAACTGTCTTTAATTACTGCTGAGTAT--------ATAACA---TAGTTGGG------AA  39366
PolarBear    -----TATGTATAATATCCAGTGC-TCATC------CCAGCAAGTGTCCT---CTTTAATG--CCCGTCATC------CA  9604
Cow          T----TGATAGAAGTATTAAGAGTTTTAGCCTGGAATCACTGGGTAATGGATAGTACAGTAGTTCCATTAAC-----AAA  36587
Dog          -----TACTTA---CATTTAACAC--CATC------TCAGTAAGTGTCCT---CTTTAATA--CCCATCACC------CA  35181
BelugaWhale  TTGTATGGTAGGTGTATGTTTAATTCTTATGTACATTTAATGCCAAACAGTT--TTCAAAAGTGCTTATACCATTTTACA  33603
Donkey       -----TTAACAAAATATGTAAGACGTCTAT-----ACCGATAACTAG----------AAGA--------CA---------  37786
Cat          -----TTGGAGTTTATTTTTGTGAATGGTG------TGAGAAAGTGGTCTAG-TTTCAACC--TTCTGCATG------TT  39702
Ferret       -----TACCTATAACACCCAGTGC-TCATC------CCAATAAGTGTCCT---CTTTAATA--TTCATCACC------CA  36644
Lion         -----TTCAAAGTGAACTCAGTG--TAATT------TATCACAATAATAGAA-TGA-AGGG--GAAAAAATG------TC  37797
Vaquita      TTGTATGGTAGGTGTATGTTTAATTCTTATGTACATTTAATGCCAAACAGTT--TTCAAAAGTGCTTATACCATTTTACA  34290
Pig          -----CCAAAGGAATCTATGGAATATTTGT-TAGGACTAATAGGTGA---AT---TCAGGA---TTGTTAA------GGA  30955
```

```
Majority     TTTATCCCATXAGTCXA-XAACTTTTCTTTTTCTGACAAXCCTCAXTT-TTX-TXAXXTGTTT------TCTXA------
                      46090     46100     46110     46120     46130     46140     46150     46160
Human        AATAGTTTGCTTTTTTTTTTTTTTTTTTTTTTTTGAGACAGAGTCTTGCTCTGTCACCAGGCTGGAATGCAGTGGCATGA  39446
PolarBear    TTTATCCCATCCCTCCA--AACTCCTC-CCTTCCCGCAACCCTCATTT----------TGTTC------TCTTA------  9659
Cow          AGGAACAAAGTAGAGAAAGTGATTTGATTTTGTTGAAAAGAAAGAACAATCTGTTAATTGTCTTCCTTTTTTTA-AAGTA  36666
Dog          TTTGGCTCATCCCTCCA--CGCTCTTAACCCTCCCGCAACCCTCAGTTGTT--TCTTATGTTC------TCTTG------  35245
BelugaWhale  TTCCCACCAGCAGTATATGAAAGTTCCAGTTGCTCAGCATCCTCACCTTGGTATTATCTTTTTATCTCTTCTAATGGGTG  33683
Donkey       ---------------------------------TGGCAAAGAGAAGTT------------------------A-------  37802
Cat          GCTGTCCAGTTCTCCCAGCACCATTTGTTAAAGAGGCTGTCTTTTTTCCATTGGATGTTCTTT------CCTGC------  39770
Ferret       TTTATCCCATCCCTCTA--CTCTTTTC-CCTTCTAGCAACCCTCAGTT----------TGTTC------TCTTA------  36699
Lion         AATAGGAGAATAGGACAGATACAATAGG-ACAGATACAGCATTCATTC-AT--GATAAAACTC------AGAAA------  37861
Vaquita      TTCCCACCAGCAGTATATGAAAGTTCCAGTTGCTCAGCATCCTCACCTTGGTATTATCTTTTTATCTCTTCTAATGGGTG  34370
Pig          TGCATTTCAATATACAAAG--ATTTTTTTTTTCTGATATTAGCAAGTTTTTGGAAAATGAAAT------TTTAA-AAGTT  31026

Majority     -AT-------TTATAAAGACTTTA------------ATGATTX-GXATGTGGXTCAGTTTTTXXG-XTTTAT--AATXAA
                      46170     46180     46190     46200     46210     46220     46230     46240
Human        TCTCAGCTTACTGCAACCTCCGCCTCCTGGGTTCAAGCGATTC---TCCTGCCTCAGCCTCCTGAGTAGCTGGAACTACA  39523
PolarBear    -ACT--------ATACAGTCTCTT------------ATGGTTT-GCCTCCT-CTCTGTTTTT------------ATCTTA  9704
Cow          TGT-------TTAGAATAACAATA------CTT--TATGACTAAAGAATGAATCAAGTCTTTTTGTCATGATGGAATAAA  36731
Dog          -TT---------ATAGATATTTCT------------GTGATTTTGCCTTCCTCTCTGTTTTTAATCAATAAA--ATACAA  35301
BelugaWhale  TGTAAGTAGTTTCTCAAGGTTTTA------GTTTTTATGACTAATGAGGTTGAGGAACTTTGCTCTTTTTTTCTAATTGA  33757
Donkey       ---------------AAGACCTAA------------ATAAATG-------------------------------AAGAGA  37824
Cat          -TTTG-----TCAAAGATGAGTTG------------GCCATAC-GTTTGTGGGTCTAGTTCTGGG-GTTTCT--ATTCTA  39828
Ferret       -AC---------ATACACTCTCTC------------ATGGTTT-GCCTTC----CTGTTTT-------------ATCTTA  36739
Lion         -ACTG-----GTAAGGGAACTTCC------------ACAGGTT-GAGTGAGGGACATCCAATAAGAGCCTAA--AACAAA  37920
Vaquita      TGTAAGTAGTTTCTCAAGGTTTTAATTTTAGTTTTTATGACTAATGAGGTTGAGGAACTTTGCTCTTTTTTTCTAATTGA  34450
Pig          CAT-------TTATAGAAACATCA------GA----AAGATGAAATATCAGAATAAATTTAACAGAATATATTCAGGCAA  31089
```

```
Majority      TTTTATT-GTTCCTTTC----XTTXCCTTGTGTAX--AXATGTTTTATTTCTTAAATTCAACAT---XA-XAATAAAXGA
                          46250     46260     46270     46280     46290     46300     46310     46320
Human         GGTGCACGCCACCACACCCGGCTAATTTTTTGTAT---TTTTAGTAGAGACTGGGTTTCGCCATG-TTGGCCAGGATGGT  39599
PolarBear     TTTTATT-TTTCCTTTC----CCTCCCCT-TGTTC--TTCTGTTTTGTTTCTTAAATTCCATAT--------ATAAGTGA  9768
Cow           GTTTCTTAAAGCCTGTT---TTTAACATAAGAAATTTATATGCATG--CAGTTTATTTTACCTTTTCTGAACAGAGGAAA  36806
Dog           TTTTTTC-GCTTTTTCC----CTTTCCCTGTGTTC--ATCTACTTTGTTTCTTAAATTCCACAT--------ATGAATGA  35366
BelugaWhale   TTAATCTAGAGCTTTTG---TGTTTCCAGGTAAATTTAAGTAATCAATTTCTTTAAAACAACAG--TAACAACAACAAAA  33832
Donkey        ---------------T-------ACACTA-TG----------------TTCATGAAT----------------TAGAAGG  37849
Cat           TTCCATT-GGTCTATGT----GTCTGTTTTTGTGCCAGAATGTTTTCTCTTTAAAAGCAGGAATG--AAGTAATGTCTGT  39901
Ferret        TTTTACT-TTTCCTTCC----CTTCCCCT-TGTTC--ATCTGTTTCATTTCTTAAATTCCACAT--------ATGAGTGA  36803
Lion          TTCCATT-CTTTAATG-----GTGAAATATTG------AATGTTTTCTCTTTAAAAGCAGGAATG--AAGTAATGTCTGT  37986
Vaquita       TTA-----GAGCTTTTG---TGTTTCCAGGTAAATTTAAGTAATCAATTTCTTGAAAACAACAG--TAACAACAACAAAA  34520
Pig           CTACCTCAGTAACTACAAGATATAACACAGAGAAATTAAAGAGCTGAATAAATGGATACACCATGTTTACAAATAGAAAA  31169

Majority      CTTCATATGTTAAA--TGTXTTTTTCTTACATAGTGXAATXAGAATAGTATATTTXTTAGT---X-------A-AT-CCA
                          46330     46340     46350     46360     46370     46380     46390     46400
Human         CTTGCTCTCTTGACGTTGTGATCTGCCCACGTCGGCCTCCCAGAGTGCTGGGATTACAGGCCTGAGCC------ACTGCG  39673
PolarBear     AATCATGG--TATT--TGTCTTTCTCTGACTTATTTTGTTTAGCATAATACACT--CTAGT----------------CCA  9826
Cow           CGTTGAGTTTTAAAATTTCTGTGTTTTTTCTAACATAGATGAGGGTAGGGTCTGTGTATATGCAGA-----CAGATGCCA  36881
Dog           CATCATAGGATATT--TGTCTTTCTCTGACTTACTTCACTTAGCATAGTATACT--CTAGT--------------T-CCA  35427
BelugaWhale   CCTGCAGGGGTGAA--TGTTTTCTTCTTAAAATGAGGAATGAAGTAAGGATGTCTGTTTCCACAATTTTTCTAGCTTTTG  33910
Donkey        CTCAATATG-TGAAGATATCGTTTTTCCCCAAATTGGAATGGAAATTGAATCTA--TAAA-----------------TCA  37909
Cat           TTTCACATTTTCTCC-TGCTTTTTGTTTACATAGGGCAAATAAAATAGCATAAAGATTAG----GAAGAAGTAAAA-CTA  39975
Ferret        TATCACA---TATT--TGTCTTTCTCTGACTTACTTTTCTTAGCATAATACACT--CTAGT----------------CCA  36860
Lion          TTTCACATTTTCTCC-TGCTTTTTCTTTACATAGGGCAAATAAAATAGCATAAAGATTAGAAAGGAAGAAGTAAAA-CTA  38064
Vaquita       CCTGCAGGGGTGAA--TGTTTTCTTCTTAAAATGAGGAATGAAGTAAGGATGTCTGTTTCCACAATTTTTCTAGCTTTTA  34598
Pig           CTCATTATGTTAAAGATGTCATTTCCCCCCAAATTGGGATG-GAATTGAATCTGTAAAT---CAGT-----CAGAT-TCA  31239
```

```
Majority     TCTA-X-TTAGGCCAATATGATTTTTAAAA--TAAAA---------AACCCAAAG---AAATCTATAA---------AAT
                     46410     46420     46430     46440     46450     46460     46470     46480
Human        TCTGAC-CTAGTTTACTATTTTCTTACGGAGTTAAACA------TTAACCTA--------TTTTATGGTCCAT----AAC  39734
PolarBear    TCCA-TGTTGTCCCAACATGATTTTTAAAA--TAAAA---------AACCCAAAG---GAATCTACAA---------AAT  9882
Cow          ATT----TGAATC-ATTAAGACCTTTGAAAACCAGTAGCTGTACTTAGCTAGAA----AAATATACAGTTGTCATCCAA-  36951
Dog          CCCA-TGTTACGTCAACATGATTTTTAAAA--TA------------AACCGACAG---AAATCTATAA---------AAT  35480
BelugaWhale  CTTACATTAAGGCGAGTAAAATAAATAAAAAGCATAAAGATCAGAAGGGAAGAAGTAAAACTGTATTATTCACAGTCAAC  33990
Donkey       GTCA-----AGCCCAATAGG-CTCTTT--------------------------------TTTTTTTT-----------  37938
Cat          TCTTCTGCTCAATAAACATAATTTTTAAAA--TAAAA---------AACCCAAAG---GAATCTAAAA---------AAT  40032
Ferret       TTCG-TGTTGTGCCAGCATGATTTTTAAAA--TAAAA---------AACCCAAAG---GACTCTCCAA---------AAT  36916
Lion         TCTT---CTCAGTAAACATAATTTTTAAAA--TAAAA---------AACCCAAAG---GAATCTAAAA---------AAT  38118
Vaquita      CTTACATTAAGGCGAGTAAAATAAATAAAAAGCATAAAGATCAGAAGGGAAGAAGTAAAACTGTATTATTCACAGTCAAC  34678
Pig          AC-------AGGC-ATTTA---CATTGAAAGTGATTA------CTTGATCTAAA----ATCTATGTGG---------AA-  31288

Majority     -----GTTTXAXAGAACTGATCATTGAXXTT------------AGAATGGTTXTATGATT--CAAGTTCAATATACAAAA
                     46490     46500     46510     46520     46530     46540     46550     46560
Human        TTCTACTTTTAGTTTTTTACCCAGTAAGAAC---ATGTTTGCTGTAAAACTTGTATGTGTATCAAAAACTTTA-CTGATA  39810
PolarBear    -----GTTTTGTAGAACTGATCATTGAATTT------------AGAAGGGTTACATAATA--CAAGTCCAATATACAAAA  9943
Cow          ---ATGTCTCAAA-AACAGACTTTACAGACA---AATATTACTAGGACATTTTTATGATTGTCAGATGCAAAATAGTATT  37024
Dog          -----GTTTTGTAAAACTAATCATTGAAT---------------G----GTTACATGATA--CAAATTCAATATATAAAA  35534
BelugaWhale  ATGATTTTTAAAATAGAAAACCCAAAGGAATCTAAATGATTTTAGAATGGTTTACAGGATAC--AGTTCACCATACAAAG  34068
Donkey       ------TTTTAAA-----GATTTTC-----------------------TTTTTTATT--------------TATTGTT  37968
Cat          -----GGTGCATAGAACTGATCAGTGAATTT------------AGAATGGTTATATGATG--CCAGTTCAGTATACAAAA  40093
Ferret       -----ATTTCATAGAACTGATCACTGAATTT------------AGAAGGGTTACATAATA--CAA-----A---ACAAAA  36969
Lion         -----GTTGCATAGAACTGATCAGTGAATTT------------AGAATGGTTATATGATG--CCAGTTCAGTATACAAAA  38179
Vaquita      ATGATTTTTAAAATAGAAAACCCAAAGGAATCTAAATGATTTTAGAATGGTTTACAGGATAC--AGTTCACCATACAAAG  34756
Pig          ---A--T---AAA-AAAGAACCTAGGAGAGT---AAAAC--------CATTTTGAAAA-----AGGAAAAAAGTTGGGAT  31343
```

```
Majority     ATAGTTTC----CTGATATTATTAGCAAATTTTTGGAAAATGAAATTTAAAAAAA----TCCATTTATAAAAATXTCAGA

                  46570     46580     46590     46600     46610     46620     46630     46640

Human        ATGGTCCCAAACTTGAAATAACCCAAATGTTTATCAACAGGAAAATGGATAAATTGTGGTATAATCATATAGTATATTAC  39890
PolarBear    ATAGTTTG----CTGATATTATTAGCATATTATTGGAAAATGAAATTTTAAAAAAA---TGCTTTTATAAAAAT------  10010
Cow          TCATTTTTAT-GTTCCAGCTTTTTCCATGTCTGTTGTAATTGACTTT----ACTA----TATAGAATTCTTGTTTCCAAT  37095
Dog          ATTGCTTC----CTGATATTATTAGCAAATTATTGAAAAATGAAATTTAAAAAAG----TCCATTTACAAAAATATGCG-  35605
BelugaWhale  ATAATTTTTT-TCTGATATTATTAGCAAAGTTTTGGAAAATGAAATTTTAAAAAA----TCCGTTTATAGAAACATCAGA  34143
Donkey       TTTTTCCC----CCTCTTCTCCCAGTACGTAGTTGTATATTTTAGTTGTGGGTCCT---TCTATTTGTGGCAT-------  38034
Cat          ATTGTTTC----CTGATATTATTAGCAAATTACTGGAAAATGAAATGAAAAAAA-----TCCATTTACAAAAAA-TACCA  40163
Ferret       ATAGTTTC----CTGATATTATTAGCTAATTATTAGAAAATGAAAATTTAAAAA-----TGCTTTTACAAAAATATGAGA  37040
Lion         ATTGTTTC----CTGATATTATTAGCAAATTCCTGGAAAATGAAATGAAAAAAA-----TCCATTTACAAAAA--TACCA  38248
Vaquita      ATAATTTTTT-TCTGATATTATTAGCAAAGTTTTGGAAAATGAAATTTAAAAAAA----TCTGTTTATAGAAACATCAGA  34831
Pig          ACCATCTGAT-TTCAAGATTGATTGTAAAGTTGAAGTAATCAAA-------ACAA----TTTAGTATTAGCACAGTTAG-  31410

Majority     XAGACA-AAATATC-AAAGAATAAATTTAACAAATAATGTGGG-AGACATGTACAXCAGTAACTATAXAAGACG--XCAA

                  46650     46660     46670     46680     46690     46700     46710     46720

Human        TACTTACTAATAACAAAAGAATGGTTAGTAGTACATGCGAAAACATGGGTGAATAGTAAGCATTGTATGAAGCA----AA  39966
PolarBear    --GAGG-AAATATC-AAGGAATAAATTTAACAAATAATGTGAG-AGACATGGACACCAGTAACTATACAAGACATAGCAA  10085
Cow          AG------CATGTC-TCAGCAGCCTTTCCCTGTCTAGACTGT--CTTCCTCTACAGCTGTATTTCTGTCAAA-G--AAAA  37163
Dog          -AGACA-GAATATC-AGAGAGTAAATGTAACAAATAATGTGGGGAGACATGTACACCAGTAACTATACAGGACA---CAA  35679
BelugaWhale  AGGACA-AAATATC-AAAGAATAAATTTAACAAAAAATGTGCA-TGATAACTACATCAATAACTGCAAGGCATG--GCAA  34218
Donkey       ---------------GTGGGATGCCTCCTCAGCATGGCTTG-----ATGAGTGCTGCTAGGTCTGTGCT---C-------  38084
Cat          GAAACACAAATATC-AAGGAATACATTTAATAAATAATTTGGG-AGACAAGTATACCAGTAACTATATAAGATGCTACAA  40241
Ferret       AAGACA-AAATATT-AAGGAATAAATTTAACAAATAGTGTGGG-AGACATTAATACCAGTAACTATACAAGACATGGCAG  37117
Lion         GAAACACAAATATC-AAGGAATACATTTAAAAAATAATTTGGG-AGACAAGTATATCAGTAACTATATAAGATGCTACAA  38326
Vaquita      AGGACA-AAATATC-AAAGAATAAATTTAACAAAAAATGTGCA-TGATAACTACATCAATAACTGCAAGGCATG--GCAA  34906
Pig          ----------TGTC-ACAGAATAGTGTTCAGAATAAACTG---CTACGTTTACAGTCAATTATTTA-C----------A  31465
```

```
Majority     AGAGATATTAXAGACCTAAATGAGTAAAAA--ACCATGTTCATGAA-TTAGAAGGCTCAATATGTT-AAAATGTCATTTT
                       46730     46740     46750     46760     46770     46780     46790     46800
Human        AGAAACCAGACAGACCTGAAAGAGTACATAG---TGTGATTCCATTTCTTGAAAGTTTAAGAGCAG------ATCATTAA  40037
PolarBear    AAAGATATTAGAGACCTAAGTGAGAAAGAA--ACTATGTTCATGAA-TTAGAAGGCTCAATATGTT-AAAATGTCATTTT  10161
Cow          AAAGAATTTTGTAAGTGGGGGAAGTTAATGTTATTAGAAACTTAGTCTTGGGGAAATAATCTTACTCAAAAC--TATTTT  37241
Dog          AGAAATATTAGAGACCTAAATGAGAAAGAA--ACCATGTTCATGAA-TTAAAAGGCTCAGTATGTT-AAAATGTCATTTT  35755
BelugaWhale  AGAGAAATTAAAGACCTAAATAAATAGATAC-ACCATGTTCATGAG--TAGAAGGCTCAATGTGTTAAAGATGTCATTTT  34295
Donkey       --AGAATCCGA--------ACCAGCAAAAC---CCTGGGTCGCCAA---AGCAGAGCCCACGGACTTAA----CCACTCA  38144
Cat          AGAGATATTAAAGACCTGAATGAGGAAGAA--ACCATGTTCATGAA-TTAGAAGGCTCAATATGTT-AAAATGTCATTTT  40317
Ferret       AGAGATATTAGAGACCTAAATGAGAAAGAA--ACCATGTTCATGAA-TCAGAAAGCTCAATATGTT-AAAATGCCATTTT  37193
Lion         AGAGATATTAAAGACCTGAATGAGAAAGAA--ACCATGTTCATGAA-TTAGAAGGCTCAATATGTT-AAAATGTCATTTT  38402
Vaquita      AGAGAAATTAAAGACCTAAATAAATAAATAC-ACCATGTTCATAAG--TAGAAGGCTCAATGTGTTAAAGATGTCATTTT  34983
Pig          AAGGTGTCTTG--------GTCATTTAATG-----GGAAA---------AGGGAAGTCTTTTTTTT--------TTTTTT  31515

Majority     CCCCTAAATTGGAT----TTGAATCTATAAGTCAGTCAAGCTCAACAGGCTTTTAAATTGAAATTGACAAG-TTGATTCT
                       46810     46820     46830     46840     46850     46860     46870     46880
Human        TTTTTTGTTTAGAA-------A-CCAGAATGTTGGAGATTAACAGAAAGGGAATATGGTGGCATTTTCTGGTGTGATGGA  40109
PolarBear    CCCCTAAATTGAATGGAGTTGAATCTATAAGTCAGTCAAGCTCAACAGGCTTTTAAATTGAAATTGACAA--TGGATTCT  10239
Cow          GTATTAAACTAGTT----TC---TTTAGAAGTTAAACATTTCAAAGACACAGCTCTAATGGATTGGAATTGGATTTTATT  37314
Dog          CCCCTAAATTGGATGGAATTAAATCTGT--GTCAGTGAAACTCAACAGGCTTTTAAAATGAAATTGACAAG-TTGGTTCT  35832
BelugaWhale  TCCCCTAATTGGGA----TGGAATTTATAAATCAGTCAAGCTGAACAGGCATTTACGTTGAAAGTGA--TGAATTGATCT  34369
Donkey       GCCGTGGCACCAAC---------CC----------CCAAGCCCAATAGGCTTTTAAATTGAAATTGACAAG-TCGATTCT  38204
Cat          CCCCCAAATTGGATGGAATTGAATCTATAAGTCACTCAAGCTCAACAGGCTTTTAAATTGAAATTGACAAG-TTGATTCT  40396
Ferret       CTCCTAAATTGAATGGAATTGAATCTTCAAGTCAGTCAAGCTCAACAGGCTTTTAAATTGAAATTGACAAG-TGGATTCT  37272
Lion         CCCCCATATTGGATGGAATTGAATCTATAAGTCAGTCAAGCTCAACAGGCTTTTAAATTGAAATTGAGAAG-TTGATTCT  38481
Vaquita      TCCCCTAATTGGGA----TGGAATTTATAAATCAGTCAAGCTGAACAGGCATTTACGTTGAAAGTGA--TGAATTGATCT  35057
Pig          GTCTTTTTGCTGTC----TC---TT---GGGCCACTCCCGCAGCATATGGAGGTCCCCAGGCTAGG----G-ATCTAATC  31580
```

```
Majority     AAAATTTA--------TGTGGAAATAAAAAAXGACCTAGATX--A-CTAAA-----CXTTTTCCAAAAGAAAAAAATAX-
                     46890     46900     46910     46920     46930     46940     46950     46960
Human        AACGTCC---------TATGTTAATAAACTAAGTTGCAAAACAAATCCAAAA-----GTTGTTTGAAGCAGTAGATTATC 40175
PolarBear    AGAATTTA--------TGTGGAAATAAAAAAGAACCTAGATG--AACTAAA---ACCATTTTCCAAAAGAAAAATGTTG- 10305
Cow          GGAAGGAA--------TGTGTAATTATTTG--AAAGTGGG------GAGGA------GGCTTGACAGTG-GTAAGATAG- 37370
Dog          AAAATTTAGAATTAGATGTGGAAATTAAAAAGGACCTAGATATAAGCTAAA---ATCATTTTCCAAAAGAAAAAAAAAA- 35908
BelugaWhale  ACACTTTA--------TATGGAAATAAAAA--GACCTAGAAT--AACTAAA------GCCATTTGAAAAAGAAAAAGAA- 34430
Donkey       AAAATTTA--------TGTGGTAATAAAAAAGGACCTGGAA------TAAA---ACCATTTTGAAAAAGAAAAAGTTGGG 38267
Cat          AAAATTTA--------TGTGGAAATAAAAA--GACCTAGATT-----AAAA----CCATTTTCCAAAAGAAAAAAAAAGT 40457
Ferret       AAATTTTA--------TGTGGAAATAAAAAAGAATCTAGATG--AACTAAATAAAACCATTTCCAAAAGAAAAAAGTCA- 37341
Lion         AAAATTTA--------TGTGGAAATAAAAAAGGACCTAGATG-----AAAA----CCATTTTCCAAAAGAAAAAAAAAGT 38544
Vaquita      ACACTTTA--------TATGGAAATAAAAA--GACCTAGAAT--AACTAAA------GCCATTTGAAAAAGAAAAAAAA- 35118
Pig          AGAGC-------------TGTAACTGCC----AGCCTACA------CCAGA------GCCACAGCAACGCGGGATCCAA- 31630

Majority     -XCAXGAT--CTGATTTCAAGATTCACTGTAAACTTGTAGTAATCAAACA--GTTTAATATTAGCATGATTAGT-TTTAC
                     46970     46980     46990     47000     47010     47020     47030     47040
Human        TCTGAGAACTCTGAATTTCCAGTA-GTTGTG-TCTTGCTGTAATGAGAAA---TATGAGATTGG---GAATGAC---TAG 40244
PolarBear    -ACATGAT--CTGATTTCAAGATTCACTGTAAACTTGTCATAATGGAACA--GTTTAATATTAGCAAGGTTAGTATTTAC 10380
Cow          -----GGT--CTTTCTTGAGTCCATTTTATATAGGTACAGAAATGAGATACCTCTTTAAGTTAGG-TATATTTC-TGCCA 37441
Dog          -TCACAA---CTGATTTCAAGATTCACTGTAAACTTGTAGTAATCAAACA--GTTTAACATTAGCAAGATTAGTTTTTAT 35982
BelugaWhale  -----GAG--TTGGAATACTATTTGATTTCAAGAGTAAAGTAATCGCAGC--AGTTTAGTATAGCATGATTAGT-GTCAC 34500
Donkey       ACACTCACTGTCTGATTCAGGATTCACTGTAAACTTGAAGTAATCAAAACA-GTTTAGTATTAGCATGATTAGT-GTCTC 38345
Cat          GACACTAT--CTGATTTCAAGATTCACTGTAAATTTGTGGTAATCAAACA--GTTTAATATTAACAGGATTAGTATTTAT 40533
Ferret       -ACACGAT--CTAATTTCAAGATTCACAGTAGACTTGTAGTAATCAGACA--GTTTAGTATTAGCAAGGTGAGTATTTAC 37416
Lion         GACACTAT--CTGATTTCAAGATTCACTGTAAATTTGTGGTAATCAAACA--GTTTAATATTAGCAGGATTAGTATTTAT 38620
Vaquita      -----GAG--TTGGAATACTATTTGATTTCAAGAGTAAAGTAATCACAGC--AGTTTAGTATAGCATGATTAGT-GTCAC 35188
Pig          -----GCC--GCATCTGCAACCTACACCACA-GCTCACGGCAACGCCCGA--TCCTTAACCCAC--TGAGCAAC-GGCAG 31697
```

```
Majority     AGAATAGTGTXCAGAAATAAACTCCTACATTTAAGGTCAGC-TGATTATTTACAAAGGTGCCTTGGTCATTCAA------
                       47050     47060     47070     47080     47090     47100     47110     47120
Human        GTGAGTCCATGTAGCAGGGGCC---TAGATGTGGGATTTGA-TGTGTGTGTGTGTGTGTGTGTGTGTGTGTGTG------  40314
PolarBear    AGAATAGCGTACAGAAATAAACTCCTACATTTAAGGTCCAC-TGATTACTTACAAAGGTGTCTTGGTCATTCAA------  10453
Cow          GTGCTGCTGTTAATCTGTAGCATCAGGATTTTGCTGAAAGTATGACTTTTTATTTCATTATGTGAATCATTTTA--CGGA  37519
Dog          AGAATAGTGTACAGAAATAAAC---TACAGTTAAGGTCAGC-TGATTACTTACAAAGGTGTCTTGGTCATTCAG------  36052
BelugaWhale  AGAACAGTATTTATAAATAAACTCCTACATTTAAGGTCAGT-TGATTATTTACAAAAGTCCCTTGGTCATTTAATGGTGA  34579
Donkey       AGAATAACGTTCAGAAATAGACTCCTACATTTAAGGTCAAT-TGATTATTTACAAAGGTGCCTTGGTCATTCAA------  38418
Cat          AGAATAGTGTGCAGAAAGAAGCTCCTATATTTATGATCAAC-TGATTATTTACAAAGGTGCCTTGGTCATTCAT------  40606
Ferret       AGAATAGAGTGCAGAAGTAAACACCTACATTTAAGGTCCAC-TGATTACTTACAAAGGTGTCTTGGTCATTCAG------  37489
Lion         AGAATAGTGTGCAGAAAGAAGCTCCTATATTTATGATCAAC-TGATTATTTACAAAGGTGCCTTGGTCATTCAT------  38693
Vaquita      AGAACAGTATTTATAAATAAACTCTTACATTTAAGGTCAGT-TGATTATTTACAAAAGTCCCTTGGTCATTTAATGGTGA  35267
Pig          GGATCG-----AACCCACAGCCTCATGGTTCC-TAGTCAGATTGGTTAACCACTGCGCCACGATGAGAACTCCA--GGGA  31769

Majority     ----------T--TGGAGAAATGGTGCXAGAAXACTXX--GAAACACATATGAAAAAAA-GAAACXCAAAC---------
                       47130     47140     47150     47160     47170     47180     47190     47200
Human        -------------TGTGTGTGTGTAGCTTGGACTTTAACAGATTTACATTTTAGAAAGA--ATTTTTGTTC---------  40370
PolarBear    ----------T--TGGAGAAATGGTGCCAGAAAACAATTGGAAACACATATGAAAAAAAAGAAACTCAAAC---------  10512
Cow          T---------TCTGTTACATGTTACTCAGAACTGTTTGA-GATATGTCTGTTTTAACTTTTATATACAGACGTTTTTGAT  37589
Dog          ----------T--TGTGGAAATGGTGCCAGAAAACAGTTGAAAACAGATATGAGAAAAA-GGAACTCAAAC---------  36110
BelugaWhale  AAGGGAAGTATTTTTAACAAGTGGTGCTAGAACAGTTG--GATACACAAATGAAAACAACAAAAAAATTAGAAACTCAA-  34656
Donkey       ---------------------TGGGG---------------AAAGAGAAGTTTTCTCAA-CAA-----------------  38444
Cat          ----------T--TGGGGAAATGGTGCCAGAAAACA----GAAACACATATGAAAAAAA-GAAAC--AAAC---------  40658
Ferret       ----------T--TGGAGAAATGGTG--------------GAAACACATATGAAAAAAA-GAAACTCAAAT---------  37533
Lion         ----------T--TGGGGAAATGGTGCCAGAAAACA----GAAACACATATGAAAAAAA-GAAAC--AAAC---------  38745
Vaquita      AAGGGAAGTATTTTTAACAAGTGGTGCTAGAACAGTTG--GATACACAAATGAAAACAACAAAAAAATTAGAAACTCAA-  35344
Pig          AG--------TCTAGCACAAATGGTGCTAGAACAATTG--GATATGCACATGAAAACTCCACATTAGAAAC---------  31830
```

```
Majority      ----T------ACTTXATACTGTA-----CAGAAAAAGT----AGATG-------AATX--TATACCTAAA-----CCTA

                        47210     47220     47230     47240     47250     47260     47270     47280

Human         ---------CATTTGTATTTCACAGG---TAGGATCAGTAT-TGTATAGAG---GGGAGAATAGTTTTAAAA----CATT  40430
PolarBear     ----T------ACTTAATACAGTA-----CAGAAATAAT----AGATG-------AATA--TATACCTAAA-----CCTA  10559
Cow           ATGATGTCATTTTATTTTATTGTAACGTTTTGAAAATATTCCAGCACGTTGTTTCAGTTTGAATCTCTATAAGGGTCCTA  37669
Dog           ----T------ACTTAATATTGTA-----CAGAAATATT----AGATG-------GATA--TATACCTAAA-----CCTA  36157
BelugaWhale   ------ACCTTACTTCATGCCATA-----CGGAAAAAG-------ATG-------AATT-ACATACCTAAA-----CCTA  34705
Donkey        -------------ATGGTG----------CTAGAACAGT-------TG-------GATA--CACGTATGAA---------  38476
Cat           ----T------ACCTGATACTGTA-----CAGAAAAGTT----AGATG-------GATG--TATATCTAAA-----CCTA  40705
Ferret        ----T------ACTTAATACAGTA-----CAGAAATAAT----AGATG-------AATA--TATACCTAAA-----CCTA  37580
Lion          ----T------ACCTGATACTGTA-----CAGAAAAGTT----AGATG-------GATG--TATATCTAAA-----CCTA  38792
Vaquita       ------ACCTTACTTCATGCCGTA-----CGGAAAAAG-------ATG-------AATT-ACATACCTAAA-----CCTA  35393
Pig           ------TCAGTCCTTCATACTGTA-----CAGAAAAAG----AGGATG-------AATT-ACATACCTAAA-----CCTC  31882

Majority      AAAAA---TAAATATACAAAGCTTTTXAGAAAAAAA-TTATAGAATAT-ATCCTCAACCTTGA--GGTAAATG----AAG

                        47290     47300     47310     47320     47330     47340     47350     47360

Human         ACGGTAGTTCATTC--CAAAGATACCAAGGTCCTGAATTTTAGGGTCTTGGAAGGAGAATGCAGGAAATGATAAAACGAG  40508
PolarBear     AGAAAC--TAAATATATAAAGCTTTTTTTAAAAAAA-TTGTAGAATAT-ATTCATGAACTTGA--GGTAAATA-------  10626
Cow           ACAACATGTCCTCCTGCATAGAGGTGATGAGAGCCT-TCCTCTAATATAGTCTGCATGCTTAGTCGGTCAGTCGTCAGTC  37748
Dog           AAAAA-------TATATAACACTTTTTA---AAAAA-TTACATTATAT-AT-CACAAACTTGA--GGTAAATGA-----T  36217
BelugaWhale   AAATC---TAAATATGCAAAGCTTTTAAGAAAAAAAATCCTAGAATGT-ATCCTCAACCTTGG--GGTAAAT----GAAG  34775
Donkey        ----------------AAAAGCTTTAAGAAAAAAAA-TCATAGACTGT-ATTCTCAACCTTAG--CATAAATG----AAG  38532
Cat           AAAAAC--TAAATACATAAAGCATTTTG---AAAAA-ATATAGAGTAT-ATATCTAACCTTCA--GGTAAATGAAGATTT  40776
Ferret        AAAAAC--AGAATGTA-AAAGCTTTTTA-AAAATAA-TTATAGAATAT-ATTCAC-AACTTGA--GGTAAAGA-------  37644
Lion          AAAAAC--TAAATACATAAAGCATTTTG---AAAAA-ATATAGAGTAT-ATATCTAACCTTCA--GGTAAATGAAGATTT  38863
Vaquita       AAATC---TAAATATGCAAAGCTTTTAAGAAAAAAAATCCTAGAATGT-ATCCTCAACCTTGG--GGTAAAT----GAAG  35463
Pig           ACAAC-TATAAATAGGCAAAGCTTTTGAGAAAAAAA-CCCTAAAATAT-GTCTTCAATCTTAAGGGGTAAAT----GAGA  31955
```

```
Majority     TTTTCT-AGAXAAGAC------ACAAAAA-CCACTAXCCATAAAGGAATAATTTATAAACTCTACAXTXCATCTAAATGA
                         47370     47380     47390     47400     47410     47420     47430     47440
Human        TTATCTGGAAGAAGACAAGTTTGGAATAA----T--ATTAAAGAGTTTTGTCTTAGAATCACTGGGTTGGTAGAAGATGA  40582
PolarBear    TTTTTT-AGAGAAGAC------ACAAAAA-CCACTACCCATAAAGCAATAATTTATAAACTAGACAGTTTACCTAAATGA  10698
Cow          ATGTCTGACTATTTGTGACCCTATGGAATGTAGCCTGCCAGGCTCTTCTGTCCTTGGAATTCTACAGGCAAGAATACTGG  37828
Dog          TTTTTT-AGAGAAGAC------AC-AAA-----CTACCCATAAAGGAATAGTTTATAAACTAGACATTTCACCTAAATTT  36284
BelugaWhale  ATTTCTTAGAAAAGGT------ACAAAAATACACTAACCATAAAGGAATACTTTACAAA--CTAGAGTCCATCTAAATGA  34847
Donkey       CTTTCTCACACAAGAC------AAAAAA---CACTGACTGTAAAGGAATAATTTATAAA--CTAGACTCCATCTAAACTA  38601
Cat          TTTTTT-AGAGAACAC------ACAAAAA-CCACTACCCATAAAAGAATAATTTATAAACTAGACATTTCATCTAAATGA  40848
Ferret       TTTATT-AGGAAAGAC------ACGAAAA-CCACTACCCATAAAGCAATAGTTTATAAACTCCACATCTTACCTAAATGA  37716
Lion         TTTTTTTAGAGAACAC------ACAAAAA-CCACTACCCATAAAAGAATAATTTATAAACTAGACATTTCATCTAAATGA  38936
Vaquita      ATTTCTTAGAAAAGGT------ACAAAAATACACTAACCATAAAGGAATACTTTACAAA--CTAGAGTCCATCTAAATGA  35535
Pig          ATTTCTGAG-ATAGGTTACC--AAAAAAACCCACTAACCAAAAAGGACTGCTTTACCAA--CTAGAGGCCATCTCAATGA  32030

Majority     XAXAA-TT--------CTCATCAAAAG-ACATTGTT-AAGACAATGAAAAGGCAA-GG-----CACAGATTGGGAAAAGA
                         47450     47460     47470     47480     47490     47500     47510     47520
Human        TG---------------CCATTAACAA-AGGGGG---AAAAGTAGGAAAAGGAGACTCG---TTTTTGGTGGGAAAAAAG  40640
PolarBear    -AAAACTT--------CTCATCAGAAG-ACATTGTT-AAGACAGTGAAAAGGCAA-GT-----CACAGATTGGAAAAAGA  10761
Cow          AGTAGGTTGCTATTTGCTCCTCCAGGGTATCTTCCC-GACCCAGGGTTGAACCCACATTTTCTCCTGTATTGGCAGGTGG  37907
Dog          TAAAAATT--------CTCATCAAAAG-ACGTT----AAGACCATGAAA-GCCAA-AT-----CACAAATTGGGAAAAGA  36344
BelugaWhale  AA-ACCTT--------CTCATCAAACG-ACATTATT-AAGAGAATGAAAAGGC-ACGG-----CACAGGCTGAGAGAAAA  34910
Donkey       GA--------------CTCATCCAAAA-ACATT----AAGACAATGAAAAGGCAA-GG-----CACAGACTGGGAGGAAA  38656
Cat          -AAAA-----------CTCATCAAAAG-TCACTGTT-AAGACAATGAACAGGCAA-GG-----CACGGGTTGGGAAAAGA  40908
Ferret       -AAACCTT--------CTCATCAGGAG-ATGTTGTTTAAGATAATGAAAAGACAA-GT-----CACAGATTGGGAAAAGA  37780
Lion         -AAAA-----------CTCATCCAAAG-TCACTGTT-AAGACAATGAACAGGCAA-GG-----CACGGGTTGGGTAAAGA  38996
Vaquita      AA-ACCTT--------CTCATCAAACG-ACATTATT-AAGAGAATGAAAAGGC-ACGG-----CACAGGCTGAGAGAAAA  35598
Pig          AA-AA-----------CTCCTCAAAAG-ACATTGTT-AAGACAATGAAAAGGTGA-GG-----CACAGACTGGAAGAAAA  32090
```

```
Majority     TAXTAATATTCACATATTTATXCATA--TXTCTGA-CAAAGGACTT----GTATCCAGAGTATGTAAAGAATTCTTT---
                      47530     47540     47550     47560     47570     47580     47590     47600
Human        AATAATCCGCTACTGTTCTGTTTTTTTTTGTTTGTTTTTTGTTTTTTATTTTTTATTGATCATTCTTGGGTGTTTCTCGC  40720
PolarBear    TACTAATATTCACATATTAATACATA--TATTTG--CAAAGGACTT----GTATGCAGAGTATATAAAGAATTCTTTCAA  10833
Cow          -ATTCTTTACAACTGAGCCACC----------TGG-GAATCCACTT--TGTTGGCCAGTCACTCAGTCACGTCCAAC---  37970
Dog          TGCTAATACTCACATATTAATACATA--TATCTAA-CAAAGGACTT----GTATGCAGATTATATACAGAATTCTTT---  36414
BelugaWhale  TATTAATATTCATTCAGATATCAATACATGTCTGA-CATAATAAATACTTATATCCAGAGTACGTAAAGAATTCTTG---  34986
Donkey       GAGCAGTATTCACATATCAATACGTA--TGTCTA--CAAAGGACTT----GTATCCAGAGTATGTACAAAATGCTTC---  38725
Cat          TCCTAATATTCACATATTTGTGCATA--TATCTGA-CAAAGGACTT----GTATCCAAAATAGCTTAAGAATTCTTT---  40978
Ferret       TACTAATATTCACATATTAATACATA--TTTTTGA-CAAAGGACTT----CTCTGCAGAATATATAAAGAATTTTCTTTC  37853
Lion         TCCTAATATTCACATATTTGTGCTTA--TATCTGA-CAAAGGACTT----GTATCCAAAATAGCTTAAGAATTCTTT---  39066
Vaquita      TATTAATATTCATTCAGATATCAATACATGTCTGA-CATAATAAATACTTATATCCAGAGTATGTAAAGAATTCTTG---  35674
Pig          TATTAATGTCCACATATTTATC----------TGA-CAAAGGACTTA-TAATACCCAGAGTATGTAAAAAATTCTTC---  32155

Majority     ------------------XACATCTXTAAATACAAACAGCCTAAT--TTTTTTAAATAGACAAAAGAAGGTATATGAATG
                      47610     47620     47630     47640     47650     47660     47670     47680
Human        AGAGGGGGATTTGGCAGGGTCATAGGACAATAGTGGAGGGAAGGTCAGCAGATAAACAAGTGAACAAAGGTCTCTGGTTT  40800
PolarBear    AAAAAGAAAGGATTCTTTCACATCTATAAATACAAACAGCCTAAG--TTTTTGAAAAAGACAAAAGAAGGTATATGAATG  10911
Cow          --------------------TGTTTG-CAGCACGCCAGGTTTCCCT-GTCCTTCACCGACTTGCAGAGTTTGCTCAAACT  38028
Dog          -----GAAAAAATTCTTTCACATCTATAATTACAAACAGCATAATT-TTTTTGAAATAGGTGAAAGAA--CATATGAATA  36486
BelugaWhale  --------------------CATGTC-AAATACAAAGAGACTAAT---TTTTTAAATGGACAGAAGAAGGTACACGAA--  35040
Donkey       -------------------ACATCAATAAATAGAAACAACCTAAT---GTTTTTAATGAGTGAAAGAAGATATACAAATA  38783
Cat          ------------------CACATCTGTAAGCACAAACAGCCTAATTTTTTTTGAAATAGACCAAAGAAGGTATATGAATG  41040
Ferret       TT--TGAAATAATTCTTTCACATCTATAAATACAAATAGCCTAA---TTTTTGAAATAGATAAAAGAAGGTATATGACTG  37928
Lion         ------------------CACATCTGTAAGCACAAACGGCCTAATTTTTTTTGAAATAGACCAAAGAAGGTATATGAATG  39128
Vaquita      --------------------CATGTC-AAATACAAAGAGACTGAT---TTTTTAAATGGACAGAAGAAGGTACACGAA--  35728
Pig          --------------------CACAT--CGGTACGAACAGCTTAATC-TTTTTTAAATGGACAAAAGAAGGTACATGAA-T  32211
```

```
Majority     -------GCT-----AATAAGCACATGAAAATAT----XTATTAGTCATCAGGAAAACACAAGTTATAACTACTGTGAGG
                       47690     47700     47710     47720     47730     47740     47750     47760
Human        TC------CTAGGCAGAGGACCCTGCGGACTTCCGC-AGTGTTTGTGTCCCTGGGTCCTTGAGATTAGGGAGTGGTGATG 40873
PolarBear    -------GCT-----GATGAGCACATGAAAATA--C--ATATTAGTCATCAAGAAAA--CACGTTATACCTACTGTGAGG 10973
Cow          CATGCCCGTTGAGTTGGTGATATCATCCAAGCATCTCATCCTCTGTCATCCCTTTCTCCTCCTTTCCCAGCATTAGGGTC 38108
Dog          -------GCTG--ATAATAAGCACATGAAAATA--T--GTATTAGTCATCAAGAAAACACGGGTTATAACTGCTGTGAGA 36553
BelugaWhale  ---------TGGCTAAATAAGCACATGAAAAGAT----TTATTAGTCATAAGGAAATCACAATTTATAGCTAATATGAGA 35107
Donkey       -------GCT-----AATAAGCACAGGAAAACAT----ACATTAGTCATCAGGAGAAAACAAGTTATAACCACTGTGGGG 38847
Cat          -------GTT-----GATAAATGCACGAAAATAA-T--ATATTTGTCATCAGGAAAACACAAGTTATAACTGCTGTGAGG 41105
Ferret       -------GCT-----AATGAGCACATGAAAATA--T--ATACTAGTCATCAAGAAAAAGCAAGTTATAATTGCTATGAGG 37992
Lion         -------GCT-----GATAAATGCACAAAAA--------TATTTGTCATCAGGAAAACACAAGTTATAACTGCTGTGAGG 39188
Vaquita      ---------TGGCTAAATAAGCACATGAAAAGAT----TTATTAGTCATAAGGAAATCACAATTTATAACTAATATGAGA 35795
Pig          -------G----GCTAATAGGCACATGTAAAGAT----TTATTAGTCATAAGGAAATCACGGTTTATAACCACTACAAGA 32276
```

```
Majority     TATCAXGTCACACCAACT---AGAATCTAGGCTAAA-ATTAAACAGATTGG-----------CAATGCTAAATGTT---G
                       47770     47780     47790     47800     47810     47820     47830     47840
Human        ACTCTTAACGAGCATGCT----GCCTTCAAGCATCT-GTTTAACAAAGCACATCTTG---CACCGCCCTTAATCCATTTA 40945
PolarBear    CGTCCTGTCATGCCAACT---AGAATCTTGGCTAAA-ATTAAACAGATTGG-----------CAACA-AAAACA-T---G 11033
Cow          TTTTCTAATAAGTTGGCTCCTTGCATCAGGAATATTCATTGGAAGGACTGATTCTGAAGCCACAGTACTGGAGCTTCCAA 38188
Dog          TAT---GTCACACCAACC---AGAATTTAGGCTCAC-ATTAAACAGATTGG-----------CAACA-TAAGCGTT---G 36611
BelugaWhale  TACCACTTTAGACCAACT---AGAATCTAGGCTAAA-GTTAAACAAATCGG-----------TAATGCCAAATGTT---G 35169
Donkey       TACCACTTCACGCCAACT---AGAATCTAGGCTAAA-GTTAAACAGATTGG-----------CAATGCCAAATGTT---G 38909
Cat          TAT---GTCACACCCACT---GGAATTTAAGCTAAA-ATTAAACGGATTGG-----------CAATG-TAAATGTT---G 41163
Ferret       TATCTTGTCATGCCAACT---AGAATTTTGGCTAAA-GTTAAACAGATTGG-----------CAACA-TAAA---T---G 38050
Lion         TAT---GTCACACCCACT---GGAATTTAAGCTAAA-ATTAAATGGATTGG-----------CAATG-TAAATGTT---G 39246
Vaquita      TACCACTTCAGACCAACT---AGAATCTAGGCTAAA-GTTAAACAAATCGG-----------TAATGCCAAATGTT---G 35857
Pig          TACCACTACAGATCAACT---AGAATCTAGGCTAAA-ATTAAACAGATTGG-----------CAGTGCCAAATGTT---G 32338
```

```
Majority     ATGAGGA----TGTXGAGCAAAT---TGGAACAATTTGCCATTGCTGGTGAXAGTGTAAAATAGTTTGGAAAACAAC---
                      47850     47860     47870     47880     47890     47900     47910     47920
Human        ACCCTGA-GTGGACACAGCACATGTTTCAGAGAGCACAGGGTTGGGGGTAAGGTCACAGATCAA-CAGGATAAGAAT---  41020
PolarBear    ATGAGGA----TATAGAGCAAA----TGGAACAGTTTACCATGGCTGGTAAAAATGTAAAATAGTTTGGTAAACAGC---  11102
Cow          ATCAGTC----CTTCCAGTGAATATTCAGAATTGATTTCCTTTAGCATTGACTGGTTTTATCTCCTTGCAGTCCAAGGGA  38264
Dog          ATGAGGA----CGTAGCGAGGAC---TGGAACAACTCACCATGGCTGGTAAAAATATAAAATAGTTTGAAAAACAAC---  36681
BelugaWhale  ATGAGGA----TGTGGAGCAGCT----GAAACTATATGCCATTGTTGGTGAGTG--TAAAATAG-TTGGAACACA-G---  35234
Donkey       ATGAGGA----TGCAGAGCACCTG---GCACTTTTTTGCCATTGTTGGTGAGAGTGTAAAATAGTTTGGAAAACA---G-  38978
Cat          ATGAGGAAGGATGTGGAGCAGT----TGGAACAACTTGCCATTGCTGGTGAAAGTGTAAAATAGTTTGGAAAACAAC---  41236
Ferret       ATGCAGA----TATAGAGCAAG----TGGAACAATTCACCATGGCTGGTAAAAGTGTAAAATAGTTTGGAAAACAGC---  38119
Lion         ATGAGGAAGGATGTGGAGCAAT----TGGAACAACTTGCCATTGCTGGTGAAAGTGTAAAATAGTTTGGAAAACAAC---  39319
Vaquita      ATGAGGA----TGTGGAGCAGCT----GAAACTATATGCCATTGTTGGTGAGTG--TAAAATAG-TTGGAACACA-G---  35922
Pig          ATGAGGA----TGTGGAGCAAAC--T-AAAACTATACGCCATTGCAGGTGAGAGTGTAAAATAGTTTGGAAAACA-G---  32407

Majority     --TTTGGCAGTATCTTATA------AAGTTAAACA---------------------------------------------
                      47930     47940     47950     47960     47970     47980     47990     48000
Human        -TTTTCTTAGTACAGAACA------AAATGAAAAGTCTCCCATGTCTACCTCTTTCTACACAGACACGGCAACCATCCGA  41093
PolarBear    --TT-GGCTGTATCTTACA------AAGTTAAACA---------------------------------------------  11128
Cow          CTCTCAGGAGCCTTTCCAACATCACAATTCAAAAG------------------C--------------------------  38300
Dog          --TT-GGCAGTATCTTATA------AAGTTAAGCAGCCTATGACCCAGTACTTCTACATCAAGGTTTTCTACCT------  36746
BelugaWhale  --TTTGGCAGTTTCTTATA------AAGTTAAAC----------------------------------------------  35260
Donkey       --TTTGGCAATTTCTTAGA------AAGTTAGACA---------------------------------------------  39005
Cat          --TTTGGCAGTATTTTATA------AAGTTAAACATGAATACAGCTTATGACCTCGTACTTTCTGGTCTAGGTTTTTTTT  41308
Ferret       --TT-GTCATTATCTTATA------AAGTTAAACA---------------------------------------------  38145
Lion         --TTTGGCAGTATTTTATA------AAGTTAAACATGAAGACAGCTTATGACCTCGTACTTTCTGGTCTAG---------  39382
Vaquita      --TTTGGCAGTTTCTTATA------AAGTTAAAC----------------------------------------------  35948
Pig          --TTTGGGAGTTTCTTAGA------AGGTCAAAA----------------------------------------------  32433
```

```
Majority     -------------------------------------------------------ATAAACA-X---------------
                      48010      48020      48030      48040      48050      48060      48070      48080
Human        TTTCTCAATCTTTTCCCCACCTTTCCCCCCTTTCT---------A------TTCCACAAAACCGCCATTGTCATCATGGC  41158
PolarBear    --------------------------------------------------------TGAACATG----------------  11136
Cow          -------------------------------------------------------ATGAATTCT----------------  38309
Dog          ----------------------------------------AG--------------TGAAAAAGA---------------  36757
BelugaWhale  -------------------------------------------------------ATAAACA------------------  35267
Donkey       --------------------------------------------------------TAA-CC------------------  39010
Cat          TTTTTTTTTTTTTTTTCCAACGTTTATTTATTTTTGGGACAGAGAGAGACAGAGCATGAACGGGGGAGGGGCAGAGAGAG  41388
Ferret       --------------------------------------------------------TAAACATG----------------  38153
Lion         --------------------------------------------------------------------------------  39382
Vaquita      -------------------------------------------------------ATAAACA------------------  35955
Pig          -------------------------------------------------------ATAAATA------------------  32440

Majority     ---------------------------------------------GCCTA-X-TXTGACCCAGXXXXT------------
                      48090      48100      48110      48120      48130      48140      48150      48160
Human        CCGTTCTCAATGAGCTGTTG---GG---------TACACCTCCCAGACGGGGTGGTGGCCGGGCAGAGGGGCTCCTCACT  41226
PolarBear    ---------------------------------------------GCCTA-----TGACCCGGTACTT------------  11154
Cow          ---------------------------------TTGGTGC--TCAGCCTTCTTTATGGTCCAAC---T------------  38339
Dog          ---------------------------------CTTGTGC----AGCCTA-----TGACCCAGTACTT------------  36783
BelugaWhale  ------------------------------------------T------GTCCTATGACCCAG-----------------  35282
Donkey       -----------------------------------------------CTACCCTACAGCCTAGTACTT------------  39031
Cat          AAGGAGACACAGAATCGGAAACAGGCTCCAGGCTCTGAGCCATCAGCCCAGAGCCTGACGCGGGGCTCGAACTCCCGGCC  41468
Ferret       ---------------------------------------------GCCTA-----TCACCCAGTACTT------------  38171
Lion         --------------------------------------------------------------------------------  39382
Vaquita      ------------------------------------------T------GTCCTATGACCCAG-----------------  35970
Pig          --------------------------------------------AATCTACCTTGTGACCTCG----T------------  32460
```

```
Majority     ----------------------------------------CT-C-----------------XTCTAGGTTTTTACCC-AG
                     48170     48180     48190     48200     48210     48220     48230     48240
Human        TCCCAGTAGGGG---CGGCCGGGCAGAGGCGCCCCTCACTCCCCAGTAGGGGCAGCCGGGCAGAGGCGCCCCTCACCTCC  41303
PolarBear    ----------------------------------------ATACA-----------------TCTAGGTTTTTCCCCCAA  11177
Cow          ----------------------------------------CT------------------CTTATCCGTACATGACT-CC  38360
Dog          ----------------------------------------CTACA-----------------TCAAGGTTTTCTACCTAG  36806
BelugaWhale  ------------------------------------------------------------TACTTCCATTTCT-------  35295
Donkey       ----------------------------------------CTAC-----------------TTCTAGGTTTTTACCC-AG  39053
Cat          CGCGAGATCGTGACCTGGCTGAAGTCGGACGCTCAACCGACTGCGCCACCCAGGCGCCCCGTTCTAGGTTTTTATCC-AG  41547
Ferret       ----------------------------------------CTACA-----------------TCTAGGTTTTTACCC-AA  38193
Lion         -------------------------------------------------------------------GTTTTTACGC-AG  39394
Vaquita      ------------------------------------------------------------TACTTCCATTTCT-------  35983
Pig          ----------------------------------------AT------------------TTCCTAGGTTTTTACCC-AT  32481

Majority     TGAAAA-X--X-C-X----AGACTTGTACAAATATGTGTATAAAAAAC------TTTATTTATGATGGTCCCAAACTTGA
                     48250     48260     48270     48280     48290     48300     48310     48320
Human        CGGATGGGGCGGCTGGCCGGGCGGGGGGCTGACCCCCCCACCTCCCTCCCGGATGGGGCAGCTGGCCGGGCGGGGGGCTG  41383
PolarBear    TGAAAA-------------GGACTTGTACAAATATGTATATAAAAAC-------TTTATTCATCATGGTCCCAAATGTGA  11237
Cow          TGGAAAAACATGCACAGCCTTAACGATACCGACCTTTGTCAGCAAAGTAACGTCTCTACTTTTTAATACACTAAGCTTGT  38440
Dog          TGAAAA-------------AGACTTGTGCAAATATGCATATAAAAAAA------TTTATTCATGATAATCCCAAACTTGA  36867
BelugaWhale  ---------------------AGTTGTACAAATGTGTGTATAAAAAA--------CTATTTATAATGGTCCCAAACTTGA  35346
Donkey       TGAAAACACGTTCCCTACAAGACTTGTGCAAATATGTGTATAAAAAAC------TTCATTCGTGATGGTCCCAAACTTGA  39127
Cat          TGTAAACATGTTCACTGTAAGACTTGTACAAA--TGTGTATAAAAAAC------TTGATTTATGATGGTCCCAAACTTGA  41619
Ferret       TGAAGG-------------AGACTTGTACAAA-ATGTATATAAAAAAA------TTTATTCATGATGGTCCCAAACTTGA  38253
Lion         TGTAAACATGTTCACTGTAAGACTTGTACAAA--TGTGTATAAAAAAC------TTGATTTATGATGGTCCCAAACTTGA  39466
Vaquita      ---------------------AGTTGTACAAATGTATGTATAAAAAAA-------CTATTTATAATGGTCCCAAACTTGA  36035
Pig          TGAAAACATGTTCACTACAAGAGTTATACAAATGTGTGTATAGAAAA--------CTATTTATAATGGTCCAAAACCTGT  32553
```

Majority	AATTACCCAAAT-GTCTGTCA------ATAGGAAAATGGGTCAATGAATTGTTG-TATAATCATACAGTXGATTACT---	
	48330 48340 48350 48360 48370 48380 48390 48400	
Human	ACCCCCCCACCT-CCCTCCCGGACGGGGCGGCTGGCCGGGCTGGGGGCTGACCCCCGCCACCTCCCTCCCGGACGGGGC-	41461
PolarBear	AATTA-CCCAGT-GTCTGTCA------GTAGGAAAATGGGTCAATGAATTGTGG-TATAATCATACAGTAGATTACT---	11305
Cow	CATTGCTT-----TTCTTCGA------AGGAGCAAGCATCTTTTAATTTCATTGCTCCAGGCACCATCTGCATTGATGTT	38509
Dog	AATTATCCAAAT-GTCTGTCA------GTAGAAAAATGGGTCAATGAATTCT------AATCATGCAGTAGATTAGT---	36931
BelugaWhale	AACAATCTAAATGTTTTATCA------ACAGGAAAATGGATTAAGAAGTTAGTCATACAG--------TGGATTACTACT	35412
Donkey	AATAA--C-----GTCTGTCA------TCAGGAAAATGGATCAGTAAATTGTTG-TGTAGTCATATAGTGGATTACT---	39190
Cat	AATTACCCAAAT-GTCTGTAA------ATAGGAAAATG--TCAGTGAATTGTAG-TATAATCAAACAGTAGATTACT---	41686
Ferret	AATTA-CCAAAT-GTCTGTGACACAGAATAGGAAAATGGGACAATGATTTGTGG-TATAATCATATAGTAGAGTACT---	38327
Lion	AATTACCCAAAT-GTCTGTAA------ATAGGAAAATA--TCAGTGAATTGTAG-TATAATCAAACAGTAGGTTACT---	39533
Vaquita	AACAATCTAAGTGTCTTATCA------ACAGGAAAATGGATTAAGAAGTTAGTCATACAG--------TGGATTACTACT	36101
Pig	ATCCAGAT-----GTCTATCA------ATAGGAAAATAA----TAAGTT----------G--------TGGATTGTT---	32597

Majority	---ACTTA-T-ATAACAAAAGAATGACTATTAATAC-------------------ATG-CAXAAACATGAGTGAATATT	
	48410 48420 48430 48440 48450 48460 48470 48480	
Human	---GGCTGGC-CGGGCTGGGGGCTGACCCCCGACCTCCTTCCCGG----------ACG--GGGCGGCTGGCCGGGCAGA	41525
PolarBear	---AATTA---GTAACAAAAGAATGACTATTAACAA-------------------ATAACAAAACCATGAGTGAAGATT	11359
Cow	GGAGCCCAAAAAGATAAAGTCTGTCACTGTTTTCATTGTTTCCCCATTTATTTGCCATG-AAGTGATAGGACCGGATGCC	38588
Dog	---AATTA---GTGACAAAAGAATGACTATTA---T-------------------ACA--A-AAACATGAGTGAATATT	36979
BelugaWhale	T--ACTCATTAAGAGCAAAAGAATTACTATTAATAC-------------------ATG-TAGCAACACAAGTGAATATT	35469
Donkey	---ACTCA---ATAATAACAGAATGACTGTCTATAC-------------------TTG-GAACAAACTGAGTGGATATT	39243
Cat	---GCTTACTCATAACAAAAGAATGATTATTA-----------------------ACA-CAAAAACATGAGTGAATATT	41738
Ferret	---AATTA---GTAACAAAAGAATGACTATTAACAA-------------------ATA-CAAAAACATGAGTGAATATT	38380
Lion	---ACTTACTCATAACAAAAGAATGATTATTA-----------------------ACA-CAAAAACATGAGTGAATATT	39585
Vaquita	T--ACTCATTAAGAGCAAAAGAATTACTATTAATAC-------------------ATG-TAGCAACACAAGTGAAAATT	36158
Pig	---ACTCATTAAGAACAAAAGAATTACTATTAATAT-------------------GTG-TAGTAGCGTGAGTGAATATT	32653

```
Majority     AA--------------AACXTTATATG--AAGC-----------------AAAAGAAGCCAGACA-----CAAAAXAGTA
                      48490      48500      48510      48520      48530      48540      48550      48560
Human        GGG-------GCTCCTCACTTCCCAGTAGGGGCGGCC--------GGGCAGAGGCGCCCCTCACCTCCCGGAAGGGGCGG  41590
PolarBear    AA--------------GACAGCATATG--AAGC-----------------AAAAGAAGCCAGACA-----GAAAACAGAA  11401
Cow          ATGATCTTTGATTTTGAATGTTGAGTTTTAAGCCAGCTTTTTCACTCTCCATCAAGAGGCTCTTTTGTTCCTCCTCATTT  38668
Dog          AA--------------GACAATATATG--AAGC-----------------AAAAGAAGCCATACC-----CAA----GTG  37017
BelugaWhale  AAG-------A-----AACGTTATATG--AAGC-----------------AAGAGAAGCCAGACG-----CAAAAGAGTA  35513
Donkey       GT------------TAAATGTTATATG--AAAC-----------------AAAGGAAGCTAGATA-----CAAAACATTG  39287
Cat          AGT------------AGACCTTCTATG--AACC-----------------AAAAGAAGCTAGACA-----CAAG--AGTA  41780
Ferret       AA--------------GACAGTATATG--AAGC-----------------ACAAGAAGCCAGACA-----GAAAACAGAA  38422
Lion         AG---------------ACCTTCTATG--AACC-----------------AAAAGAAGCTAGACA-----CAAG--AGTA  39624
Vaquita      AAG-------A-----AACGTTATATG--AAGC-----------------AAGAGAAGCCAGATG-----CAAAAGAGTA  36202
Pig          AG------------TAAACATTGCG----AAGC-----------------AACAGGAGACAGA-------CAAAAGATTA  32693

Majority     XXTACTGTG--TGATTC-----------CCATTTTTATAAAGT------ACXGATAXTTAATTTT-TAGTATAGAAACC-
                      48570      48580      48590      48600      48610      48620      48630      48640
Human        CTGGCCGGGCGGGGGTCTGACCCCCCCACCTCCCTCCTGGAT--------GGGGCGGCTGGCCTGGCGGGGGCTGACCCC  41662
PolarBear    ATCACTGAAA-TGAATCATTGTGTGATTCCATTTTTATAAAATT----AAAGAACAGATAATCAA-TTTTTAGTATA---  11472
Cow          TCTACCATA--TGGTTG---GTGTCAT-CTGCATATCTGAGGTT-----ATTGGTATTTCTTCCTGCAGTCTTGATTCC-  38736
Dog          AATACTCAAC-TGATTC-------CGT----TTTTTACAAAGTT----AAAGAACAGATAATTTT-TAGTAAAGAAACC-  37079
BelugaWhale  TATACTATG--TGATT------------CCCTTTCTGTAAAGA------GCAGATAATTAATTTT-CAGTATAGAAACC-  35571
Donkey       CATACTGTGTGTGATT------------CCATTTCTATAAAGTT-----AGGAGCAGATAATTTT-TAGTATAGAAACC-  39348
Cat          CTCACTGTG--TGGTTC------------CATTTTTGTAAAG------AACAGATAATCAAGTTT-TAGTATAGTAGCC-  41838
Ferret       ATCACTGAAA-TGAATC----TGTGATCCCATTTTTATAAAGTTTAATAAAGAACAGATAATCTA-TTTTAAGTATA---  38493
Lion         CTCACTGTG--TGATTC------------CATTTTTATAAAG------AACAGATAATCAAGTTT-TAGTATAGTAGCC-  39682
Vaquita      TATACTGTG--TGATT------------CCCTTTCTGTAAAGA------GCAGATAATTAATTTT-CAGTATAGAAACC-  36260
Pig          TATACTGTG--TGATT------------CTATTTCTCTGAAGA------GTAGATAATTAATTTT-CAGTATAGAAACC-  32751
```

```
Majority     -AAAATAATXGTTGCCTG--TGGGTGTT-----XXGATGAACAGXAAGTACAAGGX----AATTCTCTGGGGTATTGGAC
                      48650     48660     48670     48680     48690     48700     48710     48720
Human        CACCTCCCTCCCAGACGGGGTGACTGCCGGGCGGAGACGCTCCTCACTTCCCAGACGGCGTGGCTGCCGGGCGGAGGGGC  41742
PolarBear    ---AATAGTGGTTGCCTG--TGGGTGTT-----GGGATTAACAGGAAGTACAAGG-----AATTCTCTGCAGTATTGGAC  11537
Cow          -AGCTTGTGCTCCATCCAGTCCAGCATTTCTC-ATGATGTATTCTGCATATAAGTTA---AATAAACAGGGTGACAGTAT  38811
Dog          -AAAATAATGGTGGCCTG--TGGGTGTT-----GGGATTAACAGCAAGTACAAGGT----AATTCTCTGGGGTATTGGAC  37147
BelugaWhale  -AAAATAATCATTGCTTG--TGGGTGT-------TGGGGATTAATAAGTATTAGGAC---ATTTTCTGGGGTGA-GGGAC  35637
Donkey       -AAAATATTAGTTGCCTG--TGGGTGT-------TGGGAATTAGCAGGAAGGGAA----------TCTGGGCTGATGGGC  39408
Cat          -AGAATAAGGGTTGCCTG--TGGGTGTT-----CGGATGAACAGGAAGTACAAGGAAATTAATTCTCTGTGGTGTTGGAC  41910
Ferret       ---AAT----GTTGCCTA--TGGGTTTG-----AGGTTTAACAAGAAGCACAAGGG----AATTCTCTGCAGTATTGGAC  38555
Lion         -GGAATAAGGGTTGCCTG--TGGGTGTT-----CGGATGAACAGGAAGTACAAGGGAATTAATTCTCTGTGGTGTTGGAC  39754
Vaquita      -AAAATAATCGTTGCTTG--TGGGTGT-------TGGGGATTAATAAGTATTAGGAC---ATTTTCTGGGGTGA-TGGAC  36326
Pig          -AA-------------------AGTA-------ATGGTTGCCTGTGGGTATTGGGT-----ATTGACAGAA----AGGAT  32795

Majority     ATG-TTCT---ATATCAATTAACAAAGTTGTAAAACAAATCCAAAATT-----------TGTTTGAAACAGTAGGCTACC
                      48730     48740     48750     48760     48770     48780     48790     48800
Human        TCCTCACT----TCTCAGATGGGGCGGCTGCTGGGCGGAGGGACTCCTCG--------CTTCTCAGATGGGGCGGCCGGG  41810
PolarBear    ATG-TTCC---ATATCAATTAACAAAGTTGT---ACAA-TCCAAAAT-----------TTGTTTGAAACAGTAGGGTATG  11598
Cow          ACAGCATTGACATACTCCTTGGCTCAATTTTGA-ACCAGTCCGTTGTTC--------CATGTCTGGTTCTAACTGTTGCT  38882
Dog          ATA-CCCC---ATATCAATTAACAAAGTTGT---ACAG-TCTAAAAGTGGTT-----TGAAACTGGGAAAGGCTACCACC  37214
BelugaWhale  ATGTTCTT----TGTCAATGAACAAAGCTGTAAAATAAATA-ACAATT-----------TATTTGAAATAGTAGGCTACC  35701
Donkey       ATG-TTCT---GTATCAATTAACAAAGTTGTAAAGCAGATCCAAACTT-----------TGTTTGAAACAGTAGGCTGCC  39473
Cat          ATG-TTCT---ATATGGATTTACAGAGTTTTCAAACAAGTCCAAAAT-----------TAGTTTGAAACTGTAGAATACC  41975
Ferret       ATG-TTCC---ATATCAGTTAACTAAGTTGT---GCAA-TCCAAAATAAGTTGGATTTCTGTTTGAAACAGTAGGCTATC  38627
Lion         ATG-TTCT---ATATGGATTTACAAAGTTTTCAAACAAGTCCAAAAT-----------TCGTTTGAAACTGTAGAATACC  39819
Vaquita      ATGTTCTT----TGTCAATGAACAAAGCTGTAAAATAAATA-ACAAT---------------TTGAAATAGTAGGCTACC  36386
Pig          ATA-TATT--CATATCGGTGAGCAAAGTTGTAAGATAAATCCAAAATT-----------TGTTTGAAATAGAAGGCTACC  32861
```

```
Majority     TCTGGGAAC-CATGAACTACTXGT--A-------GTTTTGTCTT------GCTGGAAT--GTGAAATATGAGAAGGGGX-
                 48810      48820      48830      48840      48850      48860      48870      48880
Human        CCGAGACGCTCCTCACCTCCCAGACGGGGTCGCGGCCGGGTAGAGGCGCTCCTCACAT--CCCAGACGGGGCGGCGGGGC  41888
PolarBear    TGTGGGAAT-CATGAACTATGCCT--A-------GTTTTATTTT------GCTGGAAT--GTGAAATAAGAGAGGGGGGG  11660
Cow          TCTTGACCTGCATACAGGGTTTGC--AGAGGCAGGTAAGGTAGT------CTGGTATTTCATTCTCTTTAAGAATTTTCC  38954
Dog          TCTGGGAAC-CATGAACTACCCC---A-------GTTTTATTTT------GCTGGAAT--GTGAAGTAAGAGAGGAGGG-  37274
BelugaWhale  TGTGGAAACCCATCAACTACTTGT--AGA-----GTTCTGTCTT------TCTGCAAT--GTAAAATGTGAGAAGGG--G  35764
Donkey       TCTGGGAAG-CATGAACTGCTTGT--A-------GCTTTGTCTT------GCTAGAGT--GTAAAATAGGAGAAGGGG--  39533
Cat          TCTGGGAAC-CATGAGCTAGTCCT--A-------GTTTTATCTT------GCTGGAAT--GTGAAATATGAGAAGGGGA-  42036
Ferret       TCTGGGAAT-CAGGAACTACCCTT--A-------GTTTTTTGTTTTTTGGGCTGGAAT--GTGAAATATGAGAGGGGGG-  38694
Lion         TCTGGGAAC-CATGAGCTAGTCCT--A-------GTTTTATCTT------GCTGAAAT--GTGAAATATGAGAAGGGGA-  39880
Vaquita      TGTGGAAACCCATCAACTACTTGT--AGA-----GTTCTGTCTT------TCTGCAAT--GTAAAATGTGAGAAGGG--G  36449
Pig          TCTGGAAAC-CATCAACTATTTGT--A-------ATTTTGTATT------TTTGCAATTTGTAAAATACGAGAAGGG--G  32923

Majority     AATGACTAAAGTTGAGTCCTGATAGGXA--GGGGTTTGGATATAGG----ATTTGATT---TGTTGTACTAAGGAGCTTG
                 48890      48900      48910      48920      48930      48940      48950      48960
Human        AGCGGCGCTCCCCACATCTCAGACGATG--GGCGGCCGGGCAGAGACGCTCCTCACTTCCTAGATGGGATGGCGGCCGGG  41966
PolarBear    AATGACTTAAGTTGAGTCCTGATAGGTGTAGGGGTTTGGATATAGG----ATTTGATT---TGTTC-----AGGAGTTTG  11728
Cow          ACAGTTTGTTGT--GATCCACATAGCCA--AAGCCTTTAGTGTAGTC---AGTGAAGCAGATGTTCTTCTAGAATTCTCT  39027
Dog          AATGACTAAAGTTGAACCCTGATAGGTT--GGGGTTTGGATATAGG----ATTTGATT---TGCTG-----AGGAGCTTG  37340
BelugaWhale  AATGAGTAAAAA--TGTTCTGGTGGGCA--GGGGTCTGGATATAGG----ATTTGATT---TGTTGTACTAAGGAGATTG  35833
Donkey       AATGACTAAAGTTGAGTCACGATAGGCA--GGGATCTGGATATGGG----ATTTGATT---TGTTATACTGAGGAGCTAG  39604
Cat          -GTGACTAAAGTTGAGTTCTGATAGGTA--GAGGTTTGCATATAAG----ATTTGATT---TGTTATGTTAGGGAGCTTG  42106
Ferret       AATGACTGAAGTTGAGTCCAGATAG------GGGTTTGGATATAGG----ATTTGATT---TGTTG-----AGGACTTTA  38756
Lion         -GTGACTAAAGTTGAGTTCTGATAGGTA--GAGGTTTGCATATAAG----ATTTGATT---TGTTATGTTAGGGATCTTG  39950
Vaquita      AATGAGTAAAAA--TGTCCTGGTGGGCA--GGGGTCTGGATATAGG----ATTTGATT---TGTTGTACTAAGGAGATTG  36518
Pig          AATGGCTACCAA--AGTCCTGTTGGGCA--GGTGTCTGGATATAG-----ATTTGATT---TGTTTTACTAAGGGGTTTG  32991
```

```
Majority     GAC----TTTACCAG-------------------------------------------ATX-------------------

                       48970     48980     48990     49000     49010     49020     49030     49040

Human        AAGAGGCGCTCCTCACTTCCTAGATGGGATGGCGGCCGGGCAGAGACCTCCTTTCTTTCCAGACTGGGCAGCCAGGCAGA  42046
PolarBear    AAC----TTTACCAG-----------------------------------------------------------------  11739
Cow          TGCC--TTTTCCGTG-------------------------------------------ATC-------------------  39043
Dog          GAC----TTTACCAG-----A-TT----------TACATATTAGAAAGGATTTTCTATTTTG--T------G-GGGGTCA  37391
BelugaWhale  GAC------TTCGCG-------------------------------------------ATG-------------------  35845
Donkey       GAC----TAGACTAA-------------------------------------------AGTTG-----------------  39620
Cat          GAC----TTTGCCAGATTTACATTTTAGAAAAGATTTTTCTTCCAT---GTGTGTTTCGTGGGGTATGGAGTGAGGGTTA  42179
Ferret       AAC----TTTACCAATTTACATTTTATTTATTTTTTTTTTTTTTAAAGATTTTATTTATTTATTTGACAGAGAGAAATCA  38832
Lion         GAC----TTTACCAGATTTACATTTTAGAAAAGATTTTTCTTCCAT---TTGTGTTTCGTGGGGTATGGAGTGAAGGTTA  40023
Vaquita      GAC------TTCACG-------------------------------------------ATG-------------------  36530
Pig          AGC----TTTACCAG-------------------------------------------ATT-------------------  33005

Majority     ----TATACACXG---TAGACAG-----------------------------------------XGAXTTTTGG------

                       49050     49060     49070     49080     49090     49100     49110     49120

Human        GGGGCTCCTCACGTTCCAGACGATGGGCGGCCAGGCAGAGACGCTCCTCACTTCCCAAACGGGGTGGCGGCTGGGCAGAG  42126
PolarBear    --------------------------------------------------------------------------------  11739
Cow          ----CAAAGAATG---TTGGCGGT------------------T---------------------TGATCCCTGG------  39071
Dog          CAGCTATATATAG-GACAGTTAAAC-ACTTTCCAATAATATGCTCCAGAGATACTAAGGTGTTCTGAATTTTAGGT----  37465
BelugaWhale  ----TTTACACT----TAGAAAG------------------------------------------GATTTTTG-------  35868
Donkey       -----AGTCACGA---TAGGCAG------------------------------------------GGATCTGGA------  39644
Cat          CTACTGTACACAG-GGTAGACA----AACACTGTACTGTCATCTCCAGAGATATTAAGGTGTTCTGAATTTTAGGT----  42250
Ferret       CAAGTAGGCAGAGAGGCAGGCAGAG-AGAGAGAGAGAGGAGGAAGCAGGCTCCCTGCTGAGCAGAGAGCCCGATGCGGGA  38911
Lion         CTACTGTACACAG-GGTAGACAGTTAAACACTGTACTGTAATCTCCAGAGATATTAAGGTGTTCTGAATTTTAGGT----  40098
Vaquita      ----TTTACACT----TAGAAAG------------------------------------------GATTTTTG-------  36553
Pig          ----TATACATTT---CAGAAAG------------------------------------------GATTTTTG-------  33029
```

```
Majority     -------TXTCAG-ATTT-G------TATTXTATXXAGAAX--AG-------------CTT---X--X---A--------
                       49130     49140     49150     49160     49170     49180     49190     49200
Human        GCTGCAATCTCGGCACTTTGGGAGGCCAAGGCAGGCGGCTGGGAGGTGGAGGTTGTAACGAGCCGAGATCACGCCACTGC  42206
PolarBear    --------------------------------------------------------------------------------  11739
Cow          -------TTCCT---CTT-G------TGTTTTCTAAATCCA--A--------------CTT------GAACA--------  39104
Dog          -------TCTTAGAATATGAAGGAAATGTTGTATCAAGAAATTAGGGG------GTCCCTGG--GTGGCGCAGCGGTTTG  37530
BelugaWhale  -------TTTCA---TTT-G------CATTTTTTAGGGG-----------------------------------------  35890
Donkey       --------TATGGGATTTGA----TTTGTTATACTGAGGAGCTAG-GA----------CTTT--ACTA------G-----  39688
Cat          -------TCTTAGAATATGGAAGAAATAATGTATCAAGAAATTAGAACTTCTGAGTTATTTG--GAAGTAGACTGGTTTG  42321
Ferret       CTCG--ATCCCAGGACCCTGAGACCATGACCTGAGCCGAAGGCAGCGGCCT--AACCACTGA--GCCACCCAGGCGCCCC  38985
Lion         -------TCTTAGAATATGGAAGAAATAATGTATCAAGAAATTAGAACTTCTGAGTTATTTG--GAAGTAGACTGGTTTG  40169
Vaquita      -------TTCCA---TTT-G------CATTTTTTAGGGA-----------------------------------------  36575
Pig          -------TTCCA---TTT-G------TATTTTGTTGGGG-G--A--------------CCG------ACGGG--------  33061

Majority     ------XTTTACXTTTXGGTGAGGATT-ATTACTCXATATXTGGAXGATAGT-------X--TTGGGATGACTXTGC--A
                       49210     49220     49230     49240     49250     49260     49270     49280
Human        ACTCCAGCCTGGGCACCATTGAGCACTGAGTGAACCAGACTCCGTCTGCAATTCCGGC-ACCTCGGGAGGCCGAGGC--T  42283
PolarBear    ------ATTTACATTTTAGAAAGGATT-TTTCTTCCATTTGTATTTTGTG-------------GGGGGTCACTAACCGTA  11799
Cow          ------TTCAACGTACTATTGAAGCCT-AGCTCGGAGAATTTTGAGCATTAC----------TTAGCTTGCATGTGA--A  39165
Dog          G----CACCTGCCTTTGGCCCAGGGCG-CGA-TCCTGGAGACCCGGGATAGAATCCC--ACGTCGGGCTCCCGGTGC--A  37600
BelugaWhale  --------------GCAGAGGGGGGTT-ACTACTGTACAT----AGGACAGT----------TTAA--------------  35927
Donkey       ------ATTTACATTTTAGAAAGGATT-TTTGTTCCATTTGTGTTTTGAGGGAAGCAGAGGAGAGTCACTACTGTACATC  39761
Cat          GGAGAAGTATTCGTTTGGGCTTAGATA-ATCACTAGATTGATGGATGATAGTCCCATTAACAAAGGGAAGATCATGGAAA  42400
Ferret       C----AATTTACATTTTAAAAAGGATT-TTTCTTCCATTTGTATTTCGTGTATGTGT--ATGTGGGGGTCACTGCTC-TA  39057
Lion         GGAGAAGTATTCATTTGGGTTTAGATA-ATCACTAGATTGATGGATGATAGTCCCATTAACAAAGGGAAGATCATGGGAA  40248
Vaquita      --------------GCAGAGGGGGGTT-ACTACTGTACAT----AGGACAGT----------TTAA--------------  36612
Pig          ------GGTGGGGTGGGGGTGGGGGTT-ACTACTGTAAAATTGGAGGACAGT----------TTAA--------------  33110
```

```
Majority     AGGAGATTAGTTATAXX--XGATTTAXACAATAXTCTGCTTCXXACATTTTAAA---GTCTCATXAGXAAAXTCXAXGTA
                     49290     49300     49310     49320     49330     49340     49350     49360
Human        GGCGGATCACTCGCGGTTAGGAGCTGCAGACCAGCCCGGCCAACACAGCGAAACCCCGTCTCCACCAAAAAAATACGAAA  42363
PolarBear    CATAGGACAGTTAAA------AGTTTTACAATAATCTGCCTCGGACATATTAAG---GTATGAT-------TTTTAGGTT  11863
Cow          ATGAGTGCAATTGTGCAG-TAGTTTGAACATTGTTTGGCATTACCTTTCTTTGG---GATTGGAATGAAAACTGACCTTT  39241
Dog          TGGAGCCTGCTTCTCC-----CTCTGCCTATGTCTCTGCCTC-----TCTCTCT---CTCTCTCTCT----CTCTGACTA  37663
BelugaWhale  ---AACTTTACAATAATC-TGCTTTAGAGACACCGAGG--TCCTGAATTTTAGA---GTCTCAGAAGTAGAA--------  35990
Donkey       AGGAAGACAGCTAAA------AACTTTAAAAAGCTCTGCTCTGGAGATACTGAAG--TCTTGAATTTTAGGGTCTTAAAA  39833
Cat          AGGAGATTGGGTTTTGGT-GGAAAGAAACAATAATTTGTTACTATCTTTTTAAAA--GTATCTTTAGGAAAGACAATGTA  42477
Ferret       CATAGAGTAGTTAAGC-----ACTTTTACAATAATCTGCTCCAGAGATATTAAG---GTGTCCTCAG----TTTTATGTT  39125
Lion         AGGAGATTGGGTTTTGGT-GGAAAGAAACAATAATTTGTTACTATCTTTTTAAAA--GTATCTTTAGGAAAGACAATGTA  40325
Vaquita      ---AACTTTACAATAATC-TGCTTTAGAGACACCGAGG--TCCTGAATTTTAGA---GTCTCAGAAGTAGAA--------  36675
Pig          --------AACTTTAATC-TGCTCTAGAGATACTGAGGTGTCCTGAATTTTAGA---GTCTCAGAAGAAAA---------  33169

Majority     CTT---XGAATATGGAGAXAATGA------TGTATCAAGAAATTAGA--AXTTCTGAGX---TTATTT-XA-AG-AGAAA
                     49370     49380     49390     49400     49410     49420     49430     49440
Human        ACCAGTCAGGCGTGGCGGCGCGTG-CCTGCAATAGCAGGCACTCGGC--AGGCTGAGGCAGGAGAATCAGGCAGGGAGGT  42440
PolarBear    CTT---AGAATATGGAGGAAATGA------TGTATCAAAAAATTAGA--ACTTCTTAG----TTATTT-------GGAAA  11921
Cow          TCT--AGTCTTGTGGCCACTGCTG------AGTTTTCTAAATTTGTTGGCCCATTGAGTGCAGCACTTGAACAGCATCAT  39313
Dog          TCA---TAAATAAATAAAAATTAA------AAAAAAAAGAAATTAGA--ACTTCTGAG----TTACTT-------GGAAA  37721
BelugaWhale  ----------TATGGAGAGAATGA------TATATGAAGAAATTAGACAATTTCTGAGT----TATTTAGA-AGTAG-AC  36048
Donkey       GT----AGAATATGGAGGAAATGA------TGTATCAAGAAACTAGAAGAATTCTAAG----TTATTT-------GGAAG  39892
Cat          CTTCAGTGACTAAAGAATGATTGAGTCTTGTGTTCTGATGGAATAAA--ATTCCTTAAAGCCTTGTTTTAACATAAGAAA  42555
Ferret       CTT---AGAATATGGAGGAAATGA------TGTATCAAGAAATTAGA--ACTTTTGAG----TTATTT-------GGAAA  39183
Lion         CTTCAGTGACTAAAGAATGATTGAGTCTTGTGTTCTGATGGAATAAA--ATTCCTTAAAGCCTTGTTTTAACATAAGAAA  40403
Vaquita      ----------TATGGAGAGAATGA------TATATGAAGAAATTAGACAATTTCTGAGT----TATTTAGA-AGTAG-AC  36733
Pig          ----------TATGGAGATGTATA------TGTATCAAGAAATTAGAAGAATTCTGAGT----CATTTGGA-AGCAG-AT  33227
```

```
Majority     TTTAGTAGATTXGAAGTAGTATTAGTTTCGGC----TTAGAATC---ACTGGGTTGATGGATXGTAGTTCCATTAACAAA
                       49450     49460     49470     49480     49490     49500     49510     49520
Human        TGCAGTGAGTC-GAGATGGCAGCAGTACAGTCCAGCTTCGGCTCGTCATCAGA--GGGAGACTGTGGAAAGAGAGGGAGA  42517
PolarBear    TAGACTGGTTTGGGAGAAGTGTTAGTTTGGGC----TTAGAATC---ACTGGGTTGATGGATGATAGTCCCATTAACAAA  11994
Cow          CTTTTAGGATGTGAAATAGCTCAGCTGGAATT----CCATCACCTCCACTAGCTTTGTTCGTAGTAATGCTTCCTAAGGC  39389
Dog          TAGATTAGTTTGGGAGAAGTATTAGTTTGGGC----TTAGAATC---ACTGGGTTGATGGATGATAGTCCCATTAACAAA  37794
BelugaWhale  TTTGGTAGA-----AGTATTAAGAGTTTCGGC----CTAGAATC---ACTGGGTTGATGGATAGTAGTTCCATTAACAAA  36116
Donkey       TAGAGTGAG--AGAAGTATTAG-AGTTTTGGC----TTAGAATC---ACTGGGTTGATGGATAATAGTCCCGTTAACAAA  39962
Cat          TTTACATGCTTTCAATTTATTTTACCTTCATTCACATTGGAAAC---CCTGAGTTTTAAGATGTTTTTTCCTTAACAAAA  42632
Ferret       TAGACTAGTTTGGGAGAAGTATTAGTTTGGGC----TTAGACTT---GATGGGTTGATGGATGATAGTTCCACTTTCAAA  39256
Lion         TTTACATGCTTTCAATTTATTT-ACCTTCATTCACATTGGAAAC---CCTGAGTTTTAAGATGTTTTTTCCTTAACAAAA  40479
Vaquita      TTTGGTAGA-----AGTATTAAGAGTTTCGGC----CTAGAATC---ACTGGGTTGATGGATAGTAGTTCCATTAACAAA  36801
Pig          TTTGGTAGAAGAGAAGTATTACGAGTTTTGGC----CTAGAATC---ACTGGGTTGATGGATAGTAGTCCCCACAAAGGG  33300

Majority     GGG---AAGAXAGTGGAXAAXGGGATTTGT-X-TTTTGAT-XGXAAAGA--X----G--ATTGTCTT-----TTAAA---
                       49530     49540     49550     49560     49570     49580     49590     49600
Human        GGG----AGACCGTGGGGAGAGGAGGGAGGGGGAGGGGGAGGGGGAGGGGGAG---G--GCTGTTCTGTTTTTTTAA---  42585
PolarBear    GGG---AAGATAGTAGAAAAGGAGATTTGT---TTTTGGT--GGAAAGA----------ATTATCTG-----TTAAA---  12048
Cow          CCACTTGACTTCGCACTCCAG--GATGTCTGGCCCTAGGTGAGTGATCCCACCATCGTGGTTATCTGGGTCATGAAAATC  39467
Dog          GGG---AAAATAGTGGAAAAGGAGATTTAT---TTTCAGT--GGAAAGA----------ATTATCTG-----TTAAA---  37848
BelugaWhale  GGG---AGCAAAGTAGAGAAAGTGATTTGTTTTTGTTGAAAAGAAAGAACAAT-CTGTTACTGTTTTCCTTTTTTTTAA-  36191
Donkey       GGA----AAAAAGTGGAAAATGAGATTTGG---TTTTGAT--GGAAAGAATAATCTGTTACTGTCTT-----TTAAA---  40025
Cat          AAG---ATGAGAGTCTGTATGTGATCTCACAGACCCCAATTTGAATATG----------ATTGCTTA-----TAGGAGCA  42694
Ferret       GGG---AAGATAGTGGAAAAGGGGATTTGT---TTTTGGT--GGAAAGA----------ATTGTCTG-----TTAAA---  39310
Lion         AAG---ATGAGAGTCTGTACATGATCTCACAGACCCCAATTTGAATATT----------ATTGCTTA-----TAAGAGCA  40541
Vaquita      GGG---AGCAAAGTAGAGAAAGTGATTTGTTTTTGTTGAAAAGAAAGAACAAT-CTGTTACTGTTTTCCTTTTTTTTAAA  36877
Pig          --A----ACAAAGTGGAGAAA--GATTTGTTTTTATTGAAAAGAGAGAACAAT-CTGTTACTATCTTACTTTTTAAAA--  33369
```

```
Majority     -XGTATG-TTTAGGAAT-XTATGTAATGT---ACTTTA-------TGACTAAAGAATGAATCXAXTC--TTTGTAGTGAT
                       49610     49620     49630     49640     49650     49660     49670     49680
Human        --CTATGCTTTAGGGATGATATATAATGT---ACTTCA-------TGACCAAGGAATCAATCTAATCTTTTTGTAATGAT  42653
PolarBear    -AGCGTG-TTTGGCAGTGATACTTAATGT---ACTTTA-------TGACCAAAGAATGAATTGAGTC---TTGGAGTGAT  12113
Cow          TTTTTTG-TATAGTTCTTCTGTGTATTCTTGCCATCTCCTCTTAATGTCTTCTGCCTTTGTTAGGTCC-TTTATTGTGCC  39545
Dog          -AGTATG-TTTAAGAAT-------AATGT---ACTTTA-------TGACTGAAGAATGAATTGAATC---TTTTGGAAAT  37906
BelugaWhale  --GTATG-TTTAGGA-----ATG--ATAATGTACTTT-------GTGACTAAAGAATGAATCAAATGTTTTTGTAGTGAT  36254
Donkey       --GTATG-CTTAGAAATAATGCTTAATGT---ACTTTA-------TGACTAAAGGATGAATCGAGTCTTTTTGTAGTGTT  40092
Cat          -AACAGC-ATTAAAGACCTTTAAAAACTTGTGATTCTAC-----TTGCCTGGAAAAATATGCCATTG---TCAGGGCACC  42764
Ferret       -AGCATG-TTTAGGAATGATACTTAATGT---ATTTTA-------TGACCAAAAAATGAATTAATTC---TTGGAATGAT  39375
Lion         -AACAGC-ATAAAAGACCTTTAAAAACTTGTGATTCTAC-----TTGCCTGGAAAAATATGCCATTG---TCAGGGTACT  40611
Vaquita      --GTATG-TTTAGGA-----ATG--ATAATGTACTTT-------GTGACTAAAGAATGAATCAAATGTTTTTGTAGTGAT  36940
Pig          --GTATG-TTTAGCA-----GTG--ATCTTGTACTTTATT----ATAACTAAAGAATGAATTGAGTG--TTTGTAGTGAT  33433

Majority     GGAAT------AAAATTCCTT-----AAAGCCTTGTTTTAACXTAAGAA-----T----ATTXAXTTTXTTTTATCTTAA
                       49690     49700     49710     49720     49730     49740     49750     49760
Human        GGCAT------AAAATTCCTT-----TAAGCCTTGTTTTAATACAAGAAG-----------TCAGTTTATTTAACCTCCA  42711
PolarBear    GGAAT------AAAATTCTTT-----AAAGCCTTGTTTTAACATAAATT---TATATGCATTCAATTTATTTTACCTTTA  12179
Cow          CGTCTTTCCATGAAATGTTCCCTTGGTATCTCTAATTTTCTTGCAGAGA---TCTCTA-GTCTTTCTCCTTCTGTTTTCC  39621
Dog          GGAAT------AAAAATCCTT-----AAAGTGTTGTTTTCACATAAGAA----ATTTACATTTAACTTATTTTATCTTTA  37971
BelugaWhale  GGAGT------AAAATTTCT------TAAAGCCTGTTTCAACCTAAGAA----------GTTTATATAGATTCAATTTAT  36312
Donkey       GAAAT------AAAATTCCCT-----AAAGCCTTATTTTAGCGTAAGAAATTTGTGTACATTCAATTTATTTTACCCTAA  40161
Cat          TGGGT------GAC-TCAGTT-----AAGCAACTGACTTCAGTTCAGGT---CATGATCTCACGGTTCGTGGGTTCTTGC  42829
Ferret       GGAAT------AATATTCCTT-----AAAGCCTTGTTTT---------------------------------------  39403
Lion         TGGGT------GAC-TCAGTT-----AAGCAACTGACTTCAGCTCAGGT---CATGACCTCACGGTTCGTGGGTTCTTGC  40676
Vaquita      GGAGT------AAAATTTCT------TAAAGCCTGTTTCAACCTAAGAA----------GTTTATATAGATTCAATTTAT  36998
Pig          GGACT------AAAATGTCT------TAAAGCTCGTTTTAACGTAAGAA----------ATTTACATGCGTGCAATTTAT  33491
```

```
Majority     TTXACATTGG-----------GTTXTAAAA--XXTXAXACCTCTTATCCTAAAAT--ACXTGAGAT---TCTGTGTATGT
                      49770     49780     49790     49800     49810     49820     49830     49840
Human        TGTACAGAGGAAAT--ACTGAGTTTTAAGA--TTTTTCTATTTTTTTTCTGACA--AAAATGAAAG---TCTGCATATAT  42782
PolarBear    TTCACAGTGGAAAC--ACTGAGTTTTAAAATTTTTT--TCCCCATAACATAAAA---AGATGGGAAGAGTCTGTTTATGT  12252
Cow          TCTATTTC-------------TTTGCATTGTTCACTTAAAAAGGCTTTCTTATTTCTCCTTGCTAT---TCTTTGGTACT  39685
Dog          TTCACAGTGGATACTCACTGAGTTTTAAGATTTTTTTTCCCCCTTAACATAGAG---AGGTGAGAT---TCTGTGTATGT  38045
BelugaWhale  TTTACCTT-------------TTTGAACAA---AGGAAACATTGAGTTTTAAAATTGTTCTGTGTT---TTTCCTAACAT  36373
Donkey       TGCACAGAGGAAAC--ACTAAGTTTTAAGA--TTTTTCTGTTTTTTTCCTAACGT-AGAATGAGAG---TCTGTATATGT  40233
Cat          CCCACATTGG-----------GTTCTA----TGCTGACAGCTCTGAGCCTAGAGCCTGCTTGGGAT---TCTGTGTCTCC  42891
Ferret       --------------------------------------TCCCCTTATCATAAAA---AGATGAGAA---TCTGTTTATGT  39439
Lion         CCCGCATTGG-----------GTTCTA----TGCTGACAGCTCTGAGCCTAGAGCCTGCTTGGGAT---TCTGTGTCTCC  40738
Vaquita      TTTACCTT-------------TTTGAACAA---AGGAAACATTGAGTTTTAAAATTGTTCTGTGTT---TTTCCTAACAT  37059
Pig          TTTATCT--------------TTTGAACAG---AGGAAACATGGAATTTTAAATTT--CTTGTGAG---TCTGTG-----  33544

Majority     -TCTCATAGAXCCCAATTTGAATATTGTTGCXTAT-AAGAGCXXATAGCATAAXXGACCTTTAAAAACTAGTGAT-----
                      49850     49860     49870     49880     49890     49900     49910     49920
Human        -TCTAAGAGACACCAATTTGAATACTGTTGCTTAT-AAGAGCAAATAGCATAAAAGACCTTTAAAAATTGGTGAT-----  42855
PolarBear    -TCTCACAGAGCCTAATTTGAATATTGTTGCATATTAAGAGCAAATTGCAAAAAAGACTTTTAAGAACTAGTGAT---TG  12328
Cow          -TTGCATTTACATAGGTAAATCTTCCCTTTTCTCT-TTTGCCTTTCACTTCTC--TTCTTTTCTCAGCTATTTGT-----  39756
Dog          -TTTCACAGACCCCAATATTAATATTGTTGCTTAT-AAGAGCAGATAGCATAAAATACCTTTAAAAACCAGTGAT---T-  38119
BelugaWhale  -AGAAAGATGAGAGTCTGTGTATGTTCAAACAGAC-CCCAATTTGAATAATAA--GACCTTTAAAAACGAGTGAC-----  36444
Donkey       -TCTAACAGACCCCAATTTGAATGCTGTTGCTTAT-AAGAGCAAATAGCATAGAAGACCTTTAAAGACTAGTGAT-----  40306
Cat          CTCTCTTTCTGCCCCTCCCCACTCATGCTGTCCCTCCCTCCCTCA----------CTCTCTCAAAAATAAATAAAACATT  42961
Ferret       -TCTCA--GAGTCTGATTTGAATTTTCTTTCATAT---AAGCAAATGGCAAAAAAGACCTTTAAGAACTAGTGAT---T-  39509
Lion         CTCTCTTTCTGCCCCTCCCCACTCATGCTGTCCCTCCCTCCCTCATTCTCTCTCTCTCTCAAAAATAAATAAAACATT  40818
Vaquita      -AGAAAGATGAGAGTCTGTGTATGTTCAAACAGAC-CCCAATTTGAATAATAA--GACCTTTAAAAACCAGTGAC-----  37130
Pig          --TATGTTCAAACAGATTTGAATACTGTTGCTTAT-AAGAGCCTATAAGACC-------TTTGAAAACTAGTGAT-----  33609
```

```
Majority     --TATACTTGCCTGGAAAAATATGCCATTGTCATCCAGGTGTCTCAAAAA-GGGACTTTTACAG------ACAGTATTAC
                              49930     49940     49950     49960     49970     49980     49990     50000
Human        --TATATTTGCCTGGAAAAATACTCCA-TGTAATTCAGATGACTCAAAAG---GACGTTGGTAG------ACAATGTTAC  42923
PolarBear    ATTATACTTGCCTGGAAAAATATGCCATTGTCATCCAGGTGTCTCAAGGA---TAGTTTTATAG------ACCATGTTAT  12399
Cow          --AAGGCCTCCTCAGATAAACATTTTGCCTTTTTGCATTTCTTTTTCTTG-GGGAAGTCCCTAGCCTTCTATTATTTTCC  39833
Dog          ---ATACTTGCCTGGAAAAATATGCCACTGTCATCCAGGTGTCTCAAAAA-GGTACTTTTACAG------ACTGTATTAC  38189
BelugaWhale  --TATACTTACCTGGACAAATATGCAGTTGTCATCAAGATGGCTCAAAAA-GGGACTTTTACAG------ACAGGATTAC  36515
Donkey       --TATACTTGCCTAGGAAAATATGCTGTTGTC---CAGATGTCTCAAAAA-GGGACTTTTACAG------ACAGTATTAC  40374
Cat          AACAAAGTTTATTAAAAAAAAATGCCATTGTCATCCAAGTGTCTCAAAAA-GGTACTTTTACAG------ACAGTATTAC  43034
Ferret       ---ACACTTGCTTCGAAAAATATGCTATTGTCATCTAAGTGTCTCAAAAAAGGTACTTTTATAG------ACCATATTAT  39580
Lion         AAAAAAGTTTATTAAAAAAAAATGCCATTTTCATCCAAGTGTCTCAAAAA-GGTACTTTTACAG------ACAGTATTAC  40891
Vaquita      --TATACTTACCTGGACAAATATGCAGTTGTCATCCAGGTGGCTCAAAAA-GGGACTTTTACAG------ACAGGATTAC  37201
Pig          --TATACTTACT-GGAAAAATATATGATTGTGATAGAGATGTCTCAAAAA-TGGACCTTTATAG------ACAGTGTTAC  33679

Majority     AAGG-AAAC-TT--TTATGGTTATCAG-------ATACAG---------AACAGGATTTCATTCTTATATTCCAGCTT--
                              50010     50020     50030     50040     50050     50060     50070     50080
Human        AAGG-ACAC-TT--TTATGGTTATCAG-------ATATAGT---------ACAGGATTTCATTTTTATGTTCCAGCTTCC  42983
PolarBear    AAAG-AAAC-TT--TTATGGTTATCAG-------ATACAG---------AACAGGATTTCATTCTTATATTCTAGCCA--  12457
Cow          AAGTCATGCATAAGTTACATTTGGCAGCTAGGCTGTGCAGCTACTCAGAAATAGAGTTACAGGCAGGTTTTACAGTAA--  39911
Dog          AAGG-AAAC-TT--TTATGGTTATCAG-------ATACAG---------AACAAGATTTCA----TATATTCCAGCTA--  38243
BelugaWhale  AAGG-ACAT-TT--TTATCATTATCAG-------ATACAA---------AACAGAATTTCATTCTTACATTCCAGCTT--  36573
Donkey       AAGG-AAAC-TT--TTGTAGTTATCAG-------ATACAGC---------ACAGGATTTTGTTCTTCTGTTCCGGCTT--  40432
Cat          AAGG-AAAC-TT--TTATGATTATCAG-------ATACAG---------AAGAGGATTTCATTCTTCTATTCCAGCTT--  43092
Ferret       AAAG-AAAC-TT--CTATGGTTATCAG-------ATACAG---------AACAGCATTTCATTGTTATATTCCAGATT--  39638
Lion         AAGG-AAAC-TT--TTATGATTATCAG-------ATACAG---------AAGAGGATTTCATTCTTCTATTCCAGCTT--  40949
Vaquita      AAGG-ACAT-TT--TTATCATTATCAG-------ATACAA---------AACAGAATTTCATTCTTACATTCCAGCTT--  37259
Pig          AAGG-ATGC-TT--TTACGGTTATCAG-------ATACAG-----A---ACAGAATTTCATTCTTATATTTCAGCTC--  33737
```

```
Majority     -----TATCCAAA-ATGTTTCTTCTAATTCACTTTACTATAGAATTCTGTT----TCTAAAAGCATCTA--------TCT
                      50090     50100     50110     50120     50130     50140     50150     50160
Human        AGCTTTATCTAAA-ATATTTCTTGTAATAGATTTTACTGTAGAATT--GTT----TCCTAAGGCATCTG--------TCT  43048
PolarBear    -----TATCCAAA-ATGTTTCCTCAATTTCACTTTACTATAGAATTTGGTT----TCTAAAAG----TA--------TCT  12515
Cow          -----ACCTAGTAAGATTTTTTTTTTGTTT------------GACATCTGTTGGAATTAACTAACAGCTAAAAAGTATTCT  39974
Dog          -----TATCCAAA-ATGCTTCCTCAAATTCACTTTACTATAGAATTCGGTT----TCTAAAAGCATCTA--------TCT  38305
BelugaWhale  -----TTCCCAAA-ATGTTTCTTCTGACTT------CACTAGAATTCTTTT----TCCAACAGCATCTA--------TCT  36629
Donkey       -----TATCCAAA-ATACTTCTTCTAATTGACCTTACTCTAGAATTTTGTT----TCCTACTGAATCTG--------ACT  40494
Cat          -----TATCCAAA-ATGTTTCCTCAAAATCACTTTACTATAGAATTCTATT----TCTAAAAATGTCTG--------T-T  43153
Ferret       -----TATCCAAA-ATGTTTCCTCAAATTTACCTTACTATAGAATTTTGTT----TCTAAAAA----AA--------TCT  39696
Lion         -----TATCCAAA-ATGTTTCCTCAAAATCACTTTACTATAGAATTCTGTT----TCTAAAAATGTCTG--------T-T  41010
Vaquita      -----TTCCCAAA-ATGTGTCTTCTGACTT------CACTAGAATTCTTTT----TCCAACAGCATCTA--------TCT  37315
Pig          -----TTTCCATG-GTGTTTCTTCTAATT------------GAATTCTGTT----TTTGACAGCATCT--A------TCT  33787

Majority     C-AACAGCCTTT-CCCTTATCTGTCCAGAC----TTTCTT---A-XTACA----TACCTGTTTTTT-GTGTCAGAAAAAA
                      50170     50180     50190     50200     50210     50220     50230     50240
Human        C-AACAGCCTTT-CCCTTATCTGTGCAGAC---TTTTCTTTCTCTGTACTTGTTGAATAGGTGCTTTTTGTCAAAGAAAA  43123
PolarBear    C-AACGGCCTTT-CCCTTATCTGTCCAGAC----TTTCTTTCTAAATATA----TACCTGGTTTTT-GTGTCAAA-----  12579
Cow          CTATCAGTATAT--ACCTACATTTCTGGTTAACTGTTCTT------TGAA-----ATTTATTTTCCCATGTAGGGAT-GG  40040
Dog          C-AACAGCCTTTTCCCTTATCTGTCCAGACAGACTTTCTTTTAAAATATA----TACCTGTTTTTTTGTGTCAAAGAAAA  38380
BelugaWhale  C-AACAGCCTTT--CCCTG----TCCAGACTTTCTTTCTC------TACA------CGTATATTTGTGTCAAAGAA--AA  36688
Donkey       T-AACAGGCTTT-CCCTCCTCTGTCCAGAC----TTTGT----G--TACAG---TACCTATTTTTG-GGGTCAAAGAAAA  40558
Cat          C-AGTGGCCTTT-CCCTCATCTGTCCAGAC----TTTCTTTCTATATACA----TGCCAGTTTTTC-GTGTCAGGAAAAA  43222
Ferret       C-AGTGGCCTTT-CCGTTATGTGTCTAGAC----TTTCTTTCTAAATATACTCCTACCTGGTTTTT-GTGTCATAAAAA-  39768
Lion         C-AATGGCCTTT-CCCTCATCTGTCCAGAC----TTTCT----ATATACA----TGCCAGTTTTTT-GTGTCAGAAAAAA  41075
Vaquita      C-AACAGCCTTT--CCCTG----TCCAGACTTTCTTTCTC------TACA------TGTATATTTGTGTCAAAGAA--AA  37374
Pig          C-AACAGCTTTT--CCCTG----TCCAGACTTCCTTTGTT------TGCA-----ACCTATTTTTGAGTCAAAGGAA-AA  33848
```

```
Majority     AAAXAATTTT----AGAAGTGAGAAAAGTTAAT-GTTATTAGAAACTTAGTCTCAGGTAAATAA--------TTTATTAA
                     50250     50260     50270     50280     50290     50300     50310     50320
Human        AAATAATTTT----ACAGGTGAAAAAATTTCAT-GTTATTAGAAACTTAATCTCAGGTAAATAAATG-----TTTAATAA  43193
PolarBear    ---------------GAAGTGAAAAAAGTTAAT-GTCATTAGAACCTAAGTCTCCGGTAAATA---------TCTAATAA  12634
Cow          AAAGA-TAAG----AAAGATAAGTATGATCATTCATTTTCCTTAGCTCAGATTCTTTGTTTTTT-------TTTTTTTTA  40108
Dog          AAATAATTTT----AATAGTGAGAAAAGTTAAT-ACTGTTAGAACCTTAGTCTCAGGTAAGTA---------TCTACTAA  38446
BelugaWhale  AAAGAATTTT----AGAAGTGAGAAAAGTTAAT-GTTATTAGAAACTTAGTCTTGGGGAAATAA-------TCTTATTCA  36756
Donkey       AAATAATTTT----AGAAGTGAGAAAAGTTAAT-GTTATTAGAAACTTTGTGTCAGGTAAATA---------TTTAATAA  40624
Cat          AAAAAATTT-----AGAAGTGAGAAAAGTTAAA-GTCATTAGAATTTTAGTCTCAGGTAACTAAGAAGTCG-TGCATTAA  43295
Ferret       ---TAATTTT----AGAAGTAACCAAAGTTAAT-GTCATTAGAACCTTAGTCTCCGGTAAATA---------TCTATTAA  39831
Lion         AAAAATTTTTTTTTAGAAGTGAGAAAAGTTAAC-GTCATTAGAATTTTAGTCTCAGGTAAATAAGAAGTCCCTGCATTAA  41154
Vaquita      AAAGAATTTT----AGAAGTGAGAAAAGTTAAT-GTTATTAGAAACTTAGTCTTGGGGAAATAA-------TCTTATTCA  37442
Pig          AAAGAATTTT----AGAAATGAGGAAAGTTAAT-GTTATTAGAAACTTAGTTTTGGGGAAATGA-------TTTTATTCA  33916

Majority     A------------------------------TCAGTXAATTAAG--------------------XT-ATTTTGTAATGC
                     50330     50340     50350     50360     50370     50380     50390     50400
Human        A------------------------------TTGATACAGTAAG--------------------GACATTTTCTAATGA  43222
PolarBear    G------------------------------TCAGTGCATTAAA--------------------ATTAATTTGTAATAC  12663
Cow          A------------------------------------AGCTAGC---------------------AGCTTGTGTAACAC  40130
Dog          G------------------------------TCAGTGCATTAAG--------------------ATTATTTTGTAATGC  38475
BelugaWhale  A------------------------------------AACTA--------------------------TTTTGTAATAA  36773
Donkey       A------------------------------CCAATGCATTAAG------------------------AC-TATATTGT  40648
Cat          GAATATTTTGTAATAGAGGCCCCTGGGTGGCTCAGTCAGTTAAGCGGCCGACTTCAGCTCAGGTCATGATCTCACGGTGC  43375
Ferret       G------------------------------TTAGTGTATTAAG--------------------ATTAAATTGTAAAGC  39860
Lion         GAATATTTTGTAATAGAGGCGCCTGGGTGGCTCAGTCAGTTAAGCAGCCGACTTCAGCTCAGGTCATGATCTCACGGTGC  41234
Vaquita      A------------------------------------AACTA--------------------------TTTTGTAATAA  37459
Pig          A------------------------------------AACTA--------------------------TTTTGTAATAA  33933
```

Majority	ATTAGTTC--	
	50410 50420 50430 50440 50450 50460 50470 50480	
Human	ATTGGTTC--	43230
PolarBear	CTTAGTTC--	12671
Cow	TATGCTTT--	40138
Dog	ATTAGTTC--	38483
BelugaWhale	ACTAGTTC--	36781
Donkey	AGTAGTTT--	40656
Cat	GTGAGTTTGAGTTCCGCGTCGGGCTCTGTGCTGACAGCTCAGAACCTGGAGCCTGTTTCAGATTCTGTGTCTCCCTCTCT	43455
Ferret	GTTCCTTC--	39868
Lion	GTGAGTTTGAGCCCCGCGTCGGGCTCTGTGCTGACAGCTCAGAACCTGGAGCCTGTTTCAGATTCTGTGTCTCCCTCTCT	41314
Vaquita	ACTAGTTC--	37467
Pig	CCTAGTTT--	33941

Majority	--	
	50490 50500 50510 50520 50530 50540 50550 50560	
Human	--	43230
PolarBear	--	12671
Cow	--	40138
Dog	--	38483
BelugaWhale	--	36781
Donkey	--	40656
Cat	CTCTCTCTCTGACCCTCCCCCGTTTATGCTCTGTCTCTCTCTGTCTCAAAAATAAATAAATGTTAAAAAAATTTTTGGGG	43535
Ferret	--	39868
Lion	CTCTCT----GACCCTCCCCCGTTCATGCTCTGTCTCTCTCTGTCTCAAAAATAAATAAATGTTAAAAAAAAT--T----	41384
Vaquita	--	37467
Pig	--	33941

```
Majority     --------------------------------------------------------------------------------
                       50570     50580     50590     50600     50610     50620     50630     50640
Human        --------------------------------------------------------------------------------  43230
PolarBear    --------------------------------------------------------------------------------  12671
Cow          --------------------------------------------------------------------------------  40138
Dog          --------------------------------------------------------------------------------  38483
BelugaWhale  --------------------------------------------------------------------------------  36781
Donkey       --------------------------------------------------------------------------------  40656
Cat          GCGCCTGGGTGGCGCAGTCGGTTTAGCGTCCGACTTCAGCCAGGTCACGATCTCGCGGTCCGGGAGTTCAAGCCCCGCAG  43615
Ferret       --------------------------------------------------------------------------------  39868
Lion         ------------------------A-------------------------------------------------------  41385
Vaquita      --------------------------------------------------------------------------------  37467
Pig          --------------------------------------------------------------------------------  33941

Majority     --------------------------------------------------------------------------------
                       50650     50660     50670     50680     50690     50700     50710     50720
Human        --------------------------------------------------------------------------------  43230
PolarBear    --------------------------------------------------------------------------------  12671
Cow          --------------------------------------------------------------------------------  40138
Dog          --------------------------------------------------------------------------------  38483
BelugaWhale  --------------------------------------------------------------------------------  36781
Donkey       --------------------------------------------------------------------------------  40656
Cat          GAGCCTGTTTCCGATTCTGTGTCTCCCTCTCTCTCTGCCCCTCCCCCGTTCATGCTCTGTCTCTCTCTGTCCCCAAAATA  43695
Ferret       --------------------------------------------------------------------------------  39868
Lion         --------------------------------------------------------------------------------  41385
Vaquita      --------------------------------------------------------------------------------  37467
Pig          --------------------------------------------------------------------------------  33941
```

```
Majority     ----------------------------------------------------------------------------CTTC
                                 50730     50740     50750     50760     50770     50780     50790     50800
Human        -------------------------------------------------------------------------CTTCAAG  43237
PolarBear    ------------------------------------------------------T----------------------TCC  12675
Cow          ----------------------------------------------------------------------------GCCT  40142
Dog          ------------------------------------------------------C----------------------TTC  38487
BelugaWhale  ----------------------------------------------------------------------------CTTC  36785
Donkey       -------------------------------------------------------------------------------C  40657
Cat          AATAAACGTTGAAAAAAAAAATTTTTTTTTAAAATAAAAAAAAAATTAAAAAAAAAGAATATTTTGTAACATATTCCTTC  43775
Ferret       ------------------------------------------------------C----------------------TTC  39872
Lion         ------------------------------------AAAAAAAAA-----------GAATATTTTGTAATATATTCCTTC  41418
Vaquita      ----------------------------------------------------------------------------CTTC  37471
Pig          ----------------------------------------------------------------------------CTTT  33945

Majority     AGAAATAAAAGTGAAAC-TTTCAAAAACA----------TAGCTCXA-ACTAGT---ATTGG-AATTGGATTTTATTGAA
                                 50810     50820     50830     50840     50850     50860     50870     50880
Human        AGATATAAAAGTGAAATATTTCAAAAATA----------CAACTGTTCCTAAGTAGCATTAG-AATTTGATTTTGTTAAA  43306
PolarBear    AGAAGTAAAAGTGAAAC-ATTAAAAAAAAACCTAACCCCTAACTCCA-ACTAGT---ATTGG-GATTGGATTTTATTGAA  12749
Cow          TGAAGTCTAACTAAAAC-TTTTAGTCATA----------CAACTCAATACCAGAGAAACAAACAACTCAATGAAAGTGGG  40211
Dog          AGAAATAAAAGTGAAAC-ATTCCAAAACAA-----C---TAGCTCCA-ACTAGT---ATTGG-GGTTGGATTAT-TTGAA  38552
BelugaWhale  AGAAGCGTAACA------TTTCAAAGACA----------CAGCTCTA-ATTAGT---ATTGG-AATTGGATTTTATTGGA  36844
Donkey       TTAAATAAAAGTGAAACATTTCAAAAACA------CAGCTAGCTTCA-ATTAGT---ATTAA-AATTGAATTTTAATGAA  40726
Cat          AGAAATAAAAGTGAAAC-ATTAAATAAA--------ACCTAGCTCTG-ACTAGT---ATTGG-ATTTGGCTTTTATTGAA  43841
Ferret       AGAAATAAAAGTGAAAC-ATTAAAAAAGAA---AACAACTAGCTCCA-GCTAGT---ACTGA-GATTGGACTTCATTGAA  39943
Lion         AGAAATAAAAGTGAAAC-ATTAAAAAAATAAA-AAAACCTAGCTCCA-ATTAGT---ATTGG-ATTTGGCTTTTATTGAA  41491
Vaquita      AGAAGCGTAACA------TTTCAAAGACA----------CAGCTCTA-ACTAGT---ATTGG-AATTGGATTTTATGGGA  37530
Pig          AGAAGTGAAACA------TTTCAAAAACA----------TAGCTCTTAACTAGT---ATTAA-AATTGGATTTTATTGAA  34005
```

```
Majority     AGGAA----TTTGTAACTATTTGTCAGTGGGGAGGAGGC------TTGACAA---TTGTAAAATATGGTCTC------TT
                     50890     50900     50910     50920     50930     50940     50950     50960
Human        ---------TTTGTAACTATTTAGCAGTGACTATGAGGC------TTGACAA---TTCTAAGGTACAGTCTCTTTAA-TT  43367
PolarBear    AGGAG----TTTGTAACTGTTTGTCAGTG--AAGGAACC------TTGACAG---TTGTAAAA---------------TT  12799
Cow          AAAAAGACCTAAACAGACATTTCTCCAAAGAAGACATACAGATAGCTAATAAACACTTGAAAAGATGCTCAA------C-  40284
Dog          AGGAG----TTTTTAACTATTTGTCTGTGGGGAGGAACC------TTGACAG---CTGTAAAA---------------TT  38604
BelugaWhale  AGGAA----TTTTTAACTCTTTGGAAGTGGGGAGGAGGC------TTGACAA---TTGTAAAATATGGTCTC------T-  36904
Donkey       AGGAA----TTTGTAAGTATTTGGCAGTGGGGAGAATAC------TTAACAG---TTGTAAGATATGGTCTCTTTAG-TT  40792
Cat          AGGAA----TAACTAGTTTTTCATTGGAG---AGGAGGT------TTGACAG---TTGTAAAATATGGTCTCTTTAATTT  43905
Ferret       AGGAG----TTTGTAACAATTTGTTAGTGGGAAGGAGCC------TTGACAA---CTGTAAAA---------------TT  39995
Lion         AGGAA----TAACTAGTTTT-CATTGGGG---AGGAGGT------TTGACAA---TTGTAAAACATGGTCTCTTTAATTT  41554
Vaquita      AGGAA----TTTTTAACTCTTTGGAAGTGGGGAGGAGGC------TTGACAA---TTGTAAAATATGGTCTC------TC  37591
Pig          AGGAA----TTTGTAACTGTTTGGCAGTGGAGAGCGTGC------TTGACAA---TTTTAAGATATGGTCTC------T-  34065

Majority     TTTGAGTCATTTCTTTATAGCTGTGGAT---A-------TGGAATAT---------CCXTTAAAGTX-X--AT-------
                     50970     50980     50990     51000     51010     51020     51030     51040
Human        TTCTAGTGATTTCTTTGTAGCTACGGA----AC------TGGAATAT---------CCCTTTAGGT-----AT-------  43416
PolarBear    CTTGAGTCATTTCTTTATAGCTGTGGAT---A-------TGGAATATT--------CCTTTAA-----------------  12844
Cow          -ATCGCTCATTATTAGAGAAATGCAGATCAAAACTACAGTGAGATATCACCTCACACCATTAAGAATGGCCATAATCAAA  40363
Dog          TTTGAGTCATTTCTTAATAGCTTTGGAT---A-------TGGAATATT--------CCTCTAAGGT-----AT-------  38654
BelugaWhale  -TGAGTCCATTTTTTTATAGGTGTGGA---AA-------TGGAATAC---------CTCTTTAAGTTAGGTAT-------  36957
Donkey       TTTGAGTTATTTCTTTGCAGCTTTGGA----A------GTGGTATAT---------CACTTTAAGTTAAGTAT-------  40846
Cat          TTTGAGTCATTTCTTCATAGCTCTGGAT---A-------TGGACTGTC--------CCTTTAAAGT-----AT-------  43955
Ferret       TTTGAGTCATTTCTTTATAGCTGTGGAT---A-------TGGAATATT--------CCTTTAAGTTAAGTAAT-------  40050
Lion         TTTGAGTCATTTCTTCATAGCTCTGGAT---A-------TGGACTGTC--------CCTTTAAAGT-----AT-------  41604
Vaquita      TTGAGTCCATTTTTTTATAGGTGTGGA---AA-------TGGAATAC---------CTCTTTAAGTTAGGTAT-------  37645
Pig          -TGGATTCATTTTTTTATAGGTGTGGA---AA-------TGGAATA---------GCCCTTTAAGTTGGGTAT-------  34118
```

```
Majority     --ACATAT-----------TTCTGCCAGTGCTGCT---------AATTGTTATTCTATAAT-----AGXAGG-ATTTAA-
                      51050     51060     51070     51080     51090     51100     51110     51120
Human        --ACATAT-----------TTCTGCCAGTGGTGA------------TTGTTACTCTGTAAT-----AGCAGG-ATTTTCT  43465
PolarBear    -------------------TTCTGCCAGTGCTGCT---------AATTGTTATTCTATAAT-----AGCAGG-ATTTAC-  12889
Cow          AAATCTACAAAAAAATAAATGCTGGAGAGGGTGTGGAGAAAAGGGAATGCTCCTGCACTGTTTGTGGGAATGTAAATTGA  40443
Dog          --ATATGT-----------TTCTGCCAGTGTTGCT---------AATTGTTATTCTGTCAT-----AG-----ACTTAT-  38701
BelugaWhale  --ACATGT-----------TTCTGCCAGTGCTAC-------------TGCTTTTCTTTAAT--------------GTCAG  36997
Donkey       --ACATAT-----------TTCTGCCAGTGCTGCT---------AATTGTTATTCTGTAGT-----AGTAGG-ATTTAT-  40897
Cat          --ACATAT-----------TTCTGCCAGTAATCCT---------GATTGTTATTCTATAAT-----AGTAGG-ATTTAAA  44007
Ferret       --ATATAT-----------TTCTGCCAGTGCTGCT---------AATTATTATTCTATAAT-----AGCAGG-ATTTAT-  40101
Lion         --ACATAT-----------TTCTGCCAGTAATTCT---------GATTGTTATTCTATAAT-----AGTAGG-ATTTAAA  41656
Vaquita      --ACATGT-----------TTCTGCCAGTGCTAC-------------TGCTTTTCTTTAAT--------------ATCAG  37685
Pig          --ATGTAT-----------TTCTGGCACTGCTGC--T-------AATTGTTACTCTGCTAT--ATTGGGATTTATTTTAT  34174

Majority     TATTGCTA----AAAGTXTGACATTTTGCTTTATGTGAA----AGAACTGAAATCATTCCATAGATTCTGTTA-CATGT-
                      51130     51140     51150     51160     51170     51180     51190     51200
Human        GAAAATTATTGCTAAACATGACATTTTGCTCTACCTGAA----AGCAGTTAAACCATTCCATAGAGTCTGGAA-CATTT-  43539
PolarBear    TATTGCTA----AAAGTGTGACATTTTGCTTTGTGTGAA----AGAACTGAAATCATTCCATAGATTCTGTT---ATGT-  12957
Cow          TACAGCCACTATGGAATATGGTATGGAGATTCCTTAGAACACTAGGAATAAAAGCACCATAT-GACCCAGCAATCCCAC-  40521
Dog          TATTGCTA----AAAGT--AATATTT----TCACGTGAA----AGAACTGAAATCATTCCAAGGATTCTGTTT-GATATA  38766
BelugaWhale  TATTATTGCTG-AAAGTATGAC-TTTTTCTCTATGTAAA----AGAACTGAAATCATTTTATAGATTCTGTTA-CATGT-  37069
Donkey       TAGTGCTA----AAAGTGTGACTTTTTGCTTTATGTGAA----AGAACTGAAATCATTCTATAGGTAATGGTG-TATGT-  40967
Cat          TATTGTTA----AAAGTGTGATATTTTGCTTTATGTGAA----AAAACTGAAATCATCCCATAGATTCTGGTA-TATGT-  44077
Ferret       TATTGCTG----AAAGTGTGACATTTTGCTTTATGTGAA----AGAACTGAAATCATTCCTCAGATTCTGAT---ATATG  40170
Lion         TATTGTTA----AAAGTGTGATATTTTGCTTTATGTGAA----AAAACTGAAATCATCCCATAGATTCTGGTA-TATGT-  41726
Vaquita      TATTACTGCTG-AAAGTATGAC-TTTTTCTCTATGTAAA----AGAACTGAAATCATTTTATAGATTCTGTTA-CATGT-  37757
Pig          TATTACTG----AAAGTATGACATTTTTATTTTTGTGAA----AGAACTGAAATCATTTATA-GATTCTGTTA-CACAT-  34243
```

```
Majority     ---TACTCAGAAXTCTT---TGAGATTTTCATTTTAA--TTTXTXTAAAGACATTTT-TATXTAATGTCATTTCATTTTG
                  51210     51220     51230     51240     51250     51260     51270     51280
Human        ---TACTTAGAACTCTT--TTAAGATTTTCATTTTAA--CTTTTACAGACATATTTTTGAGGTGATGTCATTCT-TTTTG 43611
PolarBear    ---TACTCAGAACTCTT---TGGGACTTTCATTTTA-------TGTGAAGACATTTT-TATATAATGTCATTTCATTTTG 13023
Cow          ---TCCTAGGCATATATCATGAGGAAACCAAAATTGA--AAGAGACACATGTATCCC-ATTGTTGATTGCAGCACTATTT 40595
Dog          GGTTAACCAGAACTCTT---TGGGATTTTCATTTTAACTTTTATGTAAAGACATTTT-TATATAATGTCATTTCATTTTG 38842
BelugaWhale  ---TATTCAGAATTCTC---TGAGATTTCCAA-TTAA--TTTTTACATACAGACATT-TTTGATATGTTATTTCATTTTT 37139
Donkey       ---TATTCAGAAGTTTT---TGAGATTTTCATTTTAATATTTACTTGCAGACATTTTGTATATGATGTCATTTCATTTTG 41041
Cat          ---TACTCAGAATTCTT---TGAGATTTTAATTTTTA------TGTGAAGACATTTT-TATATAATGCCATTTCATTTTG 44144
Ferret       G--TACTCAAAACTCT-----GGGGTTTCATTTTAACTTCTATGTGAAGACATTTT-TATGTAATGTCATTTCATTTTG 40242
Lion         ---TACTCAGAATTCTT---TGAGATTTTAATTTTTA------TGTGAAGACATTTT-TATATAATGCCATTTCATTTTG 41793
Vaquita      ---T---CAGAATTCTC---TGAGATTTCCAA-TTAA--TTTTTACATACAGACATT-TTTGATATGTTATTTCATTTTT 37824
Pig          ---TACTCAGAACTCTT--TTAAGATTTCCAGTTTAA--TTTTTACATATTTTTTTT-GCTCTCATGTCATTTGGTTTTT 34315

Majority     TTA-TAAT----GCTTTGAGAG--------TATTCCAGCACA------T-XXTTGAG---TTX-----XXTXT---XG--
                  51290     51300     51310     51320     51330     51340     51350     51360
Human        CTA-TAAT----GTTTTGAAAG--------TATTCCAGTAGA------TTATTTCATA-GTTTTACTATTTATAAAGGTT 43671
PolarBear    TTA-TAAT----GCTTTGCGAG--------TATTCCAGCACA-----------TGAG----------------------- 13056
Cow          ACAATAGCTAGAACATGGAAGCAACCTAGATGTTCATCAACAGAGGGATGGATAAAGAAGTTATGGTACGTGTATACGAT 40675
Dog          TTA-TAAT----GTTTTGAGAA--------TTTTCCAGCACA-----------TGAG----------------------- 38875
BelugaWhale  TTG-TAAT----ACTTTGAGAC--------TATTCCAGCACG------TTGTTTCAG---TTTGAATGTTTATGAGGGTC 37197
Donkey       TTA-TAAT----GCTTTGGATAG-------TATTCCAACACA------TTGTTTCACA-GTTTGAATGTTTATG-AGGTT 41101
Cat          CTA-TAAT----GCTTTGA-AG--------TATTCCAGCACA-----------TGAG----------------------- 44176
Ferret       TTA-TAAT----GGTTTGAGAG--------TATTTCAGCACA-----------TGAG----------------------- 40275
Lion         CTA-TAAT----GCTTTGA-AG--------TATTCCAGCACA-----------TGAG----------------------- 41825
Vaquita      TTG-TAAT----ACTTTGAGAC--------TATTCCAGCACG------TTGTTTCAG---TTT----GTTTATGAGGGTC 37878
Pig          TTAATGAT----GCTTTGAGAC----T----ATTCCAAAACA------TGGTTTAAG---TTTCAGTGTTTATAAAGGGT 34374
```

```
Majority     -XAAXA--------X-XTG-A-A-X-------------G------XXCTTCTAGTTTAGTCTAGCCTTCTA----TTTT
                       51370     51380     51390     51400     51410     51420     51430     51440
Human        TTAACAACTCATCTTCTTGCATATAAAGG----TAGTGAGA-----GCCTTATTTCATAGTGTTACTATTTATGAGGGTT  43742
PolarBear    ---------------------------------------A--------CTTCTAGTTTAGTCTAGGCTTCTG----TTTT  13085
Cow          GGAATATTACTCGGCCATGAAAAGGAACACA-----TTTGAGTCAGTTCTAATGAGGTGGGATGAACCTAGAAA--CTAT  40748
Dog          ---------------------------------------A--------CTTCTAGTTTAGTCTAGGCTTCTA----TTTT  38904
BelugaWhale  CTAACAGCATGTCCTCTTGCATAGAGGTGTAGAGATAATGTGAGCCTTCCTCTAATTTAGTGTAGCCTTCTA----TATT  37273
Donkey       CTAACAACATGTCCTGTTGCAGATAGAGAGG----TAATGAGAGCCTTCCTCCAGTTTAGCCT-----TCTA----TGTT  41168
Cat          ---------------------------------------T--------CTTCTAGTTTAGCCT-----TCTA----TTTT  44200
Ferret       ---------------------------------------A--------CTTCTAGTTTAGTCTAGGTGTCTA----TTTT  40304
Lion         ---------------------------------------T--------CTTCTAGTTCAGCCT-----TCTA----TTTT  41849
Vaquita      CTAACAGCATGTCCTCTTGCATAGAGGTGTAGAGATAATGTGAGCCTTCCTCTAATTTAGTGTAGCCTTCTA----TATT  37954
Pig          CTAACA-------GTTTTGTATAGAGGTA-------TT-GAGATCCTTCCTCTAATTTAGTGTAGCTTT-------CTCT  34432

Majority     TTTCCAGCTCATGC----GTAAGGTA-----------CTTTTGGCAAGTAGGC---TATGCAG-----CTTCTXAAAAA
                       51450     51460     51470     51480     51490     51500     51510     51520
Human        TTAACAACTTATCTTCTTGTATATAAAGGTAGTGAGAGCCTTCTCTAGGTGCCT---TCTGTGTTTTTCCAGCTCATGCC  43819
PolarBear    TTTCTAGCTCATGT----GTATGGTA-----------CTTTTGGCAAGTAGGC---TATGCGG-----CTTCTTAAAAA  13141
Cow          TATACAGCACAAA-----GTAAGTCAGAGAGAGATAAATATCTTATTCTAACGCATATATACGG-----AATCTAGAAAA  40818
Dog          CT-CTAGCTCATCT----GTAAGGTA-----------CTTTTGGCAAGTAGGC---TGTACAG-----CTGCTTAAAAA  38959
BelugaWhale  TTACCAGCTCATGC----ATAAGTTA-----------CTTTTGACAACTAGGC---TATGCAG-----CTACTCAGAAA  37329
Donkey       TTTCCAACTCATGC----ATAAGGCA-----------CTTTTGGCAACTAGAC---TATGCAG-----CTACTCAGAAA  41224
Cat          TTCCTAGCTCATGC----GTAAGGTA-----------CTTCTGGCAAGTAGGC---TGTGCAGTGCAGCTTCTTGAAAA  44261
Ferret       TT-CTAGCTCACGC----GTAAGGTG-----------CTTTTGGAAAGTGGGC---TGTGCAG-----CTTCTTAAAAA  40359
Lion         TTCCTAGCTCATGC----GTAAGGTA-----------CTTCTGGCAAGTAGGC---TGTGCAGTGCAGCTTCTTGAAAA  41910
Vaquita      TTTCCAGCTCATGC----ATAAGTTA-----------CTTTTGACAACTAGGC---TATGCAG-----CTACTCAGAAA  38010
Pig          TAGACTGCTCACAC----GTAAGTTA--------C-----TTTTGGCATAAGGC---TATGCAG-----CTACTCAGAAA  34487
```

```
Majority     -------TGCAGAGTT--------------------ACAGC---CAG-TTTTTACAGTAAACCTAAGATTT---------

                       51530     51540     51550     51560     51570     51580     51590     51600

Human        -------TAATATGCTTTTG---ACAACTACACCACACAAC---TACTTGGAAATACAAGAGTTAATAGTTACC----TT  43882
PolarBear    -------TGCAG--TT--------------------ACAGC---CAG-TTTTTACAATAAACCTAAGATTT---------  13179
Cow          GTGATATTGAAGAATTTATGTACAGGGCAAAAGTGGAGAAG---TAGACATAGAGAATAAACCTACACACA---------  40886
Dog          -------TGTAG--TT--------------------ACAGCATCTAG-TTTTTACAATAAGCCTAAGATTTATTTATTTT  39009
BelugaWhale  -------TGCAGAGTT--------------------ACAGG---CAG-CTTTTACAGTAAACCTA---------------  37363
Donkey       TG-----CAAAGAGTT--------------------ATGGC---CAG-CTTTTACAGTCAGCCTAG--------------  41261
Cat          -------TGCAGAGTT--------------------ATAGC---CAG-TTTTTACATTAAATCTAAGATTT---------  44301
Ferret       -------TGCAG--TT--------------------ACAGC---CAG-TTTTTACAGTAAACCTAAGATTT---------  40397
Lion         -------TGCAGAGTT--------------------ATAGC---CAG-TTTTTACATTAAGCCTAAGATTT---------  41950
Vaquita      -------TGCAGAGTT--------------------ACAGG---CAG-CTTTTACAGTAAACCTA---------------  38044
Pig          -------TGAAGAGCT--------------------ACAGG---TAG-CTTTTATAGTAAACCTA---------------  34521

Majority     ----------------XXXXXX-TTTTTGTGTGGTGGCTGTTGGACTT--AACTAA----ACAGCTAGCAGAATAA----

                       51610     51620     51630     51640     51650     51660     51670     51680

Human        TTACGGAAACCTGG---TGAGGCTTTTTGCGTGGTGGCTGTTGCAG--------AA----ACAGCCAACAGAATAG----  43943
PolarBear    ------------------------TTT-GTGTGGTAGCTGTTGGTCTT--GGCTTA----ACAGCTAGCAGAATAA----  13224
Cow          -TGGGGAGAGGGGAGTGGAGGGTGAGATGTATGGAAAGAGTTAACATGGAAACTTACACTACCAATTGTAAAATAGATAG  40965
Dog          GTTTTTATTTTTATTTTATTTTATTTTTTTGTGGTAGCTGTTGGTCTT--GACTAA----ACAACTGTCAGAATAA----  39079
BelugaWhale  ---------GTAAGATTTTTGTGTGTGTGTGCGGTGACTGTTGGAGTT--AACTAA------AGCTAGTAGAATAA----  37422
Donkey       ----------------TAAGATTTTTTTGTGTGGTGGCTGTTGGAGTT--AGCGAA----ACGCCTATCAGGATAA----  41315
Cat          -------------------------TTTGTGTGGTGGCTGTTGGACCT--GACTAA----ACAGCTAGCAGAATAA----  44346
Ferret       ------------------------TTTTGTGTGGTGGCTGTTAGTC-------TTA----ACAGCTAGCAGAATAA----  40438
Lion         -------------------------TTTGTGTGGTGGCTGTTGGACCT--GACTAA----ACAGCTAGCAGAATAA----  41995
Vaquita      ---------GTAAGATTTTTGTGTGTGTGTGCGGTGACTGTTGGAGTT--AACTAA------AGCTAGTAGAATAA----  38103
Pig          ---------GTAAGATTTTTTCTTATTTGTGCAGCGGCTGTTGACATTTTAACTAA----ACAGCTAGTAGAATAG-TAA  34587
```

```
Majority     C--AXXAGAATTATCTATCTGTATAT-ACCTGCATTTCTGG---TTAACTCTTCTTTGGX----AXTTTCTGTTTTCCCA
                                51690     51700     51710     51720     51730     51740     51750     51760
Human        C--AGAAGTACCACCTATCTGCACCT-ACCTGCTTTTTTTCTCTTTAACTCTTCTTTGGA----A---TTTGTTT-CTCA  44012
PolarBear    C-----GGAAGTATCTTTCTGTATATTACTTGCATTTCTGG---TTAACCCTTTGG--------AATTTCTGTTTTCCCA  13288
Cow          CCAATGGGAATTTGCTGTATGGCTTA-GGAAACTCAAATGGGCTCTGCACCAACCTTGAAGGGTGGGATGCAGAGGGAGA  41044
Dog          C-----AGAAATATCTTTCTGTATAT-ACCTGCATTTCTGG---TTAACCCTTCTTTGG-----AATTTCTATTTTCCCA  39145
BelugaWhale  C--AGAAGTATTCTCTACCTGTATAT-ACCTACGTTTCTGG---TTTAACTGTTCTTTGAAATT----TCTGTTTTCCCA  37492
Donkey       C--AGAAGTACTATCTATCTGTCTAT-ACCTGCGTTTCTGG---TTAACTCTTCGTTGG-----AATTTCTGTTTTCCCA  41384
Cat          C-----AGAAGTGTCTTTCTGTATAT-ACCTGCATTTCTGG---TTAATTCTTCTTTGG-----GTTTTCTGTTTTCCCA  44412
Ferret       C-----AGAAATCTCTCTCTGAATAT-ACCTGCATTTCTAG---TTAACCTTTTTTTTTTTTTTAATTTCTCCTTTCCCA  40509
Lion         C-----AGAAGTATCTTTCTGTATAT-ACCTGCATTTCTGG---TCAATTCTTCTTTGG-----GTTTTCTGTTTTCCCA  42061
Vaquita      C--AGAAGTATTCTCTACCTGTATAT-ACCTACTTTTCTGG---TTTAATTGTTCTTTGAAATT----TCTGTTT-CCCA  38172
Pig          C--AGAAGTATTTCCTATCTGTATAT-GCCTACTT---T----TCTGGTTAGTTCTTTGAAATT----TCTATTTTCCCA  34653

Majority     TATAGGAAXGTA---AAGAG--------TATGAGCATTCATTTTTCTTAX-CTCAGATT-----X-TTTTTTTATTTTTT
                                51770     51780     51790     51800     51810     51820     51830     51840
Human        TACAGGA--------AAGAG--------TAGCAGCATTCATTTTTTTTTT-CTTAGCTTAGAGTCTTTTTATTTTGTTTT  44075
PolarBear    GATAAGAAAGTAGTAAAGTG--------TACAGACATTCATTTTTCATAA-CTCAGA-T-------TCTTTTTATTTTTT  13351
Cow          TGGAAGGAGGTTACAAAGGGAGGGGATATGTATGTATACACCTATAGCTGATTCATGTTGAGATT-TGACAGGAAACAGC  41123
Dog          GATAGGAAAGTA---AGGTG--------TACAAACATTTATTTTTAA--A-CTCCGA-T-------TCTTTTTATGTTTT  39203
BelugaWhale  TATAGAA----TGCAAAGAG--------TATGAGCATTCATTTTTCTTAG-CTCAGATTA--ACT-TTATTTTATTTTTT  37556
Donkey       ATTAGGAAAGCA---AAAAGA-------TATGAGCATTCATTTTTCTTAG-CTCAGGTTCTTTTTATTTATTTATTTTTT  41453
Cat          GATAGGAATGTA---AAGAA--------TATGAGCATTCATTTTTCGTAA-CTCAGGTT-------TATTTTTATTTTTT  44473
Ferret       AATAGGAAAGTA---TAGTG--------TACAGACATTCATTTTTCATAA-CTCATA-T-------TCTTTTGTTTTTTT  40569
Lion         GATAGGAATGTA---AAGAA--------TATGAGCATTCATTTTTCGTAA-CTCAGGGT-------TTTTTTTATTTTTT  42122
Vaquita      TATAGAA----TGCAAAGAG--------TATGAGCATTCATTTTTCTTAG-CTCAGATTA--ACT-TTATTTTATTTTTT  38236
Pig          TGTAGGAA---TGCAAAGAG--------TATGAGCATTCATTTTTCTTAGATTCTTTTT---ATT-TTATCTGTTTTTTT  34718
```

```
Majority     ATT-TTTTCTAAAGCCAXCAGC-TTCTATAACAXTXTGCTTTGCCCT------------------G-AAGTCTAACTTAA
                       51850     51860     51870     51880     51890     51900     51910     51920
Human        TTGTTTTTCTAAAGCTAGAA---------AACACT--GCTTTGCCTT------------------G-AAGT-----CTAA  44120
PolarBear    ATT-TTTT-TAAAGCCAACAGC-TTCTATAATATTATGCTTTGCCCT------------------G-AAGTCTAACTTAA  13409
Cow          AAAATTCTGTAAAGCAATTATCCTTCAATAAAAATTAAATTAATTTAAAAAATAAAACTATTTGGGAAAATAAATTTCAA  41203
Dog          AGT-TGTC-TACAGCCAGCAGC-TTCTTTAACTTTATGCTTTGCCCT------------------G-AAATCTAACTTAA  39261
BelugaWhale  TGT-TTTTCTAAAGCCAGCAGC-TTGTATAACACTGTGCTTTGCCCA------------------G-AAGTCTAACTAAA  37615
Donkey       AT--TTTTCTAAAGCTAGCAGC-TTCTATAACACTGTGCTTTGCTCT------------------GAAGTCTTAACTAAA  41512
Cat          ATT-TTTC-TAAAGCCAACAGC-TTTTATAACAT---GCTTTGCCCT------------------G-AAGCCTTAACTAA  44528
Ferret       ATT-TTTTCTAAAGCCAACAGC-TTCTATAACGTTATGCTTTTCCCT------------------G-AAGTCTAACTTAA  40628
Lion         ATT-TTTC-TAAAGCCAACAGC-TTTTATAACATTATGCTTTGCCCT------------------G-AAGC-TTAACTAA  42179
Vaquita      TGT-TTTTCTAAAGCCAGCAGC-TTGTATAACACTGTGCTTTGCCCA------------------G-AAGTCTAACTAAA  38295
Pig          G---TTTTTTAAAGCCAACAGC-TTCTGTAACACTGTGCTTTCTTTG--C----------CTT---G-AAGTCCAACTCAC  34779

Majority     AACTGCTTGGAATTAAAACTGCTTTGCCTCTCAGTTTC-ATATTTGATAAAGGAAGTTACCAGTGC-XTTTTTATAAATG
                       51930     51940     51950     51960     51970     51980     51990     52000
Human        AACTGCTTAGAATTAAAACTTCTTTGCCTCTCAGTTTC-ATATTTGATAAAGAGAGTTAGGAAAA---CTTTTATAAATG  44196
PolarBear    AACTGCTTGGAATTGAAACTGCTTTTCTTCTCAGTTTC-ACATTTGATAAAGGATGCTACCAGTGCTTTTTTTATAAATG  13488
Cow          ACATTTTTAGGATTAAAACTGCATTGCCTTTCAGTTACCATATTTGATAAATGAAGTTACTAGTGC-TTTTTTATAAGTG  41282
Dog          AACTGCTTGGAGTTGAAACCACTTTTCTACTCAGTTTC-ACATTTGATAAAGGAAGCTAGCATTA--CTTTCTATAAATG  39338
BelugaWhale  AC-TGCTTGAGAGTAAAACTGCTTTGCCTCTCAGTTAT-AT-TTCGATAAAGGAAGTTACGAGTGC--TTTTTATAAATG  37690
Donkey       AACTGCTTGGAATTAAAACTGCTTTGCCTCTCAGTCCT-ATATTTGATAAAGGAAGTTACCAATG---CTTTTATAAATG  41588
Cat          AACTGATTGGAATTGAAACTGCTTTTCCTCTCAGTTTC-ACATTTGATAA-GGATGTTACCAATGC-TTTTTTATACATG  44605
Ferret       AATTGCTTGGAATTGAAACTGTTTTTCCTCTCAGTTTC-GCGTTTGATAATGGATACTACCAGAG--TTCTTTATTAATG  40705
Lion         AACTGATTGGAATTGAAACTGCTTTTCCTCTCAGTTTC-ACATTTGATAA-GGATGCTACCAATGC-TTTTTTATAAATG  42256
Vaquita      AC-TACTTGAGAGTAAAACTGCTTTGCCTCTCAGTTAT-AT-TTCGATAAAGGAAGTTACAAGTGC--TTTTTATAAATG  38370
Pig          AC-TGCTTGGGATTAAAACTGCTTTGCCTTTCTGTTACCATATTTGATAAAGTAAGTTACTGGTG---TCTTTATAAATG  34855
```

```
Majority     CTTGAGTCTG--AAAGACTT-GGAGAATGA---------GAATGTACTTATGTTTT---GTTTAAATAGTTTTCACTGXA
                     52010     52020     52030     52040     52050     52060     52070     52080
Human        TGTGTCTCTG--AAAGACTT-GGAGAATGA---------AGATGTACCTAAGTTTT---GTC--ACTGATTTTCATTGGA  44259
PolarBear    CTTGAGTGTG--AAAGACTTAAGAGATTGA---------GAATGTACTTATTTTTTTTAGTTTTGCTAATTTCTGCTGTA  13557
Cow          CTTGTATCTG--AAAGACTT-GGAAAACGAAAATAAGGAGAGTATACTTAAGTTTT---GTTAAA-TAATTTTCACTGAA  41355
Dog          CTGGAATGTG--AATGACTTAAGAGAATGA---------CAGTGTACTAAT-------ATTTTTTTTAGTTTCCAGTATA  39400
BelugaWhale  CTTGAGTCTG--GAAGAATT-GGAGAATGA---------GAATGTACTTATATTTT---GTTAAAATAGTTTTCACTGGA  37755
Donkey       CTTTAGTCTG--AAAGACTT-GGAGAATGA---------GAATGTGCTTATGTTTTGTTACATATAT-GTCTTCA-TAGA  41654
Cat          GTTGGGTCTG--AAAGACTTTAGAGAATGA---------GAATGTACTTATCTTT----TCTTAAATAGTTTCTACTGTA  44670
Ferret       CTTGAGTGTGTGAAAGACTTAAGAGATTGA---------GAATGTACTTAC--------ATTTTGTTAGTTTCCACTATA  40768
Lion         GTTGGGTCTG--AAAGACTTCAGAGAATGA---------GAATGTACTTATCTTT----TGTTAAATAGTTTCCACTGTA  42321
Vaquita      CTTGAGTCTG--GAAGAATT-GGAGAATGA---------GAATGTACTTATATTTT---GTTAAAATAGTTTTCACTGGA  38435
Pig          CCTTGGTCTT--TAGGACTT-GGAGAATGA---------GAGTGTACTTATGTTTT---GCT----------TTCACTGGA  34911

Majority     TAGATGAXTT-----ATAAAGTTTTGAAATGTTGCACTTCAXGTTGGTATACA--GTTGAAAGTATAGAATTAGAA-TAT
                     52090     52100     52110     52120     52130     52140     52150     52160
Human        CAGAAGTA--------TAAGGTTTTGCCGTGTCGTATTTTATATTTGTACACA--GTTGAAAGTATAA-------------  44317
PolarBear    TAAATAGGTAGAAGCATAAAGTTTTGAAATGTTGCACTTCAGGTTGATAGACA--GTTGGAAGTATAGAATTAGAA-TAT  13634
Cow          TAGATGGA-------AGTAGTTTTTGAGATTTTGCACTTCAAGTTGGTATATACAGTTGGAAGTATACAATTAGAA-TAT  41427
Dog          TAAATAAGTA------TAAAGTTCTGAAGTGTTGTACTTCAAGTTGATACACA--GTTGGAA--ATAAAATTAGAG-TAT  39469
BelugaWhale  CAGATGAAGT-----AAAAAGTTTTGAGATGTTGCACTTCAAGTTGGTATATACACTTGAAAGTATAGAACTAGAAATAT  37830
Donkey       CAGATGAAGT-----ATAAAGTTTTGAAATGT----GCTCAGGTTGGTATGCA--GTTGAAAGTATAGAATTAGAT-TAT  41722
Cat          TAAATAAGTGGAAGTATAAAGTTTTGTAATGTTGCACTTCAGGCTGATG--CA--GTGGAAAGTATAGAATTAGAA-AAT  44745
Ferret       TAAATAAGTAGAAGTATAAAGTTTTGAAATGT---ACTTCAGGTTGATAAATA--GTTGGAAGTGTAGAATTAGAA-TAT  40842
Lion         TAAATAAGTGGAAGTATAAAGTTTTGTAATGTTGCACTTCAGGCTGATG--CA--GTTGAAAGTATAGAATTAGAA-AAT  42396
Vaquita      CAGATGAAGT-----AAAAAGTTTTGAGATGTTGCACTTCAAGTTGGTATATACAGTTGAAAGTATAGAACTAGAAATAT  38510
Pig          CAGATGATCT-----AAAAAGTTTAGAGACGTTGCACTTCAAGTTGGTACATG--GTTGAAAGTATAGAGTTAGAA-TAT  34983
```

Majority	TGGTGAATGAGTGGGACTAAAATAATAGATTTACAAATTAAATGAG---AAGGTGGTTTT--------------------	
	52170 52180 52190 52200 52210 52220 52230 52240	
Human	CGGTGAATGAGTAGGACTAAAATAAT----TTACAAATTATATGAG---AAAGTAGGCCAGGTGC----TG----TGGCT	44382
PolarBear	TGGTGAATGAGTGGTACTAAAAGAATAGATTTACAAATTAAATGAG---AAGGTGGTTTT--------------------	13691
Cow	TGGTAAATGAGTGAGATTAAAATAATGGGCATATAAAAAACCTTTTTCTGGAGTGTTTATATGAGCAAATAAGGATAGGA	41507
Dog	TGGTGAATGACTGTGACTAAAATAATAGATTTATGAGTTAAATGAG---AAGGTAGTTTT--------------------	39526
BelugaWhale	TGGTGAATGAGTGGGACTAAAATAATAGACTTATAAAATAG--------AAGTTGATTTT--------------------	37882
Donkey	TGGTTAATGAGTGGGACTAAAATAATAGGTTTACGGATTAAATGAC---AAAGTGATTTT--------------------	41779
Cat	TGGTGAATGAGTGGGACCAAGGTAATAGATTTACAAATTAAATGAG---CAGGTGGTTTT--------------------	44802
Ferret	TGGTGAATGAGTG--ACTAAAATAATAGATTTACAAATTAAATGAG---AAGGTGGGTTT--------------------	40897
Lion	TGGTGAATGAGTGGGACTAAGGTAATAGATTTACAAATTAAATGAG---CAGGTGGTTTT--------------------	42453
Vaquita	TGGTGAATGAGTGGGACTAAAATAATAGACTTATAAAATAG--------AAGTTGATTTT--------------------	38562
Pig	TGGTGAATGAGTGGGACTAAAGTAATAGACTTACAAAATA--G------AAGGTGATTTT--------------------	35035

Majority	------TGAAACCCAGGAC----TGGAGTGTTT-------------ATATGAGCAAGTAAAGAT----AGGACTTTGGG-	
	52250 52260 52270 52280 52290 52300 52310 52320	
Human	CATGCCTGTATTTCAGCACT--TTGGGAGGCTG-A--------GGCAGGTGGATCACTTGAGGCC---AGGAGTTCGAGA	44448
PolarBear	------TGAGACCCAAGTAC---TGGAGTGTTC-------------ACATGAGCAAGTAAAGAT----AGGACTTTGGG-	13744
Cow	AGGACATGGAGACCAGAACAACTTGGGGCACTCTAACCATTTTGAGAGGTAAATGAGTTAGCAGGTGGAGGTATGTGGGG	41587
Dog	------TGAGACCTAAGTTT---TGGGA-GTTC-------------ATATGAGCAAGTAAAGAT----AGGACTTTGGG-	39578
BelugaWhale	------TGAAACCCCTTAC----TGGAGTGTCT----------A---TATGAGCAAGTAAAAATGGGAACGACTTGAGA-	37938
Donkey	------TGAAACCCAGGTGC---TGGAGTGTTT-------------ATATGAGCAAGTAGAGATAGGAAGGACCTTGGG-	41836
Cat	------TGAGACCCAGGT------------TTT-------------ACATGAGCAAGTAAAGAT----AGGACTTTGGG-	44846
Ferret	------TGAGACAGAAGAGC---TGGAGTGTTC-------------ACATGAGCAAGTAAAGCT----AGGACTTGGGG-	40950
Lion	------TGAGACCCAGGT------------TTT-------------ACATGAGGAAGTAAAGAT----AGGACTTTGGG-	42497
Vaquita	------TGAAACCCCTTAC----TGGAGTGTCT----------A---TATGAGCAAGTAAAAATGGGAACGACTTGAGA-	38618
Pig	------TGAAACCCCTTACTGTGTGTAGTGTT-------------GATGTGAGCAAGTAAAGATGGGAAGGACCTTGGC-	35095

```
Majority     ---GCCAGAACAGCTTGGGGAXATCCCAACCATTATTGGGAATGAATGAGTAAGCAGAGGGTT-CAAAGCAGCTXT----
                               52330     52340     52350     52360     52370     52380     52390     52400
Human        CCAGCCTGGCCAACATGGTGAAACCCCATCT-CTACTGAAAATAGAAAAATTAGCTGGGTGTGGTGGTGCACACCTGAAT  44527
PolarBear    ---GCCAAAACAGCTTGAAGAACTCCCAACTATTATTGGGAATGAATATGTAAGCAGAGGGTT-TGAAGCAGCTAT----  13816
Cow          G-GGTGTGTGTGGGCGGGTGTCACTGTAACCATTTTGAGACGTAAATGAGTTAGCAGAGGGGT-CAGTGTGTGTGTGTAT  41665
Dog          ---GCCAAT-TAGCT-GTGGACCTCCCAACTATTATTGGGAATGAGTACATAAGCAGACAGTT-CAAAGGAGCTGT----  39648
BelugaWhale  ----CCAGAACACGTTGGGG-CAGTCCAACCATTTTGGGAAGTAAATGAGTTAGCAGAGGGGT-CAAAGCAGGTAT----  38008
Donkey       ---ACCAAAACAGGTTGGGG-------A-----TCTGG-----GAATGAATAAGTAGAGGGTT-CAAAGCACAGGTAT--  41893
Cat          ---GCCAGTACAGCTTGAGGAATTCCCA-CCATTACTGGGAATGCATGCTTAA-CAGAAAGTT-CAAA-CAACTAT----  44915
Ferret       ---GCCAAAACGGCTTGAGGAACTCCCAACTATTACTGGGAATGAGTACATAAGCAGAGGGTT-TGAAGCAGCTGT----  41022
Lion         ---GTCAGTACAGCTTGAGGAATTCCCA-CCATTACTGGGAATGCGTGCTTAA-CAGAAAGTT-CAAA-CAACTAT----  42566
Vaquita      ----CCAGAACACGTTGGGG-CAGTCCAACCGTTTTGGGAAGTAAATGAGTTAGCAGAGGGGT-CAAAGCAGGTAT----  38688
Pig          ---ATCAGAATAGTTGGGG--CACTCCAGCCATTTTGG-----AAATGAGTCAGCTGAGGGTC-CAAGGCAGGTGT----  35160
```

```
Majority     -CAAATGAGTCAGGGAAGTAAGXA-------AACTAGAAATGAATTGTTAA--GGATGCTGTGAGAA---------CAGA
                               52410     52420     52430     52440     52450     52460     52470     52480
Human        TCCCAGCTACATGGGAGGCTGTGGCACGA--GAATCACTTAAACCTGGGAAGTGGAGGTTGTAGTCAA-------CCAAG  44598
PolarBear    -AAAATGAATCAGGGAAGTAAGAA-------AACTAGAAATGAATTGTTAA--GGGTGTTGTGAGAA---------CAGA  13877
Cow          GCCTGCTTGTTGTGTGCACTCAGTCATGTCCAACTTTTGTGACCCCATGGGCTGTAGCCCGCCAAGCTCCTCTATCCACA  41745
Dog          -AAAATGAATAAGGGAAGTAA----------AACTAGAAAGGAATTGTTAA--GGGTATTGTGGGAA---------CAGA  39706
BelugaWhale  -CAAATGAGTCAGGGAAGTAAGGA-------AACTAGAAGTAAGTTATTAA--GGATGCTGTGAGAA---------CAGA  38069
Donkey       -CAAATGAGTCAGGGAAGTAAGAA-------AACTAGACATGAATTGTTAG--GGGTTCTGTGAGAA---------CAGA  41954
Cat          -ACAGTGAGTCAGGGAAGTAAGAA-------AACCAGATGTGAATTGTTAG--GGGTGCTGTGAGAA---------CAGA  44976
Ferret       -AAAATGAATCAGGGAAGTAAGAA-------AACAAGAAATGAATTGTTAA--GGATG---TGAGAA---------CAGA  41080
Lion         -ACAGTGAGTCAGGGAAGTAAGAA-------AACTAGATGTGAATTGTTAG--GGGTGCTGTGAGAA---------CAGA  42627
Vaquita      -CAAATGAGTCAGGGAAGTAAGGA-------AACTAGAAGTAAGTTATTAA--GGATGCTGTGAGAA---------CAGA  38749
Pig          -CAGATGAGTCGGGGGTACGGAG---------ACTTGAAAGAAGTCATTAG--GGAGACTGTGAGGC---------CAGA  35219
```

```
Majority     GAGATTT---GGTAAGGATGAGAGGGCTCACAGT--XTTTCGGCCATGGATGGATTTAACAAATT---------ACCCAG

                       52490     52500     52510     52520     52530     52540     52550     52560

Human        ATCATGCCACTGCACTCCAGCCTGGCAATACAGCAAGACTCTGTCTTAGATAGATA-GATAGATAGATAGATAGATAGAT  44677
PolarBear    GAAATTT---GGTAAGGATGAGAGGGCTCACAGT--ATTTTGACCATGGATGGATTTAACAAATT---------ACAAAT  13943
Cow          GAATTTTCCAGGCAAGAATACTGGAATGCGTTGCCATTTCCTACTCGAGGGGATCATCCTGACCCTGGGATCAAACCCGG  41825
Dog          GACATTT---GGTAGGGATGAGAGGGCTCACAGT--ATTTTGACCATGGATGAATTTAACAAATT---------ACCCAG  39772
BelugaWhale  GAGATTT---GGTAAGGTTGAAAGGGCTCACAGC--TCTTCCGCCACTGGTGGATTTAACAAATT---------ACCCAG  38135
Donkey       GACATTC---AGTAAGGCTGAGAGGGCTCACAGT--TCTTCGGCTGCGGATGGGTTTAACAAATT---------ACCTAG  42020
Cat          GATAT-----GGTAAGTATGAGAGGGCTTACAGT--ATTTTGGCCATGGATGGATTTAACAAATT---------ACCCAG  45040
Ferret       GAGATT----GGTAAGTGTGAGAGGGCTCACAAT--ATTTTGATCCTGGATGGATTTAACAAATG---------ATAAAT  41145
Lion         GATAT-----GGTAAGTATGAGAGGGCTTACAGT--ATTTTGGCCATGGATGGATTTAACAAATT---------ACCCAG  42691
Vaquita      GAGATTT---GGTAAGGTTGAGAGGGCTCACAGC--TCTTCCGCCACAGGTGGATTTAACAAATT---------ACCCAG  38815
Pig          GAGATTT---GATAAGGATAAGAGGGCTCGTAGC--TCTTCCACCAGGGGTGGATTTAATAAATC---------ACCCAG  35285

Majority     GATCTTGGTGA----AAXTTTTATGTGAAAACT----GCAGGTAAAC-ACCAGTAAAAAGCAAAGGTCAGTCAAGG-AAA

                       52570     52580     52590     52600     52610     52620     52630     52640

Human        AGA-TAGATACTGTGAAACCCAGTGCTAGAGT-----GTTTATATTTAATAAGTAAAGATAGGTAGAGTATCGGAACAAA  44751
PolarBear    TAG-TGAG-------ACCGTTTATGTAAAAGCT----GCAAGTAAAT-ACCAGTAAAGAACAAAGGTCAGTCAAGG-AAA  14009
Cow          TCTCTTGCACCT-CCTGTATTGGCAGGCAAATTCTTTACCACTGCACCACC--TGGGAAGTAGCAGAGGGTCCAAACAGG  41902
Dog          CATCTTAGTG-----AAACTTAATATGAGAACT----GCAGGTAAAC-ACTAGTAAAGAGCAAAGGTCAGTCAAGG-AAA  39841
BelugaWhale  GATCTTGGTGAG-ACCCTTTTG-TGTGAAAACT----ACAGTTAAAAAGCCAGTAGAAGACAAAAGTCAGTCAAAG-AGA  38208
Donkey       GGTCTTATAAG----GTCACTTATGTGAAAGCT----GCAGGCAGAA-GCCAGTAGAGAACAAAGGTCAGTCAAGG-AGA  42090
Cat          AATCTTGGTGA----GACTGTTACGTGAAAACT----GT-GATAAAC-ACCAGTAAAAAGCAAAGGTCAGTCAAGG-GAA  45109
Ferret       TAG-TGAGTGAGACCATTTTTTATGTCAAAACT----GTAGGTAAAT-ACCACTGAAGAGCAAAGGTCATTCAAGG-AAA  41218
Lion         AATCTTGGTGA----GACTGTTACGTGAAAACT----GT-GATAAAC-ACCAGTAAAAAGCAAAGGTCAGTCAAGG-GAA  42760
Vaquita      GATTTTGGTGAG-ACACTTTTG-TGTGAAAACT----ACAGTTAAAAAGCCAGTAGAAGACAAAAGTCAGTCAAAG-AGA  38888
Pig          GACCTTG--------------G-TGAG----------ACCCCTGTG-------TGTGAA--AACTGCAGGTAAAGGAGA  35331
```

```
Majority      AAAXAXATGGACT--GATTAAAGGTGAAGGXA-ATGTTCACTTTAGAATTGAGGTAGAAAACCACTTTTGAXXTTGGXAG
                        52650     52660     52670     52680     52690     52700     52710     52720
Human         AACAGCATGGGA---AACTAACAATTTTGGGGAATG---AATGAGTAATCAGGATTCAAAGCAAGTATCAAA-TGAGTCG  44824
PolarBear     AAAGAGATAGAGT--GATCAAAGGTGAAG----ATGTTCTCTATAGAATTGAGGTAGAAAATGACTTCTGAGTTTGGAAG  14083
Cow           TATCAAATGAATTAGGAATTAAAGAAACTGGAAATG---AGTATTGGGGTGCTGTAAGAAA-----------------AG  41962
Dog           AAG---AGAGACT--GACGAAAGGTGAAG----ATGTTCACTATAGAATTGAGGTTGAAAACTACTTTTGAGTTTGGAAG  39912
BelugaWhale   AATGAGATGGACTGGGACTGTTCAAAAGTGAAGATGTTCACTTTAGAACTGAGGTAGAAAACTTTTAAGTTTGTATTGGA  38288
Donkey        GAAGAGATGGACT--GATCAAAGGTGAAG----ATGCTCACTTTAGATTTGAAGTAGAAAACCACTTTTGAGATTGGAAG  42164
Cat           AAA---AGAGACT--GATTAAAGGTGAATGAACATGGTCACTTTAGAATTGTGGTAGAAAACCACTTTTGAATTTGGCAG  45184
Ferret        GAA----TAGACT--GATCAGAGGTGAAG----ATGTTCACTATAGAATTGAGGTAGAAAACCACTT------------G  41276
Lion          AAA---AGAGACT--GATTAAAGGTGAATGAACATGGTCACTTTAGAATTGAGGTAGAAAACCACTTTTGAATTTGGCAG  42835
Vaquita       AAAGAGATGGACTGGGACTGTTCAAAAGTGAAGATGTTCACTTTAGAACTGAGGTAGAAAACTTTTAAGTCTATATTGGA  38968
Pig           AAACAAAGG----------TCAGTAAAAGGGAAAAG---AGG--TGGACTGGTA--------------------------  35370

Majority      AG-AAGTATTCAAATAGAAATAGTTTTTGCATTTTTTTX--ACATATTATTX----T-GAATTTGAGAAGAACATCT--T
                        52730     52740     52750     52760     52770     52780     52790     52800
Human         GGAAATTAGAAAAGTAGAAAT------TGAAGTTTTTAG--GGGTGCTATTAG---AGAAATTCAGTGAGGAAGAGA---  44890
PolarBear     AG-AAGTATACAAATAGAAATAGTTTTTACATTTTTTT--AACATATTATTTG--TTTCGCATTTGAAAGAACATCT--T  14156
Cow           AG-GTTTGGTAAGATGAGAGGG-CCCATGGCTC-TTTGAC-CCATATGGAT-----T-TAACA-----AATAACCCA--G  42025
Dog           AG-AAGTATT-AAGTAGACATAGGTTTTACATTTTTTTTTTACATACTATTTT--TTCGAATTTGAGAGGAACATCT--T  39986
BelugaWhale   AT-GTTCACTTAGAAACAAGTT-TCTGTGTCTTGTTTTGA-ATTTGTGAGT-----A-GAAGAGCTCTATTAAGCCA--A  38357
Donkey        AG-AAGTGTTCAAATAGAAACAGTTTTTACGTTTTCTTTG---TTTTGAATT----TGTGAGTAGAAGAATATCTTTGCT  42236
Cat           AGGAAGTATTCAAATAGAAACAGTTTTTGCATATTTTT---ACATATTATTTTGTTTCAAATTTGAGAAGAACATCT--T  45259
Ferret        GG-AAGTATTCAAATAGAAATAGTTTTTACTTTTTTTTTTAACATATTATT-----TTGAATTTAGGAAGAACATCT--T  41348
Lion          AG-AAGTATTCAAATAGAAACAGTTTTTGCATATTTTT---ACATACTATTTTGTTTCAAATTTGAGAAGAACATCT--T  42909
Vaquita       AT-GTTCACTTAGAAACAAGTT-TCTGTGTCTTGTTTTGA-ATTTGTGAGT-----A-GAAGAGCTCCATTAAGCCA--A  39037
Pig           -----CTGATCAAATGTGAAG-----ATGTTTA-CTTTA---------GGAT-----T-GAGGT-----AGAAGGTTT--G  35418
```

```
Majority     AAGXX--AGGCTTTAAGAAGGCTGAATAATAT--GGAAAATAAT---ATTAAAGATTTCTXCAATTTATATACAACCTAT
                      52810     52820     52830     52840     52850     52860     52870     52880
Human        GGGTTCACAGTTCCTTGTTGACTGATGGATGT------GGTGATTTAAC--AAATTACCCATGATCCTTGC-CGACACCC  44961
PolarBear    AA-----AAGCCTTAAGAAGTCTGAATAACATATGGGAAATGATGTGATTAAAGATTTCTTCAATTTATATACAACCTAT  14231
Cow          AA--------TCTTGGTGAGACTTTTTTGT-----GTTAAAACT---A----CAATATAAGCCACTAG-----AAAATAA  42080
Dog          AAGCA--AAACTTTAAGAAGGCTAAATAATACATGGAAAATGATGTGATTACAGATTTCTGCAATTT--ATACAACCTGT  40062
BelugaWhale  AGG----CTTTTTTAAGAAGGCATATATGGAA---GACAGTATA---A----AGATTTCTACAATTTATATGCAGCTTAC  38423
Donkey       AAGCAAAAGGCTTTAAGAAGACCAAATTATATATGGAAAATGGTGTAATTAATGATTTCTATAATTTATATGTAACTTAT  42316
Cat          AAGCAAAAGGCTTTAAGAAGGCTGAATAATATATGGAAAATAAT---ATTAAAGGTTTCTGCAATTTATATACAACCTGT  45336
Ferret       AAGCATAAGGCTTTAAGAAGGCTGAATAACAT--GGGAAATGTTGTGATTAGAGGTTTCTGTAATTTTTATACAACCTAT  41426
Lion         AAGCAAAAGGCTTTAAGAAGGCTGAATAATATATGGAAAATAAT---ATTAAAGATTTCTGCAATTTATATACAACCTGT  42986
Vaquita      AGG----CTTTTTTAAGAAGGCATATATGGAA---GACAGTATA---A----AGATTTCTACAATTTATATGCAGCTTAC  39103
Pig          AA--------TTTGGAAGAGG-----------------AATATT---C----AAATAGAAACAGTTT---------TTAC  35457

Majority     ATGCATGAATAAAAGCATTACTAC--AGTTGTTGCXTAA---CXTAGAAATCATTTTA-ATTATTACT--GGTTTTACAT
                      52890     52900     52910     52920     52930     52940     52950     52960
Human        ATTTATG--TAAAAATTGCAGGTAAAAGCTAGTGGAGAA---C--AAAGGTCAGTCAGCAGGAGAAAAGTGATGTACTGA  45034
PolarBear    GTACATAAATAAAAGCATTACTACATTGTTGTTGCCTTAGTTCTTCGAAATCATTTTA-ATTATTACT--GATTTTACAT  14308
Cow          AC-TGTGG---AAAATTCTGAGAG--AGTTGGGAATACC--------AGACCACCTGA-CCTGCCTCTTGAGAAATCTGT  42145
Dog          ATGCATAAATAAAAGCATTACGAC---GTTGTTGCCTTA-TTCTTAGAAATCATTTTA-ATTATGACT--GGTTTTACAT  40135
BelugaWhale  ATGCATGAGTAAAAGCATTACTAC--ATCTGTTGTTGAA--------AAACAGTCTT--TAAAATTCTTTAGTTTTACAT  38491
Donkey       GTGCATAAGTAAAAGCATTACTAT---GTTGCATACATTA--CATAAGAATTATTTTA-ATTATTACT--GGTTTTACAT  42388
Cat          ATGCATAAATAAAAGTATTACTATATTGTTGTTGCCTAAGTTCTTAGAAATCATTTTA-ATTGTTACT--GGTTTTACAT  45413
Ferret       ATGCACGAATAAAAGCATTACTACATTGTTGTTGCTTTGGTTCTTCGAAATCATTTCA-ATTATTACT--GGTTTTACAT  41503
Lion         ATGCATAAATAAAAGTATTACTATATTGTTGTTGCCTAAGTTCTTAGAAATCATTTTA-ATTGTTACT--GGTTTTACAT  43063
Vaquita      ATGCATGAGTAAAAGCATTACTAA--ATCTGTTGTTGAA--------AAACAGTCTT--TAAAATTCTTTAGTTTTACAT  39171
Pig          AT-CTTG--------TCTTGA-----ATTTGTGAGTAGA--------AAAACATCC-------------------TCCCT  35496
```

```
Majority     ACACAAACCAAAAGTTTCTGGATTTACXTGGG----GAGTGGAAGGGAAAAGAT-XATG-GAAAGAAGTCCAAA-TTATT

             52970 52980 52990 53000 53010 53020 53030 53040

Human        TCAGAGGTGAAGATGCTGGCTTTAGAATTGAG------GTGAAAAACTGGTTTTGAGTTTGGAAGAGAAGTATT---CAA  45105
PolarBear    TCACAAACCAAAAGTTTGTGCGTTTACCTGGG----GAGCGGAAGGGAAAAGAT-CATG-GAAAGAAGTCCAAA-TTATT  14381
Cow          ATGCAGGTCAAGAAGCAACAGTTAGAACTGGACATGGAACAACAGACTGGTTCCAAATA-GGAAAAGGAGTACG-TCAAG  42223
Dog          TCACAAACCAAAAGTTTCTGGGTTTACCTCAG----GACTAGAAGGGAAAGATTATATA-GAAAGAAGTCCAAAATTATG  40210
BelugaWhale  ACCTAAA-CCAAAGTTTCTGTCTGGAATTACCTG-GGAAGTGTAAGGGAAAGGATTGTG-GAAAGAAGTCCAAA-TTATA  38567
Donkey       AACCAAACCAAAAGTTTCTGGACTTACCTGGG----GAGTGGAAGGGAAAGAAC-AGCA-GAAAGAAGTCCAAA-TTATT  42461
Cat          ACACAAACCAGAAGTTTCTGGATTTACTTGAG----GAGTGGAAGGGAAAAGAT-CATG-GAAAGAGGTTCAAA----TT  45483
Ferret       TCACATACCAAAAGTTTGTGGATTTACCTGGG----GAGTGGAAGGGAAAAGAT-CTTG-GAAAGAAGTCCAAG-TTATT  41576
Lion         ACACAAACCAAAAGTTTCTGGATTTACTTGAG----GAGTGGAAGGGAAAAGAT-CATG-GAAAGAGGTTCAAA----TT  43133
Vaquita      ACCTAAA-CCAAAGTTTCTGTCTGGAATTACCTG-GGAAGTGTAAGGGAAAGGATTGTG-GAAAGAAGTCCAAA-TTATA  39247
Pig          ATGCA----AAAAGGCTTTCTTAAGAA--GGA-----------AAACTAGT---ATAT--GGAAAAGGTGTAAT-TGGAG  35553

Majority     GTACTTTGTCTTCTGAAGAATCATAGATCTGTAACCCAGAGAGACCCAATCTTCCTTACTTCTCTGATAGGGTAATAAAT

             53050 53060 53070 53080 53090 53100 53110 53120

Human        GTAGAAACCATTTTAAAATTTTATTTGCTTTGAATCCGTGATTACATGAACATCTTCCCTAAGC--AAGAGGCTTTGAAA  45183
PolarBear    GTACTTTGTCTTCTGAAGAATCACAGATCTGTAACCCAGAGAGACCCAATCTTCCTTACTTCTT-----GGGTAATAAAT  14456
Cow          GCTGTATATTGTC------ATCCTGCTTATTTAATTTAT----A--------TGCAGAGTAC---ATCATGAGAAATGCT  42282
Dog          GTACTTTGTGTTCTGAAGAATCACAGATCT--AACCTAGAGAGACCCAA---TCTTTACTTCTCTGATAGGATAATAAAT  40285
BelugaWhale  GTACTTTGTCTTCTGAAGAATCATAGATCTGTAACCCAGAGAGACCCCATCTTCCTTACTTCTCTAATAGGGTCATAAAT  38647
Donkey       GTATTTTGTCTTCTGAAAAATCATAGATCTGTAACCCAGAGAGACCCAGTCTTCCTTACTTCTCTGAGAGGGTGATAAAT  42541
Cat          ATTCTTTGCCTT-TGAAGAATCCTAGATCTGTAACCCAGAGAGACCCAATCTTCCTTACTTCTCTGATAGGGTAATAAAT  45562
Ferret       GTACTTTGTCTTCTGAGAGATCACAGATCTATAACCCAGAGACACCCTGACTTCCTTACTTCTCTGATAGGCTAATAAAT  41656
Lion         ATTCTTTGCCTT-TGAAGAATCCTAGATCTGTAACCCAGAGAGACCCAATCTTCCTTACTTCTCTGATAGGGTAATAAAT  43212
Vaquita      GTACTTTGTCTTCTGAAGAATCATAGATCTGTAACCCAGAGAGACCCCATCTTCCTTACTTCTCTAATAGGGTCATAAAT  39327
Pig          -----ATTTCTCC------AACTTG--TATGCAACCCAT----A--------TGCCTA-------AGTAAAAGCATTACT  35601
```

```
Majority     GGCCTATATGTTTCATAAGCTAA---GGGCTATAXATTTTCTTGTGTCCTTTTTCXTTTCTAGGGXTGGAGTGTTTTT--
                            53130     53140     53150     53160     53170     53180     53190     53200
Human        GAGTTGAATAATGTATGGAAAATGGTGTGCTTAAAGATTTTTTACATTCATA-----TACAATGAATAAAA--TTTAT--  45254
PolarBear    GGCCTACTTGTTTCATAAGCTAA---GGGCTGTACATTTTCTTGTGTCCTTTTTCTTTTCCAGGGATGGAGTGTTTTT--  14531
Cow          GGGCTGGATGAAGCACAAGCTGG-----AATAAAGATTGCTG-GGAGAAATA-----TC--AATAACCTCAGAT------  42343
Dog          GGCCTATGTGTTTGATAAGCTAA---GGGCTATACATTTTCTTGTGTCCTTTTTC------AGGGCTGGAGTTTTTTT--  40354
BelugaWhale  GGCCTACATGTTTCATAAGTTGA----GACCACACTTTGTTTTATTTATTTATTTATTTTTGGCTGCGTTGGGTCTTCAT  38723
Donkey       GGCCTAGATGTTTTATAAGCTGA---GGACCACATGTTTTCTTATGCCTTTTTCTTTTCCAGGCCTGGGGTGTTTTT--  42616
Cat          GGCCTATGTGTTTTATAAGCTAA---GGGCTATATATTTTCTTGTGCCCTTTTTCTTTTCTAGGGCTGGAGTGTTTTT--  45637
Ferret       GGCCTATGTGTTTCATGAACTAATACAGGCTATATATTTTCTTGTGTCCTTTTTCTTTTCCAGGTATGGAGTGTTTTTCT  41736
Lion         GGCATATGTGTTTTATAAGCTAA---GGGCTATATATTTTCCTGTGCCCATTTTCTTTTCTAGGGCTGGAGTGTTTTT--  43287
Vaquita      AGCCTACATGTTTCATAAGTTGA----GGCCACACTTTGTTTTATTTATTTA----TTTTTGGCTGCGTTGGGTCTTCAT  39399
Pig          CCATTCGTTGTTGCATAAATTC-------TTAAA-ATTATT----TTAATTA-----TT--ACTAGTCT-----------  35651

Majority     --------------------------------------------------------------------------------
                            53210     53220     53230     53240     53250     53260     53270     53280
Human        --------------------------------------------------------------------------------  45254
PolarBear    --------------------------------------------------------------------------------  14531
Cow          ---------ATGCAGATGA-------------CACCACGCTTATGGCAGAA-----A-GCGAAAAAGA------------  42383
Dog          --------------------------------------------------------------------------------  40354
BelugaWhale  TGCT---GCACGCGGGCTTTCTCT-AG--TTGCGGCGAGCAAGGGGCTACTCTTCGTTGCAGTACACAGGC------TT-  38790
Donkey       --------------------------------------------------------------------------------  42616
Cat          --------------------------------------------------------------------------------  45637
Ferret       TTTTTTAATTTTTAAATTTTTTTTTAATTTTTTTATAAACATAATGTATTTTTATCCCCAGGGGTACAGTCTTTGAATTG  41816
Lion         --------------------------------------------------------------------------------  43287
Vaquita      TGCT---GCACGCGGGCTT--T--------TGCGGCGAGCAAGGGGCTACTCTTCGTTGCAGTACACAGGC------TT-  39459
Pig          ----------TGCAAAC-----------------CCAAACCAAAAGTTGCA-----T-G----ATTTA------------  35682
```

```
Majority     --------------------------------------ATATATGAAAATATGCTTTAGTXA-TATGATTXXAAAXGTCX
                      53290     53300     53310     53320     53330     53340     53350     53360
Human        --------------------------------------ATGCATAAATAAAAGCATTACCTCATGCCTTCTTAA----CT  45292
PolarBear    --------------------------------------ATATAGGAAAGTGTGCTT-AGTCG-TATGATT----------  14561
Cow          ---------------ACTAAAGAGCCT------CT-TGATGAAAGTGAAAGAGG--AGAGTGAAAAAGTT--AAATCTCA  42437
Dog          --------------------------------------ATGTATGAAAGTATGCTT-AGTCA-TATGATTTAAAAAGTCC  40394
BelugaWhale  ------------CTCATTGGACGGCTT------CTCTTATTGCAGAGCATGGGCTCTAGGTACGCAGGCTT-CAGTAGCT  38851
Donkey       --------------------------------------ATATATGAAATTAGGCTT-AGTTG-TGTGATTAAAGAAATC-  42655
Cat          --------------------------------------ATAGATGAAAGTATGCTTTAGTCG-TATGATTAAAGAAGTCC  45678
Ferret       CCAGCAGGTTTACACACTTCACAGCATGGAGTGTTTTTATATATGAAAATATGCTT-AGTCA-TATGATTAAAAA-GTCC  41893
Lion         --------------------------------------ATATATGAAAGTATGCTTTAGTCAATATGATTAAAGAAGTCC  43329
Vaquita      ------------CTCATTGGACGGCTT------CTCTTATTGCAGAGCATGGGCTCTAGGTACGCAGGCTT-CAGTAGCT  39520
Pig          ---------------CCTGGGGA---------------ATGGAAGGGAAAGGGT--GGCGGAAAGAAGTCC-AAATGATT  35729

Majority     GTATTTXCCAATTTCATGTT-TCAT-------------------------------------------------------
                      53370     53380     53390     53400     53410     53420     53430     53440
Human        GAACATAAACATTTGAATTATTACT-------------------------------------------------------  45317
PolarBear    --------------------------------------------------------------------------------  14561
Cow          ACATTCAGAAAACTAAGA---TCAT-------------------------------------------------------  42459
Dog          TCATTTCCCAATTTCATGTTCTCAT-------------------------------------------------------  40419
BelugaWhale  GTGGCATGCGGGCTCAGTAG-TTGT-------------------------------------------------------  38875
Donkey       --ATTTTCCAGTTTTGCATTCTCATAATGTCGTTATTTTAAATGAAGATTTTCATCATAT--------------------  42713
Cat          TTATTTCCCAATTTCATGTTCTCAT-------------------------------------------------------  45703
Ferret       ACATTCCCTCATTTTATGTTTTCTTTTTTGGTTTTATTTATCTATTTTTTTATTAGCATATGTATTATTTGTTTCAGGA  41973
Lion         TTATTTCCCAATTTCATGTTCTCAT-------------------------------------------------------  43354
Vaquita      GTGGCATGCGGGCTCAGTAG-TTGT-------------------------------------------------------  39544
Pig          GTACTTTGTCTTCTGAA------A--------------------------------------------------------  35747
```

Majority	--	
	53450 53460 53470 53480 53490 53500 53510 53520	
Human	--	45317
PolarBear	--	14561
Cow	--	42459
Dog	--	40419
BelugaWhale	--	38875
Donkey	--	42713
Cat	--	45703
Ferret	GTACAGGTCTGTGATTCATCAGTCTTACACAATTCACAGCGCTCACCATAGCACATACCCTTCCCAGTGTCCATCCCCTA	42053
Lion	--	43354
Vaquita	--	39544
Pig	--	35747

Majority	--	
	53530 53540 53550 53560 53570 53580 53590 53600	
Human	--	45317
PolarBear	--	14561
Cow	--	42459
Dog	--	40419
BelugaWhale	--	38875
Donkey	--	42713
Cat	--	45703
Ferret	AGCTCCCCATCCCTCCCACCACCCTCCACCCCTGAGATTGAGTCTCTTACGGTTTGTCTCCCCCTCTGGTTTTGTCTTGT	42133
Lion	--	43354
Vaquita	--	39544
Pig	--	35747

```
Majority     --------------------------------------------------------------------------------

                      53610     53620     53630     53640     53650     53660     53670     53680

Human        --------------------------------------------------------------------------------  45317
PolarBear    --------------------------------------------------------------------------------  14561
Cow          --------------------------------------------------------------------------------  42459
Dog          --------------------------------------------------------------------------------  40419
BelugaWhale  --------------------------------------------------------------------------------  38875
Donkey       ----TTAGCTATATGTGAATATTTAGTAGCCACCCTCTCTGAAGCTCTTAACTGAGGGAGTATCAGAGTGTGAAGAGAGT  42789
Cat          ------------------------------------------------------------------A-------------  45704
Ferret       TTCATTTTTCCTTTCTTCCTCTATGCTCCTCTGCCTTGTTTCTCAAATTCCACATTGATGTCTGTGAGATCTAATGTTAA  42213
Lion         ------------------------------------------------------------------A-------------  43355
Vaquita      --------------------------------------------------------------------------------  39544
Pig          --------------------------------------------------------------------------------  35747

Majority     --------------------------------------------------------------------------------

                      53690     53700     53710     53720     53730     53740     53750     53760

Human        --------------------------------------------------------------------------------  45317
PolarBear    --------------------------------------------------------------------------------  14561
Cow          --------------------------------------------------------------------------------  42459
Dog          --------------------------------------------------------------------------------  40419
BelugaWhale  --------------------------------------------------------------------------------  38875
Donkey       GTAAATTTTGGTACTTACATGGTCTTTGAGAGGAAAGGACATGTCTTTGACAGGAAAGTGTATAATATGAATTTTGTTTT  42869
Cat          -------------------------------------------------------G------------------------  45705
Ferret       TTGTCTTTCTTTGATTGACTTATTTCATTTATCATAATACCCTGTAGTTCCACCCATGTTGTTGCAAATGGCAAGATTTC  42293
Lion         -------------------------------------------------------G------------------------  43356
Vaquita      --------------------------------------------------------------------------------  39544
Pig          --------------------------------------------------------------------------------  35747
```

Majority	--	
	53770 53780 53790 53800 53810 53820 53830 53840	
Human	--	45317
PolarBear	--	14561
Cow	--	42459
Dog	--	40419
BelugaWhale	--	38875
Donkey	GGGATTCTCCTATAGTAAATTAAGAGCACAGGCTGTGGAGTCATATACTTCCTGAGTTTTAATCCTTTTTCTGCCACTTC	42949
Cat	--TCTC----------	45709
Ferret	ATTTTTTTGATGGTTGCATAACATTCTTTTTCATCTATCTATCTATCTCACATCTTCTTCATCCATTCATCTGTTGATGG	42373
Lion	--TCTC----------	43360
Vaquita	--	39544
Pig	--	35747

Majority	--	
	53850 53860 53870 53880 53890 53900 53910 53920	
Human	---G	45318
PolarBear	--	14561
Cow	--	42459
Dog	--	40419
BelugaWhale	--	38875
Donkey	CCGTCTTCACCACTTAGTAACTGTCTGACCTTGGCAACTTCACTAGCTGTCATCATAAATGGGATTGATGGGTCATGATA	43029
Cat	--	45709
Ferret	ATATCTAGGCTCTTTCCATAGTTTGGCTATTGTGGACATTGCTGCTGTAAACTTTGGGGTGCACTTGCCCCTTCTGATCA	42453
Lion	--	43360
Vaquita	--	39544
Pig	--	35747

Majority	---XGCATXC---------AATCTTAAGXG	
	53930 53940 53950 53960 53970 53980 53990 54000	
Human	---GTTTTACACGCCCAAACAAAAGTTCTGGGTT	45349
PolarBear	--	14561
Cow	---GTCATCC---------AGTCCCATCAC	42477
Dog	--	40419
BelugaWhale	---GGCTCGC---------GAGCTCTAGCG	38893
Donkey	ATAGCATCTTAGAGGTGTAAAGTAGTTAACTTCCAGAGAATGTTAAATATCTATGCAAAAGAAAAGTAGCATCTTTTCTG	43109
Cat	---ATTATCTAAACTGAA-AACTTTAATAA	45735
Ferret	CTACATCGTATCTTTGGAGTAAATACCCAGTAGTGCTGGATGAGAGATAGCTCTGTTTTCAACTTGAGGAATCTCCATGC	42533
Lion	---ATTATCTAAAATGCA-AACTTTAATAA	43386
Vaquita	---GGCTCGC---------GAGCTCTAGCG	39562
Pig	---AATCCTAGGTT	35758

Majority	TACTTXTCAAATAGAT-X---------GTA----------AXXATTAGCTGXCTTTCCTTTCTT----------------	
	54010 54020 54030 54040 54050 54060 54070 54080	
Human	TACTGGAGGAATGGA-------------------------AGGGAAAGATCTCTTTCATTCCCCC--A------------	45390
PolarBear	--	14561
Cow	TTCATGGGAAATAGATGG---------GCA----------AACAGTGGCTGACTTTATTTTTCTG---------------	42523
Dog	--	40419
BelugaWhale	TGCAGGTTCAGTAGCTGTGGT------GTA----------AGGCTTAGCTGCTCCGCGGCATGTG---------------	38942
Donkey	TATTTCTCCAAAAAACCAGGTTGTCT--TCTTTGACTGTTAAAACTATCCTACCTTCCTTGTTATTCAGCTTTTTTA---	43184
Cat	TATTTCACACCAAAATATGAATATTTAGTACTAAAGTATGAATATTTAGTAGCTACCCCTTCTA----------------	45799
Ferret	TGTTTTTCAGAGTGGCTGCATCAGCTTGCATTCCCACAACAGTATAGGAGGGTTCTCCTTTCTCTGCATCCTCGTCAACA	42613
Lion	TATTTCACACCAAAATATAAATATTTAGTACTAAAGTATGAATATTTAGTAGCTACCCCTTCTA----------------	43450
Vaquita	TGCAGGTTCAGTAGCTGTGGT------GTA----------AGGCTTAGCTGCTCCGTGGCATGTG---------------	39611
Pig	TTCAACCCAGAGAGA--------------------------------GCCCATCTTCCTTACTT----------------	35790

```
Majority     ---------------------------AGX--TCTCAACTAACAGX-G-X-XAACACATXGTGX-----CTGTATXTTT-

                       54090      54100      54110      54120      54130      54140      54150      54160

Human        ---------------------------GTAAACCTTTCCCATTCAGGATCATGGGAAGATGCTGAAGAATTGTAGAACTG  45443
PolarBear    --------------------------------------------------------------------------------  14561
Cow          ---------------------------AG----CTCCAAAATCAC-------TGCAGATGGTGA-----CTGCAGCCATG  42560
Dog          ---------------------------------------------------------A--------------------T-  40421
BelugaWhale  ---------------------------GGA--TCTCGCGGACCAGG-GCTCGAACCCATGTCCC-----CTGTATTGGCA  38987
Donkey       ----------------CTTCTGCTTTTACCACCCACTGTCCAGAAATTATCTAACACAAATCCTTAACAATAAACTATTC  43248
Cat          ---------------------------AAC--TATTAACTAACTGAGGGAATATCAGAGTGTGACCAAAGTGTAAGTTTT  45850
Ferret       TCTGTCATTTCCTGACTTGCTAATTGTAGCCATTTTGACTGGTGTGAGGTGGATCTCATTGTGGTTTTGATTTGTATTTC  42693
Lion         ---------------------------AAC--TATTAACTAACTGAGGGAATATCAGAGTGTGACAAAAGTGTAAGTTTT  43501
Vaquita      ---------------------------GGA--TCTCCCGGACCAGG-GCTCGAACCCATGTCCC-----CTGTATTGGCA  39656
Pig          ---------------------------------CTCAAATAGGAA-------AATAAATGGT-------CTGTATGTTT-  35822

Majority     X---------------------------------XXATTAXAAACAGATGXGCTCCTXGGXAAGATATATGATGACAXAT

                       54170      54180      54190      54200      54210      54220      54230      54240

Human        T-----------------------------------AACACAGGCAGACCTAATGCTTCATAAGCTA-ATGTTTCCATTT  45487
PolarBear    --------------------------------------------------------------------------------  14561
Cow          ----------------------------------AAATTAAAAGATGATG-GCCCCTTGG-AAGACAAGTTATGACCAAC  42604
Dog          ---------------------------------------------------------------------T----------  40422
BelugaWhale  GGC-------------------------------AGATTCTAAACCACTGCGCCACCAGGGAAGCCCCAAGGCCACACAT  39036
Donkey       TATTCT------------------CTTCATAATAGGTTTAGGGAGACTTTACCTTTTGGAAATCATATTCTAAAAGTCAT  43310
Cat          GATGTT---------------ACATGGTCTTTGGAAGAAAAGGACAGATCCTCTCTTGGCAATTGTGTATAATGCAATAT  45915
Ferret       CATGATGCCGAGTGATGTCGAGCACTTTTTCATGTGTTAGCCATCTGATGTCTTCTTTGGAGAAATGTCTGTTCATTCTT  42773
Lion         GATGTT---------------ACATGGTCTTTGGAAGAAAAGGACAGATCCTATCTTGGCAATTGTGTATAATGCAATAT  43566
Vaquita      GGC-------------------------------GGATTCTAAACCACTGCGCCACCAGGGAAGCCCCAAGGCCACACAT  39705
Pig          --------------------------------------CATAAGCTGAGG-GCCAC--------ACATTTTGTGCCTTTT  35855
```

```
Majority     TTGGAXX------C-T---ATTTTXCAGTGTTXGGGTACT---------------TTTATAXATGAAAGTCAGTXTAGTT
                      54250     54260     54270     54280     54290     54300     54310     54320
Human        TTGGACC------------CTTTTTCGGTGTTGCTGGAGTGTG------------TTTATATTTGAAAATAGGCTTTGTT  45543
PolarBear    --------------------------------------------------------------------------------  14561
Cow          CTAGACA-----AC-----ATATTAAAAAGCGGAGACATTA-------------CTTTGCCAGCAAAGGTCTGTCTAGTC  42661
Dog          ------------------------------------------------------------------------------C-  40423
BelugaWhale  TCAGTGT-----CCTTTCTCTTTTCCAGGGTTGGGGTACT---------------TTTATATATGAAAGTCAGCTTAGTT  39096
Donkey       TTGAAAAGAGAAGCCT----TTTTTCACCCTATATATACAGTGATAGGTGGTATCTTTATTATGGTGCTTTTTTCTCGAC  43386
Cat          TTGTCTTTGGGATCCTTGTATAGTGTGGTGGGTTAAGACCACAGGCCATGGAGTCCTATTTCCTGAGTTTCAGTCTTTTT  45995
Ferret       TTGCCCATTTCTTCCTTGGATTATTTGTTCTTTGGGTATTGAGTTCTATAAGGTCTTTGTAGATTTTGGATACTAGCCCT  42853
Lion         TTGTCTTTGGGATCCTTGTATAGTGTGGTGGGTTAAGACCACAGGCCATGGAGTCCTATTTCCTGAGTTTCAGTCTTTTT  43646
Vaquita      TCAGTGC-----CCTTTCTCTTTTCCAGGGTTCGGGTACT---------------TTTATATATGAAAGTCAGCTTAGTT  39765
Pig          TTAAA--------------GTTTTCCAAGGTTGGAGTGTT---------------TTTGTAAATGAAAGTTAGCTTAGTT  35906

Majority     XTATTTXXAGA---------AXTCATCATTTCTCAATTTGAXAG------------TTCA--CATA--------------
                      54330     54340     54350     54360     54370     54380     54390     54400
Human        ATATTATTAAAGGAGCCCCCATTCCCAGTTTCACATTCTCATAG-----------TATCATTTAT---------------  45597
PolarBear    --------------------------------------------------------------------------------  14561
Cow          AAAGATTTGGTT---TTTCCATTAGTCACGTATGGATGTGAGAG------------TTGGACCATAA-------------  42713
Dog          -------------------------------------TC-----------------------------------------  40425
BelugaWhale  TTATTTAAAGA---------AATCATCATTTCTCAATTTTACA-------------TTCT--CATA--------------  39138
Donkey       CTATTCCCAGAAGGAAAAGGACAGACTAAATTAGAA-AAAACTGAAGTTTGCTGTTTTGAGTTTCCTGATACCAAAACCT  43465
Cat          CTATTACTTCTTGTCTTTTACTACTTAATGTCTGACCTTGGTCGACTTAACTAGCTCTTATCCATAAATGGGATTGTACT  46075
Ferret       TTATCTGATATGTCATTTGCAAATATCTTCTCCCATTCTGTTGGTTGTCTTTGGTTTTGTTGACTGTTTCCTTTGCTATG  42933
Lion         CTATTACTTCCTGTCTTTTACTACTTAATGTCTGACCTTGGTCGACTTAACCAGCTCTTATCCATAAATGGGATTGTACT  43726
Vaquita      TTATTTAAAGA---------AATCATCATTTCTCAATTTTACA-------------TTCT--CATA--------------  39807
Pig          ATATTTAAAGA---------AATCATCGTTTCCCAATTTTACA-------------CTCG--CATAA-------------  35949
```

```
Majority     ATAAAXTT-----G--------------------XT----------------AGACTTACXTAATATTTAGGACTA
                       54410     54420     54430     54440     54450     54460     54470     54480
Human        TTAAAATG----A---------------------------------------AGACTTTTATAATATTTGGCACTA  45630
PolarBear    ------------------------------------------------------------------------------  14561
Cow          AGAAAGCT-----G-------------------AGCAC--------------AGAAGAATTGAATCTTTTGAACTG  42751
Dog          --AT--------------------------------------------------------------------------  40427
BelugaWhale  GTATCATTATTT-AA------------------AATTG--------------AGACTTCCATAATATTTAGCACTA  39181
Donkey       ATAACTTTAGGTTGAATTACGAAGTATTTGTTCTTGTTATTCAAACAAACGAATGAACAAGAACACAAAATGTCAGAATT  43545
Cat          ATAGAGTTGTACAGT-------------------GT-------T-------TGCAATGTAGTTAATTTGCAGGGAAT  46119
Ferret       CAAAAGCTTTTTAGCTTGATGAAGTCCCAATAGTTCATTTTGTCTTGCTTTCCTTGCCTTTGACTATGTGTCTAGGAAGA  43013
Lion         ATAGAGTTGTACAGT-------------------GT-------T-------TGGCATGTAGTTAATTTGCAGGGAAT  43770
Vaquita      GTATCATT---T-AA------------------AATTG--------------AGACTTCTATAATATTTAGCACTA  39847
Pig          GTACCACT-----G-------------------TTTA---------------AGACTTTCGTGATATTTAAGACTA  35986

Majority     AAATAT---------------XGA-------------------------------------------------------
                       54490     54500     54510     54520     54530     54540     54550     54560
Human        AGATAT--------------GAA--------------------------------------------------------  45639
PolarBear    ------------------------------------------------------------------------------  14561
Cow          TGGTGTT-------------GGAGAAG----------------------------------------------------  42765
Dog          ------------------------------------------------------------------------------  40427
BelugaWhale  AAATAT---------------GA--------------------------------------------------------  39189
Donkey       CTCTATAGACTGCTTAGCCAAGAAGAGTCCTTGGTCTTACATAAGAAGAA------------------T----------  43596
Cat          GTTAAATGT---TGATGTAGAAGAAAAG---------------------------------------------------  46144
Ferret       AATTGCTATGGCTGAGGTTGAAGAGGTTGCTGCCTGTGTTCTTCTGAAGAATTTTGATGGATTCCTGTGTCACATTTAGG  43093
Lion         GTTAAATAT---TGATGTAAAAGAAAAG---------------------------------------------------  43795
Vaquita      AAATAA---------------GA--------------------------------------------------------  39855
Pig          AAATAT---------------GA--------------------------------------------------------  35994
```

```
Majority     --------------------------------------------------------------------------------

                      54570     54580     54590     54600     54610     54620     54630     54640

Human        --------------------------------------------------------------------------------  45639
PolarBear    --------------------------------------------------------------------------------  14561
Cow          ----------------------A---------------------------------------------------------  42766
Dog          --------------------------------------------------------------------------------  40427
BelugaWhale  --------------------------------------------------------------------------------  39189
Donkey       ------------------ATGCATTTGTATGCCGATTATGTGTTTTAAGGTATATATGTATTTTTTTATTCCCTGCTTAA  43658
Cat          ---------------------TA-----------------------------------------GC--------------  46148
Ferret       TCTTTCATCCATTTTTAAATCTATTTTTGTGTGTGGTGTATGGAAGTGGTCCATTTTCATTCTTCTGCATGTGGTTTTCC  43173
Lion         ---------------------TA-----------------------------------------GC--------------  43799
Vaquita      --------------------------------------------------------------------------------  39855
Pig          --------------------------------------------------------------------------------  35994

Majority     ------------------TXTTTAAAAGACACCTT---------------------------------------------

                      54650     54660     54670     54680     54690     54700     54710     54720

Human        ------------------TACTTAGTAGTCACCT----------------------------------------------  45655
PolarBear    --------------------TTGAAAAG----------------------------------------------------  14569
Cow          -----------------CTCTTTAAGAGTCCCTTGG--------------------------------------A-----  42786
Dog          --------------------CTAAAGAGAAAACTT----T----------------------------------------  40443
BelugaWhale  -----------------ATATTTAATAGCCACCCT---------------------------------------------  39207
Donkey       CTTACATTTTCTTTCTTTTTTTAAAAAGATTTTATTTTTCCTTTTTCTCCCAAAGCCCCCCGGGTACATAGTTGTGTCT-  43737
Cat          --------------------TTCAAAAAATAAGGTTATTTATTTTCTTTGACTTTTCA---------AAACGATTCCAC-  46198
Ferret       AATTTTCCCAACACCATTTGTTGAAAAGATTGTCTTTTTTCCACTGGACATTCTTTCCTGCTTTGTCAAAGATTAGTTGA  43253
Lion         --------------------TTCAAAAAATAAGGTTATTTATTTTCTTTGACTTTTCA---------AAATGATTCTAC-  43849
Vaquita      -----------------ATATTTAATAGCCACCCT---------------------------------------------  39873
Pig          -----------------ATATTTGGTAGCCACCT----------------------------------------------  36011
```

```
Majority     ----------------------------TTTXGAAGCTATT-AACTG-------------------T-------------

                    54730     54740     54750     54760     54770     54780     54790     54800

Human        ----------------------------CATGGAAGCTATT-AACTGAG------------------------------  45675
PolarBear    --------------------------------------------------------------------------------  14569
Cow          ----------------------------CTGCAAGGAGATCCAACCAGTCCAT-----------CCTG------------  42815
Dog          -------------------------------------CACTCA-------------------------------------  40449
BelugaWhale  ----------------------------TTCTGAAGCTATT-AACGG---------------------------------  39225
Donkey       ----------------------TTTTAGTTGTGGGTCCTTCTAGTTGTGGCATGTGTGATGCAGCCTCAGCATGGCTTGA  43795
Cat          ---------------------------CTTTCCATATTATTTAACTG-----------------TTTT------------  46222
Ferret       CCATAGAGTTGAGTGTCCATTTCTGGGCTCTCTGTTCTGTTCCATTGATCTGTGTCTCTGTTTTTATGCCAGTACCATGC  43333
Lion         ---------------------------CTTTCCATATTATTTAACTG-----------------TTTT------------  43873
Vaquita      ----------------------------TTCTGAAGCTATT-AATGG---------------------------------  39891
Pig          ----------------------------TTTTGAAGCTGTT-AACTA-----------------CCTG------------  36033

Majority     -----------------------A--X-----------GAGTATCAXGTTGXCTAAAGAX-------------------

                    54810     54820     54830     54840     54850     54860     54870     54880

Human        ----------------------G---------------GAGTGTCAGGTAGTGTAAATGT--------------------  45698
PolarBear    --------------------------------------------------------------------------------  14569
Cow          -----------------------AAGGAAATCAGTCCTGAATATTCATTGGAAGGACTG---------------------  42851
Dog          --------------------------------------------------GCCG--------------------------  40453
BelugaWhale  -----------------------AGAG-----------GAGTATCAAGTAGTGTAAAGACA-------------------  39252
Donkey       TAAGCAGTGCCATGTCCGGGCCCAGGATTTGAACTGACAAAATCCTGGGTGGCGGAAGCAGA------------------  43857
Cat          -----------A-----------------CTAACGTTCACAATTCACACTGCCCAGGAAT-------------T------  46255
Ferret       TGTCTGATGATTACAGCTTTGTAATAGAGCTTGAAGTCAGGCATTGTGATGCCTGCAGCTTTGGTTTTCTTTTTCAGCCT  43413
Lion         -----------A-----------------TTAACGTTCACAATTCACACTGCCCAGGAAT-------------T------  43906
Vaquita      -----------------------AGAG-----------GAGTATCAAGTAGTGTAAAGACA-------------------  39918
Pig          -----------------------AGAG-----------GTGTATCAGGTGGTGTAAAGA---------------------  36058
```

```
Majority     ----------XXGTAAAAGTTG---------------X-TT--AXTTTCTTGGCTTTTGXTTGAAAAGAACAGATXXTCT
                      54890     54900     54910     54920     54930     54940     54950     54960
Human        -------------TGATAGTTTACT------------ACAGTAACTGGCTTGGTCTTTGGGAGAAGAGGACAGATCTTTT  45753
PolarBear    --------------------------------------------------------------------------------  14569
Cow          ----------ATGTTAAGGCTGA--------------AACTCTAATACTTTGGCCACATGACGGGAAGAACTGACTCATT  42907
Dog          ------------------------------------------------------------TTAGAAACAACAAATACCCA  40473
BelugaWhale  ----------GTGTAAATGTTG-----------------TT--AGTTACATGGCATTTGGGAGAAAAGAACAGATCTTCT  39303
Donkey       ----------GCGCGCGAACTTAACCACTCAGCCATGGGGCTGGACTTCTTAACTTAGATTTTCAATTAACAGACCCAGT  43927
Cat          ----------ACCCAACACAAATCCA----------GAACAATAAACTATCCTATTTTCTTTATAATGGGAAAATGCAGG  46315
Ferret       TTCTGTGGCCACGCAGGGGTTTTTCTGTTTCCATACAAATTTTAGGATTATTTGTTCCATTTTGTAAAAAAAAATGTTCA  43493
Lion         ----------ACCCAACACAAATCCA----------GAACAATAAACTATCCTATTTTCTTTATAATGGGAAAATGCAAG  43966
Vaquita      ----------GTGTAAATGTTG-----------------TT--AGTTACATGGCATTTGGGAGAAAAGAACAGATGTTCT  39969
Pig          ----------GTGTAAATGTTG-----------------TT--AGTTACATGGAGTTGGAGCGAAAAGGACATATCTTCT  36109

Majority     CTA---GGCCTTXATAXX--TTACATAAAGTTTGTATTT-----------------------------------------
                      54970     54980     54990     55000     55010     55020     55030     55040
Human        TTAGGCAACCTGTATG----TAATGTAATGTTTGTTTTT-----------------------------------------  45788
PolarBear    --------------------------------------------------------------------------------  14569
Cow          GGAAAAGACCCTGATGC---TGGCAAAGAGTGAAGGCAGGAC--------------------------------------  42946
Dog          CCATT-TGCTTTGACACGGAT-----------------------------------------------------------  40493
BelugaWhale  CTA---GGCCATTATATA--TTACCTGAAGTTTGTCTTTAT---------------------------------------  39339
Donkey       GATGATTTTGTTAGATAAGATGCCACAAAATAGAAAATTCACTGTCTTTCTTCTTTCTGATA-------AGTTCAGATGC  44000
Cat          CTGTTAACCTTT--TGAGGATCATATTCTAAAAATAATTT---------------------A------------------  46354
Ferret       TCACAGTACTTTGGTAGGGATTGCATTAAATGTGTAGATTGTTCTTGGTAGCATGGACATTTTCACAATATTTGTTCTTC  43573
Lion         CTGTTAACCTTT--TGAGGATCATATTCTAAAAATAACTT---------------------A------------------  44005
Vaquita      CTA---GGCCATTATATA--TTACCTGAAGTTTGTCTTTAT---------------------------------------  40005
Pig          CTA---GGCCTTCATATA--TTGCACAAAGTTTGTCTT------------------------------------------  36142
```

```
Majority     --------------------------------------------------------------------------------
                               55050     55060     55070     55080     55090     55100     55110     55120
Human        --------------------------------------------------------------------------------  45788
PolarBear    --------------------------------------------------------------------------------  14569
Cow          --------------------------------------------------------------------------------  42946
Dog          --------------------------------------------------------------------------------  40493
BelugaWhale  --------------------------------------------------------------------------------  39339
Donkey       CTCTGCACA------------------------------------------------------CAAAAATCTTCCTTAGT  44026
Cat          --------------------------------------------------------------------------------  46354
Ferret       CAATCTGTGAATATGGAACGTTTTTCCATTTCTCTGTATCTTCCTCAATTTCTTTCTATAGTTTGTGAGCACAGATTCTT  43653
Lion         --------------------------------------------------------------------------------  44005
Vaquita      --------------------------------------------------------------------------------  40005
Pig          --------------------------------------------------------------------------------  36142

Majority     --------------------------------------------------------------------------------
                               55130     55140     55150     55160     55170     55180     55190     55200
Human        --------------------------------------------------------------------------------  45788
PolarBear    --------------------------------------------------------------------------------  14569
Cow          --------------------------------------------------------------------------------  42946
Dog          --------------------------------------------------------------------------------  40493
BelugaWhale  --------------------------------------------------------------------------------  39339
Donkey       TGTGCAAGAGGAGATGTGGAGAGCTTCCTGTAGTTTTTTTGTATTTCTGGAGTGATGAAGAGCTAGTTAACAAGATTTTC  44106
Cat          --------------------------------------------------------------------------------  46354
Ferret       TTCCCCTTTGGTTAGATTTATCCCTAGGTATCTTGTGATTTTGGGTGCAATTGTATATGGGTTCAACTCCCTGCTTTCTT  43733
Lion         --------------------------------------------------------------------------------  44005
Vaquita      --------------------------------------------------------------------------------  40005
Pig          --------------------------------------------------------------------------------  36142
```

```
Majority     --------------------------------------------------------------------------------
                      55210     55220     55230     55240     55250     55260     55270     55280
Human        --------------------------------------------------------------------------------  45788
PolarBear    --------------------------------------------------------------------------------  14569
Cow          -------------------------------------------------------------------------------G  42947
Dog          -----------GG-------------------------------------------------------------------  40495
BelugaWhale  --------------------------------------------------------------------------------  39339
Donkey       ATGGAAATATATTCAGAACCAGAGCTTACTACTCTTTTGTACAAAGCCAACCTAAGGGGATTTTTAAGTGCATCAGCTGG  44186
Cat          --------------------------------------------------------------------------------  46354
Ferret       TCTTCTGTCTTGTTGGTTTATAGAAATGCAACTGCTTTCTGTGCATTGAATTTATATCCTGCCATTTTATTGAATTCTTG  43813
Lion         --------------------------------------------------------------------------------  44005
Vaquita      --------------------------------------------------------------------------------  40005
Pig          --------------------------------------------------------------------------------  36142

Majority     --------------------XAXGGGACCTCTTATAGT-TAGTGGGTTXAXAGXA-----------GACAATGGA-----
                      55290     55300     55310     55320     55330     55340     55350     55360
Human        ----------------------GAGATCCTACTATAGTGTAGTTGTTGAAGAGCAC-A--------GACTATGGA-----  45832
PolarBear    --------------------------------------------------------------------------------  14569
Cow          ----AG--------------AAGGGGATGACAGAGGATGAGATGGTTGGATGGCATCAC--CAA--GTCAATGGAC----  43001
Dog          --------------------AAGTGGA---------G-----------------------------G-------------  40504
BelugaWhale  ---------------------AAGGGTCCTCCTGTAGT--AGTGGGTTGAGACTACA---------GACTATGGA-----  39382
Donkey       CCATTCATGTATGTAAACTTGGGTGAAACTCTTAGAGTAAAACATGACTTAAGTATAACTTGACAAGTAAATTATCAAGT  44266
Cat          ----A---------------AAAGGGAAGCCTTTATGTATGCAGTAATA-----------------GATAG---TA----  46391
Ferret       TATGAGTCCTAGCAGGTTTGAAGTGGAGAATTTTGGGTTTTCCACATAAAATGTCATATATCATCTGCAAAGAATGTGAA  43893
Lion         ----A---------------AAAGGGAAGCCTTTATGTATGCAATAATA-----------------GATAG---TA----  44042
Vaquita      ---------------------AAGGGTCCTCCTGTAGT--AGTGGGTTGAGACTACA---------GACTATGGA-----  40048
Pig          ---------------------TAGGGACCTCTAATAGTGTAGTGAGTTGAGAGCAT------G---GACTATGGA-----  36187
```

```
Majority     -------------------------------------------------------------------------TXATAXT
                     55370     55380     55390     55400     55410     55420     55430     55440
Human        ------------------------------------------------------------------------ATCATGCT  45840
PolarBear    --------------------------------------------------------------------------------  14569
Cow          ------------------------------------------------------------------------ATGAGTTT  43009
Dog          ---------------------------------------------------------------------------GTATT  40509
BelugaWhale  ------------------------------------------------------------------------GTCATACT  39390
Donkey       GAGTTCACATATTATCTTCTAGAGATACCTTCTCTGATTCATCCAATAAAGTCACTATCACACCACTCTCTTCTAGTCTT  44346
Cat          -------------T-------------------------------------------C-TT---------------TATT  46399
Ferret       TTTCATGACTTCTTTGCTGATTTGGATGCTTTTTATTTCTTTTTGTTGTCTTGATTACTGAGGCTAGGACTTCTAGTACT  43973
Lion         -------------T-------------------------------------------C-TT---------------TATT  44050
Vaquita      ------------------------------------------------------------------------GTCATACT  40056
Pig          ------------------------------------------------------------------------GTCACACT  36195

Majority     CTG-TGAGTTTXAXTX-TT-TXX---------------------------------------------------GCCATT
                     55450     55460     55470     55480     55490     55500     55510     55520
Human        CC-CTGAGTTTAATCCTTTCTCT---------------------------------------------------GCTACT  45868
PolarBear    --------------------------------------------------------------------------------  14569
Cow          GAG-TGAATTTTATTGACTATGCCAA-----------------------G------------------------GCCTTT  43041
Dog          ATGCTGAGT-----------------------------------------------------------------------  40518
BelugaWhale  CC--TGAGTTTTAATCCTTTTCCT--------------------------------------------------GCCATT  39418
Donkey       CTGCTAAGTCACCTTTAATCACATCACTNAAAATCATAGATCTGTAACCCAGAG--------------------AGACCC  44406
Cat          GTGATGTAC---------------------------------------------------------------------TT  46410
Ferret       ATGTTGAATAGCAATGGTGATAGAGGACATCCCTGCCATCTTCCTGACCTTAGGGGAAAAGCTCTTGGTTTTTCTCCATT  44053
Lion         GTGATGTAC---------------------------------------------------------------------TT  44061
Vaquita      CC--TGAGTTTTAATCCTTTTCCT--------------------------------------------------GCCATT  40084
Pig          CCC-TGAGTTTTAATCCTTTTTCT--------------------------------------------------GCCACT  36224
```

```
Majority     TTCTGTCTTTACCTCTTTXTXAX---------------------------------------------------------
                              55530     55540     55550     55560     55570     55580     55590     55600
Human        TCCTGTCTATACCACTTAGTAACTAT------------------------------------------------------ 45894
PolarBear    -------------------------------------------------------------------------------- 14569
Cow          GACTGTGTGGATCACAATAAACTGGA------------------------------------------------------ 43067
Dog          -------------------------------------------------------------------------------- 40518
BelugaWhale  TTCTGTCTTTACCCCTTAGTAACTCT------------------------------------------------------ 39444
Donkey       AGTCTTCCTTACTTCTCTGAGAGGGTGATAAATGGCCTAGATGTTTTATAAGCTGAGGAC-C------------------ 44467
Cat          TTTTTTTGCTTTTTTTT--------------------------------------------------------------- 46427
Ferret       TTTTTTTTTAATTTTTTTTTTAAAGATTTTTATTATTTATTTATTTGACAGAGAGAGATCACAAGTAGGCAGAGAGGCAG 44133
Lion         TTTTTTTGCTTTTTTTTT-------------------------------------------------------------- 44079
Vaquita      TTCTGTCTTCACCCCTTAGTAACTCT------------------------------------------------------ 40110
Pig          TCCTGTCTTTACCACTTTATCTC--------------------------------------------------------- 36247
```

```
Majority     --------------------------------------------------------------------------------
                              55610     55620     55630     55640     55650     55660     55670     55680
Human        -------------------------------------------------------------------------------- 45894
PolarBear    -------------------------------------------------------------------------------- 14569
Cow          --------------A----------------------------------------------------------------- 43068
Dog          -------------------------------------------------------------------------------- 40518
BelugaWhale  --------------G----------------------------------------------------------------- 39445
Donkey       ----------------------------------------------------------A--------------------- 44468
Cat          -------------------------------------------------------------------------------- 46427
Ferret       GAAGAGAAAGAGGAGGAAGCAGGCTCCCTGCCGAGCAGAGAGCCCGATGCGGGACTCGATCCCAGGACCCTGAGATCATG 44213
Lion         -------------------------------------------------------------------------------- 44079
Vaquita      --------------G----------------------------------------------------------------- 40111
Pig          -------------------------------------------------------------------------------- 36247
```

```
Majority     --------------------------------------------------------------------------------
                      55690     55700     55710     55720     55730     55740     55750     55760
Human        --------------------------------------------------------------------------------  45894
PolarBear    --------------------------------------------------------------------------------  14569
Cow          --------------------------------------------------------------------------------  43068
Dog          --------------------------------------------------------------------------------  40518
BelugaWhale  --------------------------------------------------------------------------------  39445
Donkey       --------------C-----------------------------------------------------------------  44469
Cat          --------------------------------------------------------------------------------  46427
Ferret       ACCTGAGCCGAAGGCAGCGGCTTAACCCACTGAGCCACCCAGGCGCCCGGTTTTTCTCCATTTATAATGATATTCTCATT  44293
Lion         --------------------------------------------------------------------------------  44079
Vaquita      --------------------------------------------------------------------------------  40111
Pig          --------------------------------------------------------------------------------  36247

Majority     -----------------------------------------------------------------------XCTTTAGXA
                      55770     55780     55790     55800     55810     55820     55830     55840
Human        ---------------------------C----------TA--------------------------------ATCTTGGG  45905
PolarBear    --------------------------------------------------------------------------------  14569
Cow          -----------------------------------------------------------------------AATTCTGAA  43077
Dog          -----------------------------------------------------------------------------GAA  40521
BelugaWhale  -----------------------------------------------------------------------ACCTTGGGC  39454
Donkey       ATGTTTTCTTATGCCTTTTTTCTTTTCCAGGCCTGGGGTGTTTTTATATATGAAATTAGGCTTAGTTGTGTGATTAAAGA  44549
Cat          ------------------------------------------------------------------------CCCCAGAA  46435
Ferret       ATATGGGTTTTTCTTAGATGGCTTTTATGATATTGAGGTATGTTCCCTCTAACCCTACACTGTGGAGTTTTAATCAAGAA  44373
Lion         ------------------------------------------------------------------------CCCCAGAA  44087
Vaquita      -----------------------------------------------------------------------ACCTTGGGC  40120
Pig          -----------------------------------------------------------------------ACTTTTGGC  36256
```

```
Majority     AG-----------------------------------TTXAAXTAXC-------TCTXATCTA----TAAATGXA---
                      55850     55860     55870     55880     55890     55900     55910     55920
Human        CA-----------------------------------TTTAACTAAT-------TGTTACCTG----TAAATGGG---  45934
PolarBear    --------------------------------------------------------------------------------  14569
Cow          AGAG-------A-------------------------TGGGAATACCAGA-CCATCTGACCTGCCTCTTGAGAAACC-  43121
Dog          AGAA----------------------------------------------------------------------------  40525
BelugaWhale  AG-----------------------------------CTTAAGTACC-------TCTCATTTA----TAAATGGG---  39483
Donkey       AATCATTTTCCAGTTTTGCATTCTCATAATGTCGTTATTTTAAATGAAGATTTTCATCATATTTAGCTATATGTGAATAT  44629
Cat          AGAA----------------------------------------------AAGGACAGACTAAATTAGGAAAAACTG  46466
Ferret       AGGATGCTATACTTTGTCAAATACTTTTTCTGTATCAATTGAGAGTATCTCATGTTTCTTGTTCTTTTATTAATATATTG  44453
Lion         AGAA----------------------------------------------AAGGACAGACTAAATTAGGAAAAACTG  44118
Vaquita      AG-----------------------------------CTTAAGTACC-------TCTCATTTA----TAAATGGG---  40149
Pig          AG-----------------------------------TTTAAGTACC-------TCTCATCCA----TAAAGGGA---  36285

Majority     --ATTXATXGGTXAX-AT-------------------AGCAXTC-------TAAGAXTTGTAAATTGCATXGCAXATAGG
                      55930     55940     55950     55960     55970     55980     55990     56000
Human        --GTTGATAGGTCATAATG-----------------ATAGCAATC-------TGAGAGTTGTAAATTGCCCGGCATGTAGT  45989
PolarBear    --------------------------------------------------------------------------------  14569
Cow          -TATATGAAGGTCAGGA-------------------AGCAACAG------TTAGAACTGGACATGGAACAACAGACTGA  43174
Dog          ----------GT--------------------------------------------------------------------  40527
BelugaWhale  --ATTGATGGGTTATAAT-------------------AGCACTCT------TAAGAGTTGTAAAGTGCCTGGCATGTAGG  39536
Donkey       TTAGTAGCCACCCTCTCTGAAGCTCTT-AACTGAGGGAGTATCAGAGTGTGAAGAGAGTGTAAATTTTGGTACTTACATG  44708
Cat          AAATTTACTGAT--------ACCA-------TAACCTATAACTCCCGGGGCTCTAGATTGGATTATGAAGTATTCGTTCT  46531
Ferret       TATCATGTTGGTTGATATGTGCATGTTGAACCAACCTAGCAGTCCAGGAATAAATCCCACTTGGTTGTATTGAACAAACC  44533
Lion         AAATTTACTGAT--------ACCA-------TAACCTATAACTCCTGGGGCTCTAGATTGGATTATGAAGTATTCATTCT  44183
Vaquita      --ATTGATGGGTTATAAT-------------------AG---TCT------TAAGAGTTGTAAAGTGCCTGGCATGTAGG  40199
Pig          --ATAGTTGGGTCATGAT-------------------AGCAATC-------TTAGAGGTGTAAATTGCTTGGGACATAGG  36337
```

```
Majority     TTTCTTTCAGTGAATGGXAXATATCAA---------------------------------------------------CG
                      56010     56020     56030     56040     56050     56060     56070     56080
Human        TAATTAGCAGTGAATATTAAATATTGA---------------------------------------------------TG  46018
PolarBear    --------------------------------------------------------------------------------  14569
Cow          TTCCAAATAGGAAAAGGAATACATCAAGG------------------------------------------------CTG  43206
Dog          -----------CAGTCGGAGAA----------------------------------------------------------  40538
BelugaWhale  TTACTTTCAGTGAATGTTAAATATCAA---------------------------------------------------CG  39565
Donkey       -GTCTTTGAGAGGAAAGGACATGTCTTTGA------------------------------------------------CA  44739
Cat          TGTTATTCCAACAAATGAACAAATGAGAACACAA-------------------------------------------TCA  46568
Ferret       TTTTAATGTACTGTTGGATCCTATTGGCTAGTATTTTGCTGAGAATTTTTACATGCATGTTCATCCGGGATATCGGTCTG  44613
Lion         TGTTATTCCAACAAATGAACAAATGAGAACACAAACTG---------------------------------------TCA  44224
Vaquita      TTACTTTCAGTGAATGTTAAATATCAA---------------------------------------------------CA  40228
Pig          TTTCTTTGAGTGAATGTTAAATATCA---------------------------------------------------TTG  36366

Majority     XGAAA--GTAAAXTATCTTCTTTXCTATA-------------XTA-----------------------------------
                      56090     56100     56110     56120     56130     56140     56150     56160
Human        CAAAA---TAAAATTACATCTTTTCTATA--------------TT-----------------------------------  46046
PolarBear    --------------------------------------------------------------------------------  14569
Cow          TATATT-GTCACCCTGCTTATAACTTATATGCAGA-------GTA-----------------------------------  43243
Dog          ----------------------------G-GA------------------------------------------------  40541
BelugaWhale  CAAAA--GAAAAGTACCTTCTTTTCTACA---------------------------------------------------  39592
Donkey       GGAAAGTGTATAATATGAATTTTGTTTTGGGATTCTCCTATAGTAAA--------------------------------T  44787
Cat          GGATTCTGTACTCCGTTTGCTTTGCCAAGAAATGG-CT----GTA-----------------------------------  46608
Ferret       TGCTTCTTTTTGATAGGGTCTTTGTCGGTTTTGGGATCAAGGGTAATGTTGGCCTCAAAAAACGAGTTTGGAAGTTTTCC  44693
Lion         GGATTCTGTACTCCGTTTGCTTTGCCAAGAAATGG-CT----GTA-----------------------------------  44264
Vaquita      CAAAA--GAAAAGTACCTTCTTTTCTACA---------------------------------------------------  40255
Pig          TGAAAA-GAAAAGTATCTTCTTTTCCATA-----A-------ATC-----------------------------------  36398
```

```
Majority     --------------------------------------------------------------------------------
                      56170     56180     56190     56200     56210     56220     56230     56240
Human        -------------------------------------------------------------------------------- 46046
PolarBear    -------------------------------------------------------------------------------- 14569
Cow          -------------------------------------------------------------------------------- 43243
Dog          -------------------------------------------------------------------------------- 40541
BelugaWhale  -------------------------------------------------------------------------------- 39592
Donkey       TAAGAGCACAGG--C----------------------------------------------------------------- 44800
Cat          ----------G--------------------------------------------------------------------- 46609
Ferret       TTACATTGCTGTTTTTTGGGAACAGTTTCCAGAAAATAGGTATTAATTCTTCTTTAAAAGTTTGGTATAATTCCCTTGGG 44773
Lion         ----------G--------------------------------------------------------------------- 44265
Vaquita      -------------------------------------------------------------------------------- 40255
Pig          -------------------------------------------------------------------------------- 36398

Majority     --------------------------------------------------------------------------------
                      56250     56260     56270     56280     56290     56300     56310     56320
Human        -------------------------------------------------------------------------------- 46046
PolarBear    -------------------------------------------------------------------------------- 14569
Cow          -------------------------------------------------------------------------------- 43243
Dog          -------------------------------------------------------------------------------- 40541
BelugaWhale  -------------------------------------------------------------------------------- 39592
Donkey       ------------------------TGTGGAGTCATATACTTCCTGAGTTTTAATCCTTTTTCTGCCACTTCCCGTCTTCA 44856
Cat          ----------------------------------------T--------------------------------------- 46610
Ferret       AAGCCATCTGGCCCTGGGCTCTTGTTTGTGGGAGATTTTTTTTATTACAGCTTCAATTTCCTTGCTGATTATGGGTCTGT 44853
Lion         ----------------------------------------T--------------------------------------- 44266
Vaquita      -------------------------------------------------------------------------------- 40255
Pig          -------------------------------------------------------------------------------- 36398
```

```
Majority     ----------------------------------------CXT----AXXAG-ATXTATTTXT----------------
                      56330     56340     56350     56360     56370     56380     56390     56400
Human        ----------------------------------------TTTTCCAAGAAGTCAAGATCTTT----------------  46069
PolarBear    --------------------------------------------------------------------------------  14569
Cow          ----------------------------------------CATCATGAGAAACACTGGGCTGT--A-------------  43267
Dog          --------------------------------------------------------------------------------  40541
BelugaWhale  -----------------------------------------------------AGTTATTTAT--C-------------  39603
Donkey       CCACTTAGTAACTGTCTGACCTTGGCAACTTCACTAGCTGTCATCATAAATGGGATTGATGGGTCATGATAATAGCATCT  44936
Cat          ----------------------------------------CTTACATAAGAGTATATGTTTGT---------A------  46634
Ferret       TTAGGTTTTCTGTTTCTGCCTAGTTCAGTTTTGGTAGTTATATGTTCCTAGGAATGCATTCATTTCTTCCAGATTGTCT  44933
Lion         ----------------------------------------CTTACATAAGAGTATATGTTTGT---------A------  44290
Vaquita      -----------------------------------------------------AGTTATTTAT--C-------------  40266
Pig          ----------------------------------------CAAAAAAAAAAAAAAAAAAAACAA--A-------------  36422

Majority     ------XTXXCAATAXTTG-X-TTAA-----------------------------------------------------
                      56410     56420     56430     56440     56450     56460     56470     56480
Human        ------GTCTTATCCTTTGATTCTCAGA----------------------------------------------------  46091
PolarBear    --------------------------------------------------------------------------------  14569
Cow          -----GGAAGCACAAGCTGGAATCAA------------------------------------------------------  43288
Dog          --------------------------------------------------------------------------------  40541
BelugaWhale  -----ATCTTCTTTAACTG-------------------------------------------------------------  39617
Donkey       TAGAGGTGTAAAGTAGTTAACTTCCAGAG---------------------------------------------------  44965
Cat          -------TGCCAATTATAAGTCTTAA-AG----------------------T-------------------------  46656
Ferret       AATTTGTTGGCAATAGTTGCTCATAATAAGTTCTTGTTATTGTTTGTATTTCTTTGGTGTTGTTTGTGATTTCTTTGTGA  45013
Lion         -------TGCCAATTATAAGTCTTAA-AG----------------------T-------------------------  44312
Vaquita      -----ATCATCTTTAACT--------------------------------------------------------------  40279
Pig          -----AACACCAACAGGTG-ATTTAT------------------------------------------------------  36442
```

```
Majority     --------------------------------------------------------------------------------
                      56490     56500     56510     56520     56530     56540     56550     56560
Human        --------------------------------------------------------------------------------  46091
PolarBear    --------------------------------------------------------------------------------  14569
Cow          --------------------------------------------------------------------------------  43288
Dog          --------------------------------------------------------------------------------  40541
BelugaWhale  --------------------------------------------------------------------------------  39617
Donkey       -----------------------------------------------------------------A--------------  44966
Cat          --------------------------------------------------------------------------------  46656
Ferret       TTTCTCCTCTTTCATTCAGGATTCTTTTTGGGGGGTCCTTTATTTTTGATAGGTCTGTCTAGGGGTTTATCCATCTTATT  45093
Lion         --------------------------------------------------------------------------------  44312
Vaquita      --------------------------------------------------------------------------------  40279
Pig          --------------------------------------------------------------------------------  36442

Majority     --------------------------------------------------------------------------------
                      56570     56580     56590     56600     56610     56620     56630     56640
Human        --------------------------------------------------------------------------------  46091
PolarBear    --------------------------------------------------------------------------------  14569
Cow          --------------------------------------------------------------------------------  43288
Dog          --------------------------------------------------------------------------------  40541
BelugaWhale  --------------------------------------------------------------------------------  39617
Donkey       --------------------------------------------------------------------------------  44966
Cat          --------------------------------------------------------------------------------  46656
Ferret       AATTATTTCAAAGAATCAGCTCTTAGTTTTATTGGTCATTCTGCTGTTCTTTTGGTTTCTGTCTCGTTGATTTCTACTCT  45173
Lion         --------------------------------------------------------------------------------  44312
Vaquita      --------------------------------------------------------------------------------  40279
Pig          --------------------------------------------------------------------------------  36442
```

```
Majority     --------------------------------------------------------------------------------
                      56650      56660      56670      56680      56690      56700      56710      56720
Human        --------------------------------------------------------------------------------  46091
PolarBear    --------------------------------------------------------------------------------  14569
Cow          --------------------------------------------------------------------------------  43288
Dog          --------------------------------------------------------------------------------  40541
BelugaWhale  --------------------------------------------------------------------------------  39617
Donkey       --------------------------------------------------------------------------------  44966
Cat          --------------------------------------------------------------------------------  46656
Ferret       GATCTTTATTATTTCTCTTCTGCTGAGTTTAGGTTTTTTTGCTCTTCTTTCTCCAGCTCCTTTAGGTTTAGGGTTATATA  45253
Lion         --------------------------------------------------------------------------------  44312
Vaquita      --------------------------------------------------------------------------------  40279
Pig          --------------------------------------------------------------------------------  36442

Majority     --------------------------------------------------------------------------------
                      56730      56740      56750      56760      56770      56780      56790      56800
Human        --------------------------------------------------------------------------------  46091
PolarBear    --------------------------------------------------------------------------------  14569
Cow          --------------------------------------------------------------------------------  43288
Dog          --------------------------------------------------------------------------------  40541
BelugaWhale  --------------------------------------------------------------------------------  39617
Donkey       ---------------------------------------------ATG--------------------------------  44969
Cat          --------------------------------------------------------------------------------  46656
Ferret       TTTGAGACCTTTCTTGTTTCTTGAGAAAGGCATGTATTGCTATATACTTTCCTCTTAGAACCGCTTTGGCTGGATACCAA  45333
Lion         --------------------------------------------------------------------------------  44312
Vaquita      --------------------------------------------------------------------------------  40279
Pig          --------------------------------------------------------------------------------  36442
```

```
Majority     --------------------------------------------------------------------------------
                              56810     56820     56830     56840     56850     56860     56870     56880
Human        -------------------------------------------------------------------------------- 46091
PolarBear    -------------------------------------------------------------------------------- 14569
Cow          -------------------------------------------------------------------------------- 43288
Dog          -------------------------------------------------------------------------------- 40541
BelugaWhale  -------------------------------------------------------------------------------- 39617
Donkey       -------------------------------------------------------------------------------- 44969
Cat          -------------------------------------------------------------------------------- 46656
Ferret       AGAGTTTGAACAGTTCTGTTTTCATTTACATTTGTTTCCATGAATTTTTAAAATGAATTCTTTAAAAAGAATTTAAAATT 45413
Lion         -------------------------------------------------------------------------------- 44312
Vaquita      -------------------------------------------------------------------------------- 40279
Pig          -------------------------------------------------------------------------------- 36442
```

```
Majority     --------------------------------------------------------------------------------
                              56890     56900     56910     56920     56930     56940     56950     56960
Human        -------------------------------------------------------------------------------- 46091
PolarBear    -------------------------------------------------------------------------------- 14569
Cow          -------------------------------------------------------------------------------- 43288
Dog          -------------------------------------------------------------------------------- 40541
BelugaWhale  -------------------------------------------------------------------------------- 39617
Donkey       -------------------------------------------------------------------------------- 44969
Cat          -------------------------------------------------------------------------------- 46656
Ferret       CTTTAATCTTCTTCAGTAGGATACTCGTTAGCCTACATGTGTTTGAATTCTTTCCAATTTTCCTCTTGTGATCAAGTTCA 45493
Lion         -------------------------------------------------------------------------------- 44312
Vaquita      -------------------------------------------------------------------------------- 40279
Pig          -------------------------------------------------------------------------------- 36442
```

```
Majority     --------------------------------------------------------------------------------

                      56970     56980     56990     57000     57010     57020     57030     57040

Human        --------------------------------------------------------------------------------  46091
PolarBear    --------------------------------------------------------------------------------  14569
Cow          --------------------------------------------------------------------------------  43288
Dog          --------------------------------------------------C-----------------------------  40542
BelugaWhale  --------------------------------------------------------------------------------  39617
Donkey       --------------------------------------------------------------------------------  44969
Cat          --------------------------------------------------------------------------------  46656
Ferret       AGTTTCAAAGCATTGTGGTCTGAAAATATGCAGGGAATGATCCCAGTCTTTTGGTACTGGTTGAGACCTGATTTGTGACC  45573
Lion         --------------------------------------------------------------------------------  44312
Vaquita      --------------------------------------------------------------------------------  40279
Pig          --------------------------------------------------------------------------------  36442

Majority     --------------------------------------------------------------------------------

                      57050     57060     57070     57080     57090     57100     57110     57120

Human        --------------------------------------------------------------------------------  46091
PolarBear    --------------------------------------------------------------------------------  14569
Cow          --------------------------------------------------------------------------------  43288
Dog          --------------------------------------------------------------------------------  40542
BelugaWhale  --------------------------------------------------------------------------------  39617
Donkey       --------------------------------------------------------------------------------  44969
Cat          --------------------------------------------------------------------------------  46656
Ferret       CAGGATGTGATCTATTCTAGAGAATGTTCCATGTGCACTCGAGAAGAATGTGTATTCTGTTGCTTTGGGATGGAATGTTC  45653
Lion         --------------------------------------------------------------------------------  44312
Vaquita      --------------------------------------------------------------------------------  40279
Pig          --------------------------------------------------------------------------------  36442
```

```
Majority     --------------------------------------------------------------------------------
                     57130     57140     57150     57160     57170     57180     57190     57200
Human        --------------------------------------------------------------------------------  46091
PolarBear    --------------------------------------------------------------------------------  14569
Cow          --------------------------------------------------------------------------------  43288
Dog          --------------------------------------------------------------------------------  40542
BelugaWhale  --------------------------------------------------------------------------------  39617
Donkey       --------------------------------------------------------------------------------  44969
Cat          --------------------------------------------------------------------------------  46656
Ferret       TGAATATATCTGTGAAGTCCATCTGGTCCAGTGTGTCATTTAAAGCCTTTATTTCCTTGTTGATGTTTTGCTTGGATGAT  45733
Lion         --------------------------------------------------------------------------------  44312
Vaquita      --------------------------------------------------------------------------------  40279
Pig          --------------------------------------------------------------------------------  36442
```

```
Majority     --------------------------------------------------------------------------------
                     57210     57220     57230     57240     57250     57260     57270     57280
Human        --------------------------------------------------------------------------------  46091
PolarBear    --------------------------------------------------------------------------------  14569
Cow          ----------------G---------------------------------------------------------------  43289
Dog          --------------------------------------------------------------------------------  40542
BelugaWhale  --------------------------------------------------------------------------------  39617
Donkey       -------T-TAA--------------------------------------------------------------------  44973
Cat          --------------------------------------------------------------------------------  46656
Ferret       CTGTCCATTTCAGTGAGGGGGCTGTTAAAGTCCCCTACTATTACTGTGTTATTGTCTATGTGTTTCTTTGATTTGTTATT  45813
Lion         --------------------------------------------------------------------------------  44312
Vaquita      --------------------------------------------------------------------------------  40279
Pig          ----------------C---------------------------------------------------------------  36443
```

```
Majority     --------------------------------------------------------------------------------

                                57290     57300     57310     57320     57330     57340     57350     57360

Human        --------------------------------------------------------------------------------  46091
PolarBear    --------------------------------------------------------------------------------  14569
Cow          --------------------------------------------------------------------------------  43289
Dog          --------------------------------------------------------------------------------  40542
BelugaWhale  --------------------------------------------------------------------------------  39617
Donkey       --------------------------------------------------------------------------------  44973
Cat          --------------------------------------------------------------------------------  46656
Ferret       AATTGGCTTATATAATTGGCTGCTCCCATATTGGCATTAATATTTACAACTGTTAGAACTTCGTGTTGGATAAACCTTTT  45893
Lion         --------------------------------------------------------------------------------  44312
Vaquita      --------------------------------------------------------------------------------  40279
Pig          --------------------------------------------------------------------------------  36443

Majority     -------------------------XT---X-------------------------------------------------

                                57370     57380     57390     57400     57410     57420     57430     57440

Human        --------------------------------------------------------------------------------  46091
PolarBear    --------------------------------------------------------------------------------  14569
Cow          -------------------------ATTGCC-------------------------------------------------  43295
Dog          --------------------------------------------------------------------------------  40542
BelugaWhale  --------------------------------------------------------------------------------  39617
Donkey       -------------------------ATATCT------------------------------------------AT-----  44981
Cat          -------------------------ATACAT-------------------------------------------------  46662
Ferret       TAAGTATGATATAGTGTCTTTCCTCATCTCTTATTATTATAGTCTTTGGCTTAACTAAAATCTAATTGAACTGATATAAG  45973
Lion         -------------------------ATACAT-------------------------------------------------  44318
Vaquita      --------------------------------------------------------------------------------  40279
Pig          -------------------------TTCTTT-------------------------------------------------  36449
```

```
Majority     --------------------------------------------------------------------------------
                              57450     57460     57470     57480     57490     57500     57510     57520
Human        -------------------------------------------------------------------------------- 46091
PolarBear    -------------------------------------------------------------------------------- 14569
Cow          -------------------------------------------------------------------------------- 43295
Dog          -------------------------------------------------------------------------------- 40542
BelugaWhale  -------------------------------------------------------------------------------- 39617
Donkey       -------------------------------------------G-CAA------------------------------- 44985
Cat          -------------------------------------------------------------------------------- 46662
Ferret       GATTGCCACCCCAGCTTTCTTTTGATGTCCATTAGTATGGTAAATGATTTTCTACCCCATCACTTGAAATCTAGAGGTCC 46053
Lion         -------------------------------------------------------------------------------- 44318
Vaquita      -------------------------------------------------------------------------------- 40279
Pig          -------------------------------------------------------------------------------- 36449

Majority     --------------------------------------------------------------------------------
                              57530     57540     57550     57560     57570     57580     57590     57600
Human        -------------------------------------------------------------------------------- 46091
PolarBear    -------------------------------------------------------------------------------- 14569
Cow          -------------------------------------------------------------------------------- 43295
Dog          -------------------------------------------------------------------------------- 40542
BelugaWhale  -------------------------------------------------------------------------------- 39617
Donkey       ----------------------AA-------------------------------------------------------- 44987
Cat          -------------------------------------------------------------------------------- 46662
Ferret       AAATGAGTCTCTTGCAGACAGCATATTGATAATTCTTCTTCTTTTTTTTTTTTTTAATCCAATCTGATACCCTGTGTCTT 46133
Lion         -------------------------------------------------------------------------------- 44318
Vaquita      -------------------------------------------------------------------------------- 40279
Pig          -------------------------------------------------------------------------------- 36449
```

```
Majority     --------------------------------------------------------------------------------
                       57610     57620     57630     57640     57650     57660     57670     57680
Human        -------------------------------------------------------------------------------- 46091
PolarBear    -------------------------------------------------------------------------------- 14569
Cow          -------------------------------------------------------------------------------- 43295
Dog          -------------------------------------------------------------------------------- 40542
BelugaWhale  -------------------------------------------------------------------------------- 39617
Donkey       ----------------------------------------------------------G--------------------- 44988
Cat          -------------------------------------------------------------------------------- 46662
Ferret       TTGATTGGGGCATTTAGCCCATTTACATTCAGGGCAGCTATTGAAAGTTAGGAATTTAGTGCTATTGTATTACCTGTAAG 46213
Lion         -------------------------------------------------------------------------------- 44318
Vaquita      -------------------------------------------------------------------------------- 40279
Pig          -------------------------------------------------------------------------------- 36449

Majority     --------------------------------------------------------------------------------
                       57690     57700     57710     57720     57730     57740     57750     57760
Human        -------------------------------------------------------------------------------- 46091
PolarBear    -------------------------------------------------------------------------------- 14569
Cow          -------------------------------------------------------------------------------- 43295
Dog          ----------------------------------------------------A--------------------------- 40543
BelugaWhale  -------------------------------------------------------------------------------- 39617
Donkey       -------------------------------------------------------------------------------- 44988
Cat          -------------------------------------------------------------------------------- 46662
Ferret       TTGACTATTACTGTATATTGTCTCTGTTCCTTTCTGGTCTATGGTACTTTGAAGCTTTCTCTTTGTTTAGAGGACTACTT 46293
Lion         -------------------------------------------------------------------------------- 44318
Vaquita      -------------------------------------------------------------------------------- 40279
Pig          -------------------------------------------------------------------------------- 36449
```

```
Majority     ----T-------------------------------------------------------AXAACTXTCC-AATTTCCTT
                      57770     57780     57790     57800     57810     57820     57830     57840
Human        ------------------------------------------------------------AGTTTCCTACGTTCTTTCTG  46111
PolarBear    -------------------------------------------------------------------------------T  14570
Cow          ---GG---------------------------------------------------GAGAAATATC--AATAACCTC  43316
Dog          --------------------------------------------------------------AA-CATTA-TATGGTCTC  40559
BelugaWhale  ----T------------------------------------------------------GAAAACTGTCCCATTTACCTT  39639
Donkey       ---AAAAGTAGCATCTTTTCTGTATTTCTCCAAAAAACCAGGTTGTCTTCTTTGACTGTTAAAACTATCC-TACCTTCCT  45064
Cat          ---AT----------------------------------------------------------ATCCTTA-AAT-TCCTT  46679
Ferret       TCAATATTTCTTGTAGGGCTGGTTTGGTGATTACAAATTCTTTTAGTTTCTATTTGTCCTGGAAGCTTTT-TATATCTCT  46372
Lion         ---GT----------------------------------------------------------ATTCTTA-AAT-TCCTG  44335
Vaquita      -----------------------------------------------------------GAAAACTGTCCCATCTACCTT  40300
Pig          ---GT------------------------------------------------------TAAAACTGTCCCACCTACCTT  36472

Majority     TTTTATTCAGATXTT---X-T-XXCTTACCATCXACT--------------------TCAAGAAATTATCCAACACAAAT
                      57850     57860     57870     57880     57890     57900     57910     57920
Human        TGTTATTCAGCTATTTTACTTCTGCTTAATATCCACTA-------------------TCCAGAAAACATCCAACACAAAT  46172
PolarBear    CCTTATTT------------------------------------------------------------------------  14578
Cow          AGATATGCAGATGACACCACC-GCTATGGCAGAAAGT----G---------------AAGAGGAACTATAAAAAGCCTCT  43376
Dog          ATTCATTT---------------GGGG--------------------------------------------AATATA---  40577
BelugaWhale  TTTTACTCAGTTGTT-----T-TACTCACCATCCACT----G---------------TCAAGAAATCATCCAACACAAAT  39694
Donkey       TGTTATTCAGCTTTTTTACTT-CTGCTTTTACCACCCACT--------------G--TCCAGAAATTATCTAACACAAAT  45127
Cat          CTTTACTTACATTTTCGGCTA-ACAGACCCAG-------------------------TCATGATTTTGTTCAATAAAATG  46733
Ferret       CTTTATTTTCAGTGACAGCCT-AGCTGGGTATAGTATTCTTGGCTGCATATTTTTCTTGTTCAGTACCCTGAATATATCA  46451
Lion         CTTTACTTACATTTTCGGCTA-ACAGACCCAG-------------------------TCATGATTTTGTTCAATAAAATG  44389
Vaquita      TTTTACTCAGTTGTT-----T-TAATCACCATCCACT----G---------------TCAAGAAATCATCCAACACAAAT  40355
Pig          TATTATTCAGTCGTC-----T-GACTTACCATCCACT----G---------------TCCAGAAATTATCCAACACAAAT  36527
```

```
Majority     TCAXA---------------------------------------------------------------------------

                      57930     57940     57950     57960     57970     57980     57990     58000

Human        CC------------------------------------------------------------------------------  46174
PolarBear    --------------------------------------------------------------------------------  14578
Cow          TGATG---------------------------------------------------------------------------  43381
Dog          --------------------------------------------------------------------------------  40577
BelugaWhale  TCAAA---------------------------------------------------------------------------  39699
Donkey       CCTTA---------------------------------------------------------------------------  45132
Cat          CCACA---------------------------------------------------------------------------  46738
Ferret       TGCCAGCCCTGTTTTGTCCACCAGGTCTCTAGTTAGGTCTGCTGGCAATCTAATGTTTCTATACTTGTAGCTTACAGACC  46531
Lion         CCACA---------------------------------------------------------------------------  44394
Vaquita      TCAAA---------------------------------------------------------------------------  40360
Pig          TCAAA---------------------------------------------------------------------------  36532

Majority     --------------------------------------------------------------------------------

                      58010     58020     58030     58040     58050     58060     58070     58080

Human        --------------------------------------------------------------------------------  46174
PolarBear    --------------------------------------------------------------------------------  14578
Cow          --------------------------------------------------------------------------------  43381
Dog          --------------------------------------------------------------------------------  40577
BelugaWhale  --------------------------------------------------------------------------------  39699
Donkey       --------------------------------------------------------------------------------  45132
Cat          --------------------------------------------------------------------------------  46738
Ferret       TCTTGTCCCAAGCTGCTTTCAGGATTTTCTCTTTGTCTCTGAGATTTGTAAGTTTTACTCTTAGATGTCAGGGTGTTGAC  46611
Lion         --------------------------------------------------------------------------------  44394
Vaquita      --------------------------------------------------------------------------------  40360
Pig          --------------------------------------------------------------------------------  36532
```

```
Majority     --------------------------------------------------------------------------------
                       58090     58100     58110     58120     58130     58140     58150     58160
Human        --------------------------------------------------------------------------------  46174
PolarBear    --------------------------------------------------------------------------------  14578
Cow          --------------------------------------------------------------------------------  43381
Dog          --------------------------------------------------------------------------------  40577
BelugaWhale  --------------------------------------------------------------------------------  39699
Donkey       --------------------------------------------------------------------------------  45132
Cat          --------------------------------------------------------------------------------  46738
Ferret       CTATTTTTTACTGATTTTGAAGGGACATCTCTGTGCCTTCTGGGTTTTGATGCCTGTTTCCTTCCCCAAATTAGGGAGGT  46691
Lion         --------------------------------------------------------------------------------  44394
Vaquita      --------------------------------------------------------------------------------  40360
Pig          --------------------------------------------------------------------------------  36532

Majority     --------------------------------------------------------------------------------
                       58170     58180     58190     58200     58210     58220     58230     58240
Human        --------------------------------------------------------------------------------  46174
PolarBear    --------------------------------------------------------------------------------  14578
Cow          --------------------------------------------------------------------------------  43381
Dog          --------------------------------------------------------------------------------  40577
BelugaWhale  --------------------------------------------------------------------------------  39699
Donkey       --------------------------------------------------------------------------------  45132
Cat          --------------------------------------------------------------------------------  46738
Ferret       TTCTGCTGTAATTTGCTCCAGTATACCTTCTGTCCCCCTCTCTTTCTTCTTCTGGGATCCCAATTCTAAAATTGTTTCAG  46771
Lion         --------------------------------------------------------------------------------  44394
Vaquita      --------------------------------------------------------------------------------  40360
Pig          --------------------------------------------------------------------------------  36532
```

```
Majority     --------------------------------------------------------------------------------
                               58250     58260     58270     58280     58290     58300     58310     58320
Human        -------------------------------------------------------------------------------- 46174
PolarBear    -------------------------------------------------------------------------------- 14578
Cow          -------------------------------------------------------------------------------- 43381
Dog          -------------------------------------------------------------------------------- 40577
BelugaWhale  -------------------------------------------------------------------------------- 39699
Donkey       -------------------------------------------------------------------------------- 45132
Cat          -------------------------------------------------------------------------------- 46738
Ferret       TTTTTCTCTTGAATTCTCCCTTTGTGTTCTATTACTTGTTCATTTCTCTTTTCCTTAACTTCTTTATTTTCCATCATTTG 46851
Lion         -------------------------------------------------------------------------------- 44394
Vaquita      -------------------------------------------------------------------------------- 40360
Pig          -------------------------------------------------------------------------------- 36532

Majority     --------------------------------------------------------------------------------
                               58330     58340     58350     58360     58370     58380     58390     58400
Human        -------------------------------------------------------------------------------- 46174
PolarBear    -------------------------------------------------------------------------------- 14578
Cow          -------------------------------------------------------------------------------- 43381
Dog          -------------------------------------------------------------------------------- 40577
BelugaWhale  -------------------------------------------------------------------------------- 39699
Donkey       -------------------------------------------------------------------------------- 45132
Cat          -------------------------------------------------------------------------------- 46738
Ferret       GTCTTCTTTATCCTATTTCTCTCTCCTGCCTCATTTATCCTAGAAGTTAGAGCCTCCATTTGTTATTATACCTTATTAAT 46931
Lion         -------------------------------------------------------------------------------- 44394
Vaquita      -------------------------------------------------------------------------------- 40360
Pig          -------------------------------------------------------------------------------- 36532
```

```
Majority     ---------------------------------------AAAXTAAAC--------TCTTTTATTCTTTTAATAXX----

                      58410     58420     58430     58440     58450     58460     58470     58480

Human        ---------------------------------------AAAATAAAT--------TATTTAATTTCCTTAATAAT----  46203
PolarBear    --------------------------------------------------------------------------------  14578
Cow          ---------------------------------------AAAGTGAGAGAGGAGA-GTGAAAAAGTTCTCAACATTA---  43418
Dog          ---------------------------------------AAAAATAGTGAA-----------------------------  40589
BelugaWhale  ---------------------------------------ACAATAAAC--------TCTTGTATTCTTTTAATAAT----  39728
Donkey       ---------------------------------------ACAATAAAC--------TATTCTATTCTCTTCATAAT----  45161
Cat          ---------------------------------------AAAGAGAAAATTCAGCCTCTTTCTTCTTTCTAA--------  46771
Ferret       AACCTTTTTGATTTCAACTGTTAGATTTTATTTCTCCAGAAAGGGATTCTCTAGTTTCTTCTATGCTTTTTTCAAGCCCA  47011
Lion         ---------------------------------------AAAGAGAAAATTCAGCCTCTTTCTTCTTTCTA---------  44426
Vaquita      ---------------------------------------ACAATAAAC--------TCTTGTATTCTTTTAATAAT----  40389
Pig          ---------------------------------------ACAGTAAAC--------TCTTATATGCTCTTAATA------  36559

Majority     --------------------------------AGGTTTAGGCA----------------GGTTTTXGCCTTTTGGGAAT

                      58490     58500     58510     58520     58530     58540     58550     58560

Human        --------------------------------AGGTTTAGGCA----------------GGCTTTTGCCTTTTGGGAAT  46234
PolarBear    --------------------------------------------------------------------------------  14578
Cow          ---AGAAA--------------------ACGAAGATCATGGCATC--------------TGGTCCCATCACTTCGTGGGA  43461
Dog          --------------------------------------------------------------------------------  40589
BelugaWhale  --------------------------------AGGTTTAGGCA----------------GGTTTTTGCCTTTTGGGAAT  39759
Donkey       --------------------------------AGGTTTAGGGA----------------GA-CTTTACCTTTTGGAAAT  45191
Cat          -----------------------------------------------------------TATATTCAGGTGCTTGTGCAT  46792
Ferret       GATAGTATCTTTATAATCATCATTCTGAACTCTAGTTCCAACATCTTACTAAGTCCATGTTGATTAGGTCCCTAGCAGTT  47091
Lion         -----------------------------------------------------------TATATTCAGATGCTTGTGCGT  44447
Vaquita      --------------------------------AGGTTTAGGCA----------------GGTTTTTGCCTTTTGGGAAT  40420
Pig          --------------------------------GGTTTTAGGCA----------------GATTTTAGCCTTTTGGGAAT  36590
```

```
Majority     CATATTXTAAGAATCATTTGAAAAGGGA--AGCXTTTTTTTTC-------CTXX---X-CAGTGAATAG--GXGATAGCT
                      58570     58580     58590     58600     58610     58620     58630     58640
Human        TATTTTTGGAAAATCATTTGAAAGGGGA--AGCCTTTTTTCTC------CTTATGGGTACAGTG-ATAGGTAGTATCTTT  46305
PolarBear    --------------------------------------------------------------------------------  14578
Cow          AATAGATGGGGAAACAGTGGAAATAGTGTCAGACTTTATTTTCTTGGGCTCCAAAAACACTGCAGATGGTGATTGCAGCC  43541
Dog          ------------------------------------------------------------AGGGAATAAAGGGGAAAGGA  40609
BelugaWhale  CATATTCTAAGAATCATTTGAAAAGGGA--AGCGTTTTTTTCG-------CC-------CTGTATGTG-TAGTGATGGGT  39822
Donkey       CATATTCTAAAAGTCATTTGAAAAGAGA--AGCCTTTTTTCAC------CCTATATATACAGTG-ATAGGTGGTATCTTT  45262
Cat          GTACAAACCGTCCTTAGTTATGAAGTATAGAGGGCTTCCTTTAGTTTTTCTTATTTCTGGAGTGATTAG--GAGCTAGCC  46870
Ferret       GGTACCACCTCATTCTTTTTTTTTTTTTTTTTAAGGTTATTCCGTTTTGTCGTTTTGTCTAGAGAATAGATGAATGAGAG  47171
Lion         GTACAAATCGTCCTTAGTTATGAAGTATAGAGGGCTTCCTTTAGTTTTTCTTATTTCTGGAGTGATTAG--GAGCTAGCC  44525
Vaquita      CATATTCTAAGAATCATTTGAAAAGGGA--AGCGTTTTTTTCG-------CC-------CTATATGTG-TAGTGATGGGT  40483
Pig          CATATTTTAAGAATCATTTGAAAAGGGA--AGCCTTTTTTCTC-------C--------CTATATGTA-----TAGGGCT  36648

Majority     AGTAA-AXTTATTGTGXTTTTXTATTCXX---GXXXGAXTATGATATTCCTA-ATAGGAA-ACCXGAAATTTGCXGTTTT
                      58650     58660     58670     58680     58690     58700     58710     58720
Human        ATTGGTCTTTTTTTTTCCTTGTCTTTCTCCCAGAAGGAAAAGGACAGACCAAATTAGGAGAAACTGAAATTTGCTTTTTT  46385
PolarBear    --------------------------------------------------------------------------------  14578
Cow          ATGAA-ATTAAAAGACGCTTACTCCTTGGAA-GAAGGGTTATGACCAACCTAGATAGCAT-ATTCAAAAGCAGAGACATT  43618
Dog          GAAAA-AATGAGTGGGAAATATCAGAGAGGGAGACAGAACA-------------TGAGAG-ACTCCCAACTCTGGGA---  40671
BelugaWhale  AGTAT-CTTTATTGTGATTTTTTTTTTC----------TTGCCTTTTTCCCT-AAAGGAAAAACAGAAGTTTGCTGTTTG  39889
Donkey       ATTAT-GGTGCTTTTTTCTCGACCTATTCCCAGAAGGAAAAGGACAGACTAAATTAGAAAAAACTGAAGTTTGCTGTTTT  45341
Cat          AGCAA-GATTTTCATGGTAATATATCCAGAG-GCCAGAGCCTACTACTCTTCTGTACAAA-GC--C--ATCCAAAGGGGA  46943
Ferret       AACAA-AATGCTTAAAGCGTAACAGTGACCCCAGAAAAATACACACTTAACCAATAAGAG-ACTCAAAATTTTGGGGGGG  47249
Lion         AGCAA-GATTTTCATGGTAATATATCCAGAG-GCCAGAGCCTACTACTCTTCTGTACAAA-GC--C--ATCCAAAGAGGA  44598
Vaquita      AGTAT-CTTTATTGTGATTTTTTTTTTC----------TTGCCTTTTTCCCT-AAAGGAAAAACAGAAGTTTGCTGTTTG  40550
Pig          AGGAA-A--AACTGAAATTTGCT--------------GTTTTGAGTTTCCTA-ATACTATCACCTGTAACTCATGGGATT  36710
```

```
Majority     AXTXTXCXGGXA----ATGGCC------------------------------------------------XGTAT
                      58730     58740     58750     58760     58770     58780     58790     58800
Human        GAG-TATCCTGATACCATAACTATAACACATGGGGCT----------------------TTAGG---------TTGGAT  46432
PolarBear    --------------------------------------------------------------------------------  14578
Cow          ACTTTGCCAAGA----AAGGTC------------------------------------------------TGTCT  43641
Dog          --------------------------------------------------------------------------------  40671
BelugaWhale  AGTTTCCTGATA----TCATAA------------------------------------------------CCTAT  39912
Donkey       GAGTTTCCTGATACCAAAACCTATAACTTTAGG---------------------------------TTGAAT  45380
Cat          TTTCTAAAGGCATTGGCTGGCCA---------------------------------------------TTCCTGT  46973
Ferret       GGCACGGAAAGAGAATATGATCAGGCTAGTGAATAGAACAGAGTCACACACTAGATTTGATTTGGGATGTATTTTGGTCT  47329
Lion         TTTCTAAAGGCATTGGCTGGCCA---------------------------------------------TTCCTGT  44628
Vaquita      AGTTTCCTGGTA----TCATAA------------------------------------------------CCTAT  40573
Pig          A---T-----------A-GGT-------------------------------------------------TGTAT  36721

Majority     -XT-XAX-------------------------------------------------------------TT-XX
                      58810     58820     58830     58840     58850     58860     58870     58880
Human        TGTGAAGTA---------------------------------T----------------------------TTGTTCTC  46450
PolarBear    --------------------------------------------------------------------------------  14578
Cow          AGTCAAGGC--------T-------------------------------------------A--TGGTTTTCC  43662
Dog          --------------------------------------------------------------------------------  40671
BelugaWhale  AATTCATG-----------------------------------------------------------GGACTATAC  39929
Donkey       TACGAAGTA---T-----------------------------------------------------TTGTTCTT  45398
Cat          --------------------------------------------------------------------------------  46973
Ferret       GTTAGAAGAAACTGCCTTCCAAATTTAAAAGAAAGAAAAATGTATTTGTGTGTATATATGTATATTTGTGTGTGTGTGTA  47409
Lion         --------------------------------------------------------------------------------  44628
Vaquita      AATTCATG-----------------------------------------------------------GGACTATAC  40590
Pig          TATGAAG----------------------------------------------------------TA---TTCTT  36735
```

```
Majority     GTTAXXCA-ATATAT--XT-A-----------------------------------------------------------
                      58890     58900     58910     58920     58930     58940     58950     58960

Human        GTTATTCAAACAAACTAATGA-----------------------------------------------------------   46471
PolarBear    --------------------------------------------------------------------------------   14578
Cow          AGTAGTCACGTATGTATGTTAGAGTTGG----------------------------------------------------   43690
Dog          --------------------------------------------------------------------------------   40671
BelugaWhale  GTTGGACTGAATTATTTGTTCTTGTTA-----------------------------------------------------   39956
Donkey       GTTATTCAAACAAACGAATGAACAAGAACACA-----A------------------------------------------   45431
Cat          ---------ATATA------------------------------------------------------------------   46978
Ferret       TATATGCACACACATAGATAAACAAAAATAAGGGTAAATACAATGAAGGGATGGAATATGACTATAAAAATGAAAACTAG   47489
Lion         ---------ATATA------------------------------------------------------------------   44633
Vaquita      GTTGGACTGAATTATTTGTTCTTGTTA-----------------------------------------------------   40617
Pig          GTTATTTAAATAAATGAATGAAA---------------------------------------------------------   36758

Majority     ----------------------------------------------AAAXAAATGAATGATC-----AAGAATTAGGACT
                      58970     58980     58990     59000     59010     59020     59030     59040

Human        ----------------------------------------------ACAAGAACAAATTATC-----AAGTTTCTGTACT   46500
PolarBear    --------------------------------------------------------------------------------   14578
Cow          ----------------------------------A-----CTGTGAAGAAAGCTGAATGCTG-----AAGAATTGATGCT   43726
Dog          ----------------------------------------------A--------------------ATGAACTAGGGGT   40685
BelugaWhale  -------------------------------------------TTCAAACAAATGAATGAAC-----AAGAGCACAGTCT   39988
Donkey       ---AATGTC-------AGAATTCTCTATAGACTGCTTAGCCAAGAAGAGTCCTTGGTCTTACATAAGAAGAATATGCATT   45501
Cat          ----------------------------------------------AACTTCAGTAAAGCTCTTAGCATAAAATAGGACT   47012
Ferret       AAAAGATTTTAAAAAAAGAATTGGTAAGATAAAAAGTTAGTTGAAAAAAGAAAGGAAAAATTTAATAATAAAAAAGAGAA   47569
Lion         ----------------------------------------------AACTTCAGTAAAGCTCTTAGCATAAAATATGACT   44667
Vaquita      -------------------------------------------TTTAAACAAATGAATGAAC-----AAGAGCACAGTCT   40649
Pig          ---------------------------------------------AAGAACACAAACTGTT------AGGATTTTATACT   36787
```

```
Majority     TX-AGATTGCTTTGXTAAGXAAAGG--TTXGXA----------------------------------------------
                                  59050     59060     59070     59080     59090     59100     59110     59120
Human        C--AGACTGCTTTGTCAAAAAAAGGTCTTAGTCCT---------------------------------------------   46533
PolarBear    --------------------------------------------------------------------------------   14578
Cow          TTTGAACTGGGGTGTTGGAGAAGACTCTTGAGAGT---------------------------------------------   43761
Dog          GG-TGGAAGGGGAGGTGGGCGGG-------G-------------------------------------------------   40708
BelugaWhale  ATCAGATTGCTTTGCTAAGAAAAGGCGTTAGAAG----------------------------------------------   40022
Donkey       TGTATGCCGATTATGTGTTTTAAGGTATATATGTATTTTTTTATTCCCTGCTTAACTTACATTTTCT-----TTCTTTTT   45576
Cat          TA-AATTTAACTTAATAAGTTAA---------------------------------------------------------   47034
Ferret       TG-TGATCAGACAGGTGAATAGAACAAAGCCAACAGGTGAATAGAACAAAGCCATACACTAGACTTAGAGTATATTTTGG   47648
Lion         TA-AATTTAACTTAATAAGTTAA---------------------------------------------------------   44689
Vaquita      ATCAGATTGCTTTGCTAAGAAAAGGCGTTAGAAG----------------------------------------------   40683
Pig          C--AGGTTACTCTGCCAAGAAAAGGCCTTAGAAA----------------------------------------------   36819

Majority     -------------------------------------------------------X------------------------
                                  59130     59140     59150     59160     59170     59180     59190     59200
Human        ------------------------------------------------------------GT--A----------T----   46537
PolarBear    --------------------------------------------------------------------------------   14578
Cow          -------------------C--------------------C-CTT----------GGA----------------------   43769
Dog          -------------------------------------------------------G------------------------   40709
BelugaWhale  -------------------------------------------------------AA-----------------------   40024
Donkey       TTAAAAAGATTTTATTTTTCCTTTTTCTCCCAAAGCCCCCCGGGTACATAGTTGTGTCTTTTTAGTTGTGGGTCCTTCTA   45656
Cat          --------------------------------------------------------------------------------   47034
Ferret       TCTCTTAGAAGAAACTGTATCCCAAAATTTTAAAGAAAGAAGAACTTACATAAAAGTCCTACTAAAGACACAATAGAATA   47728
Lion         --------------------------------------------------------------------------------   44689
Vaquita      -------------------------------------------------------AA-----------------------   40685
Pig          -------------------------------------------------------A------------------------   36820
```

```
Majority     -------------------------------------------------X-------AXGTGGATGTXTGTCXGTTATAT
                     59210     59220     59230     59240     59250     59260     59270     59280
Human        ---------CA--------------GAATAATATGTGTTCCTTGTTGACTGATGCCGATTGTTGTGTGTGCCCATTATGT  46594
PolarBear    --------------------------------------------------------------------------------  14578
Cow          -------------------------------------------------CT----GCAAGGAGATCAGTCCTGGGTGTTC  43796
Dog          -----------------------------------------------------------GTGGGGGTGACTGGGTGACGG  40730
BelugaWhale  --------------------------------------------------------TATGCATTTGTATGTCAGTTATAT  40048
Donkey       GTTGTGGCATGTGTGATGCAGCCTCAGCATGGCTTGATAAGCAGTGCCATGTCCGGGCCCAGGATTTGAACTGACAAAAT  45736
Cat          -------------------------------------------------TT--------ATCGAGTGAGTTCACATATTA  47057
Ferret       GGTGATGTTTTATGTAAAACCATTTGAAGACCTTGCAGTCTATCCACTATTCCTTATTAGTGGTTCCAAATGGTCTATGG  47808
Lion         -------------------------------------------------TT--------ATCAAGTGAGTTCACATATTA  44712
Vaquita      --------------------------------------------------------TATGCATTTGTATGTCAGTTATAT  40709
Pig          ---------------------------------------------------------ATAGGCATCTGTGTGTGACTTAT  36843

Majority     GTTTTAAGGTAT--------------------ATXXTTAATXTGTTGATTCACT-------XTX---XA-CT-TGXATTA
                     59290     59300     59310     59320     59330     59340     59350     59360
Human        GTTTTAAAGTAT--------------------ATGTGTATTTTTTGGATTCTGTG------CTT---TAGCT-TACATTT  46644
PolarBear    --------------------------------------------------------------------------------  14578
Cow          TTTGGAAGGAATG-------------------ATGCTAAAGCTGAAACTCCAGTA-----CTTTGGCCACCT-CGTGTGA  43851
Dog          GCACTGAGGGGT----------------G----CACTTGACAGGATGAGC-----A--------------CT--------  40763
BelugaWhale  GTTTTAAGGTAT--------------------ATG-TGTATTTTTTGATTCCCT--------------------GCAGTA  40087
Donkey       CCTGGGTGGCGGAAGC-------AGAGCGCGCGAACTTAACC-ACTCAGCCATGGGGCTGGACTTCTTAACT-TAGATTT  45807
Cat          TTTCCTAGAGATA-----------TTCTTTAATCATCCAATAAAGTCACTAACACACCACCTTCTTCTAGTTCTCTG---  47123
Ferret       ACTAAAAGATGTTTCTGAAACTAAAACAAAGGATACTTAATAAGCTGAGAAATATACATGTACACTCCACTTGTGTATAA  47888
Lion         TTTCCTAGAGATACC---------TTCTTTAATCATCCAATAAAGTCACTATCACACCACCTTCTTCTGGTTCTCTG---  44780
Vaquita      GTTTTAAGGTAT--------------------ATG-TGTATTTTTTGATTCCCT--------------------GCAGTA  40748
Pig          GTTTTAAGGTAT--------------------ATGTTTTATTTGTTGATTCCTTC-----CTTTAAACAAAT-TACATTA  36897
```

```
Majority     TCAXTTAACAGACCXAXTGATGATTT-------------------------------------------TGTT
                      59370     59380     59390     59400     59410     59420     59430     59440
Human        TCAATTAACGGGCCTAGAGATGATTT-------------------------------------------TGTT  46674
PolarBear    ------------------------------------------------------------------------------  14578
Cow          AGAGTTGACTCATTGGAAAAGACCCTG-----------------------------------------ATGCT  43883
Dog          ---------------GG-------------------------------------------------GTGTT  40770
BelugaWhale  TCATTTAACAGACCCAATGATGATTT-------------------------------------------TGTT  40117
Donkey       TCAATTAACAGACCCAGTGATGATTT-------------------------------------------TGTT  45837
Cat          CTA--AGTCCATTTTAATCAACAACTT--------TT--------------------------------TGCT  47154
Ferret       CTTTACGTACTTCATGGTACTCCCCAAATCAAAACTACCCATAAACCTACGTGTGCTGTGTGTGTGTGTGCGCGCGTGTG  47968
Lion         CTA--AGTCTATTTTAATCAACAACAT--------TT--------------------------------TGCT  44811
Vaquita      TCATTTAACAGACCCAATGATGATTT-------------------------------------------TGTT  40778
Pig          TCATTTAACAGACCCAGTAATAATTT-------------------------------------------TGTT  36927

Majority     AGAXTATXTTX--GC-CACAA---AATAGXAAATTCAGTCTGTTXAT-T-ATTTG--AXXA----GXTGCCTGTGXXCAC
                      59450     59460     59470     59480     59490     59500     59510     59520
Human        AGATAAGATAC-----TACAA---AATGCAAAGTTCAGTCTCTTCCT----TTTGACAAATTCAGGTGCCCTGTGCGCAC  46742
PolarBear    ------------------------------------------------------------------------------  14578
Cow          GGGA-GGGATTGGGGGCAGGAGG-AGGAGGGAACGACAGAGGATGAGATGGCTGGATGGCATC--ACTGACTCAATGGAC  43959
Dog          ATTCTATATGTTGGCAAATTGAACACCAATGAAA------AATAAATTT-A---------------TT------------  40816
BelugaWhale  AAAATAAGAT---GC-CACAA---AATAGGAAATTCAGCCTGTT---------------CA----GGTGCCTGTGCACAT  40171
Donkey       AGATAAGATGC-----CACAA---AATAGAAAATTCACTGTCTTTCTTCTTTCTGATAA-GTTCAGATGCCTCTGCACAC  45908
Cat          TTGCTTTATTCATAGCAGTTAATCACTATAAGAGATATTTTGTTCATGTAAGTTAGTCCACCTCTCCTACTAGACTGTAA  47234
Ferret       TGTGTATGTATGTATATATACAACATCTATAAAATTCCAAGATCAATTTGATTTCTAAGTGTTAGGTTAGATACAGCAAA  48048
Lion         TTGCTTTATTCATAGCAGTTAATCACTATAAGAGACATTTTGTTCATGTAAGTTAGTCCACCTCTCCTACTAGACTGTAA  44891
Vaquita      AAAATAAGAT---GC-CACAA---AATAGGAAATTCAGCCTGTTTC--TGGTAGG--TTCA----GGTGCCTGTGTACAT  40843
Pig          AGAT-AAGAT---GC-CACAA---AGTATGAAATGCAACCTGATAA----ACTCG--AGTA--------CCTGTGCACAC  36985
```

```
Majority     AXAAXT-TXX-T--------------------------------------------------------------------
                              59530     59540     59550     59560     59570     59580     59590     59600
Human        ACAAATCTTTGTCAGTAGTGAAAGGACATGTGATAGCTTCCTGTAGTTTTTTCATATTTCTGGAGTGGTGAAGAG-----  46817
PolarBear    --------------------------------------------------------------------------------  14578
Cow          ATGAATTTGAGTG-------------------------------------------------------------------  43972
Dog          --------------------------------------------------------------------------------  40816
BelugaWhale  AGATGTTTTCCTT-------------------------------------------------------------------  40184
Donkey       AAAAATCTTCCTTA------------------------------------------------------------------  45922
Cat          GAG-----------------------------------------------------------------------------  47237
Ferret       GGGATAGAATATGATTTCCAGAAAGTGGCCGCTTTTCTGTTCATAATTGCTGCTTTTCTTTGATCTCCTGTTGAGTTCGT  48128
Lion         GAGA----------------------------------------------------------------------------  44895
Vaquita      AGATGTTTTCCTT-------------------------------------------------------------------  40856
Pig          ATAAATCTCCTT--------------------------------------------------------------------  36997

Majority     -----------------------------------AXTTXXGGXAGXAXXTG-----TGGAGAGCTTXCTGTAGT-X-C-
                              59610     59620     59630     59640     59650     59660     59670     59680
Human        -----CT---------------ACCCAGCCAAGATTTTCAGGGCAATATATTCAGAAGCCAGAGCTTACTCTTCTGTACA  46877
PolarBear    --------------------------------------------------------------------------------  14578
Cow          -----------------------------------AACTCCAGGAGTTGTTGA----TGGACAGGGAGGCCTGGCGTGC-  44012
Dog          --------------------------------------------------------------------------------  40816
BelugaWhale  -----------------------------------ACTTATGAAAGGAAGTG-----TGGAG-GCTTTTTGTAGT-----  40218
Donkey       ----------------------------------GTTGTGCAAGAGGAGATG-----TGGAGAGCTTCCTGTAGTTTTT-  45962
Cat          -----------------------------------CATTGGGGATCTTACTGATCATTTTGTTCACTGCTGTATCCCCCA  47282
Ferret       AGGTGCTTAGAATGGTTTGATAACTCTCTAGCTAAATTCCCGGGACCAGATGAAATCTAGGTCTCTAACTCTTCCACCCT  48208
Lion         ----------------------------------GCATTGGGGATCTTACTGATCATTTTGTTCATTGCTGTATCCCCCA  44941
Vaquita      -----------------------------------ACTTATGAAAGGAAGTG-----TGGAGAGCTTTTTGTAGT-----  40891
Pig          ------------------------------------CCTTAAAGAGGAGGTG-----TGGAGAGCTTTTTGTTAT-TTC-  37034
```

```
Majority     -------------TXCTATTTTTAGXXTCTTGAAGAGCXATXCAGCAAGATXTTTCTGATAATATXTX-XAG-----AAA
                               59690     59700     59710     59720     59730     59740     59750     59760
Human        AAGACAACCTAAGGGGATTTTTAAATGCATTGGCTTGCCGTTCAAGTATATAAACCTAAGTGAAACTCCTAGA--GTAAA  46955
PolarBear    --------------CCTCATTTCATGTTCTTATAT-CTCATCTAAAATGAAAACTTTCATAATATTTAGCAC----TAAA  14639
Cow          -------------TGCGATTCATGGGGTCGCAAAGAGTCGGACACGACTGAGCGACTGA-ACTGAACT-GACT----GAA  44073
Dog          --------------AAAAAAAGAAAACTTTCATAA---TATTTAGCACTAAAA---TAAGAATATTTAGTAC----TAAA  40872
BelugaWhale  -------------TCTTATTTTTGGAATGATGAAGAGCTAACCAGCAAGATTTTTCTGGTAATATATT-CAG-----AAG  40279
Donkey       -------------TTGTATTTCTGGAGTGATGAAGAGCTAGTTAACAAGATTTTCATGGAAATATATT-CAG------AA  46022
Cat          GAGCCTCAAACAGTGCTAAA--CATGTTCTTG-A----CACTCAGTAACTGGTTTTTTTTGATACTCAGTAA-----AAA  47350
Ferret       CTTGCTAGCCCTCCCCTCATTTCATGTTCTTATATTCTGATCTGAAATGAAAATTTTCATAATATTTA-CAC----TAAA  48283
Lion         GAGCCTCAAACAGTGCTAAAAACATGTTCTTG-A----CACTCAGTAACTGGTTTTTTTTGATACTCAGTAACTCTTAAA  45016
Vaquita      -------------TCTTATTTTTGGAATGATGAAGAGCTAACCAGCAAGATTTTTCTGGTAATATATT-CAG-----AAA  40952
Pig          -------------TTTTATTTCTGGAGTGGTAAAGAGCTAACCAGTAAGATTTTTCTGGTAATACATT-TAG-----AAG  37095

Majority     XTATAACT------TACTACTCXCTTCTATACATAGTCAAC--CXAAGGGAATTTTTXAATG-------------TATTG
                               59770     59780     59790     59800     59810     59820     59830     59840
Human        ATATGACT--TAAGTATAGCTTGAGTCCATATATTATCACT--TAGAGATACCTTCTCTGATCATCCAATAAAGTCATTA  47031
PolarBear    ATATGAGTATTTAGTAGCCACCCCTTCTAAAACTATTCACCCACCGAGGGAGTATCAG----------------------  14697
Cow          GTAGAACT------TACTAGT--TTTATGTTGAAAGTCAAC--CTACGCA-ATTTTTAAATG-------------CGTTG  44129
Dog          ATAGGAAT----AATAGCCACCCTTTCTAAAACTATTAACTGACTGAGAGAGTATCAGTATGAGGAG----T---T-TTG  40940
BelugaWhale  CCATAGCT------TACTACTCTCTTCTGTACAGAGTCAAC--TTAAGGGAATTTTTAAATG-------------TATTG  40338
Donkey       CCAGAGCT------TACTACT--CTTTTGTACAAAGCCAAC--CTAAGGGGATTTTTAAGTGCATCAGC--TGGCCATTC  46090
Cat          GAATCAGT-----GTGTAAGTGAATTTTAAAGCCACTTAGAGATCAAGTTGATCTTAGAATTT------------TATAG  47413
Ferret       ATATAAATTTTTATTAGCCACCCCTTCTAAAAATAGTCAACAACCGAGGGACTAACAGTATGAAGAGAATGTAGGTTTTG  48363
Lion         GAATCAGT-----GTGTAAATGAATTTTAAAGCCACTTAGAGATCAAATTGATCTTAGAATTT------------TATAG  45079
Vaquita      CCATAGCT------TACTACTGTCTTCTGTACAGAGTCAAC--TTAAGGGAATTTTTAAATG-------------TATTG  41011
Pig          CCAGAACA------TACGACT--CATTTGTACAGAGTCAAC--CCAAGGGGATTTTTAAATG-------------CATTG  37152
```

```
Majority     XTGCTGTTTAATCT-T--------AGTATAXAAX-----------TTGT-TAXAATGTG--ACTTXATT--GACAXTTCC
                      59850     59860     59870     59880     59890     59900     59910     59920
Human        CTACTATCAATCTTCCAGTCCTAGACAAAGTAACTT---------TTAAGCACATCACTGTTTTACCTCACAGCATATAC  47102
PolarBear    ------------------------AGTATGAA------------------------------------------------  14705
Cow          --TCTGTATAAACT-T---------GAATGCAAC------------TCT-TAAACTGTG--ACTTAATT--GACACTTCC  44180
Dog          ATGTTACATGGTCTTTGGGAGAAAAGGATACA------------GTTGTATATAATGTGATATTTTGGGGGGGTATCTTC  41008
BelugaWhale  --GCTGTTCGTTCA-T---------GTATTTCA------------CTGT-TAAAATATG--ACTTAATT--GCCACT---  40386
Donkey       ATG-TATGTAAACTT----------GGGTGAAACTCT--------TAGAGTAAAACATG--ACTTAAGTATAACTTGACA  46149
Cat          ATATTGTTGAATATATATACATACACAACACACACA-------TATAATGTTTATGGGTAGTGTTGATTTGGGGAGTGCC  47486
Ferret       ATGTTACATGATCTTTGGGAGAAAAGTATAAATCTTGGTTGGCAGTTGTATCTAATGTGATATTTGTTTTGGAGATCCTC  48443
Lion         ATATTGTTGAATATGTATACATACACAACACACACA-------TGTAATGTTTATGGGTAGTGTTGATTTGGGGAGTACC  45152
Vaquita      --GCTGTTCGTTTA-T---------GTATATAAACTTGAGTGAAACTGT-TAAAATATG--ACTTAATT--GACACT---  41071
Pig          --GCTGTCCATTCA-T---------GTATATAA----------------------------ACTTGAGT--GACACTTC-  37189

Majority     --TAAATTAT-A--X-ACTXATXAGATGAGTTC---ACAT--ATTTTCTCTGATC-ATCCAGTAXXATAACTATCAXATC
                      59930     59940     59950     59960     59970     59980     59990     60000
Human        CGTAGAAAGTATCTGGTTCAGGTA--CTAGTTA-GTCCAGCTCCCTACTATAAGGTATCAGGAATCAT-ATAATCATCTT  47178
PolarBear    --------------------------------------------------GAGAAT-AT---------------------  14713
Cow          ---AAGTTGT-AT-G-ATTTATCAGATGAGATC---ACAT--AT---CTCTGATC-ATCCAGTAAAATCACTACAATATC  44245
Dog          C-TACAGTGT-AGTGTACTAGTTAGTTGACCTTTCCCC-----CATTTCCCCAGC-ATTGGGGATCTTACCGATCATTTT  41080
BelugaWhale  ---AAGTTAT-A-----CTTATCAGGTGAATTC---ACAT--ATTATCTCTGATC-ATCCAGTAAAATAACTACCACATC  40451
Donkey       AGTAAATTATCAAGTGAGTTCACATATTATCTT-CTAGAGATACCTTCTCTGATTCAACCAATAAAGTCACTATCACACC  46228
Cat          A-TGAAGTAC-CTAAAATTA--TACACAAGTGGAGTATACATATTTCTCAGTGG--ATTGAGGATCCTTCATTTTAGTTA  47560
Ferret       C-TGTAGTGT-AGTGTACTAGTTAGTCTACCTCTACCAATAGACTGTAAGAGAAC-ATTGGGGATCTTACTAATTATCTT  48520
Lion         A-TGAAGTAC-CTAAAATTA--TACACAAGTGGAGTATACATATTTCTCAGTGTT-ATTGAGGATCCTTCATTTTAGTTA  45227
Vaquita      ---AAGTTAT-A-----CTTATCAGGTGAATTC---ACAT--ATTATCTCTGATC-ATCCAGTAAAATAACTACCACATC  41136
Pig          ---AAGTTAT-A-----CTTATCAGGTAAGTTT---ACAT--ATTATCTCTGATC-ATCCAATAAAATCACTACCACAGC  37254
```

```
Majority     XXTCTCT-TCTATXCCXCTG----CTAAGXCACTTTTAAXCACATTXCTGT-GCTTTACTTTCTXCATAGCAXTT-----

                      60010     60020     60030     60040     60050     60060     60070     60080

Human        GTTCACTCTCTGTACCCTCAGAACGTAAAACATTGCTAGACACATCCCTGAATACCCAGTAAATGTTTAAGGAATTAATG  47258
PolarBear    --------------------------------------------------------------------------------  14713
Cow          ACCCTCT-TCCAGTCCTCTG----TTATGTTACTTTTAATCTCATTACTGT-GCTTTACTTTCTCCATAGCACT------  44313
Dog          GTTCACT-CCTGTACCCCTGGAGCCTCAAATAGTGCCAAACACATTCTTGA-TACTCAGTAACAACATCATCATCATCAT  41158
BelugaWhale  ACCCTCT-TCCAGTCCTCTG----CTAAGTCACTTTTAATCACATTACTGT-GCTTTACTTTCTCCATAGCACTT-----  40520
Donkey       ACTCTCT-TCTAGTCTTCTG----CTAAGTCACCTTTAATCACATCACTCT-GCTTTACTTTCTTCATAGCTCTT-----  46297
Cat          GTTT-CA-GAAATATCCCTTAGTCTTTAGACAATTAGGAAC-TACTCTTGT-GGAATATTGGATAGATTGCAAGTCCTTC  47636
Ferret       GTTCACT-GCTGTACCCCCAGAGCCTCAAACAGTGCCAAACACGTTCTTGA-TACTAATTAACTTTTCTGTTTATGTTTT  48598
Lion         GTTT-CA-GAAATATCCCTTAGTCTTTAGACAATTAGGAAC-TACTCTTGT-GGAATATTGGATAGATTGCAAGTCCTTC  45303
Vaquita      ACCCTCT-TCCAGTCCTCTG----CTAAGTCACTTTTAATCACATTACTGT-GCTTTACTTTCTCCATAGCACTT-----  41205
Pig          ACCCTTT-TCTAATCCTCTG----CTAAGTCGCATTTAATCACATCACTCT-TCTTTACTTTCTTCATGACAGTT-----  37323

Majority     AATCACTTTXA---------------GAAATXA-TCTGGTTCGTGTACTGGGTAGT--CTXTCTCTCXCACTAGACTACG

                      60090     60100     60110     60120     60130     60140     60150     60160

Human        AGTGAATTAATTTTGAAGATACGTTGGAGACCAAGATAATCTGACAGTCTTATAGATGTTGTTGAACATACACAAACACA  47338
PolarBear    --------------------------------------------------------------------------------  14713
Cow          -ACTACTTCTA---------------GAAGTTA-TCTGGTTCATGTACTAGGTAGG--CCATCTGTCTCAGTAGACAACG  44374
Dog          CATTATTATTATTATTATTGAGAGAAAGAATGT-GAGCATGTGAGTAGTGGTGAGGGGCTGAAAGAGTGACGGGAGAGAG  41237
BelugaWhale  AATCACTATAA---------------GAAATAA-TCTGGTTCGTGTACTGGGTAGT--CCATCTCTCCCACTAGACTACA  40582
Donkey       AATCACTGTAA---------------GAAATTA-TCTGGTTCATGTACTGGTTACT--CTGCCTCTCCCGCTAG----CC  46355
Cat          AAACAGTTTTAC----------ATAAAATATCA-CCAATTTTGTGTTTTTAGTAGGACCTTTATTTTTGGTGAGGCTTTG  47705
Ferret       ACTTATTTTTA----------------GAGCAT-GCACACTCCAATGGTGGGGAGTGGCAGAGGTAGAGGGAGAGAGAGG  48661
Lion         AAACAGTTTTAC----------ATAAAATATCA-CCAATTTTGTGTCTTTAGTAGGACTTTTCTTTTTGGTGAGGCTTTG  45372
Vaquita      AATCTCTATAA---------------GAAATAA-TCTGGTTCGTGTACTGGGTAGT--CCATCTCTCCCACTAGACTACA  41267
Pig          AATCACTCTAA---------------GAAATTA-CCTGGTTCATGTCCTAGATAGT--CCACCTCTCCCACTAGATTACA  37385
```

```
Majority     GGATAACAXCAGGXATCTTAXTGCTTGCX--GTTT-----ACTGCTGTACCXT--------------------CAGAATC
                               60170     60180     60190     60200     60210     60220     60230     60240
Human        TTATA----TAGTTTATAGGTTGTGTTGAATGTTTTAGGAAATACCATAAATTACCTAAAGTTTTATACAAAATGGAGTA  47414
PolarBear    --------------------------------------------------------------------------------  14713
Cow          AGAGAACATCAGGGCTCTTATTGCTTGCT--GTC------ATTGATGTACTC---------------------CAGAATC  44425
Dog          GATCTTAAGCAGGTTCCATGCCTCGTGTGGAGTTTAACACAGGCCTGGATCTCACAATTCTGAAATCATGACCTGATATG  41317
BelugaWhale  GGACAGCATCAGGGATCTTACTGCTTACT--GTTC-----ACTGCTGTACCCT--------------------CAGAACC  40635
Donkey       ---------CAGA--TCTTATTGATCTT---GTTC-----ACTGCTGTACCCT--------------------CAGAGCC  46396
Cat          GAATAAAGGCAGGTAACTAATTTGTTG----GATAAAAATATATCAGTTTATTTTA--------------A----AGGTC  47763
Ferret       ATCTTAGAGTAGGCTCCACACCCAGTGCAGAGCCTGACCTGGGGCAGGATCTTA--------------TGACCTGAGCTG  48727
Lion         GAATAAAGGCAGGTAACTAATTTGTTG----GATAAAAATATATCAGTTTATTTTA--------------A----AGGTC  45430
Vaquita      GGACAGCATCAGGGATCTTACTGCTTACT--GTTC-----ACTGCTGTACCCT--------------------CAGAACC  41320
Pig          GGAGAGCATCCGGGATCTTACTGCTTACT--GTTT-----GCTGCTGCACCC---------------------CAGAACG  37437

Majority     XAA------AACAGT--GCCAGTCAAG-TTCTTGATX----CTCA---------X-A----GTAA-TGXTTAAAGAATTA
                               60250     60260     60270     60280     60290     60300     60310     60320
Human        TACATGTATATTTCTGAATGTGTCAAGATCCTTCATTTTAGCTAAGTCTCAGAAATATCCTTGAGATCCTAAAAGACTAA  47494
PolarBear    --------------------------------------------------------------------------------  14713
Cow          TAA------AAG-----GCCAGACATT-TTCTTGATA----CTCAAG-------A-AA---GTAACTGCTTAAAGAGTTG  44478
Dog          AAAGAAATCAGGAGTGGGACACTTAAC-TAACTGAGCCA--CCCAGGTGCCCTGGTACTC-GTAA-CTCTTACAGAATCA  41392
BelugaWhale  TAA------AGAAGT--GCCAGACATA-TTCTTGATAA---CTCA----------------GTAA-TGTTTAAAGAATTA  40686
Donkey       TAA------AACAGT--GCCAGACACA-TTCTTGATT----TTCA----------------GTGGCTGTTTAAAGAATCA  46447
Cat          AGA-A--T-AACAGTAAGTTAATGAAG-TCCATGAGTTT--T----------ATATAGATGTTGA-TAGTGACAAAATAC  47825
Ferret       AAA----TCAAGAGTCAGACACTTAAC-TGACTGAGCCT--CTCAGGTACCTCAATACTCAGTAA-CTCGTAAGGAATCA  48799
Lion         AGA-A--T-AACAGTAAGTTAATGAAG-TCCATGAGTGT--TTTATAT---TACATAGATGTTGA-TAGTGACAAAATAC  45499
Vaquita      TAA------AGAAGT--GCCAGACATA-TTCTTGATAA---CTCA----------------GTAA-TGTTTAAAGAATTA  41371
Pig          AAA------AACAGT--GCCGCTCATG-TTCTTGATA----CTCA----------------GTAACTGTTTAAAGAATTA  37488
```

```
Majority     XTGT--TTGAATAAATTTTGAAGACAC-T---AGXAGATCAAGATXXTCTTAGAXTTTTATAG-TA---T---ATXTA-X
                     60330     60340     60350     60360     60370     60380     60390     60400
Human        GAACCATTGATCTGGCATAGTAGATATACTGCAAGTCCTTCAAACAACTGTA----TTTAAATATTACCTATTTTATAGG 47570
PolarBear    -------------------------------------------------------------------------------- 14713
Cow          ATGT--TTGAATAAATTTTAAGGACAC-TTTTAGAAGATCAAGATGGTCTTAATTTTA---------------TTGTA-- 44538
Dog          GTGT--ATGAATGAATTTTAAAGACAC-T---TGGAGATCAAATTAATCTTAGAATTTTACAGATATTGTTGAATGTATA 41466
BelugaWhale  ATGT--TTGAATAAATTTTGAAGGCAC-T--TAGAAGATCCCGATGGTCTTAGAATTTTATAG--A-------TAGTA-- 40750
Donkey       GTGT--GTGAATGAGTTTTGAAGACAC-T---TGGAGATCAAGATGATCTTAGAGTTTTATAGATATTGTTGAATATATA 46521
Cat          TTAG--GTGGATTAAGT--GATAATAT-T---AGTAAATTAGGAGAGACTCA-----TTGTAGTTAAGCTGTCATGTGGA 47892
Ferret       GTGT--GTGAATGAACTTCAAAGACAC-T---TAGAAATCAAATTGATCTTGGAATTTTATAGATGTTGT---ATATATA 48870
Lion         TTAG--GTGGATTAAGA--GATAATAT-T---AGTAAATTAGGAGAGACTTA-----CTGTAGTTAAGCTGTAATGTGGA 45566
Vaquita      ATGT--TTGAATAAATTTTGAAGGCAC-T--TAGAAGATCCCGATGGTCTTAGAATTTTATAG--A-------TAATA-- 41435
Pig          ATGT--TTGAGTAAATTTTGAAGCTAC-T--TAGAAGATCAAGATAATCTTAGAGTTT---------------GTAT--- 37545

Majority     ----------------------X---------------X----------TTGAXTGTTTTGX--GGGGGAGTACTATAA
                     60410     60420     60430     60440     60450     60460     60470     60480
Human        CATTTTGAATAATATTTTTATTTTTTGGTGAAGTTTTGGAATGAAGACAGTTAACTAATTTGTTGATTGAATTATGTTAC 47650
PolarBear    -------------------------------------------------------------------------------- 14713
Cow          -------------------------------------------------TTGAGTGTTTGG---GGGTGTGTACTGTGA 44565
Dog          CACACACACACACACACACACACACACACACCCACACACATATAATGTTAATGGGTAGTGTTGATTTGGAGAATACCATGA 41546
BelugaWhale  -------------------------------------------------TTGAATGTTTTCT--GGGGGTGTACTGTAA 40778
Donkey       TTT---------------TA--TATATATAATAT-TGTTTATAGATAGTGTTGAATGTTTTG---GGGCGCATACCATAA 46580
Cat          TTGAC---------TGGATGATTATTTTTACAGCAT---TGC-------ATTCTTTGATTCCACTTTTTGAGTACAAT-- 47951
Ferret       CATACATACACACACACGCGCGCACACACACAGCACACGTAG---GTTTATGGGTAGTTTTGATTTGGGGAGTACCATGA 48947
Lion         TTGAC---------TGCATGATTATTTTTACAGCAT---TGC-------CTTCTTTGATTCCACTTTTTGAGTACAAT-- 45625
Vaquita      -------------------------------------------------TTGAATGTTTTCT--GGGGGTGTACTGTAA 41463
Pig          -------------------------------------------------CTGAATGTTTTG---GGGGGTATACTGTAA 37572
```

```
Majority     AGTACCXAAAATTATAT--AAAA-TGGAGTATACATGTATATTTCTCA-CTGTTTAAGGATTCTTCATTTTAGTTAG--T
                       60490     60500     60510     60520     60530     60540     60550     60560
Human        TTTACTTTAAATCTCACG-GTAACAGAATAAGGTAATTAAGTTCATGAGTGATTTAGAGTATATTGATGTTAAT-AG--T  47726
PolarBear    --------------------------------------------------------------------------------  14713
Cow          AGTACCAAAATTTGTATACAAAAATGGAGTATACATGTATATTTCTCT-GTGTGTAAGAGTTTTCATTTTAAT-AGATT  44643
Dog          AGTATCTAAAGTTACAT--AGTAGTGGTGTATACATGTATATTTTTCAACTTATCAAGTATCCTTCATTTTAGTTAG--T  41622
BelugaWhale  AGTACCAAAAATCATAT-CAAAA-TGGAGTATACATGCATATTTCTCA-CTGTGTAAGGATTCTTCAGGTTAGTTAGATT  40855
Donkey       AGTACCCAAAAGTATCTACAAAA-TGGAGTGTACAGGTATATTTCTCA-ATGTATAAGCATTCTTCATTTTAATGAGAAC  46658
Cat          -ATTTTCCAAATTAAAC----------------CATTAATAATTT----CTTATCA-----CCCTAATTTTTGCTAA--G  48003
Ferret       AGTACGTAAAGTTATAC--ACAAGTGGAGTGTACATGTATATTTCTCAGCTTATTAAGTATCCTTTGTTTTAGTT-----  49020
Lion         -ATTTTTCAAATTAAAC----------------CATTAATAATTT----CTTATCA-----CCCTAATTTTTGCTAA--G  45677
Vaquita      AGTACCAAAAATCATAT-CAAAG-TGGAGTATACATGCATATTTCTCA-CTGTGTAAGGATTCTTCAGGTTA--TAG--T  41536
Pig          AATACCAAAAATTTTATACAAAA-TGGAGCTTACATGTATATTTTTTCAGTGTGTAAGGATTCTTCATTTTAGTTAGGTT  37651

Majority     CTCAGAAATATCCTTGAGTTTTTAGAAGATTAXGAACCA-T-XTAAGX----C--AATXXATAGATTXTAAGATCTTTAA
                       60570     60580     60590     60600     60610     60620     60630     60640
Human        CACA-AAATATTAGGTAGTTTAGACGGCAATAGTAATATATCAGGAGAGGCTCATTGTAGATAAGCCATAATATGGATTG  47805
PolarBear    --------------------------------------------------------------------------------  14713
Cow          CTTAGAACTATCCTTGAGTCTT-----------------------AGA----T--GATATTCTGTTTATTGCATCTTTCA  44694
Dog          TTCAGAAATATCCTTTAGTCTGTAGACAGTTAGGAACCACTGATACAG----AATAAGGAATAGACTGTAAGTTCTTCAA  41698
BelugaWhale  CTCAGAAATATCCTTGAGTTCTTAGAAGATTAAGAACCA-T--CAAAA----C--ATCACATATTTTATTGATTCTTTAG  40926
Donkey       CTCAGAAATTGCTTTGAGTCCTTAGAAGTT----AACTACCAGTGTGG----AATAGTGGATAGACTGTAAGTTCTTCAA  46730
Cat          T-C--A-ATATTTCACACTTTTTGTATTTCTGTTAGTCTGTAATAA-G--------ATGATTAAATTGTAAGA--T---A  48065
Ferret       -TCAGAAACATCTTTTAGTCCATAGACCATTTGGAACCACTAATAAGG----AATAGTGGATAGACTGCAAGGTCTTCAA  49095
Lion         T-C--A-ATATTTCACACTTTTTGTATTTCTGTTAGTCTATAATAA-G--------ATGATTAAATTGTAAGA--T---A  45739
Vaquita      CTCAGAAATATCCTTGAGTTCTTAGAAGATTAAGAACCA-T--CAAAA----C--ATCACATATTTTATTGATTCTTTAG  41607
Pig          CTCAGAAATATCCTTGAATTTTTAGAAGATTTAGA-CCA-T--TAAGA----C--ATCACTTATTTTATTGCATCTTTAG  37721
```

```
Majority     AXAGTTTTGC--TTTAGAXAX-----A-TTTXTTX--TCTXTAXTX--A---TXXTTTTTGG-TGAAGC--TTTGGAATA
                      60650     60660     60670     60680     60690     60700     60710     60720
Human        ACTGCATTATCATTTTTATAAACTTTACATTCTTTGATCCCACCTTCAAGTACATTTTTCAAATCAAACAATTTATAATT  47885
PolarBear    --------------------------------------------------------------------------------  14713
Cow          TAAGTTTTGT--TTTTG------------TTTTT------------------TGTGTTTTGG-TATAGT--TTTGGA-TA  44738
Dog          ACAGCTTTGC--ATAAAACATCACCTATTTTATTGTGTCTGTAGTAGGACCTT-ATTTTTGG-TGAGGC--TTTAAAATA  41772
BelugaWhale  TAAGTTCGGA--TTTTGG----------TTTTTTG--TTTATTTTG------TGTGTTTTGG-TAAAGC--TTTGGAATA  40983
Donkey       ATAGTTTTCC--ATAAAACATCACCTATTTTATTGCCTCTTTAGTAAGATTTTTATTTTTGG-TGAAGC--TTTGGAATA  46805
Cat          ATTGTGTT-C--TTTAGCCATTGGAAAATTTCTT---TCTGAAATGGCA-----ATTTTTGC-TTGTCA--TTT---ATT  48128
Ferret       ATGGTTTTAC--ATAAAACATCACCTATTCTATTGTGTCTTTAGTAGGACTTTTATTTTTGG-TGAGGC--TTTGGAATC  49170
Lion         ATTGCGTT-C--TTTAGCCATTGGAAAATTTCTT---TCTGAAATTGCATTTTAATTTTTGC-TTGTCA--TTT---ATT  45807
Vaquita      TAAGTTTGGA--TTTTGG----------TTTTTTG--TTTATTTTG------TGTGTTTTGG-TAAAGC--TTTGGAATA  41664
Pig          TAAGTTTGAG--TTTGGG----------GCTGTT------------------TATTTTTTGG-TGAAGC--TTTGGAATA  37768

Majority     AAGGCAGGXAA--XTAA---TTTGTTGTTT-----AAAATATA-----TCACTTT-----AAAGCTCAGAGTAA--CAGA
                      60730     60740     60750     60760     60770     60780     60790     60800
Human        -TCTCAGGTAAAATTATAATCTTCCTAATTTTGGTCAAACCAGTTTTTCACTTTTTAAAAATGTTCATATTAGTTCATT  47964
PolarBear    --------------------------------------------------------------------------------  14713
Cow          AAGGTAGGCAA--TTAA---TTTGTTGTTC-----AGAATATA-----TCACTCT-----AAAGCGCAGAGTAA--CA-G  44795
Dog          AAGCCCGGTAA--CTAC---TTTGTTGATT-----AAAATATATCAGTTTACTTT-----AAAGGGCGTAGTAA--CAGA  41835
BelugaWhale  AAAGCAGGCAA--CTAA---TTTGTTGTTT-----AAAATATA-----TCACTTT-----AAAGCTCAGAGTAA--CAGA  41041
Donkey       AAAGCAGGTAACTTTAA---TTTGTT----------AAAT---------------------------AGAATAA--CAGA  46843
Cat          ACTCTTTATGT--GTG----TGTGTGTGTG-----TGTGT--------ATACCTA-----CACACACTCTGTT---CAGA  48181
Ferret       AAGGCAGATAA--CTAA---TTTTTTGATT-----AAAGTGTATCAGTTTACTTT-----AA-GGTCAGAGTAA--CAGA  49232
Lion         ACTCTTTACGT--GTG----TGTGTGTGTG-----TGTGTGTGTGTATATACATA-----CACACACTCTGTT---CAGA  45868
Vaquita      AAAGCAGGCAA--CTAA---TTTGTTGTTT-----AAAATATA-----TCACTTT-----AAAGCTCAGAGTAA--CAGA  41722
Pig          AAGACAGGCAAC-TTAA---TTTTCTGTTTG----AAAATATA-----TCACTTT-----AAAGCTCAGAGTAG-----G  37825
```

Majority	ATAAGXTAATCAAGTC-CATGA-GTGATT-TATAATATATAGATGTTGATAGTGACAAA---------------------	
	60810 60820 60830 60840 60850 60860 60870 60880	
Human	ATAAGATGATTAAATTACAAGATACAGTCATAAATCTTGGGGATAGTGATGAGGATACTCTCGGAGAAATGCATTGTTAG	48044
PolarBear	--	14713
Cow	AAAAGATAATCAAGTC-CATGA-GTGATAATATAATAGGTAGATGTTGATAGTGATAAA---------------------	44852
Dog	ATAAGTTAATCAAGTC-CATGA-GTATTT-TATAGTATATAGATGTTGAATGTTATTAG----T---GACAAAATATTTA	41905
BelugaWhale	ATAAGATAATCAAGTC-CATGA-GTGATT-TATA----GTAGATTTTGATAGTGACAAA---------------------	41093
Donkey	ATACGTTAATCAAGTC-CATGA-ATGATT-TATAGTATATAGATGTTGATAGTGACAACAT-ATTTAGATAGATTAGGTG	46919
Cat	AACATTTACCCAGTAG-TTACC-TTAATT-GGTAACATA---ATTTTATTTGATAATAATT-----------------CT	48238
Ferret	AAAAGTTAATCAAGTC-CATGA-GTGTTT-CATAGAATATAGATGTTGATAGTGACAAAAT-ATTTAGGTAGATTATGTG	49308
Lion	AACATTTACCCAGTAG-TTACC-TTAATT-GGTAACATA---ATTTTATTTGATAATAATT-GGTGGATATGATTTTTCT	45941
Vaquita	ATAAGATAATCAAGTC-CATGA-GTGATT-TATA----GTAGATTTTGATAGTGACAAA---------------------	41774
Pig	GTAAGATAATCAAGTC-CATGA-GTGATT-TATAATGGATAGATGTTGATAGTGACAAA---------------------	37881

Majority	--ATATTTT--AAAGTAGAGGAXT-X-AXX-T--GTAAGC-CATAAT-G-TGGATT-GACTGCATAATTATTTTTACAGC	
	60890 60900 60910 60920 60930 60940 60950 60960	
Human	GTAATTTTGTCACTGCATAAATGTCATAGAGTACATTTGCACAAACCTGCTACACATGTAGGCTATATGGTATATGTAGC	48124
PolarBear	--	14713
Cow	--ATATTT----AGGTAGAGGAAT----------ATAAGC-CATACT-G-TGGATT-GACTGCATAATAATTTTTAAAGC	44912
Dog	G-ATAGATAATAAATTAGGAGAGTCTCATTGTAGGTAAGC-CATAAT-G-TGGATTTGATTGCATAATTATTTTTACAGC	41981
BelugaWhale	--ACATTTT---AGGTAGAGGAAT----------GTGACC-CATAGT-G-TGGAGT-GACTGCATAATAATTTTTACAGC	41154
Donkey	GTAATTTTAACAAATTAGGAGAGGCTCATTGTAGGTAAGC-CATAATGG-TGGATT-GACTGCATGATCATTTTTACAGC	46996
Cat	CTCTCCCTTTGAAAGCCCAGTTTTTTAATTTTTAGAAATT-ATTCAG-G-TATACTCAGCTATGTTTTTTAATTTAAAAA	48315
Ferret	GTATTATGAATAAATTAGGAGAGGCTTATTGTAAGTAAGC-C----T-G-TGGATT-GACTGCATAATTATTTTTACAGC	49380
Lion	CTCTCCCTTTGAAAGCCCAGTTTTTTAATTTTTAGAAATT-ATTCAG-G-CATACTCAGCTATGTTTTTAAGTTAAAAAA	46018
Vaquita	--ACATTTT---AGGTAGAGGAAT----------GTGACC-CATAGT-G-TGGAGT-GACTGCATAATAATTTTTACAGC	41835
Pig	--ATGTTT----AAGTAGAGGAAT----------GTAAGC-CA-----G-TGGATT-GACTGCATAATTATTTTTACAGC	37937

Majority	ATTG---CCTTCTTTGATTCCATTTTTTAGTATATT----TTCCAAAT-TGAG--CAATTTAGAATTTCTCAGGTAAAAT	
	60970 60980 60990 61000 61010 61020 61030 61040	
Human	CTTTTGCTCCTAGGCAA-CAAATTTGACAGCATGTTACTGTTCTGAATACTGGAGGCAGTTGCAA---CACAGTAATAAG	48200
PolarBear	--	14713
Cow	ATTA---TCTGATTCCATT----TTTTTAGTACATT----TTCCAATT-TGAG--CAATTTAGAATTTTTCAGGTAAAAT	44978
Dog	ACTA---CCTTCTTTGCTTCCATTTTTTACTATGATAC-ATTCCAAAT-TGTGAACCATTTATAATTTCCCAGGTAAAAA	42056
BelugaWhale	ATTG---CCTTCTTTGATTCCATTTTTTAGCACATT----TTTCAAAT-TGAA--CAATTTAGAATTTCTCAGGTAAAAT	41224
Donkey	ATTG---CTTTCTTCGATCTCATTTTTGAGTAGAATA---TTTTAAAT-TGAA--CAATATATAATTTCTAAGGTAAAAT	47067
Cat	AAA----TTTTTTTTAAATGTTTATTTTTGAGAGACAG-AGACACAGC-AGGGGAGGGGT-------AGAGAGAGAGAGA	48382
Ferret	ATTG---CCTTCTTTGTTCCCAGTTTTGAGTATATT----TTCTAAAT-TG--AACCATTTATAATTTTCCCAGGTAAAA	49450
Lion	AAAAATTTTTTTTTTAAATGTTTATTTTTGAGAGACAG-AGACACAGT-GGGGGAGGGGGGGGGGGAGAGAGAGAGAGA	46096
Vaquita	ATTG---CCTTCTTTGATTCCGTTTTTTAGCACATC----TTTCAAAT-TGAA--CAATTTAGAATTTCTCAGGTAAAAT	41905
Pig	ATTG---CCTTCTTCGATTCCATTTTTCAGTGTATT----TTACAAAT-CAAAA-TAATTTAGAATTTCTCAGGTAAAAT	38008

Majority	T-ATAAT-TCCXTAATTTTTGCTAAGTCAAAATTTTCAX-TT-TTTTGTATTTTTCTXAGTCT----------XT---AC	
	61050 61060 61070 61080 61090 61100 61110 61120	
Human	TATTGATGTATCTAAACA--GAAAAGGTACAATAAAAATATGGTATTATAATCTTATGGGACCACCATTGTATATGTGGC	48278
PolarBear	--	14713
Cow	--ATAGTGTCCCTAATTTTTGCTAAGTCAAAATTTTCAC-TGATTTTGTATATTCCTTAGTCT----------------G	45039
Dog	TTACAATCTCCT-AATTTTTGCTAAGTCAAAAGTAGAGT-TTTTTTTTTTTTTTTTTGTATTT----------CTGTTAG	42124
BelugaWhale	T-ATAAT-----TAATTTTTGCTAAGTCAAAATTTTCAC-TT-TTTTGTATTTTCCTTAGTCTTAGACTAAGTCTTAGAC	41296
Donkey	T-ATGATGTCCCTACTTTTTGCTAAGTCAGAATTTTCAT-TTTTTTTGTGTTTTTGTTAGTCT----------------G	47129
Cat	GGGAGA--ACAGAATCCAAAGCAGGCTCCAAGGC--------------TCTGAGCTCTGAGC----------ACAGAAT	48435
Ferret	TTACAACCTCCTTAATTTTTGCCAGGTCAGAAGT-------TTAACTTTTTTTTTTTCCATTT----------CTGTTAG	49513
Lion	GAGAGAG-AGAGAGACAGACACAGAATCCAAAGCA--G--------TCTCCAAGGCTCTGAGC----------ACAGAGC	46155
Vaquita	T-ATAAT-----TAATTTTTGCTAAGTCAAAATTTTCAC-TT-TTTTGTATTTTCCTTAGTCTTAGACTAAGTCTTAGAC	41977
Pig	T-ATAATCTCCATAATTTTTGCTGAGTCAGATTTTTCAC----TTTTTAATTTCTTTCAGTCT----------------C	38067

```
Majority     TCC-XAXTXX-----AATXAGATGATTGAATTGTAAG-ATAATTGTGTTCTTTAGCCATTGG----ACXATTTCTTTATG

                      61130     61140     61150     61160     61170     61180     61190     61200

Human        CTGTCAATGACCAAAATGTAGTTATGTGGCACATGACTGTGATTGTATTCTTTAGTCATTGG----ACAGTTTCTTTATG 48354
PolarBear    -------------------------------------------------------------------------------- 14713
Cow          TTC----------------AGATGATTGAATTGTAAG-ATAATTGTTTTGTATAGCCACTG-----ACCATTTCTTTATG 45097
Dog          TTCATAATAA-----AATAAAATGATTAAATTGTAAG-ATAATTGTGTTCTTTAGGCATTGG----ACAATTTATT---- 42190
BelugaWhale  TCCTTATTAGTCTCTAATAAGACGATTGAATTGTAAG-ATAATTGTGTTCTATAGCCATTG-----ACCATTTCTTTATG 41370
Donkey       T--------------AATAAGATGATT-AATTGCAAA-ATAATTG--TTCTTTAGCCATTGC----ACAGTTTCTTTATG 47187
Cat          CCGACATGGG-----GCTCAAACCCACGAACCGTGAG-ATCATGACCTGAACT-GAAGTCGG----ATGCTCAACAGACG 48504
Ferret       TCAGTAATAA-----AAT-----GATTAAATTATAAG-ATAATTGTGTTCTTTAGCCATTGG----ACAATTTCTTTATG 49578
Lion         CCGACATGGG-----GCTCGAACCCACGAACCGTGAG-ATCATGACCTGAACT-GAAGTCAG----ATGCTCAACGGACG 46224
Vaquita      TCCTTATTAGTCTCTAATAAGACGATTGAATTGTAAG-GTAATTGTGTTCTATAGCCATTG-----ACCATTTCTTTATG 42051
Pig          T-------------------AATAAGTTGATTGAATC-ATAGCTGTGTTCTGTAACCATTGGACCCATCATTTCTTTATG 38127

Majority     AAATTACATTTTAATGTTTTTGATTATCATTTGTTACTCTTTACA-----------TATACTGTTCAGAAATAT--TTAC

                      61210     61220     61230     61240     61250     61260     61270     61280

Human        AGATTACATTTTAATGTTGTTGCTTATTAATAATTACTCTTTATA------------ATACTGTTAAGAAACAT------ 48416
PolarBear    -------------------------ATCATTTGTTACTTTTTACA-----------TATATTGTTCAGAAACAT--GTAT 14755
Cow          AAATTTCATTTTAATGTTTTTGATTGTCAGTTGTTACTCT--ACA-----------TATGCTATCCAGAGATGTCTCTAC 45164
Dog          AAATTGCCTTTTAATATTTTTGCTTATCATTTGTTACTCTTTATG-----------TATACAGTTCAGAAATAT--TTAC 42257
BelugaWhale  AAATTGCATTTTAATGTTTTTGATTATCACTTGCTACTCTTTACA-----------TATGCTATCCAGAAATATATTTAC 41439
Donkey       AAATTACATTTTAATATTTTTGCTTATCTCTTGTTACTTGTTGCA-----------TATACTGTTCAGAAACTTACTTAC 47256
Cat          GAGCCACCCAGGCGCCCCTTTAAAAATTTTTTTTTAATGTTTATG-----------CTTATTTTTGAGACAGAG--AGA- 48570
Ferret       AAATTGCATTTTAATATTTTTACTTATCATTTGTTATCTGTGACAGTACCAGAAACTGTTCTGTTCAGAAACAT--TTAC 49656
Lion         GAGCCACCCAGGCGCCCCTTTAAAAATTTTTTTTTAATGTTTATG-----------TTTATTTTTGAGACAGAG--AGA- 46290
Vaquita      AAATTGCATTTTAATGTTTTTGATTATCACTTGCTACTCTTTACA-----------TATGCTATCCAGAAATATATTTAC 42120
Pig          AA-TTACATTTTTATGTTTTTTATTATTGCTTGTTACTCTTTACA-----------TATACTGTCCAGAAATATATTTAT 38195
```

```
Majority     CCAATAGTAATCTTAATTGATG--GTAATTTCATTTAATAGTA------ATTGGTGGATATGA-TTTTCTCTATTXCTTT
                       61290     61300     61310     61320     61330     61340     61350     61360
Human        -TAACAGCAATCTTAAATAGTTG-ATAATTTCATTTAATAATA------ATAGGTGGATATGA--TTT---TATTCCTTT  48483
PolarBear    CTAATAATTATCTTAATTGATAATGTAATTCTATTTAATA---------ATTGGTGGATATGA-TTTTCTCTATTCCTTT  14825
Cow          CCAATAATAATCTTAATTGATG-----AGATCATTTAGTAGTA------ATTGGTGGATAAGAGTTTTTCCATTACTCT  45233
Dog          CAAATAGTGATCTTAATTGATAATGTAATTTTATTAAATTTTATTTAATAATGGTGGATATTA-TTTTCTCTATTCTTTT  42336
BelugaWhale  CCAATAGTAATCTTAATTGATG-----ATTTCATTTAGTAGTA------ATTGGTGGATATGA-TTTTCTCTGTTACTTT  41507
Donkey       CCAGTAATAGTCTTAATTAATTG-ATAATTTCATTTAATGATA------ATTGGTGGGTATGA-TTTTCTTTATTCCTTT  47328
Cat          --GACAG--ATCATGAGCGGGG--GAAGGGCGGAGAAAGA-----------GGGAGACACAG-AAAGCGAAGCAGGCTC  48631
Ferret       CTAATAGTTATCTTAATTGATAATGTAATTTTATTTAATAGTA------ATTGGTGAATATGA-TTTTCTCTATTTCTTT  49729
Lion         --GACAG--ATCATGAGCGGGG--GAAGGGCAGAGAAAGA-----------GGGAGACACAG-AAAGCGAAGCAGGCTC  46351
Vaquita      CCAATAGTAATCTTAATTGATG-----ATTTCATTTAGTAGTA------ATTGGTGGATATGA-TTTTCTCTGTTACTTT  42188
Pig          CCAATAGTAATCTTAATTGATG-----GTTTCATTTAGTAATA------ATTGGTAGATATGA-TTTTCTCCATTACTTT  38263

Majority     GAAAXCCTGGTTTTTGAATTTTTAGGAATTA-TTCAAGTATXCTCAGCTGTGTTTTTAAGTTCTTTTTTAAAXTTTG---
                       61370     61380     61390     61400     61410     61420     61430     61440
Human        GAAAGCCTGGCATTTGAATTTTCAAGAATTA-TTTAAGTATTCTCAGCTATGTTTTTAAGTTCTTTTTGGAAATTTGTAG  48562
PolarBear    GGAAGCCTGGCTTTTGAATTTTTA--------TTCAGGTGTATTCAGCTATGTTTTTAAGTTCTTTTTTAAACTTTG---  14894
Cow          GAAAACCGTGTTTGTAAATTTTTAGTA-TCA---CAGGTATGCTTAGCTATGTTTTTAAGTTCTCTGTTGAAGTTTG---  45306
Dog          -------TGCTTTTTGAATTTTTAGGAATTA-TTCAGGTCTACTCAGCTGTATATTTAAATTCTTTTTTAAA--------  42400
BelugaWhale  GCAAACCTGGTTTTTGAATTTTTAGGAAGTATCCCAAGTATGCTCAGTTCTGTTTTTAAGTTCTTTATTAAAGTTTG---  41584
Donkey       GAAAGTCCAGTTTTTGAATTTTTAGGAATTA-TTCAAGTATACTCAGCTGTGTTTTT---------GT------------  47386
Cat          -------CAGGCTCTGAGCTGTGAGCACAGA-GCCCAATGTGACCCAGGGCTCAATTAAGTTCTTTTTTAAACTTTG---  48700
Ferret       GGAAGCCTGGCTTTTGAATTTTTAGGAAATA-TTCAGGTTTACTCAGCTATGTTCTTAAGTTCTTTTTTAAACTTTG---  49805
Lion         -------CAGGCTCTGAGCTGTGAGCACAGA-GCCCAATGTGACCCAGGGCTCAATTAAGTTCTTTTTTAAACTTTG---  46420
Vaquita      GCAAACCTGGTTTTTGAATTTTTAGGAAGTATCCCAACTATGCTCAGCTCTGTTTTTAAGTTCTTTATTAAAGTTTG---  42265
Pig          GAAAACCTAGTTTTTGAATTTTTGGGAATTATC-CATTTGCACTCAGCTGTGCTTTCACATTATTTATTAAAGTTTG---  38339
```

```
Majority     ---TTTTGTG------XTTTTTT-TACACAAATGAGGAAAATTAATATAXTXA-XACTX-TTTTTXAATGTCTTTCAGCC

                              61450     61460     61470     61480     61490     61500     61510     61520

Human        TTTTTTTACACAAATAGGAAAATTCATACATTCTTTTATTGTTGATATAT-AAGGACTG-TTTTTTAATGTCTTTCAGCC   48640
PolarBear    ---TTTTGTG------GTTTTTA-TGCA-AA-TGAGGAAAATTAATAAAG-----ACT--TTTTTAAATGTCTTTCAGCC   14955
Cow          ---TTTTGTG------ATTTTTT-T--TTTTTTTTACGCACATAAGATAAACACAACTGTTTCTTTACTTTCTTTCAGCC   45374
Dog          ---TTTTGTG------GTTTTTA-TGCA-AA-TGAGGAAAATTAATAAGG-----ACT--TTCTAAAATGTTTTACAGCC   42461
BelugaWhale  ---TTTTGTG------ATTTTTT-TATGCAAATAAGGAAAATCAATATACTAAAGACTGTTTCTTTAATGTCTTTCAGCC   41654
Donkey       ----TTTGTG------TTTTTTA---TGCAAATAAGGAAAAGTAATATAATAAAGACTTCTTTTTCAATGTCTTTCAGCC   47453
Cat          ---TTTTGTGGGGTTTCTTTTTTTTACATAAATGGGGAAAATTAATAAACT----ATT--TTTTTAAATGTCTTTCAGCC   48771
Ferret       ---TTTTGTGTGG---GTTTTTAATGCA-AAATGAGGAAAATTAATAAAA-----ACT--TTTTAAAATGTCTTTCAGCC   49871
Lion         ---TTTTGTGGGGTTTCTTTTTTTTACACAAATGGGGAAAATTAATAAACT----ATT--TTTTTAAATGTCTTTCAGCC   46491
Vaquita      ---TTTTGTG------ATTTTTT-TATGCAAATAAGGAAAATCAATATACTAAAGACTGTTTCTTTAATGTCTTTCAGCC   42335
Pig          ---TTTTGTG------ATTTTTA-T--GCAAATAAGGAAAATTAATATAATAAAGATTGTTTCTTTAATGTCTTTCAGCC   38407

Majority     AATGGTAATGATAGTAAAAAATTTAAAGGAGAAGATAAAATGGATGGTGCTCCTTCTCGTGTACTTCATATTCGAAAATT

                              61530     61540     61550     61560     61570     61580     61590     61600

Human        AATGGTAATGATAGTAAAAAATTTAAAGGAGAAGATAAAATGGATGGTGCTCCTTCTCGTGTACTTCATATTCGAAAATT   48720
PolarBear    AATGGTAATGATAGCAAAAAATTTAAAGGAGAAGATAAAATGGATGGTGCTCCTTCTCGTGTACTTCATATTCGAAAATT   15035
Cow          AATGGTAATGATAGTAAAAAATTCAAAGGAGAAGATAAAATGGATGGTGCTCCTTCTCGTGTACTTCATATTCGAAAATT   45454
Dog          AATGGTAATGATAGCAAGAAATTTAAAGGAGAAGATAAAATGGATGGTGCTCCTTCTCGTGTACTTCATATTCGAAAATT   42541
BelugaWhale  AATGGTAATGATAGTAAAAAATTTAAAGGAGAAGATAAAATGGATGGTGCTCCTTCTCGTGTACTTCATATTCGAAAATT   41734
Donkey       AATGGTAATGATAGTAAAAAATTTAAAGGAGAAGATAAAATGGATGGTGCTCCTTCTCGTGTACTTCATATTCGAAAATT   47533
Cat          AATGGTAATGATAGTAAAAAATTTAAAGGAGAAGATAAAATGGATGGTGCTCCTTCTCGTGTACTTCATATTCGAAAGTT   48851
Ferret       AATGGTAATGATAGCAAAAAATTTAAAGGAGAAGATAAAATGGATGGTGCTCCTTCTCGTGTACTTCATATTCGAAAATT   49951
Lion         AATGGTAATGATAGTAAAAAATTTAAAGGAGAAGATAAAATGGATGGTGCTCCTTCTCGTGTACTTCATATTCGAAAGTT   46571
Vaquita      AATGGTAATGATAGTAAAAAATTTAAAGGAGAAGATAAAATGGATGGTGCTCCTTCTCGTGTACTTCATATTCGAAAATT   42415
Pig          AATGGTAATGATAGTAAAAAATTTAAAGGAGAAGATAAAATGGATGGTGCTCCTTCTCGTGTACTTCATATTCGAAAATT   38487
```

```
Majority     ACCTGGTGAAGTGACAGAAACTGAAGTTATTGCTTTAGGCTTACCTTTTGGTAAGGTGACCAACATCCTTATGCTGAAAG
                       61610     61620     61630     61640     61650     61660     61670     61680
Human        ACCTGGGGAAGTAACAGAAACTGAAGTTATTGCTTTAGGCTTACCTTTTGGTAAGGTGACCAACATCCTTATGCTGAAAG  48800
PolarBear    ACCTGGTGAAGTGACAGAAACTGAAGTCATTGCTTTAGGCTTACCTTTTGGTAAGGTGACCAACATCCTTATGCTGAAAG  15115
Cow          GCCTGGTGAAGTGACAGAAACTGAAGTTATTGCTTTAGGCTTACCTTTTGGTAAGGTGACCAACATCCTTATGCTGAAAG  45534
Dog          ACCTGGTGAAGTGACAGAAACAGAAGTTATTGCTTTAGGCTTACCTTTTGGTAAGGTGACCAACATCCTTATGCTGAAAG  42621
BelugaWhale  ACCTGGTGAAGTGACAGAAACTGAAGTTATTGCTTTAGGCTTACCTTTTGGTAAGGTGACCAACATCCTTATGCTGAAAG  41814
Donkey       ACCTGGTGAAGTGACAGAAACTGAAGTTATTGCTTTAGGCTTACCTTTTGGTAAGGTGACCAACATCCTTATGCTGAAAG  47613
Cat          ACCTGGTGAAGTGACAGAAACTGAAGTTATTGCTTTAGGCTTACCTTTTGGTAAAGTGACCAACATCCTTATGCTGAAAG  48931
Ferret       ACCTGGTGAAGTGACAGAAACTGAAGTTATTGCTTTGGGCTTACCTTTTGGTAAGGTGACCAATATCCTTATGCTGAAAG  50031
Lion         ACCTGGTGAAGTGACAGAAACTGAAGTTATTGCTTTAGGCTTACCTTTTGGTAAAGTGACCAACATCCTTATGCTGAAAG  46651
Vaquita      ACCTGGTGAAGTGACAGAAACTGAAGTTATTGCTTTAGGCTTACCTTTTGGTAAGGTGACCAACATCCTTATGCTGAAAG  42495
Pig          ACCTGGTGAAGTGACAGAAACTGAAGTTATTGCTTTAGGTTTACCTTTTGGTAAGGTGACCAACATCCTTATGCTGAAAG  38567

Majority     GAAAAAATCAGGTA-TACTTC-TTTCAGGACTTATAAAA--TGTTAAGCCCCAAACTATCCAGTAXGTGTGAATTAGCTA
                       61690     61700     61710     61720     61730     61740     61750     61760
Human        GAAAAAATCAGGTA-CACTTC-TTTCAGGGTTTATGAAA--TGTTAAACCCCAAACTATCCAGCAGGTGTGAATTAATTG  48876
PolarBear    GAAAAAATCAGGTA-TGCTTC-TTTCAGGACCTATAAAA--TGTTAAGCTGCAAACTACCCAGTATGTGTGAATTAGCTA  15191
Cow          GAAAAAATCAGGTA-TACTTA-TTTCTGGACTTATACCA--TGTTAAGCTCCAAACTATCCAGCAAGTG-GATTTAGCTG  45609
Dog          GAAAAAATCAGGTA-TGCTTCCTTTCAGGACCTATAAAA--TGTTAAGCCTGAAACTACCCAGTATGTGTGTATTAGCTA  42698
BelugaWhale  GAAAAAATCAGGTA-TATTTC-TTTCAGGACTTATAAAA--TGTTAAGCCCCA-ACTATCCAGCAGGTGTGAATTAGCTG  41889
Donkey       GAAAAAATCAGGTA-TACTTC-TTTCAGGACTTATATAAAACGTTAAGCCCCAAACTACCCAGCAGGTGTAAATTAGCTA  47691
Cat          GAAAAAATCAGGTA-TGCTTC-TTTCAAGACTTACAAAAC-TGTTAAGCCCCAAGCTATCCTGTGTGTGTGAATTAGCTA  49008
Ferret       GAAAAAATCAGGTAATGCTTC-TTTTAGGTTCTGTAAAA--TGTTAAGCCCCAAACTACCCTGTATGAGGGAATTAGCTA  50108
Lion         GAAAAAATCAGGTA-TGCTTC-TTTCAGGACTTACAAAAC-TGTTAAGCCCCAAGCTATCCTGTGTGTGTGAATTAGCTA  46728
Vaquita      GAAAAAATCAGGTA-TATTTC-TTTCAGGACTTATAAAA--TGTTAAGCCCCA-ACTATCCAGCAGGTGTGAATTAGCTG  42570
Pig          GAAAAAATCAGGTA-TACTTC-TTTCAGGACTTATAAAA--TGTTAAGCCCCACACTATCCAATAGGTGTGAATTAGGTA  38643
```

Majority	TTTCCTAATAAAAGGATAGA--ATATAG-----TAGTAGGTTACTTTTCTAAAAGAACATXACAGAAGTCTTATTCTGAC	
	61770 61780 61790 61800 61810 61820 61830 61840	
Human	-TA-------AAAGGAGAAA----ATAG--------TAGGTTATTTTTCTTAAAGA-----ACAGAAGTCTTATTCTCAC	48932
PolarBear	ACTCCTAATAAAAGGATAGA--ATATAG-----T----CCTGACTTTTCTAAAAGAACATAACA-AAGTCTTACTCTGAC	15259
Cow	TGGCCTGATGAAATGATAGTTTATATAATTATACATAAGGTTACTTTTCTTAAGGAACATGACAGAAGTTTTACTCTGAC	45689
Dog	ATTCTTAATAAAAGGATAGA--ATATAG-----TATGTCTTAACTTTTCTAAAAGAACATAACAGAAGTCTTATTCTGAT	42771
BelugaWhale	TTGCCTGATGAAAGGATAGT----ATA------TAGTAGGTTACTTTTCTTAAAGAACATGACAGAAGTCTTACTCTGAC	41959
Donkey	TTGCCTAATACGAGGATAGA----ATA------TTGTAGGTTGTTTTCCTAAAAGAACATAACAGAAGTCTTATTCTGAC	47761
Cat	TCTCCTAATAAAAGGGTAGA--ATATAG-----TAGGTCCTAACTTTTGTAAAAGAGTATGACAGAAGT----TCTTGAC	49077
Ferret	ATTCCTAATAAAAGGATAGA--ATATAG-----TAGGTCTTAACTTTTCTAAAAGAACATAACAGAAGTCTTATTTTGAT	50181
Lion	TCTCCTAATAAAAGGGTAGA--ATATAG-----TAGGTCCTAACTTTTGTAAAAGAGCATAACAGAAGT----TCTTGAC	46797
Vaquita	TTGCCTCATGAAAGGATAGT----ATA------TAGTAGGTTACTTTTCTTAAAGAACACGACAGAAGTCTTACTCTGAC	42640
Pig	TCTCCTGATAAAAAGGTAGT----ATT------CAGTAGAGTGCTTTTCTCAGAGAACATGACAGAAGTCTTACTCTGAT	38713

Majority	TTTTCCTGGTAGXTGTGTTTGGGAAACTAATGCTAGGGAGCCAAAATGAATGAGTCACTGTAGTTTTCAAATTTTGCCTT	
	61850 61860 61870 61880 61890 61900 61910 61920	
Human	TTTTTCGAGTAGATGTCTGTGGGAAACTAATGCTAGGTAGCCAAAGTGAGTGAATCACTATAGTTTTTAAACTTTGTCTT	49012
PolarBear	TTTTCCTGGTAATGCTGTTTGAGAAACTAATGCTAGGAAGCCAAAATGAATGAGTCACTATAGTTTTCAAATTTTGCCTT	15339
Cow	TTTTCCTGGTAGCTGTGTATAAGAAATTAATGCTAGGGAATTAAAATGAGTAAGTCACCATAGTTTTCAAATTTTGCTTC	45769
Dog	TTTTCCTGGTAATGCTGATTGAGAAACTAATGCTGTGGAGCCAGAACGAGTGATTCACTGTAGTTTTCAAATTTTGCCCT	42851
BelugaWhale	TTCTCCTTGTAGCTGTGTTTGGGAAACTAATGGTAGGGAGCTAAAATGAATAAGTCACCGTAGTTTTCAAATTTCAAATT	42039
Donkey	TTTTCCTGCTAGCTGTATGTGGTAAACTAATCCTAGGGAGCCAAAATGA--ATCTCACCATCGTTTTCAAATTTTTCCTT	47839
Cat	TTTTCCTGGTAATGGTGTTTGGGAAATTAATACTAGGGAGCCAAAATGAATGAGTTACTGTAGTTTT-AAATTTCGCCTT	49156
Ferret	TTTTCCTGGTAATGCTATTTGAGAAACTAATGCTAGGGAGCCAAAAT----GAGCCAATGTAGTTTTCACATTTTGCCTT	50257
Lion	TTTTCCTGGTAATGGTGTTTCAGAAATTAATACTCGGGAGCCAAAATGAATGAGTTACTGTAGTTTT-AAATTTTGCCTT	46876
Vaquita	TTCTCCTTGTAGCTGTGTTTGGGAAACTAATGGTAGGGAGCTAAAATGAGTAAGTCACTGTAGTTTTCAAATTTCAAATT	42720
Pig	TTTTCCTGATAGCTATGTTTGGAAAACTAAAGCTAGGGAGCTGAAATGAATAAG--------GTTTTCAAATTCTGCCAT	38785

```
Majority     TTGAATTCACTTTAGGACTCTAATTGAACAATTGTTATAATGAGATTGTGGAAAATAC----------AAATGAAGTATT
                       61930     61940     61950     61960     61970     61980     61990     62000
Human        TTGAATTGACTTTAGGGCTCTAATTGAATAATTGTT---ATGAAATTGTGGAAAATAC----------AAATGAAGTATT  49079
PolarBear    TTGAATTCGCTTTTGGA--------------TTGTTATAAAGAGATTGTGGAAAATACAAATGAATGCAAATGAAGGATT  15405
Cow          ATAAATTCACTTTAGGACTGTAGTTGAACTATTGTT-CAAGGAGATTGTGGGAAATAC----------AGATGAAATATT  45838
Dog          T-GAATTCATTTTTAGA--------------TGGTTATAATGAGATTGTGGAAAATACAAATGTATATAAATGCAGTTTT  42916
BelugaWhale  TTAAATTCCCTTTAGGACTCTAATTGAACTACTGGT-CAATGAGATTGTAGGAAATAC----------AGATGAAATACT  42108
Donkey       TTGAATTCCCTTCAGGACTCTAATTGAACAATTATTATAATAAGATT--GGAAAATAT----------AAATGAAGTATT  47907
Cat          TTGAATTCACTTT-GGACTCTAATTGAACAGTTGTTATAATGAGATTGTGGAAAATAC----------AAATGAAATATT  49225
Ferret       TTGAATTCACTTTTGGA--------------TTGTTATAATGAGATTGTGAAAAATAC----------AAATGAAGGATT  50313
Lion         TTGAATTCACTTTTGGACTCTAATTGAACAGTTGTTATAATGAGATTGTGGAAAATAC----------AAATGAAGTATT  46946
Vaquita      TTAAATTCCCTTTAGGACTCTAATTGAACTACTGGT-CAATGAGATTGTAGGAAATAC----------AGATGAAATACT  42789
Pig          TTGAATTCACTGTAGGACTCTAACTGAGCCATTGTT-CGGTAAGATTATGGGAAATAC----------AGATGAAATGTT  38854
```

```
Majority     T-CTTTTATTGTGGCTTTGCTCTTTGTAATATCAGAAATCAG--------------------------------------
                       62010     62020     62030     62040     62050     62060     62070     62080
Human        T-CTTTTACTGTGGCTCTGTTCTTTG--GTATCAGAAATCAG--------------------------------------  49118
PolarBear    T-CTTTTATTGTGACTCTACTCTTTGTAATATCAGAAAACAG--------------------------------------  15446
Cow          TTTTTTTGTTGGGGCGTTGCTCTTTGTAATATCAGAAATCAG--------------------------------------  45880
Dog          TTCTTTTATTGTGTCTCTACCGTTTGTAATATCAGAAAACGGGATACTAGTAGCTTTTTTAAATATTTATTTATTTATT  42996
BelugaWhale  TCTTTT-ATTGTGGCTTTGCTCTTTGTAATATCGGAAATCAG--------------------------------------  42149
Donkey       T-CTTTTATTGTGGCTCTGCTCTTTATAATATCAGAAATCAG--------------------------------------  47948
Cat          T-CTTTTATTGTGGCTTTG--------A-TATCAGAAAACTG--------------------------------------  49257
Ferret       T-CTTTTATTGTGGCTCTACT--TTGTAATAACACAAACTAG--------------------------------------  50352
Lion         T-CTTTCATTGTGGCTTTG--------A-TATCAGAAAACAG--------------------------------------  46978
Vaquita      TCTTTT-ATTGTGGCTTTGCTCTTTGTAATATCGGAAATCAG--------------------------------------  42830
Pig          TGT---------GGCTTTAAT-TTTGTAATTTCAGAAATTGG--------------------------------------  38886
```

```
Majority     --------------------------------------------------------------------------------
                               62090     62100     62110     62120     62130     62140     62150     62160
Human        --------------------------------------------------------------------------------  49118
PolarBear    --------------------------------------------------------------------------------  15446
Cow          --------------------------------------------------------------------------------  45880
Dog          TATTCATTTATTATTTAGTTTAATTTAATTTAATTTAATAACAGAGCACAAGCCAGGGGGAGCAGCAGGGAGAGGGAGAA  43076
BelugaWhale  --------------------------------------------------------------------------------  42149
Donkey       --------------------------------------------------------------------------------  47948
Cat          --------------------------------------------------------------------------------  49257
Ferret       --------------------------------------------------------------------------------  50352
Lion         --------------------------------------------------------------------------------  46978
Vaquita      --------------------------------------------------------------------------------  42830
Pig          --------------------------------------------------------------------------------  38886

Majority     ---------------------------------TAGGG------------------------------------------
                               62170     62180     62190     62200     62210     62220     62230     62240
Human        ---------------------------------CAGGG------------------------------------------  49123
PolarBear    ---------------------------------CGGGG------------------------------------------  15451
Cow          ---------------------------------TAGGG------------------------------------------  45885
Dog          GCGGGATCCCTATTGAGCAGGGATCCCTATGTGTGGGGCTCCATCCATCCCAGGACCTTGGGATCATGCCCTGAGCTGAA  43156
BelugaWhale  ---------------------------------TAAGG------------------------------------------  42154
Donkey       ---------------------------------TAGGG------------------------------------------  47953
Cat          ---------------------------------TGTGG------------------------------------------  49262
Ferret       ---------------------------------TGGGG------------------------------------------  50357
Lion         ---------------------------------GGTGG------------------------------------------  46983
Vaquita      ---------------------------------TAAGG------------------------------------------  42835
Pig          ---------------------------------TAGGG------------------------------------------  38891
```

```
Majority     ------------------------------------ACACTAG-TAGCATCTTATCCTTTAAGCTTATCCCATGGAAAT
                     62250     62260     62270     62280     62290     62300     62310     62320
Human        ------------------------------------GTACTAG-TCGCATCTT--TCCTTAAGCT-------------- 49149
PolarBear    ------------------------------------GCACCAG-TAGCATCTTATCCTTTAATCTTATCCCATGGAAAT 15493
Cow          ------------------------------------ACGTTAG-TATCATCTTACCTTTTAAGCTTATCCCAGGGAAAT 45927
Dog          GGCAGATGTTTAACCATTTGACCCACCCACACGCCCCACACTAG-TAGCATCTTAACCTTTAAGCTTATCTCATGGAAAT 43235
BelugaWhale  ------------------------------------ACACTAG-CAGCATCTTATCCTTTAAGCTTATCCCAGGGAAAT 42196
Donkey       ------------------------------------GCACTAAATAGCATCTTTTCCTTTAAGCTTATCCCATGGAAAT 47996
Cat          ------------------------------------ATACTAG-TAGCATCTT---CTTTAAGTTTATCTCATGGAAGT 49301
Ferret       ------------------------------------GCACTAG-TAGCATCTTATCCTTTAAGTTTATCAGATGGAAAT 50399
Lion         ------------------------------------ATACTAG-TAGCATCTT---CTCCTTTAAGTTCTCATGGAAGT 47022
Vaquita      ------------------------------------ACACTAG-CAGCATCTTATCCTTTAAGCTTATCCCAGGGAAAT 42877
Pig          ------------------------------------ACCTTAG-TAGCATCT-GTCCTTTAAGCTTATCCCAGGGAAAT 38932

Majority     ATTTATCTGATAGCATTAGAAGAAAATAGTCATTAATCCTAGATA-TTCTTCATTGAATAXATGTACCTGTAGCAGXAGG
                     62330     62340     62350     62360     62370     62380     62390     62400
Human        ---------------TTATGAGAA------------------TA---T-------------------------------- 49161
PolarBear    ATTTATCTGATAGCATTAGAAGAAAATAGTCACTAATCCTAGGTA-TTCTTCATTGAATAAATGTATCTGTGGCAGAAAG 15572
Cow          ATTTATCTGATAGCATTAGAAAAAATTAGTGTTAG-TCCCAGATATT-CTATACTGAATAGATATATCTGTAGCAGTAGG 46005
Dog          ACTTATCTGGTAGCATTAGAAGAAAATAATTATTAATCCTAGATA-TTCTTCATTGAATAAATATCCCTGTGGCAAAAAA 43314
BelugaWhale  ATTTATCTTATAGCATTAGAAGAAGATAGTCATTAGTCCCAGATATT-CTATATTGAATAGTTATACCTGTAGCAGTAGG 42275
Donkey       ATTT--CTGATAGCATTAGAGGAAAATAGTCATTAATCCTGTATA-TTTTTCATTGCATAGATGTACCTGTAGCAATAGG 48073
Cat          GTTTATCTGATAGCATTAGAAGAAAATAGTCATTAATCCTAGATAACTCTTCATTTAATAAATGTATCTGTTGCAGAGGT 49381
Ferret       ATTTATCTGATAGCATTAGAAGAAAATAGTCACTAATCCTAGGTA-TTCTTCATTGAATAAATGTACCTGTGGCAGAAAG 50478
Lion         GTTTATCTGATAGCATTAGAAGAAAACAGTCATTAATCCTAGATAATTCTTCATTTAATAAATGTATCTGTGGCAGAGGT 47102
Vaquita      ATTTATCTGATAGCATTAGAAAAAGATAGTCATTAGTCCCAGACATT-CTATATTGAATAGTTATACCTGTAGCAGTAGG 42956
Pig          ATTTATCTGATAGCATTAGAAAAAAATTGTCATTA-TCCCAGATGTTTCTGTATCGCATAGATGTACCTATAGCAATAGG 39011
```

```
Majority     GXCCAGGTATATTAAAAAX-AAATTTTGTTTTAAAATTTCTAATGATACCATTTAGAAGAATTTAT-GAAATTTAACATT
                              62410     62420     62430     62440     62450     62460     62470     62480
Human        -----G---------AAA-----TTTTATTTAAAAATGTTAAATGATACCATTTAGAAGAATTTAT-GAAATTTAACACT  49221
PolarBear    ACTCAGGGGTATTAAAAA---GATTTTGTTTTAAAATTTCTAATGATACTATTTAAAAGAATTTAT-GAAATTTAACATT  15648
Cow          GTCCAGGTGTATTAAAAATCAAATTTTGTTTTAAAGTTCCTAATAGCA---TTTAGAAGAATTTAGAGAAATTTAATGCT  46082
Dog          GCCCAGGCATATTAATAA---GATTTTGTTTTAAAATTTCTA---ATACCATTTAGAAGAAGGTGT-GAAATTTAACATC  43387
BelugaWhale  GTCCAGGTATATTAAAAATCAAATTTTGTTTTAAAGTTTCTAATGATACCATTTAGAAGAATTTAGAGAAATTTAATGTT  42355
Donkey       ACCCAGGTATATTAGAAAGGAAGTTTTGTTTTAAAATTTCTAATGATGCTATTTAGAACAGTCTGT-GAAATTTAAGATT  48152
Cat          GTCCTGGCATATTAAAAT------TTTGCTTTAAAATTTCTAATGATATCATTTAGAAGAACTTAT-GAAATTTAACATG  49454
Ferret       ATGCAGACATATTAAAAATGAGAGTTGGTTTAAAATTTCTAGTGATACCATTTAAAAGAAATTAT-GAAATTCAACATT  50557
Lion         GCCCTGGCATATTAAAAT------TTTGCTTTAAAATTTCTAAT---ATCATTTAGAAGAACTTAT-GAAATTTAACATG  47172
Vaquita      GTCCAGGTATATTAAAAATCAAATTTTGTTTTAAAGTTTCTAATGATACCATTTAGAAGAATTTAGAGAAATTTAATGTT  43036
Pig          GCCCAGGTACATTAAAAGTCAAATTT-GTTTTAATGTTTTCAGTGGTACTATTTAGGAGAATTTAGAGAAGTTTAGTGTT  39090

Majority     CTCTTAAATATAXTTAATGTCACTA-------ATAATTTGATTTTTAATTTTTTTCTTTGXCTCTXTTXAATTATAC-TG
                              62490     62500     62510     62520     62530     62540     62550     62560
Human        CCCTTTTATATACTTGAGGTTAATC------TGACATTTAATTTGTAATTTTGTTCTTTGTCTCTATATAATTATAC-TG  49294
PolarBear    CCCTTAAATATAATTAATGTCACTAATAATCTAATATTTAATTTTTAATTTTTTCCTTTGTCTCTTTTGAGTTATGC-TG  15727
Cow          CTCTTAAATATAGTTAATATCACTA-------ATAAGATGGCATTTAATTTTTTTTTCTAG------TAGATTATAAATG  46149
Dog          TA-TTAAATATAATTAATGTCACTA-------ATAATTGGATTTTTAATTTTTTTCCTTG--TCTCTTGACTTATAC-TG  43456
BelugaWhale  CTCTTAAATATAGTTAATATCACTA-------ATAATATGAAATTTAATTTTTTTCTTTGT------TAAATTGTAAATG  42422
Donkey       ATCTTAAATACAGTTAATATAACTC-------ATAAAGTGACATTTAATTTTTTTTCTT-----TGTTAAATTATAC-TG  48219
Cat          CTCTTAAATATAATGAATGTCACTA---ATCTGATATTTAATTCTTAATTTTTTTCTTT--CTCTCTTGAGTTATAC-TG  49528
Ferret       CATTTAAATATAATTAATGTCACTA---ATCTTATATTTAATTTTCAATTTTTTCC-----CTTTTCTGAGTTATAC-TG  50628
Lion         CTCTTAAATATAATAAATGTCACTA---ATCTGATATTTAATTCTTAATTTTTTTCTTT--CTCTCTTGAGTTATAC-TG  47246
Vaquita      CTCTTAAATATAGTTAATATCACTA-------ATAATATGACATTTAATTTTTTTCTTTGT------TAAATTGTAAATA  43103
Pig          CTCTTAAATATAGTTAATGTCACTT-------ATAACGTGACATTTGATTTTTTTCTTTGTCTCTTTTAAATTATAC-TG  39162
```

```
Majority     CTTTAAAAATGACTTGGAGATCTCTT---ATGTTCTCTAGGXTAGGAGTGGTAAAATTCTGATGCTAGAAAA--TGTTTA

                       62570     62580     62590     62600     62610     62620     62630     62640

Human        CTTTTAAAATGACTTGGAAATCTT-----ACATTCTCTAGGGTAGAAGTGGTAAAATTCTGATGCTAGAAAA--TGTTTA  49367
PolarBear    CTTTAAAAAAAACTCTGAGATCGCTT---ATGTTTTCTAGTATCGGAATGGTAAAATTCTGATATTGAAAAA--TGTCTA  15802
Cow          CTTTAAAAATGACTTGGAGATCTC-----ATGTTTTCTAGGTTAGGCATGGTAAAATTCTGTTTCTAGAAAA--TGTTTG  46222
Dog          CTTTAAAAATGACTTTGAGATCTCTT---ATGTTCTCTAATAGGGGAATGGTAAAATTCTAATATTAGAAAA--TGTTTA  43531
BelugaWhale  CTTTAAAAATGACTTGGTGTTCTCTT---ATGTTTTCTAGGTTAGGAGTGGTAAAATTCTGGTGCTGGAAAA--TGTTTA  42497
Donkey       CTTTAAAAATGACTTTGAGATCTCTT---ATGTTCTCTAGGTTAGGATTGGTAAAATTCTGAAGCTAGAAAAAGTGTTTA  48296
Cat          CTTTAAAAATGACT--GAT-TTTCTT---ATGT-CTTTA--A--G-AGTGGTAAAATTCTGATGTTAGAAAA--CGTTTA  49594
Ferret       CTTTAAAAATGACTTTGAAATCCCTTTTTGTGTTCTCTAGTATCGGAATGGTAAAATTCTGATACTGAAAAA--TGTTTG  50706
Lion         CTTTAAAAATGACT--AAGATTTCTT---ATGT-CTCTA--A--G-AGTGGTAAAATTCTGATGTTAGAAAA--CGTTTA  47313
Vaquita      CTTTAAAAATGACTTGGTGTTCTCTT---ATGTTTTCTAGGTTAGGAGTGGTAAAATTCTGGTGCTGGAAAA--TGTTTA  43178
Pig          CTTTAAAAATGACTTGGAGATCTCTT---ATATTCTCTAGGTTAT---TGGTAAAATACTGGTGCTAGGAAA--TATTTA  39234

Majority     GATTTTA-------------------TTTTCTCTAGCTTTTGTTGGAXGA-XTACATTTGTGGTGTATGGAAA------

                       62650     62660     62670     62680     62690     62700     62710     62720

Human        CATTT----------------------T--CTCTAGCATTTGTTGCAAGA-ATAGATTTGTGG-GTGTGAAAAATCATA  49420
PolarBear    GATTTTA-------------------TTTTCTCTAGCCTTTGTTGGA----CTACATTTGTGGTGTACAGAAA------  15852
Cow          GATTTTA-------------------TTTTCTCTAGCCTTTTTTTGAGGAGTTACATTTGTAATGTATGGAAAT-CAGA  46281
Dog          GATTTTACTTTGTCAAAAAAAAAAGATTTTTGTCTAGTTTTTGTTAGA----CTACATTTGTGGTGTGTGGAAA------  43601
BelugaWhale  GATTTTA-------------------TTTTCTCTAGCCTTTGTTGAAATGGGTACATTTGTAGTGTATGGAAA------  42551
Donkey       GACTTAA--------------------TTTTTTTAGCTTCTCTTGGAA---GTACATTTGTGGTATATGGAAAATCAGA  48352
Cat          GATTTTA-------------------TTTTCTTTAGCTTTTGTTGGGGGA-CTATATTTGTGGTATATGGAAA------  49647
Ferret       GATTTTA-------------------TTTTCGCTAGCTTTTGTTTG-----TCACATTTGTGGTGTATGGAAA------  50755
Lion         GATTTTA-------------------TTTTCTTTAGCTTTTGTTGGGGGA-CTATATTTGTGGTATATGGAAA------  47366
Vaquita      GATTTTA-------------------TTTTCTCTAGCCTTTGTTGAAATGGGTACATTTGTAGTGTATGGAAA------  43232
Pig          GATTTTA-------------------TTTTCTCTAGG-TTTATTGGGGGAAGTACATTTGCAGTGTACAGAAAG-ATCA  39292
```

```
Majority     --T-AAAGGCAGAAGT----TXTA--CGGATAXCTT----CTXAG-TT------AATTGTATATCTAAACTTXAACAAGG
                     62730     62740     62750     62760     62770     62780     62790     62800
Human        GGTGGAAGGCAGAAGCAGCGTGTACTCGGTTTTCTT----CTGAGGTTAGCTTTAACTGTATACCTAAACTTCAACAAGA  49496
PolarBear    ----ATAAG-GAAAGT--A-------AGGATAACTT------------------ATTTGAGTGTCTAAATGTTAACTGGG  15900
Cow          GATGGAAGGCAGAAATACTGTGTACGCTAAGAGCTTTCTACTAAGGTTAGCTTTAATTGTATTTCTAAGCTTCAATAA-G  46360
Dog          ----TAAGG-ATAAGT----------AGGATAACTT------------------ATTTGAGTGTCTAAATGT-ACCTGGA  43647
BelugaWhale  -ATCAGAGGTGGAAGT----TGTATTCTAAGAGCTT----CTGAGTTTAACTTTAATTGTATCTCTGAACTTCAGTAAGA  42622
Donkey       GATCGAAGGCAGAAGCACCATGTACTCAAAGAGCTTTTTTCTGAGGTTAGCTTTAGTTGTATACCTAAACTTGAATAAAG  48432
Cat          ----ATAAGGATAAGT--A-------AGGATAACTT------------------A-TTGAACATCTAAATGT-AACTGGG  49694
Ferret       ----ATA-------------------AAGATAACTT------------------ATTTGGGTGTCTAAATGT-AACTGGG  50793
Lion         ----ATAAGGATAAGT--A-------AGGATAACTT------------------ACTTGAACATCTAAATGT-AACTGGG  47414
Vaquita      -ATCAGAGGTGGAAGT----TGTATTCTAAGAGCTT----CTGAGTTT------AATTGTATCTCTAAACTTCAGTAAGA  43297
Pig          GGTGGAAGGCAGAAGTACTGTATATGCAAAGAGCTTTCTTCTGAGGTTACCTTTAATTGTGTATCAAAACTTGAATAAAG  39372

Majority     GATTTCTGATTXTACTTA-XAGXXCA---------------------T--XTTXTAGATACTGATTTTTCTAATTAGCTG
                     62810     62820     62830     62840     62850     62860     62870     62880
Human        GATTTCTAATTCTGCTTATCAGAACATTTAAAAGTAACCTGAAGGATTATATTCTAGATAATTGATTAA--AATCATCTG  49574
PolarBear    AA-----GGTTATACTC------------------------------T-------AGATATGGATTTTTCTCATTGGCTG  15938
Cow          GATTTCTGATTCTACCTATTAGGGCA------------------------ATTCTCGATAGTGATTTTT-TAATGAGCCA  46415
Dog          AA-----GGTTATACTC------------------------------T-------AGATAGTAATTTTTTTCATTAGCTG  43685
BelugaWhale  GATTTCTAATTCTACCTACTAGAGCATCTAAATGTAACTTTAAAGGTTATATTCTAGATACTGATTTTTCTAATGAACCA  42702
Donkey       GATTTCTAATTTTATTTATTAGAGCATCTAGATGTAACTTGAAAAGTTATATTCTAGATACTGATTTTTCTAGTTAGTTG  48512
Cat          AA-----GGTTATAGTC------------------------------T-------TG-TACTGATTTTTCTCATTAACTG  49731
Ferret       AA-----GGTTATACTC------------------------------T-------AGATACTGATTTTTCTCATTAGCTG  50831
Lion         AA-----GGTTATAGTC------------------------------T-------TG-TACTGATTTTTCTTATTAACTG  47451
Vaquita      GATTTCTAATTCTACCTACTAGAGCATCTAAATGTAACTTGAAAGGTTATATTCTAGATACTGATTTTTCTAATGAACCA  43377
Pig          GATTTCTGATTCTACCTACTAGAGCATCTGAATGTGACTTGACAGTTTATGTTTTAGATAGTGATTTTTCTAAGGAGCTG  39452
```

```
Majority     AATAGATTACACAGATA-TTGAAGTTTATTTGAA-TXTAATTCATAGTATTAAAATTTTTGCTATTTAAATTTTCAGGCA

                      62890     62900     62910     62920     62930     62940     62950     62960

Human        AATAGATTGCTTAGATA--TTAATTTTATTTGAA------TTTATAGTATTAAAAATTGTGCTACTTAAATTTTCAGGCA  49646
PolarBear    AATAAGTTATA--GATA-ATGAGGTTTACTTGAA-TATAATTCATAGTATTCCAATTTTTGCTATTTAAATTTTCAGGCA  16014
Cow          AATAGATTACACAGATA-TGGAAGTTTATTTGAA-TTTAATTCATAGTATTATAATTTTTGCTATTTAAATTTTCAGGCA  46493
Dog          AATAGATTACACAGATA-CTGAAGTTTACCTGAAATATAATTCCTAGTATTAAAATTTTTGCTATTTAAATTTTCAGGCA  43764
BelugaWhale  AACAGATTACACAGATA-TTGAAGTTTATTTGAA-TTTTATTCATAATATTAAGATTTTTGCTATTTAAATTTTCAGGCA  42780
Donkey       AATAGATTACACAGATA-TTGAAGTTTGTTTGAA-TTTAATTCACAGTATTAACCTTTTTGCTATTTAAATTTTCAGGCA  48590
Cat          AATATATTACACAGATA-CTTACGTTTATTTGAA-TATAATTCATGGTATTAAAATATTTGCTATTTAAATTTTCAGGCA  49809
Ferret       AATAGATTACACAGGTA-TTGAGGTTTACTTGAA-TATAATTCATAGTATTCCAATTTTTCCTCTTTAT-TTTACAGGCA  50908
Lion         AATATATTACACAGATA-CTGAGGTTTATTTGAA-TATAATTCATGGTATTAAAATGTTTGCTATTTAAATTTTCAGGCA  47529
Vaquita      AACAGATTACACAGATA-CTGAAGTTTATTTGAA-TTTTATTCATAATATTAAGATTTTTGCTATTTAAATTTTCAGGCA  43455
Pig          GATAGATTAAGCAGATAATTGAAGTTTATTCAAT-TTTAATTCATGGCAGTAAAATTTTG--TATTTAAATTTTCAGGCA  39529

Majority     TTTTTGGAACTAGCAACAGAGGAAGCAGCTATTACTATGGTTAATTACTATTCTGCTGTGACACCTCATCTTCGTAACCA

                      62970     62980     62990     63000     63010     63020     63030     63040

Human        TTTTTGGAACTAGCAACCGAGGAAGCAGCTATTACTATGGTTAATTACTATTCTGCTGTGACACCTCATCTTCGTAACCA  49726
PolarBear    TTTTTGGAACTAGCAACAGAGGAAGCAGCTATTACTATGGTTAATTACTATTCCGCTGTGACACCTCATCTCCGTAACCA  16094
Cow          TTTTTGGAACTAGCAACAGAGGAAGCAGCTATTACCATGGTTAATTACTATTCTGCTGTGACACCTCACCTTCGTAACCA  46573
Dog          TTTTTGGAACTAGCAACAGAGGAAGCAGCTATTACTATGGTTAATTACTATTCTGCTGTGACACCTCATCTCCGTAACCA  43844
BelugaWhale  TTTTTGGAACTAGCAACAGAGGAAGCAGCTATTACTATGGTTAATTACTATTCTGCTGTGACACCTCACCTTCGTAACCA  42860
Donkey       TTTTTGGAACTAGCAACAGAGGAAGCAGCTATTACTATGGTTAATTACTATTCTGCTGTGACACCTCATCTTCGTAACCA  48670
Cat          TTTTTGGAACTAGCAACAGAGGAAGCCGCTATTACTATGGTTAATTACTATTCTGCTGTGACACCTCATCTCCGTAACCA  49889
Ferret       TTTTTGGAACTAGCAACAGAGGAAGCAGCTATTACTATGGTTAATTACTATTCTGCTGTGACACCTCATCTCCGTAACCA  50988
Lion         TTTTTGGAACTAGCAACAGAGGAAGCCGCTATTACTATGGTTAATTACTATTCTGCTGTGACACCTCATCTCCGTAACCA  47609
Vaquita      TTTTTGGAACTAGCAACAGAGGAAGCAGCTATTACTATGGTTAATTACTATTCTGCTGTGACACCTCACCTTCGTAACCA  43535
Pig          TTTTTGGAGCTAGCAACAGAGGAAGCAGCTATAACTATGGTTAATTACTATTCTGCTGTGACACCTCATCTTCGTAACCA  39609
```

```
Majority     ACCAATTTATATCCAGTACTCCAATCACAAAGAACTAAAAACAGATAATACATTAAACCAAGTGAGTATGTGTAGGTACA
                      63050     63060     63070     63080     63090     63100     63110     63120
Human        ACCAATATATATCCAGTACTCGAATCACAAAGAACTAAAGACAGATAATACATTAAACCAAGTAAGTATGTGTAGGTACA  49806
PolarBear    ACCAATTTATATCCAGTACTCCAATCACAAAGAATTAAAAACAGATAATACGTTAAACCAAGTGAGTATGTGTAGATACA  16174
Cow          ACCGATTTATATCCAGTACTCCAATCACAAGGAACTAAAAACAGATAATACATTAAACCAAGTGAGTATGTGTAGGTTCA  46653
Dog          ACCAATTTATATCCAGTACTCCAATCACAAAGAACTAAAAACAGATAATACGTTAAACCAAGTGAGTATGTGTTGGTACA  43924
BelugaWhale  ACCGATTTATATTCAGTACTCCAATCACAAAGAACTAAAAACAGATAATACATTGAACCAAGTGAGTATGTGTAGGTACA  42940
Donkey       ACCAATTTATATCCAGTACTCCAATCACAAAGAACTAAAAACAGATAATACGTTAAACCAAGTGAGTATATGTAGGTACA  48750
Cat          ACCAATTTATATCCAGTACTCCAATCACAAAGAACTAAAAACAGATAATACATTAAACCAAGTGAGTATGTGTAGGTACA  49969
Ferret       ACCAATTTATATCCAGTACTCCAATCACAAAGAACTAAAAACAGATAATACATTAAACCAAGTAAGTATGTGTAGGTACA  51068
Lion         ACCAATTTATATCCAGTACTCCAATCACAAAGAACTAAAAACAGATAATACATTAAACCAAGTGAGTATGTGTAGGTACA  47689
Vaquita      ACCGATTTATATTCAGTACTCCAATCACAAAGAACTAAAAACAGATAATACATTGAACCAAGTGAGTATGTGTAGGTACA  43615
Pig          ACCGATTTATATCCAGTACTCTAATCACAAAGAACTAAAAACAGATAATACATTAAACCAAGTGAGTATATGTAGGTACA  39689

Majority     TAAATAAAATGGCCTAGAACATATTAAGAATCTCATAAATATX------AACAGAAAG----XTTAXAATTATTAAGATA
                      63130     63140     63150     63160     63170     63180     63190     63200
Human        TAAATAAAATGGCCTAGAACATATTATGAATCTCATAAACATT------AATAGGAAGGAAATGTTTACCTGTTCAGATG  49880
PolarBear    TAAATAAAATGG------ACATATTAAGAATCTCAAACTTATT------AACAGAAAG-----ATGTAACTAA--GGGCA  16235
Cow          TAAACAAAATGTCCTAGAGCGTATTAAGACTCATATTAG------------GAGAAAAT-TTTTAACCATTGAGAAGATT  46720
Dog          TAAATAAAATGGCCTAAGACATATTAAGAATCTCAAATGTATG------AATGGAAAGTAATCATTTAACCGTTTAACCA  43998
BelugaWhale  TAAATAAAATGGCCTAGAACGTATTAAGAATCTCATAAACAT--TAGCAGAAAAAAATT-TTTTTACCATTCAGAAGATT  43017
Donkey       TAAATAAAATGTCCCAGACAATACTAAGAATCTCATGAACATATT----AAAAGAAAA------TAAAATTTTTAATGTT  48820
Cat          CAAATAAAATGGCCTAGGACATATTAAGAGTCTGAAACATATT------AACAGAAAG------TAAAAATTTTTAACCA  50037
Ferret       TAAATAAAACGG------ACATATTAAGAATCTCAAACTTAT-------AACAGAAAG-----ATGTAATTAT--GGGTA  51128
Lion         CAAATAAAATGGCCTAGGACATATTAAGAGTCTGAAACATATT------AACAGAAAG------TAAAAATATTTAACCA  47757
Vaquita      TAAATAAAATGGCCTAGAACGTATTAAGAATCTCATAAACATATTAGCAGAAAAAAATT-TTTTTACCATTCAGAAGATT  43694
Pig          TCAATAAAACGGCCTAGAACATATTAAGAATCTCAT----------------AAACA---TATTAACAGAAAATAAGAAA  39750
```

```
Majority      TTT------GAAT-TTCTTGTXGTTTTTTTGTTGTT--TTTTA-GACATTTTCTTGCAGTATGTCCCTAAGTATTAAATA
                       63210     63220     63230     63240     63250     63260     63270     63280
Human         TTC-------------CTTATAGGCATTTTCTTGTTATTTTTAAGGTATTTTCATGCAGAATGTTTCTAAGTATTAAATA  49947
PolarBear     TTT-----------------------TCTTGTTATT--TTTAAAGATACTTTCTTGTAATATGTCCCTAAATATTAAGTA  16290
Cow           TTT------GTATGATCTTTTAAATTTTTTGTTGT---TGTTGCTATATTTTCTTACAGTATGTCCCTAAGTATTAAATA  46791
Dog           TTCA-----GAAGATTAACTATGGCATTTTGTTATT--TTCAA-CACATTTTCTTGTAGTACATCCCTAAATGTTAAATA  44070
BelugaWhale   TTTCTGTGAGCATTTTCTTGTTTTTTGTTTTCTGGTTTTTTTGTGGCATTTTCTTGTAGCATGT---TAAGTATTAACCA  43094
Donkey        TCAG----AAGATGTCTCTGTGGGCATTTTCTTGTTATTTTTAAGACATTTTCTTGCAGCA--T-----------TAAATA  48884
Cat           TTCA-----GAAG-CTATGGGCATTTTCTTGTTATT--TTAAA-GACATTTTCTTGCAGTATGTCCCTAAGTATTAAATA  50108
Ferret        TTT-----------------------TCTTATTATT--TTTAA-GATATTCTCTTGCAGTATGTCCCTAAATATTAAATA  51182
Lion          TTCA-----GAAG-CTATGGGCATTTTCTTGTTATT--TTAAA-GACATTTTCTTGCAGTATGTCCCTAAGTATTAAATA  47828
Vaquita       TTTCTGTGAGCATTTTCTTGTTTTTTGTTTTCTGGTTTTTTTGTGGCATTTTCTTGTAGCCTGT---TAAGTATTAACCA  43771
Pig           TT-------GAACCATTAAGAAGATTTTTCTTTGGGTATATTCTTGT-TTTTTTCGCAGTATGTTCCTAAGTATTAAATA  39822

Majority      TTATGTTAATTTACAAAAAGTATGTGCAGTCTATTATTTTCATGTT--TTAAATTGGTACTXGATAAATTAGTTAAATAG
                       63290     63300     63310     63320     63330     63340     63350     63360
Human         CTATGTCATTTT---GAA--TTCCTTTAATCCCCTGGTCAAATGTTCTTTTTCTTGTTGCAAGTTAAAATTTTTGTGTAG  50022
PolarBear     TTATGTTAATTTACAAAAAGTATGTGCAGTCTATTATTTTCATGTT--TTAAATTGGTACTTGA---ATTAAATAATT--  16363
Cow           TTATATTACTTTACAGAACATATGTGCAGTCTTTTATTTTCATATT--TTAAATTAGTACTGGATAAATTA----AATAG  46865
Dog           TTATGTTAGTTTACAAAA----TGTGCAGTCTATTATTTTCATGTT--TTAAATTGGTACTTGATGAATTAGTTAAGTAA  44144
BelugaWhale   TTATGTTAATTTACAGAAAGTATGTGTGGTCTCTTATTTTCATATT--TTAAATTAATACTGGATAAATTAGTTAAATAG  43172
Donkey        TTATGTTAATTTATAAAAAGTATGTGTGGTCTGTTATTTTCATATT--TTAAATTGGTACAGGATCAATTA---AA-TAG  48958
Cat           TTATGTTAATTTACAAAATGTATGTGAAGTCTATTATGTGCATGTT--TTAAATTGGTACTTGATGAAGTAAATAA-TA-  50184
Ferret        TTATGTTAATTTACAAAAAGTATGTACAGTCTATTATTTTCATGTT--CTAAATTGGTTCCTGATGAATTAGTTAAATA-  51259
Lion          TTATGTTAATTTACAAAATGTATGTGAAGTCTATTATGTGCATGTT--TTAAATTGGTACTTGATGAAGTA----A-TA-  47900
Vaquita       TTATGTTAATTTACAGAAAGTATGTGTGGTCTCTTATTTTCATATT--TTAAATTAATACTGGATAAATTAGTTAAATAG  43849
Pig           TTACGTTAACTTAGAAGAGATATGTACAGTCAGATGCTTTCGTATT--TTAAATTAGTACTGGGTAAATTAGTTAAATGG  39900
```

```
Majority     XATTTTTT--CAXAG-TTTTAAGGT-------------------------------------------------------

                      63370     63380     63390     63400     63410     63420     63430     63440

Human        AAACTTATCAGGTAGGATTTATTATTAGCATAAAC------------------------TGTTTAT-ACAACTCTCCTGC  50077
PolarBear    --TTTTTT--CAAAG-CTTTAAGGT-------------------------------------------------------  16383
Cow          TATCTTTT--CAGTTTTTTTAAGGT-------------------------------------------------------  46888
Dog          TACTTTTT--CAAAG-CTTTAATGT-------------------------------------------------------  44166
BelugaWhale  CATTTTTTT-CAGTGATTTTAAGGT-------------------------------------------------------  43196
Donkey       TATTTTTT--CAGTGCTTTTAAAGT-------------------------------------------------------  48981
Cat          --TTTTTT--CAAAG-CTTTAAGGT-------------------------------------------------------  50204
Ferret       --TTTTTT--CAAAG-TCTTAAGGT-------------------------------------------------------  51279
Lion         --TTTTTT--CAAAG-CTTTAAGGT-------------------------------------------------------  47920
Vaquita      CATTTTTTT-CAGTGATTTTAAGGT-------------------------------------------------------  43873
Pig          TATTTTTTTTCAGTGCTTTTAAGATCTGTCACAGTGTTCGTTTTCTTTTTTTTTTTTTTTTTTTTTTATTATTTTCCCAC  39980

Majority     --------------------------------------------------------------------------------

                      63450     63460     63470     63480     63490     63500     63510     63520

Human        TTAGAATTTAAGGTAAGAAGTTCAGTGCCTTTGGTTATTTGATTTTTTTTATAGGTCTTAATTTCTTACGGAAAATTTTT  50157
PolarBear    --------------------------------------------------------------------------------  16383
Cow          --------------------------------------------------------------------------------  46888
Dog          --------------------------------------------------------------------------------  44166
BelugaWhale  --------------------------------------------------------------------------------  43196
Donkey       --------------------------------------------------------------------------------  48981
Cat          --------------------------------------------------------------------------------  50204
Ferret       --------------------------------------------------------------------------------  51279
Lion         --------------------------------------------------------------------------------  47920
Vaquita      --------------------------------------------------------------------------------  43873
Pig          TGTACAGCAAGGGGGTCAGGTCATCCTTAGATGTATACATTGCAGTTACAGTTTTTTCCCCCACCCTTTCTTCTGTTGCG  40060
```

Sequence	63530 – 63600	
Majority	---------TCTGGTCAAAATGTTCA--TTTTCA	
Human	TTTTGAAGATGTGTTAATGGTTTTCCTGGAAA---AGAAATCTTAAGGATGAAGAATATGTGAATGTATTGATGTTTTAA	50234
PolarBear	---------TCTGGTCAAAATGTTCA--TTTTCA	16406
Cow	---------TCTGGTCAAAGTTCT--TTTTCT	46909
Dog	---------TCTGGTCAAAATGTTCA--TTT-CA	44188
BelugaWhale	---------TCTGGTCAAAGTGTTCT--TTTTCT	43219
Donkey	---------CCTTGTCAGAATGTTGG---------------------T--------------------------TTTTTT	49005
Cat	---------TCTAGTCAAAATGTTCA--TTTTCA	50227
Ferret	---------TCTGGTCAGAATGTTCA--TTTTCA	51302
Lion	---------TCTAGTCAAAATGTTCA--TTTTCA	47943
Vaquita	---------TCTGGTCAAAGTGTTCT--TTTTCT	43896
Pig	ACATGAGTATCTAGACATAGTTCTCAATGCTATTCAGCAGGATCTCCTTATAAATCTTTTCTAGGTAGTGTTCGTTTTCT	40140

Sequence	63610 – 63680	
Majority	TGTTGCAA-XTTXAAATTTTTGTATAGAAACTGT--	
Human	AAATGTAATATAGAAGATATTGTTTAAAATTAATGTATTCAAAAATAGCATTAGAATGTTTTCCGACCTACATTATA---	50311
PolarBear	TGTTGCAA-CTTCAGATTTTTGTATAGAAACTGTT---	16440
Cow	TTGTACAA-ATTAAAACTT--GTACAGAAACTGT--	46940
Dog	TGTTGTAA-GTTCAGATTTTTGTATAGGAACT--	44219
BelugaWhale	TTGTGCAA-ATTAAAGTTTTTGTATAGATACTGT--	43252
Donkey	TGTTACAA-GTTAAGATTTTTGTGTAGAAACCGTT---	49039
Cat	TGTTGCAA-GTTCAAATTTTTGTATAGAAACTGTA---	50261
Ferret	TGTTGCAA-GTTCAGATTGCTCTATAGAAACTGTT---	51336
Lion	TGTTGCAA-GTTCAAATTTTTGTATAGAAACTGCA---	47977
Vaquita	TTGTGCAA-ATTAAAGTTTTTGTATACATACTGT--	43929
Pig	TTATGCAG-ATTAAAGTTTTTGTATAGAAGCTATGAGGTAGGGACTTCAGCATAAACTTTTTTTTTTTTTTTGTCTTTTA	40219

```
Majority     --------------------------------------------------------------------------------

                     63690     63700     63710     63720     63730     63740     63750     63760

Human        ---AGACTGCTGTAATGATCTAAAACTTTAGCTGTTTTAATATAGTTTTAAAACTAATGATATCTTTCTCTGTCAGTAAA  50388
PolarBear    --------------------------------------------------------------------------------  16440
Cow          --------------------------------------------------------------------------------  46940
Dog          --------------------------------------------------------------------------------  44219
BelugaWhale  --------------------------------------------------------------------------------  43252
Donkey       --------------------------------------------------------------------------------  49039
Cat          --------------------------------------------------------------------------------  50261
Ferret       --------------------------------------------------------------------------------  51336
Lion         --------------------------------------------------------------------------------  47977
Vaquita      --------------------------------------------------------------------------------  43929
Pig          GGCATGGCACATGGAGGTTCCCAGGCTAGGGGTCCAGTCATAGCTGTAGCCACGGGCCTATGCCACAGCAACACCAGATC  40299

Majority     -----------------------------------------------------------------ATGAGATAGGACT-

                     63770     63780     63790     63800     63810     63820     63830     63840

Human        ATACAAACTTTTTCTTAATAAAAATGTAATGGAAAACTGTTCCTCATAGATTTTTGTCACTTTACAAAGTGACAAAATCA  50468
PolarBear    -----------------------------------------------------------------AGGAGATTGGGCT-  16453
Cow          -----------------------------------------------------------------CTGAGGTAGGAC--  46952
Dog          -----------------------------------------------------------------ATGAGATTGAGCT-  44232
BelugaWhale  -----------------------------------------------------------------CTGAGGTAGGAC--  43264
Donkey       ----------------------------------------------------------------TATGACGTAGGAGT-  49053
Cat          ----------------------------------------------------------------CATGAGATTGGGCT-  50275
Ferret       ----------------------------------------------------------------TATGAGATTGGGC--  51349
Lion         ----------------------------------------------------------------CATGAGATTGGGCT-  47991
Vaquita      -----------------------------------------------------------------CTTAGGTAGGAC--  43941
Pig          TGAGCCACATCTGCGACCTACACTGCAGCTTACAGCTACGCTGGATCCTAACCCACTGAGTGAGGCCAGGATAGAACCT  40379
```

```
Majority     ----------------------TACTATTAAC--------------------ATAAACTGT--ATACAACTCTGCTGCTT
                      63850     63860     63870     63880     63890     63900     63910     63920
Human        TTTTGTTAGTTTATGGAAAATAAGCTTGTAAA--A-------CTTTTTACCTAAAAGATAGGACTGAAATTTCAGCTTTT  50539
PolarBear    ----------------------CACTATTTGC--------------------ATAAACTGT--AC-----TCTGCTGCTT  16484
Cow          ------------------------TTAACTA----------------------TAAACTGTTTATACAACTTTGATT---  46983
Dog          ----------------------TGCTATTCAC--------------------ATAAACTAT--ATAGAACTCTGTTGCCT  44268
BelugaWhale  ------------------------TTAAGCA----------------------TAAACTGTTTATGCAACTCTGCTGCTT  43298
Donkey       ----------------------TTTTATTAGC--------------------ATAAACTAT--ATACAACTCTCCTGCTT  49089
Cat          ----------------------TACTATTAGC--------------------GTAAACTGT--ATA--GGTCTGTTGCTT  50309
Ferret       ----------------------CACTATTAAC--------------------ATAAACTGT--AGA--AGTCTGCTGCTT  51383
Lion         ----------------------TACTATTAGC--------------------ATAAACTGT--ACA--GGTCTGTTGCTT  48025
Vaquita      ------------------------TTAAGCA----------------------TAAACTGTTTATGCAACTCTGCTGCTT  43975
Pig          GTGTATGAATGATGGTCAGGTTCATTAACCACTGAGCCACGCCGGGAACTCCATAAACTATTTATACAGCTCTGCTTCTT  40459

Majority     AGACTTT-AAGGTATGATGTTCAATXCCTTTGGTT---ATTTGACTTTTX------------------------------
                      63930     63940     63950     63960     63970     63980     63990     64000
Human        TTAATTTGATGATGAGTTTTTAATTTCTTTTGAAAAAGAATGTATGCTTCTAATAATTTATCAAGAGGAAGAATACCAAA  50619
PolarBear    AGACTTTTAAGGTATGATGTTTAATGCTTTTAGTT---ATT---------------------------------------  16522
Cow          AGA-TTT-ACTGTGTGAAATTCAGTACCTTTGGTT---ATTTGACTTTTT------------------------------  47028
Dog          GGACTTTTAAAGTATGTT------TA----T-------ATT---------------------------------------  44292
BelugaWhale  AGACTTC-AGTGTACGAAGTTCAGTCCCTTTGGTA---ATTTGACTTTTA------------------------------  43344
Donkey       ATACTTT-AAGGTATGAAGTTCAATGCCTTTGGTT---ACTTGACTTTT-------------------------------  49134
Cat          AGAGTTTTAAGGTATGTT---TAATACCTTTAGTT---ATTAATAACTAA------------------------------  50353
Ferret       AGACTTT-AAAGTATGATGTTTAATGCCTTTAGTT---ATT---------------------------------------  51420
Lion         AGAGTTTTAAGGTATGTT---TAATACCTTTAGTT---ATTAATAACTAA------------------------------  48069
Vaquita      AGACTTC-AGTGTACGAAGTTCAGTCCCTTTGGTA---ATTTGACTTTTA------------------------------  44021
Pig          AGACTTT-AGTGCATGAAATTCAGTGCCTTTGGTT---ATTTGACTTTTTTTTTTTTTTTTTTTTGTCTTTTTAGGGCTG  40535
```

```
Majority     --------------------------------------------------------------------------------

                       64010     64020     64030     64040     64050     64060     64070     64080

Human        AGAAAATATCTGCTCTTCTTTCTTTTTACTTAGATTTTTTTGCATTTTTAACTTATTTTTAACAAATAATTGTTTATACT  50699
PolarBear    --------------------------------------------------------------------------------  16522
Cow          --------------------------------------------------------------------------------  47028
Dog          --------------------------------------------------------------------------------  44292
BelugaWhale  --------------------------------------------------------------------------------  43344
Donkey       --------------------------------------------------------------------------------  49134
Cat          -------------------------------------AATTAGAA-----------------------------------  50361
Ferret       --------------------------------------------------------------------------------  51420
Lion         -------------------------------------AATTAGGA-----------------------------------  48077
Vaquita      --------------------------------------------------------------------------------  44021
Pig          TACCTGCAGCATATGGAGGTTCCCAGGCTAGGGGGCGAATTGGAGCTATAGCTGCCGGCCTACACCACAGCCATAGCAGT  40615

Majority     ---------------------------------------T---XAX----------------------------------

                       64090     64100     64110     64120     64130     64140     64150     64160

Human        TATGGTGTACAATGTGATGTTTTAATACATATATATTATGGAATGATCAAATCTGGCTGGTTAACATATTCATCTCTTCA  50779
PolarBear    ---------------------------------------T---GAC----------------------------------  16526
Cow          --------------------------------------------------------------------------------  47028
Dog          ---------------------------------------T---GAC----------------------------------  44296
BelugaWhale  --------------------------------------------------------------------------------  43344
Donkey       --------------------------------------------------------------------------T-----  49135
Cat          ---------------------------------------ATAAAAATGTTAGAAATAAAA--------------------  50382
Ferret       ---------------------------------------TTATAAC----------------------------------  51427
Lion         ---------------------------------------ATAAAAATGTTAGAAATAAAA--------------------  48098
Vaquita      --------------------------------------------------------------------------------  44021
Pig          GCCAGGTCAGAGCCGAGTCTGACCTATACACCACAGCTCATGGCGACGCCAGATCTTTAACCCACTGAGTGAGGCCAGGG  40695
```

```
Majority     ------------TTTXTATAGGX------------------------------------------------------CAT

                     64170     64180     64190     64200     64210     64220     64230     64240

Human        GTACTTACAATTTCTTTATGGTAAGAACATTTAAAATTCCTTTAGCTATTTTGGAAGTATACAGTGGAATGTGGAAAAAC  50859
PolarBear    ------------TTTTTATAGGT------------------------------------------------------CTT  16540
Cow          ------------------------------------------------------------------------------AT  47030
Dog          ------------TTTGTATAGGT------------------------------------------------------CTT  44310
BelugaWhale  ------------------------------------------------------------------------------AT  43346
Donkey       ------------------------------------------------------------------------------AT  49137
Cat          ---------ATATTTCTATAAGAAA----------------------------------------------------CCT  50401
Ferret       ------------TTTTTATAGGA------------------------------------------------------CTC  51441
Lion         ---------ATATTTCTATAAGAAA----------------------------------------------------CTT  48117
Vaquita      ------------------------------------------------------------------------------AT  44023
Pig          ATCGAACCTGCATCCTCATGGATGCTTGTCAGATTCGTGTCTGCTGAGCCATGACAGGAACTCCTTATTTGACTTTTTAT  40775

Majority     AGGTCTTA-ATTTCTTATGGAAAAATTXTTATTGAAGATGTGTTA-ATGGTTTTCC--TGGGGGXGGG---X-----XGA

                     64250     64260     64270     64280     64290     64300     64310     64320

Human        TGTTTTTCCTCTGCTCACACCACAACAATCAACACAGAAGGCTTCTGTGGCCTCAAATGTGGGGGAATTTTTTTCCACGC  50939
PolarBear    AGGTT-------TCTCATGGAAATATTTTTATTGAAGATGTGTTA-ATGGTTTTCT--TTGGGGGG--------------  16596
Cow          AGGTTTTA-ATTTCTTATGGATAAATTCCTATTAAAGATGAATTA-ATGATTTTC----TTGG-------AG-----GGA  47092
Dog          AGGTT-------TC--ATGGAAGAATTTTTACTGCAGATGTGTTC-ATGGTCTTCC--TGGAGGAAGCAAT---------  44369
BelugaWhale  AGGTCTTA-ATTTCTTATGGAAAAACTCCTACTGAGGATGTGTTA-ATGGTTTTCC--TGGGGGGGGG-----------A  43411
Donkey       AGGTCTTA-ATTCCTTATGGAAAAATTCCTATTGAAGATATGTTA-ATGGTTTTCC--TGTGGGGA-------------A  49200
Cat          AAGACCTATAGGTCTTATGGAAATATTTTTATTGAAGATGTGTGA-ATGGTTTTCT--GGTGGGAGTGGGGGTGTTTGGG  50478
Ferret       AGGTT-------TCTCATGGAAATATTTTTATTGAAGATCTGTTA-ATGGTTTTCC--TTGGGGAGGGA-----------  51500
Lion         AAGACCTGTAGGTCTTATGAAAATATTTTTATTGAAGATGTGTGA-ATGGTTTTCT--GGTGGGAGTGGGGGTGTTTGGG  48194
Vaquita      AGGTCTTA-ATTTCTTATGGAAAAACTCCTACTGAGGATGTGTTA-ATGGTTTTCC--GGGGGGGGGGG--G-----GGA  44092
Pig          TGGTCTTA-ATTTCTTGTGGAAAAATTCCTGTTGGAGATATGTTA-ATGGTTTTCC--TTTGGGATGTCCAG-----GGG  40846
```

```
Majority     AATGTAAG-AATTGAGAATATATG---AATATATTTATTXATTTTTTAAAAATGTGATGTAGAAGATA---CTAATTATT
                              64330     64340     64350     64360     64370     64380     64390     64400
Human        CAAGCAAGCAGTCAGGTCTGCAGTGTTCTCCAACTTAGCTCAGTTTCAACACTGTCTGCCGGGAGATAGCATCAGATCCC  51019
PolarBear    -ATGTAAG-AATTGAGAATATATG---AATATATTGATTTGTTTTTTAAAAATGT-----AGAAGATA---CTGAGTATT  16663
Cow          AATAAAAG-AATTGAGAATATATG----A---ATGTGTT-ATCTTTTAAAAATGCAAGGTAGAAGTTT---CTAGTTATT  47160
Dog          -ATGTAAA-AATTGAAAATATATG---AATATTTTGATTTATTTTTTAAAA-TGTGATATAGAAGATA---TTAATTATT  44440
BelugaWhale  AATGTAAG-AATTGAGAATATTTGTTGAGAATATGTGTTGATCTTTTAAAAATGTGATGTAGAAGATA---TTAATTATC  43487
Donkey       AATGTAAG-AAGGGAGAATATATG----A---ATGTATGGATCTTTTAAGCATGTGATGTAAAAGACA---TTAATTATT  49269
Cat          AATGTAAG-AATTGAGAATGTATG---AATATATTGA---ATTTTTTTAAG-TGTGATGTAGCAGATA---CCAATTATT  50547
Ferret       -ATGTAAG-AATTGAGAGTATATA---AATATATTGATGTATTTCTTAAAAATGTGATGTAGAAGATA---CTAATTATT  51572
Lion         AATGTAAG-AATTGAGAATATATG---AATATATTGA---ATTTTTTTAAG-TGTGATGTAGAAGATA---CCAATTATT  48263
Vaquita      AATGTAAG-AATTGAGAATATATGTTGAGAATATGTGTTGATCTTTTAAAAATGTGATGTAGAAGATA---TTAATTATC  44168
Pig          GATTCAAG-AATTGAGAATATGTG----A---ATGTATTGATTTTTTAAAAACGTGATATGGAAGATA---CTG----TT  40911

Majority     TAAAATTAATATTT---TTTTXAAAAAGTAX--TATAAGA-ATGGTTTTAGACCT----AAATACA--TAGXATTGCAXT
                              64410     64420     64430     64440     64450     64460     64470     64480
Human        ACAGGTTGAGGGCTCAGTCCCACAAGATCTCCTGAGACACCAGCAAGTCAGGCCTCTGGAACTTCTGATTGACCAGCTTC  51099
PolarBear    TAAAATTAATATAT---T---------------TTTA----ATGGGTTTATACCT----AAATATA--TAGCATTGCAGA  16715
Cow          TAAAATTAATGTTT---CTTTAGAAAAGTATCATAGAAGA-GTGGTTTTAGATAT----AAACACT--TAGGATTGCAGT  47230
Dog          TAAAATTAATATAT---TTTTTTAAAAATGG--TATAAG--AACGGTTTAAGCCT----AAATACATAGAGCATTGCAAG  44509
BelugaWhale  TAAAATTATT--TT---TTTAGAAAAAGTAT-----AAGA-ATGGTTTTAGATCT----AAGTATA--TAGGATTGCAAT  43550
Donkey       TAAAATTAATATAT------TAAAAAAATAG--TATATGA-ATGGCTTTAGACCT----AAATAGG--TAGGGCTGCAAT  49334
Cat          TAAAATTAATATGTATATATTTTTTAAATAG--TATAGGA-ATGATTTTAGACCT----AAAAACA--TAGCATTTAAGG  50618
Ferret       TAAAATCAATATTT---TAA---AAAAGTAA--TATAGGA-ATGGGTTTATACCT----AAATACA--GAGCATCACAGG  51637
Lion         TAAAATTAATATGTATATATTTTTTAAATAG--CATAGGA-ATGATTTTAGACCT----AAAAACA--TAGCATTTAAGG  48334
Vaquita      TAAAATTATT--TT---TTTAGAAAAAGTAT-----AAGA-ATGGTTTTAGATCT----AAATACA--TAGGATTGCAAT  44231
Pig          TAAAATTAATATTT---TTTAGAAAAAGTAT-----AAGA-ATGGTTTTAGATAC----AAATTCA--TAGGATTACAAT  40976
```

```
Majority     AA----TGGTCTAAAAACTTTAGCTCTTTTAATATAGTTTTTAGCTAATTATAT-XTTTCTGTTTCAGTAAAATCTAAA-
                       64490     64500     64510     64520     64530     64540     64550     64560
Human        AAGTTGGGGTTCCCACAACCCCCTTTTTGGATTTCATTAATTTGCTAGA-GCAGCACACAGAACTCAAGGAAACATTTAC  51178
PolarBear    AA----TGGTCAAAAATCTTTAGCTCCTTTAATATAGTTTTTAGCTAACTAT---CTTTCTGTTTCGGGAAGATCTAAA-  16787
Cow          AAACAGTGATATAGAAACTTTAGCTCTTTTAATGTAGCTTTTAGCTAATTTTA-----TCTGTTTTAGTAAAATATAAAG  47305
Dog          AA----TGGTCTAAAATTGTAAGCTCTTTTAATACAGTTTTTAGCTAATTATATCTTTTCTGTTTCAGTAATATCTAAA-  44584
BelugaWhale  AAAAACTGGGCTAAAA------GCTCTTCAAATGTAGTTTTTAGCTAATTATA-----TCTGTTTTAGTAAAATCTAAA-  43618
Donkey       AA----TGATCTAAAAACTTTAGCTCTTTTAATATAGTTTTTAGCTAATTATAT-CTTTCTGTTTCAGTAAAATGTAAA-  49408
Cat          AA----CGGT------CTTTTAGCTCTTTTAATACAGTTTTTAGCTAATTGTATGTTTTATGTTTCAGTAAAATATAAA-  50687
Ferret       GA----AGG--AAAAAC--T-AGCTCTTTTAATATAGTTTTTAGCTAATTATATCTTTTCTGTTTCAGTAAAATCTAAA-  51707
Lion         AA----TGGT------CTTTTGGCTCTTTTAATACAGTTTTTAGCTAATTATATGTTTTATGTTTCAGTAAAATATAAA-  48403
Vaquita      AAAAACTGGTCTAAAA------GCTCTTCAAATGTAGTTTTTAGCTAATTATA-----TCTGTTTTAGTAAAATCTAAA-  44299
Pig          AAAAACTGTTCTAAAAACTTGAGCTCTTTTAATGTAGTTTTTAGCTAAGTATAC--TTTCTGTTTTAGTAAACT------  41048

Majority     CTATXTCTGGCXXTTTGTCTTA--GAAAATXTAATGGAA-AATAATTCCTCA--TAGATTTT-AACCTTCA--TAAAAAG
                       64570     64580     64590     64600     64610     64620     64630     64640
Human        TGAGATTTACTAGTTTATTATATAGGATATTAAAAAGGATAATAAGCCTCTAATCATGCCTTGATCTTTCAGGTGACCAG  51258
PolarBear    CTATATATGACCCTTTGTCTTATTGAAAATGTAATGGAG-AATAACTCCTCA--TAGATTTT-AACCATCA--TAAAAAG  16861
Cow          CTGTGTCTGGC-TTCTGCCTT-----AG--CAAATGGAA-AATAAT-CCTCA--CAAAATTT-AACCTTTG--TAGAAAA  47370
Dog          CTATATTTGGCTCTTTGTCTT---GAAAATGTAGTGGAA-TGTAATTCCTCA--TAGATTTT-AACCATCA--TAAAAAG  44655
BelugaWhale  CTGTGGCTCTT-----ATCAA-----AAATTAAATGGAA-AATAATTCCTCA--TAGATTTT-AATCTTTG--TAAAAAA  43682
Donkey       CTGTACCTGGC--TCTGTCTCACTGAAAATTTAATGGAA-AATTATTCCTTA--TAGATTTT-AACTTTCG--TAAAAAG  49480
Cat          CTAT--CTGGCTGTTTGTCTTACTGAAAATGTAATGGAA-AATAATTCCTCA--TATATTTT-AACCTTCA--TAAAAAC  50759
Ferret       GTATGTCTTACTCTTTGTCTTACAGAAAATGTCATGGACTAATAATTCCTCA--TAGATTTTTAACCATCA--TAAAAAG  51783
Lion         CTAT--CTGGCTGTTTGTCTTACTGAAAATGTAATAGAA-AATAATTCCTCA--TATATTTT-AACCTTCA--TAAAAAC  48475
Vaquita      CTGTGGCTCTTGTCTTATCAA-----AAATTAAATGGAA-AATAATTCCTCA--TAGATTTT-AACCTTTG--TAAAAAA  44368
Pig          CTGTCTCTGGT-TCATGTTGA-----AAATTAAATGGAA-AATAATTCCTTA--TGGATTTT-AACTTTTG--TAAAGAA  41116
```

```
Majority     TGAC--TXTTAAGATCATTTT---GATAGTTTATXAA-AAATAAGCATTTAAAACTTTTTGTCTAA-AGAT-------AA
             64650     64660     64670     64680     64690     64700     64710     64720
Human        TCCCCATCTTAAAGTGGGGCTGCCAGCCATCAGTCAACTCATTAGCATACAAAATATATCATTTTGGAGATTTATTAGGA  51338
PolarBear    TGACC-TTTCAAGATCATCTT---GATAGTTTATAAATAAATAAGCATTTAAAACTTTCTGTCTGA-AGAT-------AA  16929
Cow          TAAC----TTAAGATCATTTT---GATAGTATATGAA-CAATAAGCTTGTAAAACTTTTTGCCTAAAAGAT-------AA  47435
Dog          TGACT-TTTCAAGATCATTTTT--GATAGGTTATA----AATAATCATTTAAAACTTACTGTCTAA-AG-----------  44716
BelugaWhale  TAAT----TTAAGATCATTTT---GATAGTTCATGAA-AAATAAGCTTGTAAAACTTTTTGCGTGAAAGAT-------AA  43747
Donkey       TGAC----TTAAGACCATTTT---GATAATTTATGAA-CTATAAGCTTTTAAAACTTTTTATCTAAAAGAT-------AA  49545
Cat          TGACC-TTTCCAGATCATTTT---GATAGTTGATA----AATAAATATTTAAAATTTTTTGTCTAA-AGAC-------AA  50823
Ferret       TGATC-TTTCAAGATCATTTT---GATAGTTTATATGTAAATAAGCATTTAAAACTTTCTATCTGA-AGAT-------AA  51851
Lion         TGACT-TTTCCAGATCATTTT---GATAGTTGATA----AATAAATATTTAAAATTTTTTGTCTAA-AGAC-------CA  48539
Vaquita      TAAT----TTAAGATCATTTT---GATAGTTCATGAA-AAATAAGCTTGTAAAACTTTTTGCCTGAAAGAT-------AA  44433
Pig          ---------TAAGATCGTTTT---GATAGCTTATGGA-AAAGAAGCTTATAGAACTTTTTGTCTAACAGTT-------CA  41176

Majority     GGCTAAAXTT-----TCAG-X---TTGTTTTTTTT---------------------------------------------
             64730     64740     64750     64760     64770     64780     64790     64800
Human        ATTTAGAGTTGTATGCCAGGAAATGGGGTTGAGGACCAAATGTCTATTTTACAATGTCACAATACAGTATTATGCATTAC  51418
PolarBear    GGCTAAATTT-----TCCACT---TTTTTTTAAAT---------------------------------------------  16956
Cow          GGCTAACATT-----TCAG-TTTTTTGTTTTTTTT---------------------------------------------  47464
Dog          -GCTAAATTT-----TC-------TTTTTTTGTTG---------------------------------------------  44738
BelugaWhale  GGCTAAAATT-----TCAG-----TTGTTTTTTT----------------------------------------------  43771
Donkey       GGCTAAAATT-----TTAGCTGTTTTTGTTTTTTG---------------------------------------------  49575
Cat          GGCTGAATTT-----TCAG-----CTGGATTTTGT---------------------------------------------  50848
Ferret       GACTAAATTT-----TCTGCTGTTTTTTTCTTTGT---------------------------------------------  51881
Lion         GGCTGAATTT-----TCAG-----CTGGGTTTTGT---------------------------------------------  48564
Vaquita      GGCTAAAATT-----TCAG-----TTGCTTTTT-----------------------------------------------  44456
Pig          GGGTAAAATT-----TCAGGTTTTTTGTTTTTTTTAATTTGATAATAATCTTTATTTCTTTGGAAGTCCCTTGTAGCACA  41251
```

Majority	--TTTAATTTGXTAXTA-----------------------	
	64810 64820 64830 64840 64850 64860 64870 64880	
Human	AGTAGTTGCCATGCTGTGCGGTAGATCACCAGAACCTATTCTTCCTGTCTGAAACTTTGTACTTTGA-CCATCATCACCC	51497
PolarBear	--TTTAATTTGATAATA-----------------------	16971
Cow	---TTTAAGTTTGGTA--------------------------	47477
Dog	--TTTAATTTGACAATA-----------------------	44753
BelugaWhale	---------------------------------------A-----AATTTGATA--------------------------	43781
Donkey	--TTTAATTTGGTA--------------------------	49587
Cat	--TTTAATTTGGTAATA-----------------------	50863
Ferret	--TTTAATTTGCTGTTA-----------------------	51896
Lion	--TTTAATTTGGTAATA-----------------------	48579
Vaquita	---------------------------------------A-----AATTTGATA--------------------------	44466
Pig	CTGGGTTACAGGATCTGTTGTTGCCCAGCTGTGGCATAGGTTGCAAGTGTGGCGTGAGTTCATTTGATCCCTGGCCTAGG	41331

Majority	----------------------------------ATTTTCATTA-------CTTTTGAACCAGAAGTTGTACTTCTAAGA	
	64890 64900 64910 64920 64930 64940 64950 64960	
Human	CTTCCCTGTTCACTCCTCCCTCCCCCAGCCTCTGGTGACTATTTTTCTACTCTCTACTAATGTGAGTTCAGCTTTTT-TA	51576
PolarBear	----------------------------------ATTTTCATTA-------CTTTTGAACCAGAAATTGTACTTCTAAAA	17010
Cow	----------------------------------ATTTTCGTTA-------CTTTTGAACCAGAAGTTGTACTT------	47510
Dog	----------------------------------ATTTTCATTA-------CCTTTGAACCAGAAATTATGCTTCAAAAA	44792
BelugaWhale	----------------------------------ATTTTCATTT-------CTTTTGAACCAGAAGTTGTACTTCTCAGA	43820
Donkey	----------------------------------ATTTTCGTTC-------CTTTTGAACCAGAAGTTGTACTT-TAAGA	49625
Cat	----------------------------------ATTTTCATTC-------CTTTTGAACCAGAAATTGTACTTCTAGGA	50902
Ferret	----------------------------------ATTTTTATTA-------CTTTTGAACCAGAAATTGTACTTCCAAAA	51935
Lion	----------------------------------ATTTTCATTC-------CTTTTGAACCAGAAATTGTACTTCTAGGA	48618
Vaquita	----------------------------------ATTTTCATTT-------CTTTTGAACCAGAAGTTGTACTTCTCAGA	44505
Pig	AACTTTCACATGCTGCGAGTGCAGCAAAAAATATATTTTTTTTAAAAATTTCTTTTGAACTAGATGTTGTACTCA---GA	41408

```
Majority     ATTTATCATGAGGAAATATACCAAAAGAAAA-T---TATTCTATATTCXTTTTACTTAG-----------AAT------T
                              64970     64980     64990     65000     65010     65020     65030     65040
Human        GATTA-CATATGTAAGTGAGATAATGTGTTATTTGTCTTTCTTTGTTTGACTTATTCACTTAGCACACACAGTGTCCTCT  51655
PolarBear    ATTTATCTTT-GGAAATATACTAGAAGAAAA-----TATTCTATATTCATTTTACTTAG-----------AAT------T  17067
Cow          -CTTAA-------AATTTTATCAAAGGAAAA-TGCCTATTCTCTATTCTTTTTACTT-----------------------  47558
Dog          ATTTATCATT-GGAAATATACCAAAAGAAAA-----TCTTCTATACTCATTTTACTTAT-----------AAT------T  44849
BelugaWhale  ATTTATCATGAGGAAATATACCAAAAGAAAA-TATGTATTCTCTATTCTTTTTACTTAG-----------AGT------T  43882
Donkey       ATTTATCGTGAAGAAATATGCCAAAAGAAAA-TAGCTATTCCATGTTCTTTTTATTTAG-----------AAT------T  49687
Cat          ATTTATCATGAGGAAATATGCCAAAGGAAGA-----TATTCTATATTTATTTTACTCAG-----------AAT------T  50960
Ferret       ATTTA-CATT-GGAAATATTCTAAAAGAAAA-----TACTCTGTTTTCATTTTACTTAG-----------AAT------T  51991
Lion         ATTTATCATGAGGAAATATGCTAAA---AGA-----TATTCTATATTTATTTTACTCAG-----------AAT------T  48673
Vaquita      ATTTATCATGAGGAAATATACCAAAAGAAAA-TATGTATTCTCTATTCTTTTTACTTAG-----------AGT------T  44567
Pig          ATTTAGCATAAGGAATAATACCAAAAGAAAAATGTCTATTCTGTATTCTTTTTACTTTA-----------AGT------T  41471

Majority     TAAAATGGCATTTAATATCCCAATTTTATTTT----ATATXTTAGTATTTT-CAGTAAAGTAA-----------------
                              65050     65060     65070     65080     65090     65100     65110     65120
Human        TAAAATGACATTTAATATCCCATTTCTAGTTCGTTATTATTTAAGTAATTTGCAAGTACATTCTCATTTTAAAAGATTAA  51735
PolarBear    TAAAATGGTATTTA-TATTTCAGTTTTATTTT----ATATGTTAGTATTTC-TAGTAAAGTAA-----------------  17124
Cow          -AAAGTGGCATTTAATATCCCAGTTGGGTTTA----ACATATTAATATTTT-CAGTAAAGTAA-----------------  47615
Dog          TAAAATGGCATTTAGTATTTCAATTTTATTTT----ATATGTTAGTATTT--TAATAAGGTAA-----------------  44906
BelugaWhale  TAAAATGGCATTTAATATCCCAATTTTATTTT----ACATGTTAGTATTTT-CAGT-----AA-----------------  43935
Donkey       TAAAATGGCATTTAATATCCCAATTTTATTTT----ATATATTAGTATTTT-CAGTAAAGTAA-----------------  49745
Cat          TAAAATGGCATTCAGTATTTCAATTTTATTT-----ATATATTAGTATTTT-TAGTAAAGTAATTTTATTTATATATTAG  51034
Ferret       TAAAATGGTATTTA-TATATCAATTTTATTTT----ATATATTAGTATTTT-TAGTAAAGTAA-----------------  52048
Lion         TAAAATGGCATTCAGTATTTCAATTTTATTT-----ATATATTAGTATTTT-TAGTAA----------------------  48725
Vaquita      TAAAATGGCATTTAATATCCCAATTTTATTTT----ACATGTTAGTATTTT-CAGT-----AA-----------------  44620
Pig          AAAAATGGCATTTGACATCCCAGTTTTATTTT----ACATGTTAGAATTTT-CAGT-----AA-----------------  41524
```

```
Majority     -----------------TTAGTGAA--TGCATTCTCATTTTAAAAGATTCCAATGXAAA-----GAACATGAAAAGGATA
                      65130     65140     65150     65160     65170     65180     65190     65200
Human        ACGAAAGGAACATGTAAAGAATAAAGCTGGGGGCTGGGCTTGGTGGCTTATACTGGTAATCCCAGCACTTTGGGAGGCTG  51815
PolarBear    -----------------TTCATGAA--GATATTCTCATTTTAAAAGCTTCCAATGGAAG-----GAACATGAAAAGGATA  17180
Cow          -----------------TTAGTGAA--TTCATTCTCATTTTAAAAGATTGAAATTAAAA-----GGATGTTGAAAGGACA  47671
Dog          -----------------TTCATGAA--TGCATTCTCATTTTAAAAGCTTCCAGTGCAAA-----GATCATGAAAAA-ATA  44961
BelugaWhale  -----------------TTAGTGAA--TGCATTCTCATTTAAAAAGATTCAAATGAAAA-----TGATGTGGAAAGAATA  43991
Donkey       -----------------TTCATGAA--TATATTCTTTTTTTTATTGAGTTCATAATAGT-----TTATATCATTGT----  49797
Cat          TATTTTTAGTAAAGTAATTTGTGAA--TGCATTCTCATTTTAAAAGATTCCATTGCAGG-----GAACATGAAAAA----  51103
Ferret       -----------------TTCATGAA--GGCATTCTCATTTTAAAAGCTTCCAATGCAAG-----GAACATGAAAAGGATA  52104
Lion         -----------------TTTGTGAA--TGCATTCTCATTTTAAAAGATTCCATTGCAGG-----GAACATGAAAAA----  48777
Vaquita      -----------------TTAGTGAA--TGCATTCTCATTTAAAAAGATTCAAATGAAAA-----TGCTGTGGAAAGAATA  44676
Pig          -----------------TTAGTGAA--TGCATTCTCATTTTAAAAGATTGAAATGAAAA-----TGATGTGTACAGGACA  41580

Majority     A---AGATAAGGCATCTATAGAXAGATGTATGTA--------GGGGAX------GGGGATTTX---TAATTTTAAATAGG
                      65210     65220     65230     65240     65250     65260     65270     65280
Human        AGGCGGGTGGATTACCTGAGGTCAGGAGTTCAAGACCCGCCTGACCAACATGGTGAAACCCTGAGTCTACTAAAAATACA  51895
PolarBear    A---AGATAAGGCATCTTTA-------------------------------------T-------AATCTTAAATAGG  17211
Cow          A---AGAAAAAACATCTGTAGATAGATGTCTGTGG-TGGGGTGTGGAG------GGGGCCTTG---TAATTTCAAATAGG  47738
Dog          A---AGATAAGGCATCTATAGACAAATGTATGTA--------AGGAAA------GGGTATTTA---TAATTTTAAATAGG  45021
BelugaWhale  A---AGATAAGGCATCTGTAGATAGATAGATAGATATATGTAGGGGAG------GGGGATTTG---TAATTTCAAATAGG  44059
Donkey       -----GAAAGTTCAGTTGTA------------------------------------------CA---TTATTTCTTGTCTG  49828
Cat          ----AGATAAGGCATCTATAGACAAATGCATGTA--------AGGGAA------GGGGATTTA---TAATTTTAAATAGA  51162
Ferret       A---AGATAAGGCATCTTTA-------------------------------------TTTA---TGATCTTAAATAGG  52139
Lion         ----AGATAAGGCATCTATAGACAAATGCATGTA--------AGGGAA------GGGGATTTA---TAATTTTAAATAGA  48836
Vaquita      A---AGATAAGGCATCTATAGATAGATACATAGATATATGTAGGGGAG------GGGGATTTG---TAATTTCAAATAGG  44744
Pig          A---AGACAAGGCATCTATAGATAGATGTATGTA--------GGGGAG------GGGGATTAG---TAATTTTAAATAGG  41640
```

```
Majority     ATAACCAGTGAAAGCCCCATGAACA-AGTGACATTTGA--GTAACAA-----ACTTCAAAGTGATTAGXAAATAAAT-CA

                      65290     65300     65310     65320     65330     65340     65350     65360

Human        AAAAAAAAAAAAAATTAGCCAGGCATGGTGCCAGGTGCCTGTAATCCCAGCTACTTGGAGGCTGAGGCAAGAGAGAATTG  51975
PolarBear    ATAAACAGCGAAAGCCCCATGAAGA-AGTGACATTTGA--GTAACAA-----ACTTTAAAGTGGTTAGGAAATAAACGCA  17283
Cow          ATAACCAGTGGAAGCCTGATTGACA-AGTGACATTTGA--GTAACAA-----ACCTCAGAA--ATTAAAGAGTAAAA-CA  47807
Dog          ATAAACCATGAAATCTCCATGAAGA-AGTGACATTTGA--GTAACACTAATCACTTCAAAGTGATTAGGAAGTAAATGCA  45098
BelugaWhale  ATAACCAGTGGAAGCCCAACCAACA-AGTGACATTTGA--GTAACAA-----ACTTCAAAGTGATTAGAGAATAAGG-TA  44130
Donkey       TCACCACATAATGCTCCTCTTTACC-CCCTGTGTCCAC--CCCTCACCCCCCTTCCCTTGGTAACCACTGAACTGTTTTC  49905
Cat          ATACTTAGTGAAAACCCCATGAACA-GGTGACATTTCA--GTAACAA-----ACTTCAAAGTGATTTGGAAATAAATACA  51234
Ferret       ATAAACAGTGAAAGCCCCATGAAGA-AGGGACATTTGA--GTAACAA-----ACTTAAAAGTGGTTAGGAAGTAAATGTA  52211
Lion         ATACTTAGTGAAAACCCCATGAACA-GGTGACATTTCA--GTTACAA-----ACTTCAAAGTGATTTGGAAATAAATACA  48908
Vaquita      ATAACCAGTGGAAGCTCAACCAACA-AGTGACATTTGA--GTAACAA-----ACTTCAAGGTGATTAGAGAATAAGG-CA  44815
Pig          CTAACAAGTGAAAGTCCTATTAGGA-AGTGATATTTAG--GGAGCAC-----ATTTCAAAGTGGTTGGAGAATAAC--CA  41710

Majority     CTTA-----------TTCTGGTCTG-GGTTA--------------TTCCAG----ACATAGGGAACAATCAATGCAA-AG

                      65370     65380     65390     65400     65410     65420     65430     65440

Human        CTTGAACCCGGGGGGGTGGAGCTTGCGGTGATCCCAGATGCGTCACTGCACTCCAGCCTGGGTGACAG--AGCGAGACTC  52053
PolarBear    CTTA-----------TTTTGGTCTG-GGTTA--------------TTCCAG----ATTCAGGGAACAATCAATGCAA-AC  17332
Cow          CTTA-----------TTACAGTTTT-GGTTA--------------TTTTTG----ACATAGGGAATAATCAGTGCAA-CG  47856
Dog          CTTC-----------TTCTGACCT--GGTTA--------------TTCCAG----AAATAGGGAGC----AATGCAA-AG  45142
BelugaWhale  CTTA-----------CTGCAGTCTT-GGTTAT-------------GTTCCAG----ACATAGGGAGCAGTCAGTGCAG-TG  44181
Donkey       TTTG----------TCCATGTGTTT-GTTTAT-------------ATTCCAC----ATATGAGTGAAA-TCATNNNNNNNN  49957
Cat          CTTA-----------TTCTGGCCTG-GACTA--------------TTCCAG----ACATAGGGAACAGTCAATGCAACAG  51284
Ferret       CTTA-----------TTCTGGTCTA-GGTTA--------------TTCCAGGTAGATATAGGGAACGATCAATGCGA-AG  52264
Lion         ATTA-----------TTCTGGCCTG-GACTA--------------TTCCAG----ACATAGGGAACAATCAATGCAACAG  48958
Vaquita      CTTA-----------TTGCAGTCTT-GGTTAT-------------GTTCCAG----ACATAGGGAGCAGTCAGTGCAA-TG  44866
Pig          CTTAGT-------AATTACAGTTTG-GATTA--------------TTCCAG----ACACAGGGGACAATCAATACAG-TA  41763
```

```
Majority      TTGCTTCTGTTGA------AAAAAAAXAATXXAACAGCC---------------ATGTTTTTG-----------------
                          65450     65460     65470     65480     65490     65500     65510     65520
Human         TATCTCAAAAAAAAAAAAAAAAAAAAAAAGAGTAAAGCTGAGGCCTACACATGGATGGATGTGGAGGAGGGGCACTTAGG  52133
PolarBear     TTGCTTCTCTTGG----AAAAAAAAAAAACCAAAAAAAC---------------CTGGTTTT------------------  17375
Cow           TTCCTTCTGTTGA------AAAAAAGGTT----ACAGCC---------------ATATTTTTG-----------------  47894
Dog           TTGCTTCTGTTGA-----AGAAAACCTGGTTTTGTAGCC---------------ATAATTTTG-----------------  45185
BelugaWhale   TTC-TTATGTTGA------AAAAAATTTTTTTGACAGCC---------------ATATTTTTG-----------------  44222
Donkey        NNNNNNNNNNNNN------NNNNNNNNNNNNNNNNNNNN---------------NNNNNNNNN-----------------  49999
Cat           TTGCTTGTGTTGG------GAAAACAAAACAAAACAA-----------------TTGTTTTTGCAG------CCGTATTT  51335
Ferret        TTGCTTCTGTTGGGGGGAAAAAAAAACAAACAAAAAAAC---------------TTGGTTTTGCAG------CCATATAT  52323
Lion          TTGCTTGTGTTGG------GAAAACAAAACAAAACAA-----------------TTGTTTTTGCAG------CCATATTT  49009
Vaquita       TTC-TTATGTTGA------AAAAAATTTTTTTGACAGCC---------------ATATTTTTG-----------------  44907
Pig           CTCCTTCTGTTGA------AAAAACTTTTTCTGACAGCC---------------GTGTTTTTG-----------------  41805

Majority      ---ATTTTCAGAAGCCCTTT--------X-TTTTTTGATAATCTTTTTTA--GTTGC-----AXTTGGTCA-XXXXX--X
                          65530     65540     65550     65560     65570     65580     65590     65600
Human         ATGACTAGTGGAAGCCCCATTGAGAGGGGATATTTGAATAATGAACTTCAAAGTGGTTAGGGAATAAGCCACTTATTCCA  52213
PolarBear     --------------------------------------------------------------------------------  17375
Cow           ---ACTTAAAAAAATTCTT-------------CTTTGATAGCCTTTTTAT--ATTAC-----ACCTGGTCA---------  47942
Dog           ---GTTTTCAGAAGCCCTTT------AATCTTTTCTGATCATCTTTTTTAATATTAC-----AATTGGTCA-TGTTTTAT  45250
BelugaWhale   ---ATTTTCAAAAGTTCTT-----------TTCTTTGATACCCTTTTTAC--CTCGT-----AGTTGGTCA---------  44272
Donkey        ---NNNNNNNNNNNNNNNN------------NNNNNNNNNNNNNNNNNNN--NNNNN-----NNNNNNNNNNGGTGCCAT  50057
Cat           TTAATTTTCAGAAGCCCTTT------AATCTTTTTTGATAATCCTTTTTAG-GTTAC-----AATTGGTCAGTGTTTTGT  51403
Ferret        TTAGTTTTCAGAAGCCCTTT------AATCTTTTCTGATCATTTTTTTTA-TGTTGC-----AGTTCATCA-TGTTTTAT  52390
Lion          TTAATTTTCAGAAGCCCTTT------AATCTTTTTTGATAATCCTTTTTAG-GTTAC-----AATTAGTCAGTGTTTTGT  49077
Vaquita       ---ATTTTCAAAAGTTCTTC--------TTTTCTTTGATACCCTTTTTAC--GTCGT-----AGTTGGTCA---------  44960
Pig           ---ATTTTCAAAATCTCTTT----------TTCTTTGATCATCCTTTTTA--ATTGC-----AGTTAGTCA---------  41856
```

```
Majority     XXTT--------CTTTAAGGA--------TACTAATTAATAA-----TATT-TT--------------------AA--AA
                      65610     65620     65630     65640     65650     65660     65670     65680
Human        GACAGAAGGAATAATCAATGCAAAGTTCCTTCTGTTGAAAAAAATGCTTTTATAATTTTATTTTTCATCTTCTAAAGCCC 52293
PolarBear    -------------------------------------------------------------------------------- 17375
Cow          --TT--------GTTTATGGA--------TACTAATTAATGG-----TATT-TT--------------------TA--AA 47976
Dog          TATTTCACAGTTCTTTAAGG---------TACTAATTAACAA-----TATT-TT--------------------AAAAGA 45295
BelugaWhale  --TT--------CTTTACAGT--------TACTAATTAATG------TATT-TT--------------------AA--AA 44305
Donkey       GTCTG-------CGCCCAGGA--------TCTGAACCAGCAAA-----ACC-CC----------------------GGGA 50094
Cat          GATTTCACAATTCTTTAAGGA--------TACTACTAATTAATAGTATTTT-TAAAAATACCTCACTCTATCTAAATTAT 51474
Ferret       GATTTCATAGTTCTTTAAGGA--------TTCGAATTAACAGTGCTTTGTT-TTATTATTTATTTATTAGTTTAAAAAAA 52461
Lion         GATTTCACAATTCTTTAAGGA--------TACTACTAATTAATAGTATTTT-TAAAAATACCTCACTCTATCTAAATTAT 49148
Vaquita      --TT--------CTTTACAGT--------TACTAATTAATGG-----TATT-TT--------------------AA--AA 44994
Pig          --TT--------CTTTACAGT--------TAATAATTGT--A-----TTTT-TT--------------------AA--AA 41888

Majority     TAC----TT----XGCTAXATCTTTT----ATTTTCTTTAGTATTAGCTAXTATGTTTATC------TTGTTCT-GTTTA
                      65690     65700     65710     65720     65730     65740     65750     65760
Human        TTCAGTCTCCTTTGGTCATCTTTTTTTTGTAGTTGGTTATGCCTTAACAACAGTACTTTGAGGATACTAGTTACAGTATT 52373
PolarBear    -------------------------------------------------------------------------------- 17375
Cow          CAC----TC----TGCT--ATCTTTT----ACTTTCCTTAGTATTAGCT-----GTTTATC------TTGCTCT-CCTC- 48029
Dog          TAC---TAT---ATGTTAATTATCTT----ATTTTCTTCAGTGTTAGCTATTATGT-----------TTGTCTT-GTATA 45353
BelugaWhale  TAC----TT----GACT--ATCTTTT----ACCTTCTTTAGAATTAGCTGTTATGTTTATC------TTGTTCT-CTCTA 44364
Donkey       CACTGAAGC--AGAGCTCACAAACTT----AACCACTCCGCCACAGGCCAGCCCCTGT-TT------TTCTCTT-GTTAA 50160
Cat          TTCATTCTTTCAGTGTTAGCTGTTAT----GTTTATCT-TCTGTCCATCTGTCTGTCTTCCTCCCCCTAGTTGT-GTTTA 51548
Ferret       TAC---TTTTAAAAAAAAAAGATTTT----ATTTATTTATTTGAGAGGGAGAAGGCGGAGTGGGGGGCGGGTGT-GCAGA 52533
Lion         CTCATTCTTTCAGTGTTAGCTGTTAT----GTTTATCT-TCTGTCCATCTGTCTGTCTTCCTCCCCCCAGTTGT-GTTTA 49222
Vaquita      TAC----TT----GACTGTATCTTTT----ACTTTCTTTAGAATTAGCTGTTATGTTTATC------TTGTTCT-CTCTA 45055
Pig          TAC----TT----GACTGTATCTCCT----AATTTCGTTAGTATTAGCTATTATGTTTATC------TTGTTCT-CTCTT 41949
```

```
Majority     TXCATCT---CTTXCTCCXXCCCGCCCTAATTGTCTGTTT----------------------------------------
                      65770     65780     65790     65800     65810     65820     65830     65840
Human        TAAAAAATACGTCACTATATGCTGAGTTATTTTTTCAGTGTTAGCATTTATGTTTATCTTGTTCTCATTCTCTTCATCCC  52453
PolarBear    --------------------------------------------------------------------------------  17375
Cow          ----------CTTCATCCCACCCGCTCTCATTGCTTGTTTT---------------------------------------  48060
Dog          TCCATCT--GTCTATCCCTTCTCCCCCCAGTAGTCTGTT--CAT----A-------------------------------  45394
BelugaWhale  TTCTTCT---CTTCCTCCCACCCACCCTCATTGTCTGTTT----------------------------------------  44401
Donkey       TCTGTCTTTTGTCAGTCTAATTTAC---AGGCCTCAGGTGGAGAACCTAAGATAGGGAGAGGAAGAGATTTTCCCT----  50233
Cat          TATATTTTAGAATGGACAATTAGGTTTTATTGGACTGGTATGCTTTCCTCTAGAGTAAAATAAGGAGTTTGGACACTGAG  51628
Ferret       GGGAGAT--GCAGACTCCCTGCTGAGCAAGGAGCCTGATTCCAAGCTCAATCCCAGGACCACTGAGATCATGACCTGAGC  52611
Lion         TATATTTTAGAAAGGACAATTAGGTTTTATTGGACTGGTATGCTTTCCTCTAGAGTAAAATAAGGAGTTTGGACACTGAG  49302
Vaquita      TTCTTCT---CTTCCTCCCACCCACCCTCATTGTCTGTTT----------------------------------------  45092
Pig          TCCCTGT---CTCCCTCCTACCCACCCTCATTGTTTGTTT----------------------------------------  41986

Majority     -----------------ATATTTCAGAATGGACAATTAGGTTTTX----TTGXAAXTTGCATGCTTTCCTCTAXAGTAAG
                      65850     65860     65870     65880     65890     65900     65910     65920
Human        CCCTTTGTTGTCTTTTCGTGTTTCAGAATGGACAATTAGATTTTG-----TTAAAGTTGTATGCTTTCCTCTAGGATAAA  52528
PolarBear    --------------------------------------------------------------------------------  17375
Cow          -----------------ATATTTCAGAATGGGCAGTTAGGTTTTA----CTGGACTTTACATGCTTTCCTCTAGAGTAAA  48119
Dog          --------------------TTTCAGAATGGATAGTTAAGTTTTACTGTTTG--GACTGGTAGGCTTTTATTACAGTAAA  45452
BelugaWhale  -----------------ATATTTCAGAATGGACAATTAGGTTTTA----TTGGA-CTTGCATGCTTTCCTCTAGAGTAAG  44459
Donkey       ----------TCCCT--ACATCTCA--TTGGACATGTGGATATAGCTAAATAGAAGACACCAGCCTG--GGCACTGCAAT  50297
Cat          CTCT--GGGATCCCA--TAAATACGAAAGAGGTCCTTACTCTGGGCCTGTAAAACTCTCTTTAGAATTTCTTTCTTTTAA  51704
Ferret       CACCCAGGTGCCCCT--AATTAACAGTATTAAAAAAAATACTTCACTATGTGTAAATTACTGAATTTCCTTCACTGTTAG  52689
Lion         CT----GGGATCCCA--TAAATACCAAAGAGCACCTTATTCTGGGCCTGTAAAACTCTCTTTAGAATTTCTTTCTTTTAG  49376
Vaquita      -----------------ATATTTCAGAATGGACAGTTAGGTTTTT----TTGGA-CTTGCATGCTTTCCTCTAGAGTAAG  45150
Pig          -----------------ATATTTCAGATTGGATGATTAGGTCTT-----TTGACACTTGCATGTTTTCCTCTAGAGTAAG  42044
```

```
Majority     GTAATGAG------TTAXTTGGATATTAAXCTG-----------------GGAXXCTCTC-C-AAATGXCA----XA--
                            65930     65940     65950     65960     65970     65980     65990     66000
Human        GTAATGAAGAGCGTTTACTAAGCTGTTACCCCCATTCCCCCCAAATACCAAAGAGGCTCTTATTCTGGGTCAACAAAACT  52608
PolarBear    --------------------------------------------------------------------------------  17375
Cow          GTAAT----------GAGTTGGATACTAAGCTG-----------------GGATCCCCCC-C-AAATGCCAA----A--  48163
Dog          GTAATGAG------TTT---GGATATTAAGCTG-------------------GGATTCTC-C-AAATACCA---AAA--  45496
BelugaWhale  GCAACGAG-------GACTTGGATACTAACCTG-----------------GGATCCCCCC-CCAAATGCCA--------  44505
Donkey       GTTATACG------TACTTTGGA-ATGAAAGTG---------------ACTAATTAGAGT---ATATTTTAA---GA--  50346
Cat          AGGAGGAT------TTTTT-GTTTGTTTGTTTGTTTTTAATGATAAGGGAGAGAGATGATG-A-GGAAATGGGAGAAA--  51773
Ferret       CTGTTAAG------TTTATCGTGTATCCATCTGTCTGCCCTTCCTCCCCCAGTAGTCTATT-C-ATATTTCA---GAA--  52756
Lion         GGGAGGAT------TTT-------GTTGGTTTTTTTTTAATGATAAGGGAGAGAGATGATG-A-GGGAATGGGAGAAA--  49439
Vaquita      GTAATGAG-------GACTTGGATACTAACCTG-----------------GGATCCCCCC-CCAAATGCCA--------  45196
Pig          GTAATAAG-------GAGTTGGATACTAAGCTG-----------------GGATTCTCCC---AAATGCCAA----A--  42090

Majority     ----GAGGTCTTTATTC--TAGGCCTGT-----AAAACTC---CXTTTAG-----------------------------
                            66010     66020     66030     66040     66050     66060     66070     66080
Human        CCCTTTAGAATTTCTGGTACCAGGCTGTGCGTGGTGGCTCACACTTGTAATCCCAGCACTTTGGGAGGCTGAGGTGGGCG  52688
PolarBear    --------------------------------------------------------------------------------  17375
Cow          ----GAGGTCTTTACT---TAGGCCTTT-----AAAACCC---TCTTTAG-----------------------------  48198
Dog          ----AAGGTCCTTATT--CTGGGTCAGT-----AAAACTC---CCTTTAG----A---------------AT--------  45535
BelugaWhale  ----GAGGTCTTTATTC--TTGGCCTTC-----AAAACTC---CTTTTAG-----------------------------  44541
Donkey       ----TAAGGTTTTA-----TGAACTTATA----AAATATG---TCTTTGG-----------------------------  50380
Cat          ----GAGGAATTTGTTCATTAGGCAGACTTTCCATTAACC---CTTTGATTTTCGTCCTAGGACTCTGCCAGTTTCTAAT  51846
Ferret       ----TTAGGTTTTACTGGTTATACTGGT-----ATGCTTT---CCTTCAGGTTAAGTAATGAGTTTGGATACCAAGCTGG  52824
Lion         ----GAGGAATTTGTTCATTATGCAGACTTTCCATTAACC---CTTTGATTTTCATCCTAGGACTCTGCCAGTTTCTAAT  49512
Vaquita      ----GAGGTCTTTATTC--TTGGCCTTC-----AAAACTC---CTTTTAG-----------------------------  45232
Pig          ----GAAGCCTTCATTC--TTGGCCAGT-----GAAACTT---CCTTTAG-----------------------------  42126
```

```
Majority     --------------------------------------AGTTTCTGTTCXTTTAA--------GGGAGCATTTXGTTGTT
                      66090      66100      66110      66120      66130      66140      66150      66160
Human        GATCACGAGGTCAGGAGATCGAGACCATGGTGAAACCCCGTCTGTACTAAAAATACGAAAATTAGCTGGGCGTGGTGGCG 52768
PolarBear    -------------------------------------------------------------------------------- 17375
Cow          --------------------------------------AGTTTCTGTTCTTTTAA--------GGGGGTGTTTAGTTGTT 48232
Dog          ---------------------------------TCCTG--TTTCTTT----TAAA--------GGGAGCATTTTGTTGTT 45568
BelugaWhale  --------------------------------------AGTTTCTGTTCTTTTAA--------GGGAGTGTTTAGTTGTT 44575
Donkey       -------A----------------------------CCATTTTTTACTCATGTCA---------AGTGTATGCACATCTC 50416
Cat          CTGACATAATGTGGCGGTTAACAGTGCAAACCCTGTTTGTTCTGTGTTCACTAAGCTTTCTTTGTTCCCATTTGTTTCCA 51926
Ferret       GATTCCCCAGAAAGGCCTGGGCCAATAGAACTCTTTAGAATTCCTCTTTCTTTAA--------GGGAGCATTTTGTTATT 52896
Lion         CTGACATAATGTGGCGGTTAACAGTGCAAACCCTGTTTGTTCTGTGTTCACTAAGTCTTCTTTGTTCCCATTTGTTTCCA 49592
Vaquita      --------------------------------------AGTTTTTGTTCTTTTAA--------GGGAGTGTTTAGTTGTT 45266
Pig          --------------------------------------AATTTCTGTTCTTTTAA--------GGAAGCATTTGGTCATT 42160

Majority     -TTGXTTTT-GTTXTTXTTATTXAATA--ATAAAAGXGX---XTGXTGATGA----GGAGGAAGAGX--------AGCTG
                      66170      66180      66190      66200      66210      66220      66230      66240
Human        GGCGCCTGTAGTCCCAGCTACTCGGGAGGCTGAGGCAGGAGAATGGTGCAGACCCAGGAGGCGGAGCTTGCAGTGAGCCG 52848
PolarBear    -------------------------------------------------------------------------------- 17375
Cow          ---GATTTT-GTTTTTATTAAGTTG----GTTGA---------TGGTGAGGA----GGAAGAAGAGTTT------AGCTG 48285
Dog          -TTTGTTTT-TTAATGGTTAAAGGGGA-GAGAGAGATG----ATGGGGGTGG----GGTGGGGAGGC--------AGCAG 45629
BelugaWhale  GTTGCTTTT-GTTTTTATTGTTTAATA--ATAAGTTGGT----TGATGGTGA----GAAGGAAGAG----------GATG 44634
Donkey       -TAGATCTT-CTTCCCAATATCAC------TAAAAAAG----GGGAAAAAAG----AGAGAAAAA----------AGCCA 50470
Cat          -TTTATTTT-CTGCATTCCAGGAATTTTGGTAAAAGTATTCTATGCTAATGA----GGACATTCTCTCT------ATTCA 51994
Ferret       -TTTGTTTT-TTAATGGTTAAAAGAGATGATGGGGGAG----GGGAGGAAGA----GGAGTTTAATT--------ATTTG 52958
Lion         -TTTATTTT-CTGCATTCCAGGAATTTTGGTAAAAGTATTCTATGCTAATGA----GGACATTCTCTCT------ATTCA 49660
Vaquita      GTTGCTTTT-GTTTTTATTGTTTAATA--ATAAGTTGGT----TGATGGTGA----GGAGGAAGAG----------GATG 45325
Pig          ---GCCTTT-ATTTTTGCTATTTCACT--ATTAAAGGGT---GTGGGGTAGG----GGGAGAGGAACTG------AGCTG 42221
```

```
Majority     ---TTTGTTAGCTATACTTTATGTTA-ACCCTTTTA---------TTTTCATTAACT-T---TTCAXXTT-------X--

                       66250     66260     66270     66280     66290     66300     66310     66320

Human        AGATCGCGCCACTGCACTTCAGCCTGGGCGACAGAGCGAGACTCTGTCTCAAAAAAAAAAAAAAAAAAGAATTCCTGGTA  52928
PolarBear    --------------------------------------------------------------------------------  17375
Cow          ---TTTGTTAGCCAGGCTTTCTGTTA-ATCCTTTTA---------TTTTCACATTCCAT---TTCACCT-----------  48338
Dog          ---TTTACCTGTTGCTCATTAGGTAG-A---------------CTTTCCATTAACCCT---TTTATTTT-CCCCCTCT-  45684
BelugaWhale  ---TTTGTTAGCCATACTTTCTGTTA-ACCCTTTTA---------TTTTCA-----------TTCTCCTT----------  44680
Donkey       --------CAATAAAACCTTTTAAAAAAC---------------T--CCAC-AATA-----TTCAGATT----------  50508
Cat          ---GTGTTTGGGTTTATTCCATGTTGTATTAATTTATAGGCATTTTTATTTTTAATTTTAATTCCAGTGT-AGGTAACA-  52069
Ferret       ---TTCATTAGGCTGACTTTACTTTA-ACCCTTTTATCCCACCCCCTCCCCGTAACTCT---CCCAGTTT-CTCTGCCA-  53029
Lion         ---GTGTTTGGGTTTATTCCATGTTGTATTAATTTATAGGCATTTTTATTTTTAATTTTAATTCCAGTGT-AGGTAACAC  49736
Vaquita      ---TTTGTTAGCCATACTTTCTGTTA-ACCCTTTTA---------TTTTCA-----------TTCTCCTT----------  45371
Pig          ---TTTGTTAGCTAGACTTTCTGTTA-ACTCTTTTA---------ACCCTGTTAACTCT---TTCACCTT-CTCC--CT-  42281

Majority     CCTCCTGTGTACXTT----CXAATTTCTAATTTGATTCCTXTG---------GTTXCTATCAGTGCAAACCCTGTTT---

                       66330     66340     66350     66360     66370     66380     66390     66400

Human        CCAGCAGGGCACTTTGGCTCATGCCTGTAATCCCAGCACTTTGAGAAGCTGAGGTAAATTCAGGGCAG-CAGTGAACTGG  53007
PolarBear    --------------------------------------------------------------------------------  17375
Cow          CCTCCTGTGTACCTG----CCAGTTTCTAATTTGATTCCTATG---------GCTGCTACCAATGCAAATGCTGTTT---  48402
Dog          GCCCTTTGTGACTGC----CAGAT-TCTAATGT--------CAC----TCATGTAACTGTCAGTGCAGACCCTGTT----  45743
BelugaWhale  CCTCCTGTGTACCTA----CCAATTTCTAATTTGATTCCTGTG---------GATGCTACCAGTGCAAACCCTGTTTT--  44745
Donkey       ----TTGCATACTTT----AGAACTTCTGAGGTTGAGCCA--G-------ACATGAC-ATGAATAAAGTTCATCTTT---  50567
Cat          -GTGTTAATTAGTTT----TAGGTGTACAATATAGTGATTCAGCAATTCTGTATGTTACTCAGTGCTTACCATGATAAGG  52144
Ferret       TGAGCCAGGTACCAC----CTGTT-TATAATTCAGGTATTCTAC----TAGTATGATTCATGTGGCAAACCCTGTT----  53096
Lion         AGTGTTAATTAGTTT----TAGGTGCACAATATAGTGATTCAGCAATTCTGTATGTTACTCAGTGCTTATCATGATAAGG  49812
Vaquita      CCTCCTGTGTACCTA----CCAATTTCTAATTTGATTCCTATG---------GATGCTACCAGTGCAAACCCTGTTTT--  45436
Pig          CCTCCTGTGTACCTG----CCAATTTCTAATCTGATTCCTGTG---------GCTGCTAGCAGTGCAAACCCTGTTT---  42345
```

```
Majority     -XGCTATGTGTTCACTXTGTCTTCTGT-CCATGCATTT--AT----TXTC-TGXXTTXCAGXAA-TTTGATAAAATTXTT
                                66410      66420      66430      66440      66450      66460      66470      66480
Human        TCACACCAC-TGCACTCCAGCCTCAG--CAACAAAGCAAGACACAGTCTCTTAAAAAAAAAAAAAAGGAAGAAATTCTGGT  53084
PolarBear    --------------------------------------------------------------------------------  17375
Cow          --GCTATGTGTTCACTGTGTCTTCAGT-CCATGCAATT----------TC-TGTGTTCCAGGAA-TCTGATAAAATTATT  48467
Dog          -TGCTATGTGTTCATTGTGTCTTCTC----ATTTGTTTCCATTCATTTTC-TGTATACCAG-TATTTTGATAAAAGTATT  45816
BelugaWhale  --GCTATGTGTTCACTGTGTCTTCTGT-CCATGCATTT----------TC-TGAGTTTTGGGAA-TTTGATAAAATTATT  44810
Donkey       -TGCCAAAGATTTTCT-TG-CTTCCAAACAGTACTATTTAAT---------TGTGT------AAGTGTAAGCAGAATGT-  50628
Cat          GTACTCTTTAATCCCCATCACCTCATTTATATGCATTTTTCTGGGCTTTCATAAATAGAAGAGAGCCCAGTAAATATGAG  52224
Ferret       -TGCTATGTGTTCACTATGTCTTCTGTGTGATTCCTTTCTATTAATTTTG-CGTATTCCAG-GAATTTGATGAAAGTGTG  53173
Lion         GTACTCTTTAATCCCCATCACCTCATTTATAGGCATTTTTCTGGGCTTTCATAAATAGAAGAGAGTCCAATAAATATGAG  49892
Vaquita      --GCTATGTGTTCACTATGTCTTCTGT-CCATGCATTT----------TC-TGAGTTTTGGGAA-TTTGATAAAATTATT  45501
Pig          --GGTACATGTCCACTGTGTCATCTGT-CCATTTAGTTCCATTCATTTTC-TGTGTTTCAGGA--TTTGATAAAATTATT  42419

Majority     CTCAGCTAGTG-------ATGACCTT--CTCTXTTCAGTA-------TCTGCGTTTATTCCATT--------------TT
                                66490      66500      66510      66520      66530      66540      66550      66560
Human        TCCTTTAAGGGAGATTTGTTGTTGTTTTCTTCTTTTAATGGTGAGGGTACAAGTTGGTCACAGGAGGGAGAAGAGAAATT  53164
PolarBear    --------------------------------------------------------------------------------  17375
Cow          CTTGGCCAGTG-------ATGACCT-----CTCTTCAGTG-------TCTGCATTTATTCCATT--------------TT  48514
Dog          CTCAGCTGATG-------ACAACACTCTTTCTATTCAATA-------TTTGGGTTTATTCCATT--------------TT  45868
BelugaWhale  CTGAGCTAGTG-------ATAACCTT--TTCTCTTCAGTA-------TCTGCATTTATTCCATT--------------TT  44860
Donkey       -TTAG--AATA-------GCAGCTTATAAACTGTTTCCAA----------GCACCTTCGACACC--------------CT  50674
Cat          TCAACCTTGAATTCTTCCATGATCCTTCCTCAAATTACCAA--GTCTTACCAGTTCACTCTTGT--------------TA  52288
Ferret       CTCAGCCAATG-------ATGACGTTCTCTCTATTCAGTA-------TTTGGGTTTATTCCATT--------------TT  53225
Lion         TCAACCTTGAATTCTTCCATGATCCTTCCTCAAATTACCAA--GTCTTACCAGTTCACTCTTGT--------------TA  49956
Vaquita      CTGAGCTAGTG-------ATAACCTT--TTCTCTTCAGTA-------TCTGCATTTATTCCATT--------------TT  45551
Pig          CTCAGT-GATG-------ACCTTCT-----CTCGTCTGTG-------TCTGTGTTTATTCCATT--------------TT  42465
```

```
Majority     GTATTTATTTA----TAGTCATTTTX-AXTAGTGTTTTATAATTCAAGAAXAXTTAXXGAATG--TGTATXTCAATCATX
                     66570      66580      66590      66600      66610      66620      66630      66640
Human        TAGCTCTTTTGTTAATGAACTAGACTTTCTGTTCGTCTGTTTTTCACCCACTCCCTCCTCCTGTGAACCTGCCAATTTCT  53244
PolarBear    --------------------------------------------------------------------------------  17375
Cow          ----TTATTTA----TAGGCATTTT--AATAGTGTTTTATAACAAAAGAGAGA----CGAATA--TGTACATCAACCATG  48578
Dog          ATATTCATTAA----TGGGTGTTTTTAATTCA---------ATTCCAGTATAGTTAATATATGGTATTATACTAGTTTCA  45935
BelugaWhale  GTACTTATTTA----TAGTCATTTT--AATAGTGTTTTATAATAGAAGAGAGACAAATGAATG--TGTGTGTCAATCATG  44932
Donkey       CGAGTACTTCAGAAGTAGTTAATTTGCA-------T--ACAATTGATATGCTAGTTATGATTA---ATATATGAATAA--  50740
Cat          ATATCTGCCTAGTTTTCATCATCTTTCTTGCTACTTTAGTAGCTCAGACACAGTTACCTTTTGGCTGTACTGTGATAACA  52368
Ferret       GTATTGATTTA----CAGGCATTTTTTATTAAGTTTTTAAAATTCCAGTATAGTTACTATATGGGGTTATACTAGTTTTA  53301
Lion         ATATCTGCCTAGTTTTCATCATCTTTCTTGCTACTTTAGTAGCTCAGACACAGTTACCTTTTGGCTGTACTGTGATAACA  50036
Vaquita      GTACTTATTTA----TAGTCATTTT--AATAGTGTTTTATAATAGAAGAGAGACAAATGAATG--TGTGTGTCAATCATG  45623
Pig          GTATTCATTTA----TAGGCATTGT--AATAGGGTTTTGTAATAGAAGAAA------CAAATG--TGTGTGTCAACCGTG  42531

Majority     GAT-T--CTTCTAGAXCXXTT--CATXAAATCAX-CATGTXCTXXGTATTCAXTCTTCTCAGTXTCXXCTXAG-TTTCX-
                     66650      66660      66670      66680      66690      66700      66710      66720
Human        GATTTGGTTCCTGTGGCTGCTACCAGTATAACACCTGTTTGCTGTGTGTTCACTGTGTCTCCAGTCCATTTGG--TTCCA  53322
PolarBear    --------------------------------------------------------------------------------  17375
Cow          AAT-T--CTTCCACAACCTTT--TATCAAGTCA--CAAGTCGTATGAATTCACTCTTCTCAATATCTGCCCAGTTTTCAT  48651
Dog          GGTGT--ATAGTGAGTCAACA--ATTTTATACAATTTTATACTTAGTGCTCATCATA---AGTGTACTCTTTAATCCCCA  46008
BelugaWhale  AAT-T--CTTCCAGAACCTTT--CATCAAATCAG-TAGGTTCTGTGAATTCACTCTTTTCAATATCTGCCCAGTTTTC--  45004
Donkey       ----T--TCTT---ATCTATT--CATTAAATCAGTC---CCCTCAGGATTTTTATTAGTCAGCATATCGTTA--------  50798
Cat          GCTCT--CTTATTGATCTGCT--TTTTTGCTTTCCCCTCT-TGAATTCTTTCTCCCTCCCTACTTCCTCTCCC-TTCCCA  52442
Ferret       GGTGT--ACAGTATCGTGGTT--CAACAATTTTATACATTACTCATTGTTCATCATGATCAGTGTACTCTTTCATACCCA  53377
Lion         GCTCT--CTTATTGATCTGCT--TTTTTGCTTTCCCCTCT-TGAATTCTTTCTCCCTCCCTACTTCCTCTCCCCTTCCCC  50111
Vaquita      AAT-T--CTTCCAGAACCTTT--CATCAAATCAG-TAGGTTCTGTGAATTCACTCTTTTCAGTATCTGCCCAGTTTTC--  45695
Pig          AGT-T--CCTCCATAACCTTT--CATCAAATCAT-CAAGTCCATTGAATTCACTCTTCTCAGTGTCTGCCTAGATTTC--  42603
```

```
Majority     TTCAXTTTTXCTTCTTCACTTGCTCAXAAACAXTCATCTC---GCCAXAT-AATAT-XCT-AA-XAT-AATTCTTTTXT-
                     66730     66740     66750     66760     66770     66780     66790     66800
Human        TTCATTTTCTGTGTTCCAGGAATTTGATAAAATTATTCTCAGCTAATGATAACCTTCCCTCCCTTCAGTGTTTGTTTATT  53402
PolarBear    --------------------------------------------------------------------------------  17375
Cow          TTCTTTTTTTCTACTTCAGTTGCTCAGACATAGCCATGTCTTAGCTATAT-AATAATCTTACCTAAT-AACTCTCTTGAA  48729
Dog          T-CACCTCA-TTTATAGGCATTTTCATGGGCTTTCATAACAGAGGCAAATAAATATGAGTCAACCACGAATTCTTCCATG  46086
BelugaWhale  TTCTTTCTTGCTACTTCATTTGCTCAGACACAGTCATCT-----------------------------------------  45043
Donkey       ---ACTGTAAGAGTTTGAGTTGCTTAAGACTTGATA-------GCTGAATGAGTAAGGCGAAATTGTGAAATATTTTCTG  50868
Cat          TACACTTCCGCCTCTCCCCTTCCCTATTAGTCTCTTTCCCCCCACCCCGTCCCTCTCTCT-AAATATATATATTTATATC  52521
Ferret       T-CACTTCA-TTTATACGCATTTTCATGGGCATTCATAATAGAGGCAAATGAATATGAGTCAACAATGAATTCTTCTGTG  53455
Lion         TACACTTCC-CCTCTCCCCTTCCCTATTAGTCTCTTTCCCCTCACCCCGTCCCTCTCTCTTAAATATATATATTTATATA  50190
Vaquita      TTCTTTCTTGCTGCTTCGTTTGCTCAGACACAGTCATCT-----------------------------------------  45734
Pig          TTCTTTCTTGCTACTTCAACAGCTGAGATACAGTCAGCTCTTGGCTATAT-TATA--TATA----AT-AGCTCTCTT---  42672

Majority     -T-XTTX-----XTX-XXTTX---X-T-X--------------X-X---C---TTXG---TCTGCX-XGX---CX--X-
                     66810     66820     66830     66840     66850     66860     66870     66880
Human        TTATATTCATTCACTTCTAGGCATTTTAATAGAGTTTCATAATAGAAGACATGCAAATGAGTATGCATTAACCATGAATT  53482
PolarBear    --------------------------------------------------------------------------------  17375
Cow          GTGAAGCCACTCAGTCGTGT-CTGACTCTTTGGCAACCCATGGATTTTAACCTACCAG---GCTCCTCCGTCCATGGGAT  48805
Dog          ATCCTTT-----GTCAAGTTACCAAGTCTTACC---------AATTTATTCTTCTTAG-TATCTGCCTAGTTTTCATCAT  46151
BelugaWhale  --------------------------------------------------------------------------------  45043
Donkey       CTCTTTCTCGTTGTCAAATAA--------------------------AC---TTGGACATTTGCCCTTTCCTCA--G-  50914
Cat          TTTTTTTTAATGGTTGGTTTATTTATTTTGAGAGTGGGGGAGAGTGTGTGCAAGTAGGGGAGGGACAGAGAGAGCGGGAG  52601
Ferret       ATCATTC-----ATCAGATTACTAAGTCTTTTC---------AGTATACTCTTTTTAAATGTCTGCCTAGTTTTCATCAT  53521
Lion         TTTTTTTTAATAGTTGGTTTATTTATTTTGAGAGTGGGGGAGAGTGTGTGCAAGTAGGGGAGGGACAGAGGGGGCGGGGG  50270
Vaquita      --------------------------------------------------------------------------------  45734
Pig          ----------------------------------------------------ACTGG---TCTCCT-------------  42683
```

```
Majority     -------X-------AXX---X-AG-TXGXA-A-XT-----------X--ACTTXTX--TXTAX----C---AXCAG--C
                      66890     66900     66910     66920     66930     66940     66950     66960
Human        CTTTCACATCCGTTCATGAAATCACCAGGTCCTATTAGTTCACACTTTTTAATTATCTGCCTGGTTTTCTCCATCTTTCC  53562
PolarBear    --------------------------------------------------------------------------------  17375
Cow          TTTCCAAGCAAGATTACTGGAGTGG-TTGCC-ATTTCCTTCTCCAGGGGATCTTCCCAACCCAGGGATCAAACCCAGGTC  48883
Dog          C-----GTTCTTGCTACTCGCATAGCTCAAACACACACT-------ATCACCTCTTGGCTATATTA--CTATGATAGCTC  46217
BelugaWhale  --------------------------------------------------------------------------------  45043
Donkey       -------AG------ACTCTCCCAACTTGTTTG-GT--------------ACCTCTTTTTTTAGGATCCAACACCATTAC  50966
Cat          G--GGAGAGAGAGAGAGAGAGAGAGAATGAATGAATGAATGAATGAATGAATGAATGAATATATAT--CCCAACCAGGCT  52677
Ferret       C-----TTTCTTGTTACTTGAATAGCTCAGACACACACA-------GTCGCCTCTTGGCTATATTG--TTTTAATAGCTC  53587
Lion         GAGGGGGAGAGAGAGAGAGAGAGAGAGAGAGAGAGAGAATGAATGAATGAATGAATGAATATATATATCCCAACCAGGCT  50350
Vaquita      --------------------------------------------------------------------------------  45734
Pig          ------T-------------------TCGTA-ATTT--------------TCCTCT------------------------  42699

Majority     XC-TACXX---------A--TXX-TXCX-T---------CATGAXXTXT----T-CTCTCT--XAXCXAXAT----X---
                      66970     66980     66990     67000     67010     67020     67030     67040
Human        TGCTACTTGTATTGCTCAAACACACTCACCTGTTGGCTATATTACTACGACAGTTCTCTTTATAAACTACTTTCTTTTTC  53642
PolarBear    --------------------------------------------------------------------------------  17375
Cow          TCCTGC-ATTGTAGGCAGATGCTTTACTGTCTGAGCCACTAGGAAAGTTA-GTAACTCTCT--TACTGATGTTTA-ATT-  48957
Dog          TCATACTCATCT-----ACTTCTTTACTTTCCTC----TCTTGATTTCT----TTCTCCCTAGTACCTCTCTCTTCACCC  46284
BelugaWhale  --------------------------------------------------------------------------------  45043
Donkey       ATCTGCCGA--------AAATATCCACTTA---------CATAGGCCATAGAGCAATTTTTGTAGACAAAATTA------  51023
Cat          CCACACTGGCAGTGCAGAGCTCCATGTGGTGCTCGAAGTCATGAACTGTGAGATCATGACCTGAGACGAAATCAAGAGTC  52757
Ferret       TCTTACTGATCT-----ATTTTTTTACTTTCCCC----TCTTGAATTCT----TTCTCCCTAGTACCTCCCT--------  53646
Lion         CCACACTGGCAGTGCAGAGCTCCATGTGGTGTTCGAAATCATGAACTGTGAGATCATGACCTGAGACGAAATCAAGAGTC  50430
Vaquita      --------------------------------------------------------------------------------  45734
Pig          ----------------------------------T--A-------AATT------CTTTCT--TACCAGTAC----AGC-  42723
```

```
Majority     -----TCT--C----TTX-TTTCXX-X-X-XXC----XX--GX-TATXTCTTTCTAAAAXA-XXXAATCTGA-TTX--TT
                      67050     67060     67070     67080     67090     67100     67110     67120
Human        TTTTCCCTTCTGGAATTTTTTTCTCGCTAGTAC-A--GCCAGTACGTATCTTTCTCAAATGCACATATCTAAGTTA--CT  53717
PolarBear    --------------------------------------------------------------------------------  17375
Cow          -----TTTGTCTTGAATACTTTCTCCCTAGTACAAATGTGTGTGTATCTCTTTCTAAAATGCACAGATCTAACTTG--CT  49030
Dog          CACTGTCT--CCCTTTTCCTTTTCTACCTCCCCCCA--------TATGTCCTTGTAAAACA----AATCTGACTTAG-TT  46349
BelugaWhale  -----------------------------------------------------------G--------------------  45044
Donkey       ------CTAACAAAAGTATGGTTTTCATCTTATGGGGTTCAGAAAATGCTACCCCAAAATATGAGAACTTG---------  51088
Cat          AGGTATTTAACCAGCTGAGCTGCCCAGGTGCTCCTAAGTATGTATATACTTTTGTAAAACAGACAAATCTGATTTTAGTT  52837
Ferret       -----TCT--CTTCCTTTCCTTCCTCCCTTTCTCAT--------TATCTCTCTCCCTAACA----AATCTGACTTAGGTT  53707
Lion         AGGTACTTAACCAGCTGAGTCACCCAGGTACCCCTAAGTATGTATATATCTTTGTAAAACAGACAAATCTGATTTTAGTT  50510
Vaquita      -----------------------------------------------------------G--------------------  45735
Pig          -----TCTG----------TGT---------G----TGTGTGTGTCTGTCTTTCTAAA-TGCACAAATCTGAGTTA--TC  42772

Majority     XCXCTXCAGCA--------GATCCTTTTCATGCTCAGGATAAAGGCCCTXAGAAGAGATGGCTACTATTT---CTGTCTT
                      67130     67140     67150     67160     67170     67180     67190     67200
Human        CCCATGCAGTA--------GGTCCTGTTCATGCTCAGGCTAAAGGCCCTTTCT-GAGCTTACTACTTTTTGTCTTGTCTT  53788
PolarBear    ---------------------------TCATGTTCACGACAAAGGCCCTCAGAAGAGGTGGCTACTATTT---CTATTTT  17425
Cow          CCCCTGCAGCA--------GTTTGTTTTCATGCTCAGGATAAAGGTCCTTTCT-GAGATGACTACTGTTT---CTGTCT-  49097
Dog          ACTCCCCTGCAA----T-AGATCCTTTTCATGCTCAAGATAAAGGCCCCCAGAAAAGATGGCTA---TTT---CTATCTT  46418
BelugaWhale  ------CAGCA--------AATCCTTTTCATGCTCAGGGTAAAGGCCCTTT---GAGATGAGTACTGTTT---CTGTCTT  45104
Donkey       --------GCA----------TGTTGAATATCTTCAATTGAAGGATTTTAAGAAAAAGCAGAAGCAAGGA---GT-TCAC  51146
Cat          ACTCTCCTGCAG----CCATATCCTTTTCATGCTCAGGATAAAGCCCTTCAGAAGAGATGGTTACTATTT---CTGTCTT  52910
Ferret       ACTATCCTGCAG----C-AGATTCTTTTCATGTTCATGATAAAGGGCCTCAGAAGAGGTGGCTACTATTT---CTGTTTT  53779
Lion         ACTCTCCTGCAG----CCATATCCTTTTCATGCTCAGGATAAAGCCCTTCAGAAGAGATGGCTACTATTT---CTGTCTT  50583
Vaquita      ------CAGCA--------AATCCTTTTCATGCTCAGGGTAAAGGCCCTTT---GAGATGAATACTGTTT---CTGTCTT  45795
Pig          CTCCTGCAGCAAATCTTCAGATCTTTTCCTTGCTCAAGATAAAGGCCCTTTCT-GAGATGATTACTTATT---CTGTCTT  42848
```

```
Majority     AT-GGCTACTCXC-----TATTTGCCT-ATGCTTCTAXCCCAAAAGTAATTGTAAATCCTCTAACCTGGCATGCTTTCTC
                     67210     67220     67230     67240     67250     67260     67270     67280
Human        AT-GACTACTCCCACC-CTATTTGCTT-ATGCTTCTGTCCCAAAAGTAATGGTGCATCCTGTAACCTGGCATGTTTTCTC  53865
PolarBear    ATTGGCTATA--------TATTTGCCT-ATGCTTCTA-CCCAAAAGTAATTGTAA--------ACCTGGCATG-------  17480
Cow          GT-GGCTGCTCCCCCCTCCATTTTCCT-GTGCTTCTATCTCAGTAGTAATGATAGATCCTATAACTTGGCATACCTTCTC  49175
Dog          ATTGGCTATA--------TATTTGCCT-ATGCGTCTA-CCCAAAAGTAATTGTAACTCTTCTAACCTGACATTCTTTCTC  46488
BelugaWhale  AT-GGCTACTCCCACC-CTATTTTCCT-GTGCTTCTATCACAGTAGTAATTGTAGTGCCTGTAACCTGGCATGCTTTCTC  45181
Donkey       GCCAACCTCTCTC-----CGTCCCCCTCACCCTTCTTCCCTGAAACAGATCATAAAAC-TCTCATGTGAAATATGCTCTT  51220
Cat          AC-GGCTGTA--------TGTTTCCCT-GTGCTTCTA-CCCAAAAGTAATTGTAAATCCTCCAACCTGGCATGCTTTCTC  52979
Ferret       ATTTGCTATA--------TATTTGCCT-ATGCTTCTA-CCCAAAAGTAATTGTAA--------ACCTTGCATGCTTTCTC  53841
Lion         AC-GGCTGTA--------TGTTTCCTT-GTGCTTCTA-CCCAAAAGTAATTGTAAATCCTCCAACCTGGCATGCTTTCTC  50652
Vaquita      AT-GGCTACTCCCACC-CTATTTTCCT-GTGCTTCTATCCCGGTAGTAATTGTAGCGCCTATAACCTGGCATGCTTTCTC  45872
Pig          AC-GGCTCCTCCCACTGTTATTTGAGT-ATGCTTCCATCCCAATAGTAATTGTAGGTTCTGTAATCCAGTATGCTTTCTC  42926

Majority     TTATTTXXAGTCT--TTTGAGGCCACTGTGTTTCTGTGATGTCACTTTTGCCTGGAA-TATTGTGGCCTG-CCTGCCTTT
                     67290     67300     67310     67320     67330     67340     67350     67360
Human        TTGATGTCAGTCG--TTTGCTGCCATTATGTCACTGC--TCTCA---T-GCATGGAA-TATTGTGGGTTGGCCTGCCTTT  53936
PolarBear    --------------------------------------------------------------------------------  17480
Cow          TTATTTT--GTCT--TTGGAGGCCACTGT--TTCTGTGATGTAACTTTTGCCTGAAA-TATTGTGACCTG-CCTATGTTT  49247
Dog          TTACTTCTAGTAT--TTTGGGACCACTGTGTTTCTGTGATGTTAATTTTTCCTGGAA-TATTATGACCTG-CCTGCCCTT  46564
BelugaWhale  TTATTTT--GTCT--TTGGAGGCCATTGTGCTTCTGTGCTGTCACTTTTACCTGGAA-TATTGTGGCCTG-CCTGCCTTT  45255
Donkey       CTTGTACCAGGAG----GAAGGACGACATTCTTATCACCAGAGACGGGGAACTGAGGGTCAAGAAATCTG-TACAAACAA  51295
Cat          TTATTTCCAGTCT--TTTGAGGCCACTGTGTTTCTGTGATGTCACTCTTGCCTGGAA-TATTAAGGCCTG-CCTACCCTT  53055
Ferret       TTATTTCCAATCTTATTTCCAGCCACTGTGTTTCTGTGATGTTA-CTTTGCCTGGAA-TATTATGGCCTG-CCTGCTTTT  53918
Lion         TTATTTCCAGTCT--TTTGAGGCCACTATGTTTCTGTGATGTCACTCTTGCCTGGAAATATTAAGGCCTG-CCTACCCTT  50729
Vaquita      TTATTTT--ATCT--TTGGAGGCCATTGTGCTTCTGTGCTGTCACTTTTACCTGGAA-TATTGTGGCCTG-CCTGCCTTT  45946
Pig          ATATATT--GTAT--TTTGAGGCCTTTGTGTTTCTGTGCTGACACTTTTGCCTGCAC-TGTTGTGGCCCG-CCTGCCATT  43000
```

```
Majority     -GTACCACTTTTCC---AACCCACACTTCACATGGCCA-GTTGTTAGACATCTTCAGGTCTCCC-TTTAGTTX-TXX---
                     67370      67380      67390      67400      67410      67420      67430      67440
Human        TGCACCACCCTTCCTCCACCTCACACTTCACATGGCCA--GTGTTAAATATCTTGAGGTCTCCC-TTTAGCTATTTCTTC  54013
PolarBear    --------------------------------------------------------------------------------  17480
Cow          -GTGCCAAGTTTCC---ATCCCACACTTCATATGACCA-GTTGTTAGACATCTTCAAGTTTCC--ATAAGCCAGTTT---  49317
Dog          -GTGCCATTTTTCC---AACTCACATTTCACACAGCCA-TTTGCTAGACATCTTCAGGTCTCACTTTTAGTT--TCC---  46634
BelugaWhale  -GTACCACCTTTCC---ATCCCTTACTTCACATGGCCA-CTTGTTAGACATCTTCAGGTTTCCC-ATTAGTTAGTTT---  45326
Donkey       -ACCTTGTTAAACT---CACCCTTATCTT-CCTAGTTA-CTTCTTCACCACTTACTGACCCTAGCCCAAACCCCTCTG--  51367
Cat          -GTGCCACTTTTCC---AACCCACACTTGACATGGCCG-GTTGGTAGACATCTTCAGGTCTCCCTTT-AGTT--TCC---  53124
Ferret       -GTATTAGTTTTCC---AACACACACTTCACATGACCA-GTTGCTACACATCTTCACGTCTCACTTT-AGTT--TCC---  53987
Lion         -GTGCCACTTTTCC---AACCCACACTTCACATGGCCG-GTTAGTAGACATCTTCAGGTCTCCCCTT-AGTT--TCC---  50798
Vaquita      -GTACCACCTTTCC---ATCCCTTACTTCACATGGCCA-CTTGTTAGACATCTTCAGGTTTCCC-ATTAGTTAGTTT---  46017
Pig          -GCATCACCTTTCT---ATTTAGCATGTTGCATGGCCAAGTTGGTAGGCACTCTCAGGTTTCCC-ATTAGCTAGTTT---  43072

Majority     TCTTGAAAGCCATCCTCTGATCCACA--------------------AAG-------------------------------
                     67450      67460      67470      67480      67490      67500      67510      67520
Human        TCCTAAAAGCTATCCCCTGATAAGCA--------------------AAGA------------------------------  54043
PolarBear    --------------------------------------------------------------------------------  17480
Cow          CCTTGAAAGCCATCCTCTGATCTACA--------------------AAG-------------------------------  49346
Dog          TCCAGAAAGCTGTCTTTTGATCCACA--------------------AAGA------------------------------  46664
BelugaWhale  CCTTGAAAGCCATCCTCTGATCCACA--------------------GAG-------------------------------  45355
Donkey       TCTTGTCAATTGTTCACAAATTTA--------------------------------------------------------  51391
Cat          TCTAGAAAGCCATCTTTTGATCCACAGTGTGGGTGAGGTTTGCATTAAGAAACTTCGACACCGGCGCCTGGGTGGCTCAG  53204
Ferret       TCCAGAAGGCTATCTTTTGATCTATA--------------------AAGA------------------------------  54017
Lion         TCTAGAAAGCCATCTTTTGATCCACAGTGTGGGTGAGGTTTGCATTAAGAAACTTTGACACCGGCGCCTGGGTGGCTCAG  50878
Vaquita      CCTTGAAAGCCATCCTCTGATCCACA--------------------GAG-------------------------------  46046
Pig          GCCTCAAAGCCATCCTCTGATGTAGA--------------------GAG-------------------------------  43101
```

```
Majority     --------------------------TGGATTAGG-----------------------------------------TTTG
                      67530     67540     67550     67560     67570     67580     67590     67600
Human        -------------------------GTAGATTAGG-----------------------------------------TTTG  54057
PolarBear    --------------------------------------------------------------------------------  17480
Cow          --------------------------TGGATTAGG-----------------------------------------TTTG  49359
Dog          -------------------------CTGGATTAGG-----------------------------------------TTTG  46678
BelugaWhale  --------------------------TGGATTAGG-----------------------------------------TTTG  45368
Donkey       --------------------------TTGTTT-------------------------------------------CTTTG  51402
Cat          TCAGTTAAGCATCCAACTCTTGGTTTTGGCTTAGGATCTGATTTCACAGTTCAGGAGTTTGAGCCCCATGTTGGGCTTTG  53284
Ferret       -------------------------GTAGTTTAGG-----------------------------------------TTTG  54031
Lion         TCAGTTAAGCGTCCAACTCTTGGTTTTGGCTTAGGATCTGATTTCACAGTTCAGGAGTTTGAGCCCCATGTTGGGCTTTG  50958
Vaquita      --------------------------TGGATTAGG-----------------------------------------TTTG  46059
Pig          --------------------------TGAATTAGG-----------------------------------------TTTG  43114

Majority     GATT----------------------------------------------------------------------------
                      67610     67620     67630     67640     67650     67660     67670     67680
Human        GATT----------------------------------------------------------------------------  54061
PolarBear    --------------------------------------------------------------------------------  17480
Cow          GATT----------------------------------------------------------------------------  49363
Dog          CATT----------------------------------------------------------------------------  46682
BelugaWhale  GATC----------------------------------------------------------------------------  45372
Donkey       TAT-----------------------------------------------------------------------------  51405
Cat          CACTGCCAGTGGAGAGCCTGCTTGAGATTCTCGCCCTCTCTCTCTGCCCCTTCCCTGCTAGCATGTGAATGTGTATGCTC  53364
Ferret       CATT----------------------------------------------------------------------------  54035
Lion         CACTGCCAGTGGAGAGCCTGCTTGAGATTCTCTCCCTCTCTCTCTGCCCCTTCCCTGCTAGCATGTGAATGTGTATGCTC  51038
Vaquita      GATT----------------------------------------------------------------------------  46063
Pig          GATT----------------------------------------------------------------------------  43118
```

```
Majority     -----------------------------------------------------AAGAAXCTTTAAXACAAATTAAAATCAC
                     67690     67700     67710     67720     67730     67740     67750     67760
Human        -----------------------------------------------------GAGAGTGTT------AACTCCGAA----  54079
PolarBear    ---------------------------------------------------------------------------------  17480
Cow          -----------------------------------------------------AAGAACCTTTAATGCAATTTAAAATCAC  49391
Dog          -----------------------------------------------------AAGAAATCTTAACACAAATTAAAATCAC  46710
BelugaWhale  -----------------------------------------------------AAGAACCCTTAATACAAATGAAAATCAC  45400
Donkey       ------------------------------------------------------AAAAGATGTAA--CAGCTTCCTGTTCT  51430
Cat          TTTCTCTCTTTCTCTCTCTCTCAATAAATAAATAAACTTAAAAAAAAAAAAAAAGAAACTTTGACATAAATTAAAACCAC  53444
Ferret       -----------------------------------------------------AAGGAACTTCAACGCAAATTAAAATCAC  54063
Lion         TTTCTCTCTTTCTCTCTCTCTCAATAAATAAATAAACTTTAAAAAAAAAAAAAAGAAACTTTGACATAAATTAAAACCAC  51118
Vaquita      -----------------------------------------------------AAGAACCCTTAATACAAATGAAAATCAC  46091
Pig          -----------------------------------------------------AAGAACCTTTAATACAAATTAAAATCAC  43146

Majority     AGTGAGATACTGCCATGTACTAX--CAXATTGGCTAAAATTAAGACXGGTAATGCCAAGTGTTGG-AACAATGTXGAGAA
                     67770     67780     67790     67800     67810     67820     67830     67840
Human        --TGAGATACTGCTATGCACATAACCAGAGTGGCTAAAATTA--A-----------AAGGATAGGTAATA----------  54134
PolarBear    ---------------------------------------------------------------------------------  17480
Cow          G-TGAGATACTGCCATGTGCTAC--CATATTGGATACATTGA-----GGTAATTCCAAGTGTTGG-AACAATGTAGAAAA  49462
Dog          AGTGAGATACTACCATGTACTAA--TAGATTGC-TAAAATTAAGATGGATGATGCCAGGTGTTGGTAACAAGG---AGAA  46784
BelugaWhale  AGTGAGATACTGCCATGTA-TAC--CATACTGGATAAAATTAAGACCGGTAATACCAAGTATTGG-AACAATGTAGAGAA  45476
Donkey       GGT----TATT-TCAGGT-CTTTATTCTCTTGTGAAGGCTCT---CACGTACATCTAAAAATTG--AGTAACATTTGTGT  51499
Cat          ATTGAGCTACTACCATGTACTGTGTCTGATTGCCTATGATTAAGATGGGTAATGCCAGATGTTGGTAACAAGGTTGGGAA  53524
Ferret       AGTGAGATACTACCATGTACCAT--CAGATTGA-TGAGAT---GAGTGATGCCGAGTGTTGGTAACAAGGTAG---AGAA  54134
Lion         ATTGAGCTACTACCATGTACTGTGTCAGATTGCCTATGATTAAGATGGGTAATGCCAGATGTTGGTAACAAGGTTGGGAA  51198
Vaquita      AGTGAGATACTGCCATGTA-TAC--CATACTGGATAAAATTAAGACCGGTAATACCAAGTATTGG-AACAATGTAGAGAA  46167
Pig          --TGAGATATTGCTGTGTACTAC--CATATTGGCTAAAATTAAGACAGGTAATACCAAATGTTGG-AACAGTGTAGAGAA  43221
```

```
Majority     GCTTGAACTTT-XTAXATAAGTGATGGGAATGTAAATTGGTAGAATXATXTTTAAAAACTTGAAATATCTAXTAAAGCTG
                      67850     67860     67870     67880     67890     67900     67910     67920
Human        ------------------AAATACTGGTGAC--AA----------CCATTTTTGAAAACTCGAAATATCTACTAAAGTTG 54184
PolarBear    -------------------------------------------------------------------------------- 17480
Cow          GCTTGAACTCTTATAAATAAGTGATGGGAATGTAAGTTGGTAGAATAGTTTTTTAAATCTTCATTTATCAGCTAAAGCTG 49542
Dog          TCTGGAGCTTTCATATATACATT-TGGGAATGTAAATTCTTAGAATTGTGTTTAAAAACTTGAAACATATAGTAAAGCTG 46863
BelugaWhale  GCTTGAACTTC-GTAAATAAGTGATGAGAATGTAAATTGGTAGAATCATTTTTAAAACCTTGAAATATCTACTAAAGCTG 45555
Donkey       GCTTGT-CTCCTGTTAATCTGTCTTTGTCAGTTTAATTTTCAGACCCA-GCCAGAGACCCCAAGA---G-GGTGGAG--G 51571
Cat          ACTGGAACTTTCATATATACATGCTAAGAATGTAAATTAGTAGACTTATGTTTAAAAACTTGAAATATGTAGTAAAGCTG 53604
Ferret       GCTAGAACTTTTGTTTATACGTG-TGGGAATGTAAGTTCATAGAATGATGTTTAAATACTTGACATATGTAGTGAAGGTG 54213
Lion         GCTGGAACTTTCATATATACATGCTAAGAATGTAAATTAGTAGACTTATGTTTAAAAACTTGAAATATGTAGTAAAGCTG 51278
Vaquita      GCTTGAACTTC-GTAAATAAGTGATGAGAATGTAAATTGGTAGAATCATTTTTAAAACCTTGAAATATCTACTAAAGCTG 46246
Pig          ACTTGAACTC--------AAGTGATGGGAATATAAATTGATAGAATTGTTTTTAAAACCTTGAAATATCTACTTAAGCTA 43293

Majority     AGCATATACAT--ATTCTTTGACTTAGTAAATCTX--CCTAGTTA-CATACCTGAGAAAATATA-AXATATGTGCATCAA
                      67930     67940     67950     67960     67970     67980     67990     68000
Human        AGCATATAGCT--ATTCTTTGATGTAGTAAACTCGCTCCTAGTTAACATACCTGAGAACATGT--ACATAGGTATGTTAA 54260
PolarBear    -------------------------------------------------------------------------------- 17480
Cow          AGCATATTCCT--GTTCTTTGACACAGTAATTTT---ACTAGTCA-TATATCTGAGAAAATTTATATATATGTGCATCAA 49616
Dog          AGCATATACAT--ATTCTTTGACTTAGTGAATCTACTCCTAGTTA-TACAAATGTGCAT---CA-AAAGATATGTATGAA 46936
BelugaWhale  AGCATATACAT--ATTCTTTGACTTAGTAATTTTT--ACTAGTCA-CGTATCTGAGAAAATAAATACATATGTGCATGAA 45630
Donkey       AAAACTTTCTTC--CTCTCCTACCCATC---TCT----CCAGTT------CCCAGTAAAATACAAAATCAAAAATACTAA 51636
Cat          AGCATATACAT--AATCTTTGTCTTAGTAAATCTA-TCCTAGTTA-CATACCTGAGAAAATATG-TAATATGTGCATCAG 53679
Ferret       AGCATATACATGTATTCTTTGACTCAGTAAATCTACTCCTAGTTA-CATACCTGAGAAAACACA-TTGTATGTACATCAA 54291
Lion         AGCATATACAT--AATCTTTGTCTTAGTAAATCTA-TCCTAGTTA-CATACCTGAGAAAATATG-TAATATGTGCATCAG 51353
Vaquita      AGTATATACAT--ATTCTTTGACTTAGTAATTTTT--ACTAGTCA-TGTATCTGAGAAAATAAATACATATGTGCATGAA 46321
Pig          AACATACACAT--AATCTTTGATTTAGTAATTCT---ACTAGTTA-CATACCTGAGAAAAC----ACGTGTGTGTGTCGA 43363
```

```
Majority     AAGATA-TGTATXAGAAAGTTCATGGXACACTA-TATGTAATAGCCCAA--AGCTGGAAATGATCCACATGTTCAGCAGC
                              68010     68020     68030     68040     68050     68060     68070     68080
Human        AAGATA-TGTACAAGAAAGTTCATGGAACACTG-TATGTAATAGCCCAAAAAACTATAAATCATCCAAATGTTCAACAGC  54338
PolarBear    --------------------------------------------------------------------------------  17480
Cow          AAGATA-TGTATGGGCAAGTTCATCGCACACTA-TATGTAATTACCCAA--GGCTGGAAATGATCTAAATGTTGAGCAGC  49692
Dog          GAAGTT-----------CAT----GGAACACTAATACATAATAGTCCAA--AGCTGGAAATGATCCACATGTTCAGCAGC  46999
BelugaWhale  AAGATA-TGTATAAGAAAGTTCATGGCACACTG-TATGTAATAGCCCAG--AGCTGGAAATGATCCACATGTTCAGCAGC  45706
Donkey       ATCCT-------------GCTCA--ACATATTT-TCTGTAGTAACC-----ATTTGGAAGCCTTTCA----TTGAAAAAC  51691
Cat          AAGATAATGTATGAGAAAGTTCATGGAACACTAATACATAATAGTCCCA--AACTAGAAATCATCTACATGTTCAGCAGC  53757
Ferret       AAGATC-----------TGTATGAGAAAATTCATGGAACACTAATGCAA--AGATAGAAATGATTCATATGTTCAGCAGC  54358
Lion         AAGATA-CGTATGAGAAAGTTCATGGAACACTAATACATAATAGTCCCA--AACTAGAAATCATCTACATGTTCAGCAGC  51430
Vaquita      AAGATA-TGTATAAGAAAGTTCATGGCACACTG-TATGTAATAGCCCAG--AGCTGGAAATGATCCACATGTTCAGCAGC  46397
Pig          AAGATA-GGTATGAGAAAGTTGACAGCACACAC-TGTGAAA---CCCAA--AGCTGGAAGCGATCCGTACGTTCAGCAGC  43436

Majority     AGTAGAATXTGTTGTGGXATATTCATTTGTTGGAATATTXTAG--TXCAAAAAGAA------------TGAACAAGCTAC
                              68090     68100     68110     68120     68130     68140     68150     68160
Human        AGCAGAATATGTTGTGTTGTATTCATTTAGTGGAATATTGTAT--AGTAATAAAAA------------TGAACAAGCTAC  54404
PolarBear    --------------------------------------------------------------------------------  17480
Cow          AGTAGAATGTGTTGTGGTATATTTATTTGTTGGAATACTCCCT--GGCAAAAAGAA------------TGAACAAGCTAC  49758
Dog          AGTAGGGTATGCTGTGGCATATTCATTCATTAGAATTTGTTAGAATACAAAAAAAAAAAAAAAAAAATTGACCAAGCTGC  47079
BelugaWhale  TGTAGAATGTGTTGTGGTATATTTATTCGTTGGAATACTGACT--GGCAAAAAGAA------------TGAACAAGCTAC  45772
Donkey       AAACTGCGGTGCCGCAGC-------TTTGCTGGCATCCT---GAGCACAAAGTTAGCCTGAT---TATTCATTAATCCAG  51758
Cat          AGTAGAATATGTTGTGGCATATTCATTTGTTGGAATTTCTTAGAATACAAAAAGAA------------TGATCAAGCTAC  53825
Ferret       GGAA---TATGCTGTGGCATATTTATTCATTGGAATTTGTTAGAATACAAAAAAA-------------TGACCAAGCTGC  54422
Lion         AGTAGAATATGTTGTGGCATATTCATTTGTTGGAATTTCTTAGAATACAAAAAGAA------------TGATCAAGCTAC  51498
Vaquita      TGTAGAATGTGTTGTGGTATATTTATTCGTTGGAATACTGACT--GGCAAAAAGAA------------TGAACAAGCCAC  46463
Pig          AGTAGAATGTGTTGTGGTATATTCATTGACTAGGTTATTGTC---AGTAAAAAGAA------------TTAATAAGCTAC  43501
```

```
Majority     TGGTACCTGCAAAACXTGGATGAACATCATAAAAATAATGTTTATTGXAAGAAGCCA-----------------------
                      68170     68180     68190     68200     68210     68220     68230     68240
Human        TGCTGCCTGCAAAACATGGGTA------GTGTACATCATAATTATTGGAAGAAGCCA-----------------------  54455
PolarBear    --------------------------------------------------------------------------------  17480
Cow          TGGTGCATCCAAAACTTGAATGGACATCATAAAAATTATGTTTATTGGAAGAAGCCA-----------------------  49815
Dog          TGGTACCTGCAAAACATGTATGTACACCATAACAATAATATTTATTGAAAGAAGCCAAA----CCAAAAAATAAATAAAT  47155
BelugaWhale  TGGTGTCTCTAGAACTTGGATGAACATCATAAAAATTATGTTTATTGGAAGAAGCCA-----------------------  45829
Donkey       CATTAGCC-----ACTAGAGTGACCAACCTGACAAGCTTTTTCCAGGCAATGACCTGAA---------------------  51812
Cat          TTATACTTGCAAAACATGGATGTATATCATAAAAATAATGTTTAGTGAAAGAAGCCA-----------------------  53882
Ferret       TAGTACCTGCAAAACATGAATGCACATCATGAAAGTAATATTTACTGAAAGAAGCCAACCCCCCCCAAAAAGCAAAAAAC  54502
Lion         TTATACTTGCAAAACATGGATGTATATCATAAAAATAATGTTTAGTGAAAGAAGCCA-----------------------  51555
Vaquita      TGGTATCTCTAGAACTTGGATGAACATCATAAAAATTATGTTTATTGGAAGAAGCCA-----------------------  46520
Pig          TGGTGCCTCCAGAACTTGGATGATTATCATAAAAACCATGTTAATTGCAAGAAGCTA-----------------------  43558

Majority     ----------GACCAAAGTAGAACA---------TXCXATATGATTCCATTT-ATATAACTGGGAGAAAGCATGAGTGTG
                      68250     68260     68270     68280     68290     68300     68310     68320
Human        ----------AACCAAAGTAGAATACA-------TCTGATATGATTCCATTT-ATGTAACTTGGAGGAAGCGTGAGTATG  54517
PolarBear    --------------------------------------------------------------------------------  17480
Cow          ----------GACCAGAGTAGTACA-------------------TTCCATTT-ACATAACTGGGAGAAAACGTGTGTG--  49863
Dog          AAATAAGCCAGACGAAAGTAAAACA---------TACTATATGACGCCATTT-ATTTAACTGATAGAAACCATGAATGTG  47225
BelugaWhale  ----------GATCAAAGTAGAACACG-------TACTATATGGTTCCATTT-ATATAACTGGGAGAAAGCATGTGTGTG  45891
Donkey       --------CCAGCCAAAGGAGAAC---------------TGAGAGTCAGC---ACTTATCTTTAAGAACAGATGCCTATG  51866
Cat          ----------GACCAAAGTAGAACA-----------C-ATATGATGCCATTTTATATAACTAGGAGAAAGCATGAATGTG  53940
Ferret       AAAAATGCCAAAACTAAGTAGAACACATCATATGTATTCTATGATACAATTT-ATATAACTGAGAGAAAACATGAATGTA  54581
Lion         ----------GACCAAAGTAGAACA-----------C-ATATGATGCCATTTTATATAACTGGGAGAAAGCATGAATGTG  51613
Vaquita      ----------GATCAAAGTAGAACACA-------TACTATATGGTTCCATTT-ATATAACTGGGAGAAAGCATGTGTGTG  46582
Pig          ----------GACCAAAGTAGAACACA-------TACTGCAGAATTCCATTT-ATATAATTGGGAGAACGCGTGTGTGCG  43620
```

```
Majority     GCTTCTGGAGTGTTGATAATGTTCTGTTTTTTX-XCTAGATAGTCXTTTCACAGATATGTCCAATTTGTGATATTTTATT

                       68330     68340     68350     68360     68370     68380     68390     68400

Human        GCTTCTAGAGTGTTGATAATATACTGTTTTTTA-TCTGGGTAATTATTTCACAGAGATGTCCAATTTGTGGTATTTTATG  54596
PolarBear    --------------------------------------------------------------------------------  17480
Cow          GCTTTTGGAGTGTTGGTAATGTTCTTTTTTTTA-TCTCGATAGTGCTTTCAT-GATATGGTTTATTTGA--TATTTTATT  49939
Dog          GCTTTTAGAGTTTTGATAATGTTCTTTTTTTTAT--CTAGGTAGTCATTTCACAGATATGTCCAATTTGTGATGTTT----  47299
BelugaWhale  GTTTCTGGAGTGTTGGTAATGTTCTATTTTTTTATCTAGATAATCCTTTTACAGATATGTTCTATTTGTGAAATTTTATT  45971
Donkey       GTTTTTGGTACAGGGTTGGTATAATGTGACTTTACTT--GTAGCCCAGCCACAGGAATTTACAACA-GT--TACTGAATG  51941
Cat          GCCTCTAGGGTTTTGACAATGCTCAATTTTT------ATCTAGTCATTTTATAGATATGTCCAATTTGTGATGTTTTATT  54014
Ferret       GCTTTTGGAACTTTGATAATGTTCTGTTTTT-----TATCTAGTCATTTCATAGATCTGTCCAACTTGTGATGTTTTATT  54656
Lion         GCCTCTAGGGTTTTGACAATGCTCTGTTTTT------ATCTAGTCATTTTACAGATATGTCCAATTTGTGATGTTTTATT  51687
Vaquita      GTTTCTGGAGTGTTGGTAATGTTCTATTTTTTTATCTAGATAGTCCTTTTACAGATATGTTCTATTTGTGAAATTCTATT  46662
Pig          GCTTCTGGAGTGCTGGTAATGTTCTCTTTTTAAATCTAGATAGTCCTTTCATAGATATGTTCAATTTGT--AA---TATT  43695

Majority     GAGCTGTATACACTTAGGATTTGTGTCCTTTTATGTGTATATGTATG--TTCTATACXAAGTTTAATTAAAATCTAGGTT

                       68410     68420     68430     68440     68450     68460     68470     68480

Human        GAGCTTTATA--CTTAGGTTTTGTGTCCTTTTGTGTGTGTGTGTAATATTTTGATAAGAAGTTTTATTAAAATCTAGGTT  54674
PolarBear    --------------------------------------------------------------------------------  17480
Cow          GAGCTGTGTATACTCATGATTTGTGTCCTTTTAT--GTGTATGATTG-TTTCAATACAAAGTTTAATTAAAATCTAGATT  50016
Dog          ---------ACACTTAGGATTTGTGTCCTTTCATATGTGTATATATG--TGGTTTGCCAAATTAAAATCTAGGTT-----  47363
BelugaWhale  GAGCTGTATATACTTACGATTTGTGTCCTTTTATGTGTATATGATTG-TTTCAATATGAAGTTTAATTAAAATCTAGGTT  46050
Donkey       CACATGTGT-TTCTGGCTATCCA-GTCTCTCTTAGAAAATTTACATG--TGCTATCCTAGTACT--CTGATGTTTGAAAA  52015
Cat          GAGCTGTATACTCTTAGGATCTGTGTCCTTTTATATGTATATGTA-A--TAGTTCACTAAGTTCAGTTAAAATCTTTGTT  54091
Ferret       GAGCT-TATACCCTTCTGATTTGTGTCCTTTTATGTGTGTATGTA-A--TGGTTCACTAGGTTTAATTAAAACTTA----  54728
Lion         GAGCTGTATACTCTTAGGATCCGTGTCCTTTTATATGTATATGTA-A--TAGTTCACTAAGTTCAGTTAAAATCTTTGTT  51764
Vaquita      GAGCTGTATATACTTATGATTTGTGTCCTTTTATGTGTATATGATTG-TTTCAATATGAAGTTTAATTAAAATCTAGGTT  46741
Pig          GAGCTGTATACGCTTACAGTTTGTGTCCTTC-----GTTTATGATTGCTTTCAGTATGAAAGTTAAT-AAAATCTAGATT  43769
```

```
Majority     GGCAAGCCAACTCTGXTCCATGAA----TTAAAAAAATGTATGAAX-----XATAXTTG-----TTAA-TGATAXTTGAA
                                68490       68500       68510       68520       68530       68540       68550       68560
Human        TGCAAGCCAACACTGCTCCATGAATTATAAGAAAAAATATATGAAGTCATTAAAAATGAAATCATAAAGAAATGTATGAA  54754
PolarBear    --------------------------------------------------------------------------------  17480
Cow          GGCAGGTCACCTCTGCTCCATGAATATTTTTAAGACATGTATGAAA-----CATGAATGA--AGTTCTTTGTTAATAGAG  50089
Dog          GGCAAGCCAACTCTGTTCCATCAA----TTAAAAAAATACAGAAAA-----GTTATTTG-----TTAA-TGATATTTGTT  47428
BelugaWhale  GGCAAGCCAACTCTGCTCCATGAATTTTTTTTTAAAACGTATGAAG------AGGCTTCCCTGGTGGCACAGTGGTTAAG  46124
Donkey       TGTCA--CGCCCTTGTTATATGAA------AATGACAGTGCTGAGC-----TGTGCTT------TGAGTTGTTTATTCAA  52076
Cat          GGCAAGCCAACTCTGTTTCATCAG----TTAAAAAAAATATGGAAA-----GTTATTTG-----CTAA-TCATAGTAGAA  54156
Ferret       GGTTGGCAAGCACTGTTTCATCAA----CTAAAAAAATACAGAAA------GTTATTTG-----TTAA-TGGAGAACCTA  54792
Lion         GGCAAGCCAACTCTGTTTCATCAA----TTAAAAAAAATATGGAAA-----GTTATTTG-----TTAA-TGATAGTAGAA  51829
Vaquita      GGCAAGCCAACTCTGCTTCATGAATTTTTTTAAAAAACGTATGAAG------AGGCTTCCCTGGTGGCACAGTGGTTAAG  46815
Pig          GGCAAGCCATCTCTGCCCCAT-----TTTTTAAAA-ATGTATGAAG------ATACTTG-----TTAA-TGATAATGAAG  43831
```

```
Majority     A--AXCTAAT-----TGTTAAAX-A-----------GXXGATGGAGAX----------AAGAXAAAAAGTATTGGAAAGA
                                68570       68580       68590       68600       68610       68620       68630       68640
Human        GTAATTTGTTACTGATGTGAGAGAACATGATTGTTAAGCCATAAAAGGAAGATAGAGAACAATGAATAGTATTAAATAAA  54834
PolarBear    --------------------------------------------------------------------------------  17480
Cow          A--ACATAAT-----TGTTAAGGTAAAAAAAG----GAAGATGTAGAG---------AGTGAGTATA-GTATTGGAAAGA  50148
Dog          ATTTGTTAAT-----AATAGAAAGCTATTTGTTATTGATAATGGAGAACCTAATTATTAAGCCAAAAAGAAGATGGAGGA  47503
BelugaWhale  A--ATCCACC-----TGCCAATACAGGAGATA----CGGGTTCGAGCCCTGGTCCAGGAAGATCCCACATGCTGCAGAGC  46193
Donkey       A------GGC-----AGTGAAAGGTC----------AACTATCCAGTCACTCTTTTTTTCTCCTGTTCCTCTTATAAAAG  52135
Cat          A--ACCTAAT-----TGTTAA---------------GCTAA----------------------AAAGAGAAGATGGAGAA  54192
Ferret       ATTATTAAGC-----TATAAAA--------------GAAGATGGAGAA----------AAGTGAATAGTATTGGGGAAGA  54843
Lion         A--ACCTAAT-----TGTTAA---------------GCTAA----------------------AAAGAGAAGATGGAGAA  51865
Vaquita      A--ATCCACC-----TGCCAATACAGGAGATA----CGGGTTCGAGCCCTGGTCCAGGAAGATCCCACATGCTGCAGAGC  46884
Pig          A--ACATAAT-----TATTAAGGTAAAAAT------GGAGATGGAGAAC--------AATGACTA---GTATTGGAAAGA  43887
```

```
Majority     AAGTTAAACAGTAXTGAAX-CAAATACTTATAX---X-----XT-TATAGAXTATATAXA-------TACATACATXXXT
                      68650     68660     68670     68680     68690     68700     68710     68720
Human        AGTATGAATAGTATTGAATAAAAATATGAATAGTATTGAAAAAAATACAGACTAAGTATAATATTTATATATATATGCTT  54914
PolarBear    --------------------------------------------------------------------------------  17480
Cow          AAGATATGCAGTAGATAA-----GTGCTTATATG--C-----TTGTATGGAGTATATATG-------TACATACATGCTT  50209
Dog          AAGTTCAATAGTATTGGGGAAGAAAGTTTGTATA--CATCCTTTTTATAGACTAGATACA-------TATTTACTCATAT  47574
BelugaWhale  AACTAAGCCCATGCACCA--CAACTACTGAGCCT--G-----CTCTCTAGAGCCTGCAAA-------C-CACAATTAC-T  46255
Donkey       GTAACATGCATCCTTGAGAGGGATGACTCAGA--------------ATAGAGTTTATGTA-------T---TGAATGCTT  52191
Cat          AAGTG-AATAGTACTGGGGGGAAAGGTATGTA--------------ATAGACATATCGCA-------CACACACACCTAG  54250
Ferret       ATGT------ATGTTGTAGACATACACCTATGTA--C-----TTATACAGACTAGATACA-------TATATGCTTGTGT  54903
Lion         AAGTG-AATAGTACTGGGGGGAAAGGTTTGTA--------------ATAGACATATCGCA-------CACACACACCTAG  51923
Vaquita      AACTAAGCCCATGCACCA--CAACTACTGAGCCT--G-----CTCTCTAGAGCCTGCAAA-------C-CACAATTAC-T  46946
Pig          CAAGTATGCAGTAGCTAAT-CAAGTACTTATGTG--C-----TTGTGTAGAGTACATATA-------TACGTGTCTATGT  43952

Majority     G----TG-A---XATATCTCTGGAAGGATACCCA-----AGATAATATTG-TGAGGAXTGGGTAGGGGAAAGCAGACAGG
                      68730     68740     68750     68760     68770     68780     68790     68800
Human        GTAGATGTAGCTAGTATCTCTGGAAGGATATCGA-----AGATAATACTG-TGAGGGGTTGGTAGGGGAAAATAGACTGG  54988
PolarBear    --------------------------------------------------------------------------------  17480
Cow          GAATATGTAGGTGATGTCTCTGGAAGAATACCCA-----AGAT--TATTG-TGAGGG-TTCGTAGGGGAAAGCATACAGG  50280
Dog          G----TGAAGATGGTATCTCTGGAAGGATACCCA-----TGATAATATTGGTGAGGAATGTTTAGGGGAAAGCAGACAGG  47645
BelugaWhale  GAGCCCACATGCCACAACTACTGAAACCCACGCGCTTAGAGCCCGTGCTC-TGCAAC-AAGAGGAGCCACCGCAATGAGA  46333
Donkey       -------C-----CTATGTGT-GAGGCATA---------------TGCT----AGGCAGGCGGGAGACAAGGCAGGATCA  52239
Cat          G----TG--------ATATATGGAAGGGTACCCA-----TGATAATATCGGTATGGAATGGGTATGAGAAAGGATACAGG  54313
Ferret       G----TGTAGATGGTATCTCTGGAAGGATACCCA-----TGATAATATTGGTGAGGAATGCGTTTGGGAAAGCAGCTAGG  54974
Lion         G----TG--------ATATATGGAAGGGTACCCA-----TGTTTATATCGGTATGGAATGGGTATGAGAAAGGATACAGG  51986
Vaquita      GAGCCCACATGCCACAACTACTGAAACCCACGCGCTTAGAGCCCGTGCTC-TGCAAC-AAGAGAAGCCACCACAATGAGA  47024
Pig          G-----------GATGTCCCTGGAAGGATACCCA-----AGGTAATATTGGTGAGGG-TGTGTAGGGTAAAGCAGCCAGG  44015
```

Majority	G-TGACAGGGTTGCAGAGGAAACTT--AACCTTTTGTACCATTT-GXATAGTATXCC----------A-TGATCATGTGA	
	68810 68820 68830 68840 68850 68860 68870 68880	
Human	G-TGACAGAGTTGCAG-GGGAATTTTTAATCTTTAATACCACTT-TTATACCAT----------------GATCATG-GA	55048
PolarBear	--	17480
Cow	--TGAGTGAGTTGCAGAGGAAAGTT--AACTTTTTATACTGTTT-GGATAGTATGCC----------A-TGATCATGTGA	50344
Dog	G-TGATAGGGTTGCAGAGGAAAC----AACCATTTGTACCATTT-GAATAGCATACC----------A-TGATCATGTGA	47708
BelugaWhale	AGCCTGTGCACCGCGATGGAGAGTA--GCCCCTACTCGCCACAA-CTAGAGAAAGCCCGCGCACAGCAACGAAGACGCGA	46410
Donkey	GTTTGAAAAGCTATAGTGGGGGCCT---TCCTGCCTTGAC-TTTAGAATAG-ATGGT------T--CATTGGCAAATGGG	52306
Cat	G-TGACAGGGTTGCAGAGGAAACTTTTAACCTTTTGTTCCATTTTGGATAGTAT-----------------ATCATGTGA	54375
Ferret	G-TGATAGGGTTGCAGAGGAAACTT--AACCATTTGTACCATTT-GGATAATATATC----------A-TGATCATGTGA	55039
Lion	G-TGACAGGGTTGCAGAGGAAACTTTTAACCTTTTGTTCCATTT-GGATAGTAT-----------------ATCATGTGA	52047
Vaquita	AGCCCATGCACCGCGATGGAGAGTA--GCCCCTACTCGCCACAA-CTAGAGAAGGCCAGCGCACAGCAACGAAGACGCGA	47101
Pig	G-TGCCTGGGTTCCAGAAGAAACAT--AACTTTTCGTACTGTTT-GTGTAGTATTCC----------A-TGCTCATGCGA	44080

Majority	TTCCTGTTTTAAAAAG-ACA----------TTCCATTATTTGAAXTAG-A---GATGTTAGTGTTTTCCTAAG-------	
	68890 68900 68910 68920 68930 68940 68950 68960	
Human	TTCCTATTTTAGAAAACA------------TTTTATTATTTGAAGCAG------ATGTTAGTGTTTTTCTAAG-------	55103
PolarBear	--	17480
Cow	TTCCTGTTTAGAAAGGTAT-------TTGATTCAGTTATTTGAAATAGTA---GCAGA-AGGGTTGTTCTAAG-------	50406
Dog	TTCCTGTTTCAGAAAG-ACAG------TCATTCCATTATTTGAAAGAGGA---GATGTTAGTGTTTTCCTAAA-------	47771
BelugaWhale	TGCAGCCAAAAATAAATAAATAAATGTCAGTTTATTTATTTAAAAAAAAAATGGGGGAGGGAGATGCAAGAGGGAGGAGA	46490
Donkey	TTTTTATTTCTTGGTTTGTTTGTCTGCTTGCTTGTTTGTTTTTGGTAA-----------AATGTATCCACAAG-------	52368
Cat	CTCCTGTTTTAAAAAG-ACA----------TTCCATTATTTGAAGTAGGA---GATGTTAGTATTTTCCCAAG-------	54434
Ferret	TTTCTATTTTAAAAAG-ACA----------TTCCATTATTTGAAAGAG------ATGTTAGTATTTTCCTATG-------	55095
Lion	TTCCTGTTTTAAAAAG-ACA----------TTCCATTATTTGAGGTAGGA---GATGTTAGTATTTTCCTAAG-------	52106
Vaquita	TGCAGCCAAAAATAAATAAATAAATGTCATTTTATTTATTTAAAAAAAAA-TGGGGGAGGGAGATGCAAGAGGGAGGAGA	47180
Pig	TTCCTGTTTTAAAAAG-----------ACATTCCGTTATATGAAGTAGCA---AATGCTAGAGTTGTTCAAAG-------	44139

```
Majority     --TTGTGATATTTCXATGCTTATAAT-AXATAAATAT-------ATGTAAGGACATG-TTCACTAATGAAGAA-AGTAAT
                        68970     68980     68990     69000     69010     69020     69030     69040
Human        --TTGTGGTAG-TCTATGCTTGTTATTATAGATAAAT------ACAGTAAGAACATG-TTCACTAATGAAGAA-GATAAT  55172
PolarBear    --------------------------------------------------------------------------------  17480
Cow          --TTGTGATATTTCCATGCTTATTA--TAATAAATGT-------ATGTA--GACATG-TTCACTAATGAAGAA-AGTGAT  50471
Dog          --TTGTGATATTTCTTTG--TAGTAACAGATAACTAT-------ATTCAAGGACATGCTCCACTAATGAAGTA-ATTAAT  47839
BelugaWhale  TATGGTGATATATGTATATGTATAGC-TGATTCACTTTGTTATAAAGTAGAAACTAA-CACACCATTGTAAAGCAATTAT  46568
Donkey       ----AAGATGTTTACCTCACCATGGCTA---AAATAC------AAAATGTGGAT----TTCATAATTCCAAAGTCTTAAG  52431
Cat          --TTGTGATATTCCCATGCTTGCAATAACATAAATAT-------ATGTAAGGACATGCTTCACTAGTGAAGGA-AGTAAT  54504
Ferret       --TTGTGATATTTCTTTGCTTATAGTAACATAAATAT-------ATCCAAGGACATGGTTTACTAATGAAAGA-AGTAAT  55165
Lion         --TTGTGATATTTCCATGCTTGCAATAACATAAATAT-------ATGTAAGGACATGCTTCACTAGTGAAGGA-AGTAAT  52176
Vaquita      TATGGTGATATATGTATATGTATAGC-TGATTCACTTTGTTATAAAGTAGAAACTAA-CACACCATTGTAAAGCAATTAT  47258
Pig          --TTGTGATAGTTCCATGCTTGTTA--TAATAAACGTT------ATGTAAAGACATG-TTCACTAATGAAGAA-AGTGAC  44207

Majority     ATTGCAGTAAAXTACTTAAAATACT----T-----------TXAXTCA--------------------------------
                        69050     69060     69070     69080     69090     69100     69110     69120
Human        ATTGCAATGAGCTACTTAAAATATT----------------TAAAT----------------------------------  55202
PolarBear    --------------------------------------------------------------------------------  17480
Cow          ATTGCAATGAGCTACTTAAAATACTTTATTTAAATTTGTATTGGACTA---AATATGTTCAAATATTTA--AA-------  50539
Dog          ATTGCAGTACACTACTTAAAATACT----T-----------TGAGTCA--------------------------------  47872
BelugaWhale  ATGCCAATAAA-GATGTTAAACAAAAAAGCTACACATAAATGAGACCAAAAAATGTATGAAGTTACTTGTTAATGATAAT  46647
Donkey       CTTACTGTAAA-----GAAAACTCTTAATT----TTTATTGTGAAACA--------------------------------  52470
Cat          ATTGCAGTACACTACTTAAAATACT----T-----------CAAGTCA--------------------------------  54537
Ferret       ATTGCAGTACATTACTTAAGATACT----T-----------TGAGTCA--------------------------------  55198
Lion         ATTGCAGTACACTACTTAAAATACT----T-----------CAAGTCA--------------------------------  52209
Vaquita      ATGCCAATAAA-GATGTTAAACAAAAAAGCTACACATAAATGAGACCAAAAAATGTATGAAGTTACTTGTTAATGATAAT  47337
Pig          ATTGCAGTGAG-TGCTTAAAATAAT------AAATATGTATTGGGTCA---AATATATT-AGATATTTA--AAT------  44268
```

```
Majority     --------------------------------------TTTAGTCA--A--XTATATAA---------------------
                      69130     69140     69150     69160     69170     69180     69190     69200
Human        ------------ACA-----------------------TTTAAACATTTGAATAGATAC----------TGC--------  55229
PolarBear    --------------------------------------------------------------------------------  17480
Cow          ---AAAC---ATTGTTCTAGAAATACTTTCATCCCTCTTTT-ATAA----TATCTATAAG---------TCGTGTAAATG  50599
Dog          --------T-----------------------------TTTAGCCA--A---TATATAA---------------------  47889
BelugaWhale  AGAGAACGTAATTGTTAAGGTAAAAAAGGAAGATGGAGAACAGTGAATAGTATTGGAAAGAAAAGTATGCAGTAGATAAG  46727
Donkey       --------------------------------------TTTCATACAGACAATACACAA---------------------  52491
Cat          --------C-----------------------------CTCAGCCA--A---TATATAG---------------------  54554
Ferret       --------C-----------------------------GTTAGCCA--G---TATAGAA---------------------  55215
Lion         --------C-----------------------------CTCAGCCA--A---TATATAG---------------------  52226
Vaquita      AGAGAACGTAATTGTTAAGGTAAAAAAGGAAGATGGAGAACAGTGAATAGTATTGGAAAGAAAAGTATGCAGTAGATAAG  47417
Pig          --TAAAC---ATTGCTCTAGAAATCCTTTTATCTCTTTTTTTATAACTATTCTCTATAA----------TGGTATAAATG  44333

Majority     ------------------------TTTAT--------XAGGTXATAT-T-X--ATT------------------------
                      69210     69220     69230     69240     69250     69260     69270     69280
Human        ------------------------TCTAT--------AAAACTAATTTTACCAATT-----------------------T  55254
PolarBear    --------------------------------------------------------------------------------  17480
Cow          TGTCCA------TTGTATTAAATATTTAT---CATATATTTCAAAATGT-TGGATT--TTAGTCACTTTAGCTAACATAA  50667
Dog          ----------A-------------TTTGT---------AGGCCTAAT-T----ATT------------------------  47908
BelugaWhale  TACTTAACATGTTTGTATGGAGTACATATATACATATATGCTTGTGTATGTAGGGAAAGCAGACAGGGTGACTGAGTTGC  46807
Donkey       -------------------A--TGTTCATAAACATTTTAAAGAATATTAATGAAAT------------------------  52526
Cat          ----------A-------------TCTGT---------AGGTCATAT-T----ATTG-----------------------  54574
Ferret       ----------G-------------TTTAT---------AGGCCATAT-T----ATT------------------------  55234
Lion         ----------A-------------TCTGT---------AGGTCATAT-T----ATTG-----------------------  52246
Vaquita      TACTTAACATGCTTGTATGGAGTACATATATACATATATGCTTGTGTATGTAGGGAAAGCAGACAGGGTGACTGAGTTGC  47497
Pig          TATCT-------TTCTAAAAAATATTTGT---TTCAAAATGTTGGAT-T-TGAATTAATGAGTCACTTTGGCTAACTTCA  44401
```

```
Majority     ---------TCA--------------T----TAGCATTTAAATAATGAAGT-----TTAACXAGATGAG-CATGTGGTC-
                      69290     69300     69310     69320     69330     69340     69350     69360
Human        A---------CA----------------A-TTATTTTTTCAATAATAG--------T-ATCAAGATG-------------  55286
PolarBear    --------------------------------------------------------------------------------  17480
Cow          ATTTATAGGTCGTGTTT------TATTTAATTATCATGTAGATGACGAAGT-----TTGACTAGATGAG-TGTGTGGTCT  50735
Dog          ---------TCT--------------T----TAGCATTTAAATAATGAGGT-----TTAACCAGATGGG-CATGTGGTC-  47954
BelugaWhale  AGAGGACACTTAACTTTTTATACTGTTTGGATAGTACACC-ATGATCAAGTGATTCTTATTTTAAGGAGACGTTCCATTA  46886
Donkey       ---------TAA-------------------TACCACCTAGGTTAAGAAATAATGGTCAATGAAATGAGATCCTAAGT--  52576
Cat          -------TTTAA--------------T----TAGCATTTACATAATGAGGT-----TTGACCAGATGGG-CATGTGGTC-  54622
Ferret       ---------TCA--------------T----TAGCATTTAAATAGTGAGAT-----TTAACCATATAGG-CATGTGGTCC  55281
Lion         -------TTTAA--------------T----TAGCATTTAAATAATGAGGT-----TTGACCAGATGGG-CATGTGGTC-  52294
Vaquita      AGAGGAAGCTTAACTTT--ATACTGTTTGGATAGTACACC-ATGATCAAGTGATTCCTATTTTAAGGAGACGTTCCATTA  47574
Pig          GTTTACAGATCA--T-------------AATTAACATTTAGGTGGTGAAGT-----TTGACTAGATGAG-TATGTGATTT  44460

Majority     -TTTGAATXCCAAACTXTGTGATX-TTCCTGGTTATGXGAT---------------------------------------
                      69370     69380     69390     69400     69410     69420     69430     69440
Human        --TCTACTAAAATAATCTATCACATTTCAAAATGTTGAGTTTT-------------------------------------  55327
PolarBear    -----AATTCCAGACTCTGTGAT--TTCCTGGTTATGTGAC---------------------------------------  17514
Cow          CTTTGAG-ATTATGCTCTGTGATT--TCCTAGTTATCAGATCTG-------ATCAGATCAGTCGCTCAGTAGTGTCCGAC  50805
Dog          -TCTGAATTCCAAACTGTGTGATT-CCCCTGGTTATGTGTC---------------------------------------  47993
BelugaWhale  TTTAAAATAGCAGATGTTAGAGTTGTTCTAAGTTGTGATATTTCCATGCTTATTATAATAAATGTATATAAAGACATGTT  46966
Donkey       ----GAGTCGCTGGTAGCAATAACGTTCACCAGTCTGGGCC------------------------------T--------  52614
Cat          -TCTGAATTCCAAACTGCGTGAT--CTCCTGGTTATGGGAC---------------------------------------  54660
Ferret       -TTTGAATTCCAAACTCTGTGAT--CTCCTGGTTATGTGAT---------------------------------------  55319
Lion         -TCTGAATTCCAAACTGCGTGAT--CTCCTGGTTATGGGAC---------------------------------------  52332
Vaquita      TTTAAAATAGCAGATGTTAGAGTTGTTCTAAGTTATGATATTTCCATGCTTATTATAATAAATGTATATAAAGACATGTT  47654
Pig          CTTTGAGTAGCAAGCCATATGGTA--TCCTGGTTATGGAAT---------------------------------------  44499
```

```
Majority     ---------------------------------------------------------------AXTT----------------

                       69450     69460     69470     69480     69490     69500     69510     69520

Human        ---------------------------------------------------------------AATT----------------  55331
PolarBear    ---------------------------------------------------------------ATTT----------------  17518
Cow          TCTTTGCGACCCCA-TGAATCGCAACACGCCAG-GCCTCCCTGTCCATCACCAACTCCCGGAGTTCACCCAGACTCACGT  50883
Dog          ---------------------------------------------------------------ATTT----------------  47997
BelugaWhale  CACTAATGAAGAAAGTGAACAGTGAGCTACTTGCAATGAGCTACTTAAAATACTTTATTTAAATATATATTGGGTTAAAT  47046
Donkey       ----------------------------------------------------CTGGCC----GAGCT----------------  52625
Cat          ---------------------------------------------------------------ATGT----------------  54664
Ferret       ---------------------------------------------------------------AATT----------------  55323
Lion         ---------------------------------------------------------------ATGT----------------  52336
Vaquita      CACTAATGAAGAAAGTGAACAGTGAGCTACTTGCAATGAGCTACTTAAAATACTTTATTTAAATATATATTGGGTTAAAT  47734
Pig          ---------------------------------------------------------------AGTT----------------  44503

Majority     --------------------------------------------------------------------------------

                       69530     69540     69550     69560     69570     69580     69590     69600

Human        --------------------------------------------------------------------------------  55331
PolarBear    --------------------------------------------------------------------------------  17518
Cow          CCATCGAGTTAGTGATGCCATCCAG--CCATCTCATCCTCTGTCGTCCCCTTCTCCTCCTGCCCCCA-ATCCCTCCCAGC  50960
Dog          --------------------------------------------------------------------------------  47997
BelugaWhale  ATGTTCAGATGTTTAAATTAACA----TTGTTCTAGAAATCTTTTTATCCCTCTTTTTTTATAACTATATTCTTTATAGT  47122
Donkey       ------------------------------------------------------------------------------G-  52626
Cat          --------------------------------------------------------------------------------  54664
Ferret       --------------------------------------------------------------------------------  55323
Lion         --------------------------------------------------------------------------------  52336
Vaquita      ATGTTCAGATGTTTAAATTAACAAACATTGTTCTAGAAATCTTTTTATCCCTCTTTTTTTATAACTATATTCTTTATAGT  47814
Pig          --------------------------------------------------------------------------------  44503
```

```
Majority     --------------CAGTGAATTAA-------------------------------------------------------
                      69610     69620     69630     69640     69650     69660     69670     69680
Human        ---------------AGTAAGTCA------------------------T-------------------------------  55341
PolarBear    --------------CAGTGAACTA--------------------------------------------------------  17528
Cow          ATCAGGGTCTTTTCCAATGAATCAACTCTTTGCATGAGGTGGCCAAAGTACTGGAGTTTCA----------GCTTTAGCA  51030
Dog          --------------CAGTGAACTG--------------------------------------------------------  48007
BelugaWhale  AGTGTAAATATGCCCATTGTATTAAGTATTTATTGTATGTT-TCAAAATGTTGGATTTGAATTAACAAGTCACTTTAACA  47201
Donkey       --------------TAGGGGAGACAAG-----------------------------------------------------  52639
Cat          --------------CAATGAAATGAC------------------------------------------------------  54676
Ferret       --------------CATTGAACTA--------------------------------------------------------  55333
Lion         --------------CAATGAAATGAC------------------------------------------------------  52348
Vaquita      AGTGTAAATGTGCCCATTGTATTAAATATTTATTGTATGTT-TCAAAATGTTGGATTTGAATTAACAAGTCACTTTAACA  47893
Pig          --------------------------------------------------------------------------------  44503

Majority     -------------TTTTTTT--------------------------X----XT---------ATXTCCA-----ACT---
                      69690     69700     69710     69720     69730     69740     69750     69760
Human        -------------TTTGACT--A---------------------------------------ATACATA-----AAT---  55359
PolarBear    -------------TTTTTTT--------------------------T---------------GTTTCCA-----GCT---  17546
Cow          TCA----------TTCCTTCCAAAGAAATCCCAAGGCTGAT-CTCAGTGAAATGAGC-----ATCCCCT----CACC---  51087
Dog          -------------TTTCTTT--------------------------T----TT---------ATTTCTA-----GCT---  48027
BelugaWhale  TAAATTTATAGGTCATATTTTATCAGCATTTAGATGATGAAGTTTGACTAGATGAGTGTGTGATCTCTTTGAGTACCAAG  47281
Donkey       -------------GTCCTTG------------------------------------------AGCTACAGCAGAAATAA-  52663
Cat          -------------TTTTTTT--------------------------T----TT---------GTTTCCA-----GCT---  54696
Ferret       -------------TTTTTT-------------------------------------------GTTTCCA-----GCT---  55349
Lion         -------------TTTTTTT--------------------------T----TTTTTTT----GTTTCCA-----GCT---  52373
Vaquita      TAAATTTATAGGTCATATTTTATTAGCATTTAGATGATGAAGTTTGACTATATGAGTGTGTGATCTCCTTGAGTACCAAG  47973
Pig          -------------TAC------------T-------------------GAAATGACC-----ATCCTCTA---CACC---  44528
```

```
Majority     ----------------TTATTGAG-ATAAAA--TAGAT-ACA--TTGTGTAA-XTTTAAGGTATATAATGTGTTGATTTG
                      69770     69780     69790     69800     69810     69820     69830     69840
Human        ----------------TTATAGA---TAAAA-TTAAGTAGCA-TTTAAATGATTGTTTAGATATATA-TCTGGACTCTCA 55417
PolarBear    ----------------TTATTGAG-ATATAA--TTGAT-ATA--TTGTGTAA--TATAAGGTATACAGTGTGTTGACTTG 17602
Cow          ---T------------TTGTTGAG-ATAATAGACATAT-ACA--TTGTGTAAATTTTAAGATATACAACTTATTTATTTG 51148
Dog          ----------------TTATTGAG-ATAAAA--TGGAT-ATA--TTGTGTAA--TTTAAGGGATATAATGTATTGATTTG 48083
BelugaWhale  CTATATGATTTCCTGGTTATGTAATATTTCAGTGAAATGACCATCCTCCCACCTTTTTTGAGATATAATTCACATATACA 47361
Donkey       --------T-----GGCCACAGGG-ATATGG--CTGATGTCTCTTTTTTCCTTCTTCTAAGGTTGCTATGTTCTGTCTCC 52727
Cat          ----------------TTGTTGAG-ATAAAAA-TTGAT-ACA--TTGTGTAAG-TTTAAGGTATATATTGTGTTGATTTG 54754
Ferret       ----------------TTATTGAG-ATATAA--TAAAT-ATT--TTGTATAA--TTTAAGATATATAATGTGTTGATTTG 55405
Lion         ----------------TTATTGAG-ATAAAAA-TTGAT-ATA--TTGTGTAAA-TTTAAGGTATATATTGTGTTGATTTG 52431
Vaquita      CTATATCATTTCCTGGTTATGTAATATTTCAGTGAAATGACAATCCTCCCACCTTTTTTGAGATATAATTCACATATACA 48053
Pig          ---T------------TTATGGAGTATGATTGGTAGATGACC--TGGTGTGAGTTTTAAGGCCTGTAGCTTGTTGATTTG 44591

Majority     ATACATT------------------------T-ATATATTGCAAAATGATTACCACCA------TAGCTAATATGTTXXC
                      69850     69860     69870     69880     69890     69900     69910     69920
Human        AGCC---------------------------------------GTGTGATCTGTGGTT-------ATATAATATTTTAGT 55451
PolarBear    ATACACT------------------------TTATATATTTCAGAATGATTACCGCCT------TAGCAAATGTGCTGCT 17652
Cow          ATTTATTTATGTTTTG-CAAAATGAT-TTATTTATGTTTTGCAAAATGATTACCAACACCACTTTAGCTAACAGGTCCAG 51226
Dog          ATACACT------------------------T-ATATATTGCAAAATGATTACCACCA------TAACAAATGTGTTGTC 48132
BelugaWhale  TTGTGTGGGTTTTAAGGTGATTTGAT-ATATTTATATTTCGCAAAATGATTACCACCACCACATTAGTTAATAGGTCCAG 47440
Donkey       AGCTCTCAGGAGTAAATCAAACTAATCATACTTCTCTATGGTGAAATCAGGGCTCTCA--------GCTAATACAGCAGC 52799
Cat          ACACACT------------------------T-ACGGATTGCAAAATTATTTCTACCA------TAGTATATGTGCTTTC 54803
Ferret       ATACAC---------------------------ATATATTGCAGAATGTTTACCGCCA------TAGCAAATATGCTGCT 55452
Lion         ACAC-----------------------------ACGGATTGCAAAATTATTTCTACCA------TAGTATATGTGCTGTC 52476
Vaquita      TTGTGTGGGTTTTAAGGTGATTTGAT-ATATTTATATTTCGCAAAATGATTACCACCACCACATTAGTTAATAGGTCCAG 48132
Pig          ATATATT---------------------------------GCAGAATGATTACTACCACCACCATTAGTAATAGGTTCCC 44638
```

```
Majority     AAAGAA----AXCACTXXATGA-TTT---GTTTATTCTXGTA-AGTGTTGTGCTAATCTT---XTGT--T--TXCCTTTT
                      69930     69940     69950     69960     69970     69980     69990     70000
Human        AAAATG-------ACT-------TTTCTTGTTTATTCTCCTG----AGTGTCCTAATTCTTTTGTAT------TCTCTTT  55507
PolarBear    AGAGGG----AGCACTGACTATTTGT---GTTTATTCTCATA-AGTGTTGGCCTAATTCTTTTGTGTTCT---TCCTTTT  17721
Cow          AAAAAAATGTGACACTTCATGAATTTGGGGGTCATATTTGCATAGGACCATGTTAAACT----CTGTATTGTTTCAATGT  51302
Dog          AAAGGG----AGCACT-------------GTTTGTTTTCATA-AGTGTTGTCCTAATTCT-----AT-------------  48176
BelugaWhale  AAAAAAATGTGACACTTCATGATTTTGGGG-TCATATTTGCATAGGGCCATGCTAATCT----CTGTGTTGTTCCAGTTT  47515
Donkey       AACGCATGAAGGGAAAAAAAGA-------AACTTCACTAGCAAATAGTCAAGCTAAATC-----------AATCACATAT  52861
Cat          AAAGTT----ACCAGT-------TTT---GTTTATTCTCTTA-TCTTTTGTGTTCGCCTT--------------CCTTTT  54854
Ferret       AGAGGG----AGCACTGACTATTTTT---GTTTATTCTTATA-AGTGTTGGCCTAATTCTTTTGTGTTCTCCTTCCTTTT  55524
Lion         AAAGTT----ACCAGT-------TTT---GTTTATTCTCATA-TCTTTTGTGTTTGCCTT--------------CCTTTT  52527
Vaquita      AAAAAA-TGTGACACTTCATGATTTTGGGG-TCATATTTGCATAGGGCCATGCTAATCT----CTGTGTTGTTCCAGTTT  48206
Pig          CAGAAA-TGTAACACTTCATGAATTTGGGGGTTATGTTTGCATAGGTCCATGCTAATCTTCT-CTGTATTGTTCCAATTT  44716

Majority     CAGTXTTTATGCTGCTXAGTTGA-TATTXCTATTCATTTAXCAATATA---GXTTTTTT--T-----------------
                      70010     70020     70030     70040     70050     70060     70070     70080
Human        CA----TTAACCTGTTGATATAGATTTTTCAAACTGTAAACCTATGTATAGGTATTTGT--------------------  55562
PolarBear    CGTATATCATGGTGTGCAGTTGA-TATTCCTATTCATATGCCAATATA---GGTTTTTC--------------------  17776
Cow          TGGTATGTGTGCTGCTAAAGGGAGCACGGATCCTTCCTTATACATAGT---GTTCTTATTCTTTTGTGTTTGTCTTCCTT  51379
Dog          -------CATGGTGTTCAGTTGA-TATTCCTATTCATCTGCCAATACA---GCTTTTTC--------------------  48224
BelugaWhale  TAGTATATGTGCTGCTGAAGGGAACACTGATCATTCCTTATTATCATAAATGTTCTCATTCTTTTGTATCCGCCTTCCTT  47595
Donkey       AAGGATCTGAGGTGGGCTTATTAGAGTCTTTAGCCATTAAAGAGTAACTGGGACATGAC--T-----------------  52921
Cat          CAT---TTATCATGCTCAGTTTA-TATTCCTATTCATCTGCCAGTACA---GCTTTTTCTTTTTTAAAAAAAAAATTTTT  54927
Ferret       CTTTTATCAAGGTGTTCAGTTGA-TATTTCTGTTCATCTCTT--TCAA---GCTTTTTT--------------------  55577
Lion         CAT---TTATCATGCTGAGTTTA-TATTCCTATTCATCTGCCAGTACA---GCTTTTTC--------------------  52579
Vaquita      TAGTATATGTGCTGCTGAAGGGAACACTGATCATTCCTTATTATCATAAATGTTCTCATTCTTTTGTATCCGCCTTCCTT  48286
Pig          TAGTGTATGAACTGCCGAAACAAGCACTGATCATTCTTTATTCTCATA---AGTGTCCTCATTTTGTGC---TGTCCTTT  44790
```

```
Majority     --------------TXXX------------------------------------------------------------X-
                          70090     70100     70110     70120     70130     70140     70150     70160
Human        --------------------------------------------------------------------------------  55562
PolarBear    --------------------------------------------------------------------------------  17776
Cow          TTCATTTATCGCTGTGCTTTGTTTATAT-ATACGCCTGTTCGTCTGCCAGTACAACT-TCTTCAAACTGTAAAG----CA  51453
Dog          --------------------------------------------------------------------------------  48224
BelugaWhale  TTCATTCATCACTGTGCTCTGTTT-----ATATTCCTGTTCATCTGCCAATACAACT-TTTTCAAACTATAAAG----CA  47665
Donkey       --------------TGCC--------------------------------------------------------------CT  52927
Cat          TCAACGTTTATTTATTTTTGGGACAGAGAGAGACAGAGCATGAACGGGGGAGGGGCAGAGAGAGAGGGAGACACAGAATC  55007
Ferret       --------------------------------------------------------------------------------  55577
Lion         --------------------------------------------------------------------------------  52579
Vaquita      TTCATTCATCACTGTGCTCTGTTT-----ATATTCCTGTTCATCTGCCAATACAACT-TTTTCAAACTATAAAGAAAGCA  48360
Pig          TTCATTCATCACTGTGCTTTATT------AT---CCTATTCATCTGCCAGTACAGCTCTTTTCAAACTGTAGAG----CA  44857

Majority     X--XX-AGGTATTXGTCAXTATTAXAT-XAAC--TX-XXTGXCXXX-XXT--GXXXT-XXXXXXX-AGAXAAGGTAGAAA
                          70170     70180     70190     70200     70210     70220     70230     70240
Human        -------GGTATTTGTCATTGTTATATTTTAGAACTATTTGCCTTTGTAT--GTTTTTTGACCAC-AGATAAGGTAGAAA  55632
PolarBear    --------------------------------------------------------------------------------  17776
Cow          GCATAGAGGTGTTAGTTACTATTATATTTAACAGTTATTTGCCTTTATATAGGTTTTTTGACCCCCAAATAAGGTAGAAA  51533
Dog          --------------------------------------------------------------------------------  48224
BelugaWhale  ATGTAGAGGTATTTGTCATTATTATATCTAACAGTTATTTGCCTTTATATAGGTTTTCTGACCCC-AGATAAGGTAGAAA  47744
Donkey       GTGTGAAAATAAAAAATAGTAGAA-----AAC--T--------------T--G-------------AAG--AAATATAAA  52969
Cat          GGAAACAGGCTCCAGGCTCTGAGCCATCAGCCCAGAGCCTGACGCGGGGCTCGAACTCACGGACTGCGAGATCGTGACCT  55087
Ferret       --------------------------------------------------------------------------------  55577
Lion         --------------------------------------------------------------------------------  52579
Vaquita      GTGTAGAGGTATTTGTCATTATTATATCTAACAGTTATTTGCCTTTATATAGCTTTTCTGACCCC-AGATAAGGTAGAAA  48439
Pig          GTGTAGAGGTATTTGTCATCATTATATTTAACTGTTCTTTGCCTTTATATAGGTTTTCTGACCCC-AGATAAGGTGGGAA  44936
```

```
Majority     GAAAXTAXT---XCXXXTXA--GA--XXG-CA----XATAATAGXAAAGXXATAATAGTAAXCAATGCAGXAATTTAGGA
                      70250     70260     70270     70280     70290     70300     70310     70320
Human        GAAATTATTT--CCTTATCATAGA--CTGTCATCATAATAACAGGAAAGGGATAATAGTAATAAAGGCAGTCTTTTGG-A 55707
PolarBear    -----------------------------------------------------------AAACTCTAAAGCAATGTAACG 17797
Cow          ACAATTATTT--CCTTATCAGTGAATCTGTCAGAGAAATAATAGAAAAGACATAATGGTAATAAAGGCAGTGTTTCTGGA 51611
Dog          -----------------------------------------------------------AAACTATAAAGCAATGTAGCA 48245
BelugaWhale  GAAATTATT---CCTTATCAGTGAATCTGTCAGT--AATAATAGGAAAGGCATAATAGTAATAAAGGCAGTCTTTTTGGA 47819
Donkey       GAAACAA------------------------------ACATTTTCATACATAGGACAACAACCAGTGCAAGAAAAAGGGG 53019
Cat          GGCTGAAGTCGGACGCTTAACCGACTGCGCCACCCAGGCGCCCCCAGTACAGCTTTTTCAAACTGTAAAACAATGTAGTA 55167
Ferret       -----------------------------------------------------------AAACTCTACAGCGATATAACA 55598
Lion         -----------------------------------------------------------AAACTGTAAAACAATGTAGTA 52600
Vaquita      GAAATTATT---CCTTATCAGTGAATCTGTCAGT--AATAATAGGAAAGGCATAATAGTAATAAAGGCAGTCTTTTTGGA 48514
Pig          GATACTATTT--CCTTATCAGTGAATCTGCCAGT--AATAATAGGAAAGGCATAATAGTAATAAGGGTAGTCTTTTGAAG 45012

Majority     GXATTTGACATXG--XTTAGTTXACA-ATTXTCTGCAAXTAT-TGXGTTTTTTCAACTCCAGATATATA-XXX---CAXA
                      70330     70340     70350     70360     70370     70380     70390     70400
Human        GCATTTAACATAATATTTGATTGACAGATTTCCTACAAATAC-TGTGTTTATTCAATTTTATGGGTATA-TTTTTTCGCA 55785
PolarBear    GTATTTGTCATTG---TTACTTAACA-GTTATTTGCCTTTATGTAGGTTTTTT-GACTCCAGATAGGTA-------GAAA 17865
Cow          GCATTTAACATAG-ATTTAGTTGACA--TTTCCTAAAAATAC-TGTATTTTCTCAATTCTGTATATAT--TTTTTTCACT 51685
Dog          GTATTTGTCATTG---TTGTTTAATA-ATTATTTGCCATTATATAGGTTTTTTTGACTCCAGATAGGTA-------GAAA 48314
BelugaWhale  ACATTTAACATATGATTTAGTTGACAGATTTCCCCAAAATAC-TGTATTTTTTCAGCTCCATATATATAATTTTTTCACA 47898
Donkey       AAAAATGAGGTGA--ATGAGCCCTGA---TCTCTCCAGCTTTGTGCCTGTAGTCACTTTCTGGAATATGCCAAGGCAAGA 53094
Cat          CTATTTGTTGTTG---TTACTTAACA-ATTATTTGCCTTTATATAGGTTTTTT-GACCCCAGATAGGTA-------GAAA 55235
Ferret       GTATTTGTCATTG---TTACTTAACA-ATTATTTGCCTTTATGTAGGTTTTTT-GACTCCAGATAG-----------AAA 55662
Lion         GTATTTGTTATTG---TTACTTAACA-GTTATTTGCCTTTGTATAGGTTTTTT-GACCCCAGATAGGTA-------GAAA 52668
Vaquita      ACATTTAACATATGATTTAGTTGACAGATTTCCCAAAAATAC-TGTATTTTTTCAGCTCCATATATATATTTTTTTCACA 48593
Pig          GCATTTAATATA---TTTAGTTGACAGATTTCCTAAAAATTC-TGTATTTTTTCAATTCCATATGTAT--TTT---CACA 45083
```

```
Majority     TTTTAXTATCTC--AAATCAATAX----------------GATCTGTCATAGTXXXXTXAGCAATATCXTATTXXTXGTA
                      70410     70420     70430     70440     70450     70460     70470     70480
Human        TTTTAAAATCTCAGAAATTTGA-TGTGTCCTGCAATAGTCTATCTTCCATAGTTCAATTGGCAACATCTTATTAGTCATA  55864
PolarBear    AATTATTTCCTT----ATCAATG-----------------AATCTGTCACAGT------AGTAATATTCTGTT--CTGTA  17916
Cow          TTTTAAAATCTCAGAAATCAAGATATGTCTTATA--AGTTGATCTGTCATGGTTCAATTAGCAATATCTTATTAGTCCTA  51763
Dog          AATTATTTCCTT----ATCAAT---------------------CTGTCACAGT------AGGAATAGTCTATT--CTGTA  48361
BelugaWhale  TTTTAAAATCTCAGAAATCAAGATGTGTCTTATAATAGTTGATCTGTCATGGTTCAATTATCAGTATCTTCCTAGTCCTA  47978
Donkey       GTTAAGTAACACAGAATATGATA-----------------GTCTTACTAAGTTGAGGTGACAGATATCAGATTTAGGAGA  53157
Cat          TACTATTTCCTT----ACTGGTG-----------------AATCTATCATAGT------AGTAATACTCTAGT--ATGTA  55286
Ferret       AATTATTTTCTT----TTTAATGG----------------AATCTGTCACAGT------AGTAATACTCTGTT--CTGTA  55714
Lion         TACTATTTCCTT----ACTGGTG-----------------AATCTATCATAGT------AGTAATATTCTAGT--ATGTA  52719
Vaquita      TTTTAAAATCTCAGAAATCAAGATGTGTCTTATAATAGTTGATCTGTCATGGTTCAATTATCAATATCTTCCTAGTCCTA  48673
Pig          TTTTAAAATCTC--AAATCAAGAT----------------GGTCTGTCATGGTTCAATTAGCAATATCTTATTAGTCGTA  45145

Majority     XATAAXATTATTXXACATTT----------------T--X--A--XXAAAA-------------TXCTCCA-ATTTTAT-
                      70490     70500     70510     70520     70530     70540     70550     70560
Human        CATAAAACAATAGTGCATTTTATAATTAATGGTGTATTTGATACCATAAAATGTATGGGAAATATTCTTCATATTATATC  55944
PolarBear    TTT----TTTTTTCACATTT----------------T----------AAAA-------------T-CTCAG-TGATTAAA  17951
Cow          CATAAAAGGATAGTGCATTTTACCATTAATGATATATTAGAAACAATAACATGGATGGGAAAGATTCTTTATATTTTATC  51843
Dog          TGTAT-ATTTTTTCACATTT----------------T----------AAAA-------------T-CTCA----------  48390
BelugaWhale  CGTAAAATTATAGTGTATTA-------------------GATACCATAACATGTATGGAAAATATTCTTCATATTTTATT  48039
Donkey       GGCTAAATCACCTGAAATTTGA-----GGTGCTGAGAACTGAAGAGGAAAGAGAGCAGAGAAAGAGCTCCAGAAATC---  53229
Cat          TAT-----TTTTTCACATTT----------------T----------AAAA-------------T-CTCA----------  55311
Ferret       TTT----TTTTT-CACATAT----------------T----------AAAA-------------T-CTCAT-AAATTAAA  55748
Lion         TAT-----TTTTTCACATTT----------------T----------AAAA-------------T-CTCC----------  52744
Vaquita      CGTAAAATTATAGTGTATTA-------------------GATACCATAACATGTATGGAAAATATTCTT---ATTTTATT  48731
Pig          CATAAGAAAATTGTGCATTC-------A----TGTAT--GGAAAAATA--A-------------TTCTTCATATTTTATC  45197
```

```
Majority     TTGAAG--TTCCTGAAATTAAXATGGGTTTGAACTGTGGTATAXATC---TXCTGGTTTAATX-AXTAGTATTTCAGAAG
                         70570     70580     70590     70600     70610     70620     70630     70640
Human        TTGAAGTTTACCTGAGAATTTTGTGGTTTTGGACTAGACTACACGTTGGTTTCTATTCTAAAA-ATTTTGGTGTCAGAAG  56023
PolarBear    AAAAAAATTCTAAGAAATTAAGATGTGTCTCAACAGTAGTGAATCTC---TCCCGGTTTAATT-AGCAGTATCTTAGAAG  18027
Cow          TTGAAGTTTCCTGAAAATTTTATGGCTGTGTACTGTGGTATACATCGGTTTCTGTTGTAAAA-ATTTTGGTTTCAAAAG  51922
Dog          -------------GAAATTAAGATGTGTTTCAACAGTAATTGATCTG---TCCTGGTTTAATT-AGCAGTATCTTAGAAG  48453
BelugaWhale  TTGAAGTATTCCTGAAAATTTTATGGCTTTGTAGTATGGTATACATCAATTTCTGTTCTAAAA-ATTTTGGTTTCAGAAG  48118
Donkey       ---------TGCAAGAGTTACCCTAGAGTCTATTGGTGATACTGAGC---TGCCACATGCATAGGGTAAAACTCCATCAG  53297
Cat          -------------GAAATTAAGATGTGCCTTAAGTGTCGTTGATTTG---TC-TGGTTTAATT-AGTAGTATTTTAGAAG  55373
Ferret       AAAATA----TCAGAAATTAAGAGGTGTCTTAACAGTAGTACATCTC---TCCTGGTTTAATTTAATAGTATCTTAGAAG  55821
Lion         -------------GAAATTAAGATGTGTCTTAAGTGTCGTTGATTTG---TC-TGGTTTAATT-AGTAGTATTTTAGAAG  52806
Vaquita      TTGAAGTATTCCTGAAAATTTTATGGCTTTGTAGTATGGTATACATCAATTTCTGTTCTAAAA-ATTTTGGTTTCAGAAG  48810
Pig          TTGAAGCATTCCTGAAATTTCTGTGGTTTTGTACTATGGCATACATCAATTTCTGCTTTAA----TTT-AGATTCAGAAG  45272

Majority     --TTATGCTAGTATGAAAAAAGA--CTTAATXTGTAT-CTGAATATTCCTCXTTTAAGATTXTATTAGAAATAATAAGCT
                         70650     70660     70670     70680     70690     70700     70710     70720
Human        -TTAAGTGTAGTATGAAAAA----------TGTATTTTCTGAATGTTCCTATTTTAAGATTGTATTAGAAATAATAAGCT  56092
PolarBear    --TTATGCTAGTATGAAAAAGGA--CTTAATATGTATTCTGAGTATTCCTCCTTTAAGATTGTATTAGAAATAATAAGCT  18103
Cow          --TTACACTAGTATCAAAAAAAA------ATTTGTAT-CTGAATGTTTCTCTTTTAGGATT-TATTAGAAATAATAAGCT  51992
Dog          --TTACGCTAGTCTGAAAAAAGA--CTTAATATGTATCCTGAATATTCCTCCCTTAAGATTATATTAGAAATAATAAGCC  48529
BelugaWhale  --TTATGCTAGTATGAAAAAAAGA-CTTAATTTGTAT-CTGAATATTCTTCTTTTAGGATT-TATTGGAAATAATG---T  48190
Donkey       GCTGGTCAAAGAATGATTGAGGAGCTGTAAGCTGAGCAATGCCCATTGCTCATACAGAGCT-TATACAGAGCCAAA-G--  53373
Cat          --TTATGCTACTATGAAAAA-GA--CTTAATATATGTCCTGAATATTCCTCCTTTAAGATTGTATTAGAATTAATAAACT  55448
Ferret       --TTATGGTAATAT---------------------------GTATTCCTCCTTAAAGATTGTATTAAAAATAATAAGCT  55871
Lion         --TTATGCTACTATGAAAAA-GA--CTTAATATATGTCCTGAATATTTCTCCTTTAAGATTGTATTAGAAATAATAAACT  52881
Vaquita      --TTATGCTAGTATGAAAAAAAGA-CTTAATTTGTAT-CTGAATATTCTTCTTTTAGGATT-TATTGGAAATAATG---T  48882
Pig          --TTATACTAGTTTGAAGAAAAAGGCTTGATTTGTAT-CTGAATGTTCCTATTT-AGGATT-TATTAGAAATAATAAGCT  45347
```

```
Majority     TTATGGGAGTTCTTTTGGATAATCTAGTAATCCC-TTGCTGAGGTTTACTATGTGCCTTTTTT-ATGGAGT---GAATAA
                             70730     70740     70750     70760     70770     70780     70790     70800
Human        TTATGGGAGTTCTTTTGGATAATTCAGTAACCCCCTTGTTGAGATTTACAACGTACCTGTTTT-ATGGAGT---GATTAA  56168
PolarBear    TTATGGAAGTTTTTCTGGGTAATCTAGTAATC---TTGCTGAGGTTTACCATGTGCCCTTT-------------GAATAA  18167
Cow          TTATAGGTGTTCTTTTGGATAATCTAGTAATTTC-CTCTTAAGGTTTGCCATTTGCCTTTTTT-ATGGAAT---GAATAA  52067
Dog          TTATGGTAATGTTTTTTTG------GGTTATC---TTGCTGAGATTTACTATGTGCCTTTTTT-ATGGAGTAGTGAATAA  48599
BelugaWhale  TTATGGGAGTTCTTTTGGATAATCTAGTAATCCC-CT--TAAGGTTTACTATGTGCCTTTTTT-ATGGAAT---GAATAA  48263
Donkey       T-AGAGAGCCCTCCTTGAACTCCCTAGACACTCAATAG--AGATCCCAGAATGTTCACACCTTAAC--AATA--GGGCTA  53446
Cat          TTACAGGAGTTCTTTTGGATAATCTGGTAATCCCCTTGCTGAGGTTTACTGGGTACCTTTTTT-ATGGAGT---GAATAA  55524
Ferret       TTATGGGAGTTTTTTTTGGTAATCTAGTAATC---TTGCTGAGGTTTACCATGTGCCTTTTTT-ATGGAGT---GAATAA  55944
Lion         TTACAGGAGTTCTTTTGGATAATCTGGTAATCCCCTTGCTGAGGTTTACTGGGTACCTTTTTT-ATGGAGT---GAATAA  52957
Vaquita      TTATGGGAGTTCTTTTGGATAATCTAGTAATCCC-CT--TAAGGTTTACTATGTGCCTTTTTT-ATGGAAT---GAATAA  48955
Pig          TTATGGGAGTTCTTTTGGATAA---A-TCTCCCC-TTGTTGTGGTTTACCATGTGTCTTTTTTTATGGAGT---GAATAA  45419

Majority     TGTTAXATTGAAXTTTTX------TCTCTAAA----------AGTTTCTGTTTAAA--A-----CATTTTCTGAT--TAT
                             70810     70820     70830     70840     70850     70860     70870     70880
Human        TGTTAAAGTTGTATT----------CTCTAAA------------TTTCTGTTTAAA--A-----CATTTTCTGATGGTAC  56219
PolarBear    TGTTAGATTGAAGTT----------CTCTTAA----------AGTTTCTGTTTAAA--ATAA--CATTTTCTGAT--TAT  18221
Cow          TGTTAAAGTTTCATTGT-------TCTCTAAA----------AGTTTCTGTTTAAA--A-----CATTTTCTG-------  52116
Dog          TGTAAGACTGAAGTTTTATTGTTCTCTTTAGAGAAACTTTTAAGTTTCTTTTTAAA--A-----CATTTTCTGAT--TAT  48670
BelugaWhale  TGTTAAAGTTTCATTGT-------TCTCTAAA----------AGTTTCTATTTAAA--A-----CATTTTCTG-------  48312
Donkey       AACTAGCCTGCCTGTTAAGATT--GCTCTTGAC---------CATCCCTGATCAAGCTACAGAACAAATCATGAA--AGA  53513
Cat          TGTTAAGTTGAAGTTTTATT-----CTCTAAA----------AGTTTCTGTTTAAA--A-----CATTTTCTGAT--GTT  55580
Ferret       TGTTAGGTTGAAGTTTTATT-----CTCTAAA----------ATTTCCTATTTAAA--A-----GATTTTTTGAT--TAT  56000
Lion         TGTTAAGTTGAAGTTTTATTGT--TCTCTAAA----------AGTTT--GTTTAAA--A-----CATTTTCTGAT--GAT  53014
Vaquita      TGTTA--GTTTCATTGT-------TCTCTAAA----------AGTTTCTATTTAAA--A-----CATTTTCTG-------  49002
Pig          TATTAGATTAAAATTTTG------TTTCTGAA----------AGTTTCTGTTTAAA--ATAAAACATTTTCTGTT--AAT  45479
```

```
Majority     XTGAATCTGXCAAACTATATCTTG--TTCTTTTGAAAATAAATTACATTGCCTXTCTTTGCTTGTTCAATA---TTGGTC
                       70890     70900     70910     70920     70930     70940     70950     70960
Human        ATGTATTTATAAAACTACATCTTG--TTCTTTTGAGAATAAATTACATATCATACCTTTGCATGTTCAACA---TTGATC  56294
PolarBear    GTGAATTTGCCAAACTATATTTTG--TTCTTTTGAAAATAAATTATACTGCCTGTCTTTGCCTGTTCAGTA---TAGGTC  18296
Cow          ---AATCTGTCAGACTACATCATG--CTGTTTTGAGAATAAATTACATGACTTATCTAT-CTTGTTCAGCA---TTGGTC  52187
Dog          GTGAATTTGCCAAACTATATTTTG--TTCTTTTGAAAATAAACTACACTGCCTATCTTTGCTTGTTCAATA---AAGGTT  48745
BelugaWhale  ---AATCTGTCAGACTACATCTTG--TTGTTTTAAGAGTAAATTACACTGACTGTCTTT-CTTGTTCCACA---TTGGTC  48383
Donkey       GTCAAACTGATCAAAAGTAACTTAAATGCCTACCAAACAGAATTCAACACTTTTTATTTATTTATTTATTTA--TTTATT  53591
Cat          ATGAATCTGCCAAACTATATTTTG--TTCTTTTGAAAATAAGTTACGTTGCCTTTCTTCGTTTGTTCAATTATCTAGGTC  55658
Ferret       GTGAATCGGCCCAACTGTATT-----CTCTTTTGAAAATAAATTACATTGCCTGTCTTTGCTTGTTCAGTA---TAGGTC  56072
Lion         ATGAATCTGCCAAACTATATTTTG--TTCTTTTGAAAATAAATTATGTTGCCTTTCTTCGTTTGTTCCATTATCTAGGTC  53092
Vaquita      ---AATCTGTCAGACTACATCTTG--TTGTTTTAAGAGGAAATTACACCAACTGTCTTT-CTTGTTCCACA---TTGGTC  49073
Pig          ATGAGTGTGTCAAACTATATCTTG--TTCTCTTG-GGATGAATTACATGGCCTATCTTTTCTTGTTCAGCA---TTCATC  45553

Majority     CCAAACCCTAGATAATTGTTTTGAAG-----TAAATACTAGTAGAATACTTGAATTAT-AAACAGATAATACCTGGCTCA
                       70970     70980     70990     71000     71010     71020     71030     71040
Human        CCAAAGCCTGGATAATTTTTTTGAAT-----GAAATACTAGTAGAATATTTGATTTATTAAACAGGTAGTACCTGGCTCA  56369
PolarBear    CCAAACCCTAGATAATTGTTTTGAAG-----TAAATACTGGTAGAATACTTGAATTAT-AAACAGATAATACCTGGCTCA  18370
Cow          CCAAACCCTCAATAACTGTTTTGAGG-----TAAATACTAGTATGATACTTGAATTGT-AAACAGGCAATATGTGGCTTA  52261
Dog          CCAAACCCTAGATAATTGTTTTGAAT-----TAAAGATTAGTAGACTACTTGAATTAT-AAACAGATAATACCTGGTTCA  48819
BelugaWhale  CCAAACCCTAGATAATTGTTTTGAAGTAAAATAAATACTAGTATAATACTTGAATTAT-AATC--------CTTGGCTCA  48454
Donkey       TATTTATTTATTTATTTATTGTTATTTCAGATTGGCCCTGAAATAACAACTG--TTGC-CAAT---------CTTCCTCT  53659
Cat          CCAAACCTTAGATAATTGTTTTGAAG-----TAAATACCAGTAGGATACTTGAATTAT-AAACCGATAATACCTGGCTCA  55732
Ferret       CCAAACCCTAGATAATTGCTTTGAAG-----TAAATACTAGTAGAATACTTGAATTAT-AAACAGGTGGTACCTGGCTCA  56146
Lion         CCAAACCTTAGATAATTGTTTTGAAG-----TAAATACCAGTAG-ATACTTGAATTAT-AAACCGATAATACCTGGCTCA  53165
Vaquita      CCAAACCCTAGATAATTGTTTTGAAGTAAAATAAATACTAGTATAATACTTGAATTAT-AATC--------CTTGGCTCA  49144
Pig          CCAAACCTTAGATGATTATTTTGAAA-----TAAATACTAGTAGAATACTTGAGTTAT-AAACAGACAAAACCTGGCTCA  45627
```

```
Majority     TGGCAGGTGCTCAAAAGTATTTAT--TGAXTGAXTGTTAAATTTTGTTTAATCTGTATGTGTTCTAGAAAAGAAXTGAAC
                              71050     71060     71070     71080     71090     71100     71110     71120
Human        TGGCAGGTGCTTAAAAATATCTGT--TGAGGGCCATTTAATTTTGTTTAATCTGTGTAAGTTCTAGGAACAAAGTGAAC  56447
PolarBear    TGGCAGGTAATCAAAAGTATTTAT--TGAGTGACTATTAAATTTTGTTTAATCTGCATGTGTTCTAGAAAAGAAATGAGC  18448
Cow          TGGCAGGTGCTTAAAAGTGTATTTACTGAGTGAGTGTTAAATTTTATTTAATCCGTATGTGTTCTAGAAAAGAATT----  52337
Dog          TGGCAGGTGCTCA-AAGTATTTAT--TGA----CCATTATATTTTGTTTAATCTGTATGTGTTGTAGAAAAGAAATGAGC  48892
BelugaWhale  TGGCAGGTGCTCAAAAGTATTTAT------TGAGTGTTAAATTTTATTTAATCT--ATGTGTTTTAGAAAATAATT----  48522
Donkey       TTTTGCATGAGGAAGACTGC---C----ACTGAGC-TAACATCTGTGCCAATCTTCCTCTATTTTTTTTAA-GATTGGCC  53730
Cat          TGGTAGGAGCTCCAAAGTATTTAT--TGAATGATTGTTAAATTTTGTTTAATCTGTGTGTGTTGTAGAAAA-AAATGAAC  55809
Ferret       TGTTAGATGCTCAGAATTATTTAT--TGAGTGACTATTAAATTTCGTTTACTCTGTATATGTTCTAGAAAAGAAATGAGC  56224
Lion         TGGTAGGAGCTCCAAAGTATTTAT--TGAATGATTGTTAAATTTTGTTTAATCTGTGTGTGTTGTAGAAAA-AAATGAAC  53242
Vaquita      TGGCAGGTGCTCAAAAGTATTTAT------TGAGTGTTAAATTTTATTTAATCT--ATGTGTTTTAGAAAAGAATT----  49212
Pig          TGGCAGGTGCTCAAAAGTAT-----------------------TTATTTAATCTAGATATGTTCTAGGAAATAATTGAAC  45684

Majority     TAATAGTGTAACATGTAGCAAAGTATAAGT------GAAAATACTGAGTATGTTTCTAAAGTGCTTATCTTTCAATTTTC
                              71130     71140     71150     71160     71170     71180     71190     71200
Human        TAGTAGTGTAACAGGTTGCAAAGTAT--GT------GACAATAATGGGTATGTTTCTAAACTACATA-ATCTTAATTTCC  56518
PolarBear    TAATAGTGTAACATGCAGCAGAGTATAAGT------GAAAATACTGAGTATGTTTCTAAAGTGTTTATCTTTCAATTTTT  18522
Cow          TAATACTGTAGCATGTAGCAAAGTATAAGT------GAATATACCAGGAATGTTTCTAAAGTGCTTATGTTTTGATTTTT  52411
Dog          TA-----------------AAAGTATAAGT------GAAAAT--TCAGTATGTTTCTGAAGTGTTTATCTTTCAATTTTC  48947
BelugaWhale  TAATATTGTAACGTTTAGCAAAGTATAGGT------GAAAATACCAGGAATGTTTCTAAAGTGCTTATCTTTCATTTTTT  48596
Donkey       CTGAGCTAACATCTGTTGCCAATCTTTTCTTT--TTCTTGTTCTTCTTCTTCTCCCCAAAGCCCCCCTGTACATAGTT--  53806
Cat          TAATAGTGTAACATGTAGCAAAGTTTAAAT------GAAAATACAGAGTGTGTTTCTAAAGTGCTTATCTTTTAATTTTC  55883
Ferret       TAGTAGTATAACATACAGCAGAGTTTAAGT------GGAAATACTGAGTGTGTTTCTAACTTGTTTATCTTTCAATTTTC  56298
Lion         TAATAGTGTAACATGTAGCAAAGTTTAAAT------GAAAATATGGAGTGTGTTTCTAAAGTGCTTATCTTTTAATTTTC  53316
Vaquita      TAATATTGTAACGTTTAGCAAAGTATAGGT------GAAAATACCAGGAATGTTTCTAAAGTGTTTATCTTTCTTTTTTT  49286
Pig          TAATGGCGTAACATGTAGCAAAGTATATCTTCACCTAAAGATACGGATAGTGTTTCTAAGGTGCTTGTCTTTGGATTTTC  45764
```

```
Majority     AGCGTGCTCAAGCAGTTCTTCAAGCTGTGACAGCTGTCCAGACAGCAAATACTCCTCTTAGTGGCACCACAGTTAGTGAG
                      71210     71220     71230     71240     71250     71260     71270     71280
Human        AGCGTGCTCAGGCAGTTCTTCAAGCTGTGACAGCTGTCCAGACAGCAAATACTCCTCTTAGTGGCACCACAGTTAGCGAG  56598
PolarBear    AGCGTGCTCAAGCAGTTCTTCAAGCTGTGACAGCTGTCCAGACAGCAAATACTCCTCTTAGTGGCACCACAGTTAGTGAG  18602
Cow          AGCGTGCTCAAGCAGTTCTTCAAGCTGTGACTGCTGTCCAGACAGCAAATACTCCTCTTAGTGGCACCACAGTTAGTGAG  52491
Dog          AGCGTGCTCAAGCAGTTCTTCAAGCTGTGACAGCTGTCCAGACAGCAAATACTCCTCTTAGTGGCACCACAGTTAGTGAG  49027
BelugaWhale  AGCGTGCTCAAGCAGTTCTTCAAGCAGTGACAGCTGTCCAGACAGCAAATACTCCTCTTAGTGGCACCACCGTTAGTGAG  48676
Donkey       -GTATATTCTAGTTGTAGGTNNNNNNNNNNNNNNNNNNNNNNNNNNNNNNNNNNNNNNNNNNNNNNNNNNNNNNNNNNNN  53885
Cat          AGCGTGCTCAAGCAGTTCTTCAAGCTGTGACAGCTGTCCAGACAGCAAATACGCCTCTTAGTGGCACCACAGTTAGTGAG  55963
Ferret       AGCGTGCTCAAGCAGTTCTTCAAGCTGTGACAGCTGTCCAGACAGCAAATACTCCTCTTAGTGGCACCACAGTTAGTGAG  56378
Lion         AGCGTGCTCAAGCAGTTCTTCAAGCTGTGACAGCTGTCCAGACAGCAAATACGCCTCTTAGTGGCACCACAGTTAGTGAG  53396
Vaquita      AGCGTGCTCAAGCAGTTCTTCAAGCAGTGACAGCTGTCCAGACAGCAAATACTCCTCTTAGTGGCACCACAGTTAGTGAG  49366
Pig          AGCGTGCTCAAGCAGTTCTTCAAGCTGTGACGGCTGTCCAGACAGCAAATACTCCTCTTAGCGGCACCACAGTTAGTGAG  45844

Majority     AGTGCAGTGACTCCAGCCCAGAGTCCAGTGCTTAGAATAATTATTGACAATATGTACTACCCTGTAACACTTGATGTTCT
                      71290     71300     71310     71320     71330     71340     71350     71360
Human        AGTGCAGTGACTCCAGCCCAGAGTCCAGTACTTAGAATAATTATTGACAACATGTACTACCCTGTAACACTTGATGTTCT  56678
PolarBear    AGTGCAGTGACTCCAGCCCAGAGTCCAGTGCTTAGAATAATTATTGACAATATGTACTACCCTGTAACACTTGATGTTCT  18682
Cow          AGTGCAGTGACTCCAGCCCAGAGTCCAGTGCTTAGAATAATTATTGACAATATGTATTATCCTGTAACACTTGATGTTCT  52571
Dog          AGTGCAGTGACTCCAGCCCAGAGTCCAGTGCTTAGAATAATTATTGACAATATGTACTACCCTGTAACACTTGATGTTCT  49107
BelugaWhale  AGTGCAGTGACTCCAGCCCAGAGTCCAGTGCTTAGAATAATTATTGACAATATGTATTACCCTGTAACACTTGATGTTCT  48756
Donkey       NNNNNNNNNNNNNNNNNNNNNNNNNNNNNNNNNNNNNNNNNNNNNNACAACCATA-TTTTTAATTTTCAAAACCTTTTAATCTTCT  53964
Cat          AGTGCAGTGACTCCAGCCCAGAGTCCAGTGCTTAGAATAATTATTGACAATATGTACTACCCTGTAACACTTGATGTTCT  56043
Ferret       AGTGCAGTGACTCCAGCCCAGAGTCCAGTGCTTAGAATAATTATTGACAATATGTACTACCCTGTAACACTTGATGTTCT  56458
Lion         AGTGCAGTGACTCCAGCCCAGAGTCCAGTGCTTAGAATAATTATTGACAATATGTACTACCCTGTAACACTTGATGTTCT  53476
Vaquita      AGTGCAGTGACTCCAGCCCAGAGTCCAGTGCTTAGAATAATTATTGACAATATGTATTACCCTGTAACACTTGATGTTCT  49446
Pig          AGTGCAGTGACTCCAGCCCAGAGTCCAGTACTTAGAATAATTATTGACAACATGTATTACCCTGTAACACTTGATGTTCT  45924
```

```
Majority     TCACCAAGTAAGTTTAAXTTAATTTTCATGTTTACCTATAAAT-----------TGGAGAAATAATAATA-CAGTAGATA
                      71370     71380     71390     71400     71410     71420     71430     71440
Human        TCACCAAGTAAGTTTAATC-----TGCATAATTACCTATAAAT-----------TAGAGAAATAATAATA-TAGTAAATA  56741
PolarBear    TCACCAAGTAAGTTTAACTTACTTTTCATGTTTACCTATAAACATGAGAAACAATGGAGAAACAATGATA-GAGTAGATA  18761
Cow          TCACCAAGTAAGTTTAATTTCATTTGCAT-TTTATCTATAAAA-----------TGAAGAAATAATAATA-CAGTAGATA  52638
Dog          TCACCAAGTAAGTTTAACT-AATTTTCATCTTTACCTATAAAC-----------TGGAGAAATAATGATA-CAGTAGATA  49174
BelugaWhale  TCACCAAGTAAGTTTAGTTTAATTTGCATGTTTATCTATAAAA-----------TTGAGAAATAATAATA-CAGTAGATA  48824
Donkey       TTGATCATCCTTTTTACATTGTAACTGGTCGTGTTTTATGACT-----------TCACAATTTTTTAAGGATATTA-ATT  54032
Cat          TCACCAAGTAAGTTTAACTTAATTTTCGTGTTTACCTATAAAT-----------TGGAGAAATAAAGATAACAGTAGATA  56112
Ferret       TCACCAAGTAAGTTTAACTTAATTTTCATGTTTACCTATGAAC-----------TGGAGCAATAATG-TA-CAGTAGATA  56525
Lion         TCACCAAGTAAGTTTAACTTAATTTTCATGTTTACCTATAAAT-----------TGGAGAAATAAAGATAACAGTAGATA  53545
Vaquita      TCACCAAGTAAGTTTAGTTTAATTTGCATGTTTATCTATAAAA-----------TTGAGAAATAATAATA-CAGTAGATA  49514
Pig          TCACCAAGTAAGTTTAATTTAATTTCAATATTCATCTGTAAAT-----------TGGAGAAATAATAATA-TAGTAGATA  45992

Majority     AATAATATTTT-----GATTTTAATGTTTT-AACAGATATTTTCTAAGTTTGGTGCTGTATTGAAGATAATCACATTTAC
                      71450     71460     71470     71480     71490     71500     71510     71520
Human        AGTAATGTTTT-----GATTTTAATGTTTT-AACAGATATTTTCTAAGTTTGGTGCTGTATTGAAGATAATCACATTTAC  56815
PolarBear    AATAATACTT------GATTTTTATATTTT-AACAGATATTTTCTAAGTTTGGTGCTGTATTGAAGATAATCACATTTAC  18834
Cow          TAATTTGTTTT----GATTTTTAATATTTT-AACAGATATTTTCTAAGTTTGGTGCTGTATTGAAGATAATCACATTTAC  52713
Dog          AATAATACTTT-----GATTTTAATGTTTT-AACAGATATTTTCTAAGTTTGGTGCTGTATTGAAGATAATCACATTTAC  49248
BelugaWhale  AAATTTGTTTT-----GATTTTAATGTTTT-AACAGATATTTTCTAAGTTTGGTGCTGTATTGAAGATAATCACATTTAC  48898
Donkey       AATGATATTTTACAAATATTTCACTATACTTAAATTATATCCT----GTTTTCTTCAGTGTTAG---CTGTTATGTTTAT  54105
Cat          AATAATATTTT-----GATTTTAATTTTTT-AACAGATATTTTCAAAGTTTGGTGCTGTATTAAAGATCATCACATTTAC  56186
Ferret       AATAATACTTT-----GATTTTAATGTTTTTAACAGATATTTTCTAAGTTTGGTGCTGTATTGAAGATAATCACATTTAC  56600
Lion         AATAATATTTT-----GATTTTAATTTTTT-AACAGATATTTTCAAAGTTTGGTGCTGTATTAAAGATCATCACATTTAC  53619
Vaquita      AAATTTGTTTT-----GATTTTAATGTTTT-AACAGATATTTTCTAAGTTTGGTGCTGTATTGAAGATAATCACATTTAC  49588
Pig          AAATTTGTTTTAATTGATTTTTAATGTCTC-AACAGATATTTTCTAAGTTTGGTGCTGTATTGAAGATAATCACATTTAC  46071
```

```
Majority     AAAAAATAACCAGTTTCAAGCTCTTCTCCAGTATGGTGATCCAGTAAATGCTCAACAAGCAAAACTAGTAAGTCTX-AGX

                       71530     71540     71550     71560     71570     71580     71590     71600

Human        AAAAAATAACCAGTTTCAAGCTTTGCTCCAGTATGGTGATCCAGTAAATGCTCAACAAGCAAAACTAGTAAGTCT-----  56890
PolarBear    AAAAAATAACCAGTTTCAAGCTCTTCTCCAGTATGGTGATCCAGTAAATGCTCAACAAGCAAAACTAGTAAGTCTGGAGC  18914
Cow          AAAAAATAACCAGTTTCAAGCTCTTCTCCAGTATGGCGATCCAGTAAACGCTCAGCAAGCAAAACTAGTAAGTCT--AG-  52790
Dog          AAAAAATAACCAGTTTCAAGCTCTTCTCCAGTATGGTGATCCAGTAAATGCTCAACAAGCAAAACTAGTAAGTCTGGAGC  49328
BelugaWhale  AAAAAATAACCAGTTTCAAGCTCTTCTCCAGTATGGCGATCCAGTAAATGCTCAGCAAGCAAAACTAGTAAGTCT--AG-  48975
Donkey       CTT--GTTTTCTCTCTCCCCCTCTTCCCCTTTCCTCCCTTTCGTTGTCTGTCCATATTTCAGAACAGTTAGGTTTT-ATT  54182
Cat          AAAAAATAACCAGTTTCAGGCTCTTCTCCAGTATGGTGATCCAGTAAATGCTCAACAAGCAAAACTAGTAAGTCTGGAGC  56266
Ferret       AAAAAATAACCAGTTTCAAGCTCTTCTTCAGTATGGTGATCCAGTAAATGCTCAACAAGCAAAACTAGTAAGTCTGGAGC  56680
Lion         AAAAAATAACCAGTTTCAGGCTCTTCTCCAGTATGGTGATCCAGTAAATGCTCAACAAGCAAAACTAGTAAGTCTGGAGC  53699
Vaquita      AAAAAATAACCAGTTTCAAGCTCTTCTCCAGTATGGCGATCCAGTAAATGCTCAGCAAGCAAAACTAGTAAGTCT--AG-  49665
Pig          AAAAAATAACCAGTTTCAAGCTCTCCTCCAGTATGGTGATCCAGTAAATGCTCAGCAAGCAAAACTAGTAAGTCT--AG-  46148

Majority     ----TTX-XTXTTTTTTTTT------------GAGATAGXGATATTTTTCAGTGGXAGATATATATACACACATATATA-

                       71610     71620     71630     71640     71650     71660     71670     71680

Human        ------------TTCTT-TT------------GAGATGGTGATTTTTTTTTATTG-AAATGTATATGTAGAAAAATATA-  56943
PolarBear    ---ATTTTTTTTTTTTTTTTTT----------GAGATTTCGATATTTTTTAGTGGCAGATAGACATACACGCATATATA-  18979
Cow          -----------GTTTTT-TT------------GAGAGCA--ATAATTTTTAGTGGCAGATATATATACACACACATATAT  52844
Dog          CCTATTTTTTTTTTTTTTTTTTTTTTT------TACTATAGCCATATTTTTCAAAGGCAGATAGATATACACGC-TATATA-  49400
BelugaWhale  -----------CTTTTTTT-------------GAGATAGCAATAATTTTCAGTGGTAGATATGTATATACACATATATAT  49031
Donkey       GGACTTACGTGCTTTCCTGT------------AAAGTAAT-GGGGAATTGGATACTAAGCTAAGATTCCCCTCCAGATG-  54248
Cat          TTGCTTTTTTTTTTTTTTTTTTTT-------ATAGATAGTGATCTTTTTCAATGGTGGGTACACATAAACAC--ATATA-  56336
Ferret       ---ATTTCTTTTTTCTTTTG------------GATATAGCTATATTTTTCAGTGGCAGATAGACACACACACACACACAC  56745
Lion         TTGCTTTTTTTTTTTTTTTTTTTTTTTTTTTAAATAGATAGTGATCTTTTTCAATGGTGGGTACACATAAACAC--ATATA-  53776
Vaquita      -----------CTTTTTTTT------------GAGATAGCAATAATTTTCAGTGGTAGATATGTATATACACATATATAT  49722
Pig          -----------CTTTTTATT------------GAGATTATGGTAATTTTCAGCAGCCAATATTTATTTATATATATCCCC  46205
```

```
Majority     ---------------------------------TA-----GCTAATGTTAT---------TAATACTGACATAACTTAG
                      71690      71700      71710      71720      71730      71740      71750      71760
Human        ---------------------------------TA-------------TAT-----------ATGTATGTATA-CTTAA  56964
PolarBear    ---------------------------------TA-----GCTAATGTTAT---------TAATACTAACATAACTTGG  19011
Cow          ATGTAACTAATATCGTTAATATTATTATTTTACATATTAATATTAATATTATGTCAGTAACTAATATTGACATAACTTAG  52924
Dog          ---------------------------------TC-----ACGAATGTCAT---------TAATACTGACATA------  49426
BelugaWhale  GT----------------------------G--TA-----GCTAATGTTAT---------TAATACTGACATAACTTAG  49066
Donkey       ---------------------------------CCG------AAAAGGTCT---------TTGTTCTGGTCCA--TCA-  54276
Cat          ---------------------------------TT-----GCTGATGTTAC---------TAATACAGACATAACTTGG  56368
Ferret       AC------------------------------ATA-----GCTAACATTAA---------TAATACTGACATAACTTGG  56780
Lion         ---------------------------------TT-----GCTGATGTTAC---------TAATATGGACATAACTTGG  53808
Vaquita      GT----------------------------G--TA-----GCTAATGTTAT---------TAATACTGACATAACTTAG  49757
Pig          AT-------ATCTA--T---G------T---ATATA--G---CTAATGCTAT---------TAATACTGGTGTAACTTAG  46250

Majority     CAATACCATGTAGCATTTTAGATGGCACAAATTGXTGTCATTTAGATAXTATTAGAAAAGCTTAGTA----TX-TAAXAA
                      71770      71780      71790      71800      71810      71820      71830      71840
Human        TGATATCTTGTAGCATTACAGATAGTAGAAATTGATGGCAT-----TAATATTAAGAAACCTTAATATT--TGTCATAAA  57037
PolarBear    CAATACCATGTAGCATTATAGATGGTGCAAATTGATGTCATTTAGATACTATTAAGAAAGCTTAAAT----TTATGATAA  19087
Cow          CAGTACCATATAGCATTTTAGATGACACAGATTAATGTCATAGAGATAGTATTAGAAAG-CTTAGTATTTGTGATAAAAA  53003
Dog          -----CCATGTAGCTTTATGGATGGCACAAATTGGTGTCATTTAGATATTATTAGGAAAGCTTAATA----TT----TGT  49493
BelugaWhale  CGATACCATATAGCATTTTAGATGGCACACATTATTGTCATTAAGGTAATATTCGAAAAGCTTAGTATTTATGATAAAAA  49146
Donkey       -AACTTCCTGTAGAATTTCTGGTTCCTTTAAGGGAGG--ATTTTGTTAGTGTTTTTTAATTGTAA----------GCCAG  54343
Cat          CAATACCATGTAGCATTGTAGATGGCACAAATTGGTGTCATTTAGATACAATTAGAAAAGCTTAGTA----TT-TGTGAT  56443
Ferret       CAATACCTTATAGCATTAT-GATGGCACAAATTGGTGTCATTTAGATACTATTAGGAAAGCT---------------TAA  56844
Lion         CAATACCATGTAGCATTGTAGATGGCACAAATTGGTATCATTTACATACAGTTAGAAAAGCTTGGTA----TT-TGTGAT  53883
Vaquita      CGATACCATATAGCATTTTAGATGGCACACATTATTGTCATTAAGGTAATATTGGAAAAGCTTAGTATTTATGATAAAAA  49837
Pig          ---TACCGTATAGCATTTTAAATGACACAGATTGCTGTCATTAAGATAATGTTAGAAAA-ACC--------TAGTAATAA  46318
```

```
Majority     AGAAATGTTTATTTTTAAGGATAGTCATATTTGTAGTTATCTATAATATAGGCTTXT-TTATTTTGCATTTTT-GTTTGA
                       71850     71860     71870     71880     71890     71900     71910     71920
Human        AGAAATATTTATTTTTAGGAA-AATCATAGTTGTAGTTAACCATGATGTGGGTTTCTATTATTTTACTTTTTTTTTTTTT  57116
PolarBear    AGAAGTGTTTCTTTTTAAGGATAGTTATATGTGTAGTTATCTATAATATAGACTTCTCTTATTTTGCATTTTT-GTTTGA  19166
Cow          GAAA-TGTTTATTTTTAACAATTGTCATATTTGTAGTTATCTATAATATAGGCTT---ATATTCTGCATTTTT-GTTTTA  53078
Dog          GATAGTGTTTCTTTTTAAGGATAGTTACATGTGTAGTTTTCTGTAATACAGGTTTCTGTTATTTTGCACTTTG-ATTTGA  49572
BelugaWhale  AGAAATGTTTATTTTTAACA---GTCATATTTGTAGTTATATGTAATATAGGCTT---ATAGTTTGCATTTGT-GTTTGA  49219
Donkey       GGAGATGTTCTGGGGAGGGGAGGAAGGGGAGTTTAGCTGTTTGTT-TATGAGCCA----GACTTTGCATTAACCCTTTTA  54418
Cat          AGAAATGTTTATTTCTAAGGATAGTTCTATCTATAGTTATCTATAATATTGACTTCTATTACTGTCCATTTTT-----GA  56518
Ferret       AGAAGTGTTTCTTTTTAAGGATAATGGTATGTGTAGTTATCTATA---CATACTTTTGTTATTTTGCATTTTTTGTTTGA  56921
Lion         AGAAATGTTTATTTCTAAGGATAGTTCTATCTATAGTTATCTATAAT--TGATTTCTGTTACTTTCCATTTTT-----GA  53956
Vaquita      AGA--TGTTTATTTTTAA-----GTCATATTTGTAGTTATATGTAATATAGGCTT---ATAGTTTGCATTTTT-GTTTGA  49906
Pig          AAAA-TGTTTATTTTTAACAATGTACATATTTGTAGGTATCTATAATATAGGCTT---GTATTTTGTGTTTTT-GTTT--  46391

Majority     TTTAGGTCCAAA-GT-----TTTXTGCTCTTGAGGATATAGCAGGATTATATTGTGCATGCACAC-XAGTXAAGATTTTA
                       71930     71940     71950     71960     71970     71980     71990     72000
Human        TTTTAATTTAAGGACAAAAGTTTGTACTCTTAAGAAAAAAGCAGGACTATATTGTGAATGTGCACCAGCATAAGATTTGA  57196
PolarBear    CTTAGGTCCAAA-GT-----TTTAAGCTCTTGAGGATATAACAGGATTAAATTGTGCGTGCACAC-CAGTAAAGATTTTA  19239
Cow          TCTAGGTCCAAATAT-----TTTGTGCTCTTGAGGATATAGCAGGATTATATTATGCATGCATACCTGTTTAAGATTTTA  53153
Dog          TTTAGGTCTGAA-AT-----TTTAAGCTCTTGAGGATATAGCAGGATTAAATTGTGCATGTACAC-TAGTAAAGATTTTA  49645
BelugaWhale  TTTAGGTCCAGAAGT-----TTTGTGCTCTTGAGGATATATCAGGATTGTATTGTGCATGCATACCTGTATAAGATTTTA  49294
Donkey       TTTTCACTCCTTCCT-----CCTGTGAACCTGACAATTTCT--GGTCTGATTCATGTGGTTGTTATCAGTGCAAACTCTG  54491
Cat          TTTAGGTCCAAA-GT-----TTTATGCTCCTAAGGATATGGCAGGAT-ATATTGTGCATGAACAC-CAGTAAAGATTTTG  56590
Ferret       CTTAGGTCCAAA-GT-----TTTAAGCTCTTGAGGATATAGCAGGATTAAATTGTACATGCACAC-CAGTAAAGATTTTA  56994
Lion         TTTAGGTCCAAA-GT-----TTTATGCTCCTGAGGATATGGCAGGAT-ATATTGTGCATGAACAC-CAGTAAAGATTTTG  54028
Vaquita      TTTAGGTCCAGAAGT-----TTTGTGCTCTTGAGGATATATCAGGATTGTATTGTGCATGCATACCTGTATAAGATTTTA  49981
Pig          ----------------------------------GATGTA------------CATGCATACCTG--TATTTAAGATTTTA  46423
```

```
Majority     AGTGTTTTCCAGTGGGAGTTTCAAAGTAATTAATTTAGCTATCAAGATAGTTTG-GCTGAAATATATCATTACA-CATGT
                      72010     72020     72030     72040     72050     72060     72070     72080
Human        AGTGGCTTTTGCTGGGAATTTCAAAGTAATT-----AGTTTTCAAAAGAGTTTG-GCTGAAATA------TACA-GATGT  57263
PolarBear    AATGTTTTCCAGTGGGAGTTTCACAGTAATTCATTTAGCTATCAAGACAGTTTG-GCTAAAGTATATCATCACG-CATGT  19317
Cow          CA--TTTTCCAGTGGGAGTTTCAAAGTACTTAATT-AGCTGT-AAGGTACTTTG-GCTGAAATATATCAGTACT-CATGT  53227
Dog          AGTGTTTTCCAGTA--ATT--------AATT----TAGCTATCAAGACCGTTTG-GCTAAAGTATATCATCACA-CATAT  49709
BelugaWhale  CA--TTTTCCAGTGGGAGTTTCAAAGTAATAAATTTATCTAT-AAGATAGTTTG-GCTGAAATACATCAGTAGA-CATGT  49369
Donkey       TTTGCTGTGCATTCACTATGTCTT--CTGTCCATTTGCTTCTGTTCGTTTTCTGTGTTTTAGGAGTTTGATAAA---TTT  54566
Cat          AGTGTTTTCCAGTGGGAGTTTCAGAGTAATTAATTTAGCTATCAAGACACTTGG-GCTGAAATA-ATCATCACA-CACGT  56667
Ferret       AGTGTTTTCCAGTGGGAGTTTCAGAGTAATT----TAGCTGTCAAGAGAGTTTG-GCTAAAATATATCATCACA-CATGT  57068
Lion         AGTGTTTTCCAGTGGGAGTTTCAG--TAATTAATTTAGCTATCAAGACACTTGG-GCTGAAATA-ATCATCACA-CATGT  54103
Vaquita      CA--TTTTCCAGTGGGAGTTTCAAAGTAATAAATTTATCTAT-AAGATAGTTTG-GCTGAAATACATCAGTAGA-CATGT  50056
Pig          AG--TTTTACAGTAGAAGTTTCAAAGTAATTAATTTAGCTAT-AAGGTAGTTTG-GCTGAAATATATTAATACAATATGT  46499

Majority     GGCTCAGATGCT-ATGATAGGCTCTCAGTTT-ATTTAAGACTACXXTXT-TTTAATATTCTTTTTAAXGATTTTTTTX--
                      72090     72100     72110     72120     72130     72140     72150     72160
Human        GGCTCAAATGCT-ATGATAGGCTCTCAGTTT-ATTTAAAACTAT----------ATATTCTCTTTAATGTCTTTTTTTT-  57330
PolarBear    GGCTCCGATGTT-ATGATAGGCTGTCAGTTT-ATTTAAGACTACAGTATATTTAATATTCTTTTTAAAGA----------  19385
Cow          GGCTCAAATGCT-GTAATAAGCTCTCAGTTG-ATTT-------C--------CGATATTCTTTTTAAGTATTTTTT----  53286
Dog          GGCTCAGACGTT-ATGATAGGTTCTCAGTTT-ATTTAAGACTATGGTATATTTAATATTCTTTTTAAGGTTTTTTTTTT  49787
BelugaWhale  GGCTCAGTT-CT-ATGATAGGCTCTCAGCT--ATTT-------C--------CAGTATTCTTTTTAAGTATTTTTTTA--  49428
Donkey       TTCTCGGCTACTGATGATCTCCTCTCTCTTCAGTGTTTGCATTTATTCTGTTTTGTATTTATTTATAGGTGTTTTAT---  54643
Cat          GGTTCAGATATT-ATGATAAGCTCTCAGTGT-ATTTAAGACTACAGTATGTTTAATACTCTCTTTAAAG-CCTTTTTTTT  56744
Ferret       GGCTCAGATGTT-ATGATGGGCTCTCAGTTT-ATGTAAGACTACAGTATATTTAATATTCTTTTTGAAGATTGTTTTAA-  57145
Lion         GGTTCAGATATT-ATGATAAGCTCTCAGTTT-ATTTAAGACTACAGTATATTTAATACTCTCTTTAAAGGCTTTTTTTT  54181
Vaquita      GGCTCAATT-CT-ATGATAGGCTC--AGCT--ATTT-------C--------CAGTATTCTTTTTAAGTATTTTTTTA--  50113
Pig          TGCTCAGATGCT-TTTATAGGCTCTCAGTTT-ACTT-------C--------CAGTATTTTTTTAAAATATTTTT-----  46557
```

```
Majority     ----AAGATAGGTTTTTTGAATAGGTTCXTAGTCTACTTTTTATTCACATCATTTACCTTTTT-GTX-XT--TGTTATAT
                      72170     72180     72190     72200     72210     72220     72230     72240
Human        -----A--AAGGTTTTATGAATAGGTTTCTAGTC-ACTTTTTATTCATATCATTTATCTTTTTTGTCAT---TGTTACAT  57399
PolarBear    ----------GCTTTTTTGAATAGGTTCTTAGTCTACTTTTTATTTACATCCTTTATATTTTTTAT------TGTTACAT  19449
Cow          ---AAAAATAGGTTTTTTCGATACGCTCTTGGTCTCCTTTTTATTCCAATCACTTACCTTTTT-GTTGTTGTTGTTGTAT  53362
Dog          TTTAAAGAACACTGTTCTGAAT-GGTTCTTAGTCTACTTTTTATTTACAACATACATATTTTT---------TATTATGT  49857
BelugaWhale  ---AAAGATAGGTTTTTTGAATAAGTTCTTAGTCTCCCTTTTATTCACATTACTTACCTTT---GTTGTTGTTGTTATAT  49502
Donkey       --------TGGGGTTTCACAGT-AGAAGAGAGGCAAGTGAATATTTGTGTTAACCATGAATTC-----------TTCCAT  54703
Cat          T---AAGACTGCTTTTTTGAATAGATTCCTGATCTGCTTTTTATTCACATCATTTACATTTTTTTT--GT--TGTTATAT  56817
Ferret       ---AAAGACAGCTTTTTAAAATAGGTTCCTAGGCTACTTCTTATTTACATCCTTTATATTTTT-AT------TGTTATAT  57215
Lion         T---AAGACTGCTTTTTTGAATACATTCCTGATCTGCTTTTTATTTCCATCATTTACGTTTTTTTTTTGT--TGTTCTAT  54256
Vaquita      ---AAAGATAGGTTTTTTGAATAAGTTCTTAGTCTCCCTTTTATTCACATTACTTACCTTTTTTGTTGTTGTTGTTATAT  50190
Pig          ---------AAGTTTTCTCAATAAGTCCCTAGTCTCCTTTTTATTCACTTCACTTACCTTTTA-GTTGCT------ATAT  46621

Majority     ATGGAGGGTAAGAAACTATTAAAA-GAAGTAAAAT-ATCTTCCCATATCATGGCCACCATTGAAAACTGCATACCAXATC
                      72250     72260     72270     72280     72290     72300     72310     72320
Human        ATGGAGGATGTAAGGTCATTGAGA-GATGTAAAAT----TTCCC--ATCAAGGCTGCCACTGAAAACTGCGCACCACATC  57472
PolarBear    ATGGAGGGTAAGAAACTATTAAAA-GAAATAAAAT-ATGTTCTCACATCATGGCCATCCTTGAAAACTGCATACCATATC  19527
Cow          ATGGAGGGTAAGAAACTATTCAAA-GCAGCAAAAT---TTGCTCATGTTACAGCTAGCATTGAAAACTGCATACCA----  53434
Dog          ATGGAGGGTGAGAAACTATTAAAA-GAAGTAAACTTATGTTCCCACATCATGACCACCTTTCAAAACTTCGTACCGTATC  49936
BelugaWhale  ATGGAGGGTAAGAAACTGTTCAAA-GCAGTAAAAT---CTACCC--GTCATAGCTACCACTGAAAACTGCATACCACGTC  49576
Donkey       A-ACCCTTTATCAAATCATTAGGT-CCTGTCAGTTCATTCTCCTTCATATCTGTCTAGTTTTAATCTTTCTTACTACTTC  54781
Cat          ATGGAGGATAAGAAACTACTAAAA-GAAGTAAAAT-ATCTTCCCATATCATGACCACTATTGAAAACTGCATATCATATC  56895
Ferret       ATGGAGGGTAAGAAACTATTAAAA-GAAGTAAAGT-ATGTTCTCATATTATGGCCACCCTTGAAAACTACATATCCTATC  57293
Lion         ATGGAGGATAAGAAACTACTAAAA-GAAGTAAAAT-ATCTTCCCGTATCATGACCACTATTGAAAACTGCATATCATATC  54334
Vaquita      ATGGAGGGTAAGAAACTGTTCAAA-GCAGTAAAAT---CTACCC--GTCATAGCTACCACTGAAAACTGCATACCACATC  50264
Pig          TTGAAGAGTAAGAAACTATTCAAAAGTAGTAAAAT---CTACCCATGTCATGGCTATCATTAAAAACTACATACCACCTC  46698
```

```
Majority     TTTGCTTCTAAATGCCTTAATCTTCTGTTTGTGTATCATATTT-AAATCTACCTTTGXTCTAXCTCTTTAACTTCTTGTG
                      72330     72340     72350     72360     72370     72380     72390     72400
Human        TTTGCTTCCAAGTACCTTAATCTTCTGTTTG-----TATATCTTAAATCTACTTTTGCTCTGTCTCTTTAACTTTTTGTG  57547
PolarBear    TTTGCTTCTAAATGCCTTAATCTTCCATTTGTGTATCGTATTTTAAATCTACCTTTGTTCTAGCTCTTTAACTTCTTGTG  19607
Cow          -------------------GTCTT---------TGTCTTATTT-AAATCTTCCTTTG-TCT----CTTTAACTTCTTGTG  53480
Dog          TTTGCTTCTAAATGCCTTAATCTTCTGTTTCTGTATCATATTT-AATTCTACCTTTGCCCTAGCTGTTTAACTTCTTGTG  50015
BelugaWhale  TTTGTTTCTAAATGC-CTAATCTTCTGTTTGTGTATCATATTT-AAATCTACCTTTT-TCTGTCTCTTTAACTTATTGTG  49653
Donkey       TGTAATTC-AGACACAATCACCTCTTGCCTATAT-TACTATAATAGCTCTCTTGCTAATTTATTTTCTTTCCTTTCTCTC  54859
Cat          TTTGCTACCAACTGCCTTAATCTTCTATTTGTGTATCGTATTT-AAATCTACCTTTGCTCTAGCTCTTTAACGTCTTGTG  56974
Ferret       TTTGCTTCTAAATGCCTTAATCTTAAGTTTGTGTATCATATTT-AAATCTGCCTTGTATCTAGTTCTTTAAGTTCTTGGG  57372
Lion         TTTGCTACCAACTGCTTTAATCTTCTATTTGTGTATCGTATTT-AAATCTACCTTTGCTCTAGCTCTTTAACGTCTTGTG  54413
Vaquita      TTTGTTTCTAAATGC-CTAACCTTCTGTTTGTGTATCTTATTT-AAATCTACCTTTT-TCTGTCTCTTTAACTTCTTGTG  50341
Pig          TTTGCTTCTAAATGC-TTCGTCTTCTGTCTAGGTGTCTTATTT-AAATCTACCTTTT-TCTGTCGCTTTAACTTCTTATG  46775

Majority     CCATATTATCTCATTGTXATTATTATTCCATTATTAAACCATTAAATATTCATTATTAAACCATTAC-------------
                      72410     72420     72430     72440     72450     72460     72470     72480
Human        CCATAGTATCTCATTATTATTACTATTCCATTATTAAGCCATTAAAAATTCATTATTAAGCCATTATTAT---------G  57618
PolarBear    TCATATTATCTCACTGTGATTATTATTCTGTTGTTAAACCATTAAATATT----ATTAAACCATCAC-------------  19670
Cow          CTGTAGTATAATATTGTTATTTTTATTCCACTATTAAAAAATTAAATATTTATTAA------------------------  53536
Dog          TCATATTATTTCATTGTGATTAT---TCCATTGTTAAACCATTAAATATTCATTGTTAAACCATTAC-------------  50079
BelugaWhale  CTGTAGTATCTCATTTTTA------TTCCATTATTAAAACATTAAATATTTATTATTA----------------------  49705
Donkey       TTGAATTCTTTCTCCCTGGT--ACAGTCAAGTATATATCTTTCTAAAATGCACAAATCTGACTTAA--------------  54923
Cat          CCGTGTTATCTCATTGTGATTATTATTCTGTTGTTAAATGATTAAATATTCATTATTAAACCATTAC-------------  57041
Ferret       TCATATTTTTTCATTGTAATTATTATTCTGTTGTTAAACCATTACATATTCATTATTAAACCATTAC-------------  57439
Lion         CCATGTTATCTCATTGTGATTATTATTCTGTTGTTAAACGATCAAATATTCATTATTAAACCATTAC-------------  54480
Vaquita      CTGTAGTATCTCATTTTTA------TTCCATTATTAAAACATTAAATATTTATTATTA----------------------  50393
Pig          CCATATTATCTCATTGTTA---TTATTTCATTATTAAACCATTAAACGTTTATTACTATACCATTGTTATTAAACAATTA  46852
```

```
Majority     -ATCAACTGTCTXTACTTAXATTCCTTX------X--CTTACCTCTTTTTTCTCCCTC-TTACTCTTACTTTCAGACTAA
                      72490     72500     72510     72520     72530     72540     72550     72560
Human        AAACCATTTAATAAGCTTACATTTATCT------TAACTGGTTTTCTTCCTTTCATTCATT-CTTTTACTTTCAGATTAA 57691
PolarBear    -ATCAACTGTCTTTACTTATCTTCCTTTACCTTCTGCCTTACCTCTTTTT-CTCCCTT-TTACTCTTACTTTCAGACTAA 19747
Cow          --------GTGTGT--GTAAAGTGTT-------------AGTCACTT----------------------------AGTCG 53565
Dog          -ATCAACTGTCTTTACTTATCATCTTTTGCCCTTTATCTTACCTCTTTTT-CTCCCTC-TTACTCTTATTTTCAGACTAA 50156
BelugaWhale  AATCAACTGCCTGTACTTATATTTGTC-----------TTACCTTTTTTCTCCCTTTC-ACACTTCTACTTTCAGACTAA 49773
Donkey       -GTTACTCCCCTGCAGCAGGTCCCCTTCATGCTCAGGATAAAGGCCCTTTCTGAGATGGCTACTGTTTCTCCTACCCTAT 55002
Cat          -ATCAGCTGTCTT-----CCTTTACCTT--C---TGTCTTACCTCTTTTTTTTCCCTT-TTACTCATACTTTCAAA-TAA 57108
Ferret       -ATCAACTGTCTTTACTTACCTTCCAT----------CTTGACTCTTTTT-CTCCCTT-TTACTCTTACTTT-AAACCAA 57505
Lion         -ATCAGCTGTCTTATCTTCCTTTACCTT--C---TGTCTTACCTCTTTTTTCTCCCTT-TTACTCATACTTTCAAA-TAA 54552
Vaquita      AATCAACTGTCTGTACTTATATTTGTC-----------TTACCTTTTTTCTCCCTTTC-ACACTT--ACTTTCAGACTAA 50459
Pig          AATCAACTGTCTGT--TTAAATTGTTC-----------TAACCTCCTTTCTCCCTGTC-ATCCTTTTACTTTCAGAGTAA 46918

Majority     TTAT-GGTCTGTAATACTTCAGTGCTTCCTCTAGATCAAGATAGATTGT----ATGGAACCCAGACCATTTTTGATT---
                      72570     72580     72590     72600     72610     72620     72630     72640
Human        TTAT-TGTCTCTAATACTTAAATGCTTCCTCCAGATCAAGATTACTTCT----GTGGAGCCCAGACCATTTTTTACAATT 57766
PolarBear    TTAT-GGTGTGTAATACTTCAGTGCCTCCTCTAGATTAAGATAGATTGT----GTGGAACGCAGACCATTTTTGATT--- 19819
Cow          TGTC-TGACTGT--T--TGCAAC----CCCGTGGACTATAATAGCCTGC----C------CCAGGCTTCTCTG------- 53619
Dog          TTAT-GTTCTGTAATACTTCAGTGCTTCCTCTAGATTAAGATAGATTGT----ATGGAACCCAGACCATCTTTGATT--- 50228
BelugaWhale  TTAT-GGTCTAT--T----CAATACTTCCTCCAGATCAAGATAGGTTGT----ATGGAA-CCAGGCCATTTTTAATTATT 49841
Donkey       TTGCCTGTGCGTC-TATCCCAGAAGTAATTGTAGATCCTCAAACATGGC----ATGCT--TTCTCTTGTTTCAATC--- 55072
Cat          TTAT-GGTCTGTAATACTTCAGTGCTTCCTCTAGATCAAGATAGATTGT----ATTGAACCCAGACCATTTTTGATT--- 57180
Ferret       TTAT-GCTGTGTAATACTTCAGTGCTTCCTCTAGATTAAGATAGATTGATTAAATGGAACTTAGACCATTTTTGATT--- 57581
Lion         TTAT-GGTCTGTAATACTTCAGTGCTTCCTCTAGATCAAGATAGATTGT----ATTGAACCCAGACCATTTTTGATT--- 54624
Vaquita      TTAT-GGTCTAT--T----CAATACTTCCTCCAGATCAAGATAGGTTGT----ATGGAA-CCAGGCCATTTTTAATTATT 50527
Pig          TTAT-TGACTAT--TACTTCATTGCTTCCTCCAGATCAGGATAGCTTGT----TTGGTA-CCAGACCATTTTTAATTATT 46990
```

```
Majority     GTTTCATTGTXTTTCCATCCTCAGAATCTCATTTGGGGAGCAAAAATAGCTTCAGGTAGCAGAAAGXCTATACXXGTAAT
                       72650     72660     72670     72680     72690     72700     72710     72720
Human        GTTTCATTGCATTTCCATCCACACAACCTCATTTTGGGAGCAAAATTAGTTGTAGGTAGCAGAAAGGCTATACAAGTAAT  57846
PolarBear    GTTTCATTGTGTTTCCATCCTCAGAATCTCATTTGGG------------------TAGCACAAAGTCTGTACTGTTAAT  19880
Cow          --TCCATGGAATT-CTCCC-------------------GGCAAGAATA--TTGGAGTGGTTAAACCATTAAATCAGCCAT  53675
Dog          GTTTCATTGTGTTTCCATCCTCAGAATCTCATTTGGGGAGC----AAA-----AAATAGCACAAAGTCTGTACCGTTAAT  50299
BelugaWhale  GTTTCATTGTATT-CCATCCTTAGAATCTCATTTGGAGAGCAAAAATAGCTTCAGGTAGCAGAAAGGCTTTACTAGTAAT  49920
Donkey       -TTTTGAGGCCACTGTGTCTCTGTGATGTCACTTTTG--CCTAGAATA--TTGTGGCCTGCTTGCTTTTGTGCCTGTTTT  55147
Cat          GTTTCATTGTGTTTCCAACTTCAGCATATCATTTGGGGAGCAAAAATAGCTTCAGATATTTTTG---CTATACCGTTTAT  57257
Ferret       GTTTCATTGTGTTTCCATCCTCAGAATCTCATTTGGGGAGCTGATATAGCTTCAAGTAGCACAAAGTCTGTACTGTTAAT  57661
Lion         GTTTCATTGTGTTTCCAACTTCAGCATGTCATTTGGGAAGCAAAAATAGCTTAAGATATTTTTG---CTATACCGTTTAT  54701
Vaquita      GTTTCATTGTATT-CCATCCTTAGAATCTCATTTGGGGAGCAAAAATAGCTTCAGGTAGCAGAAAGGCTTTACTAGTAAT  50606
Pig          GTTTCATTGCATT-CCACCCTGAGAATCTCATTTGGGGATCCAAAATAGCTTCAGGTAGCAGAAATGCTATACTAGTAAT  47069

Majority     ATTGGC-ATAATTGAAATGG---TTTACCAAGTGCTGTTATCTCA-TTTTTTXCTAATAXX-------T-----------
                       72730     72740     72750     72760     72770     72780     72790     72800
Human        AATAGC-ATAATTGTAATAG---TTTACCAAGTGCCGTAGTCTCA-TTTTTTCTGACCACT-------------------  57902
PolarBear    ATTGGC-ATAATTGAAATGG---TTTAGCAAGTGCTGTTATCTCA-TTTTTTTCTGGTATT-------T-----------  19937
Cow          CTTC----CACTTAAAATGGT--CTTACCTCTTTTTTCTCTCTTT-CACTCTTTTACTTTCAGACTGATTATGGTATATT  53748
Dog          ATTGAC-ATAATTGAAATGG---TTTAGCAAGTGCTGTTATGTCAATTTTTTCCTGATATT-------T-----------  50357
BelugaWhale  AATAAC-ATAATTGAAATGG---TTTACCAAGTGCTGTTACCTCA-TTTTTTTCTG---------------------ACT  49974
Donkey       CTACCCTACACTTCACATGGCCAGTTGCTAGACATCTTCAGGTCTCCCCTTAGCTA------------------------  55203
Cat          ATTGGC-ATAATTGAAATG----TTTACCAAGTGCTGTTATCTCGTTTTTTTCCTAATA---------T-----------  57312
Ferret       ATTGGC-ATAATTGAAATGG---TTTAGCAAGTGCTGTTCTCTCA-TTTTTTTCTGATATT-------TC------TGAT  57723
Lion         ATTGGC-ATAATTGAAATG----TTTACCAAGTGCTGTTATCTCGTTTTTTTCCTAATA---------T-----------  54756
Vaquita      AATAAC-ATAATTGAAATGG---TTTACCAAGTGCTGTTACCTCA-TTTTTTTCTG---------------------ACT  50660
Pig          AATAAC-ATAATTGAAATGG---CTTACCCAGTGTTGTTAGCTCA-TTTTTTCTGACTATTTTGTAAATAGAACAGGATT  47144
```

```
Majority     X-T-XA--TGTTGTAAACAGAA--CAGGAATTTTTCCTG---------------------------TGTTACAAGGGGAT
                      72810     72820     72830     72840     72850     72860     72870     72880
Human        ACTCTA--TATTGTCAACAAAA--CAGGGTTTTCCCCTG---------------------------CGTTACAAGAGGAT  57951
PolarBear    ----TG--TGTTGTAAACAGAA--CAGGAATTTTTCCTG---------------------------TGTTACATGGGGGG  19982
Cow          ACTTCA-GTGCTACCTCCAGAT--TAAAATAACTTGTTT----GAAACCAGGCCATTTTTAACTACTGTTTCATTTCCAT  53821
Dog          ----TA--TGTTGTAAACAGAA--CAAGAATTTATCCTT---------------------------TGTTACAAAGGGAA  50402
BelugaWhale  ATTCCA--TGTTATAAACAGAG--CAGGAATTTTTCCTG-------------------------T--GTTACAAGGGGAT  50023
Donkey       ---------GTTTCCTCCAGAA----AGCCATCCTCTTA----------------------------TCCACAAAGAGCG  55242
Cat          ---------GTAGTCAACAGAA--CAGGAATTATTCTTG---------------------------TGTTACAAGGGGAT  57354
Ferret       ATTTTA--TGTTGTAAATAGAA--CAGGAATTTTTCCTG---------------------------TGTTACAAGGGGAG  57772
Lion         ---------GTAGTCAACAGAA--CAGGAATTACTCTTG---------------------------TGTTATAAGGGGAT  54798
Vaquita      ACTCCA--TGTTATAAACAGAG--CAGGAATTTTTCCTG-------------------------T--GTTACAAGGGGAT  50709
Pig          TTTTCTTATGTTACAAAGGGATGCTAAGATTTTTTCTTTTTTTGTCTTTTTGCCATTTCTTGG-GCCGCTGCCGCGGCAT  47223

Majority     GCTAAGATTTAAAG-----------------AGGTTAAATTACXTACA------X-------------------------
                      72890     72900     72910     72920     72930     72940     72950     72960
Human        GCTAAGATTTAAAG-----------------AGGT-GAATTACTTAC---------------------------------  57980
PolarBear    GCTAATATTTAAAG-----------------TAAATAAATTACTTATC------AT------------------------  20015
Cow          TCTTAGAATCTCA--TTTGGGGAGCAGAAGTAGCTTCAGGTAGCAGAA-AGACTTTACTAGTAATAATAGCATAAT-TGA  53897
Dog          GCTAATATTTAAAG-----------------AGGTTAAATTACTAATC------GT------------------------  50435
BelugaWhale  GCTAAGATTTAAAG--AGGCAAATTACTTATAATGTACACTATCTGTAAAGTTGACTTTTTTTTTTTTTTTTTGCG-GTA  50100
Donkey       GATTAGGTTTTGGTTAAGAACCTTTAGTAGAAATTAAAATCACCGTGA--------------------------------  55290
Cat          GCTAATATTTAAAG-----------------AGGT-AAATTACTTAC---------------------------------  57383
Ferret       GCTAATATTTAAAG-----------------AGGTAAAAACTAGAACT------TA------------------------  57805
Lion         GCTAATATTTAAAG-----------------AGGT-AAATTACTTAC---------------------------------  54827
Vaquita      GCTAAGATTTAAAG--AGGTAAATTACTTATAATGTACACTATCTGTAAAGTTGAC------------------------  50763
Pig          ATGGAGGTTCCCAGGCTAGGGGTCCAATTGGAACTGTAGCCACCGGCCTACGCCACAGCCACAGCAACACCAGATCCTTA  47303
```

```
Majority     --------------------------------------------------------------------------------
                       72970     72980     72990     73000     73010     73020     73030     73040
Human        --------------------------------------------------------------------------------  57980
PolarBear    --------------------------------------------------------------------------------  20015
Cow          AATGATTTACCAAGTGCTATTATCT------CATTCTTTTTCTGACTACTCTGTATTGTAAACT---GAACAGGAATTTT  53968
Dog          --------------------------------------------------------------------------------  50435
BelugaWhale  CGCGGGCCTCTCACTGTTGTGGCCT---CTCCCGTTGCGGAGCGCAGGCTCAGCGCCCATGGCT-C---ACGGGCCCAGC  50173
Donkey       --------------------------------------------------------------------------------  55290
Cat          --------------------------------------------------------------------------------  57383
Ferret       --------------------------------------------------------------------------------  57805
Lion         --------------------------------------------------------------------------------  54827
Vaquita      --------------------------------------------------------------------------------  50763
Pig          ACCCACTGAGCAAGGCCAGGGATCGAACCTGCAACCTCATGGTTCCTATTTGGATTCATTAACCACCGAACCACAATAGG  47383

Majority     -------------------------------------------AATXTACACTATATGTAAGGTAGACATTTTTTCAGTC
                       73050     73060     73070     73080     73090     73100     73110     73120
Human        -------------------------------------------AGTGAACACCACCTATAAGGTAGACATTTTTTCAGTC  58017
PolarBear    -------------------------------------------AATACACACTATATGTGAGGTAGACATATTTTCAGCC  20052
Cow          TCCTGTTAGCAGATACTAAGATTCAAAGAGGTAAATGATTTATAATGTACAC-ATCTGTAAAGTAGACATTTTTTCAGTC  54047
Dog          -------------------------------------------AATACACACTATGTGTAAGGGAGACATATTTTCAGCC  50472
BelugaWhale  CGCTCCGCGGCATGTGGGATCTTCCCAGACCGGGGCACGAACCCGTGTCCCCTGCATTAAAAGTTGACATTTTTTTAGTC  50253
Donkey       -------------------------------------------GATAC-TACCATGTCCTAC-CAG--ATTGGCTAAAAT  55323
Cat          -------------------------------------------AATGTTCACTATATGTAAGGTAGACATATTTTCAGTC  57420
Ferret       -------------------------------------------AAGAGGTAAATTATTTGAGGTAGACATATTTTCAGCC  57842
Lion         -------------------------------------------AATGTTCACTATATATAAGGTAGACGTATTTTCAGTC  54864
Vaquita      --------------------------------------------------------------------ATTTTTTTAGTC  50775
Pig          AACT-CCAAGGGGTGCTAAGATTTAAAGAGGTAAATTACTTATAAAATACACCATCTTTAAGGCAGACATTTTTT-AGTC  47461
```

```
Majority     TX----XTTTTCTCCAGTATGTTGCTACTCACTGGTTCTXAAGTTACA--AXCTAATCTTATTATATTTTCCTTTTCAGG
                       73130     73140     73150     73160     73170     73180     73190     73200
Human        TTAGCCTTTCTCTCCAGTATGCTGCTTCTCACTGGTTCTCGGGT-ATA--ATCTAATCTTACTGTGCTTTCCGCTTCAGG 58094
PolarBear    TG----TTTTTCTCCAGTATGTTGCTACTCACTGATTCTTAAGTTATG--ACCTAATCTTATTATATTTTCCTTTGCAGG 20126
Cow          TA----CTCTTCTCCAGTATGTTTCTACTCAGTGGTTCCCAATT-GCA-----TGATCTAATCATACTTCCCTTTTCAGG 54117
Dog          TG----T-----T----------GCTACTCACTGGTTCTTAAGTTACATGATCTAATCTTATTATATTTTCCTTTTCAGG 50533
BelugaWhale  TA----CTTTTCTCCAGCGTGTTCCTACTCACTGGTTCCCAATT-GCA-----TGATCTAATCCTATTATGTTTCCCTTT 50323
Donkey       TA----AGAGGGGTAAA-----TACCAAGTGTTGGTAATAATGTAGAGAAGCTTGAATTC-TCATATGTAAGTGATGGGA 55393
Cat          TG----ATTTTCTCCAGTGTGTTGCTACTCACTTGTTCTTAAGTTACATGATCTAATCTTATTATATTTTCCTTTTCAGG 57496
Ferret       TG----TTTTTCTCCAATATGTTGCTACTTACTGGTTCTTAAATTACATTACCTAATCTT-CTATATTTTCCTTTTCAGG 57917
Lion         TG----ATTTTCTCCAGTATGTTGCTACTCACTTGTTCTTAAGTTACATGATCTAATCTTATTATATTTTCCTTTTCAGG 54940
Vaquita      TA----CTTTTCTCCAGTGTGTTCCTACTCACTGGTTCCCAATT-GCA-----TGATCTAATCCTATTATGTTTCCCTTT 50845
Pig          TA----CTTTTCTCAG---TGTTGCTACTCACTGGTTCCCAGTTTGCA-----TGATCTTATTATATTTCTCTTTCCAGG 47529

Majority     CATACATTAACAXT--TAGAATTCTTTTTTCCAA----AAATAATGATTTC-TCTTXACATGCCCCAGCCCTCCAACTTT
                       73210     73220     73230     73240     73250     73260     73270     73280
Human        CATAGGTTAATAAT--TAGAATTCTTTTTTTTTG-TCCAAAAAATGA-T---------TATCCTCCTTTCACATGCCCT- 58160
PolarBear    CATACATTAACACT--TGGAATTCTTTTTTCCAA----AAATAATGATTTC-TCTTTACATGCCCCAGCCCTCCAACTTT 20199
Cow          AATAAATTAGCAATTATAAAGTTGTT-TTTTCAA----AAATAATCAACTT-TCCTAACATGCTTCAGCCCTTCAACTTT 54191
Dog          CATACATTAACACT--TGGAATTCTTTTTTCCAA----AAATAATGATTTC-TCTTTACATGCCCCAGTCCTCCAACTTT 50606
BelugaWhale  ------TCAGCAAT--TAGAGTTCTTCTTTAAAA----AAATAATTGTCTT-TCCTAACATGCCCCAGCCCTCCAACTTC 50390
Donkey       ---ATGTAAAT--TGATAGAATCATTTTTAAAAACTTGAAATATCTGTTAAAGCTGAGCATATACATTTTCTTTGACTTA 55468
Cat          CATACATTAACACT--TGGGATTCTTTTTTCCAA----AAATACTGATTTC-TCTTTACATGTCCCTGACCTCTAACTTT 57569
Ferret       CACACATTAACATT--TGGAATTCTTTTTTCCAA----AAATAATGATTTC-TCTTTACATGTCCCAGCCCTCCTACTTT 57990
Lion         CATACATTAACACT--TGGGATTCTTTTTTCCAA----AAATATTGATTTC-TCTTTACATGTCCCTGACCTCTAACTTT 55013
Vaquita      ------TCAGCAAT--TAGAGTTCTTCTTTAAAA----AAAGAATTGTCTT-TCCTAACATGCCCCAGCCCTCCAACTTT 50912
Pig          AATAAGTTAAC-----TAGAGTTCTT-TTTATCA----AAATAAT-------TGTAAACATGCTGCAGCCCTCCACCTTT 47592
```

Majority	GGAGA-TATTTTAT--ATTTAAAGAGTTACCAAAATCCATGTGGTATGAGGTTTGATCAGTAGCAGAC--AATCTAGAAG	
	73290 73300 73310 73320 73330 73340 73350 73360	
Human	------------------------GGCTACCAAAATCAATGTGGTATGAGGTTTTATGAATAGGAGAC--AATCTAGAAG	58214
PolarBear	GGAGA-TATTTTAT--ATTTAAAGAGTAACCAAAATCCATATGGTATGAGGTTTGATCAGTAGCA------ATCTAGAAG	20270
Cow	GGAGA-TAA-----------AAATAGATACCAAAATAATTGTGGTATGAGGTTTGATCAATAGCAGAC--AATCTAGAAG	54257
Dog	GGAGAATATTTTATGTATTTAAAGAGTAACCAAAATACATGCGGTATGAACTTTGATCAGTAGCAGAC--AGTCTAAAAG	50684
BelugaWhale	GGAGA-TATTTTAT--ATTTAAAGAGTTACTGAAATCATTGTGGTATGAGGTTTGATCAGTAGCAGAC--AATCTAGAAG	50465
Donkey	GTAAATCTCCTTCT--AGTTACATACCTGAGAAAATACATGCA-TATGTACGTCAAAAGATATGTATGGGAAAGTTCATG	55545
Cat	GGAGA-TATTTTAT--ATTTAAAGAGTAACCAAAATCCATGTGGTATGAGGTTTGATCAGTAGCAGAC--GGTCTAGAAG	57644
Ferret	GGAGA-TATTTTAT--ATTTAGAGAGTAGCCAAAATCCATGTGGTATGAGGTTTGGTCAGTAGCAGAGAAAATCTAGAAG	58067
Lion	GGAGA-TATTTTAT--ATTTAAAGAGTAACCAAAATCCATGTGGTATGAGGTTTGATCAGTAGCATAC--GGTCTAGAAG	55088
Vaquita	GGAGA-TATTTTAT--ATTTAAAGAGTTACTGAAATCATTGTGGTATGAGGTTTGATCAGTAGCAGAC--AATCTAGAAG	50987
Pig	GGAGA-TATTT-AT--ATTTAAAGGGTTACCAAAATCATTGTGGTATGAGGTTTGATCAATAGCAGAC--AGTCTAGAAG	47666

Majority	CCATGATCTTTATGGCATTTTTCACA------------ACTTCAXAACAXATTTAAA-TATAGTGCATXTTTTT-CTTAA	
	73370 73380 73390 73400 73410 73420 73430 73440	
Human	TCATGGTTTCTGTGACATTTTTCACA------------ACTTCA---AACGTTTAAA-TATAGTCTACATTTTT-CTTGG	58277
PolarBear	CCATAATCTTTATGGCATTTTTTTTTTGC--------AACTTCACAACATATTAGAA-TATAGTGCATGTTTTT-CTTAA	20340
Cow	CCATGTTCTTGATGGCATTTTTCACA------------ACTTCA-AACAAATTTAAA-TATAGTGCACATTTTT-CTTAA	54322
Dog	TCATGATCTTTATGGCATTTTTT--GC----------AACTTCACAACATATTTAAAATATAGTGTATGTTTGT-CTTAA	50751
BelugaWhale	CTGTGTTCTCGATGGCACTTTTCACA------------ACTTCA-AACAAATTTAAA-TATAGTGCACATTTTT-CTTAA	50530
Donkey	GCATACTATATGTAATAGCCCAAAGCT----------GGAAACAGTCCAAAGTCCAAATATGTTCAGCAGCAGAATATAT	55615
Cat	CCAGGATGTTTATGGCACTTTTCACA--------------GCTTCCAAATATTTAAA-TATAGTGCATGTTTTT-CTTAA	57708
Ferret	CCATGATCTTTATGGCATTTTTTTTTCCCCC------ACCATGACAACATATTTAAA-TATAGTGCAT--TTC--CTTAA	58136
Lion	CCAGGATGTTTATGGCACTTTTCACACTTCCAAATATTTCACTTCCAAATATTTAAA-TATAGTGCATGTTTTT-CTTAA	55166
Vaquita	CCGTGTTCTCGATGACACTTTTCACA------------ACTTCA-AACAAATTTAAA-TATAGTGCACATTTTT-CTTAA	51052
Pig	CCATGTTCTC---GGCAATTTTCATA------------GCTTCA-AACAAATCTAAA-TATAGTGCATGTTTTT-CTTAA	47728

```
Majority     TGTGGAATATTTT----TTGTC--ATCCCTTCTGATXCAGAGGGAA-GAGTA----TTTGGTAAAGG-ACCAATGAAATG
                               73450     73460     73470     73480     73490     73500     73510     73520
Human        TGTGGAATATTTT----CCTTG----CTCTTCTGACCCCAAAGAAA--AGTA----TTTGTTAGAGG-ACCAATTAAATG  58342
PolarBear    TGTGTAATATTTT----TTGTG--ATCCCTTCTGAGGCAGAGGGAA-GAGTA----TTTGGTAAAAG-ACCAATGAAATG  20408
Cow          TGTGGAATATTTTCTTTTTGTCA-TTCCCTTCTGATCCAGAGGAAC--ACAC----TTTAGTAAAGG-ACCAGTGAAATG  54394
Dog          TGTGGAATATTTT----CTGTC--ATCCTTTCTGATGCGGAGGGAA-GAGTA----TTTGGTAAAGG-ATCAATGAAATG  50819
BelugaWhale  TATGGAATATTTTC--TTTGTCA-TTCCCTTCCGATCCAGAGGAAG--AGTA----TTTGGTGAAGG-ACCAGTGAAATG  50600
Donkey       TGTGGTATATTCATTCATTGAA--ATTCATTGGAATACACAAAGAGTGAATAAGCTCCTAGTACCTGCAGTAATACATGG  55693
Cat          TGTGGAATATTTT----TTGTC--ATCCCTTCTGATGGAGAGGGGAAGAGTA----TTTGGTAGAAG-G-CAATGGAATG  57776
Ferret       TGTGAAATATTTT----TTTTATCACCTCTTCTGGTGCAGAGGGAA-GAGTA----TTTGGTAAAAG-ACCAATGAAATG  58206
Lion         TGTGGAATATTTT----TTGTC--ATCCCTTCTGATGGAGAGGGGA-GAGTA----TTTGGTAGAAG-G-CAATGGAATG  55233
Vaquita      CATGGAATATTTTC--TTTGTCA-TTCCCTTCCGATCCAGAGGAAG--AGTA----TTTGGTGAAGG-ACCAGTGAAATG  51122
Pig          CGTGGAATATATT---TTTGCAA-TTCCCTTCTGATCCAGAAGACT--A-------TTTGGTAACGG-ACCAATGAAATG  47794

Majority     GAATTTGATTTGGAATTATACTGCATTTGGGCTATA--ATTCAATGGGGAAAGTAGTTTATATGTGTXTTTTGTCTGAXG
                               73530     73540     73550     73560     73570     73580     73590     73600
Human        AAATTTGAGTGGGGATTATACTGTGTTTGAGCTATAATTCTTAA-GGGGAAAATTGTTTATATGTGTATCTTGTCTGAAG  58421
PolarBear    GAATTTGATTTGGAATTATACTGCACTGGGGCTATA--GTTCAGTGGGGAAAGTAGTTTATATGTGTGTTTTGTCTGAAA  20486
Cow          GAATTTGTTTCAGAATTGTACCTCATTTGGGCTATGTGATTCAATGGGGAAAGTAGTTTATATATATATTTTT---C--A-G  54468
Dog          GAATT------------ACACTGTGTTTGGGCTGTA--ATTCAGTGGGGAAAGTAGTTTATATGTGTGTTTTGTCTGAAG  50885
BelugaWhale  GAATTTGATTCAGAATTATACTGCATTTGGGCTATACAATTCAGTGGTTAAAGTAGTTTATATATATATCTTT---C--A-G  50674
Donkey       ATGTGCGTCTTAAAAATAACGTTTCTTGAAAGAAGCCAGACCAACGTAGATCACATACTAT--ATG-ATTCCATTT-ATG  55769
Cat          GAATTTAATTTGAAATTACACTGCATTTGGGGTATA--ATCCAATGGGGAAAGTAGTTTAT--GTG--TTTTGTCTGAGG  57850
Ferret       GAATTTGATTTGAAATTACACTATATTTTGGCTATA--ATTCAA-GGGGAAAGTAGTTTATGTGTGTGTTTCATCTGAAA  58283
Lion         GAATTTAATTTGAAATTACACTGCATTTGGGGTATA--ATCCAATGGGGAGAGTAGTTTAT--GTG--TTTTGTCTGAGG  55307
Vaquita      GAATTTGATTCAGAATTATACTGCATTTGGGCTATACAATTCAGTGGTTAAAGCAGTTTATATATATATCTTT---C--A-G  51196
Pig          AAATTTGAATTGGAATTATACTACATTGGGGCTATATAATTCAGTGGGGAAAGTAGTTTATATATATATCTTC---C--A-G  47868
```

```
Majority     TAATTTATAXXX--ATXAXXACAAAAGXTTTTCCTTTAATT-T-TT----CAX-X-XTTGAAGTTTT-ATTTAACCAGTC
                      73610     73620     73630     73640     73650     73660     73670     73680
Human        TAATACATAGTTACATTAAATCTCATAAAAGTACCTCAATTGTGT-----CAACTGTTTGACATTTTTGTTTA---A-TC  58492
PolarBear    TAATTTATAGTA--ATCATGACAAAAGATTATGCTTTAATAATGTTA--TCAGGTATTTGAAGTTTTCATTTAACCAATC  20562
Cow          TATTTAAT-----------------AGGTTTCATTTAACC--------------------GATCTTC--T--GCACAGA-  54507
Dog          TAATCTACAGTA--ATCATGACAAAAGGTTGTACTTTAATG---TTA--TCAGGTGTTTGAAGTTTTCATTTACTCCGTC  50958
BelugaWhale  TATTTGAT-----------------AGGTTTCTTTTTT------------------------------------------  50696
Donkey       TAACTGGGAAAA--AGCATGGGTGTGGCTTCTGGCATGTTGGTAAT--------------ATTCTTTTATCTAGATAATC  55833
Cat          TAATCTATAGTA--ATGAGAACAAAAGATTCTACTTTAATTATGTTA--TCAGATATTTGAGGTTTAAATTTAACCAGTC  57926
Ferret       TAATCTATA-----ATCATGACAAAAGATTATCTTTTAATAATCTTAAATCAGACACTTGAAGTTTT-ATTTAACCAATC  58357
Lion         TAATCTATAGTA--ATGAGAACAAAAGATTGTACTTTAATTATGTTA--TCAGATATTTGAGGTTTAAATTTAACCAGTC  55383
Vaquita      CATTTGAT-----------------AGGTTTCTTTTTTCT--------------------TTTTTTT--T----------  51228
Pig          TATTTG-------------------AGGTTTCATTTAACC--------------------AATCTTC--TCTGCATAGA-  47907

Majority     XTCTGTGTA-AGATAXTAAGCATC-AGGTATTT-GTGAAXGT-TATACACA----CTCACTCAGGTCTTTTCTACAAAGA
                      73690     73700     73710     73720     73730     73740     73750     73760
Human        TTCTGTGAACAGATACTAAGCATCCAACTATGATATTGATGTTTGTGCACAGGTATGCACTCAGGTCTTCTTGACAAAGA  58572
PolarBear    TTCTGTGTACAGATAATAAGCATC-AGGTATTT-GTGAATGT-TATATACA----CTCACTCAGGTCTTTTCTACAAAGA  20635
Cow          ----AAATA--AGCATTAAACTAC-TGGTGTTG-GTGAATGTTTACATACA----CTCA----GGTCTTCTCTACAAAAA  54571
Dog          TTCTATGTACAGATAATAAACATC-AGGTATT--GTGAACAT-TA-ATAC--------ACTCAGGGCTTTTCTGTAAAAA  51025
BelugaWhale  -----------TTTTTTTTTTTTT-GTGCGGTG--TGCGGGCCTCT-CACT----GTTGC---GGCCTCTCCCAT-----  50749
Donkey       AT-TTTACAGATATGTCCACTTTG-TGATATTTTATTGAGCTGTATACACT----TAGAATTTGTGTCCTTTTCTATATA  55907
Cat          GTCTGTGTA-GGATGATAAGCATC-AAGTATTT-GTGAGGGT-TACACTC--------AATCATGTCTTCTCTACAAAGA  57994
Ferret       TTCTATGTACAGATAATAAGCATC-AGGTATTT-GTGAATAT-TGAATACA----CTTACTCAGGTCTTTTCCACAAAGG  58430
Lion         GTCTGTGTA-GGATAATAAGCATC-AAATATTT-GTGAGGGT-TACACTC--------AATCATGTCTTCTCTACAAAGA  55451
Vaquita      ----TTTTT--TTTTTTTTTTTTT-GTGCGGTA--TGCGGGCCTCT-CACT----GTTGT---GGCCTCTCCCAT-----  51286
Pig          ----TTGCA--AGCATTAAACTAT-AGGTCTTG--TGAATGTTTATATAAA----CTCACTTGGGACTTTTCTACAAAGA  47974
```

```
Majority     TXTACAGTTGTAATTCCAAGAGAGT----AGGTAATTATTX---ATTA----TTAATTAXCTA-TTCTGGTATCTGA-AC
                               73770     73780     73790     73800     73810     73820     73830     73840
Human        TCTAAAGTTATAATTCTAAGAGAGT----AGATAATTGT-----TTTA----CTAAGTACTTA-TTCTGGTATCAGA-AC  58637
PolarBear    TACACAGTTGTAATTCCAAAAGAGT----AGGTAATTATTGAATATTA----TTAATTAACTG-TTCTGGTATCTGA-AC  20705
Cow          TCTACAG---------------------AGATAATTAC-----TTTA----TTAAGCTTTTA-TTCTGGTATCTGA-GC  54618
Dog          TGTACCATTGTAATTCCAAGAGAAT----AGGTAATTGTTTGATATTAA---TTAATTAGCTA-TTCTGGTATCTGA-AC  51096
BelugaWhale  TGCGCAG--------CAAAGGCTCC----AGACACACAGG----CTTA----GCGGCCATGGC-TCATGGGCCCAGCCAC  50808
Donkey       TGTGTAAT----GTTTCAATAAGTT-------TAATTAAAA---TCTAG--ATTGGCAAGCCAGCTCTGCTTTATGA-AT  55970
Cat          TCTACAGTTGTAATTCAGAGGGAGTTATTAGGTAATTATTTAATATTCG---TTATTTAACTA-TTCTGGTATCTGA-AC  58069
Ferret       TGTATAATTGCAATTCCAAGAGAGC----AGGTAATTATTTAGTATTAATTATTAATGAACTA-TTTTGGTATCTTA-AC  58504
Lion         TCTACAGTTGTAATTCCGAGGGAGTTATTAGGTAATTATTTAATATTCA---TTATTTAACTA-TTCTTGTATCTGA-AC  55526
Vaquita      TGCGCA---------------------------ACACAGG----CTCA----GCGGCCATGGC-TCATGGGCCCAGCCAC  51330
Pig          TCTACAATTGTTATTCTAAGAAATT----AGGTGATTATAA---TTTA----CCAAGTTTTTA-TTCTGGTATCTGA-AC  48041
```

```
Majority     TCAACTTCATTTCAC--TTATTAG-AGAAGGAAAAAAGAGTTTATTTCCCAAACTTGCTT-GCCCCATTATCTTGTAA--
                               73850     73860     73870     73880     73890     73900     73910     73920
Human        TCA----CACTTCAC--TTATTAC-AGAAGGAAAAAGAAATTTGTTTCCTAAACTTGCTTTGCCCCATTACCTTGTAA--  58708
PolarBear    TCAAGTTCATTTCACTATTATTAG-AGAAGGAAAAA---GTTTATTTCCCAGACTTGCTT-GCCCCATTATCTTATAA--  20778
Cow          TTA-CTGTATTGCAC--TTATTGGTGAAGGAAAAAAGGAATCTATTTTCTAATCTTGCTT-GTCCCATTATCTTGTAA--  54692
Dog          TCAACTTTATTTCAC--TTACTAG-AGAAGGAAAAAA-AGTTTATTTCCCAAACTTGCTT-GCTCCATTATCTTGTAA--  51169
BelugaWhale  TCTGTGACATGTGGG--ATCTTCCCGGACCGGGGCACGAACCCATGTCC----CCTGCAT-CGGCGGGCGGACTCTGAAG  50881
Donkey       TAAAAAATGTAA-GCATTTATTAATGATAATGGAGA---ACATAATTGTTAAGCTAAAAA-AGGAGGATGGAGAACAGTG  56045
Cat          TGAACATTGTTTCAC--TTATTAG-AGAAGGAAAAAAGAGGTCGTTTCCCGAACTTGCTT-GCCCCATTATCTTGTAA--  58143
Ferret       T-----TCATTTCACTATTATTAG-AGAAGGAAAAAA--GTTTATTTCCCAAACTTGC-----CCCATTATTTTATAA--  58569
Lion         TGAACATCATTTCAC--TTATTAG-AGAAGGAAAAAAGAGGTCGTTTCCCGAACTTGCTT-GCCCCATTATCTTGTAA--  55600
Vaquita      TCTGTGACATGTGGG--ATCTTCCCGGACCGGGGCACGAACCCGTGTCC----CCTGCAT-CGGCAGGCGGACTCTGAAC  51403
Pig          TCAATTATATTTCAC--ATTTTAG-AAAAGAAAAAAGGAGTTTGTTTTCTAAACTTGCTT-GCTCCATTATAATCCAGAG  48117
```

```
Majority     ------------------------CCCAGACXC-----------------------------------------------
                      73930     73940     73950     73960     73970     73980     73990     74000
Human        ------------------------TCTAGACCC-----------------------------------------------  58717
PolarBear    ------------------------CC-TGATAC-----------------------------------------------  20786
Cow          ------------------------TCCAGACT------------------------------------------------  54700
Dog          ------------------------CCCTGACAC-----------------------------------------------  51178
BelugaWhale  CACTGC---------------GCCACCAGGGAA-----------------------------------------------  50899
Donkey       AATAGT-------ATGGGAGAAAACGTGGAGTA----------------------G------------------------  56072
Cat          ------------------------TCCAGATTC-----------------------------------------------  58152
Ferret       ------------------------CCCTGACAC-----------------------------------------------  58578
Lion         ------------------------TCCAGATTC-----------------------------------------------  55609
Vaquita      CACTGC---------------GCCACCAGGGAA-----------------------------------------------  51421
Pig          CCGCACCCATGGCATACAGAAGTTCCCAGGCTAAAGGTTAATTAGAGCTGCAGCTGCTGGCCACAGCCACAGCCACGCAG  48197

Majority     --------------------------------------------------------------------------------
                      74010     74020     74030     74040     74050     74060     74070     74080
Human        --------------------------------------------------------------------------------  58717
PolarBear    --------------------------------------------------------------------------------  20786
Cow          --------------------------------------------------------------------------------  54700
Dog          --------------------------------------------------------------------------------  51178
BelugaWhale  --------------------------------------------------------------------------------  50899
Donkey       --------------------------------------------------------------------------------  56072
Cat          --------------------------------------------------------------------------------  58152
Ferret       --------------------------------------------------------------------------------  58578
Lion         --------------------------------------------------------------------------------  55609
Vaquita      --------------------------------------------------------------------------------  51421
Pig          GAACCAAGCTGCAGCTGCGACTTACACCACAGCTCATGAAAACACAGGGATCGAACCTGCATCCTCATGGATACTAGTCG  48277
```

```
Majority     ----------------------------CTTGTTAAAACATATACTTTTGATGACTCTAGTAATTTTTCTT---CATGC
                      74090     74100     74110     74120     74130     74140     74150     74160
Human        ----------------------------TTTGGTAAAATTTGCATTTTTGATAACTACAGTAATGTTCATTTTACTTGC  58768
PolarBear    ----------------------------CTTGTT-------------TTGATGACTGTAGTAATCTTTCTT---CATGC  20821
Cow          ----------------------------CCTTTTAAAACCTATACTTTGGATGACTCCAGTATTTTTTCTT---CATAC  54748
Dog          ----------------------------TTTGTTGAAACAGA--CTTTTGATGACTGTAGTAATTTTTCTT---CATGC  51224
BelugaWhale  -------------------------G-----CCCTAAACATATACTTTTGATGACTCCAGTCATTTTTCTT---CATGC  50945
Donkey       -------------------------AAATATACTTAAATG------CTTGTTTGGAACAGATGTACACATG---CATGC  56117
Cat          ----------------------------CTTGTTAAAACATATACTTTTGATGACTCTAGTAATCTTTCTT---CATGC  58200
Ferret       ----------------------------CT----------------------GACTGTAGTAATCTTTCTT---CATGC  58604
Lion         ----------------------------CTTGTTAAAACATATACTTTTGATGACTCTAGTAATCTTTCTT---CATGC  55657
Vaquita      -------------------------G-----CCCTAAACATATA------ATGACTCCAGTCATTTTTCTT---CGTGC  51461
Pig          GGTTCGTTAATACTGAGCCACGACAGGAACTCCTTCAAACATACACTTTTGATGACTCTAGTAATTTTTCTT---CATGC  48354

Majority     CTGTATCTGGACT----GTTGAGTGTACCTAGAGAATATC-ACATAATCTAG-CAGTTGGTACCAGTTTAAATTCATCAG
                      74170     74180     74190     74200     74210     74220     74230     74240
Human        CTGCATCTGGGCTCTACTTAGAGAATAT-CACATAATATT-ACATAATCTGA-CAATTGGTACCAGTTTAAATCCATCAA  58845
PolarBear    CAGTATCTGTACT----GTTGAGTATACCTAGAGAATATC-ATATAATCTAG-CAATTGGTACCAGTTGAAATTCATCAT  20895
Cow          CTT-ATCTGGACT----ATTGAGTGTAGCCAGAGAATATC-ACATAATCTAACCAGTTGATAGCAGTTTAAACTCATCAG  54822
Dog          CCATATATGGACT----GTTGAGTATACCTAGAGAATGTC-ATATAATCTAG-CAGTTGGTACCAGTTGAAATTCATCAG  51298
BelugaWhale  CTTTATCTGGACT----GTGGACTGTACCTAGAGAATATC-ACATAATCTAGCCAGT----ACTAATTTAAATTCTTCAG  51016
Donkey       TTGTGTATGTA------AATGGTTTCTCTGGAAGGATACC--CAAGATAAGGTTGGTGAGGAGTGGGTTGGGGAAAGCAG  56189
Cat          CTGTATCCAGACT----GTTGGGTGTACCTAGGGAATATC-CTGTAATCTAG-CAGTTGATACCAGTTGAAGTTTATCAG  58274
Ferret       CTGTAT-----------ATTGAGTATAC-TAGAGAATATC-CTATAATCTAG-CAATTGTTACCGGTCGAAATTCATCAT  58670
Lion         CTGTATCCAGACT----GTTGGGTGTAGCTAGAGAATATC-CTGTAATCTAG-CAGTTGATACCAGTTGAAGTTTATCAG  55731
Vaquita      CTTTATCTGGACT----GTGGACTGTACCTAGAGGATATC-ACATAATCTAGCCAGT----ACTAATTTAAATTCTTCAG  51532
Pig          TTTTATCTGGACT----GTTGAGTGTGCCTAGAGAATAACTACATAATCTAAGCAGTTGGTACCAGTTTAAATTCATCAG  48430
```

```
Majority     CATCAACTACAAATAGX-----CCCTCTACTCTGCTTGAAAAATCTTACCACATTTCTXCAGTCAXCATGCTGTCCCA--

             74250 74260 74270 74280 74290 74300 74310 74320

Human        CGTCAACTACAAGTAAG-----CCCTCTACTCTACTTTAAAAATCTCTTCCCATTTCTTCAGTCAAC-AGTTTTTCTG--  58917
PolarBear    CATCAACTACAACTAGACCAGGCCCTCTACTCTACTTGAAAAATCTTACCATGTTTCTCCAGGCAGCATGCTGTCCCA--  20973
Cow          CATTAACTACAAATAGG-----CCCTCTACTCTGCCTGCAAAATCTTACCACGTTTCTTCAGTCAGCATGTTCTCCCA--  54895
Dog          CATCAACCACAACTAGACTTGGCCCTCTACTGTACTTGAAAAATCTTACCACATTTCACCAGTCCTCATGCTGTCCCA--  51376
BelugaWhale  CATCAACTACAAATAG-----------ACTCCGCTTGCAAAATCTTACCACATTTCTTCGGTC----TGTTCTCCCA--  51078
Donkey       ACAGGGTTGCAGAGGAA------CTTTTAATCTT-TTGTACCATTTGGATAGTATACTGTGATCATAATTCTATTTTAAA  56262
Cat          CATTAATTACAAATAG-----GCCCTCTATTCTGCTTGACAAATGTCACCACATTTCTCCAG----CATGCTGTCCCA--  58343
Ferret       CTTCAACTACAACTAGACTAAGCTCTCTACTTGA----AAAAATCTTACCCTGTTTCTCCAGTCAGCATGCTGTCCCA--  58744
Lion         CATTAATTACAAATAG-----GCCCTCTATTCCGCTTGACAAATGTCACCACATTTCTCCAG----CATGCTGTCCCA--  55800
Vaquita      CATCAACTACAAATAG-----------ACTCCGCTTGCAAAATCTTACCACATTTCTTCGGTC----TGTTCTCCCA--  51594
Pig          CACCAGCTGTAAATAGG-----CCCTCTACTCTGCTTGAAAAATCTCACCATATTTCTTCAGTCAGCATGTTCTCCCA--  48503
```

```
Majority     ----TTTTTCACAAT-GACTGTATCAGAX-XAAATTT-TCATTCCACCAAGT-CTCTCTTCACTCCTCCTTATGACCTTG

             74330 74340 74350 74360 74370 74380 74390 74400

Human        ----TTTTTCACAAT-GCCTATGTCAGATAGAAATTTTCCCTTCTACCATAT-CTCTCCTCACTCTC--------ACTTG  58983
PolarBear    ----TTTTTCACAAT-GACTATATCAGA---AAATTC-TCATTCCACCAAGT-CTCTCCTTACTTCCCCTTTTGACCTTG  21043
Cow          ----TTTTCCACAATTGTCTGTATCAGATGGAAATTTGTCTTTCTACCAAGT-CGCTTTTCACTCTT----ATGACCTTG  54966
Dog          ----ATTTTCACAAT-GACTATATCAGA---AAATTC-TCATTCCACCAAAT-CTTTTCTTACTCCCC-TTATGACCTTG  51445
BelugaWhale  ----TTTTCCACAGT-GACTATATCGGATAGAAATTTCTCTTTCCACCAAGT-TTGTCTTCACTCTTTCTTACGACCTTG  51152
Donkey       AAGATATTCCATAGGGGCTGGCCCTGTGGCCGAGTGGTTGAGTTCGCGCGCTCCGCTGCAGGCGGCCCAGTGTTTCGTTG  56342
Cat          ----TTTTTCACAAA-GACTGTATCAGA---AAATT----AGTCTACCAA---CTCTCTTCACTCCTCCTTATGAGCTTG  58408
Ferret       ----TTTTTCACAGT-GACTGTATCAGA---AAATTC-TCATTCCACCAAGT-CTCTCCTTACTCCCCTTTAGGACCTTG  58814
Lion         ----TTTTTCACAAA-GACTGTATCAGA---AAATT----AGTCTACCAA---CTCTCTTCACTCCTCCTTATGAGCTTG  55865
Vaquita      ----TTTTCCACAGT-GACTATATCGGATAGAAATTTCTCTTTCCACCAAGT-CTGTCTTCACTCTTTCTTACGACCTTG  51668
Pig          ----TTTTTCACAAT-GACTGTATCAGATGGAAATTTCTCTTTGCATCAAGT-CTCTCTTCACTCTTTCTTATGACCTTG  48577
```

```
Majority     CCTACTGCTTCTCAGAAAAAATA-GAAACTATCAGATGGAAACTCCCATCAGCTTTTCTCACAAAATCTACTTATACCX-
                      74410     74420     74430     74440     74450     74460     74470     74480
Human        CTT---------GAAGAGGAAAA--------TCACATGGAAGCTTC-ATCAGCTTTTCTTGCAAAATCTGCTAATACTCA  59045
PolarBear    CCTCCCACTTCTCAGGAAAAAATA-GAAACTATCAGATGGAAACTCCCATCAGCTTTTCTCACAAAATCTACTTAAAACA-  21121
Cow          CCTACTGCTTCTCAGAAAAAAAAAAGAAACTATCAGATGGAAAGTCTTATCAGCCTTTCTCACAAAATATACTTATACC--  55044
Dog          TGTACTGCTTCTCAGGAAAAAATA-GAAACTATCAGATGGAAACTCCCATTAGCTTTTCTCACAAAATCTAATTATATCA-  51523
BelugaWhale  CCTGCTGCTTCTCAGAAAAAATA-GAAACTATCAGATGGAAACTCC-ATCAGTATTTCTCACAAAATCTACTTATACC--  51228
Donkey       GTT-CGAATCCTGGGCGCGGACATGGCACTGCTCATCGGACCACGCTGGGGCGGCGTCCCGCATGCCACAGCTGGGAG--  56419
Cat          CCTACTATTTCTCAGAAAAAATA-GAAAGTATCAGATGGAAACGCCCATCAGCTTTTCTCACAAAATCCACTTATACCA-  58486
Ferret       CCTCCTGCTTCTTAGGAAAAAATA-GG---------TGGACACTCCCATCAGCTTTTCTCACAAAATCTACTCATACCA-  58882
Lion         CCTACTATTTTTCAGAAAAAATA-GAAGGTATCGGATGGAAACTCCCATCAGGTTTTCTCACAAAATCCACTTATACCA-  55943
Vaquita      CCTGCTGCTTCTCAGAAAAAATA-GAAACTATCAGATGGAAACTCC-ATCAGTATTTCTCACAAAATCTACTTATACC--  51744
Pig          CCTACTGCTTCTCTGTAAAAATA-GAAACTGTCAAATGGAAAGTCC-ATCAGCTTTTCTCGCAAAATCTACTTATACC--  48653

Majority     CACCTATCTT--------------------------TCTCTTCTA-ATAA-AAGGCATAGATATTT------CTAAGACC
                      74490     74500     74510     74520     74530     74540     74550     74560
Human        CACTTGTCTT--------------------------TCTCTTTTA-ATAA-AAGGCATAGATGTTTAGTTATCTAAGACC  59097
PolarBear    CACCTATCTT--------------------------TCTCTTCTA-ATAA-AAGGCATAGATATTT------CTAAGACC  21167
Cow          CACACATTTC--------------------------TTTCTTCTAGATAT-AAGGCATAGATATTT------CTAAGACC  55091
Dog          CACCTATCTT--------------------------TCTCTTCTA-ATAA-AAGGCATAGATATTT------CTAAGACC  51569
BelugaWhale  CACACATATCTG------------------------TCTCTTCTAAATAA-AAGGCATAGATATTT------CTAAGACC  51277
Donkey       GACCCACAACGAAGAATATACAACTATGTACCGGGGGGCTTTGGGGACAAAAAGGAAAAAATAAAA------TCTTTAAA  56493
Cat          CACCTATCAT--------------------------TCTCTTCTA-ATAA-AAGGCATAGATAACT------TTAGGACA  58532
Ferret       CACCTATCTT--------------------------TCTCTTCTA-TTAA-AAGGCATAGCTATTT------CTAAGATC  58928
Lion         CACCTATCGT--------------------------TCTCTTCTA-ATAA-AAGGCATAGATGACT------TTAGGACA  55989
Vaquita      CACACATATCTG------------------------TCTCTTCTAAATAA-AAGGCATAGATATTT------CTAAGACC  51793
Pig          CACATAGTCTCACAAAAATCTACTTATACCCATACATATCTTCTAAATA--AAGGCATAGATATTT------CTAAGGCT  48725
```

```
Majority     AGTAAACACCAGATCCTATCCTCTTCTGCCTTTCAAGAGACTTTGCACT--CTTTCATCCCCTCTTTCTCTTATATTTTT
                          74570     74580     74590     74600     74610     74620     74630     74640
Human        AATAAACACCAAAGCCTATCGTCTTCTGCCTGTCAAGAGACTGCACTAT--CTGTCATCCC--CTTTCTCATTTATTTCT  59173
PolarBear    AGTAAACACCAGATCCTGTCCTCTTCTGCCCTCCAAGAGACTGCACGAT--CTGTTATCCCCTCTTTCTC--ATATTTTT  21243
Cow          AGTAAACACCAGACACCACCCTCTTCTGCCTCTTAGGAGTCTTTGTACT----TTTATCTCCCCTTTCTCTTGTATTTTT  55167
Dog          AGTAAATACCAGATCCTGTTCTCTTCTGCCCTCCAAAAGACTTTGCA----CTATCATCCCTTCTTTCTCTTATATTTTT  51645
BelugaWhale  AGTAAACACCAGATACCATCCTGTTCTGCCTCATAAGAGCCTTTGCATT----TTCATCCCCTCTTTCTCTTGTATTTTT  51353
Donkey       AAAAAAAAAGATATTCCATGATTT--GAAATTGCAGATGTTAGTGTTTATGAGAGTGTTTACTGTCATACATATCTTTTG  56571
Cat          AGTAAACACCAGATCCTGTCTTCTTCTCCCCTTCAAGAGACTTTGCACTATCGTTCATCCCCTCTTTCTCTTACATTTTT  58612
Ferret       AGTAAACACCCCATCCTGTCCTTTTCTGCCCTCCAAGAGACTTTGCA----CTGTCATCCACTCTTTCTC--ATATTTTC  59002
Lion         AGTAAACTCCAGATCCTGTCTTCTTCTCCCCTTCAAGAGACTTTGCACTATCGTTCATCCC-TCTTTCTCTTACATTTTT  56068
Vaquita      AGTAAACACCAGATACCATCCTGTTCTGCCTCATAAGAGCCTTTGCATT----TTCATCCCCTCTTTCTCCTGTATTTTT  51869
Pig          GGTAGACACCAGATCCCATTCTCT-CTGCCT---GAGAGCCTTTGCACTA--TCTTTCTTTCTCTCTCTGTTATATTTTT  48799

Majority     CTCTATGTTGGC-TACTTCTCACCAGTGTTTCAAGTATCCTTAAATXTCTCTCA------TTTTAAXTGAATTTAC----
                          74650     74660     74670     74680     74690     74700     74710     74720
Human        CTCTATATTGGC-CACTTTTCACCAG--TTTCAAGCATTCTCAGCTTC-TTTCA------TTTTAAATGAATTTACCTTC  59243
PolarBear    TTCTCTGTTGGC-TACTTCTCACTAGTGTTTCAAATATCCTTAAATCTCTCTCA------TTGTAAGTAAATTTAC----  21312
Cow          CTCCATGTTAGT-TACTTCTCACCAGTTTTCAAGTGTGCCTAAATGTCTCTCTCTTTTTTTTTTAATGAATTTATT---  55243
Dog          CTCTTTGTTGGC-TATTTCTCACCAGTGTTTTAAGTATCCTTATATCTCCTTCA------CTGTAATTGAATTTAC----  51714
BelugaWhale  CTCTTTGTTGGT-TACTTCTCACCAGTGTTTCAAGTGTGCTTAAGTGT-TCTCTC--ATTTTTTAAATGAATTTAC----  51425
Donkey       ATGTACACATGCATGTGTTTTCTCAGGTATGTAACTGGAAGTAGATTTACTAAG-------TCAAAGAAAATGTATA---  56641
Cat          CTCTGTGTTGGC-TACTTGTCACCAGTGTCACAAGCATCATTAAATTTCTCTCA------TTTTGAGTGAATTTAC----  58681
Ferret       CTCTCTGTTGGC-TATTTCTCACCAGTATTTCAAACATCCTTAAATCTCT--CA------TTGTATGTGAATTTAC----  59069
Lion         CTCTGTGTTGGC-TACTTGTCACCAGTGTCACAAGCATCATTAAATTTCTCTCA------TTTTGAGTGAATTTAC----  56137
Vaquita      CTCTTTGTT-----ACTTCTCACCAGTGTTTCAAGTGTGCTTAAGTGT-TCTCTC--ATTTTTTAAATGAATTTAC----  51937
Pig          CTCTACTTTGGC-CACCT------AGTATTTCAGGTATACTTAAGTGTCTCTC----ATTTTTTAAATGAATTTAT----  48864
```

```
Majority     --CCTXAAXTCCACA-------TATCTCT-----CCATCTATCCCCCTXTTXXTCTTCCTTTTCA---TTTATAXCCAGA
                      74730     74740     74750     74760     74770     74780     74790     74800
Human        AACTTTCAATCCATCCATC-TATAACTCTACGTACTTTCTATCACCCTGCCTCCCCTCTCCCTTTTCACTCATAACCAGA  59322
PolarBear    --CCTGAACTCCACA-------TAACTCT-----CCATCTATTCACCCATTCTCCCTTTTT--CA---TTTATAGCCAGA  21373
Cow          ACCCTTAACTCCATAATTCGTATATTTAT-----CCATCTAACACCTCATCTCCCTTTCTTTTCA---TTTATAACTACA  55315
Dog          --CCTGAGTTCCACA-------TCTTTCT-----CCATCTAT-CACCCTTTCTTCTCTCTTTTCA---TTTATAGCCAGA  51776
BelugaWhale  --CCTTAATTCTGTATTCCATATATCTGT-----CCATCTAACACCTTGTCTCTCTTCCGTTTCTTCATTTATAACTAGA  51498
Donkey       TGCTCAGCTTTAACAGATATTTCAAGTTT-----TTAAAAATGATTCTATCAATTTACATTCCCATCACTTATATATGAG  56716
Cat          --CCTGAACTCCACA-------T-------------ATGTAT-CACCCTTTCTTCTCCCTTTTCA---TTTATAGCCAGA  58735
Ferret       --CCTGAACTCCACA-------TAACTCT-----CCAGCTATTCCCCTATTCTTCTCTCTTGTCA---TTTATAGCCAGA  59132
Lion         --CCTGAACTCCACA-------C-------------ATGTAT-CACCCTTTCTCCTCCCTTTTCA---TTTATAGCCAGA  56191
Vaquita      --CCTTAATTCTGTATTCCATATATCTGT-----CCATCTAACACCTTGTCTCTCTTCCCTTTCTTCATTTATAACTAGA  52010
Pig          --CCTTAATTCCACA-------TATCTGT-----CCTCGTATCCCCTTGTTTCTTCTCCCTTTT----CGTATAACCACA  48926

Majority     CTTXTTAAATAA-----------------------GCTCTCTTTGTTTGCTGTT------------------------
                      74810     74820     74830     74840     74850     74860     74870     74880
Human        CTTCTTAAATAGT-----------------------CTATCTTTG-TTGTT---------------------------  59349
PolarBear    TTTATTAAATAA-----------------------GCTCTCTTTGTTTGCTGTT------------------------  21404
Cow          CTTCTTAAATAA-----------------------GTTGTCTTTGCTTATT---------------------------  55343
Dog          TTTACTAAATAA-----------------------GCTCTCTTTGTTTGCTGTT------------------------  51807
BelugaWhale  ATTCTT------------------------------------------------------------------------  51504
Donkey       AATTCAAGCTT-------------------------CTCTACATTATTACCAACACT---------------------  56748
Cat          CCTACTAAATAA-----------------------GCTCTCTTTGTTTGCTGTG------------------------  58766
Ferret       TTTACTAAATAG-----------------------GCCC--TTTGTTTGCTGTT------------------------  59161
Lion         CCTACTAAATAA-----------------------GCTCTCT--GTTTGCTTTG------------------------  56220
Vaquita      ATTCTTTTTTTTTTTTTTTTTAAACATCTTTATTGGGGTATAATTGCTTTACAATGGTGTGTTAGTTTCTGATTTATAAC  52090
Pig          CTTCTTAAGTAAA----------------------GCTGTCTTTGCTTGCTGATG-----------------------  48959
```

```
Majority     ---------------------TATACTTACTCCCTTTTT---TTT-CTCTACCTACTATAATTTGGTTTXAGCCCCCATT
                       74890     74900     74910     74920     74930     74940     74950     74960
Human        ----------------------TA-TTATCTCATTT---------CTCTACTTACTATTATTTGGTTTTAGCACT-GTT  59395
PolarBear    ---------------------TATACTTACCTCCTTTTT-------CTCTACCTACTGCAGTTTAGTTTCAACCCCCATT  21456
Cow          ----------------------ATACTTATTCCCTTCT---TATTTCTCTACTTATTGTAATTTGGTTTCAGCACCCATT  55398
Dog          ---------------------TATACTT-CTTTTTATTT-------CTCTACCTACTATAATTTGGTTTCAACCCCCTTT  51858
BelugaWhale  --------------------------------------------------------------------------------  51504
Donkey       ---------TG-----------GTATTTAC--CCCTCTTAATTTTAGCCAATCTGGTAGGACATGGTAGTGTCTCATGGT  56806
Cat          ---------------------TATACTTTTTCCCTTTTTA--TTT-CCTCACCTACTATAATTTGGTTTCAGCCCCGATT  58822
Ferret       ---------------------TATACTTACCTCCTTTTTA--TTTCCTCTACCTCTTGCATATTGGTTTTGACCCTCCTT  59218
Lion         ---------------------TATACTTTTTCCCTTTTTA--TTT-CCTCACCTACTATAATTTGGTTTCAGCCCCGATT  56276
Vaquita      AAAGTGAATCAGCTATACATATACATGTGCTCCCATGTGTCTTCCCTCTTGCGTCTCCCTCCCTCCCACTCTCCCCCTCC  52170
Pig          ----------------------ACACTTACTCTCCTCTCT-TCTTTCTTTAATGATTGTGCTTTGGTTTTAGCCTCCATT  49016

Majority     TACCCAAATTGTTCTTC-CCAGTGTCACCAGTGATCACTACAGTAGCTAAATCTCAGCAG-X-TTATTCAGTX-------
                       74970     74980     74990     75000     75010     75020     75030     75040
Human        GACCCAAATTGCTCTTCCTCAGCGTCACCAGCGATTAGTAGAGTAGCTAAATGCTAGTGATA-TGATTTAGTC-------  59467
PolarBear    TACCCAAATTGTTCTTC-CCAGTGTCACCAGCGATTGCTATAGT-GCTAAATCTCAGCG----TAATTCAATC-------  21523
Cow          TACTCAACAAGAGATCA-CTGGAGTAGCTAAATCTCATCAGAATTGTTCAGTCC----------------TT-A-----  55454
Dog          T-CCCAAATTGTTCTTTTCCAGTGTCATCAGTGATCACTACAGTAGGAAAATCTAAAAAGCA-TTATTCAGTTTAATGAA  51936
BelugaWhale  --------------------------------------------------------------------------------  51504
Donkey       GATTTTAATTTGTATTA---AAGGTTCTTAATCCCAACC----TAATCCCCTCTTAGGAG-ATGGGGATATTTCCATACT  56878
Cat          TACTCAAATTATTCTTC-CCAGTGTCCCCAG-GATCACTACACTAGGTAAATCTCACCAGCC-TTATTCAGTC-------  58892
Ferret       TACCCAAATTGTTCTTC-CTACTGTCACCAGTGATTGCCACAGT-GCTAAATCTCAACAGTA-TTATTCAATC-------  59288
Lion         TACTGAAATTATTCTTC-CCAGTGTCCCCAG-GATCACTACAGTAGGTAAATCTCACCAGCC-TTATTCAGTC-------  56346
Vaquita      CACCCCTCCAGGCTGTC-CCAAAGCCCCGAGCTTATATCCCTGTGCCTTGCGGCTGCTTCCCCCTAGCTATCT-A-----  52243
Pig          TACCCAAGTTGTTCTTC-CCAGGGTCACCAGTGATCACTGGAGTAGCCAGATGTCAGC-------AG--AATT-A-----  49080
```

```
Majority     -------CTT---A--CCTATCCTCTAGGTAACATTTG-ACACTGTG----------------------XGCCATTCTCT
                      75050     75060     75070     75080     75090     75100     75110     75120
Human        -------CTTGTCTTACTTAATACCTAGATAATATTTG-GCACTGTGC----------------------ACCATTGTCT  59517
PolarBear    -------CTGATCATCCATAT---CTAGGTAACATTTG-ACACTGTG----------------------AGCCATTCTCT  21570
Cow          ------TCTT---A--CCTAACCTCTAGGTAACCTTTG-ACACTGAATCT--------------------GTCTTTCCCT  55502
Dog          TTCAGTTCTTATCATCCCTAACCTCTAGGTAACTTTTG-ACACTGTA----------------------AGCCATTCTCT  51993
BelugaWhale  --------------------------------------------------------------------------------  51504
Donkey       TGTAATAATAGACAGGTATAG--TGTAAGGACCTGTTCACTAATGAA-----------------------GAAATAATAT  56933
Cat          ----------------CTTACCCTCTAGGTACCTTTGATACAGCGTG----------------------AGCCATTCTCT  58934
Ferret       -------CTTATTGTCCCCATTCTCTAGGTAACATTTG-ACTCTG------------------------AGCCATTCTCT  59336
Lion         ----------------CTTACCCTCTAGGTACCTTTCATACAGCGTG----------------------AGCCATTCTCT  56388
Vaquita      ------CCTTA--C--TACGTTTGTTAGTGTGTATATG-TCCATGACTCTCTCTCGCCCTGTCAAAACTCACCCTTCCCC  52312
Pig          ------TCT----G--CCTGTCCTCTAGGTAGCACTTG-ACACTGAGC-----------------------TATTCCCTT  49124

Majority     TTCTTAAAATCXTCTXTTCTCCTGGTCTCTAACACTTCTCACCTAGAT-ATATTCCACCTCTCTGXT----A--------
                      75130     75140     75150     75160     75170     75180     75190     75200
Human        T----AAAATTCTCTTTCTTCCTGGTCTCTGATACTTCTCACCTGGAT-TTATCCTACCTCTCTATTT------------  59580
PolarBear    TTCTTAAAATCATCTCTTCTCCTGGTCTCCAACACTTCTTGCTTAGAT-GTATTCTACCTCTCTGACT---A--------  21638
Cow          TTCTTAAAATCCTTTTTTCTCCTGGTCTTTAACACTTCTCACCTAGAT-ACATCCCACCTCTCTGTTC---A--------  55570
Dog          TTCTTAAAATTGTCTCTTCTCCAGGTCTTTAACACTTATCAACTGGAT-ATATTCCACCTCTCT-------A--------  52057
BelugaWhale  --------------------------------------------------------------------------------  51504
Donkey       TGCAGTGAACTGCTTAAAAAACTA--TTTAAATATGTATTAGGTTAAATATATTCAAACATTTAAATTAATA----AACA  57007
Cat          TTCTTAAAATCTTCTTTTCTCCTGGCCTC-AACACTTCTTGCCTAGAT-ATGTTCCACCTCTCT--T-------------  58997
Ferret       T----AAAATCATCTCTTCTCCTGGTCTCTAACTCTTCTCGCTTAGATTATATTTTACCTCTCTGACT---A--------  59401
Lion         TTCTTAAAATCTTCTCTTCTCCTGGTCTC-AACACTTCTTGCCTAGAT-ATGTTCCACCTCTCT--T-------------  56451
Vaquita      CTCCCCATATCCTCAAGTCCGTTCTCCAGTAGGTCTGCGCCTCTATTC-CTGTCTTATCCCTAGGTTCTTCATGACATTT  52391
Pig          TCTTAAAAATGTTCTTTTCTCCTGGTCTCTGACACTTCTCATCTAGAT-TTATCCCACCTCTCTGAC-------------  49190
```

```
Majority     -TGTCTTATCA--------------------------TATCCTTCCC----CTTCTGCCTGGGGGACTTCACCTGTTCT
                               75210     75220     75230     75240     75250     75260     75270     75280
Human        CTTCCTTGTCT--------------------------TGTAACTCCC----CTTCTACCTGGGAGGCCTCACCTGCTGT  59629
PolarBear    -TGTCTTATCA--------------------------TATCCCTCCCTACCCTTCTGCATGGGGGACTTCATCTATTCC  21690
Cow          -TTATTTGTT---------------------G----TATCCCTCCC----CTTCTTTTAGGGTGACCTCACCTGTTAT  55618
Dog          -TGTCTTATCA--------------------------TATCCCTCCC-----TTCTGCCTGGGGGACTTCATCTATTCC  52104
BelugaWhale  --------------------------------------------------------------------------------  51504
Donkey       TTGCTCTAGAAAT------------------------CTTTTTATCCTGCTTTATTTTCTGTATAACTGTATTCTCCAT  57062
Cat          -TGTCTCATCA--------------------------TATCCTTTCCCTTCTTTCTGCCTGGGGGACTTCACCTGTTCC  59049
Ferret       -TATCTTATTA--------------------------TATCCTTTCC----CTTCCGCAGGGGGGACTTAATCTGTTTT  59449
Lion         -TGTCTCATCA--------------------------TATCCTTTCTCTTCTTTCTGCCTGGGGGACTTCACCTGTTCC  56503
Vaquita      TTTCCCCTTAAATTCCATATATATGTGTTAGCATACGGTATTTGTCTT----TGTCTTTCTGACTTACTTCACTCTGTAT  52467
Pig          -TTCTTTCTT---------------------T----TTTCATCCCT----CTTCTGCCAGGGGGACCTCACCTGTCCT  49238

Majority     ------------------------------CAXAGCTCAATTACAC----CTTTGTGACTCTGACAGTGCXC---ACAT
                               75290     75300     75310     75320     75330     75340     75350     75360
Human        ------------------------------CACAGCTCCATTAAACC---TCTTATGATTCAGACAGAGTTC---ACAT  59672
PolarBear    ------------------------------CACAGCTGAATTATACTATACTTTGTGACTCTGACAGTGCCC---CCAT  21736
Cow          A-----------------------------CACAGCTTAGTTACAC-----TTTGTGATTCTGACAGTACTC---ATAT  55660
Dog          ------------------------------CGCAGCTGAGTCACAC----CTTTGTGACTCTGACAGTGCCTTTGACAT  52149
BelugaWhale  --------------------------------------------------------------------------------  51504
Donkey       A------------------------------GTAGTGTAACTGTG------TTTATTGTATTAA--ATATTTATCAAAT  57103
Cat          ------------------------------CATAGCTCAATTATAC----CTTCATGACTCTGACT-TGCCC---ACGT  59090
Ferret       ------------------------------CACCACTGAATTACAC----CTTTGTGACTCTGACAGTGTCC---ACGT  59491
Lion         ------------------------------CATAGCTCAATTATAC----CTTCATGACTGTGACA-TGCCC---ACGT  56544
Vaquita      GACAGACTCTAGGTCTATCCATCTCATTACAAATATCTCAATTTCATTT--CTTTTTAAGGCTGAGTAATATT---CCAT  52542
Pig          ------------------------------CATAGCTCAGTTGCACC----TTTGTGATTCTGACAGAACTC---ACAT  49280
```

```
Majority      GACTCT-----------TCTAGC-TCAGATCTXTTCTAAGAGACCCAGXCTAGGAACTTAGTGGACCAT-TAGGCACCTT
                       75370     75380     75390     75400     75410     75420     75430     75440
Human         CACATT----------TTCTAGC-TCAGACCTCTCCTAAGAGACCCACACTAAGAACCTGGCGGACTAA-TGAATATCTT  59740
PolarBear     GACTCT------------CTAGCATCAGATCCTTTCTAAGAGACCCAGGCTAGGAACTTAGTGGACCAT-CAGGCACTTT  21803
Cow           AACTTT--------ATTTCTAGG-TCACACATCTTCTGAGAGACCCAGGCTAGGA----------CGGT-GGAGCACCTT  55720
Dog           GATTCT------------CTAGTGTTATATCCT----AAGAGACTCAGACTAGGAACTTAGTGAACCAT-TAGGCACCTT  52212
BelugaWhale   --------------------------------------------------------------------------------  51504
Donkey        GTTTCCA-------AATATTGGGTTTGAAT--TAA-TGAGTCACTTTAGCTAACAAATTTATAGGTCAAATTATTATTTT  57173
Cat           GGCTCTT----------TCTAGC-TCAGATCTCTTCTGAGAGACCCAGACTAGGAACTTAGTGGACCAT-TAAGCACCTT  59158
Ferret        GACTCT------------CTAGCATCAGATCCTTTCTAAGAGACCCAGACTAGGAAATTAATGGACCAT-CAGGAACCTT  59558
Lion          GGCTCTT----------TCTAGC-TCAGATCTCTTCTGAGAGACCCAGAGTAGGAACTTAGTGGACCAT-TAGGCACCTT  56612
Vaquita       TGTGTATATGTGCCACATCTTCT-TTATCCATTCGTCCGATGATGGGCGCTTAGGTTCTTTCCATCTCC-GGGCTATTGT  52620
Pig           GACTC-----------TTCTAGC-TCAGACCCCTTCTAGGAGACCCAGGGTAGGAACCCTGTGGACTGT-GGGGCACTCG  49347

Majority      ---CAGACTTAGTGTGTTCAXTTCAXCTCC-TGTCACX----------------------TAC----------------
                       75450     75460     75470     75480     75490     75500     75510     75520
Human         ---CAAAC--AGTGTGTTCAATCTAGCTCC-TGTTTTTGGTGTCCGCTGTGTTAG-GAAAGTGCAAG--ATACCAACTT  59810
PolarBear     ---CAGACTTAGTGTGTTCAGTTCAACTCC-TGTAACT----------------------TA-----------------  21839
Cow           ---CAGACTTAGTGTGTTCAATTAAACTCC-TGTCACCCCCCGCCCCACCACCACCAAAAATAC--------TAAAATCT  55788
Dog           ---CAAACTTCGTGTGTT-----CAACTCC-TGTCACT----------------------TAC----------------  52244
BelugaWhale   --------------------------------------------------------------------------------  51504
Donkey        TTTTTAATTATTTTTTTTTGAGGAAGAT-----TAACCCT----------------GAACTAA----------------  57215
Cat           ---CAGACTTAGTGTGATCAGTTCAGCTCC-TGTCACC----------------------TAC----------------  59195
Ferret        ---CCAAATTAGTGTGTTTAGTTTAACTCC-TGTAACT----------------------TAA----------------  59595
Lion          ---CAGACTTAGTGTGATCAGTTCAGCTCC-TGTCACC----------------------TAC----------------  56649
Vaquita       AAATAGAGCTGCAATGAACATTTTGGTACATGACTCTTTTTGAATTTTGGTTTTCTCAGGGTATATGCCCAGTAGTGGGA  52700
Pig           AA-CAGACTCAGCATGTTCAAGTAAACTC--TCTTACCCCTGCCACCACCCTCCCCAAAGATACA-------TATAAAAA  49417
```

```
Majority     -------------------------------------------CC--------------------------TCT------

                      75530     75540     75550     75560     75570     75580     75590     75600

Human        CAGTCTCATTAACAAGCCAGTAAACATGGTATTTTTCCCTTTCCTTCCAACCTGTCATCAGATATTATTGATTATACCTC  59890
PolarBear    --------------------------------------------------------------------------------  21839
Cow          GTATTTCATATACCATTCTCTAGGTATTGATCAGGACAAAGTACCA---------------------TCAATCTAAGAAA  55847
Dog          -------------------------------------------CC--------------------------TCC------  52249
BelugaWhale  --------------------------------------------------------------------------------  51504
Donkey       ------------------------------------C---G------------------------------TCT------  57220
Cat          -------------------------------------------CC--------------------------ACT------  59200
Ferret       -------------------------------------------CT--------------------------TCC------  59600
Lion         -------------------------------------------CC--------------------------ACT------  56654
Vaquita      TTGCTGGGTCATATGGTAATTCTATTTGTAGTTTTTTAAGGAACCTCCATACTGTTCTCCATAGTGGCTGAACCAATACA  52780
Pig          ATCCTTCAACCTCTGGTATTTTCCTTTATGAAAGGTCAAGGTACCAA--------------------TCAATCTAAGAAG  49477

Majority     -----------------CTGG-----------------------CCTCCTC--CCT--------------------X---

                      75610     75620     75630     75640     75650     75660     75670     75680

Human        CTAAATTTTTGTTGAATCTGGCCTTTTTTCTCCAACTCTTGCTACCATTGCAGTCTAAGCCAGTGTTATTCAAGATGCTG  59970
PolarBear    -----------------------------------------------CCCC--CCT--------------------G---  21847
Cow          CA---AGACAGAAAACATTGAATCTTTC---T-TT------CTTCTTCCTC--CTTTCAGCCTATCACCAAATCTTACTG  55912
Dog          -----------------CTGG--------------------------CCTC--CCC--------------------G---  52261
BelugaWhale  ------------------------------------------------------------------------------AA  51506
Donkey       -------------GCTGCCAA----------------------TCCTCCTC--TTTTTG---------------CTGAGG  57248
Cat          -----------------CAGC-----------------------CTTTCTT--CCC--------------------A---  59215
Ferret       -----------------CTGG-----------------------CCACCCC--CTT--------------------A---  59615
Lion         -----------------CAGC-----------------------CTTTCTC--CCC--------------------C---  56669
Vaquita      CATTCCCACCAGCAGTGCAAGAGTGTCCCCTTTTCTCCACACCCTCTCCAG--CATTTATTGTATAACTAGAATTCTTAA  52858
Pig          C-------CAAGAAATACTAAATTTTT--------------TTTCCTCTTC--CTTTCAGCCTATCATCAAATCTTATTG  49534
```

```
Majority     XCCCCCCXXCCTX--A-TT-XTGTCAAAAXAAATCCT--X-GCCTCTATCTCTC---X------ACTGTGGTATTAACTX
                      75690      75700      75710      75720      75730      75740      75750      75760
Human        GTCTCTCAAGGTAGTAGTGTGTGTCAAACTGAAATCAGCATACTTCTAGCAACAGTATGC-----TTAATGTCAATATTG  60045
PolarBear    -CCCC----------------CCACAAAAAAAATCCT----TGCCCCAT----------------CTGTGGTGCTCACTG  21890
Cow          ATTTTAGATCCTA--AGTTTTTGTTCA-GTCTATCCTTTT-GCCTCCAACTCTTAATTGTCGTCACTGCAGCCTAAGCTA  55988
Dog          -CCCCACCA------------CCCCAAAACAAATCCT----GCCTC-AC----------------CTGTGGTGTTCCCAG  52307
BelugaWhale  ATAAGCTGTCTTT--ATTTGCTGTTTATACTTATTATCCT-GTTCTTATTTCTCTACTTAGAGTAATTTGGTTTCAGCCC  51583
Donkey       AAGACTGGCCCTG--AGCTAACGTCTGTGCCCATCTTTCTCTACTTTATATGTGGGACGCC--TACCTCAGCATGGCTTG  57324
Cat          -CCCCCCCCCC------------AAAAAAAAAAACCA----AACCCTTCCTCAC-----------CTGTGTCAGTAAAAA  59267
Ferret       -CCTCGCTCCTTC--------CCCCCAAAAAAACCCT----GCCTCTGTGTTGTT--------CACTGTGGTAGTAAGTC  59674
Lion         -CCCCCCCCCCCC--CCCCCCAAAAAAAAAAAACCCA----AACCCTTCCTCAC-----------CTGTGTCAGTAAAAA  56731
Vaquita      ATAAGCTGTCTTT--ATTTGCTGTTTATACTTATTATCCT-CTTCTTATTTCTCTACTTAGAGTAATTTGGTTTCAGCCC  52935
Pig          AGTCTAGATCCTA--AATTTCTATTGA-ATCTATCCTTTT-GTCTTCAACTCTC--TTGCCATCACTGTAGTTTAAGTTA  49608

Majority     GC-----TCAAGGTACCXA-ATTTCATGTC---AXTAACAAGCCAGAA-----X-AACATGCAT----XX------X-X-
                      75770      75780      75790      75800      75810      75820      75830      75840
Human        GCATAAATTTTGACACAGACTTCTTATATTCATGCAGAGTAGCCTGGGCTTCTGGACCGTCAATCATAATTTAAGTAGCA  60125
PolarBear    T-----------GTACCAA-ATTGGACATC---AGTAACAAGCCAGAA-------AACATGGCT----------------  21932
Cow          GTGTTTTTCAAAGTGCTAGTCTCCAATGCT---ATAAG-AAGCTTGTG-----CCAACATTCAA----ATT-----AGCT  56050
Dog          G-----------GTACCAA-ATTCTATGTC---AGTAACAAGCCAGAA-------AACATGACTT---------------  52350
BelugaWhale  CTATTTATCCATATTTTTCTCCCTCGTGTC---ACCAGTGATCACTAGAGTAGCTAAATCTCAACAGAATT-----ACTC  51655
Donkey       CCAAGCGGTGCCGTGTCCGCACTGGGGATCCAAACTGGCGAACCCTGG-GCCACCAAAGCGGAAC------------ATG  57391
Cat          GC-----TCAGGGTATCGA-ATTCCATGTC---AGTAACAAGCCAGAA-------AACATGTCT----------------  59315
Ferret       TC-------AGGGTACCAA-ATTTCACATC---AGTAACAAGCCAGAA-------AACACTTTTATT-CCA-----TTTG  59730
Lion         GC-----TCAGGGTATCGA-ATTCCATGTC---AGTAACAAGCCAGAA-------AACATGTCT----------------  56779
Vaquita      CTGTTTATCCATATTTTTCTCCCTCGTGTC---ACCAGTGATCACTAGAGTAGCTAAATCTCAACAGAATT-----ATTC  53007
Pig          GT------CAAATTTCTGGTATCTGAGGCT---ACAAG-GAGCTTGTA-----CCAAAACACAA----ATC-----AGCA  49664
```

```
Majority     TTTATTTATCT-XX-TACTTTXAG--X----CXTATCAXTAAATCTTACTX-----TTCTXATCXTCAATTTCTCTXGXA
                       75850     75860     75870     75880     75890     75900     75910     75920
Human        TTGATGTAGTAATAATTCTCTTTCATATCTCCACTTCCTTGGCCTTCTGACTGAACTCACCATTTTGACATCTTCCCCTA  60205
PolarBear    TTTATTCCTTT----TACTTTCAG-------CCTATCACCAAATCTTACTGA----TTCTCATCCTAAATTTCTCTTGAA  21997
Cow          TACTTTAAGCTATAGTTTGTTTAGTGTCAATATTGATCATAAATTCTGATACAAACTTCTTTTCATGCAAGGTGAACTGG  56130
Dog          TTTATTCCTCT----TTCTTTCAG-------CCTATCACCAAATTTTACTGA----TTCTTATCATCAATTTCTTTTGAA  52415
BelugaWhale  AGTCCTTATCTTAACTAACCTTAG-GTAACATTTGACATTGAACCTTTCTTTCCCTTTCTTAAAATCGTCTTTTCTCCTG  51734
Donkey       CTCACTTAACTGCTACACCACAAA--T--TATTTTTTATTTTATTTTAT-------TATTGTTTGCCAGTCCCTCGAAGT  57460
Cat          TTTATTTCTCT----TCCTTTCAT-------CCTACCACCAAATCTTA---------TTCTCATCCTAAATTTCTGTTGAA  59376
Ferret       TTTATTTATTTAT-TTATTTTCAG-------TCTATCAACAAATCTTACTGA----TTCTCATCCTTAATTTCTGTCATA  59798
Lion         TTTATTCCTCT----TCCTTTCAT-------CCTACCACCAAATCTTA---------TTCTCATCCTAAATTTCTGTTGAA  56840
Vaquita      AGTCCTTATCTTAACTAACCTTAG-GTAACATTTGACATTGAACCTTTCTGTCCCTTTCTTAAAATCGTCTTTTCTCCTG  53086
Pig          TATTTTTGGCTGTACTGTATTTAGTGTCGATGTTGATT-TAAATTTT---------TTTTTTTTTTTTGTCTTTTTTAGG  49734

Majority     GCTAT-------X----C----XX--------C-CTTTTGTCTC----------CCXGCTCTC----XTGCXCACCAC--
                       75930     75940     75950     75960     75970     75980     75990     76000
Human        CCTCCATTATTTTTCCACAGAGTACTAGGAATCATCTCAACGTCAAGTCATATTGCTTCTAATCACAATACCCTTTAGTG  60285
PolarBear    TCTGT---------------------------C-CTTTTGTCTC----------CCAGCTCTC----TTGC-CACCAT--  22032
Cow          GCTGCTAGTCCATGAACCA---CAGTTTGAGTAGCACTAATCTAAACCATCAAGCCGTCTCTTAAATCTGTGCCTCATTG  56207
Dog          TCTAT---------------------------T-CTTTCGTCTC----------TCAGCTCT------TGC-CACCAC--  52448
BelugaWhale  GTCTCTGACACTTCTCACC---TAG-ATATATCCCACCTCTCTGTCCCTTCCTTGTTATATTCCTCCCCTTCCACCACGG  51810
Donkey       GTTATT----TAATTAGCA---TTTAAATGATGAGTTTTGACTAGATGAGTA--CCTGGTCTC---TTTGAATCCAAG--  57526
Cat          TCTAT---------------------------C-CTTTTGTCTC----------CCAGCTCTC----TTGCACACCAC--  59412
Ferret       CCTAT---------------------------C-CTTTTGTCTC----------CCGGCTC--------GC-CACCAC--  59829
Lion         TCTAT---------------------------C-CTTTTGTCTC----------CCAGCTCTC----TTGCACACCAC--  56876
Vaquita      GTCTCTGACACTTCTCACC---TAG-ATATATCCCACCTCTCTGTCCCTTCCTTGTTACATTCCTCCCCTTCCACCACGG  53162
Pig          GCTGC----ACCTGGGGCG---TA--TTGAG-GTTCCCAGTCTA------CAGATCGAATCTCGAATCAGAGCAGCAG--  49796
```

```
Majority     ----XCTAGT--CTAAA-------CCAXGTGTTXTTCAAAGTG---------CTGGTCTCTGATTXTACCAGGAGCTTTT
                              76010     76020     76030     76040     76050     76060     76070     76080
Human        ACTTTTTTGTCCTTGCAATAAAGTCCACAG--TCCTTATCATGGCTTA----TAAGACTCTAAATCAGTCCTCAGCTTGC  60359
PolarBear    ----TCTAGT--CTAAG-------CCAGGTGTCACTCAAAGTG---------CTGGTCTCTGATGTTACCAGGAGCTTTT  22090
Cow          GCCTTCTAGT--CTAAATCCACCACTGTGAG-TTTTAAGAATGATCTCAACTTAAAGTCATATTTCTTCCTTTCTCTGTA  56284
Dog          ----TCTAGT--CTAAG-------CCAGGTGTTCTTCAAAGTG---------CTAGTCTCTGATGTTACCAGGAGCTTTT  52506
BelugaWhale  GGACCTCAGCTGTTACACACAGCTCAATTACACCTTTGTGATTCTGACTACTCACATCACTTTTTCTAGCTCAGACCTCT  51890
Donkey       -CTATATGATCCCTTGG-------TTATATGATATTT-CAGTGA--------AATATTTATTTTTTTCCCCCAGCTCTTT  57589
Cat          ----CCTCGT--CTAAA-------CCAGGTGTTATTCAAAGTG---------CTGGTCTCTGATGGTACCAGGAGCGTTT  59470
Ferret       ----CCTGGT--CTAAG-------CCACGTGTTACTCAGAGTG---------CTGGTCTCTGATGTTACCAGGAGCTTTT  59887
Lion         ----CTTCGT--CTAAA-------CCAGGTGTTATTCAAAGTG---------CTGGTCTCTGATGGTACCAGGAGCGTTT  56934
Vaquita      GGACCTCAGCTGTTACACACAGCTCAATTACACCTTTGTGATTCTGACTACTCACATCACTTTTTCTAGCTCAGACGTCT  53242
Pig          --CTGCCAGC--CT--A-----CACCACGGC-TCGTAGCAATG--CCGGATTCTTAGCCGGGATTGAACCTGTGTTCTCA  49862

Majority     GCCAAAATA--CAAGTTAGCATCCTTXTAGCTATAATGTGXTTAX----TGA-XATGTTGATAT-AXATTCTGACACA-A
                              76090     76100     76110     76120     76130     76140     76150     76160
Human        CTCTCCATT-CTCCTCTCATGCCTTTTTAGCTATATTGGACTTCTTTAAATAGCTTGCATATACTGTATTTTCTCTGA--  60436
PolarBear    GCCAAGATA--CAAGTCAGCATGCTTCTAGCTATAATGTGTG-----------ATTATTGATAC-AGATTCTGACATAGA  22156
Cow          CCCTTCAATGACTGATTTGTGTCCTTGGTATAAAAATGCTCTTTC---TTTAACATACTTATAT-AAAACTCTTAAATCT  56360
Dog          GCCAAAATA--CAAGTCTGCATGCTTCTAGCTATGATGTGTTTAG----TGT-GATATTGATAT-ATATGCTGACATAGA  52578
BelugaWhale  TCTAAGAGACCCAGATTAGGAACCCATGGACCATGGCGCACCTTCAAACTTAGCGTGTTCAATT-AACTCCTGTCACCAC  51969
Donkey       TGAGATATAATTGACATATAATATTGTGTAAGTTTAGGTGCACAA-----CC---TGTTGATTTGATACACTTATATA-T  57660
Cat          GCCAAAATA--CAAGTCAGCATACTTCTAG---TAGTGTGTTTAG----TGC-GCTGTTAATAC-AGATTCTGACACA--  59537
Ferret       GCCAAAATA--CAAGTCAACATACTTCTAGCTATAATGTGTTTAG----TGT-GATATTGATAC-AGATTCTGACATAGA  59959
Lion         GCCAAAATA--CAAGTCAGCATACTTCTAG---TAGTGTGTTTAG----TGC-GCTGTTAATAC-AGATTCTGACACA--  57001
Vaquita      TCTAAGAGACCCAGATTAGGAACCCATGGACCATGGCGCACCTTCAAACTTAGCGTGTTCAATT-AACTCCTGTCACCAC  53321
Pig          TGGACACTAGTCAGATTCGTTTCCGCTGAGCCACAATGGGAACTC---CTGA---TGTTGATAT-AAATTCTGACGCAAA  49935
```

```
Majority      CTCCTXA--TTCATGCAGATTG--XTTTGGGCTGCTA-----------------------------------------

                        76170     76180     76190     76200     76210     76220     76230     76240

Human         TGTCTCG--ATCTTACAGATTGCTGTTCCATTTGCTAATACTATTTTTTCTCTTCATCTCTTTATGCCCAACTTCTGCTT  60514
PolarBear     TTCCTTACCTTCATGCAGATAG--GTTTGGGCTGATA-----------------------------------------  22191
Cow           TGCCTCT---CCAGTCTCATCT--TACACCTCTGTAA---CCATATTGGGCTTTTTTTTTTTTTTTATGATTTTTATTG  56432
Dog           CTCCTTATCTTCATGCGGATAG--GTTTGGGCTGCTA-----------------------------------------  52613
BelugaWhale   CACCCCCC-ACCACCAAAAATA--CGAAAGCCTGTATTTTCTGTATCAATAAAGGTCAAGGTACTATGAATCTTAAGAAA  52046
Donkey        TGCAAAATGATTATCACCATAGC-ATGTGTGCTGCCG-----------------------------------------  57696
Cat           CTCCTT---TTCATGCAGAGTG--ATTTGGACAGCTA-----------------------------------------  59569
Ferret        CTCCTTATCTTTAGCCAGATAG--GTTTCGGCTGCTA-----------------------------------------  59994
Lion          CTCCTT---TTCATGCAGAGTG--ATTTGGACAGCTA-----------------------------------------  57033
Vaquita       CACCCCCC-ACCACCAAAAATA--CGAAAGCCTGTATTTTCTGTATCAATAAAGGTCAAGGTACTATGAATCT-AAGAAA  53397
Pig           CTCCTCT---TCATGCGGAGTG--ACCTGGGCTGCTA---GTGTATGAACCAGTAGTACTGTTTCAAGCCATCCTCTCTT  50007

Majority      -------------------------------------X------------------------GACCAXG---AATCAXX

                        76250     76260     76270     76280     76290     76300     76310     76320

Human         CTCTTTGTCTTTCAAGTATCAGTTTAGAAGCCTCTTCCTGAGATAAGTCTTCTGTAACATTTCAACTAGATTAGATCCCT  60594
PolarBear     ---------------------------------------------------------------GTCCATG---AATCA--  22203
Cow           TGGTAAAAGTCACG---------TAAAAAATACTATTTTAACCATATTTAACTGCACAGTTC--AGTGGC---ACTAAGC  56498
Dog           ---------------------------------------------------------------GTTCATG---AATCA--  52625
BelugaWhale   CAAGCCAGAAAACG---------TTGAATCTTCCTTTATTCCTCTTCCTTTCAGCTTATCACCAAATAGT---ACTCATT  52114
Donkey        -----AAG----------------A------------GT-----------------------AAACAAG---AATGGTT  57716
Cat           ---------------------------------------------------------------GTCCAAG---AATCC--  59581
Ferret        ---------------------------------------------------------------GGCCATG---AATCACA  60008
Lion          ---------------------------------------------------------------GTCCAAG---AATCC--  57045
Vaquita       CAAGCCAGAAAACG---------TTGAATCTTCCTTTATTCCTCTTCCTTTCAGCTTATCACCAAATAGT---ACTCATT  53465
Pig           AAATCTGTGCTTCA---------T------TGGCTTTCTGACTAAACTCA-CCACTTTGAAT--ATTAAG---AATGATC  50066
```

```
Majority     XCXT-CCGTAAAGCTCT----AATTTATCCCATCATC-----TCTTAATTCCTTACXTTAT---------TGG-------
                     76330     76340     76350     76360     76370     76380     76390     76400
Human        TCCTTTCTTAGTTCTCAGTATCACACATAAAATTTATAATT-TGTTAAATGTGTATATTTTCTGTCGGACTGTAAGTTCT  60673
PolarBear    GTTT-GAGTAAAGCACT----GATTTAAGCCATCATC-----TCTTAAATCCTTACTTCAT---------TGG-------  22257
Cow          ACATT-CATATTGTTGTGC--AACTTTCACCATCATCCATG-TCTGAATTTTCCACCTTCC----TAAACTGA-AACTCT  56569
Dog          GCTT-GAGTAAAGCCCT----AATACAAGCCATCATC-----TTTTAAATCCTTATTTCAA---------TGG-------  52679
BelugaWhale  CTAGACCCTAAATTTCTGTTGAATCTATCCTTTTGTCTCCA-ACTCTTTTGCCATCACCATAGTCTAAGCTAGTGTCATT  52193
Donkey       TATTCTCATAAGTGTCC--TAATTCTTTTGCATTCTCCTTTCTTTTTATTCATCACTGTGC------------T------  57776
Cat          -----CAGAAGGCCTCT----GACTCACCTCACCA-------CTTTGAGTTCTTCCCCTA--------------------  59625
Ferret       GCTT-GAGTAAAGCACC----AATCCAAGCCATCATC-----TCTTAAATCCTTACTTCAG---------TGG-------  60062
Lion         -----CAGAAGGCCTCT----GACTCACCTCACCA-------CTTTGAGTTCTTCCCCTA--------------------  57089
Vaquita      CTAGACCCTAAATTTCTGTTGAATCTATCCTTTTATCTCCA-ACTCTCTTGCCATCACCATAGTCTAAGCTAGTGTCATT  53544
Pig          TCAA--CTTAAAGTTGT----A-TTTCTTCCTTTCTTCCTT-TCTCAGTGCCCTTCAGTGAA---TGCTTTGG-GTTTTT  50134

Majority     -------------------CTTTTCACT------------------------------------CXAXTCATAX------
                     76410     76420     76430     76440     76450     76460     76470     76480
Human        GAGTACCATGTCTGCTTTGCTTTCTGCTGTATTTATTAAACATTGACATTCAGGGTGAAAATCAGAAGTCATAGTAACAA  60753
PolarBear    ----------C--------CTTCTGACT------------------------------------CAGTTCAC--------  22275
Cow          GTCTCTATTAAACA-CTAACTCCCCAGTTCCCCTCCCCAACTTCCACTCCCAGTCC----CCTGGCATCTACCA---GTG  56641
Dog          ----------T--------CTTCTGACT------------------------------------CCGCTCACAA-C----  52700
BelugaWhale  CAAAGTGCTAGTCT-TAGATGCTACAAGGCACTTGTGCAAAAATACAAATCAGCAT----ACTTCTAGCTATAGTTTGTT  52268
Donkey       -------------------CTGTTTA---------------------------------------TATTCAT--------  57790
Cat          -----------------------------------------------------------------CACTTAT--------  59632
Ferret       ----------C--------CTTCTCACT------------------------------------CAGCTCACAA-C----  60083
Lion         -----------------------------------------------------------------CACTTAT--------  57096
Vaquita      CAAAGTGCTAGTTT-TAGATGCTACAAGGCACTTGTGCAAAAATACAAATCAGCAT----ACTTCTAGCTATAGTTTGTG  53619
Pig          TTGTTTGTT---TG-TTTGCTTTTTGGT--CTTTTTTCAGGGCCCACCCATGGCAT----ATGAAAATTCCCAG---GTC  50201
```

```
Majority     ----TTXXXTCTX-----X--------------TTXTXCCACAX-----------------X-X-T-----------

                       76490     76500     76510     76520     76530     76540     76550     76560

Human        TCTTAATTATCAATAAAAAGTATTTTATGCGATCCTCATATTTATTAAAGAAAACACCTTAATACCTTTCAGAAAATCTT  60833
PolarBear    ----------CTA--------------------TTTTTCCAC-------------------------------------  22287
Cow          TACTTTCTGACTATGAATTGGAGTATTCTAG---GTATCTCATAAATGGAATCAAACAGTATTTGTCTGCATCTAATGTG  56718
Dog          ----TTTTACTTC--------------------TTAGCCCACAC----------------CTGTTT-----------T  52727
BelugaWhale  TAGTGTCAGTGTTGATAATAAATTTGGACACAAAGTTCTCTTCATGCAGAGTGACTTGGGTTGCTAGTCCATGAACCACA  52348
Donkey       ----TTTCATCTACCAG-T-----A--CAG---TTTTTTCAAAC-----------------------------------  57819
Cat          -----------TT--------------------TTCTCCC---------------------------------------  59641
Ferret       ----TTTGACTTC--------------------TTCCCCCACTC----------------CTGTTT-----------T  60110
Lion         -----------TT--------------------TTCTCCC---------------------------------------  57105
Vaquita      TAGTGTCAGTGTTGATAATAAATTTGGACACAAAGTTCTCTTCATGCAGAGTGACTTGGGTTGCTAGTCCATGAACCACA  53699
Pig          AGGGGTCAGATTG-GAGCTACAGCTGCTCGC---CTATGCCACAG-CAATGCCAGATCCAAGCTATGT-CAGCGAATG-A  50274

Majority     --------TTC----A-XAGAGTACTAAG-TA-----X-------TGATC-------------------------T--

                       76570     76580     76590     76600     76610     76620     76630     76640

Human        GTTTATTTATTGGAAAGTAGTGTATTAGGCAAAGATGCGTCTTCTCTTGGCTTTCTAATTCAGCTTTTATAATGTTGAGG  60913
PolarBear    ------------------AGAGTACTAA---A-------------TGATC-------------------------T--  22304
Cow          TATTGAACTTC----TTTAAATTCTTGTG-TATGCTGTGTATTCTCTGATCTCATGGGCTTACAACTTGCTATATT-CTA  56792
Dog          --------TTC----CACAGAATACTAAG-AA-------------TGATC-------------------------T--  52752
BelugaWhale  GTTTGAGTAGC----ACTGATGTAAGCCA-TCATCTCTTAAATCTGTGTTTCATTGGTCTTCTGACAAAACTCACCACTT  52423
Donkey       --------TAT----AAAGCAATGTTGAG-GTCTTTGTCA-----CTGTTATATT----------T-------------  57857
Cat          -------------------GAGCACTAAG-GA-------------TGAT--------------------------T--  59658
Ferret       --------TCC-------AGAGTACTAAG-AA-------------TGATC-------------------------T--  60132
Lion         -------------------GAGCACTAAG-GA-------------TGAT--------------------------T--  57122
Vaquita      GTTTGAGTAGC----ACTGATCTAAGCCA-TCATCTCTTAAATCTGTGTTTCATTGGTCTTCTGACAAAACTCACCACTT  53774
Pig          CACCACACCTC----ACGGCAATGCTGGA-TA--C--TTAACCCACTGAGC----AAGGTCAGGATTG----ACC-CTC  50336
```

```
Majority     ---------X----CAACTTAAAGTCXTATAACTT----CCATTCTCA-GTGCCCTTCAGTG--ACTGACTTTTGTCCTT

                      76650     76660     76670     76680     76690     76700     76710     76720

Human        AATATAATTACTTGATGCATAAAAATATCAATCTTTTTAATAGTATAATAAGACATATAATGTAATCTCCTTTTCACCTG  60993
PolarBear    --------------CAACTTAAAGTCCTATAACTT----C-ATTCTCA-GTGCCCTTCAGTG--ACTGACTTTTGTCGTT  22362
Cow          TGTGCTAAAATACTTTTCTCAAGACTATACTTTGAGGGATCATTTCTA-TAGTTCATTACTG--GA-GAAGTTTCTCTGA  56868
Dog          --------------CAACTTAAAGTTCTGTAACTT----C-ATTCTCA-GTGCCCTTCAGTG--ACT-----TTTTCCTT  52805
BelugaWhale  TGAGTTTTAAGAATGATCTCAAAGTCATATATCTT----CCTTTCTCA-GTGCCCTTCAGTG--ACTGATTTTTGTTCTA  52496
Donkey       ---------AACAATTATTTGGCTTTATATAGATTTTC-TGACCCCAG-GTAAGGTAGAAAG--A---AATTATTTCCTT  57921
Cat          --------------CAACATAAAGTCCTATTACTT----CCATTCTCA-GTGCCCTTCAGTG--ACT----TTTGTCCTT  59713
Ferret       --------------CAACTTAAAGTCCTATAACTT----CCATTCTCA-GTGCCCTTCAGTG--ACTGACTTTTGTCCTT  60191
Lion         --------------CAACATAAAGTCCTATTACTT----CCATTCTCA-GTGCCCTTCAGTG--ACTGGCTTTTGTCCTT  57181
Vaquita      TGAGTTTTAAGAATGATCTCAAAGTCATATATCTT----CCTTTCCCA-GTGCCCTTCAGTG--ACTGATTTTTGTCCTA  53847
Pig          TGTCCTGAGGGATACTAGTCGGGTTCGTTAACCACTGAGCCACGACGG-GAACTCCCT---------GATTTTTGTCCTT  50406

Majority     A-GTATAAAGTCCTCA-XTCCTTAA--CATGGCTTACAAAAC-------TCTAAATCTATCCTC----X--AGTX-TTGT

                      76730     76740     76750     76760     76770     76780     76790     76800

Human        GGATTTAAAAATTGTTTTTACATAGTCCTTACATTATAACACACATAAGTAGGAAATGGTTCTT----TTTGGTTTTGT  61069
PolarBear    A-GTATAAAGTCCTCA-GTCCTTAA--CATGGCTTACAAAAC-------TCTTAATCAATCCTC-------AGC--TTGT  22422
Cow          ACGTGTAGAGCACCCG-AAACCACG--AGGAGGAGATAAAGCGTTCTCTTCTGAGTGTGAAGTCGGCTTTTGGTG-TTGC  56944
Dog          A-GTATAAAGTCCTTA-CTCCTTAA--CAAGGCTTGCAAAAC-------TCTTCATCTATCCTC-------AGC--TTGT  52865
BelugaWhale  A-ATATAAAATCCTCC-TTCCTTAA--CATAGCTTATAAAAC---------TAAATCTTGCCTC----TCCAGTA-TCAT  52558
Donkey       ATCAGTGAATCTGTCATAGTAATAA--TAGGAAAGGCATAAGAATAA----TAAAGGCAGTCTTAAA-AGGAACATTTAA  57994
Cat          A-GTATAAACTTCTCA-GCCCTTAA--CATGGCTTACAGAAC-------TTTAAAGCAATCCTC-------AGTGTTTGT  59775
Ferret       A-GTATAAAGTGCT---GTCCTTAA--CGTGGCTTACAAAAT-------T--TAATCAGTCCAC-------AGC--TTTT  60247
Lion         A-GTGTAAACTTCTCA-GTCCTTAA--CATGGCTTACAGAAC-------TCTAAAGCAATCCTC-------AGTGTTTGT  57243
Vaquita      A-ATATAAAATCCTCC-TTCCTTAA--CATAGCTTATAAAAC---------TAAATCTTGCCTC----TCCAGTA-TCAT  53909
Pig          A-GTGTAAAGTCCTCC-TTCCTCAA--CAAAGCTGACAAAAC-------CCCAAGTCTTGACTC----TCCAGTC-TCAT  50470
```

```
Majority     CTT-------ACCTTTTACTAT-----ATTGGACTTTTTAXAAXAXTAGXXTATTTTTXXXG--------TATTTTCTCT
                     76810     76820     76830     76840     76850     76860     76870     76880
Human        TATTAGAAACAGCTTTTCAGAAACATGTCTTTATTAGGAATATCTTTAGTTGACACTGGACAGTATATTGTGTCTTCTGG  61149
PolarBear    CTC-------GCCTTTTACT-----------GACTTTTTA--AAA-TAGCTTGCAAATGCTG--------TATTTTCTCT  22473
Cow          TTCTGCAGCTGCCATTAGCCATTGATGATCGTTCTTCTTTTCCTTTTAGGAGATGAAGAGGGAAAGA--ACGTAATCTGA  57022
Dog          CTC-------ACCTTTTACTAT-----ATTGGACTTTTTA--AAA-TAGATTGCATGTGCTG--------TTTTTTCTCT  52922
BelugaWhale  CTT-----CCACCTTTAACCAT-----ATTGGACTTCTTTAAATGCTTGCATATTCTA------------TATATTCTCT  52616
Donkey       CAT-------ATGATTTAGTTG-----ACAGATTTCCTGAAAATACTGTATTTCTTCAATTCTCTAG--GTATTTTTTTT  58060
Cat          CTT-------TCCAATCTCATC-----TCTCCTCTTTTAACTAAATTGGAATTTTTTTAAAG--------TTTTTCCTTT  59835
Ferret       CTT-------GCCTTTTACTAT-----ATTGGACTTTTTA--AAAATAGCTTTCATGTGCAG--------CATTTTCTCT  60305
Lion         CTT-------TCCAATCTCATC-----TCTCTTCTTTTAACTAAATTGGAATTTTTTTAAAG--------TTTTTCATTT  57303
Vaquita      CTT-----CCACCTTTAACCAT-----ATTGGACTTCTTTAAATGCTTGCATATTCTA------------TATATTCTCT  53967
Pig          CTC-----CTACCTCTAAGTAT-----ATCAAACTTATGTTTGTGCTTGTGTATGCTG------------TATTTTCTCA  50528

Majority     GATTT--CTTAGXCTTA-CAXCTTGATGTTCCATTTGCTTA--TTCTTTTCTCTTCGTCTATTTATGCCCAAATCTTTCC
                     76890     76900     76910     76920     76930     76940     76950     76960
Human        AAGTCCTGTTTATATGATCATTTTATTTTAACATATAAGTGGGTTGTTTCTTATTC-TTTATTTCTTCCAGGAGATTCCA  61228
PolarBear    GATTT--CTCAGGCTTC-CAGCTTGATGTTCCATTTGCTTCTATTCCTTTTTCTTTGTCTTTTTATGTCC-AGCTCTTCC  22549
Cow          GTGGA--AAAAAAAAAA-TAAATAAA--------------A--TACTTTTCTCTTCATCTCTTTATGACCAACTCATTTC  57083
Dog          AATTT--CTCAGACTTA-CAGCCTCATGTTCCATTTGCTTC---TGCTTTCTCTTTGGTTTTTTATCTCC-AACTTTT--  52993
BelugaWhale  GATCT--CATGGGCTTA-CAACTTGCTGTTCTGTTTGCTAA--AACTGTTCTCTCCATCTCTTTATGCCCAACTCCTACC  52691
Donkey       GCATT--TTAAAATCTCAGAAATCAGGGTGTGTCTCACAGCAGTTCATCTGTCGTGGTTTAATTAGCAGT--ATCTTCTT  58136
Cat          TATTTATTTTAGAGAGAGTGCGTAGGTGGGGGAGTGGGACAGAGGAGGAGGAG---------GAGGAGACAGAGAATCCT  59906
Ferret       GATTT--CTCAGGCTTAACAGCTGGATGTTCCATTTGCTTCTACTGCTTTCTCTTTGTCTTTTTATGTCCCAACTTTTCC  60383
Lion         TATTTATTTTAGAGAGAGTGCGTAGGTGGGGAAGTGGGACAGAGGAGGAGGAGAAGGAGGAGGAGGAGACAGAGAATCCT  57383
Vaquita      GATCT--CATGGGCTTA-TAACTTGCTGTTCTGTTTGCTAA--AACTGTTCTCTCCATCCCTTTATGCCCAACTCCTACC  54042
Pig          GATCT--CATAAGCTCA-CAGCTTG------------------CTCTTTTACTTGCTAAAATTTTTTCTTAGCTC-TTTA  50586
```

```
Majority     TGTC---TTTCAGGTTT--------AAXTCACATCTC-AGAGAGGT-----CTTCCATAAXCTTTCAAC---TAXATTAX
                      76970     76980     76990     77000     77010     77020     77030     77040
Human        TTTCATCACTAACTGATAAT----AAAAATAAATATGAGATAAAACATTTGCAGCAGTAAGATTATGTCATTCATTTCCT  61304
PolarBear    TGTCTTCTTTCAGGTTT--------G---CAAGTCTC-AGAGAGGT-----CTTCCATAAAGTTCCAAC---TACATTAT  22609
Cow          TGTC---TTTCAGGTTTCAGTTTAGAAATCTCTTCTCCAGAGAGGT-----CTTCTATAACCTTTCAAC---TAGATTTG  57152
Dog          -GTC---TTTGAGGTTT--------A---CACATCTC-AGAGAGGT-----CTTC-GTAAACTTCTAAC---TACATCAT  53048
BelugaWhale  TCTC---TTTCAAGTATCAGTTTAGAAGTCTCTTCTC-AGAGAGGT-----CTTTCATAACCTTTCAAC---TAGATTGG  52759
Donkey       AGTCATACATGAAAAATCATACTGCATTTTGTAATTT--ATGGTGTA----TTCCTGAAAATTTTGTGCCTTTGTACCAA  58210
Cat          AAGCAGGCTCCATGCTC--------AGCACAGAGCTC-AACACGGGACTCATTTCCACAACCCTGGGAT---CATGACCC  59974
Ferret       TGTC---TTTCAGGTTT--------G---CAGGTCTC-AGAGAGGT-----CTTCCATAAACTTCCATC---TACATTAT  60440
Lion         AAGCAGGCTCCATGCTC--------AGCACAGAGCTC-AACACGGGACTCATTTCCACAACCCTGGGAT---CATGACCC  57451
Vaquita      TCTC---TTTCAAGTATCAGTTTAGAAGTCTCTTCTC-AGAGAGGT-----CTTTTATAACCTTTCAAC---TAGATTGG  54110
Pig          TGCC---CCTCAGATTT-------GAAGTCTCTTCTT-----AGAT-----CATCTGTAAACTTTGAAC---TAGATTAG  50643

Majority     ATCCC-----------TTCCTTTC--TTAGTTCTC---ACXACC---ACACATCAAATTTATAATTATTTGTTAAAT---
                      77050     77060     77070     77080     77090     77100     77110     77120
Human        TCTCACAGAGTACACATTGCTTTATGCAGATTCTTTGAGAGACCTA-ATGGAATTATCCTTTGCTAATTTCTTGGGCAAA  61383
PolarBear    ATCCC-----------TTC-------TTAGTTCTC---ACTACC---ACACATCAAATTTATAATTATTTGTTAGAT---  22662
Cow          ATCCC-----------TTCTTTTC--TTAGTTCTC---ACCACC---ATGGATCAAATTTATGATTACTTATTAAGT---  57210
Dog          AGCCC-----------TTCCTTTC--TTAGTTCTC---GCTACC---ACACATTAAATTTATTT---ATAATTACTT---  53103
BelugaWhale  ATCCC-----------TTCCTTTC--TTAGTTCTC---ATCACT---GCACATCAAACTTAAAATTACTTGTTAAGT---  52817
Donkey       AGTACAGTAGATCAATTTCGGTTC--TAAAGATTTT--GGTGTC---AGAAGTTACACTAGTATGAAAAAAAGACAT---  58280
Cat          AAGCTGAAATCAAGAGTTGGACAC--TCAAGTAACTGAGCCACCCAGGCACCCCAACTATATTGGACTTTGTAAAATA--  60050
Ferret       ATCTC-----------TTCCTTTC--TTAGTTCTC---ACTACC---ACAAATCAAATTTGTA----TTTGTTAGAT---  60494
Lion         AAGCTGAAATCAAGAGTTGGACAC--TCAAGTAACTGAGCCACCCAGGCACCCCAACTATATTGGACTTTGTAAAATA--  57527
Vaquita      ATCCC-----------TTCCTTTC--TTAGTTCTC---ATCACT---GCACATCAAACTTATAATTAGTTGTTAAGT---  54168
Pig          ATACC-----------TTCCTTTC--TTAGTTCTC---ACTGCC---ACACATTACATTTATA--TATTTGTTAAAT---  50699
```

```
Majority     GTCTG-----------------------------------------------------------------------

                     77130     77140     77150     77160     77170     77180     77190     77200

Human        GATTCTCCTTGACATCTGCTTAATTTACAATCTGTATATTTTACATTCATATTAATGGAGAATTTATGATGAAATCAGTG  61463
PolarBear    GTCTG---------------------------------------------------------------------------  22667
Cow          GTCTGAATTCTGGAGGAAGAAATGGCAACCCACTCCAGTATTCTTGCCTGGGAAATCCCACGGACAGAAGACCTGCTGGG  57290
Dog          GTCTG---------------------------------------------------------------------------  53108
BelugaWhale  GTCAG---------------------------------------------------------------------------  52822
Donkey       GTATC----------------------------------------------C--TGAATATTCCTCTATTAAGATCGTATT--  58313
Cat          GCTTG---------------------------------------------------------------------------  60055
Ferret       GTCTG---------------------------------------------------------------------------  60499
Lion         GCTTG---------------------------------------------------------------------------  57532
Vaquita      GTCTG---------------------------------------------------------------------------  54173
Pig          ATAT----------------------------------------------------------------------------  50703
```

```
Majority     ---------------------------------------------------------------TATTTTCTG-TTTTACT

                     77210     77220     77230     77240     77250     77260     77270     77280

Human        GTTTTAGGATGCTTTAGGAAAATCCTGCATAATCTACCAGGGTGGGGTTATTTGATATTGGTAAATAATTAGTCTGAAGT  61543
PolarBear    ---------------------------------------------------------------TATTTTCTG-TTTTACT  22683
Cow          CTGTAGTCCATAGGGTTGCAAAAGAGTTGGGCATGACTGAGCAACTAAACAAAAAAGTGTCTGTGTTATCTG-TTGGACT  57369
Dog          ---------------------------------------------------------------TATTTTTTG-TTTTACT  53124
BelugaWhale  ---------------------------------------------------------------TATTATCTG-TTGGACT  52838
Donkey       ----AG-----------------------------------------------------AA-------ATAATAAGGTTTA--T  58331
Cat          ---------------------------------------------------------------CATATGCTGTATTTTCT  60072
Ferret       ---------------------------------------------------------------TATTTCCAGGTTTTACT  60516
Lion         ---------------------------------------------------------------CATATGCTATATTTTCT  57549
Vaquita      ---------------------------------------------------------------TATTATCTG-TTGGACT  54189
Pig          -----------------------------------------------------------------TTTTCCG-TTGGACT  50717
```

```
Majority     XTAAGTT-----ATATAAGAGTGGGT--ATCTTGTCTGCTTTGTT-----CTCTGCTGTATTXTTAGAACCTAGCACA--

                         77290     77300     77310     77320     77330     77340     77350     77360

Human        ATTACATTAGTATTATAAGCTTAATTTTGTGTTGTTGATTTTGATAAT--TTTTCATGACTTGCCAAATATTAAAAAGTA  61621
PolarBear    GTAAGTC-----ATGTAAGAGTGGGT--ACCTTTTCTTCTTTGTT-----CTCTGCTGTATTCTTAGAACGTAGCACA--  22749
Cow          ATAAGCT-----GTATAAGAGTGGTT--GTCATGTCTGCTTTGTT-----CTCTGCTGTGTTCTTAGAACCTAACACA--  57435
Dog          CTAAGTT-----ATGTAAGAGTGAGT--ACCTTGTCTGCTTTGTTCTTTGCTCTGCTGTATTCCTAGAACATAGTGCA--  53195
BelugaWhale  ATAAGTT-----ATATATGAGTGGGT--ATCATGTCCACTTTGTT-----CCCTGCTACATTTTTAGAACCTAGCACA--  52904
Donkey       GGGAGTTCTT--TTGGATAATCTAGT--AATCCGCTTGTTAAG-------ATTTACCGTGTTTCTTTTTTATGGAGTG--  58398
Cat          CTGATTTCTCAGGCTTAGAACCTGAT--GTTTCATTTGCTTCTACTGCTTTCTCTTTGTCTTTTTATGTC-CACCTCT--  60147
Ferret       GTAAGTC-----ACATAAAAGTGGGT--ACCTTGTCTTTTTTT-------CTCTGCTGTATTCCTAGAACACAACACA--  60579
Lion         CTGATTTCTCAGGCTTAGAACCTGAT--GTTTCATTTGCTTCTACTGCTTTCTCTTTGTCTTTTTATGTC-CACCTCT--  57624
Vaquita      GTAAGTT-----ATATATGAGTGGGT--ATCATGTCTACTTTGTT-----CCCTGCTACATTTTTAGAACCTAGCACA--  54255
Pig          CCAAGTT-----ATATAAGAGTAGGT--ATCATGTCTGCTTTGTT-----CTCTGCTATGTTCCTAGAACCCGGCACA--  50783

Majority     -XTGTATGGTACATTGTAGGTATTAGAACTTTCTCAA--CATGTCACAACT--TTCCCAGCATCTGXTGTGCTACAG-AT

                         77370     77380     77390     77400     77410     77420     77430     77440

Human        GATATTTAAAGAATTTGGAAGTTAAGGTTTCTCTCAGTGTGTGTTAAATCTGATCCCCAACAATCTCTTCAAAAAAGTAA  61701
PolarBear    -GTGTATGGCACATTGTAGGTATTAGAACTTTCATCG--CATGTCACAACT--CTCCCAGCATCTGTTGTGCTAC-----  22819
Cow          -TTATA-GATAT---GAGGGCATT-----TTTTTCAA--CATGACACAACT--TAGCCGACAGCTGGTGTGCTGTAG-AT  57500
Dog          -GTGTATAGCAC----------TTAGAACTTTCATCC--CGTGTCACAACT--CTTCCAGCATCTGCTCTGCTACAG-AT  53259
BelugaWhale  -GTGTATGGTGTATTACAGGTATTAGGGCTTTTTCAACCCATGTCACAACT--TTCCCAGCACCTAGAGTGCTGTAG-AT  52980
Donkey       -AATAATGTTACATTG-AAGTTTTATTGTTCTCTGA----AAGTTTCTGTT--TTCTGATTATATGGAT--CTACCA-AA  58467
Cat          -ACCTGTCTTCCTCTTTCAGGTTTAGAAGTCTC----------TGAGAAGT--CTTCCGTAAACTTCCA-GCTACAT-CA  60212
Ferret       -ATATATGGCACATTGTTGGTATTAGAACTTTCCTCT--CATGTCACAACT--CTCCCAGCATCTGCTATGTTACAA-AT  60653
Lion         -ACCTGTCTTCCTCTTTCAGGTTTAGAAGTCTC----------TGAGAAGT--CTTCCGTAAACTTCCA-GCTACAT-CA  57689
Vaquita      -GTGTATGGTGTATTACAGGTATTAGGGCTTTTTCAACCCATGTCACAACT--TTCCCAGCATCTAGAGTGCTGTAG-AT  54331
Pig          -GTGTATGGTGTATTGTAGGTATTGGA-CTTACTCAAGCTGTGTCACAACT--TTCCCAGCATCTGGTTTGCTATAG-AT  50858
```

```
Majority     CT-CTTCXTACTTTXCAATCATCCATGT----ACATCTC-AGTXTGTAATXAAATGAGTTGGTTCTAAACTTTGTAATTC
                                 77450     77460     77470     77480     77490     77500     77510     77520
Human        GTTATCAATAGTAAACGATGAGAAAAATGGAAATAGTTTTATTTTGGCTTTTCATGTTTTCAGTCCATATGCAGTGTTTC  61781
PolarBear    --------CATTTTGCAGTCATCCTTGT----ATATCTC-AGTATATTATGGAATGAGTTGGTTCTAAACTTTGTAGTTC  22886
Cow          CT-CTTCCTACCTTTCAACCATCCATGT----ACATCTC-AGTGTGTAATAAAATGAGTTAGATCTAAACTTTGTAATTC  57574
Dog          GT-CTTCTCGTTTTGCAGTCATCCTTGT----GGATCTC-AGTATATAATGAAATAGGTTGGTTCTAAACTTTGTAGTTC  53333
BelugaWhale  C--CTTCCGACCTTTCAACCATCCATGT----ACATCTC-AGTGTATTATAAAATGAGTTGATTCTAAACTTTGTAATTC  53053
Donkey       CTGCATCTTGTTGTTTGAGAATAAAT-T----ACATTTCCTGTCTTTGCTTGTTTAGCATTGGTTTAGGCGTTGGTCTCA  58542
Cat          CA-TCCCTTCCTTTATTCTCACTACCAC----ACATCA--AATTTGTAATTACTTGTGAGATGTCTATATTTTGTTTTCC  60285
Ferret       CT-CTTCCCATTTTACAGTTATCCTTAT----ATATCTC-AGTATGTTATGGAATGAGTTGGTTCTAAACTTTGTAGTTC  60727
Lion         CA-TCCCTTCCTTTGTTCTCACTACCAC----ACATCA--AATTTGTAATTACTTGTGAGATGTCTATATTTTGTTTTCC  57762
Vaquita      C--CTTCCGACCTTTCAACCATCCATGT----ACATCTC-AGTGTATTATAAAATGAGTTGATTCTAAACTTTGTAATTC  54404
Pig          TT-CTTCACGCCTAGCAGTTATTGATGT----ACAT-T---------A-TAAAATGAGTTGGTTCTAAACTTTGTGATTC  50922

Majority     AGTT-GCTAXATTT-TTATGCTTGTT------AAAACAGTAGAGGTCATATAAAATT-ATAGGCAAAGAATAX-----AA
                                 77530     77540     77550     77560     77570     77580     77590     77600
Human        ATTGTGCTGCTTTAATTATGCTTTCTGTGCCTAATATCTTTCCTGTGTTTTGATTTCCATGTTTTAGCCATAAACTTTAA  61861
PolarBear    AGT--GCTACATTT-TTATACTTGTT------AAAACAATGGAGGTTATATAAAATT-ATAGCCAAAGAATAC-----AA  22951
Cow          AGGT-GCTACATTT-TTATGCTTGTT------AAAACAGTAGAAATCATGTAAAATTTACAGGTAAGGAATAA-----TA  57641
Dog          AGTT-GCTACATTT-TTATGCTTGTT------AAAATGGTGGAGGTTATATAAAATT-ATAGGCAAAGTATAC-----AA  53399
BelugaWhale  AGTT-GCTGTATTT-TTATGCTTGTT------AAAACAGTAGAGATCATGTAAAATTTATAGGTAAGGAATAA-----GA  53120
Donkey       AACT--CTAGATAA-TAGTTTTGAAA------TAAACACTAGTAGAATAGTTGAATT-ATAAACAGACAATACC----TG  58608
Cat          TATAAGTTATATAA-GAGTGGGTGGC------TCTTCTGCTTTGTTCCTAGAACAT--ATATTCCTAGAACAT-----AG  60351
Ferret       AGTT-GCTACATTT-TTATGCTTGTT------AAAACAATAGAAGTTATATATAATT-ATGGGCAAAGAATAC-----AA  60793
Lion         TATAAGTTATATAA-GAGTGGGTGGC------TCTTCTGCTTTGTTCCTAGAACAT--ATATTCCTAGAACAT-----AG  57828
Vaquita      AGTT-GCTGTATTT-TTATGCTTGTT------AAAACAGTAGAGATCATGTAAAATTTATAGGTAAGGAATAA-----GA  54471
Pig          AGTT-GCTATGTTT-TTATGCTTGTT------AAAGCAGTTGAGGTCATCTAAAATTTATAGATAAGGAATA-------A  50987
```

```
Majority     GTTTGGGAAGAGAATCTXAAGCCTACTTCTCCTTTTCTATTG-XTGATAGTT---CCATATGAATGAGAGA-TGAAAAAX
                               77610     77620     77630     77640     77650     77660     77670     77680
Human        ATTTACATGAAAACTTTTTTTTCTGATTGTTTTTGCTTACTGTCTAAACCTTCA-CTGTGAGTAGGCACATTTGGAAGGT 61940
PolarBear    GTTTAGAAAGAGAATCTCAAGCCTACC-----TCTCCTAGTG-ACAGTTGTT---CTATATGAATGGGAGA-TGAAAAAT 23021
Cow          GTTTGGGAAGAGAATTTCAAGCCTGCTTCTCTACTTCTATAG--TGATAGTTGA-TCAGATGATTGAGAGAATGAAAAAA 57718
Dog          GTTTAGAAAGAGAATCTCAAGCCTACCTCTACTTTTCTAGTG-GCAATTGTT---CCAAATGAATGGAAGA-TGAAAAAT 53474
BelugaWhale  ATTTGGGAAGGGAATCGTAAGCCTGCTTCTCCACTTCTATAG--TGATTGTTGT-TCAAATGAATGAGAGAATGACAAAA 53197
Donkey       GCTTGTGGCAGGTGCTCAAAAGTATTTATTGAGTGGGTGTTAAATTTTATTTAATCTATATGTGTTCTAGA--AAAGAAC 58686
Cat          CACGGTGTATAGCACATTGTAGGTATTAGAACTTTCATACTGTGTCACAACT---CTCCTAGCACTTGTGG-GGAACATC 60427
Ferret       GTTTAGAAAGAGAATCTCAAGCCTACCGT--CTCATCTTTTA-GTGACAGTT---CCATATGAGTGGGAGA-TGAAAAAT 60866
Lion         CACGGTGTATAGCATATTGTAGGTATTAGAACTTTCATACTGTGTCACAACT---CTCCTAGCACTTGCGG-AGAACATC 57904
Vaquita      ATTTGGGAAGGGAATCTTAAGCCTGCTTCTCCACTTCTATAG--TGATTGTTGT-TCAAATGAATGAGAGAATGACAAAA 54548
Pig          GTTTGTGAAGGGAACTTCAAGCCTTCCTCTCTGTTTCTGCAG--TGATAGT----TCATATGAATGAGAGAATGAAAAAA 51061

Majority     -X--------ATCT-CTTAAGCXCTGGXAATT---CAGTT-------AGCAGGCAXAGTGTACCTTTAGGTTTTG--GXG
                               77690     77700     77710     77720     77730     77740     77750     77760
Human        CTTTCTGCAAATTATCTTGAGCTTTTAATGTCAGAAATCTTTGCTCCAACAAGATCACCACATTCCTGATTGCTGAACCA 62020
PolarBear    ----------ACAT-CTTAAGCACTGGAAATT---CAGTT-------AGCCAGCAAAGTGTACCTTTAGGTTTTG--CAG 23078
Cow          -T--------ATCA-CCTATGCCTTGGGAATT---CATTT-------AGCAGGCATAGTGTATCTTTAGGTTTGGAGGGG 57778
Dog          ----------A--------AGCACTGGGAATT---CAGTT-------AGCCGACAAAATGTACCTTTACATTTTG--GTA 53524
BelugaWhale  -T--------ATTG-CTTACGCCCTGGGAATT---CATTT-------AGCAGGCATAGTGTACCTTTAGGTTTGG--GGG 53255
Donkey       AA--------ATAG-TGTAACATGTAGCAAAGTAAAAGTGC-----AAATACTGGATGTGTTTCTTAAGTGCTTA--T-- 58748
Cat          ----------ACCT-GCTGTGCTACAGATCTCTTCCTGTTTTG---CAGTCATCCTTGTATATCTCAGTATACTA--C-A 60490
Ferret       ----------ACAT-CTTAAGCACTTGGAATT---CAGTT-------AGCTGGCAAAGTGTACCTTTAGGTTT------- 60918
Lion         ----------ACCT-GCTGTGCTACAGATCTCTTCCTGTTTTG---CAGTCATCCTTGTATATCTCAGTATACTA--C-A 57967
Vaquita      -T--------ATTG-CTTACGCCCTGGGAATT---CATTT-------AGCAGGCATAGTGTACCTTTAGGTTTGG--GGG 54606
Pig          AT--------ATCA-TTTATGCACTGAATATT---CATTT-------AGCAGGCAAAGTGTACCTTTAGGTTTTT-GGGG 51121
```

```
Majority     -AAGAGAATTTATAGTXTTC-T--------TTAAAGTATGGT-----CCAGAGCCT-----AAGXAGGXTXTTGTCATGT

             77770     77780     77790     77800     77810     77820     77830     77840

Human        CAGGACTGGTTTGTGTGCTACTGCTATTTCTTGGGATTTTGCAAATACATGAGCTTGAAGTGAGCTTGCATATATTTTTT  62100
PolarBear    -AAGAGAATTTATAGTGTTC-T--------TCAAAGTATGGT-----CCAGAGCCC-----ACGTAGGTTCTTGTCATGT  23138
Cow          -AAGAGAACTTAGAGTGTTCTT--------TTAAAGTATGGT-----TCAGAACCT------------------------  57820
Dog          -AAGAGAATTTATAGTGTCC-T--------TCAGAGTATGAT-----CCAGAGCTC-----ACCTAGGTTCTTGTCCTAT  53584
BelugaWhale  -AAGAGAA-TTATAGTATTC-T--------TTAAAGTATGGT-----CCAGAGCCCATTCTTATCAGACTGTGGTCCAGA  53319
Donkey       -CTTTCAATTTTCAGCGTGC----------TCAAGCAGTTCT-----TCAAGCTGT-------GACAGCTGTCCAGACAG  58805
Cat          -AAGTGAATTGGTTCTAAAC-T--------TTGTAGTTCAGTTG---CCACATTTTT----AAAAAAGTTTTTTTAATGT  60553
Ferret       -AAGAGAATTTATAGTTTTC-T--------TCTGACTGTGGT-----CTAGAGCCC-----ACCTAGGTTCTTGTCATGT  60978
Lion         -AAATGAATTGGTTCTAAAC-T--------TTGTAGTTCATTTG---CCACATTTTT----AAAAAAGTTTTTTTAATGT  58030
Vaquita      -AAGAGAA-TTATAGTATTC-T--------TTAAAGTATGGT-----CCAGAGCCCATTCTTATCAGACT--GGTCCAGA  54668
Pig          -AAGAGAATTTATAGTA----T--------TTAAAGTATGAT-----CCAGAGCCTAT----------CT----------  51163

Majority     XAATTTCTTTTCATATAXATTACXCATAXAG------------AATGGGACTCTTAGG-TATCCTGAACTAAG-AGTCAT

             77850     77860     77870     77880     77890     77900     77910     77920

Human        AAATTTGATTTTACTCCCTTTCTTATTTGTGGTCACCTCCTTTAACCAACTATTTAAAATACCTGGATTTTTGCTCTCAT  62180
PolarBear    AGATTGCTTGTGGAAAAAAAAAAAAAAAAAAAA-----------AAAAGGACTCTTAGG-TATCCTGACCTAAGAAGTCAT  23206
Cow          -----TCTTTTCATATAGATTGCTTATAAAA------------AATGGGACTCTTAGGCTCCTCTGAGCTAAC-AGTTAT  57882
Dog          AGATTGCATCTATATATAGATATGCATATATACATATGCATCTAATGGGACTCTTAAGATATCCTGAACTAAGAAATCAT  53664
BelugaWhale  TACATTCTTATCATATAGATTGCTTACACAG------------AATGGGACTCTTAGGCCATTCTGAACTAAC-AGTTCT  53386
Donkey       CAAATACTCCTCTTAGTGGCACCACAGTGAGT--------GAGAGTGCAGTGACTCCA--GCCCAGAGTCCAGTGCTTAG  58875
Cat          TTATTTATTTTT----GAGGAAGACAGAGTGT--------GAGCAGGGGAGGTGCAGG---GAGAGAGCGAGAGACACAG  60618
Ferret       AGATTGCTTGTT----AAAAAAAACAAAAAAC-----------ATTGGGACTCTTCAG-TATCCTGAACTAAGAAATCAT  61042
Lion         TTATTTATTTTT----GAGGAAGACAGAGT----------GAGCAGGGGAGGGGCAGG---GAGAGAGCGAGAGACACAG  58093
Vaquita      TACATTCTTATCATATAGATTGCTTACACAG------------AATGGGACTCTTAGGCCATTCTGAACTAAC-AGTTCT  54735
Pig          -ACATTCTTATCATATAGATTGCTTATAAAA------------AATGGGGCTCTTAGGCCATCCTGAACTAAC-AGTCTT  51229
```

```
Majority     AATCTTTGGGGTTGGGAGCCCAGTACTCTGTTT----TTGAAAGCCCTGGGAATXAC-TGCCATGTAAAGGCAGCTTGAG
                             77930     77940     77950     77960     77970     77980     77990     78000
Human        ACACCCT-CAGCTCCTATTTCTTTCCTCTTTTG----CCAACAGATGATTAATTTACATCTTGTTTGCTTATTTCTCTAG  62255
PolarBear    AATCTTTGGGGATGGCAGCTGAGCACT--GTTT----TTGGAAGCTCTGGGAACCAC-TGCTGTGTAAAGGAAGCTTGAG  23279
Cow          AATCTTTGGGATTGGGAGCCCACTGCTCTGTTTAGTTTTCAAGCCCTGGGAATCAC-TGCCATGTAAAGGCAGCTTGAG  57961
Dog          AATCTTTGGGGGTGGGACCCCAGTACTCTGTTT----TTGAAAG--------------TGCTATGTAAAGGCAGCTTGAG  53726
BelugaWhale  AATCTTTGGGGTTGGGAGCCCACTACTCTGTTTTGTTTTGAATGCCCTGGGAATCAC-TGCCATGTAAAGGCAGCTTGAG  53465
Donkey       AATAATTATTGACAACA-TGTACTACCCTGTAACA--CTTGATGTCCT--------T-CACCAAGTAAGTTTACTTTAAT  58943
Cat          AATTT---GAAGCAGGCTCCAAGCTCTGAGCTG----TCAGCAC-----------AG-AGCCCAACATGGG--GCTTGAA  60677
Ferret       AATCTTTGGGGATGGCAGCTCAGTACTCTGTTT----TTGAAAGCTCTGGGTACCAC-TGCTACGTAAAGGCAGCTTGAG  61117
Lion         AATCT---GAAGCAGGCTCCAAGCTCTGAGCTG----TCAGCAC-----------AG-AGCCCAACATGGG--GCTTGAA  58152
Vaquita      AATCTTTGGGGTTGGGAGCCCACTACTCTGTTTTGTCTTGAATGCCCTGGGAATCAC-TGCCATGTAAAGGCAGCTTGAG  54814
Pig          TGGGAGCCCACTACTCTTTGTTTTGTCTTGTTTTGTTTTGAAAGCCCTGAGAAT--C-TGCCATGTACAGGTAGCTTGAG  51306

Majority     TGACAGAGCC--AACCAAACTCT--TTCCATATACTAG-TTAAGATXTTTGGCAGTTATTTTCAGTTAATTGGAA-TTAT
                             78010     78020     78030     78040     78050     78060     78070     78080
Human        CTCTTGTACCTTAGGTATACTTCTGCATCTTCCACCTACATAAAGTTTTTGTTT-TTGTTTTTAATGTTCAGAGGGAAAA  62334
PolarBear    TGATCGCGCC--AATGAAATTCT--TTTTTTATACTAG-TTAAAATTTTGGGCAGTTATTTTCAGTTAATTGGAAAATTT  23354
Cow          TGACATTGCC--AACCAGACTCT--TGCCATGTATTAG-TTACGATCTTTGGCAATTACTTTCAGTTAATTGGAA-TTAT  58035
Dog          GGATAGAACC--AACCAAACTCT--TATTATATA-----TTACAGTTTTTGGCAATTAATTTTAGTTAATTGGAAGATAT  53797
BelugaWhale  TGACAGAGCC--AACCAAACT----TTCCATATATTAG-TTATGATCTTTGGCAATTATTTTCATTTAATTGGAA-TTCT  53537
Donkey       TTGCATGTTT--ACCTATAAATT--GGAGAAATAATACAGTAGATAAGTTGTT--TTGATTTTGATGTTTTAACAGATAT  59017
Cat          CTCATGAACT--A-TGAGATCAT--GACCTGAGCCGAA-GTCAGGCACTCAATGGACTGAGTCACCCAGGCGCCCCTCAG  60751
Ferret       TGATAGAGTC--AATGAAATTCT--TTTTATACACTGG-TTAAAATTTTTGGCAGTTATTTTCAGTTAATTGGAAAATTT  61192
Lion         CTCATGAACC--A-TGAGATCAT--GACCTGAGCCGAA-GTCAGGCACTCAACGGACTGAGTCACCCAGGCGCCCCTCAG  58226
Vaquita      TGACAGAGCC--AACCAAACT----TTCCATATATTAG-TTATGATCTTTGGCAATTATTTTCATTTAATTGGAA-TTCT  54886
Pig          TGACAGAGCC--AACCAGACTCC--TTCCATATTCTAGGTTATGATCTTTGGCAGTTACTTTCAGTTAATTGGAA-TTCT  51381
```

```
Majority      TTCCCCCTAGCATTTT---GCTTTATAAGAGAATAACAACTCXGTAAAAAAXAGATTAACAGTGGAG--GTATTAGT---
                               78090     78100     78110     78120     78130     78140     78150     78160
Human         TTTCAAAGGGTCCTGGAGGGCTTTCTAACAATTGGCATATTCATTGTTAGATAATTTGGCAGTCGG---ACCTCAGT---  62408
PolarBear     TGCCCCCTAGCATTTT---GCTTTATAAGAGAATAACAGCT---TGGAAAA-AATTTAATAATGGAG--GTATTAGT---  23422
Cow           TTCCCCCTAGCATTTT---ATTTTATAAGAGAATAGTAACTCAGGAAAAAAAATCTTAAAAATGGAGTTGTATTAGT---  58109
Dog           TTCCCCCTAGCATTTT---GCTTTATAAGAGAATAACAACT---CAGGAAA-AGATTAACAATGGAA--GTATTAG----  53864
BelugaWhale   TTCCCCCTAGCATTTT---GCTTTATAAGAGAAGAGCAACTCGG--AAAAAAACATTAACAATGGAGTTTTATTAGT---  53609
Donkey        TTTCTA--AGTTTGGT---GCTGTGTTGAAGATAATCACATTTACAAAAAATAACCAGTTTCAAGCTCTCCTTCAGT---  59089
Cat           TTGCTAC-ATTTTTAT---GCTTATTAAGACAATGAAGGCTATATAAAAAACAGATTAACAGTGGA---GCATTTGT---  60821
Ferret        TGCCCCCTAGCATTTT---GCTTTATAAGAGAAGAACAGCT---CAGAAAA-AGACTAACAGTGGAG--GTATTAGTAGT  61263
Lion          TTGCTAC-ATTTTTAT---GCTTATTAAGACAATGAAGGCTATGTAAAAAACAGATTAACAGTGGA---GCATTAGT---  58296
Vaquita       TTCCCCCTAGCATTTT---GCTTTATAAGAGAAGAGCAACTCGG--AAAAAAACATTAACAGTGGAGTTTTATTAGT---  54958
Pig           TTCCCTGTAGCATTTT---GCTTTATAAGAGAATAGCAACTCAG---AAAACATACCAACAGTGGAGTTTTATTAGT---  51452

Majority      GAGGGTTGGAGGG-AAAAATTCAACA-ATCTCXTGAATTTGTXXTGAGGTT-GT-------------------T-X-X-
                               78170     78180     78190     78200     78210     78220     78230     78240
Human         -ATTGCTGGGA---AAGAATTCAACT--CCTAAAAAACTTAGTGTGTGTTTAGTA-ATTTTGTATCAGCACTTTTAGCAT  62481
PolarBear     GAGGGTTGGAGCG-AAAAATTCAACA-GTCCTGAGGATTCGTGTGTGTGTGTGTGT--GTTGTGTGTGTGTGTGTGTGTG  23498
Cow           GAGGGTTGGAGGGGAAAAATTTAACC-ATCACCTGAATTTGTTCTGAGGTTCGTGTATATTGCAGTCCAGTCTCTCTTAA  58188
Dog           ---GGTTGGAGGG-AAATATTCAACA-ATCTCTTGAATTT------------GT-------------------------  53901
BelugaWhale   GAGGGTTGGAGGGGAAAAATGTAACT-ATCTCCTGAATTTGTCCTGAGGTTGG-------------------CTTTAAA  53668
Donkey        -ATGGTGATCCAGTAAATGCTCAACAGGCAAAACTGGTAAGTCTAGACCTTTTT-------------------------  59142
Cat           GAGTGTTGGA-----AAAATTCAACA-ATCTCTTGAATTT------------GT-------------------------  60857
Ferret        GAGGGTTGGAAGG-GAAAATTCAACA-ATCTCTTGAATTT------------AT-------------------------  61303
Lion          GAGTGTTGGA-----AAAATTCAACA-ATCTCTTGAATTT------------GT-------------------------  58332
Vaquita       GAGGGTTGGAGGGGAAAAATGTAACT-ATCTCCTGAATTTGTCCTGAGGTTGG-------------------CTTTAAA  55017
Pig           GAGGATAGAAGGGGAAAAATGTAACCCATCTCCCAAATTTGTCCTGAGGTTGATGTCTGTTACAGTCTAGACTCTTTAAA  51532
```

```
Majority      -A-CCCTAAXGTTCA-----------------------------TAT---------------------ATA----TT-TC

                        78250     78260     78270     78280     78290     78300     78310     78320

Human         TAACCTAGATACTGTGACTCAAACCCAGCTCTTTTCTCATAAGGTATCATTTTCTCTTTGATAACATTGAAGTAGTCACC  62561
PolarBear     TATTCCTAAGGTTCA-----------------------------TAT---------------------ATA----TT-TC  23523
Cow           AACCTCTGAAGTCAGGGGACACATGTACACCCATGGCTAATTCATGTCTATGTATGGCAAAAAACATTACAATATTG-TC  58267
Dog           ----CCTAAGATTCG-----------------------------TATTC------------------TGTA----TT-TT  53925
BelugaWhale   AAGCTCTTAAGTCA----------------------------A--------------------------CA---------  53685
Donkey        --TTTATAAAGTGA-------------------------------------------------------TATT--TT-TC  59162
Cat           ----CCCAAGGTTCA-----------------------------TAT---------------------ATA----TT-TC  60878
Ferret        ----CCTAAGGTTCA-----------------------------TGT---------------------ATA----TT-TC  61324
Lion          ----CCTAAGGTTCA-----------------------------TAT---------------------ATA----TT-TC  58353
Vaquita       AAGCTCTTAAGTCA----------------------------A--------------------------CA---------  55034
Pig           AAGC-C--AAGTCGTAGTTTTTGTCATGGCTCA--GCAGA--AATGAATCTGACTAGCATTCACGAGGACA-CAGGT-TC  51603

Majority      AGTCC-----------------------AATATXTAAAXXTTXT----TXAA--------------------------AG

                        78330     78340     78350     78360     78370     78380     78390     78400

Human         TAAGAGTGAATTTTGCTACTTATCTTTTGATATTTATCTATTGT---GTAGAAACATCAGTTCTATTACTTATCTCACAT  62638
PolarBear     AGTCC-----------------------AATTTCTAAA------------AA--------------------------GG  23542
Cow           AAGTCATGATCTTTTAATTAGTTAATTAAAAATAAAAACCCTCTTAAGTCAACCAACAAGGTAATGTTACGTATATTGAG  58347
Dog           AGTCC-----------------------AATCTCTAAA------------AA--------------------------GG  53944
BelugaWhale   ---------------G------------AAAAGATAATGTATAT----TGAG---------------------------G  53707
Donkey        ATT-------------------------GGCACATAGATGTCC-----ACAA--------------------------AC  59186
Cat           AGTCC-----------------------AGTCTTCAAACGTCTGC---AAAA--------------------------AA  60906
Ferret        AGTCC-----------------------CATTTCTAAA------------AA--------------------------GG  61343
Lion          AGTCC-----------------------AGTCTTCAAAAGTCTGC---AAAA--------------------------AA  58381
Vaquita       ---------------G------------AAAAGATAATGTATAT----TGAG---------------------------G  55056
Pig           AATCCCTGGCCTT--GCTCAGTGGGTTAAGGATTTGGTGTTGCT---GTGAG---GTGTGGTG-TCTGTTGAAGACTTGG  51674
```

```
Majority     TXXXXCAAAXAGAATGGTAXTC-C--------------A-CTATTAXAATAAT-ATAGTTCTAXATTXAAATATATAATG
                      78410     78420     78430     78440     78450     78460     78470     78480
Human        TTTCCCTATAGGAATGCTACATTAGTAAATGAATACCTATCTTCAGTGCTTTCTTCACCTTTACACCAGGGTATTCAATT  62718
PolarBear    TCATGCAAA-GACGT-----------------------A-CTATTAAAATAAT-ATAGTTCTAGATTTACATCCCTAGTG  23596
Cow          GTTGGCATTTAGGGTGGTAGTCACCACCAGTAATCACCAATCATTGTAAAAATAATGATTATCAAATAAAAAGTAAATTA  58427
Dog          TCTTGCAAA-AGAATGTTGATC-T--------------A-CTATTAAAATAAT-ATTGTTCTAGATTTATATACCTAATG  54006
BelugaWhale  GTTGACATTTAGGGTGGTAATCAC----A---------AGTCATTTTAACAATCATAATTGTCAAGTAAAAAGTAAATTG  53774
Donkey       TCATATATA-CACATGTAAATA---------------TAGCTAATGTTATTAATACTGTCATAACTTAGCA-ATATCATG  59249
Cat          ACAAACAAACAAAAAAAAACTG-G--------------T-CTACTAAAGTACT-ATAGTTCTAGATGTATACATATAATG  60969
Ferret       TCATGCAAA-AACATGTTGGTC-T--------------A-CTATTAAAATCAT-ATAGTTCTAGCTTTACATACCTAATG  61405
Lion         A-AAAAAAAAAAAATGCTGGTC-T--------------A-CTACTAAAGTACT-ATAGTTCTAAATATATACATATAATG  58443
Vaquita      GTTGACATTTAGGGTGGTAATCAC----A---------AGTCATTTTAACAATCATAATTGTCAAGTAAAAAGTAAATTG  55123
Pig          CTCGG-ATCTGGTGTGGCTGTGGC----ATAGGCCAGCAGCTACTGCTCCAATTCAGCCTCTAGGCTGGAA-----A---  51741

Majority     --------GATCTTGGTXTATG-TAAAXATCA--GGCTCAA--------TCTATAXAT----------XTATTATCAAAA
                      78490     78500     78510     78520     78530     78540     78550     78560
Human        AATAGTTATGTTATTCTGGCTGATAAAGTTGATTGATACATGGCTTCCACTTTTATGTGATGATAGGTACCTTGCCTAAC  62798
PolarBear    --------GATCTTGGTCTATA-TAAACATCA--GGTTTAA--------TCTGTTGAT----------GTATTATCAGAA  23647
Cow          TTTTAAATGATCCTCACATTTATTAATGAAAA--GGCCCTAATGCCTTTTTCAGAAATCTTTTTCAGTGTATTGGCAAAC  58505
Dog          --------GATCTTGGTCTATG-TAAACATCA--AGCTTAA--------TCTCTTGAT----------ATTTAATCAGAA  54057
BelugaWhale  TTTTAAATGATCTTCACATTTATTAAAGAAAA--TGCCCTAATGCCTTTTTCAAAAATCTT-----GTTTATTGGCAAGC  53847
Donkey       T--------AGCATTATAGATGGCACAAAATG--GTATCATTAAAATATTGGAAAAGCTT-------ATTTGTGATAAAG  59312
Cat          --------GATCTTGGTCTATG-TAAACATCA--GGCTTAA--------TCTATTGAT----------CTATTATCAGAA  61020
Ferret       --------GATTTTGGTCTGTA-TAAACATCA--GGCTTAAG-------TCTATTGAT----------GTATTATCAGAA  61457
Lion         --------GATCTTGGTCTATG-TAAACATCA--GGCTTAA--------TCTATTGAT----------CTATTATCAGAA  58494
Vaquita      TTTTAAATGATCTTCACATTTATTAAAGAAAA--TGCCCTAATGCCTTTTTCAAAAATCTT-----GTTTATTGGCAAGC  55196
Pig          -----------CCTC-TATGTGCTGCAGGTGC--GGCCCC---------CCCAAAA----------AAACAAAAGCAAAA  51789
```

```
Majority     XAA------------CT---------T-----------XTXTTTATTTTCAAGGT---GGTTATAGAGC----TAAXCA-
                      78570     78580     78590     78600     78610     78620     78630     78640
Human        CCAAATGTAATTCAAATCCTGATGTGTATGAACTC--TCTTTCAGTTTTTACCAGTGCTGTATTGAAGTGGATGAATCT-  62875
PolarBear    GAA------------CTG--------T-----------CATTTCATTTTCACAGTG--ACTTATAGAGC----TAAACAT  23690
Cow          AAAAATATCATTCACTTACTGTGGCTTTCTAGTTCAGTTTCTGTATTTTCAAGGTATAGAAAGTGAAGTCGCTTAGTCAT  58585
Dog          GAA------------CTG--------T-----------TGCTTCATTTTCAACGTG--GGTTATAGAGC----TAAACAT  54100
BelugaWhale  AG---------T----------------------------GTATTGGCCAAAGAT------------------A--TCA-  53869
Donkey       AAA------------------------------------TGTTTATTTTTAAGATT--AGTCATATCTGCAGTTATCTAT  59354
Cat          GAA------------CT---------------------TGTTTCATTTTCAT------GGTGATTTAGC----TAAAC--  61055
Ferret       GAA------------CTG--------T-----------CACT-----TTTACAGTG--ATTTATAGAGG----TAAACAT  61495
Lion         GAA------------CT---------------------TGTTTCATTTTCATTTTCATGGTGATTTAGC----TAAAC--  58535
Vaquita      AG---------T----------------------------GTATTGGGCAAAGAT------------------A--TCA-  55218
Pig          AAAAA--------ACGTAGGTCAACATAAAAGGTAATGCTGTGTATGTTGAGGGT-TGGCATTTAGGGCGG--TGATCA-  51857

Majority     -----AXTCTTCAT----TTCTGAACTATTGTTTTATACAG-TAAATGTTTXTTCTTCTAAT-------GAAAAATTAA-
                      78650     78660     78670     78680     78690     78700     78710     78720
Human        -----GTGTTGAATTATTTTCTAGGTTAGTTTTTAGGTAGGTAGATTAGTCCATAAAAAAATT------CGTATATTAAG  62944
PolarBear    -----ATTTTTCAT----TTCTGAACTGTTATTTTATACAG-TAAATGTTTATTCTTCTAAT-------GAAAAATTAAC  23753
Cow          GTCCGACTCTTTGTGACCCCGTGGACTGTAGCCTACCAGGC-TCCTCCGTCCATGGGATTCTCCAGGCAAGAATACTGG-  58663
Dog          -----ATTCTTCAT----TTCTGAA---TTGTCTTATACAG-TAAATGTTTATTCT---AAT-------GAAAAATTAAC  54157
BelugaWhale  --------------------CTTAACTGTGGCTTTCTATCT-AGTTCAGTTTCTATATTTTC-------GAGAAATAGA-  53920
Donkey       AATATAGGCTT-ATATTACTTTGCATTTTTGTTTGATTTAGGTCCAGAAGTTTTGTGCTCTT----GAGGATATAGCAGG  59429
Cat          -----AGTCTTCAT----TTCTGAACTATTGTTTTATACAG-TAAATGTTTATTCTTCTAAT-------GAAAAATTAA-  61117
Ferret       -----AGTTTTCAT----TTCTGAACCATTATTTTATACAG-TAAATGTTTCTTCTTCTAAT-------AAAAAATTAAC  61558
Lion         -----ATTCTTCAT----TTCTGAACTATTGTTTTATACAG-TAAATGTTTATTCTTCTAAT-------GAAAAATTAA-  58597
Vaquita      --------------------CTTAACTGTGGCTTTCTATCT-AGTTTAGTTTCTATATTTTT-------GAGAAATAGA-  55269
Pig          ---CAAGTGATAGTAGCAATCGTAATTATCAAATAGAAAGT-AAATTGTTTTAAATGATCCT-----CACATTTATTAA-  51927
```

```
Majority     AGTATATTCC-CTTGTTCXTT------AGTTTTTTCTAGTC-----G--TTATATC----------XCAXCTGGAGTAGA
                       78730     78740     78750     78760     78770     78780     78790     78800
Human        AGAAAAGGTTTTATATTCCCAGAGGTGAGAATATTTGAATTA-AAAGCTTTATATA-----CTTAAGCAACTGTAGAGGT  63018
PolarBear    AATATATTCT-CCTGTTCTTT------AGTTTGTTTTAGGC--------TTATATC-----------CAGCTGGAGTAGA  23807
Cow          AGTGAGTTGC-CATTTCCTCCTCCAGGGGATCTTCCGGACC--CAGGGATCGAACCCAGGTCTACCGCATTTCAGGCAGA  58740
Dog          AATATATTCC-CTTGTTCTTT------AGTTTGTTCTAGTC--------TTATATC-----------CAGCCAGAGTAGA  54211
BelugaWhale  AGTAATCTCC-TTTTTACCTT------GACTTTTAAAAATT-----GTTTTATACA-----ACCCTGAAATTATAACACA  53983
Donkey       ATTGTATTGTGCATGTACACCTGTATAAGATTTTAAGTGTTTTCCAGTAGGAGTTT------CAAAGTAATTAATTTAGC  59503
Cat          --TATATTCC-CTTGTTCTTT------AGTTTCTTCTAGGC--------TTACATC-----------CAGCTGGAGTAGC  61169
Ferret       AATATATTCC-CTTGTTCCTT------AGTTTGTTCTAGCC--------TTCTATC-----------CAGCTGGAATAGA  61612
Lion         --TATATTCC-CTTGTTCTTT------AGTTTCTTCTAGGC--------TTATATC-----------CAGCTGGAGTAGC  58649
Vaquita      AGTAATCTCC-TTTTTACCTT------GACTTTTAAAAATTA-TTTGTTTTATACA-----ACCCTGAAATTATAACACA  55336
Pig          AGAAAATACC-CTAATGCCTTTTTCAGAAATCTTGTTTATTGGCAAGCAGTGTATT-GGGCAAACAAAGATTTGGGCAAA  52005

Majority     AATGG-------TGATTATATAG------X---GAGXAGXAAAAAAAA-CXCATAXTCATCT-----------TAACAGC
                       78810     78820     78830     78840     78850     78860     78870     78880
Human        TTACTTTTTCCATAACACGACATGTTTATTCTTCTAATGAAAAGTTAACAGTATGTTCACCTGTTCTCTAGTTTGTTTCC  63098
PolarBear    AATGT-------TGATTATATAG----------GACCAGGAAAAAAAA-CCCGTAATAATCA-----------TAACACC  23858
Cow          TGCTTTAACCTCTGAATCACCAGGGAAGCCCTCAAGGTATAGAAGTAGACTCCTTTTCACCCAGGATCT---TTTAAAAT  58817
Dog          AATGG-------TGATTATATAG----------GAGCAGGAAAAAAC--CCCATAATAA-------------TAACAGT  54258
BelugaWhale  CATGGTAGAAAGTAGCTGTTTTT----GTTTTTGTTAT-TAGAGACAG----CTTTTCAGGT----------GTGTTATC  54044
Donkey       CAGTG-------AGATAGTTTAG-----CT---GAAATATGTTAATATACTTGTGCTCAAAT----GCT---GTGATAGG  59561
Cat          AATTG-------TGATTATATAG----------GAGCAGGAAAAAAAC-CCCATAATAATCA-----------TAACAGC  61220
Ferret       AATGG-------TGATTATATAG----------TACCAGGAAAAAAAAACTCATAATAATCA-----------TAGCAGC  61664
Lion         AATTG-------TGATTATATAG----------GAGCAGGAAAAAAAA-ACCATAATAATCA-----------TAACAGC  58700
Vaquita      CATGGTAGAAAGTAGCTGTTTTT----GTTTTTGTTAT-TAGAGACAG----CTTTTCAAGT----------GTGTTATC  55397
Pig          CAAAG-ATTCTTTCAGTTTCCAT----ATTTTCGAGAAATAGAATTAACCTCCTTTTCACCT-GTACTT---TTAAAAGT  52076
```

```
Majority     AGCAT------A----GTCTT--A-T--------TATAA---CTCACATTGTA----XCTTTCACA-------TA-----
                      78890     78900     78910     78920     78930     78940     78950     78960
Human        AGCTTATATCCAGCTGAACTAGAAATGGTGATTATATAGGAGCAGAAAAAGTACAAGATAATCATAGCGGTAACATAGTC  63178
PolarBear    AGCAT------AT---GTCTT----T--------TATAA---CTCACATTGTA-----CTTTCACA-------TA-----  23897
Cow          TATTTA-ATTTATACAACCCTGAAAT--------TGTAA---CACACTTGGTAGAAAATTGTAACA-------CAGCTTT  58878
Dog          AGAAT------AT---GTCT--------------TATAA---CTCACATTGTA-----CTTTTGCA-------TA-----  54295
BelugaWhale  TATCT---------CTATTAGGAATG--------TGTA------CAGTTGACACTGGACAGTGACA-------CT-----  54089
Donkey       CTCTT----------GG-CTT--ATT--------TAAGA---C-TGTATCCAATGTTCTCTTTAAA-------GA-----  59604
Cat          AGCAT------G----GTCTT----T--------TATAA---CTCACATTATA-----CTTACACA-------TA-----  61258
Ferret       AGCA------------A--T--------------TATAA---CTCACATTGTA-----CTTTCACA-------TA-----  61696
Lion         AGCAT------G----GTCTT----T--------TATAA---CTCACATTATA-----CTTACACA-------TA-----  58738
Vaquita      TATCT---------CTATTAGGAATG--------TGTA------CAGTTGACACTGGACAGTGACA-------TT-----  55442
Pig          TGTTT-----TATACAGTTCTGAAGT--------TATAA---CACATGTGGTAGAAAATACCTGTT-------TG--TTT  52131

Majority     ---TGTGATTTTTTAT-----TTAATXCTCAAGGT----------AACTTTGTGAGGATCC-----------TGGCAGCC
                      78970     78980     78990     79000     79010     79020     79030     79040
Human        CTTTATAATTCATGATGTATTTTCACATATGTTGTCTTTAATTTACTCCTCTTAATAACCCTTGAAGAAGCATGACAGCC  63258
PolarBear    ---TGTGATCTTTTAT-----TTAATCCTCAAGGT----------AACCTTGTGAGGATCC-----------TGGCAGCC  23948
Cow          TAGGGGTATTATCCAC----TTTTATTAGGAATATGTGCAGTTGACACTGCACAGTGACACT---GTGTCTTTTGGAAAT  58951
Dog          ---TGTCATCTTTTAT-----TTAATCCTCAAGGT----------AACTCTGTGGGGATTA-----------TGGCAGCC  54346
BelugaWhale  --GTGTCTTTTGGAAA-----TCCTTTAATATGGT----------CACTCTGTTTTAATAT----GCAAGT---GGATTG  54145
Donkey       ---TTTTTTTTGAAAGACAGGTTTTCTGAATAGGTTGCTTGTCTATTTTTTATTCACATCA----------TTTACATTT  59671
Cat          ---TGTGATCTTTTGT-----TTAATCCTCAAAGT----------AACC-TGTGAGGATCA-----------TGGCAGCC  61308
Ferret       ---TGTGATCTTTTAT-----TTAATCCTCAAGGT----------ACTCTTGTGAGGATCC-----------TGGCAGCC  61747
Lion         ---CGTGATCTTTTGT-----TTAATCCTCAAAGT----------AACC-TGTGAGGATCA-----------TGGCAGCC  58788
Vaquita      --GTGTCTTTTGGAAA-----TCCTTTAATATGGT----------CACTCTGTTTTAATAT----GCAAGT---GGATTG  55498
Pig          ATATGTTATTAGAGACAGCTTTTCAGTAGTAGTGT-TGCTTTTTTTATTTTACCTAAGTACA---GTTAATTTAGCAGAG  52207
```

```
Majority     ATTATCATCTXTG----TTTTCCAGATTAAGAAAGATCCTGA-GATTCAGAGAGGTTAAGTAACTTGTTTAAXATGACCT
                          79050     79060     79070     79080     79090     79100     79110     79120
Human        ATTGTCATCATTC-CTGTTTTCCCGATTAAGA--AAT--TGA-GATTCAGAGAGGTCAAATAACTTGCTTAAGATGACCG  63332
PolarBear    ATTATCATCTCTG----TTTTCCAGATTAAGAAAGATCCTGA-GATTCAGAGAGGTTAAGTAACTTGTTCAAGATGACCT  24023
Cow          CCTATCATTAATGATTTCCTTCCAGGAGAATATATTTCATTAAGTTTCATTAGTGGCTTTTCCTATTTTTTATCCATATG  59031
Dog          ATTATTACCTCTG----TTTTCTAGATTAAGATAGATCCTGA-GATTCAGAGAGGTTAAGTAACTTGTTCAAGATGACCT  54421
BelugaWhale  TTTCTCATTAATGATT-CCTTCCAGGAGAGTGTATTTCATTA-GTTTCATTGGTGGCTTTTCCTGTTTTTAATCCATATG  54223
Donkey       GTTGTTGTTGTTA---TAGAGGGAGGGTAAGAAAGTATTAAAAGAAGTAGAATCATCCCATGTCATGGCT--ACCATTGA  59746
Cat          ATTACCATCTTTG----TTTTCCAGATTAAGAAAGATCCTGA-TACTCAGAGAGGTTAAGTAACTTGCTCAGTATGACGT  61383
Ferret       ATTATCTTCTCTG----TTTTCCAGATTAAGAAAGATCCTGA-GATTCAAAGAGGTTAAGTA----------GATGACCT  61812
Lion         ATTACCATCTTTG----TTTTCCAGATTAAGAAAGATCCTGA-TACTCAGAGAGGTTAAGTAACTTGCTCAATATGACGT  58863
Vaquita      TTTCTCATTAATGATT-CCTTCCAGGAGAGTGTATTTCATTA-GTTTCATTGGTGGCTTTTCCTGTTTTTAATCCATATG  55576
Pig          TGTATAATTTCTG----CTGTATAGCAGAGTG-GCTCAGTTA---TACATGTATGTTTTCTGCTGTATAGCAGAGTGGCT  52279

Majority     AATT-GAATATAGGCTTTT---TAGCTCTAGAGCCTGTACTCTTCTAT---TCCTGAGCTTTTC-------TGGGATGA-
                          79130     79140     79150     79160     79170     79180     79190     79200
Human        AAAA-GAGTCTAGGCTTTTAATTAATCCTAGACCTGGCACTCGTCAGT---TTCAGAACTTTTC-------GGGAATGA-  63400
PolarBear    AATT-GAGTGTAGGCTTT----CAGCTCTAGAGCTTGCACTCTTCTAT---TCCCAAGCTTTCC-------TGGGATGA-  24087
Cow          CAGTGTTTCATTGTGGTGT---TAATTATTCATTCTATA-TTTAACATCTTTACTGAGTTTATCATGTTTTTGACATAAA  59107
Dog          AATT-GACTATAGGCTTT----CAGTTCTAGAGTTTGCACTCTTCTAT---TCCTAAGCTTTTC-------TGGGATGA-  54485
BelugaWhale  CAATGTTT-ATTGTGTTGT---TAACTATACATTCTGTA-TTTAACATCTTTACTGAGTTTATCATGTTTTTGCCATAAA  54298
Donkey       AAACTGCATACCACATGTT---TGCTTCTAAAAGCCATAATCTTCTGT--TTGTCTGTCTTATT-------TAAATCTAC  59814
Cat          AATT-GAATGTAGACTTT----CAGCTCTAGAGCCTGTACTCTTCTAT---TCCAGAGCTTTCC-------TGGGATGA-  61447
Ferret       GATTTGAGTGTAGGCTTT----TAGATTTAGAGCTTGCACTCT---AT---TCCTGGGCTTTCC-------TGGGATAA-  61874
Lion         AATT-GAATGTAGACTTTT---CAGCTCTAGAGCCTGTACTCTTCTAT---TCCAGAGCTTTCC-------TGGGATGA-  58928
Vaquita      CAATGTTT-ATTGTGTTGT---TAACTATACATT----A-TTTAACATCTTTACTGAGTTTATCATGTTTTTGCCATAAA  55647
Pig          CAGTTATACATGTATGTTT---TTCATATTTTTGCTATGGTTTATCACAGGTATTGAGTATGT--TCCCCATGAATAGAG  52354
```

```
Majority      TGTTXTATTTTCTAA-TTCAACATTTTATTCTGATTXTGGT--TGAACACTTCAAGTTAT----GGAAATAAT------A
                      79210     79220     79230     79240     79250     79260     79270     79280
Human         CATTGGATTTGCTAATTTCATCAAGATAATCTGATTGGGGG--TGAACATTTCAGGTTAAGGAAAATACTAGTTTTTTTC  63478
PolarBear     TGTTCTATTTTCTAA-TTCAACAGTCCATTTTGATTCGGGT--TGAACACCTCAAGTTAA----GGAAATAAT------A  24154
Cow           TCTTATATTTACTAT-GAAGATATTTTTTCCTGATTGTTTTCATGTACTCTCCAAACTTTCCCTTTGCATAGA------T  59180
Dog           TGTTACATTTTCTAA-TTCAACAA---GTTTTGATTAGG----TGGACATCTCAAT---------GAAATAAT------A  54542
BelugaWhale   TCTTATACTTAGTAT-GAAAATACTTTTTCCTGATTGTTTT--TGTGTATTCCAAACCTTCCCTTTGCATAGA------C  54369
Donkey        CTTTCCTCTCTCTC--TTTAACTTCTTTGCCGTGTTATTTCATTGTTATTTTCATTCCAT-------CATTAT------A  59879
Cat           TGTTGGATTTTCTAA-TTTAAGTT---ATTTTGATTCAGGT--TGAACACCTCAAGTTAC----GGAAATAAT------A  61511
Ferret        TGTTGCATTTTCTAA-TTCAACAATCTATTTGGATCCAGGT--TCAACACCTCAAGTTAA----GGAAATAAT------A  61941
Lion          TGTTGGATTTTCTAT-TTCAAGTT---ATTTTGATTCAGGT--TGAACACCTCAAGTTAC----GGAAATAAT------A  58992
Vaquita       TCTTATACTTAGTAT-GAAAATACTTTTTCCTGATTGTTTT--TGTGTACTCCAAACCTTCCCTTTGCATAGA------C  55718
Pig           TCCCCTGTCCCCTA---AATACAGTAGGACCTTGTTAT----AGGAATATTGCCTGTTTCTATTAGGAATGTG------C  52421

Majority      GTATTTGCCXCGTTTTATXGGAATTGCTATTATGCATT-ATAGTGATAXXTXTTTGGCTTAAGGCXCTTCXTAAAATGTG
                      79290     79300     79310     79320     79330     79340     79350     79360
Human         TTTTTTGCCTCATTTTATGGGAATTGCTATTATGTGTT----AGGATA--ATTTGGTTTGAAGCAACTTTGTACAATGTG  63552
PolarBear     GTGTTTGCCCCATTTCATGGCAATCGCTATTATGCATT-ATAGTGATACTTATTTGGCTTGAGGCGCTTCATAAAATGTG  24233
Cow           ACATTTGAGAGGCTTTTTTGCAAATTAACTTGGGCTTTTACTGTCAGAAATCTTTGCTCCAGCAAGATCACTACAATCTT  59260
Dog           GTTTCTGCCCCATTTCATGGGAATTGCTCTTATGCATT-ATAGTGATA--TATTTGGCTTGAAGCACTTCATAAAATGTG  54619
BelugaWhale   ACATTTGAGAGGCCTTTCTGCAAATTAACTTGAGCTTTTACTGTGAGAAATCTTTGCTCTAGCAAGATCACTGCAATCCC  54449
Donkey        CCATTAAATA---TTCATTATTAAACCATTAATTCAATTGTCTTTACTTACCTTTGTCTTACCTCTTTTTCTTTATTTCA  59956
Cat           GTTTTTGCCCTGTTTTATGGGACTTGCTATTATGCCAT-ATAGTGATACTTACTTGGCTTGAGGCACTTCATAAAATGTG  61590
Ferret        GTGTTTGC-------------ATTACTACTATACATT-ATAGTGATACTTATTTGGCTTGAGGCACTTCATAAAATGTG  62006
Lion          GTTTTTGCCCCGTTTTATGGGACTTGCTATTATGCCTT-ATAGTGATACTTACTTGGCTTGAGGCACTTCATAAAATGTG  59071
Vaquita       ACATTTGAGAGGCCTTTCTGCAAATTAACTTGAGCTTTTACTGTGAGAAATCTTTGCTCTAGCAAGATCACTGCAATCCC  55798
Pig           ACAGTTGACG-------CTGGACATGACACTGTGTCTT-----TTGGCAGTCCTT---TTATAATGGTCACTCTTATTTT  52486
```

```
Majority      GCAT--CAAACAAAAGTTACCTTATAAAATTCXACTAATTCTTCTAAGT----ACTGCCAT-------CCAGCAAAAAAT
                                79370     79380     79390     79400     79410     79420     79430     79440
Human         GCAT--CAAATAGCAGTTACCTTCTAAAACTGTACTGAATCATATAAGT----ACTGCCAT-------CCAGCAATAAAT  63619
PolarBear     ACAT--CAAACAAAAGTTACCTTATAAAATTACACCAATTCTTCTAAGT----ACTGCCAT-------CCAGCAAAAAAT  24300
Cow           GATTGCTGAACACTGACTGACTTGTGTGCTACTGTTATTTCCTGGAAGTTG--GCAAATAACAATGAACTTAAAGTGAGC  59338
Dog           GCAT--CAAACAAAAGTTACCATATAAAATTATACCAATTTTTCTAAGT----ACTGCCAT-------CCAGCAAAAAAT  54686
BelugaWhale   GATTGCTGAACACAGACTGACTTGTGTGCTGCCATTATTTCCTGGAATTTT--GCAAACAC--ACGAGCTTGAAGTGAGC  54525
Donkey        CTCT--CAGACTAATTATGGTATGTAATACATCAGTGCTTCCTCCAAGTCAAGATAGCTTGTATCGAACCCGGACCGGTT  60034
Cat           TCAT--CAAACAAAAGTTACCTTACAAAATTCTAACAATTCTTCTAAGT----ACTGCCAT-------CCAGCAAAAAAT  61657
Ferret        ACCT--CAAACAAAAGTTATGTTCTAAAATTACACCAATTCTTCTAAGT----ACTACCAT-------CCAGCAAAAAAT  62073
Lion          TCAT--CAAACAAAAGTTACCTTACAAAATTCGAACAATTCTTCTAAGT----ACTGCCAT-------CCAGCAAAAAAT  59138
Vaquita       GATTGCTGAACACAGACTGACTTGTGTGCTGCCATTATTTCCTGGAATTTT--GCAAACAC--ACGAGCTTGAAGTGAGC  55874
Pig           AAC------ATGC-AATGGATTCATTAGTTACTAATAGTAAAAATATGTAA--ACATTAGGTAAAATATTTGCAGC-AGT  52556

Majority      XTGTATXTATAATAAAATT----------------AAAGTATTATGCAGACTATTATGGTAXGACTTTXGAXAGGACTG
                                79450     79460     79470     79480     79490     79500     79510     79520
Human         ATAT--GTATAATAAAATT-A--------------ACCTTATTGAGCAGAGTAGTGTGGTACTACTTT-GGTAGGACT-  63679
PolarBear     GTGTATGTATAATAAAACT----------------AAATTATTACGTAGACTATTATGGTGTGACTTT-GGCAGGACTG  24362
Cow           TTGCATATATAGTTTACTTTAATTTTTCTT---CTTACTTCACTTGTCATTTTACCTCAGCCAGCTGTTGAAAATGCCTG  59415
Dog           ATGTATGTATAATAA---------------------AGTATTATGCAGACCATTATGGTACAACTCT-TGTAGGACTG  54742
BelugaWhale   TTGCATATCTAGTTTACTC------ATTTG-------------TAGTCATCTTCCCTCAGCCAGCTATTGAAAGTGCCTG  54586
Donkey        TTGATT-TCTGGTTCATTGCA----TTTCCATCCTCAGAATCTCATTTGGGGAGCAAAAATAGCTTCAGGGAGCAGAAAG  60109
Cat           GTGT----ATAATAAAATT----------------AAAGTATTATGCAGACTATTATGGTATGACTTT-GACAGGACTG  61715
Ferret        GTGTATGTATAATAAAATT----------------AAAGTATTATTCAGGCTATTATGGTATGACTTTTGGCAGGACTG  62136
Lion          GTGT----ATAATAAAATT----------------AAAGTATTAAGCAGACTATTATGGTATGACTTT-GACAGGACTG  59196
Vaquita       TTGCATATCTAGTTTACTC------ATTTG-------------TAGTCATCTTCCCTCAGCCAGCTATTGAAAGTGCCTG  55935
Pig           AAGAACATGTCATTCATTTCTTCTTACTTGATACTTACTGCTTTATGCAGATTCTTTGAGAAAACTAATGAAATTTCCT-  52635
```

```
Majority     X----CTATXXTTGTG-ATCCTXX-CTTATTTTTTTCTAT-TAAATTATTTGCAGATTTTTT------TAATT------A
                      79530     79540     79550     79560     79570     79580     79590     79600
Human        -----TCCTGTTTGTG-ATCCTTA-TTAGTTTTTTTAAAACTAACCTATTTGCAATTTTTAA------AAATT------A  63740
PolarBear    A----CTATATTTGTA-ATC-----TTTATTTTTTTTAAT-TGAATTATTTGCAGATTTTTTC-----AAATT------A  24420
Cow          GATTTCTGTTATCATGTATCCTTAGCTCTTTTTCCTCTCC-CCTGTTGCTTATATACATATTGTTTCCTGATT-TCTCTA  59493
Dog          A----CTGTATTTGTT-ATC-----CTTATTTATT-AAAT-CAAATTATTTGCAGATTTTTTT-----TAATT------A  54799
BelugaWhale  GATTTCTATTCTTGTGTATCCTCAGCTCTTGTTCCTCTCG-TATGTTGCTAACAGGTAATTT------TAATT-----TA  54654
Donkey       G----CTATACTAATA-ATAATAGCATAATTTTATGCTTTACCAAGTGTTATCTCACTTTTTA-----TGACTACTGCTC  60179
Cat          -----TCATCCTTGT-----------TTATTTTTT------TAAATTATTTGCAGATATTTT------GAATT------A  61761
Ferret       A----CTATATTTGTA-ATCCTTG-TTTATTTTTTTAAAT-TGAATTATTTGCAGATTTTTTT-----GAATT------A  62198
Lion         -----TCATCCTTGT-----------TTATTTTTT------TAAATTATTTGCAGATATTTT------GAATT------A  59242
Vaquita      GATTTCTATTCTTGTGTATCCTCAGCTCTTGTTCCTCTCG-TATGTTGCTAACAGGTAATTT------TAATT-----TA  56003
Pig          --TTTCTGTTTTCTTGGGCAGATT-CTCTTTGACATCTGC-TTAATTCTGTTTAGCCTTTTTTGTGCTTAACAATCTGTA  52711

Majority     AAAGTXTTAGCATATXTGXTXAACTGGTTGTATTTTAGATTTAAG----TAGCATTTTCTATTTAAAGAA-GATAGTAT-
                      79610     79620     79630     79640     79650     79660     79670     79680
Human        AAAGTATTAGCATGGATGCTTAGCTGGTTGTATTTTAGGTTGAAG----TAGCATTTTCTATTTAAGGAA-GATAATAT-  63814
PolarBear    AAAGCATTAGCATATTTGTTTACCTGGTTGTATTTTAGATTTAAG----TAGCACTTTCTATTTAAAGAA-GATGGTAT-  24494
Cow          GCTCTTGTATCTAAGGTACAGTTTTGTGTGTGCCTTGTGTGTATACCTTGTGTGTATCTTAGGTATACCTGTGTATAATA  59573
Dog          AAAGCTTTAGTATATGTGCTTAACTGGTTGTATTTTA-----AAG----TAGCA-TTTCTATTTAAAGAA-GATAGTAT-  54867
BelugaWhale  CATATTGTTTCCTGATTTCTCTAGCTTTTGTGTCTTAGGTGTA---CTTGTGTATCTTCCATCTACATAAAGATACTATT  54731
Donkey       TATGTTGTAAACAGAATA-GGAATTTTTCCTGTATTA----CAGG----GAGATGCTAAGATTTAAAGATGTAAATTAC-  60249
Cat          AAAGCATTAGCAAATGTGTTCAACTGGTTGTATTTTAGATTTAAG----TAGCACTTTCTATTTAAAGAA-GATAGTAT-  61835
Ferret       AAAGCATTAGCATATATATTTAACTGGTTGTATTTTAGATTTAAG----TAGCATTTTCTATTTTAAAAA-GATAGTAT-  62272
Lion         AAAGTATTAGCAAATGTGTTCAACTGGTTGTATTTTAGATTTAAG----TAGCACTTTCTGTTTAAAGAA-GATAGTAT-  59316
Vaquita      CATATTGTTTCCTCATTTCTCTAGCTTTTGTATCTTAGGTGTA---CTTGTGTGTCTTCCATCTACATAAAGATACTATT  56080
Pig          TATTCCACATTCATGTCATTGGAGGATTTATGA-TGAAATTAATGATTTGGGGATGTTTTAGGAAAACCC-TGCATGAT-  52788
```

```
Majority     ---TTGTGGAAAXAAATATATGTAC-------AGCTGGGAXAXCXAAGAATGATXTATXATATTTGGTCC-CTTTCCTGG
                        79690     79700     79710     79720     79730     79740     79750     79760
Human        --TTGGGGGAAAGTAATACATGGAC-------AGCTGGAACAGCAAAATTTGATATATGAAATTTGAATCCTTTTCCTGG  63885
PolarBear    ---TTGTGGAAACGAATACATGTAC-------AACTGGGACAACAAAGAATGATGTATGGTATTTGATCC-CTTTCCTGG  24563
Cow          C-CTACATAAAGGTGGTTTTTTTTTTTTTTTTGGTTAAAGGTTTCTAGACAGCTTTCAAGCAGTTAGCAT-AATCATT-A  59650
Dog          ---TTGTGGAAACAGATACATGTAC-------AGCTGGGATA----ATAATGATACATGCTATTTGATCC-CTTTCCTGG  54932
BelugaWhale  TTTTAATTAAAGGAAGAAT--------------GTCAAAGGCTTCTAGAGGGCTTTCTAGCAGTTAGCAT-ATTCATAGT  54796
Donkey       ---TTATCCTAATGCACAT-TGTCTGT----AAGGTAACTAGCCAGAAAATAGGAGTCATCCTTTATTCCTCTTCCTTTC  60321
Cat          ---TTGTGGAAACAAATA-ATGTAC-------AGCTGGGATAACGAAGAATGATGCATGATACTTGGTCC-CTTTCCTGG  61903
Ferret       ---TTGTGGAAACAAATACATGTAC-------AACTGGGATAACAAATAATAATGTATAATATGTGATCC-CTTTCCTGG  62341
Lion         ---TTGTGGAAACAAATA-ATGTAC-------AGCTGGGATAACGAAGAATGATGCATGATACTTGGTCC-GTTTCCTGG  59384
Vaquita      TTTTAATTAAAGGAAGAAT--------------GTCAAAGGCTTCTAGAGGGCTTTCTAGCAGTTAGCAT-ATTCATAGT  56145
Pig          --CTAATCTGGGGTGGTATTTATGTTGGG--AAGTAAATTGGTCCAAAGTATTATTATTATTATTATTTTTAATTTTT-T  52863

Majority     TGTCTTTTATXTGCAGGTCTTCATTGTTGGGTTTTA-----TTGTCAAAGTCATA---TTTCTTXTTAATTTTCATTCCT
                        79770     79780     79790     79800     79810     79820     79830     79840
Human        TAGCTTTTAAGTTCGAGTTTTTGTTGTTGAGTTTTA-----TTACTAAAATTATACTTTCACTAATTTTTATTTTTTGTT  63960
PolarBear    TGTCTTTTATGTGCAGGTCTTCATTGTTGGGTTTTA-----TTGTCGAAGTCCTG---TTTCTTGCTAATTTTCATTCCT  24635
Cow          AGATATTTGGCAGT-CAACCTCTTTGATAGAACCTG-----GCCCCAAAGAAACT---GCTTGTTTTGAAGTTAGTCATT  59721
Dog          TGTTTTTTATGTGCAGGTCTTCATTGTTGGGTTTTA-----TTGTCAAAGTCCTG---TTTCTTGATAATTTTCATTCCT  55004
BelugaWhale  AGATATTTGGCAGT-CAACCTCATTGAAAGAACTCA-----GCCCCAAAGAAACTCT-GCTTATTGTGAAGTTAGTGATT  54869
Donkey       AGCCTATCACCAAA-TCTCATTGATTCTAGATCCTAAATTTCTATTGAATCTATCCT-TTTGTCTTCAACTCTCTTGCCA  60399
Cat          TGGCTTTTATGTGCAGGTCTTCAATATTGGGTTTTA-----TTGTCGAAATCCTA---TTTCTTGCTAATTTTCATTCCT  61975
Ferret       TGTCTTTTATATGCAGGTCTTCATTGCTGGGTTTTG-----TTGTCGAAGTCCTG---TTTCTTATTAATTTTCATTCCT  62413
Lion         TGGCTTTTATGTGCAGGTCTTCAATATTGGGTTTTA-----TTGTCGAAATCCTA---TTTCTTGCTAATTTTCATTCCT  59456
Vaquita      AGATATTTGGCAGT-CAACCTCATTGAAAGAACTCA-----GCCCCAAAGAAACTCT-GCTTATAGTGAAGTTAGTGATT  56218
Pig          TATTTTCCCACTGTACAGCAAGGGGGTCAGGTTATC-----CTCACATGTATACA---TTACAATTACAGTTTTCCCCCC  52935
```

```
Majority     TAAGACTGCTGATATACTTTATTCAC---TTTTACAGGCCCTAGATG-------GTCAGAATATTTATAA-----TGCCT
                     79850     79860     79870     79880     79890     79900     79910     79920
Human        TAAGATTGCTGATATGCTTTATTCAC---TTTTACAGGCCCTAGATG-------GTCAGAATATTTATAA-----TGCCT  64025
PolarBear    TAAGACTGCTGATATACTTTATTCAC---TTTTACAGGCCCTAGATG-------GTCAGAATATTTATAA-----TGCTT  24700
Cow          TTGTATCAGTACATTACTTCTTGCAT---TAATCGAGGTACT--ATGATCCAAAGTCAGGTCTTTTCTTA-----TATCA  59791
Dog          TAAGACTGCTGATATACTTTATTCAC---TTTTACAGGCCCTAGATG-------GTCAGAATATTTATAA-----TGCTT  55069
BelugaWhale  TTGTGTCAGTACATTGCTTTTTGCAT---TAATTTAGATAAT--ATGACCCAAAGCCAGGTCTTTTATTACAAGATATCA  54944
Donkey       CCACCCTAGTCTAAGCCAGTGTTACT-CATAGTCCTGGTCTCTGATGCTAC--AACAAGCTTTTGCCAAAATACAGATCA  60476
Cat          TAAGACTGCTGATATACTTTATTCAC---TTTTACAGGCCCTAGATG-------GTCAGAATATTTATAA-----TGCTT  62040
Ferret       TAAGACTGCTGATATACTTTATTCAC---TTTTACAGGCCCTAGATG-------GTCAGAATATTTATAA-----TGCTT  62478
Lion         TAAGACTGCTGATATACTTTATTCAC---TTTTACAGGCCCTAGATG-------GTCAGAATATTTATAA-----TGCTT  59521
Vaquita      TTGTGTCAGTACATTGCTTTTTGCAT---TAATTTAGATAAT--ATGACCCAAAGCCAGGTCTTTTCTTACAAGATATCA  56293
Pig          ACCCTTTGTTCTGTTGCAACATGAGTATCTAGACAAAGTTCTCAATGCTATTCAGCAGGATCTCCTTGTA-----TATCT  53010

Majority     GCTGTACCCTAAGGAT--TGATTTTTCCAAACTTGTGAATTTGA--------------A-TGTAAAATACAACAAT--GA
                     79930     79940     79950     79960     79970     79980     79990     80000
Human        GCTGTACCCTAAGGAT--TGATTTTTCCAAACTTGTGAATTTGA--------------A-TGTAAAATACAACAAT--GA  64086
PolarBear    GCTGTACCCTAAGGAT--TGATTTTTCCAAACTTGTGAATTTGA--------------A-TGTAAAATACAACAAT--GA  24761
Cow          GATTTT--TTATATA---TTATTGAAATAGTCACCTAAGAGTGA--------------ATTTTGACATTCATGTATAAGG  59852
Dog          GCTGTACCCTAAGGAT--TGATTTTTCCAAACTTGTGAATTTGA--------------A-TGTAAAATACAACAAT--GA  55130
BelugaWhale  GATTTTT-TTGTATGATGTTATTGAAATAGTCACCTAAGAGTGAATTTTGCTACTCATCTTTTGACGTTCATATCTTGGG  55023
Donkey       GCATAGTTCCAGCCACAGTATGTTTAGGGTTGGTGTTAACGTAA--------------ATTCTGACACAGACTCCTTACC  60542
Cat          GCTGTACACTAAGGAT--TGATTTTTCCAAACTTGTGAATTTGA--------------A-TGTAAAATACAACAAT--GA  62101
Ferret       GCTGTACCCTAAGGAT--TGATTTTTCCAAACTTGTGAATTTGA--------------A-TGTAAAATACAACAAT--GA  62539
Lion         GCTGTACACTAAGGAT--TGATTTTTCCAAACTTGTGAATTTGA--------------A-TGTAAAATACAACAAT--GA  59582
Vaquita      GATTTTT-TTGTATGATGTTATTGAAATAGTCACCTAAGAGTGAATTTTGCTCCTCATCTTTTGATGTTCATATATTGGG  56372
Pig          ATTCTAGGTTGTGTC---TGATAAGCCCAAGCTCCCGATCCCTCC--C----------ACTCCCTCCCCCTCCCATCAGG  53075
```

```
Majority     TAAAAGTAGGG--ATTATACT---CGACCTG--ATCTTCCATCTGGGGATGGACA------------ACC-TGCATTGGA
                      80010     80020     80030     80040     80050     80060     80070     80080
Human        TAAAAGTAGGG--ATTATACT---CGACCTG--ATCTTCCATCTGGGGATGGACA------------ACC-TGCATTGGA  64146
PolarBear    TAAAAGTAGGG--ATTATACT---CGACCTG--ATCTTCCATCTGGGGATGGACA------------GCC-TGCATTGGA  24821
Cow          TAGAGACATAG--ATTCTATTACATGACTCGCCATTTCCCCTATAGGAATGAGTA-----AATGAAGACC-TATTTTCGG  59924
Dog          TAAAAGTAGGG--ATTATACT---CGACCTG--ATCTTCCATCTGGGGATGGACA------------ACC-TGCATTGGA  55190
BelugaWhale  GAGAGATATAG--ATTCTATTACTTGGCTCAACATTTCCCCTATAGGAATGAGTA-----AAAGAAGACC-TGTTTTCAA  55095
Donkey       TTAATGCAGAGTGACCTGGGCTGCTAGTCTATGAATCACAGTTTGAGTAGCACTGATCTAAGCCATCATCATCTCTTAAA  60622
Cat          TAAAAGTAGGG--ATTATACT---CGACCTG--ATCTTCCATCTGGGGATGGACA------------ACC-TGCATTGGA  62161
Ferret       TAAAAGTAGGG--ATTATACT---CGACCTG--ATCTTCCATCTGGGGATGGACA------------GCC-TGCATTGGA  62599
Lion         TAAAAGTAGGG--ATTATACT---CGACCTG--ATCTTCCATCTGGGGATGGACA------------ACC-TGCATTGGA  59642
Vaquita      GAGAGATATAG--ATTCTATTACTTGGCTCAACATTTCCCCTATAGGAATGAGTA-----AAATAAGACC-TATTTTCAA  56444
Pig          CAGCCACAGTC--TTTTCTCCAAGTCCATGATTTTCTTTTCTGAGGAGATGTTCATTTGTGCTGGATATT-AGATTCCAG  53152

Majority     CCC---AGCTATTGCTGCAGCAT------TTGCCAAGGAGACTTCTCTC---TTAGGTATGATT----------------
                      80090     80100     80110     80120     80130     80140     80150     80160
Human        CCC---AGCTATTGCTGCAGCAT------TTGCCAAGGAGACATCCCTC---TTAGGTATGATTT---------------  64199
PolarBear    CCC---AGCTATTGCTGCAGCAT------TTGCCAAGGAAACATCCCTC---TTAGGTATGATT----------------  24873
Cow          TGCTTTGTTTGCTTTTACAGCAG--AGTATTGAATTAATTACTTATTAGGTATTGAATAAGAAT---TAATTACTTATGA  59999
Dog          CCC---AGCTATTGCTGCAGCAT------TTGCCAAGGAGACCTCCCTC---TTAGGTATGATT----------------  55242
BelugaWhale  TGCTTTGTTTGTCTTTACAGAAG--AGTATTGAATTAAT-ACTTATATTACTCTGGCTGAGAG-----------------  55155
Donkey       TCTCTGTTTCATTGGCCTTCTGACTAAACTCACCGCTTTGACTTCTTCCTTCACACCTGTTGCT------TTTCCACAGA  60696
Cat          CCC---AGCTATTGCTGCAGCAT------TTGCCAAGGAGACCTCTCTC---TTAGGTATGATT----------------  62213
Ferret       CCC---AGCTATTGCTGCAGCAT------TTGCCAAGGAGACATCCCTC---TTAGGTATGATT----------------  62651
Lion         CCC---AGCTATTGCTGCAGCAT------TTGCCAAGGAGACCTCTCTC---TTAGGTATGATT----------------  59694
Vaquita      TGCTTTGTTTGTCTTTACAGAAG--AGTATTGAATTAAT-ACTTACATTACTCTGGCTGAGAG-----------------  56504
Pig          TAATAAGTGATATCATATGGTATTTATCTTTGTCTTTCTGGCTCATTTCACTCAGGATGAGATTCTCTAGTTCCATCCAT  53232
```

```
Majority      -----T----TTATTGTCTATACCACTTT------------T-----CTCCCATTT---TGCCAX-ATGGGAA-AGTGCC
                       80170     80180     80190     80200     80210     80220     80230     80240
Human         ----------TTATTGTCTTAACCACTTT---------------T--CTCCCATTT---TGCCAA-ATGGAAA---AGTA  64245
PolarBear     -----T----TTATTGTCTATACCACTTT------------T-----CTCCCATTT---TGCCAA-ATGGGAA-GGTGCC  24922
Cow           AGTATTG-AATTAATATTTATATTACTCTGCCTGAGAGAGTTGATACTTCCCATTCTCATGTGATGATAGGTGCTTTCCC  60078
Dog           -----T----TTATTGTCTATACCACTTT------------T-----CTCCCATTT---TGCCAA-ATGGGAA-AGTGCC  55291
BelugaWhale   --------AGTTGATACT-----------------------T---GGTTTTCATTCTCATATGATGATAGGTACATTGCC  55201
Donkey        GT-------ACTAAGAATGATCTCAACTAAAAATCGTCTTTCTTCCATTCTCACTGCCCTTCAGTGACTGAGT-CTTGTC  60768
Cat           -----T----TTATTGTCTATACCACTTT------------T-----CTCCCATTT---TGCCAA-ATGGGAA-AGTGCC  62262
Ferret        -----T----TTATTGTCTATACCACTTT------------T-----CTCCCATTT---TGCCAC-GTGGAAA-GGTGCC  62700
Lion          -----T----TTATTGTCTATACCACTTT------------T-----CTCCCATTT---TGCCAA-ATGGGAA-AGTGCC  59743
Vaquita       --------AGTTGATACT-----------------------T---GGTTTTCATTCTCATATGATGATAGGTACATTGCC  56550
Pig           GTTGCTGCAAATGGCATT-ATGTCATTCTTTTTTA---TG--GCTGAGTGCTATTC-CATGGTGT-ATATATACCACATC  53304

Majority      T------------ATTXAAATCCCAGTAAGTATAATGAATCCTTC----ATTTTGGAATTTAXGAAATT---GTGTTAGG
                       80250     80260     80270     80280     80290     80300     80310     80320
Human         C----------------------CAGTAAGTATAATGAATCCCCCC---ATTTTGGACCTTACCAAATT---GTGTTAGG  64297
PolarBear     T------------GTTAAACTCC-AGTAAGTATAATGAATCCCCC----ATTTTGGAATTTAAGAAATT---GTGTTAGG  24982
Cow           TAATTCAAATAGAATTCAAATCCTGATGTGCATGTAGTCTCTTTATGTGAATATAGAAATGGGGAAACT--TGTGTTGAA  60156
Dog           T------------GTTAAACTCC-AGTAAGTATAATGAATCCCTC----ACTTTGTAATTTAGCAAATT---GT--TGGG  55349
BelugaWhale   TAATTCAAATATAATTCAAATTCTGATGTGTATGTAGTCCCTTTAAGTGAGTTT----TCATCAGTAAT----TATTGAA  55273
Donkey        TTTAGTATAAAGTCCTCATTCCTTAACATGGTTTACAGAACTTTAAATCAATCCTCAGTTTGCCTCTCTG--GTCTCATC  60846
Cat           T------------ATTAAAACTACAGTAAGTATAATAAATCCTCC----ATTTTGGAATTTAAGAAATT---GTGTTAGG  62323
Ferret        T------------GTTAAACTCCCAGTAAGTATAACGAATACCCC----ATTTTGGAATTTAAGAAATT---GTGTTAGG  62761
Lion          T------------ATTAAAACTACAGTAAGTATAATAAATCCTCC----ATTTTGGAATTTAAGAAATT---GTGTTAGG  59804
Vaquita       TAATTCAAATATAATTCAAATCCTGATGTGTATGTAGTCCCTTTAAGTGAGTTT----TCATCGGTAAT----TATTGAA  56622
Pig           T--TCCGAATCCAAT-CATCTGTCGATGGACATTTGGGTTGTTTCCATGTCTTGGCTATTGTGAATAGTGCTGCAATGAA  53381
```

```
Majority     T-X-----TTTAATT-T-----TT---TTT----TTTTTTTA-XTAAGATG-CATATGAATGCTGTTGGGAGA-------
                               80330     80340     80350     80360     80370     80380     80390     80400
Human        TTT-----CGTGAGTTT----------TCT----TTTTCTCAAGTGAGAAGGCATATGAATACTGTTAAGAAA----AAC  64354
PolarBear    T-------TTTATTTTTA--TTTTTACTCT----TTTTTTTAGATAAGAGG-CATTTGAAAGCTGTTGGGAGG-------  25041
Cow          T-TGTGTTTTTAAGT-TAGCTTTTAGCTTGGTAAATATATTCACTTAAAT--CTTAATTTTGTATGTTAAAGG--AAAGG  60230
Dog          T-------TTTATTTAT------------------TTTTTTAGATAAGAGG-CATATGAATGCTGTTGGGAGA-------  55396
BelugaWhale  ATTG-----GTGAAC-------TTGGGTTG--AATTGTGTTT--TTAGGT----TAGTTTTTAGCTTGGTAAA-------  55326
Donkey       TCCCATCTTTTAATTATATTGAACTTCTTTAAATACTTTGCATATACTGTA-TTTTTTCTCTGATCTCTCAGG-------  60918
Cat          T-------TATTTTT---------------------TTTTT---TATGATG-TATATGAATGCTGTTGGGAGA-------  62364
Ferret       T-------TTTACTTTT---TTTTTTTTTT----TTTTTTAAGATAAGGAG-CATTTCAATGCTGTTGGGAGA-------  62819
Lion         T-------TATTATTAT----------TAT-----TTTTTT---TATGATG-CATATGAATGCTGTTGGGAGA-------  59851
Vaquita      ATTG-----GTGAAC-------TTGGGTTG--AATTGTGTTT--TTAGGT----TAGTTTTTAGCTTGGTAAA-------  56675
Pig          CATGCGGGTGCATGTGTCTCTTTTAAGTAG--AGTTTTGTCCAGATAGATG-CCCAAGAGTGGATTGCGGGGTCATATG  53458

Majority     -------AAAACTTAAAGTGTTAX-------TAAAAATTCTATCX------GCTXTAT-TCTTTTCATC----------A
                               80410     80420     80430     80440     80450     80460     80470     80480
Human        CCAAAGTATTCTTTATAAGTCTAA-------TTAAAATTATATGT----TAGTTTTGTTCCTTTTAATACCTTCAG---C  64420
PolarBear    -------AAAACCTAAAGTGTTAA-------TAAAAAATCTATTA------GCTGTAT-TCCTTTCACC----------A  25090
Cow          GTTTTCCTCTTTTCACAGAGTGGGAGTATTTTAAATATAATATTT----TAACTATAA-ACTTTATATACAAAGCAGCTA  60305
Dog          -------AAAACCTAAAATGTTA--------TGAAAATTCTGTCA------GCTGTAT-TCCTTTCACT----------G  55444
BelugaWhale  -------TATATTCACA--------------TAAATCTTAACTTT-------TTTTGA-ATTTTATTTT----------A  55367
Donkey       ----TTTGCAACTTGCTAACCCA---TTTGCTGATATTGCTTTCT-------CTTCATCTCTTTATGCCC--A------G  60976
Cat          -------AAAGCTTAAAGTGTTAA-------TTACAGTTCTATCA------GCTATAT-TCCTTTCATC----------A  62413
Ferret       -------AAAACCTAAAGTGTTA--------TGAAAATTCTGTCA------GCTGTAT-TCCTTTCACC----------G  62867
Lion         -------AAAGCTTAAAGTGTTAA-------TTACAGTTCTATCA------GCTATAT-TCCTTTCATC----------A  59900
Vaquita      -------TATATTCACA--------------TAAATCTTAACTTT-------TTTTGA-ATTTTATTTT----------A  56716
Pig          GAAGTTCTATGTATAGATTTCTAAGGTATCTCCAAACTGTTCTCCATAGTGGCTGTACCAGTTTACATTCCCACCAGCAG  53538
```

```
Majority     TXCTCA----TTTTCTTXTTCCAAAATAAAAG--------AAGAXAAGT---------------------TCT-------
                      80490     80500     80510     80520     80530     80540     80550     80560
Human        TTCTCTTCCATGCCACTCTCCCAGAACAAAAG--------AAGAAAAGC---------------------TCT-------  64464
PolarBear    TCCTCA----TTTTCTC-TCCCAAAATGAAAG--------AAGAAAAGC---------------------TCT-------  25129
Cow          TTGAGG----TTTCCTTTTTCCATAACAGAAC-ACTCTTGACTATTTTTAAGTGTTTCAGTTACGTTCCTTCT--CCAAT  60378
Dog          TCCTCA----TTTTTTTCTCCCAAAATGAAAG--------AAGAAAAGT---------------------TCT-------  55484
BelugaWhale  TTTAGT---------TTTTTGTACAGCAGGTT-----CTTATTATCTGT---------------------TTT-------  55405
Donkey       CTCCTCCTTCCCTTCCTCTTTCAAGTTTCAGT---------TTAGAAGT--------------------CTCT-------  61020
Cat          TCCTCA----GTTTCTC-TGTCAAAATAAAAG--------AAGA---GC---------------------TCT-------  62449
Ferret       TCCTCA----TGTTCTT-TCCCAAAATAAGAG--------AAGAAAAGC---------------------TCT-------  62906
Lion         TCCTCA----GTTTCTC-TGT-----TAAGAG--------AAGA---GC---------------------TCT-------  59931
Vaquita      TTTAGT---------TTTTTGTACAGCAGGTT-----CTTATTATCTGT---------------------TTT-------  56754
Pig          TGCAGGAGGGTTCCCTTTCTCCACAGCTCCTCCAGCACTTGTTATTTGTGGATTTATTAATGATGGCCATTCTGACTGGT  53618

Majority     ---------------AATGAAGAAATACT-----X-TGTA---AAGTXATTTTTACACGTT------------AAATTTG
                      80570     80580     80590     80600     80610     80620     80630     80640
Human        ---------------AATGAAGAAATACTGTTGAATTCTT--AATGCCATATTTACACTTT------------AAACTTG  64515
PolarBear    ---------------CATGAAGAAATACT--------GTT---GAGTTCTTATTTCACGTT------------AAATTTG  25171
Cow          A--ATACAGCCATTAAAATAGT-CATACTGAAGTAATATA-AAAATTCTCTTTCAAACATC---AAGTGTGAAAAATACG  60451
Dog          ---------------AGAGAAGAAATACT--------GTT---GAGTT--TATTTCACGTT------------GAACTTG  55524
BelugaWhale  ---A-----------------TACATATT-----AGTGTATACATGTCAATCCCAATCTCCC------------AGTTCA  55448
Donkey       ---------------------T--ATC------------AGAGAGGTCTTTTCATAACCTTT--C---------AACTAG  61054
Cat          ---------------AATAAAGAAATACT--------GTT---GAGTT--ATTTACACGTT------------AAACTTG  62489
Ferret       ---------------AATGAAGAGATACTA---TACTGTA---GAGTTGTTATTTCATGTT------------AAATTTG  62953
Lion         ---------------AATAAAGAAATACT--------GTT---GAGTT--ATTTACACGTT------------AAACTTG  59971
Vaquita      ---A-----------------TACATATT-----AGTGTATACATGTCAATCCCAATCTCCC------------AATTCG  56797
Pig          GTGAGGTGGTATCTCATGGAACACACCCTCACACCATGCACAAAAATAAACTCAAAATGGCTGAAAGACTTAAATATACG  53698
```

```
Majority     ATXTCACACTGTCTAGGT----XT--X----------CCCAGGTCTAXAGTCCTTGTGCATAT-----------------
                              80650     80660     80670     80680     80690     80700     80710     80720
Human        ATGTCACTATGCCTGAGT--------------------CTAGGCCTAAAGTTCTTGTGTGTGT-----------------  64558
PolarBear    ATGTCACACTGTCTACGT----CTA-AG---------CCCAGGTTTAGAGTCCTTGTGCATAT-----------------  25220
Cow          TTTGTCTACTATTAATATAGTTCTATATTTACACACCTAGTAGATCTTGGTCTACATAGATATCAGGCTTGATCTATTTA  60531
Dog          ATGTCAGACTGTCTAGGT----CTG-AG---------CCTAGGTCTAGACTC--TGTGCATAT-----------------  55571
BelugaWhale  TCCCACCACCACCACCA--------------------CCCCCAACACTTTCCCTCTTGGATA------------------  55490
Donkey       ATTGGATCCTGTCCATTCTTAGTTCTCATCACCACACATCAAATTTATAATTATTTGTTACAT-----------------  61117
Cat          ATTACACACTGTCTTGGT--------------------CTATGTCTAGAGTCTTTGTGCATAT-----------------  62532
Ferret       ATGTCACACTGTCTAGGTT---CTA-AG---------CCCAGGTTTAGAGTCCTTGTGCATAT-----------------  63003
Lion         ATTACACACTGTCTTGGT--------------------CTATGTCTAGAGTCTTTGTCCATAT-----------------  60014
Vaquita      TCCCACCACCACCACCA--------------------CCCCCAACACTTTCCCTCTTGGATA------------------  56839
Pig          ACAGGACACCATCAA---ACTCCTAGAAGAAAACATAGGCAAAACACTCTCTGACATCAACATCATGAAT-ATTTTCTCA  53774

Majority     --------------------------T----CTTTAG------------AGCTAATCTGATTGTTTTAATTTAGCACTGC
                              80730     80740     80750     80760     80770     80780     80790     80800
Human        ------------------------------TTTTCAGT------------GCTGATCTGATTGTTTTAATTTAGCATTGC  64596
PolarBear    --------------------------T----CTTTAG------------GGCTAATCTGATTGTTGTAATCCAGCACTGT  25258
Cow          TGTATTGTTACCAGAAGAATTGTTGTTTTATTTTTGA--AGTGAATTAGAGCTAAACATGTTCTTCA-TTTCTGAACTGT  60608
Dog          --------------------------T----CTTTAA------------GGATAATCTGATTGTTTTAATTTAGCAGTGC  55609
BelugaWhale  -----------------------------AATCTTAA-------------------------------T-TTTGTAT---  55506
Donkey       ---------------------GTGTATTTTCTTTTGGACTGTAAGTTATATACGAGT-GA--GTACCATGTCTGCTTTGT  61173
Cat          --------------------------T----CTTTAGT-------CTTTAGCAATTCTGATTGCTTTAATTTAGCACTGC  62575
Ferret       --------------------------T----CTTCAG------------GGCTAATCTGAATGTTATAATTTAGCACTGC  63041
Lion         --------------------------T----CTTTAGT-------CTTTAGCAATTCTGATTGCTTTAATTTAGTACTGC  60057
Vaquita      -----------------------------AATCTTAA-------------------------------T-TTTGTAT---  56855
Pig          GGTCAGTCTCCCAAAGCAATAGAAATTAGAGCAAAAATAAACCCATGGGACCTAATCAAACTGAAAAGCTTTTGCACAGC  53854
```

```
Majority     ----------ATAGAAACAXTGAAAAGTXAACAGATAAAACAT----GTAATAGTATT-AGTCATAX-----------GG

             80810 80820 80830 80840 80850 80860 80870 80880

Human        ----------ATGGAAACAGTGAAATGTTAAGAGACAAAACAT--ACATAATGGGTGG-AGGCATATG----------GG  64653
PolarBear    ----------ATAGAAACAGTGAAAAGTGAACAGGTAAAACAT----GTAATGGAAGT-AGTCATAA-----------TG  25312
Cow          TACT----TTATAAAGAAAATGTTTGTTCTTCTAATAAAAAATCAACAATATATTCTCCTGTTGTGTAGTTTGTTCCAAG  60684
Dog          ----------ATAGAAGCAATGAAAAGTGAATAGACCAAAC------GTAACAGGAGT-AGTCATAG-----------GG  55661
BelugaWhale  ------A----TTAAAGGAACGGGTTTTCTTTTTTTAGAGTGG-----GAATATTTT--AATTAT-------------AA  55556
Donkey       --------TCTCTGCTACTTTCCTAGAACCTAGCATAAGGTAT----GGCATATTGTA-AATATTA------------GG  61228
Cat          ----------ACAGAAACGGTGAAAAGTGAACAGGTGAAACAT----GTAATGGGATT-AGTCATAT-----------GG  62629
Ferret       ----------ATAGAAACAGTGAAAAGTGAAGAGGCAAAACAC----GTAACGGAAGT-GGTCATA------------TG  63094
Lion         ----------ACAGAAACGGTGAAAAGTGAACAGGTGAAACAT----GTAATGGGATT-AGTCATAT-----------GG  60111
Vaquita      ------A----TTAAAGGAACGGGTTTTCTTTTTTTAGAGTGG-----GAATATTTT--AATTAT-------------AA  56905
Pig          AAAGGAAACCAAAAAGAAAACAAAAAGACAACTTACAGAATGG-GAGAAAATAGTTTCAAATGATGCAA--CGGACAAGG  53931

Majority     GTTTTTTCT--ATA--ATATTATAGCCTGTCTCTTAAAAAA-----------------CAATTTA------AAC--G---

             80890 80900 80910 80920 80930 80940 80950 80960

Human        ATTTTTTTT--GAAGCATATTGTAGTGGCCTTTTAAAAAAA-----------------CAATTTA------AACTAGA--  64706
PolarBear    GATTTT---------------ATAGCCTGTCTGTTGAA--------------------CAATTTA------AACTGGG--  25349
Cow          CTTCTGTCTAGCTGGAGTATAAATGGCGATTATATAGGAAGAGAAAAAAACATAACAGCAGCATATTTTTTTATATGCTT  60764
Dog          TTTTTTTCA--ACAC-ATATTATAGCCTATCTCTTAAAAAG-----------------CAATTTA------AACTAGG--  55713
BelugaWhale  GCTTTATAT-------ACATAAGGAGCTGTTATAGAGGTT----------------------------------------  55589
Donkey       GTTTTTTTC--ATCCCATGTCATAGCTTTCCCAGCACCTGG--TG-------------TGCTGCA---------------  61276
Cat          GTTTTTTCA--ATG--ATATTATAATCTGTCTCTTAAAAAA-----------------CAATTTA------AAC--G---  62677
Ferret       GATTTTTTT--CAAT-ATATTATAGCGTGTCTCTTAAAAAA-----------------CAATGTA------AACTAGG--  63146
Lion         GTTTTTTCA--ATG--ATATTATAATCTGTCTCTTAAAAAA-----------------CAATTTA------AAC--G---  60159
Vaquita      GCTTTATAT-------ACATAAGGAGCTATTATAGAGGTT----------------------------------------  56938
Pig          GCTTAATCT--CTAGAATATATAAACAACTTACACAATCCAACAGCAAAAAAGCCAATCAATCAATGGAAAAATGGGCAA  54009
```

```
Majority     -AAGAATTTTCTAT--------AAAATTTATTA------TGXXTTATTXAXATTTTGTC--------TTA----------
                       80970     80980     80990     81000     81010     81020     81030     81040
Human        -AGAAATTTTCTGT--------AAAATTAATTT----TGCTATTTCTTTGCATTTTGTC--------TTA----------  64755
PolarBear    -AAGAATTTTCTAT--------AAAATTTTTTT------TG---TATTCACATTTTGTC--------TTA----------  25393
Cow          TATATGCCTTTTTCTGTGCTTTATAATTCATGATACTTGCACACATGTGGTCTTTTATC--TAATCCTTACAATAATCCT  60842
Dog          -AAGAATTTTCTAT--------AAAATTTATTT------TGGTTTTCTCTTTTTTTGTC--------TTA----------  55760
BelugaWhale  ----TACTTTTTCC--------ATAACAGGGCA------CTCTTGATTATATTTTAGTC---------------------  55630
Donkey       GATAGGTCTCTTCCCA--CCTTTCAACTAGCCATGAACATGTCAGTATATTATGGAATGAGTTGGTTCTA----------  61344
Cat          -AAGAATTCGCTATG-------AAAATTTATTC------TGG--TATTCACATTTTGTC--------TCA----------  62723
Ferret       -AAGAATTTTCTAT--------TAAATTTATTT------TGG--CATTTATGTTTTTTC--------TTA----------  63191
Lion         -AAGAATTCGCTATG-------AAGATTTATTC------TGG--TATTCACATTTTGTC--------TCA----------  60205
Vaquita      ----TACTTTTTTC--------ATAACAGGGCA------CTCTTGATTATATTTTAGTC---------------------  56979
Pig          AAGACAT-TTCTCCAAAGATATACAGATGGCCA-ACAAACACATGAAAAAATGCTCATCATTGCTGATTATAAGAGAAAT  54087

Majority     -------------ATCATTTATATTTTA-----CCTTGTTCTACAATG-GA--TTTGAC---TCACCTXACATA------
                       81050     81060     81070     81080     81090     81100     81110     81120
Human        -------------ATCATTTATATTTTA-----CTTTGTACCACAATG-GA--CTTGAG---CCACCTAATA--------  64803
PolarBear    -------------ATCATTTATATTTCA-----CCTCATTCTACAATG-GA--TTTGAC---TCA---------------  25434
Cow          GCAAG--GAAGCATGGCAGCCATTGTCA---TCTGTCTTCCAGATTCTGAGATTAGAAAATATTAAATAACTTGTTCAAG  60917
Dog          -------------ACCATTTATATTTCA-----CTTTGTTCTCCAGTG-GA--TTTGAC---TCACCACACACACACAGA  55816
BelugaWhale  ------T------GTTTCAGTTACTTT------CCTTCTTCAACAGTGTGA------------CAATTAAAATAGTCATA  55680
Donkey       ---------AACTTTGTAAATCAGTTCCTACGTTTTTATGCTTATTA-AAA--TGGTC-ATGTAAAATTAAAGGCAAGGA  61411
Cat          -------------ATCATTTATATTTCA-----CCTTGTTCTACAATG-GA--TTTGAG---TCACCTCCCATA------  62773
Ferret       -------------TTCATTTATTTTTCA-----CCTCATTCTGCGATG-GA--TTTGAC---TTACCTCACAGAA-----  63242
Lion         -------------ATCATTTATATTTCA-----CCTTGTTCTACAATG-GA--TTTGAG---TCACCTCCCATA------  60255
Vaquita      ------T------GTTTCAGTTACTTT------CCTTCTTCAACAGTGTGA------------CAATTAAAATATTCATA  57029
Pig          GCAAATTAAAGTATTATTAATATAATGAAACTTTTGTTCTTGATTTTGAAATTTTTCATGACTAGCTGAAGTTAGATAT  54167
```

```
Majority     ---A---A-X--AAAAATAXT-----GCAAA-----AATTTGCAACXTCTAGTTGATXGAGTAAAGATGAAGA-------
                      81130     81140     81150     81160     81170     81180     81190     81200
Human        -------A----AAATACAGT-----GCAAAC-----ATATGCAACTTATAGTTGATAGCGTGGAATCAAGATGAAGA--  64860
PolarBear    --------------AGAAAAT-----GCAAA-----AATTTGCAACTCCTAGTTGATGGAGTGAAAATGAAGA-------  25483
Cow          GTGACCTAATTGAGTGGTCTTTCAACTCTAGAGCATAAGTATTACCATCCAGCAAAAAATATATGTGTAATAAAATTAAC  60997
Dog          CACACACACACGAAAAACAAT-----GCAAA-----AATTTTTAACTACTGGTTGATCGAGTCGAGATGAAGA-------  55879
BelugaWhale  CTGAGGTAATACAAAAATTCTCT----------------TTCAAACATCAAATGTGAAAAATATGT--------------  55730
Donkey       ATAAGAGTTTGAGAAGAGAATC----TCAGG--CCTACCTCTCCACTTACAGTGATAAGTGTTCAAATGAATG-------  61478
Cat          ------------AAAAAAACC-----GCAAA-----AATTTGCAACAACTAGTTGATGGAGTAAAGATGAAGATAATAGG  62831
Ferret       -----------GAAAAACAAT-----GCAAA-----AATTTGCAACTACTGGTAGATGGAGTCAAGATGAAGA-------  63294
Lion         ------------AAAAAAAT------ACAAA-----AATTTGCAACAACTAGTTGATGGAGTAAAGATGAAGATAATAGG  60312
Vaquita      CTGAGGTAATACAAAAATTCT------------------TTCAAACATCAAATGTGAAAAATACGT--------------  57077
Pig          TCAAAGAATTTAAAAGATTCCCTTGAGCAAGTGCTAAACCTTTAACCCCCACCCCCAGCAAT-CGC-CAAGAAAATTGAT  54245

Majority     -----AAAAATAAGGGAAA------ATGTTAC--------TTT--------------------GCAAGXAAAA-------
                      81210     81220     81230     81240     81250     81260     81270     81280
Human        ----AAAAAATAAGGAAAA-------TCTTAC--------TTT--------------------GTAAGTAAAA-------  64894
PolarBear    -----AAAAATAAGGGAAA------ATGTTAT--------TTT--------------------GCAAATAAAA-------  25517
Cow          TTTTAA-CTATTGTGCAAACTATTGATATTACAGTACTA-TTTTGGCAGGATGAACTATATTTGTGGCCTTTGTTTTTTT  61075
Dog          -----AAGAATAAGAGAAA------ATGTTAT--------TTTT------------------TGCAAGTTATA-------  55915
BelugaWhale  ----------------------------------------T------------------------GGTCTAC--------  55738
Donkey       ----AAAAATTTAAAAAAAGTATCTAGGCACTGGGAATTCCTTTA------------------GCAGGCAGTG-------  61529
Cat          GAATGAAGAATAAGGGAAA------ATGTTAC--------TTT--------------------GTAAGTAAAA-------  62870
Ferret       -----AAAAATAAGGGAAA------GTGTTAT--------TTT--------------------GCAAAAAAAAA------  63329
Lion         GAATGAAGAATAAGGGAAA------ATGTTAC--------TTT--------------------GTAAGTAAAA-------  60351
Vaquita      ----------------------------------------T------------------------GGTCTAC--------  57085
Pig          ACATAGGTTATAGTAAACATTGAC-ATATAACGGAAGTAGTTTCAATGTGGCTTTTCCTGTTTTCAGTCTATATGCCGTG  54324
```

```
Majority     ---T----------------------------X-XTGAA-TGATTTATAACTT---AAGATGAAATTTTACAGTTTT---
                      81290     81300     81310     81320     81330     81340     81350     81360
Human        -----------------------------T--GTGCAAAGTGATTTATAAGTT---AAGATGGAATTTTAGAGTTTCCTT  64940
PolarBear    ---T----------------------------GAGTGAA-TGATTTATAATTT---AAGATGAAATTTTACAGTTTT---  25559
Cow          TAATTAAAATATTTGCAGGTTTTTGAAATTGAAAGTATATTAGCACATATATTTAACTGGTTATATTTTATATTTAAGTA  61155
Dog          ---T----------------------------GAGTAAAGTGATTTATAAC-----AAGATGAAATTTTACAGTTTT---  55956
BelugaWhale  ---T----------------------------------ATTAATATAGGTCT---------GGTTTTACACACTTAA---  55769
Donkey       ----------------------------------------T-GCCTTCAAGTTTTGAGGAAGAAAATTTACGGTATT---  61565
Cat          -----------------------------------TGAG-TGATTTATAACTT---GGGATGAAATTTT--AGTTTT---  62906
Ferret       ---T----------------------------GAGTGAAGTGATCTATAACTT---AAGATGCAATTTTACAGTTCT---  63372
Lion         -----------------------------------TGAG-TGATTTATAACTT---AGGATGAAATTTT--AGTTTT---  60387
Vaquita      ---T----------------------------------ATTAATATAGGTCT---------GGTTTTACACACTTAA---  57116
Pig          T--TTCACTCTGTTGTTAACTATGCGTCCTATGTCTTTACT-GTGTTTATCTTTTCCTGATGTTTTTGTGTACTCTTCAA  54401

Majority     --ATTTGCTGTGTTTAGGAAXX-----------TCTTGATATCTAATAT-----------XTGTAA---------GATT
                      81370     81380     81390     81400     81410     81420     81430     81440
Human        TTATTTGATGTATTTCTTGA---------------TT----TCTAGTAT-----------TGGTAA---------GATT  64980
PolarBear    --ATTTGCTGTGTTTGGGAAAT-----------TCTTGATTTCTTATATA---------TTTGTAA---------GATT  25607
Cow          GCACTTTCTATTTAAAGACA-------GTAGTATTTGTGGAAAACAATATACATACAGCTAGTGTAGCAAAGAAAAGAGT  61228
Dog          --ATTTGCTGTGTTTGGGAAAT-----------TCTTGATTTCTGACAT-----------GTGTAA---------GATT  56002
BelugaWhale  --TCTTGGTCTGCATAGATA--------------------------------------------T-CAG-------------  55791
Donkey       --CTTCAAAGTGTGGTCCTA-TG----------TTCTTGTTATATAGATTGC-------TTATACAA----------AAT  61615
Cat          --ATTTGCTGTGTTTCAGAAGT-----------TCTTGACATTTGATAT-----------TTGTAA---------GATT  62952
Ferret       --ATTTGCTGTGTTTGGGAAA---------------TTGATTTCTTATATA---------TGTGTAA---------GATT  63417
Lion         --ATTTGCTGTGTTTCAGAAAT-----------TCTTGACATTTGATAT-----------TTGTAA---------GATT  60433
Vaquita      --TCTTGGTCTGCATAGATA--------------------------------------------T-CAG-------------  57138
Pig          ACTTTCCCTTTGCATAGACAAATTCGAGAAGTCTTTCTGCAAATTAACTTGAGCTTTAACTGT-CAG--AAATATTGCAT  54478
```

```
Majority     AT---CTTTAAATCATX------------TTTGTGTXCATATGGAACCC-----AATATTGATGAXTTATAAACT-AGCT
                      81450     81460     81470     81480     81490     81500     81510     81520
Human        AT---CTTTAAGTCATG-----------ATTTTTATATATATGGAACTC-----AAAATTGATAGTTGACAAACT-AGCT  65040
PolarBear    AT---CTTTAAATCAGA-----------GTTTCTGTCCATATGGAAACC-----AAAATTGATGAGTTATAAACT-AGCT  25667
Cow          ATGGAATTTGATCCCTT---TCCGGGCAACTTTTA-TTATGTACAGCTTTTC--ATTGTTGA--GTTTTTTGTCAGAGT-  61299
Dog          AT---GTTTAAATCAG-------------TTTCTGTCCATATGGAACCC-----AAGATTGATGAATTACAAACT-AGTT  56060
BelugaWhale  -----GCTTGATCTATT-------------------T-ATGT------------ATTGTTAT------------------  55816
Donkey       GGGAGTCTCAGACCAT-------------CCTGAATTAACTTAGCAT-------AATTTTGGGGATTGGGAGCCCAATAC  61675
Cat          TT---CTTTAAATCAG-------------AGTGTGTCCATATGGAACCC-----AAGATTGAGGAGTTAAAAACT-AGCT  63010
Ferret       AT---CTTTAAATCAGC-----------ATTCCTGTCCATATGGAACCC-----AAGATTAATGAGTTATAAACT-AGCT  63477
Lion         TT---CTTTAAATCAG-------------AGTGTGTCCATATGGAACCC-----AAGATTGAGGAGTTAAAAACT-AGCT  60491
Vaquita      -----GCTTGATCTATT-------------------T-ATGT------------ATTGTTAT------------------  57163
Pig          ATATAGTTTAATTTTTTCTGTTTAGCTCATTTGTAGTCATCTTAACCTCCACCAACTATTGACAGTACCTAGACTTCTTT  54558

Majority     CTTACAGTTT--AGTTTATGTCCCTCAGTC--A---AGAAAAAXCATTTTATAATTAAGTAGTTCTTGGA-------AAT
                      81530     81540     81550     81560     81570     81580     81590     81600
Human        TTTACAATTT--AGTTTATGTCTGTCATTTCAA--AAGAAAAAATAG--TATAATTAAGTAGTTATTGGA-------AAT  65107
PolarBear    CTTAGAGTTC--AGTTTATGTCTCTCAGTCAGA-AAAAAAAAAGCATATTATAATAAAGTAAT-CTTGCA-------AGT  25736
Cow          CGTACTTTTTCTAATTTTTATCTCT---TAAAATTGCTGATATACTTTATTCACTTTTACAGGCCCTAGATGGTCAGAAT  61376
Dog          CTTACAGTTC--AGTTTATATACCTCAGTCAGATAAAGAAAAACCATCTTATAATGAAGTAATTCTTGGA-------AGT  56131
BelugaWhale  --------------------------C----------AGAAGAATTGTTTTCATTTTTGAGGT-----------------  55843
Donkey       TCTGTTGTTTTTTGTTTTTTGTTTTGAAGTTGT---GGGAACCACAGC-TATATCAAAGCAGCTT---GAGTGACAGAGC  61748
Cat          CTTACAGTTT--AGTTTGTGTCCCTCAGTC------AGAAAAAGCAT--TATAATGAAGTAATTCTTATA-------AAT  63073
Ferret       CTTAGAGTTT--AGTTTATGCCCCTCAG---AA-AAAGAAAAAGTGTATTATAATGAAGTAATTCTTGCA-------AGT  63544
Lion         CTTACAGTTT--AGTTTGTGTCCCTCAGTC------AGAAAAAGCATATTATAATGAAGTAATTCTTAGA-------AAT  60556
Vaquita      --------------------------C----------AGAAGAATTGTTTTCATTTTTGAGGT-----------------  57190
Pig          CCTTATGTTTTCAGCTCTTGTTCCTCTCTTCTGTTGCTAATAGGCAGTTTTCATTTACATTGTTTGCTGATTTCTCTAAC  54638
```

```
Majority     AGATATA-TATTTACTGTA------------GATATGTAACAXTTGTGXTTTC-------AATTATAAATATGTAGTA--
                      81610     81620     81630     81640     81650     81660     81670     81680
Human        ATTTA-----CTTCCC--A-------------ATATATAACAGTCATGCCATC------AGTTTGTGAATATGAAATC--  65159
PolarBear    AGATACA-TATTTACTATA------------GATATATAACAATTGTGCTGT-----------TATAAATATGTAGTAAA  25792
Cow          ATTTATAATGCTTGCTGTACCCTAAGGATTGATTTTTCCAAGCTTGTGAATTTGAATGTAAAATACAACAATGATAAAAG  61456
Dog          AGATATA-TATTTACTGTA------------GATATGTAACAGTTCTGCTATC-------TGTTGTAAATATGTAGTAA-  56190
BelugaWhale  ------------------------------------------------------G-----AATTATAGAGCT--------  55856
Donkey       CAACCAAACTCTTTCCATA------------------TGCTAGTTGTGTTCTTTAGCAATTACTTCAGATAATTGG-AGT  61809
Cat          AGATTTA-TATTTATTGTG------------GATATGTAACAATGGTGTTTTC-------CA--TTGAAAGTGCAGTA--  63129
Ferret       AGATATA-TATTT-TTGTA------------GATATGTAACACTTGAACTATC-------CATTATAAATATGTAGTAA-  63602
Lion         AGATTTA-TATTTATTGTG------------GATATGTAACAATGGTGTTTTC-------CA--TTGAAAGTGCAGTA--  60612
Vaquita      ------------------------------------------------------G-----AATTATAGAGCT--------  57203
Pig          TCTTGTA-TCTTTGTTATACTATGTATCTTCGGTCTACATAATTT-TGTTTTT---TTTTAATTAGAAGAATGTCGAATG  54713

Majority     T-----TAA--------GTGATAT---ATCTXTGAACAGA----------GACACC------------TAATGC-----A
                      81690     81700     81710     81720     81730     81740     81750     81760
Human        T----GTAA--------GTGGTAT---ATATGTAGAGAGAGATACTCTAAGGCAGTTGG---TATT--TGATTCTGGTAA  65219
PolarBear    T----ATAAA----TATGTAGTAAT--AAACATGAACAGA----------GACACC------------TTATGC-----A  25835
Cow          TAGGGATTATACTCGACCTGATCTTCCATCTGGGGATGGACAACCTGCATTGGACCCAGCTATAGCTGCAGCATTTG---  61533
Dog          T----GTAA--------GTGATAC---ATCCATGAACAAA----------GACACC------------TGATGC-----A  56228
BelugaWhale  ------------------------------------------------------------------------------A-  55857
Donkey       TTCTTCCC--------CTTAGCATTTTGCTTTATAAGAAA-----------AGAGC---------AACTCA---------  61852
Cat          ------TAA--------GTGATAC---ATCTGTGAACAGA----------GACACCC-----------TAATGC-----A  63166
Ferret       T----GTAA--------GTGATAA---ATCCATTAACAGA----------GACACC------------TTATACTATAAA  63645
Lion         ------TAG--------GTGATAC---ATCTGTGAACAGA----------GACACCC-----------TAATGC-----A  60649
Vaquita      ------------------------------------------------------------------------------A-  57204
Pig          TTTCTAGAAGGCTCT-CTTAACATTTAGCGTATTCATTGCAGA--TACTCGGCACTCAGCCTCTTTAAGAGAATTCGGCT  54790
```

```
Majority     CAAGAGAAACAATTGATTTTTGAAAT--TAATTCTG-----GTA----ACT----------------------TCAGACA
                      81770     81780     81790     81800     81810     81820     81830     81840
Human        CTAA--TAAGAATTAACTACC----TGTTAATTCTG-----GCA----GCT----------------------GCAAACA  65262
PolarBear    CAAGAGAAGCAATTGATGTTTGAAAT--TAATTCTG-----GTA----ACT----------------------TCAGACA  25882
Cow          CCAAGGAAACATCTCTCT-TAGGTATGATTTTTAT-TGTCTGTACCAC-TTCTGCCAAATGGGCAAGTGCCTGTTAAACT  61610
Dog          CAAGAGAAGCAATTGATACTCAGAAT--TAATTCTG-----GTA----ACT----------------------TGAGAAA  56275
BelugaWhale  -------AACATGTTCTT-------------------------------C------------------------------  55869
Donkey       -GAAAAAAACAGATTAACATGGGAGACTTAGTGAGGTTTGGAGG----------------------------GAAAAATA  61903
Cat          CAAGAGAAGCAGTTGACATTTGAAAT--TAATTCTG-----GTG----ACT----------------------TCAGACC  63213
Ferret       CAAGAGAAGCAATTGATATTTGAAAT--TAATTCTG-----GTT----AT-------------------------AGACA  63689
Lion         CAAGAGAAGCAGTTGACATTTGAAAT--TAATTCTG-----GTG----ACT----------------------TCAGACC  60696
Vaquita      -------AACATGTTCTT-------------------------------C------------------------------  57216
Pig          CCAAAGAAACTCCTTGTTGTGAAGATAGTAATTCTGTATCAGTACTTTACTCTTTGCATTGATCTAGGTCCTATGACCCC  54870

Majority     AAACTGACCCTTTA-----------TTCTAATTTTCTAATA-XATCTAGCAXA-------------AGCCATAAATGTTT
                      81850     81860     81870     81880     81890     81900     81910     81920
Human        AATCTGACCCTTTA-----------TTCGAATTTTCTGGTGTATCCTAGCAGA-------------AGTCATAG-TGTTT  65317
PolarBear    AAATTGACCCTTTA-----------TTTTAATTATCTAATA-CATCTAGCACA-------------AGCCATAATTGTTT  25937
Cow          CCAGTAAGTATAATGAATCTCCCATTTTGAATTTTAAGAAATTATGTTAGATCCCCCCACCCCCTCAGGTAAGAATGTAT  61690
Dog          AAACTGGCTCTTT-------------TCTAATTTTCTAATA-TATCTAGCACA-------------AGCCATAATTGTTT  56328
BelugaWhale  -----------------------------ATTTCTGGACTGTTATTTTATAT-----------------AGTAAATGTTT  55903
Donkey       CAACAATCTCTTTAAAAACCTCGTCAAAAAACTATTGGTCTACCTTTAAAATA---------ATATAGTTTTAGATGTAC  61974
Cat          AAACTGACCCTTTA-----------TTCTAATGTTCTAACA-CATCTAGCACA-------------AGCCCTAACTGTCT  63268
Ferret       AAATTTACCCTTTAA----------TTCTAATTTTCTAATA-CATCTAGCACA-------------AGCCATAATTGTTT  63745
Lion         AAACTGACCCTTT----------------AATGTTCTAACA-CATCTAGCACA-------------AGCCCTAACTGTCT  60746
Vaquita      -----------------------------ATTTCTGGACTGTTATTTTATAT-----------------AGTAAATGTTT  57250
Pig          AAACCAGGTCTTTTCTTACATCAGATTTTTTTTGTATGATACTAT-TGAAAT------AGTTACCCAAGAGTGAATTTTT  54943
```

```
Majority     ATCATGAX--------------------TTT-AACAGATTCTTAAGGTTGTX--A-------------------ATATTC
                     81930     81940     81950     81960     81970     81980     81990     82000
Human        ATTATGAC--------------------TGT-AGTAGATGCTTTAGGTCACT---------------------TACACTC 65355
PolarBear    ATCATGAT--------------------TTT-AACAGATTGTTCAGGTTAAT--A-------------------ACATTC 25975
Cow          ATGAATACTGTTGGAAGAAAAACCTAAATTCATAAGTATAATTAAAGTTGTGTCACATTGCTTTTACCTATCTCATCTTC 61770
Dog          ATCATGAT--------------------TTT-AACAGATTCTTTAG----------------------------CATTC 56358
BelugaWhale  ATTCT-----------------------T-CTAATGAAAAATTAACAGTGTG-------------------------TTC 55934
Donkey       ACACCTAATGAGTCTTGG------TGTCTGTAAACATCAGGCTTGATCTATG------------------CGTGTGTTG 62029
Cat          ATCATGAC--------------------TTTTAACAGAGTCTTAAGGTTGGT--A-------------------ATATTT 63307
Ferret       ATCATGAT--------------------TTT-AA-AGATTCTTTAGGTTAAT--A-------------------ACATTC 63782
Lion         TTCATGAC--------------------TTT-AACAGAGTCTAAAGGTTGGT--A-------------------ATATTT 60784
Vaquita      ATTCT-----------------------T-CTAATGAAAAATTAACAGTGTG-------------------------TTC 57281
Pig          CTGCTCATCTTTCGAT----------A-TTCGTATATTGGGTCGAGACAGTG--ATTCTGTTAGGTGCCTTAGCATTTTC 55010

Majority     TTTTCTGACAG----------AATCTCTA-T-------CACTGTG----------------------------------
                     82010     82020     82030     82040     82050     82060     82070     82080
Human        TTTCCTGACAG----------AATCTCTACTAC-----CTCTGTG---------------------------------- 65385
PolarBear    TTTTCTGACAG----------AATCTCTTAT-------CACTGTG---------------------------------- 26003
Cow          TCTCCCAAAATAGAAGAGGAAAAGCTCTAATGAAAAAATACTGTTGAGTTCTTAATAAAACATTTACACTTTAAAGTCAT 61850
Dog          TTTTCTGACAG----------AATTTCTA---------TACTGTG---------------------------------- 56384
BelugaWhale  CC-------------------------------------CTGTT----------------------C------------ 55942
Donkey       TTATCAGA-AG----------AAGTGTTGTTTCATTTATACAGTAATTTATAGATCTAAACATGT-----------TCAT 62087
Cat          TTTTCTGACAG----------AATCTCTA-T-------CACAGTG---------------------------------- 63334
Ferret       TTTTCTGACA-----------ATTCTCTTAT-------CACTGTG---------------------------------- 63809
Lion         TTTTCTGACAG----------AATCTCTA-T-------CACAGTG---------------------------------- 60811
Vaquita      CC-------------------------------------CTGTT----------------------C------------ 57289
Pig          CCTATAGGAAT-GAGTAAGGGAAGACCTATTTTCAGTGCTTTGTTTGCCTTTACAGCAGAGAATTG-AATTAATACTTAA 55088
```

```
Majority     ----T--GG--------GTTTATTTTAAGCCTTTATTCCCTC-------------AACTTGGCTTGTTX------AXTT-
                     82090     82100     82110     82120     82130     82140     82150     82160
Human        ----T--GT--------GTCTAGCTTAATTCGTAATT--C---------------AAGTTAATAAGATT-----------  65423
PolarBear    ----T--GC--------GCTTAGTTTAAACCTTTATTCCCTC-------------AACTTGGCTTGTT------------  26044
Cow          GCTATCTAGAAGCCATTGTGCATATTCAGTCTTAATTGTTTCAGTTAGATTGGAAAACCAATCTGATT--GTTTCAGTTT  61928
Dog          ----T--GG--------GCTTAGTTTAAGCCTTTATTCCCTC-------------AACTTGGCTTGTTA------AGTT-  56430
BelugaWhale  ----TCTAG---------TTCGTTCCAGGTTTCTATC-------------------------------------------  55966
Donkey       CATTTCTGACTG---TTGTATACAGTAAGTCTTTGTTCTTCTAAT-------GAAAAATTAATACATTC---CCCTGTT-  62153
Cat          ----T--GG--------GCCTACTTTAAGCCTTTACTCCCTT-------------AACTTGGCTTCTTA------AATT-  63380
Ferret       ----T--GG--------GCTTAATTTAAACCTTTATTTCCTC-------------AGCTTGGCTTGTTA------AGTT-  63855
Lion         ----T--GG--------GCCTACTTTAAGCCTTTACTCCCTT-------------AACTTGGCTTCTTA------AATT-  60857
Vaquita      ----TCTAG---------TTCGTTCCAGGTTTCCATC-------------------------------------------  57313
Pig          TTACTCTGGCTGAGAGAGTTGGTCCTCGGCTTTCATT-CTCATGTGATGATAGGTACCTTCCCTAATTCAAATATAATTC  55167

Majority     --------------------------------------------GAXTATTTCGTAGTGTGC-CATTTTTA-XT------
                     82170     82180     82190     82200     82210     82220     82230     82240
Human        --------------------------------------------GAATATTTCACAGTGTGC-AGTTTTTA---------  65449
PolarBear    --------------------------------------------GACTATTTCATAGTGTAC-CGTTTTTA---------  26070
Cow          AGCCCTGCACAAAAA---AAACCCAGAAAGTTAACAGAGGC-AGCATATAATGGGAGTAGGTATATGGATTTTTCAAAAC  62004
Dog          --------------------------------------------GACTATTTCATAGTGTAC-CATTTTTA---------  56456
BelugaWhale  ------------------------------------------C------AGCTGGAGTAG---CATTGGTGATT------  55989
Donkey       --------------------------------------------CTCTAGTTTGTTCCAGGCCTATATCCAGCT------  62183
Cat          --------------------------------------------GATTATTTCGTAATGTAC-CATTTTAA---------  63406
Ferret       --------------------------------------------GACTGTTTCATAATGTGC-CATTTTTATTTATTTAT  63890
Lion         --------------------------------------------GATTATTTCGTAATGTAC-CATTTTAA---------  60883
Vaquita      ------------------------------------------C------AGCTGGAGTAG---CATTGGTGATT------  57336
Pig          AAATCCTGATGTGTATGTAATCCTTTTAAGTGAGTTTTCACCAGTATTGTATTGAACTAGGTGAACTTGTGTTTTTAGAT  55247
```

```
Majority     ------------------AAAGGT-------X-X-----------X-A-XAA----------------------------
                      82250     82260     82270     82280     82290     82300     82310     82320
Human        -------------------AAGGC--------------------------AA----------------------------  65456
PolarBear    -------------------AAGGT--------------------------AA----------------------------  26077
Cow          ATATCGTAGTCTTAAAACACAGGTTTAACTAGGA---AGAATTTTATATAAAATTTTTTTTGGTATTTATTTGCATTTTG  62081
Dog          -------------------AAGGT--------------------------AA----------------------------  56463
BelugaWhale  ----------------ATATAGGAACAGAAAAAA-----------ACATAA-----------------------------  56013
Donkey       -------------GGAGTAGAAATGATGATTATATAGAATCAGTGGGAAAAAACATAAT---------------------  62229
Cat          --------------------AGGT--------------------------AA----------------------------  63412
Ferret       TTATT-TATTTATTTTTTAAAGATTTTATTTATT-----TATTTGACAGAGA----------------------------  63936
Lion         --------------------AGGT--------------------------AA----------------------------  60889
Vaquita      ----------------ATATAGGAACAGAAAAAA-----------ACATAA-----------------------------  57360
Pig          TAGTTTTTAGCTTGGTAAATATATTCATGTAAAACTCAGTTTTGTATATTAAAGAGTTTTCTTTCAGAGTGGGAATATT-  55326
```

```
Majority     ----AGTCAX----------------GAGTAX--X-XGAGACATTTA--GTGATA-------------------------
                      82330     82340     82350     82360     82370     82380     82390     82400
Human        ----AGTCAG----------------GAAT-------GAAAGA------GTGAAA-------------------------  65478
PolarBear    ----AGTCAA----------------GAGT-------GAGACAATCA--GTGATA-------------------------  26103
Cow          TCTTAGTTATTATATTTCACCTCATTCAGCAATGGGTTTGACCCACCTAATAAAAAAAACACAAATATTTGCAACTAATA  62161
Dog          ----AGTCAA----------------GAGT-------GAGACAATGA--GTGATAA------------------------  56490
BelugaWhale  --T-AATCAT-----------------AACAGCAGCACAGTCCTTTT--ATAATTC------------------------  56047
Donkey       ----AATCAT-----------------AACAGCAGCATAGTCTTTT---ATAATTT------------------------  62261
Cat          ----AGGCAA----------------GAGT-------GAGACA------GTGATA-------------------------  63434
Ferret       ----GATCAC----------------AAGTAGACGAAGAGGCAGGCA--GAGAGAGAGAGAGAGAGAGG-GAAGCAGGCT  63993
Lion         ----AGGCAA----------------GAGT-------GAGACAATGA--GTGATA-------------------------  60915
Vaquita      --T-AATCAT-----------------AACAGCAGCACAGTCCTTTT--ATAATTC------------------------  57394
Pig          --TTAATTAC-AAGCTCTATATACTTAAGCAGTTATAGAGGCTTTTTCCATAACAG-------GACATTCTTGACTATTT  55396
```

```
Majority     ------------------------------------XX-XX----------CATGATCTTTGA-----------------
                       82410     82420     82430     82440     82450     82460     82470     82480
Human        ---------------------------------------------------CATGGTTTTTGC-----------------  65490
PolarBear    ---------------------------------------------------CATGATCTTTGC-----------------  26115
Cow          GTTGACAGAGTCAAGATGAAGAAAAAAATAACAGAAAATGTTATTTTGTAAGTAAAATAAGTGATTTATAACTTCAGATGA  62241
Dog          ---------------------------------------------------CAAGATCTTTGT-----------------  56502
BelugaWhale  ----ATATATCTTACAT-------------------ATGTG----------------------------------------  56065
Donkey       ---------------------------------ATGATGTGCTTTCACATATGTGATCTTTTGG-T----------T---  62294
Cat          ---------------------------------------------------CATGATCTTTGA-----------------  63446
Ferret       TCCTGCCGAGCAGAGAGCCCGATGCGGGACTCGATCCCAGGACCCTGAGATCATGACCTGAGCCGAAGGCAGCGGCTTAA  64073
Lion         ---------------------------------------------------CATGATCTTTGA-----------------  60927
Vaquita      ----ATATATTTTACAT-------------------ATGTG----------------------------------------  57412
Pig          TTAAATTTGCTTTAGTT--ACTTTATTTGGTCAACAATATG----GCAATTAAAATA-GTCATACAGGG---GAGTTCC  55465

Majority     --------------------------------------------------------------------------------
                       82490     82500     82510     82520     82530     82540     82550     82560
Human        --------------------------------------------------------------------------------  65490
PolarBear    --------------------------------------------------------------------------------  26115
Cow          CATTTTAGTTTCCTTTTGATGAATTCAGGAAATTCTTGGTTTCTGAAGTTTGTAAGATT-ATCATTAAATGAATGT-TTC  62319
Dog          --------------------------------------------------------------------------------  56502
BelugaWhale  --------------------------------------------------------------------------------  56065
Donkey       --------------------------------------------------------------------------------  62294
Cat          --------------------------------------------------------------------------------  63446
Ferret       CCCACTGAGCCAGCC---AGGCGCCCCATAATGTGCCATTTTTAAAGGG----AAAGTT-AAGAGTGAGACAATGAGTGA  64145
Lion         --------------------------------------------------------------------------------  60927
Vaquita      --------------------------------------------------------------------------------  57412
Pig          CATCCTGGCTCAGCGGAAATGAATCC-GA-----CTAGCTTCCATGAAGACGCAGGTTTGATGCCTGATTCAGTGGGTTA  55539
```

```
Majority     --------------TTATAATTATTA--CTGTAGA----TTCTTA-AATT---TTTACTGATA------------GAAAX
                      82570     82580     82590     82600     82610     82620     82630     82640
Human        --------------TTATAATTATTA--CTATAGA----TTATTA-AACTC--TTTACTGATA-----------------  65530
PolarBear    --------------TTATAATTATTA--CTGTAGA----TTCTTA-CATT--ATTTACTGATA------------GAAAT  26160
Cow          TGGACGTATGGAACCCAAGATTGATGAATCGCAAACTTGTTCTTAGAGTTTAGCTTATTGCTATTATTCAGTTGCTAAGT  62399
Dog          ---A--------ATTCTTACTTGCT----TGTAGA----TTCTTA-AGTT--ATTTACAAATA------------GAAA-  56547
BelugaWhale  --------------------------------------------------------------T-----------------  56066
Donkey       ------------AATCCTCATAATAACCCTGTGAG--GATTATGGCAGCCATTATCATCTCTGT-------TTTCCAGAT  62353
Cat          --------------TTATAATCCTTA--CTGTAGA----TTCTTC-AATT--CTTTACTGATA------------GAAAT  63491
Ferret       TACAA-------GGTTATAGTTATTA--CTGTAGA----TTCTTA-AATT--ATTTACTGATA------------GAAA-  64196
Lion         --------------TTATAATCATTA--CTATAGA----TTCTTC-AATT--CTTTACTGATA------------GAAAT  60972
Vaquita      --------------------------------------------------------------T-----------------  57413
Pig          AGGATCTGGCGTGGCTGTGGCTGTGG---CGTAGGCCAGTGGTTACAGCT---CTGATTGA-ACTCCTAGCTTGGGAACC  55612

Majority     ---ATATXATTTTG-TATAG----AAAAA-----------AAAAAT--------CAACATTTATTG------------AA
                      82650     82660     82670     82680     82690     82700     82710     82720
Human        -------GACATTGTTTTAG-------A------------AAGAAT--------CAAAATTTATTG------------AG  65564
PolarBear    T--GTATTATTTTG-TACAAGAGAAAAAA-----------AAAAAT--------CAACATTTATTG------------AA  26206
Cow          ATTATCCAACTCTT-TGTGTCCCCATGGACTGTAGCATGCCAGGCTCCTCTATCCACTGTCTCCCGGAGTTTGATGAACA  62478
Dog          ------TTATTTTA-CAGAA----GAGAA-----------AAAAAT--------CAACATTTATTG------------AA  56585
BelugaWhale  ---AAAATATACATATGTGCT------------------------------------T----------------------  56085
Donkey       TAAGAAAGATTTTG--AGATTCAGAGAAG-----------TTAAGTAACTTGCTCAACGTGACCTG------------AC  62408
Cat          G--ATACGATTTT--TACAG---AAGAGG-----------AAACAT--------CAACATTTATCA------------AG  63533
Ferret       ------TTATTTTA-TATAG----AAAAA-----------AAAAAT--------CTACATTTATTG------------AA  64234
Lion         G--ATATGATTTT--TACAG---AAGAGA-----------AAACAT--------CAACATTTATCG------------AG  61014
Vaquita      ---GAAATATACATATGTGCT------------------------------------T----------------------  57432
Pig          TCCATATGCCACAGGTGTGGCCCTAAAAA----GATTTTTAAAAATATAGTGGGCA-TGTCCATTGTGGCCCTTTGGAAA  55687
```

```
Majority     TAACT----CCTAGGTCC-TAGG------------------CATTCTACTAGGCGC-X--X----------GXX--X---
                      82730     82740     82750     82760     82770     82780     82790     82800
Human        TAGCT----TCTAGGTC-ATA------------T------TCATTCAGCTAGGCAC------------------------  65597
PolarBear    TAACT----CCCAGGTCC-TAGG------------------CATTGTACTAGGCACAG-TA----------GTT--C---  26247
Cow          TAAGTTGAGCTTACATTCATCAATGTAAAAAGAAAAAGCATCACTT-ATTTAGTGTGGATATGTAACATGTGTTATC-CA  62556
Dog          TAACT----CTTAGATC--TAGG------------------CATTCTACTAGGCGCAG-TA----------GTT--C---  56625
BelugaWhale  --------------------------------------------------------------------------------  56085
Donkey       TGAGT-G----TAGGCTTACAAATCT-------TGAGCTTGCACTCTTCTATTCCAGAACTTTTCTGGGATGATGTTGGA  62476
Cat          TAGCT----TCTAGGTCC-TAGG------------------CATTCTAATAAGCGC------------------------  63566
Ferret       TAATT----CCCAGGTCC-TAGG------------------CATTCTGCTAGGCACAG-CA----------GTC--C---  64275
Lion         TAGCT----TCTAGGTCC-TAGG------------------CATTCTACTAGGC--------------------------  61045
Vaquita      --------------------------------------------------------------------------------  57432
Pig          CGAATCTGACTAGGAACCCTAAGGTTGCAGGTTTGATCCCTGGCCTCGCTCAGTG-GGCTAAGGGTCCAGCGTTGCCATG  55766

Majority     --T----XCTGGATXAGATACATTATTTA-ATCCTTAAT---------------------X-------GTAG--------
                      82810     82820     82830     82840     82850     82860     82870     82880
Human        --------TTGG--CAGATACATTACT----TTGTTAA-----------------------------AATAGAT---GG-  65630
PolarBear    --T----ACTGGATCAGATACGTTATTTA-ATCCTTAGC------------A--------CAA----AGTAG--------  26288
Cow          TTTATAA-ATATGTTATATAAGTGA--TACATCATCTATGAACAGAGAGACACTAATGCACAAGAGAAGCATTTGATATT  62633
Dog          --T----GCTGGAG-AGATAAATTATTTA-ATCCTTA--------------A--------T-------GTAG--------  56660
BelugaWhale  --------T-----TACATATGTGA------TCTTTTAT-----------------------------------------  56105
Donkey       TTT----GCTA-ACTCAGCAAGTTATTCTGATTCACGTT-----------------------------GAACCTCAGGTT  62522
Cat          -------ACTGTAC-AGTCACATTATTTA-ATCCTTA-----------------------A---------A---------  63596
Ferret       --T----ACTAGATCAGATACATTATTTA-ATCCTTAAC------------A--------CAA----AATAG--------  64316
Lion         -------ACTGTTC-AGTCACATTATTTA-ATCCTTA-----------------------A---------A---------  61075
Vaquita      --------T-----TACATATGTGA------TCTTTTAT-----------------------------------------  57452
Pig          GCTGTGGTGTAGGTCACAGACACAGCTTGGATCCCGTGTTGCTGTGTCTGTAGCATAGGCCAGCAGCTGTAGCTCCAGTT  55846
```

```
Majority     --A----TAACCTCAXAATTTTACATATA----------XTTTCTGAGAAGTTTAAAAATXCACATGGAACTCATTAAA-
                      82890     82900     82910     82920     82930     82940     82950     82960
Human        -------TCTCATCAAGATTTTACATATA----------GTTTCTAAGAAGTTGAAAAATTCACATGCAACTCATCAAAG 65693
PolarBear    --G----TAACATCAAGATTTTACATACA----------GTTTCTGAGAAGTTTAAAAATTCACATCCAGCTCTTTAAA- 26351
Cow          TGAAATTAATTCTGATAATTTTAGACAAAACTTAACCTTA-TTCTGATTGTCTAATAACAACTTGCAGAAATTATAGCAG 62712
Dog          --A----TGACATCAAGATTTTATGTCTA----------GTTTCTGAGAAGTTTAAAAATGCACATCCAACTCCTTAAA- 56723
BelugaWhale  -----CTAATCCTCATAAT--------------------AACCCTG---------CAAGGAAACATGGCAGCCAT----- 56146
Donkey       AAG----GAAGATGATAATTTTTGCCCCA----------TTTCATGGGAA--TTGCTATTATGCATCATAGTGATATTTA 62586
Cat          --A----TAACCAAAGCAGATGACGTCTC----------TTTTTTGAGAAATTTAAAAATTCACATGGAACTCGTTGAA- 63659
Ferret       --G----TAACATAAAGATTTTACATACA----------GTTTCTGAGAAGTTCAAAAATTCACCTGCAGCTCATTAAA- 64379
Lion         --A----TAACCAAAGCAGATGACGTCTC----------TTTTTTGAGAAGTTTAAAAATTCACATGGAACTCGTTGAA- 61138
Vaquita      -----CTAATCCTCATAAT--------------------AACCCTG---------CAAAGAAACATGGCAGCCAT----- 57493
Pig          GGACCCCTAGCCTGGGAACCTCC-ATATGCCTTGGGTGCAGCCCTAAAAAAGCAAAAAAAAAAGAAAAAAGTCATACAAA 55925

Majority     ---CAACTTAA----------------------------------------------------------GTGTAAAA---
                      82970     82980     82990     83000     83010     83020     83030     83040
Human        AAACAATTTAAAGA------------------------------TAAAATACCAAGAAACTCCAGCTAAAGATAAAA--- 65740
PolarBear    ---CAACTTAA----------------------------------------------------------GTGTAAAA--- 26367
Cow          TT-TGTCATAATAAATTTATCATGATAAATTTATCATAATGAATTCTTTAGGTTGATAACATTCTTTCC-TGAGAGAATC 62790
Dog          ---CAACTTAA----------------------------------------------------------GTATAAAA--- 56739
BelugaWhale  ---TGTCGTA------------------------------------------------------------T--------- 56154
Donkey       TT-TGGCTTGCAGCA----------------------------------------------CCT------CATACAA--- 62610
Cat          ---CAATTTAA----------------------------------------------------------GGGTAAAA--- 63675
Ferret       ---CAACTTAA----------------------------------------------------------GTATAGAA--- 64395
Lion         ---CAATTTAA----------------------------------------------------------GGGTAAAA--- 61154
Vaquita      ---CGTCGTA------------------------------------------------------------T--------- 57501
Pig          TG-TGATGTA-TATATTGGTC-TACTTTTTATATAGTTCTGTGTTTATGTACCTAATAGGTCTTGGTCTGTGTAAGCATC 56002
```

```
Majority     ----T-----TGTC----------------AAGAA--ATTCC-------AGCTAGCAT-CAAGCATA-GACCATAATAAT

                      83050     83060     83070     83080     83090     83100     83110     83120

Human        ----------TACC----------------AAGAA--ACTCC-------AGCTAGTAT-TAACAATG-GACTACAATAAT  65783
PolarBear    ----T------GTC----------------AAGAA--ATTCC-------GGGTAGCAT-CAAGCACAAGACCATAACAAT  26411
Cow          TCCATCACTCTGTGG--GCTTAGTTT---AAAGCTTTATTTC---CTCAACTTGGCTTGTAAG--TA-AATAATATTAAC  62859
Dog          ----T------GTC----------------AAGAA--ATTC--------AGCTAGCAT-CAAGCACAGGATCATAATAAT  56782
BelugaWhale  ---------CTGT--------------T-----------TTC-----------------CAGA-----------------  56166
Donkey       ----------TGTG-----------------------GCGTCAAACAGAAGTTACCTTATAAAACTAGACCAATTCTTAT  62657
Cat          ----T------ACC----------------AAGAA--ATTCC-------AGCTAGTGA-CAAGCATAGGACCATAACAAT  63719
Ferret       ----T------ATC----------------AAGAA--ATTCC-------AGTTACCAT-CAAGCACA-GACCTTAACAGT  64438
Lion         ----T------ACC----------------AAGAA--ATTCC-------AGCTAGTGA-CAAGCATAGGACCATAACAAT  61198
Vaquita      ---------CTGT--------------T-----------TTC-----------------CAGG-----------------  57513
Pig          AGCGTTGATCTACGCATGTTCTGTTATCAGAAGAACTGTTTCATTTTTGAGGTGATTTATAGAGCTA-AAACATGTTCTT  56081

Majority     CACTTCC--TTGGACT------------------------------CGAAAGAXTX--------------TX-----T--

                      83130     83140     83150     83160     83170     83180     83190     83200

Human        CACTTCT--TTGGACC------------------------------CTACTGAGGT------------------------  65807
PolarBear    CACTTCC--TTGTACT------------------------------CGAAGGAGTT------T-------TG-----TGC  26441
Cow          AATTTTATGGTGTACCATTTTTTTAAAGTCAAGAATGAGACAGTAATGAATGAATCATGGCCTTTGCTTGTAATTGTTAC  62939
Dog          CACTTT---TTGGACT------------------------------CTACAGAGT-------------------------  56804
BelugaWhale  ---TTCA----------------------------------------GAAAGA---------------------------  56176
Donkey       AAGTTAT-GCT--ATCC----------------------------ATCAAAAAATA------------------------  62682
Cat          CAGTTCC--TTGGACT------------------------------CTTAAGGAGT------T-------TG-----TG-  63748
Ferret       CACTTCC--TTGGACT------------------------------CAGAGGAGT---------------TG-----TGC  64466
Lion         CAGTTCC--TTGGACT------------------------------CTTAAGGAGT------T-------TG-----TG-  61227
Vaquita      ---TTCA----------------------------------------GAAAGA---------------------------  57523
Pig          CATTTC----TGAACTGTTGTTTGACACAGTAAATGTTTATTCTAATGAAAAATTAACAGTGTATTCCCCTGTTCTCTAG  56157
```

```
Majority     TTT---TTGCXCATTTGGGTATAGTTAG----AT---AXGGCTXAGTGTGTTTGGATTCTGGATGG-AGTCCXGGT--TA

                     83210     83220     83230     83240     83250     83260     83270     83280

Human        ------GTGTGTATTTGCCCAT--CTGG----AT---ATACTTAGATGTGCTTGGATCCTGGGTGGGAGGCTTGGT--TA  65870
PolarBear    TTT---TTGCCCATTTGGATATAGTTAG----AT---GTGTCTGTGTGTGTTTGGATTCTGGGTGG-AGTCCTGGT--TA  26508
Cow          TATGAATTCTTAATTTTTTACTGATAGAACTTATAGAAAAGACAAAAATCAACATTTATTGAATAG-CTTCTAGATCCTA  63018
Dog          TTT---GTGCCCATTTACGTATAGTTAG----AT----AAGCTGCGTGTGTTTGGATTCTGGGTAG-AGTCCTGGT--TA  56870
BelugaWhale  ------TTCTGAGATTC---------------A--GAAAGGTTAAATA---ACTTGTTCAAGATGA-CCTTTCAACTCTA  56229
Donkey       CATGTATGATAAAATTAACTTTAA--AG--T-ATCGTGCAGGCTACTATGGTACTACTTTGGCAGG-ACTGACTATATTT  62756
Cat          TGT---GTGCTCATTTGGGTATAGTTAG----AT---GTGCCTCTGTGTGTTTGGATTCTGGATGG-AATACAGGT--TA  63815
Ferret       ATT---TTGCCCATTTGGGTATAGTCA-----------TGTCTGTGTGTGTGTGGATTCTGGGTGG-AGTCCTGGT--TA  64529
Lion         TGT---GTGCTCATTTGGGTATAGTTAG----AT---GTGCCTCTGTGTGTTTGGATTCTGGATGG-AATACAGGT--TA  61294
Vaquita      ------TTTTGAGATTC---------------A--GAAAGGTTAAATA---ACTTGTTCAAGATGA-CCTTTCAACTCTA  57576
Pig          TTTG--TTCCAGGCTTCTGTCCAGCTGGA---ATAGAAATGGTGATTATATAGGAACAGAAAAAAA-CATAAAAGTTGTA  56231

Majority     GAAGTCATG-TXXATTCTTCTAGGG-AATTCATCACTGTTTTCCTTGT----AACCTCAT-----GGCCCTGGTGAAATT

                     83290     83300     83310     83320     83330     83340     83350     83360

Human        GAAGTCACG-TGGATTCTTCTAGGG-AATTTATCACCATTTTTCTTGT----GATCTTAT-----GACCTTGCTGAAACT  65939
PolarBear    GAAGTCACT-TG-GGTTCTTCAGGG-AGTTCATCACTGTTTTCCTTGT----AACCTCAT-----GGCCCTGGTGAAATT  26576
Cow          GGGGTTCTG-CAAGGTACTTGGCAG--ATACATTATTTAATTCTTAAA--GTAACCAAAGTAGAAGATGATACGAAGATT  63093
Dog          GAAGTCAGC-TGGATTCTTCTAGGG-AATTCATCACTGTTTTCCTTGC----AATTTCAT-----GGCCCTGGTGAGATT  56939
BelugaWhale  GAGGT---------GTACTCCTCCT--ATTCCAGA---ACTTTCCTGG-----------------GATGACGTTGAATTT  56278
Donkey       GTGGCCCTTACTAATTTTTTTAAATTAAACTATTTGCAGGTTTTTTGA-----ATTAAA------AGTATTAGCACATTT  62825
Cat          GAAGTCATG-AAGATTCTTCTAGGG-AGTTCATCACTGTTTTCCTTGT----AACCTCAT-----AGCCCTGATGAAATT  63884
Ferret       GAAGTCACC-TG-ATTCTTCTAGGG-AATTCATCACT--CTTCCTTGT----AACCTCAT-----GGCCCCAGTGAAATT  64595
Lion         GAAGTCATG-AAGATTCTTCTAGGG-AGTTCATCACTGTTTTCCTTGT----AACCTCAT-----AGCCCTGATGAAATT  61363
Vaquita      GAGGT---------GTACTCCTTCT--ATTCCAGA---ACTTTCCTGG-----------------GATGACATTGAATTT  57625
Pig          ACAG--CAG-CATAGTCCTTTATA---ATTCATGATGCACTTTTCCATATGTGATCTTTTATCTAGACCTCAGTAACCCT  56305
```

```
Majority     TTT--------TTTAAGTAATAXTAAAATX--TGTTTTT--CTCAT---------------AAAXATTATGAAG-----
                      83370     83380     83390     83400     83410     83420     83430     83440
Human        TTT----------AAAACAATAATAAAGTCATTTTTTTT--CTCTTT---------------GAGAT--GGAATCTCAC  65989
PolarBear    TCT--------TTTAAATAATAATGAAAT----GTCTG-----------------------AATAATAATGA-------  26613
Cow          TTATAGTCATTTCTAAGGAGTTTAAAATTT--TACTTTGAACTCATTAAAGAAACAGTGGTAAAAGACCGAGAAATTTCA  63171
Dog          TTTATTTTTTTTTTAAGTAATAATGAAATAAATGTCTTT--TTCATA--------------AAAGATTATGAAG-----  56997
BelugaWhale  GCTTA------TTTAGCAAGTTA---TTC--TGATTC-------------------------------------------  56304
Donkey       GCTTAGCTGGTTTTATTTTAGGTGTATGTAG-CACTTTCTATTTAAAGAAGA--------T-AATATTTGTGGAAACT-A  62894
Cat          TTT---T----TTTAAGTAATAGTAAAAC----ATTTTT--CTCAT---------------AAAAAATATGAAG-----  63930
Ferret       TCT--------TTTAAGTAATAATGAAAT----GTCTTT--CTCATT--------------AAAGATCACGAGT-----  64641
Lion         TTT---T----TTTAAGTAATAGTAAAAC----ATTTTT--CTCAT---------------A---AATATGAAG-----  61406
Vaquita      GCTTA------TTCAGCAAGTTA---TTC--TGATTC-------------------------------------------  57651
Pig          GTGAGAAAGCATGGTAGCTATTGTCATCTC--TGTTTT---CTAGCTGCAGAAAGATT--CTAAGATTCAGAAAGGTTAA  56378

Majority     -C--XCTG---GC--A-----TAATTA-ACAT----GTATCTTCCTCTATACTTTCATTATTCATTTTTCAGGTTTTCTG
                      83450     83460     83470     83480     83490     83500     83510     83520
Human        TCTGTCGCCCGTGTCAGAGTGTGGTGGCGCAG---TCTCGGCTCACTGCAACTTCCGCCTCCCGGGTTCAAGCGATTCTC  66066
PolarBear    ----------------------ATT----------GTATCTTCCTCTATACTTTATTAGTCATTTTTCAGGTGTTCTG  26660
Cow          GCTAGTTTCAAACATAGTACTTCCTTAGACCC--GTAATCACTTCCTGGGACCCTAAGAGCTATGTGTATTTGCCTATTG  63249
Dog          -C--CCTG---GC--A-----TAATTA-ACTT----GTATCTTC-TCTATACTTTTATCATTCATTTTTCAGGTGTTCTG  57058
BelugaWhale  ---------------A------GGTTGAACAA---CCTAGGTTAAGGAAGATAATATTGGATTGGCCAAAATGTTTGTTG  56360
Donkey       ACACATGTACAGCT-GG-----GATCACAAAGAACGATGTATATGAAATTTCTTTCATTCCTTTCCTGGTGGCTTTTATG  62968
Cat          -C--ACTG---GC--A-----TAATTA-ACTT----GTGTCTTCCTCTGTACTTTCATTATCCATTTTTCATGTATTGTG  63992
Ferret       -C--CCTG---GC--A-----TAATT----------TTATCTTCCTCTATACTTTTATTATTAATTTTTCAAGTGTTCTG  64698
Lion         -C--ACTG---GC--A-----TAACTA-ACTT----GTGTCTTTCTCTGTACTTTCATTATCCATTTTTCAGGTATTGTG  61468
Vaquita      ---------------A------GGTTGAACAA---CCTAGGTTAAGGAAGATAATATTGGATTGGCCAAAATGTTTGTTG  57707
Pig          ACAACTTGCTCACG-ATGACCTAATTGAATGTAGGCTTTCAACTCCATGGATTGCGCTCTTCTATTCCAAAACTTTCCTG  56457
```

```
Majority     XXTT-------------TXTTTGCACTAAAAGTXTATGAGTAAAGTTCA-----TTCACTAATTAATAAA--------TT
                      83530     83540     83550     83560     83570     83580     83590     83600
Human        CCGC---------C---TCAGCCTCCTGAGCATCTGGGACTACAGGCGTGTACCACCACCCCTGGCTAA-------CTTT  66127
PolarBear    CATT-------------T-TTTGCATTAAAAGCAT--GAGTAAAGTT-G-----TTCACTAA---ATAAA--------TT  26707
Cow          GGGGGGTAGTTGATGAATTTATGTAT---GTTTTTTGGATTCTGGTTGGAGTCATGGATTTGTCTATGGAGTTTATCATT  63326
Dog          CATG-------------T-TTTGCACTAAAAGCAT--GAGTTAAGTT-G-----TTCACTACTTAATAAA--------TT  57108
BelugaWhale  GGGT-------------TTTCCTTAA--GATGTTATGGA---AAACCCAA---ATGAACTTATTGGCCAACCCAAGAGTT  56419
Donkey       TGTAGG-----------TTTTCACTGTTGAGTTTTATTGTTAAAGTC--------ATACCTTTTTCTGG---------TT  63020
Cat          TAT--------------T-TTTGCACTAAAAGCATATGAGTAAAGTTCA-----CTCCTTAATAAATAAATGTGGCT-AG  64051
Ferret       CATT-------------T-TTTGCATTAAAAGCATATGAGTAAAGTT-G-----CCCACTACTT-ATAAA--------TT  64749
Lion         TAT--------------T-TTTGCACTAAAAGCATATGAGTAAAGTTCA-----CTCCTTAATAAAT----GTGGCT-AG  61523
Vaquita      GGGT-------------TTTCCTTAA--GATGTTATGGA---AAACCCAA---ATGAACTTATTGGCCAACCCAAGAGTT  57766
Pig          GGATGACATAGGACTTTTTTATTCAACGGGTTTTTCTGATTCAGGTCCA--CCATTG-CAGGTTAAGGAAAATAATAGTT  56534

Majority     TGTCTTCX------XTCTTCG-XCTCTATTTTAAGTATTTTAGTAXA-------TCTTTXTTTCTGCACTTT-XTTTAAT
                      83610     83620     83630     83640     83650     83660     83670     83680
Human        TGTCTTTT------TTCGTAGAGATGAGATTTCACCATGTTGGCCAG--GCTGGTCTGGAACTCCTGATCTC-AGGTGAT  66198
PolarBear    TGTCTTCA-------TCTTCTAGCTCTATTCTGAATATTTCAGTATA-------TCTTTATTTCTGCAGTGT-GCTTGAT  26772
Cow          TTTCTTGTAATGGCCTTGTTGGAATTCTTTTTTAGAGTAATAATAAATAAGAAATCTTTCTCATAAAAGATT-ATACAAT  63405
Dog          TGTCTTCA-------TCTTCTAGCTCTATTTTGAATATTTTAATATA-------TCTT-GTTTCTGCACTCT-GATTAAA  57172
BelugaWhale  TTTGCTCA-----TTTCATGGTAGTTGCTGTTATGCATCATAGTGAT--ATTTAGTTGTCTTGAGGCACCTC-ATTTAAT  56491
Donkey       TTTATTCCTTGAGATTGCT---GATATACTTTATTCCCTTTTACAGGC------CCTAGATGGTCAGAATAT-TTATAAT  63090
Cat          TGTCTTCT-------CCTTC--ACTCTGTTTTGACTATTTTAATATA-------TCTTTGTTTCTGCACTCTTGCTTGGT  64115
Ferret       TGTCTTCA-------TCT------TCTATTTTAAATATTTCAGTATA-------TCTTTATTTCTTCACTCT-GCTTGAT  64808
Lion         TGTCTTCT-------CCTTC--GCTCTGTTTTGACTATTTTAATATA-------TCTTTGTTTCTGCACTCTTGCTTGAT  61587
Vaquita      TTTGCTCA-----TTTCATGGTAGTTGCTATTATGCATCATAGTGAT--ATTTAGTTGTCTTGAGGCACCTC-ATTTAAT  57838
Pig          TTTGCTCT---G-TTTCATAGGAGTTGCTATCATGTATCATAGTAAT------ATTTGTCTTGAGGCACCTT-ATTTAAT  56603
```

```
Majority     G--TGGXATXA-XXAAATGTTGXCTTGT---GAACATATGTGAATTTCAXCTTGTTTTACTA-AATGACTTCATXGTTXA
                       83690     83700     83710     83720     83730     83740     83750     83760
Human        C--TGCCCGCCTCAGCCTCCCAAAGTGCTGAGATTACAGGTGTGAGCCACCATGCCTGGCCGCTTTTGTT-CATTTTTTG  66275
PolarBear    A--AGAAA-----------GATGAA-GGT---GAAGATATATGAAT-TCACCTTTTTTTACTA-AATAACTTCATC-----  26829
Cow          CC-TGGCATAACTTAACTTTTGTCTTATTCAGTGCTTTTATCATTTCTCAAGTGTTCTACAT-TTTTGCATTAAAAGA-A  63482
Dog          G--AGAAAA--------CGTTGTGTGGT---GAAGATATGTGAAT-TCACCTTCCTTTACTA-AATGACTTCATCATTTA  57237
BelugaWhale  G--TGGCATCAAACAGAAGTTACCTTAT-----ACCTATACCAATTTTTATAAGCGTTACCA-TACAGCA--AAAGAT-A  56560
Donkey       GCTTGCTGTACCCTAAGGATTGATTTTT-C--TAAACTTGTGAATTTGAATGTAAAATACAACAATGATAAAAGTAGGGA  63167
Cat          A--AGGAAAAA-GGAAATGTTTTGTAGT---GGAAATATGTGAAT-TCACCTT---TTACTA-AATGATTTCATCGTTTA  64184
Ferret       A--AGAAA-----------GTTGAA-AGT---GAAGATATGTGAACACCTTTTTTTTTTCTA-AATGACTTTATTGTTTA  64871
Lion         A--AGGAAAAA-GGAAATGTTTTGTAGT---GGAAATATGTGAAT-TCACCTT---TTACTA-AATGATTTCATCGTTTA  61656
Vaquita      G--TGGCATCAAACAGAAGTTACCTTAT-----ACCTATACCAATTTTTATAAGTGTTACCA-TACAGCA--AAAGAT-A  57907
Pig          G--TGGCAT---CGAGAAGTTACCTTAT-----ACCTATAACAATTTTTACAAGTATTACCA-TCCAGCA--AAAAAT-A  56669
```

```
Majority     T-GTGTTTTAT-TGXTTTTACATTTTAGTA---------T-AATTTGGTTTTACTXT------------X---TAAGTAT
                       83770     83780     83790     83800     83810     83820     83830     83840
Human        A---GCGTTGTACATTTTTACATTAAAGGCGT------GTAAATTAAGTTGCTCGCT-----------TTGCCAAAAAAT  66335
PolarBear    ---------------------------------------------------------ATAT-----------------TAAGTAT  26840
Cow          T-GTGAGTAAA-GTTGCTCAGTTTTTAGTAA-------ATTAATTTGGTGTCACTGT----------TCTCTCTTGAAGA  63543
Dog          C-CTGTTTTAA-TGGTTTTGCATCTTGTGA---------T-AACCAGGTTTTTATAC----------------TAAGTAT  57289
BelugaWhale  T-GTGTATAAT-AAAATTAACTTTAAAGTAGTGTGCAGACTATTATGGTACTACTTTGGCAGCACTGACTATATTTGTGA  56638
Donkey       TTATACCCGACCTGATCTCCCATCTGGGGATGGACAACCTGCATTGGATCCTGCTATTGC-----TGCAGCATTTGCCAA  63242
Cat          T-CTATTTCAG-TGGTTTTGCATTTTGTTA---------T-AACTGGGTTTT--GTT----------------TAAGTAT  64234
Ferret       T-ATGTTTTAA-TGGTTTTGCATCTTATTA---------T-AATTTGGTTTTTATAT----------------TAAGTAT  64923
Lion         T-CTATTTCAG-TGGTTTTGGATTTTGTTA---------T-AACTGGGTTTT--GTT----------------TAAGTAT  61706
Vaquita      C-GTGTATAAT-AAAATTAACTTTAAAGTATTGTGCAGACTATTATGGTACTACTTTGGCAGCACTGACTATATTTGTGA  57985
Pig          G-GTGTATTAT-AAAGTTTACTTTAAAGTA---------TTATGCAAATGTTAGGGT----------ACTGCTTTGGTGA  56728
```

```
Majority     CCCCAGATATTTTTTGAATGTTAGATGAGTTGCAAGTTTC-AAAAATCAAA--TAATXGTATGGACTTTGAXAGTTTTGA

                           83850     83860     83870     83880     83890     83900     83910     83920

Human        TGTCTAGTGTCTTTTTCCACTT--CTGTGTTGAATTTTTTAGTATAACGCATTTTTGTTTCTTCATCTCTGCCTCATAAG  66413
PolarBear    CCCCAGATATTTTTTGAATGTTAGATGAGTTAAAAGTTTC-AAAAATCAAA--TAATGGTATGGACTCTGGTAGTTTTGA  26917
Cow          TTTCAGTATAATATTCTGGCGTACCTTGTTTCAATGTGCTTCACAGATAACTGTGTTCTTTTACAGATTGAAGGTTTGTG  63623
Dog          CCCCAGATATCTTTTGAATGATAGGTGAGTTGTAAGTTTC-AAAAATCAAA--TAATGGTATGGACTCTGGTAGTTTTGA  57366
BelugaWhale  CCCTTGTTAATTTTTTTAAATTAAAATATTTGCAGGTTTT-TAAAATTAAAAGTATTAGCACATATGTTTAA---CTGGT  56714
Donkey       GGAGACATCCCTCTTAGGTATGATTTCTGTTGTCTATACC----ACTTTTCTCCCATTTTACCAAATGGGAAAGTGCCTT  63318
Cat          CCCCAGATATTTTTTGAATGTTAGGGGAGTTACAAGATTC-AAAAATCAGA--TAATGGAATGGACTCTGATAGTTTTGA  64311
Ferret       CCTCAGATAGTTTTTGAATGTTAGATGAGTTGAAAGTTTC-AAAAATCAAA--TGATAGTATGGACTTTGGTAGTTTTGA  65000
Lion         CCCCAGATATTTTTTGAATGTTAGGGGAGTTACAAGATTC-AAAAATCAGA--TAATGGAATGGACTCTGATAGTTTTGA  61783
Vaquita      CCCTTGTTAATTTTTTTAAATTAAAATATTTGCAGGTTTT-TAAAATTAAAAGTATTAGCACATATGTTTAA---CTGGT  58061
Pig          CTCTTGTTAGTTTTTTTGTATTAAAATATTTGCAGATTTTGTAAAATTAAAGGTATTAGCACATATGTTTAA--CT-GAA  56805

Majority     TA-ATTTAXAAXAGG-XTAACXATTX--CTATTTTTTGXTCTTATTXTCTGTGTAAATAAAT----ATTTTTGTACAA-A

                           83930     83940     83950     83960     83970     83980     83990     84000

Human        AAAAAGGAAAAAGTGTTTAGCCTTTCCTAAGTGACTTCTTTTTTTTATCTGTTTTAATGTTTTTGCATCTTGTTATAACT  66493
PolarBear    TA-ATTTACAAGAGGGGTAACCATT--------TTTTC-TCTTATTTTCTATGTAAATACATT---ATTTTTGTACAA-A  26983
Cow          GCAGCCCAGCATTGTCATGATAGTTA--GCATTTTTTAGCAATAAAGTATTTTGTAATAACAGTATATACTTTTTTAA-A  63700
Dog          TA-ATTTAAAAGAGG--TAACCATT--------TTTTCCTCTTATTTTCTATGCAAATACGT----ATTTTTGTACAA-A  57430
BelugaWhale  TACATTTTAGATTTAGATAACACTTTT-CTATTTAAAGAAGATAGTATTTGTGGAAACCAATATATAT-TGGTTGGAATA  56792
Donkey       TTAAACTCCAGTAAGTACAATGAATCCCCTGTTTTGGAATTTAAGAAATTGTATTAGGTTTT----ATCTTTCTCAGGTG  63394
Cat          TC-ATTTATAAGAGG--TAACCACTGTTAATATTTTTGCTCTTATTTTCTATGTAAATATGT----ATTTTTGTACAA-A  64383
Ferret       TA-ATTTACAAGAGG--TAACAATT--------TTTT----TCATTTTCTGTGTAAAAACAT----ATTTTTGTACAA-A  65060
Lion         TC-ATTTACAAGAGG--TAACCACTGTTAATATTTTTGCTCTTATTTTCTATGTAAATAAGT----ATTTTTGTACAA-A  61855
Vaquita      TACATTTTAGATTTAGATAACACTTTT-CTATTTAAAGAAGATAGTATTTGTGGAAACCAATATATAT-TGGTTGGAATA  58139
Pig          TATATTTTGGATTAAAGTAGCACTTT--CTATTTAAGGGAGGTAACATTTGTGGAAACCAATGTACGTATAGCTGGGATA  56883
```

```
Majority     ATAAAAATCA-XXTATX-A-TTCAGT-----TCTXTGTXATGTTTTTTTXTAATTGTT-ATTTTT--GTAAXAATTTTT-
                      84010     84020     84030     84040     84050     84060     84070     84080
Human        GGGCTTTTTA-ACTATA---CTCAAAAATAGGTTAAAAGTAGGTGAGTTAAAGGTTTTAAAAATCAGATAATGGACCCTG 66569
PolarBear    ATTAAAACCACACTGT--G-TGCAGT-----TCT-----GTGTTTTATTATAATTGTT-ATTTTA--GTAAGAATTTAA- 27046
Cow          ACATAATACTATTTGCACACTTAAAC----TACCATATAGTGTCAACGTAGAAAAAT--ATTTTGGGGTGAAAATATGTG 63774
Dog          TTAAAAATCACTCTGTAAG-CGTAGT-----TCT-----GTGTTTTATTGTAATTGTT-ATTTTA--GTAAGAATTTTTT 57496
BelugaWhale  GTAAAGAACAGGGTATGGAATTTGATCCCTTTCTTGGTGACTTTTATGTGCAGTTTTTCATCGTTGAGTTTTATTGTCAA 56872
Donkey       AGAAGGCGTA-TGAATGCTGTTGGGAGAAAAACC-TAAAGTGTTCTTC-ATAAGCGTAATTTTGTCTGTTATATTCCTTT 63471
Cat          ATTAAAATCA---CAT----TGCAGT-----TTTGTG--ATATGTTCT---AATTGTT-ATTTT---GTAAGAATTTTT- 64441
Ferret       ATTAAAATTA--CTAT--G-TGCAGT-----TCTTTATTATGTTTTATTGTAATTACT-ATTTTA--GTAAGAATTTTT- 65126
Lion         ATTAAAATCA---CAC----TGCAGT-----TTTGTG--ATATGTTGTTATAATTGTT-ATTTT---GTAAGAATTTTT- 61916
Vaquita      GTAAAGAACAGGGTATGGAATTTGATCCCTTTCCTGGTGACTTTTATGTGCAGTTTTTCATCGTTGAGTTTTATTGTCAA 58219
Pig          GCAAAGAACAAAGTATGGAATTTGATCCCTTTCCGGGTGACTTTCATGTGCAGGTTTTCATTGTTGAGTTTTATTGTCAA 56963
```

```
Majority     --X---AATAXTTTTAATAACTX-X-XTTATTXTTTXAGATTTCTAATATXTTTGAAAGTACCTTTACXX--CGACATTT
                      84090     84100     84110     84120     84130     84140     84150     84160
Human        GTTTTGATAATTCACAAGAGATAACCATT-TATTTTCATATTTTTTATGTAAATACATGTTTCTGTACAAAATTAAAATT 66648
PolarBear    -----AAAAAAACTTAATAGCTTCTTAGCATGCTAGTGGATTTGTAGTACTTGAGAAAGCACCTTTATC---AGACATTT 27118
Cow          AATTCATCTTCCTTTACTAAATGAC-TTCATTGTTTACATGTTTTAACGGGGATTTTTAAATTTTTAAATATCCTCAAAT 63853
Dog          TGAA-AAATAATTTTAATAGCTATCTAGCATGCTGGTGGATCTGTAATACTTTTGAAAGTACTTTTACTTCCAGACGTTT 57575
BelugaWhale  AGT---CATACTTTTTCTAATT----TTTATTTCTTAAGATTGCTGATATAT-TTTGTTCACTTTTACAGGCCCT-AGAT 56943
Donkey       ------CACTACCTCACCTTTT--CTCCTATTCTCCTAGTCTCCCAAAATAG--AAGAAAAGCTCTACTGA-AGAAATAC 63540
Cat          -------------TTGAAAACTA----------------ATTT-TAATATTTTTGAAAGTACCTTTGTT---GGACATTG 64488
Ferret       -----AAAAAA-TTTAATAGCTGTCTAGCATGCTAGTGGATTTGTAGTACTTTTGAAAGTACCTTTATT---GGACATTT 65197
Lion         -------------TTGAAAACTA----------------ATTT-TAATATTTTTGAAAGTACCTTTGTT---GGACATTG 61963
Vaquita      AGT---CATACTTTTTCTAATT----TTTATTTCTTAAGATTGCTGATATAT-TTTGTTCACTTTTACAGGCCCT-AGAT 58290
Pig          AGT---TATACTTTTATTAATT----TTTATTTTTTAAGATTCCTGATACACCTTTGTTTACTTTTACAGGCCCT-AGAT 57035
```

```
Majority     AXTCT--ATTTTTATAXTTTTTX---XTATACXXTAAAXATGGCXT-----X-AXTGTXXAAXATACAATGTAAAAAACA
                      84170     84180     84190     84200     84210     84220     84230     84240
Human        GCTCTGTATTTGCAGATTTGCATTATCT-TTTATTGTAATTGACATT-------TTACTTAGAATTTGTTTTAAAAGATA  66720
PolarBear    AATCT--AGTTCCATACTTTTTATTTATATA---TATATATGGC---------ATTTTGATAAACACCCTGTACATAACT  27184
Cow          AGTT----TTTTAAAGGTGAGTT---ATAAATTTTAAAAATCAAAT-----AAAGGAATATATTTATAATTCATAAGATA  63921
Dog          TATTA--ATATATGTGGCATTGTGACAAATACCCTGTACATAACTTTTTTGTTATTGGGTCAAAGAAAATGTTAAAGGCT  57653
BelugaWhale  GGTCAGAATATTTATAATGCTTG---CTGTACCCTAAGGATTGATTTTTCCAAACTTGTGAATTTG-AATGTAAAATACA  57019
Donkey       TGTTGAGTTCTTAATAAAATATT----TAAACTTCAAACTTGATGT----CACACTATCTAGGTCTAAGTCTAGGGCTCT  63612
Cat          AATCT--TCTTCTATACTTTTTA----T-TAG--TATATATGGC----------ATGTGACAGATACCCTGTACATAACT  64549
Ferret       AACTT--ACTTCCATACTTTTTA---AAATA---TAAACATGGC---------ATTGTGGCAAACACCCTGTGAATAACA  65260
Lion         AATCT--TCTTCTATACTTTTTA----T-TAA--CATATATGGC----------ATGTGACAGATACCCTGTACATAACT  62024
Vaquita      GGTCAGAATATTTATAATGCTTG---CTGTACCCTAAGGATTGATTTTTCTAAACTTGTGAATTTG-AATGTAAAATACA  58366
Pig          GGTCAGAATATTTATAATGCTTG---CTGTACCCTAAGGATTGATTTTTCCAAACTCGTGAATTTG-AATGTAAAATACA  57111

Majority     TCTTTG-TATTTCTXGAGATTATTCTCTX-------------A-ACTAGTXX-XTATCTAGAGXTAG----------ATT
                      84250     84260     84270     84280     84290     84300     84310     84320
Human        GCTTTAATACCTGCGTAGCATGTTCATGG---------------GTATGCCATACCTTTGAAAGTGG----------ACA  66775
PolarBear    TTTTTG-TCTTTC---GGGTTATTTCCTG-------------AGACTAG----ATTCCTAGTGCTAG----------ATT  27233
Cow          ACTACC-TATATTTTGA---TATATTTTT---------------TCTTATTTTCTATGTAAATACATAT--------TTG  63974
Dog          CCTGAT-TCTTCCTGTAAATTTCTTTCTGGAAAGATGATATTAAACTAGTATGGTATTTGAGGCTGGCCATTCT---ACT  57729
BelugaWhale  ACAATGATAAAAGTAGGGATTATACTCGA---------------CCTGATCTTCCATCTGGGGATGGACAACCTGCATTG  57084
Donkey       TGTGCA-TATTCTTAGGGCTAATCTGATTG--------TTTTAATTTAACA--CTGCATAGAAACAG------------T  63669
Cat          TTTT-G-TCTTTC---AAATTATTCCCTT-------------AAACTAG----ATGCCTAGAGCTAG----------AAT  64597
Ferret       TTTTTA-TCTTTC---AGATTATTTCCTG-------------AGACTAG----ATTTCTAGAGCTAG----------ATT  65309
Lion         TTTT-G-TCTTTC---AAATTATTCCCTT-------------AAACTAG----ATGCCTAGAGCTAG----------AAA  62072
Vaquita      ACAATGATAAAAGTAGGGATTATACTCGA---------------CCTGATCTTCCATCTGGGGATGGACAACCTGCATTG  58431
Pig          ACAATGATAAAAGTAGAGATTATACTCGA---------------CCTGATCTCCCATCTGGGGATGGACAGCCTGCATTG  57176
```

```
Majority     TACTAA-XXA-XXTCAAAGAXTTTXX--X----AAATXTTTGTGAXTTXTGA---TTTTTACTGCAAATTTCTTTTTAGA
                         84330     84340     84350     84360     84370     84380     84390     84400
Human        TGTAGGCT--ATTCCATACTTTTTATTAA-A--ACACCCTTGTACTTTTTTG----CCT--CTCAAATTATTTTCTTAGA  66844
PolarBear    TATTGG------GTCAAAGA-------------AAATGTTAGAGACTCCTGA---TTCTTACTGCAAATTTCTTTTTAGA  27291
Cow          TAAAAAATTAAAATCACACTGTTTGC-------AGTTTTGTGTTTGTCATTT---TATT-GAGGGTTTTTTTTTTTTTCC  64043
Dog          TTTTTGCCTACCAGCATTGATTTTTTTTTTTTTCAAATATTTGTCATTTTTAAAAATTTTTTTTTCTTTTTTTTTTTTATG  57809
BelugaWhale  GACCCAGCTATTGCTGCAGCATTTGCCAAGGAGACATCTCTCTTAGGTATGA---TTTTTATTGTCTATACCACTTTTCT  57161
Donkey       GAAAAGTTAACAGACAAAACATGTAGTGAGAGTAGGCATATGGGTTTTTTCAAAACATG---TGATAGCCTCTCTTTTAA  63746
Cat          TACTAA------ATCAAAAA-------------AAGTGTTAAAGACTCCTGA---TTCTTACTGCAAATTTCTTTCTAGA  64655
Ferret       TATTGG------ATCAAAGA-------------AAATGTTAGAGACTCCTGA---TTCTTACTGCAAATTTATTTCTAGA  65367
Lion         TACTAA------ATCAAAAA-------------AAGTGTTAAAGACTCCTGA---TTCTTACTGCAAATTTCTTTCTAGA  62130
Vaquita      GACCCAGCTATTGCTGCAGCATTTGCCAAGGAGACATCTCTCTTAGGTATGA---TTTTTATTGTCTATACCACTTTTCT  58508
Pig          GACCCAGCTATTGCTGCAGCATTTGCCAAGGAGACATCTCTCTTAGGTATGA---TTTT-ATTGTCTGTACCACTTTTCT  57252

Majority     XAXATXATACTAAATTAGAAAGAGXGTGTTA--X------AAATAAGTATAATAAGACTCTXAXTTTGX---X-X--X--
                         84410     84420     84430     84440     84450     84460     84470     84480
Human        CTAGATTCCTAGAATTAGAATTATTGGGTCA---------AAGAAAATGTTAAAGCCTGTTTACTTTA------------  66903
PolarBear    AGGATGATACTAAATTA----------------------ATATGAATGGTATATGAGGCTGGCTAT-------------  27335
Cow          CGAAAAATGGTAATTGTAATAG--CAAGATAGACTG---CTAATGGATATAT-AATACTTTTGTATTTATGTTGAACGTT  64117
Dog          ATAGTCACACAGAGAGAGAGAGAGAGAGAGAGGCA--GAGACATAGGCAGAGGGAGAAGCAGGCTCCA------------  57875
BelugaWhale  CCCATTTTGCCAAATGGGAAAGTGCCTGTTAAACT----CCAGTAAGTATAATGAATCTCCCATTTTGGAATTTAAGAAA  57237
Donkey       GAAAACAATTTAAGCTAGAAGGAATTTTCTA---------TAAAATTTATTTTGTTATTTGCCTTTTTGTCCTAATCATT  63817
Cat          AAGGTGATATTAAACTAATATGAATGG-----------------------------------------------------  64682
Ferret       AAGATGATACTAAACTA----------------------ATATGAATGGTATATGAGACTGGCTAT-------------  65411
Lion         AAGGTGATATTAAACTAATATGAATGGGGGGCGCCTTGGTGGCTCAGTCGGTTAAGCGTCTGACTTCGGCTCAGGTCATG  62210
Vaquita      CCCATTTTGCCAAATGGGAAAGTGCCTGTTAAACT----CCAGTAAGTATAATGAATCTCCCATTTTGGAATTTAAGAAA  58584
Pig          CCCATTTTGCCAAATGGGAGAGGGCCTGTTAAATT----CCAGTAAGTATA--ATGACTCTCATTTCGGAATTTAAGAAA  57326
```

```
Majority     X--X------XX------------------------A-TGCAC-XTGXXX-C-X-X----XXX------XXXAATTCAT-

                     84490     84500     84510     84520     84530     84540     84550     84560

Human        ------------------------------------TTTGCA------------------------------AATTTCTT  66917
PolarBear    --------------------------------------TCCACT------------------------------------  27341
Cow          TAGTTTCCCTTCATACTTTTCATT-----AATATAAATGGCATTGTGACCACCAGTACCTGTATGTAACGTTTTTGTCCT  64192
Dog          --------------------------------------TGCACCGGAAGCCCGACG-------------TGGGATTCGA-  57903
BelugaWhale  TTATGTTAGGTTTCTCCCCGCATC-----AGGTAAGAAAGCAT-ATGTATGCTGTTGGAAGAAAAC--CCTAAGTTCATA  57309
Donkey       TATGTTTCATTTTT----------------------ATTCTACAATGGATTTGACCCACTTAATAAAAACACAATGCAAA  63874
Cat          --------------------------------------------------------------------------------  64682
Ferret       --------------------------------------TCCACT------------------------------------  65417
Lion         ATCTCACGGTCTGTGAGTTCGAGCCCCGCGTCGGGCTCTGTGCTGACAGCTCAGAGCCTGGAGCCTGTTTCAGATTCTGT  62290
Vaquita      TTATGTTAGGTTTCTCCCCGCATC-----AGGTAAGAAAGCAT-ATGTATGCTGTTGGAAGAAAAC--CCTAAGTTCATA  58656
Pig          TTGTTACTTTTTTTCCCTCTCA-------GGTA-AGAAGGCAT-ATGAATGCTATTACAAGAAAAAAACCTAAACTCATC  57397

Majority     X-X-TX----AXATTATATCAXCTAGAATCXTX-T-AT-X---------------------------X---XAX-----X

                     84570     84580     84590     84600     84610     84620     84630     84640

Human        CATCTAA---AGGTGATATTAACTTAAACTAA---CAT-------------------TA---------------------  66951
PolarBear    ------------TTTTTGCCTACCAGCATTG-------------------------------------------------  27360
Cow          TTATTTACTTAGACTATAG-AACTAGAATCATTGT-GTCAGAGAAATGTTAAAGCCTTGTGATTCTTGTTTGAAATTTCT  64270
Dog          ------------TCCCGGGTCTCCAGGATCGC-GCCCT------------------------------------------  57928
BelugaWhale  AGTATAATGAAAATTATATCAGTTATATTCTTTTTCACCACTCTCATCTTCTCTCCCAGAATAGAAGAGGAAAAGCTCTA  57389
Donkey       AATCTG---CAACTAGTAGTTGATAGAGTCAAGATGAAGA---------AAGAAT-----------AAGGGAAAATGTTA  63931
Cat          --------------------------------------------------------------------------------  64682
Ferret       ------------TTCTTGTTTACCAGCATTG-------------------------------------------------  65436
Lion         GTCTCCCTCTCTCTCTGACCCTCCCCCGTTCATGCTCTGTCTCAAAAATAAATAAACATTTAAAAAAAAAAAACAAAAAA  62370
Vaquita      AGTATAATGAAAATTATATCAGTTATATTCTTTTTCACCACTCTCATCTTCTCTCCCAGAATAGAAGAGGAAAAGCTCTA  58736
Pig          AGAATAGTTAAAATTATGTCAGTTATATTCCTTTT-ACTA--------CTCTCATCTTCTCTCCCAAAACAAAAGCTGTA  57468
```

```
Majority     -X---------AT--TATTTAATXCTGGATAAAATAXTTTTA---CCTTCAAGTXTACXTXATGXTTTTTTGGATTTGCT
                      84650     84660     84670     84680     84690     84700     84710     84720
Human        -----------ATTATATATGAGGCTGGGTGTCCTACTTTTTCC-CCTGCCAGCATAC------TATTTTTTAATTTCAC  67013
PolarBear    -----------------------------------AGTATTA-----------------------TTTTTTGGATTTGAT  27382
Cow          TTCCAGAATG-GTGATAATTAATTTAACATACATGGTATGTGAGGCTGGCTATTCTACTTTTCCTGTTCAGCATTGAGT  64349
Dog          ---------------GGGCCAAAGGCAGGCACCAAACTGCTGCG-CCACCCAGGGATCCCCTAT-TTTTTTTGATTTGCT  57991
BelugaWhale  ATGAAGAA---ATACTGTTGAGTTCTTAATAAAATATTTACA----CTTTAAATTGAAGTCATGCTGTCTAGGGCCCCTT  57462
Donkey       TT---------TTG-TAAGTAAAATGAGTGAAGTGATTTATAG---CTTAAGATGAAATTTTAGTTCCTTTTGTTTGAT  63998
Cat          ---------------TACATGAGGCTGGCTATTCCACTTTTTTC-CCTACTAGCATTGAGTATTATTTTTTTGATTTACT  64746
Ferret       -----------------------------------ACTATTA-----------------------TTTTTTGGATTTGCT  65458
Lion         AACTAATATGAATGGTATATGAGGCTGGCTATTCCACTTTTTTC-CCTACTAGCATTGAGTATTATTTTTTTGATTTACT  62449
Vaquita      ATGAAGAA---ATACTGTTGAGTTCTTAATAAAATGTTTACA----CTTTAAATTGAAGTCATGCTGTCTAGGACCCCTT  58809
Pig          ATGAAGAA---ATACTGTTGAGTTTTGAATAAAATATTTATA---CTTTAAAGTTGACGTCATGTTGTCTAGGGCCCTGT  57542

Majority     -TGGATATTAATTTG---GGCATAGGXATXTGTCAGATTXTA---TTTAATTTXTATT---TA-TTTATGAGCTAXCTTA
                      84730     84740     84750     84760     84770     84780     84790     84800
Human        ---AAGATTAATTTG------ATAGGCAAATGCC----TAT----TTTAATTTCTGT---------TAAGATTTAGTTTA  67067
PolarBear    -AGGATATTAATTTGGTAGGAATAGGAATGTGTCAAATTAT----TTTAATTTTTAT----TATTTTATGAGCTAGCTTA  27453
Cow          ATTAGTATTTTTATT---TGCTAGGGTATTAATAAGGATGTCAGATTATTTTAATTTTTATCATTTTATGAACTAGTTTA  64426
Dog          TAGAATATTAATTTGATAGGAATAGGAATGTGTCAAGTTTATTATTTAACTTTTATGAGCTAACTTATTAGACATTTTT  58071
BelugaWhale  GTGCATATTCTTTAG---GGCCAATCTAATTGTTTCAATTTAGCACTGCACAGACAATGAAAAGTTAACAGATGCAACAT  57539
Donkey       GTGTTTGGGAATTCT-TGATTTCTGATGTTTGTAAGATTCTC---TTTAAATCACAGTGTCTA-TCCATATG-GAACCCA  64072
Cat          --GGATATTAATTTG------ATAAGAATGTGTCAGGTTATT----TTAATTTTT-TA-------TTATGAGCTAGCTTG  64806
Ferret       -AGGATATTAATTAGATAGGAATAGGAATGTGTCAAATTGT----TTTAATCTTTAT----TATTTTATGAGCCAACTTA  65529
Lion         --GGATATTAATTTG------ATAAGAATGTGTCAGGTTATTAT-TTTAATTTTT-TA-------TTATGAGCTAGCTTG  62512
Vaquita      ATGCATATTCTTTAG---GGCCAGTCTAATTGTTTCAGTTTAGCACTGCACAGACAATGAAAAGTTAACAGATGCAACAT  58886
Pig          AC--ATATTCTTTAG---GGCTAATCTAATTGTTTTAATTTAACACTGCACAGAAATCAGAAAGTTAACAGACCCAACAA  57617
```

```
Majority     XAATTTTTAAGTGCTTATTAGACATTT---AAGTCTCTGAATTGGTTGTCTAGXTTCTTTCTXXTGGGATGTACA---AT
                             84810     84820     84830     84840     84850     84860     84870     84880
Human        CAATCTTTAAATGTTTATTTGATAGTCAC-AATTTGCTAAATTGCTTGCCTGCATTCTTTCTGTTAGGATGTACGGTAAT  67146
PolarBear    TAATTTTTAAGTGCTCATTAGACATTTAC-AATTCCCTGAATTGGTTGTCTAGGTTCTTTCTATTGGGATGTACA---AT  27529
Cow          TAATTTCTAAATGCTCATCAGATATTTA---CAAATCTGAATTGGTTGTGTGGATTCTTTCTTTTGGAGTATACA---AG  64500
Dog          AAATTTTTAAATTCTTATTAGACATTTAC-ACGTCTCTGAATTGGTTGTCTAGGTTTTT------GGGATATACA---AT  58141
BelugaWhale  ATAATGG-GAGTAGGTGTATAGAATTT--------TTTCAAAGCATATTATACTCTCT--TTTAAACAAT-TTTA---AT  57604
Donkey       AGATTGTTGAGTTGCAAAGTAGCTCTT-----ACAGTTTAGTTTATG-TCTA---TCGATAAAAAAAGAAAAAGC---AT  64140
Cat          TAATTTTTAAATGCTTACTAGACAG-----AAGTCTCTGAATTGGTT-TCT---TTCTAT----TGGGATGTAC------  64867
Ferret       GAATTTTTAAGTGCTTATTAGACATTTTCCAAGTCTCTGAATTGGTTGTCTAGGTTCTTTCTGTTGGGATGTACA---AT  65606
Lion         TAATTTTTAAATGCTTATTAGACAG-----AAGTCTCTGAATTGGTTGTCT---TTCTAT----TGGGATGTAC------  62574
Vaquita      ATAATGG-GAGTAGGTGTATAGAATTT--------TTTCAAAGCATATTATACTCTCTCTTTTAAACAAT-TTTA---AC  58953
Pig          GTATTGG-GAGTAGGTATAAGGATTTT--------TTTCAAAGCATATTATAGCTTCTCTTAAAAACAAT-TTTA---AC  57684

Majority     TXATATAAAXTGTXTATTAAGGTTATTX--ATATT---------T---CXTATGAGCTGAGCCTTCAACTTGATXC----
                             84890     84900     84910     84920     84930     84940     84950     84960
Human        TCTTATAAAATGCATAGTAAGGATATTAATATGCATATT--------TTGGATGAGCCAAGCCTTCAGCTTTATTCACTT  67218
PolarBear    ACATATAAATTGTGTATTAAGGGTATTC--ATACT---------T---CATATGAGCTGAGTCTTCAACTTGAT------  27589
Cow          ACATGTTAAATGTATATTAAGGATATTTTGCTCCT-----------ATTTTATGAGCTGAGCATTCAGCTTTATAC--TG  64567
Dog          ATATGTAAAATGTGTATTAAGGATATAC--ATATT---------T---CATATGAGCTGAGTCTTCAACTTGAT------  58201
BelugaWhale  TAGAA----TTTCATATAAAATTTATTTTGGTATTTATCAGTATTTGCGTTTTGTCTTAATCATTGTATTTCACCTCATT  57680
Donkey       TATAATGAAGTAATTCTTGGAAATAGATAATTATTTACCGTGGAT--ATGTAACAATTGTGCTGTCCATTCGTTAA----  64214
Cat          --ATGTAAAATGTGTTTTAAGGTTATCC--ATATT---------T---CATATAAGCTGAGTCTTCAACTTGATTC----  64927
Ferret       ACATGTAAAGTGTGTATTAAGGGTATTC--ATACT---------T---AGTATAAGCTGAGTCTCCAACTTGATA-----  65667
Lion         --ATGTAAAGTGTGTTTTAAGGTTATCC--ATATT---------T---CATATAAGCTGAGTCTTCAACTTGATTC----  62634
Vaquita      TAGAAAGAATTTTATATAAAATTTATTTTGGTATTTATCAGTATTTGCATTTTGTCTTAATCATTGTATTTCACCTCATT  59033
Pig          TAGAAAGAATTTTATAGAAAATTTATTTTGGTATT-----------GCATTTTGTCTTGAACATCACATTTTGCCTG-TT  57752
```

```
Majority     ---TTACAATATTTTXGACXAACCTXTTTATAGAAAAATT---------ATTTTTAAATAATT--TATCTCAATCCAAAA
                      84970     84980     84990     85000     85010     85020     85030     85040
Human        ATGTTAGGTCAGTTTTTGGAAAAGTATTTTTCAATAATCTCT-------CTTTTTAAATAATT--GATCTCAATCCCAAA  67289
PolarBear    ---TCACAATATTTTAGACTAGCCAGTTTATAGAAAAATT---------ATTTTTAAATAGTT--TATCTCAATCCCAAA  27655
Cow          ---TTACTGTAGATTGG--CCAGTTA--TGGAAAAAAAT----------GTTTTAAAATAATT--CATCATAATCCAAAC  64628
Dog          ---TCACAGTATTTTAGAC--ACCAGCTTATAGATGAGTT---------ATTTTGAAATAATT--TATCTCAATCCAAAA  58265
BelugaWhale  ---CTACAATGGATTTGACCCACCTAATCAAAAACAAAACAAAACACAGATTTGCAACTAATAGTTGACAGAGTCAAGAT  57757
Donkey       ---ATATGTTATAAGTGATAAACATCTATGTGGGACACCT---------GCCACACCACAGGT--TGGCTTGAT-AAGCA  64279
Cat          ---ACACAGTATTTTACACTAGCCAGTTTATAGAAAAATT---------ATTTTTAAAT---T--TATCTCAATCCAAAG  64990
Ferret       ---TTACAATATTTAAGACTAGCCAGTTTATAGAAAAATT---------ATTTTTTAATAATT--TATCTCAATCCAAAA  65733
Lion         ---ACACAGTATTTTACGCTAGCCAGTTTATAGAAAAATT---------ATTTTTAAAT---T--TATCTCAATCCAAAG  62697
Vaquita      ---CTACAATGGATTTGACCCACCTAATCAAAAACAAAACAAAACACAAATTTGCAACTAATAGTTGACAGAGTCAAGAT  59110
Pig          ---CTACAATGGATTTGACCCACCTAACCAAAAATAAAA----------ATTTGAAACTAATAG-TGACAGAGTCAAGAT  57818
```

```
Majority     GTATCCATTTATAGGACATGAAGTTAATTTTTTAC-C---------CCXACACATTTGTGGX--ATATTGTAATCTTTAT
                      85050     85060     85070     85080     85090     85100     85110     85120
Human        GTATTCATTTGTAGGACATGAAGACACAATTTTCT-----------TCTTCATACGTTTGGTGAATATTTTGATCTC---  67355
PolarBear    GTATCCATTTATTGGACATGAAGTTAACTTTTTC-C---------CCCACACATTTGTGG---CTATTGTGATCTTTAT  27722
Cow          ATTTCCATTTATAGGACATTAAGGTAACTTTGCAC-CCTCGCCCGCTTCACTCGCCTTTGGTGGATAGTATAATCTTCAT  64707
Dog          ATACCCATTTATAGGACTTGAAGTTAACTTTTTC-C---------CCCACACATTTGTGG---ATATTGTGATCTTTAT  58332
BelugaWhale  GAAGAAAAAATAAAGGAA--AATGTTATTTTGTA------AGTAAAATAAGTGATTTATAATTTAAGATGAAATTTT---  57826
Donkey       GTGTGTATGTCCGCGCCCGGGATCCAAACCAGTGAATCCTGGGTTGCCGAAGCAAGAGTGCATGAACTTTTAGCTGCTAC  64359
Cat          ATATCTATTTATCGGACATGAAGTTAATTTTTTCC-C---------CC---ACATTGGTGG---ATATTGTCATCTTTAT  65054
Ferret       ATATCCATTTATGGAACATGAAGTTAACTTTTTC-C---------CC-ATACATTTTTGG---ATATTGTCATCTTTAT  65799
Lion         ATATCTATTTATCGGACATGAAGTTAATTTTTTCC-C---------CT---ACATTGGTGG---ATATTGTCATCTTTAT  62761
Vaquita      GAAGAAAAAATAAAGGAA--AATGTTATTTTGTA------AGTAAAATAAGTGATTTATAATTTAAGATGAAATTTT---  59179
Pig          GAAAAAAATAAGGGGA----AATGTTATTTTGTAA-CGAAAGTAAGTGAAGTGATTTGTAATCTAAGATGAAATTTT---  57890
```

```
Majority      -A--XGAGTCACTTAXTATTTTXCCTTTT---AAAGTAGTTTTCAAAC--ACCGTTTATAG-G--TAACATCCAAT----
                        85130     85140     85150     85160     85170     85180     85190     85200
Human         ------AGTTTC----CACTTCTCTTTTT--------GCTTTTTAAAC--ATTGTTTATGG----TGATATCCAAT----  67407
PolarBear     AA--GGAACCTCTTA-TGCTTTGCCTTTT---AAAGTAGTTTTCAAAC--ATCCTTTATAG-TAATAACATCCAGT----  27789
Cow           GA--AGAATCACTTACTATTTTACTTTTTT--AAAGTAGTGATCAGAC--GCTGTTTGTAGTGACTAGTGACATGC-AGT  64780
Dog           TA--GGAACCACTTA-TATTTTGCCTTTTTTAAAAGTTGTTTTCAAAC--ACCGTTTATAA-A---GACCTCCAAT----  58399
BelugaWhale   ----ACAGTT-----TTATTTGATGTATTC--AGGAAATTCTTGGTTTCTGAAGTTTGTAA-GATTATCATTAAATGAGT  57894
Donkey        -ACCACCATGCCAGCCTCTTTAATTTTTC----ACATAGTCTTCAAAC--ACCCTGTATAG----TGACATCCAGT----  64424
Cat           AA--GGAGCCACTTACTATTTTGCCTTTT---ATAGTAGCTTTCAGGC--ACCCTTTATAG-C---AACATCCAAT----  65119
Ferret        AAAAGGAACCATCTA-TGTTTTGCCTTTT---AAAGTAGTTTTCAAAC--ACCCTTTATAG-T---AACATCCAGT----  65865
Lion          AA--GGAGCCACTTACTATTTTGCCTTTT---ATAGTAGCTTTCAGGC--ACCCTTTATAG-C---AACATCCAAT----  62826
Vaquita       ----ACAGTT-----TTATTTGATGTATTC--AGGAAATTCTTGGTTTCTGAAGTTTGTAA-GATTATCATTAAATCAGT  59247
Pig           ----ATAGTTTCCTTTTATTTGATGTATTC--AGGAAATTTTTGGTGCCTGAAGTTTGTGAAACTTATCTTTAAGTGAGA  57964

Majority      -ATTACTTTAGTXAGGTCATGCCTTCTGGAATGGTAACTAAATCTTTTCTAT-GTTTA-TTTGCCTCCAXTGATATTTAA
                        85210     85220     85230     85240     85250     85260     85270     85280
Human         -ATTGCATGAGTGAGGTCATGCCTTTTAGAATAATAACTGAATCTTTTC-----T----------TCTAATTAT--TTAA  67469
PolarBear     -ATTACTTTAGTGAGGTCATGTCTTCTGGAATGGTAACTAAATCTTTTCTAT-GTTTA-TTTGCCTCCAGTGAAATT-AA  27865
Cow           -ATTGTTTTAGTAAGGTCATACCTTCTGGAATGGTGACTAAATTTTTTTCTCTGTTTA-TTTGCTTCCATTGATATCTAA  64858
Dog           -ATTACTTTTAATA------GCCTTCTGGAGTGGTAACTAAATTTTTCCTAT-GTTTA-TTTTCCTCCAATGATATTTAA  58470
BelugaWhale   TGTTTCTATACATATGGAACCCAAGATTGATGAGTCACAAACTAGTTCTTACAGTTTAGTTTATGTTTATCAAT---TAA  57971
Donkey        -ATTGCTTTAGGGAGGTCCTGCTGTCTGGAATGGTAACCAAATCTTTTCTAT-GTTTA-TTTGCCTCCAGTGATACGTAA  64501
Cat           -ATTACTTTAGTAAGGTCATGCCCTCTGGAATGGTAACTAAATCTTTTCTAT-GTTTA-TTTGCCTGCAATGATATTTAA  65196
Ferret        -ATTACCTTAGTGAAGTCATGTTTTCTGGAATGGTAACTAAATCTTCTCTAT-GCTTA-TTTGTCTCCAGTGAAATTTAA  65942
Lion          -ATTACTTTAGTAAGGTCATGCCCTCTGGAATGGTAACTAAATCTTTTCTAT-GTTTA-TTTGCCTGCAATGATATTTAA  62903
Vaquita       TGTTTCTATACATATGGAACCCAAGATTGATGAGTCACAAACTAGTTCTTACAGTTTAGTTTATGTTTATCAAT---TAA  59324
Pig           -ATTTCTGTACATATGTAACCCAAGATTGATGAGTCACAAACTGGTTCTTAGAGTTTAGTTTATGTTCATCAAT---TAA  58040
```

```
Majority     T--GTCAGGTGACTTCAAAATACAAC-AAACTAXTATTGAT-GXXA--GATCCATTTCCTGCTGAGTCTTXCTCAAGCAG

                       85290     85300     85310     85320     85330     85340     85350     85360

Human        T----CAGATGACTTCA----GCA----AACTAATATTGGTGGG-----ATTAACTTCCTGCTCAGTCTTCCAGTAACAG  67532
PolarBear    TAAGTCAGGTGACTTTCAAA---------------------------------------------------------CAG  27888
Cow          T----CAGGTGACTTCAGATTACAAC-AAACTAATATTGAT-AAGATCCATTTTCAGCTTG---AGTCTTTCTTAAGCTG  64929
Dog          TAAGTCAGGTGACTTTGACATACAAC-CAACTAGTATC-AT-TG----GATCCATTTCCTGCTGAGTCTTCTTCAAACAG  58543
BelugaWhale  A----AAAGAAAAAGC--ATTATAAT-GAAGTAATTTTTGGAAATAGATATTTACTTACTGT-GAATATATAGCATGTGT  58043
Donkey       TAAGTCAGATGACTTCAAAGTATGACCAAACTAGTATTGATCCA-----ATCCATTTTCTGCTAAGTCTTTCTCAAACAA  64576
Cat          T--GTCAGGTGACTTCAACATACAAC-AAACTGGTATTGAT-GGTATTGATCCGTTTCCTGCTGAGACTTCCTCAAGCAG  65272
Ferret       TAAGTCAGGTGACTTCAGCATACAAC-TCCCTAGTATCGAT-GG----GATCCATTTCCTGCTGAGTCTTCCT----CAG  66012
Lion         T--GTCAGGTGACTTCAACATACAAC-AAACTGGTATTGAT-GGTATTGATCCATTTCCTGCTGAGACTTCCTCAAGCAG  62979
Vaquita      A----AAAGAAAAAGC--ATTATAAT-GAAGTAATTTTTGGAAATAGATATTTACTTACTGT-GAATATATAGCATGTGT  59396
Pig          A----AAAGAAAAAGC--ATTATAAT-GAAGTAATTCTTA--GATATTTATGTAGATATGT---AACATTT-----GTGT  58103

Majority     TACTTGTTCAAAATGAAATAATTGATAG------XTGACCCTTTATTTCAAA--GATTCTGTAAGAACACAAGTACA--A

                       85370     85380     85390     85400     85410     85420     85430     85440

Human        TACTTGTTCAAAATGAAATAATTGATA---------GAGTATTTATTTCAAA--AATTTT-TAAGAACACAAATATA--A  67598
PolarBear    TACTTGTTCAAATGGAAATAATTGAT----------CTCCCTTTATTTTAAA--GATTCTGTAAGAACACAAGTACT--A  27954
Cow          TACTTGTTCAAAGTGAAATAATTTATAG------GTATCCCTGTGTTTCAAA--AATTCTGTAAGAACACAGATAGAAAA  65001
Dog          TACTTGTTCAAAATAAAATAATTGATAGCTAGCTATCTCTCTTTATTTCAAA--GATTCT-TAAGAACACAAGTACT--A  58618
BelugaWhale  TATCCATTTATAAATATGTAA--TATAA------GTGATAC-ATCTATGAACTGAGACACACTAATGCACAAGAGAAGCA  58114
Donkey       TACTTGTTCAAAATGAAATAATTGATGG------ATATCCCTTTATTTCAAA--GATTCTGTAAGAACACAAGTACA--A  64646
Cat          TACTTGTTCAAAATGAAATAATTGATAG------ATGACCCTTTATTTCGAA--GATTCTGTAAGAACACAAGTACT--A  65342
Ferret       TACTTGTTCAAAATGAAATAATAGAT----------CTTCCTTTGTTTCAAA--GATTGTATAAGAACACAAGTACTT-A  66079
Lion         TACTTGTTCAAAATGAAATAATTGATAG------ATGACCCTTTATTTCGAA--GATTCTGTAAGAACACAAGTACT--A  63049
Vaquita      TATCCATTTATAAATATGTAA--TATAA------GTGATAC-ATCTATGAACTGAGACACACTAATGCACAAGAGAAGCA  59467
Pig          TAGCCGTTTGTAAATAGGTAA--TATAA------GTGATTC-ATCTTTGAAC--AGAGATACTAATGTACAAGAGAAGCA  58172
```

```
Majority     AXTGATAC------TTXTTTTCTGCAAATATTTAGGGG---GATTCACTTTACCTTGTAT-------AATTTTT---ATA
                               85450     85460     85470     85480     85490     85500     85510     85520
Human        GGTGATAC---------TCTTTTGCTAAAATTTGTGGGGG-GCTTCACTTTACCTTGTAT-------GATTTTTATATTA  67661
PolarBear    AGTGATCC------TTCTTTTCTGCAAATATTTAGGGG---GATTAACTTTACCTTGTAT-------AATTTTT---ATA  28015
Cow          GAAAGTAAGTGAAGTCG--CTCAGTAGT-ATCCAACTCTTTGCGACCCCATGGACTGTAGCCCACCAGGCTCCT-CTATC  65077
Dog          GATGGTCC------TCCTTTTCTGCAAATATTTGGGGG---AATTTCCTTTACCTTGTAT-------AATTTTT---ATA  58679
BelugaWhale  TTTGACATGTGAAATTAATTCTGGTAAT--TTTAGACAAAACTGACCCTTTATTCTA-----------GTTGTC-TAATA  58180
Donkey       GTTGATAAT-----TCTTTTCTGCTAAATATTTAGGGG---GATTCACTTTGCCTTGTAT-------AATTTTT---ATA  64708
Cat          AATA-------------TTTTCTGCAAATATTTAGGG----AATTCACTTTACCTCGTAT-------AATTTT----ATA  65394
Ferret       AGTGATGC------TTCTTTTCTGCAAATATTTAGGGG---GATTCACTTTTCCTTGTAT-------AATTTTT---ATA  66140
Lion         AATA-------------TTTTCTGCAAATATTTAGGG----AATTCACTTTACCTCGTAT-------AATTTT----ATA  63101
Vaquita      TTTGATATGTGAAATTAATTCTGGTAAT--TTTAGACAAAACTGACCCTTTATTCTA-----------GTTGTC-TAATA  59533
Pig          ATTGGTATTTGAAATTAATTTTGGTAATTATTTATCTATTTGTTTGTCTTTTTGCCATTTCTTGGGCTGCTGCTGCGGTA  58252

Majority     TCTAAGG---ACTTGGCTGAATTT--GTAATCAAXAXX--XTAATA--CATTTTAATTGGTTTATTTTACAXAGTGATTT
                               85530     85540     85550     85560     85570     85580     85590     85600
Human        GATCTAG-CTACTTGACTGAATTTAATTACTTTAGAATCAGTACTATAAATTTTAATTGATTTATTTTACACAGTAATTT  67740
PolarBear    TTTAAGA---ACTTGACTGAATTT--GCAATCAGTA-----TAATA--CATTTTAAT-GGTTTATTTTACACTGTAATTT  28082
Cow          CGTTGGATTTTCCAGGCTAGAGTA--CTGTAGTGGGTT--GCCATTTCCTTTTCCAGGGGAATCTTCCCGATCCAGGGAT  65153
Dog          TCTGAGA---ACTTGACTGAATTT--GGAATCAATA-----TAGTA--CATTTTAATTGATTTGTTTTACACAGTAATTT  58747
BelugaWhale  CATCTAG---CACAAGTCATAGCAG-TTTATCATGACC--ATAATAGATTCTTTAGGTTGGTAACATTCTTTCTTGAGA-  58253
Donkey       TCTAAGT---ACATGACTGAATTTAATTACTTTGGAATCAGTATTATAAATTTTAATTGATTTATTTTACACAGTAATT-  64784
Cat          TCTAAGG---ATTTGGCTGAATTC--GGAATCAATA-----TTATA--CATTTTAATTGGTTTATTTTACATAATGGTTT  65462
Ferret       TCTAAGA---ACTTGACTGAATTT--GGAATCAATACA--ATAATA--CATTTTAATTGGTTTATTTTACACAGTAATTT  66211
Lion         TCTAAGG---ATTTGGCTGAATTC--GGAATCAATA-----TTATA--CATTTTAATTGGTTTATTTTACATAATGGTTT  63169
Vaquita      CATCTAG---CACAAGTCATAGCAG-TTTATCATGACC--ATAATAGATTCTTTAGGTTGGTAACATTCTTTCTTGAGA-  59606
Pig          TATGGAGGTTCCCAGGCTAGGGGT--CTAATCGGAGCT--GTAGCCGCCGGCCTATGCCACAGCCCCAGCAACGCGGGAT  58328
```

```
Majority     X----TTAAGACT--AGCTTTTATACTTGGCTTCTXTT-------TT-ATTTTCT---------X-----TTAGTGA---
                       85610     85620     85630     85640     85650     85660     85670     85680
Human        T----TTAAGACT--AACATTTATGTCTGGCTTTTCTG-----------TTTCCT---------------TTAATG----  67784
PolarBear    T----TTAAGACT--AGCTTTTATACCTGGCTTTTTTTATACCTTTTCTTTTTCT---------T-----TTAGTAA---  28139
Cow          CGAACCCGGGTCT----CCCACATTGTAGGCAGCCACTTCACCATCTGAGCCACCAGGGAAACAGATAACTAGGTGAT--  65227
Dog          C----TTAAGACT--AGCCTTTATACCTGGCTCCTCTT----------CTTTTTC---------T-----TTTATTA---  58794
BelugaWhale  -------GAATCT----CTGTCACTGTGTGGGGCTAATTTAAGGCTTTATTCCCT-----------CAACTTGG------  58305
Donkey       -------------------TT--T--TAAGCTTCTAAT-----------TTCTTT----------------TAGTG----  64810
Cat          A----TTAAGACT--AGCATTTATACTTGGCTTCTCTT-----T-TTCTTTTAGT---------G-----CTAGGGC---  65513
Ferret       TA---TTAAGACT--AGCTTTTATACTTAGCTTCTCTT-----------TTTTCT---------T-----TTGGTAA---  66258
Lion         A----TTAAGACT--AGCATTTATACTTGGCTTCTCTT-----T-TTCTTTTAGT---------G-----CTAGGGA---  63220
Vaquita      -------GAATCC----CTGTCACTGTGTGGGGCTAATTTAAGGCTTTATTCCCT-----------CAACTTGG------  59658
Pig          CCAAGCCACATCTGCAACCTACACCACAACAGCTCATGGCAACGCCGGATCCTCAACCCACTGAGCAAGGCCAGGGATTG  58408

Majority     ---------C-TA-AGGATCACAGT--GTCTCAGAA---AATGTAXTTTTTTC-AAGAAGGXAGACTGAGTG--CTTTGT
                       85690     85700     85710     85720     85730     85740     85750     85760
Human        ---------C-TA-GGTATCACAGT--GTCTCAGAA---AATGTAATTTGTTCAAAGAAAGGAGACTGAGTG--CTTTGT  67846
PolarBear    ---------C-TA-GGGATTACAGT--GTCTCAGAA---AATGTAATTTTTTC-AAGAAGGAAGACTGAGGG--CTTTGT  28200
Cow          AATTCTTTTCTGCCAAATATTTAGGGGGATTCACTTTATTTTGTATCATTTTTGTATCTAAGCAGTTGACTGAGTTTAGT  65307
Dog          ---------C-TA-GGGATTACCGT--GTTTCAGAA---AATGTAACTTTTTC-AAGAAGGAAGACTGAGTG--CTTTGT  58855
BelugaWhale  ---------CTTGTAAGTAGATAAT---ATTGACTA--TGGTGTACCATTTTTTAAAGTCAAGAGT----------GAGA  58361
Donkey       ---------C-TA-GGGATCACTGT--GTCTCAGAA---AATGTAATTTATTC-CAGAAGGGAGACCGAGTG--CTTTGT  64871
Cat          ---------T-TACAGTGTCTCAGT--GTCTCAGAA---AATGTACTTCTTTC-AAGAAGGGAGACCTAGTG--CTTTGT  65575
Ferret       ---------C-TA-AGGATTACAGT--GTCTCAGAA---AATAGAATTTTTTCCAGGAAGGAAGACTGAGGG--CTTTGT  66320
Lion         ---------T-TACAGTGTCTCAGT--GTCTCAGAA---AATGTACTTCTTTC-AAGAAGTGAGACCTAGTG--CTTTGT  63282
Vaquita      ---------CTTGTAAGTAGATAAT---ATTGACTA--TGGTGTACCATTTTTTAAAGTCAAGAGT----------GAGA  59714
Pig          AACTCGCAACCTCCTGGTTCCTAGTCGGATTCGTTAACCATTGAGCCATGACGGGAACTCCTAATTTTGGTAACTTGAGA  58488
```

```
Majority     AATT----AATTAGAAATCTGACATCTAAATA-CATAATC-AC-AAATCXGGCAGACTATTAGTTATT-----CACATAT
                       85770     85780     85790     85800     85810     85820     85830     85840
Human        AATG----AGTTTAAAATCTGACATCTAAAATACATAATC-AC-AAATGTGGCCGACTATTAGTCATT-----CACATAT  67915
PolarBear    AATT----AATTAGAAATCTGACATCTAAATA-CATAATC-AC-AAACCTGTCAGACTATTAGTTATT-----CATATAT  28268
Cow          CATTTTGGAATCAATATTATAAATTTTAATTGGTTTATTTTACACAGTAAGTTTTTATGTGGCTTCTCTGCTTCTTTCTT  65387
Dog          TATT----AATTAGAAATCTGACATCTAAATA-CATAATC-AC-AAACCCGGCCAACTATTAGTTATT-----CACGTAT  58923
BelugaWhale  CA----GTAATGAGTGATACA-----TGGCCT--TTGCTT------GTAATTGTTACTGTGAATTCTTA---AATTTTTT  58421
Donkey       AATT----AGTTAGAAATCTGACGTCTAAATA-CATAATC-AC-AAACGTGGCAGACTATTAGTTATT-----CACATAT  64939
Cat          AAAT----AATTAGAAATCTGACATCTAAATA-CATAATC-AC-AAATCCAGCAGACTATTAGTTATT-----CACGTAT  65643
Ferret       AATT----AATTGGAAATCTGACATCTAAATA-CATAATC-A--AAACCTGGCAGAGTATTAATTATT-----CACATAT  66387
Lion         AAAT----AATTAGAAATCTGACATCTAAATA-CATAATC-AC-AAATCCAGCAGACTATTAGTTATT-----CACGTAT  63350
Vaquita      CA----GTAATGAGTGATACA-----TGGCCT--TTGCTT------GTAATTGTTACTGTGAATTCTTA---AATTTTTT  59774
Pig          CA----A-AATTAACCTTTTA--TTCTAATTT-TCTGATACATCTAGCACACGTCATAGTAGTTTATC--ATGACCATAT  58558

Majority     TCTACTTCAGAGAACAAXGTGAT---AAAAATCCTTGAGATT-TATAAATTGAGACATCGTTATTATG-ATCATTTTA--
                       85850     85860     85870     85880     85890     85900     85910     85920
Human        TCTGTTTCAAAGAACACAGTGAT--CAAAAATCCTTCAGATT--ATAAATTGAGACATCATTATAATGGATCATTTTA--  67989
PolarBear    TCTACTTCATATAACAAAGTGAT---AAAAATCCTTGAGATT-TATAAATTGAGACATCGTTACTATG-ATCATTTTA--  28341
Cow          TCAGAGTTAGGAATCACTGTGTCTTCAAAAATGCAGTTTATT-TAAGAATGGAGATGAGTGCTTTGTA-ATTAGTTAGAA  65465
Dog          TCTACTTCATAGAACAAAGTGAT---AAAAATCCTTGAGATT-TATAAATTGAGACATCATTACTATG-ATCATTTTA--  58996
BelugaWhale  TAACTGATAGAAATTATTTTATA-GAAAAGACAAAAATCAGCATTTACTGAATAGTTTCTATGTCATA-GGCATTCTGCA  58499
Donkey       TCTACTT-------CAAGGTGAT--CAGAAATCCTTGAGATT--ATAAATTGAGACATCATTATAATG-ATCATTTTA--  65005
Cat          TCTACTTCAAAGAACAAGGTGAT---AAAAATCCTTGAGATT--ATAAATTGAGACGTTGTTACTATG-ATCATTATA--  65715
Ferret       TCTACTTCATAGAACAAGATGAT---AAAAATTCTTGAGATT-TATAAATTGAGACATTGTTACTATG-ATCATTTTA--  66460
Lion         TCTACTTCAAAGAAAAAGGTGAT---AAAAATCCTTGAGATT--ATAAATTGAGATGTCGTTACTATG-ATCATTTTA--  63422
Vaquita      -AACTGATAGAAATTATTTTATA-GAAAAGACAAAAATCAGCATTTACTGAATAGTTTCTATGTCATA-GGCATTCTGCA  59851
Pig          TCATCTTGAGATAACATTCTTCCCGAGAGAATCTGTGTCACCGTGTGGGCGTAATTTAAGCCTTTAT---TCCCTTA---  58632
```

```
Majority     --TCA-TGTTTAAGAAGTGTAXTT--AATTTATTAAGG------------GGTGGATCACTTTTAGAAAAA-T-------
             85930     85940     85950     85960     85970     85980     85990     86000
Human        --TCA--TGTTAAGAGGTGTAGTT--AATTTATTAAGG------------GGTAGATCACTTTTAGAAAAA------T--  68043
PolarBear    --TCA-TGTTTAAGAAGTGTAGTT--AATTTATTAAGG------------GGTGGATCACTTTTAGAAAAA-T-------  28396
Cow          ATCTGATGTTTAAATA-CATAATCACAAACCTGGCAGACTATTAGTTATTCATATTCTACATCAAAGAACAAGGTGATCA  65544
Dog          --TCA-TGTTTAAGTA------T---AGCTTATTAAGG------------GGTGGATCGATTTTAGAGAAG---------  59043
BelugaWhale  AGTCACTTAGCAGATA-CATTATT-TAATCCTTAAA-----------A----TA-ACCAAAGTAGAAGATGAC---AACA  58558
Donkey       --TCA-TGTTTAAAAAGTGTAGTT--AATTTATTAAGG------------GGTGGATCACTTTTAGAAAAAT--------  65060
Cat          --TCA-TGTTTAAGAAGTGTATTT--AATTTATTAAGG------------GGTGGAACACTTTTAGAAAAA-T-------  65770
Ferret       --TCA-TGTTTAAGAAGTGTAGTT--AATTTATTAAGG------------GGTGGCTCACTTTTAGAAAAAAT-------  66516
Lion         --TCA-TGTTTAAGAAGTGTATTT--AATTTATCAAGG------------GGCGGATCACTTTTAGAAAAA-T-------  63477
Vaquita      AGTCA-TTAGCAGATA-CATTATT-TAATCCTTAAA-----------A----TA-ACCAAAGTAGAAGATGAC---AACA  59909
Pig          ACTTAGCTTCTAAGTT-AATGATAATGATTCTTTCA-------------TAATGTACCATTTTTGAAAATATTTTTTTAA  58698

Majority     ----TGCTAGAAGTAATTTTTCATGATCATGTTGTC--------------------------TTACATTTAAAAAATTAG
             86010     86020     86030     86040     86050     86060     86070     86080
Human        ----TGCTGGAAGTAATTTTTCATGATCATGTTATC---------------------------TACATTCTAAAAATTAG  68092
PolarBear    ----TGCTAGAAGTAATTTTTCATGATCATGTTGTC--------------------------T-ACCTTTTAAAACTTAG  28445
Cow          AAAATCCTTGAGATTATAAATTGAGATGTCGTTATAATG-ATCATTTTATCATGTTTAAGAAGTATAGTTAATTTATTAA  65623
Dog          ----TGCTAGAAGTAATTTTTCATGCTCATGTTGTC--------------------------T-ACATTTTAAAAATTAA  59092
BelugaWhale  AGATTTTTTATAGTAGTTTCTAAGAAGTTTAAAATA--------------------------TTACATGCAACTCATTAA  58612
Donkey       ----TGCTAGAAGTAATTTTTCATGATCTTGTTGTC---------------------------TACATTTAAAAAATTAG  65109
Cat          ----TGCTAGAAGTAATTTTTCATGATCATGTTGTC--------------------------T-ACATTTAAAAAATTAG  65819
Ferret       ----TTCTAGAAATAATTTTTCATGATCATGTTGTC--------------------------T-ACCTTTTAAAAATTAG  66565
Lion         ----TGCTGGAAGTAATTTTTCATGATCATGTTGTC--------------------------T-ACATTTAAAAAATTAG  63526
Vaquita      AGATTTTTTATAGTAGTTTCTAAGAAGTTTAAAATAATT--ACATGCAAAAAAAAAAAAATATTACATGCAACTCATTAA  59987
Pig          TCTATCTATGTATTTATTTTTATGGCTGCACCTACAGCATATGGACATTCCTGGGCCAGGGATTGAATCCAAGCCAC--A  58776
```

```
Majority     GGAA-------------GATAATGTGTACAAAGAXTACTTAA--TGTTAGAGCTTTCCATGTATTTTGAGT----------
                      86090     86100     86110     86120     86130     86140     86150     86160
Human        GAGA-------------GAGACTGTGTACAAAGAGTGTTT-A--TTTTAGAGCTTTCCTTGTATTTCAAAT----------  68147
PolarBear    GGAA-------------GATAATGTGTACAAAGAATACTTAA--TGTTAGAGCTTTCTATGCATTTTGAGT----------  28501
Cow          GGGGTGGATCACTTTTAGAAAATTGCTAAAAGTAATTTTTCA--TGATCACGCTATCTACATTTTTTAAATTAATGCAGT  65701
Dog          GAAA-------------GATAATGTGTACAAAGAATACTTAA--TGTTAGAACTTTCTATGCATTTTGAGT----------  59148
BelugaWhale  AGAA------ACAAGGGTAAAATACCAAGAAA-----CTTCA---GCTAGCGTCAAACATATGACCGTAGTAATCACTTT  58678
Donkey       GGCA-------------GATAATGTATAAAAAGAATACTTA---TTTTAGAACTTTCTAAGTATTTTGAGT----------  65164
Cat          GGAA-------------GGTGCCGTGTACAAAG--TACTT----TG---GAGCTTTCCATGCATTTTGAGT----------  65868
Ferret       GGAA-------------GATAATATATACAAGGA-TACTTA---TATTAAAGCTTTCTGTGCATTTTGAGT----------  66619
Lion         GGAA-------------GGTACTGTGTACAAAGAGTACTT----TA---GAGCTTTCCATGCGTTTTGAGT----------  63577
Vaquita      AGAA------ACAAGGGTAAAATACCAAGAAA-----CTTCA---GCTAGCGTCAAACATATGACCGTAGTAATCACTTT  60053
Pig          CCTGCGACCTACATCGCAGCTGTGGCAACACTGGGTCCTTTAACCCACTGCACTGGGC-CATGGGTTGAACCCGTGCCTC  58855

Majority     -----TGAAT----AAAGTA-------XXGTCAGCCATCTCATCATAT-GGCCTTAACAGAAAGGCAAAACAAACTTTTT
                      86170     86180     86190     86200     86210     86220     86230     86240
Human        -----TGAAT------------------AACAGGCATTCTCATCATAAAGTTTTAAAAGAAAGGCAAAGCAGACTTTCT  68204
PolarBear    -----TGAAT----AAAGTGG------CAGTCAGCCATTTCATCATAT-GGCCTTAACAGAAAGGC-AAACAAATTTTTT  28564
Cow          T--AATACATTCCAAGAATAGAGCCTTCTGTGTGGCAGCCTTTCTCAT-CATATAGTTTAAAAAGAAAAGCAAACTTTGT  65778
Dog          -----TGAAT----AAAGTAA------GAGCAAGGTATCTCATCATAT-GACCTTAACAGAAAGGCAGACCAAATTTTTC  59212
BelugaWhale  CT---TGGACCCTAAGAGC-------TATGTGTGTTTGCTTATTTGG---GTGTA----------GTTAGATGAG---TTT  58733
Donkey       -----TGAAT----AAAGTA------TTAGTCAGCCATTC--TCAAAG-GATCTTAGCAGAAAGGCAGAGCAGACTTTTT  65226
Cat          -----TGAAT----AAAGTAA------TTGTCATCCATTTCTTCATAT-GGCCTTAACAAAAGGCAATACAAAACTTTTT  65932
Ferret       -----TGAAT----AAAGTAG------CAGTCAGCCATCT-----TAT-GGCCTTAACAGAAAGGCTAAACAAGTTTTTT  66678
Lion         -----TGAAT----AAAGTAA------TTGTCATCCATTTCTTCATAT-GGCCTTAACAAAAGTCAATACAAAACTTTTT  63641
Vaquita      CT---TGGACCCTAAAAGC-------TATGTGTGTTTGCTTATTTGG---GTGTA----------GTTAGATGAG---TTT  60108
Pig          TGCAGTGGATTCCTAACCCACTGCACCACA-GTGGAAACTCCTAGTGT-ACCATA-TTTAAAAGTAAAGTCAAA---AGT  58929
```

```
Majority     ATAXGAAATCT-TTGATGTTAAAATAATTGATAATTGAGAACAGATATAACAAACATTT-XTTCATAAAGGTAAATCTGT
                       86250     86260     86270     86280     86290     86300     86310     86320
Human        GTAGGAAATCA-TTGACGTTAAAATAG-TTATAATTGTGAACAGATACAACATTTATTC-----ATGAAGGTAAACATGT  68277
PolarBear    ATGAGAAATCTATTGACCTTAAAATAATTGATAATTGAGAACAGTTATAACAGACCTTTTGTTCATAAAGGTAAATCTGT  28644
Cow          ATAAGATATTT--TGATGTTAAAATAATTGATGATTGAGAATAGATGTAACAACCATCT--------A-GATAAATCTGT  65847
Dog          ATGAGAAATCTGTTGATCTTAAA-----TAATAATTGAGAACAGATATTACAAACATTT-GTTCATTAAGGTAAATCTGT  59286
BelugaWhale  GTATGTGTTTT--TGATTCTGGGTGGAGTCATGGTTA-GAGGGAATTCATCA------T--------C-GTTTTCCTTGT  58795
Donkey       ATAGGCAATTTCTTGGTGTTAAAATGTTTGATAATTGAGAACAGATATAACAAACATGT-ATTCATAAAGGTAAATATGT  65305
Cat          ATAGGAAATCTGTTGATGTCAAAATAATTGATAATTGAGGACCGATCTAACAAACATTT-GTTCATAAAGGTAAATCTGT  66011
Ferret       ATGAGATATATGTTGACCTTAAAATAATTGATAATGGAGA--AAGTATAATAAACCTTT-GTTCGTAAAGGCAAATCTGT  66755
Lion         ATACGAAATCTGTTGATGTCAAAATAATTGATAATTGAGGACCGATCTAACAAACATTT-GTTCATAAAGGTAAATCTGT  63720
Vaquita      GTATGTGTTTT--TGATTCTGGGTGGAGTCATGGTTA-GAGGGAATTCATCA------T--------C-GTTTTCCTTGT  60170
Pig          GAGGGAATAAT--GAATGACACATGGCCTTGTTACTG----TAGATTCTTAAATTCTTT--------A-TTTTTTTTTTT  58994

Majority     AGTTCTATXGGAXCAGTGTCTCT--AAATTTTTCTGCACXTXGAAATCACCAGGC---CCTACCCTAG-ATTGAGAATCA
                       86330     86340     86350     86360     86370     86380     86390     86400
Human        AGGTCTTATAGAATATTGTTTCTCAAAATTTTGCTGCACGTTGAAGTCACCAGAT---TCTACCCCAG-GCTGAGAATAG  68353
PolarBear    A----CATAGAA-CAGTATCTC---CA--TTTTCTGCACATTGAAATCACCAGGCGGCCCTACCCTAGACT-GAGAATCA  28713
Cow          AATCCTTATATATCTGTGCTTCT--CAGTTTTCCTGCACATCAAAGTCACCAGGC---CCTACCCTAG-ACTGAAAATCA  65921
Dog          AATTCTATAGAAACAGTGTCTC---AGGTTTTTCTGTACA------TCACCAGGC---------------T-GAGAATCA  59341
BelugaWhale  AATCTCATGGCTTTGATG--------GAATTTTTTTAAAGTAATAA------AGT---CTC--------ATAAAAGATTA  58850
Donkey       AGTCCTTATAGAACACGTTTTCTC-AAA--CTACTGTACATGGAAATCACCACAGAGGCCCTATCCTGGACTGGGAATCA  65382
Cat          AGTTCTGTAGGA-CAGTGTCTCTC-AAATTTTTCTGCATGTCGAAATCACCAAGC---CCTACCCTTACGCTGAAAATAA  66086
Ferret       AGTTCCATTTAA-TAGTGTCTT---AGGTTTTTCTGCACACAGAAATCACCAGGC---CCTACCCTAGACTTGAGAATCT  66828
Lion         AGTTCTGTAGGA-CAGTGTCTCTC-AAATTTTTCTGCATGTGGAAATCACCAAGC---CCTACCCTTACACTGAAAATAA  63795
Vaquita      AATCTCATGGCTTTGATG--------GAATTTTTTAAAAGTAATAAT----AAGT---CTC--------ATAAAAGATTA  60227
Pig          TTTTTTTTGTCTTTTGTTGTTGT--TGTTGTTGTTGCTATTTCTTG--GGCTGCT---CCCGCGGCAT-ATGGAGGTTCC  59066
```

```
Majority     TXAG-GAGTGXGTCCCAGCA-GTTTGTAGTTTTTTAAGA-TCCCAAGATGGTTTTACTATGTAGTGGGAGTTGATAATTA
                       86410     86420     86430     86440     86450     86460     86470     86480
Human        TTAAAGAGTGGGTCACAGGC-ATTCCTATGTTTTTAGGA-TCTCAAGATGGTTTTACTATGTAGCAAGAGTTGAGAATTA  68431
PolarBear    TAAG-TAGTGAGTCCTAGGA-GTTTGTAGTTT---AAGA-TCTCAAGATGGTTTTACTGTGTAATGAGAGTTGATAATTA  28787
Cow          CTGC-AAGTGGGGTTCCGTGCATTTGTAATTGTTTAAGT-TTCCAAGATGGTTTTACTATGTAGTGGGAGTTGAGCATTA  65999
Dog          TGTG-GAGTGAGTCCTAGCA-GTCCATAGTTTCTTAAGA-TCTCAAGATGCTTTTACTGTGTAATGGGAGCTGATAATTA  59418
BelugaWhale  TAC-------AGTTACACCATAACT--AACTTCT--GTC-TTATTCAGTGCTTTTATCATTTTTCAAGTGTTCTACATTT  58918
Donkey       TTAG-GAGCGGGTCCCAGAC-GTTCGTATTTTTTTAAGA-TCCCCAGATGGT-TTACCATGCAGTGGGAGTTGAGAACTA  65458
Cat          TGAG-GAGTGTGTCCCAGTA-GTTTGTAGTTTTTCAAGA-TCCCAAGATGGTTTTACTCTGTAGTGG-AGTTGATAAATA  66162
Ferret       TGAA-TAGAGTCTCCTAGGA-GTTTGTAGTTTTTTAAGA-TCTCAAGATGGTTTTACTATGTAATGTGAATTGATAATTA  66905
Lion         TGAG-GAGTGTGTCCCAGTA-GTTTGTAGTTTTTCAAGAATCCCAAGATGGTTTTACTGTGTAATGGGAGTTGATAAATA  63873
Vaquita      TAA-------AGTTACACCATAACT--AACTTCT--GTC-TTATTCAGTGCTTTTATCATTTTTCAAGTGTTCTACATTT  60295
Pig          CAGG-CTAGGGGTTGAATCGGAGCTGTAGCCACC-GG---C-CTACGCCAGAGCCACAGCAACGTGGGATCCGAGCCGCG  59140

Majority     -CTGCTATAGAGAAAAGTTXGTAA--TTTTTAGATGCTAAATTT-AGCTTTTTA----GCAAAGTTAATGGTGTTCTCAA
                       86490     86500     86510     86520     86530     86540     86550     86560
Human        -CTGCTACAGAAAA-G----------------------------------------------------------AGCTTGT  68452
PolarBear    -CTGTTACAGAGAAAAGTTTGTAA--TTCTTAGATGCTAAATTTTAGCTTTTTAGCTAGCAAAGTTAAGGGTGTTCTCAA  28864
Cow          -CTGCTATAGAGAAAGTATGTAAT--ATTTTAGATGCTTTATTTTAGCTTTTTA----GCACAGTTATTAGTGTTCTCAT  66072
Dog          -CTGCTATAGAGAAAAGTTCGTAA--TTTTTAGATGGTAAATTTTAGCTTTTTA----GCAAAGTTAATGGTGTTCTCAA  59491
BelugaWhale  -TTGC-ATTAAAAGACTATG------AGTAAAGTTGCTCAGTTC---TTAGTAA----ATAAATTTGGTGTCTTCTTCAG  58983
Donkey       -CTGCTATAGAGAAAAGTTTGTAATTTTTTTAAGTTCTAAATTTTAGCTTTTAG-----CAAAGTTATTGGTGTTCTCGT  65532
Cat          -C------AGAGAAAAGTTAGTAA--TTTTTAGATGCTAAATTT-AGCTTGTTA----GCAAAGTTAACAGAATTCTCAA  66228
Ferret       -CTGCTATTGAGAA--GTTTGTAA--TTTTTAGATCCTAAGTTTTAGCTTTTTA----GCAAAGTTAAGAGTGTTCTCAA  66976
Lion         -C------AGAGAAAAGTTAGTAA--TTTTTAGATGCTAAATTT-AGCTTTTTA----GCAAAGTTAACAGAATTCTCAA  63939
Vaquita      -TTGC-ATTAAAAGATTATG------AGTAAAGTTGCTCAGTTC---TTAGTAA----ATAAATTTGGTGTCTTCTTCAG  60360
Pig          TCTGCAACCTACACCACAGCTCAC--GGCAACGCCGGATCGTTA-ACCCACTGA----GCAAGGCAGGGACGGAACCCG  59213
```

```
Majority     AAAAAX-TTCTTTATAGCTATTGA-ATAGTATTTTAAT-ACTTTT----TTTATTCATGCCAAATAA-ATATATXTTGGT
                               86570     86580     86590     86600     86610     86620     86630     86640
Human        AA-------CTTAATGTCATTTAA--------------------------------------------ATCTATTTTGGT  68481
PolarBear    AA-AAACTTCTTTATAGCTATTGA-ACAGTATTTTA-T-ACTTTG----TTTATTCATGCCAAGTAA-ATATATCTTGGT  28935
Cow          AAAAATATTCTTCATACCTGTTGACCCAGTATTTTAAT-ACTTTTCT--TTTATTCATGCCTTGTAA-CTCTGCCTTGAT  66148
Dog          AAAAACACTCTTCATAGCGATTAA-ATGGTATGTTAAT-ACTG------TTAATTCATGCCAA-----ATATATCTTGGT  59558
BelugaWhale  CTCTG----TCTTGAATATTTTAATATAATATGCTGGC-ATATCTCG--TTTCATTGTGCTTCACAGATAATGTTTTGTT  59056
Donkey       AAAAACGTTATTTATAGCTATTGA-GCAGTATTTTAAT-ACTTTC----TGTATTCATGCCATATGA-ACTTATCTTGGT  65605
Cat          AAAAAA-TTCTTTATAGCTATTGA-ACAGTATTTTAAT-ACTTT--------ATTCATGCCAAATAA-ATTTATTTTGGC  66296
Ferret       AAGAAACTTCTTAATAGCTATTGA-ATAGTATTTTA-T-ACTTCTACGTTTATTCATGCCAAATAA-ATATATCTTTGT  67052
Lion         AAAAAAATTCTTTATAGCTATTGA-ATAGTATTTTAAT-ACTTTG----TTTATTCATGCCAAATAA-ATATATTTTGGC  64012
Vaquita      CTCTG----TCTTGAATATTTTAATATAATATGCTGGC-ATATCTCG--TTTCATTGTGCTTCACAGATAATGTTTTGTT  60433
Pig          CA--A----CCTCATGGTTCCTAGTCGGATTCATTAACCACTGCGCC--ACGACGGGAACTCCTTAAATTCTTTATTGAT  59285

Majority     A-AXTTTTTCTGATAGAATA-TTGTTTAGCTGTTAAATTGAXTTTA---------TTTTTTTCTATG-TGTTTGTGATAC
                               86650     86660     86670     86680     86690     86700     86710     86720
Human        ACAGTTCTCCTGGCACAATT-TTAATTAGCTGTTAAACTGAATTTA---------TAATTTTCTATA-TGTTTGTGATAC  68550
PolarBear    ATAATTGTTCTGATAGACTA-TTGTTTAGCTGTTAAATTGAATTTG---------TTTTTTTCTATG-TGTC----ATAC  29000
Cow          ACAGTTCCACTGATAGATGTTCAGTTTAACTGTTATATTGACTTTA--CTTTGGTTTTCTTTTTATG-TGTTTATGATGT  66225
Dog          ATAATTGTTCTAATGGAATA-TTGTTTAGCT-TTAAATTGATTTGT---------TTTTTCTCTATG-TGTT--------  59618
BelugaWhale  -----TTTGTTTTT-AAAAACAAATTGAAGGTTTGTGGCAACCCTG---------CATTGTCAGATGATGGTTAGCATTT  59121
Donkey       ACAGTTCTTCTGACAGAATA-CTGTATAGCTGTTAAATTGAGTTTACTTTGTT-TTTCTTTTATATG-TGTCTGTGGTAG  65682
Cat          A---TTTTTCTGATAGAATG-TTGTTTAGCTGTTAAATTGA---GG---------TTTTTTTCC----TGTTTGTGATGC  66356
Ferret       ATAATTGTTCTGATTAA----TTGTTTAGCTGATAATTTGAATTTA---------TTTTTTTCTATG-TGTTCATGATAC  67118
Lion         A---TTTTTCTGATAGAATG-TTGTCTAGCTGTTAAATTGA---GT---------TTTTTTCCTGTT-TGTTTGTGATGC  64075
Vaquita      -----TTTGTTTTTTAAAAACAAATTGAAGGTTTGTGGCAACCCTG---------CATTGTCAGATGATGGTTAGCATTT  60499
Pig          AGAAATTATTTTATAGAAAACATCAGCATTTATTGAATAGCTTCTAGATTCTAGGCATTCTGAAAGG-TACTTGGCAGAT  59364
```

```
Majority     ATAAAATTAACAAGTAAACTTTTAAAT----ATTCTTTTAAAATTATTTTTAAGTGATTTCTGAAAGCTT----------
                                86730     86740     86750     86760     86770     86780     86790     86800
Human        ATAAAATTAACTGGAAAACTT-----------TGATTT------TCTTTCTTTG----T--T------------------  68589
PolarBear    ATAAAATTAATAAGTAAACTTTAAAAT----ATTCTTTTAATGTTATTTTTAAGTGATTTCTGAAGC-------------  29063
Cow          ATAAAATTAACAAGCAAACTTGTTTTT-TAAATATTTTTAAAAACAATATTTTAAAACTTTAAATGACTTGTGAAC---T  66301
Dog          ATAAGATTAACATGTAAACTT--AAAT----ATTTTTTAAAAATTATTTTTAAGTGGTTTCTGAAATCTTTGGTTTT-CT  59691
BelugaWhale  ------TTTAGCAATAAAGTATTTTGT---AATTAAGGTATATACATTTTTTTTAGACATAATGATGTTTACACAC---T  59189
Donkey       TTAAAATCAACAAGTAAACTTATTTTAAACTTAATTTTAAAACTTATTTTAAAAAGAGTTCTGAAAACTTTTGTTTTTCT  65762
Cat          ATAAAATCAACAAGTAAACTTTAAAAC----ATTCTTTTAAAATTATTTTTAAGTAATTTCTGAAAGCTT----------  66422
Ferret       ATAAAATTAACAAATAAGCTTTGAAAT----TTTCTTTGAAAGCTATTTTTAAATGATTTCTGACAC-------------  67181
Lion         ATAAAATCAACAAGTAAACTTTAAAAT----ATTCTTTTAAAATTATTTTTAAGTAATTTCTGAAAGCTT----------  64141
Vaquita      ------TTTAGCAATAAAGTATTTTGT---AATTAAGGTATATACATTTTTTTTAGACATAATGATGTTTACACAC---T  60567
Pig          CATTATTTAATCCTTAAAATAACCAAAGCAGATGGCAACAAAAATT-TATTTCATAGTTTCTGAGAGGTTTAAAA-----  59438

Majority     TTGTTACTTTTGAATGGGTTATTCACATGGTAATTTTTAAAAAC-TTTTTACTATCAXAGATTTCAGTCATCATATACTA
                                86810     86820     86830     86840     86850     86860     86870     86880
Human        -----ACATTTGTATGGGTTATTCATTTGGTAATATTT----ATTAATTTATTATTAGAGATTTCA---GCTATTTGCTA  68657
PolarBear    TTGTTACTTTTGAATGGGTTATTCACATGGTAATTTTTAAGAGC-TTTTTACTGTTAAAG--TTCAGTCATCATATAGCA  29140
Cow          CTTTTTTTTTTGAATGGGTTATTTACATGGTGATTTTTTAA-AAGCTTTTATTAAAGTTTTTTTTTTTTATCATATACTG  66380
Dog          TTGTTACTTTTGAATGGATTATTTGCGTAGTAATTTT---AAAC-TTTTTACTATGAAAG--ATTT-CAGTCATATACTA  59764
BelugaWhale  ----TAATAGACTACAGTATACTGTAAACATAGCTTTTGTA-TGCACAGGAAAATGATAAAATTTGTGTGACTCACTTTA  59264
Donkey       TTGTTACTTTTGAATGGATTATTCACATGGTAATTTTTTAAAAC-TTTTTATCATCAGAGATTTCAGTCATCATATACTA  65841
Cat          TTGTTATTTTCGAATGGGTTATTCATATGGTAGGTTTTAAAAAC-TTTTTACTATCAAAGATTTCAGTCATCATATACCA  66501
Ferret       TTGTTACTTTTGAGTGGGTTAT---CATGGTAATTTTTAAAAAAGTTTCTACTGTCAGAG--TTCAGTCATTATATAGCA  67256
Lion         TTGTTATTTTCGAATGGGTTATTCATATGGTAGGTTTTAAAAAC-TTTTTACTATCAAAGATTTCAGTCATCATATACCA  64220
Vaquita      ----TAATAGACTACAGTATACTGTAAACATAGCTTTTGTA-TGCACAGGAAAATGATAAAATTTGTGTGACTCACTTTA  60642
Pig          -----TTTGATGTGCAGGTCATTAAAGAAACAGTTTAAGGGTAAAATAGCAAGAAACTTCAGCTAGTGTCAAACATAGGA  59513
```

```
Majority     ATATAATGAATCCTACATTCXCATCATCTTGATTCAGTAGTTTCXACACTT-GTTTTATCTGTT-------CATTTTTTC

                      86890     86900     86910     86920     86930     86940     86950     86960

Human        ATATAATGAATCCCATACACCTGTCATCTTGGTTCAATAGCGCTGACACTTAGTTTTATCTATT-------CAGTTTTTC  68730
PolarBear    ATAAAGTGAATCCCACATTCGCATCATCTTGATTTAGTAGTTTCTACACTT-GTTTTATCTTTT-------CATTTTTTC  29212
Cow          ATGTAATAAATCCTATATATTCATCATCTCCATTCACTAGTTTCCACACTT-GTTATATCTGTT-------CATTTTTTC  66452
Dog          ATGTAGTGAATCCTACAT-----TCATCTCAATTTAGTAGTTTTTACACTT-GTTTTATCTTTTATCTGCTCATTTTTTC  59838
BelugaWhale  TTGCAATACTTGATTTATTGTGGTGGTCTGGAACCA-AAGCTGCAATATCT-CCAAG--------------------GTA  59322
Donkey       ATATAATAAATCCA-CATATCC-ACATCTCAATTCAGTAGTTCCCACACTT-GTTTTATCTGTT-------CATTTTTTC  65911
Cat          GTACAGTGAATCCTACATTCCCATCATCTTGATTCAATAGTTTCCACACTT-GTTTTATCTGTTCA----TCATTTTTTC  66576
Ferret       ATATAGTGAATCCCACATTCTCATCATCTTGATTTAGTAGTTTCTACACTT-GTTTCATCTGTT-------CATTTTTTC  67328
Lion         GTATAGTGAATCCTACATTCCCATCATCTTGATTCAATAGTTTCCACACTT-GTTTTATCTGTTCA----TCATTTTTTC  64295
Vaquita      TTGCAATACTTGCTTTATTGTGGTGGTCTGGAACCA-AAGCTGCAATATCT-CCAAG--------------------GTA  60700
Pig          GTGTAATAATGACT-TCCTTGGACCCCAGTGAGCCATGTGTGTTTACACAT-TTGAGTATAGTT-------AGATGAGTT  59584

Majority     TXT-TGTATTTTGAAGCAAATCCTTGACATCATGTCATGTCXXCTCAAACATTAXCATAATATCTTTAAAATAATGACAT

                      86970     86980     86990     87000     87010     87020     87030     87040

Human        TTT-TGTGTTCTAAAGCAAATCCTTGACATTGTGACATGTCTTCTCAAATATTACCATAGTATCTCTAAAATAATTACAT  68809
PolarBear    TAT-TGTATTTTTAAGCAAATCCTTGACATCATGTCAT-----CTCAAACATTATCATAGTATCTTTAAAATAATGACAT  29286
Cow          TGT-TATATTTTAAAGCAAATCCTCAACATTATGTCAT-----CTCAAACATTTTCATAATTTCTCTAAAATAATGACAT  66526
Dog          TAT-TGTATTTTGAGGCAAATCCTCGACATCATGTCATGTCATCTCAAACATTATCATAATATCTTTAAAATAATGACAT  59917
BelugaWhale  TGTCTGTATTTTTGT-------TTCTGTGCTCTGCC-T-----GATAAGAACAAGGAAAATATTTT----GTGGTGAAAC  59385
Donkey       TGT-TGTATTTTGAAGCAAGTTCTTGACATCATGTCATGTCAACTCAAGCATTACCATAATATCTTTAAAATAATGACAT  65990
Cat          TAT-TGTATTTTGAAGCAAATCCTTGACATCATGTCATGTCATCTCAAACATTACCATAATATCTTTAAAATAATGACAT  66655
Ferret       TAT-TGTATTTTGAAGCACATCCTTGACATCATGTCATGTCATCTCAAACATCATCATAATATCTTTAAAATAGTGACAT  67407
Lion         GAT-TGTATTTTGAAGCAAATCCTTGACATCATGTCATGTCATCTCAAACATTACCATAATATCTTTAAAATAATGACAT  64374
Vaquita      TGTCTGTATTTTTGT-------TTCTGTGCTCTGCC-T-----GATAAGAACAAGGAAAATATTTT----GTGGTGAAAC  60763
Pig          TGTATGTGATTTGGA-------TTCTGGGTGGAGTCAT-----GTTTAGAAGTCACTTAGATTCTTCTAGGTAATTCATT  59652
```

```
Majority     TTTCTT-----------------------ACCAAACCATAATATGATTATCACACXXATCAAATTTGACAATGATTTCTT

             87050     87060     87070     87080     87090     87100     87110     87120

Human        TTTCTT-----------------------ACCAAACCATAATATGATTATTACACTTACCAGAATTGACAATGATTTCTT  68866
PolarBear    TTTCTT-----------------------ATCAAACCATAATATGATGATCATACCCATCAAGTTTGACAATGATTTCTT  29343
Cow          TTTCTTA----GCAACCATACCTGATGGAAGTTGATTAAAACCTGATCATAAAACTTACCAAAATTGAGAATGGTCTCTT  66602
Dog          TTTCTTTTTTTTAATAATGACATTTTCTTATCAAACCATAATATGATTATCACACTCATCAAATTTGACAGTGATTTCTT  59997
BelugaWhale  TAT--------GTGAATTCACCTTCCTTTACCAAAT---GACTTCATCG-----TTTACCTGTTTT---AATGGTTTTAC  59446
Donkey       TTTCTT-----------------------ACCAAACCATAATGTGATTATCA-ACCTATCAAATTTTTCAATGATTTCTT  66046
Cat          TTTCTT-----------------------ACCAAACCATAATATGATTATCACACCCATCAAATTTGACAGTGACTTCTT  66712
Ferret       TTTCTT-----------------------ATCAAACCATAATATGATTATCATACCCATCAAATTTGACAGTGATTTCTT  67464
Lion         TTTCTT-----------------------ACCAAACCATAATATGATTATCACACCCATCAAATTTGACAGTGACTTCTT  64431
Vaquita      TAT--------GTGAATTCACCTTCCTTTACCAAAC---GACTTCATCG-----TTTACCTGTTTT---AATGGTTTTAC  60824
Pig          TTTTTCC----TTGAAAGCTCATGGCCTTGGTGGATTTTTTTTTAAGTA-ATAACAAGTCTTTTTGAAGAAAGATTATGA  59727

Majority     AATGTCATTGAATACCCAATCCATCTTCAAATTTCCCCAGTTGTCTCAAAAAA-TGTXTTTTAAGXGTTGATTTGTTCAG

             87130     87140     87150     87160     87170     87180     87190     87200

Human        AATGTCCTCAAATACCCAGCTTACATTAAAATTTCCCCAATTATTTTGAAAAT---GCTTTTAAAGTTTGATTTGTTCAG  68943
PolarBear    AATGTCATTGAATACCCAATCCATGTTCAAATTTCCCCAGTTGTCTCAAAAAA-TGTATTTTAAGAGTTGATTTGTTCAG  29422
Cow          AATGTTG--A-ATACCCAGTTCATCTTGAA-TTTCCCCAATTGTCTCAAAAAA-TGTTTTTTAAGGGTTGATTTGTTCAG  66677
Dog          AATGTCACTGAATACCCAATCCATCTTCAAATTTCCCCAGTTGTCTCAACA-----TATTTTAAGACTTGATTTGTTCAG  60072
BelugaWhale  ATCTT------ATAACTGGGTTTTTTTA-----TTTTAAATGTCTGCAGATAG-TTTTTGAAAATGGGTGAGTTATAAGT  59514
Donkey       AATGTCGTTGAATACCCAGTCCATCTTCAAATGTCCCCAGTTGTCTCAAGAAATGTTTTTTTAAGGGTTTATTTGTTCAA  66126
Cat          AATGTCATTGAGTACTCAATCCATCTTCAAATTTCCCTGGTTTTCTCAAAAAA-TGTATTTTAAGAGTTGCTTTGTTCAG  66791
Ferret       AATGTCATTGAATACCCAATCCATCTTCAA-TTTCCCCAGTTGTCTCAAAAAAATGTATTTTAAGAGTTGATTTGTTCAG  67543
Lion         AATGTCATTGAGTACTCAATCCATCTTCAAATTTCCCCGGTTTTCTCAAAAAAATGTATTTTAAGAGTTGCTTTGTTCAG  64511
Vaquita      ATCTT------ATAACTGGGTTTTTTTA-----TTTTAAATGTCTGCAGATAG-TTTTTGAAAATGGGTGAGTTATAAGT  60892
Pig          AGCCCTG--ACATAACTAACTTGTGTC-----TTGTTCAGTGCTTTTATAAGT-CATTTTTCAAGTACTGCACATTTTGC  59799
```

```
Majority     AATAGGATCCTCTCAAGGGCCATATATAGCATTTGGTTGTTATTTCTCXTAAGTCTCTATTAATCTG--TAXTA-GTCCT
                         87210     87220     87230     87240     87250     87260     87270     87280
Human        AACAAGGTCCTATGGAGGGCCGCACATAGTATTTGGTTGTTACATCTCATTAGTCTGTTTCAGTCTG--TAATA-GTCCT  69020
PolarBear    AATAGGATCCTCTCAAGGGCCATACATAGCATTTGGTTGTTATTTCTCCTAAAACTCTATTAATCTG--TGGTA-GTCCT  29499
Cow          AATAGGATCTTTTCAAGAGCCACATACTGCGTTTGGTAACTGTGTCTCCTGAGTGTCTATTATTCTGAAGATTA-GTGAT  66756
Dog          AATAGGATCTTCTCAAGGGCCATAGATTCCTTGTGGTTGTTATTTTTCCTAAGACTC--TAAATCTG--TGGTA-GTCAT  60147
BelugaWhale  TTTAAAAATCAAATAATGGA-ATTTATA--ATTT----ATGAGATAACTTATATTTTGATTTTTT-----CTTATTTTCT  59582
Donkey       AATAGGATCCTCTCAAGGGCCACACATGTCATTTGGTTGTTACGCCTCCTAAATCTCTCTTAATCTA--TAACA-GTCCT  66203
Cat          AATA---TCCTCTCAGGGGCCATACATGACATCTGGT---TATTTCTCTTAAGACCCTGGTAATCTG--TAGTA-GTACT  66862
Ferret       AATAGGATCTTCTCAAGGGCCATAGATAGCATTTGGTTGTTATTTCTTCTAAGACT--GTTAATCTG--TGGTA-GTCCT  67618
Lion         AATA---TCCTCTC--GGGCCATATATGACATCTGGT---TATTTCTCTTAAGACCCTGGTAATCTG--TA-------CT  64574
Vaquita      TTTAAAAATCAAATAATGGA-ATTTATA--ATTT----ATGAGATAACTTATATTTTGATTTTTTT----CTTATTTTCT  60961
Pig          TTTAAAAGAATGTGAATAAA-GTTTGCTCCATTC--CCAATAAATAAATTTGGTGTCTTCT-TCCAGGCTCTTATGTAGA  59875

Majority     CTCCC--TTCATCTTCACCCTCA----XTTCCATTGACTTAAT--------------GAAGAAACCTAATTATTTT----
                         87290     87300     87310     87320     87330     87340     87350     87360
Human        CTCCC---TCTCTCTACTCCCAA--TCTCTCATTCATTTACTT---------A--TGAAGTGATGTTTCCTTTT-----  69078
PolarBear    CTCCC--TTCATCTTCACCCTCA----GTTCCACTGACTTAAT--------------GAAGAAACCTAATTATTTT----  29555
Cow          ATAGA-GGTTATTTTTATATAGAGATTATTAAAGTCATCTTCTTCCACCCAACCCTCAGTTTCATTGACTTATTTATTAT  66835
Dog          CTCCC--TTCATCCCCACCCTCTC--CATTCCATTGACTTAAT--------------GAAGAAACCTAGAAATTT-----  60204
BelugaWhale  ATGTAAATACATATTTGTACAAA----ATTAAAATCACACTATT------------TGTTTTATTTGAATTATTCT----  59642
Donkey       CCCTC----CATCCTCACCTTCA----GATAGATTGACTTACT--------------GAAGAAACCAAGTTATTCTTTT-  66260
Cat          CTCCC--TTCATGCCCACCCTCA----GATCCATTGACTTAAT--------------GAGGAAACCTAATTATTTT----  66918
Ferret       CTCC------ATCTTCACCCTCA----GTTCCACTGACTTAAT--------------GAAGAAACCTAGTTATTTT----  67670
Lion         CTCCC--TTCATGCCCACCCTCA----GATCCATTGACTTAAT--------------GAGGAAACCTAATTATTTT----  64630
Vaquita      ATGTAAATACATATTTGTACAAA----ATTAAAATCACACT----------------GTTTTATTTGAATTATTCT----  61017
Pig          ATATTTGAATATTTTAATATATAC-TGACACGCCTCATTTTATTGTGCTTTGCAGATGCTTTTTTTTTTTTTTTTTTTTT  59954
```

```
Majority     ---GAGAAAGTCCTACAGTCTGGATTGGTGTGGCTGTTT----CTTTGTAGTGTCATCTT------AACTTGTTCCTC--
                              87370     87380     87390     87400     87410     87420     87430     87440
Human        ----AGAAAGTTTCACAGATTAGACTAGTCTGGCTGTTT----CTTTAGAGTGTCATGTT------AACTTGTTCCTC--  69142
PolarBear    -GAGAAAAAGCCCTACTGTCTTAATTGGTGTGGCTGTTT----CTTTGTAGTGTCACCTTT-----AACTTGTTCCTC--  29623
Cow          AGAAACCTAGTTATATCCTTTTAGAAAATGCCACAGTCTG--GATTGGTCTGGCTATAGTAGCATTAACTTGTTCTT---  66910
Dog          --GGGGAAAGTCCTACAAACTTGATTGGTGTGGCTGTTT----CTTTGTAGTGTCATCTT------AACTTGTTCCTC--  60270
BelugaWhale  ----ACTGAG--AATTTTTTTGAAAATTGATAATTATAATA-GCTTCATAACATTCTAGTGGATATATAATACTTTTG--  59713
Donkey       ----AGAAACTTCCACAGTCTGGATTG-TCTGGCTGTTT----CCTTGTAGTGTCATCTT------AACTTGTTCTTC--  66323
Cat          ---GAGAAAGTCCTACAGTCTGGATTGGTGTGGCTCTTT----TTTTGTAGTGTCATCTT------AACTTGTTTCC---  66982
Ferret       -GAGAAAAAATCCTTTAGTCTTGATTGGTGTGGCTGTT-----CTTTGTAGTGTCATCGT------AACCTGTTCCTC--  67736
Lion         ---GAGAAAGTCCTACAGTCTGGATTGGTGTGGCTCTTT----TTT-GTAGTGTCATCTT------AACTTGTTTCC---  64693
Vaquita      ----ACTGAG--AATTTTTTTGAAAATTGATAATTA------GCTTTATAACATTCTAGTGGATATATAATACTTTTG--  61083
Pig          GCAAATTGA---AGGTTCCATGGCAACCCTCCCTTGTCAGATGATGGGTAGCATTCTTTTAGCAGTAATATGTTTTTAAC  60031

Majority     -CATCCAT-TG-CATTTTTTATGTAAACCAGAAGTTAGCTCCAAAAGCTTGATTAGATTTAGATTCTGT----TATT---
                              87450     87460     87470     87480     87490     87500     87510     87520
Human        -TATCTGC-TG-TGTTTCTTATATAAACTTAAAGTTAGTTCCAAAGGCTTGATAAGATTTAGAGCCTTT-----------  69208
PolarBear    -CATCCAT-TG-CATTTTTTATGTAAACCAGAAGTTAGCTCCAAAAGCTTGATTAGATTTAGGTTCTGT----TATTTAT  29696
Cow          CAATCCAC-TG-CATCTTTTATATAAATAAGAAATTAGCTCCAAAAGCTAGATTAGATTCAGATTCTGT----TATT---  66981
Dog          -CATCCAC-TG-CATATTTTATGTAGACCAGAAGTTCGCTTCAAAAGCTTGATTAGATTTATGTCCTGT----TA-----  60338
BelugaWhale  -GAAGTAT-----GTTGGACATTTAGTC-------TACTTTCATAC-----------TTTTCAT-TAGTA---TAAA---  59762
Donkey       -CATCCACTGCGTTTTTTTAATGTAAACTAGAAGTTAGCTCCAAAAGCTTGATGAAGTTTAGGTTTTGTTATTTGTTTGC  66402
Cat          ----------A----------TGTGAACCAGAAGTTAACTCCAAAAGCTTGATTAAATTTAGATTCTGT----TATT---  67035
Ferret       -CATCCAT-TGGCATTTTTTATGTAAACCAGAATTTAGCTCCAAAAGCTTCATTAGATTTAGGTTCTGT----TATTTAT  67810
Lion         ----------A----------TGTGAACCAGAAGTTAACTCCAAAAGCTTGATTAAATTTAGATTCTGT----TATT---  64746
Vaquita      -GAAGTAT-----GTTGGACATTTAGTC-------TACTTTCATAC-----------TTTTCAT-TAGTA---TAAA---  61132
Pig          TAAGGTATATA-CATTGTTTTTTTAGACATAATGCTGTTTGCACA--CTTAAC-AGATTACAATATAGTG---TAAA---  60101
```

```
Majority     -TATATTTTTCAA--------XCAAGAATACTTAATAG---XTXTTCTTGTGTTTCATATTGTATXT--GTTGGAAGCAT
                      87530     87540     87550     87560     87570     87580     87590     87600
Human        -TTTTTTTTTGA------------------------AG---TGTTTCTTGTGCTTCCTATTGTGTCTCAGTAGGAGACAT  69260
PolarBear    TTATATTTTTCAA--------GCAAGAGTACTTTATAG---GTGTTCTTGTGTTTCATATTGTATCTCAGTTGGAAGCAT  29765
Cow          -TACATTTTTAAA--------GCAAGAATATGGAATAT-GATGTTCCTTGTGATCTCAGTTGGAAGC--GTAAAATATAT  67049
Dog          -CATTTTTTTCAA--------GCAGGAATACTTTATAGTAGGTGTTCTTGTGTTTCATGTTGTATCTTAATTGGAAACAT  60409
BelugaWhale  -TGGTGCTGTGAC--------AAACACCTGTACA-------TAACTTTTTTGTCTCTTATT------------TACTTAG  59814
Donkey       TTATATTTTTAAA--------GCAAGAATACTTTATAG--GTGTTCCTTGTGCTTCATATTGTATCTTGGTTGGAAGCAT  66472
Cat          -TCTATTTCTCAAGAATATTCATAGGAATACTTCATAG---GTGTTCTTGT--TTCATGTTGTATGTCTATTGGAAGCAT  67109
Ferret       TCATATTTTTCA---------GCAAGAATACTTCATAG---GTATTCTTGTGTTTCATA---------------------  67857
Lion         -TCTATTTCTCAAGAATATTCATAGGAATACTTCATAG---GTGTTCTTGT--TCCATATTGTATGTCTATTGGAAGCAT  64820
Vaquita      -TGGTGCTGTGAC--------AAACACCTGTACA-------TAACTTTTTTGTCTCTTATT------------TACTTAG  61184
Pig          -CATAGTCTTTAT--------ATGC-ACTGGGAAACTA-AACAAATTGTATGACTCACGTTATG-----GCAATGCTCAC  60165

Majority     AAAACATCTGGTTATCCCAC---------TTTTAGTGATGCTTTAAGATAGATTG-TGGTTCAGGTGGTGACATTCTGAT
                      87610     87620     87630     87640     87650     87660     87670     87680
Human        CTAATATCTGGTTGCCCCACTTTTAGTGATTTTAATGATGCTTTAAGACTGATTGTGGATTTGGGTGTTGACATTCTGAT  69340
PolarBear    AAAATATCTGGTTATCCCAC---------GTTTGGTGATGCTTCAAGATGAATTG-TGGTTCAGGTGGTGACATTCTGAT  29835
Cow          GGTTGGGTCACTTTTAGTGA---------TTTTAGTGATACTTTAAGATTGT----GGTTTCAGGTGGTGACATTCTGAT  67116
Dog          AAAACATCTGGTTATCCCAC---------ATTGAGTGTTGCTTTAAGACAGATTG-TGGTTCAGGTGGTGACATTCTGAT  60479
BelugaWhale  ACTAG----ATTCCTAGAAC---------TAGAATCATTGGGTCAAAGAAAA----TGTTAAAGCCTTCTGAATCTTGTT  59877
Donkey       AAAACGTCTGATTATCCCACTTTCAGTGATTTTAGTGATGTTTTAAGATTGATTGTCGTTTCAGGTAGTGACATTTTGTT  66552
Cat          AAAACATCTGGTTATCCCAC---------TTTTAGTCATGTTTTAAAATAGATTG-TAGTTTAGGTG------TTATGAT  67173
Ferret       -AAACATCTGGTTATCCCAT---------GTTTAGTGATGCTTTAAGATGGATTG-TGGTTCAGGTGGTGACATTCTGAT  67926
Lion         AAAACATCTGGTTATCCCAC---------TTTTAGTCATGTTTTAAAATAGATTG-TAGTTTAGGTG------TTATGAT  64884
Vaquita      ACTAG----ATTCCTAGAAC---------TAGAATCATTGGGTCAAAGAAAA----TGTTAAAGCCTTCTGAATCTTGTT  61247
Pig          TTTCCTATGATACTTGCT-----------TTATTGTGGTGGTCCGGAACCAA----AGCTGCAGTATCTGAAGTATTCCT  60230
```

Majority	CCTTCCATTA-TATTTTTC-TACTAATGGTTTXAGTATCCACTAATGATTGCTGC-CTTAATTXATTTCATTTGGGGGTT	
	87690 87700 87710 87720 87730 87740 87750 87760	
Human	CCTTTCGTTACTTTTTTTTCTACTAATGATTTTAGTATCCAATAATGGTTGCTGC-CTTAATTAATTTCACTAGTGGGTT	69419
PolarBear	CCTTCCATTG-TATTTTTC-TACTAATGGTTTGACTTTCCACCAGTGATTTGTGC-CTTAGTTTATTTTGTTTGGGGTTT	29912
Cow	CCTTCCATTT-TATTTTTC-TATTAATGGTTTTAGTATCAACTAATGGCTGCTGC-CTTTATT----TCATTTGGAGGTT	67189
Dog	CTTTCCATTA-TATTTTTC-TACTAATGGTTTGAGTTTCCACCAGTGATTGGTGC-CTTAATT-ATTTCATTTGGTGTTT	60555
BelugaWhale	GCAGATTT-----CTTTCTAGAATGGTGATATTAATTTAAACTAACATGAATTGTATATGA--------GGCTGGCTATT	59944
Donkey	CCTTCCATTA-TATTTTTC-TAGTAATGGTTTTAGTATCCCCTAATGATCGCTGC-CTTAATTTATTTCATTTTGGGGTT	66629
Cat	CCTTC-ATTA-TATTGATC-TA---ATGGTTTGAGAATCCACCAGTAATTGCTGC-CTTAATTTATTCTATATGGGGGTT	67246
Ferret	CCTTCCATTG-TATTTTTC-TACTTATGGTTTAAGTATCCACCAATGAATTGTGC-CTTAATTTATTTCATTTGGGAGTT	68003
Lion	CCTTC-ATTA-TATTGATC-TA---ATGGTTTGAGAATCCACCAGTGATTGCTGC-CTTAATTTATTCTATATGGGGGTT	64957
Vaquita	GCAGATTT-----CTTTCTAGAATGGTGATATTAATTTAAACTAACATGAATTGTATATGA--------GGCTGGCTATT	61314
Pig	GCAGTTTCTG-CACTCTTCCTGATGAGGACAAGGAAAGTATTTTGTGGCGAAAACACGTGAAT----TCATCTTCTTTTA	60305

Majority	GCAAATG-TTGCCTT----TCCCCCCTAATTCACATATTTC---TCTACATTAATTAGTTGAAAATCTTTATA-----GA	
	87770 87780 87790 87800 87810 87820 87830 87840	
Human	GCAAATA-TTGCTTTT---TCTCCCCTAATTCACATATTCCTTCTCTACATTAATTAGCTGAAAATCTTTATAAAGTAGA	69495
PolarBear	GTGAATG-TTGCCTTCCCACCCTCCCCAATTCACGTATTTC---TCTACATTAATTAGTTGAAAATCTATATA-----GA	29983
Cow	GCAAATT-TTGGATT----TTTCTTCTGATTTAAACATTT--CTTCTACAATAATTGGTTGAAAGTGTTTAGTG----TT	67258
Dog	GCAAATG-TTGCCTT----TCCCCCCTAATTGACATATTTC---TCTACATTAATTAGTTGAAAATCTTTATA-----GA	60622
BelugaWhale	GTACTTT-TTCCTCT----ACCGGCTTATTATAATTGTTAT-TATTTTATGAACTAGTTTATAATTTTTCATTG----CT	60014
Donkey	GCAAGTG-TTCCTTTTT--TTCCCCCTAATTCACACATTCC--TTCTGTATTAATTAGTTGGGAATCTTTATAAAGTAGA	66704
Cat	GCAAATG-TTGCCTTC---CTCCTCCCCATTCACATATCCC---TTTCCATTAATTAGTTGAAAATCTTTGTA-----GA	67314
Ferret	GCGAATG-TTGCCTT----TCCCCCCTAATTCACATATATC---TTTACA----TTAGTTGAAAATCTTTAAAA----GA	68067
Lion	GCAAATG-TTGCCTTC---CTCCTCCCCATTCACATATCCC---TTTCCATTAATTAGTTGAAAATCTTTGTA-----GA	65025
Vaquita	GTACTTT-TTCCTCT----ACCAGCTTATTAT----GTTAT-TATTTTATGAACTAGTTTATAATTTTTCATTG----CT	61380
Pig	GTAAATAACTTCACC----ATTTACCTGTTTTTATGGTTTTACATCCCATATAAATGGGTTTAATTATTTATTT----TT	60377

```
Majority     ATTTTCC--------TTCATCAACT--------AGGXCTGTTTACCTTGTAATTXAATTTGTTCAXGAC---T---AGTT
                      87850     87860     87870     87880     87890     87900     87910     87920
Human        ATTTTCC--------TTCATCAACT--------AGAATTATCAACCTTGTAATTCAATTTGTGCAGGACAATT---AGTT  69556
PolarBear    GTTCTCC--------TTCATCGACT--------AGGGCTGTTTACCTTGTAATACAGTTTGTACAGGAC-------AGTT  30040
Cow          CCTTTGTCAACTAGGGCTATTTACAGTTTGTGCAGGACAGTTAGTTAAGTTCTTGAATTTATTTGCTAATTTTTA---GT  67335
Dog          GTTTTCC--------TTCATCAACT--------AGGACTGTTTACCTTGTAATGCAATTTGTGCAGGAC-------AGTT  60679
BelugaWhale  TATTAG----------GTACTTACA---------AGTCTCTGAATTGGTTGTCTGGATTCTTTCTAT-----TAGGACGT  60070
Donkey       ATTTTCC--------TTCTTCAACT--------AGGGCTGTTTACCTTGTAAT-TGGTTTCTGCAGGACAGTT---AGTT  66764
Cat          ATTTTCC--------TTCATCAACT--------AAGGCTGTTTACCTTATAAT-TGGTTTGTTCTAGAC-------AGTT  67370
Ferret       GTTTTCC--------TTCATCAACT--------AGGTCTATTTACTTTGTAATACAATTTGTACAGGAC-------AGTT  68124
Lion         ATTTTCC--------TTCATCAACT--------AAGGCTGTTTACCTTATAAT-TGGTTTGTTCTAGAC-------AGTT  65081
Vaquita      TATTAG----------GTACTTACA---------AGTCTCTGAATTGGTTGTCTGGATTCTTTCTAT-----TAGGATGT  61436
Pig          AATTTTTA---TGGCTATACTCACAGCAGGTGGAAGTTC-CCAGGCCAGGGATTGAATCTGAGCCACAGCTGTGGCATGT  60453

Majority     --AAATTCTTGCATTTATTTTXT---------------TGGTTTTAGGTGGAAGAAATTGATGCCCAGCTTT--------
                      87930     87940     87950     87960     87970     87980     87990     88000
Human        --AAATTCTTGCATTTGTTTGCT------------------------AATTTTAAGTTGGTGCCCAGCTTT--------  69601
PolarBear    --AAATTCTTGCATTTATTTTTT---------------TGGTTTTTGGTGGAAGAAATTGATGCCCAGCTTT--------  30095
Cow          GGAAGAAGTTATGTCCAGATTTTGCTTTTAGTTTATTATG-TGTGAGTGGGTACAGACTAGATTCTTCCCTACTTCTGCT  67414
Dog          --GAATTCTTGCAATTATTTGTT-----------------AGTTTTGGTGGAAGAAATTGACGCCCAGCTTT--------  60732
BelugaWhale  ACAAAACGTAAAATGTATATTA-------AGGATATTATGTTCATATTTGGTATGAACTA-AGCATTCA--------ACT  60134
Donkey       --AAATTCTTGCATTTATTTGCT----------------AGTTTTGAGTGGAAGAAGTTGATTCCCAGCTT---------  66817
Cat          --AAATTCTTGCGTTTATTTACT----------------AGTTTTAGGTGGAAGAAATTGATGCCCAGCTTT--------  67424
Ferret       --AAATTGTTGCATTTATTTGTTGGA---TTTATTTGTTGGTTTTTCCTGGAAGAAATTGATGCCCAGCTTTG----TTT  68195
Lion         TTAAATTCTTGCATTTATTTACT----------------AGTTTTAGGTGGAAGAAATTGATGCCCAGCTTT--------  65137
Vaquita      ACAAAACGTAAAATGTATATTA-------AGGGTATTATGTTCATATTTGGTATGAACTA-AGCATTCA--------ACT  61500
Pig          GATGTACGCTGCATGCTGGAT---CCTTTAACCCACTGTGCTGTGTTGGGGATCAAACTCTTGCCTTCCCCTGCAGTGAC  60530
```

```
Majority     -TGTTTTT-----------T-ATTAATTATTTAAACCAGTTT--A-XTGAATGGGTACAX----CCTAGATTCTTCCCAA
                     88010     88020     88030     88040     88050     88060     88070     88080
Human        -TGTTTTCA------------GGTAAGTACTTAAACCAGCTT----ATGAACAGGTACAG----TATAGATTCTTCCCAC  69660
PolarBear    -TGTTTTT-----------TCATTAATTATTTAAACCAGTGT--GTATGAATGGGTACAG----CCTAGATTCTTCACAA  30157
Cow          TTAAACTTGAAACTCTTAGTTACTTCCAGGTTTTGATAGCCCTAGAGTGGTTTGAGATGGTGACCTTACCCTAGAAGTGA  67494
Dog          -TGTTTTT----------TTCATTTATTATGTAAACCTTTTC--A--TGAATGGGTACAA----CCTAGATTCTTTCCAA  60793
BelugaWhale  TTATACTTATA--TTTTAG--ACTGGCTAGTTACGGAA------AAATATTTTAAAATAA-----TTCATCACAATCCAA  60199
Donkey       -TGGTTTT---------------TACTAAACCAATTTA---------TGAAAGGATCAAG----CCTAAATTCTTCCCAG  66868
Cat          -TGTTTTT-----------T-GTTAATTATTTAAACCAGTTT--A--TGAATGGGTACAA----CCTAGATTGTTCCCAA  67483
Ferret       TTGTTTTTGTTTTTGTTTTTTATTAATTATTTAAACCAGTAT--G--TGAATTGGTACAG----CCTAGATTCTTCCTAG  68267
Lion         -TGTTTTT-----------T-GTTAATTATTTAAACCAGTTT--A--TGAATGGGTACAA----CCTAGATTCTTCCCAA  65196
Vaquita      TTATACTTATA--TTTTAG--ACTGGCTAGTTACGGAA------AAATATTTTAAAATAA-----TTCATCACAATCCAA  61565
Pig          CCAAGCCGCTGCATTTGGATTCTTAACCCACTGTGCCACAGTGGGAATGCCCAGATTTTTAAATTTTAAATATCCTCAAA  60610

Majority     XTTCTGCT---------T---TTAAACTTTAGAAACT------GTAGGTTGTTTCX------------------------
                     88090     88100     88110     88120     88130     88140     88150     88160
Human        TGTCTGCT-------------TTAAACTTTTGAAACT------GTCAGTTGTTTGC------------------------  69697
PolarBear    -TACTGCT---------T----TAAACTTTAGAAACT------GCAGGTTGCCTCC------------------------  30193
Cow          GTGTTTTT-----TAATTTATTTATGTTGTTATTAT----TA-TTTGATCTTCAGTTCAGTTCAGTCGCTCAGTCGTGTC  67564
Dog          -CTACAGT---------T----TAAACTTCAGAAAGTAAGTAAGTAAGTTGCCTCT------------------------  60835
BelugaWhale  ACGTTTCC-----------ATTTACAGGACATTAAG-------GTGGCTTTTTTTTACCCCCCACACCTTTGGTGG----  60257
Donkey       CTTCTGCT-------------TTAAACTTTAGAAACT------GTTGGTTGTTTCC------------------------  66905
Cat          CTTCTGCT---------T----TAAACTTCAGAAACT------GTAGGTTGCTTCTGCTTTAAACTTCAGAAACTG----  67540
Ferret       -TACTCCT---------T----TAAACTTTATAGACT------GCAGGTTGCCTTC------------------------  68303
Lion         CTTCTGCT---------T----TAAACTTCAGAAACT------GTAGGTTGCTTCC---T--------------------  65234
Vaquita      ACGTTTCC-----------ATTTACAGGACATTAAG-------GTGGCTTTTTTTTACCCCCCACACCTTTGGTGG----  61623
Pig          TAGTTTTTGGAAGTAGGTAAGTTATGTTTTAAAAATCAAATA-GTGGAATTTAAGAGATAGCCACTTATATTTTGATGTC  60689
```

```
Majority     -AGGTT--CTGGTAGCCCCAGA-GTGA-------------T---TTGAGATAGTGACCTTAGTCCAGAGGTGA-XCAGTT
                       88170     88180     88190     88200     88210     88220     88230     88240
Human        -AGGTT--CTGGTAGCCCAGAG--TGG----------------TTTGAAATGGTGACCTTAGCCCAGAAGT----------  69747
PolarBear    -AGGTT--CTGGTAGCCCCAGA-GTGA-------------T---TTGAGATAGTGACTTTCGTCCAGAGGTGA-GCTGTT  30252
Cow          CAACTC--TTGGCCACCCCATGAGTCACAGCACGCCAGGCCTCCCTGT--CCATCACCATCTCCCAGAGTTCACTCAAAC  67640
Dog          -AG-TG--CTGGTAGCCTCAAATGTGG-------------T---TTGAGATAGTGACCTTATTCCAGATGTGA-GCTGTC  60894
BelugaWhale  GTACCG--TTATCTTTATGAGGAACCA-------------CTTACTGT-----TTTGCTTTTTAAAGTAGTCA-TCAA--  60314
Donkey       -AGGTT--CTGATAGCCCCAGA-GTGA----------------TTTGAGATAGTGACCTTAGCCTAGAGGTGA-TCAGTT  66964
Cat          TAGGTT--CTGGTAGCCCCAGA-GTGG-------------T---TTGAGATAATGGCCTTAGCCCAGAGGTGA-GCAGTT  67600
Ferret       -AGGTT--CTGGTAGCCCCAGA-GTGA-------------T---TTGAGATAGTGACTTTAGTCCAGAGGTGA-GTAGTT  68362
Lion         --GGTT--CTGGTAGCCCCAGA-GTGG-------------T---TTGAGATAATGGCCTTAGCCCAGAGGTGA-GCAGTT  65292
Vaquita      GTACCG--TTATCTTTATGAGGAACCA-------------CTTACTGT-----TTTGCTTTTTAAAGTAGTCA-TCAA--  61680
Pig          GTTCTTATTTTTCTATGTAAATAGATATTTTGTACAGAATCACCCTGTATTTACTATTTTATTTGAACTGTCACTCTA-C  60768
```

```
Majority     T--TXTTTAT----GTAATAATGATTTT-----TTATTTTG-GTCTTAGCCAGTGGXCCAAAAAAGGGAAAX--AGTGGT
                       88250     88260     88270     88280     88290     88300     88310     88320
Human        --------AT-------GTAAT--TATT------TAATTTG-GCCTTGACCAGTGGCCCAAAAAAGGAAGG---GGGTGG  69800
PolarBear    T--TGTTTATTAACGTAATAATTATTTT-----TTATTTTG-GTCTTAGCCAGTGGTCCAGAAAAGGGAAT----G-GGT  30319
Cow          TCACGTCCATCGAGTCAGTGATGCCATC--CAGCCATCTCATCCTCTGTCGTCCCCTTCTCCTCCTGCCCCC--AATCCC  67716
Dog          T--GGCTTATCAATGTAATAATGTTTTT-----TTATTTTA-GTCTTAGCCAGTGGCCCAAAAGAGGGAAGC--AG-GGT  60963
BelugaWhale  --ACACTCTA------AATAGTGACATACACTGTTATGTTA-GTGAGGTCATGCCTTC--TGGAATGGTGACTAAATTTT  60383
Donkey       T--GATTTAT-------GTGA--ATTTTGGGGGTTTTTTTG-GACTTGCCCAATGGCCCAAAACAGGAACG-------GG  67025
Cat          T--TATTTAT----GTAATAATAATTTT-----TTATTTTG-GTCGTAGCCAGTGGCCCAAAAAAAGAAAAG--GGTGGT  67666
Ferret       T--TGTTTATTAATGTAATAATTATTTT-----TTATTTTG-GTCTTACTTAGTGGTCCAAAAAAGGGAAT----G-GGT  68429
Lion         T--TATTTAT----GTAATAATAATTTT-----TTATTTTG-GTCATAGCCAGTGGCCTAAAAAAAGAAAAG--GGTGGT  65358
Vaquita      --ACACTCTT------AATAGTGACATACACTGTTATGTTA-GTGAGGTCATGCCTTC--TGGAATGGTGACTAAATTTT  61749
Pig          TGAGGATTTTTGGGAAAATGATAATTCTAATAGCTGCATAGCATGGTAATGGGTATATAACATAATACTTTTGGAAGTAT  60848
```

```
Majority     GGTGGATAAGGGACTAAA---CAGAGTTAATTAA-AATTTAGATATGTTTCT--------CTCAAAAAAXAATT-CATCX
                       88330     88340     88350     88360     88370     88380     88390     88400
Human        GGTGGATAAAGGACTAAA---CAAAATTAATTAA-AATTTAGAAATTTTTCT--------CTCAGAAGGAA-TT-CATGC  69866
PolarBear    GGTGGATAAGGGACTAAA---CAGAGTTAATTAA-AATTTAGATCTGTTTCT--------CTCAAGAAGGAATTTCATCC  30387
Cow          TCCCAGCATCAGAGTCGTTTCCAA--TGAGTCAGCTCTTTGCATGAGGTGGCCA-AAGTACTGGAGTTTCAGTTTTAGCA  67793
Dog          GGTGAATAAGAGACTAAA---CAGAGTTAATTAA-AACTCAGATATGTTTCT--------CTCAAAAAAGAATT-CATCC  61030
BelugaWhale  TTTCTCTGTTTA-TTTGCTTCTAGTGATATTTAGTAAGTCAGGTGACTTCA----GAGTACAACAAACTAA-TATTGATA  60457
Donkey       CCTGGATAAAGGACTAAG---TAGAGCTAATTAA-AATTTAGATATGTTTTT--------CTCACAAAAGAATT-TATCC  67092
Cat          GGTGGATAAG-GACTAAA---CAGAGCTAATTAA-AATTTAAATATGTTTCT--------CCCCAAAAATAATA-CATCA  67732
Ferret       GGTAGATAAGGGACTAAA---CAAAGTTAATTAA-AATTTAG-TATGTTTCT--------CTCAGGAAGGAATT-CATCC  68495
Lion         GGTGGATAAG-GACTAAA---CAGAGCTAATTAA-AATTTAAATATGTTTCT--------CCCCAAAAATAATA-CATCA  65424
Vaquita      TTTCTCTGTTTA-TTTGCTTCTAGTGATATTTAGTAAGTCAGGTGACTTCA----GAGTACAACAAACTAA-TATTGATA  61823
Pig          GTTGGACATTTAGTCTACTTTCATACTTTTTTATTAATATAAATGGCACTGTGACAAACGCTTAAAATAACTTTTTTGTT  60928

Majority     CCCTAAAATA------AAGAACCAGG-------------AAGCATTAGTATTTCCTTACTAAATACCAATAGAATATAAA
                       88410     88420     88430     88440     88450     88460     88470     88480
Human        CCATAAAACA------AA-GACCAGG-------------ATACATTCGTATTTTATCGGTAAATACCAATAGATTATAAA  69926
PolarBear    CCCTAAAATA------AAGAACCAGG-------------AAGCATTAGTATTTCCTTACTAAACACCAATAGAATGTAAA  30448
Cow          TCATTCCTTCCAA-AGAACACCCAGTCTTAATGATCCCAAAAAGGAAGAGGGGAGTGGTTAAAGGACTAAAGAGAACTAA  67872
Dog          CCCTAAAATA------AAGAACCAGGAATGCACCAGG--AAGCGTTAGTATTTCCTTACTAAATACCAACAGAATATAAA  61102
BelugaWhale  GGATCCATTT-----------CCTGCTTGAGTCTTCCTCAAACAGTACTTGTGCAAAATGAAATAATTGCTCGGTATCCT  60526
Donkey       TCCTAAGATA------AAGAACAAG---T----------AAGCATTAATATTTTCTCACTAAGTACCAGTAGACTATAAA  67153
Cat          CCCTAAAATA------AAGAACTGGG-------------AAGCATTAGTGTTTCCTTACTAAATACCAAAAGAATATAAA  67793
Ferret       TCCTAAAATA------AAGA-------------------AAGCAGTAGTATTTCCTTACTAAATACCAATAGAA-ATAAA  68549
Lion         CCCTAAAATA------AAGAACTGGG-------------AAGCATTAGTGTTTCTTTACTAAATACCAAAAGAATATAAA  65485
Vaquita      GGATCCATTT-----------CCTGCTTGAGTCTTCCTCAAACAGTACTTGTGCAAAATGAAATAATTGCTCGGTATCCT  61892
Pig          TCTCAGATTAGATTACTAGAACTAGACTCATTGGGTC--AAAACAAGTGTTAACATCTTCAGATGCTTTTTGCAAATTTC  61006
```

```
Majority     TCACTATTCTCXTGXAGCTAATTGTGAAAXAAAAAAXAACCTACA-GATA-X-----AGTGTGAGTTGTTTTCTCAAAGG
                      88490     88500     88510     88520     88530     88540     88550     88560

Human        TCACTTTTCTCGTATACATAATTGTGAAAAAAAAAA-ACCATCCA-GGTA-TGC----ATGTTATTTATTCATTGAGAGG 69999
PolarBear    TCCGTTCTCTCTTGTAGCTAATTGTGAGAAAAAAAAT-ATCTACA-GATAAG-----AGTGTGAGTTGTTTTCTCAAAGG 30521
Cow          T--CAAATTTAGGTATATTTATCTCAAACAAGAATTCATCCCATGAAATAACCAGGAAGCATAAAAACTTTTTTCGTAAA 67950
Dog          TCACTTTTCTCTTACAGCTATTTGTGAGGGAAAAAATGATCCACA-AATAATG----AGAATGAGTTGTTTTCTCAAAGG 61177
BelugaWhale  T--TTATTTCAAAGAT-TCTGT-----AAGAACACAGATACTAGGTGATA--------------ATTCTTTTCTGCTAAAT 60585
Donkey       TCACT--TCT--TATAGCTAATTGTG-AGAAAAAAATAACCTCCA-GACA-TG----AGTGTGAGTTATTTACTCAAAGG 67222
Cat          TCACT--TCTCCTGCAGCTACTTATGAGGG-AAAAAAGACCCACA-GATATG-----AGTGTGAGTTGTTTTCTCAAAGG 67864
Ferret       TCTCTCCTCTCTTGTAGCTAAGTGTGAGAAAAAATG--ATCTACA-GATCA-------GTGTGAGTTGTTTTCTCAAAGG 68619
Lion         TCACT--TCTCCTGCAGCTACTTATGAGGGGAAAAAAGACCCGCA-GATATG-----AGTGTGAGTTGTTTTCTCAAAGG 65557
Vaquita      T--TTATTTCAAAGAT-TCTGT-----AAGAACACAGATACTAGGTGATA--------------ATTCTTTTCTGCTAAAT 61951
Pig          TTTCTAGTATGGCGATGTTTAT-TTAAACTAACATAAATGTTATATGATG-CTGACTATTCTACTTTTTCTCCTACCAGG 61084

Majority     ATTAAXATCCCTTAGGTGTTCTTTT-CCTTGTATCTXTGT-----GTATGTGCAGTCT------------------GTCT
                      88570     88580     88590     88600     88610     88620     88630     88640

Human        ATATACATCTCTCAGGTGTTTTTTG---TCGTATGTGTAC-----TTTTGTGTAGTCT------------------GCCT 70053
PolarBear    ATTAAAATCCCTTAGGTGGTGTTTT-CCTTGTATCCGTGT-----GTATGTGTA----------------------GTCT 30573
Cow          ACCTAGTGG-ACTAAAATCACTTCTCTATTAAAACTAACTGTGGCGGGGGTGGGGGGCCCCCTCCACCAGTGTGTTATTA 68029
Dog          ATTAAAGTCCCTTAGGTGATGTTTC-CCTTGAATCCTTGT-----GTATATGCAATCT------------------GTCT 61233
BelugaWhale  ATTTAGGGGGATTCACTTTACTTTG-TATGATTTTTATAT-----CTAAGCACTTGACTA--------AATTTA--ATTA 60649
Donkey       ATTAACATCTCCCAGGTGTTCTTTT--TCTTTACCTGTGT-----GTATGTGCAGTCTG----------------TGCCT 67279
Cat          ATTAACATCCCTTAGGTGTTCTTTT-CCTTGTATCTGTGG-----GTATGTGCAGTCT------------------GTCT 67920
Ferret       ATTAAAATACCTCAGGTGGTGTTTTTCCTTGTATTCATGT-----GTATTTGTA----------------------GTCT 68672
Lion         ATTAACATCCCTTAGGTGTTCTTTT-CCTTGTATCTGTGG-----GTATGTGCAGTCT------------------GTCT 65613
Vaquita      ATTTAGGGGGATTCACTTTACTTTG-TATGATTTTTATATTATATCTAAGCACTTGAC--------------TA--ATTA 62014
Pig          ATTGAGTATTATTATTTTCTACTTGCTACAATATTAATTTTCTAAGAATGTGTCAGGTTATTACTTTTAATTT--TATTG 61162
```

```
Majority     GTTTA------TACTTTTTAAATAT--GATTCTATAAGA-TATGCAGTAAAAACAX--------ACATCTTTTGTGAAAC
                               88650     88660     88670     88680     88690     88700     88710     88720
Human        GTTTA------TTCTTTTTGAATA-------GGATGATA-TGTACAATTAAAAAAA----------ATCTTTTGTGAAAT  70109
PolarBear    GTTTA------TACTTTTTAAATAT--GATTCTATAAGA-TGTGCAGTAAAAACAA--------ACATCGTTAGTGAAAC  30636
Cow          ATTCAGTCAAAAAGTTAGCATCTTTTTCCTTGTATCCATATGTATGTTTGTTCTGTGTCTTTTTATTTTTTTAATATGAT  68109
Dog          GTTTA------TACTTTTTAACTGT--GATTCTATAGGT-TATGCAGTAAAAACA------------TCTTTTGTGAAAC  61292
BelugaWhale  ATTTG-GAATCCGTATTATAAATTTTAATTGGTTTATTT-TACACAGTAATTTT-----TTAAGACTAGCATTCTTGGCT  60722
Donkey       GTTTA------TTCTTTTTGAATAT--GATTCTATAACA-TATTCAATAGAAACA------------TCTTTTGTGAAAC  67338
Cat          GTTTA------TACATTTTTAATAT--GGTTCTATAAGC-TATGCAGTAAAAACAA--------ACATCTTTTATGCAAC  67983
Ferret       ATTTA------TACTTTTTAAATA-----TTCTGTGGGA-TGTGCAGTAAAAACAA--------ACATCTTTTGTGAAAC  68732
Lion         GTTTA------TACATTTTTAATAT--GGTTCTATAAGA-TATGCAGTAAAAACAA--------ACATCTTTTATGCAAC  65676
Vaquita      ATTTG-GAATCCGTATTATAAATTTTAATTGGTTTATTT-TACACAGTAATTTT-----TTAAGACTAGCATTCTCGGCT  62087
Pig          TTTTA-TGAGCTAGTTTATAATTTTTAAATGCTTATTAGATGTTTACAAGTCCT-------TG-AGTTGGTTGTCTGGAT  61233

Majority     ATTGCTTTCTTA-TATATGCTATTTCA------GTAGAGTGATGAG-GGGGAAAACTACAAAXCTAGACTAATTGGAACT
                               88730     88740     88750     88760     88770     88780     88790     88800
Human        GTGGTTTTCTTA-AATACATTATTGCATTTCATACACAGTGATGAA-GGGAAAAACTACAAAGCTAGACAGATTGCAA--  70185
PolarBear    ATTGCTATCTTA-TATATGCTATTTCA------GTAGAGAGATGAG-GGGGAGAACTGCAAAGCTAGACTAATTGGAA--  30706
Cow          TCCGTAGGTGTGCAGTAAAAAACATCTTCT---GTGAAACATGTCT-CATGTATCCCATTACAATAGAGCAATGGGGGCA  68185
Dog          ATTGCTATCTTA-CATGTCTTATTTCA------GTAGAGTGATGAT-GGGGAAAACTACAAAGCTAAACTAATTGGAAAT  61364
BelugaWhale  TCTTTTTTTTAGTGCTAGGGACCAC------------AGTGTCTC--CAAAAATGTAATTTATTCAAG--AATGGAGACT  60786
Donkey       AGGGCTATCTTA-TGTATGCAATTACA-C-----TAGACTGATGAGTGGGGGAAAAAACAAAGGCAGACTAATTGGAAAT  67411
Cat          ATTGC------A--ATATGCTGTTTCA------GTAGGGTGATGAG-GGGGAAAACTACAAATCCAGACAAATTGGAAAT  68048
Ferret       ATTGCTGTCTTACTGTATGCTATTTCA------GTAGATTGGTGAG-G----AAACTGCAAAGCTAGACTAATTCGAACT  68801
Lion         ATTGC------A--ATATGCTGTTTCA------GTAGGGTGATGAG-GGGGAAAACTACAAATCCAGACAAATTGGAAAT  65741
Vaquita      TCTTTTTTTTAGTGCTAGGGACCAC------------AGTGTCTC--CAAAAATGTGATTTATTCAAG--AATGGAGACT  62151
Pig          TCTTTCTATGGGATGTGCAAAACAT--------GTAAAATGTGTAT-TTAGGATATTACATTTATATT-TAGTATGAGCT  61303
```

```
Majority     TA---------ATTAGTATGT-AGTAGTGAGCTAATAGAGCAGTACTCAXAT-ATTTTXTTATA-----ATTTAAGTGAT
                       88810     88820     88830     88840     88850     88860     88870     88880
Human        -----------ATTAGTATTGCAGTAGTGAGGTAATAGAGCAATACCCAAATGTTTTCTTAAAA--TATATGTAGGTGAT  70252
PolarBear    -----------ATTAGTATGT-AGCAGTGAGCTGTTAGAGCAGTATTCATAT-ATTTTCTTATA------TTTAAGTGAT  30767
Cow          GGGAACTACAAAGTTGAAAATTAGTATTGAGCTAATAGAGCAGTAATCGAATATTGTCTTAACAAATATATTTAAGTGCT  68265
Dog          TA---------ATTAGTATGTTAGCAGTGAGCTAATCGAGCAGTACTCATATTATTTCCTTATA------TTTAAGTGAT  61429
BelugaWhale  GAGGGCTTTTTAATTAGTTAGAAATCTGACATTTAAA------TACGTAATCACAAACTTGGCA-----GACTATTAGTT  60855
Donkey       TA---------ATTAGTATTGTAGTAGTGAGCTAATAGAGTGGTACTCTCTT-TTCTTACAGTAT-----ATTTAATGAT  67476
Cat          TA---------ATTAGTATGT-CGCAATGAGC-AATAGACCAATACTCATCT-ATTTTCTTATAA-TATATTTAAGTGAT  68115
Ferret       TA---------ATTAGTAGGT-AGCAGTGAGCTAATAGAGCAGTACTCATAT-ATTTTCTTATA------TTTAAGTGAT  68864
Lion         TA---------ATTAGTATGT-CGCAATGAGC-AATAGACCAATACTCATCT-ATTTTCTTATAA-TATATTTAAGTGAT  65808
Vaquita      GAGGGCTTTTTAATTAGTTAGAAATCTGACATTTAAA------TACATAATCACAAACTTGGCA-----GACTATTAGTT  62220
Pig          AA-AGCTTCAACTTTATACATATATTTTAGACTGGC----CATTTATGGAAAAAAATTTTAAAA----TAATTCATCCCA  61374

Majority     GCCTATGTTTTCC-CATTTGTGTAT--------TTTTAGAAAATAAA-ATATTCATT----ATTGTC--TTTTAGATATT
                       88890     88900     88910     88920     88930     88940     88950     88960
Human        GCCTGTGTTCTTTACTTTTGTGTGTGTGTATGTGTTT-TTAAACAAG---ATTCATT----GTAGTC--TTTTAGATATA  70322
PolarBear    GCCTTTGTTTTCC-CACGTGTGTGT--------TTTTAGAAAATAAG-ATATTTACTC---GTTGTC--TTTTAGATATT  30832
Cow          GCCTGTGATTTTC-CTTTTGTATATG-------TTTTAGAAAATAAA-ATATATT-----TAGTGTTGTTTGTAGATACT  68331
Dog          GCCTGTGTTTTGC-CATGTGTGTAT--------TTTTAGGAAATAAG-ATGCTCATT----GTTGTG--TTTTAGGTATT  61493
BelugaWhale  ATTCACATATTCT-ACATCAGAGAA----------CAAGGTGATCAA-AAATCCTTGA--GATTATAAATTGAGATGTCA  60921
Donkey       GCCTATATGTTTG-CTTTTGTGTGCATATTTCTTTTTAGAAAATAGATACATTTATTCACTGTTGTC--TTTTAGATAGT  67553
Cat          GCCCATGTTTTCC-CGTGTGTG-GT--------TTT---AAAATGAG-ATACTCATT----ATTGTC--TTTTAGATATT  68175
Ferret       ACCTATGTTTTCC-CATGTGTGTAT--------TTTTAGAAAATAAA-ACATTCATT----A-------T--TAGGTA--  68919
Lion         GCCCATGTTTTCC-CATGTGTG-GT--------TTT---AAAATAAG-ATACTCATT----ATTGTC--TTTTAGATATT  65868
Vaquita      ATTCACATATTCT-ACATCAGAGAA----------CAAGGTGATCAA-AAATCCTTGA--GATTATAAATTGAGATGTCA  62286
Pig          ATCCAAAAGTTTT-CATTTATAAAAC-------ATCAACACTTTTTA-CCCTCTTCCTT-TGGTGTGAATAGTATGGTCT  61444
```

```
Majority     ATTTAATAAATTTAGGCTGTTTAAG---T------TATCCTCCAAAGGTGAATGCTATTTGGAAATTC-----TTXCTTG
                        88970     88980     88990     89000     89010     89020     89030     89040
Human        ATTTAATAAATTTAGGCTGTTCAGGGTTTC--A-GTATCCTACAAAGAAGAACGCTGCTTCTAAGTTTTTGGGTTTCTTG 70399
PolarBear    ATTTAATAAATTTAGGCTGTTTCAG----------TATCCTCCAA-GGTGAATGCCATTTGGAAATTC-----TTACTTT 30896
Cow          TTGTAATAAATTTAAGCTATTCATGGTTTCT---GTATCCTCCAAAGATGAATGTTATTTGGAAATTC----TTGTTTCA 68404
Dog          ATTTAGTAAATTTAGGCTGTTTCAG----------TAGCCTCCAA-GGTGAATGCCATTTGGAAATTC-----TT----- 61552
BelugaWhale  TTATAATGATTTTATCATGTTTAAGAAGTGTAGTTAATTTATTAAGGGGTGGTCACTTTTAGAAAAAC------TGCTAG 60995
Donkey       ATTTAATAAATTTAGGCTGTTGAAGGTTTC--A-GTATCCTCCAAAGGTGAATGTTATTTGGAAATTCT--TGTATTTTG 67628
Cat          ACTTAATAAATTTAGGTTGTTACAG----------TATCCTCCAAAGTTGAATGCTATTTAGAAATTC-----TTGTGTG 68240
Ferret       -TTTAGTAAATTTAAGCTGTTTCAG----------TATCTTCCAA-GTTGAATATCATTTGGAAATTC-----TTACTT- 68981
Lion         ACTTAATAAATTTAGGTTGTTACAG----------TATCCTCCAAAGGTGAATGCTATTTAGAAATTC-----TTGTGTG 65933
Vaquita      TTATAATGATTTTATCATGTTTAAGAAGTGTAGTTAATTTATTAGGGGGTGGTCACTTTTAGAAAAAC------TGCTAG 62360
Pig          TTGCAAGGACCACTTGCTGTTAATGCTTTCTAAAGTAGTCTTCAAGTACTCTTCATAGTATGACATACA--GTATTGCTG 61522

Majority     -----GTXTTTTTACT--------GAACCATTCAGTTGXT-CAGAGCTTAGGAAGAATX----AAATTATCAXTTGGTAT
                        89050     89060     89070     89080     89090     89100     89110     89120
Human        AACCAGAGCTTAGAAAGAACAAAAATATGGCATGGTATTTTCATGGTTTAGTAAGGCTACTTTTAGTTAATTATTGTGAT 70479
PolarBear    -----GTTTTTCTACT--------GAACCATTCAGTAGCCACAGAGCTTAGGAAGAATA----AAATTATCACTTGGTAT 30959
Cow          -----GTGTTTTTGCT--------GAACCATTCAGTAGCACCAGAGCTTAGAAAGAAT----AAAATTGCCACTTGGTAT 68467
Dog          -----GTTTTTCTACT--------GAACCATTCAAT--------AGCTTAGGAAGAACA----AAATTATCAGTTGGTAA 61607
BelugaWhale  -----AAGTAATTAAT--------TTT-CATGATCATGTTGTCTACATTTTAAAAATT------AAT--GCAGATAATAT 61053
Donkey       GG--AGTTTTTTTACCCCCTGCTGAAACTATTCAGTTGCTCCAGAGCTCAGGAAGAACA----AAATTATCACTTGGTAT 67702
Cat          -----GTTTTTTTTTT--------GTTTTTGTTTTT-------GTTTTTTTTCTGAAC---------CAT---TCAGTAG 68288
Ferret       -----GTTTTTCTACT--------GAACAATTCAGTGTCCACAGAGATTAGGAAGAACA----AAATTATCACTTGGTAT 69044
Lion         --------TTTTTTTT--------TTT-----------------------CTGAAC---------CAT---TCAGTAG 65960
Vaquita      -----AAGTAATTAAT--------TTTTCATGATCATGTTGTCTACATTTTAAAAATT------AAT--GCAGATAATAT 62419
Pig          -----GAGTGGTGACT--------AAATTATTTTTCTGTTTATTTGCCTAAGAAGACTTT--AAAGTAAGCAGATGATAT 61587
```

```
Majority      T------------TTCAX-----------GAATTT------XX-X-GTA---------A---GGC---------------

                       89130     89140     89150     89160     89170     89180     89190     89200

Human         GTAGGCCATTAAGTAGACTTTTCTAAAACGAAACCCCAGAGGAGAGGGGTTTTGAAGATACATATATACTGGTTTCTAAG  70559
PolarBear     T------------TTCAT-----------GAATTT-----------GTA---------A---GGC---------------  30978
Cow           ATTG-TAAGGCCACTTAAAGTTCATCTTTATGATATAGGTAAATAAATACTTTTGAGAAGGAAATGGC---ATTCCACTC  68543
Dog           T------------TTCAT-----------GAATTT-----------GTA---------G---GGC---------------  61626
BelugaWhale   GTAC-AAAGAATACTTTAGAGCCTTCTGTGTATTTTGAGTTAATAAGTA-----------GCAGC---------------  61106
Donkey        T------------TTCAT-----------GAGTTT-----------GTA------------AGGC---------------  67721
Cat           C------------CTCA------------GAGCTT-----------------------A---GTC---------------  68303
Ferret        T------------TTCAA-----------GTATTTCTCAGTATTTTATA---------A---GGC---------------  69074
Lion          C------------CTCA------------GAGCTT-----------------------A---GTC---------------  65975
Vaquita       GTAC-AAAGAATACTTTAGAGCCTTCTGTGTATTTTGAGTTAATAAGTA-----------GCAGC---------------  62472
Pig           -----TGATGGCATCCAT--TTCCTGCTTGAGTCCTCCTTAAACAGTACTTGTTCAAAATGAAAT------AATTGATAG  61654

Majority      ---TXXTTTCATTTAATTATTXTGATXXGTAG---------GCCAT------------TAA-----------GTAGACTT

                       89210     89220     89230     89240     89250     89260     89270     89280

Human         AACTAATCTTTTTGGATCATTGGTATTGGTAGAAGGGATTCGCTATGTTTTCTGCCACCATTGGATCTCTGTATAAAGTT  70639
PolarBear     ---TGCTTTCAGTTAATTATTGTGCTGTGTAA---------GCCAT------------TAA-----------GTAGGCTT  31023
Cow           CAGTATTCTTGCCTGGAGAATTCCATGGACAG---------AGAAGCCTGGCGGGCTACAGTCTACAGGGTTGCAAAGAG  68614
Dog           ---TACTTTCAGTTAATTATTACGAT--GTAG---------GCCAT------------TAA-----------GGAGACTT  61669
BelugaWhale   ---CGTTCTCATTATACGGTTTTAACAGAAAA---------GCAAA-----------------------G---CAAACTT  61148
Donkey        ---TACTTTCAGTTAATTATTGCGAT--GTAG---------GCCGT------------TAA-----------ACAGACTT  67764
Cat           ---TGATTTCATTTAATTATTATGAT--GTAG---------GCCAT------------TAA-----------GTAGACTT  68346
Ferret        ---TATTTTCAGTTAGTTATTGTGATACGTAG---------GCCAT------------TAA-----------GTAGACTT  69119
Lion          ---TGATTTCATTTAATTATTATGAT--GTAG---------GCCAT------------TAA-----------GTAGACTT  66018
Vaquita       ---CGTTCTCATCATATGGTTTTAACAGAAAA---------GCAAA-----------------------G---CAAACTT  62514
Pig           GTATTCCTTTGTTTCAAAGATTCTGTAAGAAC---------ACAGA--TGTTAGGTGATAATCCTTTT--CTGTTAAATA  61721
```

```
Majority     T----------TTXA--------AAAAGA-------XTXCTTGTGGA----------AATA--X-GAG--------TCTT
                      89290     89300     89310     89320     89330     89340     89350     89360
Human        TGGCTCCACTATCAAAATGCTTAGATAGATGCCTTTGCAATAGTTGCC-ACCCTAGGAATAGCTCAAAAATTATTAACTT 70718
PolarBear    T----------TCTA--------AAAAGAA------ATGCTGGTTGA----------AAT-----GAG--------TCTT 31056
Cow          T----------CTGACACAACT-GAAGGACTCACACTTTCTAGAAAGAAATGGTGGTGAAA-AAGGAG--------TCTT 68674
Dog          T----------TCTA--------AAAAGAT------ATGCTTGCTGA----------AATCCTTAAAG--------TCTT 61707
BelugaWhale  T----------GTA------T----AGGA-------TATCTTTTGAG-----GTGAAAATA-GTTGAT--------AATT 61187
Donkey       T----------TCTA-----C---AAAGAA------ATGCTGATAAA----------AAAA----GAG--------TCTT 67798
Cat          T----------TTAA--------AAAAGA--------TCCTTGTGGA----------AAG-----GAG--------TCTT 68377
Ferret       T----------CCTA--------AAAAGAA------ACACTGGTAGA----------AAT-----GAG--------TTTT 69152
Lion         T----------TTTA--------AAAAGA--------TCCT-GTGGA----------AAG-----GAG--------TCTT 66048
Vaquita      T----------GTA------T----AGGA-------TATCTTTTGAG-----GTGAAAATA-GTTGAT--------AATT 62553
Pig          T----------TTA---------GGGGGATTCCCTTTACCTTGTGTG--ATTCTTTTATG-TTATA----------CCTG 61770

Majority     AAGAATACA---TTAXA-CCAGTTTCT-----AAGAAGT-GGGXXTTTTTAGATGATTGGTATTGATAGAAG-GGAT-TA
                      89370     89380     89390     89400     89410     89420     89430     89440
Human        AATACTGGTTACTTAAATTCCACCTCTGTTGCTTGGAGTTGTGCTTTTTTTTTTTTTTTTTTTTTTTGAGATAGAGTCTG 70798
PolarBear    AAGGATACT---TTGTA-CCAGTTTCT-----AAAAAGT-GGGGTCTTTTAGATGATTGTTATTGATAGAAG-GAAT-TA 31124
Cow          AAAAATACA---CATAGACCAGTTTCT-----AAGGAGT-GAGATTTTTTGGATAATTGGTATTGATAGAAGGGGGT-TA 68744
Dog          AAGGATAC----TTACA-CCAG------------------GGGTCTTTTTATGTGATTGGTATTGATAGAAG-ATAT-TA 61762
BelugaWhale  GAGAACAGA---TATAA--CAACCATT-----TATTCAT-AAGG----GTAAATATGTAGTTCTTATAGA------T-CA 61245
Donkey       AAGATATGC---ATATA-CCAGTTTCT-----CAGAAGTGGGATCTTTCTGGATGATTGATATTGATAGAAG-GGAT-TA 67867
Cat          TAGGATACT---TTATA-CCACTTTCT-----AAGAAGTGGGGTCTTTTTGGATGATGGGTATTGACAGAAG-AGAT-AA 68446
Ferret       AAGGGTACA---CTATA-CCAGTTTGT-----AAAAAGT-GGGGTCTTTTAGATGATTGGTGTTGGTGGAAG-GAAT-TA 69220
Lion         GAGGATACT---TTATA-CCACTTTCT-----AACAAGTGGGGTCTTTTTGGATGATGGGTATTGACAGAAG-AGAT-AA 66117
Vaquita      GAGAACAGA---TATAA--CAACCATT-----TATTCAT-AAGG----GTAAATATGTAGTTCTTATAGA------T-CA 62611
Pig          AGCACTTGA---TTAAATTTAATTACT-----TTAGAGT-CAG---TATTACAAGTTTTGATTTATTTTACACAG---TA 61835
```

```
Majority     GCTGTTTTTTC--TACCA---------------CTATTTGCTCTCCCTATAAA-------AATTTGGCTC----CACTAT
                      89450      89460      89470      89480      89490      89500      89510      89520
Human        GCTCTGTCACCCAGGCTGGAGTGCAGTGGCATGCAATCTCGGCTCACTGCAAGCTCCGCCTCCTGGGTTCACGCCATTCT  70878
PolarBear    GCTGTTTTTTC--TACCA---------------CTATTTGCTTTCTCTATAAA-------AATTTGTATC----CACTGT  31176
Cow          GCTTTTTTTCT---ACCA---------------CTGTTTTCTCTCCCTATAAA-------AGTTTGGCTC----CACTAT  68795
Dog          GCTGTTTTTTCC-TACCA---------------CTATTTGCTTTCCCTATAGG-------AATTGGGCTC----CACTAT  61815
BelugaWhale  GTTTTTCTCAA---ACT------------------TTCCTGCATATCAAAAT-------TACCAGGC-------CTTAC  61289
Donkey       GCTGTTTTTTC--TACCA---------------CCATTTGCTCTCCCTATAAA-------AATTTGGCT-----TAACAT  67918
Cat          GCTGTTTTTTTCCTAACA---------------CTATTTGCTTTCCCTAGAAA-------AATATGGCTC----CACTGT  68500
Ferret       GCTGTTTTTTC--TACCA---------------CTATTTGTTTTGCCTATAAA-------AATTTGACTG----CACTGT  69272
Lion         GCTGTTTTTTTCCTAACA---------------CTATTTGCTTTCCCTAGAAA-------AATATGGCTC----CACTGT  66171
Vaquita      GTTTTTCTCAA---ACT------------------TTCCTGCACATCAAAAT-------TACCAGGC-------CTTAC  62655
Pig          ATTTTTTTTTT---CTTT---------------TTTTTCCCTCTTTTTGCTAT-------TTCTTGGG-C----CGCTCC  61885

Majority     CA----------------------A-A----ATXCCTAGATCTCT----TTGGG----------GTAATTCCTATGATA
                      89530      89540      89550      89560      89570      89580      89590      89600
Human        CCTGCCTCAGCCTCCCAAGTAGCTGGGACTACAGGCGCACGCCACCACGCCCAGCTAATTTCTTCATATTTTTTTAGTA  70958
PolarBear    CA----------------------A-A----ATGCCTAGATCTTT----TGGGG----------GTAATTCCTGTGATA  31214
Cow          CA----------------------AAGTGCCTTAGGGAGATCCTT---TTTGGG----------ATAGTTCCTACCACA  68839
Dog          AA----------------------A-A----ATGCCTACATATTT----TGGGG----------GTAGTTCCTGTGATA  61853
BelugaWhale  C-----------------------------CTAGACTGAGAATCCC--------------------TAATGATTAGTGGG  61320
Donkey       CA----------------------AAATG-CATAGATAGATCTCT----CTGGG----------ATAGTTCCTTTCATA  67960
Cat          CA----------------------A-A----ATGCCTAGA--TCT----TTTGC----------ATAATTCCCATGATA  68536
Ferret       C-------------------------------------------T-T----TAGGG----------GTCATTCCTGTGATA  69295
Lion         CA----------------------A-A----ATGCCTACA--TCT----TTTGG----------GTAATTCCCATGAGA  66207
Vaquita      C-----------------------------CTAGACTGAGAATCCC--------------------TAATGATTAGTGGG  62686
Pig          C---------------------------GCGTCATATGGAGGTTCCCAGGCTAGGG----------GTCAAATCAGAGCTG  61929
```

```
Majority     GGAATAAATATTTAGAAAATTAT------TAGTTTAAA----------------------------AGT------AGTTA

                     89610     89620     89630     89640     89650     89660     89670     89680

Human        GAGACGGGGTTTTACTGTGTTAGCCAGGATGGTCTTGATCTCCTGACCTCCTGATCCTCCTGCCTCGGCCTCCCAAAGTG  71038
PolarBear    GGAATAAATATTTAGAAAATTAT------TAGTTTAAA----------------------------AGG------AGTTG  31254
Cow          GGAATATCTTTTCAGAAAAGTAG------TT---TAAA----------------------------AGT------AATTA  68876
Dog          GGAATAAATATTTACAAAGTTAT------TAGTTTAAA----------------------------AGT------AATTA  61893
BelugaWhale  TTTCTAATTTTTTAAGATTCCAG------GA---TTCC----------------------------AAG------ATGTA  61357
Donkey       GGAATAACTATTTAGAAATGTAT------TAATTTAAA----------------------------AGT------AGTTA  68000
Cat          GGAATAAATATTTAGAAAATTAT------TAGTTTAAA----------------------------AGT------AGTTA  68576
Ferret       GGAATAAATACTTAGAAAATTAC------TAGTTTGAA----------------------------AGG------AGTTA  69335
Lion         GGAATAAATATTTAGAAAATTAT------TAGTTTAAA----------------------------AGT------AGTTA  66247
Vaquita      TTTCTAATTTTTTAAGATTCCA---------------------------------------------AG------ATGTA  62715
Pig          TAGCCACCGGCCTACGCCAGAGC------CA---CAGC----------------------------AGC------GCGGG  61966

Majority     CTTAGA-TTCCACCTTGA----------------TTGTTACAGTTCTTCATTTCATATATTTTGTXGCAAAAACATTTAA

                     89690     89700     89710     89720     89730     89740     89750     89760

Human        CTGGGATTACGGGCGTGAGCCACCGCGCCTAGCCTGCTTAGAGTTTTTTATGTAATT-ATTTTGTGACACAAACATTTAA  71117
PolarBear    CTTAGG-TGCCACCTTAA----------------TTGTTACAATTC---ATTTCATATATTTTGTAGGAAAAACACATGA  31314
Cow          TTTAGATTCCACCTTTGT----------------TGCTTACAGTTCTTCATTTCATACATTTTGAGGCAAAAACATTTAA  68940
Dog          CTTAAGATTCCACCTTAA----------------TTGCTACAGTTC-----TCCATATATTTTGTAGCAAAAACACATGA  61952
BelugaWhale  GTGGGAG-------TTGA----------------GAATTAAC--------TGCTATA------GAGGAAAGTTTGT--AA  61398
Donkey       CTTAGATTACACCTCTGT----------------TGCTTACAGTTCTTCATTTCACATATTTTGTGGCAAAAACATTTGA  68064
Cat          CTTAGA-TTCCACCTATA----------------TTGCTGCAGTTCTTCATTTCATTTATTTTGTAGCAAAAATATTTGG  68639
Ferret       CTTAGG-TGCCACCTTAA----------------TTGCTACAGTTCTTTATTTCATATATTTTGTAGCAAAAA---ACAA  69395
Lion         CTTAGA-TTCCACCTATA----------------TTGCTGCAGTTCTTCATTTCATTTATTTTGTAGCAAAAATATTTGA  66310
Vaquita      GTGGGAG-------TTGA----------------GAATTA-C--------TGCTATA------GGGGAAAGTTTGT--AA  62755
Pig          ATCCGAG-CCGCATCTGC----------------AACCTACA---CCACAGGTCACA-G--C-AACGCCAGATCGTT-AA  62021
```

```
Majority     AATACTATGGCTTAGAATGAAATT-----GTCTCA-T-CC-GGAX----AAAGTTTGTCTGATGCATATGGATACTAAT-
                                89770     89780     89790     89800     89810     89820     89830     89840
Human        AATACTGTAGCTTAGAATGAAATT-----GTCTCAATCCTAGGA-----GCCAAAAGTTTGATGAATATGAATACTAAT-  71186
PolarBear    AATACTCTGGCTTAGAATGAAATT-----GTCTCAGTGCCTGGAACC-AAAAGGTTATCTGATGCATATGGATACTAAT-  31387
Cow          AATATTGTGCTTTAGAATGGAATT-----GTCCCA-ATCCCAGAA----ACCAAAAGGTAGATGCATATGGGTATTGC--  69008
Dog          AATACTATGGCTTAAAATGAAATT-----GTCTCAATGCCGGGAACCCAAAAGGTTGTCTGATGCATATGGATACTAAT-  62026
BelugaWhale  TTTTTTAGGT---------GCTTT-----ATTTTA-------------GTTTTTTAGCAAAGTTAT--TAGTGTTCT--  61446
Donkey       AATACTACAGTTTAGAATGGAATTCTAAGGAACCAGTTCCAGGAACC-AAAAGTTTGTCTGATGTATATGGATACTAATA  68143
Cat          AATACTATGGCTTAGAATGAAAT------------------------------TGTCTGATGCATATGGATACTAAT-  68686
Ferret       AATACA-TGGCTTAGAATGAAATG-----GTCTCAATGCCTGGAACC-AAAAGTTTGTCTGATACATATGGATACTAAT-  69467
Lion         AATACTATGGCTTAGAATGAAAT------------------------------TGTCTGATGCATATGGATACTAAT-  66357
Vaquita      TTTTTTAGGT---------GCTTT-----ATTTTA-------------GTTTTTTAGCAAAGTTAT--TAGTGTTCT--  62803
Pig          CCCACTGAGCAAGGGCAGGGACCG-----AACCCGCAACCTCATG----GTTCCTAGTCGGATTTGT-TAACCACTGCG-  62090

Majority     CAXTAACCAT----TTXTTGAATATGTATTATGT--GCCAGG-CATTGTTCTTAGCC-----XTTATGAGTTAATCCTCA
                                89850     89860     89870     89880     89890     89900     89910     89920
Human        -ATTAACCAT-----------TTTTGTGTTCTGT--GTCAGG-CACTGTTCTTAGCCC----TTTACAAGTAAGTTAATA  71247
PolarBear    CATTAGCCAT----TTCTTGAATATGTATTAAGT--GCCTA--CATCATTCTTAGCC-----CTTGTGAGTTAATCCTCA  31454
Cow          ---TAATAGT----CTATCATTTATTGAGTATAT--GCCAATGCATCATTCTTAACC-----CTCTTGTGAGTTAATCCT  69074
Dog          CACTAACCAT----TTCGTGAATATGTATGAAGT--GCCAGG-CATCATTCTTAGCC-----TTTGTGAGTTAATCCTCA  62094
BelugaWhale  -CATAAAAACA-------TACTT---ACCTAT-TGAGCCAGTATTCTAATACTTTCC-----TTTATTCATGCCATGTAA  61509
Donkey       TAATAACTGACTGTTTATCGAGTATATATTATGT--TCCAGG-CGTTGTTCTTAGCCATTTGCTTGTGAGTTAACCTTCA  68220
Cat          CACTAGCCAT----TAATTGAATATGCGTTATGT--GCCAGG-CATTGTTCCTAACC-----CTTATGAGTTAGTCCTCA  68754
Ferret       CACTAGCCAT----TTCTTGGCTATGTATTAAGT--GCCTG--TATCATTCTTAGCC-----ATTATGAGTTAATCCTTA  69534
Lion         CACTAGCCAT----TGATTGAATATGCGTTATGT--GCCAGG-CATTGTTCCTAACC-----CTTGTGAGTTAGTCCTCA  66425
Vaquita      -CATAAAAACA-------TACTTTATACCTAT-TGAGCCAGTATTCTGATACTTTCC-----TTTATTCATGCCATGTAA  62869
Pig          CCACGACAGGAA--CTCCTACACAGTAATTTTATAAGGCGAGCATTTGTCCCTGGCT-----TCTCTTACTGCTAGGGAT  62163
```

```
Majority     CATTATCCCTAAATGAAGTTA-CTCTTAT---TGCXCCCXXTTTATAGATTTAGAAACTGGGACACAAAAAGATTAGCAG
                      89930     89940     89950     89960     89970     89980     89990     90000
Human        CATCATCTCTAAATGTAGTTA-CCATTAT---CGATCCCATTTTATAGATTTGGAAAGTAGGGT--ACAAAGAACAG---  71318
PolarBear    CATTATCCCTAAATGAAGTTA-CTCTTA--------CCCTCTTCATAGATTTAGAAATTGGGACACGAAAAGATTAGCAG  31525
Cow          CACCATTCTTAAATGAATTTA-TCCTTAT---TGCCCCCATTTTACAGATTTAAAGCCTGGAACACAAACTGATTAGCAG  69150
Dog          CATTATCCCTAAATGTAGTTA-TTCTTAT-TGTCC-CTCACTTCATAGGTTTAGAAACTGGGACACACAAAGATTAGCAG  62171
BelugaWhale  ATCTGTCTTGGTATCGGTACA-ATTCCAC---TGATACTGTTTAGCTGTT----------------AAATTGAAT-----  61564
Donkey       CATCATCCTTAAATGAAGTTA-CCCTTAT---TGCCCCGTTTTACAGATTTAAAAACTGGGACACAAAGAGATTAGC--  68294
Cat          CATTGTCCATAAATGAAGTTAACCCTTAACTGTCCGCCTGCTTTATCGACTTAGAAACTGGAATACAAAAAGATGAGCAG  68834
Ferret       CATTATCCCTAAAAGAAGTTA-TTCTTAT-TGTCC-CCCACTTCATAGATTTGGGAAGTGGGACACAAAAAGATAAGCAG  69611
Lion         CATTGTCCGTAAATGAAGTTAACCCTTAACTGTCCGCCTACTTTATCAGTTTAGAAACTGGAATACAAAAAGATGAGCAG  66505
Vaquita      ATCTGTCTTGGTATCGGTACA-ATTCCAC---TGATACTGTTTAGCTGTT----------------AAATTGAAT-----  62924
Pig          CACAGTATCTCCAAAAATCTA-ATTTATT---CAAGAGTGGATAGAATACTTCTA-TAAAGAATGGAGACTGAGT-GCTT  62237

Majority     TTTGCTCAGATTCCTATAATTAGTGAGTGGCTGAGXXAX-------X-AX-CXGGCAGTCTGGCTCCAA-AGTTCATACT
                      90010     90020     90030     90040     90050     90060     90070     90080
Human        TTTGCCCAGATTCCTTTAACTTTTGAGTGGCCGAGCCCTAGCACTGAAAGCCAGGTAGTCTGGTTCCAA-AGTCCGTACT  71397
PolarBear    TTTGCTCAGATTCCCATAATTAGTGAGTGGCCGAGACCG-------GCATTCTGGCAGTCTGGCTCCCA-AGTTCATGCT  31597
Cow          TTTGCTCAAATTCCCATCATTAGTGAGGGCCTGAGCCAG-----------CCAGGCAGTATGGCTCCAACAATCTGTCCT  69219
Dog          TTTGCTCCGATTCCCATAATTAGTGAGTGGTTGAGATAG-------GCATTCTGGCAGTCCGGCTTCAA-AGTTCATACT  62243
BelugaWhale  -----TTGGTTTTCTTTTCTA--TGTGTTTATGATGCA-------------TAAAATTAACAAGTA----AACTTGTTTT  61620
Donkey       -----------------AATTA--GAGTGGGTGAGAT--------------GGGTAGTCTGGCCCCAA-AGTCCATGCT  68339
Cat          TTTGCTCAGATTCTTATAGTTAGTGGGTGGCTGAGGTATT---CAGGGATGCTGGCAGTCTGGCTCCAG-AGTTCATACT  68910
Ferret       TTTGCTCATAGTCCCATAATTAGTGAGTGGCTGAGATAGT---TAGGCATTCTGGCAGTCTGGCTCCAA-AGTTCATGCT  69687
Lion         TTTGCTCAGATTCTTATAATTAGTGGGTGGCTGAGGTATT---CAGGGATGCTGGCAGTCTGGCTCCAG-AGTTCATACT  66581
Vaquita      -----TTGGTTTTCTTTTCTA--TGTGTTTATGATGCA-------------TAAAATTAACAAGTA----AACTTGTTTT  62980
Pig          TGTAATTAGAAATCTGACATC--TAAATACATAATCA--------------CAAACCTGGCGGATT--ATTAATTATTTA  62299
```

```
Majority     CCTAACAAACAA--------TAGAATAAAATATACTATAGATTAGXTGGGCCTTTTAGCCTGACTTCTTTTC--------
                               90090     90100     90110     90120     90130     90140     90150     90160
Human        CCTGACTAATAA---------ATAGTGGAATAAAAT-TAGATTAGCTTGACATTTTAGCCTGTCGTCTTCTC--------  71459
PolarBear    CCTAACCAACGA--------TTGAATAAAATATACTATAGATTAGCTGGGCTTTTAGCCTGTCTTCTTTC----------  31660
Cow          TCTAATAAACAT--------TAAAATGAAATGTGCTACAGATTAGCTAAGGCTTTTAGCCTACCTTTTTTCCCCCTATGG  69291
Dog          CCTAACAAACAAATTTGAATTTGAATAAAATATACTATAGATTAG----GCCTGTTAGCCTGCCTTCTTTTT--------  62311
BelugaWhale  TAAAAT--TTTT--------TAAAACTTACTTTAAACTTAATATTTTAAAATTTATTTTTAAATGACTTCTGAAAGCTTT  61690
Donkey       CCTAACTAATAAAC----AACAGAATAAAATGTACTTTAGATTAGCTGGGCCTTTTGGCATGCCTTTTCCC---------  68406
Cat          CCTAACAGACAA--------TTGAATAAAATATGCAGTAGATTAGTTGGGCCTCTTAGTCTGACTAAACTCCCCCCCCTC  68982
Ferret       CCTAACAAACAA--------TTGAATAAAATATACTACAGATTAGCTGGGCCTTTTAGCCTGTCTCCTTTT---------  69750
Lion         CCTAACAGACAA--------TTGAATAAAATATGCAGTAGACTAGTTGGGCCTCTTAGTCTGACTAAACTCC--------  66645
Vaquita      TAAAATATTTTT--------TAAAACTTACTTTAAACTTAATATTTTAAAATTTATTTTTAAATGACTTCTGAAAGCTTT  63052
Pig          CATATTCTACAT--------CAAAGAACAAGGTGATCAAAAATCCTTGAGGTTATAAATTAAGATGTTTTTATAATCGTT  62371

Majority     -CCCTATGGAGTTAA-AATA-GAAXAATGTCATACATATAGTAAATATGTAAGTCCTATAATTTTAATTTXCTTT-----
                               90170     90180     90190     90200     90210     90220     90230     90240
Human        -ACCTGTAGAATAGTTATCAAGAATAGTAATATACATATAGTAAAAATTTAAGTCCCATCATTTTAGCTTGCTTT-----  71533
PolarBear    -CCCTATGGAGTTAATAATA-GAACAATGTCATACATCTAGTAAATACGTAAGTCCTATAATTTTAATTTCCCCCCACAT  31738
Cow          AGTTCATGTAGAATA-ATGT-CATGCATATAGTAAATT-GGTAAGCCTGTAGTTTTAAT-TTCCTTCTTTTTTATATAGT  69367
Dog          -CCCTATGGAGTTAATAATA------ATGTCATATATGTAGTAAATACATACGTCCTATAATTTTAATTTCCCTT----T  62380
BelugaWhale  TGTTTTTGTT-----------------ACTTTTGAATG-AGTTATTTACATGGTGTTTTTTTTTAAGTTTTTATTATC--  61750
Donkey       ---CTGTGGAGTTAACAGGT-CA---ATGTCATACATGTAGTAAATACGTAAGTCCTATTATTTCAATTTCCTCC-----  68474
Cat          CCCCCATGGAGTTAA-AATG-GAATAATGTCATACATATAGTAAATATGTAAGTCCTATAGTTTTAATTTCCTTT-----  69055
Ferret       -CTCCCTGGAGCTAATAATA-GAACAGTGTTATACATGTAGTCAATATGTAAGTGCTATAATTTTAATTTTCCCCCATAT  69828
Lion         CCCCCATGGAGTTAA-AATA-GAATAATGTCATACATATAGTAAATATGTAAGTCTTATAGTTTTAATTTCCTTT-----  66718
Vaquita      TGTTTTTGTT-----------------ACTTTTGAATA-AGTGATTTACATGGTGTTTTTTTTTAAGTTTTTATTATC--  63112
Pig          TTATCATGTTTAAGA-AGTA-TAGTTAATTAAGAGATA-GGTCACTTTTTAGAAAGACTGCTAGAAGTAATTAATTTTTC  62448
```

```
Majority     ---AATTTTXTCTCC-----------------------TATACTAAGGTGTTTTCTGTTGCCAAGAXA-XTTGTTTTTT
                     90250     90260     90270     90280     90290     90300     90310     90320
Human        -------TTTTTTAC------------------------ATACTAAGATATTTTTTGTTACCAA--------------- 71566
PolarBear    ACTAAACCCCCCCCC---------------------CATATACTAAGGTGTTTTCTGTTGCCAAGAAAGATTGTTTTTT 31796
Cow          G-GGATGTTTTCTGTT------GCTATGACAGATTTTTTTGTTGCCAAGTCCATCCAGTGTTTAACCTG-GTTATTTCAT 69439
Dog          TTTAATTTCCTCTCCC--------------------CATATACTTAAATGTTTTCTGTTACCAAGATG--TTG------ 62431
BelugaWhale  ---AAAGACTTCAGTT------ATCATATCCATTATCTCAATTCACTAGTTCCCACACTTGTTTT-----ATCTGTTCAT 61816
Donkey       -------TTTTTTAT------------------------ATAATAAGATGTTTTCTGCTGCCATGACAGATTATTTTTT 68522
Cat          -----TTGTCCCTCC-----------------------TATACTAAGGTGTTTTGTGTTGCCAAGACA--TTGGCTTTT 69104
Ferret       ACTAATTTCCCCCCATATACTAATTTCCTTTTTCCCGCATATACTAAGGTATTTTCTGTTGCTAAGACA--TTGTTTTTT 69906
Lion         -----TTGTCCCTCC-----------------------TATACTAAGGTGTTTTGTTTTGCCAAGACA--TTGGCTTTT 66767
Vaquita      ---AGAGACTTCAGTT------ATCATATCCATTATCTCAATTCACTTGTTCCCACACTTGTTTT-----ATCTGTTCAT 63178
Pig          A-TGATCATGTCTAC-------ATTTTAAAAATTAATGCAGATAATATGTACAAAGAATACTTCA---G-AGCTTTCCAT 62516
```

```
Majority     TGTTGCCAAGTCCATTAXGTGTTTAACCTGXTTATXATA-TGATTGCAA-A----AACXAAAXGTATTGTTCAT------
                     90330     90340     90350     90360     90370     90380     90390     90400
Human        ---------GTCCATTAAGTATTTAGCCTGATTATCTTATTGATTAAAATAC--CAAAAATGTGTATTGTTCAT------ 71629
PolarBear    TGTTGCCAAGTCCATTCAGTGTTTAACCTGGTTATTATA-TGATTGCAA------AACCAAAAGTATTGTTCAT------ 31863
Cow          GATTACAAAACCAAATATGAGTATACCTACTCTATAAT--TGGATCCAGAATAGGAGCTGTGTGC-CTGTGTATTTATAT 69516
Dog          --TTACCAAGTCCATTTAGTGTTTAACCTGGTTATTATA-TGATTACAA------AGCCAGAAGTACTGTTTAT------ 62496
BelugaWhale  TTTTTCTGTTGTATTTTGAGGCAAATCCTTAACATCAAG-TCATCTCAA-ACATTACCATAATTT-CTCTAAAATAA-TG 61892
Donkey       -GTTGCCAAGTCCACTCACTGTTTAACCTGGTTTCATA-TGAGTACAAAAC--CAAAAGTGGGTATTGTTCAT------ 68592
Cat          TGTTGCCCAGCCCATTAGGTGTTGAACGTGCTTATTATA-TGATTGCAA------AAACAAAAGTATTGTTCAT------ 69171
Ferret       -GTTGCCCAGCCCATTCCATGTTTAACCTGATTATTTTA-TGATTGCAA------AACCAAGGGTATTGTTCAT------ 69972
Lion         TGTTGCCCAGCCCATTAGGTGTTGGACCTGGTTATTATA-TGATTGCAA------AAACAAAAGTATTGTTCAT------ 66834
Vaquita      TTTTTCTGTTGTATTTTGAGGCAAATCCTTAACATCAAG-TCATCTCAA-ACATTACCATAATTT-CTCTAAAATAA-TG 63254
Pig          GTATTTTGAATTAATAATGTAGCAGCCAGTCTCATCATA-TGGTTTTAACAGAAAGGCAAAACAAACTTTGTATG---GG 62592
```

```
Majority     ATATCCGTTCAAAATTGGATACAAAATA--AGATATATATATA-ACATG-----------CAAACCAAAATA--------
                      90410     90420     90430     90440     90450     90460     90470     90480
Human        GCATTGATTCAAAATTGGGTACAAAACG---GGTAGATATGTA-ACATG-----------CA----AA------------ 71678
PolarBear    ATGTCCCTTCAAAATTGAATCCAAAATAG-AGATATATATA---ACATA-----------CAAACCAAAATA-------- 31920
Cow          ATATATGATCATTCATATATATATGATATATGTAAACCAATTATATATGGTCATT-CATATACATGTGTATATTATATGA 69595
Dog          ATGTCCATTTAAAATTGAATAGAAAATAGGAAATACATATATA-ATATG-----------CAAACCAAAATA-------- 62556
BelugaWhale  ACATTTTCTTAC-CAAGCATACCTGAT---CAAAATTGATTAAAACCTGATTAT--CATACCTATCAG------------ 61954
Donkey       ACATCCATTTAAAATTGGATCCAAATTAA-GGATGTGTATATA-GCATG-----------CAAACCAAAATA-------- 68651
Cat          ATATCCGTTCAAAAGTGGATCCAAAATA---------------------------------------------------- 69199
Ferret       ATGTCCCTTCAAAATTGAATACAAAATAT-ATATATATATATGTATATATATATATGTTGCAAACCAAAATA-------- 70043
Lion         ATATCCGTTCAAAAGTGGATCCAAAATAG-AGATGTATGTA---TCATG-----------GAAACCAAAACA-------- 66891
Vaquita      ACATTTTCTTAC-CAAGCATACCTGAT---CAAAATTGATTAAAACCTGATTAT--CATACCTATCAA------------ 63316
Pig          ATGT-TGCCGATGTGAACATAATTGATAATTAGAATAGATACACAAACATTTATT-CATAAAGGTAAATGTGTAGTCCT- 62669

Majority     TAGTAAACTGAATTTATATTTTTATGTTTTT-ACAAAATAXACATTAAAACTTAAATACCAAAATCTTAATTTTAGCAAA
                      90490     90500     90510     90520     90530     90540     90550     90560
Human        TAATAAATTGAACTTATATTTTTATCTTTTTGACAAACAACACGTTAAAATTT--ATGTCA--ATCTTATTTTTTCAAAA 71754
PolarBear    TAGTAAACTGAATTTATATTTTTATGTTTTC-ACAAAATGTACACTAAA-CTTAAAAACCAAAATCTTAATTTTAGCAAA 31998
Cow          TAGTAAACTGAATTTATATTTTTATTTTTTC-ACAAAATATACATTAAAACTTACATACCAAAGTCTTAAACTTAGCAAA 69674
Dog          TAGTAAACTGAATTTATATTTATATGTTTTC-ACGAAGTGTACACTAAAACTTAAATGCCAGAATCTTAATTTTAGCAAA 62635
BelugaWhale  -AATTGATGATGATT---TCTTAATGTTGCT-G---AATACCCAGTCAATCTTGCATTTCC--CCAGTTGTCTCAAAAAA 62024
Donkey       TAGTAAACTGAGATTATATTTTTATCTTTTC-ACAAAATATACATTAAAACTTAGATACCAT-ATCTTAATTTTAGTAAA 68729
Cat          TAGCAAACTGAATTTCTACTTTTATGTTTTT-ACAAAATACACATTAAAACTTAAATACCAAAATCTTAATTTTAGCAAA 69278
Ferret       TAGTAAACTGAATTTGTATCTT--TGTTTTC-ACAAAATGTACATTAAAACTTAAAAACCAAAATCCTAATTTTTGCAAA 70120
Lion         TAGCAAACTGAATTTGTATTTTTATGTTTTT-ACAAAATACACATTAAAACTTAAATACCAAAATCTTAATTTTAGCAAA 66970
Vaquita      -AATTGATGATGATT---TCTTAATGTTGCT-G---AATACCCAGTCAATCTTGCATTTCC--CCAGTTGTCTCAAAAAA 63386
Pig          TACTCAACAGTGTTT---CTCAAACTTCCCT-GCAAATCAGAATTGCCAGGCCCCACCCTGGACACGGAGAATCATAAGA 62745
```

```
Majority     ----GXCCTAATCTATAATATTC-----TAAGACT----AATTATAGCAAAGAAAATT--TGGTCATAA-AATCAAACCA
                      90570     90580     90590     90600     90610     90620     90630     90640
Human        -----TCCTTTTCTATATTTTAT-----TAAGACT----AATTATAGCAAATAATA------GCCATAA-AATTAAACCA  71813
PolarBear    ----GCCCTAATCTATAATATCCCATTATAAGACT---GCATTATAGCAAAGAAAATT----GTCATAA-AATCAGACTG  32066
Cow          ----ATCCTAATCTATAATATTC-----TAAGACT----AATTATAGCAAAGA-CAGTTATGGCCAGAA-AATCACACCA  69739
Dog          ----GCTTTAATCTATAA-------------GACT---ACATTATAGCAAAGAAAATTT-TGGTTGTAA-AATCAAACTT  62693
BelugaWhale  TGT-TTTTTAAGCATTGATTTGT-----TCAGAAT--A----------------G---------------GATCTTCTCA  62065
Donkey       ----ACACTAATGTATAATATTCCATTATAAGACT----GATTATGGTAAAGGAAATTA-TGGCCATAAAAATCAAACCA  68800
Cat          ----GCCT--GTCTATAATATTCCATTATAAGACT---GAATTATAGCAAAGAAGATTC-TGGTCATAA-AATCAAGCCA  69347
Ferret       ----AGCCTAATCTATAATATCCCATTATAAGACT---GCATTATAGCAAAGAAAATTA-TGGTCATAA-AATCAGACTG  70191
Lion         ----GCCT--GTCTATAATATTCCATTATAAGACT---GAATTATAGCAAAGAAAATTC-TGGTCATAA-AATCAAGCCA  67039
Vaquita      TGT-TTTTTAAGCATTGATTTGT-----TCAGAAT--A----------------G---------------GATCTTCTCA  63427
Pig          TGGGGTCCCTTGCATTTATTTTT-----TAAGATTCCAAAATGATTTTACTGAGTAGTGGAAGTTGAGA-ACTACTACTA  62819

Majority     ----AATAATTCATCTACTGCAGACACATTTGAAAGTTATCGXCCAACAAAAAX-XAAATAATXACAAATAXAXA---AA
                      90650     90660     90670     90680     90690     90700     90710     90720
Human        ----AAAAACTCATCTACTGCAGACATATTTGAAAGCTGTCCCCCAAGAAAGACCAAAGTAATCACACATACCTA---AA  71886
PolarBear    ----AATAATTCATCTACTGCAGGCACATTTACAAGTCATTGTCCAACAAA-----------------------------  32113
Cow          ----AATAATTCATGTATTGCAGTCACATTTGAAAGCTCCAAGAGACCAAAAT---AATTACTCACAAATATGAA---AA  69809
Dog          ----ACTAATCTA-CTGCAG--A-CACATTTGCAAATCATCCTCCAACAAAAACTAAAATGATTACAAATATACA---AA  62762
BelugaWhale  ------GGGGCCACACATGGCATTTGGTAGTTATGTCTCCTGAATGTCTAT--------TACTCTGTTATAC------TC  62125
Donkey       ----AATAACTCATATACTGCAGACGCAGTTGAGAACTATCCTCCAAGAAAAGCCTAAATAATTATATACAAATACACAA  68876
Cat          ----AATAACTCATTTACTGCAGACACATTTGCAAGTCATCACCCAACAAAGACTAAGAGAATTACAAGTACACA---CA  69420
Ferret       ----AATAATTCATCTGTTA----CACATTTGTGAGTCATTGTCCAACAAAAACTAAATT---TACAAATACACA---AA  70257
Lion         ----AATAACTCATTTACTGCAGACACATTTGCAAGTCATCACCCAACAAAGATTAAGATAATTACAAGTATACA---CA  67112
Vaquita      ------GGGGCCACACATGGCATTTGGTAATTATGTCTCCTGAATGTCTAT--------TACTC----------------  63477
Pig          TAGAGAAAATTTGTTTTTAGATGCTTTATTTTAGCCTTTTAGCAGAATTATTA---GTGTCCTCATAAAAAT--A---TT  62891
```

```
Majority     CATTCTAGAAATATTTCC---CAGAAATATTAAAC-TACATAGATTTTTAAAGTGTATCACTGAATGCCATAA--CAXCA
                     90730     90740     90750     90760     90770     90780     90790     90800
Human        CATGCTAG---TATTTCC---CAGAAATATTAAAAATACATAGATT-TTGAAGCACATTGCTGAATGCCATAG--TAACA  71957
PolarBear    CATTTTAGAAATATTTCC---TAGAAATATTAAAC-AACATAGATTTTTAGAGTGTATCACTGAATGCCATAA--CATTA  32187
Cow          CATCCTAGAAATATTTCC---TGGAAGTACTGAAC-CACACCCATT-TTAAAGTATATCACTGAGTCTCAAAAC-TAACA  69883
Dog          CATTCTAGAAATACTTT----TAAAAATATTAAAC-TCCATGGATTTTTAGAGTGTATCACTGAATGCCATAA--CATTT  62835
BelugaWhale  TGTTAAAGTCCTCTCCCTCTGCCCCAACCCTCAGT-TCCATTGAC--TTATTGTGGAAACCTAGTTATATTATT-TAAGA  62201
Donkey       CATCCTAGGAATATTTCC---CAGAAATATTAAAC-TACGTACATTTTTAAACCATATCACTTAATGCCATAA--CACCA  68950
Cat          CATTCTAGAAATATTTCC---CAGAATTATGAAAC-TACATAAATCTTTAAAGTGTATCGGTGAATATCATGA--CATCA  69494
Ferret       CATTTTAGAAATATTTCC---TAGAAATATTAAAC-TACATAGATTTTTAGAGTGTATCACTGAATGCCATAAAACATTA  70333
Lion         CATTCTAGAAATATTTCC---CAGAAATATGAAAC-TACATAAATCTTTAAAGTGTATTGGTGAATGTCATGA--CATCA  67186
Vaquita      TGTTGAAGTCCTCTCCCTCTGCCCCAACTCTCAGT-TCCATTGAC--TTATTGTGGAAACCTAGTTATATTCTT-TAAGA  63553
Pig          CTTCATAGCTATTGATCAGTATTTTAATACTTTTC-TTTATTCATG-CTGT-GTAATGCTCTTGGTACAATTCT-TGATA  62967

Majority     AAATTTTTTTAAAAAACCATATAATTAGAAAAGC-AGT--ATTTTATCATGTTCAACATAAATCAT------ATTT----
                     90810     90820     90830     90840     90850     90860     90870     90880
Human        CCAAACT--AAAAAATACATGTAAATTAAAAATT-AAT--ATTTTATT---TTCAACATAAATCAG---AAATACTTGG  72025
PolarBear    AAATTTTTTTAAAAAGCCATATAATTAGAAAACC-AGT--ATTTTATCATGTTCAATATCAATCAT------ATTTTTGG  32258
Cow          AAAGTTTTTAAAA--ACTACATAATTTAAAAGCC-AGT--ATTTTATCATTTTCAACATAAATCAG------AATT----  69948
Dog          GCAATTTTTTAAAAAGCCATA-AATTAGAAAAGC-AGT--ATTTTGTCATGTTCAATA-AAATCAA------ATTTTTCG  62904
BelugaWhale  AAATGCC--------ACAATCTGGATTG---GTCTGGCT--ATTTATTTCCTTATAGTGTCATCTT------TTT-----  62257
Donkey       AAACTTCCTTTAAAAAAATCATAACTAAAAAGGC-AGT--ATTTTATCATGTTCAGCATAAATAAATCATAAATTTTTGG  69027
Cat          AAATTTTTTTAAAAA-CCATATAATTAGAAAAGC-AGT--ATTTTATCATATTAGACATAAAT-----------------  69553
Ferret       AAAAAAAATTTTAAAGCCATATAATTAGAAAAGC-AGT--ATTTTATCATGTTCAGTAGAAATTAT------ACTTTTGG  70404
Lion         AAAATTTTTTAAAAA-CCATATAATTAGAAAAGC-AGT--ATTTTATCATATTAGACATAAAT-----------------  67245
Vaquita      AAATGCC--------ACAATCTGGATTG---GTGAGGCT--ATTTATTTCCTTATAGTGTCATCTT------TTTA----  63610
Pig          TCGTGGTTTGATATTATAATATTGTTTAGCCGTTGAGTTGAGTTTATTTTGGTTTACTTTTCTATG------TGTT----  63037
```

```
Majority     -CATAAGTCACATGAAAATACCTGTA-TAATTCACTGTCCCATAACTTTGGCTTTCTCATTTCATTTCTGTTTTAA--CA
                      90890     90900     90910     90920     90930     90940     90950     90960
Human        TTAGAGGTCACATGATAATACCTGT----CCTAACTGTCCCATAACTTCGGCTTTCTCATTTCAT--GTGTTTTAA--TA  72097
PolarBear    CCAGAAGTCACATGAAAATACCTGTA-TAACTCAATGTTCCGTAACTTTGGCTTTCTCATTTTATTTCTGTTTTAA--CG  32335
Cow          ---TTTTTCATCCAAAGTTCATGAAA--ATACCTGTCCAATA--AATTTGATTTTCTCATTTTGTTTCTGTTTTAA--CA  70019
Dog          TCATAACTCACACGAAAAT-CTTGTACTAACTCACTGTCCCATAACTTTGGCTTTCTCATTT-----CTGTTTTAA--TG  62976
BelugaWhale  -----TTTTTTTTTAAGATCACGT----GTTCTTGTGTA--------TTCATATT--------ATTCATTTTTAAAA---  62309
Donkey       TCAGAAGTCACATGAAATTACCTGTACTAATTCATTGTCCCATAA--CTGGTTTCCTCATTCCATTTCTGTTTCAA--CG  69103
Cat          -CATAAATCATATGAGAATATCTGTTCTAATTCACTGTCCCATATCTTTGGCTTTCTCATTTCATTTCTGTCTTAA--CA  69630
Ferret       TCAGAAGTCCTATGAAAATACTTGTACTAACTCACTGTTCCATAACTTCGGCTTTCTCATTT-ATTTCTATTTTAA--CA  70481
Lion         -CATAAATCACACGAAAATACCTGTTCTAATTCACTGTCCCATATCTTTGGCTTTCTCATTTCATTTCTGTCTTAA--CA  67322
Vaquita      ---TTTTTTTTTTTAAGATCACGT----GTTCTTGTGTA--------TTCATATTTA---TTTATTCATTTTTAAAA---  63669
Pig          ---TATGATGCATAAAATCAACAATG--ATTTTTTTAAAACAGCAGTTTAAAACTTACTTTTAAATGACTTCTGAAAGCG  63112

Majority     TCTAAGAAGTAAGAAACTATAGTTGA-ACGTGCCTACTTCTCTATGTGTGCATTCTTAGAAAATGATXTTGAGTTC-TTT
                      90970     90980     90990     91000     91010     91020     91030     91040
Human        TCTAAGAAGT---AAAAAATAGTTGG----AGCATGCCTCTTTATATGTGCAATTTT---AAATTATGTTGACTTC-TTT  72166
PolarBear    TGTAAGAAGTAAGAAAGTATAGAAGTTAAGTTGAAACTTCTCTACATGTGTATTCTTAGAAAATGATGTTGAGTTC-TTT  32414
Cow          TCTAAGAGTTTAGAAACTGTAGTTGA-GCTTGCCTATTTTTCCATGTGTGCATTTTTAGAAAACG---TTGAGTTC-TTT  70094
Dog          TGTAAGAAGTAGGAAACTATAGAAGTTAGGTTGAAACTTCTCTACATGTGCATTCTTAGAAAATAATACTGAGTTAATTT  63056
BelugaWhale  -------------AATATTTATTTGG-CTGTGTCGAGTCTTAGTTGCG-GCCTGC-----GGGCT---CTAAGTTC-CCT  62365
Donkey       TTTTGGAATTAAGAAGCTATTGTTGA-GCATGCCTACTTCTCCATGTGTGCATTTTTAGAAAATGATGTTGAGTTC-TTT  69181
Cat          TCTAAGAAGTAAGAAACTACGGTTGA-ACATGCCTATCTCCCTATGTGTGCATTCTTAGAAAATGATCCCGAGTCT--TT  69707
Ferret       TATAAGAA----GAAACTGTAGAAGTTGAGTTGAAACTTCTCT---TATGTATTATTAGAAAATGATGTTGAATTC-TTT  70553
Lion         TCTAAGAAGTAAGAAACTACGGTTGA-ACATGCCTATCTCCCTATGTGTGCATTCTTAGAAAATGATCCTGAGTCT--TT  67399
Vaquita      -------------AATATTTATTTGG-CTGTGTCGAGTCTTAGTTGTG-GCCTGC-----GGGCT---CTAAGTTC-CCT  63725
Pig          TTTGTCGCTTTTGAGGGGGTTATTTA--CATGGTAATTTTTTAAAA---ACCTTTTGTTAAAGAT---TTCAGTTA-TC-  63182
```

```
Majority     GGATTATTGAACTATCCTCXATTTTGCTATGGCTGAATTTTXAAAATATTAA---TTTTTCAGTCTTTAAAATGTTTCTT
                       91050     91060     91070     91080     91090     91100     91110     91120
Human        GGATTATTGAACTGTCCTCTGCTTTGCTATAGCTGAATTTTTAAAATGTTAA----TTTTGAAGTCTTAAAATGTGTCTT  72242
PolarBear    GGATTATTGAACTATCCTCCATTTTGCTATGGCTGAATTTTTAATATATTAA---TTTCTCAGTCTTTAAAATGTTTATT  32491
Cow          GGATTATTAAAGTATCCTCTATTTTTCTATGGCTGAATTTTAAAAATACTGG-GTTTTTCCAGTTTTTAAAATATCTCTT  70173
Dog          GGATTATTAAACTAACC--AATTTTGCTGTGGCTGAGTTTTTAAAGTATTAA---TTTCTCAGTCTTTAAAATACTTCTT  63131
BelugaWhale  G-ACCA--GGGATCAAACCCAGGCCCCCTGCATTGGGAGCACAGAGTCTTAA-CCACTTTCTGTCTTTAAAATGTCTCTT  62441
Donkey       GAATTATTCAACTGCCT-CTATTTTGCT-TGGCTGAATTTTAAAAATACTAA----TTTTTTGTCTTTAAAATGTCTTTT  69255
Cat          GGATTATTGAACTATCCTCTGTTTTCTCAGGGCTGAATTTTCAAAATATTAA---TTTCTCAGTCTTTAAGATGTTTCTT  69784
Ferret       GGATTATTGACCTATCCTCCATTTTGCTGTGGCTAAATTTTAAAAATATT-------TCTCAGTCTTTAAAATGTTTACT  70626
Lion         GGATTATTGAACTATCCTCTATTTTCTCAGGGCTGAATTTTTAAAATATTAA---TTTCTCAGTCTTTAAGATGTTTCTT  67476
Vaquita      G-ACCA--GGGATCAAACCCAGGCCCCCTGCATTGGGAGCACAGAGTCTTAA-CCACTTTCTGTCTTTAAAATGTCTCTT  63801
Pig          --ACTAATACAGTGAATCCCACATATTCATCATTTCAATTCACTAGTTCCCATACTTGTTTTATCTGTTAAGTTTTGTTC  63260

Majority     TTAGAA----AXGAAGTTXXXAGAGCTAXAATAGTTCXATTTXAAATTTXTAACXGTGGATAAAA--TTTTAACTTXTX
                       91130     91140     91150     91160     91170     91180     91190     91200
Human        TTGGAA----AAAACATTGGAAGAGCTAAAGTAGATAGAGTTGTTCATTTAAATTGTTTATTTTTTATTTTATTTTATT  72317
PolarBear    TTAGAA------GTAGTGAGCTGAGATAGAATTGTTC-ATTTAAAATACCTGTCAGTGGACAAAA--TTTTAACTTCTA  32561
Cow          CTAAA----AAGGAAGTTGCAAGAGCTGA---AGTTGAATTGTTCATTTTTAAATTTGGATAA-AA-TTTTCACTTGTG  70243
Dog          TCAGAA----AAGAAGTAATCTGAGATAAAATTGTTC-ATT-AAAATAACTGTCAGTAGATAAAAGTTTTTAACCTCTA  63204
BelugaWhale  TTAAA----GAGGAAGTTGCAAGAGCTGG---GGTAGAATTGTTCATTTTAAAATTTGGATTA-CA-TTTTTACTTGTG  62511
Donkey       AGAAAAGAAGTTGGAAG--GGCTGAGGTAGAGTTGTTCATTTTAAAATTTGT--CAGTAGATAAAA--TTTTAAC-----  69324
Cat          TTAGAA----AAGAAGTAATGAGAGCTGAGATAGA---ATT--AAGTCCC-----------AGAA----TTGATT----  69835
Ferret       TTAGAAA--ATAAGTAATGAGCTGAAATAGAATTGTTT-ATTTTAAATACCTGTCAGTGGATAAAA--TTTTAACTTCTA  70701
Lion         TTAGAA----AAGAAGTAATGAGAGCTGAGATAGA---ATT--AAGTCCC-----------AAAA----TTGATT----  67527
Vaquita      TTAAA----GAGGGAGTTGCAAGAGCTGG---GGTAGACTTGTTCATTTTAAAATTTGGATTA-CA-TTTTTACTTGTG  63871
Pig          TTATATTTTGAAGCAAATCCTTAATGTCATGTCATCTCAAACAACCATTATTTCTCTAAAATAGTGA-CATCTTCTTAAC  63339
```

```
Majority     AATCATAAATTAACTTGAAGCAATAACCTTTAGA---GAGTTTAATTXTXTG----A-GTXCATTTTAAATAATATTT--

                      91210     91220     91230     91240     91250     91260     91270     91280

Human        TTTTTTAGTTGGAGTACTGGCTCTGTTGCCTGGGCTGGAGTGCAGTGGCGGGATCTCTGCTCACTGCAAGCTCCGCCTCC  72397
PolarBear    AATTGTAAATTAACTTGAAGCAGTAACCTTTAGA---AAGTTTAACTCT--G----A-GTCCTTTTTAAATAATATTT--  32629
Cow          AATCACAAATTAACTTGAAGCAGTGACCTTTTAA--AGAGTTTATTTAATTCTGTTA-GTATATTTTAAATAATATTT--  70318
Dog          AATCATGAATTAACTTGAAGCAATAATCTTTAGA---GAGTT-AACTCTTTG----A-GCCCTTTTAAAATAATATTT--  63273
BelugaWhale  AATCATGAATTAACTTGAAGCAATAAGCTTTAAA---GAATTTATTTAACTC--CAA-GTACATTTTATATAATATTT--  62583
Donkey       -ATCTCAAATTAACTTGAAGCAATAACGTTTAGA---AAGTTTATTTAAACTCTTTGTGTCCGTTTTAAATAATGTTT--  69398
Cat          -GTAGTGTATTGGCTCAAGATTATAG---TGAAA---ATGCTGGATCCT------------------AAAAAATAAAG--  69888
Ferret       AGTCAAAAATTAACTTGAAACAATAACCTTTAGA---GAGTTTAACTCTTTG----T-GCCCTTTTAAAATAATATTT--  70771
Lion         -GTAGTGTATTGGCTCAAGATTATAG---TGAAA---ATGCTGGATCCT------------------AAAAAATAAAG--  67580
Vaquita      AATCATGAATTAACTTGAAGCAATAAGCTTTAAA---GAATTTATTTAACTC--CAA-GTACATTTTATATAATATTT--  63943
Pig          AAGCATAT-CTGATCAGAATTGGTCACAGCCTGATCAAAGCCTGATTATCATGCCTG-TCAAACTTGAGAATGAGTTC--  63415

Majority     -GAATTAATGT-TTTTAAATCTGAAA--TTATTTTTGTCTTCTXAGCAAAGGAAATGTGTT-GGTGGTAATTGCATACTT

                      91290     91300     91310     91320     91330     91340     91350     91360

Human        TGGGTTCACGC-CATTCTGCC--GCCTCAGCCTCCTGAGTAGCTGGGGCTACAGGTGCCCTCCACCACACCTGGCTAATT  72474
PolarBear    -GAATGAATGTGTGTTAAATCTGGAA--TTATTTTTGTTTTCTCAGCAAGAGAAATGTGTT-GGTGGTAGTAGCATACTT  32705
Cow          -GAATTAACAT-TTTAAACCTGGAAAATTTTTTTTTGTCTTCTGAGCAAATGAAATG-GT--------AATAGCATACTT  70387
Dog          -GAATGAATATATGTTAAATCTGGAA--CG--T---GTTTCTTAGCAAAGGAAATCTGTT-AATGGTAGTTGCCTACTT  63344
BelugaWhale  -AAATTAATATGTTTTAAATCTGAAA--ATTTTTTTGTCTTCTCAGCAAAGGAAATA-GTT-GGTGGTAATTGCATACTT  62658
Donkey       -GAATGAATGTGTTTTATGTCTGGAA--TTATTTTTATCTTCTTAGGAAAGGAAATGTGTCTGGTGGTAGTTGTGTACTT  69475
Cat          -AGGTACTTGT---TTAGATC--A---------------------CT--GTGAGAACTACA-AGGGATTATTGT-C----  69933
Ferret       -GAATGAATGTATCTTAAATCTGGAA--TTATTTTTGTTTTCTTGGCAAGGGAAAGGTGTT-GGTAGTAGTTGCATGCTT  70847
Lion         -AGGTACTTGT---TTAGATC--A---------------------CT--GTGAGAACTACA-AGGGATTATTGT-C----  67625
Vaquita      -GAATTAATATGTTTTAACTCTGAAA--ATTTTTTTGTCTTCTCAGCAAAGGAAATA-GTT-GGTGGTAATTGCATACTT  64018
Pig          -TTAATGACAT-TGGTTACTCAGA---TCTATCTTTGAATTTCCC-CAGTTGTCTCA-AA--------AATAGTGTTCTT  63480
```

```
Majority     -------TGGAGGTATAATGGGCCACAGATATGAGG-GA----TAGA-GAAACAATXTTA---TXTCXGTXCATATTTAC

                       91370     91380     91390     91400     91410     91420     91430     91440

Human        TTT--TTTGTAGTTTTAGTAGA-GACAGGCTTTCACCGTG--TTAGCCAGGATGGTCTCA--ATCTCCTGACCTCGTGAT  72547
PolarBear    -------CGGAAGTATAACGGGCCTCAGGTGTGATATGAACATTAGAAGAAATAACGTTACCATGTCGGTGCATATTTGC  32778
Cow          -------TGAAGATACAATGGGCGA-AAACATTGGG--------AGA-AAAACATTAATG---CATCAGTTTGCATTTAC  70447
Dog          -------TGGAAATATAATAGGTCACAGATGTGATATGAATGTTAGA-GAAATAATGTTACCATGTCCATGCACATTTAC  63416
BelugaWhale  CATACTTTGGAGGTATAGTGGGCCATAAACATTAGG--------AGA-AATACATTATTA---CATCAGTTCATATTTAC  62726
Donkey       -------TGGAGGTGTAATGGGCCACAGATATAAAC--A---TTAGGAGAAGTAT-GTTATTACATCAGTTTGTGTTTAT  69542
Cat          ----------AAGCTCA------CACAGAAATGGGGGGGG----GCAGGGAACAA--------TGTAGGGGCAGAGCTAG  69985
Ferret       -------TGGAGGTATAACAGGCCACAGATGTGATATGAACATTAGAAGAAATAACATTTTCATGTTGGTGCATATTTAC  70920
Lion         ----------AAGCTCA------CACAGAAATGGGGGGG------CGGGGAACAA--------TGTAGGGGCAGAGCTAG  67675
Vaquita      CATACTTTGGAGGTATAGTGGGCCATAAACATTAGG--------AGA-AATACAGTATTA---CATCAGTTCATATTTAC  64086
Pig          -------TAAGGATTGATTTATTCA-GCATATGATCCTC----TGGA-GGGCCGCAGATGG--CATTTGGTAGT-CTCCC  63544

Majority     TTATTTGCCAGCATTCTCAAATGGAATAAGCTTATTXCTGTAGGACTAXA--ATGT-TATTCATAGGCTGCCTTTTAACT

                       91450     91460     91470     91480     91490     91500     91510     91520

Human        CCGCCCACCTTGGTCTCCCAAAGTGCTGGGATTACAGTCAT-GAGCCACCGTGCCTGGCCTCATTTAAAATTTTTTAACA  72626
PolarBear    TTATTCGCCAGCATTCTCAGATA-----------TTCCTGTTGGGCTA--AAATGT-TATTCATAGACTGCCTTTTAACT  32844
Cow          TCATGCACCAGCATTCTCAAATGGAATAAACTTGTAACTGTAGTACTAAA--ATGTAAATTCATAGGCCGCTTTTTAACT  70525
Dog          TTATTTGCCAGCATTTTCAAATAGGATAAGTTTATTCCTGTAGGGCTATAAGATGT-TGTTCATAGACTGCCATTTAACT  63495
BelugaWhale  TTATTTACCAGCCTTCTCAAATGGAACAAGCTTATAACTGTAGGACTAAA--ATGT-TATTCATAGGCTGCTTTTTAACT  62803
Donkey       T--T--ATCAGCATTTTTAAATGGAATAAGCTTGTTATGATAGGACTAAA--ATGC-TATTCATAGGCCACCTTTTAACT  69615
Cat          A-----GCCAGAA--CTGAAGTCG-----------TCATTT-----------GTGT-AGTCTATATTCTTTCCTCTCACC  70035
Ferret       TTATTTGCTAGCATTCTCAAATAGTATAAGCTTATTCTTGTAGGACTA--AAATGT-TATTCATAGATTGCCTTTTAACT  70997
Lion         A-----GCCAGAA--CCGAAGTCG-----------TCATTT-----------GTGT-AGTCTATATTCTTTCCTCTCACC  67725
Vaquita      TTATTTACCAGCCTTCTCAAATGGAACAAGCTTACAACTGTAGGACTAAA--ATGT-TATTCATAGGCTGCTTTTTAACT  64163
Pig          TCCTGAGTCAGGCCTCTCCCTCCACCCTCAGTC-CCATTGAGTTATTAGAG-AAATCTAGTTATAGGTAGTCTTTGAGAA  63622
```

```
Majority     AT--CCAGGGTA-AAATTTTCATXTAAA-GTTCTTAATGGA--TGATCATATAGTCTTTXCTTGAACTAATXTGTT-ATG
                              91530     91540     91550     91560     91570     91580     91590     91600
Human        CT-----GGGTA-AAATTTTTATTTTTGAGTCACAAATTAATTTGAAGCTGTGTCTTTTAGAGAGCTTATTCAACTCTTA  72700
PolarBear    AT--TCAGGGTA-AAATTTTCATATGAA-GTTCTTAATGGA--TGATCATGTAGACTTTGCTTGAACTAATCTGTT-AGG  32917
Cow          AT--CTTGGATGCAAATTTTCATTCCCAAGTTCCTAATGGG--TGATAATATAGTCTTTGCTTAACTGATTTTGTT-ATG  70600
Dog          GT--CCAAGGTA-ATATTTTCATATAAA-GTTCTTAATGGA--TAATCCCATAACCTTTGCTTGAACTA---TGTT-AGG  63565
BelugaWhale  AT--CCAGGGTACACATTTTCATTCAGA-GTTCTTAATGGT--TGATTTTATAGTCCTAAACTGA-----TTTGTT-ATG  62872
Donkey       AT--CCGGGGGCAAAAGTTTTATATAAA-ATTCTTAGTGGA--TGACGATGTAGTCTTTACTTAAACTGATCCATT-ATA  69689
Cat          AT--T--------GTGTTTTTATGAAAA-AAAGGAAAAGGG--GAAACAGAT-----------GAAGGAA-------GGG  70084
Ferret       AT--CCGAGGTA-AAAATTTCATATAAA-GTTCTTAATGGA--CGATCACATAGCTTTTGCTTGAACTAATCTGTT-AGG  71070
Lion         AT--T--------GTGTTTTTATGAAAA-AAAGGAAAAGGG--GAAACAGAT-----------GAAGGAA-------GGG  67774
Vaquita      AT--CCAGGGTACACATTTTCATTCAGA-GTTCTTAATGGT--TGATTTTATAGTCCTAAACTGA-----TTTGTT-ATG  64232
Pig          AATGCCTCAGTCTGAATTGGTCTGGCT--GTTCCTTATAG---TGTCAACTTGTTCCTCCATCACCTCTGCTTTAT-ATA  63696

Majority     XTCACCCATTATATTGTTCTCAGCTA--TA-GXTTTAA----GAAAT----TTTATTTATX-XX------X-XXXXT-CT
                              91610     91620     91630     91640     91650     91660     91670     91680
Human        ATTCCATTATAAATAATATTTGAATGAATGTGTTTTACATCTGGAATGATTTTTAT-CATCTGAGTAAAGGAAATATGTT  72779
PolarBear    TTCACCCGTTATATT---CTCAGCTA--TG-GTTTTAA----GAAAT----TTTATTTAC-----------------CT  32965
Cow          GTCATTGATTATATTGTTCTCAGCTG--TA-GCTTTAA----GGAAT----TTCTTTTACTAATCCCGTATTTACCTTCT  70669
Dog          TTCACTCATCATATTGTTCTCAGCTG--TA-GTTTTAA----GAAAT----TCTATTTAC-----------------CT  63616
BelugaWhale  GTCATCCATTCTATTGTTCTCAGCTA--TA-GCTTTAA----GAAAT----TTCTTTTATTAATCCTACGTTTACCTCCT  62941
Donkey       TTCACCCATTGTATTATTCTCAGCTA--TA-GTTTTAA----GAAATGTCTTCCTTTTGTTAATCCCATGTTTACCTTAT  69762
Cat          AGGACTTCTGAAATTG--GTTGG--A--AA-ACCTGAG----GGGAT----TCTG---GT-----------------CT  70128
Ferret       TTCACCCATTGTATTATTCTCGGCTA--TA-GTTTTAA----GAAAT----TTTATTCAC-----------------CT  71121
Lion         AGGACTTCTGAAATTG--GTTGG--A--AA-ACTTAAG----GGGAT----TCTG---AT-----------------CT  67818
Vaquita      GTCATCCATTCTATTGTTCTCAGCTA--TA-GCTTTAA----GAAAT----TTCTTTTATTAATCCTACGTTTACCTCCT  64301
Pig          AACTACGCATTAGCTCCAAAAGCTTGATTA-GATTCAG----ATTCA----TTCATTCATTTATTTGGC-TGTGCCTGCA  63766
```

```
Majority     CCATTTTCAT-GATAGTCTGTCAGATATTTGAATCTATT--X-XTAGTXATXCTGX--XTCATATTTTTAAAGCTAAACA
                      91690     91700     91710     91720     91730     91740     91750     91760
Human        TGATGGTAGTTGGGAACTTTGAAGGTATTACGGGCTACAGATGAGAAAAAAATGTTACTACGTCATTTCATGTTTACATA 72859
PolarBear    TCATTTTCAA-GACAGTCTGTCAGATATTTGAATTTATC----CTAGTGTTACTAT---CCATATTTTTCAGGCTAAACA 33037
Cow          CCTTTTCCAT-GATAGCCTGTCACATATTTGAAAACATTTTTCCTATTTATTCTGG-CTTCGTATTTTTAAAGCTAAACA 70747
Dog          TCATTTCCAA-GACAGTCTGTCAGATAGTTGAATTTATTTATTCTAGTGTTACTGT---TCATATTTCTCGGGCTACACA 63692
BelugaWhale  CCTTTTCCAT-GATACCCTGTCACACATTTGAAAAC----CTTATATTTATCCTGGTGTTCATATTTTTAAAGCTAAACA 63016
Donkey       CCTTTTTCCT-GACAGTCTGTCACTTACCTCTCTCG--------TGTTCATCCTAGTGTTCATATTTTTAAGGCTAAACA 69833
Cat          CAATTTT-------AGACTTTCTGA-ACTTAAGTCTGGT------GG----------------ATTTGTAGAG------- 70171
Ferret       TCATTTTCAA-GATAGTCTGTCAGATATTTGAATTTATC----CTAGTGTTATTGT---TCATATTTTTCAGGCTA---- 71189
Lion         CAATTGT-------AGACTTTCTGA-ACTTAAGTCTAGT------GG----------------ATTTGTAGAG------- 67861
Vaquita      CCTTTTCCAT-GATACCCTGTCACACATTTGAAAAC----CTTATATTTATCCTGGTGTTCATATTTTTAAAGCTAAACA 64376
Pig          GTATATGGAA-GTTTCTGGGCCAGGGATT-GAACCCATACCACAGCAGCAACCTGA-GCCACTGCAGTAACAATGGCCGG 63843

Majority     GC----C--------TTAATTCAGTG-G-TAATCAACTTC-AGCCTTTGGATATAGAAGATAGXTTTAAATTAA------
                      91770     91780     91790     91800     91810     91820     91830     91840
Human        CT--GACCAGCATTTTCAATGAAATAAGCTTATTACTATAGGACTATAAAATGTTTTTCAAAGGTTTAACTGTCCAGGGC 72937
PolarBear    GC----C--------TGAATTTAGTA-G-TAATCAACACT-AGCCTTTGGATATAAAAGGTAGGTTTAAAGTAA------ 33096
Cow          GC----C--------TGAGTTCAGTG-G-TAATCAACTTC-CGCCTTTGGATATAGAAGATAATTCTAAATCAA------ 70806
Dog          GC----C--------TTAATTCAGTA-G-TAATCAGTGCT-AGCCTTGGAATATAAAAGGTAAGTTTAAATTAA------ 63751
BelugaWhale  GCTAAACAACAGAAATTGATTCAGTG-G-TAATCAACTTC-AGCCTTTGGATGTAGAAGACAGATTTAAATTAA------ 63087
Donkey       GC-------T-----TGACTTCAGTG-G-TAATCAACTCC-AGCCTTCAGATACAGAAGGTAGATTTAAATTAAGATTTA 69898
Cat          -----------------------------------------AACTCTTGAAAATAGATGAGGAAGTCGGCTGAT------ 70204
Ferret       ---------------------------G-TAATCAATTTT-AGCCTTTGGATATAAAAGGTAGGTTTAAATTAA------ 71234
Lion         -----------------------------------------AACTCTTGAAAATAGATGAGGAAGTCAGCTGAT------ 67894
Vaquita      GCTAAACAACAGAAATTGATTCAGTG-G-TAATCAACTTC-AGCCTTTGGATGTAGAAGACAGATTTAAATTAA------ 64447
Pig          AT----C--------CTAACCTGCTG-AACCATCAAGGGC-CTCATTTCTACCTTGAAAACAAGAATAAATGTC------ 63903
```

```
Majority     ------ATCAGACTGTAGATTTAATCTTGAAAAAATTTT-----GGT-T-TTAAGAGTTGA-CAGTTATGT-X-----TG
                       91850     91860     91870     91880     91890     91900     91910     91920
Human        AGATACTTAAGACTATCTGATCATCCATTAAAAACTTTTCACATAGTCTTGCTTAAATGGATCCATTATGTTTACCCATT 73017
PolarBear    ------ATCAGACTGTAGATTTATTC-TGAAAAAGTGTT-----GGTTTTT-AAGAGTTGA-CAGTTATAG--------T 33154
Cow          ------ATCAGATGGTAAATTTAATCTTAAAAAAATTTT-----GCT-T-TTAAGAGTTGA-CACTTATGTATAT--GTG 70870
Dog          ------ATCAGACTGTAAATTTTACTTTCAAAAAGTGTT-----TTTTTTTTAAGAGTTGA-CAGTTATG---------T 63810
BelugaWhale  ------ATCATACTGTAGATTTAATCTTGTAAAATTT-------------TTAAGAGTTGA-CAGTTATGTAT----GTG 63143
Donkey       AATTAGATCAGGCTGTAGATTTTACTTTGAAAATATTTT-----GTGTTTTTAAAAGTTGA-CAGTTATATAT----ATG 69968
Cat          ------GTTA------AA---------TAACAAAATGTT-----TG--T-----GAAT----CAGTTTTGG--------G 70239
Ferret       ------ATCAGACTCTAGATTTTCAAATGAAAAAGTGTT-----GGTTTTT-AAGAGTTGA-CAGTTACAT--------T 71293
Lion         ------GTTA------AA---------TAACAAAATGTT-----TG--T-----GAAT----CAGTTTTGG--------G 67929
Vaquita      ------ATCATACTGTAGATTTAATCTTGTAAAATTT-------------TTAAGAGTTGA-CAGTTATGTAT----GTG 64503
Pig          ------TAGTGGTTTCAGCGATGCTTTAAAATAGATTGT-----GGG-T-TCA-GTTCAGG-TGGTGGCATTCTG--ATC 63966

Majority     GTATXG-------CTAAAACAAA--GAAAAGCCTTCTA-------AAGTATATTACT-GTTGCTTCTGTAATTAATAGAA
                       91930     91940     91950     91960     91970     91980     91990     92000
Human        ATATTGTTCTCAGCTGTAGTTTTAAGAAATTTATTTCATTTGTTAATACTTACTTTCCATTACCTTCCCCTTTTCTGTGA 73097
PolarBear    ATAT---------CTAAGACAAAG-GAAAGCCTTTCTA-------AAGTATGTTACT-ATTGCTTCTGTGACTGATAGAA 33216
Cow          GTGCAA-------CTAAAACAAA--GAAAAGCCTTCTA-------AAGTATATTACT-ACTGTCTCTGCAATTAGTAGAA 70933
Dog          ATAT----------TGA---AAAC-AAAAGCTTTTCTA-------AAGTGTATTACT-GCTGCTTTTATAATTGACAGAA 63868
BelugaWhale  GTATGG-------CTAAGACAAA--GTAAAGCCTTCTA-------AAGTATATTACT-GCTACCTCTGCAATTAGTAGAA 63206
Donkey       GTATGG-------TTAAAACAAA--GAAAAGCCTTCTA-------AAGTATATTATT-GCTGCTTCTGCAATTAGAAGAA 70031
Cat          GTAT-----------------------------GTT---------ATGTGTTCTGTG-TTCACTCTAATACTTAACCACA 70280
Ferret       GTATGG-------CTAAACCAAAG-AAAAACCTTTCTG-------AAGTATGTTACT-GTTGTTTCTATAATTAACAGAA 71357
Lion         GTAT-----------------------------GTT---------ATGTGTTCTGTG-TTCACTCTAATACTTAAGCACA 67970
Vaquita      GTATGG-------CTAAGACAAA--GTAAAGCCTTCTA-------AAGTATATTACT-GCTACCTCTGCAATTAGTAGAA 64566
Pig          CTTCCA-------TTATATTTTTCTGTTAATAGTTTTA-------GTATCTACTAATGATTGCTGCCTTAATTTATTTCA 64032
```

```
Majority     GAAAAACTXTGTA-TTGTGACCATAGTATAAAATGTGCCTCTATCTAAAGGAGGTAAA---AATTAGGCAGAAGATTTTC
                               92010     92020     92030     92040     92050     92060     92070     92080
Human        CAATCCGTTGATACTTGAAGACCTCTCTTGTATTCATCTTAGGGTTAATATTTTTAAGGCCAAACAGCCTAAATTCTATG  73177
PolarBear    GAATAACTGTGT--TTGTGACCACAGTCTAAAATGTGCCTCTATCTAAAGCAGGTAAA---AATTAGG----AGATTTTC  33287
Cow          TAAAAAGTATGTATTTGTGACCAAATATGAAATGCTCCTCTTATCTGAAGGAGGTAAA---TATTATACAGAAGGTTTTG  71010
Dog          GAAAAACTG----------ACTGCAGTATAAAATGTGCCTCTATCTAAAGTAAGTAAA---AATTAGGCAGAAAATTTTC  63935
BelugaWhale  GAAACACGATGCATTTGTGACCATAGTATAAAATGTGCCTCTATCTGAAGGAGGTAAA---AGTTATGCAGGAACTTTTC  63283
Donkey       GAAAAACTGTATATGTTTGACCATAGTATAAAATGTGCCTGTATCCAAAGGACGTAAA---AATTACACAGAAGATTTTC  70108
Cat          TGAAAGTTT---------AGCCAGAGTACAAAAT-TGGATTTATCCAA-----G---------TCTGGCA----------  70326
Ferret       GAATAACTGTGT--TCGTGACCACAGTATAAAATGTGCCTCTATCTAAAGCAGGTAAA---AATTAGGCAGAAGATTTTC  71432
Lion         TGAAAGTTT---------AGCCAGAGTACAAGAT-TGGATTTATCCAA-----G---------TCTGGCA----------  68016
Vaquita      GAAACACTATGCATTTGTGACCATAGTATAAAATGTGCCTCTATCTGAAGGAGGTAAA---AGTTATGCAGGAACTTTTC  64643
Pig          TTTGGAGCTTGCAAATGTTGTTTTTTTCCTTCTAATTCTCCTAT----AGTAATTGAA---AAATATTTATAATGTAGTG  64105

Majority     ACT-----CTCTAAAGATXCACCAAGAAAXCAATAGATA-CATATAGG---AAATTT-AT------------T-TTTTGA
                               92090     92100     92110     92120     92130     92140     92150     92160
Human        GTAATCAACTCCAGCCTTGTGTAATGAAATCTTCTGCATAAAGATAGGTTTAAATCAAATCAGATTGCAGATTTTATTGA  73257
PolarBear    ACTAGTTCCTCTAAAGATACACCAAGAAAACAGTAGATAATATATAGG---AAATCTCATTTCGATTCTTC---TCTTGA  33361
Cow          AGT-----CTCTAAAGATGCACCAAGAAAGCAATAGATA-CATATAGG---AAATTT---------------CATATAGA  71066
Dog          ACTAGTTGCTCTAAAGATAGAGCAAGAAAACAAAAGATG-CATATAGG---AAATCTCATTTTAATTCTTC---TCTTGA  64008
BelugaWhale  AGT-----CTCTAAAGATGCACCAAGAAAGCAATAAATA-CATACAGG---AAATTTTAG------------TGTTTTTT  63342
Donkey       ACTAGCTCCTCTAAAGATACATCAAGAAAGCAGTAGATT-CATATAGG---AAATTTTATTTAGATTTT---TTTCTTGA  70181
Cat          ----------TTGAAGA---------ATATCA-------------AAG---AAA--------------------------  70345
Ferret       ACTACTTCCTCTAAAGATACACCTAGAAAACA----------TATAGG---AAATCTCATTTGGATTCTCCCTTTTTTGA  71499
Lion         ----------TTGAAGA---------ATATCA-------------AAG---AAA--------------------------  68035
Vaquita      AGT-----CTCTAAAGATGCACCAAGAAAGCAATAGATA-CATACAGG---AAATTTTAG------------TGTTTTTT  64702
Pig          TTC-----CTTCATCAACTAAGGCTATTTATTGTTTGTG-CAGGACAG---GTTTTT---------------TGTTCACA  64161
```

```
Majority     TXTTTTTTTT--CTGAGTTCTTCCTAACTTCATATTTA-TTTCCCTTTTAGXTTCTATA-----------------TTTT
                              92170     92180     92190     92200     92210     92220     92230     92240
Human        AGAAATTGTGTTTTTAAGAGTTGACAAATATATGTTGTATGGCTAAAACAAAGAAAATACTTCTGTTGCTTCTGCATTTA 73337
PolarBear    TTTTTTTTCC--CTAAGTTCTTTCTAACTTCATATTTTGCTTCCCTTTTAGCTTCTGTG----------------CTTTT 33423
Cow          TTTTTTTTGT--GTGAGTTCTTCCTAGCTTCATATTTC-TCTCTCCATTTGTC--AATA-----------------TTTC 71124
Dog          TCTTTTTTTTCCCTGAGCTCTTCCTAATTTCATATTTA-CTTCCCTTTTAGCTTTTGTGATTTTTTTGTGGGTTTTTTTT 64087
BelugaWhale  TTTTGTTGTT--CTGAGCTCCTCCTAACTTCATATTTC-TCTTCCTTTTAATTTCTATA-----------------TTTT 63402
Donkey       CCTTTTTTTT--CTGAGTTCTTGCTAACTTCATATCTA--TTCGTTTTTAGTTT-TACA------------------TTT 70238
Cat          ---------T-------------------------TTA-----------GGCTACATTG--------------------- 70359
Ferret       TCTTTTCCCCT-CTGAGTTCTTTCTAACTTCATGTTTAGCTTCCCTTTTATCTTCTATA----------------CTTTA 71562
Lion         ---------T-------------------------TTA-----------GGCTACATTG--------------------- 68049
Vaquita      TTTTTTTTTTT-CTGAGCTCCTCCTAACTTCATATTTC-TCTTCCTTTTAATTTCTATA-----------------TTTT 64763
Pig          CCTTGTATTT--GGAAGGCAGGGGAAGAGTCGGAGTCC-TACCTTATGTAATT--CATG-----------------ATTT 64219

Majority     TT-X----TTTCCTGTTGATCTTGAT--TTCAATTTAAAXATGGTATT-X-XCTCTGA-TCTAC-CTTTXCTTTXTGCCC
                              92250     92260     92270     92280     92290     92300     92310     92320
Human        GTAGAAGAAAAACTATATATGTTTGTGACCAAAGTATAAAATATGATTCTTTCCAGGGAGGTAAAGGTT-ATGCAGAAGA 73416
PolarBear    TTTTT---TCCCCTGTTGATATTGAT--TTCAGTATAAAGGTGTTACT----TTTTGAGTTTAC-GTTTCCTCTATGCCC 33493
Cow          TT------TTTCCTGTTAATCTT--------AATTTTAAAATGGTATTTTATCTCTGA-GTCTT-CCTTTCCTTTTTTGC 71188
Dog          TTTTT---GTGCCTGTTGATCTTGAT--TTCAATTTAAAGGTGTTACT----CTCTGA--GTAC-CTTTCCTATATGCCC 64155
BelugaWhale  TTCTT---TTTCCTGTTAATCTTGAT--TTTAATTTAAAAATGGTATTCTGTCTCTGAATCTAC-CTCTCCTTTTTGCCC 63476
Donkey       TCTTG---TTTCCTGTTGATCTTGAT--TCTAATTTAAAAGTGGTATCCTCTCTCTCAGAATCTGCCTCTCTATTTGCCC 70313
Cat          ----------------TGAAGTCAAG--TCCAG---GAAGA-----------A------------------------- 70380
Ferret       TTTT-----CC-CTGTTGATCTTGAT--TTCAGATTAAAGATACTATT----CT-----TCTAC-CTTTTCTCTATGCCC 71624
Lion         ----------------TGAAGTCAAG--TCCAG---GAAGA-----------A------------------------- 68070
Vaquita      TT------TTTCCTGTTAATCTTGAT--TTTAATTTAAAAATGGTATTCTGTCTCTGAATCTAC-CTCTCCTTTTTGCCC 64834
Pig          TG------TACCACGATGGCCTG--------AATATGCACCTCCAAGCACACCTCCAATGATAA-AATTTATTTGCTCAT 64284
```

```
Majority     CACTTTCTAGATATTTCTTATA-C-----TTAAGTAATTGCCATGAGCATTTCAXATCCCTAGAAG--GATTCTGCCTCA

                       92330     92340     92350     92360     92370     92380     92390     92400

Human        TTTTCACTAGCAGCTCTAAAAGGCTACCCTCAATTAATTGCCATGAACATTTCATAGCCCTAGAAG--GATGTTGGCTCA  73494
PolarBear    CACTTCCTAGATATTTCTGATA-CCCTTATTAAGTAATTACCATGAGCATTTCATATCTGTGGAAG--AATTCTGCCTCA  33570
Cow          ACCCTTGTGAATTTTTCTTACC-C-----TTAAGTAATTGCACTGAGCTTTTCACATTCCTTGAAG--GATTCTGCCTCA  71260
Dog          TGCTTTCTAGATATTCCC-ATA-ACTTTGTTAAGTATTTGGCATGAGCATTTCATAGCCCTGAATG--GATTCTGCCTCA  64231
BelugaWhale  CACTTTCTGAATTTTTCTTACC-C-----TCC-TTAATTGCTGTGAGCTTTTCACATTCCTAGAAG--GATTCTACCTCA  63547
Donkey       CACTTTCTAATATCCTTC---------------TTAATTGCCATGAGCATTTCACATCCCTAGCAA--GATTCTGCTTCA  70376
Cat          -------TAGAAAGGGCTAAAA--------TAAATAAAGGCCACAAG--T---------------G--GATT---C----  70419
Ferret       CACTTCCTAGATATTTCTGATA-CCCTTGTTAAGTAATTGC-ATGAGTATTTCATATCCCTGATAG--GATTCTGCCTCA  71700
Lion         -------TAGAAAGGGCTAAAA--------TAAATAAAGGACACAAG--T---------------G--GATT---C----  68109
Vaquita      CACTTTCTGAATTTTTCTTACC-C-----TCC-TTAATTGCTGTGAGCTTTTCACATTCCTAGAAG--GATTCTACCTCA  64905
Pig          TTTTTAGTGGAAGAAGTTTATG-C-----CTA-GTTTTTGT-TTGCTTTTAATTAATTATTTAAACCCATTTATGAGTGG  64356

Majority     CCTCAGTG-GCATCCTGGTTTATTCATTCATTGAATTATTCAGCAGTCATTTTAGCACTXTGCTAGGTACTGTAGGGAXT

                       92410     92420     92430     92440     92450     92460     92470     92480

Human        TTTCAGTG-TCATCCTGGTTTATTC-TTTATTGTATTATTCAGCAGTCATTTTAACACTATGCTAGACACTTTAGAGATT  73572
PolarBear    CCTCAGTG-GCATCCTGGTTAATTCATTCATTGAATTATTCAGCATTCATTTTAGCACTATGCTAGGCACTG--------  33641
Cow          ACTCAGTG-GCATCCTGCTTTATTCATTCATTGCATTTTTTG---ATGGTTTTAGCACTGTGCTGGGTACTGTAGGGTCT  71336
Dog          TGTCAGTG-ACATCCTGGTTAATTCATTCATTGACTTATTCAGCAGTCATTTTAGCACTGTGCTAGGCACTGCAGAGATT  64310
BelugaWhale  CCTCAGTG-GCATCCTGCTTTATTCATCAATTGCATTTTTCAGCAGTCATTTTAGCACTGTGCTGGGTACTGTAGGGGCT  63626
Donkey       CCCCAGTG-GCACCCTGGTTTATTCATTCATTGTATTATTCAGCAGTCATTTTAGCACTGTGCTAGGTACTATAGGGACT  70455
Cat          ----AGTG-G-------------------------TTGTATGGGGTAGAATTGAGAAAGATGGAAG--AATG--------  70459
Ferret       CCTCAGTG-GCATCCTGGTTAATTCATTTATTGAATTATTCAGCATTCATTTTAGCACTATCTTAGGCACTGCCAGAATT  71779
Lion         ----AGTG-G-------------------------TTGTATGGGGTAGAATTGAGAAAGATGGAAG--AAGG--------  68149
Vaquita      CCTCAGTG-GCATCCTGCTTTATTCATTAATTGCATTTTTCAGCAGTCATTTTAGCACTGTGCTGGGTACTGTAGGGGCT  64984
Pig          GTACAGCCTGGATTCCTCCCAACCTCTGCCTTAAACTTTTGG---AACTATTGGTTACT---TCCAGTTTTTGTAGCTC  64430
```

```
Majority     CAGAA-AATTACAAGGTTTC-----------TGTGCTTATAATCTGGTAGAGG----AAACATATAAAT-AGCTCTAATG
                      92490     92500     92510     92520     92530     92540     92550     92560
Human        CAGAAGAGTAACAGGGTTTCTG---------TTCTCATGAAGCTTATCAGAGACAGAAAACATATGAGTTAGATCTAATT  73643
PolarBear    ----------------------------------GTTTAAAATCTGGTGGAGGCAACAAACATCTGAGT-AGCTCTAATG  33686
Cow          CAGAGAAATTACACAATTTC-----------TGTGCTTATAATCTGGCAGCAA----GCATCTGAATAT-ATATCTATTA  71400
Dog          CAG---AATTACAAGGTT------CTATAACTTGATTTACAATCTGGTGGAAGCAGCAAACATCTGAGT-AGCTCTAATT  64380
BelugaWhale  CAGAAAAATTACAAGGTTTC-----------TGTGCTTGTAATCT-------------TGTTTATA------CTCTGGT-  63675
Donkey       CAGAAGAATTACAAGATTTC-----------TGTTCTCATAATCTGGTGGAGGCAGCAAACATCTGAGT-GGCTTTAATT  70523
Cat          ----------------------------------------AAGTCGGGAAAGG-----A-GATTTAAAT-GTTAGAAATG  70492
Ferret       CAGAAGAATTACAGGGTTTCTGTTCTGTAATCTGGTTTACAATCTGGTGGAGGCAGCAAACAAATGAAT-AGCTCTAATG  71858
Lion         ----------------------------------------AAGTTGGGAAAGG-----A-GATTTAAAT-GTTAGAAATG  68182
Vaquita      CAGAAAAATTACAAGGTTTC-----------TGTGCTTGTAATCT-------------TGTTTATA------CTCTGGT-  65033
Pig          CAGAATGGTTTGAGA--TA-------------GTGACTTTAGCCTGGAAGCTA----GTGTTTTTA-AT-TTATTTAT--  64487

Majority     TGAGGCAAACTGAAA--TATAATAGAGTTGGTGTAGT-ATTAGAGCATAATGAAATG------X-GATTCAT-ATCX-AA
                      92570     92580     92590     92600     92610     92620     92630     92640
Human        GGAGGCAAACTGAAATATATAGTGGAGTTAGTGTGGTTATCAGCACATAAATGAGTG---------ATCCATCAACAAAA  73714
PolarBear    TGAGGCAAAGTGAAATCTGTAATAGAGTTGGTGTGGT-ATTAGAGCATAATGAAATGCATAACAAGATTCATTATCTGAA  33765
Cow          TGAGGCAAACTGAAA--TATGTAATAGTTATTATAGT-ATTAGAGCATAATTAAACG----G-GCACTTTATCATCCAAA  71472
Dog          TGAGGCAAAGTGAAATCTGTAACAGAGTTGGTGTGGT-ATTAGAGCATAATGAGATACACAGTGAGATTCAT---CTGAA  64456
BelugaWhale  ----GGAGGCAGCA---TATGCCTGAATATCTCTA----------------------------------ATGTGAGGC---  63712
Donkey       CGAGGCAAATTGAAATACATAATATAGTTAGTGTAGT-ATCAG--CACAATGAAATGA-----GTAATTCATCATCCAAA  70595
Cat          GATGGCAAAACAAAA-----AGAAAAAAAGATGTAGC-AAGAGAAGTTGCTGAAATG--------GATTGAT--------  70550
Ferret       TGAGGCAAAGTGAAATCTGTAATAGAGTTGGTGTGGT-ATTTGAGCATAATGAAATGCATGATGAGATTCATTATCTGAA  71937
Lion         GATGGCAAAACAAAA-----AGAAAAA--GATGTAGC-GAGAGAAGTTGCTAAAATG--------GATTGAT--------  68238
Vaquita      ----GGAGGCAGCA---TACGCCTGAATATCTCTA----------------------------------ATGTGAGGC---  65070
Pig          ----GTAATTATTAT--TATGTTTTAGTCTTTCTA---AGTGGCCCAAAAAGGAAAG----A-GGGGTAGAT-AGCT--A  64550
```

```
Majority     -GGAAAAATTGGGAGXGTTT-XTGGGC---------------AAA-----------AATAGXXX----------------
                      92650     92660     92670     92680     92690     92700     92710     92720
Human        -GGAGAAATTGGGAGGGTTTTATGGGC------------CAAAAACAGCATGATTAAATGTGATAGAGTATATGTCATGT  73781
PolarBear    -GGAGTAATTGGGAGGGTTTTATGGGC--------------CAAA-----------------------------------  33795
Cow          AGAGAAAGTTGAAGGGCATT-GTAGGC---CAAAAGCCATTTGATTGGGATAAAAAGACAGGACTTATTTATTTTTACTT  71548
Dog          -GGAGATATTGGGAATGTTTTATGGCCATTTGATTTGGATACAAATAAAAAGGGATGCCTGGGTGGCTCAGTGATTGTCT  64535
BelugaWhale  -----AAACTGAAATACAT-------------------------------------AATAGAGT----------------  63734
Donkey       -GGAAAAATTGGGAGGGTTTTATGGGA---CAAAGAGCATTTGATTTGGATAAG--AATAAAAAGGATTAAAAAAACCAC  70669
Cat          -GTAAAAATTATCAAAG-----T-------------------AGA-----------------------------------  70570
Ferret       -GGAGAAGTTGGGAGGGTTTAATGGGC--------------CAAA-----------------------------------  71967
Lion         -GTAAAAATTATCAAAG-----T-------------------AGA-----------------------------------  68258
Vaquita      -----AAACTGAAATATAT-------------------------------------AATAGAGT----------------  65092
Pig          -GTAAAAATTCAGATATGTT--CATCT---CAAAAAATAATTCATTCCATAAAATAACTAGGACGCATT-ACTATTATTC  64623

Majority     ------------X----TT-AGTTX----------T--X-X-XXGT-T-A--GC--------------------------
                      92730     92740     92750     92760     92770     92780     92790     92800
Human        TTTAGGTGTGATGAACATTCAGTTAT------GTGTGACGAATAGGATAATTG-------------------------A-  73829
PolarBear    ------------G-CCATTTGATTT-------GGAT--------------------------------------------  33811
Cow          ACTTTTTGTTTTGTT--TTTAGCTATGTCG-AGTCTTCATTGCTGTGTGAGGGCTTTCTCCAGTTTGCAGTGAGCGAGGG  71625
Dog          GCCTTCAGCTCAGGTCATCAAGTTCTGCATCAAGCTGCCCCACAGGGAACTTGCTTCACCCTCTGCCTATGTCTCTGCCT  64615
BelugaWhale  -----------------------------------T-TAATGTAGTATTAGAGC--------------------------  63752
Donkey       AGCATGGGGGCCAGCCACTGAGTGCTAA---AAGTTCTGTGCCCCTCACTCTGCT-------------------------  70721
Cat          --------------------AGTTT-------------------------------------------------------  70575
Ferret       ------------GGCCATTTGATT--------GGTT--------------------------------------------  71983
Lion         --------------------AGTTT-------------------------------------------------------  68263
Vaquita      -----------------------------------T-TAATGTAGTATTAGAGC--------------------------  65110
Pig          TCATTAAAACTA-----CTAGACTATAA-------TTCACTTCTATCTTAAAGCTAATTGTAAGAAAAAATAACCTCCGG  64691
```

```
Majority     -XX-------------XXAGTGAATAAATX----------------XXXXX-XAGX-TXAXCXXXTGT-ATAAAGCATAG

                               92810     92820     92830     92840     92850     92860     92870     92880

Human        -AAA------------AATATGAAAGGCTATG---------ATGCCAGAAAGTATTATGGGACAAGATCTTAAAACCAGT  73887
PolarBear    -------------------ACGAATAAGAAT---------------AAAAAACAGCATGAGCAGATGTGATAAAGTATAT  33857
Cow          CTA----CTCTTCATCGGAGTGTGCGACTTC------------TCCTTGTGGTGGC-T--TCTCTTGTTGTGGAGCATGG  71686
Dog          CTCTCTGTGCCTCTCATGAATGAATAAATATATTCTTAAAAAGAATAAAAATTAGTGTGAGCAGATGTGAGAAAGTATAT  64695
BelugaWhale  ATAA-----------TGAAGTGAGTAATTT-----------------------------A----------TCATCCAAAG  63782
Donkey       -------TTGGTGGCCTAGGTGCGCAGGTTCAGATCC----TGGCACAGACCTACTCCACTCATCAGCCATGCTGTGGAG  70790
Cat          ------------------------TAAA--------------------------------------G----AAA------  70583
Ferret       -------------------ATGAATAAA------------------AAATAAGAGCATGAGCAGATGTGATAAAATATAT  72026
Lion         ------------------------TAAA--------------------------------------G----AAA------  68271
Vaquita      ATAA-----------TGAAGTGAGTAATTT-----------------------------A----------TCATCCAAAG  65140
Pig          ATAAAAATTTTCTTTCGAAGTTGATGATTACAT--------AGTCTTTGCTTAAGC-TGATTTGTTGTCATCATCCATTG  64762

Majority     GT----TTTAGAG---AG-XXCAAXXAT--AGTGATTTATAAGCCATA--------------------------------

                               92890     92900     92910     92920     92930     92940     92950     92960

Human        GTTACCCAGGGAGTATGAATTTAATA---TGGGAATTCTTAAACTCC------------T--TTATGACTGGAAGATGAG  73950
PolarBear    ATCATGTTTAGGG---AGTGACAAGCATTCAGTTATTAGTAAGCTCTA--------------------------------  33902
Cow          GC----TCTAGAGTTCAGGCTCAATAGT--TGGGGTTCACGGACTT------AGTTGCTCTGCACCGTGTGGGATCTTCC  71754
Dog          ATCACATTTAGGG---AG-GACAA-CATTGAGTTATGATTAAGCCATAGGGTAATTGGTAAACTGTGAAAGGCAGTGGTA  64770
BelugaWhale  G--------AGA---------AAGTGGA--AGGGCTTTATGGGCCA----------------------------------  63809
Donkey       GCATCCCACATAGAGGGTGGAGGAGGATTGACACAAATGTTAGCTCTGGG----CTAATCTGC-----------------  70849
Cat          ------TTGTGAG---AC---CAG------AGTGTTAGACATATCACA--------------------------------  70613
Ferret       ATCATGTTTAGAG---AGTGACAAGCATTCAGTTATTAATAAGCCATA--------------------------------  72071
Lion         ------TCTCGAG---AT---CAG------AGTGTTTGACATATCACA--------------------------------  68301
Vaquita      G--------AGA---------AAGTGGA--AGGGCTTTATGGGCCA----------------------------------  65167
Pig          TT----TTCAGC-TCTAGCTTCAAGAAA--TTTTTTTTCTTAACCCTATA--ATTTACCTTCTTTTCTATGGTAGCCTGC  64833
```

```
Majority     ----------XTGAAGACCA--X-------------------------------TGGAGACTT-X-----XAATAAAATA

                      92970     92980     92990     93000     93010     93020     93030     93040

Human        CATCAGAGTGTCTGCGACCA--T-------------------------------TTGATGATATTA--TGTACCAAGTTT  73995
PolarBear    ----------TTGAAGAC------------------------------------TGGAGACTATTAGTCTAAATAAGATA  33936
Cow          TGGACCAGAGATTGAACCCATGTCC----CC-TGTATTGAC------------AGGCAGATTTGTAA--CCACTGGACCA  71815
Dog          CCAGAAAGAACTTGAGACAAAATCTTAGAACTGTTGTTAGCCAAGGGAGTATAGTGGAGACTATTAGTCTAAATAAAATA  64850
BelugaWhale  ---A-----------AGGCATTT-----------------------------------GATTT-----------------  63823
Donkey       ----------CTCAAGCCAAAA----------------A-------------AAAAGAAGCGGAACATTGGCAAGGGATG  70890
Cat          -----------GGATGATAA----------------------------------TAGGGGCT---------GAGAGAGGA  70639
Ferret       ----------TTGGAGAC------------------------------------TGGAGACT------CTAAGTAAGATA  72099
Lion         -----------GGATGATAA----------------------------------TAGGGGCT---------GAGAGAGGA  68327
Vaquita      ---A-----------AGGCATTT-----------------------------------GATTT-----------------  65181
Pig          CA---CATATTTGAAAACCTCTCTC----AC-ACTCCTGGT------------GTTTATATTT-TGA--AAGCTAAACAG  64890

Majority     X-X--GG-GGATCAAAACCAGGGACTTAAAAAA-CAAAGXAXXCAAXAXATGX---GAAAAAAAATATAXCAG-AAXXAT

                      93050     93060     93070     93080     93090     93100     93110     93120

Human        TAGATGTTTGGCTTTTTTCAGGTTATGAAAGCGGGGGATGAGTTAAGAACCACTGCTGTGAAGGATTCATCA---ACTAT  74072
PolarBear    TAGTTGTTGAATCTAAACCGGGGACATGAGA-ATGAAAAGAAACAA-ATATGGA-AAAAAGTAAACATAAAAGTAATAGT  34013
Cow          CCAG-GGAAGTCCAGAAGAAAGGATTTTGAAAA-CATTGCATG-AGCAGATGT---GAAGGAAAATATATC---TTGCAT  71886
Dog          CAGTTGG-GAATCTAAACTGGGGACATGAAATGAGAATAAAAGAGACAAATACATGGAAAAAAAACATAAAGGTAATAGT  64929
BelugaWhale  --------GGATAAAAATAAAGGATTTAAAAAA-CACAGCATG--AGAAATGT---GATGAAGAATATATCA---TGCAT  63886
Donkey       TTAGCTCAGGGTGAATCTTACTCACCAAAAAAAACAAAAAAAACACAGCATGAA--CAAATGTGATAAAGTATATATCAT  70968
Cat          -----GGAGGAATAAAGCCAGGTGCTTAAAA---------AGTCTATATAT--------A------ATGAGTGAAAATGT  70691
Ferret       CCGTTGT-GAGTCTAATCCAGGGACATGAAATGTGAATGAAAACCACATATGGA-AAAAAGTAAACATAAAAG-AATAGT  72176
Lion         -----GGAGGAATAAAGCCAGGTGCTTAAAA---------AGTCTATATA----------------ATGAGTGAAAATGT  68377
Vaquita      --------GGATAAGAATAAAGGATTTAAAAAA-CACAGCATG--AGAAAT-T---GATGAAGAATATGTCA---TGCAT  65243
Pig          CCCG----AATTCAGTGGTAATCAACTTCAGC--CTTTGGATATAGAAGATA----GGTTTAAATTAAATCAGACTGTAG  64960
```

```
Majority     ATTTAG-AACTTGATXATAAXX---XT-X--AXXA-------------------------------------GG--XXT-
                              93130     93140     93150     93160     93170     93180     93190     93200
Human        TTTTAGGCAGTTGGGTAAAA-----------AT---------------------------------------GACCAATT  74102
PolarBear    ACTTGGCACCTTATTGAT--------------------------------------------------------------  34031
Cow          ATTTAGGAAGTTGACAAGTATTCAGTTGTGAATGAG---------GCA---------------------TAGGG-GCATA  71935
Dog          ACTTGGCATCTTATTGATACAAGTACTTAGAAAGAATTAAACTGGGGATCCCTGGGTGGCTCAGCGGTTTAGCGCCTGCC  65009
BelugaWhale  GTTTAG-GAATTGACAAGTATTCAGTTGTGAATGAG---------GCA---------------------TAGGGTAATTG  63935
Donkey       GTTTAG-GGAGTGACAAGCATTCAGTTATGAATGAGGCA------------------------------TAGGGTAATTG  71017
Cat          CCC-GGTAACCTGATGGT--------------------------------------------------------------  70708
Ferret       ACTTGACATTTTATTGATA-------------------------------------------------------------  72195
Lion         CCCCGGTAACCTGATGGT--------------------------------------------------------------  68395
Vaquita      GTTTAG-GAAGTGACAAGTATTCAGTTGTGAATGAG---------GCA---------------------TAGGGTAATTG  65292
Pig          ATTTA--ATCTTGAAAAAAATTTGCTTTT----AA-------------------------------------GA-GTTGA  64996
```

```
Majority     XXA--XXATGAAAGGXA-TGXXG--X-AAAGAA--XAGTXXXXX--AAGATXX------------X--X--X-XGCTAXX
                              93210     93220     93230     93240     93250     93260     93270     93280
Human        TAGTTTTAAGAAACTGACTGTGGCTCCAGAGTATGTTGGAGAAGTGAAAATG----------------------GAGACT  74160
PolarBear    --------------------------------------------------------------------------------  34031
Cow          GGAAAACATGAAAGGCAGTGAGGAGAAAAAGAATTAAGTTTGGGTCAGGATCTT-----------AGAATGGATGCTATC  72004
Dog          TTTGGCCCAGGGTGCGATCCTGGAGTCCCAGGATCGAGTCCCACGTCAGGCTCCCGGCATGGAGCCTGCTTCTCCCTCCT  65089
BelugaWhale  GGAAAACATGAAAGGCAGTGATGTCAGAAAGAAC-TAGTTTGAGAGAAGATCTT-----------AGAACTGATGCTATC  64003
Donkey       GGAGTATATGAAAGGCAATGATGCCGGAAAGAAC-TAGTTCGGGACAAGATCTT-----------AGAACAGATGCTATC  71085
Cat          --------------------------------------------------------------------------------  70708
Ferret       --------------------------------------------------------------------------------  72195
Lion         --------------------------------------------------------------------------------  68395
Vaquita      GGAAAACATGAGAGGCAGTGATGTTAGAAAGAAC-TAGTTTGAGAGAAGATCTT-----------AGAACTGATGCTATC  65360
Pig          CAATCCTGTGAATAGTATGGCTGAAACAAAGAA--AAATCTTG--CAAAATGT------------ATTACTGCTGCTTCT  65060
```

```
Majority     CCAAXXAGTAXACXGGAXACTATTAGTCTAAXTAXGATAAAATTATGAATCTAAXCTAGXXA-A-XAAATXAXAATG---
                      93290     93300     93310     93320     93330     93340     93350     93360
Human        AGGAATAACAGGTGGGAGACTATTAGTCTAATTAAGATGTAATTATAAATCTAAGCTAGGAACGTAAAATGAGAATGCAA  74240
PolarBear    --------------------------------TATGA-GTACTTATG---------------------------------  34045
Cow          CTAAGGAGTATACTGGAGACTATTAGTCTAAGTAAGATAAAATTGTGAATCTACACTAGGGATA-GAAATGAGAGTG---  72080
Dog          CCTGTCTCTGCCCCTCTCTCTCTCCCTATGTCTATCATAAATAAATAAATCAATCTGAAAGAAA-AAGAAAGAAAGA---  65165
BelugaWhale  CCAAGGAGTATACTGGAGACTATTAGTCTAAGTAAGGTAAAATTGTGAATCTAAACTAGGGACA-GAAATGAGAATG---  64079
Donkey       CCAAGGAGTATACTGGGAACAATTAGTCTAAGTAATATATAATTGTGAATATAAATTAGGGACATGAAATAATAATG---  71162
Cat          --------------------------------TGTT-----TTGATG---------------------------------  70718
Ferret       --------------------------TGTCAATATCA-ATATTGATATGTCAAC--------------------------  72222
Lion         --------------------------------TGTT-----TTGATG---------------------------------  68405
Vaquita      CCAAGGAGTATACTGGAGACTATTAGTCTAAGTAAGGTAAAATTGTGAATCTAAACTAGGGACA-GAAGTGAGAATG---  65436
Pig          GCAGTCAGTAGAAGAAAAAGTTTTTGACCA--TTGTATAAAAT-GTGCCTCTGTCCAAA----A-CAGGTAAAAATT---  65129

Majority     -GAAAGAAAXAAA-ATGGGXAAAATTAAA-XTAAAAGTAXXAXGAXTTXGXX-XT-A---TAXGATXXXTXAT-------
                      93370     93380     93390     93400     93410     93420     93430     93440
Human        AGTAAGAAACAAATATGGGGAAAATTATATGTAAAAGTAATAGGACTTGGCATCTTAC--TGATGTGATTGAT-------  74311
PolarBear    --------------------------------------------------------------------------------  34045
Cow          -GAAAGAAAGAAACATGAGGAAAATTAAACATAAAAGTAGTAAGACTTGGAA-TTGATGATATGATGACTCATGAGGAAT  72158
Dog          -AAGAAAGAAAGAAAGAAAGAAAGAAAGAAAGAAAGAAAGAAAGAAAGAAAGAAAGAAAGAAAGAAAGAAAGAAAAGAAA  65244
BelugaWhale  -GAAAGAAAGAAACATGGGAACAATTAAATGTAAAAGTAATAGGACTTGGCACCTTACGATATGATGACTCATGAAGAAT  64158
Donkey       -GAAAGAAACAAATAAGGGAAAAATTAAACATAAAAGTAAGAGAATTTAGCATCTAATTGATATGTTGCTTAC-------  71234
Cat          --------------------------------------------------------------------------------  70718
Ferret       --------------------------------------------------------------------------------  72222
Lion         --------------------------------------------------------------------------------  68405
Vaquita      -GAAAGAAAGAAACATGGGAAAAATTAAATGTAAAAGTAATAGGACTTGGCACCTTACAATATGATGACTCATGAATAAT  65515
Pig          -GCACAGAAGGT-TTTCGGGCTCTCTAAAGATACACCAAGAAAGCAATAGA--TAAATAATAAGAAATTTCATTTAGGTT  65205
```

```
Majority     --------------------------------------------------------------------------------
                       93450     93460     93470     93480     93490     93500     93510     93520
Human        --------TATGA------------------------------------------------------------------- 74316
PolarBear    -------------------------------------------------------------------------------- 34045
Cow          TAAACTTGAGAAG---TCGACCTAGACAGTAGGATAT--TCATTCCTAGGACTTAGGAAAGAGTT--CTTTGGGGTTGAG 72231
Dog          GAAAGAAAAAAGAAAAAGAAAAAGAAAAAAAAAGAAAAGAAAAAAGAAAAAAGAAAAGAAAAAGAAAGAAAAAGGAAAAA 65324
BelugaWhale  TAAACTTGTGGAGGAGTCCACCTAGACAGTAGGAAAT--TCCGTC-TAGGACTTAGGCAGGAGTT--CTGTGGGGTTGAG 64233
Donkey       -------------------------------------------------------------------------------- 71234
Cat          -------------------------------------------------------------------------------- 70718
Ferret       -------------------------------------------------------------------------------- 72222
Lion         -------------------------------------------------------------------------------- 68405
Vaquita      TAAACTTGTGGAGGAGTCCACCTAGA----------T--TCAGCC-TAGGACTTAGGCAGGAGTT--CTGTGGGGTTGAG 65580
Pig          T----TTTTGAGC---TCTTCCTAACTTCTTATTTCTCTTCAATTTTAAATGTTTCTATATTTTTTTCTTTCCTATCGTT 65278

Majority     --------------------------------------------------------------------------------
                       93530     93540     93550     93560     93570     93580     93590     93600
Human        -------------------------------------------------------------------------------- 74316
PolarBear    -------------------------------------------------------------------------------- 34045
Cow          AATTTGAAAGACCAGAGGATTGATACCTAAAACTTTGGTTTAAAGAGATCAGATCAGACAGA----GGGCTTGTGGATGG 72307
Dog          AAAAGAAAAGAAAAAAAAGGAAAAAGAAAAGAAAAAAAAAAAAAAAAAAGAAAGAAAGAAAGAAAGAAAGAAAGAAAGAAA 65404
BelugaWhale  AATTTGAAAGGCCAGAGGAGTGTTGTTT-TGGTTTAGAGAGATCAGATTTGTAGTTGCCAGACACAGGGCTTGGGGGTGG 64312
Donkey       -------------------------------------------------------------------------------- 71234
Cat          -------------------------------------------------------------------------------- 70718
Ferret       -------------------------------------------------------------------------------- 72222
Lion         -------------------------------------------------------------------------------- 68405
Vaquita      AATTTGAAAGGCCAGAGGAGTGTTGTTT-TGGTTTAGAGAGATCAGATTAGTAGTTGCCAGACACAGGGCTTGGGGGTGG 65659
Pig          CATTTAGAATGGTATTTTATCTCTGAATACACCTTTTCTTTTTGCCCCACATGCTAGATTTTTCTTACCCTCATGTAATT 65358
```

```
Majority     --------------------------------XAAAXATCAAACTAXGGGATAAXTCX-XTAGAXAGTAXGAAATTTTGT
                      93610     93620     93630     93640     93650     93660     93670     93680
Human        ------------------------G--------AAAAATGAAGCATGTGGAGGAGTCCACTGGACAGTAGGAAATTCAGC 74364
PolarBear    ---------------------------------AAGAATTAAACTTGGGGAGAAGTCCACTAGACAGTAGGAAATTCTGC 34092
Cow          GGAGAAATGGGATAAAGGTGGACAAAAAAATACAAACGTCTGGTTATAGAATAAATA--GTACTAGGGTTATAAAGTATT 72385
Dog          GAAAGAAAGAAAGAAAGAAAGAAAGAAAGAAAGAAAGAAAAAAGGAAGGAAGGAAGGAAGAAGAAAGAAAGAAAACTTGT 65484
BelugaWhale  GGAGAAATTGGATAAAGGTGGTCAACAGG-TACAAACTTCCAATTATAAGATAAATAA-GTACTAGGG--ATGTAATGTA 64388
Donkey       --------------------------------TAAGAATTAACCTTGTAGATGAGTCCACTGGACAGTAGGAAATTCGGT 71282
Cat          ----------------------------------------------AGAAA----------------T--GA--TTTCGT 70732
Ferret       --------------------------------AAAAAATTAAACTAGGGGAGAAGTCCACTAGACAGTAGGAAATTCTGC 72270
Lion         ----------------------------------------------AGAAA----------------T--GA--TTTCAT 68419
Vaquita      GGAGAAATTGGATAAAGGTGGTCAACAGG-TACAAACTTCCAGTTATAAGATAAATAA-GTACTAGGG--ATGTAATGTA 65735
Pig          GCTGTGA-GCATTTCACATTCCTAGAAGGATTCGGCCTCCTGTCATTGGCATCCTGT--TTATTGATTCATCCCATTTCT 65435

Majority     CCAGXAXTXXT-TGAXTAATAG----TTCTGTGAGATAGTGAATT----GGGAGAAXAAATGTGTGGTATTTTAAAAXCX
                      93690     93700     93710     93720     93730     93740     93750     93760
Human        CTAAGACT----TGGGTAAGAG----TTCTGTGGAGTTGTGAATTC----AGAGGCCAGAGATGTGATAT-TTAAAATTT 74431
PolarBear    CTACAACT----TGGACAATAG----TTCTGTGAGCTTGTGAATT----GGGAGAACAGATGTGTGATATATTAAAAGCT 34160
Cow          ACGTGATAAATATGATTTATACTGCTGTATGTTATATATGAAA----------GAGTAAATCCTAAGAGTTCTCATTACA 72455
Dog          GGAGAAGTCCACTGGGTAGTAGGAAATTCTGTGAGCTTGTGAATT----GGAAAAACAG--GTGTGATATTTTAAAAGCT 65558
BelugaWhale  ACATGGTACATATAACGAACACTGCTCTATGTTATATAAGAAGGTTGTTAGGAGAGTAAATCCTAAGAGTTCTCATCACA 64468
Donkey       CTAGGACT----TGGGCAAGAG-----TTCGTGGGGTTGTGAATTT----AGAGGCTAGAGATGTGATATTTAAAACTTT 71349
Cat          GCAGAAGCTGTCTTAATTGATG-----ATGGTGGGACAGCAGTCT----GAAAGAAACACTGGGTGGTGGGGTAGAATGA 70803
Ferret       CTACAACT----TGGACAGTAG----TTCCATGAACTTGTGAATT----GGGAGAACAGATGTGTGATATTTTAAAAGCT 72338
Lion         GCAGAAGCTGTCTTAATTGATG-----GTGGTGGGACAGCAGTCT----GAAAGAAACACTGGGTGGTGGGGTAGAATGA 68490
Vaquita      ACATGGTACATATAACGACCACTGCTCTATGTTATATAAGAAGGTTGTTAGGAGAGTAAATCCTAAGAGTTCTCATCACA 65815
Pig          TCAACATTCACTTTAGC-ACTGTGTTTTGTACTATATAGGGACTT--------GGGAAAATCACAAGAAA-CAAATAGGG 65505
```

```
Majority     TGGATAAXAGATTCTTTAGGTATAAGXTGGCAXGAGGATTAA--TGAXXTGAAAA-T----T-----GAAACTTAA--GC
                      93770     93780     93790     93800     93810     93820     93830     93840
Human        TGGTTCA-AGATTTCCCAGGTATAAGAAAGCAAGAGGATTAA---AGCATTGTAA------T-----TAAACTTTAA-GC  74495
PolarBear    TGGCTTA-AGATTCTTCAGGTATAAGAGGGCAAGAGGATTAA--TGACTTGAAAAGTAAAATT----GAAACTTAA--GC  34231
Cow          GGA--GAAAAATTTTTTTCCTGTTTCTTCT-TGTT--ATCTG--TGAGGTGATGGATGTTCA----CTATACTTAACTGT  72524
Dog          TGGCTTA-AGATTCTTTAGGTGTAAGAGGGCAAGAGGATCAA--TGGCTTGAAAT-TACAATT----GAAACTTAA--GC  65628
BelugaWhale  AGGAGAAAAAATTTTTTTCCTATTTCTTTAATGTTGTATCTGTATGAGATGATGGATGTTCA----CTAAACTTAT-TGT  64543
Donkey       GGTTTTTAAGATTCCTCAGGTATAAAAAAGCAGGGCGGTTAA--AGACTTGAAAT-----------TGAAACTTTAA-GC  71415
Cat          TGAATAATACATCCTTTCTCTTTCCTTTGGGGCAGGGTTTAG--GGAGGTAAAAA------T-----GACTTTTTG--AA  70868
Ferret       TGGCTTA-AGATTCTTCAGGTATAAGAGGGCAAGAGGATTAA--TGGCTTGAAAT-TAAAATT----GAGACTTGA--GC  72408
Lion         TGAATAATACATCCTTTCTCTTTCCTTTGGGGAAGGGTTTAT--GGAGGTAAAAA------T-----GACTTTTTG--AA  68555
Vaquita      AGGAGAAAAAATTTTTTTCCTATTTCTTTAATGTTGTATCTGTATGAGATGATGGATGTTCA----CTAAACTTAT-TGT  65890
Pig          AAA--AATTAAGCATAAAAGTAATAGGACT-TGCC--ATCTTACTGATACGATGACTTACGAAGAATTAAACTTACATAG  65580

Majority     AGTGAXCA-TTATATTGTGGGTAAA--AATTAAATXA-----TTAGGGATGAAGTAAACX---TTAAAATTAXAAGTTXC
                      93850     93860     93870     93880     93890     93900     93910     93920
Human        AGTGCATATTTATGTTATAGATAAGATAAACAAGAAA----TCTAGGGATCAAATAGGA----TTAAAATTAGTAGTGAT  74567
PolarBear    AGTAAACATTTATATTGTGGGGAAA--AAATAAGAAA----TTGAGGGATGAAATAAATAGGATTAAAATTAGAAGTTCC  34305
Cow          GGTGATCA-TCTCATGGTGTATG----AGTCAAATCA-----TTATGCTGTCTGTACACC---TTAAATTTATACAGTGC  72591
Dog          AGTGCACAATTATATTGTGGG-AAA--AATTAAAC-------TTAGGGATGAGATAAATAGCATTAAAATTAAAAGTTCC  65698
BelugaWhale  GATAATCA-TCTCATGGTGTATGTA--AGTCAAATCA-----TTATGCT----GTACACC---TTAAATTTATACAGTGC  64608
Donkey       AGTGCACATGTCTATTGTGGGAAAAATAAAGAAGCAAAAAACTGAGAGGTGAAATACATAGGATTAAAATTAGAAGTGCC  71495
Cat          AGGG----------TTATAAG-AGA--AGTTCCAT---------GAGGACAAAGCAAGC----TTTTAATTAAAA-----  70917
Ferret       AGTGCCCATTTATATTGTGGGGGAA--AAATAAGCAAGAACTTGAGGGATGAAATAAATAGGATTAAAATTTGAAGTTCC  72486
Lion         AGGG----------TTATAAG-AGA--AGTTCCAA---------GAGGACAAAGCAAGC----TTTTAATTAAAA-----  68604
Vaquita      GATAATCA-TCTCATGGTGTATGTA--AGTCAAATCA-----TTATGCT----GTACACC---TTAAATTTATACAGTGC  65955
Pig          AGGAATC---CACCTGGACGGGA----AATTGAGTC-------TAGGCTTTAGGCA-ACC---GT-----TCTGTGGGGT  65637
```

```
Majority     TGTTCAGTCCTXTAGTTTCCATAAGGTTAATCATXAAAAX-----T-------------XGGTATAAA----TCCAATTA
                      93930     93940     93950     93960     93970     93980     93990     94000
Human        CATTCAGTACAGTAGTTAC-GTACTGTTATTCACAAGAGT---------------------ATATAAA----TCAAATTA  74621
PolarBear    TGTTCAGTTCCGTAGTTCCCATATGGTTATTCATGAGGG------T-------------GGGTATAAA----TCAAATTA  34362
Cow          TGTATGTACATTATCTCTCAATAAAACTGAAAGAAAAAAA----AC-------------T-TTTTAAGATT-CCCAACTG  72652
Dog          TACTCAGTTCAGTAGTTCCCATAAGGTTATTCATGAAGG------T-------------GGGTATAAA----TCAAATTA  65755
BelugaWhale  TGTATGTCCATTATATCTCAGTAAAACTGAAAGAAAAAAA---CTT-------------TGGTTTAAGATT-CTCAGGTA  64671
Donkey       TGTTCAGTTCAATAGTTACCATTTGCTTATTCACAAGAGATGTGATATTTAAAACTTTGGTTTTTAAGATTCCTCAGGTA  71575
Cat          -------------AGTTTCTCTAAGGAACACGTTGAAAA----------------------AGAAAAA----TTTGATTG  70958
Ferret       TGTTCAGT-----AGTTCCCATAAGGTTATTCATGAGGGG-----T-------------GGGTATAAA----TCAAATTA  72539
Lion         -------------AGTTTCTCTAAGGAACACGTTGAAAA----------------------AGAAAAA----TTTGATTG  68645
Vaquita      TGTATGTCCATTATATCTCAGTAAAACTGAAAGAAAAAAAAAACTT-------------TGGTTTAAGATT-CTCAGGTA  66021
Pig          T-TGAGAACTTAGAAGGCCCAGAGGAGTGATGTTTAAAAC----TT-------------TGCTTTAAAATT-CCCAGGTA  65698

Majority     TAAXAGATXXAAGAGGATTAAAGXCTTGAGAAXG-AX--ACTTTAAGCATTGAAGATTTGTGTTGTAGXAAA--XXAAGA
                      94010     94020     94030     94040     94050     94060     94070     94080
Human        CAAGGAATTAA-GGATATAAACGTGATAAGAAAGTATGCACTGTACTCTTTGAGGAAGTTTGCCATAGAAAG---GAAGA  74697
PolarBear    TTATGGATTAA-GGGTA---------TAAGAAAG-AT--TTATTACTCTTTGAAGAAGTGTGATGTAATAAA--GGAAAG  34427
Cow          TAAAAG-GGCAGGAGGGCTAAAGTCTTGAAA---------CTTTAAGCAGTGTACATTTATGTTGTGGGAAAA-----GA  72717
Dog          TGATGGATTAAAGGGTATAAATATGATAAGAAAGCAT--TTATTACTCTTTGAAGAAGTGTACTATAAAAAAA-GGAAAG  65832
BelugaWhale  TAAGAG-AGCAAGAGGGCTAAAGACTTGAAATTG-AA--ACTTTAAGCACTACACATTTATGTTGTGGGAAAG-TAAAGA  64746
Donkey       TAAAAAA-GCAGGGCGGTTAAAGACTTGAAATTG-AA--ACTTTAAGCGGTGCACATGTCTATTGTGGGAAAAATAAAGA  71651
Cat          TTAATGATAGAAAAGAATGGATT---CCAGCAGGCAC--A-ATAAGGAATTGAAGGGTAGGTTTGTAGAAGA------GG  71026
Ferret       AAATGGATTAA-GGATAGAAATGTGATAAGAAAGCAT--TTATTATTCTTTGAAGAAGTGTGCTATAAAAAA--GGAAAA  72614
Lion         TTAATGATAGAAAAGAATGGATT---CCAGCAGGTAC--A-ATAAGGAATTGAAGGGTAGGTTTGTAGAAGA------GG  68713
Vaquita      TAAGAG---CAAGAGGGCTAAAGACTTGAAATTG-AA--ACTTTAAGCACTACACATTTATGTTGTGGGAAAG-TAAAGA  66094
Pig          CAAGAGAGGGAACAGGGTTAAAGACTTGAAAATA-AA--ACTTTAAGCAGTGCATATTTATGTTGTGGGAAAACTAAAGA  65775
```

```
Majority     AGAAATGGATTGAGGGXTG------------------AAATAAATAGGXTTAXA-TTAGAGGTGCXTGTXCAGTTTXGTA
                      94090      94100      94110      94120      94130      94140      94150      94160
Human        AGAAATAGGATGGTAGATC------A-----G-----AAGTAAAGCAGGACCCAGTGGGGGGAGTGTTTGCAGTGAGGCA  74761
PolarBear    AGAAATGGAATGGTAGTTG--GAGTAGAGCAGGGCCAGGGAGAA-ATGTTTGCA-TTGAGGAACCATGCATAACTGTATG  34503
Cow          AGCAAGAGATCAAGGGATA------------------AAATAA----GATTAAAATTAGAAGTGCTTTTTCAGTTTAGTA  72775
Dog          AGAAATGGAATG-TAGTTG--------------------AGAAA--TGGTTGTA-TTGAGGGACTGTGTATAATTGTATG  65888
BelugaWhale  AGTAAGGGATTGAGGGATG------------------AAATAAATAGGGTTAAA-TTAGAAGTGCCTATTCAGTTTAGTA  64807
Donkey       AGCAAAAAACTGAGAGGTG------------------AAATACATAGGATTAAAATTAGAAGTGCCTGTTCAGTTCAATA  71713
Cat          AGGCATGGATTGAGGATAG---------------------AATAGTGTTTCTCA-TTTTTGTCAAAAGCAAAATTTTT--  71082
Ferret       AGAAATGGACTGGTAGTTGAGGAGTAAAGCAGGGTGGGAGGGGATGTGTTTGCA-CCGAGGAACTATATATAATCATAGG  72693
Lion         AGGCATGGATTGAGGATAG---------------------AATAGCGTTTCTCA-TTTTTGTCAAAAGCAAAATTTTT--  68769
Vaquita      AGTAAGGGATTGAGGGATG------------------AAATAAATAGGATTAAA-TTAGAAGTGCCTATTCAGTTTAGTA  66155
Pig          AGCAAGAAATTGAAGGATG------------------AAATAAATAGGATTAAATTTAGAAGTGCCTTTTCAGTTTAGTA  65837

Majority     GTTTCCATAATC-TTXATXXTTATTGTTXAGAATXGGTXTAAATCTCATTGTAATGGAXTCGAAATXT-----XAATATG
                      94170      94180      94190      94200      94210      94220      94230      94240
Human        GTATGTATAATCATTTAAAACATGGGTTTGGAGTCCTCTCAGGTTCCATGTT--TGTAATGGACATA----ATGATAATA  74835
PolarBear    GGCTCCAAAATC-CAGATGTCCTGAGTTCAAAATCTGTCTCAACTTCACTGTCTTGGCCTCCAAATTTGTAATGGATATA  34582
Cow          GTGACCATAAAG-TTTATTCTTATTCATAAGAATGGGTATAAATCAAATTATAAAGAATTAGGAATAT-----AAATATG  72849
Dog          GGCTCCAGA-TC-CAGGTGTCATGAGTTCAGAATTCATCTTAGCTTCACTGTCTTAGCCTCCCCATGTGTAGTGGTTATA  65966
BelugaWhale  GTTACCATAAAG-TTTATTCTTATTCGTAAGAATGGGTATAAATCAAATTATAATGGATTAGGAATGT-----AAATTTG  64881
Donkey       GTTACCATT--------TGCTTATTCACAAGAGTGGGTATAAATCTACTTTTAATGGATC---AA-------------TG  71769
Cat          AGTTTCAAA-TC-AGGAAATAAAATGTTCTAAAACCTCC---TCCCCACTGA---AAACCCAATACTT--------TATG  71146
Ferret       GGCTCCAAA-TC-CAGATGTCCTGAGTTCAAAATCTGTCTCAGCTTCACTGTCTTGACCTCCATATTTGTAATGGATATA  72771
Lion         AGTTTCAAA-TC-AGGAAATAAAATGTTCTAAAACCTCCC--TCCTCACCGA---AAACCCAATACTT--------TATG  68834
Vaquita      GTTACCATAAAG-TTTATTCTTATTCGTAAGAATGGGTATAAATCAAATTATAATGGATTAGGAATGT-----AAATTTG  66229
Pig          GTCACCATAAGG-TTTATTCTTTT----AAGAATGGATATGAATCCAATTAAAATGGATTAAGGATGC-----AAATATG  65907
```

```
Majority     ATAX-XAXGTTATAAGGCCXTTTTGA-GTAXTGXATT--TGGTAGXAAGXAAAAA---TTGGATAGTACTTGAXATGCAA
                       94250     94260     94270     94280     94290     94300     94310     94320
Human        ATCCCTTTCATTTAAGGCTGTTGTGA-GGATTAAATG--TGTTAATGTGCAAATAACTTTACACAGTGCCTGGTATATAA  74912
PolarBear    ATGACAGTGTTACAAGGCTATTGTGA-GGATTAAATG--TGTTAA---GTGAAGT---TTGTATAGTGCTTGGCATATAA  34653
Cow          ATA---AAATATTTACTCCTCTTTGAAGTAATGCACTCTTGGAAGGAAGGAAAAA---TGGGATGGTAGTTGAAAAGCAA  72923
Dog          ATGACAGTGTTACAAGGGTATTATGA-GTATTAAATG--TGTTAATAAGTATAGT---TTGTATAGTGCTTGGCATATAA  66040
BelugaWhale  ATAAGAAAGTACTTACTACTCTTTGAGTAAGTGCTTT--AGGAAGGAAGGAGAAA---TGGGATGGTAGTTGAGGAGCAA  64956
Donkey       T---------------ACTACTCTGA---AGTGTGCTGTAGAAAGGAAGGAGAAA---TGGGATGGTAATTGAGAAGTAA  71828
Cat          A-------ATTAGAAGGCCCTTCCAATATACTGTGTT--CAGTAGTATGTGAGAC---TTGAA-ACTACCAGATGTCCTA  71213
Ferret       ATAACAGTGTTGCAAGGCCATTGTGA-AGATTAAGTG--TATTAATAAGTGCAGT---TTGTATAGTGCTGGGCATACGA  72845
Lion         A-------ATTAGAAGGCCCTTCCAGTATACTGTGTT--CAGTAGTATGTGAGAC---TTAAA-ACTACCAGATGTCCTA  68901
Vaquita      ATAAGAAAGTACTTACTACTCTTTGAATAAGTGCTTT--AGGAAGGAAGGAGAAA---TGGGATGGTAGTTGAGGAGCAA  66304
Pig          ATA---AAGTATTGACTACTTTTTGAAGAAGTGCACA-TAGCAAAGAAGGAGAAA---TGGGATGGTAGTTGAAGAGCAA  65980

Majority     -TXAAGGCTCAAGAGGTAATGACTAXXATAAGGAAXTXTGCATAAT---CATTTTGAGTATTGGTTXC-------AAATC
                       94330     94340     94350     94360     94370     94380     94390     94400
Human        -TAAATGCTTGCTACCTATTAACTAGTATTTGTTTCTAAGGCTAATTT-AAGTCCTAGAATTGATTGC--------AA--  74980
PolarBear    -TGAATGTTCACTTCATATTAACTAGTATTTGGGTTTT---GTGGG--GTTTTTTGAAGATTTATTTATTTTAGAGAGCA  34727
Cow          -------------------------------GGAAGTATGCATAAT---CATTTTGAGTGTAGGCTCA-------AAATC  72962
Dog          -TGAATGCTCATTTCATATTAACTAGTATTTGTTTTTT---GTGGG---ATTTTGGGGGA--GACTTA-------GAGCA  66104
BelugaWhale  ATCAGGGCCCAAGAGGAAATGTTTACAGTAAAGGAGTATGCATAAT---CATTAAGAGTATGTGGTCC-------AAATC  65026
Donkey       AGCAAGACCCAAGGGGAAGTGTTTTCAGTACGGAAGTATGCATAAT---CATTAAGAGTATTGGCTCC-------AAATC  71898
Cat          -AACAGGGTAAAGAAGTAA-GACCAAGAGAAATAATTTA--ATGGA-----CTTTGGATATAAGTTTT---------A--  71273
Ferret       -TGAATGCTCAATTTATA--AACTAGTATTTGGTTTTTTTTGTGGATTGTTTTTTGAAGATTTATTTATTTTAGAGAACA  72922
Lion         -AACAGGGTAAAGAAGTAA-GACCAAGAGAAATAATTTA--ATGGA-----CTTTGGGTATAAATTTT---------A--  68961
Vaquita      ATCAGGGCCCAAGAGGAAATGTTTACAGTAAAGGAGTATGCATAAT---CATTAAGAGTATGTGGTCC-------AAATC  66374
Pig          AT-AGGGCCCGAGGGGAAATGTTTGCAGCGAGCAAGTGTGCATAAT---CATTAAGAGTATGGGCTCC-------AAATC  66049
```

```
Majority     CAXATGXGATCAGTACAAAA-CTGGCACAGXTTAACTGTCGCAGXTTCCACATTTATCATGGA---TAAAATGX-XXTAA
                           94410     94420     94430     94440     94450     94460     94470     94480
Human        -GGATTAGATCAGGA---------GTATAGTGGACATGTTGGGATTTAAATATTTA-AATATA----GAGATG-------  75038
PolarBear    CAAGTGGGAGGGGGAGAAGAAGAGGAAGAGAGAATCTCAAGTAGACTCCGCGTTGATCGTGGAGCCTGACACAGGGCTCG  34807
Cow          CAGACATCCTCAATTCAAAATCTGGCTCAGCTTTGCTGTCTTAGATTTCACATTTGTCATGGA---TATGAT---AGTAA  73036
Dog          CAAGTGGGAAGGGGA------GAGGGAGAGAGAATCTCAAGCAAACTGCTCACTGAGCATGGAGCCCAACACAGGGCTCA  66178
BelugaWhale  CAGACATCCTCAGTTCAAAAGCTGGCTCAACTTCACTGTCTCAGCTTTCACATTTGTCATGGA---TATAATGATGGTAA  65103
Donkey       TAGGTGTCCTGAGTTCAAAATCTAGCTCAGCTTCACTGTCTGAGCTTCCAAATTTGTAATGGA---TGTAATGGCATTAA  71975
Cat          -GAATGGGAAATGAA--------GGGATAAAACAAATTGTGCA---TCCTTAAAAATCATGGG---TAAAATAAT-----  71333
Ferret       TGAATGGGAGGGGGAAGAGAGGAGGAAGAGAGAATCTTAAGCAGACTCCATGTTAAGTGCGGAGCCTGACACGGG--TTG  73000
Lion         -GAATGGGAAATGAA--------GGGATAAAACAAATTGTGCA---TCCTTAAAAATCATGGG---TAAAA---------  69017
Vaquita      CAGACATCCTCAGTTCAAAAGCTGGCTCAACTTCACTGTCTCAGCTTTCACATTTGTCATGGA---TATAATGATAGTAA  66451
Pig          CACACATCCTCAGTTCAGAATCTGGCTCAGCTTCGCTGTCTCTGCTTTCACATTTGTAATGGA---TATAACGA-AGTTA  66125

Majority     XTCCCATGTX-TXAGGCT-XXG-----XAGGAXXACAT-XTTTTAACXXAAACAAAGAATTTT----GATGCTTGACAAA
                           94490     94500     94510     94520     94530     94540     94550     94560
Human        -CTT--T---------TT----------AGGA--CCATTGTTAGAACC--AGAAGAGATTTTT------TACCAAGTTCA  75086
PolarBear    ATCTCAAGAACTGAGCCTCGTGACCTGAGAGAGATCACGACCTGAGCTGAAACCAAGAGTTG-----GA-GCTTAACCAA  34881
Cow          TCCCCATGTTCTAAAGCTAGTGTTGT-GAGGATTGAATTATGTTAATGCATACAGAGAACTTTTCACAATGCCTGACATA  73115
Dog          ATCTCACAC-----CCTT----------GAGT--TCATCTCCTGATCCAAAACCAAGAATTG-----GATGCTTAACCAG  66236
BelugaWhale  TCCCCATGTTTTAAGGCTAGCGT----GAGGATTAAAT-GTTTTAAT----ACAGAGAACTTTTCACAGTGTCTGGCATA  65174
Donkey       TTCCTGTGTTCTAAGGCTCTTGC----GAGGATTACAT-GTGTTAAC--GTGTAAAGAAATTT--GCTGTGCTTGGCAGA  72046
Cat          ---------------CCT----------AAGG----ACAAGTCAAGAGAAGCCTAAGGGGAT-----GAGACATGACTAA  71379
Ferret       ATCTCAAGA-----CCCT----------GAGA--TCATGACCTGAGCCAAAACCAAGAGTTG-----GATGCTTAACCAA  73058
Lion         ------------------------------GG----ACAAGTCAAGAGAAGCCTAAGGGGAT-----GAGACATGACTAA  69058
Vaquita      TCCCCATGTTTTAAGGCTAGTGT----GAGGATTAAAT-GTTTTAAT----ACAGAGAACTTTTCACAGTGTCTGGCATA  66522
Pig          ATTCCATGTTGTAAGGCTAGTGC----GAGGATTAAAT-GCGTTAATACGTACAAAGAATTTTGCACAGTGCTTGGCATA  66200
```

```
Majority     CAXTGAATGCTAGTTTCAT--TXGCTAGTATTTGTTTTTAAGGCTAGCTTAAGTCCTAGAATTGATTGTAGTXGATTGGA
                      94570     94580     94590     94600     94610     94620     94630     94640
Human        CACAGAAATGTAGGTGCAT--TGGCTGGGCATGGTGGCTCACACCTGC---AATCCCAGCAC------------------  75143
PolarBear    CTGTGCCACCGAGGCATCC-----CTGGTGTTTATTTTTAAGGCTAGCTTAAGTCCTAGAATTGATGGTAGAGGATTGGG  34956
Cow          TAATGAGTGGAAATTTCATATTAGCTAGTATTTGTTTTGAAGGCTAGCTTAAATCCTAGAATTGATTGTAGTGAATTGGA  73195
Dog          CTGTGCCACCCAGTCACCC-----CTAGTATTTATTTTTAAGGCTAGCTTCAGTCCTAGAATTGAATGTA----------  66301
BelugaWhale  GAATGAATGCTAACTTCATATTAGCTAGTATTTGTTTTGAAGGCTAGCTTAAGTCCTAGAATTGATTGTAGTAGATTGGA  65254
Donkey       TAATGAATGCTCATTTCATATTAGGTAGTATTTGTTTTTAAGACTAGCTTAAGTCCTAGAGTTGATTGTAGTTTGTTGGA  72126
Cat          ATGTATT----GGAGGTTC-----TTGGAACAAAAAAG-AATATTAGATGCACACCAAGGA---AATCTG----------  71436
Ferret       CTGTGCCATCCAGGCACCC-----CTAGTATTTGTTTTTAAGGCTAGCACAAGTCCTAGAACTGATTGTAGAGGATTGGT  73133
Lion         ATGTATT----AGAGGATC-----TTGGAACAAAAAAG-AATATTAGATGCACACCAAGGA---AATCTG----------  69115
Vaquita      GAATGAATGCTAACTTCATATTAGCTAGTATTTGTTTTGAAGGCTAGCTTAAGTCCTAGAACTGATTGTAGTAGATTGGA  66602
Pig          TAATGAATGCTTATTTCATGTTAGCTGGTGTTTGTTTTGAAGGCTAGCTTAAGTCCTAGAATTGATTATGGTGGATTGGA  66280

Majority     TCXAGAXTATAGTGGAAATGTTGGGGCCTAGXAAATAAAGAGA-----TACTT-TTTAGGACCATTGTGA-GAACTGGAA
                      94650     94660     94670     94680     94690     94700     94710     94720
Human        -----------TTGGGAAGGCTGAGGCAGAAGAACTGCTTGAGGC---CAACATTTTGAGACCAGCCTGGGCAACATATT  75209
PolarBear    TCTAGAGTATAGTGAAAATGCTGGGGTCTAAAAAATAA----------TGCTTGCTTAGGATCATTGTGA-GAACTAGAA  35025
Cow          TTGAGAATA--ATGGAAGTGTTGAGGCA----AAATATATAGA-----AAATT-TTTAGGACCATTGTGA-GAACTAAAA  73262
Dog          ---------TAGTGAAAATGCCAAGGCCTAGAAAATAGAA--AGG---TACTTGTTGAGGATCATAATGAAGAACTGGAA  66367
BelugaWhale  TCAAGAATATAGTGGAAATGTTGGGGCCTACGAAATACAGAGA-----TACTT-TTCAGGACCATTGTGA-GAACTGGAA  65327
Donkey       TCAAGAGTGTAGTGGAAATGTTGGCATCTAGAAAATATAGAGA-----TACTT-TTTAGGACCATTGTGA-GAACTAGAA  72199
Cat          -------------ACAAAGTATGGACTTCAGTTGATGA----------TAATATATCAGTATTAGT-TGATTAATTGCAA  71492
Ferret       TCTAGAGTATAGTTTAAATGCTGGAGCCTAAAAAAATAGAAAAGTACTTGCTTGCTTAGGATCATCCTGA-GAACTAGAA  73212
Lion         -------------ACAAAGTGTGGACTTCAGTTGATGA----------TAATATATGAGTATTAGT-TGATTAATTGCAA  69171
Vaquita      TCAAGAATATAGTGGAAATGTTGGGGCCTACGAAATACAGAGA-----TACTT-TTCAGGACCATTGTGA-GAACTGGAA  66675
Pig          TCAATAATACAGTGGAAAGGTTGGGGGGTAGGAAATATAGAGA-----TACTT-TTTACAACCATGGTGA-GAACTGGGA  66353
```

```
Majority      GGGATTTTT-ATCAAGCTCACATAGAA----------------ATTTAGGGGCAGAGCTGGAGCCAGAACTGAAGTCATC
                       94730     94740     94750     94760     94770     94780     94790     94800
Human         AAGACCCCGTCTCCACCAAAAAAAAAAAAAAAAG---------AAGTAGGTGCAGAGCTGGAAGCAGAACCGAAATCATC  75280
PolarBear     GGGATTATC-ATCAAGCTCACATAGAA----------------ACGTAGGGGCAGAGCTGGGGCCAGAACTGAAGTCATC  35088
Cow           GGAACTTCT-GTCAAGCTCACATAGAA----------------ATTTAGGAGCAGAGTTGGAGCCAGAACTGAAGTTGTC  73325
Dog           AGGATT----AGCAAGCTCTTATAGAAATGTAGGGGAAGGAAAATGTAGGGGCAGAGCTGGAGCCAGAACTGAAGTCATC  66443
BelugaWhale   GGAATTTTT-ATCAAGCTCACAGAGAG----------------ATTTAGGGGCAGAGCTGGAGCCAGAACTAAAGTCATC  65390
Donkey        GGGATTTTT-ATTAAGCTCACATAGAA----------------ATTTAGGGGCAGAGCTGGAGCCAGAACTGAAATCATT  72262
Cat           TAATT--------ATACTAACGTAAAA-----------------------TGTTAA-TGGGAAAAGGAAGTG----TGCA  71536
Ferret        GGGATTATT-ATCAAGCTCACATAGAATTGTCGGGGGA----AATGCAAGGGTGGAGTGGGGGCCAGATCTGAAGTTATC  73287
Lion          TAATT--------ACACTAACGTAAAA-----------------------TGTTAA-TGGGAAAAGGAAGTG----TGCA  69215
Vaquita       GGAATTTTTTATCAAGCTCACAGAAAG----------------ATTTAGGGGCAGAGCTGGAGCCAGAACTAAAGTCATC  66739
Pig           TGGATTTTT-TTCAAGCTCCCCTAGAA----------------ATTTTGGAACATGGCTGTAGCCCGACCTGAAGTCATC  66416

Majority      AGCTTT--ATAGTCCATATTCTTT-CTTCTCACCATTTTGTGTCTTTAT---------GXAGAAAGGGGAACAGATGAAA
                       94810     94820     94830     94840     94850     94860     94870     94880
Human         AGTGTT--ACAGTCATTATTCTTT-CCTGTCACCATTATATGTCTTTAT-GAAGCAAGGGAGAAAGAAGAACAGATGAAA  75356
PolarBear     AGCTAT--GTAGTCCATGTTCTTT-CCTCTCACCATTCTATGTTTTTTCTGAAGAAAGGGAAGAGGGAAAACAGATGAAA  35165
Cow           AGTTTTTTACAGTCTGTATTCTTT-CTTCTCACCATTT-GTATCTTCAT---------AAAGAAAGGGGAACAGATGAAA  73394
Dog           AGTTAT--GTAGCCCATATTCTTTTCTTCTCACCATTCTGTGTTTTTAT-GAAGAAAGGAAAAGGGGGAACAGATGAAA  66520
BelugaWhale   AGCTTTTGATAGTCCATATTCTTT-CTTCTCACCATTT-GTATCTTTAT---------GAAGAAAGGGGAACAGATGAAG  65459
Donkey        AGCTTT--ATCGTCCATATTCTTG--TTCTCACCATCATGTATCTTTAT-GAAGAAAGACAGGAAGGGGAACAGAGGAAA  72337
Cat           GGTTAT--ATAG---GAACTCTGT-------AGAAGCTTCAGTTTTTCT----------GTAAATCTAAAACTGTTCTAA  71594
Ferret        AGCTGT--GTAGTCCATATTCTTT-CTAATCACCATTCTTTGGTTTTATGAAGAAAGGGAGAAAGGGGAAACAGATTAAA  73364
Lion          GGTTAT--ATAG---GAACTCTGT-------AGAAGCTTCAGTTTTTCT----------GTAAATCTAAAACTGTTCTAA  69273
Vaquita       AGCTTTTGATAGTCCATATTCTTT-CTTCTCACCATTT-GTATCTTTAT---------GAAGAAAGGGGAACAGATGAAG  66808
Pig           AGCTTTTTATAGTCTACGTATATA-TAGTCTACCATTTTGTATCTTTGT---------AGAGAAAGGGGAATAAATGTAA  66486
```

```
Majority     GAAGGGAGX--ACTTTTGAAGTTGGTTGAAAATTTTGAG-----------------------------------------

                     94890     94900     94910     94920     94930     94940     94950     94960

Human        GAAGTGAGGA---TTTTGAAGTTGGTTGAAAGATTTGAT-----------------------------------------  75392
PolarBear    GAAGGGAGT--ACTTTTGAAGTTGGTTGAAAAATTTGAG-----------------------------------------  35202
Cow          GAAGGGAGA--ACTTTTGAAGTTGGTTGAACATTTTAAG-----------------------------------------  73431
Dog          GAAAGGA-----CTTTTGAAGTTGGTAGAAAATTTTGAG-----------------------------------------  66554
BelugaWhale  AAAGAGAGAGGACTTTTGAAGTTGATTGAAAATTTGGGG-----------------------------------------  65498
Donkey       AAAAGGAGG--ACTTTTGAAGTTGGTTGAAAATTGTAAG-----------------------------------------  72374
Cat          AATAAAA------------ACCTTATTAAAAACCATGGG-----------------------------------------  71621
Ferret       GAAGGGAGT--ACTTTTTAAGTTGGTTGAGAAACTTGAAATTCTTTTTTTTTTTTTTTGTTTATATTTATTTATTTGACA  73442
Lion         AATAAAA------------ACCTTACTAAAAACCATGGA-----------------------------------------  69300
Vaquita      AAAGAGAGAGGACTTTTGAAGTTGATTGAAAATTTGGGG-----------------------------------------  66847
Pig          GAAGGAAGG--CCTTCTGAAGTTGGTTGAAAATTTTGGA-----------------------------------------  66523

Majority     --------------------------------------------------------------------------------

                     94970     94980     94990     95000     95010     95020     95030     95040

Human        --------------------------------------------------------------------------------  75392
PolarBear    --------------------------------------------------------------------------------  35202
Cow          --------------------------------------------------------------------------------  73431
Dog          --------------------------------------------------------------------------------  66554
BelugaWhale  --------------------------------------------------------------------------------  65498
Donkey       --------------------------------------------------------------------------------  72374
Cat          --------------------------------------------------------------------------------  71621
Ferret       GAGATGACAGAGAGGCAGAAAGGCAGGCCAGAGAGAGAGGAAGGGAAGCAGGCTTAGCAGAGAGCCCTATGTGGGGCTTA  73522
Lion         --------------------------------------------------------------------------------  69300
Vaquita      --------------------------------------------------------------------------------  66847
Pig          --------------------------------------------------------------------------------  66523
```

```
Majority     ------------------------------------------------------------------------------GG
                      95050     95060     95070     95080     95090     95100     95110     95120
Human        ------------------------------------------------------------------------------TG  75394
PolarBear    ------------------------------------------------------------------------------GA  35204
Cow          ------------------------------------------------------------------------------GG  73433
Dog          ------------------------------------------------------------------------------GG  66556
BelugaWhale  ------------------------------------------------------------------------------GG  65500
Donkey       ------------------------------------------------------------------------------GG  72376
Cat          ------------------------------------------------------------------------------TA  71623
Ferret       ATCCTAGGAACTGGGATCATGACCTGAGCCAAAGGCAGAGGCTTTAACCCACTGAGCCACCCAGATGCCCCAAAACTTGA  73602
Lion         ------------------------------------------------------------------------------TA  69302
Vaquita      ------------------------------------------------------------------------------GG  66849
Pig          ------------------------------------------------------------------------------GA  66525

Majority     AATTCTGATCTCATTCTTAGA---XACTTGCTGAACATAACAAGATTGAGAGTAGGATATAGGACTTAAG--TCTAGTGG
                      95130     95140     95150     95160     95170     95180     95190     95200
Human        AATTCTGATCTAAAAATTATAAGGCACTT----G-TTTAACA-AGTTGAAAGTAGGAAAGTAGACATAAGACTCTACTAG  75468
PolarBear    AATTCTGATCTTATTCTTAGA-----CTTTCTGAACATAACAAGATTGAGAGTAGGATGTGGGGCTTAAG--TCTGGTGG  35277
Cow          AATTCTCATCTCAGTCTTCCAAGATATTTGCTGAACATAA---GAATGAGGGTCGGTA-TAGGA-----G--TCTAGTGG  73502
Dog          AATTCTGATGCCATTCTCAGA-----CTTTATGAATATAACAAGATTGAGAGTAGGATGTGGGACATAAA--TCTCGTAA  66629
BelugaWhale  AATTCTGATCTCAGTCTTACAAGATACTTGCTGAACATAACAAGGTTGAGAATAGGGTACAGGACTTAAG--TCTAGTGG  65578
Donkey       AATTCTGATCTCAATCTTACAAGATACTTGCGGAACATAACAAGATTGAGAGTAGGATACAGGGCTT--G--TCTAGTGG  72452
Cat          AATTTGTAAATTATTTAAAG-----------GAAGTTAACAAAA---AGGTTAGTTTTTTGGGGC--AG--CTGGGTGG  71684
Ferret       AATTCTGATCTCATTCTTAGA-----CTTTCTGAACATAACAAGATTGAGAGTAGGATGTGGCAGTTGAG--TCTGGTGG  73675
Lion         AATTTGTAAATGATTTAAAG-----------GAAGTTAACAAAA---AGGTTAGTTTTTGGGGGC--AG--CTGGGTGG  69363
Vaquita      AATTCTGATCTCAGTCTTACAAGATACTTGCTGAACATAACAAGGTTGAGAATAGGGTACAGGACTTAAG--TCTAGTGG  66927
Pig          AATTCTGATCTCGGTCTTT-----TATTTGCTGAACATGA---GATTGGGAGTAGGAAATAGGACTTAAG--TCTAGTGG  66595
```

```
Majority     ATTTXTAGGGAATTCTTGAAAATAGACTAGGAAAT-CAGCTAATGTTA------AATAACAAA--XTTTGTGGAATCAAT
                            95210     95220     95230     95240     95250     95260     95270     95280
Human        ATTTG-GGGAAACTCTCAAAAATGGACTGGAAATT-CAGCTAAAAGTG------GATAACAAAATATTTCTAGAATTA--  75538
PolarBear    CTTTGTAGGAAACCTT-GAAAGTAGACTCAGATAT-CAGGTAATGTTA------CATACCAAAATGTTTATGGAATCAAT  35349
Cow          ATTTTTAGGGAATTCTTGAAAACAGACTAGGAAAT-CAGCTAATGTTG------AATAACAAA----ATGTGGACTCATA  73571
Dog          ATTTGTAGGGAACCCTTGGAAGTAGACTCCAAAAT-CGGCTAACGTTAGATTTAAATAGCAAAATGTTTATGGAATCAGT  66708
BelugaWhale  ATTTTTAGGGAATTCTTGAAAATAGAATAGGAAATGCGGCTAACATCA------AATAACAAA----ATGTGGAATCAGT  65648
Donkey       ATTTTTAGGGAATTCTTGAAAAGAGACTAGGAAAT-TAGCTGATGTTG------AACAACAAA----ATGTGGAATCA--  72519
Cat          TTCAGTCAGTAAGC--------A----TCCAACTT-CAGCTCAGGTCAT-----GATCTCACA--GTTCGTGAGTTCAAG  71744
Ferret       ATTTATAGGGGACCT--GAAAATAGACTTGGATAT-CAGCTAGTGTTA------AATACCAAAATGTTTGTGGAATAAAT  73746
Lion         CTCAGTCAGTAAGC--------A----TCCGACTT-CAGCTCAGGTCAT-----GATCTTACA--GTTCGTGAGTTCAAG  69423
Vaquita      ATTTTTAGGGAATTCTTGAAAATAGAATAGGAAATGCGGCTAACATCA------AATA---AA----ATGTGGAATCAGT  66994
Pig          GTTTTTAGGGAATTCTTGAAACTACACTAGGAAAT-CCACTAATGTGG------AATAACAAA----TTGTGGAATCA--  66662

Majority     TT-XGGTTGT---GGTGTGTGTGTGTGT---XG-GX----------G--X-TGTTTTCACTTTAXTATTTTACAAACCC
                            95290     95300     95310     95320     95330     95340     95350     95360
Human        ----GCATTT---GTGGGGTGTGTGTGT--------------------------TTTCACTCTAGTATTTGTCAAGCCC  75584
PolarBear    TTTTGGGTGTGCGTATGTGTGTGTTCACTCTAGTGCTTGCCTCGGCAGCACATATACTAAAGTTGGAATGTTTTCACTCT  35429
Cow          T---GCTTTT---TGAGTGTGTGTGTGT----G--------------------TTTTCACTTTAATATTTTACAAACCC  73620
Dog          TTTGGGGTGT----GTATGTGTTTTCACTCTAGTGCTCACTTCGGCAGCACATGTACTAAAATTGGAATATTTTTACTTT  66784
BelugaWhale  TTGGGGTTGT---AGGGTGTGTGTGTGTGTGTGTGTG--T-----GTGTGTGTTTTCACTCTAATATTTTACAAACCT  65718
Donkey       -----GTTTT---GGGGTGCGTGTGTGT------------------------GTTTTCTCTCTAATACTTTACAAACCC  72566
Cat          CCCCACATCA--GGCTCTGTGCTTACAGCTCAGAGCCTG--------GAGCCTGCTTCAGATTCTGTGTCTCCCTCTCTC  71814
Ferret       TTTGGGGTGT-TGTATGTGTTTTTTCACTCTAGTGCTCACTTGGGCAGCACATACACTAAAATTGAAATGTTTTCACTCT  73825
Lion         CCCCACATCA--GGCTCTGTGCTGACAGCTCAGAGCCTG--------GAGCCTGCTTCAGATTCTGTGTCTCCCTCTCTC  69493
Vaquita      TTGGGGTTGT---AGGGTGTGTGTGTGT----G--------------------TTTTCACT--AATATTTTACAAACCT  67044
Pig          -----GTTTT---GCATTGTGTATATGT----G--------------------TTTTCACTCTATCACTTTACAAACCC  66709
```

```
Majority     AG------------ATGAAAGCATAGCCAAAATAXAAGATTGGATTTATCCAAGTCTGGCATTGTAGAACATTAA-XGAT
                            95370     95380     95390     95400     95410     95420     95430     95440
Human        AG------------ATGAAAGCATAGACAGAATGTAAGACTGGATTTATCTAAGTCTGGAATTGTGTAACATTAAAGGAA  75652
PolarBear    AATACTTA-CCGAGATGAAAGCTTAGCCAAAATACAAGATTGGACTTATCTATGTCTGGCATTGAAGAACATC--------  35501
Cow          AG------------ATGAAGACATGTCCACAATATAAGATTTAATTTATCCAAGCCTGGCACAGTAGAGCATTAACAGAT  73688
Dog          AATACTTA-CCTAGATGAAAGCTTAGCAAAAATACAAGATTGGATTTATCCAAGCCTGGCGCTGAAGAACATC--------  66856
BelugaWhale  AG------------ATGAAAGCATATCCAAAATATAAGATTTGATTTATCCAAGTCTGGCATTGTAGAGCATTAATGGAT  65786
Donkey       AG------------ATGAAAGTGTAGCCAAAATATAAAATTTAATTTATCCAAGTGTGGCATTGTAGAGCATTAAAAGAT  72634
Cat          TCTGCC----------------CCTACCGCACTCACACG-CTGTCTCTCTC----------ATAAATACATACAT-------  71862
Ferret       AGTACTTAACCCCGATGAAAGTTTAGCTAGAATACAAGATTGGATTTATCCATGTCTGGCACTGAAGAACACTTAAAAAA  73905
Lion         TCTGCT----------------CCTACCCCACTCACACA-CTGTCTCTCTCT----CAAAAATAAATAAATACAT-------  69547
Vaquita      AG------------ATGAAAGCATATCCAAAATATAAGGTTTGATTTATCCAAGTCTGGCATTGTAGAGCATTAATGGAT  67112
Pig          AG------------ATGAAAGCATAGCCAAAAAA--ATTCTCGATTTATCCAAGTCTAGCATTGTAGAGCATTAACAAGT  66775

Majority     TAGAX-XXAX----------------CXXGCCAAAAT----GCTTAAGCTACATTGTAAAX-----TCAAGCCAAGGGA
                            95450     95460     95470     95480     95490     95500     95510     95520
Human        TAGTAGCAAATGAGCAGAGTGTTGGCTCAAGCCTAAGCTTGAGCCTAAGCTTGACTCTATGGTAAAGTCAAGTCAAGGGA  75732
PolarBear    -AAA----------------------------AAAAC----ACTTAGCCTACATTGTAAA-----GTCAAGCCAAGAAA  35542
Cow          GAGTAGCGAG----------------CAAGCCAAAAT----GCTCCAGCTACATAGTAAG------TCTAATCAAGGGA  73741
Dog          -AAA----------------------------AAAAC----ACTTAGGCTACATTGTAAATAAA-GTCAAGCCAAGAAA  66901
BelugaWhale  TAGGAACAAG----------------CAAGCCAAAAT----GCTCTAG--ACATAGTATAG-----TCTAGCCAAGGGA  65838
Donkey       TAGTAGGAAG----------------CGAGCCAAAAC----GCTTAGTCTACATTGTAAAC-----TCAAGCCAAGGGA  72688
Cat          -ACA----------------------------TACAT----ATATACA-TACATAATA-----------------TAAA  71890
Ferret       AAAA----------------------------AAAAT----ACTTGGGCTATACTGTAAA-----ATCAAGCCAAGGAA  73947
Lion         -AAA----------------------------TACAT----AAATATA-TACATAATAAATAA--ATACTAAATATAAA  69590
Vaquita      TAGGAACAAG----------------CAAGCCAAAAT----GCTCTAG--ACATAGTATAG-----TCTAGCCAAGGGA  67164
Pig          TAGTCACAAG----------------CAGGCCAAAAT----GCTC-AGCTGCATTGTAAGG-----TCTAGCCAAGGGA  66828
```

```
Majority     GAATAGAAAGGGAA--X---TXATCATAAAGGCCAAAAGTGG----GTTCAGTGGTTG-TATGGXAGTAGGAGGATC-A-
                           95530     95540     95550     95560     95570     95580     95590     95600
Human        GAATAGAAAGGGGG------TCACCATAAAGGTCAAAAGTGG----GTTTAGTGGTTG-TGTGGGAATAGGCAGATC---  75798
PolarBear    AAACAG----GGAACTAG--TCAACAGAGAGGCCAAAAGTGG----GTTCAGTGATTG-TGTGG-AGTAGGAGAATTGAG  35610
Cow          GAATAGAAAGGGAAGTGGATTTGTCATATAGTCTAAAAATGG----CTTAAGTGGTTGGTATAGAAGTAGAA---TCAA-  73813
Dog          AAACAG----------AA--TTAGCATAAAGCCCAAAAGTGG----GTTCAGTAATTG-CATGG-AATAGGAGAATTAAG  66963
BelugaWhale  GAACAGAAAGGGAAG-G---TTATCATATAGGCTAAAAATGGGTTGGTTCAGTGGCTGGTATGGGAGTAGGAGGATCTA-  65913
Donkey       GAATAGAAAGGGAGG-------GCCATAAAGGCCAAAAGTGG----GTTCAGTGGTTG---TGGGAGTAGGAAGATCAA-  72753
Cat          AAACAA---------------------AAAAAT--AAATTAG----GTTTTTTAATTC-TTCTG-AGCAAGA---TT---  71935
Ferret       AAATAGAAAGGGAACTAG--TCAACATAGAGGCCAAAAGTGG----GTTCAGTAATTG-TATGT-AGTAGGAGAATT---  74016
Lion         AAATAA---------------------AAAAAT--AAATTAG----GTTTTTTAATTC-TTCTG-GGCAAGA---TT---  69635
Vaquita      GAGCAGAAAGGGAAA-G---TTATCATATAGGCTAAAAATGGGTTGGTTCAGTGGCTGGTATGTGAGTAGGAGGATCTA-  67239
Pig          GAATAGAAAGGTAATTG--CTCGTCATATAGGCTAAAGGTGG----GTTCAGTGGTTG-TATGGGAGTAGGAGGATCGA-  66900

Majority     AAAGATGGAAGAATGAAGTCAGGAAA-GGGGATTTXAATGTTAGAAATGAATGG----ACAAA---AGAAAAAXAAAAA
                           95610     95620     95630     95640     95650     95660     95670     95680
Human        -AAGA--AAAGAATGAAGTTAGGAAA-GGAGATATAAGTGTTG--AATGACCATTACAAAAAGAGACAGAGGAAAGAAAA  75872
PolarBear    AAAGATGAAAGAATGAAGTCAGGAAAGGGGATTTTAAATACTGGAAATGAATGGCA-TAACAGATAAAGAGAAAGAAAAA  35689
Cow          GAAAATGGAAGAATGGAGTCAGGAAA-GAGGATTTAAATGTTAGCAATGAATGG-----TGAA---AGAAG----AAAG  73879
Dog          AAAGATGGGAAAAT---GTTGGGAAA--G-----------CAGAAATGAATGGCA-AAACACAAAAAGAGAAAGAAAAC  67025
BelugaWhale  TAAGATGGAAGAACGAAGTCAGGAAA-GGAGATGT-AATGTTAGAAATGAATGG-----CAAA---GAAAGAAAAAAAA  65982
Donkey       GAAGATGGAAGGATGATGTCAGGAAA-GGGGATTGAAATGTTGGAAATGAATGG-----CAAAAAAAAGAAAAGAAAAA  72826
Cat          AAGGATGTCTGTGTGACCTTAAGATA------------------AATAAGTAGTA-TCACTTC---ATCCCACTGGACT  71992
Ferret       -AAGATGGAAGAATAAAGTCAGGAAAGGGGATTTTAAATACTAGAAATGAATGG---TGACAAA-AAAGAGAAAGAAAAA  74091
Lion         AAGGATGTCTGTGTGACCTTAAGATA------------------AATAAGTAATA-TCACTTC---ATCTCACTGGACT  69692
Vaquita      TAAGATGGAAGAACGAAGTCAGGAAA-GGGGATGT-AATGTTAGAAATGAATGG-----CAAA---GAAAGAAAAAAAA  67308
Pig          GAAGATGGAAGAAAAAAGTCAGGAAAAGGGGATGTTAATATGAGAAATGAATAG-----GAAA---GAAAGGA-AAAA  66970
```

```
Majority     TCAAAGATGTAGCAAAAGAAGTTGCTAAAATGGA-TTGATGT-AGTAAAAATTGTCAAAGTAGAAGTTTTXAAGAAATTX
                     95690     95700     95710     95720     95730     95740     95750     95760
Human        ATGAAGATGTATCAAAAGAAGTTGCTAATATGGA-----TGG-------------CAAAGTAGATGTTTTTAAGAAATCA  75934
PolarBear    GCAAAGATGTAGCAAGAGAAGTTGCTAAAATGGA-TTGATGT-AGTAAAAGTTGTCAAAGTAGAAGTTTTAAAGAAATTG  35767
Cow          TCAAAGATGTAGCAGAAGAAGTTGATAAAATAGA-TTGATAT-AGTAAAA----TCAAGGTAGAAGTT---AAGGAATCA  73950
Dog          TCAAAGATGTAGCAAGAGAAGTTGCTAAAATGGA-TTGATGT----AAAAATTGTCAAAGTAGAAGTTTTAAAGAAATTT  67100
BelugaWhale  TCAAAGATGTAGCAAAAGAAGTTGGTAAAACAGA-CTGATAC-AGTAAAAATCATCAAAGTAGAAGTTTTTAAGAAATAG  66060
Donkey       TCAAAGAGGTAACAAGAAAAGTTGCTAAAAAGGA-TTGATGT-AGTAAAAATTGTCACA--AGTAGTTTTTAAGAAACTT  72902
Cat          TTGAACCTGTATCAT-ATACATGGCTG---TGTG-TTCATCC--------GTTTCAGTGGTGGAAGGGTTAGTAATGTGT  72059
Ferret       TCAAAGATGTGGCAAGAGAAGTTGCTAAA-TGGA-TTAATGT-AGTAAAAATTGTCAGAGTAGAAGTTTTAAAGAAATTA  74168
Lion         TTGAACCTGTATCAT-ATACATGGCTG---TGTG-TTCATCC--------ATTTCAGTGGTGGAAGGGTTAGTAATGTGT  69759
Vaquita      TCAAAGATGTAGCAAAAGAAGTTGGTAAAACAGA-CTGATAC-AGTAAAAATCATCAAAGTAGAAGTTTTTAAGAAATAG  67386
Pig          TCAAAGGTGTAG--AAAAAAGT---TAAAATAGGCTTGATATTAGTAAAAATCATCAAAGTAGACATTTTTAAGAAATTG  67045

Majority     TGAGACCAGAGTGTXAGATCTATXAXAXGATGATGA---GAGCTGAGA-AGAAGGAGGAAATAAAGCCAGGTGCTGAA--
                     95770     95780     95790     95800     95810     95820     95830     95840
Human        TGAGACCAGAGTCTTGGAAAAGTCATAGGATGATGCAGGGAATGGAGA----AGAGGGAAATAAAGCCAGGTGCTGAA--  76008
PolarBear    TGAACCAAGGGT-TGT---TTGACATAAGATAACGA---G--CTGGTAGAGAAGGAGGAAATAAAGCCAGGTGCTAAA--  35836
Cow          TGAGACCAGCATGTCGGACAGATTGCAGGATGACAA---GAGCTGAGC---AAGGAAAAGGAAA--TAAGGTGTGAAA--  74020
Dog          TGAGCCAAGAGTGTTTGACATATCATAGGTTGATGACAGGGTCTGGGAGAGAAGGAAGAAATAAAGCCAGGTGCTGTATG  67180
BelugaWhale  TGAGACCAGTGTGTCAGATC-ACAGGATGAGGACAA---GAGCTGAGATAGAAGGAAGAAATAAAGCCAGGTGCCAAA--  66134
Donkey       TGAGGCTAGAGTGTTGGACAGATCACAGGGTGATGAC--GGGCTGAGA---GAGATGGAAATAAAGGCACATGCCTAA--  72975
Cat          CAGAACTAACATGAGAAATCTATAA--AATTGCTGA---G--TCAACTCAAAATATCTGGGTGAT-CCACCTGATGAA--  72129
Ferret       TGGACTAAGGGTGTGTGACCTATCACAAGATGATGATGGG--CTGGGAGAGAAGGAGGAAATAAAGCCAGGTGCTGAA--  74244
Lion         CAGAACTAACATGAGAAATCTATAA--AATTGCTGA---G--TCAACTCAAAATATCTGGGTGAC-CCACCTGATGAA--  69829
Vaquita      TGAGACCAGTGTGTCAGATC-ACAGGATGAGGACAA---GAGCTGAGATAGAAGGAAGAAATAAAGCCAGGTGCCAAA--  67460
Pig          TGAGACCAGAGTGTCAGGTC-ACAGGATGATGACAG---GAGCTGAGA---GAGGAGGAGATAAATCCAGGTGCTGAA--  67116
```

```
Majority     --GTCTTTATGTAATGAGAGAAAATGTCCCAGTGACCTGATGGCTGTTTTGATGA----------GAAATGATTGGATGG
                       95850     95860     95870     95880     95890     95900     95910     95920
Human        --GTCTTTATGTAATGGGAGGAGATGTTCCAGTAATCCAATGGCTATTTTGATGGGAAAGAGTGTGGTATGATTGGGTGG  76086
PolarBear    --GTCTTTCTGTGATGAGAGAAAATGTTCTAGTGACCTGATGGTGGTTTTGATGA----------GAAATGATTGGATGC  35904
Cow          --GTCTTTATGTTGTGAGAGACAATGTCCCAGTGAC----TGACTGTTTTGATGA----------GAGATGTTTACATGA  74084
Dog          TAGTCTTTCTGTAATGAGAGAAA-TGTCCCAGTAATCCGATGGTTCTTTTGATGATGA-------GAAATGATTGGATGA  67252
BelugaWhale  --GTCTTAATGTAATGAGAGAAAATGTCGCAGTGACCTAATGGCTGTTTTGATGA----------GAAATGATTGCATGG  66202
Donkey       --GTCTTTATGTAATGAGAGAGAATGTCCTGGTGACCTGATGGCTATTTTGATGA----------GAATTGATTACATGA  73043
Cat          -GATCACTTTAT-ATGAAAAGGATTCCTGTTACACCTTTGTGTGGCAGTTTATGA------------AGCTCTTTGACAT  72195
Ferret       --GTCTTTCTGTGATGAGAGAAAATGTTCTAGTGGCCTGATGGTTGTTTTGATGA----------GAAATGATTGGATGC  74312
Lion         -GATCACTTCAT-ATGAAAAGGATTCTTGTTACACCTTTGTGTGGCAGTTTATGA------------AGCTCTTTGACAT  69895
Vaquita      --GTCTTAATGTAATGAGAGAAAATGTCCCAGTGACCTAATGGCTGTTTTGATAA----------GAAATGATGGCATGG  67528
Pig          --GTCTTTATGTAATGA-AGAAAATGTCCCAGTGACCCGATGGCTGTTTTGATGA----------GAAATGATTGTATGG  67183

Majority     TA--XAC-TCX-AAGCXATCCTAATTGATGATGGTAGAACAGCAGT-CTGAAAGTAACATTGGGTGGTGGGXTAGXGTGA
                       95930     95940     95950     95960     95970     95980     95990     96000
Human        CATTGACATCGGAAGCCATCCTCATTGATGGTGGTGGAACAGCAGT-TTGAAAGTAACATTGTGCGGTGAGGTAGAGTGG  76165
PolarBear    AG----------AAT--GTCTTAATTGATGGTGGTAGAACTGCAGT-ATGAAAGTAACATTGGGTGGTAGGGTAGAATGA  35971
Cow          TATTAACCTCA-AAGGCATTCTAATTGGTGATAGAAGAATAGCAGT-TGGAAAGTAACATTGGATGGTGGGATAGTAGGA  74162
Dog          AG----------AATCTGTCTTAATTGATGGTGGTGGGACTGCAGT-CTGAAAGTAACATTGGGTGGTAGGGTAGAATGA  67321
BelugaWhale  TA-CGACCTCA-AAGACATTCTAATTGGTGATGGCGGAACAGCAGT-CTGAACGTAACATTGGGTGGTGAGATAGTGGGA  66279
Donkey       CATTGACATCAGAAGCTCTTCTAATTGATGGTGGTGGAACAGCAAT-CTGAAAGTAACATGGGGTAGGG---TAGGGTGA  73119
Cat          TA----------TTT-TATCCTTAT-AACAATCCTAGAAGTAGAAC-----AGGCATTTTTATGTCTCA--------TCA  72250
Ferret       AG----------AATCTGTCTTAATTGATGGTGGTAGAACTGCAGTTATGAAAGCAACATTGGGTGTTAGGGTAGAATGA  74382
Lion         TA----------TTT-TATCCTTAT-AACAATCCTAGAAGTAGAAT-----AGGCATTTTTATGTCTCA--------TCG  69950
Vaquita      TA-CGACCTCA-AAGACATTCTAATTGGTGATGGCGGAACAGCAGT-CTGAAAGTAACATTGGGTGGTGAGATAGTGGGA  67605
Pig          TATCGACCTCA-AAGGCCTTCTGATTGGTGACGGTAGAACAGCAGT-CGGAAGGTAACATTGGGTGGTGGGATAGTGGGA  67261
```

```
Majority     TGAATAATGCATCCTTTTTTTT------TCCTTTGGGGAAGGGTTTAGGGAGATAAAAA-TGACTTTTTGAGAGGGTTAT
                      96010     96020     96030     96040     96050     96060     96070     96080
Human        CACATGATGCATCCTTATTCTT------ACCTTTGGAGAAAAGTTGAGGGAGACCAAAAATGACTTTTTGAGGGAATTGT  76239
PolarBear    TGAATAATGCATCCTTTTTCCT------TCCTTTGGGGAAGGGTTTAGGGAGATAAAAA-TGACTTTTTGAGAGGGTTAT  36044
Cow          TGAATAATACATCCCTTATTT-------CCCTTTGGGGAAGAGTTTAGGGAGATAAAAA-TGA----------GGGTTAT  74224
Dog          TGAATAATATATCCTTTTTCTT------TCCTTTGGGGAAGGGTTTATGAAGATAAAAAATGACTATTTGAGAGGGTTAT  67395
BelugaWhale  TGAATAATGCACCCTTTACTT-------TCCTTTGGGGAAGGGTTTAGGGAGGTAAAAA-TGACTTTTTGAGAGGGTTAT  66351
Donkey       TGAATAATGTATCCTTTTCCTT------CTCTTTGGGGAAGGGTTTAGGGAGATAAAAA-TGACTTTTTGAGAGTATTGT  73192
Cat          TGAACA-TGTGTCAATATTTTT------TTCCA-GCATACCTGCTGCCATAGCTAAAGATGTACGTTATTAAA-----AT  72317
Ferret       TGAATAATGCATTCTTTTTCCT------TCCTTTGGGGAAGGGTTTAGGGAGATAAAAA-TGACTTTTCAAGAGAGTTAT  74455
Lion         TGAACA-TGTGTCAATATTTTT------TTCCA-GCATACCTGCTGCCATAGCTAAAGATATACAGTATTAAA-----A-  70016
Vaquita      TGAATAATGCACCCTTTACTT-------TCCTTTGGGGAAGGGTTTAGGGAGGTAAAGA-TGACTTTTTGAGAGGGTTAT  67677
Pig          TGAATGATGCATCCTTTACTTTTTACTTCCCTTTGGGGAAGGGGATGGGGAGAGGAAAA-TGACTTTTTGAGAGGGTTAT  67340

Majority     AAGAAAAGTTTCATTAGGA-TAAAGCAAGCTTTTAATTAAAAAGTTTCTCTAAGGAACACATTGAATAAAAATGC-AATT
                      96090     96100     96110     96120     96130     96140     96150     96160
Human        AG---AAGTTTCATTAGAAGAAAAGTAAGTTTTTAATTAAAAAGTTAATCTGAGGAACAGGTAGAATAAAAGTGT-AGTT  76315
PolarBear    GAGAAAAGTTTCATTAGGA-TAAAGCAAGCCTTTAATCAAAAAGTTTCTCTAAGGAACACATTGAATAAAAATGC-AATT  36122
Cow          AAGAGAAGTTTCATTAGGA-TCAAGCAAGCTTTTAGTTAAACGTTTTCTCTAAGGAACACATTGACTAAAAGTGA-AATT  74302
Dog          AGGAAAAGTTTCATTAGGA-TAAAGCAAGCCTTTAATTAAAAAGTTTCTCTAAGGAACAGATTGAATAAAAATGCTATTT  67474
BelugaWhale  AAGATAAGTTTCATTAGGA-CCAAGCAAGCTTTTAATTAAATTTTTTCTCTAAGGAACACATTGAATAAAA-TGC-AATT  66428
Donkey       AAGAGAAGTTTCATTAGGA-CAAGAAAAGCTTTTAATTAAAAAGTTTCTCTAAGGAACATGTTGGAAAAAAATGC-AATT  73270
Cat          TAAAAGTATTTCTACAACT-AGAAATAA---TTTG-CT--GGAGCCAATGTATATAACATGTTTTATGTAA--------T  72382
Ferret       AAGAAAAGTTTCATTGGGA-TAAAGCAAGCTTTTAATTAAAAAGTTTCCCTAAGGAACACATTGAATAAAAATGC-AGTT  74533
Lion         -----GTATTTCTACAACT-AGAAATAA---TTTG-CT--GGAGCCAATGTATATAACATGTTTTATGTAA--------T  70076
Vaquita      AA-----GTTTCATTAGGA-CCAAGCAAGCTTTTAATTAAAATTTTTCTCTAAGGAACACATTGAATAAAA-TGC-AATT  67749
Pig          AGGAGAAGTTTCATTAGGA-TCATGGAAGCTTTTAATTAAAATTTTTCTCTAAGGAACACATGTAATAAAAATGC-GATT  67418
```

```
Majority     GTTAATGATAGAAAAG-AAAATGGATTCCAGAGGGXAAAATAAGAATTGAAGGGAAGGTTGGTGGAAGAGGAGGAA---T
                      96170     96180     96190     96200     96210     96220     96230     96240
Human        GTTAGTGGTAGAAGAG---AATGGATTCCATAGGGCAAAATAAGAACTCAAGGGAAGGTTGGTGGAAGAGGAAGAGGATT  76392
PolarBear    GTTAATGATAGAAAAG-AGCATGGATTCCAGCAGGCAAAATAAGGAATTGAAAGGAAGCTGGTAGAAGAGGAGGAA---T  36198
Cow          GTTAATGACAAAAGA--AAAATGGGTTCTAGAGGCTAAAATAGAAATTGAAGGGAAAGTTGGTGGGAGAGGAGGAA---T  74377
Dog          GTTAATGATAGAAAAG-AGAATGGATTCCAGCAGGCAAAATAAAGA-----AAGGAAGGTGGTACAAGAGGAGGAA---T  67545
BelugaWhale  GTTAATGATAGAAAAGAAAAATGGGTTCTAGAGGGTAAAATAAAAATTGAAGGGAAGCTTGGTGGAAGAGGAGGAA---T  66505
Donkey       GTTAATTATAGAAAAG-AAAATGGATTCCAGAGGGCAAAATAGGAATTGAAGAGAAGGTTAGTGAAAGAGGAAGAA---T  73346
Cat          TTTTATGA-AGGGGCG-ATTGTAAATTTGTTGATTTACAACTAGGA-----GTTAACATTAATATAAATATACAGA---A  72452
Ferret       GTTAATGATAGAGGAG-G--ATGGATTCCAGCAGACAAAATAAGGAATTGAAAGGAAGGTGGTAGAAGAGGAGGAG---T  74607
Lion         TTTTATGA-AGGGGAG-ATTGTAAATTTGTTGATTTACAACTAGAA-----GTTAACATTAATATAAATATACAGA---A  70146
Vaquita      GTTAATGATAGAAAAGAAAAATGGGTTCTAGAGGGTAAAATAAAAATTGAAGGGAAGCTTGGTGGAAGAGGAGGAA---T  67826
Pig          GTTAATGATAGAAAAG-AAAATAGGTTTTCGAGGGGAAAATAGAAATTTAAGGGAAGGTTGGTGGAAAAGGAGAAA---T  67494

Majority     GGATTGTTTCAGGAAAGAATA---GCATTTGTCATTCTTGT---------------------------------------
                      96250     96260     96270     96280     96290     96300     96310     96320
Human        GAATTGTTTCAAGAAAGAATA---GCAGTTGTCATCCTTATGAAAAGTAAAATTTTTATTTTCAAATCAGGAAATGTAAA  76469
PolarBear    GGATTGTTTCAGGAAAGAATA---TCGTTTGTCATTCTTGTCAAAAGTGAAAGTTTCAGTTTCAAATCATGAAGTACATG  36275
Cow          GGGTTGTTTCAGGAAAGAATA---GCAGTTGTCATTCTTGT---------------------------------------  74415
Dog          GGATTGTTTCAGGAAAGAATAATAGTGTTTGTCATTATTGT---------------------------------------  67586
BelugaWhale  GGATTGTTTGAGGAAAAAATA---GCAGGTGTGATTCTTGT---------------------------------------  66543
Donkey       GAATTATTTCAGGAAAGATTA---GCAGTTGTCATTCTTGTCAAAAGTGAAATTTCTTTTTTCAAATCAGGAAGTAAAAT  73423
Cat          TTATT-TTTCAG--AA-----------TTTAT---TATGG----------------------------------------  72475
Ferret       GGATTGTTTCAGGAAAGAATA---GTGTTTGTCATTTTTG----------------------------------------  74644
Lion         TTATT-TTTCAG--AA-----------TTTATCATTATGG----------------------------------------  70172
Vaquita      GGATTGTTTGAGGAAAAAATA---GCAGCTGTCATTCTTGT---------------------------------------  67864
Pig          GGATTGTTTCAGGACAGAATA---AAGTCTGTCATTCTTGT---------------------------------------  67532
```

```
Majority     --------------------------------------------------------------------------------
                              96330     96340     96350     96360     96370     96380     96390     96400
Human        ATGTGCCTTCCAGACCCCTTGGTGGTATACATGGGAGATTGGTTCTAGGACACACACAGTCCCATCCCCCACCCTCTGAC  76549
PolarBear    TTGTAAAAAACTGCCCTTCCCCACCCAAGGCCCTGTACTTTATGAATTAGAAAGGGCCTCGAAGTCCCACCCCCTGTCCT  36355
Cow          --------------------------------------------------------------------------------  74415
Dog          --------------------------------------------------------------------------------  67586
BelugaWhale  --------------------------------------------------------------------------------  66543
Donkey       TTAGAAACTGCCCTCCCCACCCAGGACCCTATAATTTATGAATTAGAAGGGGCCTCTAAGACTCACCACCAGACCTCTGT  73503
Cat          --------------------------------------------------------------------------------  72475
Ferret       --------------------------------------------------------------------------------  74644
Lion         --------------------------------------------------------------------------------  70172
Vaquita      --------------------------------------------------------------------------------  67864
Pig          --------------------------------------------------------------------------------  67532

Majority     --------------------------------------------------------------------------------
                              96410     96420     96430     96440     96450     96460     96470     96480
Human        CCCATACACCCCCTGGATACTCAAATCCACTGATGCTCAAGTTCCTTGCATAAAATGGTATAGTGTTTGCATGTGACCTA  76629
PolarBear    CTGTTCAGTAATACATGAGACAACTACCAGATGTCTTGAACAGGGTAAAGAAGTAAGACCAAAAGAAAT---GTAATCAA  36432
Cow          --------------------------------------------------------------------------------  74415
Dog          --------------------------------------------------------------------------------  67586
BelugaWhale  --------------------------------------------------------------------------------  66543
Donkey       TCAGTAGCGTGTGAGACTAATAACTGTCAGATGTCTTGAACAGAGAAAAGAAGTAAGACCAAAGGAAATCATGTGATTGA  73583
Cat          --------------------------------------------------------------------------------  72475
Ferret       --------------------------------------------------------------------------------  74644
Lion         --------------------------------------------------------------------------------  70172
Vaquita      --------------------------------------------------------------------------------  67864
Pig          --------------------------------------------------------------------------------  67532
```

```
Majority     --------------------------------------------------------------------------------
                               96490     96500     96510     96520     96530     96540     96550     96560
Human        TACACAACCTCTTATGTGTACTTTAAATCATCTCTAGATTACTTATATTACCCAGTACAATATAAATGTTATGTAAATGG  76709
PolarBear    CTTTGGATAGAAGTTTTAGAATGGGAAATGAAGGGATAAAATAAATTCTGTATCTTTAAAAATCATGGATAAAATTGTCA  36512
Cow          --------------------------------------------------------------------------------  74415
Dog          --------------------------------------------------------------------------------  67586
BelugaWhale  --------------------------------------------------------------------------------  66543
Donkey       CTTTGGATTGAAGTTTTAGAATGGGAAATGAAGGGATAAAACAAATTCTGCATCCTTAAAAATGATGGATAAAACTGTCA  73663
Cat          --------------------------------------------------------------------------------  72475
Ferret       --------------------------------------------------------------------------------  74644
Lion         --------------------------------------------------------------------------------  70172
Vaquita      --------------------------------------------------------------------------------  67864
Pig          --------------------------------------------------------------------------------  67532

Majority     -----------------------------------------------------CAAAAG------------TG-------
                               96570     96580     96590     96600     96610     96620     96630     96640
Human        TTGTTATAGTGTATTGTTTAGGGAATAATGACAAGAACAACTTTCTATACATTTGCAGTACACCATTGTTTTAC--CCCC  76787
PolarBear    TAATCAAGAGGAGCCTAAGGAGACATGACTACATGTAATATGAGATCCTGGAACAGAAAA-GGGTATTAGATGCACACCA  36591
Cow          -----------------------------------------------------CAAAAG------------TA-------  74423
Dog          -----------------------------------------------------CAAAA-----G-------TG-------  67594
BelugaWhale  -----------------------------------------------------CAAAGG------------TA-------  66551
Donkey       TAGTCAAGAGGACCCTAAGGAGACATGACTAAATGTAATATGGGGTCCTGGAACAAAAAAAGGACATCAGATGCAAACTA  73743
Cat          -----------------------------------------------------------------------TG-------  72477
Ferret       ----------------AAGGAGACATGACTAAATGTAATATGGGATCCTGGAACAAAAAA-GGATATTAGATGCACACCA  74707
Lion         -----------------------------------------------------------------------TG-------  70174
Vaquita      -----------------------------------------------------CAAAGG------------TA-------  67872
Pig          -----------------------------------------------------CAGCAG------------TA-------  67540
```

```
Majority     ---AAATTTT--------TXTTTTCTAGTCAAGAAXTAAAATATTA---AXACTGXCTTCTTXACTCAAGXXCCTATACT
                              96650     96660     96670     96680     96690     96700     96710     96720
Human        A-AATATTTTTGATCCAAGGTTGGTTGAATCGGAACCCAGAGATAC---AGAGGGCTGACTATACTTTAAGAATTAGAAT  76863
PolarBear    AAAAAGTCTA----------GACTATAGTTAATAA-TAATATATCA---GTATTGGCTGATTAATTGCAGCAACTATACC  36657
Cow          ---AAATTTT--------TATTTTCAAATCAGGAAATA---TATTGTAAAAACTGCCCTCCCCACCCAGGACCCTATACT  74489
Dog          ---AAACTTA----------GTTTCAAGTCAGGAAATAAAATGTAA---AAACCGTTCTCCCCACCCAAGCCCCTGTACT  67658
BelugaWhale  ---AAATTTT--------TATTTTCAAGTCAGGAAATAAAATATTGTAAAAACTGCCCCCCTCACCCAGGACCCTATACT  66620
Donkey       AAGAAATCTGA---AGTATGGACTTTAGTTAATAA-TAATGTGTCA---GTATTGGCTGATGAATTGCAACAAGCTTACC  73816
Cat          --TAAGTAAA-----------T---AGTGCATCA-TGAAATATT-----TTGTAGGTTTTATTTATTTTATTTTTATT  72534
Ferret       AATAAATCTGATGAAGTATGGGCTTTAGTTAATAA-TAATATATCA---GTTTTGGCTGATTAATTGGAGCAACTATACT  74783
Lion         --TAAATAAA-----------T---AGTGCATCA-TGAAATATT-----TTGTAGGTTTTATTT------TTTTTATT  70225
Vaquita      ---AAATTCT--------TATTTTCAAATCAGGAAATAAAATATTGTAAAAACTGCCCCCCTCACCCAGGACCCTATACT  67941
Pig          ---AAGTTTT--------TGTTTTCTAATCAAGAAATAAAATGTTACAAAAACTGCCTTCCCCACCCAGGACCTTATATT  67609

Majority     AT------------TATGAAT-----TAGA-------------------------AXGAACCTCATAG-TCCCXCCACT
                              96730     96740     96750     96760     96770     96780     96790     96800
Human        TAGCTGGGTGTGGTGGTGGGTGCCTGTAGTCCCAGCTACTCGGGAGGCTGACGCAGGAGAAAGGCGTGAACCCGGGAGGT  76943
PolarBear    ATACTAATGTAAGATGTTAATA---ATAGAGGGAAAAGGAAGTACCCAGGTTATATAGGAACTGTATAG-AATCTTCAAT  36733
Cow          AC-------------ATGAAT-----TAGG-------------------------ATAAACCTCAAAAGTCCCACCACC  74525
Dog          TT------------TATGAAT-----TAGA--------A----A--------------GAGCCTCAAAG-TCCCACCACC  67694
BelugaWhale  AC-------------ATGAAT-----TATA-------------------------ACAGGCCTCAAAG-TCCCACCACC  66655
Donkey       ATACTAA-----GGTGTTAATA---ATGGGGGGGAAATGGAATGCCTGGGTTATGTAGGAACTCTGTAGAACCTTCTATT  73888
Cat          TT------------TATTTTT-----TTTA----------------------------A-CGTTTTAT-TTTTTTTATT  72566
Ferret       ATGCTAATGTAAGATGTTAATA---ATAGAGGGAATAGGAAGTACCCAGGTTATATAGGAACTCTGTAG-AATCTTCAGT  74859
Lion         TT------------TATTTTT-----TTTA----------------------------A-CGTTTTAT-TTTTTTTATT  70257
Vaquita      AC-------------ATGAAT-----TATA-------------------------ACAGGCCTCAAAG-TCCCACCACC  67976
Pig          GC-------------ATGAAT-----TAGA-------------------------ACAGGCCTCAAAA-TCCCACCACC  67644
```

```
Majority     TATTCTTCTXTTCAGXXATAX------------------------------T-------------------------
                                96810      96820      96830      96840      96850      96860      96870      96880
Human        GGAGCTTGCAGTGAGCCGAGATCGTGCCACTGCACTCCAGCCTGGGCGACAGAGCGAGACTCTGTCTCTAAAAAAAAAAA 77023
PolarBear    TTTTCTGTAAATCTAAAATATAAACCTTATTTTTTTTAAAAGATTTTATTTATTTATTTGACAGAGACAGCCAGCGAGAG 36813
Cow          AAATCCTCTGTTGAGTCATATG-----------------------------T------------------------- 74548
Dog          TATCCT------CTAA-----------------------------------T------------------------- 67705
BelugaWhale  AA-TCCTCTGTTCAGTCGTATG-----------------------------T------------------------- 66677
Donkey       TT-TCTGTAAATCTAAAACT---------------------------------------------------------- 73907
Cat          TATTTT------------------------------------------------------------------------ 72572
Ferret       TTTTCTGTAAATCTAAAATATAAAC-----------------------TTTATTT------------------------ 74891
Lion         TATTTT------------------------------------------------------------------------ 70263
Vaquita      AA-TCCTCTGTTCAGTCGTATG-----------------------------T------------------------- 67998
Pig          AG-TCCTCTGTTCAGTAATA---------------------------------------------------------- 67663

Majority     --X-----------------------AGAATAACAACCA------TX----AGATGTCTTGAGTAAGX-------------
                                96890      96900      96910      96920      96930      96940      96950      96960
Human        AAAAAAATTAGAAGGGGCTGTCAGGCGTGGTGTC-TCATGCCTATAATCCCAGTCATGCCTGTAATCCCAGCACTTTGGG 77102
PolarBear    AGGGAACACAAGCAGGTGGAGTGGGAGAGGAAGAAGCAGGCTCCTAGCGGAGGAGCCTGATGTAGGGCTCGATCCCAGAA 36893
Cow          --G-----------------------AGAATAACAATCA------AT----AGATGTCTTGAACAGGGA--A--------- 74583
Dog          -------------------------------CAGAAGTA------------------T----GTGAG-------------- 67719
BelugaWhale  --G-----------------------AGAATAACAACCA------CC----AGATGTCTTGAACAGGGA--A--------- 66712
Donkey       --GTTCTAAAAAAGTAGTTTATTAAAAAAGCATAGATAAA---ATTTGTAAATGATTTAAAGTAAG-------------- 73968
Cat          ---------------------------------------------------------------TGAG-------------- 72576
Ferret       --------------------------AAAGTCATAGATAAACTTGTA----AATGGTTTAAAGTAAG-------------- 74928
Lion         ---------------------------------------------------------------TGAG-------------- 70267
Vaquita      --G-----------------------AGAATAACAACCA------CC----AGATGTCTTGAACAGGGA--A--------- 68033
Pig          --------------------------AGAATAACAACCA------CC----AGATGGCTTGAACAGGGA--A--------- 67697
```

```
Majority     ------------------------------------AAXAA--A------------------------------------
                      96970     96980     96990     97000     97010     97020     97030     97040
Human        AGGCTAAGGTGGGCATATCACTTGAGATCATGAGTTCAAAACCAGCCTGGCCAA-----CATGGTGAAACACTGTCTCTA  77177
PolarBear    CGCCAGGATCACGCCCTGAGCCAAAGGCAGACGCTTAACGACTGCGCCACCCAGGCGCCCCCCCTAAAATATAAACCTTA  36973
Cow          ------------------------------------AAGAAGTA------------------------------------  74591
Dog          -------------------------------------ACAA---------------------------------------  67723
BelugaWhale  ------------------------------------AAGAAACAAAACAGGGGA--------------------------  66730
Donkey       ----------------------------------TTAACAAAAACGGTTTAATTCTTCAGTTGAAATGGGCAAGGTTAGG  74014
Cat          ----------------------------------------A---------------------------------------  72577
Ferret       ----------------------------------TTAACAA-----------A---------------G----AGTTTCA  74944
Lion         ----------------------------------------A---------------------------------------  70268
Vaquita      ------------------------------------AAGAAACAAAACAGGGGA--------------------------  68051
Pig          ------------------------------------AGGAAGTA------------------------------------  67705

Majority     --------------------------------------------------------XCTAAGXX-XAAA-AAGATGAXTT
                      97050     97060     97070     97080     97090     97100     97110     97120
Human        CTAAAAATACAAATATTAGCCAGGTGTGGTGGTGGGCGCCTGTAATCTTAGCTACTCAGGAGGCTGAGTCAGGAGAATTG  77257
PolarBear    TTAAAAATCATAGATAAATGTGTGAATGATTTAAAGTTAACAAAAGGTTTAATTCTTCTGAGCAGGATTAAGGATGTCTA  37053
Cow          ----------------------------------------------------------TAAGATGAAAAGATAATGGTAT  74613
Dog          ---------------------------------------------------------CTACC---------ACATGTCT-  67736
BelugaWhale  --------------------------------------------------AAGAACTTTAAGACCAAAAGA-TATGATGT  66759
Donkey       AC-------T-----------------------------------------GCCTGTGCGCATCAGCAAAAATATGACTT  74046
Cat          ---------------------------------------------------------CCAAG---------AGAGAGCA-  72590
Ferret       TT-------C---------------------T----------------TTAATTCTTCTGAG-------AAAAATGTTTA  74973
Lion         ---------------------------------------------------------CCGAG---------AGAGAGCA-  70281
Vaquita      --------------------------------------------------AAGAACTTTAAGACCAAAAGA-TATGATGT  68080
Pig          ----------------------------------------------------------CAGAATCAAAA---AATGAGGT  67724
```

```
Majority     AATTGACCTT--GGATAGAGGTTTAAGAAXX-GGAAATXAAAXCAT---A-----------XTCAXAAACAAG-------
                      97130     97140     97150     97160     97170     97180     97190     97200
Human        CTTGAACCCAGGAGGTGGAGGTTGCAGTGAGCTGAGATCATGCCATTGCACTCCAGCCTGTGTGACAGAGTGAAACTCCA  77337
PolarBear    ATGTGACCTTAAGGGAAGAGGGATAGGTA-G-TTATATCACTTCATGCCACTGGACTTTGTATCATATACATGGCTGTGT  37131
Cow          AATTGACATT--GGATGGAAGTTTTAGAATA-GGAAATGAAAGGAT------------------A-AAACAAG-------  74664
Dog          ---TGAGC--A-GGGTAAAGAAGTAAGAC---CAAAAGAAATTCAATCAACT----------TTGGATACAAG-------  67790
BelugaWhale  AATTGACTTT--GGATAGATGTTTCAGAATA-GGAAATGAAAGGAC------------------A-GAACAAA-------  66810
Donkey       GA-AGAGGTGGTAGATACATAATTTATATCA-CTTCATCCTGCTGGTC--TTTGAATCTTTATCAGATAAAGG-CTGTGT  74121
Cat          ---TGAAC-----GGGGGAGGGTCAGAGA----GAGGGAGACACAG---A-----------ATCTGAAACAGG-------  72637
Ferret       -TGTGACCTTAAGGAATGAGGGATAAGTAAG-TTAAATCACTTCATCCCACTGGACTTCATATCATATACATGGCTGTGT  75051
Lion         ---TGAAT-----GGGGGAGGGTCAGAGA----GAGGGAGACACAG---A-----------ATCTGAAACAGG-------  70328
Vaquita      AATTGACTTT--GGATCGATGTTTCAGAATA-GGAAATGAAAAGAT------------------A-GAACAAA-------  68131
Pig          AATTGACTTC--AGATAGAAATTTTAGAGTG-GGAAATGAAAGGAT------------------A-AAACAAA-------  67775

Majority     ---------TTCTG----X--XAGGXTXTGAAATAATA---TXTCAGAA----CCAAX--------------------XT
                      97210     97220     97230     97240     97250     97260     97270     97280
Human        TCTCAAAAATACAAAAAGAATTAGAAAGGGCTTTAGAGTCCCACCACTAATGCTCTTCTGTTCAGTAGTACACAAGACTA  77417
PolarBear    GTTCATCCATTTTAGTGGAGGAAGGGTTAGTAATAATA---TGTCAGAA----CTAACATGAGAAATTCATTAAATTGCT  37204
Cow          ---------TTATG------------------------------------------------------------------  74669
Dog          ---------TTT------T--A-GAATGTGAAATGAAG---TTACAAAA----CCAA------------------T-TCT  67826
BelugaWhale  ---------TTCTG------------------------------------------------------------------  66815
Donkey       ATCCATCAGTTTTGGTGGAGGAAGGGTTAGTAATAATAATTTATCAAAA----CTAACA---GAAATTTATAAAATTACT  74194
Cat          ---------CTCC---------AGGCTCTGAGCTGTCA---GCACAGAG----CCCAA---------------------T  72671
Ferret       GTTCATCCATTTCCGTGAAGGAAGGGTTAGTAATAATAATGTGTCAGAA----CTAAAATGAGAAATTTATAAACTTGCT  75127
Lion         ---------CTCC---------AGGCTCTGAGCTGTCA---GCACAGAG----CCCGA---------------------C  70362
Vaquita      ---------TTCTG------------------------------------------------------------------  68136
Pig          ---------TTCTAA---AATAAATAAATAAATTAATT---AATTAAAA----TAAAAAACAAAAAAACCCCCAAATTCT  67836
```

```
Majority     GCATCTCTXAAAATCA-TGGAT----------AAAAAGATCATAGXCAAGAGGAG-----CCT--AAXGAGACX------
                      97290     97300     97310     97320     97330     97340     97350     97360
Human        ATAACTACGAGATGTTATGAACAGGGAAAAGAAGTAAGACCAAAAGAAATCATAAATTA-CTTTGGATGGAACTTTTAGA  77496
PolarBear    GAATCACTCAAAATATCTGGGTGGCCCACCTGATGAAGATCATTTCACATGGAAT-----CCC--TGTGATACCTTT---  37274
Cow          -CATCTTTAAAAAGCA-TAGAC----------AAAACTGCCATAGTCAAGAGGAG-----CCTT-AAGGAGAC-------  74724
Dog          GCATCTTTAAAAATCA-TGGAT----------AAAAAGGTCATAGTCAGGAGGAG-----CCT--AAAGAGAC-------  67881
BelugaWhale  -CATCTCTAAAAAGCA-TGGAT----------AAAACTGTCATAGTCAAGAGGAG-----CCT--AAGGAGGT-------  66869
Donkey       GAATCACTCAAAATGTCTGGGTGGCCCAGCTGATGAAGATCATTTCACGTGGAAAGGATCCCTGTGTTACTACCTTCCTT  74274
Cat          GTGGGGGTCGAACTCA-CAGACCC---------GCGAGATCATGACC----TGAG-----CC---GAAGTCAGC------  72723
Ferret       GAATTACTCAAAATGGCTGGATGGCCCACCTAATGAAGATCATTTCACATGGAAAATTAACCC--TGTGATACCTTT---  75202
Lion         GCGGGGGTCAAACTCA-CGGACC----------GCGAGATCATGAC-----TGAG-----CC---GAAGTCGGC------  70412
Vaquita      -CATCTCTAAAAAGCA-TGGAT----------AAAACTGTCGTAGTCAAGAGGAG-----TCT--AAGGAGGT-------  68190
Pig          CCATCTTTAAAAAGCA-TGGGT----------AAAACTATCATGGTCAAGAGTAG-----CCT--AAAGAGGC-------  67891

Majority     -----------ATGACTAAAT---------------XTA--ATAATXG----X--CATCCTGGAACAAA--AAXAGGX-A
                      97370     97380     97390     97400     97410     97420     97430     97440
Human        ATGGCAGATGGAGGGATAAATTTGACATTGTTCAAAATCATAGAGTAAACTCTCAAAAGTAAGGGGAACCTAAGGAGACA  77576
PolarBear    ---------ATATGGTAAACT-----GCCATACAACATTATTTTATCGTTACAATAATCCTGGAGGTAG--AACAGGC-A  37337
Cow          -----------ATGACTAAAT---------------TTA--ATAATAG-------CATCCTGAAATAAA--AAAC-ATGA  74766
Dog          -----------ATGACTAAAT---------------GT---ACTATGG-------GATCCTGGAACAAA--AAAAGG---  67920
BelugaWhale  -----------ATGACTAAAT---------------CTA--ATAACGG-------CATCCTGAAACAAA--AA------A  66906
Donkey       TTCTTTAGCATATGGTGGTTTATGAAGCTCTTTAATGATGTTTTATCCTTATA-CTGTCCTGGAAGCAG--AACAGGC--  74349
Cat          ------------CGCTCAACC-------------------G--ACTG----AGCCACCCAGGCGCCCC--AATGTAG-G  72762
Ferret       ---------GTATAGCAGTTTATGAAGCTCTTCAACATTATTTTATTGTTCTAACAATCCTGGAAGTAG--AATAGGC-G  75270
Lion         ------------CACTCAACT-------------------G--ACTG----AGCCACCCAGGTGCCCC--AATGTAG-G  70451
Vaquita      -----------ATGACTAAAT---------------CTA--ATAACGG-------CATCCTGAAACA------------A  68223
Pig          -----------ATGACTAGCT---------------CTA--ATAACAG-------GATCCTGGAACAAA--AAAAGAAAA  67934
```

```
Majority     ATGXCATTATATGAAXATXAA-------XAATAAT--------XA--XX---------TTTAGCTAAAXATGTAAATXXT
                     97450     97460     97470     97480     97490     97500     97510     97520
Human        AGATGATTAAATGTAATAGGAGATACTGGAACAACAAAAAAGGGACATTAGATGCAATTTGAGGAAATCTGATAATGGAC  77656
PolarBear    TTTTTAATCTCTTATCATCAACATGCATTAGTAATGTTTTTTCCAGCATGCCTACAGCCCTAGCTAAACATGTAAATCTT  37417
Cow          ATG-CATTCTGTGCTCAGTCATGT---CTGACTCT-----TTGCA--ACCCCATGGACTGTAGCCCACCAGGCTCCTCTC  74835
Dog          ------ACAGATAAACACCAA--------AGAAAT-----------------------CTGACAAAG--TGTGGACTTT  67960
BelugaWhale  AGGACATTAGATGCAAATTAA---------------------------------------------AGAAATCTGAATAC  66941
Donkey       ATTTTTATCCCCCATTGTCAATA----CTCATAGTGTTTTCTCCAGCATACCTGCTGCCTTAGCTAAAGATTTAAATCGT  74425
Cat          TT-----GTTTTTAATGTAGA--------AGACAT----------------------TTAGGTTAAA-CTGAAATTTTT  72805
Ferret       TTTTTAATCTCTCATCATCAAAATGCATTAATTAT-TTTTTTCCAGCATACCTGATGTCTTAGCTAAA-ATGTAAATCAT  75348
Lion         TT-----GTTTTTAATGTGGA--------AGACAT----------------------TTAGGTTAAA-CCGAAATTTTT  70494
Vaquita      AGGACATTAGATGCAAATTAA---------------------------------------------AGAAATCTGAATAC  68258
Pig          ATGACATTATTTACATAATGGGCT---TTAATTAA-----CATCA--ATATTGGCTGATTAAGTGCAACAACTATATCAT  68004

Majority     TXTAXTXAAA-----------GTT-ATTAATAATAA-X---XXG--AXTGTATGT-TATTAGXTTCT-----------
                     97530     97540     97550     97560     97570     97580     97590     97600
Human        TTTTGTTGATTTATCGATATTGGTTGATTAATTGCAAGAACTAATAAATTGTAATACCATACTAATGTATGATGCTAATA  77736
PolarBear    CAAAATTACA-----------GCT-AAAAATAATTT-ACTGGAGCCAATGTATGT-AACATGTTTTTATACATTTTTTA  37482
Cow          TCCATGGAAT-----------TTT-CCAGGCAAGAATACTGGAG--AGGGTTTGC-CATTTCCTTCTC-----------  74888
Dog          ----A----------------GTT-AATAATTATAT-------GTCAGTAT---------------------------  67983
BelugaWhale  TTTATGGACC-----------TTA-ATTAATAATAA--------------CATAT-TAATAGTGGCT-----------  66981
Donkey       TAAAATTAAAATAATTTCTACAGCT-AGAAATAATCC-ATCGGAGCAAATGTATATATAACATGTTTCATATAATTTTTA  74503
Cat          T-------------------------------------------------------------------------------  72806
Ferret       TAAAATTAGA-----------GCT-AAAAATAATTTTATTGGAGCCAGTGTATGT-AATATGCTTTAA-ACAGTTTTTA  75413
Lion         T-------------------------------------------------------------------------------  70495
Vaquita      TTTATGGACC-----------TTA-ATTAATAATAA--------------CATAT-TAATACTGGCT-----------  68298
Pig          ACTATTGTAA-----------GAT-GTTAATAAGGGGGGGAGGG--TGCCCAGGT-TATTAGGAACTT-----------  68057
```

```
Majority     -----------TXG--XATTTAXTGA----------A-C----AGTTA------------AAATACAATXCA--------
                      97610     97620     97630     97640     97650     97660     97670     97680
Human        ATGGGGGAACTGCGGTTTCCAGGTTATATAAGAACTCTGTAATGTCTTAAATTTGTTTGTAAATCTAAACCTTTTAAAAT  77816
PolarBear    CAAAGGGAAGATAGTAAGTTTATTGACCTA-----TAGCTAGGAGTAAATATGA-----AAAATACAATACAG---AATT  37549
Cow          ---------CAGGG--GATCTTCCC-----------AACCCAGAGATC------------GAACCCGTGTCTC---CTGC  74931
Dog          -----------TGGCTGATTAATTG----------TAAC--------AGT-----------AACACCATAC---------  68014
BelugaWhale  ----------------GATT-----------------------AGTTG------------GAACATATATCA--------  67002
Donkey       CAAAAGGAAGATGATAAACTTACTGTCTTCCATTGTAGCTTGGAGTTAACAGTAATATGAAAATAGAATACAGAATTTTC  74583
Cat          ------------------TTTCCTGA------------------------------------------------------  72814
Ferret       CAAAGGGAAGATGGTAAGTTTATTGACTTAA----TAGCTAGGAGTTAATATGA-----AAAATACAATACAG---AACT  75481
Lion         ------------------TTTCCTGAC-----------------------------------------------------  70504
Vaquita      ----------------GATT-----------------------AGTTG------------GAACATATATCA--------  68319
Pig          ---------TCTAG--AATCTTTTTTA-TA------AATGTAAAGCTGTTCT-------AAAATAAAAGTTTA---TTTA  68109

Majority     XXX---X-----TA--AXTXTXTTATTAATGTTTC-----G-AA------------------------A-GAAXA----X
                      97690     97700     97710     97720     97730     97740     97750     97760
Human        AGCTAAAAATTATAGATACAATTTCTAAATGATTTAATTAACAAAACCAGTTTAATTCTTCAGTTGAAATGAGCAAGGTT  77896
PolarBear    TTTGGTATGGTATAACATTATGGTATAAATAGTGCATCATGAAGTATTTTGTAGGTTGTGTTTATTGTA-GAAGACATTT  37628
Cow          TTTGGCAG---GCAG-ATTCT-TTACCACTGCGCTACCTGGGAAG--------------------CCAA-GAAAA----G  74981
Dog          ------------TAATACAATGTTAATAATAGAG------GGAAA-----------------------A-GGAAA-----  68047
BelugaWhale  -----------------------TACTATTGTATG---------------------------------------------  67014
Donkey       TTTTTCAGGATTTATCATTGTGATATAAATAATGCACCATGAAATATTTTGTAGCTTTGTATATTTTTA-TTGTAG---C  74659
Cat          -----------------------TAGTAATTTTTC-------AC------------------------A-GAAGA----G  72835
Ferret       TTT--CAGAATTTACCATTATGGTATAAATAGTGCATTATGAAATATTTTGTAGGTTGTTTTTGTTGTA-GGAAACATTT  75558
Lion         --------------------TGATAGTAATTTTTC-------AG------------------------A-GAAGA----G  70528
Vaquita      -----------------------TACTATTGTATG---------------------------------------------  68331
Pig          TTTATTTA---TTTG-TCTTT-TTGG-GCTGTACC-CACAGCATA--------------------TGGA-GGTTC----C  68157
```

```
Majority     AG----XACTXAG-XTTTTA--------------------------------------------XXX-ATTGTAGA-ATC
                      97770     97780     97790     97800     97810     97820     97830     97840
Human        AGGGATACCTGTGTGTTTCAGTGTAATATCTGAAAGGAAAAGAATAGATACATAATTTATATCACTTCTCTCTGGACTTA  77976
PolarBear    AAGTTAAACCTAA-TTTTTT------TTTCCTGATA---ATTTTTCAGAGAAGAACGTACTGAGAGAAATTGTAGA-ATC  37697
Cow          AG----GACATTA-GATGCA--------------------------------------------AATTAAGGAAGTCTGA  75012
Dog          -G----GACCCAG-GTTATA--------------TA---------------------------GGAACTCTGTAGA-ATC  68079
BelugaWhale  -----------------------------------------------------------------------------ATA  67017
Donkey       AGACACCATTAAG-ATTAAAGCTGAAAAACATTTTTACCTGATTTCTGA---GAGTATACTGAGGGAAATTGTGGA-ATA  74734
Cat          TG----TACTGAG-AAATTA------------------------------------------------ATTGTAGA-ATC  72861
Ferret       AGGTTAAACCTAA-GTTTTAAAAAAATTTTCTGATAGTAATTTTTAAAAAAAGAGCATACTGAGAGAAATTGCGGA-ATC  75636
Lion         TG----TACTGAG-AAATTA------------------------------------------------ATTGTAGA-ATC  70554
Vaquita      -----------------------------------------------------------------------------ATA  68334
Pig          AA----GCCTAGG-AATTGG--------------------------------------------AGCCGTAGCTGCCGTC  68188

Majority     XTTCATTAGAG-CTTTAXAXGXCCAGTAAAAAXTCAAGACTA-TTCCAGTGA-TTTAGAACTA-GTXAAGATGGCTTAA-
                      97850     97860     97870     97880     97890     97900     97910     97920
Human        GGGTCTCTTGTCATATACATGACTGGGCATCCATCAGCTTCAGTGGAGGAAAGGTTAGGAATAATAGTGTCAGAACTAAC  78056
PolarBear    TTTCATTAGAGGCTTTAAAAGTCCAGTAAAAACTCAAGACTAATTCCAGTGA-TTTAGAACTGTGAATAGATGGCTTAA-  37775
Cow          ATACTTTATGGACTTTA-----ATAATAACATATTAATACTG--GCTGATGA-ATTATAGCAA-GT-ATATCATACTACT  75082
Dog          -TTCATT------TTT------TCTGTAAA-----------------------TCTAAAT-----------------A--  68105
BelugaWhale  GTACAATATAT---CTA-----TTTATAATATAT--------------ATGA-TATATAACT-----ATATCATGCTATA  67069
Donkey       TGTTCTTAGAGGCTTTAAAAGTCCAGTAAAAACTC-AGACTA-TTCTAGTGA-TTGAGAACTACATAAAGATGGCTTAA-  74810
Cat          TGTCGTTAGAGACTTTAAAAGTCCAGTAAAAACTCAAGACTAATTCCAGTGATTTTAGAACTATGTAAAGATGGCTTAAG  72941
Ferret       GTTCATTAGAGG-TTTAAAAGTCCAGTAAAAACTGAAGACTAATTCCAGGGA-TTTAGAACTGTGTAAAGATGGCTTAA-  75713
Lion         TGTCGTTAGAGACTTTAAAAGTCCAGTAAAAACTCAAGACTAATTCCAGTGATTTTAGAACTATGTAAAGATGGCTTAAG  70634
Vaquita      GTACAATATAT---CTA-----TTTATAATATAC--------------ATGA-TATATAACT-----ATATCATGCTATA  68386
Pig          CTACACCACAG-CCGCA-----GCAACACCATATCCAAGCCG-TGTCTGCAA-CCTACACCA---C-AGCTCACAGCAAT  68256
```

```
Majority     GTAACTTTTTTAX---------GX--GAGTGAGGAAATAGTGGXAGXXAAGTAXATGCTAACXGCATXAAAGACAXXGAC
                      97930     97940     97950     97960     97970     97980     97990     98000
Human        ATGAGAATTTTAGGAGATTACTGAGTCACTCAAAATGTCTGGGTGACCCAGCTGATGAGGGCCATTTCACATGAAAAAGA  78136
PolarBear    ATAACTTTTCTAAGTTATCA--GCCTGGGGGAGGAAATAGTGGAAGAAAAGTATATGCTAATGGCATCAAAGACAGTGAC  37853
Cow          GCAAGATGTTAA--------------TAATGGGGGGACAGGGGTGCCCAGG-TTATGGGAACTTTGGAGAATGTTTAATT  75147
Dog          TAAACTTTATTAA---------------------AAATCAT---AGATAGGGACAC-CTATCAGCAGTTGAG----TGCC  68156
BelugaWhale  GTAAGATATTAA--------------TAATGGGGGAAAAGGGGTGCCCAGGATTATAAGAACT--GGAGAATTCTTAATT  67133
Donkey       GTAGCTTTTCTA---------------AGTTATCAGCCCTGAGGGACGAAATACATATTAGTGACATTAAGGACAGTGAC  74875
Cat          TAAGCTTTTCTAT-----CA--GCCTGGGGGAGGATATAGTGGAAGAGAACTACTTGCTAGTGGCATTAAAGACA--GAC  73012
Ferret       ATAGCTTTTCTAAGTTTTCA--GCCTGGGGGAGGAAATAGTGGAAGAAAAGTACATGCTAATTGCATCAAGGACAATGAC  75791
Lion         TAAGCTTTTCTAT-----CA--GCATGGGGGAGGATACAGTGGAAGAGAACTACTTGCTAGTGGTATTAAAGACA--GAC  70705
Vaquita      GTAAGATATTAA--------------TAATGGGGGAAAAGGGGTGCCCAGG-TTATAAGAACT--GGAGAATTTTTAATT  68449
Pig          GCTAGATCCTTAA---------GC--CACTGAGTGGGGCCTGGGATTGAAC-CTGTGTCCTCAT-GGATACTAGCAGATT  68323

Majority     TGCCTTCATCTCTTAAAXXXCTXTXAC-----GCCATAXTCTGGGCTTCCTAAAATTA----------AXAAAXAX----
                      98010     98020     98030     98040     98050     98060     98070     98080
Human        TTCCTGCAACACCTTCTTTTCTTTAGCACATGATCGTTTATGAAGCTCTTTTACATTGTTTTATCTTTATAGCAATCCTC  78216
PolarBear    TGCCCTAATCTCTCAAAGTACTCTTAC-----ACCATACTCTGGGCTTCCTAAAATGA----------AGAAAGAT----  37914
Cow          TTTCTACAAATCTAAAA--------CT-----GTTCTAAAATAAGCTTATTAAAAATT----------ACAGATAA----  75200
Dog          TGCCTTCAGC-CC------------AG-----AGCATGATCCTGGAGTCCTGGGATCG----------ATCAA-------  68201
BelugaWhale  TTTCTGTAAATCTAAAA--------CT-----GTTCTAAAGTAAGTTCATTAAAAATT----------ATAGATAA----  67186
Donkey       TGCCTTCATCTCTTAAAGCACTCTTGC-----ACTGTACTCCGGGCTGCCTAAAATGA----------AGAAGGATC---  74937
Cat          TGCCCTCATCTCTTAAAGTACTCTTAG-----GCCATACCCTGGGCTACCTAAAATGA----------AGAAAGAT----  73073
Ferret       TGCCCTAATCTCTTAAAGTACTCTTAC-----ACCATACTCTGGGCGTCCTAAAATAA----------AAAAAAAAAAAA  75856
Lion         TGCCCTCGTCTCTTAAAGTACTCTTAG-----GCCATACCCTGGGCTACCTAAAATGA----------AGAAAGAT----  70766
Vaquita      TTTCTGTAAATCTAAAA--------CT-----GTTCTAAAGTAAGCTTATTAAAAATT----------ATAGATAA----  68502
Pig          CGTTT-CCG--CTCA-----------------GCCATGATGGGAACTCCTAAAAAAT------------CAGATAA----  68367
```

```
Majority     -AATTGG--CAXAXGA-TTTAXGATAACTTGTCAXA--AGCACAGTGGTCC-TT--------T------CTGXTXA--AA
                      98090     98100     98110     98120     98130     98140     98150     98160
Human        AGAATAGATCAAGCTTTTTTTTTTTTTTTTTTTGAGATGGAGTCTTGCTCTGTCGCCCAGGCTGGAGTGCAATGGTGCGA  78296
PolarBear    GACTTGG--TAGGA---TATATCATAACCTGACACACTAACACAGTGGCCC-AGATTATAAATGGATAAGTGATAATAAA  37988
Cow          -AATTGG--CAAGTGG-TTTAAGAGAAGTTATCAAA--AACAGTTTAATTC-TTTG------TT-----CTGTTGT--GA  75260
Dog          --------------------ATCCCACTTCGGGCTCTCTGCACGG-AGCC------------------------------  68230
BelugaWhale  -AATTTG--CAAATGA-TTTAAGATAAGTTATCAAG--AACAGTTTAATTC-TT---------------CAGTTGT--AA  67242
Donkey       -ATTTGA--CGGAAGACT---AGATCACCTGACTTAGTAGTATAGTGGTCTGCTGTTATCACCACTCTGGTGATGATAGA  75011
Cat          GATTTGG--CAGAATA--ACGTTATCACCTGCCAT---AGCACAGTGGCCC-AGAT------TG-----GTGATAA---A  73131
Ferret       GACTTGG--TAGAAGAATATATCATAACCTGACATACTAGCACAGTGGCC-------A----T--------GATAATAAA  75915
Lion         GATTTGG--CAG----------TATCACCTGCCAT---AGCACAGTGGCCC-AGAT------TG-----GTGATAATAAA  70819
Vaquita      -AATTTG--CAAATGA-TTTAAGATAAGTTATCAAG--AACAGTTTAATTC-TT---------------CAGTTGT--AA  68558
Pig          -AATTTG--TAAATGA-TTGAAGATAAATTATCAAA--AACAGTTTAATTT-TT---------------CAGTTAA--AA  68423

Majority     T---GG------AXAAGTTGGGA----GAXGT-XGTGTX--------GCXTCTGXA----TAGTAGTAACXGXXXXXGG-
                      98170     98180     98190     98200     98210     98220     98230     98240
Human        TCTCGGCTCACTGCAAACTCGGCCTCCCGGGTTCGTGCCATTCTTCTGCCTCAGCCTCCCGAGTAGCTGCTACTACAGGT  78376
PolarBear    T---GG------AAAAGTATGGA----GATAT-GAAGTA--------ACATTTGGA----TAGTAGTAACAATAGTGGG-  38041
Cow          T---GG------ACAAGG---------AATGTCTGTGT---------GCCTCAGCA----GAATGTGACCTG--A--GG-  75304
Dog          ------------------------------T--G--------------CTTCT---------------------------  68237
BelugaWhale  T---GG------ACAAGGTTAGG----GATGTCTGTGT---------GCCTCAGCA----AAATATGACCTG--A--GG-  67291
Donkey       T---GG------AAAAGTTGGGA----GACTATGAAATA--------GCATTTGGA----CAGTAGTAAGAACAGTGGG-  75065
Cat          T---AG------AAAAGTGGGGA----GACGT----GTA--------TCATTTGGA----TAGTAGTAACAGCAGTGAA-  73181
Ferret       T---GA------AAAAGTGGGGA----GACATTGAAGTA--------GCATTTGGA----TTGTAGTAACAACAGTGGG-  75969
Lion         T---AG------AAAAGTGGGGA----GACGT----ATA--------TCATTTGGA----TAGTAGTAACAGCAGTGAA-  70869
Vaquita      T---GG------ACAAGGTTAGG----GATGTCTGTGT---------GCCTCAGCA----AAATATGACCTG--A--GG-  68607
Pig          T---GG------ACAAAG---------GG-GTGTCTGT---------GCCTCAGCA----GAATAGGATTTG--A--GG-  68466
```

```
Majority     GATCTCAXXAAXTTACAXTTXAXTTTTTTCTAA---TCXCXTT-AT----CTCTGTAG-CT-XXXXX-GXTX--TTGXAT
                              98250     98260     98270     98280     98290     98300     98310     98320
Human        GCCTGCCACCACGCCCAGCTAATTTTTTTGTATGTTTAGTAGAGATGGGGTTTCATCGTGTTAGCCAGGATGGTCTCGAT  78456
PolarBear    GATCTCA-CAGCTTACATTCCACTTTTTGCTAAGCCTCCCATT-AC----CTCCTCACCTTAGAAGGAGGTAAATTGCAT  38115
Cow          GGATAGAGGG----ATAGATGTATAACTTAAAA---TCACTTT-AT----CCCCATGGACT-------------TTGAGT  75359
Dog          -CTCTCT-GCCTGTGTATCTGCCTTCTCTCT---CCTTCCTCT-CT----CTCTCTC-----------------TC---T  68287
BelugaWhale  GG-TAGAGGGATAGATAGATGCATAATTTGTAA---TCACTTC-AT----CCCTGTGGACT-------------TTGAAT  67349
Donkey       GACCTCC-------CCGCTTACATTTGTGCTAAACTTCCCATT-AC----TTCCATAA--TAGAAGGAGGTAAATTGCAT  75131
Cat          GATCTCAACAGCTTACATTCCACTTTTTGTT-----TCCCGTT-AC----CTCTGTA--CTAGAAGGCGGTAG-TTGCAT  73248
Ferret       GCTCTCA-CAATTAACATTTCACTTTTTGCTAAGCTTCACATT-AT----CTCTTTA--TAGGAAGGAGGTAAATTGTAT  76041
Lion         GATCTCAACAGCTTACATTCCACTTTTTGTT-----TCCCGTT-AC----CTCCGTA--CTAGAAGGAGGTAG-TTGCAT  70936
Vaquita      GA-TAGAGGGATAGATAGATGCATAATTTGTAG---TCACTTC-AT----CCCTGTGGACT-------------TTGAAT  68665
Pig          GG-TAGAGG------TAGATAAATAGTTTATA----TCAC-----------CCTGTGGGCG-------------TTGAGT  68511

Majority     CTCCT--------------TCXAXCXCATT-TT--CTCACTTGAAAATX-GX----AGTCTTXXXATTACCAAA--GCAG
                              98330     98340     98350     98360     98370     98380     98390     98400
Human        CTCCTGACCTTGTGATAGATCAAGTATTTTTATCCCTCTCATCAACATGTGTAAACAGTTTTTTCCTCACATATCTGCTG  78536
PolarBear    CTCCT--------------TTGAGCCCATT-TT--CTCACTAGAAAATTTGC----AGTCTTATTGTTACTAAAGGGCAA  38174
Cow          CTCTA--------------TCAGATAGA---------TATGTGGCAGT--GT----TGCCAT--CATTTTCAGT--GGGG  75406
Dog          CTCTC--------------TCTCTCTCTCT-CT--CTCATGTGAATA---------AATTTT-------TAAAA------  68328
BelugaWhale  CTCTA--------------TCAGATAGATA-GGTACATACATGGCAGT--GT----GGCCAT--CAGTCTCAG---GGAG  67403
Donkey       CTACT--------------TTGAACCCAAAGTTTTCTCATTAGAAGATTAGC----AGTCTTATTTCTTCTAAAACGCAA  75193
Cat          CTCCT--------------TTGAGCCCATT-TT--CTCACTAGAAAATTAGC----AGTCTTATTTTTACCAAAGTGCAA  73307
Ferret       CTCCT--------------TTGAGCCCATT-TTT-CTCACTAGAAAATTTGC----AGTCTTACTGTTACTAAAGTGCAG  76101
Lion         CTCCT--------------TTGAGCCCATT-TT--CTCACTAGAAAATTAGC----AGTCTTATTTTTACCAAAGTGCAA  70995
Vaquita      CTCTA--------------TCAGATAGATA-GGTACATACATGGCAGT--GT----GGCCAT--CAGTCTCAG---GGAG  68719
Pig          CTCCA--------------TCAGATAT----------TACATGGCTGT--GT----GTCCAT--CATGTTCAGT--GGAG  68557
```

```
Majority     TGTTGXXTAATAATAXG-----TGACATTT-----------------AATAAAAA--AAXCTTTCAGATCTGTCACACT-
                      98410     98420     98430     98440     98450     98460     98470     98480
Human        CCTTAGCTAAAGATGCAAATCATTAAAGTTAAAATTATAATTTATGTAGCTAAAAGTAATCTGTTAGAGCCAGTGTATAC  78616
PolarBear    TGTTGTTTAATAAAGGG-----TGGCATTTCCTGGCATTTAAAAAAAAAAAAAAA--AGACTTGCAGATCTGTCACAGT-  38246
Cow          GAGTGGCTAGTAATGTG-----TCAGAACT-----------------ATTACAGG--AAATTTATAAAATGGCTGAATC-  75461
Dog          --------AATCATAGA-----TAA-ATTT-------------------GTAAAA--CATCTTTCAGATCTATCACACC-  68372
BelugaWhale  AAGGGGCTAGTAATATG-----TCAGAACT-----------------AATACAAG--AAATTTATAAAATTGCTGAATC-  67458
Donkey       TGTTGTTTATTAAAATA-----TGACATTCTCTGGCATTT--------TTTTTTA--AGGTCTCCGG-TCTATCACACTG  75257
Cat          TGTTGTTTGTTAAAAGA-----TGGCACTCCCTGGCAT-----------TTAAAA--AGTCTCTCAGATCTGTCACACT-  73368
Ferret       TGTTGTTTAATTAAAGG-----TGGCATTT------------------AAAAAAA--AGTCTTGCAG-TCTGTCACACT-  76154
Lion         TGTTGTTTGTTAAAAGA-----TGGCATTCCCTGGCAT-----------TTAAAA--AGTCTCTCAGATCTGTCACACT-  71056
Vaquita      AAGGGGCTAGTAATATG-----TCAGAACT-----------------AATACAAG--AAATTTATAAAATT---------  68766
Pig          GAAGGGCTAATACTGTG-----TCAGAACT-----------------AATACAAG--AAATTTGTAAAATTGCTGAACT-  68612

Majority     AAAA-----AAXATTCTAG-----ACTTT----------------TXATTTT---CAATTACCATTTTAXATTAATTTCA
                      98490     98500     98510     98520     98530     98540     98550     98560
Human        AATATGTGTAATATGCTTTCTATAACTTTCTCAAAGGGCCCTTGGTAAGTTTATTGTTTTCTCATTTAGCTGGGAGTTAA  78696
PolarBear    AAAA-----AAGACTGCAGCCTGGACTTT----------------TAATTTT---CAGTTACTATTTTAAATGAGTTTCA  38302
Cow          ACTC-----AAAATGCTGG-----GTGGC----------------CCAGCTAA--TGAAGATCATTTCACATAGAAAGG-  75512
Dog          AAAA-----AATACTCCAG-----ACTTT----------------TCATTTT---CAGCTACCATTTTAAATTAATTTCA  68423
BelugaWhale  ACTC-----AAAATTCTGG-----GTGGC----------------CCAGCCGA--TGAAGATCATTTCACATAGAAGGGG  67510
Donkey       AAAA-----AGGACTTTAACCTAGACACT----------------TAATTCT---CAATTACCATTTTAAATTAATTTTA  75313
Cat          AAAA-----AA-AGACTAG-----ACTTT----------------TTTTTT----CAATTTCCATTTTAAATTAATTTCA  73417
Ferret       AAAA-----AGGATTCCAGCCTGGACTTT----------------TAATTTT---TAGTTAATATTTTAGATTAATTTCG  76210
Lion         AAAA-----AA-AGACTAG-----ACTTT----------------TTTTTTTTTTCAATTTCCATTTTAAATTAATTTCA  71109
Vaquita      ---------------CTGG-----GTGGC----------------CCAGCCAA--TGAAGATCATTTCACATAGAAGGGG  68808
Pig          ACTC-----AAAA-----------ATGTT----------------CGGGC-------------GGTTCACATAGAAAGG-  68646
```

```
Majority     GXTCTCTGTTAAXTCT-CAGTGATCATTTTTCCATGAT----A-GTCTTGATGTTTTAAATAATTXTGTGCC-XCAAGTT
                                 98570     98580     98590     98600     98610     98620     98630     98640
Human        GAACAGAACTATTTTCTAGAATGTATTAATTATATAAATAGTGCATCATGAAATATTTTGTAGGTTTTATATTTTTATT  78776
PolarBear    GTTCTCTTTTAATTCT-CAGTGATCATTTTTCCATAA-------GTTTTGTTGTTTTAAATAATATTGTGCCACCAAGTT  38374
Cow          -ATCCCTGTCACATCT-TCCTTTTCTTTAGCATCTGGT----AGTATATGAAGCTCTTTGATGTTACTTGAT-TC---TT  75582
Dog          --TTTCTCTTAATTCT-CAGTGATCATTTTTCCATGAT------GTCTTGATGTTTTGAATAATATTGTGCC---AAGTT  68491
BelugaWhale  GATCCCTGTGACACCT-T------CCTTTTCATGTGGC----AGTTTATGAAGTTCTTTGACATTATTTGAT-TC---TT  67575
Donkey       ATTATTCTTTTAATTTTCAGTGATCGTTTTTCCATGAT------GTCCTGATGCTTTAAATAATATGGTGCCACCAAGTT  75387
Cat          GTTCTCTTTTAATTCT-CAGTGATCATTTTTCCATGAT----AAGTCTTCATGTTTTAAATAA---TGTGCCACCAAGTT  73489
Ferret       GTTCTTTCTTAAGTCTTCAGTGATCATTGTTCCATAA-------ATCTTAATGTTCTAAAAAATACTGTGCCTCCAAGTT  76283
Lion         GTTCTCTCTTAATTCT-CAGTGGTCATTTCTCCATGAT----AAGTCTTCATGTTTTAAATAA---TGTGCCACCAAGTT  71181
Vaquita      GATCCCTGTGACACCT-T------CCTTTTCATGTGGC----AGTTTATGAAGCTCTTTGACGTTATTTGAT-TC---TT  68873
Pig          -ATCCCTGTGACACCC-T-----TCTTTGGTATATGAC----ACTT--TGAAGCTCTGTCAT--TATTGGAC-TC---TC  68707

Majority     AC-----TACATTATAXAACCCAGATTTCAAA------------AAGAAATGTATTTCAAA-ACAXCATTATTTTGXATA
                                 98650     98660     98670     98680     98690     98700     98710     98720
Human        GCA-GCAGACAGCATTTAGATTAAACCTAAAAATACCTGATTGACAAAGTAGTTTTTCAGACAAAAGTATATTGAGGGAA  78855
PolarBear    ACCTACCTTCGTTATATAACCCAGATTTCAAC------------AAGAAATGTACTTCAAA-ACAATTATGTCTTGAATA  38441
Cow          AC-----AACAATAGCGGTTCTAGAAGTAAAA------------CAGGTGTTTTTATCCC--CCATCATCAGTATGCATA  75643
Dog          AC----CCCCGTTTTA-AACTCAGATTTCAAG------------AAGAAAAGTATTTCAAA-A-ACCATTGCCTTTAATA  68552
BelugaWhale  AC-----AACAATA------CTGAAAGTAAAA------------CA--CGTTTTTATCCC--CCATC-TCAATATGCATA  67627
Donkey       GT----CTCCATTATAAAACCCAGATTTCAAG------------AGGAAATGTATTTTTAAAACAATTATGTCTTGAATC  75451
Cat          AC-----TCCATTATAACATCCAGATTTCAAT------------AAGAAATGTATTTCAAA-A-ACCATTATGTCT--TG  73548
Ferret       ACCTATCTTCATTATGAAACCCAAATTTCAAC------------AGAAAATGTATTTCAAA-AAACAATTGTCTTGAATA  76350
Lion         AC-----TCCATTATAACATCCAGATTTCAAT------------AAGAAATGTATTTCAAA-A-ACCATTATGTCG--TG  71240
Vaquita      AC-----AACAATA------CTGAAAGTAAAA------------CA--CGTTTTTATCCC--CCATC-TCAATATGCAT-  68924
Pig          AT-----AACAAT--C----CTGGGAGTAAA-------------CAGGCATTTTTTTTCTG-CCATCATCATTATGCATA  68762
```

```
Majority     AATAAAGTTTCTTTGAACAAATTTTCTATC-------ACCTTTGAATATTXXGGTGAAGTTTT-CA---T-XAGATG-X-
                           98730     98740     98750     98760     98770     98780     98790     98800
Human        ATTGGAGAATCTTCAGAGGCTTTAAAAGTCCAGGAAAATCTTAGACTACTCCAGTGATTTAGAACGGTGTAAAGATAGCC  78935
PolarBear    TTTAAAGTTTCTTTGAACAAATTTCCTATC-------ACCTTTGAATATTTTGGTGAAGTTTT-CA---T-GAGATTGTT  38509
Cow          GACAGTGTTTTTT--------------------------ACAGCATATT----TGCTACCTTAA-------AGATG---  75682
Dog          TTTAAAGTTTCTTTGAACAAATTTTCTATC-------ACCTTTGGATAC---GGTGAAGTTTTTCA---T-GAGGTGGTT  68618
BelugaWhale  AATAGTGTTTTTT--------------------------ACAGCATACC----TGCTGCCTTGA-------AGATG---  67666
Donkey       TCTAAAGTTTCTTTGAACAAATGTTCCATC-------ACCTTTGAATATTTTGGTAAAGTTTCAT---T--AGATTATT  75519
Cat          AATGAAGTTTCTTTGAACAAATTTTCTATC-------ACCTTTGAGTATATTGGTGAAGTTTT-CA---T-TAGATCATT  73616
Ferret       TTTAAAGGTTCTTTGAAAATATTTTCTGTC-------ACCTTTGAATATTTTGGTGAAATTTT-CA---T-GAGATT---  76415
Lion         AATGAAGTTTCTTTGAACAAATTTTCTATC-------ACCTTTGAGTATATTGGTGAAGTTTT-CA---T-TAGATCAT-  71307
Vaquita      ---AGTGTTTTTT--------------------------ACAGCATACC----TGCTGCCTTGA-------AGATG---  68960
Pig          AAGAGTGTTTTTTTGTTGTTTTTTTTTTTT-------TTTACAACATACC----CACTTTCTTAAG---TAGAAATG---  68825

Majority     --A-TTGTATATTGACATTGTA------GAGATGTATTTXAGAAGAAGCAXTTCTGTAXAAATGAAGTATTAAACTTAGA
                           98810     98820     98830     98840     98850     98860     98870     98880
Human        TAAGTAGTTTTTCTAAGTTATCAGCCGCGGGGAGGAAATAATGAAAGAGAAATACATACGAGTGGCATTAAGGACAGGTG  79015
PolarBear    GAA-TTGTATATTGACATTGTA------GGGATGTATTTCAGAAGAAGCTTTTCTGCAAAAATGAAGTATTAAGCTTAGA  38582
Cow          ----TTAATCATTCAAATT-----------GTTTTC-ACAGCTAAAAGTAATTCATTAGAGGCAATGTGTATACCATGTT  75746
Dog          GAA-TTATATATTGACATTGTA------GAGATGTATTTCAGAAGACGCTTTCTTGCAAAAATTAAGTATTAAACTTAGA  68691
BelugaWhale  ----TAAATCATTAAAATTAAA------ATAATTTCTACAGCTAAAAGTAATCCATTAGAGCCAGTGTGCATAACGTGTT  67736
Donkey       TAA-TTGTATATTGGCATGGTA------GAGATATATTTTAGAAGAA--ACTTCTGCAGAGATGAAGTGTTAACCTTAGA  75590
Cat          GAA-TTGTATATTGACATTGTA------GAGGTGTATTTCAGAAGAAGCTTTTTTGAAAAAATGAAATATTAAGCTTAGA  73689
Ferret       --A-TTGTATATTGACATTGTA------GAGATGTATTTCAGAAG---CTTT-CTGCAAAAATGAAGTATTAAGCTTAGA  76482
Lion         -----TGTATATTGACATTGTA------GAGGTGTATTTCAGAAGAAGCTTTTTTGAAAAAATGAAATATTAAGCTTAGA  71376
Vaquita      ----TAAATCGTTAAAATTAAA------ATAATTTCTACAGCTAAAAGTAATCCATTAGAGCCAGTGTGCATAACGTGTT  69030
Pig          ----TAAATCGTTAAAATT-----------AATTTCTACAGCTAAAAGTAATCCATTAGAGCCAATGTATAGAACATGTT  68890
```

```
Majority     CTGTCTTGTTTXXXTTTATATATCGAATTTAGGTAAATTGXTATTTTAT----A------TTTCATG--AATTAATAATT
                       98890     98900     98910     98920     98930     98940     98950     98960
Human        GTGGCTTCTTTTACCTATTAAAGCATTCTTACACCATACTGTGAACTGTGTAAAAGGAAGGATCATTGGACATAATAGAT 79095
PolarBear    CTGTCGTGTTTAGATTGATATATCAAATTTAGGTAGATTGGTGTTTTAT----A------TTTCATG--AAGTAATAATT 38650
Cow          CTATATGTTTT---TTTACAAAGAGAAGATAGTGAATGTATTGTTTT------C------TCTCAT---GGCTAAAAGTT 75808
Dog          CTGTCG--T-----TTAATCTATCAAATTTAGGTAAATTGGTATTTTAT----A------TTTCATG--AATTAATAATT 68752
BelugaWhale  TTATATGTTTT---TTTACAAAGGGAAGATAGTAAATGTATTTTC---------------TCTCAC---AGCTAGGAGTT 67795
Donkey       CTGTTGTGTTTAGATTGACATATCGAATTTAGGGAGATTGATATTTTAT----ATTATTGTTTCGTG--AATTAGTAACT 75664
Cat          CTGTCATGTTTAGATTTATATATTGAATTTAGGTAGATTGATATTTTAT----A------TTTCATG--AATTAATAATA 73757
Ferret       CTGTCTTGTTTAGATTGATATGTCAAATTTAGGTAGATTGGTATTTTAT----A------TTTCATG--AATTAATAATT 76550
Lion         CTGTCATGTTTAGATTTATATATTGAATTTAGGTAGATTGATATTTTAT----A------TTTCATG--AATTAATAATA 71444
Vaquita      TTATATGTTTT---TTTACAAAGGGAAGATAGTAAATGTATTTTC---------------TCTCAC---AGCTAGGAGTT 69089
Pig          TTATATGTTTT---TTTTTTTTTTTTTTTTTTTTACAAGAAATG----------------TGTTAC---AGCTAGGAGTT 68949

Majority     TT-CTTGAAATGTCAACAGGGAAAGACGA-GTAATAX-------GATGACTTTXAACTTAXATTCTGXAAGTGTCAGTAG
                       98970     98980     98990     99000     99010     99020     99030     99040
Human        TATCTGACAGGGTGGTCCGGAATGGTGGAAGTAAATCGACAGGTGAGGACACTGAATTAGCATTCAGACAGTGGGGACCT 79175
PolarBear    TTCCTTGAAATGTCAACAGGGAAAGACGA-GTAATA--------GATGACTTAGAACCTACGTTCT--AAGTGTCAGTAG 38719
Cow          -------AA-CATTACTATGAAAAATACA-ATACAGA-------ATTTCTTTCCAGAGTTTATTATTACAGTATAAATAG 75872
Dog          TTTCTTGAAATGTCAACAGGGAAAGATAA-GTATAAC-------AAGGACTTAGAACTTA-ATTCTGAAAGTGTCAGTAG 68823
BelugaWhale  -------AA-CATTAATATGAAAAATACA-GTGCAGA-------ATTCCTTTTCAGAATTCATTAT---------AATAG 67850
Donkey       TTTCTTGAATTGTCAAAAGGGAAAGAGGA-GTAGTAG-------GTGACGTAGAACTGTAAGTTTGGAAGTGTCAGTAG 75736
Cat          TTGCTTGAAATGTCAACAAGGAAAGACGA-GTAATA--------GATGACTTCAAACTTA-ATCCTGGAAGTGTCAGTAC 73827
Ferret       TTCCTTGAAATGTCCACAGGGAAAGACGA-GTAATA--------GATGACTTGGAACTTA-ATTCTGGAAGTGTCAGTAG 76620
Lion         TTGCTTGAAATGTCAACAAGGAAAGACGA-GTAATA--------GATGACTTCAGACTTA-ATCCTGGAAGTGTCAGTAC 71514
Vaquita      -------AA-CATTAATATGAAAAATACA-GTGCAGA-------ATTCCTTTTCAGAATTCATTAT---------AATAG 69144
Pig          -------AA-TGTTAACATGAAAAA------TACTGA-------ATTCCTTTTCAGAATTTATCATTATGGTGTAAATAG 69008
```

```
Majority     TGTGTAATXTAT-TGT-ATXTCTXA---GGTTTTTXTT---X------------------------TTTT----------
                      99050     99060     99070     99080     99090     99100     99110     99120
Human        CACACCTTGTAT-TCCCTTTTTTGGTAAGATTTTTATTACCTCCTTAGAAAGAGGTAAATTCATCTATTTCGAGCCCAAA  79254
PolarBear    TGTGTAATCTATCTGC-ATGTCTCA---CGTTCTTTTTTTTCTTA---------------TCTTTTTTTT----------  38770
Cow          TGCATCATGAAA---T-ACTTTGTA---GGTTTTTGT------------------------------TTT----------  75905
Dog          TGTGTAATCTTGATA--ATGTCTCA---CGTTAAAAAA------------------------------------------  68856
BelugaWhale  TACATCATGAAA---T-ACTTCGTA---GGTTTTTATA----------------------------TTTT----------  67885
Donkey       TGTGTAATATACCTGTCATATATTAGAGCATTTCTTTACCTTTCAAATAAATGCTTCTTTTTAATTTCTT----------  75806
Cat          TGTGTAATCTAT-TGC-ATGTCTCA---TTCTTTGTGTGTGTGTGTGTGTGTG---------TGTGTG----------  73883
Ferret       TTTGTAATCTGTCTGT-GTGTCTCG---CATTCTTTTTTCTTTTTCTTTCTTTCTTTCTTTTTTTTTTTT----------  76686
Lion         TGTGTAATCTAT-TGC-ATGTCTCA---TTCTT-GTGTGTGTGTGTGTATCCAT----------CATGTT----------  71568
Vaquita      TACATCATGAAA---T-ACTTTGTA---GGTTTTTATA----------------------------TTTT----------  69179
Pig          TGCATCATGAAA---T-ATTTTTTA---GGTTTATAT------------------------------TTT----------  69041

Majority     --------AATG------TAGCXGT-CTATAT-TAX---------AGATTATTCTTTXAAATA-AATA-A-XTTATCTTT
                      99130     99140     99150     99160     99170     99180     99190     99200
Human        TTTTTCTGTCTAGAAGATTGGTAGTCTTATTTTTACTAAAGTACAAGATTATTTAGTAAAAGGTAGCATTCTCTGGCTTT  79334
PolarBear    --------AACA------TAGCCATACTTTAT-TA----------AGCATTTTCTTTGAAATG-AATA-AATGTATTTTT  38823
Cow          --------TATG------TAGCAG----ACGT-TG----------AGATT-----------------------TACCTGG  75933
Dog          --------AATA------TACCCAT-TTATAT-TAC---------AGCATTTTCTTTGAAATG-AATA-A---TTTTTTT  68906
BelugaWhale  --------TGTG------TAGCAGA-CAACAC-TG----------AGATTAAACCTAAAAAAA-AAAA-A--TTACCTTA  67935
Donkey       --------AGTACTTTTTTGAGCATCGTATGTGTAC---------GAAGCATCATACCAAGTA-TACGTGTAGTGGTTCG  75868
Cat          --------TGTG------TGTCCATCATATGT-TAC---------AGCATTTTCTTTTTTGGA-AGTG-AATTCTTTTTT  73937
Ferret       --------AATA------TACCCTAACTCTAT-TAC---------AGCATTTTCTTTGAAATG-AATA-AATGTATCTTT  76740
Lion         --------ACAG------CATTT-TCTTTTTT-GGA---------AGTGAATTCTTTTTTTTT-TTTT-TTTTTTTTTTT  71621
Vaquita      --------TGTG------TAGCAGA-CAACAC-TG----------AGATTAAACCAAAAAAAA-AAAA-A--TTACCTTA  69229
Pig          --------AATG------TAGCAGC-CAACAT-TG----------AGATTATTAAACCTAAGA-AAAA-A--CTACCTGA  69091
```

```
Majority     TT-XATTTXTT----TATTTTTTGAGA--------ACCAXATATX---TGAAX---GAXATCGTGGCAAXTATXCXTAG-
                      99210     99220     99230     99240     99250     99260     99270     99280
Human        TTTTTTTTTTTTTTTTTAATGTTTCAGATCTGTCACACTAAAAAGGCTTTAGACCAGGTGTTGTGGCTCACACCTGTAAT  79414
PolarBear    TTTAATTCCTTAG--TATTTCTCAAGA--------ACCACATATGT-ACAAAA---AACATCATACCAAGTATACATGG-  38888
Cow          TT-GACAGAAT------TTTTA-GAGA--------AGAATATAC----TGAAA---GAAATTGTGGGATCTATCTTCAG-  75989
Dog          TTTAATTCCTTAG--TATTTCTTGAGA--------AACATACAT------A-------CATCATACCGAATATACATGG-  68962
BelugaWhale  TT-GACGAAAT------TTTTTAGAGA--------AGAATATAC----TGAAG---GAAATGGTGGAAACTATCCTTAG-  67992
Donkey       CAAAATATTGTCTACAGATCTTTGAGA--------GCCCCTGAGACCNTGAGG---GAAATTGTGGAATATGTTCTTAG-  75936
Cat          TTCCGATTCTT----TATTTATTGATA--------ACCACAGATGT-ACAAA-----GCATCATACCAAGAATACATGG-  73998
Ferret       TT-AATTCTCTTG--TATTTCTTCAGA--------ACCACATATGA-ATAAA-----ACATCATATGAAGTATACATGG-  76802
Lion         TCCCAATTCCT----TATTTATTGATA--------ACCACAGATGT-ACAAA-----GCATCATACCAAGAATACATGG-  71682
Vaquita      TT-GACAAAAT------TTTTTAGAGA--------AGAGTATAC----TGAAG---GAAATGGTGGAAACTATCCTTAG-  69286
Pig          CT-GACAAAGT------GATTT-TTTT--------AGAGTATAC----TGAGA---GAAATTGTGGAATCTATCCTTAG-  69147

Majority     --CAGCTXT-C--------------ACAAGXTCXCTAGAGAXC-----TCTX-GACTATTCTAAT----------AGATT
                      99290     99300     99310     99320     99330     99340     99350     99360
Human        CCCAGCACTTTGACAGGTCAAGGTGGGAGGATTGCTGGAGGCCAGGAGTTTGAGACCAGCCTGAGCAACATAGTGAGATC  79494
PolarBear    --CAGTAGT-C--------------ACAAAATATCTAGAGATC-----TCTCGGACTG---TAACAGT------TAAAAT  38937
Cow          --AGGCTTT-T--------------AAAGGTCCAGTAAAAACT-----GCAA-GACTATTCTAGT-----------GACT  76035
Dog          --CAGTGGTTC--------------ACAAAGTATCTGGAGATC-----TCTCCGGCTC---TAAC----------AAAGT  69008
BelugaWhale  --AGGCTTT----------------AAAAGTCCAATAGAAACC-----TCAA-GACTATTCTAGT-----------GATT  68037
Donkey       --AGGCTTT----------------AAAAGTCCAGTAAAAA-C-----TCA--GACTATTCTAGT-----------GATT  75979
Cat          --CAGTGGTTC--------------ACAAAATGTTTAGAGATC-----TCTGGAGGCC-TCTTAGGGTTTCTAACAA-GT  74055
Ferret       --CAGTGGTTC--------------ACGGAATATCTGGAGATC-----TCTCAGACTC---TTAG---------CAAAAT  76849
Lion         --CAGTGGTTC--------------ACAAAATGTTTAGAGATC-----TCTGGAGGCCCTCTTAGGGTGTCTAACAAAGT  71741
Vaquita      --AGGCTTT----------------AAAAGTCCAATAGAAACC-----TCAA-GACTATTCTAGT-----------GATT  69331
Pig          --AGGCTTT----------------AGAAGTTTAGTAAAAACC-----TCA--GATTATTCTAGT-----------GATT  69191
```

```
Majority     XAAAACTXTAT---XAX--AAGATTACTAA--------AATAG----TGTTT-----CTAAGCTAT----TAGTCTCAX-
                      99370     99380     99390     99400     99410     99420     99430     99440
Human        TCAGTCTCTACTAAAAATAAAAAAAATTAGCCATGCCTGGTGGCATGTGCCTGTAGTCCCAGCTACTTAGTAGGCTGAGG 79574
PolarBear    CAAAACTATAT--TCAT--GACAATACTAA--------AATATTATTTGTTT-----TTTGACTCT----TACTCTCAC- 38995
Cow          TAGAACTGCAA--------AAGATGACTGA--------AATAG----TTTTT-----CTAAGTTAC----CAGCTTGGGA 76086
Dog          CAAAACTGTAT--TCAT--GACAATATTAA--------AATGTTATTTGTCT-----TTCGACTTT----TACTTTCAC- 69066
BelugaWhale  TGGAACTGTGA--------AAGATGACTGA--------AATAG----TTTTT-----CTAAGTTAC----CAGACTAGGA 68088
Donkey       GAGAACTACATA-------AAGATGGCTTA--------AGTAG----CTTTT-----CTAAGTTAT----CAGCC----- 76026
Cat          CCAAACTATATATCCAT--AACACTACTAA--------AATGT----TGTCT-----TTTGACT------TATTCTCAC- 74109
Ferret       CAAAACTATAT--TCAT--GATAATACCAA--------AATATTATTTGTCT-----TTTGACTCT----TACTCTCACC 76908
Lion         CCAAACTATATATCCAT--AACACTACTAA--------AATGT----TGTCT-----TTTGACT------TATTCTCAC- 71795
Vaquita      TGGAACTGTGA--------AAGATGACTGA--------AATAG----TTTTT-----CTAAGTTAC----CCGACTAGGA 69382
Pig          TAGAACTGCAA--------AAGGTAGCGGA--------AACAG----TTTTT-----CTAAGTTTT----CAGCT-GGGA 69241

Majority     TAGXATA-----TAXTGXTCXAAAGGTAAATA--------------GXTAATGXGATTAXGXATA-------GTGACTG-
                      99450     99460     99470     99480     99490     99500     99510     99520
Human        TGGGAGGATTGCCTGAGCCCAGAAGATAGAAACAGCAGTGAGCCATGATCATGCCACTGCACTCTAGCCTGGGTGACAGA 79654
PolarBear    TAGCATA-----CATTGCTCTGAAGGCTAATA--------------GCATATGTGATCATGTGTT-------CTTGT--- 39046
Cow          GAAGGAA-----TAGTGAAGGAGAAGTACAC---------------GCTAGTGGCACTAAGGACA-------GTGACT-- 76137
Dog          TACCATA-----TGTTAGTCTGAAGGCTAATA--------------GATAATA-GACTATGTGAT-------CATATGT- 69118
BelugaWhale  GAAGAAA-----TAGTGGAAGAGAAATACAT---------------GCTAGTGGTGCTAAGGATA-------GTGACTG- 68140
Donkey       ----C-------TGAGGGACGAAATACATAT-----------------TAGTGACATTAAGGACA-------GTGACTG- 76070
Cat          TAGCATA-----CATTGCTTTAAAGGATAATA--------------GAATATGTGATCATGTATA-------CTTGT--- 74160
Ferret       TAGCATA-----CATTTCTCTGAAGGCTAATA--------------GAATATATGATCATGTGTT-------CTTGTATG 76962
Lion         TAGCATA-----CATTGCTTTAAAGGATAATA--------------GAATATGTGATCATGTATA-------CTTGT--- 71846
Vaquita      GAAGAAA-----TAGTGGAAGAGAAATACAT---------------GCTAGTGGTGCTAAGGACA-------GTGACTG- 69434
Pig          GCAGAAA-----TGGTGGGAGAAAAGTACAT---------------GCTAATGGCTTGAAGGGTG-------GTGACTG- 69293
```

```
Majority     ----CCXTCXTCTCTTAAAAXAGTTXTT-----XACTTTX-----XXCTCTGGTCTGTCAAAAATGAAGA-AAXATXATT
                     99530     99540     99550     99560     99570     99580     99590     99600
Human        GCAAACCTTGTCTCAAAAAATAAAGGGTGGGGAAGCTTTAGCCTAGGCTTTTAATTCTCAATTACCATTATAAATTTATT  79734
PolarBear    ---------TCTTTGTAAAACAGGTCTT-----GGCTTTT-----ATTTCTGATATGTTAAATATCAGTT-GATATAACC  39106
Cow          -------CCATCTCTGACAGTATTTTT------CACCAT-------GCTCTGGACTGCCTAAAATGAAGG-AAGATCATT  76196
Dog          --TCTTTTTTTTTTAAATAAAAGTTCTT-----GATTTTTT----GTTTCTGGTGTGTTAAATATCAGTT-GATATACCC  69186
BelugaWhale  ----CCCTCATCTCTTGAAGTACTTTTG-----CATCAT-------GCTCTGGGCTGCCTAAAATGAAGA-AAGAACATT  68203
Donkey       ----CCTTCATCTCTTAAAGCACTCTTG-----CACTGT-------ACTCCGGGCTGCCTAAAATGAAGA-AGGATCATT  76133
Cat          ---------TCTTTTTGAAACAGTTCTT-----GGCTTTA-----ATTTCTGATATGTTAA-----------ATACAACT  74210
Ferret       TGTTCTTGTTCTTTTTAAAACAGTTCTT-----GGTTTTT-----ATTTCTGATGTGTCAA-------TT-GATATAACT  77024
Lion         ---------TCATTTTAAAACAGTTCTT-----GGCTTTA-----ATTTCTGATATGTTAA-----------ATACAACT  71896
Vaquita      ----CCCTCATCTCTTGAAGTACTTTTG-----CATCAT-------GCTCTGGGCTGCCTAAAATGAAGA-AAGAACATT  69497
Pig          ----CCCTCATCTCTGA-AGTACTTTTG-----CACCATA-----TGCTCCGGGCTGCCTAAAATGAAGA-AAGATCATT  69357

Majority     TXACXAXATAAXAGATCATTTGTCTTC----ATTAGTAATTT-CT--------XAG--XXGTGXAAAXXGGTGGXGAXAC
                     99610     99620     99630     99640     99650     99660     99670     99680
Human        TTAATTATTAACTAATTACTTTAATTC-TCAATTACTATTTTTCCATGAAAAGGCTTGATGTTCTAAACAGTATTATACC  79813
PolarBear    CAAATAAACAAAAGGTC-TTTGTGATC----CTCAGTCATTT-CT--------AAG---AGTGTAAAGGGGTCCTGAGAC  39169
Cow          TGGCAAGATGATAGATCATCTGTCTT-----AGTACCAAGTGATC--------CAGCATGGTAAGAATAAATGGAGAAAT  76263
Dog          CAAATAAACAAAAGCTC-TTTGTGGTC----CTCAGTAATTTTCT--------AAG---AGTGTAAAGGGGT---GAGAC  69247
BelugaWhale  TGGCCAGAGGATAGATCATTTGACTTT----AGTAGCAAGTGACC--------CAGCATGGTGAGAATAAATGGAGAAGT  68271
Donkey       TGACGGAAGACTAGATCACCTGACTTAGTAGTATAGTGGTCTGCTGTTATCACCACTCTGGTGATGATAGATGGAAAAGT  76213
Cat          CATT----TAAAAGCTG-TTTGTATTC----TTCAGTAAATT-TT--------ACG---AGTATAAAGGGGTCCTGAGAC  74269
Ferret       CAAA----CAAAAGGTC-TTTGTGTTC----CTCAGTAATTT-CT--------AAG---AGTGTAAAGGGGTCCTGAGAC  77083
Lion         CATT----TAAAAGCTG-TTTGTATTC----TTCAGTAAATT-TT--------ACG---AGTATAAAGGGGTCCTGAGAC  71955
Vaquita      TGGCAAGAGGATAGATCATTTGACTTT----AGTAGCAAGTGACC--------CAGAATGGTGAGAATAAGTGGAGAAGT  69565
Pig          TGGCAGGAAAGTAGATCATCTGACTT-----GGTAGCAAGTAGCC--------CAG-ATGGTGAGAATAGATGGAGCCGT  69423
```

```
Majority     AXXAAG--------ACTXTXAAAC----ATTTTXAGXATGGXAATACGAAXTTTGGGAAXA-TXATAGCTTAC-XATTXX
                      99690     99700     99710     99720     99730     99740     99750     99760
Human        ACCAAGTTATCTCTACTGGAAAACCCAGATTTCAAGAAAAAAAAAATGTATTTTGAAAACAATTATGTCTTAAATATTTA  79893
PolarBear    CAAAAA--------GATTGAGAAC----TTTTTCAGTGTGGGGATACAAAGTTCAAGATAA-TTCTATTAAATAAATAGG  39236
Cow          ATGGAG--------ACTGTGAAGT----AGCATTTGGGCAGTAGCAACAACATTGGGGATG-TCACAGCTTAC--ATTCC  76328
Dog          CAAAAA--------GATTAAAAGC----TTTTTCAGTATGGGGATACGAAGTTCAGGAT--------TTTCA--AGTA--  69303
BelugaWhale  AGGGAG--------ATTATGAAGT----AGCATTTGGATAGTAGCAAGAACAGTGGGGACC-TCATAGCTTAC--ATTCC  68336
Donkey       TGGGAG--------ACTATGAAAT----AGCATTTGGACAGTAGTAAGAACAGTGGGGACC-TCCCCGCTTAC--ATT--  76276
Cat          TAAAA---------------GAAC----ATTTTCATTGTGGG-ATACAAAGTTCAAGACTA-TTCTAGTAGATAAGTAGG  74328
Ferret       CTAAAA--------GATTGAGAAC----TTTTTCAGTGTGGGGATACAAAGTTGAAGACAA-TTCTATTAGATAAATAGG  77150
Lion         TAAAA---------------GAAC----ATTTTCATTGTGGG-ATACAAAGTTCAAGACTA-TTGTAGTAGATAAGTAGG  72014
Vaquita      AGGGAG--------ATGATGAAGT----AGCATTTGGATAGTAGCAAGAACAGTGGGGACC-TCATAGCTTAC--ATTCC  69630
Pig          GGGGAG--------ACTG--AAGC----AGCATTCGGACGGTAGCAGCAGCAGTGGGGATC-TCACAGCTCAC--ATTCC  69486

Majority     A---CT-TTTTAATAAACTTTAXATXGTCXTCATACTAGA-AGG-AXGTAAAXTGAATA-TXCTTGXGAXCXAXAAAATT
                      99770     99780     99790     99800     99810     99820     99830     99840
Human        AAATTCTTTTAAACATGTTTTTAACCATCTTTAAATTTTTTTGGTAAGTAATTTTCATTAGATTGTTAAAATGTATAGTG  79973
PolarBear    A---GTATTTTAATAAACAGAAGATGGCGGTAAGAGTCAG-AAG-ATATAAAGGGAAAA-TGCTAGGGATATAGGGAATT  39310
Cow          A---CT-TTTTGCTAAGCTTTCCATTGCTGCTTTACTAGG-AGG-AGGTAAATTGTATT-TGCTTT-GAGTCACAAAATT  76400
Dog          -------TTTTAATAAACAGAAGATGGTGATAAGAGTT---GAG-ATGTACAGAGAAAA-TGCTAGGAAT-TAGGAAATT  69370
BelugaWhale  A---CA-TTTTGCTGAGCTTTCCATTATCTCCTTACTAGA-AGG-AGATAAATTGTATC-TACTTG-GAGCCACAAAATT  68408
Donkey       --------TGTGCTAAACTTCCCATTACTTCCATAATAGA-AGG-AGGTAAATTGCATC-TACTTTGAACCCA-AAGTTT  76344
Cat          A---GTATTTTAATGAACA--AGATGGCAGTGAAGAGTA--GAG-ATGTACAGGGAAGA-TGCTAGGGAT-TAGG-TCAT  74397
Ferret       A---TTGTTTTAATAAACAGAAGACGGTGGTAAGAGTCAA-GAG-ATTTACAGGGAAAAATGTTAGGGATTTAGG-AATT  77224
Lion         A---GTATTTTAATGAACA--AGATGGCAGTGAAGAGTA--GGG-ATGTACAGGGAAAA--------------------T  72066
Vaquita      A---CA-TTTTGCTGAGCTTTCCATTATCTCCTTACTAGA-AGG-AGATAAATTGTATC-TACTTG-GAGCCACAAAATT  69702
Pig          T---CT-CTTTGCTAAGCTTTTCACTATCTTCTTACTAGA-AGGGAGGTAAAATGTATC-TACTT--GAGCCACAAAATT  69558
```

```
Majority     TCTCAXA-----TTAATXTTAGTAXATAATTATGTTTXATTTTT-ACTAAAGTGTTATGXT----TTAGTAAAACCTGAA

                       99850     99860     99870     99880     99890     99900     99910     99920

Human        GCACGGTAGGGTTGTATCTCAAGATATGAAGCAGTTTTTCATAAAAATAAAGTATTAACCTTAGGCTGGTATGTTTAGAT  80053
PolarBear    TTTTAGA-----TTAATTTTGGTATCTAAATATAATTAATT-----GTTCAGTTGTTTGGT----TTAGTGAGCCCTGAA  39376
Cow          TCA---------------TGAGAAAATTATTA-GTCTCATTTTT-ACTAAAGTGTAATGTT-----TATTAAAACCTGGC  76458
Dog          TTTTAGA-----TGAATTTTGGTAACTAATAACAATTAATTGTTCAGTTAATTTTTGGGGGG---TTAGTCTACCCTGAA  69442
BelugaWhale  TCACAAA-----TATTT-TCACAAGAAAATTAAGTCTTATTTTT-ACTAAAGTGTAATGTTGT--TTATTAAAACGTGGC  68479
Donkey       TCTCA-------------TTAGAAGATTAGCA-GTCTTATTTCT-TCTAAAACGCAATGTTGT--TTATTAAAATATGAC  76407
Cat          TTTTATA-----TTAATGTTGGTAACTAATTCT--TCAGTT-------AAATTTTTCTGGC----TTAGTGGGCCCTGAA  74459
Ferret       TTTAATA-----TTAATTTTGGTATCTAATTATAATTAGTTC---AGTAAATTATTTTGGG----TTAGGAAGACCTAAA  77292
Lion         TTTTATA-----TTAATGTTGGTAACTAATTCT--TCAGTT-------AAATTTTTCTGGC----TTAGTGGGCCCTGAA  72128
Vaquita      TCACGAA-----TATTT-TCACAAGAAAATTAAGTCTTATTTTT-ACTAAAGTGTAATGTTGT--TTATTAAAACGTGGC  69773
Pig          TCACTAA-----TTTTTTTCAGTAGAAAATT--GTTTTATTTTT-ACTGAAGAGTAATGTT-----TATTAAAACAGGAA  69625

Majority     AATX-------TTTTTTATTAGG-----TXGTATTGXAAX-XCAGAAA--XGACTTTGACCTAATXTTTT----------

                       99930     99940     99950     99960     99970     99980     99990    100000

Human        CAATACTGTATTTAAATAGATTGATAATTTTTTATATATTTTATGAATTTATACTTTTTCCTGAGATTTCAGGAGGGAAA  80133
PolarBear    AA----------TTGGTAG-AAT-----TGGAAATGAAA--CCAGAAA--TTAAACTGATTTAATTTTTTTAA-------  39429
Cow          ------------CTTAAGTCAGG-----TTTTTTCACACT-TAAAAGG--GGGCTTTAGCCTAGACTTT-----------  76507
Dog          AATC------GTTTGGTA--AGT-----TGAAAATGAAA--CCAGAAA--TTAAACTGACTTAATTTTTT-AT-------  69497
BelugaWhale  ATTC--------TTTTTTTTAAA-----GTCTTTCACATT-TCAAAAA--GGGCTTTAGCCTAGTCTCTT----------  68533
Donkey       ATTCTCTGGCATTTTTTTTTAAGGTCTCCGGTCTATCACACTGAAAAA--GGACTTTAACCTAGACACTT----------  76475
Cat          AATT------GTTTCTTGTCTGG-----TAGAATTGAAAAACCAGAAA--TTTAGCTGATTTAATTTTTT----------  74516
Ferret       AAGT------GTTTGGTAG-AGT-----TGGAAATGAAA--CCAGAAA--TTAAACTGATTTAATTTTTG-GT-------  77348
Lion         AATT------GTTTCTTGTCTGG-----TAGAATTGAAAAACCAGAAA--TGTAGCTGATTTAATTTTTT----------  72185
Vaquita      ATTC--------TTTTTTTTAA------GTCTTTCACACT-TCAAAAA--GGGCTTTAGCCTAGTCTCTT----------  69826
Pig          ------------TACGAATTCGG-----CATTCTCTCA------------GGACTTTCACCTAGACTTTT----------  69666
```

```
Majority     ----AGTXCXAAATXACTG----CTTTXAAATTTCT----TTAATTAATTTAATA------TAAAAAAATTATTTTATXC

                      100010    100020    100030    100040    100050    100060    100070    100080

Human        AATGAGTAGGAGATGACTGAGAGCTTAAAGTTTGGGAGTGTCAATTAACTCAGCATTCTTTTAAAAAACATGTGTCATAT  80213
PolarBear    ----TGTGGACAAGAGCTG----CT--CAGATGTCTTTCTTTGATGGATTAAA---------AAAAAAA-TCTTTTATTC  39489
Cow          ----AATTCTTAATTACTA----TTTTA-AATTAATT---TTACTTATTCTTTTAA-----TTCTCTGATTATTTT-TCC  76569
Dog          ----TGTGGAAAAGAGATG----CT--CAAATGTCT----TCT-TAGATTTAA---------AAAAAAAATCTTTTATTC  69553
BelugaWhale  ----AATTCTTAATTACCA----TTTTTTAATTAATG---TTAATTATTTTAATT------CTCAGTGATTATTTT-TCC  68595
Donkey       ----AATTCTCAATTACCA----TT-TTAAATTAATT---TTAATTATTCTTTTAA-TT--TTCAGTGATCGTTTT-TCC  76539
Cat          -----GTG-AAAGGAGCTG----CTTTCAGATGTCT----TTGATGGATTAAACAAACAAACAAAAAAAGTCTTTTATTC  74582
Ferret       ----TGTGGAGAAGAGCTG----CT--TAGGTGTCT----TTGATGGATTTTT---------AAAAAA--TATTTTATTC  77403
Lion         -----GTG-AAAGGAGCTG----CTCTCAGATGTCT----TTGATGGATTTAA-AAAAAAAAAAAAAAAGTCTTTTATTC  72250
Vaquita      ----AATTCTTAATTACCG----TTTTTTAATTAATG---TTAATTATTTTAATT------CTCAGTGATTATTTT-TCC  69888
Pig          ----AATTCTCGATTACTG----TTTTA-AATT-------TTAATTATTTTAATA------TTTGCTGAGTATTTT-TCC  69723

Majority     ATXATAG-G--ATTTGATGXTTXAAAT--G-AAXATTGTTCTTCTACXTTCCCT-----GTATTATAAATCCTATATXT-

                      100090    100100    100110    100120    100130    100140    100150    100160

Human        ATTACAGCATTTTCTTTTATTTGAAGTGAGTAAATGTATCTTTTTAAATTCCTTAGTAATTTTTGAGCACTCCATATGTA  80293
PolarBear    TTAAAGG-A--ATTTGAGGATTGATAAATG-AACTTTTTTTTTCTGCGTCCCAT-----GTATTGTTGTTGGTGTTTGT-  39559
Cow          ATGATAA-G--GCTTGATGATTTAAGT----AATATTGTACTACCAAGGTATCT-----CCATTATAAATCCTAGACTT-  76636
Dog          TTAAAAG-AGAATTTGAGGATTGATAC-TG-AACTTTTTTCTTCTGCATGCCCT-----GTATTGTCATTGGTGTTTG--  69623
BelugaWhale  ATGAT---G--TCTTGATGTTTTAAAT----AATATTGTGCTACCACGGTATCT-----CCATTACAAATCCCAGACTT-  68660
Donkey       ATGAT-G----TCCTGATGCTTTAAAT----AATATGGTGCCACCAAGTTGTCT-----CCATTATAAAACCCAGATTTC  76605
Cat          CTAAAAG-GGAATTTGAGGGTTGATAT-TG-ACC-----TCTTCTGCATACCAT-----GTATTTTCAATAGTATTTGT-  74648
Ferret       TTAACGA-A--ATTTGAGAATTAATAC-CG-AACTTTTTTCTTCTGCACGCCAT-----GTATTGTTGCTGGTATTTAT-  77472
Lion         CTAAAAG-GGAATTTGAGGGTTGATAT-TG-ACC-----TCTTCTGCATACCAT-----GTATTTTCGATAGTATTTGT-  72316
Vaquita      ATGAT---G--TCTTGATGTTTTAAAT----AATATTGTGCTACCACGGTATCT-----CCATTACAAATCCCAGACTT-  69953
Pig          ATGATAA-G--GCTTAATGTTTTAAAT----AATAATGTGCTACCAATGTATCT-----TCATTAGGAATTCCACATTT-  69790
```

```
Majority     ------------------------------------------AAXGTGTCATTG--ACTGAA-XTTCCATTAAGA--CCA
                      100170    100180    100190    100200    100210    100220    100230    100240
Human        TAAAGCATGTGAATATTTGGTAGCATTTTACAAATGTCCAGAGATTTGTGAGAGTTCCTGAGATCTTCATAGGGGGCCCA  80373
PolarBear    ------------------------------------------TTTGGGCCACCG--ACCGGA--TCCCATTAAGA--CCA  39591
Cow          ------------------------------------------AAAGTTTCTTTG--AACAAAATTTCCATCATCT--TTG  76670
Dog          ---------------------------------------------GGGCCATGG--ACTGGA--TCCCATTAAGA--CCA  69652
BelugaWhale  ------------------------------------------AAAGTTTCTTTG--AATAAATTTTTCATCAT-----TG  68691
Donkey       AAGAGGAAATGTATTTTTAAAACAATTATGTCTTGAATCTCTAAAGTTTCTTTG--AACAAATGTTCCATCACCT--TTG  76681
Cat          ------------------------------------------TTTGGGCCATGG--ACTGGA--TCCCATTAAGA--CCA  74680
Ferret       ------------------------------------------CTTAGGCCACTG--ACTGGA--TCCCATTAAGA--CCA  77504
Lion         ------------------------------------------TTTGGGCCATGG--ACTGGA--TCCCATTAAGA--CCA  72348
Vaquita      ------------------------------------------AAAGTTTCTTTG--AATAAATTTTTCATCAT-----TG  69984
Pig          ------------------------------------------AAAGTTTC------AGCAAATTTTCCATTGA-T--CTG  69819

Majority     AATATTTTAGTAAAATTTTX-------------AAGT-TATT-XX-----------TTTTCTATTGAX-----ATAGTA
                      100250    100260    100270    100280    100290    100300    100310    100320
Human        CAAAGTTTAGTATTACTTTTCACGGTAATACTAAAGTGTATTTTGCCTCTTTTTACTTTTTCTCTTAATAGCATACAGTG  80453
PolarBear    AGGAACTTAACAGAATTTT--------------AAGT-TACT---------------TTTTCCACTGA------AGAGTA  39635
Cow          AATATTTTGGTGAAGTTGTA-------------TGAT-TGTTTAA------------TTGTGTATTGGC-----ATGGTA  76719
Dog          AGCAACTTAACAAAATTTTT-------------AAGT-TACT---------------TTTTCTGTTGA------AGAGTA  69697
BelugaWhale  AATATTTTGGTGAAGTATTA-------------TGAT--GTTTAA------------TTGTATATTGGC-----ATGGTA  68739
Donkey       AATATTTTGGTAAAGTTTTCA-----------TTAGAT-TATTTAA-----------TTGTATATTGGC-----ATGGTA  76733
Cat          AGAATCTTAACAGAATTTT--------------AAGT-TACT---------------TTTTCCCCTGA------AAAGTA  74724
Ferret       AGGAATTTAACAGAATTTT--------------AAGT-TATT---------------TTTTCTACTGA------AGAGTA  77548
Lion         AGAATCTTAACAGAATTTT--------------AAGT-TACT---------------TTTTCCACTGA------AAAGTA  72392
Vaquita      AATATTTTGGTGAAGTATTA-------------TGAT--GTTTAA------------TTGTATATTGGC-----ATGGTA  70032
Pig          AATATTTCGGTAAAATTTTA-------------TGAT-TGTT-AA------------TTGTATATTGGC-----ATGGTA  69867
```

```
Majority     GAGA---XATGTTXXAGAAAAAAGGGXX----TTCTTTAAAAA-------------------TGAAGTG-------CCTX

                      100330    100340    100350    100360    100370    100380    100390    100400

Human        GTAACTGAAGGCTAATAGTATGTGTGTTTATGTGCTTTAAAAAGTTCGTGGTTTTGGCCAGGCGCAGTGGCTCAGACCTG  80533
PolarBear    GCCAATTAAAGGCAGGGAAACAAGGGAA----TGATTTGACAA-------------------TGTAATG-------CCTA  39685
Cow          GAGA---TATATTTTAGAAGAAACCATT----TTCTATAGAAA-------------------TGAAGCA-------TTTG  76766
Dog          GT--------------GAAACAAGGGAA----TGATTTGACAG-------------------CGTAATG-------CCTA  69733
BelugaWhale  GAGA---TGTATTTTAGAAGAAACCGTT----TTCTGCAAAAA-------------------TGAAGTG-------TTT-  68785
Donkey       GAGA---TATATTTTAGAAGAAAC--------TTCTGCAGAGA-------------------TGAAGTG-------TTAA  76776
Cat          GCCAATTAAAGTCAGGGAAATGAGGAAA----TAATTTGACAG-------------------TGTAATG-------CCTG  74774
Ferret       GCCAATGAAACACAGGGAAACAAGGGAA----TGACTTGACAA-------------------TGCAATG-------CCTA  77598
Lion         GCCAATTAAAGTCAGGGAAATGAGGAAA----TAATTTGACAG-------------------TGTAATG-------CCTG  72442
Vaquita      GAGA---TGTATTTTAGAAGAAACCGTT----TTCTGCAAAAA-------------------TGAAGTG-------TTT-  70078
Pig          GAGAG-ATGTGTTTCAGAAGAAACTGTT----TTCTGCAAAAA-------------------CGAAGTG-------TTC-  69915

Majority     C--ATCTAGAGT-TTXTG---CCAAGATTGAXTAATXGXGTT--------------------XTGCTAXTTTAGTT--GA

                      100410    100420    100430    100440    100450    100460    100470    100480

Human        TAATCCCAGCACTTTGGGAGGCTGAGGCAGGTGGATCACCTGAGGTCAGGAGTTCAAGACCAAAACCAGCCTGGCC---A  80610
PolarBear    C--ATCTGGAGT-TTCTG---CCACTGTCGAATAATTGTGTT---------------------ATCAACTTTAGTT--GA  39736
Cow          TTTACCTAGGCTGTTGTG---TGTAGACTGATATAGCAAATGTA------------------TTGATGTTTTATAC--AT  76823
Dog          ---ATCTGGAGT-TTCTG---CCACGATTGAATAATTGTGTTTATTTACAATAACAGCACAATAACAGCTTTAGTT--GA  69804
BelugaWhale  -------AGACTGTTGTG---TTTAGACTGATAAATCAAATGTA------------------TTGATGTTTTATAT--GT  68835
Donkey       C---CTTAGACTGTTGTG---TTTAGATTGACATATCGAATTTA------------GGGAGATTGATATTTTATATTATT  76838
Cat          C--ATCTGGAGT-TTCTG---CCAGTGTGAAATAATTGTGTT---------------------GTCAACTTTAGTT--GA  74825
Ferret       C--CTCTAGAGT-TTCTG---CCACTATTGAATAATTGTGTT---------------------ACCGACTTAAGTT--GA  77649
Lion         C--ATCTGGAGT-TTCTG---CCAGTGTGAAATAATTGTGTT---------------------GTCAACTTTAGTT--GA  72493
Vaquita      -------AGACTGTTGTG---TTTAGACTGATAAATGTATTGA--------------------TGTTTTTTTATAT--GT  70126
Pig          -------A-----------------GATTTATTTATCAACT---------------------TTGTTATTTTAT----GT  69946
```

```
Majority     ATXTXATGAAXXXTXAXTTT-GCXACAACTATCAAAA------AAGAAXXATA-AAGGTA-----------XGCTACTTA
                       100490    100500    100510    100520    100530    100540    100550    100560
Human        ACATGGTGAAACCCCATCTCTACTAAAAATACAAAAATTAGCCAGGCATGGTAGTGGGTGCCTGTAGTCTCAGCTACTCG 80690
PolarBear    ATGCTATGTACATTCA-----GCCACAACTATCTAAA------AAATACTTTC-AAGGAA-----------TGCTAATTA 39793
Cow          GTTTCATGAATTACTAGTTTTTCTTGAATTAGCAACA------AAGAAAAATG----ATA-----------AACTACTTA 76882
Dog          TTGCTATGTACATTGA-----GCCACAACTATCTGAA------AA-TACTG---AAGGAA-----------TGATATTTA 69858
BelugaWhale  GTTTCATGAATTAGTAATTTTTCTTGAAATATCAACA------GAGAAAGACAAATGATA-----------AGTAACTT- 68897
Donkey       GTTTCGTGAATTAGTAACTTTTCTTGAATTGTCAAAA------GGGAAAGAGGAGTAGTA-----------GGTGACGTA 76901
Cat          ATGGTATGTACATTCA-----GCCACAGCTAT---------------ACTT---AAGGAA-----------TACTATATT 74871
Ferret       ATGCTATGTACATTCATTCA-GCCACAACTATCTGAA------AAATACTTTC-AAGAAA-----------TGATAATTA 77710
Lion         ATGGTATGTACATTCA-----GCCACAGCTATCTGAA------AA-TACTT---AAGGAA-----------TACTATATT 72547
Vaquita      GTTTCATGAATTAGTAATTTTTCTTGAAATATCAACA------GAGAAAGACAAATGATA-----------AGTAACTTA 70189
Pig          ATTTCATGAATTAGTAATTT----AAAAATATCAACA------GGGAAAGACA----ATA-----------AGTAACTTA 70001

Majority     A-------A---ATAAG----AXTTGGAGGGG-----------------------------------------------
                       100570    100580    100590    100600    100610    100620    100630    100640
Human        AGATGCTGAGGCAGGAGAATCACTTGAAGCTGGGAGGTGGAGGTTGCAGTGAGCCAAAATCTCGCCATTACACTCCAGCC 80770
PolarBear    A-------A---GGAGG----AGGGGGAGGG------------------------------------------------ 39810
Cow          G-------AACTATAA-----TTTGGGAACCT----------------------------------------------- 76902
Dog          A-------AG--AAAGG----ACGAGGAGGGG----------------------------------------------- 69877
BelugaWhale  ------------TATAAG----TTTTGGAATCTAA-------------------------------------------- 68916
Donkey       G-------AACTGTAAG----TTTTGGAAGTGTCAGTAGTGTGTAATATACCTGTCATATATTAGAGCATTTCTTTACCT 76970
Cat          T-------A---AAGAA----AGGATGAGGGG----------------------------------------------- 74889
Ferret       A-------A---AGAGG----AGGGGGAGGGG----------------------------------------------- 77728
Lion         T-------A---AAGAA----AGGATGAGGGG----------------------------------------------- 72565
Vaquita      A-------AACTATAAG----TTTTGGAATCTAA-------------------------------------------- 70212
Pig          G-------G------------TATTAGAATCT----------------------------------------------- 70014
```

```
Majority     --------------------TCATTTCXTA--A-----------------GAGTATGCAAAAACAAAATXAC---TGTGA
                       100650    100660    100670    100680    100690    100700    100710    100720
Human        TGGGGACAAGAGCAAGACTCCATCTCAAAAAAAAAAAAAAAAAAAGTTATTGGTTTTAATTTTGGAAATGACAACCATTA  80850
PolarBear    --------------------CCATTTTCTA--A-----------------GAGTGTGCAAAACCAAAATTAC---TGTGA  39848
Cow          --------------------ACGTATCTCA--------------------GAATATGCAGGAACAAAATGAC---TGTGA  76939
Dog          --------------------TCATTTTCTA--A-----------------GAGTATGAAGAAACAA---TAC---TGTGA  69912
BelugaWhale  ------------------CTACATGTCTAATAA-----------------GAGTATGCAAGATCAAAATGAC---TGTGA  68958
Donkey       TTCAAATAAATGCTTCTTTTTAATTTCTTAGTACTTTTTGAGCATCGTATGTGTACGAAGCATCATACCAAG---TATAC  77047
Cat          --------------------TCACTTTCTA--A-----------------CAGTATTCAAAAACAAAATTAC---TATCA  74927
Ferret       --------------------TCATTTTCTA--A-----------------GAGTGTTCAAAAACAAAATTAC---AGTGA  77766
Lion         --------------------TCACTTTCTA--A-----------------CAGTATTCAGAAACAAAATTAC---TGTTA  72603
Vaquita      ------------------CTACATGTCTAATAA-----------------GAGTATGCAAGATCAAAATGAC---TGTGA  70254
Pig          --------------------ACAGGTCTAA--------------------GAGTATGCAAAAACAAAACG-------TGA  70047

Majority     GTGCA-----------------------------TXCCTA----------------------------------------
                       100730    100740    100750    100760    100770    100780    100790    100800
Human        ATAGAACCCACATTAGGCCGGGCGCGGTGGCTCATGCCTGTAATCCTAGCACTTTGGGAGGCCGAGACGGGTGGATCACG  80930
PolarBear    GTG-----------------------------------------------------------------------------  39851
Cow          GTACA-----------------------------TACCTA----------------------------------------  76950
Dog          GTGCA-----------------------------TGTCTA----------------------------------------  69923
BelugaWhale  GTACA-----------------------------TACCTA----------------------------------------  68969
Donkey       GTGTAGT-------------GGTTCGCAAAATATTGTCTACAGATCTTTGAGAGCCCCTGAGACCCTCTCTAGAGGTCCA  77114
Cat          GTGCA-----------------------------TATCTA----------------------------------------  74938
Ferret       GTGTA-----------------------------TGTGTA----------------------------------------  77777
Lion         GTGCA-----------------------------TACCTA----------------------------------------  72614
Vaquita      GTACA-----------------------------TACCTA----------------------------------------  70265
Pig          GTACA-----------------------------TGCTTA----------------------------------------  70058
```

```
Majority     -XG------AATTAGGAATGTAT-------------------CXGTXTXCTTG-------------------------AC
                              100810    100820    100830    100840    100850    100860    100870    100880
Human        AGGTCAGGAGATCAAGACCATCTTGGCTAACACGGTGAAACCCCGTTT-CTACTAAAAATACAAAAAATTAGCCGGGCGT  81009
PolarBear    -----------------ATTTA----------------------------------------------------------  39856
Cow          -GG------AATTAGGAATGTAT--------------------------CTTT-------------------------AC  76972
Dog          -AG------AACGGGAAATACGT-------------------CTGTATACTTG-------------------------TC  69952
BelugaWhale  -GG------AATTAGGAATGTAT--------------------------C-TT-------------------------AC  68990
Donkey       CAA------AATCAAAACTATTTTCATGATAATACTAAAATGTTGTTTGCCTTTTGACTCTCATTTTCTCAATAGAATAC  77188
Cat          -AG------AATTGGGAATGTAT-------------------CCTCATACTTG-------------------------CC  74967
Ferret       -AG------AATTGGTAATATAT-------------------CTGTATACTTG-------------------------TC  77806
Lion         -AG------AATTGGGAATGTAT-------------------CCTCATACTTG-------------------------CC  72643
Vaquita      -GG------AATTAGGAATGTAT--------------------------C-TT-------------------------AC  70286
Pig          -GG------AATTACGAATTTATAT-----------------CTGTGTACTTG-------------------------AC  70089

Majority     ATTGG----------------CAGCAAATT---AA---GAT---------------------------------------
                              100890    100900    100910    100920    100930    100940    100950    100960
Human        GTTGGCGGGCGCCTGTAGTCCCAGCTACTTGGGAGGCTGAGGCAGGAGAATGGCATGAACCTGGGAGGCGGAGCTTGCAG  81089
PolarBear    --------------------------------------------------------------------------------  39856
Cow          ATTGG----------------CAGCATTTT---AA---GAT---------------------------------------  76991
Dog          ATTGG----------------CAGCAAATT---AA---GAT---------------------------------------  69971
BelugaWhale  AGTGG----------------CAGCATTTT---AA---GAT---------------------------------------  69009
Donkey       GTTGGAGTTTTCTACATCACTCTGCAAACT---AACAGGATGTGTGATCTTGTGTTCCTGTGCTTTAAAGTTCTTGGTTT  77265
Cat          TTTGG----------------CAGCAAATT---AA---GAT---------------------------------------  74986
Ferret       ATTGG----------------CAGCAAATT---AA---GAT---------------------------------------  77825
Lion         ATTGG----------------CAGCAAATT---AA---GAT---------------------------------------  72662
Vaquita      AGTGG----------------CAGCATTTT---AA---GAT---------------------------------------  70305
Pig          TTGGG----------------CAGCATATT---AA---GAT---------------------------------------  70108
```

```
Majority     ---------ATC----XATTGAATGT------------------TAAAT--------GGAAAC-----------------
                     100970    100980    100990    101000    101010    101020    101030    101040
Human        TGAGCCGAGATCACGCCACTGCACTCCAACCTGAGAGACACAGCGAGACTCCGTCTCGAAAAAAAAAAAAAAAAAAAAAA  81169
PolarBear    --------------------------------------------------------------------------------  39856
Cow          ---------AGC----CATTGAATGT------------------TAAAT--------GGAAAC-----------------  77015
Dog          ---------ATC----TTTTAAATGT------------------TAAAT--------GGAAGT-----------------  69995
BelugaWhale  ---------ATC----CATTGAATGT------------------TAAAT--------GGAAAC-----------------  69033
Donkey       TAATTTCTGATA----TTTTAAACGTCAGTTGATAGAACCCAAGTAAAC--------AAAAACTCTTTGCAGTCCTCAGT  77333
Cat          ---------ATC----TATTGAATGT------------------TAAAT--------GGAAAC-----------------  75010
Ferret       ---------ATA----CATTGAATGT------------------TAAAT--------GGAAGC-----------------  77849
Lion         ---------ATC----TATTGAATGT------------------TAAAT--------GGAAAC-----------------  72686
Vaquita      ---------ACC----CATTGAATGT------------------TAAAT--------GGAAAC-----------------  70329
Pig          ---------ATG----TTTTCAATGT------------------TAAAT--------GGAAAC-----------------  70132

Majority     -AACCTATA----------------------------------AAG--TXTAA---------------------T--GAG
                     101050    101060    101070    101080    101090    101100    101110    101120
Human        GAACCCACATTAACAAAAACTCCTTGTGATTCTCCATAGTTTTAAGACTATAAAGGGGAGCTGAGACCAAAAAGTTTGAG  81249
PolarBear    --------------------------------------------------------------------------------  39856
Cow          -AACCTATG----------------------------------AAG--TATAA---------------------C--GGG  77035
Dog          -AACCTATA----------------------------------AAG--TATAA---------------------T--GAG  70015
BelugaWhale  -AGCCTATC----------------------------------AAA--TGTAA---------------------T--GAG  69053
Donkey       AAGTCTTAA----------------------------------GAG--TGTAAAGGGGTCCTGAGACCAAAAAGTTTGAG  77377
Cat          -AACCTATG----------------------------------AAG--TGTAA---------------------TCAGAG  75032
Ferret       -AACCTGTA----------------------------------AGG--TATAA---------------------T--GAG  77869
Lion         -AACCTATG----------------------------------AAG--TGTAA---------------------TCATAG  72708
Vaquita      -AGCCTATC----------------------------------AAA--TGTAA---------------------T--GAG  70349
Pig          -AACCTATA----------------------------------AAG--TATAA---------------------C--GAG  70152
```

```
Majority     AGCTA--------TGG------------------------------------------------TATTT-AACA------

                         101130    101140    101150    101160    101170    101180    101190    101200

Human        AACTTTTTCACTGTGGGATACAAAGTTGAAGACAATTATAGTTAAATAAGCAGGAGATACAGAGTATTTTAATGAACAGT  81329
PolarBear    -----------------------------------------------------------------ATTT-AACA------  39864
Cow          ATCTA--------TGG------------------------------------------------TATTT-AACA------  77052
Dog          AGCTA--------TGG------------------------------------------------TATTT-ACT-------  70031
BelugaWhale  AGCTA--------TTA------------------------------------------------TATTT-AACA------  69070
Donkey       AACTACTTCAATGTGGGATACAAAGTTTAAGAGAATTCTAATAGATAAGTAGGAGATAGAGGGTTTTTT-AATAAACAGG  77456
Cat          GGCTA--------TAG------------------------------------------------TATTT-GGCA------  75049
Ferret       AACTA--------CAG------------------------------------------------TATTT-AAAA------  77886
Lion         GGCTA--------TAG------------------------------------------------TATTT-GACT------  72725
Vaquita      AGCTA--------TTA------------------------------------------------TATTT-AACA------  70366
Pig          AGCTC--------TGG------------------------------------------------TACAT-AACG------  70169

Majority     -------TGGGGAAGTTAGTAATACTATAGA-------------------------------------------------

                         101210    101220    101230    101240    101250    101260    101270    101280

Human        AGATGGTTATAAGAGTCAAGAGATGTACAGAGAAAATGCTAGTGATTAG---AGTTTTTAGATTATTTTAATTTTGGTAA  81406
PolarBear    -------TGGGGAAGTTTGACATGCTGTAGA-------------------------------------------------  39888
Cow          -------TGGGGAAGTTAGTAATACCATAGA-------------------------------------------------  77076
Dog          --------GG--AAGTTTGTAATGCTGTAGA-------------------------------------------------  70052
BelugaWhale  -------TTGAGAAGTTAGTAATAACATAGA-------------------------------------------------  69094
Donkey       AGATGG-TGGTTGAGTCAAGAGCTATATAGAGAAAATGCTAGGAATTAGGAGACTTTTGAGATTATTTTAATTTTGGTAA  77535
Cat          -------TGGGGAAGTTTGTAACACTTTAGA-------------------------------------------------  75073
Ferret       -------TGGGGAAGTTCGACATGCTGTAGA-------------------------------------------------  77910
Lion         -------TGGGGAAGTTTGTAACACTGTAGA-------------------------------------------------  72749
Vaquita      -------TTGAGAAGTTAGTAATACCATAGA-------------------------------------------------  70390
Pig          -------TGC---AGTTAGTAATGCTATAGA-------------------------------------------------  70190
```

```
Majority     ------ATAGTT-----AGTTAAAAAGATTCACACTAATGGGTCCTAAAAGGCATTAAAGAC---T----ACTAA--XX-
                             101290    101300    101310    101320    101330    101340    101350    101360
Human        CTAATTATAATTC-CTCAGTTAAGATTTTTTGGCTTAGTGGGCCCTTAGAATTACTTCTTCC-TGTTAGAATTAAAAGTG  81484
PolarBear    ------ACAGTT-----GGTTAAAAAGATCCACACTGGTGGATCCAAAAAGGCATCAAAGAC---T----ACAAA-----  39945
Cow          ------ATA--------AAGTGGTA-----GATCCTAAAAGGTGCTAAAGATTACTTAGTATGTAT----AATAATGATT  77133
Dog          ------ATAATT-----GGTTAAAAAGATCCACACTGGTGGATCCAAAAAGGCATCAAAGAC---T----ACTAA-----  70109
BelugaWhale  ------ACAGTTA----AAGTGGTG-----GATCCTAAAAGGCACTAAAGTCTATTATGCATG-------AGTAATTATT  69152
Donkey       CTAATTATAGTTAGCTCAGTTAAGTTTTTCTAGTTTAGTGGGCCCTGAGAATCACTTGTTGCCTGTTAGAACTGAAAATG  77615
Cat          ------ATAATT-----GGTTAAAAAGATTCACACCGATGGATCCAAAAAGGCATCAAAGA------------A------  75124
Ferret       ------ATAGTT-----GGTTACAAAGATTCACACTAGTGAATCCAAAAAGGCATCAAAGAC---T----ACTAA-----  77967
Lion         ------ATAATT-----GGTTAAAAAGATTCACACCGATGGATCCAAAAAGGCATCAAAGA------------A------  72800
Vaquita      ------ACAGTTA----AAGTGGTG-----GATCCTAAAAGGCACTAAAGTCTATTATGCATG-------AGTAATTATT  70448
Pig          ------ATAGTTG----AAGCTCCACCAGTGGCTCTAAAAGGCATTAAAGATTGCTGTGCAT---T----AATAATTACT  70253

Majority     AGACATGAGA-------------AGTTTCT-ATT---------------TTAAAATT------AGATCCTTXTTTCT-GG
                             101370    101380    101390    101400    101410    101420    101430    101440
Human        AAACCAGAAATTAAAGTGATTTAATTTTTTTATTGTGGCAAAGATCTAGTCCAAATATCTTTGATGGACTT-TTTGAAAA  81563
PolarBear    G-ACATGACA-------------AGTTTTT-ATT---------------TTAAAATT------AGATACTTGTTTCTGGG  39989
Cow          AGACATGAGG-------------AGTTTCT-GTG---------------TTAAAATT------AGATCCTTATTTCT-GC  77177
Dog          AGACATGAGA-------------AGTTTTC-CTT---------------TAATA-TT------AGATCCTTGTTTCTTGG  70153
BelugaWhale  AGACGTGAGA-------------AGTTTCT-ATT---------------TTAAAATT------AGATCCTTATTTCT-GG  69196
Donkey       AAACCAGAAATTAAACTGATTTTATTTTTT-ATTGTGGAAAAGACGTGGTCAAATTTCTTTGATGGCTTTTTTTTTTTAG  77694
Cat          ---CATGAGA-------------AGTTTCT-ATT---------------TTAAA--------------------------  75146
Ferret       ---CGACATA-------------AGTTTTT-ATT---------------TTAAA-TA------T--------TTTCTGGG  78000
Lion         ---CATGAGA-------------AGTTTCT-ATT---------------TTAAA--------------------------  72822
Vaquita      AGACGTGAGA-------------AGTTTCT-ATT---------------TTAAAATT------AGATCCTTATTTCT-GG  70492
Pig          AGACATGAGA-------------AGTGTCT-TT-----------------TAAAACT------AGGCCCTTATTTCT-GG  70295
```

```
Majority     A----TTTACTTTTAAAA-TAAAATTGGTGAAT-----------------------------------------------

                     101450    101460    101470    101480    101490    101500    101510    101520

Human        CAGACTTTATTGTTAAAAAGGGAATTTGAGGCTTGGTACTGAACTTCTTCTGCATGCCATATTGAAGTTGGTGGTTTTTT  81643
PolarBear    A----TTTACTTTTAAAA-TAAAA-GGATGAAA-----------------------------------------------  40016
Cow          A----TTTACTTTAAAAA-TGAAATTGATGAAT-----------------------------------------------  77205
Dog          A----TTTACTTTTAAAA-TAAAATTGTTGAAA-----------------------------------------------  70181
BelugaWhale  A----TTTACTTTAAAAA-TGAAATTTGTGAGT-----------------------------------------------  69224
Donkey       TCTTTTTTATTCTTAAAAGTCAATTTGAGGAATGATACTGAACTTTTTTCTTCTGCATCACATGAATTTGCAGATGGTGT  77774
Cat          ---------------AG--TAAAATGGGTGAAT-----------------------------------------------  75162
Ferret       A----TTTACTTTTAAAA-TAAAAAGGATGAAAGA---------------------------------------------  78030
Lion         ---------------AG--TAAAATGGGTGAAT-----------------------------------------------  72838
Vaquita      A----TTTACTTTAAAAA-TGAAATTTGTGAGT-----------------------------------------------  70520
Pig          A----TTTACTTTAAAAAATAAAACTGGTGAGT-----------------------------------------------  70324

Majority     -----------------------------------------XAAATGXAACA----------------------------

                     101530    101540    101550    101560    101570    101580    101590    101600

Human        GTTTGTTTGCCATCAGTG-----GGATCTCATACAGACTATGGAATTTAATAGAATTTTTAAGATACTGTTT--TTC---  81713
PolarBear    --------------------------AAATAATAAATACATAAAATGGATGAGTGGAAGGAAC-----------------  40053
Cow          ------------------------------------------AGATGAAACA----------------------------  77215
Dog          --------------------------AAATAAATAATAATTAAAATGAATGA----------------------------  70207
BelugaWhale  ------------------------------------------AGATACAACA----------------------------  69234
Donkey       TTATTTGCTTGTTTGTTTTGGGCCATCAACTGGATCCCATTAAGACGAAGGAACTTAACAGAATTTTAAGTTACTTTTTC  77854
Cat          -----------------------------------------GGA-TGGAACA----------------------------  75172
Ferret       ------------------------ATAAATAATAAATACATAAAATGGATGAGTGGATGGAAC-----------------  78069
Lion         -----------------------------------------GGA-TGGAACA----------------------------  72848
Vaquita      ------------------------------------------AGATACAACA----------------------------  70530
Pig          ------------------------------------------AGATGAAACA----------------------------  70334
```

```
Majority     ------AGATTGGCCA------------------------TGAGTTAATAAT--------------------------
                      101610    101620    101630    101640    101650    101660    101670    101680
Human        --CTAAAAGGTAGCTAACTAAAGGCAGGA----AAATAGAATGATGTAACAATATAATGACTACATCTGGAGTTTCTGC  81786
PolarBear    -----AAGATTGGCCAT----------------GAGTGCCATGAGTTAATACT--------------------------  40085
Cow          ------AGATTGGCCT------------------------TGAGTTAGTAAT--------------------------  77237
Dog          ------AAATTGGCCAC----------------CACTACCATGAGTTAATAAT--------------------------  70238
BelugaWhale  ------CGATTGGCCA------------------------TGAGTTAATAAT--------------------------  69256
Donkey       TACTAAAAGGTAGCCAATTAAAGGCAGAAAGAAACAAGGGAATGATTTGATAATGTAAGGCCTACATCTGGAGTTTCCAC  77934
Cat          ------AGATTGGCCA------------------------TAAATTGGTAAT--------------------------  75194
Ferret       -----AAGATTGGCCAT----------------GAGTGCCATGAGTTAATACT--------------------------  78101
Lion         ------AGATTGGCCA------------------------TAAGTTGGTAAT--------------------------  72870
Vaquita      ------CGATTGGCCA------------------------TGAGTTAATAAT--------------------------  70552
Pig          ------AGATTAGCCA------------------------TGAGTTAATAAT--------------------------  70356

Majority     ---TGTTAAACAATTGTGATGGXTACXTGGG------GTTXGTTATTCT------------------------------
                      101690    101700    101710    101720    101730    101740    101750    101760
Human        TACTAATAAATAATTGTATTGTCAACTTTAGTCAAATATTCTGTGCATTCAGTCAGAGCTATCTGAAAATACTGACGAAT  81866
PolarBear    ---TGTTAAACCAGTGTGATGGGTATGTGGG------GTTTGTTATTCT------------------------------  40125
Cow          ---TGTTAAACAGTTATGATGGATACATGGG------GCTCATTATTCT------------------------------  77277
Dog          ---TGTTAAACCAGCGTGATGG---------------TTTGTTATTCT------------------------------  70268
BelugaWhale  ---TGGTAAACAATTGTGGTGGATACATGGG------GCTCATTATTCT------------------------------  69296
Donkey       CACTGTTAAATAATTGTGGTGTCAACTTTAGTTGAATGCTAGTTGCTTTCAGCCAAAGCTATTTGAAAATACTGAAGAAC  78014
Cat          ---TGGTAAACCAGTGTGATGGGTATGTGGG------ATTTGTTATTGT------------------------------  75234
Ferret       ---TGATAAACCAGTGTGATGGGTATGTGGG------GTTTGTTATTCT------------------------------  78141
Lion         ---TGGTAAACCAGTGTGATGGGTATGTGGG------ATTTGTTATTGT------------------------------  72910
Vaquita      ---TGGTAAACAATTGTGGTGGATACATGGG------GCTCATTATTCT------------------------------  70592
Pig          ---TGTTAAACAATTGTGATGGATACACTGG------GCTCAT---TCT------------------------------  70393
```

```
Majority     ----------------------AGTCTCT-X-----------ACT-------------------------------TTTG

                      101770    101780    101790    101800    101810    101820    101830    101840

Human        AGTATTACAATATGGG----GAGGTCACTTCTC------AAGACTGCAAGAAAAAAAATTGTTGTGTGTAGGAATGTTTG  81936
PolarBear    ----------------------AGTCTTT-----------TTTTT-------------------------------TTTT  40141
Cow          ----------------------AGTCTCTGT-----------ACT-------------------------------TGTG  77293
Dog          ----------------------AGTGTCT---------------C-------------------------------TTTT  70280
BelugaWhale  ----------------------AGTCTCT-------------ACT-------------------------------TTTG  69310
Donkey       AATGTTTAAAGAAAGGAGAAGAGGTCGCTTTCTAAGAACAAAATTACTGTGAAAGCATGCCTAGGAGTTAAGACTGTATC  78094
Cat          ----------------------AGACTCTCT-----------CTT-------------------------------TTTT  75250
Ferret       ----------------------AGTGTGTGTCTCTCTCGGTCTCT-------------------------------CTCT  78168
Lion         ----------------------AGACTCTC--------------T-------------------------------TTTC  72923
Vaquita      ----------------------AGTCTCG-------------ACT-------------------------------TTTG  70606
Pig          ----------------------AGTCCCTCT-----------ATT-------------------------------TTTG  70409

Majority     TATATG--TTGGAXATTTTC----CAXAACAA----------------AAAGTAAAA-----------TCTAAAAA----

                      101850    101860    101870    101880    101890    101900    101910    101920

Human        CCTAGGAATTAGGAATGTATACTTGACAACAAGTGAACGTATCCATTGAATGTTAAATGAGAACAACTTATGGGGAGGAT  82016
PolarBear    TTAATG--TTGGATATTTTC----CATAACAA----------------AGAGTAAAA-----------TCTAAAAA----  40184
Cow          TATATG--TTGGAAATTTTC----CAAAACAA----------------AGAGTGAAAA------A---TCTAAAAA----  77338
Dog          TTTATG--TTGACTATTTTC----CGTAACAA----------------AGAGTAAAA-----------TCTAAAAA----  70323
BelugaWhale  TATATG--TTGGAAAATTTC----CAAAACAA----------------AAAGTAAAA-----------TCTAAAAA----  69353
Donkey       TTTATA--TTTGACATTGGCAG--CAAATTAAG-----ATATCCACTGAGTGTTAAATGAAGCAA---CCTATAAAGCTT  78162
Cat          TCTGTG--TTGGATATTTTC----CATCACAA----------------AAAGTAGAA-----------TCTAAAAA----  75293
Ferret       TTAATG--TTGGATAATTTC----CATAACA------------------GAGTAAAA-----------TCTAAAAA----  78209
Lion         TATGTG--TTGGATATTTTC----CATCACAA----------------AAAGTAGAA-----------TCTAAAAA----  72966
Vaquita      TATATG--TTGGAAAATTTC----CAAAACAA----------------AAAGTAAAA-----------TCTAAAAA----  70649
Pig          TATATG--TTGGAAATTTTC----CAAAATAA----------------AAAGTTAAA-----------TCTAAAAA----  70452
```

```
Majority     -ATAAAAX---------------TGGAGAAATAAG-----------------------AAAA--------GGGGGGAATC
                      101930    101940    101950    101960    101970    101980    101990    102000
Human        AGTGAGAGGTATAGTATTTAACATGGAAAAGTTAGTAATATAGTAGAATATTTGGTTGAAAAGATCCACATTGGTGTATT  82096
PolarBear    -AGAAAA----------------TAGAGAAATAA-------------------------AAA--------AAGGGGAATC  40214
Cow          -ATAAAAT---------------TGAAGAAATAAT-----------------------AAAA--------AGGGGGGACT  77371
Dog          -AGAAAA----------------TGGAGAAAGAAG------------------------AAA--------AAAAGGAATC  70354
BelugaWhale  -ATAAAAG---------------TGGAGAAATAAG-----------------------AAAAAAAGATGGGGAGGGAATC  69394
Donkey       AATGAGAGCTATGGTATTTAACATGGAGAAGTTAGTAATGCCCTGGAATAGTCAGTTAAAAAGATCCACACTGGTGGATC  78242
Cat          -ATAAGA----------------TGGAGAAATAAA------------------------AAA--------GGGGGAAATC  75324
Ferret       -AGAAAA----------------TGGAGAAATAAG-----------------------AAAA--------AAGGGGAATC  78241
Lion         -ATAAGA----------------TGGAGAAATAAA------------------------AAA--------GGGGGAA-TC  72996
Vaquita      -ATAAAAG---------------TGGAGAAATAAA-----------------------AAAAGATGGGGGGGAGGGAATC  70690
Pig          -AGGAAAG---------------TGGAGAGATAAA-----------------------AAAG--------GCGGGGAACC  70485

Majority     CCAAAAA-----------------G--TGGGTAA----------------------A-T-AAGXACT-------------
                      102010    102020    102030    102040    102050    102060    102070    102080
Human        CAAAGACACACTGAAGACTATTGTGCATTAGTAAGTATTAAACATGAGAATTCTTAATTTTAAAATTGGACCCTAATTTC  82176
PolarBear    TGGAAAA-----------------G--TGGGCAA----------------------A---AAGCACT-------------  40237
Cow          CCCAAAA-TA--------------T--TGTAT--------------------------TGGGGGAG--------------  77394
Dog          TCGAAAA-----------------G--TGGGTAG----------------------A---AAGTACT-------------  70377
BelugaWhale  CCCAAAAATG--------------G--TATGTGGAGTGG----------------TGGTGGGGGACT-------------  69429
Donkey       CAAACAGACTGAAGACTACTATGCG--TTAGTAAATACCAAACATGAAAGGTTTCTATTTTAAAATTAGATCCTTATTCA  78320
Cat          TCAAAAA-----------------A--TGGGCAA----------------------A---AAGCACT-------------  75347
Ferret       TGGAAAA-----------------G--TGGGCAA----------------------A---AGGCATT-------------  78264
Lion         TCAAAG---------------------TGGGCAA----------------------A---AAGCACT-------------  73017
Vaquita      CCCAAAAATG--------------G--TATGTGGAGTGG----------------TGGTGGGGGACT-------------  70725
Pig          CCAAAAAATG--------------G--TGTGTAGAAAAAAACCTATAAAATAAACTCATAGGGGAGTTCCCATTGTGGCG  70549
```

```
Majority     ----A-AAAT---------------------------A---AXCTC----------------------------------
             102090    102100    102110    102120    102130    102140    102150    102160
Human        TGAGATTTACTTTAAGAATAAAAGGGGATGAAACGAGATTGGCCATGAGTTGATCATTGTTAAAGTTGGGTGATGGCTAC 82256
PolarBear    ----A-AAG----------------------------------CTC---------------------------------- 40244
Cow          ----AAAAAC-----------------------CTGTA---AACTC---------------------------------- 77410
Dog          ----A-AAGT---------------------------A---AGCTC---------------------------------- 70388
BelugaWhale  ----ATAAAA-----------------------T---A---AACTC---------------------------------- 69442
Donkey       TGGGATTTACTTTAAAAATAAAATGGGTGAGTGGATGA---AACAAGATTGGCCATTAGTTAATAGTTGTTAAACCAGTG 78397
Cat          ----A-AAAT---------------------------A---AGCTT---------------------------------- 75358
Ferret       ----A-AAA----------------------------------CTC---------------------------------- 78271
Lion         ----A-AAAT---------------------------A---AGTTT---------------------------------- 73028
Vaquita      ----ATAAAA-----------------------T---A---AACTC---------------------------------- 70738
Pig          CATCAGAAATGAATCTGACTAGGACCCATGAGGTTGCA---AGTTCGATCCCTGGCCTCATTCGGTGGGTTAAGGATCCA 70626

Majority     --------------------------------------------------------------------------------
             102170    102180    102190    102200    102210    102220    102230    102240
Human        TTAGAGTTGCATTATTTTAGTCACTTTTACTTTTGTATATGTTGGAAATCTTCCAAAACAAAATGTATAGCCATAAAAAA 82336
PolarBear    -------------------------------------------------------------------------------- 40244
Cow          -------------------------------------------------------------------------------- 77410
Dog          -------------------------------------------------------------------------------- 70388
BelugaWhale  -------------------------------------------------------------------------------- 69442
Donkey       TGATGGGTACATGGGGGTTCATTATTCAAGTCTCTTTACTTTTATGTATGTTGAAAGTTTTCCAAAACAAAAAGTAAAAT 78477
Cat          -------------------------------------------------------------------------------- 75358
Ferret       -------------------------------------------------------------------------------- 78271
Lion         -------------------------------------------------------------------------------- 73028
Vaquita      -------------------------------------------------------------------------------- 70738
Pig          GCATTGCTGTGAGCTGTGGTGTAGGTCACAGCTGTGGCTTGGATCCTATGTTGCTGTGGCGTAGACCGGCAGCTACAGCT 70706
```

```
Majority     ------------------------------------------------------------------------ATCCAGAG
                     102250    102260    102270    102280    102290    102300    102310    102320
Human        AAGGGAGCAAAGTAAGAAAAAAAGGAGAGCCTAAAAAAAAAGGTACAGGCAAAAAGGTGTGAAAAGAAACTCATCAAGAG 82416
PolarBear    ------------------------------------------------------------------------ATCCTCAG 40252
Cow          ------------------------------------------------------------------------ACCCAGAG 77418
Dog          ------------------------------------------------------------------------ATCCACAG 70396
BelugaWhale  ------------------------------------------------------------------------ACACAGAG 69450
Donkey       CTAAAAAGTGGAAAAATAGGGAAAAAAGGGGGATACCCAAAAATGGTGTTGGCAAAACTGTAAAATAAGCTTGTTTAGAG 78557
Cat          ------------------------------------------------------------------------ATCCACAG 75366
Ferret       ------------------------------------------------------------------------ATCCACAA 78279
Lion         ------------------------------------------------------------------------ATCCACAG 73036
Vaquita      ------------------------------------------------------------------------ATACAGAG 70746
Pig          CCGATTAGACCCCTAGCTTGGGAACCTCCGCATGCCACAGGTGCAGCCCTAAAAAAAAAACAAAAAAATAAAACACAGAG 70786

Majority     CAAGATTGTAAAAAAGAAA--TATCATTCCACTGTTGGAGTGCTTACT---CAGAAAAGATAAA---AAGAAATTGG-AG
                     102330    102340    102350    102360    102370    102380    102390    102400
Human        CAAGAGTATTAAATGAGAAA-CAT-ATGCCACTGACAGAGTTCTCAGAAGAGATAAAGGCTGAATTAAAAAGAGATGGAG 82494
PolarBear    AAAGATTGAAAAAA------TATTATTCCGCTGTTGTACTGCTCAGA---GAGAAGAGATAAA---AAGAAATTGG-AG 40318
Cow          CTTGAACATATATGAGAAA--CACCATTCCACTGATGA--AAGGTACT---CAGTAAAGATCAA---AGGAAATCTG-AG 77487
Dog          AAAGATTGCAACAAA------TATAATTCCACTGTTGAACTGCTCATA---GAGAAGAGAT------AAGAAATTGG-AG 70460
BelugaWhale  CAAGAATGTAAGTGAGAAA--CACCATTCTACTGATGGGGGAGGTACT---CAGAAAAGATAAA---AGGAGATTGGGAG 69522
Donkey       CAAGATTGTAAAGGAGAAACATATCATTCCACTGAAGAA---T--ATT---CAGAAAGGATAAA---AGGAGATTGG-AG 78625
Cat          AAAGATTGAGAAAAA------TATCTTTCCACTGTTGGAGTGCTTA-------GAAGAGATACA---AAGAAATTGG-AG 75429
Ferret       AAAGATTGGAAAAAAAAAAG-TATTATTCCACTGTTGAACTGCTTAGA---GAGAAAAAATAAA---AAGAAATTGG-AG 78351
Lion         AAAGATTGAGAAAAA------TATCTTTCCACTGTTGGAGTGCTTA-------GAAGAGATACA---AAGAAATTGG-AG 73099
Vaquita      CAAGAATGTAAGTGAGAAA--CACCATTCTACTGATGGGGGAGGTACT---CAGAAAAGATAAA---AGGAGATTGGGAG 70818
Pig          CAAGAATATAAATTAGAAA--CATCATTCCAATGTTAG--GAA-TACT---CAGAAAAGATAAA---AGGAGATTGG-AG 70854
```

```
Majority     GGAAAAATTTTGTAAGTCAAAAACTXAAGAGGTTAAGAATTGTGAATCTT-AAGAACCCTTCAGACTGCCAATGAAAGGT
                      102410    102420    102430    102440    102450    102460    102470    102480
Human        GGGAAAGTTTTATAAGTCAAAAACTTA---GGTTAAGGTTTGTGAATATTAAAGAGCGCTTCAGATTGCCAATGAAAGGT  82571
PolarBear    GGAAAAAATTTGTAAGTCAGAAACTGAAGAGATTAAGAATTTTGAATCTT----AACCCTTCAGACCATCAATGAAA---  40391
Cow          GGAAAATTTTTACAAGTCGAAAACTAAAGAGGTTAAGAATTGTCAATCCTGAAGAAGC-TTCAGCCTGCCAATAAAAGGT  77566
Dog          GGGGAAAATTTGTAAGTTAGAAACTGAAGAGATTAAGATTTGTGAATCTT----AACCCTTCAGACTATCAATGAAAGAT  70536
BelugaWhale  GGAAAAATTTTGTAAGTCAAAAACTAAAGAAGTTAAGAATTGTCAGTCTTCAAGAGCCGTTCACCCTGCCAATGAAAGGT  69602
Donkey       GTAAAAATTTTGTAAGTCAAAAACTACAGAGGCTAAGAAAAGTGGCTCTTAAAGAACCATTCAGACTGCTAATGAAAGCC  78705
Cat          GGAAAAA--TTAGAAGTCAGAAACTGAAGAGATTAAGAATTGTGAATCTT----AACCTTTTAGACTGTCAGTGAAAGGC  75503
Ferret       GGAAAAA-TTTGTA-----GGAACTGAAGAGATTAGGAATTGTGAATCTC----AACCCTTCAGACTGTCAGTGAAA---  78418
Lion         GGAAAAA--TTAGAAGTCAGAAACTGAAGAGATTAAGAATTGTGAATCTT----AACCTTTTAGACTGTCAGTGAAAGGC  73173
Vaquita      GGAAAAATTTTGTAAGTCAAAAACTAAAGAAGTTAAGAATTGTCAGTCTTCAAGAGCCGTTCACCCTGCCAATGAAAGGT  70898
Pig          GAGAAAATTTTATAAGTC-AAAACTAA-GAGGTTAAGAATTGTTGATCTTCAAGAACCCTTCATACTGCCACTGAAAGGT  70932

Majority     CATXATAGTTTCTATAAAGTAAAAAGATTX-AAAAAA------------CTTGGCT-AAAAGATTAA-GXGATCTGAAAC
                      102490    102500    102510    102520    102530    102540    102550    102560
Human        CATAGTAGTTTCTATAAAGTCAAAAGATC--AAAAA-------------CTTGACTTAAAAGAGTAA-GATCTCTAAAAC  82635
PolarBear    --TCATAGTTTCTATAAAGTAAAAAAAGAAAAAAAAAACCAAA------CTTGGCC-AAAATACTA----GATCTGAAAC  40458
Cow          CATAGTAGTTTCTGTAAAGTAAAAACAT-----AAA-------------CGTGGCT-AAAAGAGTAA---GATCTGACAC  77624
Dog          CATCATAGTTTCTATAAAGTAAAAAGATTAAAAAAAAAA-AAA------CTTGGCT-AAAATATTAA-GATACCTGAAAC  70607
BelugaWhale  CATAGT---TTCTGTAAAGTAAAAAGATAA-ACAAA-------------CTTGGCT-AAAAGAGTAA---CATCTGAAAC  69661
Donkey       ATTTATAGTTCCTATAAAGTAAAAAGAT---AAAAAA------------CTTGGCT-AAAAGAATAA-GGTGTCTGAAAC  78768
Cat          CATCATAGTTTCTATAACATAAAAAGACTTAAAAAAAAAAAAAA-----CTTGGCT-AAAATATTGA-GAGATCTGAAAC  75576
Ferret       --TCATAGTTTCTATAAAGTAAAAAGATTTTAAAAAAA-----------CTTGGCT-AAAATATTAAAGAGATCTGAAAC  78484
Lion         CATCATAGTTTCTATAACATAAAAAGACTTAAAAAAAAAAAAAAAAAAACTTGGCT-AAAATATTGA-GAGATCTGAAAC  73251
Vaquita      CATAGT---TTCTGTAAAGTAAAAAGATAA-ACAAA-------------CTTGGCT-AAAAGAGTAA---CATCTGAAAC  70957
Pig          CATAGTAGTTTCTGTAAAGTAAAAGGATTTTAAAAA-------------CTTGGCT-AAAAGATTAA---GATCTGAAAC  70995
```

```
Majority     CAAAGGTAACA-GCACATCCTTATGACATATACAAGAAGGCT---GGAAAGAGAAAAGAAGAAATAAAACATGAACTTAT
                       102570    102580    102590    102600    102610    102620    102630    102640
Human        CAAAGGTAACA-GCAGATTCTTATGAAATGCACCAGAAAGCT---GGAAAG---AAAGAAGAAATAAAATGTGAACTTAT  82708
PolarBear    CAAAGGCAACA-GCACATCCTTGTCACATACACAAGAAGGCTGGTGGAAAGAGAAAAGAAGAAATAAAACATGAACTTAC  40537
Cow          CAGAGTTAACA-GTACATCCTTATAACATGTACAAGAAAGCT---GAAAAGAAGAGATAAAACAT-------GAATTTAT  77693
Dog          CAAAGGCAACAAGCACATCCTTTTGACATACACAAGAAGGCTGGTGGAAAGAGAAAAGAAGAAATAAAACGTGAACTTAC  70687
BelugaWhale  CAGAGGTAACA-GCACATCCTTATGACATGTACAAGAAAGCT---GGAAAGAGAAAAGAAGAAATAAAACATGAACTTAT  69737
Donkey       CAAAGGTAACA-GCACATCTTTTTGACATACACAAGAAGGCT---GGAAAGAGAAAAGAAGAAATAAA-CGTGAACTTAT  78843
Cat          GAAAGGCAACA-GCACATCCTTATGACATAGACAAGAAGGCTGATGGAAAGAGAAAAGGAGAAATAAAACATGAACTTTT  75655
Ferret       CAAAGGCGACA-GCACATCCTTATGACATATACAAGAAGGCTGGTGGAAAGAGAAAAGAAGAAATAAAACATGAACTTAA  78563
Lion         CAAAGGCAACA-GCACATCCTTATGACATAGACAAGAAGGCTGATGGAAAGAGAAAAGAAGAAATAAAACATGAACTTTT  73330
Vaquita      CAGAGGTAACA-GCACATCCTTATGACATGTACAAGAAAGCT---GGAAAGAGAAAAGAAGAAATAAAACATGAACTTAT  71033
Pig          CAGAGGTAACA-GCACATCCTTATGACATGTACAAGAAGGCT---GGAAAGAGAAAAGAAGAAATAAAA---GAACTTAT  71068

Majority     GGG-ACAACAGTACTAAGGGCCAGTTCTACCCAGGTATGTCATGAA-TTAACTTCATGTAGGTTGGATGTTTXT--CCTT
                       102650    102660    102670    102680    102690    102700    102710    102720
Human        GGA-ACAAGAGTGCTAAGGGCCTGTTCTACCCAGGTATGTTATGAAATCAAATTCATTTAGTTTGGGTGTTTGT-TCCTT  82786
PolarBear    CGG-ACAACAGTACTAAGGGCCATTTCTATCCAGGTATGTCATGAA-TTAACTTCGTGTAGGTTGGATGTTT----CATT  40611
Cow          GGG-ACAACAGTACTAAGGGCCAGTTCTACCCAGGTATGTCATGAA-TTAATTTTATGTAGGTTGAATGTTTATCTCCTT  77771
Dog          GGG-ACAACAGTCCTAAGGGCCATTTCTATACAGGTATGTCATGAA-TTAACTTCATGTAGGTTGGTTGTTT----CTTT  70761
BelugaWhale  GGG-ACAGCAGTACTAAGGGCCAGTTCTACCCAGGTATGTCATGAA-TTAACTTCATGTAGGTTGAATGTTTGTCGCCTT  69815
Donkey       GGG-ACGACAGTGCTAAGGGTCAGTTCTACCCAGGTATGTCATGAA-TTAACTTCATTTAGATTGGATATTTGTTTCCTT  78921
Cat          GGG-ACAACAGTACAAAGGGCCATTTTGATCCAGGTATGTCACGGA-TTAACTTCATGTAGGTTGGATGTTT----CCTT  75729
Ferret       CGGGACAGCAGTACTCAGGGCCATGTCTATCCAGGTATGTCATGCA-TTAACTTCACGTAGGTTGGATGTTT----CCTT  78638
Lion         GGG-ACAACAGTACAAAGGGCCGTTTTGATCCAGGTATGTCACGGA-TTAACTTCATGTAGGTTGGATGTTT----CCTT  73404
Vaquita      GGG-ACAGCAGTACTAAGGGCCAGTTCTACCCAGGTATGTCATGAA-TTAACTTCATGTAGGTTGAATGTTTGTCGCCTT  71111
Pig          GGG-ACAGCCGTACTAAGGGCCAGTTCTACCCAGGTATGTCATGAA-TTAACTTTATGTAGGTTGAATGTTTGTTTCCTT  71146
```

```
Majority     TATCATGGGAAGAAXAAAAAA-AACATATAATTCAT--TTCTTTAGTGAACTAATATTTATTGAGAAATAAGCTGG-TAT
                       102730    102740    102750    102760    102770    102780    102790    102800
Human        TATGTTGGGAAGA-TAAAAAAGAACATATAATTCGT--TTCTTGAGTGAACCAGTATTTAT-GAGAAATAAGCTGGGTGT  82862
PolarBear    TTTCATGGAAGGA------GA-AAAATATAATTCAT--T-----AGTGAACTAGTATTTATTGAGATATAAACTGG-TAT  40676
Cow          TATCATGGAAAGAAAAAAAAA---CATGTAATTTGTTTTTTTTTAGTGAACTAATATTTATTAAGAAATAAGTTAG-TAT  77847
Dog          TTAATGGGAAGGA------GA-AAAATACAATTCAT--TTCATTAGTGAACTAACATTTATTGAAATATAAGCTGG-TAT  70831
BelugaWhale  TATCATGAGAAGGAAAAAAAAGAACATATAACTCAT--TTCTTTAGTAAACTAATATTTATTGAGAAATAAGCTGG-TAT  69892
Donkey       TATCATGGGAAGGAGAAGAAG-TACATGTAATTCAT--TTCTTTAGTGAACTAA----TATTGAGAAATAAGCTGG-TAT  78993
Cat          TTTCATGGGAAGAATAAAAGT-AACATACAATTTAC--TTCATTAGTGAACTAATATGTATTGAGGAATCAGCTGG-TAT  75805
Ferret       TTTCATGGGAAG-------GA-GAAAAATAATTGAT--T-----AGTGAACTAGTATTTATTGAGATATAAACTGG-CAT  78702
Lion         TTTCGTGGGAAGAATAAAAGT-AACATACAATTTAC--TTCATTAGTGAACTAATATGTATTGAGGAATCAGCTGG-TAT  73480
Vaquita      TATCATGAGGAGGAAAAAAAAGAACATATAACTCAT--TTCTTTAGTAAACTAATATTTATTGAGAAATAAGCTGG-TAT  71188
Pig          TATCATGGGAAGAAGAAAAAT---CGTGTAA-------TTCTTTAGTGAACTAGTATTTATTGAGA----AGCTGG-TAT  71211

Majority     GTAAGAATTTAAGGGATGAAAGGATTCCXAGTTTATTXTT--AATTGAAATGTGGATAAAAGATGCTGAGACAAAAGG-A
                       102810    102820    102830    102840    102850    102860    102870    102880
Human        GTAAGAATTTAAGGGATGAAAATATTCCAAGTTGATTGGT--AATTGAAAAGTAGATGAACGGTGCTAACACAAAAGGGA  82940
PolarBear    GTAAGAATTGAAGGGAGGGAAGGATTTTGAGTTTATTCTT--AATTGTAATGTGGATAAAAGATGGTGCGACAAAAGA--  40752
Cow          GTAAGAATATAAGGAATGAAAGGAATCAAAGTTTATTAAT--TGTTGAAAAGTAGATTAAAGATGCTGAGACAAAGGG-A  77924
Dog          GTAAGAATTGAAGGGATGGAAGGATTTTGAGTTTATTCTT--AATTAGAATGTGAATAAAAGATGGTGAGACAAAAGA--  70907
BelugaWhale  GTAAGAATTTAAGGGATGAAAGGAATCCAAGTTTATTGAT--AATTGAAATACGGATAAAAGATGCTGAGACAAAGGG-A  69969
Donkey       GTAAGAATTTAAGGAATGAAAGAATTCTGA-TTGCTT-----AATTGAAAAGTGAATAAAA-ATGTTGAGACAAAAGGGA  79066
Cat          GTAAGAATTTCAGGGATGAAAGGATTTAGAGTTCATTCTTTTAATTGGAACGTGGATAAAAGATGCTGAGACAAAAGAGA  75885
Ferret       CTAAGAATTGAAGGGAGGGAAGGATTTGTGGTTTATT-----AATTACAATGTGGATAAAAAAGGTTGAGACAAAA----  78773
Lion         GTAAGAATTTCAGGGATGAAAGGATTTAGAGTTCATTCTTTTAATTGGAACGTGGATAAAAGATGCTGAGACAAAAGAGA  73560
Vaquita      GTAAGAATTTAAGGGATGAAAGGAATCCAAGTTTATTGAT--AATTGAAATACGGATAAAAGATGCTGAGACAAAGGG-A  71265
Pig          GTAAT----TAA-----GAAAGGAATCCAAGTTTATTGGT--AATCGAAAAGTGAATAAAAGATGCTGAAACAAAAAT-A  71279
```

```
Majority     ATTCTXTCTAGAA--TAAATGAA------GTATCTTAAAGATGATAGAACAACAGTAAGACTGGAAAGTAGAACAATAGA
                              102890    102900    102910    102920    102930    102940    102950    102960
Human        ATTCTGTCTAGAA--TAAATGAAAATATGGTGACTTAAAGATAGTAGGACAGCAATAAGACTGGAAATTAGAACAATA-A  83017
PolarBear    ATTCTTTCGTGAA--TAAATGACTGGC--ATATCTTAAAGATGATAGAAGAACAGTAAGACTGGAAAATAGAACAGTAGA  40828
Cow          ATTCTGT--GGAA--TAAATGAA------GAATCCTGAAGATGACAGAGCAATAGTAAGACTGGAAAGTAGAACAGTAGA  77994
Dog          ATTCTCTCTAGAGAGCAAATGATTAGC--ATATTTTAAA----------CAACAG----ACTGGAAAACA--ACAATAGA  70969
BelugaWhale  ATTCCGT--GGAA--TAAATGAA------GAATCCTGAAGATGACAGAACAACAGTAAGACTGAAAAGTAGAACAGTAGA  70039
Donkey       ATTCTTTGTAGAA--TAAGTGAA------GTATCTCAAAGATGATAAAACAACAGCAAGACTGGAAAGTAG---AATTGA  79135
Cat          ATTCTCTCTAGAG--TGAA-GACTGGC--ATATCTTAAAGATGATAGAACAACAGTAAGACTGGAAAATAGAACAATAGA  75960
Ferret       -TTCTCTCTAGAA--TAAATGAA------GTATCTTAAAGATGCTAGAACAACAGTAAGACTGGAAAGTAGAGCAACAGA  78844
Lion         ATTCTCTCTAGAG--TGAA-GACTGGC--ATATCTTAAAGATGATAGAACAACAGTAAGACTGGAAAATAGAACAATAGA  73635
Vaquita      ATTCCGT--GGAA--TAAATGAA------GAATCCTGAAGATGACAGAACAACAGTAAGACTGGAAAGTAGAACAGTAGA  71335
Pig          ATTCT----TGAA--TAAATGAG------GAACCCTGAAGATGATAGAACAACAGTAAGACTGGAAAGTAGAACAGTAGA  71347

Majority     AATAAGAAGGAAATGTTAAAA-CTGGTAGGTAAAGGATAGG-----AAAATACTGAAAGAGAAAGCXTGTGTGATA-CAG
                              102970    102980    102990    103000    103010    103020    103030    103040
Human        AATAAGAAGGAAATGATAACA-TAGATTGGGAAAGGATAGG-----AAAATACTGAA--ACAAAATCTGTGTTGTA-TAG  83088
PolarBear    AAT---AAGGAAATGTTAAAA-CTGGTAGGCAAAAGATAGG-----AAAAGACTGAAAGAGAAAGCTTGCATGTTAACAG  40899
Cow          AATAAGAAGGAAATGTTCAAA-CTGGTAGGTAAAGGATAGG-----AAAATACTGG-------------TGTGCTA-TAT  78054
Dog          AAT---AAAGAAATGTTAAAA-CTGGTAGGGGAAAGGAAAA-----GA----CTGAAAGAGAAAGCATGCATGATA-CAG  71035
BelugaWhale  AATAAG---GAAATGTTAAAA-CTGGTAGGTAAAGGATAGG-----AAAATATTGAAAGAGAAAGCTTGTGTGATA-TAG  70109
Donkey       AATAAGAAGGAAATGTTAAAG-TTGGTAGGTTAGGTAAAGGGTAGAAAAATACTGAAAGAGAAAA--TGTGTGATA-CGG  79211
Cat          AATGAGAAGGAAATGTTAAAA-CTGGTAGGCAAAATGTGGGG----AAAATACTGAAGGAGAAAGCCTGTGTGGATACGG  76035
Ferret       AAT---AATGAAATGTTAAAA-CTGGTAGGCTAAAGATAGG-----AA------GAAAGAGAAAGCTTGTGTATTAACAG  78909
Lion         AATGAGAAGGAAATGTTAAAA-CTGGTAGGCAAAATGTGGG-----AAAATACTGAAGGAGAAAGCCTGTGTGGATACGG  73709
Vaquita      AATAAG---GAAATGTTAAAA-CTGGTAGGTAAAGGATAGG-----AAAATATTGAAAGAGAAAGCTTGTGTGATA-TAG  71405
Pig          AATAAG---GACATGTTAAAAACTGATGATTAAAGGATAGG-----ACCATTCTGAAAGAGAAAGCCTATGCGTTA-CAG  71418
```

```
Majority     TGA-CXTTTTCTCATTCTTTCTACTGGGGAAATGTTXTGTTTGAGAAACTTG---TGTTCAT--TATAACTACTTGAGTG
                       103050    103060    103070    103080    103090    103100    103110    103120
Human        TGA-TGGTTTCTTGTTCTTTCAACCGGGAGAGTGTTGTGTTGAAGGAACTTG---TATTCAT--TTTAATTACTTGAGTG  83162
PolarBear    TGA-CATTTTCTCAATCTTCCTATTGGGGAAATGTTGTATTTGAGAGATTTGTTGTGTTCAT--TATAACTACTTGAGTG  40976
Cow          TGA-CTTTCTCTTATTCTTTCTGTGGGGCAAATGTTGTGTTTGAAAGACTTA---TATT------ACACCTACCTGAGTA  78124
Dog          TGA-CGTTTTCTCAATCTTCCTACTGGGGAAAT-------TTGAGAAATTTGTTGTGTGTGTGTTACAACTACATGAGTG  71107
BelugaWhale  AGA-CTTTTTCTCATTCTTTCTACGGGGGAAGTGTTGTAATTGAGAAACTTG---TGTT------ATACCTATTTGAGTG  70179
Donkey       TGA-TGTTTTCTCATTCTTCCTGCTGGGAAAGTATCATGTTTGAGAAACTTG---CGTTCAT--TATAACTACTTGAATG  79285
Cat          TGA-CGTTTTCTCATTCCTTCTACTGGGGAAATG---TGTTTGAGAAATTTGTTGTGTTCAT--TATGACTGCTTGAGTA  76109
Ferret       ----------------------ACTGGGGAAATG---TGTTTGAGAAATTTGTTGTGTTCAT--TATAACTACTTGAGTG  78962
Lion         TGA-CATTTTCTCATTCCTTCTACTGGGGAAATG---TGTTTGAGAAATTTGTTGTGTTCAT--TATGACTACTTGAGTA  73783
Vaquita      AGA-CTTTTTCTCATTCTTTCTACGGGGGAAGTGTTGTAATTGAGAAACTTG---TGTT------ATACCTATTTGAGTG  71475
Pig          TGAACTTTTTCTCATTGTTTCTACTGGAGGAGTGTT---TTTCAGAAACTTA---TACTTAG--TATAACTACTCAAGTG  71490

Majority     TGGAATTAAG---GCAAACAAATTTTGGTAAGATTTTCTTTAGGTCATTTGAATGAATTA----AATAATG-ATGTTCAG
                       103130    103140    103150    103160    103170    103180    103190    103200
Human        TATAATTAAGAAGGCAAGCACATTTTGCTAAGATTTTCTTT-----ATTTGCATGAATTA----AATAATG-ATGTTCAG  83232
PolarBear    TGAAATTAAG---GCAGATAAACTTG-GTAAGATTTTCTTTAGGTCATTTGAATGAATTAATTAAATAATG-ATGTTCCG  41051
Cow          TGGAATTAAG---GAAAACAAATTTTGGTAAGATTTTCTTTAGGTCAGTTGAATACATTA----TATAATGTATGTTCAA  78197
Dog          TGAAATTAAG---GCAAACAAACTTTGGTAAGGTTTTCTTCAGGTCATTTGAATGAATTA----AATAAT------TCAG  71174
BelugaWhale  TGGAATTAAG---GAAAACAGATTATGGTAAGATTTTCTTTAGGTCACTTGAATTAATTA----AATAATG-ATGTTCAG  70251
Donkey       TGGAATTCAG---GCAAACAAATTCTGGTAAGATTTTCTTTAGGTCATTTGAATGAATTA----AATAATG-ATGTTCAT  79357
Cat          TGGAATTAAG---GCAAACAAACTTCTGTAAGATTTTCTTTAAGTCATTTGAATGAA--------ATAATG-ATGTTCAG  76177
Ferret       TGAAATTAAG---GCAAATAAACTTTCGGAAGTTTTTCTTTAGGTCATTTGAATGAATTA----AATAATG-ATGTTCCG  79034
Lion         TGGAATTAAG---GCAAACAAACTTCTGTAAGATTTTCTTTAAGTCATTTGAATGAATTA----AATAATG-ATGTTCAG  73855
Vaquita      TGGAATTAAG---GAAAACAGATTATGGTAAGATTTTCTTTAGGTCACTTGAAT----TA----AATAATG-ATGTTCAG  71543
Pig          TGGAATTAG-----AA--CAAATTTTG-TAAGGTTTTCTTTAGGTCACTTAAATGAATTA----AATAGTG-AGGTTCAA  71557
```

```
Majority     GGTGATTT-GTCTCTAAGAACTGAAGGACTTAC--TATATTTGTTTCATAAGCTGTAATT-XTXTG---A-TTTCAAGTA
                       103210    103220    103230    103240    103250    103260    103270    103280
Human        AGTGATTT-GTCTCTAGGAGCTGAAAGAC-TAC--TACCTTTGTTTCATAAGCTGTAATT-ATTTG----ATTTCAAGTG  83303
PolarBear    GGTGATAT-GTCTCCAAGAACAGAAGAACTTAC--TATATT-GTTTCATAAGCTGTAATT-GTCTG--------CAAGTA  41118
Cow          GGTGATTT-GTTTCTAAGAAT-GAAGAATTTGC--TATATATGTTTTGTGAGCTATAATT-GTCTG---A-TTTCAAGTA  78268
Dog          GGTGATGT-GTCTCCAAGAGCTGAAGAACTTAC--GATATGTGTTTCAGAAGCTGTAATT-GTCTG---A-T--TTCAAG  71244
BelugaWhale  GGTGATTTTGTCTCTAAAAAT-GAAGGACTTAC--TATATATGTTTTGTAAGCTGTAATT-ATTTGTCTAATTTCAAGCA  70327
Donkey       GGTGATTC-GTCTCTAGGAACTGAAGGACTTAAC-TATATTTGCTTCATAAGCTACTATTTATTTG----ATTTCAAGTG  79431
Cat          GGTGATAG-GTCTCCAAGAACTGAAGGACTTA---ACTGTTTGTTTCATAAGCTGTAGCT-GTCCG---G-TTTCAAGTA  76248
Ferret       GGTGATAT-GTCCCCAAGAACTGAAGAACTTAT--TATATTTGTTTCATAAGCTGTAAT---------------CACATG  79096
Lion         GGTGATAG-GTCTCCAAGAACTGAAGGACTGACTTACTGTTTGTTTCATAAACTGTAGCT-GTCTG---G-TTTCAAGTA  73929
Vaquita      GGTGATTTTGTCTCTAAAAAT-GAAGGACTTAC--TATATATGTTTTGTAAGCTGTAATT-ATTTGTCTAATTTCAAGCA  71619
Pig          GAAGATTT-GTCTCTT--AAC-GAAA----TAC--TGTGTATGTTTTTTAAGCTGAAATT-ATTTG---A-TTTCAAGTA  71622

Majority     TGAGTTTATGAAGAAGATAGCACTTGTATAAAAAAXTGT-GAGTTACTGTTXTGTTGAAGATGATXTTTATTTTAGATGT
                       103290    103300    103310    103320    103330    103340    103350    103360
Human        TGAGTTTATGAAGAAGATAGCACTTGCGGAAAAAAGT---GAATTACCATTATGTCGAAAGTGATACTTTTTT-ACATTT  83379
PolarBear    TGAGTTTATGAAGAAGATAGCACTTGTATAACAATGTATTGAGTTA-----TTGTTGAAGATGAT----ATTTTAGATGT  41189
Cow          --AGTTTATGAAGAAGATAGCACTTGTT--------------ATTTTTTT-----TAGATTTGACTCCTC-----ACTAT  78322
Dog          TGAGTTTATGAAGAAGATAGCACTTGTCTAAAAAAGTGTTGAGTTA-----CTGTTGAAGATGATACTTATTTTGGATGT  71319
BelugaWhale  C--GTTTATAAAGAAGGTAGCACTTGTATAAAAAA--GT-GAGTTACTGTTATGTTGAAGATGATTTTTTTTTTAGATTT  70402
Donkey       TGAGTTTATGAAGA---TAGCACTTGTGTAAAACAAT---GAGTTACTGTTATGTTGAAGATGATA--TTTTTTAGCTTT  79503
Cat          TGAGTTTATGAAGATGATAGCACTTGTAGAAAAAAGTGT-GAGTTACTGTTTTGTTGAAGATGATATTTATTTTAGATGT  76327
Ferret       TGAATTTATGAAGAAGATAGCATTTGTATAGTA---TGTTGAGTTA-----TTGTTGAAGATATTT---ATTTTAGATGT  79165
Lion         TGAGTTTATGAAGATGATAGCACTTGTAGAAAAAAGTGT-GAGTTACTGTTTTGTTGAAGATGATATTTATTTTAGATGT  74008
Vaquita      C--GTTTATAAAGAAGGTAGCACTTGTATAAAAAA--GT-GAGTTACTGTTATGTTGAAGATGATTTTTTTTT-AGATTT  71693
Pig          TGAGTTTATGAAGAAGACAGCATTTGTTTTAAAAA--GT-GAGTTACTAT-----TGAAGATGACTATTG-----AATAT  71689
```

```
Majority     GGCTTCTXGCTATAAACCATGGAAGTGAATTTTTCAA-----GTCTCTGACTTGGAAATX-------ATATTCTTTA-TG

                     103370    103380    103390    103400    103410    103420    103430    103440

Human        GGCCTTTTACTGTAAATCGTGGAAGTGAATTTTCCAA-----GCCTGTGACTTGGAATT--------ATATTTTTCA-TG  83445
PolarBear    GCCTTCTTGCTATAAACCATGGAAGTGAATTTTTCAA-----GTTTCTGACTTAGAAAA-------TATATTCTTTA-TG  41256
Cow          G-----------TGAACCATGGAAGTGAATTTTATAA-----GTCTCTGACTTGGAAGT--------ACATTCTTTT-TG  78377
Dog          GATTTCTTGCTGTAAACCATGGAAGTGAATTTTTCAA-----GTTTCTGACTTAGAAATA-------TCATTCTTTA-TG  71386
BelugaWhale  GGCTCCTCACTATGAACCATGGAAGTGAATTTTTCAA-----GTCTCTGACTTGGAGGT--------ATATTCTTTA-TG  70468
Donkey       GGCTTCTCACTGTAAACCATGTAAGTGAATTTTTCAA-----GTCTCTGACTTGGAAATAT-----TATATTCTTTG-TG  79572
Cat          GGCTTCTTGCTATAAACCGTGGAAGTGAATTTTTCAA-----ATCTCTGATTTAGAAATAT-----TACATCCTTTA-TG  76396
Ferret       GCCTACTCGGTATAAACCATGGAAGTGAATTTTTCAG-----GTTTCTGACTTAGAAATAT-----CATATTCTTTA-TG  79234
Lion         GGCTTCTTGCTATAAACCGTGGAAGTGAATTTTTCAA-----ATCTCTGATTTAGAAATAT-----TACATCCTTTA-TG  74077
Vaquita      GGCTCCTCACTATGAACCATGGAAGTGAATTTTTCAA-----GTCTCTGACTTGGAGGT--------ATATTCTTTA-TG  71759
Pig          GGCGCCTCCCTATAAACCACAGAAGTGAATTTTTCAAAGTCAGTTTTCCAGTTGAAAGGTTGGAGAAATGTTTTTTTATG  71769

Majority     CTTTGTTTGCTTTTCCATAGGAAATGGAATATTGTATATTTAATTTTTT--AACATAATACAGGTATGTACCTTAGTTCT

                     103450    103460    103470    103480    103490    103500    103510    103520

Human        CTTTGTTTGCTTTTCCAGAGGAAATAGAGTATTATATATTTGATATTTT--AACATAATACAGGTATGTACTTTAGTTCA  83523
PolarBear    CTTTGTTTGCTTTTCCATAGGAAATGGAATATTGTATATTTAATTTTTT--AGCATACTACAGGTATGTACCTTAGCCCT  41334
Cow          CTTTGTTTGCTTTTCCATAGGAAATGGAATGTTATATATTTAATTTGT---GACATAATACAGGTATGTACCTTGGTTCT  78454
Dog          CTT----TGCTTTTCCATAGGAAATGGAATATTGTATATTTAATTTTTT--AACATACTACAGGTATGTACCTTAGCCCT  71460
BelugaWhale  CTGTGTTTGCTTTTCCATAGGAAATGGAATGTTGTATATTTAATTTTTTTTAACATAATACAGGTATGTACCTTAGTTCT  70548
Donkey       TTTTGTTTGCTTTTCCTCAGGAAATGGAATGTTGTGTATTTAATTTTTT--AACATAATACAGGTATGTACCTTAGTTCT  79650
Cat          CTTTGTTTGCTTTTTCATAGGAGATGGAATATGGTATATTTAATTTTTT--AACATA---CAGGTATGTACCTTAGCCCT  76471
Ferret       CTTTGTTTGCTTTTCCATAGGAAATGGAATATTGTGTATTTAATTTTTT--AGCATACTACAGGTATGTACCTTAGCCCT  79312
Lion         CTTTGTTTGCTTTTTCATAGGAGATGGAATATGGTATATTTAATTTTTT--AACATA---CAGGTATGTACCTTAGCCCT  74152
Vaquita      CTGTGTTTGCTTTTCCATAGGAAATGGAATGTTGTATATTTAATTTTTTTTAACATAATACAGGTATGTACCTTAGTTCT  71839
Pig          TTTTGTTTGCTTTTCCATAGGAAGTGGAATGTTGTATATTTAATTTTTT--AACATAATACAGGTATGTACCTCAGTTCT  71847
```

Majority	AATATTTAATGATTTCATTGA-AACCAGTTAATT-TTCTCT----GCTAC-TTATCAGCAAACCATTTTTCTAAATGTTA	
	103530 103540 103550 103560 103570 103580 103590 103600	
Human	AATAATTAATGGTTTTGTTGA-AATCAATT-ATTTTTCTTT----ACTAC-ACATCAGCAAACAATTTTTCTAAACACTA	83596
PolarBear	AATATTTAATGATTTCATTGA-AACCAGTTAATT-TTCTCTAACTGCTACATTATGAGCAAGCCATTTTTCTAAATGTTA	41412
Cow	AATACTTAATGATTTCATTGA-AACCAGTGAATT-TTCCCT----GCTAC-TTA------AACAGTTTTTCTAAATGTTA	78521
Dog	AATATTTAATGATTTCATTGATAACCAGTTAATT-TTCTCTAATTGCTATATTATCAGCAAACCGTTTTTCTAAACGTTA	71539
BelugaWhale	AATATTTAATGATTTCATTGA-AACCAATT-----TTATCT----ACTTA-TCAGCA---ACTAATTTTTCTAAATGTTA	70614
Donkey	AATAGTTAATGATTTCATTGA-AACCAGTTTATTTTTCTCTAACTGCTAC-ACATCAGCAAGTCATTTTTCTAAATGTTA	79728
Cat	AATATTTAATGATTTCATTGA-AACCAGTTAATT-TTCTCT----GATGCATTATCAGTAAACCATTTTTCTAAATGTTA	76545
Ferret	AATATTTAATGATTTCATTGA-AACCAGTTAATT-TTCTCT----GCTACATTATTAGCAAACCATTTTTCTAAATGTTA	79386
Lion	AATATTTAATGATTTCATTGA-AACCAGTTAATT-TTCTCT----GATGCATTATCAGTAAACCATTTTTCTAAATGTTA	74226
Vaquita	AATATTTAATGATTTCATTGA-AACCAATTAATT-TTATCT----ACTTA-TCAGCA---ACTAATTTTTCTAAATGTTA	71909
Pig	AATATGTATTGATTTCACTGA-AACCAGTTGATT-TTCTTT----ATTAC-TTACCACCAAACAATTTTTCTAAATATCA	71920

Majority	TAAAAATTAACTTTCAGGAACCATTTGGAATGGTCAGCCCTAAGCCATGCAGCCATCGTGCTTAAATTGGTTTTTGTAAA	
	103610 103620 103630 103640 103650 103660 103670 103680	
Human	TAAAAATTAACTTTCAGGAACCATTTGGAATGGTCAGCCGTAAGCCATGCAGCCATCGTGCTTAAATTGGTTTTTGTAAA	83676
PolarBear	CAAAAATTAACTTTCAGGAACCATTTGGAATGGTCAGCCCTAAGCCATGCAGCCATCGTGCTTAAATTGGTTTTTGTAAA	41492
Cow	CAAAAATTAACTTTCAGGAACCATTTGGAACGGTCAGCCCCAAGCCAT-CAGCCATCGTGCTTAAATTGGTTTT-GTAAA	78599
Dog	TAAAAATTAATTTTCAGGAACCATTTGGAATGGTCAGCCCTAAGCCATGCAGCCATCGTGCTTAAATTGGTTTTTGTAAA	71619
BelugaWhale	TAAAAATTAACTTTCAGGAACCATTTGGAACGGTCAGCCCTAAGCCGTGCAGCCATCGTGCTTAAATTGGTTTTTGTAAA	70694
Donkey	TAAAAATTAACTTTCAGGAACCATTTGGAACGGTCAGCCCAAAGCCATGTGGCTATCGTGCTTAAATTGGTTTTTGTAAA	79808
Cat	TAAAAATTAACTTTCAGGAACCATTTGGAATGGTCAGCCGTAAGCCATGCAGCCATCGTGCTTAAATTGGTTTTTGTAAA	76625
Ferret	TAAAAATTAACTTTCAGGAACCATTTGGAATGGTCAGCCCTAAGCCACGCAGCCATCGTGCTTAAATTGGTTTTTGTAAA	79466
Lion	TACAAATTAACTTTCAGGAACCATTTGGAATGGTCAGCCGTAAGCCATGCAGCCATCGTGCTTAAATTGGTTTTTGTAAA	74306
Vaquita	TAAACATTAACTTTCAGGAACCATTTGGAACGATCAGCCCTAAGCCGTGCAGCCATCGTGCTTAAATTGGTTTTTGTAAA	71989
Pig	TAAAAATTAATTTTCAGCAACCATTTGGAACGGTCAGCCCTAAGCCAAGCAGCCATCGTGCTTAAAATGGTTTTTGTAAA	72000

```
Majority     CGACTTGTGTGTGTTTCGTAAAATATGCTTTATTTTAAAATTAGGGCTTCCTGTTGCAGCTGTTCCAGGAGCTCTGAGTC

             103690    103700    103710    103720    103730    103740    103750    103760

Human        CGACTTGTGTGTGTTTCGTAAAATATGCTTTATTTTAAAATTAGGGCTTCCTGTTGCAGCTGTTCCAGGAGCTCTGAGTC  83756
PolarBear    CGACTTGTGTGTGTTTCGTAAAATATGCTTTATTTTAAAATTAGGGCTTCCTGTTGCAGCTGTTCCAGGAGCTCTGAGTC  41572
Cow          CGACTTGTGTGTGTTTCGTAAAATATGCTTTATTTTAAAATTAGGGCTTCCTGTTGCAGCTGTTCCAGGAGCTCTGAGTC  78679
Dog          CGACTTGTGTGTGTTTCGTAAAATATGCTTTATTTTAAAATTAGGGCTTCCTGTTGCAGCTGTTCCAGGAGCTCTGAGTC  71699
BelugaWhale  CGACTTGTGTGTGTTTCGTAAAATATGCTTTATTTTAAAATTAGGGCTTCCTGTTGCAGCTGTTCCAGGAGCTCTGAGTC  70774
Donkey       CGACTTGTGTGTGTTTCGTAAAATATGCTTTATTTTAAAATTAGGGCTTCCTGTTGCAGCTGTTCCAGGAGCTCTGAGTC  79888
Cat          CGACTTGTGTGTGTTTCGTAAAATATGCTTTATTTTAAAATTAGGGCTTCCTGTTGCAGCTGTTCCAGGAGCTCTGAGTC  76705
Ferret       CGACTTGTGTGTGTTTCGTAAAATATGCTTTATTTTAAAATTAGGGCTTCCTGTTGCAGCTGTTCCAGGAGCTCTGAGTC  79546
Lion         CGACTTGTGTGTGTTTCGTAAAATATGCTTTATTTTAAAATTAGGGCTTCCTGTTGCAGCTGTTCCAGGAGCTCTGAGTC  74386
Vaquita      CGACTTGTGTGTGTTTCGTAAAATATGCTTTATTTTAAAATTAGGGCTTCCTGTTGCAGCTGTTCCAGGAGCTCTGAGTC  72069
Pig          CGACTTGTGTGTGTTTCGTAAAATATGCTTTATTTTAAAATTAGGGCTTCCTGTTGCAGCTGTTCCAGGAGCTCTGAGTC  72080

Majority     CTTTGGCTATTCCAAATGCTGCTGCAGCAGCTGCTGCAGCTGCTGCTGGCCGAGTGGGTATGCCTGGAGTCTCAGCTGGT

             103770    103780    103790    103800    103810    103820    103830    103840

Human        CTTTGGCCATTCCAAATGCTGCTGCAGCAGCTGCTGCAGCTGCTGCTGGCCGAGTGGGTATGCCTGGAGTCTCAGCTGGT  83836
PolarBear    CTTTGGCTATTCCAAATGCTGCTGCAGCAGCTGCTGCAGCTGCTGCTGGCCGAGTGGGTATGCCTGGAGTCTCAGCTGGT  41652
Cow          CTTTGGCTATTCCAAACGCTGCTGCAGCAGCTGCTGCAGCTGCTGCTGGCCGAGTGGGTATGCCTGGAGTCTCAGCTGGT  78759
Dog          CTTTGGCTATTCCAAATGCTGCTGCAGCAGCTGCTGCAGCTGCTGCTGGCCGAGTGGGTATGCCTGGAGTCTCAGCTGGT  71779
BelugaWhale  CTTTGGCTATTCCAAATGCTGCTGCAGCAGCTGCTGCAGCTGCTGCTGGCCGAGTGGGTATGCCTGGAGTCTCAGCTGGT  70854
Donkey       CTTTGGCTATTCCAAATGCTGCTGCAGCAGCTGCTGCAGCCGCTGCTGGCCGAGTGGGTATGCCTGGAGTCTCAGCTGGT  79968
Cat          CTTTGGCTATTCCAAATGCTGCTGCAGCAGCTGCTGCAGCTGCTGCTGGCCGAGTGGGTATGCCTGGAGTCTCAGCTGGT  76785
Ferret       CTTTGGCTATTCCAAATGCTGCTGCAGCAGCTGCTGCAGCTGCTGCTGGCCGAGTGGGTATGCCTGGAGTCTCAGCTGGT  79626
Lion         CTTTGGCTATTCCAAATGCTGCTGCAGCAGCTGCTGCAGCTGCTGCTGGCCGAGTGGGTATGCCTGGAGTCTCAGCTGGT  74466
Vaquita      CTTTGGCTATTCCAAATGCTGCTGCAGCAGCTGCTGCAGCTGCTGCTGGCCGAGTGGGTATGCCTGGAGTCTCAGCTGGT  72149
Pig          CTTTGGCTATTCCAAACGCTGCTGCAGCAGCTGCTGCAGCTGCTGCTGGCCGAGTGGGTATGCCTGGAGTCTCAGCTGGT  72160
```

```
Majority     GGCAATACAGTCCTGTTGGTTAGCAATTTAAATGAAGAGGTTAGTAAAATAATTTTCTAATGTTTATTCTTT-AACTCCA
                               103850    103860    103870    103880    103890    103900    103910    103920
Human        GGCAATACAGTCCTGTTGGTTAGCAATTTAAATGAAGAGGTTAGTAAAATAATCT-CTAATGTTTATTCTTT-AACTCCA  83914
PolarBear    GGCAATACAGTCCTGTTGGTTAGCAATTTAAATGAAGAGGTTAGTAAAATAATTTTCTAATGTTTATTCTTT-AACTCCA  41731
Cow          GGCAATACAGTCCTGTTGGTTAGCAATTTAAATGAAGAGGTTAGTAAAATAATTTTCTGATGTCTATTTTTT-AACTCCA  78838
Dog          GGCAATACAGTCCTGTTGGTTAGCAATTTAAATGAAGAGGTTAGTAAAATAATTTTCTAATGTTTATTCTTT-AACTCCA  71858
BelugaWhale  GGCAATACAGTCCTGTTGGTTAGCAATTTAAATGAAGAGGTTAGTAAAATAATTTTCTAGTGTTTATTTTTT-AACTCCA  70933
Donkey       GGCAATACAGTCCTGTTGGTTAGCAATTTAAATGAAGAGGTTAGTAAAATAATTTTGTAATGTTTATTCTTT-AACTCCA  80047
Cat          GGCAATACAGTCCTGTTGGTTAGCAATTTAAATGAAGAGGTTAGTAAAATAATTTTGTAATGTTTATTCTTT-AACTCCA  76864
Ferret       GGCAATACAGTCCTGTTGGTTAGCAATTTAAATGAAGAGGTTAGTAAAATAATTTTCTAATGTTCATTTTTTTAACTCCA  79706
Lion         GGCAATACAGTCCTGTTGGTTAGCAATTTAAATGAAGAGGTTAGTAAAATAATTTTGTAATGTTTATTCTTT-AACTCCA  74545
Vaquita      GGCAATACAGTCCTGTTGGTTAGCAATTTAAATGAAGAGGTTAGTAAAATAATTTTCTAGTGTTTATTTTTT-AACTTCA  72228
Pig          GGCAATACGGTCCTGTTGGTTAGCAATTTAAATGAAGAGGTTAGTAAAATAATTTTCTAGTGTTTATTTTTT-AACTCCA  72239

Majority     TTTCATTTGTGAAAGTTTTTCATGTTTATTTCATTTTGCACTTGCCTTTCTTTTTATGTACATTCATTAGTCAAAGTATT
                               103930    103940    103950    103960    103970    103980    103990    104000
Human        TTTCATTTGTGAAAGTTTTTCATGTTTATTTCATTTTGCACTTGCCTTTCTTTTTATGTACATTCATTAGTCAAAGTATT  83994
PolarBear    TTTCATTTGTGAAAGTTTCTCATGTTTATTTCATTTTGCACTTGCCTTTCTTTTTATGTACATTCATTAGTCAAAGTATT  41811
Cow          TTTCATTTGTGAAAGTTTTTCATGTTTATTTCATTTTGCACTTGCCTTTCTTTTTATGTACATTCATTAGTCAAAGTATT  78918
Dog          TTTCATTTGTGAAAGTTTCTCATGTTTATTTCATTTTGCACTTGCCTTTCTTTTTATGTACATTCATTAGTCAAAGTATT  71938
BelugaWhale  TTTCATTTGTGAAAGTTTTTCATGTTTATTTCATTTTGCACTTGCCTTTCTTTTTATGTACATTCATTAGTCAAAGTATT  71013
Donkey       TTTCATTTGTGGAAGTTTTTCATGTTTATTTCATTTTGCACTTGCCTATCTTTTTATGTACATTCATTAGTCAAAGTATT  80127
Cat          TTTCATTTGTGAAAGTTTCTCATGTTTATTTCATTTTGCACTTGCCTTTCTTTTTATGTACATTCATTAGTCAAAGTATT  76944
Ferret       TTTCATTTGTGTAAGTTTCTCATGTTTATTTCATTTTGCACTTGCCTTTCTTTTTATGTACATTCATTAGTCAAAGTATT  79786
Lion         TTTCATTTGTGAAAGTTTCTCATGTTTATTTCATTTTGCACTTGCCTTTCTTTTTATGTACATTCATTAGTCAAAGTATT  74625
Vaquita      TTTCATTTGTGAAAGTTTTTCATGTTTATTTCATTTTGCACTTGCCTTTCTTTTTATGTACATTCATTAGTCAAAGTATT  72308
Pig          TTTCATTTGTGGAAGTTTTTCATGTTTATTTCATTTTGCACTTGCCTTTCTTTTTATGTACATTCATT-G--AA-GTATT  72315
```

```
Majority     TTCTGTATGTTTCTACTTCTGAATAAAATCTATGTACTTGTTTAGGTAATAXXTTATTTCCTTAGACAGTCATATCT---
                      104010    104020    104030    104040    104050    104060    104070    104080
Human        TTCTGTATGTTTCTACTTCTGAATAAAATCTACATA-TTGTTTAGGTAATACTTTATTTCCTTAAACAGTCATGTCT---  84070
PolarBear    TTATGTATGTTTCTACTTCTGAATAAAATCTATGTACTTGTTTAGGTAATA-----TTTCCTTAG----TCATATCT---  41879
Cow          TTCTGTGTGTTTCTACTTCCAAATAAAATCTATGTACTTGTTTAGGTAATACTTTATTTCTGTAGCTAGTCATATCT---  78995
Dog          TTCTGTATGTTTCTACTTCTGAATAAAATCTATGTACTTGTTTAGGTAATA-----TTTCCTTAG----TCATATCT---  72006
BelugaWhale  TTCTGTGTGTTTCTACTTCCGAATAAAATCTGTGTACTTGTTTAGGTAATACTTTATTTCCGTAGACAGTCATATCT---  71090
Donkey       TTCTGTATGTTTCTACTTCTGAATAAAATCTATGTACTTGTTTAAGTAATATTTTATTTCTTTAGACAGTCATATCT---  80204
Cat          TTCTGTACGTTTCTATTTCTGAATAAAATCTATGTATTTGTTTAGGTAATA-----TTTCCCTAGGCAGTCATATCT---  77016
Ferret       TTCTGTATGTTTCTACTTCTGAATAAAATCTATGTATTTGTTTAGGTAATA-----TTTCCTTAGACAGTCATATCTATC  79861
Lion         TTCTGTACGTTTCTATTTCTGAATAAAATCTATGTATTTGTTTAGGTAATA-----TTTCCCTAGGCAGTCATATCT---  74697
Vaquita      TTCTGTGTGTTTCTACTTCCGAATAAAATCTATGTACTTGTTTGGGTAATACTTTATTTCCGTAGACAGTCATATCT---  72385
Pig          TTCTGTGTGTTTCTACT-CTGAATAAAATCTATGTACTTGTTTAGGTAATACCTTATTTCCTTAGATGGTCATATCT---  72391

Majority     ---CTTTTTATATATATAT----ATATTTATTXT-----TT----------------TTAACCTAACTACCTATTTTTCC
                      104090    104100    104110    104120    104130    104140    104150    104160
Human        -----CTTTATATCTATATCTATATATGTATATT-----TT----------------TTAACCTAA---CCTATTTTTCC  84121
PolarBear    --------TATATATATATA--TATAATTATTATTATCATT----------------TTAACCTAACTACCTATTTTTCC  41933
Cow          ---CTTTTAATATATATAT----ATATATAT--------TT----------------TTAACCTAACTACCTATTTTTCC  79044
Dog          ---CTTTTTATATATATAAT--TATTATTATAATTATTATTATTATT---------TTTAATGTAACTACCTATTTTTCC  72072
BelugaWhale  ---CTTTTTATATATATAT----ATATATATA-------TT----------------TTAACCTAACTACCTATTTTTCC  71140
Donkey       -----CTTTATATATATAT----ATA--TAT--------TT----------------TTAACCTAATTACCTATTTTTCC  80249
Cat          ---CTTTATATATATATAT----ATATTTATTATTATTATA-TTATTATTAT----TTTAACCTAACTACCTATTTTTCC  77084
Ferret       TATCTATCTATATATATATA--TATATTTACTATTATTATTATTATTATTATTATTTTTAACCTAACTACCTATTTTTCC  79939
Lion         ---CTTTATATATATATATTATTATTATTATTATTATTATT-ATATTATTAT----TTTAACCTAACTACCTATTTTTCC  74769
Vaquita      ---CTTTATATATATATAT----ATATTTTTT-------TT----------------TTAACCTAACTACCTATTTTTCC  72435
Pig          ---CTTTTTATATATATAT----AT---TTT--------TT----------------TTAACCTAACTACCTATTTTTCC  72437
```

```
Majority     TAAGAAAAATATGGTGAAGTACTATAGTXTGGCTTTTT--CTTTTTGTTGTTCTCAAA-GGCAAATGTGATCTAATTTGC
                              104170    104180    104190    104200    104210    104220    104230    104240
Human        TAAGAAAAATATGGTGAAGTACTGTAGTTTGGCTTATC---TTTTTGTTGTTCTCAAA-GGCAAATGTGATCTAATTTGC  84197
PolarBear    TAAGAAAAATATGGTGAAGTACTATAGTATGGCTTTTT--CTTTTTGTTGTTCTCAAA-GGCAAATGTGATCTAATTTGC  42010
Cow          TAAGAAAAATATGGTGAAGTACTATAGTTTGGCTTTTTATCTTTTTGTTGTTCTCAAA-GGCAAAT---ATCTAATTTGC  79120
Dog          TAAGAAAAATATGGTGAAGTACTATAGTATGGCTTTTT--CTTTTTGTTGTTCTCAAA-GGCAAATGTGATCTAATTTGC  72149
BelugaWhale  TAAGAAAAATATGGTGAAGTACTGTAGTCTGGCTTTTTATCTTTTTGTTGTTCTCAAAAGGCAAATGTGATCTAATTTGC  71220
Donkey       TAAGAAAAATATGGTGAAGTACCATAGTTTGGCTTTTTATCGTTTTGTTACTCTCAAA-GGCAGATATGATCTAATTTGC  80328
Cat          TAAGAAAAATATGGTGAAGTACTGTAGTATGGCTTTTT--CTTTCTGTTGTTCTCAAA-GGCAAATGTGATCTAATTTGC  77161
Ferret       TAAGAAAAATATGGTGAAGTACTATAGTATTGCTTTTT--CTTTTTGTTGTTCTCAAA-GGCAAATGTGATCTAATTTGC  80016
Lion         TAAGAAAAATATGGTGAAGTACTGTAGTATGGCTTTTT--CTTTCTGTTGTTCTCAAA-GGCAAATGTGATCTAATTTGC  74846
Vaquita      TAAGAAAAATATGGTGAAGTACTGTAGTTTGGCTTTTTATCTTTTTGTTGTTCTCAAAAGGCAAATGTGATCTAATTTGC  72515
Pig          TAAGAAAAATATGGTGAAGTACTATAGTTTGGCTTTTTATCTTTTTGTTGTTCTCAAA-GGCAAATGTGATCTAATTTGC  72516

Majority     AGATTT-AACTGTAAAACAATTTTGCTCTTTGCCTTTTCTAATTATTTGCTTGTTCATTTCATGCTTATGTGTCATTGCA
                              104250    104260    104270    104280    104290    104300    104310    104320
Human        AAATTT-AACTGTAAAACAATTTTGCTCTTTGCCTTTTCTAATTATTTGCTTGTTCATTTCATGCTTATATGTCATTGCA  84276
PolarBear    AGATTT-AACTGTAAAACAATTTTGTTCTTTGCCTTTTCTAATTATTTGCTTGTTCATTTCATGCTTATGTGTCATTGCA  42089
Cow          AGATGT-AACTGTAAAACAATTTTGCTCTTTGCCTTTTCTAATTATTTGCTTGTTCATTTCATGCTTATGTGTCATTGCA  79199
Dog          AGATTTTAACTGTAAAACAATTTTGTTCTTTGCCTTTTCTAATTATTTGCTTGTTCATTTCATGCTTATGTGTCATTGCA  72229
BelugaWhale  AGATGT-AACTGTAAAACAATTTTGCTCTTTGCCTTTTCTAATTATTTGCTTGTTCATTTCATGCTTATGTGTCATTGCA  71299
Donkey       AGATTT-AACTGTAAAACAATTTTGCTCGTTGCCTTTTCTAATTATTTGCTTGTTCATTTCATGCTTATGTGTCATTGCA  80407
Cat          AGATTT-AACTGTAAAACAATTTTGTTCTTTGCCTTTTCTAATTATTTGCTTGTTCATTTCATGCTTATGTGTCATTGCA  77240
Ferret       AGATTT-AACTGTAAAACAATTTTGTTCTTTGCCTTTTCTAATTATTTGCTTGTTCATTTCATGCTTATGTGTCATTGCA  80095
Lion         AGATTT-AACTGTAAAACAATTTTGTTCTTTGCCTTTTCTAATTATTTGCTTGTTCATTTCATGCTTATGTGTCATTGCA  74925
Vaquita      AGATGT-AACTGTAAAACAATTTTGCTCTTTGCCTTTTCTAATTATTTGCTTGTTCATTTCATGCTTATGTGTCATTGCA  72594
Pig          AGATGT-AACTGTAAAACAATTTTGCTCTTTGCCTTTTCTAATGATTTGCTTGTTCATTTCATGCTTATGTGTCATTGCA  72595
```

```
Majority     TTTTTTT-AAATCTTATTTTATGTGAACTACTCTAATACATTTTTCTTTGAAGGTTCTTCCAAAGATCTTGACGAGGCAC

             104330    104340    104350    104360    104370    104380    104390    104400

Human        TTTTTTTTAAATCTTATTTTATGTGAACTACTCTAATACATTTTTCTTTGAAGGTTCTTCCAAAGATCTTGACGAGGCAC  84356
PolarBear    TTTTTTT-AAATCTTATTTTATGTGAACTACTCTAATACATTTTTCTTTGAAGGTTCTTCCAAAGATCTTGACGAGGCAC  42168
Cow          TTTTTTT-AAATCTTATTTTATGTGAACTACTCTAATACATTTTTCTTTGAAGGTTCTTCCAAAGATCTTGACGAGGCAC  79278
Dog          TTTTTTT-AAATCTTATTTTATGTGAACTACTCTAATACATTTTTCTTTGAAGGTTCTTCCAAAGATCTTGACGAGGCAC  72308
BelugaWhale  TTTTTTT-AAATCTTATTTTATGTGAACTACTCTAATACATTTTTCTTTGAAGGTTCTTCCAAAGATCTTGACGAGGCAC  71378
Donkey       TTTTTT--AAATATTATTTTATGTGAACTACTCTAATACATTTTTCTTTGAAGGTTCTTCCAAAGATCTTGACGAGGCAC  80485
Cat          TTTTTTT-AAATCTTATTTTATGTGAACTACTCTAATACATTTTTCTTTGAAGGTTCTTCCAAAGATCTTGACGAGGCAC  77319
Ferret       TTTTTTT-AAATCTTATTTTATGTGAACTACTCTAATACATTTTTCTTTGAAGGTTCTTCCAAAGATCTTGACGAGGCAA  80174
Lion         TTTTTTT-AAATCTTATTTTATGTGAACTACTCTAATACATTTTTCTTTGAAGGTTCTTCCAAAGATCTTGACGAGGCAC  75004
Vaquita      TTTTTTT-AAATCTTATTTTATGTGAACTACTCTAATACATTTTTCTTTGAAGGTTCTTCCAAAGATCTTGACGAGGCAC  72673
Pig          TTTTTTT-AAATCTTATTTTATGTGAACTACTCTAATACATTTTTCTTTGAAGGTTCTTCCAAAGATCTTGACGAGGCAC  72674

Majority     TCTTCCAGTCTTTCTTAGTAATTTTTTCTTTGCAGTTATTAGTCATTGTCTTCTAAAAATATTTTTCAACTTTATCCCCC

             104410    104420    104430    104440    104450    104460    104470    104480

Human        TCTTCCCGTCTTTCTTAGTAATTTTTTCTTTGCAGTTATTAGTCATTGTCTTCTAAAAATATTTTTTAACTTTATTCCCC  84436
PolarBear    TCTTCCAGTCTTTCTTAGTAATTTTTTCTTTGCAGTTATTAGTCATTGTCTTCTAAAAATATTTTTCAACTTTATCCCCC  42248
Cow          TCTTCCAGTCTTTCTTAGTAATTTTTTCTTTGCAGTTATTAGTCATTGTCTTCTAAAAATATTTTTCAACTTTACCTCTC  79358
Dog          TCTTCCAGTCTTTCTTAGTAATTTTTTCTTTGCAGTTATTAGTCATTGTCTTCTAAAAATATTTTTCAACTTTATCCCCT  72388
BelugaWhale  TCTTCCAGTCTTTCTTAGTAATTTTTTCTTTGCAGTTATTAGTCATTGTCTTCTAAAAATATTTTTCAACTTTACCCCTC  71458
Donkey       TCTTCCAGTCTTTCTTAGTAATTTTTTCTTTGCAGTTATTAGTCATTGTCTTCTAAAAATATTTTTCAACTTTATTCCCC  80565
Cat          TCTTCCAGTCTTTCTTAGTAATTTTTTCTTTGCAGTTATTAGTCATTGTCTTCTAAAAATATTTTCCAACTTTATTCCCC  77399
Ferret       TCTTCCAGTCTTTCTTAGTAATTTTTTCTTTGCAGTTATTAGTCATTGTCTTCTAAAAATAATTTTCAACTTTATCCCCC  80254
Lion         TCTTCCAGTCTTTCTTAGTAATTTTTTCTTTGCAGTTATTAGTCATTGTCTTCTAAAAATATTTTTCAACTTTATTCCCC  75084
Vaquita      TCTTCCAGTCTTTCTTAGTAATTTTTTCTTTGCAGTTATTAGTCATTGTCTTCTAAAAATATTTTTCAACTTTACCCCTC  72753
Pig          TCTTCCAGTCTTTCTTAGTAATTTTTTCTTTGCAGTTATTAGTCATTGTCTTCTTAAAATATTTTTCAACTTTATCCCCC  72754
```

Majority	CC----C-------------CC-------CCCTGTTAAATAAAATTTATTGCTGAGTTATTTTCTTTAGTTGTACATTTT	
	104490 104500 104510 104520 104530 104540 104550 104560	
Human	CACCCCTTCCTTCCTTACCACCCCCTCCACCCAGTTAAATAAAATTTATTGCTAAGTTATTTTCTTTAGTTGTACATTTT	84516
PolarBear	CC----T-------------T------------GTTAAATAAAATTTATTGCTGAGTTATTTTCTTTAGTTGTACATTTT	42299
Cow	CCCAAAC-------------CC-CTCCCACCCTGTTAAATAAAATTTATTGCTGAGTTATTTTCTTTAGTTGTACATTTT	79424
Dog	CC----C-------------CC-------C-TTGTTAAATAAAATTTATTGCTGAGTTATTTTCTTTAGTTGTACATTTT	72443
BelugaWhale	CCTGAAC-------------CC-CTCCCACCCCCTTAAATAAAGTTTATTGCTGAGTTATTTTCTTTAGTTGTACATTTT	71524
Donkey	CC----------------------------CTTGTTAAATAAAATTTATTGCTG--TTATTTTCTTTAGTTGTACATTTT	80615
Cat	CT----C-------------CC-------CCTTGTTAAATAAAATTTATTGCTGAGTTATTTTCTTTAGTTGTACATTTT	77455
Ferret	CC----T-------------CC-------C-CCGTTAAATAAAATTTATTGCTGAGTTATTTTCTTTAGTTGTACATTTT	80309
Lion	CT----C-------------CC-------CCTTGTTAAATAAAATTTATTGCTGAGTTATTTTCTTTAGTTGTACATTTT	75140
Vaquita	CCTGAAC-------------CC-CTCCCACCCCCTTAAATAAAGTTTATTGCTGAGTTATTTTCTTTAGTTGTACATTTT	72819
Pig	CCCGACC-------------CCACCCCCACCCTGTTAAATAAAATTTATTGCTGAGTTATTTTTTTTAGTTGTACATTTT	72821

Majority	ATGTCTGGTATATTTTATTTTGATTGCTATGAAAGCTGGTATGAAATGTGGGAAG---CTCAATGTATCAGTTTATAATG	
	104570 104580 104590 104600 104610 104620 104630 104640	
Human	ATGTCTGGTATATTTTATTTTGAATGCTATGAAAGCTGGTATGAAATGTGGGAAG---CTCAGTGTATCAGTTTATGATG	84593
PolarBear	ATGTCTGGTATATTTTATTTTGATTGCTATGAAAGCTGGTATGAAATGTGGGAAG---CTCGATGTATCAGTTTACAATG	42376
Cow	ATGTCTGGTATATTTTATTTTGATTGCTATGGAAGCTGGTATGAAATGTGGGAAG---CTCAATGTATCAGTTTATGATG	79501
Dog	ATGTCTGGTATATTTTATTTTGATTGCTATGAAAGCTGGTATGAAATGTGGGAAG---CTCAGTGTATCAGTTTACAATG	72520
BelugaWhale	ATGTCTGGTATATTTTATTTTGATTGCTATGGAAGCTGGTATGAAATGTGGGAAG---CTCAGTGTATCAGTTTATGATG	71601
Donkey	ATGTCTGGTATATTTTATTTTGATTGCTATGAAAGCTGGTATGAAATGTGGGAAG---CTAAATGTATTAGTTTACAATG	80692
Cat	ATGTCTGGTATATTTTATTTTGATTGCTATGAAAGCTGGTATGAAATGTGGGAAG---CTCAGTGTATCAGTTTATAATG	77532
Ferret	ATGTCTGGTATATTTTATTTTGATTGCTATGAAAGCTGGTATGAAATGTGGGAAG---CTCAATGTATCAGTTTACAATG	80386
Lion	ATGTCTGGTATATTTTATTTTGATTGCTATGAAAGCTGGTATGAAATGTGGGAAG---CTCAGTGTATCAGTTTACAATG	75217
Vaquita	ATGTCTGGTATATTTTATTTTGATTGCTATGGAAGCTGGTATGAAATGTGGGAAG---CTCAATGTATCAGTTTATGATG	72896
Pig	ATGTCTGGTATATTTTATTTTGATTGCTGTGAAAGCTGGTATGAAATGTGGGAAGAAGCTCAATGTATCTGTTTCTGATG	72901

```
Majority     TCTAATTTGAATATTTGTTTCATTTTTAATTGGCCTCTGTTAATACGAACATTAAGCTTATTTTATC-ATTTAATACTAT

                      104650    104660    104670    104680    104690    104700    104710    104720

Human        TCTAATTTGAATATTTGTTTCATTTTTAATTGGCCTCTGTTAATATGAACATTA-GCTTATTTTATCCATTTAATACTAT  84672
PolarBear    TCTAAAT---------ATTTCATTTTTAATTGGCCTCTGTTAATACGAACATTAAGCTTATTTTATC-ATTTAATACTAT  42446
Cow          TCTAATTTGAA----TATTTCATTTTTAATTGGCCTCTGTTAATACGAACATTAAGCT-ATTTTATC-ATTTAATACTAT  79575
Dog          TCTAATTTGAATATTTGTTTCATTTTTAATTGGCCTCTGTTAATACGAACATTAAGCTTATTTTATC-ATTTAATACTAT  72599
BelugaWhale  TCTAATTTGAA----TATTTCATTTTTAATTGGCCTCTGTTAATATGAACATTAAGCT-ATTTTATC-ATTTAGTACTAT  71675
Donkey       TCTAATTTGAGTATTTGTTTCATTTTTGATTGGCCTCTGTTAATATGAACATTAAGCTTATTTTATC-ATTTAATACTAT  80771
Cat          TCTAATTTGAATATTTGTTTCATTTTTAATTGGCCTCTGTTAATACGAACATTAAGCTTATTTTATC-ATTTGATACTAT  77611
Ferret       TCCAATTTTAATATTTGTTTCATTTTTAATTGGCCTCTGTTAATACGAACATTAAGCTTATTTTATC-ATTTAATACTGT  80465
Lion         TCTAATTTGAATATTTGTTTCATTTTTAATTGGCCTCTGTTAATACGAACATTAAGCTTATTTTATC-ATTTGATACTAT  75296
Vaquita      TCTAATTTGAA----TATTTCATTTTTAATTGGCCTCTGTTAATATGAACATTAAGCT-ATTTTATC-ATTTAGTACTAT  72970
Pig          TCCAATTTGAA----TATTTCATTTTTAATTGGCCTCTGTTAATATGTACATTAAACT-ATTTT----ATTTACTACTGT  72972

Majority     TGTCATTCACAGTTCTGCATGCTAAAATGTTTGAATCAGAGTGCCTTTGTTTTTCTTTTAAGAATTTTGCCTGCATTTCA

                      104730    104740    104750    104760    104770    104780    104790    104800

Human        TATCATTCACAGTTCTGCATGCTAAAATGTTTGAATCAGAGTGCCTTTGTTTTTATTTTAAGACTTTTGCCTGCATTTCA  84752
PolarBear    TGTCATTCACAGTTCTGCATGCTAAAATGTTTGAATCAGAGTGCCTTTGTTTTTCTTTTAAGAATTTTGCCTGCATTTCA  42526
Cow          AGTCATTCACAGTTGTGCATGCTAAAATGTTTGAATCAGAGTGCCTTTGTTTTTCTTTTAAGAATTTTGCCTGCATTTCA  79655
Dog          TGTCATTCACAGTTCTGCATGCTAAAATGTTTGAATCAGAGTGCCTTTGTTTTTCTTTTAAGAATTTTGCCTGCATTTCA  72679
BelugaWhale  TGTCATCCACAGTTCTGCATGCTAAAATGTTTGAATCAGAGTGCCTTTGTTTTTCTCTTAAGAATTTTGCCTGCATTTCA  71755
Donkey       TGTCATTCACAGTTCTGCATGCTAAAATGTTTGAATCAGAGTGCCTTTGTTTTTCTTTTAAGAATTTTGCCTGCATTTCA  80851
Cat          TGTCATTCACAGTTCTGCATGCTAAAATGTTTGAATCAGAGTGCCTTTGTTTTTCTTTTAAGAATTTTGCCTGCATTTCA  77691
Ferret       TGTCATTCACAGTTCTGCATGCTAAAATGTTTGAATCAGAGTGCCTTTGTTTTTCTTTTAAGAATTTTGCCTGCATTTCA  80545
Lion         TGTCATTCACAGTTCTGCATGCTAAAATGTTTGAATCAGAGTGCCTTTGTTTTTCTTTTAAGAATTTTGCCTGCATTTCA  75376
Vaquita      TGTCATCCACAGTTCTGCATGCTAAAATGTTTGAATCAGAGTGCCTTTGTTTTTCTCTTAAGAATTTTGCCTGCATTTCA  73050
Pig          TGTCATTCACAGTTCTGCATGCTAAAATGTTTGAATCAGAGTGCCTTTG------TTACAAGAATTTTGCCTGCATTTCA  73046
```

```
Majority     TAACCAGCCATGCTTATGCAGTT-AAAGTTCAAAGTTT-AAAATTCCTGTGCATGCTTT-CCTTCCCTATGTTGAGATGA
                      104810    104820    104830    104840    104850    104860    104870    104880
Human        TAACCAGCCATGCTTATGCAGTT-AAAGTTCAAAGTTT-AAAATTCCTATGCATGCTTT-CCTTCCCTATGTTGAGATGA  84829
PolarBear    TAACCAGCCATGCTTATGCAGTT-AAAGTTCAAAGTTT-AAAATTCCTGTGCATGCTTT-CCTTCCCTATGTTGAGATGA  42603
Cow          TAACCAGCCATGCTTATGCAGTTAAAAGTTCAAAGTTT-AAAATTCCTATGCATGCTTC-CCTTCCCTATGTTGAGATGA  79733
Dog          TAACCAGCCATGCTTATGCAGTT-AAAGTTCAAAGTTT-AAAATTCCTGTGCATGCTTTTCCTTCCCTATGTTGAGATGA  72757
BelugaWhale  TAACCAGCCATGCTTATGCAGTT-AAAGTTCAAAGTTT-AAAGTTCCTGTGCATGCTTT-CCTTCCCTATGTTGAGATGA  71832
Donkey       TAACCAGCCATGCTTATGCAGTG--AAGTTCAAAGTTTTAAAATTCCTATGCATGCTTT-CCTTCCCTATGTTGAAATGA  80928
Cat          TAACCAGCCATGCTTATGCAGTT-AAAGTTCAAAGTTT-AAAATTCCTGTGCATGCTTT-CCTTCCCTATGTTGAGATGA  77768
Ferret       TAACCAGCCATGCTTATGCAGTT-AAAGTTCAAAGTTT-AAAATTCCTGTGCATGCTTT-CCTTCCCTATGTTGAGATGA  80622
Lion         TAACCAGCCATGCTTATGCAGTT-AAAGTTCAAAGTTT-AAAATTCCTGTGCATGCTTT-CCTTCCCTATGTTGAGATGA  75453
Vaquita      TAACCAGCCATGCTTATGCAGTT-AAAGTTCAAAGTTT-AAAGTTCCTGTGCATGCTTT-CCTTCCCTATGTTGAGATGA  73127
Pig          TAACCAGCCATGCTTATGCAGTTTAAAGTTCAAAGTTT-AAA-TTCCTATGCATGCTTT-CCTTCCCTATGTTGAGATGA  73123

Majority     AATGCTGTAATTTACTCTTTGATATAGATGTACTTTACCCATATTTGTCTTGGATGACTACATTTTACTCAGTTTTTCTT
                      104890    104900    104910    104920    104930    104940    104950    104960
Human        AATGCTGTAATTTACTCTTTGATATAGATGTACTTTACCCATATTTGTCTTGGATGACTACATTTTACTCAGTTTTTCTT  84909
PolarBear    AATGCTGTAATTTACTCTTTGATATAGATGTACTTTACCCATATTTGTCTTGGATGACTACATTTTACTCAGTTTTTCTT  42683
Cow          AATGCTGTAATTTACTCTTTGATATAGATGTACTTTACCCATATTTGTCTTGGATGACTACATTTTACTCAGTTTTTCTT  79813
Dog          AATGCTGTAATTTACTCTTTGATATAGATGTACTTTACCCATATTTGTCTTGGATGACTACATTTTACTCAGTTTTTCTT  72837
BelugaWhale  AATGCTGTAATTTACTCTTTGATATAGATGTACTTTACCCATATTTGTCTTGGATGACTACATTTTACTCAGTTTTTCTT  71912
Donkey       AATGCTGTAATTTACTCTTTGATATAGATGTACTTTACCCATATTTGTCTTGGATGACTACATTTTACTCAGTTTTTCTT  81008
Cat          AATGCTGTAATTTACTCTTTGATATAGATGTACTTTACCCATATTTGTCTTGGATGACTACATTTTACTCAGTTTTTCTT  77848
Ferret       AATGCTGTAATTTACTCTTTGATATAGATGTACTTTACCCATATTTGTCTTGGATGACTACATTTTACTCAGTTTTTCTT  80702
Lion         AATGCTGTAATTTACTCTTTGATATAGATGTACTTTACCCATATTTGTCTTGGATGACTACATTTTACTCAGTTTTTCTT  75533
Vaquita      AATGCTGTAATTTACTCTTTGATATAGATGTACTTTACCCATATTTGTCTTGGATGACTACATTTTACTCAGTTTTTCTT  73207
Pig          AATGCTGTAATTTACTCTTTGATATAGATGTACTTTACCCATATTTGTCTTGGATGACTACATTTTACTCAGTTTTTCTT  73203
```

```
Majority     GTACAGTACATAAACCAACCATTTTCTGACCAAATTCCTGCATTTCCTATGTACTGACCTATATTTTATTTTTTTTTT-G
                      104970    104980    104990    105000    105010    105020    105030    105040
Human        GTACAGTACATAAACCAACCATTTTCTGACCAAATTCCTGCATTTCCTATGTACTGACCTATATTTTATTTTGTTTTT-G  84988
PolarBear    GTACAGTACATAAACCAACCATTTTCTGACCAAATTCCTGCATTTCCTATGTACTGACCTATATTTTATTTTTTTTTT-G  42762
Cow          GTACAGTACATAAACCAACCATTTTCTGACCAAAT-CCTGCATTTCCTATGTACTGACCTATATTTTATTTTTTTTTTTG  79892
Dog          GTACAGTACATAAACCAACCATTTTCTGACCAAATTCCTGCATTTCCTATGTACTGACCTATATTTTATTTTTTTTTT-G  72916
BelugaWhale  GTACAGTACATAAACCAACCATTTTCTGACCAAAT-CCTGCATTTCCTATGTACTGACCTATATTTTATTTTTTTTTT-G  71990
Donkey       GTACAGTACATAAACCAACCATTTTCTGACCAAATTCCTGCATTTCCTATGTACTGACCTATATTTTATTTTTTTTTTTG  81088
Cat          GTACAGTACATAAACCAACCATTTTCTGACCAAATTCCTGCATTTCCTATGTACTGACCTATATTTTATTTTTTTTTT-G  77927
Ferret       GTACAGTACATAAACCAACCATTTTCTGACCAAATTCCTGCATTTCCTATGTACTGACCTATATTTTATTTTTTTTTT-G  80781
Lion         GTACAGTACATAAACCAACCATTTTCTGACCAAATTCCTGCATTTCCTATGTACTGACCTATATTTTATTTTTTTTTT-G  75612
Vaquita      GTACAGTACATAAACCAACCATTTTCTGACCAAAT-CCTGCATTTCCTATGTACTGACCTATATTTTATTTTTTTTTT-G  73285
Pig          GTACAGTACATAAACCAACCATTTTCTGACCAAAT-CCTGCATTTCCTATGTACTGACCTATATTTTATTTTTTTTTTTG  73282

Majority     TTCCCCAATTCCTTATTTTTTTTCTTCTGCATTGCTGTTTCCCTTCCCCATTTCATCCTTT-TCCCTGTGTGTTCACCTTC
                      105050    105060    105070    105080    105090    105100    105110    105120
Human        TTCCCCAATTCCTTATTTTTTTTCTTCTGCATTGCTGTTTCCCTTCCCCATTTCATCCTTT-TCCCTGTGTGTTCACCTTC  85067
PolarBear    TTCCCCAATTCCTTATTTTTTTTCTTCTGCATTGCTGTTTCCCTTCCCCATTTCATCCTTTTCCCCTGTGTGTTCACCTTC  42842
Cow          TTCCCCGATTCCTTATTTTTTTTCTTCTGCATTGCTGTTTCCCTTCCCCATTTCATCCTTT-TCCCTGTGTGTTCACCTTC  79971
Dog          TTCCCCAATTCCTTATTTTTTTTCTTCTGCATTGCTGTTTCCCTTCCCCATTTCATCCTTTTCCCCTGTGTGTTCACCTTC  72996
BelugaWhale  TTCCCCAATTCCTTATTTTTTTTCTTCTGCATTGCTGTTTCCCTTCCCCATTTCATCCTTT-TCCCTGTGTGTTCACCTTC  72069
Donkey       TTCCCCAATTCCTTATTTTTTTTCTTCTGCATTGCTGTTTCCCTTCCCCATTTCATCCTTT-TCCCTGTGTGTTCACCTTC  81167
Cat          TTCCCCAATTCCTTATTTTTTTTCTTCTGCATTGCTGTTTCCCTTCCCCATTTCATCCTTT-TCCTTGTGTGTTCACCTTC  78006
Ferret       TTCCCCAATTCCTTATTTTTTTTCTTCTGCATTGCTGTTTCCCTTCCCCATTTCATCCTTT-CCCCTGTGTGTTCACCTTC  80860
Lion         TTCCCCAATTCCTTATTTTTTTTCTTCTGCATTGCTGTTTCCCTTCCCCATTTCATCCTTT-TCCCTGTGTGTTCACCTTC  75691
Vaquita      TTCCCCAATTCCTTATTTTTTTTCTTCTGCATTGCTGTTTCCCTTCCCCATTTCATCCTTT-TCCCTGTGTGTTCACCTTC  73364
Pig          TTCCCCGATTCCTTATTTTTTTTCTTCTGCATTGCTGTTTCCCTTCCCCATTTCATCCTTT-TCCCTGTGTGTTCACCTTC  73361
```

Majority	CCTTTCCTTGTCTTTTCCCAAATGCCCATTCCCTTCCCTGTCTTATCCTTTATTTTCCTTGTCCTTGTCTTCATTCCCTG	
	105130 105140 105150 105160 105170 105180 105190 105200	
Human	CCTTTCCTTGTCTTTTCCCAAATGCCCATTCCCTTCCCTGTCTTATCCTTTATTTTCCTTGTCCTTGTCTTCATTCCCTG	85147
PolarBear	CCTTTCCTTGTCTTTTCCCAAATGCCCATTCCCTTCCCTGTCTTATCCTTTATTTTCCTTGTCCTTGTCTTCATTCCCTG	42922
Cow	CCTTTCCTTGTCTTTTCCCAAATGCCCATTCCCTTCCCTGTCTTATCCTTTATTTTCCTTGTCCTTGTCTTCATTCCCTG	80051
Dog	CCTTTCCTTGTCTTTTCCCAAATGCCCATTCCCTTCCCTGTCTTATCCTTTATTTTCCTTGTCCTTGTCTTCATTCCCTG	73076
BelugaWhale	CCTTTCCTTGTCTTTTCCCAAATGCCCATTCCCTTCCCTGTCTTATCCTTTATTTTCCTTGTCCTTGTCTTCATCCCCTG	72149
Donkey	CCTTTCCTTGTCTTTTCCCAAATGCCCATTCCCTTCCCTGTCTTATCCTTTATTTTCCTTGTCCGTGTCTTCATTCCCTG	81247
Cat	CCTTTCCTTGTCTTTTCCCAAATGCCCATTCCCTTCCCTGTCTTATCCTTTATTTTCCTTGTCCTTGTCTTCATTCCCTG	78086
Ferret	CCTTTCCTTGTCTTTTCCCAAATGCCCATTCCCTTCCCTGTCTTATCCTTTATTTTCCTTGTCCTTGTCTTCATTCCCTG	80940
Lion	CCTTTCCTTGTCTTTTCCCAAATGCCCATTCCCTTCCCTGTCTTATCCTTTATTTTCCTTGTCCTTGTCTTCATTCCCTG	75771
Vaquita	CCTTTCCTTGTCTTTTCCCAAATGCCCATTCCCTTCCCTGTCTTATCCTTTATTTTCCTTGTCCTTGTCTTCATCCCCTG	73444
Pig	CCTTTCCTTGTCTCTTCCCAAATGCCCATTCCCTTCCCTGTCTTATCCTTTATTTTCCTTGTCCTTGTCTTCATTCCCTG	73441

Majority	TCTCCATTCCCTATGTTCATGCTTCTGTGCTTGAACAAATGTTCCTCGGACCAACTTGCCCCAATTAACCGCCTTGAACC	
	105210 105220 105230 105240 105250 105260 105270 105280	
Human	TCTCCATTCCCTATGTTCATGCTTCTGTGCTTGAACAAATGTTCCTCGGACCAACTTGCCCCAATTAACCGCCTTGAACC	85227
PolarBear	TCTCCATTCCCTATGTTCATGCTTCTGTGCTTGAACAAATGTTCCTCGGACCAACTTGCCCCAATTAACCGCCTTGAACC	43002
Cow	TCTCCATTCCCTATGTTCATGCTTCTGTGCTTGAACAAATGTTCCTCGGACCAACTTGCCCCAATTAACCGCCTTGAACC	80131
Dog	TCTCCATTCCCTATGTTCATGCTTCTGTGCTTGAACAAATGTTCCTCGGACCAACTTGCCCCAATTAACCGCCTTGAACC	73156
BelugaWhale	TCTCCATTCCCTGTGTTCATGCTTCTGTGCTTGAACAAATGTTCCTCGGACCAACTTGCCCCAATTAACCGCCTTGAACC	72229
Donkey	TCTCCATTCCCTATGTTCATGCTTCTGTGCTTGAACAAATGTTCCTCGGACCAACTTGCCCCAATTAACCGCCTTGAACC	81327
Cat	TCTCCATTCCCTATGTTCATGCTTCTGTGCTTGAACAAATGTTCCTCGGACCAACTTGCCCCAATTAACCGCCTTGAACC	78166
Ferret	TCTCCATTCCCTATGTTCATGCTTCTGTGCTTGAACAAATGTTCCTCGGACCAACTTGCCCCAATTAACCGCCTTGAACC	81020
Lion	TCTCCATTCCCTATGTTCATGCTTCTGTGCTTGAACAAATGTTCCTCGGACCAACTTGCCCCAATTAACCGCCTTGAACC	75851
Vaquita	TCTCCATTCCCTGTGTTCATGCTTCTGTGCTTGAACAAATGTTCCTCGGACCAACTTGCCCCAATTAACCGCCTTGAACC	73524
Pig	TCTCCATTCCCTATGTTCATGCTTCTGTGCTTGAACAAATGTTCCTCGGACCAACTTGCCCCAATTAACCGCCTTGAACC	73521

Majority	ATGATCCATGACCACCTCACCATTCTGCGGGAACCACCCTTCGTTATGGATGATCTGTTCATCTCCGCTCTTCCTCGACT	
	105290 105300 105310 105320 105330 105340 105350 105360	
Human	ATGATCCATGACCACCTCACCATTCTGCGGGAACCACCCTTCGTTATGGATGATCTGTTCATCTCCGCTCTTCCTCGACT	85307
PolarBear	ATGATCCATGACCACCTCACCATTCTGCGGGAACCACCCTTCGTTATGGATGATCTGTTCATCT-------TCCTCGACT	43075
Cow	ATGATCCATGACCACCTCACCATTCTGCGGGAACCACCCTTCGTTATGGATGATCTGTTCATCTCCGCTCTTCCTCGACT	80211
Dog	ATGATCCATGACCACCTCACCATTCTGCGGGAACCACCCTTCGTTATGGATGATCTGTTCATCTCCGCTCTTCCTCGACT	73236
BelugaWhale	ATGATCCATGACCACCTCACCATTCTGCGGGAACCACCCTTCGTTATGGATGATCTGTTCATCTCCGCTCTTCCTCGACT	72309
Donkey	ATGATCCATGACCACCTCACCATTCTGCGGGAACCACCCTTCGTTATGGATGATCTGTTCATCTCCGCTCTTCCTCGACT	81407
Cat	ATGATCCATGACCACCTCACCATTCTGCGGGAACCACCCTTCGTTATGGATGATCTGTTCATCTCCGCTCTTCCTCGACT	78246
Ferret	ATGATCCATGACCACCTCACCATTCTGCGGGAACCACCCTTCGTTATGGATGATCTGTTCATCTCCGCTCTTCCTCGACT	81100
Lion	ATGATCCATGACCACCTCACCATTCTGCGGGAACCACCCTTCGTTATGGATGATCTGTTCATCTCCGCTCTTCCTCGACT	75931
Vaquita	ATGATCCATGACCACCTCACCATTCTGCGGGAACCACCCTTCGTTATGGATGATCTGTTCATCTCCGCTCTTCCTCGACT	73604
Pig	ATGATCCATGACCACCTCACCATTCTGCGGGAACCACCCTTCGTTATGGATGATCTGTTCATCTCCGCTCTTCCTCGACT	73601

Majority	CTTCTCTCTTCTTGTCTTACGCTGCTTGCTCTTCTCTCCTTCTAAAGATGGTTACGCCCCAAAGTCTGTTTACCCTCTTC	
	105370 105380 105390 105400 105410 105420 105430 105440	
Human	CTTCTCTCTTCTTGTCTTACGCTGCTTGCTCTTCTCTCCTTCTAAAGATGGTTACGCCCCAAAGTCTGTTTACCCTCTTC	85387
PolarBear	CTTCTCTCTTCTTGTCTTACGCTGCTTGCTCTTCTCTCCTTCTAAAGATGGTTACGCCCCAAAGTCTGTTTACCCTCTTC	43155
Cow	CTTCTCTCTTCTTGTCTTACGCTGCTTGCTCTTCTCTCCTTCTAAAGATGGTTACGCCCCAAAGTCTGTTTACCCTCTTC	80291
Dog	CTTCTCTCTTCTTGTCTTACGCTGCTTGCTCTTCTCTCCTTCTAAAGATGGTTACGCCCCAAAGTCTGTTTACCCTCTTC	73316
BelugaWhale	CTTCTCTCTTCTTGTCTTACGCTGCTTGCTCTTCTCTCCTTCTAAAGATGGTTACGCCCCAAAGTCTGTTTACCCTCTTC	72389
Donkey	CTTCTCTCTTCTTGTCTTACGCTGCTTGCTCTTCTCTCCTTCTAAAGATGGTTACGCCCCAAAGTCTGTTTACCCTCTTC	81487
Cat	CTTCTCTCTTCTTGTCTTACGCTGCTTGCTCTTCTCTCCTTCTAAAGATGGTTACGCCCCAAAGTCTGTTTACCCTCTTC	78326
Ferret	CTTCTCTCTTCTTGTCTTACGCTGCTTGCTCTTCTCTCCTTCTAAAGATGGTTACGCCCCAAAGTCTGTTTACCCTCTTC	81180
Lion	CTTCTCTCTTCTTGTCTTACGCTGCTTGCTCTTCTCTCCTTCTAAAGATGGTTACGCCCCAAAGTCTGTTTACCCTCTTC	76011
Vaquita	CTTCTCTCTTCTTGTCTTACGCTGCTTGCTCTTCTCTCCTTCTAAAGATGGTTACGCCCCAAAGTCTGTTTACCCTCTTC	73684
Pig	CTTCTCTCTTCTTGTCTTACGCTGCTTGCTCTTCTCTCCTTCTAAAGATGGTTACGCCCCAAAGTCTGTTTACCCTCTTC	73681

Majority	GGTATGTTATTGTTAGCACTATACTTTTATTATTGATTTGATTTTTGTTTCACCTTAATTCTTATTTGTAGCTAGCACTT	
	105450 105460 105470 105480 105490 105500 105510 105520	
Human	GGTATGTTATTGTTAGCACTATACTTTTATTATTGATTTGATTTTTGTTTCACCTTAATTCTTATTTGTAGCTAGCACTT	85467
PolarBear	GGTATGTTATTGTTAGCACTATACTTTTATTATTGATTTGATTTTTGTTTCACCTTAATTCTTATTTGTAGCTAGCACTT	43235
Cow	GGTATGTTATTGTTAGCACTATACTTTTATTATTGATTTGATTTTTGTTTCACCTTAATTCTTATTTGTAGCTAGCACTT	80371
Dog	GGTATGTTATTGTTAGCACTATACTTTTATTATTGATTTGATTTTTGTTTCACCTTAATTCTTATTTGTAGCTAGCACTT	73396
BelugaWhale	GGTATGTTATTGTTAGCACTATACTTTTATTATTGATTTGATTTTTGTTTCACCTTAATTCTTATTTGTAGCTAGCACTT	72469
Donkey	GGTATGTTATTGTTAGCACTATACTTTTATTATTGATTTGATTTTTGTTTCACCTTAATTCTTATTTGTAGCTAGCACTT	81567
Cat	GGTATGTTATTGTTAGCACTATACTTTTATTATTGATTTGATTTTTGTTTCACCTTAATTCTTATTTGTAGCTAGCACTT	78406
Ferret	GGTATGTTATTGTTAGCACTATACTTTTATTATTGATTTGATTTTTGTTTCACCTTAATTCTTATTTGTAGCTAGCACTT	81260
Lion	GGTATGTTATTGTTAGCACTATACTTTTATTATTGATTTGATTTTTGTTTCACCTTAATTCTTATTTGTAGCTAGCACTT	76091
Vaquita	GGTATGTTATTGTTAGCACTATACTTTTATTATTGATTTGATTTTTGTTTCACCTTAATTCTTATTTGTAGCTAGCACTT	73764
Pig	GGTATGTTATTGTTAGCACTATACTTTTATTATTGATTTGATTTTTGTTTCACCTTAATTCTTATTTGTAGCTAGCACTT	73761

Majority	TGGCTTAAAGTTGAATAGTAAATCTTTTGCTATTTTTCTTTGCTATTTAAAACTCTCCATAGACACAAAATTTGTTTTAA	
	105530 105540 105550 105560 105570 105580 105590 105600	
Human	TGGCTTAAAGTTGAATAGTAAATCTTTTGCTATTTTTCTTTGCTATTTAAAACTCTCCATAGACACAAAATTTGTTTTAA	85547
PolarBear	TGGCTTAAAGTTGAATAGTAAATCTTTTGCTATTTTTCTTTGCTATTTAAAACTCTCCATAGACACAAAATTTGTTTTAA	43315
Cow	TGGCTTAAAGTTGAATAGTAAATCTTTTGCTATTTTTCTTTGCTATTTAAAACTTTCCATAGACACAAAATTTGTTTTAA	80451
Dog	TGGCTTAAAGTTGAATAGTAAATCTTTTGCTATTTTTCTTTGCTATTTAAAACTCTCCATAGACACAAAATTTGTTTTAA	73476
BelugaWhale	TGGCTTAAAGTTGAATAGTAAATCTTTTGCTATTTTTCTTTGCTATTTAAAACTCTCCATAGACACAAAATTTGTTTTAA	72549
Donkey	TGGCTTAAAGTTGAATAGTAAATCTTTTGCTATTTTTCTTTGCTATTTAAAACTCTCCATAGACACAAAATTTGTTTTAA	81647
Cat	TGGCTTAAAGTTGAATAGTAAATCTTTTGCTATTTTTCTTTGCTATTTAAAACTCTCCATAGACACAAAATTTGTTTTAA	78486
Ferret	TGGCTTAAAGTTGAATAGTAAATCTTTTGCTATTTTTCTTTGCTATTTAAAACTCTCCATAGACACAAAATTTGTTTTAA	81340
Lion	TGGCTTAAAGTTGAATAGTAAATCTTTTGCTATTTTTCTTTGCTATTTAAAACTCTCCATAGACACAAAATTTGTTTTAA	76171
Vaquita	TGGCTTAAAGTTGAATAGTAAATCTTTTGCTATTTTTCTTTGCTATTTAAAACTCTCCATAGACACAAAATTTGTTTTAA	73844
Pig	TGGCTTAAAGTTGAATAGTAAATCTTTTGCTATTTTTCTTTGCTATTTAAAACTCTCCATAGACACAAAATTTGTTTTAA	73841

```
Majority     TGCATGCTGATTTATTTTGCATGGTCTTTAATTTAAT-ATCATTCACATAGCTTTGAGGGTTTATCAAAAATT-ATTCTT

                              105610    105620    105630    105640    105650    105660    105670    105680

Human        TGCATGCTAATTTATTTTGCATGGTCTTTAATTTAAT-ATCATTCACATAGCTTTGAGGGTTTATCAAAAATT-ATTCTT  85625
PolarBear    TGCATGCTGATTTATTTTGCATGGTCTTTAATTTAAT-ATCATTCACATAGCTTTGAGGGTTTATCAAAAATT-ATTCTT  43393
Cow          TGCATGCTGATTTATTTTGCATGGTCTTTAATTTAAT-ATCATTCACATAGCTTTGAGGGTTTATCAGAAATT-ATTCTT  80529
Dog          TGCATGCTGATTTATTTTGCATGGTCTTTAATTTAAT-ATCATTCACATAGCTTTGAGGGTTTATCAAAAGTT-ATTCTT  73554
BelugaWhale  TGCATGCTGATTTATTTTGCATGATCTTTAATTTAAT-ATCATTCACATAGCTTTGAGGGTTTATCAGAGATT-ATTCTT  72627
Donkey       TGCATGCTGATTTATTTTGCATGGTCTTTAATTTAAT-ATCATTCACATAGCTTTGAGGGTTTATCAGAAATT-ATTCTT  81725
Cat          TGCATGCTGATTTATTTTGCATGGTCTTTAATTTAAT-ATCATTCACATAGCTTTGAGGGTTTATCAAAAATT-ATTCTT  78564
Ferret       TGCATGCTGATTTATTTTGCATGGTCTTTAATTTAAT-ATCATTCACATAGCTTTGAGGGTTTATCAAAAATT-ATTCTT  81418
Lion         TGCATGCTGATTTATTTTGCATGGTCTTTAATTTAAT-ATCATTCACATAGCTTTGAGGGTTTATCAAAAATT-ATTCTT  76249
Vaquita      TGCATGCTGATTTATTTTGCATGATCTTTAATTTAAT-ATCATTCACATAGCTTTGAGGGTTTATCAGAGATT-ATTCTT  73922
Pig          TGCATGCCGATTTATTTTGCATGGTCTTTAATTTAATTATCATTCACATAGCTTTGAGGGTTTATCAAAAATTTATTCTT  73921

Majority     TTCAAAATTCACTGTTCAAAATCTTGATCTTCTTTATTCATTTGTGAGAATGATGAGTTTGAATGAGTGATCATGTTGAT

                              105690    105700    105710    105720    105730    105740    105750    105760

Human        TTCAAAATTCACTGTTCAAAATCTTGATCTTCTTTATTCATTTGTGAGAATGATGAGATTGA----GTGATCATGTTGAT  85701
PolarBear    TTCAAAATTCACTGTTCAAAATCTTGATCTTCTTTATTCATTTGTGAGAATGATGAGTTTGAATGAGTGATCATGTTGAT  43473
Cow          TTCAAAATTCACTGTTCAAAATCTTGATCTTCTTTATTCATTTGTGAGAATGATGAGTTTGAATGAGTGATCATGTTGAT  80609
Dog          TTCAAAATTCACTGTTCAAAATCTTGATCTTCTTTATTCATTTGTGAGAATGATGAGTTTGAATGCGTGATCATGTTGAT  73634
BelugaWhale  TTCAAAATTCACTGTTCAAAATCTTGATCTTCTTTATTCATTTGTGAGAATGATGAGTTTGAATGAGTGATCATGTTGAT  72707
Donkey       TTCAAAATTCACTGTTCAAAATCTTGATCTTCTTTATTCATTTGTGAGAATGATGAGTTTGAATGGGTGATCATGTTGAT  81805
Cat          TTCAAAATTCACTGTTCAAAATCTTGATCTTCTTTATTCATTTGTGAGAATGATGAGTTTGAATGAGTGATCATGTTGAT  78644
Ferret       TTCAAAATTCACTGTTCAAAATCTTGATCTTCTTTATTCATTTGTGAGAATGATGAGTTTGAATGAGTGATCATGTTGAT  81498
Lion         TTCAAAATTCACTGTTCAAAATCTTGATCTTCTTTATTCATTTGTGAGAATGATGAGTTTGAATGAGTGATCATGTTGAT  76329
Vaquita      TTCAAAATTCACTGTTCAAAATCTTGATCTTCTTTATTCATTTGTGAGAATGATGAGTTTGAATGAGTGATCATGTTGAT  74002
Pig          TTCAAAATTCACTGTTCAAAATCTTGATCTTCTTTATTCATTTGTGAGAATGATGAGTTTGAATGAGTGATCATGTTGAT  74001
```

```
Majority     GTCTGAATGTTTCATTAATATGTCAGAAAGATAATCTTTAGCTGACTTGCACGTAAAA-TTAATTCCATTTTTGGATTAC
                       105770    105780    105790    105800    105810    105820    105830    105840
Human        GTCTGAATGTTTCATTGATATGTCAGAAAGATAATCTTTAGGTGACTTCCACATAAAA-TTAATTCCATTTTTGGATTAC  85780
PolarBear    GTCTGAATGTTTCATTAATATGTCAGAAAGATAATCCTCAGCTGACTTGCATGTAAAA-TTAATTCCATTTTTGGATTAC  43552
Cow          GTCTGAATGTTTCATTAATATGTCAGAAAGATAATCTTTAGCTGACTTGCACATAAAAATTAATTCCATTTTTGGATTAC  80689
Dog          GTCTGAATGTTTCATTAATATGTCAGAAAGATAATCTTGAGCTGACTTGCATGTAAAA-TTAATTCCATTTTTGGATTAC  73713
BelugaWhale  GTCTGAATGTTTCATTAATATGTCAGAAAGATAATCTTTAGCTGACTTGCACGTAAAA-TTAATTCCATTTTTGGATTAC  72786
Donkey       GTCTGAATGTTTCATTAATGTGTCAGAAAAATAATCTTTAGCTGACTTGCACATAAAA-TTAATTCCATTTTTGGATTAC  81884
Cat          GTCTGAATGTTTCATTAATATGTCAGAAAGATAATCTTCAGCTGACTTGCATGTAACA-TTAATTCCATTTTTGGATTAC  78723
Ferret       GTCTGAATGTTTCATTAATATGTCAGAAAGATAATCTTCAGCTGACTTGCATGTAAAA-TTAATTCCATTTTTGGATTAC  81577
Lion         GTCTGAATGTTTCATTAATATGTCAGAAAGATAATCTTCAGCTGACTTGCATGTAACA-TTAATTCCATTTTTGGATTAC  76408
Vaquita      GTCTGAATGTTTCATTAATATGTCAGAAAGATAATCTTTAGCTGACTTGCACGTAAAA-TTAATTCCATTTTTGGATTAC  74081
Pig          GTCTGAATGTTTCATTAATATGTCAGAAAGATAAACTTTCCCTGACTTGCACATAAAA-TTAATTCCGTTTTTGGATTAC  74080

Majority     CTCTCTGTGGCTCCAAAA-AAGGTGTTTATGGAGATGTGCAGCGTGTGAAGATTTTGTACAATAAGAAAGACAGTGCTCT
                       105850    105860    105870    105880    105890    105900    105910    105920
Human        CTCTCTGTGGCTCCAAAA-AAGGTGTTTATGGAGATGTGCAGCGTGTGAAGATTTTATACAATAAGAAAGACAGCGCTCT  85859
PolarBear    CTCTCTGTGGCTCCAAAA-AAGGTGTTTATGGAGATGTGCAGCGTGTGAAGATTTTGTACAATAAGAAAGACAGTGCTCT  43631
Cow          CTCTCTGTGGCTCCAAAA-AAGGTGTTTATGGAGATGTGCAGCGTGTGAAGATTTTGTACAATAAGAAAGACAGTGCTCT  80768
Dog          CTCTCTGTGGCTCCAAAA-AAGGTGTTTATGGAGATGTGCAGCGTGTGAAGATTTTGTACAATAAGAAAGACAGTGCTCT  73792
BelugaWhale  CTCTCTGTGGCTCTAAAATAAGGTGTTTATGGAGATGTGCAGCGTGTGAAGATTTTGTACAACAAGAAAGACAGTGCTCT  72866
Donkey       CTCTCTGTGGCTCCAAAA-AAGGTGTTTATGGAGATGTGCAGCGTGTGAAGATTTTGTACAATAAGAAAGACAGTGCTCT  81963
Cat          CTCTCTGTGGCTCCAAAA-AAGGTGTTTATGGAGATGTGCAGCGTGTGAAGATTTTGTACAATAAGAAAGACAGTGCTCT  78802
Ferret       CTCTCTGTGGCTCCAAAA-AAGGTGTTTATGGAGATGTGCAGCGTGTGAAGATTTTATACAATAAGAAAGACAGTGCTCT  81656
Lion         CTCTCTGTGGCTCCAAAA-AAGGTGTTTATGGAGATGTGCAGCGTGTGAAGATTTTGTACAATAAGAAAGACAGTGCTCT  76487
Vaquita      CTCTCTGTGGCTCTAAAATAAGGTGTTTATGGAGATGTGCAGCGTGTGAAGATTTTGTACAACAAGAAAGACAGTGCTCT  74161
Pig          CTCTCTGTGGCTCCAAAA-AAGGTGTTTATGGAGATGTGCAGCGTGTGAAGATTTTGTACAATAAGAAAGACAGTGCTCT  74159
```

```
Majority     AATACAGATGGCTGATGGAAACCAATCACAACTTGGTAAGATTAAACTATGTGTTATCTAT-ACATCTTTAAATCTGATT
                      105930    105940    105950    105960    105970    105980    105990    106000
Human        AATACAGATGGCTGATGGAAACCAATCACAACTTGGTAAGATTAAACTATGTTTTATCTAT-ACATCTTCACTTCTGCTT  85938
PolarBear    GATACAGATGGCCGATGGAAACCAATCACAACTTGGTAAGATTAAGCTATGTGTTATCTAT-ACATCTTTAAGTCTGAAT  43710
Cow          AATACAGATGGCTGATGGAAACCAGTCACAACTGGGTAAGAATAAACTATGTGTCATCTAT-ACGGCTTCA-ATCTGATT  80846
Dog          GATACAGATGGCTGATGGAAACCAATCACAACTTGGTAAGATTAAACTATGTGTTATCTATTACATCTTTAA------AT  73866
BelugaWhale  AATACAGATGGCTGATGGAAACCAATCACAACTGGGTAAGATTAAATTATATGTTATCTGT-ACATCTTTAAATCTGATT  72945
Donkey       AATACAGATGGCTGACGGAAACCAATCACAACTTGGTAAGATTAAACTGCGTGATATCTAT-ATGTCTTTAAATCTGATT  82042
Cat          GATACAGATGGCTGATGGAAACCAATCACAGCTTGGTAAGGTTAAACA--GT--TACCCAT-ACGTCTATAAATCTGAAT  78877
Ferret       GATACAGATGGCTGATGGAAACCAATCACAACTTGGTAAGATTAAACTGTGTGTTAGCTAT-ACATCTTTAAATCTGAAT  81735
Lion         GATACAGATGGCTGATGGAAACCAATCACAGCTTGGTAAGGTTAAACA--GT--TACCCAT-ACGTCTATAAATCTGAAT  76562
Vaquita      AATACAGATGGCTGATGGAAACCAATCACAACTGGGTAAGATTAAATTATATGTTATCTAT-ACATCTTTAAATCTGATT  74240
Pig          AATACAGATGGCGGATGGAAACCAGTCACAACTGGGTAAGA-TAGTTTTTGTGTTGCTTAT-ACATCTTCAGATCTGATT  74237

Majority     TCAAATATATAA-XTAAACCTGTGAATAGAAATAAAXACX------TTT------------------ATATTAGTXAGA-
                      106010    106020    106030    106040    106050    106060    106070    106080
Human        TCAAATGCATAATGTGAATGTGCGAATAAAAATAAACTCC-------TTT-----------------ACATCAGTAAGA-  85993
PolarBear    CTGTTAATAAAA-ACAAACCCCTTTATGGCAGTAAGAA-------------------------------ACTATTT----  43754
Cow          TCAAATGTATAATGTAAACCTGTGAATAAAAACAAACTCC------TTT------------------ATATCAGTAAGA-  80901
Dog          CTGTTAATAAAA-AAAAACCCTATTATGGCAGTAAGTG-------------------------------ATTCTTT----  73910
BelugaWhale  TCAAATATATAATCTAAACCTGTGAATATAAACAAACTC-------TTT------------------ATATTAGTAAGA-  72999
Donkey       TCAAATATATAATGTAAATCTGTGAATAGTAAGAAACCATTTGTTTTCTTAATAGCCAGGGCTCAAAATGTCATGTTAAT  82122
Cat          CTGTTAATAAAA-TCAAACCCCTTTATGGAAGTAAGAACCTTTATTTAC------------------TCATTCATTGATT  78938
Ferret       CTGTTAATAAAA-ACAAACCCCTTTATGGAAGTAGAAA-------------------------------ATCATTC----  81779
Lion         CTGTTAATAAAA-TCAAACCCCTTTATGGCAGTAAGAACCTTTATTTAC------------------TCATTCATT----  76619
Vaquita      TCAAATATATAATCTAAACCTGTGAATATAAAC-------------TTT------------------ATATTAGTAAGA-  74288
Pig          TCAAATATATAA-----GTCTATGAATAAAGA-AAAGCCC------TTT------------------ATATCAGTAAGA-  74286
```

```
Majority     -----AXCXATTTTTTT---------------------------------------------------------------
                      106090    106100    106110    106120    106130    106140    106150    106160
Human        -------A-ATTATTTT---------------------------------------------------------------  86002
PolarBear    ----------TTTTTT----------------------------------------------------------------  43760
Cow          -----AGCTGTTTATT----------------------------------------------------------------  80912
Dog          ----------TTTTTTTT--------------------------------------------------------------  73918
BelugaWhale  -----AGCCATTTATT----------------------------------------------------------------  73010
Donkey       TTTCATGTTGTCTTTTTGGAGGAAGATTAGCCCTGAGCTGACTACTGCCACTCCTCCTCTTTTTGCTGAGGAAGACTGGC  82202
Cat          CATTCATTCATTTATTTA--------------------------------------------------------------  78956
Ferret       ----------TTTTTT----------------------------------------------------------------  81785
Lion         CATTCATTTATTTATTTA--------------------------------------------------------------  76637
Vaquita      -----AGCCATTTATT----------------------------------------------------------------  74299
Pig          -----AACCATTTTTTT---------------------------------------------------------------  74298

Majority     --------------AATAGCCAG-------------------------------------GGCTCAA-----AATGTCA
                      106170    106180    106190    106200    106210    106220    106230    106240
Human        --------------AATGGCCAG-------------------------------------GGCTCAA-----AATGTCA  86025
PolarBear    ---------------ATAGCTG--------------------------------------GGCTCAA-----AATGTCA  43781
Cow          --------------AATAGCCAG-------------------------------------GGTTCAC-----AGTGTCT  80935
Dog          ------TTTTTTTTGATAGCCG--------------------------------------GGTTCAA-----AATGTCA  73948
BelugaWhale  --------------AATAGCCAG-------------------------------------GGTTTAA-----AATGTCA  73033
Donkey       CCTGAGCTAACATCCATACCCATCTTCCTCTACTTTATATGTGGGATGCCTACCACAGCATGGCTTGACAAGTGATGCCA  82282
Cat          ------TTATTTTGGATAGCCAA-------------------------------------GGCTCAA-----AATGGCA  78987
Ferret       ---------------ATAGCTG--------------------------------------AGCTCAA-----AATGTCA  81806
Lion         ------TTATTTTGGATAGCCAA-------------------------------------GGCTCAA-----AATGGCA  76668
Vaquita      --------------AATGGCCAG-------------------------------------GGTTCAA-----AATGTCA  74322
Pig          --------------AATAGCCAG-------------------------------------AGTTCAA-----GATGTCA  74321
```

Majority	TGT---	
	106250 106260 106270 106280 106290 106300 106310 106320	
Human	TTT---	86028
PolarBear	TGT---	43784
Cow	TGT---	80938
Dog	TGT---	73951
BelugaWhale	TGT---	73036
Donkey	TGTCCGCACCTGGGATCCGAACCGGCGAACCCTGGGTCACCAAAGCGGAACATGCAAACTTAACATCTGCGCCACTGGGC	82362
Cat	CGT---	78990
Ferret	TGT---	81809
Lion	CGT---	76671
Vaquita	TGT---	74325
Pig	TTT---	74324

Majority	-------TAATTTTCATATTTTAATGTGAXTXGTTGTACATGATGAACATATTTGTATTTTTCTGCTGTCCTCTTA-CCA	
	106330 106340 106350 106360 106370 106380 106390 106400	
Human	----------------TAATGTGA-ATA----GTTGTATGTGACGAACATATTTGTATTTTTCTGCTATCCTCCTA-CTG	86086
PolarBear	-------TAATTGTCATATTTTAATGTAT----TTATACATGATAAACATGTTTGTA-TATTCTGCTGTCCTCTTA-CCT	43851
Cow	-------TAATTTTCATATTTTAATATGTATAGTTGTACATGATGAACATATTTGTATTTTTGTGCTATTCTCTTA-CCA	81010
Dog	-------TAATTTTCATATTTTAACATAT----TTATACACGATGACCATGTTTGTA-TTTTCTCCTGTCCTCTTA-ACA	74018
BelugaWhale	-------TAATTTTCATATTTTAACATGAATAGTTGTACACGATGAACATATTTGTATTTTTCTGCTGTCCTCTTAACCA	73109
Donkey	TGGCCCTTAATTTTCATGTTCTAATGTGAATAGTTGTACATGATGAACATATTTATATTTTTCTGCTATCCTTTTC-CTG	82441
Cat	-------TAATTTTCGTATTTTAATGC-------------ATGATGAATATGTTTGTGGTTTTCTGCTGTCCTCTTA-CCA	79050
Ferret	-------TAATTTTCTTATTTTAATGTAT----TTATACATGATGAACATGTTTGCA-TATTCTGCTGTCCTCTTA-CCA	81876
Lion	-------TAACTTTCGTATTTTAATGCGAGTCATTATACATGATGAATATGTTTGTGGTTGTCTGCTGTCCTCTTA-CCA	76743
Vaquita	-------TAATTTTCATATTTTAACATGAATAGTTGTACATGATGAACATATTTGTATTTTTCTGCTGTCCTCTTAACCA	74398
Pig	-------TAATTTTCATATTTTAATGTGAATAGTTGTACATGATGAACATATTTGTATTTTTCTGCTTTCCTCTTA-CCA	74396

106410 106420 106430 106440 106450 106460 106470 106480

Majority	TTCCTTAAGTACAXTC-TAATGGAAAAAGGTTTT-GGCTCCAAAATTGCAATAACAATTTCACATAAGAATT-ATGACAT	
Human	TTTTTCAAATGCACTCATAATAGGAAGAGATTTT-AGCTTCAAAATTGTAATAATAGTTTCACATGAGAATT-ATATCAT	86164
PolarBear	AGCCTCAGGTACAATC-TAATGGAAAAAGGTTTG-GGCTCCAAAATGGCAGTAACAGTTTCACATAATAATG-ATAGCAT	43928
Cow	TTTCTTGCTCGGAGTT-TAATGGAAAAAGGTTTT-GGCTTCAAGATTGAAATAACAGTTTCACCTAAGAATT-ATGATAT	81087
Dog	ATCCTCAAGTACAATC-TAATGG---AAGATTTG-GCCTCCAAAATTGTAGTAATCGTTTCACATAAGAAGG-GTAGCGT	74092
BelugaWhale	TTTCTTGTGTGAAGTC-TAAGGAAGAAATGTTTT-GGCTTCAAAATTACAATAACAATTTCACATAAGAATT-ATGACAT	73186
Donkey	TTTCTTAGGTGCAGTC-TAACGGAAAAAGGTTTC-GGCTCCAGAATTGCAGTAACAATTGCACATAAAGAATTATGATGT	82519
Cat	TTCCTCAAGTACAATC-CAGTGGAAAAAGGTTTA-GGCTCCAGAATTGCAATAATAATTTCACATAAGAATT-ATGACAT	79127
Ferret	ATCCTTAAGTACAATC-TAATGGAAAAAGGTTTG-GAGTCCAAAATTGCAGTAACAG--T-----AATAATG-ATACTAC	81946
Lion	TTCCTCAAGTACAATC-CAGTGGAAAAAGGTTTA-GGCTCCAGAATTGCAATAATAATTTCACATAAGAATT-ATGACAT	76820
Vaquita	TTCCTTGTGTGAAGTC-TAAGGAAGAAATGTTTT-GGCTTCAAAATTACAATAACAATTTCACATAAGAATT-ACGACAT	74475
Pig	ATTTTTGGGCAGAGTC-TGATGGTAAAAGGTTTTTGGCTCCAAGATTGCAGTAACAATTTCACGGAAGAATTTATAACAT	74475

106490 106500 106510 106520 106530 106540 106550 106560

Majority	TTATATTAAA-GATCCTTAGTGTTTTTCATTCCTGGTTACTCCCTGT-AATAGAAGTTAACTGTTC---------ATATA	
Human	TTATATTGAA-GATTCATAGTGATTTTTATT-CTAGGTACTCCTTGTTAATAGAGGCTAACTGTTC---------ATACA	86233
PolarBear	TTATATTAAA-GATCCTTAGTGTATTTCTTTCCTGGTTACTCTTTAC-AATAGAAGTTAACTGTTC---------ATTTG	43997
Cow	ATATATTAAAAGATTCTTAGCATTTTTCATTTCTGGTTACTCCCTAT-AATTGAAGTTAACTGTTC---------ATATA	81157
Dog	TTATATTAAA-GATCCTTAGTATCTTTCTTTCCTGCTTACTCCCTGT-AATAGAAGGTAACTGTTC---------ATATA	74161
BelugaWhale	ACATATTAAA-GATCCTTAGTGTTTTTCATTCCTGGATACTCCTTGT-AATCAAAGTTAACGGTTC---------ATATA	73255
Donkey	TTAAATTAAT-CATCTTTAGTGTTTTTCATTCCTGGTTACTGCCTGT-AATATAAGTTAACTGTTTCTGTCGTCCATATA	82597
Cat	TTGTATTAAA-GATCCCTACTGTTTTTCATTCCTACTTACTCCCTGT-GATAGAAGTTAACTGCTATGCTAA---ATATA	79202
Ferret	TTACATTAAA-GATCTTTAGTATTTTTCTTTCCTGGTTACTCTCTAT-AATAGAAGTTAAGTGTTC---------ATATA	82015
Lion	TTGTATTAAA-GATCCCTAGTGTTTTTCATTCCTACTTATTCTCTGT-GATAGAAGTTAACTGCTAGGCTAA---ATATA	76895
Vaquita	ACATATTAAA-GATCCTTAGTGTTTTTCATTCCTGGATACTCCTTGT-AATCAAAGTTAACGGTTC---------ATATA	74544
Pig	TTATATTAAA-GATCCTTAGTGGTTTTCATTCCCAGTTACTCCCTAT-AGTCAAAGTTAACTGTTC---------ATGTA	74544

```
Majority     GTCCATGGAATCCTGATTAATGTTATGTTXTTTTAATTCTTCTAAATTTTTXTXTAAXXTTTXTACT-XAA---A-X--X
                      106570    106580    106590    106600    106610    106620    106630    106640
Human        GTCCATAGAATCCTTCTTAATGCCATGTT----------------ATTT-----TAATTCTTCTA---------------  86277
PolarBear    GTCCATGGAATCCTGGTT----TTAATT-------ATTCT---AAGTATTTGTCTAAACTTAGTAATGGAA---A-----  44055
Cow          GTCCATGGGATCCTGATTAACGCCATGTTAATTTAATTCTTCTAAAGTCATTTGAAAGTATTTGA---------ACTTAG  81228
Dog          GTCCATGGAATCCTGATTAATGTGAAATT---TTAATTCA---AAATATTTGTCTAAACCTAGTATTGGAA---AATGGA  74232
BelugaWhale  GTCCACGGAATCCTGATTAACGCTGTGTTATTTTAATTCTTCTAAAGTCATTTGCAAGTATTTAACT--AA---ACTTAG  73330
Donkey       GTCCATGGAATCTTGATTAATGTCATGTT-TTTTAATTCTTCTAAATCG-TTTGTAAGGTTTAGGCTAAACTGTGTAATG  82675
Cat          GTCCATGGAGTCCTGATTAATGTGGTGTTATCTTAATTCTCCTAGATTTTTG-CTAAACTTAATG---GAA---A-----  79270
Ferret       ATCTATGGAATCCTGATT----TTAATT-------ATTCT---AAGTATTTGTCTAAACTTAGTAACGGAG---A-----  82073
Lion         GTCCATGGAGTCCTGATTAATGTGGTGTTATCTTAATTCTCCTAGATTTTTG-CTAAACTTAATAATAGAA---A-----  76966
Vaquita      GTCCATGGAATCCTGATTAACGCTGTGTTATTTTAATTCTTCTAAAGTCATTTGCAAGTATTTAACT--AA---ACTTAG  74619
Pig          GTCCATGGAATCCTGATTAACAC--TGTTGTTTCAATTCTTCTAAATTC-CTTGTAAGTGTTCGACT--AA---ATTTAG  74616

Majority     -AAXGAGTCACATGGAAAATGTATTTTAGAGATACCAGGTTXTT-ACAAAATGAAAAACTTGTGCCAAATACA-----CC
                      106650    106660    106670    106680    106690    106700    106710    106720
Human        ----AAGTCTTGCG-----------------ATACCAGGTTCTT-AGAAAATGAAAAGCTTGTGCTAAAAACA-----CT  86330
PolarBear    ----GAATCACATGGAAAATGTATTTTAGAGGTACCAGGTTTCT-ACAAAATGAAAAACTTGTGCCACATACA-----CC  44125
Cow          TAAAGAGTTACATG---AGTGTATTTTAGAGATAGCAGGTTACT-ATAAAATGAAAAACTTAAACTAAATGCC-----CC  81299
Dog          AAATGAGTCACATGGAAAATGTATTTTAGAGATACCAGATTTTTTACAGAATGAAGAACTTGTGCTAGACAGG-----CT  74307
BelugaWhale  TAAAGAGTCACATGGGAAATGTATTTTAGAGATACCAGGTTCTT-ATAAAATGAAAAATTTACACCAAATACC-----CC  73404
Donkey       GAAAGAGTCAAATGGAAAATGTGTTTTAGAGATACCACGTTCTT-ACGAAATGAAAAATTTACACCAAGTACA-----CC  82749
Cat          ----GAGTCACATGGAAAACGTATTTTAGGGGTACTAGGTTTTT-ACAAAATGAAAACCTTGTGCCACATACAATACACC  79345
Ferret       ----GAGTCACATGTAAAATGTATTTTAGAGATACTGGGTTTTT-ACAAAATGAAAAACTTGTGCCACATATA-----CC  82143
Lion         ----GAGTCACATGGAAAACGTATTTTAGGGGTACTGGGTTTTT-ACAAAATGAAAACCTTGTGCCACATACAATACACC  77041
Vaquita      TAAAGAGTCACATGGGAAATGTATTTTAGAGATACCAGGTTCTT-ATAAAATGAAAAATTTACACCAAATGCC-----CC  74693
Pig          TAAAGAATCACATGG--AAAATATTTTAAAGATACCAGGTTCTT-ATAGAAAAGAAAACGTGCTCAAAGTACA-----TC  74688
```

```
Majority     ATTGT--TAATCCCTTTTGGAATCTTAAAATGTACTTXAGTCAAGTTACACTT-ATATTATTA-CTTAXXTA---AATAA
             106730 106740 106750 106760 106770 106780 106790 106800
Human        GTTGT--TAATCCCTTTGGGAATCTTAAAAGGCACTTTAGTCAGGTTACACTTTATATCATTA-CTTAAATAAGTAATTG 86407
PolarBear    ATTGT--TAATCCCTTTTGGAATCTTAAAATGTACTTGAGTCAAATTACACAT-ATATTCTTT-CTTAGGTA---AATAT 44198
Cow          ACTTT--AATCCTTTTT-GGAACCTTAAAATGTACTTCA-ACAAGTTTCACTT-ACATTATTA-GATAAATAAGTGATCA 81373
Dog          GTTGTGTTAATCCCTTTTGGAATCTTAAAATGTACTTAAGTCCA-TTATACTT-CCATTAAATATTTACGTT---AATAA 74382
BelugaWhale  ACTGT--TAATCCTTTTTGAAATCTTAAAATGTACTTCAGACAAGTTACACTT-ATACTATTA-GATAAATAATTGATGA 73480
Donkey       ATTGT--TAATCCCATTTGGAATCTTAAAATGTACTTTAGTCACGTTACACTT-AAATTATTA-TTTAGATAAATGATTG 82825
Cat          ATTGT--TAATCCCTTTTGGAATCTTAAAATGTACTGTAGTCAAATTACACTT-ACATTCTTAACTTAGGTA---AGTAT 79419
Ferret       ATTGG--TAATCCCTTTTGGAATTTTAAAATGTACTTGAGTCAAATTACACAT-ATATTCTCTACTTAAGTA---AATAA 82217
Lion         ATTGT--TAATCCCTTTTGGAATCTTAAAATGTACTGTAGTCAAATTACACTT-ACATTCTTAACTTAGGTA---AGTAT 77115
Vaquita      ACTGT--TAATCCTTTTTGAAATCTTAAAATGTACTTCAGACAAGTTACACTT-ATACTATTA-GATAAATAATTGATGA 74769
Pig          ACTGT--TAATCCCTTT-GCAATCTTAAAATTTACTTCAGACAGGTTACACTT-TTCTTATTA-GATAGTT----GATGA 74759

Majority     TTTAAT-----TAA---TATXTCATTTGACTTCATACACAAAGTCAGATCACATAAATCTGGAATACAXCTGCATATTCA
             106810 106820 106830 106840 106850 106860 106870 106880
Human        ATTATTTTAATTAAACTTATGTCGTTTGACTTCATACAT---CCTCGAATACATAAATCTAGAACACACCTGCACATTCA 86484
PolarBear    TTTAA------------TGTACCATTTGACTTTAGATACAAAGTCAGATCACATAAATTTGGAATACAACTGCATATTCA 44266
Cow          CTTAAT-----TAG---CATCTCATTCAACTTCATGCATAAAGTCAAATCACATTAATCCAGAAGGTAGTT--------- 81436
Dog          --GAA------------TGTATCATTTGATTCTATACACGAAGTCAGATCGCATAAATATGGTATACAGCTATGTATTCA 74448
BelugaWhale  CTTAAT-----TAA---CATCTCGTTTGAATTCATGCACAAAGGCAAATTACATAAATCTAGAATGTAGCTGCATAGTCA 73552
Donkey       ATGATTTTAATTAA---TATTGCATTTGACTTCATACACAAAGTCAAATCACATAAATCTAGAATACAACTGCAAACTCA 82902
Cat          TTTAATTAAATTAA---TGCATCATTTGCCTTCATACACAAAGTCAGATCACA--AATCTGGAATACAACGACACATTCA 79494
Ferret       TTCA-------------TGTTTCATTTGTCTTTATATACAAAGTCAGATCACATGAATCTGGAATACAACTGCATATTCA 82284
Lion         TTTAATTAAATTAA---TACATCATTTGCCTTCATACACAAAGTCAGATCACATAAATCTGGAATACAACTACACATTCA 77192
Vaquita      CTTAAT-----TAA---CATCTCGTTTGAATTCATGCACAAAGCCAAATCACATAAATCTAGAATGTAGCTGCATATTCA 74841
Pig          TTTAAT-----TAA---TATCTCATATGACTTCCTCTGCAAAGGCTGATCAC-TAAATCTGTAGAGTAGCTGCATAATCA 74830
```

```
Majority     CAGT-GTGAT-TGACTGTAAATXTCAGTATGTTCTT-AGCTTATAATTCTGTTCATTGAGATTCAGGATGATGGTGCCT
                     106890    106900    106910    106920    106930    106940    106950    106960
Human        CACCAGTGAT-TGACTGTAAATGCCAGGATGTT------CTTACATTTCTGTTCATTGAGATTCAGGATAATGGTGCCT  86557
PolarBear    CAAT-GTGGTAAGACTGTAAATATCA---TGTTCTT-AGCTTATAATTCTGTTCATTGAGATTAACGGATGATGATGCCT  44341
Cow          -AGT-GTGAT-TGACTGTAAATGTCAGTATGATGTT-AGCTTATAATTCTGTTCATTAAGATTTGGGGATGATGGTACCT  81512
Dog          CAAT-GTGATACGACTGTAAATATTA---TGTTCTT-AGCTTATAATTCTGTTCATTGAGATTTGGGCATGATGATGGCT  74523
BelugaWhale  TGGT-GTGAT-TGACTGTAAA-GTCAATATGTTCTT-AGCTTATAATTCTGTTAGTTGAGATTTGGCGATGATGGTACTT  73628
Donkey       CAAT-GTGAT-TGACTATCAG--TGG----GTTCTC-AGCTTACAGTTCTCTTCATGGAGATTCAGAGATTATGGTGCCT  82973
Cat          CAGT-GTGATAAGACTGTAAATATCAGTATGATCTTTAGCTTGTAATTCTGTTCATTGACATTC--GG-------TGCCT  79564
Ferret       CAGT-GAGCTAAGACTGTAAATATCA---TGTT------CTTATAATTCTTTTCATTGAGATTCAGGATGATGATGCCT  82354
Lion         CAGT-GTGAGAAGACTGTAAATATCAGTATGATCTTTAGCTTGTAATTCTGTTCATTGACATTC--GG-------TGCCT  77262
Vaquita      TGGT-GTGAT-TGACTGTAAA-GTCAATATGTTCTT-AGCTTATAATTCTGTTAGTTGAGATTTGGCGATGATGGTACTT  74917
Pig          CAGT-ACAAT-TGACTATAAATGTCAGTATGTTCTT-AGATAATAATTCTGTTCATTGAGATTCAGGATGATAGTACCT  74907

Majority     CAGAXTGGTTGTXTTCACXTTGTTAATTTTGAAATTTGCCATTTTTGATGTAAGGACTAGTGTATTTTTGCTCTTA--TA
                     106970    106980    106990    107000    107010    107020    107030    107040
Human        CAGACTGATTGTATTCACATTGTTAATTTGTAAATTTGCCATTTTTGATGTAAGGACGAGTGTATTTTTGCTCAGAGATG  86637
PolarBear    CATATTGGTTGTTTCCACGTTGTTAATTCTGAAATTCGTTATTTTTGATGTAAGGGCGG-TGTATTTTTGCTCTTAA-CA  44419
Cow          CAGACTGTGTGTACTCACATCATTAATTTTGAAATTTGCCATTTTG----------------------TGCTGTTA--TA  81568
Dog          CAAATTGGTTGCTTTCACTTTTTT---TTTGAAATTTGTCACTTTTGATGTAAGGAC---TGTATTTTTGCTATTA--CA  74595
BelugaWhale  TAGATTGGGTGTACTCACGTTATTAATTTTGAAATTTGCCATTTTTGGGGTAAAAACTAGTGTATTTTTGCTATTA--TA  73706
Donkey       CAGACTA---GTGTTCACAGTGTTAATTTTGAAAGTTGCTATTTTTGATGTAAGGACTATTGTATTTTTGCTATTA--CA  83048
Cat          CAGGGTGGTTGTTTTCACGTT---AATTTCGAAATTTGTCACTTATGACGTAATGACTAGTGTATTTTTGCTCTTA--TG  79639
Ferret       CATGTTGCTTGTTTTCACATTGTTAATTCTGAAATTTGTCATTTTTCATGTGAGGGCTA-TGTATTTTTCCTCTTAG-CA  82432
Lion         CAGGTTGGTTGTTTTCACGTTGTTAATTTCGAAATTTGTCACTTATGACGTAAGGACTAGTGTATTTTTGCTCTTA--TG  77340
Vaquita      TAGACTGGGTGTACTCACGTTATTAATTCTGAAATTTGCCATTTTTGGGGTAAAGACTAGTGTATTTTTGCTGTTA--TA  74995
Pig          CAGACTAGATATACTCACATCATTAATTTTGAAGTTCGCCATTTTCGATGTAAAAACTAGT-TGTTTTTGCTGTTA--TA  74984
```

```
Majority     XCCAGTGAGACTCAX-AAACTAG-AACATGAT-ATCTGGACCTTTTGGCCTTTGGXAT--CAGATAGAX---TAAGTTTG
                       107050    107060    107070    107080    107090    107100    107110    107120
Human        AACAACTTGCTATATTAGACCAATTTTGGCCTTTGACATCACATAGAAAATTTTGAATTTCAAACACAT-AATAGGTTTG  86716
PolarBear    CCCAGTGAAACTCA---AACTTG-AACA-----GTGTGATATATTTGGCCTTTGGTAA--CAGA------AGTAAGTTTG  44482
Cow          TCCAGTGAGATTCAC-AGACTAACAATATGATAAACCAGACCTTTTAGCCTTTGGCAT--CAGATAGAA------GTTTG  81639
Dog          CCCAATGAAATTCAC-AGACTTG-AACG-----GTATGATATATTTGGTCTTTG--------GA---------AAGTTTG  74651
BelugaWhale  TCCAGTGAGATTCAC-AAACTAAGAACATGATGAACTAGACCTTTTAGCCTTTGGAGT--CAGAAGTAA------GTTTG  73777
Donkey       TCCAATGAGACTCAT-AAATTAACAACATGAAATACTGGACCTTTTGGCCGTTGGAGT--CAGAAGTA-----AGTTTTA  83120
Cat          CCCAGTGGAGGTCAT-AAACTTG-AACATGAC-ATATGGACCTTTTGGCGTTTGGTAT--CAGATAGA--AGTAAATTTG  79712
Ferret       CCCAGTTAAACTCAT-AAACTTG-AACA-----GTGTGATATATTTGGCCTTTGGTAG--CAGATAGAAAAGTAAGTTTG  82503
Lion         CCCAGTGAAGGTCAT-AAACTTG-AACATGAC-ATATGCACCTTTTGGCATTTGGTAT--CAGATAGA--AGTAAATTTG  77413
Vaquita      TCCAGTGAGACTCAC-AAACTAAGAACATGATGAACTAGACCTTTTAGCCTTTGGAGT--CAGAAGTAA------GTTTG  75066
Pig          TTCTGTGAGACTCAC-AAGCCAGCAACATGATGACCTAGACCTTTTAGCCTTTGGGAT--CAGACAGAAA--TGAGTTTG  75059

Majority     AATTTCAGTGTTTCTTACCAGCTATCATGGTAGCTCAGACAAXGTATAAAACT--ATACATCTGGCTCTCXACTTTTTAA
                       107130    107140    107150    107160    107170    107180    107190    107200
Human        AATTTTAGTGTTTCTTGGCAATAGTCTTAGCAACTCAGATGAGGTATAAAACT--ATCCATCTGGCTGTCCA-TTTTTAA  86793
PolarBear    AATTTCATTGTTTCTTACCAGCTATCATGGTAGCTCAGACAACATATAAAACT--ATACATCTGGCTCTCCACTTTTTAA  44560
Cow          AAATTCAGT--TTCTTACCAGCTATTAGGGTAGCCCAGGCAATG-----AATTGTCGGCAGCCCACAC-CAGTACTCTTG  81711
Dog          AATTTTCTTGTTTCTTACCAACTGTCATGGTAGCTCAGACAACACATAAAACT--ATACATCTGGCTCTTCACTTTTTAA  74729
BelugaWhale  AAATTCTGTGTTTCTTACCAGCTATTATC-CGTCTT---TAGTG---GAAGTTATATAAAAGT-----------------  73833
Donkey       AATTTCAGTG--T--AACCAGCTATTGTGGTAGCTTAGACAGTGTATAAAGCTTTATACATCTGGCTGTCTATTTTTTAA  83196
Cat          AATTTTATTATTTCTTACCAGCTTTCATGGTAGCTCAGACAACGTATAAAACT--AGACCTCTGGCTCTCTGCTTTTTAA  79790
Ferret       AATTTCATTATTTCCTACCAGCTATCATGGTAGCTCAGACAACATGTAAAACT--ATACATCTGGCTCTCTACTTTTTAA  82581
Lion         AATTTTATTATTTCTTACCAGCTTTCATGGTAGCTCAGACAACGTATAAAACT--AGACCTCTGGCTCTCTGCTCTTTAA  77491
Vaquita      AAATTCTGTGTTTCTTACCAGCTATTATC-CATCTT---TAGTG---GAAGTTATATAAAAGT-----------------  75122
Pig          AAATTCAGTATTTCTTACCAGCTATTACAGTAGCTCAGACAGTGTATAAAACTTTATACGTCTGGCTCTCCACTTTGTAA  75139
```

```
Majority     AATGGAGATATCCATCTTTA-----------------ATGAAAGATATXTAA-------A-----AGTGCTAATCATGTC
                     107210    107220    107230    107240    107250    107260    107270    107280
Human        AATGGTGGTATGTGTTTT-------------------A-AAGAGATACGA---------A-----AGTGCTGATTATGTC 86839
PolarBear    AATGGAGAGATCTATCTTCA-----------------ATGAAAGATTTATAA-------A-----AGTGTTAATCATGTC 44611
Cow          GCTGGGAAATCCCATGGAAG-----------------GAGGAACCTGGT----------A-----GGCTCTGGTC----C 81755
Dog          AATGGAGATACCCATCTTTA-----------------ATGAAAGATATATAA-------ATAAAGAGTGCTAATCAGGTC 74785
BelugaWhale  GCTGATGATGTC-------------------------ATAGATGCTAATTAA-------A-----TCTTTTCTTCT---- 73872
Donkey       AATACAGATATCTGTCTTTAGAAATGTAAAGTGCTATATGAAAGATATGTATGAAAGATATGTAAAGCGCTGACCATATC 83276
Cat          AATGGAGATACCCGTCTTTA-----------------ATGAAAGATCTGTAA-------A---AAAGTGCTAATCATGTT 79843
Ferret       AATGGAGATATCCATCTTCA-----------------GTGAAAGATTTATAA-------A-----AGTGCTAATCATGTC 82632
Lion         AATGGAGATACCCATCTTTA-----------------ATGAAAGATGTATAA-------A---AAAGTGCTAATCAAGTT 77544
Vaquita      GCTGATGATGTC-------------------------ATAGATGCTAATTAA-------A-----TCTTTTCTTCT---- 75161
Pig          AATGGAGATACCTATCTTTA-----------------AAGGAACTTATTTAA-------A-----CGTGCTGATCTTGTC 75190

Majority     CTAGATG-CCTAGTAAATX--------TCCTTCTG-XTGTGTACACTTCTGTACTCTGGTAGGGGTAGTCATTGCCATTG
                     107290    107300    107310    107320    107330    107340    107350    107360
Human        A-AGATA-CTTAGTACATATTTT----CCTCTTTCTCTGTGTGCACTTCTGTACTCTGGTTGGAATAGTCATTGCTGTTG 86913
PolarBear    CTAGATG-CCTACC-------------TTCTGCTG-CTTTGTATACTTCTGTGCTCTGGTAGGGGTAGTCATGGCCATTG 44676
Cow          ATG-GTG-TT-GCTAAGAG--------TC-----------AGACACGACTG---------------AGCGACTTCC---- 81794
Dog          CTAGATG-CCTAGTAAA-----------TCTTTTC-TTCTTTTGCTGTGTGTGCTCTGGTAGAGGTAGTCATGGCCATTG 74852
BelugaWhale  ---------------------------TCCT-CTGCTTGTGTACACTTCTGTACTCTGGTGGGTGTAGTCATTGCCATTG 73924
Donkey       ATAGATG-CTTAGTAAATC--------TTTCTCTGCTTGTGTACACTTCTGTACTCTGCTGGGGGTAGTGATTGCCATT- 83346
Cat          CTGGGTGTCCTCGTAAATCTTTTTTTCTCCCTCTG-CTGTGTATACTTCTGTACTCTGGTAAGGGTAGTCATGGGCATTG 79922
Ferret       CTAGATG-CCTAATAAATGT-------TTCTTCTG-CTGTGTGTATGTCTGTGCTCTGGTAGGGGTAGTCATGGCCATT- 82702
Lion         CTGGGTGTCCTTGTAAATCTTTTTTTCTCCCTCTG-CTGTGTATACTTCTGTACTCTGGTAAGGGTAGTCATGGCCATTG 77623
Vaquita      ---------------------------TCCT-CTGCTTGTGTACACTTCTGTACTCTGGTGGGTGTAGTCATTGCCATTG 75213
Pig          ATAAGTG-CTCATTAAATC--------TCCTTCTGGTTGTATACACTTCTGTACTCTGGTAGA-GTAGTCATTGCCATTG 75260
```

```
Majority     TTTTTATATACXTTGTAAGTAGCATTTTA-------X--TXCAAAGT-GXXAAAGCATTCTCAAATGTTTCATTTTTTT-
                            107370    107380    107390    107400    107410    107420    107430    107440
Human        TTTTCATATATGTTGTAAGTAGCACTTAA---CA-GTACTTCATAATAG---GAGTATTCTCAAATATGTCATTTTTTT-  86985
PolarBear    TTTTTATGTACATTGTAAATAGCATCTTA------------GAAAGC-ATTAAAGCTTTCTCAAATGTTTCATTTTTTTT  44743
Cow          CTTTCA---CTTTT--------CACTTT----CATGCATTGTAGAAG-GAAATGGCAACCT-----ACTCCAGTGTTCT-  81852
Dog          TTTTTATATACGTTGTAAGAAGCATTTAA------------CAA---------AGCTTTCTCAACTGTTTCATTTTTTTT  74911
BelugaWhale  TTTTTATAAACTTTGCGAGTAGCATTTTACAGTTTTTGTTTCATAGTTGTCAAAGCATTCTTAAATGTTTGATTTTCTT-  74003
Donkey       -TTTTATATACATTGTAAGTAGCATTTTA---CA-GTGCTTCATAGTTAACAAAGCATTTTCAAATATTTTTTTTTCCT-  83420
Cat          CTTTTATGTACATTGTAAGTAGCATTTAA------------AAAA--------ATATTTCTCACATGTTTCATTTTTTT-  79981
Ferret       -------------TGTAAGTAACATTTTA------------CAAAGC-ATTAAAGCTTTCTCAGATGTTTCATTTTTTC-  82755
Lion         CTTTTATGTACATTGTAAGTAGCATTTAA------------AAAA--------ACATTTCTCAAGTGTTTCATTTGTTT-  77682
Vaquita      TTTTTATAAACTTTGCGAGTAGCATTTTACAGTTTTTGTTTCATAGTTGTCAAAGCATTCTTAAATGTTTGATTTTCTT-  75292
Pig          TTTTTATATCCTTTGTGAGTAGCATTTT----CCCATGCTTCAGAGTTGACAAAACAGTCTCAAATATTTAAATTTTTT-  75335

Majority     ---CATCACT----GTGTGAGCTGTGGGTGGCATTACCCT-CAT----ATAAAGGATAXACAGTCATTATGGTTGAGAAG
                            107450    107460    107470    107480    107490    107500    107510    107520
Human        --CCCTAATT----GTTTGAGGTATGGGTGGCATTGTGTC--ATTTAAATAAGGGATGCATAGTCTTTATGGTTGGTAAT  87057
PolarBear    -CTCATCACCCACAGTGTGAGCTGTGGGTGGCATTACCCTTCAT----ATAAAGTATATACAAGCATCATGGTTGAGAAG  44818
Cow          ---TGCC--T----GGAGAAACCCAGGGACGGAGGAGCCT-GGT--------GGGCTGC----------CGTCTGTGGGG  81904
Dog          TCTCATCAGT----ATGGGAGCTGTGGG-GGTATTACCCT-CAT----ATAAAGGATATATAGTGGTTATGGTTGGGAAG  74981
BelugaWhale  ---CCTCATT----GTGTGAGCTGTGGGTGGCATTACCCT-CATGTAAATAAAGGATACGTAATCATTACAGTTGAGAAG  74075
Donkey       ---CATCACC----TTATGATCTGTGGGTAACATTTCCCT-CATATAAATAAGGGAT-------CATTATGGTTGAGAAG  83485
Cat          -CTCATCACT----GTGTGAGCTGTGGATGACATTACCCT-CAT----GTAAAGGATTTACAGTTATCATGGTTAAGAAG  80051
Ferret       ---CATCACT----GTGTGAGCTGTGGGTGGCATTACCCTTCAT----ATAAAGGATATATAGGCATCATGGTTGAGAAG  82824
Lion         -CTCATCACT----GTGTGAGCTGTGGATGACATTACCCT-CAT----GTAAAGGATTTACAGTTATCATGGTTAAGAAG  77752
Vaquita      ---CCTCATT----GTGTGAGCTGTGGGTGGCATTACCCT-CATGTAAAGAAAGGATACGCAATCATTACAGTTGAGAAG  75364
Pig          ---TCTCATT----GTGTGAGCTATGGGTAGCATTACCCT-CATATAAATAAGGGATGCACAACTATTATGTTTGAGAAG  75407
```

```
Majority      TACCTGAGGTAGAATTT-AACCAAATTCTTCTGGATATTTTT-TGTTTTX-----TXTAAGGACTATTTT----AAGTGA

                        107530    107540    107550    107560    107570    107580    107590    107600

Human         TACCTGTGGTAGAATT--ACCTCAGATCTTCCAGATAGTTTT-TGTTTTA-----TGTAAAGACTGTTTT----ATGTAA  87125
PolarBear     TACCTGAGGTAGAATGT-AACCAAACTCTTCTGGATACTTTT-TATATA----------AGGACTATTTTAAATAAGTCA  44886
Cow           TCGCACAG----------AGTCAGACACAACT--GAAG-----GGACTTA------------GCAGCATC----AGGCAA  81951
Dog           TACCTGAGGTAGAATTT-AACCAAGCTTTTCTGGATATTTTT-TATGTA----------AGGACTA--------------  75035
BelugaWhale   TACCTGAGGTAGAATTT-AACCAAATGTTTCACGATATTTTT-GGTTTTGTTTTATGTAAGGACTAGAT-----AACTGA  74148
Donkey        TACCTGAGGTTAGAATTTAAACAAATTCTTCTGGGTATTTTTTGGTTTTGGTTTATGTAAGGACTATTTTAAATAAGTGA  83565
Cat           TACCTCAGGTAGAATTT-AATCAAATTCTTCTGGATATTTTG-TATATATAC--ATATAATGACTATTTTAAATAAGTGG  80127
Ferret        TACCTGATGTAGAATTT-AACCAAACTCTTCTGGATATTTTT-TATATA----------AGGACTATTTTATATAAGTTG  82892
Lion          GACCTCAGGTAGAATTT-AATCAAATTCTTCTGGATATTTTA-TATATA--------TAATGACTATTTTAAGTAAGTGG  77822
Vaquita       TACCTGAGGTAGAATTT-AACCAAATGTTTCACGATATTTTT-GGTTTTGTTTTATGTAAGGACTAGAT-----AACTGA  75437
Pig           TACCTAAGGTAGAATTTTAATCAAATTCTTCT--GTATTTTT-GGTTTTGTTTTATGTAAGGACTATTTT----AACTGT  75480

Majority      TATTTTAAT-TTTTACTATACTTGTATTAGTCTGTTTGGGCTGCTATAACAAAATA-CCACA------------------

                        107610    107620    107630    107640    107650    107660    107670    107680

Human         TATTTTAATTTTTTGCCATGCTCGT---AGTCTGCTTGGGCTGCTGTAACCAAAATACCATAGACTGAGTAGATGAAACA  87202
PolarBear     CATTTTAAT-TTTTGTTATAGTTCTATTAGTTTCCTTG--CTGCTAAAACAAAATA-CCGCA------------------  44944
Cow           TGTATAAAA-TTTTA---------------TATATCTGGCTTTCCATTTTAAAAAA-TGG--------------------  81994
Dog           ----------TTT-------------------------------------------------------------------  75038
BelugaWhale   TACTTTAAT-TTTTGCTATACTTGTATTAGTCTGTTTGGGCTGCCATAACAAAATA-CCACAGACTGAGTGGCTAAAACA  74226
Donkey        TATTTTAAT-TTTTA-TATACTTATGTTAGTCTGCTTGGGCTGCTGTAACAAAATAACCACAGACTGAGTGGCTTAAACA  83643
Cat           CATTTTAGT-TTTTACTATACTTACATCAGTTTGT----GCTGCTAAAACA--ATA-CCACA------------------  80181
Ferret        CATTTTAAT-TTTTACTATAATTGTATTAGTTTGTTTGGGCTGCTAAAACAAAATA-TCACA------------------  82952
Lion          CATTTTAGT-TTTTACTATACTTACATCAGTTTGT----GCTGCTAAAACA--ATA-CCACA------------------  77876
Vaquita       TACTTT-------TGCTATACTTGTATTAGTCTCTTTGGGCTGCCATAACAAAATA-CCACAGACTGAGTGGCTAAAACA  75509
Pig           TATTTTAAT-TTTTACTGTACTTGTATTAGTCTGCTTGGGCTGCCGTAACAAAATA-CTGTAGGCTTATT-----AAACA  75553
```

```
Majority     --AXAAGTTTATXTTXA-ACAXTXCTGAAAGTTAX-TAXTX------AGG----------------TGCCAGTAGATTTX

                       107690    107700    107710    107720    107730    107740    107750    107760

Human        ACAAAAATGTATTTCTTCAAAGTTTTGAGAAATAAAACTTTGGGGTCTAGAAGTCCCAGATGAAACTGCTAGCAGATT--  87280
PolarBear    --GACTGCATGCCTTAA-AAAAAACAGAAATTTACTTTCTC------AGG----------------TGCCAGCAGATTCA  44999
Cow          --AGATGTCTATCTT-------TAGTGGAAGTTC--TATAA------AAG----------------TGCTGATTGTGTCA  82041
Dog          --------------------A-----------------------------------------------------------  75039
BelugaWhale  ACAGAAATTTATTTTCTTATAGTTCTGGAGGCCAG-TAGTT------AGG----------------TGCCAATAGATTTG  74283
Donkey       ACAGAAATTTATTTTCTCACAGTTCTGAAGGCTATTAGTCCCAGACAAGG----------------TGCCAGTAGATTT-  83706
Cat          --GA--G--TGGCTTAA-ACA--ACAGAAATTTTTTTTCTC------AGG----------------TGCCAGTAGATTGA  80230
Ferret       --GATTGAGTGACTTAA-ACA--ACAGAAACTT----------------G----------------TGCCAGCAGATT--  82993
Lion         --GA--G--TGGCTTAA-ACA--ACAGAAATTTTTTTTCTC------AGG----------------TGCCAGTAGATTGA  77925
Vaquita      ACAGAAATTTATTTTCATATAGTTCTGGAGGCCAG-TAGTT------AGG----------------TGCCAATAGATTTG  75566
Pig          ACAGAAGTTTATTTTCTTACAGTTCTGGAGGCTAG-TAGTT------AGG----------------TGCCAGCAGTTTTG  75610

Majority     GTTTCTGGTAAGGACTTTCTTCCTGGCTTGCAGAXXGCTACCTTCTCCXTGTGTCCTCACA------------TGGCCTT

                       107770    107780    107790    107800    107810    107820    107830    107840

Human        GTTTCTGGTGAGG---TCTCTCTTGGCTTGCAGCCAACCATCTTCCCCCTGTGTCCTCATG------------TGGCCTT  87345
PolarBear    GTTTCTGATAAGGGCTTTTTTCCTGACTTGCAGATGGCTAACTTCTCCATGTGTCCTCCCT------------TGGACTT  45067
Cow          TTGTGTG--------------------------------------TGTGTGTGTGTGT---------------TAGTCAT  82068
Dog          ---------A-GGACTTCTGTCCT-------------------------TCTG---------------------------  75057
BelugaWhale  GTTTCTGGTGAGGACTTTCTTCCTGGCTTTCAGACAGCTCCATTCTCCATATGTCCTCACATGGCCTTTCCTCTGAGCCT  74363
Donkey       GTTTCAGGTGAGAACTTTCTTCCTGGTTTGCAGGCAGCTTCCTTCTTTTAGTGTCTGCAAA------------TGGCCTT  83774
Cat          GTTTCTGATAAGGGCTTTCTTCCTGGCTTACAAATGGCCACCTTCTCTGCATCTCCTCACA--------------GCCTT  80296
Ferret       GTTTCT---AAGGGCTTTCTTCCTGGCTTGCAGTTGACTTACTTCTTCGTGTGTCCTCACA------------TGGACTT  83058
Lion         GTTTCTGATAAGGGCTTTCTTCCTGGCTTGCAAATGGCCACCTTCCCCGCATCTCCTCACA------------TAGCCTT  77993
Vaquita      GTTTCTGGTGAGGACTTTCTTCCTGGCTTTCAGACAGCTCCATTCTCCATATGTCCTCACATGGCCTTTCCTCTGAGCCT  75646
Pig          GTTTCTGGTTAGGAC--TCTTCCTGGCT-----------------TGCATGTGTCCTC---------------TGATCTT  75656
```

```
Majority      TC-TCTGAGCTCTCTG-----XGGTTTCTCTT---CTTCTTATAAGGACACTAGTCTTATTGGATXAGGGCCTCAXCCTT
                       107850    107860    107870    107880    107890    107900    107910    107920
Human         TCCACTGTGCAAGCACGTTAAGAGTCTCTT-----CCCCTTGTAAGGATCCTAGTCCTGTTGAATCAGGGCTTTACCCTT  87420
PolarBear     TCCTCTGAGTTCTCTG------GGTTTCTCTT---CCTCTTATAAGGACACTAGTCTAATTGGATGAGGGCCTCATCCGC  45138
Cow           T-----CAGTCCTATA-----TGACTCTTTGC----AACT----------CCA-------TGGACTATAGCCT-------  82110
Dog           --------------T--------------------CTTCTTATAAGGACACTAGTCTAATTAGATGAGGGCCTCATCCTT  75103
BelugaWhale   TT-TCTCAGCCTCTGAGGCTCTGATTTCTCTTCCTGTACTTATAAGGACACCAGTCTTATTGGATTAGGGCCTCACCCAT  74442
Donkey        TCCTCTGAGCTCTCTGG----TATCTTTTCTC----TTTTTATAAGGACACCAGTCTTACTGGATTAGAGCTC-ACCCTT  83845
Cat           TCCTCTGAGCTCTCTG------GGTTTCTCTTCCTCTTCTTCTAAGGACACTGGTCTTACTGGATGAGGGCCTCATCCTT  80370
Ferret        T----TGAGCTCACTG------GGTTTCTCTC---CCTCTTATAAGGACACTGATCTAACTGAATGAGGACCTCATCCTT  83125
Lion          TCCTCTGAGCTCTCTG------GGTTTCTCTTCCTCTTCTTCTAAGGACACTGGTCTTACTGGATGAGGGCCTCATCCTT  78067
Vaquita       TT-TCTCAGCCTCTGAGGCTCTGATTTCTCTTCCTGTACTTATAAGGACACCAGTCTTATTGGATTAGGGCCTCACCCAT  75725
Pig           TT-GCTCAGCCTTCGA-----TAGCTCTTCTC----TACTTGCAAGAACACCAGTCCTGTTGGATTAGGGCCTCACCCTT  75726

Majority      A-TGACCTCATTTAACCTTAATTACCTCCTT-AAAGGCCCTGTCTCCAGATA--GTCACATTG--GGATTAGAGCTTCAA
                       107930    107940    107950    107960    107970    107980    107990    108000
Human         AATGACCACATTTAACCTTAATTACCTCCTT-AAAGGTCCTGTTTTTAAGTACAGTTACATTG-GGGGTTAGGACTTCAA  87498
PolarBear     A-TGACCTCATTTAGCCTTCATTATCTCTTTAAAAGTCCTGGTCTTTTGATG--GTCATATTGTAGGATTAGAGCTTCAG  45215
Cow           -------------------ACCAGGCTCCT---------CTGTCCCTGAAAT--GTTCCA-------------------  82140
Dog           A-TGACCTCATTTAACTTTCATTATCTCCCTTAAAGTCCCTGTCTTCAGATG--GTCATTTTGCAGGATTAGAGCTTCAG  75180
BelugaWhale   G-TGACCTCATTTAACCTAAATTACCTCCTT-AAAGGCCCTGCCTCCAGGTA--GTCACATTGAGGGATTAAAGCTTCCA  74518
Donkey        A-TGACCTCATTTAACCTTAATTACTTCCTT-GAAGGCCCTGTCTCCAAATA--GTCATGTTGAAGGGTTAGAGATTTAT  83921
Cat           G-TGACCTTATTTAACCTTCA-----------------CTATCTCCT--------------------TAGAGCTTCAA  80410
Ferret        A-TGACCTCATTTAACTTTCA------------AAGTCCCTGTCTTCT-----------A-----GGATTAGAGTTTCAG  83176
Lion          G-TGACCTTATTTAACCTTCA-----------------CTATCTCCT--------------------TAGAGGTTCAG  78107
Vaquita       G-TGACCTCATTTAACCTAAATTACCTCCTT-AAAGGCCCTGCCTCCAGGTA--GTCACATTGAGGGATTAAAGCTTCCA  75801
Pig           A-TGACCTCATTTAACCTAAATTGCCTCCTT-AAAGGCCCTGTCCCCAGATA--GTCACATTGACAGGTTAGAGCTTTAA  75802
```

```
Majority     TATATGAATTTTGGGAGAATACAGTTCAGTGCGTAACAGTACTTA-AATTTGT--AAATCTTTTATCCCT----XCTTTT
                       108010    108020    108030    108040    108050    108060    108070    108080
Human        CATATAAATTTGGGGAGGACACAATTCAGT---TAATAGTACTTA-AATTTGT--AAATCATTTATCCC-----AAATTT  87567
PolarBear    TATATGACTTTTGGGAGAATACAGTTCAGTGCATAATAGTATTTA-CATTTGT--AAATCTTTTATGGCT----GCTTTT  45288
Cow          GGCAAGAATACTAG-------------AGTGGGTTGCCGTTCCC---------------TTCTCCA--------------  82178
Dog          TATAGGACTTTTGGGAGAACACAGTTCAATGCATAATAGTACTTATAAAAAGC--AGGGGTAAAAGATTT----GCAAAC  75254
BelugaWhale  TATATCACTTTTGGGAGAATACAGTTCAGTGCCTAACAGTACTTA-ACATTGAT-AAATCTTTTACCCCT----AATTTT  74592
Donkey       CATCTGAATTTTGAGAGAATACAGTTCAGTGTGTAACAGTACGTA-CATTTGT--AAATCTTATACCCCTA---ATTTTT  83995
Cat          TATATGAATTTTGGGAGAATACAGCTCAGTGCGTAACAGTACTTATGTTTTACTAGAGCCTTGACTGTCTAATAGCATGA  80490
Ferret       TATATGACTTTTGGGAGAATACAATTCAGTGCATAACAGTACTTAGCATTTGT--AAATCTTTTATGCCT----GCTTCT  83250
Lion         TATATGAATTTTGGGAGAATACAGCTCAGTGCGTAACAGTACTTATATTTTACTAGAGCCTTGACTGTCTAATAGCATGA  78187
Vaquita      TATATCACTTTTGGGAGAATACAGTTCAGTGCCTAACAGTACTTA-ACATTGAT-AAATCTTTTACCCCT----AATTTT  75875
Pig          GATAGGAATTTTGGGAGAATATAGTTCAGTGTGTGACAGTACTTC-GTTTTAT---AATTCTTTACCCCT----AATTTT  75874

Majority     TATAAGATCTTTTTACT-------TGACGTACTAAXAG-ATXATTTATAGTTTTTGAACTATATGTAGTGGT--------
                       108090    108100    108110    108120    108130    108140    108150    108160
Human        TATAAGATATTGTCACTAAAGTTTTGACTTGCTAGTAG---GGCTTATAATTTTTGGACTATCTGTAGTGGTAAATA---  87641
PolarBear    TATAAGATCTTTTTTAA-------TAAAGCTTTGACGT-G--ATTTATAGTTTTTAAACTATA----ATAGT--------  45346
Cow          ---GGGATATTTCTGAC-------CCGGGGATCAAAGGCAGGTCTC----CTCCTGCATTGCAGGCAG------------  82232
Dog          T-TAAGATCTTTGA-AA-------TAAAGCTTTGACAT-G--ATTTACAGTGTTTAAACTATTT--TACAGT--------  75312
BelugaWhale  TATAAGATTTTTTTACTAAAGCTTTGACTTACTAATAGCATAATTTATAGGTTTCAAACTATATGTAGTGGCAGATA---  74669
Donkey       AGTAAGATCTTTTCACTAAATCTTTGACTTACTAATGGCATGATTTATAGTTTTTGGACTATATCTAGTGGCAGATA---  84072
Cat          TTTGTAGTTTTTAAACC-------ATATGTAATAGCAA-ATAATTTATTTTTTCTCATTGCTTT--TAGGGT--------  80552
Ferret       TTTAAGGCCTTTTT-AA-------CAAAGCTTTGACA-----AATTGTATTTTTTGAACTATATGTAATAGT--------  83309
Lion         TTTGTAGTTTTTAAACC-------ATATGTAATAGCAA-ATAATTTATTTTTTCTCATTGCTTT--TAGGGT--------  78249
Vaquita      TATAAGATTTTTTTACTAAAGCTTTGACTTACTAATAGCATAATTTACAGGTTTCAAACTATATGTAGTGGCAAATA---  75952
Pig          TATAAGATCTTTCTGCTAAAGCTTTCACTTACTAATAGCATGATTTATAGTTTTTGGACTATACGTAGTGTCAGGTAGAG  75954
```

```
Majority     -ATXTATTATTTCTCAT--GXTGCXAGATTAG----TGC-TTTCTTGAAGTATX------------TATGT---------
                      108170    108180    108190    108200    108210    108220    108230    108240
Human        -ATTCATCATTTCTCATT-GCTGCTAGGTAGA-TTTTGC-TTTCTTGAACTGTGTGACCATAAAAGTATGTACTGACACC  87717
PolarBear    -AAATAACATTTTTCAT----TGCTAGATTT-----TGC-TTTTTTGAAGTAG--------------ATGA---------  45392
Cow          -ATTTTTTACCATCTGA--GCCACCAGGGAAG--------CTCCTTGAT-----------------TATGT---------  82275
Dog          -AAAT--TATTTCTCAT----GGCTAGATTT-----TGC-TTTTTTGAAATCCT------------TATGA---------  75358
BelugaWhale  -ATTTATTATTTCTCATT-GCTGCCAGGTTAGATTTTGC-TTTCTTGAAGTATATGTCCATAAAAATATGTACTTGCACC  74746
Donkey       -ATGTATTCTTTCTCCTT-GTTGCTAGGGTAGATTTTGCTTTTCTTGAAGTATGTGACCATAAAAATATGTGCTTACACC  84150
Cat          -AGATTTTGAAGTATAT----GACCAGAAAAA----TAT-GTATTCATACCATT------------AATGC---------  80601
Ferret       -AAATA-TATTTCTCAT----TGCTAGATTT-----TGC-TTTTCCAAAGTAG--------------ATGA---------  83354
Lion         -AGATTTTGAAGTATAT----GACCAGAAAAA----TAT-GTATTCACACCATT------------AATGC---------  78298
Vaquita      -ATTTATTATTTCTCATT-GCTGCCAGGTTAGATTTTGC-TTTCTTGAAGTATATGTCCATAAAAATATGTACTTGCACC  76029
Pig          AATTTATTATTTCTTAATTGCTGCAAGAGTAGATTTTGC-TTTCTTGAAGTATAGGTCCATAAAAATATGTATTCGTACT  76033

Majority     --------CTTTAAAACAC--ACACACAXATACTCAAATCCXATACAAAACTAXGGTGTXCTATTGAAAAAAATTAXXXA
                      108250    108260    108270    108280    108290    108300    108310    108320
Human        ATCATTTCCTTTAAAACACACACACACAAACACACACA--C-ACACACG-----CATACACTCCCACACAAAAC------  87783
PolarBear    --------CCATAAAA-----ATATGTAGATGACCATA--AAAATAATGGCACACCATTAATGCCTTTAATATTGAAAAA  45457
Cow          ------------------------CATAGATGCTCAT-------------------------------------------  82288
Dog          --------TTCTAAAA-----ATACGC----ACTCATA--CCATTGATG-----CCTTTAATATTGA-AAAAATTAGTGA  75413
BelugaWhale  TTTAATGCCTTTATAGCACACA--CACAAACACACAAATCCTATACAAAACTAAGGTGCGCCATTGAAAAAAATAAGTGA  74824
Donkey       ATCTATGCCTTTCTCACAC--ACACATGCTCACTCAAATCTTACACAAAACTAGGATGTACTATTAGAAAGAAT------  84222
Cat          --------TTTTAAAATAC--ACACCCACATACTCAAATCCCACAGAAAACTAAGGTGTGCTGTTGAAAAAAATT----A  80667
Ferret       --------CCATAAAT-----ATATGT---TACTCACA--CCATTAATA------CCTTTATATTGAAAAAAATTAGTGA  83410
Lion         --------CTTTAAAATTC--ACACCCACATACTCAAATCCCACAGAAAACTAAGGTGTGCTGTTGAAAAAAATT----A  78364
Vaquita      TTTAATGCCTTTATAGCACACA--CACAAACACACAAATCCTATACAAAACTAGGGCGCGCTATTGAAAAAAATAAGTGA  76107
Pig          GTTAATGCCTTTAAAACACACAAACACAGATGCACAAATCCTATGCAAAACTAGGGTGTCCTATTGAAAAAGATAAGTGA  76113
```

```
Majority     GAATTTTAAAAATTTAGTATGCTTTATTTCTGGGCTCTGTTCTCATTT----TGGTTTTCATAGGACTCTATTTGGAAAT

                      108330    108340    108350    108360    108370    108380    108390    108400

Human        ------T----CTATGGTGTACT--ATTTAGTA-TGGTGTACTAA-----AAAGATTATCA---AACTTGGTATTTAAAA  87842
PolarBear    GAATTTTTAAAATTTAATATGCTTTATTTCTGGGCACTGTTCTTTT--------GTTTTCATACGACT--GTTTGGAAAT  45527
Cow          -----------------TATTCTTTTCTTCT---TTTTCTGCTTATGTACACTTGTGT-------ACTCTGGTTAGGGAT  82341
Dog          GACATTTGAAAATGTAATATGCTTTATTTTTGGGCCCTATTTTCCTTT----TGGTTTTCATAGGACTCTATTTGGAAAT  75489
BelugaWhale  AATTTTTTTAAATGTAGTATACGTTTTTTATGGGCTCTGTTCTCATTCTTTTTGATTTTCACGGGACTCTGTTTGGAAAT  74904
Donkey       GACTTAAAAAATTTTAGTATGCTTTGTTTCTGGGCTCTGTTCTC--------TGATTTTCATGGAACTCTATTTGGAATT  84294
Cat          GAATTTAAAAAATTTAGTATGCTTTGTTTTGGGGCCCTGTTCTCCCTT----TGGTTTTCATAGGACTCTATTTGGAAAT  80743
Ferret       GAATTTTAAAAATTTAATATGCTTTATTTCTGGGCACTGTTCTCCTTT----TGGTTTTCATAGGACTCTATTTGGGAGT  83486
Lion         GAAT--AAAAAATTTAGTATG-----TTTTGGGGCCCTGTTCTCCCTT----TGGTTTTCATAGGACTCTATTTGAAAAT  78433
Vaquita      AATTTTTTAAAATGTAGTATACGTTTTTTATGGGCTCTGTTCTCATTCTTTTTGGTTTTCATGGGACTCTGTTTGGAAAT  76187
Pig          AACTTAA-AAAATGTAATATGCTTTGTTTCTGGGCTCTGTTCTCATTCTTACTGGTTTTTATGGGATTCTATTTAAAAAT  76192

Majority     ACTCATTGTTTTATGATATAGGCAGA-------------------TXTTTTTAT----------GCTTAGAAA-------

                      108410    108420    108430    108440    108450    108460    108470    108480

Human        AGTTA--GCTATAT-----A-------------------------C-TTTTTACAAGA-----GACATGTAAAGCAGA--  87882
PolarBear    ACTCATTGTTTCATGATACAGGTAG-----T------------TTTTTTTTTAT----------GCTTAGGCA-------  45573
Cow          AGTCATTGTCAT---------------------------------TATTTTTAT----------G---------------  82363
Dog          AACT--CATTTCATGATATAGGCAGGTTTTT------------TTGTTTTTTGT----------TTTTTAATGCCTAGGA  75545
BelugaWhale  ACTCATTGTTTTATAATATAGGCAGA-------------------TGTTTTTAT----------GCTCAGCAA-------  74948
Donkey       ACTCATTGTTTTGTAATTTAAGCAGA-------------------TGTTTTTAT----------GCTTAGGAA-------  84338
Cat          ACTCATTGTTTTATGGTATAGGCAGATAAATTTTTTTTTTAATTTTTTTTTTAACATTTATTTATTTTTGAGACAGAGAG  80823
Ferret       ACAA-------CATGAAGGAGCTA-------------------CTACTCTTTGA----------ACTTTAGTA-------  83523
Lion         ACTCATTGTTTTATGGTATAGGCAGATATATATTTAAAAAAAAATTTTTTTTAACATTTATTTATTTTTGAGACAGAGAG  78513
Vaquita      ACTCATTGTTTTATAATATAGGCAGA-------------------TGTTTTTAT----------GCTCAGAAA-------  76231
Pig          ACTCATTGTTTTATGATAGAGGCAAA-------------------TGTTTTTAT----------GCTCAGGAA-------  76236
```

```
Majority     ----GTAGTATGGG--------------------AGAAATAAACAGCATGXTAAAGAGAT-CATAG--GTATTTAAGTGT

                      108490    108500    108510    108520    108530    108540    108550    108560

Human        ----AGAATATGAA--------------A--GATTGAAAGGAAAAGGATG-TAAAGAGATACTAAT--ATACTGGACAGA  87939
PolarBear    ----GTGGTATGGG--------------------GGAAATAAC-AGAATT---AAGAGAT-CATA---GTGTTTAATCGT  45621
Cow          -----TACTTT---------------------------ATAAGAAGCCTTTTACAGTGTTTCATAG--TTGACAAAGCAT  82409
Dog          C---ATAGTATGGG--------------------GGAAATAAGGAGAATT---AAAAGAT-ACTAT--GGGTTTAAATGT  75596
BelugaWhale  ----GTAGTATGGG--------------------AGAAATAAACGGCAAGGTATAGT----CAGTA--GTATACAAGTGT  74998
Donkey       ----GTAGTATGGG--------------------AGAAATAAACAGCAAGGAAAACTAAGAGACAATAGTGTTTAAATGT  84394
Cat          A--CAGAGCATGAGCAGGGGAGGGTCAGAGAGAGAGGGAGACACAGAATCTGAAACAGGC-TCCAG--ACTCTGAGCTGT  80898
Ferret       ----GAAGAATT------------------------------------TT---AACAGA---ATA---CCATTTAAGGAT  83554
Lion         AGACAGAGCATGAGCAGGGGAGGGTCAGAGAGAGAGGGAGACACAGAATCCGAAACAGGC-TCCAG--GCTTTGAGCTGT  78590
Vaquita      ----GCAGTATGGG--------------------AGAAATAAACGGCAAGGTATAGT----CAGTA--GTATACAAGTGT  76281
Pig          ----GTAATATGGG--------------------AGGAATAAACAGCATGGAATATTGATACATAG--TTATTCAAATGT  76290

Majority     GAAT---GAGATTGTTGCAAATCTTAAAG--------------------------GCC--------ATGTTAAATCATTT

                      108570    108580    108590    108600    108610    108620    108630    108640

Human        TAGT-----AAGCAAGACAAAACTTAAAT-------------------------------------ACCTTTATTAA---  87974
PolarBear    GAAT---TAGATTGTTGCAAACCTCAGAG--------------------------GCC--------ATGTTAAATCAT--  45662
Cow          ---------------------TCTCAAA---------------------------------------TGTTTGATTTCTT  82429
Dog          GAAT---GAGATTGTTGCAAACCTTGGAG--------------------------GCC--------ATGTTAAATTGGAT  75639
BelugaWhale  GAAT---GGGATTGTTGCAAATCTTAGAG--------------------------GCC--------ATGTTAAATCATTT  75041
Donkey       GAAT---GGGATTGTTGCAGTCCTCTAAG--------------------AGA--AGCC--------ATGTTAAATGATTT  84441
Cat          CAGCCCAGAGCCCGATGCGGGGCTCGAACTCAGGGACCGCAAGATCATGACCTGAGCCGAAGTCGGGCACCCAACTGACT  80978
Ferret       AACA---TATACAAATT--ATTCTTAAA--------------------------------------ACATCGAAT-----  83586
Lion         CAGCCCAGAGCCCGATGCGGGGCTCGAACTCATGGACCGCGAGATGATGACCTGAGCCGAAGTCGGACACCCAACCGACT  78670
Vaquita      GAAT---GGGATTGTTGCAAATCTTAGAG--------------------------GCC--------ACGTTAAATCATTT  76324
Pig          GAAT---GGGATTGTTGAAAATCTTAAAG--------------------------GCC--------ATGTTAAATCATCC  76333
```

```
Majority     T--------------------------------A-TTTTAAAAXXTA------------TAAACATTAAGATGTTTCAAT
                      108650    108660    108670    108680    108690    108700    108710    108720
Human        ------------------------------------CTTAAGACAGAAT----------TGATAAAGGATTTAGTTCACT 88008
PolarBear    ------------------------------------TTGAGACCACAGTT---------TTAAAATTTGCAAATATTGAT 45697
Cow          T----------------------------------TTTCTCATTGTG------------TGAACTGTGGGTGGTGTCTGT 82463
Dog          G-----------------------------------TTTTAAGAATAATT---------TGTGTATTATTTTAAAATGAC 75675
BelugaWhale  TA--------------------GA-----CTAGAGTTTTAAAATTTG------------CAAACATTAAGTTGTTTCAAT 75084
Donkey       C--------------------------A----GAGTTTCAAAGTTTG------------TAAATGTTAAGATGTTTCTTT 84479
Cat          G----------GCGCCCCTAGGCAGATATTTTTATGTTTCGGAAGTAGTATGGGGGAAATAAAGAACATTAAGAGATAGT 81048
Ferret       ------------------------------------TTAAGAAAACA------------CTAAAATAGGCATGGTAGAAT 83618
Lion         GAACCACCCAGGCGCCCCTAGGCAGATATTTTTATGTTTAGGAAGTAGTATGGGGGAAATAAAGAACATTAAGAGATAGT 78750
Vaquita      TA--------------------GA-----CTAGAGTTTTAAAATTTG------------CAAACATTAAGATGTTTCAAT 76367
Pig          TA--------------------GA-----CCAGAGTTTTACAACTTG------------CCAACATTGAGATGTTTCATT 76376

Majority     T---------TTTAACCT-XTTTAATTXATATXXTAGXGTTTXX-XTAAATTATTTATTTXXXGAXTXATAAAATTTGTA
                      108730    108740    108750    108760    108770    108780    108790    108800
Human        A----------GAAAGATATAACAGTTTATGTATATGTGTGCCTAGTGAAATAGCC------------TCAAAATATATA 88066
PolarBear    T---------TTTT-------TTAATGAAGATTCTACCATCTA--GTGTTTTCTT----------------AAATTGG-- 45741
Cow          T------------------GTTCAGTCGCTA-AGTCGTGTTC------AGTTCTCTGCATGCCAGGCTCCTCTGTCT-TC 82517
Dog          C---------TTTAACAGAATTCTGATAAAATCCCAAGGAGTA--GTAGTTTTTCATG-----------TTAAACTG--- 75731
BelugaWhale  TTCT----ACCACAACCT-ATTTAATTGATA--CTGGAATTTTC-TTAAATTGGATATTTTAGGAATAATATAATTTGTA 75156
Donkey       A-GTAAACTTTTTACCATAGCTTATTTAATTGATACAGTATTTTCTTAAATCAGATATTTTAGGAATAATATAATTTGTA 84558
Cat          A---------TGTGAACGAGATTGCTGCAAATCTCAGAGACCATGCTAAATCATTTTAGACCAGAGTTTTAAAATTTGCA 81119
Ferret       G---------TTTA-------TTTAAAAAATTCTTAATGTTT---GCAAATCTTT----------------AAACTGT-- 83661
Lion         AGTGTTTAAATGTGAACGAGATTGCTACAAATCTCAGAGGCCATGCTAAATCATTTTAGACCAGAGTTTTAAAATTTGCA 78830
Vaquita      TTCT----ACCATAACCT-ATTTAATTGATA--CTGGAATTTTC-TTAAATTGGATATTTTAGGAATAATATAATTTGTA 76439
Pig          TCG-----ACCATAGCCT-ATTTAATTGATACACTCGTGTTTTC-TTAAATTGGCTATTTTAGGAGTAATACAATCTGTA 76449
```

```
Majority     AATTATCTTTTGATXTAXT-ATACTGATTCTAAAGGX-T-X----------X--X---AAXXTACCAAAGAGTTGATCXT
                      108810    108820    108830    108840    108850    108860    108870    108880
Human        AATAATAAGTAGATAGAACTACAGGTAGACTTGAGCA---------------------AATCTATTATAGTGGTGG----  88121
PolarBear    -ATGTTTTAATAATTTGTGTATATTATCTTTAAATGA-----------------A----CTTTAACAGAAGTTTAA-CAA  45798
Cow          CACTATCTACCAGAGT----TTGTTCAAATTCACG---TCCTTTAAATCAGT--------GATGCTAATGATCTCGTCCT  82582
Dog          -GTTTTTATTTAATGTATAGCTGCTTTCTCAAAAGTA-----------------A----AGTTACTTAGTTCTTCA-TAA  75788
BelugaWhale  AATTATGTTTTGAAGTAGT-GTACTGATCTTCAATGGTTAAAGTAAATGAGTTCTAAGGAAATGTCAAAGAGTAGTTCCT  75235
Donkey       AATTATCTTTAAAAATAGT-ATACTGGTCCTTGAAGACTTA--------GATTCTAGCAAAATGCCAAAGAGTAGTTCCT  84629
Cat          AACTTTAAGGTGTTTCTTTAATAATCTTTCTACCGTGGCTT-------------ATTTAATTTAGCGTTTTCTTAAATTG  81186
Ferret       -GGTCTCAAGTGATTTA---ACATGGCCTCTAAGG--------------------------TTTGCAACAATCTCA-TTC  83710
Lion         AACTTTAAGGTGTTTCTTTAATAATCTTTCTACCATGGCTTTCTACCATGGCTTATTTAATTTAGCGTTTTCTTAAATTG  78910
Vaquita      AATTATGTTTTGAAGTAGT-GTACTGATCTTCAACGGTTAAAGTAAATGAGTTCTAAGGAAATGTCAAAGAGTAGTTCCT  76518
Pig          AATTATCTTTTAAAATAGTATAACTGATCTTCAAG---TAAAATAAATCGATTATACCAAAATACCAAAGAGTAGTTCTT  76526

Majority     T--TGTTAAAAX-AATA--CTAGTTTCTCCXA-TXATATTTTCAA---CTGX--ACAAXAA-XXXX---CCATXXTTATT
                      108890    108900    108910    108920    108930    108940    108950    108960
Human        --ATTTCA-----A-----TTCACTTTTCTGAATTACATGTCAAA---CTGACCAAAAAAAGA-------CAAAATCAAC  88179
PolarBear    A--ATACCAAAG-AGTA--GTAGTTCCTTC-------ATGTTAAA---CTGG--TTTTTAT----------------TTA  45845
Cow          C--TGTCA-----------CCCCCTTCTCC---TTTTGCTTTCA------------------GTCTTT-CCACATTACCC  82627
Dog          T--AGTCCTTGA-TCTA--CTAAGTTATCT-------CTCT-------C-----ACAAAAA----------------GAT  75828
BelugaWhale  ---TGTTAAAAT-AATTATCTTGTTTCTCCAATTCATATTTTCAAGAACTACTTACAAGAAAGCCTCAACTGTTCTTATT  75311
Donkey       TCATGTTAAAAACAATTATCTGGCTTCTCCAATTAATATTTTTAAGCACTGTTTACAAAAATACTC---CTGTTTTTATT  84706
Cat          T--TGTTTTAAG-AGTA--ACGTGTTATCCTA-AATGAACTTTAA---CAGA--ATTTTAACAAAATA-CCAAAGAGTAT  81254
Ferret       A--CATTTAAAC-ACTA--CTA--TCTTTT-------AATT-------C-----TCTGTAT----------------TTC  83748
Lion         T--TGTTTTAAG-AGTA--ACATATTATCCTA-AATGAACTTTAA---CAGA--ATTTTAACAAAATA-CCAAAGAGTAT  78978
Vaquita      ---TGTTAAAAT-AATTATCTTGTTTCTCCAATTCATATTTTCAAGAACTACTTACAAGAAAGCCTCAACTGTTCTTATT  76594
Pig          T--TGTTAAAAT-AATTGTCTTGTTTATCCAGCTCATATTTTCAAGAACTACTTA-AAAAAAGCCTTT-TCTTTTTTATT  76601
```

```
Majority     TXATATATAXCTGCCATCTCAAAAGTAAACTTX----GT--TXTXTAACAGA-C---ATCTACTAAATTATATCT-GTCA
                    108970    108980    108990    109000    109010    109020    109030    109040
Human        AAAGATACAAATGATAC----AATACAATTA-----------CCTTAACA-----------G---AATTATGTGT--CTA  88228
PolarBear    TTATATATAGCTGTCATCTCAAAAGTAAATTT----AGTTCTTCTTAACAGATCTTTATCTACTTAATTATATCTTATCA  45921
Cow          TTATAAATAAGGGATAC----ACAGTCATTATG----GT--TGAAAAGTA--------CCTGTTCAATCAAATTC-TTCT  82688
Dog          TTCAAGAGATCTTCCTTCCCAAGAGGAATT------AGCAAGGAGGGAAATAGCAA-ATGTTATGGAATATAATG-GGGA  75900
BelugaWhale  TAATATGTAGCTGCCATCTCAAAAGTAAACTTA----CT--TCTTCTTTAA--------CTACTAAATTATATCT-AGCG  75376
Donkey       TAATATATAGTTG-CATCTCAGACATAAACTTACTTGGTTCTTCGTAGCAGACC----TTTACT-TACTAAATTT-ATCA  84779
Cat          TTTTATATAACTGCCATCTCAAAAGTAAACTTATTTAGTTCTTTATAACAGACCTTTAGCCGCTATATTACATCT-GTCA  81333
Ferret       TCTCATACTACT----TCCTAAGCATAAA-----------------AAA--A-----ATCT----ACTTATATCATGAAA  83796
Lion         TTTTATATAACTGCCATCTCAAAAGTAAACTTACTTAGTTCTTTATAACAGACCTTTAGCCACTATATTACATCT-GTCA  79057
Vaquita      TAATATGTAGCTGCCATCTCAAAAGTAAACTT----------CTTCTTTAA--------CTACTAAATTATATCT-AGCG  76655
Pig          TAGTATATAGGTGTCATCTCAAAAGTAAACTTA----GT--TCTATAATAGACCTTTACCTACTAAATTATATTT-GGCA  76674

Majority     AAGTGTGCTGTAAAG-X-GTGGAAATC-TG-TAACTCTGGTGTTA-TGATATTTGAGCAATTTTTAAGTATXTGTATATA
                    109050    109060    109070    109080    109090    109100    109110    109120
Human        AGTTATGCATC--------------C-----AACACAGTTTTAAACGCATATGGAACATTTTTGAAAAA----------  88279
PolarBear    AAGTGTGCTGTGA-----GTGGAAATCCTG-TAACTCTGTTACGA----TATTTGAATAATTTTTAAGTATGGTTATATA  45991
Cow          GGATGCTCT-------------------------TTGGTT----TCTTATATATGGACTGTTTAAATAA----------  82728
Dog          AACTATTTACTTA-----CTTACTTACTTACTTACTTACTTACTTAC--TTATTTATTTAT-TATTTATTTATTTATGAT  75972
BelugaWhale  AAGTGTGCTGTAAAGGGAGTAGGAATCATG-TAACTCTGGTGTTG-TGATATTTGAGCAGTTTTTAAGTATGTGTATATA  75454
Donkey       AAGTGTGCTGTAAAGTGAGTGGAAATCATA-TAACTCTGCTGTTA--GATATTTGAGCAGTTTTTAAGTATATGTATGTA  84856
Cat          AAGTGTGCTGTAAAGTGCATGGAAATCCTG-TAACTCTAGTGTTAATGATATTTGAGCAATTTTTAAGCGTATGTCTATA  81412
Ferret       CAATAT-TTCCAA-----GTGG------TA-TGACACAATTACCT----TAACAGAATTATACATATATAGATTTATGCA  83859
Lion         AAGTGTGCTGTAAAGTGCGTGGAAATCCTG-TAACTCTAGTGTTAATGATATTTGAGCAATTTTTAAGTGTGTGTCTATA  79136
Vaquita      AAGTGTGCTGTAAAGGGAGTAGGAAT--TG-TAACTCTGGTGTTG-TGATATTTGAGCAATTTTTAAGTATGTGTATATA  76731
Pig          AAGTGTGCTATAAAGGAAATAGAAATCATG-TAACTCTGATG----TGATATTTGAGCAATTTTTAAGTATT--------  76741
```

```
Majority     TTGGCTTTCACATX--XACX-X--XXTGXATAGTGAAGAACTCT-AAGTCGCACTTXGAGGTATTGCTGXTCTCACAAXA
                               109130    109140    109150    109160    109170    109180    109190    109200

Human        --------------------------------------------------------------------------------  88279
PolarBear    TTGGCTTTCACAGAACTACCCTATTCTGAATAGTGAAGAATTCT-CAGTCACACTTTGAGGTCTTGCTGGTCTCCCAAAA  46070
Cow          --------------------------------------------------------------------------------  82728
Dog          TTTATTTATTCATGA-GACACA--CACAGAGAGAGAGAGAGATA-GAGAGGCA----GAGACACAGGCAAAGAGAGAAGC  76044
BelugaWhale  TTGGCTTTCACTTCTTCATTAC--TGTGAGTAATGGAGAACTCT-AAGTCGCACTTTGAGGTATTGCTGGTCTCATAGAA  75531
Donkey       TTGGCTTTCACTTC--TACTGT--TATGAATAATGAAGAACTCTTAAGTTGTACTTTGAGGCTTTGCTGGTCTCACAAAA  84932
Cat          TTGGCTTTCACAGAGGAACTGT--CATGCATAGTGAAGAACTCT-AAGTCACACTTAGAGGTATTGCTGAGATTTCAAGA  81489
Ferret       TTTGTCTTT-TAAG--TGC----------ATATGGGAATATTTA-CAAAAGCA------GGCTTCAGTAAAGCAAAGAGT  83919
Lion         TTGGCTTTCACAGAGGAACCGT--CACACATAGTGAAGAACTCT-AAGTCACACTTTGCGGTATTGCCGAGATTTCAAGA  79213
Vaquita      TTGGCTTTCACTTCTTCATTAC--TGTGAGTAATGGAAAACTCT-AAGTCGCACTTTGAGGTATTGCTGGTCTCATAGAA  76808
Pig          --------------------------------------------------------------------------------  76741

Majority     GAG-----TTCAAXAGAXCXTXTXCXXX-----------GA-------------AATAGCAAATGXTATGGAGX-TAATG
                               109210    109220    109230    109240    109250    109260    109270    109280

Human        ------------------------------------------------T-------------------------------  88280
PolarBear    GAGA---TTTCAAGAGATCCCCTCCTTCCCCCAAGGAGGGA-------------AATAGCAAATTGTATGGAGTATAATG  46134
Cow          --------------------------------------------------------------------------------  82728
Dog          AGGCTCCACTCAGGGAGCCTGATGCAGGACTTGATCCCAGG-------------ACTCCAGGATCACCTAGGGA-TCCCC  76110
BelugaWhale  GGGAGC-TCTCAATAGACCTTCCACCCAAAAAAAGTCAAAAAAAAGGAGGGAAAAATAGCAAATGATATGGAGA-AAATG  75609
Donkey       GAGAA--TTTCAAGAGGTCTTTCCTCTTAAAGGAAAGGAGG---------GGAAAATAGCAAATGAAATGATGAAAAATA  85001
Cat          GA------TTCCCCTCATCCAATGGAGG-----------GA-------------AATAGCAAATGGTATGGAGTATAATG  81539
Ferret       AAGT---ATCCTGTAGACCCTATTCTCT--------------------------GTTGCTCAATT---TAGAGT-TGATA  83966
Lion         GA------TTCCCCCCATCCAGTGGAGG-----------GA-------------AATAGCAAATGGTATGGAGTATAATG  79263
Vaquita      GGGAGC-TCTCAATAGACCTTCCCCCAAAAAAAAGTCAAAAGAAAGGAGGGAAAAATAGCAAATGATATGGAGA-AAATG  76886
Pig          ---------------------------------------AA---------------------------------------  76743
```

```
Majority     GGGGAAAA-ATAAATTTAAAA-TTATGATA-XAXXTAAGTCCCTATGCATCAATAATAA---CAA------ATGTAAAGG
                     109290     109300     109310     109320     109330     109340     109350     109360
Human        -----------------------------------------------------------CA----------GTCACAT  88289
PolarBear    AGGAAACA-ATAAATTTAAAAATTATGATAGAATCTAAATCCCTATGTAGCAATAATAACAACAA------ATGTAAAGG 46207
Cow          ------------------------------------------------------------------------ATGATGC  82735
Dog          AGGAAATA-ATAAATTTAAAATTTATGATAGAATCTAAGTCCCTATGCACCAGTAATAATAGCAACAACA-ATGTAAAGG 76188
BelugaWhale  GGGGAAAATAGAAATTTGAAAGTAATGATA-GAATCAAGTCCAGATGTATCAATAATAA---CAG------ATGTAAAGG 75679
Donkey       GGGGAAAATATGAATTAAAA--------------TGAAGTCCAGATGCATTAATAATAG---CAA------ATATAAAGG 85058
Cat          GGGGAAAATATAAATTTTAAAATTAAGATAGAATCTAAGTCCCTATACATCAATAATAACAACAACAGCAAATGTAAAGT 81619
Ferret       ACAAAAAG-ATATATTTAGAATTTCT-----CAATTAAAGTACTATACA--A--AGTAG----AT------ATGTATAGT 84026
Lion         GGGGAAAATATAAATTTTAAAATTATGATAGAATCTAAGTCCCTATACATCAGTAATAACAACAACAACAAATGTAAAGT 79343
Vaquita      GGGGAAAATAGAAATTTGAAAGTAATGATA-GAATCAAGTCCAGATGTATCAATAATAA---CAG------ATGTAAAGG 76956
Pig          -----------------------------------------------------TAA---CAG------ATGTAATGG 76758

Majority     AGTAAATTCATCAGTTAAAA--------GATXXGTTTTTTXAT--AG-CTGATTTT-----TTAAAAGAGTGTTAT----
                     109370     109380     109390     109400     109410     109420     109430     109440
Human        ACTAAA--------CCATAA--------AGCAAGCTTC-------AG-CTAATTTC--------AAAGAGTATCAT----  88333
PolarBear    AGTAAATGCATCAGTGAAAA--------GACAGGTTGTCAGAT-----CTGAGTTT-----TTAAAAGTTTATTAC----  46265
Cow          TTTAATTT------TTACTAT--------ACTTGTTTT-------AGTCTGCTTG------------GGCTGCCAT----  82778
Dog          AGTAAGTGCATCAGTCAAAA--------GATAGGTTGTCAGATC-TGATTTTTTTT-----TTTTTAACTGGTTGC----  76250
BelugaWhale  AGTAAATTCATCAATTAAAAGA----CAGATTTGATTTTTTAAA-AGCAACCTATT-----CTTTACAAGAGATGTGAGT 75749
Donkey       AGTAAGTTCATCAGTTAAAAGATTGTCAGATTTGATTTTTAAAATGCTATCTCTTTGCTATTTACAAGAGACATATAAAG 85138
Cat          AGTAAATGCATCAGTCAAAA--------GACAGGTCGTCAGAT-----CTGATTTT-----TAAAAAGTTCTTTAC----  81677
Ferret       A----------CAGTGAAAGT-------GGTGGGTTTTTTTTT-----TTTTTTTT-----ATAAACATCTGTT------  84073
Lion         AGTAAATGCATCAGTCGAAA--------GGCAGGTCGTCAGAT-----CTGATTTT-----TAAAAAGTTCTTTAC----  79401
Vaquita      AGTAAATTCATCAATTAAAAGA----CAGATTTGATTTTTTAAA-AGCCAC-TATT-----CTTTACAAGAGATGTGAGG 77025
Pig          ATTAAATTCATCAGTTAAAAG--------ATTTGCTTTTTTAAAAAGTTAGCTGTT-----ATTTACGAGTGACATGAAG 76825
```

```
Majority      ----AAGA-----A-AGATTTAAAGCACAAGGATATAGAAAXGTTTAAATGAXAAGG---XATAAACAAAAGAXAACTTT

                       109450    109460    109470    109480    109490    109500    109510    109520

Human         -------------ACAGACCTCATTCTCTATTGCACAAGTAAGTTAGAATTAATAAC-------AAAAAGATG-------  88386
PolarBear     ----AAGA-----A-ACACATAAAGCACAAGA-TGTGGAAAGGTTGAAAGTAAAAGG---GATATAAAAAAGATAAATTT  46331
Cow           -----------------------AGCAAA-----ATACAACAAACTGAGTGACTAA--------AACAACAGAAA-----  82817
Dog           ----GAGA-----A-ACTTAAAAAGCACAAGGATGTGGACAGGTTGAAAGAAAAGGGATATATATATAAAAGATAAATTT  76320
BelugaWhale   TGCAAAGATGTAGAAAGATTGAAAGTAAAGGGATATAAAAAGGA-TAATTGACAGAT---AATAAGCAAAAGAAAACTTA  75825
Donkey        TGCAAGGATGTGGAAAGGTTTATAGTAAAGGGATATAAAAAAGATACATTGGGCAAA--TAGTAAGCAAAGAAAAACATA  85216
Cat           ----AAGA-----A-GCATATAAAGCACAAGAATGTGGAAAGATTGAAAGTAAAGGGATATAGAAAAGATAGATTAATTT  81747
Ferret        -------------------TTTAT-CCCCAGG-GGTACAGGTCTGTGAATCACCAGG---TTTACACACTTCATAGCACT  84129
Lion          ----AAGA-----A-GCATATAAAGCACAAGAATGTGGAAAATTTGAAAGTAAAGGGATATAGAAAAGATAGATTAATTT  79471
Vaquita       TGCAAAGATGTAGAAAGATTGAAAGTAAAGGGATATAAAAAGGA-TAATTGACAGAT---AATAAGCAAAAGAAAACTTA  77101
Pig           TTCAAAGA-----A-AGATTGAAAGTAAAGG-ATATAGAAAAGATTTATTGGCAAGT---AGTGAGCAAAAGAAAACTTC  76895

Majority      XG-AX---CAXCAAX--AAGATAAAAX------------------TTTXTTTA---------TG-----TGXTTTTAXGX

                       109530    109540    109550    109560    109570    109580    109590    109600

Human         --------CAT---------ATAAAA-------------------TCTGTAT---------------------CCTG---  88406
PolarBear     AG-AT---CACCAGG--AAGATAAAA-------------------GTTTTTTAT-------GTG-----TGTGTGTA---  46371
Cow           ---------------------------------------------TTTATTT--------------------TCTTAAGT  82832
Dog           AG-AT---CACCAGG--AAGATAAAAAG----------------AGTTTTTGGT-------GTG-----TGTATGTTTGG  76366
BelugaWhale   TGTACCTATATTCATTTAAGATAAAGATTATTGATAAAAGATTTATTAGTTCACCAAGAAGATG-ACAAG--TTTTATGT  75902
Donkey        TG--CCCATATTAATTGAAGGTAAAGATTATTGAGCACAG----ATTTAGTTCAAAAGTAAGTTCGAACAGGTTTTATGT  85290
Cat           AG-AT---CACCAGG--AAAACAAAA-------------------GTTTTATG----------------TGGGTGTG---  81783
Ferret        --------CACCAA---AGCACATAC-------------------CCTCCCCA----------G-----TGTCCATA---  84161
Lion          AG-AT---CACCAGG--AAAATAAAAC-----------------AGTTTTATG----------------TGGGTGTG---  79509
Vaquita       TGTACCTATATTCATTTAAGATAAAGATTATTAATAAAAGATTTATTAGTTCACCAAGAAGATG-ACAAG--CTTTATGT  77178
Pig           TGCACCTATATTAATTTAAGATAAAGATTATTCATAAAAGAT---TTTGTTTACCAGGAAGATGGACAACGTTCTTATGT  76972
```

```
Majority     -X-X--GTGCCTAATGAAATAGCCTCAAAATATGTAAATCATAAATGAATAGA----ACTGXA-GGGAGAATTGAGCAAA
                      109610    109620    109630    109640    109650    109660    109670    109680
Human        -------AGATTTAAAAATAAGTCTCAGTATT------GCATGAGTCAGAAGT-----TTG-----TTTGTTTG------ 88457
PolarBear    ------CTCCCTAATGAAATAGCCTCAAAATATGTAAATCATAAATGAATAGA----ACTAGA-GGGAGACTTGAGCAAA 46440
Cow          -TCTG-GAGACTAGT-AATTAG------------------GTGAC--AACAGA----TTTG-----GTTTCTAGAGAGGA 82880
Dog          GGGGATGCCCCTAATGAAATAGCCTCAAAATATATCAATCATAAATGAATAGATAGAACTAGA-GGGACTCTTGAGGAAA 76445
BelugaWhale  GTATGTGTGCCTAATGAAATAGCTTCAAAATGTGTAAATTATAAATGGATAGA----ACTGCA-GGGAGAATTGAGCAAA 75977
Donkey       ATATATGTGCCTCATGAAATAGCCTCAAAAGATATAAATCCTAAATGGATAGA----TCTACA-GGGAAAATGGAGCAAA 85365
Cat          -------CCCCTAATGAAATATCCTCAAAATATGTAAATCATAAATGAATAAA----ACTGGA-GGAAGAATTG------ 81845
Ferret       ------ATCCCNNNNNNNNNNNNNNNNNNNNNNNNNNNNNNNNNNNNNNNNNNNN----NNNNNN-NNNNNNNNNNNNNNNN 84230
Lion         -------CCCCTAATGAAATATCCTCAAAATATGTAAATCATAAATGAATAGA----ACTGGA-GGAAGAATTGAGCAAA 79577
Vaquita      GTATGTGTGCCTAATGAAATAGCTTCAAAATGTGTAAATTATAAATGGATAGA----ACTGCA-GGGAGAATTGAGCAAA 77253
Pig          ATCTGTGTGCCTAATGAAATAGCCTCAAAAACCTCCCACTGTGGT--GGTAGA----TTTGCTCAAATCTCCCTGGAGTT 77046

Majority     TXXTCC-TTATTGAGGGAGATTTCAACACACCTTTCTGAATTATGTCAGGCAGA-----------CAAAAAAA----CAA
                      109690    109700    109710    109720    109730    109740    109750    109760
Human        ---TTTGTTTTTGAGCCAG----T-----GTCTTGCTCTGTCCCCCAGGGTGGAG----------TGCAGTGG---T--G 88510
PolarBear    TCTTCTCTTATACAGAGAAATTTCAACACACCTTTCTGAATTATGTCAGGCAGA-----------CAAAAAGAGTATCAA 46509
Cow          CTTTC--TTCCTAGCTT--------------------------------GCAGA-----------CCAG----------- 82904
Dog          TCTTCTCTTATAGGGAGAGATCCCAACACACCTTTCTGAATTAAGTCAGGCAGA-----------CATAATA-----CAA 76509
BelugaWhale  TTCACC-ACGATAATGGAGGTTTTTATACACCTTGCTGGATTATGTCAGGCAGAAACCCC---CTCCCAAAAA----TCA 76049
Donkey       TCCACCATCATAGTGGGGGATTTCA--ACCCCTTTCTGAATTATGTCAAGCAGAA----------CAAAAAAAAAATCAA 85433
Cat          ----------------------TCAACACACCTTTCTGAATTATTTCAGGCATA-----------CAAAATGATGATCAA 81892
Ferret       NNNNNNNNNNNNNNNNNNNNNNNNNNNNNNNNNNNNNNNNNNNNNNNNNNNNNN-----------NNNNNNNNNNNNNNN 84299
Lion         TCTTCCATCACA----GAGATGTCAACACACCTTTCTGAATTATTTCAGGCATA-----------CAAAATGAT---CAA 79639
Vaquita      TTCACC-ACGATAATGGAGGTTTTTATACACCTTGCTGAATTATGTCAGGCAGAAACCCCTCCCCCCCAAAAA----TCA 77328
Pig          CTCTCCATTTATGGTTTATATATTTTAACATTTT-CTGAATTATGTTAGGCAGA-----------CAAAAAAG----TTA 77110
```

```
Majority     CAAAGATACAAATGXXACAACACAATT----------------------------TTATGCAT---AXAXTTACACAT-X
                      109770    109780    109790    109800    109810    109820    109830    109840
Human        CTATTGT------GGCTCACTGCAGCC-----------------------------TCGACCTCCCAGGCTTAAGCA---  88552
PolarBear    CAAAGATACAAATGGTAGGACACAATT----------------------------ACCTTAAC---AGAATTGCACATGT  46558
Cow          ----------------------CTCCC----------------------------TTCTCCAT---ATGTCCTCACAC--  82929
Dog          CAAAGATACAGTTACCTTAAAATTAC-----------------------------ACATGTAT---AGAACTACACACTT  76557
BelugaWhale  GCAAGACGCAAATGATACAGCACAATTAGATTAACAGAATTACACATGTATAGAATTAGGCATCTAATAGTTACACATCC  76129
Donkey       GAAAGATACAACTGACAGAATACGATTACCTTAACACAGTTACATATGTACAGAATTATGCATCTAACAGTTATATATCC  85513
Cat          CAAGGATACAAATGGTACAACACAAATG-------------------------ACACATGTAT---AGAATTATACATGT  81944
Ferret       NNNNNNNAGGGAGATCATATGATAGTT----------------------------GTCTTTCT---CTGCTTG-------  84341
Lion         CAAGGATACAAATGGTACAACACAAATG-------------------------ACACATGTAT---AGAATTATACATGT  79691
Vaquita      GCAAGACGCAAATGATACAGCACAATTAGATTAACAGAATTACACATGTATAGAATTAGGCATCTAATAGTTATACATCC  77408
Pig          ACAAGATACAAATGACATAACACACTT----------------------------TTATTCATCTAATAGTCACACAT--  77160

Majority     A-------AXXCTTTT-----ACACTAXGAACXTTTAXGAACATTG-------A-------XTAAAGCAGGCTTCAGTAA
                      109850    109860    109870    109880    109890    109900    109910    109920
Human        ----------GTCCTT--------------GCACTTAAG--CCTC-----------------CAAAGTAG--C----TAG  88583
PolarBear    ATAGAATTACACATTTAA--CACATTAAGTACATATGGGAACACTT-------A-------TGAAAGCAGGCTTCAATAA  46622
Cow          ---------TGCCTTTC-------CTGGGAGCCTTCCTCAGCCTC----------TAAGGTGCTGA-TATCTTTTCGTGT  82982
Dog          A-------ACACTGTT------TTTTAAGTACATTGGGGAACATTT-------A-------CTAAAGCAGGCTTCAGTAA  76610
BelugaWhale  A---ACACATTCTTTTTAAGTACACAAGGAATGCTTACAGAAAGTGATCACAAACTAAACTATAAAGCAAGCTTCAGTAA  76206
Donkey       A---ACACATTCTTTTTAAGTACACATGGAACGTTTATAG-AACTGATCACATACTAAATTGTAAAGCAGGCTTCAGGAA  85589
Cat          A-------ACACATTC-----TTTTTAAGTACA-TAGGGAACATTT-------A-------CTAAAGCAGCCCTCTATAA  81997
Ferret       --------ACTTATTT------CGCTAAGCATG-ATACG--CTCTA-------G-------TTCATCCATGTTGTCGCAA  84390
Lion         A-------ACACATTC-----TTTTTAAGTACA-TAGGGAACATT--------A-------CTAAAGCAGCCCTCCATAA  79743
Vaquita      A---ACACATTCTTTTTAAGTACACAAGGAATGCTTACAGAAAGTGATCACAAACTAAACTATAAAGCAAGCTTCAGTAA  77485
Pig          ---------TCCTTTTTAAGTACACTGGGAACGTTTACTGAAATTGATCG-----TAAACTATAAA-CAAGCTTTGGTAA  77225
```

```
Majority      ATXTXAGAG-AAGTATCATATAGACCATATTCTCTG----TTGCACAATTAAGTTA--GAGTTA----------------
                             109930    109940    109950    109960    109970    109980    109990    110000
Human         GACTAAAAGCATGCACCACCTG--CCCGGCTAATT--------TATTATTA--TTA-----TTA----------------  88630
PolarBear     AGCAAAAAGT--ATCCTATAGA--CCCTATTCTCTG----TTGCACAATT---TAA--GAGTTA----------------  46673
Cow           ACTTACAAG-GA-CATCA-----------GTCTT--------------ACTGGATTA--GGGCC-----------------  83017
Dog           AGCAGAGAGTAAGTAACATATA--TTCTATTCTCTG----TTGCACAATT---T----GAGTTA----------------  76661
BelugaWhale   ATTTCAGA-----AAGTATACAGACCAAATTCTCTGACTACTGTGCAATTAAGTTAAAGAGTTAATAACAGAAAGATACA  76281
Donkey        ATTTCAGAGTAAGCGTCATATAGACCAAATTATCTGACTATTGCACAATTAAGTT---GAGTTAATAATA-AAAAAAACA  85665
Cat           GGCAAAGAGGAAGTATCATGTAGACCATATTCACTG----TTGCACAGTCAAGTTA--GAGCTA----------------  82055
Ferret        ATGGCAAG----ATTTTAT-----TTCTTTTGATGG----CTGCATAGTA---T-------TTC----------------  84431
Lion          GGCAAAGAGGAAGTATCATGTAGACCATATTCACTG----TTGCACAGTCAAGTTA--GAGCTA----------------  79801
Vaquita       ATTTCAGAG-AGTAAGTATACAGACCAAATTCTCTGACTACTGTGCAATTAAGTTAAAGAGTTAATAACAGAAAGATACA  77564
Pig           ATTTCAAAA-AG-TATCATATAGACCA-GTTTTCTGGCTATTGCACAACTAAGTTG--GAGTTAATAACA--AGTATACA  77298

Majority      ------A-----TXATATXTAGAXACXTATAAAT-TTTCCCAATATCTCATGGATCAAAGAAGTTATTTTAATGAGAATT
                             110010    110020    110030    110040    110050    110060    110070    110080
Human         ------------TTTTTTGTAGAGAT-----GAGTTCTCACTGTGTTGCCCAG---ACAGAAGTTATTTTAATGGGAATA  88690
PolarBear     ------A-----TAACAAAAAGATATATTTAGAA-TTTCTCAGTCTCTCATGGATCAAGCAAGTTACTTTAA---TAATT  46738
Cow           -TCAC--CCTTATGACCTCTTTTAACCTATATTC-CGTCCGTAAAGGCCCTGCCTCCAAATAGTCACATTGAGGGGT---  83090
Dog           ------A-----TAACAAAAAGATACATTTAGAA-TTTCCTAATATCTCATGGATCGAGGAAGTTACTTTAATGAGAATT  76729
BelugaWhale   TTTAAAATCTCCAGGTATCTGGAAATTGAAAAAT-AATTCCAATATCTCATGTCTCAAAGAAGTTATTTTAATGGGAATT  76360
Donkey        TTTAAAATTTCCGTATATCTGGAAATTTAAAAATAAGTTCTGATGTCTCATGGATCAGAGAACTTATTTTAATGAGAATT  85745
Cat           ------G-----TAACAAAAAGATACACTTATA--TTTCCCAGTATTTCATGGATCAAAGAAGTTACTTTAATAAGAATT  82122
Ferret        ------A-----TTGTGTATATATAC---CACAT-CTTCTTGATCTC-CACGG--TAGCCAAACTATGGAAA---GAACC  84490
Lion          ------G-----TAACAAAAAGATACACTTATA--TTTCCCAGTATTTCATGGATCAAAGAAGTTACTTTAATAAGAATT  79868
Vaquita       TTTAAAATCTCCAGGTATC--GAAATTGAAAAAT-AATTCCAATATCTCATGTCTCAAAGAAGTTATTTTAATGGGAATT  77641
Pig           TTTAA--TCTCCAGGTATCTGGAAACTGAAAAAT-AATCTTAATACTTCACGTATCAAAGAAGTTGTTTTAATGAGAATT  77375
```

```
Majority     TAGAATXCTTTAAAACTAAAATGATAATAAAAGTACTATACA-AA-TTAGATATGTGTGGTACAGTGAAAGTGGTGTTTT
                       110090    110100    110110    110120    110130    110140    110150    110160
Human        TAAAATACTTAAAA--TTAAGCGATAA----AGTACTATATACAAATTAGATATATGTGGTACAGTGAAAGAGAAGTCTT  88764
PolarBear    TAGAATACTT------TAAAATGATAATGAAAGTACTATACA-AA-GTAGATATGTGTAGTACAGTGAAAGTGGTGTTTT  46810
Cow          ----------TAAAGCTTCAGT-------------------A-TA-TGAAGTTTG----------GGATAATGCAATT-C  83128
Dog          TAGAATACTTAAAAC-TAAAATGATAATGAAAGTACT-TACA-AA-TTAGATATGTGTAGTACAGTGAAAGTGGTGTTTT  76805
BelugaWhale  TTCAAATATGTAAAACT-AAATGGTAATAAAAGTACTGTACA-TA-TTAG-TATGTGTGGTACAGTGAAAGTGATGTT-T  76435
Donkey       GGAAAATATTTAAAACT-AAAGGATAATGAAGGTACTATACA-AATTTAGATATGTGTGGTACAGTGAAA--GGTGTTTT  85821
Cat          TAGAATACTTAAAAG-TAAAATAATGAAACTACTATACTA-A-AA-TTAGGTATGTGTAGTACAGTGAAAGCAGTGTTGT  82198
Ferret       TAGATGTCCA------TCAACAGATGA-------------------ATGGAT----------CAA-GAAAGTGGTGTTTT  84534
Lion         TAGAATACTTAAAAACTAAAATAATGAAACTACTGTACTA-A-AA-TTAGGTATGTGTAGTACAGTGAAAGCAGTGTTGT  79945
Vaquita      TTCAAATATGTAAAACT-AAATGGTAATAAAAGTACTGTACA-TA-TTAG-TATGTGTGGTACAGTGAAAGTGATGTT-T  77716
Pig          TAAAAGTATGTAAAACTTAAATGGTTTTAAAAGTACTATACA-TA-TTAGATGTGTGTGGTACAGGGAAAGTGGTATT-T  77452

Majority     GACAGGAGGTTA--------------------------------------------------------------------
                       110170    110180    110190    110200    110210    110220    110230    110240
Human        GACGGGAAGTTTTA------------------------------------------------------------------  88778
PolarBear    GACAGG---TTA--------------------------------------------------------------------  46819
Cow          ACTG----------------------------------------------------------------------------  83132
Dog          GACAGGAGGTTA--------------------------------------------------------------------  76817
BelugaWhale  TACAGAATGTTTTAACTGTTAAGAATAATGGTATAGGGACTTCCCTGCTGGCACAGTGGTTAAGAATCCACCTTCCTGGG  76515
Donkey       GACAGAAGGTTTTA------------------------------------------------------------------  85835
Cat          GACAGGAGGTTA--------------------------------------------------------------------  82210
Ferret       AACAGGAAGTTA--------------------------------------------------------------------  84546
Lion         GACAGGAGGTTA--------------------------------------------------------------------  79957
Vaquita      TACAGAATGTTTTAACTGTTAAGAATAATGGTATAGGGACTTCCCTGCTGGCACAGTGGTTAAGAATCCACCTTCCTGGG  77796
Pig          AATAGAATGTC----C---T------------------------------------------------------------  77465
```

Majority	--	
	110250 110260 110270 110280 110290 110300 110310 110320	
Human	--	88778
PolarBear	--	46819
Cow	--	83132
Dog	--	76817
BelugaWhale	CTTTCCTGGTGGTGCAGTGGTTAAAAATCTGCCTGCCAATTCAGGAGACACAGGTTCAAGCCCTGGTCCAGGAAGATCCC	76595
Donkey	--	85835
Cat	--	82210
Ferret	--	84546
Lion	--	79957
Vaquita	CTTTCCTCGTGGCGCAGTGGTTAAAAATCTGCCTGCCAATTCAGGAGACACAGGTTCAAGCCCTGGTCCAGGAAGATCCC	77876
Pig	--	77465

Majority	--	
	110330 110340 110350 110360 110370 110380 110390 110400	
Human	--	88778
PolarBear	--	46819
Cow	--	83132
Dog	--	76817
BelugaWhale	ACGTGCTGAGGAGCAACTAAGCCCGTTCGCCCTAGAGCCCGTGCTCTGCAACAAGAGAAGCCACCGCAATGAGAAGCCCG	76675
Donkey	--	85835
Cat	--	82210
Ferret	--	84546
Lion	--	79957
Vaquita	ACGTGCTGAGGAGCAACTAAGCCCGTTCACCCTAGAGCCCGTGCTCTGCAACAAGAGAAGCCACCGCAATGAGAAGCCCG	77956
Pig	--	77465

Majority	--	
	110410 110420 110430 110440 110450 110460 110470 110480	
Human	--	88778
PolarBear	--	46819
Cow	--	83132
Dog	--	76817
BelugaWhale	TGCACTGCAGTGAAGATACTGCCCCCACTCGCTCCAACTAGAGAAAGCCCACATGCAGTAACAAAGACCCAACGCAGCCA	76755
Donkey	--	85835
Cat	--	82210
Ferret	--	84546
Lion	--	79957
Vaquita	TGCACTGCCGTGAAGATACTGCCCCCACTCGCTCCAACTAGAGAAAGCCCGCATGCAGTAACAAAGACCCAACGCAGCCA	78036
Pig	--	77465

Majority	--	
	110490 110500 110510 110520 110530 110540 110550 110560	
Human	--	88778
PolarBear	--	46819
Cow	--	83132
Dog	--	76817
BelugaWhale	AAAATAAATAAATAAATTTATAAGAAAAAAGAACCTGCCTGCCAATGCAGGGAACACGGGTTTGAGCCCTGGTCTGGGAA	76835
Donkey	--	85835
Cat	--	82210
Ferret	--	84546
Lion	--	79957
Vaquita	AAAATAAATAAATAAATTTATAAGAAAAAAGAACCCGCCTGCCAATGCAGGGGACACGGGTTTGAGCCCTGGTCTGGGAA	78116
Pig	--	77465

```
Majority     ------ACTT----------------------------------------------------------------TA----

                      110570    110580    110590    110600    110610    110620    110630    110640

Human        ------ACTT----------------------------------------------------------------TA----  88784
PolarBear    ------ACTT----------------------------------------------------------------TA----  46825
Cow          -------CCT-----------------------------------------------------------------A----  83136
Dog          ------ACTT----------------------------------------------------------------TA----  76823
BelugaWhale  GATCCCACGTGCCGGGGAACAACTGAGCCCATGCGCCACAACTATTGAGCCTGCGCTCTAGAGCCTGTGAGCCACAACTA  76915
Donkey       ------ACTTTT--------------------------------------------------------------TA----  85843
Cat          ------ACTT----------------------------------------------------------------TA----  82216
Ferret       ------ACTT----------------------------------------------------------------TA----  84552
Lion         ------ACTT----------------------------------------------------------------TA----  79963
Vaquita      GATCCCACATGCCGGGGAACAACTGAGCCCATGAGCCACAACTATTGAGCCTGCGCTCTAGAGCCTGTGAGCCACAACTA  78196
Pig          -------TTT-----------------------------------------------------------------A----  77469

Majority     --------------------------------------------------------------------------------

                      110650    110660    110670    110680    110690    110700    110710    110720

Human        --------------------------------------------------------------------------------  88784
PolarBear    --------------------------------------------------------------------------------  46825
Cow          --------------------------------------------------------------------------------  83136
Dog          --------------------------------------------------------------------------------  76823
BelugaWhale  CTGAGCCAACGTGCCACAACTACTGAAGCCTGCATGCCTAGAGTCCATGCTCTGCAACAAGACACTGCAATGAGAAGCCT  76995
Donkey       --------------------------------------------------------------------------------  85843
Cat          --------------------------------------------------------------------------------  82216
Ferret       --------------------------------------------------------------------------------  84552
Lion         --------------------------------------------------------------------------------  79963
Vaquita      CTGA---------------------A-GCCTGCATGCCTAGAGTCCATGCTCTGCAACAAGACACTGCAATGAGAAGCCT  78254
Pig          --------------------------------------------------------------------------------  77469
```

```
Majority     --------------------------------------------------------------------------------

                     110730    110740    110750    110760    110770    110780    110790    110800

Human        --------------------------------------------------------------------------------  88784
PolarBear    --------------------------------------------------------------------------------  46825
Cow          --------------------------------------------------------------------------------  83136
Dog          --------------------------------------------------------------------------------  76823
BelugaWhale  GCGCACGTCAACAAAGAGTAGCCCCCTGCTCGCCGCAACTTGAGAAAGCCCGCACACGGCAACGAAGACCCAACACAGCC  77075
Donkey       --------------------------------------------------------------------------------  85843
Cat          --------------------------------------------------------------------------------  82216
Ferret       --------------------------------------------------------------------------------  84552
Lion         --------------------------------------------------------------------------------  79963
Vaquita      GCGCACGGCAACAAAGAGCAGCCCCCTGCTCGCCGCAACTTGAGAAAGCCCGCACACGGCAACAAAGACCCAACACAGCC  78334
Pig          --------------------------------------------------------------------------------  77469

Majority     ------------------------------AGAATAATGGTATAAACCCAAGGAAAGGA---GATAATAA----AGACAA

                     110810    110820    110830    110840    110850    110860    110870    110880

Human        ------------------------------AGAATAACAGTGTTACCCCCAGGGAAGGA---GATATTAA----AGACAA  88827
PolarBear    ------------------------------TGAATGTTGGTATAAACCTAAGAAAAGGAAATGGTAATAA----AGACAA  46871
Cow          ------------------------------ACAGTATTTATTTACATTTTA-----------------------------  83157
Dog          ------------------------------AGAATGATGGTATAAACCCAAGGAAAGG--ATAATAATAA----AGATAA  76867
BelugaWhale  AATAAGTAAATAAATAATTTAGAAAAAAAAAGAATAATGGCATAAACCCAAGGAAAAGA---GATAATAA----AGACAA  77148
Donkey       ------------------------------AGAGTAACAGTATAAACCGGGGGAAAGGA---GATGATAA----AGACAA  85886
Cat          ------------------------------AGAATGATGGTGTAAACCCAAGGAAAGAA---GAA--TAA----AGACCA  82257
Ferret       ------------------------------T--ATGTTGGTATAAACCCAAGGAAAGGAGATGATGATAATAATAGACAA  84600
Lion         ------------------------------AGAATGATGGTGTAAACCCAAGGAAAGAA---GAAGATAA----AGACCA  80006
Vaquita      AATAAGTAAATAAATAATTTAGAAAAAAAAAGAATAATGGCATAAACCCAAGGAAAAGA---GATAATAA----AGACAA  78407
Pig          ------------------------------AGAATAATGGTATAAATTCAAGGAAAAGA---GATAATAA----AGACAC  77512
```

```
Majority     GACTAGAAATCAXTGGTATAGAAGACAGTTGAGAGAAAAAAA---AAAGCTAAAAGTTGGGTCTTTXGATAAXAXTAATA
                     110890    110900    110910    110920    110930    110940    110950    110960
Human        GACTGGAAATCAATGGTATAAAAGACAGTTGAGAGAAAAAAAC---ATGCCACAAGTTGGATTCTTTGAAAAGACTAAAA  88904
PolarBear    GACTAGAAATCATTGGTATAGAAGACAGTTGAGAGAAAAAA----AAAGCTAGAAGCTGGGTCTTTGGATAG-ATTGATA  46946
Cow          ---------------------------------------------------AAATGTTTTATCCCT----AATTTTTATA  83182
Dog          GACTAGCAATCACTGGTATAGAAGACAGTTAAGAGAAAAAAC---AGAGCTAGAAGGTGGGTCTTTGAATAA-ATTAATA  76943
BelugaWhale  GACTAGAAATCAATGGTATAGAAGACAGTTGAGAGAAAAAAAAATAAAGCTGAAAGTTGGGTCCTTTGACAAGACTAATA  77228
Donkey       GACTAGAGATCAGTGGTATAGAGGACAGTTGAGAAAAAAATA--TGAAGCTAAAAGTTGGGTCTTTTGAAAAGACTAATT  85964
Cat          GACTAGAAATCACTGGTATAGAAGACAGTTGAGAGAAAGAAAGA-AAAGCTAGAAGCTGGGTTTTTGAATA---------  82327
Ferret       GACTAGAAATCATTGGTATAAAAGACAGTTGAGAGAAAAAA----AAA-----AAGCTGGGTTTTTGAGTAG--TTAATA  84669
Lion         GACTAGAAATCACTGGTATAGAAGACAGTTGAGAGGAAGAAAGA-AAAGCTAGAAGCTGGGTTTTTGAATAG-ATTAATA  80084
Vaquita      GACTAGAAATCAATGGTATAGAAGACAGTTGAGAGAAAAAAAAA--GAGCCAAAAGTTGGGTCCTTTGACAAGACTAATA  78485
Pig          GACTGGAAATCAGTGGTATAGAAGACAGTTAAGAGAAAAAAA------GCTAAAAGTCAAATCCTTTGACAAGACTAATA  77586

Majority     AAATAGACAAGTAGATGTTAAGATTCATTTTTTTAAA-AATGAGAGAGGCAACACAAATAA--T--TTTGAATTATAAAG
                     110970    110980    110990    111000    111010    111020    111030    111040
Human        AAATAGACAAACATCTA--AAGATTG-ATT--AAAA--AATGAGAGTGGTAACACAAAAAAATTTTTTTGAATTATAAAG  88977
PolarBear    AAATAGACAAGTGGATGTTGAGATTCATTTTTTAAAA-AATGAGAGG--CAACACAAATAA--TACTTTGAATTATAAAG  47021
Cow          AATTAAAAAGATAAA-------ATTT-TTATCTTT---------------------------------------------  83209
Dog          AAATAGACAAGTGAGTATTGAACTTGATTTTTTAAAA-AGTGAGAGA--CAACACAAATAA--TACTCGGAATTATAAAC  77018
BelugaWhale  AA-----CAAGTGGATATCAAGATTCATTTTTTTTTA-AATGGGAGAGGCAGCACAA-TAT-----TTTTAATTATAAAG  77296
Donkey       AATTAC-CAAGTGGATGTTGAGTTTCGTTTTTAAAAA--ATGGGAGAGTCGACACAA-TAT-----TTTGAATTATGAAG  86035
Cat          AAATAGACAAGTAGATGTTAAGATTCATTTTTTGGAATAACGAGAGAGGCAACACACCTAAG-TTTTTTGAATTATAAAG  82406
Ferret       AAATAGAGAAGTAGATGTTGAGATTCATTTTTTTAAA-AATGAGAGA--CAACACAAACAA--TACTTTGAATTATAAAG  84744
Lion         AAATAGACAAGTAGATGTTAAGATTCATTTTTTGGAATAATGAGAGAGGCAACACACCTAAG-TTTTTTGAATTATAAAG  80163
Vaquita      AA-----CAAGTGGATATCAAGATTCATTTTTTTTTA-AACGGGAGAGGCAACACAA-TAT-----TTTTAATTATAAAG  78553
Pig          AAATAGACAAGTAGATGTCAAGATTT-TTTTTTTCAA-AATGTGAGAGGCAACACAAATAT-----TTTGAATAATAAAG  77659
```

```
Majority     GGG----ATAATTATTGATATAGCTCCAGTT-CCACATTATATAAAC---TCCTGTA--------------TCTATXTAT
                       111050    111060    111070    111080    111090    111100    111110    111120
Human        GGG----AGAAGCATTGATAAAGCTCCAGTTTCCACATCATATAAACAC-CACTATG--------------TCTGTAAAT  89038
PolarBear    GGG----ATAATTGTTGATATAGCTCCAGTT-CCACATTTTATAAAC---TCCTCC---------------TGTATAAAT  47078
Cow          ---------TCACTAA------AGCTTTGCTT-ACGTACCATGA-----------TGTA---------------GTTTTTGGA  83250
Dog          TTCTCCTGTATATACTGTTATTCATTCATAT-CTTTTTTTGGTAAACAT-TTCTTTATTT-TCT-------TTTATTTAT  77088
BelugaWhale  GGG----ATAATTATTGATATAGTTCTAGTT-CCACATTATGTAAAC------TGTA---------------CGTATTTTG  77351
Donkey       GAG----ATAACTATTGATGTAGCTCCAGTT-CCACACTATATAAACTCTTCCTGTA---------------TCTTTAAA  86095
Cat          GGT----ATAATTGTTGACATAGCTCCATTT-CCACAT---ATAAACTTGTCCTGTGGCTCTCTCTCTCTCTCTATATAT  82478
Ferret       GAG----ATACTTACTGATA-AACTTCAGTT-CCACATTTTATAAAC---TCCTGTATAA-AAA-------TCTATAAAT  84807
Lion         GGT----ATAATTGTTGACATAGCTCCATTT-CCACAT---ATAAACTCGTCCTGTAGCT---------------ATATAT  80221
Vaquita      GGG----ATAATTATTGATAGAGTTCTAGTT-CCACATTATGTAAAC------TGTA---------------CGTTTTTTT  78608
Pig          GAG----ATAATTAT------AGCTCCAGTT-TCACATTATATAAGC---ATCTGTA---------------TCTATCTAT  77711

Majority     TTTTXTATA-------------------------------------------------------------TTTT-
                       111130    111140    111150    111160    111170    111180    111190    111200
Human        TTCTTGAGG--------------------------------------------------------------TCTT  89051
PolarBear    TATTTTTTA------------------------------------------------------------CCATCTT  47094
Cow          CTATATGTA--------------------------------------------------------------GT---  83261
Dog          TTTTCTGAAAGATAAATAAAGCAGAGGGAGCAGCAGAGGGAGGGGGTGAAGCAGGAAGCCCTGACATGGGGCTCTACCCC  77168
BelugaWhale  TTTACCATC-------------------------------------------------------------TTTT-  77364
Donkey       GTTTTTACA--------------------------------------------------------------TTT-  86107
Cat          ATATATATAA----------------------------------------------C------TTTTTTT  82496
Ferret       TTTTTTTAA------------------------------------------------------------CCATCTC  84823
Lion         ATATATATA--------------------------------------------------------------TT---  80232
Vaquita      TTTACCATC-------------------------------------------------------------TTTT-  78621
Pig          CTATCTCTC-------------------------------------------------------------GTTTT  77725
```

```
Majority     X-AAXCTTXTXXTTATT--------------------CATTAATATCTTC--TTCAGAGATTAAGGGAAAAGTAX---AA
                      111210    111220    111230    111240    111250    111260    111270    111280
Human        TTAAAATTTTTATTATT-----------------CGTTAATAATCTCTTTCATAGATGGATTAAGGGAAATGTAA---AA  89111
PolarBear    TGA--CTTTCCGTTATT--------------------CATTAATATCTTC--TTTAGAGGTTAAGGGAAAAGTAA---AA  47147
Cow          ---GGCA------AATT--------------------TCTCATTGCT-------GATAGGTTAG----------------  83289
Dog          AGAACCCTTAACCCACTGAGCCCCACCCAGACGCCCCCATTAATACCTTC--TTCAGAGATAAAGGGAAAAGTAA---AC  77243
BelugaWhale  ---AATTTCTCGTTATT--------------------CATTACTATT-----TTCAGAGATAAAGGGAGAGGTAG---AA  77413
Donkey       ---AACTTTTCATTATT--------------------CATTAGTATTTTC--TTCAGAGATGGATTAAGGGAAAG--GTA  86160
Cat          TTAACCATCTTCTCATTG---------------TT--CATTAATATCTTC--TTCAGAGATTAAGAGAAAAGTAG---AA  82554
Ferret       -------TTGAATTTGT--------------------TTTTAATATCTTC--TTCAGAGATTAAGGGAAAAGTAA---AA  84871
Lion         TTAACCATCTTCTCATTG---------------TT--CATTGATATCTTC--TTCAGAGATTAAGGGAAAAGTAG---AA  80290
Vaquita      ---AATTTCTCGTTATT--------------------CATTACTATT-----TTCAGAGATAAAGCGAGAGGTAG---AA  78670
Pig          TAAACCATCTTTTAATT--------------------TCTCATTATTCA---TTGATGGATTAAGGGAAAAGTAATGAAA  77782

Majority     AATTATATTCAACCAAAAAT-GCCATTTCCACTGTGCAACATTGACTTTTTXCTCCTTT-----ATCATTGATTGCTTT-
                      111290    111300    111310    111320    111330    111340    111350    111360
Human        AATTACATTAAATCAAAAAT-GTCATTTCAATTGTGAAACATTGACTTTTTCCT-----------TTATT-ATTGCCTT-  89177
PolarBear    AATTGTATTCAACCAAAACTTGCCATTTACACTGTGCAACATTGGCCTTTTCCTCTTTT-----ATCATTGATTGCTTT-  47221
Cow          -ATTTTGCTTTCTTGAAGT---ATATTTCCATTA---AATATGGACTCAC--------------ACCATTAAT-GCCTT-  83346
Dog          AATTACATTCAAACAAAAGT-GCCATTTCCATTGTGCAACATTGGCCTTTTCCTCTTTT-----ATCATTGATGGCTTT-  77316
BelugaWhale  AATAATACTCAACCAAAAAT-GACGTTTCCACCATGCAGCATTGACTTTTTTCTTCTTG-----ATCACTGATTGCTTT-  77486
Donkey       AATTATATTTAACCAAAAAT----------GCCGT------TTGACTTTTTCCTCCTTT-----ATCATTGATTGCTTTT  86219
Cat          AATTATACTCAACCCAAAGT-GCCATTTCCACTGTGCGACATTGGCCTTTTTCTCTTTTTATCGTTCATTGATTGTTTT-  82632
Ferret       GATTATTTTCCACTGAAAGT-GCCATTTCCACTGTGCAACATTGGCCATTTCCTCCTTT-----ATCATTGATTGCTTT-  84944
Lion         AATTATACTCAACCCAAAGT-GCCATTTCCACTGTGCGACATTGGCTTTTTTCTCTTTT-ATCGTTAATTGATTGTTTT-  80367
Vaquita      AATAATACTCAACCAAAAAT-GACGTTTCCACTATGCAGCATTGACTTTTTTCTTCTTG-----ATCATTGATTGCATT-  78743
Pig          TATAATGTTCAACCAAAAAT-GACATTTCCACTGC--GATATTGACTTTCCTCCTCT-------ATCGTTGATTGCCTT-  77851
```

```
Majority     GTCCATTTATGCCATATTTCTTGXAACAG---ATTTCAAGATAT-AGAGTCCTGTTATTT-AAACTTGATTCATTTTAAA
                                   111370    111380    111390    111400    111410    111420    111430    111440
Human        GTCCATTTATGCCATATTTCTTGGAATAGGAGAGTTCAAG-TGTAAGATTCACATTGTTTAAAATTTTATTCATTTAAAA  89256
PolarBear    GCCCATTTACACCATATTTCTTATAACTG---ATTTCAAGATA---GAGTCCTGTTATTT-ATACTTGAATC---TTAAA  47291
Cow          ---------------------TAAAACAC---ACAC-------------------------AAATCC----TATGCAAAA  83373
Dog          GTCCATTTACACCGTATTTCTTGTAACCA---GTTTCAGGATATCAGAGTCCTGTTATTT-AAACTTGGTTCATCTTAAA  77392
BelugaWhale  GTTCATTGATGCCGTATTTCTTGGAACAG---ATTTCAAGATATTAGAGTCCTGTTATTT-AAACTTGATTCATTTAAAA  77562
Donkey       GTCCATTCATGCCGTATTTTTTGGAATAG---ATTTCAAGATAT--GAGTCCC-TTTTTT-TAACTTGATTCATTTTGAA  86292
Cat          GTCCATTTGTACCATATTTCTTGTAACAG---GCTTCAAGATATCAGAGTCCCAATATTT-AAGCTTGATTCATCTTAAA  82708
Ferret       GCCCATTTACCCCATATTTCTTATAACCG---ATTTCAAGGTA---GAATCCTGTTATTT-AAACTGGATTCAGCTTAAA  85017
Lion         GTCCATTTATGCCATATTTCTTGTAACAG---GCTTCAAGTTATCAGAGTCCCAGTATTT-AAACTTGATTCATCTTAAA  80443
Vaquita      GTTCATTGATGCCATATTTCTTGGAACAG---ATTTCAAGATATTAGAGTCCTGTTATTT-AAACTTGATTCATTTAAAA  78819
Pig          GTTCATTGATGACCTATTTCTTAGAGCAG---ATTT-------------------------AAACTTGATTTATTTAAAA  77903

Majority     TTAG-TAATTCAAGTACTCT--XG---TGTTTTGTTTAATCATTATG--T-TXCCTCAGTTTTTGACAAGXXT----TTT
                                   111450    111460    111470    111480    111490    111500    111510    111520
Human        TTAG-TAATTCAGGTAATTTCCTTC--TGCTCTGTTTACTCCTTGTG---TACTTTCAGTTTTTGACAAAGCTTTTGTTT  89330
PolarBear    TTAG-CAATTCAAATAATCCCTGG---CAGTTTGTTTAATCATTACG--TGTGCTGCAGTTTTTGAC----------TTT  47355
Cow          CTAG-GA-----TGTGCTAT-------TGAAAAA-------AATA-------AGTGAAACTTTTTTTAA--------TAT  83418
Dog          TTAG-TAGTTCAAATAATCTACTG---TTTCTTGTTTAATCATTATG--TCTGCTTCAGTTTTTGACAAG-------CTT  77459
BelugaWhale  TTTTTCTAATCATTTCCTTC-------TGCTTTGTTTAATCAATATA----TACCTCCATTTTTGACGAGCTT----TTT  77627
Donkey       TTAT-TAATTCAAATACTCTCTTGCCTTGTTTTGTTTAATCATTGTA--TGTACTTAACTTTTTGACAGGC------TTT  86363
Cat          TTAG-TAATTCAAGTACTCTTCTG---CTTTTTGCTTAATCGTTATG--TATGCCTTAGTATTTGACAAGGTTTTCTTTT  82782
Ferret       TTAG-TAATTCAAGTAATCT---G---CTTTTTGTGTAATAATTATTATTGTGCCTCAGTTTTTGACAAG-------CTT  85083
Lion         TTAG-TAATTCAAGTACTCTTCTG---CTTTTTGCTTAATCATTATG--TATGCCTTAGTATTTGACAAGGTTTTCTTTT  80517
Vaquita      TTTTTCTAATCATTTCCTTC-------TGCTTTGTTTAATCAATATA----TACCTCCATTTTTGACGAGCTT----TTT  78884
Pig          TTAT-CAATTTATTTCCTTT-------TGCTTTATTTACTCAATA-------ACCTCAATTTTTGACAAGGTT----TAT  77964
```

```
Majority     XTTTTTTXA-----A-T-CTTAAAAACCCTGGTATTCTA-----CCCCTCTTCTTCAXCCCTCATCCACTTTAGATTACX
                              111530    111540    111550    111560    111570    111580    111590    111600
Human        TCTTTTTTA---------CTTAAAAATTTTGGT--------A---TTCTAATTATCATCCCCCTTCCCCTTTAGATCACT  89390
PolarBear    TTTTGTT------------TTTTAAACCGTGATACTCGA-----CCCCTCATCTTCA-CCCTCATCCACTTGAGATTACT  47417
Cow          ACTTTGTT------------TATGG-------------------ACTCTGTTCTT---------TTTGGTTTTCATGGG-  83457
Dog          TTTTTAAAAA---AAGTGTTTAGAAATTGTCACACTCAA-----CCCCTCTTCTTCAGCCCTCATCCATTTGAGATTCCA  77531
BelugaWhale  ATTTTTTAA----TACC-CTTAAAAGCCCCGGTATTCTAATT--ACCCCTTTCCTTAACCCCCATCCACTTTAAATTTAC  77700
Donkey       CTTTTTTAA---------CTTAAAAATCCTGGTATTCTAATTGTCCCTCCTCCCCCAACCCTCATCCACTTTAGATC---  86431
Cat          CTTTTTTTT----GAATACTTAAAAACCCTGATATTCTA-----CCCCTCAACTTCGGCCCTCATCCACTTCATATT---  82850
Ferret       CTTTTTTAAA---AA-TACTTAAAAACCATGATGCTTGA-----CTCCTCATCTTCAGCCCTCATCCACTTGAGATTACT  85154
Lion         CTTTTTTTTTTTTGAATACTTAAAAACCCTGGTACTCTA-----CCCCTCAACTTCGGCCCTCATCCACTTCATATTACT  80592
Vaquita      ATTTTTTAA----TACC-CTTAAAAGCCCTGGTATTCTAATT--ACCCCTTTCCTTAACCCCCATCCACTTTAAATTTAC  78957
Pig          ATTTTTTTA----GCAT-TGTAAGGG------TATTCTAATT--ACCCCGTTTCTCAACCTCCATCCACTTTACGTGGG-  78030

Majority     ----ATXCTTTTGTCAGGAA--AGCTGACTAGA-ATXTCTCTCATTACCTGGCTACTTCCTGAGXTAGCTGTTTGGTTAT
                              111610    111620    111630    111640    111650    111660    111670    111680
Human        TCAGATCCTATTGTCAGCAATCAGCTGGCTAGA--ACTCTCTTGTTACCTGGCTACTTCCTGAGGTAGCTGTTTGGTTAT  89468
PolarBear    TCAGATTCTTTTGTCAAGTC--AGCTGACTAGG-ATACTTCTCATTACCTGACT-CTTCCAGAGTTAGCTCTTTGGTTAT  47493
Cow          ----A--CTCTATTTGGGAA------------------------ATACTCAGTTGTTTTATAAGCAAAT---------AT  83498
Dog          ----ATTGTTTTGTCAAGTC--CGATGACTAGA-ATATTTCTCATTACCT-A---TTTCCAGAGGTAGCTCTCTGGTTAT  77600
BelugaWhale  TCACATCCTTTTATCAGCAATCAGCTGACTAGA-ATCTCCCTTATTACGTGGTTACTTTCTGAGTTAGCTGTTTGGTTGT  77779
Donkey       ------TCTTTTGTCAGAAATCAGCTGACTAGA-ATCTCTCTCATAATCTGGCTATGTCTTGAGGTAGTTGTTTGATTAT  86504
Cat          -------CTTTTGTCAGGTC--AGCTGACTCGACATACTTCTCATTACCTGACTACTTCTAGAGATAGCTTTTTGGTTAT  82921
Ferret       --------------------------GACTAGA-ATACCTCTCATTACCTGACTACTTCCAGAGTTAGCTCTTTGGTTAT  85207
Lion         TCATATTCTTTTGTCAGGTC--AGCTGACTTGACATACTTCTCATTACCTGACTACTTCTAGAGATAGCTTTTTGGGTTT  80670
Vaquita      TCACATCCTTTTATCAGCAATCAGCTGACTAGA-ATCTCCCTTATTACGTGGTTACTTTCTGAGTTAGCTGTTTGGTTGT  79036
Pig          ----ATCCTTTACTCAGCAGTCAGCTGACTAAA-ATCTCTTTCTGTACCTGGTTACTTTCTAAGGGAGTTGTTTGGTTAT  78105
```

```
Majority     TTTGCTTTATTTT-AATCCCATTTCTCT-ATCATCCATCTCCTCTTGGTGCAGGTAC-------------TATGCCTAGG

                            111690    111700    111710    111720    111730    111740    111750    111760

Human        TTAGCTTTATTTT-AATCCCATTCCTCT-ATCACTCATCTGTTCTGTATGCAGTTAC-------------TATGCCTGGG  89533
PolarBear    TTTGCTTTGTTTT-AATACCATTTCTCT-ATCATCCATCTCCTCTTGGTACAGGTAC-------------TATGCCTAGG  47558
Cow          TTT-------------------------------------------------------------------TATGTTCAGA  83511
Dog          TTTGCTTTATTTT-AATCCCATTTCTCT-ATCATCCATCTCCTCTTGGTACAGGTAC-------------TGTCCCTAGG  77665
BelugaWhale  TTTGCTTTATTTTTAACCCCATTTCTCT-ATCATCCATCTCCTCTTGGTGCAGTTACTTA---------CTATGCCTAGG  77849
Donkey       TTTGCTTTGTTTT-AACCCCATTTCTGT-ATCATCTATCTCCTCTTTGTACAGTCAC-------------TATGCCTAAG  86569
Cat          TTTGCTTTATTTT-AATCCCATTAGTCT-GTTATCCACCTCCTCTTGATGCAGGTGC-------------TATTCCTAGG  82986
Ferret       TTTGCTTTATTTA-AATCCCAGTTCTCTTATCATCCATCTCCTCTTCGTACAGGTACATAGTAGAAGTACTGCGCCTAGG  85286
Lion         TTTGCTTTATTTT-AATCCCATTAGTCT-ATTATCCATCTCCTCTTGGTGCAGGTGC-------------TATTCCTAGA  80735
Vaquita      TTTGCTTTATTTTTAACCCCATTTCTCT-ATCATCCATCTCCTCTTGGTGCAGTTACTTA---------CTATGCCTAGG  79106
Pig          TTTGCTTTATTTT-AATCCCATTGTTCT-CTCATCTGTCTCTCCTTGATGCA----TTTA---------CTGTGTCTAGG  78170

Majority     ACTTTTT--TGTTTTCTTATTTCTGCCCTTCAGTATCTCAAAGCTCAGCT--GTTTGTGCAGTCTTTTCATAACTTTTCA

                            111770    111780    111790    111800    111810    111820    111830    111840

Human        ACTT------ATTTTCTTATTCTTTCCCTCCATTATCTCAAAGCTCTGCT--GTTTGTGCTGTCTTTTTAT---------  89596
PolarBear    ACTTT-----GTTTTCTTATTTCCGCCCTTCAGTATATCAAAACTCAGCT--ATTTGTGCAGTCTTTTCATAACTTTTCA  47631
Cow          AAGT--------------------------AGTATGGGAGAAATAAA------TGGCAAGGTATAGTCAGTAATAGTCA  83558
Dog          ACTTT-----TTATTGTTATTTTCACCCTTCAGTGTATCACAGCTCAGCT--GATTGTGCAATCTTTTCATAACTTTACA  77738
BelugaWhale  ACCTCTTG-T--TTTGTTATTTGTGCCCTTCAGTATCTCAAAGCTCAACTCTGTTTGTGTAGTATTTTTATAACTTCTCA  77926
Donkey       ACTTACTGTTTTTTTTTTATTCCCGCCCTTCAGTATCTCAAAGCTCAGCT--GTTTGCGCAGTCTTTCCCTAATTTTCCA  86647
Cat          ACTTTTTATTGTTTTCTTATATCTGCTCTTCAGTATCTCAAAGCTCAGCT--GTTTATGCCGTCTTTTCATGACTTTTCG  83064
Ferret       AGTTTAT--TGTTTTCTTATTTCTGCCCTTCATTATATCAAAACTCAGCT--GTTTGTGCAGTCTTTTCATAACTTTTCA  85362
Lion         ACTTTTTATTGTTTTCTTATATCTGCTCTTCAGTATCTCAAAGCTCAGCT--GTTTATGCCGTCTTTTCATGACTTTTTG  80813
Vaquita      ACCTCTTG-T--CTTGTTATTTGTGCCCTTCAATATCTCAAAGCTCAACT--GTTTGTGTAGTATTTTTATAACTTCTCA  79181
Pig          ACTTTTTG-TGTTTTCTTATTTGTGCTCTTCAGTATCGCAAAGTTCAGCT--CTTTGTGCGGTGTTTTAAAACTTTCAA  78247
```

```
Majority     AA-GCTGAACTAAGACAGCTTTXTTXATXACAACCTT-AGTCTACTTCTTTATACCACTTTATTCCCCAGACTCATTCCT
                      111850    111860    111870    111880    111890    111900    111910    111920
Human        ---------TTA-GACAGCTTTTTTTCTGACAACGTTAAGTCTACTTATTTATATCACTTTATTTCTCAAACTCACACTT  89666
PolarBear    AA-GCTGAACTAAGATAGCTTT----ATAAGAGTCTT-TGTTTACTTCTTTATATCACTTTATTTCCCAGATTCATTTCT  47705
Cow          A--GTGTGAATGGAAT--TATTGCAAATCTTAG-----AGGC-------C-ATGTTAAATCATTTT--AGACC-------  83612
Dog          AA-GCTGAACTAAGCATTCTTT----ATAACAG-------TGTGCTTCCTTATACCACTTTATTCCCTAAATTCATTCCT  77806
BelugaWhale  AAAGCTGCACTAATACACCTTTTCTTATGACAACCTT-AGACTACTTCTTTATGCCACTTTATTCCCAAAACTCATTCCT  78005
Donkey       AA-GCTAC-CTAGGACAGCTTTCTT-ATGACAGCCTT-AGTGTGCT---TTATACCACTTCATCCCCAAGACGTATGCCT  86720
Cat          AA-GATGAACAAAGATAGCTTTCTT-ATAACAACCTT-AGTCTACTTCTTTATACCACTTCATTCCC-AGATTCATTCCT  83140
Ferret       AA-GCTGAACTAAGACAGCTTTCTTTATAACAGCCTT-AATCTACTTCTTTAAACCACTTTATACCCTAGATTCATTCCT  85440
Lion         AA-GATGAACAAAGATAGCTTTCTT-ATAACAACCTT-AGTCTGCTTCTTTACACCACTTCATTCCC-AGATTCATTCCT  80889
Vaquita      AAAGCTGCACTAATACACCTTTTCTTATGACAACCTT-AGACGACTACTTTATGCCACTTTATTCCCCAAACTCATTCCT  79260
Pig          A--GTTGCACTAAAACACCTTTTCTTATGACAA-----GGTC-------T-ATGTCACATTATTCTCCAAACTCATTCCT  78312

Majority     TATAACACAT--TTTGTATGAGGTAATAGCACTTTATCATAAGCAAAGTTCTGTTCACTTAA---AATA------ATAGA
                      111930    111940    111950    111960    111970    111980    111990    112000
Human        AATAACACAT--TTTATCTGAGGTAGT------TTATC-TAAACAAAAGTTTGTTCACTTAAAATAGTT------ATAGA  89731
PolarBear    TATAACACAT---TTGTATGAGCTAAT----------CATAAGCAA-------------TAG---AATG------ATAGA  47750
Cow          ------AGAA--TTTTT--AAATTTGCAG-ACATT---------AAAGATGTTTCCATTTCT----AC----T--ATAG-  83661
Dog          TATAATGCAT---TTGTATGAGCTAAGAGTACTTTATCATAAGTAA-------------TAG---AATG------ATAGA  77861
BelugaWhale  TATAACACAT--TTTGTTTGAGGTAATAGCACTTTATTATAAGCAAAGTTCTGTTCACTTAAAACAACA--ATTAATAGA  78081
Donkey       TAAAAAACAT--TTTGTGGGATGGGGCCGGCCCCGTGGCCGAGTGG-------TTAAGTTCGCGCGCTCCGCTGCAGGCG  86791
Cat          TATAATCCATGCCTTATATGATGTAGTAGCACTTTTTCATAAGCAAAGTACTGTTCACTTAA---AACA------ATAGA  83211
Ferret       TAAAACACAT---TTGTGTATGCTAATAGCTCTTTATCATAAGCAA-------------CAG---AATG------ATAGA  85495
Lion         TATAACCCATGCCTTATATGATGTAGTAGCACTTTTTCATAAGCAAAGTACTGTTCACTTAA---AACA------ATAGA  80960
Vaquita      TATAACACAT--TTTGTATGAGGTAATAGCACTTTATTATAAGCAAAGTTCTGTTCACTTAAAACAACAGAATTAATAGA  79338
Pig          TATCATAGAT--ACTGT--GAACTAATAGCACTTTCTAATAAGAAAAGTTCTGTTCATTTAATAGAAT----T--ATAGA  78382
```

```
Majority     ACTTTAAAAATGT--------TTTATAATTCAAGTAAGTTTTATTATAATAG---TTTAT--------ATACCATCTCTA
                      112010    112020    112030    112040    112050    112060    112070    112080
Human        ACTTTAAAATT-----------TTATAATTC-AGTGGGTTTTATTGTAAAAGGATTTTAT--------GTACTGTCACTG  89791
PolarBear    ACTTTAAAAATGT--------TTTGTAATTCAAGTAAGTTTTATTATAATAG---TGTAT--------GTACCATCTCT-  47810
Cow          -CCT-----------------------ATTTAATTAA-------TATACTAGAATTTTC--TTAA---ATTAGGTAGTTT  83705
Dog          ACTTTAAAAATGT--------TTTGTAATTCAAGTAAGTTTTA---TAATAG---CTTAT--------ATACCATGTCTA  77919
BelugaWhale  ACCTTAAAAATAT--------TTTATAATTCAAGTAAGCTTTATTATAAAAG---TTTAC--------ATACCATCACTA  78142
Donkey       GCCC----AGTGTTTCGTCAGTTCG-AATCCTGGGCACAGACATGGCACTG----CTCAT-------CAAGCCACGCTTA  86855
Cat          ATTCTAGAACTTT------ATTTTATAAATCAAATAAGTTTTATAAAAGGAG---TTTAT--------GTACCATCTCT-  83273
Ferret       ACTTTAAAAGTGT--------TTTGCAATTCAAGTTAATTTTA--ATAGT-----TTTAT--------GTACCATCTCTA  85552
Lion         ATTCTAGAACTTTGAAAATATTTTATAATTCAAATAAGTTTTATAAAAGGAG---TTTAT--------GTACCATCTCT-  81028
Vaquita      ACCTTAAAAATAT--------TTTATAATTCAAGTAAGCTTTATTATAAAAGGAGTTTAC--------ATACCATCACTA  79402
Pig          ACCTTAAAAATAT--------TTTATAATTCAAGTAAATTTTATTATAAAAGGAGTTTACATTAACACACTGGATAATTT  78454

Majority     TGAAT----AATT------AXTT-GATTTTATAAGTGXX---GAAATTTAGXX-AX-------------XG---------
                      112090    112100    112110    112120    112130    112140    112150    112160
Human        TAGAT----AATT-----------GGTTTTACAGGTGTAAGCGTAAATTTGCA-AG------------------------  89831
PolarBear    -GAAT----AATT-----------GATTTTATAAGTG-----AAAATTTAGAAGTATATGT----GATGTGCAGGGG---  47862
Cow          AGG------AAT-------AACAGAATTTGTAAATTATGTTTGAAAT--AGTGTA-------------CTGA--------  83749
Dog          TGAGT----AATTG-----AATTGAATTTTGTAAGTG-----AAAATTT-------------------------------  77954
BelugaWhale  TGGAT----AATT------GAGTTGATTTTATAAGTATATGGGAAATACAGTGTAAGGAGGTTAAGGCCTGACAGC--TT  78210
Donkey       AGAATATACAACTATGTACCGGGGGGCTTTGAGGAGAAAAAGGAAAAAAAAAAAAAAGCATTTTGTGGGAGGTAATTGCA  86935
Cat          -GAAT----AATGC-----AGTT-GATTTTGTAAGTG-----AAAATTT-------------------------------  83306
Ferret       TGAAT----AAT------------GATTTTATAAGTG-----AAAATT--------------------------------  85579
Lion         -GAAT----AATGC-----AGTT-GATTTTGTAAGTG-----AAAATTT-------------------------------  81061
Vaquita      TGGAT----AATT------GAGTTGATTTTATAAGTATATGCGAAATACAGTGTAAGGAAGTTAAGGCCTGACAGC--TT  79470
Pig          GAGTT----GATTTTATTAAGTGTAAGTTTTTAAATGTATGTGAAATGCAGTGTAAGAAAG----GGCCTGGAGATGATT  78526
```

```
Majority     --TTTXX-TAXTXXX-X---TXAAA--TXXAAXXAX-X---X-TACXAAX-X-XAXX-XTXX-XTAAX-XX---X-----
                       112170    112180    112190    112200    112210    112220    112230    112240
Human        --TATATGAAATTAAGTATGTGAAATGCAGTGTGAGAAACAAATGAGAAAATTCAGACTTGTGAGAAGAT----------  89899
PolarBear    CGCCTGGGTAGCGCAGTTGTTAAAGCGTCTGCCTTAGGCTCGGGGCGTGATCCCGGCGTTCCGAGATCGAGTCCCACATC  47942
Cow          TCTTCAGCGATTAAA------TGAATTCTCAAGAAG-------TACCAAAAAGTAGTTCTTTGTTAAAGTAATTA-----  83811
Dog          --------------------------------------------------------------------------------  77954
BelugaWhale  ATTTTTTTTATTAGCCTCAGTTAAAAATCTAAGAAAAAACTATTACAAATGTATAACACTGCAGTAAATCAGGAAGAGTA  78290
Donkey       CTTTATCGTAAGCAAAGTTCTATTCACTTGAAACAGTAGTTAATAGAACTTTGAAAATATTTTATAATTCAGATAAATTT  87015
Cat          --------------------------------------------------------------------------------  83306
Ferret       --------------------------------------------------------------------------------  85579
Lion         --------------------------------------------------------------------------------  81061
Vaquita      ATTTTTTTTATTAGCCTCAGTTAAAGATCTAAGAAAAAACTATTACAAATGTATAACACTGCAGTAAATCAGGAAGAGTA  79550
Pig          TTTTTTTCTATTAACCTCAGTTAAAAATCTAAGAAAAAA-TAATACAAATACATAACACTGCAATAAAACAATTAGAATA  78605

Majority     -----TXTT-TTTC--TXCXXXT-------------------------------------------------------ATT
                       112250    112260    112270    112280    112290    112300    112310    112320
Human        -TG--TTTTGTTTTTTTTCTG-T--------------------------------------------------GCTT  89922
PolarBear    GGGCTCCTCCGCTGGGAGCCTGCTTCTTCCTCTCTCACTCGCCTGCTGTGTTCCCTC-------TCTCGCTGGCTGTCTC  48015
Cow          -----TCTTATTTC--TCCAATT--------------------CGTATTTTCAA--------------------GAAC  83842
Dog          --------------------------------------------------------------------------------  77954
BelugaWhale  AA--CTTTTTTTTCCTTCCAGTTTTGAGATATAATTGACATACAGCACTGTGTAAGAGGTACAGCATTAATGATTTGATT  78368
Donkey       TATTAAACAGTTTCTGTACCATC-------------------------------------------------------ACT  87041
Cat          --------------------------------------------------------------------------------  83306
Ferret       --------------------------------------------------------------------------------  85579
Lion         --------------------------------------------------------------------------------  81061
Vaquita      AA--CTTTTTTTTCCTTCCAGTTTTGAGATATAATTGACATACAGCACTGTGTAAG--------CATTAATGATTTGATT  79620
Pig          AAAACTTTTCTTTCCTTCCAGTTTTGAGA--------------GGCACAGCATAAT--------------------AATT  78651
```

```
Majority     T--XT--X--X----------ATXXXXATAAAXX-X-X-X-TATXTGTGAAATGCA--X-GTATAACAAGTGAGAAA---

                       112330    112340    112350    112360    112370    112380    112390    112400

Human        AGCCT-----------------TGGCGAAAAA---------TCTGAGAAAACTTAAA-ATATATAACACTGGAGTGAC--  89973
PolarBear    TCTGTCACATAAATAAATAAAATCTTAAAAAAAAAA-AAAGTATATGTGAAATGCA----GTGGAACTAGTGAGAAA---  48087
Cow          TACTTACA--AAAGAGCCTCAACTGTTTTTAGTTCA-TATGTAGCTGCCATCTTAA--AAGTATAGTTTTTCATAAT---  83914
Dog          ---------------------------AGAAG---------TATGTGTGAAACGCA----GTAGAACTAGTGAGAAA---  77991
BelugaWhale  TGTATACATCATGAGTTGATTATCACAGTAAATTTAGTGAACATCCATCATCTCATATACTTATAAAATAAAAGAAATAG  78448
Donkey       GTGAATAATTGAGCTGTTTTTATAAGTGTAAATTTG-TAAGTATATGTGAAATGCAGTGTAAGAAAGTAGTGAGAAA---  87117
Cat          ---------------------------AGAAG---------TATATGTGAAATGCA----GTAAAACAAGTGAGAAA---  83343
Ferret       ---------------------------AAAAG---------TAT--GTGAAATGCA----GTGGAACTAGTGAGAA----  85613
Lion         ---------------------------AGAAG---------TATATGTGAAATGCA----GTAAAACAAGTGAGAAA---  81098
Vaquita      TGTATACATCATGAGTTGATTATCACAGTAAATTTAGTGAACATCCATCATCTCATGTACTTATAAAATAAAAGAAATAG  79700
Pig          TACATACATCAAGAAGTGATTATCACAATAAATTTAGTGAACATCCATCATCTCAT--AGTTACAAAATGAAAGAAATAG  78729

Majority     --AAATTCAGGCCTGAGAT---TACTTTTXT----T--TCTXTTAGCCTX-AXTT-------ACAAAACTAAXAAAAATA

                       112410    112420    112430    112440    112450    112460    112470    112480

Human        ---AATCAGGAAGAGTGG-----ACTCTTTT----T--TTTTTTAATTTT--TTT-------AAACAAATTAGGGAAGTA  90030
PolarBear    --GAATTCAGGACTAAGA----GGATTTTTT-------TCTTTTAGTCTC-AGTT-------AAAAATCTAAGAAAAATA  48146
Cow          --AGATCTTTACCTACTCT-----CTAGCAAAATGTGCTGTAGAGGGAGT-ATTTATAAAATGCAGCAGTGTTAA--ATA  83984
Dog          --GAATTCAGGCCTGAGAG--ATGATTTTTT-------TCTGTTCGCCTC-GGTTGAAAAAAAAAAAACTAAGAAAAAAG  78059
BelugaWhale  AAAAAATTTT-TGT--TATGAGAACTCCTAGGGTTTAGTCTCTTAACTTTCATGTATAACATACAGCAGTGTTAAAATTA  78525
Donkey       --AAATTCAGGCCTGAGAG----AATTTTTT-------TCTGTTAGCCTC-AGTT-------GAACATCTAAGGAAAAAG  87176
Cat          ----ATTCAGGCCTGAGAT--TTTTTTTTGTTTGTTTTTCTGTTGGCCTC-AGTT-------GCAAATCTAAAGAAA---  83406
Ferret       ---AATTCGGGACTCAGA----TGATTTT---------------------------------------------------  85635
Lion         --GAATTCAGGCCTGAGAT--TTTTTTTT---------TCTTTTGGCCTC-GGTT-------GCAAATCTAAAGAAA---  81154
Vaquita      AAAAATTTCT-TGTGTTATGAGAACTCCTAGGGTTTAGTCTCTTAACTTTCATGTATAACATACAGCAGTGTTAAAATTA  79779
Pig          AAAAATTTTTGTGTGATGAGAAAACTCTTAGGATTTACTCTCTTAACTTTCATGTGTAACATACATCATTGTTAA--TTA  78807
```

```
Majority     TATTACAAATXTXT-AACACTACAXXCAXAATAATT---------------GGAAGAGTAAACTTGTTTTTACAAATTAG
                      112490    112500    112510    112520    112530    112540    112550    112560
Human        AGTTC--AATGGCTAGTGGTTA---TCATAATA-----------------AGAAAAAT-----------------TTAA  90070
PolarBear    CATTACAAATACATTAACACTACAGTAAAAACAGTC--A------------GGAAGAGTAAACTTGTTTTTAAAAATTAG  48212
Cow          TATTTGTCATGT-TGTACATTACATCCTTAGTACCTCTTTATCTTATAACTGGAAGAGTAAATTTGATTTTACAAATTAG  84063
Dog          CATTACAAATATAT-AACACTACAGTAAAAACAGTC--A------------GGAAGAGTAAGCTTGTTTTTACAAATTAG  78124
BelugaWhale  TATTTATCATGT-TGTACGCTACATCCTTAGTATTT----ACCTTATAACTGGAATAGTAAACTTATTTTTACAAATTAG  78600
Donkey       TATTAAAAATATAT-AGCACTGCA-CTAAAACAATTA--------------GAAAGAGGAAGCT---TTTTACAAATGAG  87237
Cat          -ATTACAAATACAT-AACACTACAGTAAAA-TAATT--G------------GGAAGAGTAAACTTGTTTTTACAAATTAG  83469
Ferret       --------------------------------------------------------------------------------  85635
Lion         -ATTACAAATACAT-AACACTACAGTAAAA-GAATT--G------------GGAAGAGTAAACTTGTTTTTACAAATTAG  81217
Vaquita      TATTTATCATGT-TGTACGCTACATCCTTAGTATTT----ATCTTATAACTGGAATAGTAAACTTATTTTTACAAATTAG  79854
Pig          TTTTTCTCATGT-TGTACATTACATCCCTAATACTTAAATATCTTATAACTGGAAGAGTAAACTTGTTTTTACAATTTAA  78886
```

```
Majority     GGAAGCTATAAGTGAAA-AA------TACXXTTGATTCTTTTCTGAATGGTACCTTA-TACTTXTATAGCATTTTCCTTT
                      112570    112580    112590    112600    112610    112620    112630    112640
Human        GGAAGTTATAGGTAAAATAAGAATTTTACTTTTGTTTCTTTTCTGAATGGTACCTCA-TATTTTGACAGCATTATCCTTT  90149
PolarBear    GGAAGCTGTAAGTGAAA-AAAGAACATACTTTTGATTCTTTTCTGAATGGTACCTTA-TACTTTTATAGCATTTTCCTTT  48290
Cow          GGAAGCTATAAATGAAA-AA-------------GATTTTTC--AGAATGGTACCTTA-TACTTTTATAGCATTTTCCTTT  84126
Dog          AGAAGCTACAAGTGAAA-AAAGAAAATACTGTTGGTTCTTTTCTGAATTGTACCTTA-TACTTTCATAGCATTTTCCTTT  78202
BelugaWhale  GGAAGCTATAAATGAAA-AA-------------GATTTTTTT-GGAATGGTACCTTA-TACTTCTATAGCATTTTCCTTT  78664
Donkey       GAAAGCTGTAAGTGAAA-AA----TTTACGTTTGATTCTTTTCTGGATGGTACCTTA-TACTTCCACGGCATTTTCCTCT  87311
Cat          AGAAGCTGTAAGTGAAA-TAAGAATTTACTTTTGAATCTTTTCTGAATGGTACCTTAACACTTCTACAGCATTTTCCTTT  83548
Ferret       ----------------------------------------------------------A---------------------  85636
Lion         GGAAGCTGTAAGTGAAA-TAAGAATTTACTTTTGAATCTTTTCTGAATGGTACCTTAACACTTCTACAGCATTTTCCTTT  81296
Vaquita      GGAAGCTATAAATGAAA-AA-------------GATTTTTT--GGAATGGTACCTTA-TACTTCTATAGCATTTTCCTTT  79917
Pig          GGAAGCTATAAGTGAAA-AA-------------GATTTTTT--GGAATGGTACCTTA-TACTTTTATAGCATTTTCCTTT  78949
```

```
Majority     TCAGACCTGTTTTAT---------ATTTAACTGGTTTA--TTCTATACAAGTAAGTATGACAAGATGCATGGATTTAACA

                       112650    112660    112670    112680    112690    112700    112710    112720

Human        TCAGATCTGTTTTAT---------ATTTAATTGGTTTA----TTATACATGTAAGTATGATTAGATTCATGGATTTAACC  90216
PolarBear    TCAGACCAATTTTAT---------ATTTAACTGGTTTATATTCTATACAAATAAGTATGACAGGATGCACAGATTTAATA  48361
Cow          TCAGACCTGTTTTAT---------ATTTAACTGGTTTG--TTCTATACAAGTAAGTATGACAAGATGCATGGATTTAACA  84195
Dog          TCAGACCTGTTTTAT---------ATTTAACTGATTTATGTTCTATACAGGTAAGTATGATAAGATGCATGGGTTTAATA  78273
BelugaWhale  TCAGACCTGTTTTAT---------ATTTAACTGGTTTA--TTCTATACAAGTAAGTATGACAAGATGCATGGATTTAACA  78733
Donkey       TGAGGCATGTTTTAT---------ATTTAACTAGTTTA--GTCTATGCAAGTAAGTATGTCAAGATGCATGGTTTTAACA  87380
Cat          TCAGAACTGTTTTATATTTAACTGGTTTAACTGGTTTATATTCTATGTGAGTAAGTAGGAAAAGATGCTTGGATTTAATA  83628
Ferret       ---------------------------------------------------------------GATG-------------  85640
Lion         TCAGAACTGTTTTAT---------GTTTAACTGGTTTATATTCTATGTGAGTAAGTAGGAAAAGATGCTTGGATTTAATA  81367
Vaquita      TCAGACCTGTTTTAT---------ATTTAACTGGTTTA--TTCTATACAAGTAAGTATGACAAGATGCATGGATTTAACA  79986
Pig          TCAGACCTGTTTTAT---------GTTTAACTGGTTTA--TTCTATACAAGTAAGTATGACAAGATGCATGGATTTAACA  79018

Majority     AATTCTGGGT-ATCTCCTGTGTCAGACTGTGTACTGTAGTATACTAATCAAATTAAAA-CATT-TGAGAGTAGGGATTTT

                       112730    112740    112750    112760    112770    112780    112790    112800

Human        AGTTCTTTGT-ATCTTCTGTGTCAAAATGTATACTGTAGTATACTGTTTATATTAGAAACTCTTTAAAAGTGGATGTTCT  90295
PolarBear    AATTCTGTGT-ATCTCCAGTGTCAGACTGTGTACTGCAGTATACTAATCAAATTAAAACATTT-TGAGAGTAGGGATTTT  48439
Cow          AGTTCTGGGT-ATCTCCTGTGTCAGACTGTGTACTGTAGTGTACTAATCAAATTAAAAACATT-TGAGAGTAGGGATTTT  84273
Dog          AATTCTGTGT-ATCTCTGGTATCAGACTATGTGCTGCAGTATACTAATCAAA---AAACATTT-TGAGAGTAGGGGTTTT  78348
BelugaWhale  AATTCCGGGT-ATCTCCTGTGTCAGACTGTGTACTGTAGTATACTAATCAAATTAAAA-CATT-TGAGAGTAGGGATTTT  78810
Donkey       AATTCTGACT-ATATCCTATGTCAGACTGTGTAGTGTGGTCTACTCATCAAATTAAAAAGATTCTGAGGGTAGGAACTCT  87459
Cat          AATTGGGCAT-ATCTCTGGTGTCAGACTGTGTAC------GTACTG----------------T-TGTGAGTAGG-ATTTT  83683
Ferret       --------------------------------------------------------------------------------  85640
Lion         AATTGTGCAT-TTCTCTGGTGTCAGATTGTGTAC------GTACTG----------------T-TGTGAGTAGG-ATTTT  81422
Vaquita      AATTCCGGGT-ATCTCCTCTGTCAGATTGTGTACTGTAGTATACTAATCAAATTAAAA-CATT-TGAGAGTAGGGATTTT  80063
Pig          AATTTTGGATTATCTCCTGTGTCAGACTGTGTACTGTAGTATACTAATCAAATTAAAAACATT-TGAGAGTAGGGATTTT  79097
```

```
Majority     GTTCTCAXTTCCCTGCTCTTCCA-XATTGCCTTTAXCTCTC-TGCTGTTAATACTCTAATACTTTGA-----GTAGTCAA
             112810 112820 112830 112840 112850 112860 112870 112880
Human        ATTCTTAATTTCCTGATCTTCCA-CATTGTCTTTATCTGTAACTCTACTGTTTTTTGTTGGTTTTTGTTTTTGTTTTGAG 90374
PolarBear    GTTCTCATTTCCCTGCTCTTCCA-CATTGCCTTTATCTCTC-TGCTGTTAATACTCTAATACTTTGA-----GTAGTCAA 48512
Cow          GTTCTCAGTTCCCTGCTCTTCCA-TATTGTCTTTACCTCTC-TGTTGTTAATACTCTAATACTTTGATTTGAGTAGTCAA 84351
Dog          ATTCTCATTTCCCTGCTCTTCCA-TATTGCCTTTATCTCTC-TACTGTTAATACTCTAATACTTTGA-----GTAGTCAA 78421
BelugaWhale  ATTCTCAATTTCCTGCTCTTCCA-TACTGCCTTTACCTCTC-TGTT--TAATACTCTAATACTTTGA-----GTAGTCAA 78881
Donkey       --TCTCAGTTCCCTGCCCTTTCA-CATT-CCTTTACCTCTC-TGCTCTTAATACTCTAATACTGTGATTTGAGCAGTCAA 87534
Cat          GTCCTCATTTCCCTTTTCTTCCA-CCTTGCCTTTATCTCTC-TGCTGTTAATACAC--AAACTTGGA-----ATAGTCAA 83754
Ferret       -------------------------------------------------------------------------------- 85640
Lion         GTCCTCATTTCCCTTTTCTTCCA-CCTTGCCTTTATCTCTC-TGCTGTTAATACAC--ATACTTGGA-----GTAGTCAA 81493
Vaquita      ATTCTCAATTTCCTGCTCTTCCA-TACTGCCTTTACCTCTC-TGTT--TAATACTCTAATACTTTGA-----GTAGTCAA 80134
Pig          GTTCTCAATTCTCTGCTCCTCCAATATTGCCTTTACCTCTC-TGTTGTTAATACTCTAATACTTTGATTTGAGTAATCAA 79176

Majority     AT-AGACCTTCAGTTT---AGCXAXACTATATGAC-TTATGAAATGGTCTTCTTTAGTTGT------GACTAGT-GAXTA
             112890 112900 112910 112920 112930 112940 112950 112960
Human        AC-AGAGTCTCGCTCTGTCACCCAGGCTGGAGTGCACTGTGGCACCATCTCAGCTCACTGTAACCTCCGCCTCCTGGGTA 90453
PolarBear    ATCAGACCTTCAGTTT---AA--AGTATATATGAC-TTTTGAAATGGTCTTAAGTTGTTGT------GACTAGT-GAGTA 48579
Cow          AT-AGACGTTCAGTTT---AGCTAAACTATATGAC-TTATGACATGGTTTTCTTCAGTTGT------GACTACTTGAATA 84420
Dog          ATCAGACGTCCAGTTT---AA--AGTATAT--GGC-TTACGAAATGGTCTTCAGTTGTTCT------GACTAGT-GAGTA 78486
BelugaWhale  AT-AGACTTTCAGTTT---AGCTAAACTATATGAC-TTATGACATGGTTTTCTTCAGTTGT------GACTAGT-GAATA 78949
Donkey       ATCAGACCTTCTGTTT---AGCTCAAGCATATGAC-TTATGAAATAGTCTTTTTTAGTTGTTGT---GACTAAT-GAATA 87606
Cat          ATCAGACCTTCAGTTT---AA--AGTATAT--GAC-TTATAAAATGGTCGTAAGTTGTTGT------GGCTAGT-GAGTA 83819
Ferret       -------------------------------------------------------------------------------- 85640
Lion         ATCAGACCTTCAGTTT---AA--AGTATAT--GAC-TTATAAAATGGTCGTAAGTTGTTGT------GACTAGT-GAGTA 81558
Vaquita      AT-AGACCTTCAGTTT---AGCTAAACTATATGAC-TTATGACATGGTTTTCTTCAGTTGT------GACTAGT-GAATA 80202
Pig          AT-AGACCTTCAGTTT---AGCTAAACAGTATGAC-TTATGAAATGGTTTTCTTCAGTTGT------GACTAGT-GAATA 79244
```

```
Majority     AAACATAAAACXTATA--ACTGTTCTGTAATCTCAGACCA-GTTCATTTAGTTTAAAAATAACCCAACTGCTXCCTCAAG
                               112970    112980    112990    113000    113010    113020    113030    113040
Human        CAAGCAATTCTGCCTCAGCCTCCTGAGTAA-CTGGGACCACAGGCACGAGCCACCACGCCCAGCTAATTTTTGTATTTTT  90532
PolarBear    AAA-------CATAGAGAACTGTTCTGTAATCTCAGATCA-GTTCATTTAGCTTAAAAATAATCCAACTGCTACTTCAAG  48651
Cow          AAACATAAAACGTATA--ACTGTTCTCTAATCTCAGACCA-GTTTATTTAGTTCAAAAGTAACCCAACTGCTGCCTCAAG  84497
Dog          AAATGTGAAACGTAGAGAACTGTTCTGTAATCTCAGGCCA-GTTCATTTAGCTTAAAAATAACCCAACTGTTACTTCAAG  78565
BelugaWhale  AAACATAAAACGTATA--ACTGTTCTCTAATCTCAGACCA-GTTCTTTTAGTTGAAAAGTAACCCAACTGCTGCCTCAAG  79026
Donkey       AAACATATAACATGTT-AACCGTTCTTGAATCTCAGACAA-GTCCATTTAGTTTAAAAATAACCCAACCAGTACCTCAAG  87684
Cat          AAACATAAAACATGTA--ACTTATCTGTAACCTCAGACCA-GTTCGCTTAGTTTAAAAATAACCCAACTGCTACCGCAGA  83896
Ferret       --------------------------------------------------------------------------------  85640
Lion         AAACATAAAACATGTA--ACTTATCTGTAACCTCAGACCA-GTTTGCTTAGTTTAAAAATAACCCAACTGCTACCGCAGA  81635
Vaquita      AAACATAAAACGTATA--ACTGTTCTCTAATCTCAGACCA-GTTCTTTTAGTTGAAAAGTAACCCAACTGCTGCCTCAAG  80279
Pig          GAACATAAAACATATATAACTGTTCTCTAATCTCAGACCA-GTTCATTTAGTTCAAAAATAACCCAGCTGCTGCCTCAAG  79323

Majority     AAGTAGGCATGTXAT---GTT-CTGTGTTTAGGCACTGTGCTGTGTGCTG-GGGTAGAGACXGTGGTTCCTGCCTTCATG
                               113050    113060    113070    113080    113090    113100    113110    113120
Human        A-GTAGAGATGGGGT---GTTACCATATT--GGC--CAGGCTG-GTCTCGAACTCCTAGACCTTGTGATCCGCCCACCTC  90603
PolarBear    AAGTAGGCATGTAAT---GTT-TTGTGTTTAAGCACTGTACTTTGTGCTG-GAATAAAGACCGTGGTGCCTGCCTTCATG  48726
Cow          AAGTAGGCATGTGAT---GTT-CTGTGTTTAGGCACTGTGCTGTGTGCTG-GGGTAGAGACACTGGTTCCTGCTTTCATG  84572
Dog          AATTAGGTATGTAAT---GTT-CTGTGTTTAGGCACTGTGCTGTGTGCTG-GGATAGAGACCGTGGTTCCTGCCTTCATA  78640
BelugaWhale  AAGTAGCTATGTGAT---GTT-CTGTGTTTAGGCACTGTGCTGTGTGCTGTGGGGAGAGACACTGGTTCCTGCTTTCATG  79102
Donkey       AAGTAGCCACATAAT---GTT-CTGTTTTTAGGCATTGTGCTGTGTGCTG-GTGTAGAGACAGTGGTTCCTGCCCTCATG  87759
Cat          AAGTAGGCATGTAATAATGTT-CTGTGTT-AGGCACTGTGCTGTGTGCTG-GGATAGAGACCGGGGTTCCTACCTTCATG  83973
Ferret       --------------------------------------------------------------------------------  85640
Lion         AAGTAGGTATGTAATAATGTT-CTGTGTT-AGGCACTGTGCTGTGTGCTG-GGATAGAGACCGGGGTTCCTACCTTCATG  81712
Vaquita      AAGTAGCTATGTGAT---GTT-CTGTGTTTAGGCACTGTGCTGTGTGCTGTGGGGAGAGACACTGGTTCCTGCTTTCATG  80355
Pig          AAGTAGCCATGTGAT---GTT-CTGTGTTTAGGCACTGTGCTGTGTGCTG-GGGTAGAGACACTGGTTCCTGCTTTCATG  79398
```

```
Majority     TACTTTXCAGTCTGAAGGAGGATXX-X-AGATAAT-----AAAAT-GTGATGAATTTAXAAAAAAGAAX------A-X--
                              113130    113140    113150    113160    113170    113180    113190    113200
Human        AGCCTCCCAAAGTGTTG--GGATTACAGGCGTGAGCCAACGCGCCCGGCATCTACTGTTAATACTTTATTATCTAGTTGA 90681
PolarBear    TACTCTGCAGTCGGAAAGAGGAT---TTAGATAAAA----TAAATTGTGATGAATTTAAAAAGAAGTAA------A---- 48789
Cow          TATTTTACAGTCTGAAGGAGGATG------ATAAT----TAAAAT-GTGATGAATTTATAAAAAGAAATAGA----ACTG 84637
Dog          TACTTTGCAGTGGGAAGGAGGAT---TTAGATAAT-----TAAATTGTGATAAATTTTAAAAAGAAGTA------A---- 78702
BelugaWhale  TATCTTACAGTCTGAAG-AGAATAT---AAAT-------TAAAAT-GTAATGAATTTATGAAAATAAGTAAAGGGTACTG 79170
Donkey       TAGTTTACAGAGTGCAGGAGGATAATTAAAAT---------AAATTGTGATGAATTTAAAAAGAAGTAAAGG---ATCCT 87827
Cat          TCCTTTGCAGTCGGAAGGAGGAGTAGTTAGATAATTAAAATAAATTGTGATGAATTTAAGAAGAAGAAA------A---- 84043
Ferret       -------------------------------------------------------------------------------- 85640
Lion         TCCTTTGCAGTCGGAAGGAGGAGGAGTTAGATAATT----------GTGATGAATTTAAGAAGAAGAAA------A---- 81772
Vaquita      TATCTTACAGTCTGAAGGAGGATAT---AAATAAT----TAAAAT-GTAATGAATTTATGAAAATAAGTAAAGGGTACTG 80427
Pig          T-TACCTTAGTCTGAAGGAGGGTGT---GCAGAAT----TAAAATCATAATGAATTTATAAAAAGGCTT--------CTA 79462

Majority     --TXGGG-AXTATCTAXTGAGGTTCTGAACCTTT-CXAGAGGATGAAGAAAAGGCCTTXTT-AGXAAXTAACACT-----
                              113210    113220    113230    113240    113250    113260    113270    113280
Human        TTATAGTCATTTAGACCTTCAGTTTAGCCAAAGTGCATGACATATTAGAAATGGTCTTTTTTAGTTATTTTGACT----- 90756
PolarBear    --GGATG-AATATCTAGTGAGGTTCTGAACCTTT-CCAGAGGATGAAGGAAAGGCCTTTTC-AGAGAAGAACGAT----- 48859
Cow          TATGGGG-ACTATCTACTAAGGTTCTGAACCTTTTCTGAAGAGTGCAGAAAGGGTCCTCTTAAGGAAATAACACT----- 84711
Dog          ---------------------------------------AGGGTGAAGGGAAGGCCTTTCC-AGAG---AATGAT----- 78734
BelugaWhale  CATGGGGGAATATCTAGTGAGGTTCTGAACCTTTTCTAGAGGATGAAGAAAAGGCCCTCTTGAGGAAGTAATACTAAA-G 79249
Donkey       TCATGGGGAATACCTAATGAGGTTCTGAACCTTT-CCAGAGAATAAAGG-AAGGTCTTCCTGAGTAAGTAACATCAAAAG 87905
Cat          ------------------------------GAGT-TCTGCATGGGGAATATGAACCTTTCC-AGAGAAGAACAGT----- 84086
Ferret       ----------------------------------------------------------------------A--------- 85641
Lion         --T--------------------------CGAGT-GCTGCATGGGGAATATGAACCTTTCC-AGAGAAGAACAGT----- 81817
Vaquita      CATGGGGGAATATCTAGTGAGGTTCTGAACCTTTTCTAGAGGATGAAGAAAAGGCCCTCT-GAGGAAGTAATACTAAA-G 80505
Pig          CACAGGG-ACTGTCTAGTGAGGGTCTGAACCTTT-CTAGAGGATGAACACAAGGCCTTCTT---GAAGTAAGACT----- 79532
```

```
Majority     GAXAA-TAGAXTAGGAGGGATAGTGAAGGAGATGXGAAATTGTTATAXATAGAACA------------------------

                         113290    113300    113310    113320    113330    113340    113350    113360

Human        GGTGAATAAAACAT--ATAACTTTCTAGCATCAGACCAGTTCATTTAGTTTAAAAATGATCCA-----------------  90817
PolarBear    GAGAA--AGGTTAGGAAAGATAACGAACAAGTTGGGAAATTATTATAGATAGAACA------------------------  48913
Cow          GACAAATAGCTTAGGAAAGATTGTGAAGGAGATAGGAAATCATTATAAGTAGAAAA------------------------  84767
Dog          GAGAA--AGGCTAGGAGGGATAATGAACAAGATGGGAAACTGGTATAGAT----CA------------------------  78784
BelugaWhale  GATAAATAGATTAGGGGGGATAGTGAAGGAGATAAGAAATTGTTATAAATAGAAAATAGTGAAGGAGATAAGACATTGCT  79329
Donkey       GATGTGTAGACCAGGAGGGACAGTGAAGGAGAT---AAATTGTTACAAATAGACCA------------------------  87958
Cat          GAGAA--AGACTAGGAGGGATAATGACCA-GATGAAAAATTATTGTAGCTAGAACA------------------------  84139
Ferret       -------------------------------TTGGGAAATTGTTATACATAGAACA------------------------  85666
Lion         GAGAA--AGACTAGGAGGGATAATGACCA-GATGAAAAATTATTGTAGCTAGAACA------------------------  81870
Vaquita      GATAAATAGATTAGGGGGGATAGTGAAGGAGATAAGAACTTGTTATAAATAGAAAA------------------------  80561
Pig          GAAGAGTAGTAGATTAGGGATGGTAAAGGAGATAGGAAATTGTTATAAATAGAAAA------------------------  79588

Majority     ------------GCATGCACACAGGGCTAAA-GGTAAGAAA---AACXXXCACGTTAAXGAAAATAGG-TTTTGTATAGC

                         113370    113380    113390    113400    113410    113420    113430    113440

Human        ------------GCAGCTATGTCAAGAAATA-GTCATGTAATGTTCTGTTTTCACACACTGCACTATGTTCTGGGGTAGA  90884
PolarBear    ------------GCATGCACACAGTGCTAAA-GGTAAGAAA---AACAAACATGTCAAGGA-GATAGG-TTTTATATAGC  48975
Cow          ------------GCATG--CACCAGGCTAAA-GGTTAAATA-CTAGCATTTGT-TTAAAAAAAAAATG-TTTTGTACACC  84829
Dog          ------------GCATGCATACAGGGCTAAA-GGTAAGAAA---AACTAGCACTTTAAGGA-GATAGG-TTTTATGTAGT  78846
BelugaWhale  ATAAATAGAAAAGCATGCACACGGGGCTAAA-GGTAAAAAA----AA-CTAGTGTTTAAGAAAATACA-TTTTGTACACC  79402
Donkey       ------------GCATGCACAGGGGACTAAA-GGGAAGAAA---AACTGGCAC-TTAAAGCAACCATG-TTTTGTATAGC  88020
Cat          ------------GCATGCACACAGGGCTAAAAGGTAAGAAA---AATTGGCACTTCAAGGAAGATAGGGTTTTGTATAGC  84204
Ferret       ------------GCATACACACAGGGTTAAA-GGTGGGAAA---AACGAGCATGTCAAGGA-GATAGG-TTTTATATAGC  85728
Lion         ------------GCATGCACACAGGGCTAAAAGGTAAGAAA---AACTGGCACTTCAAGGAAGATAGGGTTTTGTATAGC  81935
Vaquita      ------------GCATGCACACGGGGCTAAA-GGTAAAAAA----AAACTAGTGTTTAAGAAAATACG-TTTTGTACACC  80623
Pig          ------------GCACAGACACCGGGCTAAA-GGTAAAAAAACTAGCATTTTCGTTTAAGGAAATAGG-TTTTGTACAGC  79654
```

```
Majority     TGGAAC--TTAAAGTXCAAGGA-GTATAGTACTAAXATACTGT-AAGTTCC-------------------------ATGA
                      113450    113460    113470    113480    113490    113500    113510    113520
Human        GACAAAGGTTTGGATTTTGGAATATTT-GCATTATACCAAT-TGAGCTTCCCA--------------------AATCTGA  90942
PolarBear    TGGAGC--TTAAAGTGCAAGGAAGTACGGTACTAAAACACCCT-AAGTTCC-------------------------ATGA  49027
Cow          TGAAAA--CTTAAGTACAAGGA-GGATAGTACTAAGATACTGT-AAGTTCC-------------------------ATGA  84880
Dog          TCTAAC--TTAAAGTGCAAAGAAGTATAGTACTAAAAT-CTAT-AAGTTCC-------------------------ATGA  78897
BelugaWhale  TGGAAC--ATTAAGTACAAGGA-GTACAGTACTAAGATACTGT-AGGTTCC----------------------ATGAGGA  79456
Donkey       TGGAGTG-TTTAAGTGCAAGGA-ATATAGTACTGAGATACCAT-AAGTTCC-------------------------ATGA  88072
Cat          TGGAGG--GTAAAGTTCAAAGA-GTACAGTACTAAAATACTGT-AAGTTCC-------------------------ATGA  84255
Ferret       TGAAGC--TTAAAGTGCAAAGAAGTATAGTACTAAAAAACCAC-AAGTTCC-------------------------ATGA  85780
Lion         TGGAGG--GTAAAGTTCAAAG---------------------T-----TCC-------------------------ATGA  81962
Vaquita      TGGAAC--ATTAAGTACAAGGA-GTACAGTACTAAGATACTGT-AGGTTCC----------------------ATGAGGA  80677
Pig          TGGAAC--ATAAAGTACAAGGA-GTCTAGTACTGAGATACTCTTAAGTTCTTACACAAGGAGTCTAGTACTAAGATACTA  79731

Majority     GGGGT-AGGATGGTTCTTG-TCTTACCACTTGATTCTCAG---CACTAACAAAGTAGTTGGTATTGTATAACATCATCAA
                      113530    113540    113550    113560    113570    113580    113590    113600
Human        AATGTTCCAATGAGCATTTCCCTTGA-GCATCATGTTGGCCCTCAGTTTTGGATTTTGGAACATTTCAG-ATTTC----A  91016
PolarBear    GGGGT-AGGATGGTTCTTG-TCTTAC-ACTTGATTATCGG---CACTAACAAAGTAGTTGGTATTGTCATACATCATCAA  49101
Cow          GGGTC-AGGATAGTTTTTA-TATTACCATTTGATTCTCAA---CACTAACAAAGTTACTAGTATTATAAATCATCAATAA  84955
Dog          GGGGGTAGGATGGTTCTTA--CT----ACTTGATTATCAGCACTACTAACAAAGTAGTTGGTATTGTTGTACATCCTCAA  78971
BelugaWhale  GGGCT-AGGATGGTTCTTG-TCTTACCACTTGATTCTCAG---CATTAACAAAGTGGTTAGTATTGTATATCATCAAT-A  79530
Donkey       GGGCT-AGGATGGTTCTTG-TCTTACCTCTTGATTCTCAG---CACTAA-AAAGTGGTTGGTATTGTACATCATCAGT-A  88145
Cat          GGGGT-GAGATGGTTCTTG-TCTTACCACTTGATTCTCAG---CGCTAACAAAGTAATTGGTTTTGT---ATATCATCAA  84327
Ferret       GGGGTTAGGATGGTT-TTG-TCTTAC-ACTTGATTATCAG---TACTAACAAAGTAGTGGGTATTGTTTTACATCATCAG  85854
Lion         GGGGT-GAGATGGTTCTTG-TCTTACCACTTGATTCTCAG---CACTAACAAAGTAATTGGTTTTGT---ATATCATCAA  82034
Vaquita      GGGCT-AGGATGGTTCTTG-TCTTACCACTTGATTCCCAG---CATTAACAAAGTGGTTAGTATTGTATATCATCAAT-A  80751
Pig          GGGCT-AGGGTGATTCTTAATCTTAGCATTAGCTTCTCAG---CAGTAACAAAGCATTTAGTATTCTATAATACC----A  79803
```

```
Majority     AATATATG-TTCAGAGAGGCTGGAGAGAT-ATXTTAGGGTCAGACTGXGAAAXXTXAAXAXGGCATGTTAAAXXTC---T

                        113610    113620    113630    113640    113650    113660    113670    113680

Human        GATTTCTGCATTAAGGATACTCAACCTGT----------ACAGACTGGGAAAGGT--ATAAGCCATGTTAAAGAA----T  91080
PolarBear    GAAATAC--TTCCAAGAGGCTAGGGAGAT----------------TTCATT-CTTTAACATGGCTT-ATACTTTTC---C  49158
Cow          AATATTTA-TTCAGGGAGGCTTGAGAAAG-ATGTTAGGGTCAGACTGAGAAATAT--AGAAGACATGTTGAA-------T  85024
Dog          GAAGTATG-TTCAAAGAGGCTGGGGAGAT----------------TTTATT-CTTTAACATGGTTT-TTGCCTTTC---C  79029
BelugaWhale  AATGTTTA-TTCAGGGAGGCTTGATAGAG-ATGTTAGGGTCAGACTGAGAAAGGCGCATAAGCCATGTTAAAGAAAGAGT  79608
Donkey       AATATCTG-TTCAAAGAGGCTGGAGAGATAAT-TTAGGGTCAGACTGGGAAAGGT--ACAAGCCATGTTAAAGAA----T  88217
Cat          TAAATATG-TTCAGAGATGCTGGGGAGATCAGGATATAGTCCAAATTCATTTCTTCAACATGGCTTTATACCTTTC---C  84403
Ferret       GAAATATG-TTCAAAGAGGCTGAGGAGAT----------------TTCATT-CTTTAATATGGCTT-ATACCTTTC---C  85912
Lion         TAAATATG-TTCAGAGATGCTGGGGAGATCAGGATATAGTCCAAATTCATTTCTTTAACATGGCTTTATACCTTTC---C  82110
Vaquita      AATGTTTA-TTCAGGGAGGCTTGATAGAG-ATGTTAGGGTCAGACTGAGAAAGGCGCATAAGCCATGTTAAAGAAAGAGT  80829
Pig          AATATCTG-TCCAGGGAAGCTTGAGAGGA-ATATTAGGGTCAGACTGAGAAAGGT--TTA-GTCATGCTAAAGA----GT  79874

Majority     TAGTTTGAACTTX-TCCCTAAGGATTTTAAG--CAGGTAATGACAT---CAGATTTGTAATTT-ATAGAGGTCATCCAGC

                        113690    113700    113710    113720    113730    113740    113750    113760

Human        GAGTTTCAACTTTGTCCTTAAGGGTTTTAAG--CAGGTATTGACGTGATCAGATTTGTAATTT-ATGAAGGTCATCAAGC  91157
PolarBear    TAGTCTGA-------CCTCAAGGATTTTAAG--CAGATAATGACAT---CAGATTTGTAGTTT-ATAGAGTTCATCCAGC  49225
Cow          AAGTTGAGGCTTCATCCTTAAGGATTTTAAG--CAGGTAGTGACAT----AGATTTGTA-TAGAACGGAGG---------  85088
Dog          TAGTCTGATCT--GACCTTAACGATTTTAAG--CAGGTAATGACAT---CAGATTTGCAATTT-ATAGGGCTCATCCAGC  79101
BelugaWhale  AAGTTTAGACTTTATCCCTAAGGGTTTTAAG--CAGGTAGTGACAT----AGATTTGTA-TAGAATGGATTG--------  79673
Donkey       GAATTTGGACTTTGTCTCTAAGGGTTTTAAG--CAGGTAATGACATGATCAGATTTGCAATTT-ACAGAGGTCATCCAGC  88294
Cat          TAGTCTGA-------CCCTAAGGATTTTAAG--CAGGTAATAACAT---GAGATTTGCAATTT-ATAGAGGTCATCCAGC  84470
Ferret       TAGTGTGA-------CCTTAAGGATTTTAAG--CAGGTAATGACAT---CAGATTTGTAATTT-ATAGAGTTCATCTAGC  85979
Lion         TAGTCTGA-------CCCTAAGGATTTTAGGGTCAGGTAATAACAT---GAGATTTGCAATTT-ATAGAGGTCATCCAGC  82179
Vaquita      AAGTTTAGACTTTATCCCTAAGGGTTTTAAG--CAGGTAGTGACAT----AGATTTGTA-TAGAATGGATTG--------  80894
Pig          AAGTTTAGACTTTTATCCATAAGAGTTTAAG--CAGATAGCGACTTGGTCAGATTTGTAATTTTACAGAGGTCTTCTGGC  79952
```

```
Majority     TGCT--GTGTA---GAATGGATTGGAGAAAA-GTAGAGAAAAA-TCC-AACAGGAAAATAAATT------------GAGT
                     113770    113780    113790    113800    113810    113820    113830    113840
Human        TGCTGTATAGA---AAATGGATTGGAAAAATTATCCAACAGGAAGACAAATATGAGACT-----------------GAGT  91217
PolarBear    TGCT--GTGTGT---AATAGAT-GGAGAAAA-GTAGAAAAAAA-TGC-AACAGGAAGATAAATTAGGAGACT----AGGT  49292
Cow          ---------------AACA---TAGAGAAAA-ATCTAGCAAGA-TGA-ATTGGGAAACTGATTT------------GAGT  85135
Dog          TGCTATGTGTAATAGAATGGATTGGAGAAAA-GTAGAGCAAAG-TCC-AACAG--------------------------T  79152
BelugaWhale  --------------GAGAAAAGTAGAGAAAA-ATCCAACAAGA-CAA-ATTAGGAGACTGATT-------------GAGT  79723
Donkey       TGCTGTGTTTA---GAATGGATTGGAAGAAA-GTAGAGAATAA-TCC-AACAGGAGTAGAAATTAGGAGACTAAGTGAGT  88368
Cat          TGCTCTGTGTAATAGGATGCATTTGAAAAAA-GTAGGGAAAAA-TCCCAACAGGAAGACAAATTGGGAGACA----GAGT  84544
Ferret       TGCT--GTATAATAGAATGGGTTGGAGAAAA-GTAGAAAAAA--TCC-AACAGGAAGATAAATTAGGAGACT----GAGT  86049
Lion         TGCTCTTTGGAATAAGATGCATTTGAAAAAAAGTAGGGAAAAA-TCC-AACAGGAAGACAAATTGGGAGACA----GAGT  82253
Vaquita      --------------GAGAAAAGTAGAGAAAA-ATCCAACAAGA-CAA-ATTAGGAGACTGATT-------------GAGT  80944
Pig          TGCT--GTGTAT--GAATGGATTGGAGAAAA-GTAGAGAAAAA-TCC-AACAAGACA--AATTT------------GGAG  80011

Majority     TATGCAGATCAAAAAATXATGXCAGTGAAAAGTGGAGAAXX-GAGA-GATGAATTTGAGAGATAATTAGGA---AGAAGA
                     113850    113860    113870    113880    113890    113900    113910    113920
Human        TATCCAGATAAAAAA-TAATGTCAGCGAGAAGTAGAGAAAC--AGATCATGAATTTGAGGGACAATTAGGA---GGAAGG  91291
PolarBear    TATGCAGATCAAAACATTGTGACAGTGAAAAGTGGAGAATG-GAGA-GATGAAT-TGAGGGATAATTAGGA---AGAAAA  49366
Cow          TATACAGATAAAAAG-TAATGGCAGTGAAAAGTGTAGAAATAGA-----TGAATTTGAGGGATAATTAGA------AAGG  85203
Dog          TTTGCAGGTCAAAACATTGTGACAGTGTAAAGCAGAGAATG-GAGA-GCTGTAT-TGAGGGATAATTAGGAGTAAGAAGA  79229
BelugaWhale  TATCCAGATAAAAAA----TGGCAGTGAAAAGTGTAGAAATT-AGAAGATGAATTTGAGAGTAATTA---G----GATGG  79791
Donkey       TATCCAGATCAAAAA-TAATGGCAGTGAAAAGTGGAGAGGGAGACAGTATGAATTTGAGAGATACTTATAGG-AGGAATA  88446
Cat          TA-GCAGATCAAAAAAATTGTGACCGTGAAAAGTAGAGAAT---------------TGAGAGATAATTAGGAA-GAAAAGA  84607
Ferret       TATACAGATCAAAACATTTTTACAGTGAAAAGTGGAGAATA-GAGA-GATGAAT-TGAGGGATAATTAGGA---AGAAGG  86123
Lion         TA-GCAGATCAAAAAAATTGTGAC--TGAAAAGTAGAGAAT---------------TGAGAGATAATTAGGAA-GAAAAGA  82314
Vaquita      TATCCAGATAAAAAAATAATGGCAGTGAAAAGTGTAGAAAT--AGAAGATGAATTTGAGAGTAATTAAGAG----GATGG  81018
Pig          ACTGAAGATAAAAAA-TAATGGCAGTGAAA-GTGTAGAAACAGAGAAGATGGATTTGAGAGATAATAGGAG----GAAGA  80085
```

```
Majority     GTATCATAGGACTTGGCAATTAGGATATGGGGCAGAAGGGAGAAAAAGTT--ACAGATGAGTACTAAGTTTCTTTCT-TG
                       113930    113940    113950    113960    113970    113980    113990    114000
Human        CTATCACAATATTTAGCAATTAGGATGTGTAACAGAAGGGAGAAAAAGAGCTAGGGATGCTTATTAAGTTTCTTTCTTGT 91371
PolarBear    GTATTCTAGGACTTGGCA----GGTTGTGGGGCAGAAGGGGGAAAAAGTT--ACAGATGAGTACTAAGTTCCTTTCTGCA 49440
Cow          GTTACATAGGACTTGGTAATTAGGATATGGAGCAGAAGGGAGAAAAAGTT--AAAGTTTCTA-------AAGTTTCT-TG 85273
Dog          GTATCGTAGGACTTGGGAATTAGGTTATGGGGCAGAAGGGAGAAAAAGTTTTATAGATAAGTACTAAGTTTATT----CA 79305
BelugaWhale  GTATGGTAGGACTTGGCAATTAGGATATAGATCAGCAGGGAGAAAATGTT--ACAGATGAGTATTAAGTTTCTTTCT-TG 79868
Donkey       GAGTCATAGGACTTGGCAATTGGGATATGAGGCAGAAAGAAGAAGAAGTT--ACAGATGAGTACTAAGTTTCTTTCTTGG 88524
Cat          TTATCGTGGGACTTGGCAGTTAGGTTATGGGGCAGGAGAGAGAAGTTGTT--ACAGATGAGTACTAAGTTTCTTTCTGTG 84685
Ferret       GTATTCTAGGACTTGGCAATTAGGTTGTGGGGCAGAAGGGAGAAAAAGTT--ATAGATGAGTACGAAGTTTCCTTCTGCA 86201
Lion         TTATCATAGGACTTGGCAGTTAGGTTATGGGGCAGGAGAGAGAAGTTGTT--ACAGATGAGTACTAAGTTTCTTTCTGTG 82392
Vaquita      GTATGGTAGGACTTGGCAATTAGGATATAGATCAGCAGGGAGAAAATGTT--GCAGATGAGTACTAAGTTTCTTTCT-TG 81095
Pig          GTATTATAGGACTTGGCAATTAGGATATGGAGCAGAAGCGAGCAAAGGTT--ACAGATGAGT-------TTCTTTCT-TG 80155

Majority     GCAACTAAXT-TATGATTTAT---CAGGATTGTCAAGGAAGAGAGAGATTCATAATCTGGATTTTCATTCTATGAAAGAA
                       114010    114020    114030    114040    114050    114060    114070    114080
Human        GTAGCTCACTATACGATTTGTTAGCAGGATTGTTGAGGAAGATAGATATTCATAATCTGGATTTTCATTCTATGAAAGTA 91451
PolarBear    GCAACTAAGTGTATGACTTA---GCAGGATTGGCAAGGGAGAGATTGATTCATAATCAGGATTTTCATCCTATGAAAGAA 49517
Cow          GGCATTA----------TCAG---CAGGACTGTC--------AAGAAACTCAT--TCTGGGTTTTCATTCTATGAAAGAA 85330
Dog          GCAACTAAGTTTATGATTTAT---CAGGATTGGCAAGGAAGAGAGAGATTCATAATCAGGATTTACATTTTATGAAAGAA 79382
BelugaWhale  GGCATT------------TAG---CAGGACTGTCAAGGAAGAGAGAGACTCACATTTTGGATTTTCGTTCTATAAAAGAA 79933
Donkey       GCATCTAAGTATATGATTTATCAACAGGATTGTCAAAAAAGAGAGATATTTATAATCTGGATTTTCATTCTATGGGAGAA 88604
Cat          GCATCTA------TGATTTAT---CAGGATTGACAAGGAAGAGA-AGATTCATAATGAGGATTTGCATTCTGTGAAAGAG 84755
Ferret       GCAACTAAGTATATGACTTATCAGCAGGATTGGCAAGGAAGAGAGAGATTCATAATCAGGATTTTCATCCTATGACAGAA 86281
Lion         GCATCTAAGTATATGATTTAT---CAGGATTGGCAAGGAAGAGA-AGATTCATAATGAGGATTTTCATTCTGTGAAAGAG 82468
Vaquita      GGCATT------------TAG---CAGGACTGTCAAGGAAGAGGGAGACTCACATTTTGGATTTTCGTTCTATAAAAGAA 81160
Pig          GGCATTA----------TCGT---CAGGACTGTCTAGGAAGAGAGAAATTCATA-TCTAGATTTTCATTCTGTGGAAGAA 80221
```

```
Majority     G-CTGGCTTTTTT-GCCAAAATGCCTTGTCTAGTGCAAATXAATXTXXATX---GTCTTGTCACCAGTGGCTGTTGTAAA
                       114090    114100    114110    114120    114130    114140    114150    114160
Human        G-CTGGCTTTTTT-GCCAGAATGCCTTGTCTAGTTCAAATGAAAATGTACTTAAGTCTTATCACCAGTGGCTATGGTAAA  91529
PolarBear    A-CTGGCTTTTTC-GCCAGAATGCTTG-TCTATTGCAAATAAATTTAAATC---ATCTTGTCACCAATGACTGTTGTAAA  49591
Cow          G-CTGGCTTCTTT-GCCTCAGTGCCTTGTTGAGTGCAAATGAAT-----TT---GTTTTGTCACCAGTGGCTATTGTAAA  85400
Dog          A-CGAACTTTTTT-GTCAAAATACTT----TAGTGCAAATAAATTTAAATC---ATCTTGT---CAGTGGCTGTTATAAA  79450
BelugaWhale  G-CTGGCTTTTTT-GCCTCAGTGCCTTGTCTAGTGCAAATGAAC-----TT---GTTTTGTCACCAGTGGCTGTTGTAAA  80003
Donkey       G-CTGGCTTTTTT-GCCAAAGTGCCTTGTCTAGTGCAAATTAA-----------GTCTTGTCACCAAAGGCTATTGTGAA  88671
Cat          G-CTGGCTTTTTTTGCCAAAATGGCC----TAGGGCAAATAAATTTAAATC---ATCTTGTCACCAGTGACTGTTGAAAA  84827
Ferret       AACTGGCTTTTTT-GCCTGAATGCTTG-TCTAGTGCAAATAAATTTAAATC---ATCTTGTCACCAAGGGCTGTTGTAAA  86356
Lion         G-CTGGCTTTTTT-GCCAAAATGGTC----TAGGGCAGATAAATTTAAATC---ATCTTGTCACCAGTGACTGTTGAAAA  82539
Vaquita      G-CTGGCTTTTTT-GCCTCAATGCCTTGCCTAGTGCAAATGAAC-----TT---GTTTTGTCACCAGTGGCTGTTGTAAA  81230
Pig          G-CTGGCTTTTTT-GCCTAAATGCCTTGACTCATGCAAATG---------T---GTCTTGTCACCAGTAGCTAGTGTAAA  80287

Majority     CTCATGATTTATTT------------------------------------------------------------------
                       114170    114180    114190    114200    114210    114220    114230    114240
Human        TTGGTGATTTATTT------------------------------------------------------------------  91543
PolarBear    CTTACGATTTATTT------------------------------------------------------------------  49605
Cow          TTCGTGATTTATTT------------------------------------------------------------------  85414
Dog          CTTGCCATTTATTT------------------------------------------------------------------  79464
BelugaWhale  TTCATGATTTATTT------------------------------------------------------------------  80017
Donkey       CTCATGATTTATTT------------------------------------------------------------------  88685
Cat          CTCAAGATTTATTTAAAAATCATCGGGTGCCTAGGTGGCTCAGTAGGTTAAGTGTCCAAGTCTTGGTTTTGATTCAGGTT  84907
Ferret       CTTAAGATTTATTTT-----------------------------------------------------------------  86371
Lion         CTCAAGATTTATTTAAAAATCATGGGGCGCCTGGGTGGCTCAGTAGGTTAAGTGTCCAAGTCTTGGTTTTGATTCAGGTT  82619
Vaquita      TTCATGATTTATTT------------------------------------------------------------------  81244
Pig          TTCATGATTT----------------------------------------------------------------------  80297
```

```
Majority     --------------------------------------------------------------------------------
                       114250    114260    114270    114280    114290    114300    114310    114320
Human        --------------------------------------------------------------------------------  91543
PolarBear    --------------------------------------------------------------------------------  49605
Cow          --------------------------------------------------------------------------------  85414
Dog          --------------------------------------------------------------------------------  79464
BelugaWhale  --------------------------------------------------------------------------------  80017
Donkey       --------------------------------------------------------------------------------  88685
Cat          GTGATCTCACGGTTCGTGAGTCCCAGCCCCTCACTGTCAGCATGCAGCCAGCCTCTCTTTGCCCCTCACCCTTTCATGTT  84987
Ferret       --------------------------------------------------------------------------------  86371
Lion         GTGATCTCACGGTTCGTGAGTCTCAGCCCCTCACTGTCAGCATGGAGCCAGCCTCTCTTTGCCCCTCGCC-TTTCATGTT  82698
Vaquita      --------------------------------------------------------------------------------  81244
Pig          --------------------------------------------------------------------------------  80297

Majority     ----------------------------------------------------TAAAATCATAG-AAT--TCAGTTATTTC
                       114330    114340    114350    114360    114370    114380    114390    114400
Human        ----------------------------------------------------TAAAATCATAAGAAAATTCAAATTTTTA  91571
PolarBear    ------------------------------------------------A----AAAATCATAG-AAT--TTGGTTATTTC  49630
Cow          ----------------------------------------------------TAAAACCATAGCAAAA-TTAGGTATTTC  85441
Dog          ------------------------------------------------A----AAAATCATAG-AAT--TCAGTTATTTT  79489
BelugaWhale  ----------------------------------------------------TAAAATCATAACAGAATTCAGATATTTC  80045
Donkey       ----------------------------------------------------TAAAATCATAAGAGAATTCAGAT-TTTG  88712
Cat          CTCGCTCAACATAAATAAGCTTAACCAAAAAAAAAAAAAAAAAAAATT-ATTTAAAAATCATGG-AAT--TCAGTTATTTC  85063
Ferret       ------------------------------------------------A----AAAATCATAG-AAT--TCAGTTATTTC  86396
Lion         CTCTCTCAACATAAATAAGCTTAACCAAAAAAAAAAAAAAAAAGATTTATTTAAAAATCATAT-AAT--TCAGTTATTTC  82775
Vaquita      ----------------------------------------------------TAAAATCATAACAGAATTCAGATATTTC  81272
Pig          ----------------------------------------------------TAAAATCATAGCAAT--TCAGGTATTTC  80323
```

```
Majority     AGCTATTCTAAAAGA-----AATTATTTTTGCTTTGAACTTTG--AATATTGTTTTTTAATAGGCAT---TTCTTTCTTT
                       114410    114420    114430    114440    114450    114460    114470    114480
Human        AACTGTTACGTTAAAAG---AATTATGTTTGTTTTGAGCTTTG---ATATTGTTTGTTAATAGACAT----GCTTTCTTT  91641
PolarBear    TGTTCTAAAAGAAGA------ATTTTTTTTTGCTTTGGACTTTGTTAATACTGTTTTTTAATAGGCAT---TTTTT-CTTT  49700
Cow          AACTATTCTAAGAATTATTGTAATATTTTTGCTTTGGATGTTG--A-TATTGTTTATTAATAGGCAT---T-CTTCCTCT  85514
Dog          AACTATTCTA--AAA------A-TTATTTTGCTTTGAACTGTGTTAATA--TTTTTTTAATAGGCAT---TTTTTTCTTT  79555
BelugaWhale  AGCTTATCTAAAA-TGA--GAATTATTTTTGCTTTGAACCTTG--A-TACTGTTTTTTAATAGGCAT---C-CTTCCCCT  80115
Donkey       AACTATTCTAAAATATG---AATTACTTTTGCTTTGAACTTTGTTAATGTTG-TTTTTAATAGGCAT----TCTTTCTTT  88784
Cat          AGCTATTCCAAAAGAAG---AATTCTTTTTGTTTTGAACTTTGTTAATATTGTTTTTTAATAGGCATATTTTTTTTCTTT  85140
Ferret       TGCTATTCTA-AAGA------ATTCTTTTTGCTTTTGGACTT--------TGTTTTTTAATAAGCAT---TTTTT-CTTT  86457
Lion         GGCTATTCCAAAAGA------ATTCTTTTTGTTTTGAACTTTGTTAATATTGTTTTTTAATAGGCAT-TTTTTTTTCGTT  82848
Vaquita      AGCTTATCTAAAA-TGA--GAATTATTTTTGCTTTGAACCTTG--A-TACTGTCTTTTAATAGGCAT---T-CTTCCCCT  81342
Pig          AGCTCCTCTAAAA-------TAATATTTTTGCTTTAAACCTTG--A-CTTT--TTAA-AATAAGCAT---T-CTTTTTCT  80386

Majority     TTATAGCCATGAACCATCTTAATGGACAGAAAATGTATGGAAAAATTATTCGTGTTACTCTGTCTAAACATCAGACTGTA
                       114490    114500    114510    114520    114530    114540    114550    114560
Human        TTATAGCCATGAATCATCTTAATGGACAGAAAATGTATGGAAAAATTATTCGTGTTACTCTGTCTAAACATCAGACTGTA  91721
PolarBear    TTACAGCCATGAACCATCTTAATGGACAGAAAATGTATGGCAAAATTATTCGTGTTACTCTGTCTAAACATCAGACTGTA  49780
Cow          TTATAGCCATGAATCATCTTAATGGACAGAAGATGTACGGAAAAATTATTCGTGTTACTCTGTCTAAACATCAGACTGTA  85594
Dog          TTACAGCCATGAACCATCTTAATGGACAGAAAATGTATGGTAAAATTATTCGTGTTACTCTGTCTAAACATCAGACGGTA  79635
BelugaWhale  TTATAGCCATGAATCATCTTAATGGACAGAAGATGTATGGAAAAATTATTCGTGTTACTCTGTCTAAACATCAGACTGTA  80195
Donkey       TTATAGCCATGAACCATCTTAATGGACAGAAGATGTATGGAAAAATTATACGTGTTACTTTATCTAAACATCAGACTGTA  88864
Cat          TTATAGCCATGAACCATCTTAATGGACAGAAAATGTATGGCAAAATTATTCGTGTTACTCTGTCTAAACATCAGACTGTA  85220
Ferret       TTACAGCCATGAACCATCTTAATGGACAGAAAATGTATGGCAAAATTATTCGTGTTACTCTGTCTAAACATCAGACTGTA  86537
Lion         TTATAGCCATGAACCATCTTAATGGACAGAAAATGTATGGCAAAATTATTCGTGTTACTCTGTCTAAACATCAGACTGTA  82928
Vaquita      TTATAGCCATGAATCATCTTAATGGACAGAAGATGTATGGAAAAATTATTCGTGTTACTCTGTCTAAACATCAGACTGTA  81422
Pig          TTATAGCCATGAATCATCTTAATGGACAGAAAATGTATGGAAAAATTATTCGTGTTACTCTGTCTAAACATCAGACTGTA  80466
```

```
Majority     CAACTACCTCGAGAGGGACTTGATGATCAAGGGCTAACAAAAGATTTTGGTAATTCCCCATTGCATCGTTTTAAGAAACC

                     114570    114580    114590    114600    114610    114620    114630    114640

Human        CAGCTACCTCGAGAGGGACTTGATGATCAAGGGCTAACAAAAGATTTTGGTAATTCCCCATTGCATCGTTTTAAGAAACC  91801
PolarBear    CAACTACCTCGAGAGGGACTTGATGATCAAGGGCTAACAAAGGATTTTGGTAATTCCCCATTGCATCGTTTTAAGAAACC  49860
Cow          CAACTACCTCGAGAGGGACTTGATGATCAAGGGCTAACAAAAGATTTTGGTAATTCCCCATTGCATCGTTTTAAGAAACC  85674
Dog          CAACTACCTCGAGAGGGACTTGATGATCAAGGGCTAACAAAGGATTTTGGTAATTCCCCATTGCATCGTTTTAAGAAACC  79715
BelugaWhale  CAACTACCTCGAGAGGGACTTGATGATCAAGGGCTAACAAAAGATTTTGGTAATTCCCCACTGCATCGTTTCAAGAAACC  80275
Donkey       CAGCTACCTCGAGAGGGACTTGATGATCAAGGGCTAACAAAAGATTTCGGTAATTCCCCATTGCATCGTTTTAAGAAACC  88944
Cat          CAACTACCTCGAGAGGGACTTGATGATCAAGGGCTAACAAAGGATTTTGGTAATTCCCCATTGCATCGTTTTAAGAAACC  85300
Ferret       CAACTACCTCGAGAGGGACTTGATGATCAAGGGCTAACAAAGGATTTTGGTAATTCCCCATTGCATCGTTTTAAGAAACC  86617
Lion         CAACTACCTCGAGAGGGACTTGATGATCAAGGGCTAACAAAGGATTTTGGTAATTCCCCATTGCATCGTTTTAAGAAACC  83008
Vaquita      CAACTACCTCGAGAGGGACTTGATGATCAAGGGCTAACAAAAGATTTTGGTAATTCCCCACTGCATCGTTTCAAGAAACC  81502
Pig          CAGCTACCTCGAGAGGGACTTGATGATCAAGGGCTAACAAAAGATTTTGGTAATTCCCCATTGCATCGTTTTAAGAAACC  80546

Majority     TGGATCCAAAAATTTTCAGAACATTTTTCCTCCTTCTGCAACCCTTCACCTATCCAATATCCCGTAAGTATTAAAGCTGG

                     114650    114660    114670    114680    114690    114700    114710    114720

Human        TGGATCCAAAAATTTTCAAAACATTTTTCCTCCTTCTGCCACCCTTCACCTATCTAATATCCCGTAAGTATATAAGCTAG  91881
PolarBear    TGGATCCAAAAATTTTCAGAACATTTTTCCTCCTTCTGCAACCCTTCACCTATCCAATATCCCGTAAGTATGAAAGTTGG  49940
Cow          TGGATCCAAAAATTTTCAGAATATTTTCCCTCCTTCTGCAACCCTTCACCTATCCAATATCCCGTAAGTATTAAAGATGG  85754
Dog          TGGATCCAAAAATTTTCAGAACATTTTTCCTCCTTCTGCAACCCTTCACCTATCCAATATCCCGTAAGTACTAAGCT-AG  79794
BelugaWhale  TGGATCCAAAAATTTTCAGAACATTTTTCCTCCTTCTGCAACCCTCCACCTATCCAATATCCCGTAAGTATTAAAGCTGG  80355
Donkey       TGGATCCAAAAATTTTCAGAACATTTTTCCTCCTTCTGCAACCCTTCACCTATCCAATATCCCGTAAGTATTAAAGCTAG  89024
Cat          TGGATCCAAAAATTTTCAGAACATTTTTCCTCCTTCTGCAACCCTACACCTATCCAATATCCCGTAAGTATTACAGCTGG  85380
Ferret       TGGATCCAAAAATTTTCAGAACATTTTTCCTCCTTCTGCAACCCTTCACCTATCCAATATCCCGTAAGTATGAAACTTCT  86697
Lion         TGGATCCAAAAATTTTCAGAACATTTTTCCTCCTTCTGCAACCCTACACCTATCCAATATCCCGTAAGTATTAAAGCTGG  83088
Vaquita      TGGATCCAAAAATTTTCAGAACATTTTTCCTCCTTCTGCAACCCTCCACCTATCCAATATCCCGTAAGTATTAAAGCTGG  81582
Pig          TGGATCCAAAAATTTTCAGAACATTTTCCCTCCTTCTGCAACCCTTCACCTATCCAATATCCCGTGAGTATTAAAGCTGG  80626
```

```
Majority     AGT---TTATTAA--GGATATACATTTTAGGAATAAAATATAAAAATTATTATTAATCTT--TACTCTTTTTTC--CATC
                        114730    114740    114750    114760    114770    114780    114790    114800
Human        AGT---GTATTGA---GATACATTCTATTTTGATAAAATATGAAATTTATTCTTAATCTT--CACTTTTTCTTC-CCATT  91952
PolarBear    AGT---TTTTTAA--GGGTATACATTTTAAGAATAAAACATAAAATTTATCATTAATCTTT-ACTTTTTTTTTCCCCATC  50014
Cow          AGTAGTTTATTAA--GGATATACATTTTAGGAATAAAATAAAAAAATTTTTAATAATGTTATTACTCTTTTTTTTCCATC  85832
Dog          AGT---TTTCTAA--GGGTATACATTATAGAAATAAAACATAAAATTTATTATTAATCTT----CTTTTTTTTC--CATC  79863
BelugaWhale  AGTAGTTTATTAA--GGATATACATTTTAGGAAGAAAATATAAACATTTTTTATTA-ATTGTTACTCTTTTTCT-----C  80427
Donkey       AGG---TTATTGA--GGATATGTGTTTTAAGGATAAAATGTAAAGTTTATTATTAATATT--TACCTTTTTTTC--CATC  89095
Cat          AGA---TTTTTAAATGGGTATACATTTTAGAAATAAAATTTGAAAATTATTATTAATCTT----TACTTTTTTC--CATC  85451
Ferret       AGT---TTTTTAA--GGGAGTACATTTTAAGAGTAAAACATAAAATGTATTATTAATCTTT-ACCCTTTTTTTC--CATC  86769
Lion         AGA---TTTTTAAATGGGTATACATTTTAGAAATAAAATTTGAAAATTATTATTAATCTT----TACTTTTTTC--CATC  83159
Vaquita      AGTAGTTTATTAA--GGATATACATTTTAGGAAGAAAATATAAACATTTTTTATTA-ATTGTTACTCTTTTTCT-----C  81654
Pig          GGT---TTATTAA--GGATATACATTTTAGGACTAA-----AAAAAATGTTATTCATCTT--TACTCTTTCTTT-CCGTC  80693

Majority     CAATTTTCCTAGTCCTTCAGTAGCAGAAGAGGATCTACGAACACTGTTTGCTAACACTGGGGGCACTGTGAAAGCATTTA
                        114810    114820    114830    114840    114850    114860    114870    114880
Human        CAATTTTCCTAGTCCATCAGTAGCAGAAGAGGATCTACGAACACTGTTCGCTAACACTGGGGGCACTGTGAAAGCATTTA  92032
PolarBear    CAATTTTCCTAGTCCTTCAGTAGCAGAAGAGGATCTACGAACACTGTTTGCTAACACTGGGGGCACTGTGAAAGCATTTA  50094
Cow          TAATTT-CCCAGTCCTTCAGTAGCAGAAGAAGATCTCCGAACACTGTTTGCTAACACTGGGGGCACTGTGAAAGCATTTA  85911
Dog          CAATTTTCCTAGTCCTTCGGTAGCAGAAGAGGATCTACGAACACTGTTTGCTAACACTGGGGGCACTGTGAAAGCATTTA  79943
BelugaWhale  TAATTTTCCCAGTCCTTCAGTAGCAGAAGAGGATCTGCGAACACTGTTTGCTAACACTGGGGGCACTGTGAAAGCATTTA  80507
Donkey       CAATTTTTCTAGTCCTTCAGTAGCAGAAGAGGATCTACGAACACTGTTTGCTAACACTGGGGGCACTGTGAAAGCATTTA  89175
Cat          CAATTTTCCTAGTCCTTCAGTAGCAGAAGAGGATCTACGAACACTGTTTGCTAACACTGGGGGCACTGTGAAAGCATTTA  85531
Ferret       CTGTTTTCCTAGTCCTTCAGTAGCAGAAGAGGATCTGCGAACACTGTTTGCTAACACTGGGGGCACTGTGAAAGCATTTA  86849
Lion         CAATTTTCCTAGTCCTTCAGTAGCAGAAGAGGATCTACGAACACTGTTTGCTAACACTGGGGGCACTGTGAAAGCATTTA  83239
Vaquita      TAATTTTCCCAGTCCTTCAGTAGCAGAAGAGGATCTACGAACACTGTTTGCTAACACTGGGGGCACTGTGAAAGCATTTA  81734
Pig          TAATTTTCCCAGTCCTTCAGTAGCAGAAGAGGATCTCCGAACGCTGTTTGCTAACACTGGGGGCACTGTGAAAGCATTTA  80773
```

```
Majority     AGTTTTTTCAGTAAGCAAGCTTT-CTTGTCTTTAAATTAGTGACTTGATAG-AAGTAAAAGTTTTCTTCA-AGTAGTTTT

             114890    114900    114910    114920    114930    114940    114950    114960

Human        AGTTTTTTCAGTAAGCAAGCTTC-CTTATCTTTAAATTAGTGACCT-------GATAAAA----TTTTTA-AGTAGTTTT  92099
PolarBear    AGTTTTTTCAGTAAGCAAGCTTT-CTTGTCTTTAAATTAGTGACTTCATAGAAATTAAAAGTTGTCTTCATAGTAGTTTT  50173
Cow          AGTTTTTTCAGTAAGCAAGCTTT-CTTGTCTTTAAATTAGTGACTTGATAG-AAGTAAAAGTTTTCTCCA-AGTAGTT--  85986
Dog          AGTTTTTTCAGTAAGCAAGCTTT-CTTGTCTCTAAATTAGTGACTTTG-----ACAAAAAGTTTTCTTCA-AGTAGTTTT  80016
BelugaWhale  AGTTTTTTCAGTAAGCAAGCTTT-CTTGTCTTTAAATTAGTGACTTGATAG-AAGTAAAAGTTTTCTTCA-AGTAGTTC-  80583
Donkey       AGTTTTTTCAGTAAGCAAGCTTTTCTTGTTTTTAAATTAGTAACTTGATAG-AAGTAAAAATTTTCTTCA-AGTAAATTT  89253
Cat          AGTTTTTTCAGTAAGCAAGCTTT-CTTATCTTTAAATTAGTGACTTGATAGAAGTTAAAAATTTTCTTCA-AGTAGTTTT  85609
Ferret       AGTTTTTTCAGTAAGCAAGCTTT-CTTGTCTTTAAATTAGTGACTTCATATAAGTTAAAAACTGTCTTCATAGTAATTTT  86928
Lion         AATTTTTTCAGTAAGCAAGCTTT-CTTATCTTTAAATTAGTGACTTGATAGAAGTTAAAAGTTTTCTTCA-AGTAGTTTT  83317
Vaquita      AGTTTTTTCAGTAAGCAAGCTTT-CTTGTCTTTAAATTAGTGACTTGATAG-AAGTAAAAGTTTTCTTCA-AGTAGTTC-  81810
Pig          AGTTTTTTCAGTAAGCAAGCTTTTCTTGTCTCTAAATTA--G----------AAGTCAAAGTTTTCCTAA-AGTAGTTTT  80840

Majority     TATT----------CTTTTTGTTTATAG-AAATTTTT-GATTCTGGAATGGGCAAAAA-TTCAAGTATTTAA---GCCCT

             114970    114980    114990    115000    115010    115020    115030    115040

Human        TGTT----------CTTTTCGTTTATAG-AAATTTTT-GATTCCGGAATGGCCAAAA--TTCAAGTATTTAATAAGCCCT  92165
PolarBear    TATC----------CTTTTTGTTTATGG-AAATT----GATTCTGGAATGGGTAAAAA-TTCAAGTATTTAA---GCCCT  50234
Cow          --------------GTTTTCATTTCTAG-ATATCTTTTGATCCTGAAATGAGCAAAAG-T------ATTTAA---GCCAT  86041
Dog          TATC----------TTTTTTGTTTATG--AAATT----AATTCTGGAATAGGTGAAA--TTCAAGTATTTAA---GCACT  80075
BelugaWhale  ---------------TTTTTGTTTCTAG-ACGTCTTT-GATTCTGGAATGGGCAAAAG-TT-AAGTATTTAA---GCCCT  80641
Donkey       TTTTTTTATTTTATTTTTTTATTTTTATTGAAGTTTTTGATTCTGCAATGGGGAAAAAATTCAAGTATTTAA---GCCCT  89330
Cat          TATT----------CTTTTTGTTTATGG-AAATTTTT-GATTCTGTAGTGGGCAAAAA-TTCAAGTATTTAA---GCCCT  85673
Ferret       TACC----------CTTTATGTTTATGG-AAATT----GATTCTAGAATGGGTAAAAA-TTCAAGTATGTAA---GCCCT  86989
Lion         TATT----------CTTTTTGTTTATGG-AAATTTTT-GATTCTGTAGTGGGCAAAAA-TTCAAGTATTT----------  83374
Vaquita      ---------------TTTTTGTTTCTAG-AAGTCTTT-GATTCTGGAATGGGCAAAAG-TT-AAGTATTTAA---GCCCT  81868
Pig          TATG----------ATTTTTGTTTATAG-ACATCTTT-GATTCTGGAATAGGCAGAAG-TT-AAATATTTAA---GCCCT  80903
```

Majority	TCCTAATTTTTATGAAGTATCTAATTTCATAATTTTGTTTCAGAAGAGATCACAAAATGGCTCTTCTTCAGATGGCAACA	
	115050 115060 115070 115080 115090 115100 115110 115120	
Human	TTCTAATTTGTATGAAGTGTCTAATTTTATAATTTTGTTTCAGAAGAGATCACAAAATGGCTCTTCTTCAGATGGCAACA	92245
PolarBear	TCCTAATTTTTATGAAGTATCTGATTTCATAATTTTGTTTCAGAAGAGATCACAAAATGGCTCTTCTTCAGATGGCAACA	50314
Cow	TC-TGATTTTTATGAAGTATCTAATTTCATAATTTTGTTTCAGAAGAGATCACAAAATGGCCCTTCTTCAGATGGCGACA	86120
Dog	TCCTAATTTTTATGAAGTATCTAATTTCATAATTTTGTTTCAGAAGAGATCACAAAATGGCTCTTCTTCAGATGGCAACA	80155
BelugaWhale	TCCTAATTTTTATGAAGTATCTAATTTCATAATTTTGTTTCAGAAGAGATCACAAAATGGCTCTTCTTCAGATGGCAACA	80721
Donkey	TTCTAACATTTATGAAGTATCTAATTTCATAATTTTGTTTCAGAAGAGATCACAAAATGGCTCTTCTTCAGATGGCAACT	89410
Cat	TCCTAATTTTTATGAAGTATCTAATTTTATAATTTTGTTTCAGAAGAGATCACAAAATGGCTCTTCTTCAGATGGCAACA	85753
Ferret	TCCTAATTTTTATGAAGTATCTAATTTCATAATTTTGTTTCAGAAGAGATCACAAAATGGCTCTTCTTCAGATGGCAACA	87069
Lion	------TTT-TATGAAGTATCTAATTTTATAATTTTGTTTCAGAAGAGATCACAAAATGGCTCTTCTTCAGATGGCAACA	83447
Vaquita	TCCTAATTTTTATGAAGTATCTAATTTCATAATTTTGTTTCAGAAGAGATCACAAAATGGCTCTTCTTCAGATGGCAACA	81948
Pig	TCCTAATTTTTATGACATATCTAATTTCATAATTTTGTTTCAGAAGAGATCACAAAATGGCTCTCCTTCAGATGGCAACA	80983

Majority	GTGGAAGAAGCTATCCAGGCCTTGATTGATCTTCATAATTATAACCTTGGTGAAAACCATCATCTGAGAGTGTCTTTCTC	
	115130 115140 115150 115160 115170 115180 115190 115200	
Human	GTGGAAGAAGCTATTCAGGCCTTGATTGATCTTCATAATTATAACCTTGGAGAAAACCATCATCTGAGAGTGTCTTTCTC	92325
PolarBear	GTGGAAGAAGCTATCCAGGCCTTGATTGATCTTCATAATTACAACCTTGGTGAAAACCATCATCTGAGAGTGTCTTTCTC	50394
Cow	GTGGAAGAAGCTATTCAGGCCTTGATTGATCTTCATAATTATAACCTTGGTGAAAACCATCATCTGAGAGTGTCTTTCTC	86200
Dog	GTGGAAGAAGCTATCCAGGCCTTGATTGATCTTCATAATTACAACCTTGGTGAAAACCATCATCTGAGAGTGTCTTTCTC	80235
BelugaWhale	GTGGAAGAAGCTATTCAGGCCTTGATTGATCTTCATAATTATAACCTTGGTGAAAACCATCATCTGAGAGTGTCTTTCTC	80801
Donkey	GTGGAAGAAGCTATCCAGGCCTTGATTGATCTTCATAATTATAACCTTGGTGAAAACCATCATCTGAGAGTGTCTTTCTC	89490
Cat	GTGGAAGAAGCTATCCAGGCCTTGATTGATCTTCATAATTATAACCTTGGTGAAAACCATCATCTGAGAGTGTCTTTCTC	85833
Ferret	GTGGAAGAAGCTATCCAGGCCTTGATTGATCTTCATAATTACAACCTTGGTGAAAACCATCATCTGAGAGTGTCTTTCTC	87149
Lion	GTGGAAGAAGCTATCCAGGCCTTGATTGATCTTCATAATTATAACCTTGGTGAAAACCATCATCTGAGAGTGTCTTTCTC	83527
Vaquita	GTGGAAGAAGCTATTCAGGCCTTGATTGATCTTCATAATTATAACCTTGGTGAAAACCATCATCTGAGAGTGTCTTTCTC	82028
Pig	GTGGAAGAAGCCATCCAGGCCTTGATTGATCTGCATAATTATAACCTTGGTGAAAACCATCATCTGAGAGTGTCTTTCTC	81063

```
Majority     CAAGTCAACAATTTAAAAA--GGGGGAGAT-GAAGATTGAGGGTGAATCACATTGTTTGGTGTCATCACCTATT-GACTG
                        115210    115220    115230    115240    115250    115260    115270    115280
Human        CAAGTCAACAATTTAAAAA--TGGGAAGAT-GAAGATTGGGGGTGAATCACATTGTTCAATGTCATCACCTATTTGACTG  92402
PolarBear    CAAGTCAACAATTTAAAAA--GGGGGAGGT-GAAGAATAAGGGTGTATCACATTGTTTGGTGTCATCACCTATT-GACTG  50470
Cow          CAAGTCAACAATTTAAAAAAGGGGGGAGAT-GAAGATTGAGGGTGAATCACATTGTTTGGTGTCATCACCTATT-GACTG  86278
Dog          CAAGTCAACAATTTAAAAA--GGGGGAGGT-GAAGATTGAGGGTGTATCACATTGTTTGGTGTCATCACCTATT-GACTG  80311
BelugaWhale  CAAGTCAACAATTTAAAAA--AGGGGAGAT-GAAGATTGGCGATGAATCACATTGTTTGGTGTCAACACCTATT-GACTG  80877
Donkey       CAAGTCAACAATTTAAAAA--GGGGGAGAT-GAAGATTGGGGGTGAATCACATTGTTTGGTGTCATCACCTATT-GACTG  89566
Cat          CAAGTCAACAATTTAAAAA--GGGGGAGGT-GAAGATGGAGGGTGTATCACATTGTTTGGTGTCATCACCTATT-GACTG  85909
Ferret       TAAGTCAACAATTTAAAAA--GGGGGAGGT-GAAGCTCGAGGGTATATCACATTGTTTGGTGTCATCACCTATT-GACTG  87225
Lion         CAAGTCAACAATTTAAAAA--GGGGGAGGT-GAAGATGGAGGGTGTATCACATTGTTTGGTGTCATCACCTATT-GACTG  83603
Vaquita      CAAGTCAACAATTTAAAAA--AGGGGAGATTGAAGATTGGCGATGAATCACATTGTTTGGTGTCAACACCTATT-GACTG  82105
Pig          CAAGTCAACAATTTAGAAA--GGGGGAGAT-GAAGATTGGGGGTGAATCACATTGTTTGGTGTCATCACCTATT-GACTG  81139

Majority     TTCAGAAAAGTGGGGACCAGAGTTTGATTTTTTTT-TTGTTGTC---------TTTTTTTTTTTTTTCCATGCTGTTATC
                        115290    115300    115310    115320    115330    115340    115350    115360
Human        TTCAGAAAAGTGGGGACCAGAGTTTGATTTTTTTTGTTTTTGTTTTTTTGGGGTTTCTTTTTTTTTTCCATGCTGTTATC  92482
PolarBear    TTCAGAAAAGTGGGGACCAGAGTTTGATTTTTTTG-TTGTTTCC---------TTTTTTTTTTTTTCCCATGCTGTTATC  50540
Cow          TTCAGAAAAGTGGGGACCAGAGTTTGATTTTTTGT--TTTTG-------------TTTTTGTTTTTTCCATGCTGTTATC  86343
Dog          TTCAGAAAAGTGGGGACCAGAGTTTGATTTTTTTG-TTGTTGTTGTTG----TTTCCTTTTTTTTTCCATGCTGTTATC  80385
BelugaWhale  TTCAGAAAAGTGGGGACCAGAGTTTGATTTTTTTTGTTGTTTTCTTTTT----TCTTTTCTTTTTTCCCATGCTGTTATC  80953
Donkey       TTCAGAAAAGTGGGGACCAGAGTTTGATTATTTTG----TTG-----------TTTCCTTTTTTTTTTTCATGCTGTTATC  89631
Cat          TTCAGAAAAGTGGGGACCAGAGTTTGATTTTTTTT-TTTGTTTC---------CTTTTTTTTTTTTT-CATGCTGTTATC  85978
Ferret       TTCAGAAAAGTGGGGACCAGAGTTTGATTTTTTTG-TTGTTGTT---------TCCTTTTTTTTTCCCCATGCTGTTATC  87295
Lion         TTCAGAAAAGTGGGGACCAGAGTTTGATTTTTTTT-TTGTTTCC---------TTTTTTTTTTTTTTTCATGCTGTTATC  83673
Vaquita      TTCAGAAAAGTGGGGACCAGAGTTTGATTTTTTTTGTTGTTTTCTTTTT----TCTTTTCTTTTTTCCCATGCTGTTATC  82181
Pig          TTCAGAAAAGTGGGGACCAGAGTTTGATTTTTTTC--TGTTGG-TTTTC----TTTCTTTTTTTTTTCCATGCTGTTATC  81212
```

```
Majority      ATTCCTTGGTTATAAAA-TGAAATGGCATATGTAAAGGCAGAGTTGTTAACTGCTXTATTTCATCTGTTCTATAGGGAAG
                        115370    115380    115390    115400    115410    115420    115430    115440
Human         ATTCCTTGGTTATAAAA-TGAAATGGCATATGTAAAGGCAGAGTTGTTAACTGCTATATTTCATCTGTTCTATAGGGAAG  92561
PolarBear     ATTCCTTGGTTATAAAA-TGAAATGGCATACATAAAGGCAGAGTTGTTAACTGCTGTATTTCATCTGTTCTATTGGGAGG  50619
Cow           ATTCCTTGGTTATAAAA-TGAAATGGCGTATGTAAAGGCAGAGTTATTAACTGCTCTCTTTCATCTGTTCTGTAGGGAAG  86422
Dog           ATTCCTTGGTTATAAAA-TGAAATGGCATACATAAAGGCAGAGTTGTTAACTGCTGTATTTCATCTGTTCTATAGGTAGG  80464
BelugaWhale   ATTCCTTGGTTATAAAA-TGAAATGGCATATGTAAAGGCAGAGTTATTAACTGCTCTTTTTCATCTGTTCTGTAGAGAAG  81032
Donkey        ATTCCTTGGTTATAAAA-TGAAATGGCATATGTAAAGGCAGAGTTATTAACTGCTATATTTCATCTGTTCTATAGGGAAG  89710
Cat           ATTCCTTGGTTATAAAA-TGAAATGGCATACATAAAGGCAGAGT-GTTAACTGCTATATTTCATCTGTTCTATAGGGAAG  86056
Ferret        ATTCCTTGGTTATAAAAATGAAATGGCATACGTAAAGGCAGAGTTGTTAACTGCTGTATTTCATCTGTTCTATAGGGAGG  87375
Lion          ATTCCTTGGTTATAAAA-TGAAATGGCATACATAAAGGCAGAGT-GTTAACTGCTATATTTCATCTGTTCTATAGGGAAG  83751
Vaquita       ATTCCTTGGTTATAAAA-TGAAATGGCATATGTAAAGGCAGAGTTATTAACTGCTCCTTTTCATCTGTTCTGTAGAGAAG  82260
Pig           ATTCCTTGGTTATAAGA-TGAAGTGGCATATGTAAAGGTAGGGTTATTAACTGCTCTATTTCATCTGTTCTGTAGGGAAG  81291

Majority      CCATTTTTATTGTCTGTTTAAAATTTTAGTTTA-------ATTTTACTTTCCTTTTT---------CAACTTAGTTGACA
                        115450    115460    115470    115480    115490    115500    115510    115520
Human         CCATT-TT---GTCTGTTTAAAATTTCAGTTTAATTTTGCTTTTTTTTTTTTTTTTTTTTTCCTTTCAACTTAGTTGACA  92637
PolarBear     CCATTTTTATTGTCTGTTTATAATTTTAGTTTA-------ATTATACTTTCCTTTTT---------CAACTTAGTTGACA  50683
Cow           CCATTTTT---GTCTGTTTGAAATTTTAGTTGA-------ATTTTACTTTCCTTTTT---------CAACTTAGTTGACA  86483
Dog           CCATTTTTATTGTCTGTTTATAATTTTAGTTTA-------ATTATACTTTCCTTTTT---------CAACTTAGTTGACA  80528
BelugaWhale   CCATTTTTATTGTCTGTTTAAAATTTTAATT------------TTACTTTCCTTTTT---------CAACTTAGTTGACA  81091
Donkey        CCATTTTTATTGTCTGTTTAAAATTTAAGTTTA-------ATTTTACTTTCCTTTTT---------CAACTTAGTTGACA  89774
Cat           CCATTATTTTTGTCTGTTTAAATTTTTAGTTTA-------ATTATACTTTCCTTTTT---------CAACTTAGTTGACA  86120
Ferret        CCATTTTTATTGTCTGTTTATGATTTTAGTTTATAGTTTAATTATACTTTCCTTTTT---------CAACTTAGTTGACA  87446
Lion          CCATTTTTATTGTCTGTTTAAATTTTTAGTTTA-------ATTATACTTTCCTTTTT---------CAACTTAGTTGACA  83815
Vaquita       CCATTTTTATTGTCTGTTTAAAATTTTAATT------------TTACTTTCCTTTTT---------CAACTTAGTTGACA  82319
Pig           CCATTTTTATTGTCTGTTGAGAATTTTAGTT-A-------ATTTTACTTTCCTTTTT---------CAACTTAGTTGACA  81354
```

```
Majority     TA-CGTGCCTTAAAAAGGAAAACTAGTGTTGCTATTGTGCATTTACTAGAAAAAAGGAATTGGTTGTCTAGGGCACATTG
                     115530    115540    115550    115560    115570    115580    115590    115600
Human        TA-CGTGCCTTAAAAAGGAAAACTAGTGTTGCTATTGTGCATTTACTAGAAAAAAGGAATTGGTTGTTTAGGGCACACTG  92716
PolarBear    TA-CGTGCCTTAAAAAGGAAAACTAGTGTTGCTATTGTGCATTTACTAGGAAAAAGGAATTGGTTGTCTAGGGCACATTG  50762
Cow          TA-CGTGCCTTAAAAAGGAAA-CTAGTGTTGCTATTGTGCATTTACTAGAAAAAAGGAATTGGTTGTCTAGGGCACATTG  86561
Dog          TA-CGTGCCTTAAAAAGGAAAACTAGTGTTGCTATTGTGCATTTACTAGGTAAAAGGAATTGGTTGTCTAGGGCACATTG  80607
BelugaWhale  TA-CGTGCCTTAAAAAGGAAAACTAGTGTTGCTATTGTGCATTTGCTAGAAGAAAGGAATTGGTTGTCTAGGGCACATTG  81170
Donkey       TAACGTGCCTTAAAAAGGAAAACTAGTGTTGCTATTGTGCATTTACTAGAAAAAAGGAATTGGTTTTCTAGGGCACATTG  89854
Cat          TA-CGTGCCTTAAAAAGGAAAACTAGTGTTGCTATTGTGCATTTACTAGGAAAAAGGAATTGGTTGTCTAGGGCACATTG  86199
Ferret       TA-CGTGCCTTAAAAAGGAAAACTAGTGTTGCTATTGTGCATTTACTAGGAAAAAGGAATTGGTTGTCTAGGGCACATTG  87525
Lion         TA-CGTGCCTTAAAAAGGAAAACTAGTGTTGCTATTGTGCATTTACTAGGAAAAAGGAATTGGTTGTCTAGGGCACATTG  83894
Vaquita      TA-CGTGCCTTAAAAAGGAAAACTAGTGTTGCTATTGTGCATTTGCTAGA-GAAAGGAATTGGTTGTCTAGGGCACATTG  82397
Pig          TA-CGTGCCTTACAAAGGAAAACTAGTGTTGCTATTGTGCATTTACTAGAAAAAAGGAATTGGTTGTCTAGGGCACACTG  81433

Majority     TTATATGGGAATTA-CAATATGTTTAGGCAGGGGTGTGTAAAAAGG-TTAAGTTTTTGTTTC-TCCTGCTTGGAACTTAT
                     115610    115620    115630    115640    115650    115660    115670    115680
Human        TTATATGGGAATTA-AAATATGTTTAGGCAGGGGTGTGTAAAAAGG-TTAAGTTTTTGTTTC-TCCTGCTTGGAACTTAT  92793
PolarBear    TTATATGGGAATTA-CAATATGTTTAGGCAGGGGTGTGTAAAAAGG-TTAAGTTTTTGTTTC-TCCTGCTTGGAACTTAT  50839
Cow          TTAAATGGGAATTAACAATATGTTTAGGCAGGGGTGTGTAAAAAGG-TTAAGTTTTTTTTTCCTCCTGCTTGGAACTTAT  86640
Dog          TTATATGGGAATTA-CAATATGTTTAGGCAGGGGTGTGTAAAAAGG-TTAAGTTTTTGTTTC-TCCTGCTTGGAACTTAT  80684
BelugaWhale  TTAAACGGGAATTAACAATATGTTTAGGCAGGGGTGTGTAAAAAGGGTTAAGTTTTTGTTTC-TCCTGCTTGGAACTTAT  81249
Donkey       TTATATGGGAATTA-TAATATGTTTAGGCAGGGGTGTGTAAAAAGG-TTAAGTTTTTGTTTC-TCCTGCTTGGAACTTAT  89931
Cat          TTATATGGGAATTA-CCATATGTTTAGGCAGGGGTGTGTAAAAAGG-TTAAGTTTTTGTTTC-TCCTGCTTGGAACTTAT  86276
Ferret       TTATATGGGAATTA-CAATATGTTTAGGCAGGGGTGTGTAAAAAGG-TTAAGTTTTTGTTTC-TCCTGCTTGGAACTTAT  87602
Lion         TTACATGGGAATTA-CCATATGTTTAGGCAGGGGTGTGTAAAAAGG-TTAAGTTTTTGTTTC-TCCTGCTTGGAACTTAT  83971
Vaquita      TTAAATGGGAGTTAACAATATGTTTAGGCAGGGGTGTGTAAAAAGGGTTAAGTTTTTGTTTC-TCCTGCTTGGAACTTAT  82476
Pig          TTAAATGGAGATTA-CAATATGTTTAGGCAGGGGTGTGTAAAAAGG-TTAAGTTTTTGTTTC-TCCTGCTTGGAACTTAT  81510
```

```
Majority     TTTGAATTATTGGCTTGTCACATTTCTTTCTATTTAATCAAATAAGATACATGATACTGAAAGAAT-AAAGCAGCATTTT

                       115690    115700    115710    115720    115730    115740    115750    115760

Human        TTTGAATTACTGGCTTGTCACCTTTTTTTCTATTTAATCAAATAAGATACATGATATTGAAAGAAT-AAAGCAGCATTTT  92872
PolarBear    TTTGAATTATTGGCTTGTCACATTTCTTTCTATTTAATCAAATAAGATACATGATACTGAAAGAATTAAAGCAGCATATT  50919
Cow          TTTGAATTATCGGCTTGTCATATTTTTTT--ATTTAATCAAATAAGATACATGATACGGAAAGAAT-AAAGCAGCTTTTT  86717
Dog          TTTGAATTATTGGCTTGTCACATTTCTTTCTATTTAATCAAATAAGATACATGATACTGAAAGAATTAAAGCAGCATTTT  80764
BelugaWhale  TTTGAATTATTGGCTTGTCACATTTCTTTCTATTTAATCAAATAAGATACATGATACTGAAAGAAT-AAAGCAGCATTTT  81328
Donkey       TTTGAATTATTGGCTTGTCACATTTCTTTCTATTTAGTCAAA---GATACATGATATTGAAAGAAT-AAAGCAGCATTTT  90007
Cat          TTTGAATTATTGGCTTGTCACATTTCTTTCTATTTACTCAAATAAGATACATGATACTGAGAGAATTAAAGCAGCATTTT  86356
Ferret       TTTGAATTATTGACTTGTCACATTTCTTTCTATTTAATCAAATAAGATACATGATACTGAAAGAATTAAAGCAGCATTTT  87682
Lion         TTTGAATTATTGGCTTGTCACATTTCTTTCTATTTACTCAAATAAGATACATGATACTGAGAGAATTAAAGCAGCATTTT  84051
Vaquita      TTTGAATTATTGGCTTGTCACATTTCTTTCTATTTAATCAAATAAGATACATGATACTGAAAGAAT-AAAGCAGCATTTT  82555
Pig          TTTGAATTATTGGCTTGTCACATTTCTTTCTATTTAATCAAATAGGATACATGATA----AAGAAT-AAAGCAGCATTTT  81585

Majority     TT-----------AGTTTTTACTACCTTAGGCTTTATTGCTTTAAAAAAA-CATTGGCCTTTTGTATCTCACAATTCTGG

                       115770    115780    115790    115800    115810    115820    115830    115840

Human        T------------AGTTTTTACTACCTTAGGCTTTATTGCTTTGAAAACAACATTGGCCTTTTGTATCTCACAATTCTGG  92940
PolarBear    TT-----------AGTTTTTACTACCTTAGGCTTTATTGCTTTAAAAAAA-CATTGGCCTTTTGTATCTCACAATTCTGG  50987
Cow          TTTTTTTTTTTCCAGTTTCTACTACCTTAGGCTTTATTGCTTTAAAAAAAACATTGGCCTTTTGTATCTCACAATTCTGG  86797
Dog          TT-----------AGTTTTTACTACCTTAGGCTTTATTGCTTTAAAAAAA-CATTGGCCTTTTGTATCTCACAATTCTGG  80832
BelugaWhale  TTT----------AGTTTTTACTACCTTAGGCTTTATTGCTTTAAAAAAAACATTGGCCTTTTGTATCTCACAATTCTGG  81398
Donkey       TT-----------AGTTT-TACTACCTTAGGCTTTATTGCTTTAAAAAAA-CATTGGCCTTTTGTATCTCACAAT-CTGG  90073
Cat          TT-----------AGTTTTTACTACCTTAGGCTTTATTGCTTTAAAAAAA-CATTGGCCTTTTGTATCTCACAATTCTGG  86424
Ferret       TTTT---------AGTTTTTACTACCTTAGGCTTTATTGCTTTAAAAAAA-CATTGGCCTTTTGTATCTCACAATTCTGG  87752
Lion         TT-----------AGTTTTTACTACCTTAGGCTTTATTGCTTTAAAAAAA-CATTGGCCTTTTGTATCTCACAATTCTGG  84119
Vaquita      TTT----------AGTTTTTACTACCTTAGGCTTTATTGCTTTAAAAAAAACATTGGCCTTTTGTATCTCACAATTCTGG  82625
Pig          T------------AGTTTTTACTACCTTAGACTTTATTGCTTTAAAAAAA-CATTGGCCTTTTGTATCTCACAATTCTGG  81652
```

```
Majority     TCTAGATTCAGTTATGAATGTAGGCATTAGTTAXXXXXXXXXXXXXXXXXXXXXXXXXXXXXXXXXXXXXXXXXXXXXXX

                      115850    115860    115870    115880    115890    115900    115910    115920

Human        TCTAGATTCAGTTATGAATGTAGGCATTAGTTAAAATTAACAAGATGCAGAGTATTAATTTCTTAAGACAACAAAGTGAT  93020
PolarBear    TCTAGATTCAGTTATGAATGTAGGCATTAGTTAAAATTAACAAGATGCAGAGTATTAATTTCTTAAGACAACAAAGTGAT  51067
Cow          TCTAGATTCAGTTATGAAT                                                               86816
Dog          TCTAGATTCAGTTATGAATGTAGGCATTAGTTAAAATTAACAAGATGCAGAGTATTAATTTCTTAAGACAACAAAGTGAT  80912
BelugaWhale  TCTAGATTCAGTTATGAAT                                                               81417
Donkey       TCTAGATTCAGTTATGAATGTAGGCATTAGTTA                                                 90106
Cat          TCTAGATTCAGTTATGAATGTAGGC                                                         86449
Ferret       TCTAGATTCAGTT                                                                     87765
Lion         TCTAGATTCAGTTATGAATGTAGG                                                          84143
Vaquita      TCTAGATTCAGTTATGAATGTAGGCATTAGTTAAAATTAACAAGATGCAGAGTATTAATTTCTTAAGACAACAAAGTGAT  82705
Pig          TCTAGATTCAGTTATGAATGTAGGCATTAGTTAAAATTAACAAGATGCAGAGTATTAATTTCTTAAGACAACGAAGTGAT  81732

Majority     XXXXXXXXXXXXXXXXXXXXXXXXXXXXXXXXXXXXXXXXXXXXXXXXXXXXXXXXXXXXXXXXXXXXXXXXXXXXXXXX

                      115930    115940    115950    115960    115970    115980    115990    116000

Human        TTCTGTAAGTTTGAGCCCTATGTGGAAAGCATTGTGGAATCTTAACCTTTTCGTACACACTCTTGTGGGACGTATCAT-A  93099
PolarBear    TTCTGTAAGTTTGAGCCCTATGTGGAAAGCATTGTGGAATCTTAACCTTTTTGTACACACTCTTGTGGGACGTATCAT-A  51146
Cow                                                                                            86816
Dog          TTCTGTAAGTTTGAGCCCTACGTGGAAAGCATTGTGGAATCTTAACCTTTTTGTACACACTCTTGTGGGACGTATCAT-A  80991
BelugaWhale                                                                                    81417
Donkey                                                                                         90106
Cat                                                                                            86449
Ferret                                                                                         87765
Lion                                                                                           84143
Vaquita      TTCTGTAAGTTTGAGCCCTATGTGGAAAGCATTGTGGAATCTTAACCTTTTTGTACACACTCTTGTGGGACGTATCAT-A  82784
Pig          TTCTGTAAGTTTGAGCCCTATGTGGAAAGCATTGTGGAATCTTAACCTTTTTGTACACACTCTTGTGGGACGTATCATTA  81812
```

```
Majority     XXXXXXXXXXXXXXXXXXXXXXXXXXXXXXXXXXXXXXXXXXXXXXXXXXXXXXXXXXXXXXXXXXXXXXXXXXXXXXXX

                      116010    116020    116030    116040    116050    116060    116070    116080

Human        TAAATGTCAGCACTAAGTAATGTCTTGTTTGTGGCTGAATATTTTTCGTAGATGTTTTTGAAGTTGACATGACTTACGTG  93179
PolarBear    TAAATGTCAGCACTAAGTAATGTCTTGTTTGTGGCTGAATATTTTTCGTAGATGTTTTTGAAGTTGACATGACTTACGTG  51226
Cow                                                                                           86816
Dog          TAAATGTCAGCACTAAGTAATGTCTTGTTTGTGGCTGAATATTTTTCGTAGATGTTTTTGAAGTTGACATGACTTACGTG  81071
BelugaWhale                                                                                   81417
Donkey                                                                                        90106
Cat                                                                                           86449
Ferret                                                                                        87765
Lion                                                                                          84143
Vaquita      TAAATGTCAGCACTAAGTAACGTCTTGTTTGTGGCTGAATATTTTTCGTAGATGTTTTTGAAGTTGACATGATTTATGTG  82864
Pig          TAAATGTCAGCACTAAGTAATGTCCTGTTTGTGGCTGAATATTTTTCGTAGATGTTTTTGAAGTTGACATGACTTATGTG  81892

Majority     XXXXXXXXXXXXXXXXXXXXXXXXXXXXXXXXXXXXXXXXXXXXXXXXXXXXXXXXXXXXXXXXXXXXXXXXXXXXXXXX

                      116090    116100    116110    116120    116130    116140    116150    116160

Human        CATTTAAATATATATTGCCATCCTTAGTTTGTAATTAAGATTTGGAATATGGTTGTGGATTTCTGAGCATGTGCAGACTG  93259
PolarBear    CATTTAAATATATATTGCCATCCTTAGTTTGTAATTAAGATTTGGAATATGGTTGTGGATTTCTGAGCATGTGCAGACTG  51306
Cow                                                                                           86816
Dog          CATTTAAATATATATTGCCATCCTTAGTTTGTAATTAAGATTTGGAATATGGTTGTGGATTTCTGAGCATGTGCAGACTG  81151
BelugaWhale                                                                                   81417
Donkey                                                                                        90106
Cat                                                                                           86449
Ferret                                                                                        87765
Lion                                                                                          84143
Vaquita      CATTTAAATATATATTGCCATCCTTAGTTTGTAATTAAGATTTGGAATACGGTTGTGGATTTCTGAGCATGTGCAGACTG  82944
Pig          CATTTAAATATATATTGCCATCCTTAGTTTGTAATTAAGATTTGGAATATGGTTGTGGATTTCTGAGCATGTGCAGACTG  81972
```

Majority	XXXXXXXXXXXXXXXXXXXXXXXXXXXXXXXXXXXXXXXXXXXXXXXXXXXXXXXXXXXXXXXXXXXXXXXXXXXXXXXX	
	116170 116180 116190 116200 116210 116220 116230 116240	
Human	GTCTAGCTAGTTCAGGAACTGGTGCATGTATTTTTCAAAGATAAA-GAAAGTGTACTGCGAAAATATGCAGGAAGATTAA	93338
PolarBear	GTCTAGCTAGTTCAGGAACTGGTGCATGTATTTTTCAAAGATAAA-GAAAGTGTACTGCGAAAATTTGCAGGAAGATTAA	51385
Cow		86816
Dog	GTCTAGCTAGTTCAGGAACTGGTGCATGTATTTTTCAAAGATAAA-GAAAGTGTACTGCGAAAATTTGCAGGAAGATTAA	81230
BelugaWhale		81417
Donkey		90106
Cat		86449
Ferret		87765
Lion		84143
Vaquita	GTCTAGCTAGTTCAGGAACTGGTGCATGTATTTTTCAAAGATAAAAGAAAGTGTACTGCGAAAATTTGCAGGAAGGTTAA	83024
Pig	GTCTAGCTAGTTCAGGAACTGGTGCATGTATTTTTCAAAGATAAAAGAAAGTGTACTGCGAAAATTTGCAGGAAGATTAA	82052

Majority	XXXXXXXXXXXXXXXXXXXXXXXXXXXXXXXXXXXXXXXXXXXXXXXXXXXXXXXXXXXXXXXXXXXXXXXXXXXXXXXX	
	116250 116260 116270 116280 116290 116300 116310 116320	
Human	TTTTGTGGCAGTTTTCTAAAACTGACAACCAGGTGGGACCAAAGTTTATGTGCCTTTAGTCTTAATTTACCTTGCATTGT	93418
PolarBear	TTTTGTGGCAGTTTTCTAAAACTGACAACCAGGTGGGACCAAAGTTTATGTGCCTTTAGTCTTAATTTACCTTGCATTGT	51465
Cow		86816
Dog	TTTTGTGGCAGTTTTCTAAAACTGACAACCAGGTGGGACCAAAGTTTATGTGCCTTTAGTCTTAATTTACCTTGCATTGT	81310
BelugaWhale		81417
Donkey		90106
Cat		86449
Ferret		87765
Lion		84143
Vaquita	TTTTGTGGCAGTTTTCTAAAACTGACAACCAGGTGGGACCAAAGTTTATGTGCCTTTAGTCTTAATTTACCTTGCATTGT	83104
Pig	TTTTGTGGCAGTTTTCTAAAACTGACAACCAGGTGGGACCAAAGTTTATGTGCCTTTAGTCTTAATTTACCTTGCATTGT	82132

```
Majority     XXXXXXXXXXXXXXXXXXXXXXXXXXXXXXXXXXXXXXXXXXXXXXXXXXXXXXXXXXXXXXXXXXXXXXXXXXXXXXXX

                      116330    116340    116350    116360    116370    116380    116390    116400
Human        AATATTCAGTTTTAATAAATCTTCAAAATATTTTGTATTTAGGAATAGATCTGAC------TTTAATAAAAACATGGCTC  93492
PolarBear    AATATTCAGTTTTAATAAATCTTCAAAATATTTTGTATTTAGGAATAGATCTGAC------TTTAATAAAAACATGGCTC  51539
Cow                                                                                           86816
Dog          AATATTCAGTTTTAATAAATCTTCAAAATATTTTGTATTTAGGAATAGATCTGAC------TTTAATAAAAACATGGCTC  81384
BelugaWhale                                                                                   81417
Donkey                                                                                        90106
Cat                                                                                           86449
Ferret                                                                                        87765
Lion                                                                                          84143
Vaquita      AATATTCAGTTTTAATAAATCTTCAAAATATTTTGTATTTAGGAATAGATCTGACTTTGACTTTAATAAAAACATGGCTC  83184
Pig          AATATTCAGTTTTAATAAATCTTCAAAATATTTTGTATTTAGGAATAGATCTGAC------TTTAATAAAAACATGGCTC  82206

Majority     XXXXXXXXXXXXXXXXXXXXXXXXXXXXXXXXXXXXXXXXXXXXXXXXXXXXXXXXXXXXXXXXXXXXXXXXXXXXXXXX

                      116410    116420    116430    116440    116450    116460    116470    116480
Human        AGAATCTACAGGTCAAATTAATTTGAACAGTTCTTGTCAATCCGAATTGTTGATTCTGTTTAAATGACCAATACTTTTTG  93572
PolarBear    AGAATCTACAGGTCAAATTAATTTGAACAGTTCTTGTCAATCTGAATTGTTGATTCTGTTTAAATGACCAATACTTTTTG  51619
Cow                                                                                           86816
Dog          AGAATCTACAGGTCAAATTAATTTGAACAGTTCTTGTCAATCTGAATTGTTGATTCTGTTTAAATGACCAATACTTTTTG  81464
BelugaWhale                                                                                   81417
Donkey                                                                                        90106
Cat                                                                                           86449
Ferret                                                                                        87765
Lion                                                                                          84143
Vaquita      AGAATCTACAGGTCAAATTAATTTGAACAGTTCTTGTCAATCTGAATTGTTGATTCTGTTTAAATGACCAATACTTTTTC  83264
Pig          AGAATCTACAGGTCAAATTAATTTGAACAGTTCTTGTCAATCTGAATTGTTGATTCTGTTTAAATGACCAATACTTTTTG  82286
```

Majority	XXXXXXXXXXXXXXXXXXXXXXXXXXXXXXXXXXXXXXXXXXXXXXXXXXXXXXXXXXXXXXXXXXXXXXXXXXXXXXXX	
	116490 116500 116510 116520 116530 116540 116550 116560	
Human	AAATTGATGTACTTAGTTTCAAGATTCATAGATTCTGTTATCTATGTAGACAGAATGGTCATGTATATTTTCTATTAGTT	93652
PolarBear	AAATTGATGTACTTAGTTTCAAGATTCATAGATTCTGTTATCTATGTAGACAGAATGGTCATGTATATTTTCTATTAGTT	51699
Cow		86816
Dog	AAATTGATGTACTTAGTTTCAAGATTCATAGATTCTGTTATCTATGTAGACAGAATGGTCATGTATATTTTCTATTAGTT	81544
BelugaWhale		81417
Donkey		90106
Cat		86449
Ferret		87765
Lion		84143
Vaquita	AAATTGATGTACTTAGTTTCAAGGTTCATAGATTCTGTTATCTATGTAGACAGAATGGTCATGTATATTTTCTATTAGTT	83344
Pig	AAATTGATGTACTTAGTTTCAAGATTCATAGATTCTGTTATCTATGTAGACAGAATGGTCATGTATATTTTCTATTAGTT	82366

Majority	XXXXXXXXXXXXXXXXXXXXXXXXXXXXXXXXXXXXXXXXXXXXXXXXXXXXXXXXXXXXXXXXXXXXXXXXXXXXXXXX	
	116570 116580 116590 116600 116610 116620 116630 116640	
Human	GAGTTTTTACATCTTTAGAAATGTAAAATTCAGTATAGTTTGAAAGCGGCACAATTAAAAATTAATTTTCTAACAAAGTT	93732
PolarBear	GAGTTTTTACATCTTTAGAAATGTAAAATTCAGTATAGTTTGAAAGTGGCACAATTAAAAATTAATTTTCTAACAAAGTT	51779
Cow		86816
Dog	GAGTTTTTACATCTTTAGAAATGTAAAATTCAGTATAGTTTGAAAGCGGCACAATTAAAAATTAATTTTCTAACAAAGTT	81624
BelugaWhale		81417
Donkey		90106
Cat		86449
Ferret		87765
Lion		84143
Vaquita	GAGTTTTTACATCTTTAGAAATGTAAAATTCAGTATAGTTTGAAAGCGGCACAATTAAAAATTAATTTTCTAACAAAGTT	83424
Pig	GAGTTTTTACATCTTTAGAAATGTAAAATTCAGTATAGTTTGAAAGCGGCACAATTAAAAATTAATTTTCTAACAAAGTT	82446

```
Majority     XXXXXXXXXXXXXXXXXXXXXXXXXXXXXXXXXXXXXXXXXXXXXXXXXXXXXXXXXXXXXXXXXXXXXXXXXXXXXXXX
                     116650    116660    116670    116680    116690    116700    116710    116720
Human        GGGAGGTTTGATGGTTGTTTAATTTCATTTTGTGTGTACTCTGCTTACCCCTGTAGCATGCTCAATAAACACTTCTGTAG  93812
PolarBear    GGGAGGTTTGATGGTTGTTTAATTTCCTTTTGTGTGTACTCTGCTTACCTCTGTAGCATGCTCAATAAACACTTCTGTAG  51859
Cow                                                                                          86816
Dog          GGGAGGTTTGATGGTTGTTTAATTTCCTTTTGTGTGTACTCTGCTTACCTCTGTAGCATGCTCAATAAACACTTCTGTAG  81704
BelugaWhale                                                                                  81417
Donkey                                                                                       90106
Cat                                                                                          86449
Ferret                                                                                       87765
Lion                                                                                         84143
Vaquita      GGGAGGTTTGATGGTTGTTTAATTTCCTTTTGTGTGTACTCTGCTTACCTCTGTAGCATGCTCAATAAACACTTCTGTAG  83504
Pig          GGGAGGTTTGATGGTTGTTTAATTTCCTTTTGTGTGTACTCTGCTTACCTCTGTAGCATGCTCAATAAACACTTCTGTAG  82526

Majority     XXXXXXXXXXXXXXXXXXXXXXXXXXXXXXXXXXXXXXXXXXXXXXXXXXXXXXXXXXXXXXXXXXXXXXXXXXXXXXXX
                     116730    116740    116750    116760    116770    116780    116790    116800
Human        CTCTATATTCACCTTTT-CTGTCTTTCTCTGCTGCCTTTTCTCTCTCCTCTTC-TTTGTTTTCACTCCACTGTGCTTCTG  93890
PolarBear    CTCTGTATTCACCTTTT-CTGTCTTTCTCTGCTGCCTTTTCTCTCTCCTCTTCTTTGTTTTTCACTCCACTGTGCTTCTG  51938
Cow                                                                                          86816
Dog          CTCTGTATTCACCTTTT-CTGTCTTTCTCTGCTGCCTTTTCTCTTTCCTCTTCTTTGTTTTTCACTCCACTGTGCTTCTG  81783
BelugaWhale                                                                                  81417
Donkey                                                                                       90106
Cat                                                                                          86449
Ferret                                                                                       87765
Lion                                                                                         84143
Vaquita      CTCTGTATTCACCTTTT-CTGTCTTTCTCTGCTGCCTTTTCTCTCTCTTC----TTTGTTTTCACTCCACTGTGCTTCTG  83579
Pig          CTCTGTATTCACCTTTTTCTGTCTTTCTCTGCTGCCTTTTCTCTCTCTTCCTC-TTTGTTTTCACTCCACTGTGCTTCTG  82605
```

```
Majority    XXXXXXXXXXXXXXXXXXXXXXXXXXXXXXXXXXXXXXXXXXXXXXXXXXXXXXXXXXXXXXXXXXXXXXXXXXXXXXXX
                      116810    116820    116830    116840    116850    116860    116870    116880
Human       AATTCATGTTTATTCTCTGCCAGGGTGGGAAAGGAGTAATAA-TATTACAATTCTATGGCTTTATACCATAAATAAATCT  93969
PolarBear   AATTCATGTTTATTCTCTGCCAGGGTGGGAAAGGAGTATTAA-TATTACAATTCTATGGCTTTATACCATAAATAAATCT  52017
Cow                                                                                           86816
Dog         AATTCATGTTTATTCTCTGCCAGGGTGGGAAAGGAGTAATAA-TATTATAATTCTATGGCTTTATACCATAAATAAATCT  81862
BelugaWhale                                                                                   81417
Donkey                                                                                        90106
Cat                                                                                           86449
Ferret                                                                                        87765
Lion                                                                                          84143
Vaquita     AATTCATGTTTATTCTCTGCCAGGGTGGGAAAGGAATAATAA-TATTACAATTCTATGGCTTTATACCATAAATAAATCT  83658
Pig         AATTCATGTTTATTCTCTGCCAGGGTGGGAAAGGAATAATAAATATTACAATTCTATGGCTTTATACCATAAATAAATCT  82685

Majority    XXXXXXXXXXXXXXXXXXXXXXXXXXXXXXXXXXXXXXXXXXXXXXXXXXXXXXXXXXXXXXXXXXXXXXXXXXXXXXXX
                      116890    116900    116910    116920    116930    116940    116950    116960
Human       AGATGCTGTGAAAATATACCAGCTGGTTTTTTTTA--ATTTAAA-------------------AGATGGTAACTGCTTT  94027
PolarBear   AGATGCTATGAAAATATACCAGCTGGTTTTTTTTTTAATTTAAA-------------------AGATAGCAACTGCTTT  52077
Cow                                                                                           86816
Dog         AGATGCTGTGAAAATATACCAGCTGGTTTTTTTTT-AATTTAAA-------------------AGATAGTAACTGCTTT  81921
BelugaWhale                                                                                   81417
Donkey                                                                                        90106
Cat                                                                                           86449
Ferret                                                                                        87765
Lion                                                                                          84143
Vaquita     AGATGCTGTGAAAATATACCAGCTGGTTTTTTTTTAATTTGAA--------------------AGATGGTAACTGCTTT  83717
Pig         AGATGCTGTGAAAATATACCAGCTGTTTTTTTTTTTTGTTTTGTTTTTTGGTTTTTTTTAATTTGAAAGATGGTAACTTT  82765
```

```
Majority     XXXXXXXXXXXXXXXXXXXXXXXXXXXXXXXXXXXXXXXXXXXXXXXXXXXXXXXXXXXXXXXXXXXXXXXXXXXXXXXX

                     116970    116980    116990    117000    117010    117020    117030    117040

Human        TCAGGAGGACACATATTAAACATTTCCCACCCTGTTATAATCTACTGCTTTAAAGACATAACTTTTATTGTAGCTTGTTA  94107
PolarBear    TCAGGAGGACACATATTAAACATTTCCCACCCTGTTA                                             52114
Cow                                                                                            86816
Dog          TCAGGAGGACACATATTAAACATTTCCCACCCTGTTATAATCTACTGCTTTAAAGACATAACTTTTATTGTATCTTGTTA  82001
BelugaWhale                                                                                    81417
Donkey                                                                                         90106
Cat                                                                                            86449
Ferret                                                                                         87765
Lion                                                                                           84143
Vaquita      TCAGGAGGACACATATTAAACATTTCCCACCCTGTTATAATCTACTGCTTTAAAGACATAACTTTTATTGTATCTTGTTA  83797
Pig          TCAGGAGGACACATATTAAACATTTCCCACCCTGTTATAACCTACTGCTTTACAGACATAACTCTTACTGTATCTTGTTA  82845

Majority     XXXXXXXXXXXXXXXXXXXXXXXXXXXXXXXXXXXXXXXXXXXXXXXXXXXXXXXXXXXXXXXXXXXXXXXXXXXXXXXX

                     117050    117060    117070    117080    117090    117100    117110    117120

Human        ATTCTATCTCTTTTGTTCTTGTTGTTTTTTTTTTTTTTTCCAGTAGATTTATGCACTAATAGATCTTTTGGATTTGCCATG  94187
PolarBear                                                                                      52114
Cow                                                                                            86816
Dog          ATTCT------ATCTCTTTTGTTGTTGTTGTTTTTTTTCCAGTAGATTTATGCACTAATAGATCTTTTGGATTTGCCATG  82075
BelugaWhale                                                                                    81417
Donkey                                                                                         90106
Cat                                                                                            86449
Ferret                                                                                         87765
Lion                                                                                           84143
Vaquita      ATTCTTTCT--ATCTCTTTTGTTGTTGTTGTTTTTTTTCCAGTAGATTTATGCACTAATAGATCTTTTGGATTTGCCATG  83875
Pig          ATTCTTTCT--ATCTCTTTTGTTGTTGTTGTTTTTTTTCCAGTAGATTTATGCACTAGTAGATCTTTTGGATTTGCCATG  82923
```

```
Majority     XXXXXXXXXXXXXXXXXXXXXXXXXXXXXXXXXXXXXXXXXXXXXXXXXXXXXXXXXXXXXXXXXXXXXXXXXXXXXXXX
                       117130    117140    117150    117160    117170    117180    117190    117200
Human        CTCTCTTGCTGCAGTTTCATCTTTCATCTTTTGTGTCTGCTAAAGATTTCTTACTAATCTTAGACTACCTTGTGAGTTAA  94267
PolarBear                                                                                      52114
Cow                                                                                            86816
Dog          CTCTCTTGCTGCAGTTTCATCTTTCATCTTTTGTGTCTGCTAAAGATTTCTTACTAATCTTAGA------------TTAC  82143
BelugaWhale                                                                                    81417
Donkey                                                                                         90106
Cat                                                                                            86449
Ferret                                                                                         87765
Lion                                                                                           84143
Vaquita      CTCTCTTGCTGCAGTTTCATCTTTCATCTTTTGTGTCTGCTAAAGATTTCCTACCTTGTGAGTT------------TTAA  83943
Pig          CTTTCTTGCTGCAGTTTCATCTTTCATCTTTTGTGTCTGCTAAAGATTTCCTATATTGTGAGTT------------TTAA  82991

Majority     XXXXXXXXXXXXXXXXXXXXXXXXXXXXXXXXXXXXXXXXXXXXXXXXXXXXXXXXXXXXXXXXXXXXXXXXXXXXXXXX
                       117210    117220    117230    117240    117250    117260    117270    117280
Human        CAAAAAGAACTTGATATTACTGGGAAGAAAGAGCAGCATATCTGCATATCAGCATATCTGCTTTTGCCTTGGGTGGGAAG  94347
PolarBear                                                                                      52114
Cow                                                                                            86816
Dog          CTTGCGATTTTTAACAAAGGGTATTAGGTGTTATTG-----------------GGAAGAAACTTTGTCTTGGGTGGGCAG  82206
BelugaWhale                                                                                    81417
Donkey                                                                                         90106
Cat                                                                                            86449
Ferret                                                                                         87765
Lion                                                                                           84143
Vaquita      CAAAGGGTACTTGATGTTACTGGGAAGAAACAGTA----------------TACCATCTGCTTTTGCCTTGGGTGGGAAG  84007
Pig          CAAAGGGTACTTGATGTTTGGGAAGAGGCAGTATCAT---------------CCCCATCTGCTTTTGCCTTGGGTGGTAAG  83057
```

Majority	XXXXXXXXXXXXXXXXXXXXXXXXXXXXXXXXXXXXXXXXXXXXXXXXXXXXXXXXXXXXXXXXXXXXXXXXXXXXXXXX	
	117290 117300 117310 117320 117330 117340 117350 117360	
Human	AATGATATACATTCAAGTATTTAAAAACTTAGAGTAATTTGCATTTAACAACTGAGGATGTTACTACTGTAACTTGTTAA	94427
PolarBear		52114
Cow		86816
Dog	AAGATACATTCAGGTAATTTGTGATGTTATAACTTCCACTTTTTTTTTTTTTTAAAGGAGTTCAGTTAGGGGGCTATTTG	82286
BelugaWhale		81417
Donkey		90106
Cat		86449
Ferret		87765
Lion		84143
Vaquita	GAAGATAC	84015
Pig	GACGATACACATGCAGATACTGAAAAACCTAAGAGGTATTCCGTGAGGTTACGATTTTTACTTTTTTAAAAGATTGCAAT	83137

Majority	XXXXXXXXXXXXXXXXXXXXXXXXXXXXXXXXXXXXXXXXXXXXXXXXXXXXXXXXXXXXXXXXXXXXXXXXXXXXXXXX	
	117370 117380 117390 117400 117410 117420 117430 117440	
Human	GCAGTTACAATTAGGGTGCTATTTATATTCAAAATATTTCGGTCAGTTTTTCTAAACAGATATATTGGTATCAAATAACT	94507
PolarBear		52114
Cow		86816
Dog	TGTTATTCATATTTTTGGTTA	82307
BelugaWhale		81417
Donkey		90106
Cat		86449
Ferret		87765
Lion		84143
Vaquita		84015
Pig	TAGGATGTTCATTTGTATTACTCAGAATATTTTTGGGGGGATCACATTTTTTGAAACAGGCATATTGGTATCAGATAACT	83217

Majority	XXXXXXXXXXXXXXXXXXXXXXXXXXXXXXXXXXXXXXXXXXXXXXXXXXXXXXXXXXXXXXXXXXXXXXXXXXXXXXXX	
	117450 117460 117470 117480 117490 117500 117510 117520	
Human	TTGGAGTTACACATGTTTCTTAGGTTTGTTCTGTTATAAAATATCCCACACTTATGCAAAACTTACACATAGGATAATAA	94587
PolarBear		52114
Cow		86816
Dog		82307
BelugaWhale		81417
Donkey		90106
Cat		86449
Ferret		87765
Lion		84143
Vaquita		84015
Pig	CTGCAGTTATACATGTTCCCTAGGTTTGTTCTGTTACAAAATAACTGAA--TTATGCAAAACTCATACATCAGGAAAAGT	83295

Majority	XXXXXXXXXXXXXXXXXXXXXXXXXXXXXXXXXXXXXXXXXXXXXXXXXXXXXXXXXXXXXXXXXXXXXXXXXXXXXXXX	
	117530 117540 117550 117560 117570 117580 117590 117600	
Human	AAAGTAAATGAAGGAACATAAAATATGTTTTACTATACTTAATGTAGTAATTCAGTTACCTCCAAGAAAGAAGAATATAA	94667
PolarBear		52114
Cow		86816
Dog		82307
BelugaWhale		81417
Donkey		90106
Cat		86449
Ferret		87765
Lion		84143
Vaquita		84015
Pig	AAAAAGAAAGGGAGTAAATAATACATTTAAGTGTATATTTAATGTATTAATATAATTTTTTTGGTTTTTTTTTTTTTTTT	83375

Majority	XXXXXXXXXXXXXXXXXXXXXXXXXXXXXXXXXXXXXXXXXXXXXXXXXXXXXXXXXXXXXXXXXXXXXXXXXXXXXXXX	
	117610 117620 117630 117640 117650 117660 117670 117680	
Human	TAAGTAAAATAATTTTGTTTACACTAGTGTCACTGTTGTCATCTGAAGAAGAACCATTAGAGTCAACAGTCTGGGATGAT	94747
PolarBear		52114
Cow		86816
Dog		82307
BelugaWhale		81417
Donkey		90106
Cat		86449
Ferret		87765
Lion		84143
Vaquita		84015
Pig	TTTTTTTTTTTTTTTTTGTCCTTTTAGGGCCGCACCTGTGGCATAGGGAGGTTCACCAGGTTAGGGGTCTAATTGGAGCT	83455

Majority	XXXXXXXXXXXXXXXXXXXXXXXXXXXXXXXXXXXXXXXXXXXXXXXXXXXXXXXXXXXXXXXXXXXXXXXXXXXXXXXX	
	117690 117700 117710 117720 117730 117740 117750 117760	
Human	TTGGAAGCTAGGTGATCTGTGTTTTTTTCTTAAAATAACATCGTAGCACAGAGGAACTCTACCAGCATCAGATAAGTCTA	94827
PolarBear		52114
Cow		86816
Dog		82307
BelugaWhale		81417
Donkey		90106
Cat		86449
Ferret		87765
Lion		84143
Vaquita		84015
Pig	GTGGCTGCCGGCCTG	83470

```
Majority     XXXXXXXXXXXXXXXXXXXXXXXXXXXXXXXXXXXXXXXXXXXXXXXXXXXXXXXXXXXXXXXXXXXXXXXXXXXXXXXX

                      117770    117780    117790    117800    117810    117820    117830    117840

Human        AAGTCTAAAAAACCAATACCAGGGTTAGTGAATAGTAATTGAACAAAGCTTCTCAAGGTGAAGTTTTTCTCAGTAATTAG  94907
PolarBear                                                                                     52114
Cow                                                                                           86816
Dog                                                                                           82307
BelugaWhale                                                                                   81417
Donkey                                                                                        90106
Cat                                                                                           86449
Ferret                                                                                        87765
Lion                                                                                          84143
Vaquita                                                                                       84015
Pig                                                                                           83470

Majority     XXXXXXXXXXXXXXXXXXXXXXXXXXXXXXXXXXXXXXXXXXXXXXXXXXXXXXXXXXXXXXXXXXXXXXXXXXXXXXXX

                      117850    117860    117870    117880    117890    117900    117910    117920

Human        TGGTGGCTATAATATTTGAAAGTAGGTTCTGTCACCTTTTCAGGTGCTCATTGTGGGTGGGATTGCCTTGGGAAGGAGAA  94987
PolarBear                                                                                     52114
Cow                                                                                           86816
Dog                                                                                           82307
BelugaWhale                                                                                   81417
Donkey                                                                                        90106
Cat                                                                                           86449
Ferret                                                                                        87765
Lion                                                                                          84143
Vaquita                                                                                       84015
Pig                                                                                           83470
```

Majority	XXXXXXXXXXXXXXXXXXXXXXXXXXXXXXXXXXXXXXXXXXXXXXXXXXXXXXXXXXXXXXXXXXXXXXXXXXXXXXXX	
	117930 117940 117950 117960 117970 117980 117990 118000	
Human	ACTAAGTCCTGAGCTGTGTGTGTGGACTTAGTTTGTGCCAGTTGCTAAGCTGGTGGGAGTTCCCAGTCTCCCTAGTGGGA	95067
PolarBear		52114
Cow		86816
Dog		82307
BelugaWhale		81417
Donkey		90106
Cat		86449
Ferret		87765
Lion		84143
Vaquita		84015
Pig		83470

Majority	XXXXXXXXXXXXXXXXXXXXXXXXXXXXXXXXXXXXXXXXXXXXXXXXXXXXXXXXXXXXXXXXXXXXXXXXXXXXXXXX	
	118010 118020 118030 118040 118050 118060 118070 118080	
Human	GATAACCAGAAACCAGCTCTTCCTGCCTAAGGACTACAGCAGATTCTATACTGTCTTACAGTATGCCCTAATACGGCCTT	95147
PolarBear		52114
Cow		86816
Dog		82307
BelugaWhale		81417
Donkey		90106
Cat		86449
Ferret		87765
Lion		84143
Vaquita		84015
Pig		83470

Majority	XXXXXXXXXXXXXXXXXXXXXXXXXXXXXXXXXXXXXXXXXXXXXXXXXXXXXXXXXXXXXXXXXXXXXXXXXXXXXXXX	
	118090 118100 118110 118120 118130 118140 118150 118160	
Human	CCTTCCAAAGCACCAAGTCACTAAAGGGGGATTTTGACATTCTAGAATATTTTTAATTCAGTACATCAAATTAACCCAGG	95227
PolarBear		52114
Cow		86816
Dog		82307
BelugaWhale		81417
Donkey		90106
Cat		86449
Ferret		87765
Lion		84143
Vaquita		84015
Pig		83470

Majority	XXXXXXXXXXXXXXXXXXXXXXXXXXXXXXXXXXXXXXXXXXXXXXXXXXXXXXXXXXXXXXXXXXXXXXXXXXXXXXXX	
	118170 118180 118190 118200 118210 118220 118230 118240	
Human	CCTTGGCATATAGTGAATTGCTTATTAAAAGTTAGGTAAAAATAAAAACAGCTTTGTCGTATATTAGTACTCTATGACTC	95307
PolarBear		52114
Cow		86816
Dog		82307
BelugaWhale		81417
Donkey		90106
Cat		86449
Ferret		87765
Lion		84143
Vaquita		84015
Pig		83470

Majority	XXXXXXXXXXXXXXXXXXXXXXXXXXXXXXXXXXXXXXXXXXXXXXXXXXXXXXXXXXXXXXXXXXXXXXXXXXXXXXXX	
	118250 118260 118270 118280 118290 118300 118310 118320	
Human	TTAGAGTGAACAAGCTAACAAGTATTCTTAGGACTTAGCTGCTTTGTTAATAGAGGTCTTAACTTGAATATTTAGAAATA	95387
PolarBear		52114
Cow		86816
Dog		82307
BelugaWhale		81417
Donkey		90106
Cat		86449
Ferret		87765
Lion		84143
Vaquita		84015
Pig		83470

Majority	XXXXXXXXXXXXXXXXXXXXXXXXXXXXXXXXXXXXXXXXXXXXXXXXXXXXXXXXXXXXXXXXXXXXXXXXXXXXXXXX	
	118330 118340 118350 118360 118370 118380 118390 118400	
Human	ATCAGAAAAATTATTTTTTGGTATTGAAGATGTATTTTGGAAATGGGTTTTAAAGTACCTACTTAAGGAAGAGATATCTA	95467
PolarBear		52114
Cow		86816
Dog		82307
BelugaWhale		81417
Donkey		90106
Cat		86449
Ferret		87765
Lion		84143
Vaquita		84015
Pig		83470

Majority	XXXXXXXXXXXXXXXXXXXXXXXXXXXXXXXXXXXXXXXXXXXXXXXXXXXXXXXXXXXXXXXXXXXXXXXXXXXXXXXX	
	118410 118420 118430 118440 118450 118460 118470 118480	
Human	CAGTTTATATTTAACGTTAGCATTTGAAAGCAATGAAGAAAATTTTTCCTTAACCAGGAAGTGTAATGAATCATTATATT	95547
PolarBear		52114
Cow		86816
Dog		82307
BelugaWhale		81417
Donkey		90106
Cat		86449
Ferret		87765
Lion		84143
Vaquita		84015
Pig		83470

Majority	XXXXXXXXXXXXXXXXXXXXXXXXXXXXXXXXXXXXXXXXXXXXXXXXXXXXXXXXXXXXXXXXXXXXXXXXXXXXXXXX	
	118490 118500 118510 118520 118530 118540 118550 118560	
Human	AGAGATTAAATTAACACTTTGGAAGCATTTTAGCTCCTAGAGTTAAGGTTAGTATAGTGAAATTATGGTGATTAGCTTAA	95627
PolarBear		52114
Cow		86816
Dog		82307
BelugaWhale		81417
Donkey		90106
Cat		86449
Ferret		87765
Lion		84143
Vaquita		84015
Pig		83470

```
Majority     XXXXXXXXXXXXXXXXXXXXXXXXXXXXXXXXXXXXXXXXXXXXXXXXXXXXXXXXXXXXXXXXXXXXXXXXXXXXXXXX

                       118570    118580    118590    118600    118610    118620    118630    118640

Human        TTCATTCATGTACATCTATTTGTATCACCATATAATTACTAGGGAAAGGACAAATCACTTTTCAAAGCCTCAATGTTATT  95707
PolarBear                                                                                      52114
Cow                                                                                            86816
Dog                                                                                            82307
BelugaWhale                                                                                    81417
Donkey                                                                                         90106
Cat                                                                                            86449
Ferret                                                                                         87765
Lion                                                                                           84143
Vaquita                                                                                        84015
Pig                                                                                            83470
```

```
Majority     XXXXXXXXXXXXXXXXXXXXXXXXXXXXXXXXXXXXXXXXXXXXXXXXXXXXXXXXXXXXXXXXXXXXXXXXXXXXXXXX

                       118650    118660    118670    118680    118690    118700    118710    118720

Human        TGTAAAATGGAGGCAATAAGTTCTGCTCTGCTCCCCTCACAGGATTATTACAGAGGCTCAAATAGGATAAGTGAAGGAAT  95787
PolarBear                                                                                      52114
Cow                                                                                            86816
Dog                                                                                            82307
BelugaWhale                                                                                    81417
Donkey                                                                                         90106
Cat                                                                                            86449
Ferret                                                                                         87765
Lion                                                                                           84143
Vaquita                                                                                        84015
Pig                                                                                            83470
```

```
Majority     XXXXXXXXXXXXXXXXXXXXXXXXXXXXXXXXXXXXXXXXXXXXXXXXXXXXXXXXXXXXXXXXXXXXXXXXXXXXXXXX
                      118730    118740    118750    118760    118770    118780    118790    118800
Human        GCTATGCAATTAGTTAACTAATGCCAGTTCCCAGACTGCCAGATTTGCCCACAGTAAACTTCATTATCCACAATACATGT 95867
PolarBear                                                                                     52114
Cow                                                                                           86816
Dog                                                                                           82307
BelugaWhale                                                                                   81417
Donkey                                                                                        90106
Cat                                                                                           86449
Ferret                                                                                        87765
Lion                                                                                          84143
Vaquita                                                                                       84015
Pig                                                                                           83470

Majority     XXXXXXXXXXXXXXXXXXXXXXXXXXXXXXXXXXXXXXXXXXXXXXXXXXXXXXXXXXXXXXXXXXXXXXXXXXXXXXXX
                      118810    118820    118830    118840    118850    118860    118870    118880
Human        TATCCTAATATGTCTTTTTGCTACAAAGAAATACTAATGTTTTAAAATTATTTACTAGTTTCATAAAGCAGAAAGATGTA 95947
PolarBear                                                                                     52114
Cow                                                                                           86816
Dog                                                                                           82307
BelugaWhale                                                                                   81417
Donkey                                                                                        90106
Cat                                                                                           86449
Ferret                                                                                        87765
Lion                                                                                          84143
Vaquita                                                                                       84015
Pig                                                                                           83470
```

Majority	XXXXXXXXXXXXXXXXXXXXXXXXXXXXXXXXXXXXXXXXXXXXXXXXXXXXXXXXXXXXXXXXXXXXXXXXXXXXXXXX	
	118890 118900 118910 118920 118930 118940 118950 118960	
Human	AAATGCAGTTATAGAGAGCATCCCAAGCCAGATGAAAAGAAACCATGGATTTAGCTTTATATTTTCTGTTTGAACTTGAT	96027
PolarBear		52114
Cow		86816
Dog		82307
BelugaWhale		81417
Donkey		90106
Cat		86449
Ferret		87765
Lion		84143
Vaquita		84015
Pig		83470

Majority	XXXXXXXXXXXXXXXXXXXXXXXXXXXXXXXXXXXXXXXXXXXXXXXXXXXXXXXXXXXXXXXXXXXXXXXXXXXXXXXX	
	118970 118980 118990 119000 119010 119020 119030 119040	
Human	AGCCCAATCATAAAATGCACATATTTAATTATAGACTGCACTTAAGATTTCATATGTATAAAAAAGTTGCTGTGAAACAG	96107
PolarBear		52114
Cow		86816
Dog		82307
BelugaWhale		81417
Donkey		90106
Cat		86449
Ferret		87765
Lion		84143
Vaquita		84015
Pig		83470

Majority	XXXXXXXXXXXXXXXXXXXXXXXXXXXXXXXXXXXXXXXXXXXXXXXXXXXXXXXXXXXXXXXXXXXXXXXXXXXXXXXX	
	119050 119060 119070 119080 119090 119100 119110 119120	
Human	GTAATTTTAACACTCAGGAATTTCAAGGCTAAGGAAGAAAGACATACTCTCATGAGCAGCTCTGAACTTCAAACAGGATT	96187
PolarBear		52114
Cow		86816
Dog		82307
BelugaWhale		81417
Donkey		90106
Cat		86449
Ferret		87765
Lion		84143
Vaquita		84015
Pig		83470

Majority	XXXXXXXXXXXXXXXXXXXXXXXXXXXXXXXXXXXXXXXXXXXXXXXXXXXXXXXXXXXXXXXXXXXXXXXXXXXXXXXX	
	119130 119140 119150 119160 119170 119180 119190 119200	
Human	TTCTGGCTGCCTTAATATATTAAAATGGAGCCTAAATCTGAAAACGTTTTTTTCATTCAGAAGAAAGTACAGAATTCAGT	96267
PolarBear		52114
Cow		86816
Dog		82307
BelugaWhale		81417
Donkey		90106
Cat		86449
Ferret		87765
Lion		84143
Vaquita		84015
Pig		83470

```
Majority     XXXXXXXXXXXXXXXXXXXXXXXXXXXXXXXXXXXXXXXXXXXXXXXXXXXXXXXXXXXXXXXXXXXXXXXXXXXXXXXX

                      119210    119220    119230    119240    119250    119260    119270    119280

Human        GAAATTGAGTATACTAGGAAATCTGATGGCAAGAATTCAGCAGAAAGTCTTAATTTTAAACCGTAATATTCATTGATTAT  96347
PolarBear                                                                                      52114
Cow                                                                                            86816
Dog                                                                                            82307
BelugaWhale                                                                                    81417
Donkey                                                                                         90106
Cat                                                                                            86449
Ferret                                                                                         87765
Lion                                                                                           84143
Vaquita                                                                                        84015
Pig                                                                                            83470

Majority     XXXXXXXXXXXXXXXXXXXXXXXXXXXXXXXXXXXXXXXXXXXXXXXXXXXXXXXXXXXXXXXXXXXXXXXXXXXXXXXX

                      119290    119300    119310    119320    119330    119340    119350    119360

Human        GCAAAACCTTAGTTTTATAGTTATTCTTTGTTATTTTGAATAGCTTCCCTTTAAATTTTCTAGTTTCAGATTTTGCAAAT  96427
PolarBear                                                                                      52114
Cow                                                                                            86816
Dog                                                                                            82307
BelugaWhale                                                                                    81417
Donkey                                                                                         90106
Cat                                                                                            86449
Ferret                                                                                         87765
Lion                                                                                           84143
Vaquita                                                                                        84015
Pig                                                                                            83470
```

Majority	XXXXXXXXXXXXXXXXXXXXXXXXXXXXXXXXXXXXXXXXXXXXXXXXXXXXXXXXXXXXXXXXXXXXXXXXXXXXXXXX	
	119370 119380 119390 119400 119410 119420 119430 119440	
Human	AAAAGTGTACATGTTAAAGTTATAGAGTATTAGATTATTCACATTAAGAACTGTTCAAATACGCGGAAAAATAAAACCGT	96507
PolarBear		52114
Cow		86816
Dog		82307
BelugaWhale		81417
Donkey		90106
Cat		86449
Ferret		87765
Lion		84143
Vaquita		84015
Pig		83470

Majority	XXXXXXXXXXXXXXXXXXXXXXXXXXXXXXXXXXXXXXXXXXXXXXXXXXXXXXXXXXXXXXXXXXXXXXXXXXXXXXXX	
	119450 119460 119470 119480 119490 119500 119510 119520	
Human	AATGAACACCTATGCACTTCACTCAACTATATCACTACCTAACATATTCCATATTTTAAACGTAATTATATATTAGTTGA	96587
PolarBear		52114
Cow		86816
Dog		82307
BelugaWhale		81417
Donkey		90106
Cat		86449
Ferret		87765
Lion		84143
Vaquita		84015
Pig		83470

Majority	XXXXXXXXXXXXXXXXXXXXXXXXXXXXXXXXXXXXXXXXXXXXXXXXXXXXXXXXXXXXXXXXXXXXXXXXXXXXXXXX	
	119530 119540 119550 119560 119570 119580 119590 119600	
Human	AAGAAAACGTTACAGAATGAACTGAAATCCCATATGAACCTCTTTCTGATCTTAGTTCAAACCTTATTCTTTCCCAAGAG	96667
PolarBear		52114
Cow		86816
Dog		82307
BelugaWhale		81417
Donkey		90106
Cat		86449
Ferret		87765
Lion		84143
Vaquita		84015
Pig		83470

Majority	XXXXXXXXXXXXXXXXXXXXXXXXXXXXXXXXXXXXXXXXXXXXXXXXXXXXXXXXXXXXXXXXXXXXXXXXXXXXXXXX	
	119610 119620 119630 119640 119650 119660 119670 119680	
Human	GTAATTGTTACCTTCAGTTTAGTATTTTATTATTCAGTGGTTGCTTTTCAAGAGGAATTTGTCCTATATTCTGTAGGCTA	96747
PolarBear		52114
Cow		86816
Dog		82307
BelugaWhale		81417
Donkey		90106
Cat		86449
Ferret		87765
Lion		84143
Vaquita		84015
Pig		83470

Majority	XXXXXXXXXXXXXXXXXXXXXXXXXXXXXXXXXXXXXXXXXXXXXXXXXXXXXXXXXXXXXXXXXXXXXXXXXXXXXXXX	
	119690 119700 119710 119720 119730 119740 119750 119760	
Human	AATTTCTTGTTTTGCCTAGTTATAAAAATGGCAAATAATATGATATTGGGTGTTTAAATAGTACAATATTGTTCAAAACA	96827
PolarBear		52114
Cow		86816
Dog		82307
BelugaWhale		81417
Donkey		90106
Cat		86449
Ferret		87765
Lion		84143
Vaquita		84015
Pig		83470

Majority	XXXXXXXXXXXXXXXXXXXXXXXXXXXXXXXXXXXXXXXXXXXXXXXXXXXXXXXXXXXXXXXXXXXXXXXXXXXXXXXX	
	119770 119780 119790 119800 119810 119820 119830 119840	
Human	CACTTTATAATTTTTAACAGACTGTTTGGACATTGATAATTCCCCTATCAAATCTCATGACATTTAACCAATTTTTAACA	96907
PolarBear		52114
Cow		86816
Dog		82307
BelugaWhale		81417
Donkey		90106
Cat		86449
Ferret		87765
Lion		84143
Vaquita		84015
Pig		83470

```
Majority     XXXXXXXXXXXXXXXXXXXXXXXXXXXXXXXXXXXXXXXXXXXXXXXXXXXXXXXXXXXXXXXXXXXXXXXXXXXXXXXX

                      119850    119860    119870    119880    119890    119900    119910    119920

Human        AGGATTTTAAATTGTGTTTAAGAAATAAGTCATTTGGGCCTCAGGTTTCTCTTAAGGATGTAAACAAGGAGCTATATCAT  96987
PolarBear                                                                                      52114
Cow                                                                                            86816
Dog                                                                                            82307
BelugaWhale                                                                                    81417
Donkey                                                                                         90106
Cat                                                                                            86449
Ferret                                                                                         87765
Lion                                                                                           84143
Vaquita                                                                                        84015
Pig                                                                                            83470

Majority     XXXXXXXXXXXXXXXXXXXXXXXXXXXXXXXXXXXXXXXXXXXXXXXXXXXXXXXXXXXXXXXXXXXXXXXXXXXXXXXX

                      119930    119940    119950    119960    119970    119980    119990    120000

Human        TGGTATTTTAGACGCTGAATTATGTGTAAGGTCCTTAAATACAAACATACCCTTTGTAGCTTCACAGAGCCACCCGTTTT  97067
PolarBear                                                                                      52114
Cow                                                                                            86816
Dog                                                                                            82307
BelugaWhale                                                                                    81417
Donkey                                                                                         90106
Cat                                                                                            86449
Ferret                                                                                         87765
Lion                                                                                           84143
Vaquita                                                                                        84015
Pig                                                                                            83470
```

Majority	XXXXXXXXXXXXXXXXXXXXXXXXXXXXXXXXXXXXXXXXXXXXXXXXXXXXXXXXXXXXXXXXXXXXXXXXXXXXXXXX	
	120010 120020 120030 120040 120050 120060 120070 120080	
Human	GCACTTCTCTCGCTTTTCTTCCTCCCTCTTTGGCTCCCATTTCAATGGACAGAAAGACTATGTGGCATCTCTATTTTCAT	97147
PolarBear		52114
Cow		86816
Dog		82307
BelugaWhale		81417
Donkey		90106
Cat		86449
Ferret		87765
Lion		84143
Vaquita		84015
Pig		83470

Majority	XXXXXXXXXXXXXXXXXXXXXXXXXXXXXXXXXXXXXXXXXXXXXXXXXXXXXXXXXXXXXXXXXXXXXXXXXXXXXXXX	
	120090 120100 120110 120120 120130 120140 120150 120160	
Human	TCACTTGGTTTATTGTCTTGGTATGTTGTATAGGTTTTTGAATCAGAAAATTTTAACATTTTTAGATATTTCAAACATAC	97227
PolarBear		52114
Cow		86816
Dog		82307
BelugaWhale		81417
Donkey		90106
Cat		86449
Ferret		87765
Lion		84143
Vaquita		84015
Pig		83470

```
Majority     XXXXXXXXXXXXXXXXXXXXXXXXXXXXXXXXXXXXXXXXXXXXXXXXXXXXXXXXXXXXXXXXXXXXXXXXXXXXXXXX

                       120170    120180    120190    120200    120210    120220    120230    120240

Human        AAAAAATAGAAGAATATTTAACAAACCCTTGTCAGCTTCAGTTAGCAACTTAGATCTTCCTCGTTTCCTCCACCCAATTC  97307
PolarBear                                                                                      52114
Cow                                                                                            86816
Dog                                                                                            82307
BelugaWhale                                                                                    81417
Donkey                                                                                         90106
Cat                                                                                            86449
Ferret                                                                                         87765
Lion                                                                                           84143
Vaquita                                                                                        84015
Pig                                                                                            83470

Majority     XXXXXXXXXXXXXXXXXXXXXXXXXXXXXXXXXXXXXXXXXXXXXXXXXXXXXXXXXXXXXXXXXXXXXXXXXXXXXXXX

                       120250    120260    120270    120280    120290    120300    120310    120320

Human        TCATCTCCTTTATTAATGCAGATCCCAGATATATTTTATCAAGAAATTTTGTAGCATATATTTGTGTTGTTTTATTCGTG  97387
PolarBear                                                                                      52114
Cow                                                                                            86816
Dog                                                                                            82307
BelugaWhale                                                                                    81417
Donkey                                                                                         90106
Cat                                                                                            86449
Ferret                                                                                         87765
Lion                                                                                           84143
Vaquita                                                                                        84015
Pig                                                                                            83470
```

```
Majority     XXXXXXXXXXXXXXXXXXXXXXXXXXXXXXXXXXXXXXXXXXXXXXXXXXXXXXXXXXXXXXXXXXXXXXXXXXXXXXXX
                      120330    120340    120350    120360    120370    120380    120390    120400
Human        GTCATAGTTGCTTTCTCTAATATCTAGACTAGCCTAGCTGGTCAATACTACTTTTCTTCAAATCCTAGAACCTCCCTATT  97467
PolarBear                                                                                      52114
Cow                                                                                            86816
Dog                                                                                            82307
BelugaWhale                                                                                    81417
Donkey                                                                                         90106
Cat                                                                                            86449
Ferret                                                                                         87765
Lion                                                                                           84143
Vaquita                                                                                        84015
Pig                                                                                            83470

Majority     XXXXXXXXXXXXXXXXXXXXXXXXXXXXXXXXXXXXXXXXXXXXXXXXXXXXXXXXXXXXXXXXXXXXXXXXXXXXXXXX
                      120410    120420    120430    120440    120450    120460    120470    120480
Human        AAAAATATAGTCACCAGACCAGTGGCAAATTAGTATCACCTAGGAGTTTATTAGAAACTCAGTCTATGAGCCTACTGAAT  97547
PolarBear                                                                                      52114
Cow                                                                                            86816
Dog                                                                                            82307
BelugaWhale                                                                                    81417
Donkey                                                                                         90106
Cat                                                                                            86449
Ferret                                                                                         87765
Lion                                                                                           84143
Vaquita                                                                                        84015
Pig                                                                                            83470
```

```
Majority     XXXXXXXXXXXXXXXXXXXXXXXXXXXXXXXXXXXXXXXXXXXXXXXXXXXXXXXXXXXXXXXXXXXXXXXXXXXXXXXX

                      120490    120500    120510    120520    120530    120540    120550    120560

Human        CTACATTTTGACAAGATTGGCAGGTGATGCATTTGGACATAAGTTGGAGAAGCACTCAATGCATTTTTTCAGTTCTATAA  97627
PolarBear                                                                                      52114
Cow                                                                                            86816
Dog                                                                                            82307
BelugaWhale                                                                                    81417
Donkey                                                                                         90106
Cat                                                                                            86449
Ferret                                                                                         87765
Lion                                                                                           84143
Vaquita                                                                                        84015
Pig                                                                                            83470

Majority     XXXXXXXXXXXXXXXXXXXXXXXXXXXXXXXXXXXXXXXXXXXXXXXXXXXXXXXXXXXXXXXXXXXXXXXXXXXXXXXX

                      120570    120580    120590    120600    120610    120620    120630    120640

Human        AAATCAGCTTCAAAGTTTTTTCCTGAGTGTGGAAATTATTTCATTAGTAATTTTAAGTTATGCAGATATAGTTAATACCA  97707
PolarBear                                                                                      52114
Cow                                                                                            86816
Dog                                                                                            82307
BelugaWhale                                                                                    81417
Donkey                                                                                         90106
Cat                                                                                            86449
Ferret                                                                                         87765
Lion                                                                                           84143
Vaquita                                                                                        84015
Pig                                                                                            83470
```

Majority	XXXXXXXXXXXXXXXXXXXXXXXXXXXXXXXXXXXXXXXXXXXXXXXXXXXXXXXXXXXXXXXXXXXXXXXXXXXXXXXX	
	120650 120660 120670 120680 120690 120700 120710 120720	
Human	AGAAATAATTGAACTGAAAAGGAACCCGGATATCTAGTTTTAACCAGCTGTTTTAAAGATAAACAGTTCTAGAGATCAAA	97787
PolarBear		52114
Cow		86816
Dog		82307
BelugaWhale		81417
Donkey		90106
Cat		86449
Ferret		87765
Lion		84143
Vaquita		84015
Pig		83470

Majority	XXXXXXXXXXXXXXXXXXXXXXXXXXXXXXXXXXXXXXXXXXXXXXXXXXXXXXXXXXXXXXXXXXXXXXXXXXXXXXXX	
	120730 120740 120750 120760 120770 120780 120790 120800	
Human	TGAATAGTTTAGCAACATATTTATTGTAACTTACCTGTAGTTAGAATCCAAGTCCCTGGCTTTCCAATCCAGTCTTCAAT	97867
PolarBear		52114
Cow		86816
Dog		82307
BelugaWhale		81417
Donkey		90106
Cat		86449
Ferret		87765
Lion		84143
Vaquita		84015
Pig		83470

Majority	XXXXXXXXXXXXXXXXXXXXXXXXXXXXXXXXXXXXXXXXXXXXXXXXXXXXXXXXXXXXXXXXXXXXXXXXXXXXXXXX	
	120810 120820 120830 120840 120850 120860 120870 120880	
Human	GTATAATACTGATCAAAAGAAATACAGGCCTGCTATTATTCATAAATTTAAACTATTATGTACAAGTTGATAGCTTATAA	97947
PolarBear		52114
Cow		86816
Dog		82307
BelugaWhale		81417
Donkey		90106
Cat		86449
Ferret		87765
Lion		84143
Vaquita		84015
Pig		83470

Majority	XXXXXXXXXXXXXXXXXXXXXXXXXXXXXXXXXXXXXXXXXXXXXXXXXXXXXXXXXXXXXXXXXXXXXXXXXXXXXXXX	
	120890 120900 120910 120920 120930 120940 120950 120960	
Human	TAGAAATCAGTATGATTAGTGTTAAAAGTCAAATTTAGTCTGACAGGGAAGGAATGTCCATGTGAACTGATTTTTTTTAA	98027
PolarBear		52114
Cow		86816
Dog		82307
BelugaWhale		81417
Donkey		90106
Cat		86449
Ferret		87765
Lion		84143
Vaquita		84015
Pig		83470

```
Majority     XXXXXXXXXXXXXXXXXXXXXXXXXXXXXXXXXXXXXXXXXXXXXXXXXXXXXXXXXXXXXXXXXXXXXXXXXXXXXXXX
                      120970    120980    120990    121000    121010    121020    121030    121040
Human        TAGAATGCAAATTAATACTCAATTCCTGATTTGTTTATTACATTTTGTGGTTTAATGAAATGATACTCAGGTCATTTTCA 98107
PolarBear                                                                                     52114
Cow                                                                                           86816
Dog                                                                                           82307
BelugaWhale                                                                                   81417
Donkey                                                                                        90106
Cat                                                                                           86449
Ferret                                                                                        87765
Lion                                                                                          84143
Vaquita                                                                                       84015
Pig                                                                                           83470
```

```
Majority     XXXXXXXXXXXXXXXXXXXXXXXXXXXXXXXXXXXXXXXXXXXXXXXXXXXXXXXXXXXXXXXXXXXXXXXXXXXXXXXX
                      121050    121060    121070    121080    121090    121100    121110    121120
Human        TAAATAGCTTTTCTATTAATGCTTCCTCAGTCACATTCTCTATTCCTATTGCATAGCACAGTCTAACAGTTGAAATGCCT 98187
PolarBear                                                                                     52114
Cow                                                                                           86816
Dog                                                                                           82307
BelugaWhale                                                                                   81417
Donkey                                                                                        90106
Cat                                                                                           86449
Ferret                                                                                        87765
Lion                                                                                          84143
Vaquita                                                                                       84015
Pig                                                                                           83470
```

```
Majority     XXXXXXXXXXXXXXXXXXXXXXXXXXXXXXXXXXXXXXXXXXXXXXXXXXXXXXXXXXXXXXXXXXXXXXXXXXXXXXXX
                       121130    121140    121150    121160    121170    121180    121190    121200
Human        AAGCCTTACTGAATGATGACTGATATTCTCTGTGAGAGTTAGAGCTGTACTATAAATATACATTACCTGTGCAAAGTATG  98267
PolarBear                                                                                      52114
Cow                                                                                            86816
Dog                                                                                            82307
BelugaWhale                                                                                    81417
Donkey                                                                                         90106
Cat                                                                                            86449
Ferret                                                                                         87765
Lion                                                                                           84143
Vaquita                                                                                        84015
Pig                                                                                            83470

Majority     XXXXXXXXXXXXXXXXXXXXXXXXXXXXXXXXXXXXXXXXXXXXXXXXXXXXXXXXXXXXXXXXXXXXXXXXXXXXXXXX
                       121210    121220    121230    121240    121250    121260    121270    121280
Human        CCTTCAGGGGCTATTCCCCATAACCTTAGCAAAATTTTAAAATATTCTTATGGCTAGCAATCTTATCTATTGTCATGTCT  98347
PolarBear                                                                                      52114
Cow                                                                                            86816
Dog                                                                                            82307
BelugaWhale                                                                                    81417
Donkey                                                                                         90106
Cat                                                                                            86449
Ferret                                                                                         87765
Lion                                                                                           84143
Vaquita                                                                                        84015
Pig                                                                                            83470
```

Majority	XXXXXXXXXXXXXXXXXXXXXXXXXXXXXXXXXXXXXXXXXXXXXXXXXXXXXXXXXXXXXXXXXXXXXXXXXXXXXXXX	
	121290 121300 121310 121320 121330 121340 121350 121360	
Human	TACTATTTCCTGCTTACCACCCCCATTTCTCTCTCAACCTTGTTGCTGCATTAGCTGTCCATCACTTAACACTGTTTATT	98427
PolarBear		52114
Cow		86816
Dog		82307
BelugaWhale		81417
Donkey		90106
Cat		86449
Ferret		87765
Lion		84143
Vaquita		84015
Pig		83470

Majority	XXXXXXXXXXXXXXXXXXXXXXXXXXXXXXXXXXXXXXXXXXXXXXXXXXXXXXXXXXXXXXXXXXXXXXXXXXXXXXXX	
	121370 121380 121390 121400 121410 121420 121430 121440	
Human	TCTCTGTTTTTCTTCCATCATTCTCTTCTTTGCAACTAAATGATTTAGACTTTTTATAACCATAATCAACCAGTTAGATT	98507
PolarBear		52114
Cow		86816
Dog		82307
BelugaWhale		81417
Donkey		90106
Cat		86449
Ferret		87765
Lion		84143
Vaquita		84015
Pig		83470

```
Majority     XXXXXXXXXXXXXXXXXXXXXXXXXXXXXXXXXXXXXXXXXXXXXXXXXXXXXXXXXXXXXXXXXXXXXXXXXXXXXXXX
                        121450    121460    121470    121480    121490    121500    121510    121520
Human        CTTTTTATTTGTGGTTATTGAGTTGATAAATGATAGTGCATTTTATGTAATATTCAGGTGTTATGCTATTTTTATCTGGT  98587
PolarBear                                                                                      52114
Cow                                                                                            86816
Dog                                                                                            82307
BelugaWhale                                                                                    81417
Donkey                                                                                         90106
Cat                                                                                            86449
Ferret                                                                                         87765
Lion                                                                                           84143
Vaquita                                                                                        84015
Pig                                                                                            83470

Majority     XXXXXXXXXXXXXXXXXXXXXXXXXXXXXXXXXXXXXXXXXXXXXXXXXXXXXXXXXXXXXXXXXXXXXXXXXXXXXXXX
                        121530    121540    121550    121560    121570    121580    121590    121600
Human        TCACTTTAAAACCTCCCTCCCCCCACTCCCTTTTTTTTGTGGGTTTTTTTTTTATTATTATTATTTTCCAGTTTGTGTTG  98667
PolarBear                                                                                      52114
Cow                                                                                            86816
Dog                                                                                            82307
BelugaWhale                                                                                    81417
Donkey                                                                                         90106
Cat                                                                                            86449
Ferret                                                                                         87765
Lion                                                                                           84143
Vaquita                                                                                        84015
Pig                                                                                            83470
```

Majority	XXXXXXXXXXXXXXXXXXXXXXXXXXXXXXXXXXXXXXXXXXXXXXXXXXXXXXXXXXXXXXXXXXXXXXXXXXXXXXXX	
	121610 121620 121630 121640 121650 121660 121670 121680	
Human	TTTTGCTTTTTAACTTCTGAGATTGCAGTGGTTCCAGATGGTTGAAATCACTAGTAGTGATTTACATGAAGCATTCATGT	98747
PolarBear		52114
Cow		86816
Dog		82307
BelugaWhale		81417
Donkey		90106
Cat		86449
Ferret		87765
Lion		84143
Vaquita		84015
Pig		83470

Majority	XXXXXXXXXXXXXXXXXXXXXXXXXXXXXXXXXXXXXXXXXXXXXXXXXXXXXXXXXXXXXXXXXXXXXXXXXXXXXXXX	
	121690 121700 121710 121720 121730 121740 121750 121760	
Human	ATTTATTACTGGTATATTCAACTATGGTGGAGGGAAATTGGTCGAAGAATTACGGTTAAAGAGCTCCTTTAAAAAGGAGA	98827
PolarBear		52114
Cow		86816
Dog		82307
BelugaWhale		81417
Donkey		90106
Cat		86449
Ferret		87765
Lion		84143
Vaquita		84015
Pig		83470

Majority	XXXXXXXXXXXXXXXXXXXXXXXXXXXXXXXXXXXXXXXXXXXXXXXXXXXXXXXXXXXXXXXXXXXXXXXXXXXXXXXX	
	121770 121780 121790 121800 121810 121820 121830 121840	
Human	TTTGTAGAATTCTAAAATGTGATTCTGATTCATATAGTCATGATTCTTAGAAATTAGCTACAGGAAGAGATAGAAACATT	98907
PolarBear		52114
Cow		86816
Dog		82307
BelugaWhale		81417
Donkey		90106
Cat		86449
Ferret		87765
Lion		84143
Vaquita		84015
Pig		83470

Majority	XXXXXXXXXXXXXXXXXXXXXXXXXXXXXXXXXXXXXXXXXXXXXXXXXXXXXXXXXXXXXXXXXXXXXXXXXXXXXXXX	
	121850 121860 121870 121880 121890 121900 121910 121920	
Human	GCTATAGTTGTCTTTGCCCTAAGTTTGGAGTTTTATAGTGTAATATATATATAATAGCTCTAATTATAAATACACTTTTA	98987
PolarBear		52114
Cow		86816
Dog		82307
BelugaWhale		81417
Donkey		90106
Cat		86449
Ferret		87765
Lion		84143
Vaquita		84015
Pig		83470

```
Majority     XXXXXXXXXXXXXXXXXXXXXXXXXXXXXXXXXXXXXXXXXXXXXXXXXXXXXXXXXXXXXXXXXXXXXXXXXXXXXXXX

                       121930    121940    121950    121960    121970    121980    121990    122000

Human        CTCTGGAAAAAGCATAGACATTGACATAAGAGACATTTAAAAACCATTATTTTCTCATCTGTAAAATGGAGAGAATTACT   99067
PolarBear                                                                                       52114
Cow                                                                                             86816
Dog                                                                                             82307
BelugaWhale                                                                                     81417
Donkey                                                                                          90106
Cat                                                                                             86449
Ferret                                                                                          87765
Lion                                                                                            84143
Vaquita                                                                                         84015
Pig                                                                                             83470

Majority     XXXXXXXXXXXXXXXXXXXXXXXXXXXXXXXXXXXXXXXXXXXXXXXXXXXXXXXXXXXXXXXXXXXXXXXXXXXXXXXX

                       122010    122020    122030    122040    122050    122060    122070    122080

Human        TCATATGTTCACTTATAAGGATTATGTGAAATACTGTTCCATTCCATTAGTAGTTTTTCTTTTCCTCTAATTGGGGGTGG   99147
PolarBear                                                                                       52114
Cow                                                                                             86816
Dog                                                                                             82307
BelugaWhale                                                                                     81417
Donkey                                                                                          90106
Cat                                                                                             86449
Ferret                                                                                          87765
Lion                                                                                            84143
Vaquita                                                                                         84015
Pig                                                                                             83470
```

Majority	XXXXXXXXXXXXXXXXXXXXXXXXXXXXXXXXXXXXXXXXXXXXXXXXXXXXXXXXXXXXXXXXXXXXXXXXXXXXXXXX	
	122090 122100 122110 122120 122130 122140 122150 122160	
Human	GGGCAGTTTTTGGCATTCTATTTATGGAAGGTGTTTTTTTCCCTAAGATCATTTTGATGTCTCAGAAGACATTTGCTATG	99227
PolarBear		52114
Cow		86816
Dog		82307
BelugaWhale		81417
Donkey		90106
Cat		86449
Ferret		87765
Lion		84143
Vaquita		84015
Pig		83470

Majority	XXXXXXXXXXXXXXXXXXXXXXXXXXXXXXXXXXXXXXXXXXXXXXXXXXXXXXXXXXXXXXXXXXXXXXXXXXXXXXXX	
	122170 122180 122190 122200 122210 122220 122230 122240	
Human	TACGTTGGACATTTGCCATGTACATTGTAGAGAATACAAAGATAAACAGAACCACAGTACAGCTGGTTCTATTAGACATT	99307
PolarBear		52114
Cow		86816
Dog		82307
BelugaWhale		81417
Donkey		90106
Cat		86449
Ferret		87765
Lion		84143
Vaquita		84015
Pig		83470

Majority	XXXXXXXXXXXXXXXXXXXXXXXXXXXXXXXXXXXXXXXXXXXXXXXXXXXXXXXXXXXXXXXXXXXXXXXXXXXXXXXX	
	122250 122260 122270 122280 122290 122300 122310 122320	
Human	GAAAATGCATATTCTAGCATATTTAATATATTAGGGAACAATTTAAGCATAATGTGAATTTCATGTTTACTGAAGATTTC	99387
PolarBear		52114
Cow		86816
Dog		82307
BelugaWhale		81417
Donkey		90106
Cat		86449
Ferret		87765
Lion		84143
Vaquita		84015
Pig		83470

Majority	XXXXXXXXXXXXXXXXXXXXXXXXXXXXXXXXXXXXXXXXXXXXXXXXXXXXXXXXXXXXXXXXXXXXXXXXXXXXXXXX	
	122330 122340 122350 122360 122370 122380 122390 122400	
Human	ATCCTGAGAAACAAATGAAAGCAGAAAATTACAACCAACTGAACCAGCTGTGTAAGAAATATATATATGGTGTGCACACA	99467
PolarBear		52114
Cow		86816
Dog		82307
BelugaWhale		81417
Donkey		90106
Cat		86449
Ferret		87765
Lion		84143
Vaquita		84015
Pig		83470

Majority	XXXXXXXXXXXXXXXXXXXXXXXXXXXXXXXXXXXXXXXXXXXXXXXXXXXXXXXXXXXXXXXXXXXXXXXXXXXXXXXX	
	122410 122420 122430 122440 122450 122460 122470 122480	
Human	TGTACATACCTCAGACATCTATTAGCATTCTCAGTTTTCCACAAGTTAAAAGCCACGCTCATCCATATCTGGTGCTAATA	99547
PolarBear		52114
Cow		86816
Dog		82307
BelugaWhale		81417
Donkey		90106
Cat		86449
Ferret		87765
Lion		84143
Vaquita		84015
Pig		83470

Majority	XXXXXXXXXXXXXXXXXXXXXXXXXXXXXXXXXXXXXXXXXXXXXXXXXXXXXXXXXXXXXXXXXXXXXXXXXXXXXXXX	
	122490 122500 122510 122520 122530 122540 122550 122560	
Human	ACTTCCTATCCAATTTCAGATAGCTGTTTTCAACACATCTGACACCTTCTTTTTTTCAAGCAAACTTCAGGTCTTTTTCA	99627
PolarBear		52114
Cow		86816
Dog		82307
BelugaWhale		81417
Donkey		90106
Cat		86449
Ferret		87765
Lion		84143
Vaquita		84015
Pig		83470

```
Majority     XXXXXXXXXXXXXXXXXXXXXXXXXXXXXXXXXXXXXXXXXXXXXXXXXXXXXXXXXXXXXXXXXXXXXXXXXXXXXXXX
                     122570    122580    122590    122600    122610    122620    122630    122640
Human        AGATAAAGTCATTTATAGTAGTGTCTATATATTTCTTAACCATTTAACATGTAAACTGTGCTGCCTTTTAAGAAATTAAT   99707
PolarBear                                                                                       52114
Cow                                                                                             86816
Dog                                                                                             82307
BelugaWhale                                                                                     81417
Donkey                                                                                          90106
Cat                                                                                             86449
Ferret                                                                                          87765
Lion                                                                                            84143
Vaquita                                                                                         84015
Pig                                                                                             83470

Majority     XXXXXXXXXXXXXXXXXXXXXXXXXXXXXXXXXXXXXXXXXXXXXXXXXXXXXXXXXXXXXXXXXXXXXXXXXXXXXXXX
                     122650    122660    122670    122680    122690    122700    122710    122720
Human        TCTATCCTTTTTAGTGTCAGAGTTGAAGATTTTGAGTGTTGTGCCCTAACCTTATCATTTTCCTCATAAGTCCTGTGGTT   99787
PolarBear                                                                                       52114
Cow                                                                                             86816
Dog                                                                                             82307
BelugaWhale                                                                                     81417
Donkey                                                                                          90106
Cat                                                                                             86449
Ferret                                                                                          87765
Lion                                                                                            84143
Vaquita                                                                                         84015
Pig                                                                                             83470
```

Majority	XXXXXXXXXXXXXXXXXXXXXXXXXXXXXXXXXXXXXXXXXXXXXXXXXXXXXXXXXXXXXXXXXXXXXXXXXXXXXXXX	
	122730 122740 122750 122760 122770 122780 122790 122800	
Human	TTTATTGTATGATTTTGCATAGTGTGGCAATTCTTGGAAATGCTTATGTTGTGTTATAGTATAACTGACTTACTACTTTC	99867
PolarBear		52114
Cow		86816
Dog		82307
BelugaWhale		81417
Donkey		90106
Cat		86449
Ferret		87765
Lion		84143
Vaquita		84015
Pig		83470

Majority	XXXXXXXXXXXXXXXXXXXXXXXXXXXXXXXXXXXXXXXXXXXXXXXXXXXXXXXXXXXXXXXXXXXXXXXXXXXXXXXX	
	122810 122820 122830 122840 122850 122860 122870 122880	
Human	AAATAGCTTCTAGTCTGATTCATGTTATGACAGTATACAGTATAGCAAAATAAGGACATCAGGGAACAGGAGGCTGAGCA	99947
PolarBear		52114
Cow		86816
Dog		82307
BelugaWhale		81417
Donkey		90106
Cat		86449
Ferret		87765
Lion		84143
Vaquita		84015
Pig		83470

Majority	XXXXXXXXXXXXXXXXXXXXXXXXXXXXXXXXXXXXXXXXXXXXXXXXXXXXXXXXXXXXXXXXXXXXXXXXXXXXXXXX	
	122890 122900 122910 122920 122930 122940 122950 122960	
Human	TATCACCATTTACCAATGGTAGCTTGAGCAAGGCACTTCATTTATCGAAGTCCATTTTCTCATCTGTACAATGGAGGTGA	100027
PolarBear		52114
Cow		86816
Dog		82307
BelugaWhale		81417
Donkey		90106
Cat		86449
Ferret		87765
Lion		84143
Vaquita		84015
Pig		83470

Majority	XXXXXXXXXXXXXXXXXXXXXXXXXXXXXXXXXXXXXXXXXXXXXXXXXXXXXXXXXXXXXXXXXXXXXXXXXXXXXXXX	
	122970 122980 122990 123000 123010 123020 123030 123040	
Human	TGATAGTACATGTCTTTTCTGTCTTGTGTTATTTGTAGTTTCAAGTAAGATATGAAATATGCTGTAGAAATACAGGGTTG	100107
PolarBear		52114
Cow		86816
Dog		82307
BelugaWhale		81417
Donkey		90106
Cat		86449
Ferret		87765
Lion		84143
Vaquita		84015
Pig		83470

```
Majority     XXXXXXXXXXXXXXXXXXXXXXXXXXXXXXXXXXXXXXXXXXXXXXXXXXXXXXXXXXXXXXXXXXXXXXXXXXXXXXXX

                      123050    123060    123070    123080    123090    123100    123110    123120

Human        GGAATATCTAGTGGTACAAAAATCAACTTTTGGCCCAATTTTAAGGTGAGATAATTATGAGACATTTTTCGACCTCAAAG  100187
PolarBear                                                                                      52114
Cow                                                                                            86816
Dog                                                                                            82307
BelugaWhale                                                                                    81417
Donkey                                                                                         90106
Cat                                                                                            86449
Ferret                                                                                         87765
Lion                                                                                           84143
Vaquita                                                                                        84015
Pig                                                                                            83470

Majority     XXXXXXXXXXXXXXXXXXXXXXXXXXXXXXXXXXXXXXXXXXXXXXXXXXXXXXXXXXXXXXXXXXXXXXXXXXXXXXXX

                      123130    123140    123150    123160    123170    123180    123190    123200

Human        TCTTTGTTCTTTTCTCTCTACACCATTGTGAATAACACAGTGTATATATGAGATTAATTTATTACCTCGACTTTCCATTT  100267
PolarBear                                                                                      52114
Cow                                                                                            86816
Dog                                                                                            82307
BelugaWhale                                                                                    81417
Donkey                                                                                         90106
Cat                                                                                            86449
Ferret                                                                                         87765
Lion                                                                                           84143
Vaquita                                                                                        84015
Pig                                                                                            83470
```

Majority	XXXXXXXXXXXXXXXXXXXXXXXXXXXXXXXXXXXXXXXXXXXXXXXXXXXXXXXXXXXXXXXXXXXXXXXXXXXXXXXX	
	123210 123220 123230 123240 123250 123260 123270 123280	
Human	TTGTTTTGTAAAGTAGTTTAATTTTTTATATCTATTTATAAAGTTAACTGCAGGAGTGTACCTGCAGTTAACTTTATACA	100347
PolarBear		52114
Cow		86816
Dog		82307
BelugaWhale		81417
Donkey		90106
Cat		86449
Ferret		87765
Lion		84143
Vaquita		84015
Pig		83470

Majority	XXXXXXXXXXXXXXXXXXXXXXXXXXXXXXXXXXXXXXXXXXXXXXXXXXXXXXXXXXXXXXXXXXXXXXXXXXXXXXXX	
	123290 123300 123310 123320 123330 123340 123350 123360	
Human	GTTAAGAAGAGGTTGGTTTCAGTTTTAAAGATTGTATGTCAGTGTAAAACATCCATTATTATGCTTTCAAGTAAAATAGC	100427
PolarBear		52114
Cow		86816
Dog		82307
BelugaWhale		81417
Donkey		90106
Cat		86449
Ferret		87765
Lion		84143
Vaquita		84015
Pig		83470

Majority	XXXXXXXXXXXXXXXXXXXXXXXXXXXXXXXXXXXXXXXXXXXXXXXXXXXXXXXXXXXXXXXXXXXXXXXXXXXXXXXX	
	123370 123380 123390 123400 123410 123420 123430 123440	
Human	TCCTAAAAATATGGATTACTATCAACTTTCAAGGAAAATAGCTCCTAAAAATATGGATTACTACCAACTTTCAAGGAAAA	100507
PolarBear		52114
Cow		86816
Dog		82307
BelugaWhale		81417
Donkey		90106
Cat		86449
Ferret		87765
Lion		84143
Vaquita		84015
Pig		83470

Majority	XXXXXXXXXXXXXXXXXXXXXXXXXXXXXXXXXXXXXXXXXXXXXXXXXXXXXXXXXXXXXXXXXXXXXXXXXXXXXXXX	
	123450 123460 123470 123480 123490 123500 123510 123520	
Human	TAGCTCCTAGAAATATGGATTACTAGCCACATAAATTGCTAACATTTGCTTTTATTATTATTATTATTATTATTTTAGAC	100587
PolarBear		52114
Cow		86816
Dog		82307
BelugaWhale		81417
Donkey		90106
Cat		86449
Ferret		87765
Lion		84143
Vaquita		84015
Pig		83470

Majority	XXXXXXXXXXXXXXXXXXXXXXXXXXXXXXXXXXXXXXXXXXXXXXXXXXXXXXXXXXXXXXXXXXXXXXXXXXXXXXXX	
	123530 123540 123550 123560 123570 123580 123590 123600	
Human	ACAGTCTTGCTCTGTCACCCAGGCTGGAGTGCGGTGGTATTATCTCGGCTCACTGCAACCTCCACCTCTCGGGTTCAAGC	100667
PolarBear		52114
Cow		86816
Dog		82307
BelugaWhale		81417
Donkey		90106
Cat		86449
Ferret		87765
Lion		84143
Vaquita		84015
Pig		83470

Majority	XXXXXXXXXXXXXXXXXXXXXXXXXXXXXXXXXXXXXXXXXXXXXXXXXXXXXXXXXXXXXXXXXXXXXXXXXXXXXXXX	
	123610 123620 123630 123640 123650 123660 123670 123680	
Human	AATTCTCCTGCCTCAGCCTTCCGAGTAGCTGGAATTACAGGCACCCACCACCACACCCAGCTAATTTTTGTATTTTTAGT	100747
PolarBear		52114
Cow		86816
Dog		82307
BelugaWhale		81417
Donkey		90106
Cat		86449
Ferret		87765
Lion		84143
Vaquita		84015
Pig		83470

Majority	XXXXXXXXXXXXXXXXXXXXXXXXXXXXXXXXXXXXXXXXXXXXXXXXXXXXXXXXXXXXXXXXXXXXXXXXXXXXXXXXXX	
	123690 123700 123710 123720 123730 123740 123750 123760	
Human	AGAGACAGGGTTTTACCATGTGACCAGGCTGGTTTCGAACTCCTGACCTCAAATGATCCACCTGCCTCAGCCTCCCAAAC	100827
PolarBear		52114
Cow		86816
Dog		82307
BelugaWhale		81417
Donkey		90106
Cat		86449
Ferret		87765
Lion		84143
Vaquita		84015
Pig		83470

Majority	XXXXXXXXXXXXXXXXXXXXXXXXXXXXXXXXXXXXXXXXXXXXXXXXXXXXXXXXXXXXXXXXXXXXXXXXXXXXXXXXXX	
	123770 123780 123790 123800 123810 123820 123830 123840	
Human	TGCTGGGATTACAGGTGTGAGCCACCGCTCCCAGCCTATTTGCTAATATTTAACCTCTTGAGAGTCTTTAATTCTTTTTT	100907
PolarBear		52114
Cow		86816
Dog		82307
BelugaWhale		81417
Donkey		90106
Cat		86449
Ferret		87765
Lion		84143
Vaquita		84015
Pig		83470

Majority	XXXXXXXXXXXXXXXXXXXXXXXXXXXXXXXXXXXXXXXXXXXXXXXXXXXXXXXXXXXXXXXXXXXXXXXXXXXXXXXX	
	123850 123860 123870 123880 123890 123900 123910 123920	
Human	TCAACAAGTGTTCATTACCTGCTATGTGCCAGCTTAGATGCTTTGGATGAAATACAGGTTGGTGCGAAAGTAATTGCGAT	100987
PolarBear		52114
Cow		86816
Dog		82307
BelugaWhale		81417
Donkey		90106
Cat		86449
Ferret		87765
Lion		84143
Vaquita		84015
Pig		83470

Majority	XXXXXXXXXXXXXXXXXXXXXXXXXXXXXXXXXXXXXXXXXXXXXXXXXXXXXXXXXXXXXXXXXXXXXXXXXXXXXXXX	
	123930 123940 123950 123960 123970 123980 123990 124000	
Human	TTTTGCCGTTAAAATTAGAAAAACCACAATTACTTTTGCACCAACCTAAATAACTAAAACACAATTTCTTCCTATCATGG	101067
PolarBear		52114
Cow		86816
Dog		82307
BelugaWhale		81417
Donkey		90106
Cat		86449
Ferret		87765
Lion		84143
Vaquita		84015
Pig		83470

Majority	XXXXXXXXXXXXXXXXXXXXXXXXXXXXXXXXXXXXXXXXXXXXXXXXXXXXXXXXXXXXXXXXXXXXXXXXXXXXXXXX	
	124010 124020 124030 124040 124050 124060 124070 124080	
Human	GGCTTTCAGTTCAGTATGAGATAAGTAAAGAATTATATGGGTTGATAAATTTTCTGGAGGAACTATTGGGTGTTATTACA	101147
PolarBear		52114
Cow		86816
Dog		82307
BelugaWhale		81417
Donkey		90106
Cat		86449
Ferret		87765
Lion		84143
Vaquita		84015
Pig		83470

Majority	XXXXXXXXXXXXXXXXXXXXXXXXXXXXXXXXXXXXXXXXXXXXXXXXXXXXXXXXXXXXXXXXXXXXXXXXXXXXXXXX	
	124090 124100 124110 124120 124130 124140 124150 124160	
Human	GTGTGCTCAATCTTTAGCAGTTAGGAAAGGATTCTTGAAGGTAGTTGTATTACCTGGGTTGGAGGTAAGTGTTGGGGACA	101227
PolarBear		52114
Cow		86816
Dog		82307
BelugaWhale		81417
Donkey		90106
Cat		86449
Ferret		87765
Lion		84143
Vaquita		84015
Pig		83470

```
Majority     XXXXXXXXXXXXXXXXXXXXXXXXXXXXXXXXXXXXXXXXXXXXXXXXXXXXXXXXXXXXXXXXXXXXXXXXXXXXXXXX
                      124170    124180    124190    124200    124210    124220    124230    124240
Human        CAAGTGTTAGAAACAGAACAGTTTGTTTAAAAGTTGGGGACAAAAAAGGACCAGAATCAAGATTACGGCACCCTGGAGAA  101307
PolarBear                                                                                      52114
Cow                                                                                            86816
Dog                                                                                            82307
BelugaWhale                                                                                    81417
Donkey                                                                                         90106
Cat                                                                                            86449
Ferret                                                                                         87765
Lion                                                                                           84143
Vaquita                                                                                        84015
Pig                                                                                            83470

Majority     XXXXXXXXXXXXXXXXXXXXXXXXXXXXXXXXXXXXXXXXXXXXXXXXXXXXXXXXXXXXXXXXXXXXXXXXXXXXXXXX
                      124250    124260    124270    124280    124290    124300    124310    124320
Human        AGTTAAGCATGGCTCGGTCATAAATTAGGAGACAAGGAACAAATAGAGAAATACGTTTGGATAGAATAGTCAGATTATAG  101387
PolarBear                                                                                      52114
Cow                                                                                            86816
Dog                                                                                            82307
BelugaWhale                                                                                    81417
Donkey                                                                                         90106
Cat                                                                                            86449
Ferret                                                                                         87765
Lion                                                                                           84143
Vaquita                                                                                        84015
Pig                                                                                            83470
```

Majority	XXXXXXXXXXXXXXXXXXXXXXXXXXXXXXXXXXXXXXXXXXXXXXXXXXXXXXXXXXXXXXXXXXXXXXXXXXXXXXXX	
	124330 124340 124350 124360 124370 124380 124390 124400	
Human	GCTGTGCTAAGTTGTTTGGGCTCCACTAGGGTTTCTAAATGCAGTTATTGGTAAACTGGACTTAGGAGAAGGGACAGGGT	101467
PolarBear		52114
Cow		86816
Dog		82307
BelugaWhale		81417
Donkey		90106
Cat		86449
Ferret		87765
Lion		84143
Vaquita		84015
Pig		83470

Majority	XXXXXXXXXXXXXXXXXXXXXXXXXXXXXXXXXXXXXXXXXXXXXXXXXXXXXXXXXXXXXXXXXXXXXXXXXXXXXXXX	
	124410 124420 124430 124440 124450 124460 124470 124480	
Human	TGACATGACAAATCTAGTGAATTTATGTTCATTAAAATATCCCTTTTGAATTGATTTAGTTTTTGTATGTTTCTTTTTTA	101547
PolarBear		52114
Cow		86816
Dog		82307
BelugaWhale		81417
Donkey		90106
Cat		86449
Ferret		87765
Lion		84143
Vaquita		84015
Pig		83470

Majority	XXXXXXXXXXXXXXXXXXXXXXXXXXXXXXXXXXXXXXXXXXXXXXXXXXXXXXXXXXXXXXXXXXXXXXXXXXXXXXXX	
	124490 124500 124510 124520 124530 124540 124550 124560	
Human	TAATTCCAAAAAATCATACAAATAAGTTAAACTTATCCATCTAAAAGCTGCACACAAAACTTGATGTTAGATTCTTTTAA	101627
PolarBear		52114
Cow		86816
Dog		82307
BelugaWhale		81417
Donkey		90106
Cat		86449
Ferret		87765
Lion		84143
Vaquita		84015
Pig		83470

Majority	XXXXXXXXXXXXXXXXXXXXXXXXXXXXXXXXXXXXXXXXXXXXXXXXXXXXXXXXXXXXXXXXXXXXXXXXXXXXXXXX	
	124570 124580 124590 124600 124610 124620 124630 124640	
Human	AATTACAAAATTAAGACTTCCAAAAGAATCTTGTTGAATAAGAGTAAAAGAAAGACCCTTACTCAGTAAAGCTGCGTAAT	101707
PolarBear		52114
Cow		86816
Dog		82307
BelugaWhale		81417
Donkey		90106
Cat		86449
Ferret		87765
Lion		84143
Vaquita		84015
Pig		83470

```
Majority     XXXXXXXXXXXXXXXXXXXXXXXXXXXXXXXXXXXXXXXXXXXXXXXXXXXXXXXXXXXXXXXXXXXXXXXXXXXXXXXX
                      124650    124660    124670    124680    124690    124700    124710    124720
Human        ACTTTGGCAGTTTGAACCCATGGCCATGCCTCTCTGTGTGATAAGGATTTGGCAAAACCATGGAAGAAACATGCATGTTT  101787
PolarBear                                                                                      52114
Cow                                                                                            86816
Dog                                                                                            82307
BelugaWhale                                                                                    81417
Donkey                                                                                         90106
Cat                                                                                            86449
Ferret                                                                                         87765
Lion                                                                                           84143
Vaquita                                                                                        84015
Pig                                                                                            83470

Majority     XXXXXXXXXXXXXXXXXXXXXXXXXXXXXXXXXXXXXXXXXXXXXXXXXXXXXXXXXXXXXXXXXXXXXXXXXXXXXXXX
                      124730    124740    124750    124760    124770    124780    124790    124800
Human        TTGAATTGTCTTTCCATGGTAACTTAAAACATTTTTTCATGAAACATGGATATATATCCTTAGAAAACTGCAAAACATAA  101867
PolarBear                                                                                      52114
Cow                                                                                            86816
Dog                                                                                            82307
BelugaWhale                                                                                    81417
Donkey                                                                                         90106
Cat                                                                                            86449
Ferret                                                                                         87765
Lion                                                                                           84143
Vaquita                                                                                        84015
Pig                                                                                            83470
```

Majority	XXXXXXXXXXXXXXXXXXXXXXXXXXXXXXXXXXXXXXXXXXXXXXXXXXXXXXXXXXXXXXXXXXXXXXXXXXXXXXXX	
	124810 124820 124830 124840 124850 124860 124870 124880	
Human	AGCTTGTAAGTTATAAGACAAGATTTATCTCTTTTTTTTTTTTTTCTGAGAGAGAGTCTTGCTCTGTTGCCCAGGCCGGA	101947
PolarBear		52114
Cow		86816
Dog		82307
BelugaWhale		81417
Donkey		90106
Cat		86449
Ferret		87765
Lion		84143
Vaquita		84015
Pig		83470

Majority	XXXXXXXXXXXXXXXXXXXXXXXXXXXXXXXXXXXXXXXXXXXXXXXXXXXXXXXXXXXXXXXXXXXXXXXXXXXXXXXX	
	124890 124900 124910 124920 124930 124940 124950 124960	
Human	GTGCAACAGTGTGATCTCTGCTCACTGCACCCTCCGCCTCCCGGGTTCAAGCAATTCTCCTGCCTCAGCCTCCTGAGTAG	102027
PolarBear		52114
Cow		86816
Dog		82307
BelugaWhale		81417
Donkey		90106
Cat		86449
Ferret		87765
Lion		84143
Vaquita		84015
Pig		83470

Majority	XXXXXXXXXXXXXXXXXXXXXXXXXXXXXXXXXXXXXXXXXXXXXXXXXXXXXXXXXXXXXXXXXXXXXXXXXXXXXXXX	
	124970 124980 124990 125000 125010 125020 125030 125040	
Human	CTGGGACTACAGGCGTGTGCCGCCAAGCCCAGCTAATTTTTTTGTATCTTTAGTAGAGACGGTTTCACCATGTTGGCCAG	102107
PolarBear		52114
Cow		86816
Dog		82307
BelugaWhale		81417
Donkey		90106
Cat		86449
Ferret		87765
Lion		84143
Vaquita		84015
Pig		83470

Majority	XXXXXXXXXXXXXXXXXXXXXXXXXXXXXXXXXXXXXXXXXXXXXXXXXXXXXXXXXXXXXXXXXXXXXXXXXXXXXXXX	
	125050 125060 125070 125080 125090 125100 125110 125120	
Human	GCTGGTCTCGAACTCCTGACCTCAAGTGATCTACCTGCCTCAGCCTTCTAAAGTGTTGGGATTACAGGCATGAGCCATGG	102187
PolarBear		52114
Cow		86816
Dog		82307
BelugaWhale		81417
Donkey		90106
Cat		86449
Ferret		87765
Lion		84143
Vaquita		84015
Pig		83470

Majority	XXXXXXXXXXXXXXXXXXXXXXXXXXXXXXXXXXXXXXXXXXXXXXXXXXXXXXXXXXXXXXXXXXXXXXXXXXXXXXXX	
	125130 125140 125150 125160 125170 125180 125190 125200	
Human	CACCCGGCCAAGATGAAATGTCAAGGCCATTTTAGCCACTATGCTTTTAAGACTACTTAAAGCTGTGCTTTCAGGTCTCC	102267
PolarBear		52114
Cow		86816
Dog		82307
BelugaWhale		81417
Donkey		90106
Cat		86449
Ferret		87765
Lion		84143
Vaquita		84015
Pig		83470

Majority	XXXXXXXXXXXXXXXXXXXXXXXXXXXXXXXXXXXXXXXXXXXXXXXXXXXXXXXXXXXXXXXXXXXXXXXXXXXXXXXX	
	125210 125220 125230 125240 125250 125260 125270 125280	
Human	CCAGGAATACTTGTTCACTCTATACTATTGCTGCTGCTGTTCTGGCTTGAGAATTTTGAGAATCACTAAGATACAATTTT	102347
PolarBear		52114
Cow		86816
Dog		82307
BelugaWhale		81417
Donkey		90106
Cat		86449
Ferret		87765
Lion		84143
Vaquita		84015
Pig		83470

Majority	XXXXXXXXXXXXXXXXXXXXXXXXXXXXXXXXXXXXXXXXXXXXXXXXXXXXXXXXXXXXXXXXXXXXXXXXXXXXXXXX	
	125290 125300 125310 125320 125330 125340 125350 125360	
Human	AAGTTTTGACATGTAAATTATTTGGTTGAGTAGAAAGAATTAATGTAGATAAGCAAAAACCCCTTTATAAAAAATGCTTA	102427
PolarBear		52114
Cow		86816
Dog		82307
BelugaWhale		81417
Donkey		90106
Cat		86449
Ferret		87765
Lion		84143
Vaquita		84015
Pig		83470

Majority	XXXXXXXXXXXXXXXXXXXXXXXXXXXXXXXXXXXXXXXXXXXXXXXXXXXXXXXXXXXXXXXXXXXXXXXXXXXXXXXX	
	125370 125380 125390 125400 125410 125420 125430 125440	
Human	CCCTTTAAAAAATGTTGAATCCTCACAGTATGATTAAATACTACTTGTAAAAGTGTCTGTTAACCATAATGTATTTTTTA	102507
PolarBear		52114
Cow		86816
Dog		82307
BelugaWhale		81417
Donkey		90106
Cat		86449
Ferret		87765
Lion		84143
Vaquita		84015
Pig		83470

Majority	XXXXXXXXXXXXXXXXXXXXXXXXXXXXXXXXXXXXXXXXXXXXXXXXXXXXXXXXXXXXXXXXXXXXXXXXXXXXXXXX	
	125450 125460 125470 125480 125490 125500 125510 125520	
Human	GATAATTTGAGTAGAATTATACGTGCAATATATTCTTGAGTTTGTGACCAACAAAGAAACAGATTAATGATTGAAAATTC	102587
PolarBear		52114
Cow		86816
Dog		82307
BelugaWhale		81417
Donkey		90106
Cat		86449
Ferret		87765
Lion		84143
Vaquita		84015
Pig		83470

Majority	XXXXXXXXXXXXXXXXXXXXXXXXXXXXXXXXXXXXXXXXXXXXXXXXXXXXXXXXXXXXXXXXXXXXXXXXXXXXXXXX	
	125530 125540 125550 125560 125570 125580 125590 125600	
Human	ACCATACATTACTACCTATGTCTCTCCACCTGCTATCTACTTCCAATTTTTTATATATATAAAAGTCATTAAAGTCATTT	102667
PolarBear		52114
Cow		86816
Dog		82307
BelugaWhale		81417
Donkey		90106
Cat		86449
Ferret		87765
Lion		84143
Vaquita		84015
Pig		83470

```
Majority     XXXXXXXXXXXXXXXXXXXXXXXXXXXXXXXXXXXXXXXXXXXXXXXXXXXXXXXXXXXXXXXXXXXXXXXXXXXXXXXX
                      125610    125620    125630    125640    125650    125660    125670    125680
Human        GTAATTGAGTTTGTTTTTTTTTCTTGAGACAGGGTCTCACTCTGTCACCCAGGCTGGAGTGCAAATGGCATGATCACAGC  102747
PolarBear                                                                                      52114
Cow                                                                                            86816
Dog                                                                                            82307
BelugaWhale                                                                                    81417
Donkey                                                                                         90106
Cat                                                                                            86449
Ferret                                                                                         87765
Lion                                                                                           84143
Vaquita                                                                                        84015
Pig                                                                                            83470

Majority     XXXXXXXXXXXXXXXXXXXXXXXXXXXXXXXXXXXXXXXXXXXXXXXXXXXXXXXXXXXXXXXXXXXXXXXXXXXXXXXX
                      125690    125700    125710    125720    125730    125740    125750    125760
Human        TCACTGCAGCCTCAACCTCCCAGGTTCGATAGATCCTCCCACCTCAGTCCCCCACAGGCATGCATCACCATGCCTGACTA  102827
PolarBear                                                                                      52114
Cow                                                                                            86816
Dog                                                                                            82307
BelugaWhale                                                                                    81417
Donkey                                                                                         90106
Cat                                                                                            86449
Ferret                                                                                         87765
Lion                                                                                           84143
Vaquita                                                                                        84015
Pig                                                                                            83470
```

Majority	XXXXXXXXXXXXXXXXXXXXXXXXXXXXXXXXXXXXXXXXXXXXXXXXXXXXXXXXXXXXXXXXXXXXXXXXXXXXXXXX	
	125770 125780 125790 125800 125810 125820 125830 125840	
Human	GTTTTTGTGTTTTTTTGTAGAGACAAGGTTTTGCCATGTTGCCCAGACTGGTCTCAAACTTGGGCCAAAGCCATCCACCT	102907
PolarBear		52114
Cow		86816
Dog		82307
BelugaWhale		81417
Donkey		90106
Cat		86449
Ferret		87765
Lion		84143
Vaquita		84015
Pig		83470

Majority	XXXXXXXXXXXXXXXXXXXXXXXXXXXXXXXXXXXXXXXXXXXXXXXXXXXXXXXXXXXXXXXXXXXXXXXXXXXXXXXX	
	125850 125860 125870 125880 125890 125900 125910 125920	
Human	GCCTCGGCTTCCCAATGTGCTGGGATTACATGTGTGAGCCACCACACCCAGTCATGAGAATTATTTTAAATCAGGTAAAC	102987
PolarBear		52114
Cow		86816
Dog		82307
BelugaWhale		81417
Donkey		90106
Cat		86449
Ferret		87765
Lion		84143
Vaquita		84015
Pig		83470

Majority	XXXXXXXXXXXXXXXXXXXXXXXXXXXXXXXXXXXXXXXXXXXXXXXXXXXXXXXXXXXXXXXXXXXXXXXXXXXXXXXX	
	125930 125940 125950 125960 125970 125980 125990 126000	
Human	AAAATGTCTGTGGGAATATTGAGCTTTATGATAGATACCTAAGTTTTCCCAAAAATAATTGTGTTAGCATTTCAGTGAAT	103067
PolarBear		52114
Cow		86816
Dog		82307
BelugaWhale		81417
Donkey		90106
Cat		86449
Ferret		87765
Lion		84143
Vaquita		84015
Pig		83470

Majority	XXXXXXXXXXXXXXXXXXXXXXXXXXXXXXXXXXXXXXXXXXXXXXXXXXXXXXXXXXXXXXXXXXXXXXXXXXXXXXXX	
	126010 126020 126030 126040 126050 126060 126070 126080	
Human	CACTATACTTAACCTAGAAATCTAGGTTACCAATAACAGTCTCTTTCAGGTTTTTTTCTTTTCCTGTGTCTTTCTTTTTA	103147
PolarBear		52114
Cow		86816
Dog		82307
BelugaWhale		81417
Donkey		90106
Cat		86449
Ferret		87765
Lion		84143
Vaquita		84015
Pig		83470

Majority	XXXXXXXXXXXXXXXXXXXXXXXXXXXXXXXXXXXXXXXXXXXXXXXXXXXXXXXXXXXXXXXXXXXXXXXXXXXXXXXX	
	126090 126100 126110 126120 126130 126140 126150 126160	
Human	CTAAAATCATTCATAACTGGGATGACTTATGGAGTTGATACCTTAGGTCAAATCCCAATTTAGAAACTCCCAAAAGCCTG	103227
PolarBear		52114
Cow		86816
Dog		82307
BelugaWhale		81417
Donkey		90106
Cat		86449
Ferret		87765
Lion		84143
Vaquita		84015
Pig		83470

Majority	XXXXXXXXXXXXXXXXXXXXXXXXXXXXXXXXXXXXXXXXXXXXXXX	
	126170 126180 126190 126200	
Human	TCAAATTTTGACACATACTTTAAATGAACTGTTATTGAGTAGATATA	103274
PolarBear		52114
Cow		86816
Dog		82307
BelugaWhale		81417
Donkey		90106
Cat		86449
Ferret		87765
Lion		84143
Vaquita		84015
Pig		83470
